# Gene expression profiling identifies pathways involved in seed maturation of *Jatropha curcas*

**DOI:** 10.1186/s12864-020-6666-1

**Published:** 2020-04-09

**Authors:** Fatemeh Maghuly, Tamás Deák, Klemens Vierlinger, Stephan Pabinger, Hakim Tafer, Margit Laimer

**Affiliations:** 1 Plant Functional Genomics, Department of Biotechnology, BOKU-VIBT, University of Natural Resources and Life Sciences, Muthgasse 18, 1190 Vienna, Austria; 2 Department of Viticulture, Szent István University, Villányi út 29-43, 1118 Budapest, Hungary; 3 Center for Health and Bioresources, Molecular Diagnostics, Austrian Institute of Technology (AIT), Giefinggasse 4, 1210 Vienna, Austria; 4 Austrian Center of Biological Resources (ACBR), Department of Biotechnology, BOKU-VIBT, University of Natural Resources and Life Sciences, Muthgasse 18, 1190 Vienna, Austria; 5 Plant Biotechnology Unit, Department of Biotechnology, BOKU-VIBT, University of Natural Resources and Life Sciences, Muthgasse 18, 1190 Vienna, Austria

**Keywords:** Biofuel, Gene expression, High-throughput quantitative real-time PCR, Metabolic pathways, Microarray, Next generation sequencing

## Abstract

**Background:**

*Jatropha curcas,* a tropical shrub, is a promising biofuel crop, which produces seeds with high content of oil and protein. To better understand the maturation process of *J. curcas* seeds and to improve its agronomic performance, a two-step approach was performed in six different maturation stages of seeds: 1) generation of the entire transcriptome of *J. curcas* seeds using 454-Roche sequencing of a cDNA library, 2) comparison of transcriptional expression levels using a custom Agilent 8x60K oligonucleotide microarray.

**Results:**

A total of 793,875 high-quality reads were assembled into 19,382 unique full-length contigs, of which 13,507 could be annotated with Gene Ontology (GO) terms. Microarray data analysis identified 9111 probes (out of 57,842 probes), which were differentially expressed between the six maturation stages. The expression results were validated for 75 selected transcripts based on expression levels, predicted function, pathway, and length.

Result from cluster analyses showed that transcripts associated with fatty acid, flavonoid, and phenylpropanoid biosynthesis were over-represented in the early stages, while those of lipid storage were over-represented in the late stages. Expression analyses of different maturation stages of *J. curcas* seed showed that most changes in transcript abundance occurred between the two last stages, suggesting that the timing of metabolic pathways during seed maturation in *J. curcas* occurs in late stages. The co-expression results showed that the hubs (CB5-D, CDR1, TT8, DFR, HVA22) with the highest number of edges, associated with fatty acid and flavonoid biosynthesis, are showing a decrease in their expression during seed maturation. Furthermore, seed development and hormone pathways are significantly well connected.

**Conclusion:**

The obtained results revealed differentially expressed sequences (DESs) regulating important pathways related to seed maturation, which could contribute to the understanding of the complex regulatory network during seed maturation with the focus on lipid, flavonoid and phenylpropanoid biosynthesis. This study provides detailed information on transcriptional changes during *J. curcas* seed maturation and provides a starting point for a genomic survey of seed quality traits. The results highlighted specific genes and processes relevant to the molecular mechanisms involved in Jatropha seed maturation. These data can also be utilized regarding other *Euphorbiaceae* species.

## Background

Environmental protection and proper land use are some of the main concerns of mankind. Significant emission levels of carbon dioxide (CO_2_) and other greenhouse gases into the atmosphere as a consequence of burning petroleum products for various human activities and its impact on global climate is quite obvious [1]. Actions to mitigate, reduce the effects of climate change (https://www.apha.org/topics-and-issues/climate-change) offer an excellent opportunity to provide innovative methods in order to control air pollution and greenhouse gas emission. Therefore, fuel derived from organic materials (e.g. biofuel crops) receive more attention in the process of shifting from crude fossil oil to more sustainable resources [2]. However, using food crops as first generation biofuels caused the food prices to increase globally, which culminated in a worldwide food crisis. Therefore, methods of biofuel production had progressed from first to second generation, and this novel approach utilizes only non-food crops. Among the second generation biofuels, *J. curcas* is a promising arable crop, which is frequently mentioned as the best option for marginal quality soils. This plant can be successfully cultivated on soils with low nutrient levels and low water reserves, even in areas that are considered unsuitable for agricultural production.

*J. curcas* naturally grows in tropical and subtropical climates [3] between sea level and 1800 m of altitude, and is well adapted to semi-arid, arid conditions and regions with an annual rainfall ranging between 250 and 3000 mm [4]. *J. curcas* is a rapidly growing tree that can be propagated easily, and can be used as a multi-purpose plant for biodiesel supply, medicinal uses, veterinary purposes and livestock feed [5, 6]. The oil quality obtained from the Jatropha crop is very similar to the values of conventional diesel fuel and can be used without any modification in diesel engines currently in operation [7].

*J. curcas* seed contains non-edible oils, which are traditionally used for soap production and medicinal uses [8]. In addition, its solvents are used due to its therapeutic characteristics by people suffering from various skin diseases and sensitivity to regular soap [9]. All traits mentioned above make *J. curcas* one of the best candidate as a profitable biofuel crop species for restoring wastelands and improving employment chances and subsistence in rural areas [10, 11]. Additionally, the *J. curcas* seed cake, which is a waste by-product of the biodiesel trans-esterification process, can be used for the production of various supplies such as organic fertilizer, high-quality paper, energy pellets, soap, cosmetics, toothpaste, embalming fluid, pipe joint cement and cough medicine [12].

*J. curcas* seed kernels are rich in oil (54–58%) and protein (20–28%) compared to the shell, and several efforts were made to make use of cake or kernel meal that remains after oil extraction [6]. Furthermore, it contains a variety of phenolic, flavonoid and diterpenic compounds showing notable anti-oxidant, anti-microbial, and anti-inflammatory activities [5]. However, its toxins and anti-nutritional compounds render the seed cake and oil unsuitable for use as animal feed and human consumption [13]. Therefore, efforts are required to increase oil yield and composition by improving the ability of the plant to produce favorable fruits/seeds with suitable compounds.

Breeding efforts of this biofuel crop will be accelerated by the in-depth knowledge of seed transcripts of *J. curcas* for obtaining functional genomics information to discover genes that encode enzymes involved in the biosynthesis of oil and toxin precursors and to describe their relevant metabolic pathways [14–16]. Therefore, it is necessary to establish a reliable method to characterize the temporal shifts in gene expression being in the background of the biochemical and metabolic processes which take place during seed maturation. Furthermore, such data could help to identify, characterize and – if necessary – modify the possible transcripts of interest. In Jatropha, transcriptome studies generated data describing seed development and seed germination from manually pollinated plants, with the emphasis on differentially expressed genes related to lipid biosynthesis and toxic compounds [14–18]. In addition, whole genome sequencing was applied to identify protein-encoding genes, which could help in improving the traits of interest (e.g. oil composition) of *J. curcas* [19–24]. Considering that Jatropha flowering and fruit-bearing are practically continuous, the simultaneous presence of mature and immature fruit on the same plant provides a unique opportunity to collect seeds in different stages of maturation of this open-pollinated plant. Therefore, to obtain an overview of transcripts associated with all the seed maturation stages that are potentially involved in seed maturation under the same environmental condition, we performed the following analyses: 1) generation of the entire transcriptome of six stages of *J. curcas* seed maturation using 454-Roche sequencing from pooled samples; 2) comparison of transcriptome expression in seeds of six stages of seed maturation using custom Agilent 8x60K oligonucleotide gene expression microarrays.

## Results

### Whole seed transcriptome sequencing

To cover the entire *J. curcas* seed transcriptome, total RNA was extracted from six stages of seed maturation, and equal amounts of total RNA from each sample were pooled together. From this pool, mRNA was isolated and reverse transcribed into cDNA. Normalized cDNA libraries were generated and sequenced using the GS FLX Titanium. Sequencing of cDNA libraries yielded a total of 793,875 high quality (HQ) reads with an average read length of 358 nucleotides and 262,096,927 total number of bases (SRX4559398).

After trimming and cleaning, a total of 603,459 HQ reads were assembled into 19,841 contigs (unique transcripts) containing 13,171,840 bases. Out of them, 48,978 reads were identified as singletons. The size of contigs ranged from 100 to 4088 bases, with 1035 bases as N50 contig size. All contigs can be accessed at http://short.boku.ac.at/jatropha_contigs. After removing all contaminant sequences, 19,382 unique contigs have been retained. Assembled contigs over 200 bp have been deposited at DDBJ/EMBL/GenBank under the accession GIKD00000000. The version described in this paper is the first version, GIKD01000000.

In addition, the data were compared with the Jatropha genomic sequences of Kazusa DNA Research Institute (JAT_r4.5, ftp://ftp.kazusa.or.jp/pub/Jatropha/) [25, 26] and Chinese Academy of Sciences (JatCur_1.0, ftp://ftp.ncbi.nih.gov/ genomes/Jatropha_curcas/) [27, 28] (Table S1). In total 84,6% (16,397 contigs), 3.9% (753 contigs), 1.8% (351 contigs) showed sequence similarity to both, only to JAT_r4.5 or only to JatCur_1.0 databases, respectively. However, 9.7% (1881 contigs) were found additionally in the current study. These transcripts are most probably new genes, non-jatropha or non-plant genes, or possibly sequencing artefacts. To assess the quality of the assembled transcripts, the library was compared to the reference transcriptome of JatCur 1.0 available from NCBI. The seed specific de novo RNA library of this study represented about 44.1% of the 35,788 reference RNA coding sequences with an average blast High Scoring Pairs (HSP) coverage of 93% (S.D. 16.3%), suggesting a low amount of potential chimeric contigs.

To further assess the extent of represented transcripts in the seed transcriptome, contigs of core eudicot genes were identified using BUSCO. Of the total 2326 core eudicot gene groups, 547 (23.5%) were found in a complete form in our dataset (528 of which were single copy representations suggesting low redundancy in the library). Two hundred sixty fragmented groups were also identified, while 1519 core genes were missing from our dataset. Selected core transcripts were also subject of a phylogenetic analysis, showing that the transcripts are most closely related to *J. curcas*, *Hevea brasiliensis*, *Manihot esculenta* and *Ricinus communis*, all belonging to the *Euphorbiaceae* family (Figure S1).

### Functional annotation of whole transcript sequencing data

All 19,382 unique contigs were analyzed by Blast2GO [29] and aligned using BLASTX [30]. Search in the NCBI non-redundant nucleotide database using an E-value threshold of 1e-6 identified 14,753 unique contigs. Approximately 3% (553 contigs) of the transcripts showed top BLAST hits with uncharacterized/predicted proteins, and 24% (4629 contigs) had no significant similarity to any sequence in the public dataset. The average length of annotated and unannotated contigs was 800 and 400 bp, respectively.

GO terms classification identified 13,507 *Jatropha* unique transcripts received at least one GO annotation (Table S2). The highest percentage of GO terms was found in the category BP, containing 2409 GO terms, followed by 1841 in MF and 510 in CC (Table S2, Figure S2). The most abundant GO terms in the BP category were genes involved in oxidation-reduction processes (5.1%), DNA-templated regulation of transcription (3.1%) and response to cadmium (2.4%). Within MF, the largest content of functionally assigned ESTs were related to ATP binding (6.5%), zinc ion binding (5%) and DNA binding (3.6%). In CC, the most representative categories were nucleus (14.2%), plasma membrane (9.1%) and chloroplast (7.4%).

Out of the 13,507 sequences annotated with GO terms, 4313 contigs were assigned with 5593 EC numbers representing 1008 unique enzymes, 799 of which are assigned to one or more KEGG pathways (Table S3). Additionally, of the 19,382 contigs, 37.6% were also annotated based on homology to sequences in the InterPro database (Table S3).

Moreover, 968 different contigs were identified belonging to 20 transporter classes (Table S3). Of them 223 and 236 contigs belong to the transporter classes 2A (porters) and 3A (p-p-bond-hydrolysis- driven transporters), respectively.

Protein domain characteristics for Resistance Gene Analogs (RGAs) have been identified in 85 contigs, with 70 carrying a kinase domain, 35 of which also harbored an additional serine/threonine (Ser-Thr) site. RGAs that contain Ser-Thr domain can phosphorylate serine and threonine residues, which are involved in plant development, signaling and defense [31]. However, some RGAs like the *Pto* gene from tomato encode only Ser-Thr protein kinase. Four contigs with a nucleotide-binding site (NBS-ARC) domain and eight contigs with a leucine-rich repeat (LRR) domain were found. Both domains are abundantly present in plants and have an ATPase activity [32].

In addition, 600 contigs could be identified as Transcription factors (TFs), belonging to 52 different TF classes (Table S3). The most abundant TF families were MYB-related, MYB, bZIP, AP2, ERF and RAV, represented by 66, 54, 49, 49, 49 and 46 contigs, respectively.

**Fig. 1:**
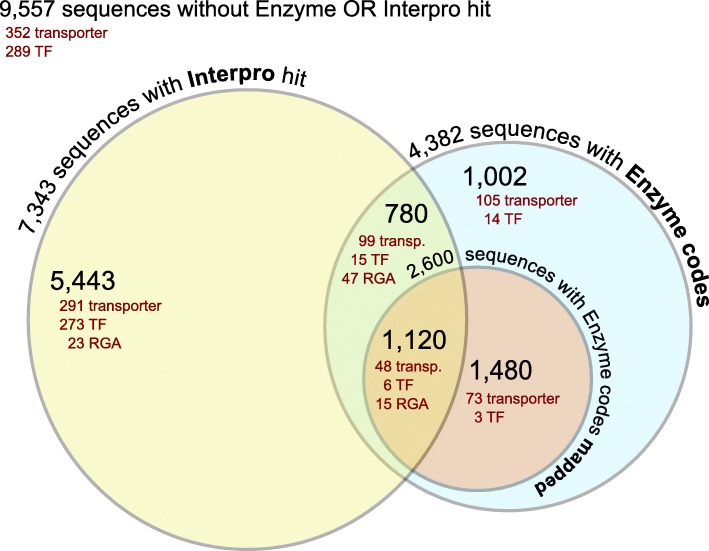
Venn diagram summarizing the functional annotation process of whole seed transcriptome sequence data. Enzyme codes originate from the Blast2Go annotation, while Interpro hits are resulting from InterProScan (With Interpro hit). Enzyme codes have been checked for mapping on the pathways of the KEGG (With Enzyme codes mapped). Results of the manual annotation for transporters, TFs and RGAs in all classes are also indicated

Annotated sequences were mapped to KEGG pathways showed 2591 contigs located on 143 pathways (Fig. 1, Table S4). Using the KEGG classifications allowed us to identify that the most highly represented pathways were purine metabolism (315 contigs), followed by starch and sucrose metabolism (226), pyrimidine metabolism (154) and phenylalanine metabolism (138). Further, glycolysis (129), pyruvate metabolism (111), flavonoid biosynthesis (111), glycerolipid metabolism (103) and phenylpropanoid biosynthesis (96) were also found in the top 20 highly represented pathways.

### Genome-wide variation in transcript expression during seed maturation

An 8x60k oligonucleotide microarray containing 57,842 unique probes was produced from 19,841 transcriptome contigs. In total 31,875 specific probes and 2604 cross-hybridizing probes (Xhyb) in sense direction, as well as 21,680 specific probes and 1683 Xhyb in antisense direction were designed.

The microarray data were normalized; differential expression patterns were identified, classified, and categorized by their possible molecular function and involvement in metabolic pathways. Principle components analysis (PCA) on transcript expression (abundance) of 57,842 probes showed a clear separation of the six different maturation stages along the first principle component (PC1); which explained 53% of total variation, and was associated mostly with variation in transcript expression over the maturation stages, where expression from stage IV and V were closer to each other (Figure S3). Significant changes in transcript expression (abundance) were observed in the early and late stages, suggesting a higher physiological differentiation in these stages. In addition, biological replicates of each stage clustered together, suggesting a minimal variation between replicates.

To identify changes in gene expression patterns during seed maturation linear models were calculated and revealed large changes in gene expression over the six stages of seed maturation. A total of 9111 probes (16% of the total probes) from 7299 contigs (38% of the total contigs) were differentially expressed with a *P*-value <1e-8 (Table S5 and Figure S4).

**Fig. 2:**
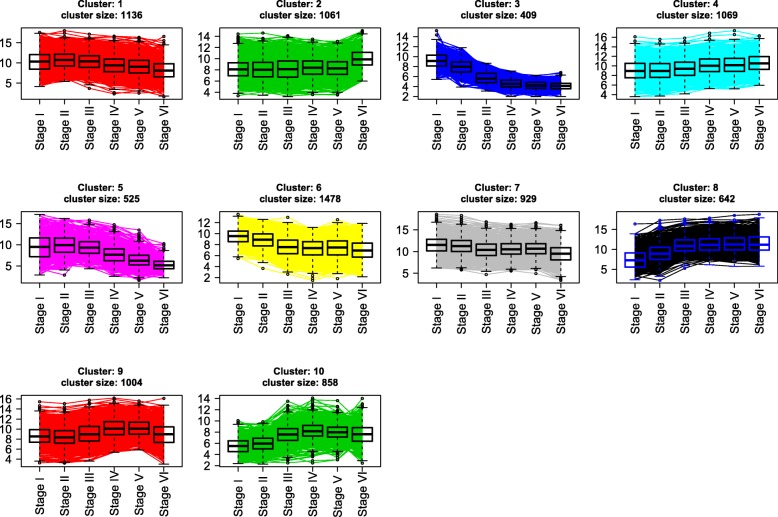
Cluster analysis of DESs based on their expression patterns across seed maturation stages. The analysis was performed using normalized and filtered data according to their expression profiles between seed maturation stages. Normal mixture modelling for model-based clustering (expectation-maximization) was performed with *P*-value <1e-8

**Fig. 3:**
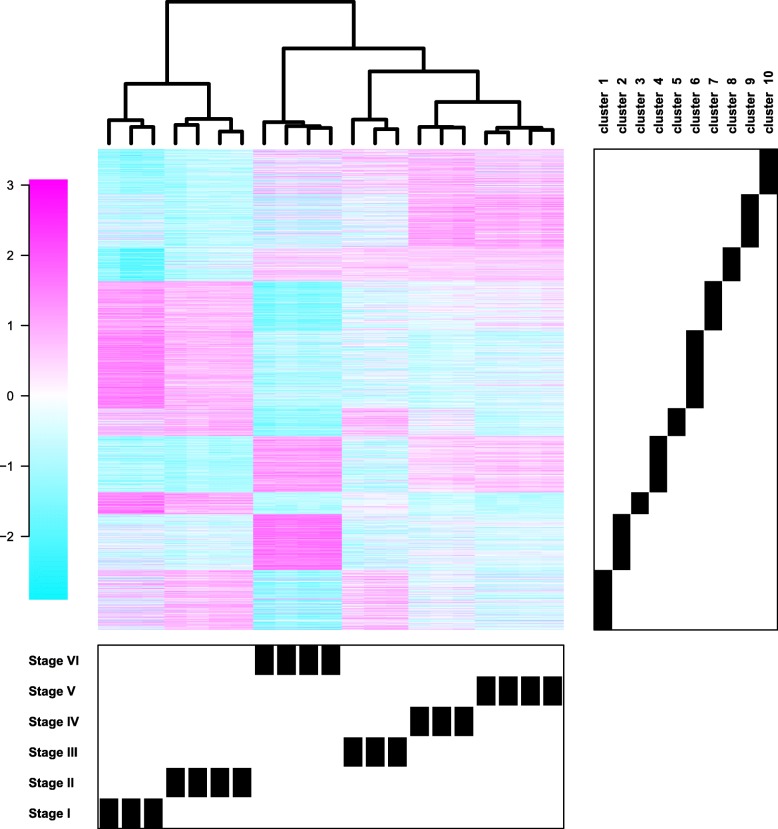
Global gene expression heat map and cluster analysis of the DESs during seed development. Cluster analysis on y-axis represents similar expression patterns among the expressed sequences, while cluster analysis on the x-axis indicates the relatedness of DESs profiles among the different seed maturation stages and the biological replications. The log_2_ of relative gene expression levels represented by the heat map on the left. Clusters of DESs according to Fig. 2, are represented on the right. Seed development stages and their biological replication are clustered at the bottom

The cluster analysis showed that gene different expression patterns could be classified into ten different clusters (1–10) (Fig. 2, 3) of co-expressed genes. Up-regulated transcripts, whose expression was increased during seed maturation, are displayed in clusters 2, 4, 8, 9 and 10 (group A), while down-regulated transcripts were displayed in clusters 1, 3, 5, 6 and 7 (group B).

Besides, we annotated manually the identified differentially expressed sequences (DESs) and patterns with respect to their specific function. Based on the Transporter Classification Database, 431 differentially expressed transcript (699 probes) were assigned to 92 transporter subfamilies, 16 subclasses and 7 classes, distributed among all clusters, representing the intense activities during the maturation process, which requires transport of metabolites within the cell and between different parts of the seed. The highest number (184) of transcripts related to transport activities were identified in class 2 (Electrochemical potential-driven transporters), followed by class 3 (Primary active transporters) with 179 transcripts. Thirteen ranscripts were classified to be transporter subfamily 1.A.33, which is related to heat shock protein (Hsp) 70 (Table S5). Furthermore, different kinds of sugar transporters (2.A.1) and ATP/ADP transmembrane transport (2.A.29) represent the role of transporters to provide necessary energy metabolism during seed maturation. Among 24 transcripts that were classified as ABC transporters (3.A.1), subfamilies A, C, E, F, G and I were identified (Table S5).

**Fig. 4:**
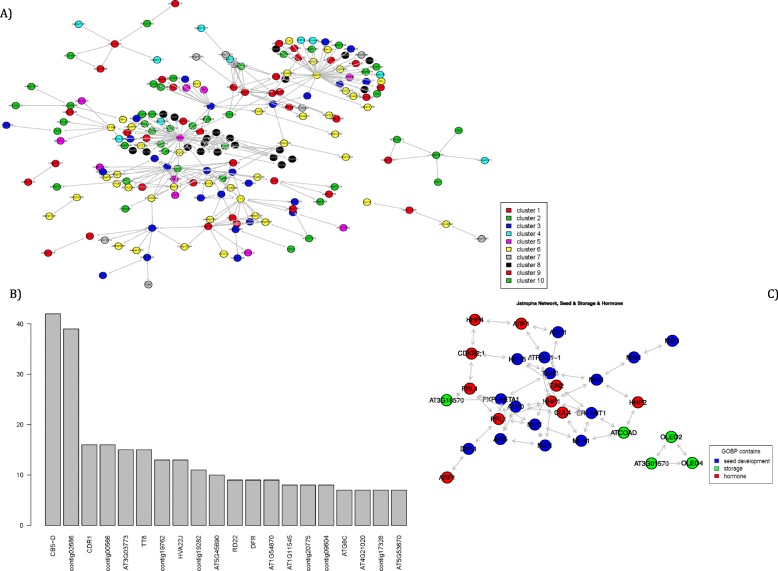
Co-expression networks based on partial correlations. **a** A network of the 300 most significant edges between contigs differentially expressed during seed development stages. Node colouring represents cluster membership (see also Fig. S3). **b** Histogram representation the top 20 of contigs differentially expressed (x-axis) with the highest number of edges (y-axis). Two major hubs could be identified with ~ 40 edges and a broad range of nodes display between 10 and 15 edges. **c** Co-expression networks based on GOs of biological processes related to seed storage, seed development, and hormones cross-talking

In addition, 47 families of TFs showed differential expression between the six seed maturation stages, involving all expression pattern clusters (Table S5). The highest number of transcripts related to TFs were found in cluster 1, followed by cluster 6, while the least number of TFs were found in cluster 3. The most abundant TF families were identified as AP2/ERF-RAV. Furthermore, we explored the co-expression patterns using partial correlation networks. We identified two intermodular hubs with around 40 edges and a broad range of nodes displaying between 15 and 10 edges (Fig. 4a-b). The CB5-D hub showed the highest number of edges followed by unannotated contig02686, CDR1 (aspartic proteinase), unannotated contig00566, TT8 (Transparent Testa 8), unannotated contig19762, and HVA22 (Fig. 4a-b). HVA22J, connected CB5-D (cytochrome B5 isoform D), to CDR1, Dihydroflavonol reductase (DFR) and TT8 (Fig. 4a-b).

To focus on processes expected to be involved in seed storage and seed developments as well as hormone pathway, the GO terms from BP category were extracted. The co-expression results showed a high degree of connectivity between seed development and hormone pathways, while seed storage is less connected to the other two pathways (Fig. 4c).

In addition, based on pairwise comparison between seed maturation stages, the highest number of differentially expression transcripts related to 889 and 272 contigs (1122 and 1673 probes), which were found between stages V and VI (Table S6).

### GO enrichment analyses of DESs

**Fig. 5:**
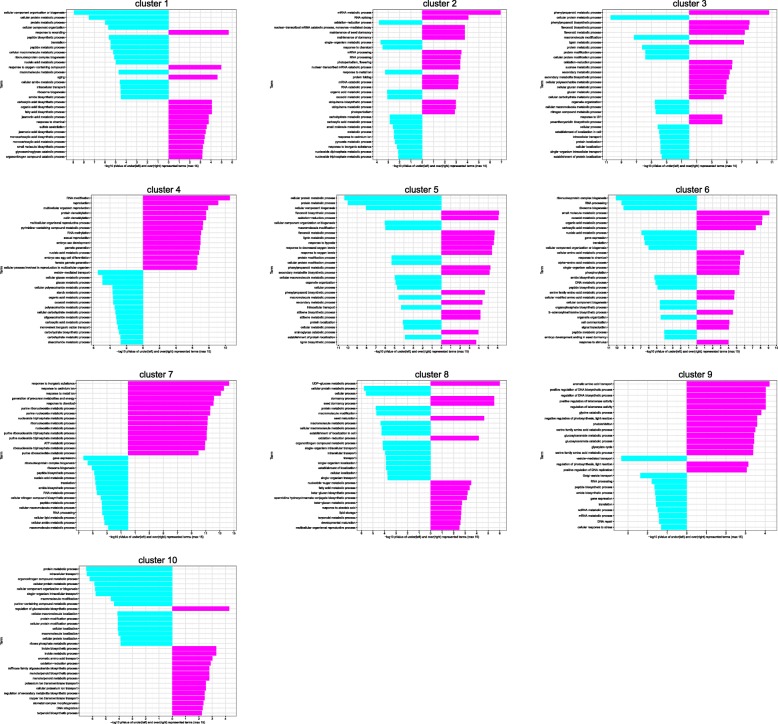
Plot of the top 15 significantly enriched GO terms in category BP, for all DESs in the 10 identified clusters (Fig. 2). Turquoise bars show under-represented and magenta bars over-represented GO terms. The x-axis indicates the statistical significance of the enrichment

GO enrichment analyses in BP categories for each cluster indicated that the most significantly over- and under-represented DESs were found in cluster 6 (Top 15 GO terms and detailed information for each cluster are shown in Fig. 5, Figure S5, Table S7 and Table S8).

Besides, visualization of enriched GO terms related to BP category showed that the GOs related to fatty acid metabolism (e.g. unsaturated fatty acid, linoleic acid), lipid storage, dormancy process, aromatic acid transports, monoterpenoid metabolism, and UDP-glucose metabolism were significantly over-represented in cluster group A, with higher DES level during late stage of seed maturation. Furthermore, Raffinose family oligosaccharides (RFOs), which are associated with late maturation in *Arabidopsis*, *Brassica napus* and *Medicago trunculata* [33–35] and transcripts related to biosynthesis of serine and glycine, Embryo sac development, RNA modification, methylation, maintenance of seed dormancy, protein folding and RNA modification were over-represented in this group.

In contrast, GOs related to phenylpropanoid and flavonoid metabolism and biosynthesis, as well as cell wall modification and carbohydrate metabolism in cluster group B, were significantly enriched with high expression levels during the early stage of seed maturation (Figure S4). Transcripts involved in hormone transporters, signaling, ATP hydrolysis coupled protein transport and purine ribonuclease metabolism were significantly over-represented in this group.

GOs involved in glucan and beta-glucan biosynthesis playing a key role in regulating seed coat-imposed dormancy [36] were over-represented in two clusters (3 and 8, respectively). Also, GOs involved in translation, RNA processing, and gene expression were under-represented in different clusters (6 and 9). They represent a different pattern of gene expression during seed maturation, indicating the involvement of two different groups of genes.

### KEGG enrichment analysis of DESs

To further understand the biological function of DESs during seed maturation, enriched KEGG pathways with *P*-value < 0.05 in the set of DESs was assessed (Table S8 and S9, Figure S4 and S6).

### Pathway enrichment in DESs related to lipid metabolism

In the plant, pathways contributing to lipid biosynthesis can be divided into three steps and cell compartments; a) fatty acid biosynthesis in the plastids, b) triacylglycerol (TAG) biosynthesis in the endoplasmic reticulum (ER), and c) oil body formation in the cytoplasm [37]. Altogether, 97 contigs and 55 enzymes distributed among 13 pathways were enriched as being involved in these steps (Figure S7).

In the first step, we could identify DESs related to 3-oxoacyl-ACP reductase (KAR, EC:1.1.1.100), containing various DESs (five contigs in clusters 1 and 8). In addition, Beta-ketoacyl-ACP synthase I (KASI, EC:2.3.1.41, two contigs in clusters 5 and 8) with DESs were identified showing different regulation patterns. The elongation from 16∶0-ACP or 18∶1-ACP [38] occurs in the plastid and is catalyzed by acyl-ACP desaturase (AAD, EC:1.14.19.2), which was represented in current study by four DESs (in clusters 5 and 8). Additionally, we identified Oleoyl-ACP hydrolase (OAH, EC:3.1.2.14, two contigs in clusters 1 and 5), removing acyl group from ACP, and Acyl-CoA synthetase (EC:6.2.1.3, two contigs in cluster 8) engaged in glycerophospholipid metabolism and fatty acid elongation.

We also identified key enzymes involved in triacylglycerol (TAG) production such as phospholipid: diacylglycerol acyltransferase (PDAT1, EC:2.3.1.158, two contigs in cluster 8), lysophosphatidic acid acyltransferase (LPAAT, EC:2.3.1.51, five contigs in clusters 2 and 4), and PA phosphatase (PAP, EC:3.1.3.4, one contig in cluster 2). Two DESs in cluster 10, encoding diacylglycerol O-acyltransferase (DGAT, EC:2.3.1.20), and two DESs corresponding phospholipid diacylglycerol acyltransferase (PDAT, EC:2.3.1.158) in cluster 8 were identified. Furthermore, triacylglycerol lipase (EC:3.1.1.3, six contigs in clusters 4 and 8), which modifies TGA into fatty acids was identified. All DESs involved in TAG production process were classified in clusters 2, 4 and 8, showing their expressions increase during seed maturation.

Contigs encoding enzymes like diacylglycerol kinase (ATP, EC:2.7.1.107, two contigs in cluster 4), aldehyde dehydrogenase (NAD+, EC:1.2.1.3, three contigs in clusters 2, 4 and 8) and glycerate 3-kinase (EC:2.7.1.31, two contigs in cluster 2), were found in cluster 2, 4 and 8, showing an increase during the last stage of *J. curcas* seed maturation. As expected, the synthesis of fatty acids requires a high amount of energy during seed maturation, which results in increased expression of enzymes related to photosynthesis as an energy supply [39].

Furthermore, two important enzymes involved in alpha-linolenic acid metabolism and biosynthesis of unsaturated fatty acids were identified in cluster 1: acyl-CoA oxidase (EC:1.3.3.6, one contig) and enoyl-CoA hydratase/3-hydroxyacyl-CoA dehydrogenase (EC:4.2.1.17, two contigs).

### Pathway enrichment in DESs related to phenylpropanoid biosynthesis

Among the significantly enriched pathways, the phenylpropanoid biosynthesis pathway contained 30 over-represented contigs and 11 enzymes located in clusters 3, 5 and 8 (Figure S8). In this pathway, we identified two over-expressed transcripts (contig05064, and contig05269) related to phenylalanine/tyrosine ammonia-lyase (PTAL, EC:4.3.1.25), the expression of which decreases during seed maturation as shown in clusters 3 and 5. Furthermore, one transcript corresponding to trans-cinnamate 4-monooxygenase (C4H, EC:1.14.13.11, one contig in cluster 5) was identified, which converts cinnamic acid to P-coumaric acid. Finally, P-coumaric acid can be conjugated by 4-coumarate: CoA ligase (4CL, EC: 6.2.1.12) and enriched to coenzyme A to form p-coumaroyl-CoA, which is the precursor for the synthesis of flavonoids, stilbenes, and other phenylpropanoids [40]. For this enzyme we also identified five transcripts, enriched in clusters 3 and 5.

Caffeoyl-CoA O-methyltransferase (CCoA-OMT; EC:2.1.1.104) with four transcripts was over-represented in clusters 3, 5, and 8. Finally, 11 transcripts in clusters 3 and 5 for peroxidase (EC:1.11.1.7) were significantly enriched and over-represented.

### Pathway enrichment in DESs of flavonoid biosynthesis-related pathways

After oil extraction, the *Jatropha* seed cake contains high amounts of polyphenols and pigments as a result of flavonoid biosynthesis. In this study, 42 DESs were annotated and enriched in clusters 1, 3 and 5. They encoded 16 enzymes involved in flavonoid, flavone and flavonol biosynthesis and isoflavonoid biosynthesis (Figure S9). Two differentially expressed transcripts (cluster 1) were identified as 6′-deoxychalcone synthase (EC:2.3.1.170) and three transcripts (clusters 3 and 5) as naringenin-chalcone synthase (CHS, EC:2.3.1.74), an important enzyme catalyzing the conversion of cinnamoyl-CoA to pinocembrin chalcone. One transcript was annotated for chalcone isomers (CHI, EC:5.5.1.6, cluster 3) that catalyzes the conversion of pinocembrin chalcone to pinocembrin, a substrate for galangin synthesis [41]. Four transcripts were identified as flavanone 3-dioxygenase or naringenin 3-dioxygenase (F3H, EC:1.14.11.9, in cluster 5), which is involved in highly conserved pathways in plants to convert naringenin into dihydrokaempferol. It is an important intermediate product, that can be converted to kaempferol by flavonol synthase (EC:1.14.11.23), identified in the current study with 10 differentially expressed transcripts (in clusters 1, 3 and 5). The presence of different expression patterns (from different clusters) of one enzyme could be explained by the existence of different isoenzymes and possibly by the interaction with other genes involved in flavonoid biosynthesis at multiple loci [42].

### Validation of microarray data using qRT-PCR

A total of 70 contigs from the DESs represented transcripts in seeds, and three housekeeping genes were selected (Table S10 and Table S11) and used for independent validation using a 48.48 chip (Fluidigm) to confirm that the changes in expressions as indicated by microarray data were authentic and reliable. Candidates for qPCR were chosen based on expression levels, known function, clusters and length of contigs. Additionally, some contigs of unknown function were selected. The corresponding primers are listed in Table S10.

The expression patterns obtained by qRT-PCR correlate strongly to moderately with data from the microarray analyses (about half of the contigs correlate with the microarray data at a Pearson correlation < − 0.8), thus confirming the reliability of the chosen approach (Figure S10).

## Discussion

The understanding of transcriptional variation during different seed maturation stages is of utmost importance for breeding strategies in *J. curcas*; especially for low anti-nutritional, high-quality oil, and bioactive component levels, which could make the crop suitable for biodiesel, animal feed and pharmaceutical use. In this study, genome-wide transcriptome analysis was used to identify the global gene expression pattern of *J. curcas* seeds at six different developmental stages, collected at the same time point on one plant. Even though *J. curcas* is an important oil crop, this is the first study of profiling genome-wide transcript expression during seed maturation of an open-pollinated plant.

The sequencing of the whole seed transcriptome of *J. curcas* revealed 19,382 unique contigs, of which 14,753 contigs were aligned through Blast search; however, 4,629 contigs could not be annotated since they did not have any BLAST hits. The high number of unannotated transcripts might be an indication of potential limitations in transcriptome assembly and annotation. The unannotated sequences could include both novel transcripts and technical artifacts from the sequencing technology (library preparation and/or sequencing machine). Additionally, the applied BLAST parameters are optimized for complex full-length RNA sequences, which does not favor BLAST searches of short (150–200 bp) sequences of low complexity. Furthermore, the comparison of contigs with different Jatropha genome sequences [19–22] revealed additional 1881 contigs in the transcript data set of the current study.

On the other hand, use of whole seed transcripts of different maturation stages allows us to design a robust and high coverage microarray platform (in *J. curcas*) to compare a constant large number of genes for the expression evaluation of different genotypes, organs, and tissues. Although RNA-Seq has some superior benefits for quantitative transcriptomics, microarray is still a common method of choice, since in compared to RNA-seq, microarray is cost effective, fast and provides concordant results. Besides, bioinformatics and statistics practices for microarrays are well established and straightforward in comparison to RNA-seq data, which is more complex [43].

The examination of genome-wide variation in transcripts at selected seed developmental points could allow us to identify DESs with the high number of edges that might be associated with lipid and flavonoid biosynthesis as well as unknown functions. It was also noteworthy to find that all DESs with high number of connectivity belong to cluster group B showing a decrease in their expression during seed maturation. The co-expression patterns showed that the CB5-D hub has the highest number of edges, followed by unannotated contig02686 (Fig. 4a-b). The CB5, a cytochrome B5 isoforms, and small tail-anchored membrane proteins play an essential role in many cellular processes, including lipid biosynthesis. It is well known that CB5-D in different morphotypes of *Brassica rapa* provide electrons for various enzymes located in the endoplasmic reticulum (ER), including fatty acid desaturase (FAD), FAD-like proteins, and are also physiologically important for p450 protein family [44–48]. Hwang et al. [45], combined in vivo and in vitro assays to show that CB5-D are targeted exclusively to mitochondrial outer membranes, while the other isoforms of CB5 (A, B, and C) are targeted to the ER. In addition, our result showed a direct connection between CB5-D from cluster 5 and CDR1 from cluster 3 (Fig. 4a). Although proteins with hydrolase activity like CDR1 do not imply the production of seed oil, overexpression of microsomal DGAT1 – a key enzyme of triacylglycerol production – resulted in differential regulation of CDR1 expression in transgenic and untransformed control in *Brassica* [49]. Furthermore, CDR1 and CB5-D, show the highest expression in the early stage of seed maturation, suggesting their indirect roles in fatty acid biosynthesis in the early stages.

Besides, the contig02686 (unannotated) from cluster 6 also contains a high number of edges, which may be connected to some hypothetical proteins. There is a need for functional analysis of this contig, which might support important biological cell functions and could potentially serve as targets for further studies.

Along with the hub with the high number of edges, TT8 from cluster 6 has an essential role in the regulation of flavonoid biosynthesis and the formation of seed coat colour. However, Chen et al. [50] reported that TT8 also affect FA biosynthesis in seeds of Arabidopsis maternally, which also inhibits FA accumulation by down-regulating of the expression of a carboxylase biotin carboxylase subunit (CAC2), beta-ketoacyl-acp synthetase II (KASII), mosaic death1 (MOD1), fatty acid biosynthesis2 (FAB2), acyl-acp thioesterase (FatA), fatty acid elongation1 (FAE1), FAD2 and FAD3, all playing an important role in FA biosynthesis during seed maturation. The TT8 also represses the expression of leafy cotyledon1 (LEC1), LEC2, FUSCA3 (FUS3), and cytidine diphosphate diacylglycerol synthase2 (CDS2), which are critical to embryonic development [50]. On the other hand, TT8 influences DFR expression, which commits phenolics to proanthocyanidins synthesis responsible for seed coat and quality germplasm of canola [51]. In Arabidopsis, At4g09820 (TT8) encodes a protein, which is important in the expression of DFR, while DFR can give rise to flavonoids [52], representing their important role in flavonoid pathways. The high connectivity and similar expression patterns on both annotated transcripts in the current study might suggest the same functions and roles in *J. curcas*.

Eventually, HVA22-like protein - a stress-inducible gene [53] from cluster 3, playing a role as a hub connection among DFR, TT8, and CDR1, represents its effect on fatty acid and flavonoid biosynthesis pathways in *J. curcas*. HVA22 is identified to be an ER- and Golgi-localized protein and is able to regulate gibberellin-mediated vacuolation negatively [53].

Considering the intense metabolic activity during seed maturation, which requires the regulation of target genes and the exchange of metabolites and proteins between different locations in seed and within the cell, it is important to identify transcripts related to transport machinery and TF. Among the differentially expressed transcripts that were classified as a transporter, subfamily 1.A.33 were related to *Hsps*, which perform diverse biological functions in collaboration with chaperons either in stress or non-stress conditions. In the absence of heat stress, *Hsp* genes are accumulated during the late stage of seed maturation [54]. Several plant cytosolic Hsp70 were identified during development, maturation, and germination of seeds of pea and Arabidopsis [55, 56]. In this study, three transcripts were identified as homologues to Arabidopsis *BiP1* of plant Hsp70 family. These genes appeared to be highly expressed in the early stage of seed maturation (cluster 6), which is in concordance with previous results, where BiPs (*BiP-1 *and *BiP-2*) showed higher expression during the early stage of seed development, which decreased toward the end of seed maturation. These are related to the BiP roles in rapid cell expansion, accumulation of seed storage protein, and seed maturation [56–58].

In the group of ABC transporters (3.A.1) which are involved in plant development, nutrition, stress response and phytohormones and primary metabolites transports [59], one transcript showed homology to AtABCG14 (cluster 5 and 10), described to be involved in translocation of cytokinins between the root and the shoots in *Arabidopsis* [60, 61]. This transcript could be essential for long-distance communication between root-shoot-fruit as well. It was also reported that the *tgd1* (trigalactosyldiacylglycerol) mutants showed a decrease in ER-derived plastid lipids, and accumulation of oligogalactoglycerolipids (TGDG) and TAG in leaf tissues [62], showing that *TGD1/ AtABCI14* encodes a membrane-spanning protein [59].

We also identified one ABCG reporter transcript in cluster 10 homologous to *Arabidopsis* AtABCG25, reported to act as a carrier to export ABA from the vascular tissues, where it is mainly produced [63]. In addition, one homolog of AtABCD1 found in cluster 4 and cluster 9 facilitated the transport of lipidic metabolites in *Arabidopsis* [64]. Moreover, transcripts associated with transporters AtABCC2 and AtABCI17 expressed in cluster 7 and 9, respectively, were reported to be involved in transport of toxic compounds in Arabidopsis. AtABCC2 is tolerant to metals and act as chlorophyll catabolic transporter, while AtABCI17 is expressed in roots and is highly sensitive to Aluminium [59]. It is clear that plant ABC transporters play an important role for survival of plant and seed maturation; however, many questions remain to be answered since only a few of the plant ABC transporters were functionally analyzed (22 out of 130 in Arabidopsis) [59, 64].

Transcripts related to TFs are distributed among all clusters with different expression patterns. Among TFs related to seed oil accumulation, we could identify differentially expressed transcripts that showed homology to Arabidopsis AP2 type TFs, WRI1 and HD-ZIP type, GLABRA2 (Table S5). The WRI1 has been studied extensively in the regulation of the transcription levels of genes in lipid biosynthesis pathways in the Arabidopsis, rapeseed, maize, potato, Siberian apricot kernel, and Jatropha seeds [24, 65–69]. It was reported that over-expression of *JcWRI1* not only increased the lipid content but also the seeds mass. In addition, over-expressing *JcWRI1* in *Jatropha* seeds increased oleic acid (C18:1) level compared to linoleic acid (C18:2), which also increased the expression level of enzymes related to oleic acid production such as BCCP2, KASI, KASIII, FATA, ACP1, and DGAT1 [24]. In the current study, WRI1 and KASI of cluster 5 presented similar expression patterns and were highly expressed in the early stage of seed maturation. This could indicate that the function of both genes closely correlates with fatty acid biosynthesis in the early stage of seed maturation. This is also in agreement with the previous report in Arabidopsis which found that WRI1 targeted many genes from FA synthesis [70].

Furthermore, GLABRA2 (GL2), a member of the HD-ZIP family, identified in cluster 3, exhibited a down-regulated expression pattern during seed development, which is in contrast with the expression pattern of identified enzymes related to lipid biosynthesis pathway, where their expression increased with development of the seed. Based on previous studies, GL2 was a negative regulator of seed oil content [71], which is in agreement with the current data.

Since the major goal of seed oil crop research is focused on oil quality and quantity, it is necessary to understand the processes involved in seed metabolism [5]. On the other hand, phenolic compounds, which are produced under optimal and suboptimal conditions, could influence and improve seed development, germination, metabolism, and biomass accumulation [72]. Furthermore, Synthesis of phenols involved in several pathways, such as flavonoid and phenylpropanoid biosynthesis pathways, could help the plant to cope with different stress conditions [73]. Therefore, focusing on DESs related to enzymes involved in lipid, flavonoid, and phenylpropanoid biosynthesis pathways is of great interest.

In the current study, most of the enzymes involved in lipid biosynthesis in *J. curcas* were identified based on the annotation of the seed transcripts. Among key enzymes involved in fatty acid concentration in the plastid, KASI was identified with two quite different expression patterns during seed maturation stages, suggesting their functional differentiation during seed maturation. However, Jiang et al. [15] and Xu et al. [74] found that the expression of the KASI gene increased during seed maturation in *J. curcas*. On the other hand, among key enzymes involved in TAG synthesis, LPAT, DGAT, and PDAT were identified in our dataset, showing that they were upregulated during seed maturation, indicating their essential roles in the synthesis of TAG. These genes were also represented by a different number of transcripts, expression level, and pattern, showing that they contain various isoforms with different functions.

A previous study reported five genes for the LPAT in the Arabidopsis genome and demonstrated different expression patterns and functions. Three of them (LPAT1, LPAT2, and LPAT3) are essential to normal plant development, where over-expression of LPAT2 improved accumulation of TAG in seeds [75].

It is also well known that DGAT, a key enzyme to the synthesis of TAG, could improve the oil content in *Arabidopsis*, *Brassica napus*, soybean, and maize seeds [76, 77], and therefore, this step has received the most attention to increasing amount of TAG [77].

In plants, DGATs encode by two genes (*DGAT1* and *DGAT2*), which were also identified in Jatropha [5, 14]. However, genetic studies with mutants showed that the disruption of the *DGAT1* gene reduces 70–80% of oil content in Arabidopsis seeds. Besides, the function of DGAT2 is unclear in the Arabidopsis mutant [77, 78]. The expression patterns of the two DGAT were also studied in developing seeds of soybean, Euphorbia, castor bean, and Jatropha [74, 79]. It was reported that in Jatropha, DGAT2 mainly expressed in leaf and poorly expressed in developing seeds. In contrast, the castor bean showed the expression of DGAT2 at a higher level compared to DGAT1 in developing seeds [77].

PDAT has various isoforms, but PDAT1 showed to have an important role in seed TAG content in Arabidopsis [80]. Using various RNAi silencing of *PDAT1* and *DGAT1* showed that both genes have an overlapping role in the synthesis of TAG in oil-seed plants. However, their expression was reported to be different in various plants. For instance, in sesame PDAT showed higher expression compared to DGAT, while in castor bean the DGAT showed a higher expression level compared to PDAT [14, 81, 82]. In Jatropha, PDAT showed lower expression compare to DGAT1, as reported by Xu et al. [74]. However, Ha et al. [14] described a higher expression of PDAT compared to DGAT. In the current study, PDAT and DGAT showed the highest expression in the last stage of seed maturation. However, the expression of PDAT starts to increase from the middle stages of seed maturation, while considering DGAT, the expression starts to increase in the late stages (Table S5, Figure S4), indicating that DGAT has more important role in later stages compared to PDAT.

The transcripts involved in the expression of the triacylglycerol and FA desaturation biosynthesis processes increased during middle to late stages of seed maturation, something previously reported in *Brassica rapa* [83], which is also in agreement with the current results. This could be explained by the rise of storage lipid production, which is also confirmed by previous research studies [17, 18, 84–86].

In this study, we also identified the expression pattern of various key enzymes involved in phenolic compounds during seed maturation of *J. curcas*. It is noteworthy to point out that, in many cases, they are present in multiple copies with different expression patterns. For instance, previous reports showed that 4CL genes contain four isoforms which differ in terms of localization and activity in Arabidopsis. In Arabidopsis, the 4CL3 has shown to be expressed in a broad range of cell types, and is mainly co-expressed with flavonoid biosynthesis pathways, while 4CL1, 4CL2, and 4CL4 are associated with lignin biosynthesis genes [79, 87, 88]. The results are in agreement with our data, where 4CL were identified with 5 DESs.

The flavonoid biosynthesis pathway is well conserved among plants [89]. Considering that the expression level of most transcripts related to flavonoid biosynthesis was more than two times down-regulated in the last stage compared to early stages in all three clusters (1, 3, 5) (Fig. 2), it is suggested that the genes involved in flavonoid biosynthesis may be essential during the early stages of seed development [42]. Six DEGs involved in flavonoid biosynthesis pathways were significantly enriched in male and female flower buds of *J. curcas*, and all of them were up-regulated in male vs. female flowers. The expression pattern of major flavonoid biosynthesis genes was also down-regulated during seed development in *Arabidopsis thaliana* [90]. However, the expression of each of these contigs did not follow a similar pattern during later stages, and even three contigs of the enzyme flavonoid 3′, 5′-hydroxylase (EC: 1.14.13.88), present in two different clusters (1 and 3) that may indicate the presence of different isoenzymes and functions. Therefore, since flavonoids are involved in protective function [91], it is important to understand the functional role of these transcripts.

## Conclusions

Different approaches were used to identify sets of genes with various transcript abundance during seed maturation. First, to obtain an overview of the variation in seed maturation stages, PCA was carried out using all transcripts present in the microarray (Fig. S3). The first principle component (PC1: 53% explained variation) analysis captured mostly temporal variation in transcript abundance, which is supported by previous studies in *Brassica rapa* [83], and *Arabidopsis thaliana* [92, 93], where seed developmental stages are the major source of transcriptional and metabolic variation.

Second, the center of our attention was directed to transcripts related to Jatropha seed maturation to correlate co-expression patterns within pathways and to anticipate putative regulatory elements of the metabolisms of interest (Fig. 4). The co-expression analysis showed that the CB5-D had the highest number of edges, connected directly to CDR1. The co-expression result showed a high degree of connectivity between seed development and hormone pathways, while seed storage is less well connected to the other two pathways.

Third, a subset of probes with variation in transcript abundance patterns between maturation was selected for further analyses. This subset of genes was present in different clusters, which were enriched in various metabolic pathways such as fatty acid biosynthesis, flavonoid biosynthesis, glucan metabolic biosynthesis, seed maturation and dormancy, sucrose and hormone metabolic processes.

Fourth, pairwise transcript expression analyses of different maturation stages of *J. curcas* seeds showed that most changes in transcript abundance occurred between stages V and VI with brown and black epicarp, respectively (Table S6), suggesting that the timing of metabolic pathways during seed maturation in *J. curcas* is in late stages. The expression results were validated for 75 putative transcripts.

Finally, cluster analyses were used to discover particular seed maturation-dependent patterns of gene expression. Transcripts related to fatty acid, flavonoid, and phenylpropanoid biosynthesis were over-represented in the early stage, while lipid storage in the late stage. Generally, the expression of the most over-represented transcripts decreases in the last stage of seed maturation.

## Methods

### Plant material

Seeds of a selected *J. curcas* plant in Kamisse, Ethiopia, were collected at six maturation stages (I-VI) and characterized according to the color of epicarp and endocarp [green-white (I), green-brown (II), green-black (III), yellow-black (IV), brown-black (V), dry-black (VI)] [94] at the same time. Three biological replicates were used for each sample, immediately frozen in liquid nitrogen and stored at − 80 °C.

### Total RNA extraction

Total RNA was extracted from six stages of seed maturation of *J. curcas* using plant RNA purification reagents (Invitrogen) according to the supplier’s instructions. The quality and concentration of total RNAs were determined using NanoVue Spectrophotometer (GE Healthcare Life Sciences) and gel electrophoresis. All RNA samples showing A260/280 ratios between 2.0 and 2.15 were selected and analyzed for RNA integrity using an Agilent 2100 Bioanalyzer (Agilent Technologies). RNA samples with an integrity number above 7.0 were used for further analyses.

### cDNA synthesis for sequencing

Equal amounts of extracted RNA from different seed maturation stages were pooled and used for cDNA library construction. To purify mRNA from 5 μg total RNA, the mRNA-Only Eukaryotic mRNA Isolation Kit (Epicentre) was used by applying exonuclease digestion followed by LiCl precipitation. One μg mRNA was used for the synthesis of the first-strand cDNA by the Mint-Universal cDNA Synthesis Kit (Evrogen). The Trimmer Kit (Evrogen) was used for normalization reaction using 800 ng amplified cDNA, which was re-amplified by 18 cycles.

### Size selection and cloning of cDNA

Two μg of normalized cDNA were digested by ten units of the *SfiI* restriction enzyme (New England Biolabs) for 2 h at 48 °C. Fragments (> 800 bp) isolated from an LMP agarose gel were purified using the MinElute Gel Extraction Kit (Qiagen). The Fast Ligation Kit (New England Biolabs) was used for ligation of 200 ng purified cDNA fragments to 100 ng *SfiI* using dephosphorylated pDNR-lib Vector (Clontech). The product was desalted by ethanol precipitation and re-dissolved in 10 μl water. Of this, 1.5 μl was used to transform NEB10b competent cells (New England Biolabs). To verify the success of normalization, 96 clones were randomly selected and sequenced.

### cDNA library preparation and sequencing using Roche 454 FLX

One million clones were plated on LB-Cm agar plates, collected and stored in glycerol stocks at − 70 °C. Half of the cells were inoculated to a 300 ml Terrific Broth/Cm culture and were grown for 5 h at 30 °C. One hundred Units SfiI digested 200 μg of purified plasmid DNA (Qiagen) for 2 h at 48 °C. LMP-Agarose/MinElute Gel Extraction Kit was used to purify inserts, which were ligated to high-molecular-weight DNA using a Sfi-linker.

The library for the Roche 454 FLX sequencing was generated according to the manufacturer’s protocols (Roche/454 Life Sciences). The concatenated inserts were sheared to fragments ranging from 400 to 900 bp. The two 454 A and B adaptors were ligated to the ends of the emulsion PCR and sequencing. The library was sequenced on one picotiter-plate of the GS FLX using the Roche/454 Titanium chemistry.

### Assembly of the sequence reads to transcripts

At first, the reads were screened for the Sfi-linker used for concatenation and linker sequences were removed. The Roche/454 Newbler software (454 Life Sciences Corporation, Software Release 2.3) at default setting was used to assemble clean reads to individual transcripts. All unique sequences with an average length of > 100 bp were used for oligonucleotide microarray design.

The seed transcriptome has been assessed using the BUSCO 4.0.2 [95] software package by identifying core eudicot genes (eudicots_odb10) in the dataset. Twenty-three of the identified core genes have been selected and aligned to homologous sequences from *Jatropha curcas*, *Hevea brasiliensis*, *Manihot esculenta*, *Ricinus communis*, *Populus trichocarpa* and *Vitis vinifera* using clustalw [96]. Phylogenetic analysis has been carried out using Beast v. 2.6 [97].

### GO annotation of whole seed transcripts

Blast2GO was used to obtain the GO information. The initial blast search was carried out using BLASTX (maximum e-value of 1e-6, gap open penalty 11, gap extension penalty 1). Maximum 20 blast hits were retained per contig, blast hits have been submitted to the blast2go annotation database for further analysis. Furthermore, the functional annotation was used to refine annotation, and specific GO terms were labeled with their putative Biological Process (BP), Molecular Function (MF), and Cellular Component (CC). Furthermore, GO IDs were used to assign enzyme commission (EC) numbers and Kyoto Encyclopedia of Genes and Genomes (KEGG) pathways [98] to contigs.

### Manual annotation of specific functions

The obtained sequences were annotated using the pipeline version of Blast2Go v2.5.0 [29]. Additional information was added to the annotation database from an InterProScan v5RC6 analysis of the sequences [99]. For protein-based similarity search, a protein sequence database of the reads was set up. Amino acid sequences for each read were defined as the most extended open reading frame of the sequence.

Specific homology searches were carried out for three distinct molecular functions of special interest: transcription factors, transporters and resistance gene analogues.

To identify transcription factors, DNA binding domain alignments were obtained from the Plant Transcription Factor (TF) Database [100]. Hidden Markov Models (HMMs) were built based on the alignment, and sequence reads were searched for these DNA binding domain models using HMMER3 [101].

Transporters were predicted based on sequence homology search using BLAST [102] against sequence entries for the Transporter Classification (TC) Database [103]. Sequence hits with an E-value lower than 1e-100 were considered as transporters of the respective class.

Reference R-gene sequences (112 genes) were acquired from the plant resistance genes (PRG) database [104] and InterPro (IPR) domains were identified for the reference sequences with InterProScan. To predict resistance gene analogue (RGA) sequences, Blast2GO and InterProScan annotation tables were filtered for these IPR domains

Further genes or functions of interest were analyzed using text-based searches (curcins, storage proteins) in the Blast2Go/InterProScan annotation or based on the enzyme codes also included in a Blast2Go annotation table.

### Microarray oligonucleotide probe design

The probes were designed (Genotypic Technology LTD.) for an 8 × 60 K oligonucleotide gene expression microarray (Agilent Technologies) using all unique sequence of whole seed transcript (contigs) from the transcriptome of different maturation stages, using the Agilent’s eArray software (https://earray.chem.agilent.com/earray/). The probes were designed in sense and antisense direction with an average probe spacing of 250 bp (500 bp sense + 500 bp antisense). A set of unique sequences was established as a database, and the probes were designed by tiling the contig sequences against the database. Probes specific to each transcript were selected for cross-hybridization when showing a hit with at least 30 bp and > 84% identity. Best probes were considered those showing single hits in the BLAST results.

### Probe labeling, hybridization, and detection

RNA labeling, hybridization onto Agilent 8x60K oligonucleotide microarrays as well as scanning and raw data analysis was carried out according to the One-Color Microarray-Based Gene Expression Analysis Protocol provided by Agilent Technologies. Total RNA (200 ng) from each sample was used to synthesize cyanine-3 labeled cRNA using the QuickAmp Labeling kit, one Color and RNA Spike-In kit one Color (Agilent Technologies). The cyanine labeled cRNA was transcribed and purified by a T7 polymerase and RNeasy mini kits (Qiagen), respectively. Samples labeled with Cy3 (825 ng) were hybridized for 17 h at 65 °C and 10 rpm in the hybridization oven using the Gene Expression Hybridization kit (Agilent Technologies). The arrays were washed according to supplier’s instructions and scanned on an Agilent G2505C scanner at 3 μm resolution. Data were acquired using Agilent Feature Extraction software version 10.5.1.1. The microarrays were hybridized with probes of six stages of seeds maturation, each with three to four biological replicates.

### Microarray data analyses

The R statistical (http://www.R-project.org) and Bioconductor software [105] were used to perform the pre-processing analyses of Agilent 8x60K oligonucleotide gene expression microarrays data. Fluorescence signal intensities from each spot were quantified. Background correction was performed using Agilent spatial detrending background estimate, followed by averaging of replicate spots, log_2_-transformation, KNN (K nearest neighbor) imputation of missing values and quantile normalization. The linear modeling functions of the LIMMA package were used for inference statistics [106]. Statistical significance was determined by *t-*statistic for seeds and corresponding *P*-values. Genes with Benjamini-Hochberg false discovery rate (FDR) corrected *P*-value <1e-8 were considered as significantly differentially expressed in different stages of seed maturation and leaf samples. Clustering was performed using normalized and filtered data. The differentially expressed sequences (DESs) were clustered according to their expression patterns across seed maturation stages. Normal mixture modeling for model-based clustering (expectation-maximization) was performed with *P*-value <1e-8 [107]. The obtained microarray data in this study were stored in the Gene Expression Omnibus (GEO) (GSE109931).

### GO set enrichment analyses

Gene set enrichment analyses were carried out according to the GO/KEGG terms using the Bioconductor GOstats package [25]. Since *Jatropha* is not a supported model organism, the complete GO/KEGG categories for the differentially expressed genes were identified, using the Blast2GO annotation file of the whole seed transcriptome. After building the gene-set collection, the corresponding parameter object was created followed by hyper-geometric testing. The differentially expressed genes of different maturation stages of seeds or clusters were analyzed for both over- and under-representation of GO terms, where KEGG and each GO category (BP, CC, and MF) were analyzed separately.

Results of the GOstats analyses were plotted as flipped bar charts displaying each identified term using the negative log10 *P*-value for the top 15 terms. Both over- and under-represented GO terms were combined in one graph, showing the *P*-value of the over-represented term on the right side and the under-represented term on the left side. In addition, heatmaps were created using the negative log10 *P*-value.

### Co-expression network analysis

Co-expression networks based on partial correlations were calculated using the DESs with a *P*-value <1e-8 (f-statistics of the model) as described above [26, 27]. Co-expression network was constructed from the 300 most significant edges. Nodes were colored according to the cluster membership from cluster analyses of DESs, and the number of connections for the top 20 nodes with the highest number of connections to other nodes was constructed as bar plots.

Further, the genes that were expected to be involved in seed development, seed storage, and hormone cross-talking were extracted by searching the available GO annotations of each contig. A partial correlation network was constructed, and a network of the top 50 most significant edges was extracted.

### Quantitative real-time (qRT)-PCR using BioMark

Primers for selected contigs from the microarray and housekeeping genes were designed using Primer3 software [28]. cDNA synthesis was performed on a total of 48 samples, including 42 test samples, four standard control samples, and two nuclease-free water (negative control) samples. For standard control, a reference sample was prepared, consisting of an equivalent pool of all test samples. In each 20 μl reaction 100 ng of total RNA per test sample as well as 800, 200, 50 and 12.5 ng of reference RNA sample (as standard control) and only water in the two negative control samples were reverse transcribed, using the SuperScript III First-Strand Synthesis System Kit (Invitrogen) according to the manufacturer’s protocol. The RT reactions were diluted 1:3, and 1.25 μl of each dilution was applied to 4 different 5 μl pre-amplification reactions, each containing 1x Qiagen PCR buffer, 0.8 mM of dNTPs, 0.25 μl of DMSO, 0.15 Unit of HotStarTaq DNA polymerase (Qiagen) and a pool of 48 different primer pairs (200 nM each). Cycling conditions for pre-amplification were 15 min at 95 °C and 14 cycles of 40 s at 95 °C, 40 s at 60 °C and 80 s at 72 °C. The cycle ended with a final step of 7 min at 72 °C. After pre-amplification, products were diluted 1:5 in nuclease-free water. QPCR amplification was performed using the BioMark system (Fluidigm) and 48.48 Dynamic Arrays. For each qPCR run 6 μl sample mix were prepared, consisting of 1x Qiagen PCR Buffer (including 1.5 mM MgCl_2_), 0.4 mM MgCl_2_, 0.96 mM dNTPs, 0.3 μl DMSO, 1x EvaGreen Binding dye, 0.18 units HotStarTaq Polymerase (QIAGEN HotStarTaq™ PCR), 0.004 μl ROX, 1x DNA binding dye (Fluidigm) and 1.5 μl of pre-amplified and 1:5 diluted samples. In parallel, six μl of assay mix were prepared, including three μl of 2x Assay Loading reagent (Fluidigm), 0.3 μl nuclease free water and 2.7 μl of 200 nM primer pair pool used for pre-amplification of test samples. 48.48 Dynamic Arrays were primed, sample mix as well as assay mix were loaded with the integrated fluidic circuit (IFC) controller MX (Fluidigm) and qPCR was performed using the BioMark system (Fluidigm) according to the manufacturer’s instructions. Cycling conditions were 15 min at 95 °C and 40 cycles of 40 s at 95 °C, 40 s at 60 °C and 80 s at 72 °C. A final step of 7 min at 72 °C ended the cycle. Ct values were calculated using the Fluidigm Real-Time PCR Analysis Software 4.1.2. Similar to microarray data analyses, qPCR data were normalized using quantile normalization, and linear models were calculated using the LIMMA package.

## Supplementary information

**Additional file 1: Figure S1.** Phylogenetic relationship of the seed specific transcriptome in the *Euphorbiaceae* family based on the sequences selected 23 core genes. Hbr: *Hevea brasiliensis* (rubber tree), Mes: *Manihot esculenta* (cassava), Jcu_own: *Jatropha curcas* sequences identified in this study, Jcu_ref: *J. curcas* NCBI reference sequences, Rco: *Ricinus communis*, Ptr. *Populus trichocarpa*, Vvi: *Vitis vinifera*.

**Additional file 2: Figure S2.** GO annotation classification of whole seed transcript sequencing data. Results are summarized for three main GO categories (BP, MF, and CC). The x-axis indicates the most abundant GO terms, and y-axis represents the number of each GO term.

**Additional file 3: Figure S3.** PCA analysis explains the variance in gene expression of the six different seed maturation stages (I-VI) with their biological replications.

**Additional file 4: Figure S4.** The expression profiles of each microarray probe during seed developmental stages. The x-axis represents different developmental stages, and the y-axis represents the log_2_ intensity values.

**Additional file 5: Figure S5.** TreeMap view in REVIGO of the enriched BP of the six different seed maturation stages of all DESs. All terms are adjusted *P*-value cutoff at 0.05 from the enrichment analysis.

**Additional file 6: Figure S6.** Plot of the top 15 significantly enriched categories of KEGG pathways in the 10 identified clusters (Fig. 2). The turquoise bars show over-represented pathways and the magenta bars show under-represented pathways. The x-axis indicates the statistical significance of the enrichment.

**Additional file 7: Figure S7.** Overview of significantly enriched and over-represented pathways and enzymes related to lipid metabolism identified in different clusters. Figures generated by the pathview package to paint the gene of interests into KEGG pathways.

**Additional file 8: Figure S8.** Overview of significantly enriched and over-represented phenylpropanoid biosynthesis biosynthesis pathways and related enzymes identified in different clusters. Figures generated pathview package to paint the gene of interests into KEGG pathways.

**Additional file 9: Figure S9.** Overview of significantly enriched and over-represented flavonoid, flavone and flavonol biosynthesis and isoflavonoid biosynthesis pathways and related enzymes identified in different clusters. Figures generated by the pathview package to paint the gene of interests into KEGG pathways.

**Additional file 10: Figure S10.** Correlation between microarray and qRT-PCR of some DESs during seed development. The y-axis represented the log_2_ intensity values from microarray analysis and the x-axis represented the Ct values from the qPCR analysis.

**Additional file 11: Table S1.** Sequence comparison between obtained contigs and the Jatropha transcriptomic sequences of Kazusa DNA Research Institute (JAT_r4.5, ftp://ftp.kazusa.or.jp/pub/Jatropha/), as well as Chinese Academy of Sciences (JatCur_1.0, ftp://ftp.ncbi.nih.gov/ genomes/Jatropha_curcas/).

**Additional file 12: Table S2.** GO classification of annotated transcripts of whole seed transcript sequencing data. Results are summarized for three main GO categories (BP, MF, and CC).

**Additional file 13: Table S3.** Annotation of whole transcript sequencing data according to Blast2Go, InterProScan, and KEGG. Manual annotation was carried out to predict RGA, TF and transporter sequences using the Plant Resistance Genes, the Plant Transcription Factor, and the Transporter Classification Databases.

**Additional file 14: Table S4** Mapping of the identified enzymes and their corresponding contigs to KEGG pathways.

**Additional file 15: Table S5.** Relative transcript expression value (log_2_) of all DESs (with a cut-off of *P*-value <1e-8) in each developmental stages and related clusters.

**Additional file 16: Table S6.** Pairwise comparison of transcripts expression values (log_2_) between different maturation stages (with a cut-off of *P*-value <1e-8).

**Additional file 17: Table S7.** The number of over- and under-represented GO terms of each category (BP, MF and CC), and related contigs in each cluster.

**Additional file 18: Table S8.** GO and KEGG enrichment analysis of significantly over- and under-represented GO terms of each category (BP, MF, and CC) and KEGG pathways for each cluster.

**Additional file 19: Table S9.** The enriched pathways of all significantly DESs for each cluster.

**Additional file 20: Table S10.** The list of 75 selected transcripts from the DESs represented in seeds and three housekeeping genes, which were used for qRT-PCR.

**Additional file 21: Table S11.** Relative expression value of all selected transcript from DESs represented in seeds and three housekeeping genes, which were used for qRT-PCR.

## Data Availability

The whole dataset generated and analyzed during the current study is available from the corresponding authors on request. Raw sequence reads are accessible in the NCBI SRA database (SRR7701127), assembled transcripts have been uploaded to TSA under the accession GIKD00000000. The version described in this paper is the first version, GIKD01000000.

## Abbreviations

GO: Gene ontology
DESs: Differentially expressed sequences
CO2: Carbon dioxide
*J. curcas*: *Jatropha curcas*
TF: Transcription factor
HMMs: Hidden Markov Models
IPR: InterPro
RGA: Resistance gene analogue
BP: Biological process
MF: Molecular function
CC: Cellular component
EC: Enzyme commission
KEGG: Kyoto encyclopedia of genes and genomes
KNN: K nearest neighbor
FDR: False discovery rate
GEO: Gene expression omnibus
HQ: High quality
Xhyb: Cross-hybridizing probes
PCA: Principle components analysis
S.D.: Standard deviation
CDR1: Aspartic proteinase
DFR: Dihydroflavonol reductase
TT8: Transparent Testa 8
ER: Endoplasmic reticulum
CAC2: Carboxylase biotin carboxylase subunit
KASII: Beta-ketoacyl-acp synthase II
MOD1: Mosaic death 1
FAB2: Fatty acid biosynthesis 2
FatA: Fata acyl-acp thioesterase
FAE1: Fatty acid elongation1
FAD2: Fatty acid desaturase2
LEC1: Leafy cotyledon1
DGAT1: Diacylglycerol acyltransferase 1
FUS3: FUSCA3
CDS2: Cytidinediphosphate diacylglycerol synthase 2
ACC: Acetyl-CoA carboxylase
MCMT: Malonyl-CoA acyl carrier protein (ACP) transacylase
KAS III: Beta-ketoacyl-ACP synthase III
KAR: 3-oxoacyl-ACP reductase
KASI: Beta-ketoacyl-ACP synthase I
KASII: Beta-ketoacyl-ACP synthase II
AAD: Acyl-ACP desaturase
OAH: Oleoyl-ACP hydrolase
FAT: Acyl-ACP thioesterase
DAG: Diacylglycerol
TAG: Triacylglycerol
PDAT1: Phospholipid: diacylglycerol acyltransferase 1
G3P: Glycerol-3-phosphate
GPAT: G3P acyltransferase
LPA: Lysophosphatidic acid
PA: Phosphatidic acid
LPAAT: LPA acyltransferase
PC: Phosphatidylcholine
DGAT: Diacylglycerol O-acyltransferase
PDAT: Phospholipid diacylglycerol acyltransferase
ATP: Diacylglycerol kinase
NAD: Aldehyde dehydrogenase
PAL: Phenylalanine ammonia-lyase
4CL: 4-Coumarate: CoA ligase
COMT: Caffeic acid 3-O-methyltransferase
CCoA-OMT: Caffeoyl-CoA O-methyltransferase
CCR: Cinnamoyl-CoA reductase
ACC: Aminocyclopropanecarboxylate
