# Supplementary figures and images for: Gene expression profiling identifies pathways involved in seed maturation of Jatropha curcas
Source: BMC Genomics. 2020 Apr 9;21:290. doi: 10.1186/s12864-020-6666-1 (PMC7146973; doi:10.1186/s12864-020-6666-1)

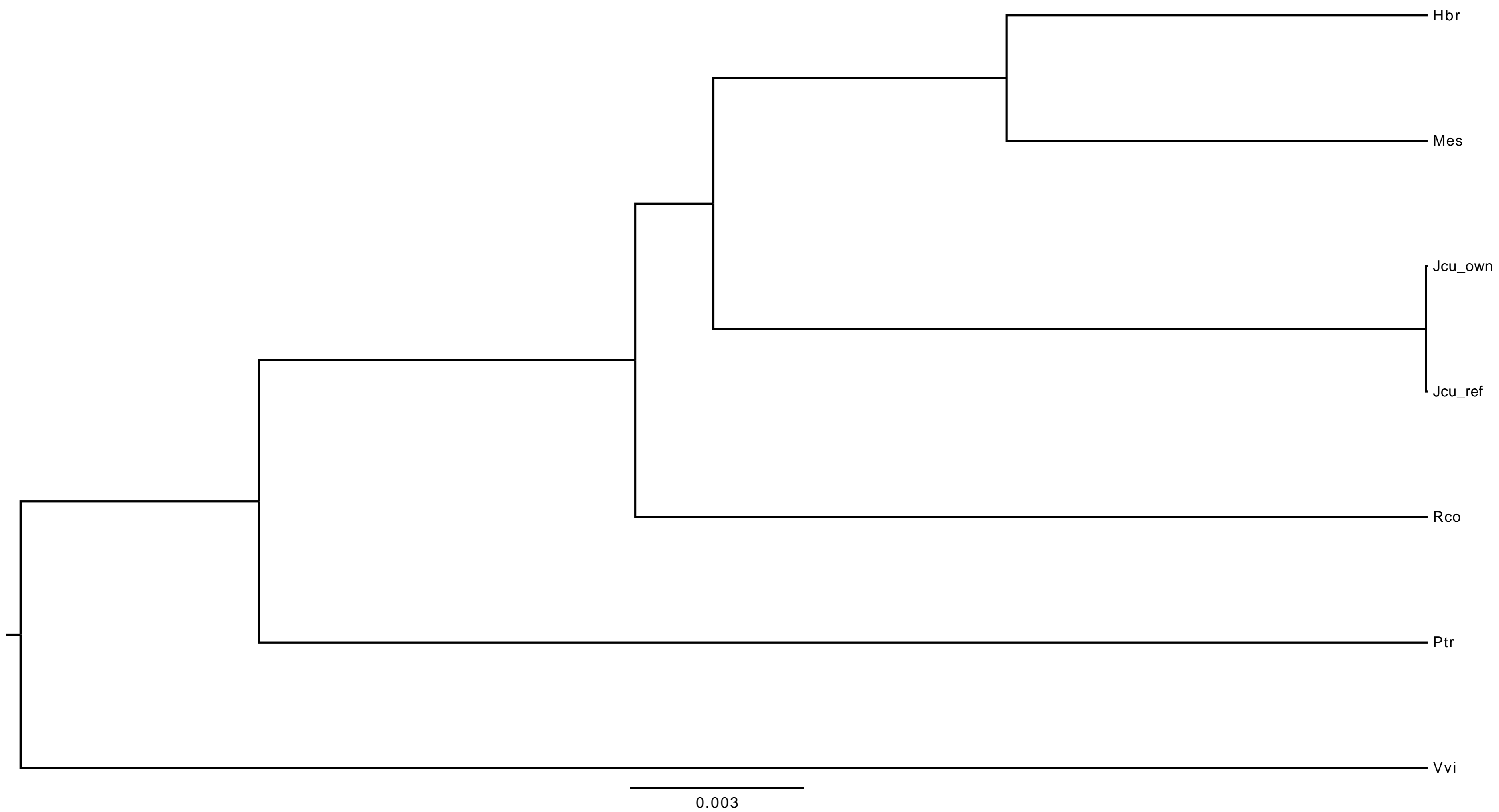

Supplement: Supplementary file 1 — Additional file 1: Figure S1. Phylogenetic relationship of the seed specific transcriptome in the Euphorbiaceae family based on the sequences selected 23 core genes. Hbr: Hevea brasiliensis (rubber tree), Mes: Manihot esculenta (cassava), Jcu_own: Jatropha curcas sequences identified in this study, Jcu_ref: J. curcas NCBI reference sequences, Rco: Ricinus communis, Ptr. Populus trichocarpa, Vvi: Vitis vinifera. [file 12864_2020_6666_MOESM1_ESM.pdf]

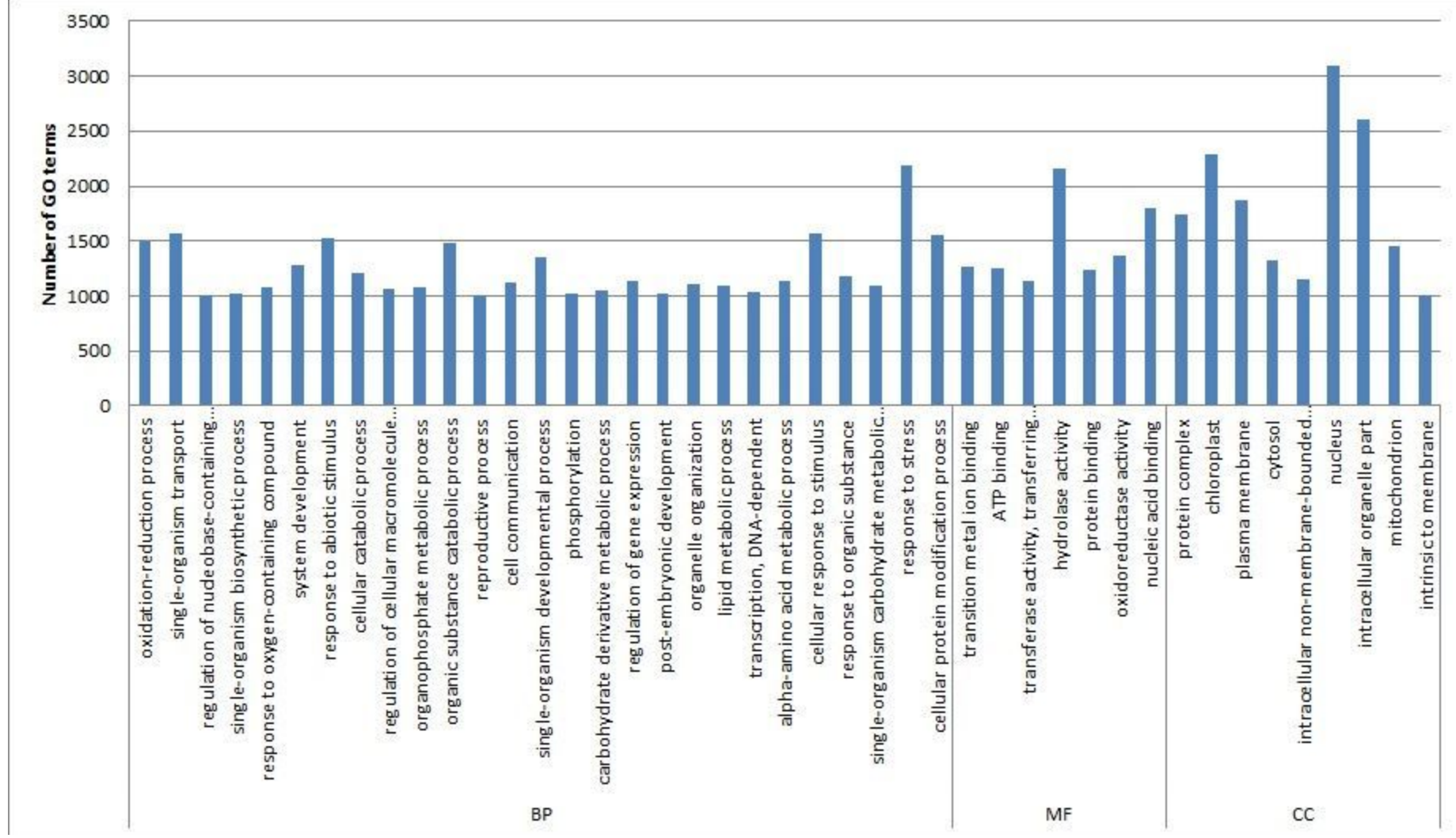

Supplement: Supplementary file 2 — Additional file 2: Figure S2. GO annotation classification of whole seed transcript sequencing data. Results are summarized for three main GO categories (BP, MF, and CC). The x-axis indicates the most abundant GO terms, and y-axis represents the number of each GO term. [file 12864_2020_6666_MOESM2_ESM.pdf]

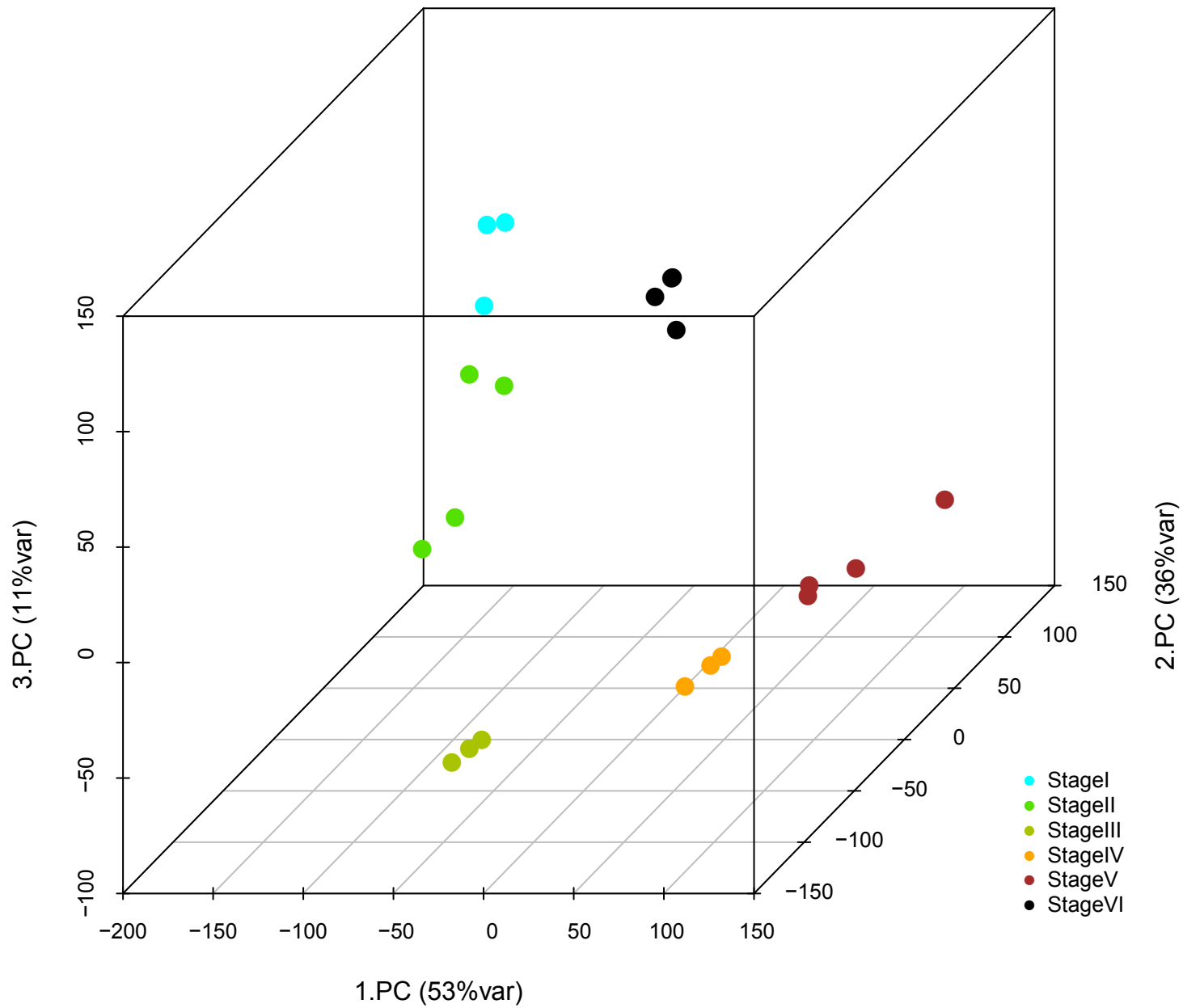

Supplement: Supplementary file 3 — Additional file 3: Figure S3. PCA analysis explains the variance in gene expression of the six different seed maturation stages (I-VI) with their biological replications. [file 12864_2020_6666_MOESM3_ESM.pdf]

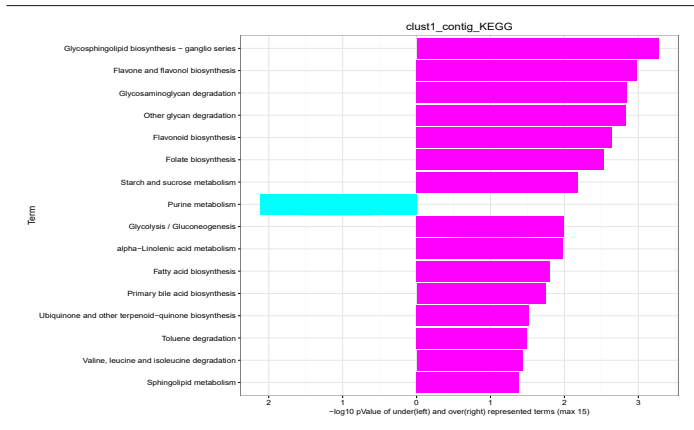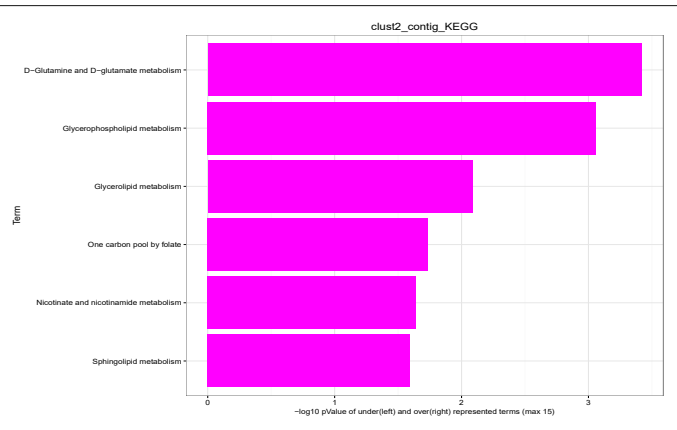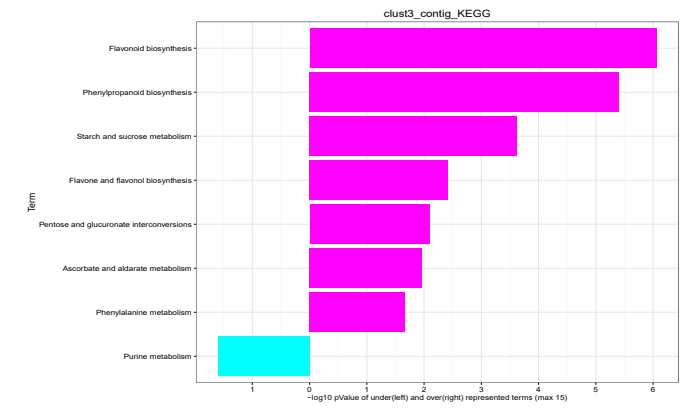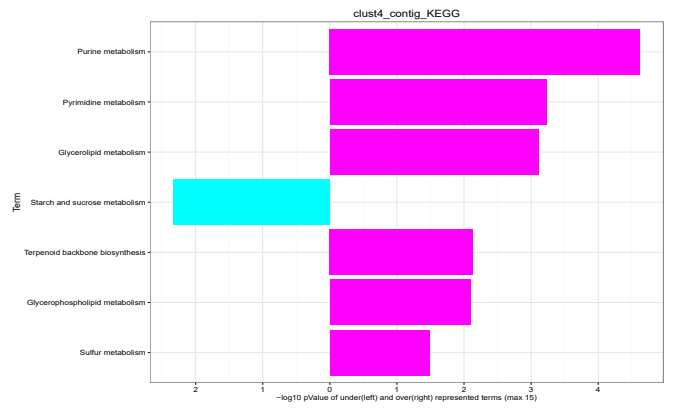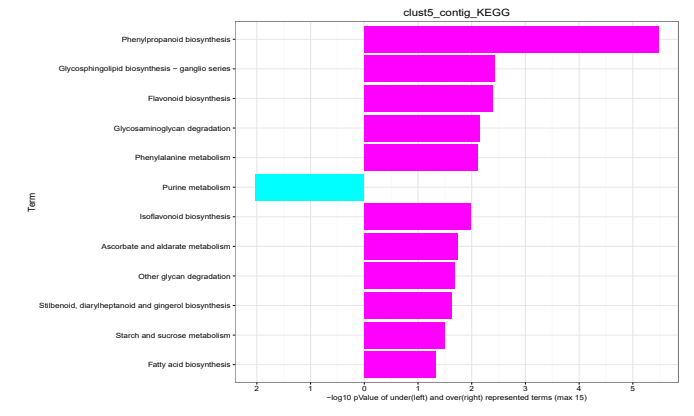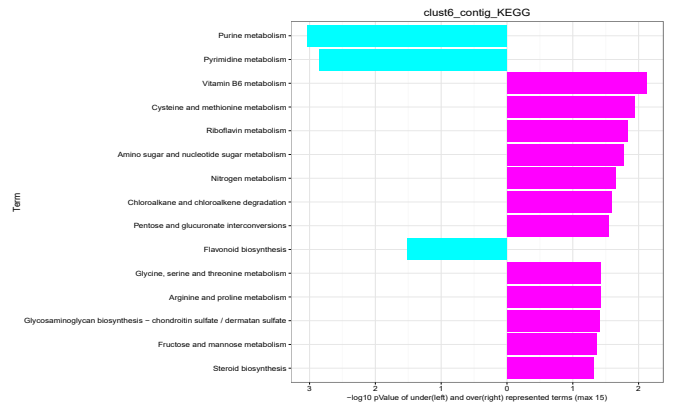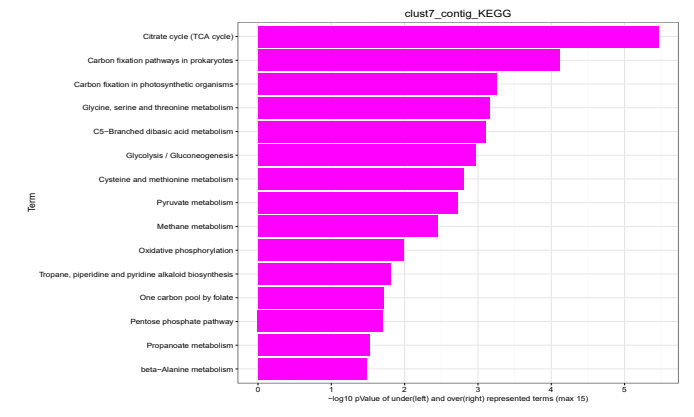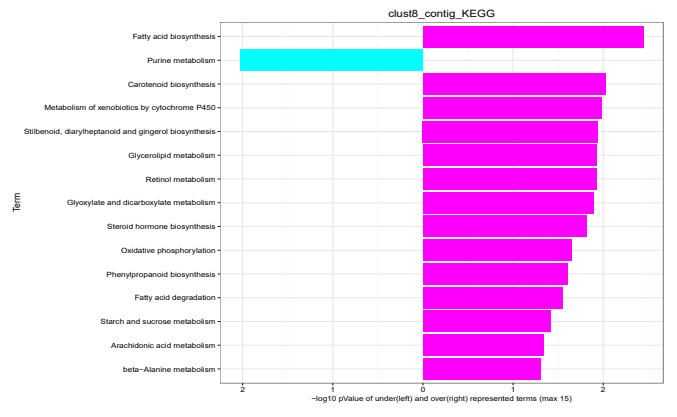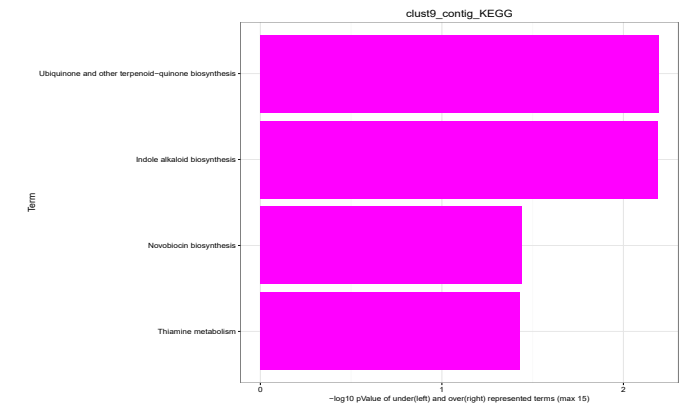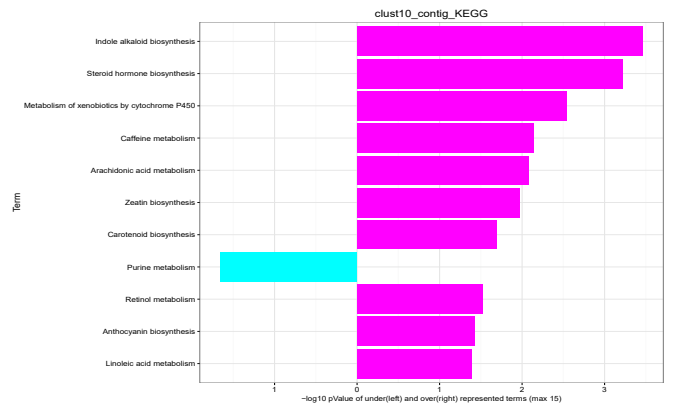

Supplement: Supplementary file 6 — Additional file 6: Figure S6. Plot of the top 15 significantly enriched categories of KEGG pathways in the 10 identified clusters (Fig. 2). The turquoise bars show over-represented pathways and the magenta bars show under-represented pathways. The x-axis indicates the statistical significance of the enrichment. [file 12864_2020_6666_MOESM6_ESM.pdf]
