# Supplementary material for: Gene expression profiling identifies pathways involved in seed maturation of Jatropha curcas
Source: BMC Genomics. 2020 Apr 9;21:290. doi: 10.1186/s12864-020-6666-1 (PMC7146973; doi:10.1186/s12864-020-6666-1)

Cluster 1_BP

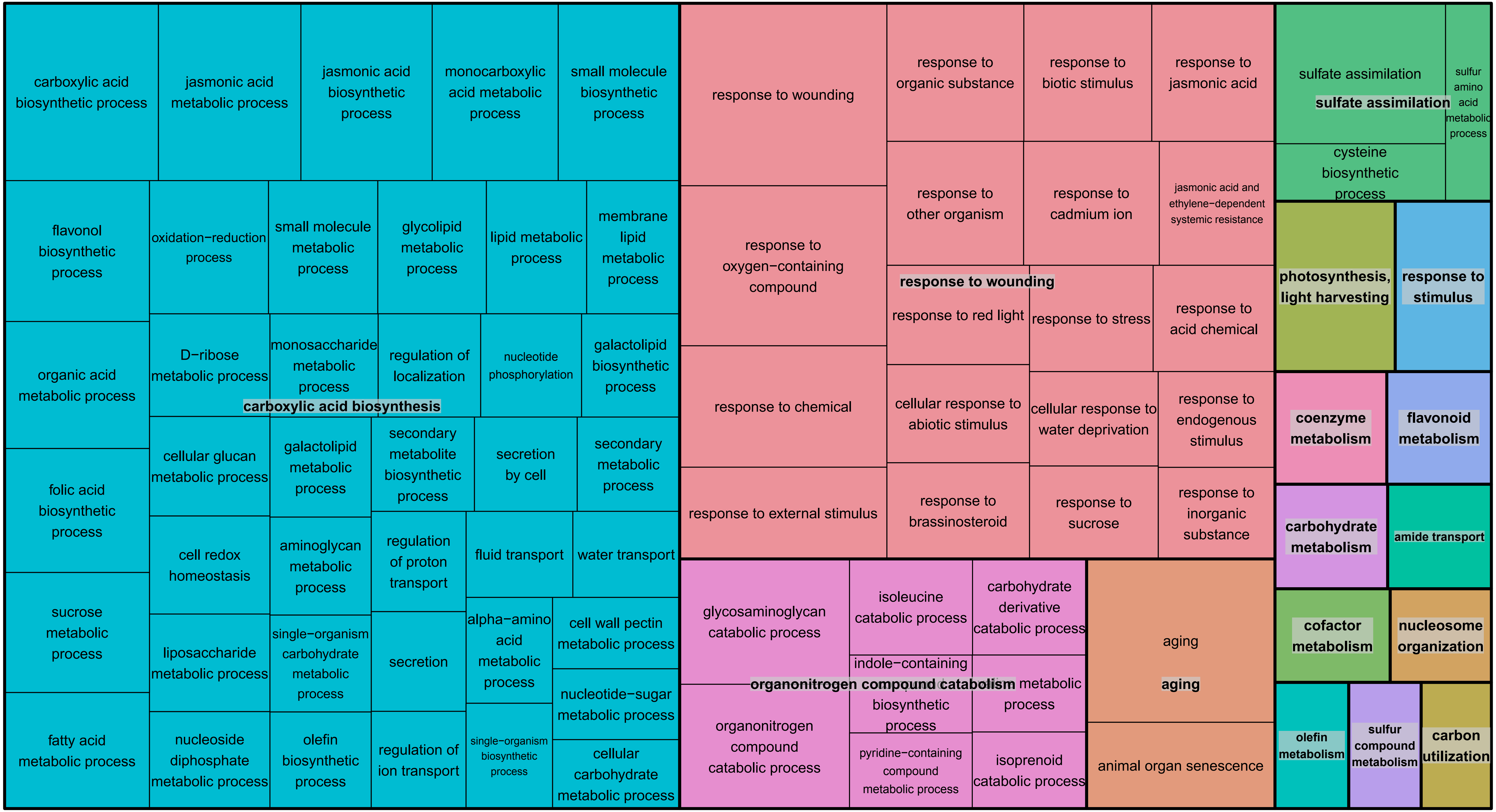
carboxylic acid biosynthetic process
jasmonic acid metabolic process
jasmonic acid biosynthetic process
monocarboxylic acid metabolic process
small molecule biosynthetic process
flavonol biosynthetic process
oxidation−reduction process
small molecule metabolic process
glycolipid metabolic process
lipid metabolic process
membrane lipid metabolic process
organic acid metabolic process
D−ribose metabolic process
monosaccharide metabolic process
regulation of localization
nucleotide phosphorylation
galactolipid biosynthetic process
carboxylic acid biosynthesis
folic acid biosynthetic process
cellular glucan metabolic process
galactolipid metabolic process
secondary metabolite biosynthetic process
secretion by cell
secondary metabolic process
cell redox homeostasis
aminoglycan metabolic process
regulation of proton transport
fluid transport
water transport
sucrose metabolic process
liposaccharide metabolic process
single−organism carbohydrate metabolic process
secretion
alpha−amino acid metabolic process
cell wall pectin metabolic process
nucleotide−sugar metabolic process
fatty acid metabolic process
nucleoside diphosphate metabolic process
olefin biosynthetic process
regulation of ion transport
single−organism biosynthetic process
cellular carbohydrate metabolic process
response to wounding
response to organic substance
response to biotic stimulus
response to jasmonic acid
response to oxygen−containing compound
response to other organism
response to cadmium ion
jasmonic acid and ethylene−dependent systemic resistance
response to wounding
response to red light
response to stress
response to acid chemical
response to chemical
cellular response to abiotic stimulus
cellular response to water deprivation
response to endogenous stimulus
response to external stimulus
response to brassinosteroid
response to sucrose
response to inorganic substance
sulfate assimilation
sulfate assimilation
sulfur amino acid metabolic process
cysteine biosynthetic process
photosynthesis, light harvesting
response to stimulus
coenzyme metabolism
flavonoid metabolism
carbohydrate metabolism
amide transport
cofactor metabolism
nucleosome organization
olefin metabolism
sulfur compound metabolism
carbon utilization
glycosaminoglycan catabolic process
isoleucine catabolic process
carbohydrate derivative catabolic process
indole−containing compound biosynthetic process
organonitrogen compound catabolism
metabolic process
organonitrogen compound catabolic process
pyridine−containing compound metabolic process
isoprenoid catabolic process
aging
aging
animal organ senescence

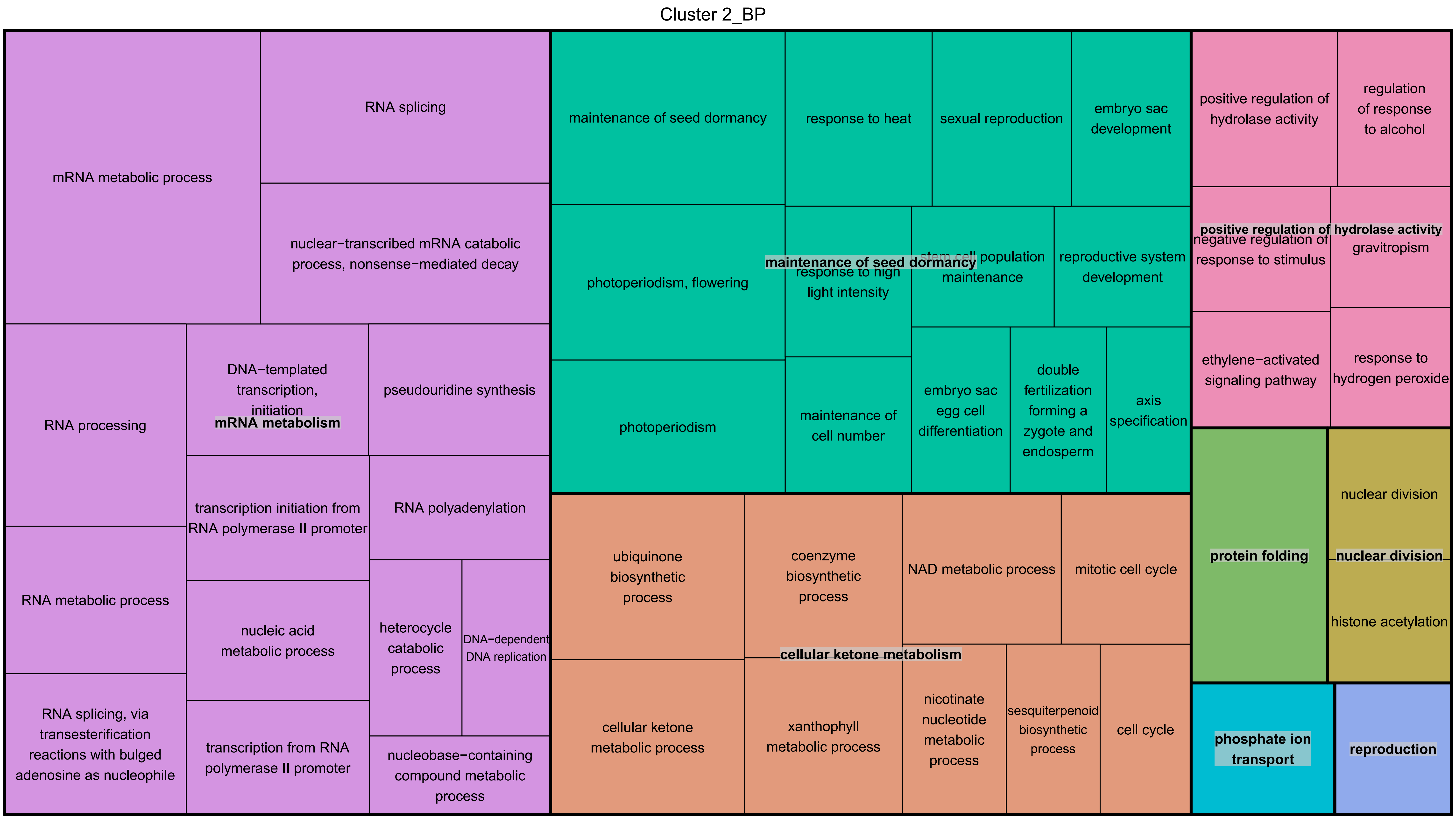
Cluster 2_BP
mRNA metabolic process
RNA splicing
nuclear−transcribed mRNA catabolic process, nonsense−mediated decay
RNA processing
DNA−templated transcription, initiation
mRNA metabolism
pseudouridine synthesis
RNA metabolic process
transcription initiation from RNA polymerase II promoter
RNA polyadenylation
nucleic acid metabolic process
heterocycle catabolic process
DNA−dependent DNA replication
RNA splicing, via transesterification reactions with bulged adenosine as nucleophile
transcription from RNA polymerase II promoter
nucleobase−containing compound metabolic process
maintenance of seed dormancy
response to heat
sexual reproduction
embryo sac development
photoperiodism, flowering
maintenance of seed dormancy
response to high light intensity
stem cell population maintenance
reproductive system development
photoperiodism
maintenance of cell number
embryo sac egg cell differentiation
double fertilization forming a zygote and endosperm
axis specification
ubiquinone biosynthetic process
coenzyme biosynthetic process
NAD metabolic process
mitotic cell cycle
cellular ketone metabolism
cellular ketone metabolic process
xanthophyll metabolic process
nicotinate nucleotide metabolic process
sesquiterpenoid biosynthetic process
cell cycle
positive regulation of hydrolase activity
regulation of response to alcohol
positive regulation of hydrolase activity
negative regulation of response to stimulus
gravitropism
ethylene−activated signaling pathway
response to hydrogen peroxide
protein folding
nuclear division
nuclear division
histone acetylation
phosphate ion transport
reproduction

Cluster 3_BP

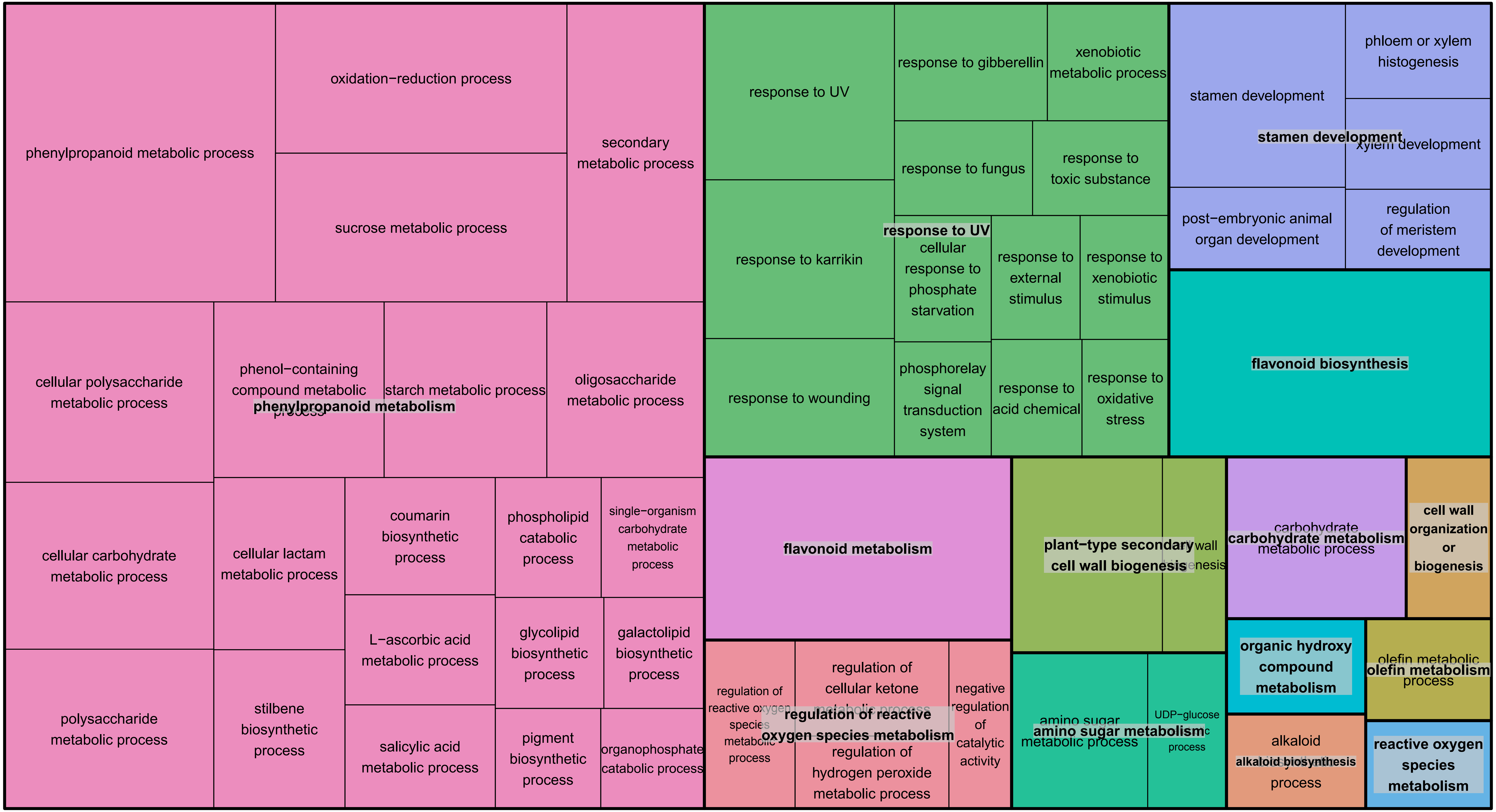
phenylpropanoid metabolic process
oxidation−reduction process
sucrose metabolic process
secondary metabolic process
cellular polysaccharide metabolic process
phenol−containing compound metabolic process
phenylpropanoid metabolism
starch metabolic process
oligosaccharide metabolic process
cellular carbohydrate metabolic process
cellular lactam metabolic process
coumarin biosynthetic process
phospholipid catabolic process
single−organism carbohydrate metabolic process
L−ascorbic acid metabolic process
glycolipid biosynthetic process
galactolipid biosynthetic process
polysaccharide metabolic process
stilbene biosynthetic process
salicylic acid metabolic process
pigment biosynthetic process
organophosphate catabolic process
response to UV
response to gibberellin
xenobiotic metabolic process
response to fungus
response to toxic substance
response to karrikin
response to UV
cellular response to phosphate starvation
response to external stimulus
response to xenobiotic stimulus
response to wounding
phosphorelay signal transduction system
response to acid chemical
response to oxidative stress
stamen development
phloem or xylem histogenesis
stamen development
xylem development
post−embryonic animal organ development
regulation of meristem development
flavonoid biosynthesis
flavonoid metabolism
plant−type secondary cell wall biogenesis
carbohydrate metabolic process
carbohydrate metabolism
cell wall organization or biogenesis
regulation of reactive oxygen species metabolic process
regulation of cellular ketone metabolic process
regulation of reactive oxygen species metabolism
regulation of hydrogen peroxide metabolic process
negative regulation of catalytic activity
amino sugar metabolic process
amino sugar metabolism
UDP−glucose process
organic hydroxy compound metabolism
olefin metabolic process
olefin metabolism
alkaloid process
alkaloid biosynthesis
reactive oxygen species metabolism

Cluster 4_BP

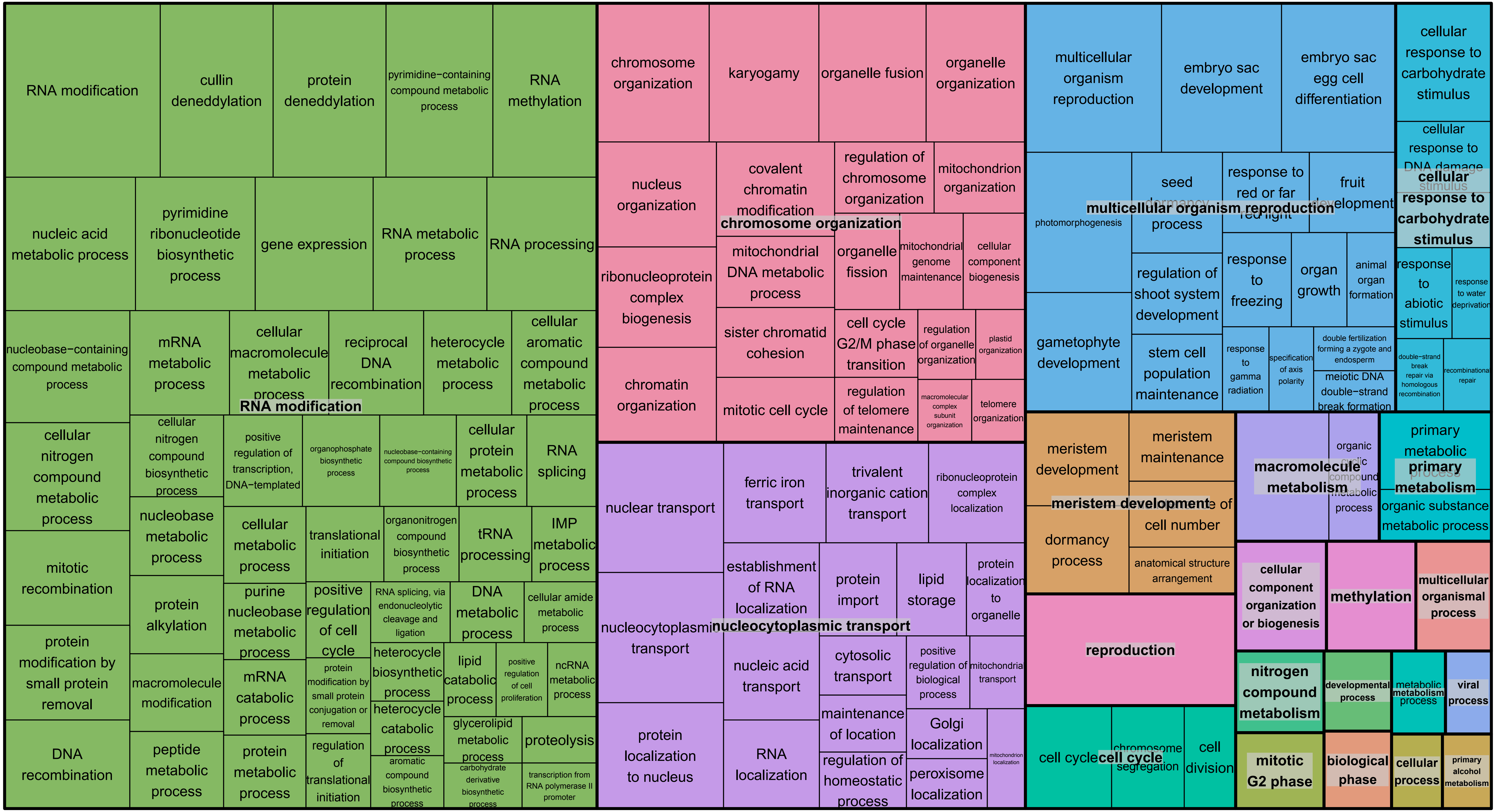
RNA modification
cullin deneddylation
protein deneddylation
pyrimidine−containing compound metabolic process
RNA methylation
nucleic acid metabolic process
pyrimidine ribonucleotide biosynthetic process
gene expression
RNA metabolic process
RNA processing
nucleobase−containing compound metabolic process
mRNA metabolic process
cellular macromolecule metabolic process
reciprocal DNA recombination
heterocycle metabolic process
cellular aromatic compound metabolic process
RNA modification
cellular nitrogen compound metabolic process
cellular nitrogen compound biosynthetic process
positive regulation of transcription, DNA−templated
organophosphate biosynthetic process
nucleobase−containing compound biosynthetic process
cellular protein metabolic process
RNA splicing
nucleobase metabolic process
cellular metabolic process
translational initiation
organonitrogen compound biosynthetic process
tRNA processing
IMP metabolic process
mitotic recombination
protein alkylation
purine nucleobase metabolic process
positive regulation of cell cycle
RNA splicing, via endonucleolytic cleavage and ligation
DNA metabolic process
cellular amide metabolic process
protein modification by small protein removal
macromolecule modification
mRNA catabolic process
protein modification by small protein conjugation or removal
heterocycle biosynthetic process
lipid catabolic process
positive regulation of cell proliferation
ncRNA metabolic process
heterocycle catabolic process
glycerolipid metabolic process
proteolysis
DNA recombination
peptide metabolic process
protein metabolic process
regulation of translational initiation
aromatic compound biosynthetic process
carbohydrate derivative biosynthetic process
transcription from RNA polymerase II promoter
chromosome organization
karyogamy
organelle fusion
organelle organization
nucleus organization
covalent chromatin modification
regulation of chromosome organization
mitochondrion organization
chromosome organization
ribonucleoprotein complex biogenesis
mitochondrial DNA metabolic process
organelle fission
mitochondrial genome maintenance
cellular component biogenesis
sister chromatid cohesion
cell cycle G2/M phase transition
regulation of organelle organization
plastid organization
chromatin organization
mitotic cell cycle
regulation of telomere maintenance
macromolecular complex subunit organization
telomere organization
nuclear transport
ferric iron transport
trivalent inorganic cation transport
ribonucleoprotein complex localization
nucleocytoplasmic transport
nucleocytoplasmic transport
establishment of RNA localization
protein import
lipid storage
protein localization to organelle
nucleic acid transport
cytosolic transport
positive regulation of biological process
mitochondrial transport
maintenance of location
Golgi localization
protein localization to nucleus
RNA localization
regulation of homeostatic process
peroxisome localization
mitochondrion localization
multicellular organism reproduction
embryo sac development
embryo sac egg cell differentiation
cellular response to carbohydrate stimulus
seed dormancy process
response to red or far red light
fruit development
multicellular organism reproduction
photomorphogenesis
cellular response to DNA damage stimulus
cellular response to carbohydrate stimulus
regulation of shoot system development
response to freezing
organ growth
animal organ formation
response to abiotic stimulus
response to water deprivation
gametophyte development
stem cell population maintenance
response to gamma radiation
specification of axis polarity
double fertilization forming a zygote and endosperm
meiotic DNA double−strand break formation
double−strand break repair via homologous recombination
recombinational repair
meristem development
meristem maintenance
meristem development
regulation of cell number
dormancy process
anatomical structure arrangement
macromolecule metabolism
organic cyclic compound metabolic process
primary metabolic process
primary metabolism
organic substance metabolic process
reproduction
cellular component organization or biogenesis
methylation
multicellular organismal process
cell cycle
chromosome segregation
cell division
cell cycle
nitrogen compound metabolism
developmental process
metabolic process
metabolism
viral process
mitotic G2 phase
biological phase
cellular process
primary alcohol metabolism

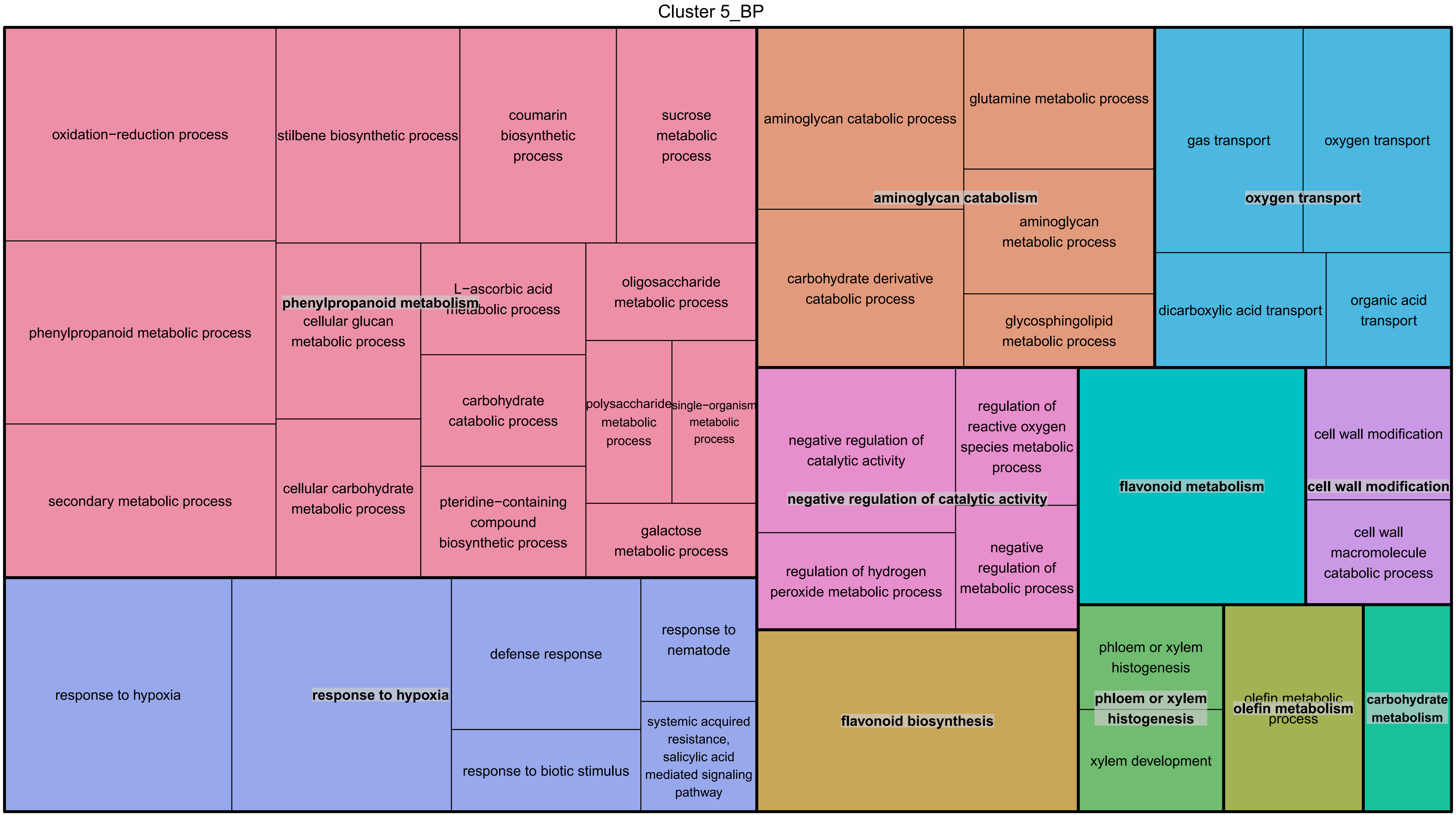
Cluster 5_BP
oxidation−reduction process
stilbene biosynthetic process
coumarin biosynthetic process
sucrose metabolic process
phenylpropanoid metabolic process
phenylpropanoid metabolism
cellular glucan metabolic process
L−ascorbic acid metabolic process
oligosaccharide metabolic process
carbohydrate catabolic process
polysaccharide metabolic process
single−organism metabolic process
secondary metabolic process
cellular carbohydrate metabolic process
pteridine−containing compound biosynthetic process
galactose metabolic process
aminoglycan catabolic process
glutamine metabolic process
aminoglycan catabolism
aminoglycan metabolic process
carbohydrate derivative catabolic process
glycosphingolipid metabolic process
gas transport
oxygen transport
oxygen transport
dicarboxylic acid transport
organic acid transport
negative regulation of catalytic activity
regulation of reactive oxygen species metabolic process
negative regulation of catalytic activity
regulation of hydrogen peroxide metabolic process
negative regulation of metabolic process
flavonoid metabolism
cell wall modification
cell wall modification
cell wall macromolecule catabolic process
response to hypoxia
response to hypoxia
defense response
response to nematode
response to biotic stimulus
systemic acquired resistance, salicylic acid mediated signaling pathway
flavonoid biosynthesis
phloem or xylem histogenesis
phloem or xylem histogenesis
xylem development
olefin metabolic process
olefin metabolism
carbohydrate metabolism

Cluster 6_BP

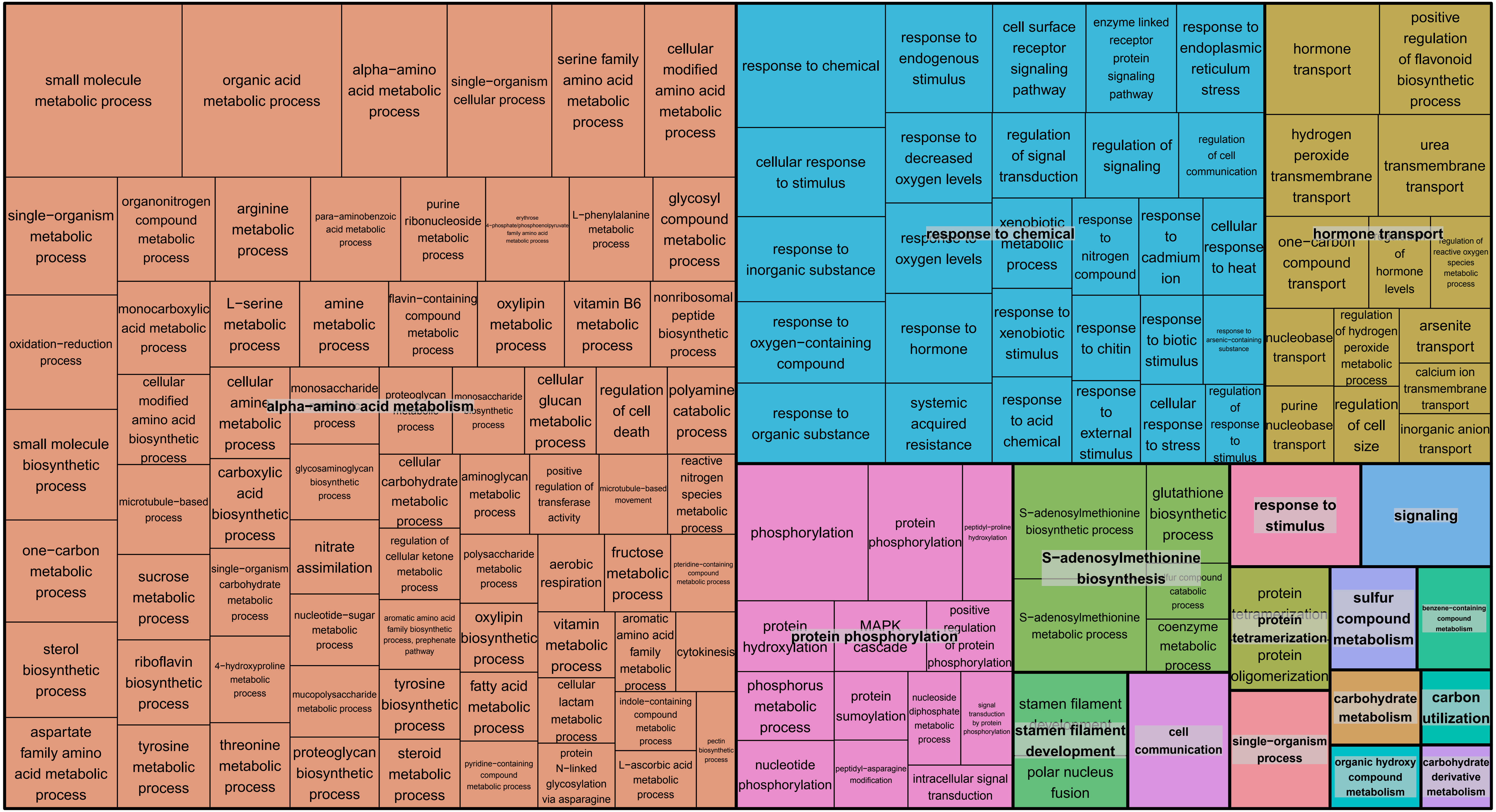
small molecule metabolic process
organic acid metabolic process
alpha−amino acid metabolic process
single−organism cellular process
serine family amino acid metabolic process
cellular modified amino acid metabolic process
single−organism metabolic process
organonitrogen compound metabolic process
arginine metabolic process
para−aminobenzoic acid metabolic process
purine ribonucleoside metabolic process
L−phenylalanine metabolic process
glycosyl compound metabolic process
oxidation−reduction process
monocarboxylic acid metabolic process
L−serine metabolic process
amine metabolic process
flavin−containing compound metabolic process
oxylipin metabolic process
vitamin B6 metabolic process
nonribosomal peptide biosynthetic process
small molecule biosynthetic process
cellular modified amino acid biosynthetic process
cellular amine metabolic process
alpha−amino acid metabolism
monosaccharide metabolic process
proteoglycan metabolic process
monosaccharide biosynthetic process
cellular glucan metabolic process
regulation of cell death
polyamine catabolic process
microtubule−based process
carboxylic acid biosynthetic process
glycosaminoglycan biosynthetic process
cellular carbohydrate metabolic process
aminoglycan metabolic process
positive regulation of transferase activity
microtubule−based movement
reactive nitrogen species metabolic process
one−carbon metabolic process
sucrose metabolic process
single−organism carbohydrate metabolic process
nitrate assimilation
regulation of cellular ketone metabolic process
polysaccharide metabolic process
aerobic respiration
fructose metabolic process
pteridine−containing compound metabolic process
sterol biosynthetic process
riboflavin biosynthetic process
4−hydroxyproline metabolic process
nucleotide−sugar metabolic process
aromatic amino acid family biosynthetic process, prephenate pathway
oxylipin biosynthetic process
vitamin metabolic process
aromatic amino acid family metabolic process
cytokinesis
mucopolysaccharide metabolic process
tyrosine biosynthetic process
fatty acid metabolic process
cellular lactam metabolic process
indole−containing compound metabolic process
aspartate family amino acid metabolic process
tyrosine metabolic process
threonine metabolic process
proteoglycan biosynthetic process
steroid metabolic process
pyridine−containing compound metabolic process
protein N−linked glycosylation via asparagine
L−ascorbic acid metabolic process
pectin biosynthetic process
response to chemical
response to endogenous stimulus
cell surface receptor signaling pathway
enzyme linked receptor protein signaling pathway
response to endoplasmic reticulum stress
cellular response to stimulus
response to decreased oxygen levels
regulation of signal transduction
regulation of signaling
regulation of cell communication
response to inorganic substance
response to oxygen levels
xenobiotic metabolic process
response to nitrogen compound
response to cadmium ion
cellular response to heat
response to oxygen−containing compound
response to hormone
response to xenobiotic stimulus
response to chitin
response to biotic stimulus
response to arsenic−containing substance
response to organic substance
systemic acquired resistance
response to acid chemical
response to external stimulus
cellular response to stress
regulation of response to stimulus
hormone transport
positive regulation of flavonoid biosynthetic process
hydrogen peroxide transmembrane transport
urea transmembrane transport
one−carbon compound transport
regulation of hormone levels
regulation of reactive oxygen species metabolic process
nucleobase transport
regulation of hydrogen peroxide metabolic process
arsenite transport
calcium ion transmembrane transport
purine nucleobase transport
regulation of cell size
inorganic anion transport
phosphorylation
protein phosphorylation
peptidyl−proline hydroxylation
protein hydroxylation
MAPK cascade
positive regulation of protein phosphorylation
phosphorus metabolic process
protein sumoylation
nucleoside diphosphate metabolic process
signal transduction by protein phosphorylation
nucleotide phosphorylation
peptidyl−asparagine modification
intracellular signal transduction
S−adenosylmethionine biosynthetic process
glutathione biosynthetic process
S−adenosylmethionine biosynthesis
sulfur compound catabolic process
S−adenosylmethionine metabolic process
coenzyme metabolic process
stamen filament development
polar nucleus fusion
cell communication
response to stimulus
signaling
protein tetramerization
protein oligomerization
sulfur compound metabolism
benzene−containing compound metabolism
single−organism process
carbohydrate metabolism
carbon utilization
organic hydroxy compound metabolism
carbohydrate derivative metabolism

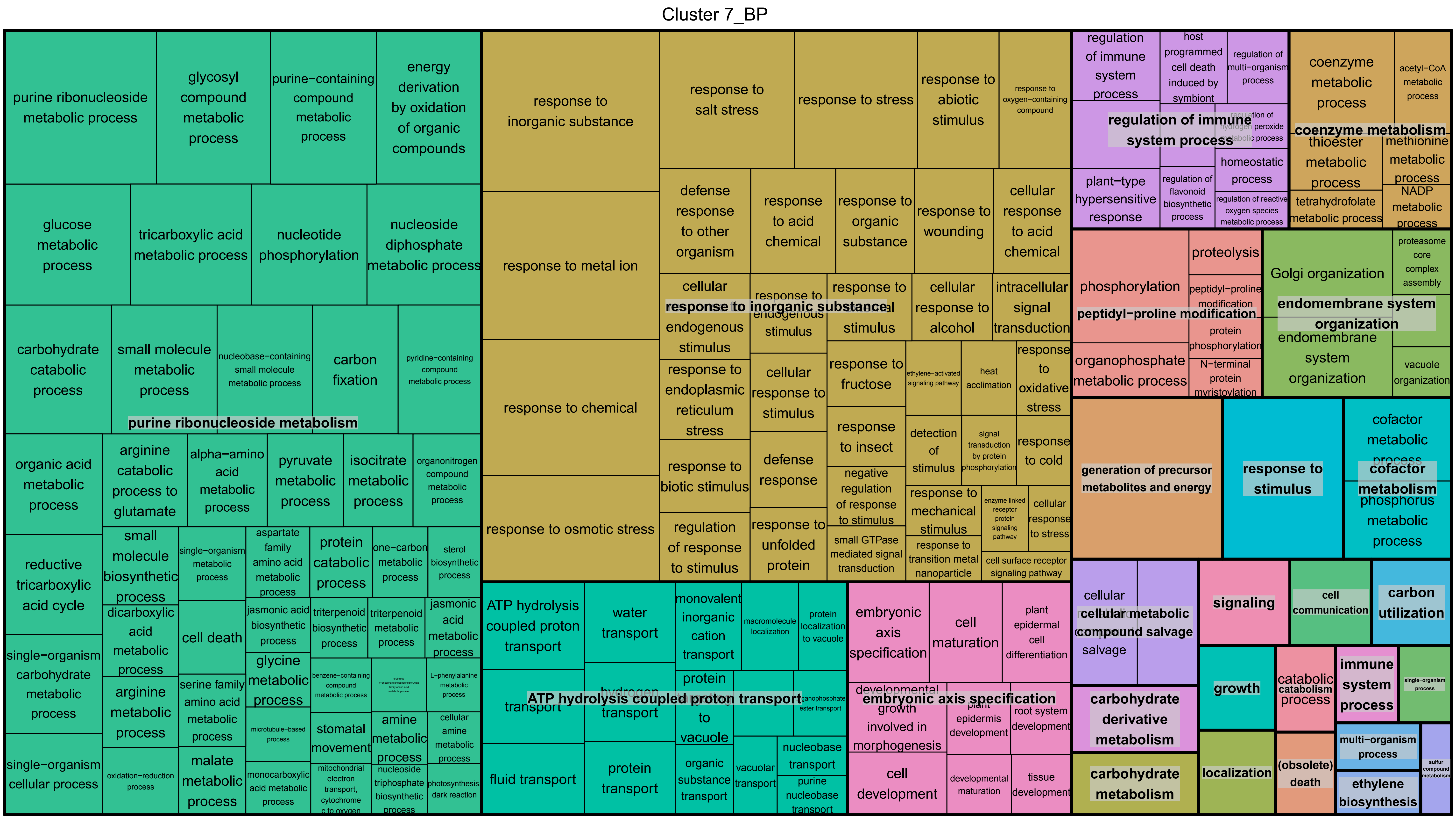
Cluster 7_BP
purine ribonucleoside metabolism
response to inorganic substance
ATP hydrolysis coupled proton transport
embryonic axis specification
regulation of immune system process
coenzyme metabolism
peptidyl−proline modification
endomembrane system organization
generation of precursor metabolites and energy
response to stimulus
cofactor metabolism
cellular metabolic compound salvage
signaling
cell communication
carbon utilization
growth
catabolism
immune system process
carbohydrate derivative metabolism
carbohydrate metabolism
localization
(obsolete) death
multi−organism process
ethylene biosynthesis

Cluster 8_BP

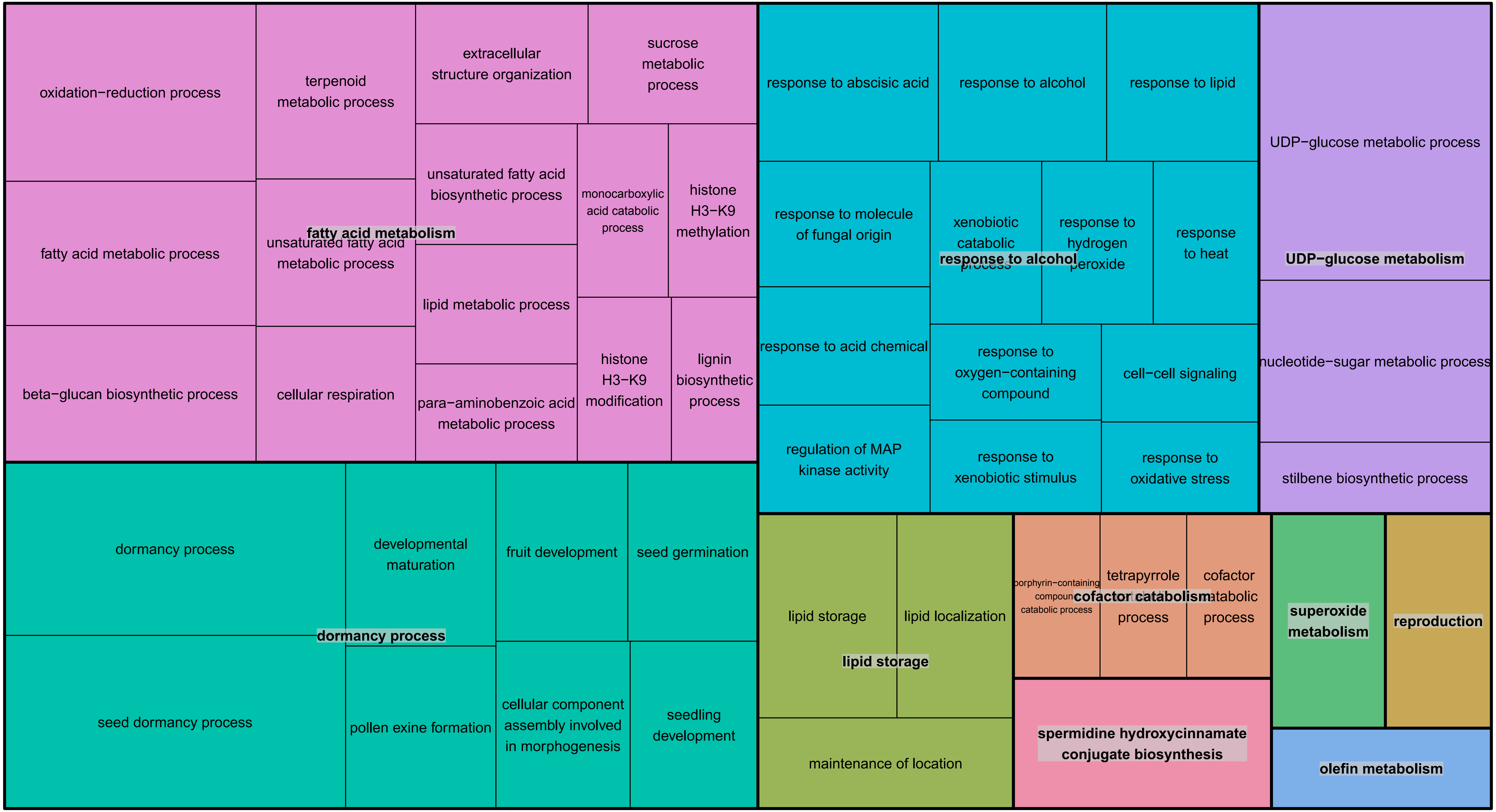

oxidation−reduction process
terpenoid metabolic process
extracellular structure organization
sucrose metabolic process
fatty acid metabolic process
unsaturated fatty acid metabolic process
fatty acid metabolism
unsaturated fatty acid biosynthetic process
monocarboxylic acid catabolic process
histone H3−K9 methylation
lipid metabolic process
beta−glucan biosynthetic process
cellular respiration
para−aminobenzoic acid metabolic process
histone H3−K9 modification
lignin biosynthetic process
response to abscisic acid
response to alcohol
response to lipid
response to molecule of fungal origin
xenobiotic catabolic process
response to hydrogen peroxide
response to heat
response to alcohol
response to acid chemical
response to oxygen−containing compound
cell−cell signaling
regulation of MAP kinase activity
response to xenobiotic stimulus
response to oxidative stress
UDP−glucose metabolic process
UDP−glucose metabolism
nucleotide−sugar metabolic process
stilbene biosynthetic process
dormancy process
developmental maturation
fruit development
seed germination
dormancy process
seed dormancy process
pollen exine formation
cellular component assembly involved in morphogenesis
seedling development
lipid storage
lipid localization
lipid storage
maintenance of location
porphyrin−containing
catabolic process
tetrapyrrole
process
cofactor catabolism
cofactor
process
spermidine hydroxycinnamate conjugate biosynthesis
superoxide metabolism
reproduction
olefin metabolism

Cluster 9_BP

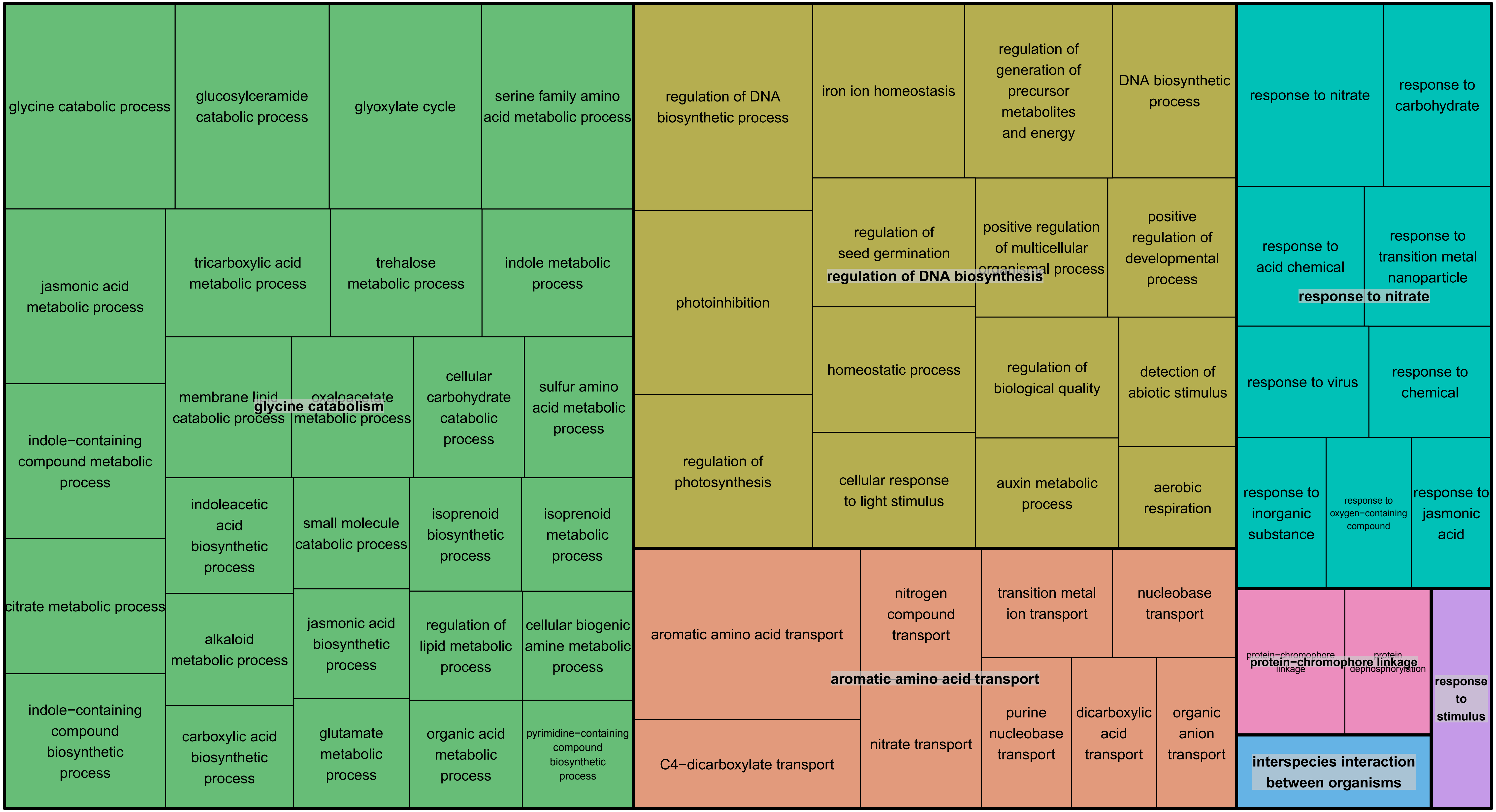

glycine catabolic process
glucosylceramide catabolic process
glyoxylate cycle
serine family amino acid metabolic process
jasmonic acid metabolic process
tricarboxylic acid metabolic process
trehalose metabolic process
indole metabolic process
membrane lipid catabolic process
oxaloacetate metabolic process
glycine catabolism
cellular carbohydrate catabolic process
sulfur amino acid metabolic process
indole−containing compound metabolic process
indoleacetic acid biosynthetic process
small molecule catabolic process
isoprenoid biosynthetic process
isoprenoid metabolic process
citrate metabolic process
alkaloid metabolic process
jasmonic acid biosynthetic process
regulation of lipid metabolic process
cellular biogenic amine metabolic process
indole−containing compound biosynthetic process
carboxylic acid biosynthetic process
glutamate metabolic process
organic acid metabolic process
pyrimidine−containing compound biosynthetic process
regulation of DNA biosynthetic process
iron ion homeostasis
regulation of generation of precursor metabolites and energy
DNA biosynthetic process
photoinhibition
regulation of seed germination
regulation of DNA biosynthesis
positive regulation of multicellular organismal process
positive regulation of developmental process
homeostatic process
regulation of biological quality
detection of abiotic stimulus
regulation of photosynthesis
cellular response to light stimulus
auxin metabolic process
aerobic respiration
aromatic amino acid transport
nitrogen compound transport
transition metal ion transport
nucleobase transport
aromatic amino acid transport
C4−dicarboxylate transport
nitrate transport
purine nucleobase transport
dicarboxylic acid transport
organic anion transport
response to nitrate
response to carbohydrate
response to acid chemical
response to transition metal nanoparticle
response to nitrate
response to virus
response to chemical
response to inorganic substance
response to oxygen−containing compound
response to jasmonic acid
protein−chromophore linkage
protein dephosphorylation
protein−chromophore linkage
response to stimulus
interspecies interaction between organisms

Cluster 10_BP

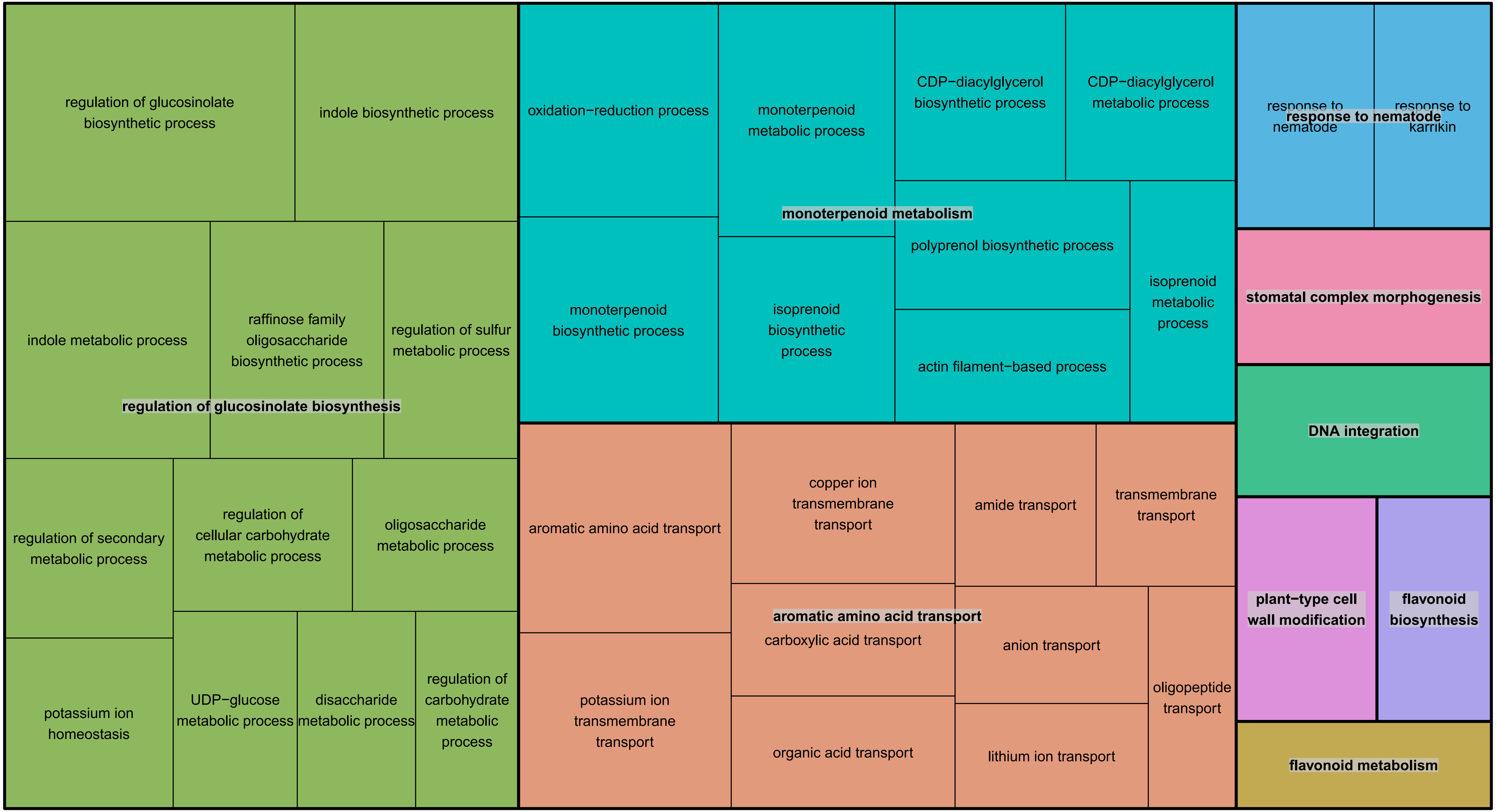

Supplement: Supplementary file 5 — Additional file 5: Figure S5. TreeMap view in REVIGO of the enriched BP of the six different seed maturation stages of all DESs. All terms are adjusted P-value cutoff at 0.05 from the enrichment analysis. [file 12864_2020_6666_MOESM5_ESM.pdf]
