# Supplementary material for: Gene expression profiling identifies pathways involved in seed maturation of Jatropha curcas
Source: BMC Genomics. 2020 Apr 9;21:290. doi: 10.1186/s12864-020-6666-1 (PMC7146973; doi:10.1186/s12864-020-6666-1)

Cluster 1

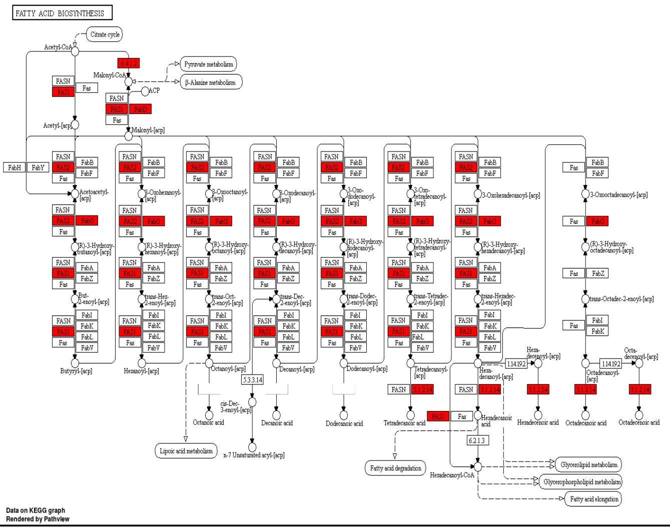

Cluster 5

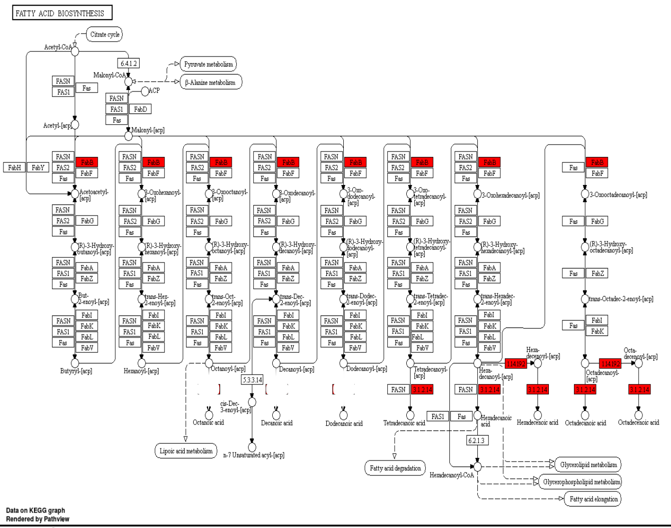

Cluster 8

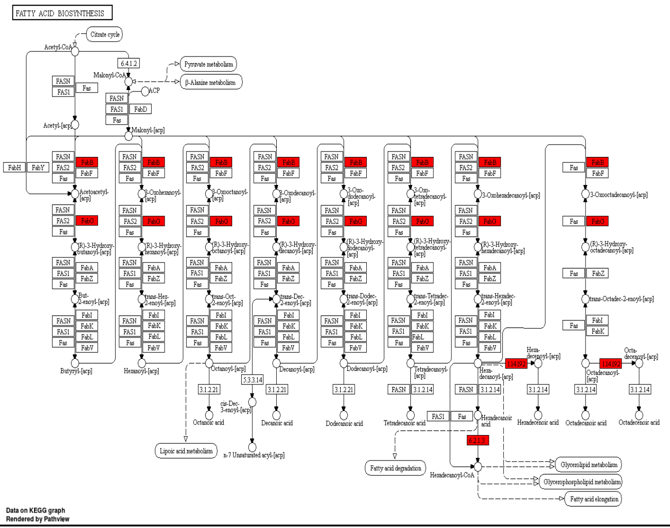

Cluster 8

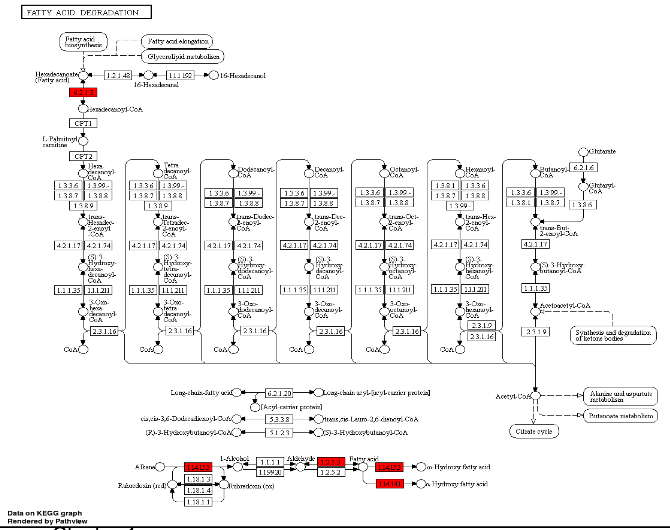

Cluster 6

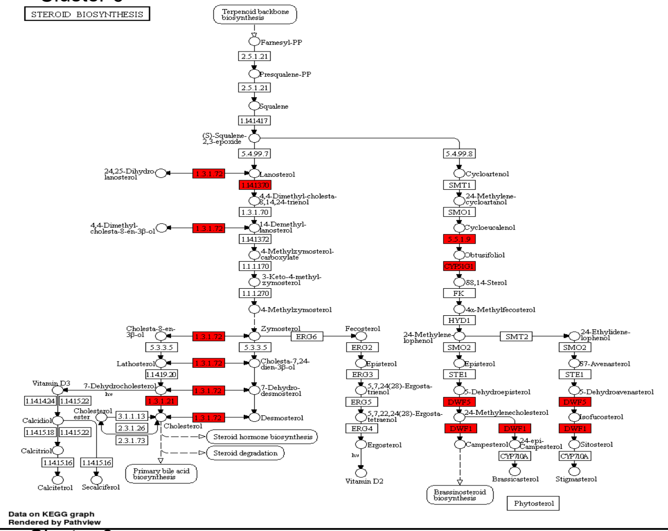

Cluster 1

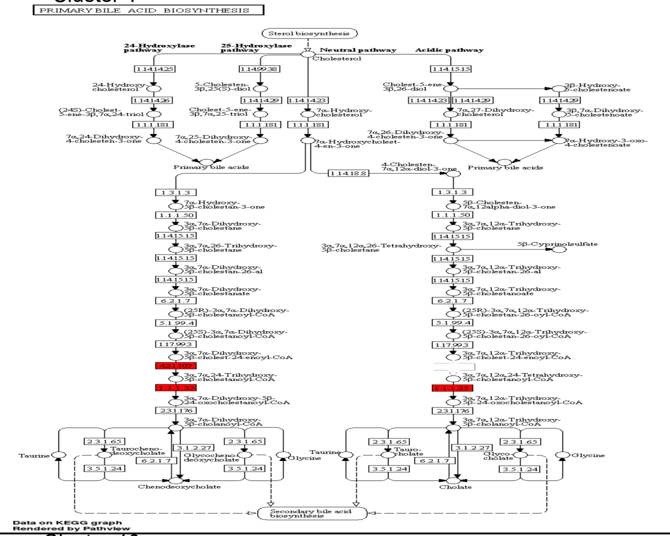

Cluster 8

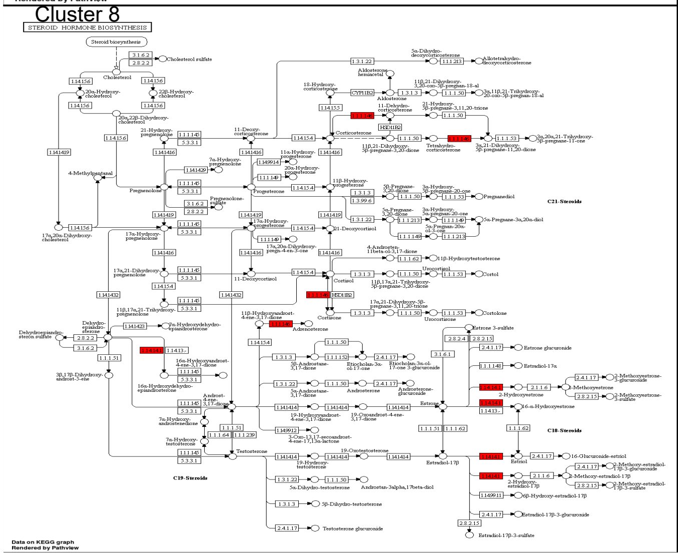

Cluster 10

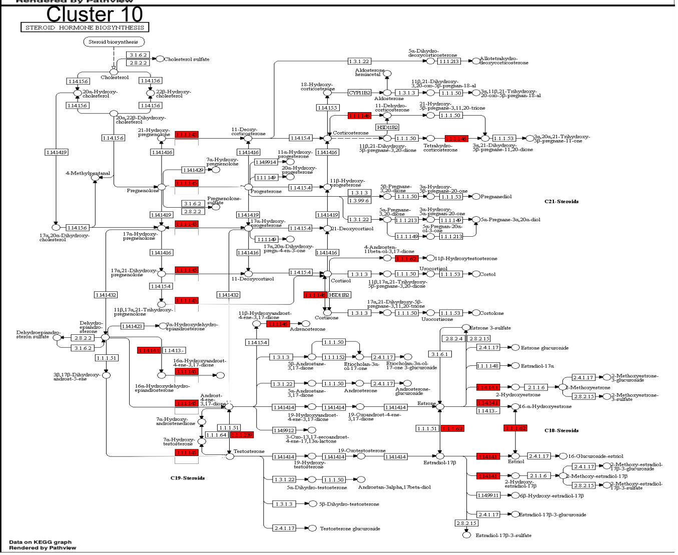

Cluster 2

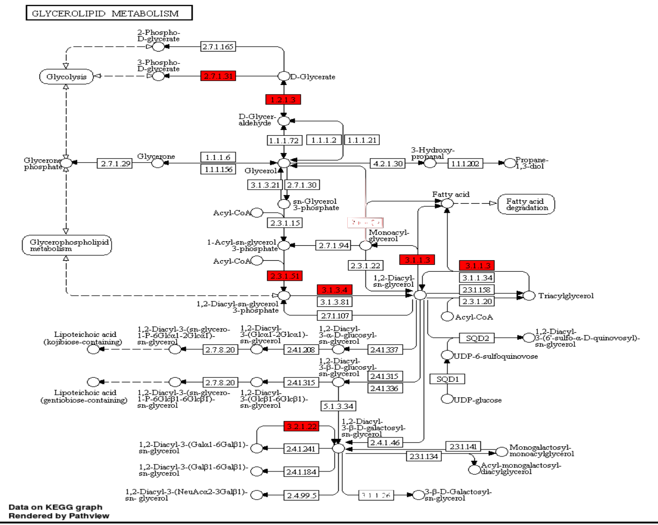

Cluster 4

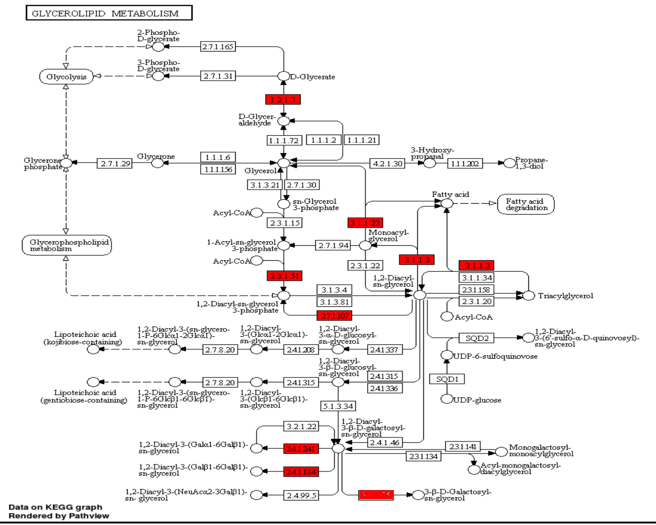

Cluster 8

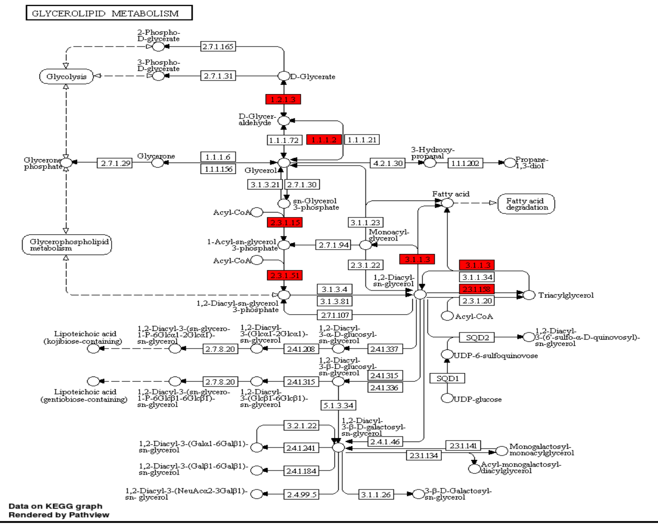

Cluster 2

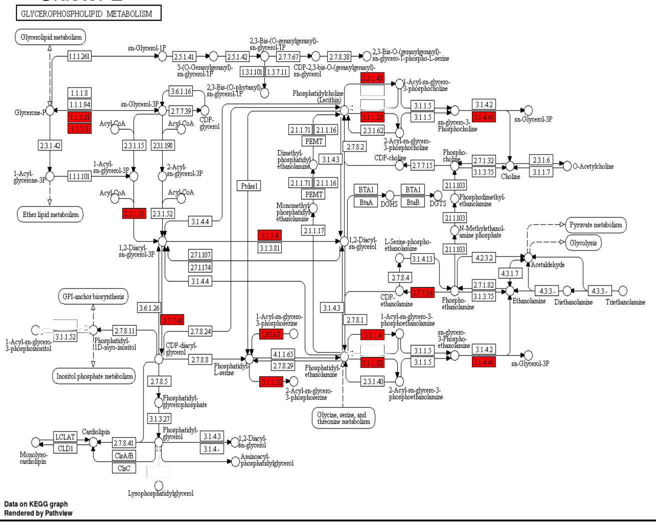

Cluster 4

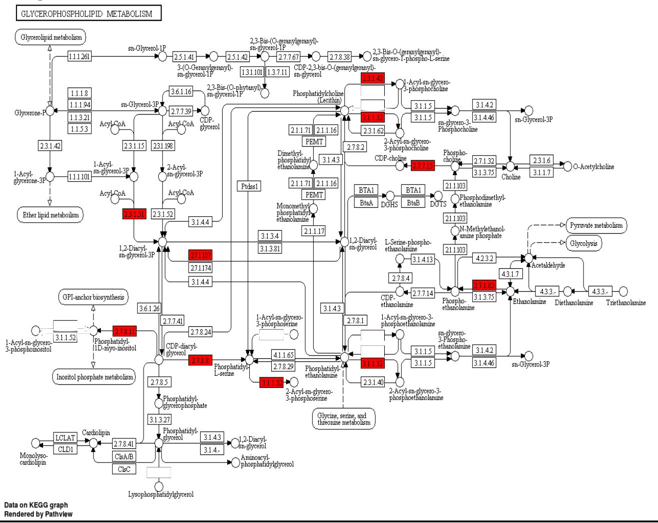

Cluster 8

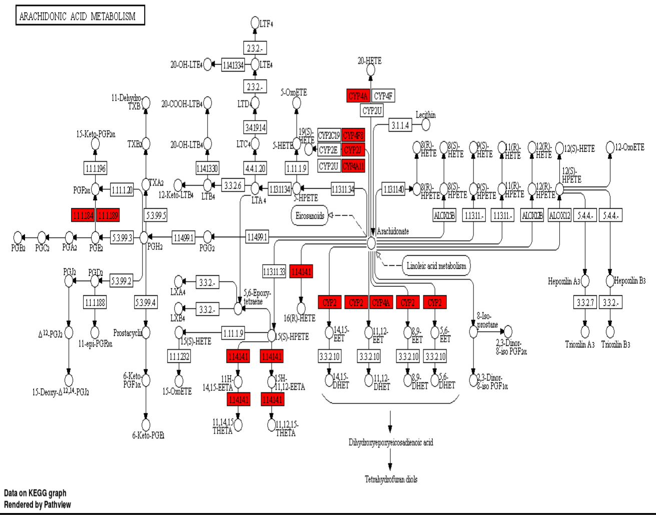

Cluster 10

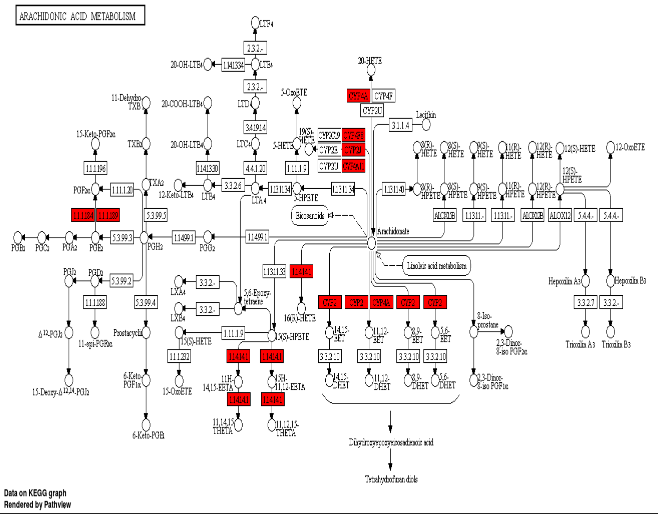

Cluster 10

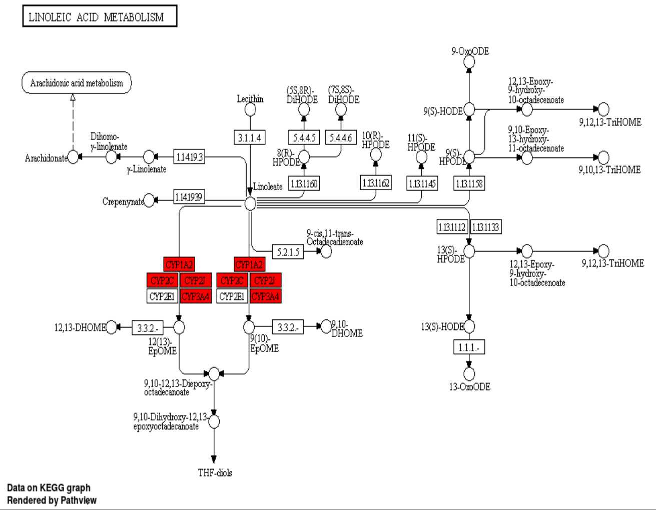

Cluster 1

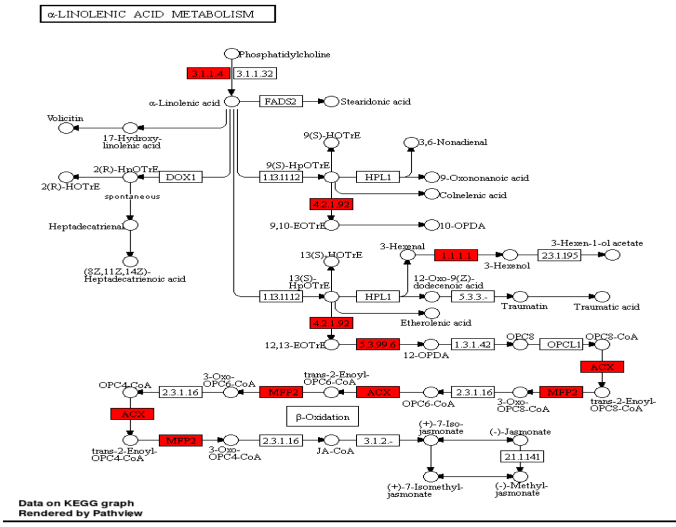

Cluster 1

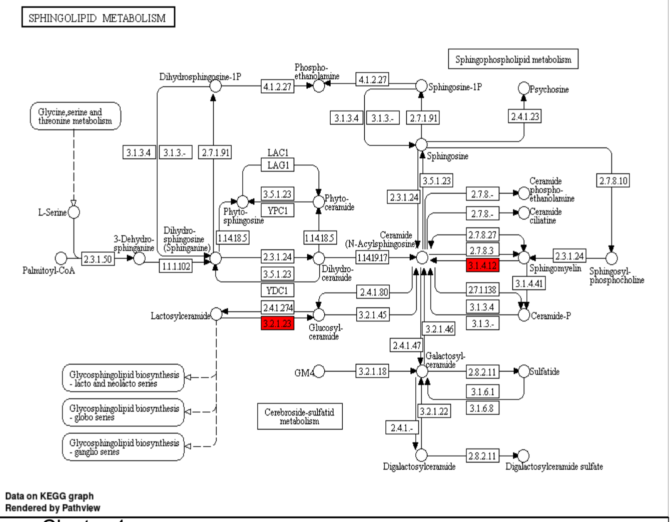

Cluster 2

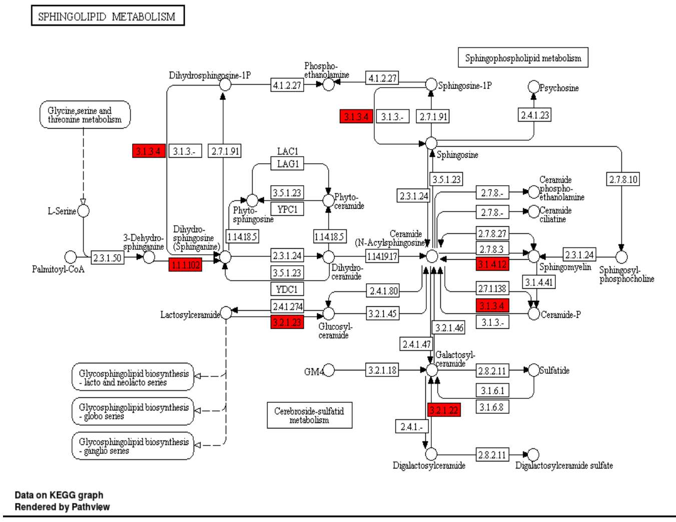

Cluster 1

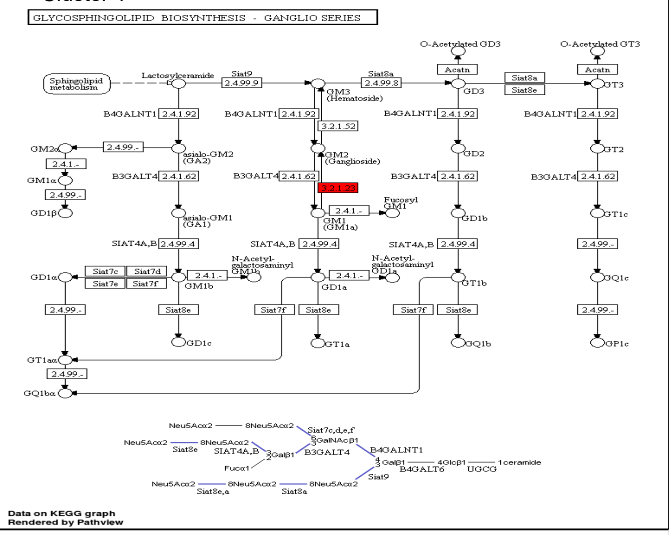

Cluster 5

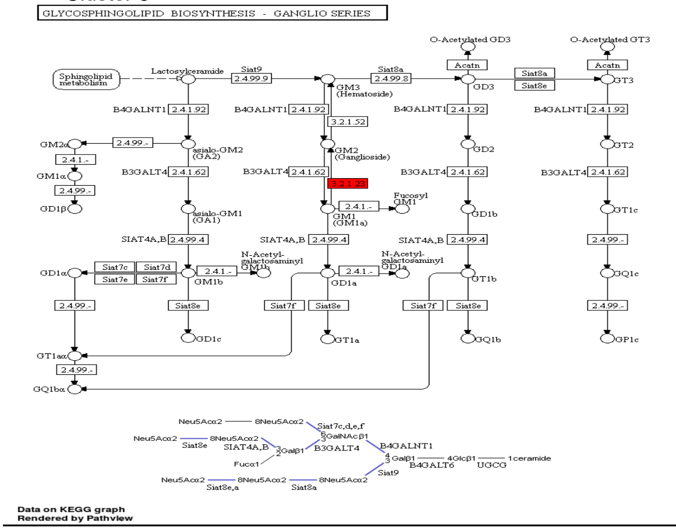

Cluster 1

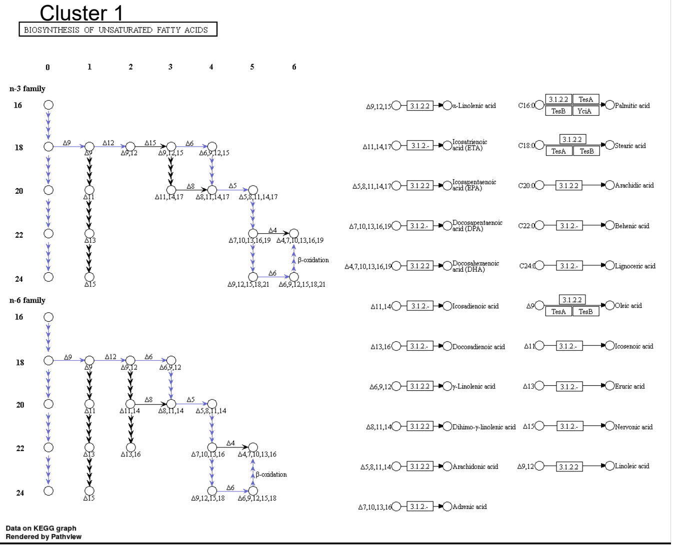

Supplement: Supplementary file 7 — Additional file 7: Figure S7. Overview of significantly enriched and over-represented pathways and enzymes related to lipid metabolism identified in different clusters. Figures generated by the pathview package to paint the gene of interests into KEGG pathways. [file 12864_2020_6666_MOESM7_ESM.pdf]
