# Supplementary material for: Gene expression profiling identifies pathways involved in seed maturation of Jatropha curcas
Source: BMC Genomics. 2020 Apr 9;21:290. doi: 10.1186/s12864-020-6666-1 (PMC7146973; doi:10.1186/s12864-020-6666-1)

Cluster 3

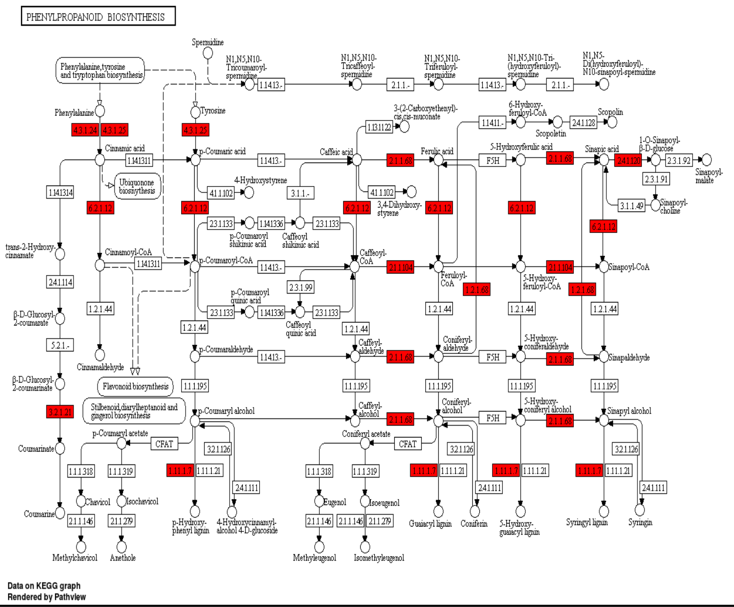

Cluster 5

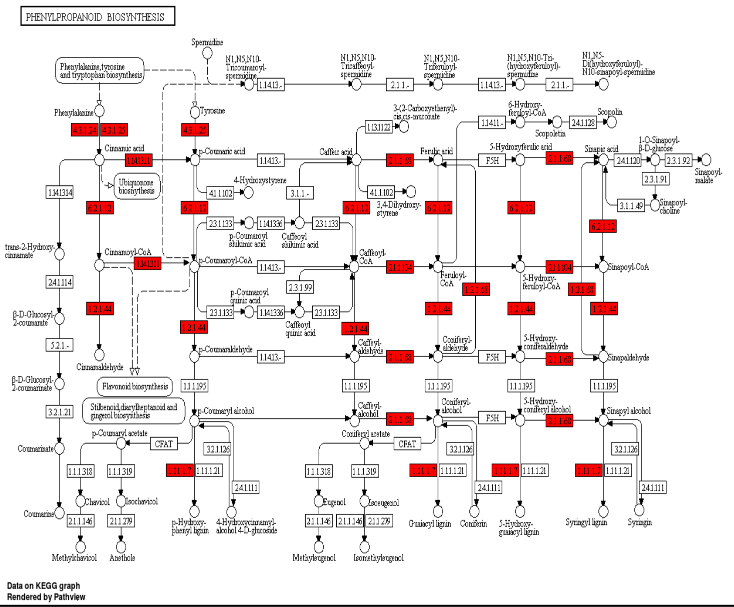

Cluster 8

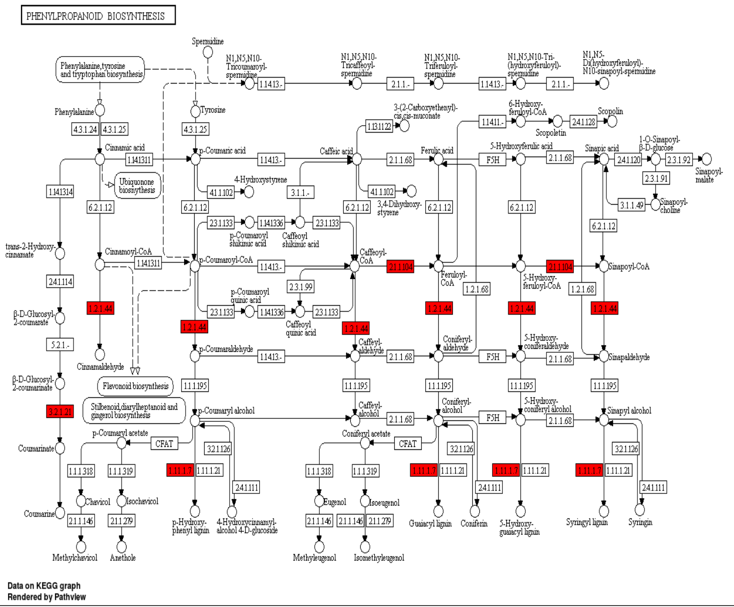

Supplement: Supplementary file 8 — Additional file 8: Figure S8. Overview of significantly enriched and over-represented phenylpropanoid biosynthesis biosynthesis pathways and related enzymes identified in different clusters. Figures generated pathview package to paint the gene of interests into KEGG pathways. [file 12864_2020_6666_MOESM8_ESM.pdf]
