# Supplementary material for: Gene expression profiling identifies pathways involved in seed maturation of Jatropha curcas
Source: BMC Genomics. 2020 Apr 9;21:290. doi: 10.1186/s12864-020-6666-1 (PMC7146973; doi:10.1186/s12864-020-6666-1)

Cluster 1

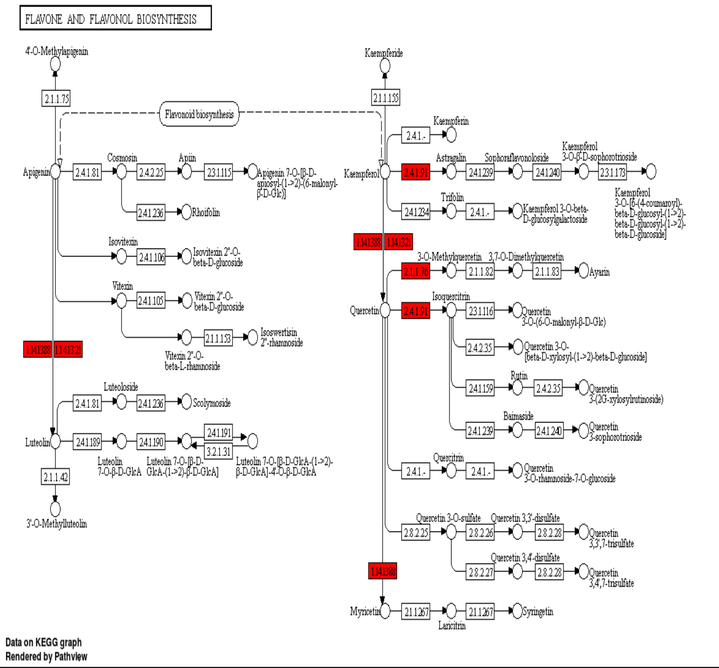

Cluster 1

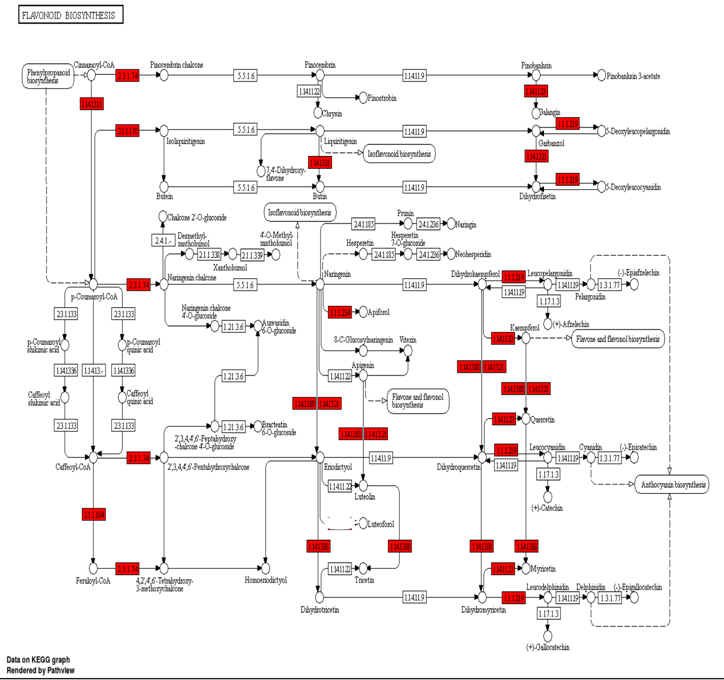

Cluster 3

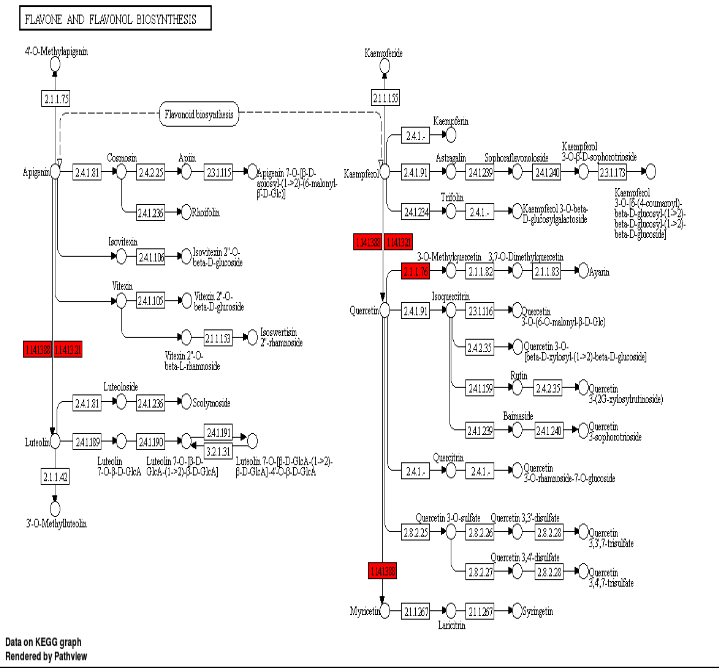

Cluster 3

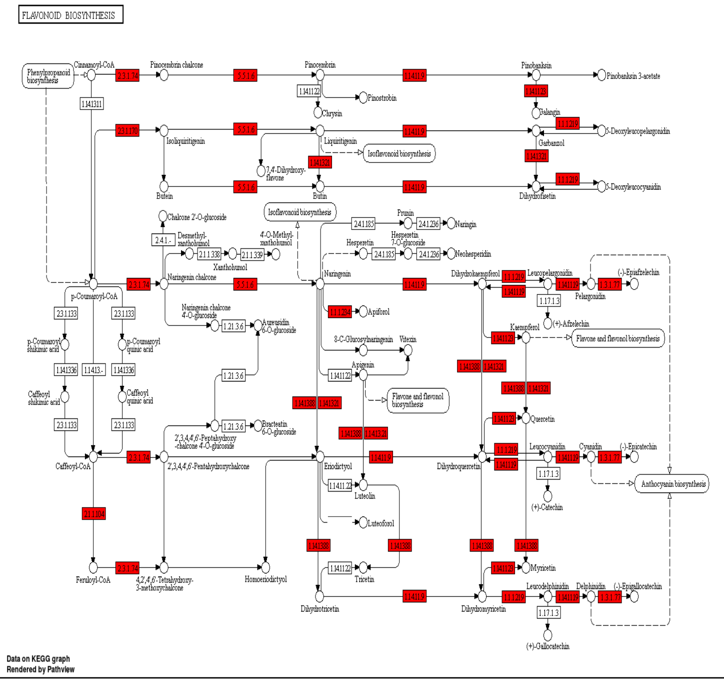

Cluster 5

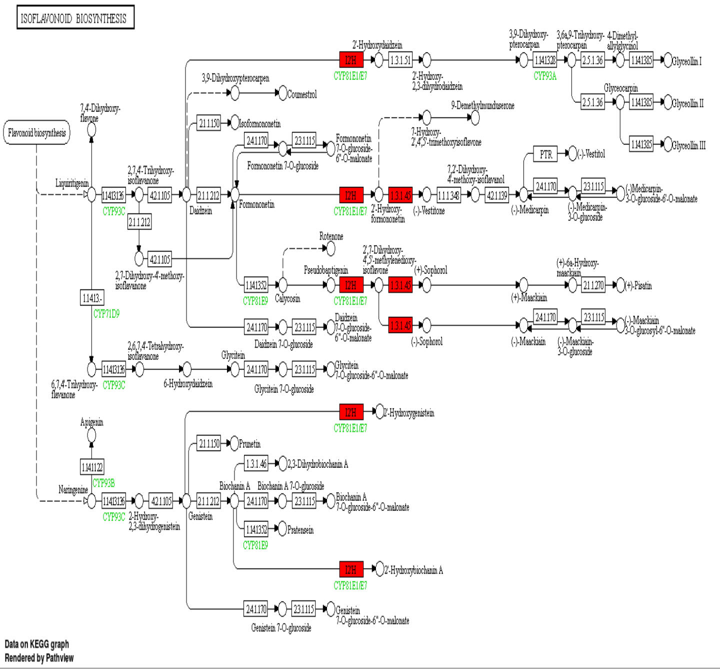

Cluster 5

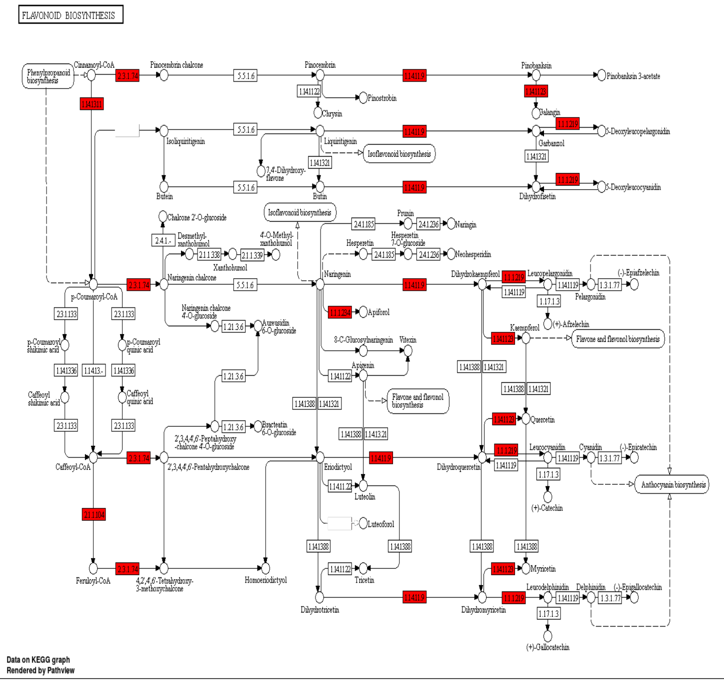

Supplement: Supplementary file 9 — Additional file 9: Figure S9. Overview of significantly enriched and over-represented flavonoid, flavone and flavonol biosynthesis and isoflavonoid biosynthesis pathways and related enzymes identified in different clusters. Figures generated by the pathview package to paint the gene of interests into KEGG pathways. [file 12864_2020_6666_MOESM9_ESM.pdf]
