# Supplementary material for: Gene expression profiling identifies pathways involved in seed maturation of Jatropha curcas
Source: BMC Genomics. 2020 Apr 9;21:290. doi: 10.1186/s12864-020-6666-1 (PMC7146973; doi:10.1186/s12864-020-6666-1)
Supplement: Supplementary file 10 — Additional file 10: Figure S10. Correlation between microarray and qRT-PCR of some DESs during seed development. The y-axis represented the log2 intensity values from microarray analysis and the x-axis represented the Ct values from the qPCR analysis. [file 12864_2020_6666_MOESM10_ESM.pdf]

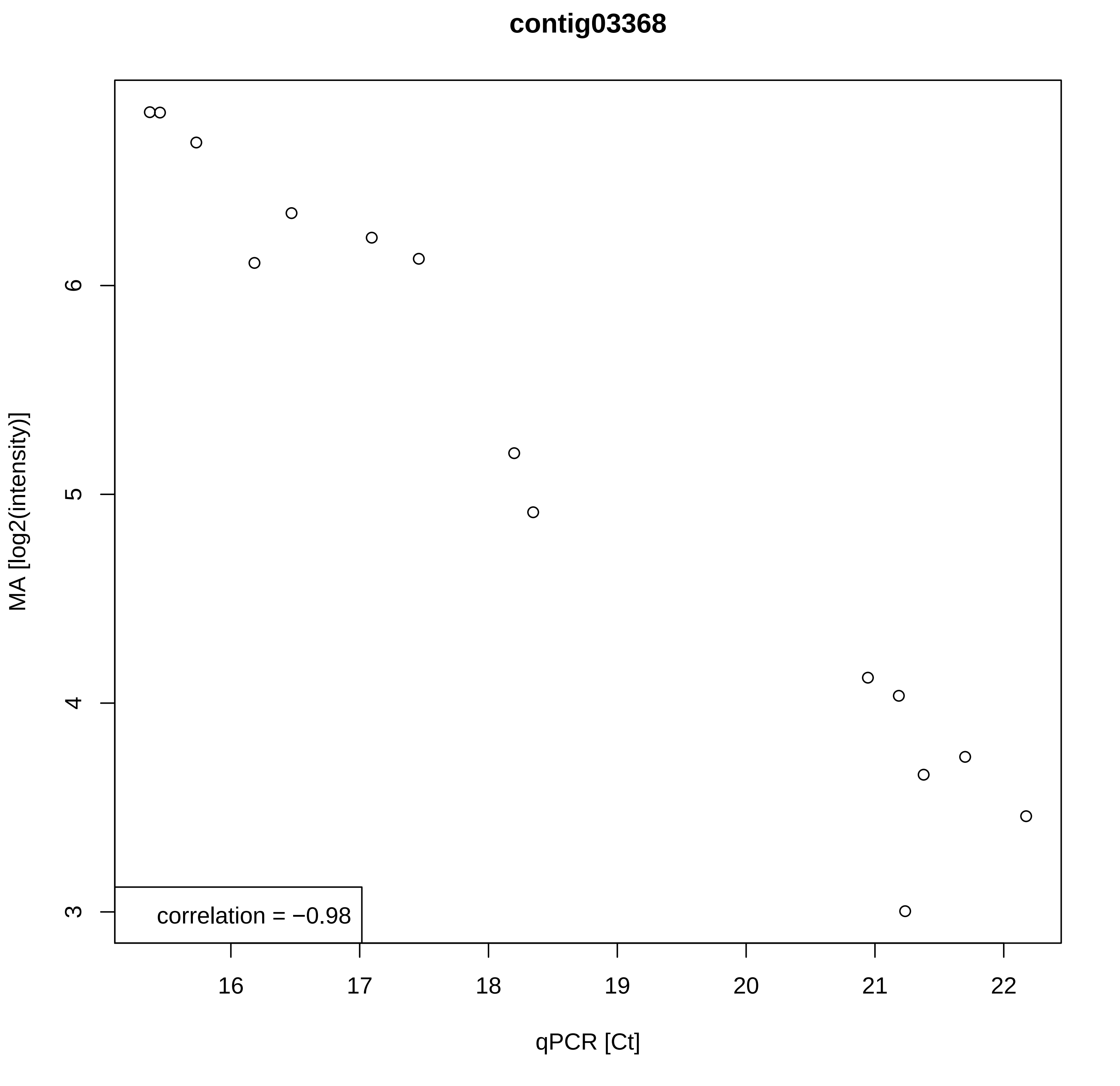
contig03368
MA [log2(intensity)]
qPCR [Ct]
correlation = −0.98
3
4
5
6
16
17
18
19
20
21
22

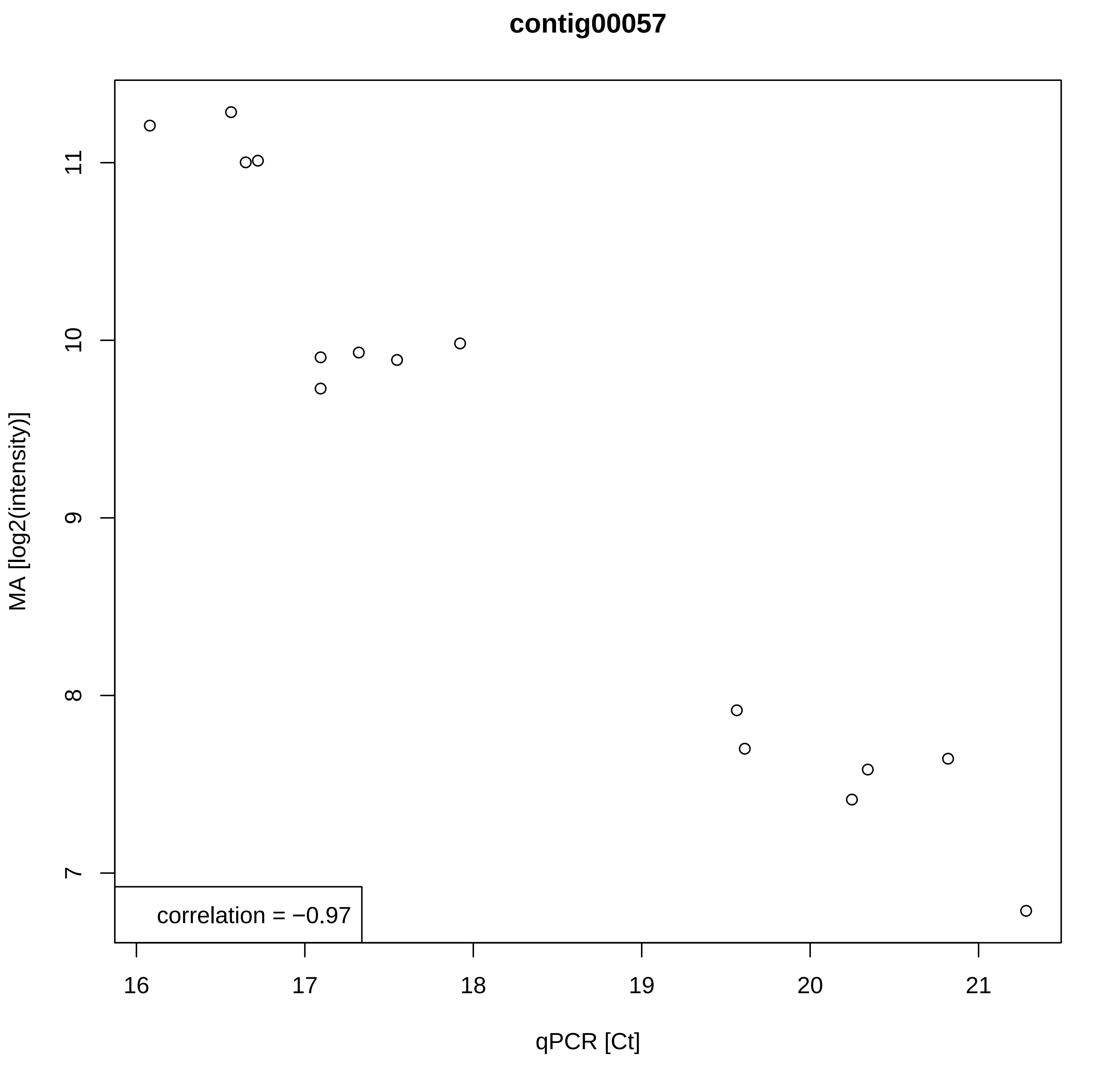

contig00057
MA [log2(intensity)]
qPCR [Ct]
correlation = −0.97
7
8
9
10
11
16
17
18
19
20
21

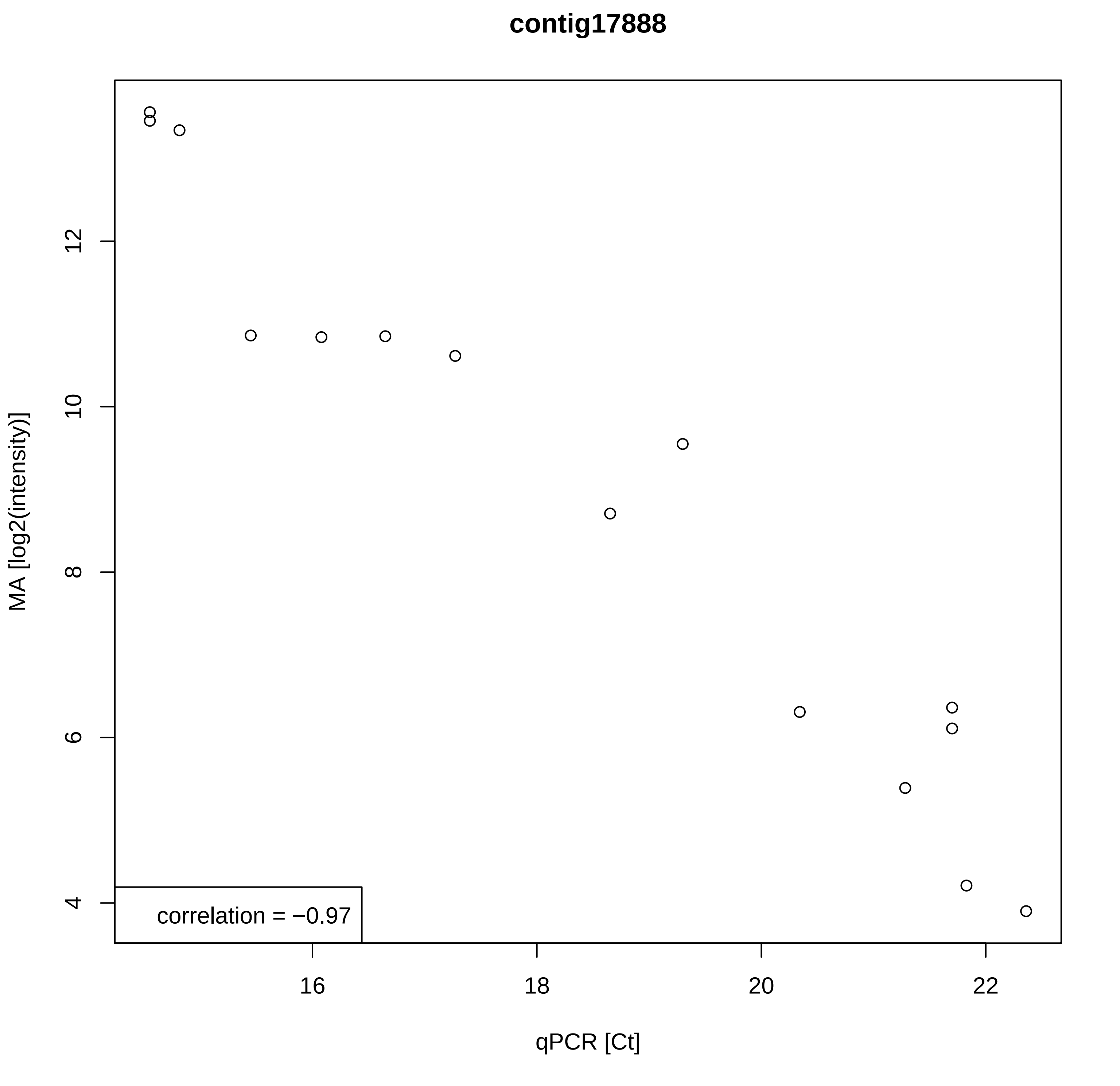

contig17888
MA [log2(intensity)]
4
6
8
10
12
correlation = −0.97
16
18
20
22
qPCR [Ct]

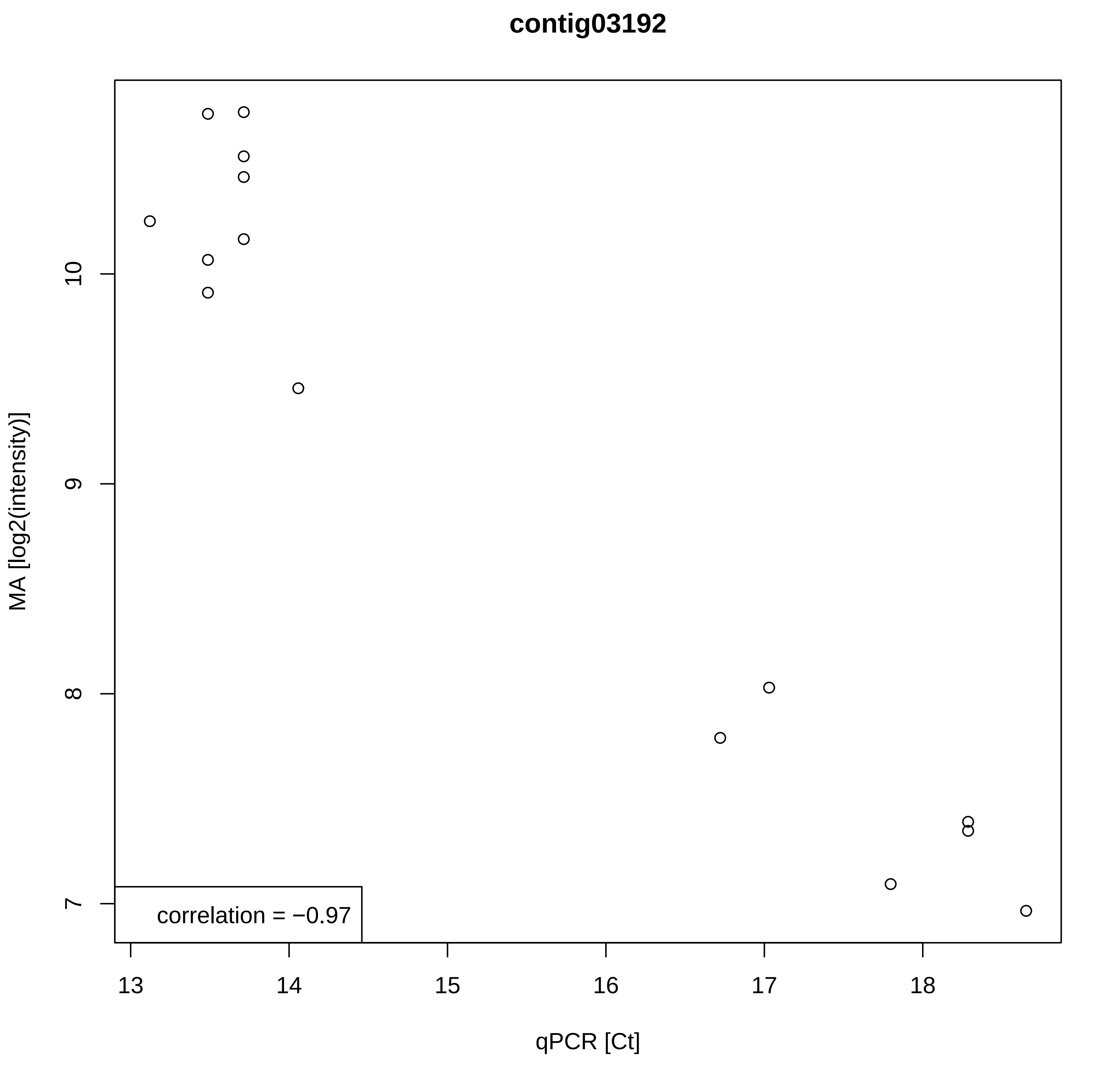
contig03192
MA [log2(intensity)]
qPCR [Ct]
correlation = −0.97
7
8
9
10
13
14
15
16
17
18

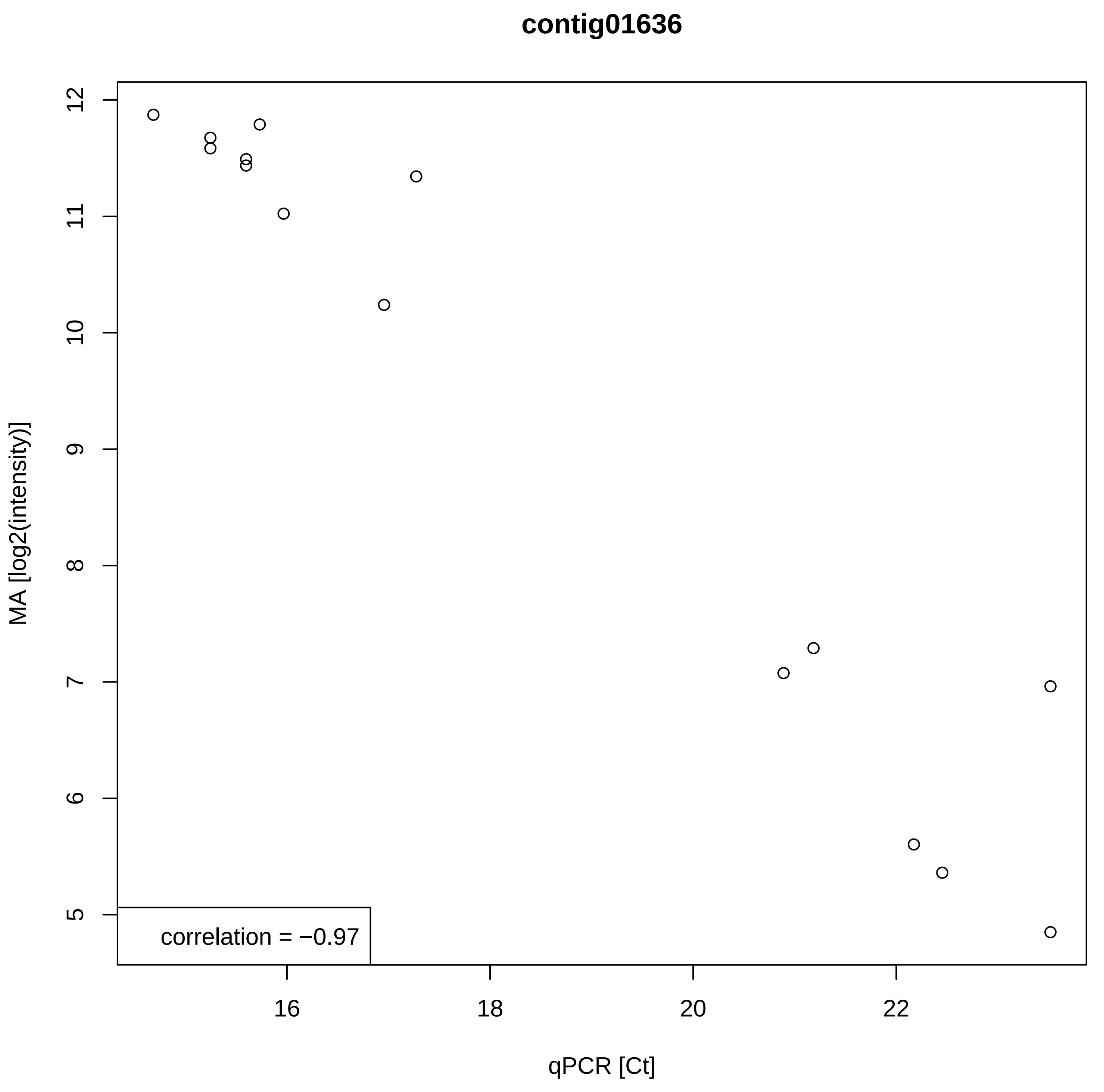
contig01636
MA [log2(intensity)]
12
11
10
9
8
7
6
5
correlation = −0.97
16
18
20
22
qPCR [Ct]

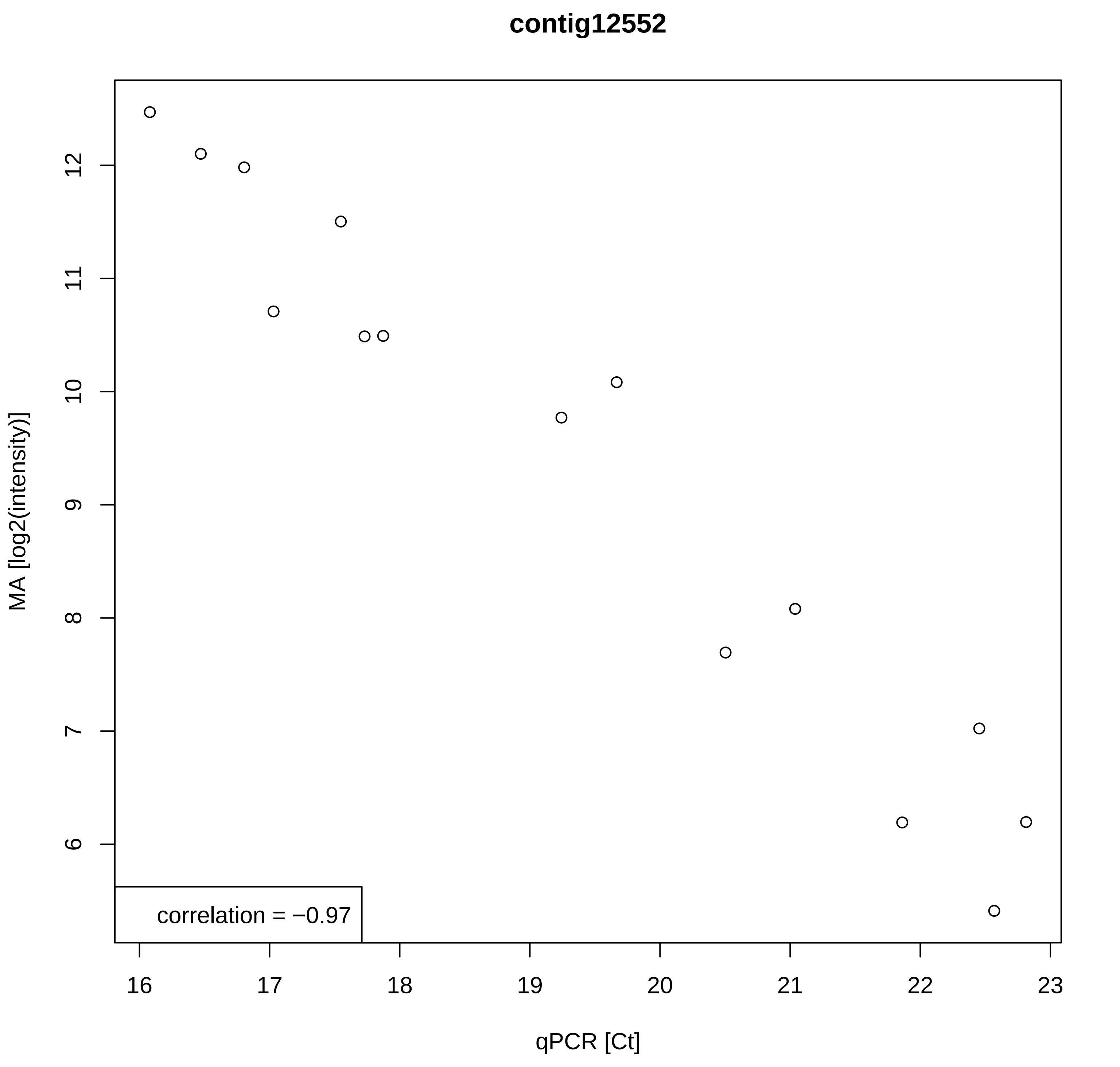
contig12552
MA [log2(intensity)]
qPCR [Ct]
correlation = −0.97
6
7
8
9
10
11
12
16
17
18
19
20
21
22
23

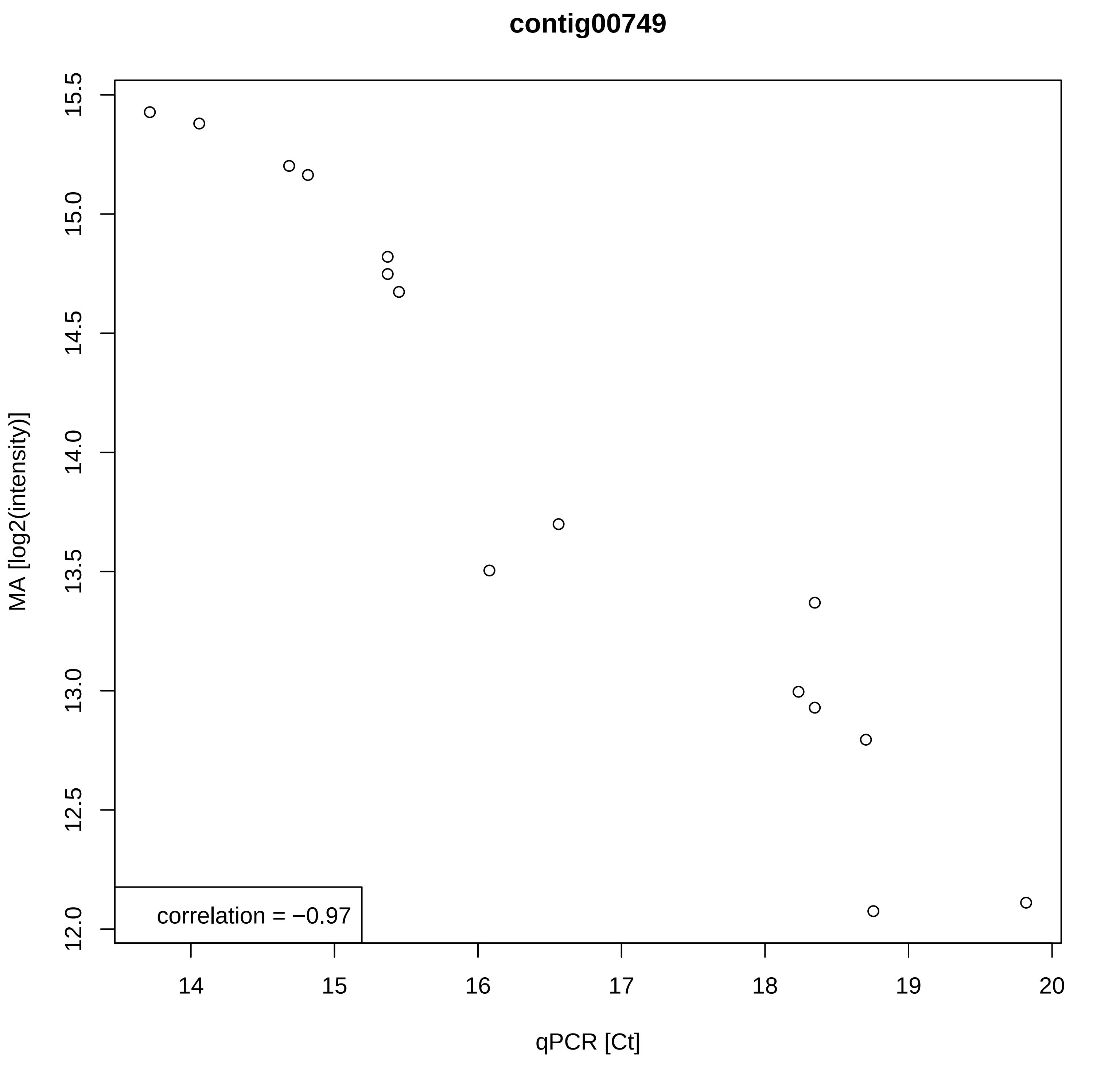

contig00749
15.5
15.0
14.5
14.0
13.5
13.0
12.5
12.0
MA [log2(intensity)]
correlation = −0.97
14
15
16
17
18
19
20
qPCR [Ct]

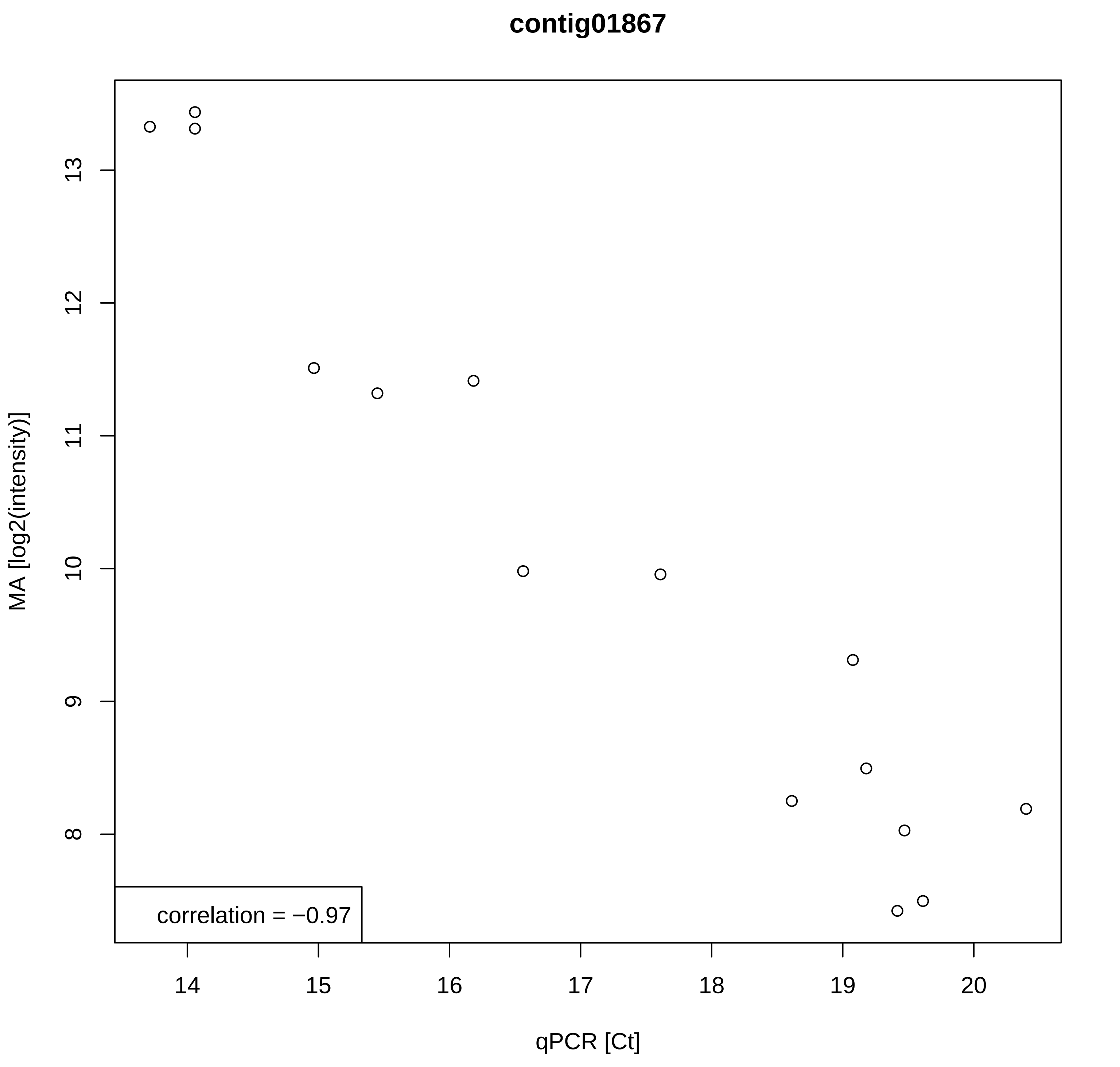
contig01867
MA [log2(intensity)]
qPCR [Ct]
correlation = −0.97
8
9
10
11
12
13
14
15
16
17
18
19
20

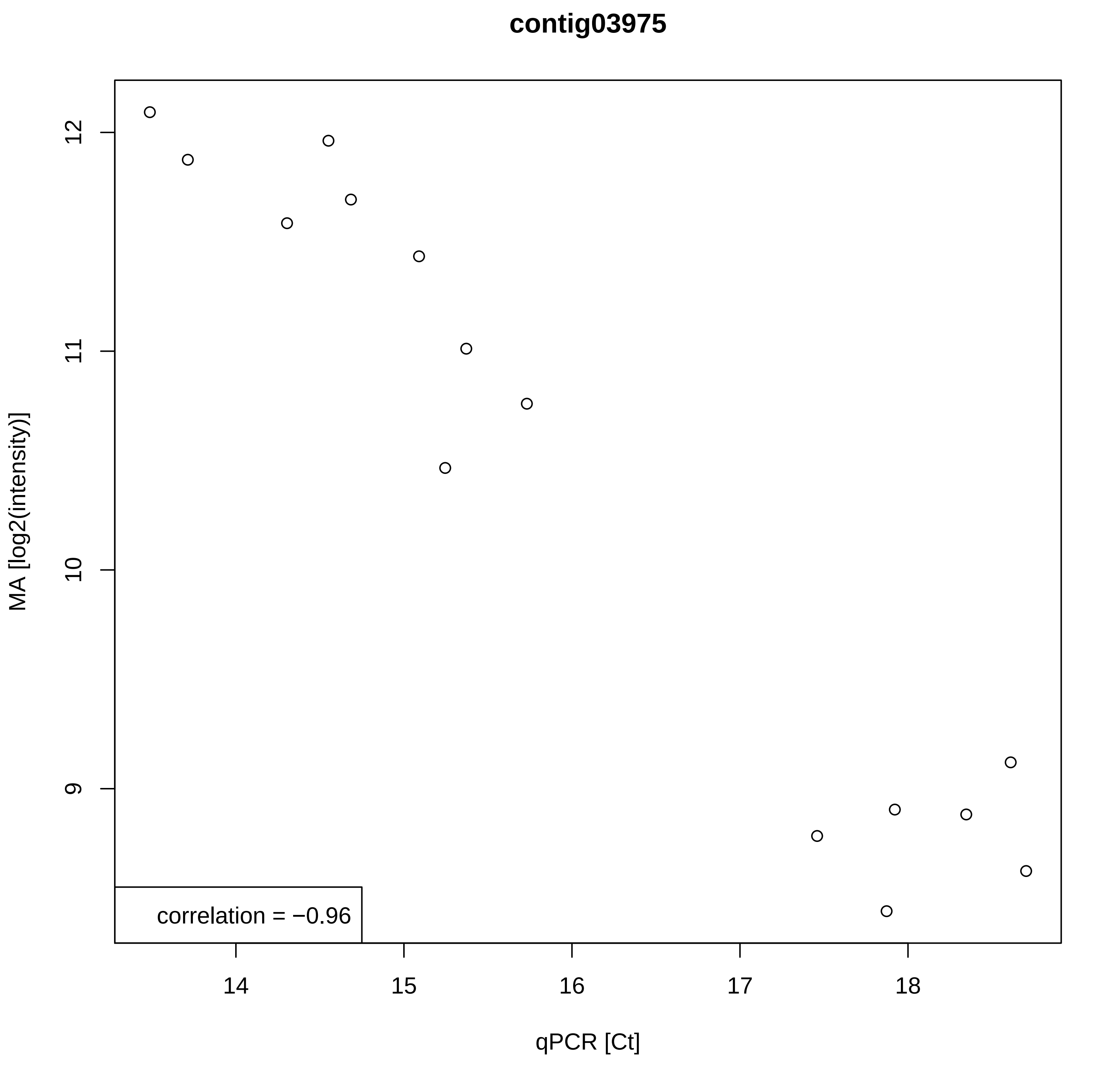
contig03975
MA [log2(intensity)]
12
11
10
9
correlation = −0.96
14
15
16
17
18
qPCR [Ct]

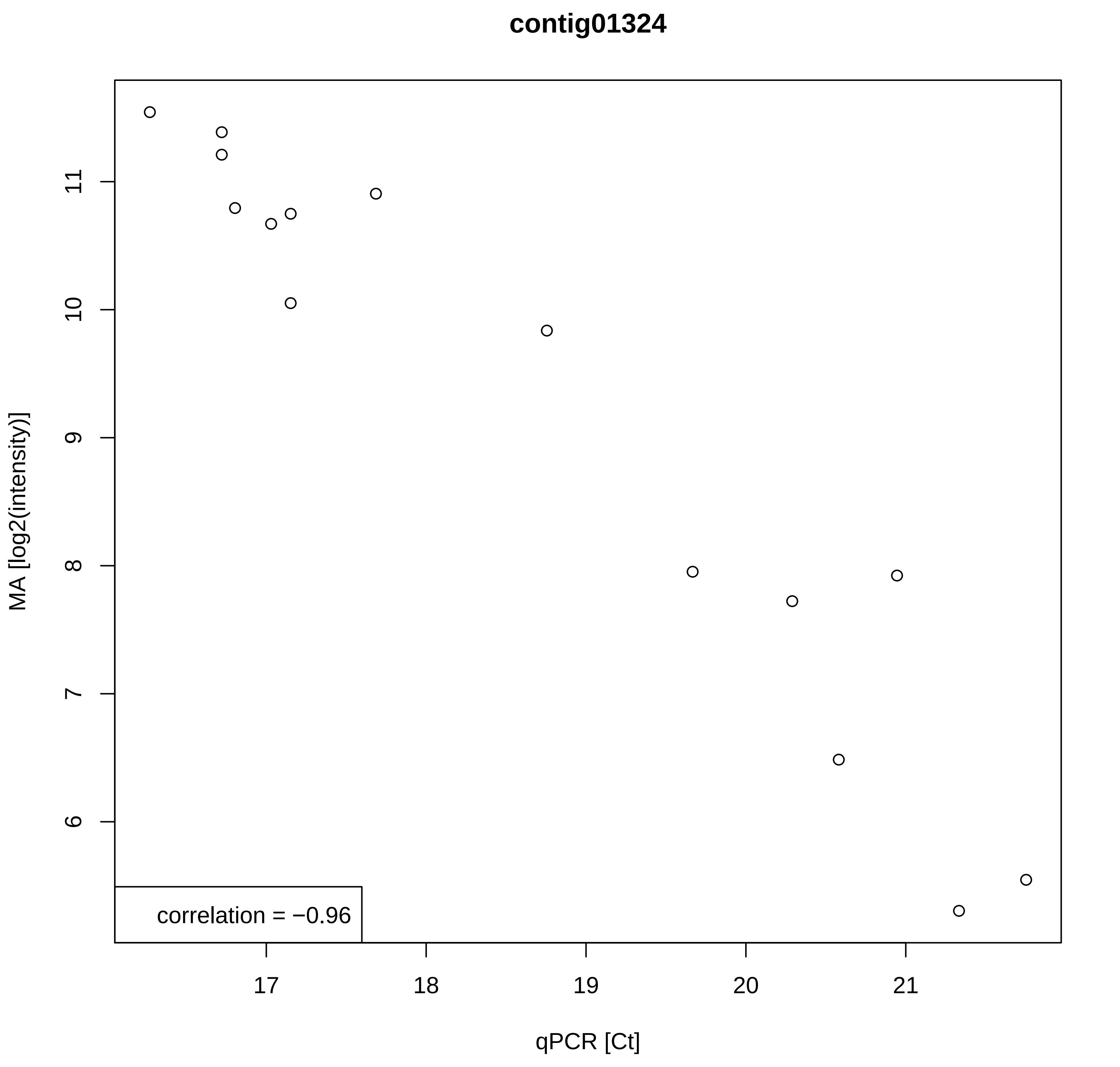
contig01324
MA [log2(intensity)]
6
7
8
9
10
11
correlation = −0.96
17
18
19
20
21
qPCR [Ct]

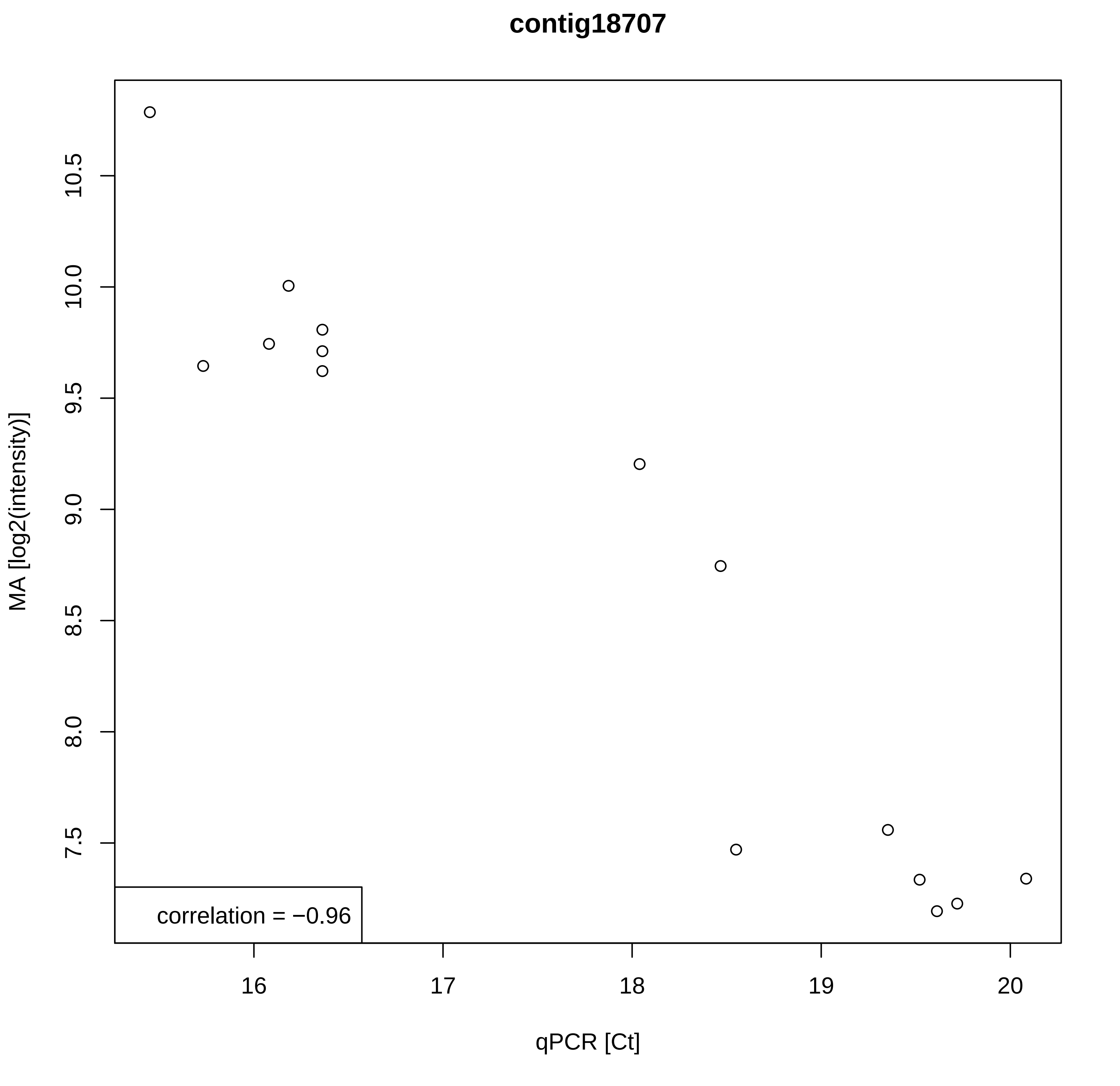
contig18707
MA [log2(intensity)]
10.5
10.0
9.5
9.0
8.5
8.0
7.5
qPCR [Ct]
16
17
18
19
20
correlation = −0.96

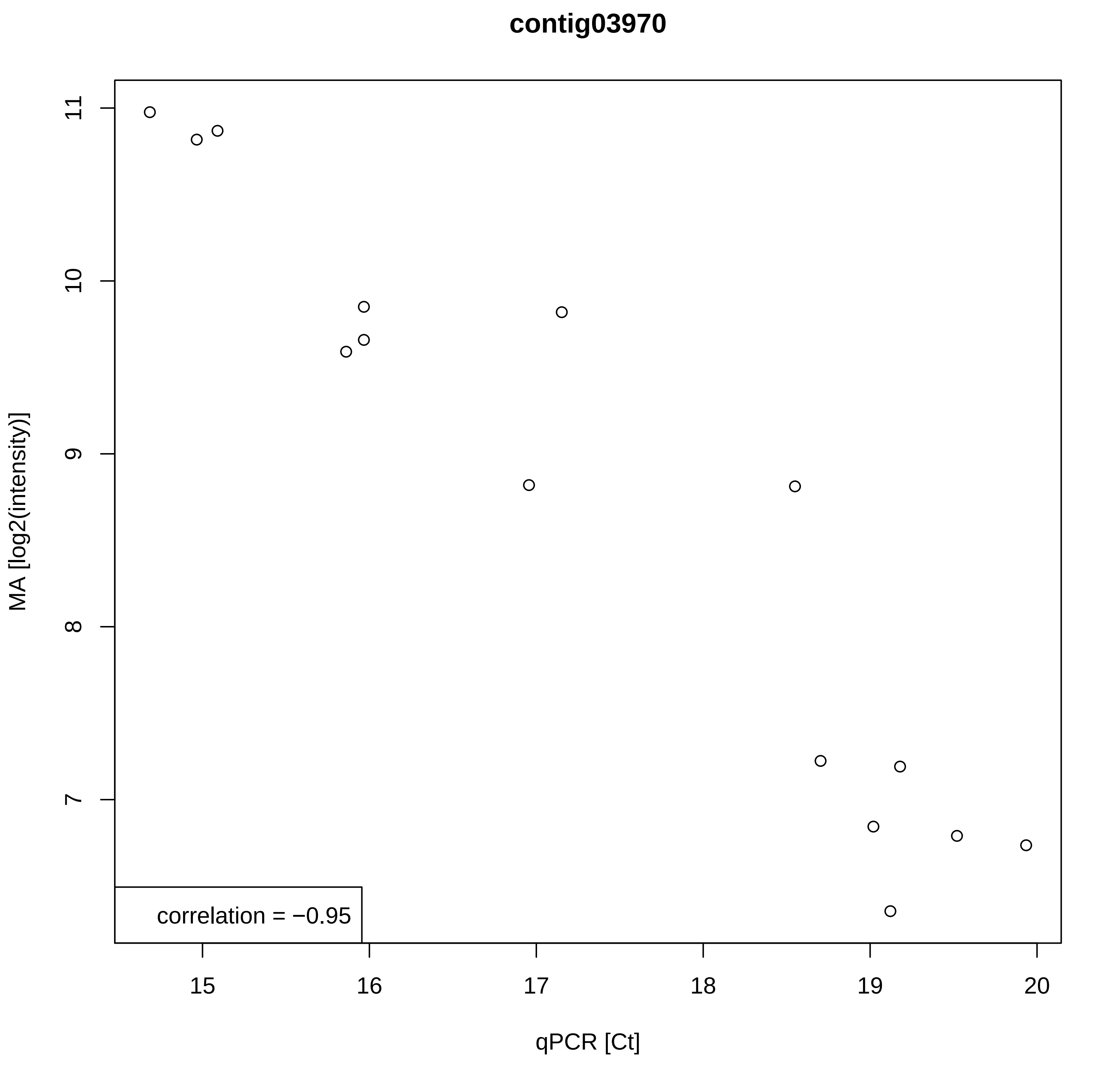

contig03970
MA [log2(intensity)]
qPCR [Ct]
correlation = −0.95
11
10
9
8
7
15
16
17
18
19
20

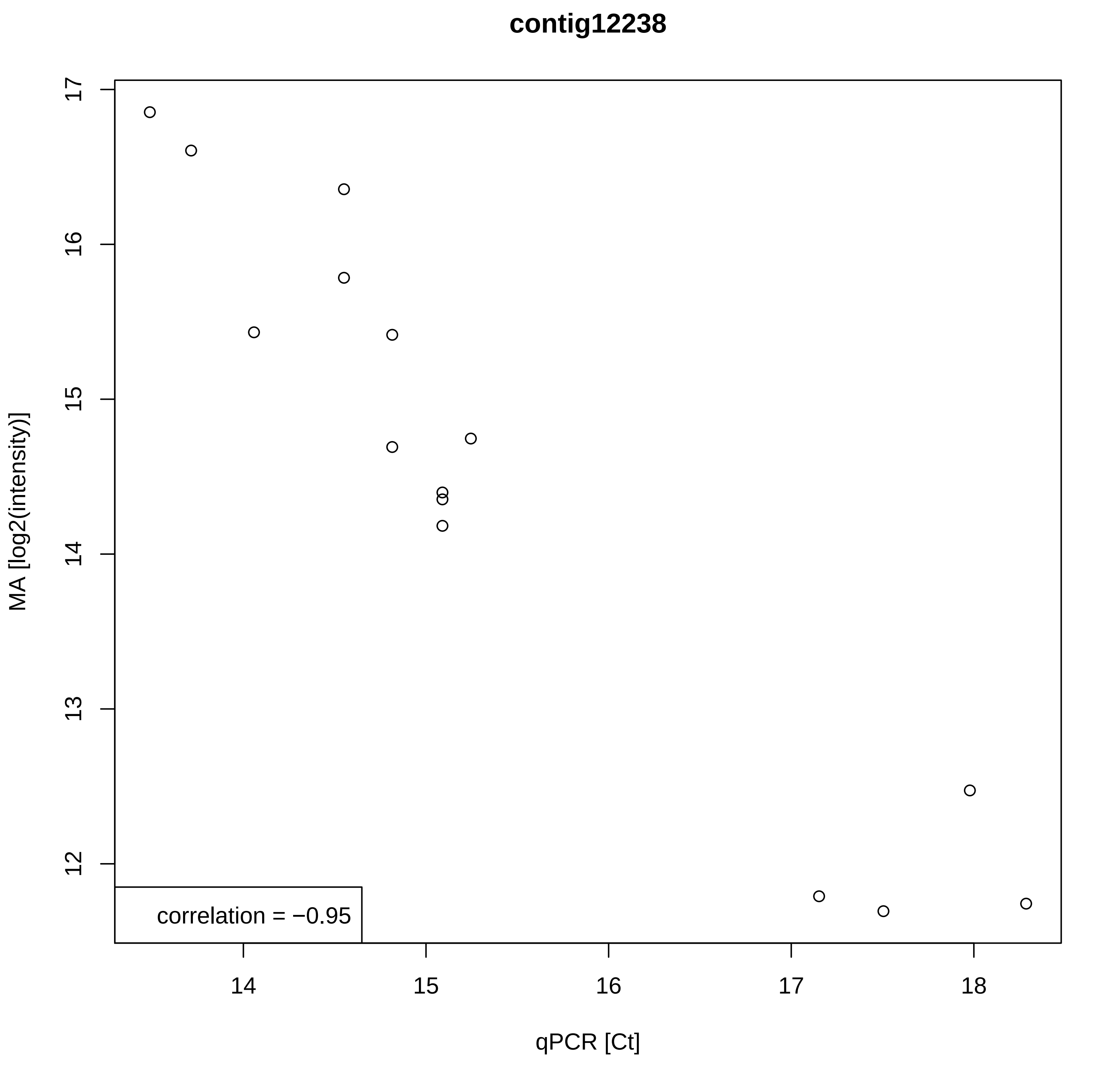
contig12238
MA [log2(intensity)]
qPCR [Ct]
correlation = −0.95
17
16
15
14
13
12
14
15
16
17
18

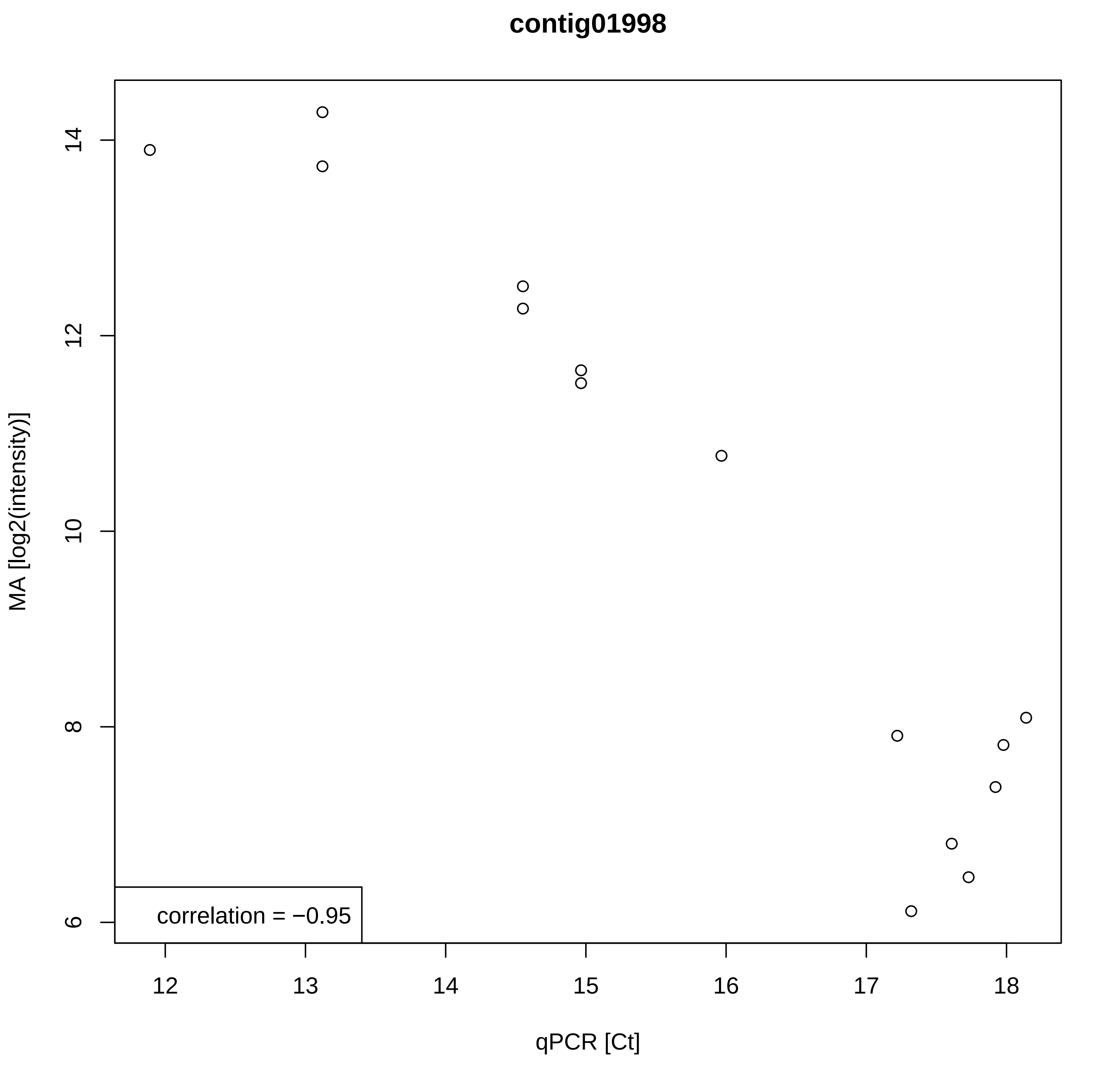

contig01998
MA [log2(intensity)]
qPCR [Ct]
correlation = −0.95
6
8
10
12
14
12
13
14
15
16
17
18

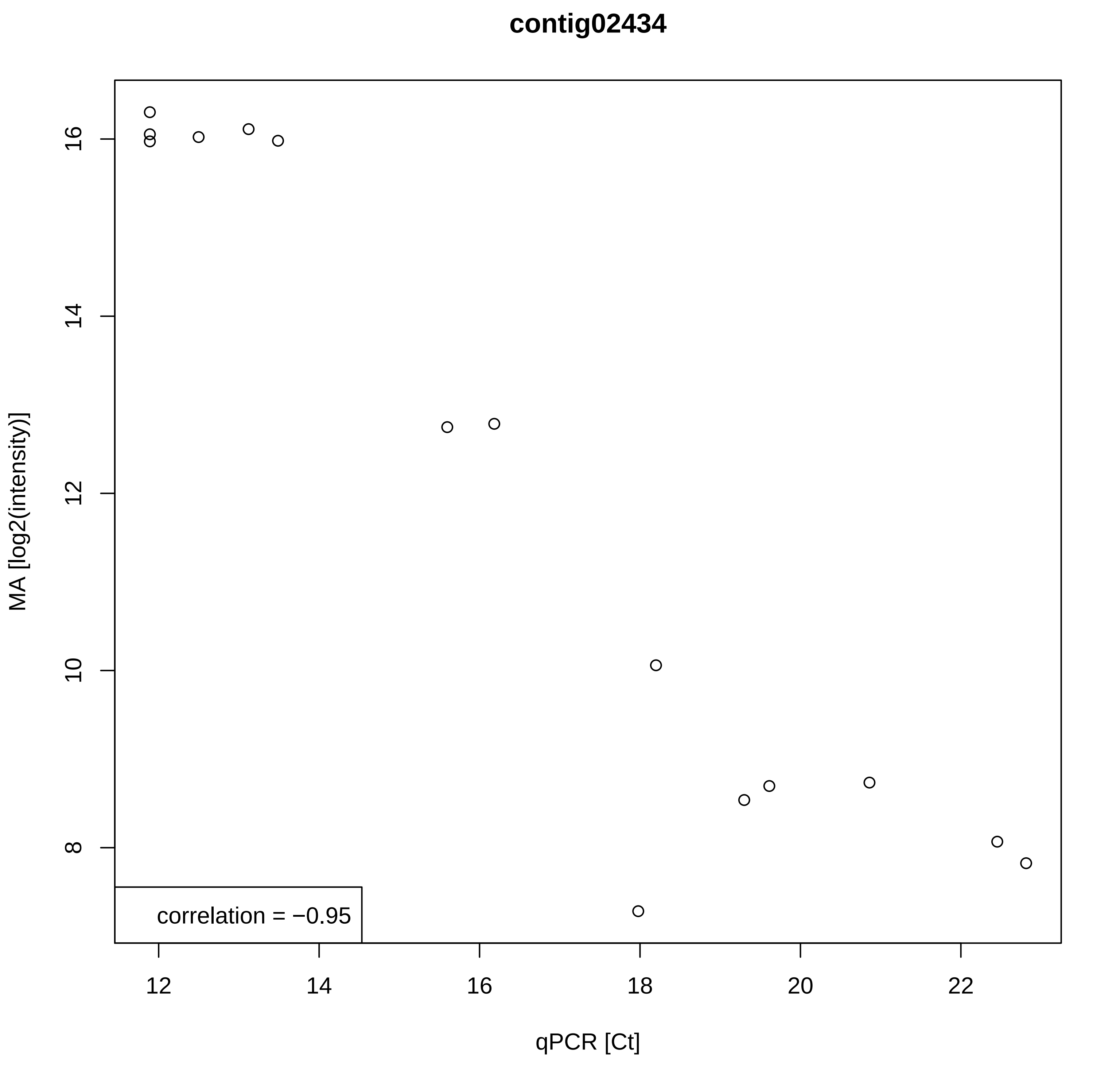
contig02434
MA [log2(intensity)]
8
10
12
14
16
qPCR [Ct]
12
14
16
18
20
22
correlation = −0.95

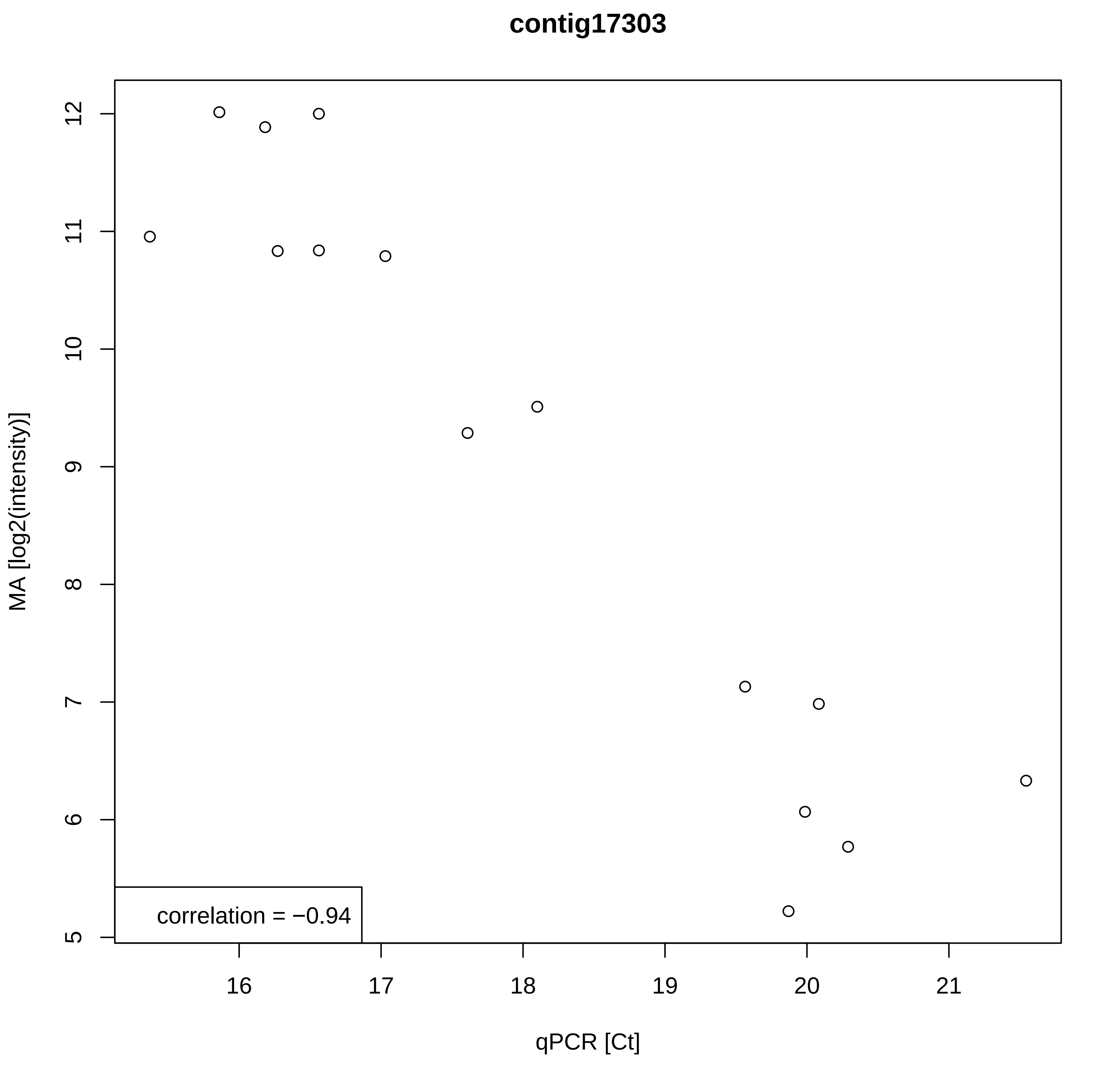
contig17303
MA [log2(intensity)]
qPCR [Ct]
correlation = −0.94
5
6
7
8
9
10
11
12
16
17
18
19
20
21

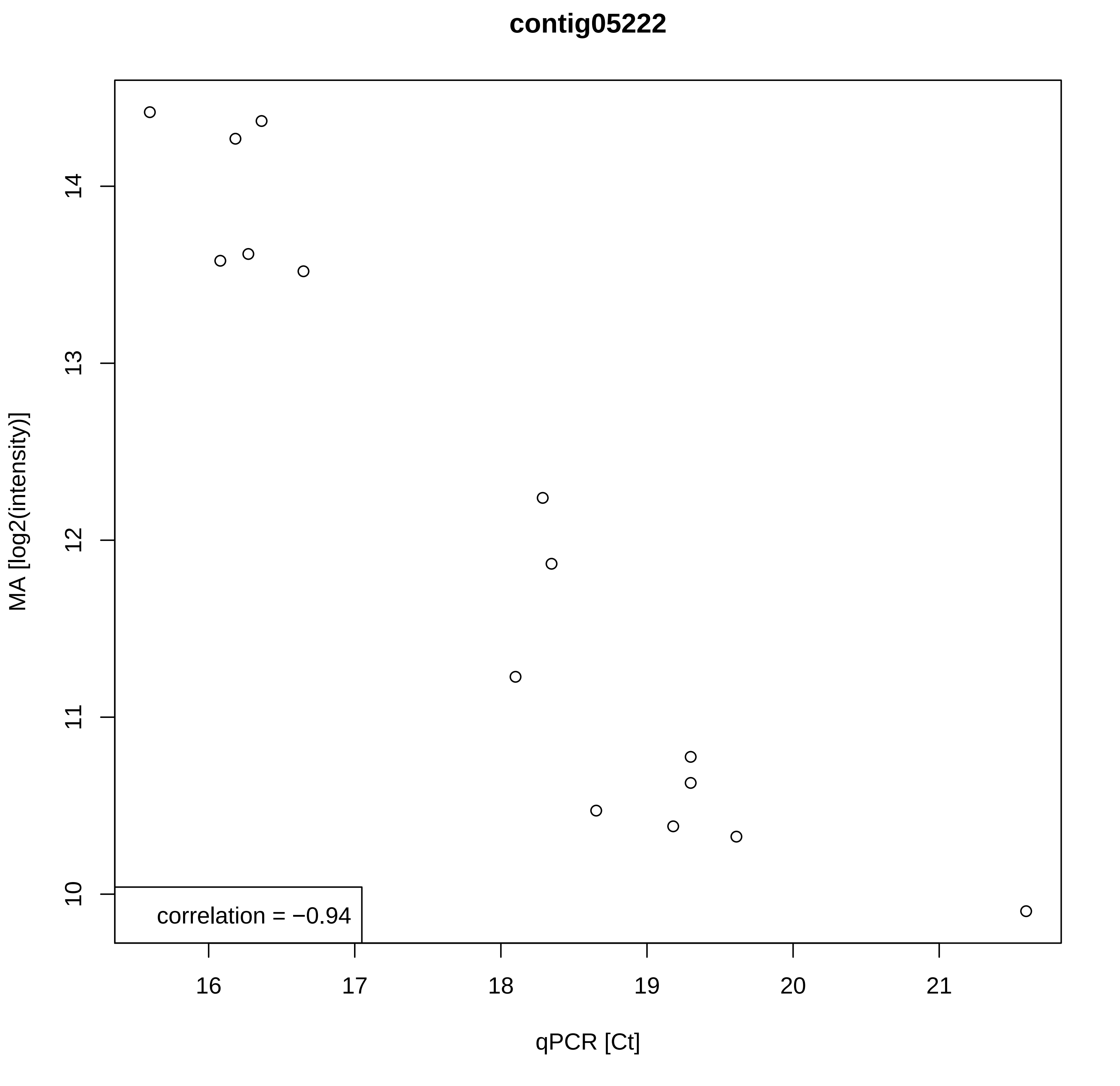
contig05222
MA [log2(intensity)]
qPCR [Ct]
correlation = −0.94
10
11
12
13
14
16
17
18
19
20
21

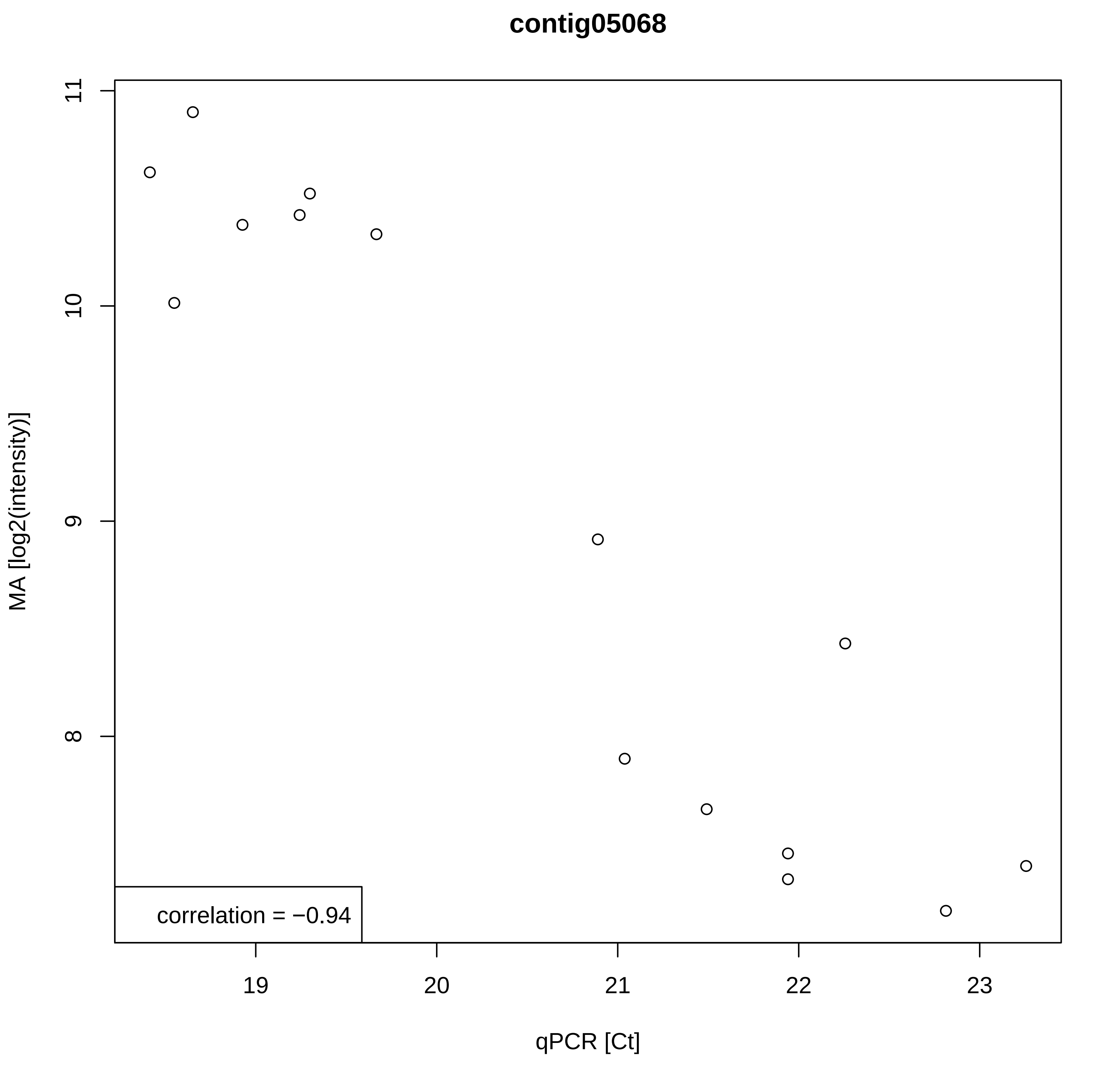
contig05068
MA [log2(intensity)]
11
10
9
8
correlation = −0.94
19
20
21
22
23
qPCR [Ct]

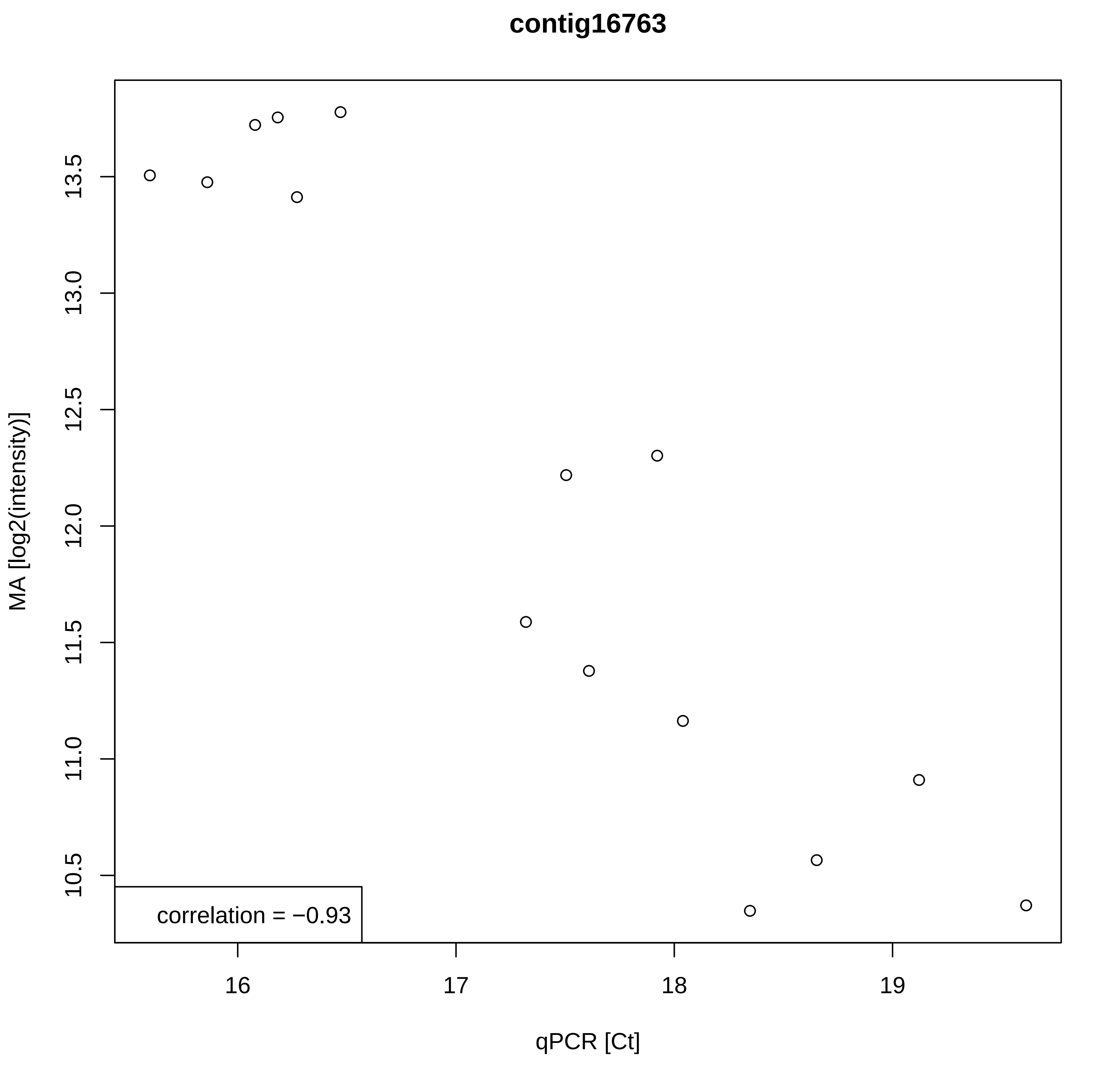
contig16763
MA [log2(intensity)]
13.5
13.0
12.5
12.0
11.5
11.0
10.5
16
17
18
19
qPCR [Ct]
correlation = −0.93

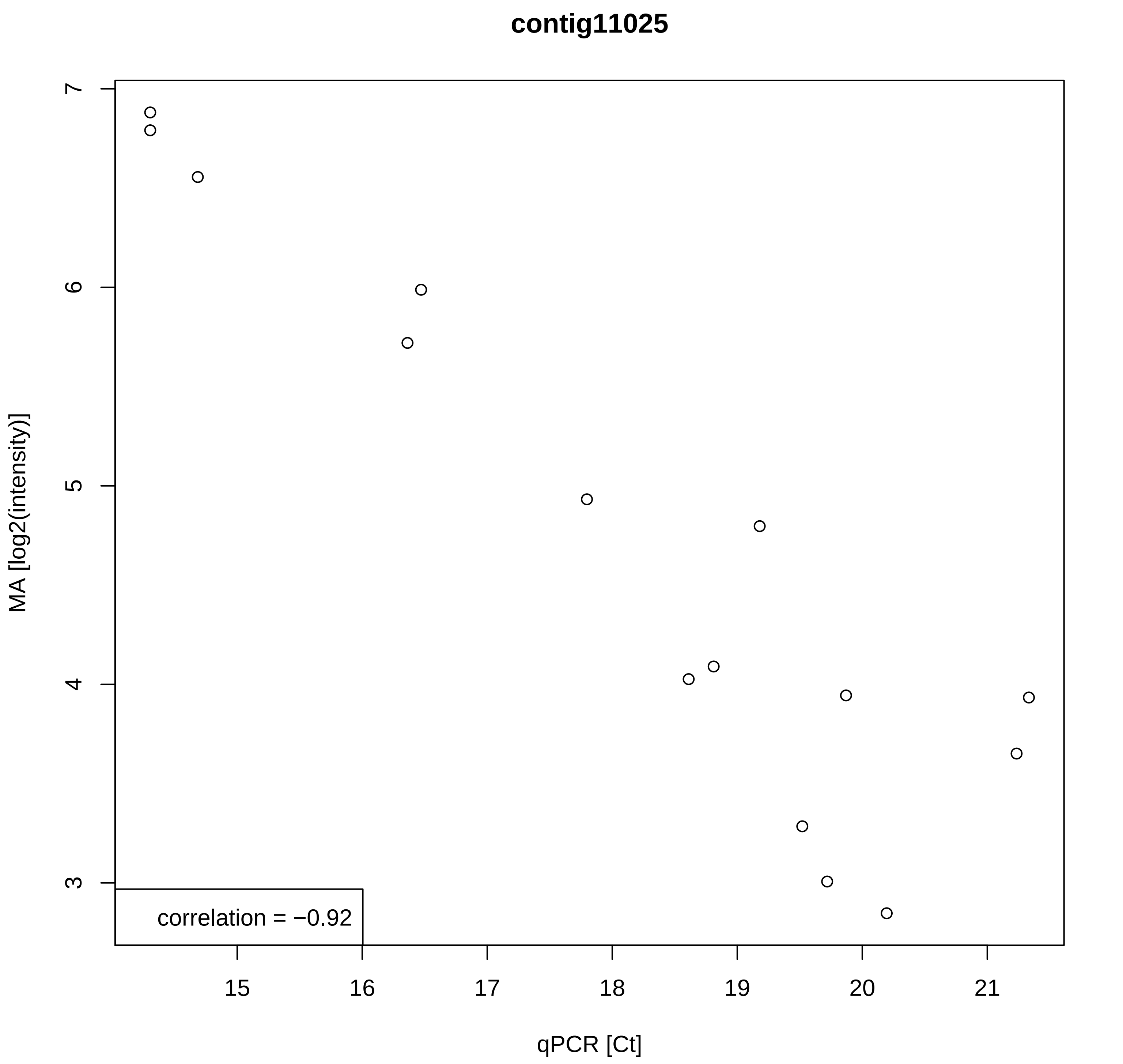

contig11025
MA [log2(intensity)]
qPCR [Ct]
correlation = −0.92
3
4
5
6
7
15
16
17
18
19
20
21

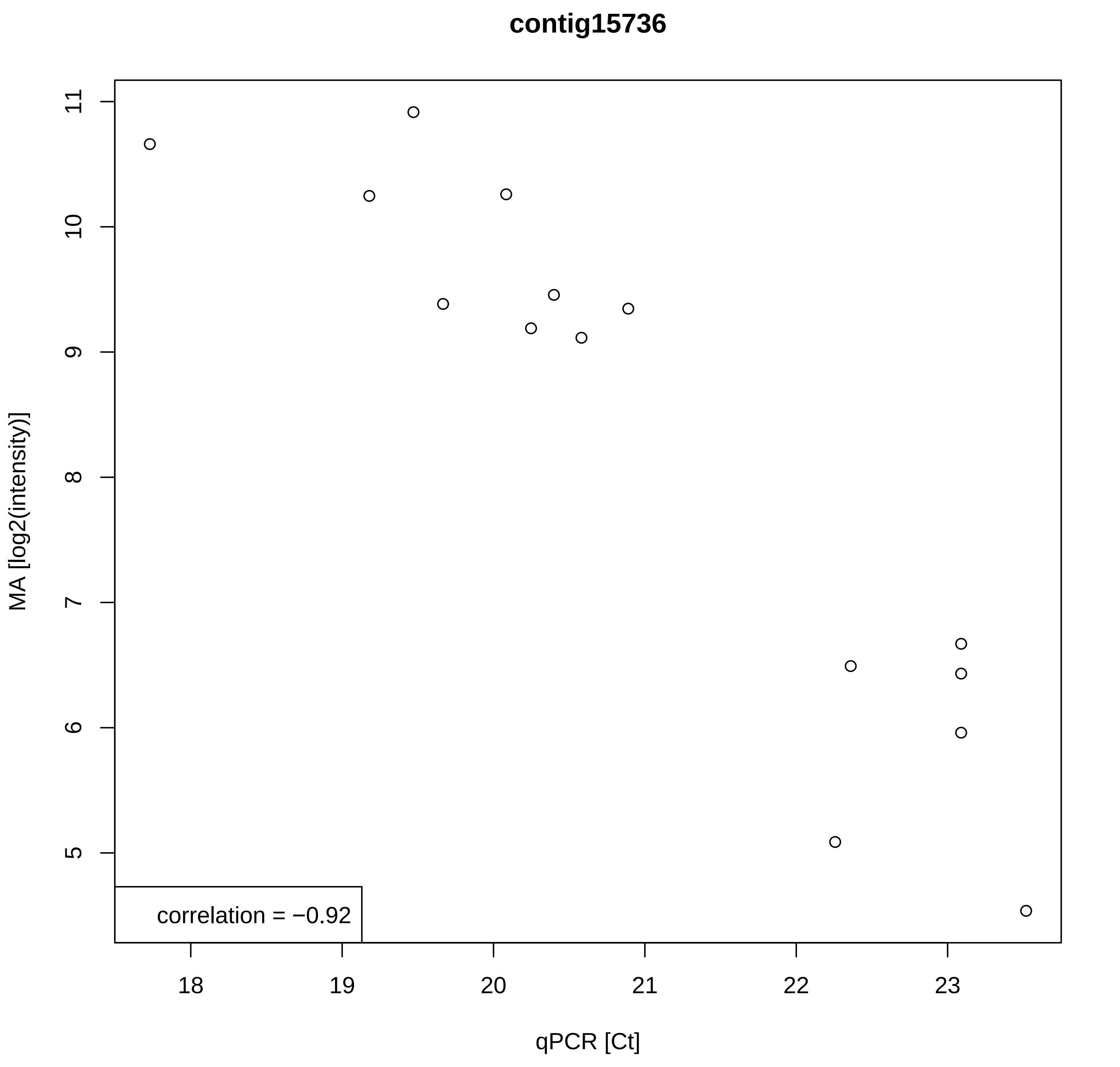

contig15736
MA [log2(intensity)]
qPCR [Ct]
correlation = −0.92
11
10
9
8
7
6
5
18
19
20
21
22
23

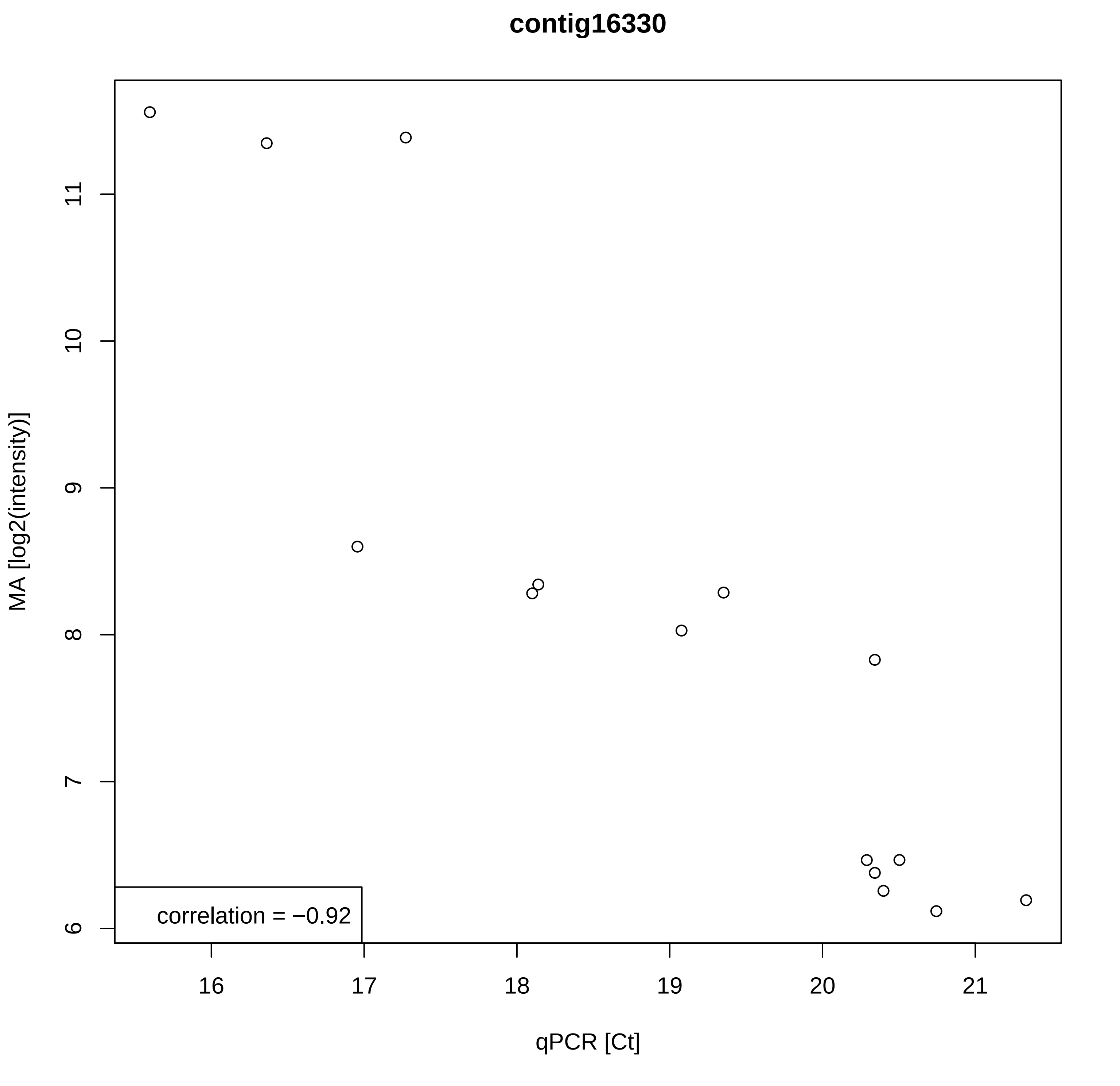
contig16330
MA [log2(intensity)]
qPCR [Ct]
correlation = −0.92
6
7
8
9
10
11
16
17
18
19
20
21

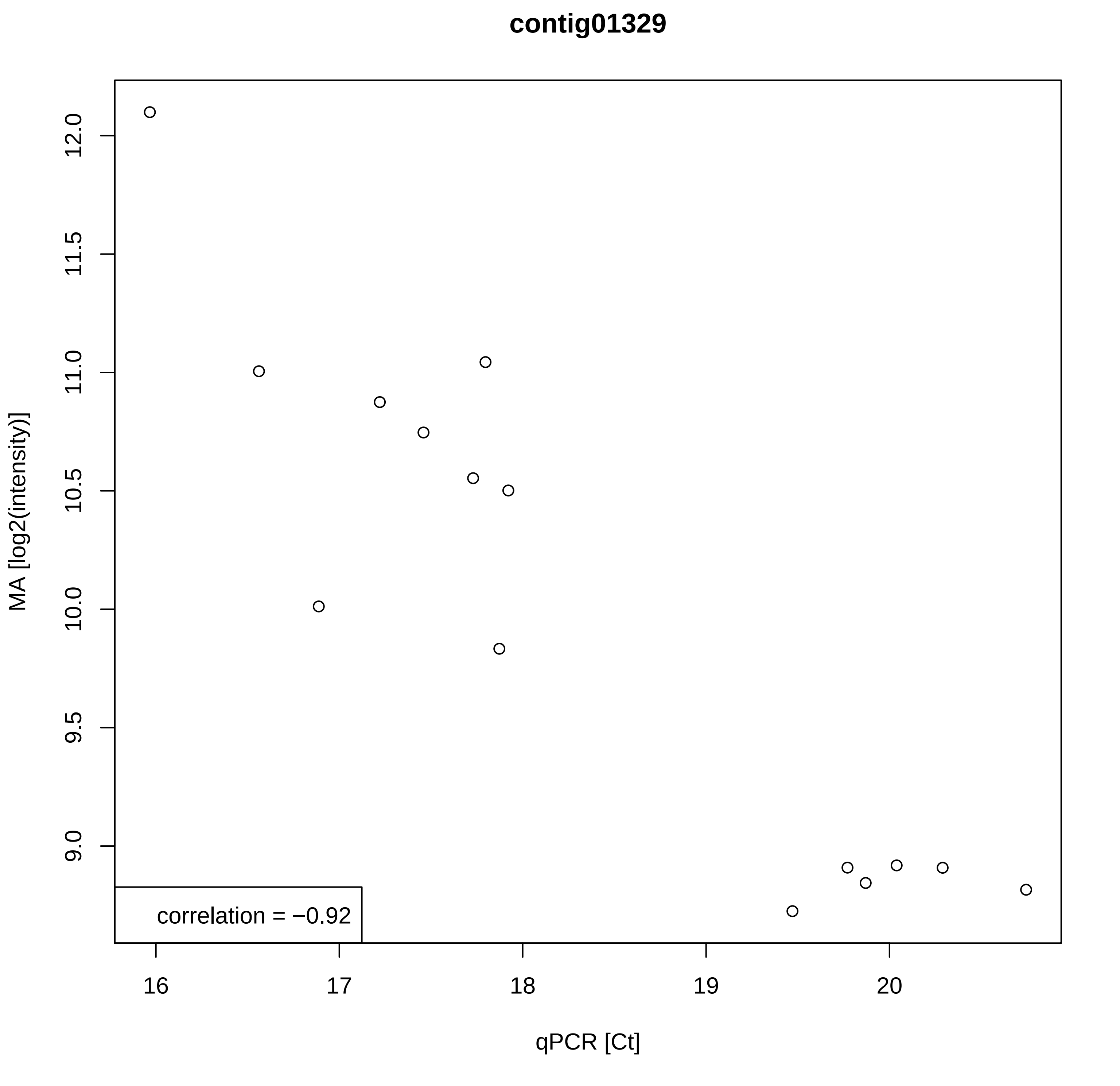
contig01329
MA [log2(intensity)]
12.0
11.5
11.0
10.5
10.0
9.5
9.0
correlation = −0.92
16
17
18
19
20
qPCR [Ct]

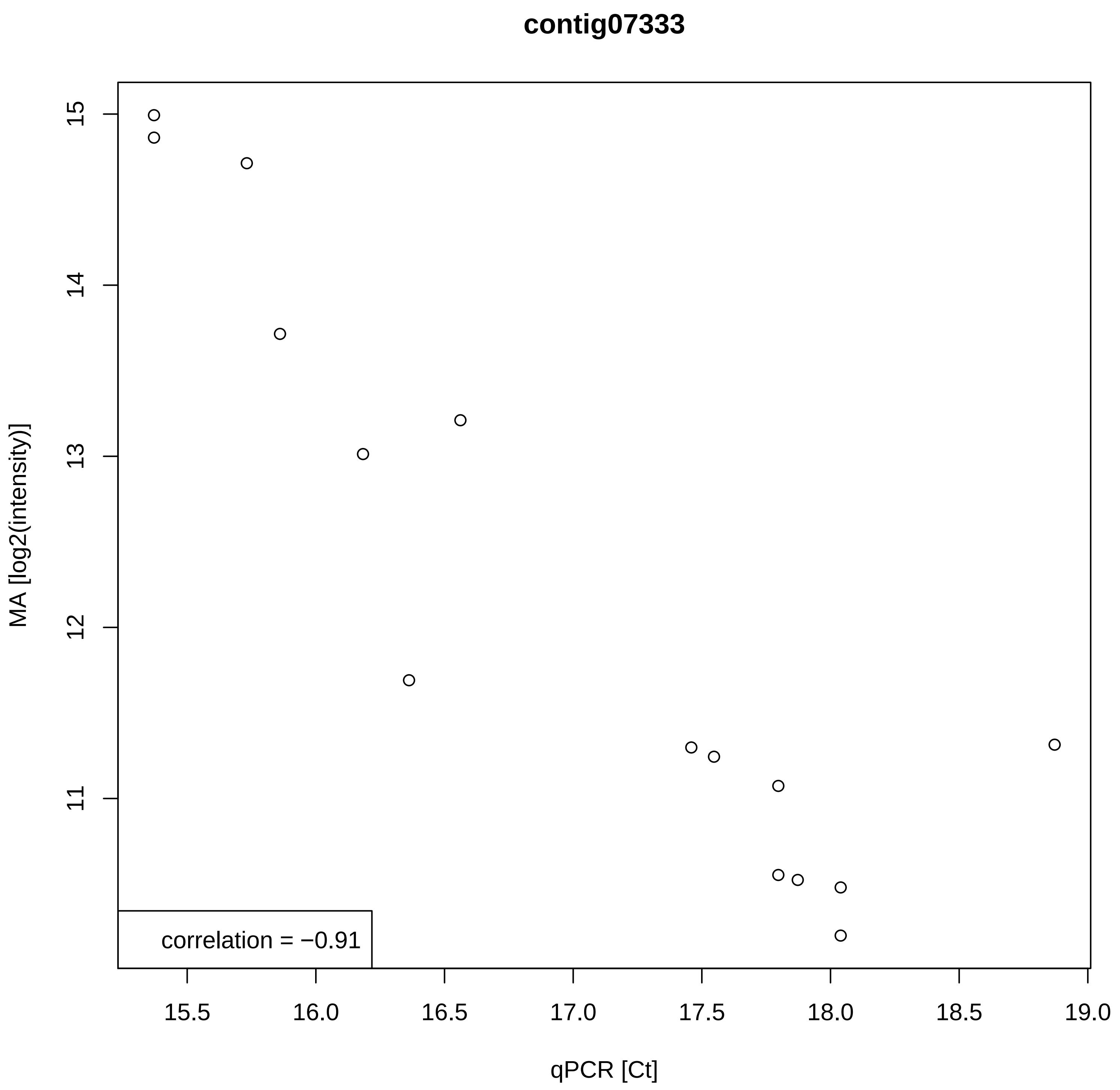

contig07333
MA [log2(intensity)]
15
14
13
12
11
correlation = −0.91
15.5
16.0
16.5
17.0
17.5
18.0
18.5
19.0
qPCR [Ct]

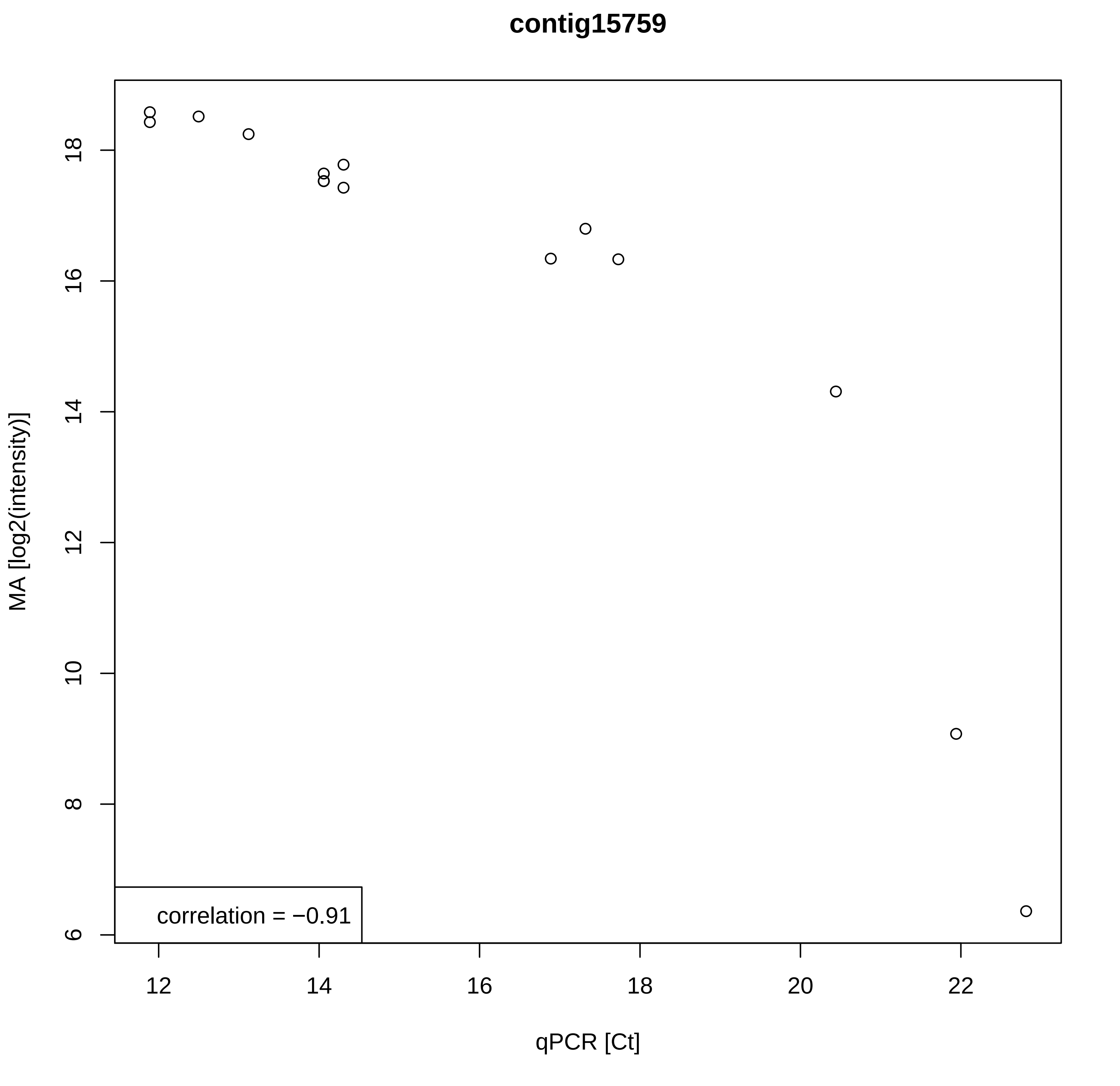

contig15759
MA [log2(intensity)]
qPCR [Ct]
correlation = −0.91
6
8
10
12
14
16
18
12
14
16
18
20
22

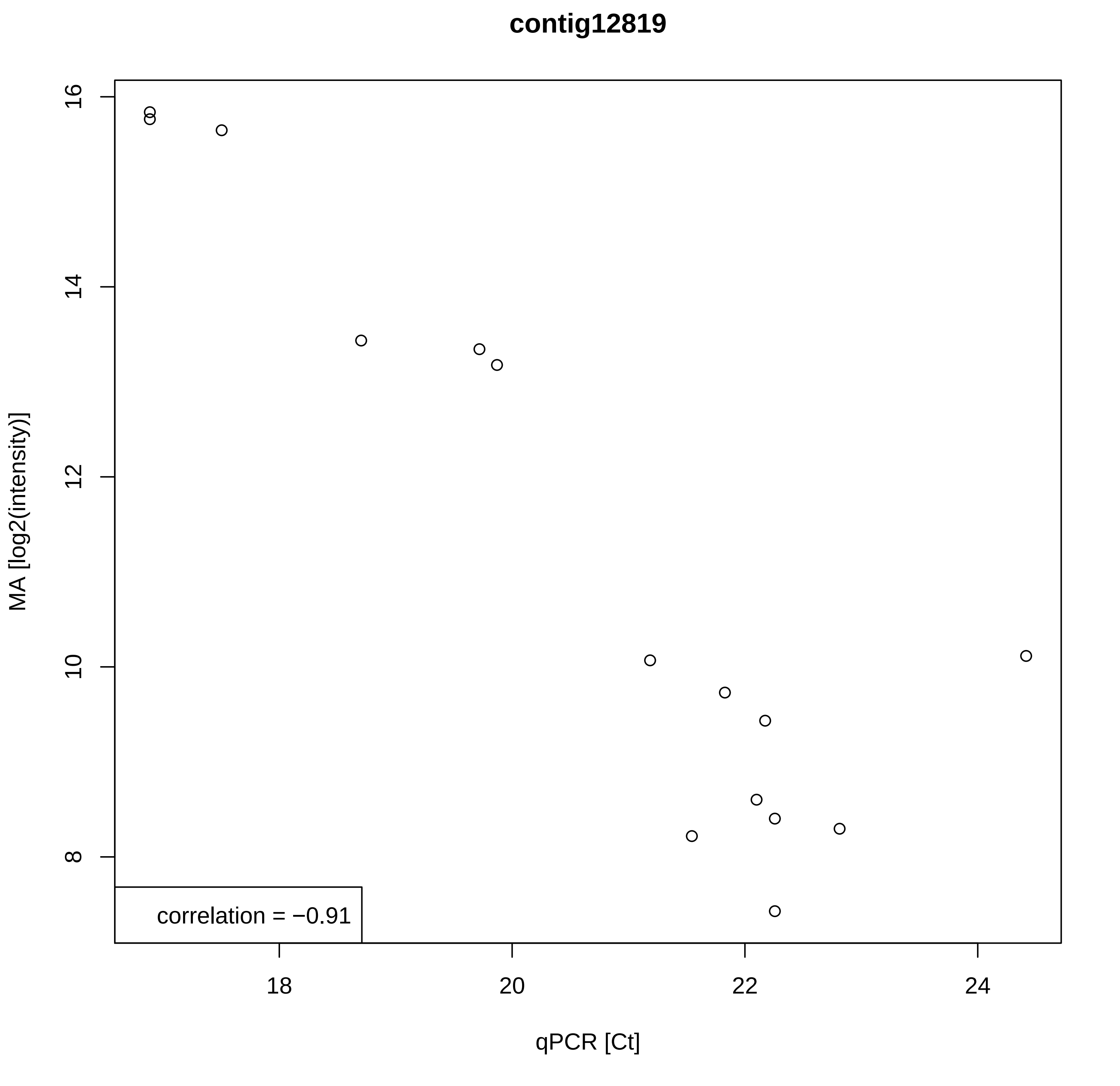

contig12819
MA [log2(intensity)]
qPCR [Ct]
16
14
12
10
8
18
20
22
24
correlation = −0.91

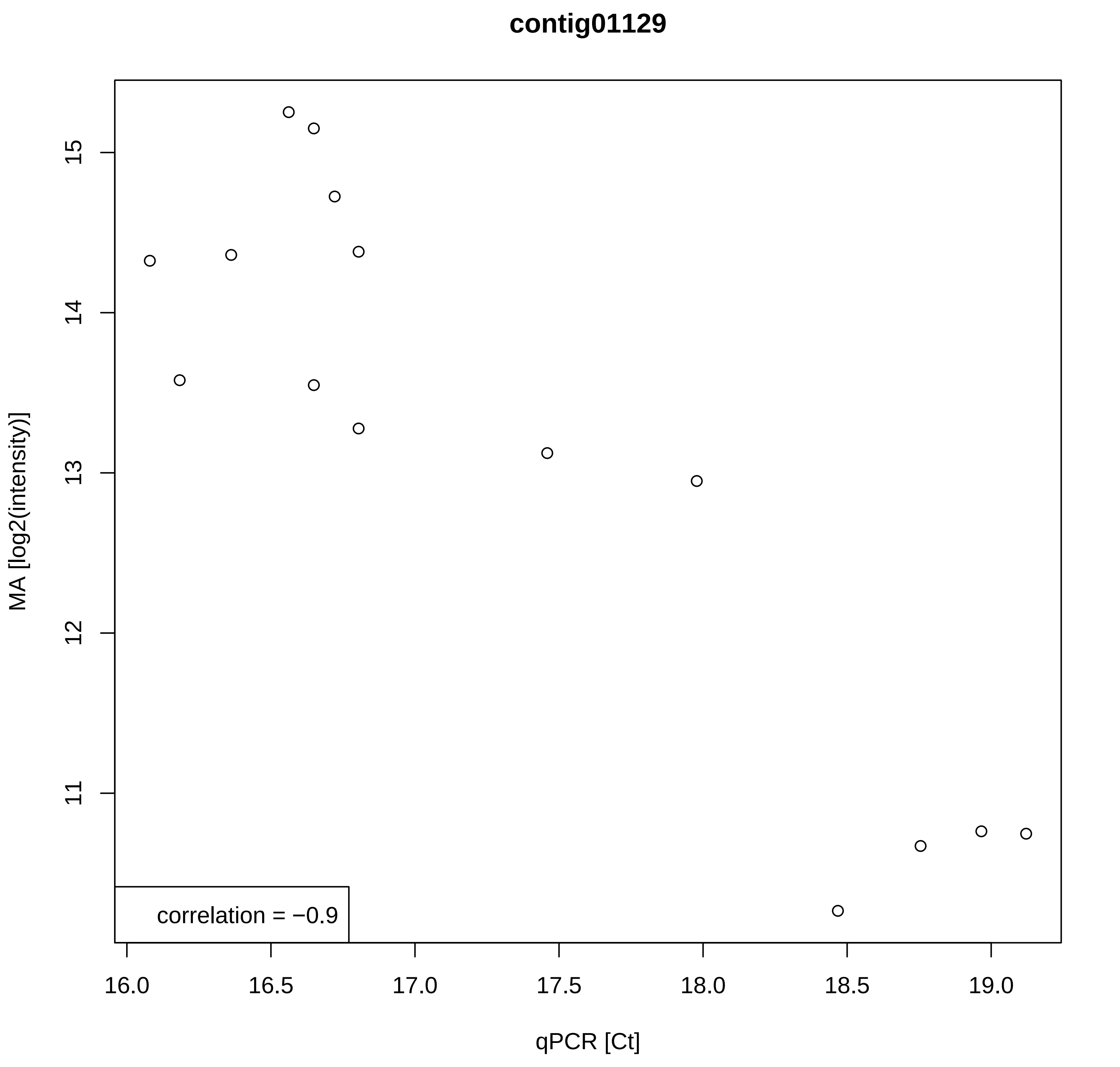
contig01129
MA [log2(intensity)]
15
14
13
12
11
correlation = −0.9
16.0
16.5
17.0
17.5
18.0
18.5
19.0
qPCR [Ct]

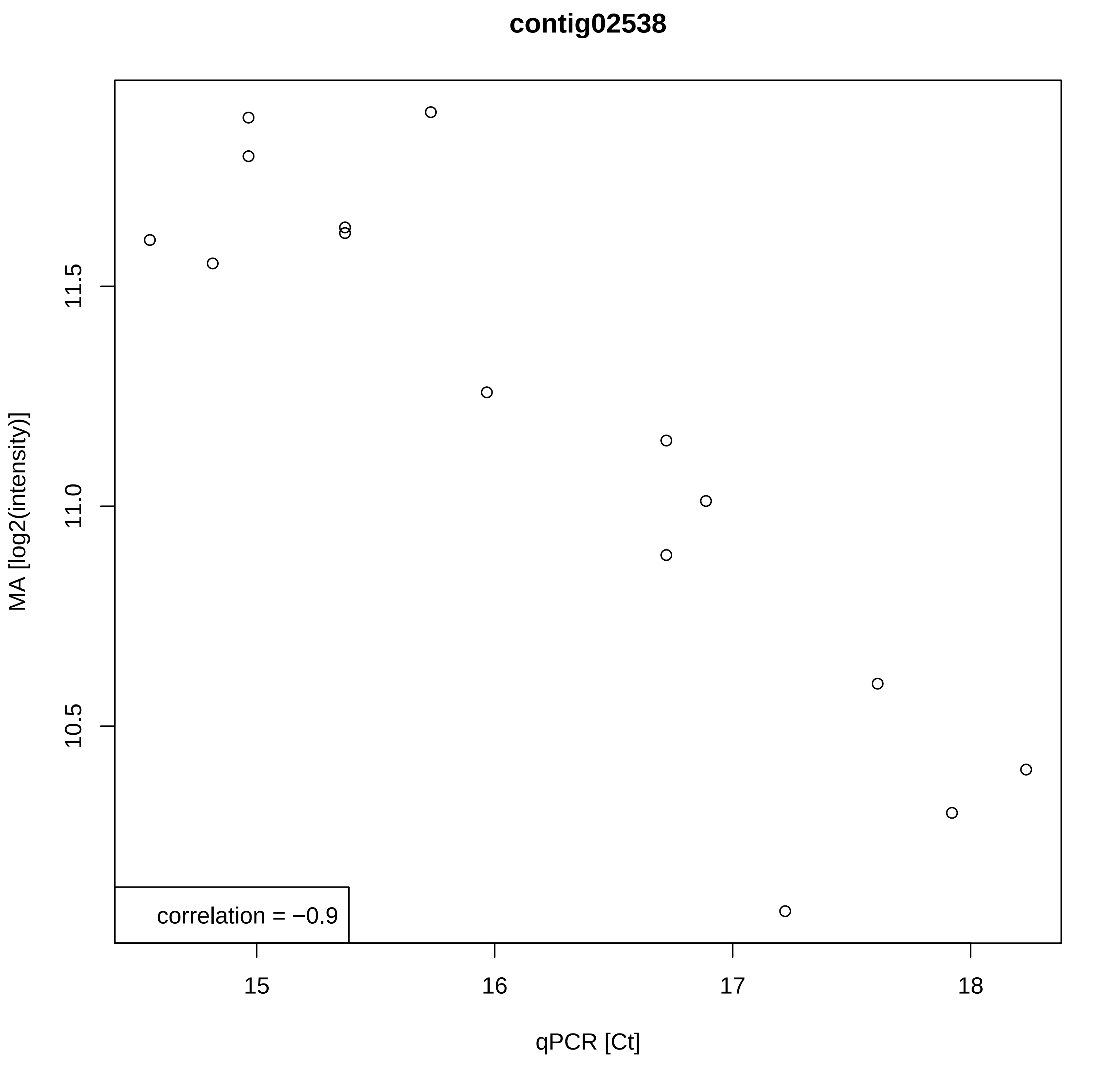

contig02538
MA [log2(intensity)]
11.5
11.0
10.5
correlation = −0.9
15
16
17
18
qPCR [Ct]

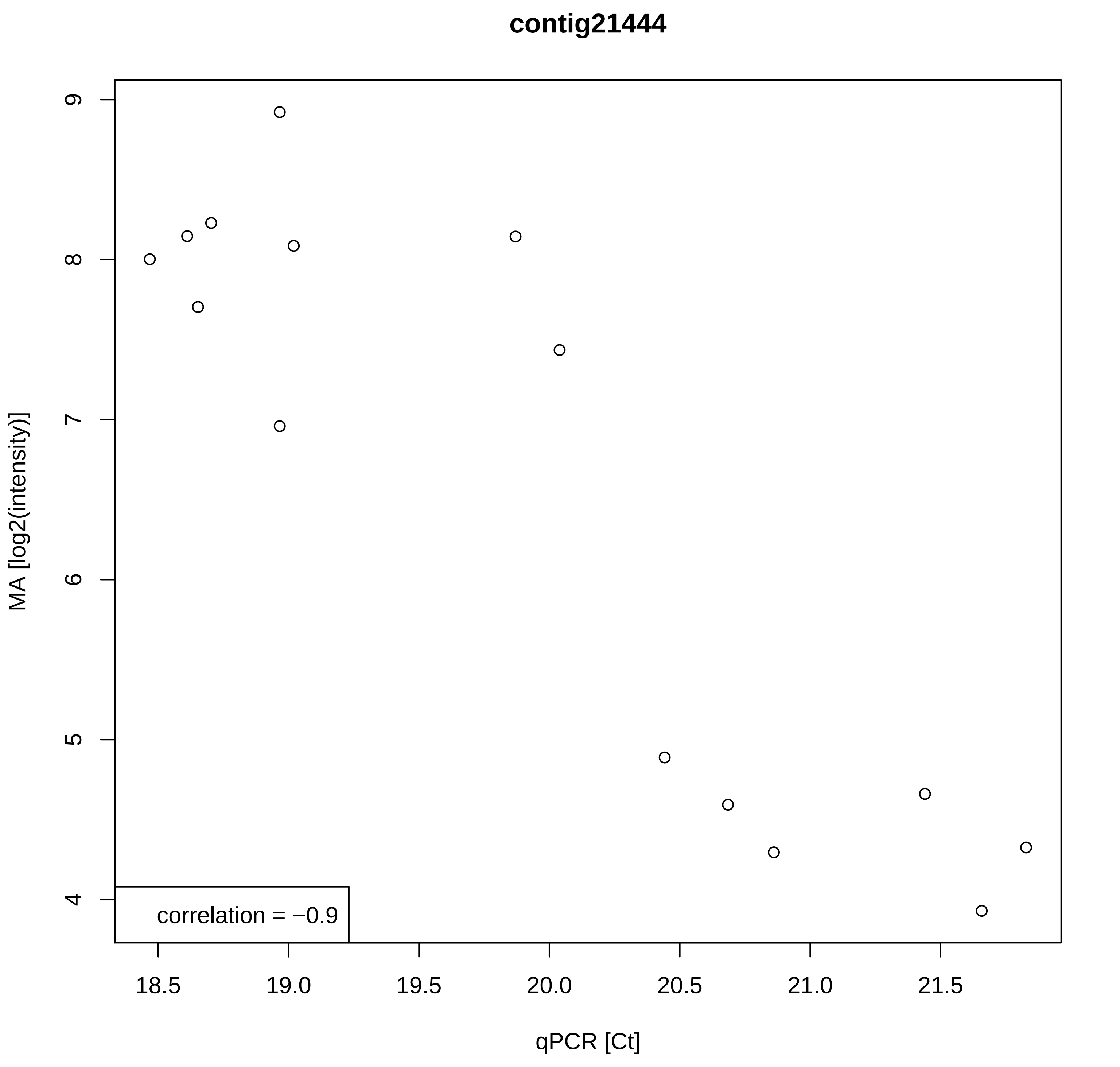
contig21444
MA [log2(intensity)]
qPCR [Ct]
correlation = −0.9
9
8
7
6
5
4
18.5
19.0
19.5
20.0
20.5
21.0
21.5

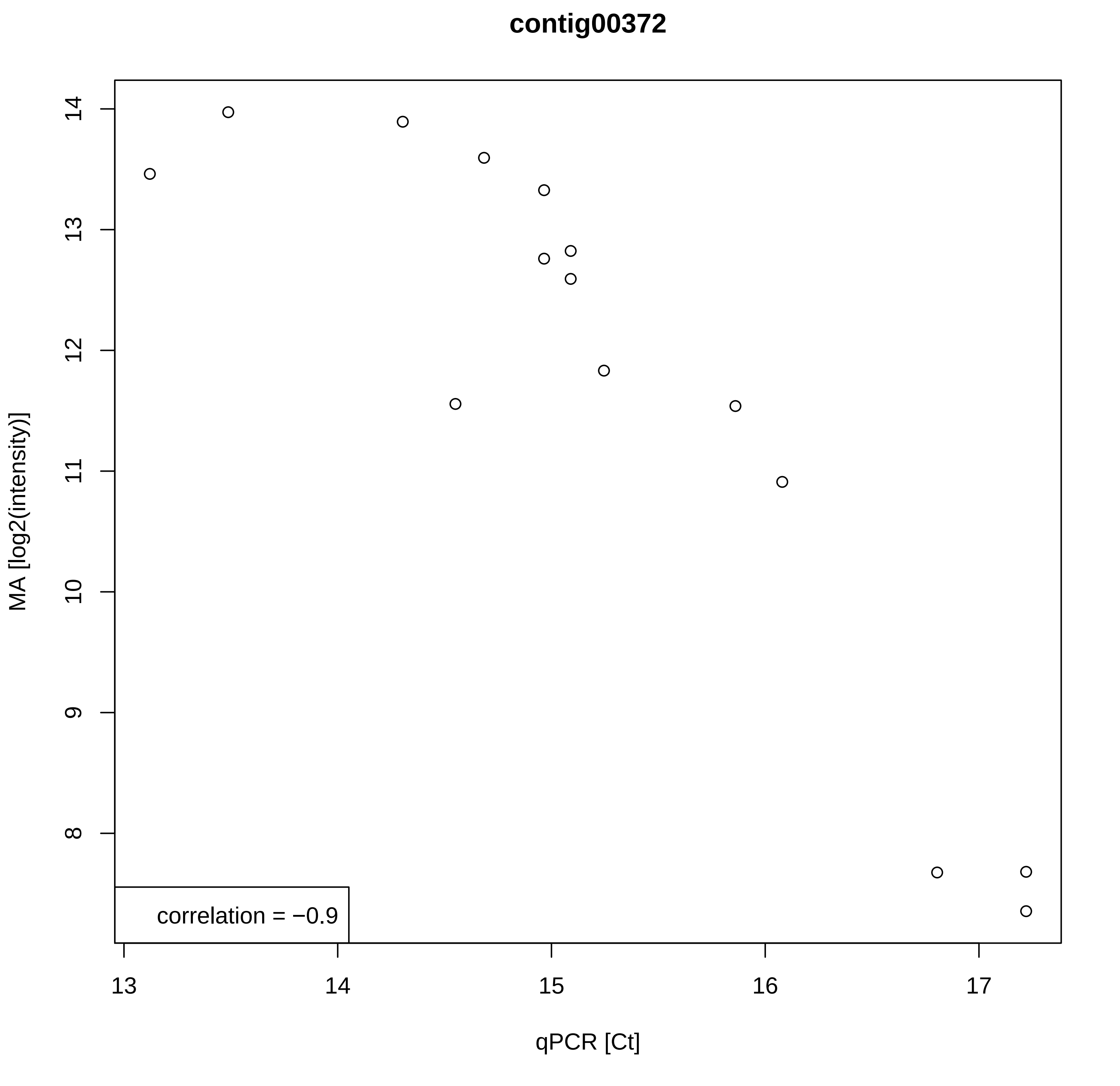
contig00372
MA [log2(intensity)]
qPCR [Ct]
correlation = −0.9
14
13
12
11
10
9
8
13
14
15
16
17

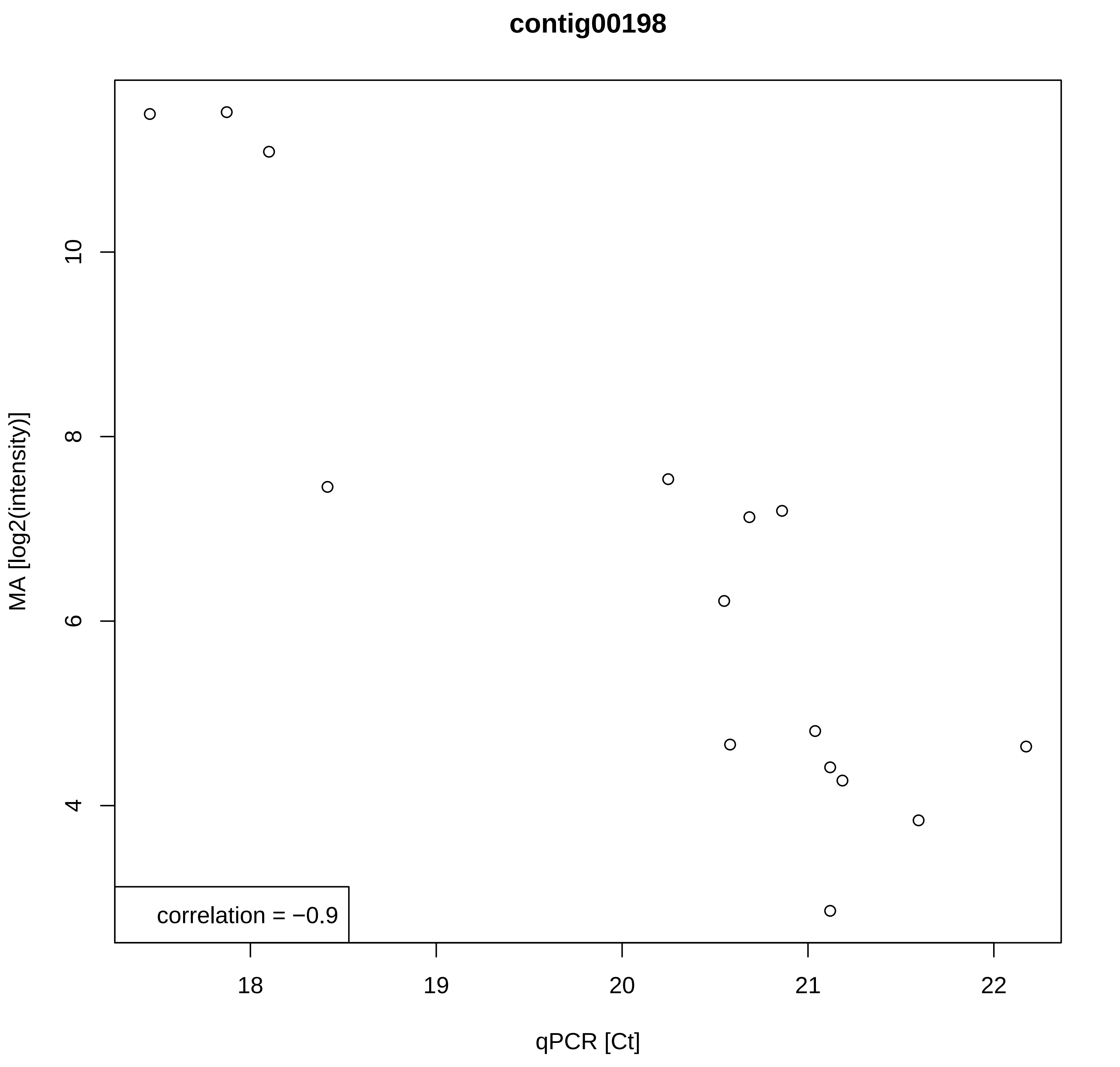
contig00198
MA [log2(intensity)]
4
6
8
10
qPCR [Ct]
18
19
20
21
22
correlation = −0.9

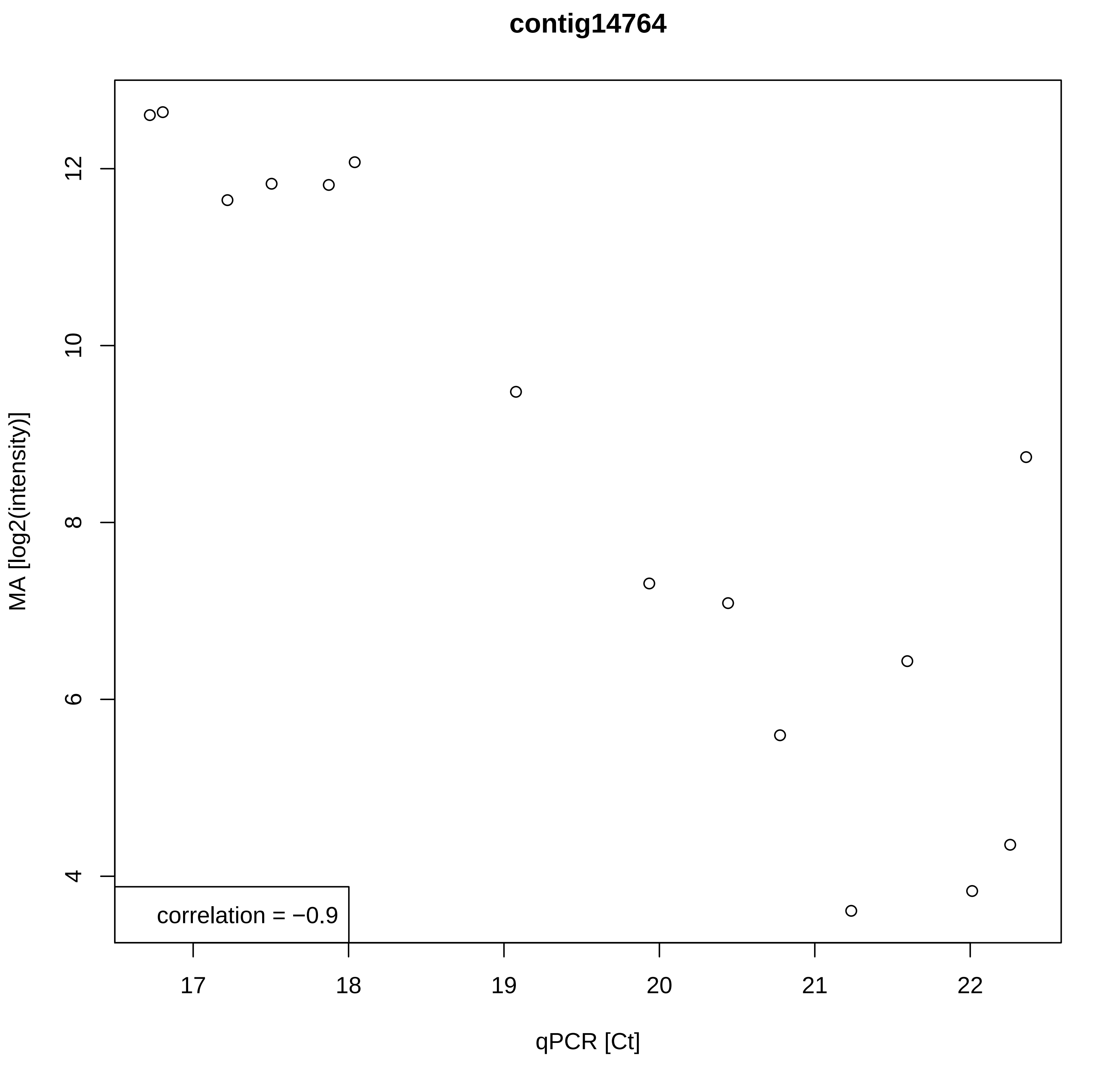
contig14764
MA [log2(intensity)]
12
10
8
6
4
17
18
19
20
21
22
qPCR [Ct]
correlation = −0.9

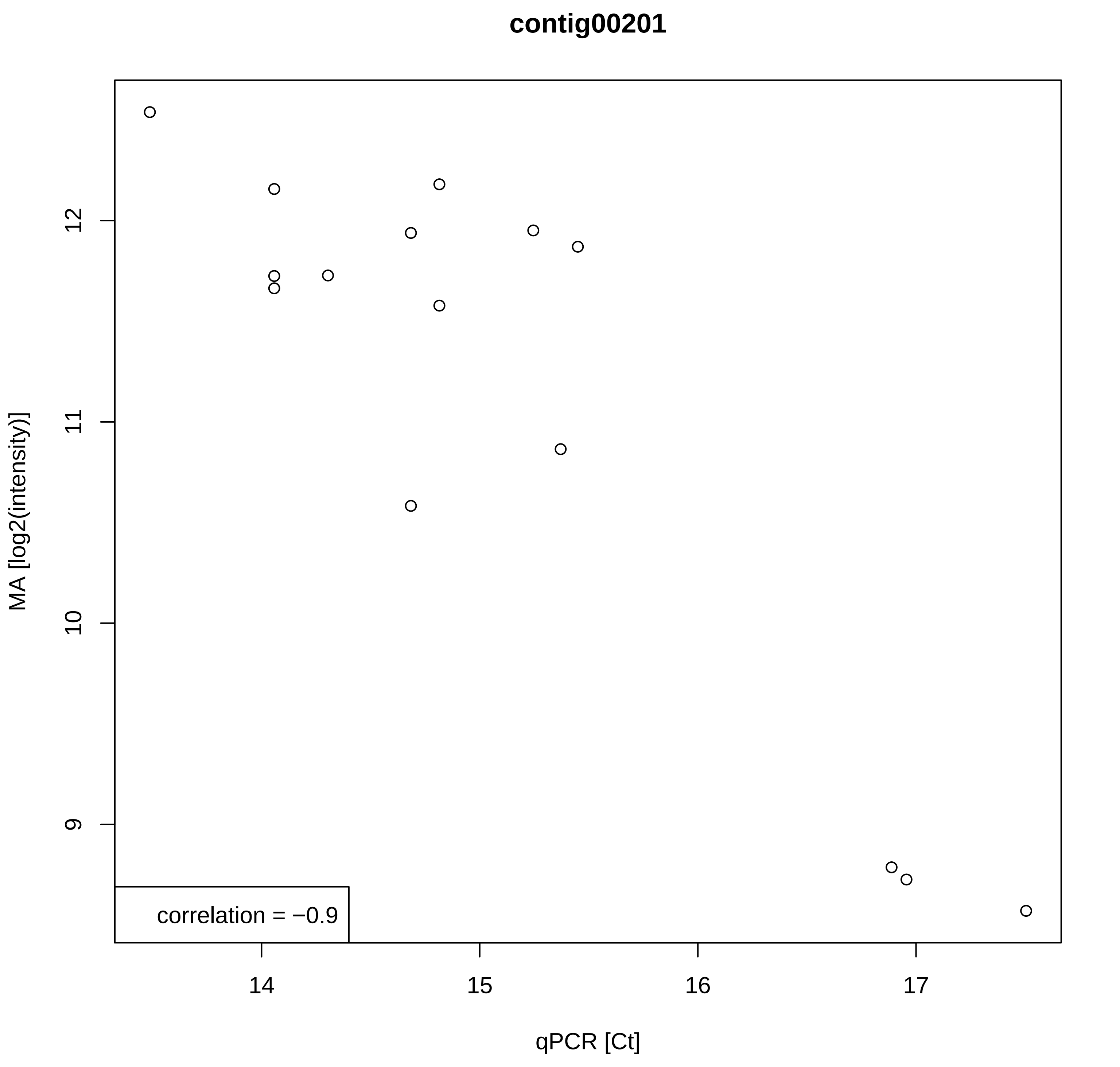

contig00201
MA [log2(intensity)]
12
11
10
9
14
15
16
17
qPCR [Ct]
correlation = −0.9

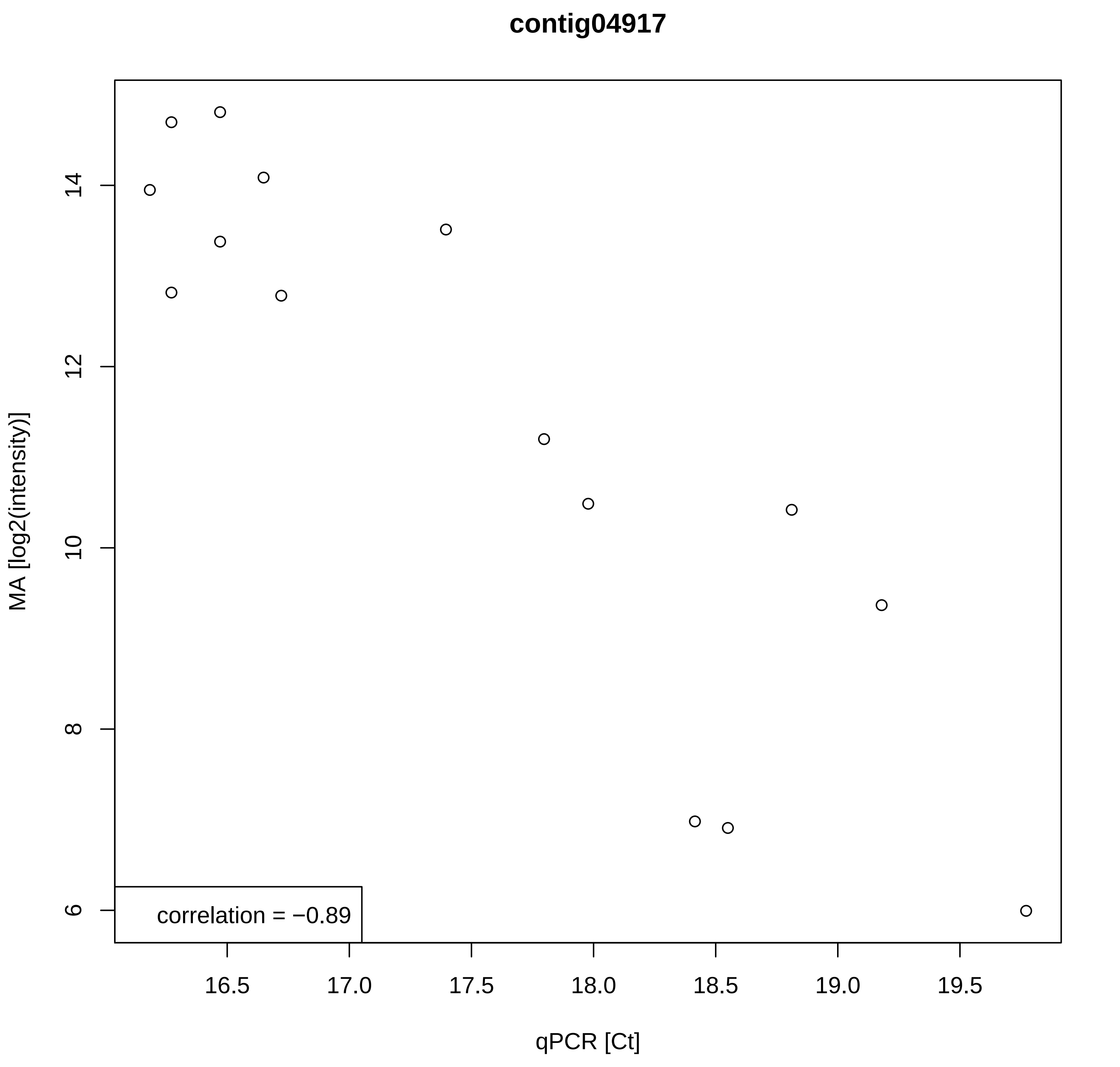
contig04917
correlation = −0.89
qPCR [Ct]
MA [log2(intensity)]
16.5
17.0
17.5
18.0
18.5
19.0
19.5
6
8
10
12
14

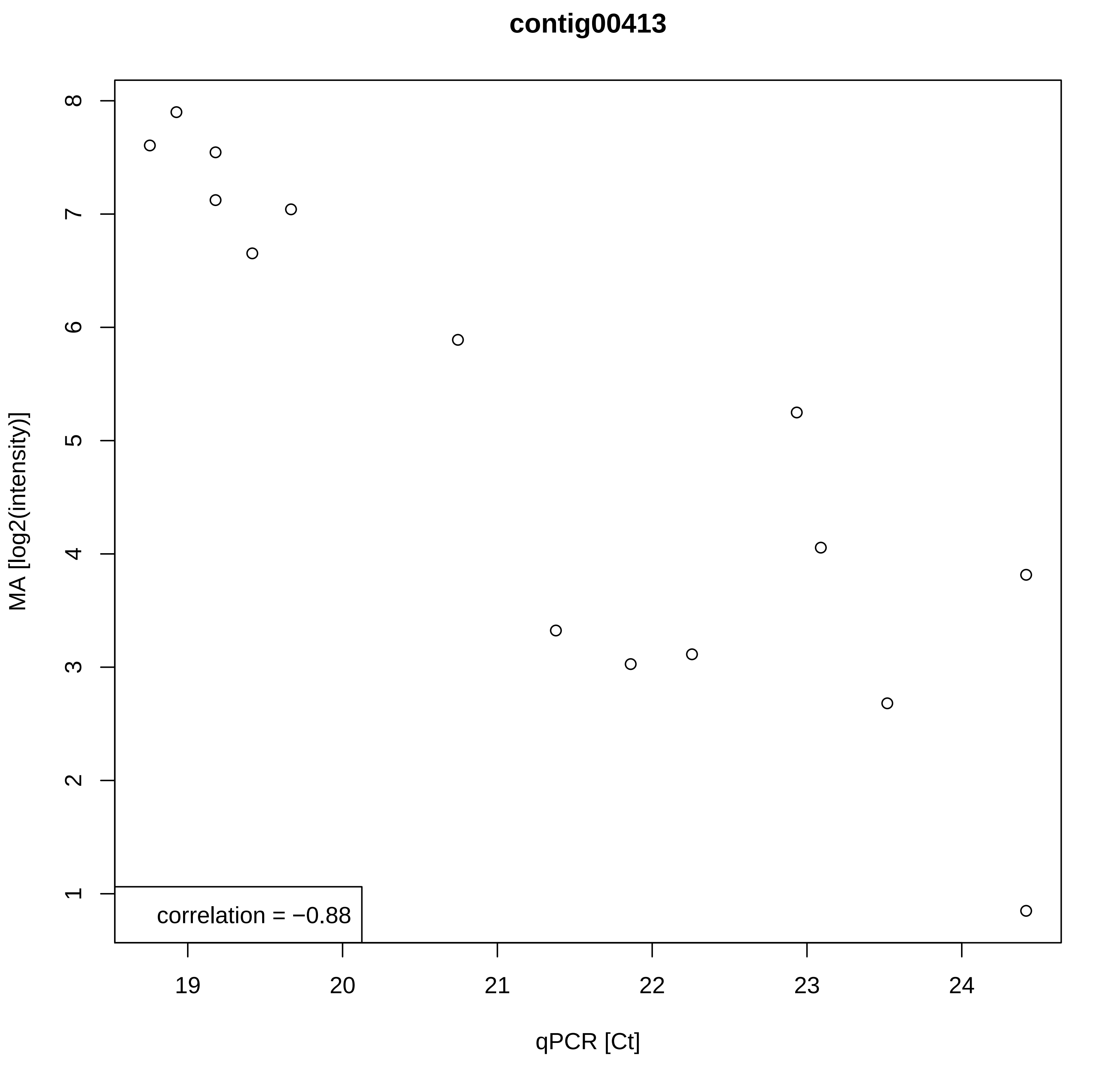
contig00413
MA [log2(intensity)]
qPCR [Ct]
correlation = −0.88
1
2
3
4
5
6
7
8
19
20
21
22
23
24

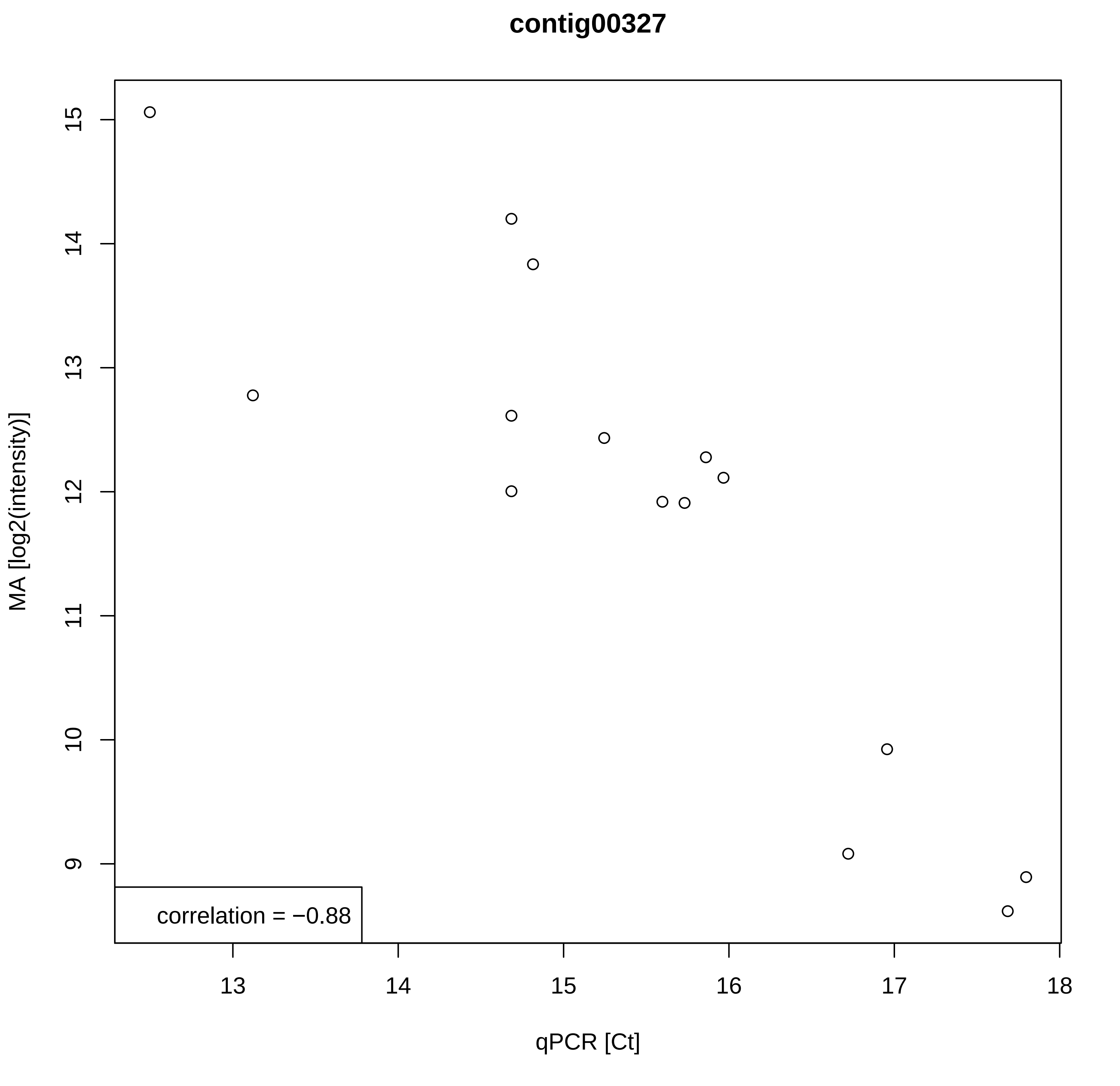
contig00327
MA [log2(intensity)]
15
14
13
12
11
10
9
correlation = −0.88
13
14
15
16
17
18
qPCR [Ct]

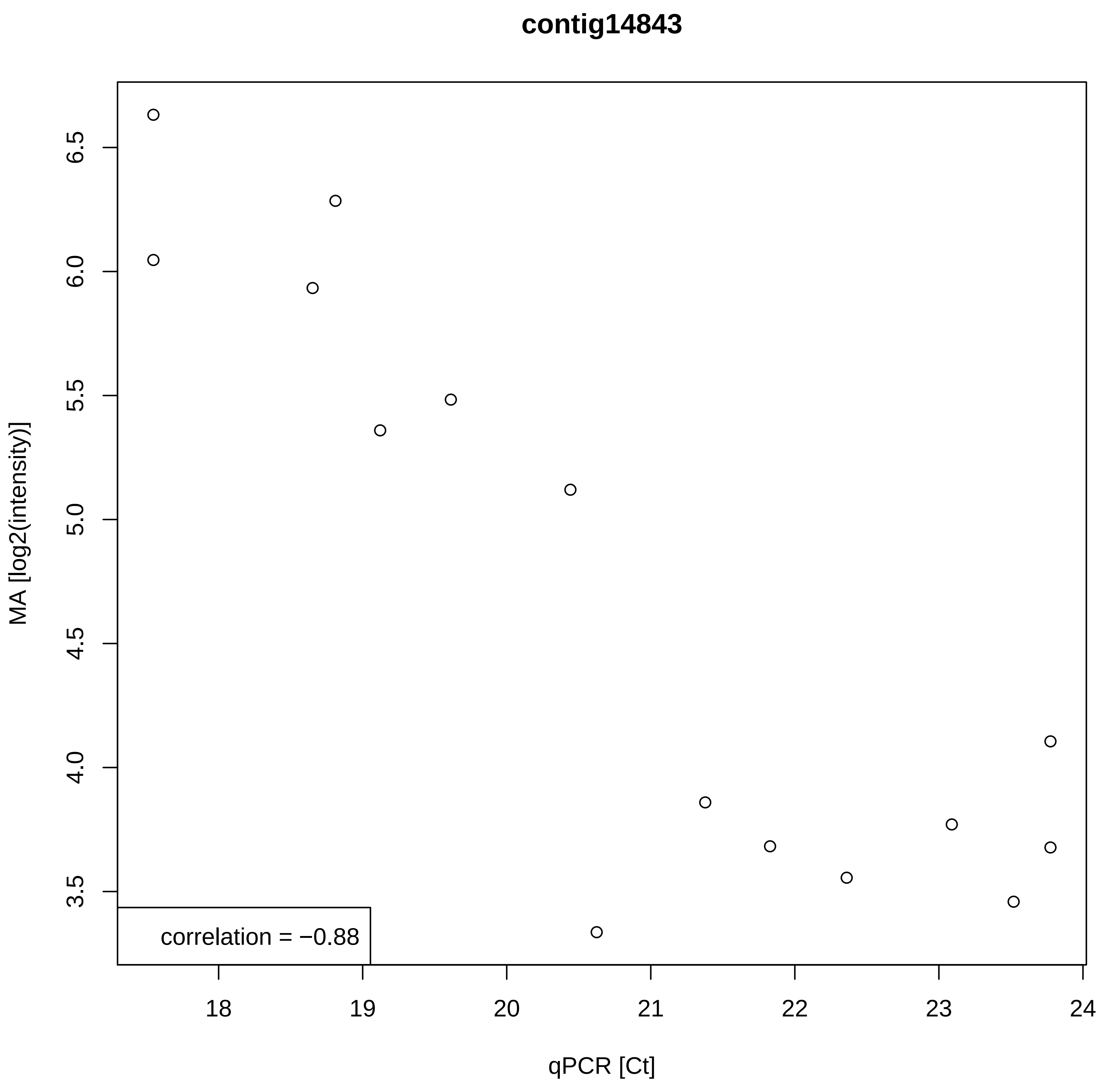
contig14843
MA [log2(intensity)]
qPCR [Ct]
correlation = −0.88
3.5
4.0
4.5
5.0
5.5
6.0
6.5
18
19
20
21
22
23
24

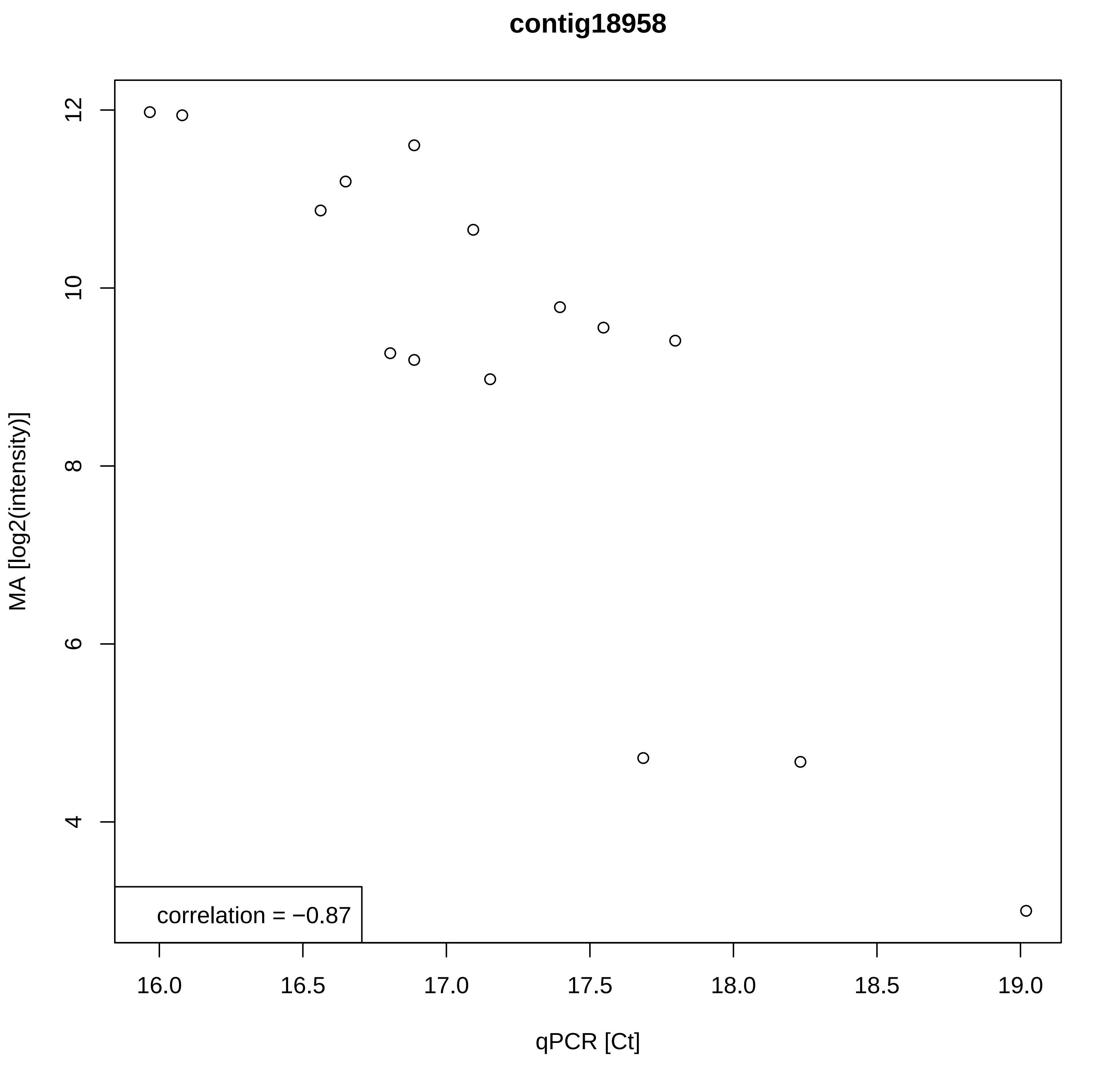
contig18958
MA [log2(intensity)]
qPCR [Ct]
correlation = −0.87
4
6
8
10
12
16.0
16.5
17.0
17.5
18.0
18.5
19.0

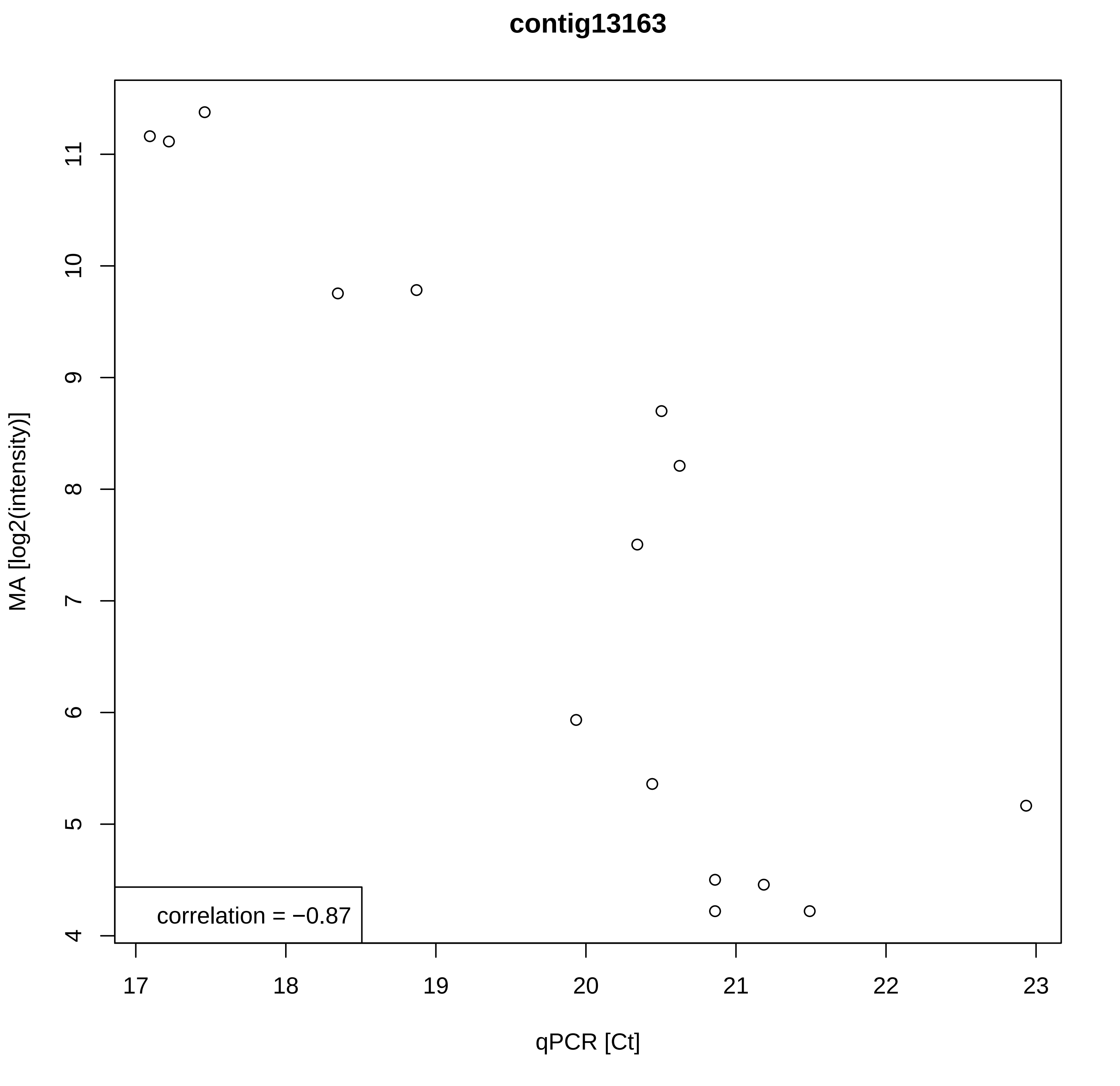
contig13163
MA [log2(intensity)]
qPCR [Ct]
correlation = −0.87
4
5
6
7
8
9
10
11
17
18
19
20
21
22
23

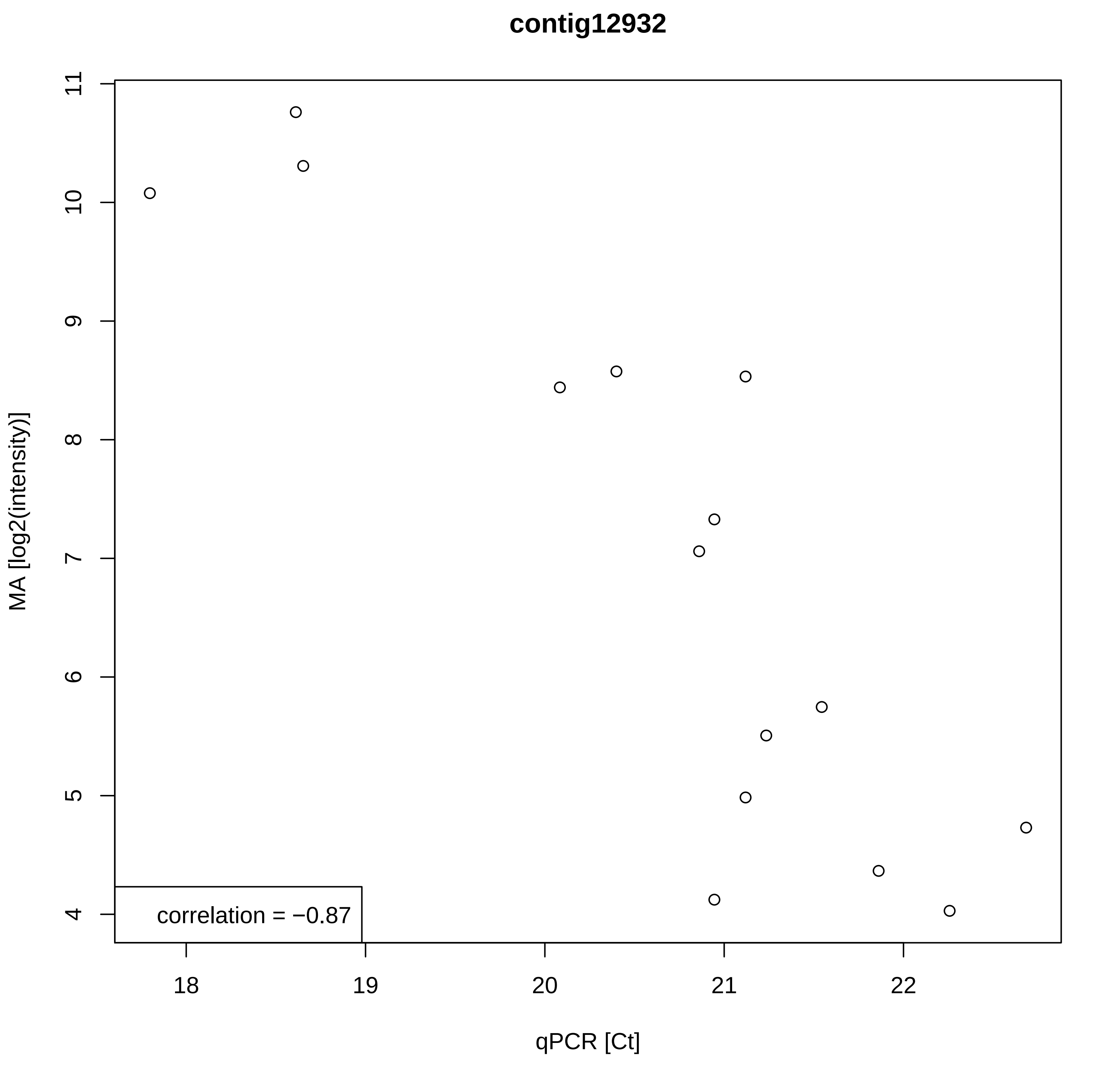
contig12932
MA [log2(intensity)]
qPCR [Ct]
correlation = −0.87
11
10
9
8
7
6
5
4
18
19
20
21
22

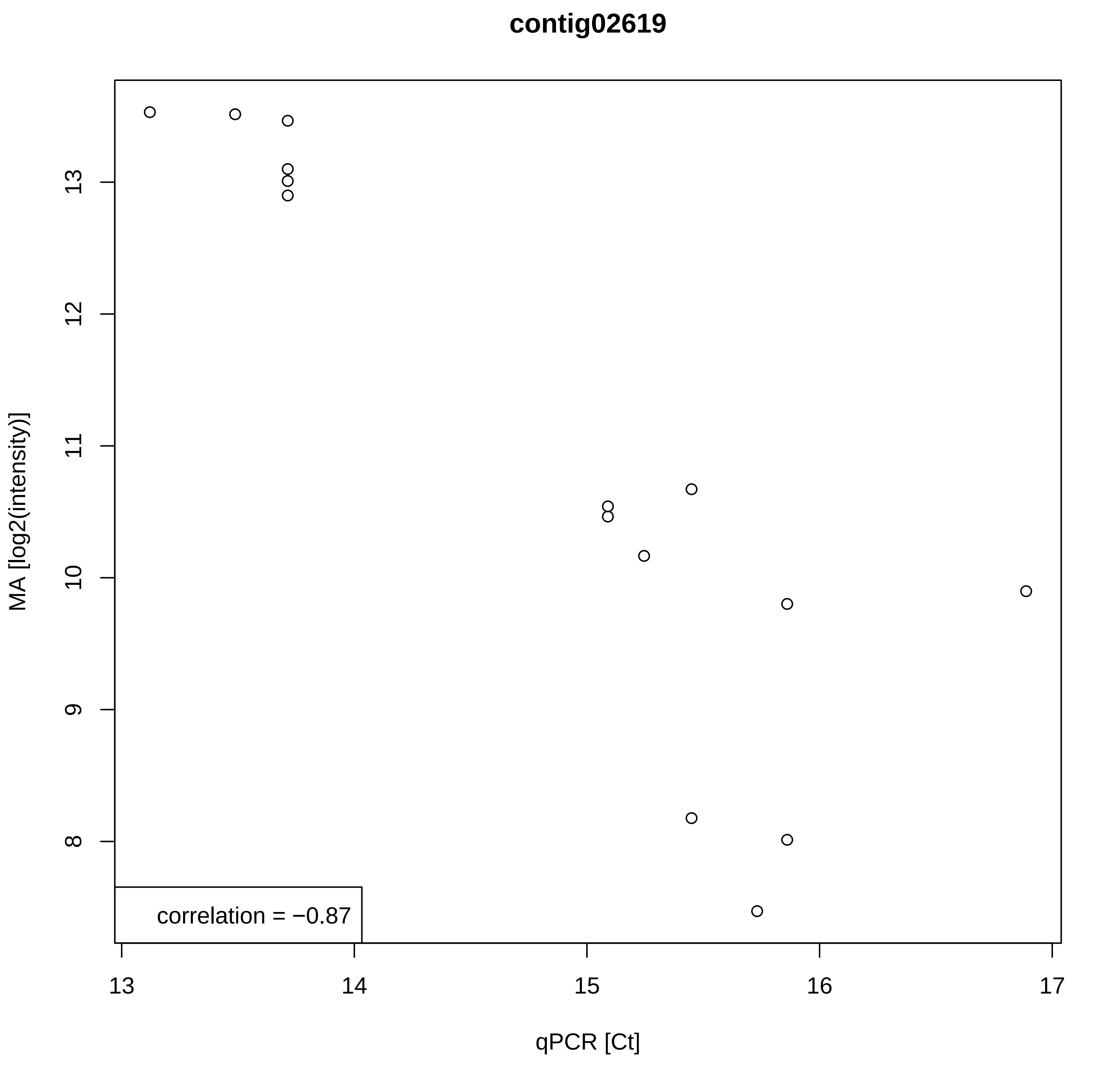
contig02619
MA [log2(intensity)]
qPCR [Ct]
correlation = −0.87
8
9
10
11
12
13
13
14
15
16
17

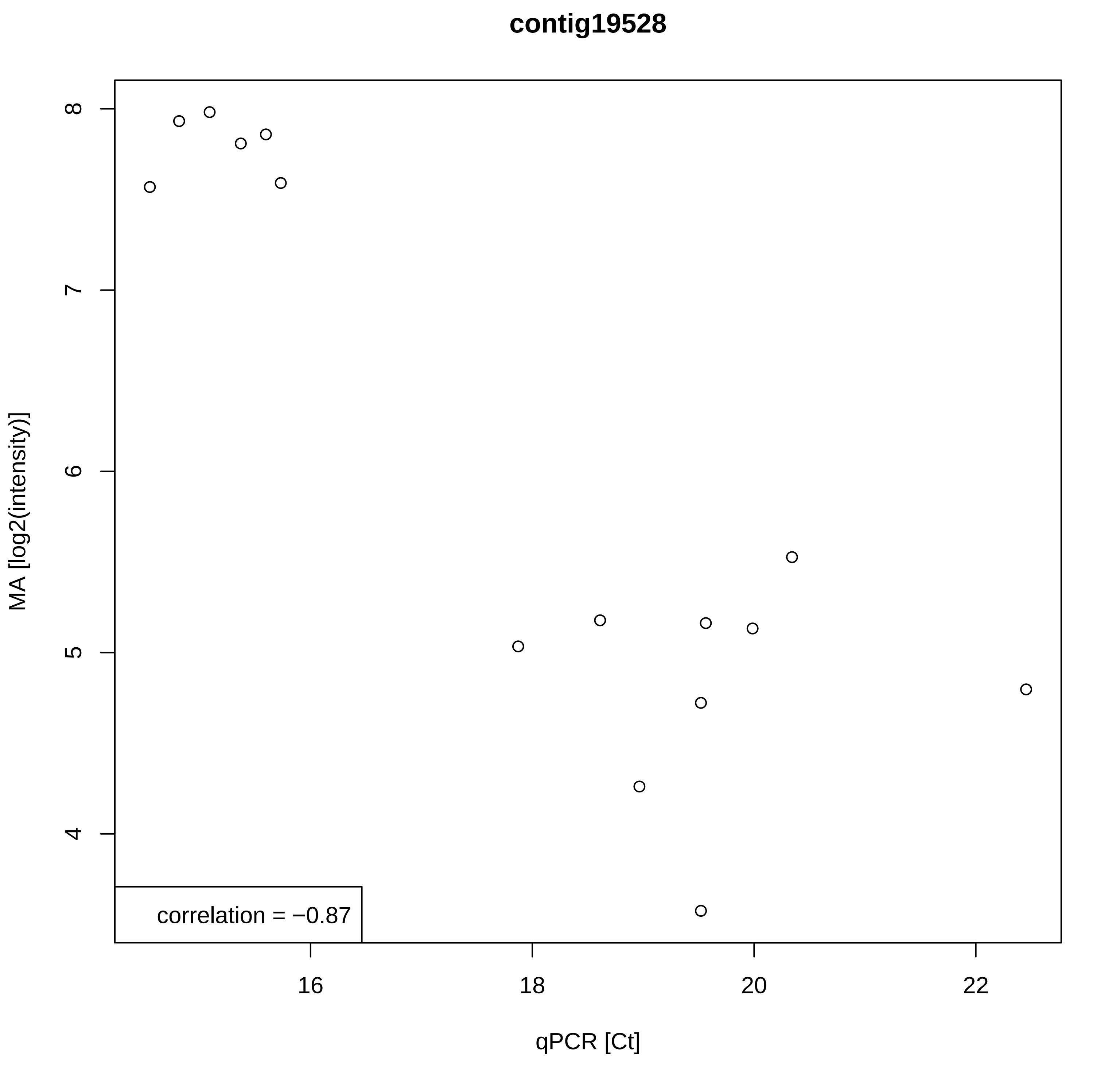
contig19528
MA [log2(intensity)]
qPCR [Ct]
8
7
6
5
4
16
18
20
22
correlation = −0.87

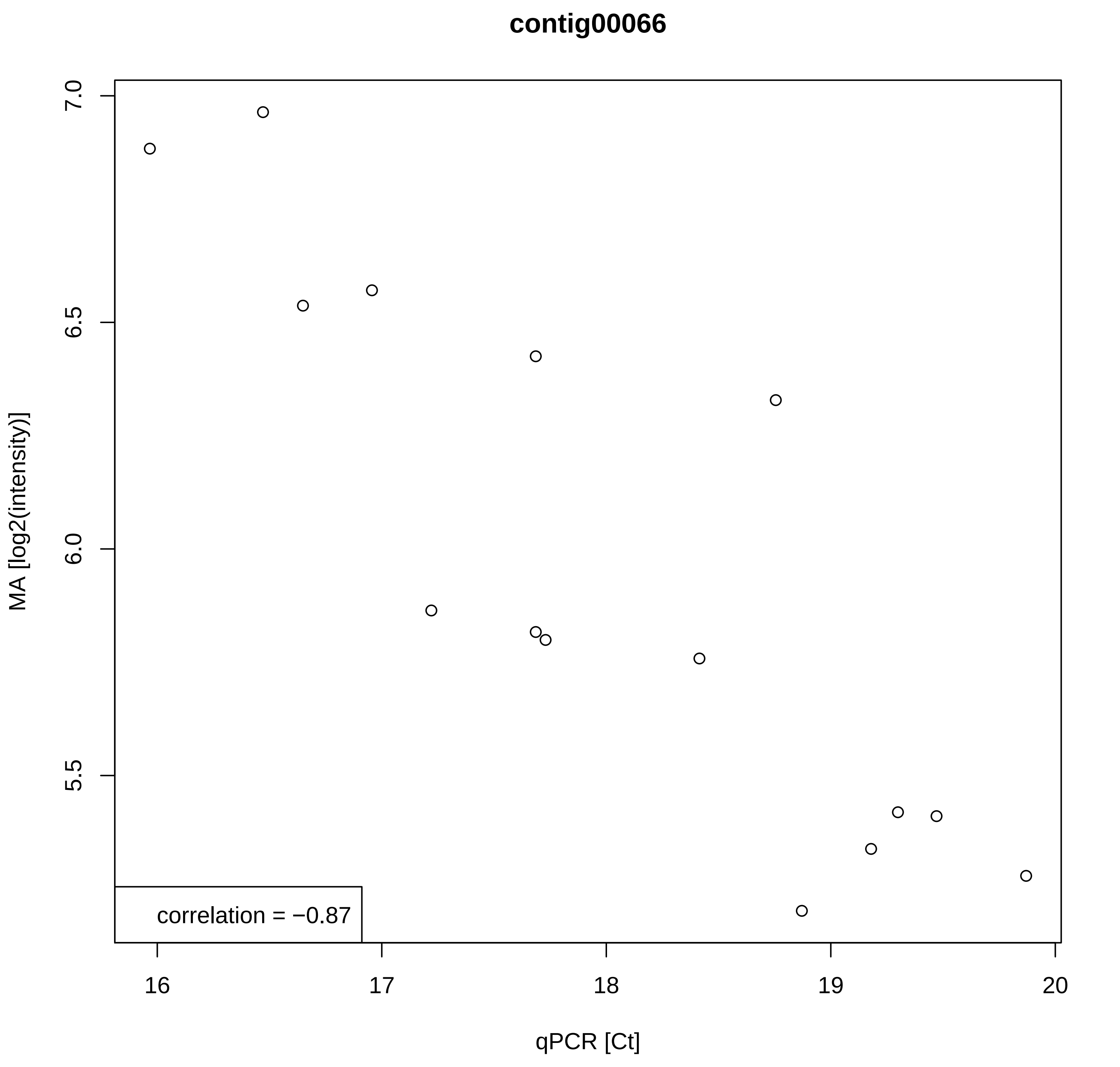

contig00066
MA [log2(intensity)]
7.0
6.5
6.0
5.5
16
17
18
19
20
qPCR [Ct]
correlation = −0.87

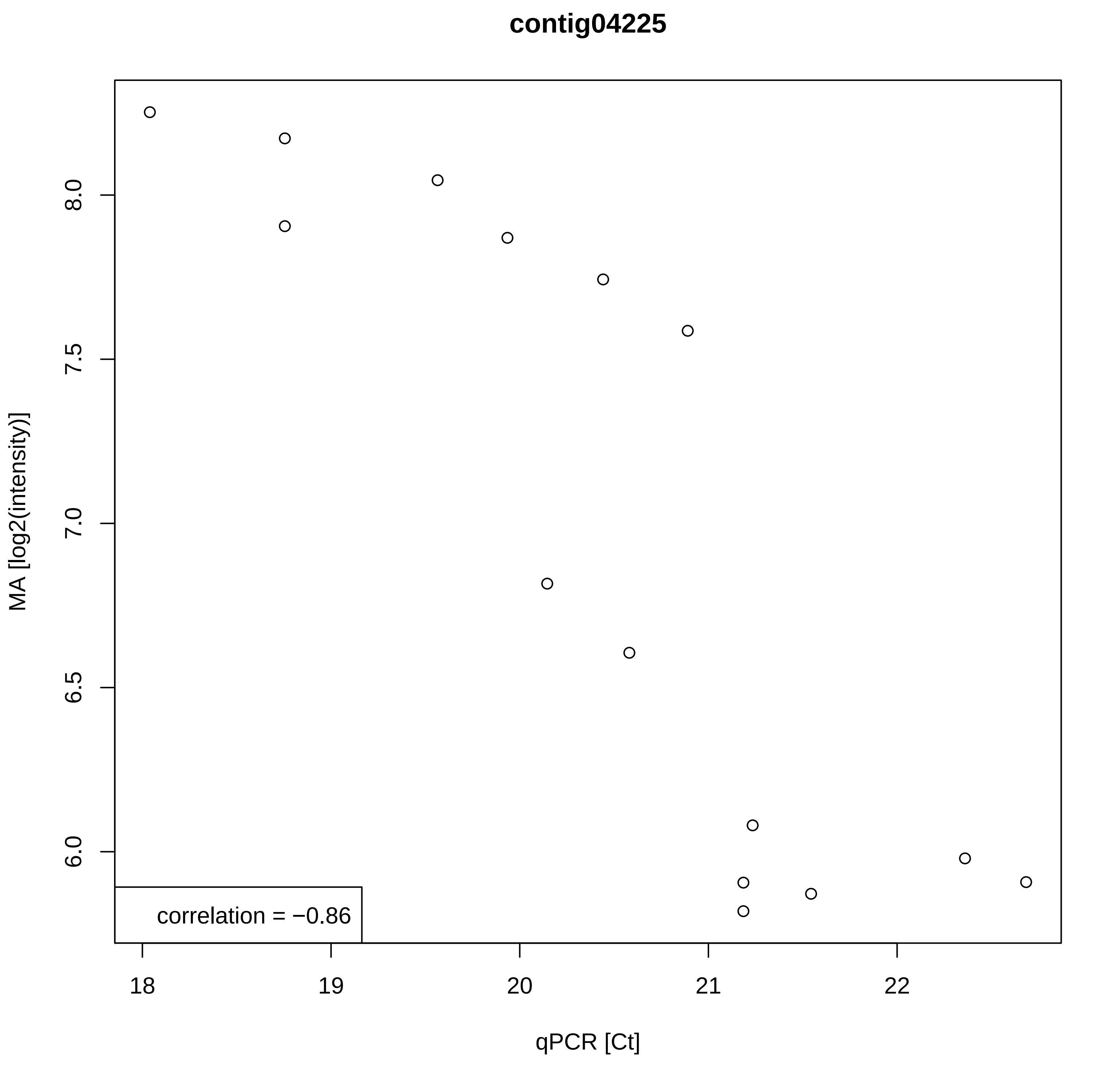

contig04225
MA [log2(intensity)]
8.0
7.5
7.0
6.5
6.0
correlation = −0.86
18
19
20
21
22
qPCR [Ct]

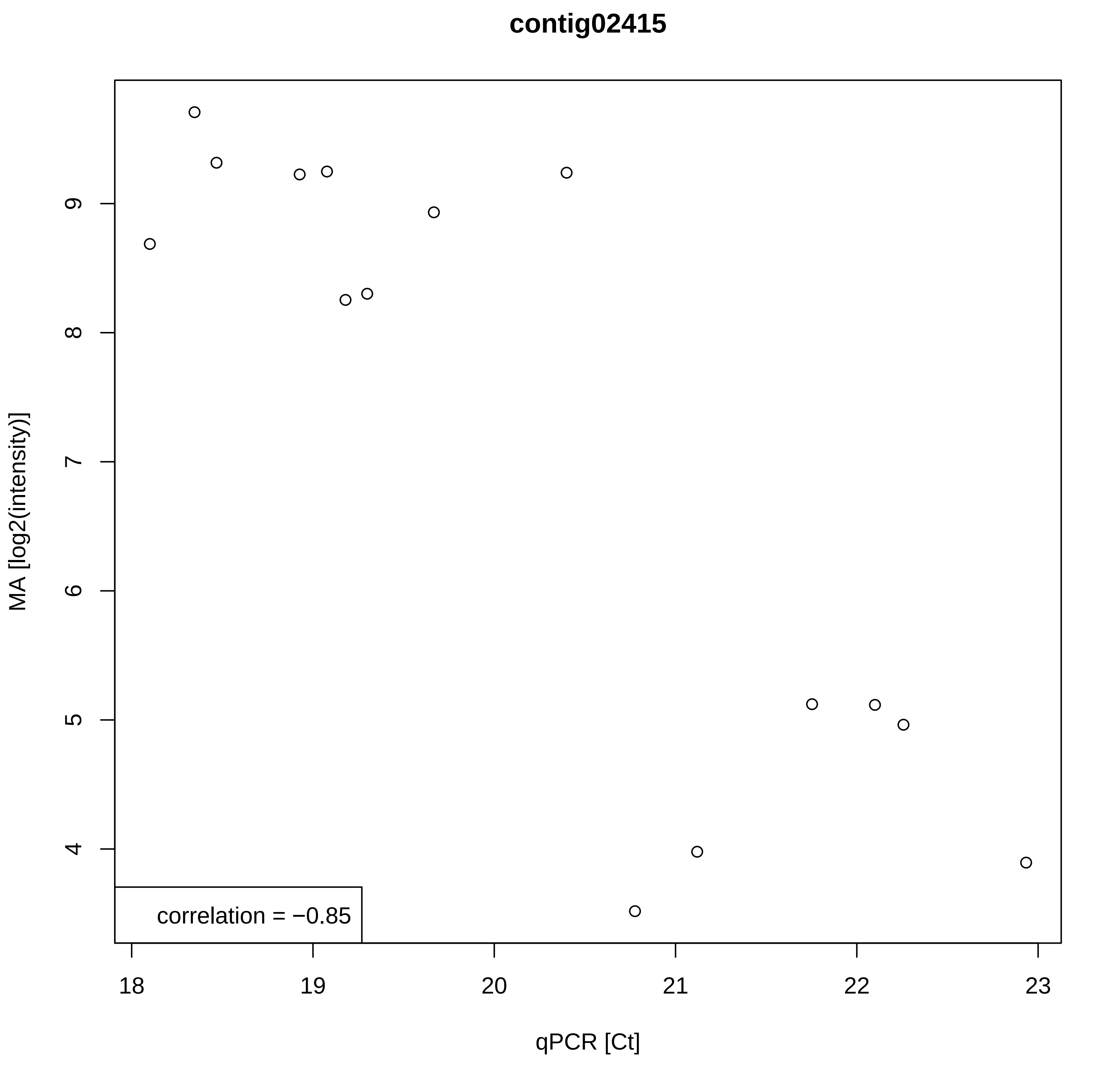
contig02415
MA [log2(intensity)]
9
8
7
6
5
4
correlation = −0.85
18
19
20
21
22
23
qPCR [Ct]

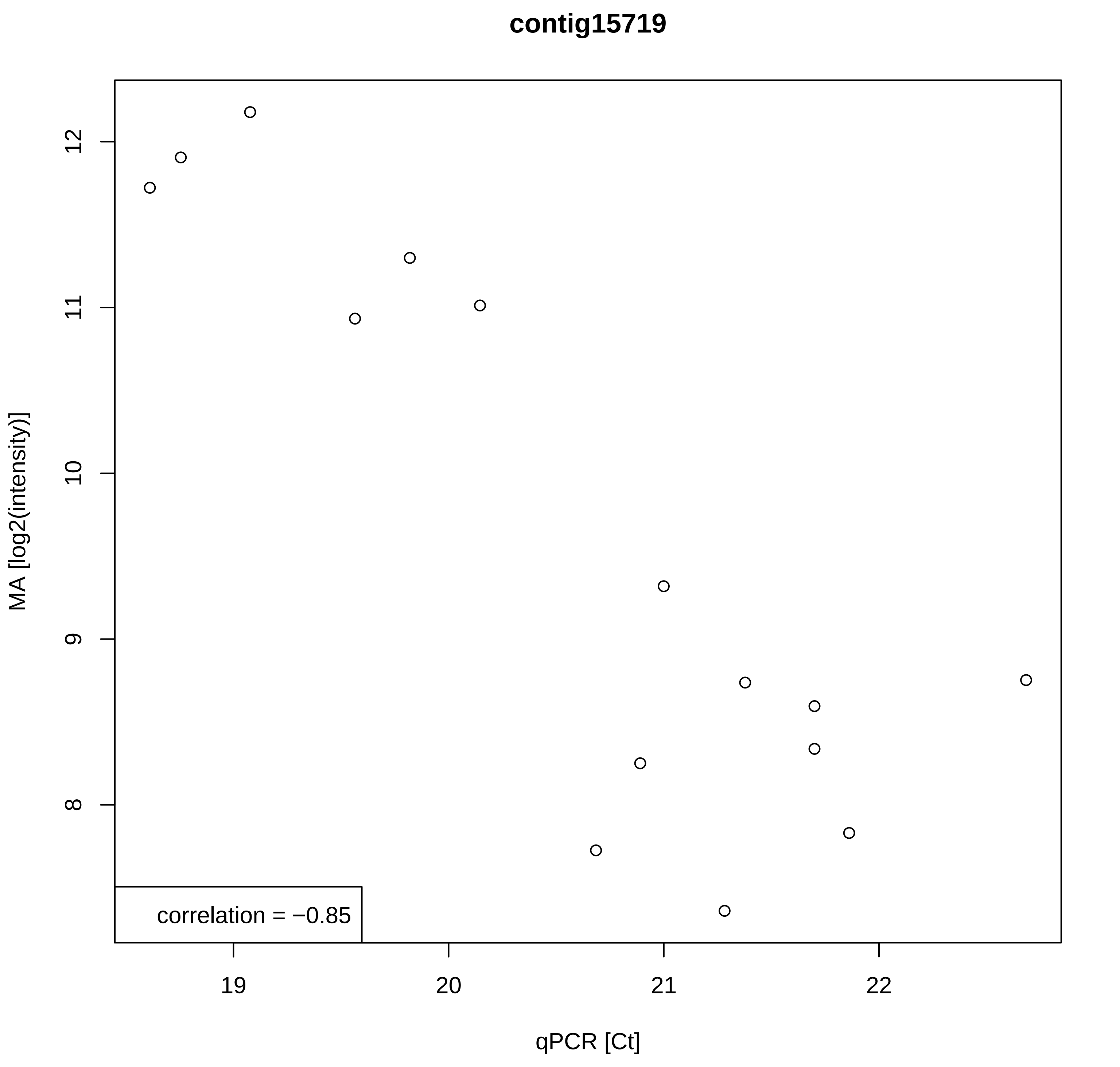
contig15719
MA [log2(intensity)]
12
11
10
9
8
correlation = −0.85
19
20
21
22
qPCR [Ct]

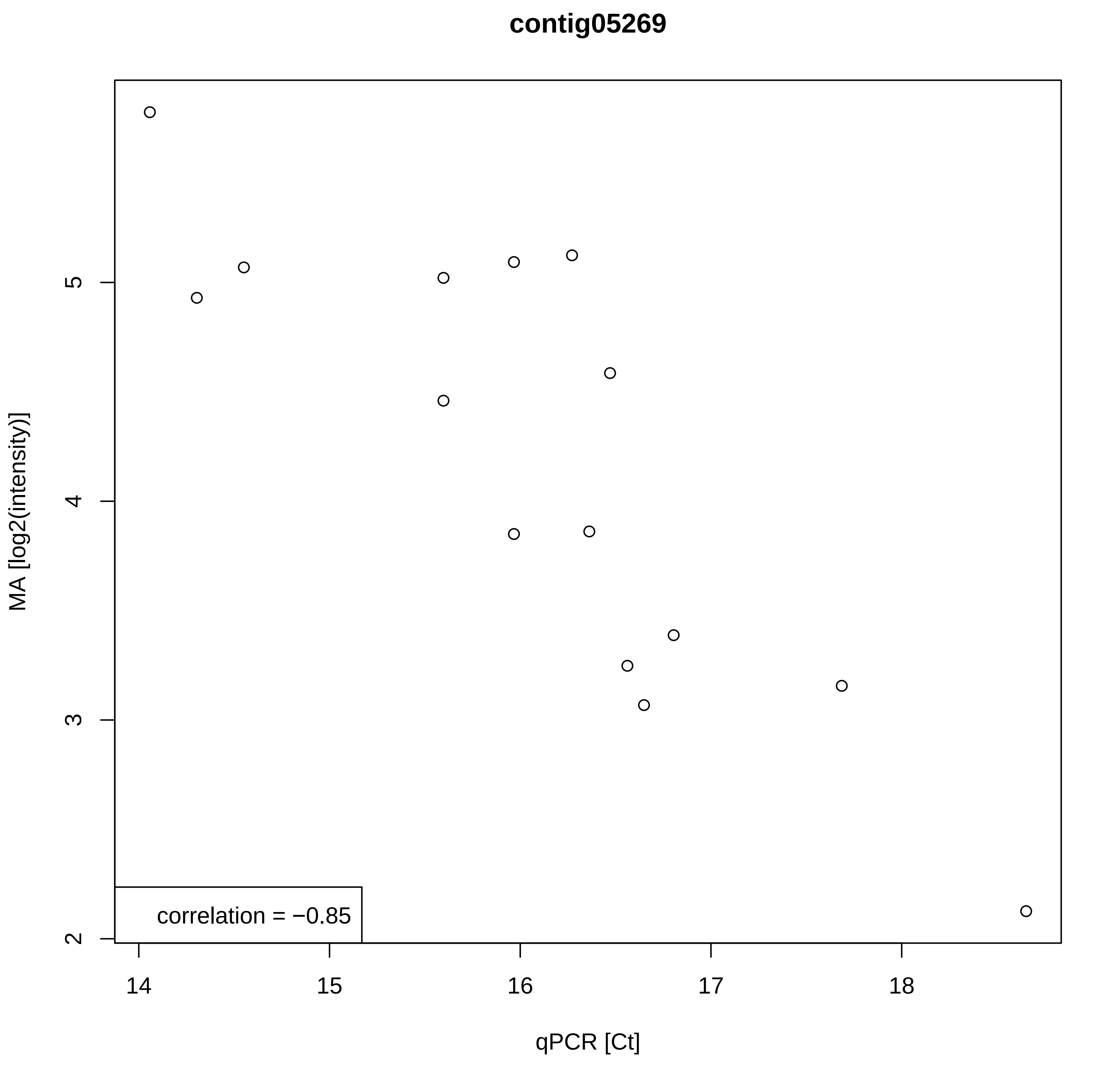
contig05269
MA [log2(intensity)]
qPCR [Ct]
correlation = −0.85
14
15
16
17
18
2
3
4
5

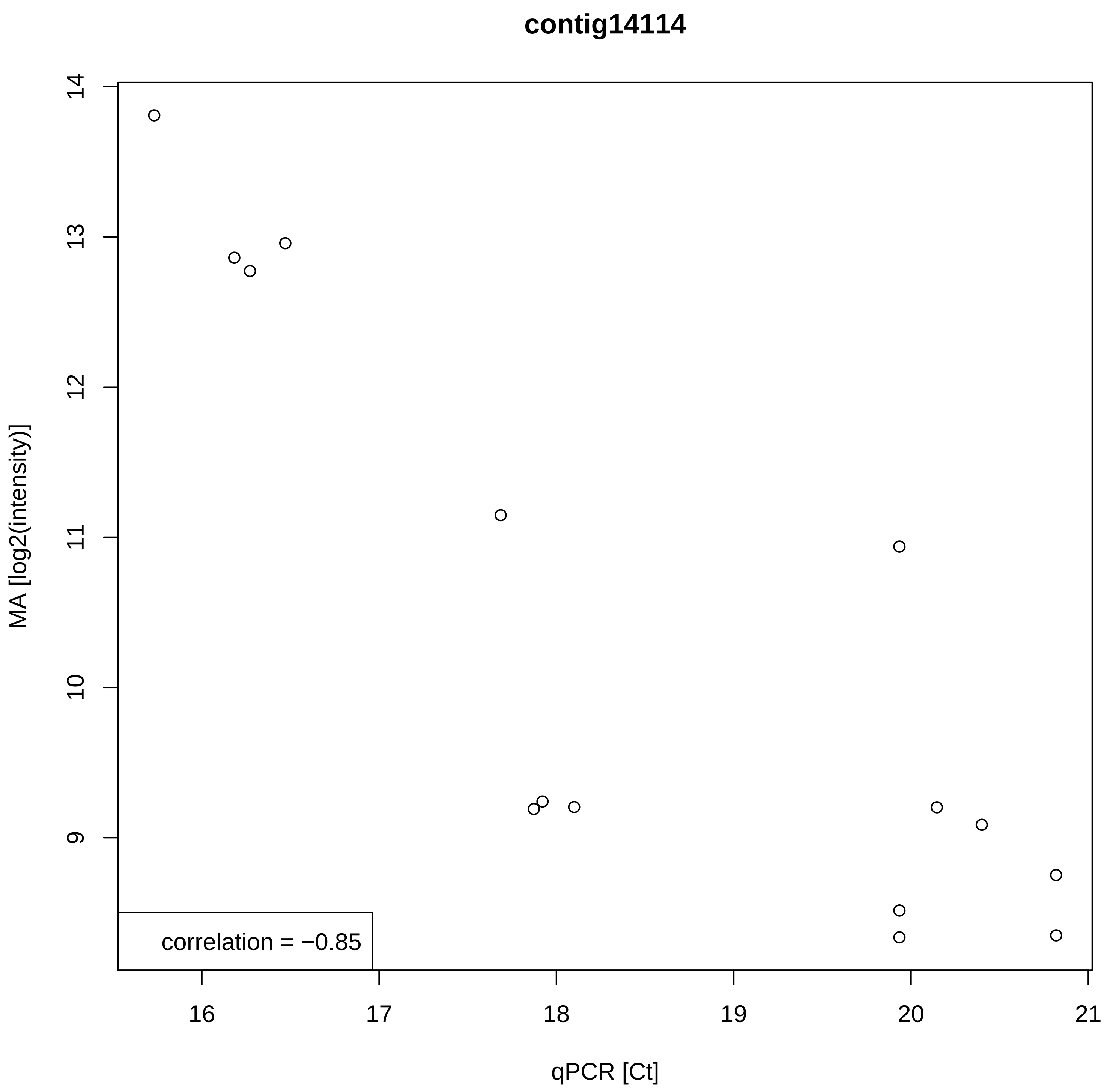
contig14114
MA [log2(intensity)]
qPCR [Ct]
correlation = −0.85
14
13
12
11
10
9
16
17
18
19
20
21

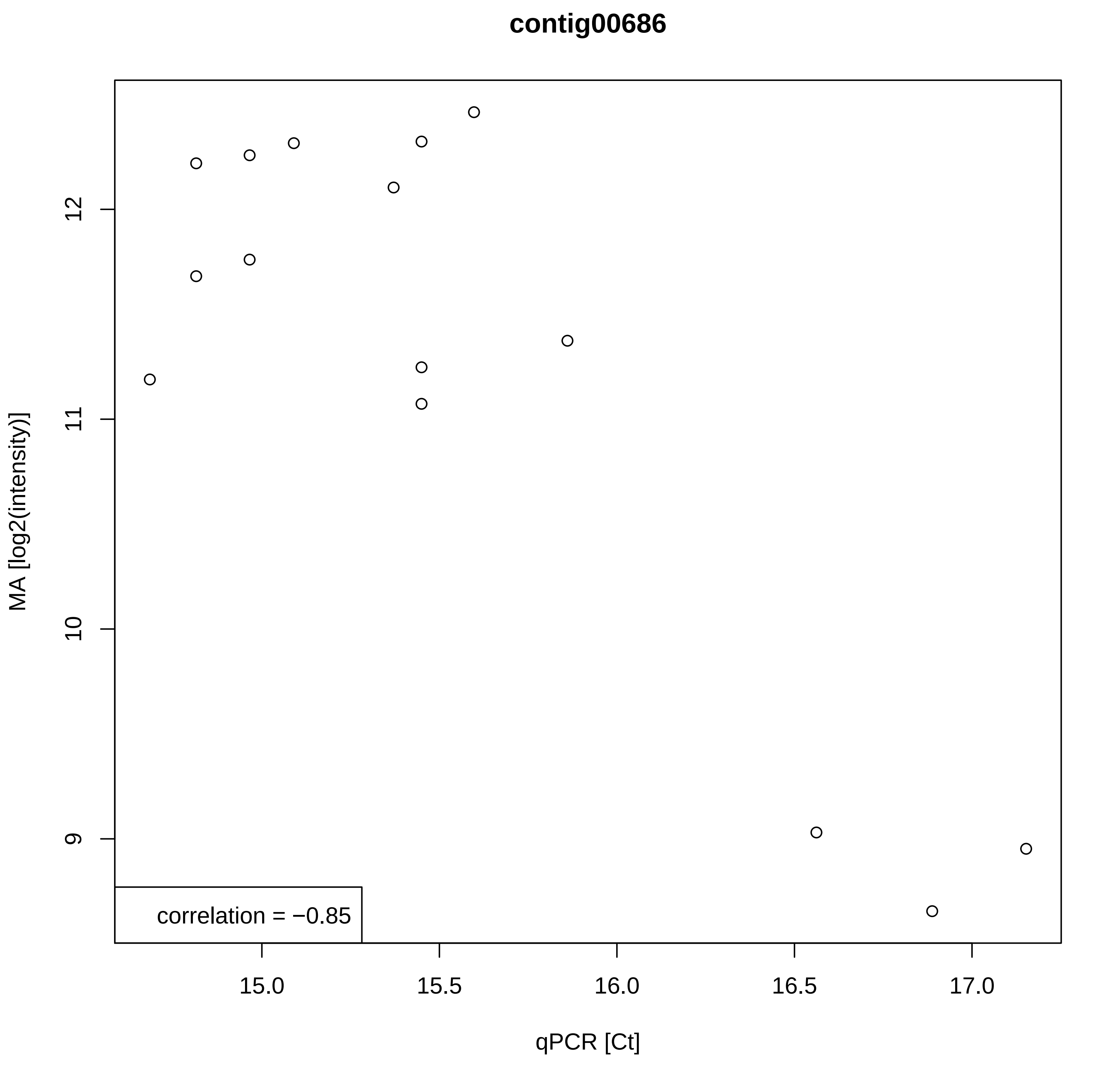
contig00686
MA [log2(intensity)]
12
11
10
9
correlation = −0.85
15.0
15.5
16.0
16.5
17.0
qPCR [Ct]

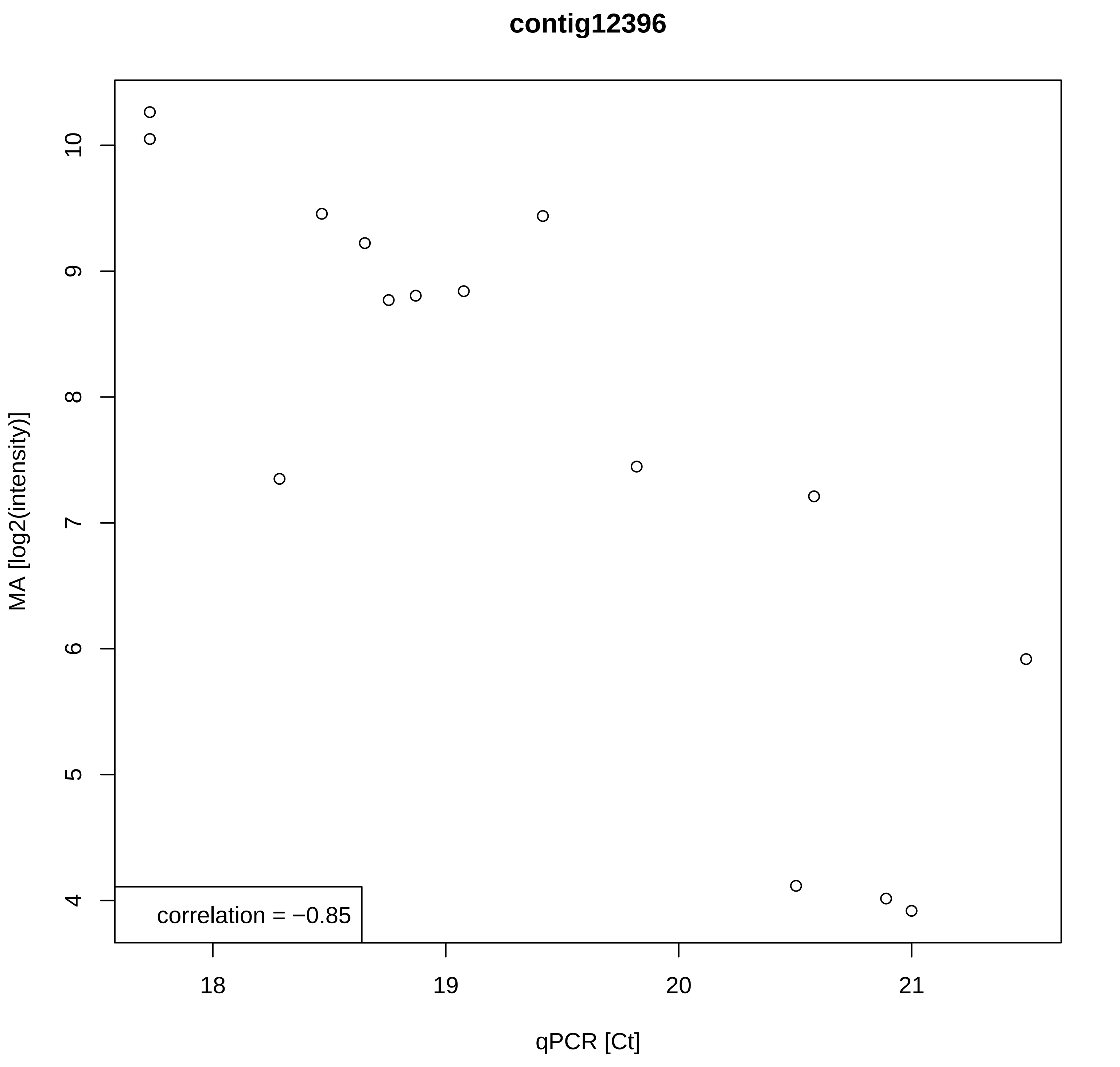

contig12396
correlation = −0.85
MA [log2(intensity)]
qPCR [Ct]
4
5
6
7
8
9
10
18
19
20
21

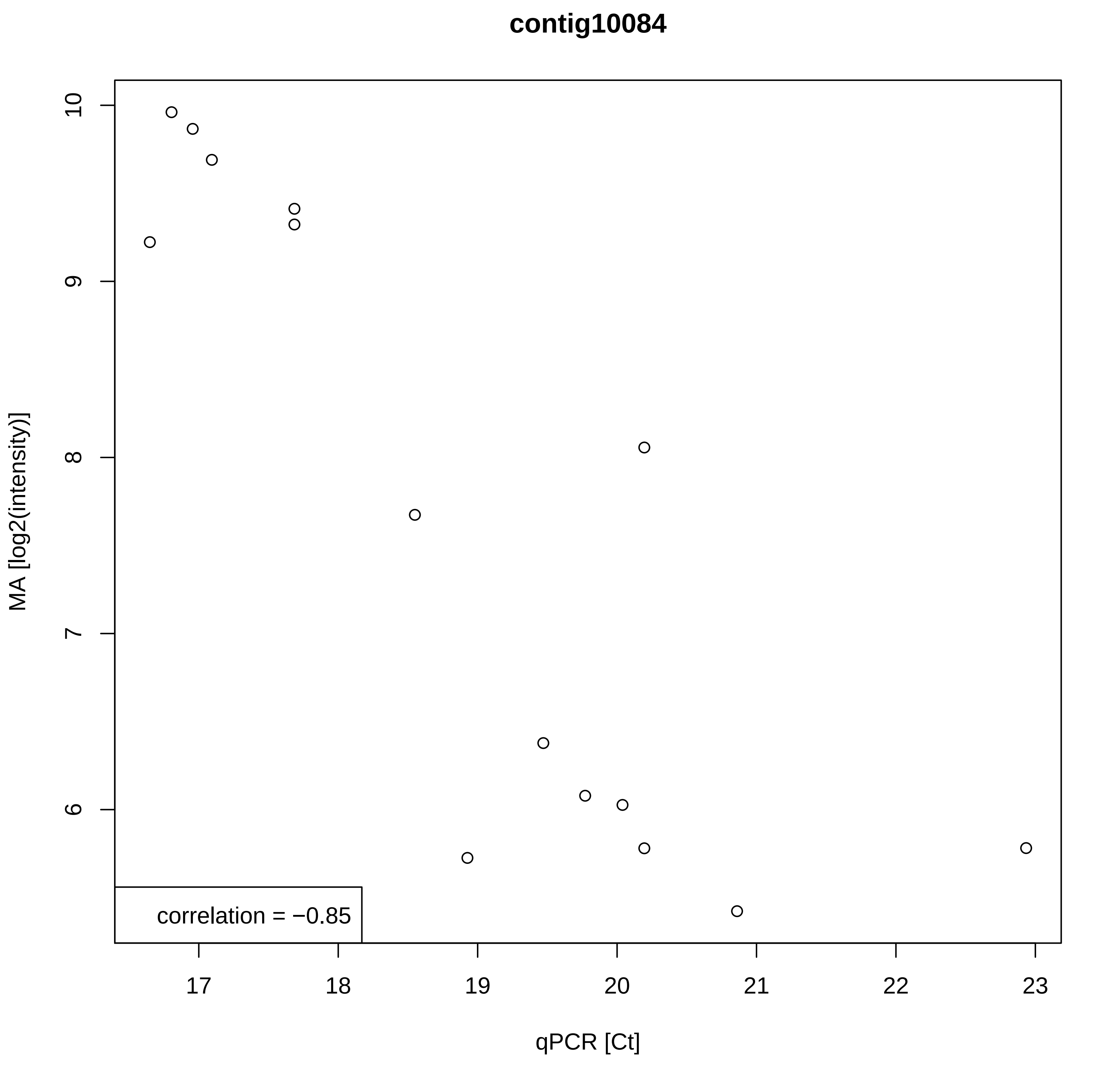
contig10084
MA [log2(intensity)]
qPCR [Ct]
correlation = −0.85
10
9
8
7
6
17
18
19
20
21
22
23

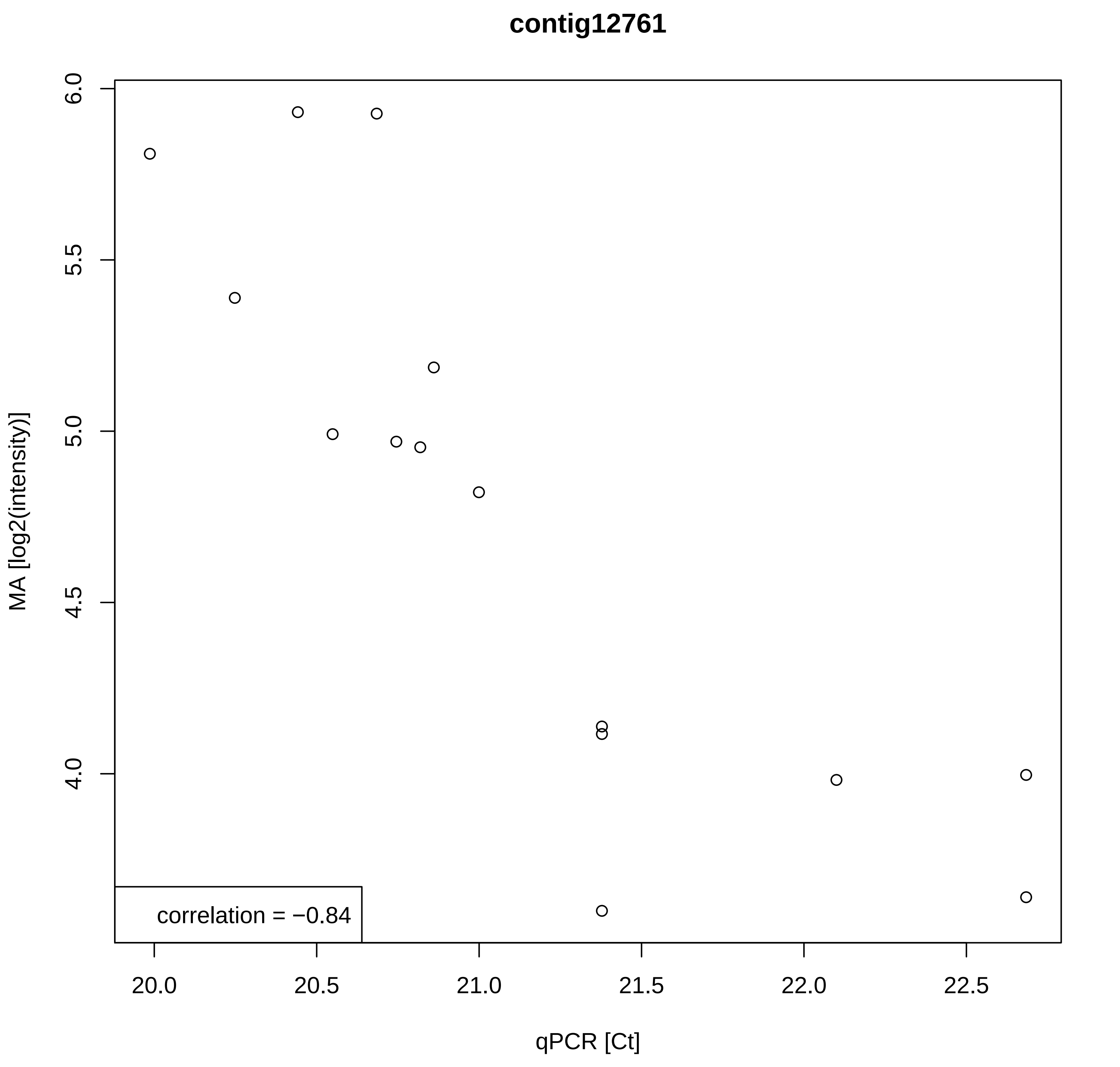
contig12761
MA [log2(intensity)]
6.0
5.5
5.0
4.5
4.0
correlation = −0.84
20.0
20.5
21.0
21.5
22.0
22.5
qPCR [Ct]

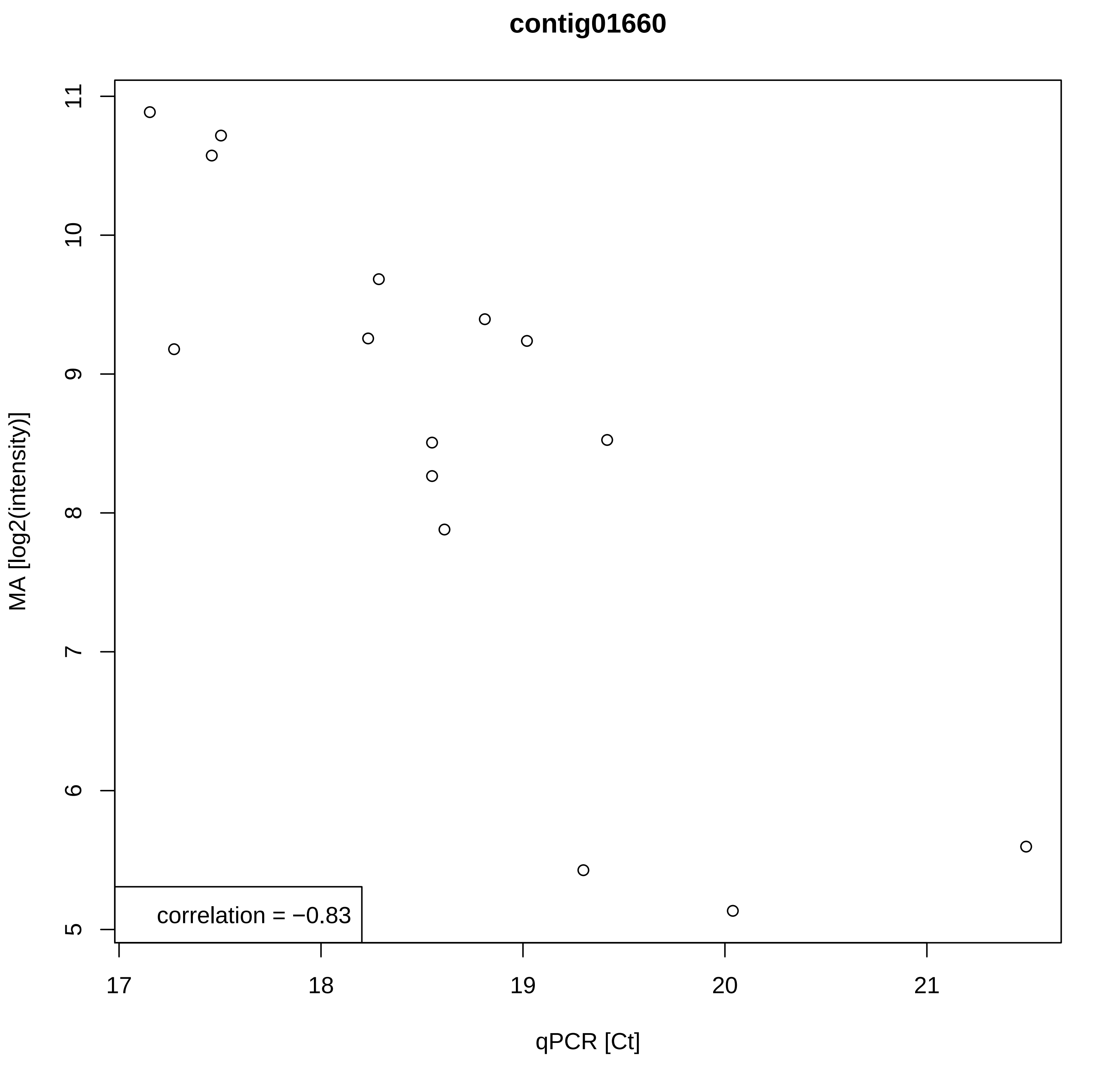
contig01660
MA [log2(intensity)]
qPCR [Ct]
correlation = −0.83
11
10
9
8
7
6
5
17
18
19
20
21

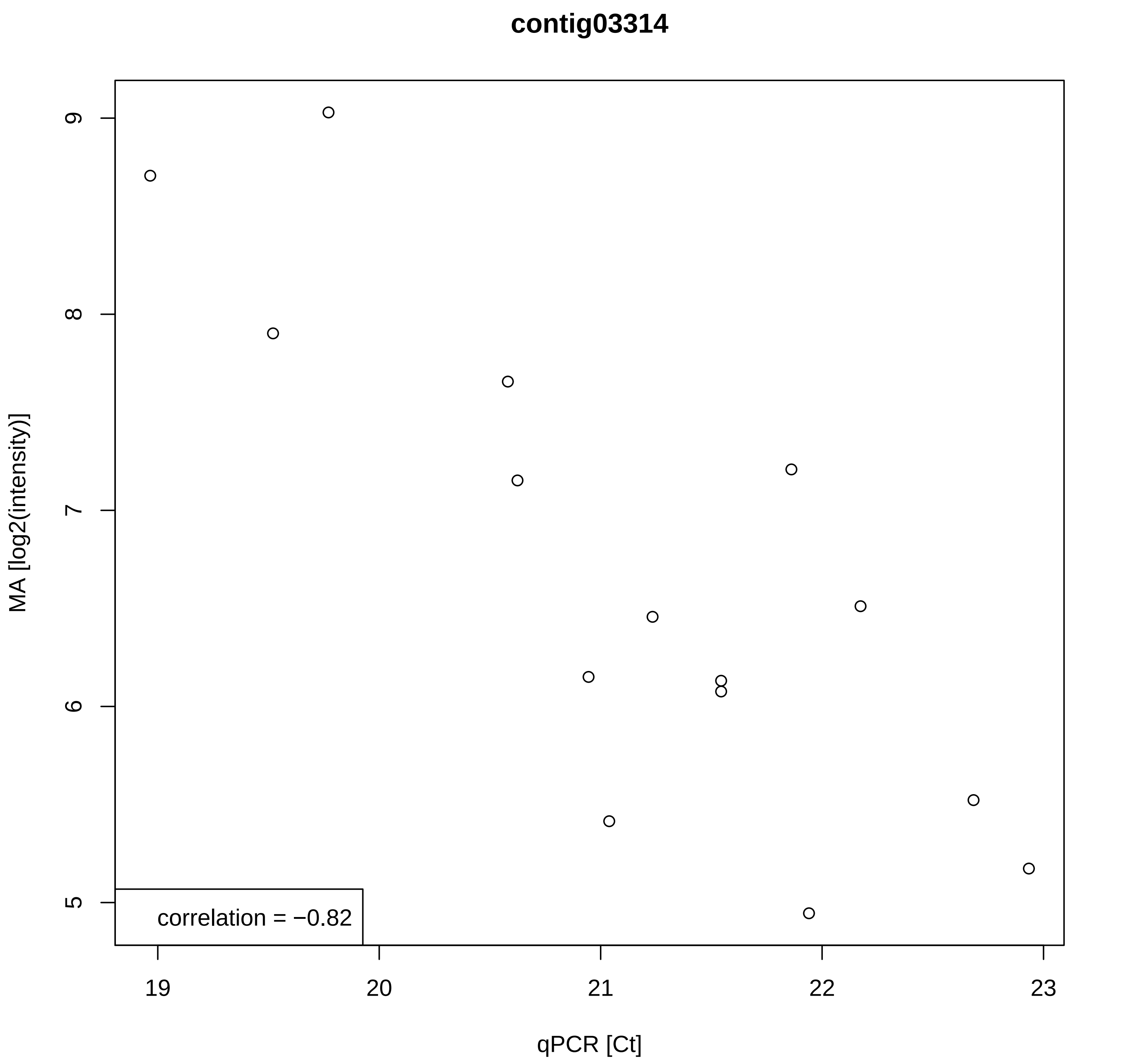
contig03314
MA [log2(intensity)]
qPCR [Ct]
correlation = −0.82
5
6
7
8
9
19
20
21
22
23

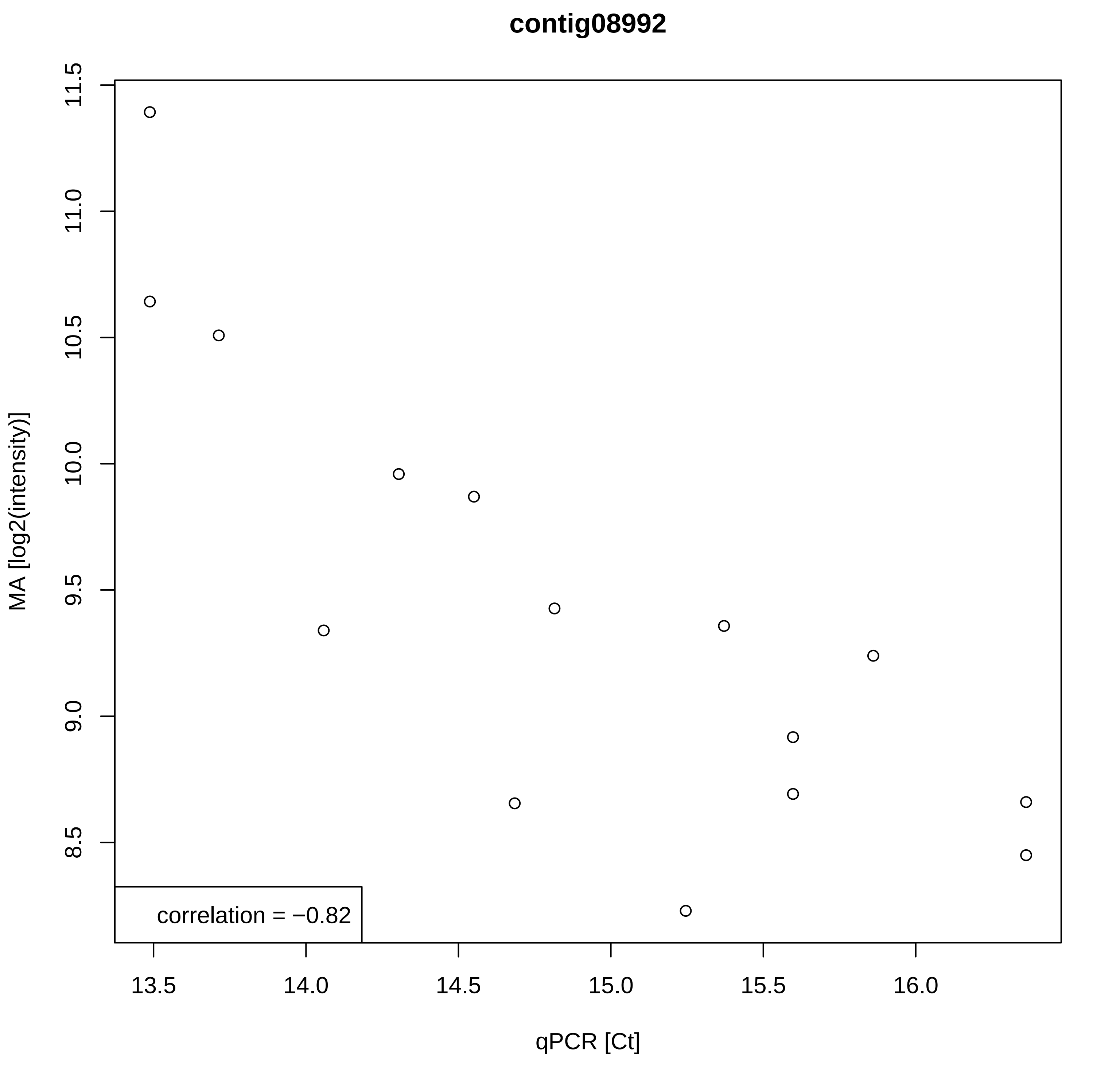
contig08992
MA [log2(intensity)]
qPCR [Ct]
11.5
11.0
10.5
10.0
9.5
9.0
8.5
13.5
14.0
14.5
15.0
15.5
16.0
correlation = −0.82

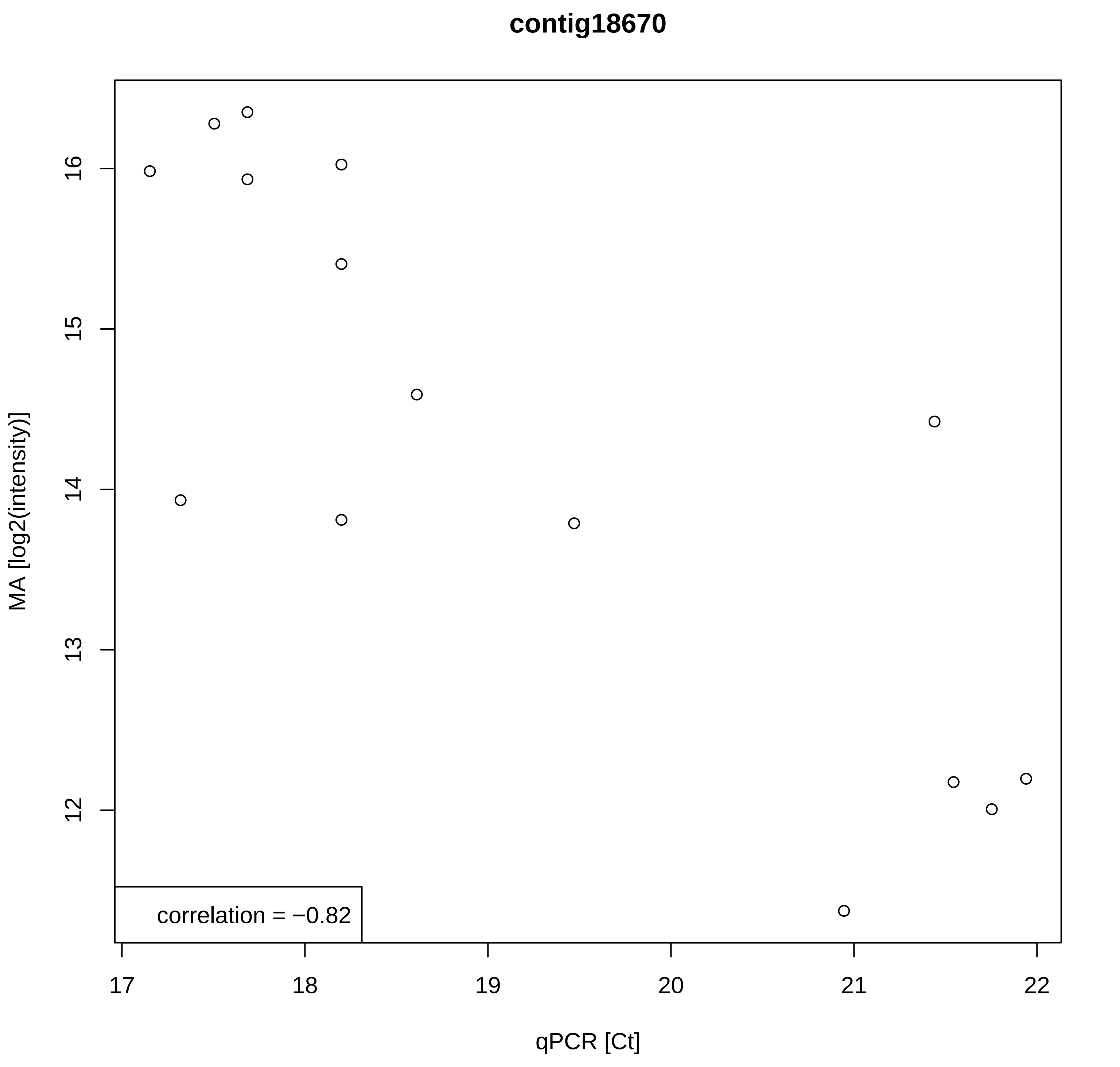

contig18670
MA [log2(intensity)]
qPCR [Ct]
correlation = −0.82
12
13
14
15
16
17
18
19
20
21
22

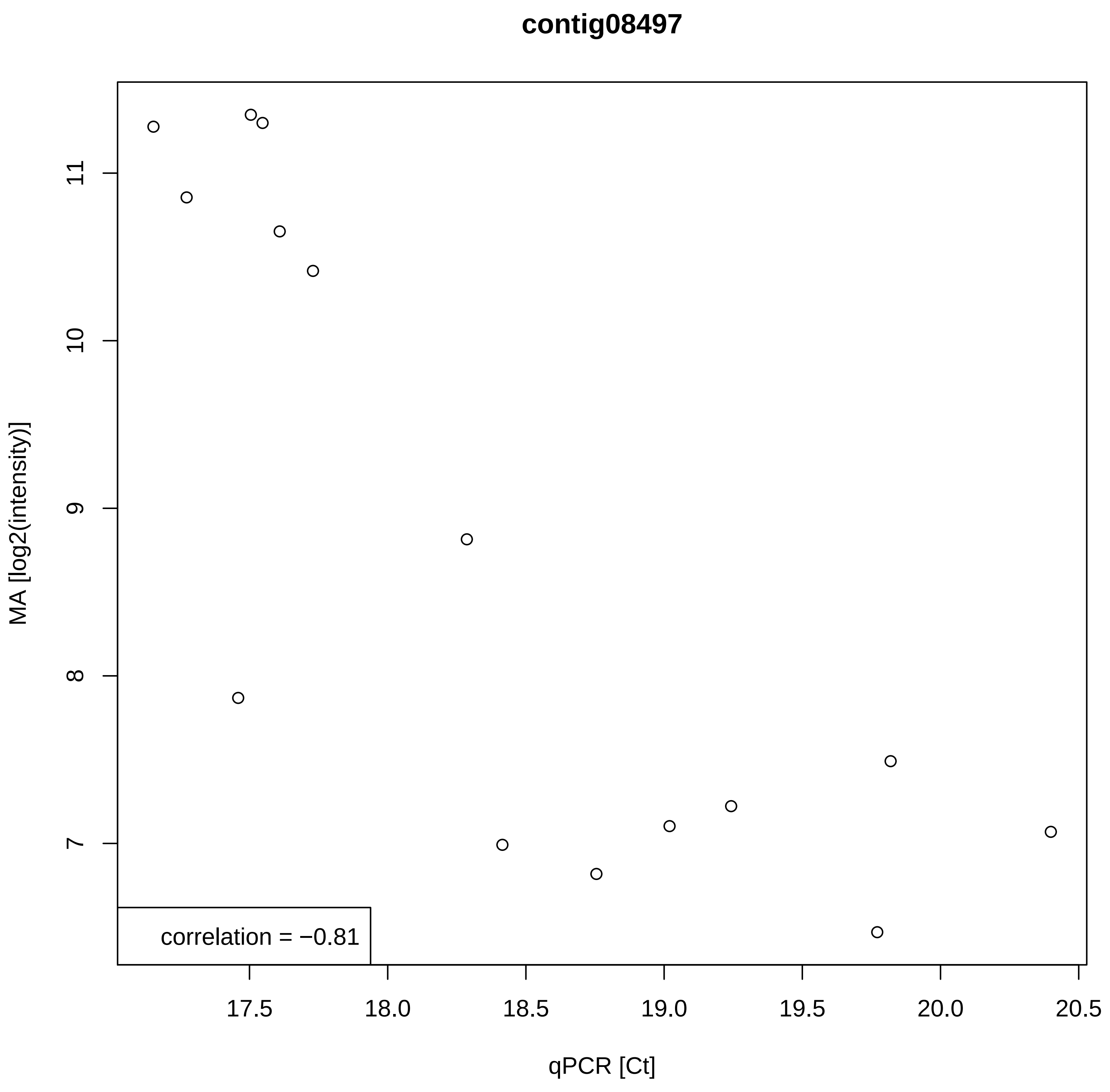
contig08497
MA [log2(intensity)]
qPCR [Ct]
correlation = −0.81
7
8
9
10
11
17.5
18.0
18.5
19.0
19.5
20.0
20.5

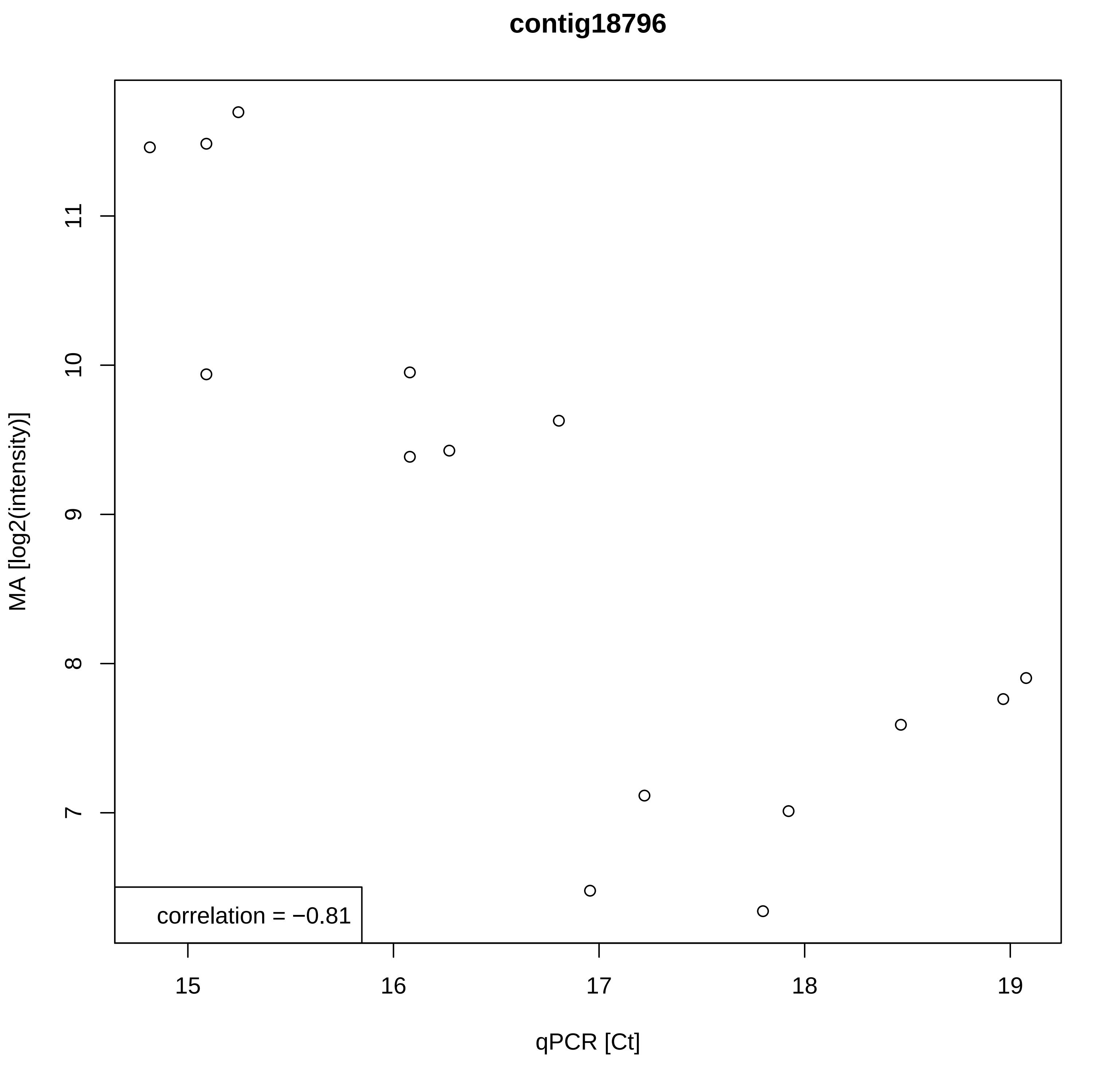
contig18796
MA [log2(intensity)]
qPCR [Ct]
correlation = −0.81
7
8
9
10
11
15
16
17
18
19

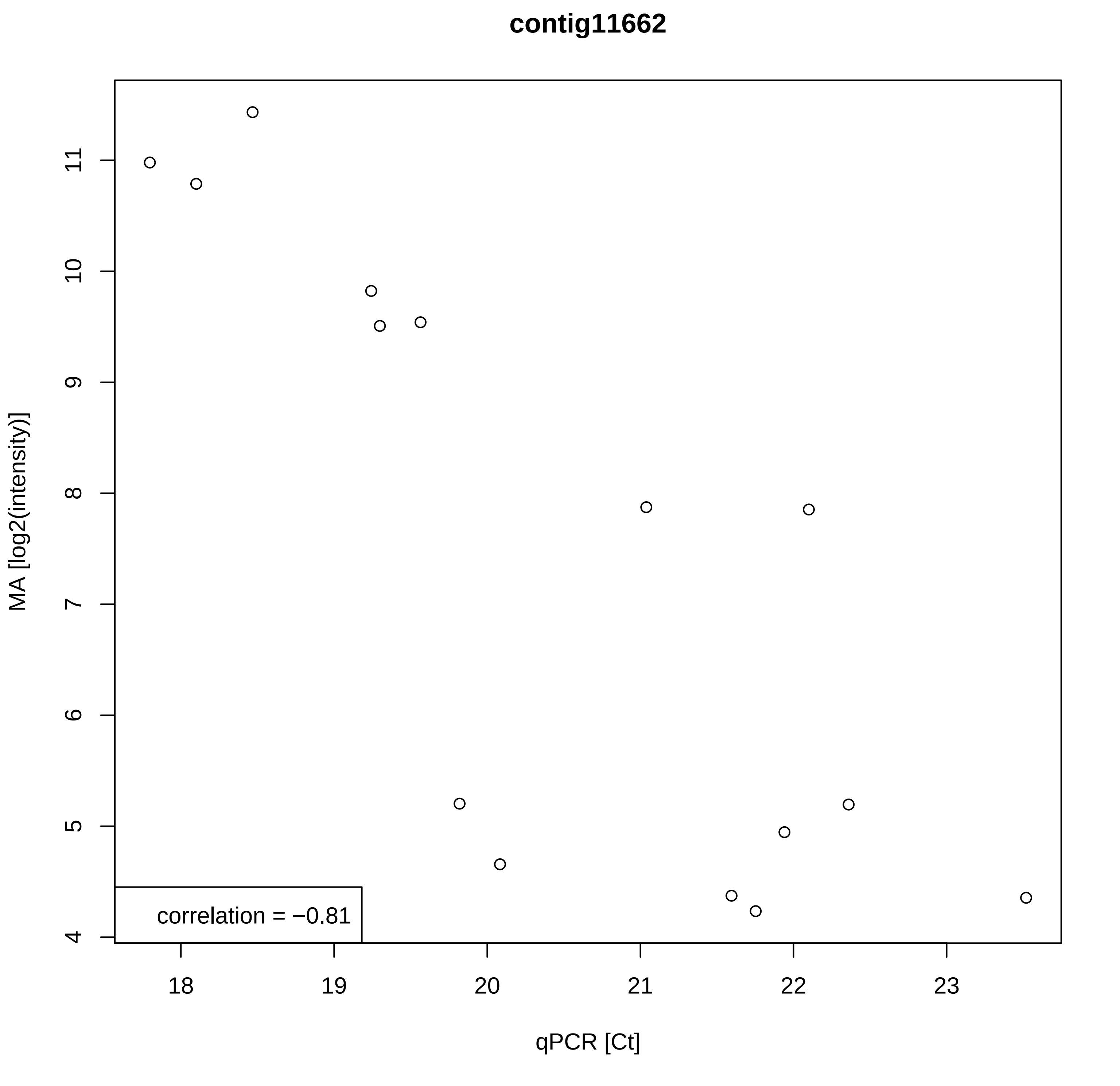
contig11662
MA [log2(intensity)]
qPCR [Ct]
correlation = −0.81
4
5
6
7
8
9
10
11
18
19
20
21
22
23

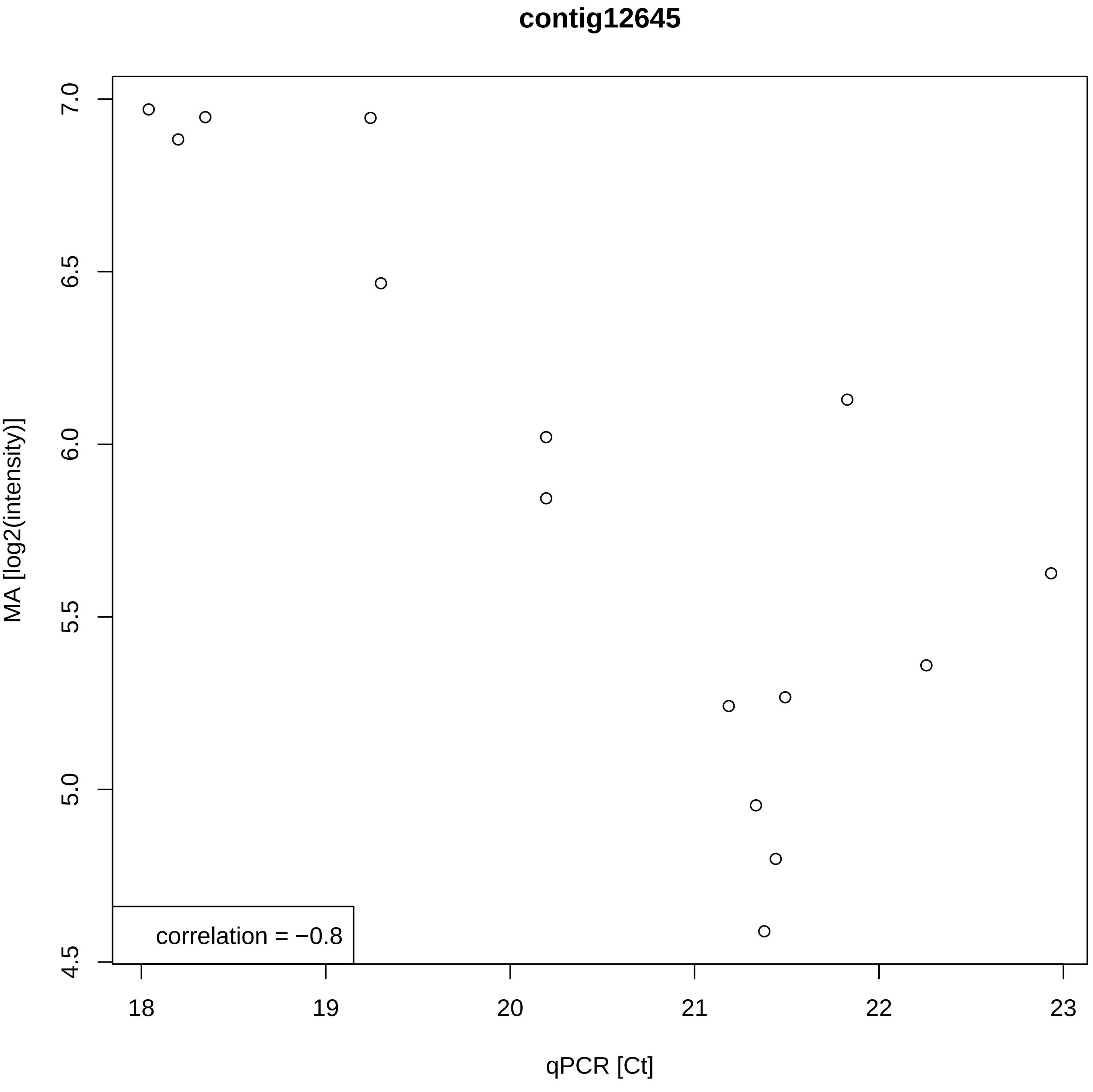

contig12645
MA [log2(intensity)]
qPCR [Ct]
7.0
6.5
6.0
5.5
5.0
4.5
18
19
20
21
22
23
correlation = −0.8

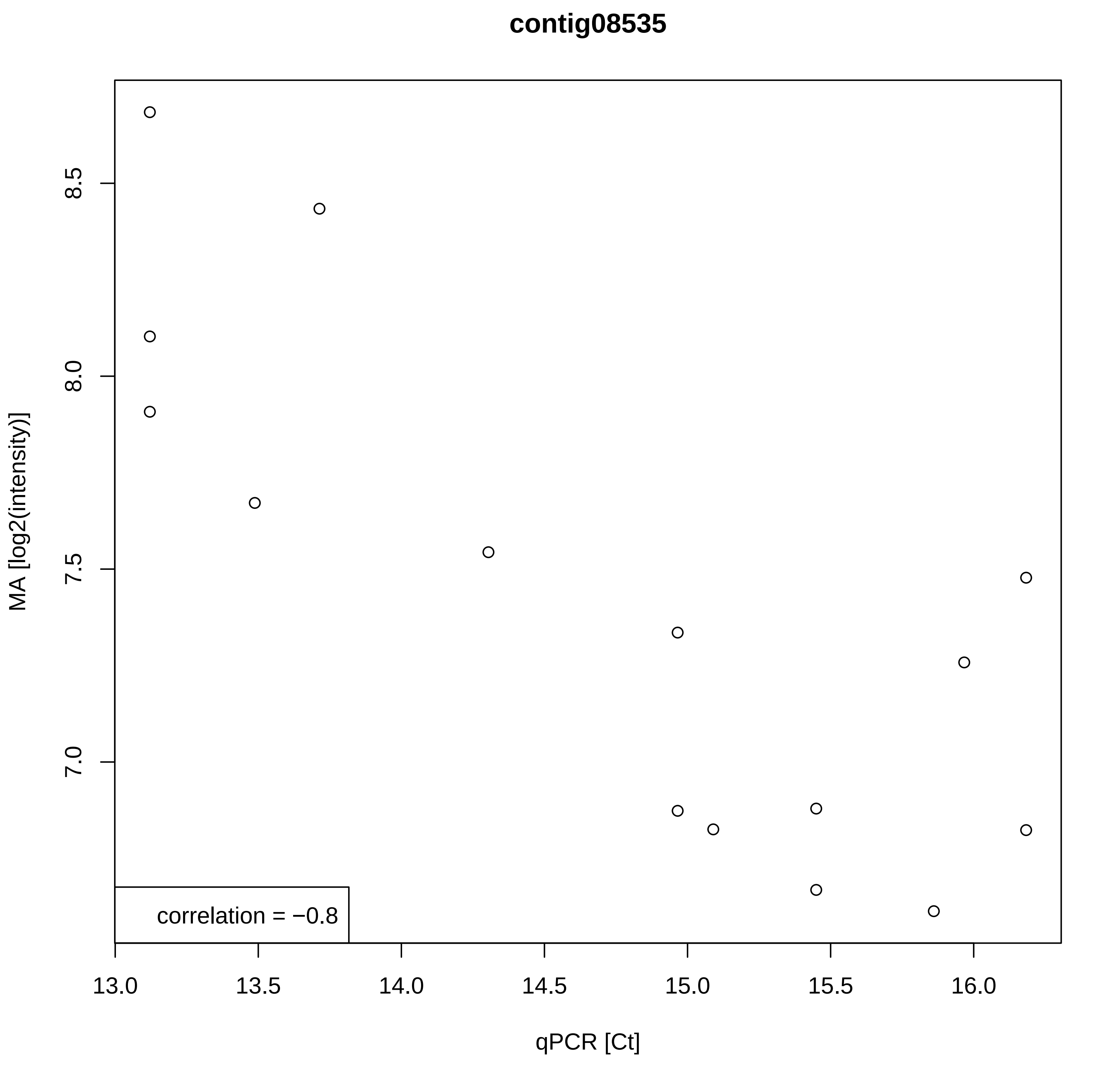
contig08535
MA [log2(intensity)]
8.5
8.0
7.5
7.0
13.0
13.5
14.0
14.5
15.0
15.5
16.0
qPCR [Ct]
correlation = −0.8

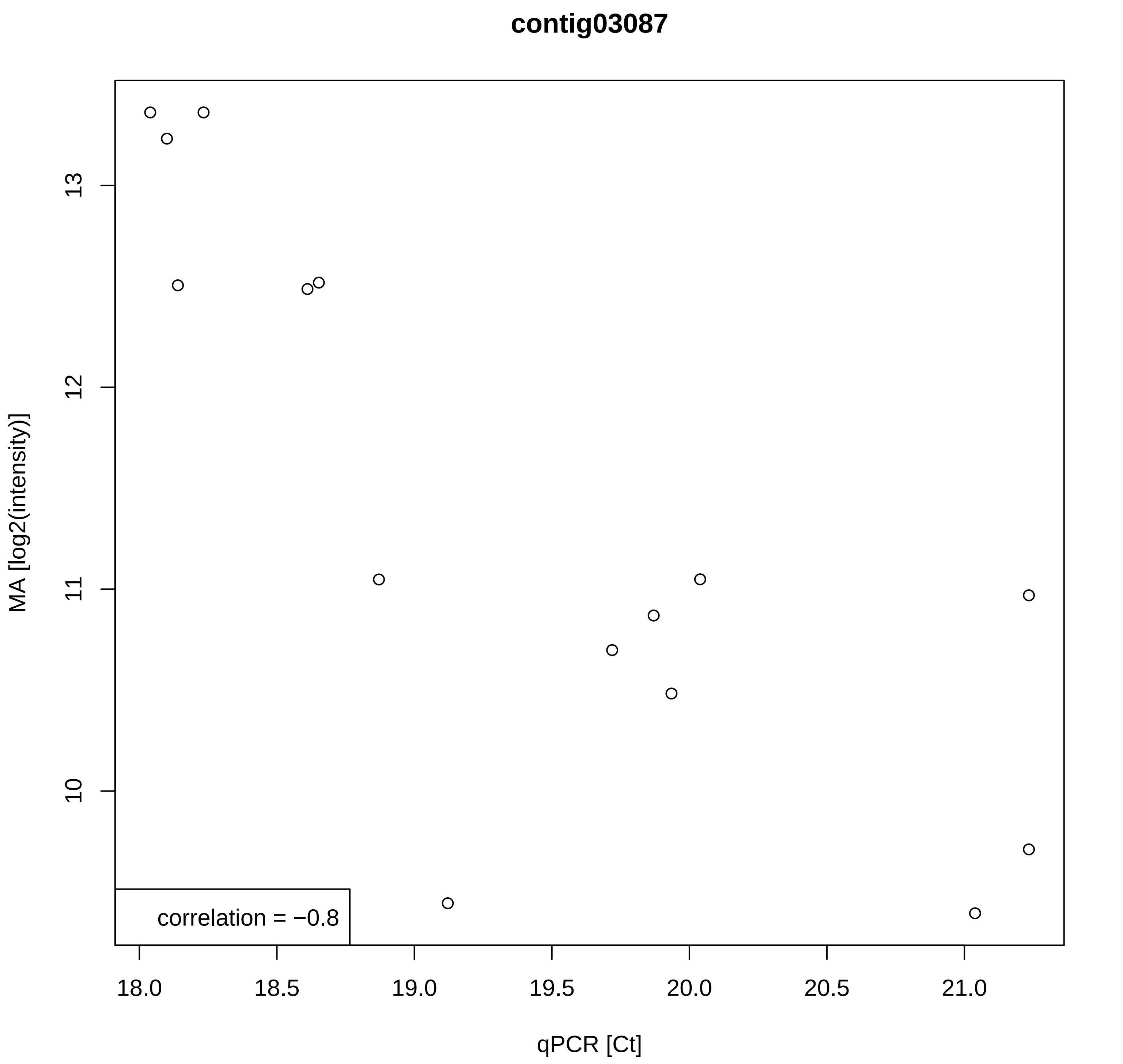
contig03087
MA [log2(intensity)]
13
12
11
10
correlation = −0.8
18.0
18.5
19.0
19.5
20.0
20.5
21.0
qPCR [Ct]
