# Supplementary material for: Gene expression profiling identifies pathways involved in seed maturation of Jatropha curcas
Source: BMC Genomics. 2020 Apr 9;21:290. doi: 10.1186/s12864-020-6666-1 (PMC7146973; doi:10.1186/s12864-020-6666-1)
Supplement: Supplementary file 4 — Additional file 4: Figure S4. The expression profiles of each microarray probe during seed developmental stages. The x-axis represents different developmental stages, and the y-axis represents the log2 intensity values. [file 12864_2020_6666_MOESM4_ESM.pdf]

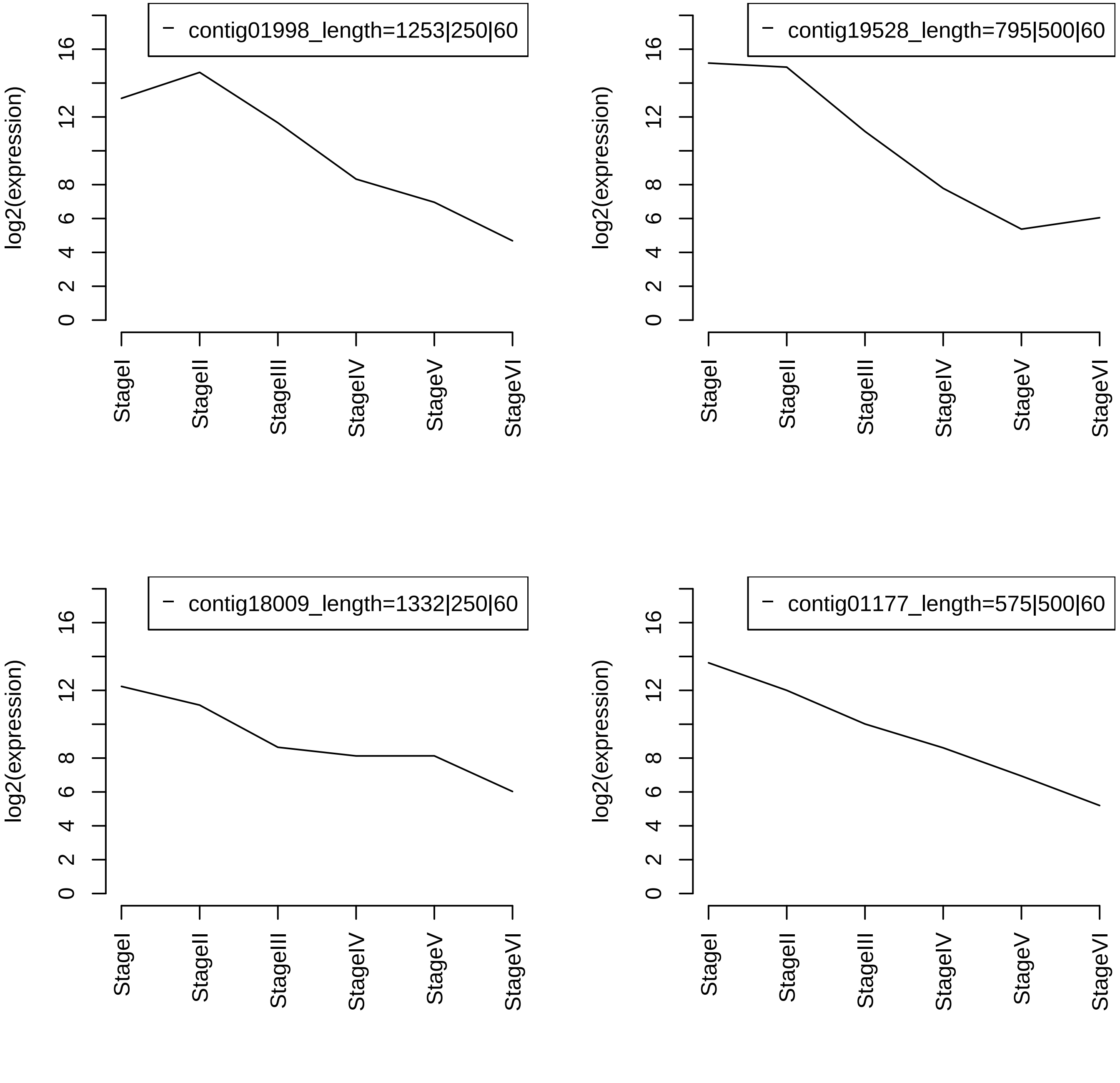

contig01998_length=1253|250|60
contig19528_length=795|500|60
contig18009_length=1332|250|60
contig01177_length=575|500|60
log2(expression)
0
2
4
6
8
12
16
StageI
StageII
StageIII
StageIV
StageV
StageVI

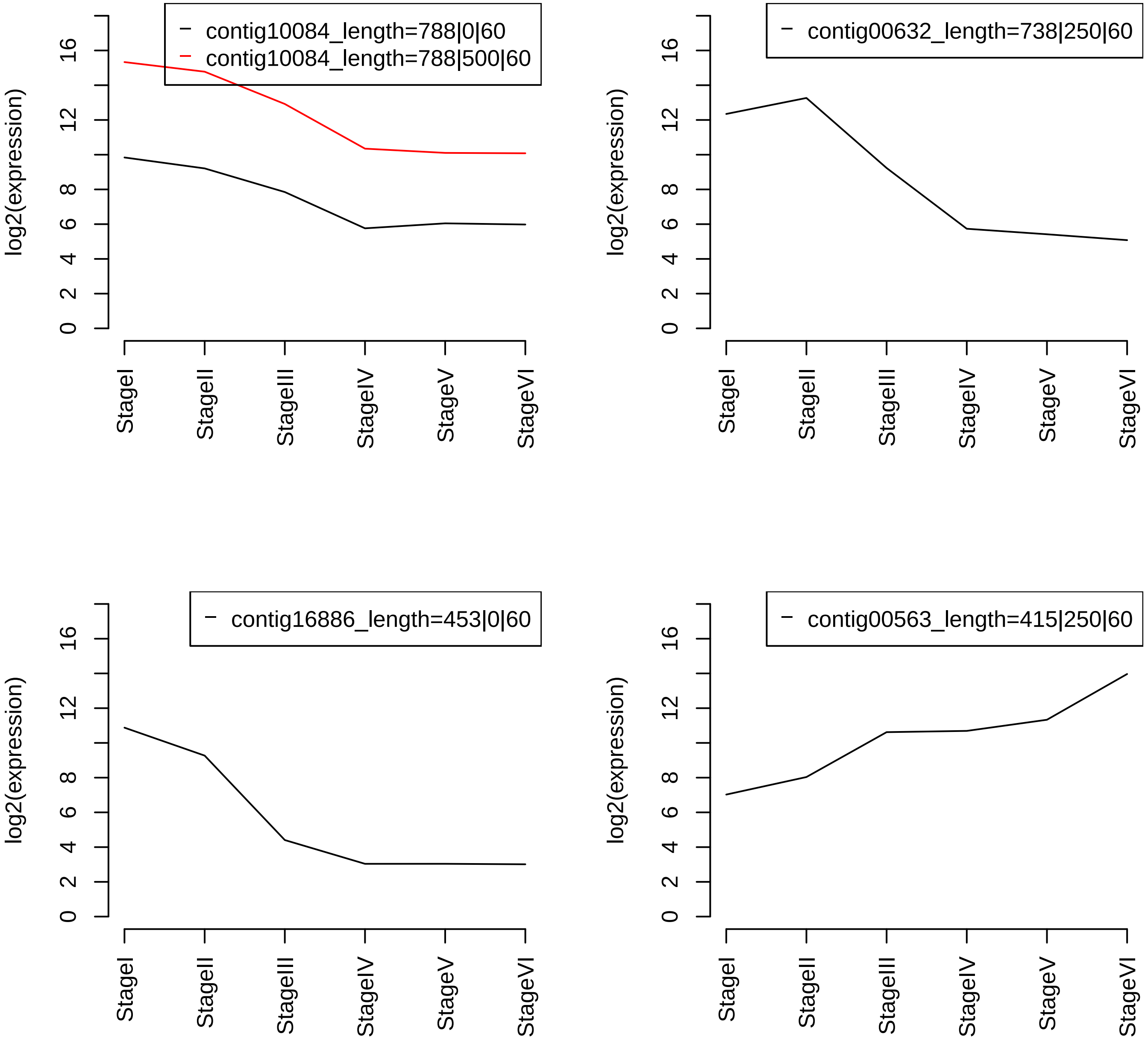
contig10084_length=788|0|60
contig10084_length=788|500|60
contig00632_length=738|250|60
contig16886_length=453|0|60
contig00563_length=415|250|60
log2(expression)
0
2
4
6
8
12
16
StageI
StageII
StageIII
StageIV
StageV
StageVI

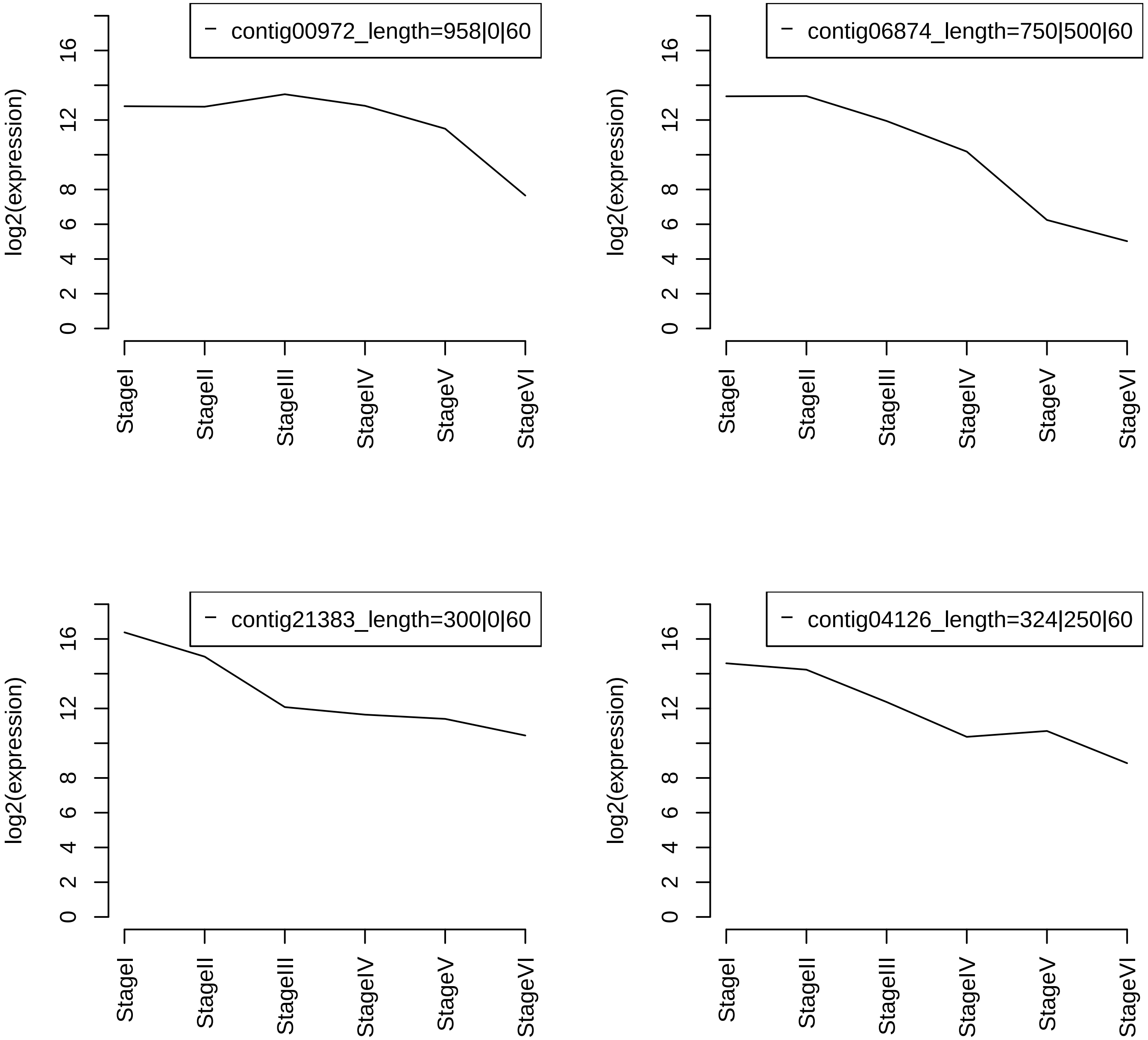
contig00972_length=958|0|60
contig06874_length=750|500|60
contig21383_length=300|0|60
contig04126_length=324|250|60
log2(expression)
0
2
4
6
8
12
16
StageI
StageII
StageIII
StageIV
StageV
StageVI

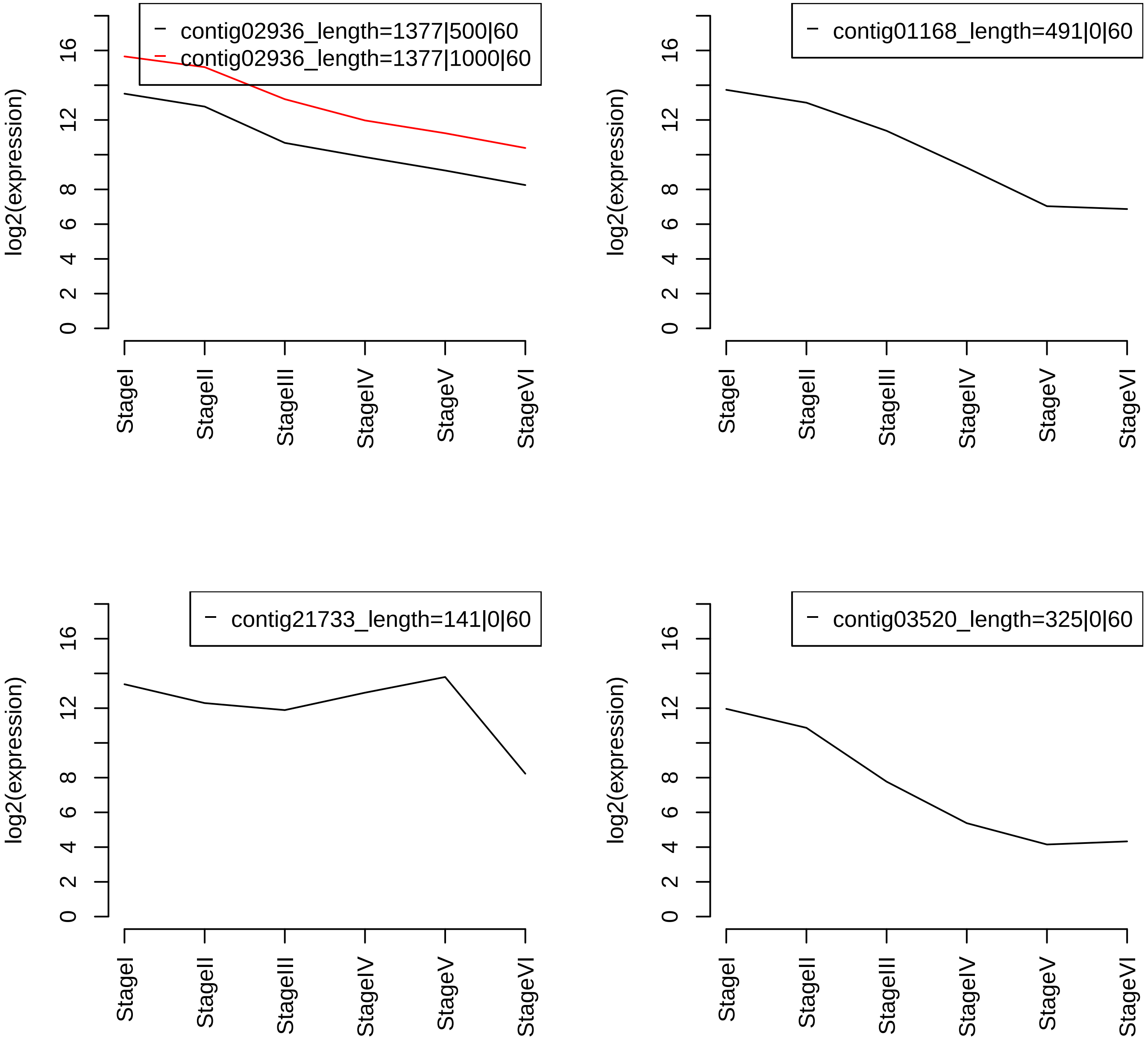

contig02936_length=1377|500|60
contig02936_length=1377|1000|60
contig01168_length=491|0|60
contig21733_length=141|0|60
contig03520_length=325|0|60
log2(expression)
0
2
4
6
8
12
16
StageI
StageII
StageIII
StageIV
StageV
StageVI

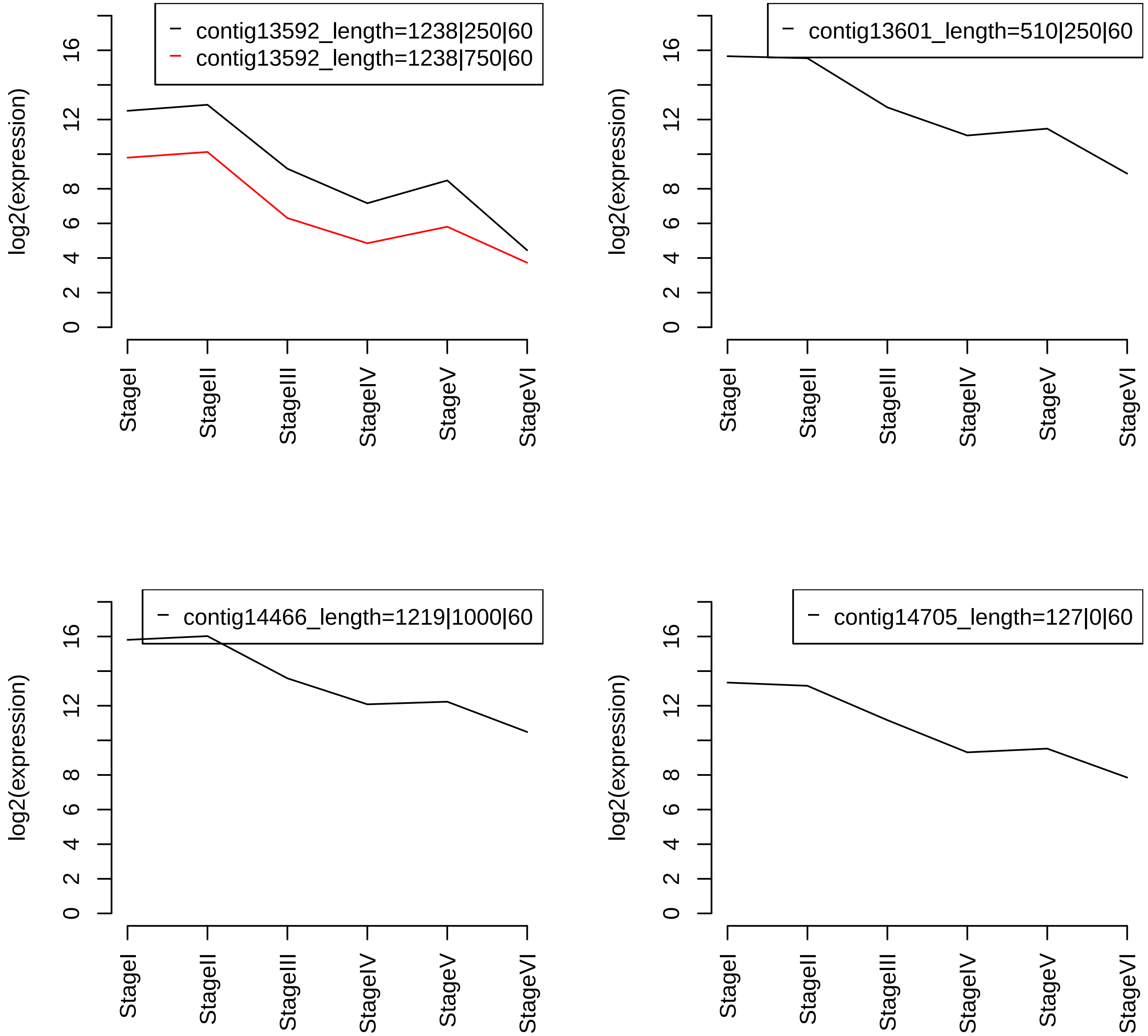
contig13592_length=1238|250|60
contig13592_length=1238|750|60
contig13601_length=510|250|60
contig14466_length=1219|1000|60
contig14705_length=127|0|60
log2(expression)
0
2
4
6
8
12
16
StageI
StageII
StageIII
StageIV
StageV
StageVI

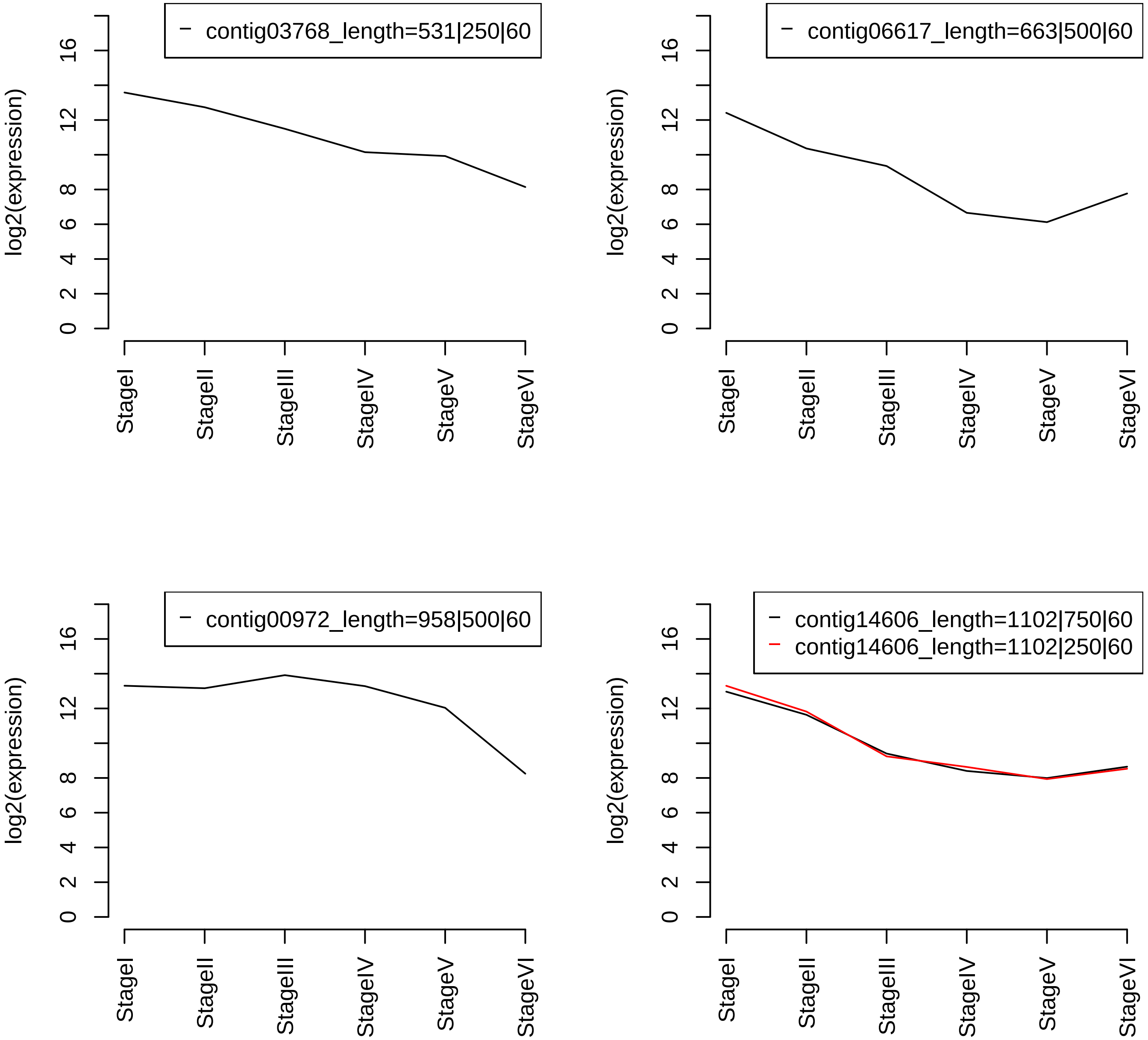
contig03768_length=531|250|60
contig06617_length=663|500|60
contig00972_length=958|500|60
contig14606_length=1102|750|60
contig14606_length=1102|250|60
log2(expression)
0
2
4
6
8
12
16
StageI
StageII
StageIII
StageIV
StageV
StageVI

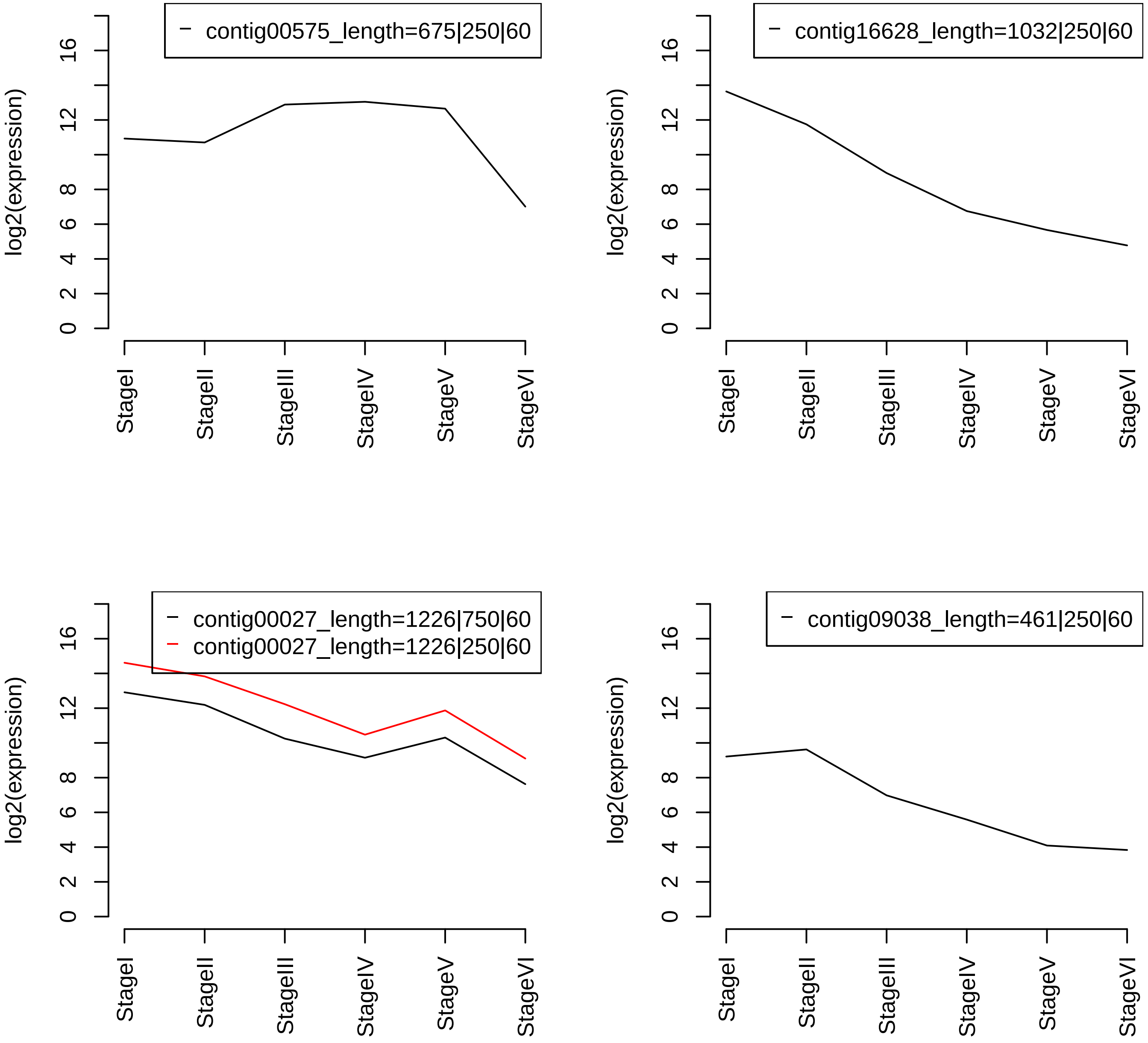

contig00575_length=675|250|60
contig16628_length=1032|250|60
contig00027_length=1226|750|60
contig00027_length=1226|250|60
contig09038_length=461|250|60
log2(expression)
0
2
4
6
8
12
16
StageI
StageII
StageIII
StageIV
StageV
StageVI

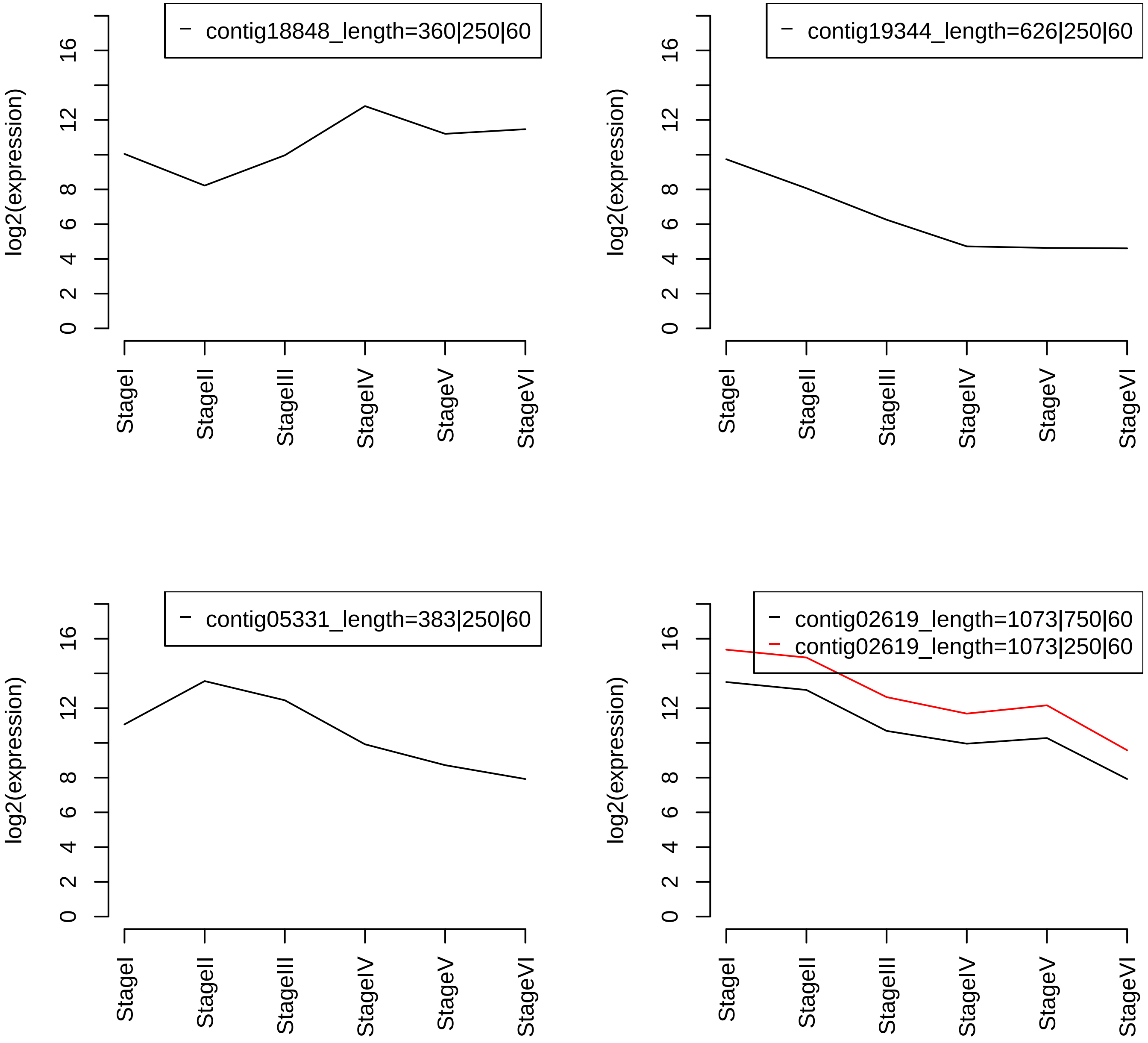

contig18848_length=360|250|60
contig19344_length=626|250|60
contig05331_length=383|250|60
contig02619_length=1073|750|60
contig02619_length=1073|250|60
log2(expression)
0
2
4
6
8
12
16
StageI
StageII
StageIII
StageIV
StageV
StageVI

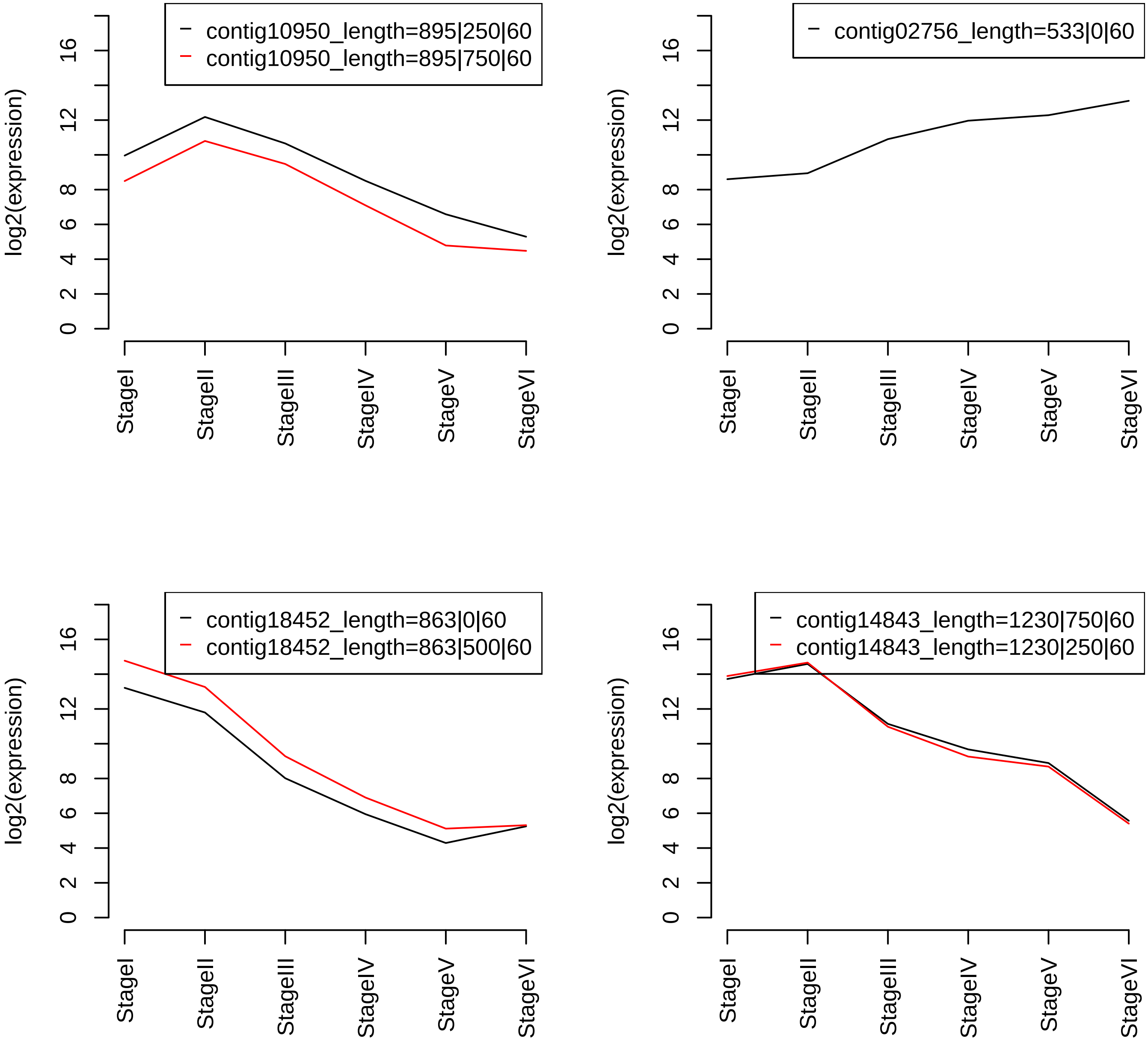
contig10950_length=895|250|60
contig10950_length=895|750|60
contig02756_length=533|0|60
contig18452_length=863|0|60
contig18452_length=863|500|60
contig14843_length=1230|750|60
contig14843_length=1230|250|60
log2(expression)
0
2
4
6
8
12
16
StageI
StageII
StageIII
StageIV
StageV
StageVI

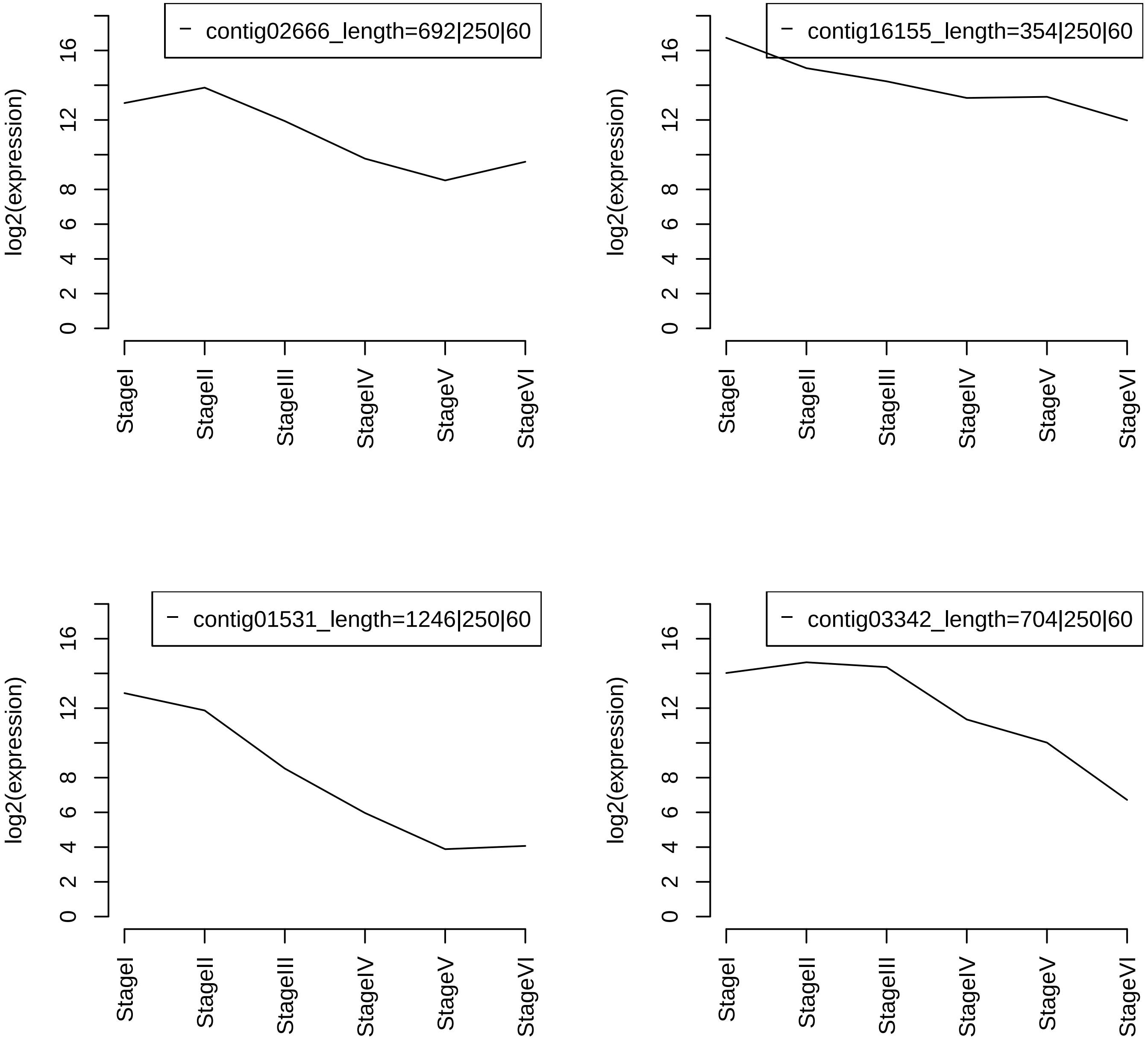
contig02666_length=692|250|60
contig16155_length=354|250|60
contig01531_length=1246|250|60
contig03342_length=704|250|60
log2(expression)
0
2
4
6
8
12
16
StageI
StageII
StageIII
StageIV
StageV
StageVI

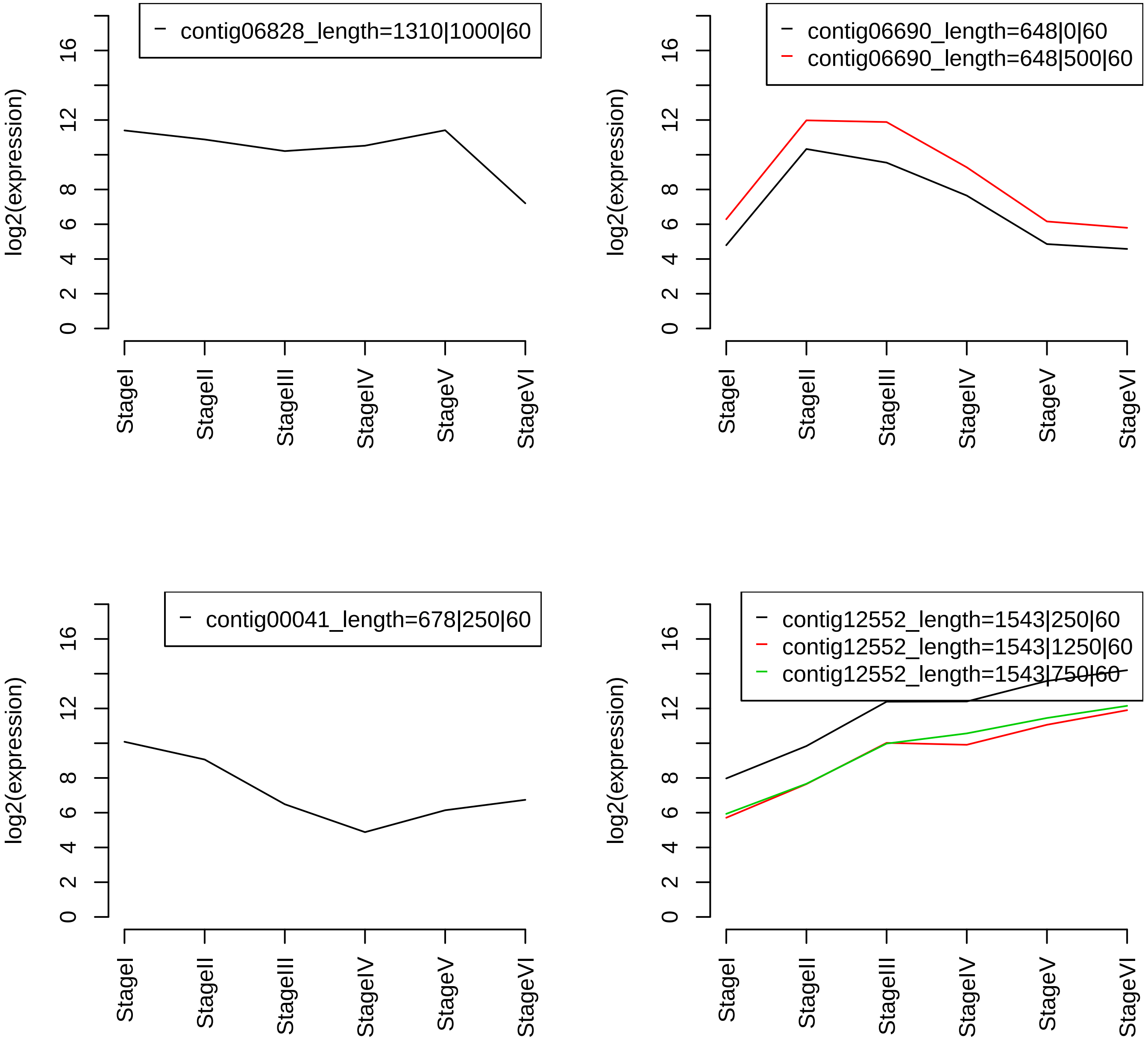

contig06828_length=1310|1000|60
contig06690_length=648|0|60
contig06690_length=648|500|60
contig00041_length=678|250|60
contig12552_length=1543|250|60
contig12552_length=1543|1250|60
contig12552_length=1543|750|60
log2(expression)
0
2
4
6
8
12
16
StageI
StageII
StageIII
StageIV
StageV
StageVI

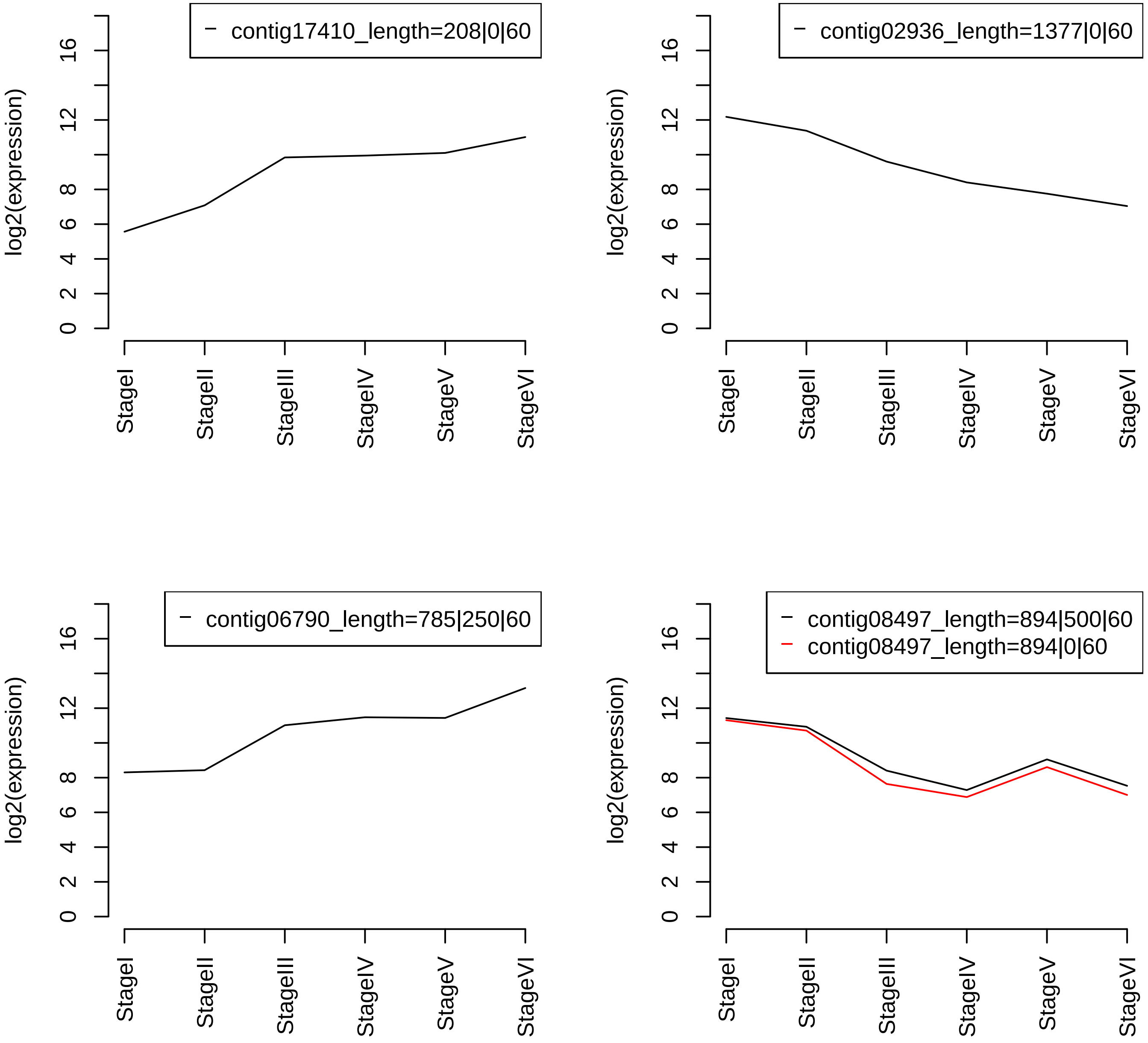
contig17410_length=208|0|60
contig02936_length=1377|0|60
contig06790_length=785|250|60
contig08497_length=894|500|60
contig08497_length=894|0|60
log2(expression)
0
2
4
6
8
12
16
StageI
StageII
StageIII
StageIV
StageV
StageVI

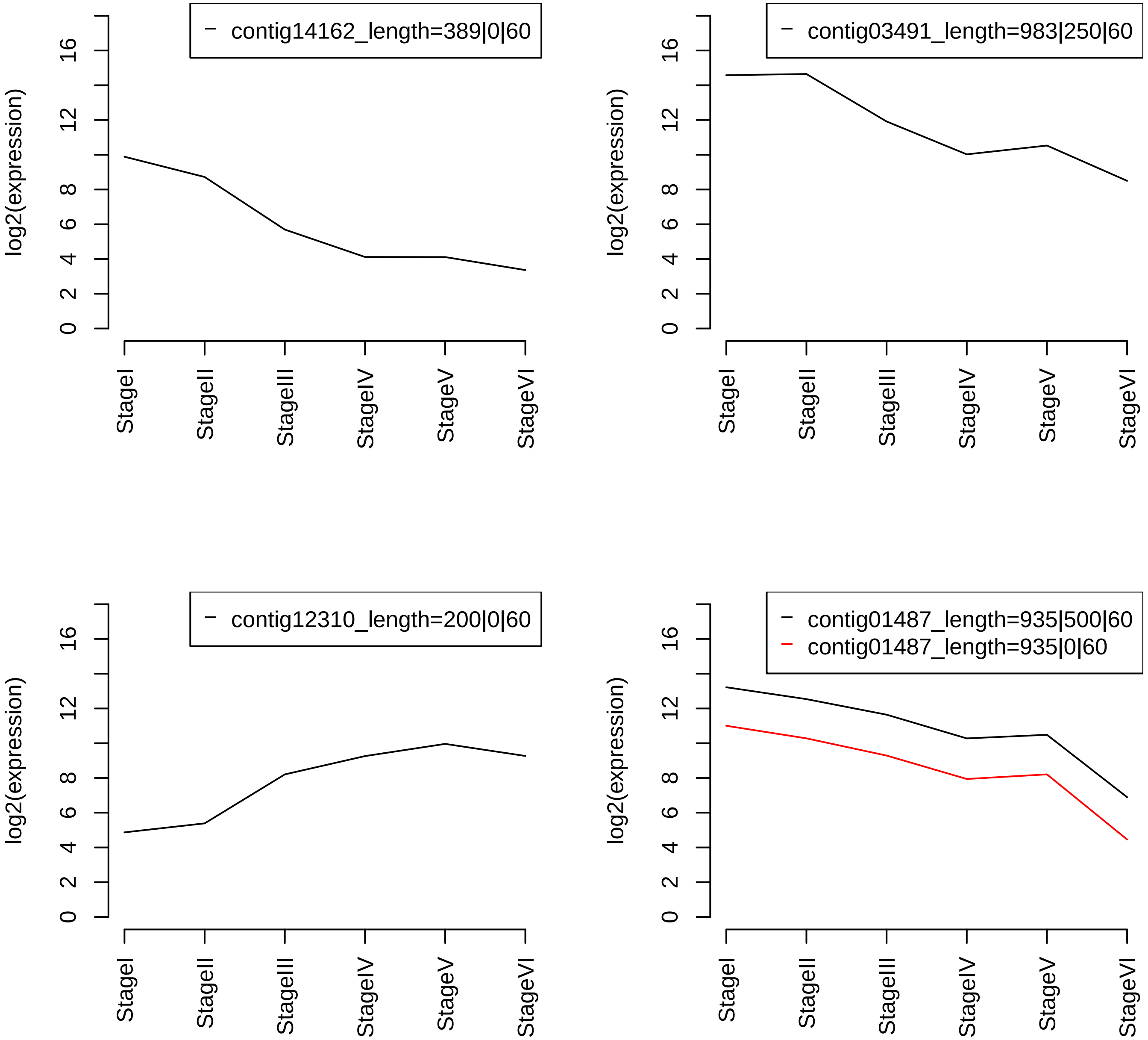
contig14162_length=389|0|60
contig03491_length=983|250|60
contig12310_length=200|0|60
contig01487_length=935|500|60
contig01487_length=935|0|60
log2(expression)
0
2
4
6
8
12
16
StageI
StageII
StageIII
StageIV
StageV
StageVI

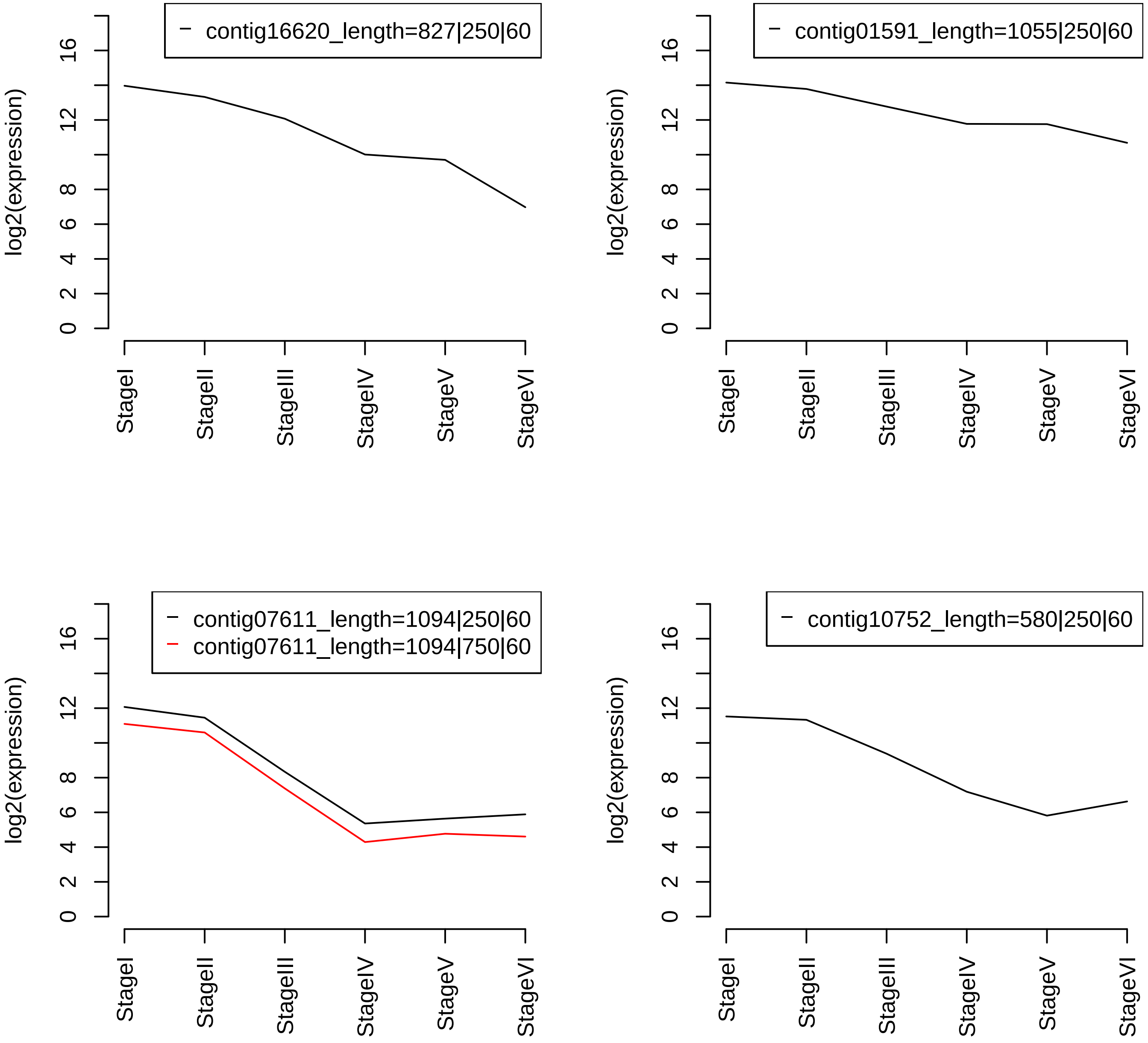

contig16620_length=827|250|60
contig01591_length=1055|250|60
contig07611_length=1094|250|60
contig07611_length=1094|750|60
contig10752_length=580|250|60
log2(expression)
0
2
4
6
8
12
16
StageI
StageII
StageIII
StageIV
StageV
StageVI

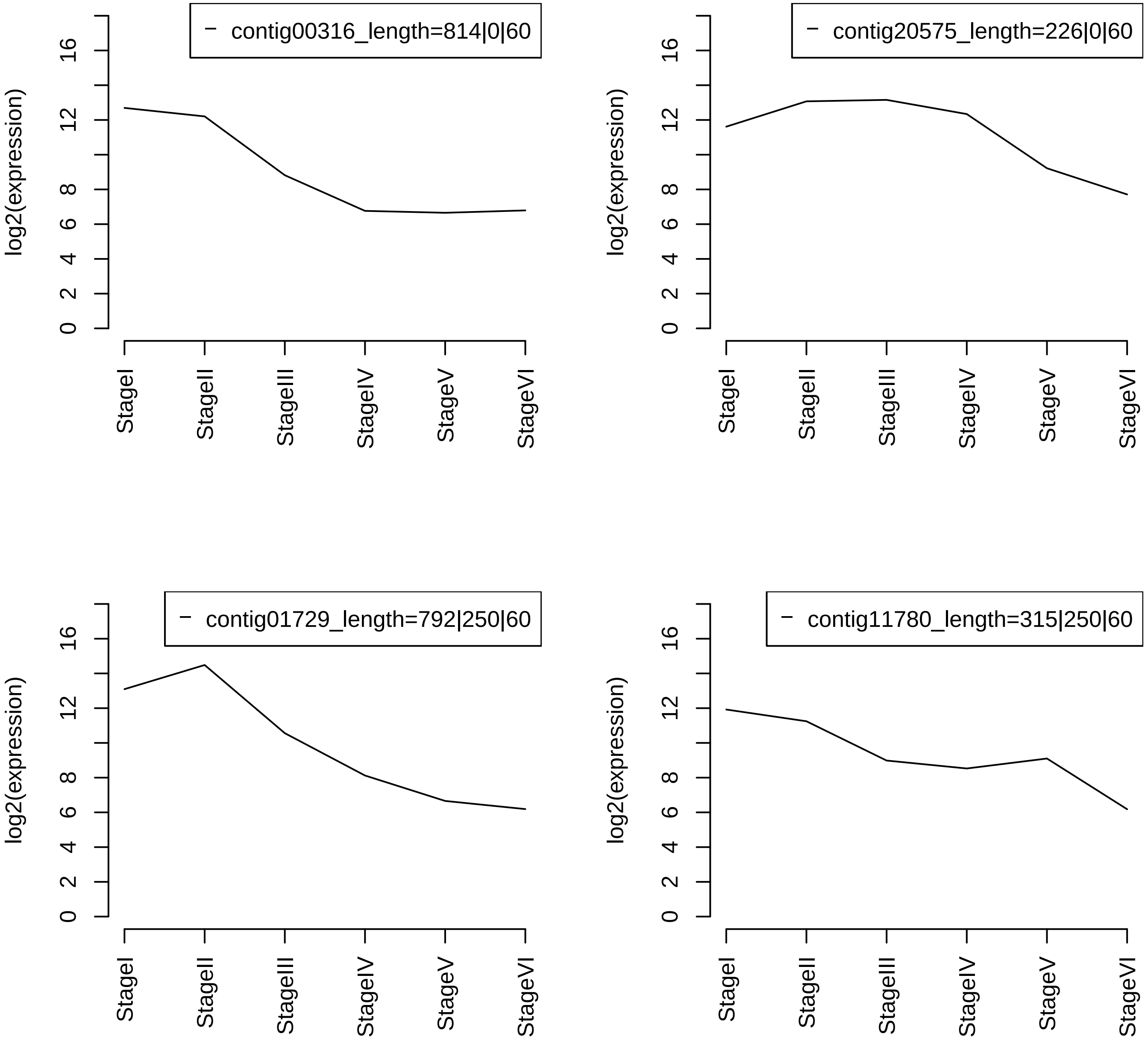
contig00316_length=814|0|60
contig20575_length=226|0|60
contig01729_length=792|250|60
contig11780_length=315|250|60
log2(expression)
0
2
4
6
8
12
16
StageI
StageII
StageIII
StageIV
StageV
StageVI

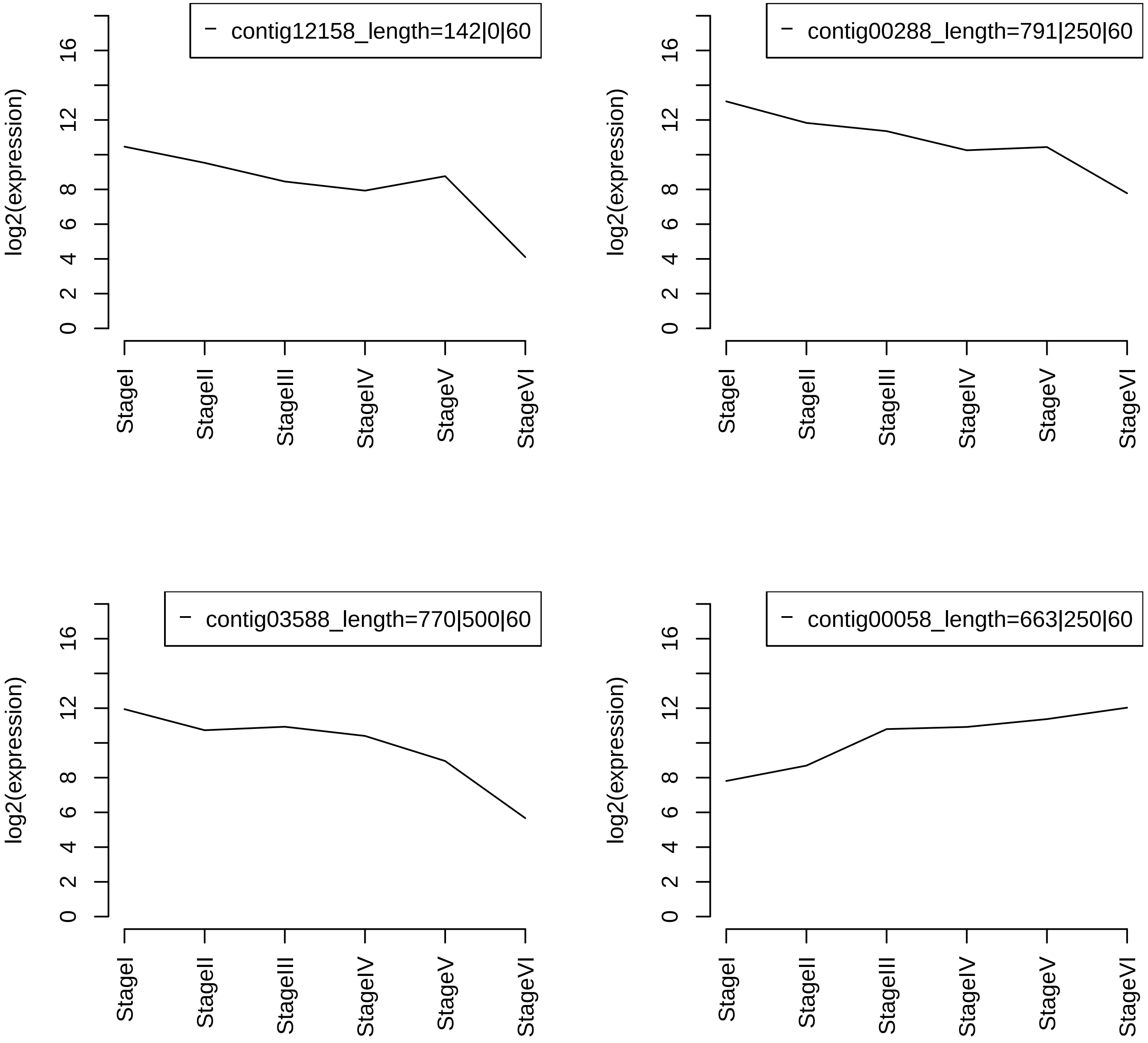

contig12158_length=142|0|60
contig00288_length=791|250|60
contig03588_length=770|500|60
contig00058_length=663|250|60
log2(expression)
0
2
4
6
8
12
16
StageI
StageII
StageIII
StageIV
StageV
StageVI

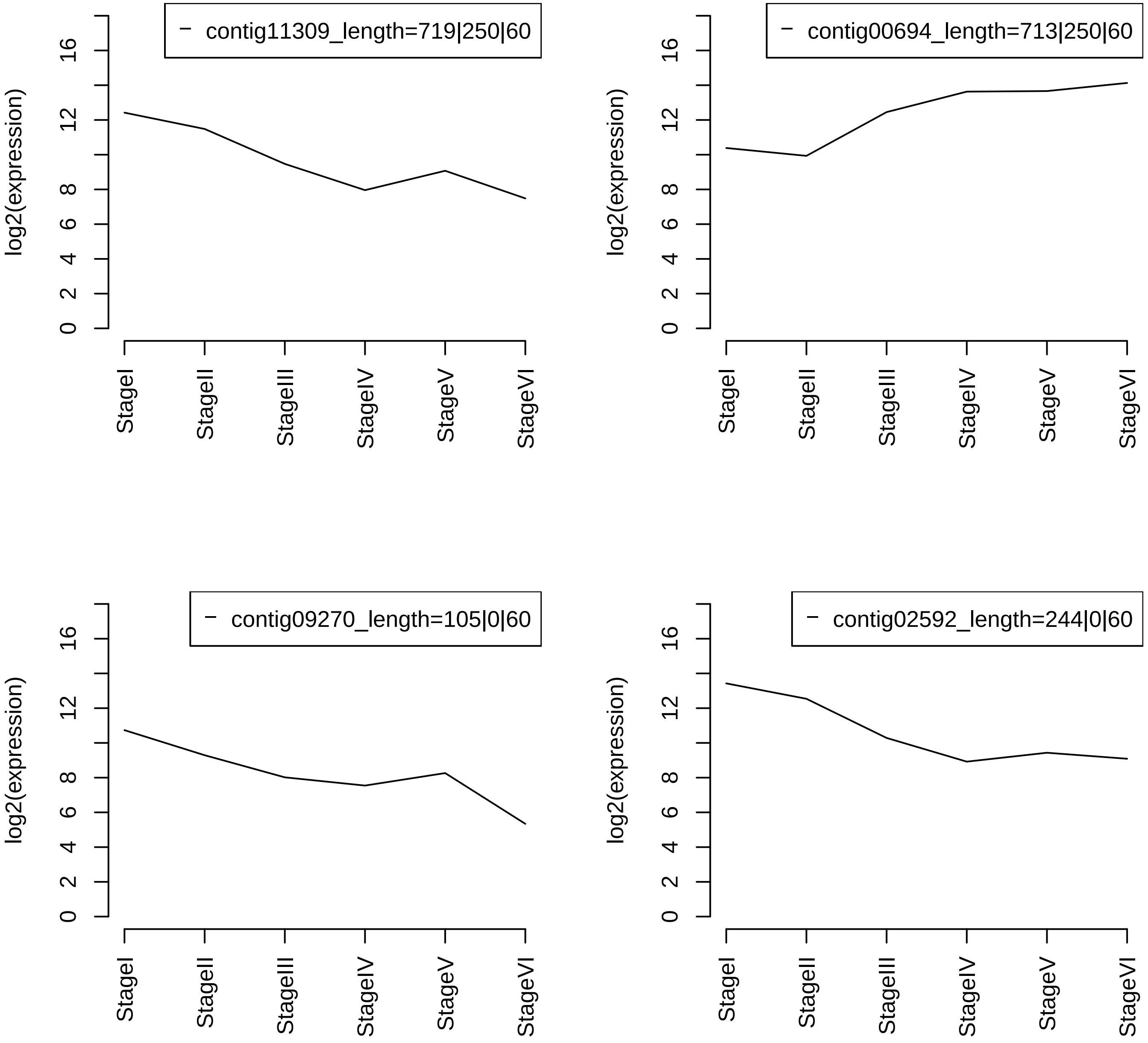
contig11309_length=719|250|60
contig00694_length=713|250|60
contig09270_length=105|0|60
contig02592_length=244|0|60
log2(expression)
0
2
4
6
8
12
16
StageI
StageII
StageIII
StageIV
StageV
StageVI

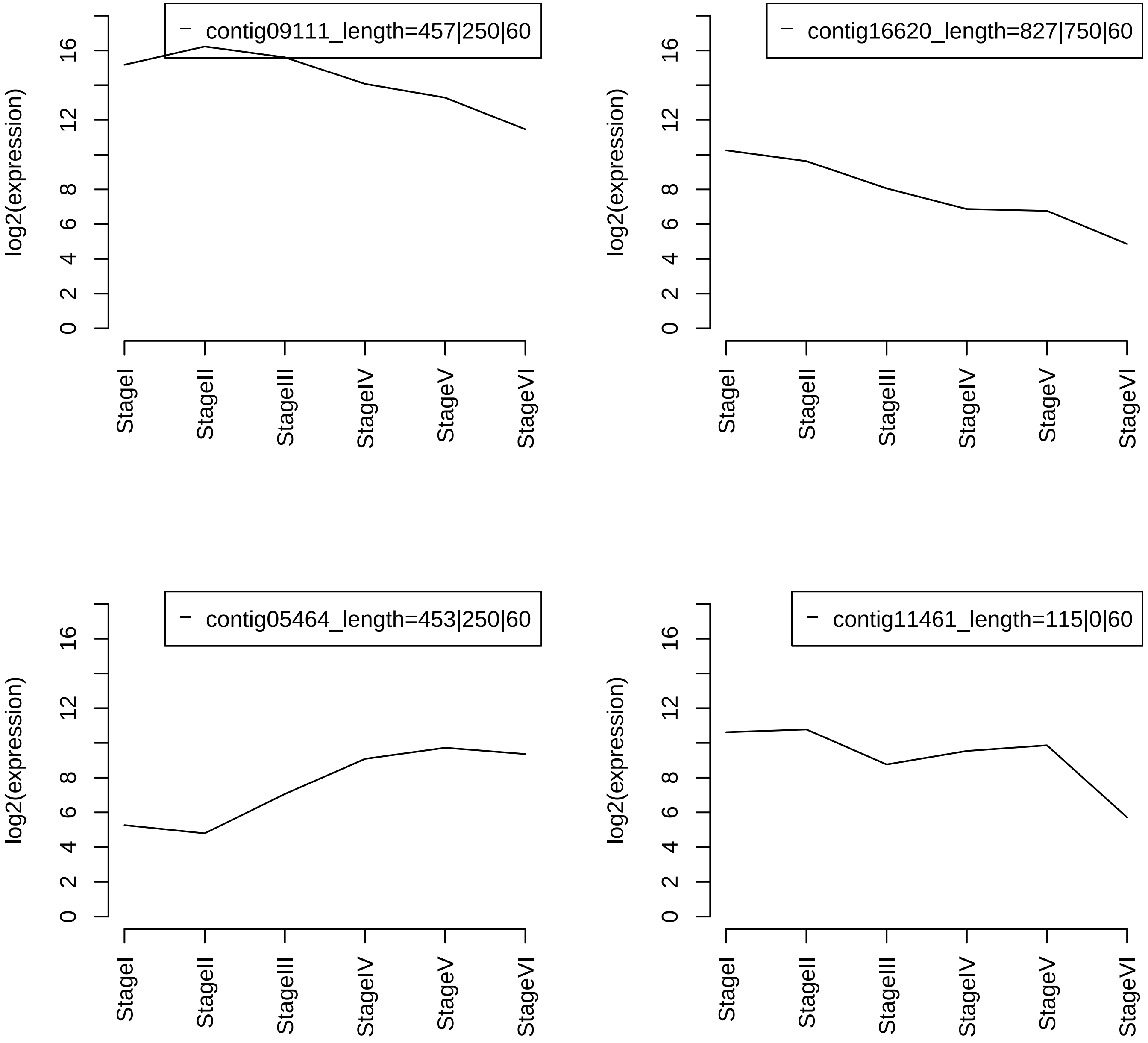
contig09111_length=457|250|60
contig16620_length=827|750|60
contig05464_length=453|250|60
contig11461_length=115|0|60
log2(expression)
0
2
4
6
8
12
16
StageI
StageII
StageIII
StageIV
StageV
StageVI

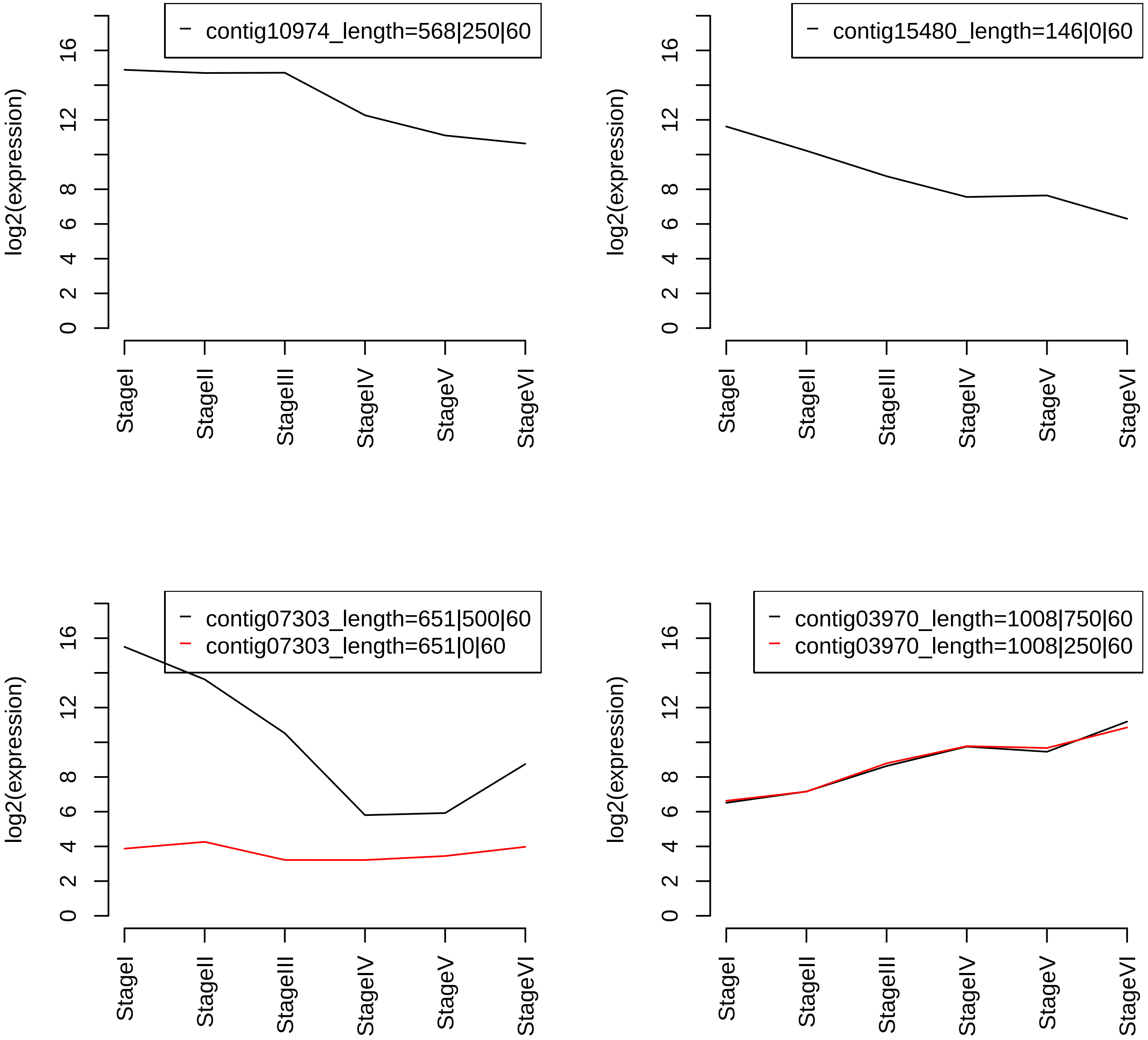
contig10974_length=568|250|60
contig15480_length=146|0|60
contig07303_length=651|500|60
contig07303_length=651|0|60
contig03970_length=1008|750|60
contig03970_length=1008|250|60
log2(expression)
0
2
4
6
8
12
16
StageI
StageII
StageIII
StageIV
StageV
StageVI

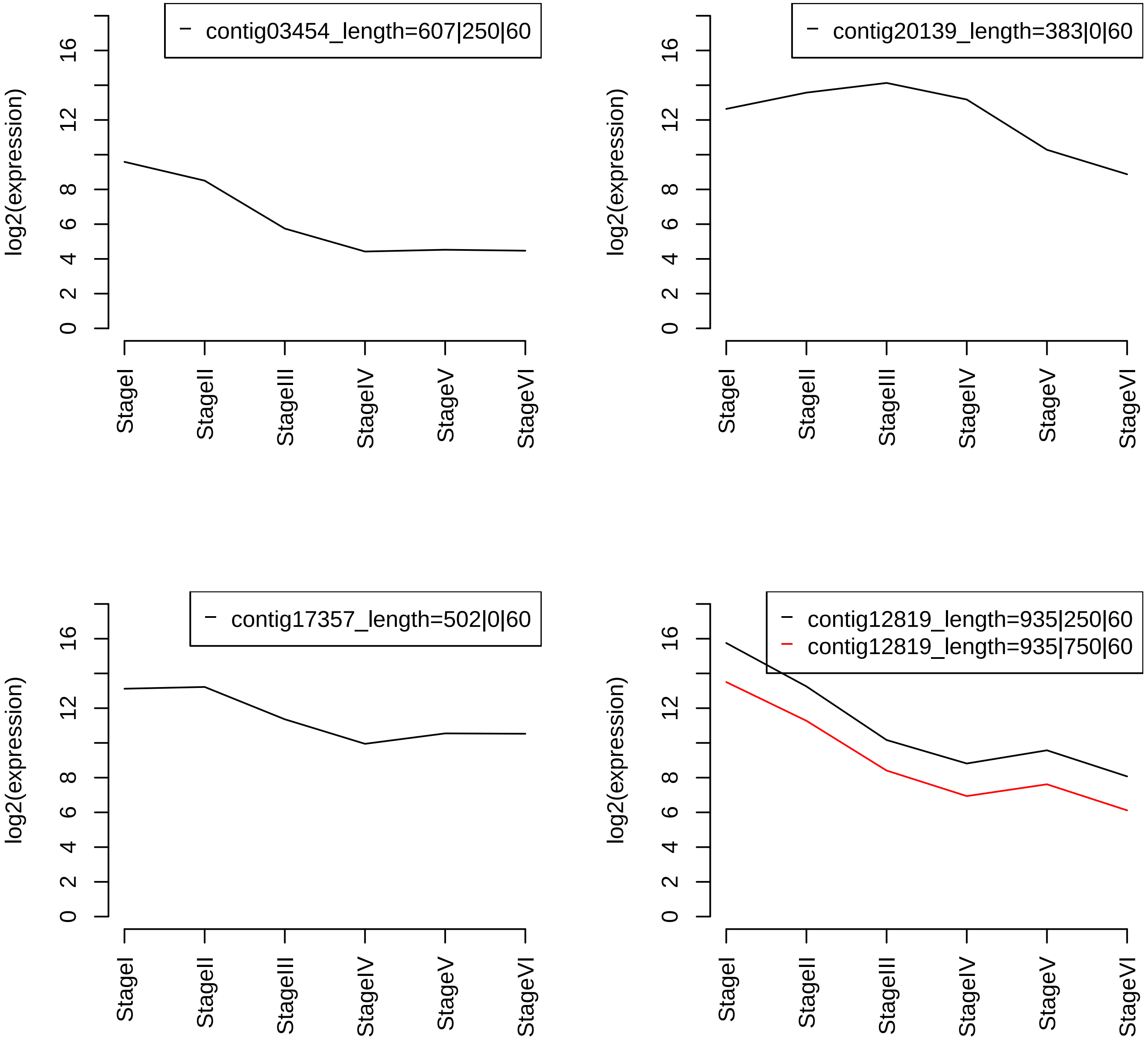

contig03454_length=607|250|60
contig20139_length=383|0|60
contig17357_length=502|0|60
contig12819_length=935|250|60
contig12819_length=935|750|60
log2(expression)
0
2
4
6
8
12
16
StageI
StageII
StageIII
StageIV
StageV
StageVI

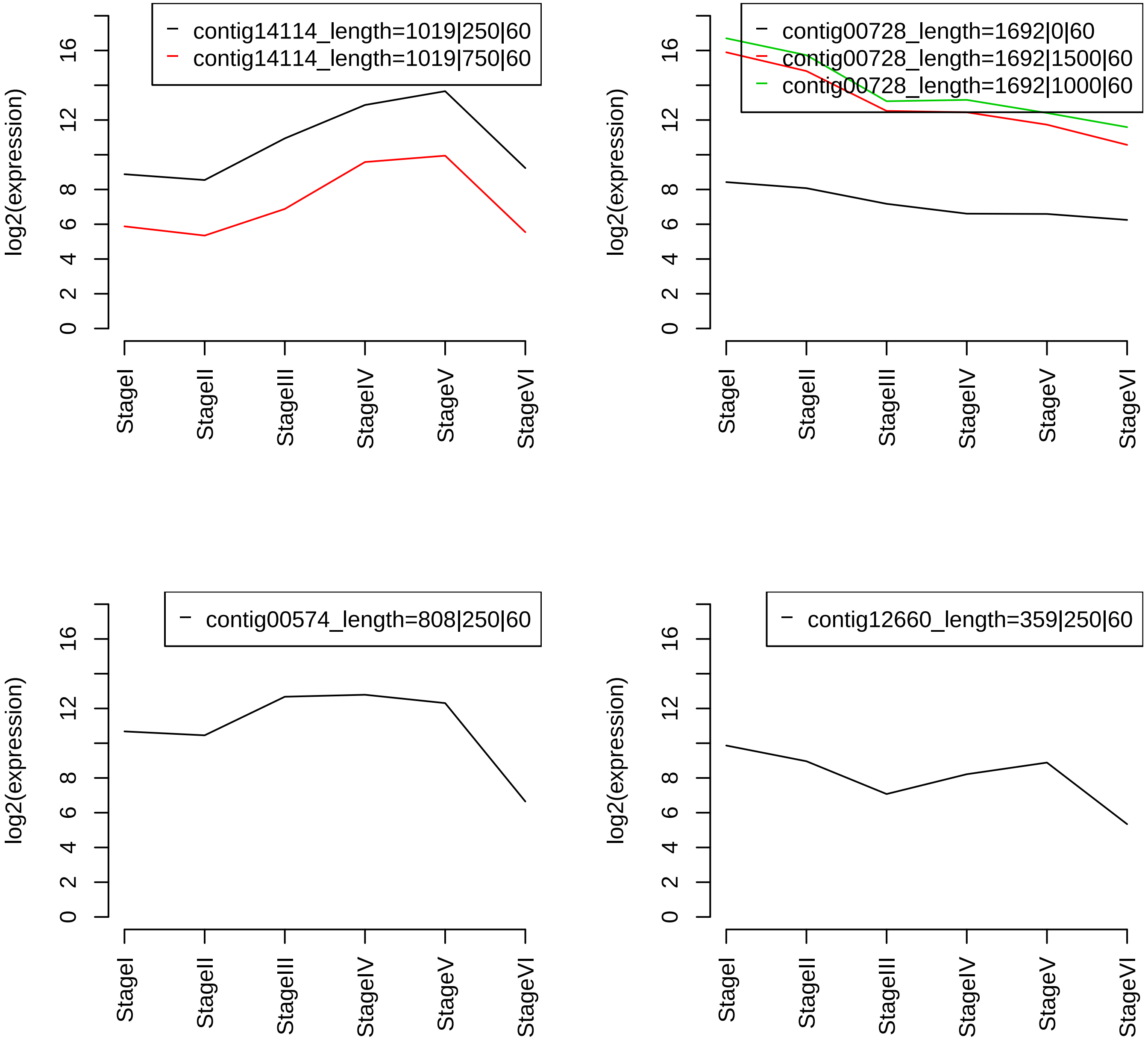

contig14114_length=1019|250|60
contig14114_length=1019|750|60
log2(expression)
0
2
4
6
8
12
16
StageI
StageII
StageIII
StageIV
StageV
StageVI
contig00728_length=1692|0|60
contig00728_length=1692|1500|60
contig00728_length=1692|1000|60
log2(expression)
contig00574_length=808|250|60
log2(expression)
contig12660_length=359|250|60
log2(expression)

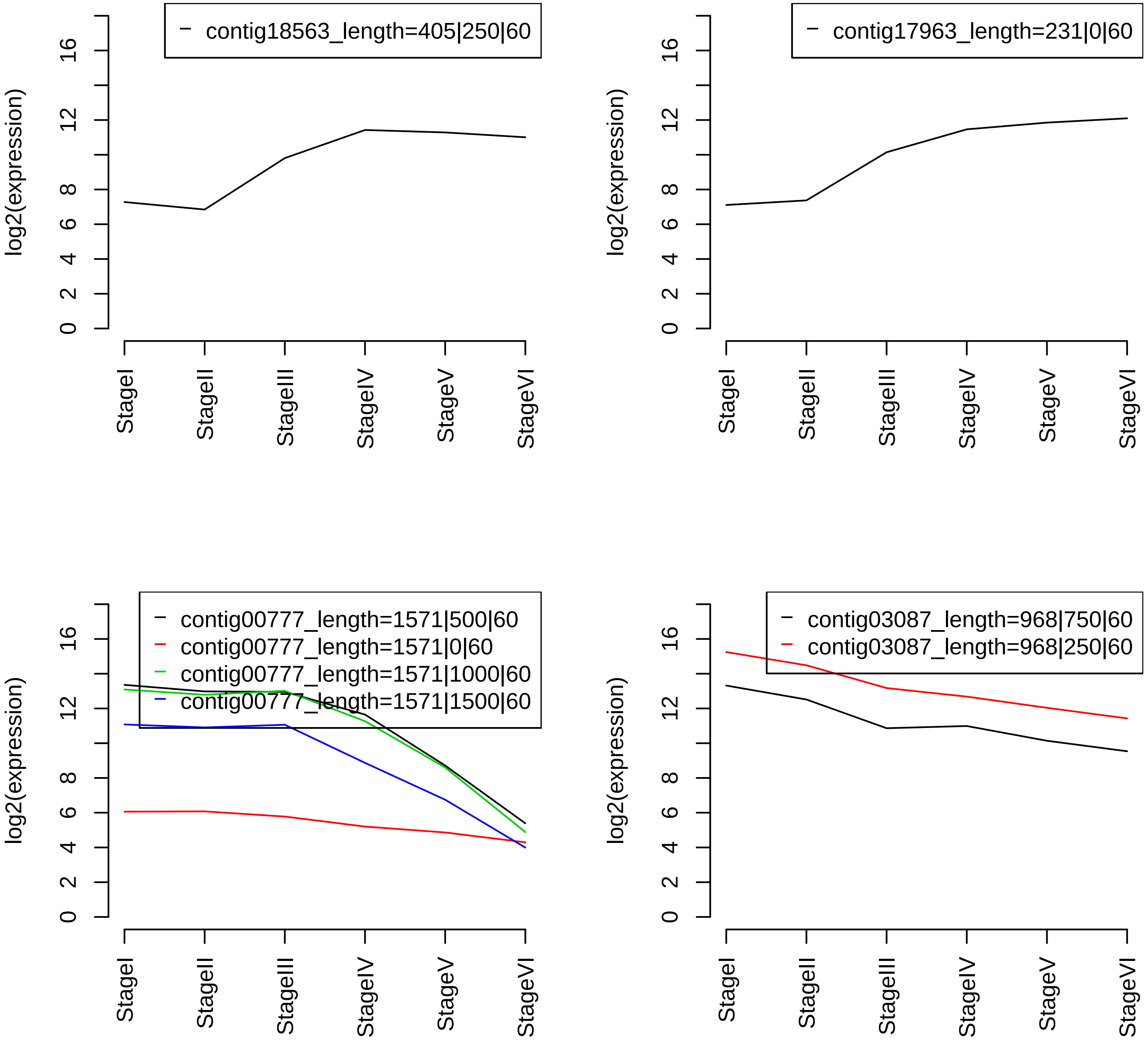

contig18563_length=405|250|60
contig17963_length=231|0|60
contig00777_length=1571|500|60
contig00777_length=1571|0|60
contig00777_length=1571|1000|60
contig00777_length=1571|1500|60
contig03087_length=968|750|60
contig03087_length=968|250|60
log2(expression)
StageI
StageII
StageIII
StageIV
StageV
StageVI

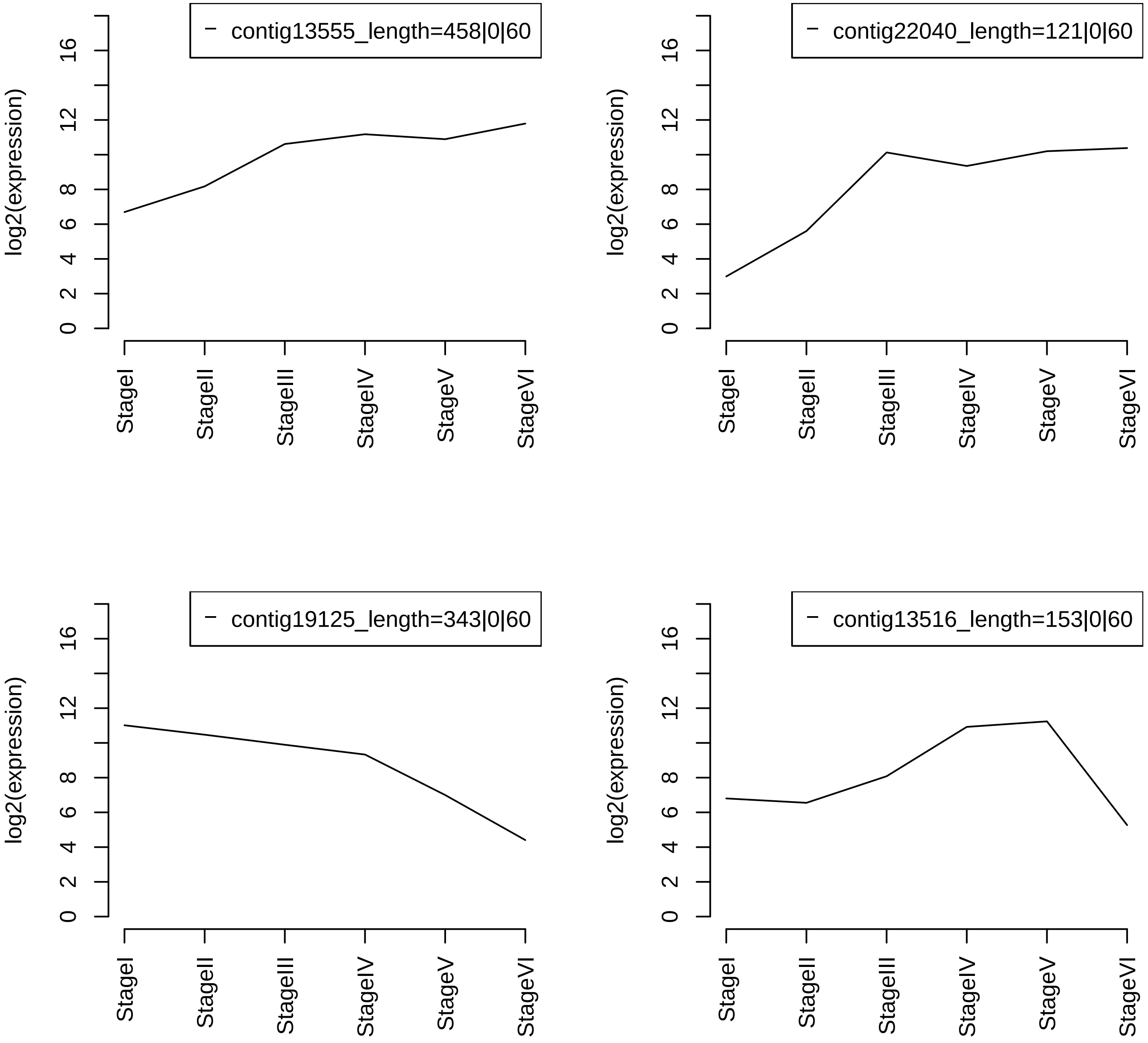

contig13555_length=458|0|60
contig22040_length=121|0|60
contig19125_length=343|0|60
contig13516_length=153|0|60
log2(expression)
0
2
4
6
8
12
16
StageI
StageII
StageIII
StageIV
StageV
StageVI

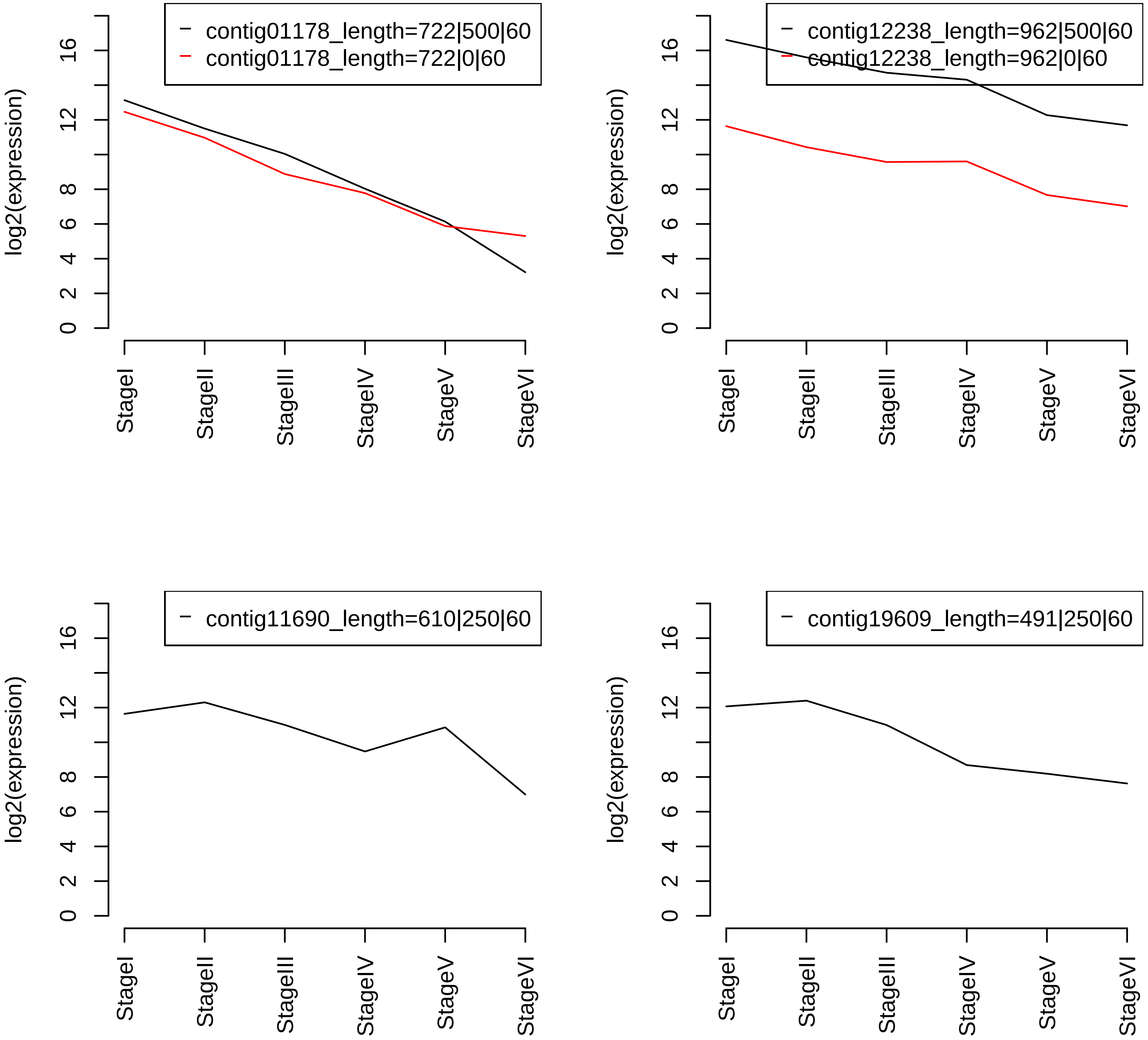
contig01178_length=722|500|60
contig01178_length=722|0|60
contig12238_length=962|500|60
contig12238_length=962|0|60
contig11690_length=610|250|60
contig19609_length=491|250|60
log2(expression)
0 2 4 6 8 12 16
StageI StageII StageIII StageIV StageV StageVI

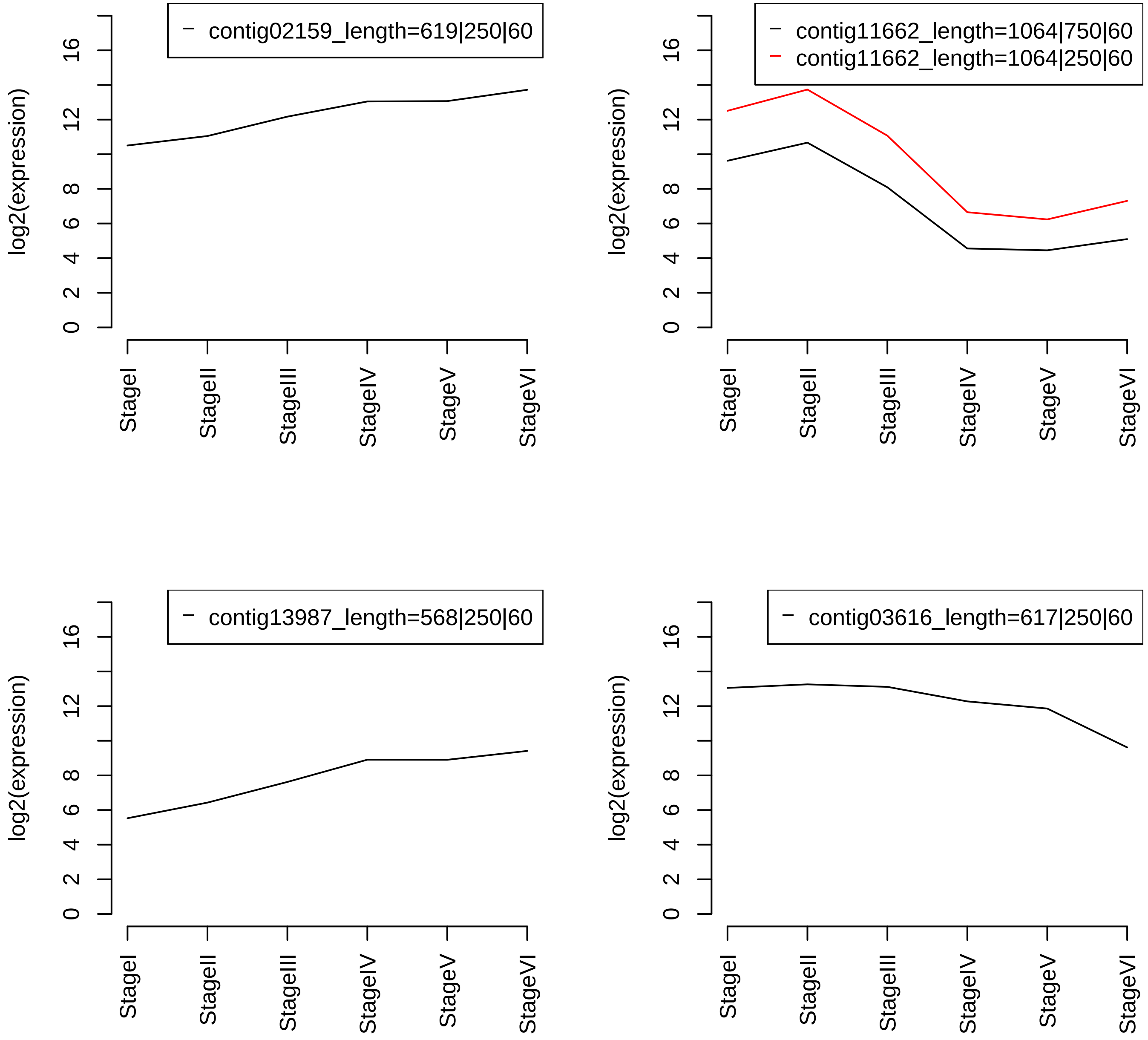

contig02159_length=619|250|60
contig11662_length=1064|750|60
contig11662_length=1064|250|60
contig13987_length=568|250|60
contig03616_length=617|250|60
log2(expression)
0
2
4
6
8
12
16
StageI
StageII
StageIII
StageIV
StageV
StageVI

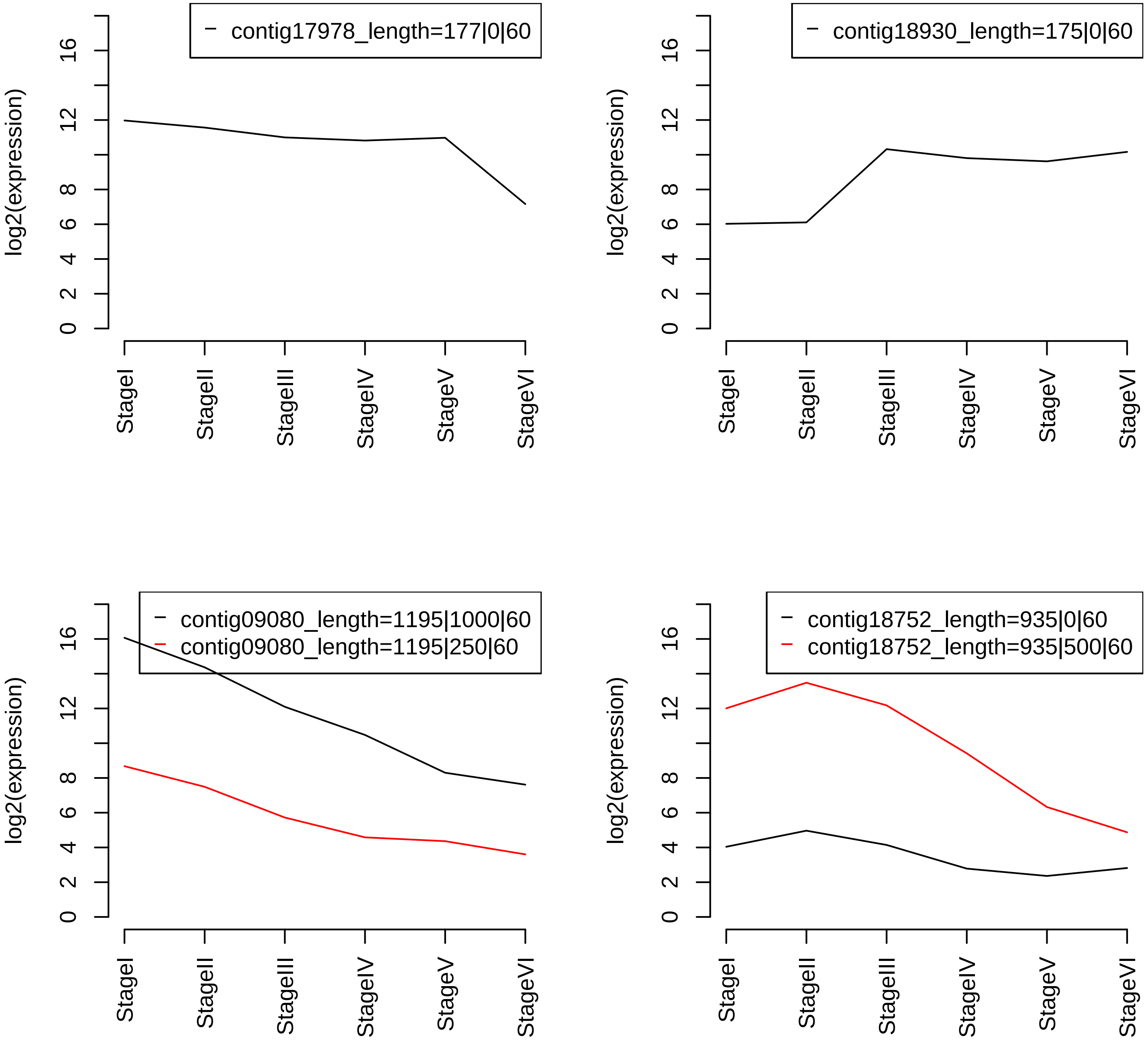

contig17978_length=177|0|60
contig18930_length=175|0|60
contig09080_length=1195|1000|60
contig09080_length=1195|250|60
contig18752_length=935|0|60
contig18752_length=935|500|60
log2(expression)
0 2 4 6 8 12 16
StageI
StageII
StageIII
StageIV
StageV
StageVI

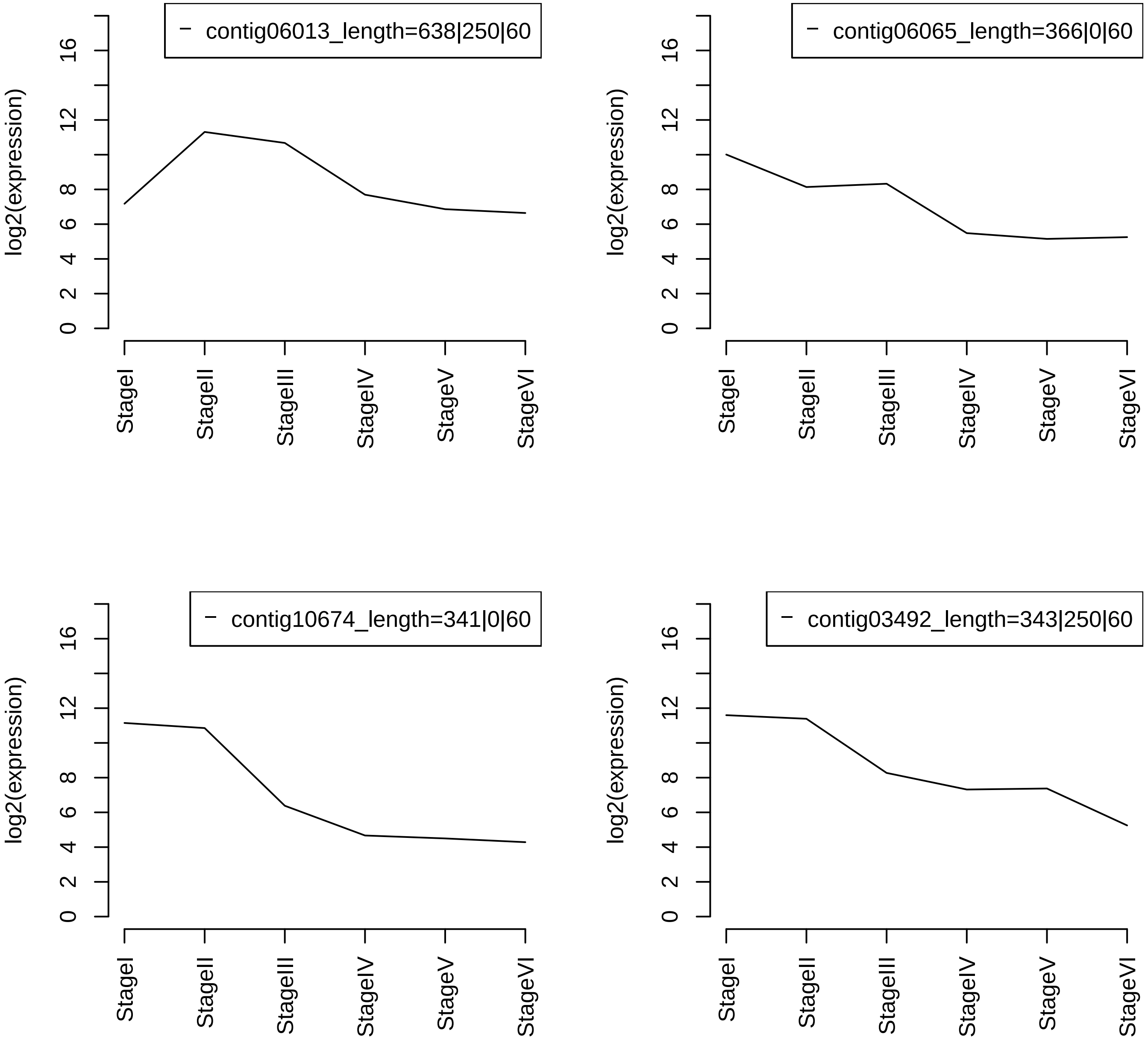
contig06013_length=638|250|60
contig06065_length=366|0|60
contig10674_length=341|0|60
contig03492_length=343|250|60
log2(expression)
0
2
4
6
8
12
16
StageI
StageII
StageIII
StageIV
StageV
StageVI

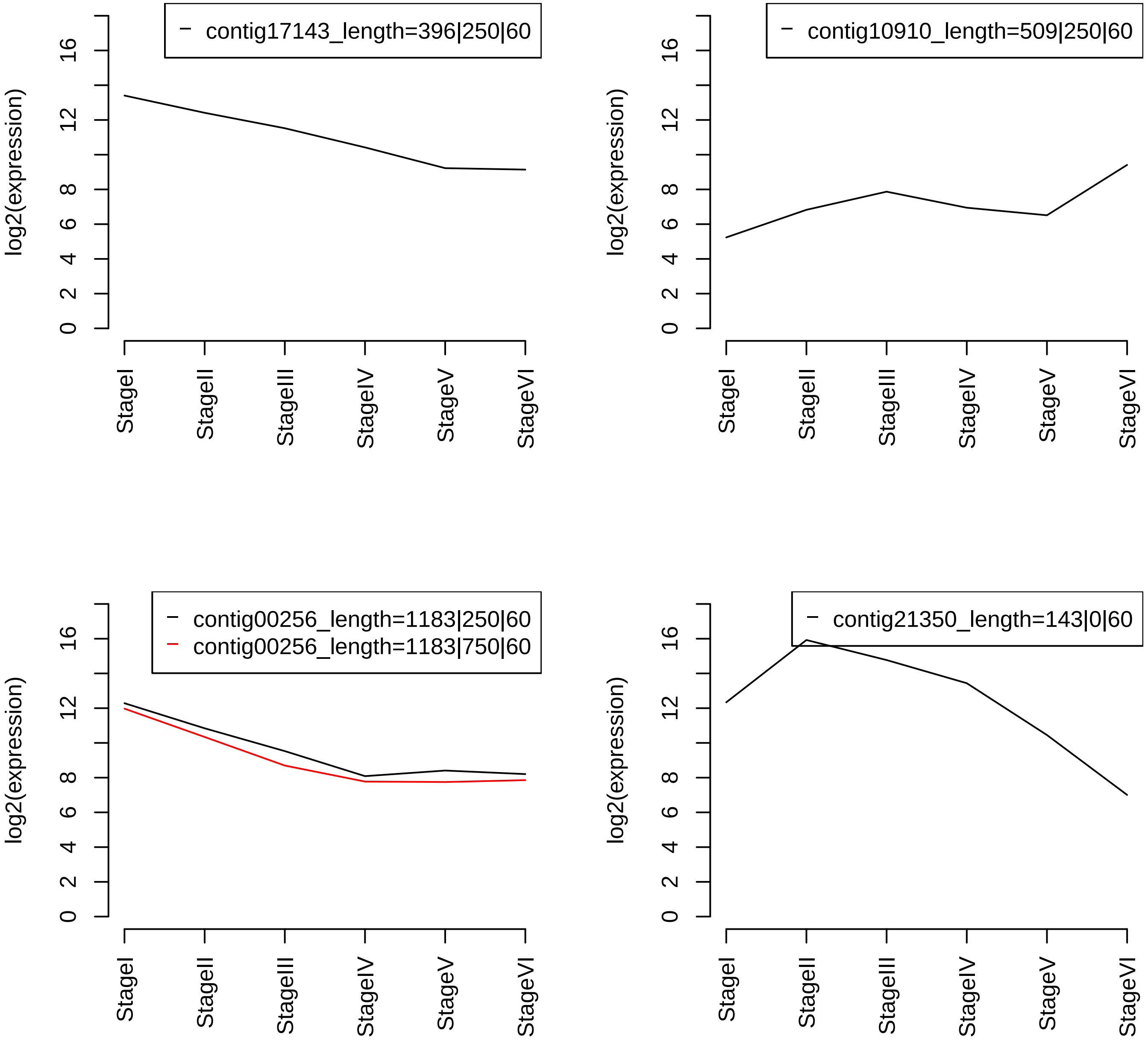

contig17143_length=396|250|60
contig10910_length=509|250|60
contig00256_length=1183|250|60
contig00256_length=1183|750|60
contig21350_length=143|0|60
log2(expression)
0
2
4
6
8
12
16
StageI
StageII
StageIII
StageIV
StageV
StageVI

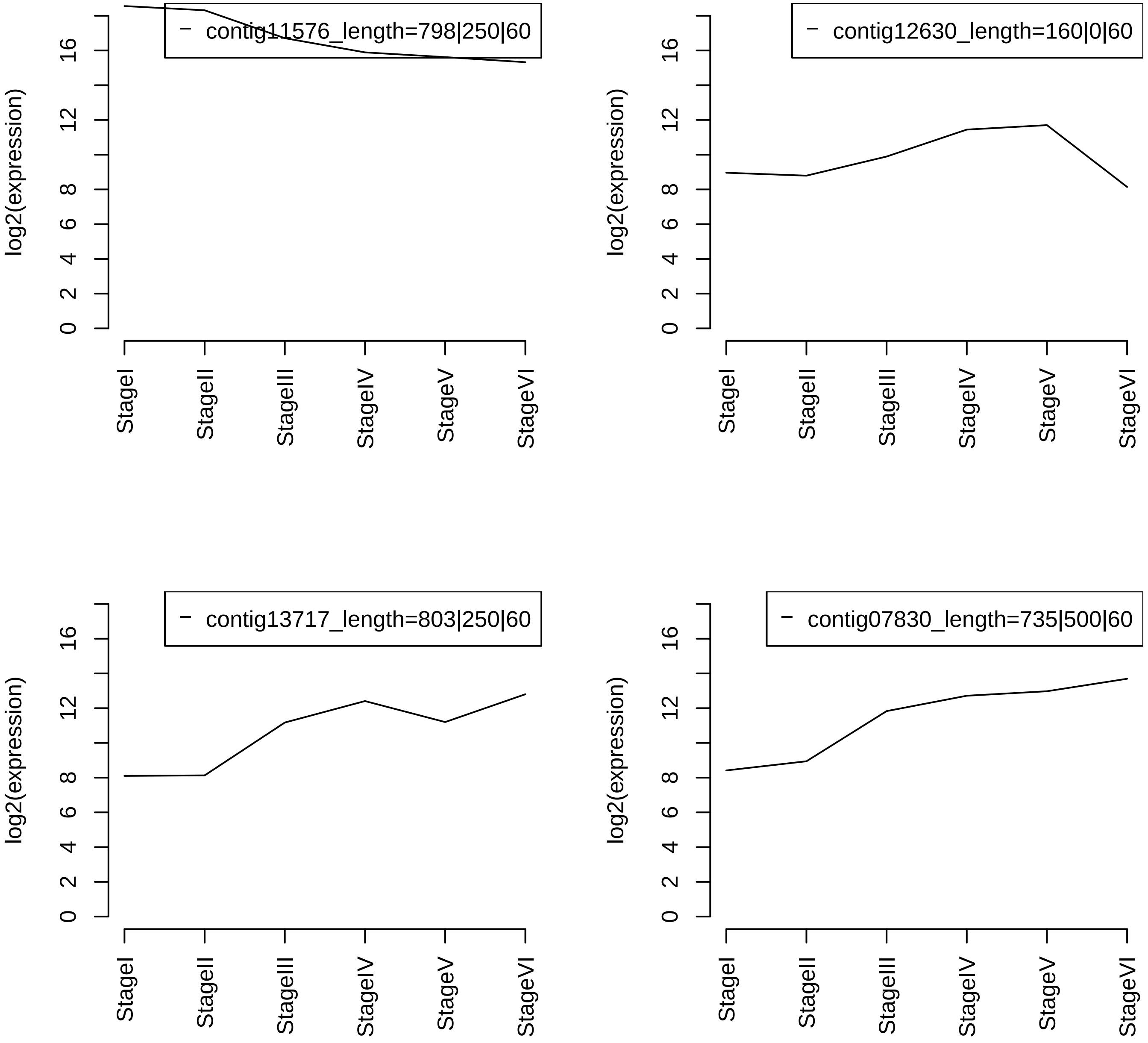

contig11576_length=798|250|60
contig12630_length=160|0|60
contig13717_length=803|250|60
contig07830_length=735|500|60
log2(expression)
0
2
4
6
8
12
16
StageI
StageII
StageIII
StageIV
StageV
StageVI

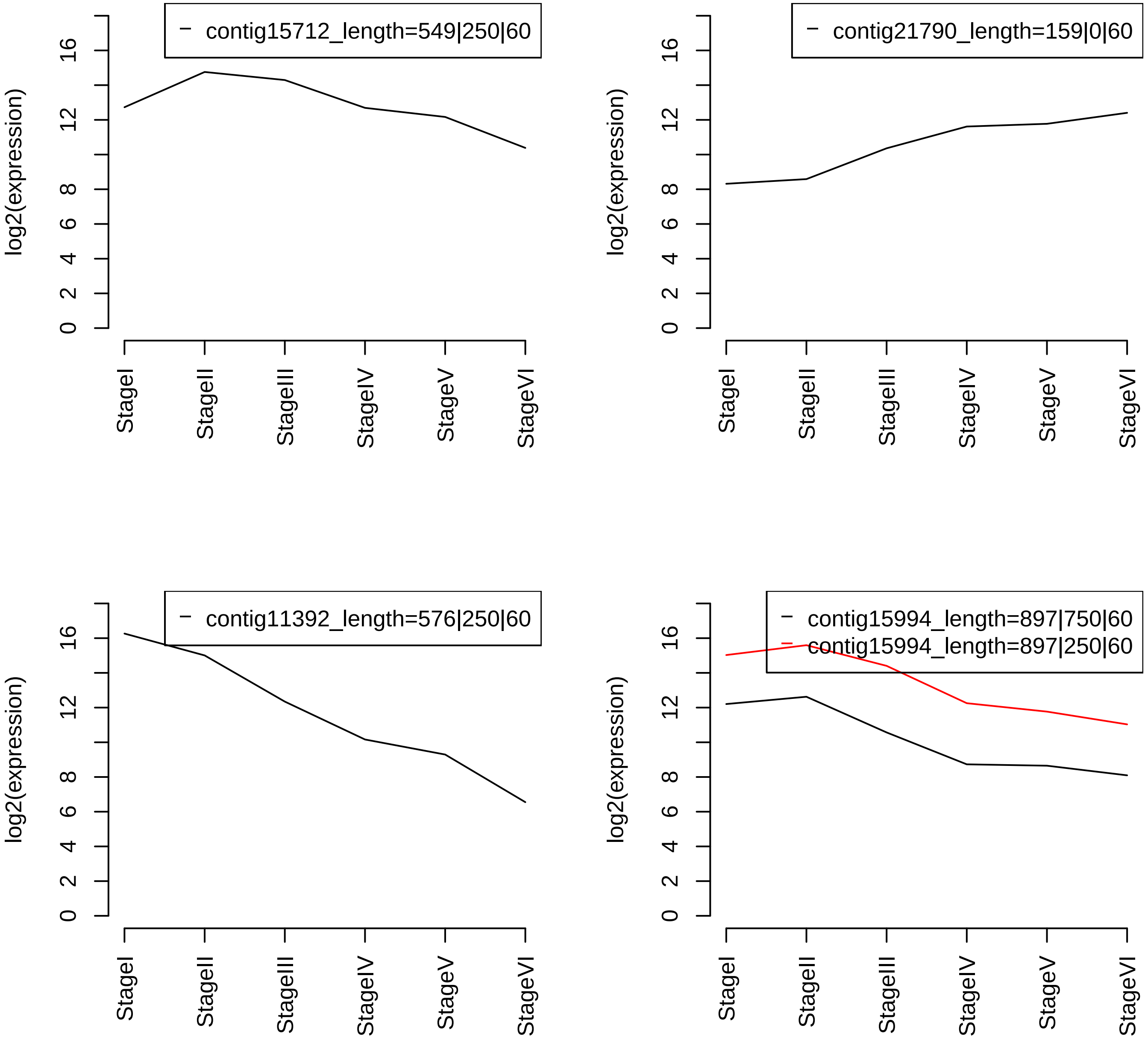
contig15712_length=549|250|60
contig21790_length=159|0|60
contig11392_length=576|250|60
contig15994_length=897|750|60
contig15994_length=897|250|60
log2(expression)
0
2
4
6
8
12
16
StageI
StageII
StageIII
StageIV
StageV
StageVI

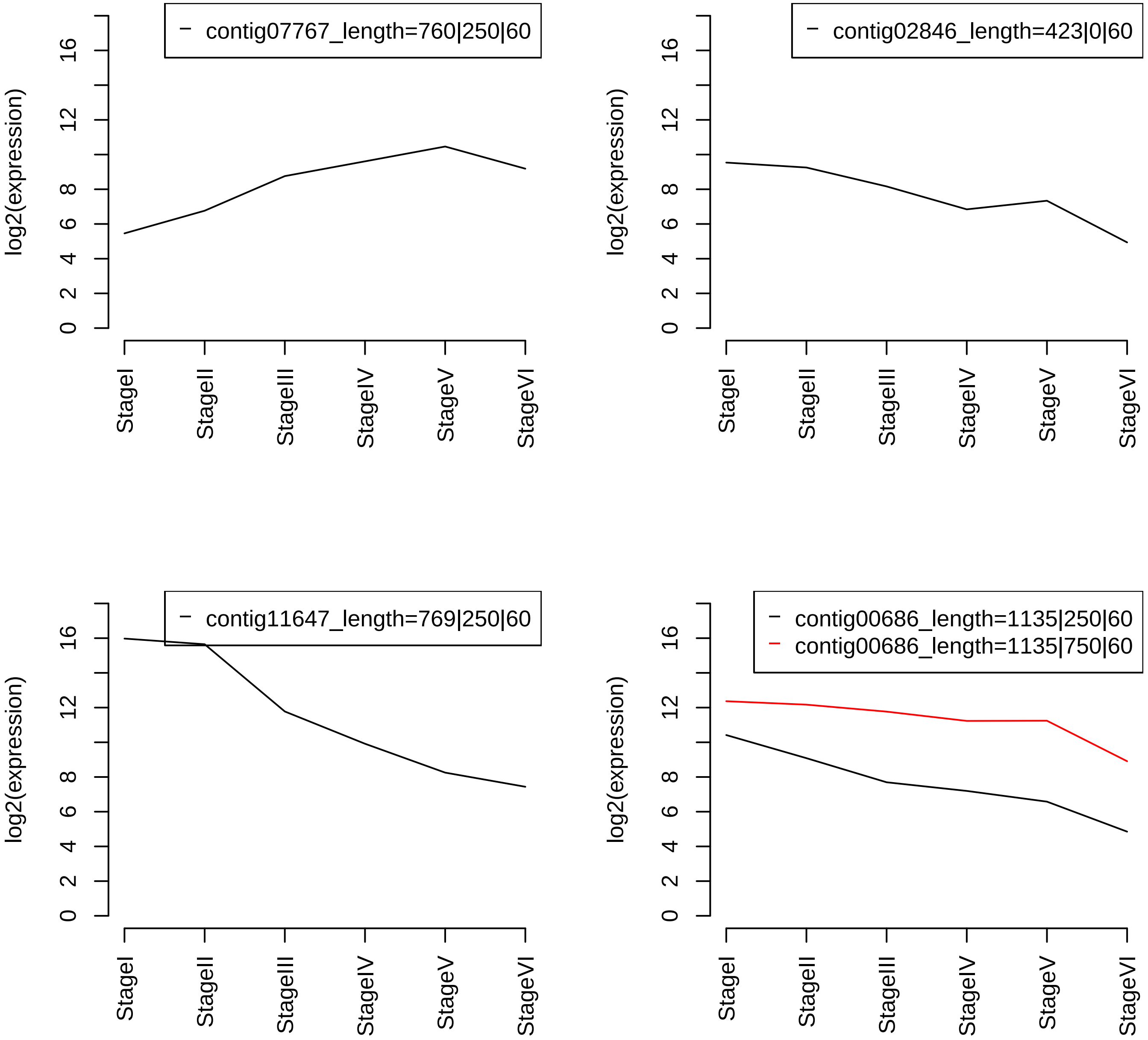
contig07767_length=760|250|60
contig02846_length=423|0|60
contig11647_length=769|250|60
contig00686_length=1135|250|60
contig00686_length=1135|750|60
log2(expression)
0
2
4
6
8
12
16
StageI
StageII
StageIII
StageIV
StageV
StageVI

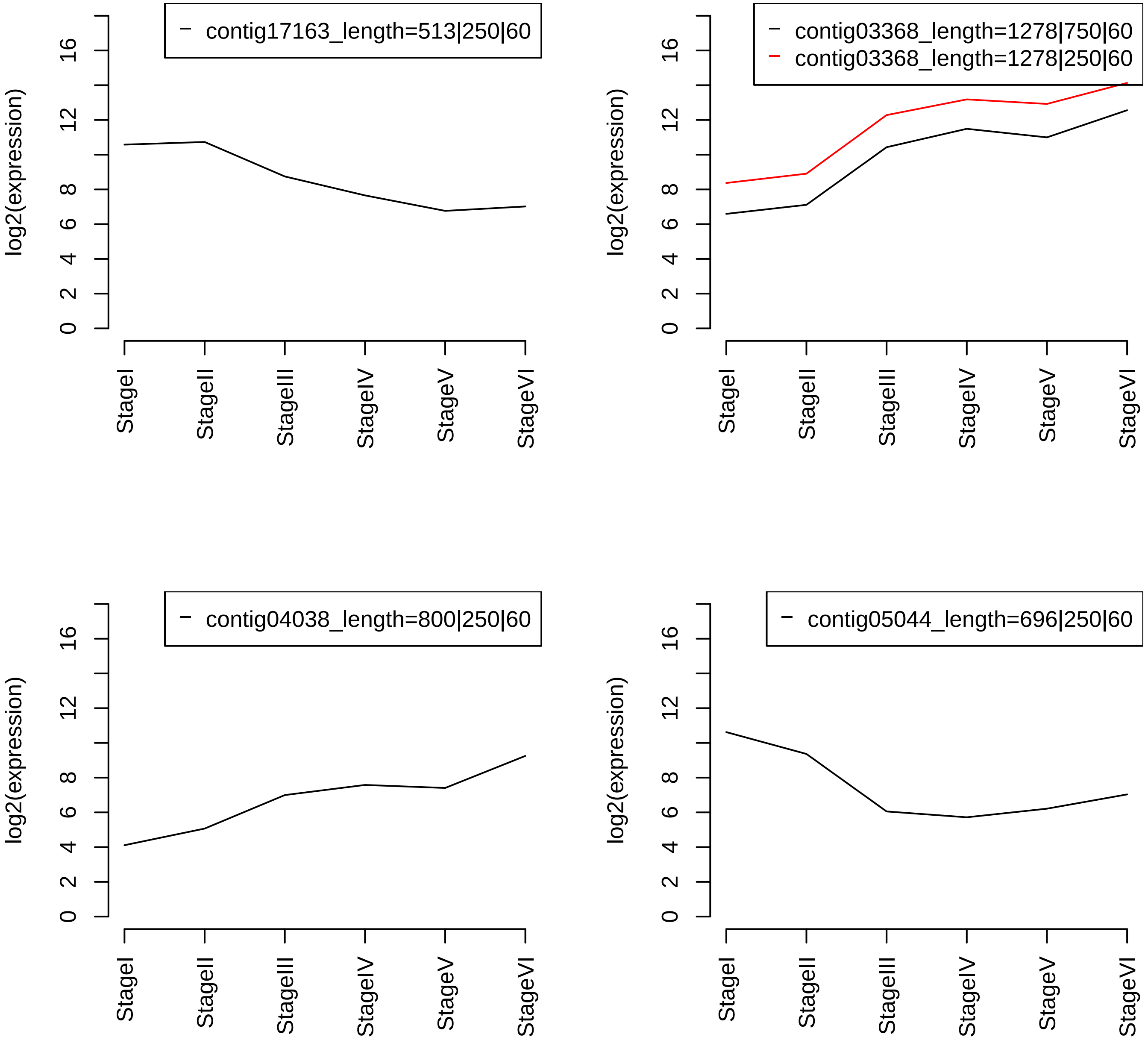
contig17163_length=513|250|60
contig03368_length=1278|750|60
contig03368_length=1278|250|60
contig04038_length=800|250|60
contig05044_length=696|250|60
log2(expression)
0
2
4
6
8
12
16
StageI
StageII
StageIII
StageIV
StageV
StageVI

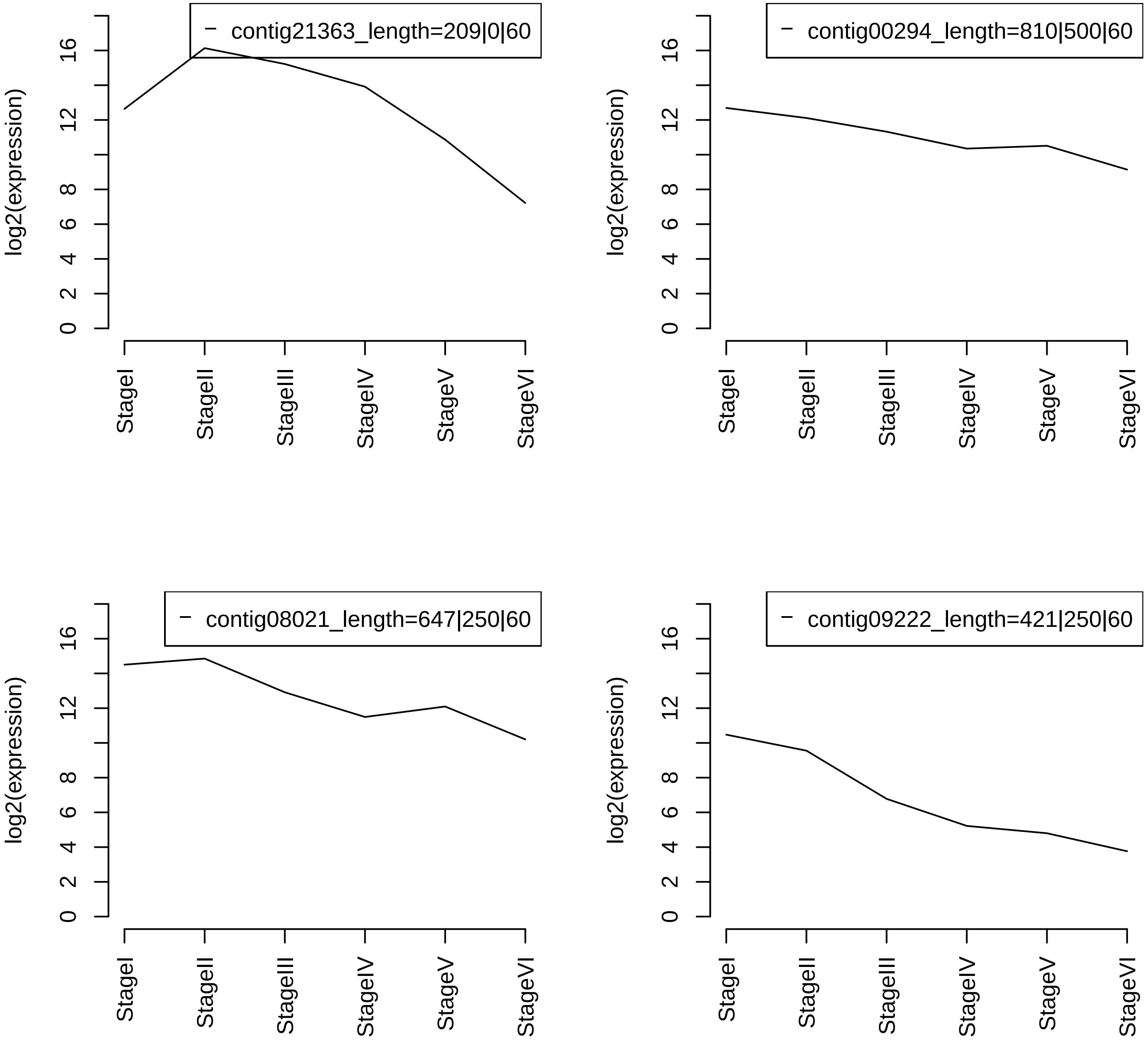

contig21363_length=209|0|60
contig00294_length=810|500|60
contig08021_length=647|250|60
contig09222_length=421|250|60
log2(expression)
0
2
4
6
8
12
16
StageI
StageII
StageIII
StageIV
StageV
StageVI

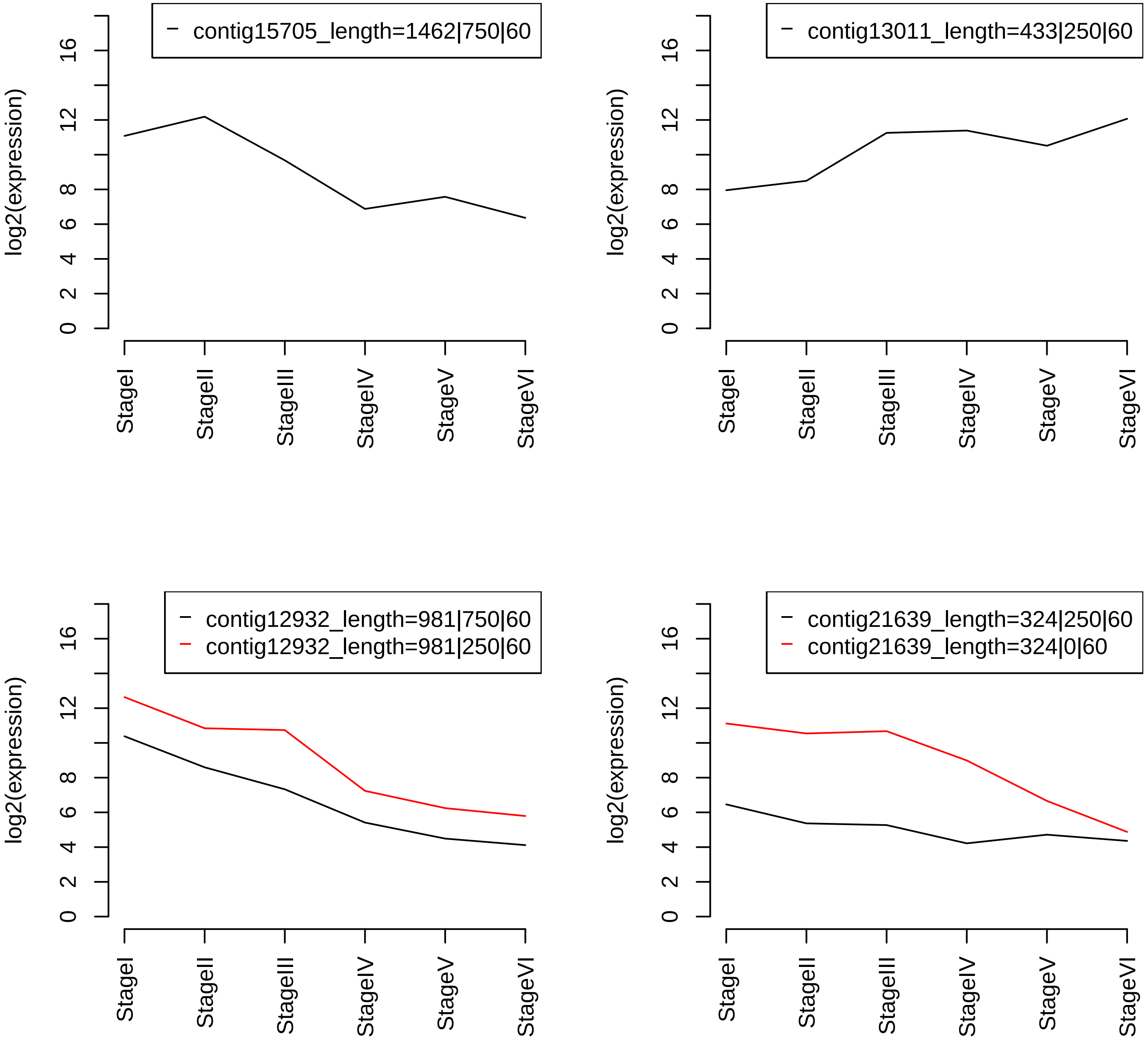
contig15705_length=1462|750|60
contig13011_length=433|250|60
contig12932_length=981|750|60
contig12932_length=981|250|60
contig21639_length=324|250|60
contig21639_length=324|0|60
log2(expression)
0
2
4
6
8
12
16
StageI
StageII
StageIII
StageIV
StageV
StageVI

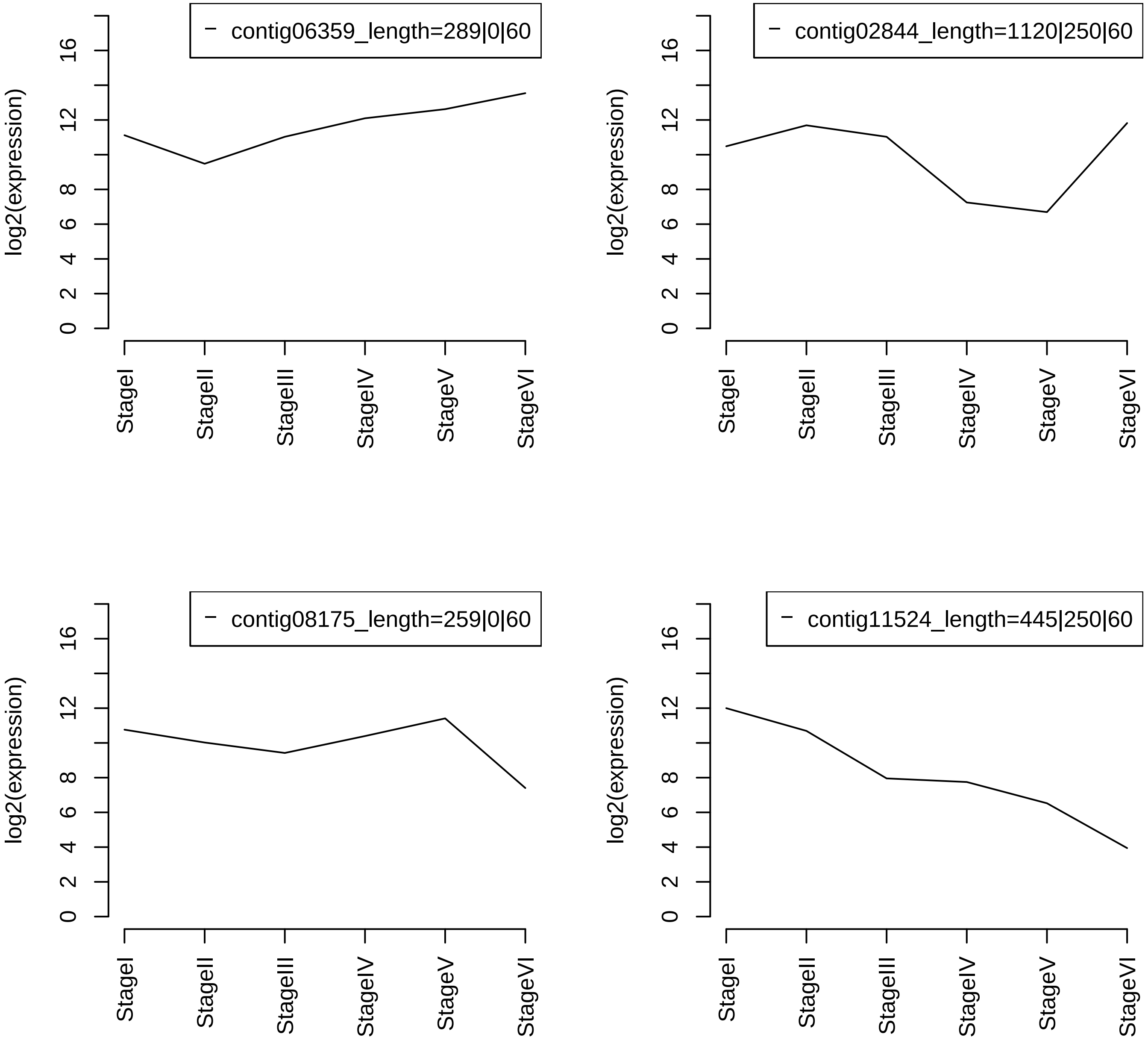
contig06359_length=289|0|60
contig02844_length=1120|250|60
contig08175_length=259|0|60
contig11524_length=445|250|60
log2(expression)
0
2
4
6
8
12
16
StageI
StageII
StageIII
StageIV
StageV
StageVI

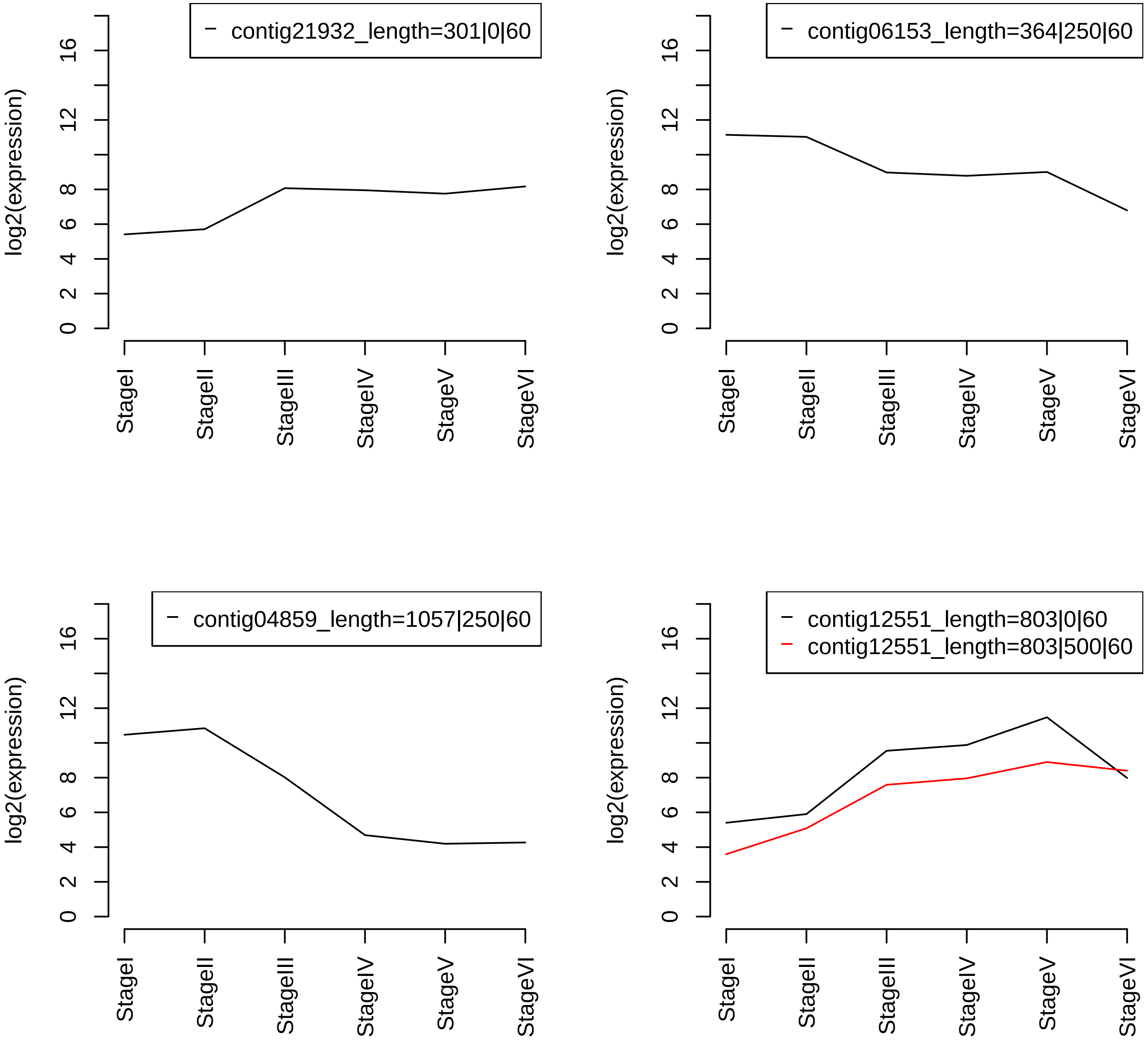

contig21932_length=301|0|60
contig06153_length=364|250|60
contig04859_length=1057|250|60
contig12551_length=803|0|60
contig12551_length=803|500|60
log2(expression)
0
2
4
6
8
12
16
StageI
StageII
StageIII
StageIV
StageV
StageVI

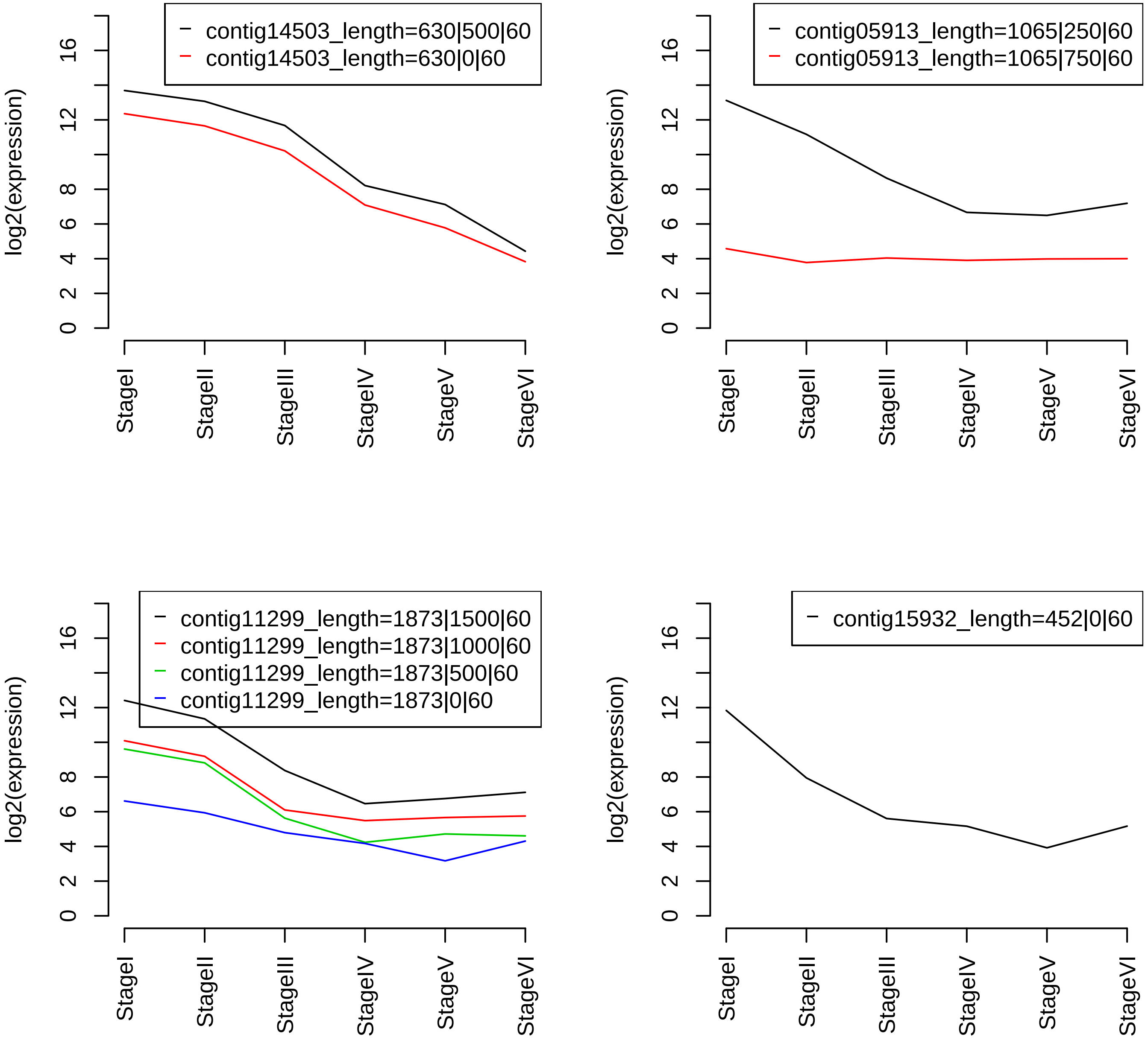
contig14503_length=630|500|60
contig14503_length=630|0|60
contig05913_length=1065|250|60
contig05913_length=1065|750|60
contig11299_length=1873|1500|60
contig11299_length=1873|1000|60
contig11299_length=1873|500|60
contig11299_length=1873|0|60
contig15932_length=452|0|60
log2(expression)
0
2
4
6
8
12
16
StageI
StageII
StageIII
StageIV
StageV
StageVI

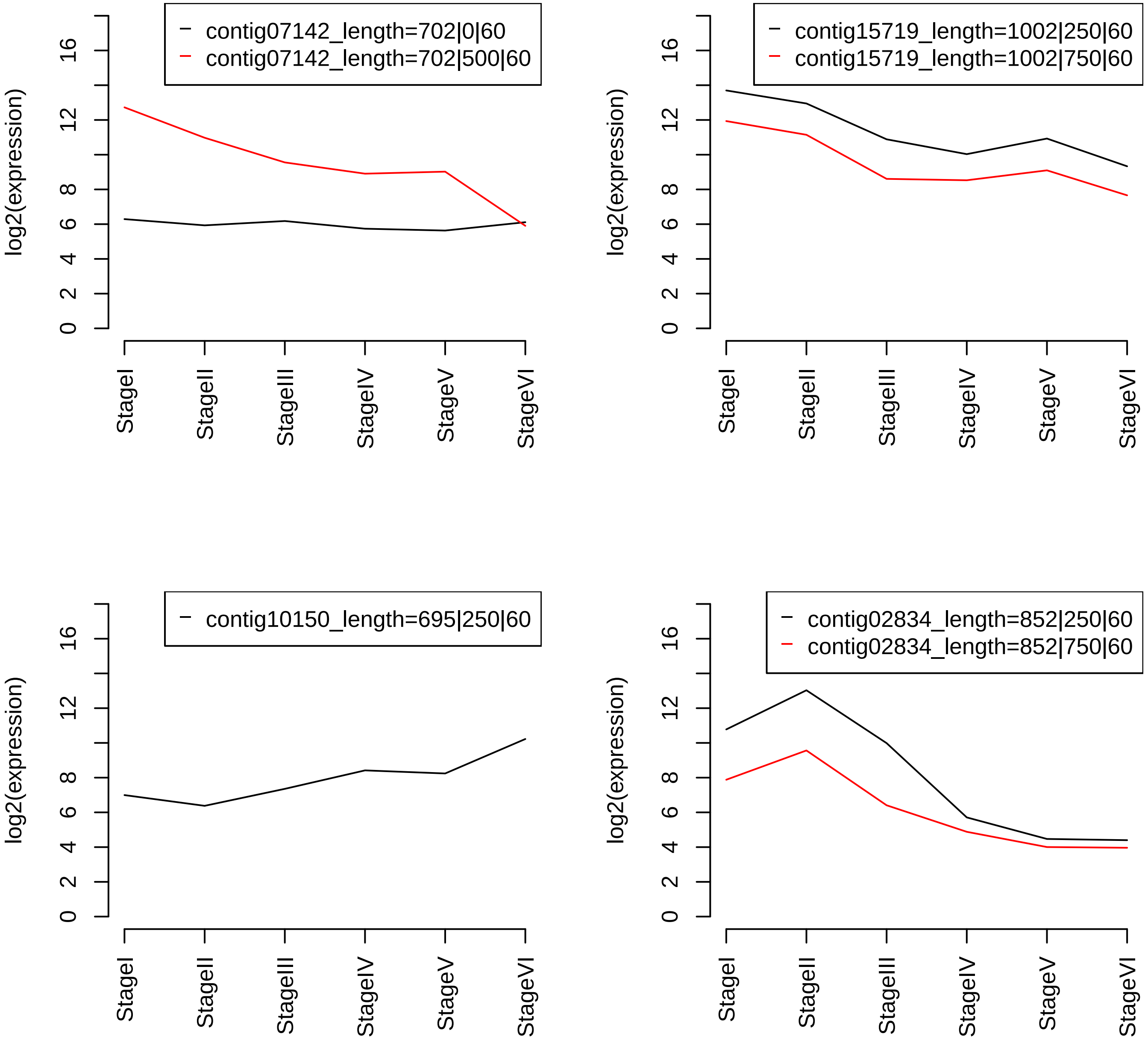

contig07142_length=702|0|60
contig07142_length=702|500|60
contig15719_length=1002|250|60
contig15719_length=1002|750|60
contig10150_length=695|250|60
contig02834_length=852|250|60
contig02834_length=852|750|60
log2(expression)
0 2 4 6 8 12 16
StageI
StageII
StageIII
StageIV
StageV
StageVI

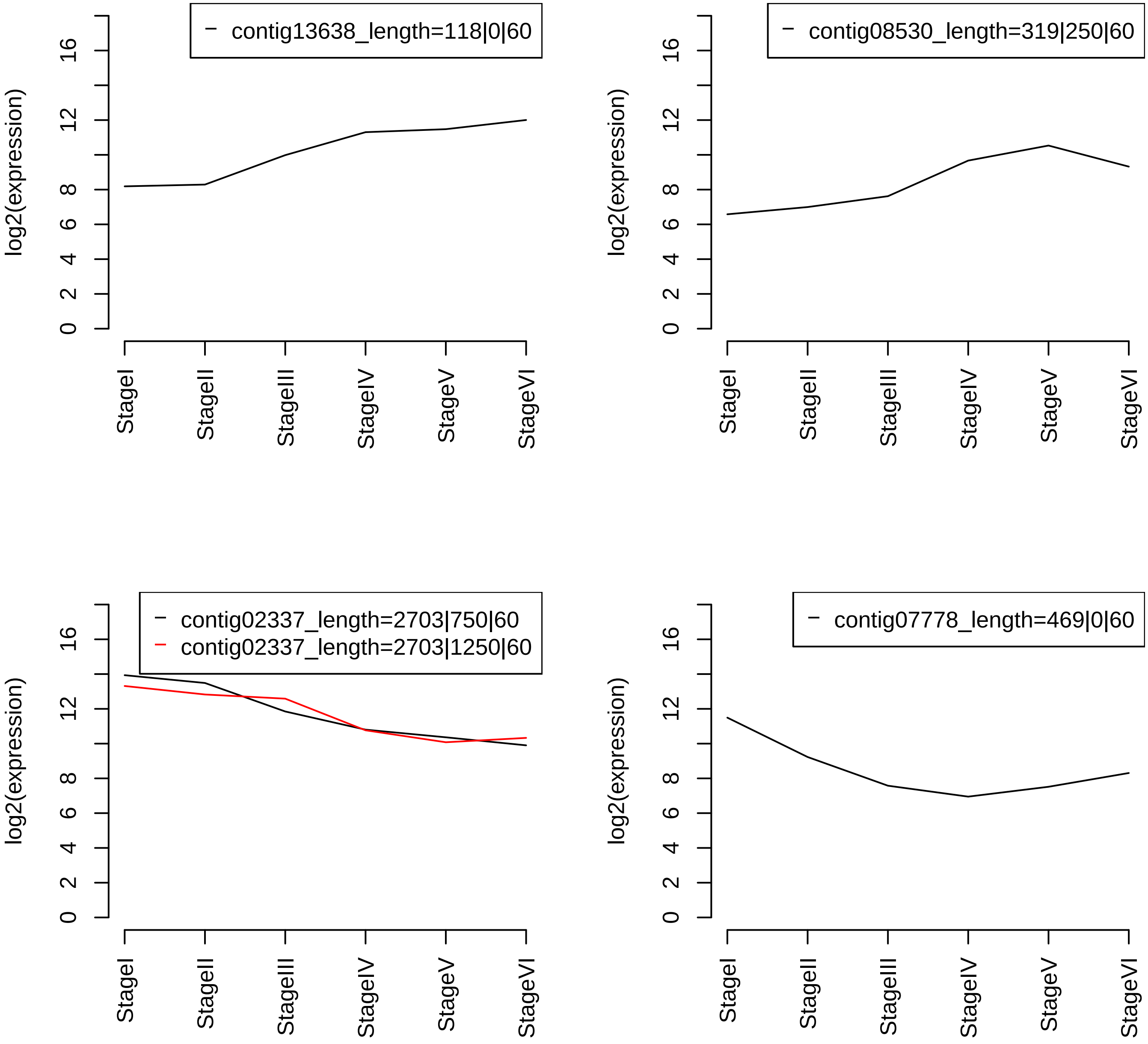
contig13638_length=118|0|60
contig08530_length=319|250|60
contig02337_length=2703|750|60
contig02337_length=2703|1250|60
contig07778_length=469|0|60
log2(expression)
0 2 4 6 8 12 16
StageI StageII StageIII StageIV StageV StageVI

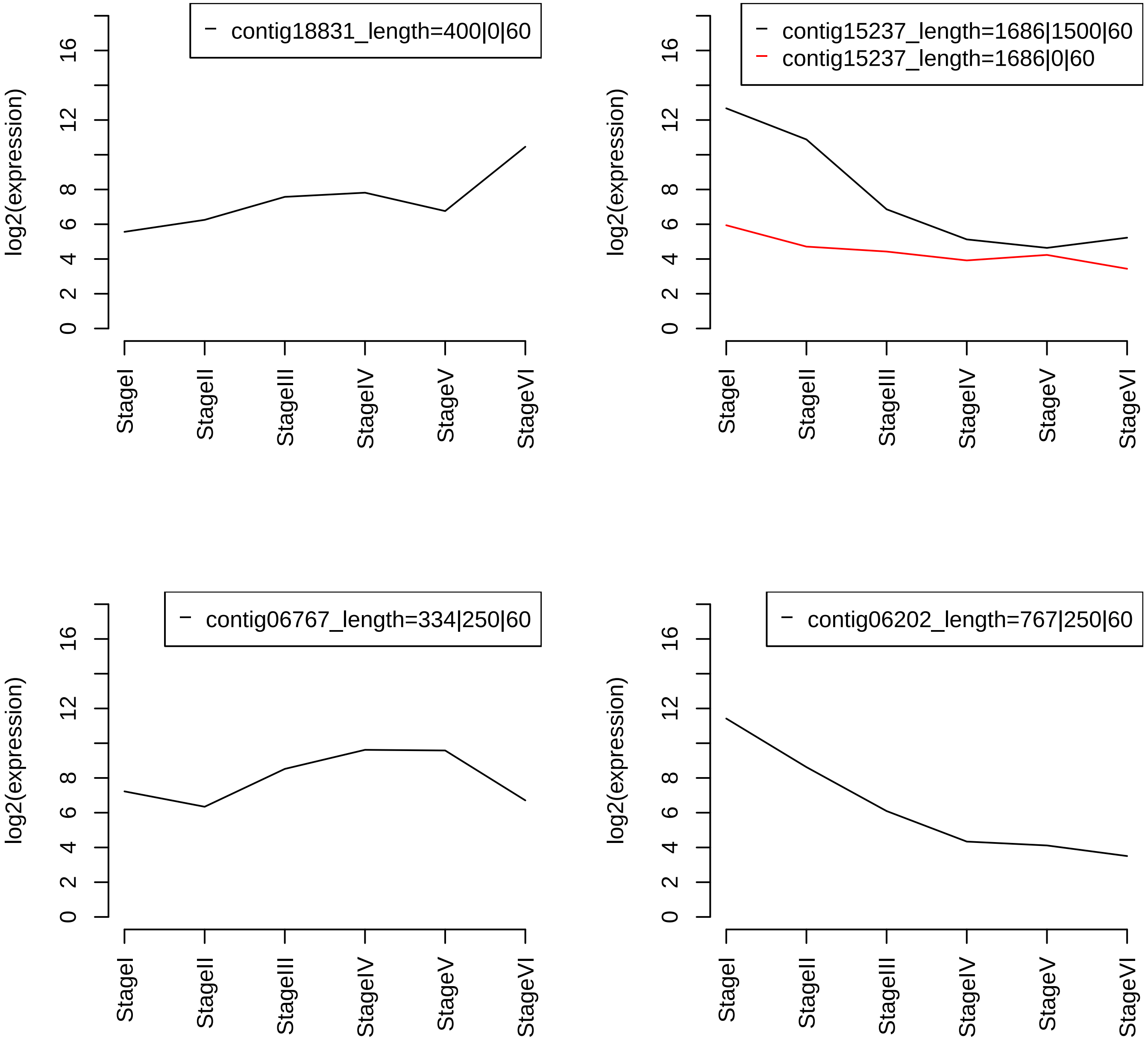

contig18831_length=400|0|60
contig15237_length=1686|1500|60
contig15237_length=1686|0|60
contig06767_length=334|250|60
contig06202_length=767|250|60
log2(expression)
0
2
4
6
8
12
16
StageI
StageII
StageIII
StageIV
StageV
StageVI

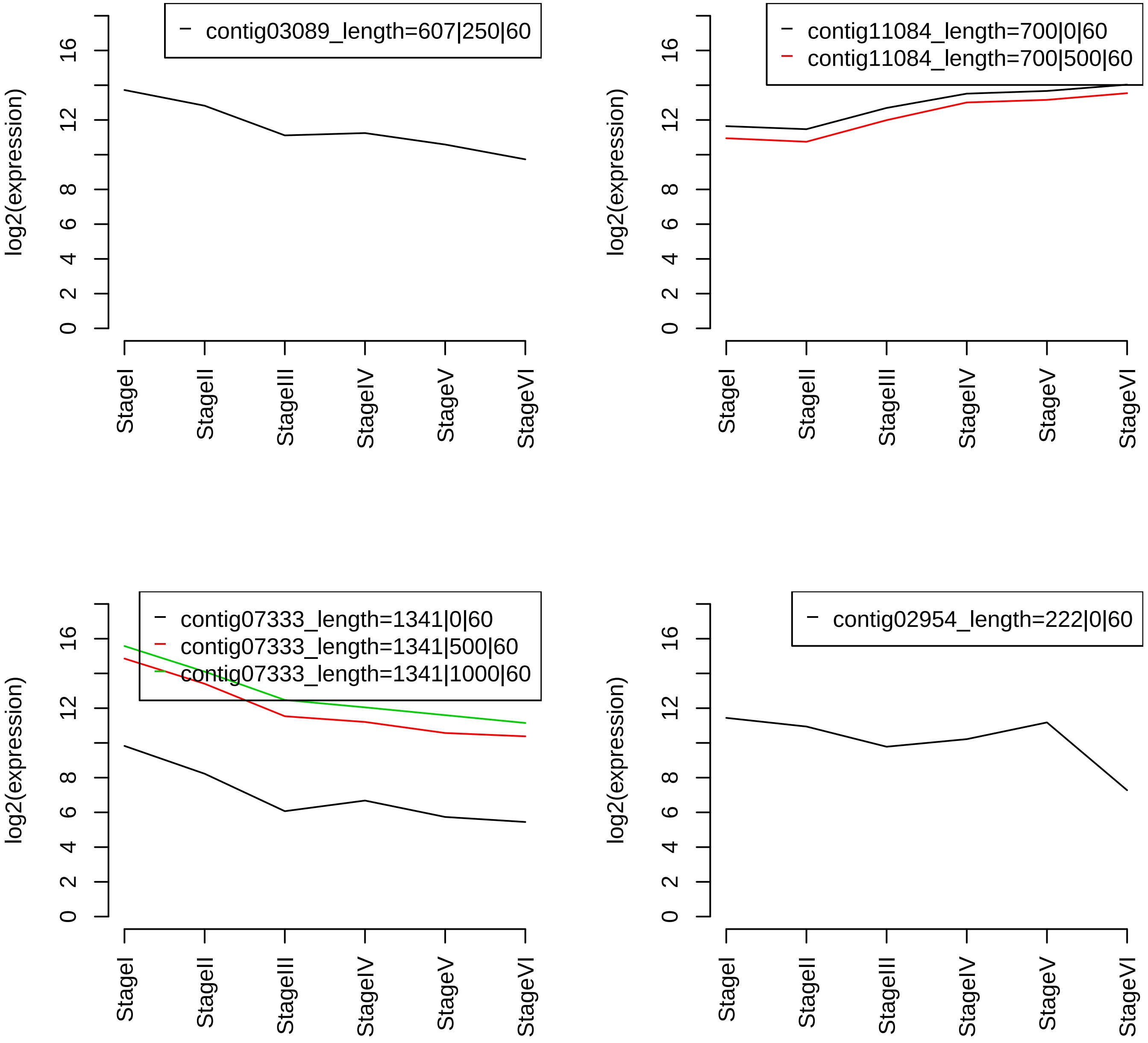

contig03089_length=607|250|60
log2(expression)
0 2 4 6 8 12 16
StageI StageII StageIII StageIV StageV StageVI
contig11084_length=700|0|60
contig11084_length=700|500|60
log2(expression)
0 2 4 6 8 12 16
StageI StageII StageIII StageIV StageV StageVI
contig07333_length=1341|0|60
contig07333_length=1341|500|60
contig07333_length=1341|1000|60
log2(expression)
0 2 4 6 8 12 16
StageI StageII StageIII StageIV StageV StageVI
contig02954_length=222|0|60
log2(expression)
0 2 4 6 8 12 16
StageI StageII StageIII StageIV StageV StageVI

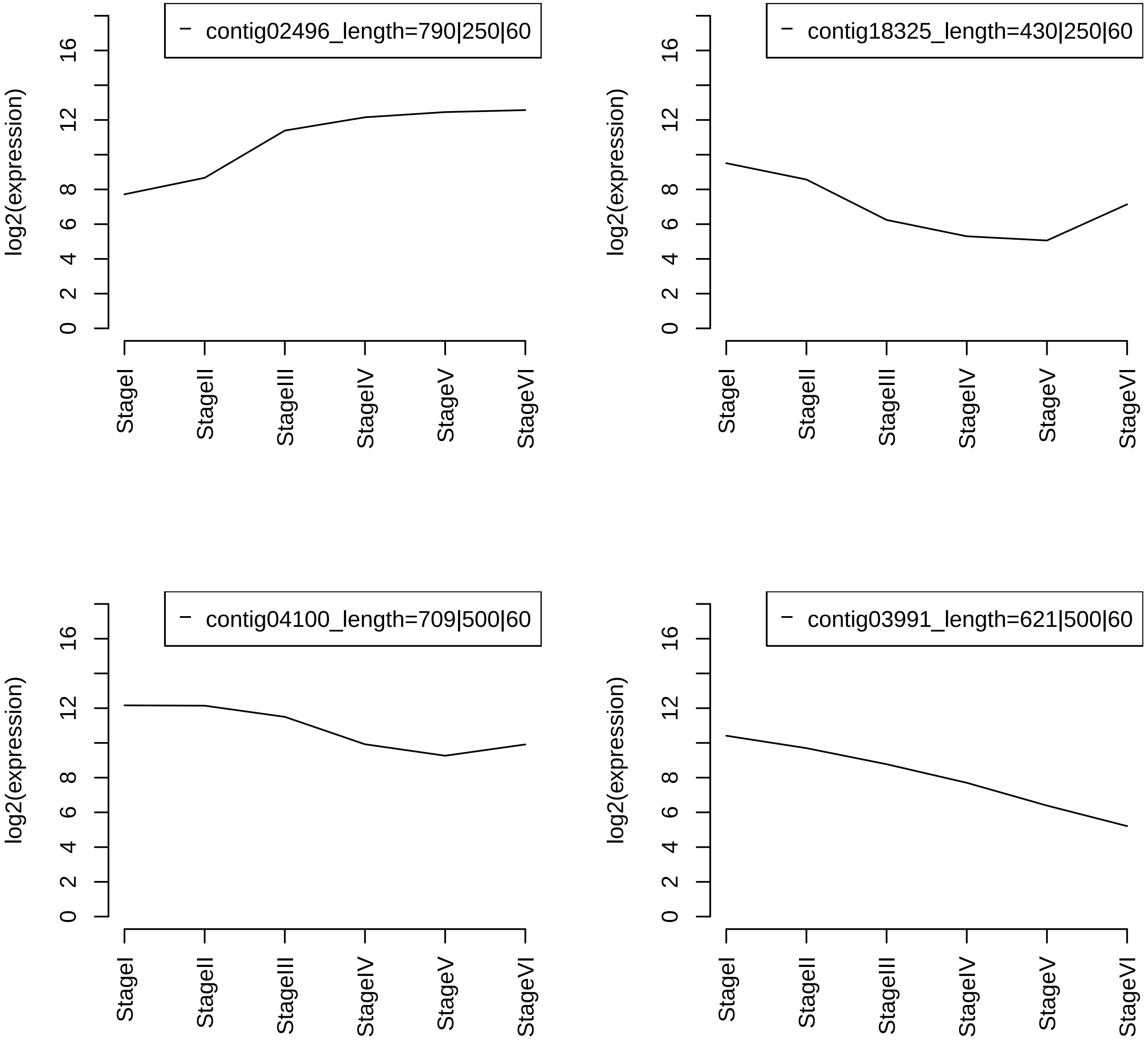
contig02496_length=790|250|60
contig18325_length=430|250|60
contig04100_length=709|500|60
contig03991_length=621|500|60
log2(expression)
0
2
4
6
8
12
16
StageI
StageII
StageIII
StageIV
StageV
StageVI

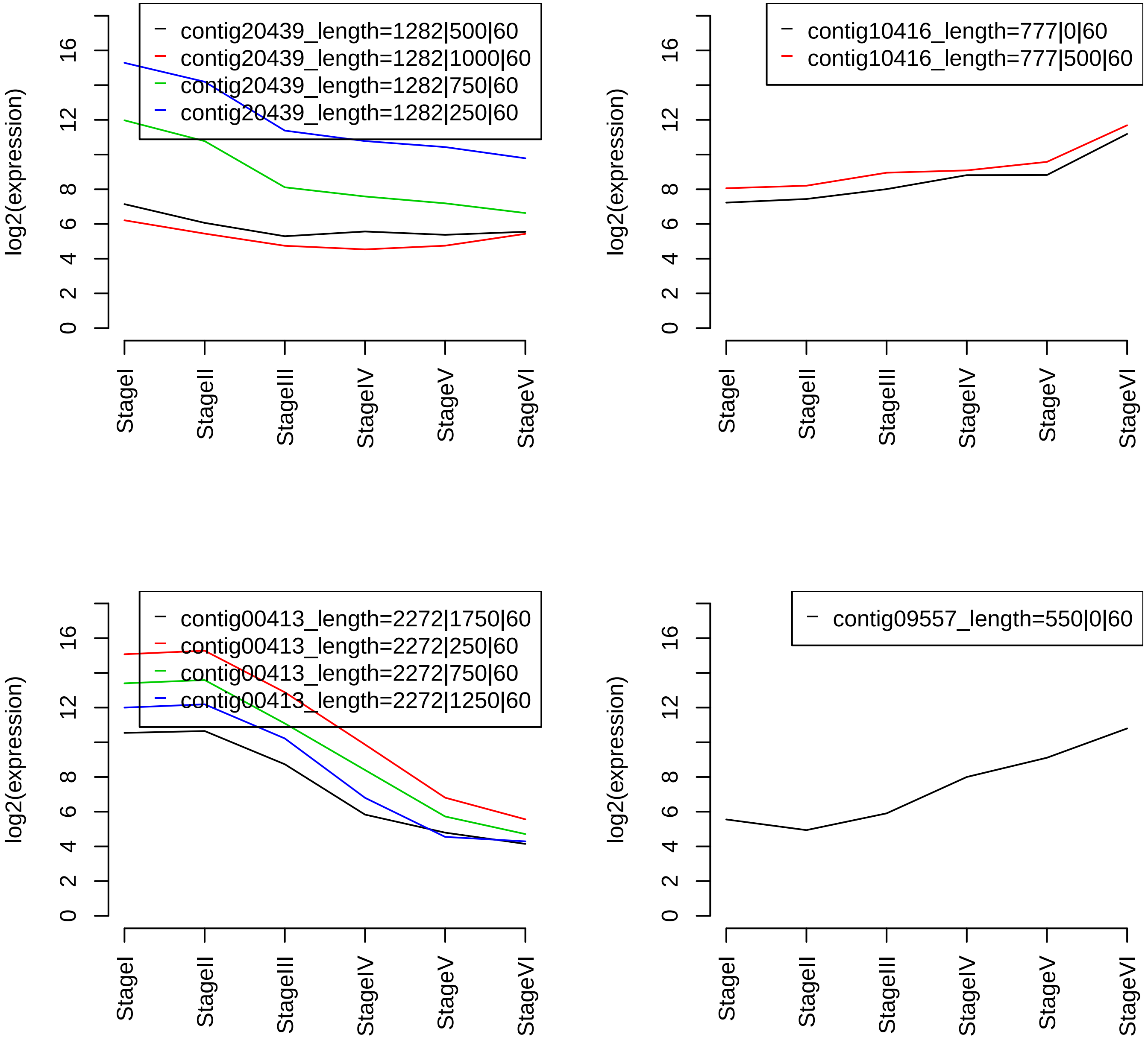
contig20439_length=1282|500|60
contig20439_length=1282|1000|60
contig20439_length=1282|750|60
contig20439_length=1282|250|60
contig10416_length=777|0|60
contig10416_length=777|500|60
contig00413_length=2272|1750|60
contig00413_length=2272|250|60
contig00413_length=2272|750|60
contig00413_length=2272|1250|60
contig09557_length=550|0|60
log2(expression)
0 2 4 6 8 12 16
StageI
StageII
StageIII
StageIV
StageV
StageVI

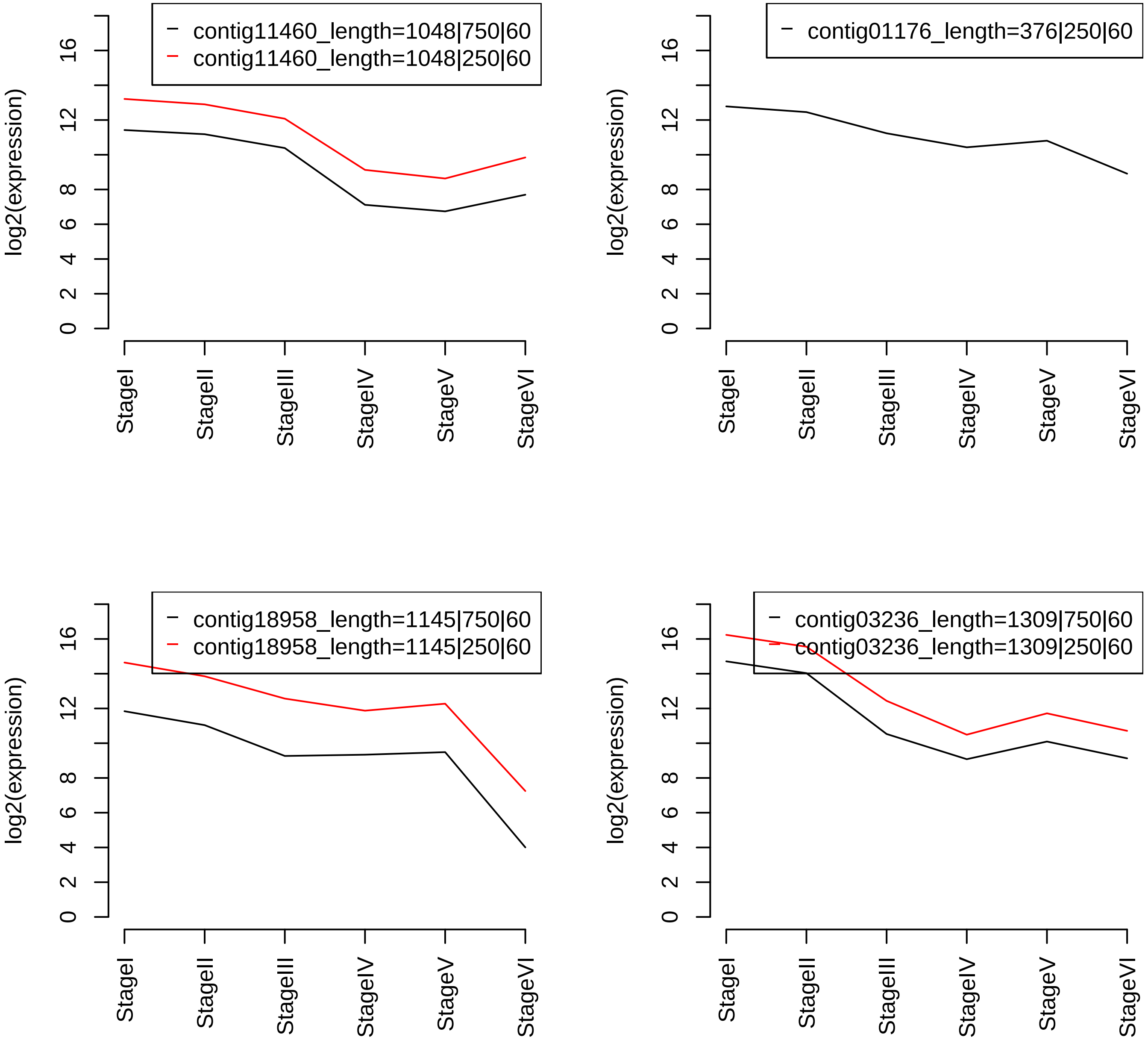

contig11460_length=1048|750|60
contig11460_length=1048|250|60
contig01176_length=376|250|60
contig18958_length=1145|750|60
contig18958_length=1145|250|60
contig03236_length=1309|750|60
contig03236_length=1309|250|60
log2(expression)
0
2
4
6
8
12
16
StageI
StageII
StageIII
StageIV
StageV
StageVI

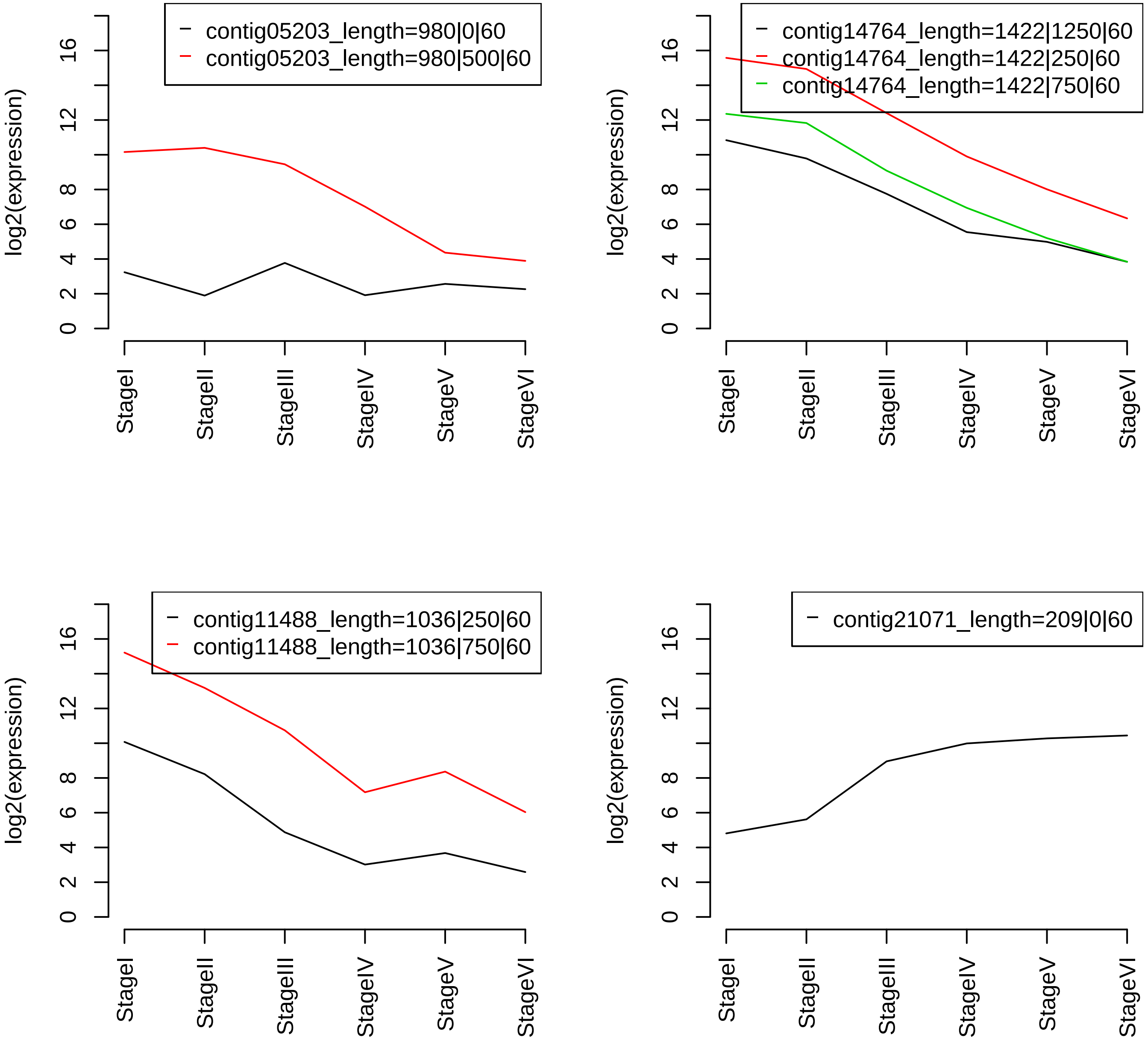
contig05203_length=980|0|60
contig05203_length=980|500|60
contig14764_length=1422|1250|60
contig14764_length=1422|250|60
contig14764_length=1422|750|60
contig11488_length=1036|250|60
contig11488_length=1036|750|60
contig21071_length=209|0|60
log2(expression)
0
2
4
6
8
12
16
StageI
StageII
StageIII
StageIV
StageV
StageVI

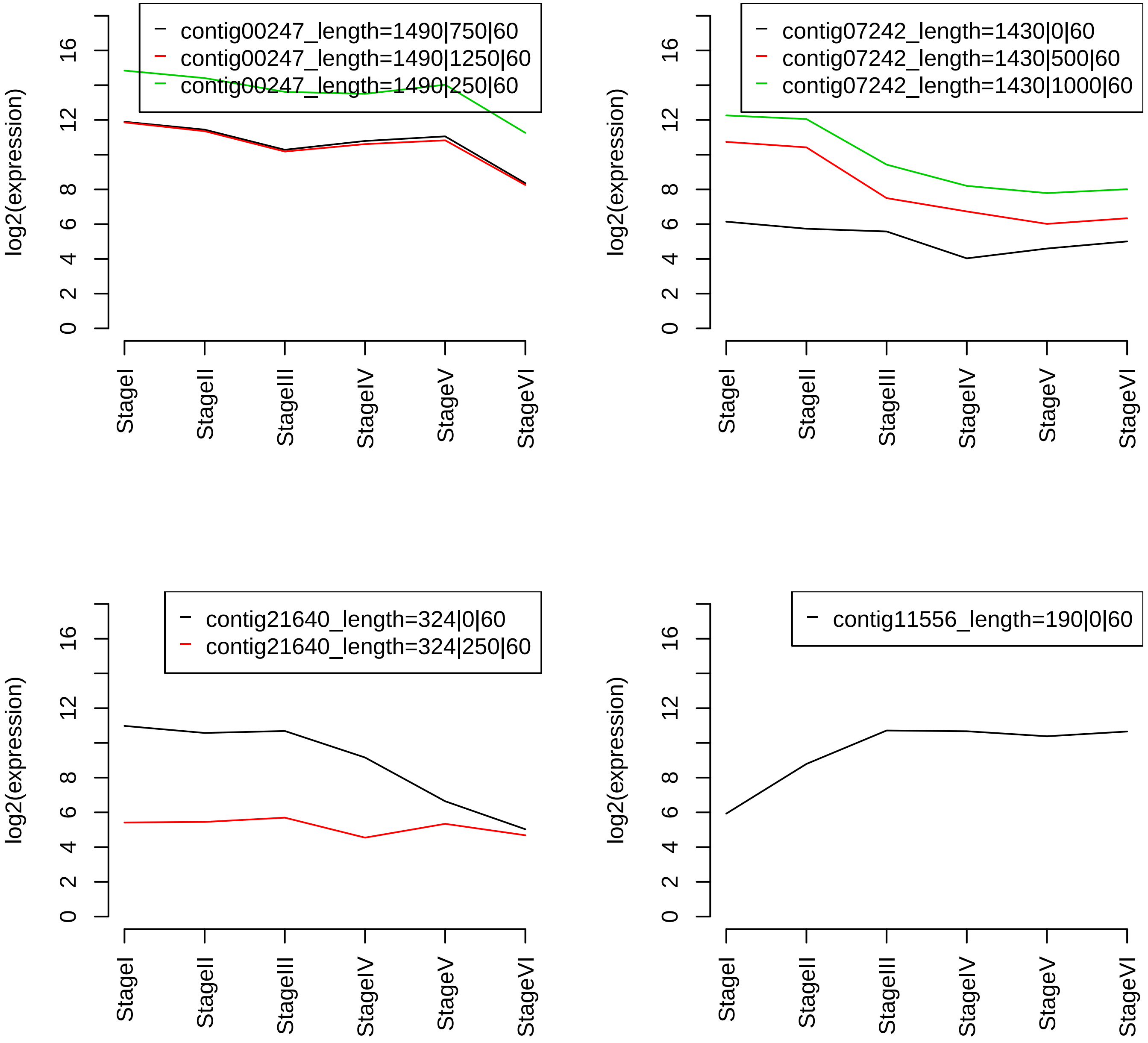

contig00247_length=1490|750|60
contig00247_length=1490|1250|60
contig00247_length=1490|250|60
contig07242_length=1430|0|60
contig07242_length=1430|500|60
contig07242_length=1430|1000|60
contig21640_length=324|0|60
contig21640_length=324|250|60
contig11556_length=190|0|60
log2(expression)
0
2
4
6
8
12
16
StageI
StageII
StageIII
StageIV
StageV
StageVI

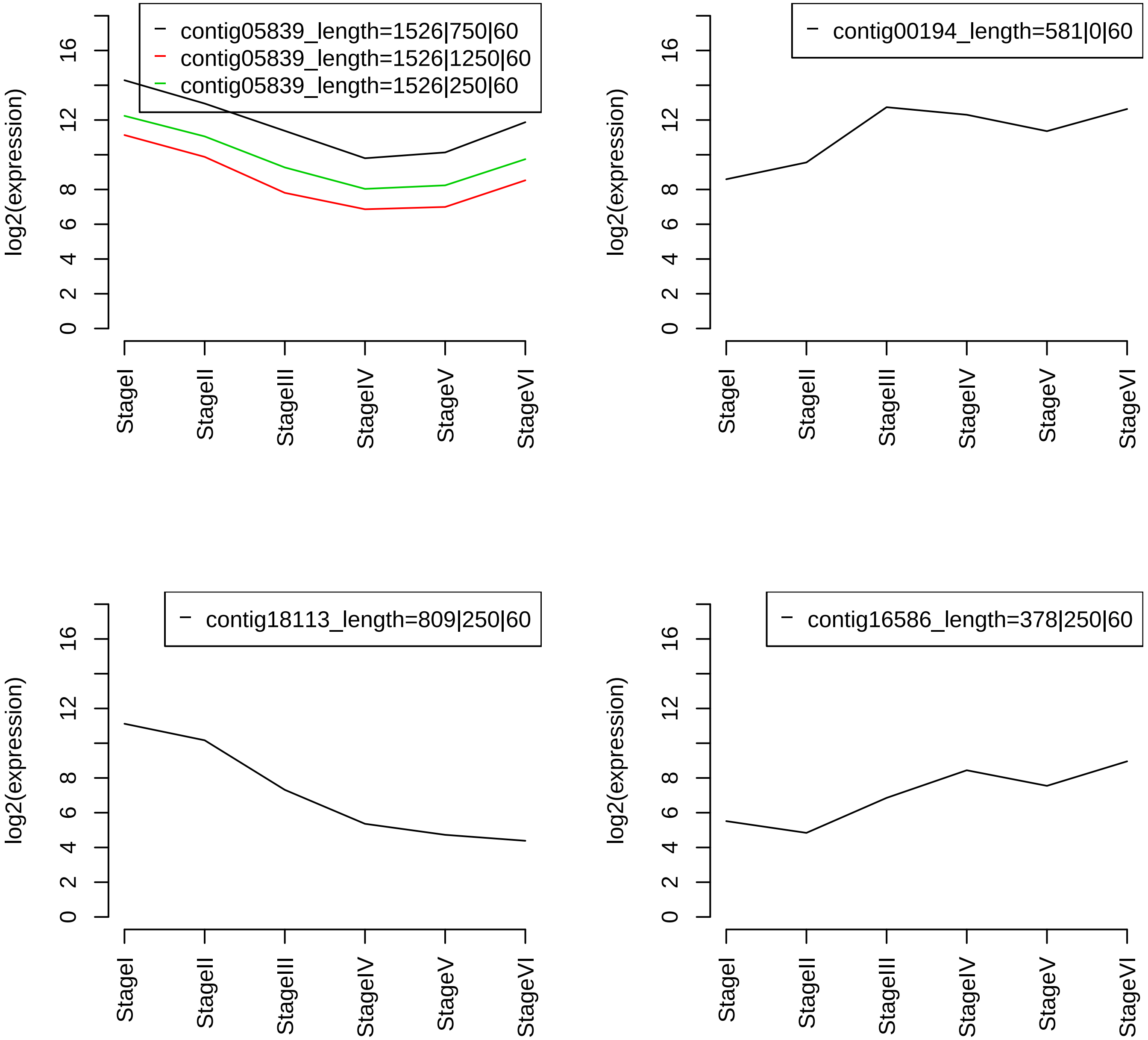
contig05839_length=1526|750|60
contig05839_length=1526|1250|60
contig05839_length=1526|250|60
contig00194_length=581|0|60
contig18113_length=809|250|60
contig16586_length=378|250|60
log2(expression)
0
2
4
6
8
12
16
StageI
StageII
StageIII
StageIV
StageV
StageVI

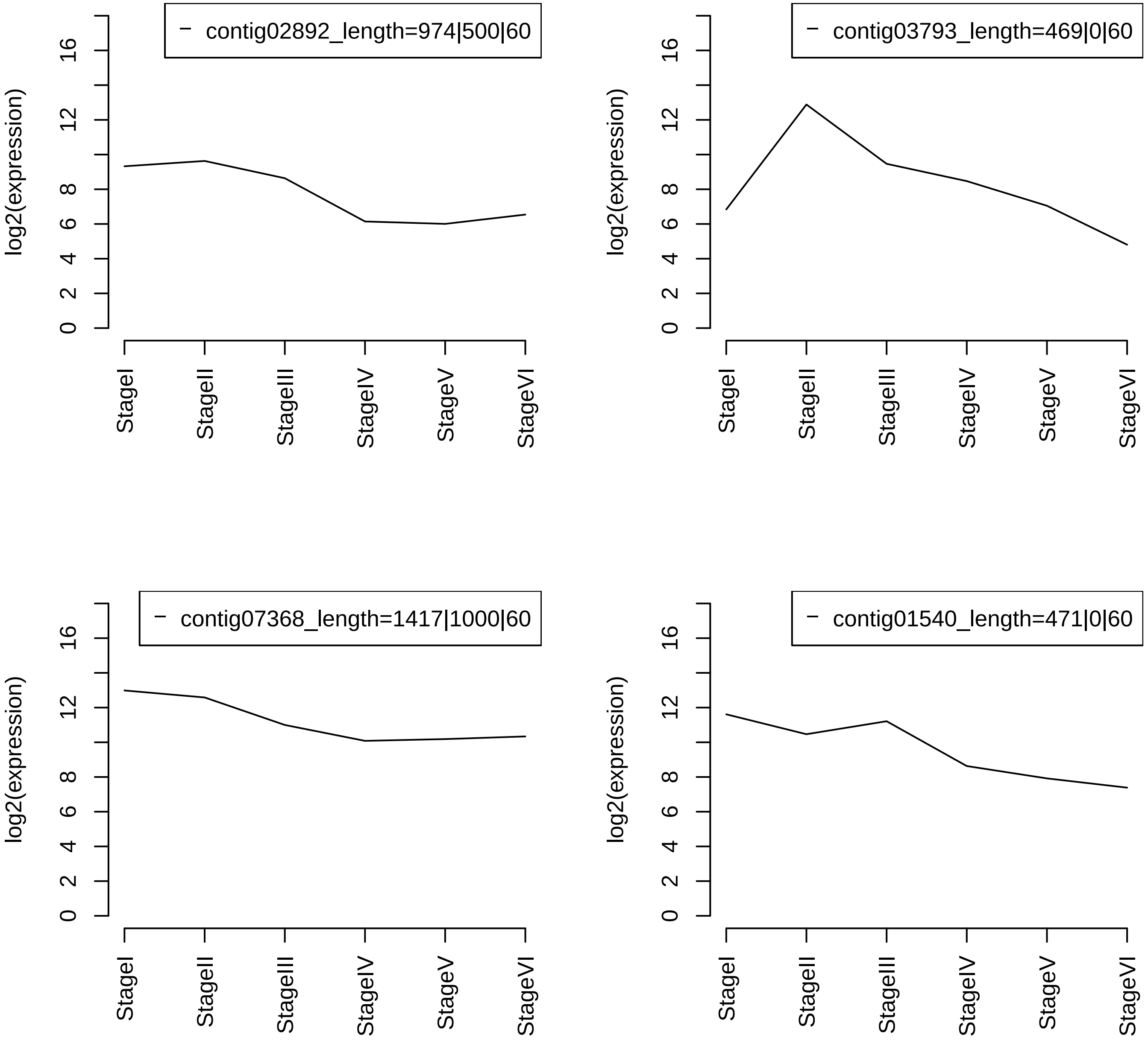
contig02892_length=974|500|60
contig03793_length=469|0|60
contig07368_length=1417|1000|60
contig01540_length=471|0|60
log2(expression)
0
2
4
6
8
12
16
StageI
StageII
StageIII
StageIV
StageV
StageVI

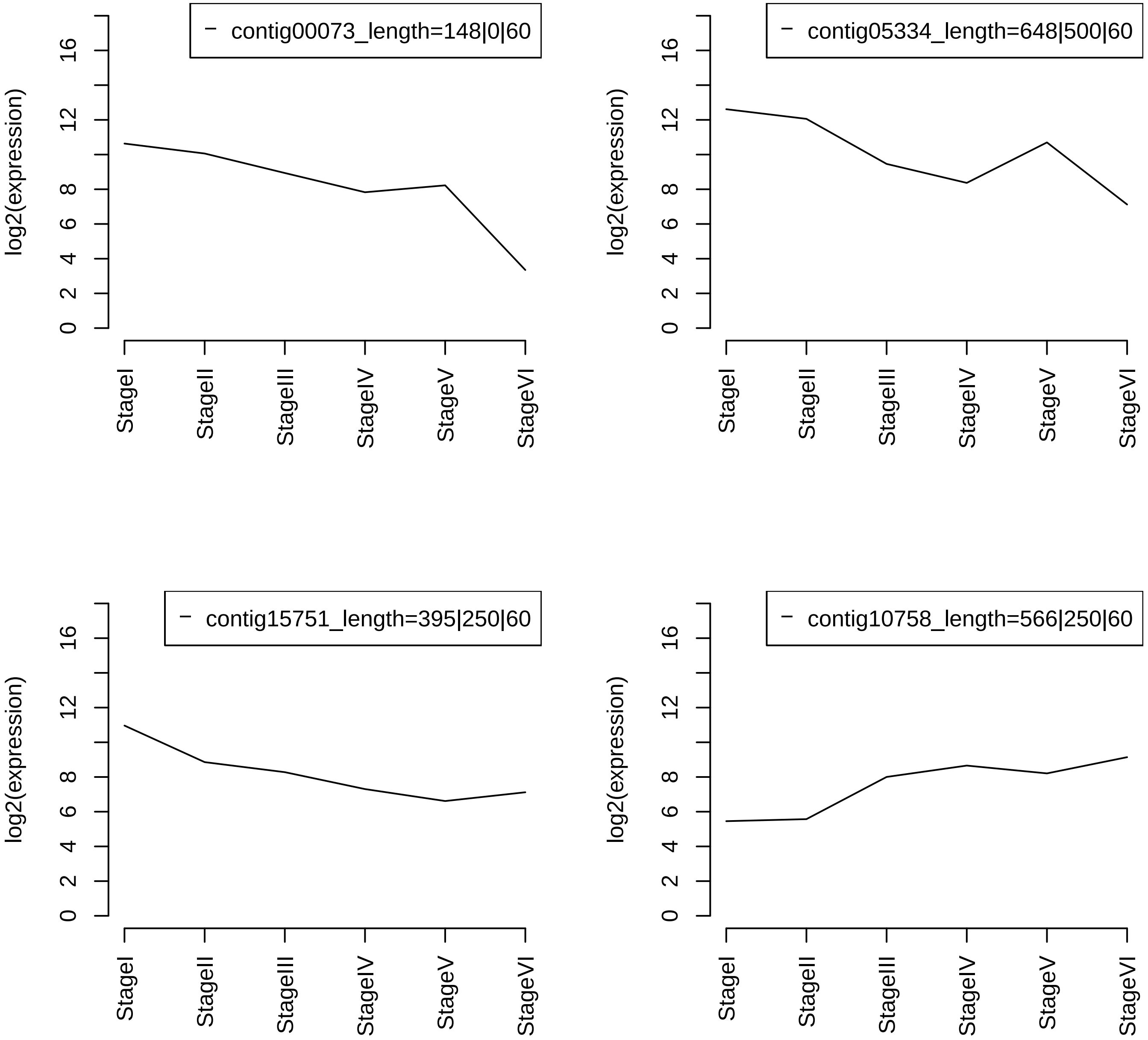
contig00073_length=148|0|60
contig05334_length=648|500|60
contig15751_length=395|250|60
contig10758_length=566|250|60
log2(expression)
0
2
4
6
8
12
16
StageI
StageII
StageIII
StageIV
StageV
StageVI

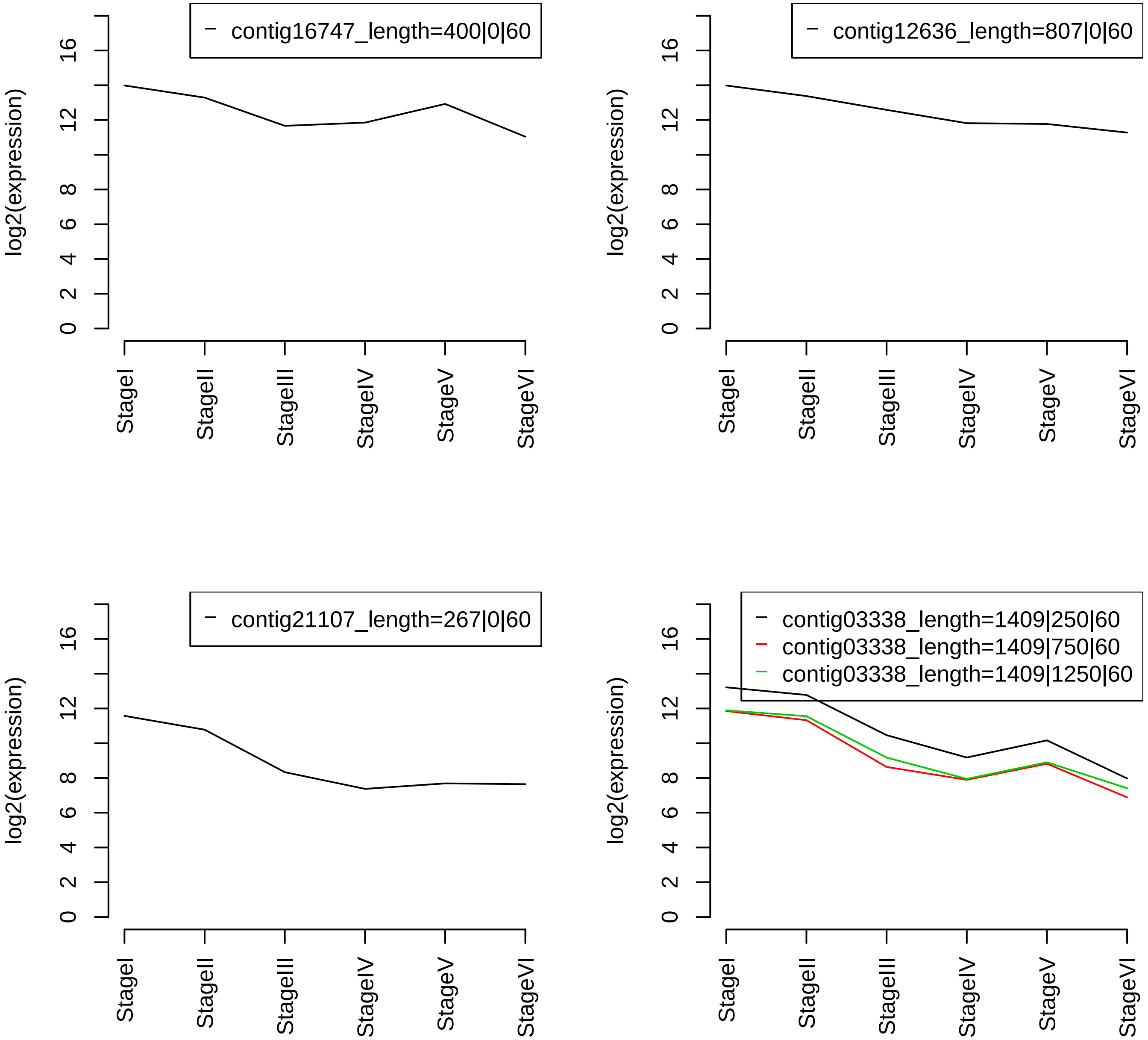
contig16747_length=400|0|60
contig12636_length=807|0|60
contig21107_length=267|0|60
contig03338_length=1409|250|60
contig03338_length=1409|750|60
contig03338_length=1409|1250|60
log2(expression)
0 2 4 6 8 12 16
StageI
StageII
StageIII
StageIV
StageV
StageVI

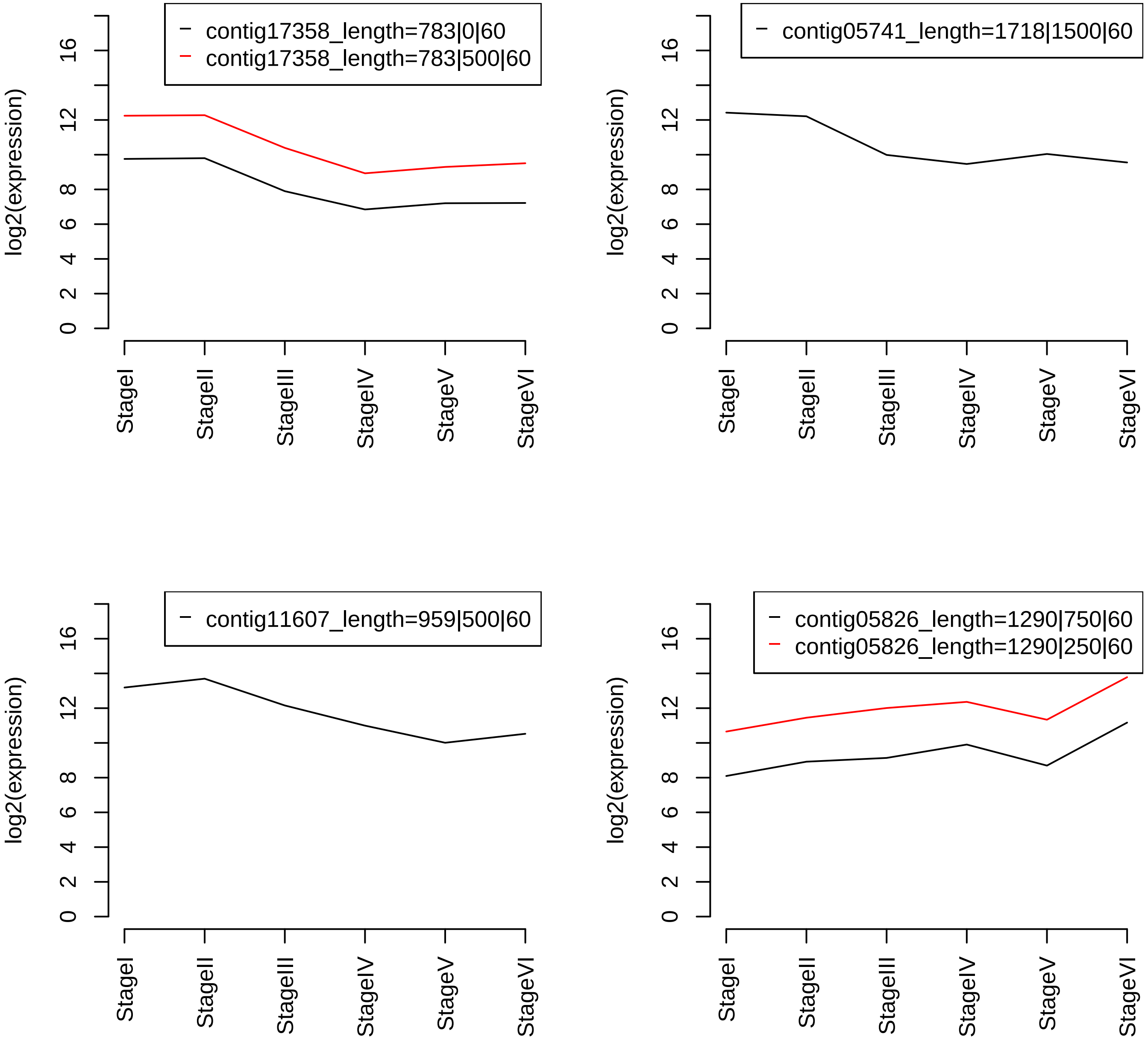

contig17358_length=783|0|60
contig17358_length=783|500|60
contig05741_length=1718|1500|60
contig11607_length=959|500|60
contig05826_length=1290|750|60
contig05826_length=1290|250|60
log2(expression)
0
2
4
6
8
12
16
StageI
StageII
StageIII
StageIV
StageV
StageVI

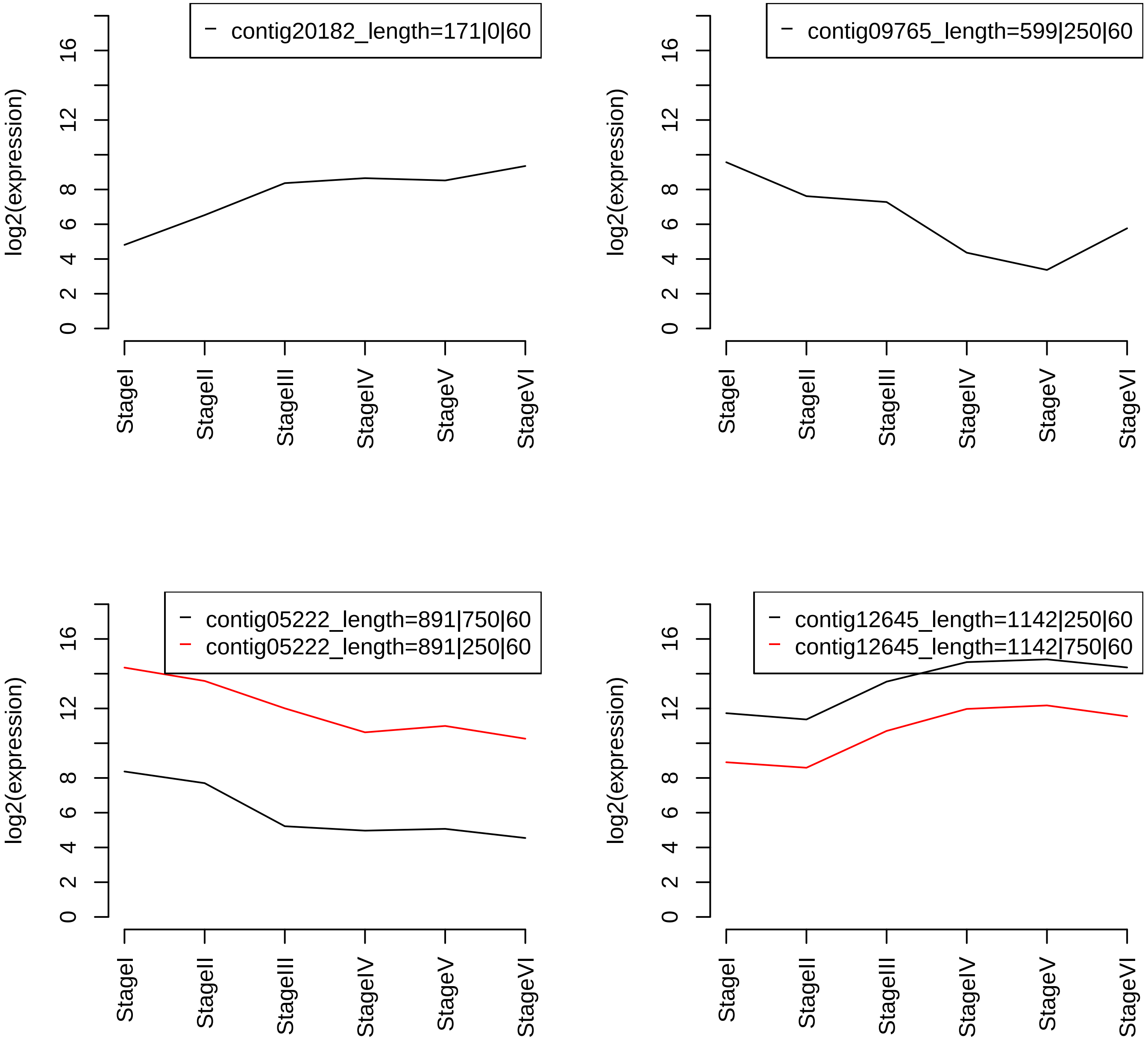
contig20182_length=171|0|60
contig09765_length=599|250|60
contig05222_length=891|750|60
contig05222_length=891|250|60
contig12645_length=1142|250|60
contig12645_length=1142|750|60
log2(expression)
0
2
4
6
8
12
16
StageI
StageII
StageIII
StageIV
StageV
StageVI

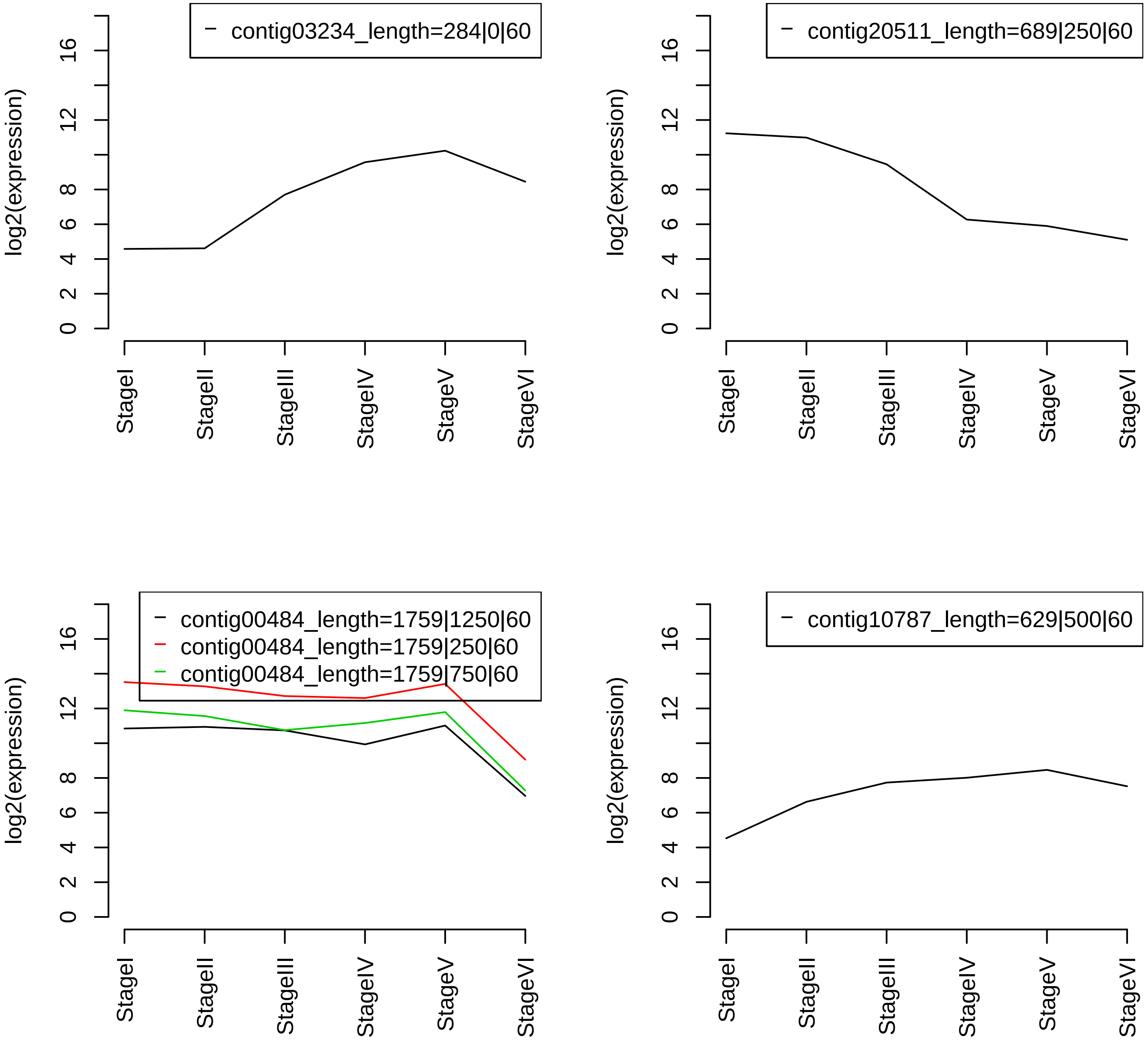
contig03234_length=284|0|60
contig20511_length=689|250|60
contig00484_length=1759|1250|60
contig00484_length=1759|250|60
contig00484_length=1759|750|60
contig10787_length=629|500|60
log2(expression)
0
2
4
6
8
12
16
StageI
StageII
StageIII
StageIV
StageV
StageVI

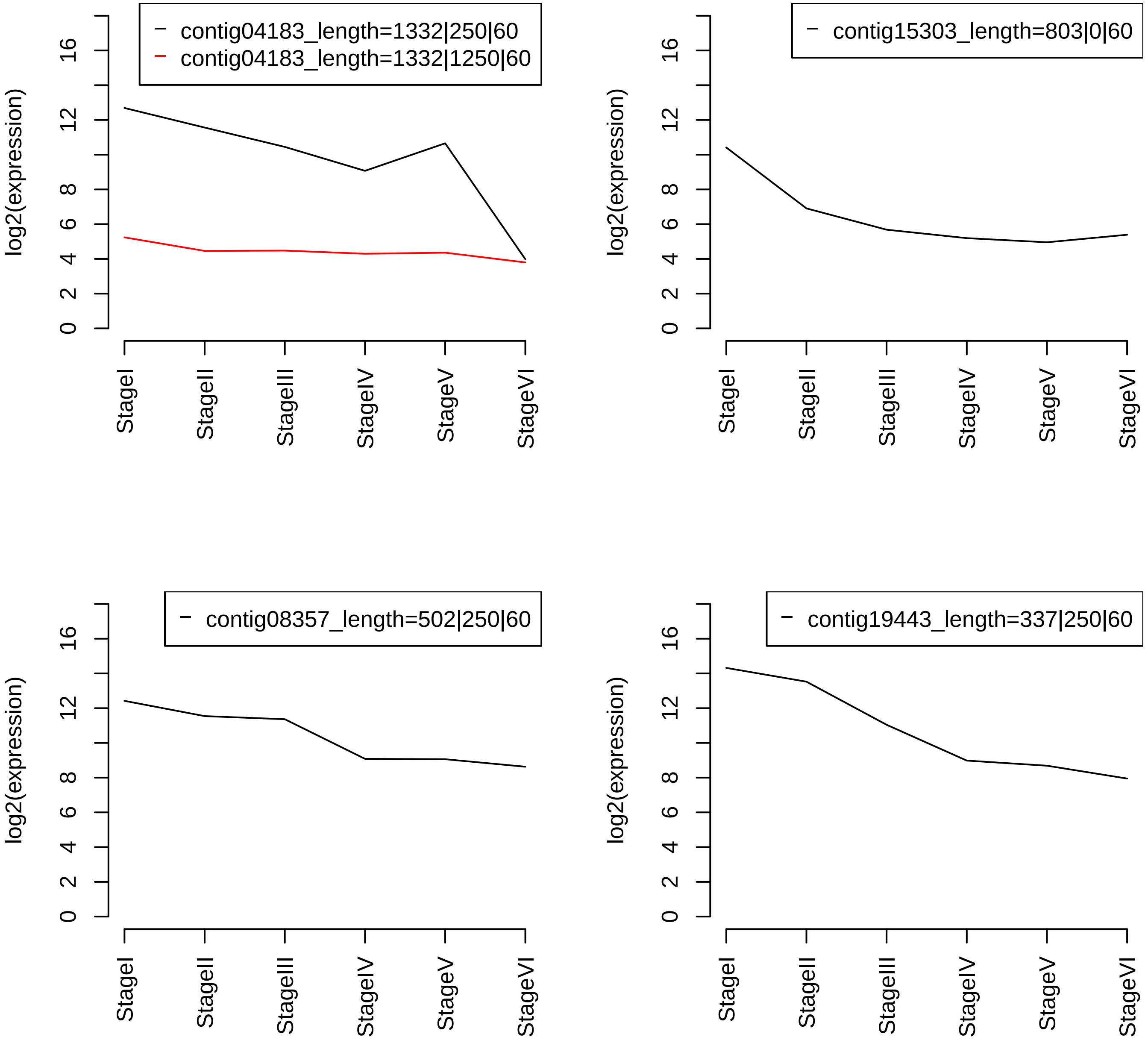
contig04183_length=1332|250|60
contig04183_length=1332|1250|60
contig15303_length=803|0|60
contig08357_length=502|250|60
contig19443_length=337|250|60
log2(expression)
0
2
4
6
8
12
16
StageI
StageII
StageIII
StageIV
StageV
StageVI

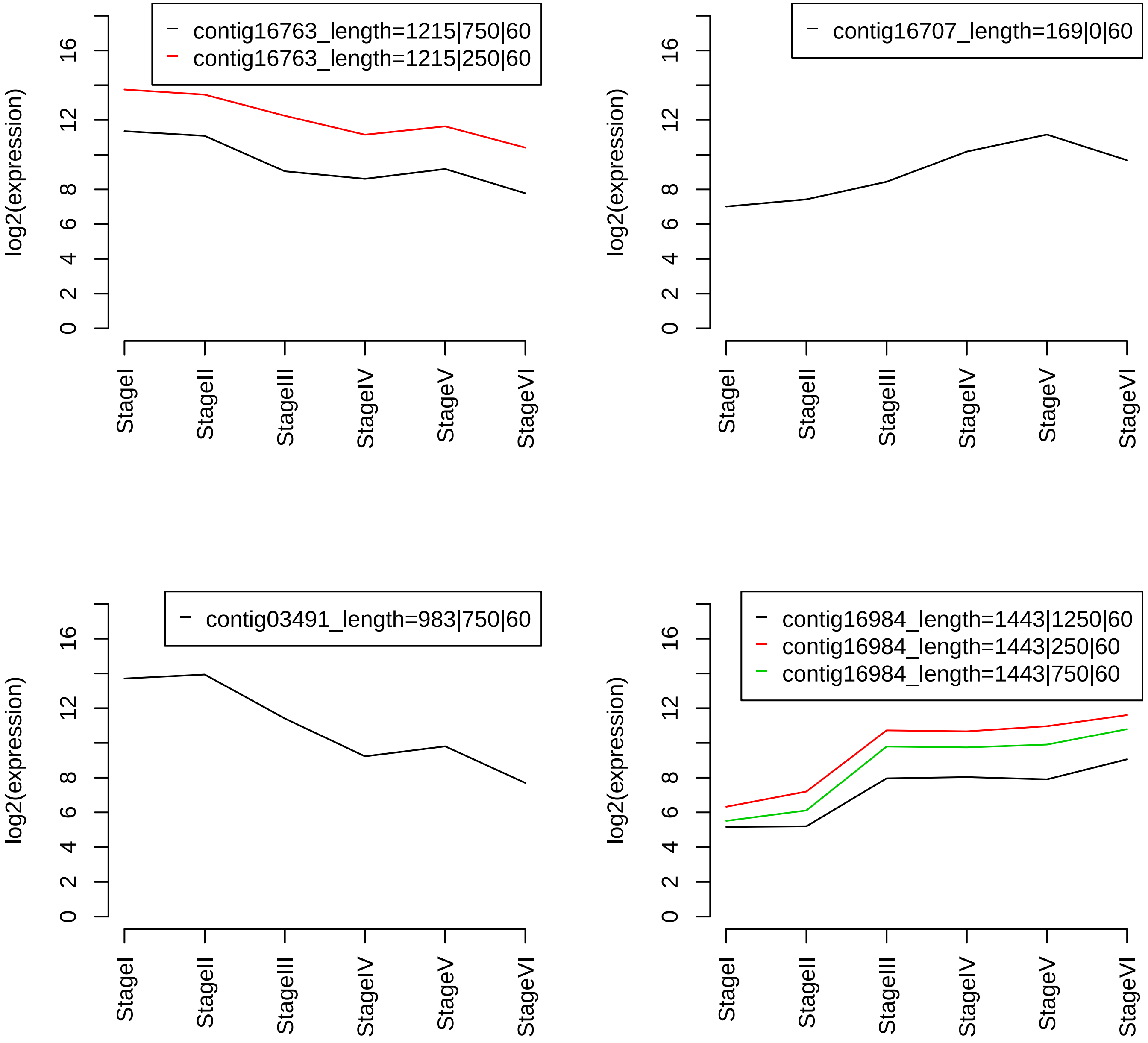

contig16763_length=1215|750|60
contig16763_length=1215|250|60
contig16707_length=169|0|60
contig03491_length=983|750|60
contig16984_length=1443|1250|60
contig16984_length=1443|250|60
contig16984_length=1443|750|60
log2(expression)
0
2
4
6
8
12
16
StageI
StageII
StageIII
StageIV
StageV
StageVI

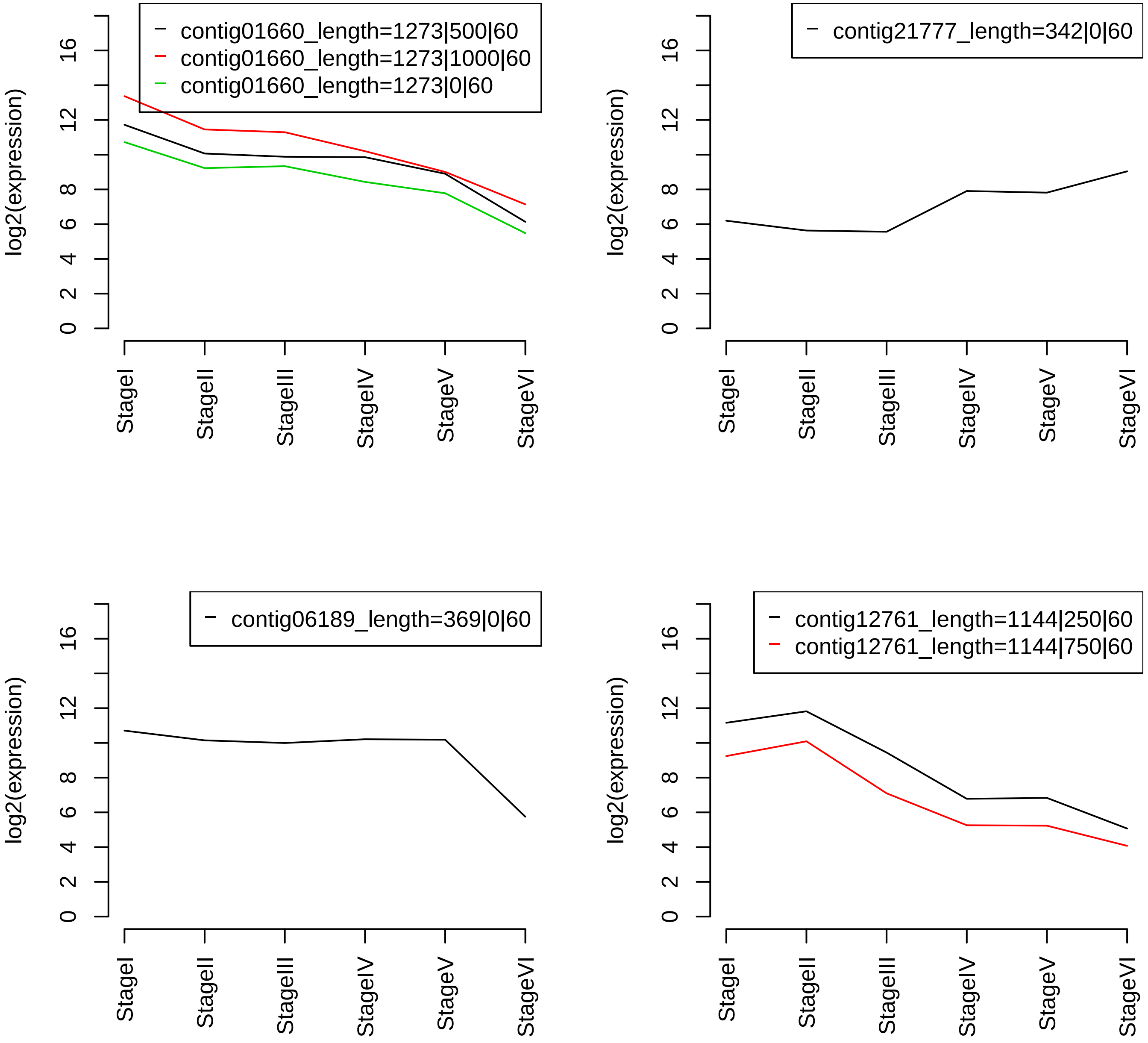

contig01660_length=1273|500|60
contig01660_length=1273|1000|60
contig01660_length=1273|0|60
contig21777_length=342|0|60
contig06189_length=369|0|60
contig12761_length=1144|250|60
contig12761_length=1144|750|60
log2(expression)
0
2
4
6
8
12
16
StageI
StageII
StageIII
StageIV
StageV
StageVI

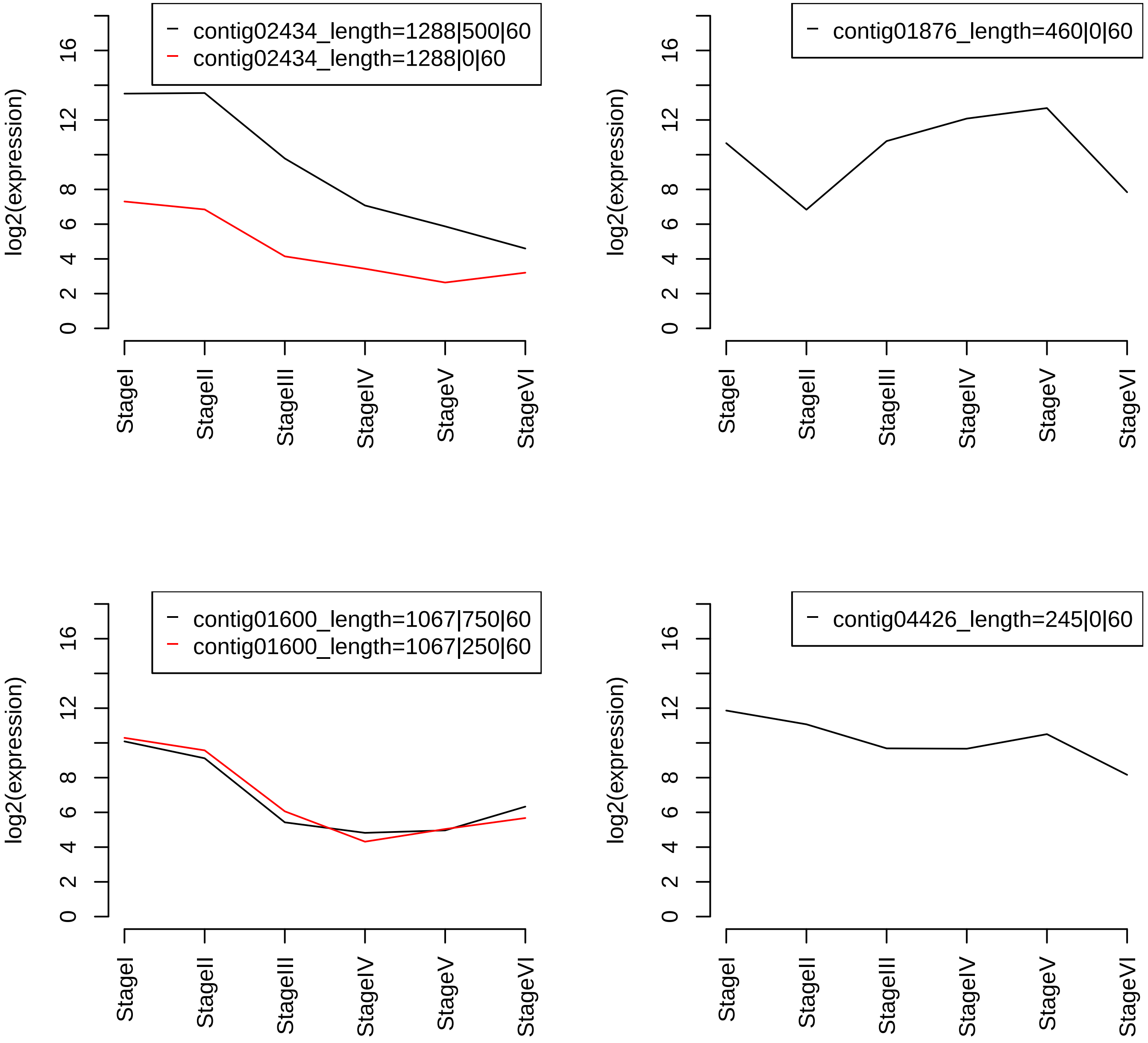

contig02434_length=1288|500|60
contig02434_length=1288|0|60
contig01876_length=460|0|60
contig01600_length=1067|750|60
contig01600_length=1067|250|60
contig04426_length=245|0|60
log2(expression)
0
2
4
6
8
12
16
StageI
StageII
StageIII
StageIV
StageV
StageVI

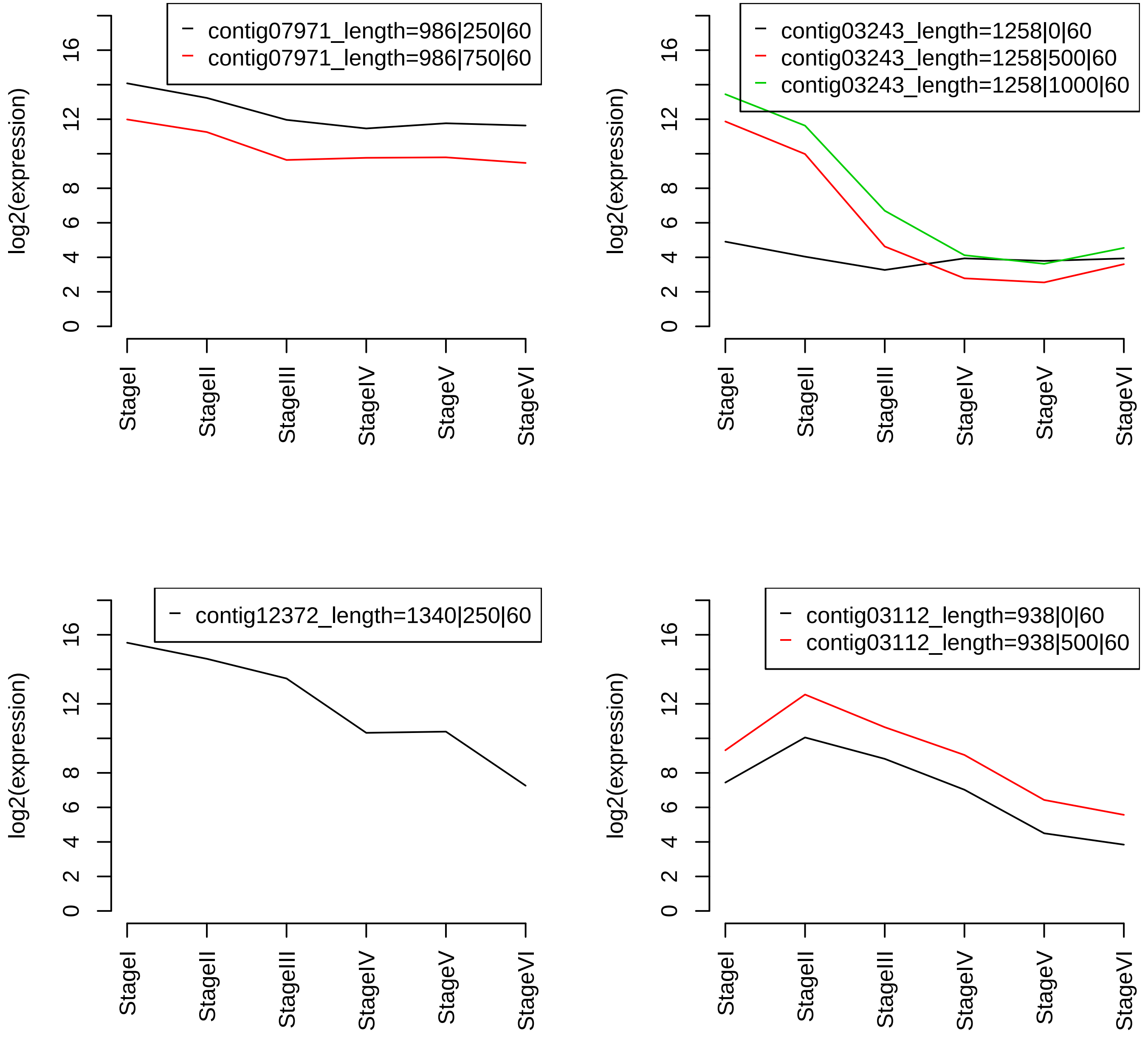
contig07971_length=986|250|60
contig07971_length=986|750|60
log2(expression)
0
2
4
6
8
12
16
StageI
StageII
StageIII
StageIV
StageV
StageVI
contig03243_length=1258|0|60
contig03243_length=1258|500|60
contig03243_length=1258|1000|60
contig12372_length=1340|250|60
contig03112_length=938|0|60
contig03112_length=938|500|60

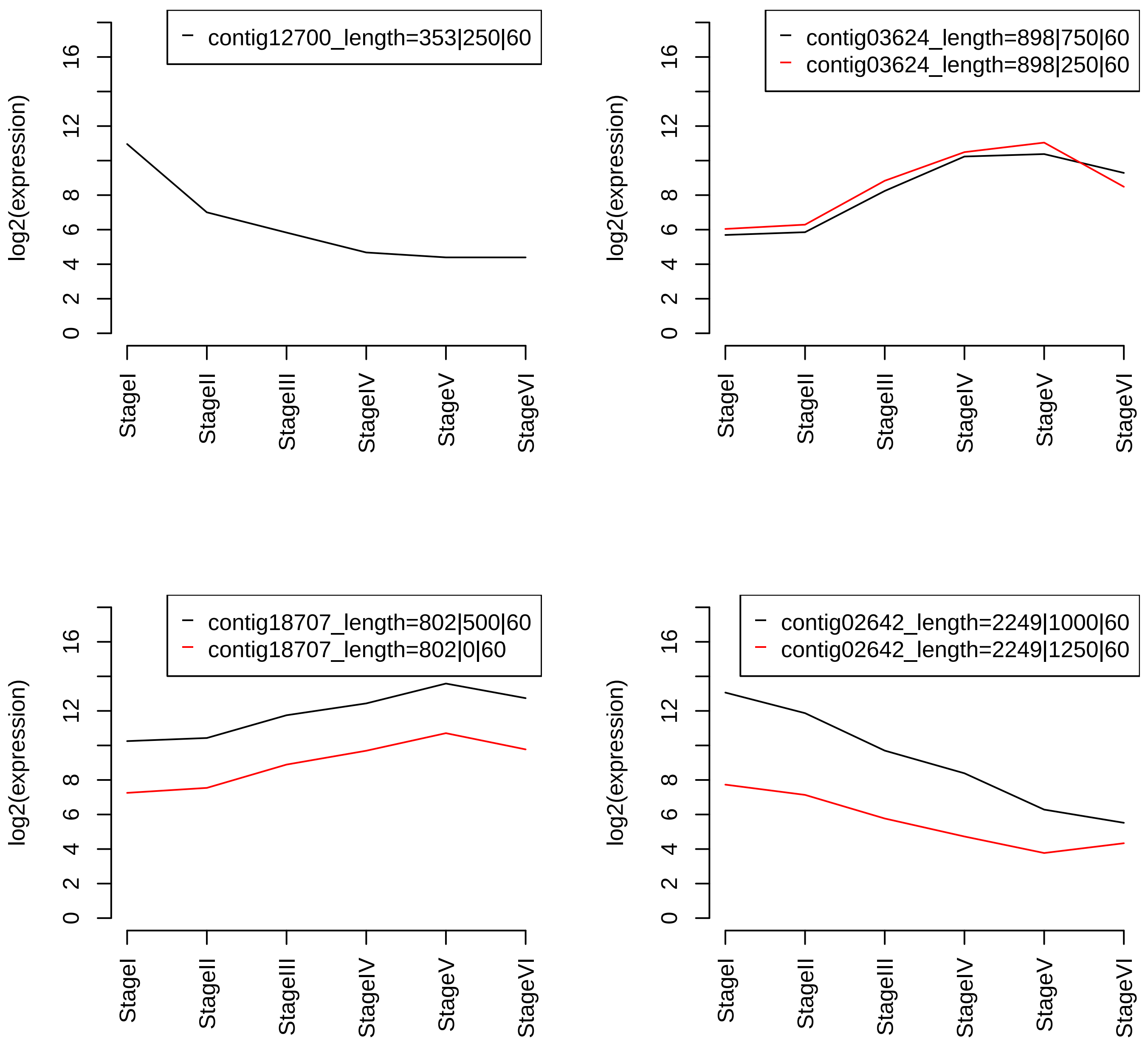
contig12700_length=353|250|60
contig03624_length=898|750|60
contig03624_length=898|250|60
contig18707_length=802|500|60
contig18707_length=802|0|60
contig02642_length=2249|1000|60
contig02642_length=2249|1250|60
log2(expression)
0
2
4
6
8
12
16
StageI
StageII
StageIII
StageIV
StageV
StageVI

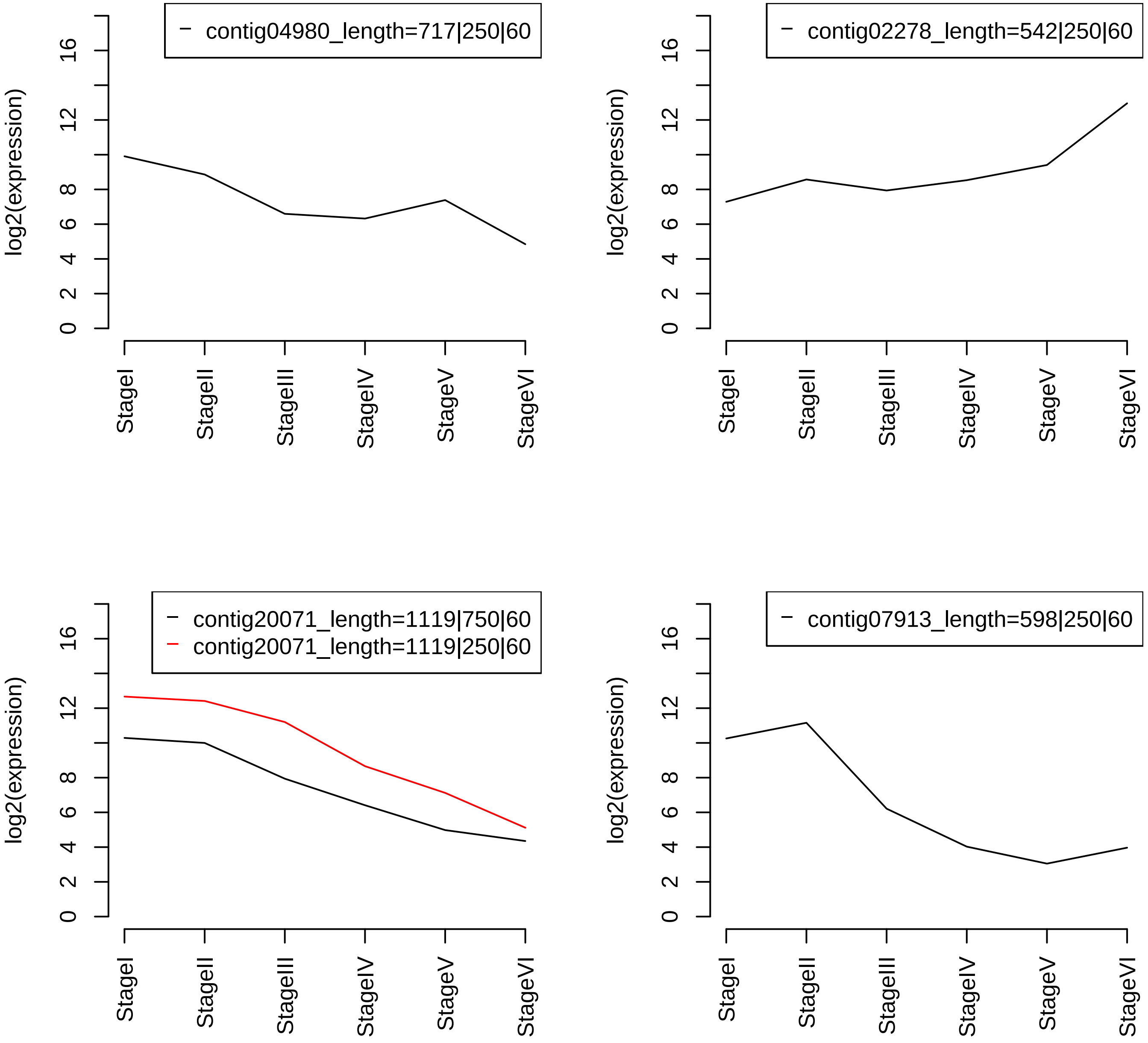
contig04980_length=717|250|60
contig02278_length=542|250|60
contig20071_length=1119|750|60
contig20071_length=1119|250|60
contig07913_length=598|250|60
log2(expression)
0 2 4 6 8 12 16
StageI StageII StageIII StageIV StageV StageVI

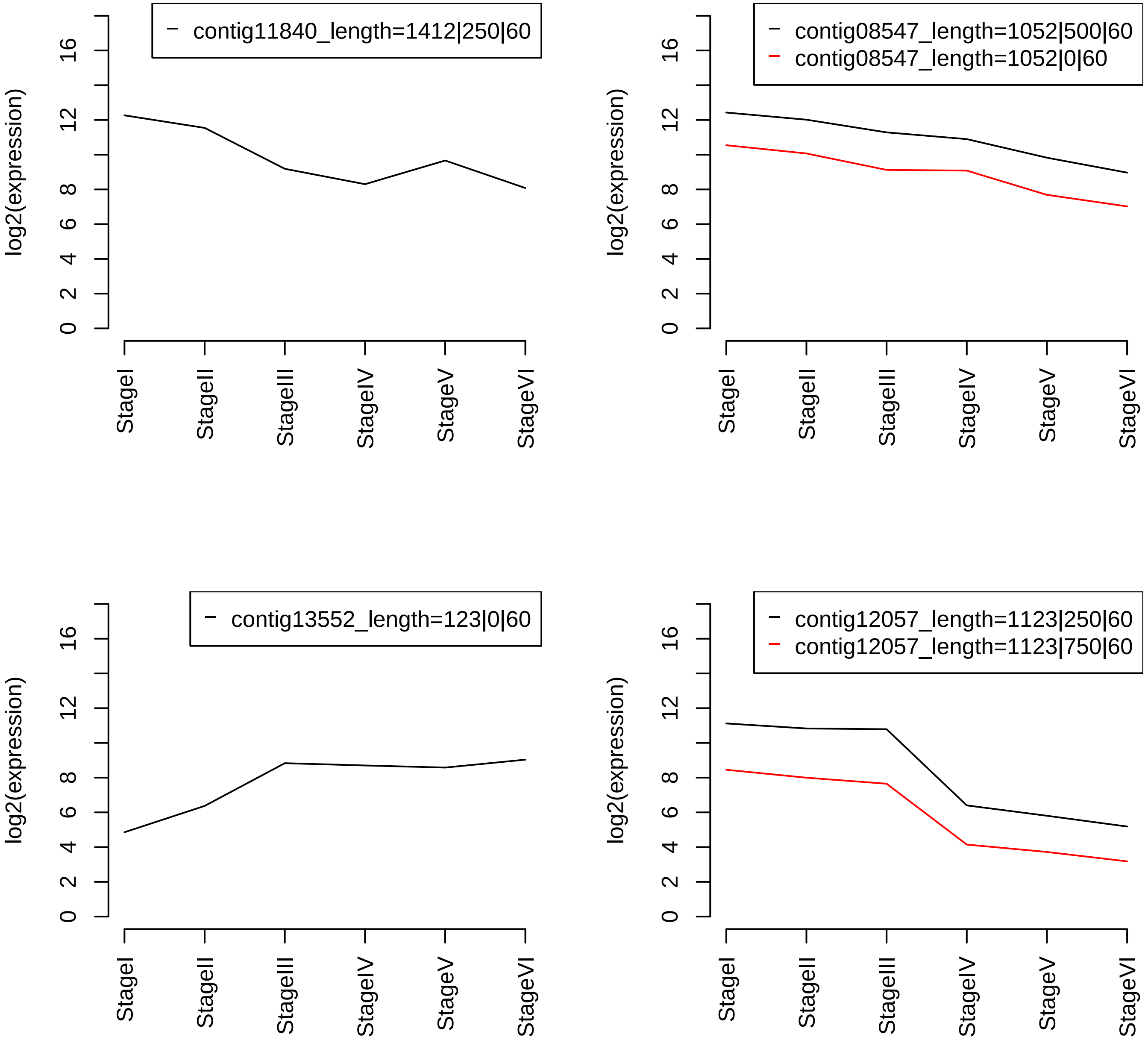

contig11840_length=1412|250|60
contig08547_length=1052|500|60
contig08547_length=1052|0|60
contig13552_length=123|0|60
contig12057_length=1123|250|60
contig12057_length=1123|750|60
log2(expression)
0
2
4
6
8
12
16
StageI
StageII
StageIII
StageIV
StageV
StageVI

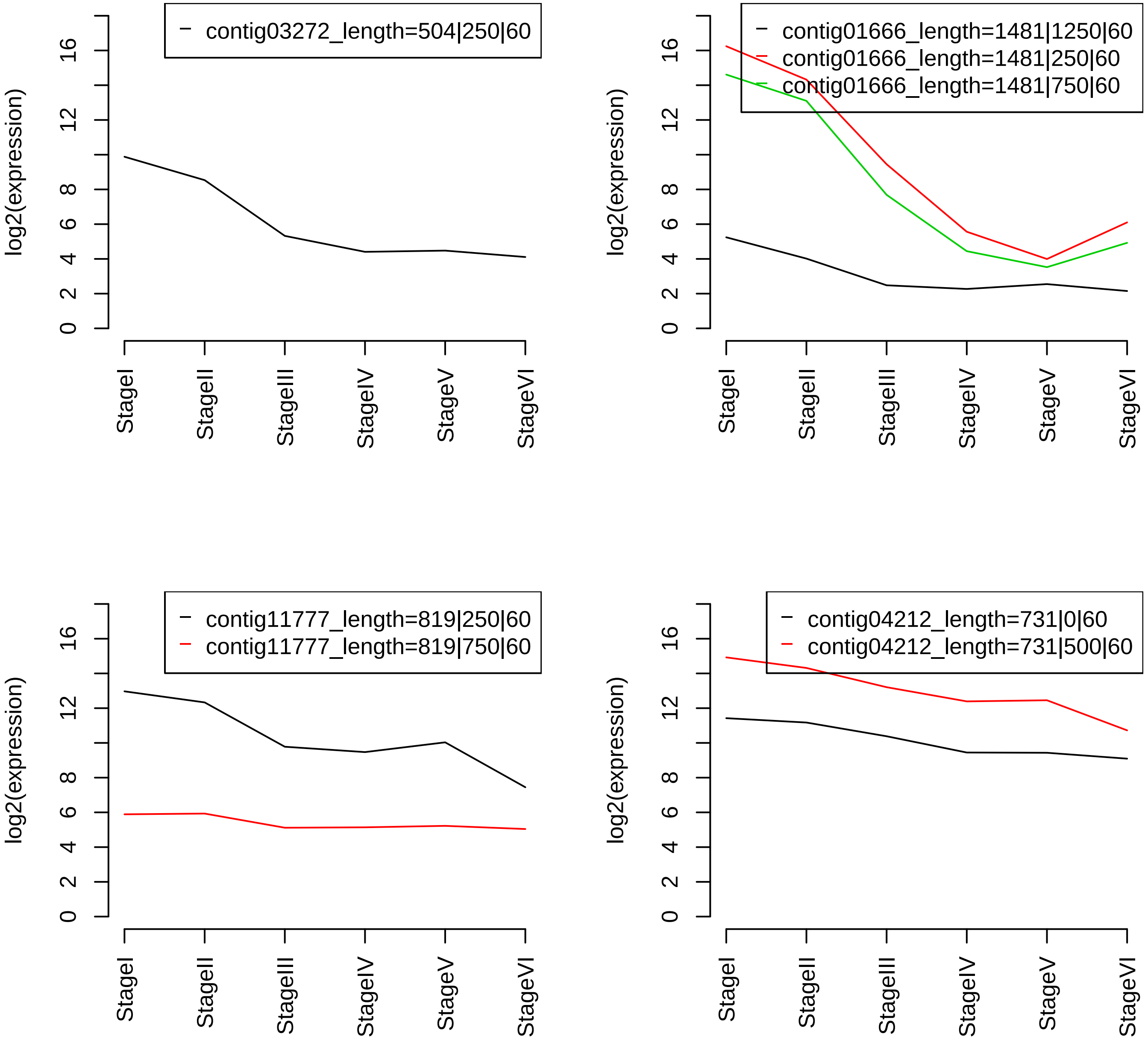
contig03272_length=504|250|60
contig01666_length=1481|1250|60
contig01666_length=1481|250|60
contig01666_length=1481|750|60
contig11777_length=819|250|60
contig11777_length=819|750|60
contig04212_length=731|0|60
contig04212_length=731|500|60
log2(expression)
0
2
4
6
8
12
16
StageI
StageII
StageIII
StageIV
StageV
StageVI

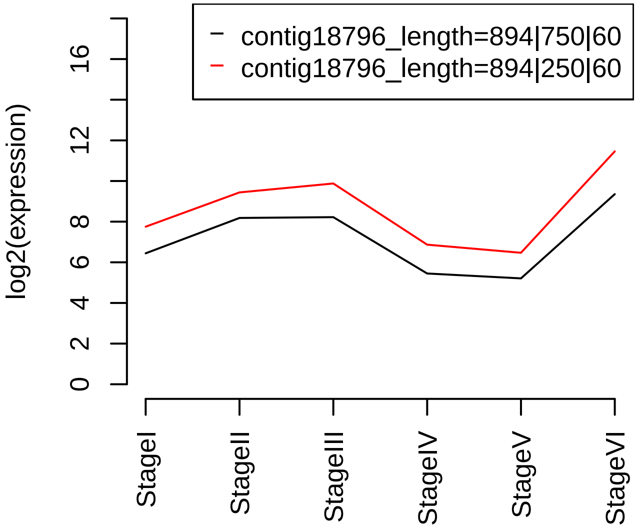
contig18796_length=894|750|60
contig18796_length=894|250|60
log2(expression)
0 2 4 6 8 12 16
StageI StageII StageIII StageIV StageV StageVI

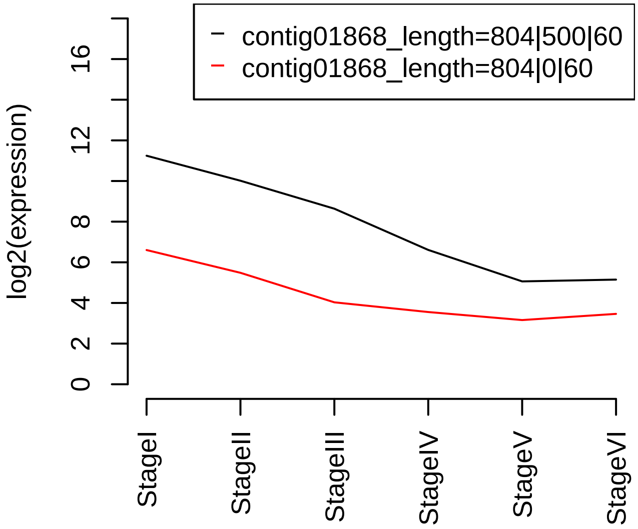
contig01868_length=804|500|60
contig01868_length=804|0|60
log2(expression)
0 2 4 6 8 12 16
StageI StageII StageIII StageIV StageV StageVI

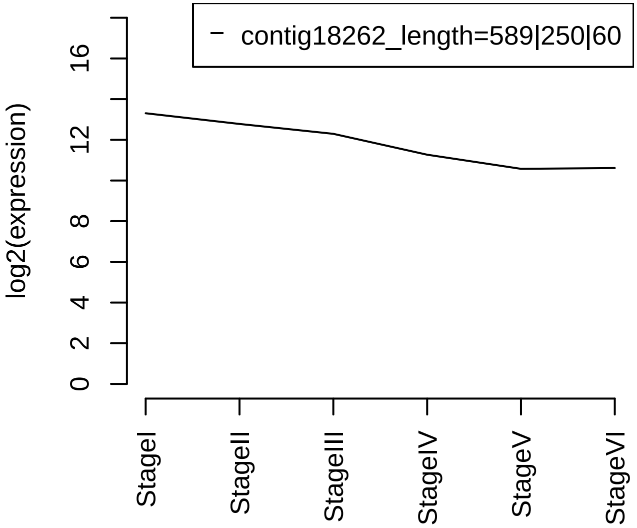
contig18262_length=589|250|60
log2(expression)
0 2 4 6 8 12 16
StageI StageII StageIII StageIV StageV StageVI

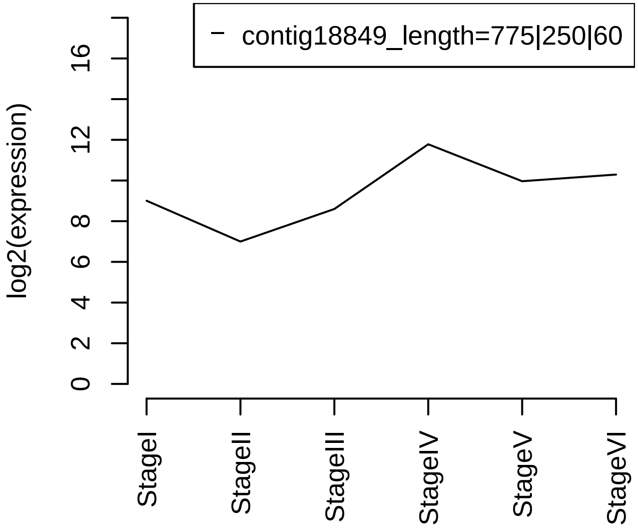
contig18849_length=775|250|60
log2(expression)
0 2 4 6 8 12 16
StageI StageII StageIII StageIV StageV StageVI

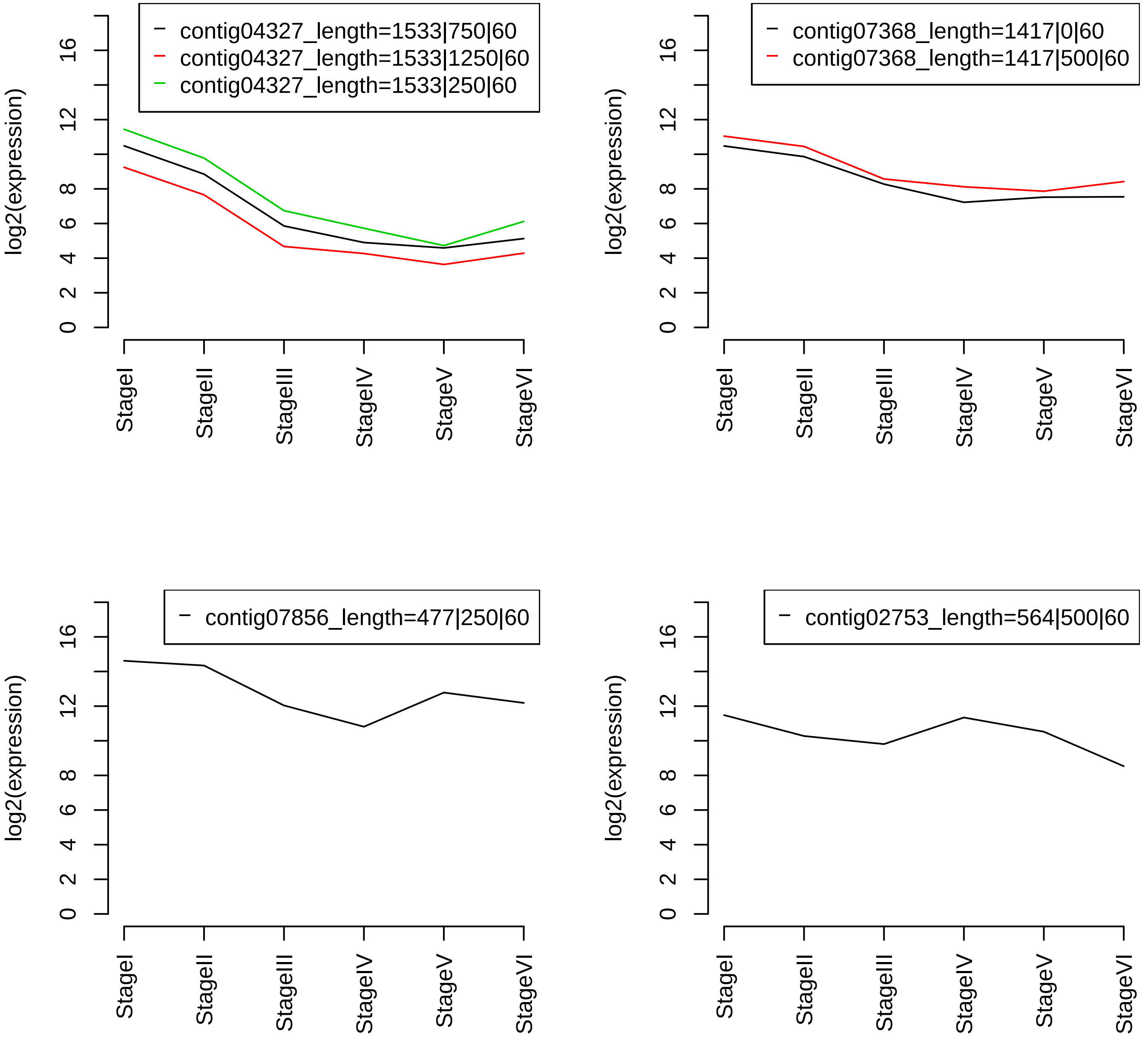
contig04327_length=1533|750|60
contig04327_length=1533|1250|60
contig04327_length=1533|250|60
contig07368_length=1417|0|60
contig07368_length=1417|500|60
contig07856_length=477|250|60
contig02753_length=564|500|60
log2(expression)
0
2
4
6
8
12
16
StageI
StageII
StageIII
StageIV
StageV
StageVI

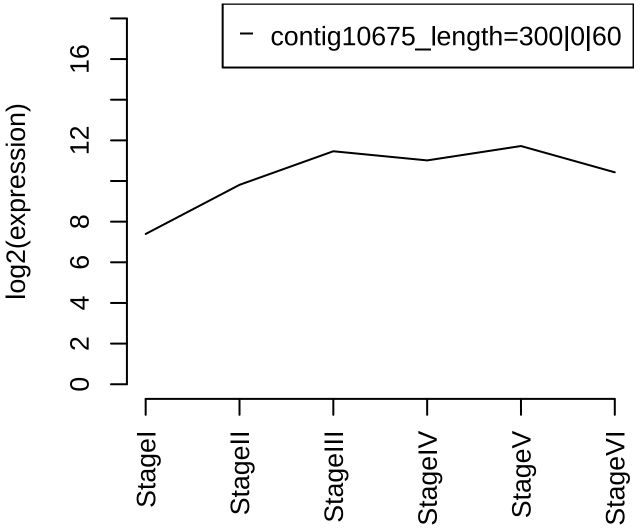

contig10675_length=300|0|60
log2(expression)
0 2 4 6 8 12 16
StageI StageII StageIII StageIV StageV StageVI

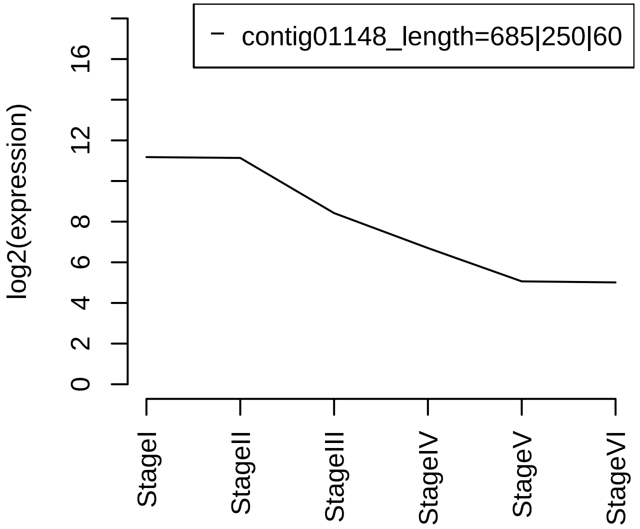

contig01148_length=685|250|60
log2(expression)
0 2 4 6 8 12 16
StageI StageII StageIII StageIV StageV StageVI

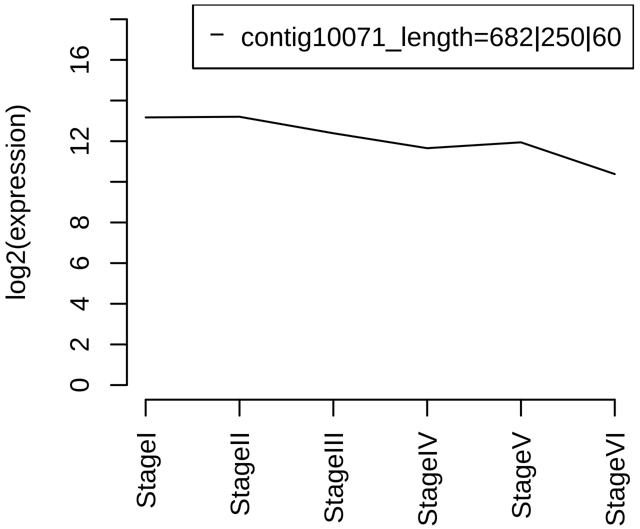

contig10071_length=682|250|60
log2(expression)
0 2 4 6 8 12 16
StageI StageII StageIII StageIV StageV StageVI

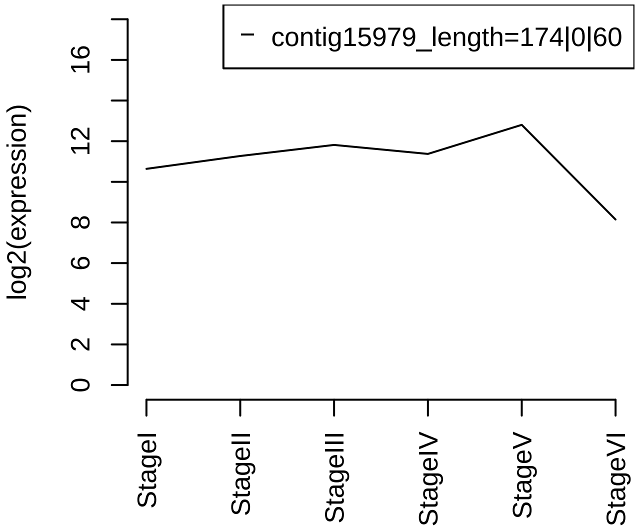

contig15979_length=174|0|60
log2(expression)
0 2 4 6 8 12 16
StageI StageII StageIII StageIV StageV StageVI

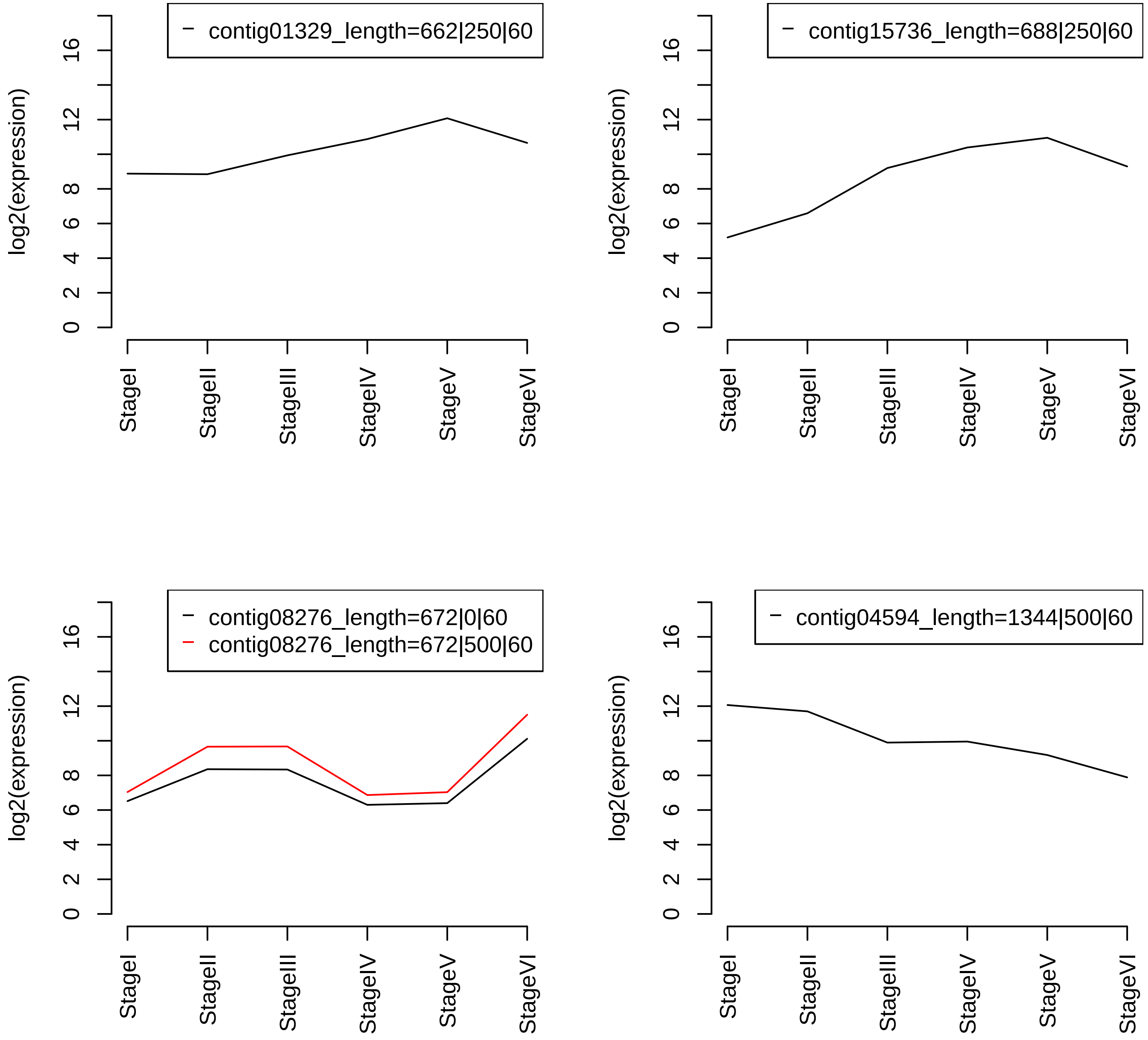

contig01329_length=662|250|60
contig15736_length=688|250|60
contig08276_length=672|0|60
contig08276_length=672|500|60
contig04594_length=1344|500|60
log2(expression)
0
2
4
6
8
12
16
StageI
StageII
StageIII
StageIV
StageV
StageVI

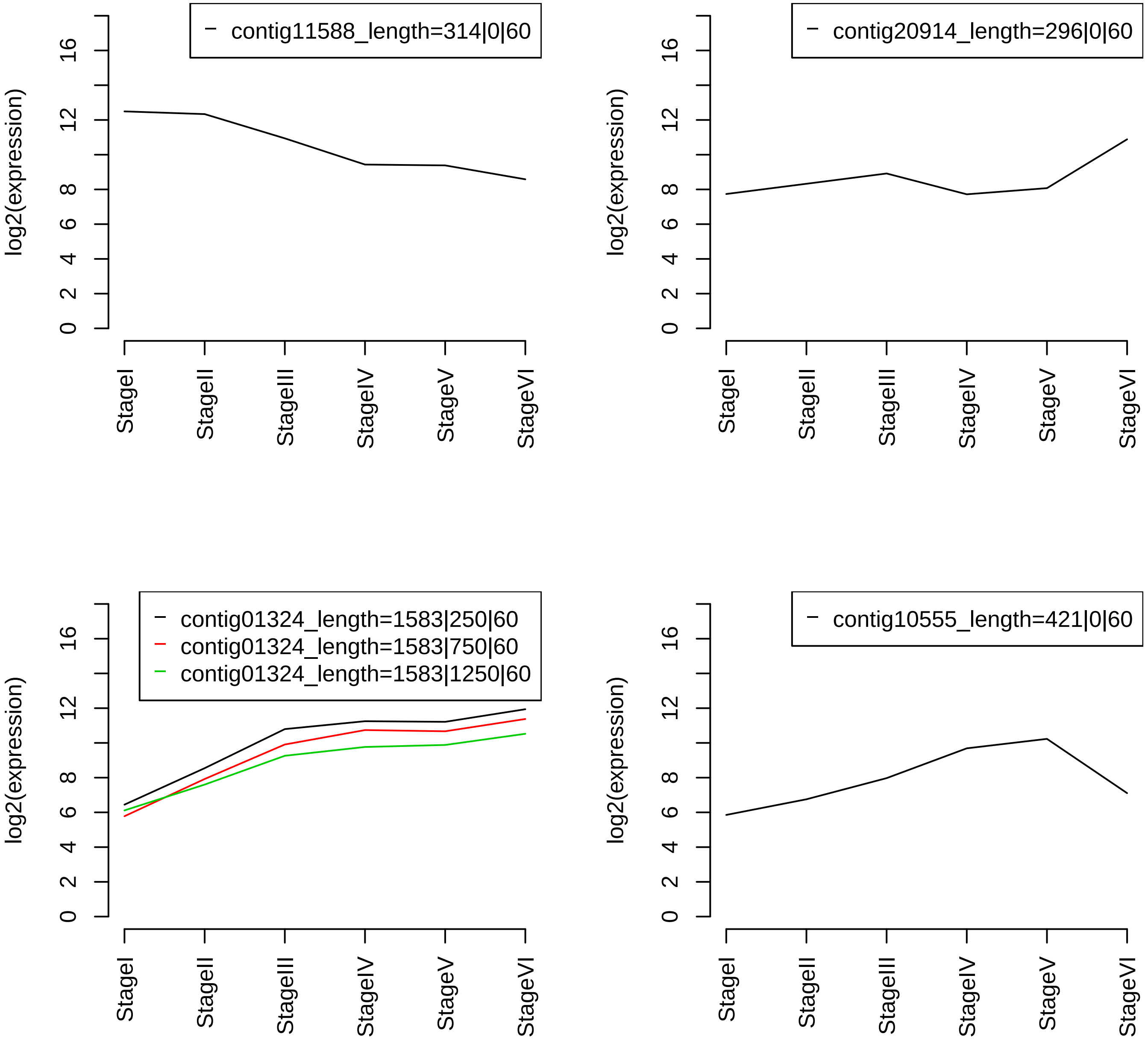
contig11588_length=314|0|60
contig20914_length=296|0|60
contig01324_length=1583|250|60
contig01324_length=1583|750|60
contig01324_length=1583|1250|60
contig10555_length=421|0|60
log2(expression)
0
2
4
6
8
12
16
StageI
StageII
StageIII
StageIV
StageV
StageVI

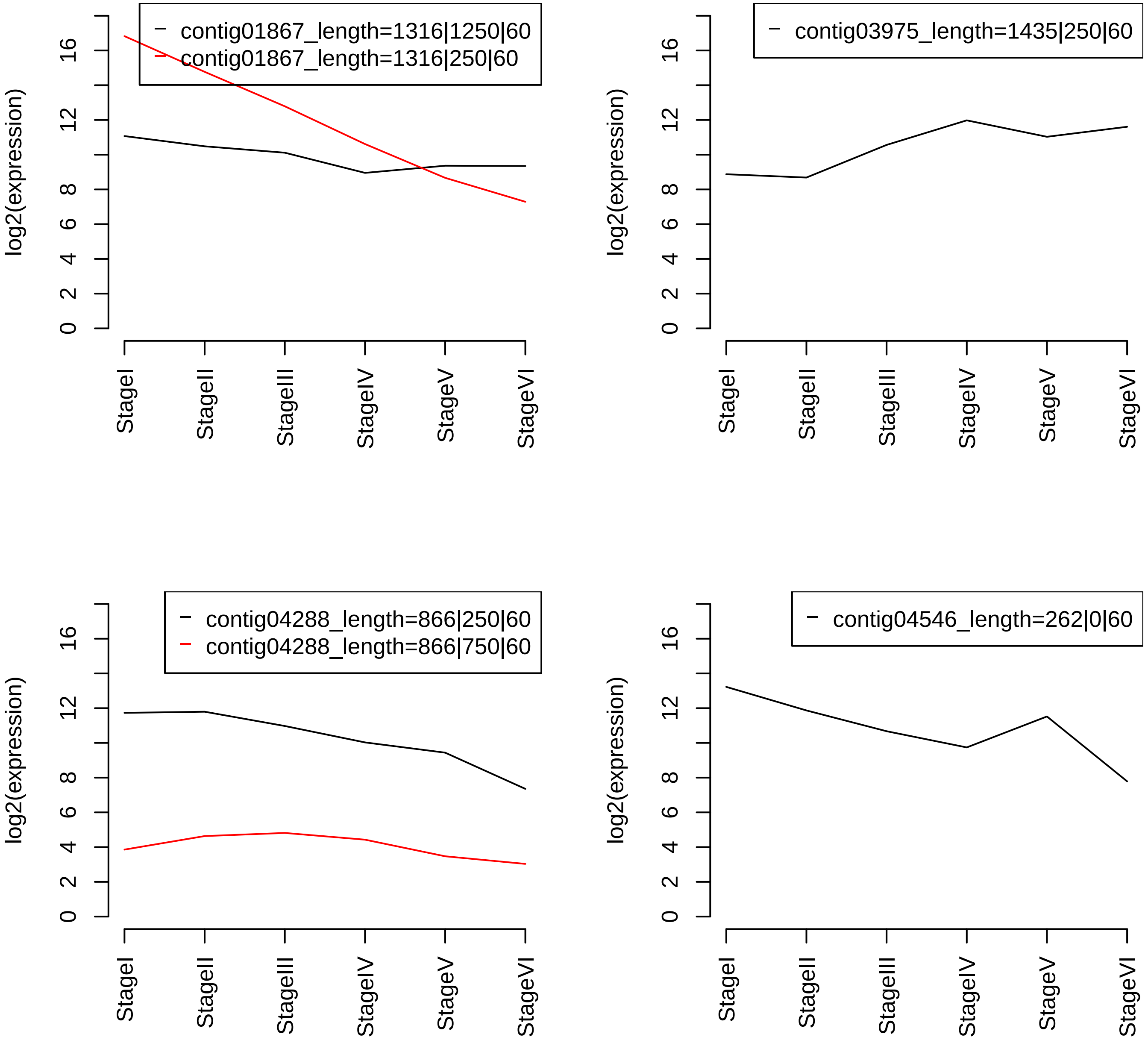
contig01867_length=1316|1250|60
contig01867_length=1316|250|60
contig03975_length=1435|250|60
contig04288_length=866|250|60
contig04288_length=866|750|60
contig04546_length=262|0|60
log2(expression)
0
2
4
6
8
12
16
StageI
StageII
StageIII
StageIV
StageV
StageVI

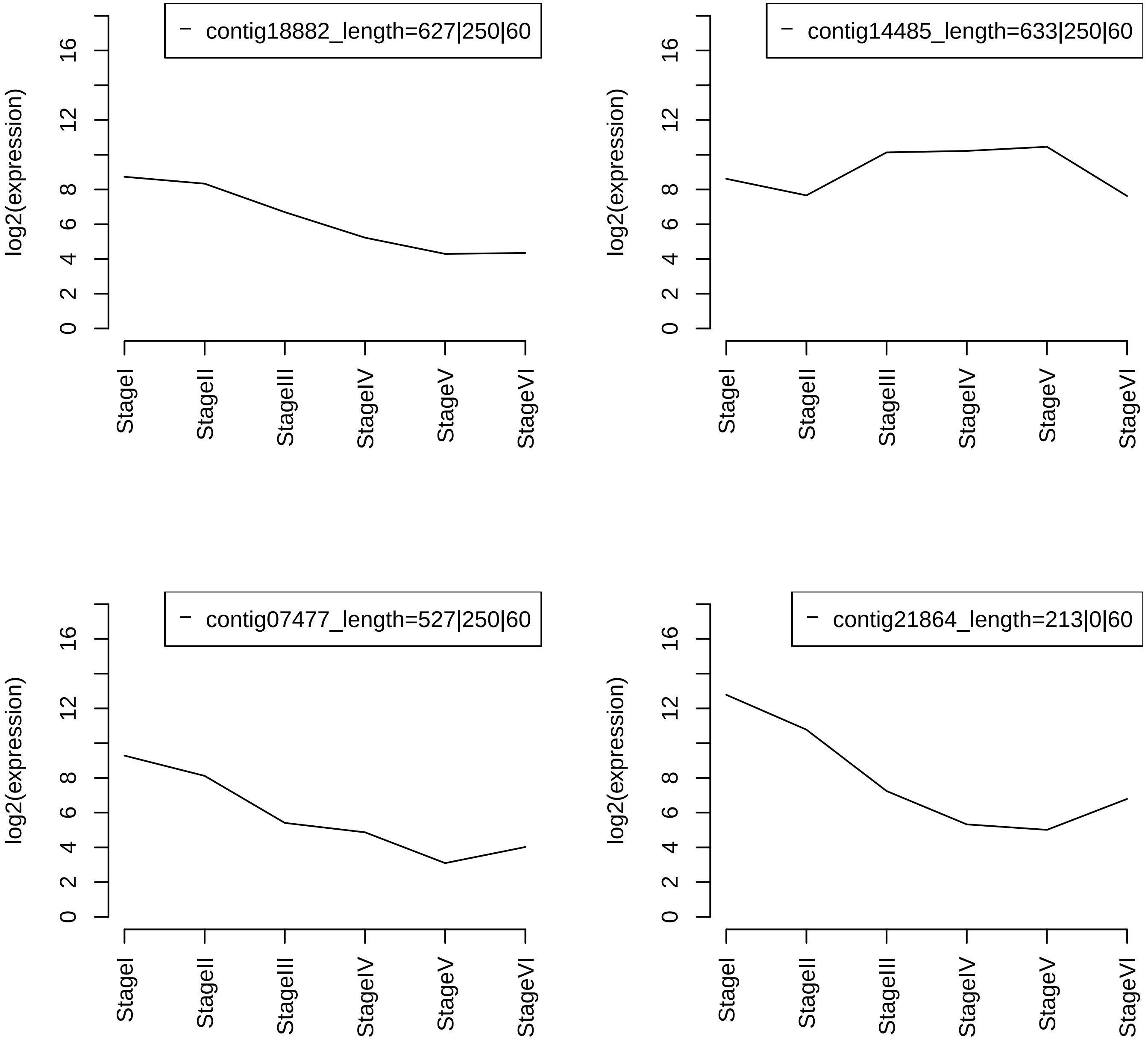

contig18882_length=627|250|60
contig14485_length=633|250|60
contig07477_length=527|250|60
contig21864_length=213|0|60
log2(expression)
0
2
4
6
8
12
16
StageI
StageII
StageIII
StageIV
StageV
StageVI

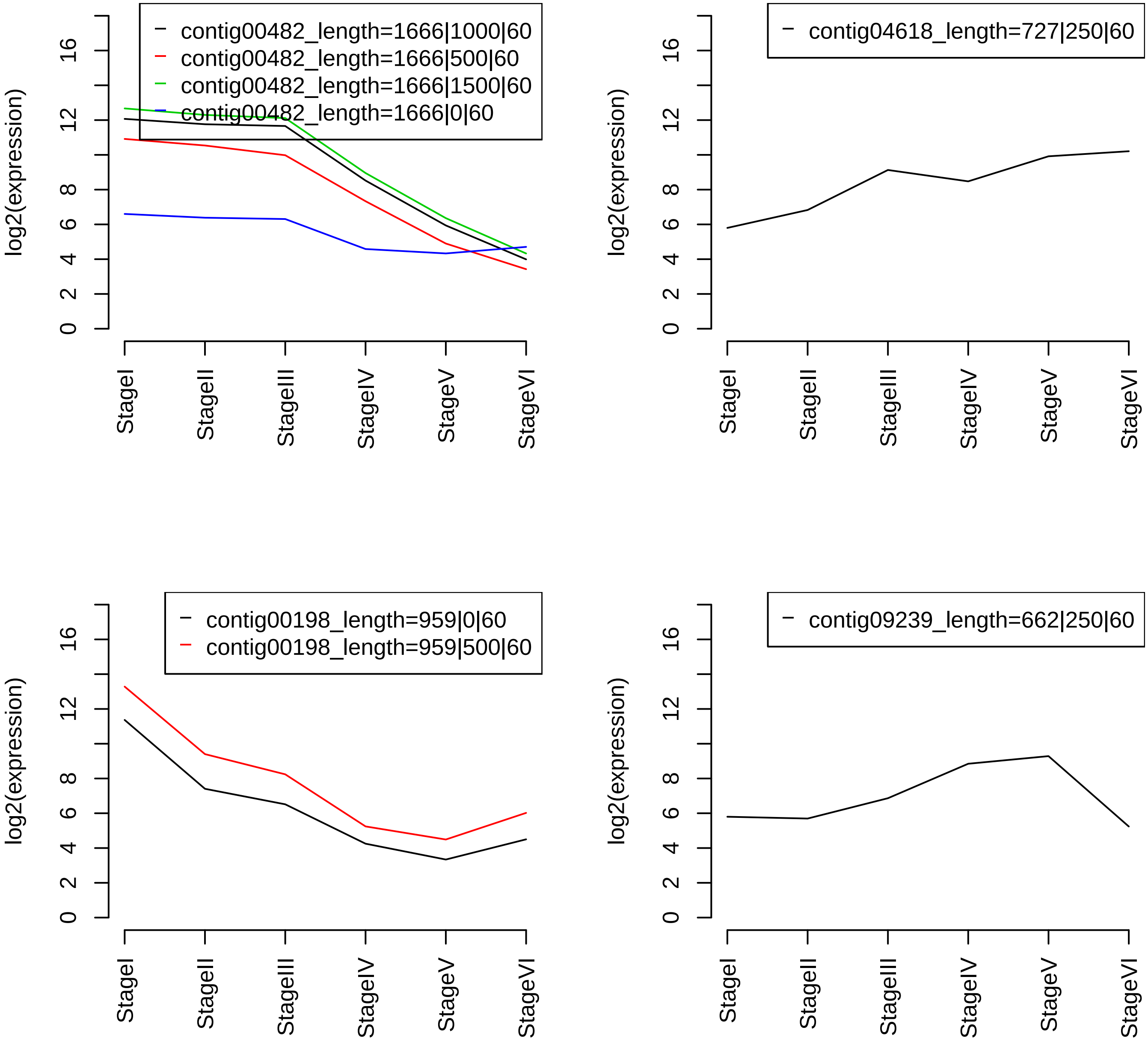
contig00482_length=1666|1000|60
contig00482_length=1666|500|60
contig00482_length=1666|1500|60
contig00482_length=1666|0|60
contig04618_length=727|250|60
contig00198_length=959|0|60
contig00198_length=959|500|60
contig09239_length=662|250|60
log2(expression)
0
2
4
6
8
12
16
StageI
StageII
StageIII
StageIV
StageV
StageVI

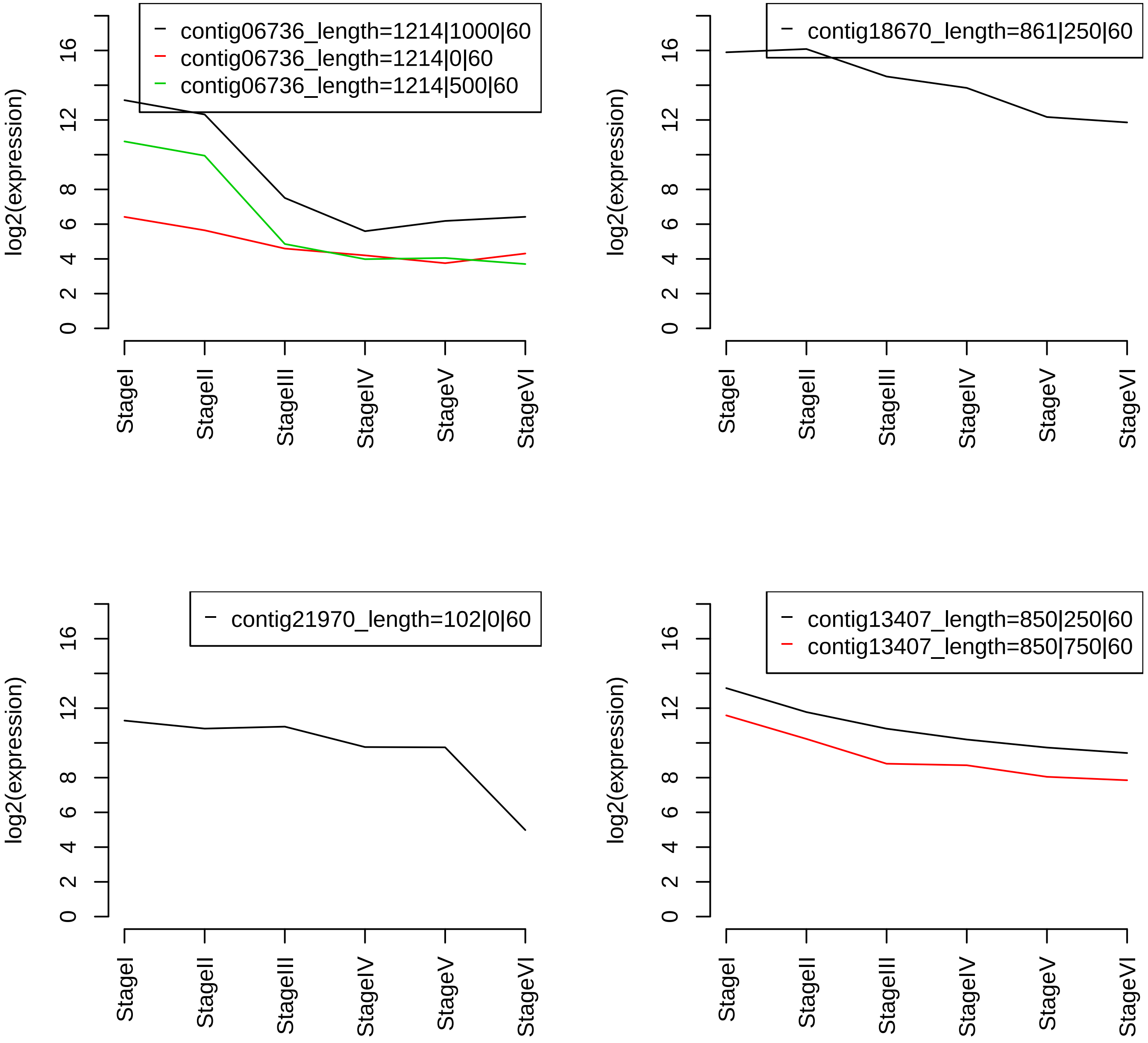

contig06736_length=1214|1000|60
contig06736_length=1214|0|60
contig06736_length=1214|500|60
contig18670_length=861|250|60
contig21970_length=102|0|60
contig13407_length=850|250|60
contig13407_length=850|750|60
log2(expression)
0
2
4
6
8
12
16
StageI
StageII
StageIII
StageIV
StageV
StageVI

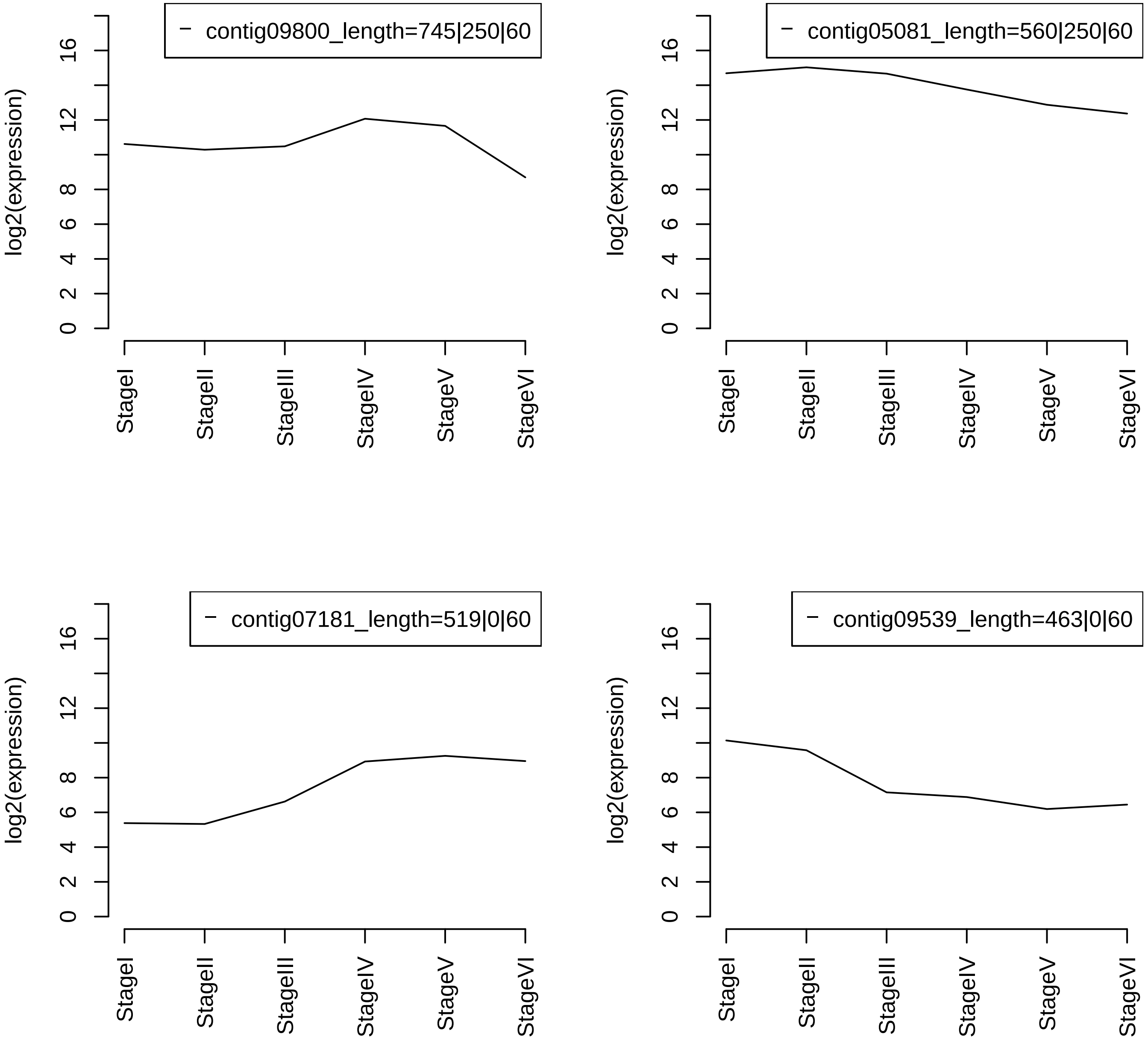

contig09800_length=745|250|60
log2(expression)
0
2
4
6
8
12
16
StageI
StageII
StageIII
StageIV
StageV
StageVI
contig05081_length=560|250|60
log2(expression)
0
2
4
6
8
12
16
StageI
StageII
StageIII
StageIV
StageV
StageVI
contig07181_length=519|0|60
log2(expression)
0
2
4
6
8
12
16
StageI
StageII
StageIII
StageIV
StageV
StageVI
contig09539_length=463|0|60
log2(expression)
0
2
4
6
8
12
16
StageI
StageII
StageIII
StageIV
StageV
StageVI

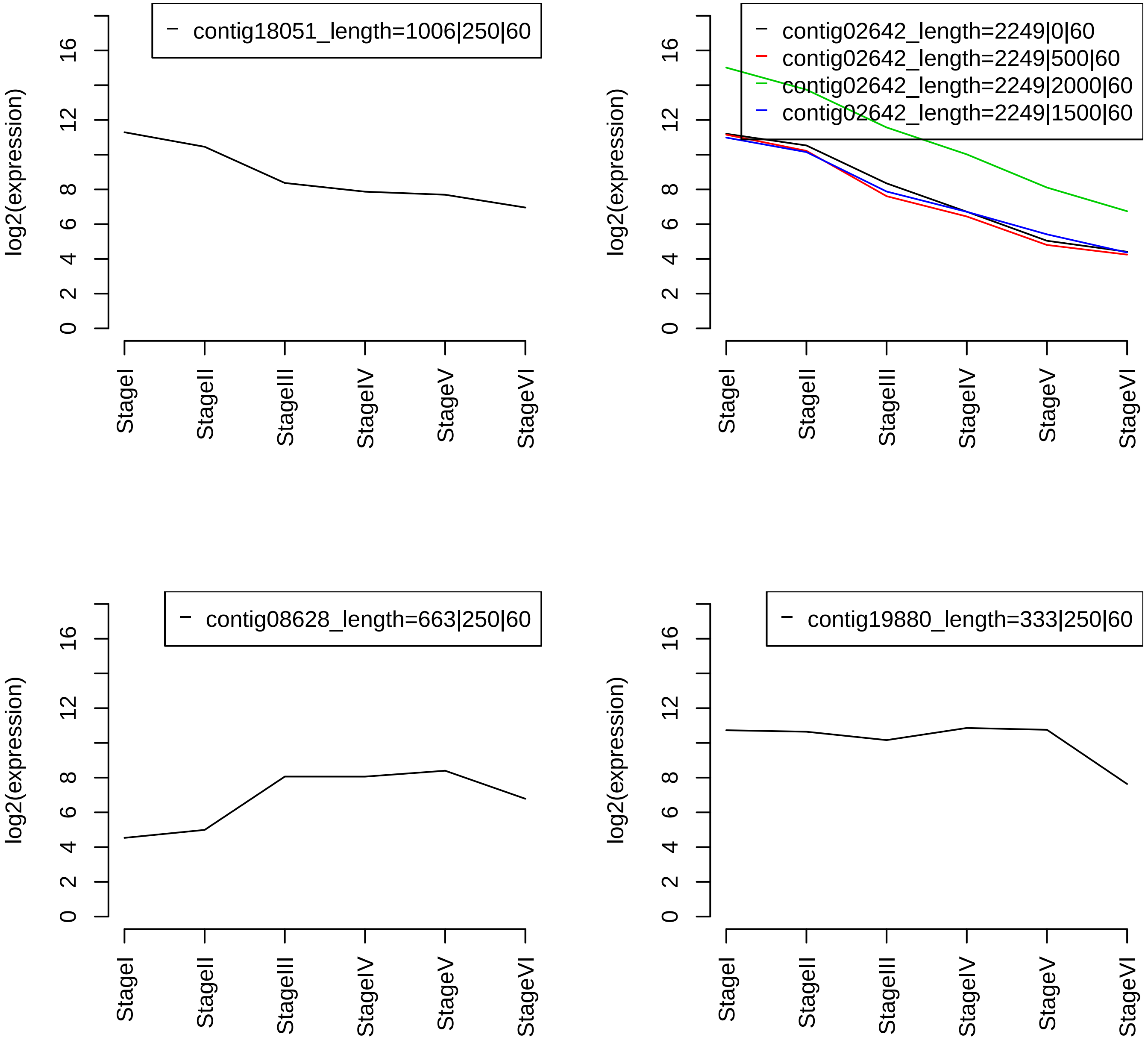
contig18051_length=1006|250|60
contig02642_length=2249|0|60
contig02642_length=2249|500|60
contig02642_length=2249|2000|60
contig02642_length=2249|1500|60
contig08628_length=663|250|60
contig19880_length=333|250|60
log2(expression)
0
2
4
6
8
12
16
StageI
StageII
StageIII
StageIV
StageV
StageVI

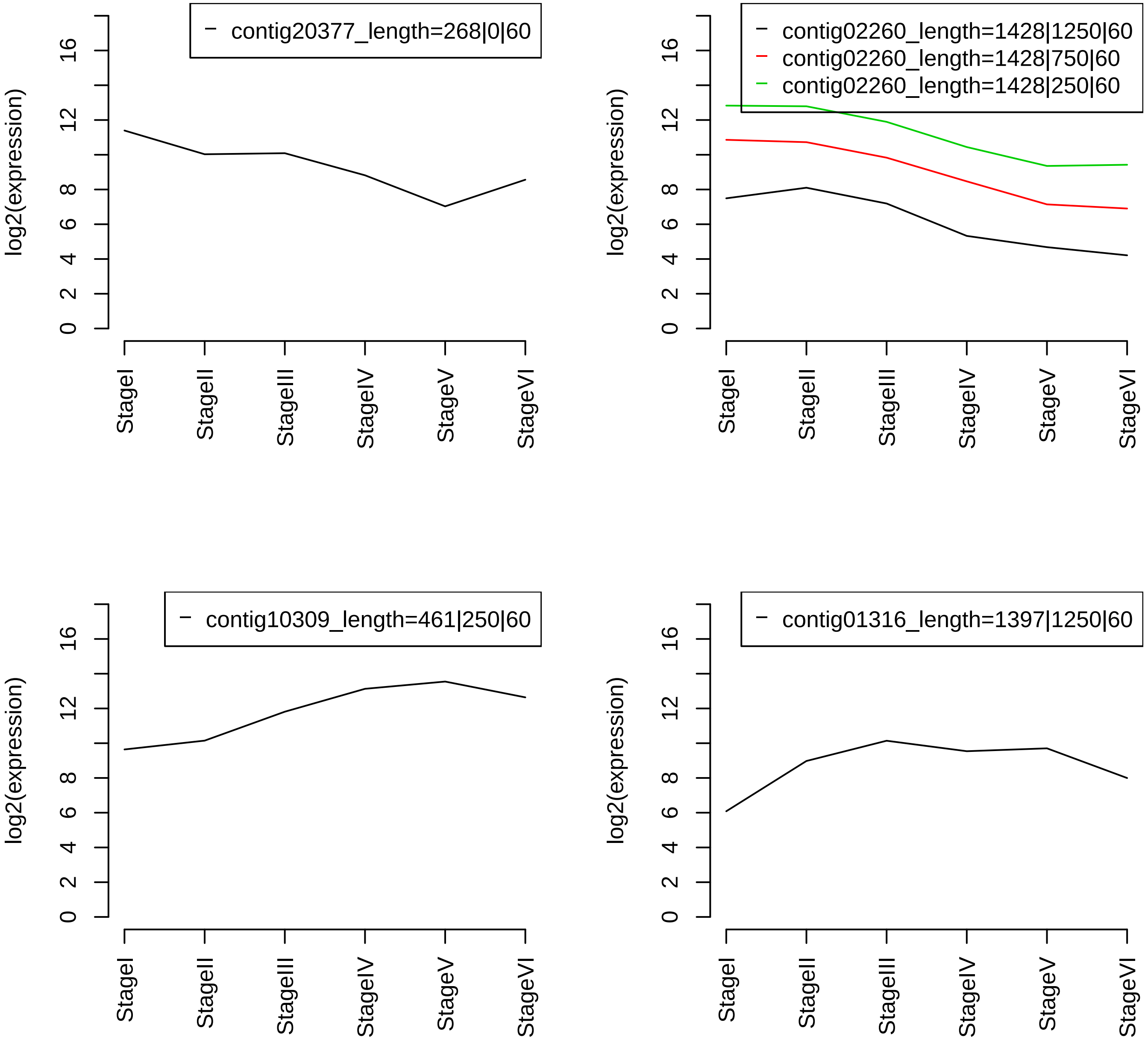
contig20377_length=268|0|60
contig02260_length=1428|1250|60
contig02260_length=1428|750|60
contig02260_length=1428|250|60
contig10309_length=461|250|60
contig01316_length=1397|1250|60
log2(expression)
0
2
4
6
8
12
16
StageI
StageII
StageIII
StageIV
StageV
StageVI

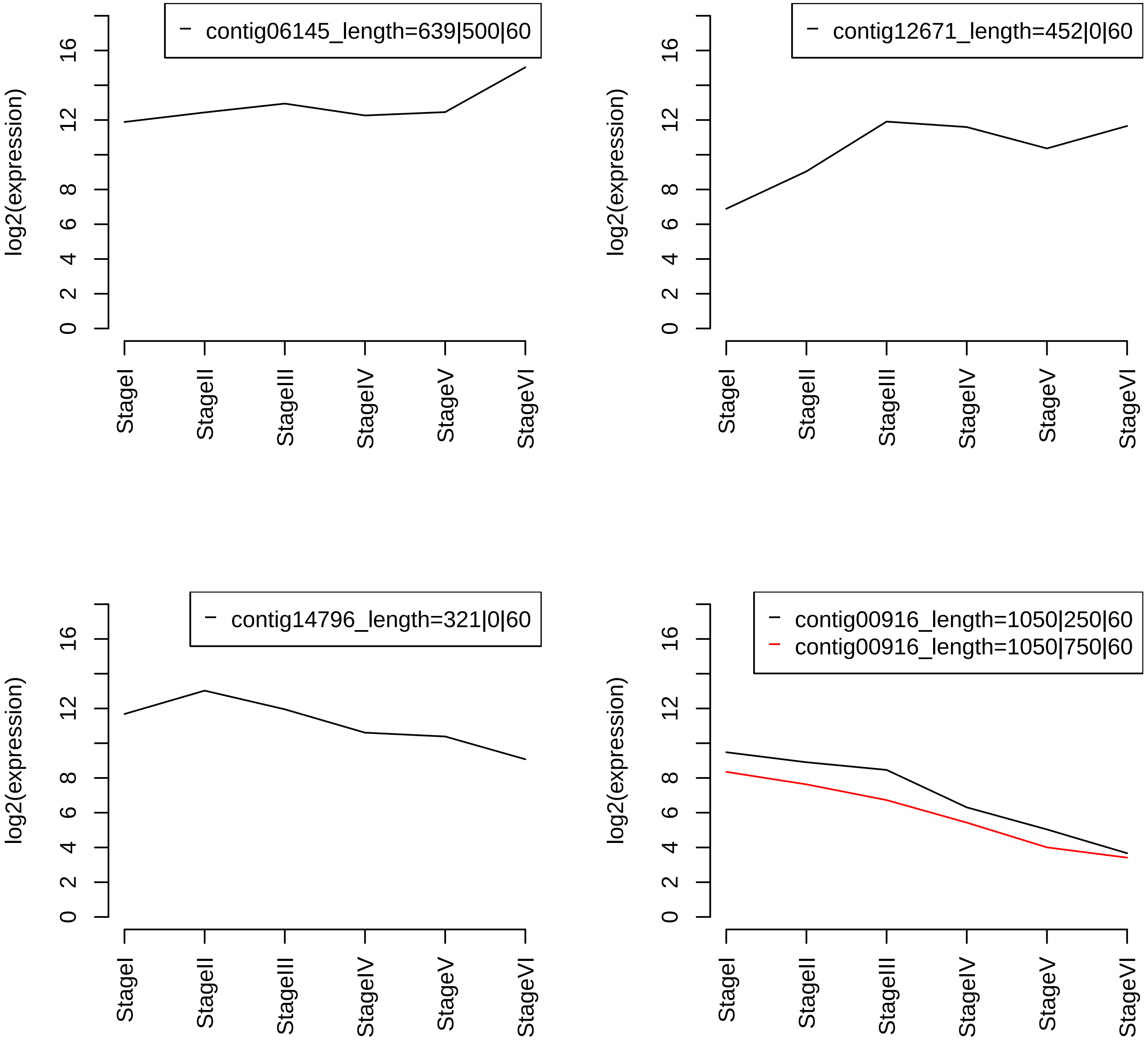

contig06145_length=639|500|60
contig12671_length=452|0|60
contig14796_length=321|0|60
contig00916_length=1050|250|60
contig00916_length=1050|750|60
log2(expression)
0
2
4
6
8
12
16
StageI
StageII
StageIII
StageIV
StageV
StageVI

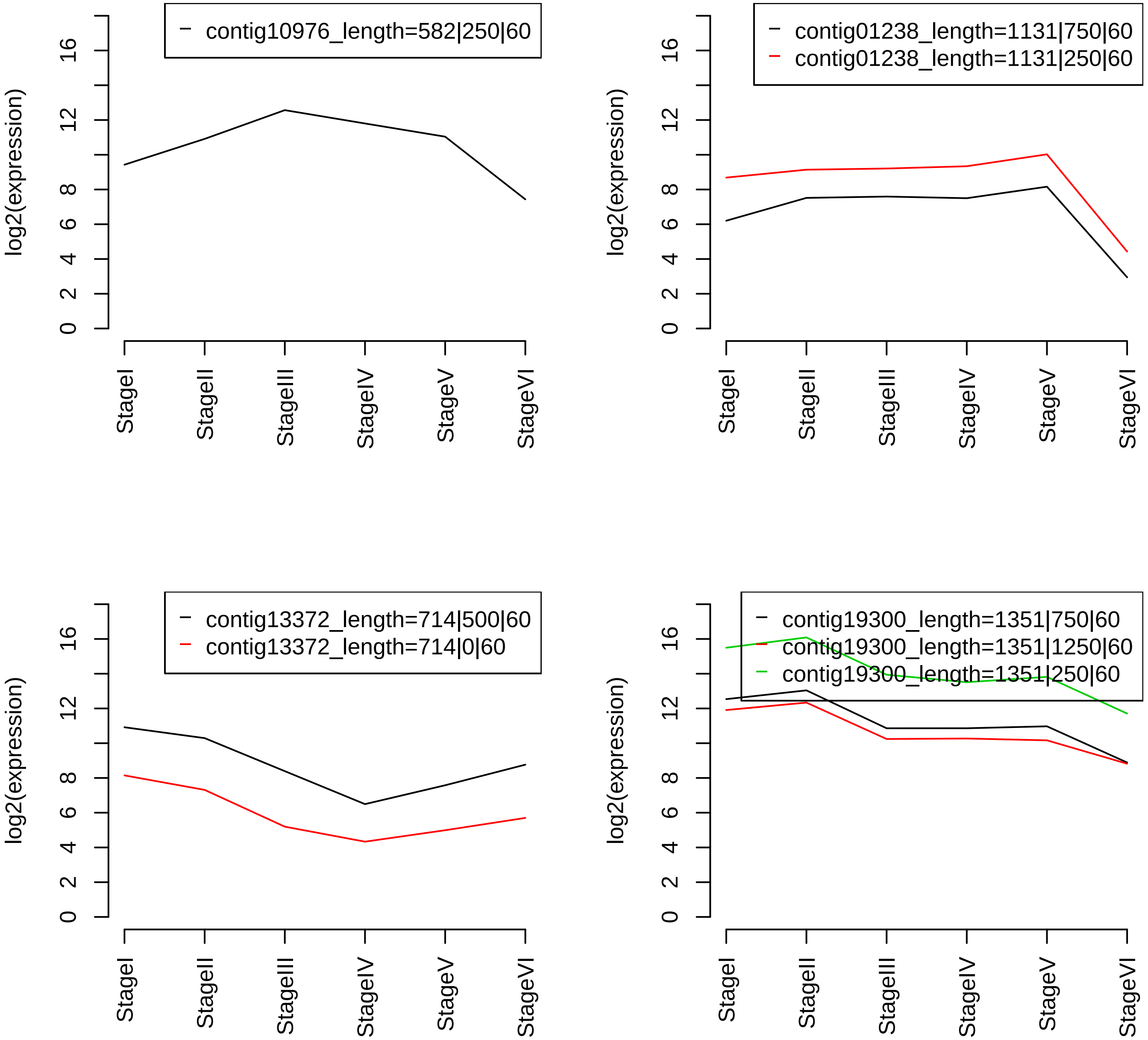

contig10976_length=582|250|60
contig01238_length=1131|750|60
contig01238_length=1131|250|60
contig13372_length=714|500|60
contig13372_length=714|0|60
contig19300_length=1351|750|60
contig19300_length=1351|1250|60
contig19300_length=1351|250|60
log2(expression)
0
2
4
6
8
12
16
StageI
StageII
StageIII
StageIV
StageV
StageVI

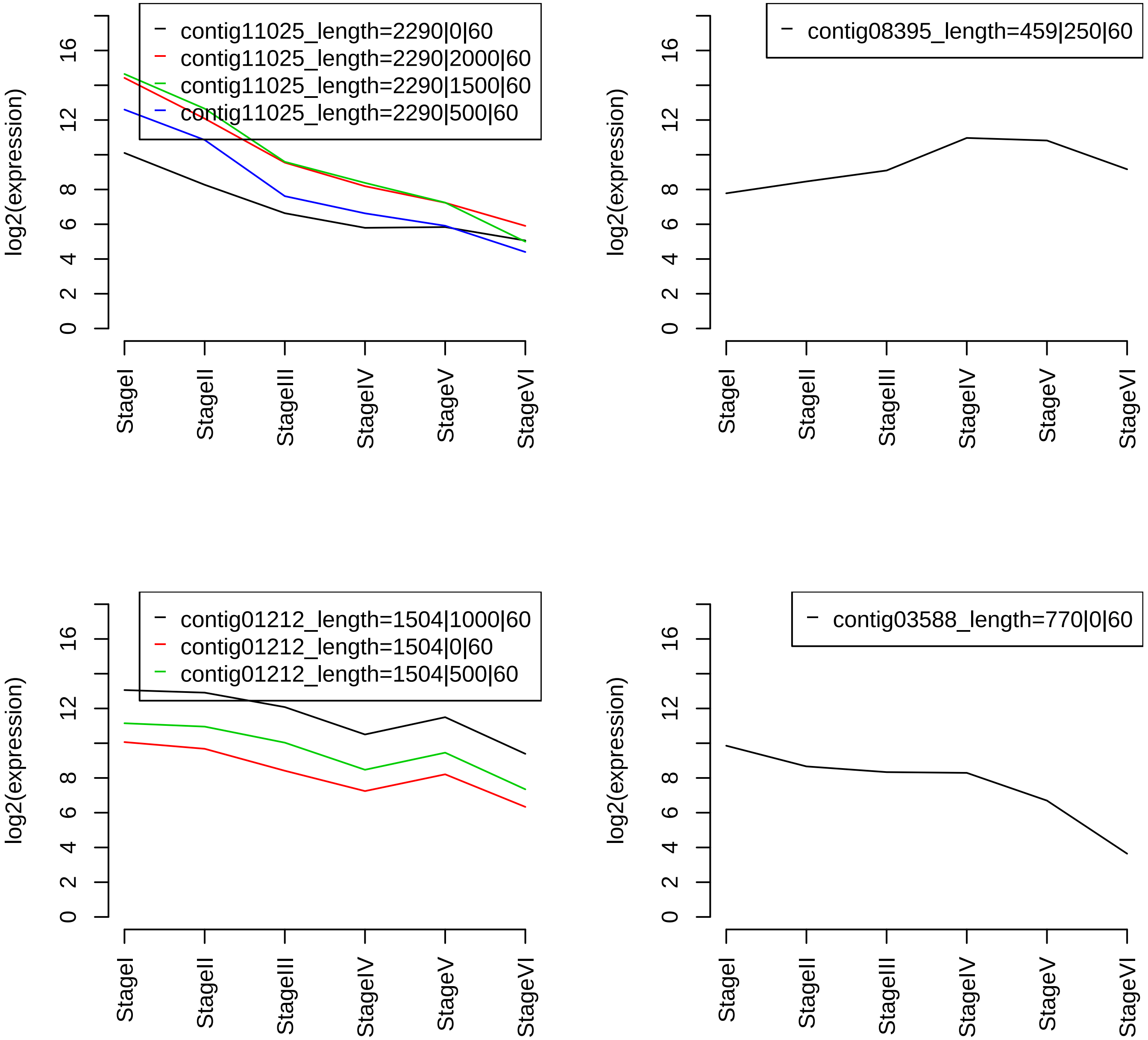
contig11025_length=2290|0|60
contig11025_length=2290|2000|60
contig11025_length=2290|1500|60
contig11025_length=2290|500|60
contig08395_length=459|250|60
contig01212_length=1504|1000|60
contig01212_length=1504|0|60
contig01212_length=1504|500|60
contig03588_length=770|0|60
log2(expression)
0
2
4
6
8
12
16
StageI
StageII
StageIII
StageIV
StageV
StageVI

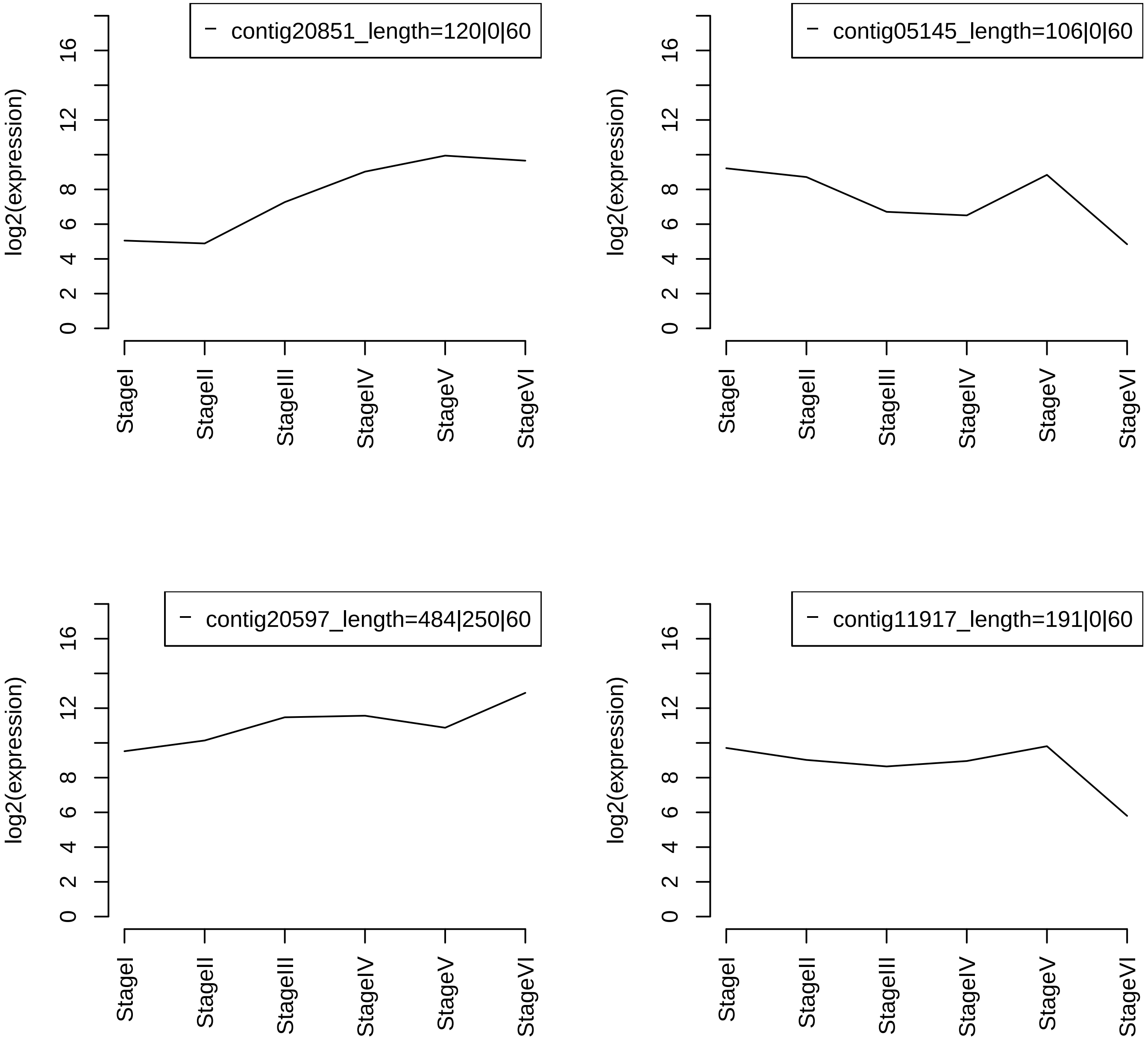
contig20851_length=120|0|60
contig05145_length=106|0|60
contig20597_length=484|250|60
contig11917_length=191|0|60
log2(expression)
0
2
4
6
8
12
16
StageI
StageII
StageIII
StageIV
StageV
StageVI

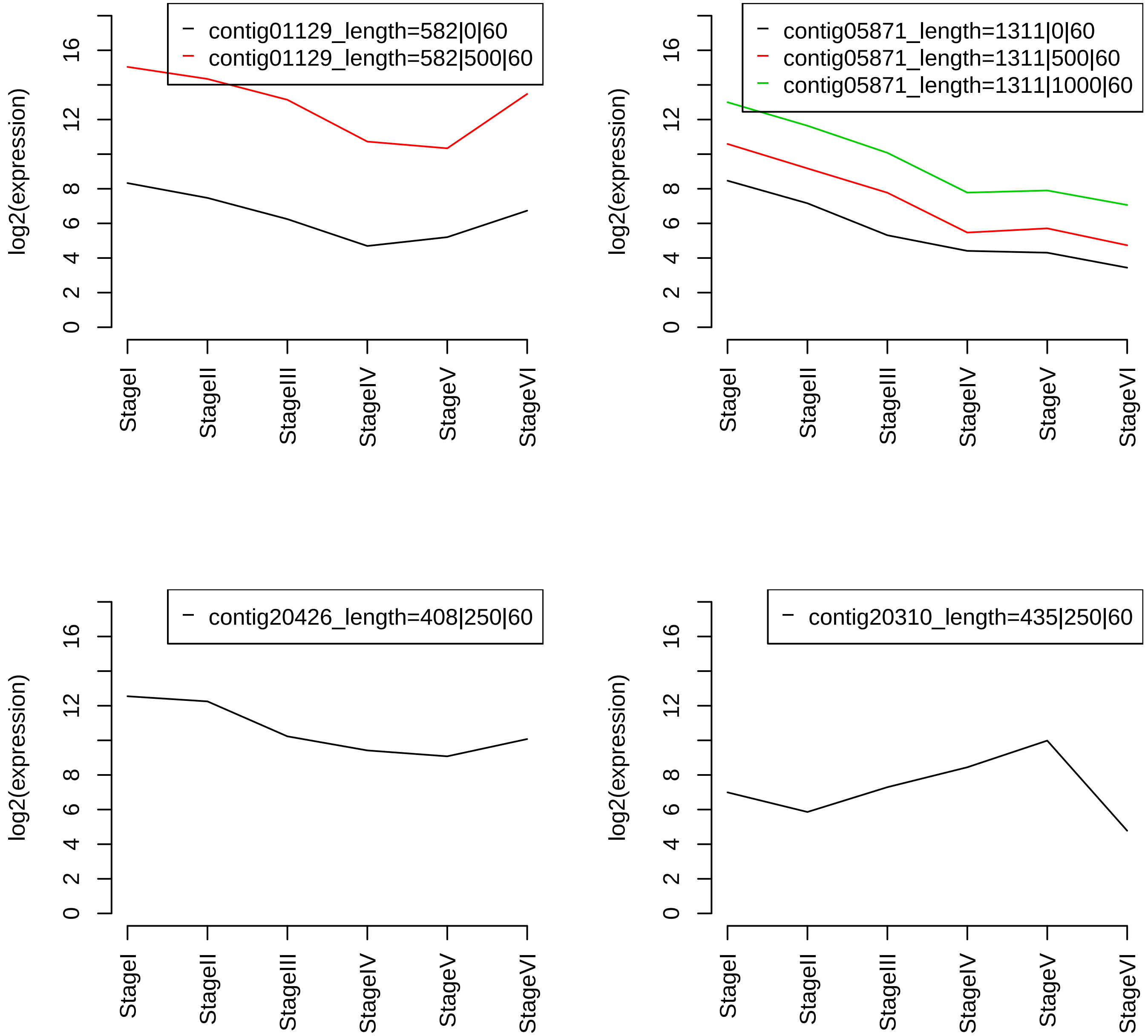
contig01129_length=582|0|60
contig01129_length=582|500|60
log2(expression)
0
2
4
6
8
12
16
StageI
StageII
StageIII
StageIV
StageV
StageVI
contig05871_length=1311|0|60
contig05871_length=1311|500|60
contig05871_length=1311|1000|60
contig20426_length=408|250|60
contig20310_length=435|250|60

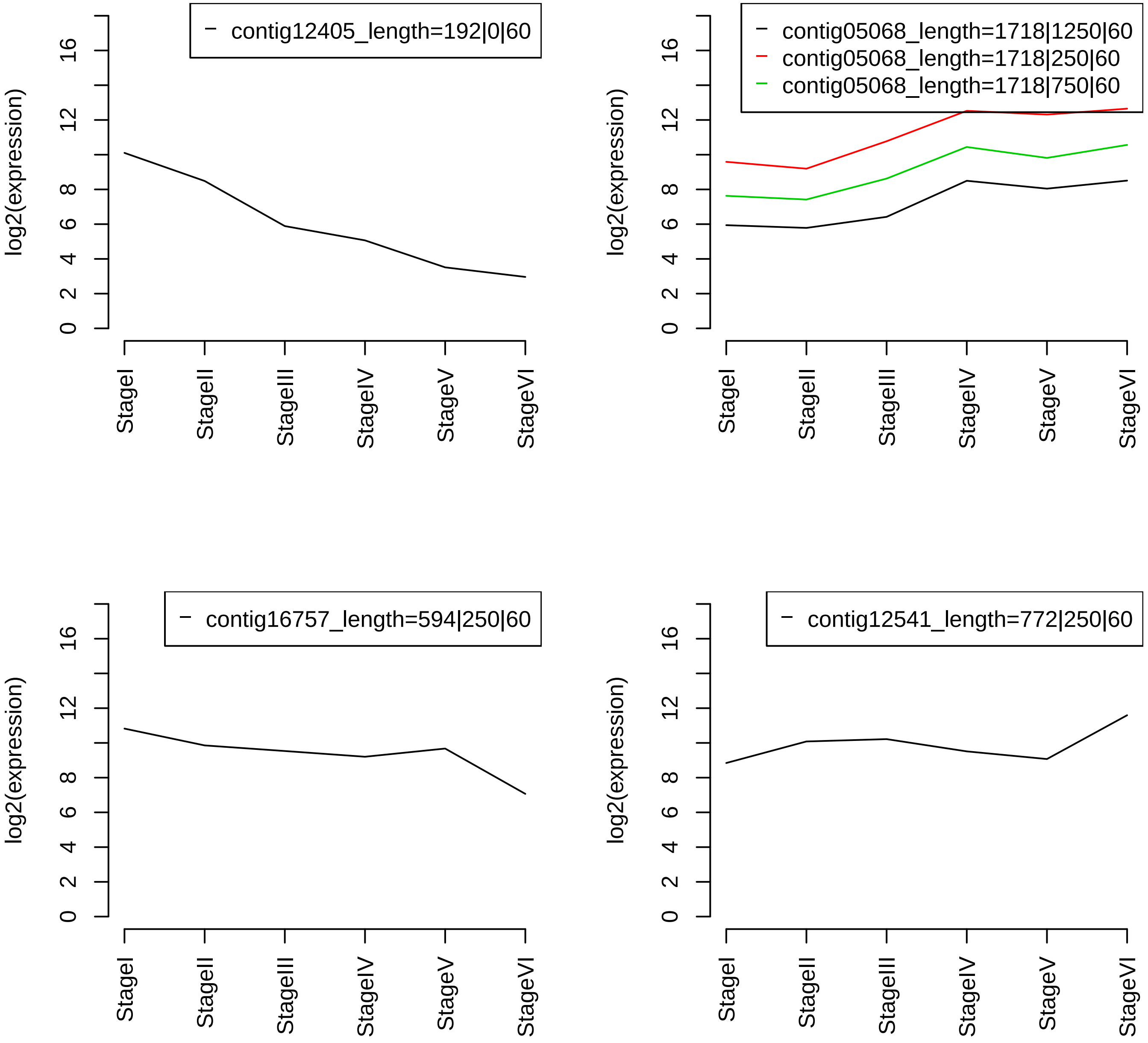
contig12405_length=192|0|60
contig05068_length=1718|1250|60
contig05068_length=1718|250|60
contig05068_length=1718|750|60
contig16757_length=594|250|60
contig12541_length=772|250|60
log2(expression)
0
2
4
6
8
12
16
StageI
StageII
StageIII
StageIV
StageV
StageVI

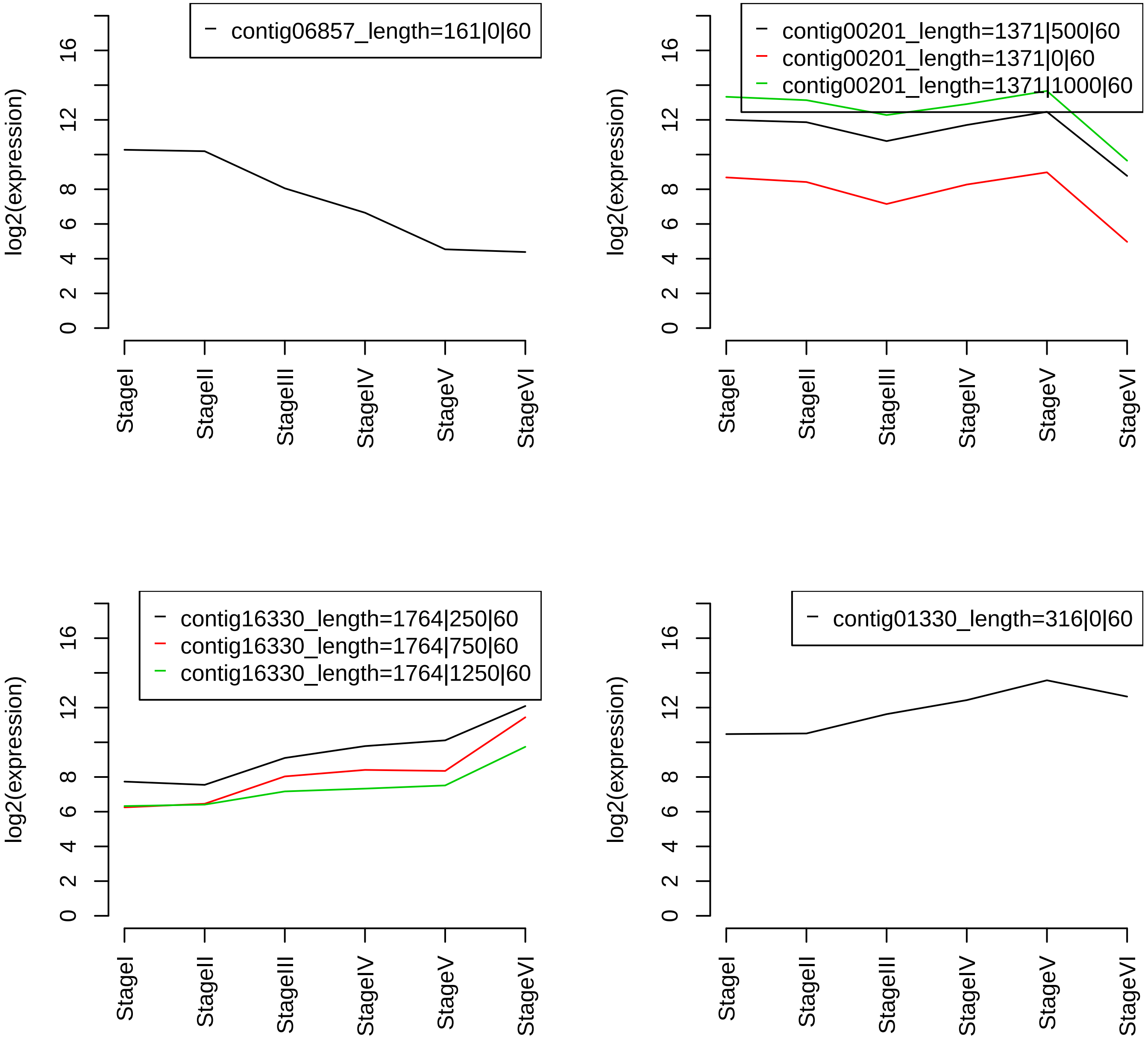
contig06857_length=161|0|60
contig00201_length=1371|500|60
contig00201_length=1371|0|60
contig00201_length=1371|1000|60
contig16330_length=1764|250|60
contig16330_length=1764|750|60
contig16330_length=1764|1250|60
contig01330_length=316|0|60
log2(expression)
0
2
4
6
8
12
16
StageI
StageII
StageIII
StageIV
StageV
StageVI

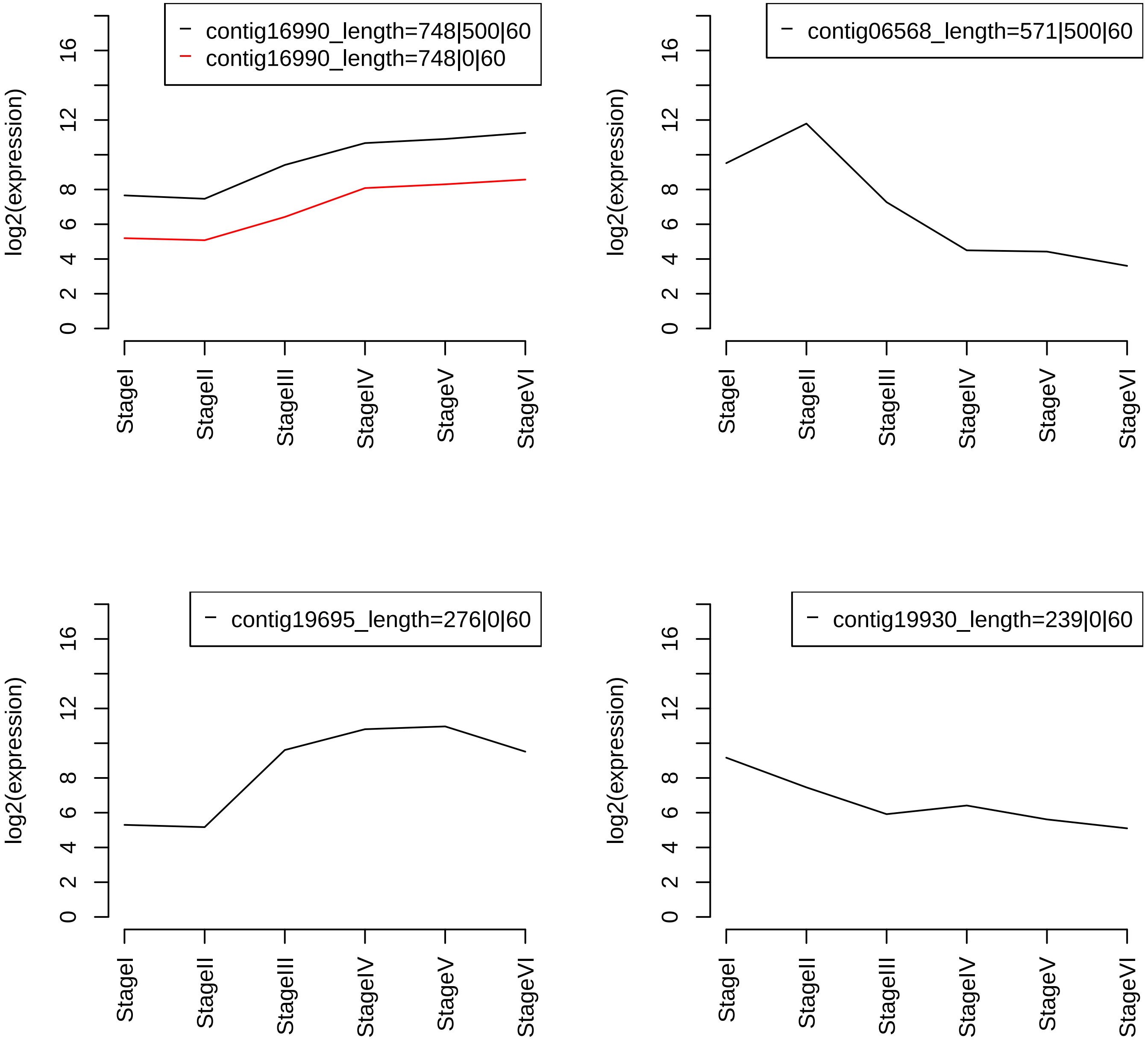

contig16990_length=748|500|60
contig16990_length=748|0|60
contig06568_length=571|500|60
contig19695_length=276|0|60
contig19930_length=239|0|60
log2(expression)
0
2
4
6
8
12
16
StageI
StageII
StageIII
StageIV
StageV
StageVI

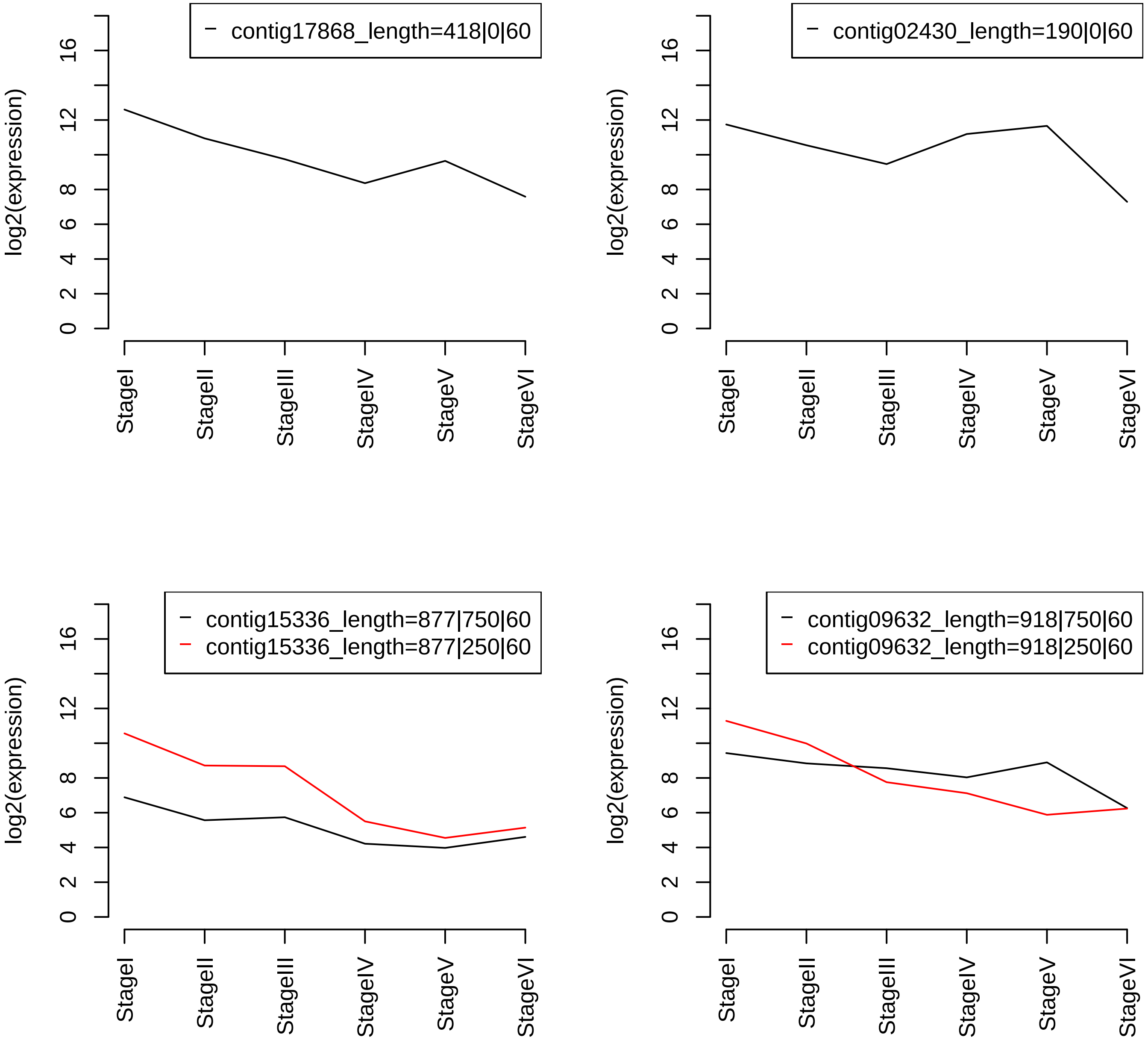

contig17868_length=418|0|60
contig02430_length=190|0|60
contig15336_length=877|750|60
contig15336_length=877|250|60
contig09632_length=918|750|60
contig09632_length=918|250|60
log2(expression)
0
2
4
6
8
12
16
StageI
StageII
StageIII
StageIV
StageV
StageVI

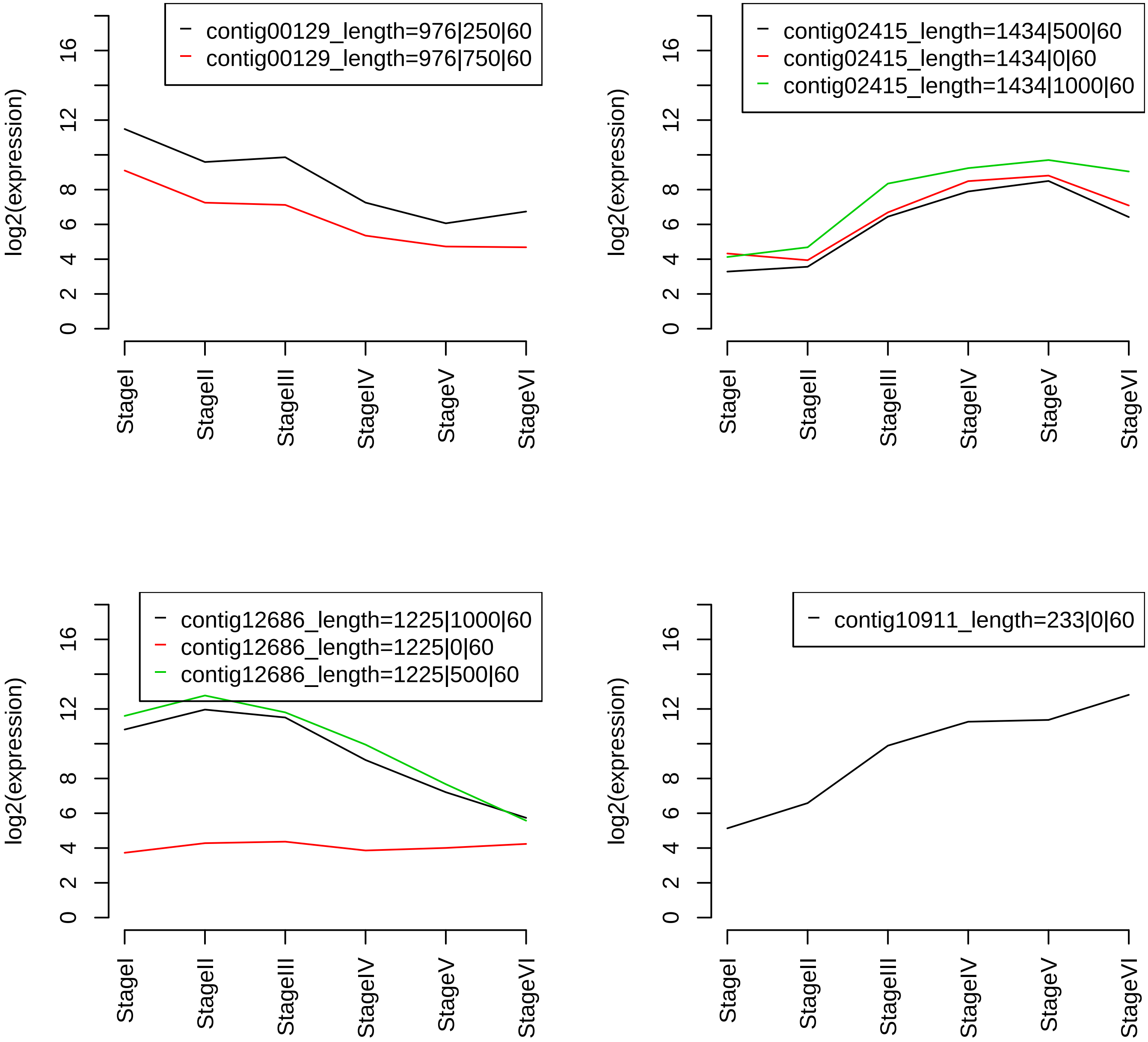
contig00129_length=976|250|60
contig00129_length=976|750|60
contig02415_length=1434|500|60
contig02415_length=1434|0|60
contig02415_length=1434|1000|60
contig12686_length=1225|1000|60
contig12686_length=1225|0|60
contig12686_length=1225|500|60
contig10911_length=233|0|60
log2(expression)
0 2 4 6 8 12 16
StageI
StageII
StageIII
StageIV
StageV
StageVI

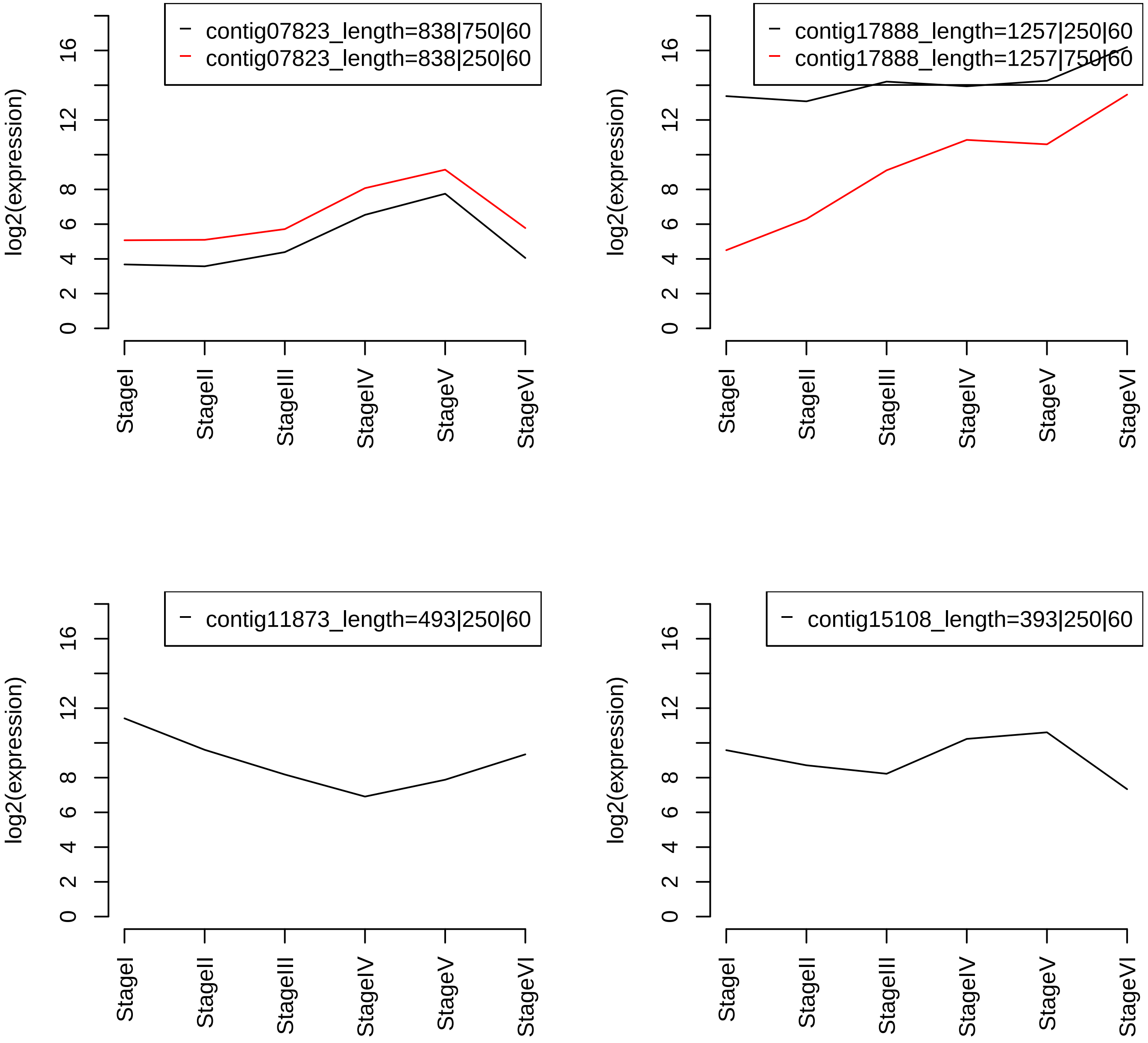

contig07823_length=838|750|60
contig07823_length=838|250|60
log2(expression)
0
2
4
6
8
12
16
StageI
StageII
StageIII
StageIV
StageV
StageVI
contig17888_length=1257|250|60
contig17888_length=1257|750|60
contig11873_length=493|250|60
contig15108_length=393|250|60

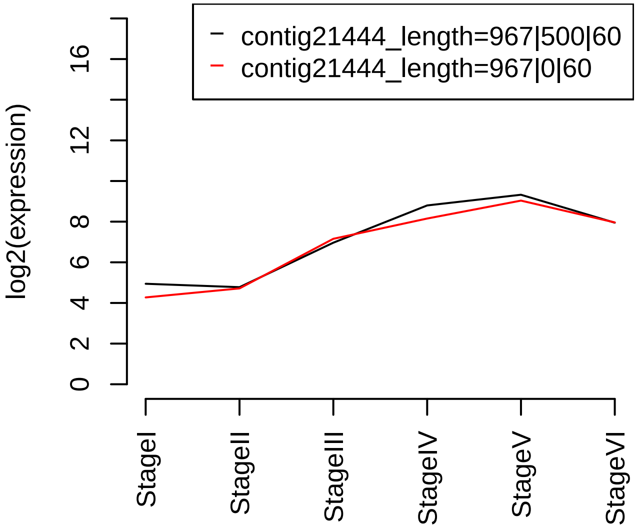
contig21444_length=967|500|60
contig21444_length=967|0|60
log2(expression)
0
2
4
6
8
12
16
StageI
StageII
StageIII
StageIV
StageV
StageVI

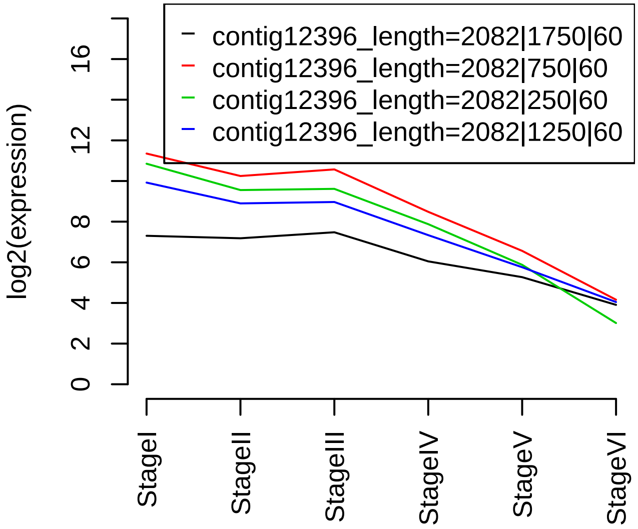
contig12396_length=2082|1750|60
contig12396_length=2082|750|60
contig12396_length=2082|250|60
contig12396_length=2082|1250|60
log2(expression)
0
2
4
6
8
12
16
StageI
StageII
StageIII
StageIV
StageV
StageVI

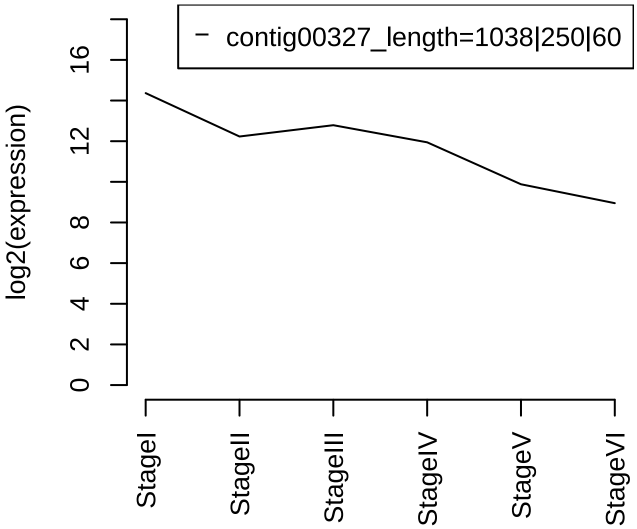
contig00327_length=1038|250|60
log2(expression)
0
2
4
6
8
12
16
StageI
StageII
StageIII
StageIV
StageV
StageVI

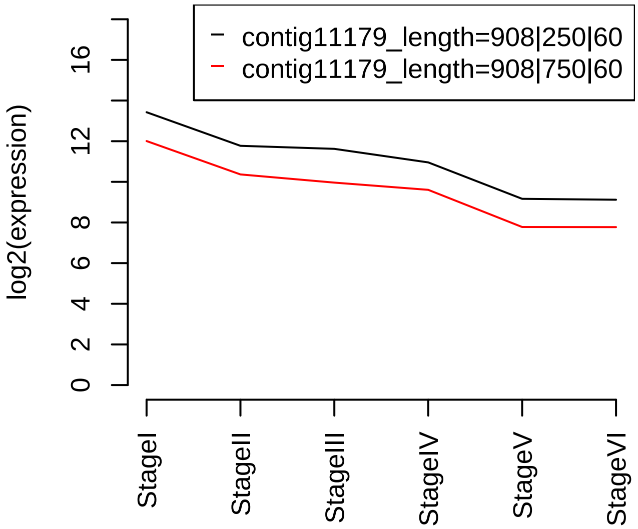
contig11179_length=908|250|60
contig11179_length=908|750|60
log2(expression)
0
2
4
6
8
12
16
StageI
StageII
StageIII
StageIV
StageV
StageVI

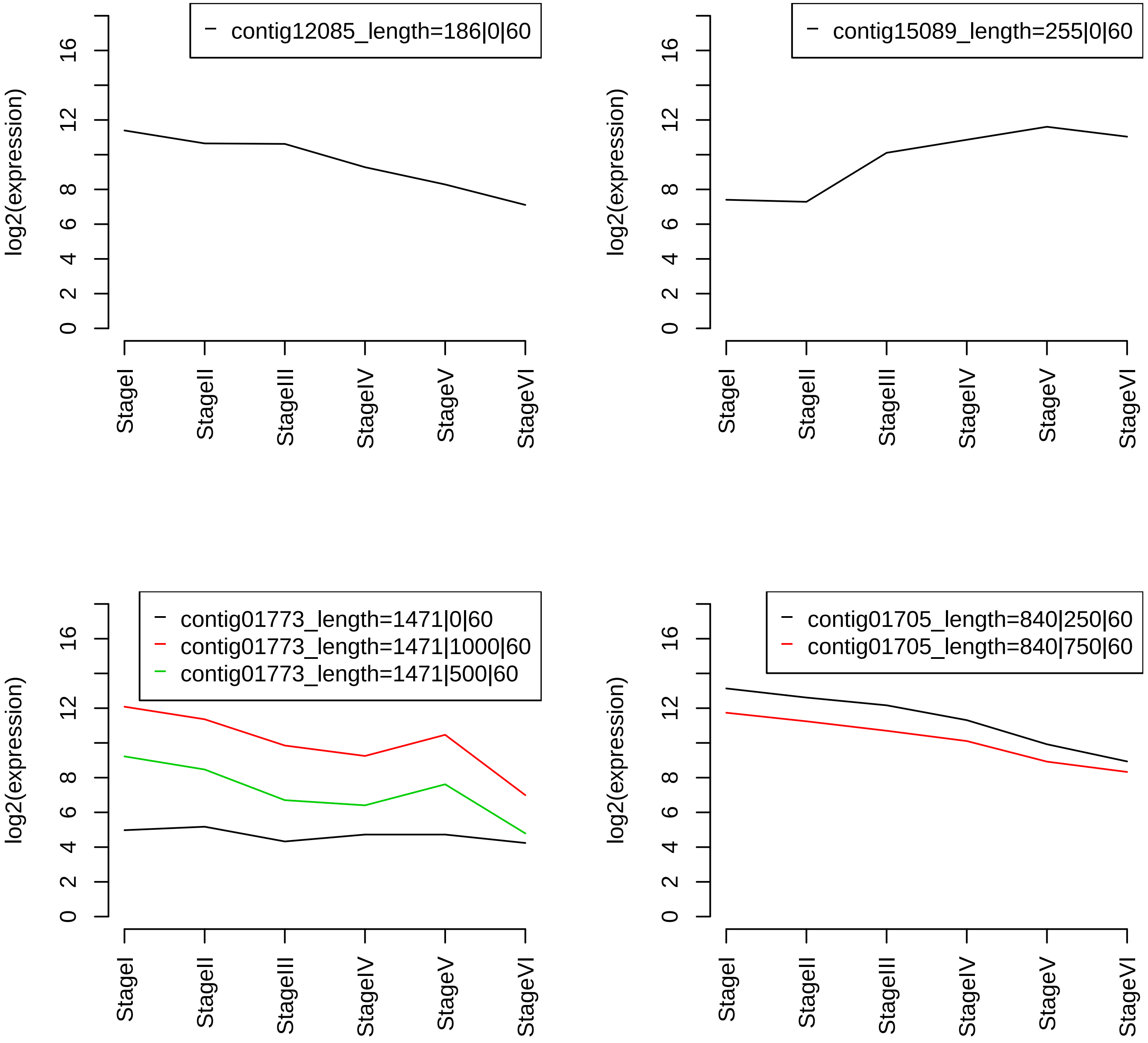
contig12085_length=186|0|60
contig15089_length=255|0|60
contig01773_length=1471|0|60
contig01773_length=1471|1000|60
contig01773_length=1471|500|60
contig01705_length=840|250|60
contig01705_length=840|750|60
log2(expression)
0
2
4
6
8
12
16
StageI
StageII
StageIII
StageIV
StageV
StageVI

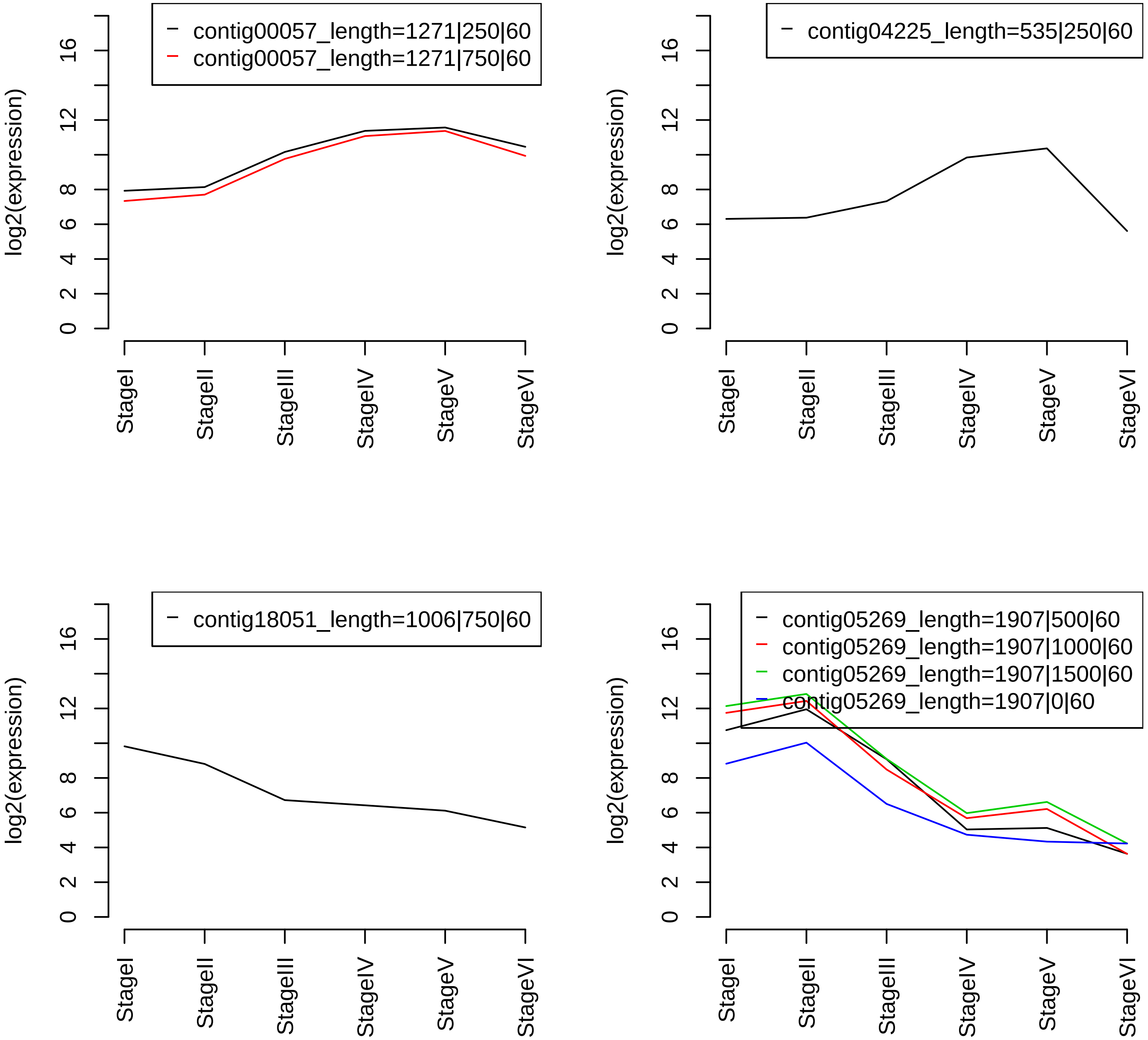
contig00057_length=1271|250|60
contig00057_length=1271|750|60
contig04225_length=535|250|60
contig18051_length=1006|750|60
contig05269_length=1907|500|60
contig05269_length=1907|1000|60
contig05269_length=1907|1500|60
contig05269_length=1907|0|60
log2(expression)
0
2
4
6
8
12
16
StageI
StageII
StageIII
StageIV
StageV
StageVI

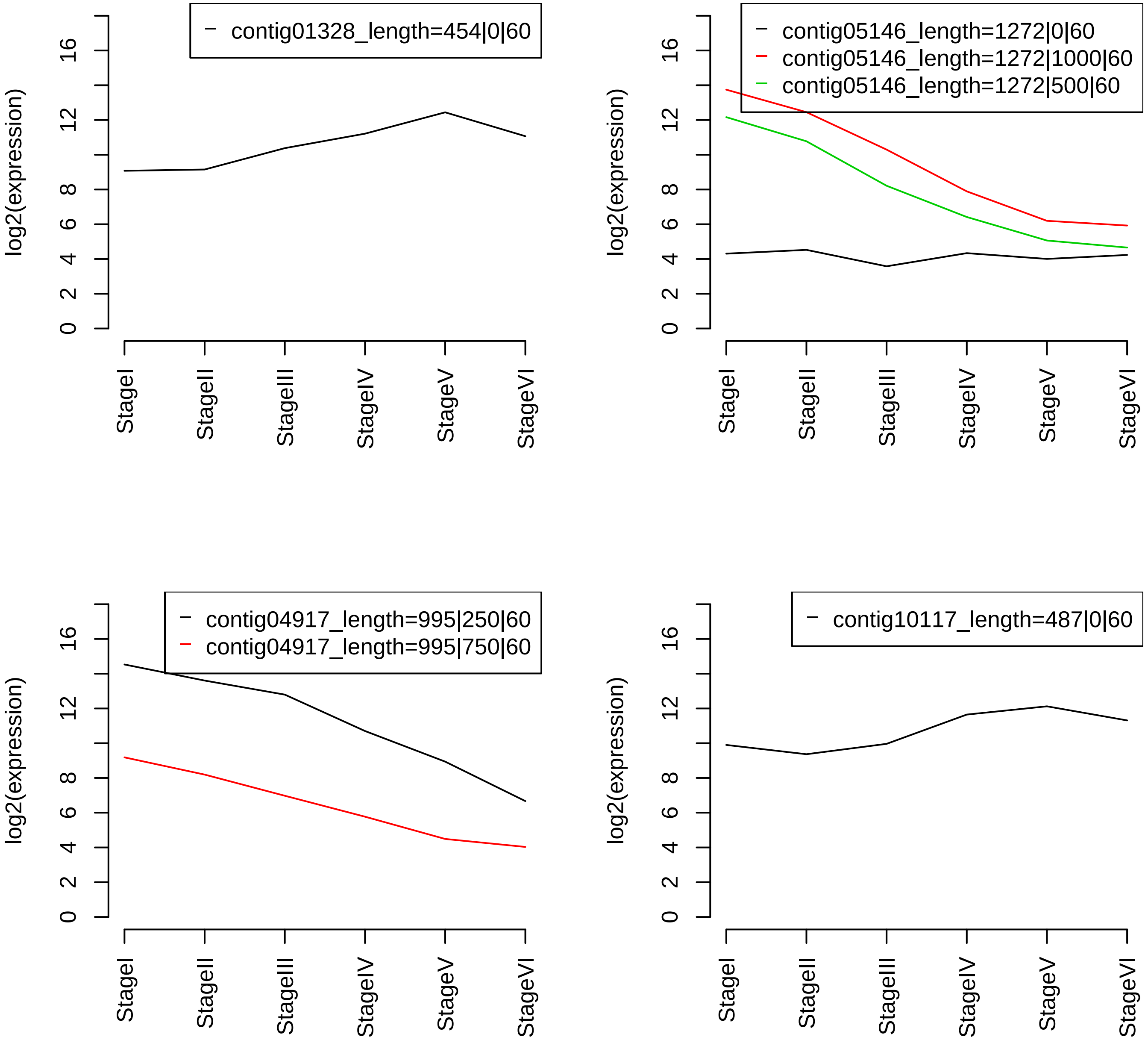
contig01328_length=454|0|60
contig05146_length=1272|0|60
contig05146_length=1272|1000|60
contig05146_length=1272|500|60
contig04917_length=995|250|60
contig04917_length=995|750|60
contig10117_length=487|0|60
log2(expression)
0
2
4
6
8
12
16
StageI
StageII
StageIII
StageIV
StageV
StageVI

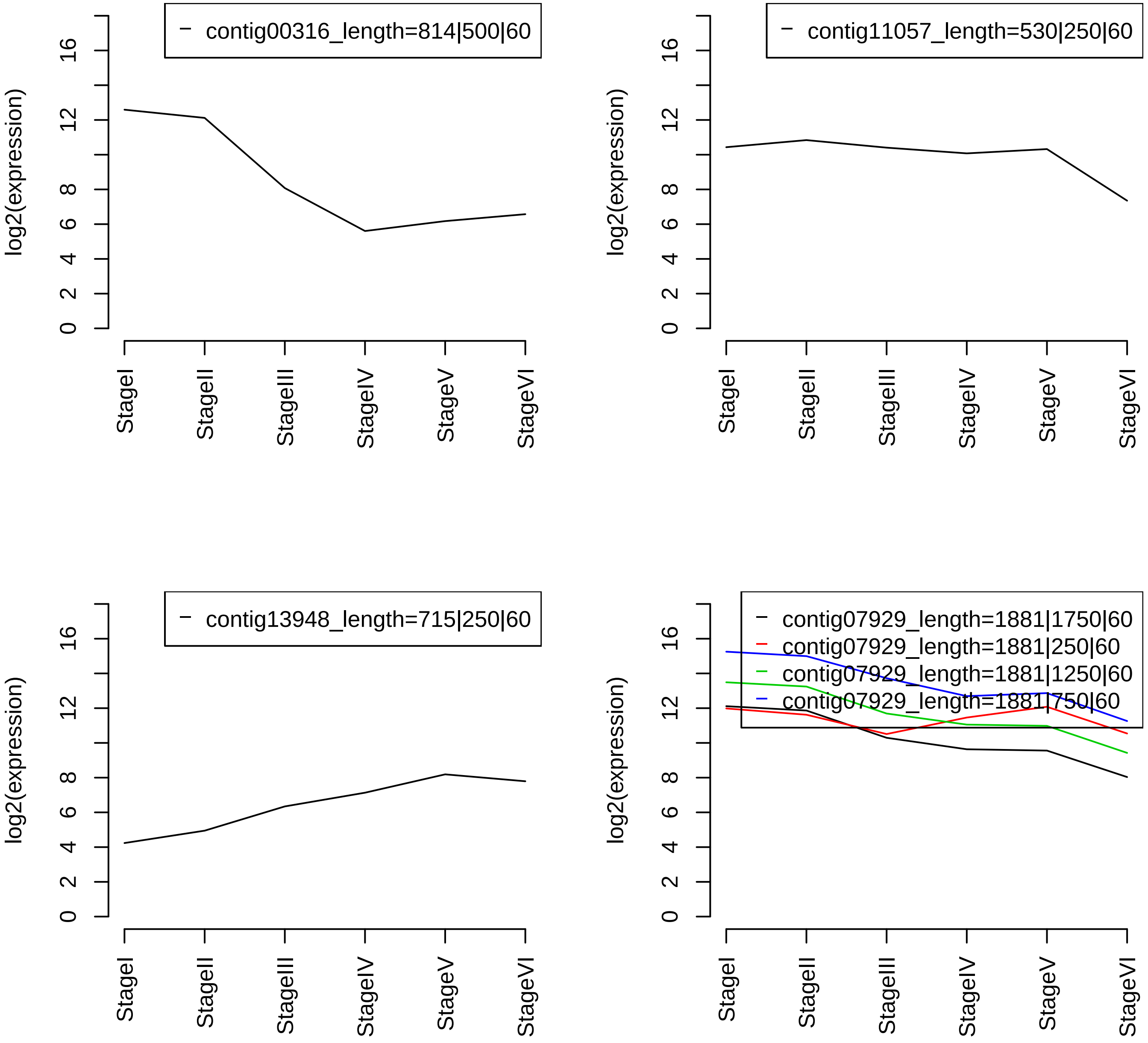

contig00316_length=814|500|60
contig11057_length=530|250|60
contig13948_length=715|250|60
contig07929_length=1881|1750|60
contig07929_length=1881|250|60
contig07929_length=1881|1250|60
contig07929_length=1881|750|60
log2(expression)
0 2 4 6 8 12 16
StageI StageII StageIII StageIV StageV StageVI

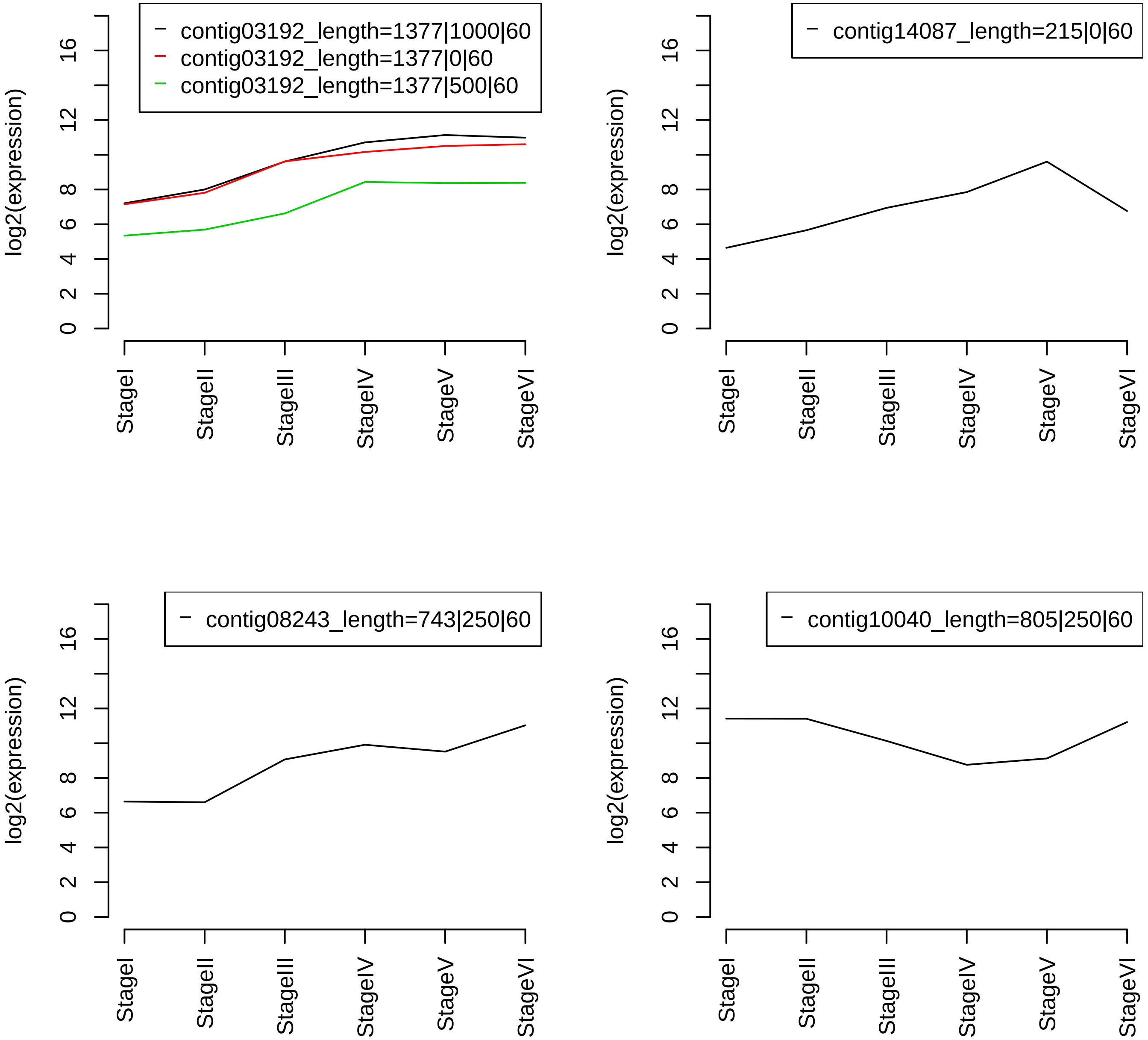

contig03192_length=1377|1000|60
contig03192_length=1377|0|60
contig03192_length=1377|500|60
contig14087_length=215|0|60
contig08243_length=743|250|60
contig10040_length=805|250|60
log2(expression)
0 2 4 6 8 12 16
StageI StageII StageIII StageIV StageV StageVI

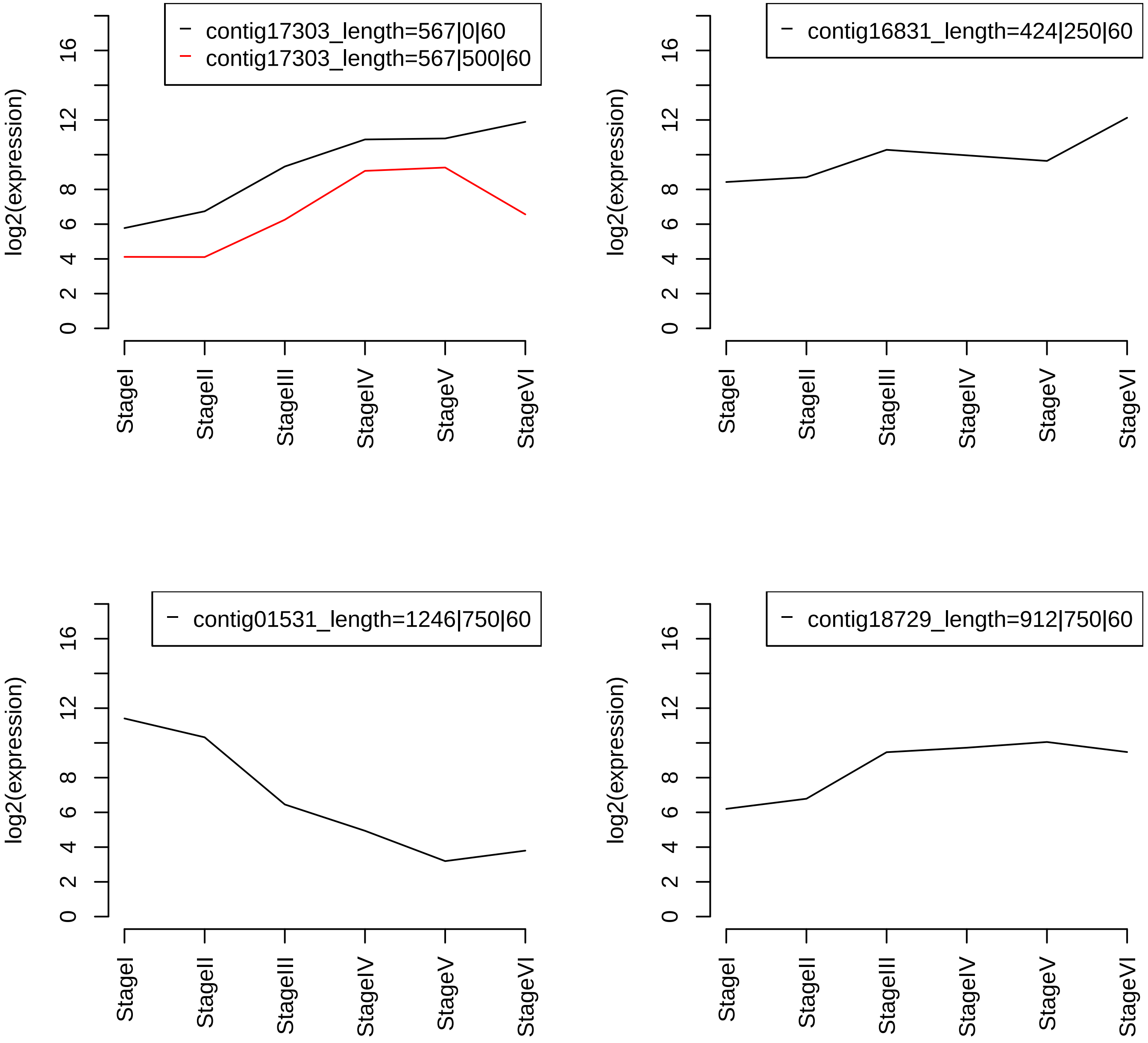
contig17303_length=567|0|60
contig17303_length=567|500|60
contig16831_length=424|250|60
contig01531_length=1246|750|60
contig18729_length=912|750|60
log2(expression)
0
2
4
6
8
12
16
StageI
StageII
StageIII
StageIV
StageV
StageVI

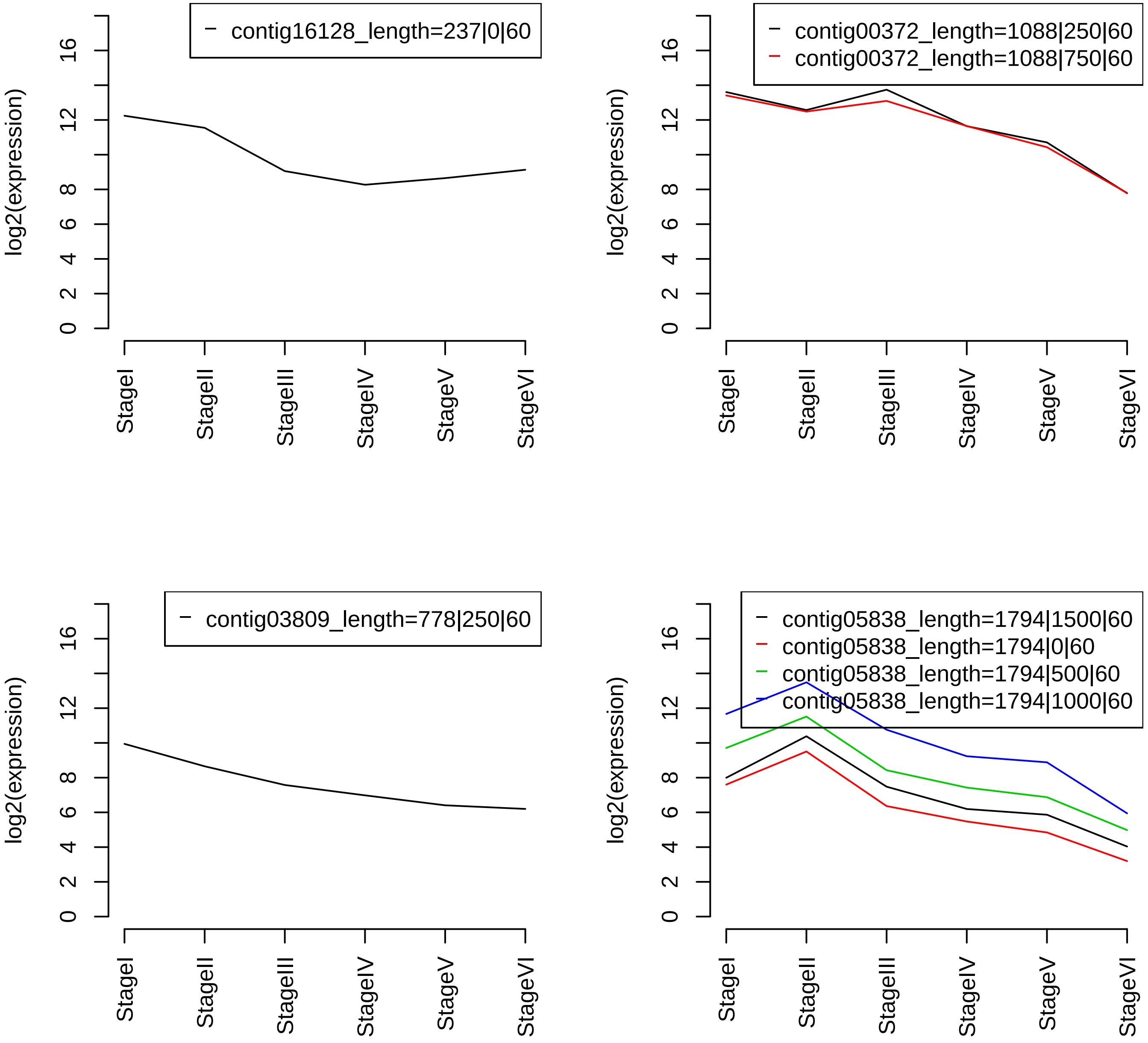

contig16128_length=237|0|60
contig00372_length=1088|250|60
contig00372_length=1088|750|60
contig03809_length=778|250|60
contig05838_length=1794|1500|60
contig05838_length=1794|0|60
contig05838_length=1794|500|60
contig05838_length=1794|1000|60
log2(expression)
0
2
4
6
8
12
16
StageI
StageII
StageIII
StageIV
StageV
StageVI

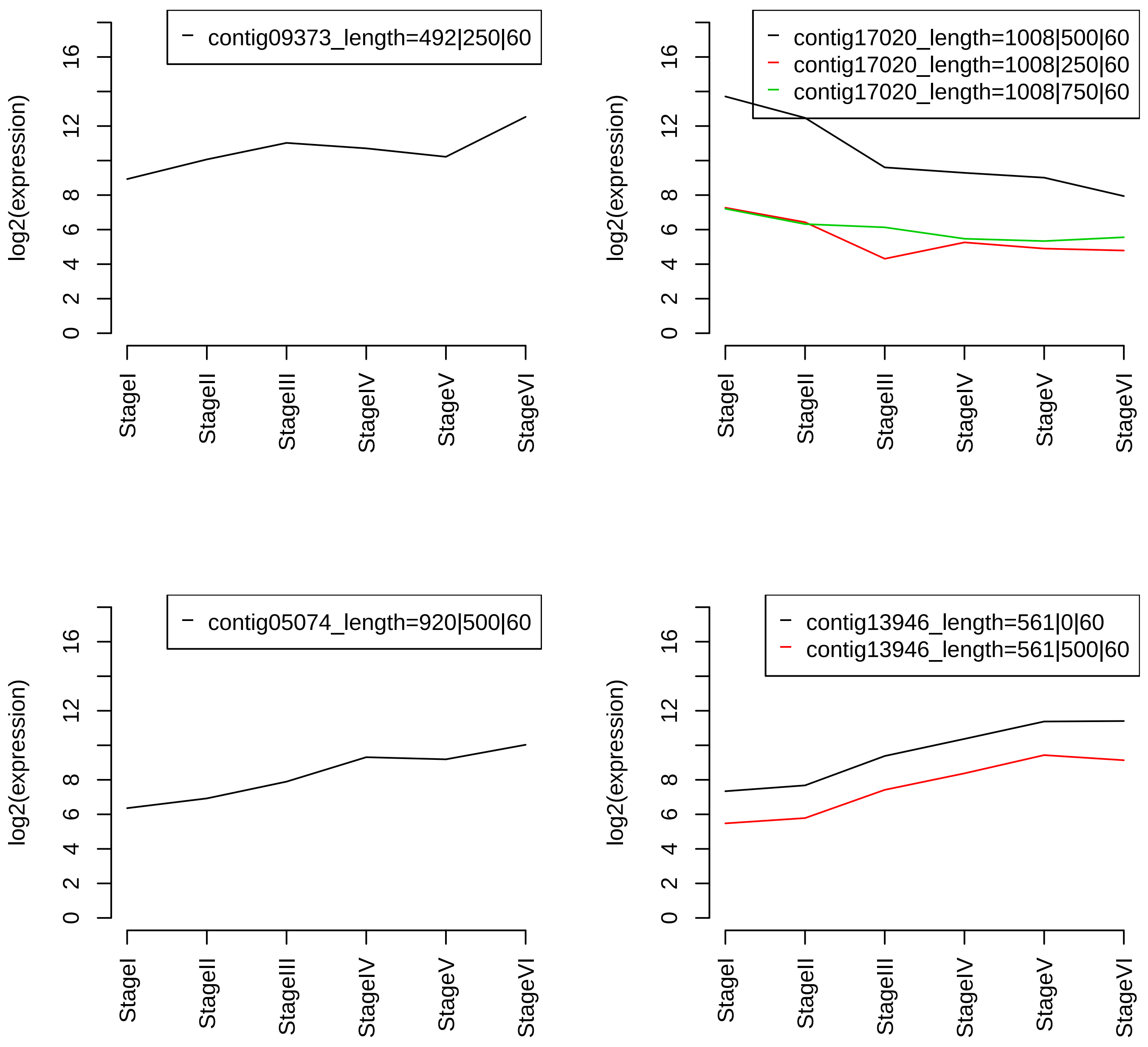

contig09373_length=492|250|60
contig17020_length=1008|500|60
contig17020_length=1008|250|60
contig17020_length=1008|750|60
contig05074_length=920|500|60
contig13946_length=561|0|60
contig13946_length=561|500|60
log2(expression)
0
2
4
6
8
12
16
StageI
StageII
StageIII
StageIV
StageV
StageVI

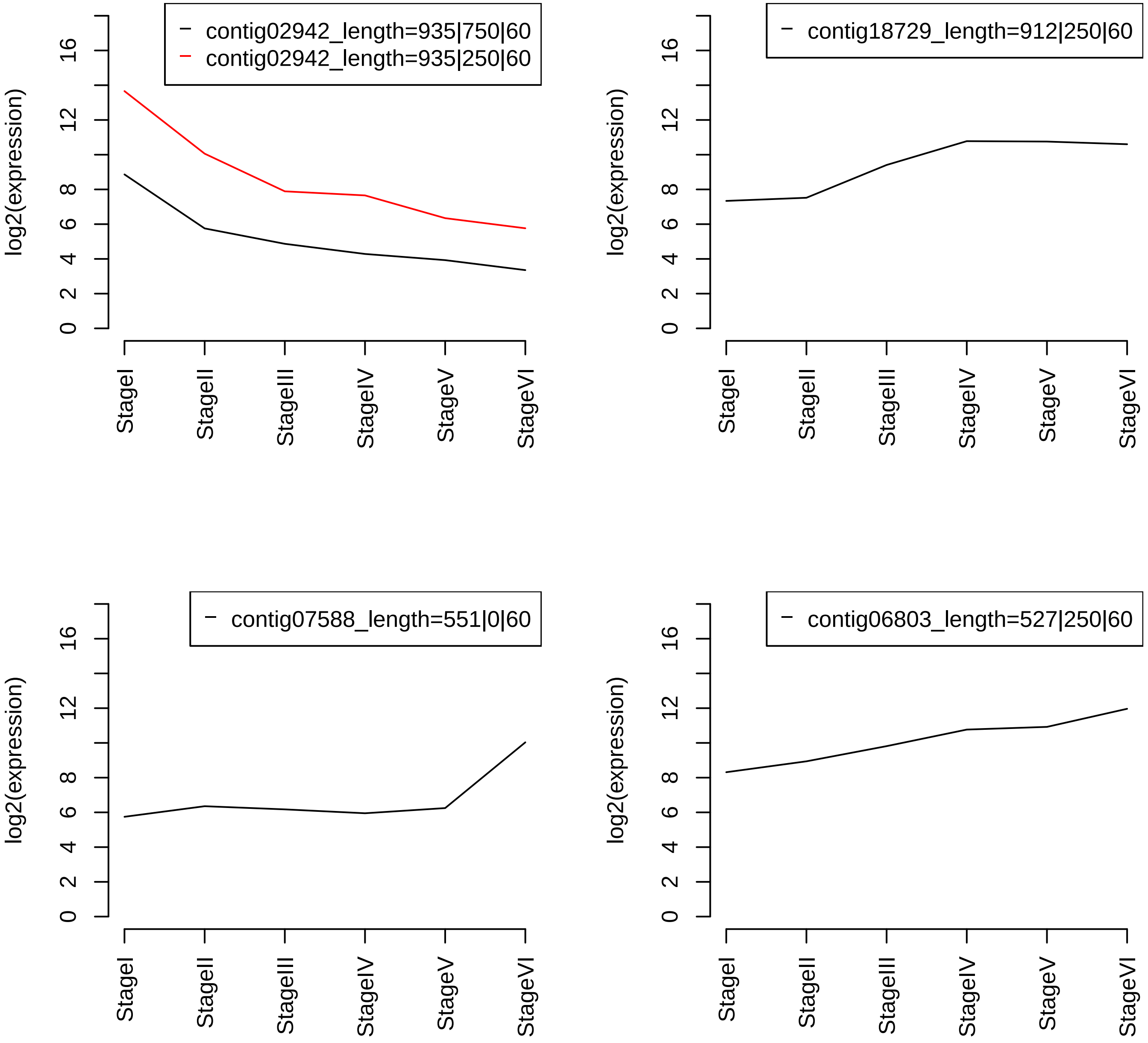
contig02942_length=935|750|60
contig02942_length=935|250|60
contig18729_length=912|250|60
contig07588_length=551|0|60
contig06803_length=527|250|60
log2(expression)
0
2
4
6
8
12
16
StageI
StageII
StageIII
StageIV
StageV
StageVI

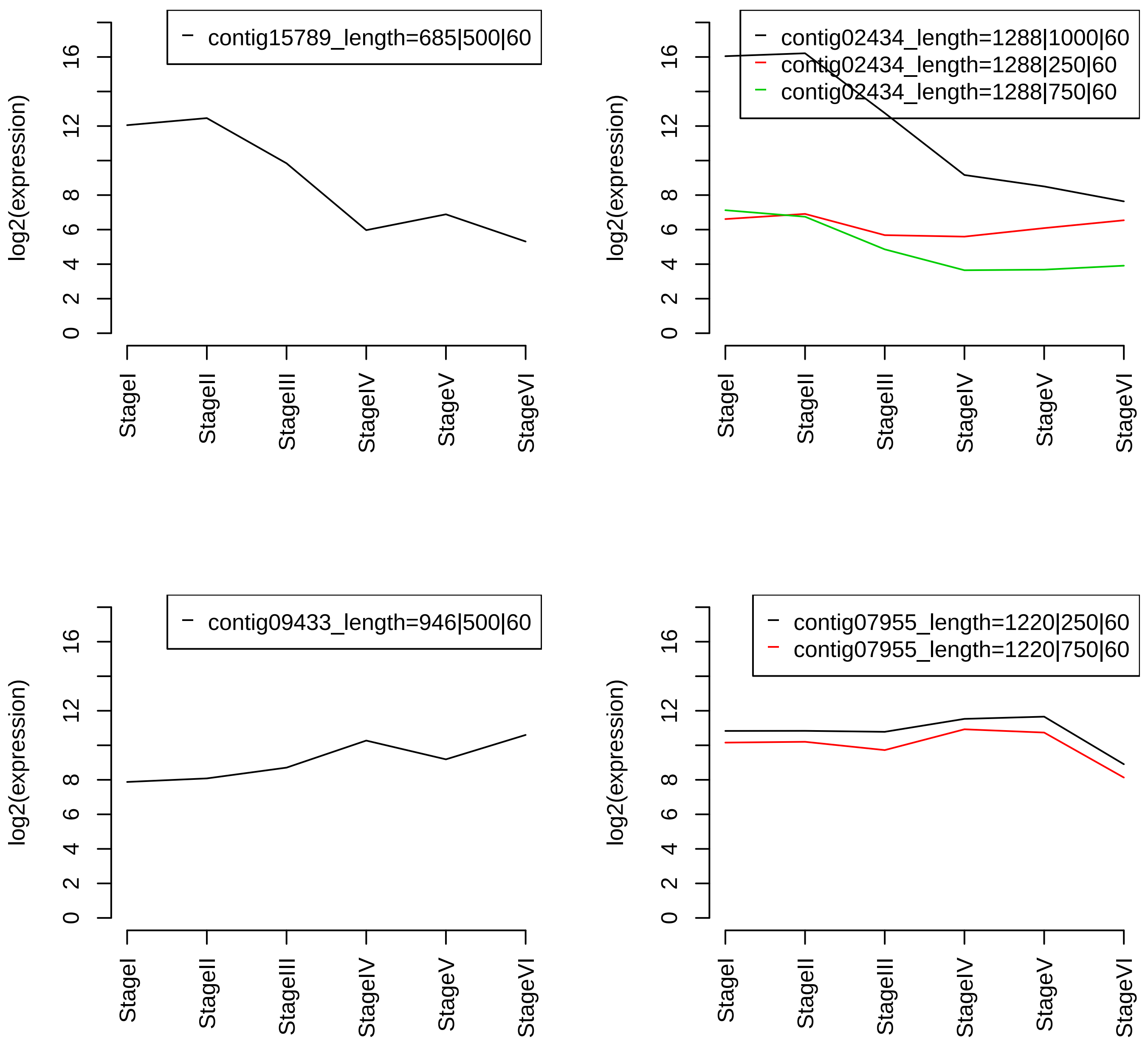
contig15789_length=685|500|60
contig02434_length=1288|1000|60
contig02434_length=1288|250|60
contig02434_length=1288|750|60
contig09433_length=946|500|60
contig07955_length=1220|250|60
contig07955_length=1220|750|60
log2(expression)
0
2
4
6
8
12
16
StageI
StageII
StageIII
StageIV
StageV
StageVI

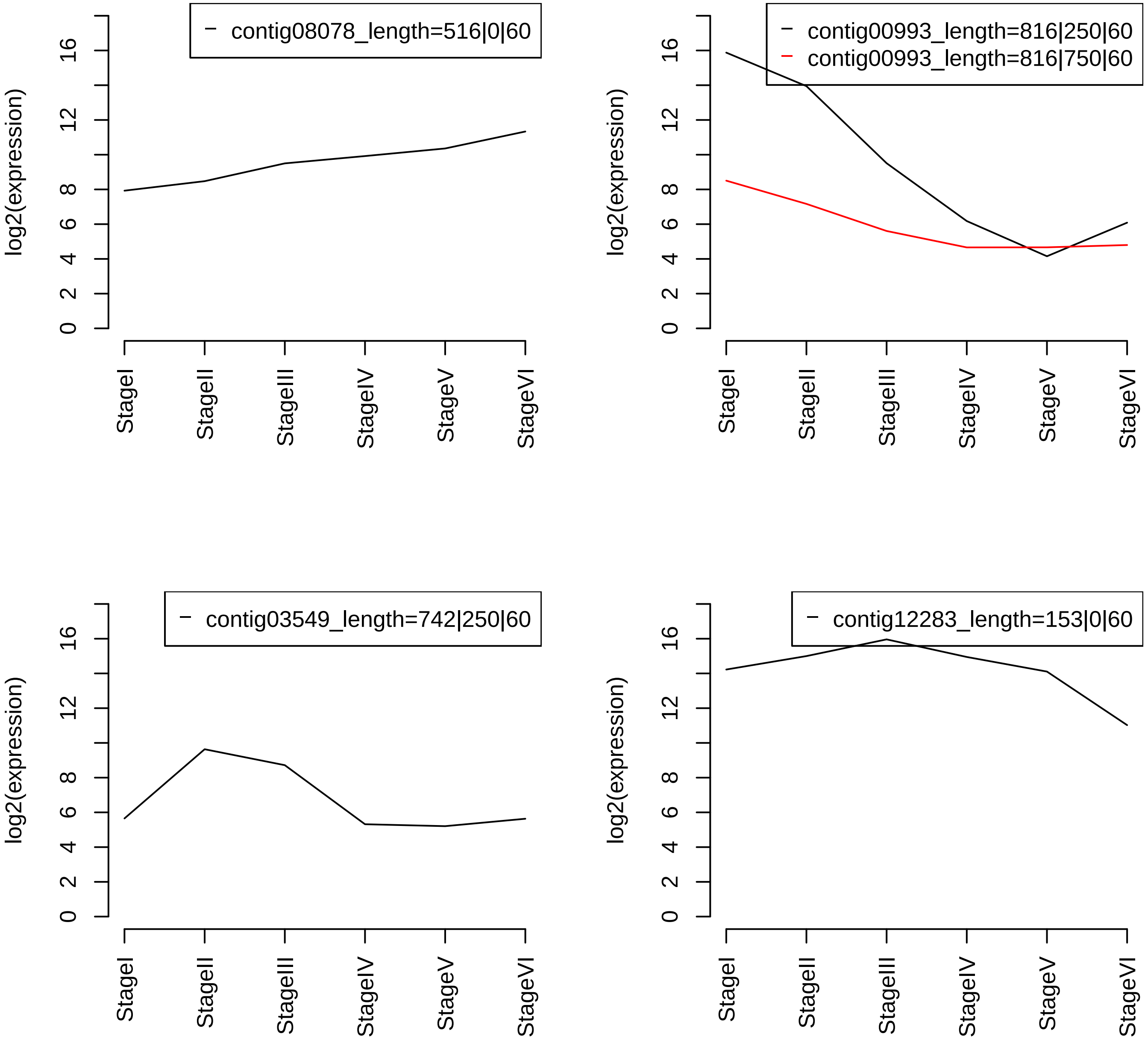

contig08078_length=516|0|60
contig00993_length=816|250|60
contig00993_length=816|750|60
contig03549_length=742|250|60
contig12283_length=153|0|60
log2(expression)
0
2
4
6
8
12
16
StageI
StageII
StageIII
StageIV
StageV
StageVI

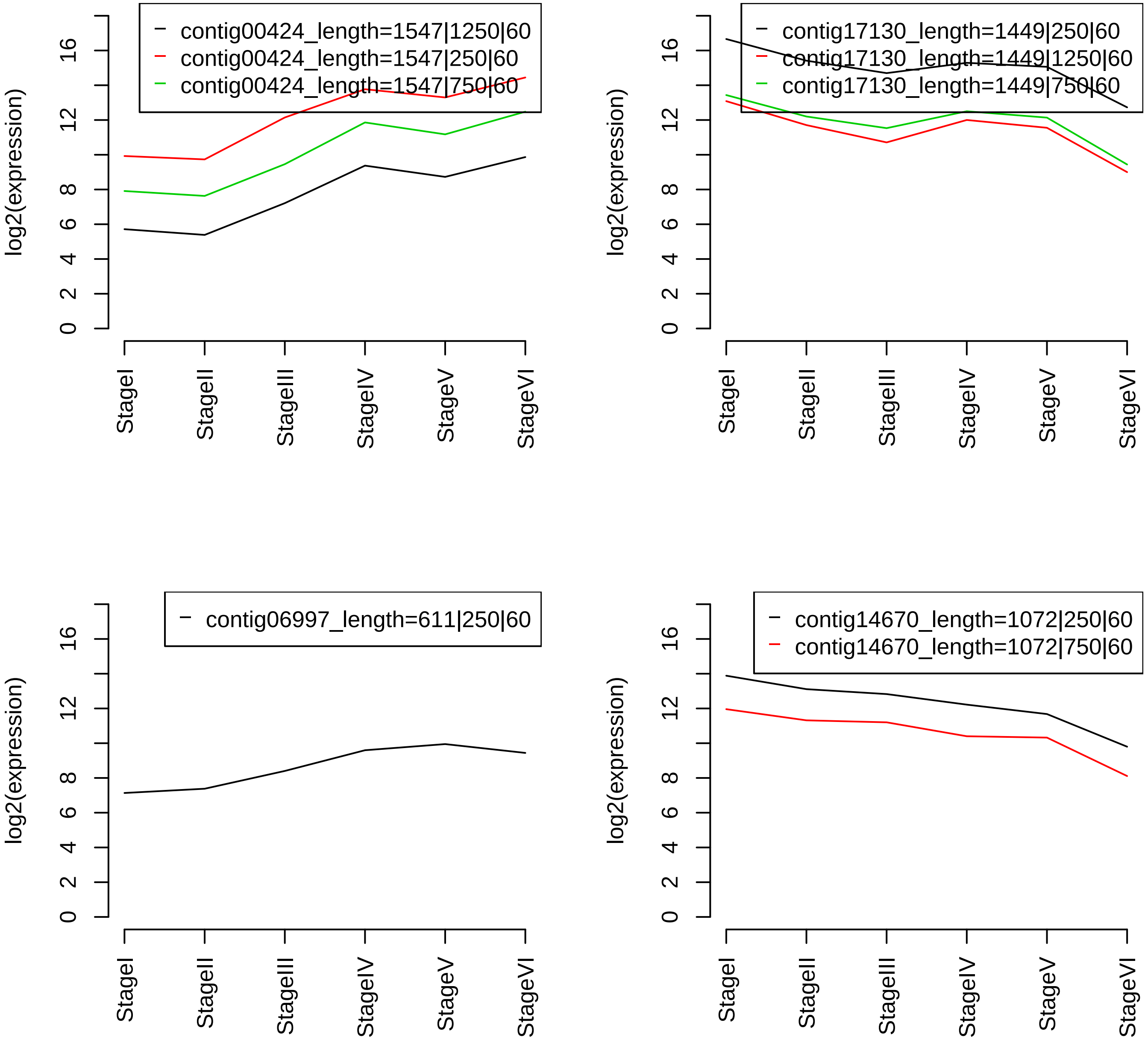
contig00424_length=1547|1250|60
contig00424_length=1547|250|60
contig00424_length=1547|750|60
contig17130_length=1449|250|60
contig17130_length=1449|1250|60
contig17130_length=1449|750|60
contig06997_length=611|250|60
contig14670_length=1072|250|60
contig14670_length=1072|750|60
log2(expression)
0
2
4
6
8
12
16
StageI
StageII
StageIII
StageIV
StageV
StageVI

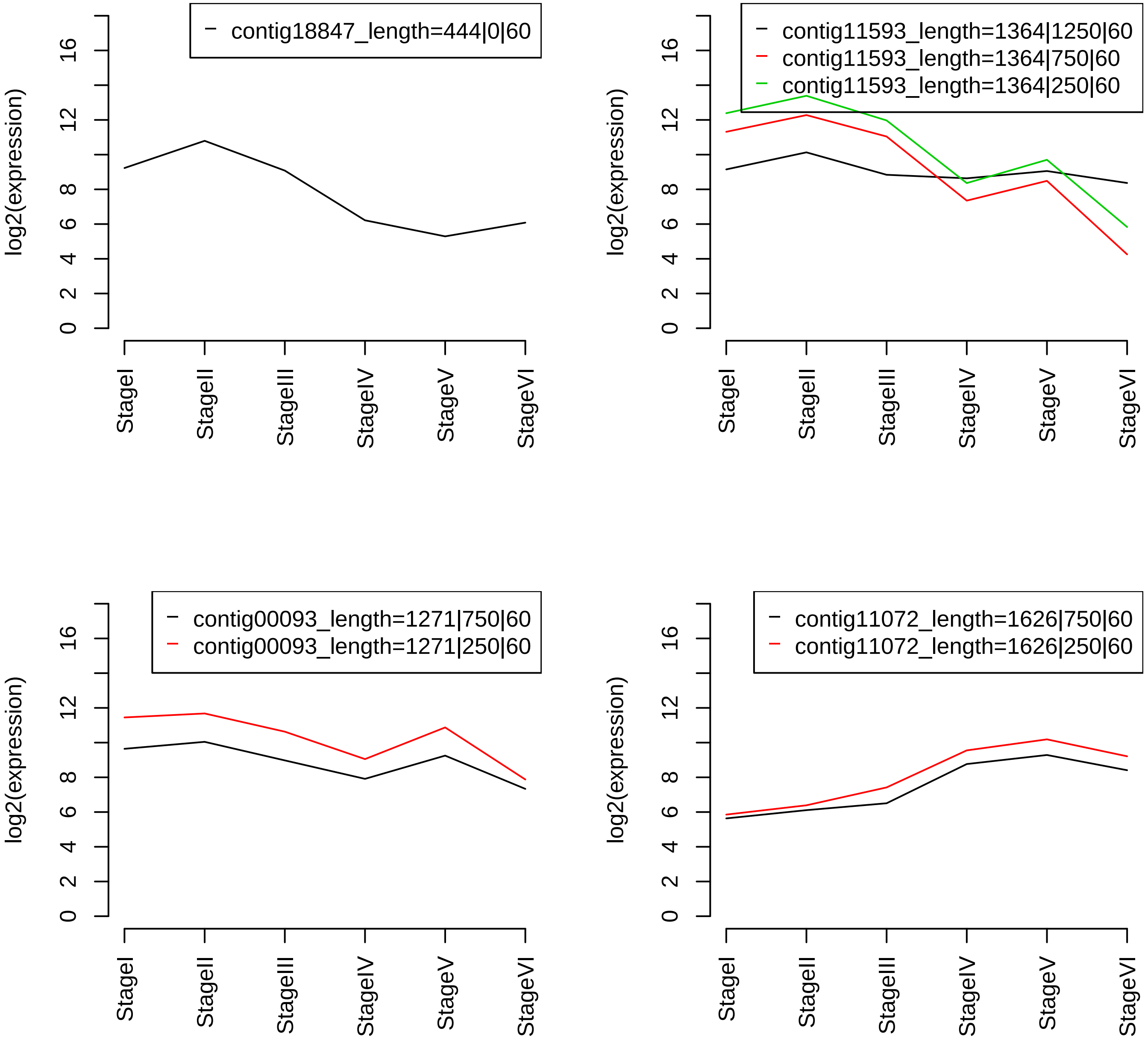

contig18847_length=444|0|60
contig11593_length=1364|1250|60
contig11593_length=1364|750|60
contig11593_length=1364|250|60
contig00093_length=1271|750|60
contig00093_length=1271|250|60
contig11072_length=1626|750|60
contig11072_length=1626|250|60
log2(expression)
0
2
4
6
8
12
16
StageI
StageII
StageIII
StageIV
StageV
StageVI

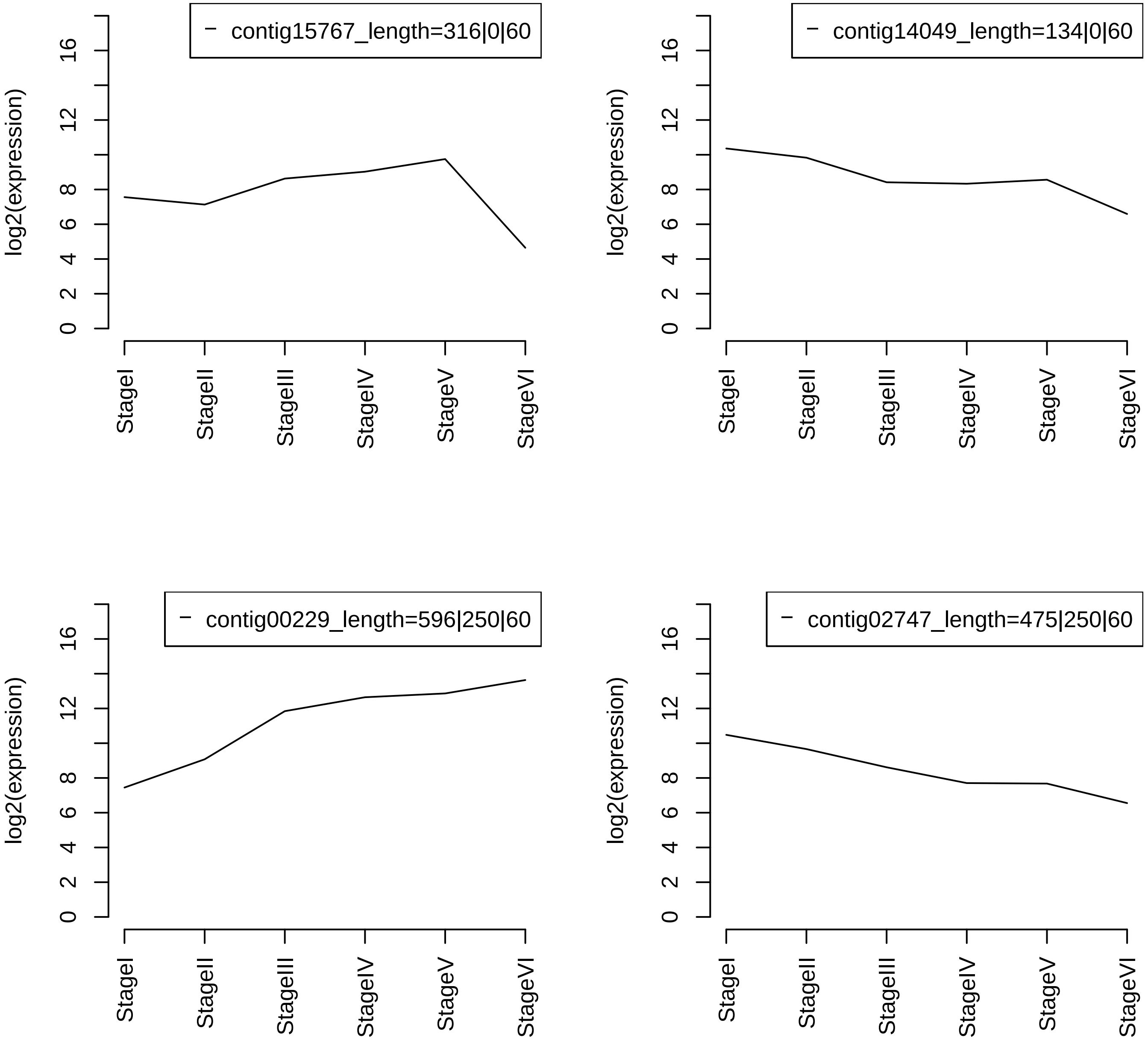
contig15767_length=316|0|60
contig14049_length=134|0|60
contig00229_length=596|250|60
contig02747_length=475|250|60
log2(expression)
0
2
4
6
8
12
16
StageI
StageII
StageIII
StageIV
StageV
StageVI

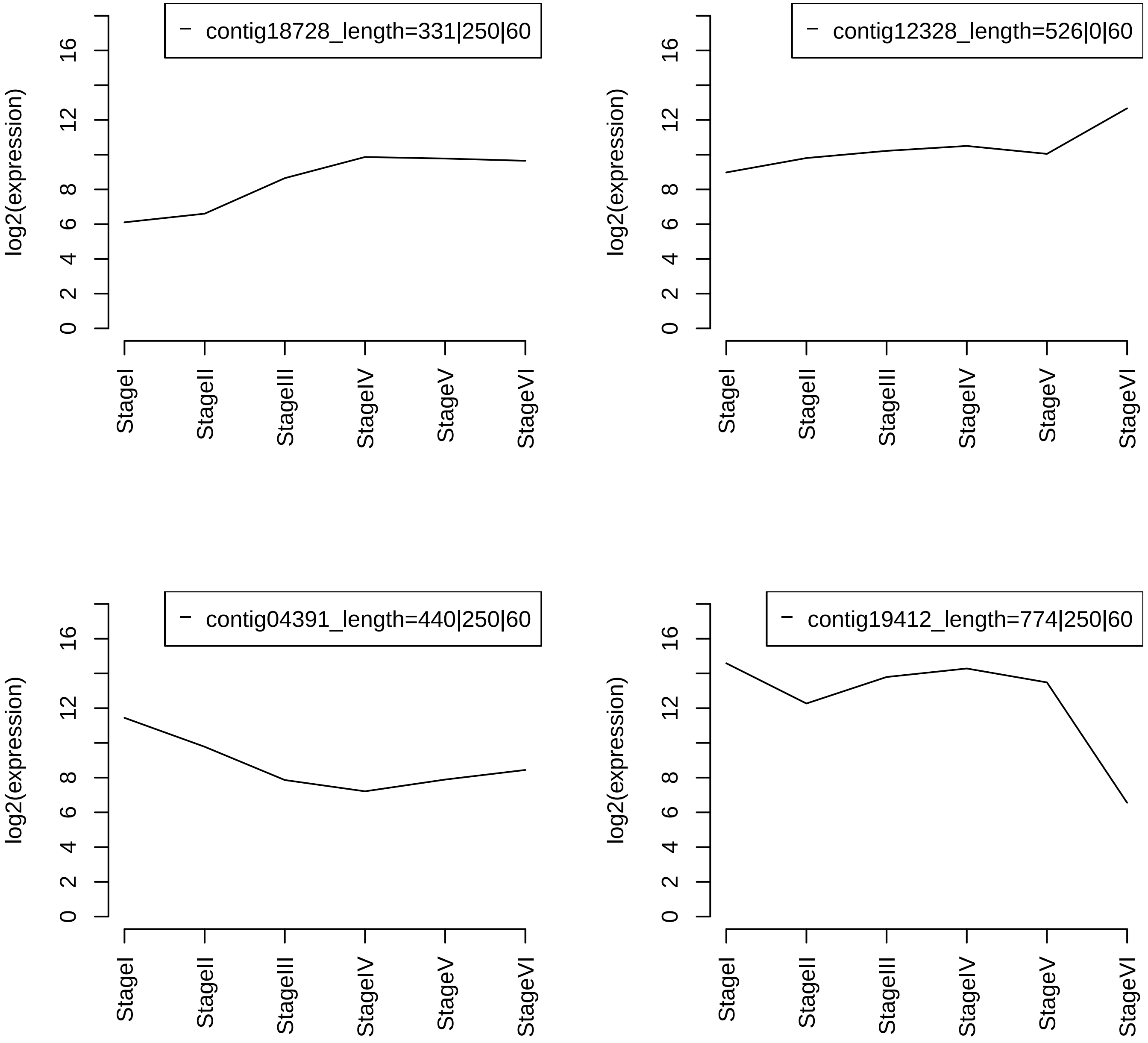

contig18728_length=331|250|60
contig12328_length=526|0|60
contig04391_length=440|250|60
contig19412_length=774|250|60
log2(expression)
StageI
StageII
StageIII
StageIV
StageV
StageVI

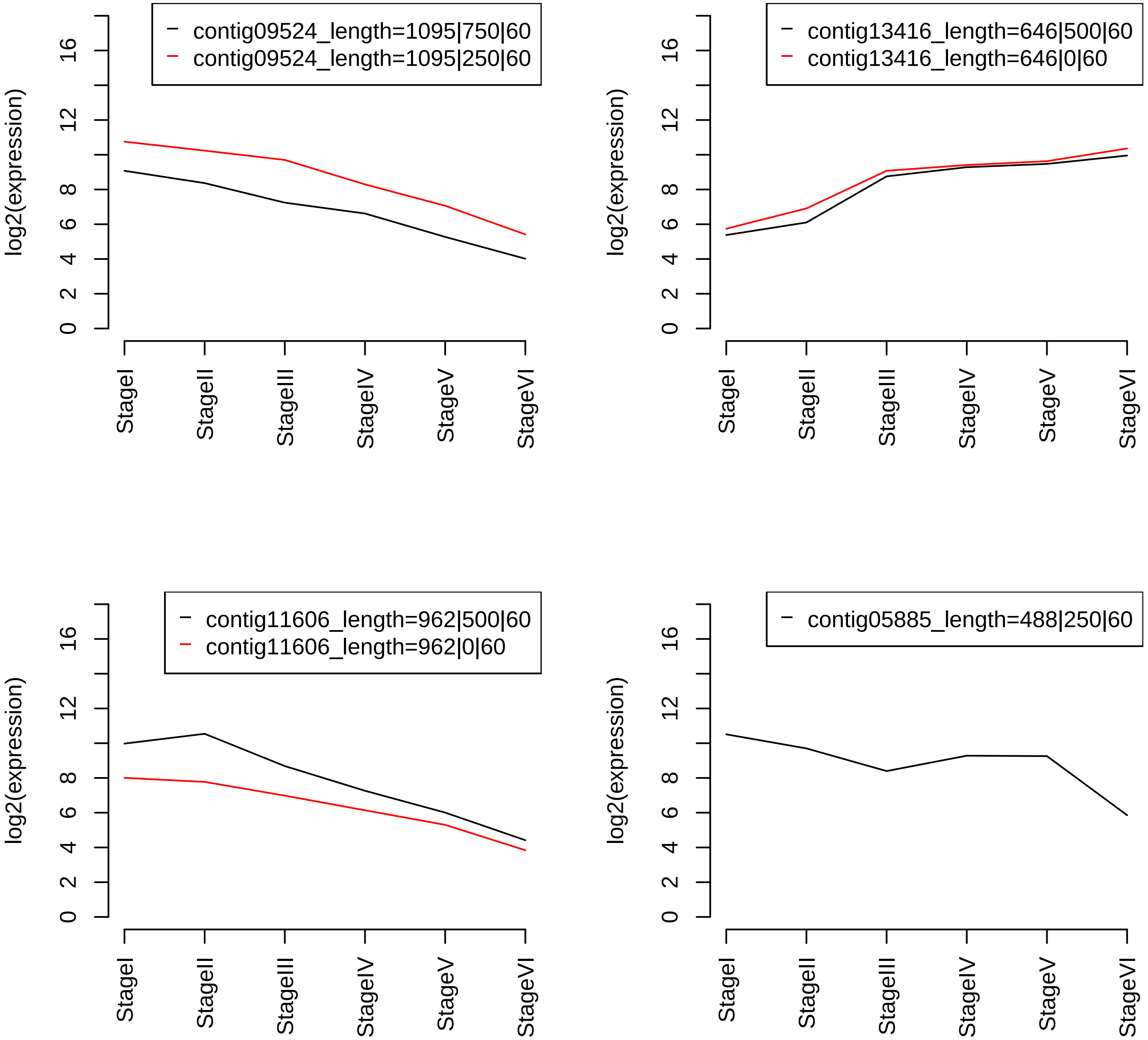
contig09524_length=1095|750|60
contig09524_length=1095|250|60
contig13416_length=646|500|60
contig13416_length=646|0|60
contig11606_length=962|500|60
contig11606_length=962|0|60
contig05885_length=488|250|60
log2(expression)
StageI
StageII
StageIII
StageIV
StageV
StageVI

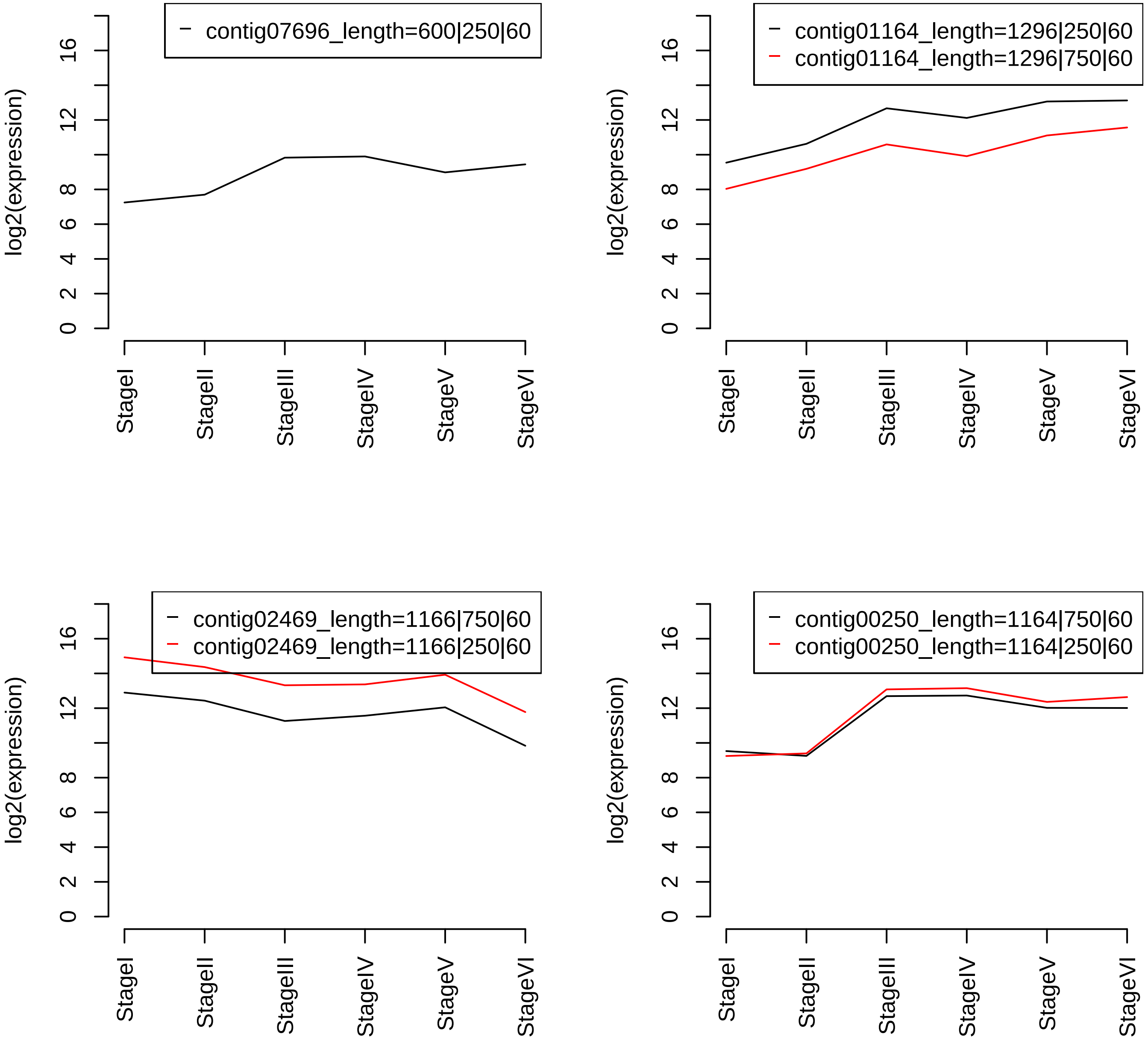
contig07696_length=600|250|60
contig01164_length=1296|250|60
contig01164_length=1296|750|60
contig02469_length=1166|750|60
contig02469_length=1166|250|60
contig00250_length=1164|750|60
contig00250_length=1164|250|60
log2(expression)
StageI
StageII
StageIII
StageIV
StageV
StageVI

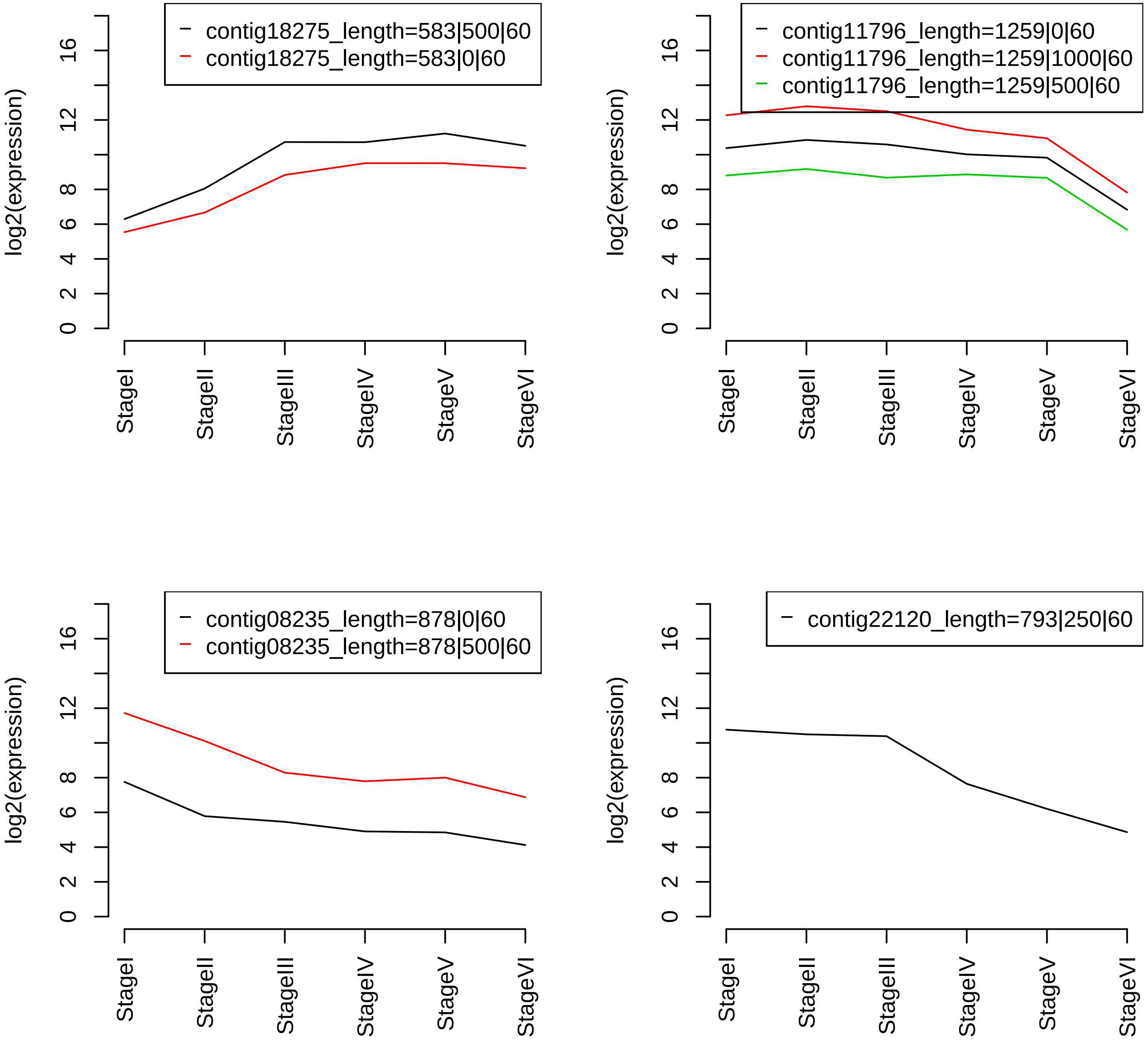

contig18275_length=583|500|60
contig18275_length=583|0|60
contig11796_length=1259|0|60
contig11796_length=1259|1000|60
contig11796_length=1259|500|60
contig08235_length=878|0|60
contig08235_length=878|500|60
contig22120_length=793|250|60
log2(expression)
0
2
4
6
8
12
16
StageI
StageII
StageIII
StageIV
StageV
StageVI

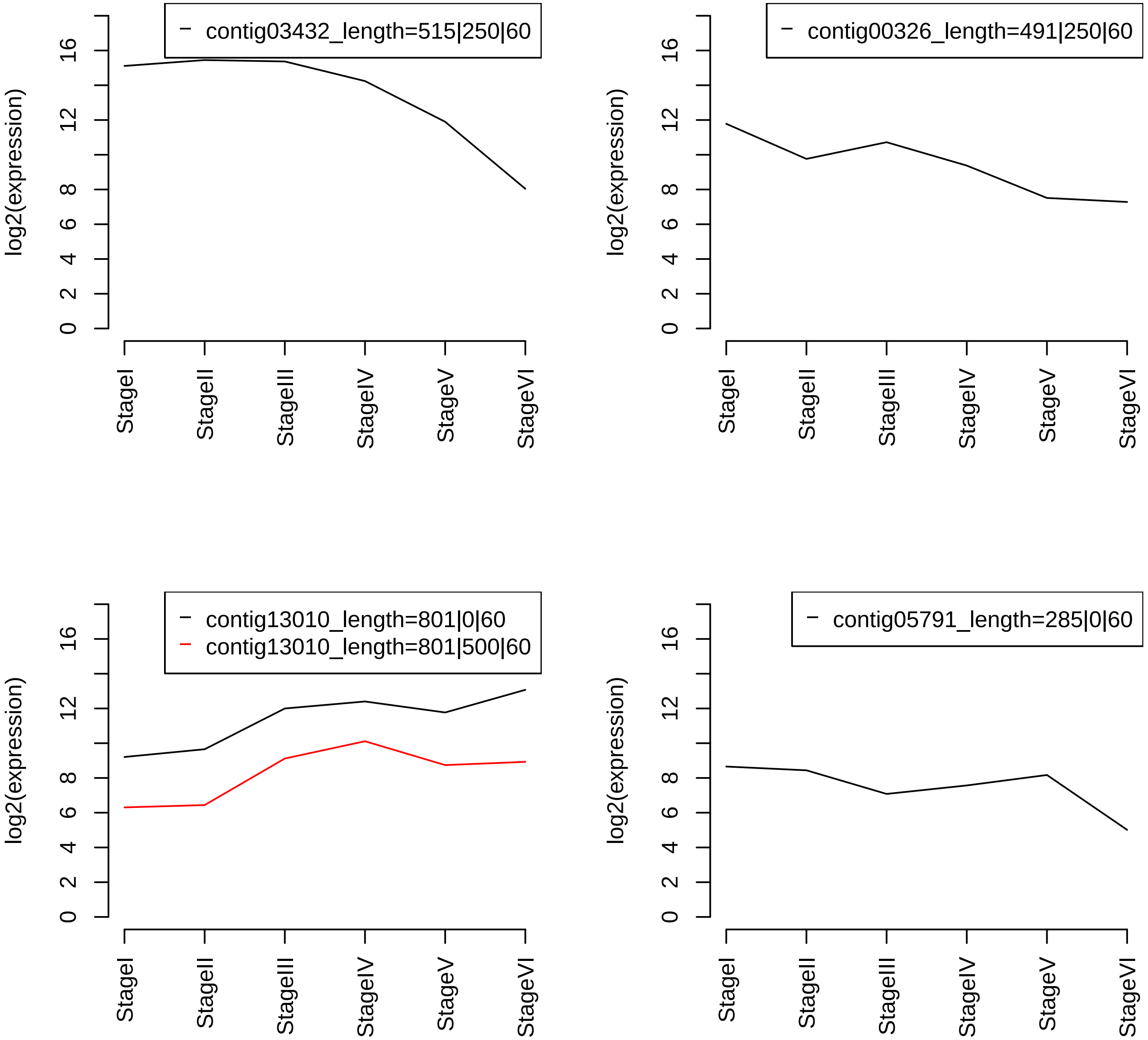
contig03432_length=515|250|60
contig00326_length=491|250|60
contig13010_length=801|0|60
contig13010_length=801|500|60
contig05791_length=285|0|60
log2(expression)
0
2
4
6
8
12
16
StageI
StageII
StageIII
StageIV
StageV
StageVI

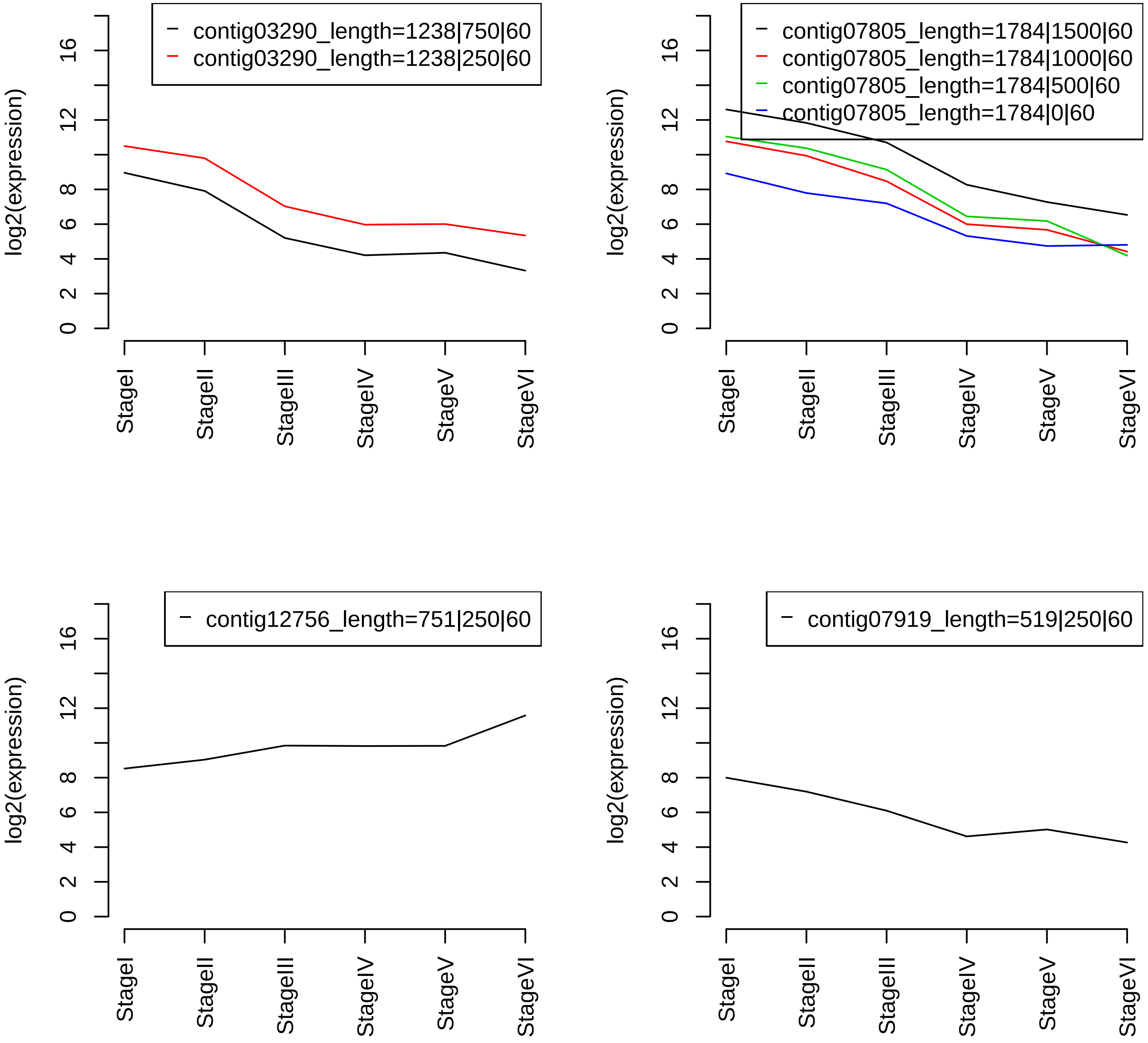
contig03290_length=1238|750|60
contig03290_length=1238|250|60
contig07805_length=1784|1500|60
contig07805_length=1784|1000|60
contig07805_length=1784|500|60
contig07805_length=1784|0|60
contig12756_length=751|250|60
contig07919_length=519|250|60
log2(expression)
0
2
4
6
8
12
16
StageI
StageII
StageIII
StageIV
StageV
StageVI

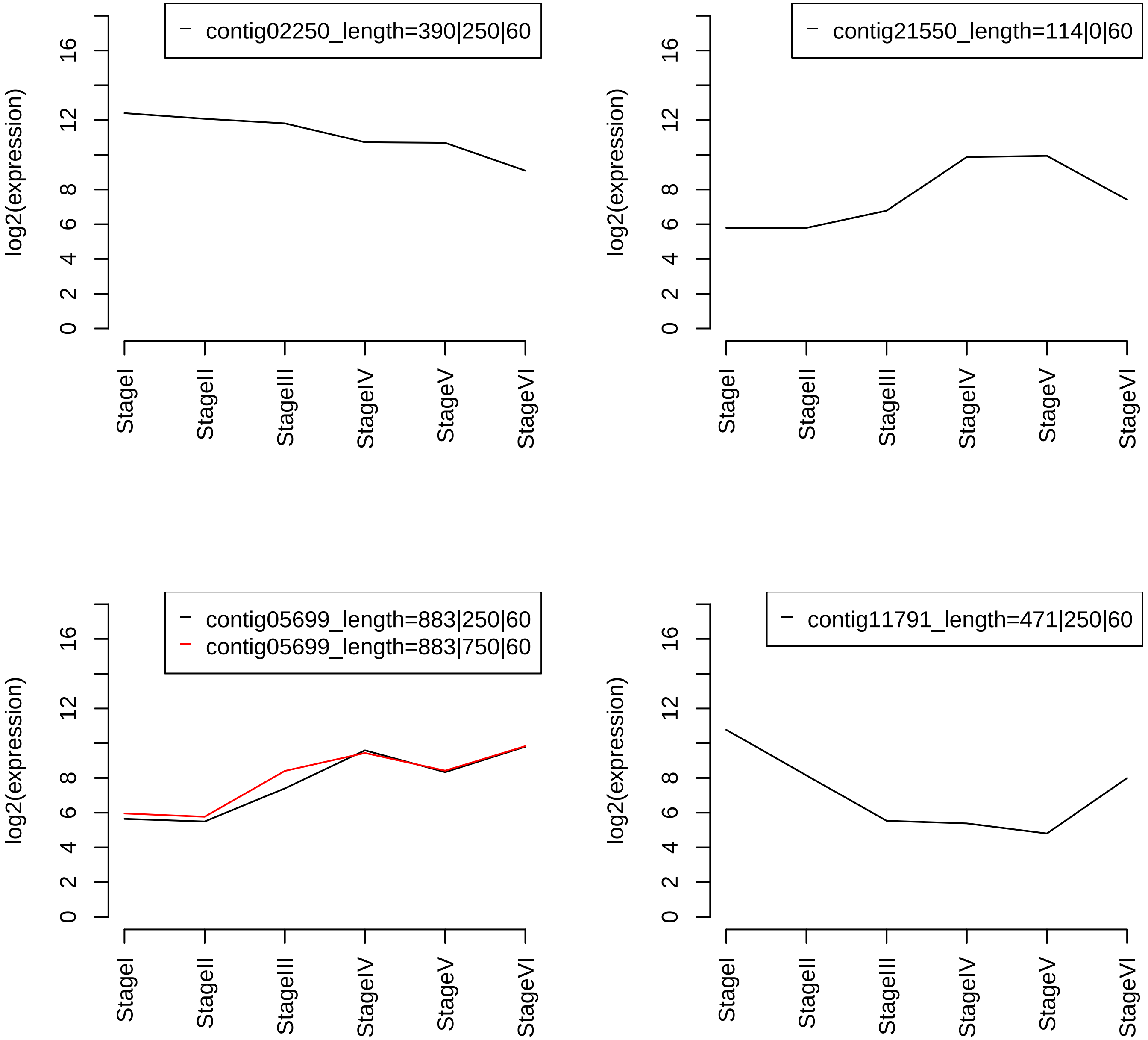

contig02250_length=390|250|60
contig21550_length=114|0|60
contig05699_length=883|250|60
contig05699_length=883|750|60
contig11791_length=471|250|60
log2(expression)
0
2
4
6
8
12
16
StageI
StageII
StageIII
StageIV
StageV
StageVI

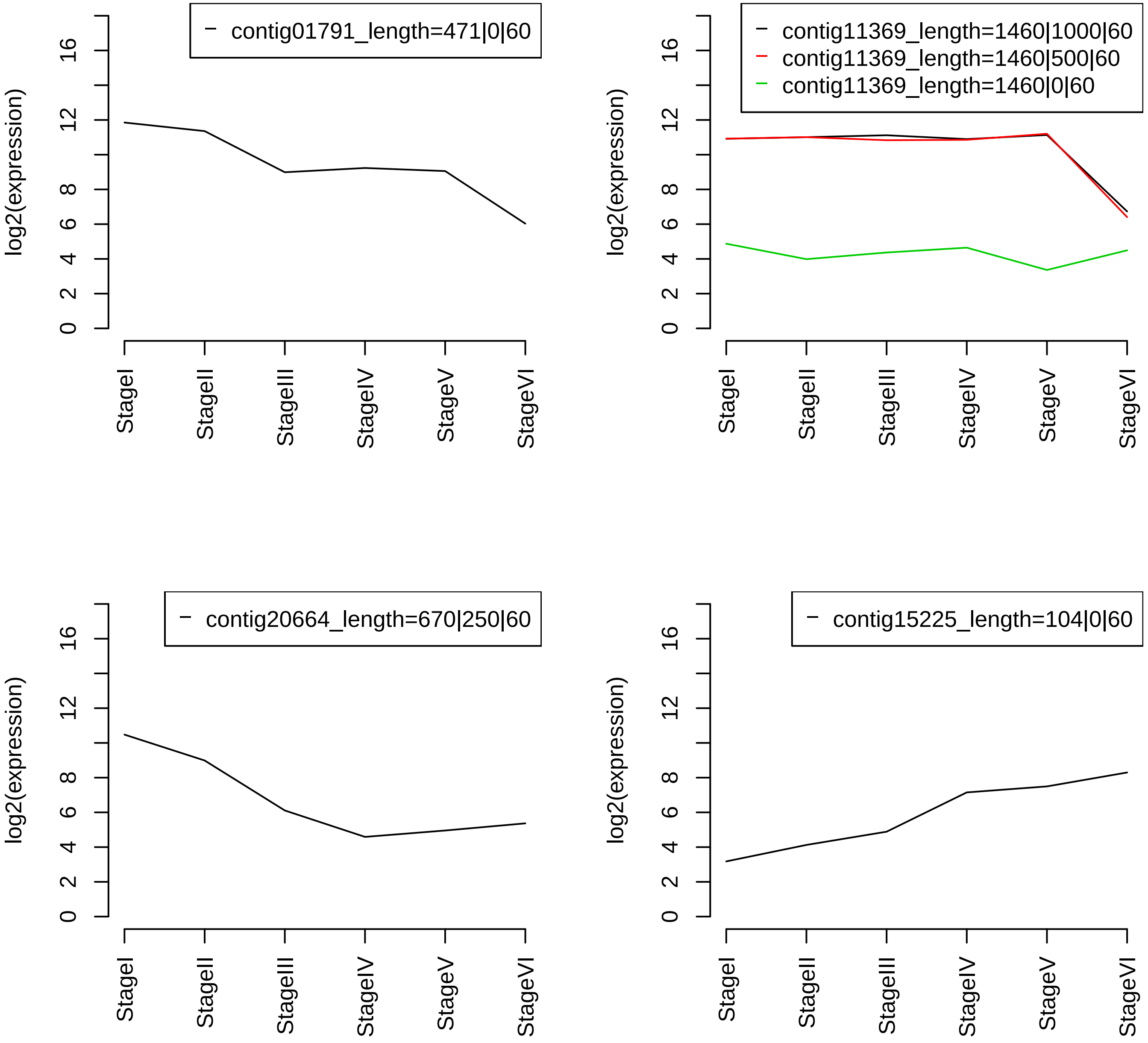

contig01791_length=471|0|60
contig11369_length=1460|1000|60
contig11369_length=1460|500|60
contig11369_length=1460|0|60
contig20664_length=670|250|60
contig15225_length=104|0|60
log2(expression)
0
2
4
6
8
12
16
StageI
StageII
StageIII
StageIV
StageV
StageVI

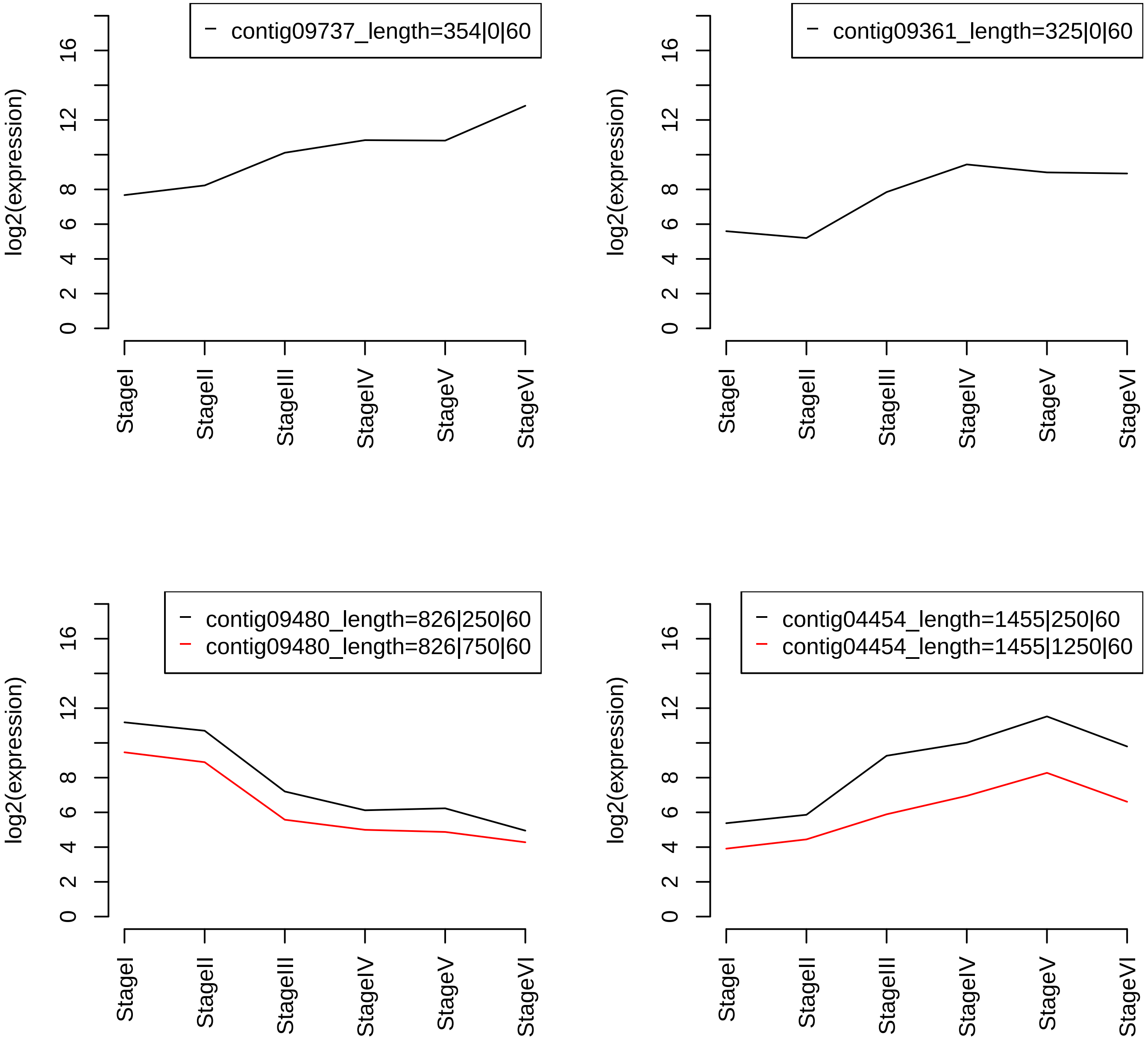
contig09737_length=354|0|60
contig09361_length=325|0|60
contig09480_length=826|250|60
contig09480_length=826|750|60
contig04454_length=1455|250|60
contig04454_length=1455|1250|60
log2(expression)
0
2
4
6
8
12
16
StageI
StageII
StageIII
StageIV
StageV
StageVI

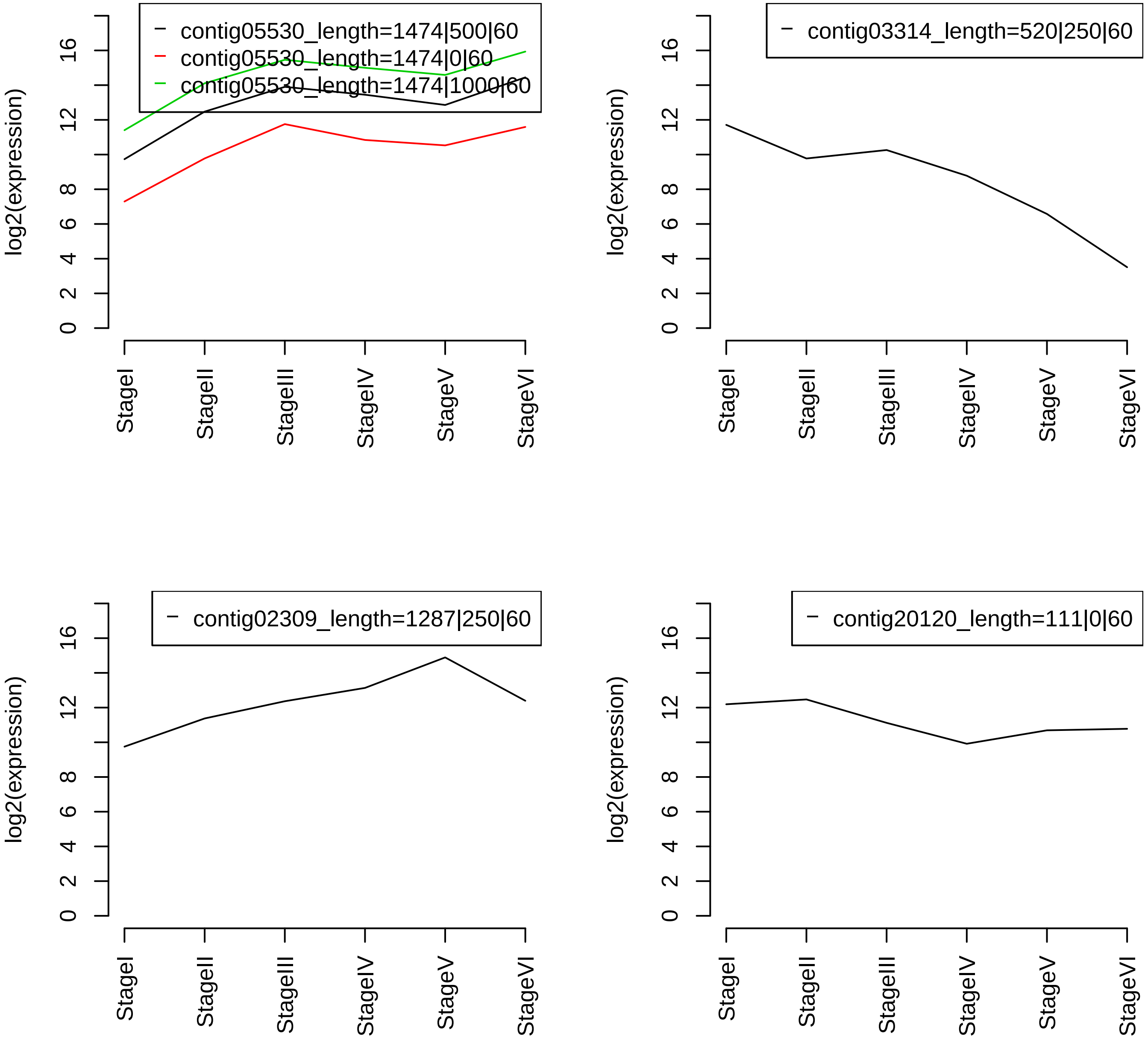

contig05530_length=1474|500|60
contig05530_length=1474|0|60
contig05530_length=1474|1000|60
contig03314_length=520|250|60
contig02309_length=1287|250|60
contig20120_length=111|0|60
log2(expression)
0
2
4
6
8
12
16
StageI
StageII
StageIII
StageIV
StageV
StageVI

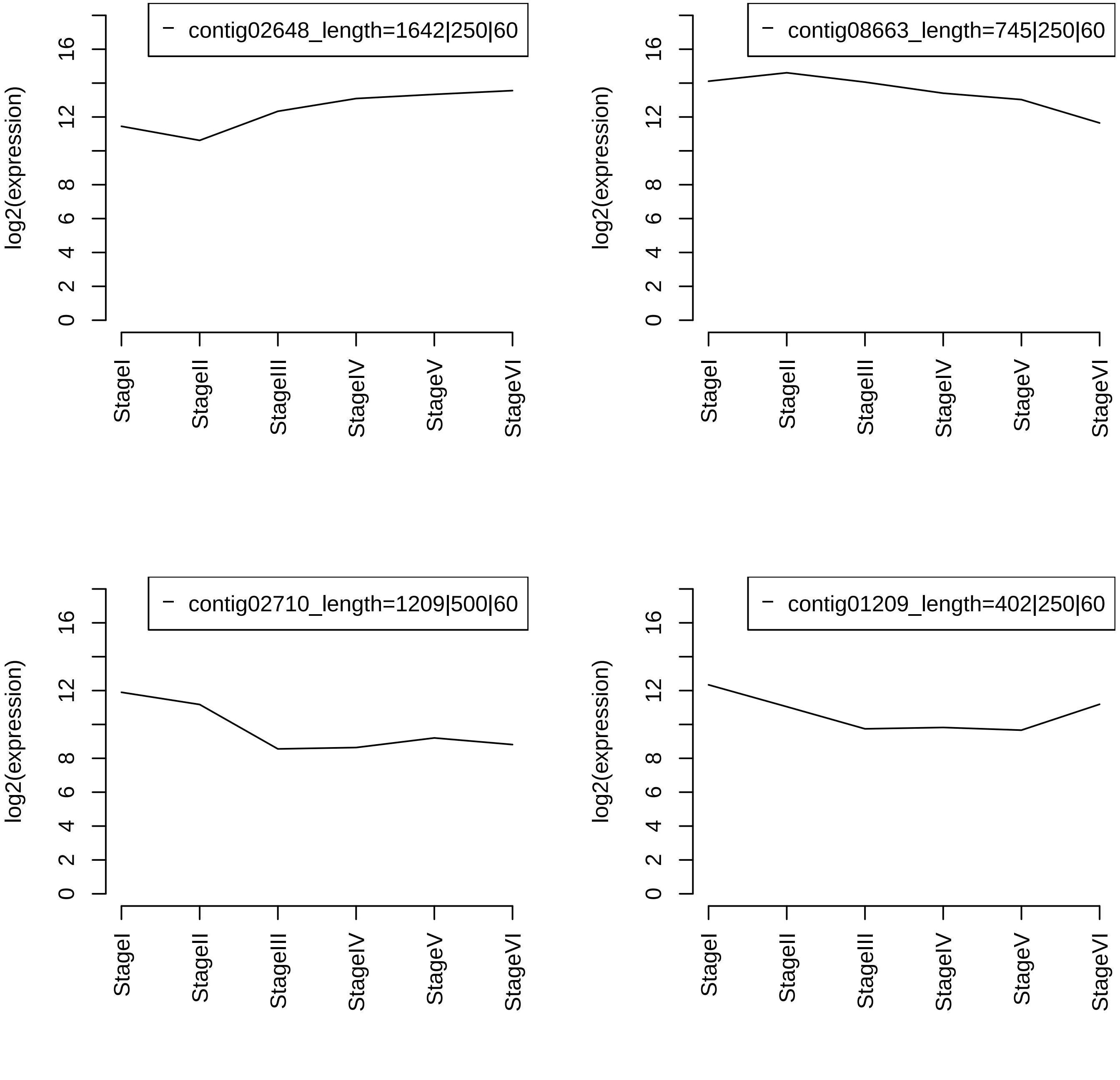
contig02648_length=1642|250|60
contig08663_length=745|250|60
contig02710_length=1209|500|60
contig01209_length=402|250|60
log2(expression)
0
2
4
6
8
12
16
StageI
StageII
StageIII
StageIV
StageV
StageVI

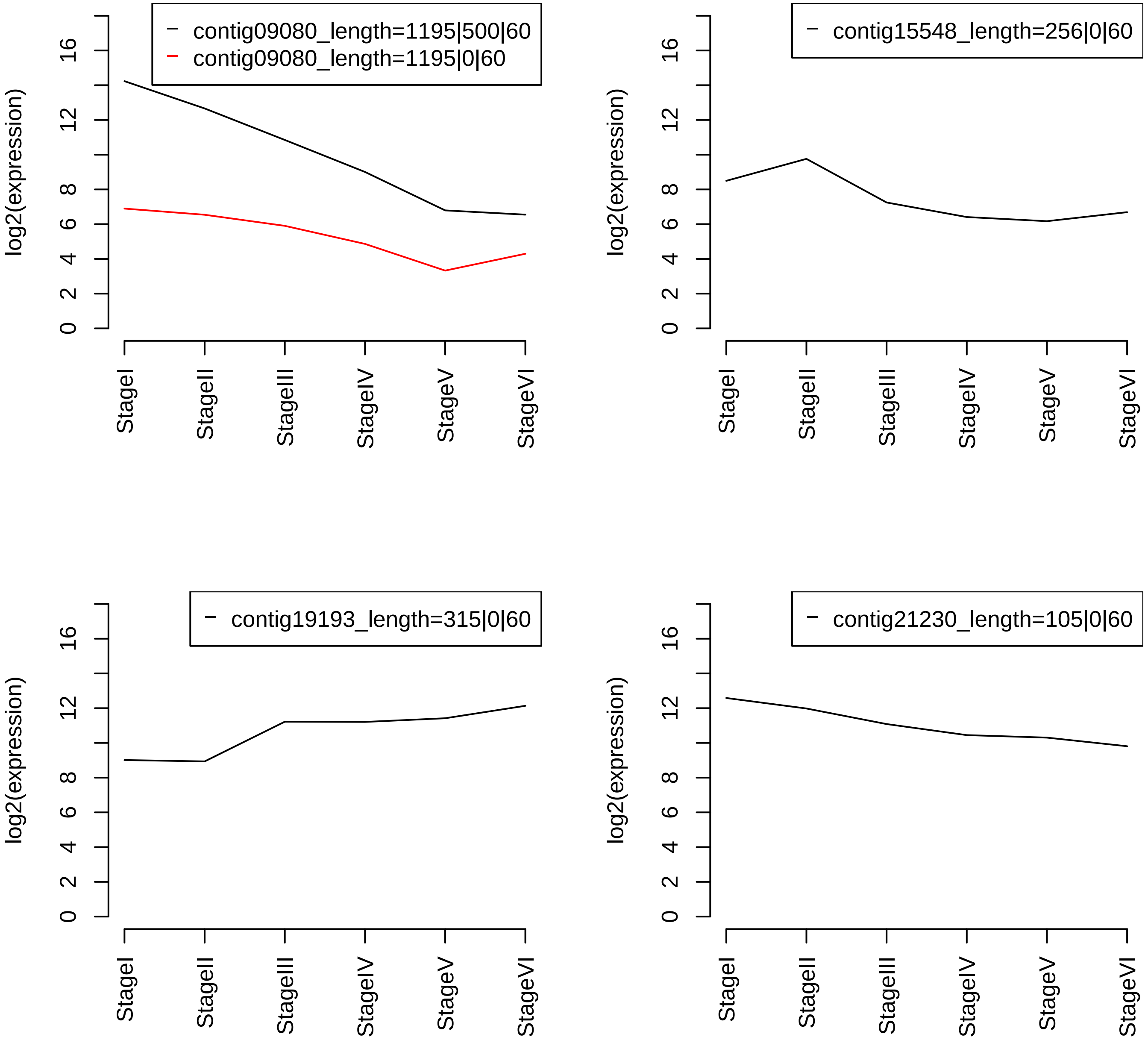
contig09080_length=1195|500|60
contig09080_length=1195|0|60
contig15548_length=256|0|60
contig19193_length=315|0|60
contig21230_length=105|0|60
log2(expression)
0
2
4
6
8
12
16
StageI
StageII
StageIII
StageIV
StageV
StageVI

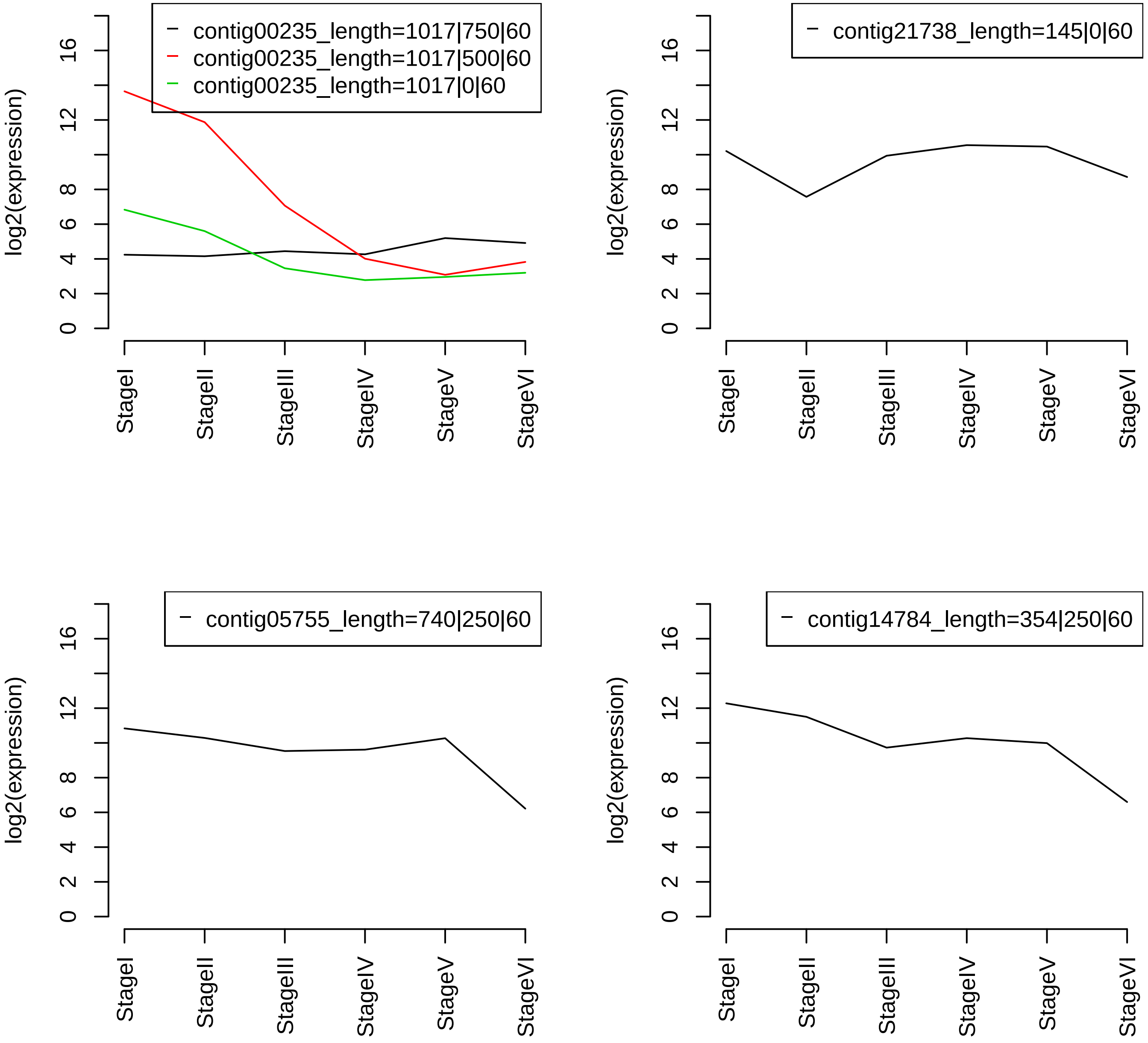
contig00235_length=1017|750|60
contig00235_length=1017|500|60
contig00235_length=1017|0|60
contig21738_length=145|0|60
contig05755_length=740|250|60
contig14784_length=354|250|60
log2(expression)
0
2
4
6
8
12
16
StageI
StageII
StageIII
StageIV
StageV
StageVI

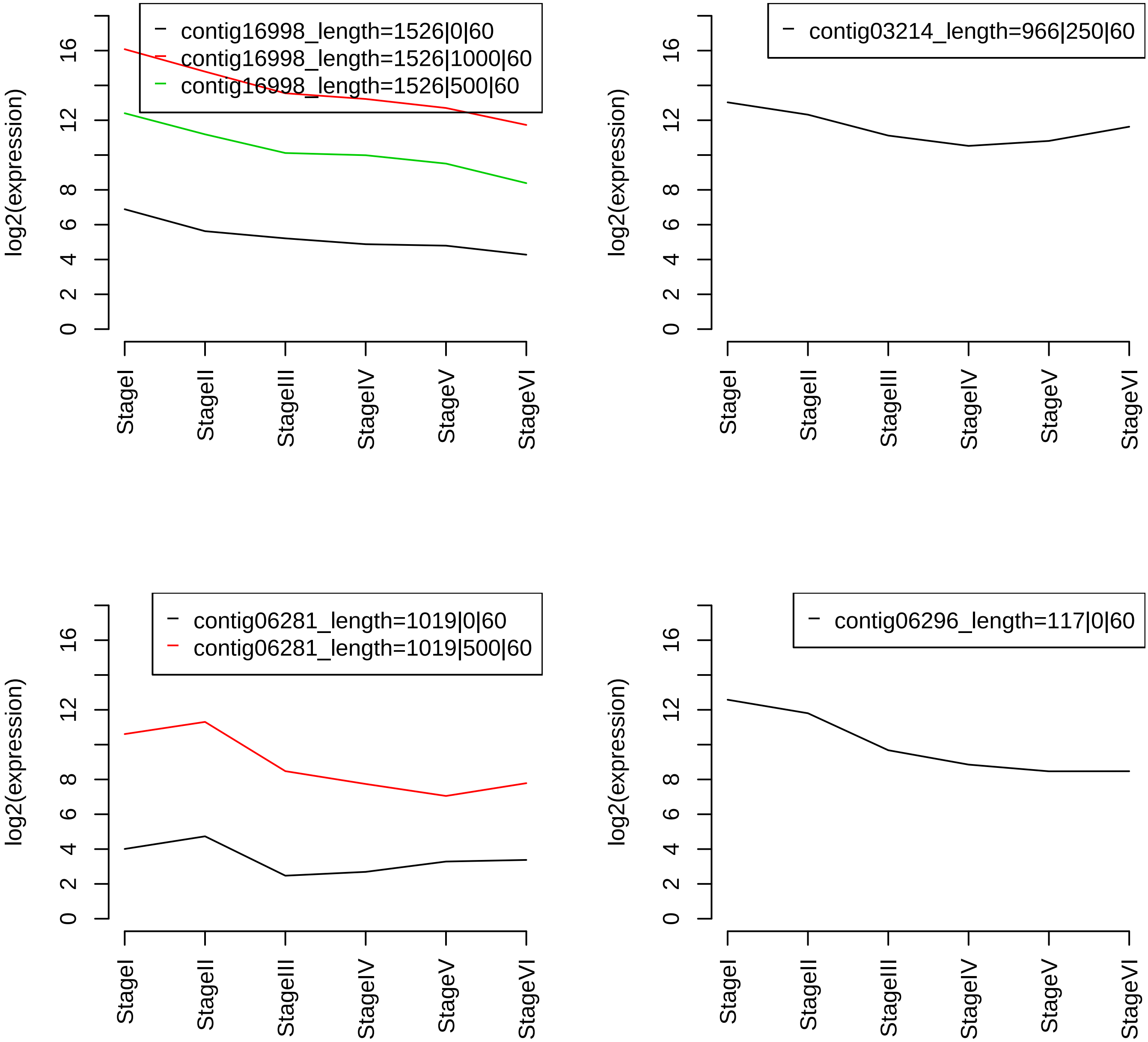
contig16998_length=1526|0|60
contig16998_length=1526|1000|60
contig16998_length=1526|500|60
contig03214_length=966|250|60
contig06281_length=1019|0|60
contig06281_length=1019|500|60
contig06296_length=117|0|60
log2(expression)
0 2 4 6 8 12 16
StageI StageII StageIII StageIV StageV StageVI

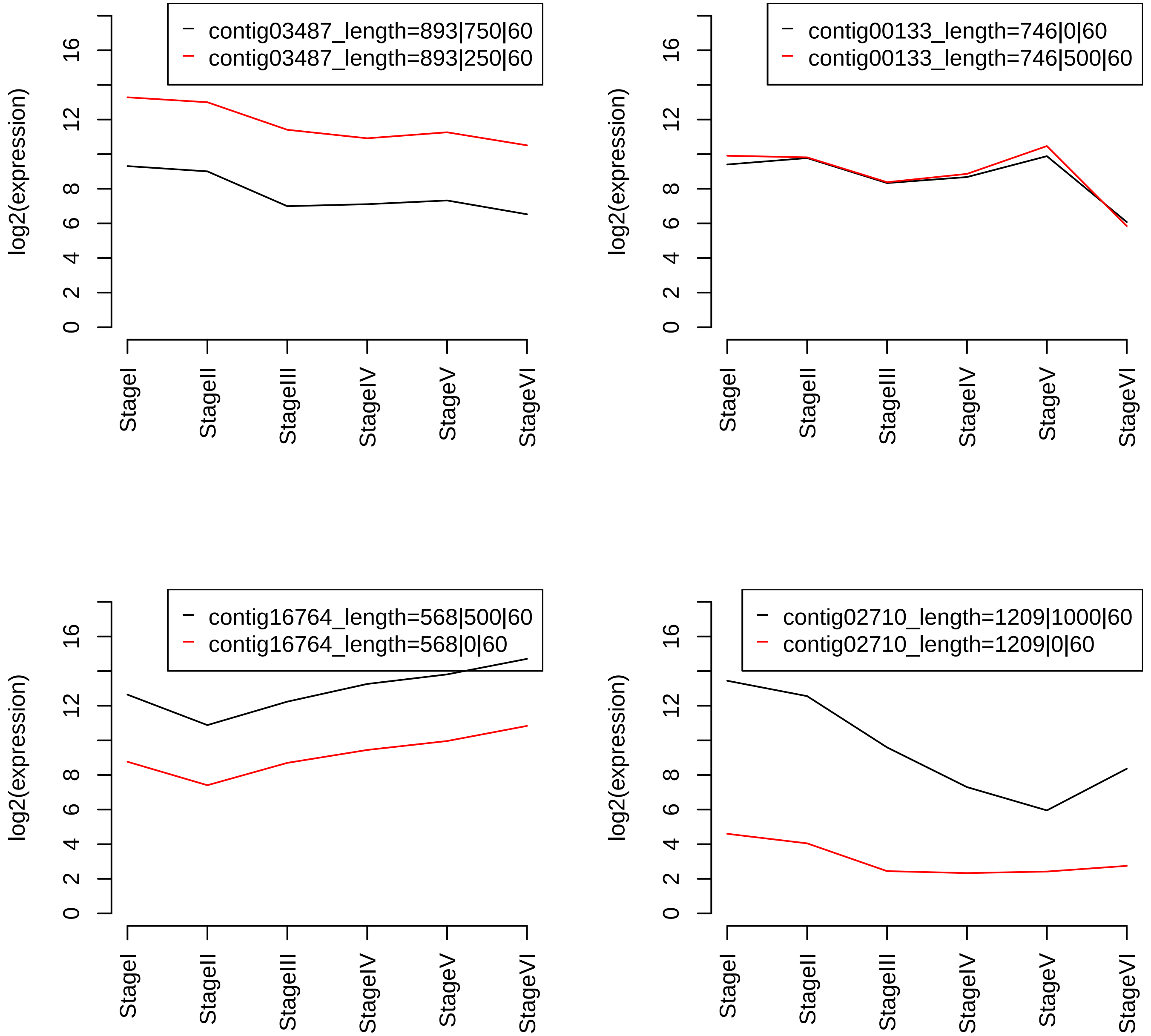
contig03487_length=893|750|60
contig03487_length=893|250|60
contig00133_length=746|0|60
contig00133_length=746|500|60
contig16764_length=568|500|60
contig16764_length=568|0|60
contig02710_length=1209|1000|60
contig02710_length=1209|0|60
log2(expression)
0 2 4 6 8 12 16
StageI StageII StageIII StageIV StageV StageVI

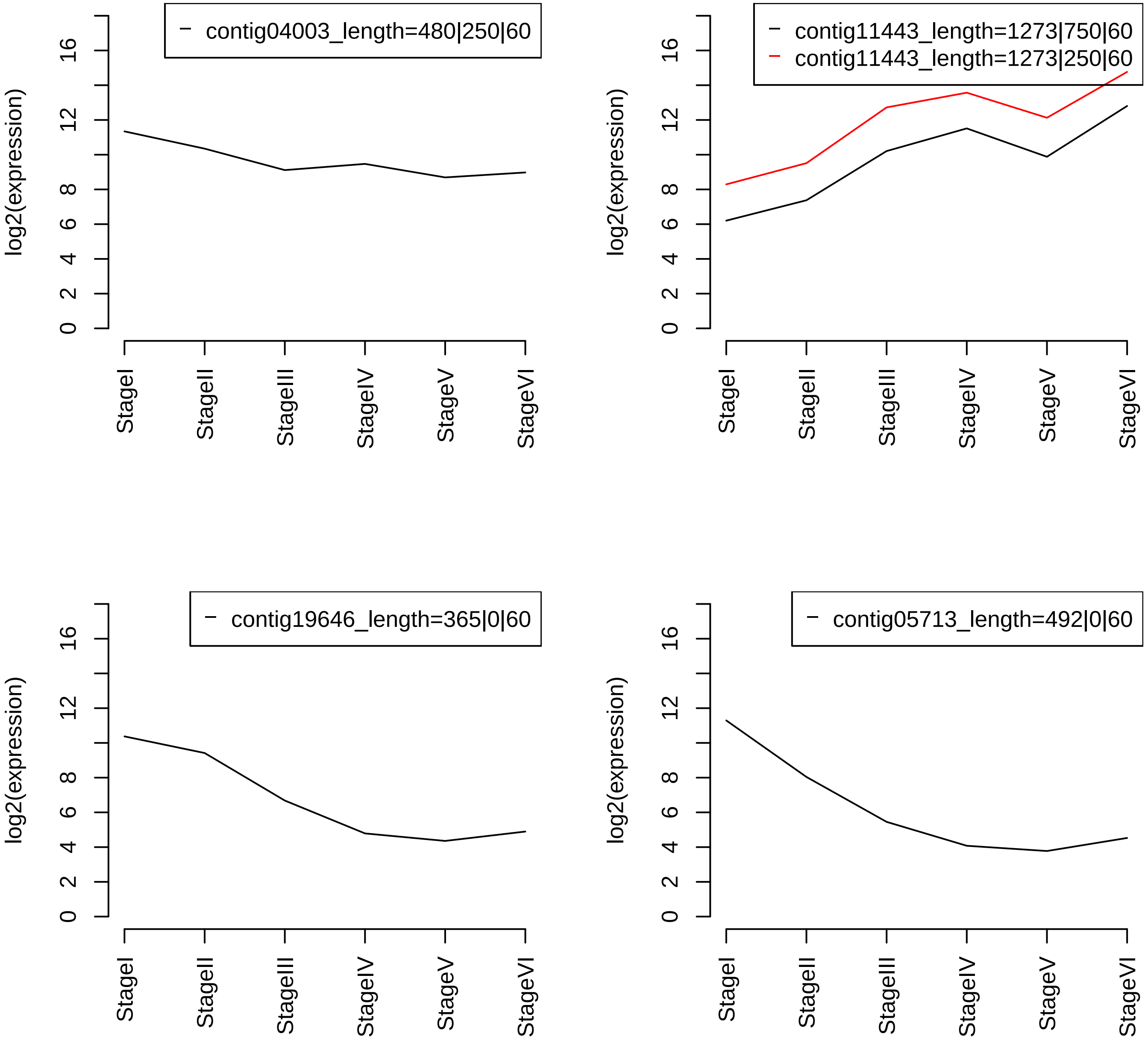
contig04003_length=480|250|60
contig11443_length=1273|750|60
contig11443_length=1273|250|60
contig19646_length=365|0|60
contig05713_length=492|0|60
log2(expression)
0
2
4
6
8
12
16
StageI
StageII
StageIII
StageIV
StageV
StageVI

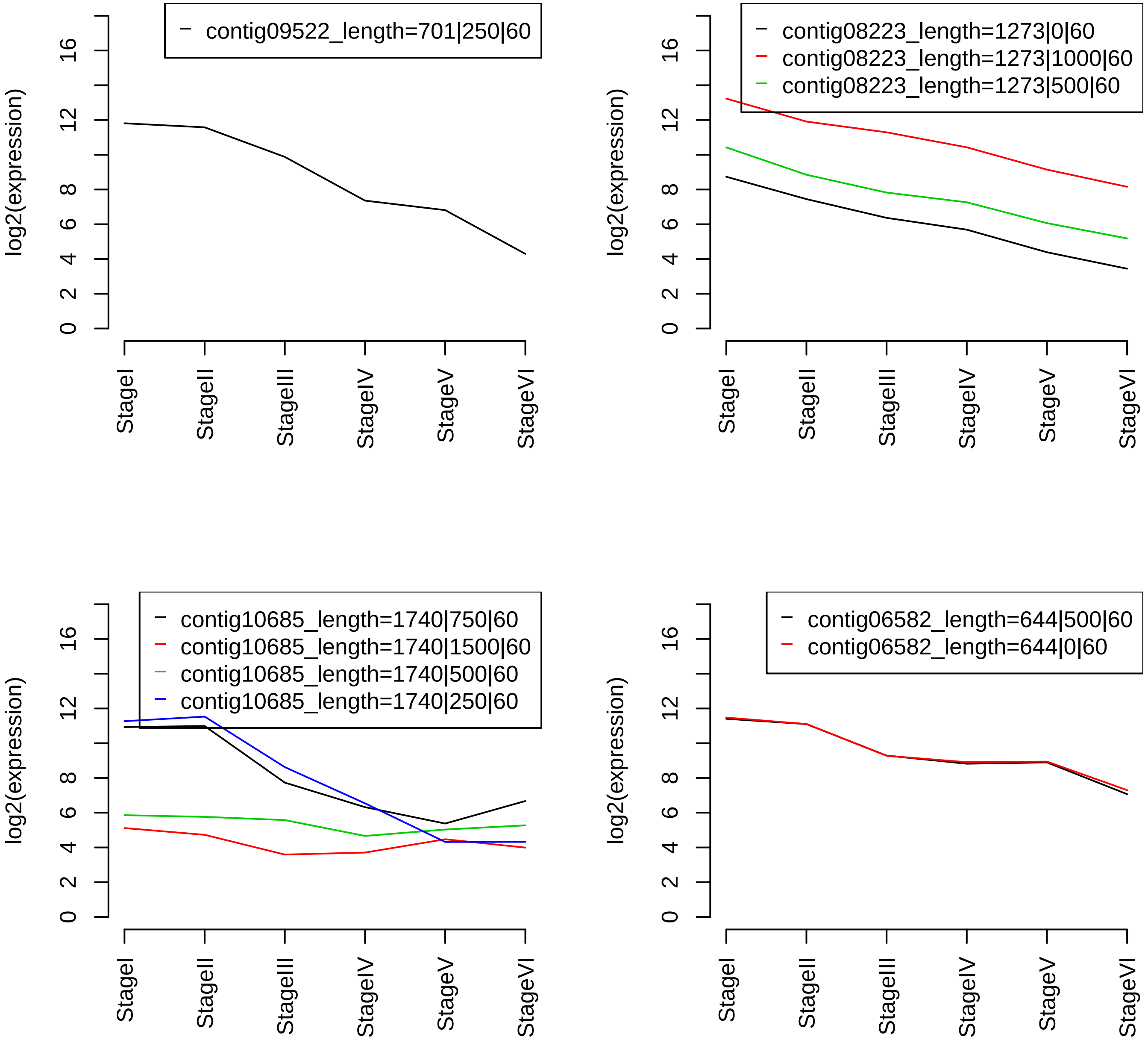
contig09522_length=701|250|60
contig08223_length=1273|0|60
contig08223_length=1273|1000|60
contig08223_length=1273|500|60
contig10685_length=1740|750|60
contig10685_length=1740|1500|60
contig10685_length=1740|500|60
contig10685_length=1740|250|60
contig06582_length=644|500|60
contig06582_length=644|0|60
log2(expression)
0 2 4 6 8 12 16
StageI StageII StageIII StageIV StageV StageVI

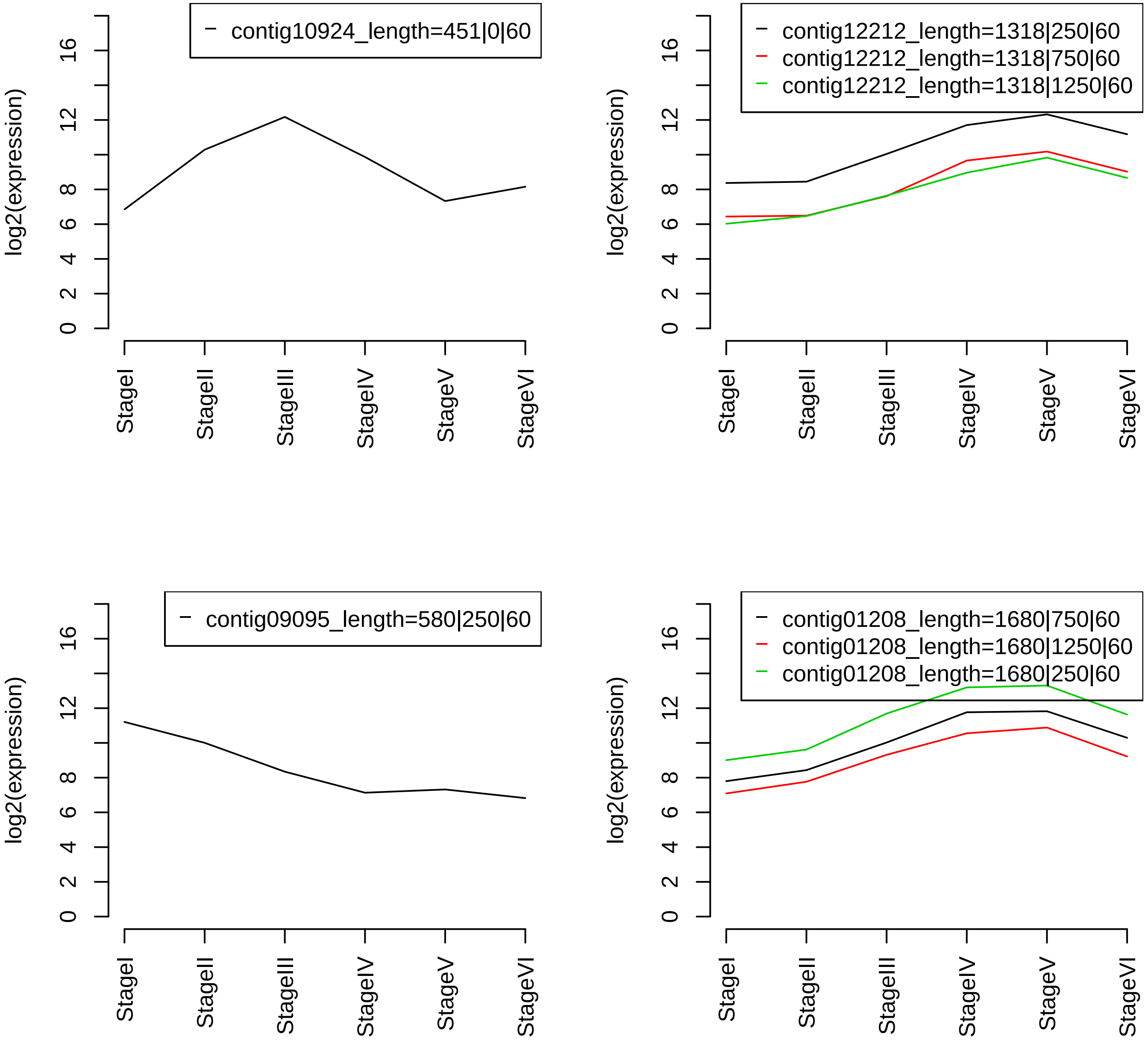
contig10924_length=451|0|60
contig12212_length=1318|250|60
contig12212_length=1318|750|60
contig12212_length=1318|1250|60
contig09095_length=580|250|60
contig01208_length=1680|750|60
contig01208_length=1680|1250|60
contig01208_length=1680|250|60
log2(expression)
0
2
4
6
8
12
16
StageI
StageII
StageIII
StageIV
StageV
StageVI

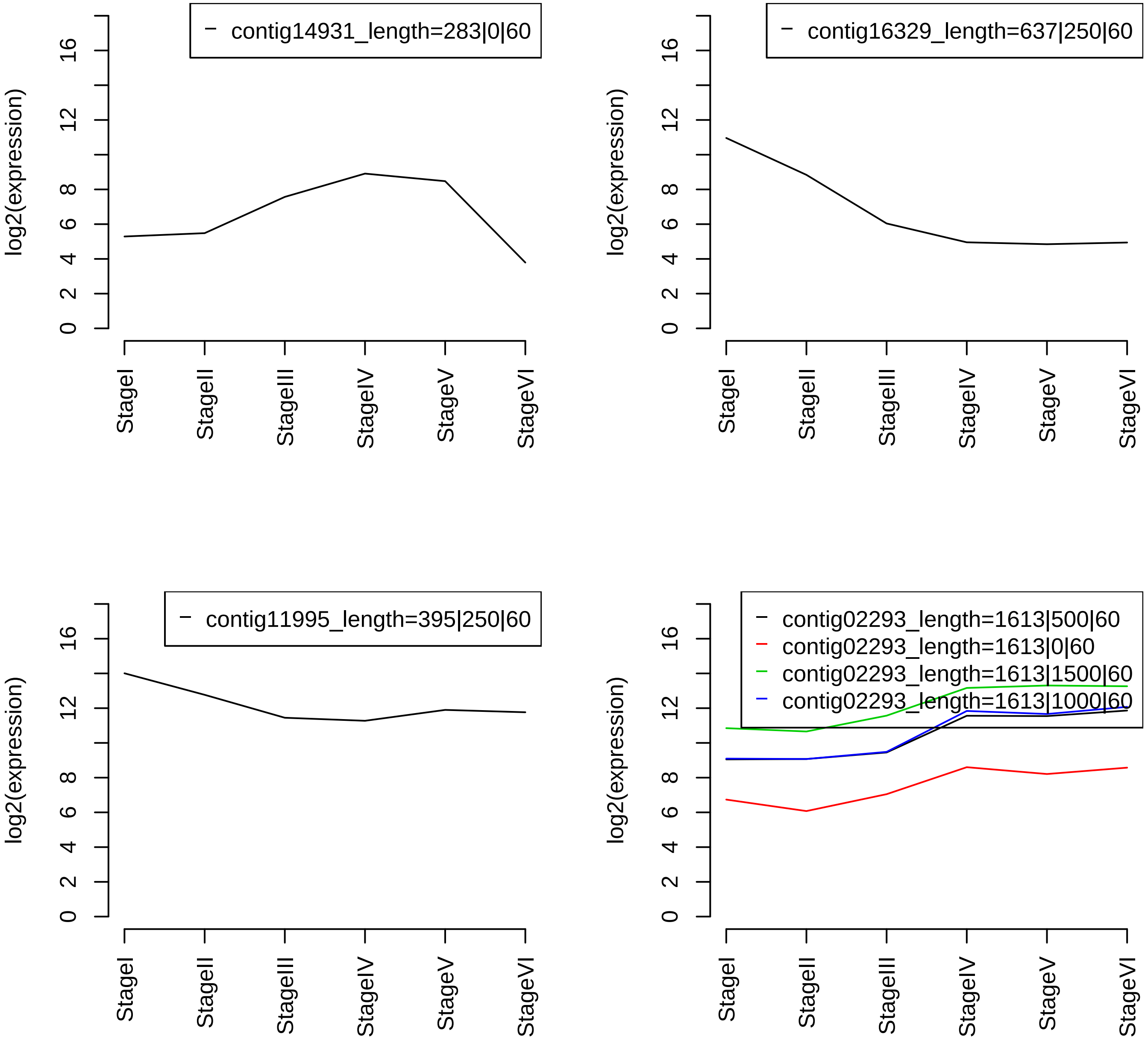

contig14931_length=283|0|60
contig16329_length=637|250|60
contig11995_length=395|250|60
contig02293_length=1613|500|60
contig02293_length=1613|0|60
contig02293_length=1613|1500|60
contig02293_length=1613|1000|60
log2(expression)
0
2
4
6
8
12
16
StageI
StageII
StageIII
StageIV
StageV
StageVI

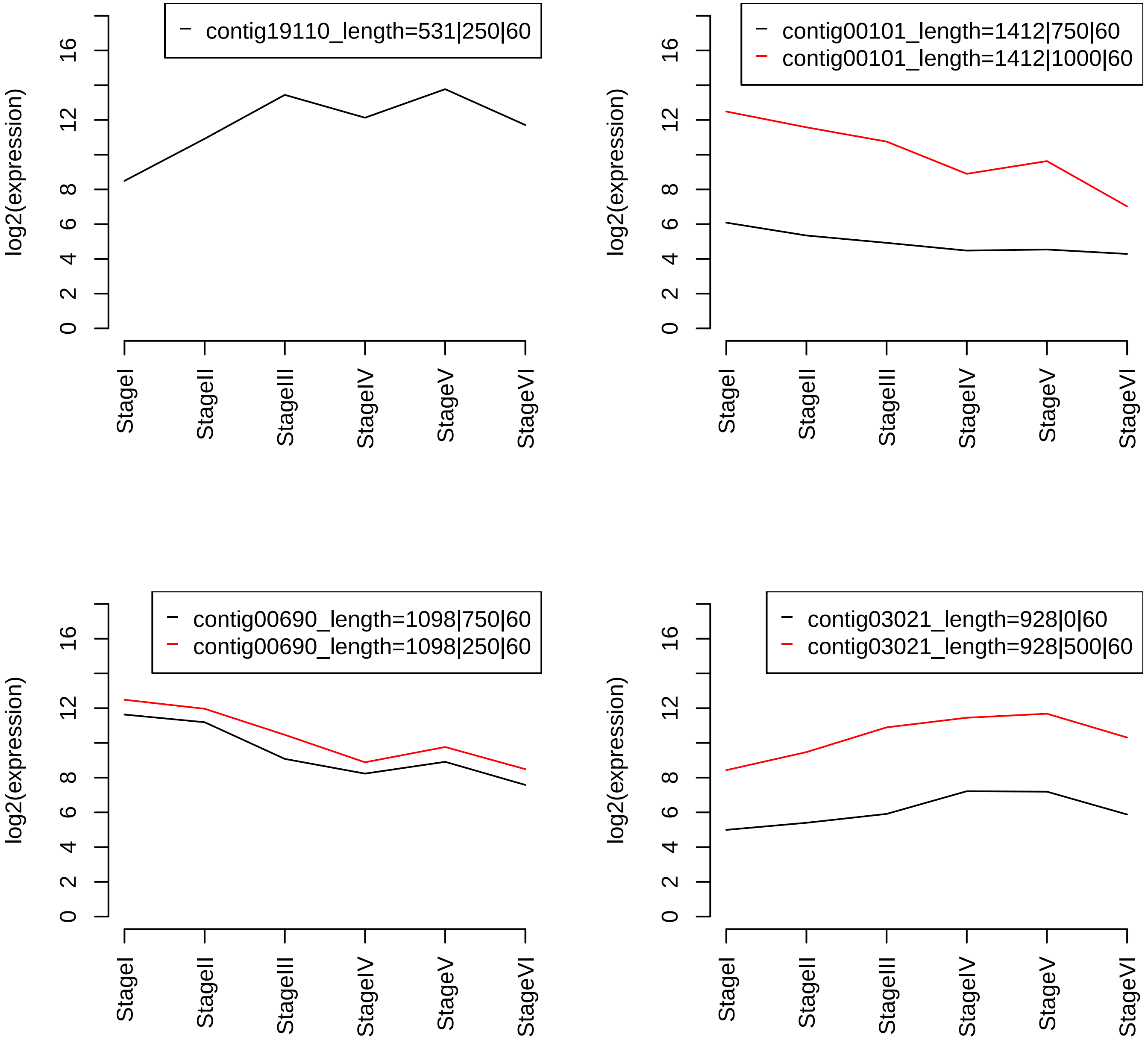

contig19110_length=531|250|60
contig00101_length=1412|750|60
contig00101_length=1412|1000|60
contig00690_length=1098|750|60
contig00690_length=1098|250|60
contig03021_length=928|0|60
contig03021_length=928|500|60
log2(expression)
0
2
4
6
8
12
16
StageI
StageII
StageIII
StageIV
StageV
StageVI

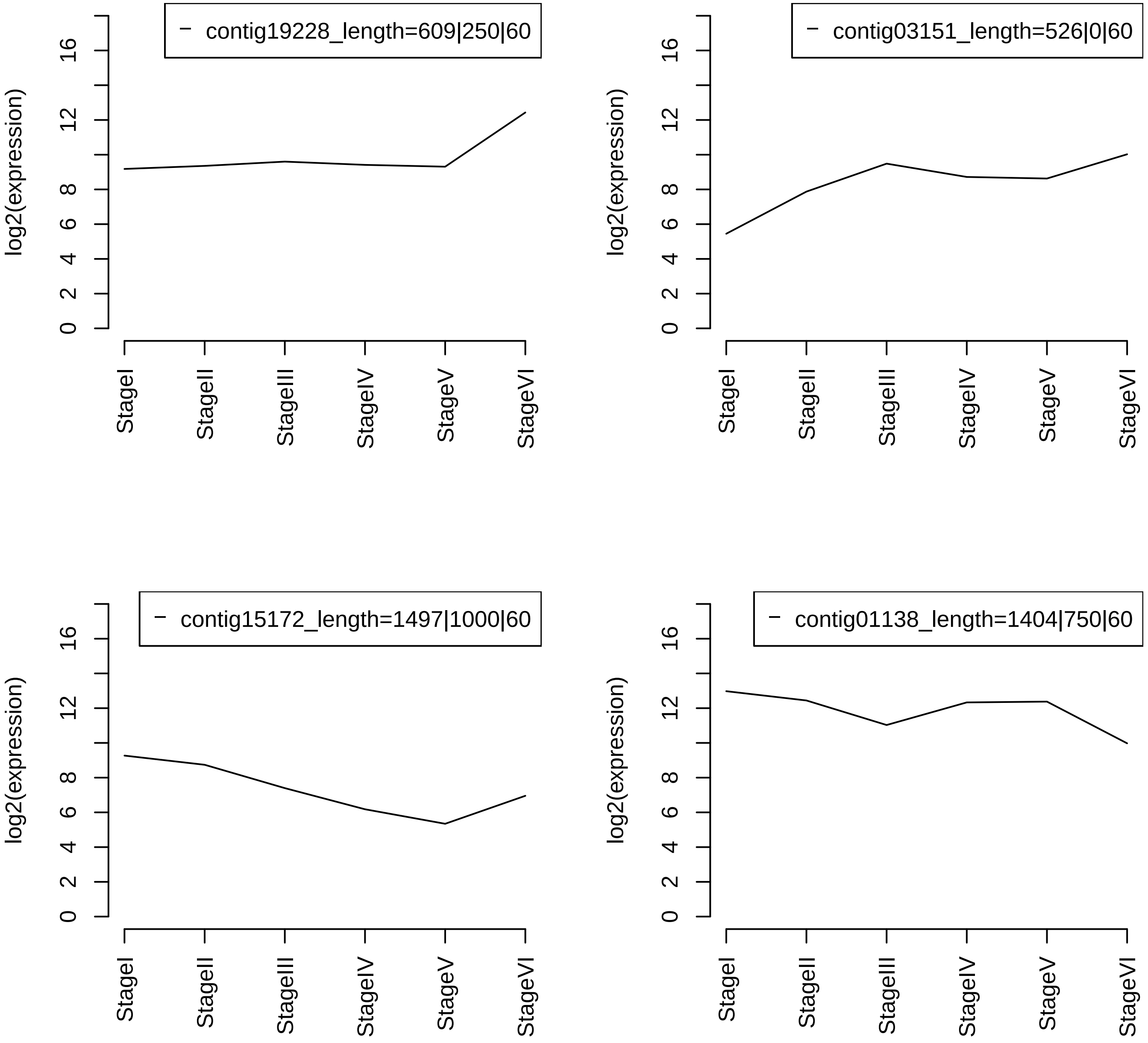
contig19228_length=609|250|60
contig03151_length=526|0|60
contig15172_length=1497|1000|60
contig01138_length=1404|750|60
log2(expression)
0
2
4
6
8
12
16
StageI
StageII
StageIII
StageIV
StageV
StageVI

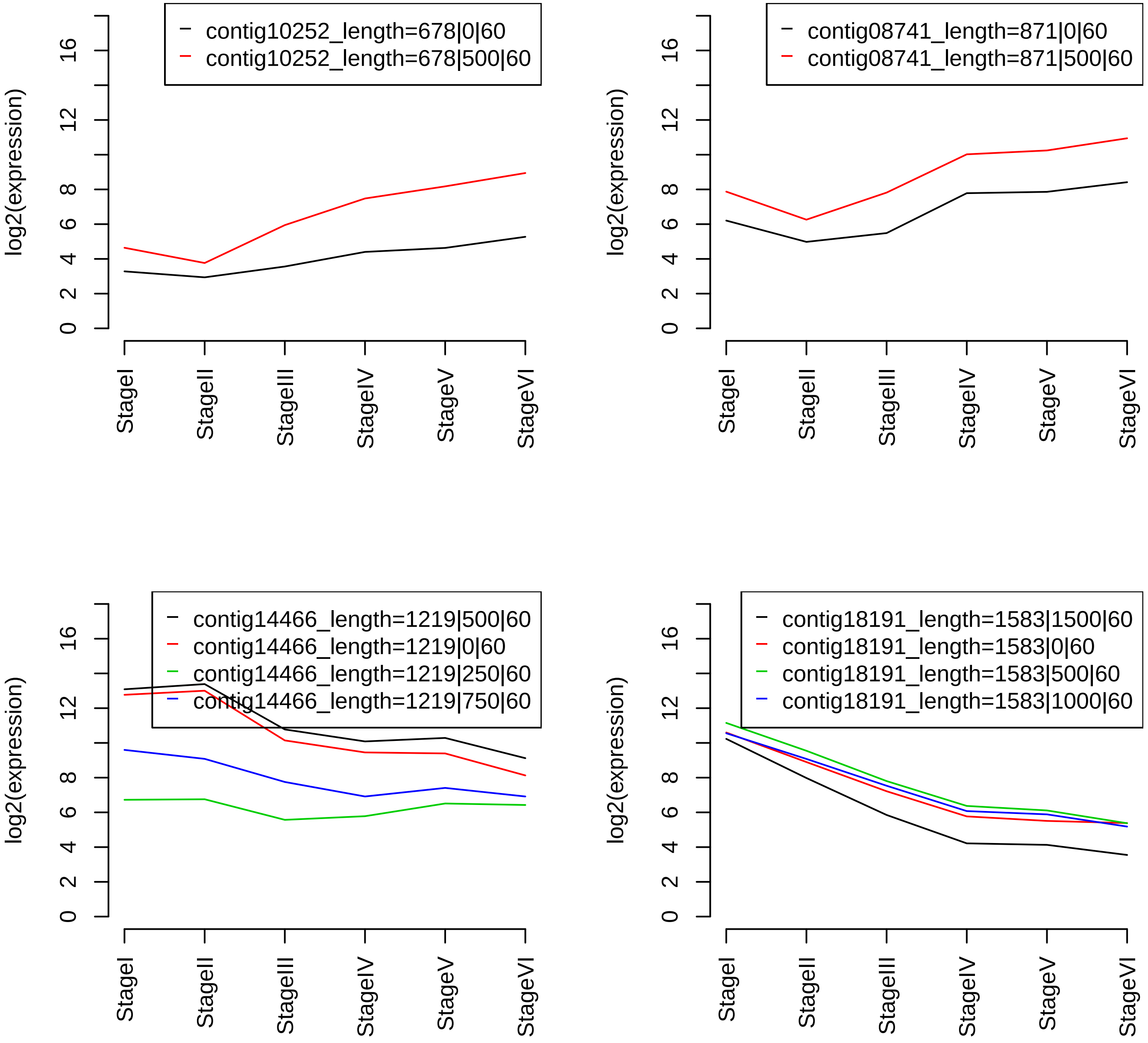
contig10252_length=678|0|60
contig10252_length=678|500|60
contig08741_length=871|0|60
contig08741_length=871|500|60
contig14466_length=1219|500|60
contig14466_length=1219|0|60
contig14466_length=1219|250|60
contig14466_length=1219|750|60
contig18191_length=1583|1500|60
contig18191_length=1583|0|60
contig18191_length=1583|500|60
contig18191_length=1583|1000|60
log2(expression)
StageI
StageII
StageIII
StageIV
StageV
StageVI

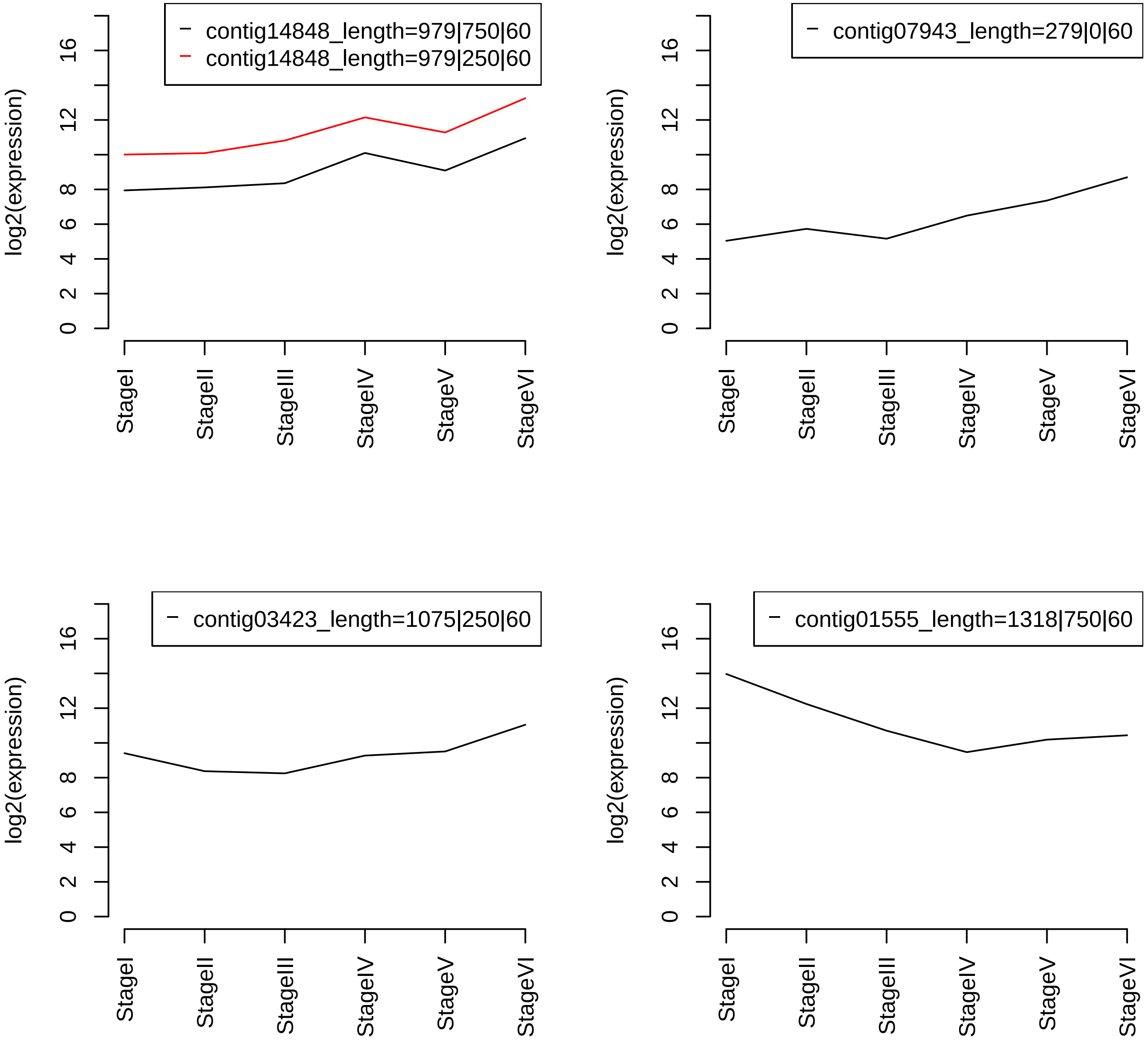
contig14848_length=979|750|60
contig14848_length=979|250|60
contig07943_length=279|0|60
contig03423_length=1075|250|60
contig01555_length=1318|750|60
log2(expression)
0
2
4
6
8
12
16
StageI
StageII
StageIII
StageIV
StageV
StageVI

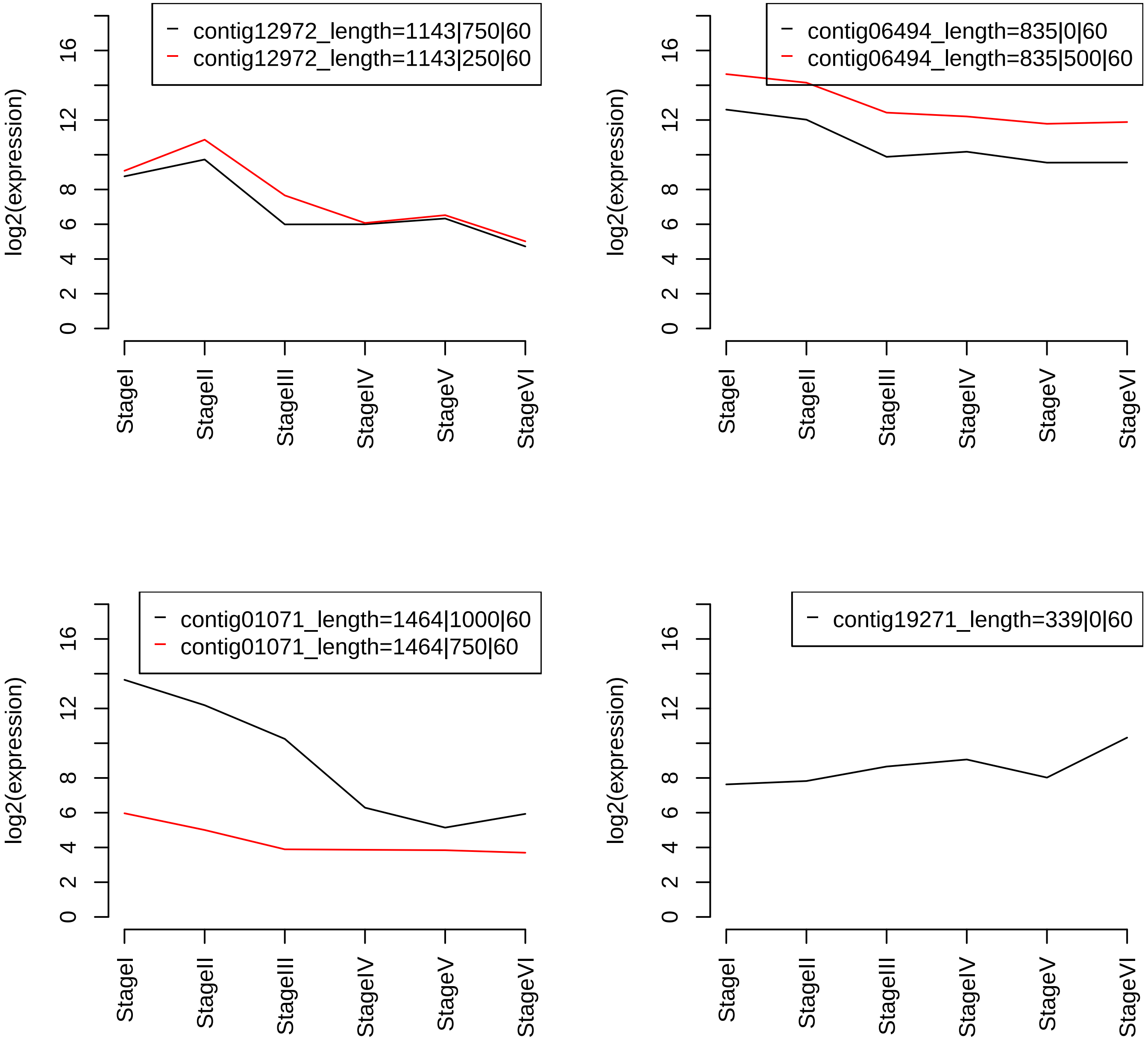
contig12972_length=1143|750|60
contig12972_length=1143|250|60
contig06494_length=835|0|60
contig06494_length=835|500|60
contig01071_length=1464|1000|60
contig01071_length=1464|750|60
contig19271_length=339|0|60
log2(expression)
StageI
StageII
StageIII
StageIV
StageV
StageVI

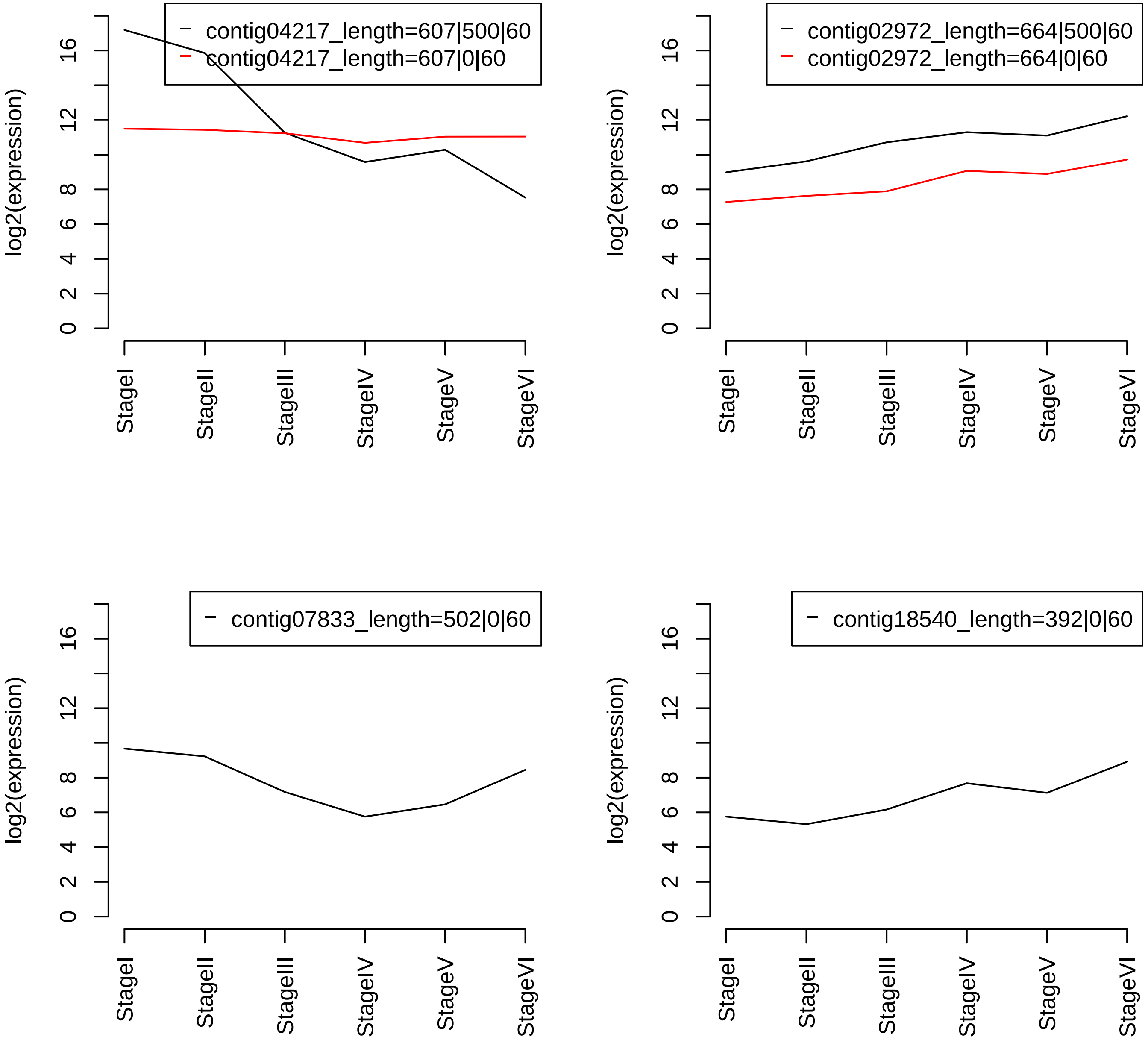
contig04217_length=607|500|60
contig04217_length=607|0|60
contig02972_length=664|500|60
contig02972_length=664|0|60
contig07833_length=502|0|60
contig18540_length=392|0|60
log2(expression)
0
2
4
6
8
12
16
StageI
StageII
StageIII
StageIV
StageV
StageVI

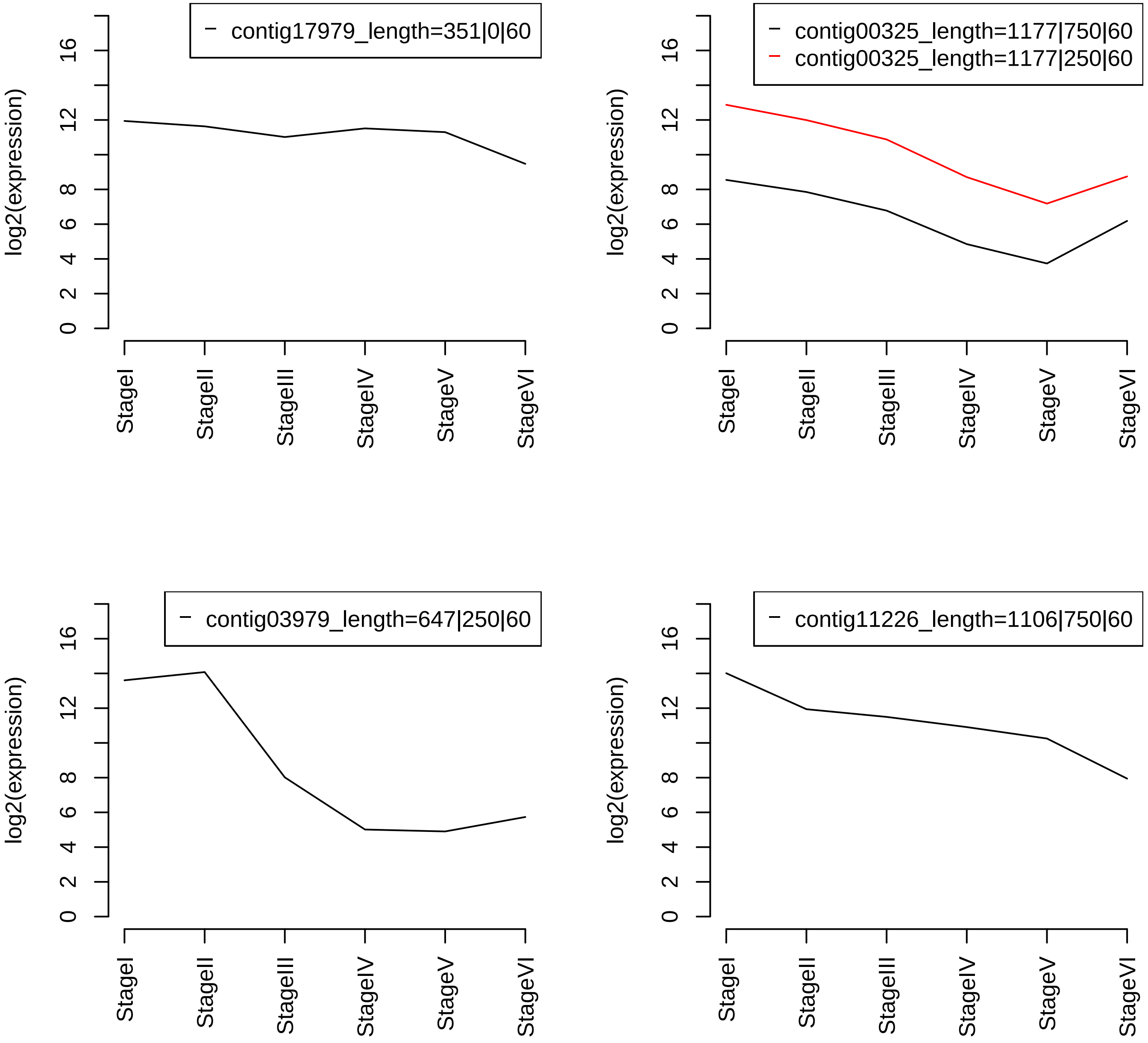

contig17979_length=351|0|60
contig00325_length=1177|750|60
contig00325_length=1177|250|60
contig03979_length=647|250|60
contig11226_length=1106|750|60
log2(expression)
0
2
4
6
8
12
16
StageI
StageII
StageIII
StageIV
StageV
StageVI

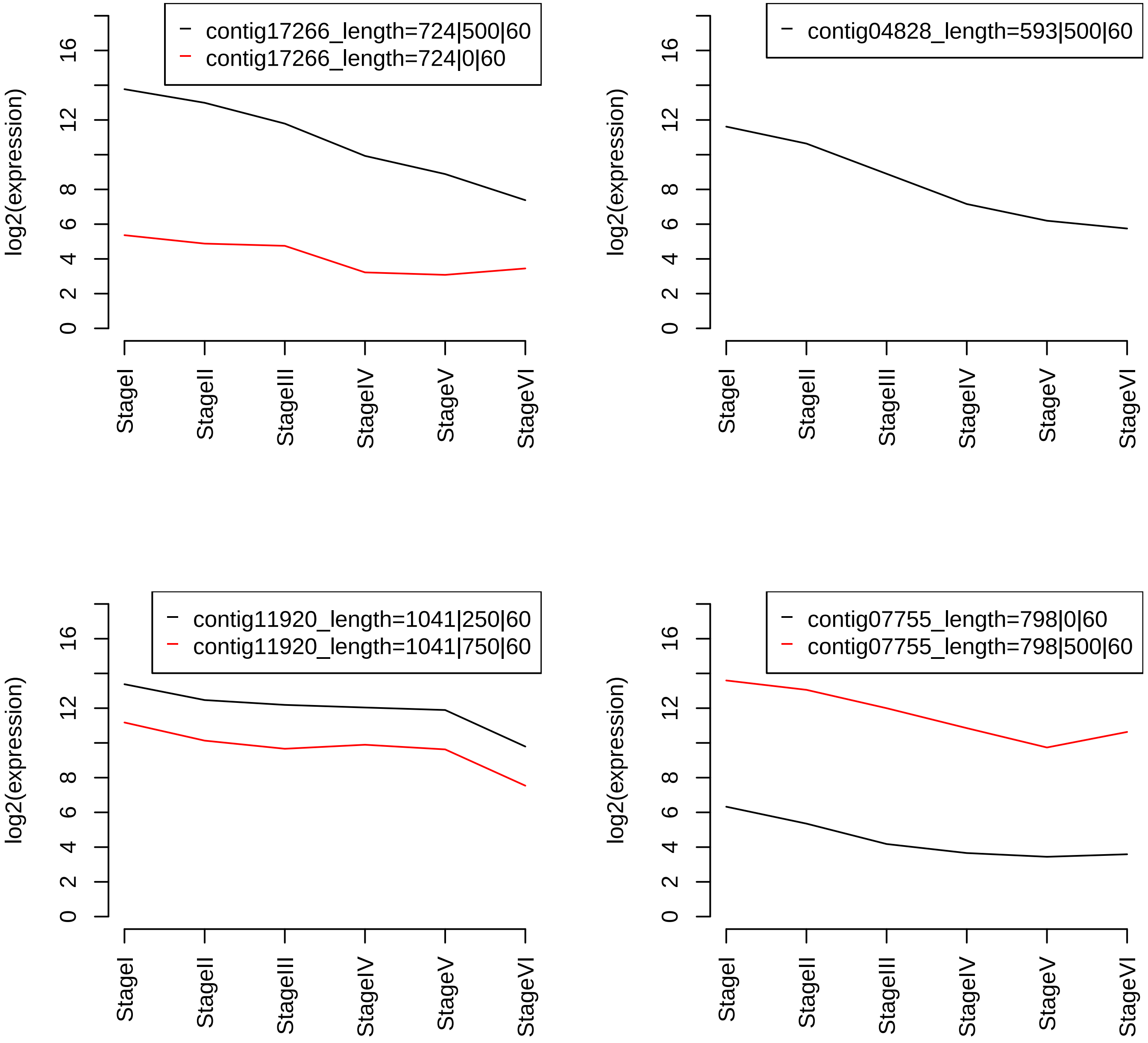

contig17266_length=724|500|60
contig17266_length=724|0|60
contig04828_length=593|500|60
contig11920_length=1041|250|60
contig11920_length=1041|750|60
contig07755_length=798|0|60
contig07755_length=798|500|60
log2(expression)
0
2
4
6
8
12
16
StageI
StageII
StageIII
StageIV
StageV
StageVI

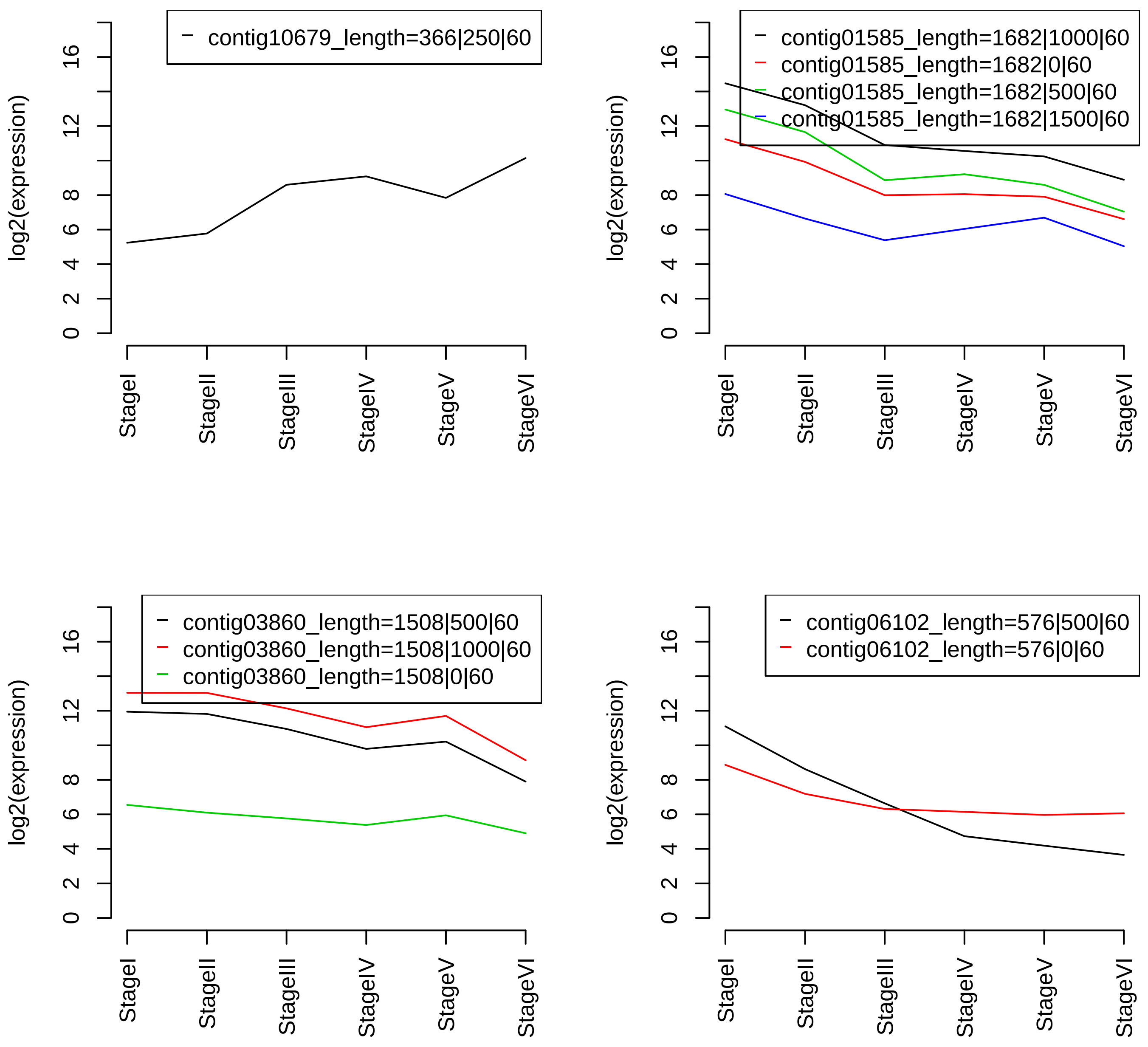

contig10679_length=366|250|60
contig01585_length=1682|1000|60
contig01585_length=1682|0|60
contig01585_length=1682|500|60
contig01585_length=1682|1500|60
contig03860_length=1508|500|60
contig03860_length=1508|1000|60
contig03860_length=1508|0|60
contig06102_length=576|500|60
contig06102_length=576|0|60
log2(expression)
0
2
4
6
8
12
16
StageI
StageII
StageIII
StageIV
StageV
StageVI

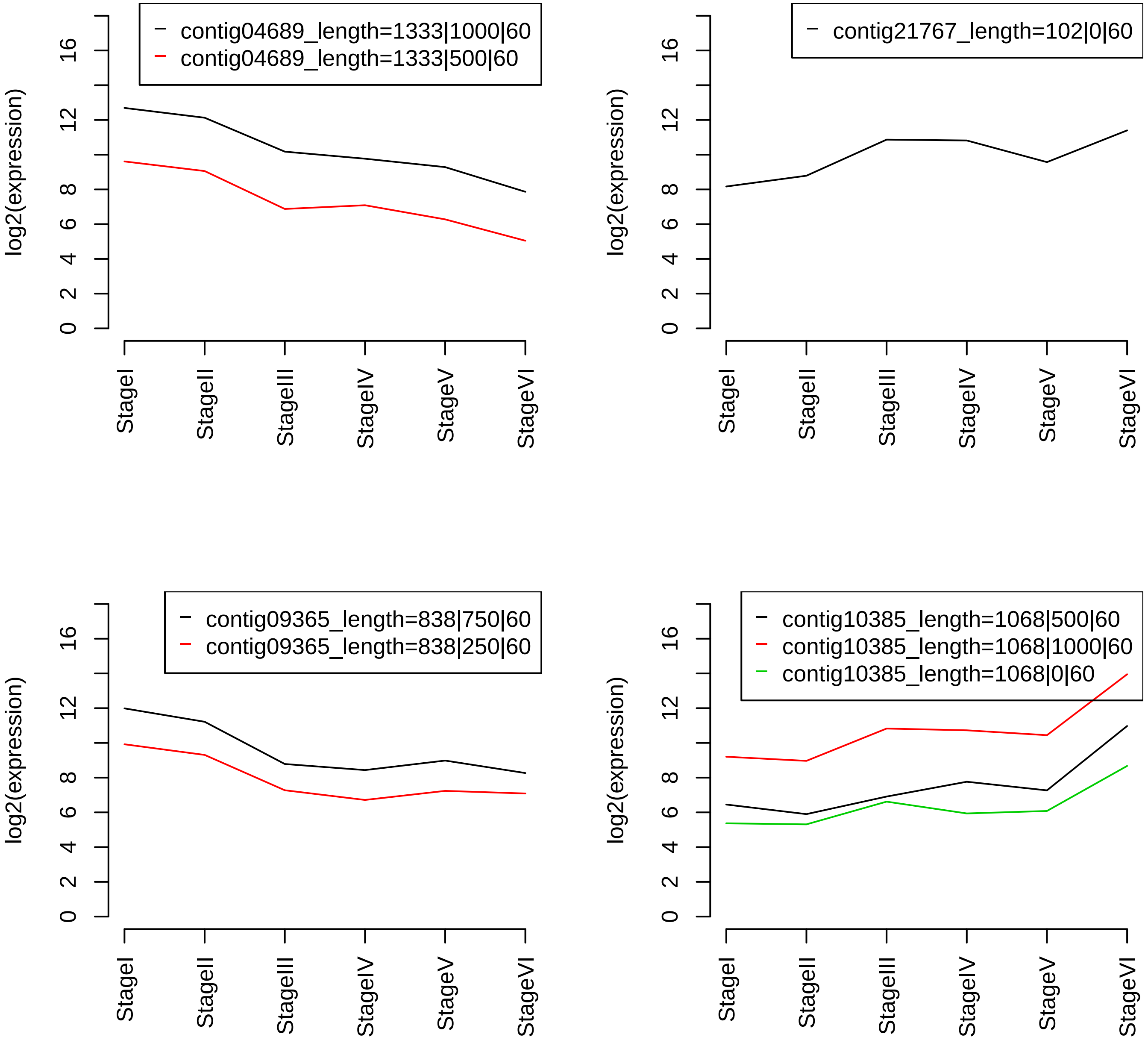
contig04689_length=1333|1000|60
contig04689_length=1333|500|60
contig21767_length=102|0|60
contig09365_length=838|750|60
contig09365_length=838|250|60
contig10385_length=1068|500|60
contig10385_length=1068|1000|60
contig10385_length=1068|0|60
log2(expression)
0 2 4 6 8 12 16
StageI StageII StageIII StageIV StageV StageVI

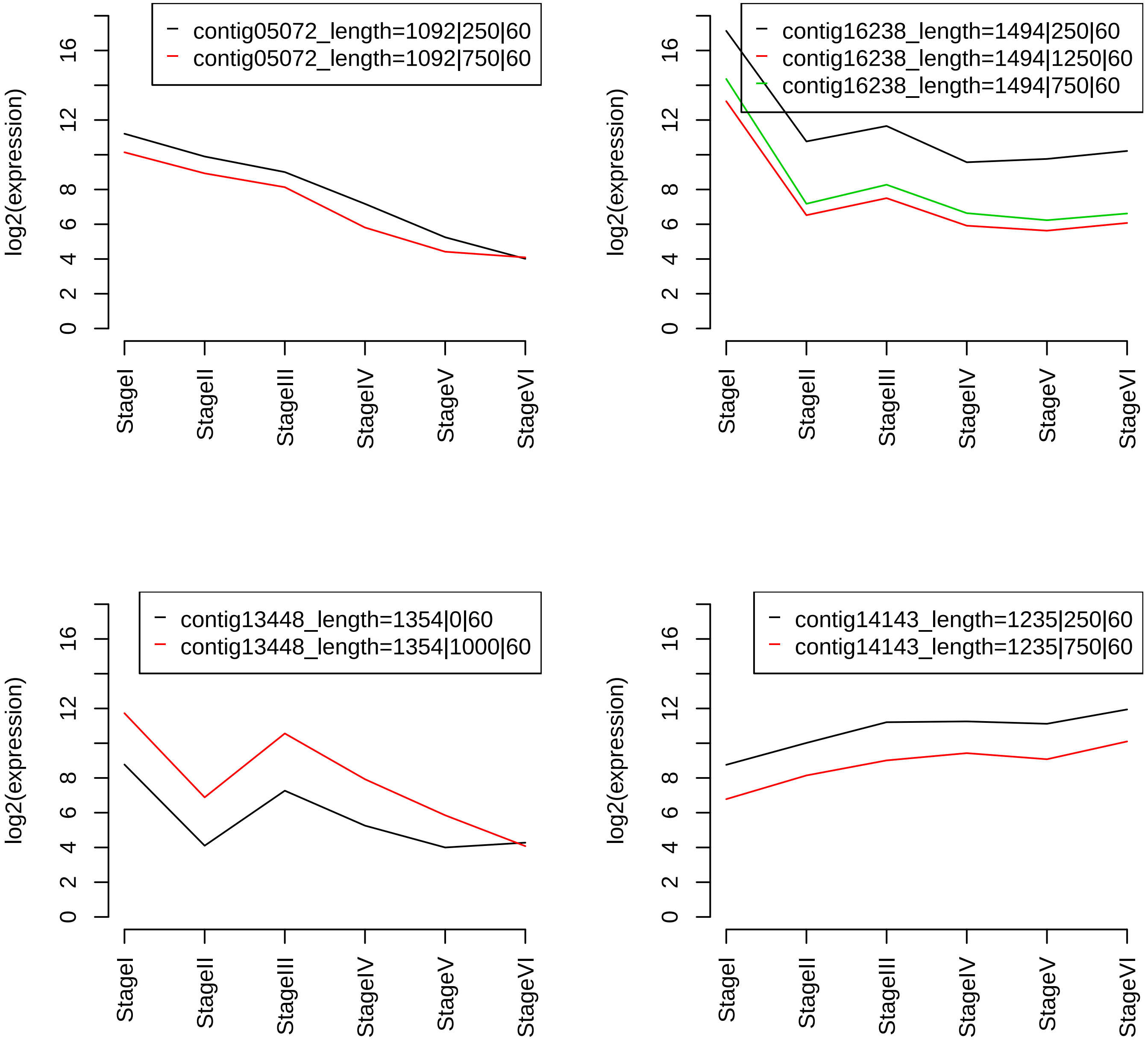
contig05072_length=1092|250|60
contig05072_length=1092|750|60
contig16238_length=1494|250|60
contig16238_length=1494|1250|60
contig16238_length=1494|750|60
contig13448_length=1354|0|60
contig13448_length=1354|1000|60
contig14143_length=1235|250|60
contig14143_length=1235|750|60
log2(expression)
0
2
4
6
8
12
16
StageI
StageII
StageIII
StageIV
StageV
StageVI

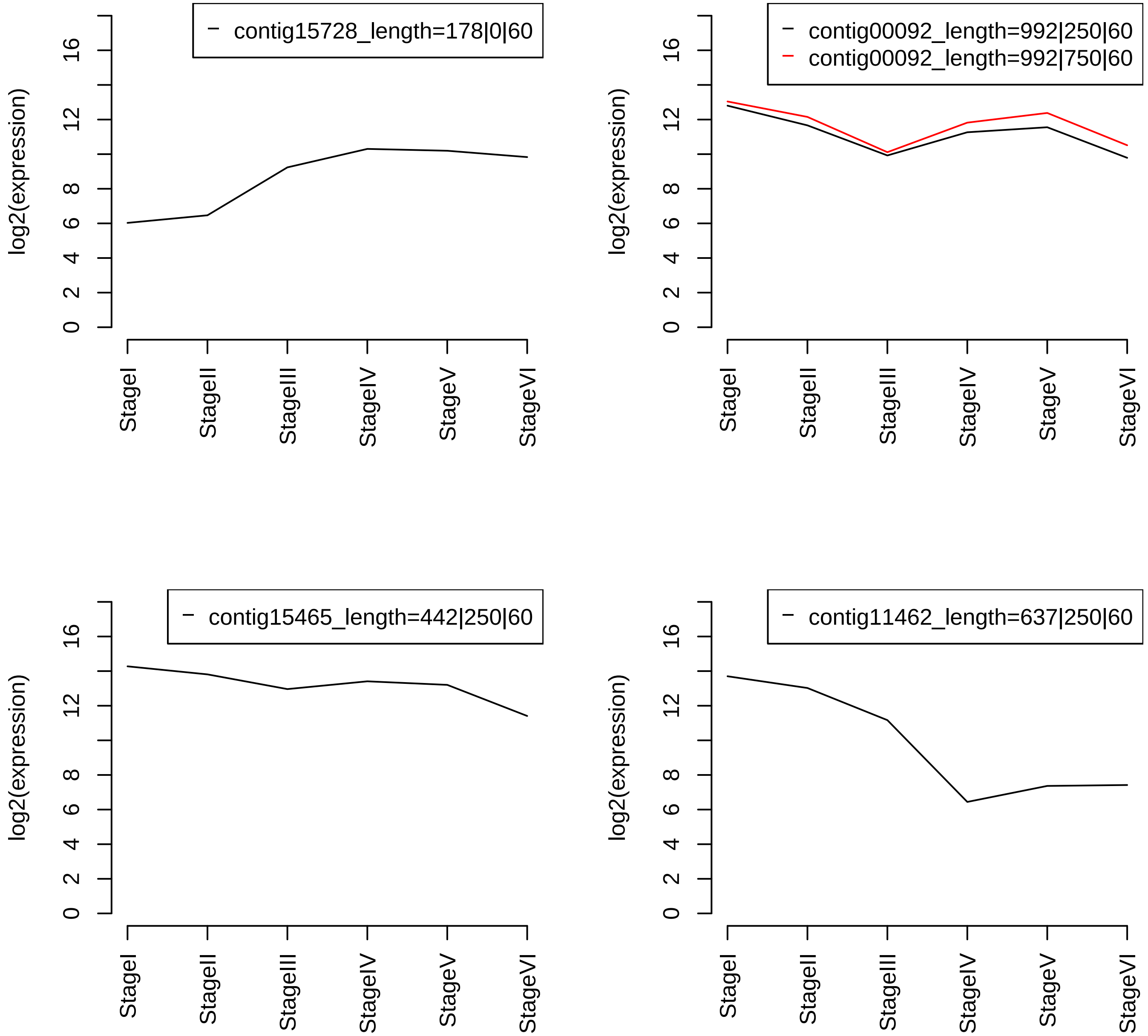

contig15728_length=178|0|60
contig00092_length=992|250|60
contig00092_length=992|750|60
contig15465_length=442|250|60
contig11462_length=637|250|60
log2(expression)
0
2
4
6
8
12
16
StageI
StageII
StageIII
StageIV
StageV
StageVI

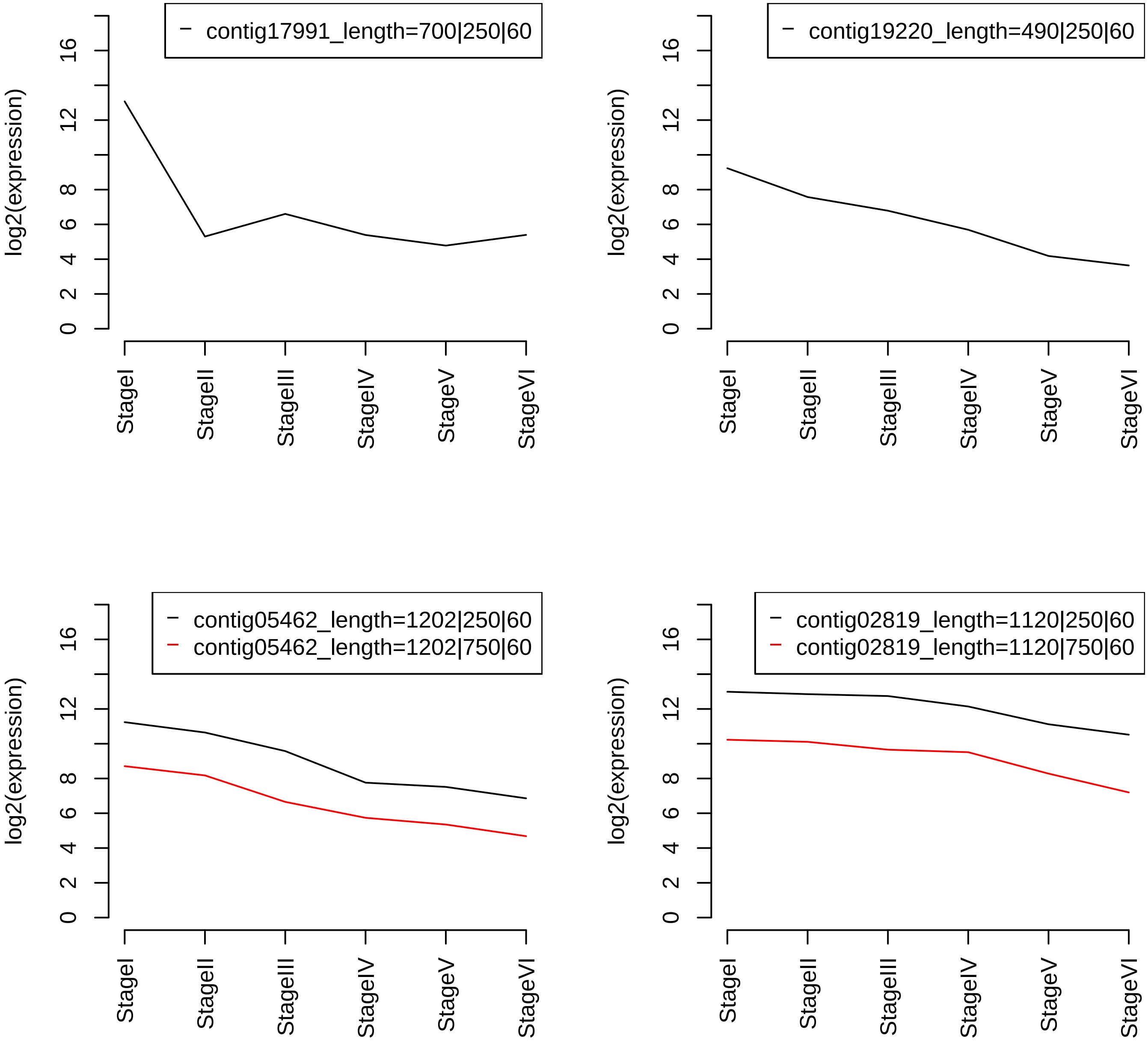
contig17991_length=700|250|60
contig19220_length=490|250|60
contig05462_length=1202|250|60
contig05462_length=1202|750|60
contig02819_length=1120|250|60
contig02819_length=1120|750|60
log2(expression)
0
2
4
6
8
12
16
StageI
StageII
StageIII
StageIV
StageV
StageVI

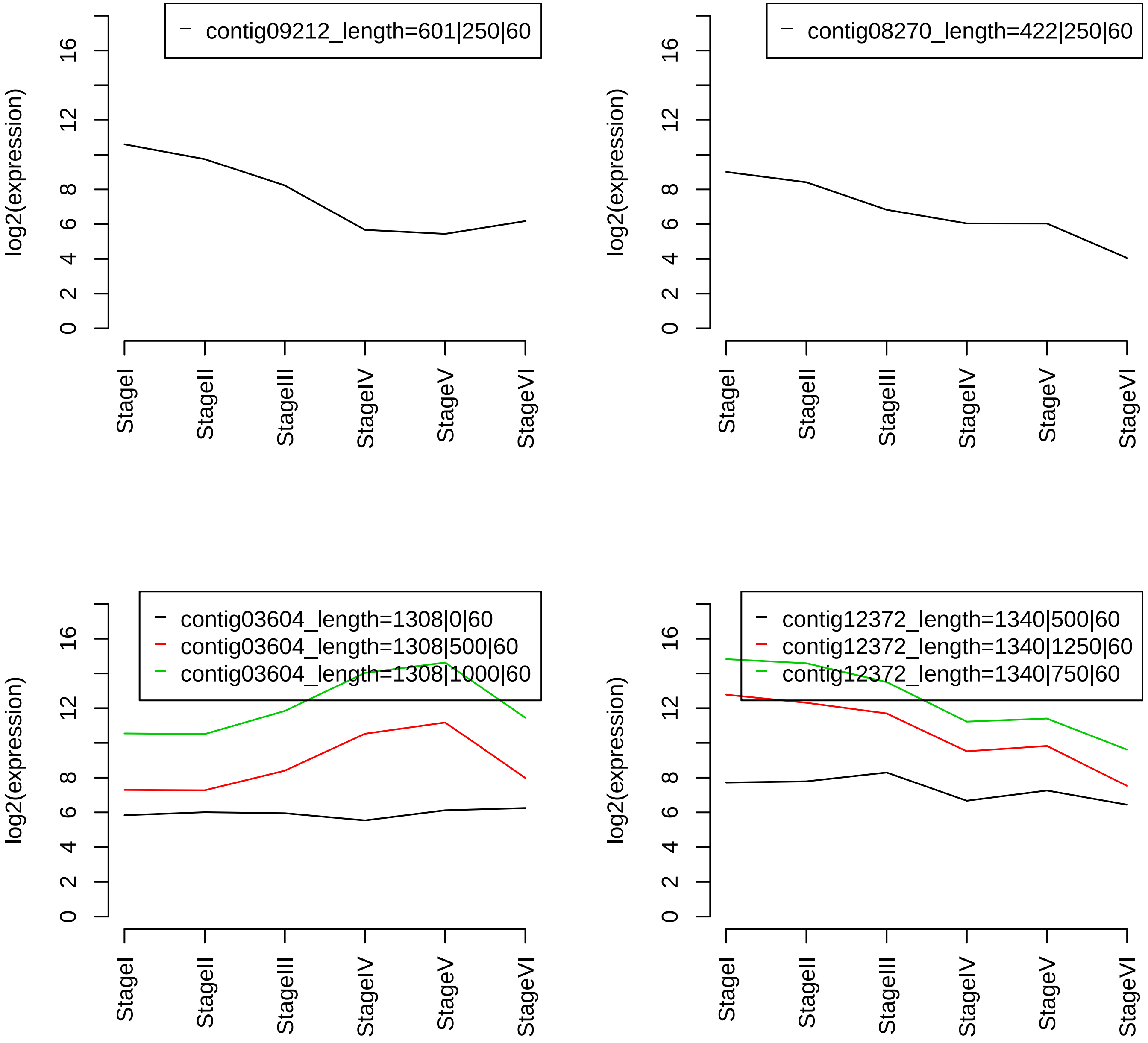
contig09212_length=601|250|60
contig08270_length=422|250|60
contig03604_length=1308|0|60
contig03604_length=1308|500|60
contig03604_length=1308|1000|60
contig12372_length=1340|500|60
contig12372_length=1340|1250|60
contig12372_length=1340|750|60
log2(expression)
0
2
4
6
8
12
16
StageI
StageII
StageIII
StageIV
StageV
StageVI

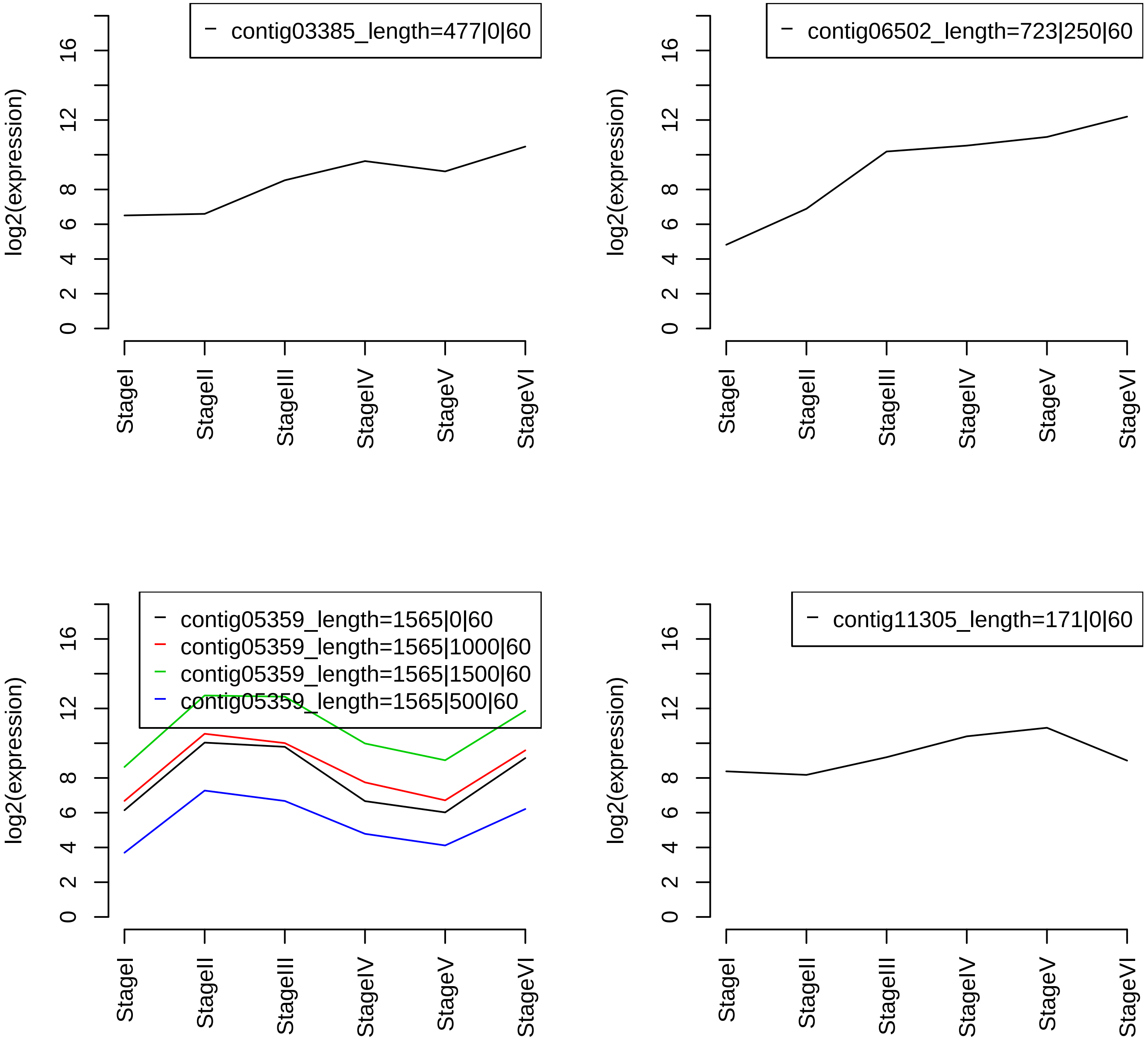
contig03385_length=477|0|60
contig06502_length=723|250|60
contig05359_length=1565|0|60
contig05359_length=1565|1000|60
contig05359_length=1565|1500|60
contig05359_length=1565|500|60
contig11305_length=171|0|60
log2(expression)
0
2
4
6
8
12
16
StageI
StageII
StageIII
StageIV
StageV
StageVI

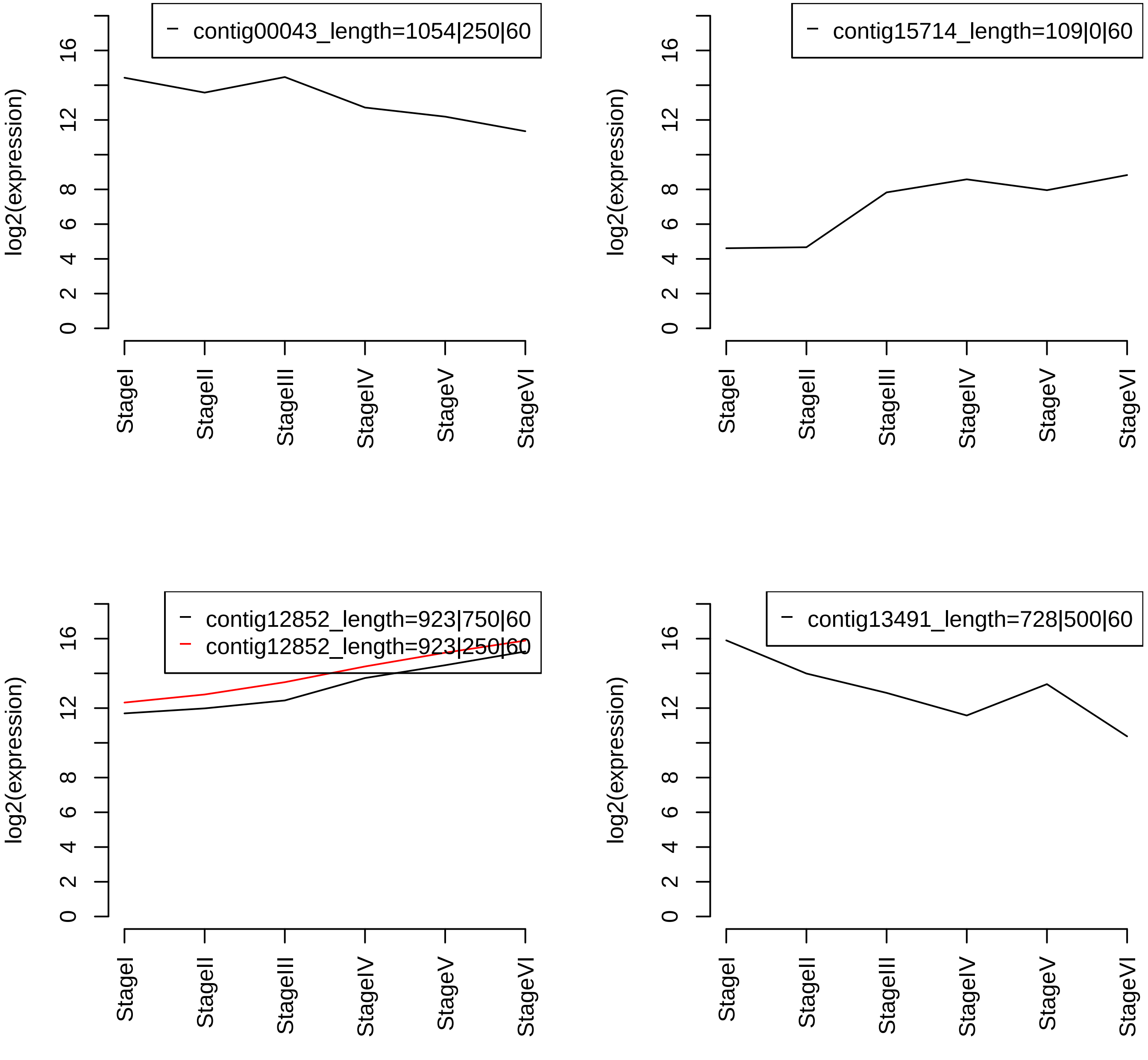
contig00043_length=1054|250|60
contig15714_length=109|0|60
contig12852_length=923|750|60
contig12852_length=923|250|60
contig13491_length=728|500|60
log2(expression)
0
2
4
6
8
12
16
StageI
StageII
StageIII
StageIV
StageV
StageVI

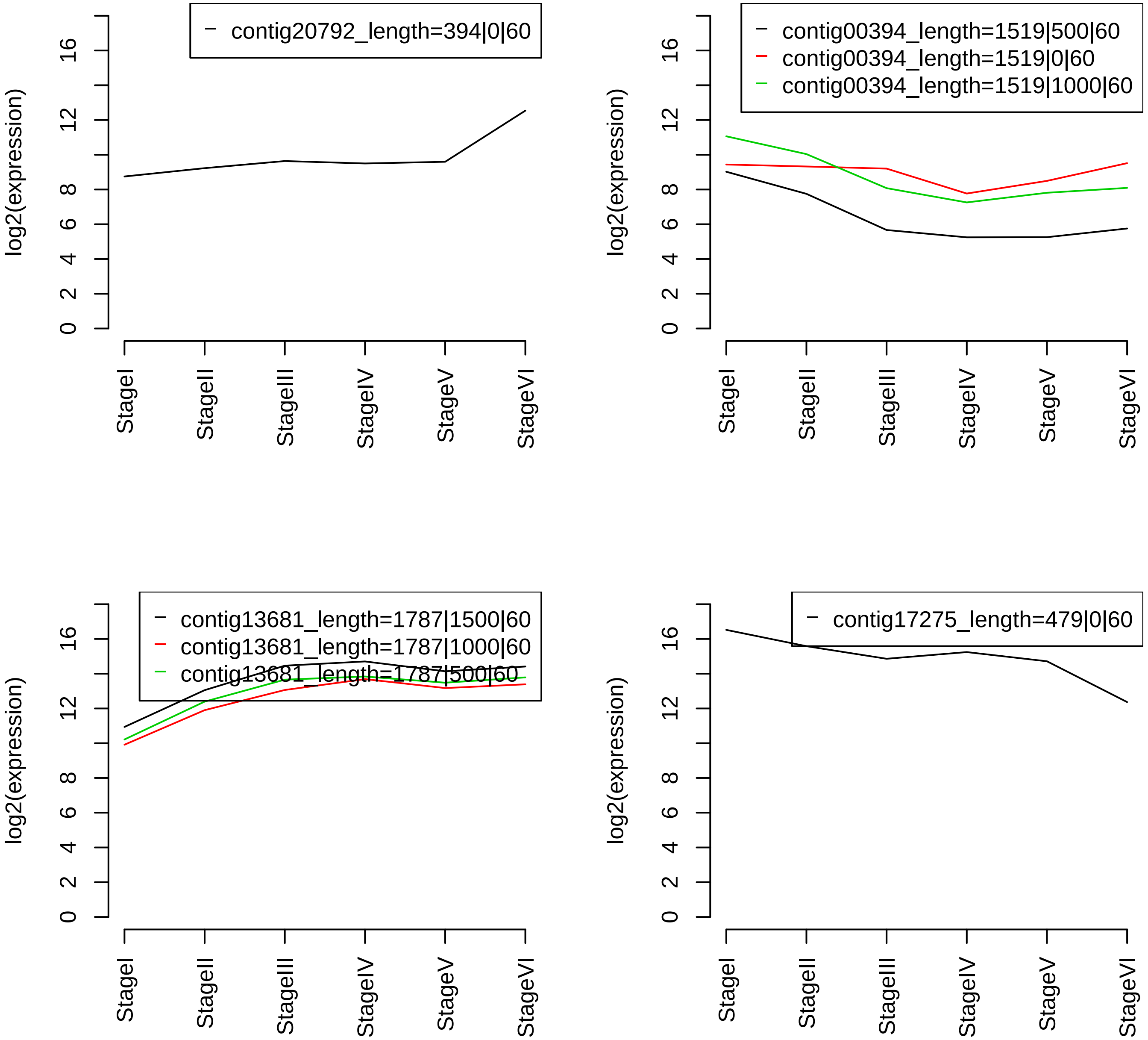
contig20792_length=394|0|60
log2(expression)
0
2
4
6
8
12
16
StageI
StageII
StageIII
StageIV
StageV
StageVI
contig00394_length=1519|500|60
contig00394_length=1519|0|60
contig00394_length=1519|1000|60
log2(expression)
contig13681_length=1787|1500|60
contig13681_length=1787|1000|60
contig13681_length=1787|500|60
log2(expression)
contig17275_length=479|0|60
log2(expression)

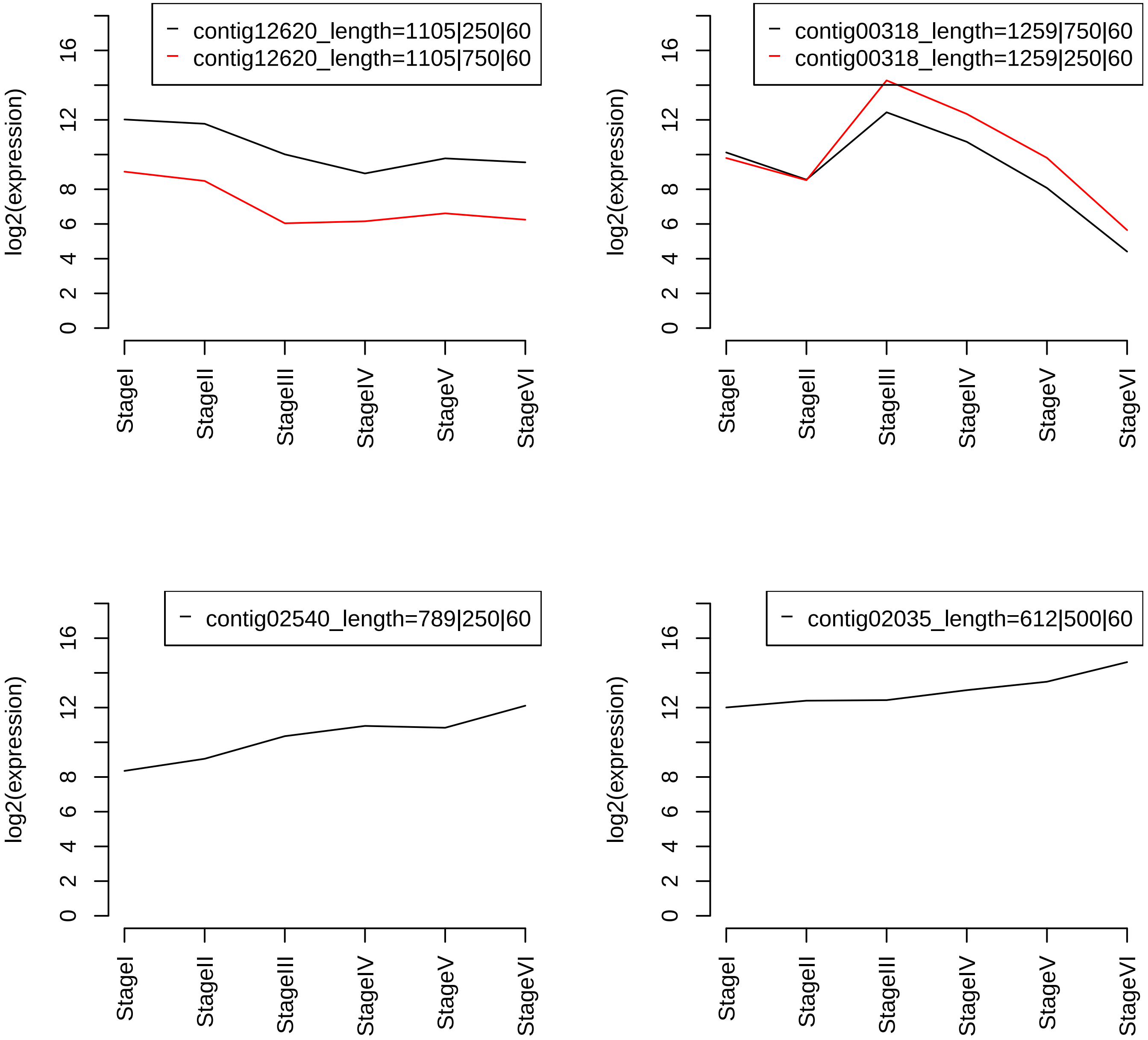
contig12620_length=1105|250|60
contig12620_length=1105|750|60
contig00318_length=1259|750|60
contig00318_length=1259|250|60
contig02540_length=789|250|60
contig02035_length=612|500|60
log2(expression)
0
2
4
6
8
12
16
StageI
StageII
StageIII
StageIV
StageV
StageVI

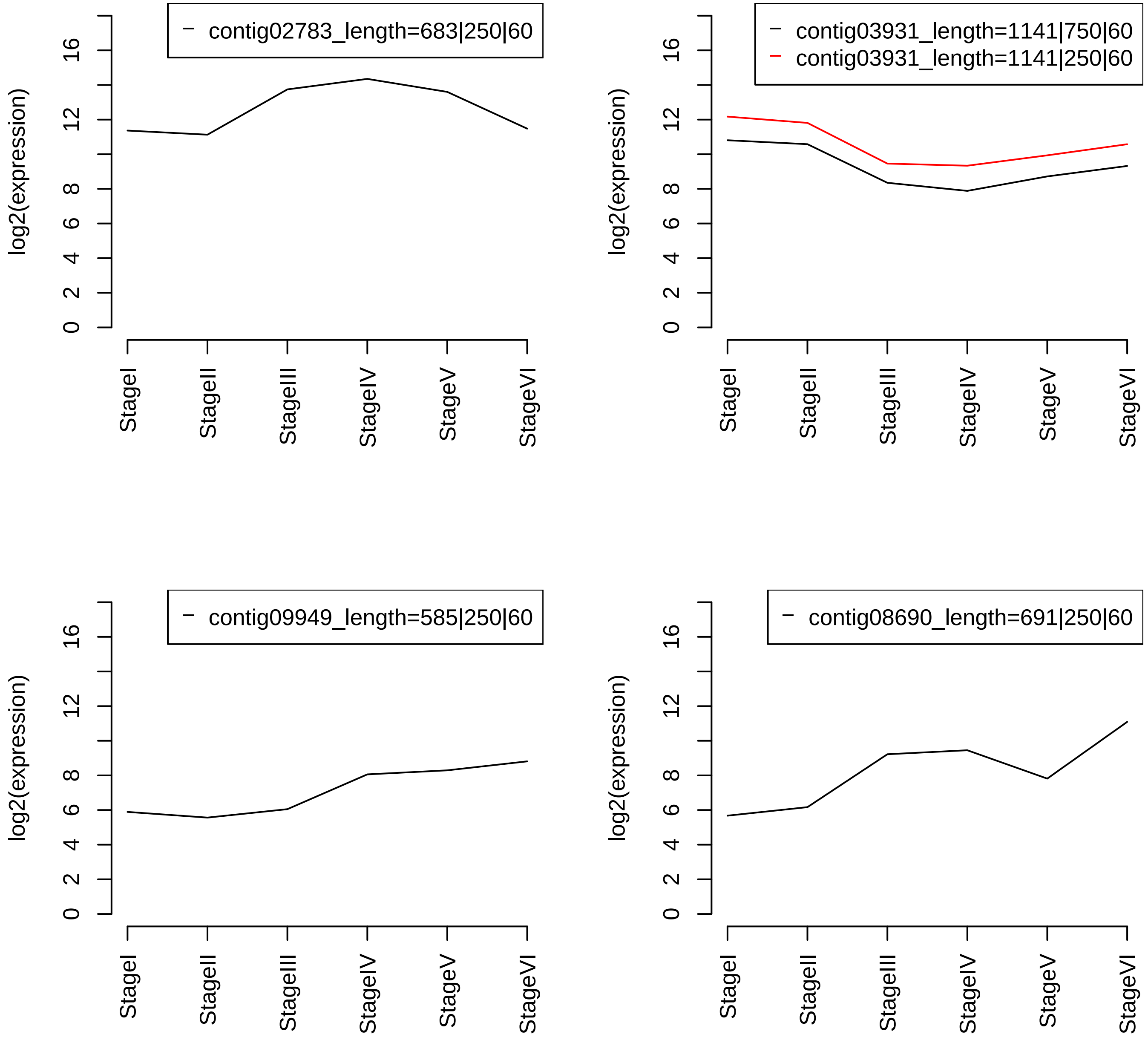

contig02783_length=683|250|60
contig03931_length=1141|750|60
contig03931_length=1141|250|60
contig09949_length=585|250|60
contig08690_length=691|250|60
log2(expression)
0
2
4
6
8
12
16
StageI
StageII
StageIII
StageIV
StageV
StageVI

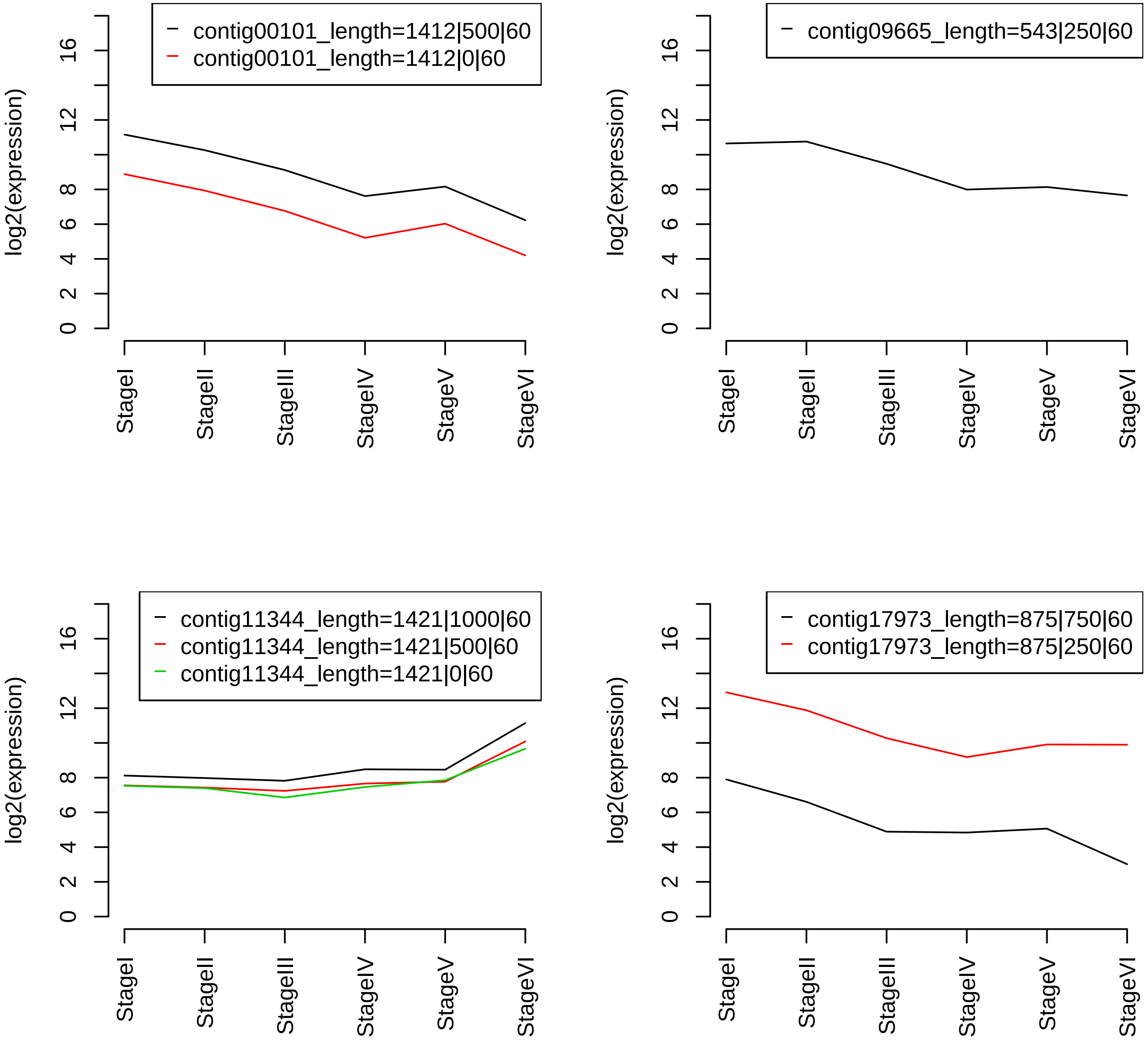
contig00101_length=1412|500|60
contig00101_length=1412|0|60
contig09665_length=543|250|60
contig11344_length=1421|1000|60
contig11344_length=1421|500|60
contig11344_length=1421|0|60
contig17973_length=875|750|60
contig17973_length=875|250|60
log2(expression)
0
2
4
6
8
12
16
StageI
StageII
StageIII
StageIV
StageV
StageVI

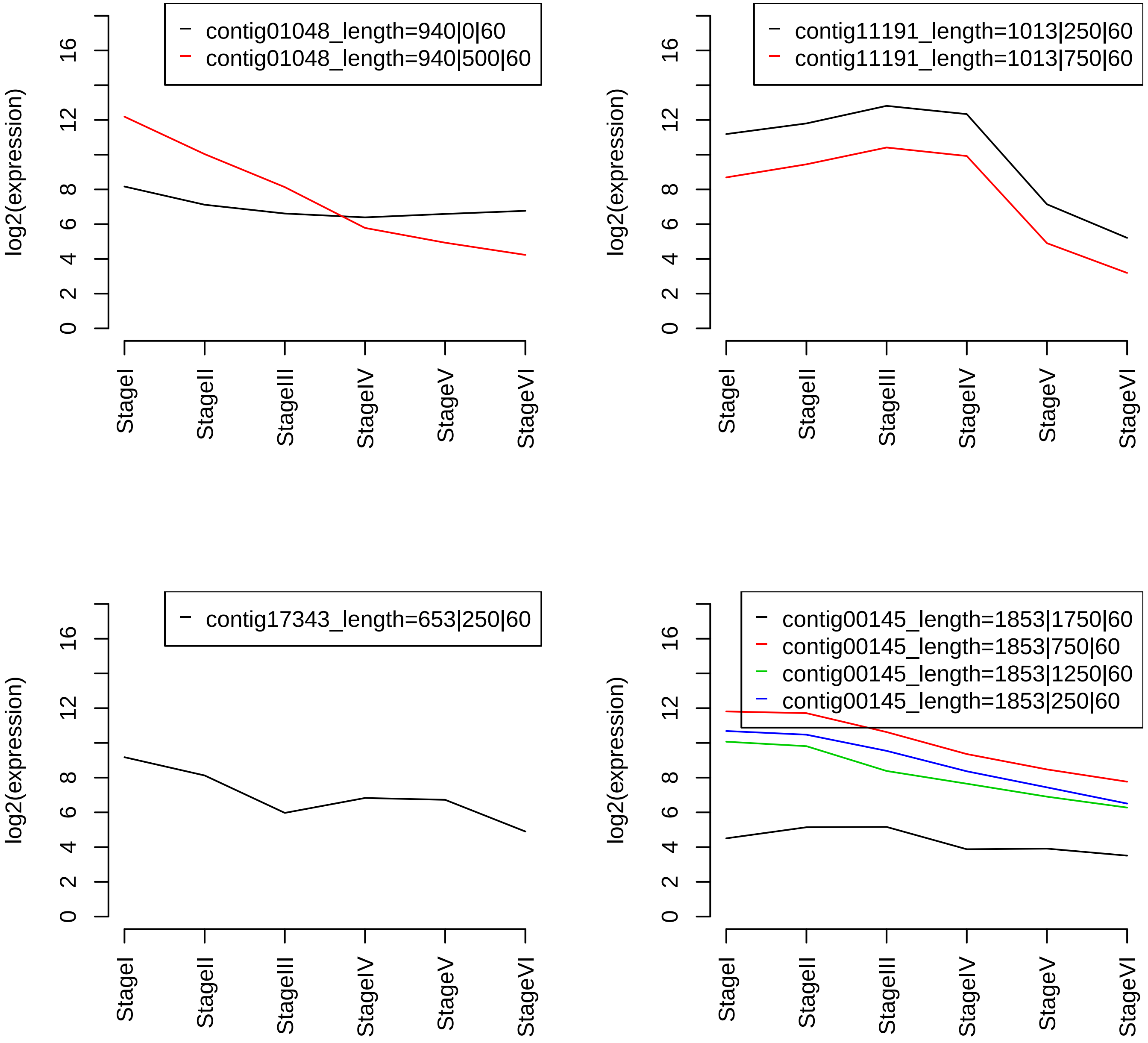
contig01048_length=940|0|60
contig01048_length=940|500|60
contig11191_length=1013|250|60
contig11191_length=1013|750|60
contig17343_length=653|250|60
contig00145_length=1853|1750|60
contig00145_length=1853|750|60
contig00145_length=1853|1250|60
contig00145_length=1853|250|60
log2(expression)
StageI
StageII
StageIII
StageIV
StageV
StageVI

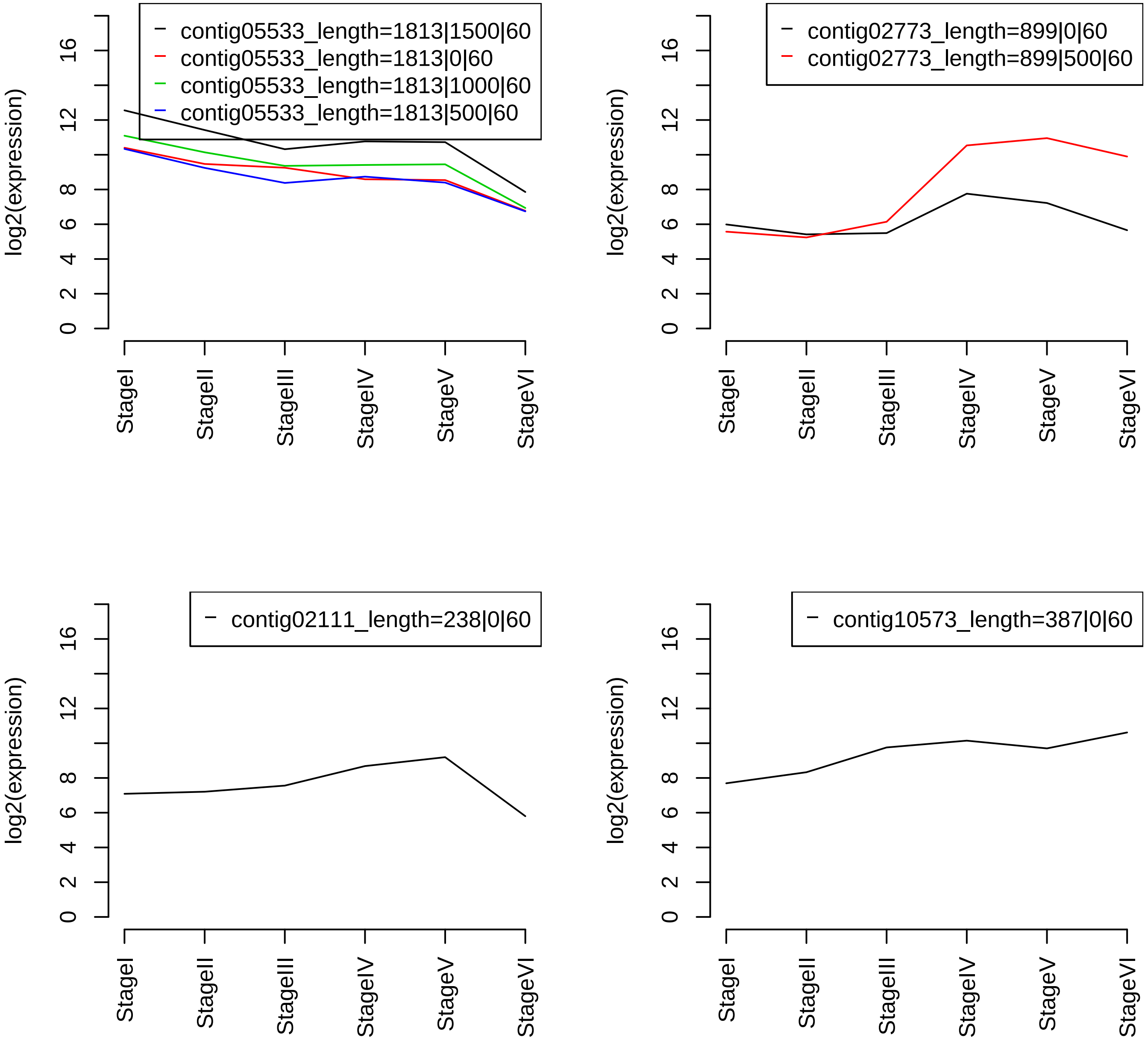
contig05533_length=1813|1500|60
contig05533_length=1813|0|60
contig05533_length=1813|1000|60
contig05533_length=1813|500|60
contig02773_length=899|0|60
contig02773_length=899|500|60
contig02111_length=238|0|60
contig10573_length=387|0|60
log2(expression)
0
2
4
6
8
12
16
StageI
StageII
StageIII
StageIV
StageV
StageVI

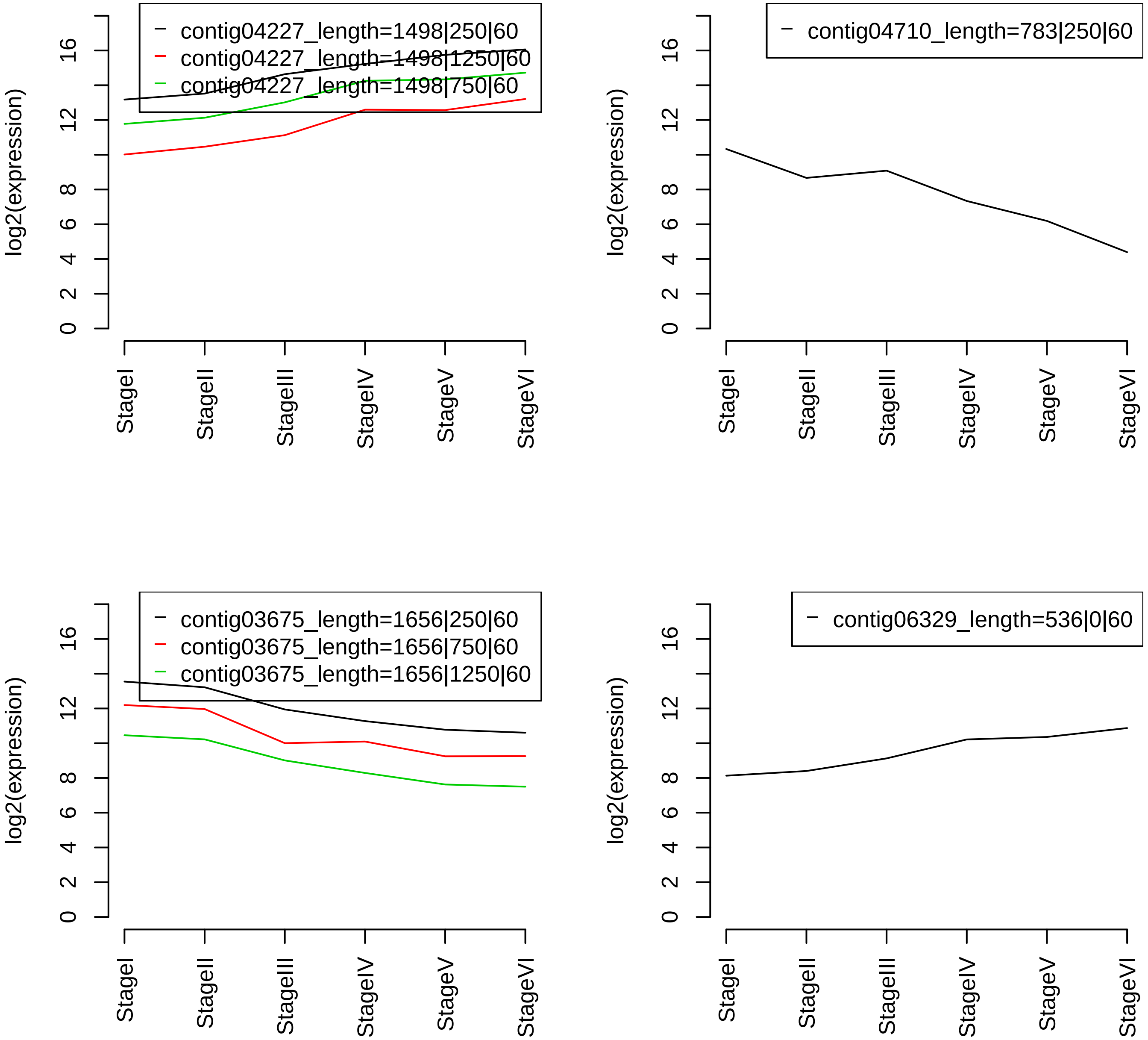

contig04227_length=1498|250|60
contig04227_length=1498|1250|60
contig04227_length=1498|750|60
contig04710_length=783|250|60
contig03675_length=1656|250|60
contig03675_length=1656|750|60
contig03675_length=1656|1250|60
contig06329_length=536|0|60
log2(expression)
StageI
StageII
StageIII
StageIV
StageV
StageVI

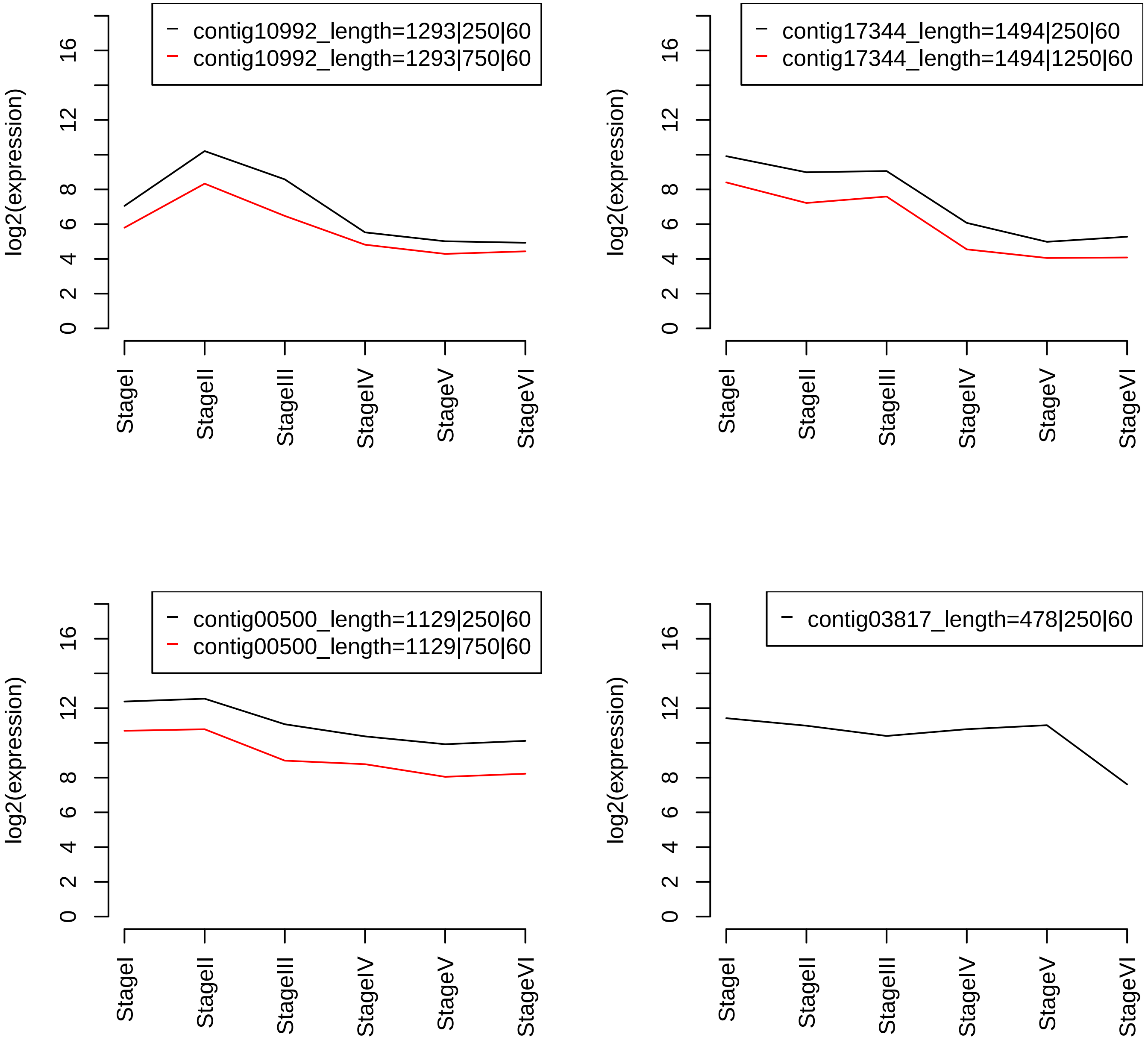

contig10992_length=1293|250|60
contig10992_length=1293|750|60
log2(expression)
0
2
4
6
8
12
16
StageI
StageII
StageIII
StageIV
StageV
StageVI
contig17344_length=1494|250|60
contig17344_length=1494|1250|60
log2(expression)
contig00500_length=1129|250|60
contig00500_length=1129|750|60
log2(expression)
contig03817_length=478|250|60
log2(expression)

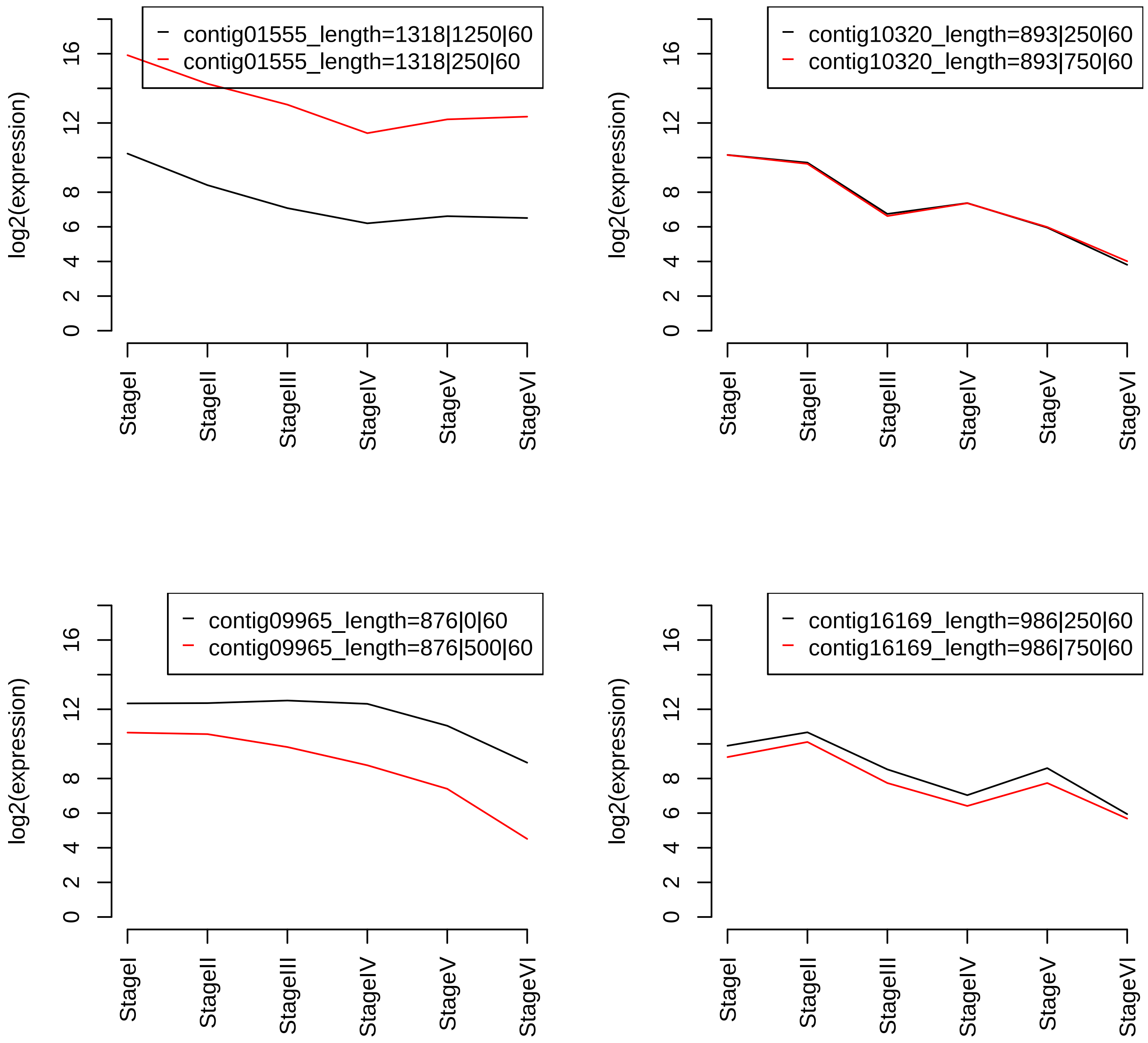

contig01555_length=1318|1250|60
contig01555_length=1318|250|60
contig10320_length=893|250|60
contig10320_length=893|750|60
contig09965_length=876|0|60
contig09965_length=876|500|60
contig16169_length=986|250|60
contig16169_length=986|750|60
log2(expression)
0
2
4
6
8
12
16
StageI
StageII
StageIII
StageIV
StageV
StageVI

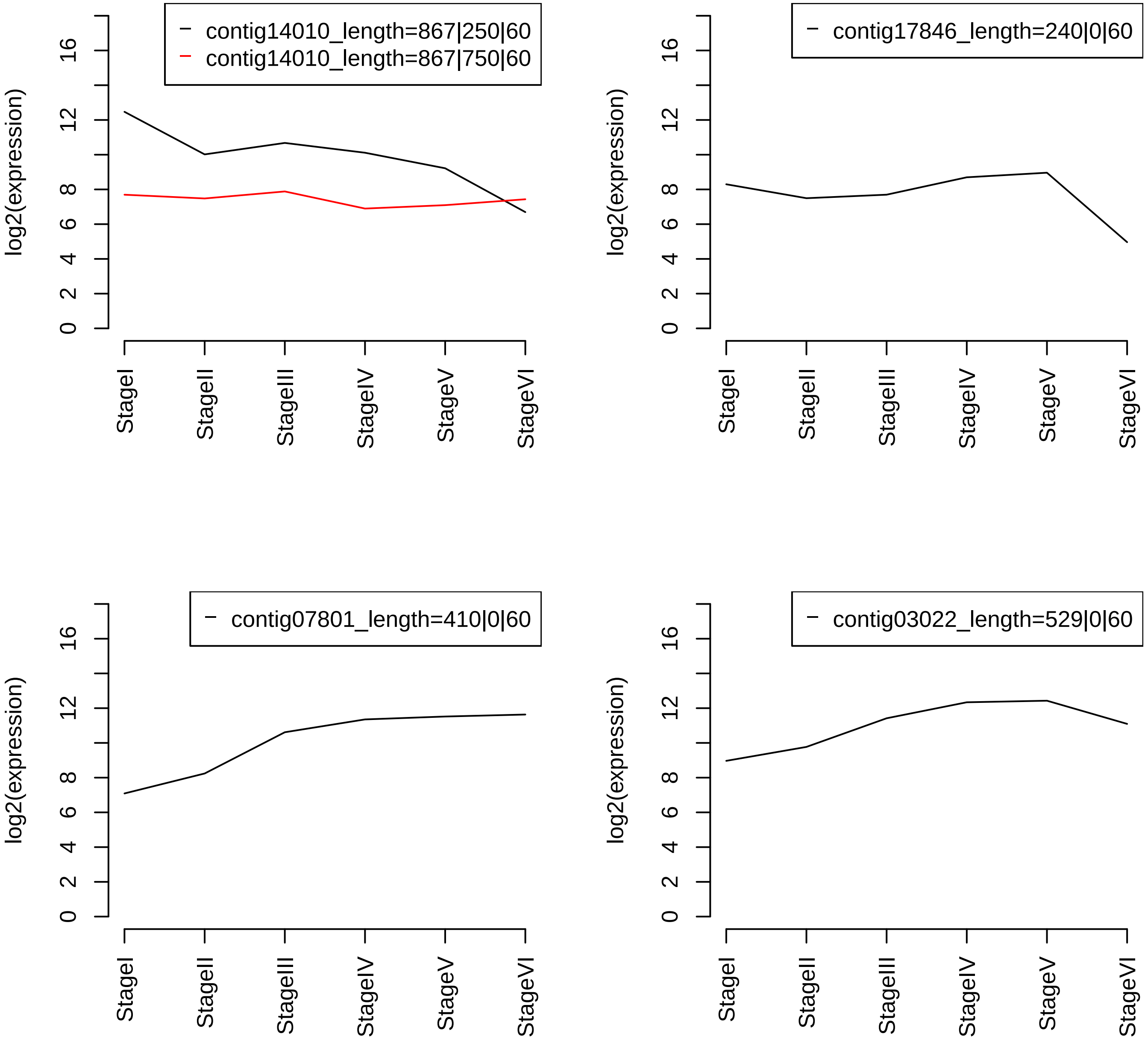

contig14010_length=867|250|60
contig14010_length=867|750|60
contig17846_length=240|0|60
contig07801_length=410|0|60
contig03022_length=529|0|60
log2(expression)
0
2
4
6
8
12
16
StageI
StageII
StageIII
StageIV
StageV
StageVI

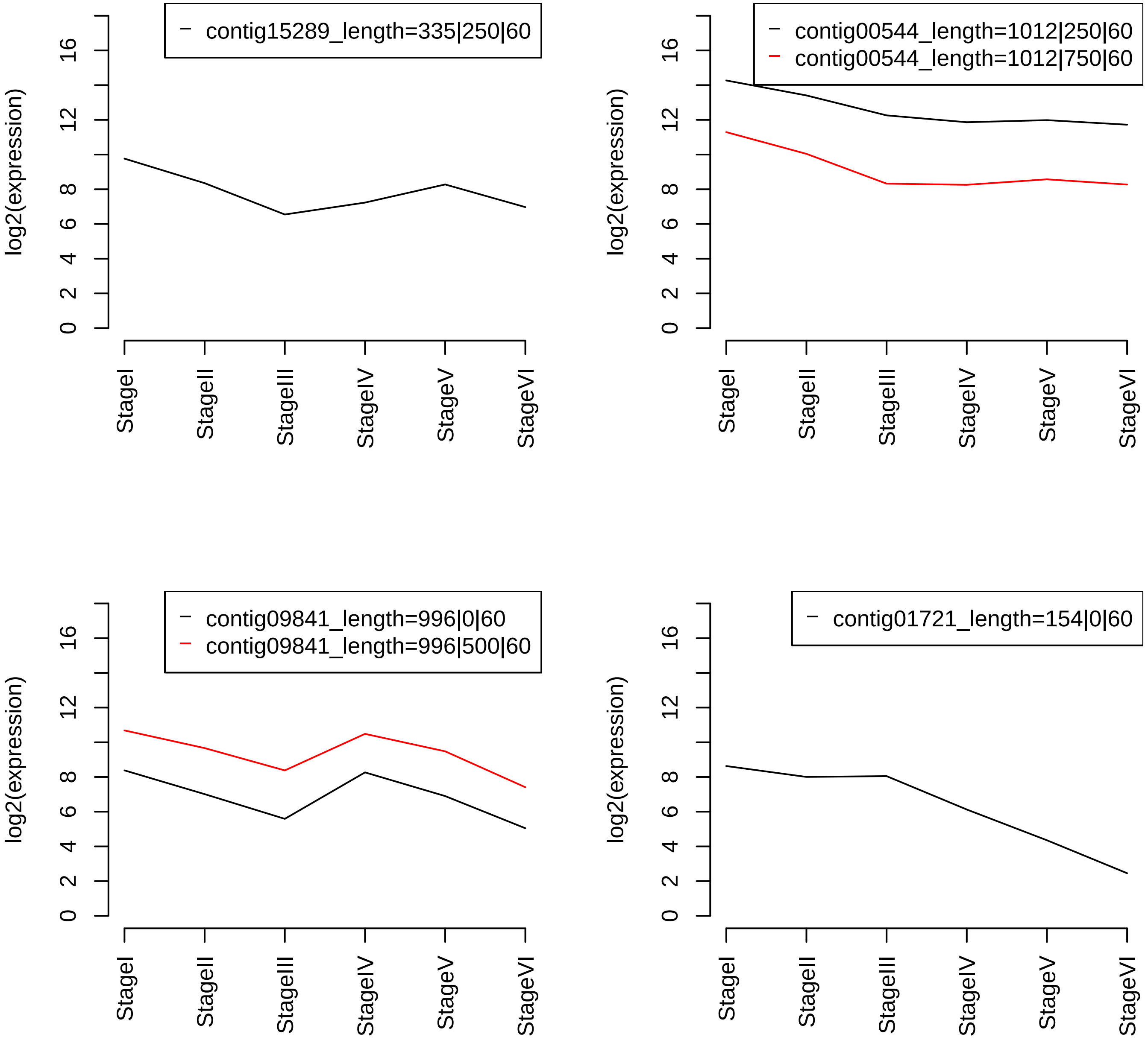

contig15289_length=335|250|60
contig00544_length=1012|250|60
contig00544_length=1012|750|60
contig09841_length=996|0|60
contig09841_length=996|500|60
contig01721_length=154|0|60
log2(expression)
0 2 4 6 8 12 16
StageI
StageII
StageIII
StageIV
StageV
StageVI

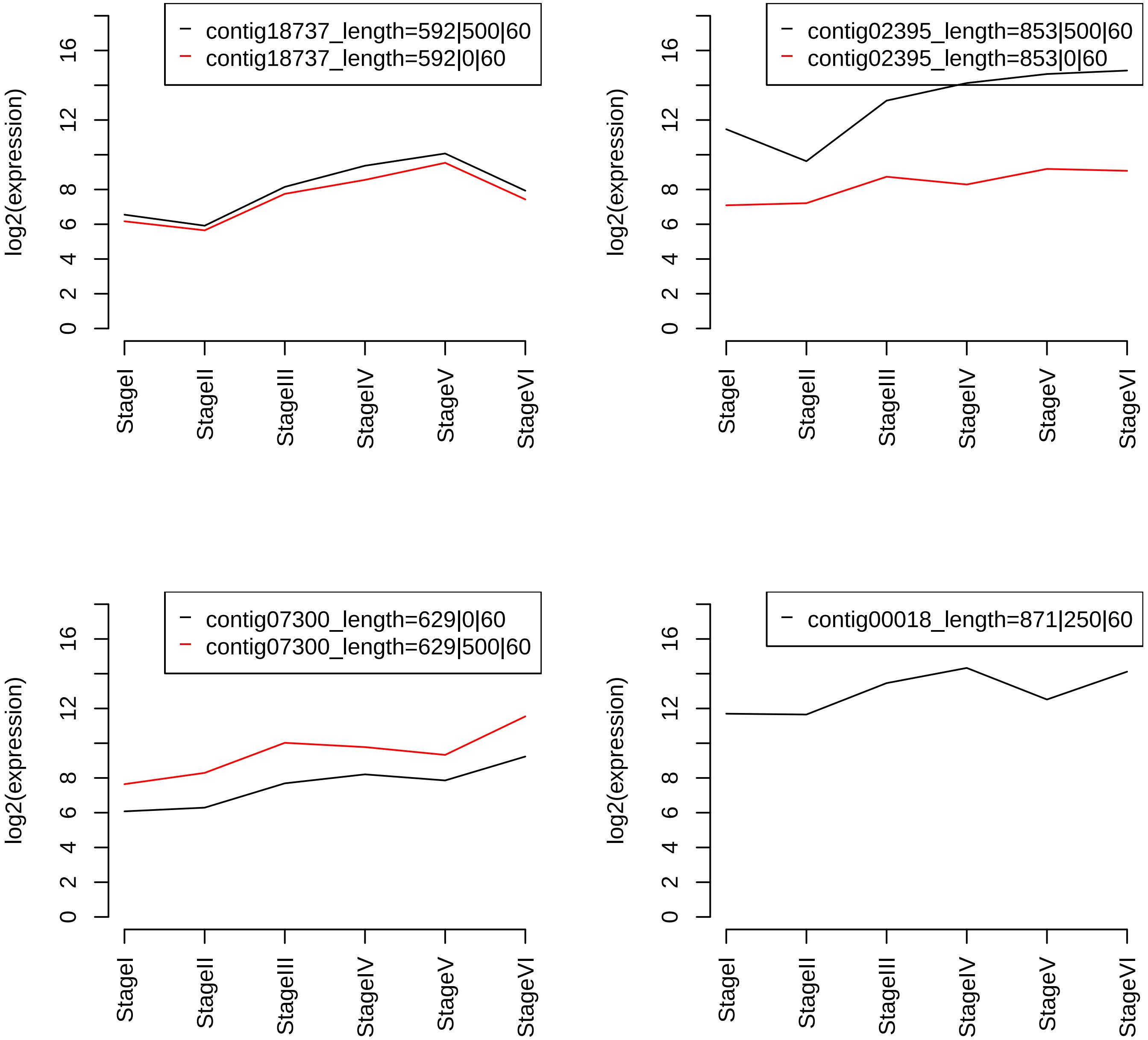
contig18737_length=592|500|60
contig18737_length=592|0|60
contig02395_length=853|500|60
contig02395_length=853|0|60
contig07300_length=629|0|60
contig07300_length=629|500|60
contig00018_length=871|250|60
log2(expression)
0 2 4 6 8 12 16
StageI StageII StageIII StageIV StageV StageVI

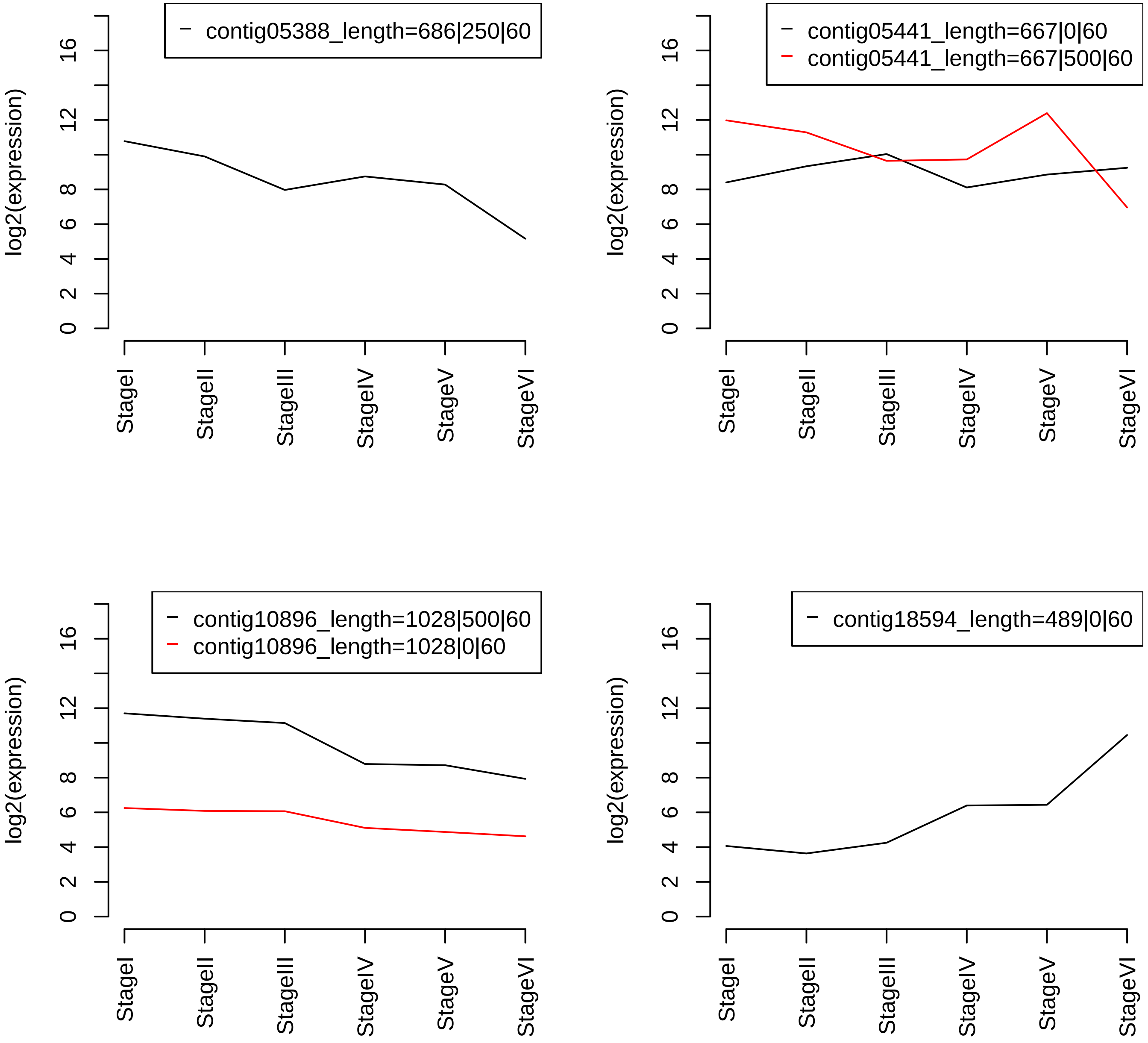
contig05388_length=686|250|60
contig05441_length=667|0|60
contig05441_length=667|500|60
contig10896_length=1028|500|60
contig10896_length=1028|0|60
contig18594_length=489|0|60
log2(expression)
0
2
4
6
8
12
16
StageI
StageII
StageIII
StageIV
StageV
StageVI

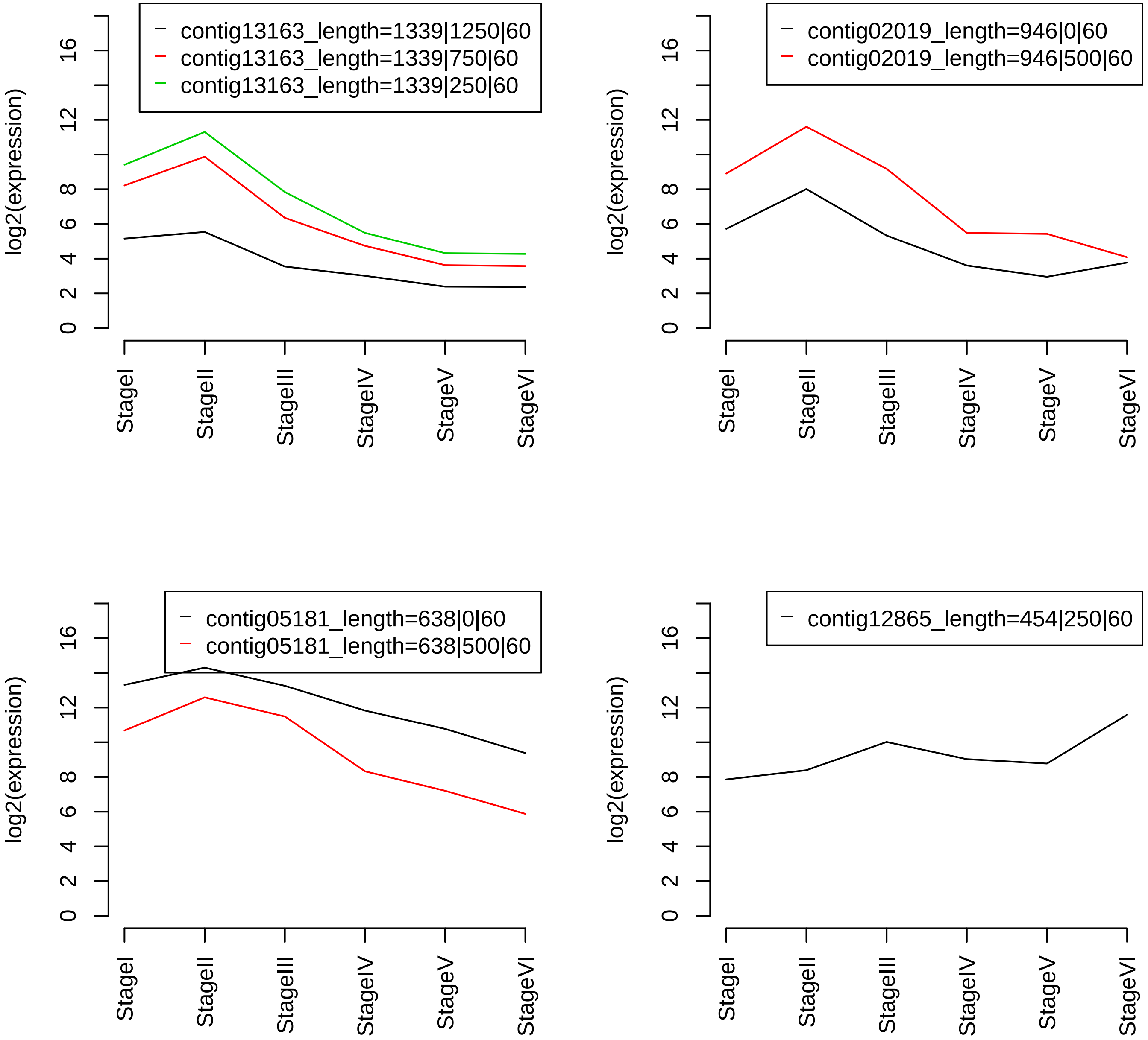

contig13163_length=1339|1250|60
contig13163_length=1339|750|60
contig13163_length=1339|250|60
contig02019_length=946|0|60
contig02019_length=946|500|60
contig05181_length=638|0|60
contig05181_length=638|500|60
contig12865_length=454|250|60
log2(expression)
0
2
4
6
8
12
16
StageI
StageII
StageIII
StageIV
StageV
StageVI

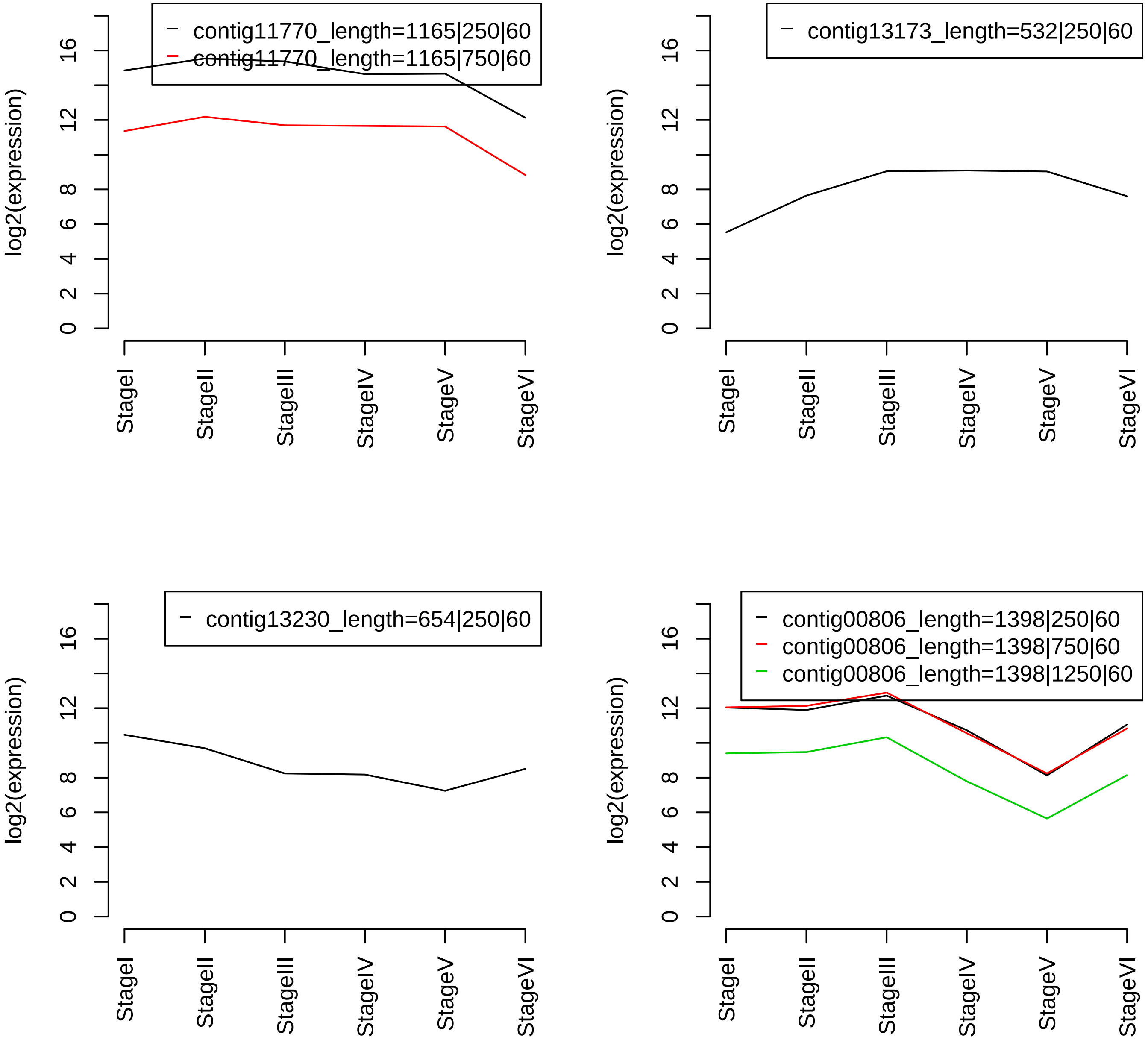
contig11770_length=1165|250|60
contig11770_length=1165|750|60
contig13173_length=532|250|60
contig13230_length=654|250|60
contig00806_length=1398|250|60
contig00806_length=1398|750|60
contig00806_length=1398|1250|60
log2(expression)
StageI
StageII
StageIII
StageIV
StageV
StageVI

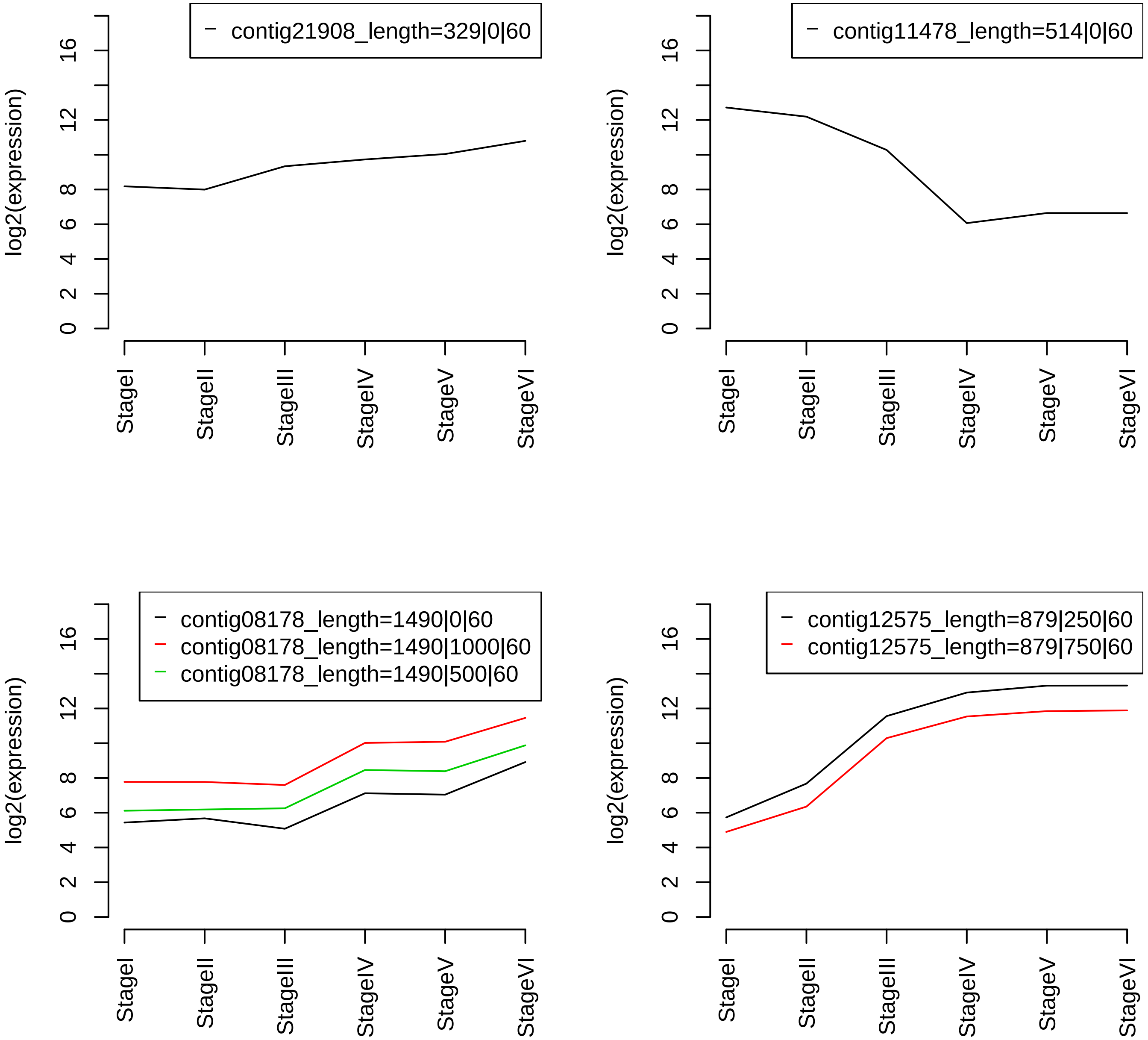

contig21908_length=329|0|60
log2(expression)
0
2
4
6
8
12
16
StageI
StageII
StageIII
StageIV
StageV
StageVI
contig11478_length=514|0|60
log2(expression)
0
2
4
6
8
12
16
StageI
StageII
StageIII
StageIV
StageV
StageVI
contig08178_length=1490|0|60
contig08178_length=1490|1000|60
contig08178_length=1490|500|60
log2(expression)
0
2
4
6
8
12
16
StageI
StageII
StageIII
StageIV
StageV
StageVI
contig12575_length=879|250|60
contig12575_length=879|750|60
log2(expression)
0
2
4
6
8
12
16
StageI
StageII
StageIII
StageIV
StageV
StageVI

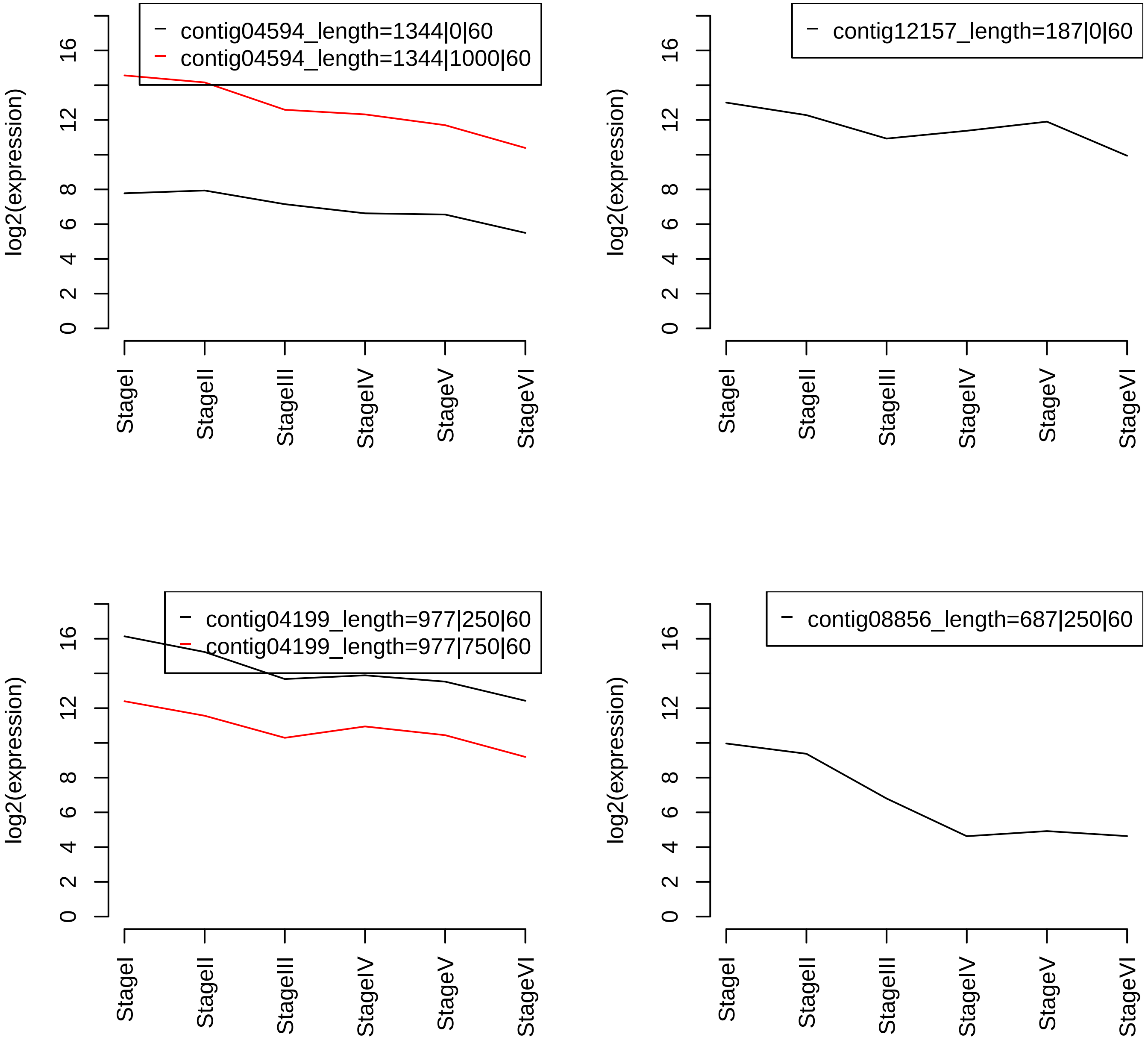
contig04594_length=1344|0|60
contig04594_length=1344|1000|60
contig12157_length=187|0|60
contig04199_length=977|250|60
contig04199_length=977|750|60
contig08856_length=687|250|60
log2(expression)
StageI
StageII
StageIII
StageIV
StageV
StageVI

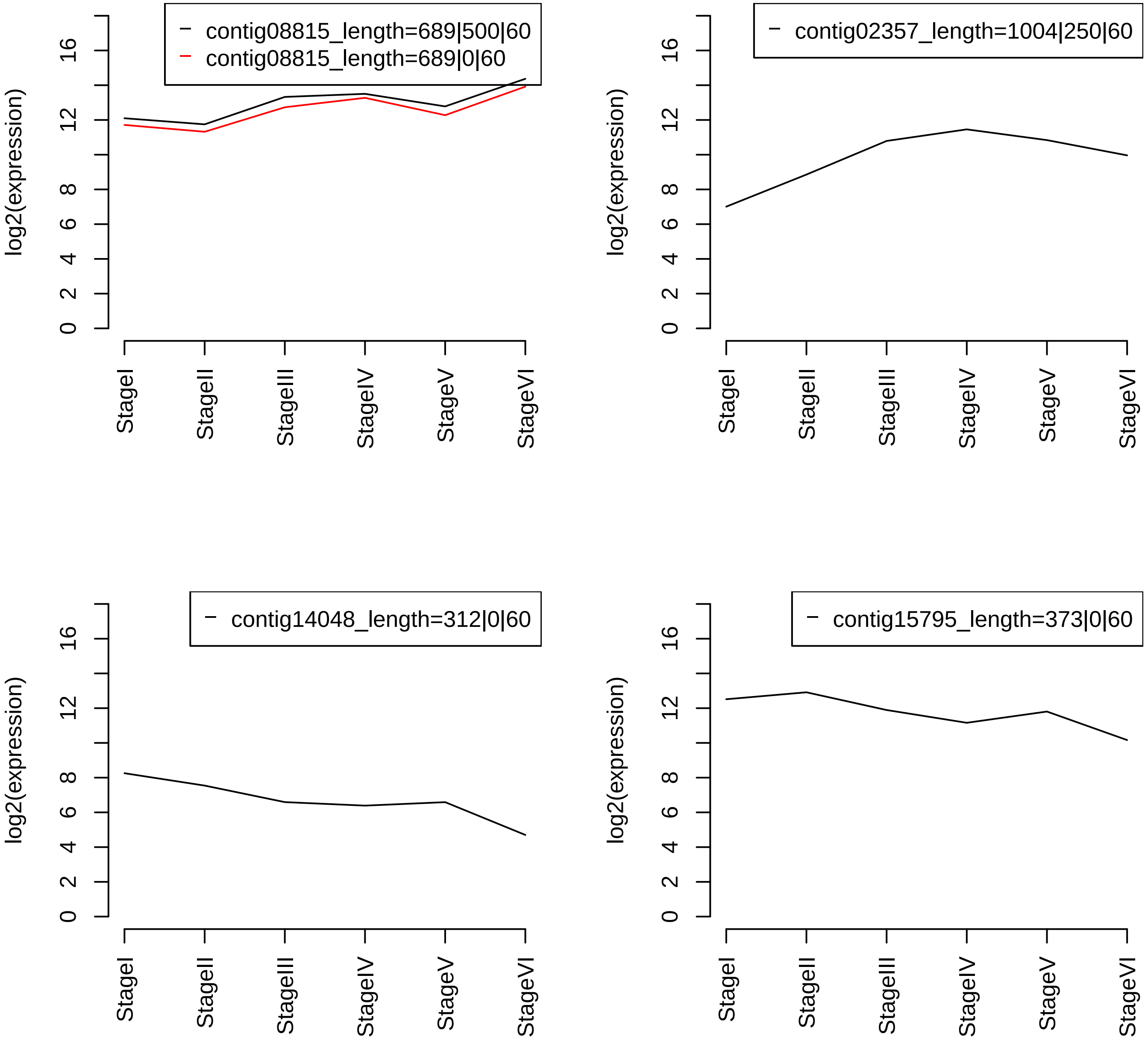
contig08815_length=689|500|60
contig08815_length=689|0|60
contig02357_length=1004|250|60
contig14048_length=312|0|60
contig15795_length=373|0|60
log2(expression)
0
2
4
6
8
12
16
StageI
StageII
StageIII
StageIV
StageV
StageVI

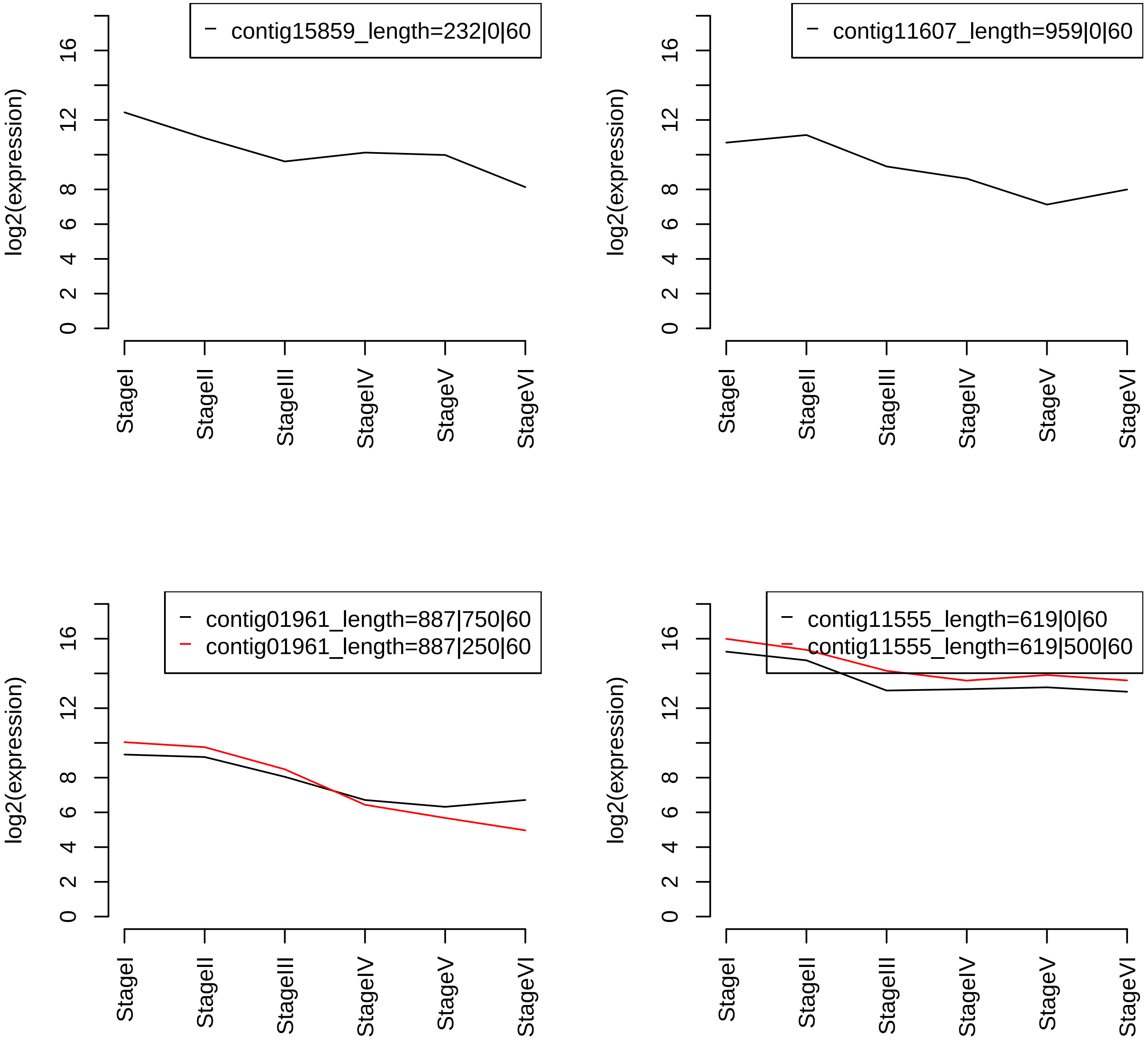
contig15859_length=232|0|60
contig11607_length=959|0|60
contig01961_length=887|750|60
contig01961_length=887|250|60
contig11555_length=619|0|60
contig11555_length=619|500|60
log2(expression)
0
2
4
6
8
12
16
StageI
StageII
StageIII
StageIV
StageV
StageVI

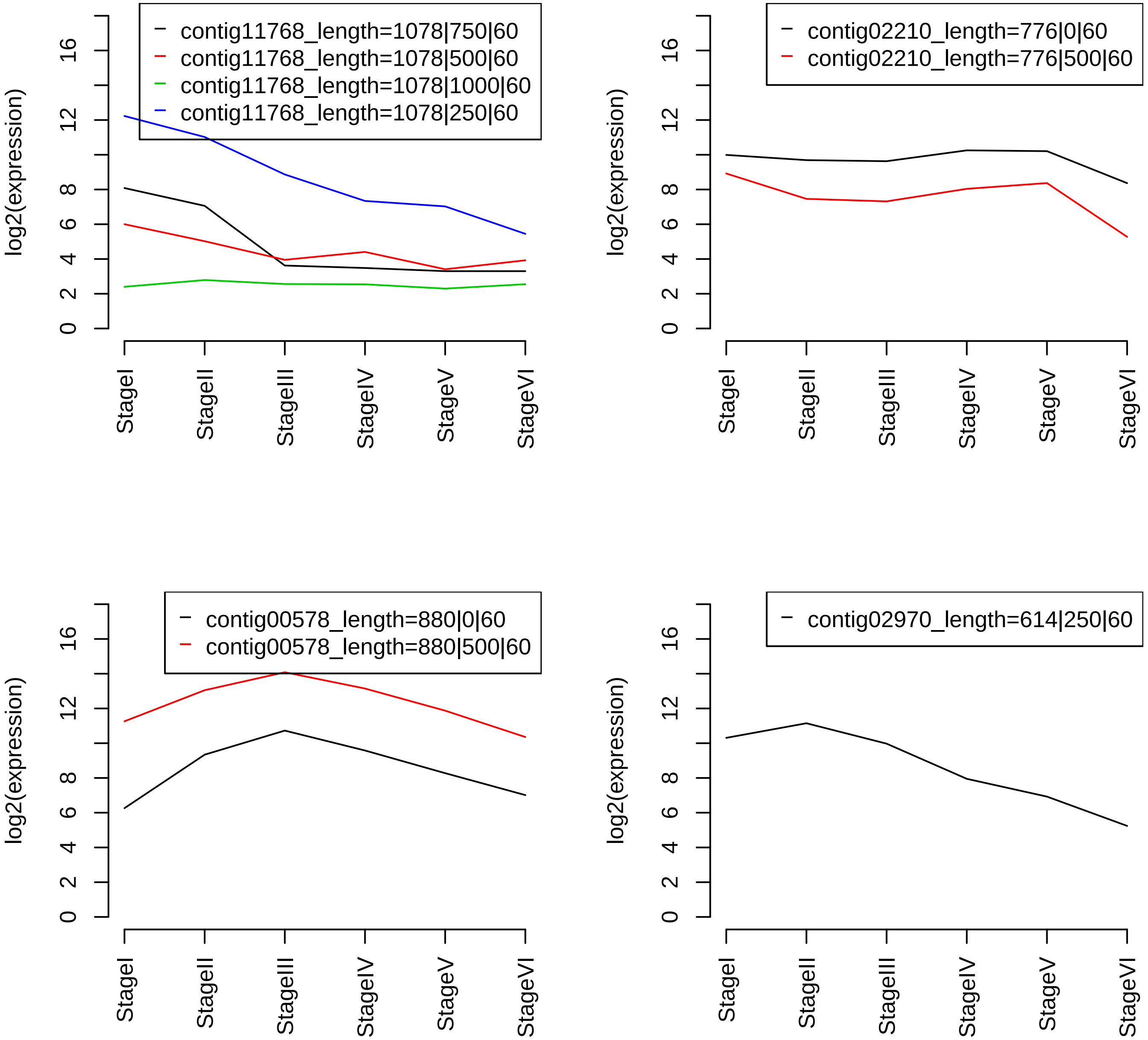

contig11768_length=1078|750|60
contig11768_length=1078|500|60
contig11768_length=1078|1000|60
contig11768_length=1078|250|60
contig02210_length=776|0|60
contig02210_length=776|500|60
contig00578_length=880|0|60
contig00578_length=880|500|60
contig02970_length=614|250|60
log2(expression)
0 2 4 6 8 12 16
StageI StageII StageIII StageIV StageV StageVI

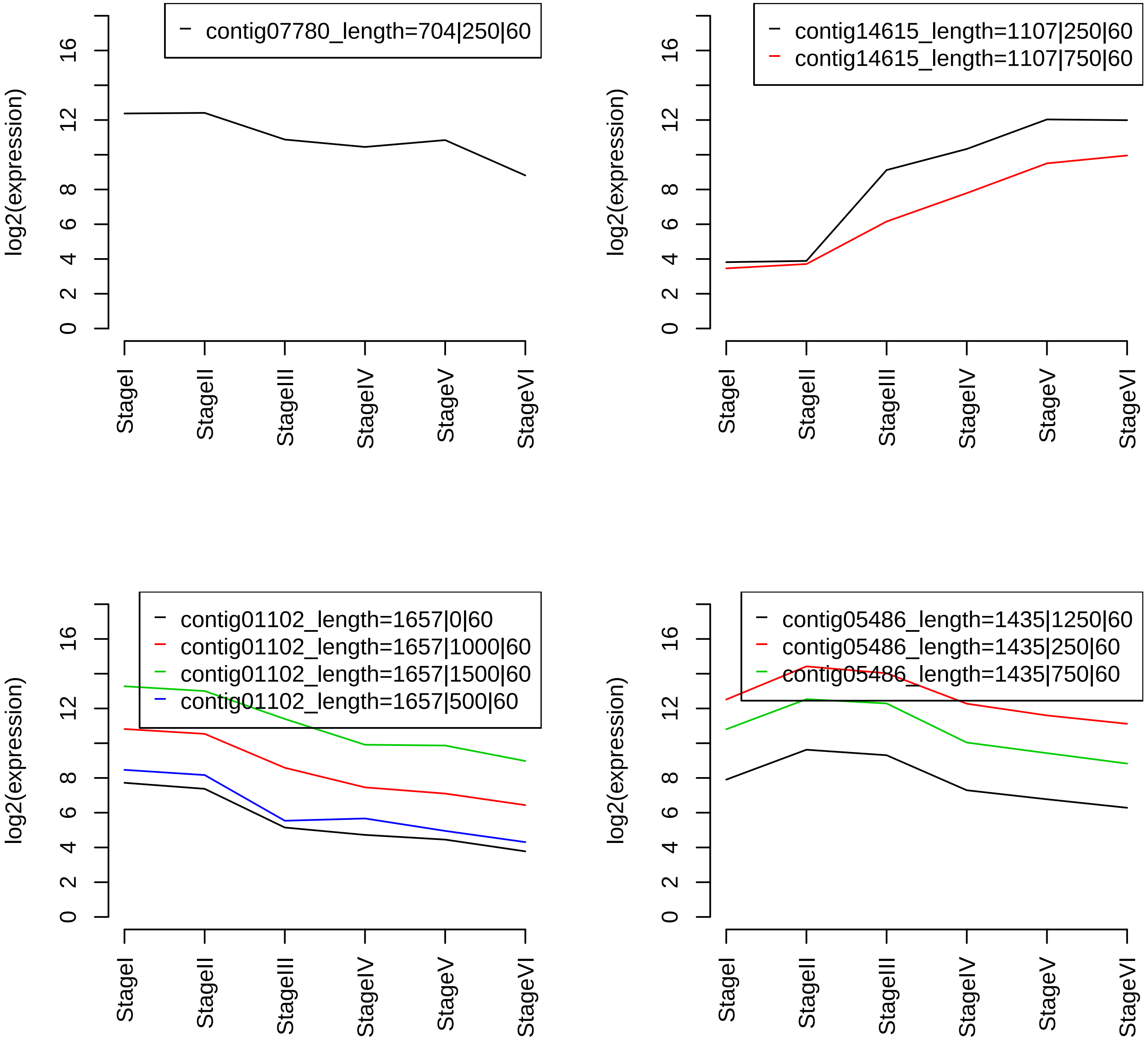
contig07780_length=704|250|60
contig14615_length=1107|250|60
contig14615_length=1107|750|60
contig01102_length=1657|0|60
contig01102_length=1657|1000|60
contig01102_length=1657|1500|60
contig01102_length=1657|500|60
contig05486_length=1435|1250|60
contig05486_length=1435|250|60
contig05486_length=1435|750|60
log2(expression)
0
2
4
6
8
12
16
StageI
StageII
StageIII
StageIV
StageV
StageVI

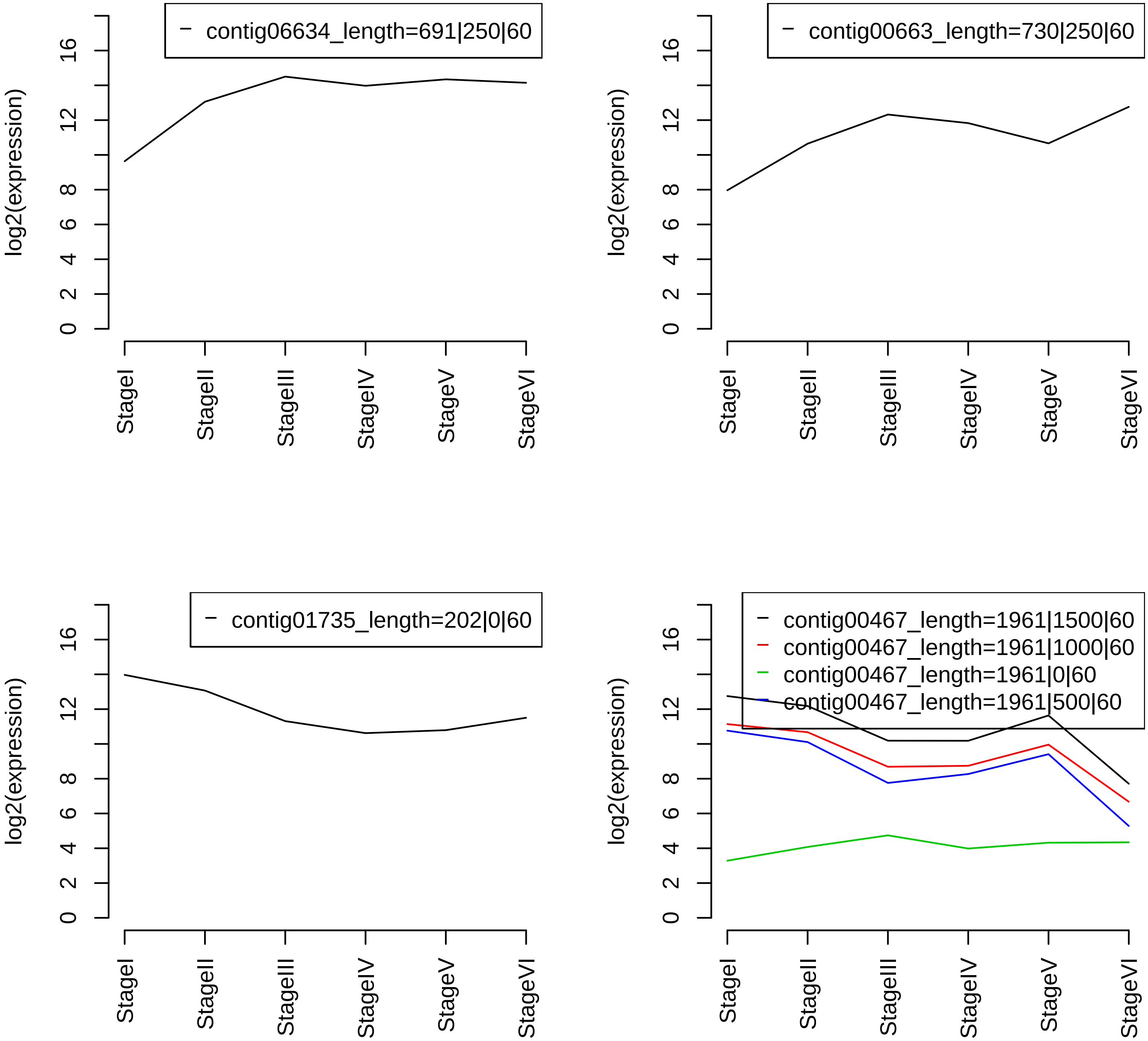

contig06634_length=691|250|60
contig00663_length=730|250|60
contig01735_length=202|0|60
contig00467_length=1961|1500|60
contig00467_length=1961|1000|60
contig00467_length=1961|0|60
contig00467_length=1961|500|60
log2(expression)
0
2
4
6
8
12
16
StageI
StageII
StageIII
StageIV
StageV
StageVI

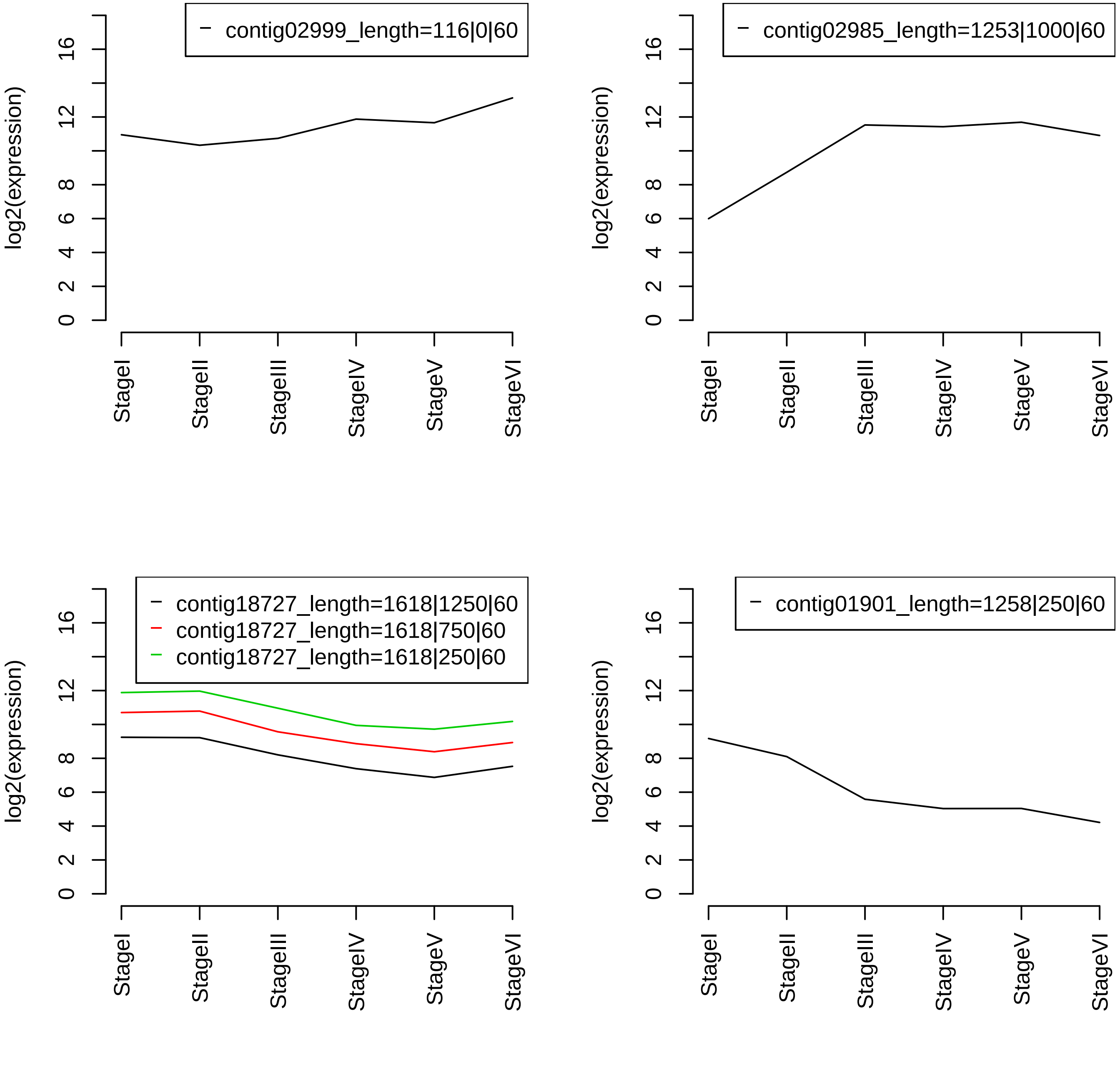
contig02999_length=116|0|60
contig02985_length=1253|1000|60
contig18727_length=1618|1250|60
contig18727_length=1618|750|60
contig18727_length=1618|250|60
contig01901_length=1258|250|60
log2(expression)
0
2
4
6
8
12
16
StageI
StageII
StageIII
StageIV
StageV
StageVI

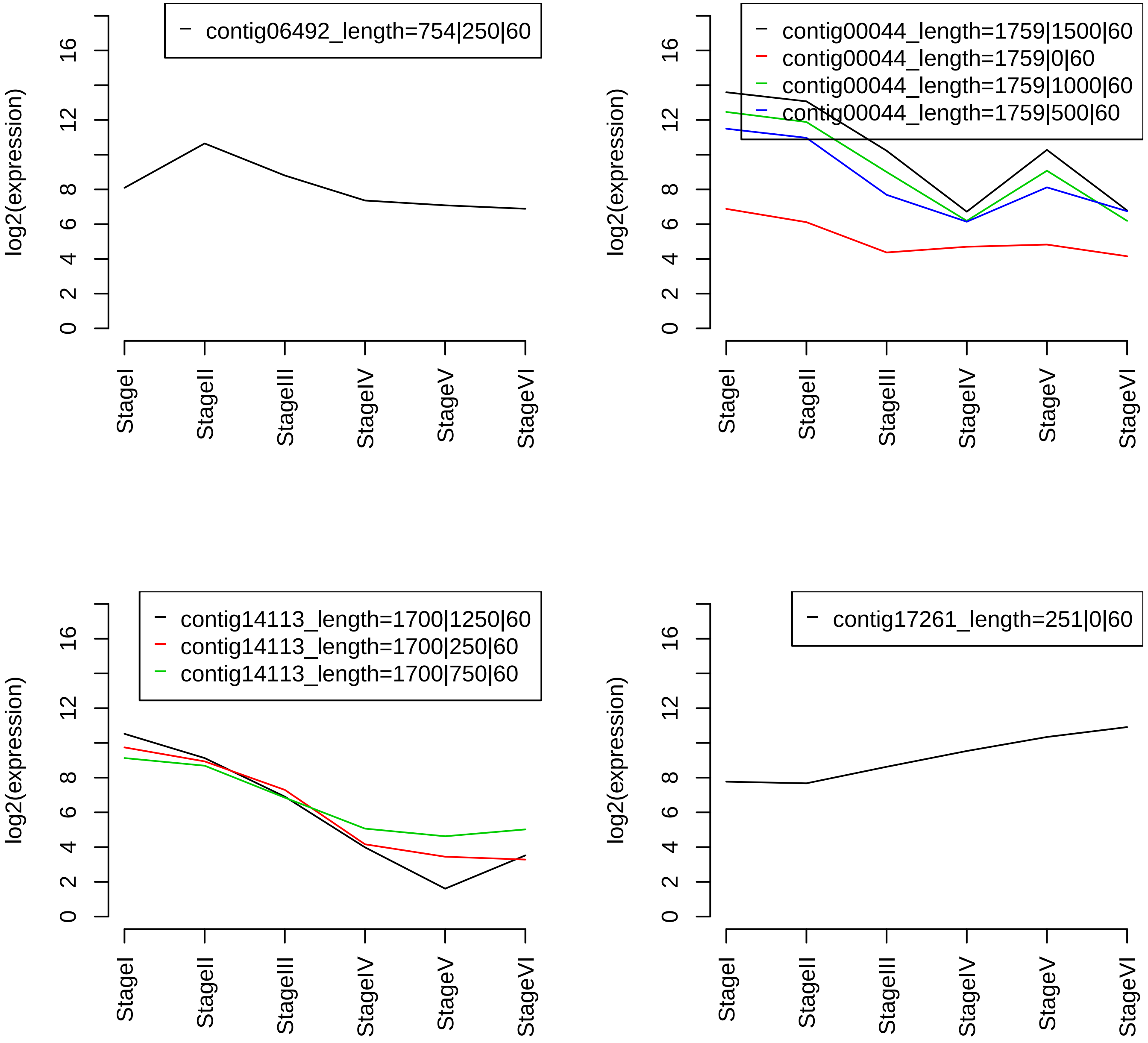
contig06492_length=754|250|60
contig00044_length=1759|1500|60
contig00044_length=1759|0|60
contig00044_length=1759|1000|60
contig00044_length=1759|500|60
contig14113_length=1700|1250|60
contig14113_length=1700|250|60
contig14113_length=1700|750|60
contig17261_length=251|0|60
log2(expression)
0 2 4 6 8 12 16
StageI StageII StageIII StageIV StageV StageVI

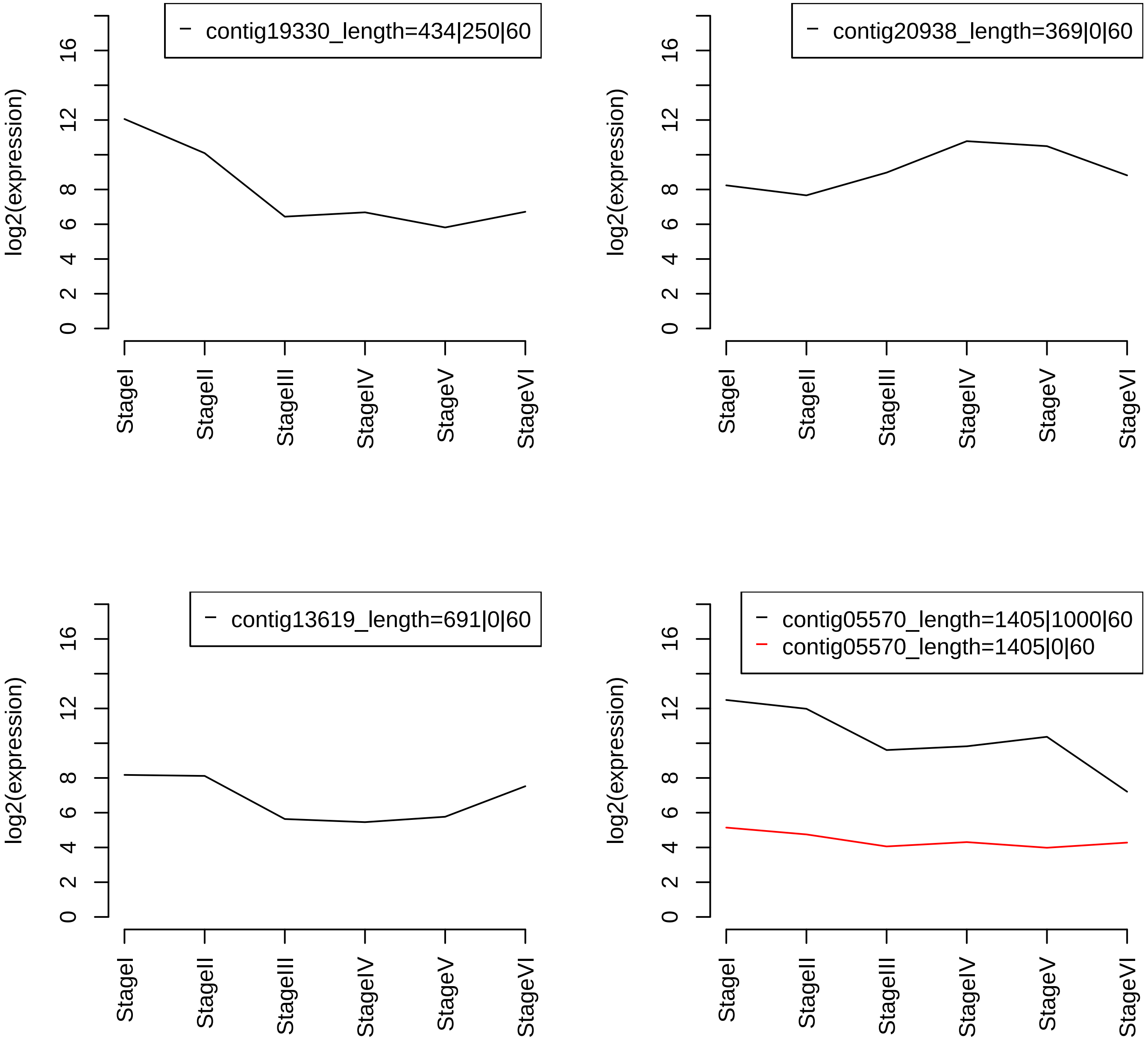
contig19330_length=434|250|60
contig20938_length=369|0|60
contig13619_length=691|0|60
contig05570_length=1405|1000|60
contig05570_length=1405|0|60
log2(expression)
0
2
4
6
8
12
16
StageI
StageII
StageIII
StageIV
StageV
StageVI

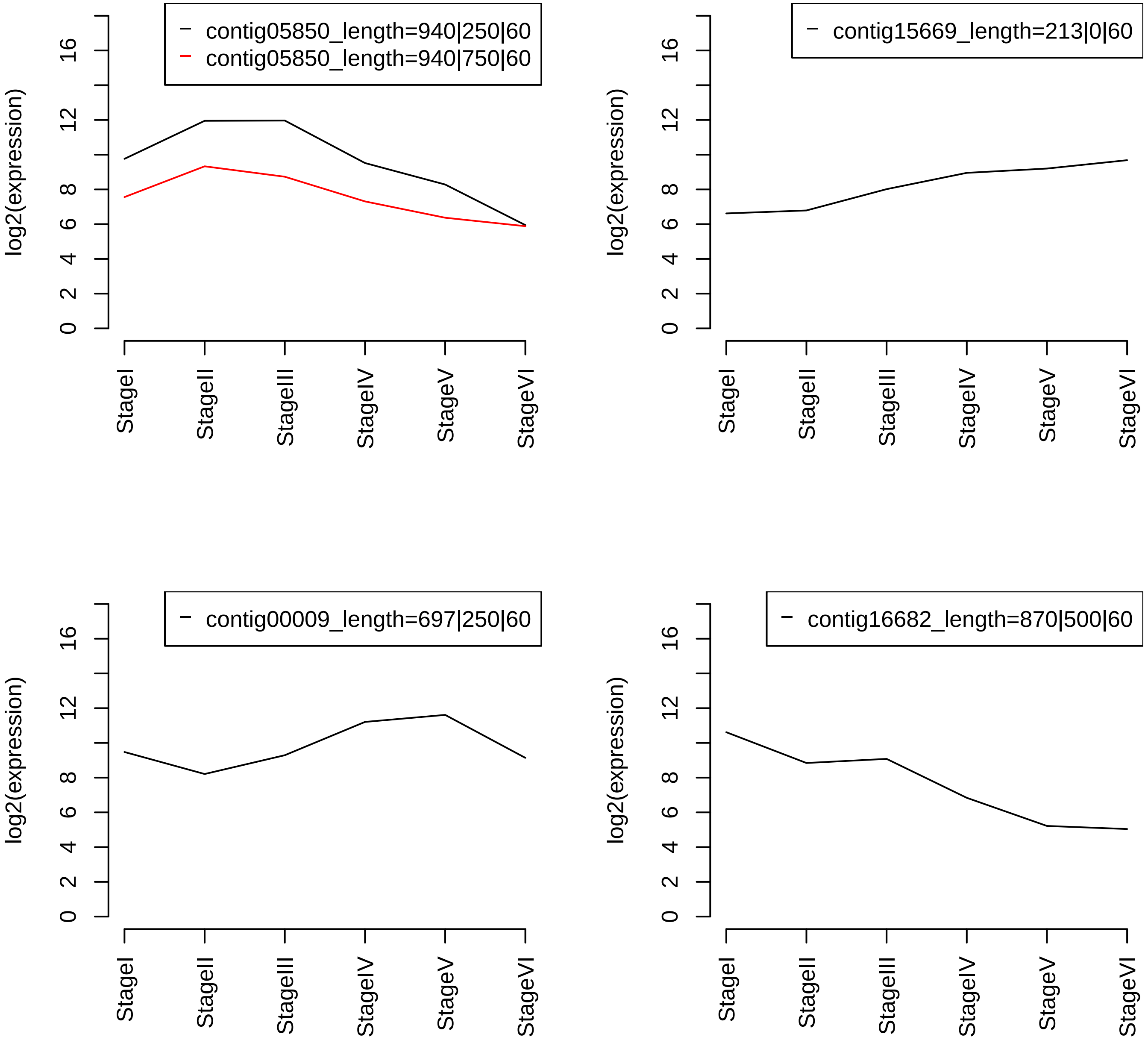

contig05850_length=940|250|60
contig05850_length=940|750|60
contig15669_length=213|0|60
contig00009_length=697|250|60
contig16682_length=870|500|60
log2(expression)
0
2
4
6
8
12
16
StageI
StageII
StageIII
StageIV
StageV
StageVI

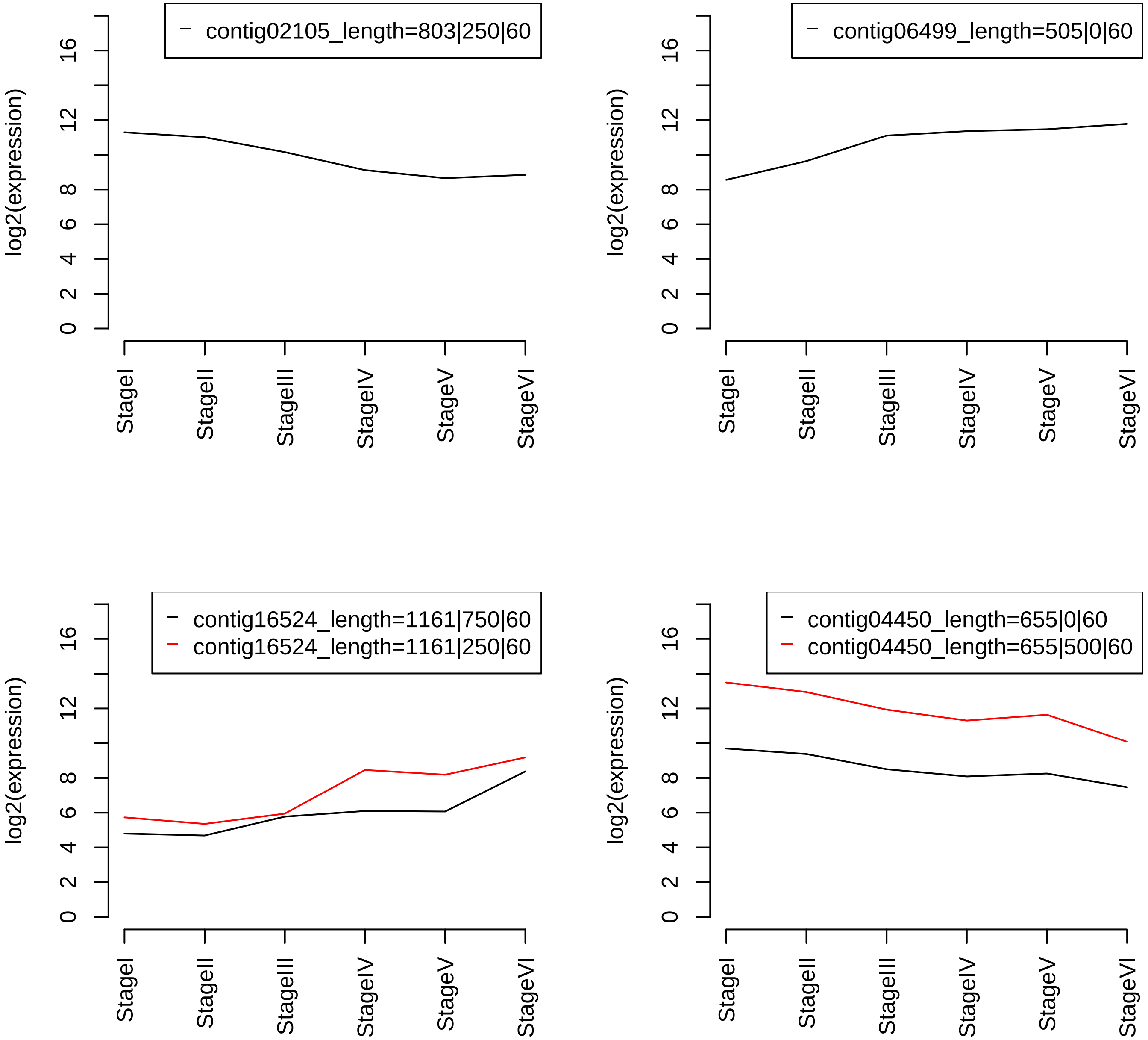
contig02105_length=803|250|60
contig06499_length=505|0|60
contig16524_length=1161|750|60
contig16524_length=1161|250|60
contig04450_length=655|0|60
contig04450_length=655|500|60
log2(expression)
0
2
4
6
8
12
16
StageI
StageII
StageIII
StageIV
StageV
StageVI

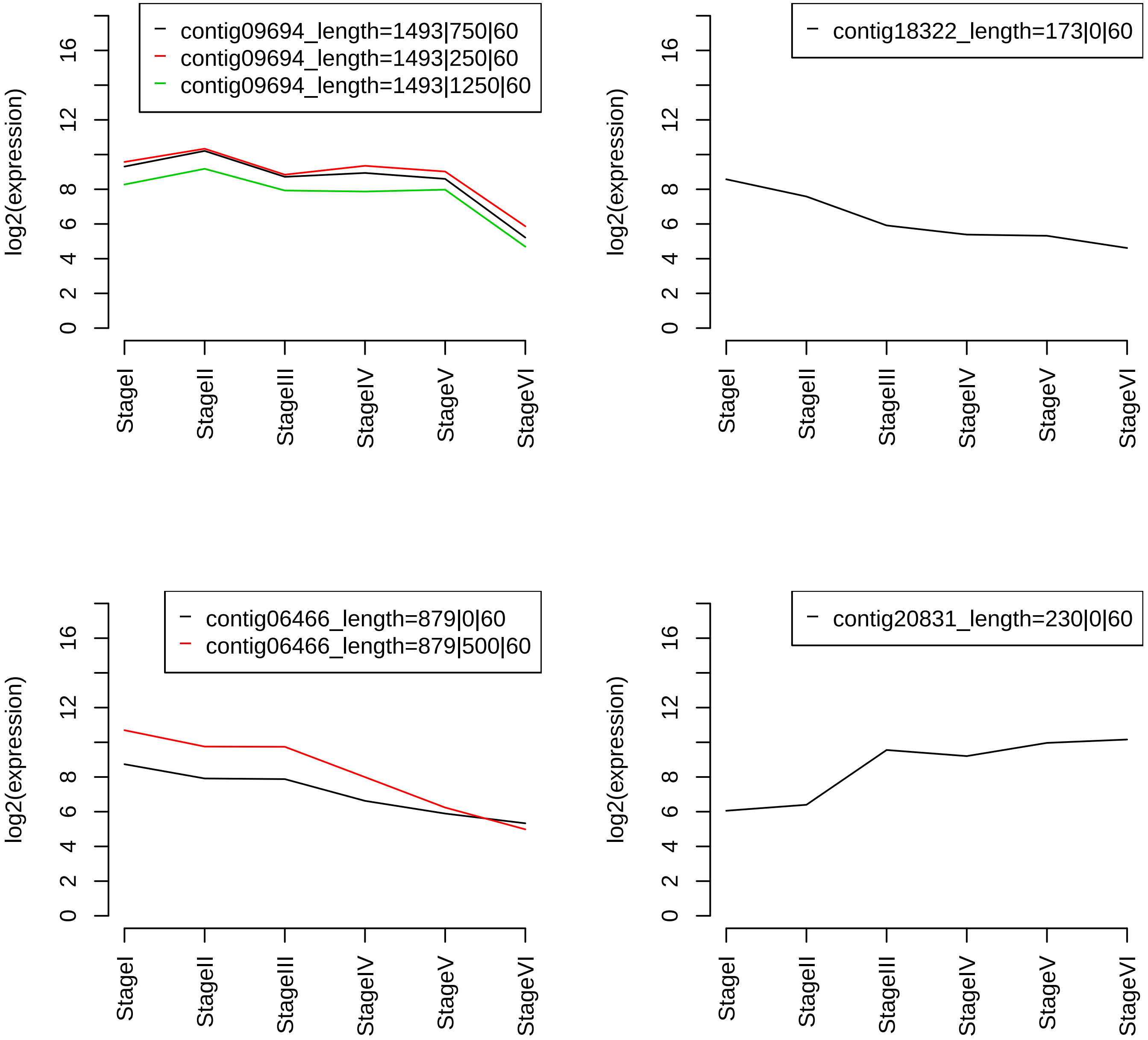

contig09694_length=1493|750|60
contig09694_length=1493|250|60
contig09694_length=1493|1250|60
log2(expression)
0
2
4
6
8
12
16
StageI
StageII
StageIII
StageIV
StageV
StageVI
contig18322_length=173|0|60
contig06466_length=879|0|60
contig06466_length=879|500|60
contig20831_length=230|0|60

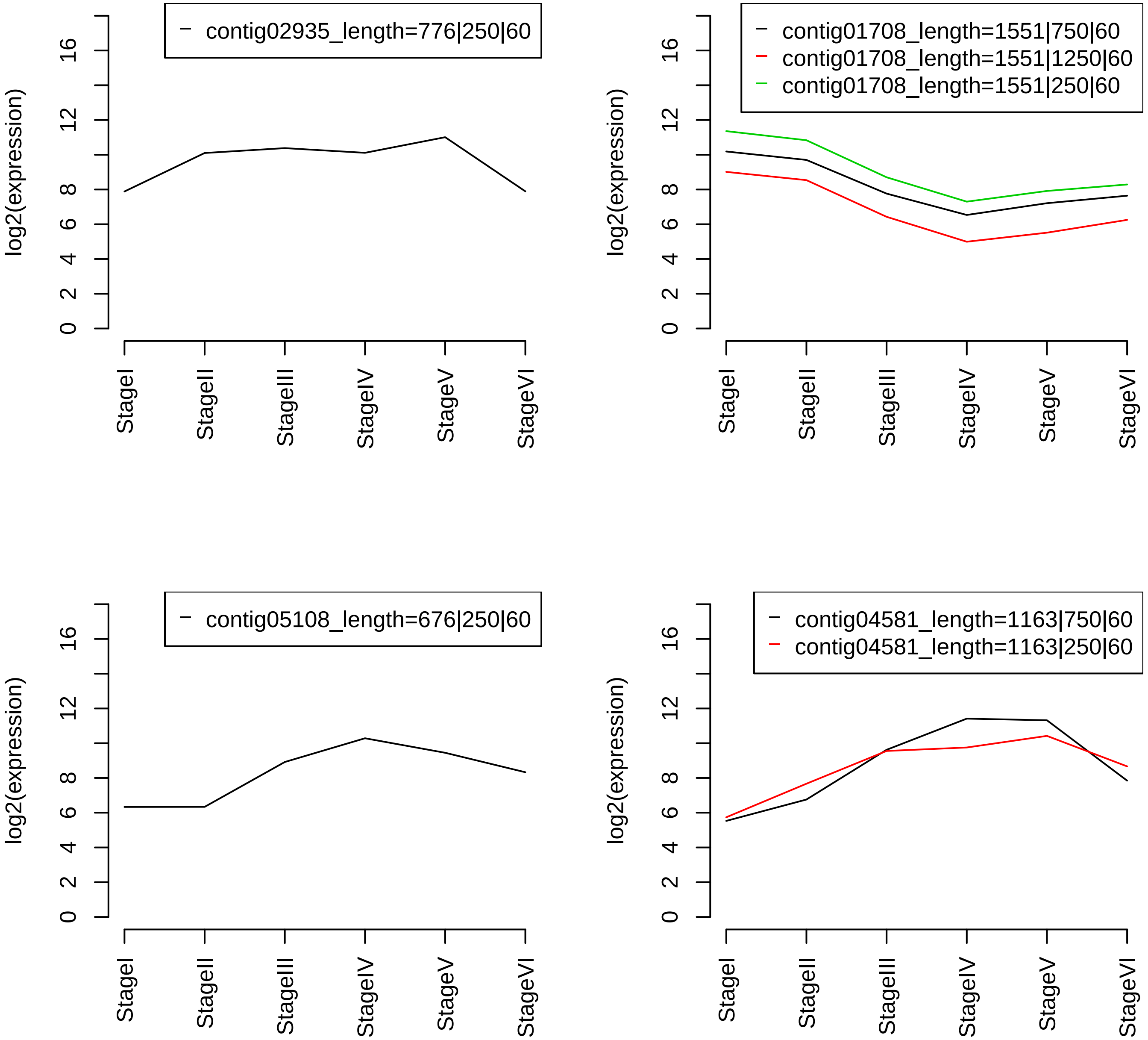
contig02935_length=776|250|60
contig01708_length=1551|750|60
contig01708_length=1551|1250|60
contig01708_length=1551|250|60
contig05108_length=676|250|60
contig04581_length=1163|750|60
contig04581_length=1163|250|60
log2(expression)
0
2
4
6
8
12
16
StageI
StageII
StageIII
StageIV
StageV
StageVI

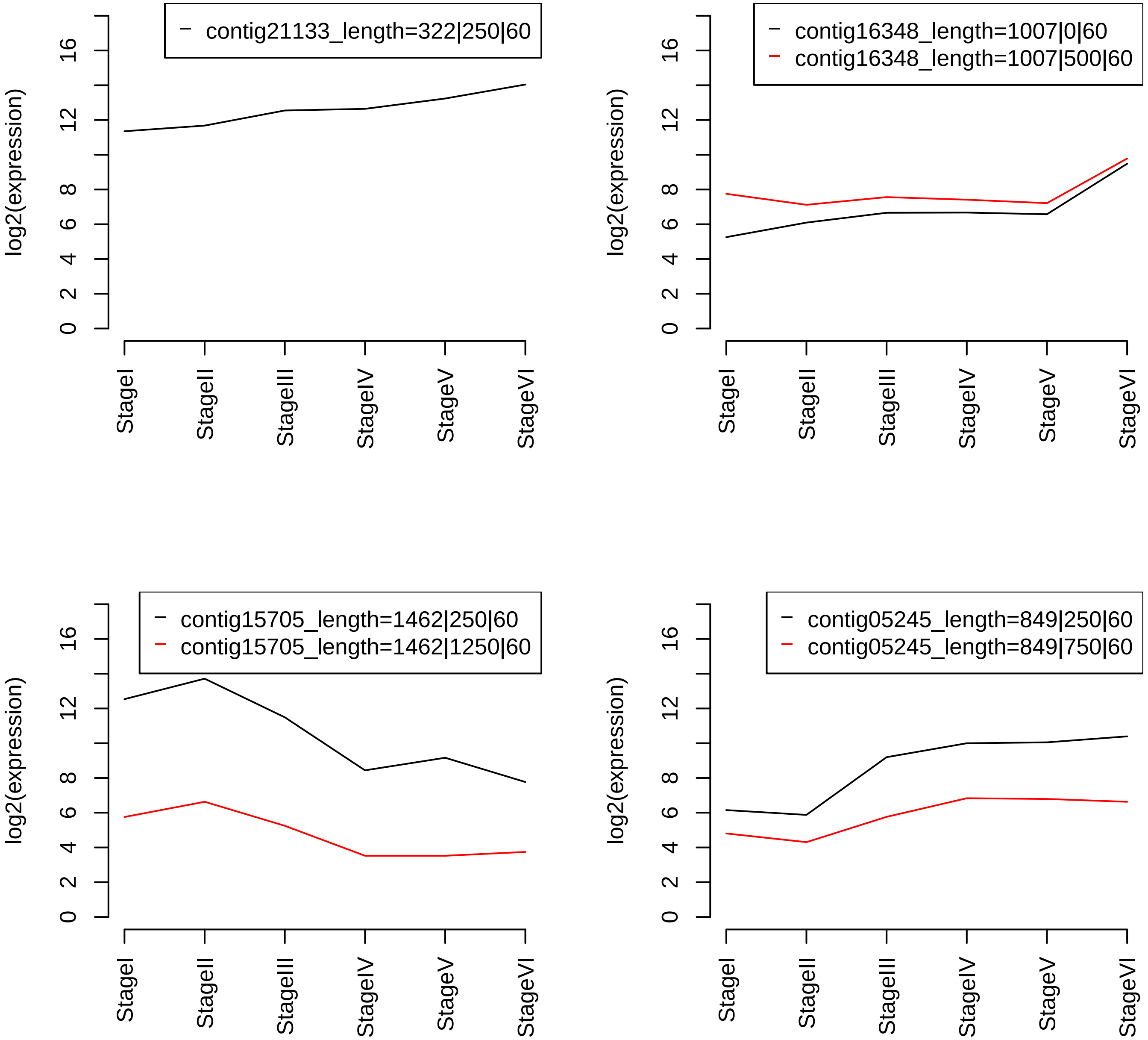
contig21133_length=322|250|60
contig16348_length=1007|0|60
contig16348_length=1007|500|60
contig15705_length=1462|250|60
contig15705_length=1462|1250|60
contig05245_length=849|250|60
contig05245_length=849|750|60
log2(expression)
0 2 4 6 8 12 16
StageI StageII StageIII StageIV StageV StageVI

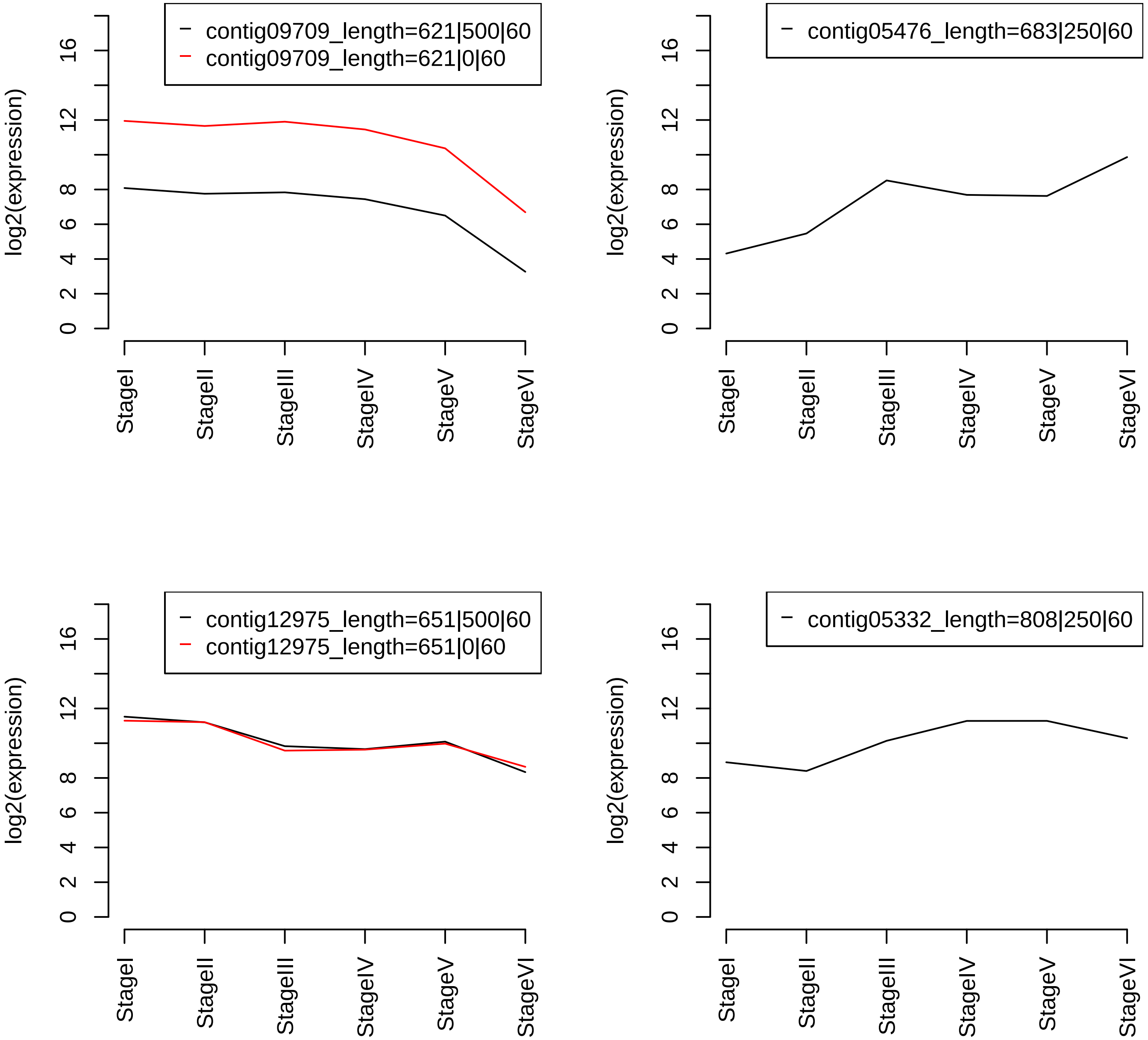
contig09709_length=621|500|60
contig09709_length=621|0|60
contig05476_length=683|250|60
contig12975_length=651|500|60
contig12975_length=651|0|60
contig05332_length=808|250|60
log2(expression)
0 2 4 6 8 12 16
StageI
StageII
StageIII
StageIV
StageV
StageVI

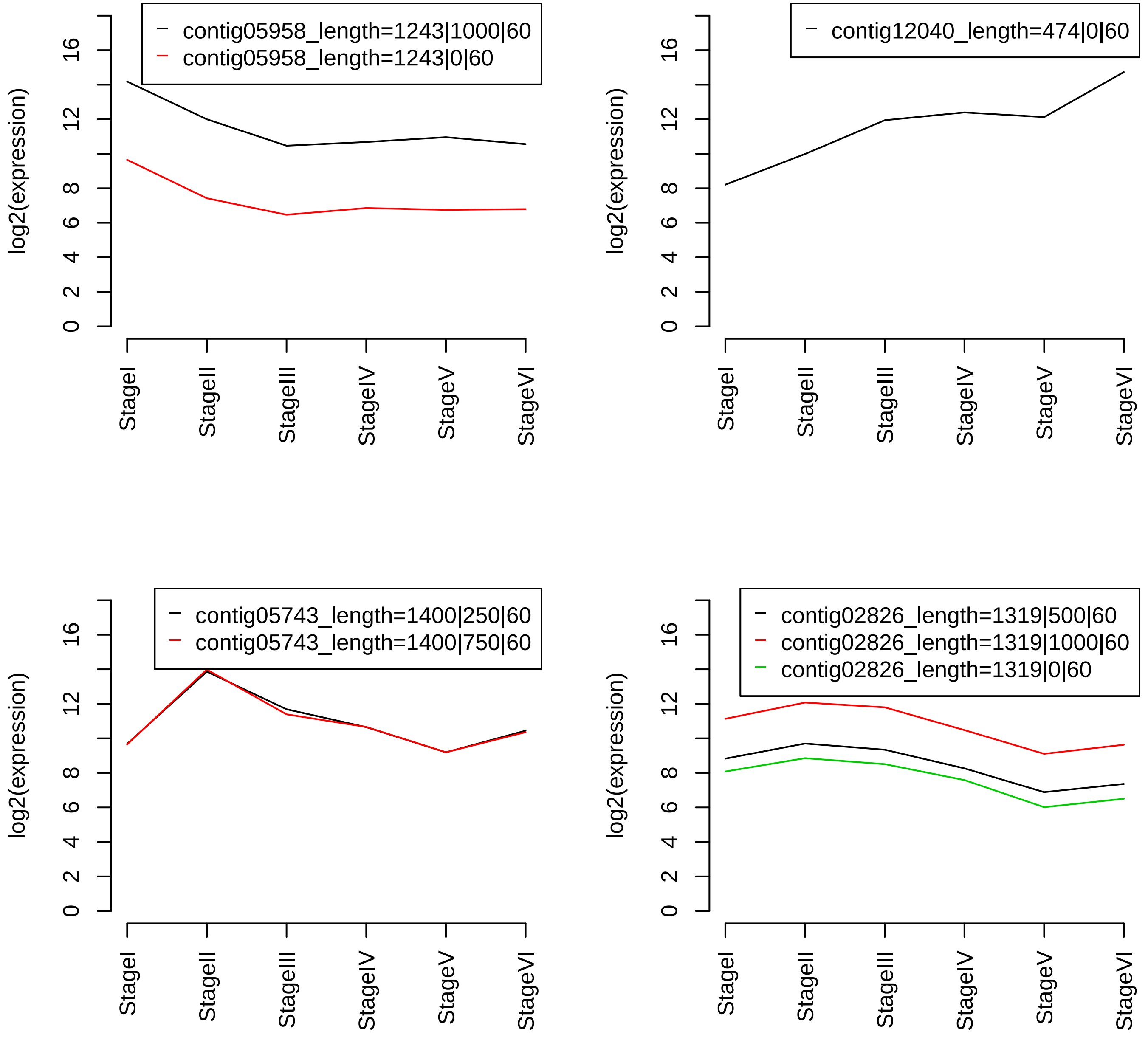
contig05958_length=1243|1000|60
contig05958_length=1243|0|60
contig12040_length=474|0|60
contig05743_length=1400|250|60
contig05743_length=1400|750|60
contig02826_length=1319|500|60
contig02826_length=1319|1000|60
contig02826_length=1319|0|60
log2(expression)
0
2
4
6
8
12
16
StageI
StageII
StageIII
StageIV
StageV
StageVI

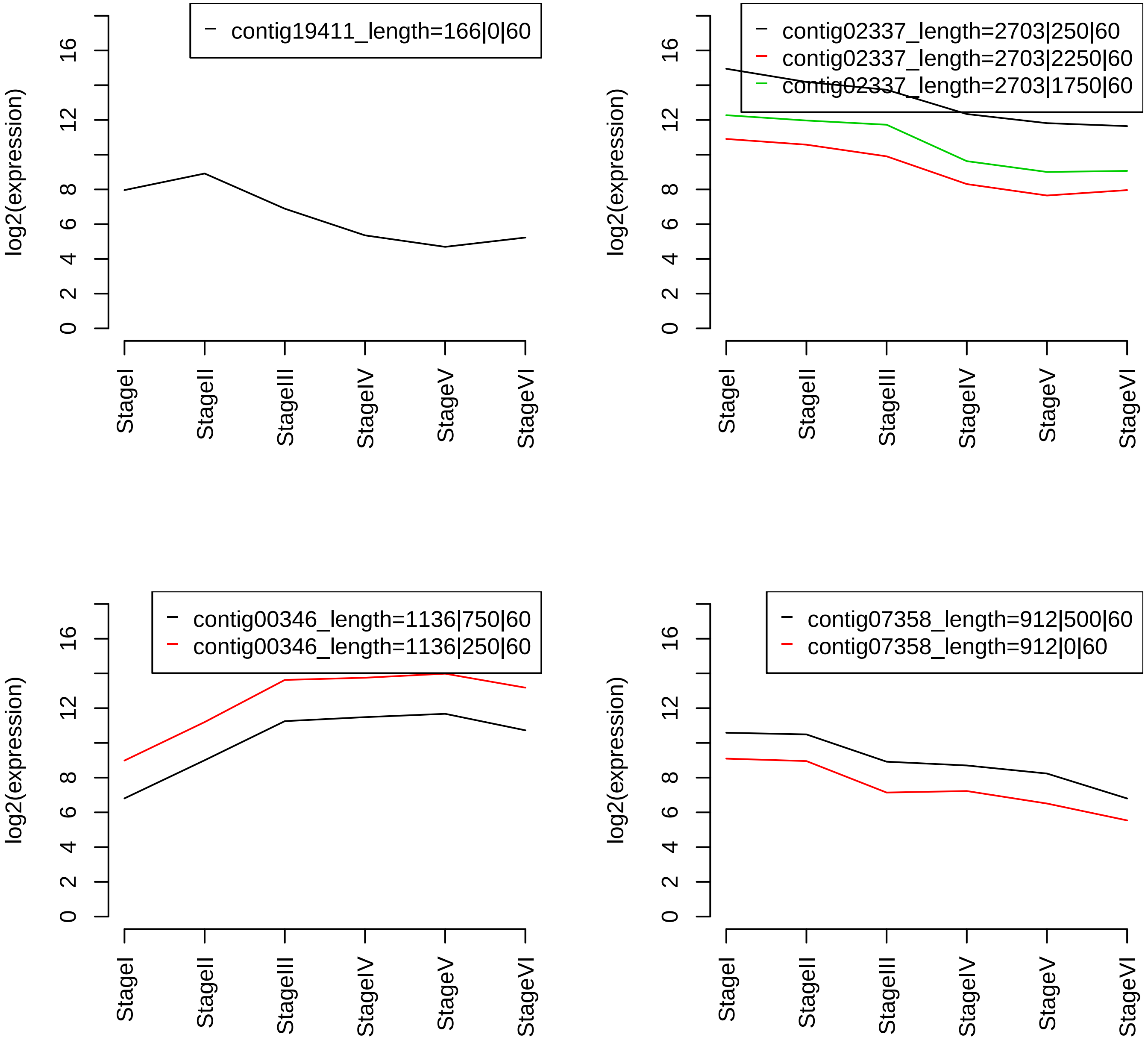
contig19411_length=166|0|60
contig02337_length=2703|250|60
contig02337_length=2703|2250|60
contig02337_length=2703|1750|60
contig00346_length=1136|750|60
contig00346_length=1136|250|60
contig07358_length=912|500|60
contig07358_length=912|0|60
log2(expression)
0
2
4
6
8
12
16
StageI
StageII
StageIII
StageIV
StageV
StageVI

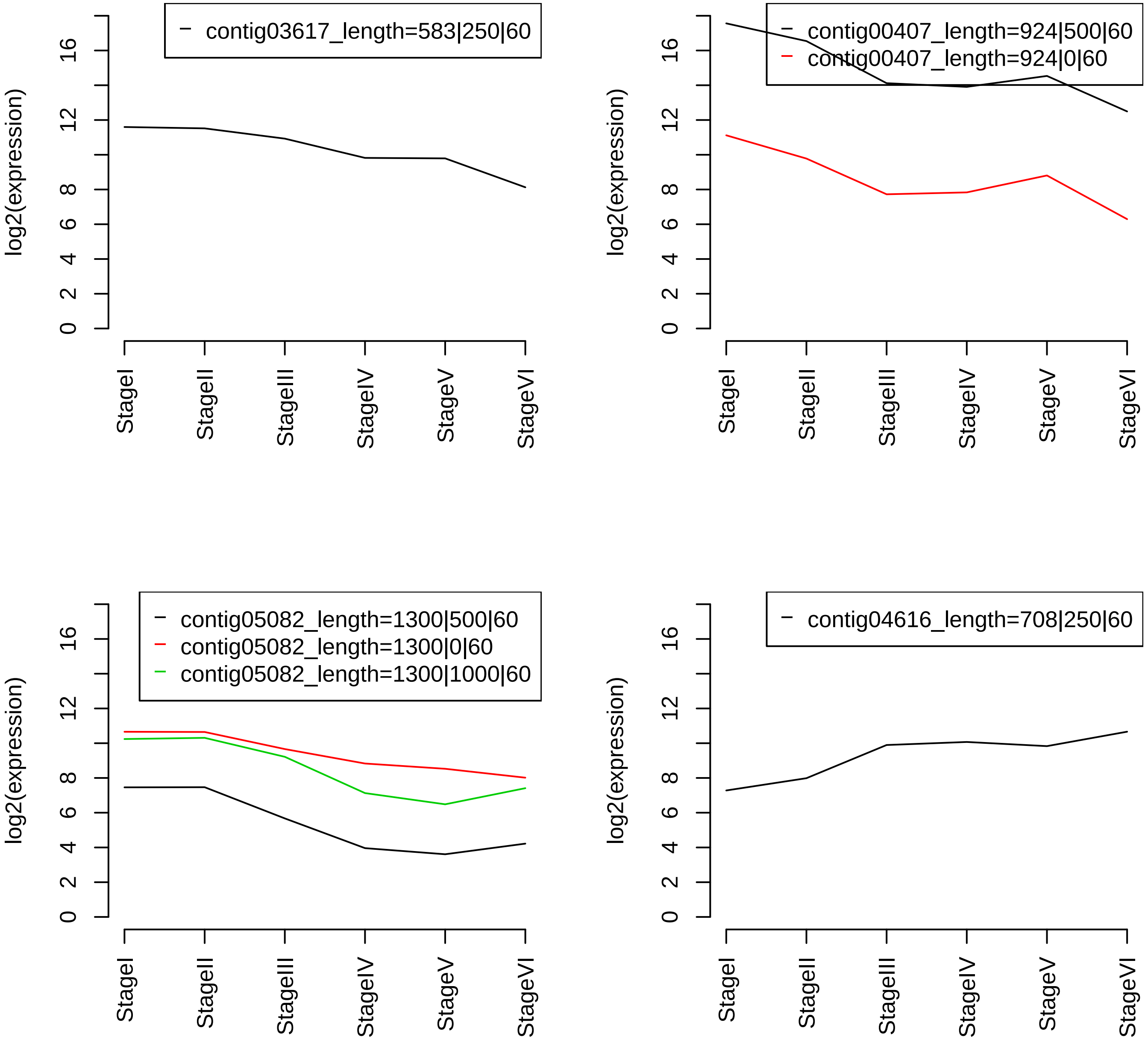

contig03617_length=583|250|60
contig00407_length=924|500|60
contig00407_length=924|0|60
contig05082_length=1300|500|60
contig05082_length=1300|0|60
contig05082_length=1300|1000|60
contig04616_length=708|250|60
log2(expression)
0 2 4 6 8 12 16
StageI StageII StageIII StageIV StageV StageVI

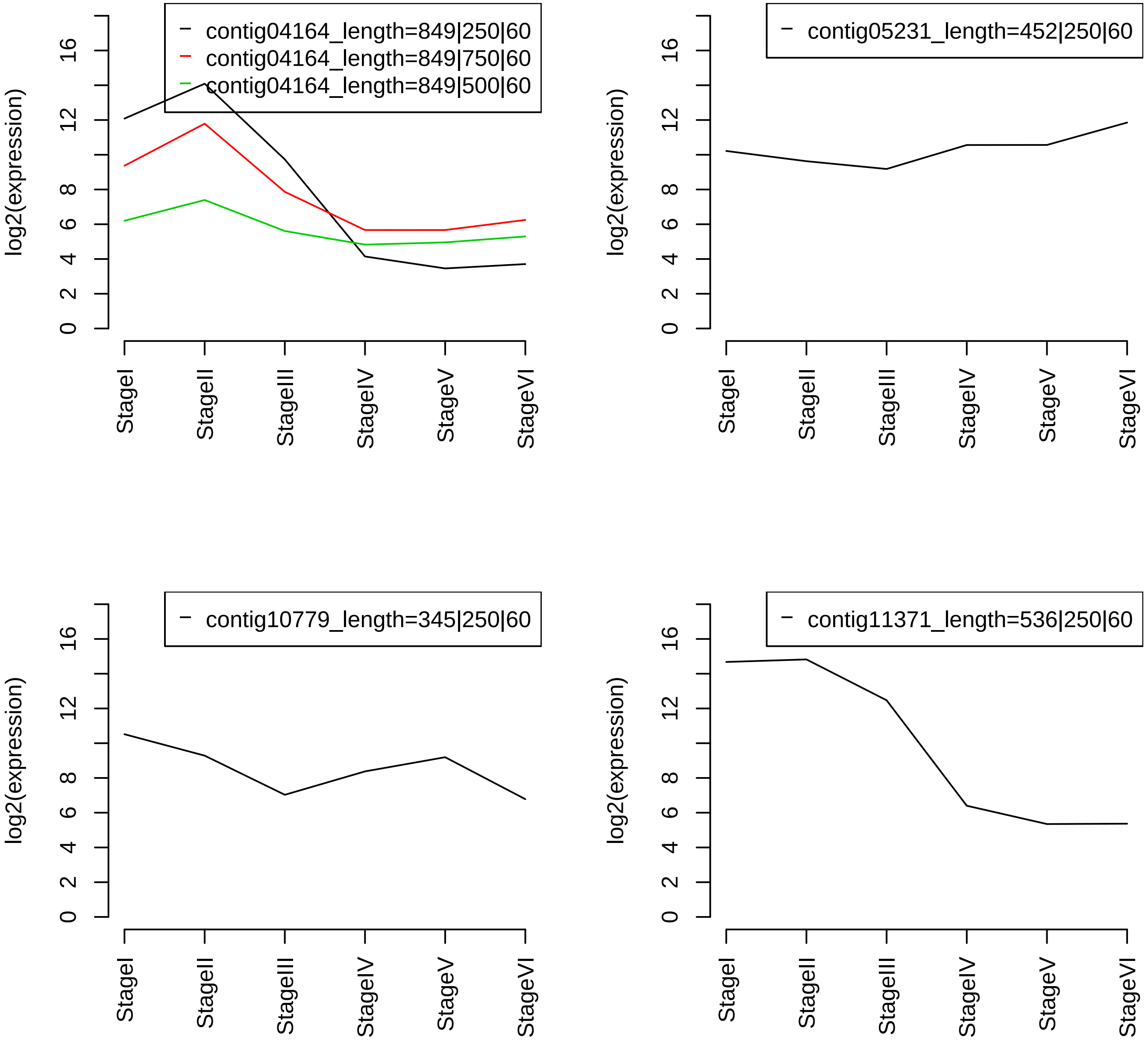

contig04164_length=849|250|60
contig04164_length=849|750|60
contig04164_length=849|500|60
contig05231_length=452|250|60
contig10779_length=345|250|60
contig11371_length=536|250|60
log2(expression)
0
2
4
6
8
12
16
StageI
StageII
StageIII
StageIV
StageV
StageVI

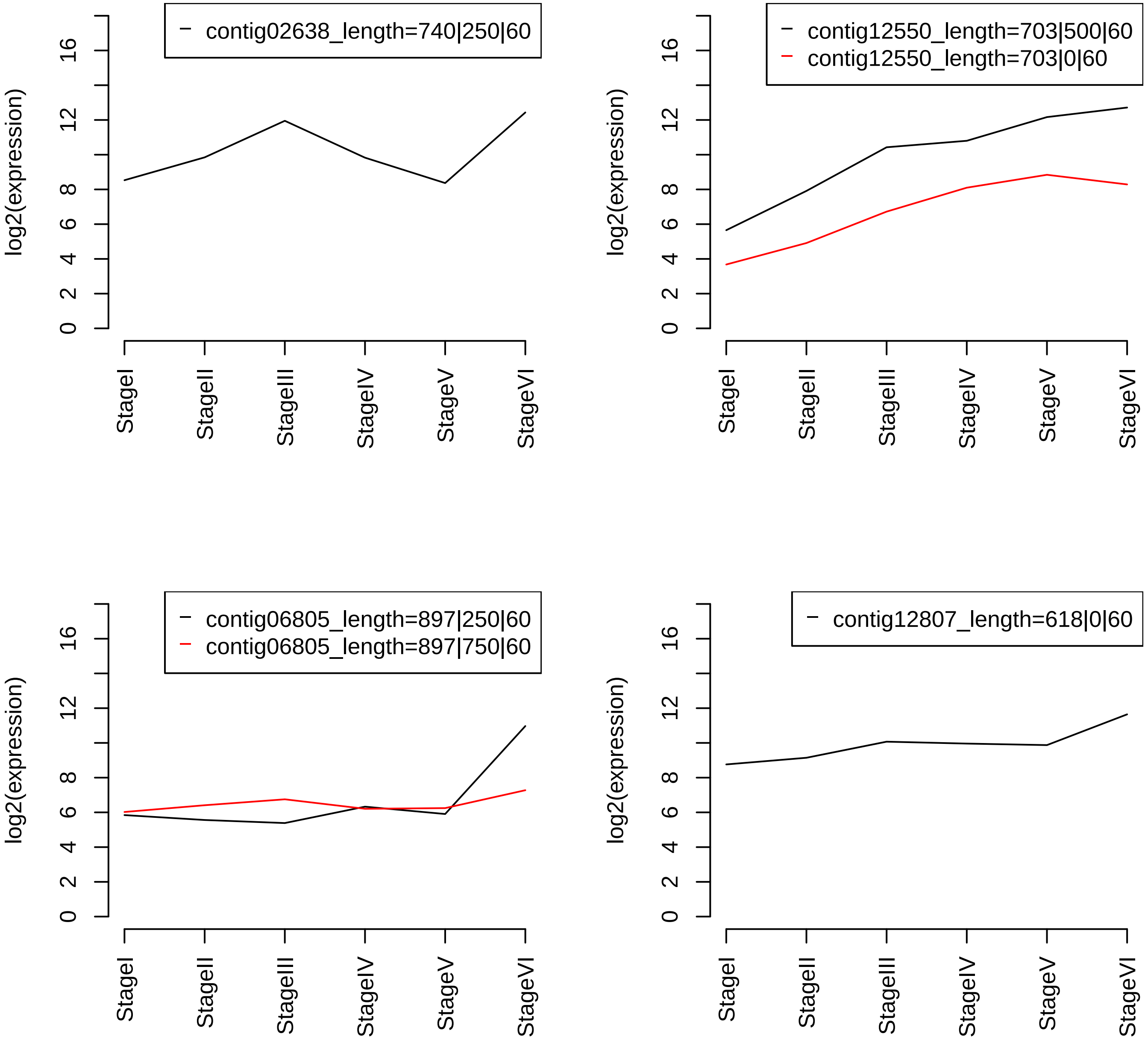
contig02638_length=740|250|60
contig12550_length=703|500|60
contig12550_length=703|0|60
contig06805_length=897|250|60
contig06805_length=897|750|60
contig12807_length=618|0|60
log2(expression)
0 2 4 6 8 12 16
StageI StageII StageIII StageIV StageV StageVI

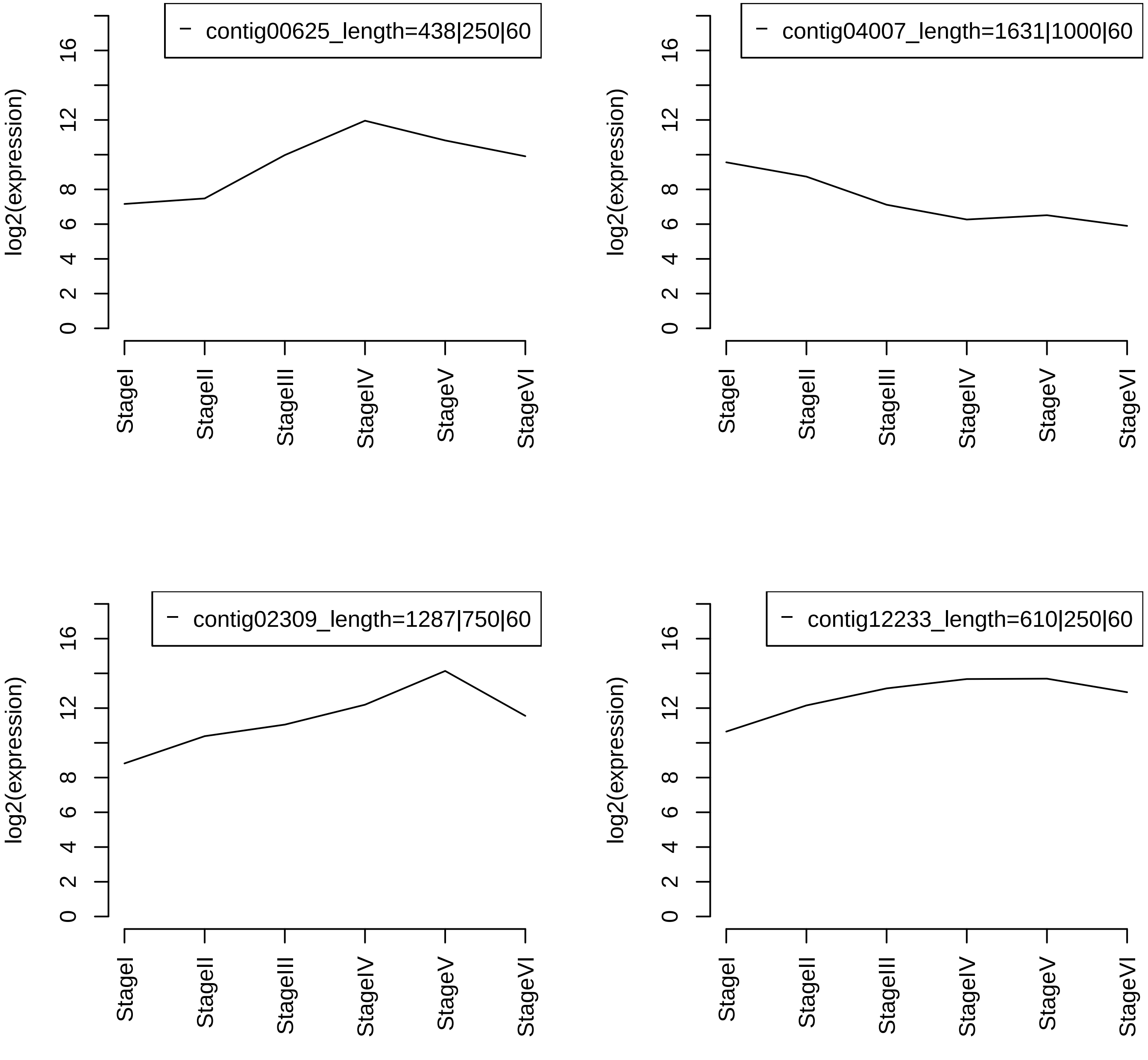
contig00625_length=438|250|60
contig04007_length=1631|1000|60
contig02309_length=1287|750|60
contig12233_length=610|250|60
log2(expression)
0
2
4
6
8
12
16
StageI
StageII
StageIII
StageIV
StageV
StageVI

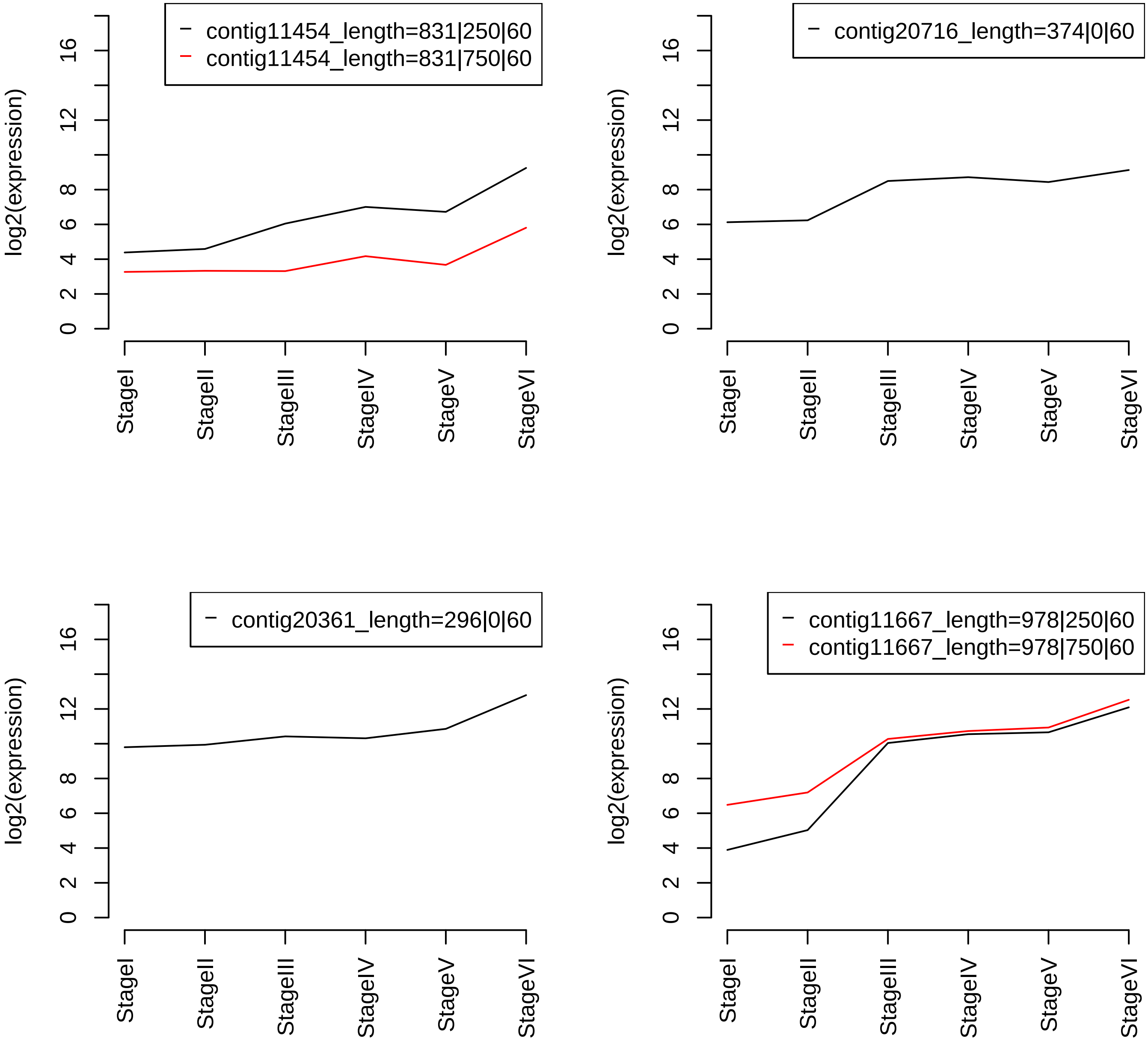
contig11454_length=831|250|60
contig11454_length=831|750|60
contig20716_length=374|0|60
contig20361_length=296|0|60
contig11667_length=978|250|60
contig11667_length=978|750|60
log2(expression)
0
2
4
6
8
12
16
StageI
StageII
StageIII
StageIV
StageV
StageVI

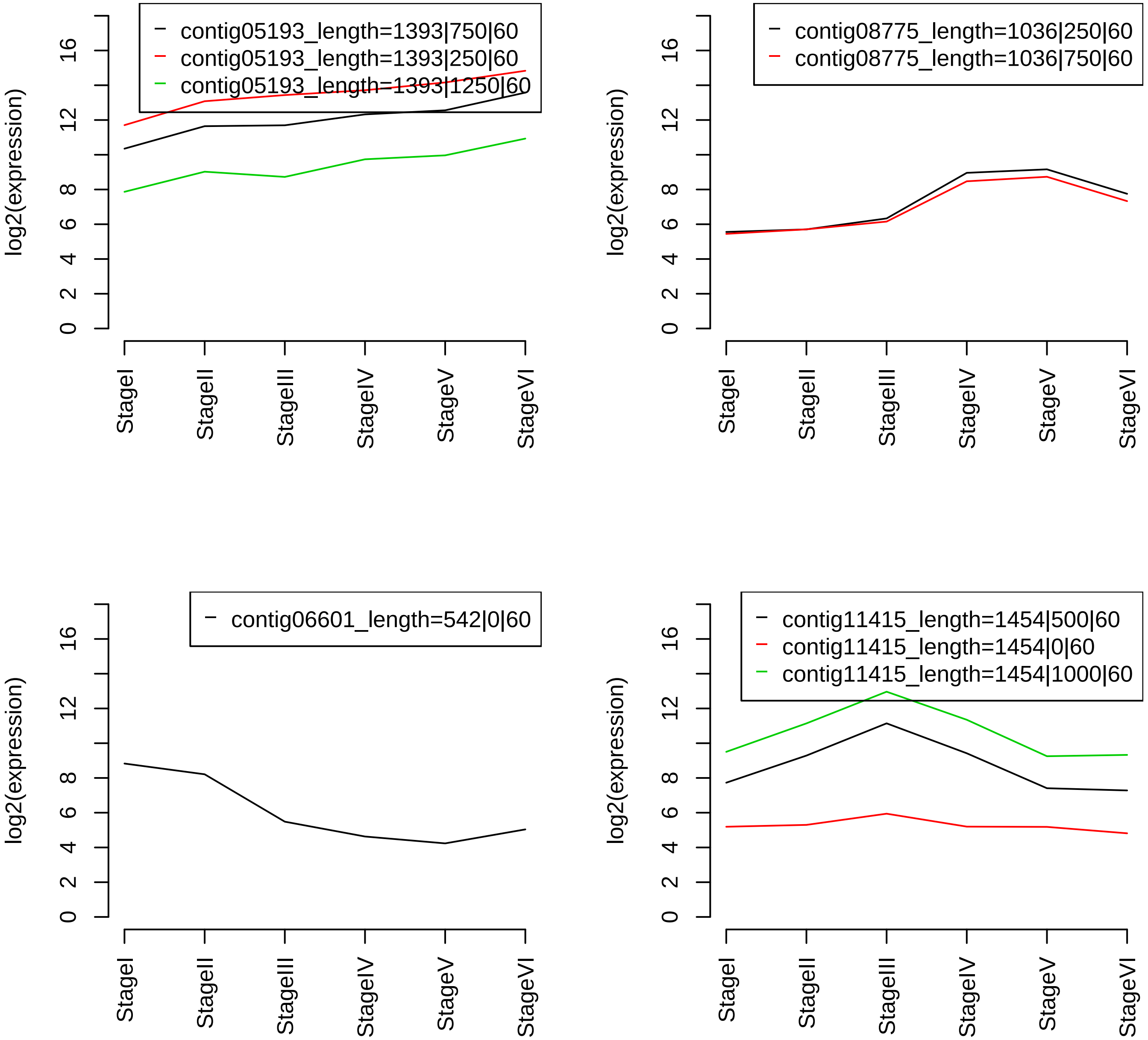

contig05193_length=1393|750|60
contig05193_length=1393|250|60
contig05193_length=1393|1250|60
contig08775_length=1036|250|60
contig08775_length=1036|750|60
contig06601_length=542|0|60
contig11415_length=1454|500|60
contig11415_length=1454|0|60
contig11415_length=1454|1000|60
log2(expression)
0
2
4
6
8
12
16
StageI
StageII
StageIII
StageIV
StageV
StageVI

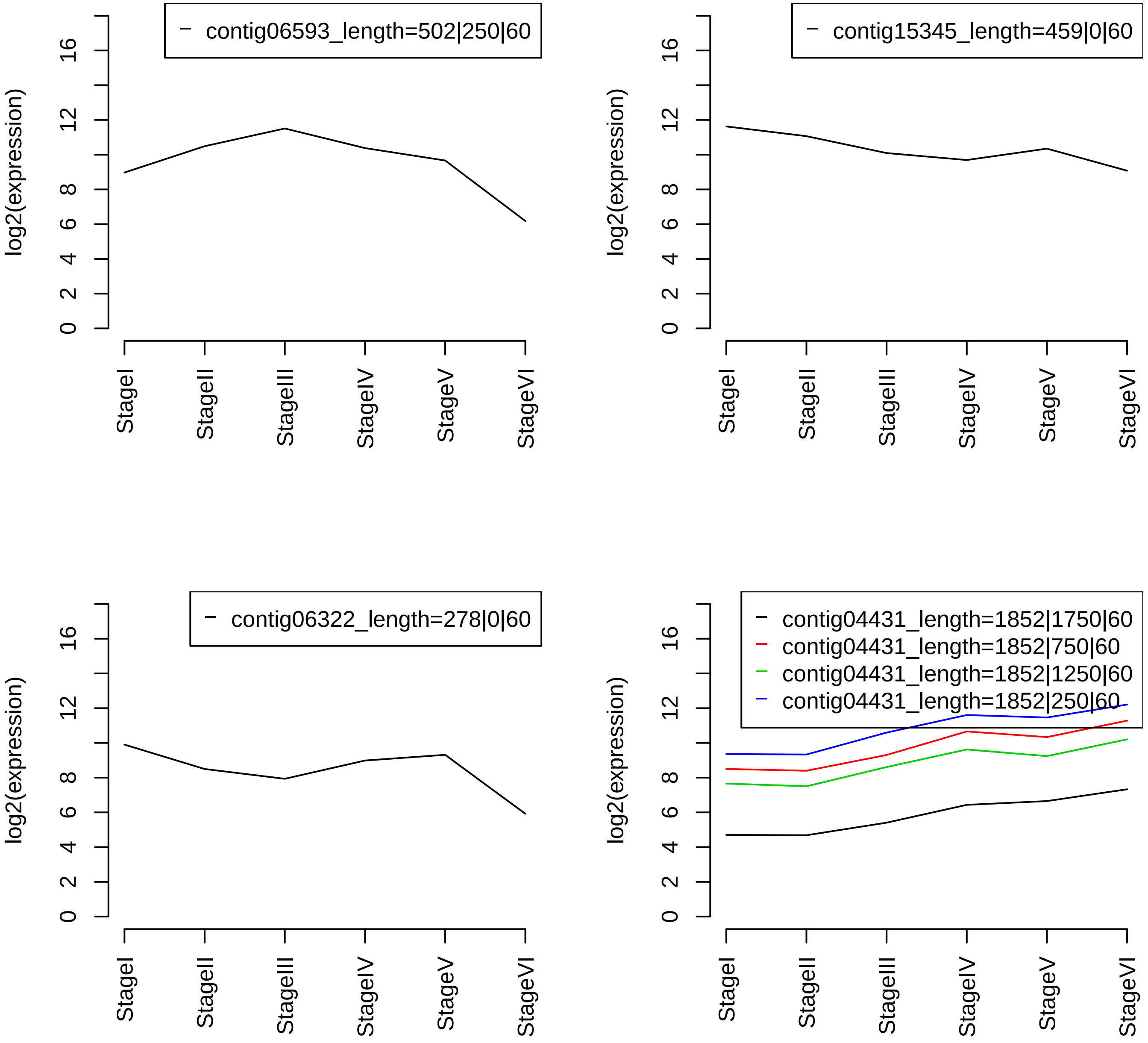

contig06593_length=502|250|60
contig15345_length=459|0|60
contig06322_length=278|0|60
contig04431_length=1852|1750|60
contig04431_length=1852|750|60
contig04431_length=1852|1250|60
contig04431_length=1852|250|60
log2(expression)
0
2
4
6
8
12
16
StageI
StageII
StageIII
StageIV
StageV
StageVI

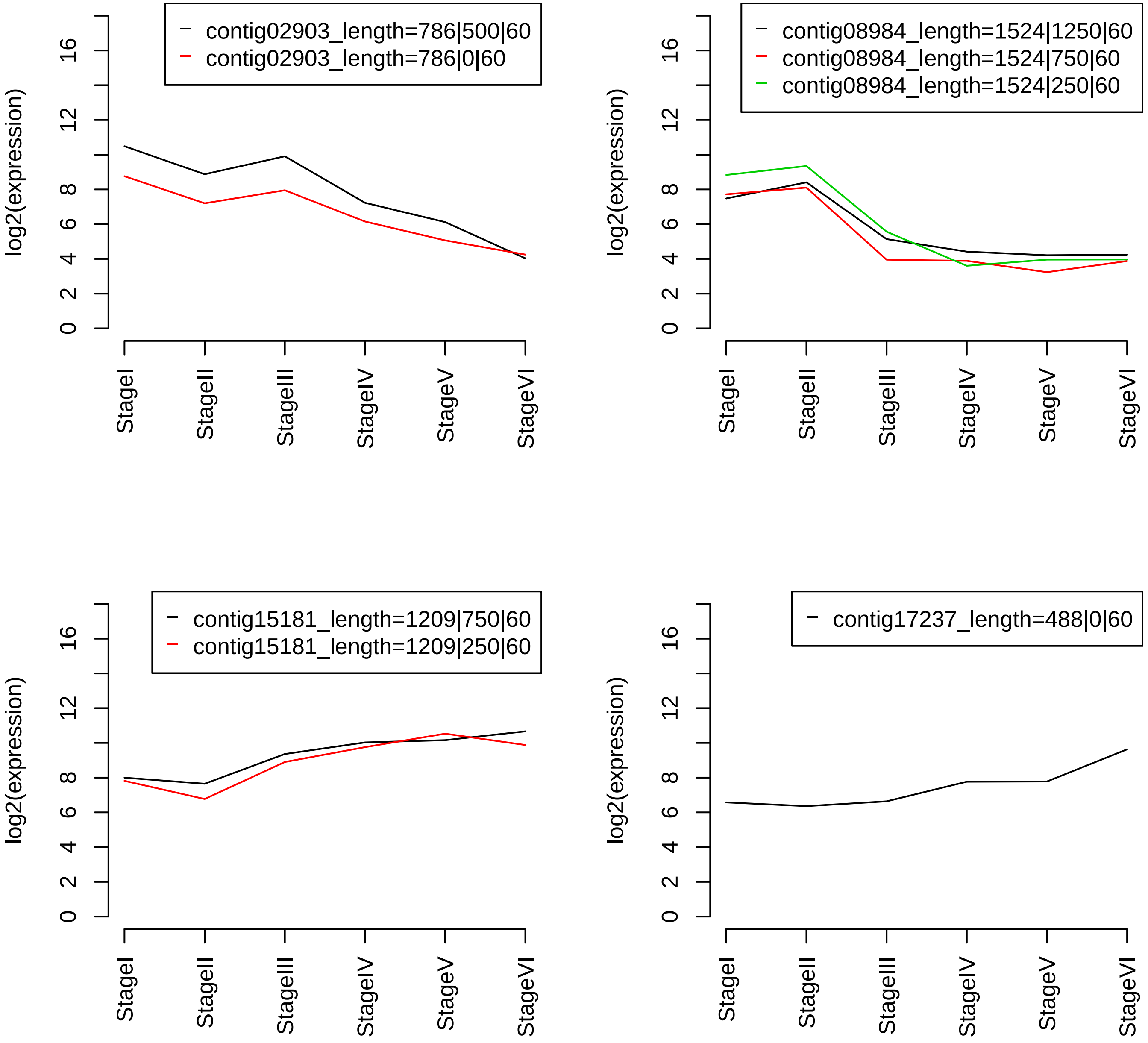
contig02903_length=786|500|60
contig02903_length=786|0|60
contig08984_length=1524|1250|60
contig08984_length=1524|750|60
contig08984_length=1524|250|60
contig15181_length=1209|750|60
contig15181_length=1209|250|60
contig17237_length=488|0|60
log2(expression)
0 2 4 6 8 12 16
StageI StageII StageIII StageIV StageV StageVI

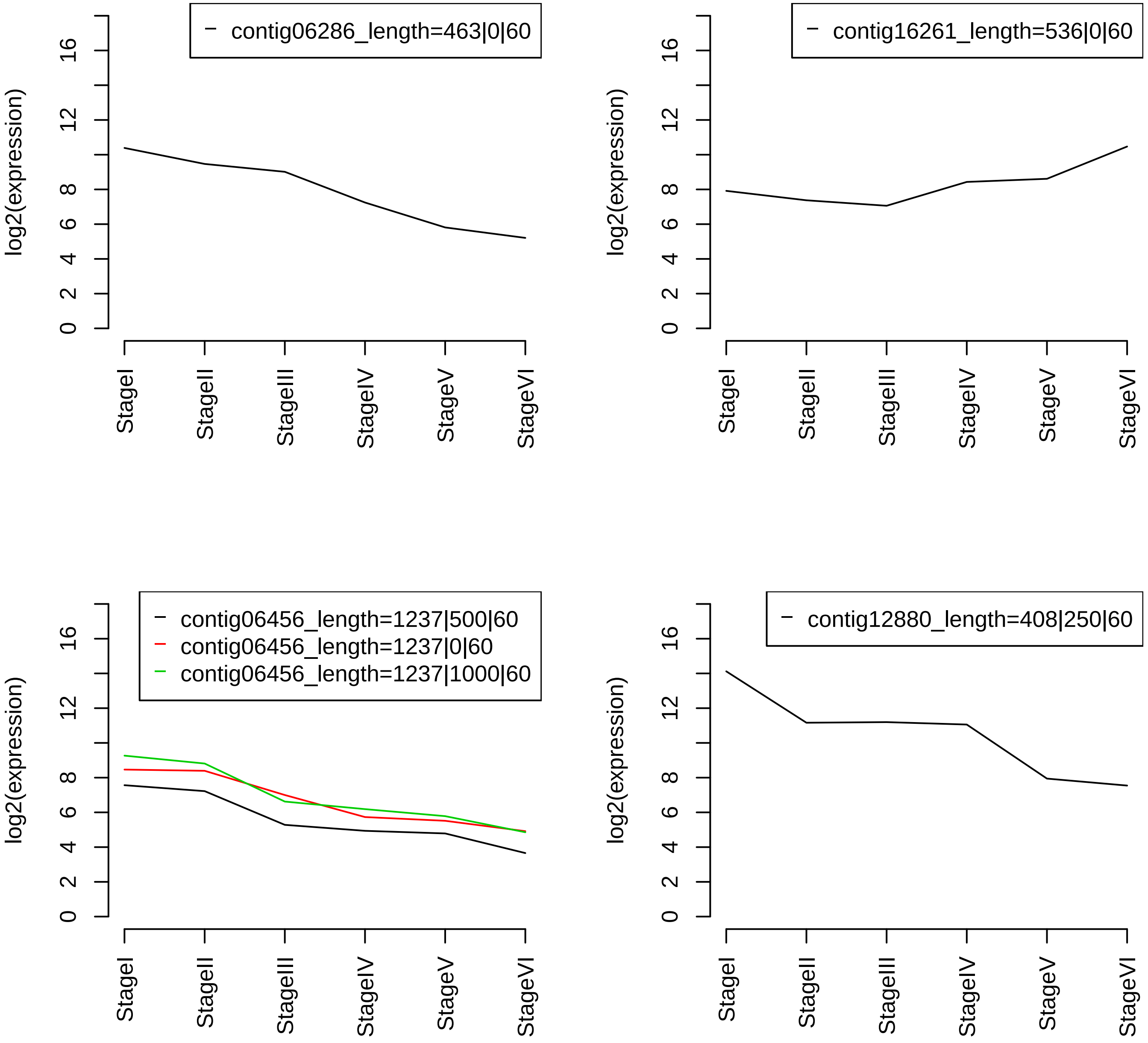
contig06286_length=463|0|60
log2(expression)
0
2
4
6
8
12
16
StageI
StageII
StageIII
StageIV
StageV
StageVI
contig16261_length=536|0|60
log2(expression)
0
2
4
6
8
12
16
StageI
StageII
StageIII
StageIV
StageV
StageVI
contig06456_length=1237|500|60
contig06456_length=1237|0|60
contig06456_length=1237|1000|60
log2(expression)
0
2
4
6
8
12
16
StageI
StageII
StageIII
StageIV
StageV
StageVI
contig12880_length=408|250|60
log2(expression)
0
2
4
6
8
12
16
StageI
StageII
StageIII
StageIV
StageV
StageVI

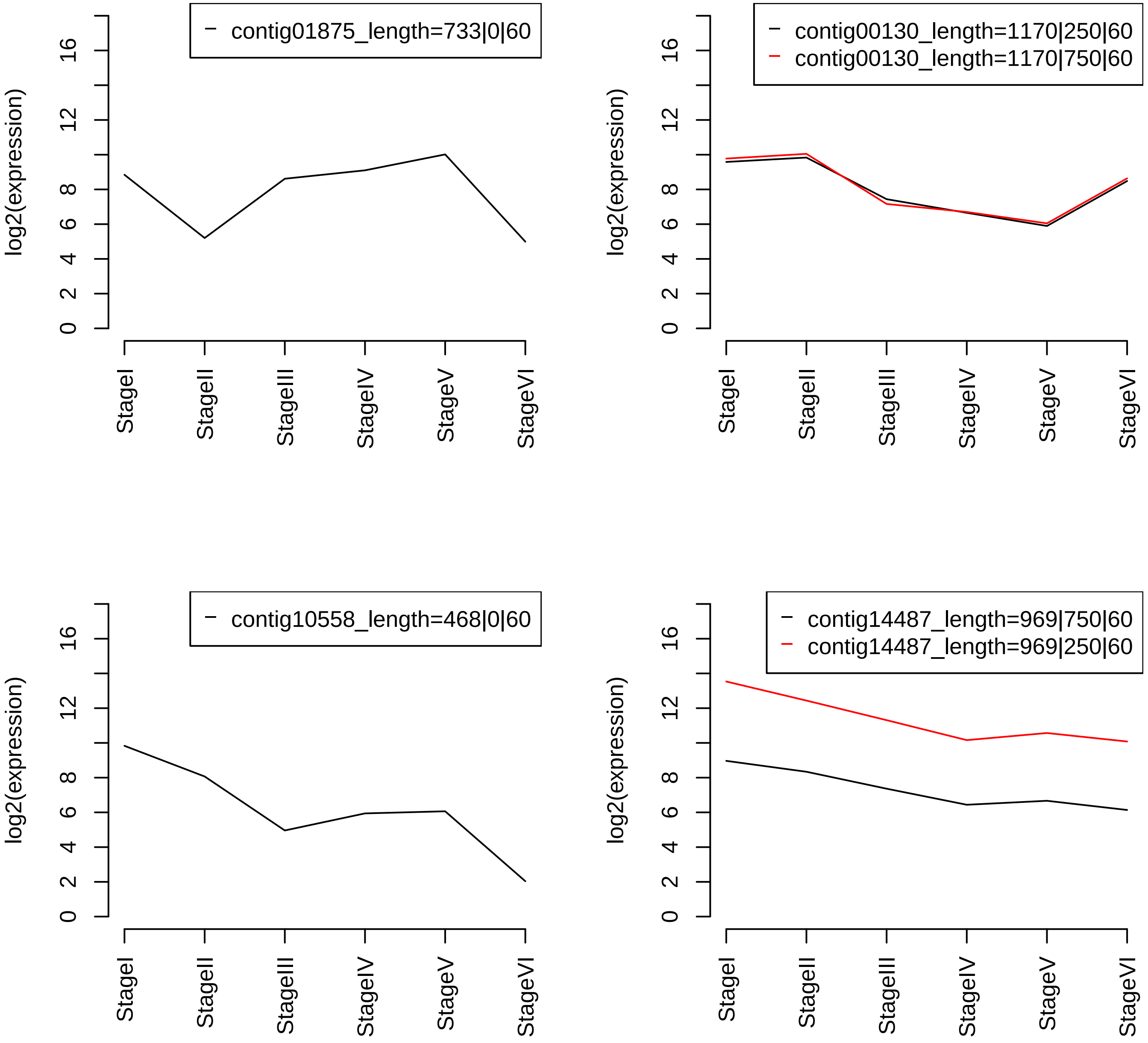

contig01875_length=733|0|60
contig00130_length=1170|250|60
contig00130_length=1170|750|60
contig10558_length=468|0|60
contig14487_length=969|750|60
contig14487_length=969|250|60
log2(expression)
0
2
4
6
8
12
16
StageI
StageII
StageIII
StageIV
StageV
StageVI

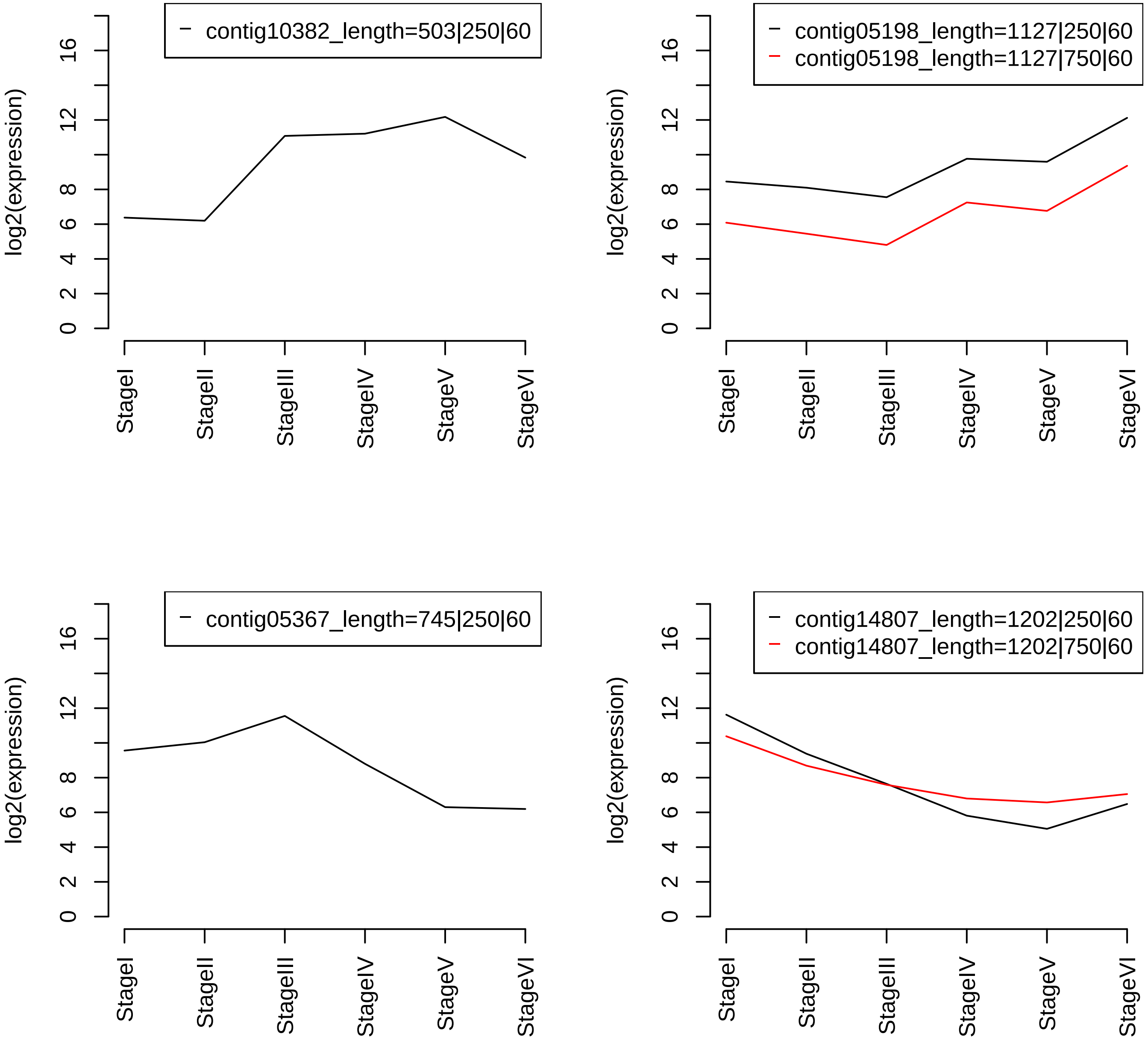
contig10382_length=503|250|60
contig05198_length=1127|250|60
contig05198_length=1127|750|60
contig05367_length=745|250|60
contig14807_length=1202|250|60
contig14807_length=1202|750|60
log2(expression)
0
2
4
6
8
12
16
StageI
StageII
StageIII
StageIV
StageV
StageVI

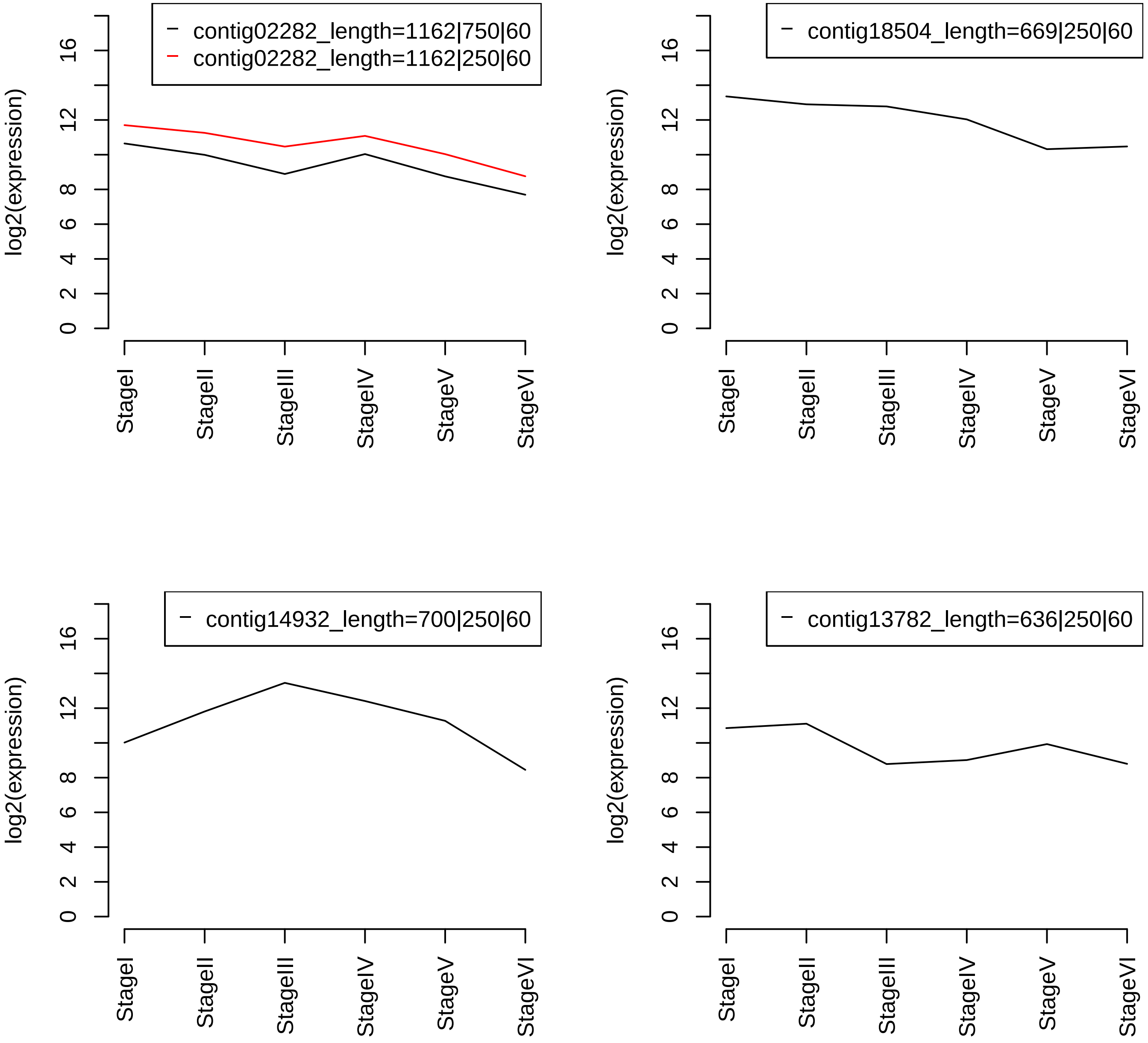
contig02282_length=1162|750|60
contig02282_length=1162|250|60
contig18504_length=669|250|60
contig14932_length=700|250|60
contig13782_length=636|250|60
log2(expression)
0
2
4
6
8
12
16
StageI
StageII
StageIII
StageIV
StageV
StageVI

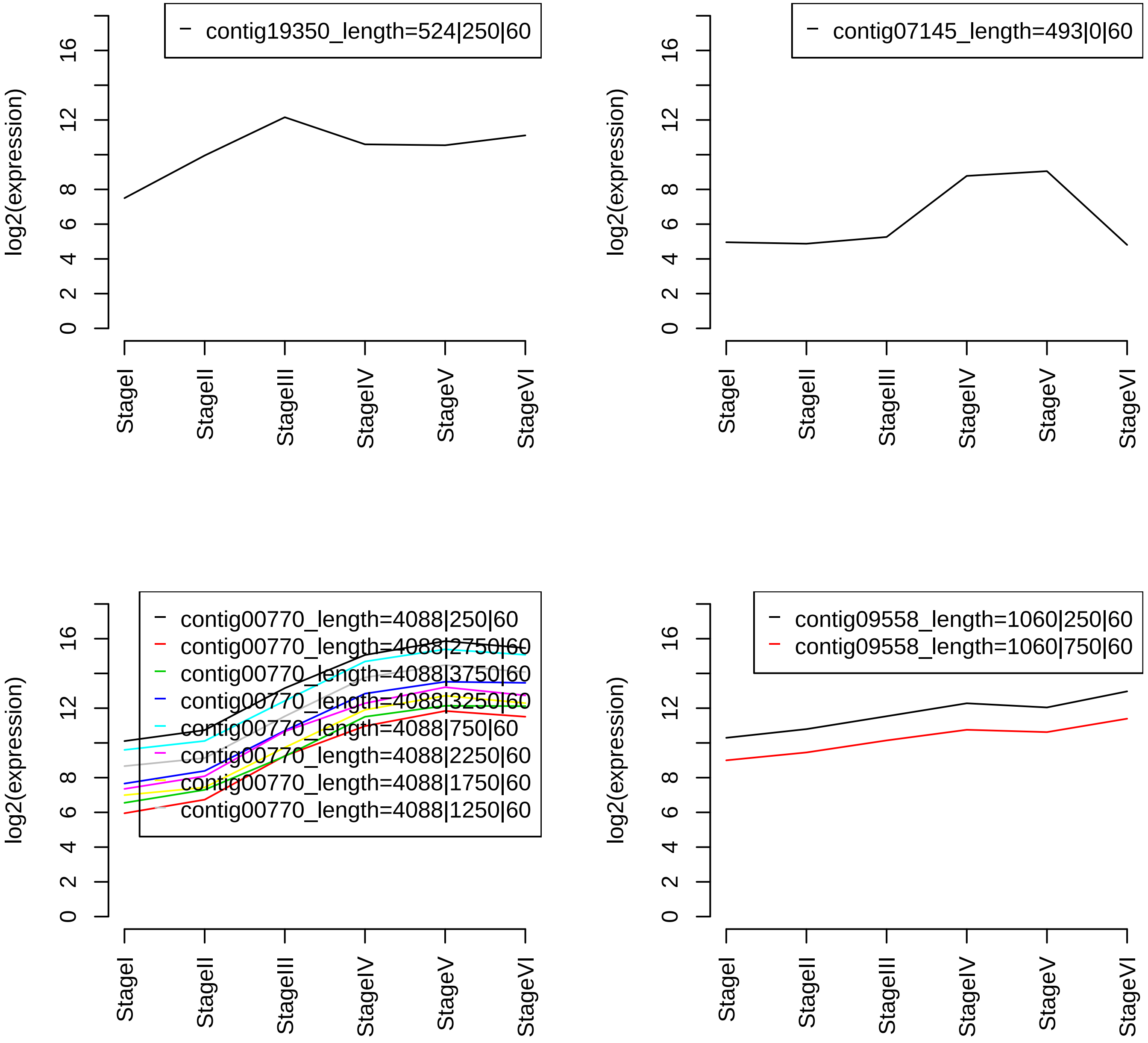
contig19350_length=524|250|60
contig07145_length=493|0|60
contig00770_length=4088|250|60
contig00770_length=4088|2750|60
contig00770_length=4088|3750|60
contig00770_length=4088|3250|60
contig00770_length=4088|750|60
contig00770_length=4088|2250|60
contig00770_length=4088|1750|60
contig00770_length=4088|1250|60
contig09558_length=1060|250|60
contig09558_length=1060|750|60
log2(expression)
0 2 4 6 8 12 16
StageI StageII StageIII StageIV StageV StageVI

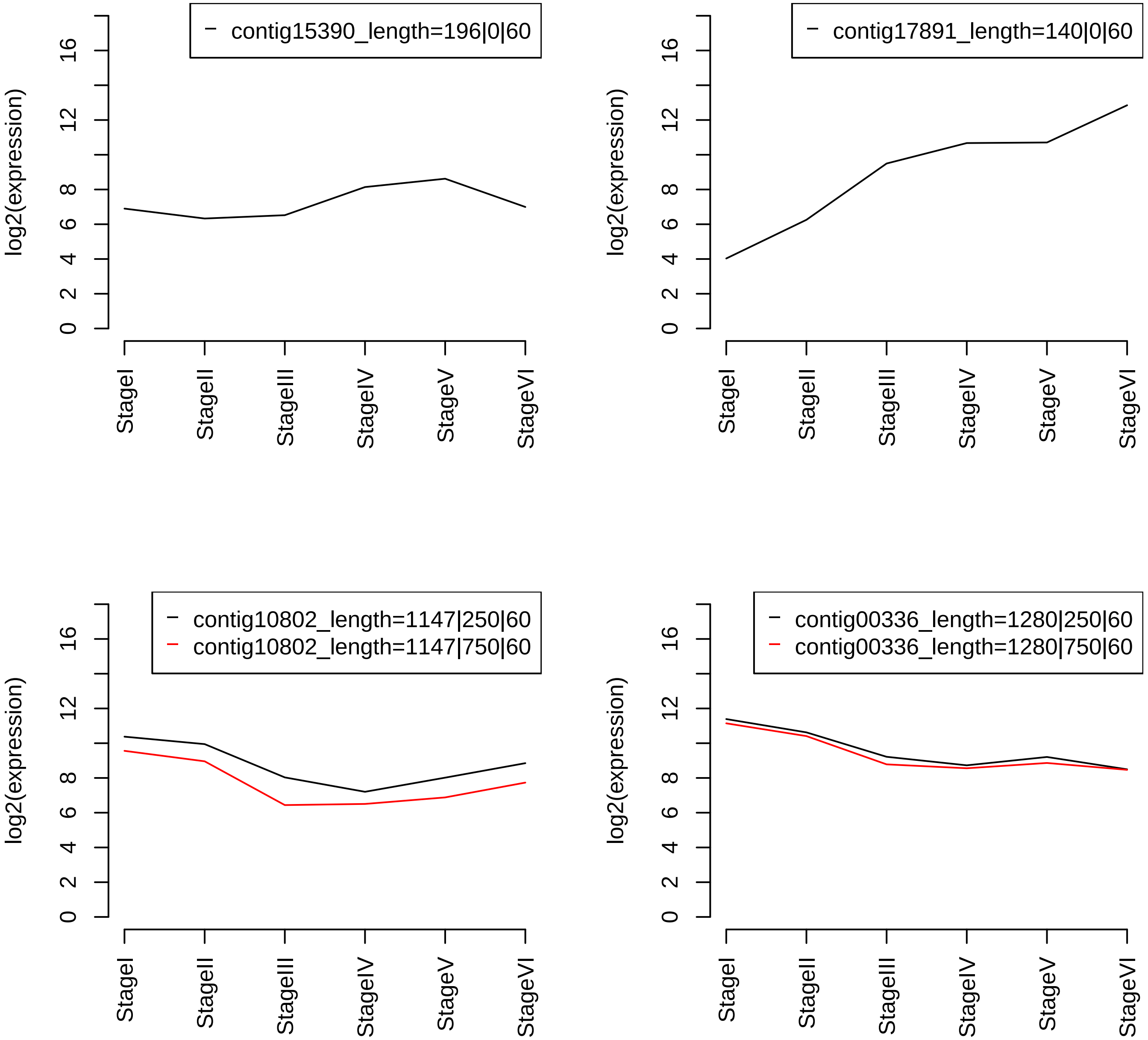

contig15390_length=196|0|60
contig17891_length=140|0|60
contig10802_length=1147|250|60
contig10802_length=1147|750|60
contig00336_length=1280|250|60
contig00336_length=1280|750|60
log2(expression)
0
2
4
6
8
12
16
StageI
StageII
StageIII
StageIV
StageV
StageVI

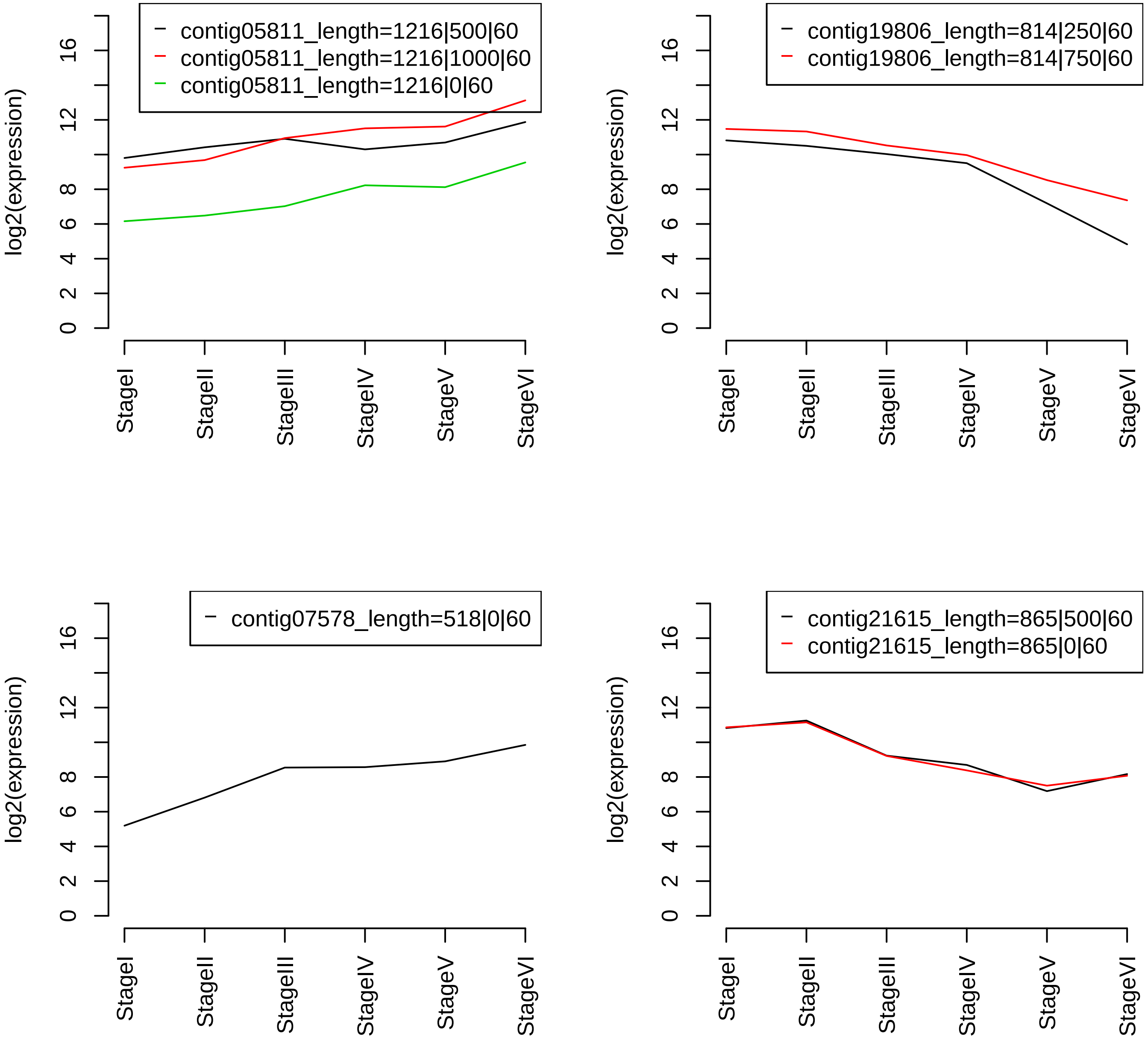
contig05811_length=1216|500|60
contig05811_length=1216|1000|60
contig05811_length=1216|0|60
contig19806_length=814|250|60
contig19806_length=814|750|60
contig07578_length=518|0|60
contig21615_length=865|500|60
contig21615_length=865|0|60
log2(expression)
0 2 4 6 8 12 16
StageI StageII StageIII StageIV StageV StageVI

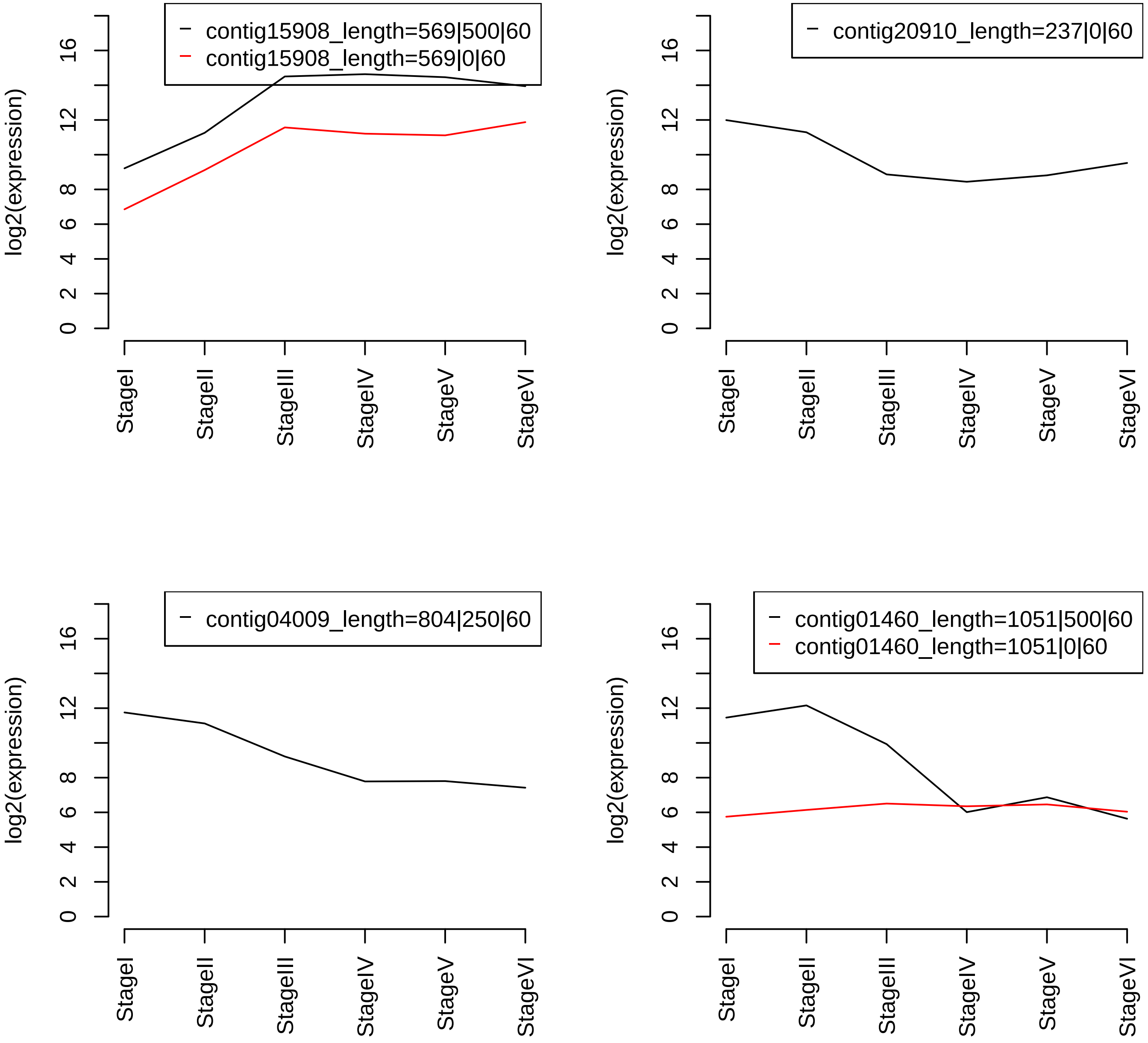
contig15908_length=569|500|60
contig15908_length=569|0|60
contig20910_length=237|0|60
contig04009_length=804|250|60
contig01460_length=1051|500|60
contig01460_length=1051|0|60
log2(expression)
0
2
4
6
8
12
16
StageI
StageII
StageIII
StageIV
StageV
StageVI

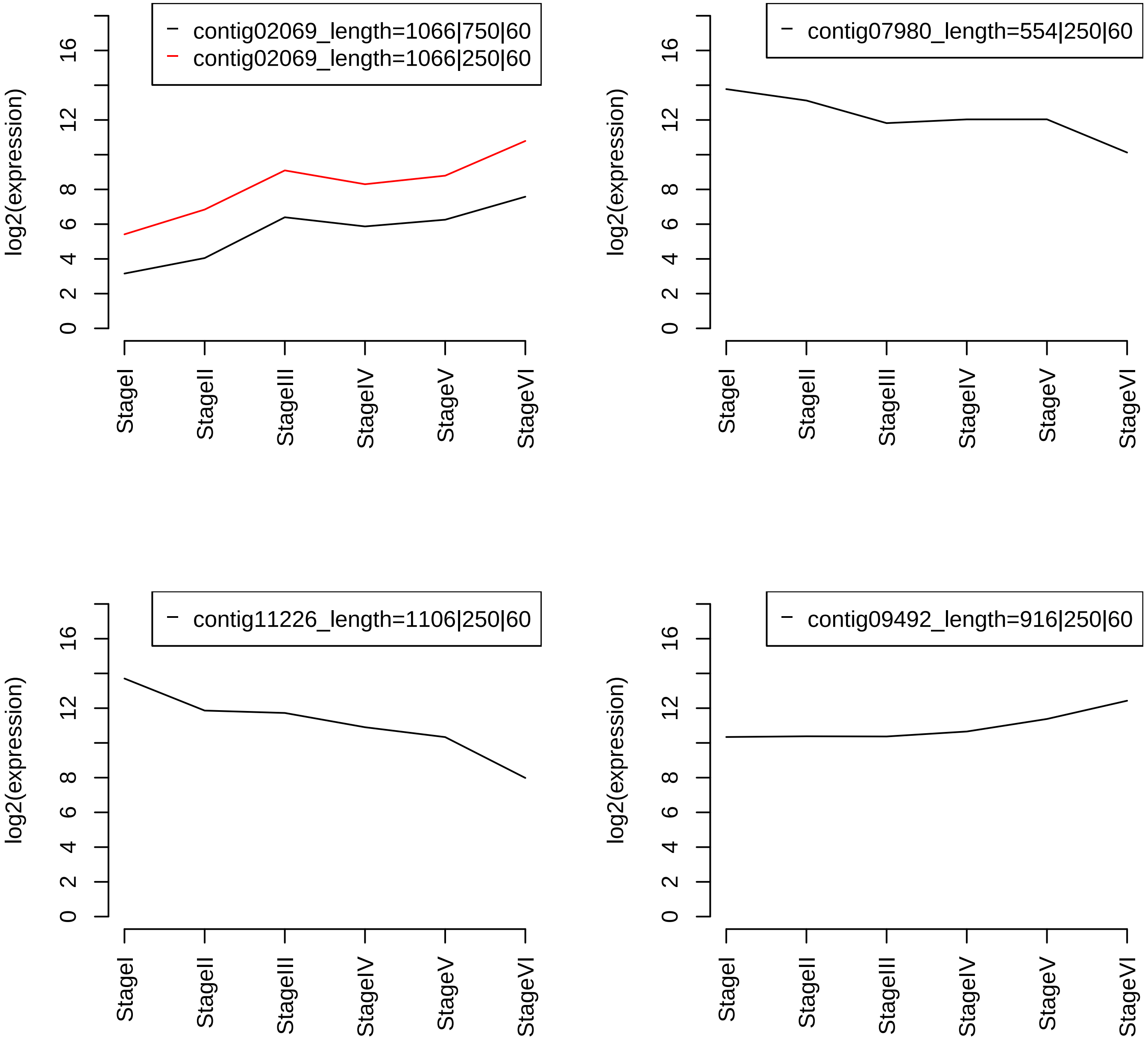

contig02069_length=1066|750|60
contig02069_length=1066|250|60
contig07980_length=554|250|60
contig11226_length=1106|250|60
contig09492_length=916|250|60
log2(expression)
0
2
4
6
8
12
16
StageI
StageII
StageIII
StageIV
StageV
StageVI

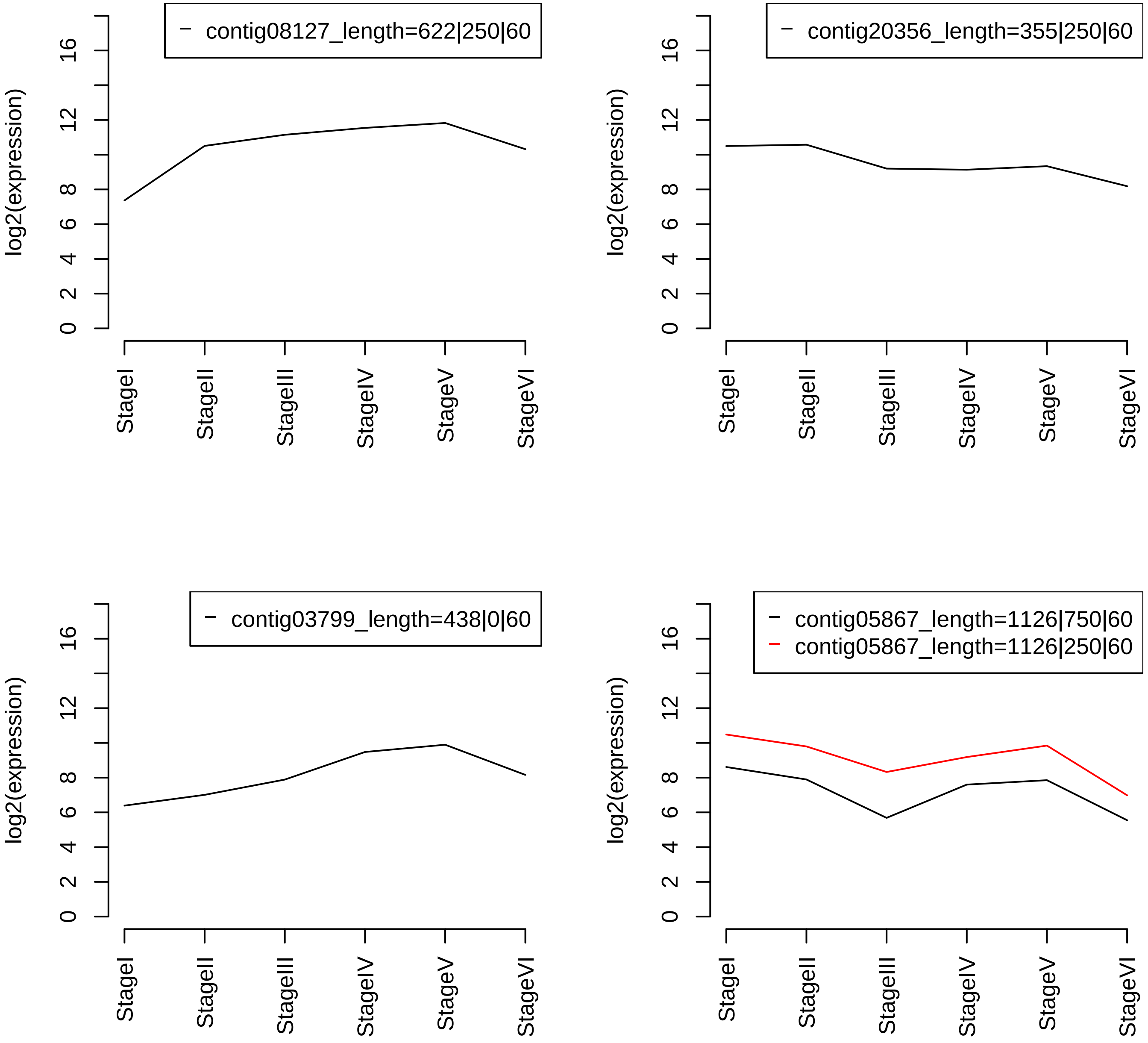
contig08127_length=622|250|60
contig20356_length=355|250|60
contig03799_length=438|0|60
contig05867_length=1126|750|60
contig05867_length=1126|250|60
log2(expression)
0
2
4
6
8
12
16
StageI
StageII
StageIII
StageIV
StageV
StageVI

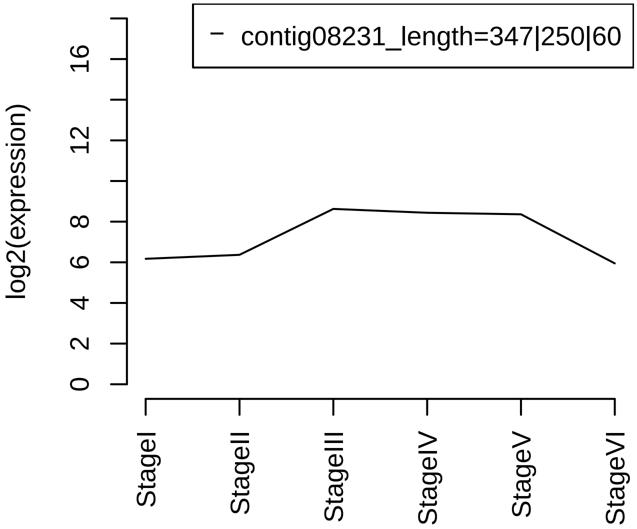
contig08231_length=347|250|60
log2(expression)
0
2
4
6
8
12
16
StageI
StageII
StageIII
StageIV
StageV
StageVI

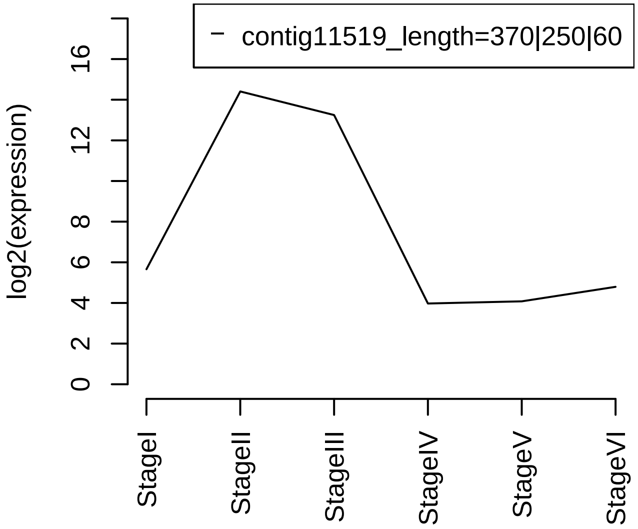
contig11519_length=370|250|60
log2(expression)
0
2
4
6
8
12
16
StageI
StageII
StageIII
StageIV
StageV
StageVI

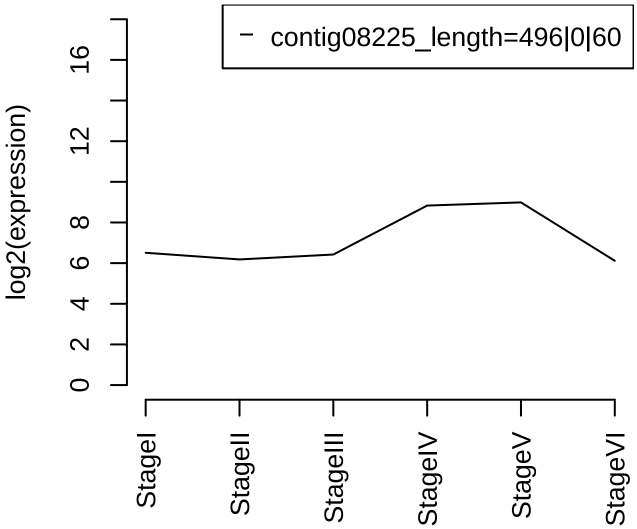
contig08225_length=496|0|60
log2(expression)
0
2
4
6
8
12
16
StageI
StageII
StageIII
StageIV
StageV
StageVI

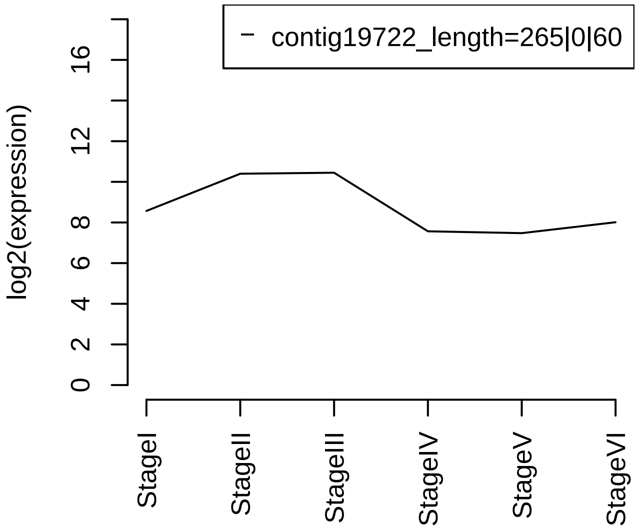
contig19722_length=265|0|60
log2(expression)
0
2
4
6
8
12
16
StageI
StageII
StageIII
StageIV
StageV
StageVI

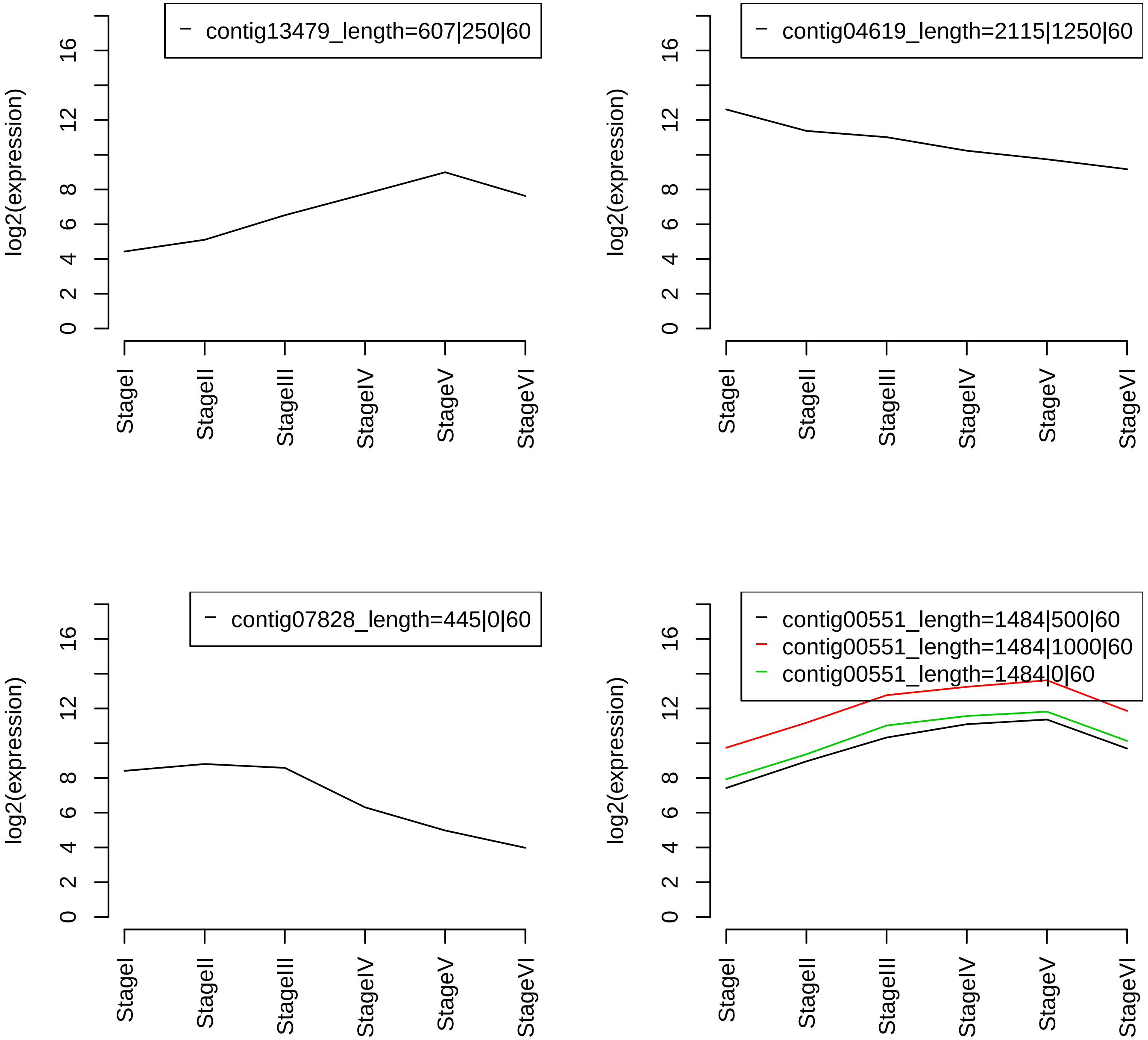

contig13479_length=607|250|60
contig04619_length=2115|1250|60
contig07828_length=445|0|60
contig00551_length=1484|500|60
contig00551_length=1484|1000|60
contig00551_length=1484|0|60
log2(expression)
0 2 4 6 8 12 16
StageI StageII StageIII StageIV StageV StageVI

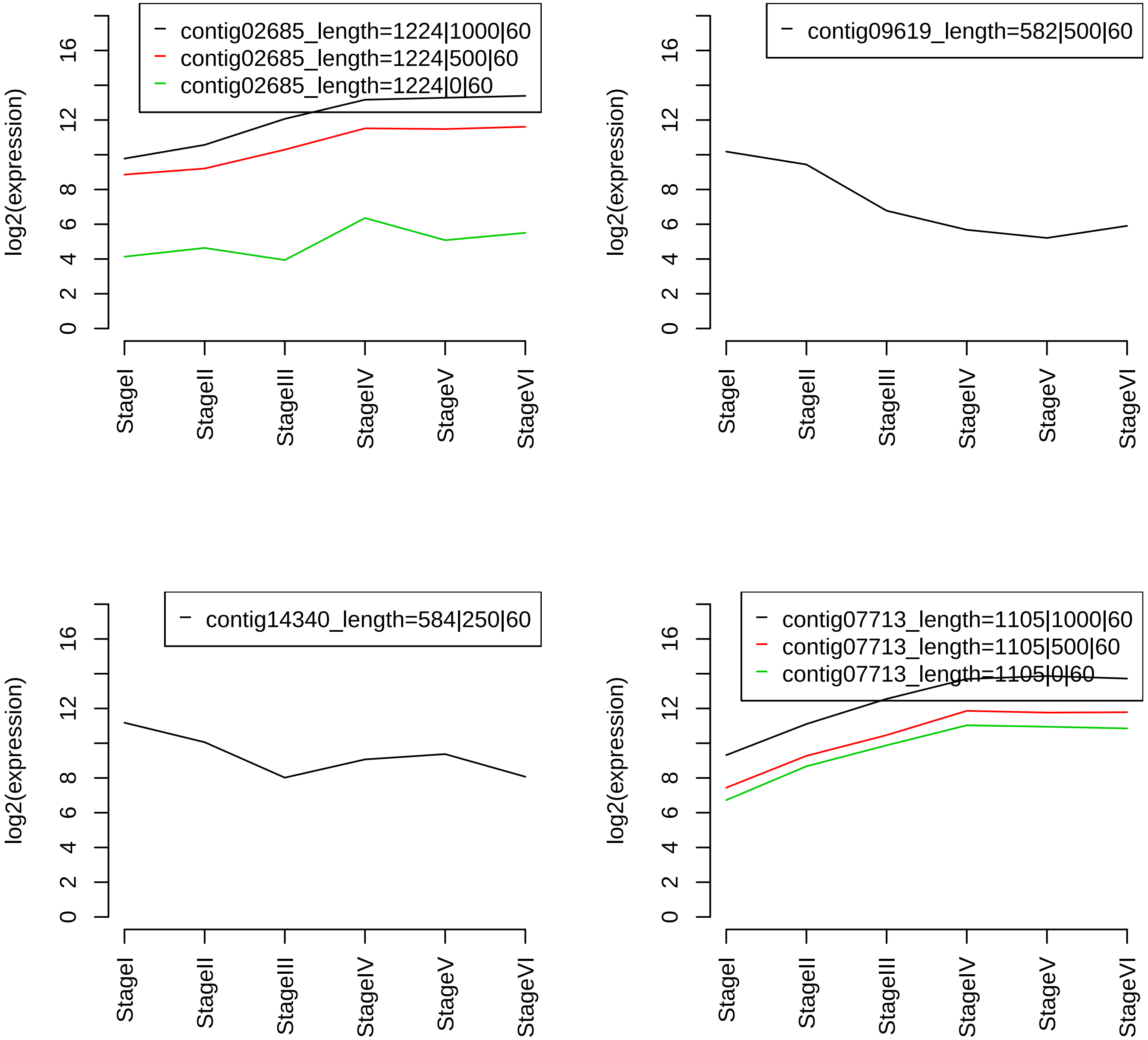

contig02685_length=1224|1000|60
contig02685_length=1224|500|60
contig02685_length=1224|0|60
contig09619_length=582|500|60
contig14340_length=584|250|60
contig07713_length=1105|1000|60
contig07713_length=1105|500|60
contig07713_length=1105|0|60
log2(expression)
0
2
4
6
8
12
16
StageI
StageII
StageIII
StageIV
StageV
StageVI

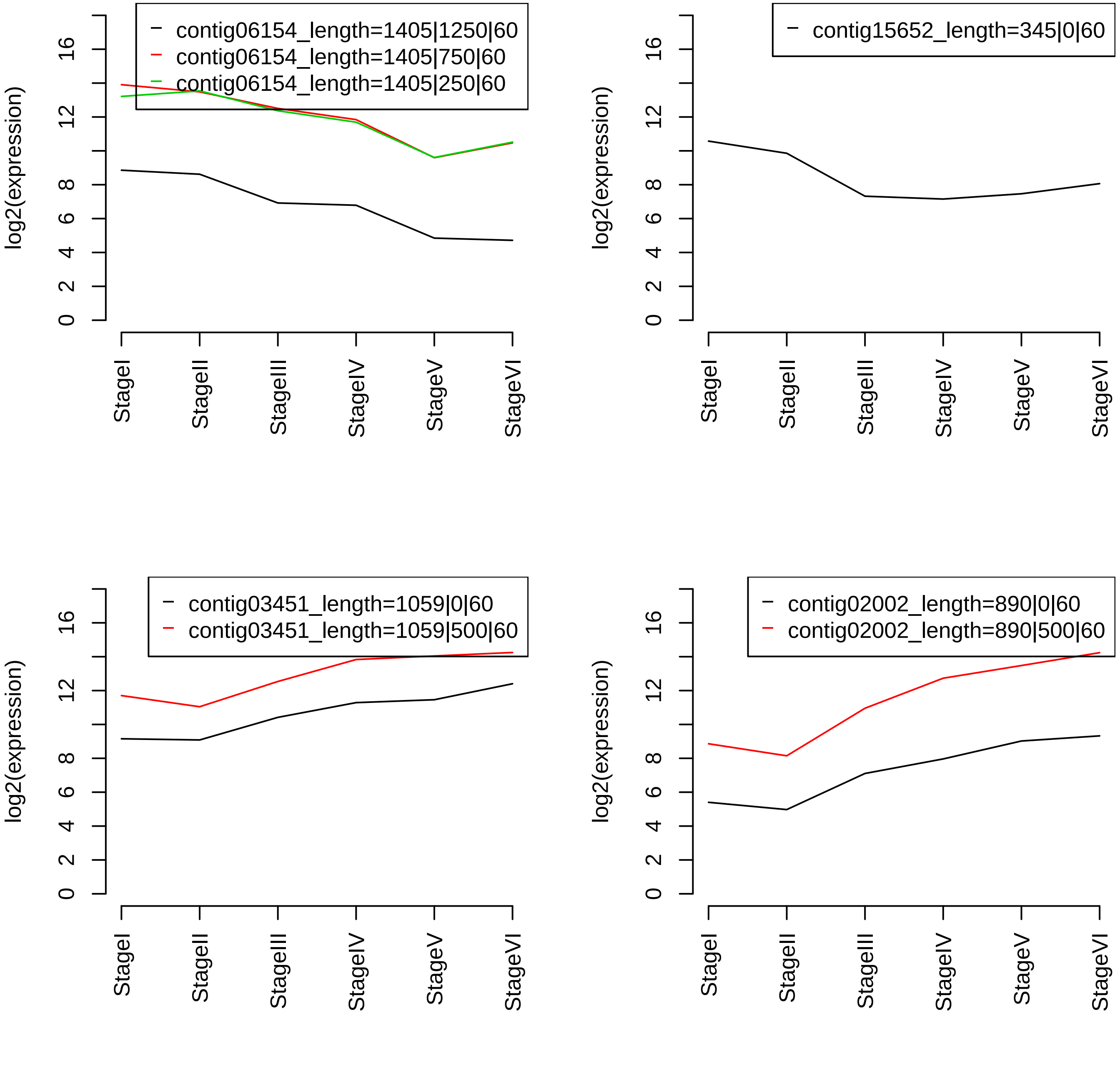

contig06154_length=1405|1250|60
contig06154_length=1405|750|60
contig06154_length=1405|250|60
contig15652_length=345|0|60
contig03451_length=1059|0|60
contig03451_length=1059|500|60
contig02002_length=890|0|60
contig02002_length=890|500|60
log2(expression)
0 2 4 6 8 12 16
StageI StageII StageIII StageIV StageV StageVI

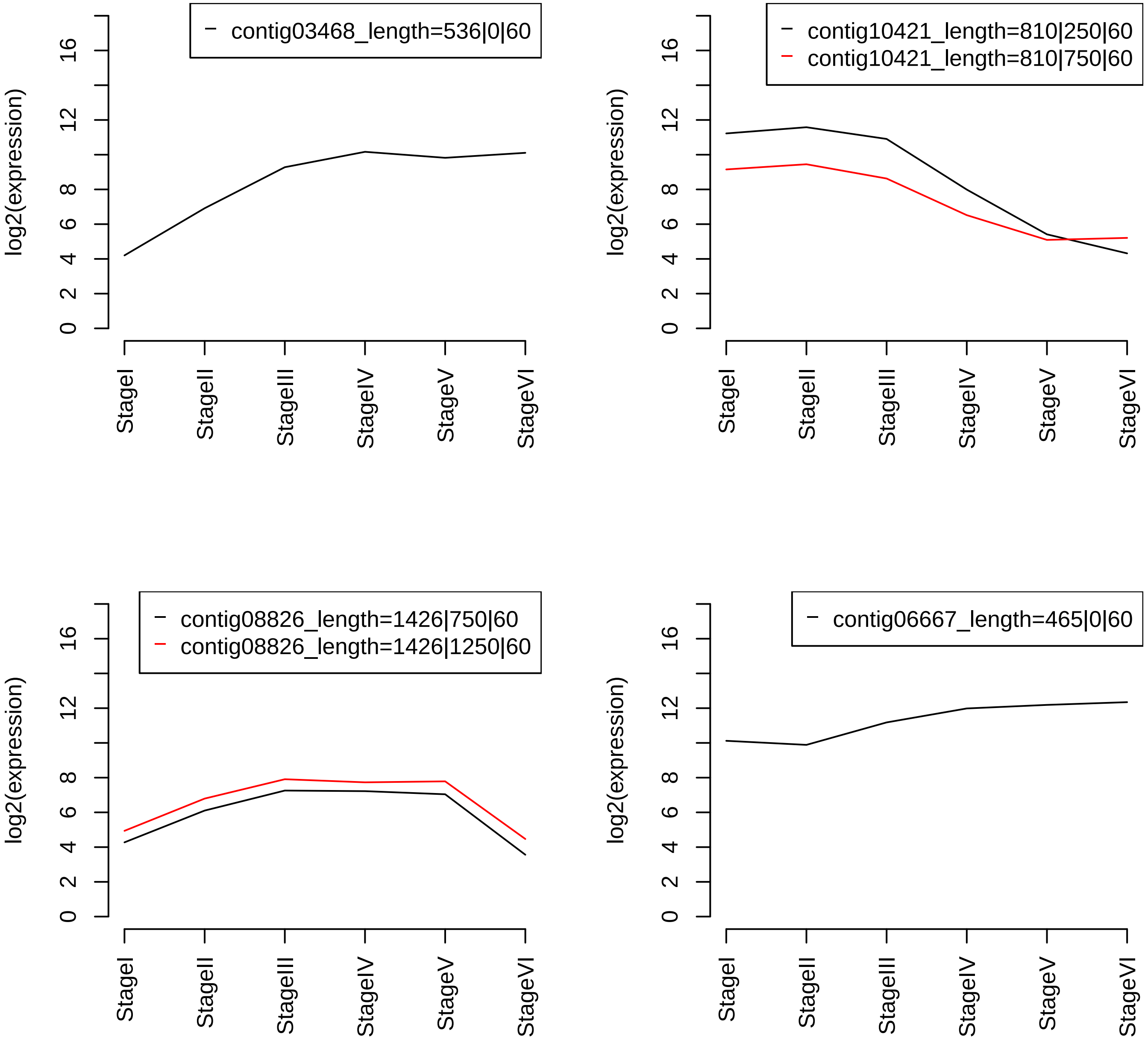

contig03468_length=536|0|60
contig10421_length=810|250|60
contig10421_length=810|750|60
contig08826_length=1426|750|60
contig08826_length=1426|1250|60
contig06667_length=465|0|60
log2(expression)
0
2
4
6
8
12
16
StageI
StageII
StageIII
StageIV
StageV
StageVI

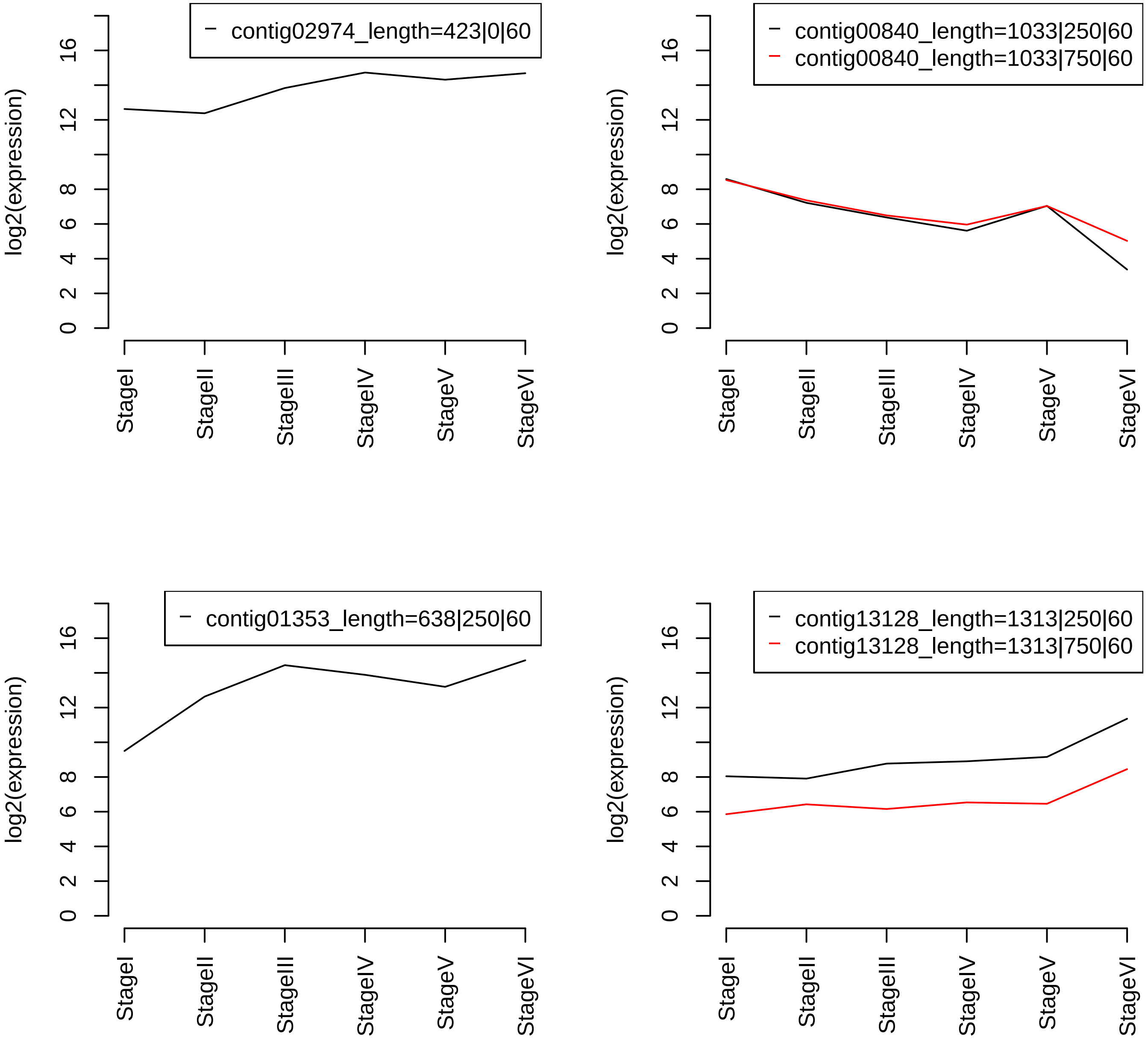
contig02974_length=423|0|60
contig00840_length=1033|250|60
contig00840_length=1033|750|60
contig01353_length=638|250|60
contig13128_length=1313|250|60
contig13128_length=1313|750|60
log2(expression)
0 2 4 6 8 12 16
StageI StageII StageIII StageIV StageV StageVI

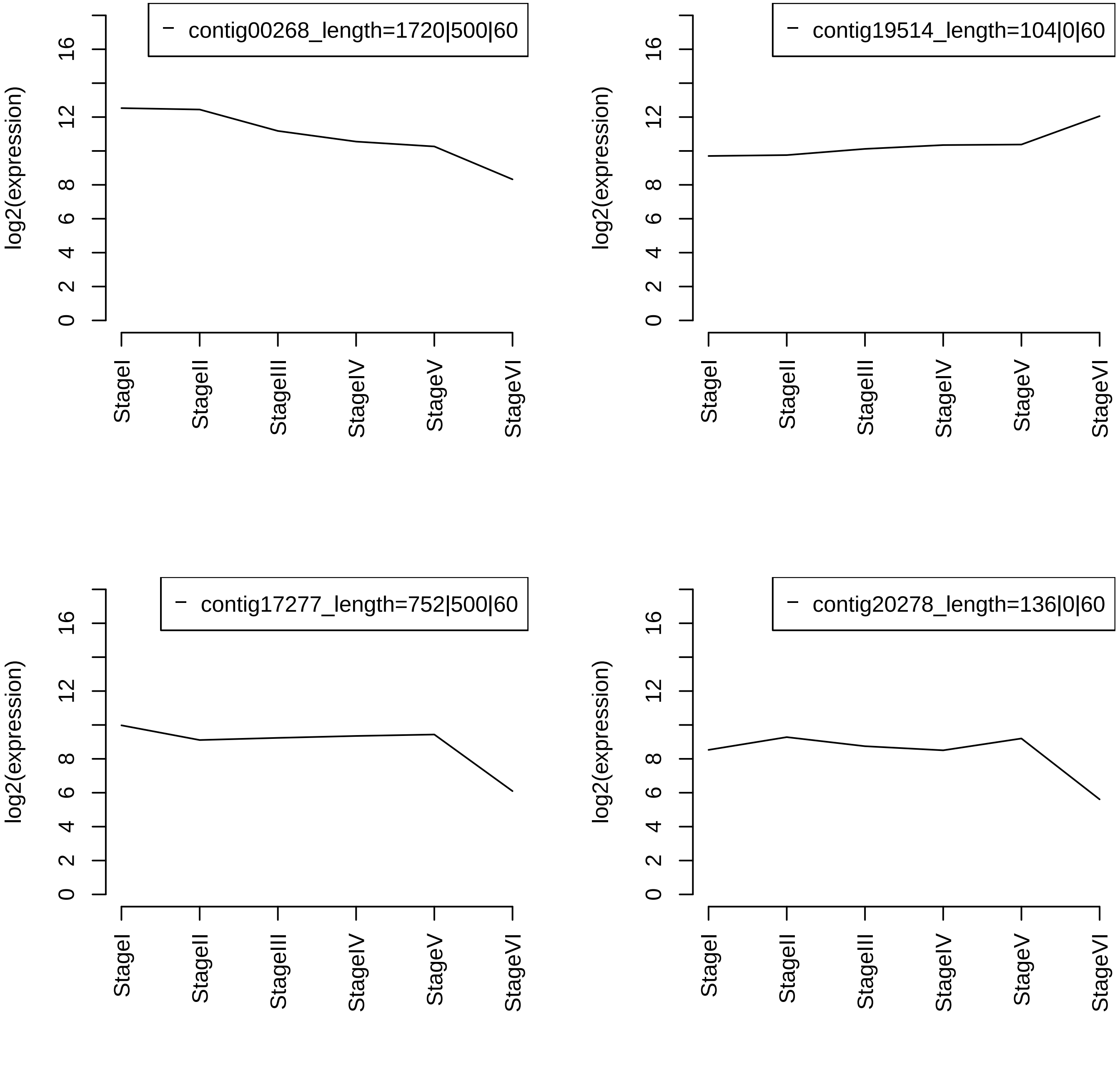
contig00268_length=1720|500|60
contig19514_length=104|0|60
contig17277_length=752|500|60
contig20278_length=136|0|60
log2(expression)
0
2
4
6
8
12
16
StageI
StageII
StageIII
StageIV
StageV
StageVI

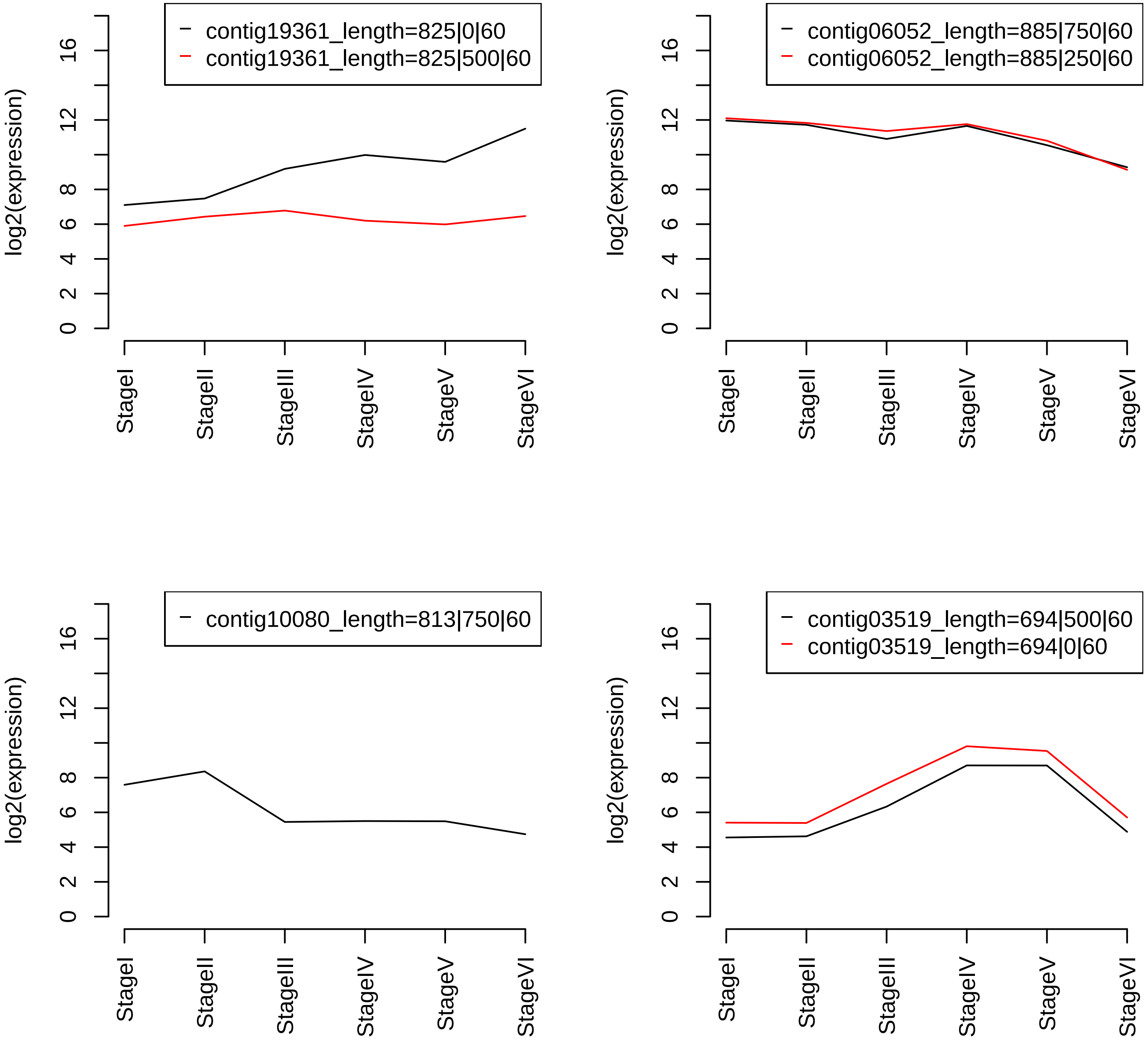

contig19361_length=825|0|60
contig19361_length=825|500|60
contig06052_length=885|750|60
contig06052_length=885|250|60
contig10080_length=813|750|60
contig03519_length=694|500|60
contig03519_length=694|0|60
log2(expression)
StageI
StageII
StageIII
StageIV
StageV
StageVI

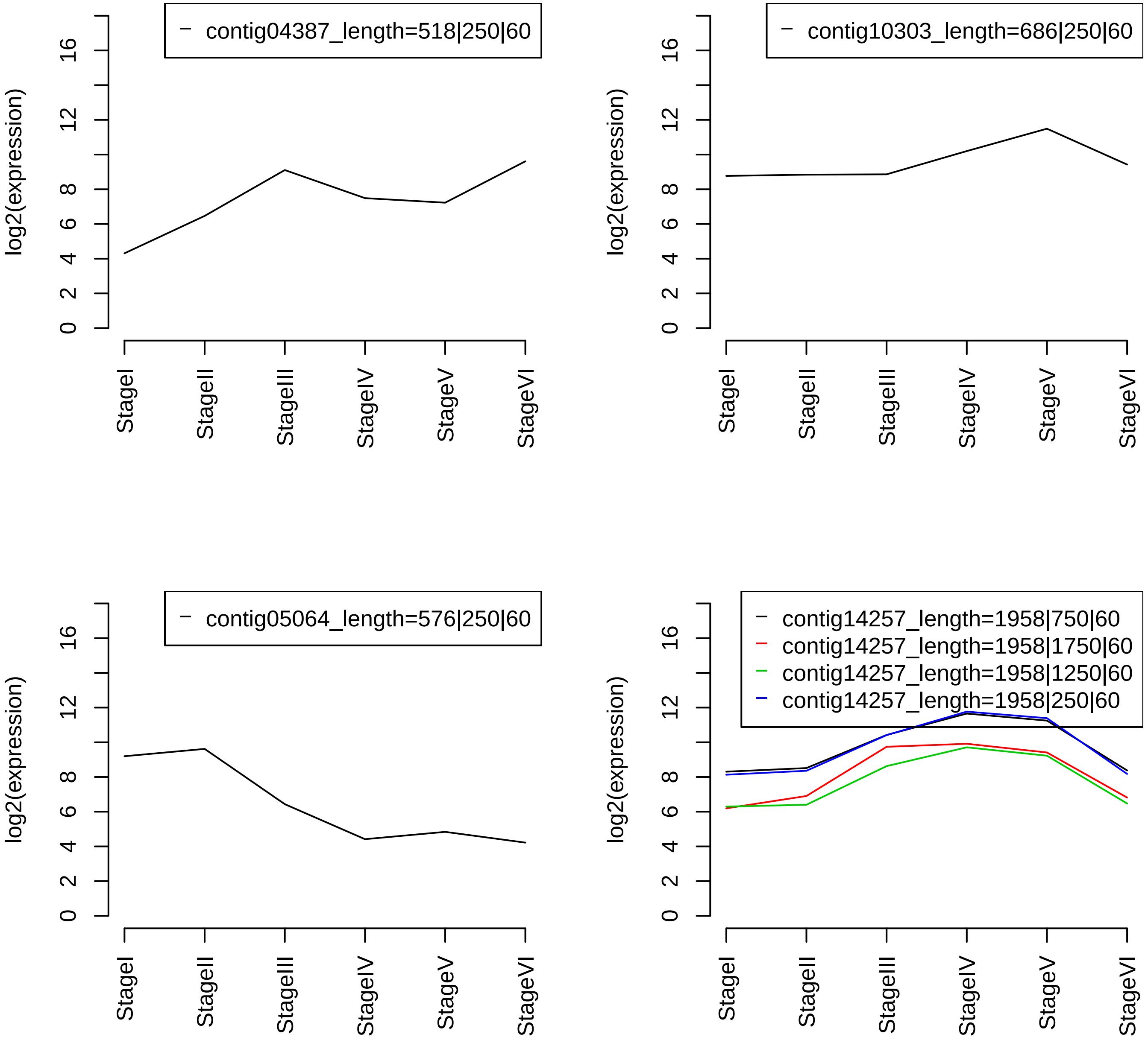
contig04387_length=518|250|60
contig10303_length=686|250|60
contig05064_length=576|250|60
contig14257_length=1958|750|60
contig14257_length=1958|1750|60
contig14257_length=1958|1250|60
contig14257_length=1958|250|60
log2(expression)
0
2
4
6
8
12
16
StageI
StageII
StageIII
StageIV
StageV
StageVI

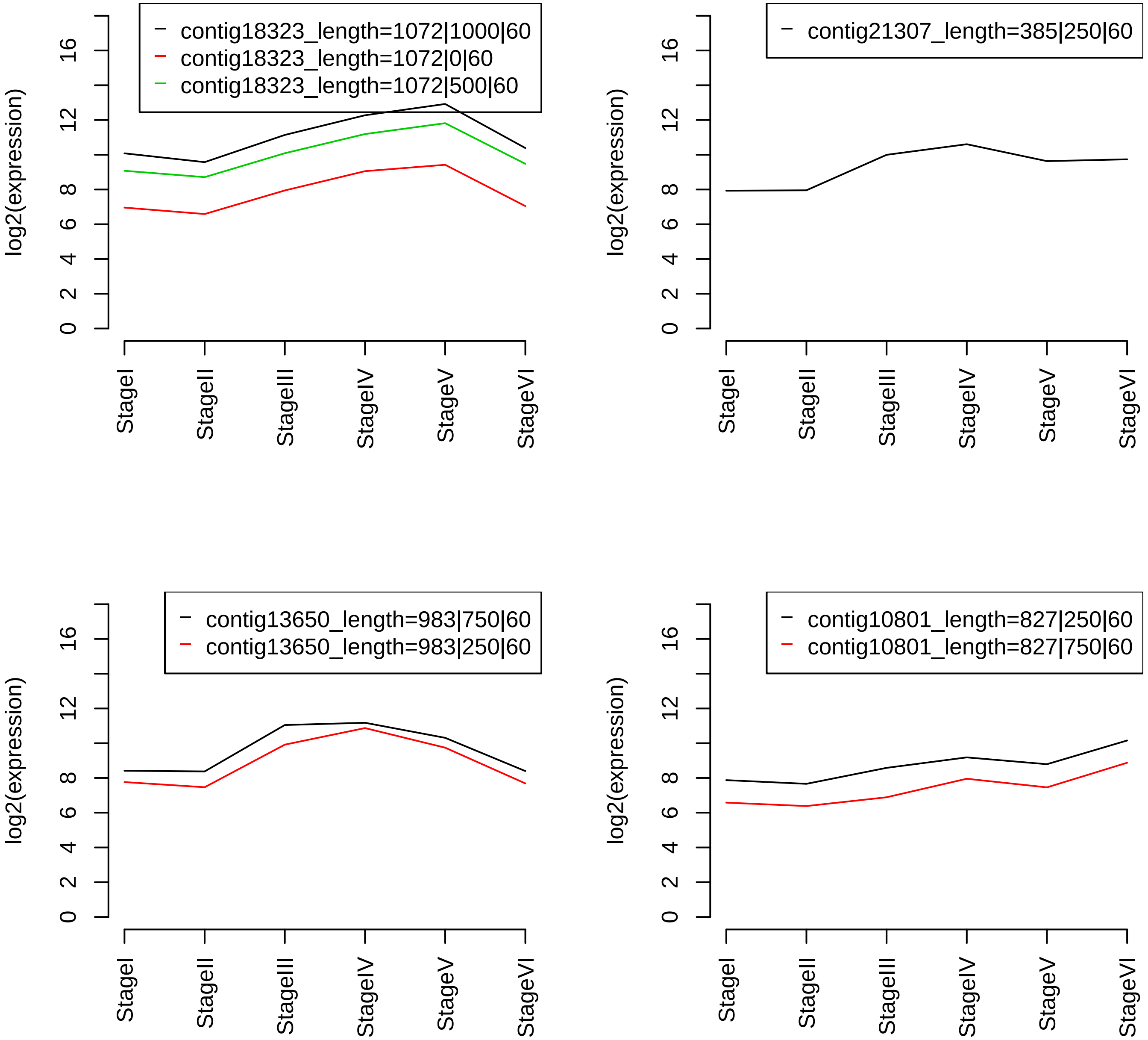
contig18323_length=1072|1000|60
contig18323_length=1072|0|60
contig18323_length=1072|500|60
log2(expression)
0
2
4
6
8
12
16
StageI
StageII
StageIII
StageIV
StageV
StageVI
contig21307_length=385|250|60
contig13650_length=983|750|60
contig13650_length=983|250|60
contig10801_length=827|250|60
contig10801_length=827|750|60

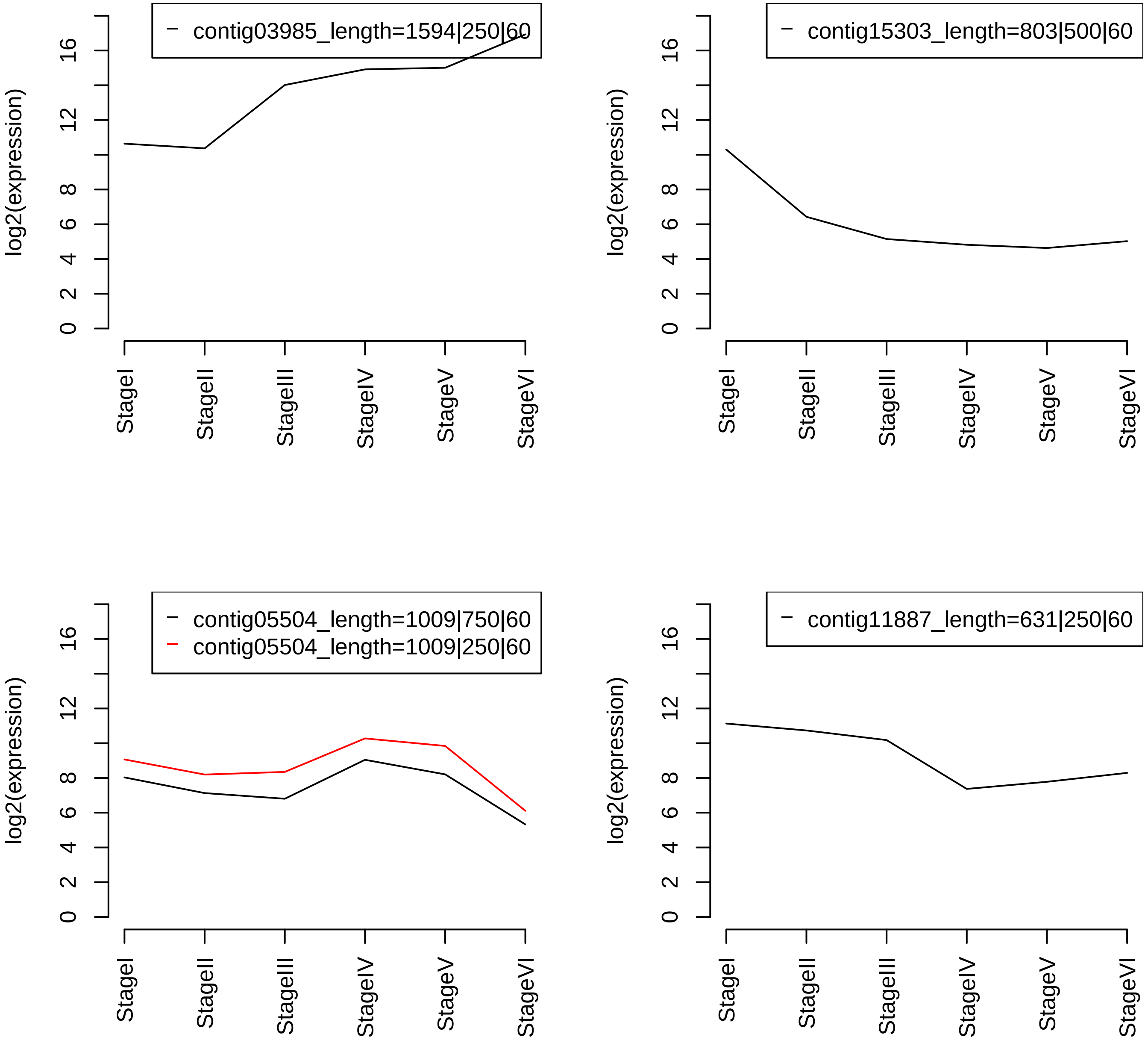

contig03985_length=1594|250|60
contig15303_length=803|500|60
contig05504_length=1009|750|60
contig05504_length=1009|250|60
contig11887_length=631|250|60
log2(expression)
0
2
4
6
8
12
16
StageI
StageII
StageIII
StageIV
StageV
StageVI

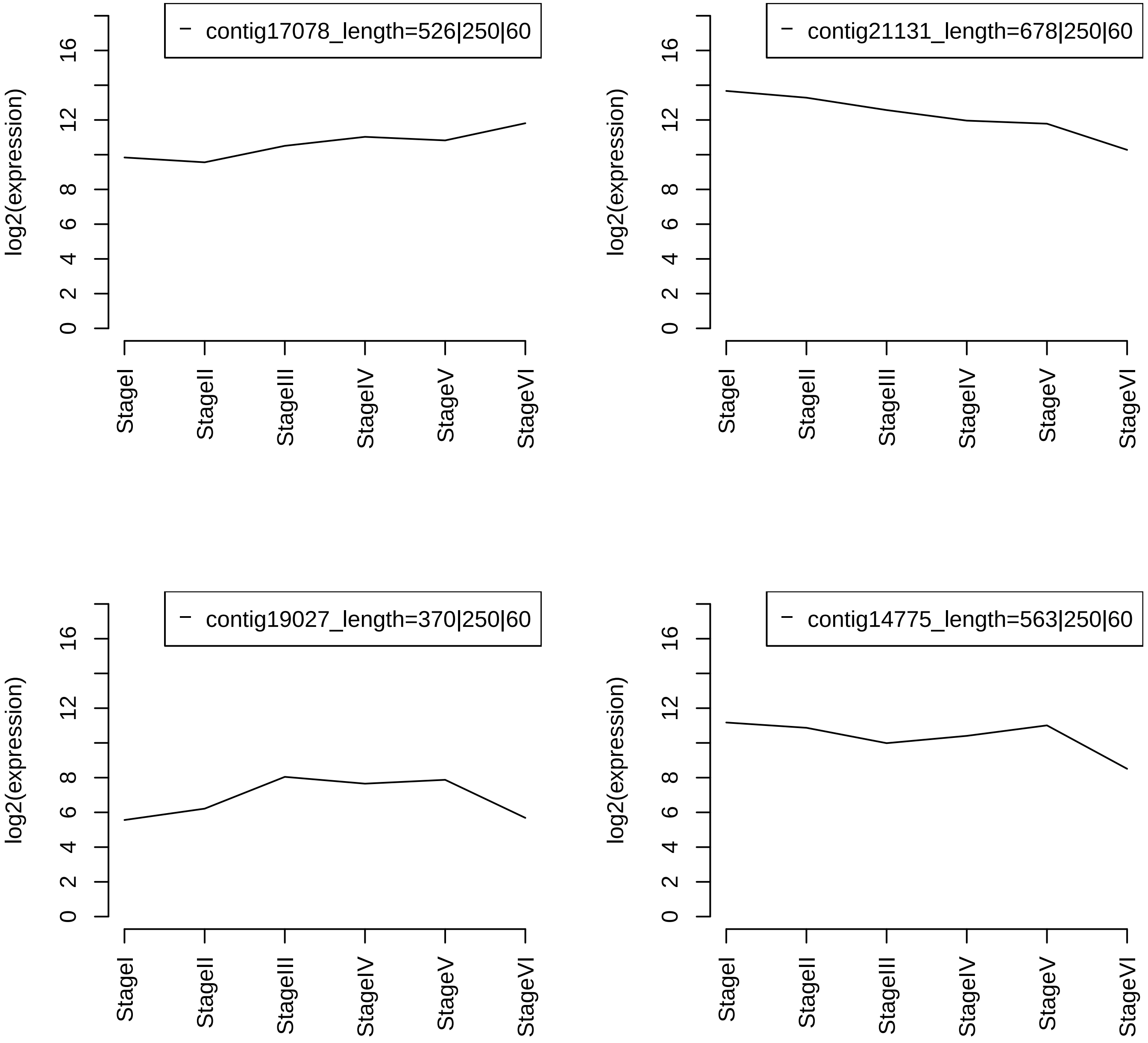

contig17078_length=526|250|60
contig21131_length=678|250|60
contig19027_length=370|250|60
contig14775_length=563|250|60
log2(expression)
0
2
4
6
8
12
16
StageI
StageII
StageIII
StageIV
StageV
StageVI

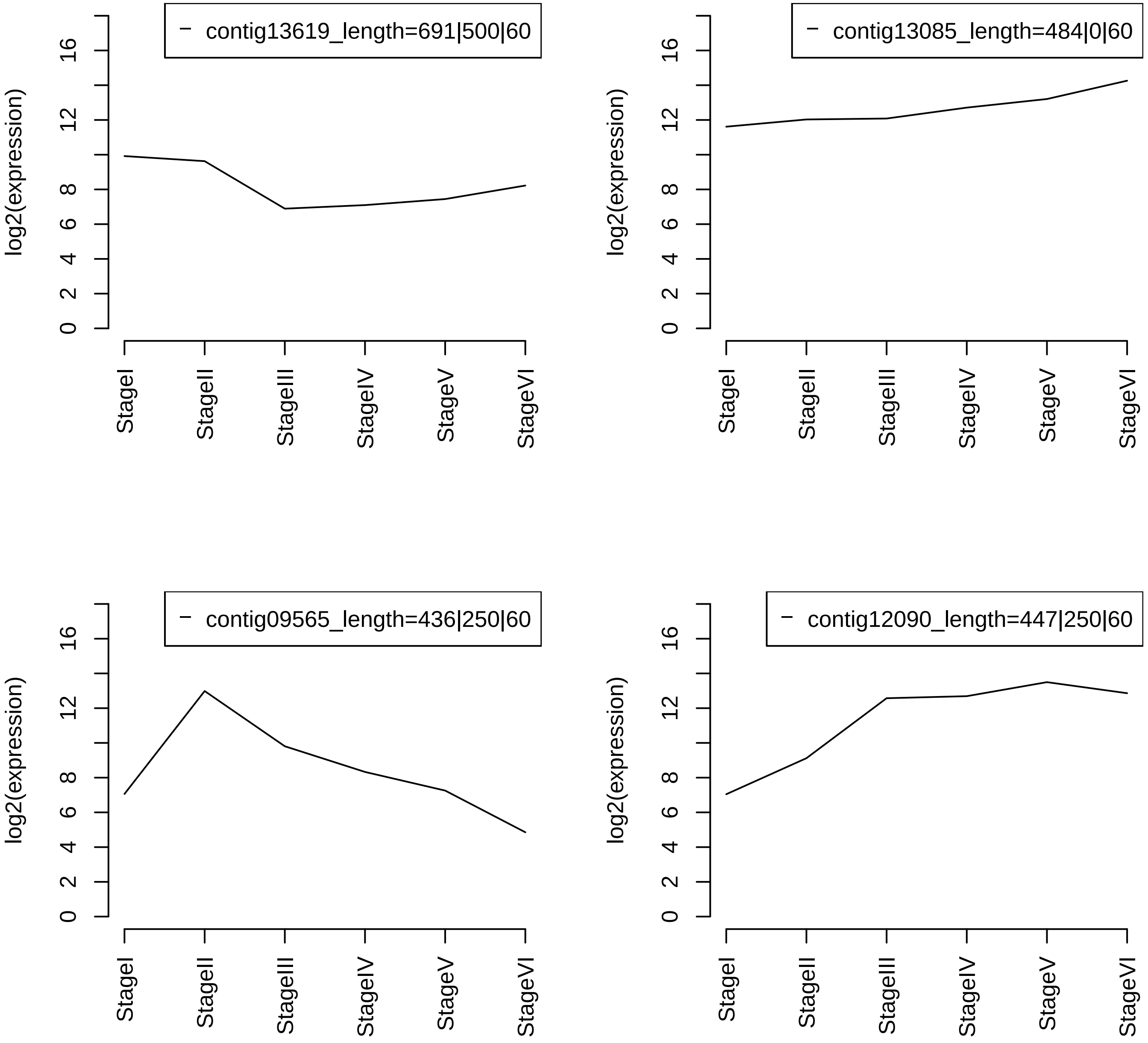
contig13619_length=691|500|60
contig13085_length=484|0|60
contig09565_length=436|250|60
contig12090_length=447|250|60
log2(expression)
0
2
4
6
8
12
16
StageI
StageII
StageIII
StageIV
StageV
StageVI

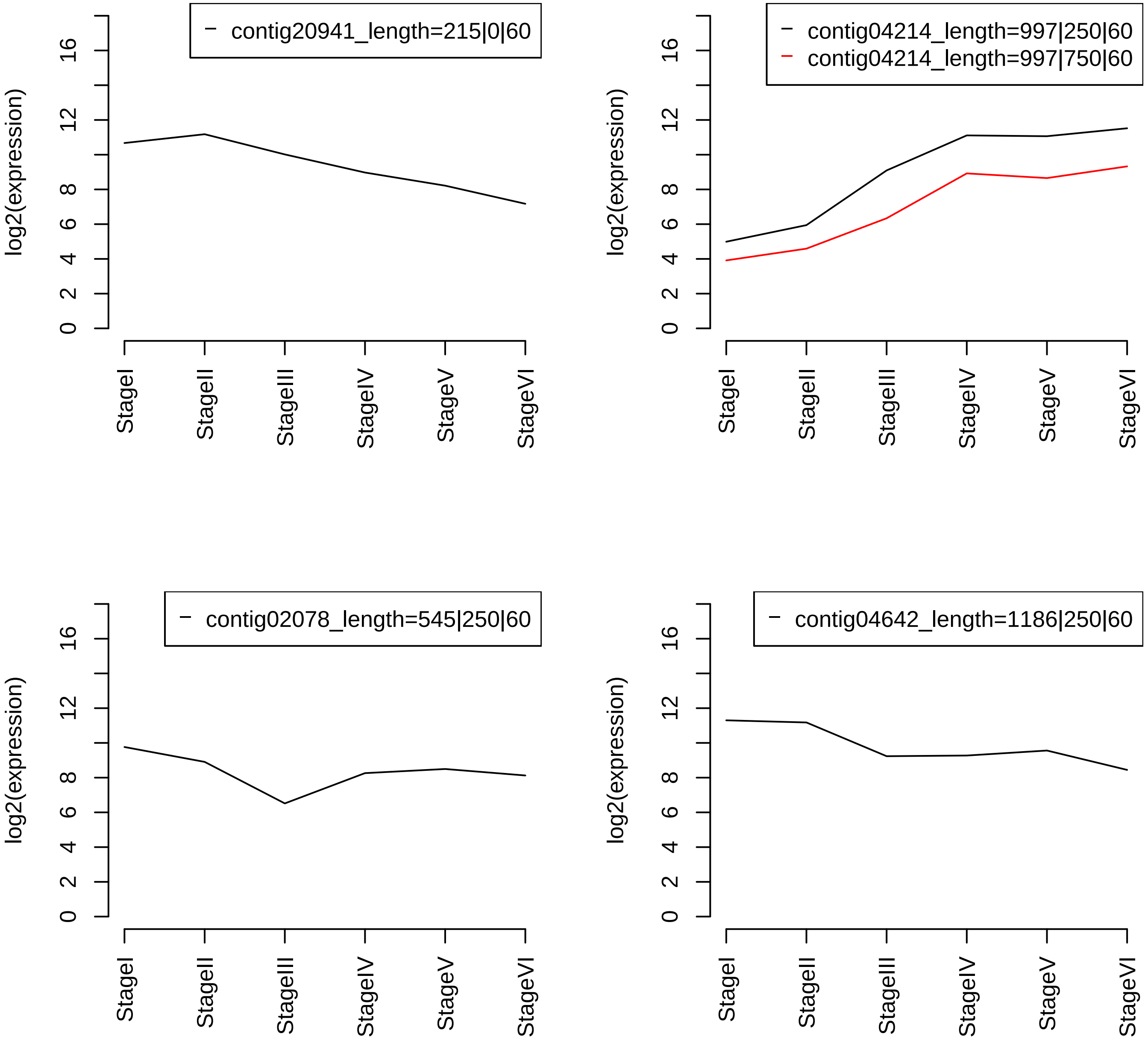
contig20941_length=215|0|60
contig04214_length=997|250|60
contig04214_length=997|750|60
contig02078_length=545|250|60
contig04642_length=1186|250|60
log2(expression)
0
2
4
6
8
12
16
StageI
StageII
StageIII
StageIV
StageV
StageVI

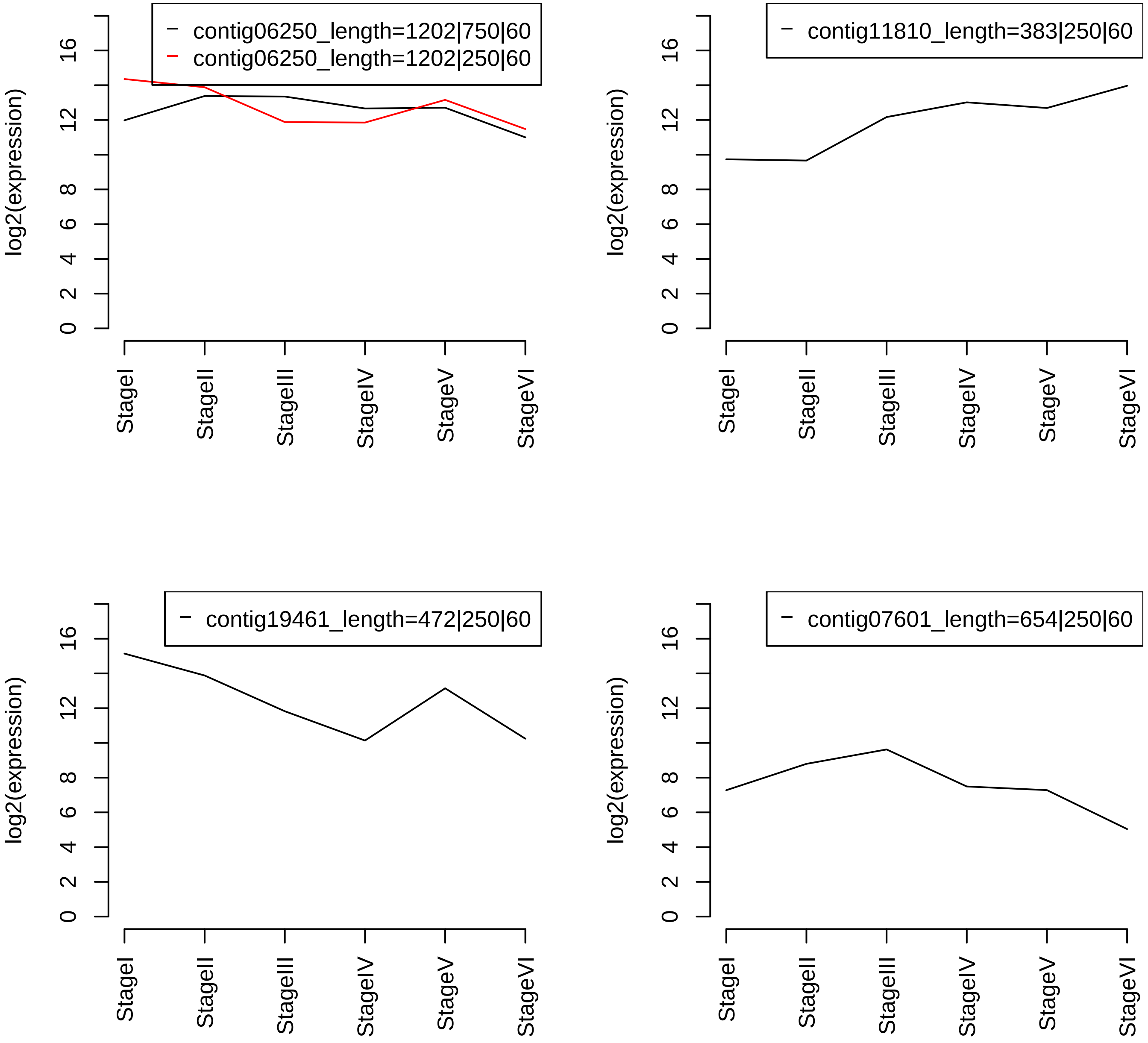

contig06250_length=1202|750|60
contig06250_length=1202|250|60
log2(expression)
0
2
4
6
8
12
16
StageI
StageII
StageIII
StageIV
StageV
StageVI
contig11810_length=383|250|60
contig19461_length=472|250|60
contig07601_length=654|250|60

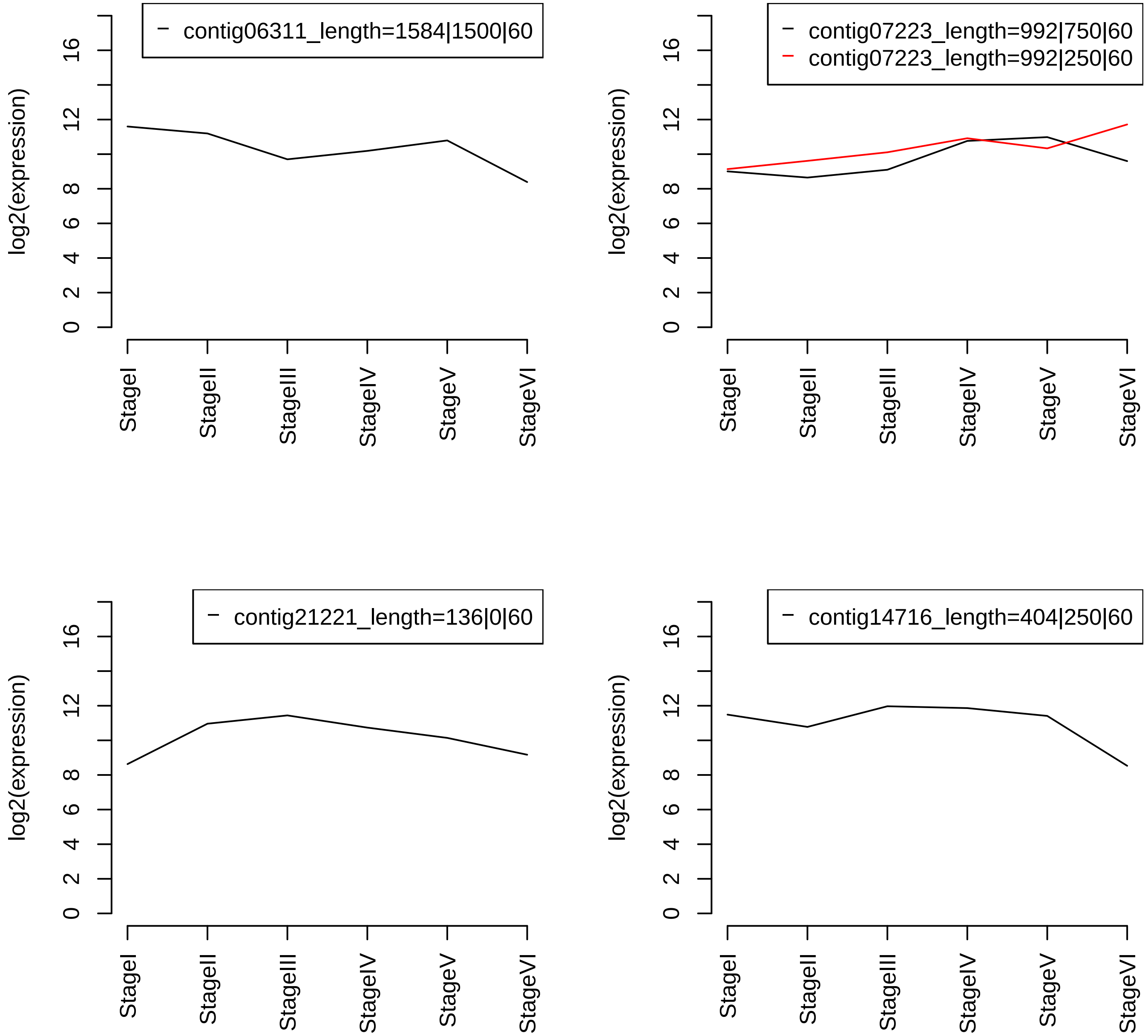

contig06311_length=1584|1500|60
contig07223_length=992|750|60
contig07223_length=992|250|60
contig21221_length=136|0|60
contig14716_length=404|250|60
log2(expression)
0
2
4
6
8
12
16
StageI
StageII
StageIII
StageIV
StageV
StageVI

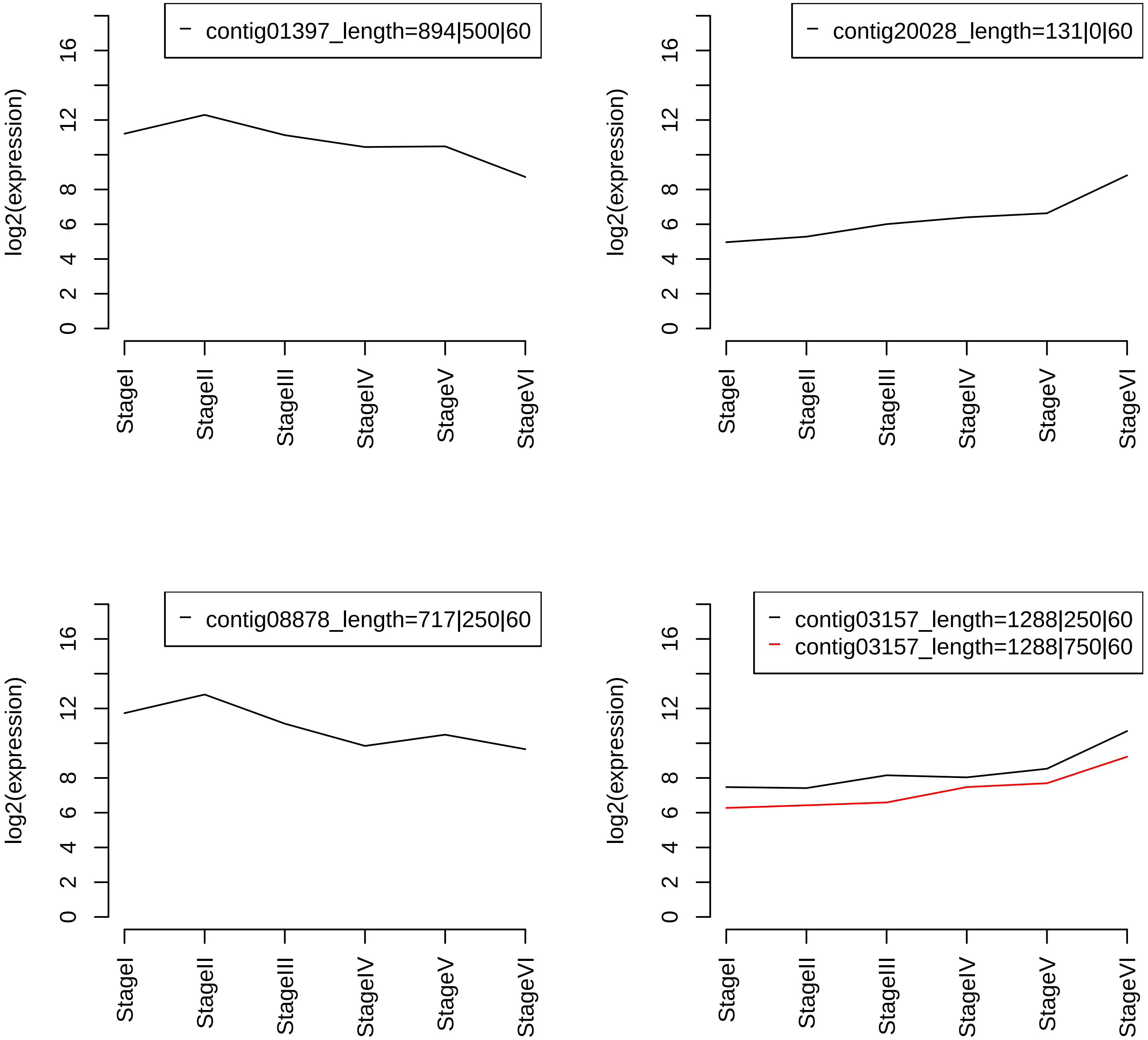
contig01397_length=894|500|60
contig20028_length=131|0|60
contig08878_length=717|250|60
contig03157_length=1288|250|60
contig03157_length=1288|750|60
log2(expression)
0
2
4
6
8
12
16
StageI
StageII
StageIII
StageIV
StageV
StageVI

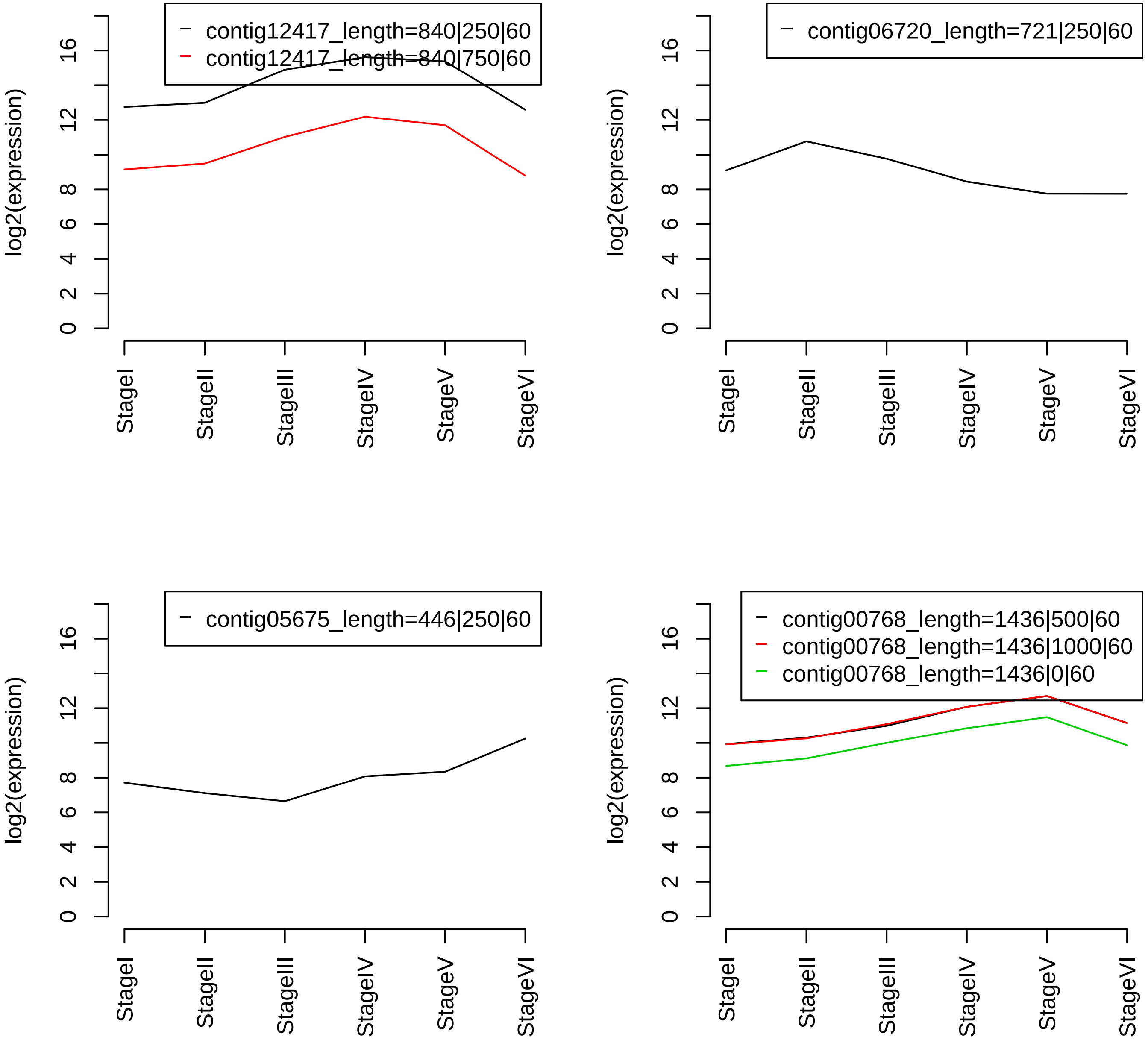
contig12417_length=840|250|60
contig12417_length=840|750|60
contig06720_length=721|250|60
contig05675_length=446|250|60
contig00768_length=1436|500|60
contig00768_length=1436|1000|60
contig00768_length=1436|0|60
log2(expression)
StageI
StageII
StageIII
StageIV
StageV
StageVI

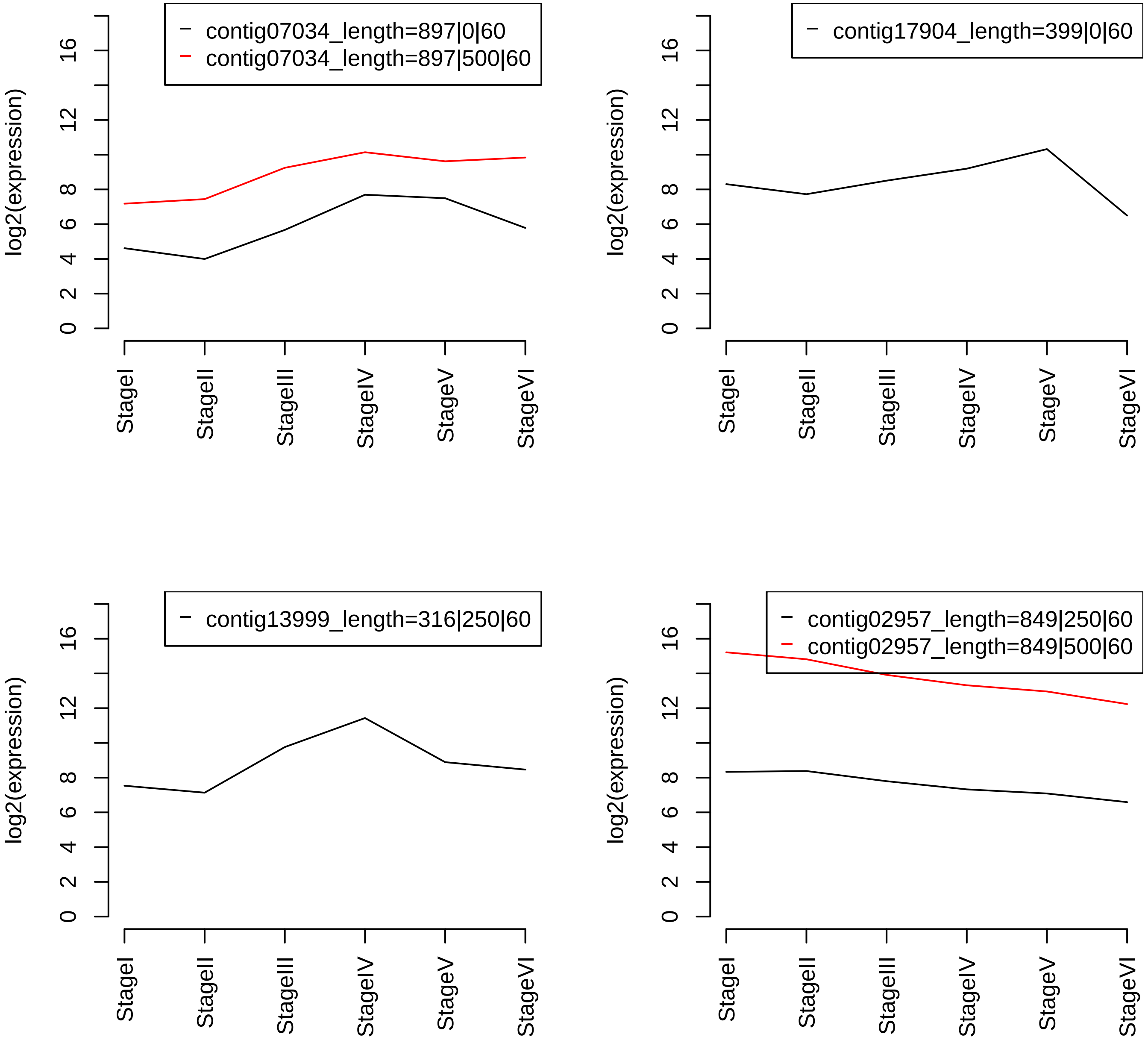

contig07034_length=897|0|60
contig07034_length=897|500|60
contig17904_length=399|0|60
contig13999_length=316|250|60
contig02957_length=849|250|60
contig02957_length=849|500|60
log2(expression)
0 2 4 6 8 12 16
StageI
StageII
StageIII
StageIV
StageV
StageVI

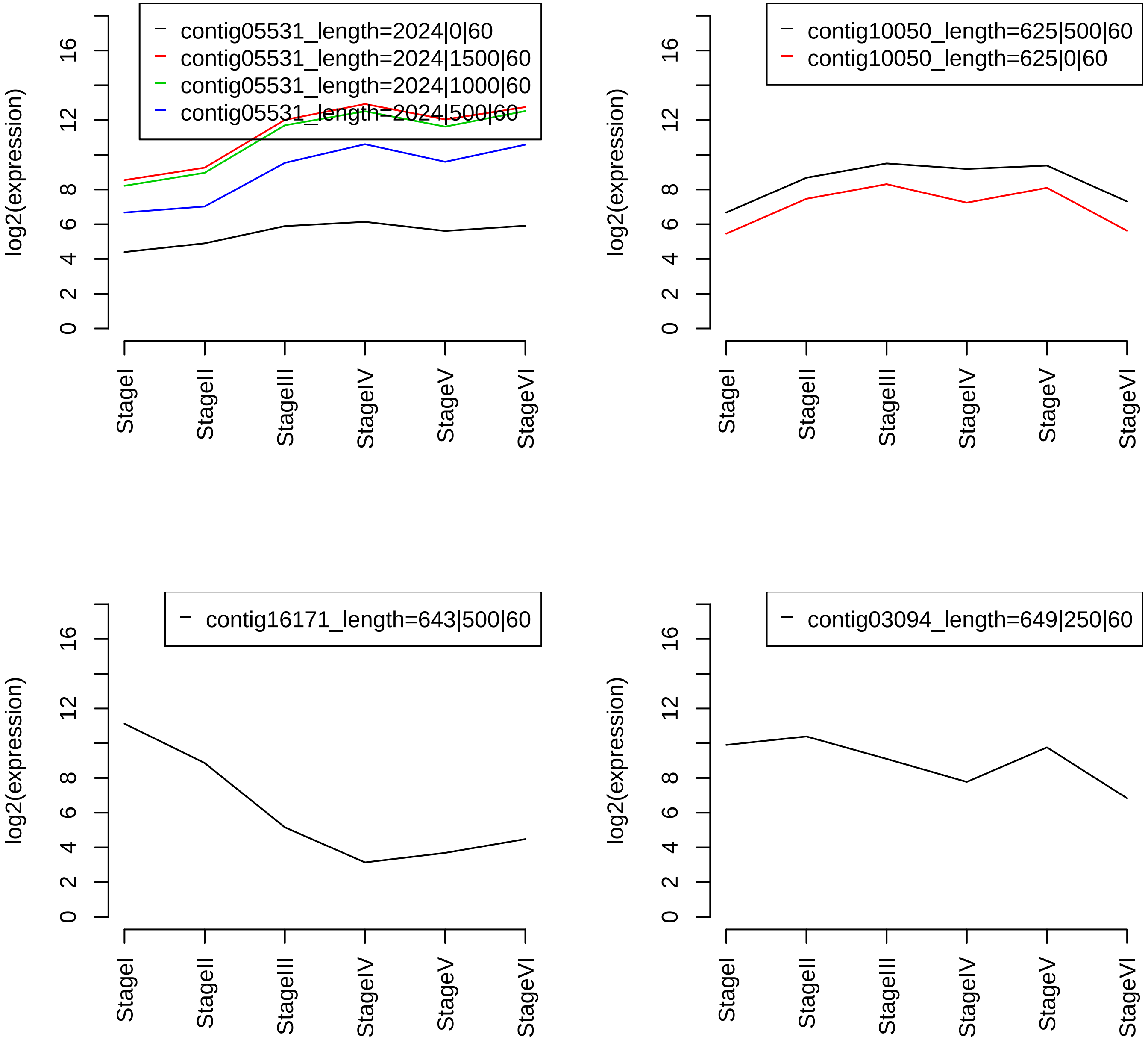

contig05531_length=2024|0|60
contig05531_length=2024|1500|60
contig05531_length=2024|1000|60
contig05531_length=2024|500|60
contig10050_length=625|500|60
contig10050_length=625|0|60
contig16171_length=643|500|60
contig03094_length=649|250|60
log2(expression)
0
2
4
6
8
12
16
StageI
StageII
StageIII
StageIV
StageV
StageVI

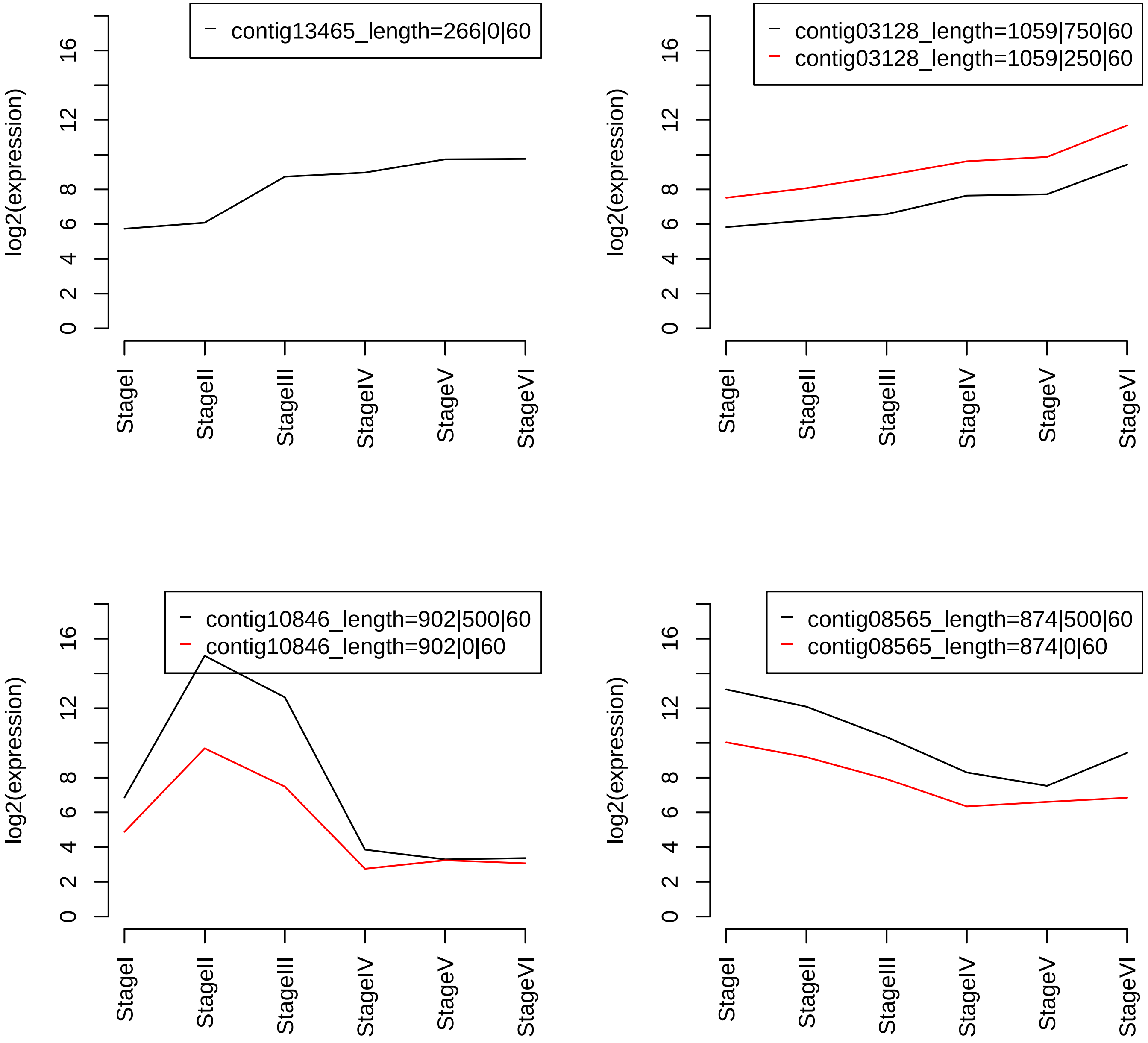
contig13465_length=266|0|60
contig03128_length=1059|750|60
contig03128_length=1059|250|60
contig10846_length=902|500|60
contig10846_length=902|0|60
contig08565_length=874|500|60
contig08565_length=874|0|60
log2(expression)
StageI
StageII
StageIII
StageIV
StageV
StageVI

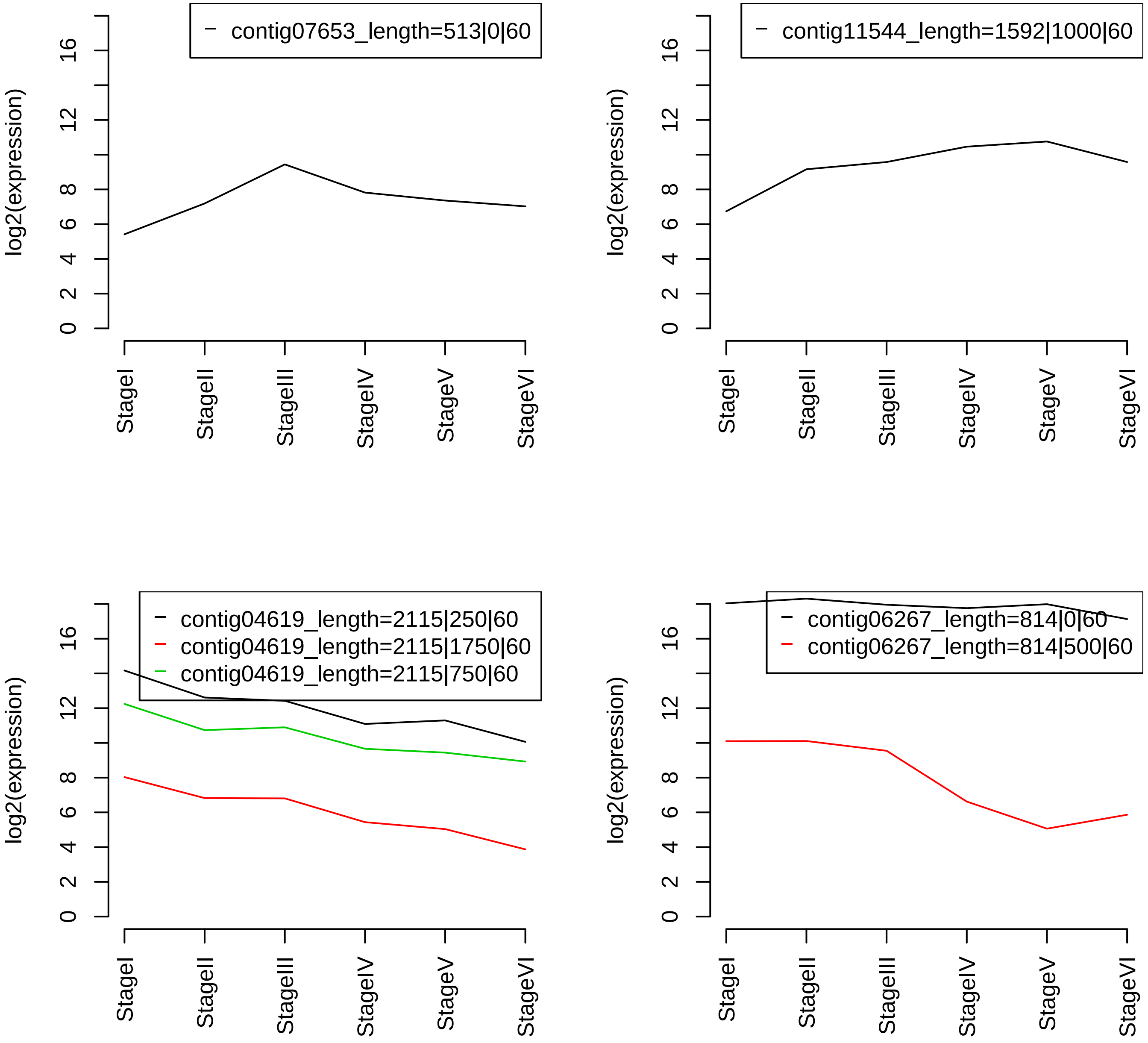

contig07653_length=513|0|60
log2(expression)
0 2 4 6 8 12 16
StageI StageII StageIII StageIV StageV StageVI
contig11544_length=1592|1000|60
log2(expression)
0 2 4 6 8 12 16
StageI StageII StageIII StageIV StageV StageVI
contig04619_length=2115|250|60
contig04619_length=2115|1750|60
contig04619_length=2115|750|60
log2(expression)
0 2 4 6 8 12 16
StageI StageII StageIII StageIV StageV StageVI
contig06267_length=814|0|60
contig06267_length=814|500|60
log2(expression)
0 2 4 6 8 12 16
StageI StageII StageIII StageIV StageV StageVI

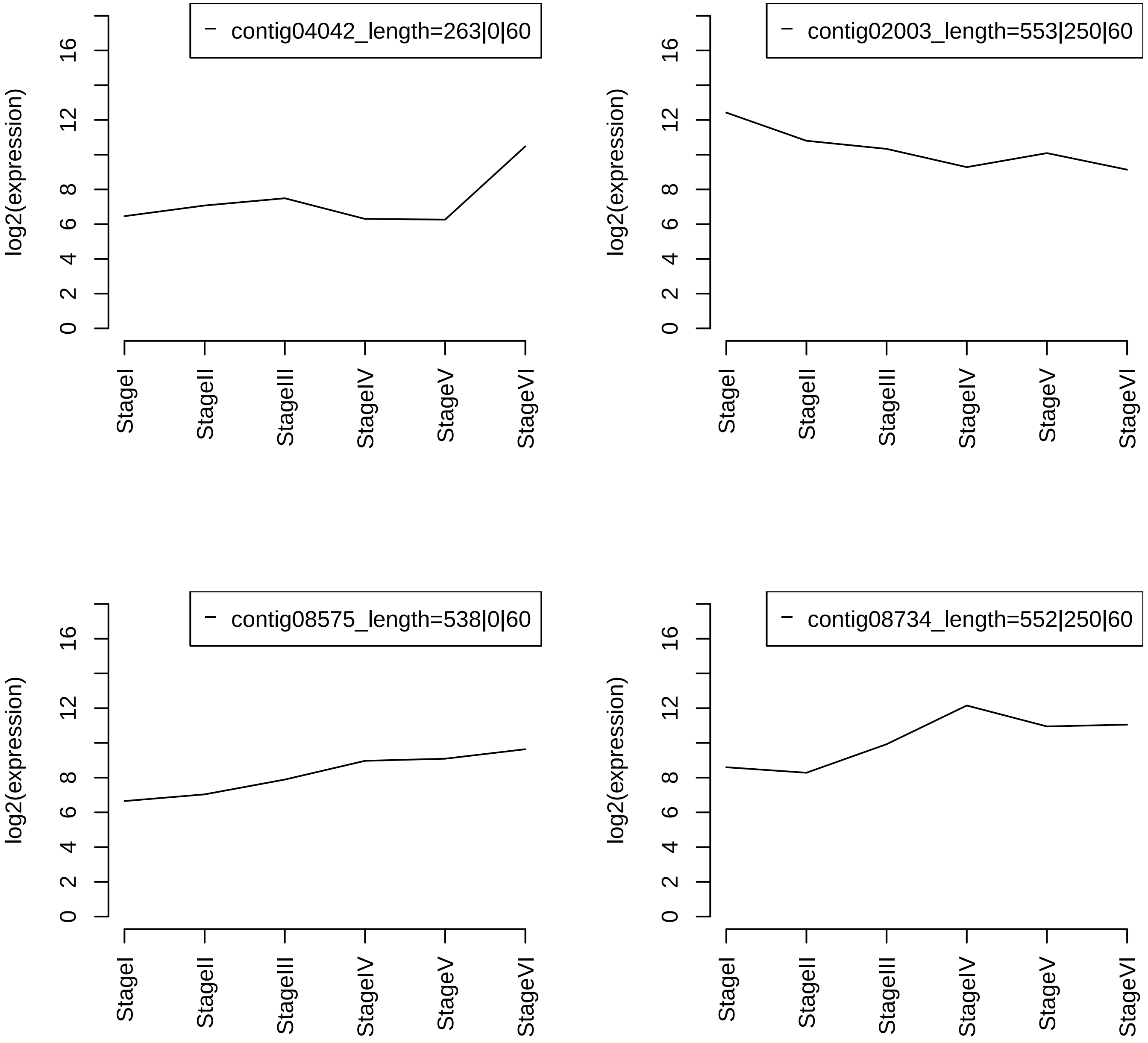
contig04042_length=263|0|60
contig02003_length=553|250|60
contig08575_length=538|0|60
contig08734_length=552|250|60
log2(expression)
0
2
4
6
8
12
16
StageI
StageII
StageIII
StageIV
StageV
StageVI

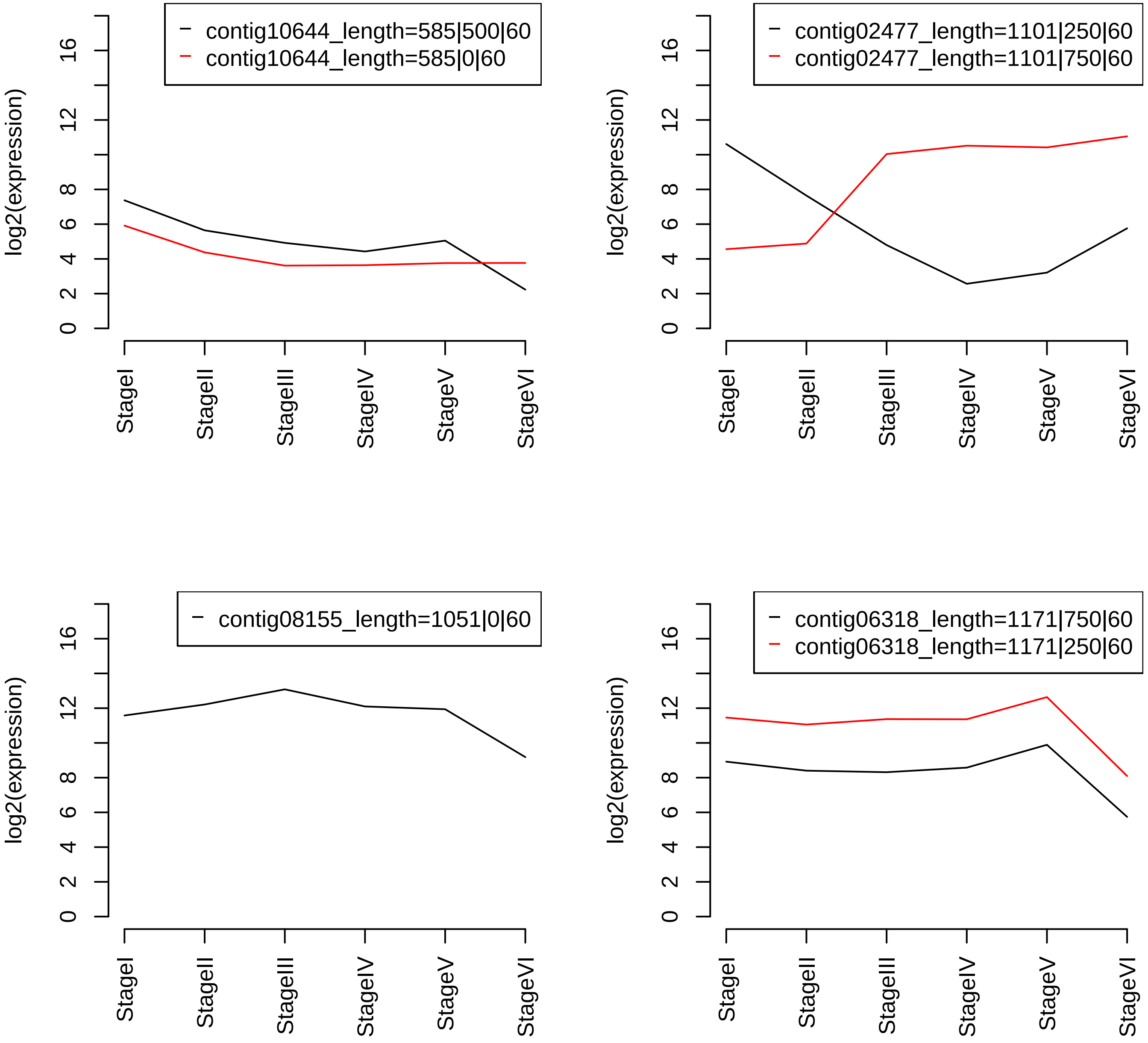
contig10644_length=585|500|60
contig10644_length=585|0|60
contig02477_length=1101|250|60
contig02477_length=1101|750|60
contig08155_length=1051|0|60
contig06318_length=1171|750|60
contig06318_length=1171|250|60
log2(expression)
0 2 4 6 8 12 16
StageI StageII StageIII StageIV StageV StageVI

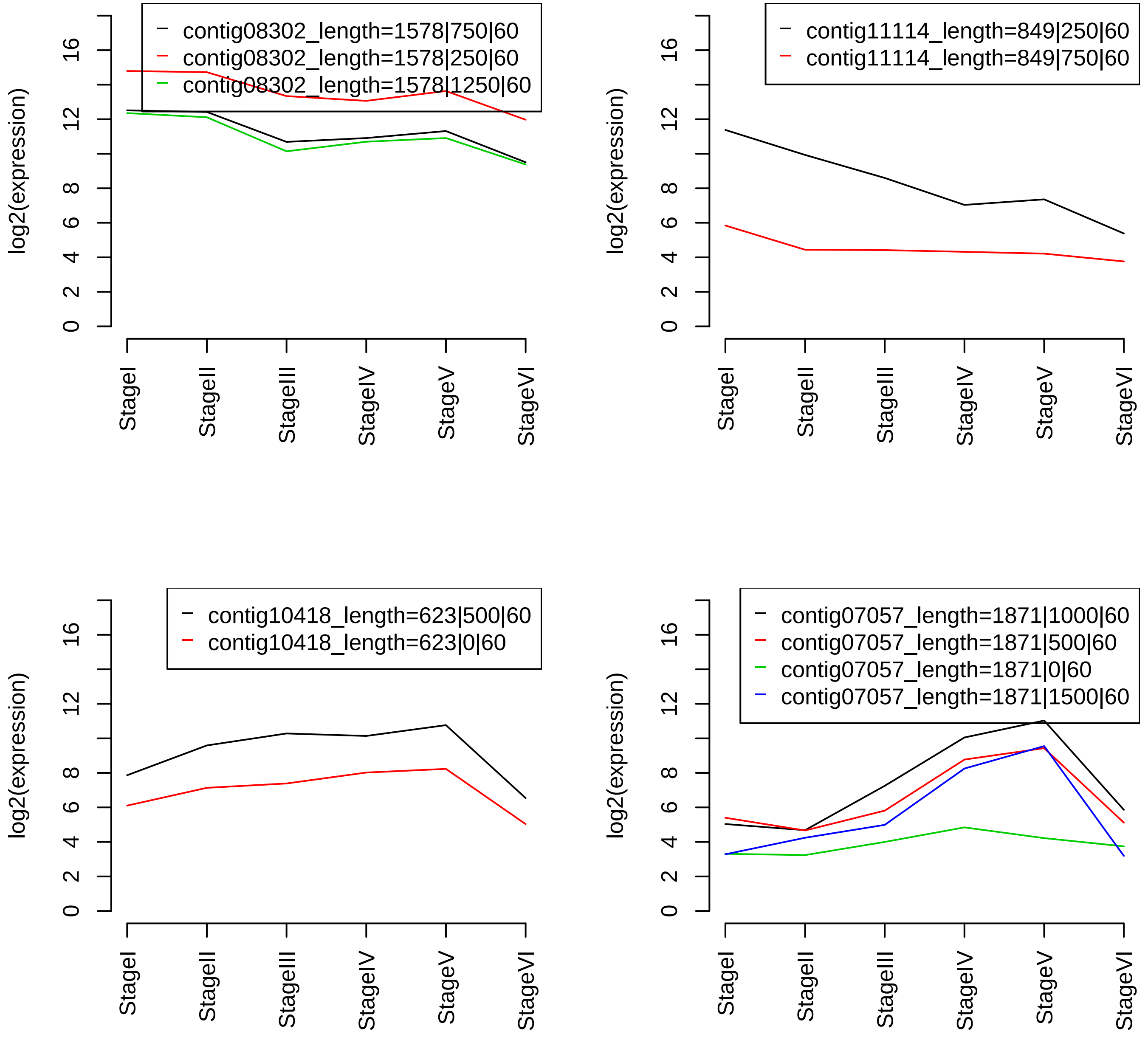
contig08302_length=1578|750|60
contig08302_length=1578|250|60
contig08302_length=1578|1250|60
contig11114_length=849|250|60
contig11114_length=849|750|60
contig10418_length=623|500|60
contig10418_length=623|0|60
contig07057_length=1871|1000|60
contig07057_length=1871|500|60
contig07057_length=1871|0|60
contig07057_length=1871|1500|60
log2(expression)
0
2
4
6
8
12
16
StageI
StageII
StageIII
StageIV
StageV
StageVI

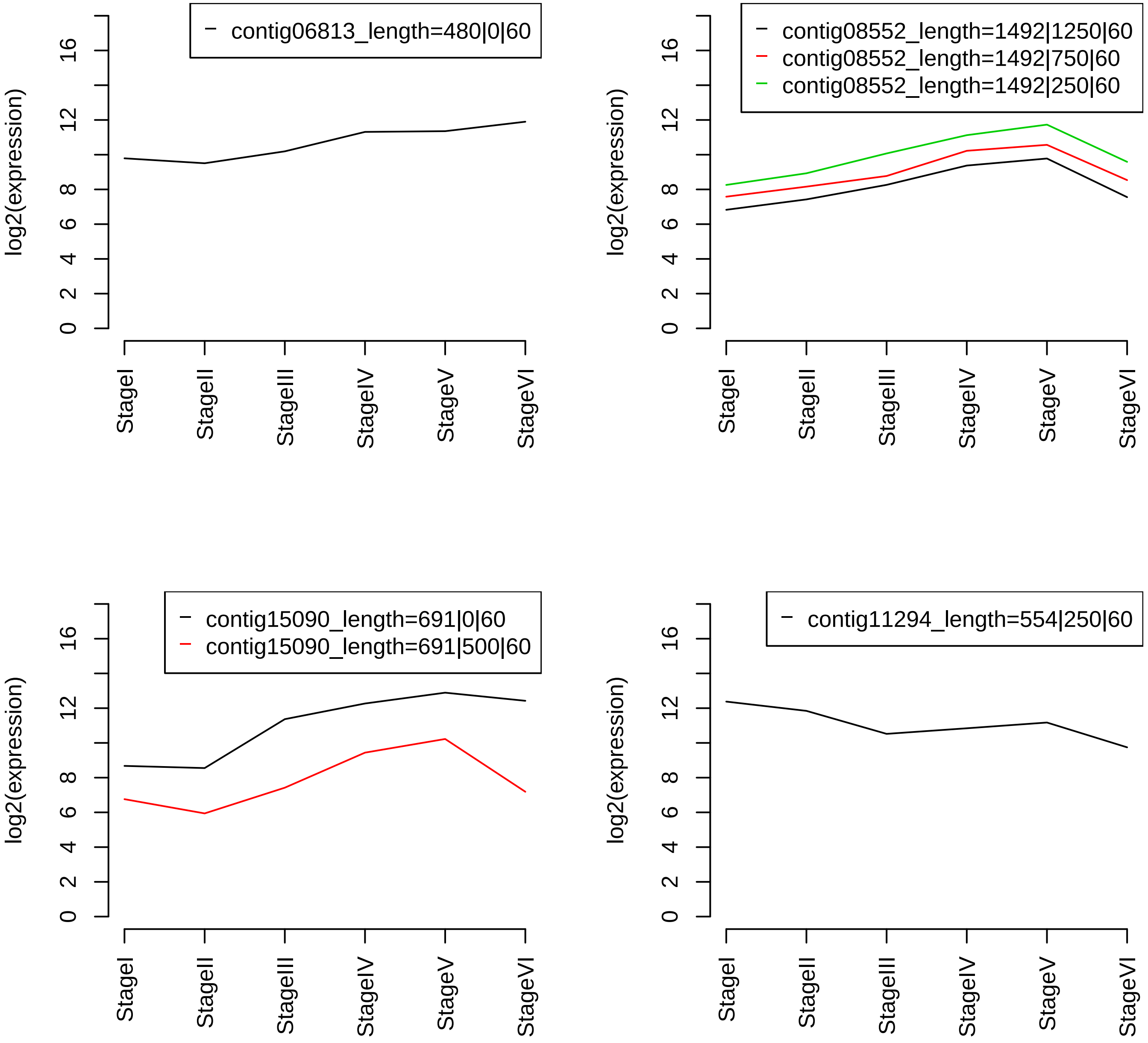
contig06813_length=480|0|60
contig08552_length=1492|1250|60
contig08552_length=1492|750|60
contig08552_length=1492|250|60
contig15090_length=691|0|60
contig15090_length=691|500|60
contig11294_length=554|250|60
log2(expression)
0
2
4
6
8
12
16
StageI
StageII
StageIII
StageIV
StageV
StageVI

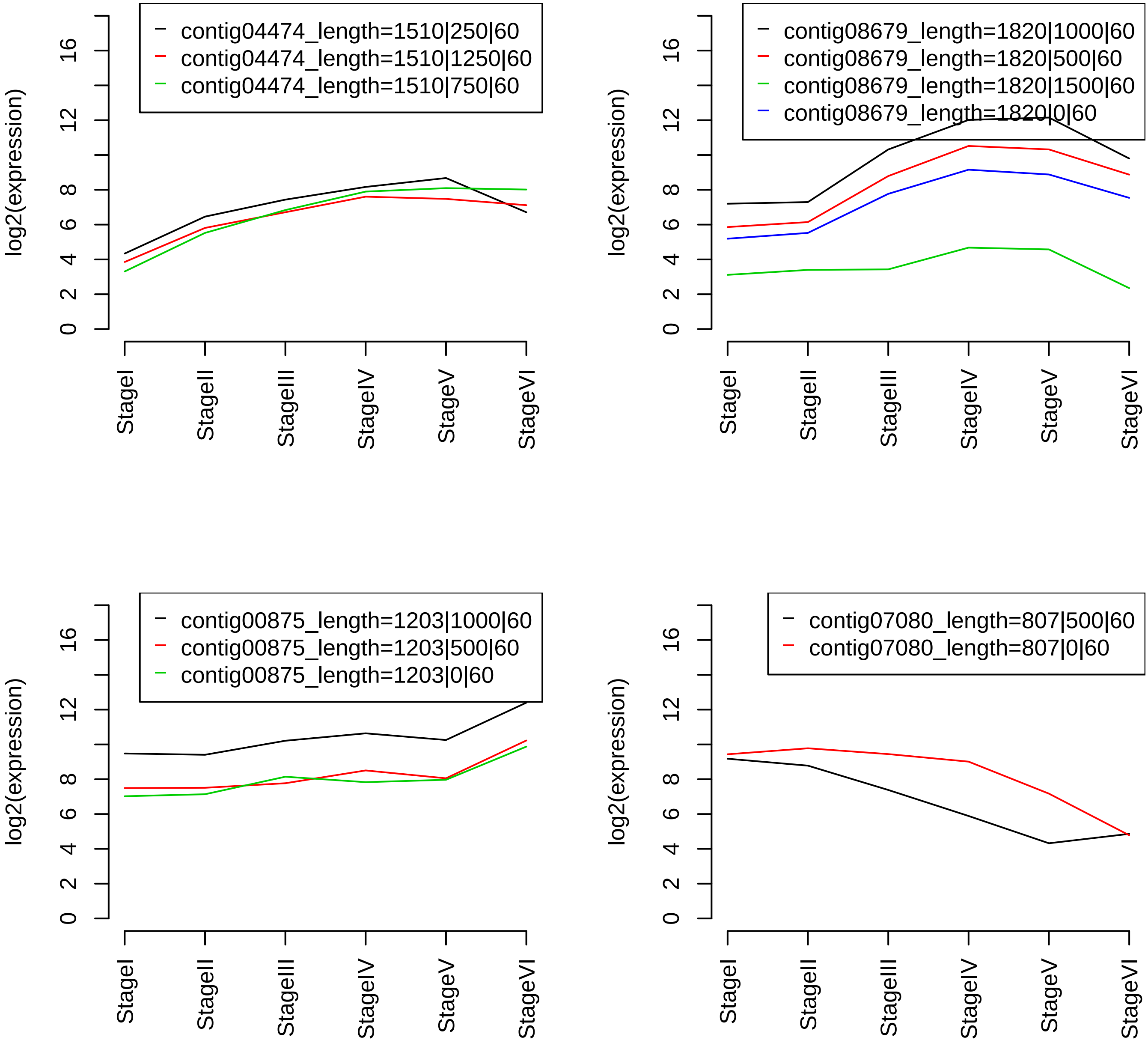
contig04474_length=1510|250|60
contig04474_length=1510|1250|60
contig04474_length=1510|750|60
contig08679_length=1820|1000|60
contig08679_length=1820|500|60
contig08679_length=1820|1500|60
contig08679_length=1820|0|60
contig00875_length=1203|1000|60
contig00875_length=1203|500|60
contig00875_length=1203|0|60
contig07080_length=807|500|60
contig07080_length=807|0|60
log2(expression)
0
2
4
6
8
12
16
StageI
StageII
StageIII
StageIV
StageV
StageVI

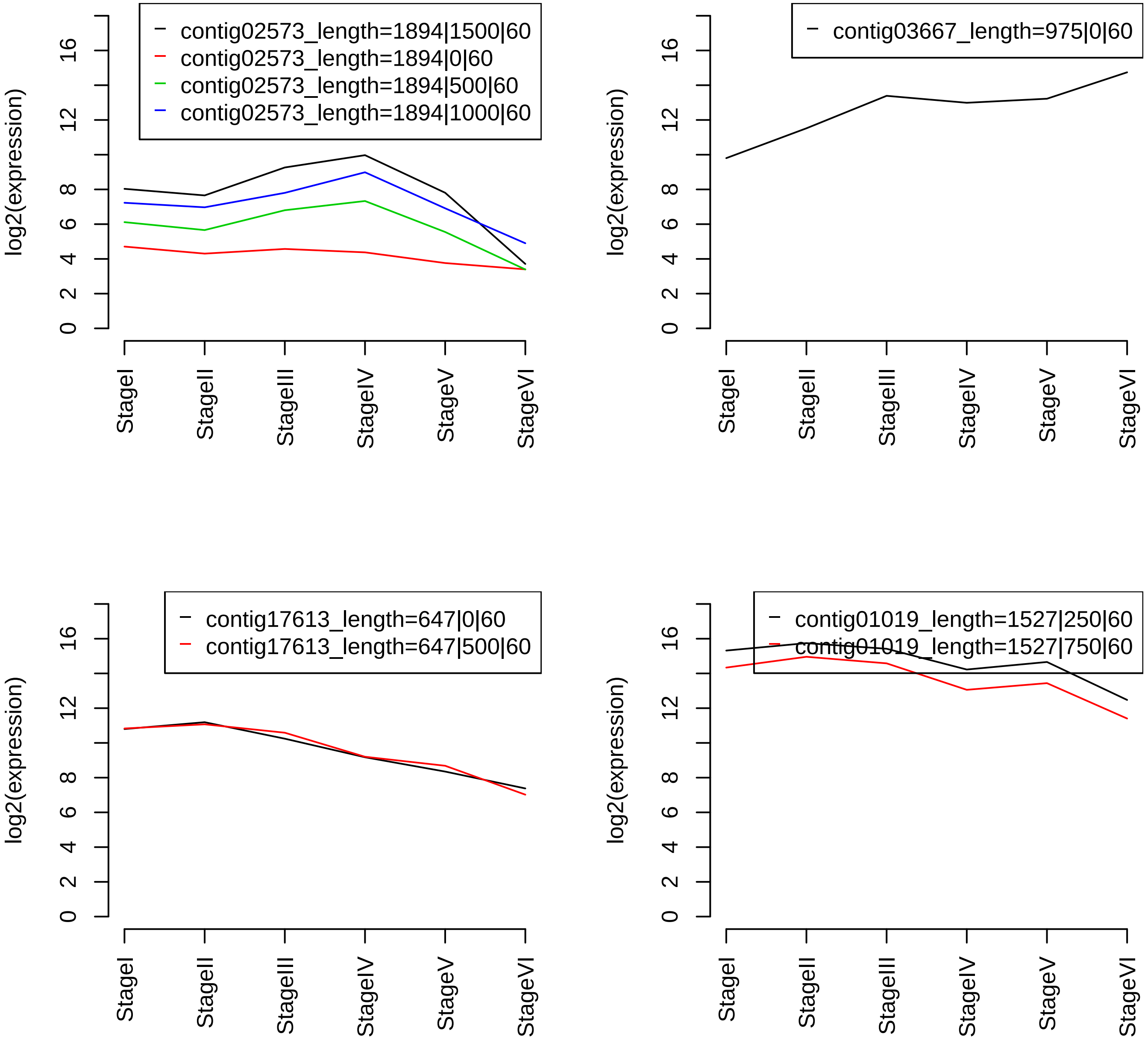
contig02573_length=1894|1500|60
contig02573_length=1894|0|60
contig02573_length=1894|500|60
contig02573_length=1894|1000|60
contig03667_length=975|0|60
contig17613_length=647|0|60
contig17613_length=647|500|60
contig01019_length=1527|250|60
contig01019_length=1527|750|60
log2(expression)
0
2
4
6
8
12
16
StageI
StageII
StageIII
StageIV
StageV
StageVI

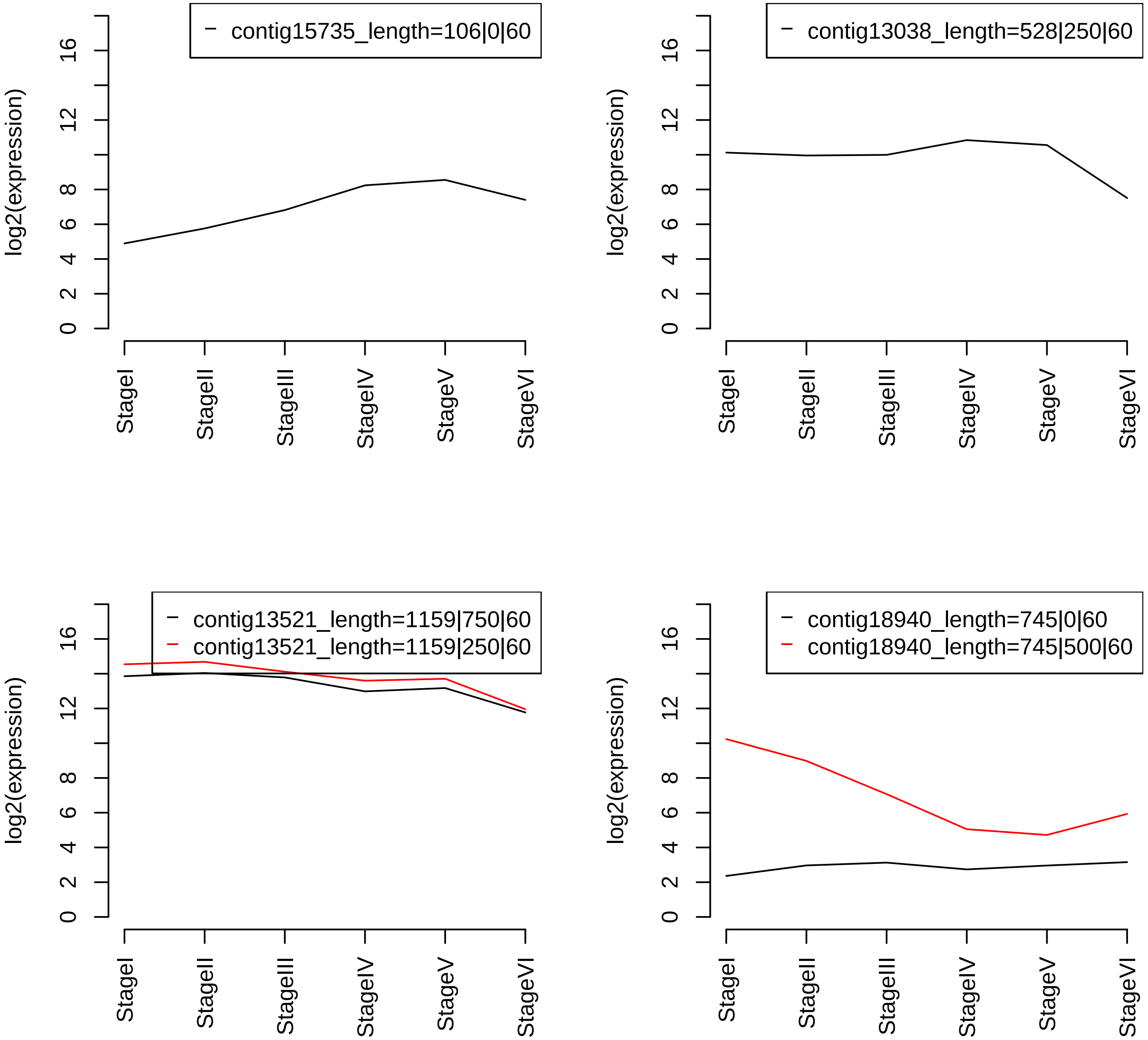
contig15735_length=106|0|60
contig13038_length=528|250|60
contig13521_length=1159|750|60
contig13521_length=1159|250|60
contig18940_length=745|0|60
contig18940_length=745|500|60
log2(expression)
0 2 4 6 8 12 16
StageI StageII StageIII StageIV StageV StageVI

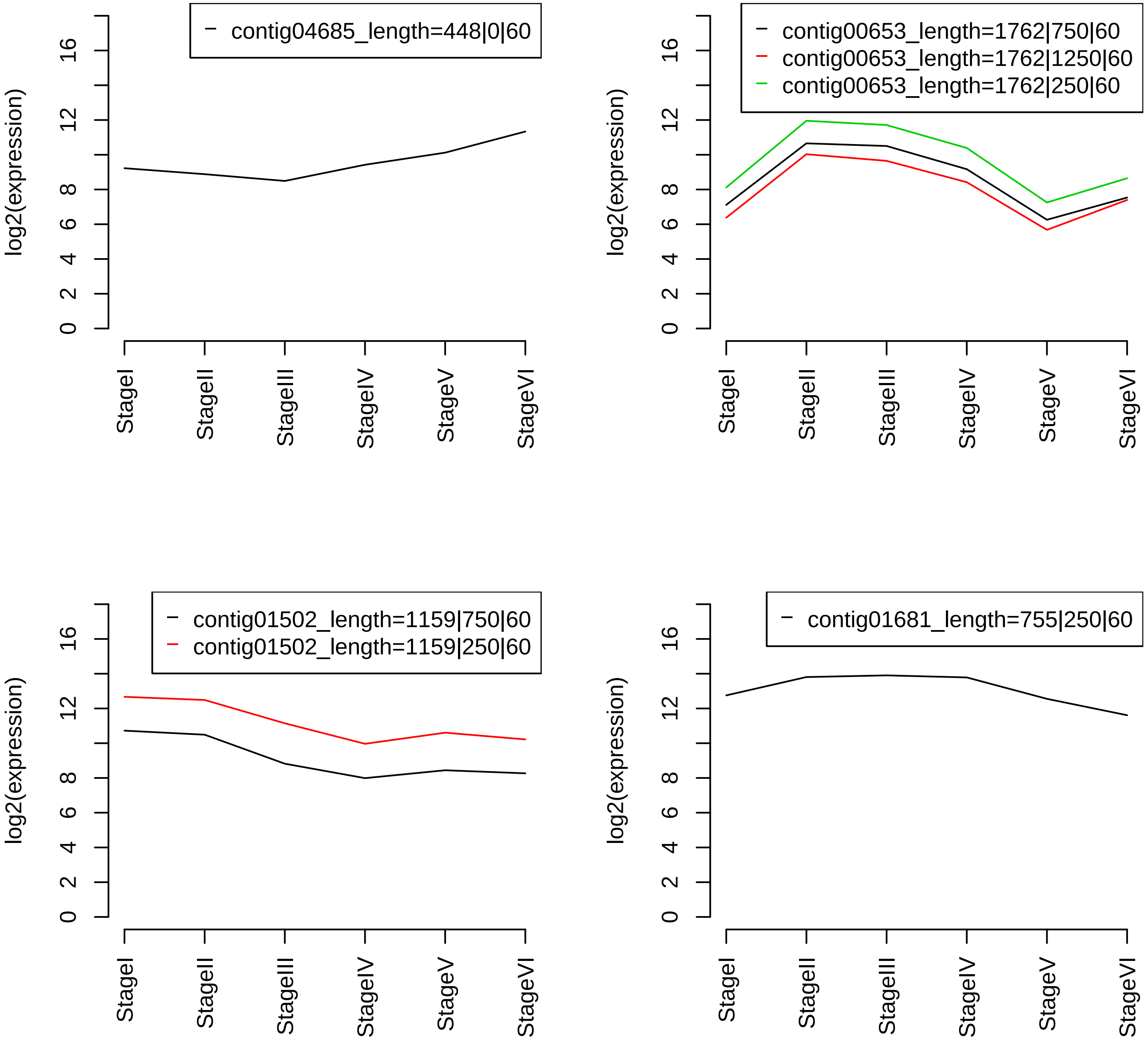
contig04685_length=448|0|60
contig00653_length=1762|750|60
contig00653_length=1762|1250|60
contig00653_length=1762|250|60
contig01502_length=1159|750|60
contig01502_length=1159|250|60
contig01681_length=755|250|60
log2(expression)
0
2
4
6
8
12
16
StageI
StageII
StageIII
StageIV
StageV
StageVI

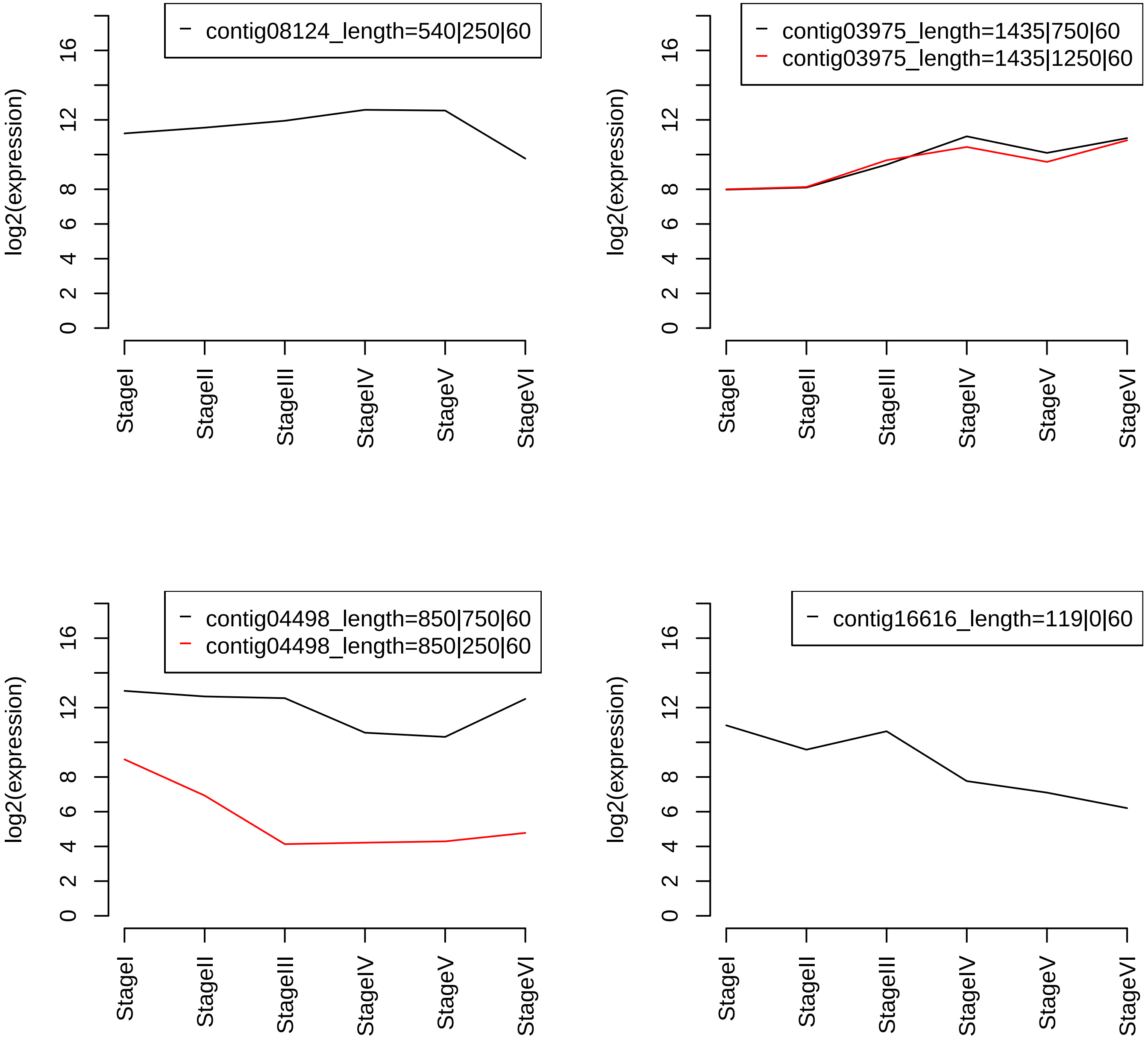

contig08124_length=540|250|60
contig03975_length=1435|750|60
contig03975_length=1435|1250|60
contig04498_length=850|750|60
contig04498_length=850|250|60
contig16616_length=119|0|60
log2(expression)
0
2
4
6
8
12
16
StageI
StageII
StageIII
StageIV
StageV
StageVI

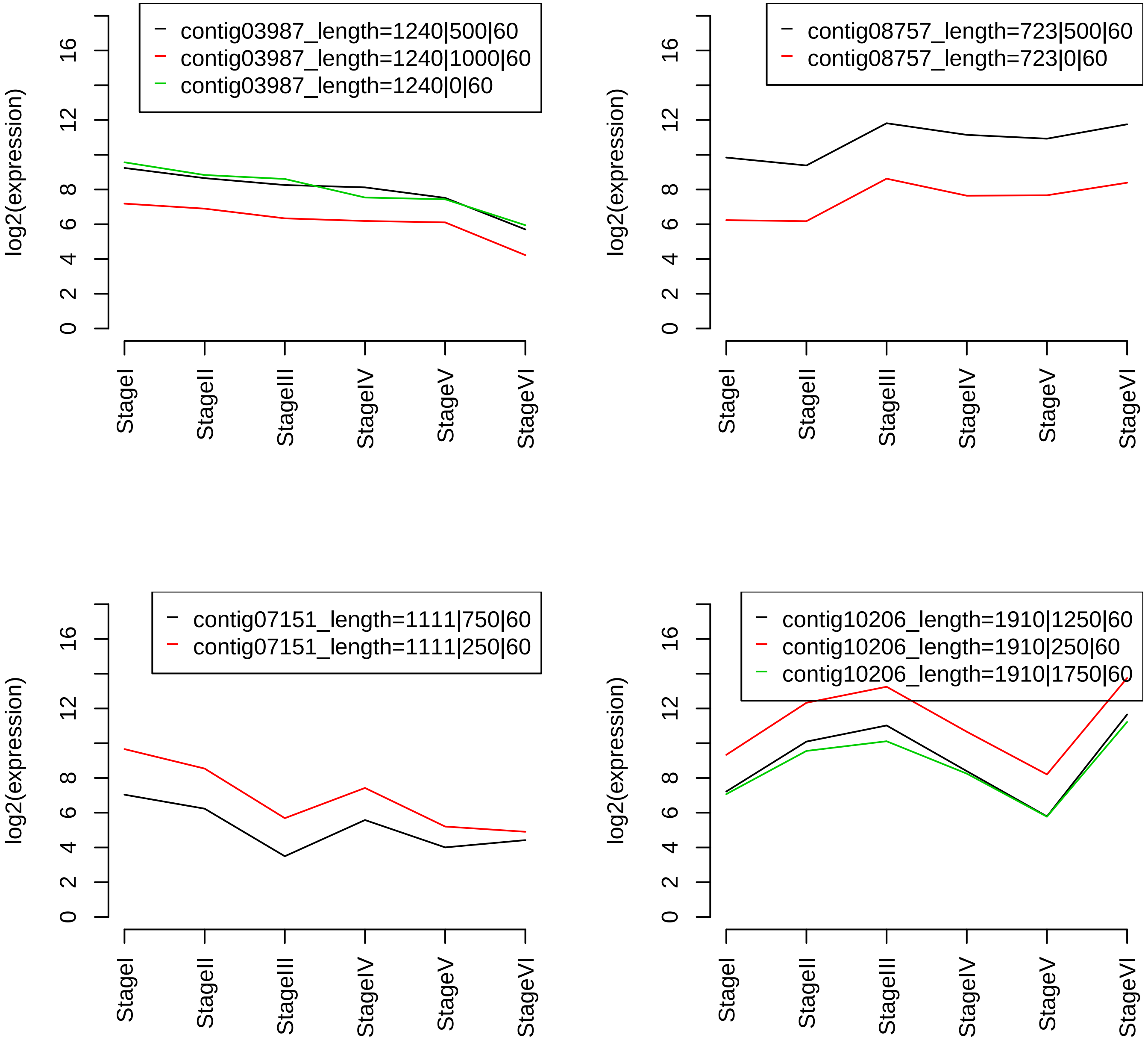
contig03987_length=1240|500|60
contig03987_length=1240|1000|60
contig03987_length=1240|0|60
contig08757_length=723|500|60
contig08757_length=723|0|60
contig07151_length=1111|750|60
contig07151_length=1111|250|60
contig10206_length=1910|1250|60
contig10206_length=1910|250|60
contig10206_length=1910|1750|60
log2(expression)
0
2
4
6
8
12
16
StageI
StageII
StageIII
StageIV
StageV
StageVI

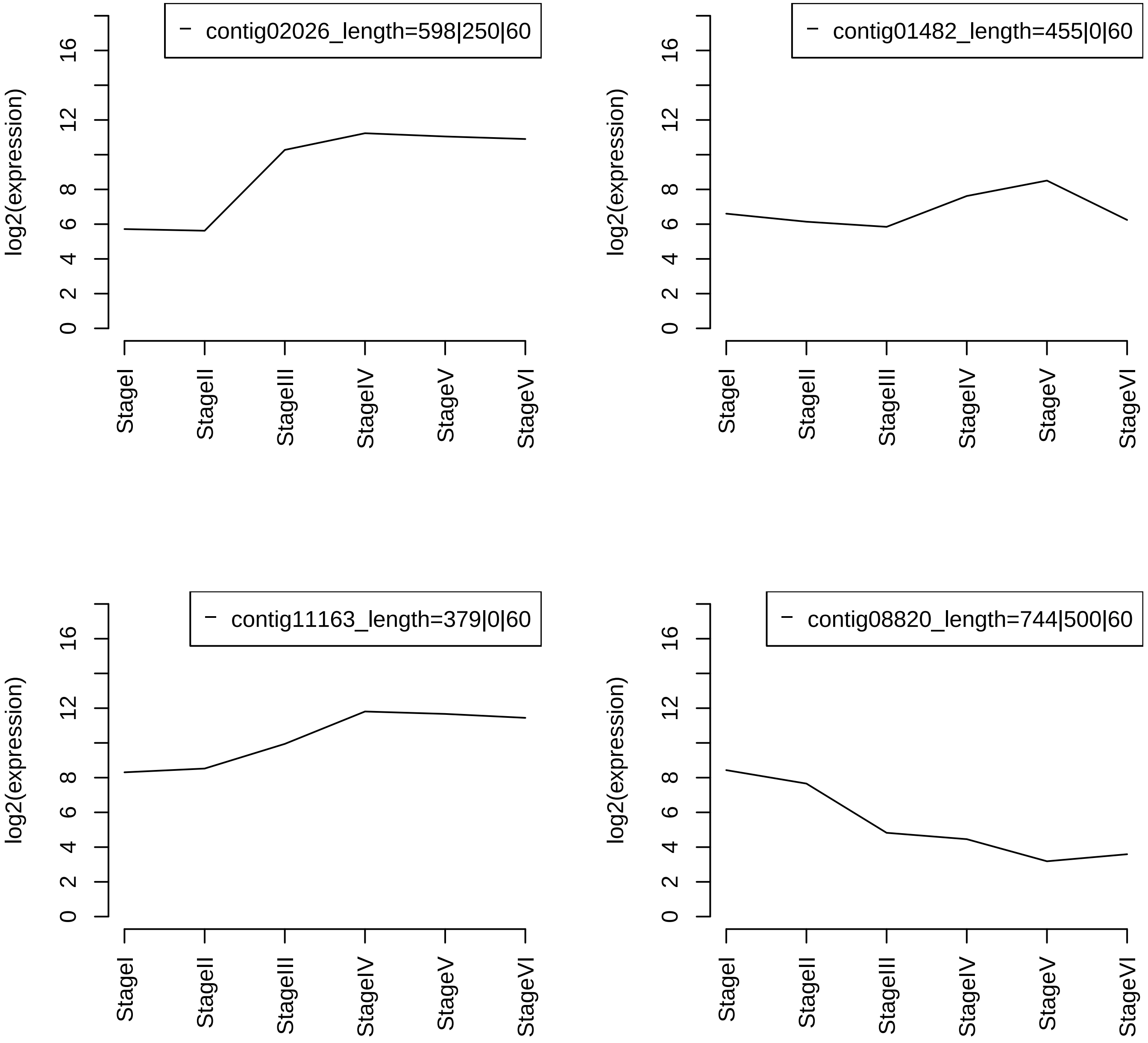
contig02026_length=598|250|60
contig01482_length=455|0|60
contig11163_length=379|0|60
contig08820_length=744|500|60
log2(expression)
0 2 4 6 8 12 16
StageI
StageII
StageIII
StageIV
StageV
StageVI

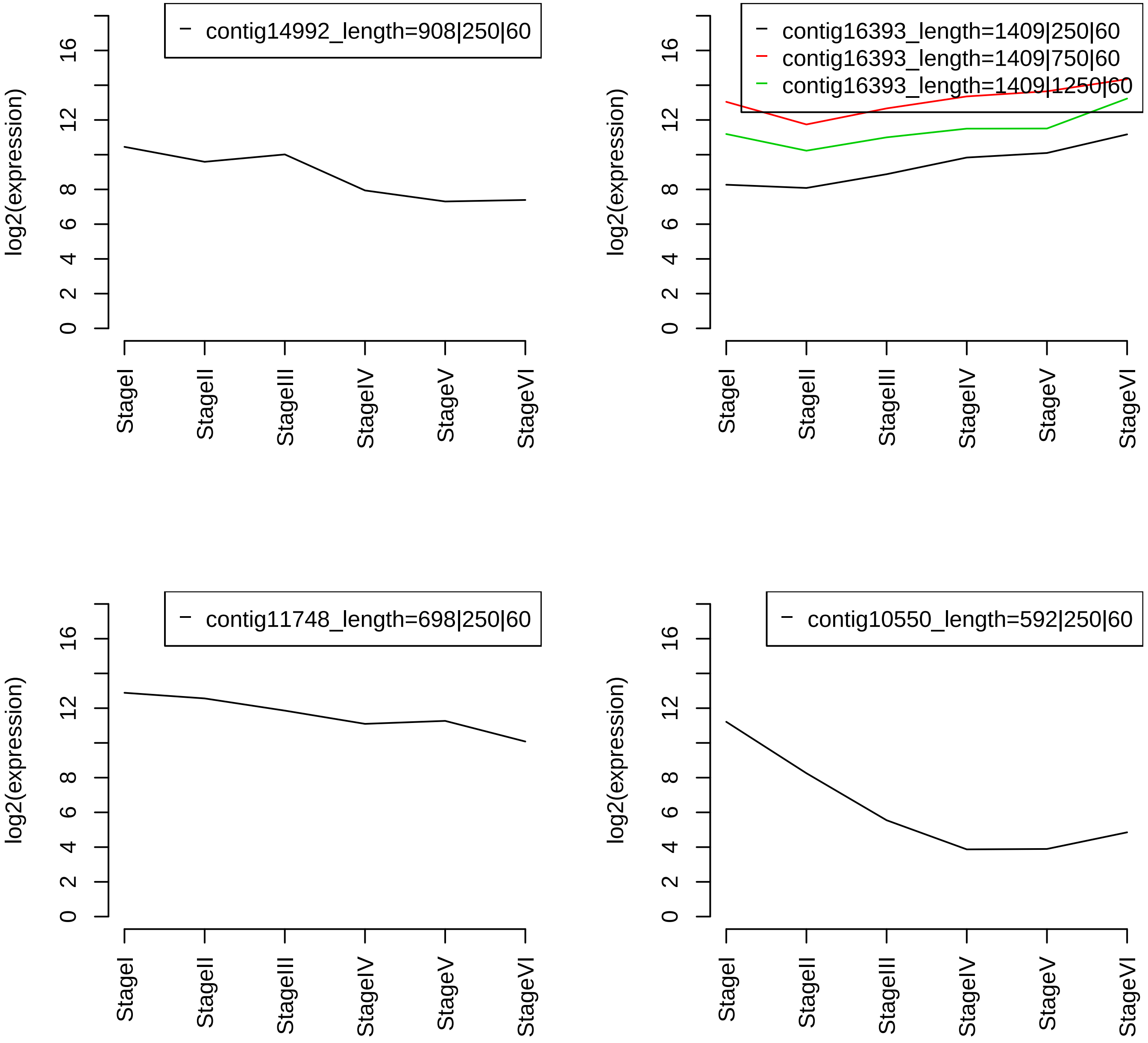
contig14992_length=908|250|60
contig16393_length=1409|250|60
contig16393_length=1409|750|60
contig16393_length=1409|1250|60
contig11748_length=698|250|60
contig10550_length=592|250|60
log2(expression)
StageI
StageII
StageIII
StageIV
StageV
StageVI

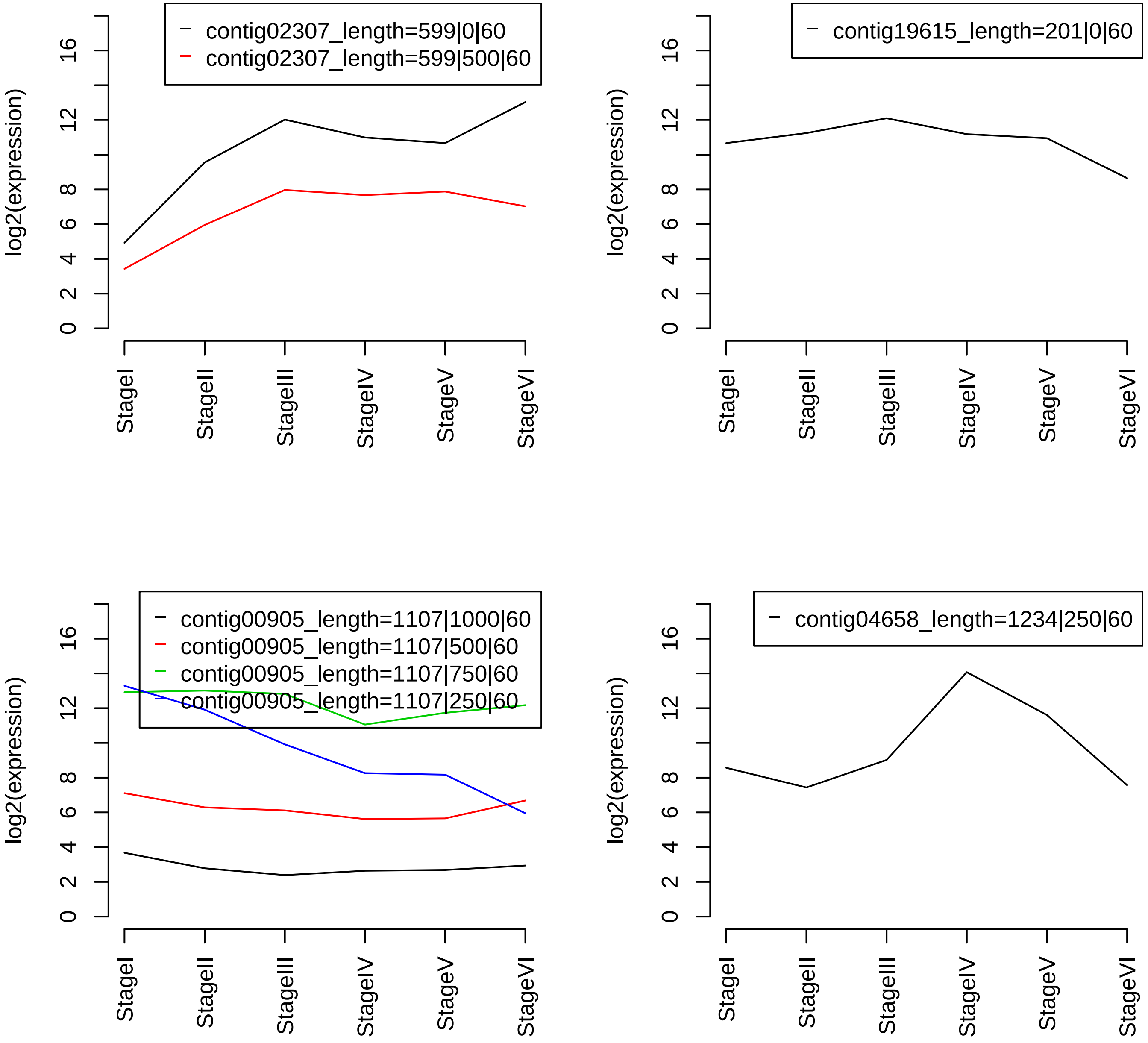

contig02307_length=599|0|60
contig02307_length=599|500|60
contig19615_length=201|0|60
contig00905_length=1107|1000|60
contig00905_length=1107|500|60
contig00905_length=1107|750|60
contig00905_length=1107|250|60
contig04658_length=1234|250|60
log2(expression)
StageI
StageII
StageIII
StageIV
StageV
StageVI

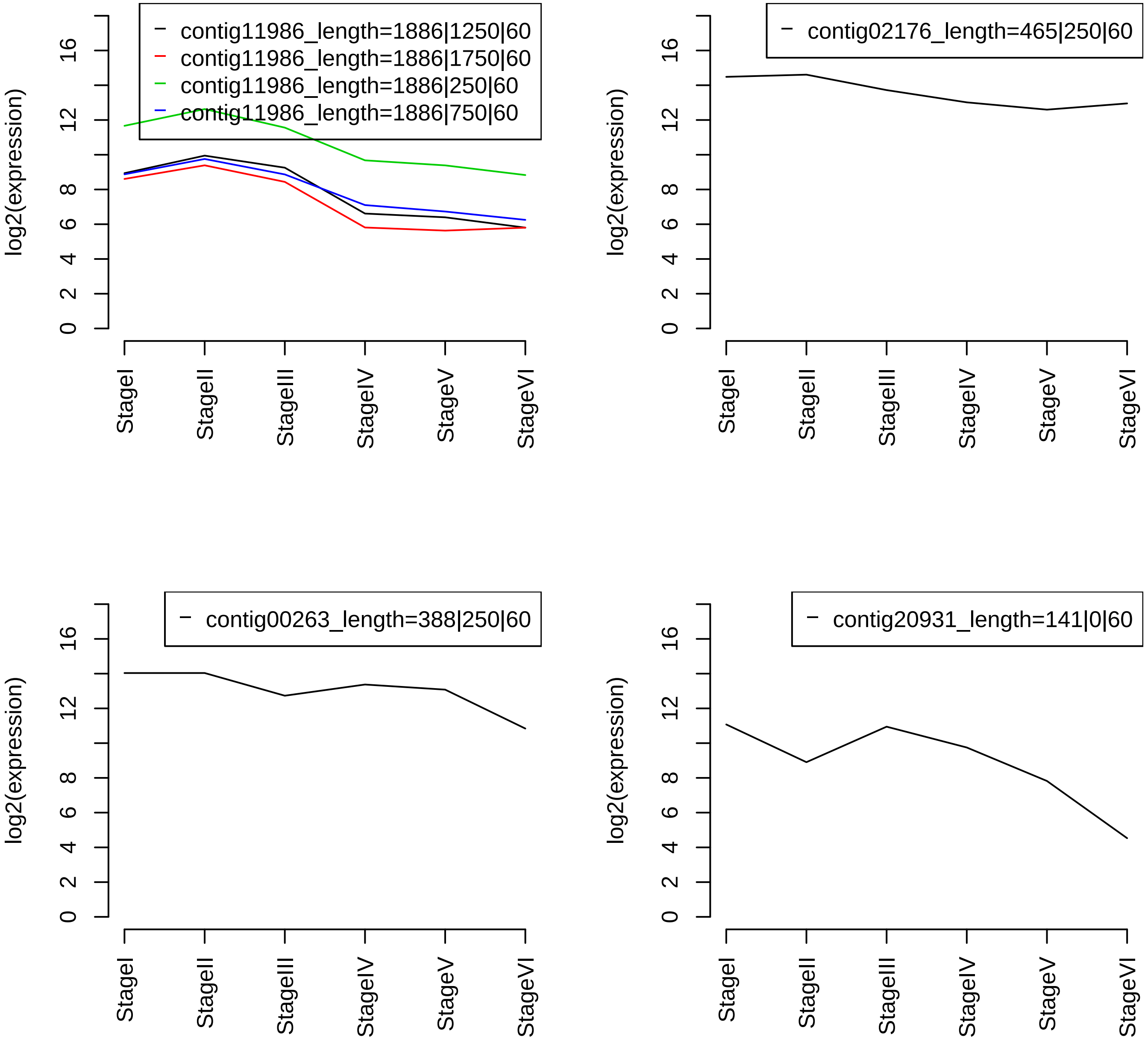
contig11986_length=1886|1250|60
contig11986_length=1886|1750|60
contig11986_length=1886|250|60
contig11986_length=1886|750|60
contig02176_length=465|250|60
contig00263_length=388|250|60
contig20931_length=141|0|60
log2(expression)
0
2
4
6
8
12
16
StageI
StageII
StageIII
StageIV
StageV
StageVI

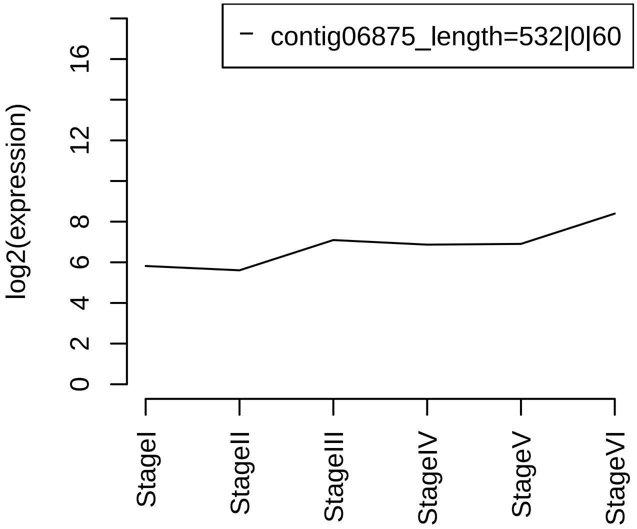
contig06875_length=532|0|60
log2(expression)
0
2
4
6
8
12
16
StageI
StageII
StageIII
StageIV
StageV
StageVI

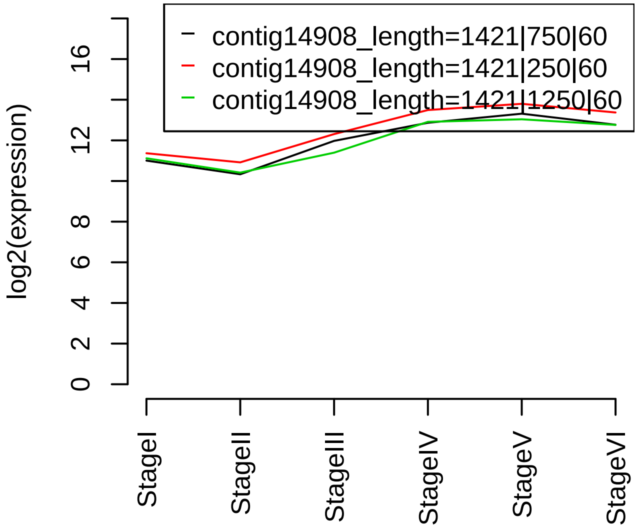
contig14908_length=1421|750|60
contig14908_length=1421|250|60
contig14908_length=1421|1250|60
log2(expression)
0
2
4
6
8
12
16
StageI
StageII
StageIII
StageIV
StageV
StageVI

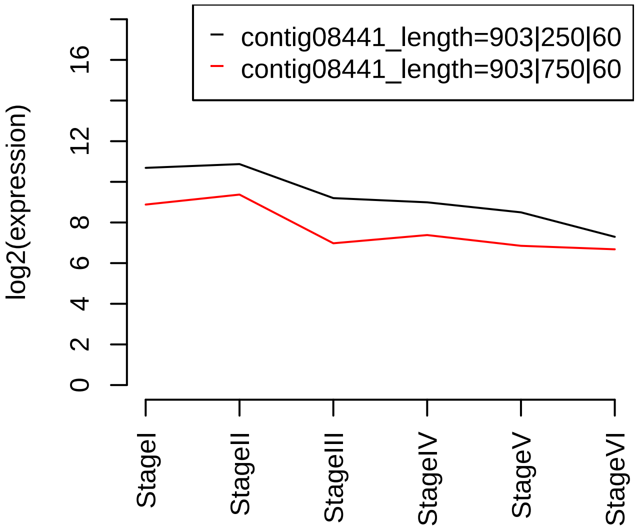
contig08441_length=903|250|60
contig08441_length=903|750|60
log2(expression)
0
2
4
6
8
12
16
StageI
StageII
StageIII
StageIV
StageV
StageVI

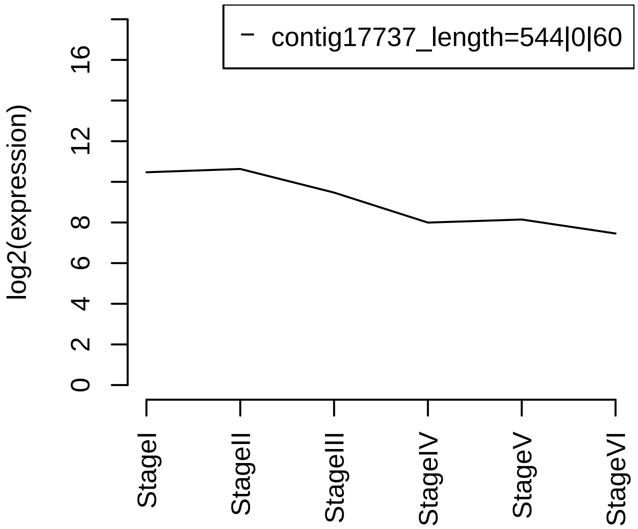
contig17737_length=544|0|60
log2(expression)
0
2
4
6
8
12
16
StageI
StageII
StageIII
StageIV
StageV
StageVI

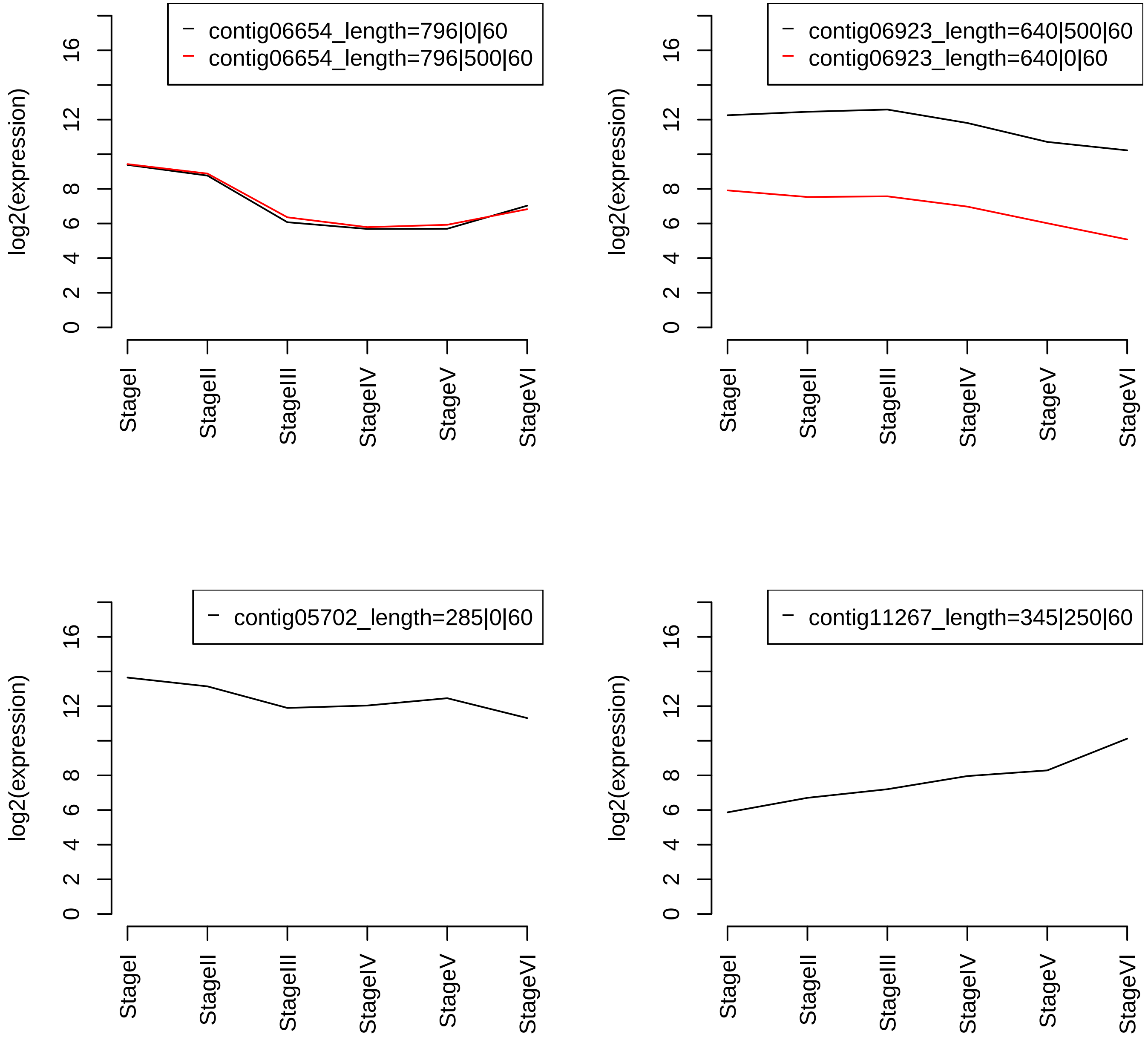
contig06654_length=796|0|60
contig06654_length=796|500|60
contig06923_length=640|500|60
contig06923_length=640|0|60
contig05702_length=285|0|60
contig11267_length=345|250|60
log2(expression)
0
2
4
6
8
12
16
StageI
StageII
StageIII
StageIV
StageV
StageVI

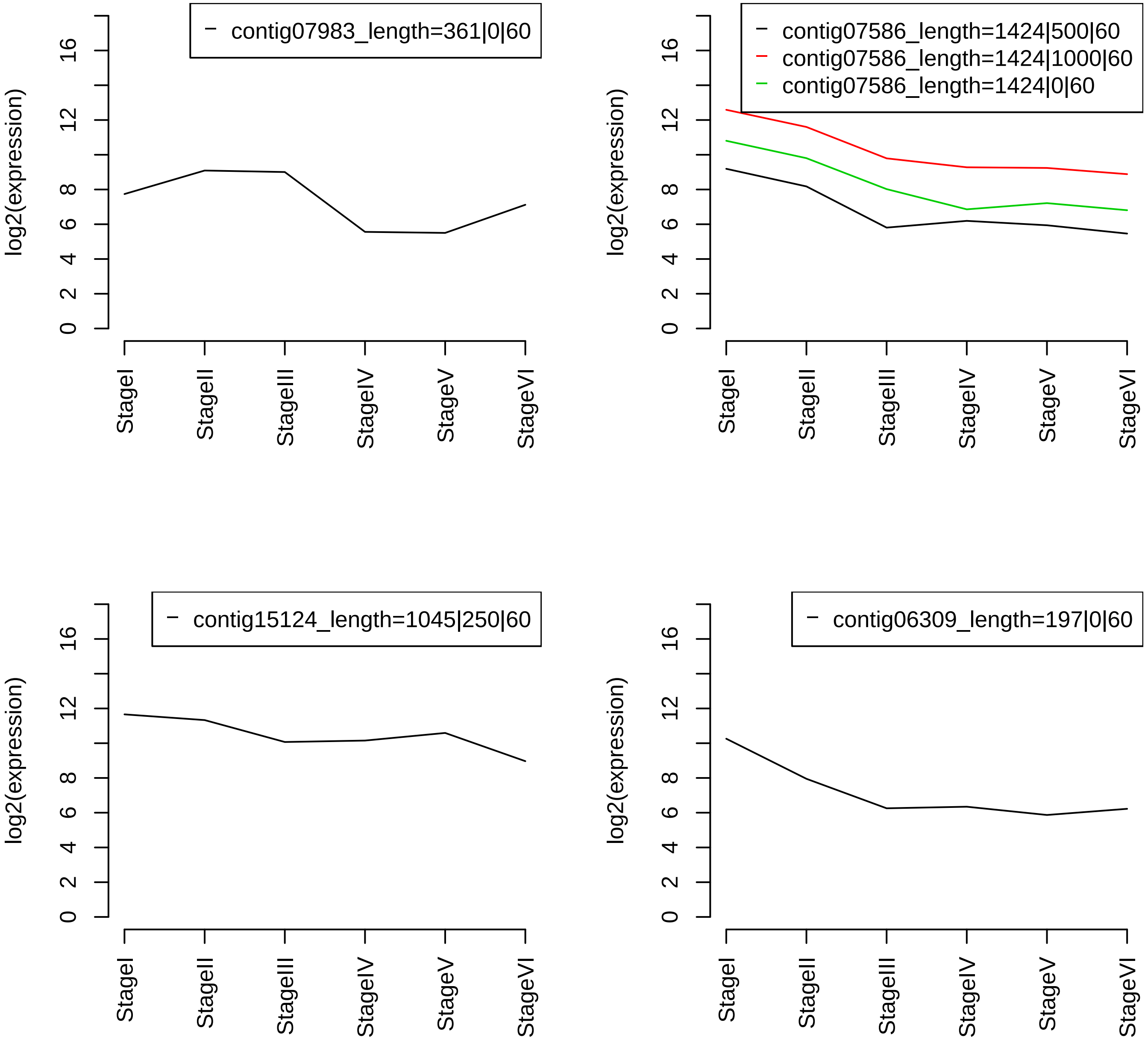
contig07983_length=361|0|60
contig07586_length=1424|500|60
contig07586_length=1424|1000|60
contig07586_length=1424|0|60
contig15124_length=1045|250|60
contig06309_length=197|0|60
log2(expression)
StageI
StageII
StageIII
StageIV
StageV
StageVI
0
2
4
6
8
12
16

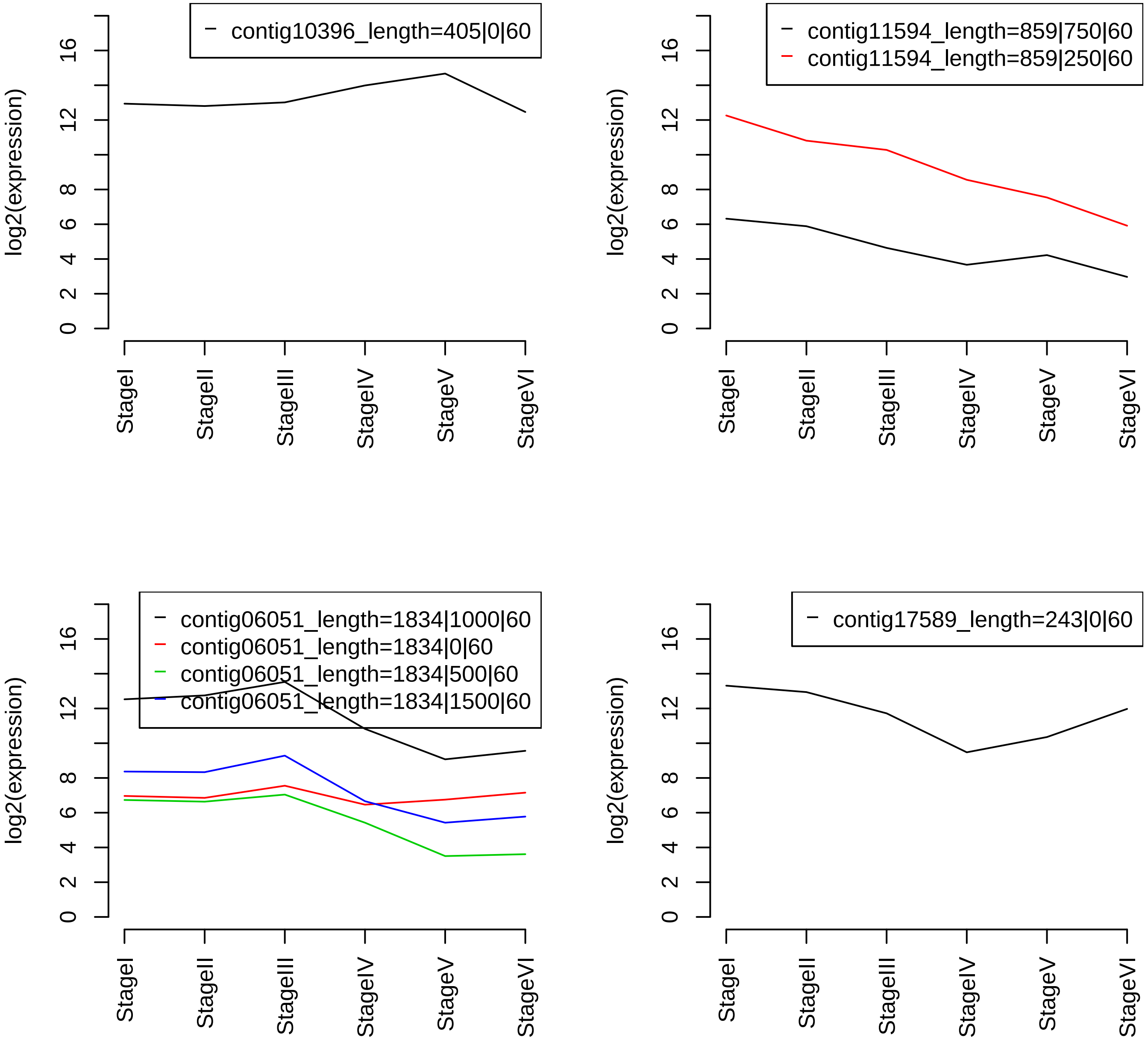

contig10396_length=405|0|60
contig11594_length=859|750|60
contig11594_length=859|250|60
contig06051_length=1834|1000|60
contig06051_length=1834|0|60
contig06051_length=1834|500|60
contig06051_length=1834|1500|60
contig17589_length=243|0|60
log2(expression)
StageI
StageII
StageIII
StageIV
StageV
StageVI

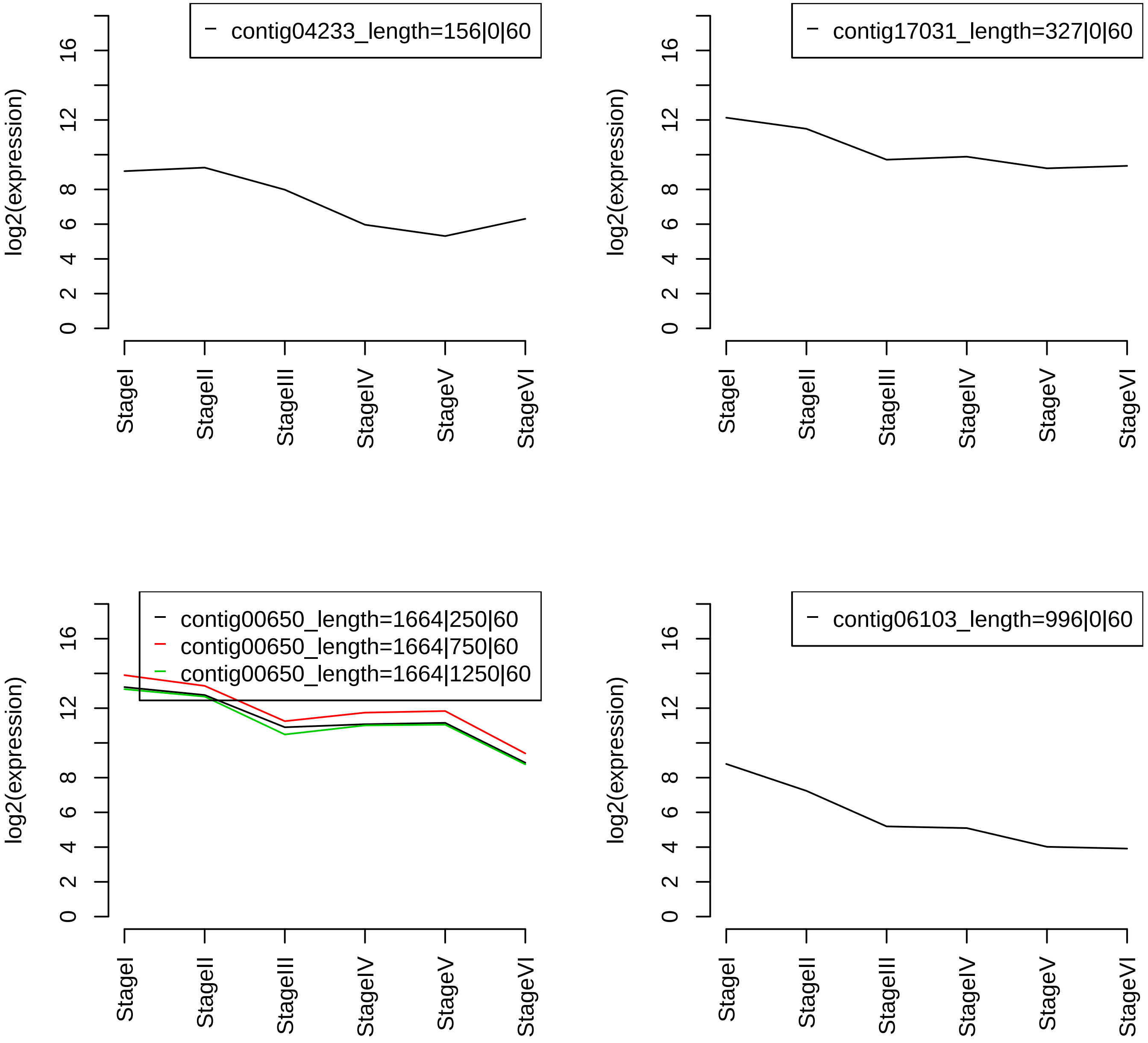

contig04233_length=156|0|60
contig17031_length=327|0|60
contig00650_length=1664|250|60
contig00650_length=1664|750|60
contig00650_length=1664|1250|60
contig06103_length=996|0|60
log2(expression)
0 2 4 6 8 12 16
StageI
StageII
StageIII
StageIV
StageV
StageVI

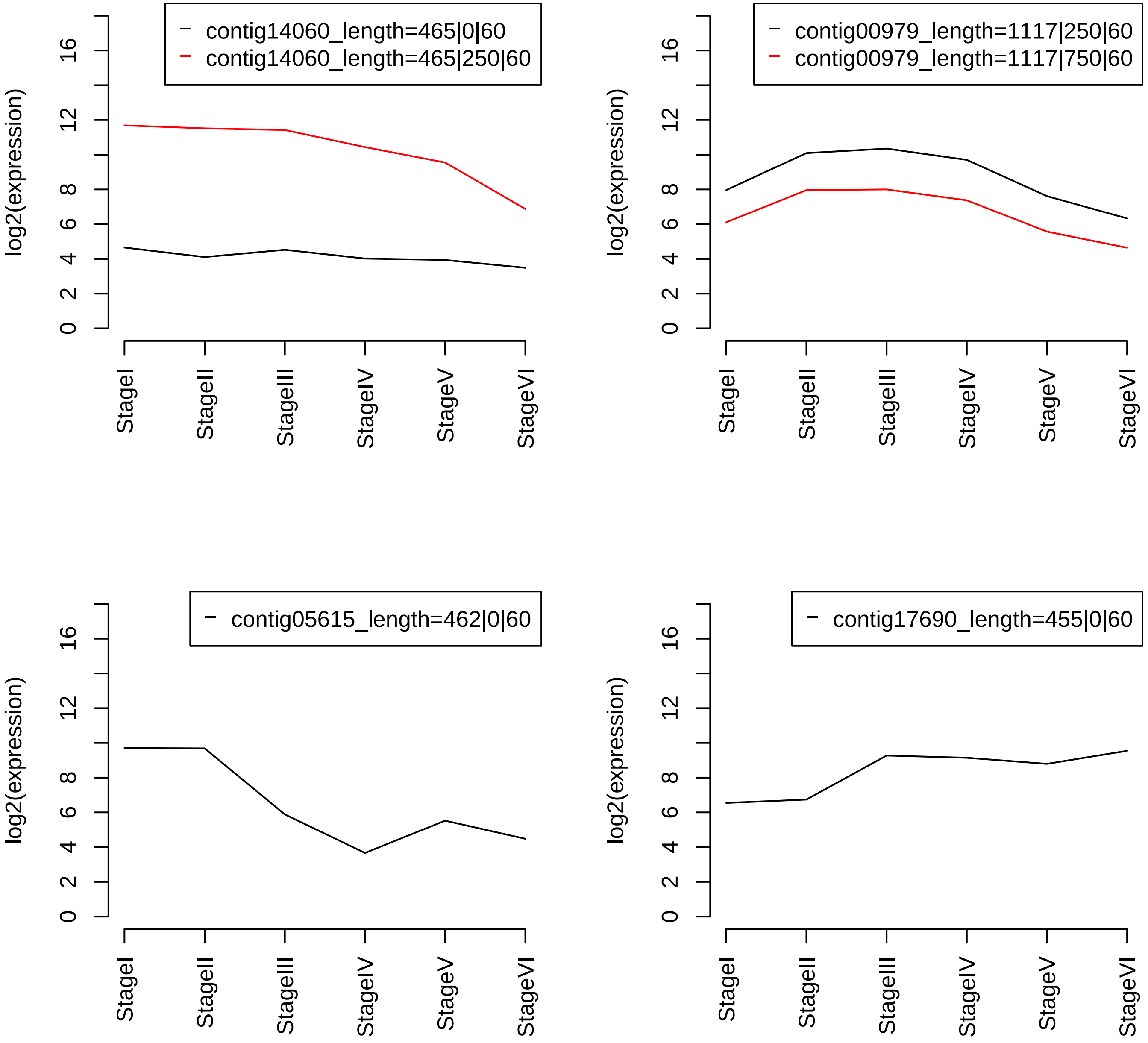

contig14060_length=465|0|60
contig14060_length=465|250|60
contig00979_length=1117|250|60
contig00979_length=1117|750|60
contig05615_length=462|0|60
contig17690_length=455|0|60
log2(expression)
0 2 4 6 8 12 16
StageI StageII StageIII StageIV StageV StageVI

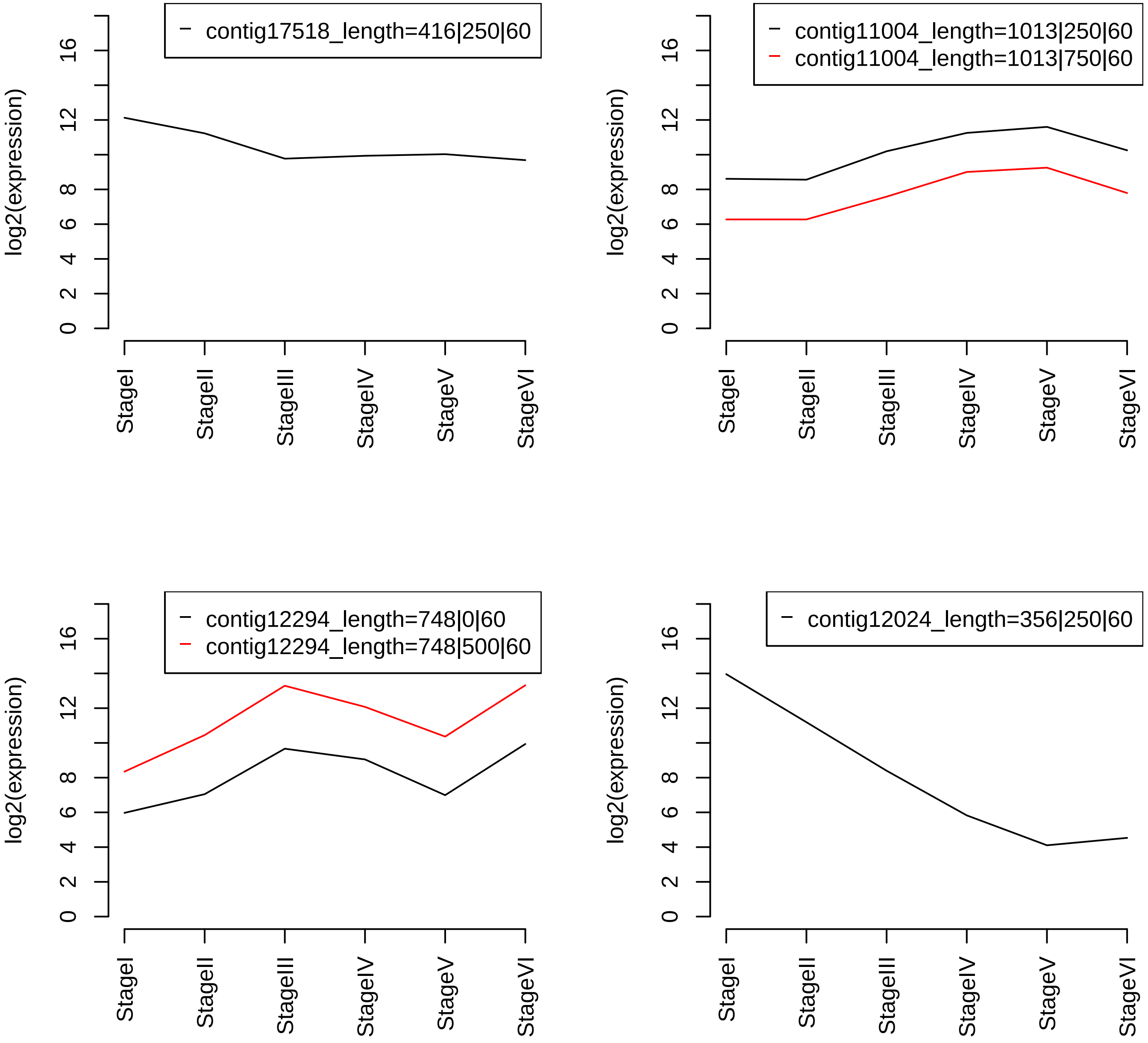
contig17518_length=416|250|60
contig11004_length=1013|250|60
contig11004_length=1013|750|60
contig12294_length=748|0|60
contig12294_length=748|500|60
contig12024_length=356|250|60
log2(expression)
0 2 4 6 8 12 16
StageI StageII StageIII StageIV StageV StageVI

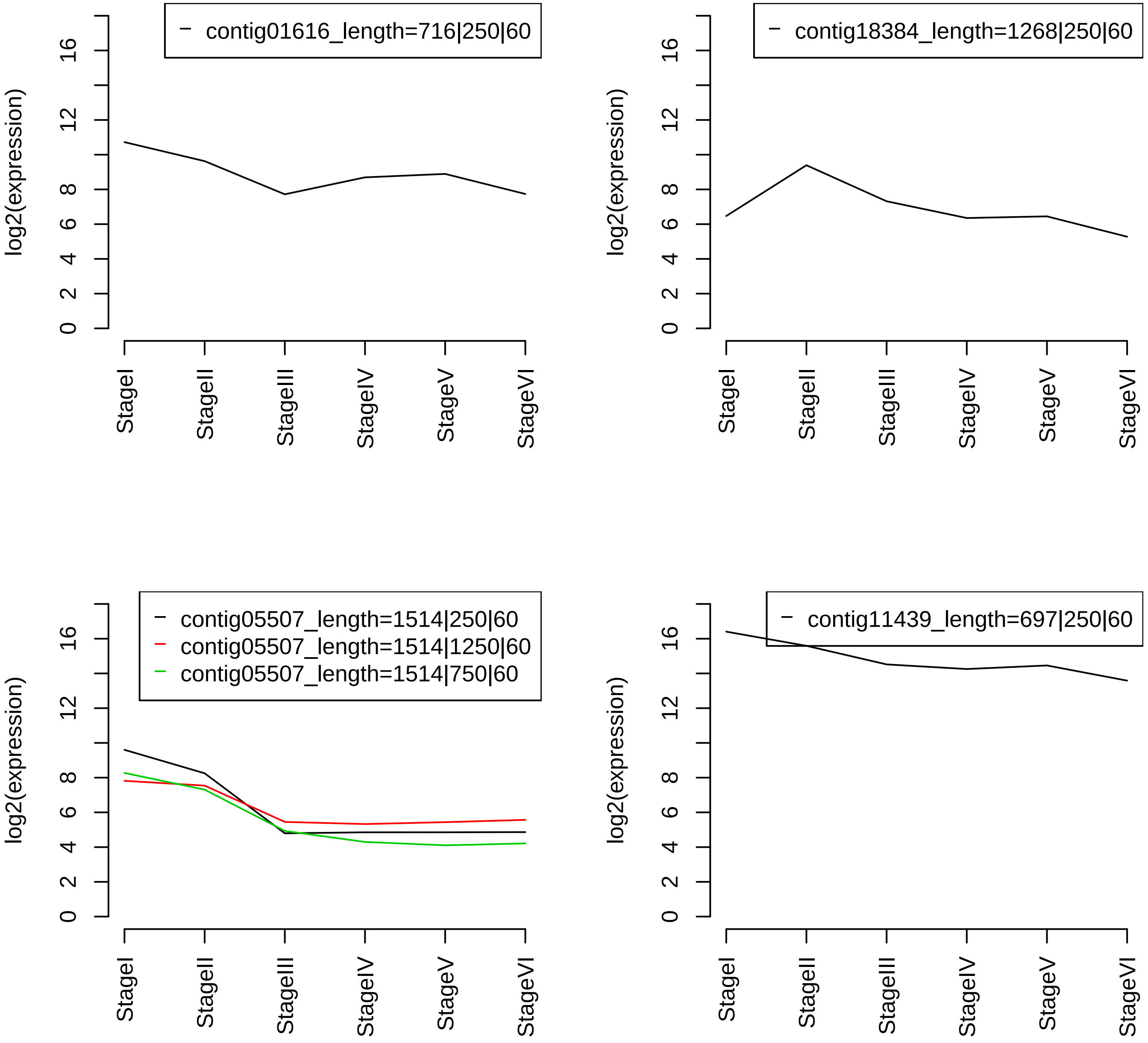
contig01616_length=716|250|60
contig18384_length=1268|250|60
contig05507_length=1514|250|60
contig05507_length=1514|1250|60
contig05507_length=1514|750|60
contig11439_length=697|250|60
log2(expression)
0 2 4 6 8 12 16
StageI
StageII
StageIII
StageIV
StageV
StageVI

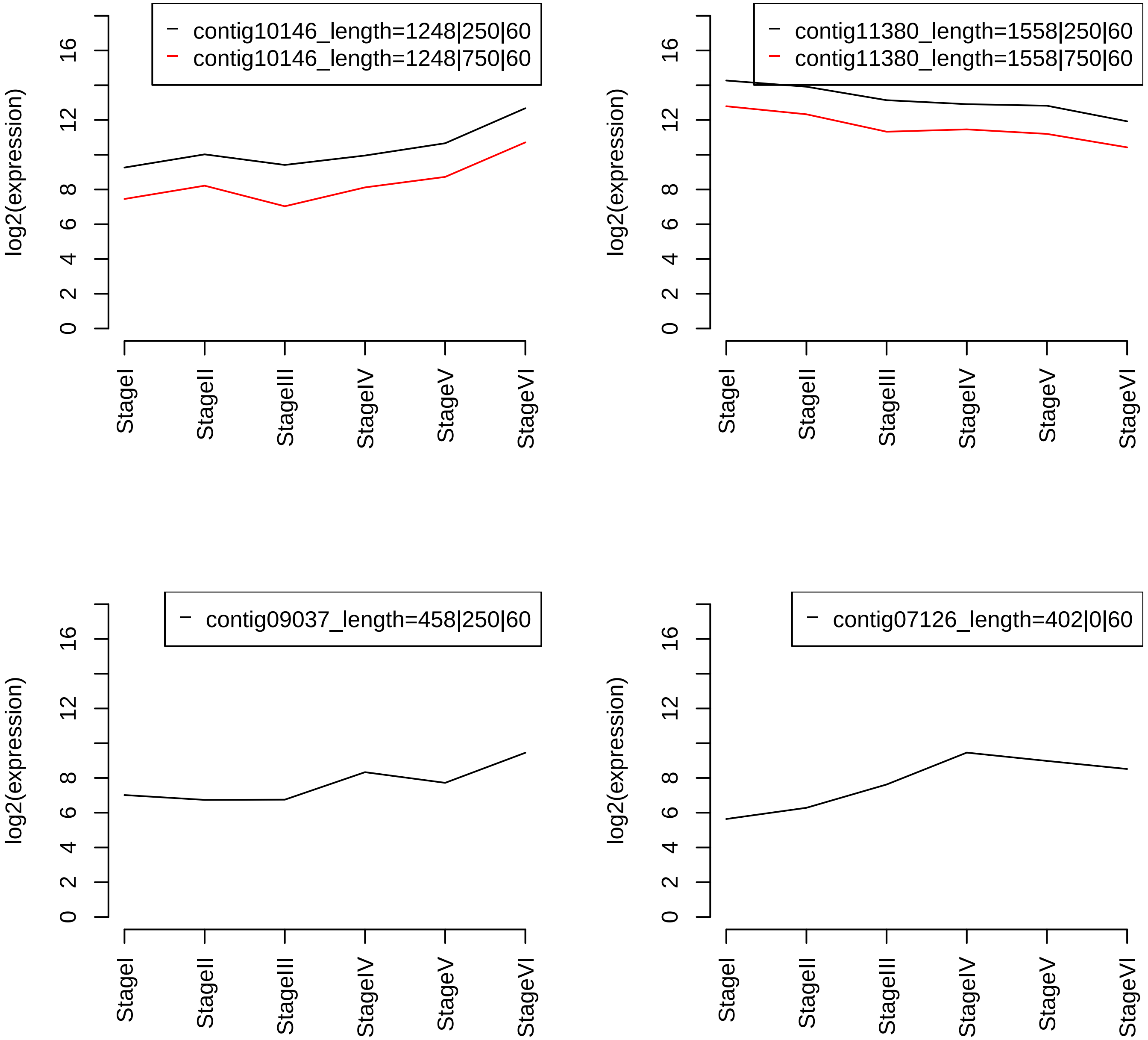
contig10146_length=1248|250|60
contig10146_length=1248|750|60
contig11380_length=1558|250|60
contig11380_length=1558|750|60
contig09037_length=458|250|60
contig07126_length=402|0|60
log2(expression)
0
2
4
6
8
12
16
StageI
StageII
StageIII
StageIV
StageV
StageVI

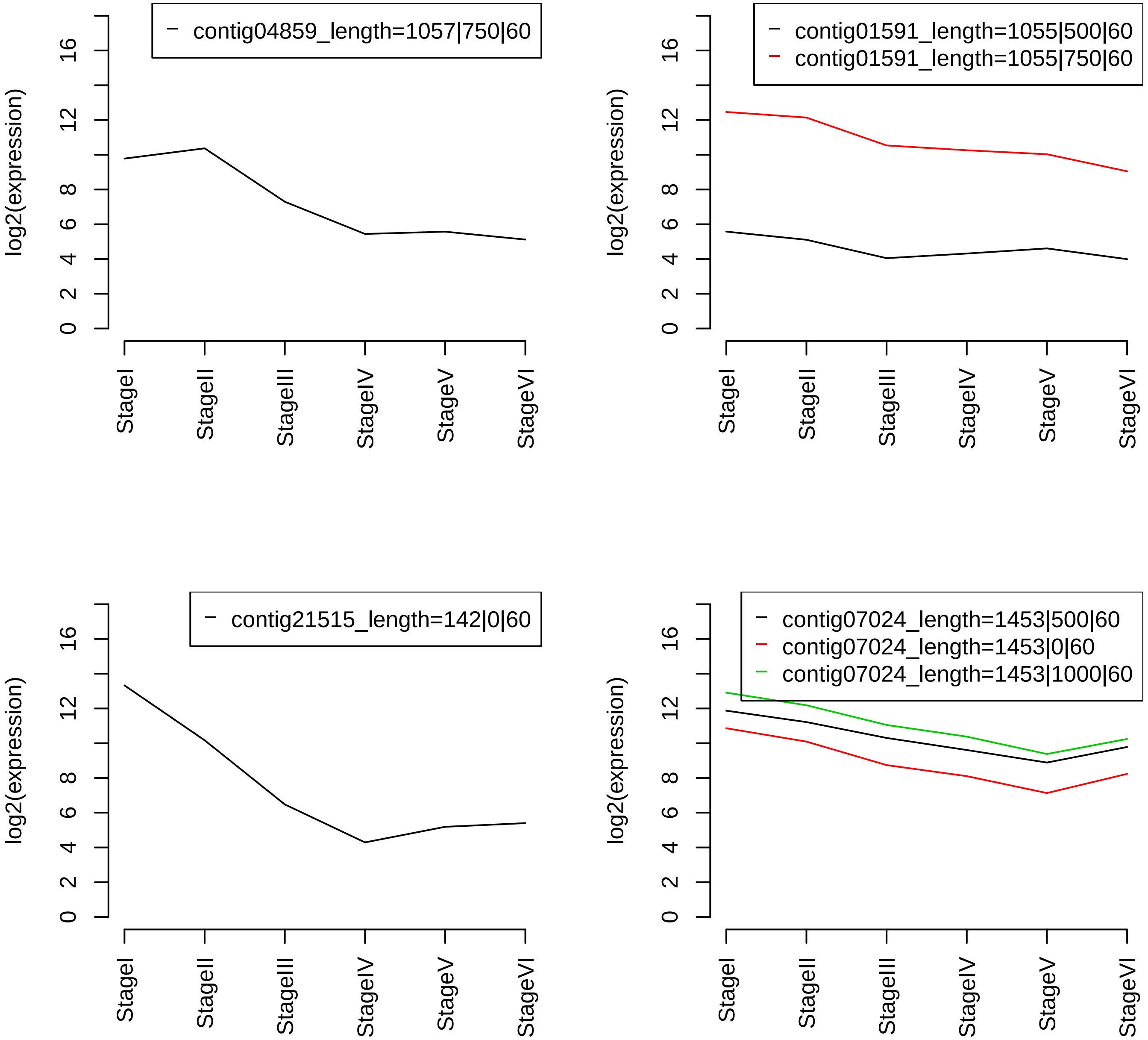

contig04859_length=1057|750|60
contig01591_length=1055|500|60
contig01591_length=1055|750|60
contig21515_length=142|0|60
contig07024_length=1453|500|60
contig07024_length=1453|0|60
contig07024_length=1453|1000|60
log2(expression)
0
2
4
6
8
12
16
StageI
StageII
StageIII
StageIV
StageV
StageVI

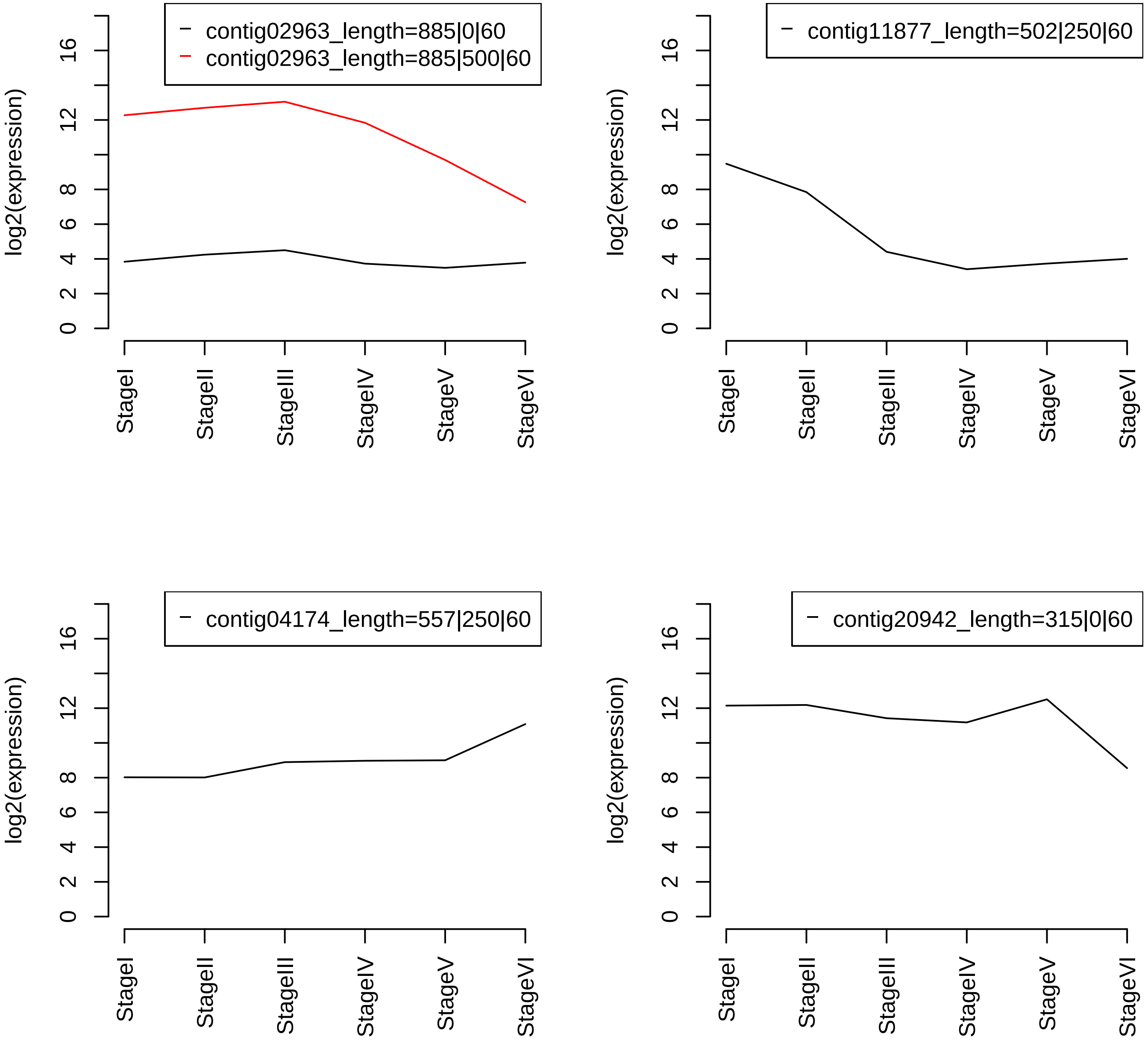
contig02963_length=885|0|60
contig02963_length=885|500|60
contig11877_length=502|250|60
contig04174_length=557|250|60
contig20942_length=315|0|60
log2(expression)
0
2
4
6
8
12
16
StageI
StageII
StageIII
StageIV
StageV
StageVI

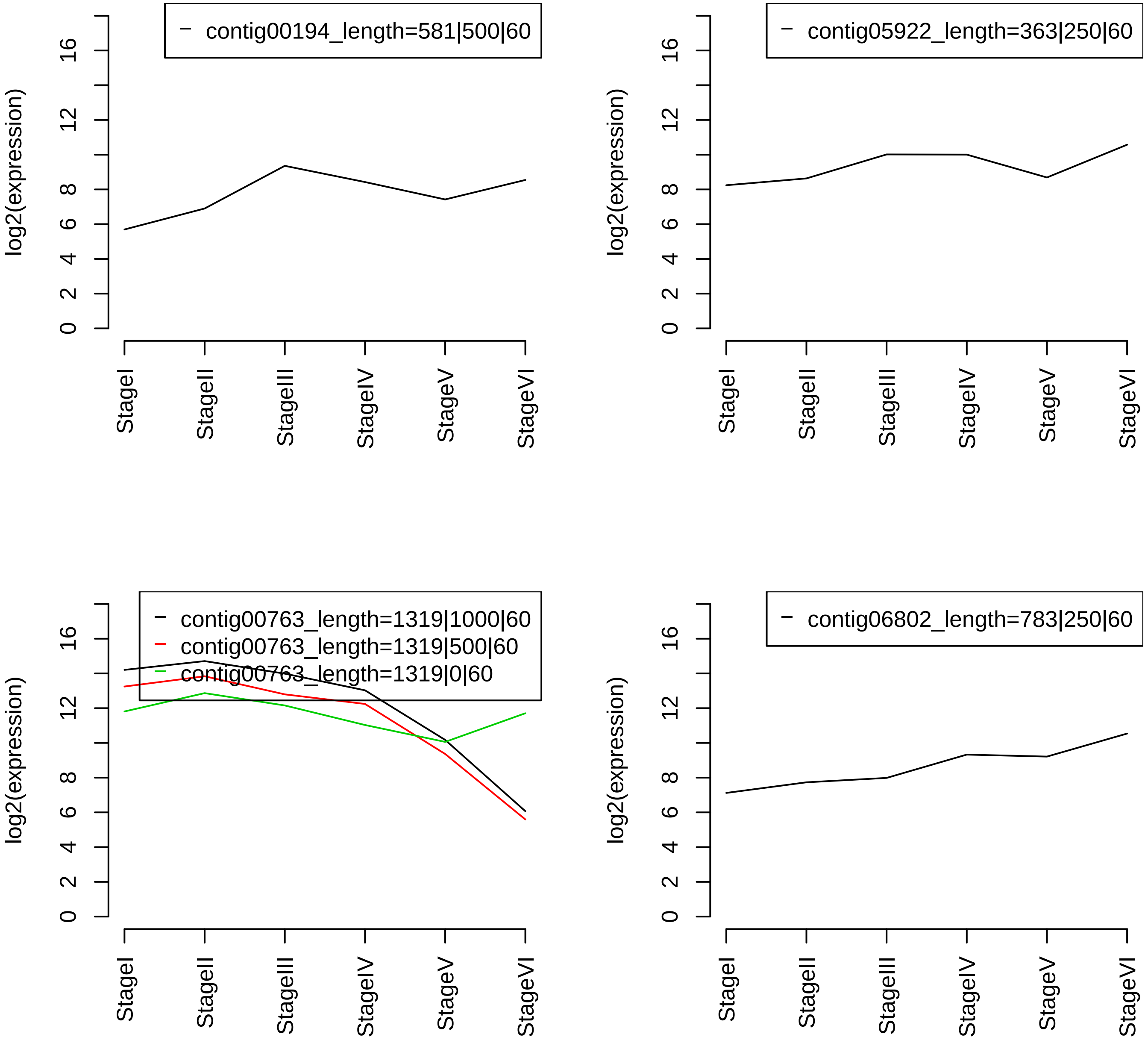
contig00194_length=581|500|60
log2(expression)
0
2
4
6
8
12
16
StageI
StageII
StageIII
StageIV
StageV
StageVI
contig05922_length=363|250|60
log2(expression)
0
2
4
6
8
12
16
StageI
StageII
StageIII
StageIV
StageV
StageVI
contig00763_length=1319|1000|60
contig00763_length=1319|500|60
contig00763_length=1319|0|60
log2(expression)
0
2
4
6
8
12
16
StageI
StageII
StageIII
StageIV
StageV
StageVI
contig06802_length=783|250|60
log2(expression)
0
2
4
6
8
12
16
StageI
StageII
StageIII
StageIV
StageV
StageVI

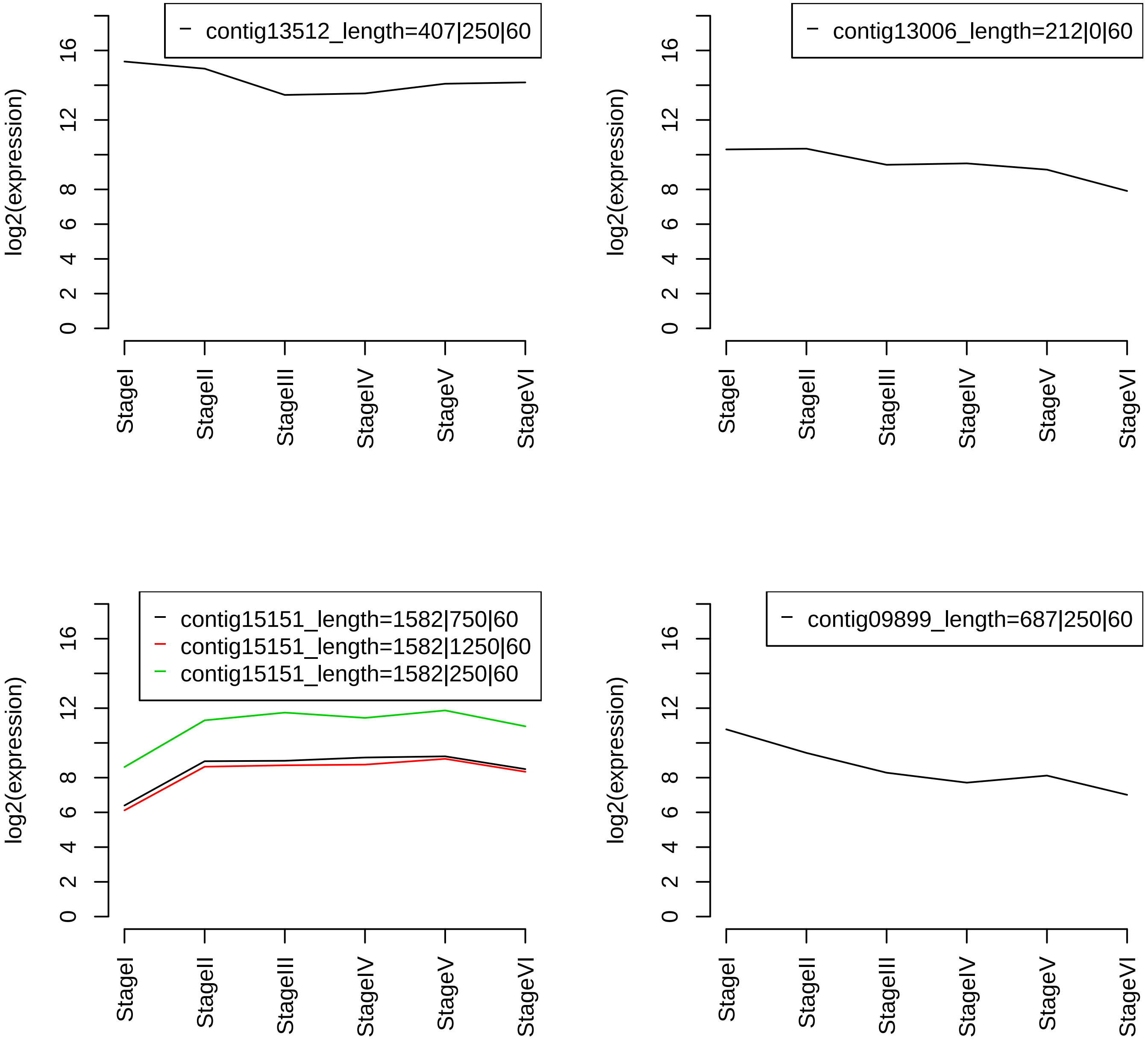
contig13512_length=407|250|60
contig13006_length=212|0|60
contig15151_length=1582|750|60
contig15151_length=1582|1250|60
contig15151_length=1582|250|60
contig09899_length=687|250|60
log2(expression)
0 2 4 6 8 12 16
StageI
StageII
StageIII
StageIV
StageV
StageVI

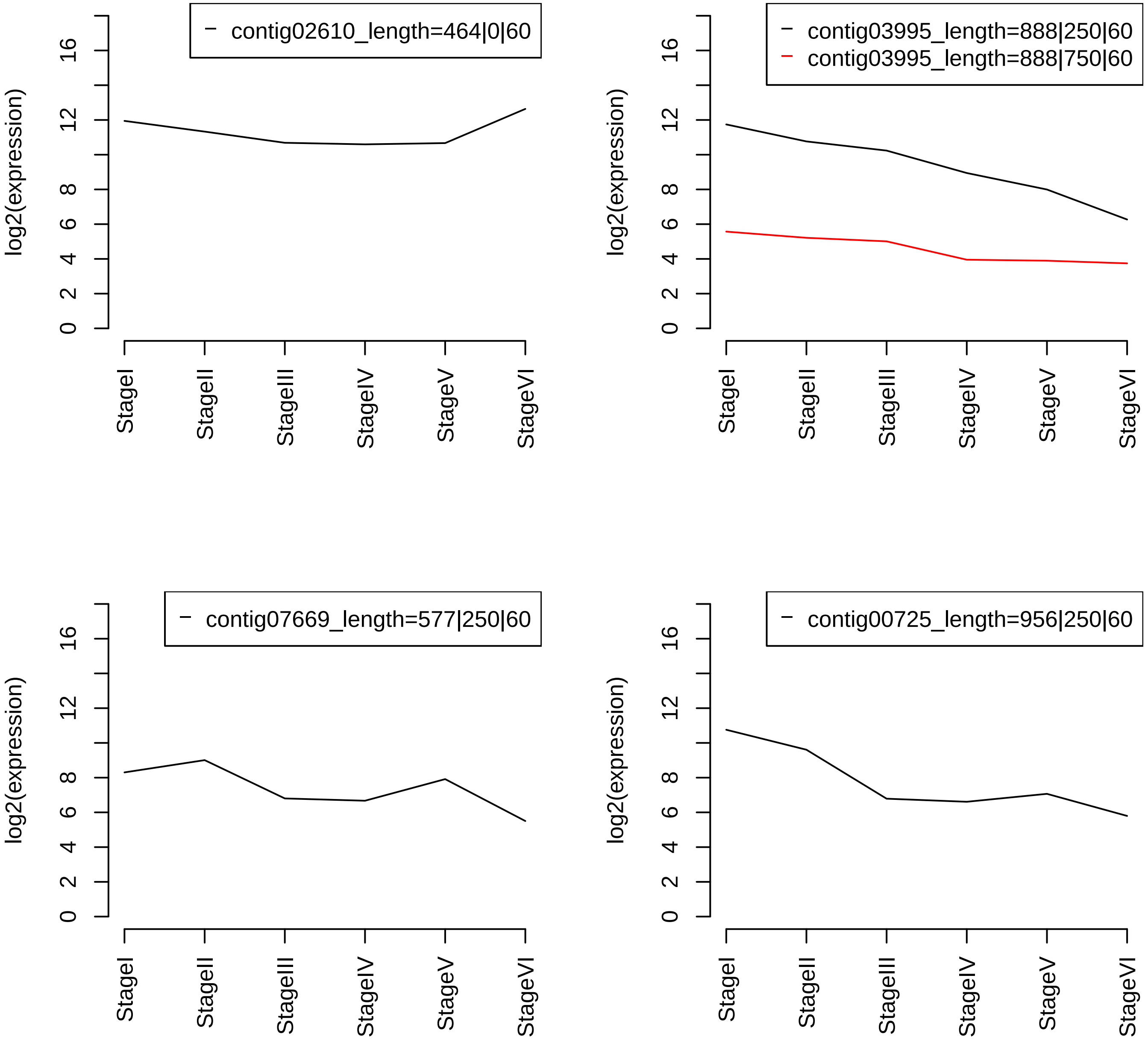
contig02610_length=464|0|60
contig03995_length=888|250|60
contig03995_length=888|750|60
contig07669_length=577|250|60
contig00725_length=956|250|60
log2(expression)
0
2
4
6
8
12
16
StageI
StageII
StageIII
StageIV
StageV
StageVI

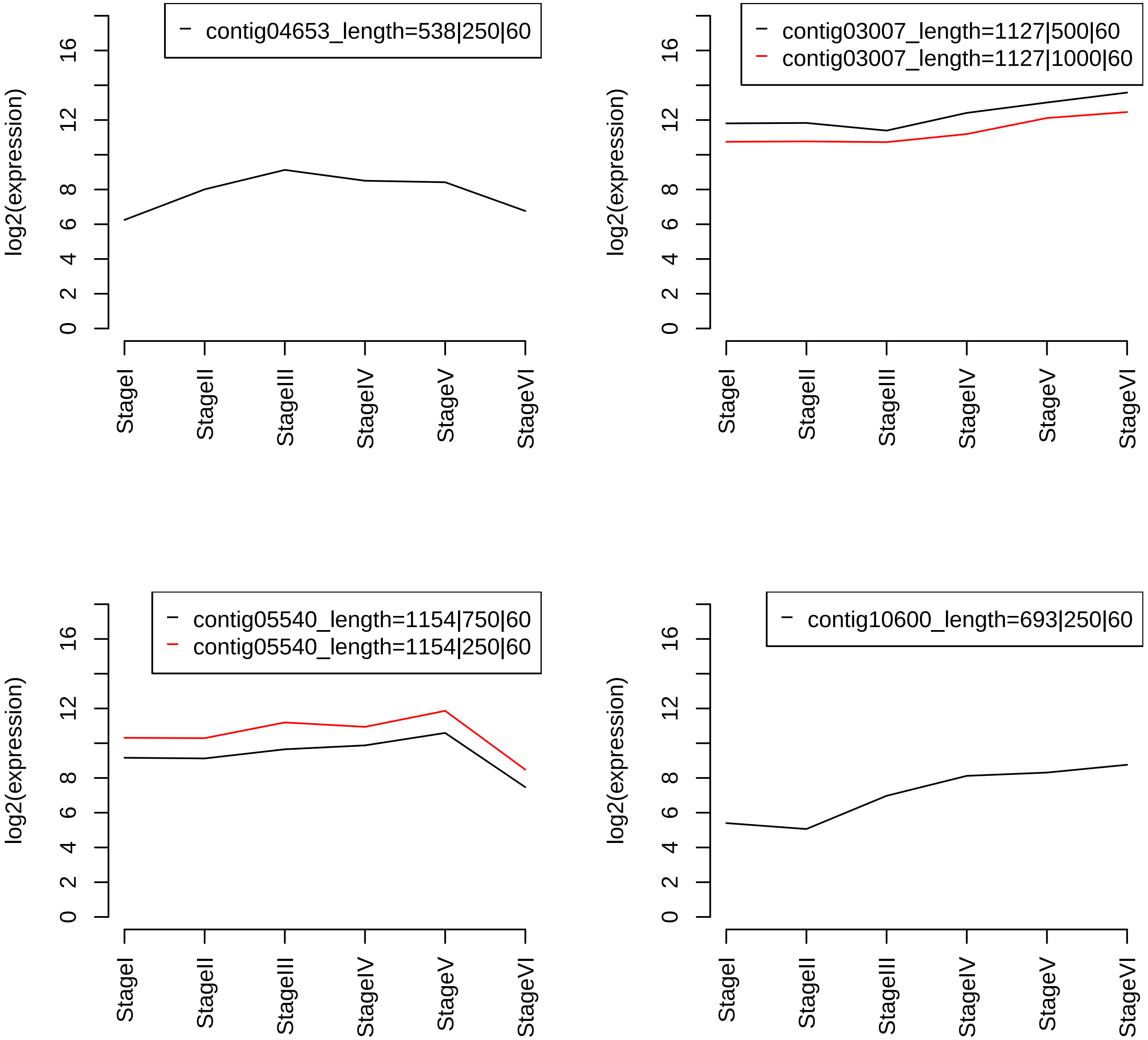

contig04653_length=538|250|60
contig03007_length=1127|500|60
contig03007_length=1127|1000|60
contig05540_length=1154|750|60
contig05540_length=1154|250|60
contig10600_length=693|250|60
log2(expression)
0
2
4
6
8
12
16
StageI
StageII
StageIII
StageIV
StageV
StageVI

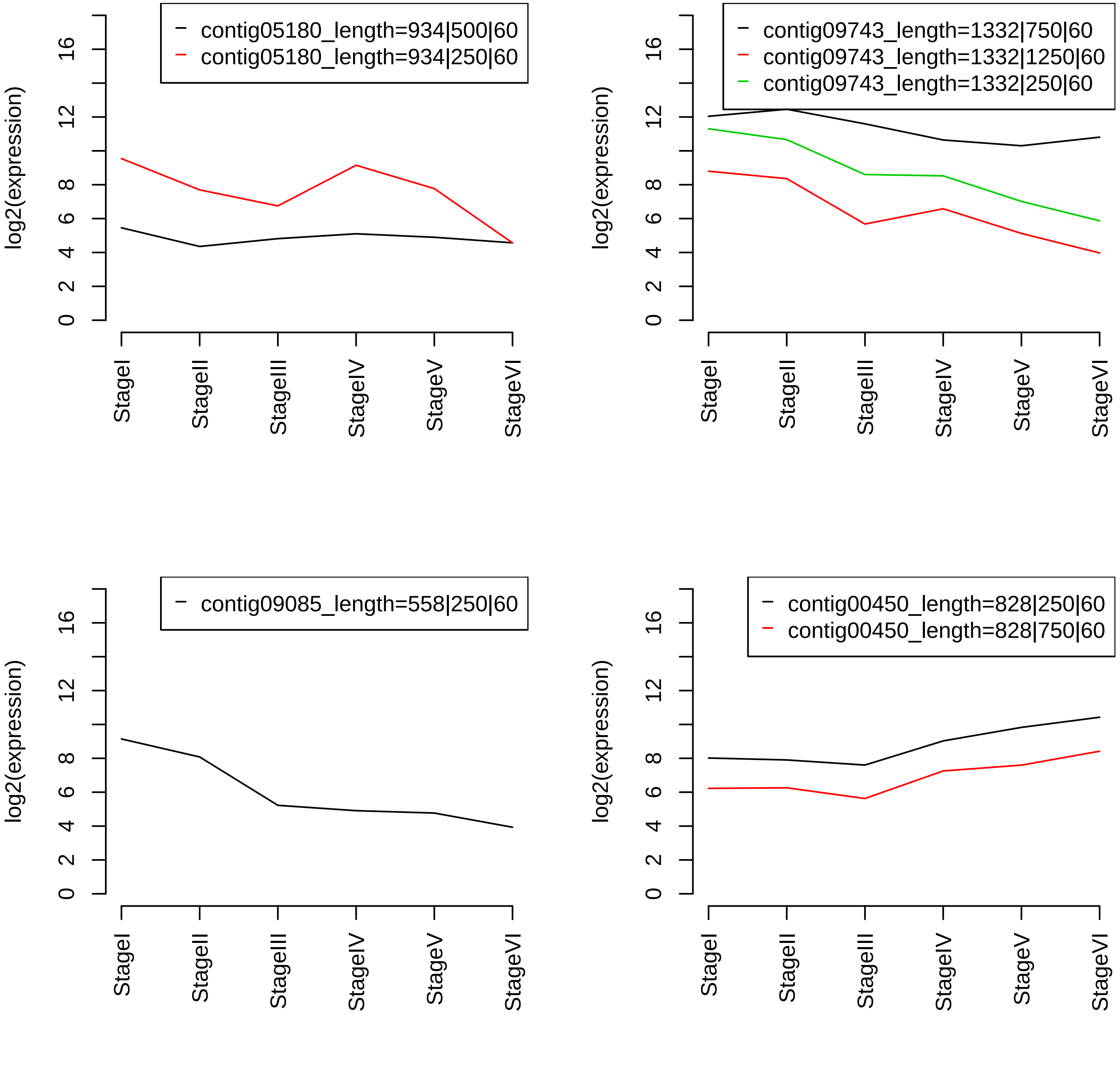
contig05180_length=934|500|60
contig05180_length=934|250|60
contig09743_length=1332|750|60
contig09743_length=1332|1250|60
contig09743_length=1332|250|60
contig09085_length=558|250|60
contig00450_length=828|250|60
contig00450_length=828|750|60
log2(expression)
0 2 4 6 8 12 16
StageI StageII StageIII StageIV StageV StageVI

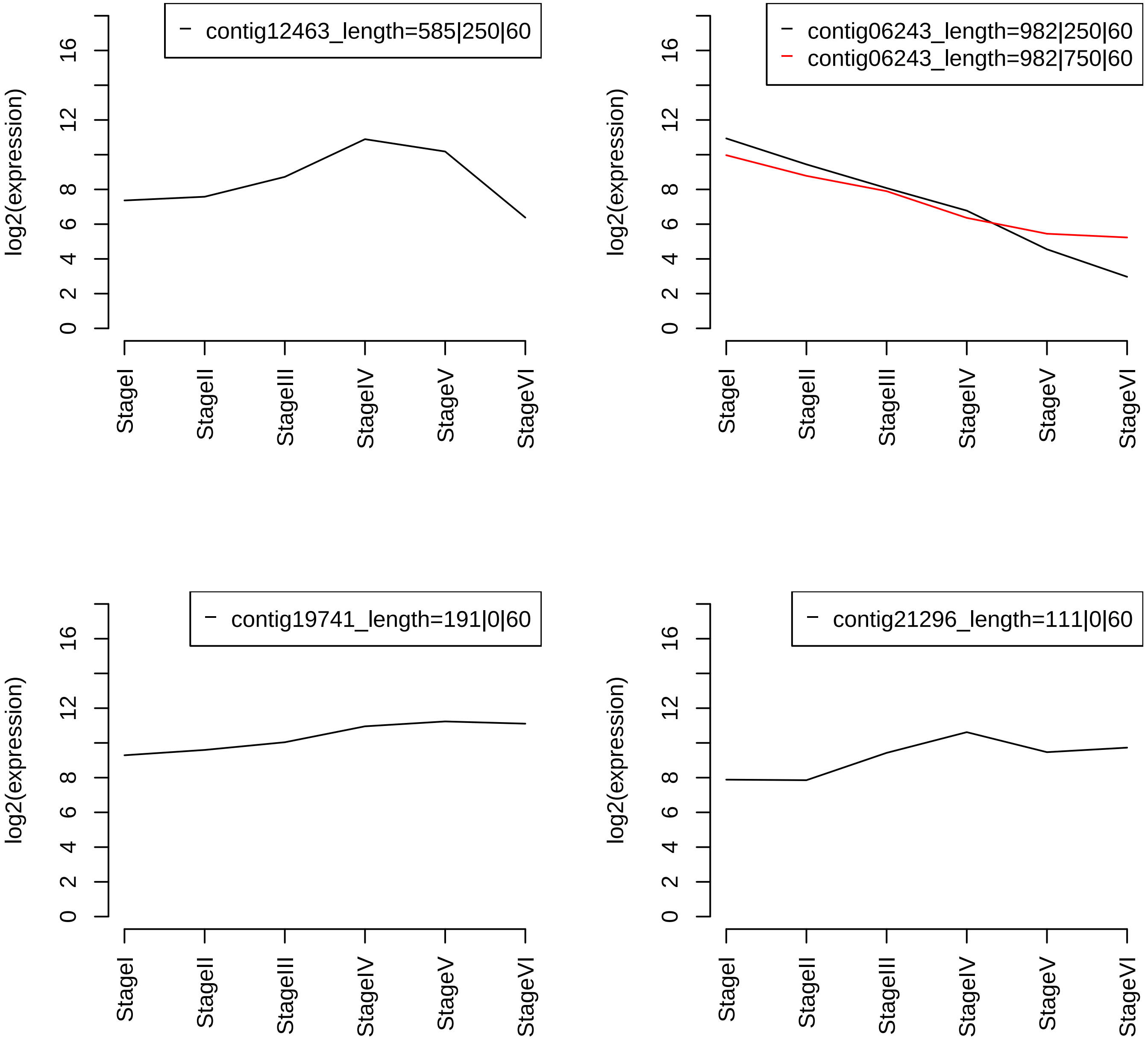

contig12463_length=585|250|60
contig06243_length=982|250|60
contig06243_length=982|750|60
contig19741_length=191|0|60
contig21296_length=111|0|60
log2(expression)
0
2
4
6
8
12
16
StageI
StageII
StageIII
StageIV
StageV
StageVI

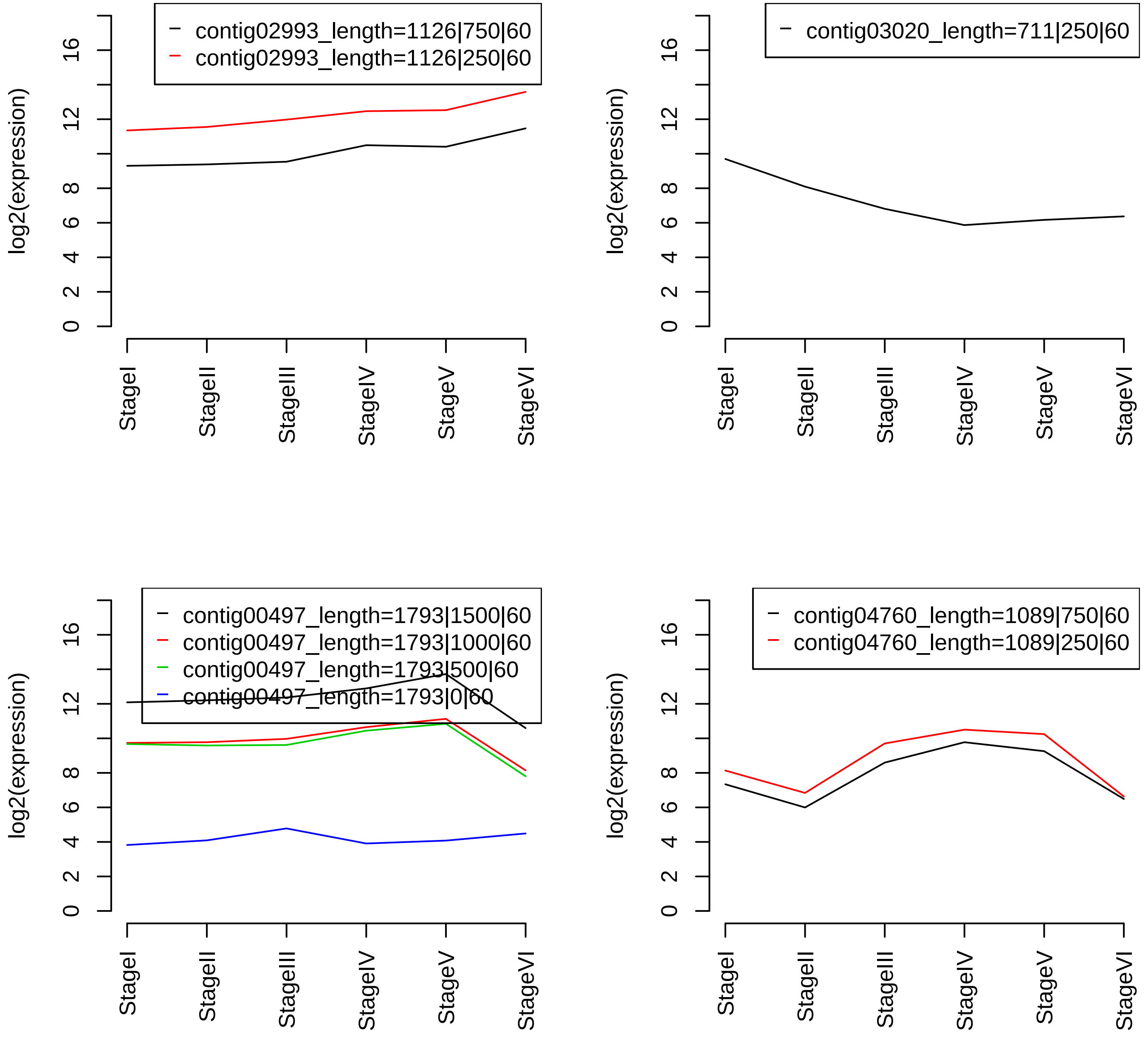

contig02993_length=1126|750|60
contig02993_length=1126|250|60
log2(expression)
0
2
4
6
8
12
16
StageI
StageII
StageIII
StageIV
StageV
StageVI
contig03020_length=711|250|60
contig00497_length=1793|1500|60
contig00497_length=1793|1000|60
contig00497_length=1793|500|60
contig00497_length=1793|0|60
contig04760_length=1089|750|60
contig04760_length=1089|250|60

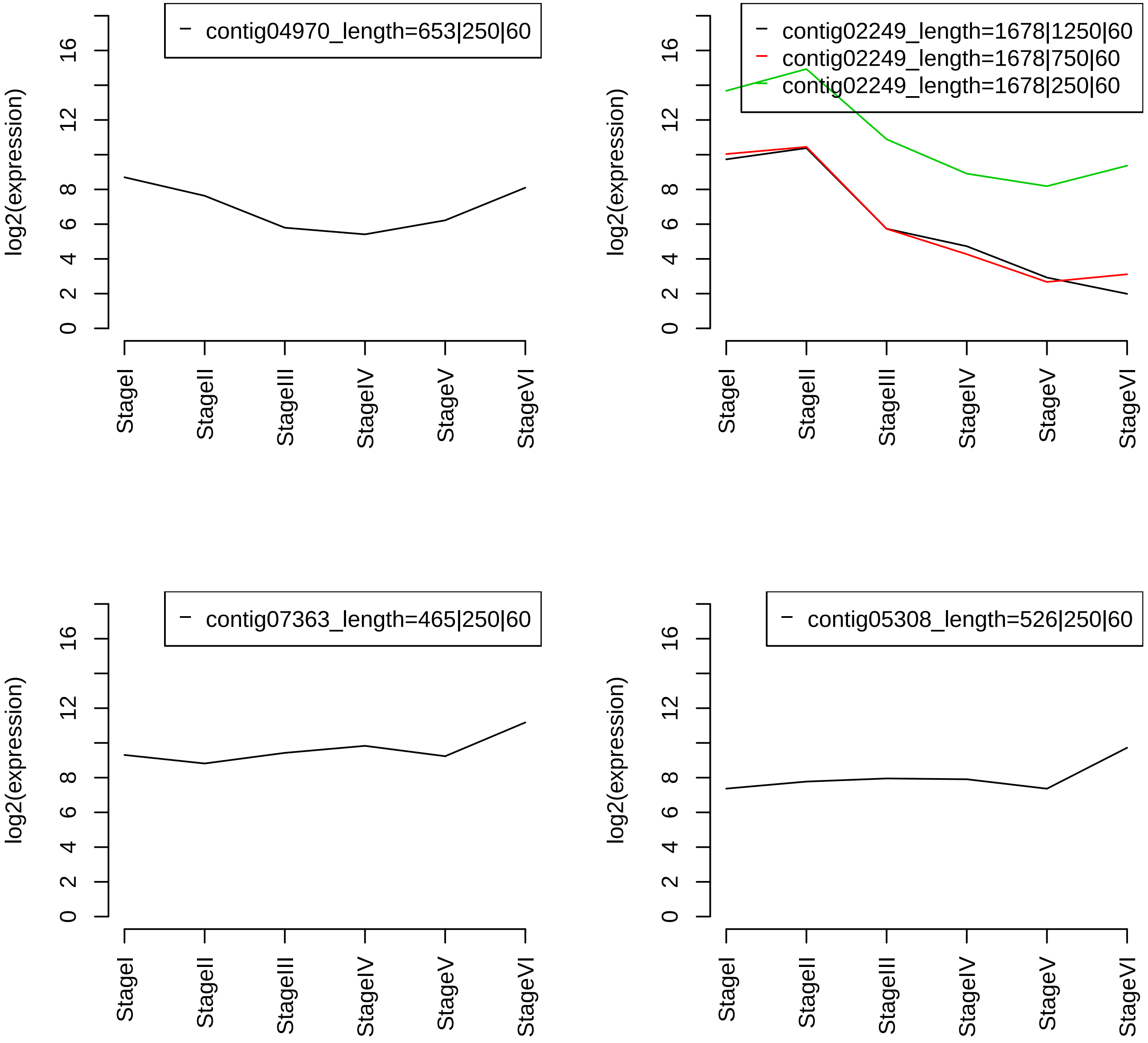
contig04970_length=653|250|60
contig02249_length=1678|1250|60
contig02249_length=1678|750|60
contig02249_length=1678|250|60
contig07363_length=465|250|60
contig05308_length=526|250|60
log2(expression)
0 2 4 6 8 12 16
StageI StageII StageIII StageIV StageV StageVI

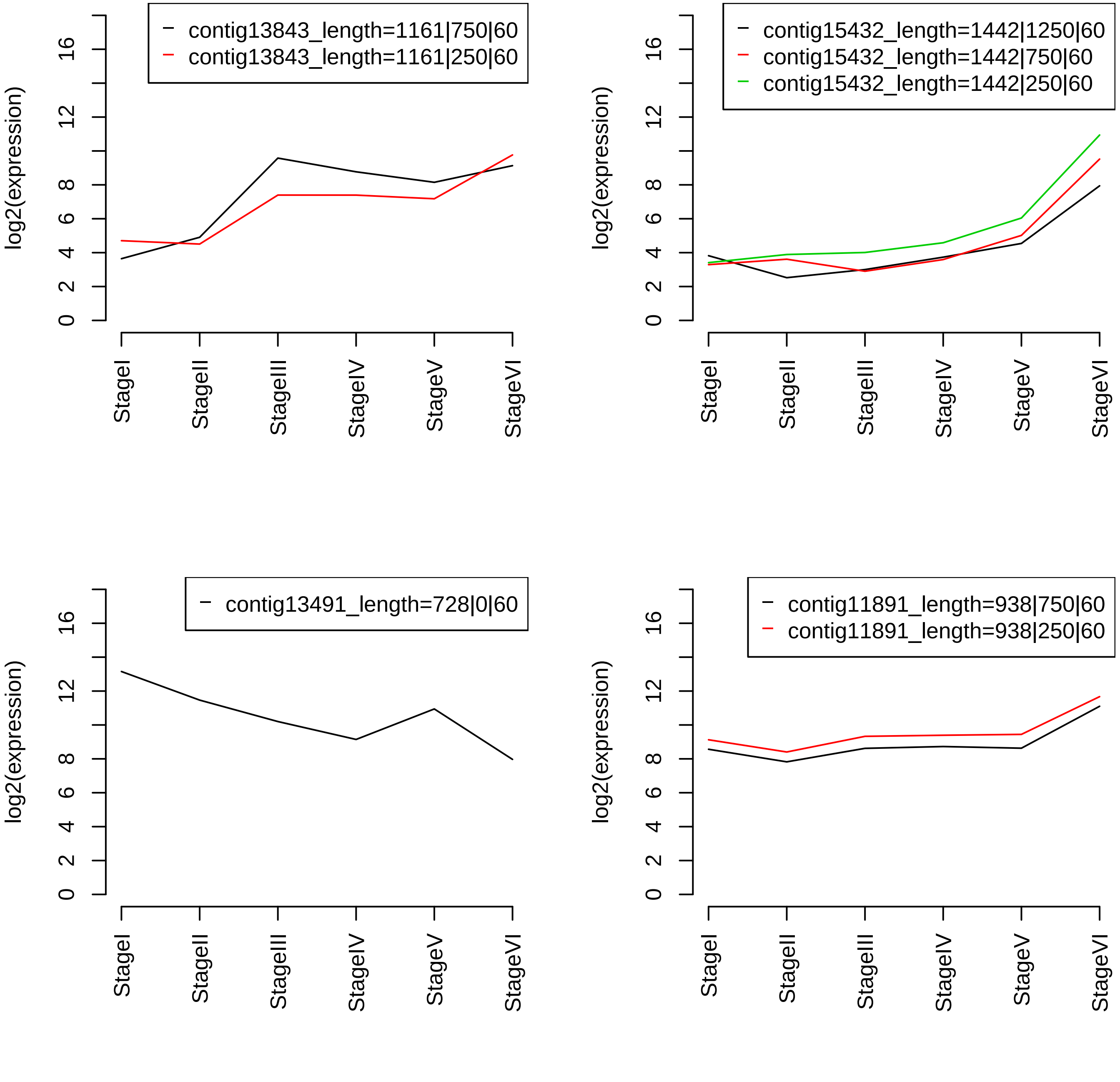
contig13843_length=1161|750|60
contig13843_length=1161|250|60
contig15432_length=1442|1250|60
contig15432_length=1442|750|60
contig15432_length=1442|250|60
contig13491_length=728|0|60
contig11891_length=938|750|60
contig11891_length=938|250|60
log2(expression)
0 2 4 6 8 12 16
StageI StageII StageIII StageIV StageV StageVI

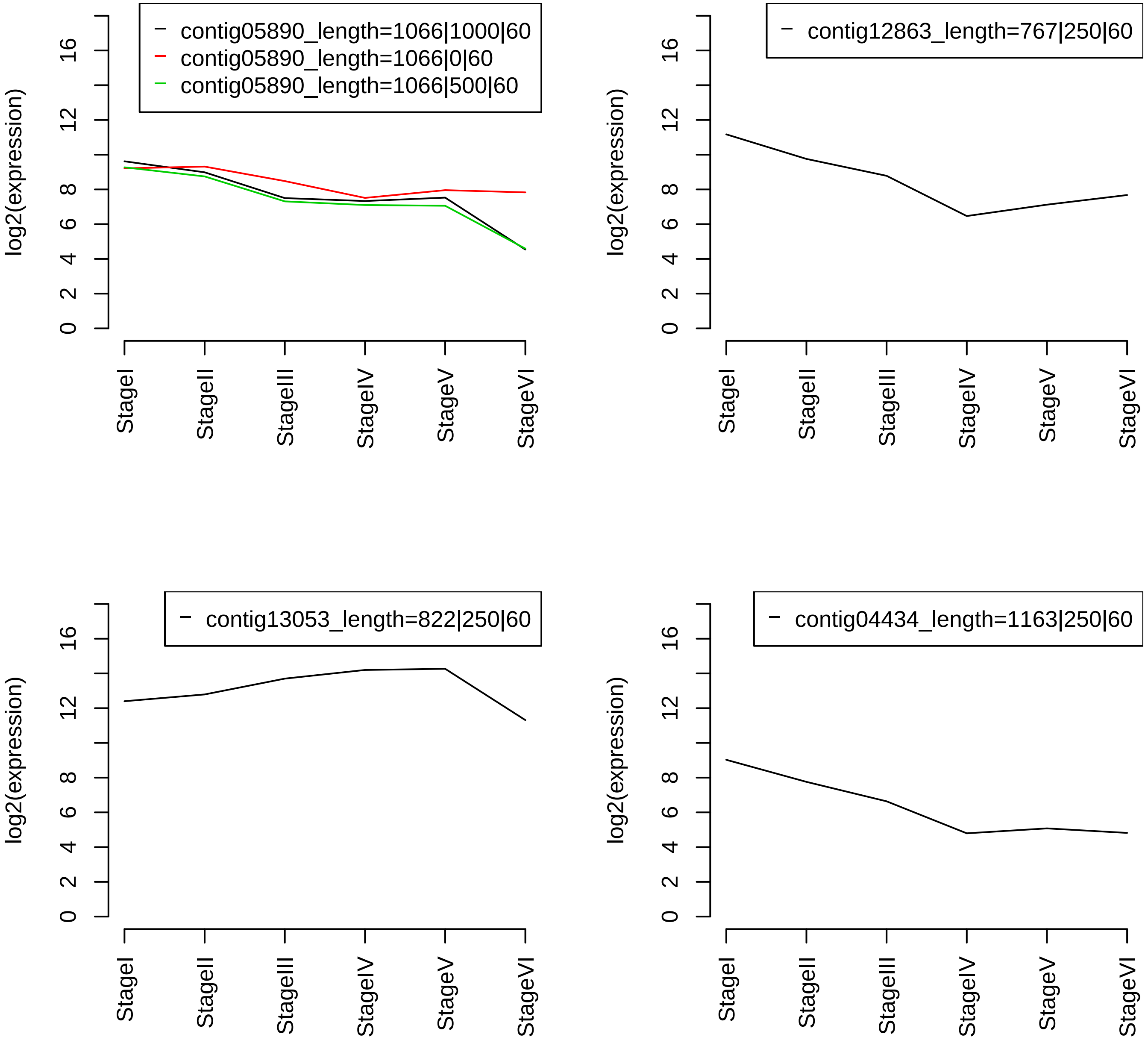
contig05890_length=1066|1000|60
contig05890_length=1066|0|60
contig05890_length=1066|500|60
contig12863_length=767|250|60
contig13053_length=822|250|60
contig04434_length=1163|250|60
log2(expression)
0
2
4
6
8
12
16
StageI
StageII
StageIII
StageIV
StageV
StageVI

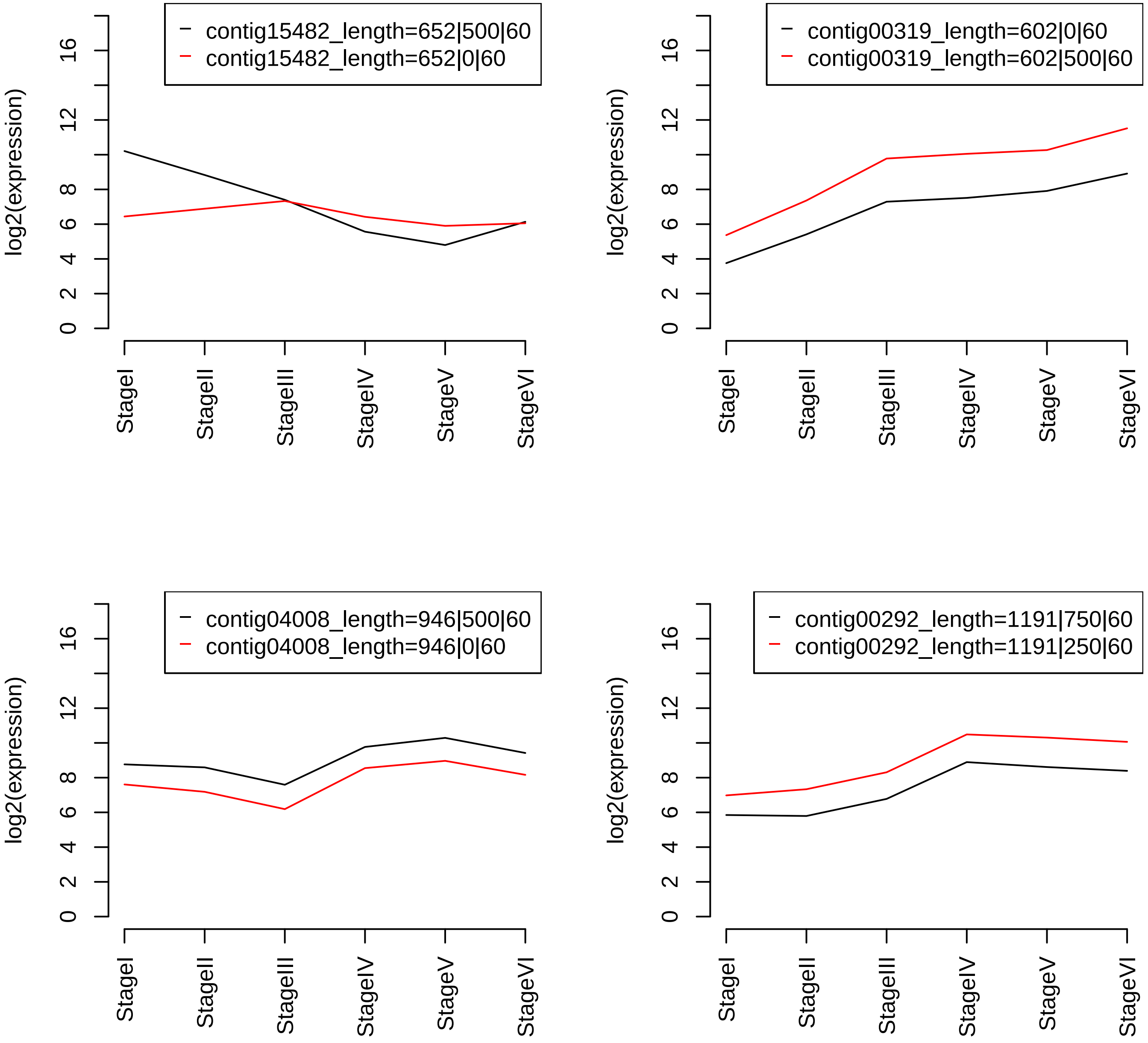
contig15482_length=652|500|60
contig15482_length=652|0|60
log2(expression)
0
2
4
6
8
12
16
StageI
StageII
StageIII
StageIV
StageV
StageVI
contig00319_length=602|0|60
contig00319_length=602|500|60
log2(expression)
0
2
4
6
8
12
16
StageI
StageII
StageIII
StageIV
StageV
StageVI
contig04008_length=946|500|60
contig04008_length=946|0|60
log2(expression)
0
2
4
6
8
12
16
StageI
StageII
StageIII
StageIV
StageV
StageVI
contig00292_length=1191|750|60
contig00292_length=1191|250|60
log2(expression)
0
2
4
6
8
12
16
StageI
StageII
StageIII
StageIV
StageV
StageVI

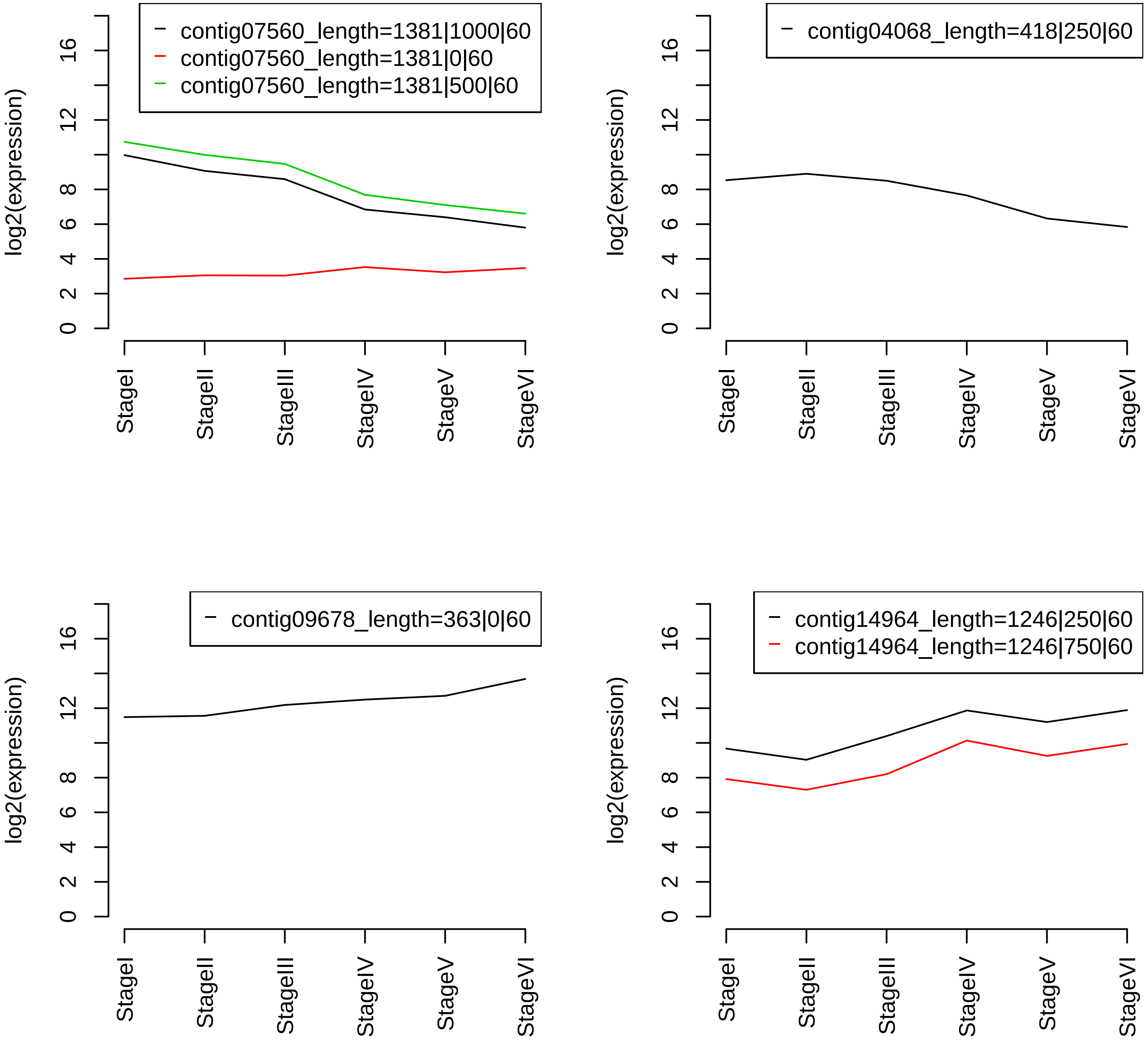
contig07560_length=1381|1000|60
contig07560_length=1381|0|60
contig07560_length=1381|500|60
contig04068_length=418|250|60
contig09678_length=363|0|60
contig14964_length=1246|250|60
contig14964_length=1246|750|60
log2(expression)
StageI
StageII
StageIII
StageIV
StageV
StageVI
0
2
4
6
8
12
16

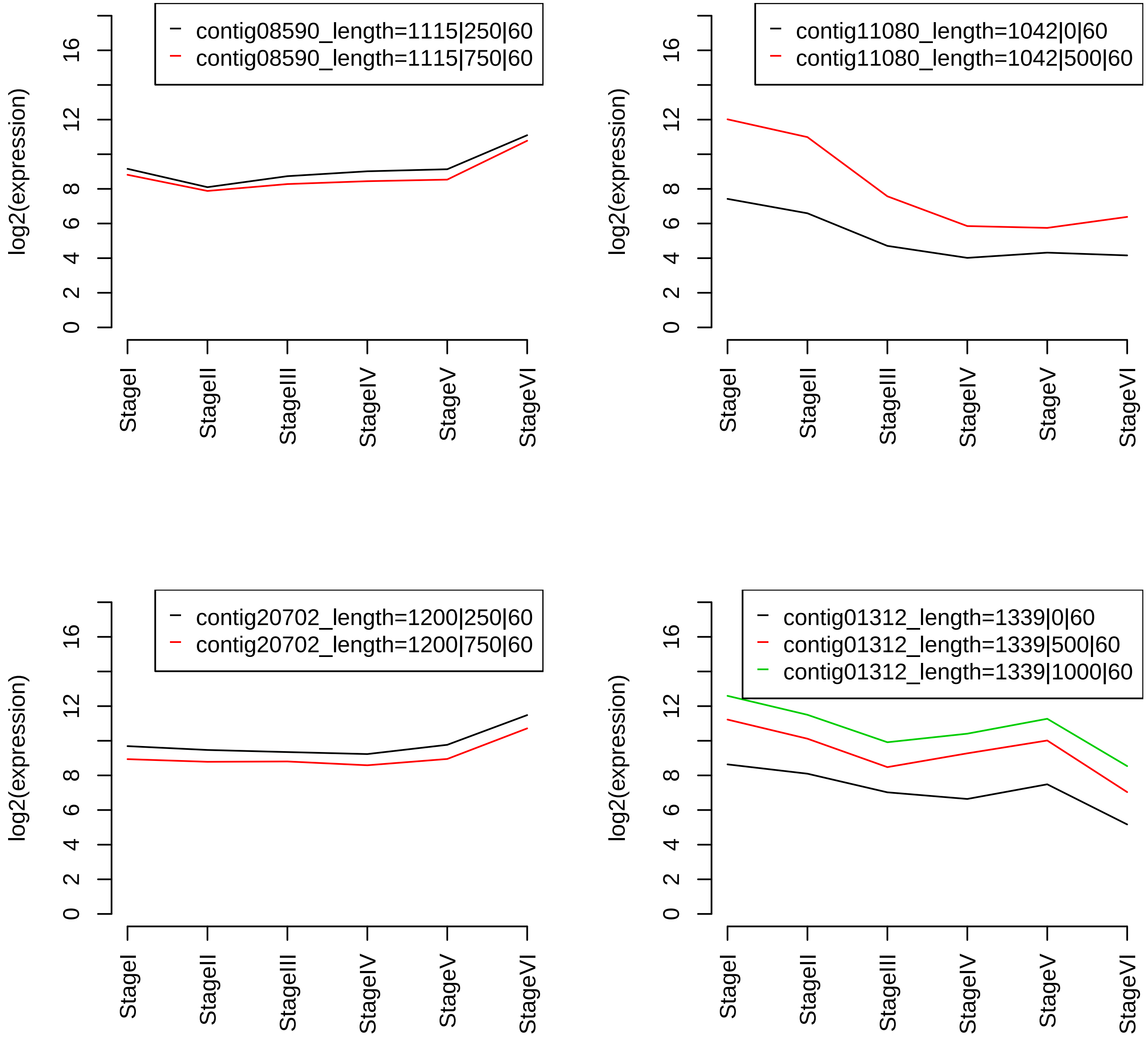

contig08590_length=1115|250|60
contig08590_length=1115|750|60
contig11080_length=1042|0|60
contig11080_length=1042|500|60
contig20702_length=1200|250|60
contig20702_length=1200|750|60
contig01312_length=1339|0|60
contig01312_length=1339|500|60
contig01312_length=1339|1000|60
log2(expression)
0
2
4
6
8
12
16
StageI
StageII
StageIII
StageIV
StageV
StageVI

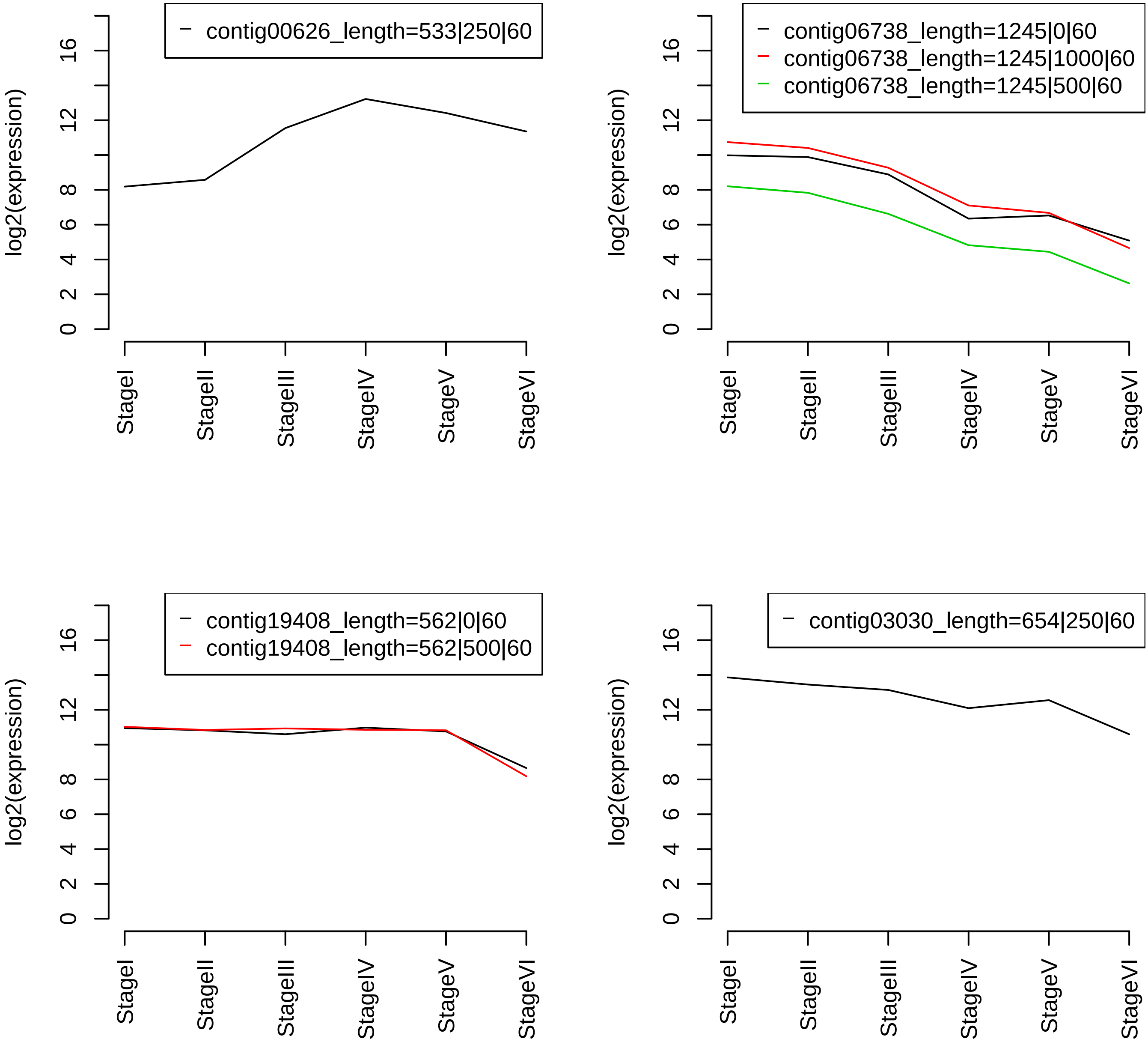
contig00626_length=533|250|60
contig06738_length=1245|0|60
contig06738_length=1245|1000|60
contig06738_length=1245|500|60
contig19408_length=562|0|60
contig19408_length=562|500|60
contig03030_length=654|250|60
log2(expression)
0 2 4 6 8 12 16
StageI StageII StageIII StageIV StageV StageVI

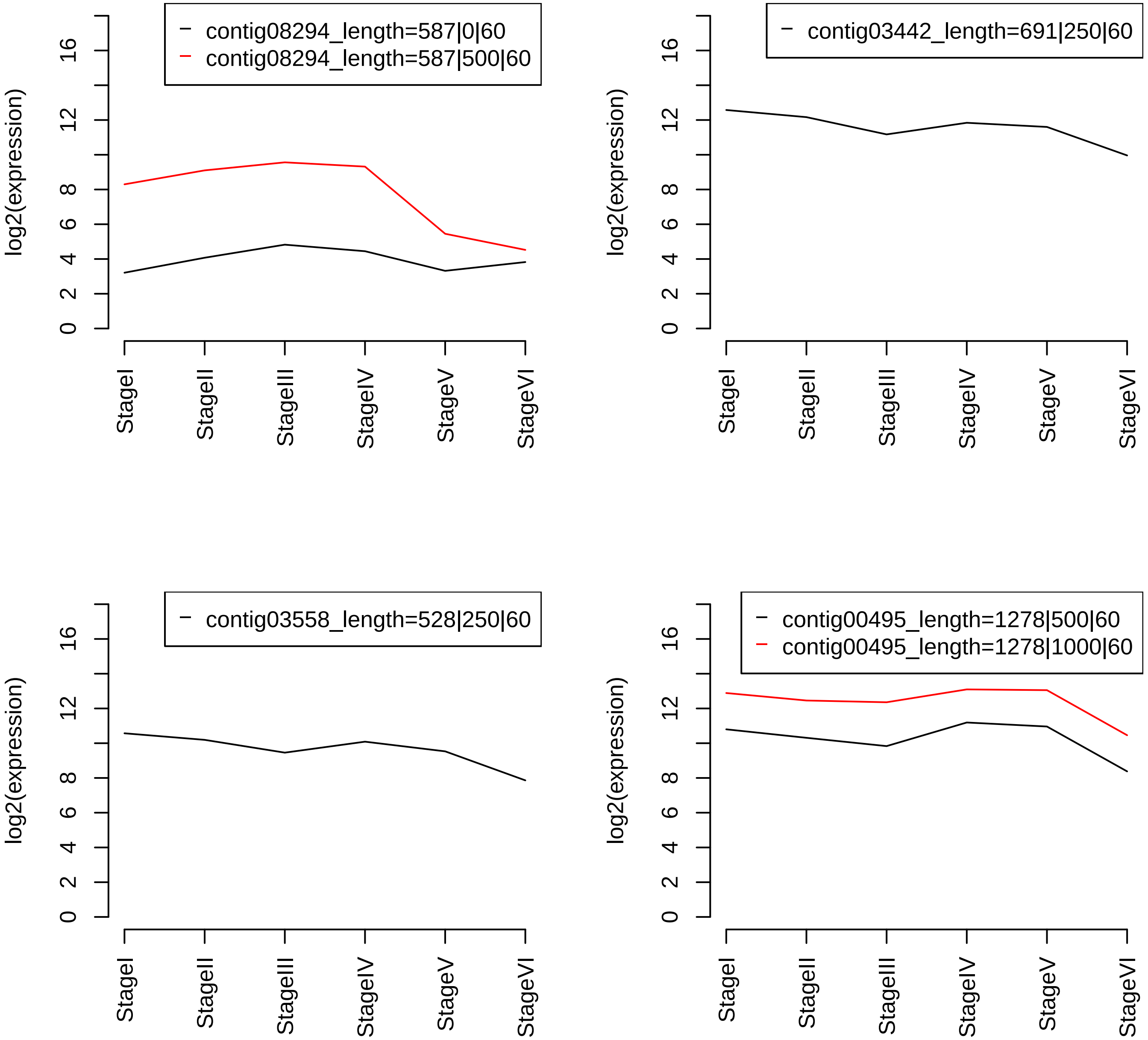

contig08294_length=587|0|60
contig08294_length=587|500|60
contig03442_length=691|250|60
contig03558_length=528|250|60
contig00495_length=1278|500|60
contig00495_length=1278|1000|60
log2(expression)
0
2
4
6
8
12
16
StageI
StageII
StageIII
StageIV
StageV
StageVI

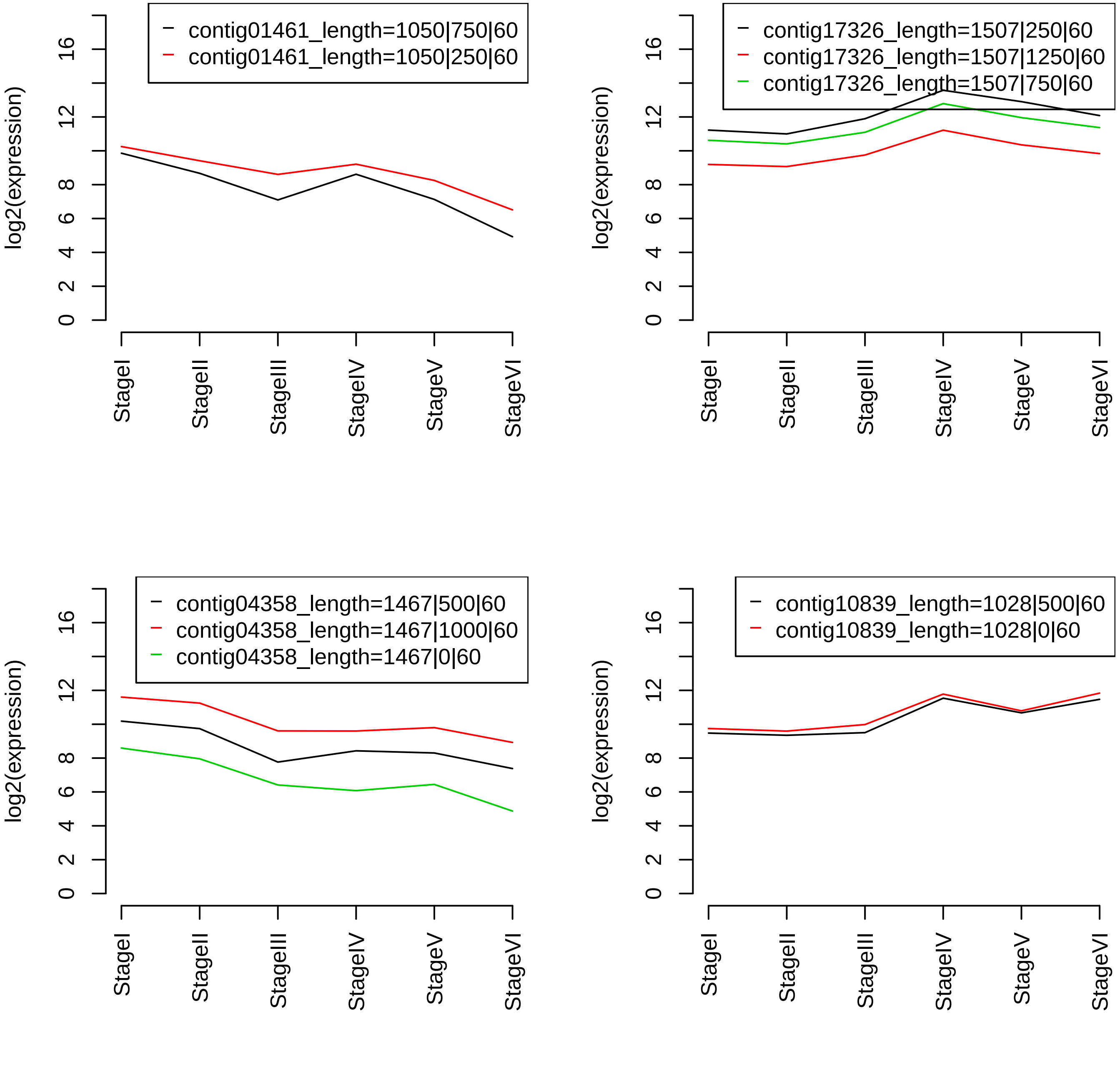
contig01461_length=1050|750|60
contig01461_length=1050|250|60
contig17326_length=1507|250|60
contig17326_length=1507|1250|60
contig17326_length=1507|750|60
contig04358_length=1467|500|60
contig04358_length=1467|1000|60
contig04358_length=1467|0|60
contig10839_length=1028|500|60
contig10839_length=1028|0|60
log2(expression)
0
2
4
6
8
12
16
StageI
StageII
StageIII
StageIV
StageV
StageVI

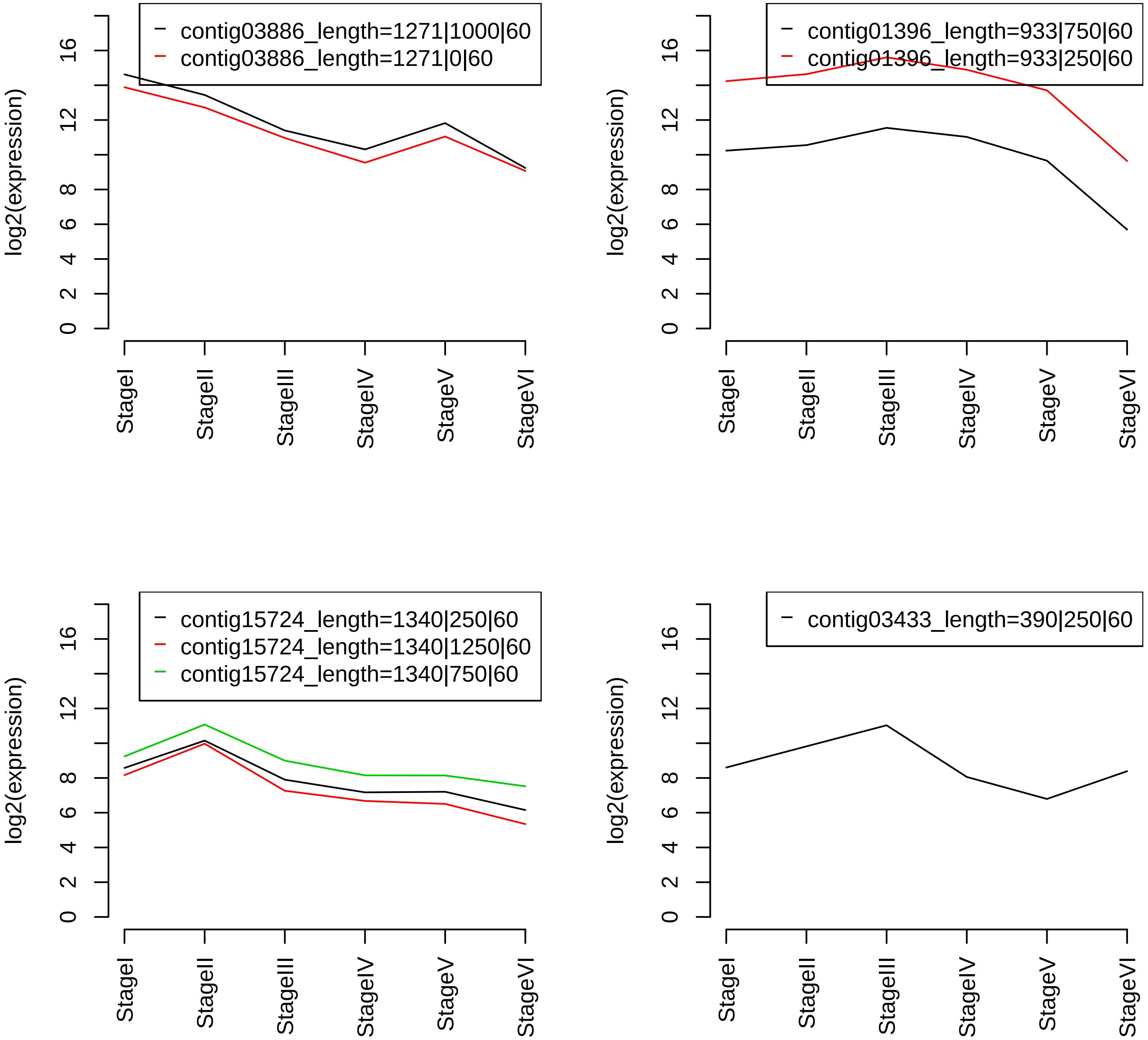

contig03886_length=1271|1000|60
contig03886_length=1271|0|60
contig01396_length=933|750|60
contig01396_length=933|250|60
contig15724_length=1340|250|60
contig15724_length=1340|1250|60
contig15724_length=1340|750|60
contig03433_length=390|250|60
log2(expression)
0
2
4
6
8
12
16
StageI
StageII
StageIII
StageIV
StageV
StageVI

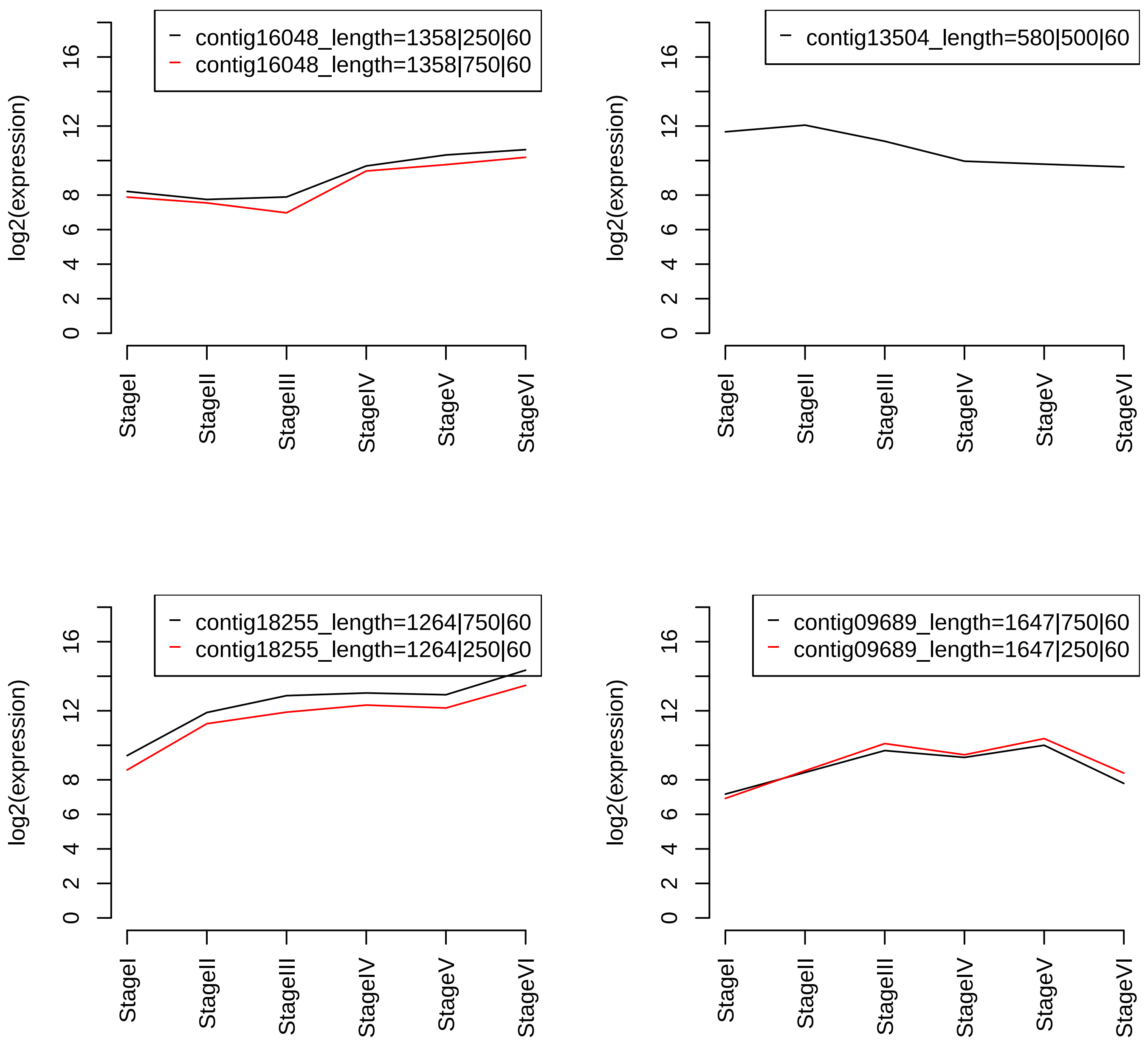
contig16048_length=1358|250|60
contig16048_length=1358|750|60
contig13504_length=580|500|60
contig18255_length=1264|750|60
contig18255_length=1264|250|60
contig09689_length=1647|750|60
contig09689_length=1647|250|60
log2(expression)
0
2
4
6
8
12
16
StageI
StageII
StageIII
StageIV
StageV
StageVI

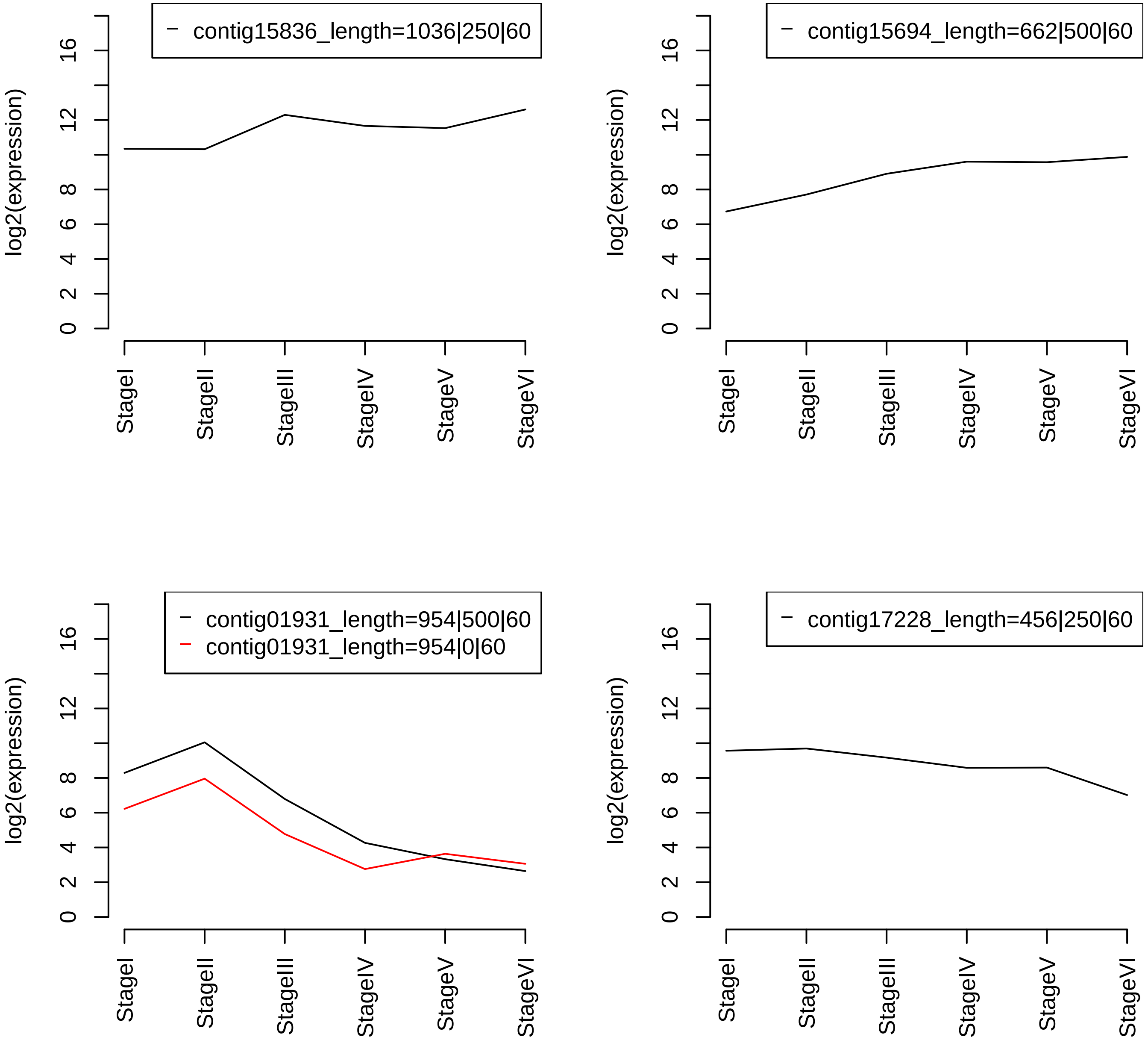
contig15836_length=1036|250|60
contig15694_length=662|500|60
contig01931_length=954|500|60
contig01931_length=954|0|60
contig17228_length=456|250|60
log2(expression)
0
2
4
6
8
12
16
StageI
StageII
StageIII
StageIV
StageV
StageVI

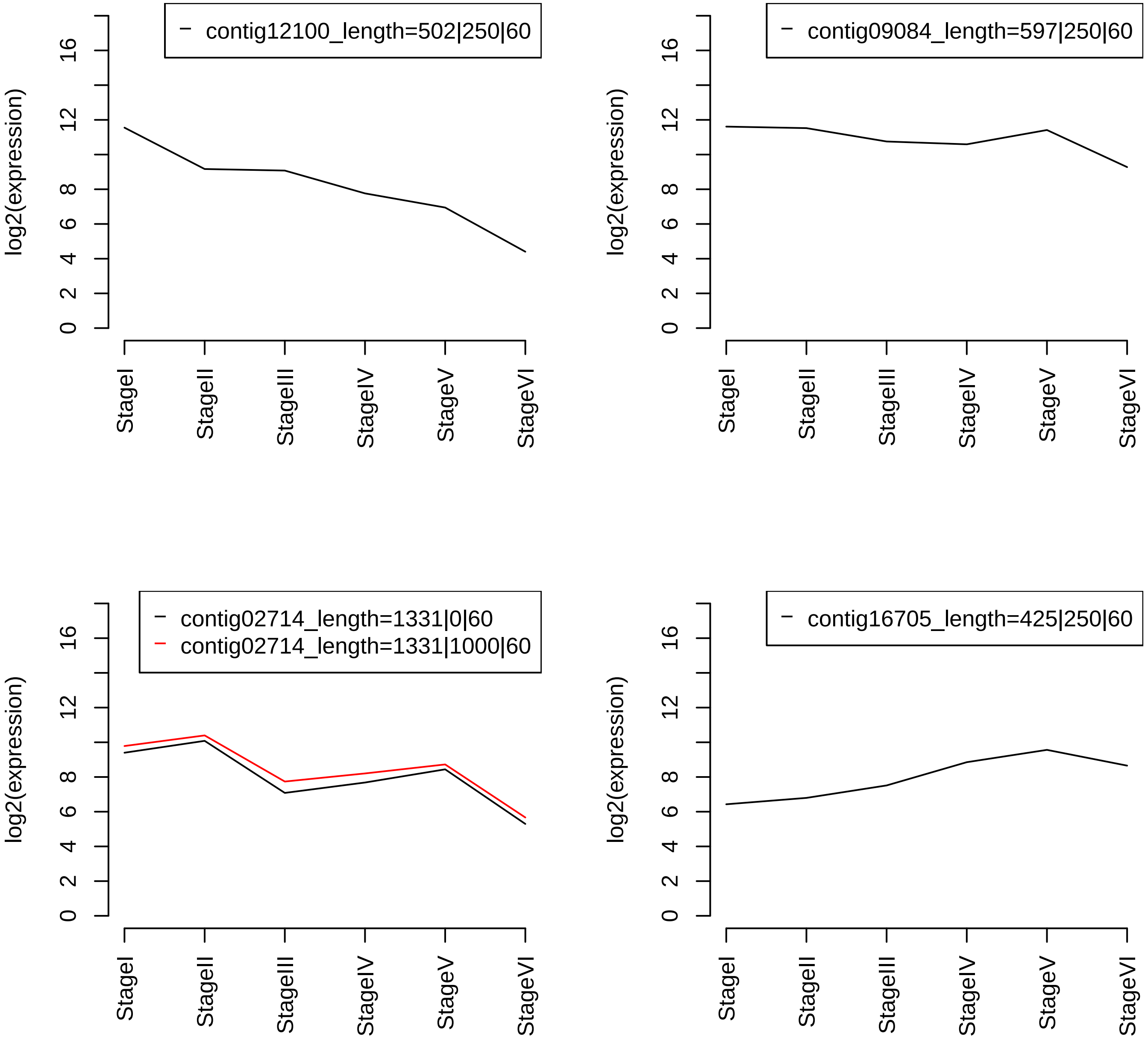
contig12100_length=502|250|60
contig09084_length=597|250|60
contig02714_length=1331|0|60
contig02714_length=1331|1000|60
contig16705_length=425|250|60
log2(expression)
0 2 4 6 8 12 16
StageI StageII StageIII StageIV StageV StageVI

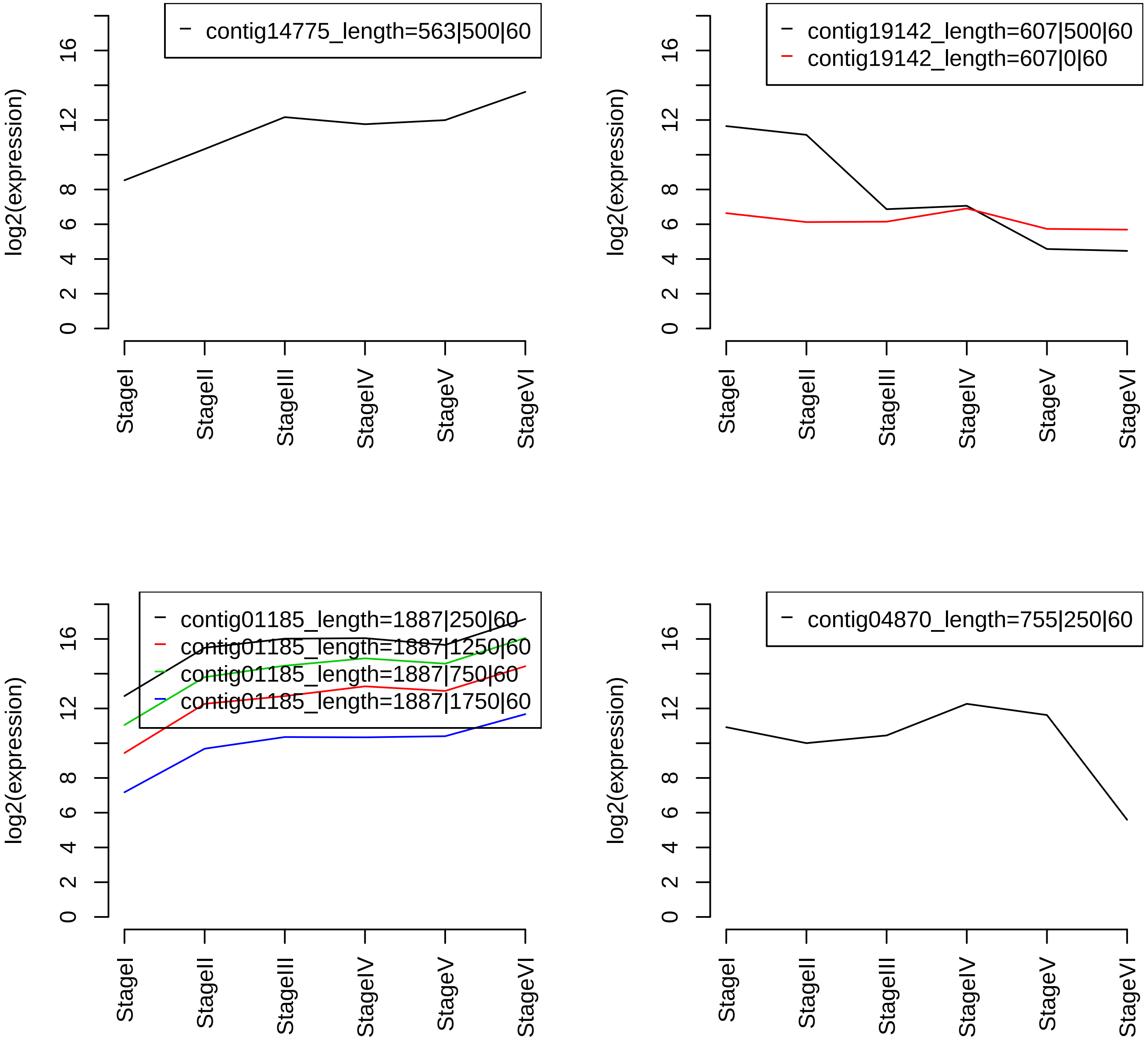

contig14775_length=563|500|60
contig19142_length=607|500|60
contig19142_length=607|0|60
contig01185_length=1887|250|60
contig01185_length=1887|1250|60
contig01185_length=1887|750|60
contig01185_length=1887|1750|60
contig04870_length=755|250|60
log2(expression)
0
2
4
6
8
12
16
StageI
StageII
StageIII
StageIV
StageV
StageVI

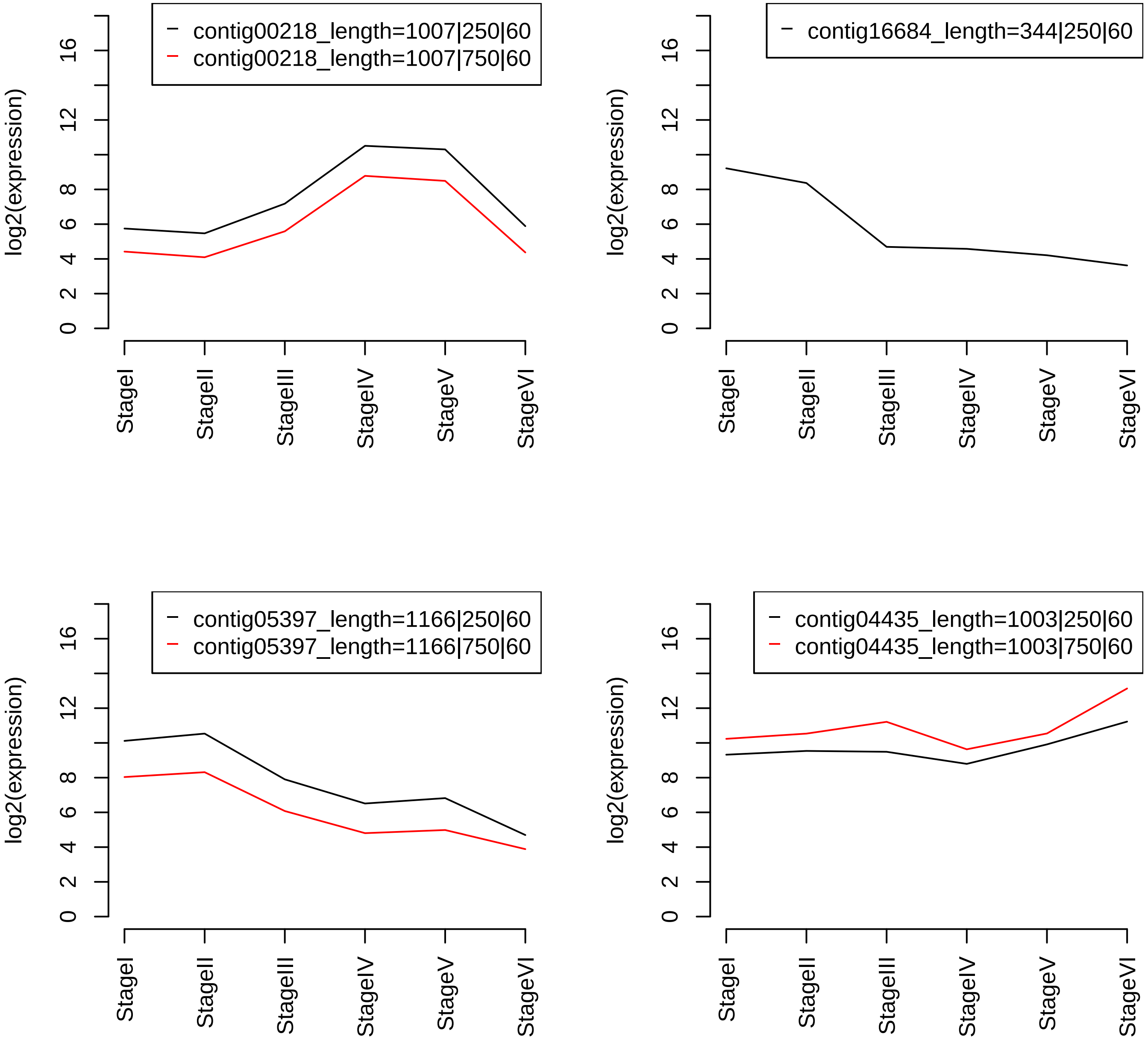

contig00218_length=1007|250|60
contig00218_length=1007|750|60
contig16684_length=344|250|60
contig05397_length=1166|250|60
contig05397_length=1166|750|60
contig04435_length=1003|250|60
contig04435_length=1003|750|60
log2(expression)
0
2
4
6
8
12
16
StageI
StageII
StageIII
StageIV
StageV
StageVI

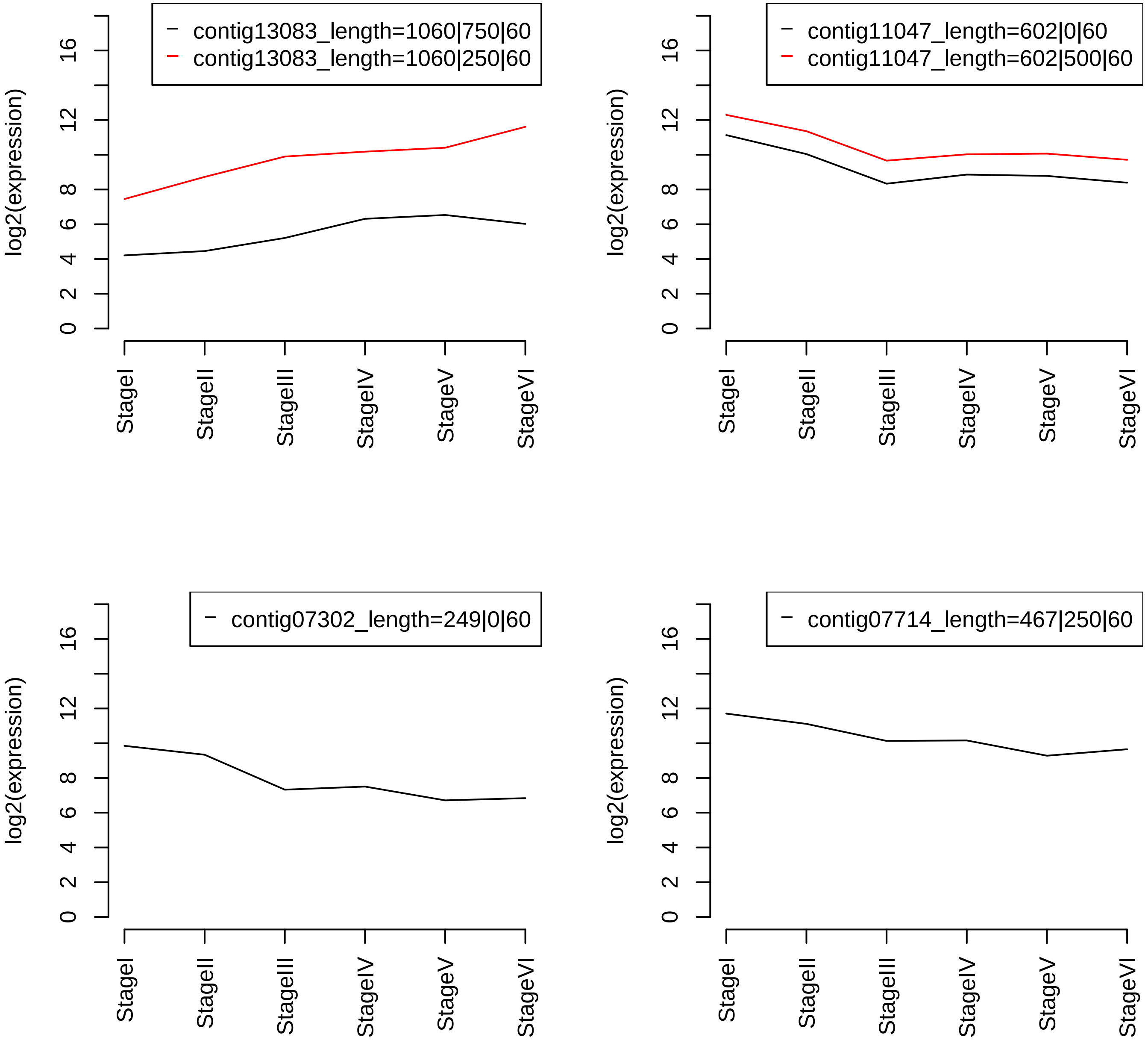
contig13083_length=1060|750|60
contig13083_length=1060|250|60
contig11047_length=602|0|60
contig11047_length=602|500|60
contig07302_length=249|0|60
contig07714_length=467|250|60
log2(expression)
0
2
4
6
8
12
16
StageI
StageII
StageIII
StageIV
StageV
StageVI

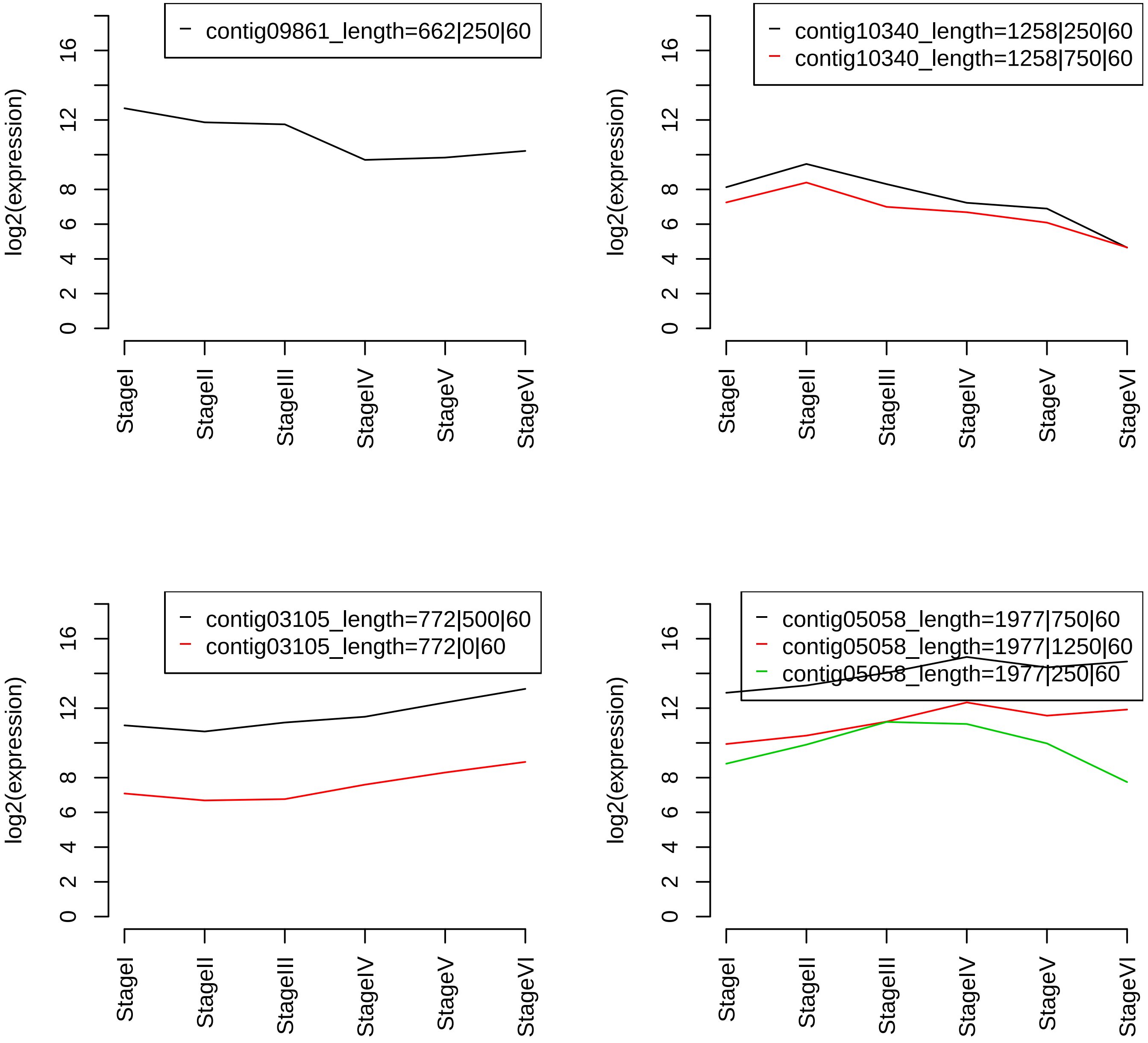

contig09861_length=662|250|60
contig10340_length=1258|250|60
contig10340_length=1258|750|60
contig03105_length=772|500|60
contig03105_length=772|0|60
contig05058_length=1977|750|60
contig05058_length=1977|1250|60
contig05058_length=1977|250|60
log2(expression)
0
2
4
6
8
12
16
StageI
StageII
StageIII
StageIV
StageV
StageVI

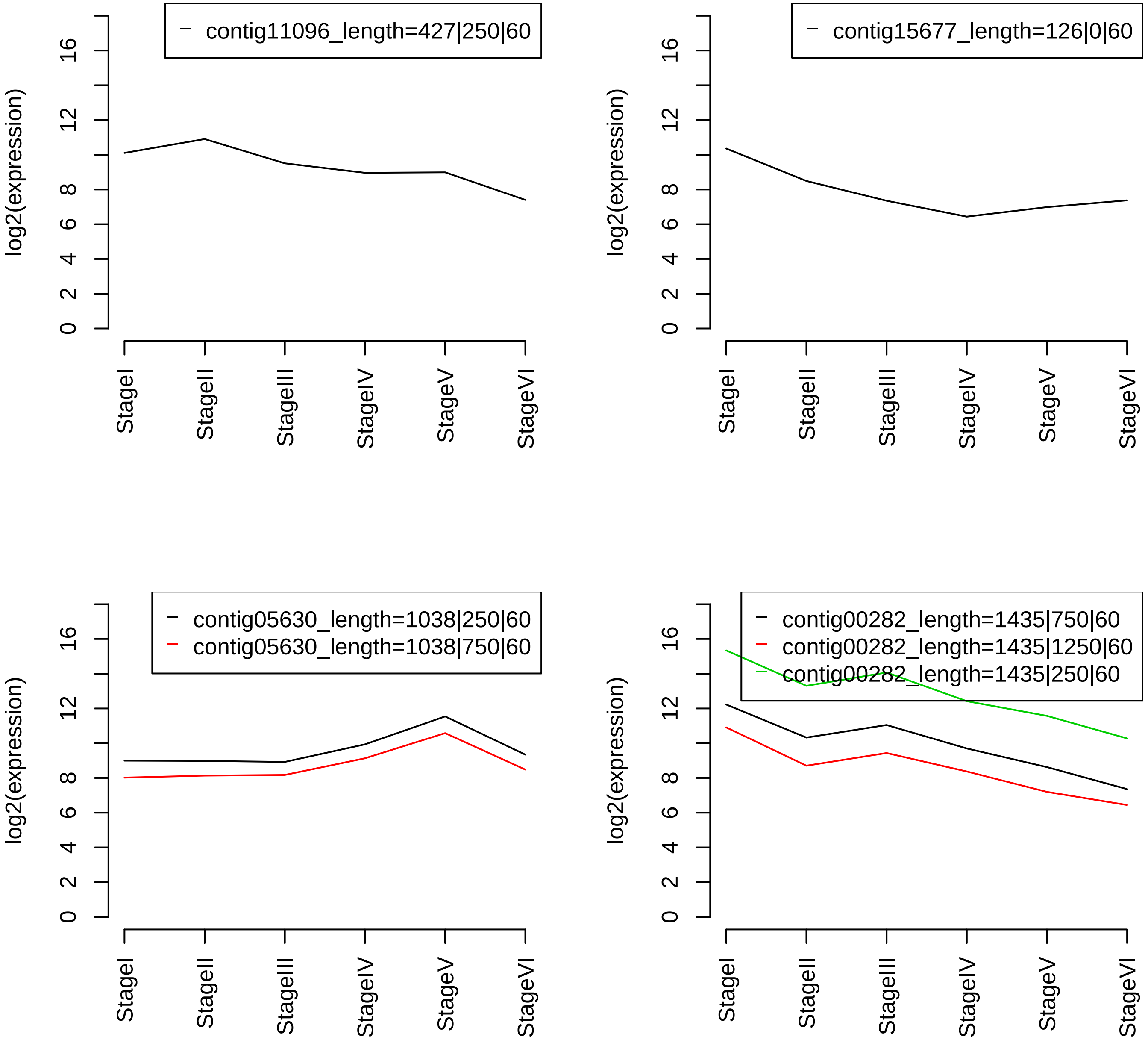

contig11096_length=427|250|60
contig15677_length=126|0|60
contig05630_length=1038|250|60
contig05630_length=1038|750|60
contig00282_length=1435|750|60
contig00282_length=1435|1250|60
contig00282_length=1435|250|60
log2(expression)
0
2
4
6
8
12
16
StageI
StageII
StageIII
StageIV
StageV
StageVI

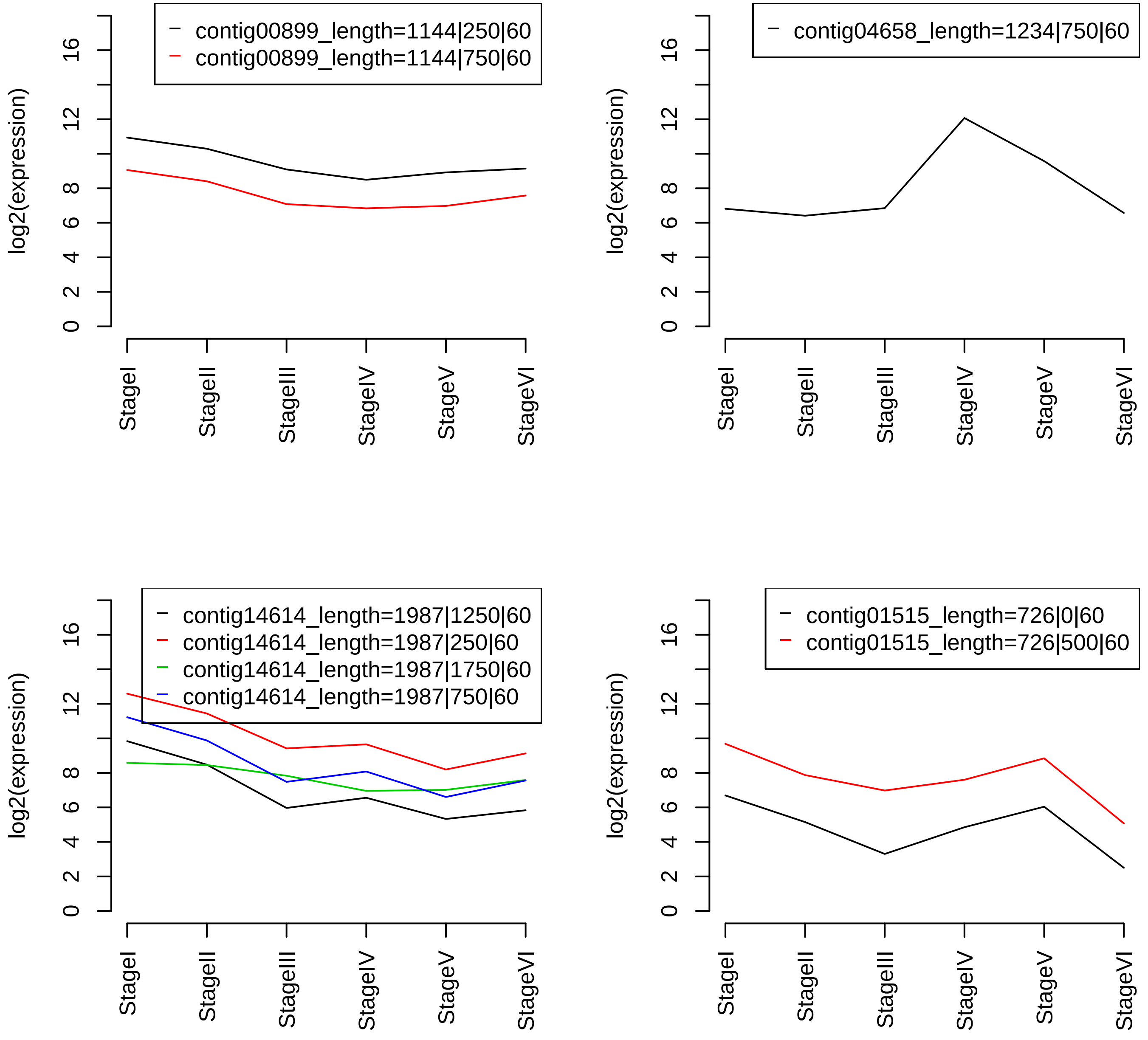
contig00899_length=1144|250|60
contig00899_length=1144|750|60
contig04658_length=1234|750|60
contig14614_length=1987|1250|60
contig14614_length=1987|250|60
contig14614_length=1987|1750|60
contig14614_length=1987|750|60
contig01515_length=726|0|60
contig01515_length=726|500|60
log2(expression)
0 2 4 6 8 12 16
StageI StageII StageIII StageIV StageV StageVI

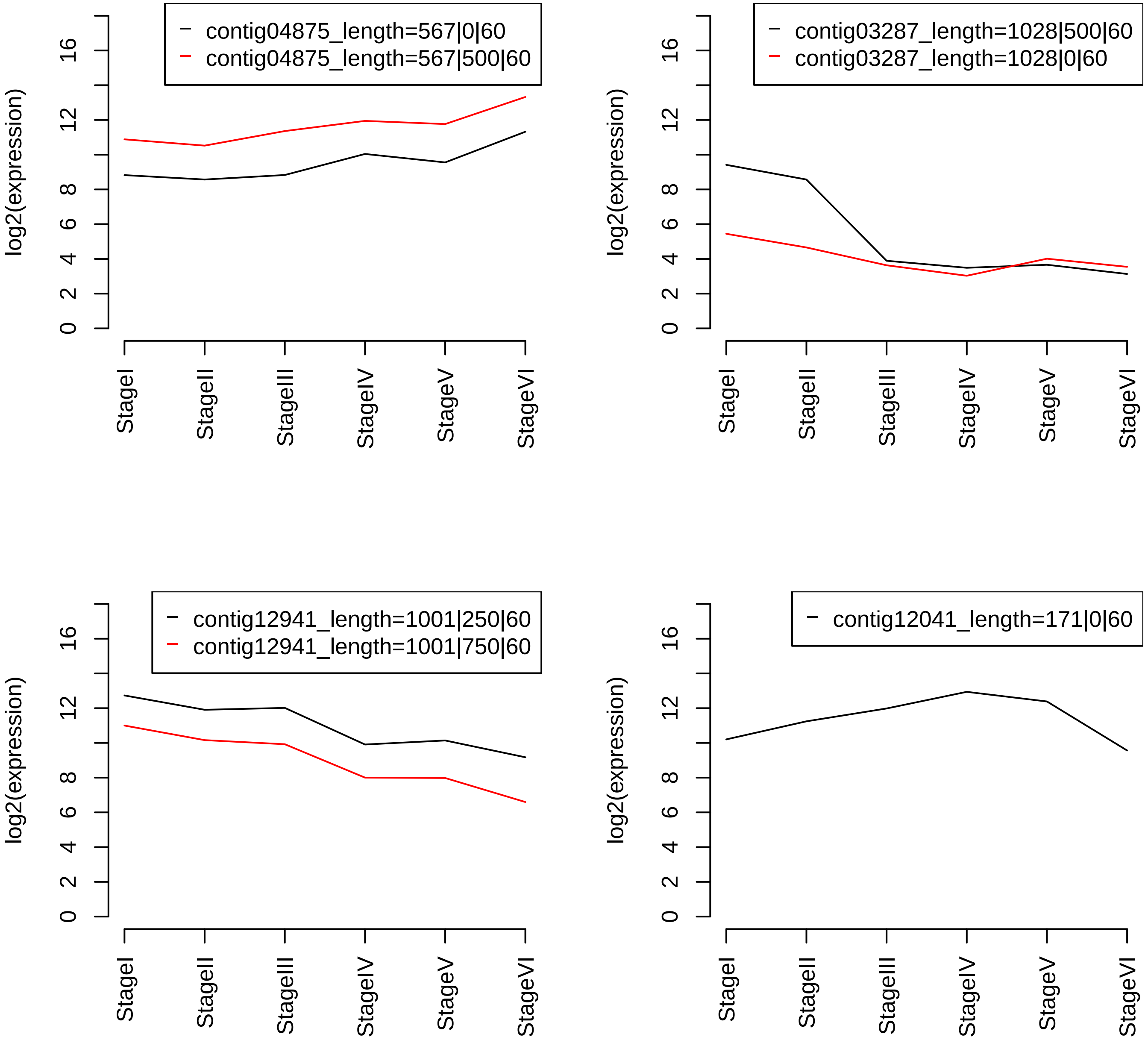
contig04875_length=567|0|60
contig04875_length=567|500|60
contig03287_length=1028|500|60
contig03287_length=1028|0|60
contig12941_length=1001|250|60
contig12941_length=1001|750|60
contig12041_length=171|0|60
log2(expression)
0 2 4 6 8 12 16
StageI StageII StageIII StageIV StageV StageVI

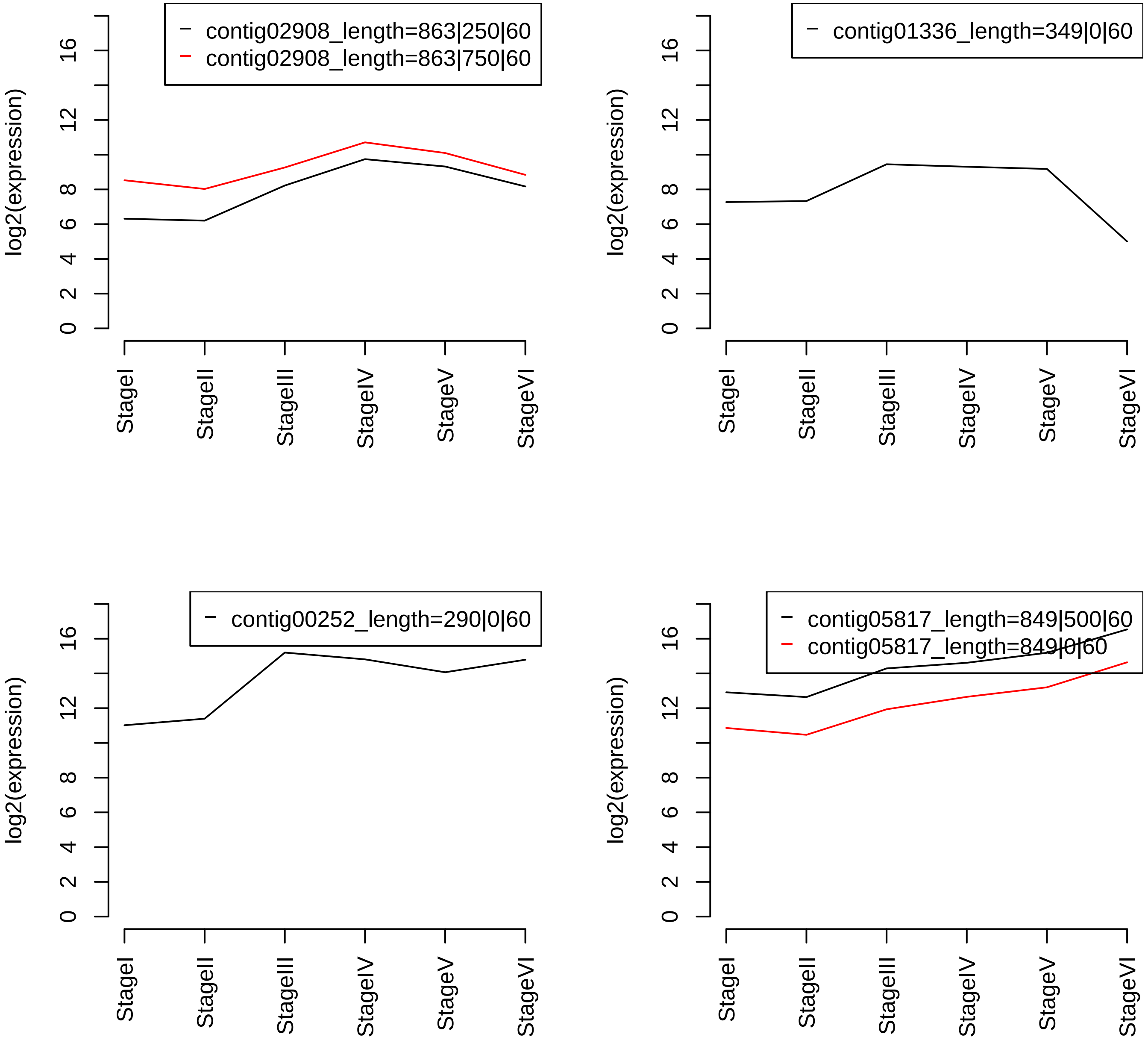
contig02908_length=863|250|60
contig02908_length=863|750|60
contig01336_length=349|0|60
contig00252_length=290|0|60
contig05817_length=849|500|60
contig05817_length=849|0|60
log2(expression)
StageI
StageII
StageIII
StageIV
StageV
StageVI

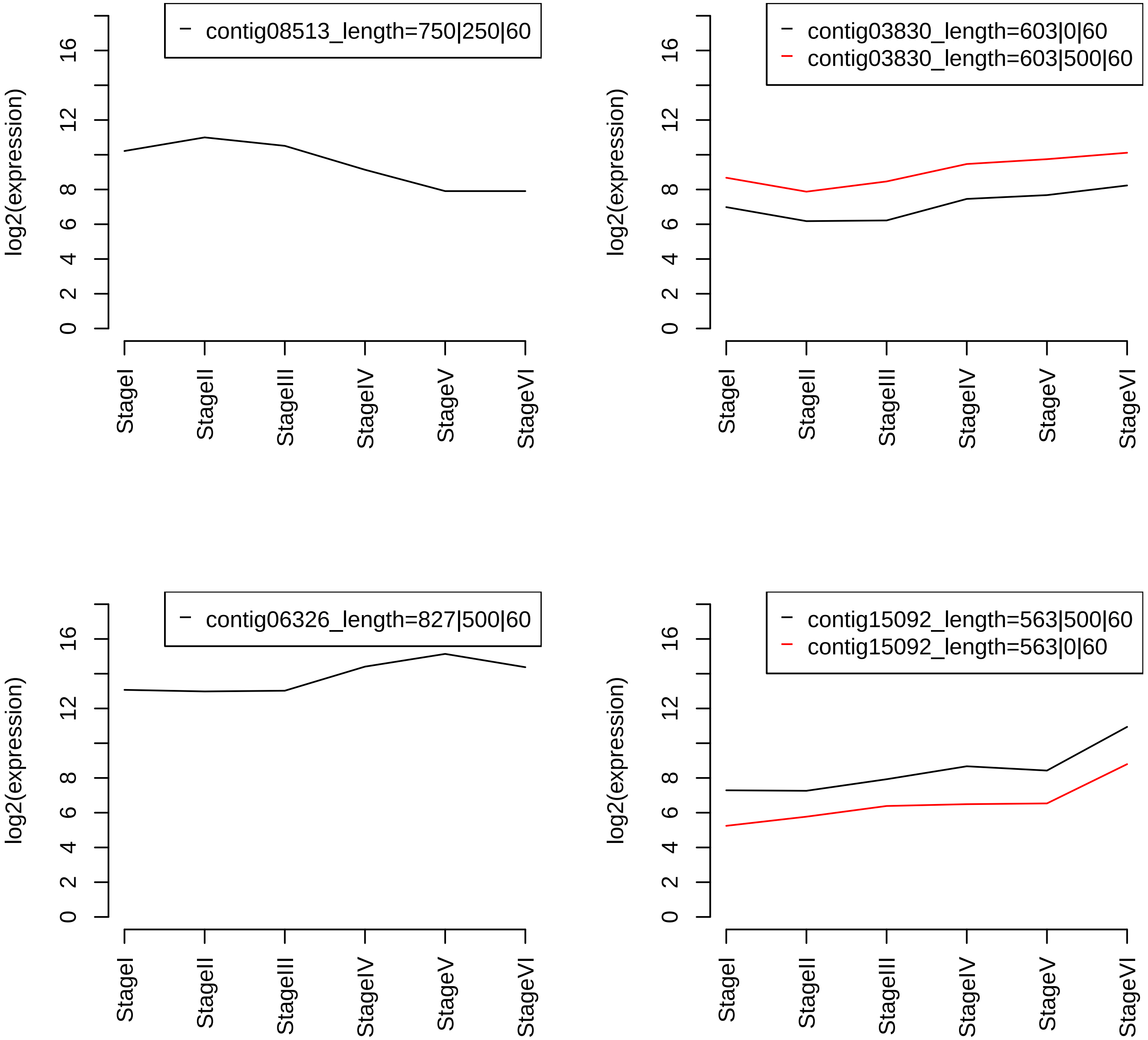

contig08513_length=750|250|60
contig03830_length=603|0|60
contig03830_length=603|500|60
contig06326_length=827|500|60
contig15092_length=563|500|60
contig15092_length=563|0|60
log2(expression)
0
2
4
6
8
12
16
StageI
StageII
StageIII
StageIV
StageV
StageVI

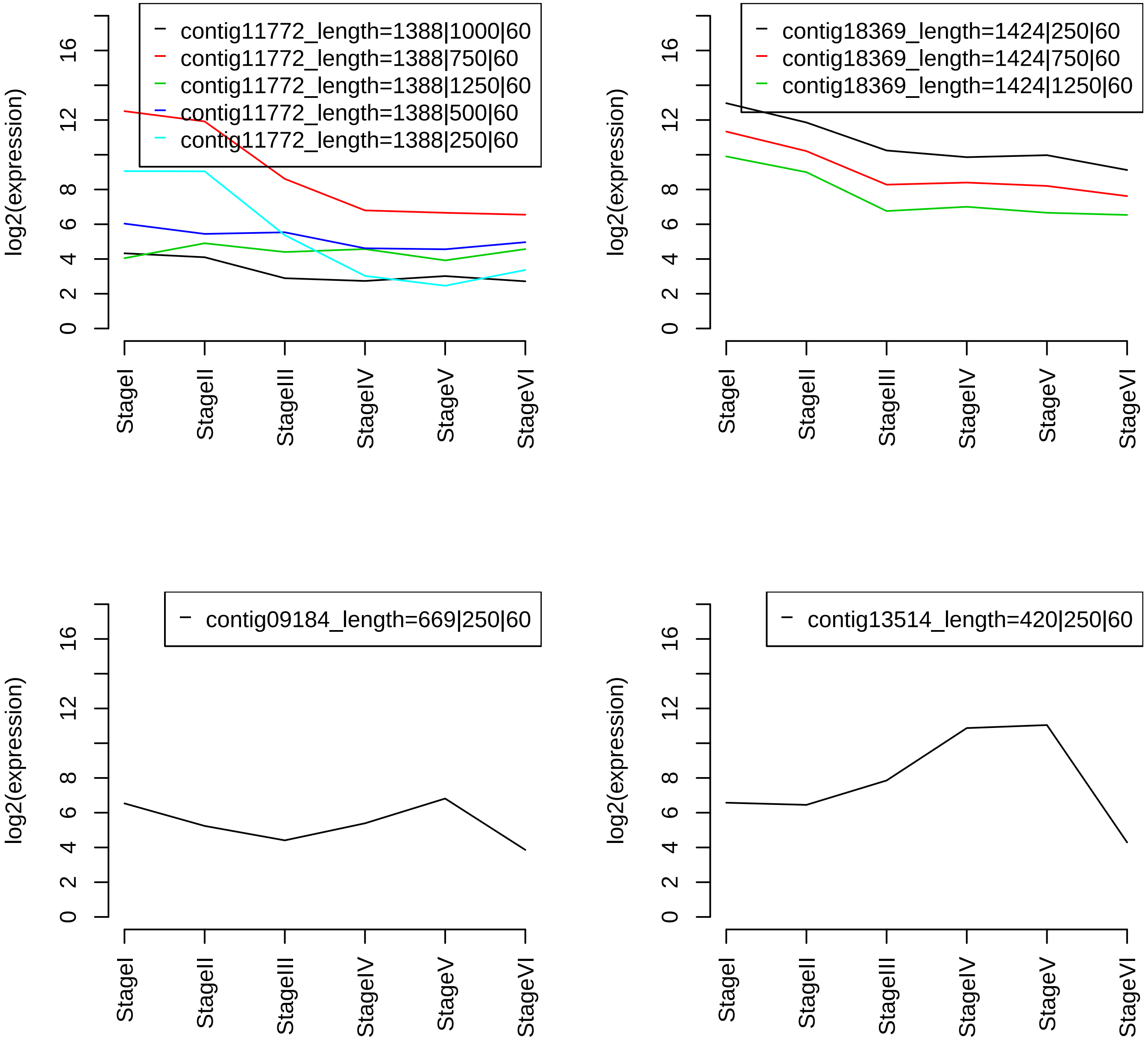

contig11772_length=1388|1000|60
contig11772_length=1388|750|60
contig11772_length=1388|1250|60
contig11772_length=1388|500|60
contig11772_length=1388|250|60
contig18369_length=1424|250|60
contig18369_length=1424|750|60
contig18369_length=1424|1250|60
contig09184_length=669|250|60
contig13514_length=420|250|60
log2(expression)
StageI
StageII
StageIII
StageIV
StageV
StageVI

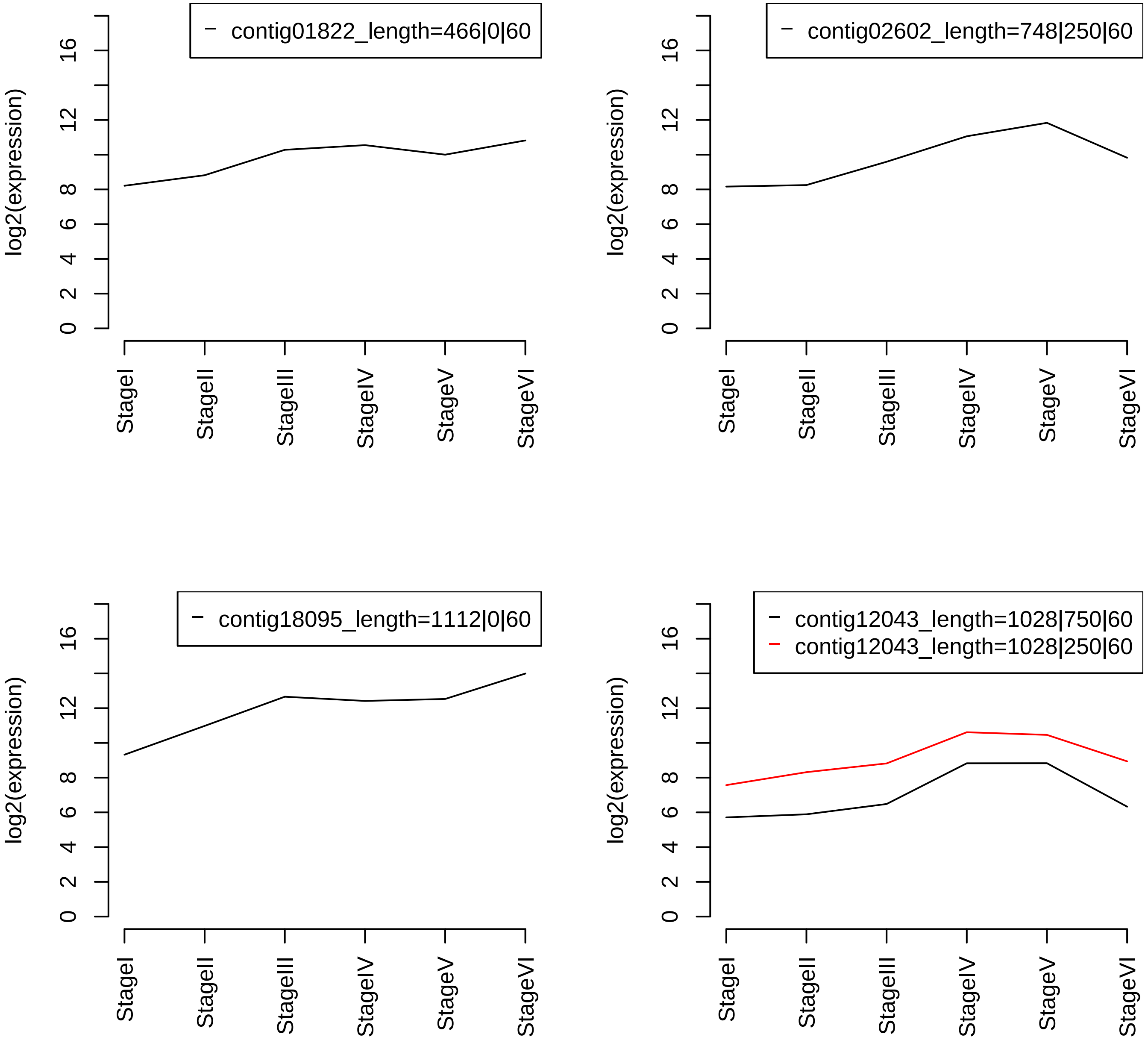

contig01822_length=466|0|60
contig02602_length=748|250|60
contig18095_length=1112|0|60
contig12043_length=1028|750|60
contig12043_length=1028|250|60
log2(expression)
0 2 4 6 8 12 16
StageI
StageII
StageIII
StageIV
StageV
StageVI

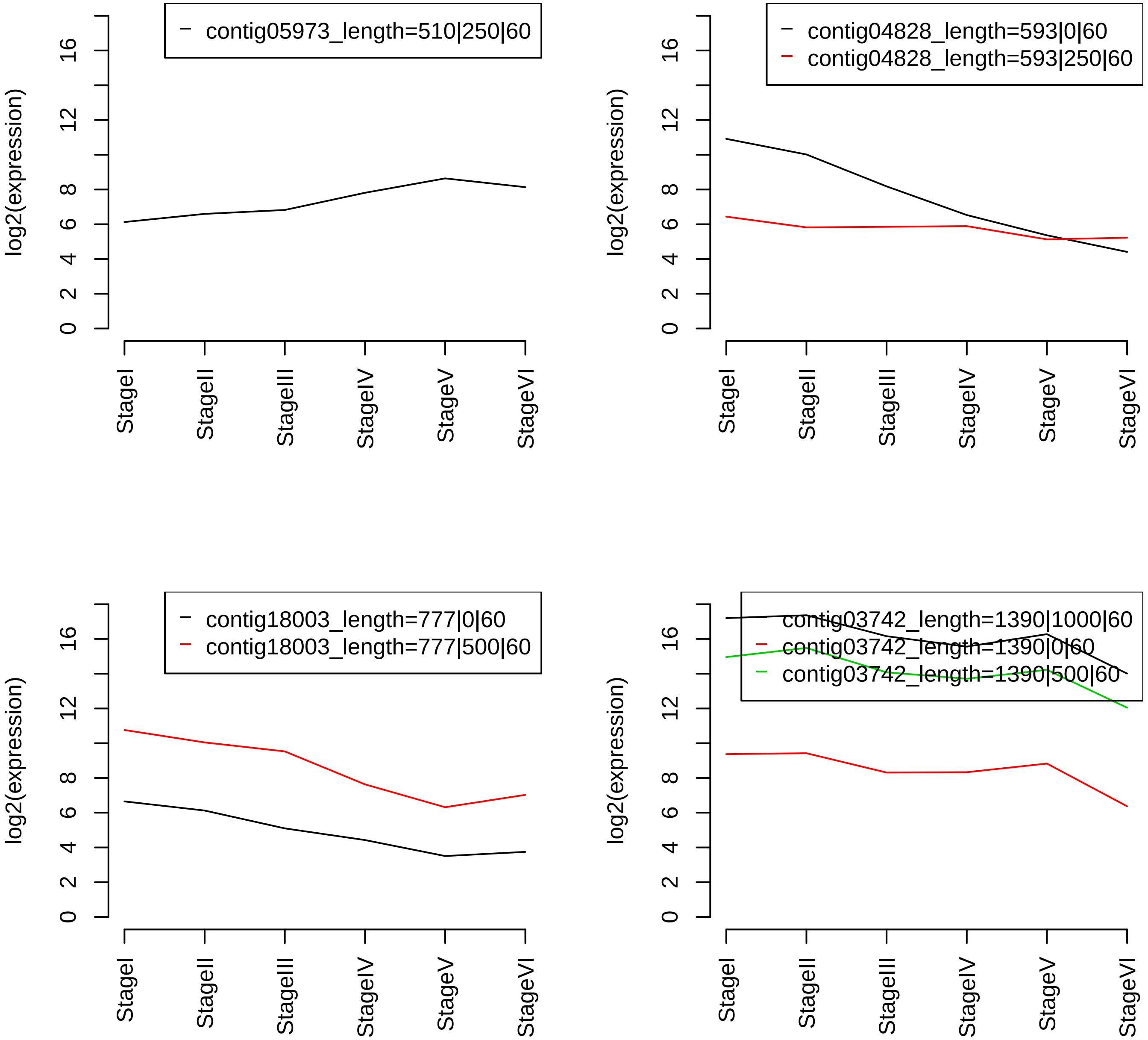

contig05973_length=510|250|60
contig04828_length=593|0|60
contig04828_length=593|250|60
contig18003_length=777|0|60
contig18003_length=777|500|60
contig03742_length=1390|1000|60
contig03742_length=1390|0|60
contig03742_length=1390|500|60
log2(expression)
StageI
StageII
StageIII
StageIV
StageV
StageVI

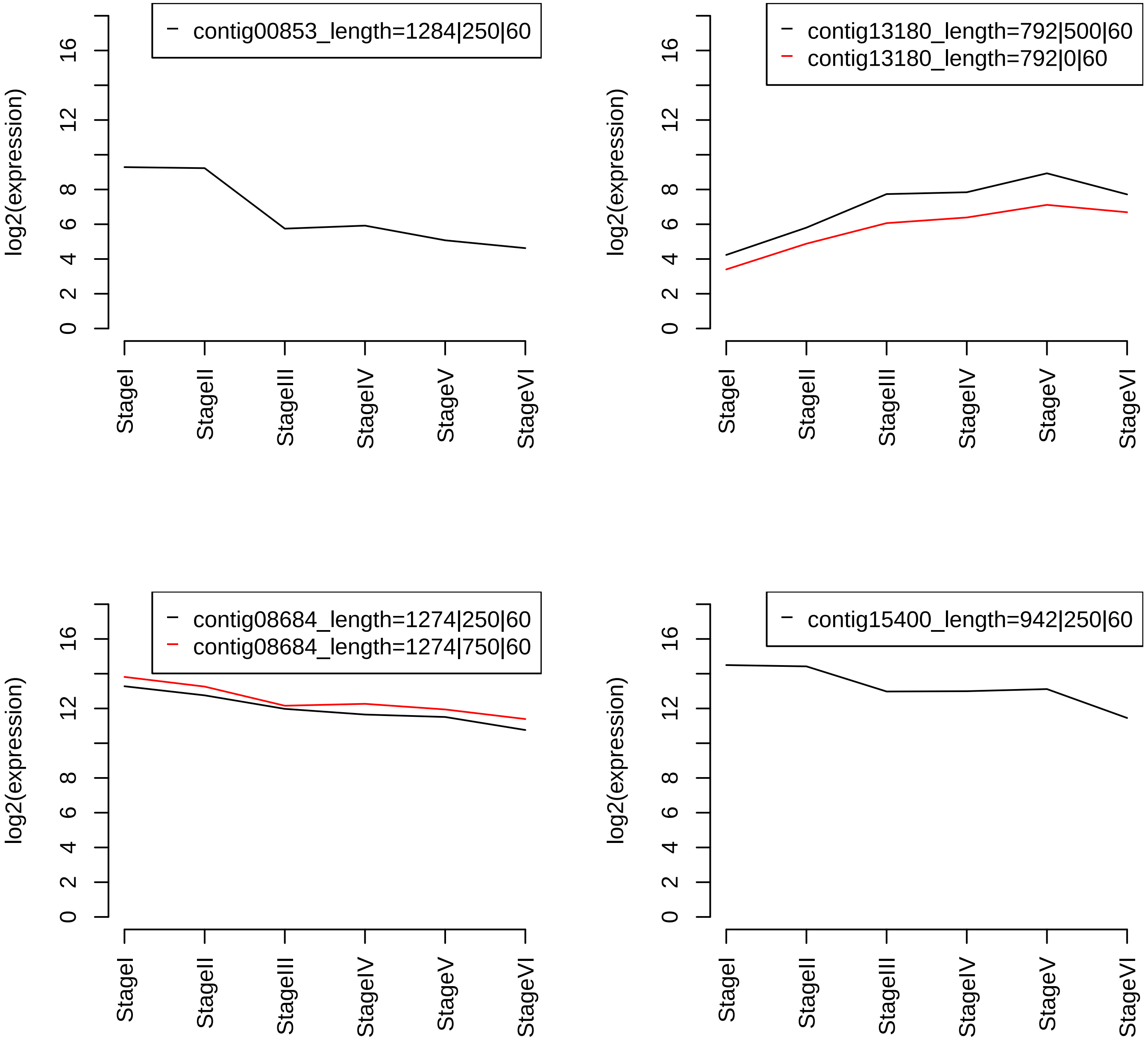

contig00853_length=1284|250|60
contig13180_length=792|500|60
contig13180_length=792|0|60
contig08684_length=1274|250|60
contig08684_length=1274|750|60
contig15400_length=942|250|60
log2(expression)
0
2
4
6
8
12
16
StageI
StageII
StageIII
StageIV
StageV
StageVI

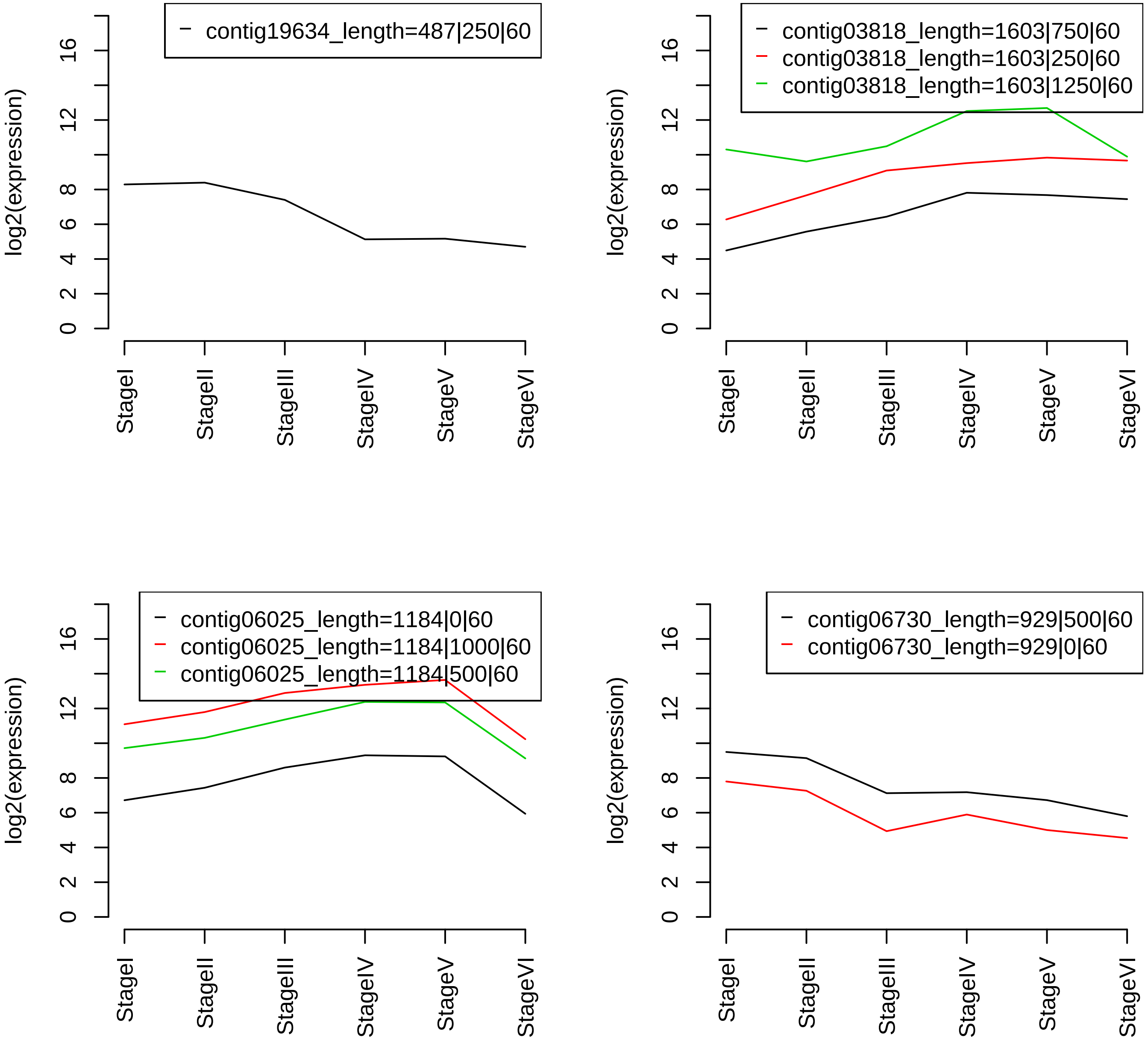
contig19634_length=487|250|60
contig03818_length=1603|750|60
contig03818_length=1603|250|60
contig03818_length=1603|1250|60
contig06025_length=1184|0|60
contig06025_length=1184|1000|60
contig06025_length=1184|500|60
contig06730_length=929|500|60
contig06730_length=929|0|60
log2(expression)
0 2 4 6 8 12 16
StageI
StageII
StageIII
StageIV
StageV
StageVI

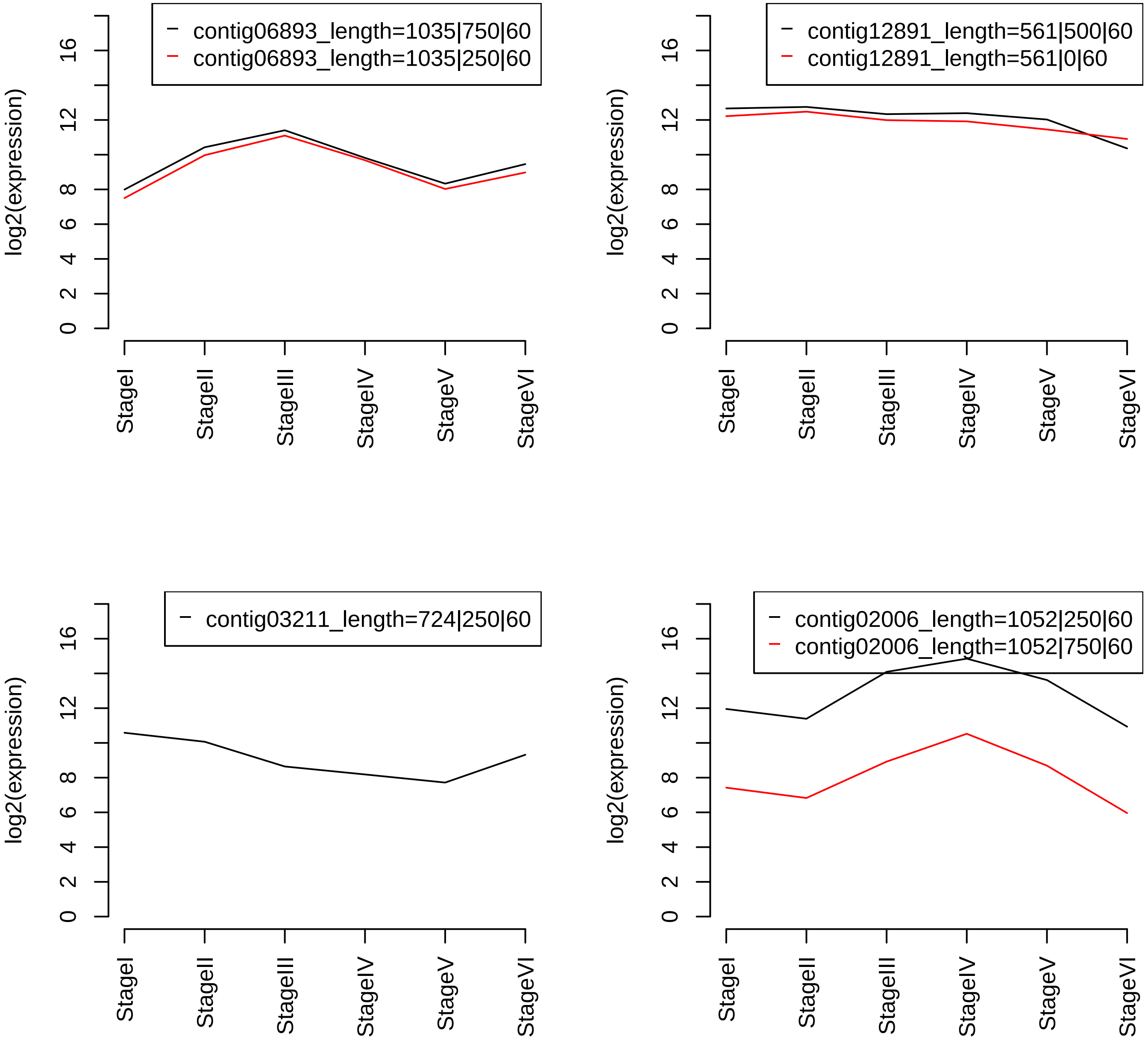
contig06893_length=1035|750|60
contig06893_length=1035|250|60
contig12891_length=561|500|60
contig12891_length=561|0|60
contig03211_length=724|250|60
contig02006_length=1052|250|60
contig02006_length=1052|750|60
log2(expression)
0
2
4
6
8
12
16
StageI
StageII
StageIII
StageIV
StageV
StageVI

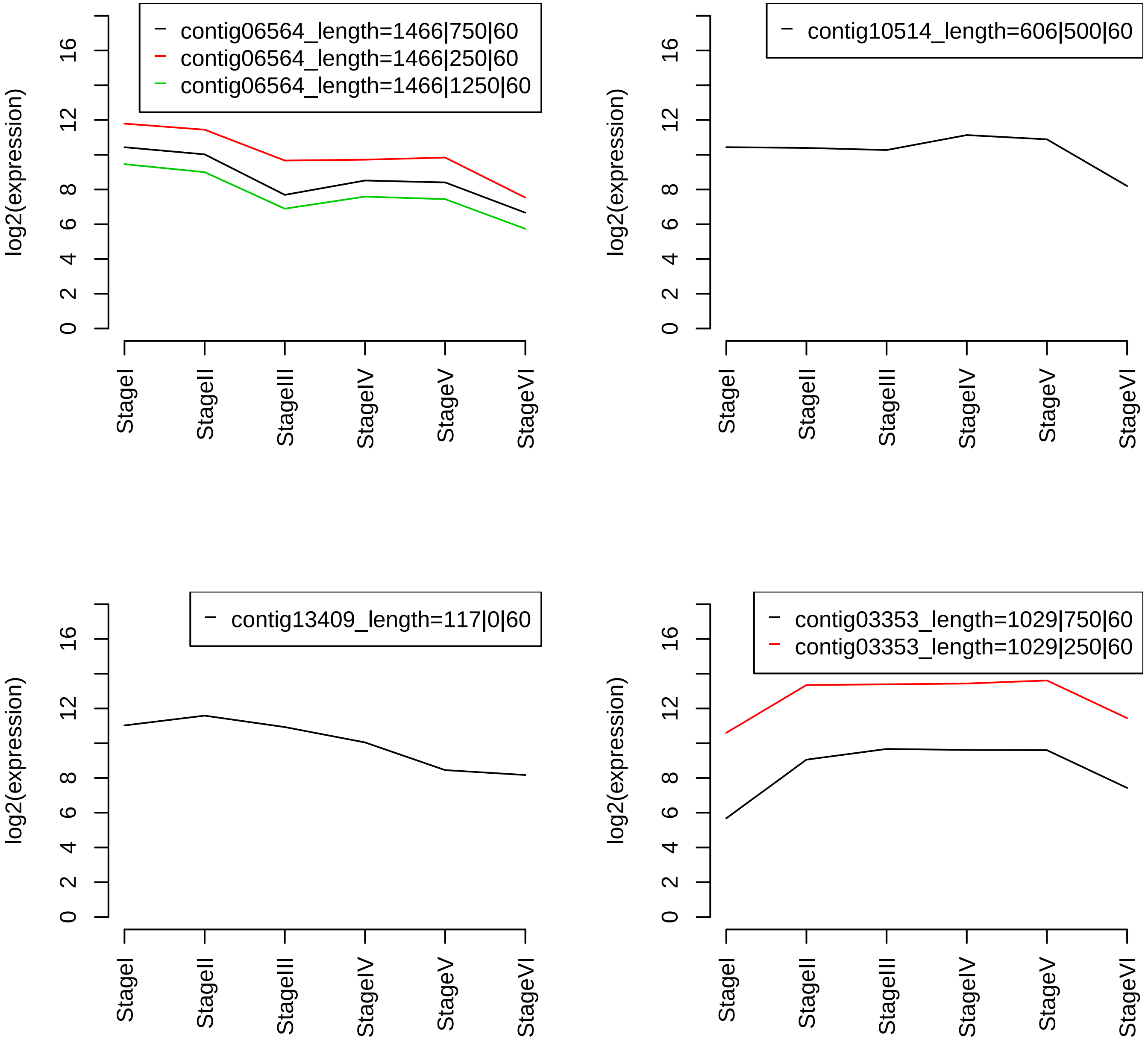

contig06564_length=1466|750|60
contig06564_length=1466|250|60
contig06564_length=1466|1250|60
contig10514_length=606|500|60
contig13409_length=117|0|60
contig03353_length=1029|750|60
contig03353_length=1029|250|60
log2(expression)
0 2 4 6 8 12 16
StageI StageII StageIII StageIV StageV StageVI

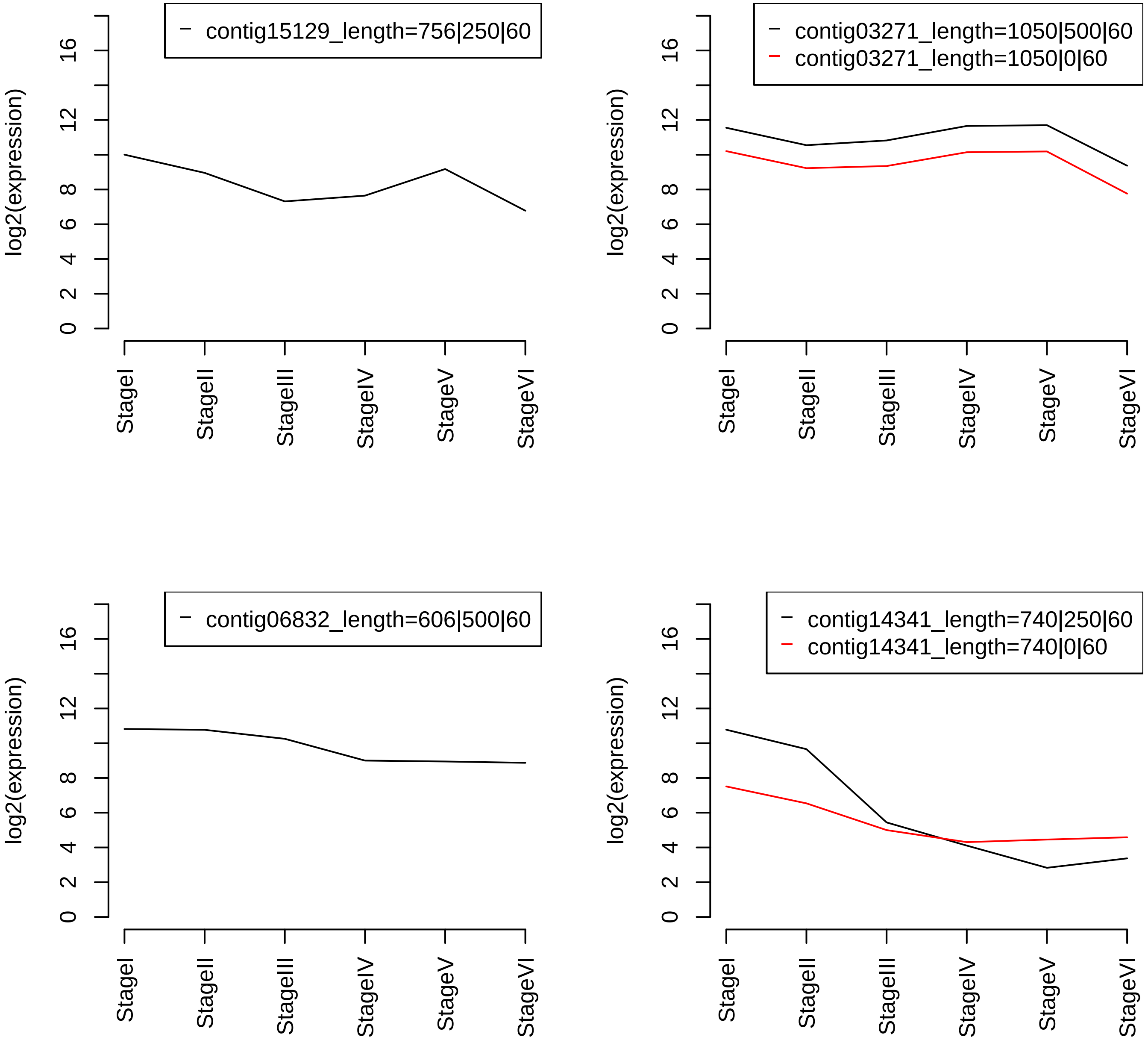

contig15129_length=756|250|60
contig03271_length=1050|500|60
contig03271_length=1050|0|60
contig06832_length=606|500|60
contig14341_length=740|250|60
contig14341_length=740|0|60
log2(expression)
StageI
StageII
StageIII
StageIV
StageV
StageVI

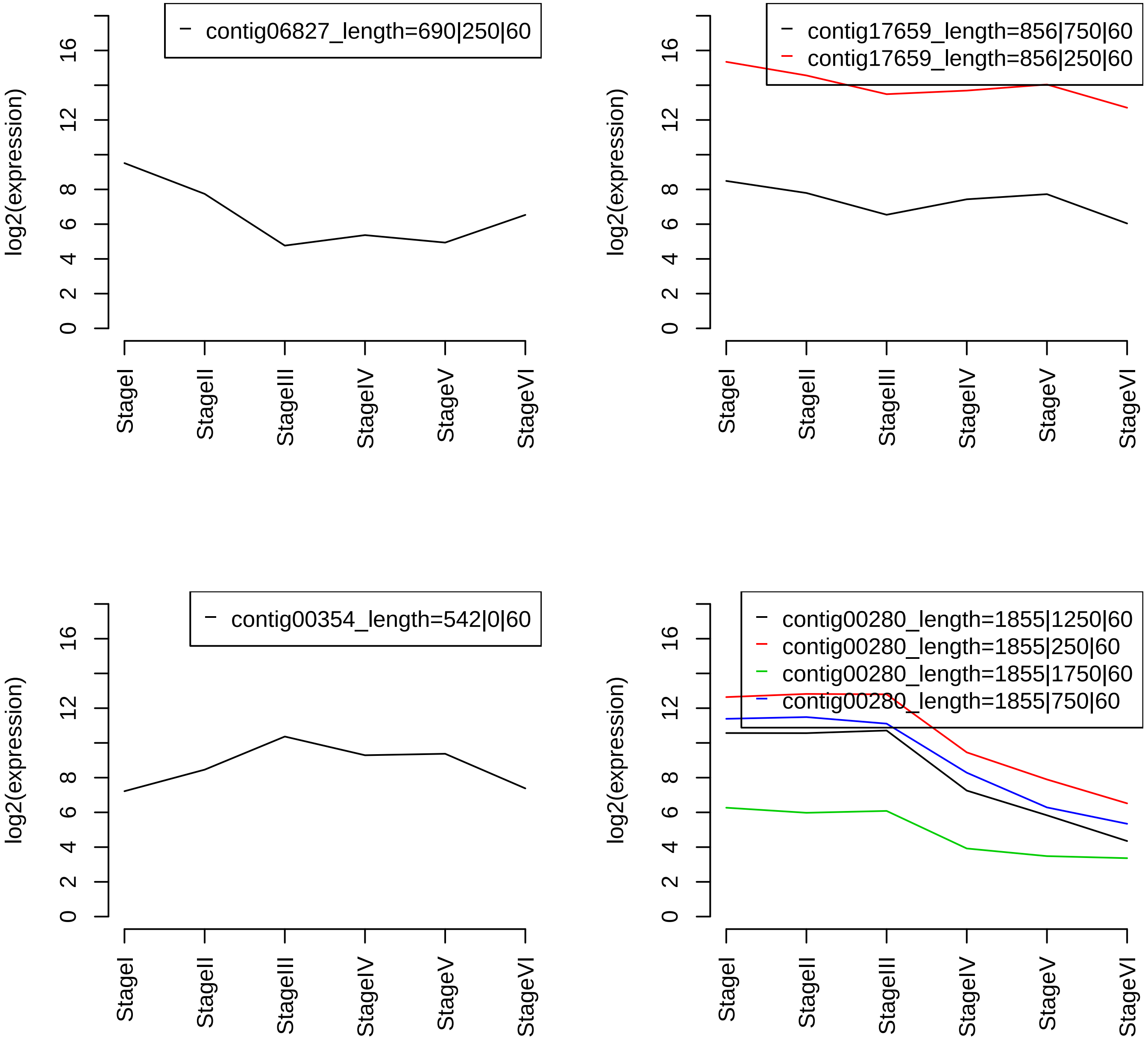
contig06827_length=690|250|60
contig17659_length=856|750|60
contig17659_length=856|250|60
contig00354_length=542|0|60
contig00280_length=1855|1250|60
contig00280_length=1855|250|60
contig00280_length=1855|1750|60
contig00280_length=1855|750|60
log2(expression)
StageI
StageII
StageIII
StageIV
StageV
StageVI

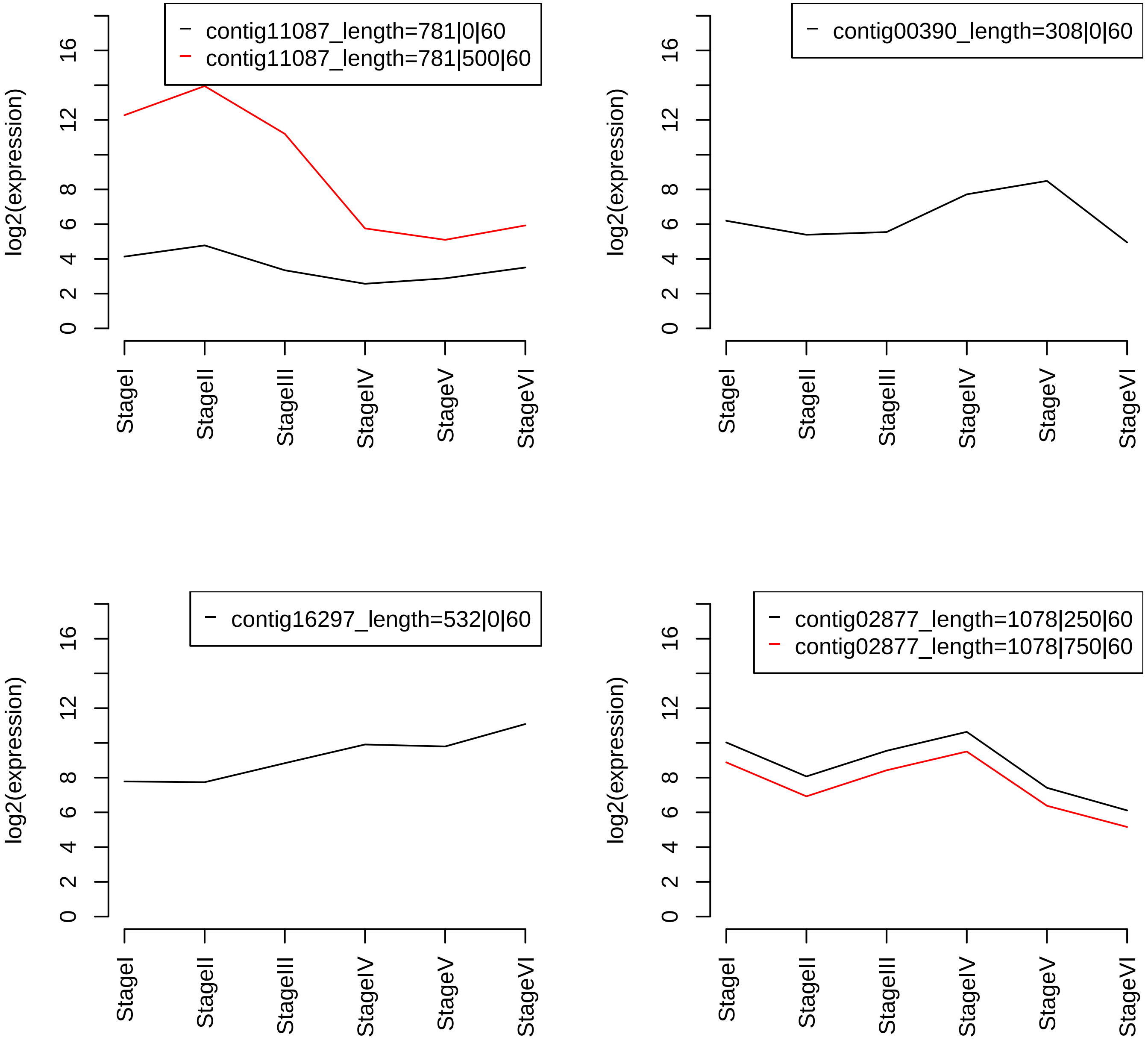
contig11087_length=781|0|60
contig11087_length=781|500|60
contig00390_length=308|0|60
contig16297_length=532|0|60
contig02877_length=1078|250|60
contig02877_length=1078|750|60
log2(expression)
0
2
4
6
8
12
16
StageI
StageII
StageIII
StageIV
StageV
StageVI

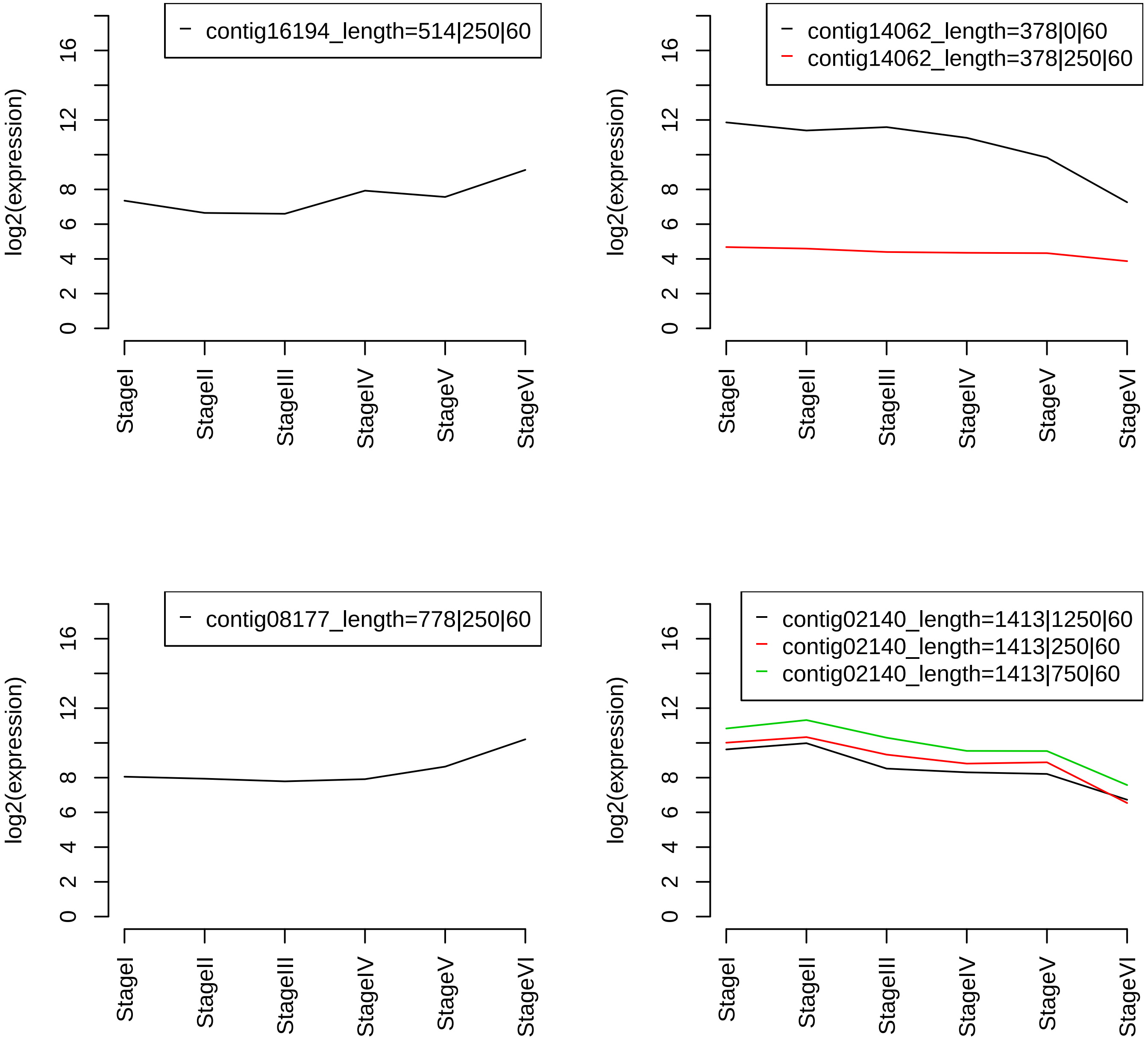

contig16194_length=514|250|60
contig14062_length=378|0|60
contig14062_length=378|250|60
contig08177_length=778|250|60
contig02140_length=1413|1250|60
contig02140_length=1413|250|60
contig02140_length=1413|750|60
log2(expression)
0 2 4 6 8 12 16
StageI StageII StageIII StageIV StageV StageVI

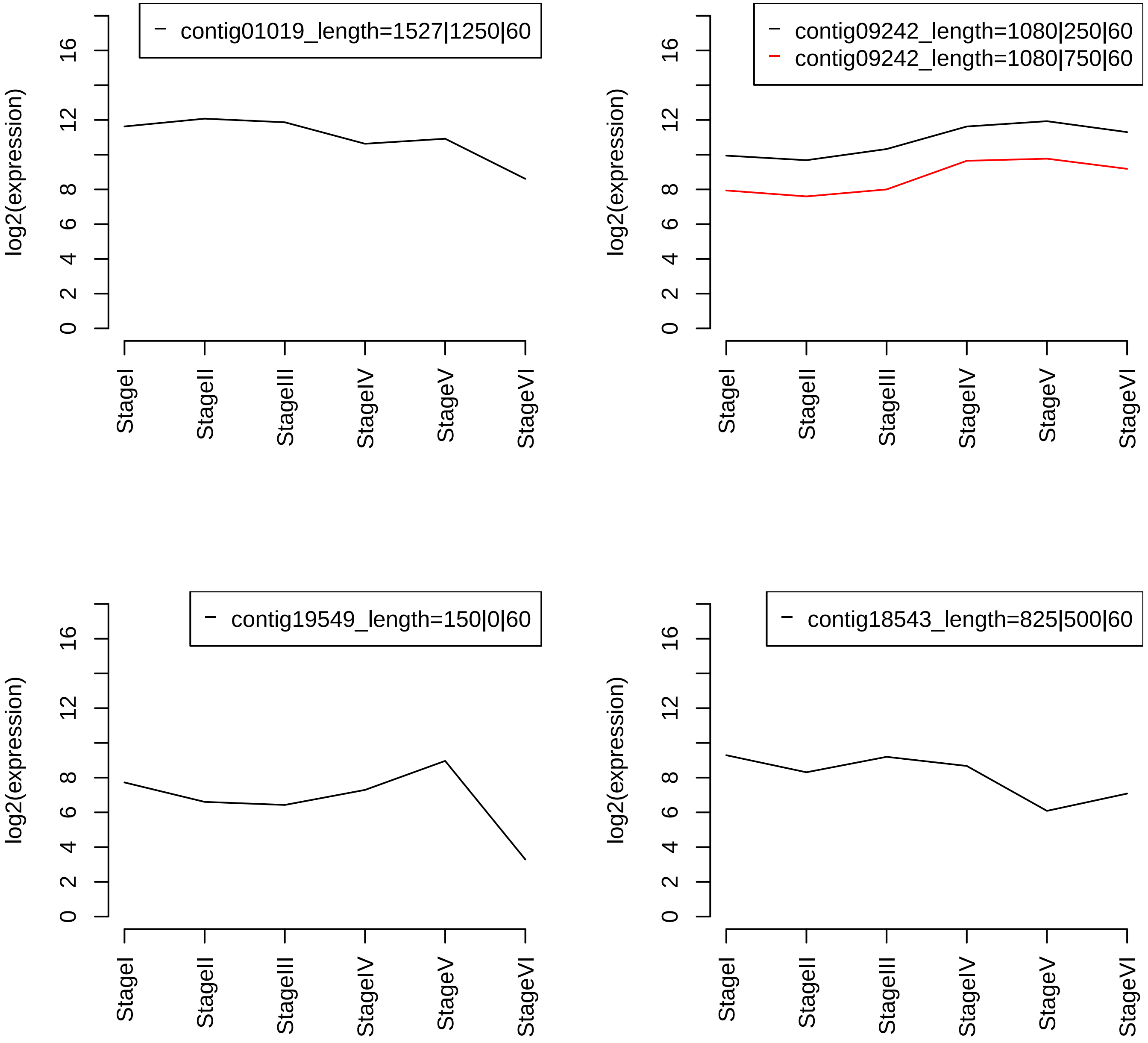
contig01019_length=1527|1250|60
contig09242_length=1080|250|60
contig09242_length=1080|750|60
contig19549_length=150|0|60
contig18543_length=825|500|60
log2(expression)
0
2
4
6
8
12
16
StageI
StageII
StageIII
StageIV
StageV
StageVI

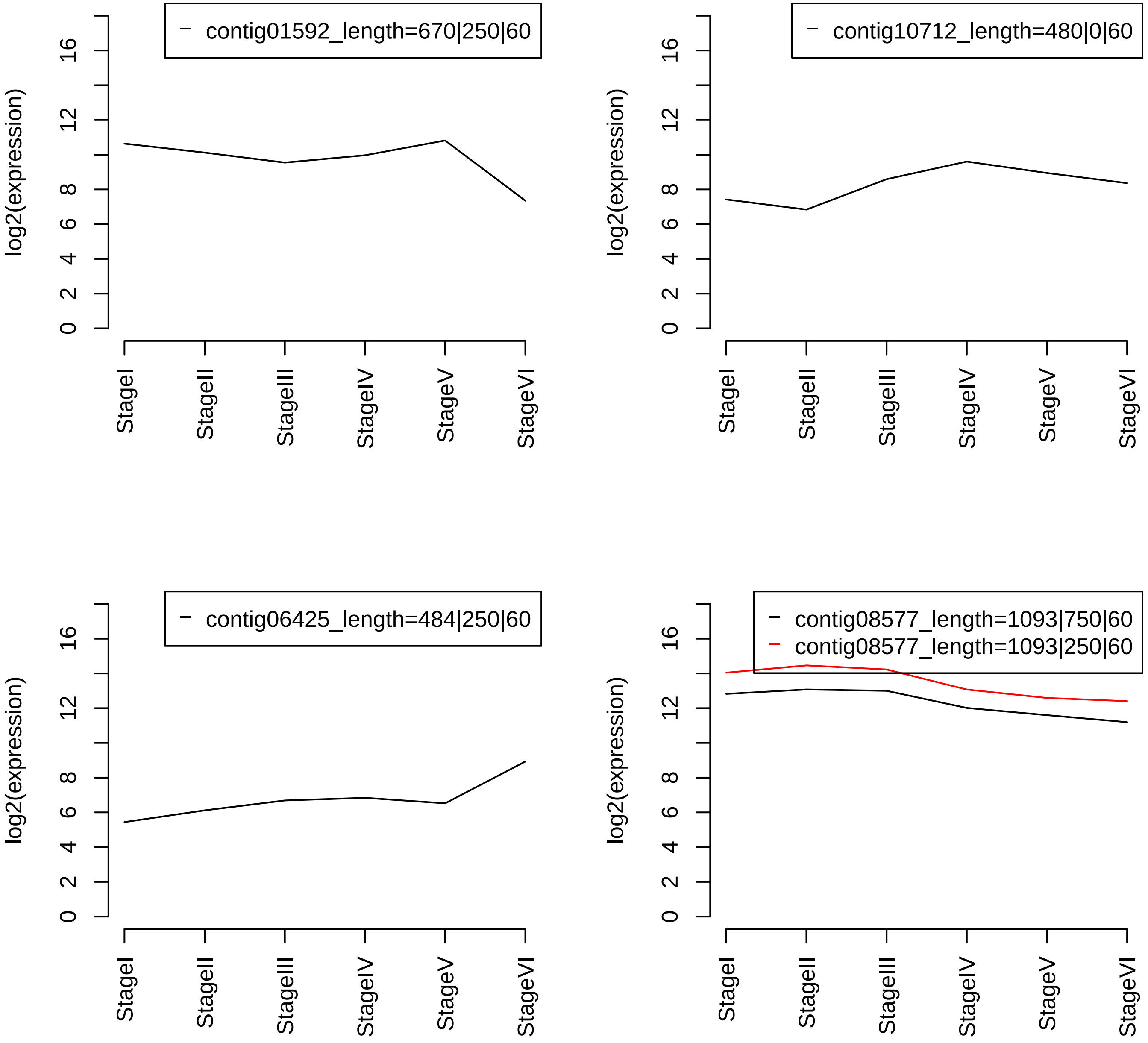
contig01592_length=670|250|60
contig10712_length=480|0|60
contig06425_length=484|250|60
contig08577_length=1093|750|60
contig08577_length=1093|250|60
log2(expression)
0 2 4 6 8 12 16
StageI StageII StageIII StageIV StageV StageVI

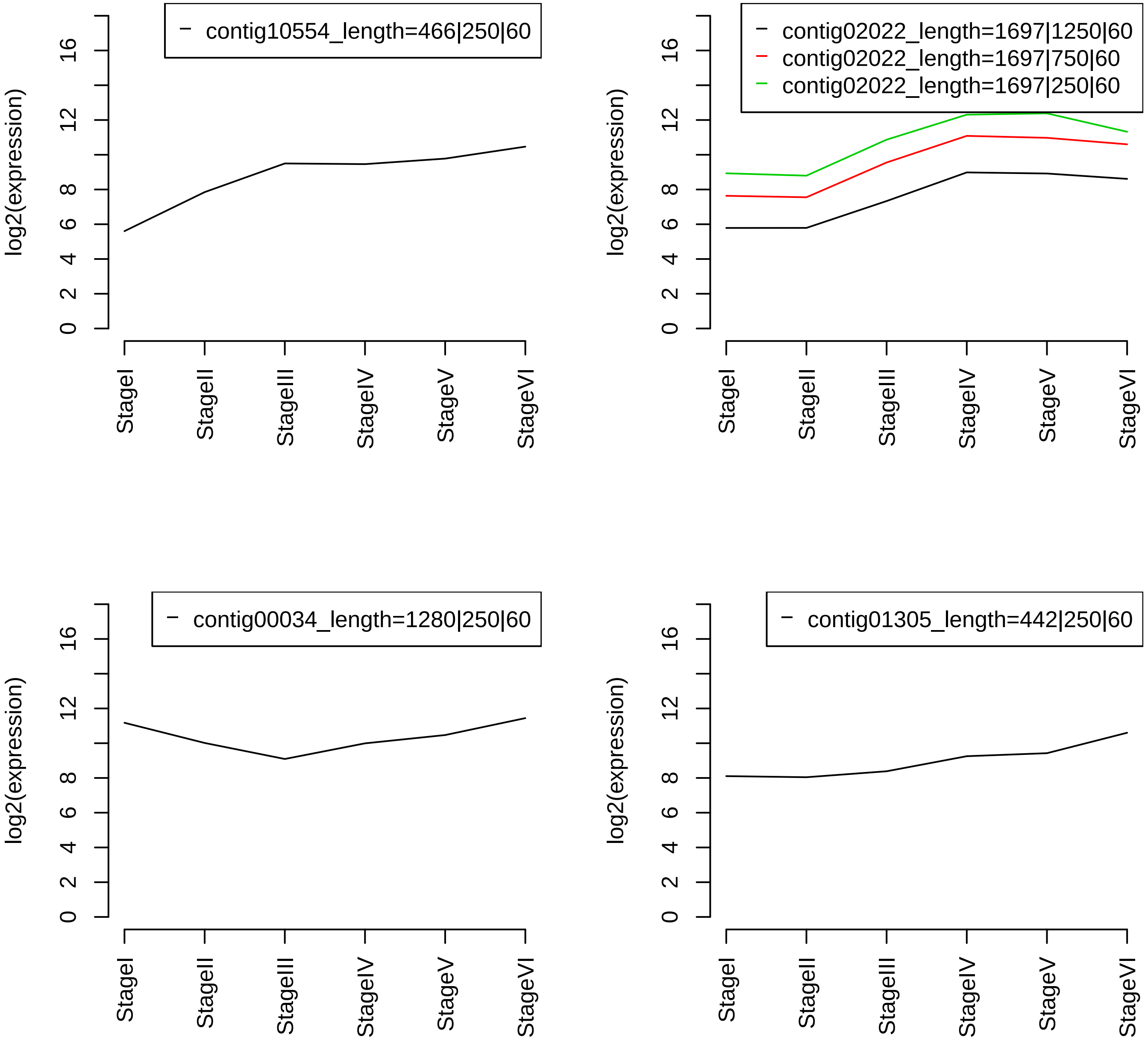

contig10554_length=466|250|60
contig02022_length=1697|1250|60
contig02022_length=1697|750|60
contig02022_length=1697|250|60
contig00034_length=1280|250|60
contig01305_length=442|250|60
log2(expression)
0
2
4
6
8
12
16
StageI
StageII
StageIII
StageIV
StageV
StageVI

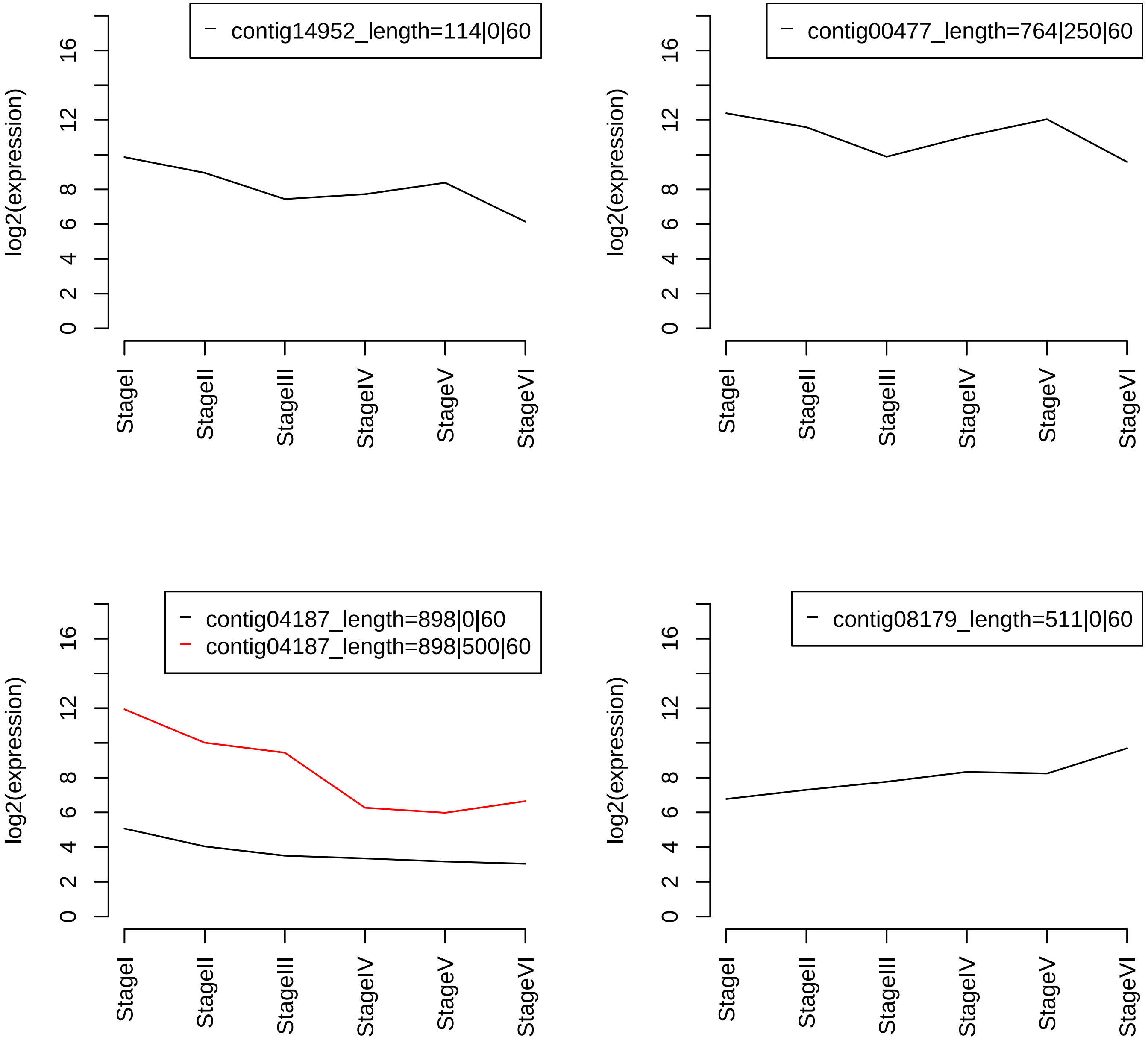
contig14952_length=114|0|60
contig00477_length=764|250|60
contig04187_length=898|0|60
contig04187_length=898|500|60
contig08179_length=511|0|60
log2(expression)
0
2
4
6
8
12
16
StageI
StageII
StageIII
StageIV
StageV
StageVI

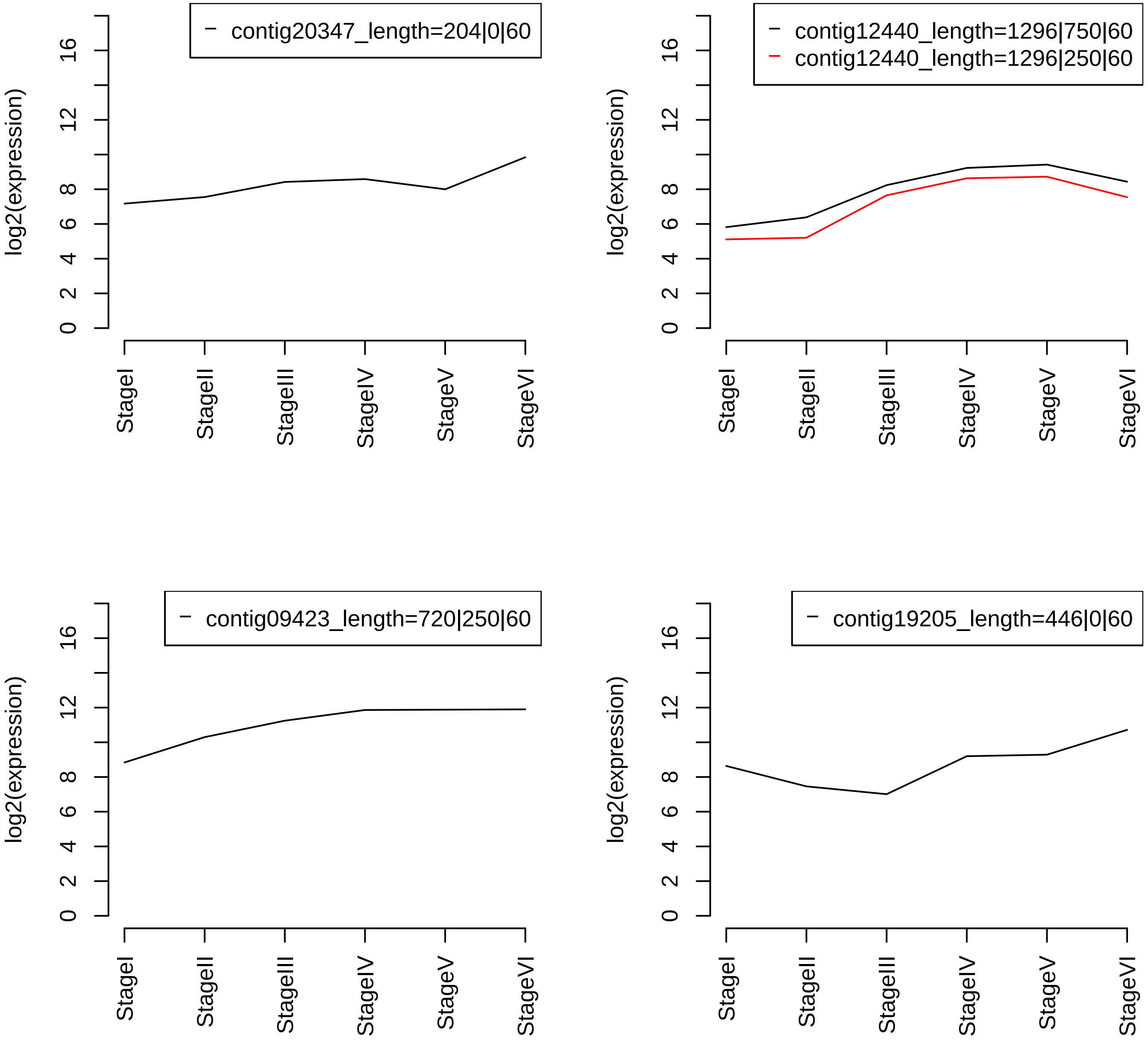
contig20347_length=204|0|60
contig12440_length=1296|750|60
contig12440_length=1296|250|60
contig09423_length=720|250|60
contig19205_length=446|0|60
log2(expression)
0
2
4
6
8
12
16
StageI
StageII
StageIII
StageIV
StageV
StageVI

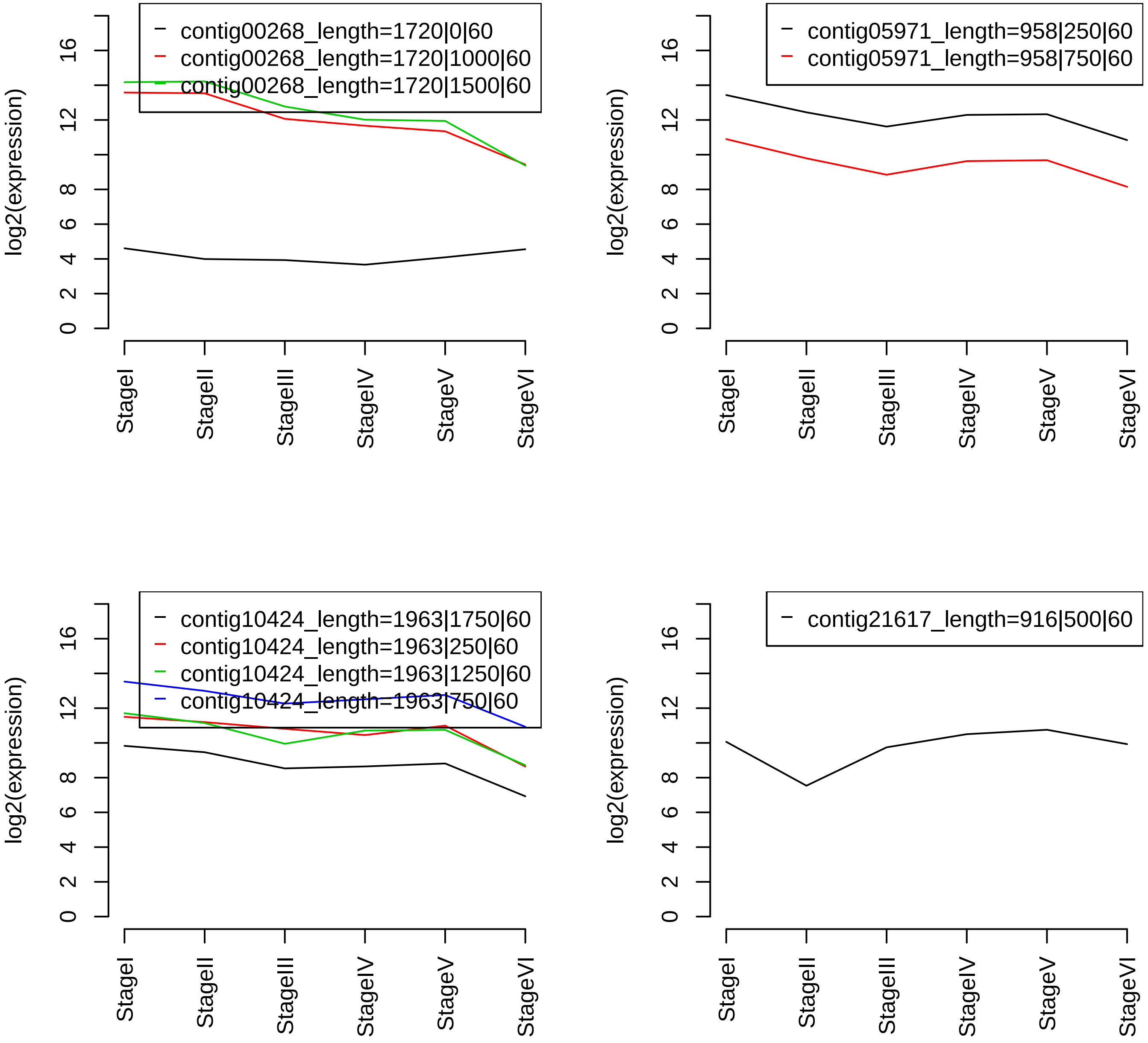
contig00268_length=1720|0|60
contig00268_length=1720|1000|60
contig00268_length=1720|1500|60
contig05971_length=958|250|60
contig05971_length=958|750|60
contig10424_length=1963|1750|60
contig10424_length=1963|250|60
contig10424_length=1963|1250|60
contig10424_length=1963|750|60
contig21617_length=916|500|60
log2(expression)
0
2
4
6
8
12
16
StageI
StageII
StageIII
StageIV
StageV
StageVI

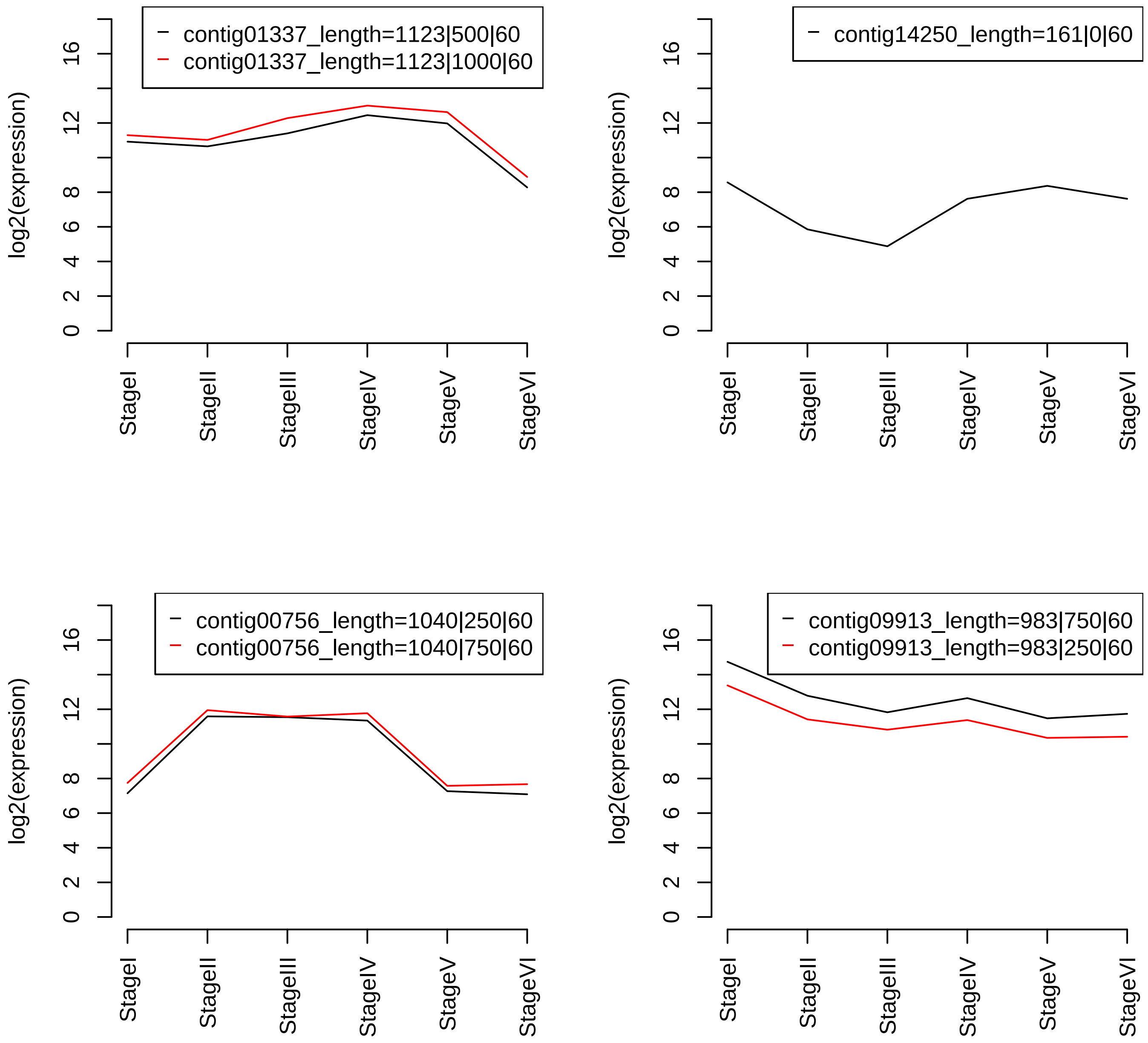

contig01337_length=1123|500|60
contig01337_length=1123|1000|60
contig14250_length=161|0|60
contig00756_length=1040|250|60
contig00756_length=1040|750|60
contig09913_length=983|750|60
contig09913_length=983|250|60
log2(expression)
0
2
4
6
8
12
16
StageI
StageII
StageIII
StageIV
StageV
StageVI

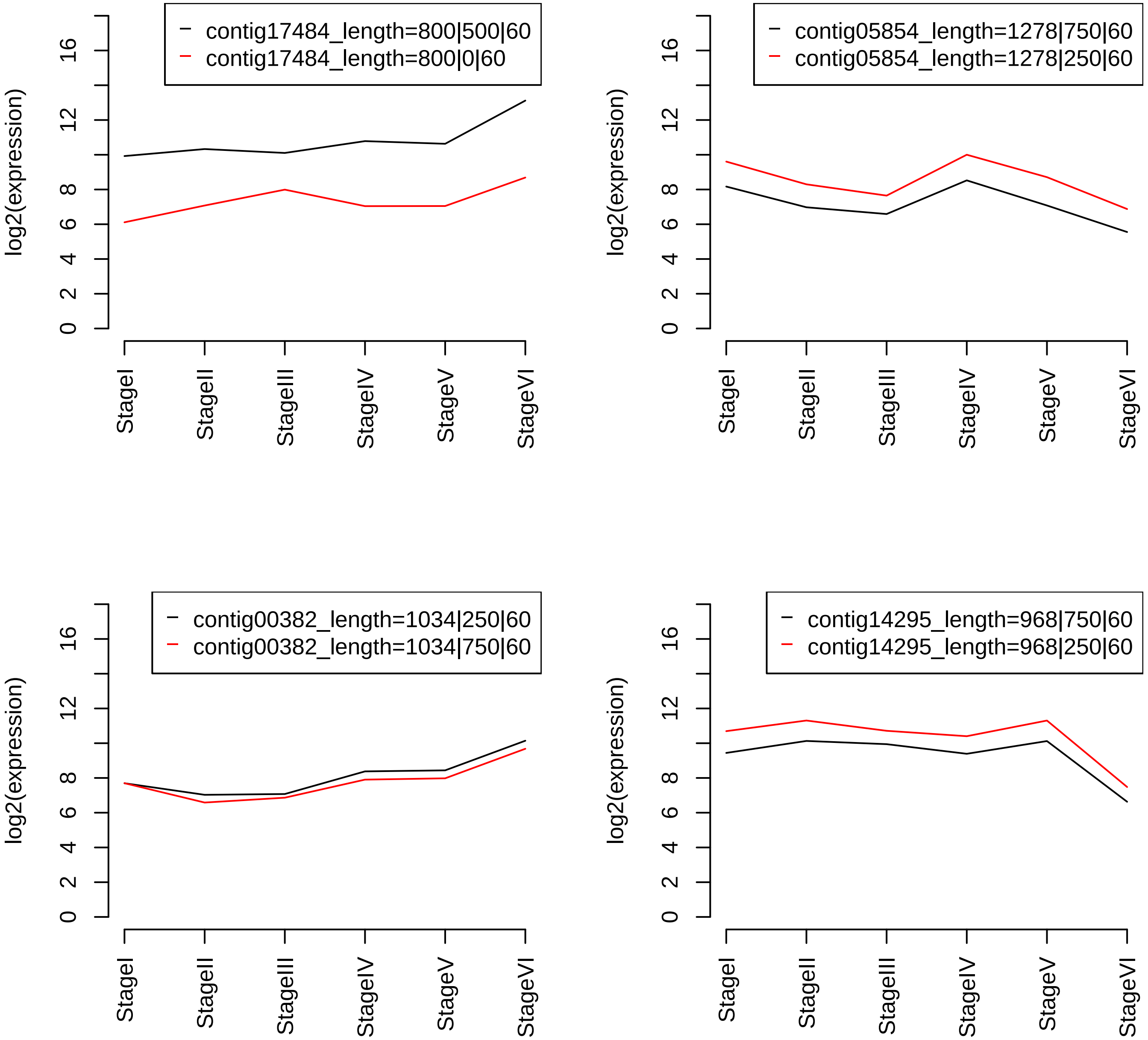
contig17484_length=800|500|60
contig17484_length=800|0|60
contig05854_length=1278|750|60
contig05854_length=1278|250|60
contig00382_length=1034|250|60
contig00382_length=1034|750|60
contig14295_length=968|750|60
contig14295_length=968|250|60
log2(expression)
0
2
4
6
8
12
16
StageI
StageII
StageIII
StageIV
StageV
StageVI

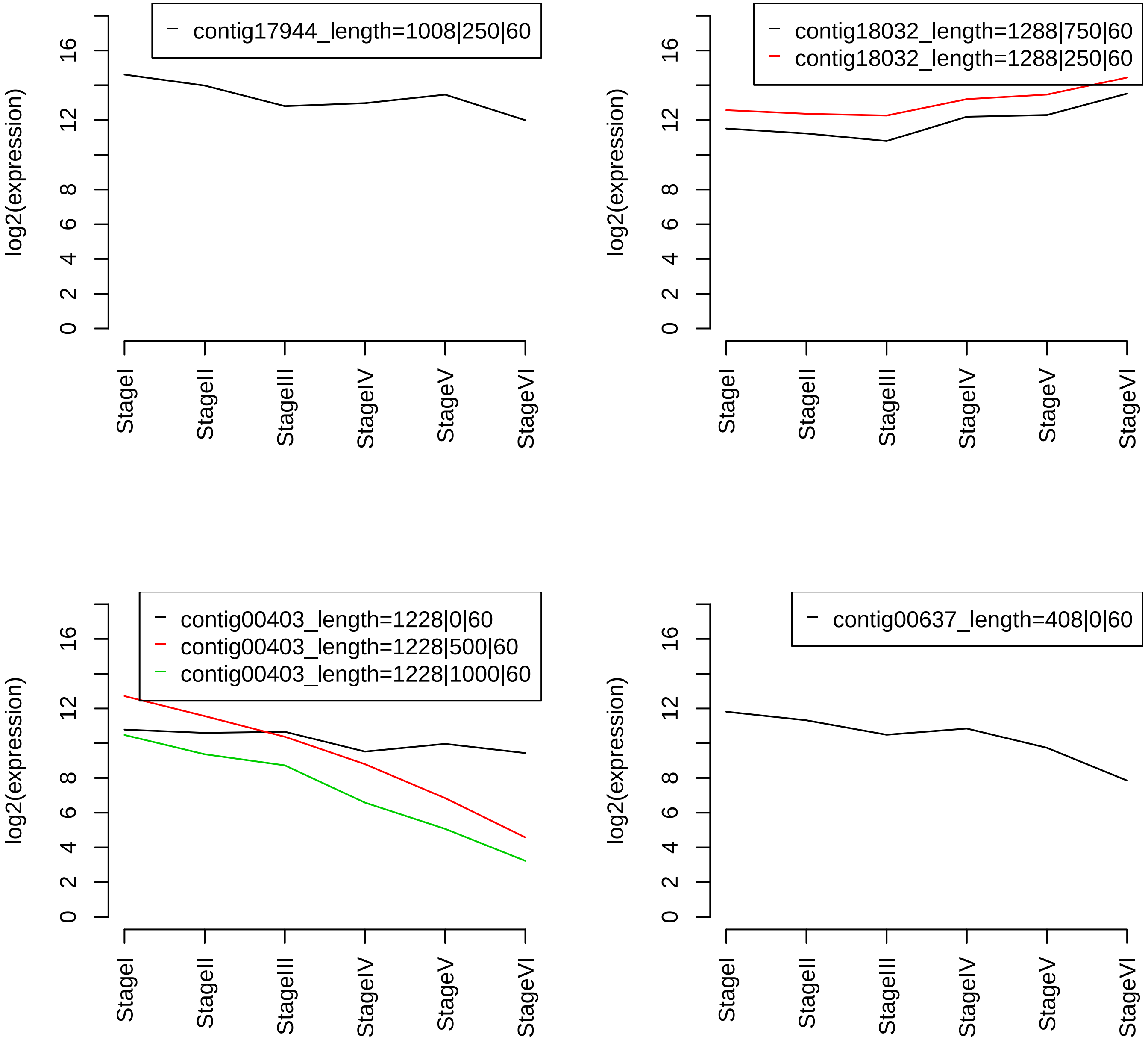
contig17944_length=1008|250|60
contig18032_length=1288|750|60
contig18032_length=1288|250|60
contig00403_length=1228|0|60
contig00403_length=1228|500|60
contig00403_length=1228|1000|60
contig00637_length=408|0|60
log2(expression)
0
2
4
6
8
12
16
StageI
StageII
StageIII
StageIV
StageV
StageVI

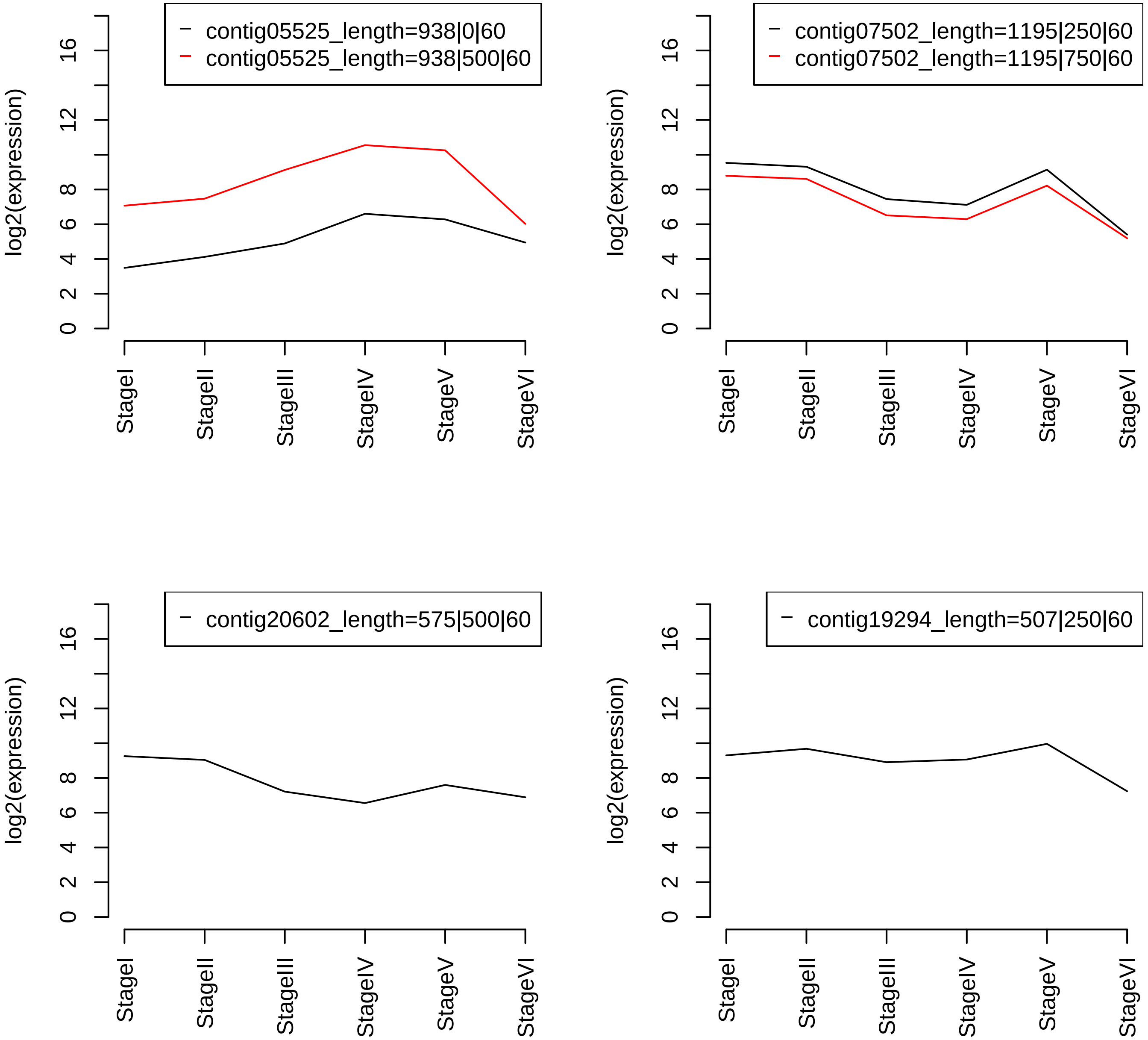
contig05525_length=938|0|60
contig05525_length=938|500|60
contig07502_length=1195|250|60
contig07502_length=1195|750|60
contig20602_length=575|500|60
contig19294_length=507|250|60
log2(expression)
0
2
4
6
8
12
16
StageI
StageII
StageIII
StageIV
StageV
StageVI

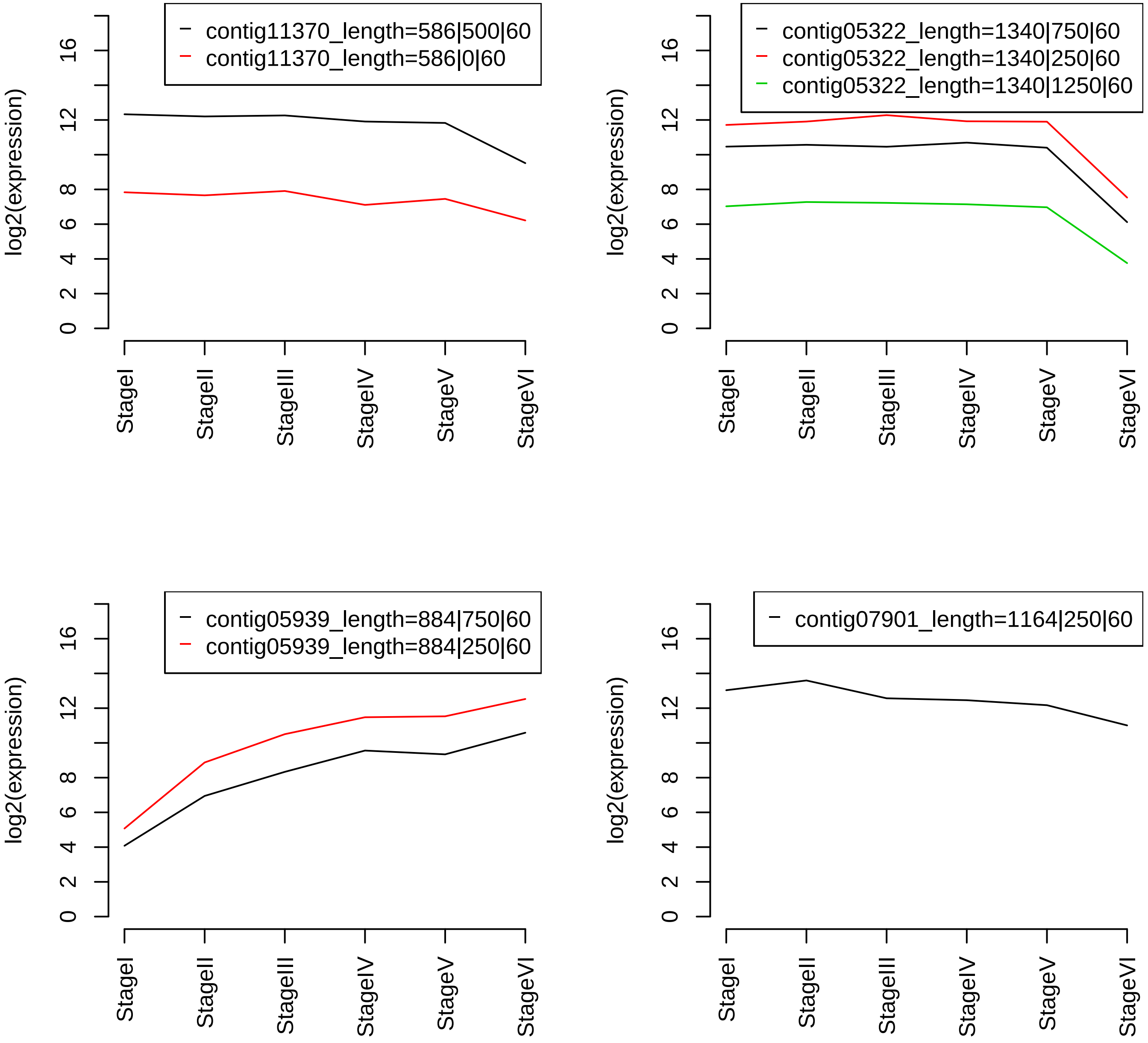
contig11370_length=586|500|60
contig11370_length=586|0|60
contig05322_length=1340|750|60
contig05322_length=1340|250|60
contig05322_length=1340|1250|60
contig05939_length=884|750|60
contig05939_length=884|250|60
contig07901_length=1164|250|60
log2(expression)
StageI
StageII
StageIII
StageIV
StageV
StageVI

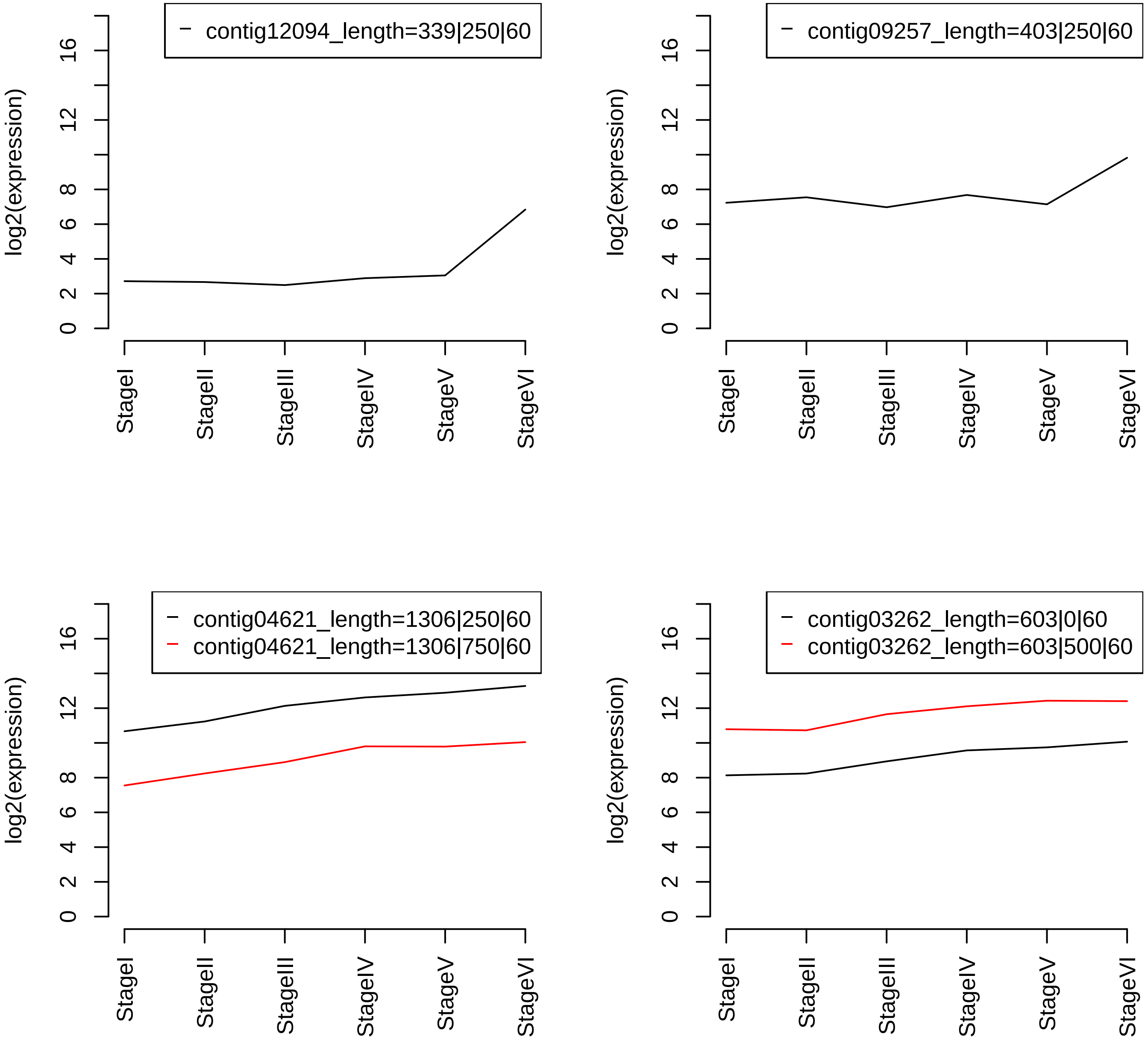

contig12094_length=339|250|60
contig09257_length=403|250|60
contig04621_length=1306|250|60
contig04621_length=1306|750|60
contig03262_length=603|0|60
contig03262_length=603|500|60
log2(expression)
0
2
4
6
8
12
16
StageI
StageII
StageIII
StageIV
StageV
StageVI

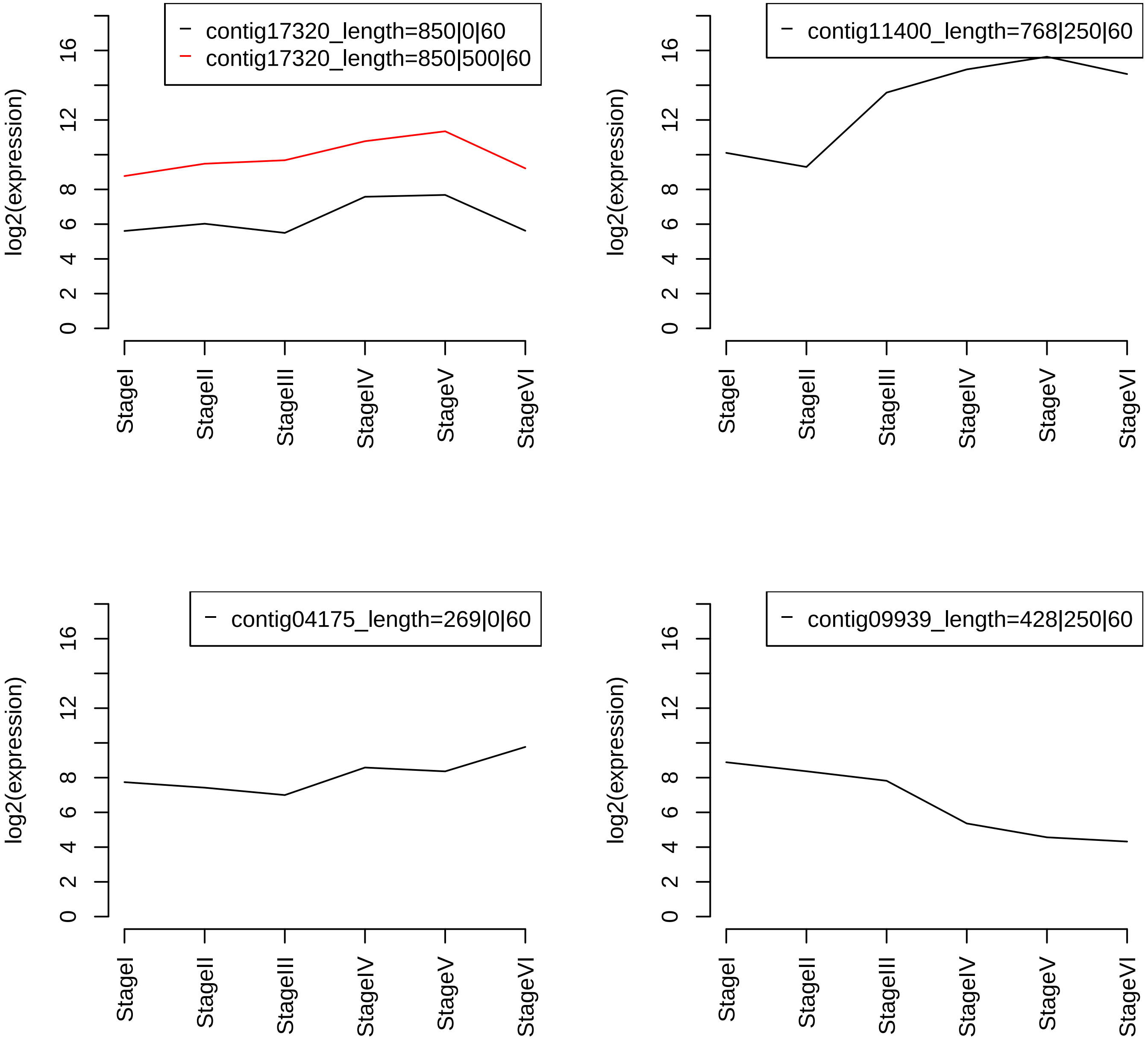

contig17320_length=850|0|60
contig17320_length=850|500|60
contig11400_length=768|250|60
contig04175_length=269|0|60
contig09939_length=428|250|60
log2(expression)
0
2
4
6
8
12
16
StageI
StageII
StageIII
StageIV
StageV
StageVI

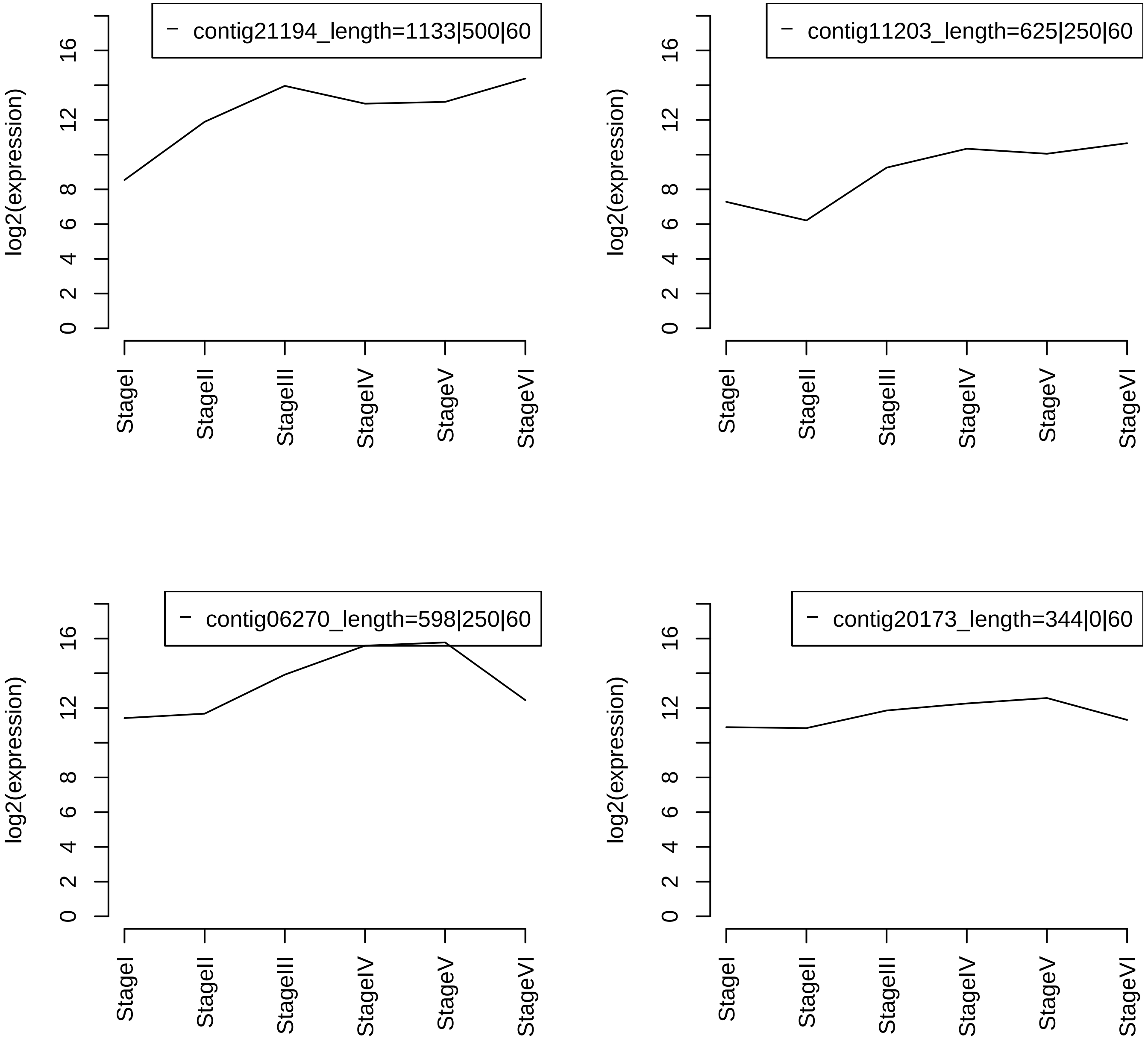

contig21194_length=1133|500|60
contig11203_length=625|250|60
contig06270_length=598|250|60
contig20173_length=344|0|60
log2(expression)
0 2 4 6 8 12 16
StageI
StageII
StageIII
StageIV
StageV
StageVI

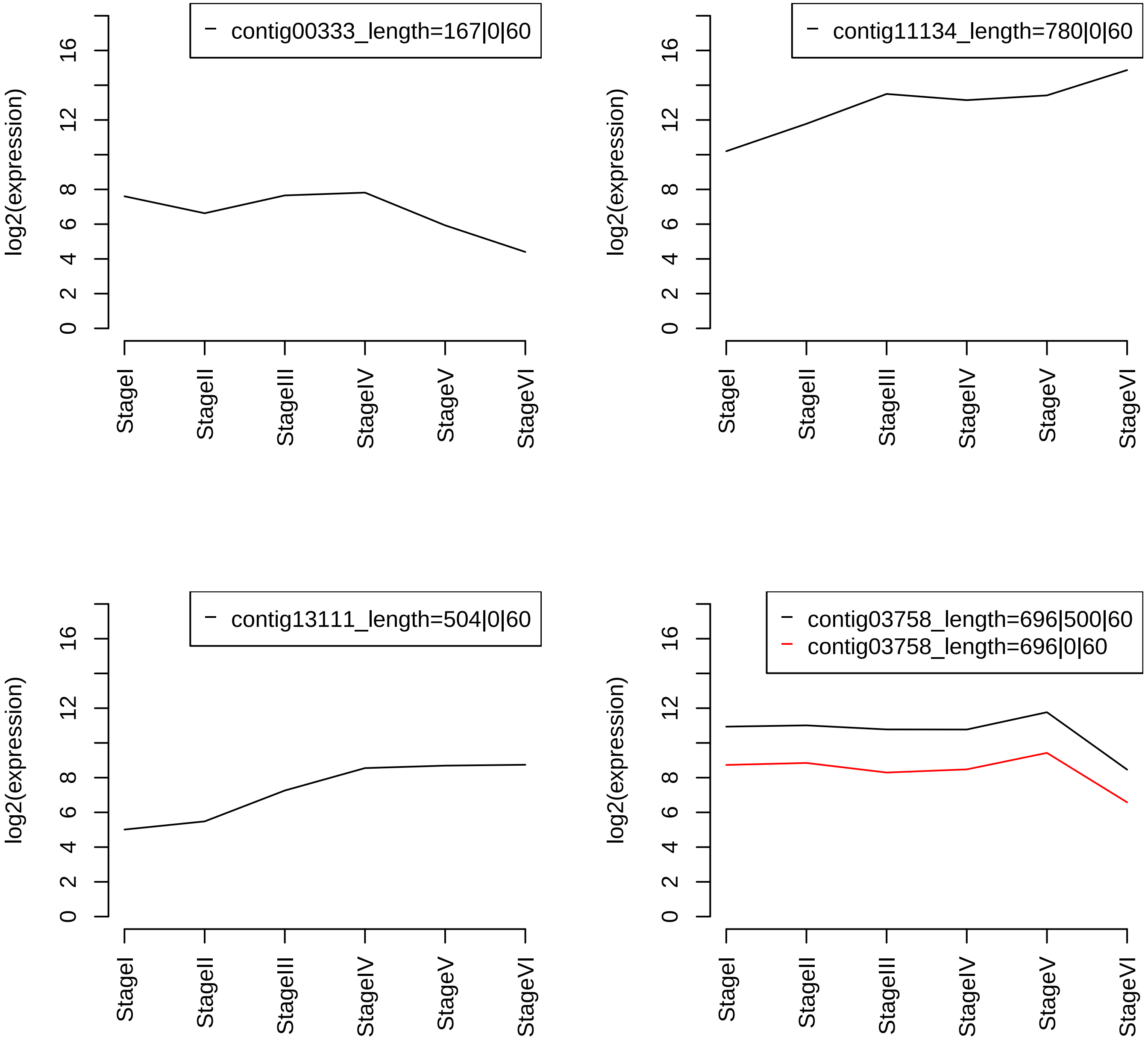

contig00333_length=167|0|60
contig11134_length=780|0|60
contig13111_length=504|0|60
contig03758_length=696|500|60
contig03758_length=696|0|60
log2(expression)
0
2
4
6
8
12
16
StageI
StageII
StageIII
StageIV
StageV
StageVI

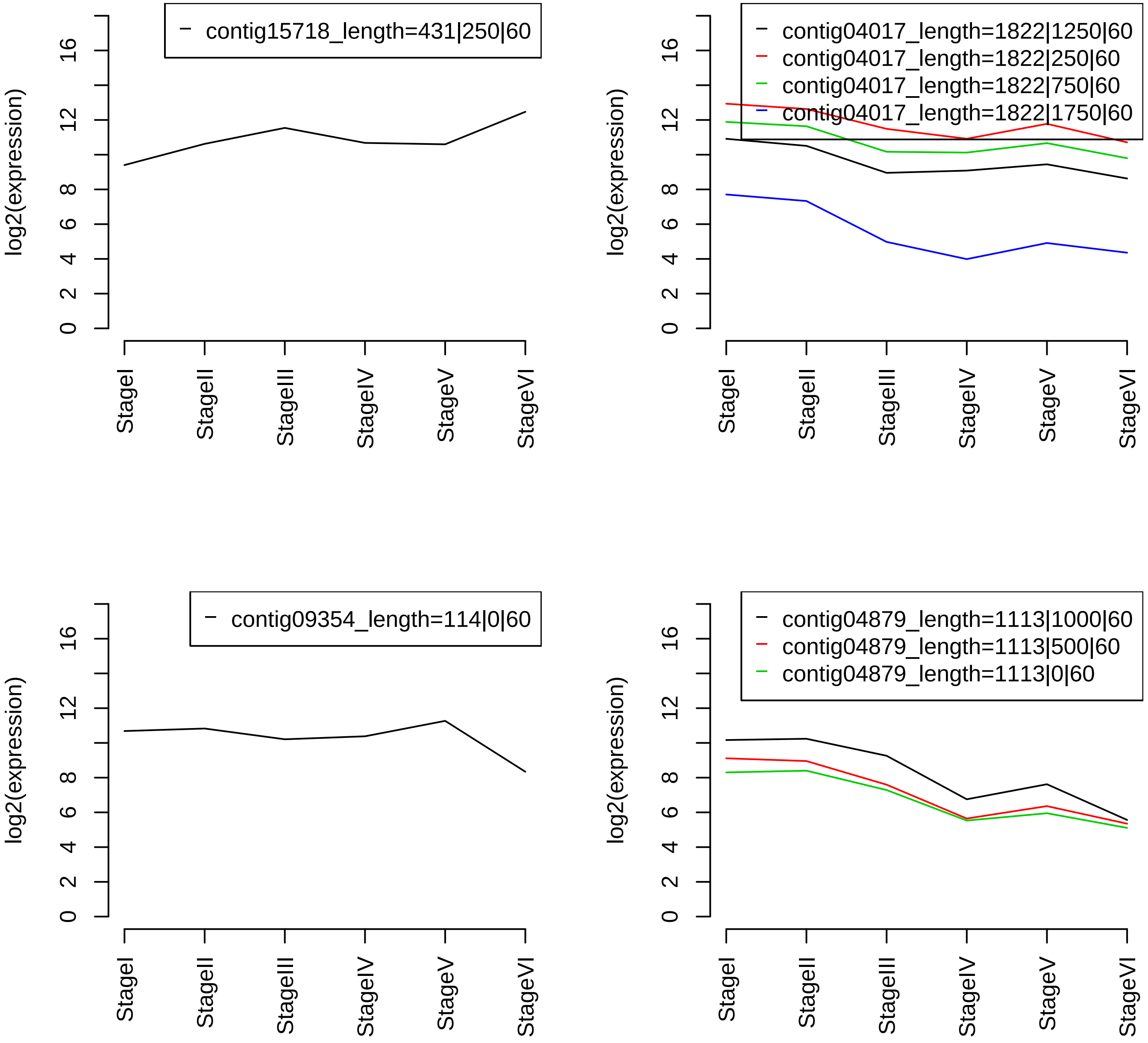

contig15718_length=431|250|60
contig04017_length=1822|1250|60
contig04017_length=1822|250|60
contig04017_length=1822|750|60
contig04017_length=1822|1750|60
contig09354_length=114|0|60
contig04879_length=1113|1000|60
contig04879_length=1113|500|60
contig04879_length=1113|0|60
log2(expression)
0
2
4
6
8
12
16
StageI
StageII
StageIII
StageIV
StageV
StageVI

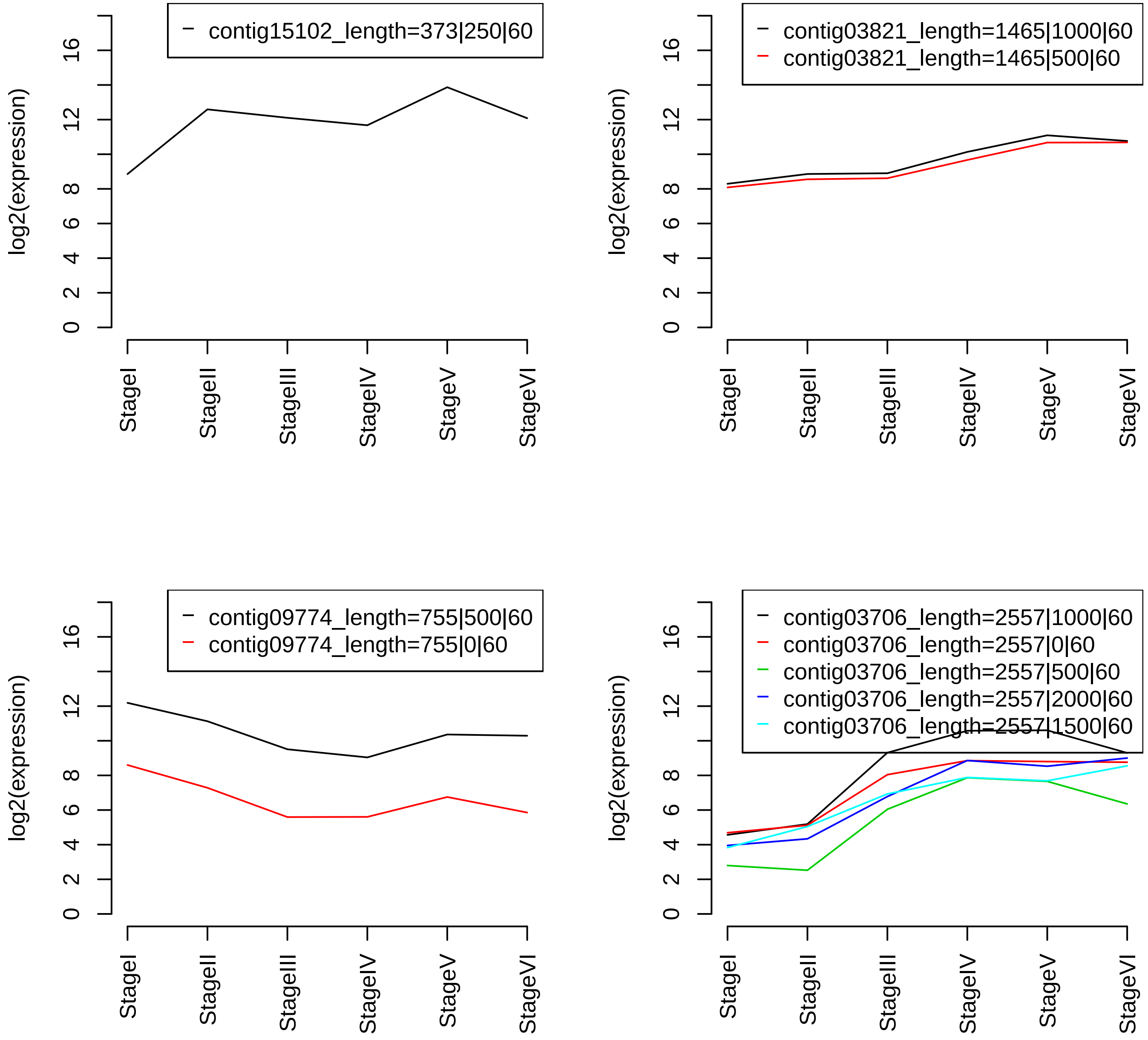
contig15102_length=373|250|60
log2(expression)
0
2
4
6
8
12
16
StageI
StageII
StageIII
StageIV
StageV
StageVI
contig03821_length=1465|1000|60
contig03821_length=1465|500|60
contig09774_length=755|500|60
contig09774_length=755|0|60
contig03706_length=2557|1000|60
contig03706_length=2557|0|60
contig03706_length=2557|500|60
contig03706_length=2557|2000|60
contig03706_length=2557|1500|60

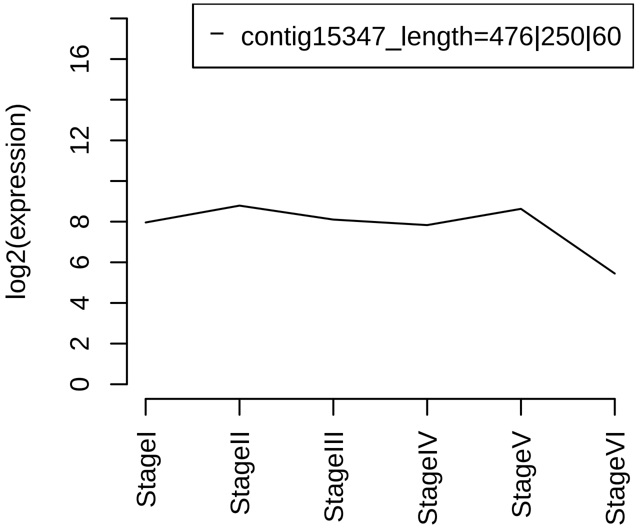
contig15347_length=476|250|60
log2(expression)
0
2
4
6
8
12
16
StageI
StageII
StageIII
StageIV
StageV
StageVI

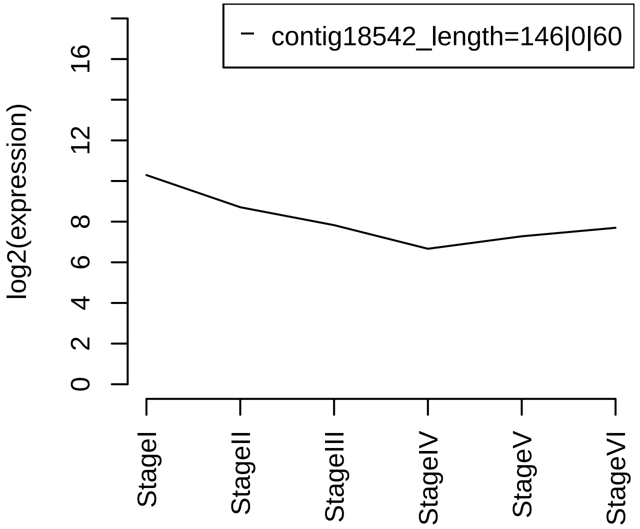
contig18542_length=146|0|60
log2(expression)
0
2
4
6
8
12
16
StageI
StageII
StageIII
StageIV
StageV
StageVI

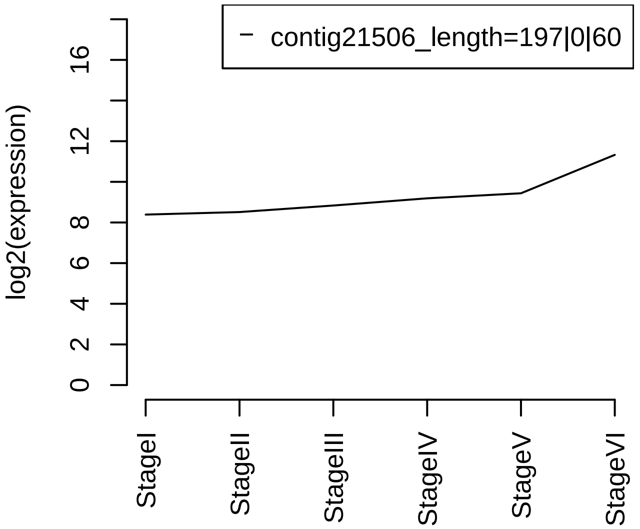
contig21506_length=197|0|60
log2(expression)
0
2
4
6
8
12
16
StageI
StageII
StageIII
StageIV
StageV
StageVI

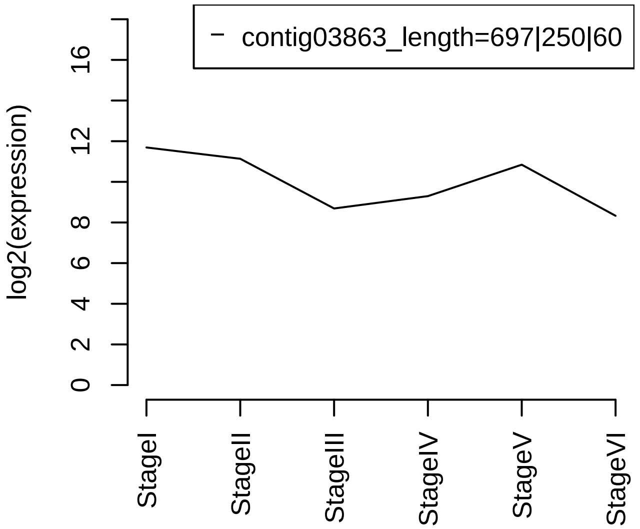
contig03863_length=697|250|60
log2(expression)
0
2
4
6
8
12
16
StageI
StageII
StageIII
StageIV
StageV
StageVI

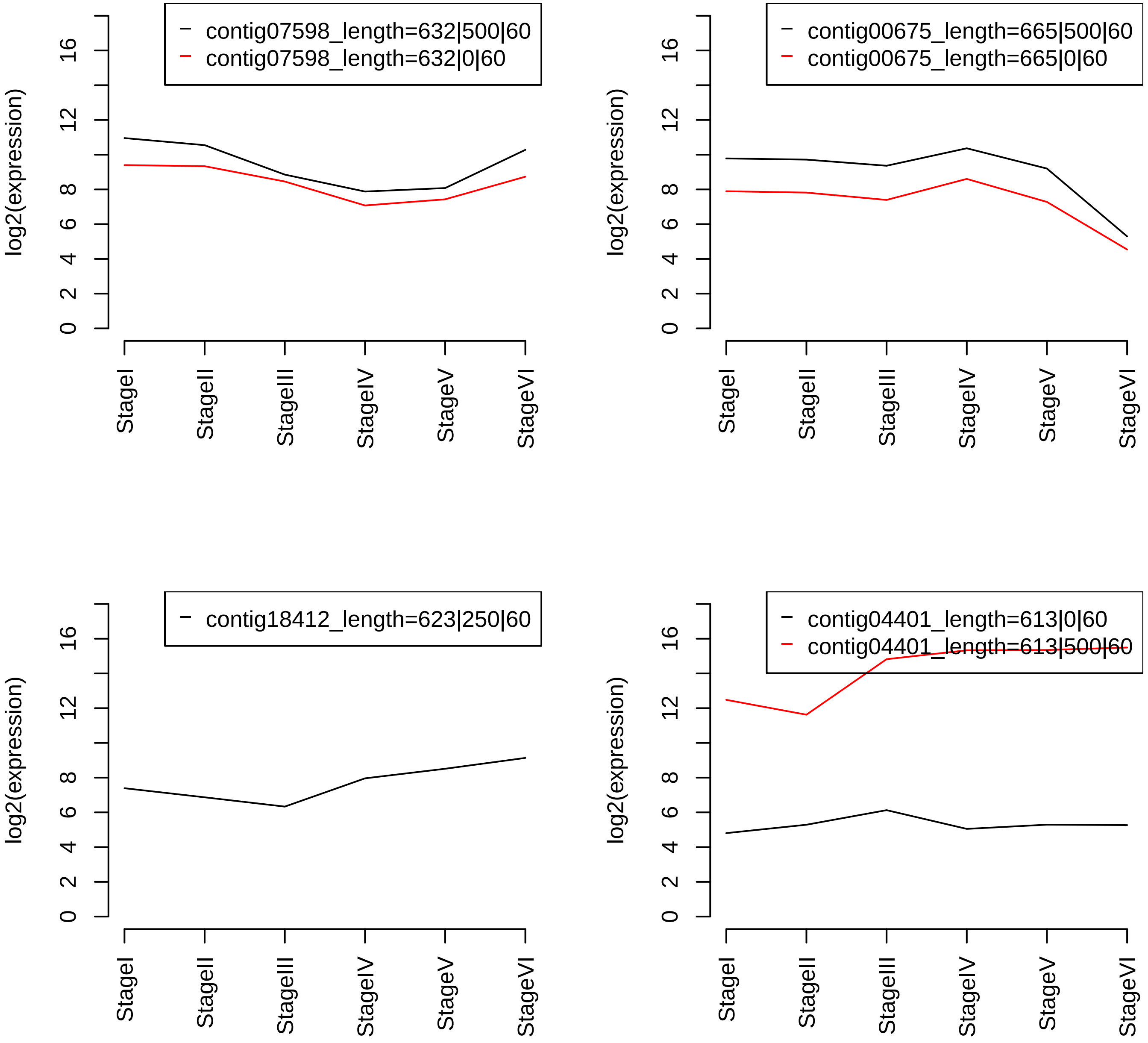
contig07598_length=632|500|60
contig07598_length=632|0|60
contig00675_length=665|500|60
contig00675_length=665|0|60
contig18412_length=623|250|60
contig04401_length=613|0|60
contig04401_length=613|500|60
log2(expression)
0 2 4 6 8 12 16
StageI StageII StageIII StageIV StageV StageVI

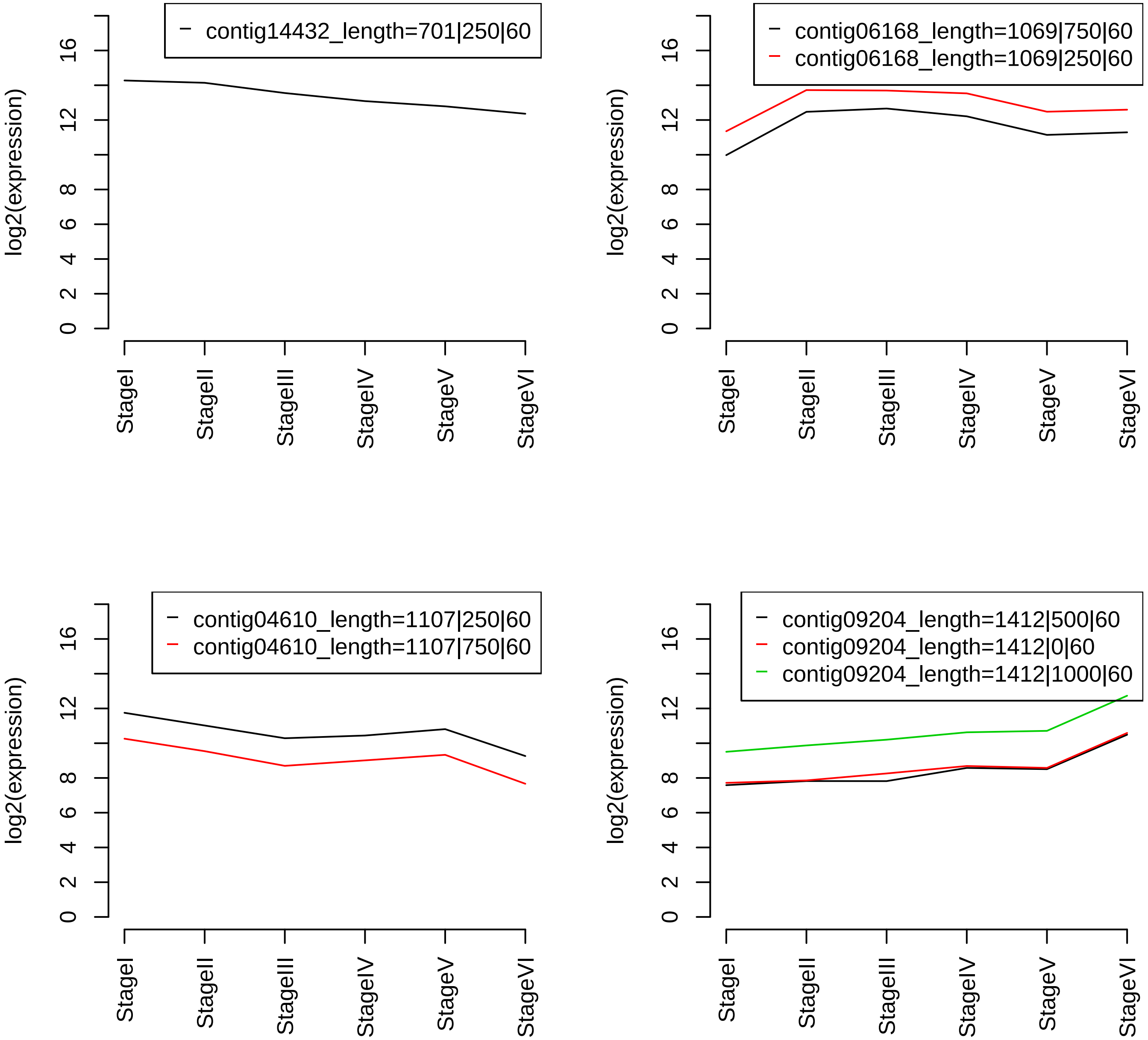

contig14432_length=701|250|60
contig06168_length=1069|750|60
contig06168_length=1069|250|60
contig04610_length=1107|250|60
contig04610_length=1107|750|60
contig09204_length=1412|500|60
contig09204_length=1412|0|60
contig09204_length=1412|1000|60
log2(expression)
0 2 4 6 8 12 16
StageI StageII StageIII StageIV StageV StageVI

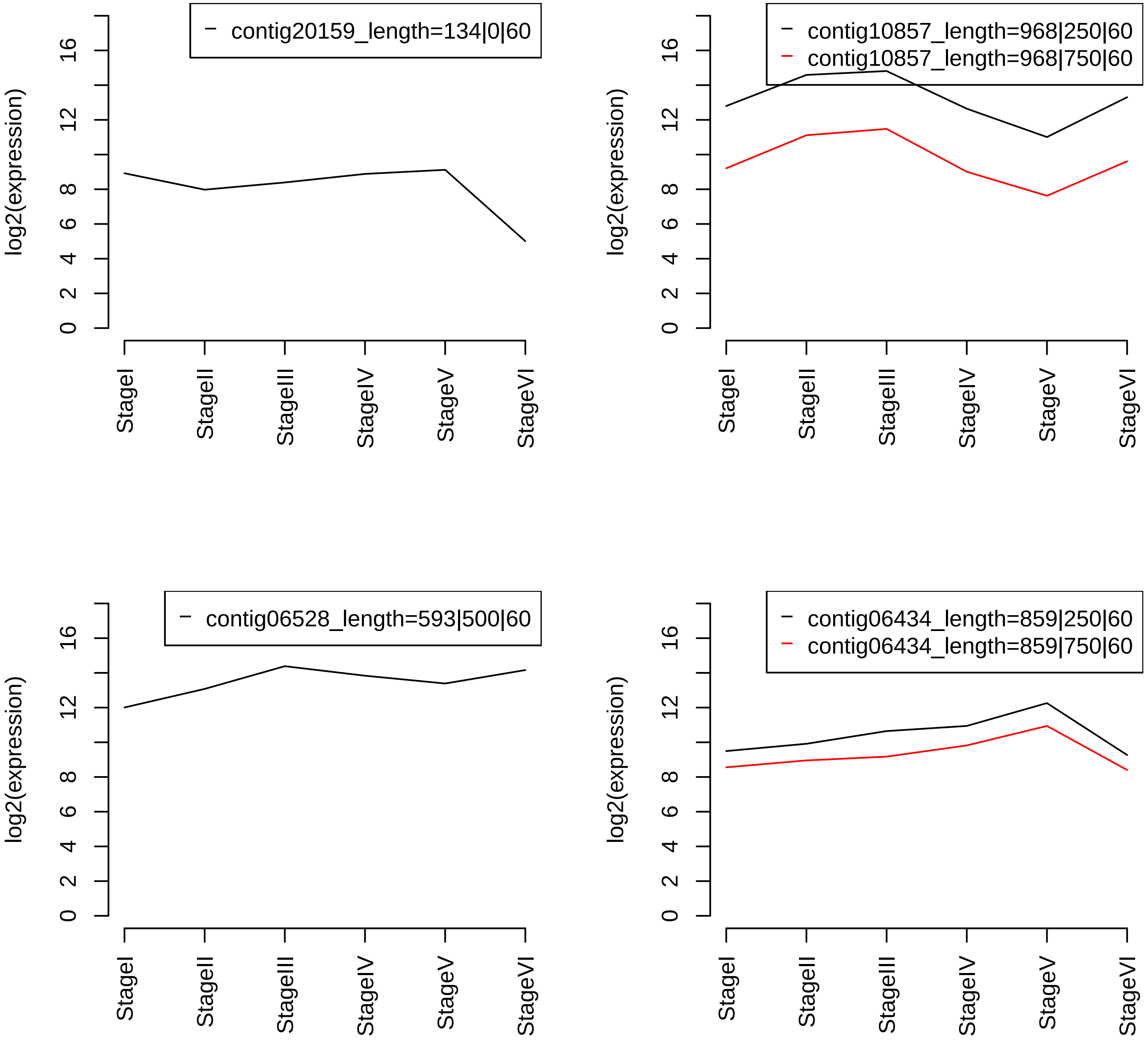

contig20159_length=134|0|60
contig10857_length=968|250|60
contig10857_length=968|750|60
contig06528_length=593|500|60
contig06434_length=859|250|60
contig06434_length=859|750|60
log2(expression)
0
2
4
6
8
12
16
StageI
StageII
StageIII
StageIV
StageV
StageVI

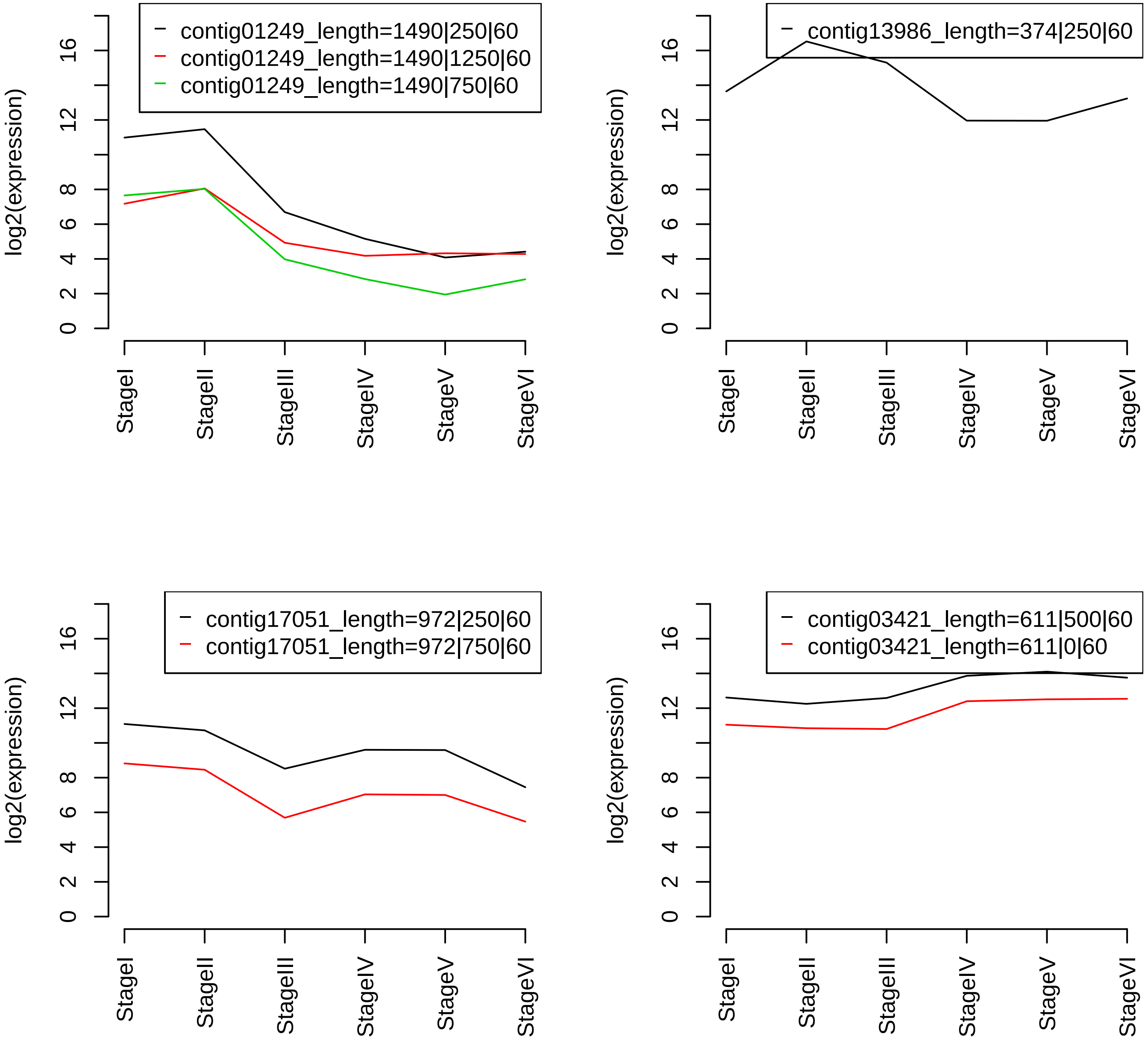

contig01249_length=1490|250|60
contig01249_length=1490|1250|60
contig01249_length=1490|750|60
contig13986_length=374|250|60
contig17051_length=972|250|60
contig17051_length=972|750|60
contig03421_length=611|500|60
contig03421_length=611|0|60
log2(expression)
0
2
4
6
8
12
16
StageI
StageII
StageIII
StageIV
StageV
StageVI

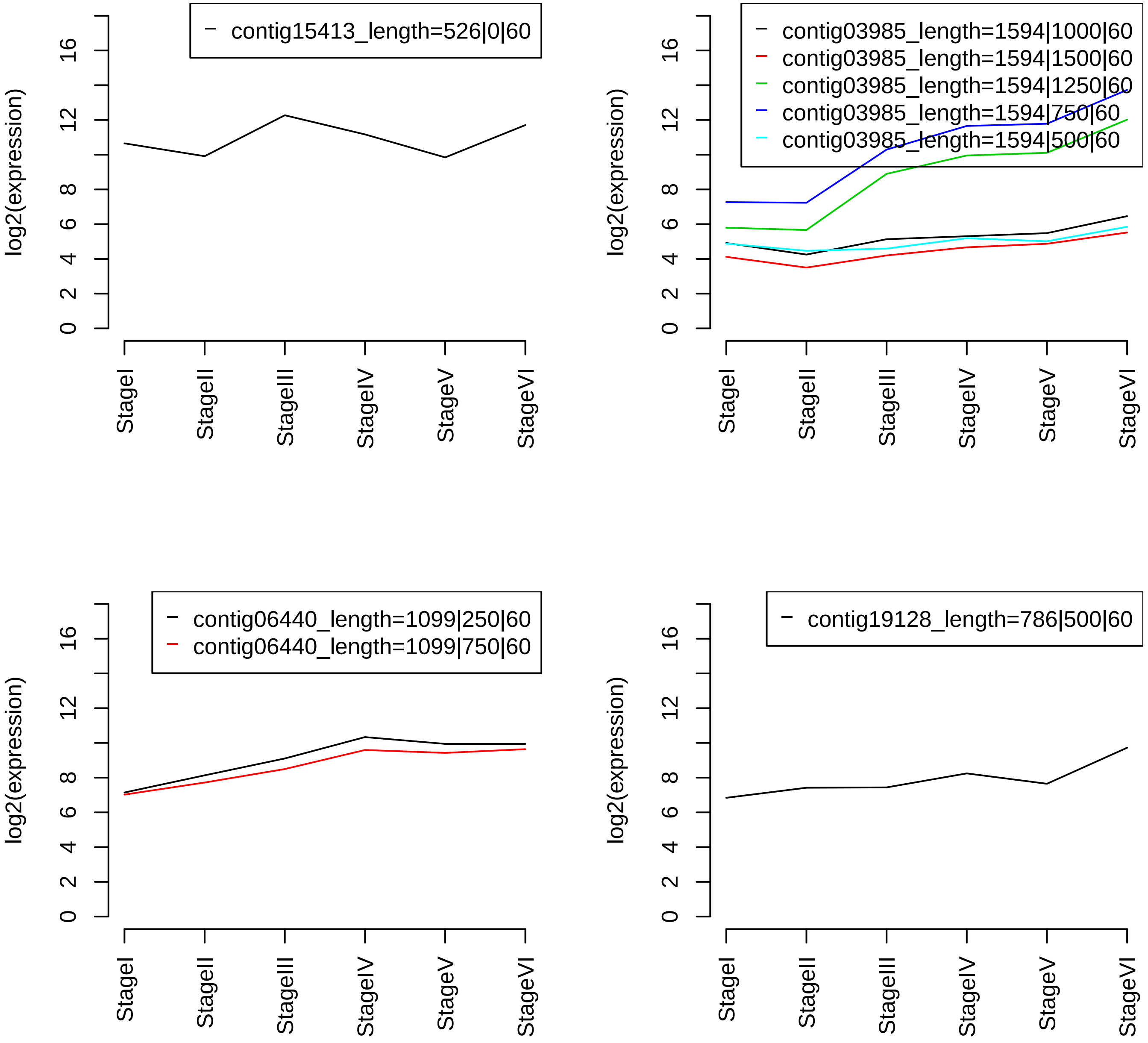

contig15413_length=526|0|60
contig03985_length=1594|1000|60
contig03985_length=1594|1500|60
contig03985_length=1594|1250|60
contig03985_length=1594|750|60
contig03985_length=1594|500|60
contig06440_length=1099|250|60
contig06440_length=1099|750|60
contig19128_length=786|500|60
log2(expression)
0 2 4 6 8 12 16
StageI StageII StageIII StageIV StageV StageVI

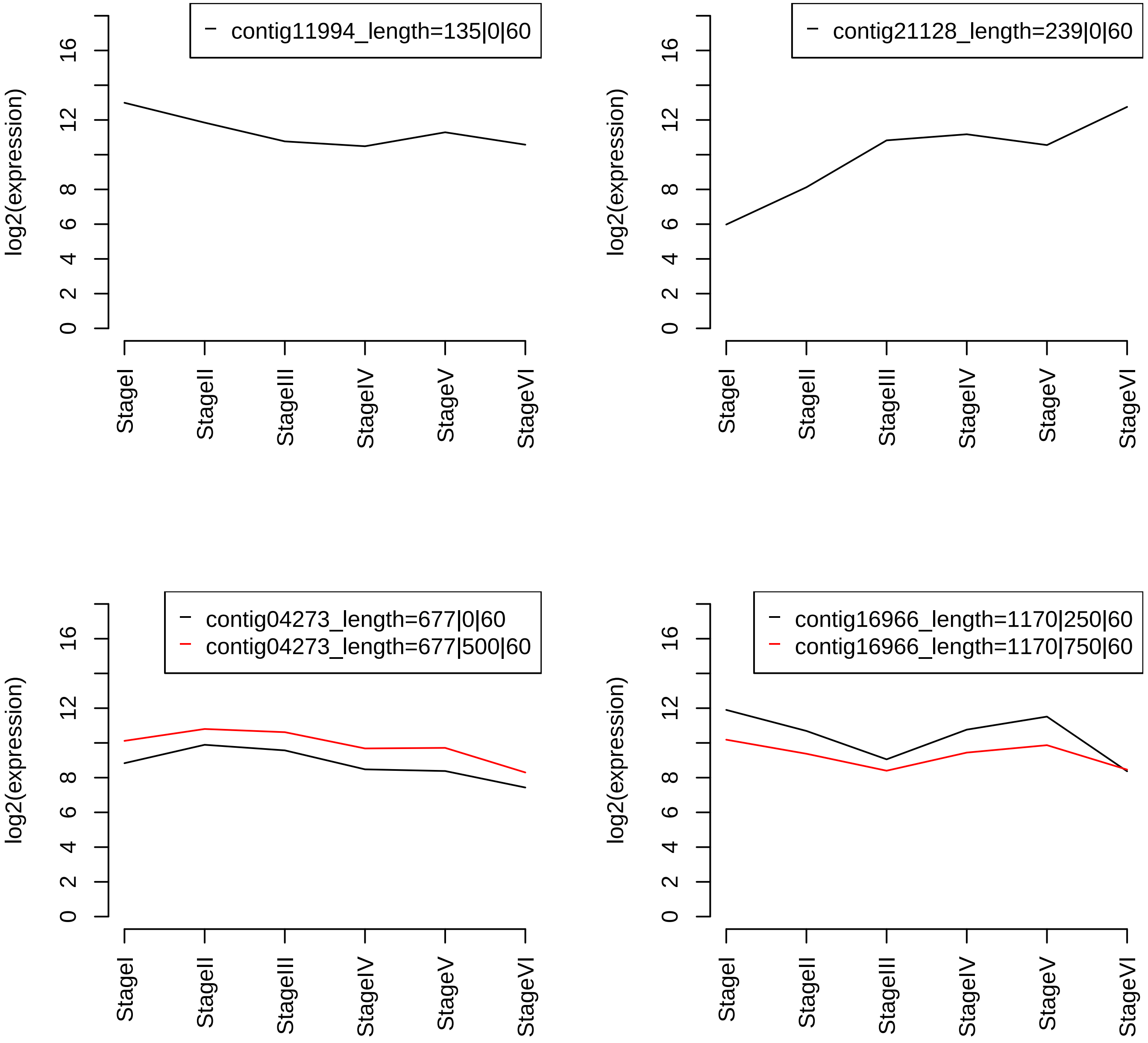

contig11994_length=135|0|60
contig21128_length=239|0|60
contig04273_length=677|0|60
contig04273_length=677|500|60
contig16966_length=1170|250|60
contig16966_length=1170|750|60
log2(expression)
0
2
4
6
8
12
16
StageI
StageII
StageIII
StageIV
StageV
StageVI

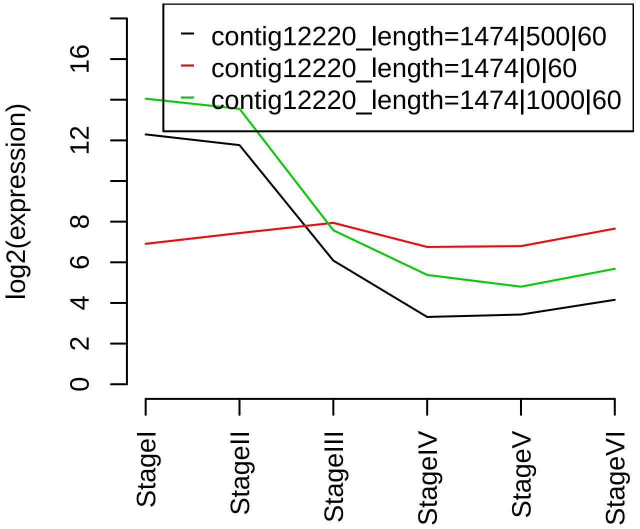

contig12220_length=1474|500|60
contig12220_length=1474|0|60
contig12220_length=1474|1000|60
log2(expression)
0
2
4
6
8
12
16
StageI
StageII
StageIII
StageIV
StageV
StageVI

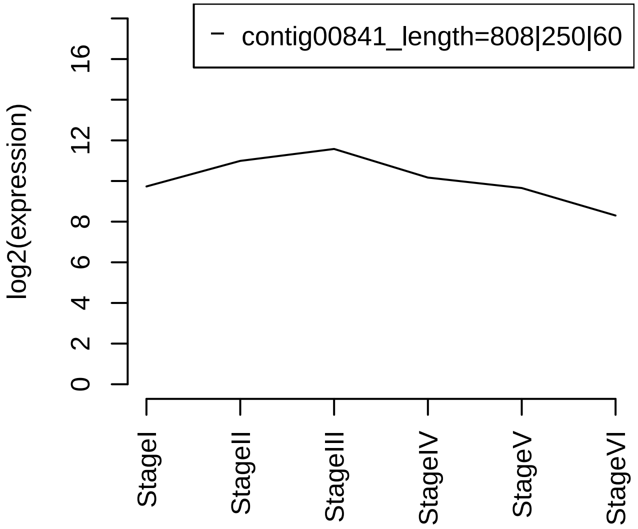

contig00841_length=808|250|60
log2(expression)
0
2
4
6
8
12
16
StageI
StageII
StageIII
StageIV
StageV
StageVI

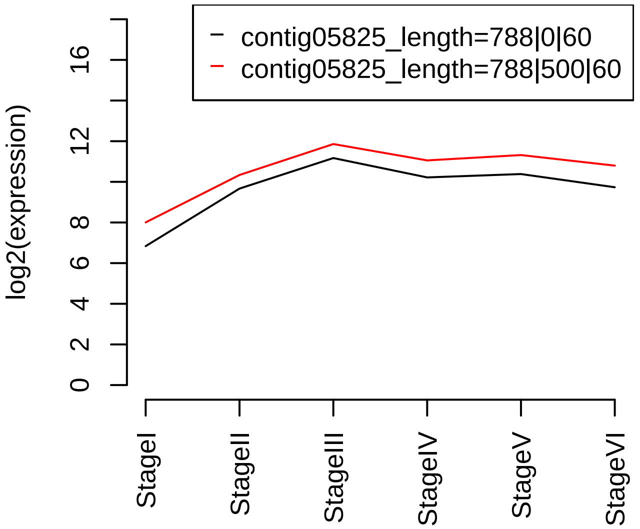

contig05825_length=788|0|60
contig05825_length=788|500|60
log2(expression)
0
2
4
6
8
12
16
StageI
StageII
StageIII
StageIV
StageV
StageVI

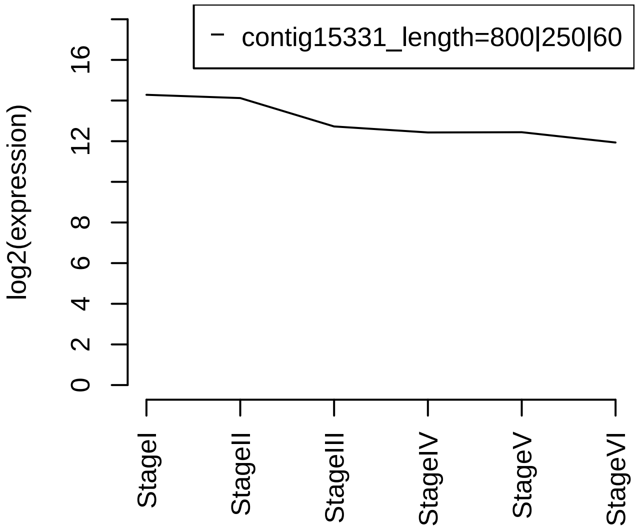

contig15331_length=800|250|60
log2(expression)
0
2
4
6
8
12
16
StageI
StageII
StageIII
StageIV
StageV
StageVI

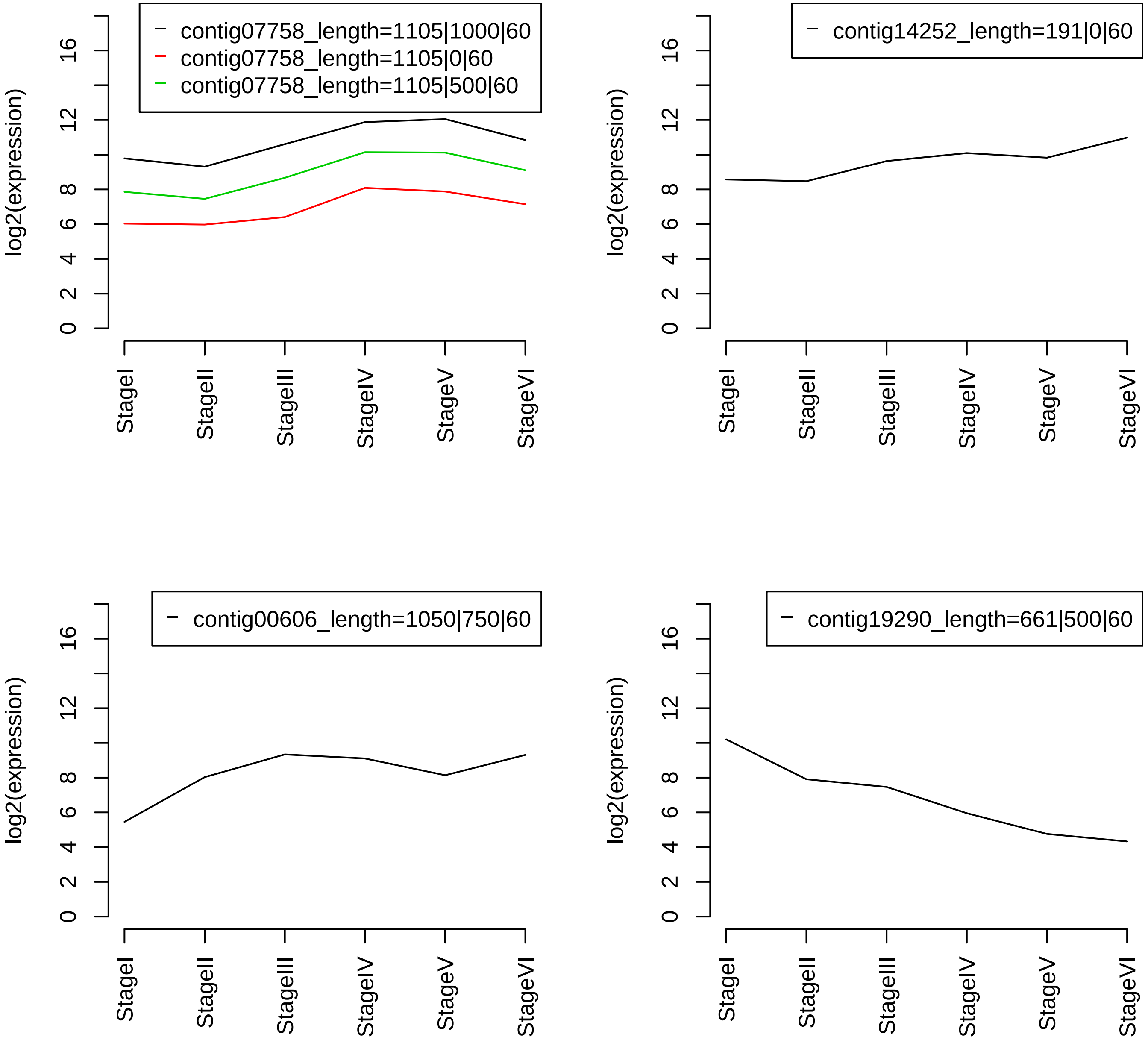
contig07758_length=1105|1000|60
contig07758_length=1105|0|60
contig07758_length=1105|500|60
log2(expression)
0
2
4
6
8
12
16
StageI
StageII
StageIII
StageIV
StageV
StageVI
contig14252_length=191|0|60
contig00606_length=1050|750|60
contig19290_length=661|500|60

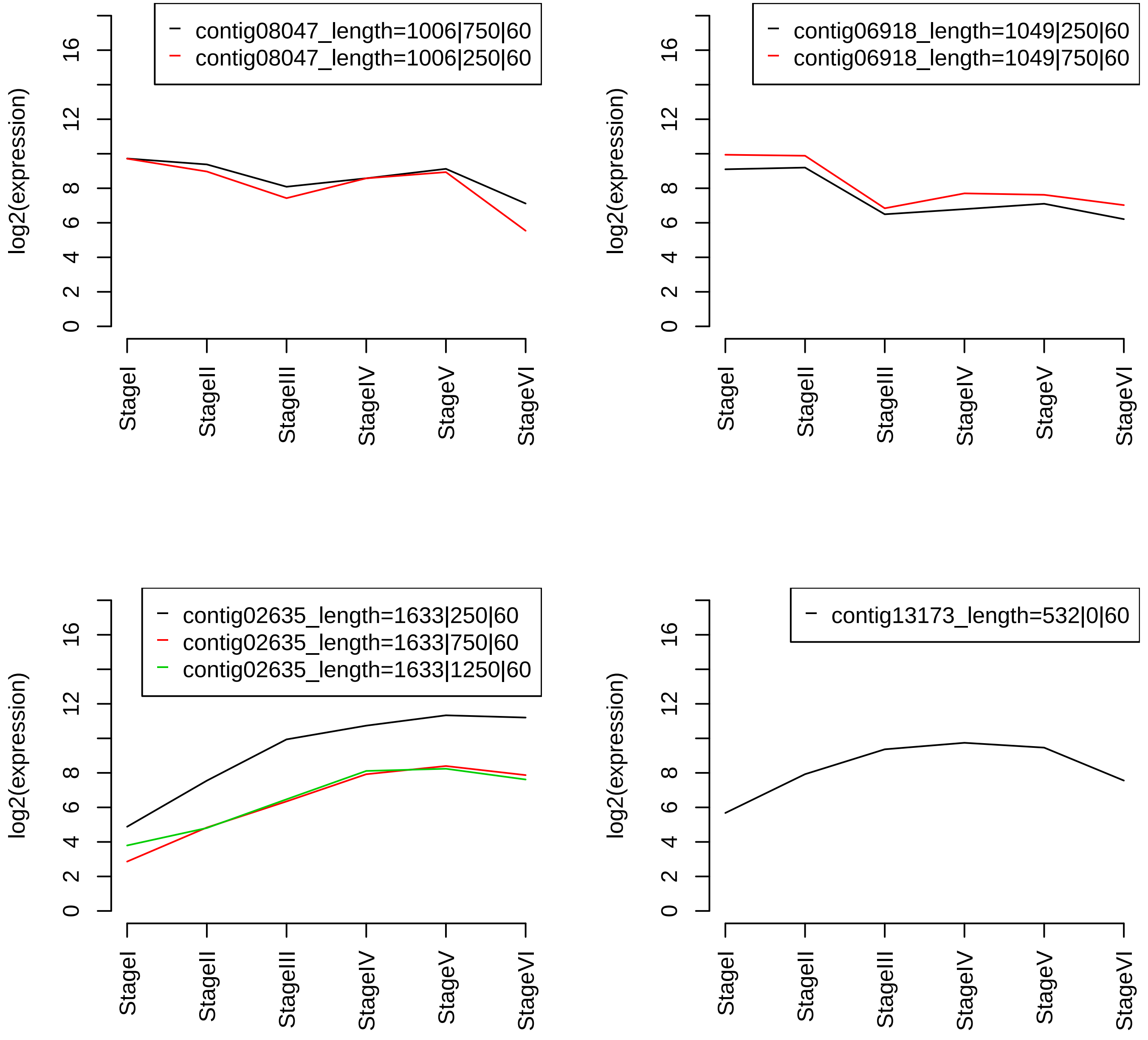
contig08047_length=1006|750|60
contig08047_length=1006|250|60
contig06918_length=1049|250|60
contig06918_length=1049|750|60
contig02635_length=1633|250|60
contig02635_length=1633|750|60
contig02635_length=1633|1250|60
contig13173_length=532|0|60
log2(expression)
0 2 4 6 8 12 16
StageI StageII StageIII StageIV StageV StageVI

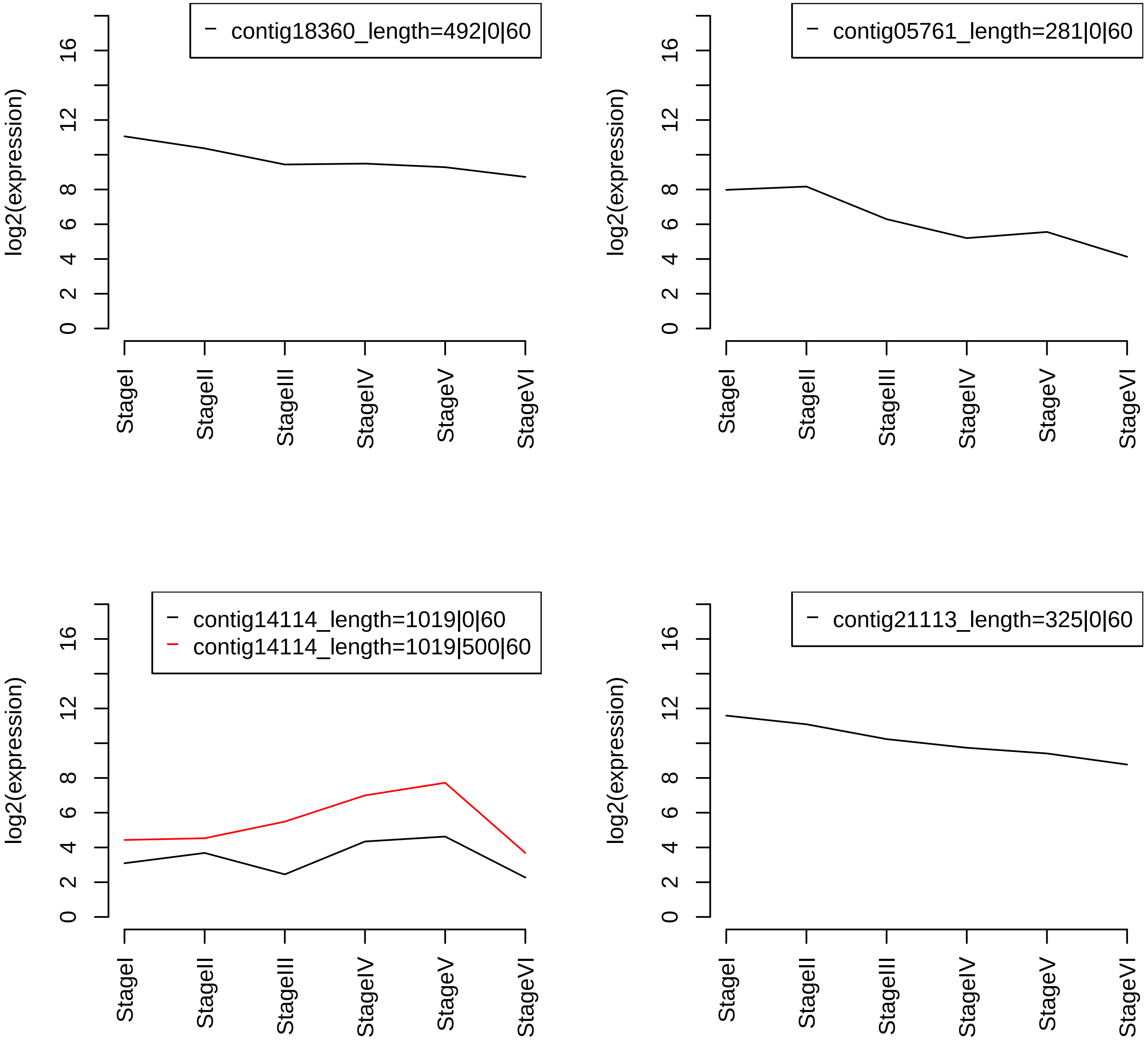

contig18360_length=492|0|60
contig05761_length=281|0|60
contig14114_length=1019|0|60
contig14114_length=1019|500|60
contig21113_length=325|0|60
log2(expression)
0
2
4
6
8
12
16
StageI
StageII
StageIII
StageIV
StageV
StageVI

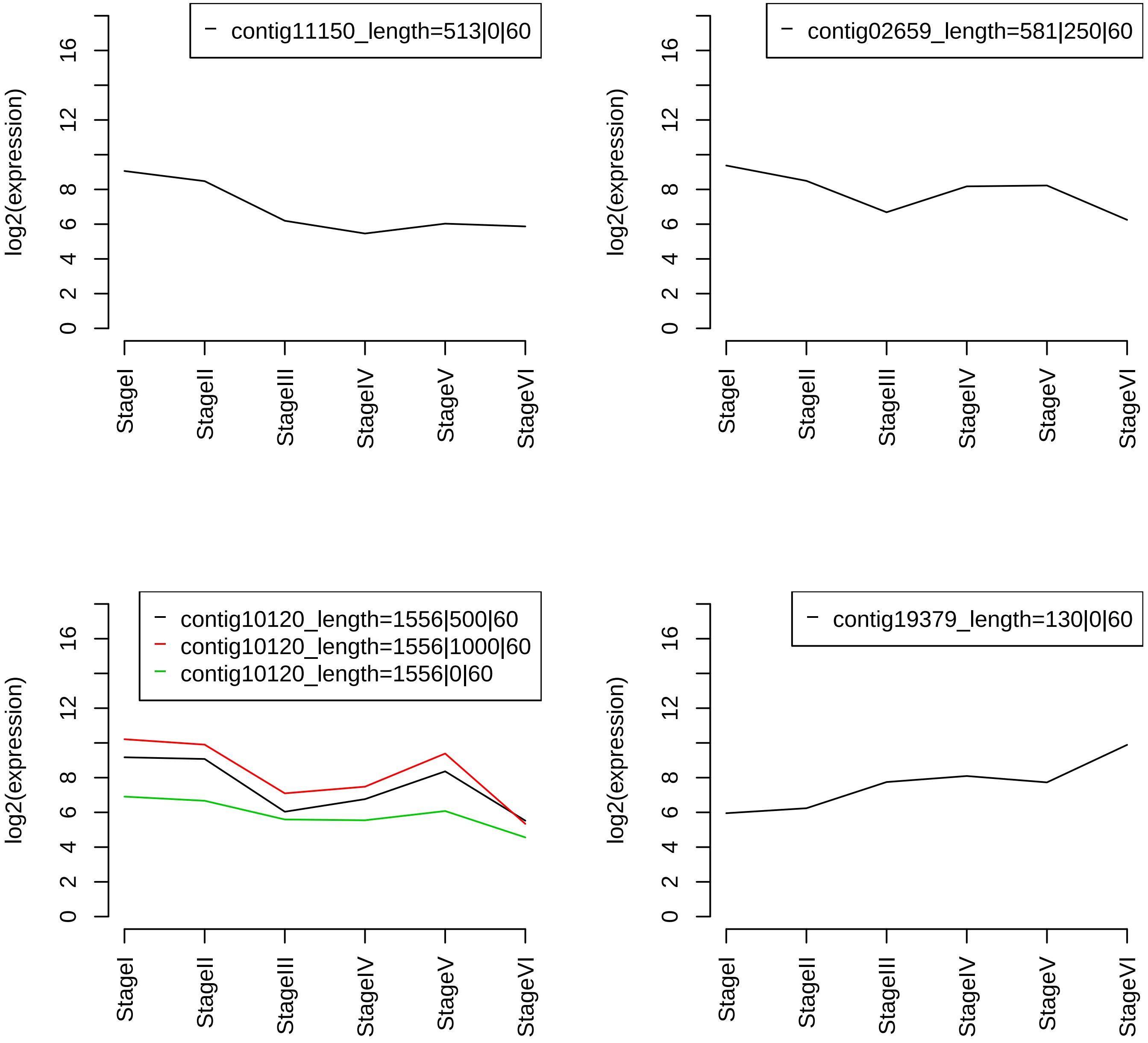

contig11150_length=513|0|60
contig02659_length=581|250|60
contig10120_length=1556|500|60
contig10120_length=1556|1000|60
contig10120_length=1556|0|60
contig19379_length=130|0|60
log2(expression)
0 2 4 6 8 12 16
StageI StageII StageIII StageIV StageV StageVI

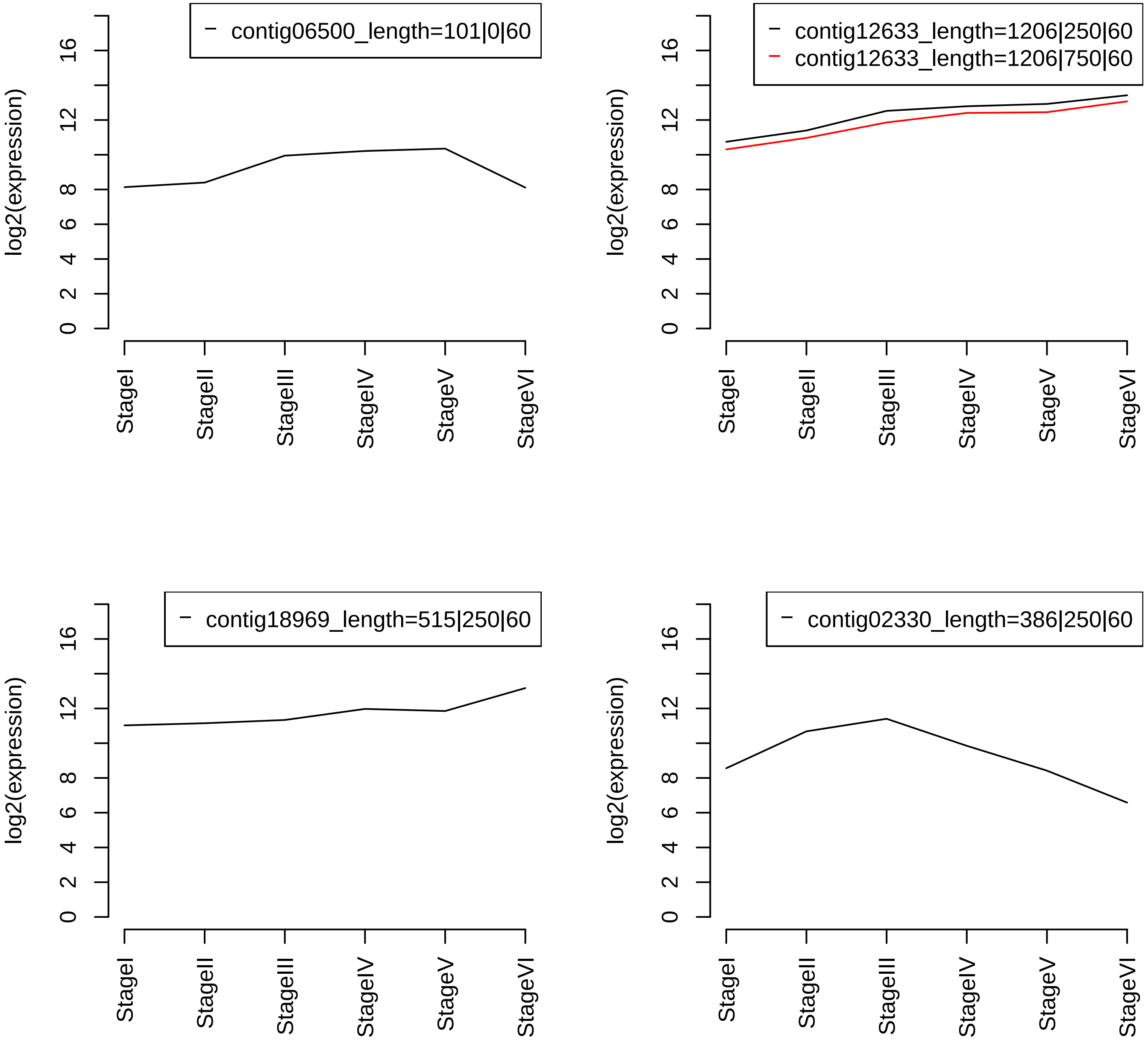
contig06500_length=101|0|60
contig12633_length=1206|250|60
contig12633_length=1206|750|60
contig18969_length=515|250|60
contig02330_length=386|250|60
log2(expression)
0
2
4
6
8
12
16
StageI
StageII
StageIII
StageIV
StageV
StageVI

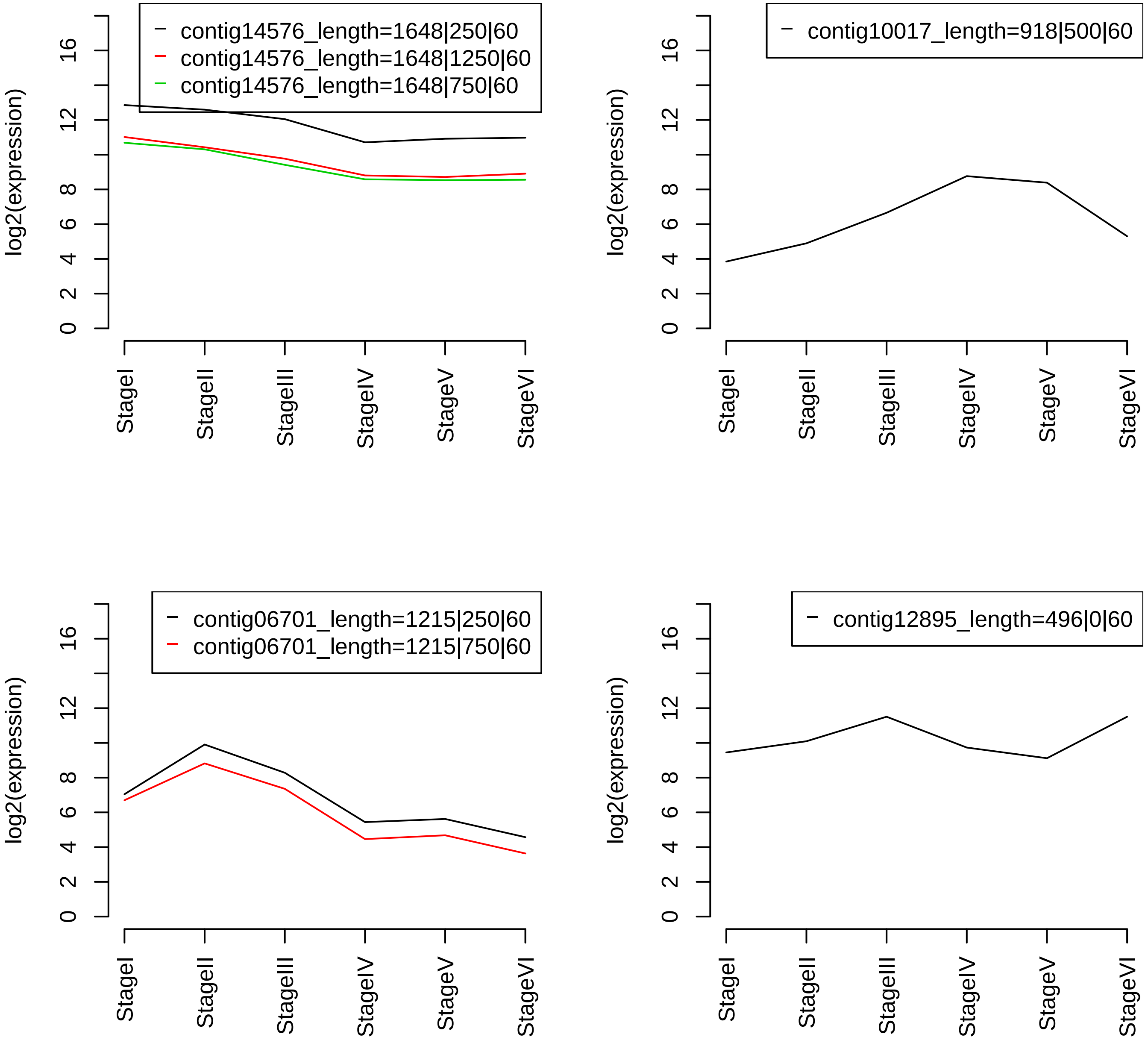
contig14576_length=1648|250|60
contig14576_length=1648|1250|60
contig14576_length=1648|750|60
contig10017_length=918|500|60
contig06701_length=1215|250|60
contig06701_length=1215|750|60
contig12895_length=496|0|60
log2(expression)
StageI
StageII
StageIII
StageIV
StageV
StageVI

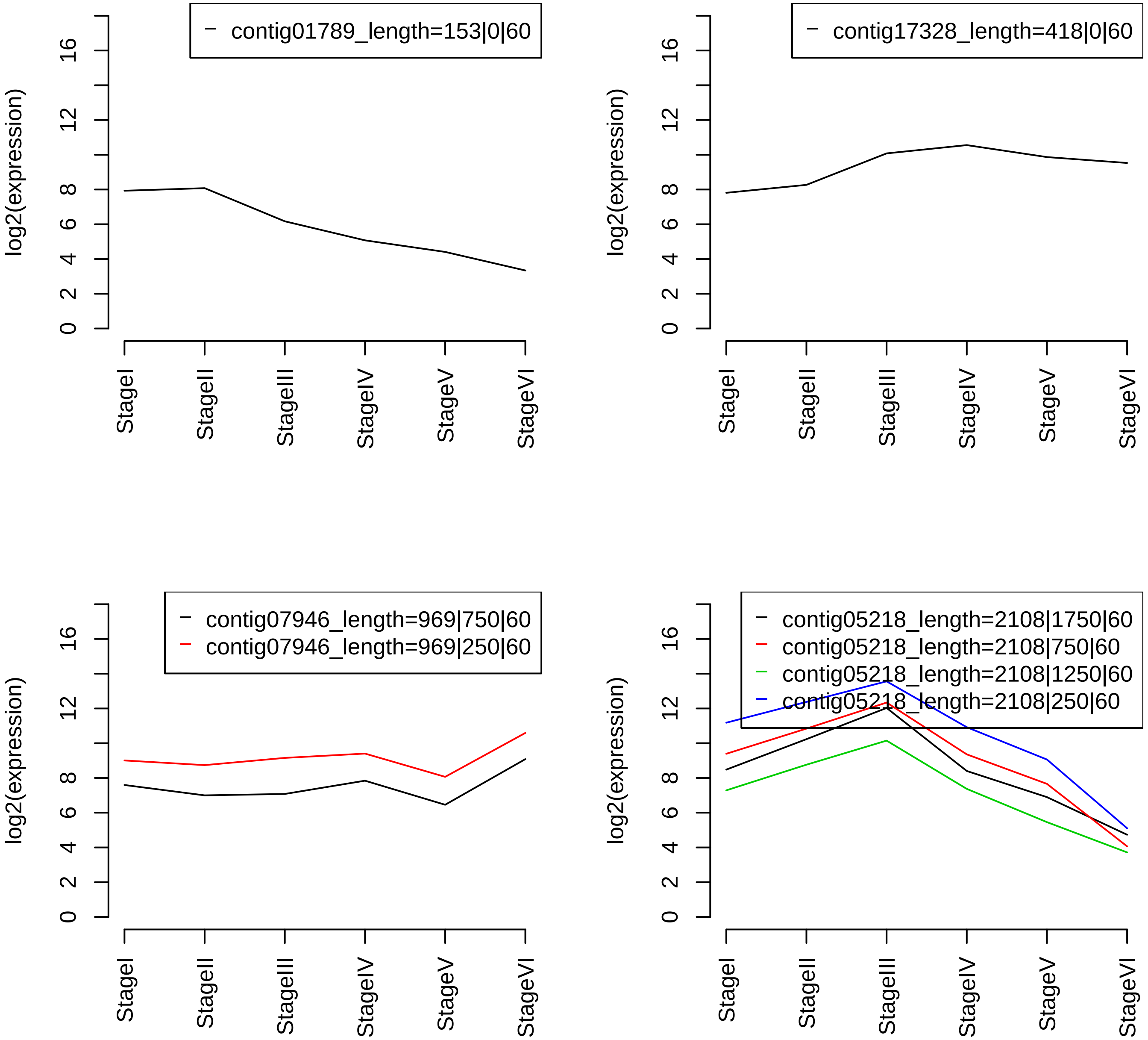

contig01789_length=153|0|60
contig17328_length=418|0|60
contig07946_length=969|750|60
contig07946_length=969|250|60
contig05218_length=2108|1750|60
contig05218_length=2108|750|60
contig05218_length=2108|1250|60
contig05218_length=2108|250|60
log2(expression)
0
2
4
6
8
12
16
StageI
StageII
StageIII
StageIV
StageV
StageVI

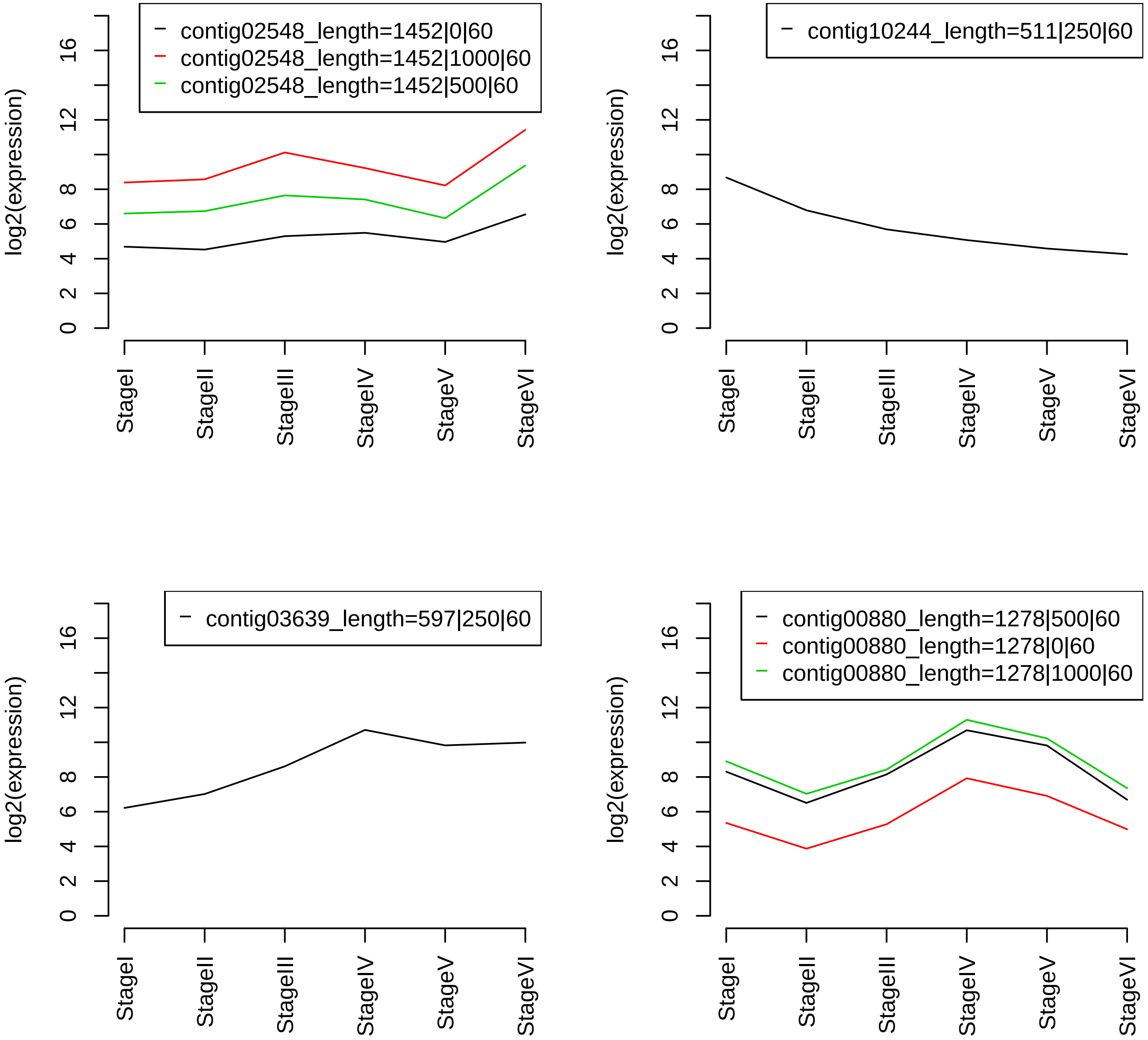
contig02548_length=1452|0|60
contig02548_length=1452|1000|60
contig02548_length=1452|500|60
contig10244_length=511|250|60
contig03639_length=597|250|60
contig00880_length=1278|500|60
contig00880_length=1278|0|60
contig00880_length=1278|1000|60
log2(expression)
0
2
4
6
8
12
16
StageI
StageII
StageIII
StageIV
StageV
StageVI

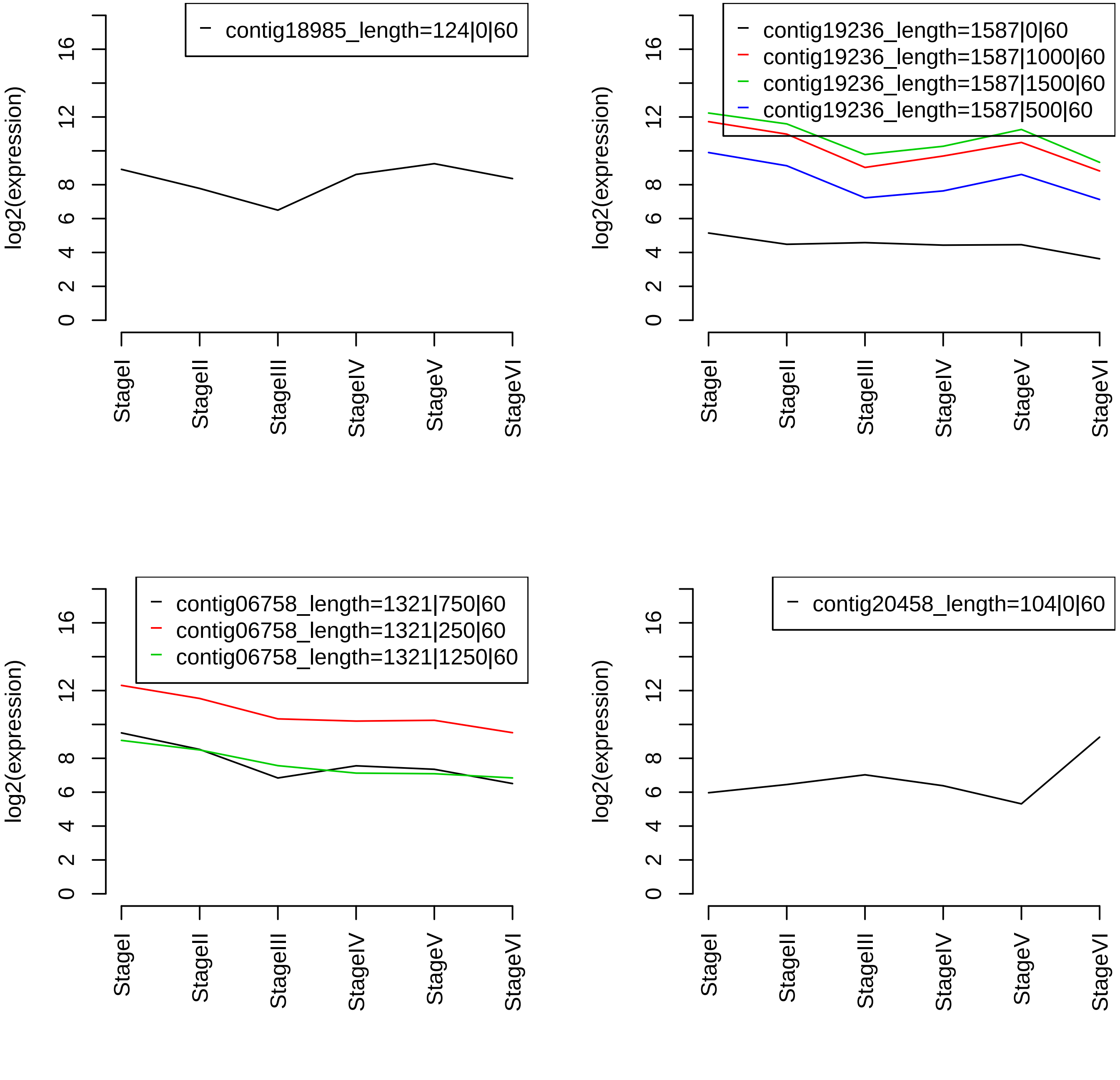
contig18985_length=124|0|60
contig19236_length=1587|0|60
contig19236_length=1587|1000|60
contig19236_length=1587|1500|60
contig19236_length=1587|500|60
contig06758_length=1321|750|60
contig06758_length=1321|250|60
contig06758_length=1321|1250|60
contig20458_length=104|0|60
log2(expression)
0 2 4 6 8 12 16
StageI StageII StageIII StageIV StageV StageVI

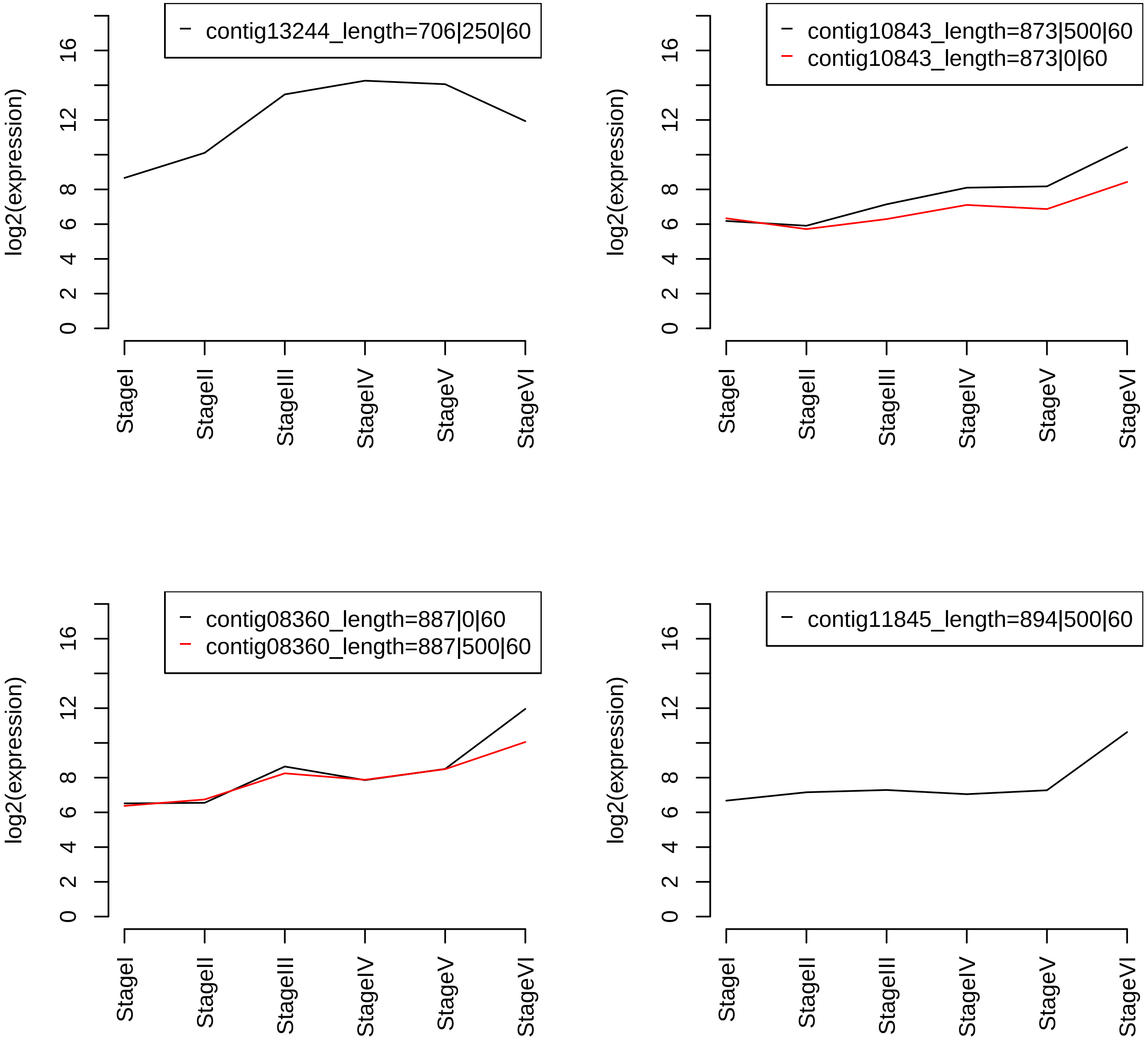
contig13244_length=706|250|60
contig10843_length=873|500|60
contig10843_length=873|0|60
contig08360_length=887|0|60
contig08360_length=887|500|60
contig11845_length=894|500|60
log2(expression)
0
2
4
6
8
12
16
StageI
StageII
StageIII
StageIV
StageV
StageVI

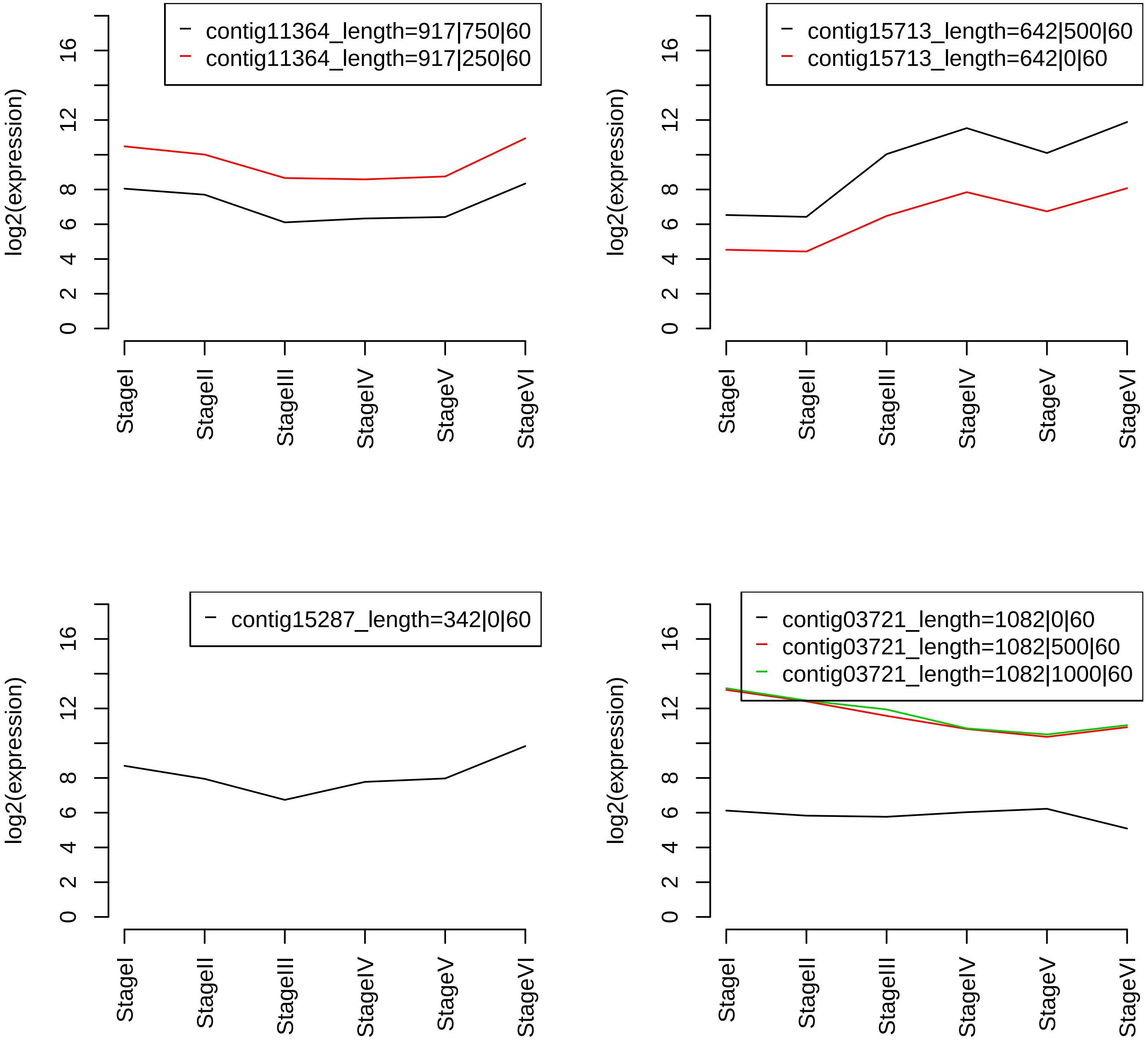
contig11364_length=917|750|60
contig11364_length=917|250|60
contig15713_length=642|500|60
contig15713_length=642|0|60
contig15287_length=342|0|60
contig03721_length=1082|0|60
contig03721_length=1082|500|60
contig03721_length=1082|1000|60
log2(expression)
0 2 4 6 8 12 16
StageI StageII StageIII StageIV StageV StageVI

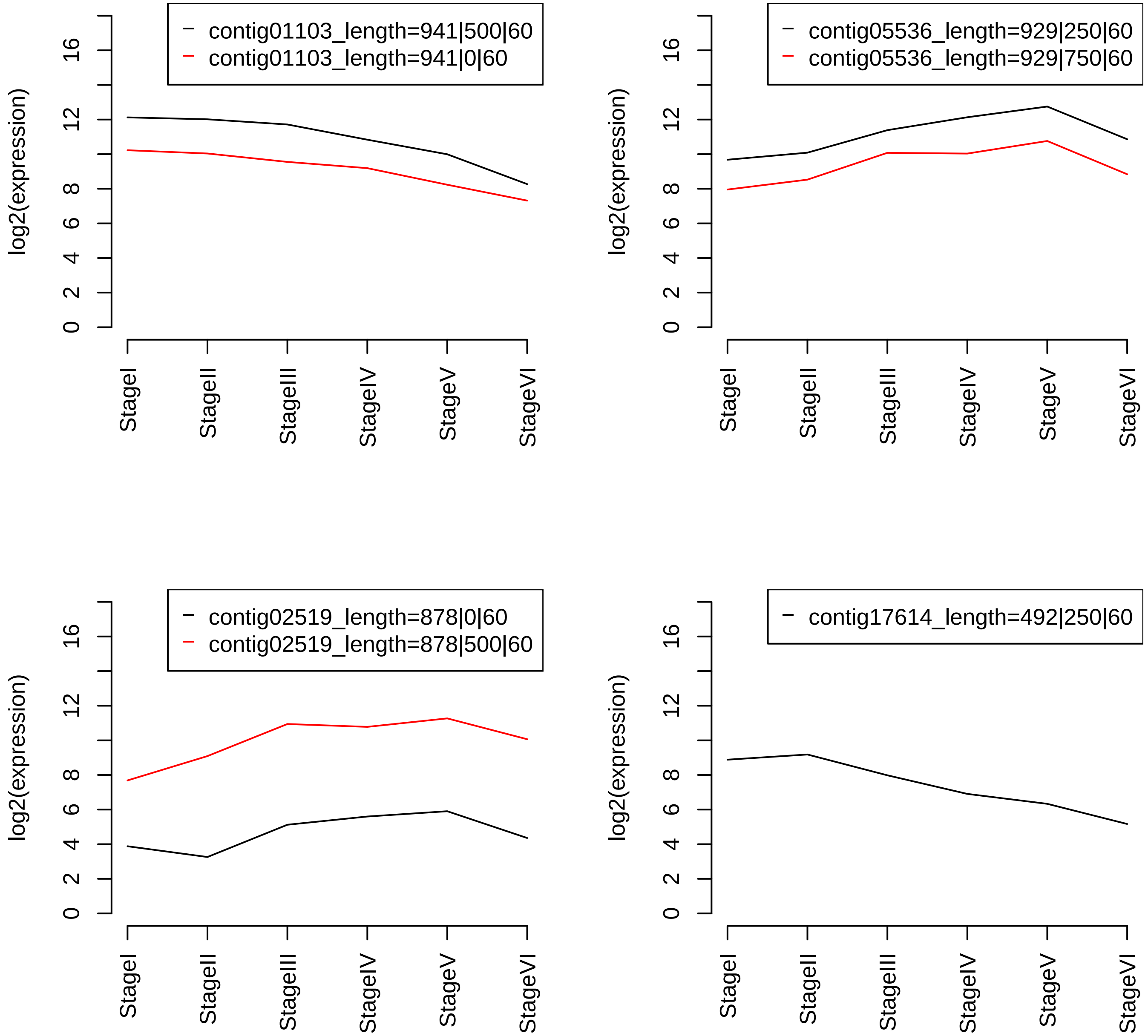
contig01103_length=941|500|60
contig01103_length=941|0|60
contig05536_length=929|250|60
contig05536_length=929|750|60
contig02519_length=878|0|60
contig02519_length=878|500|60
contig17614_length=492|250|60
log2(expression)
StageI
StageII
StageIII
StageIV
StageV
StageVI

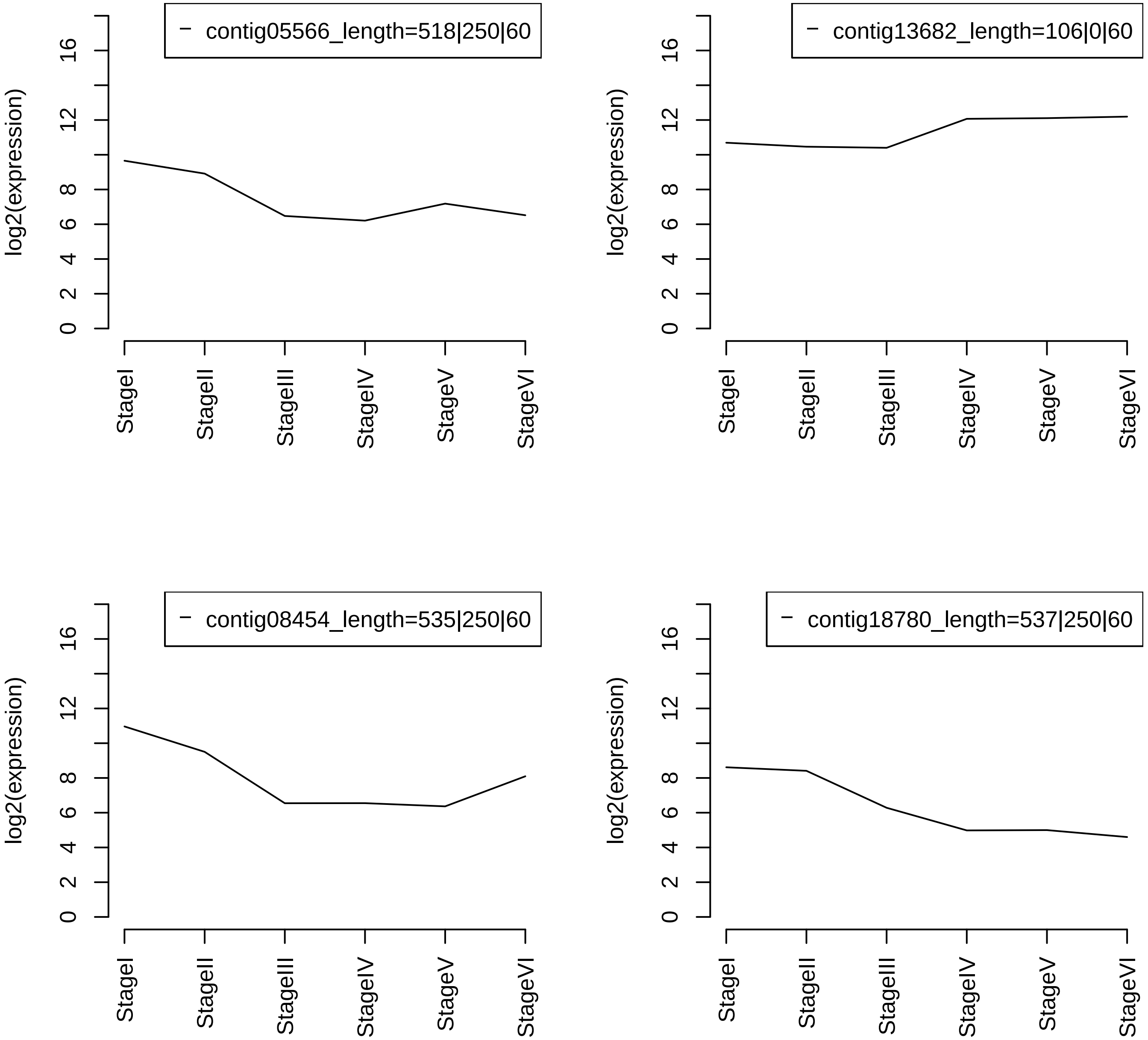
contig05566_length=518|250|60
contig13682_length=106|0|60
contig08454_length=535|250|60
contig18780_length=537|250|60
log2(expression)
0
2
4
6
8
12
16
StageI
StageII
StageIII
StageIV
StageV
StageVI

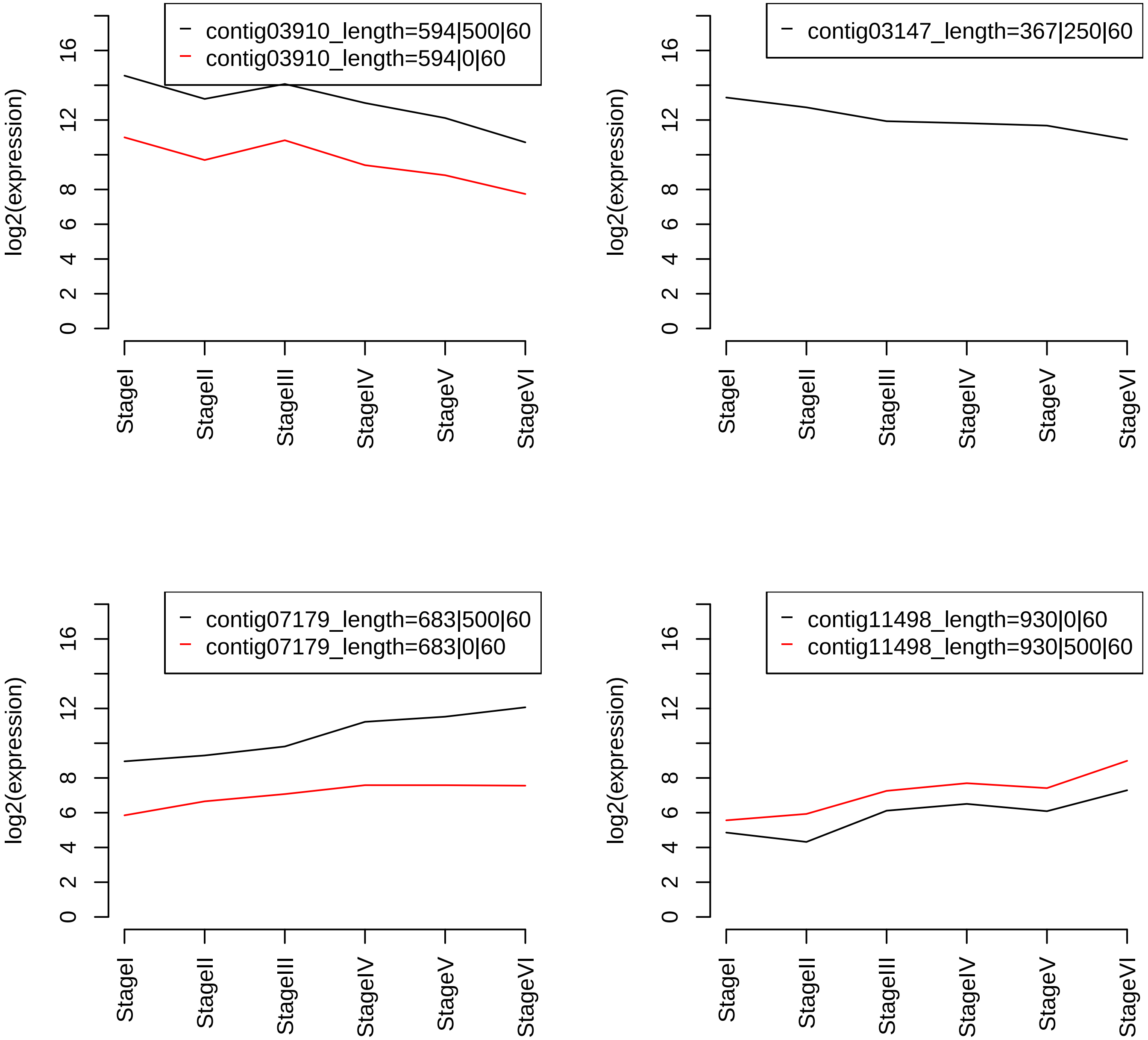
contig03910_length=594|500|60
contig03910_length=594|0|60
contig03147_length=367|250|60
contig07179_length=683|500|60
contig07179_length=683|0|60
contig11498_length=930|0|60
contig11498_length=930|500|60
log2(expression)
StageI
StageII
StageIII
StageIV
StageV
StageVI

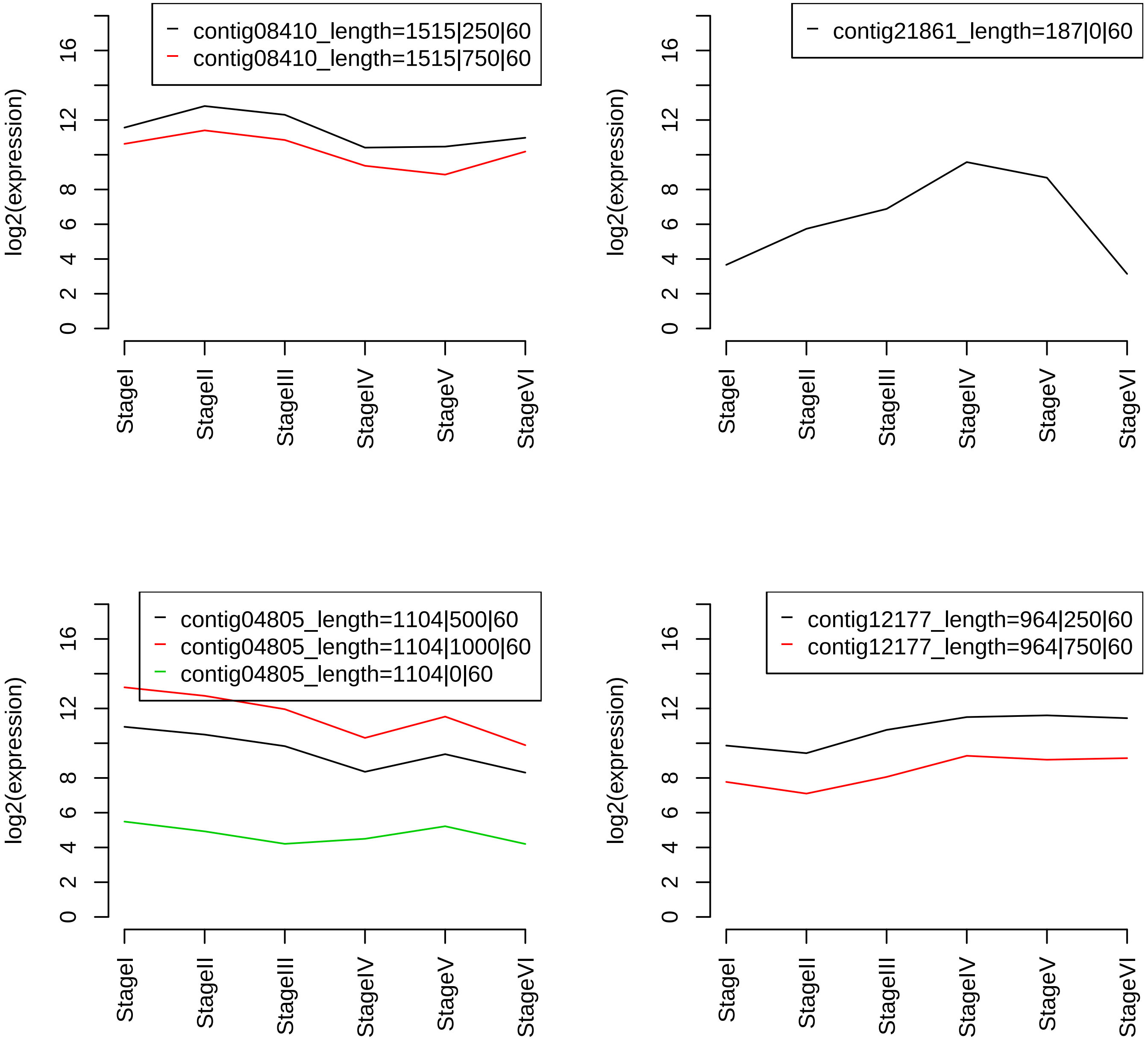
contig08410_length=1515|250|60
contig08410_length=1515|750|60
contig21861_length=187|0|60
contig04805_length=1104|500|60
contig04805_length=1104|1000|60
contig04805_length=1104|0|60
contig12177_length=964|250|60
contig12177_length=964|750|60
log2(expression)
0 2 4 6 8 12 16
StageI
StageII
StageIII
StageIV
StageV
StageVI

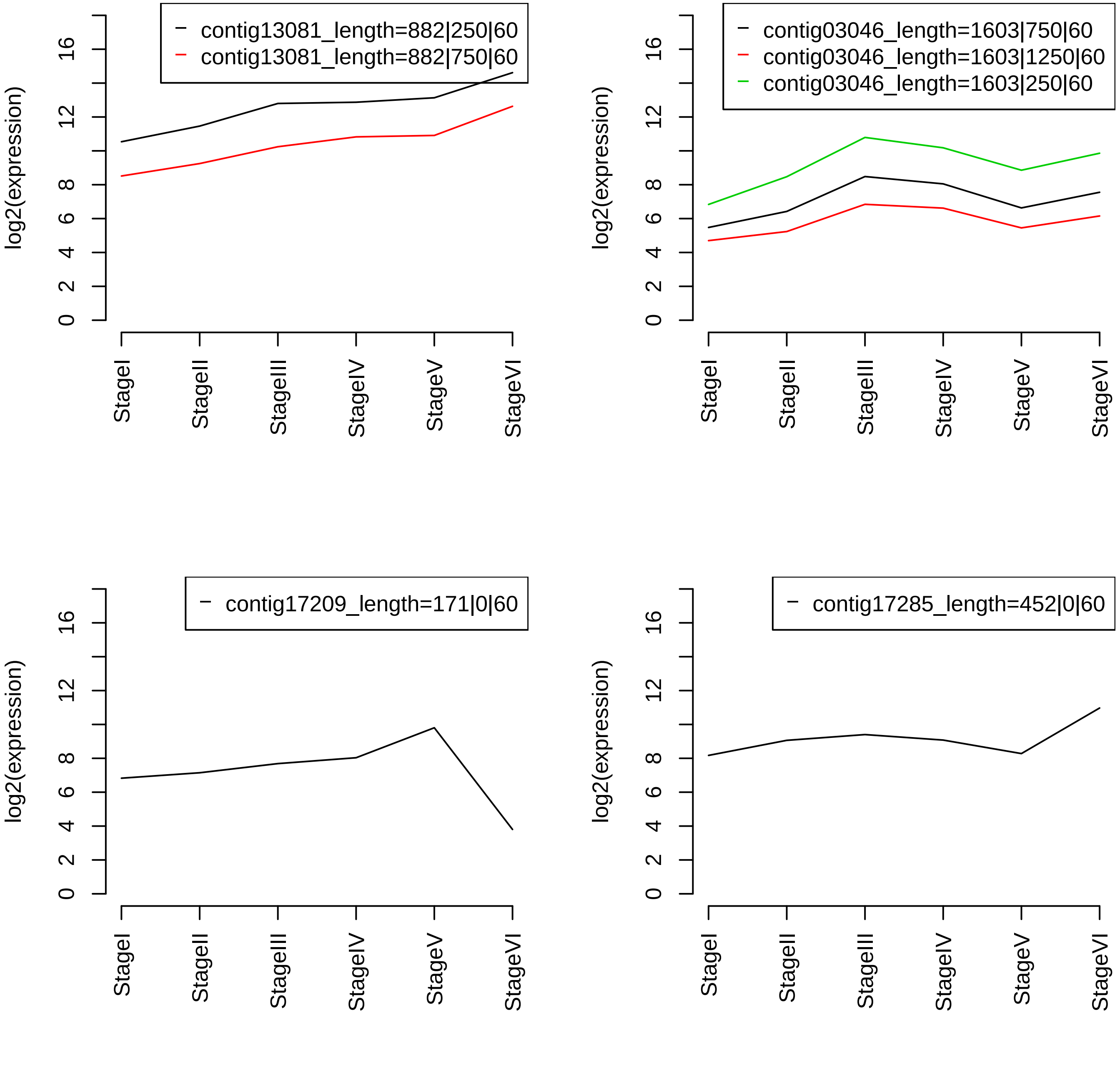

contig13081_length=882|250|60
contig13081_length=882|750|60
contig03046_length=1603|750|60
contig03046_length=1603|1250|60
contig03046_length=1603|250|60
contig17209_length=171|0|60
contig17285_length=452|0|60
log2(expression)
0 2 4 6 8 12 16
StageI StageII StageIII StageIV StageV StageVI

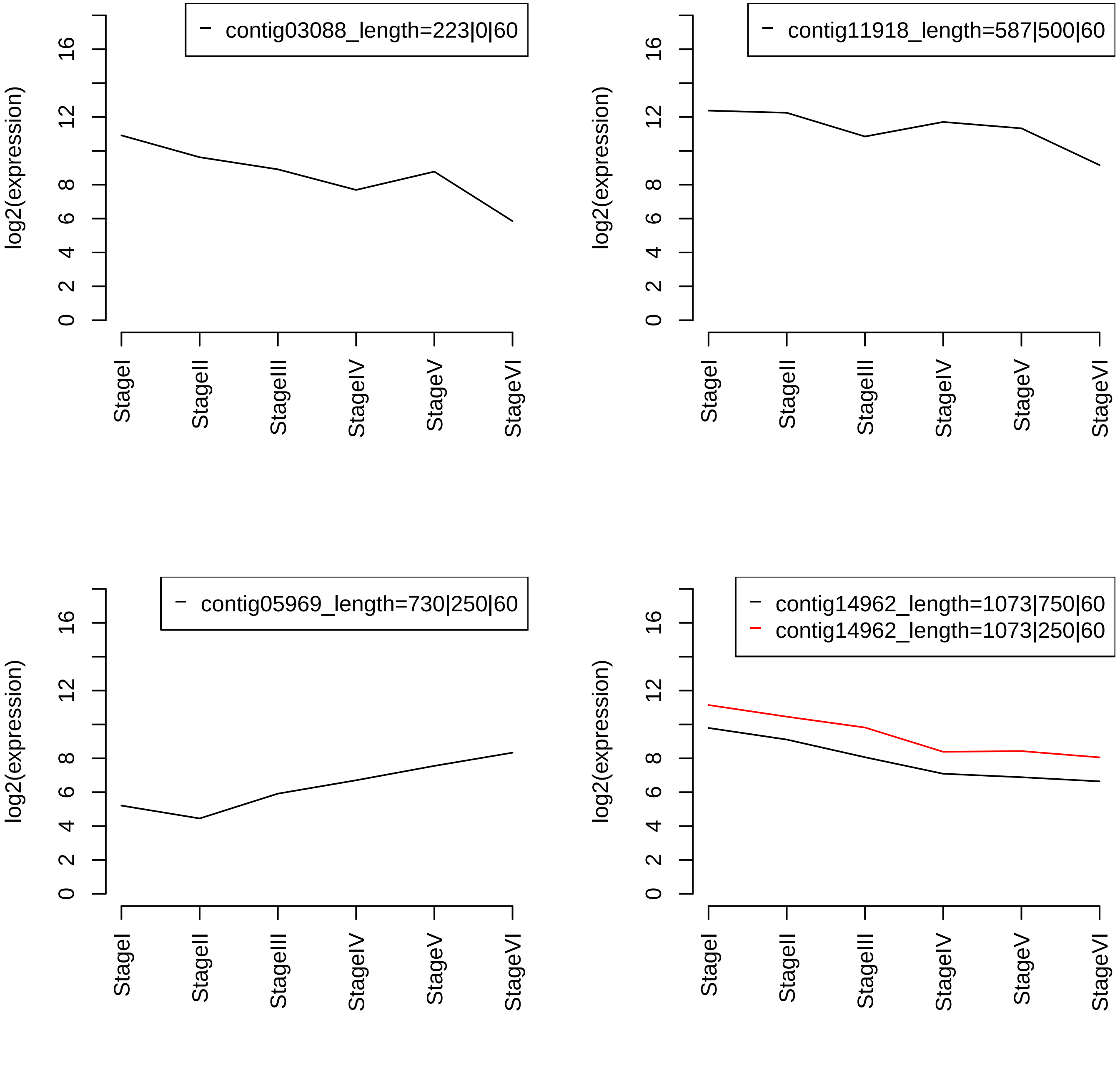
contig03088_length=223|0|60
contig11918_length=587|500|60
contig05969_length=730|250|60
contig14962_length=1073|750|60
contig14962_length=1073|250|60
log2(expression)
StageI
StageII
StageIII
StageIV
StageV
StageVI

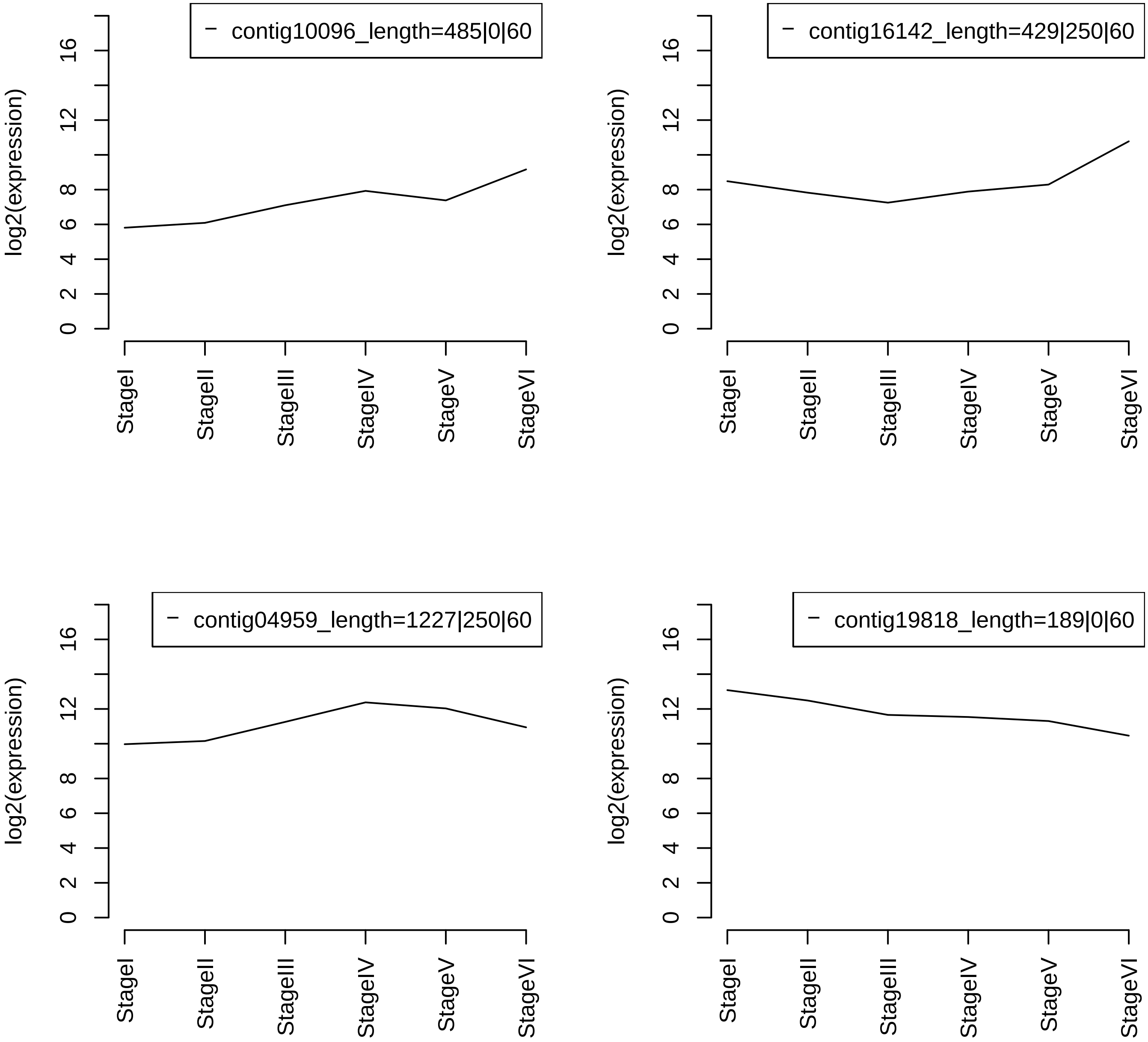

contig10096_length=485|0|60
contig16142_length=429|250|60
contig04959_length=1227|250|60
contig19818_length=189|0|60
log2(expression)
0
2
4
6
8
12
16
StageI
StageII
StageIII
StageIV
StageV
StageVI

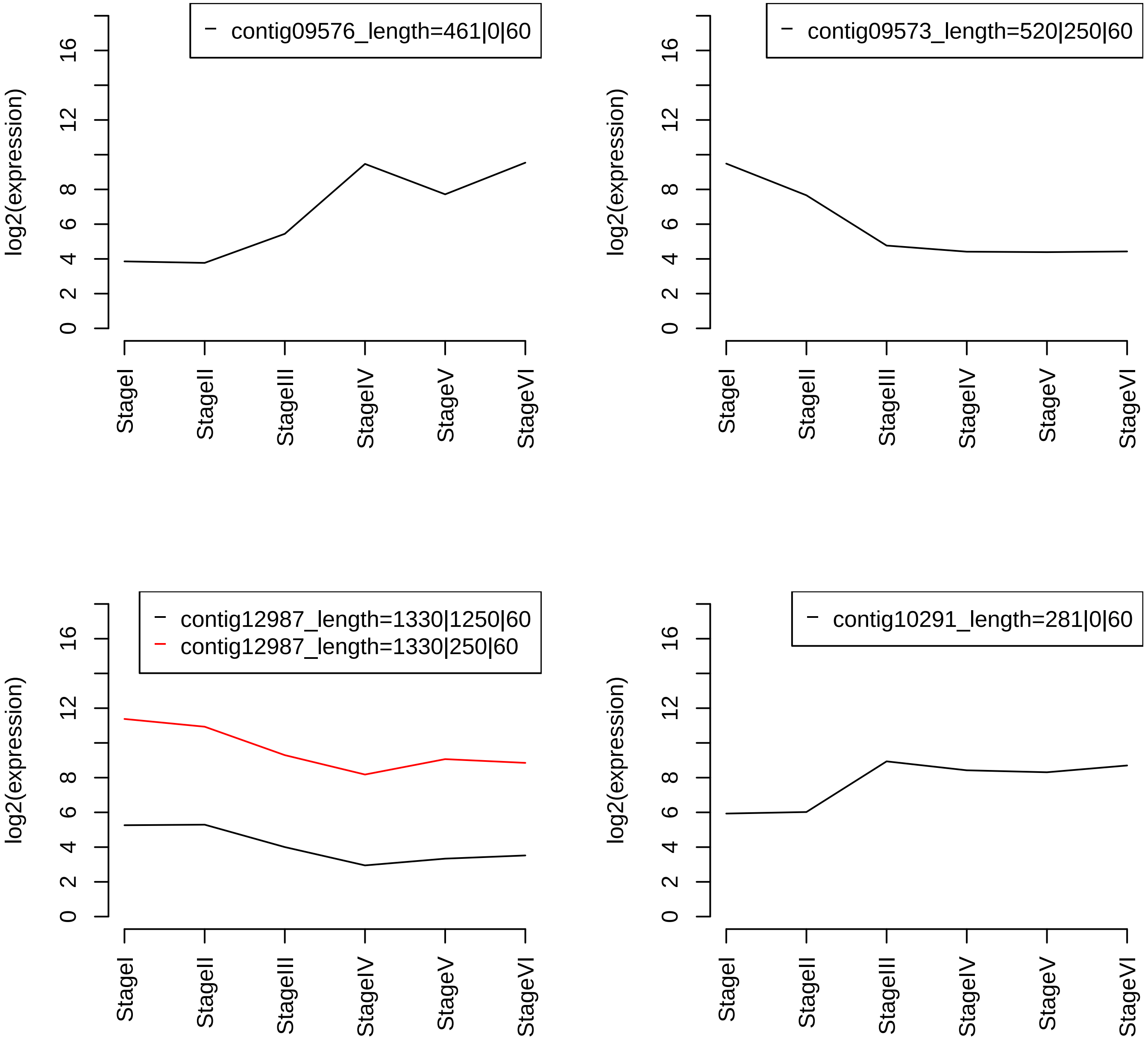
contig09576_length=461|0|60
contig09573_length=520|250|60
contig12987_length=1330|1250|60
contig12987_length=1330|250|60
contig10291_length=281|0|60
log2(expression)
0 2 4 6 8 12 16
StageI StageII StageIII StageIV StageV StageVI

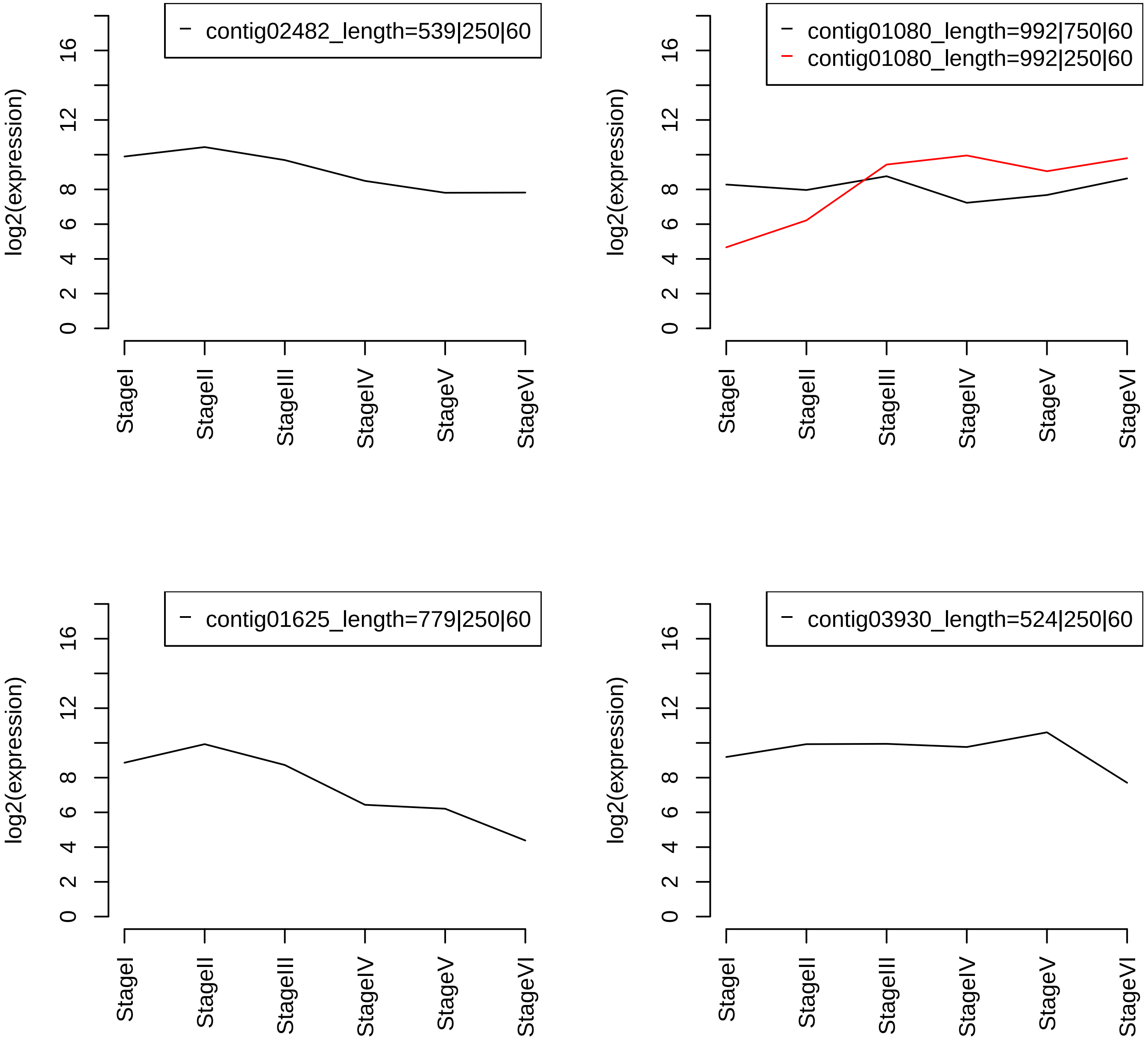

contig02482_length=539|250|60
contig01080_length=992|750|60
contig01080_length=992|250|60
contig01625_length=779|250|60
contig03930_length=524|250|60
log2(expression)
0
2
4
6
8
12
16
StageI
StageII
StageIII
StageIV
StageV
StageVI

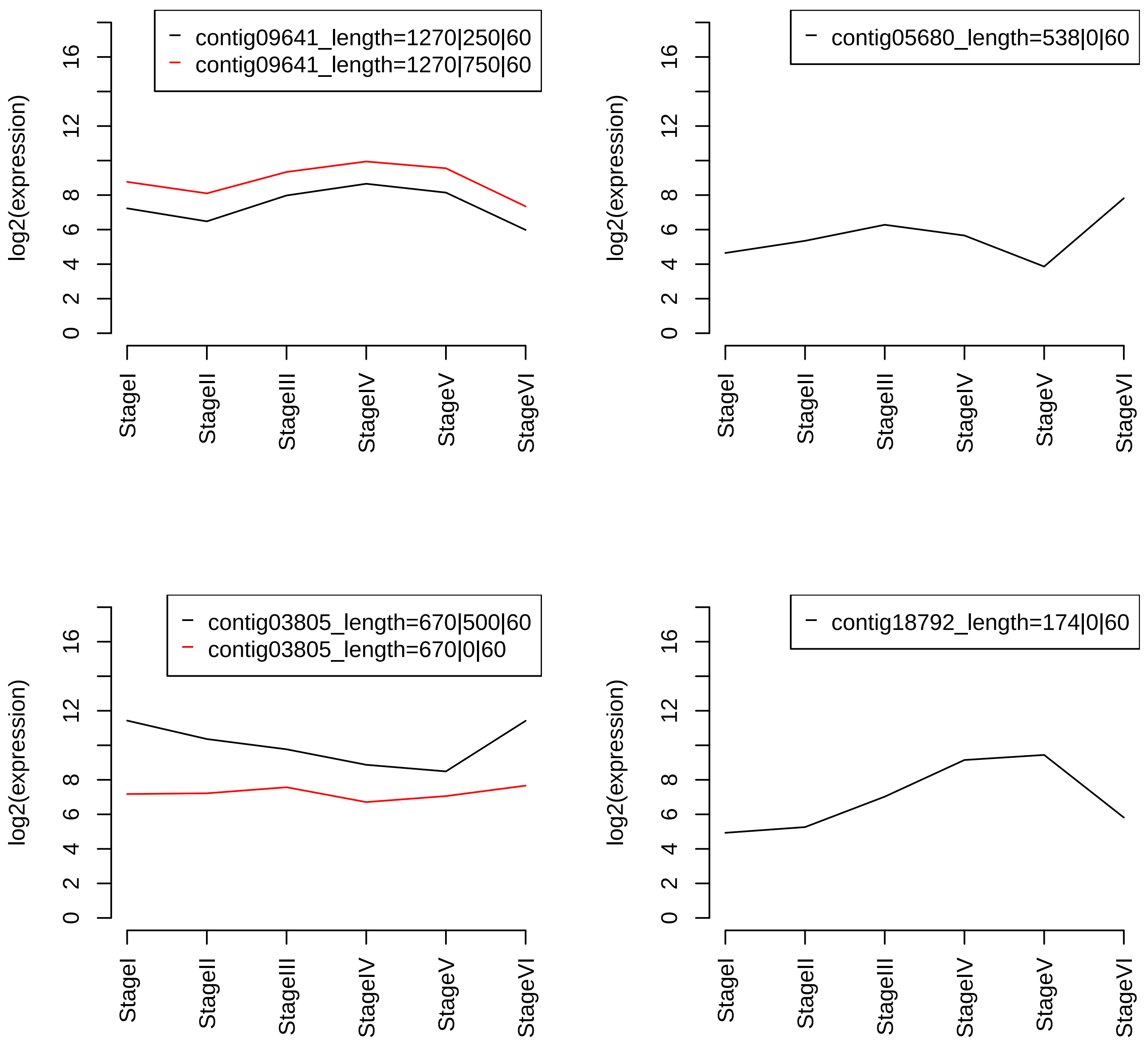

contig09641_length=1270|250|60
contig09641_length=1270|750|60
contig05680_length=538|0|60
contig03805_length=670|500|60
contig03805_length=670|0|60
contig18792_length=174|0|60
log2(expression)
0
2
4
6
8
12
16
StageI
StageII
StageIII
StageIV
StageV
StageVI

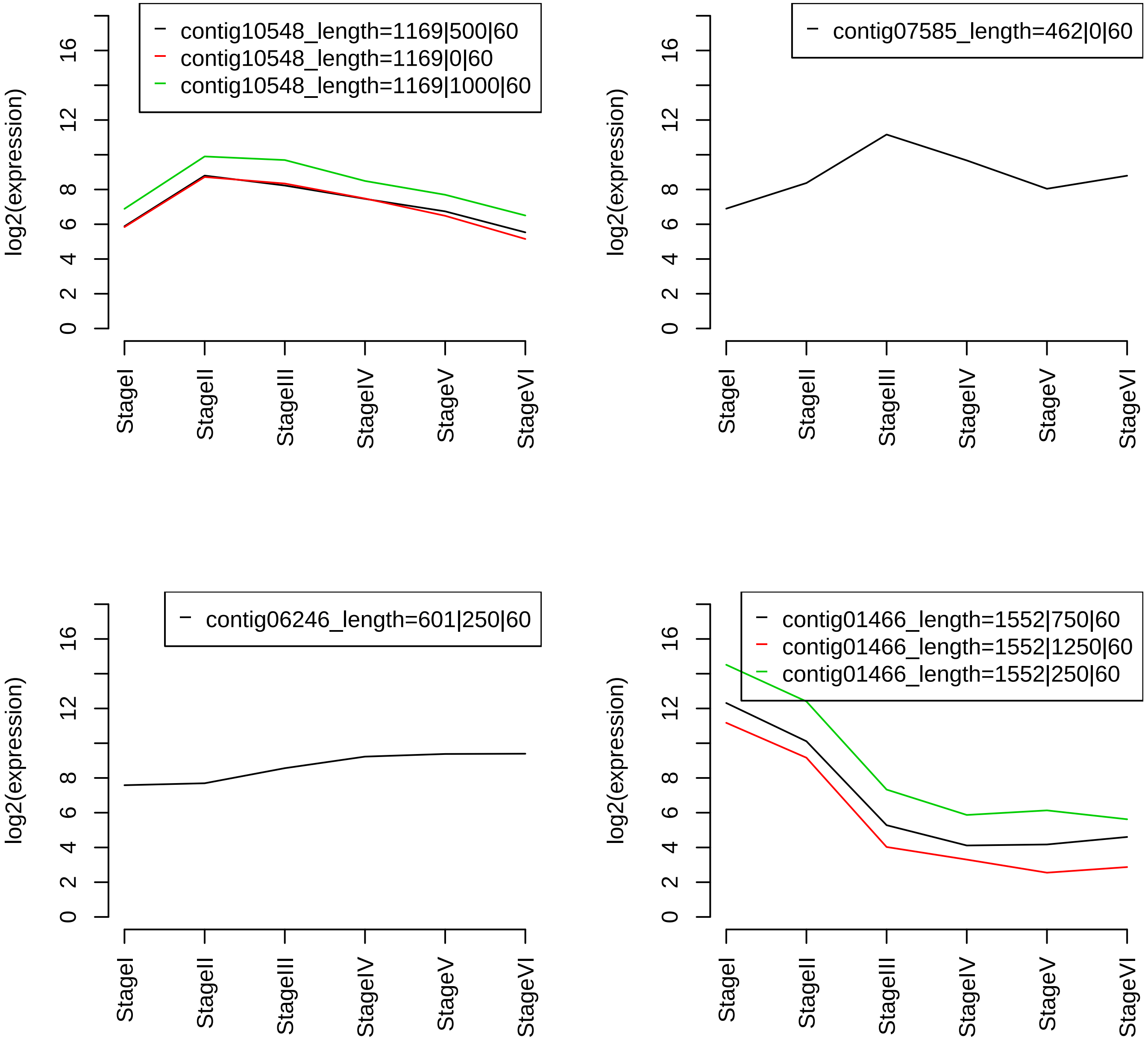

contig10548_length=1169|500|60
contig10548_length=1169|0|60
contig10548_length=1169|1000|60
contig07585_length=462|0|60
contig06246_length=601|250|60
contig01466_length=1552|750|60
contig01466_length=1552|1250|60
contig01466_length=1552|250|60
log2(expression)
0
2
4
6
8
12
16
StageI
StageII
StageIII
StageIV
StageV
StageVI

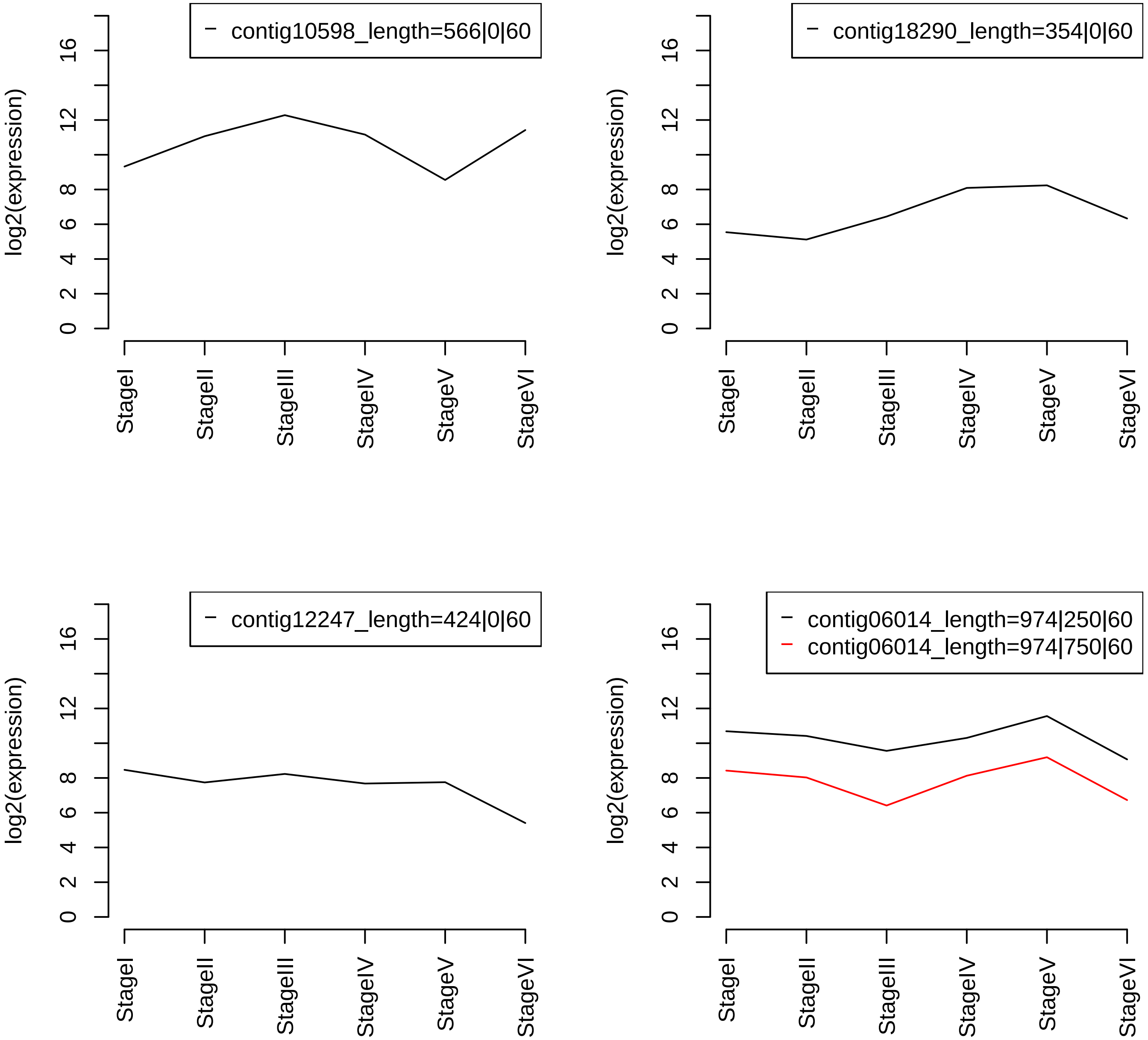

contig10598_length=566|0|60
contig18290_length=354|0|60
contig12247_length=424|0|60
contig06014_length=974|250|60
contig06014_length=974|750|60
log2(expression)
0
2
4
6
8
12
16
StageI
StageII
StageIII
StageIV
StageV
StageVI

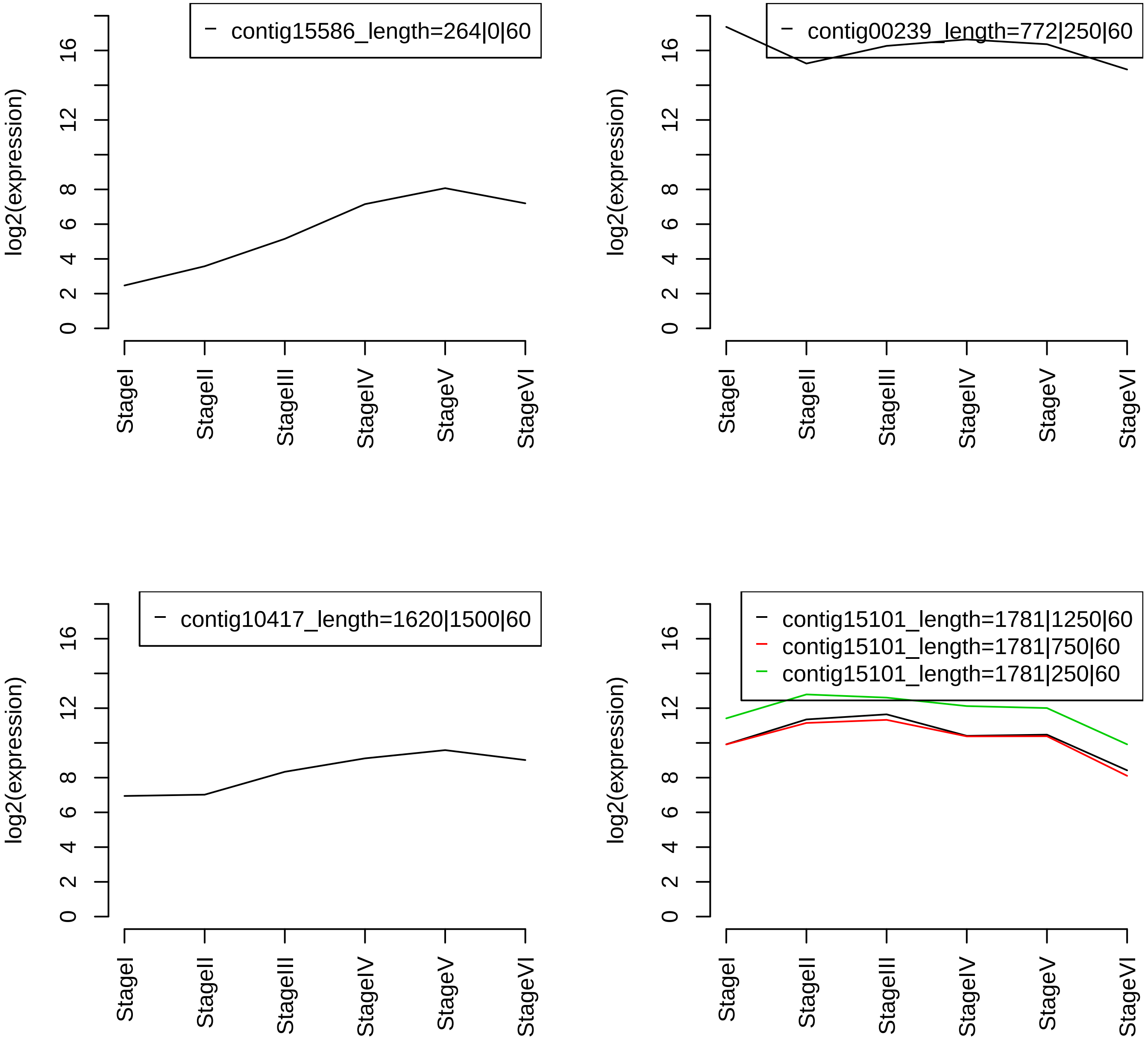
contig15586_length=264|0|60
contig00239_length=772|250|60
contig10417_length=1620|1500|60
contig15101_length=1781|1250|60
contig15101_length=1781|750|60
contig15101_length=1781|250|60
log2(expression)
0
2
4
6
8
12
16
StageI
StageII
StageIII
StageIV
StageV
StageVI

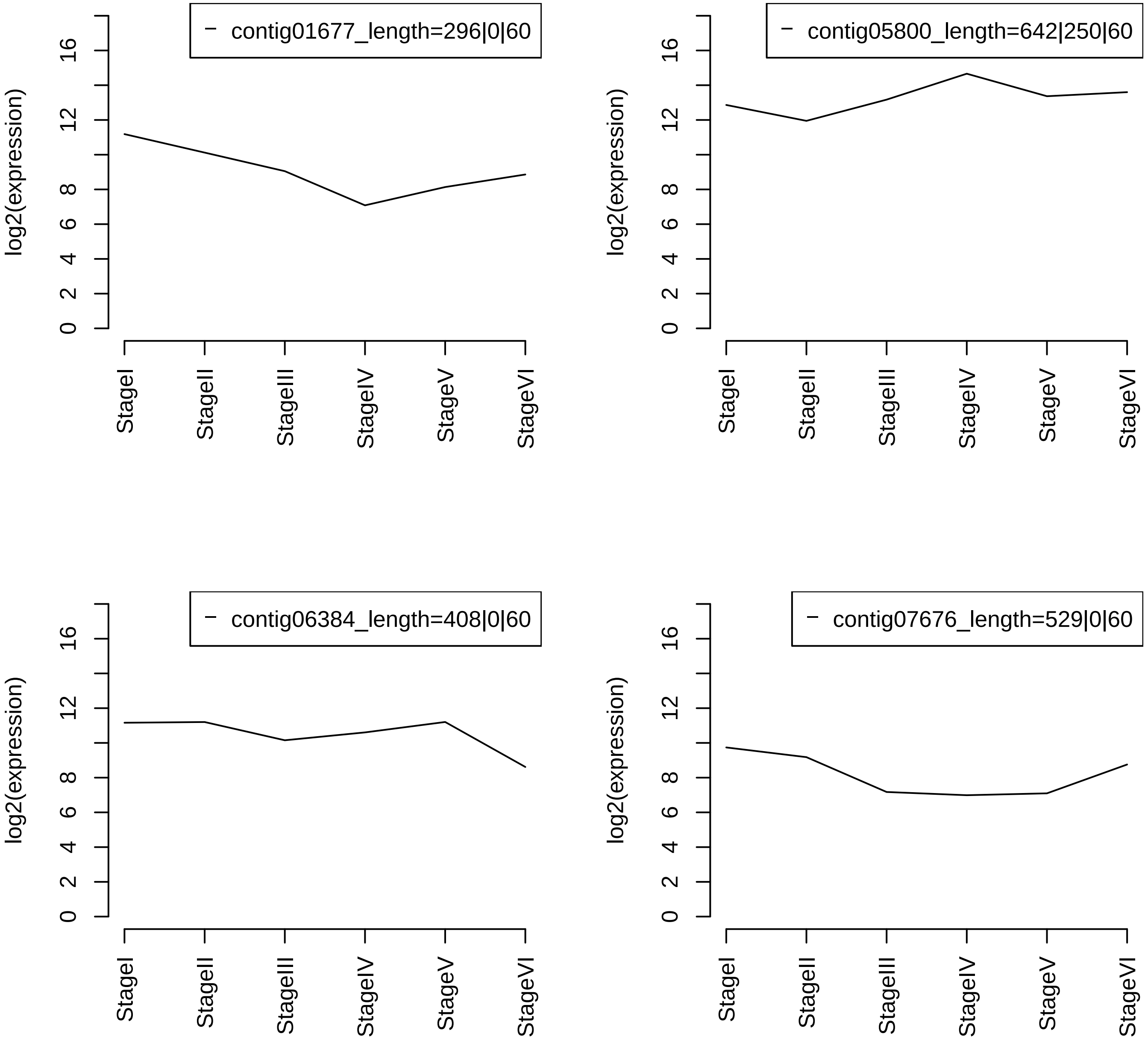
contig01677_length=296|0|60
contig05800_length=642|250|60
contig06384_length=408|0|60
contig07676_length=529|0|60
log2(expression)
0
2
4
6
8
12
16
StageI
StageII
StageIII
StageIV
StageV
StageVI

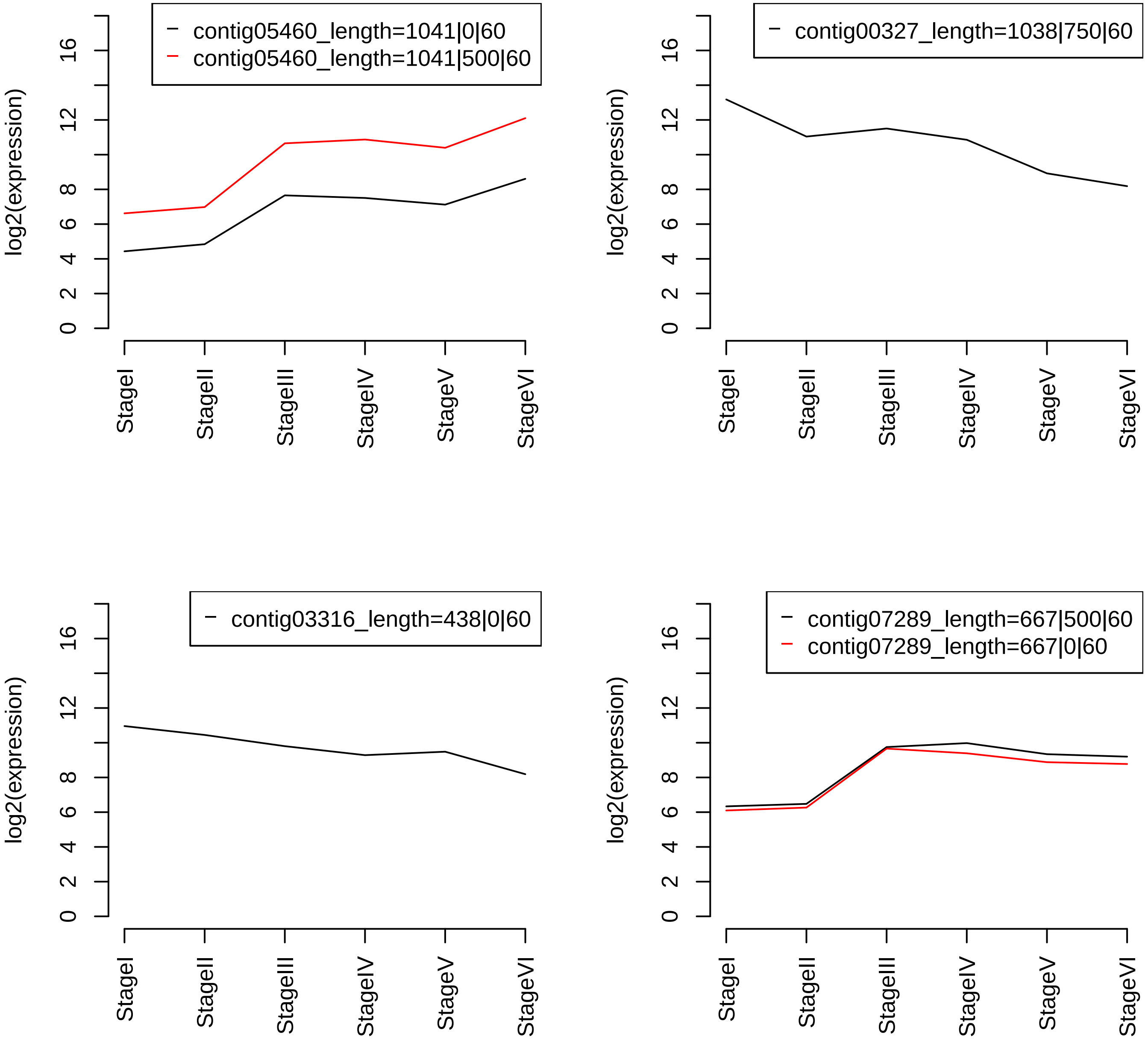
contig05460_length=1041|0|60
contig05460_length=1041|500|60
contig00327_length=1038|750|60
contig03316_length=438|0|60
contig07289_length=667|500|60
contig07289_length=667|0|60
log2(expression)
StageI
StageII
StageIII
StageIV
StageV
StageVI

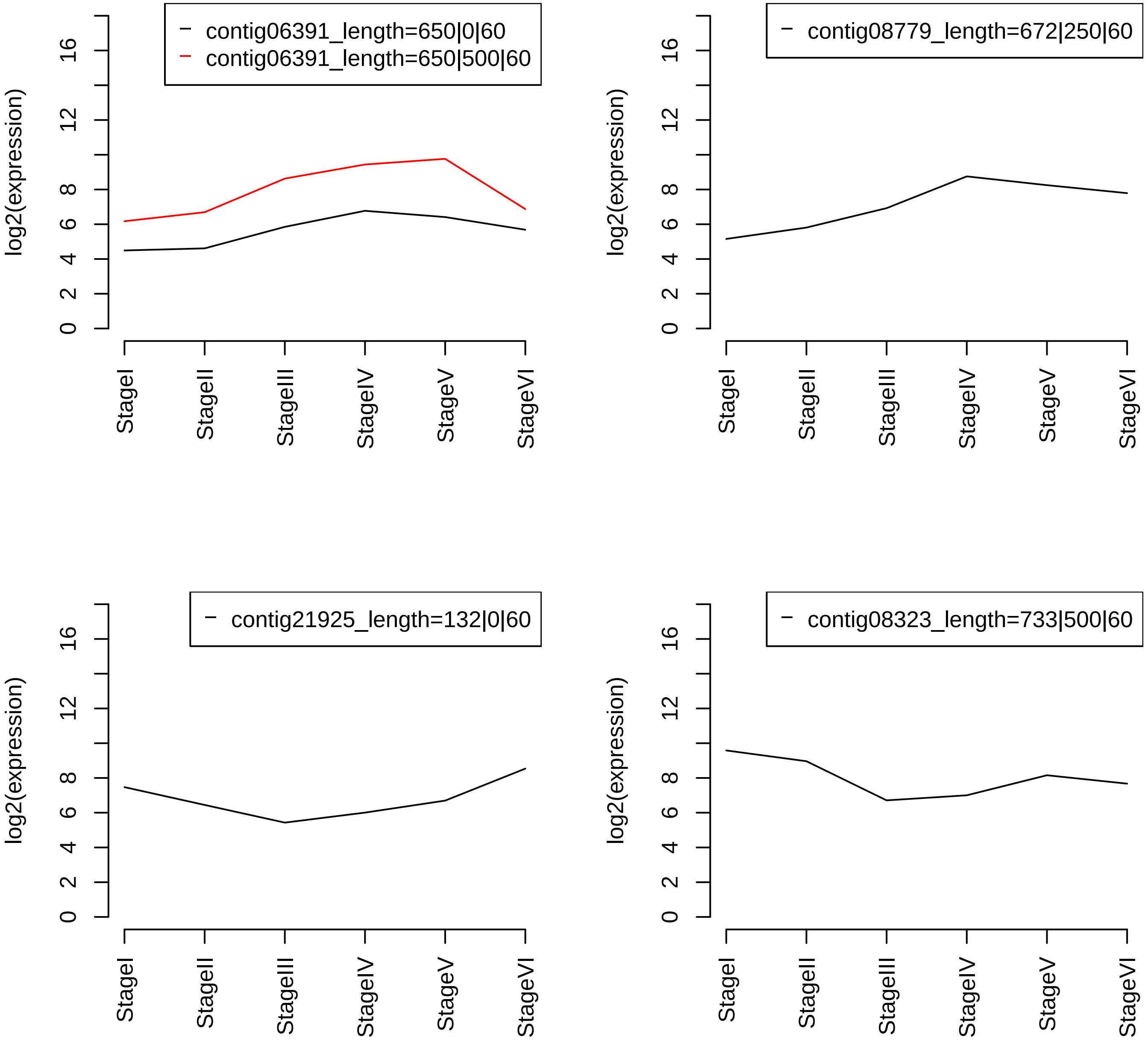

contig06391_length=650|0|60
contig06391_length=650|500|60
contig08779_length=672|250|60
contig21925_length=132|0|60
contig08323_length=733|500|60
log2(expression)
0
2
4
6
8
12
16
StageI
StageII
StageIII
StageIV
StageV
StageVI

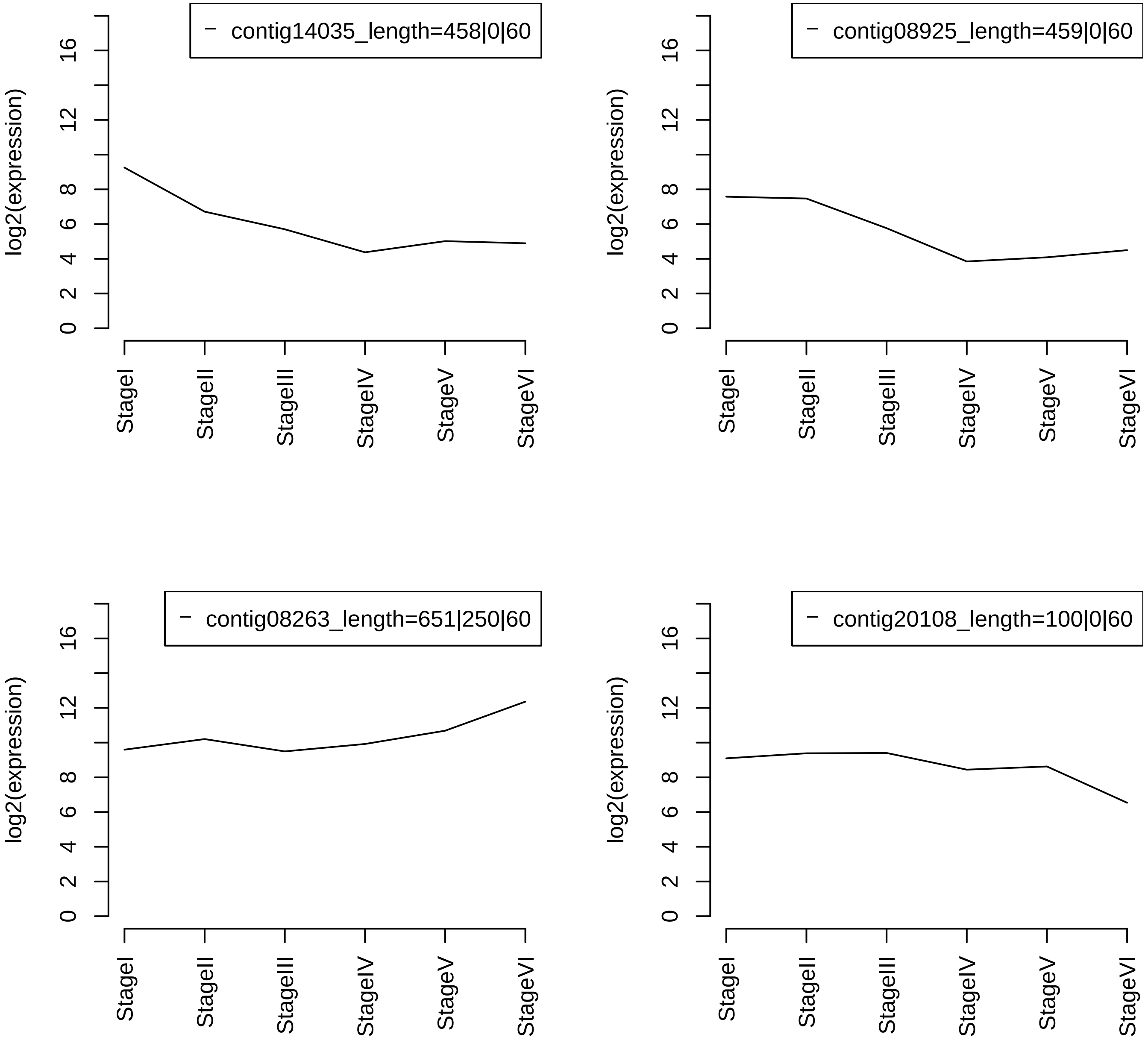

contig14035_length=458|0|60
contig08925_length=459|0|60
contig08263_length=651|250|60
contig20108_length=100|0|60
log2(expression)
0
2
4
6
8
12
16
StageI
StageII
StageIII
StageIV
StageV
StageVI

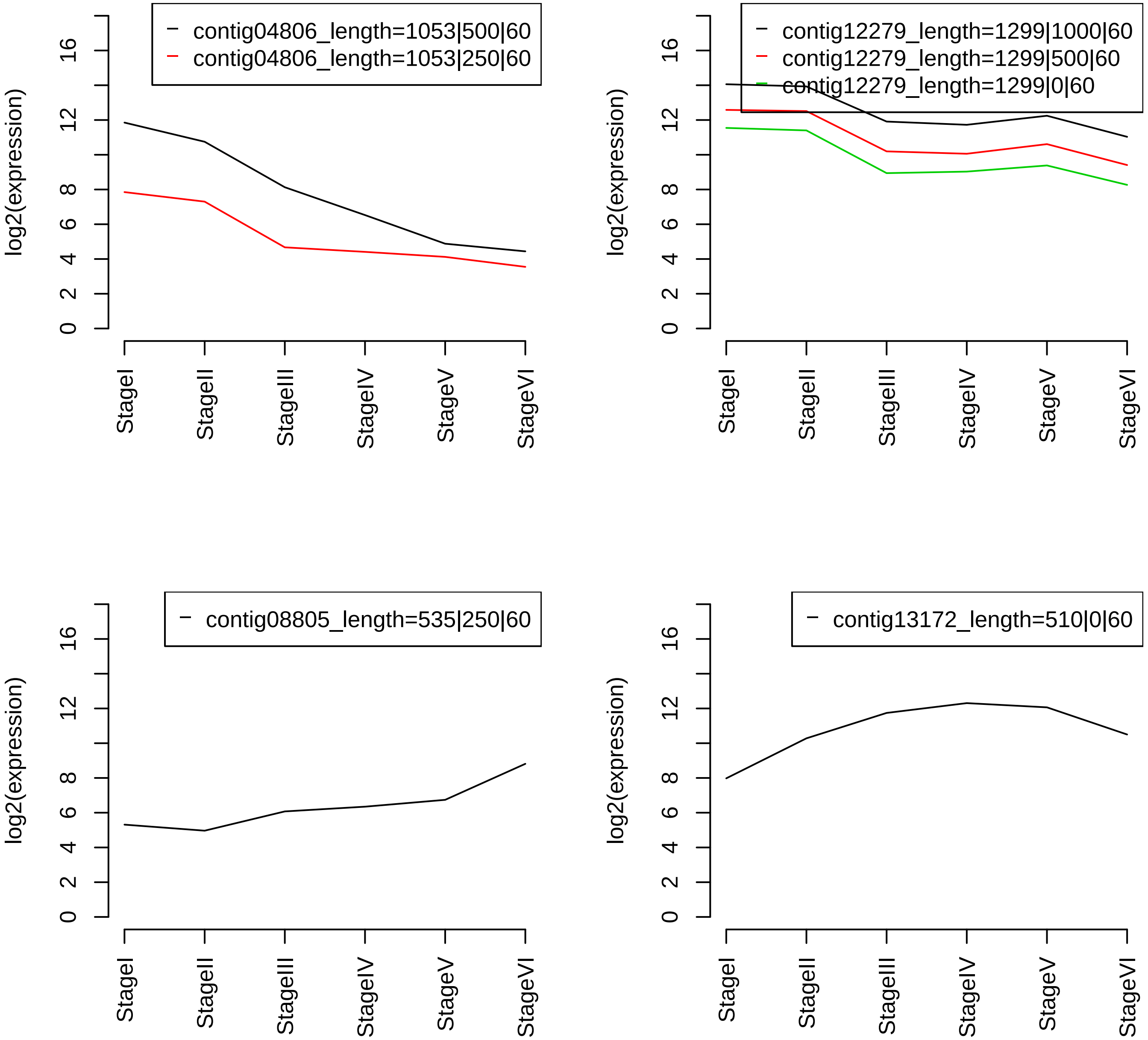

contig04806_length=1053|500|60
contig04806_length=1053|250|60
contig12279_length=1299|1000|60
contig12279_length=1299|500|60
contig12279_length=1299|0|60
contig08805_length=535|250|60
contig13172_length=510|0|60
log2(expression)
0 2 4 6 8 12 16
StageI StageII StageIII StageIV StageV StageVI

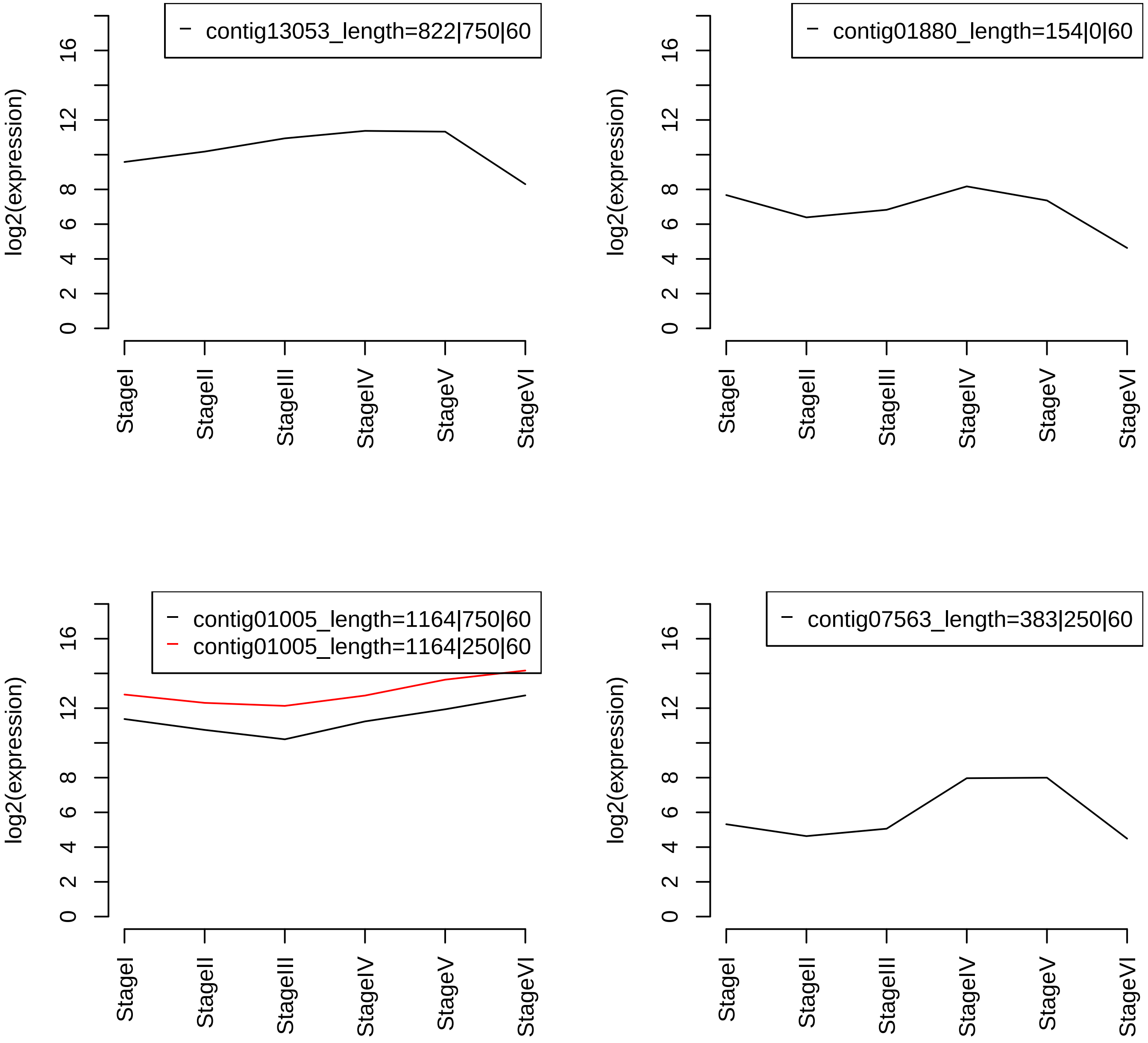

contig13053_length=822|750|60
contig01880_length=154|0|60
contig01005_length=1164|750|60
contig01005_length=1164|250|60
contig07563_length=383|250|60
log2(expression)
0
2
4
6
8
12
16
StageI
StageII
StageIII
StageIV
StageV
StageVI

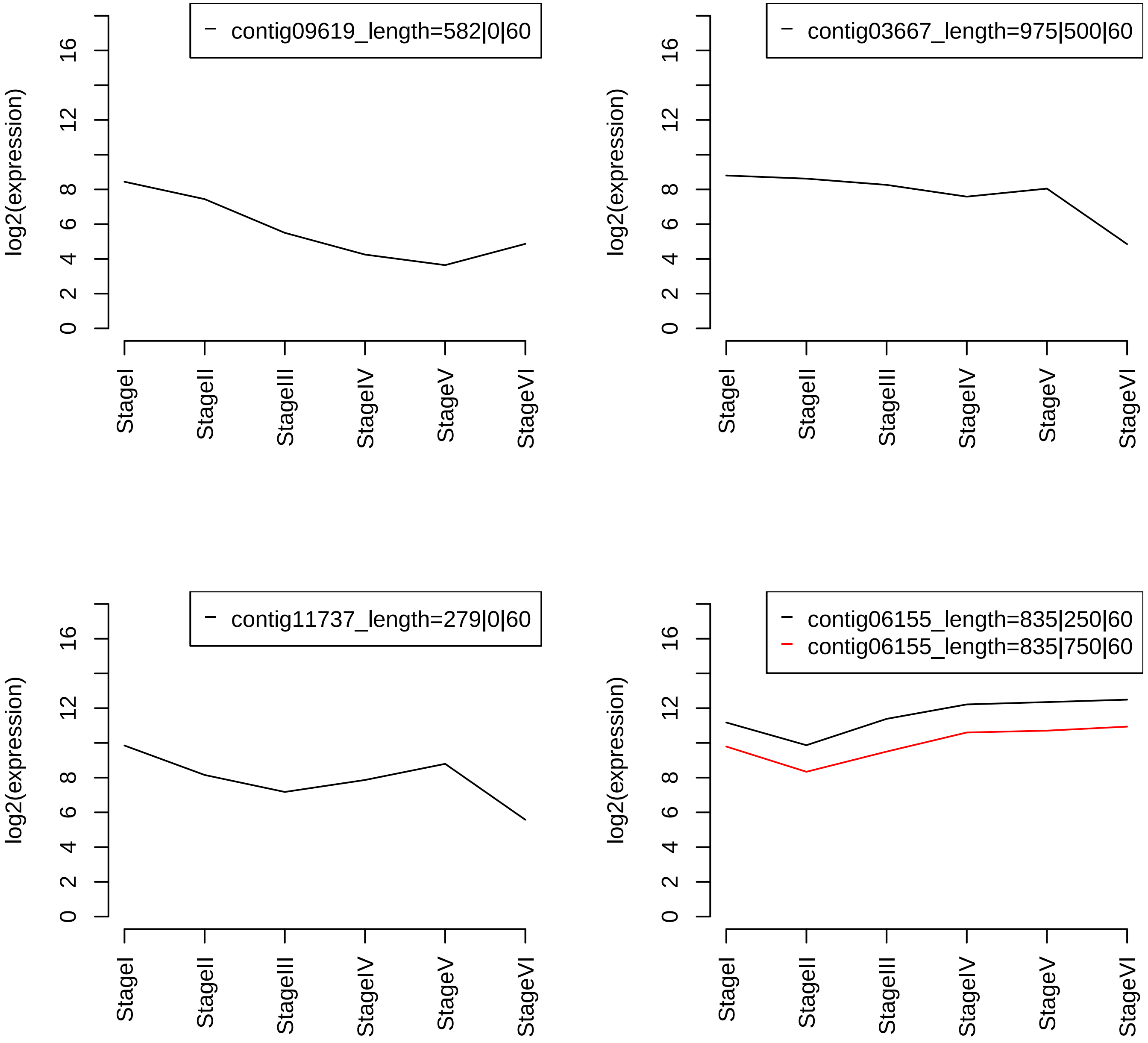
contig09619_length=582|0|60
contig03667_length=975|500|60
contig11737_length=279|0|60
contig06155_length=835|250|60
contig06155_length=835|750|60
log2(expression)
0
2
4
6
8
12
16
StageI
StageII
StageIII
StageIV
StageV
StageVI

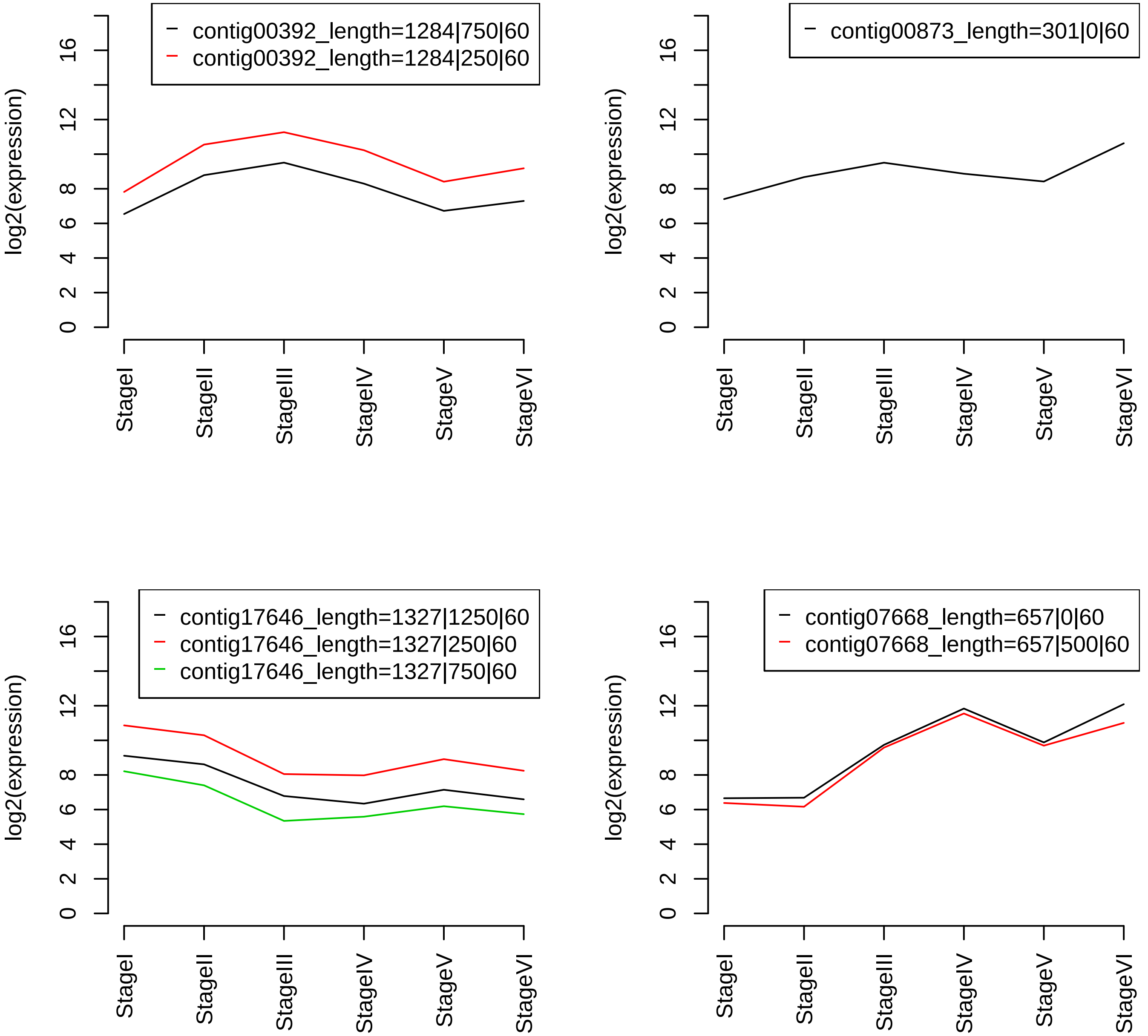

contig00392_length=1284|750|60
contig00392_length=1284|250|60
contig00873_length=301|0|60
contig17646_length=1327|1250|60
contig17646_length=1327|250|60
contig17646_length=1327|750|60
contig07668_length=657|0|60
contig07668_length=657|500|60
log2(expression)
0
2
4
6
8
12
16
StageI
StageII
StageIII
StageIV
StageV
StageVI

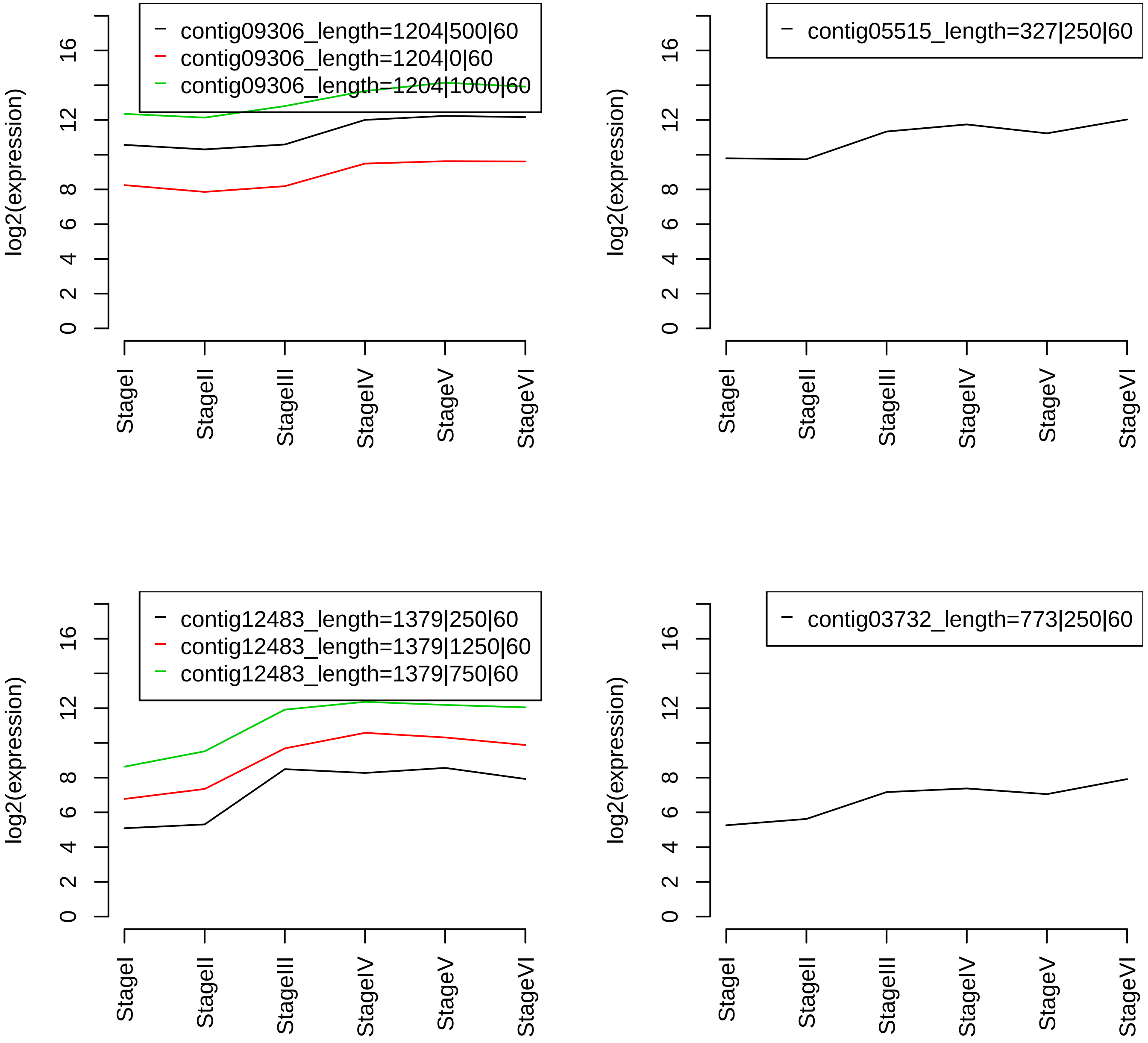
contig09306_length=1204|500|60
contig09306_length=1204|0|60
contig09306_length=1204|1000|60
contig05515_length=327|250|60
contig12483_length=1379|250|60
contig12483_length=1379|1250|60
contig12483_length=1379|750|60
contig03732_length=773|250|60
log2(expression)
0 2 4 6 8 12 16
StageI StageII StageIII StageIV StageV StageVI

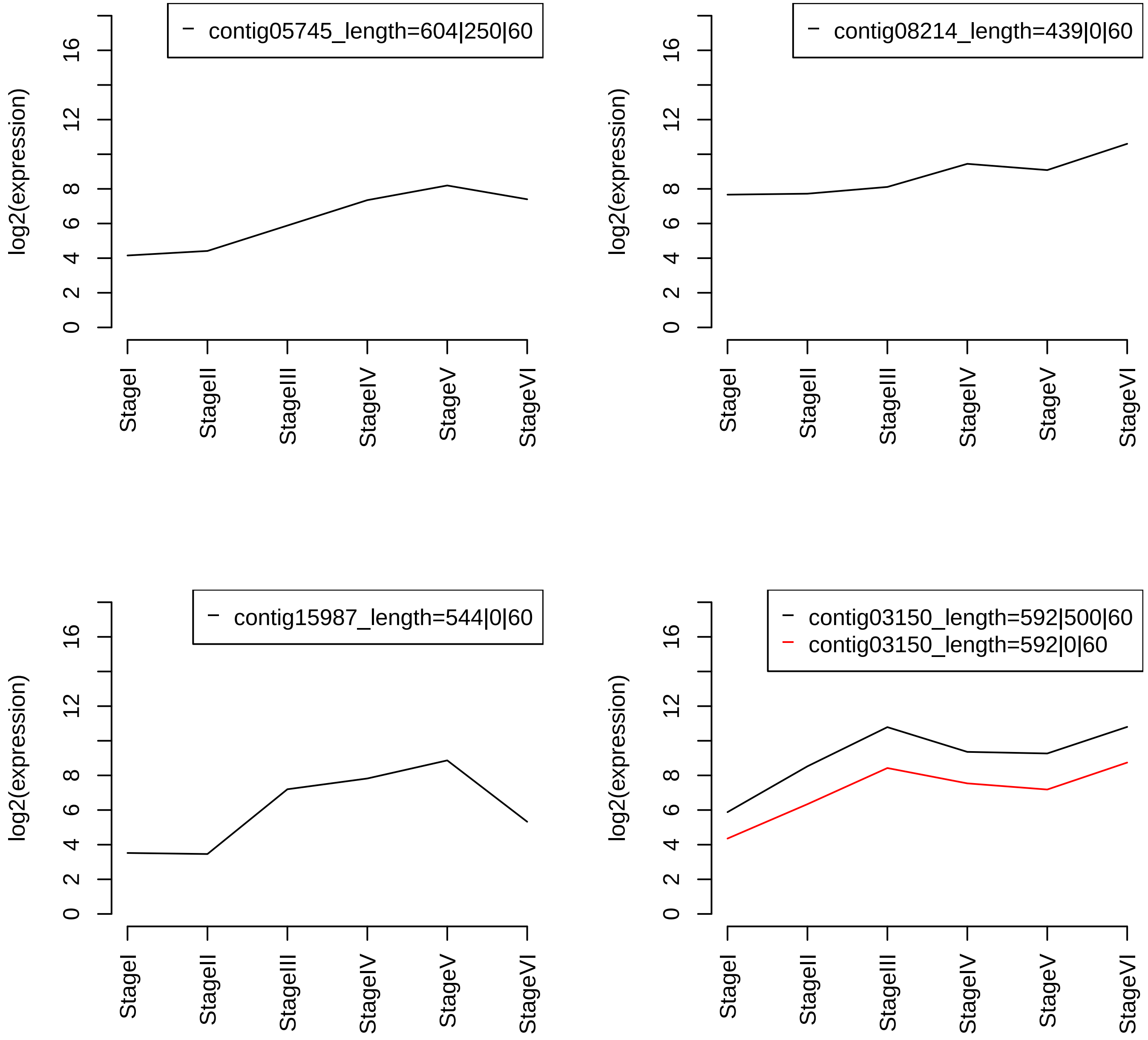
contig05745_length=604|250|60
contig08214_length=439|0|60
contig15987_length=544|0|60
contig03150_length=592|500|60
contig03150_length=592|0|60
log2(expression)
0
2
4
6
8
12
16
StageI
StageII
StageIII
StageIV
StageV
StageVI

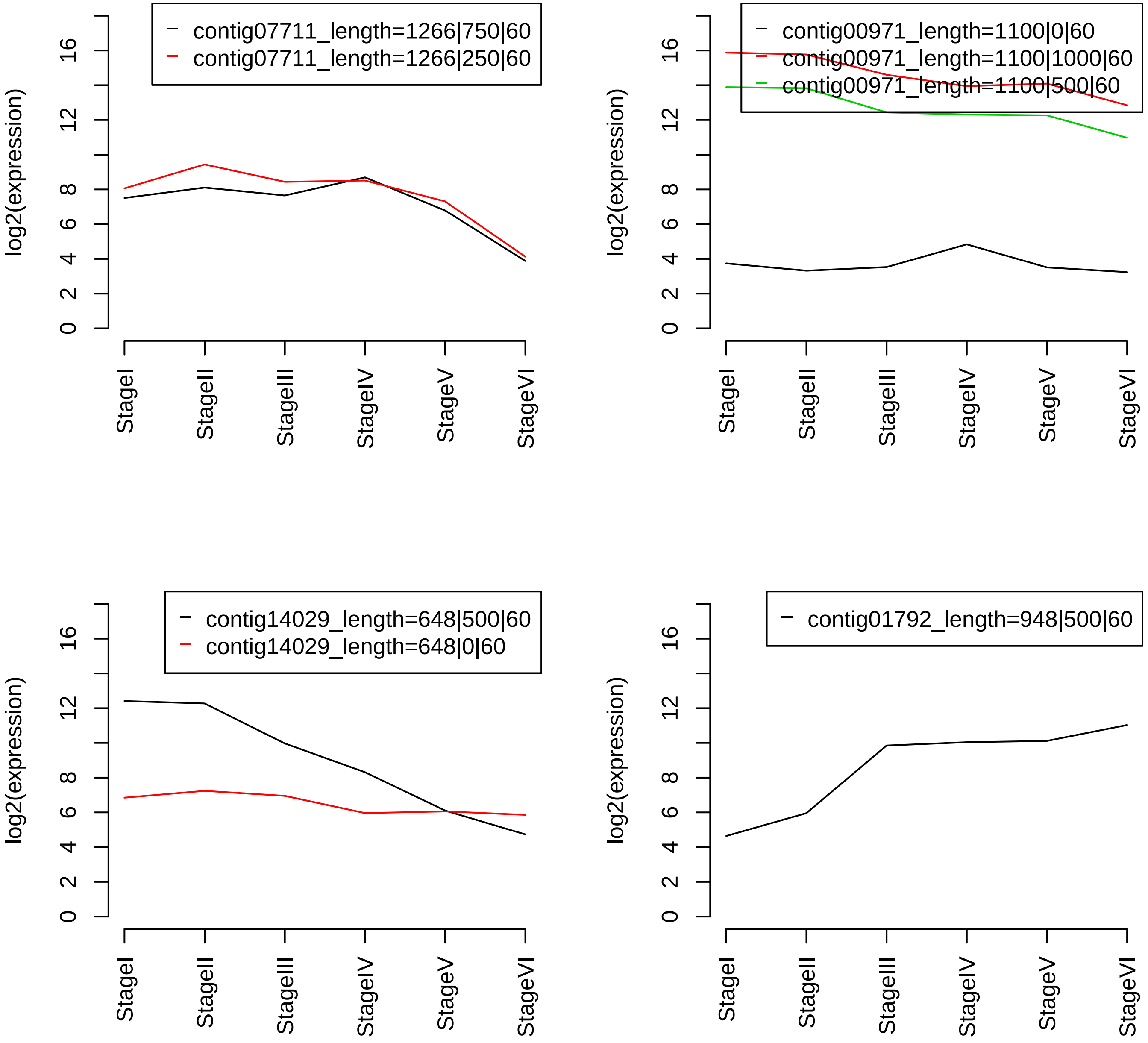
contig07711_length=1266|750|60
contig07711_length=1266|250|60
log2(expression)
StageI
StageII
StageIII
StageIV
StageV
StageVI
contig00971_length=1100|0|60
contig00971_length=1100|1000|60
contig00971_length=1100|500|60
log2(expression)
contig14029_length=648|500|60
contig14029_length=648|0|60
log2(expression)
contig01792_length=948|500|60
log2(expression)

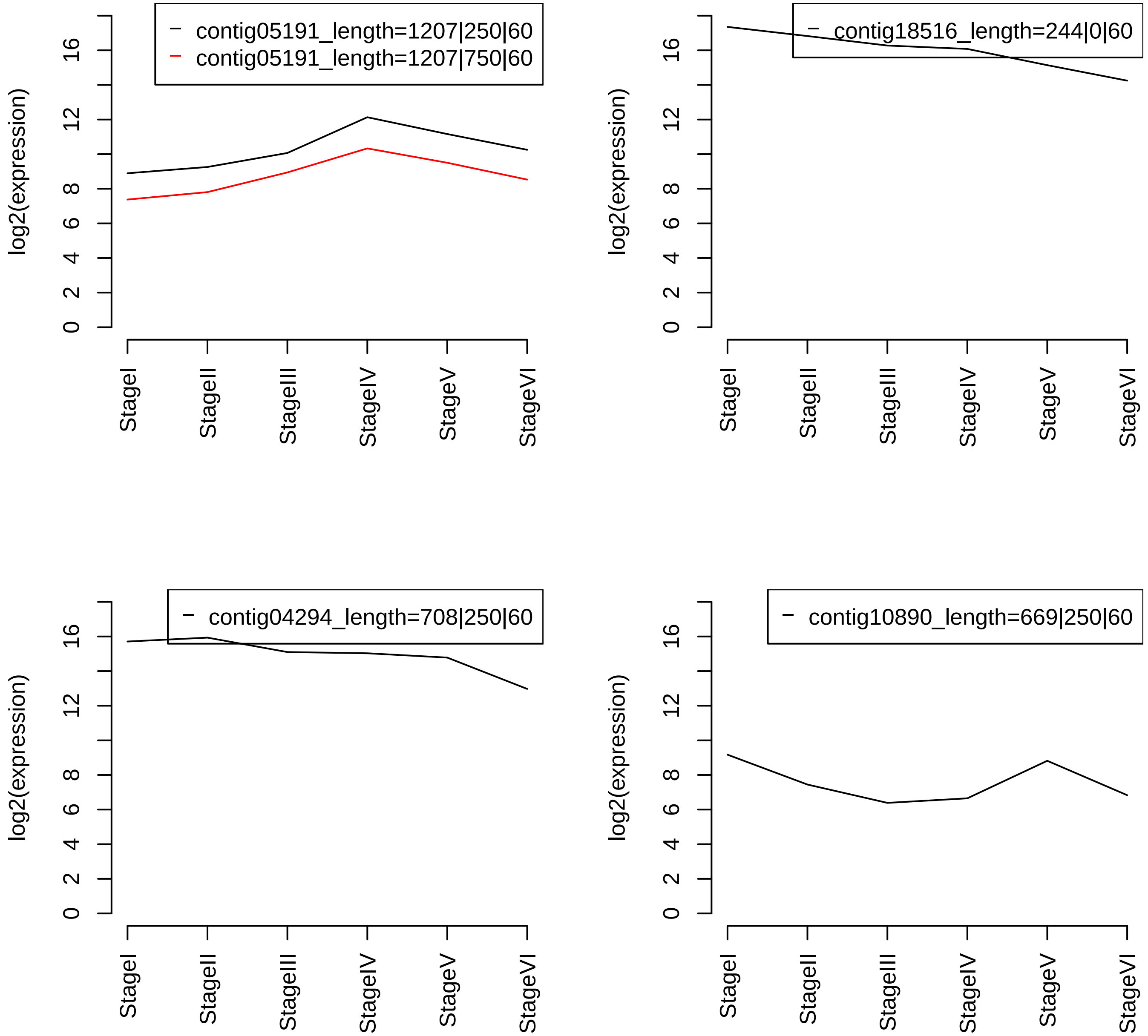
contig05191_length=1207|250|60
contig05191_length=1207|750|60
contig18516_length=244|0|60
contig04294_length=708|250|60
contig10890_length=669|250|60
log2(expression)
0
2
4
6
8
12
16
StageI
StageII
StageIII
StageIV
StageV
StageVI

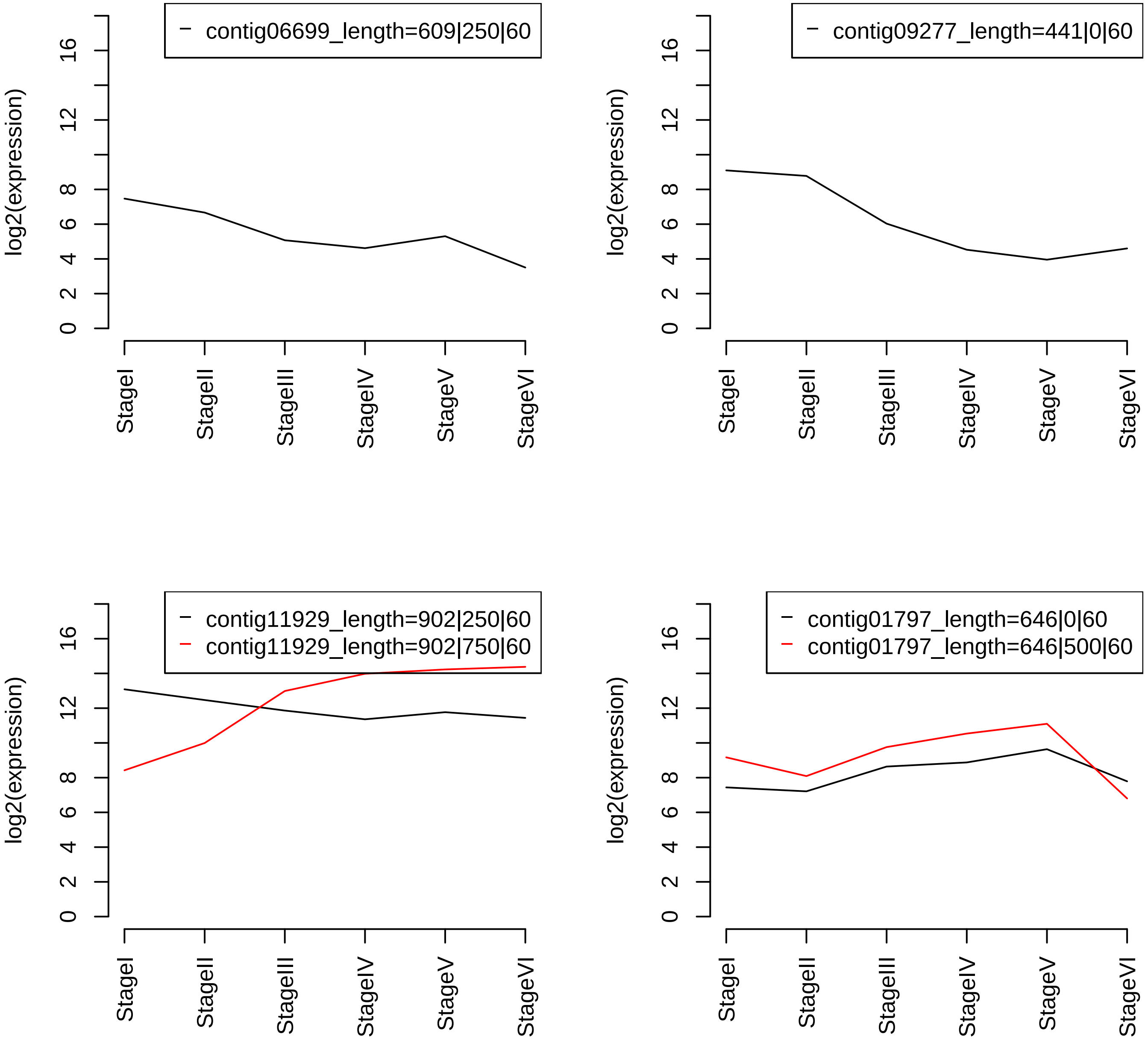
contig06699_length=609|250|60
contig09277_length=441|0|60
contig11929_length=902|250|60
contig11929_length=902|750|60
contig01797_length=646|0|60
contig01797_length=646|500|60
log2(expression)
0
2
4
6
8
12
16
StageI
StageII
StageIII
StageIV
StageV
StageVI

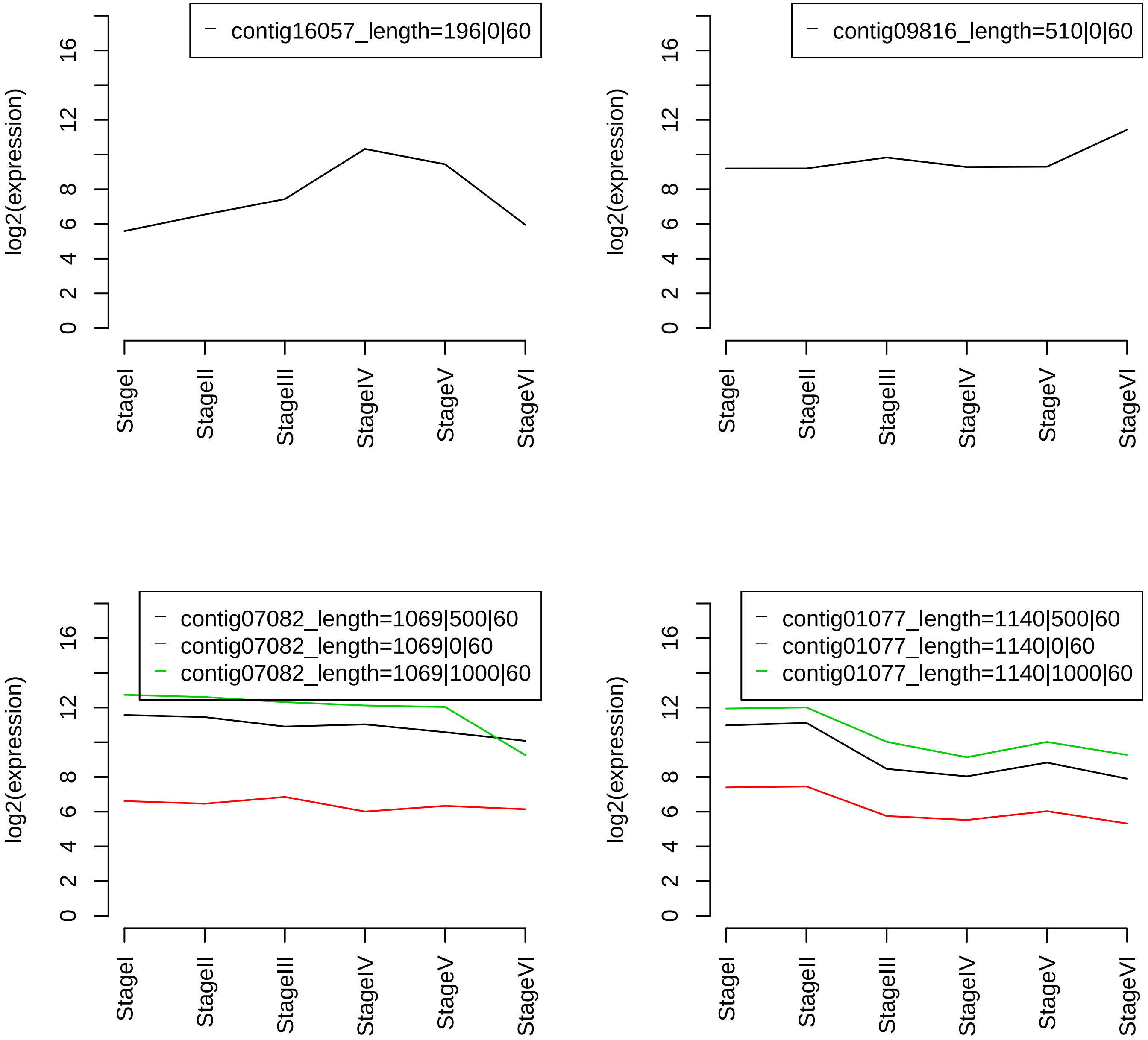

contig16057_length=196|0|60
contig09816_length=510|0|60
contig07082_length=1069|500|60
contig07082_length=1069|0|60
contig07082_length=1069|1000|60
contig01077_length=1140|500|60
contig01077_length=1140|0|60
contig01077_length=1140|1000|60
log2(expression)
0
2
4
6
8
12
16
StageI
StageII
StageIII
StageIV
StageV
StageVI

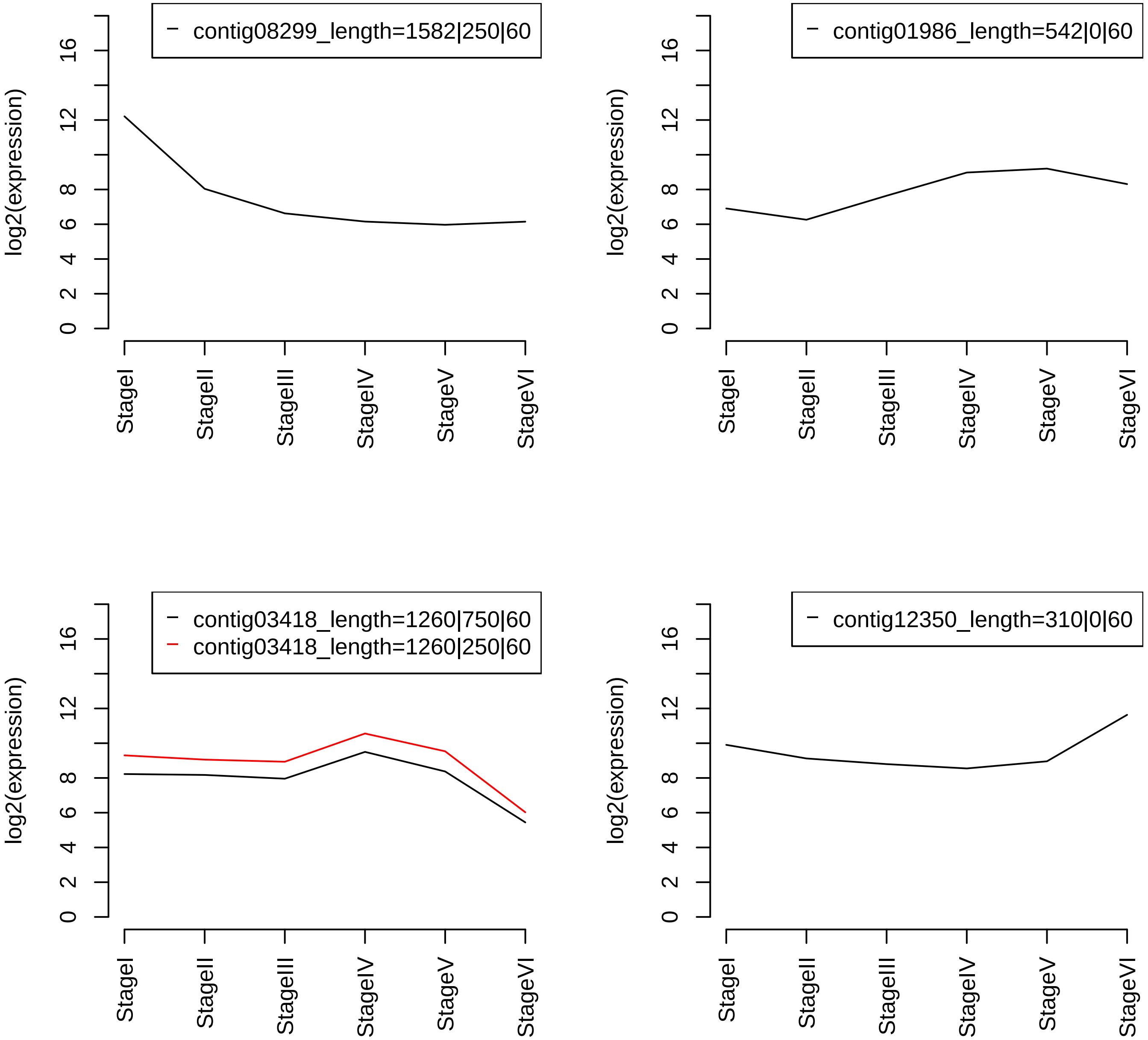
contig08299_length=1582|250|60
contig01986_length=542|0|60
contig03418_length=1260|750|60
contig03418_length=1260|250|60
contig12350_length=310|0|60
log2(expression)
0 2 4 6 8 12 16
StageI StageII StageIII StageIV StageV StageVI

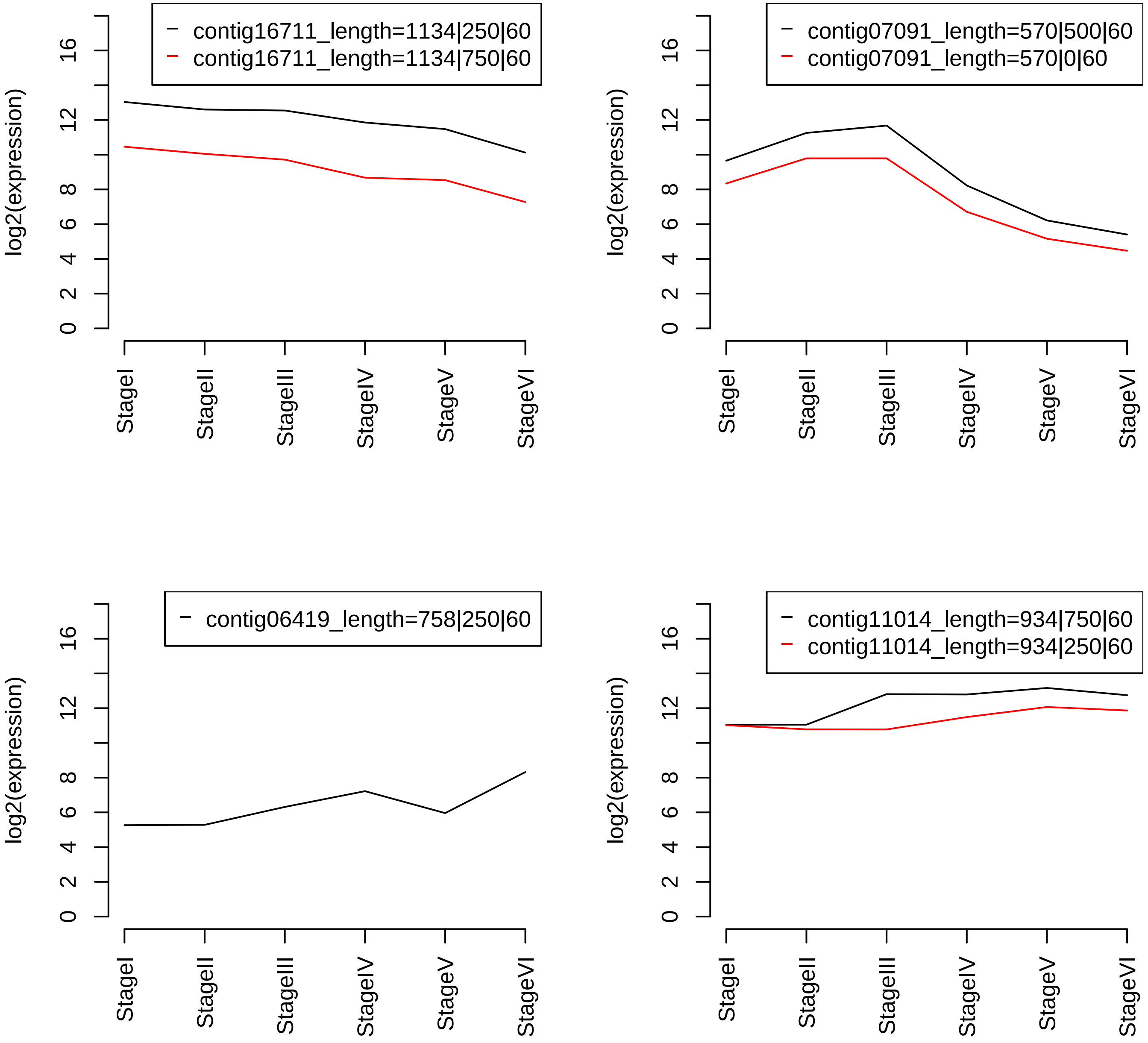
contig16711_length=1134|250|60
contig16711_length=1134|750|60
log2(expression)
StageI
StageII
StageIII
StageIV
StageV
StageVI
contig07091_length=570|500|60
contig07091_length=570|0|60
contig06419_length=758|250|60
contig11014_length=934|750|60
contig11014_length=934|250|60

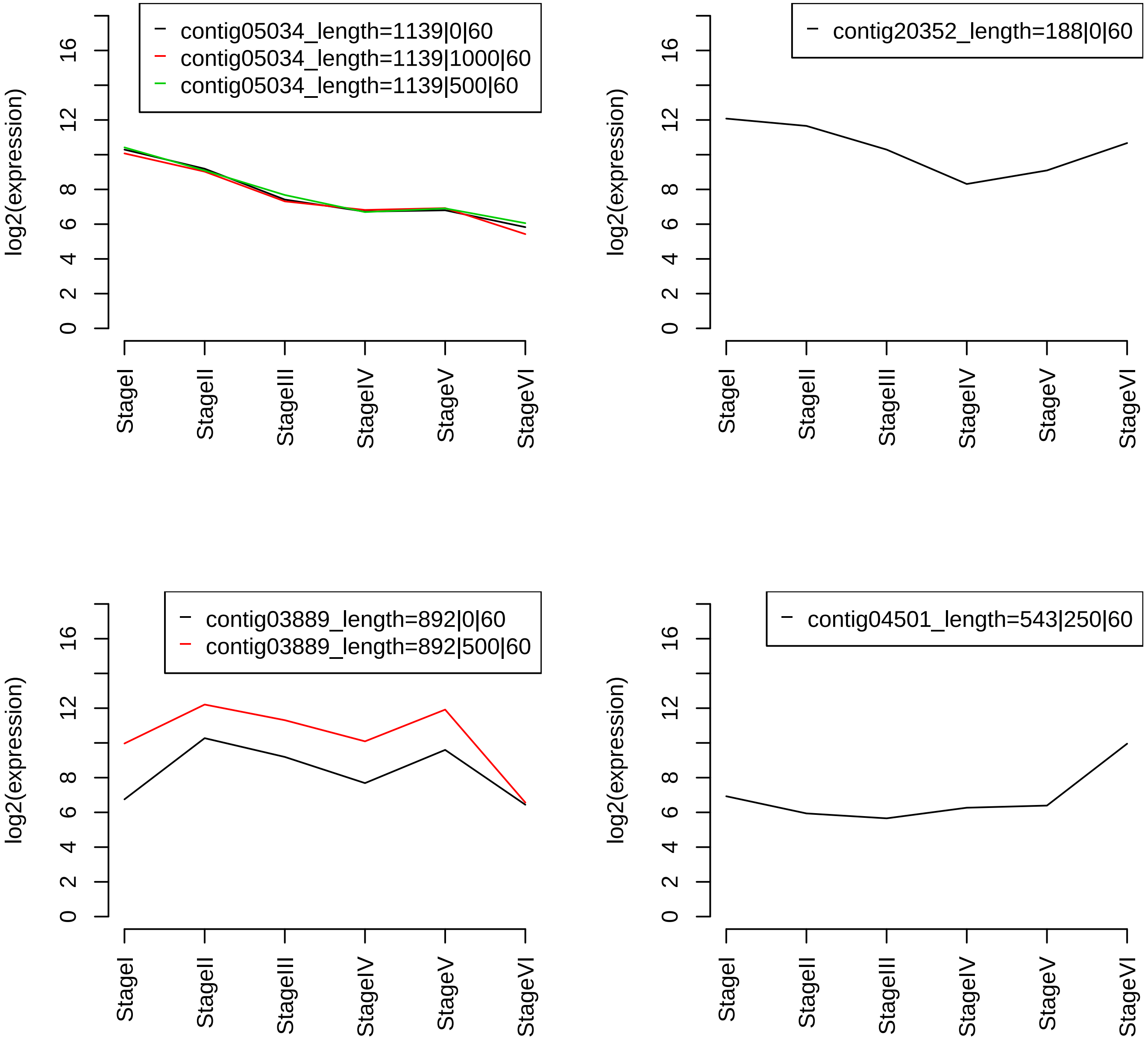
contig05034_length=1139|0|60
contig05034_length=1139|1000|60
contig05034_length=1139|500|60
contig20352_length=188|0|60
contig03889_length=892|0|60
contig03889_length=892|500|60
contig04501_length=543|250|60
log2(expression)
0 2 4 6 8 12 16
StageI StageII StageIII StageIV StageV StageVI

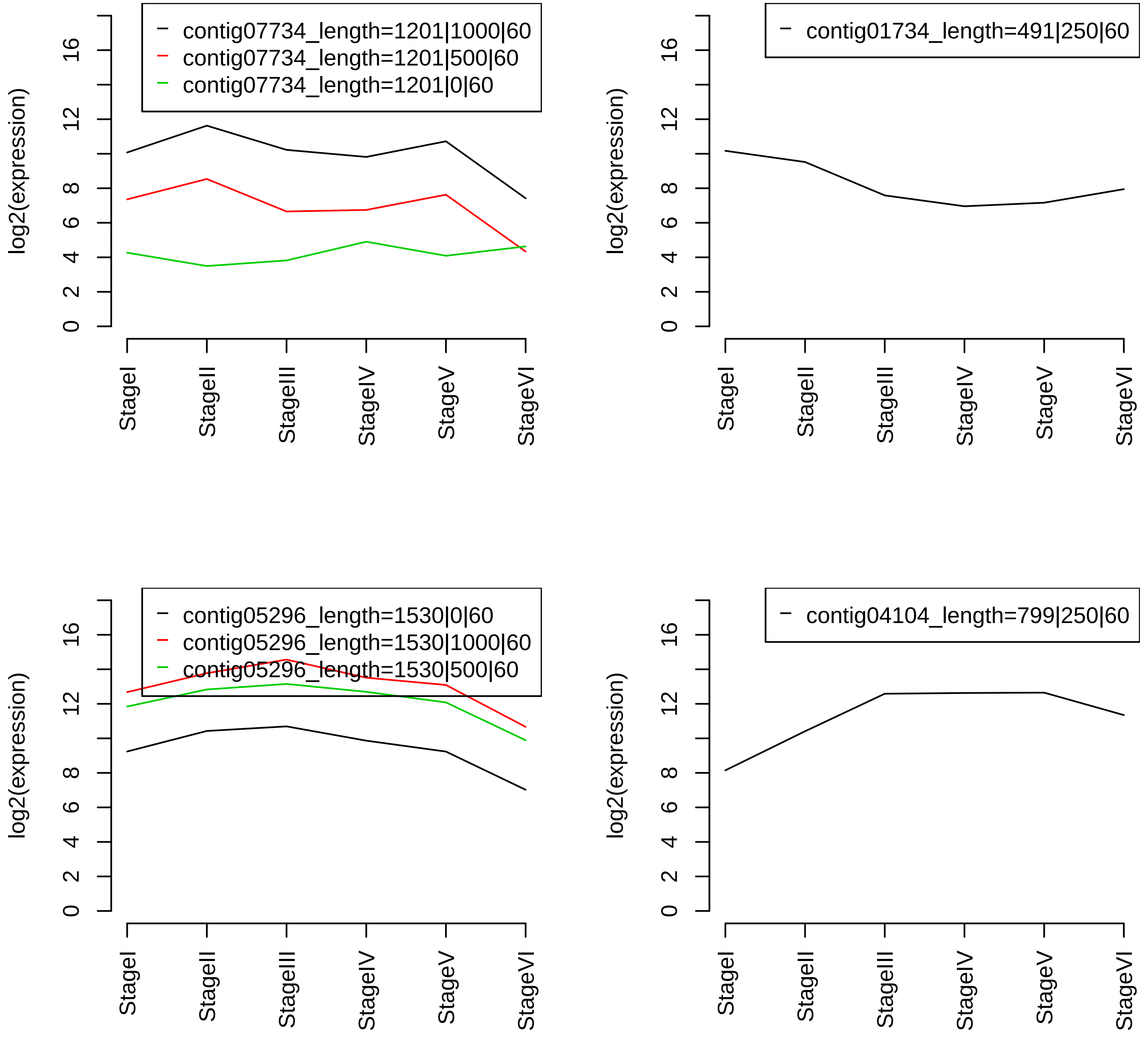

contig07734_length=1201|1000|60
contig07734_length=1201|500|60
contig07734_length=1201|0|60
contig01734_length=491|250|60
contig05296_length=1530|0|60
contig05296_length=1530|1000|60
contig05296_length=1530|500|60
contig04104_length=799|250|60
log2(expression)
0
2
4
6
8
12
16
StageI
StageII
StageIII
StageIV
StageV
StageVI

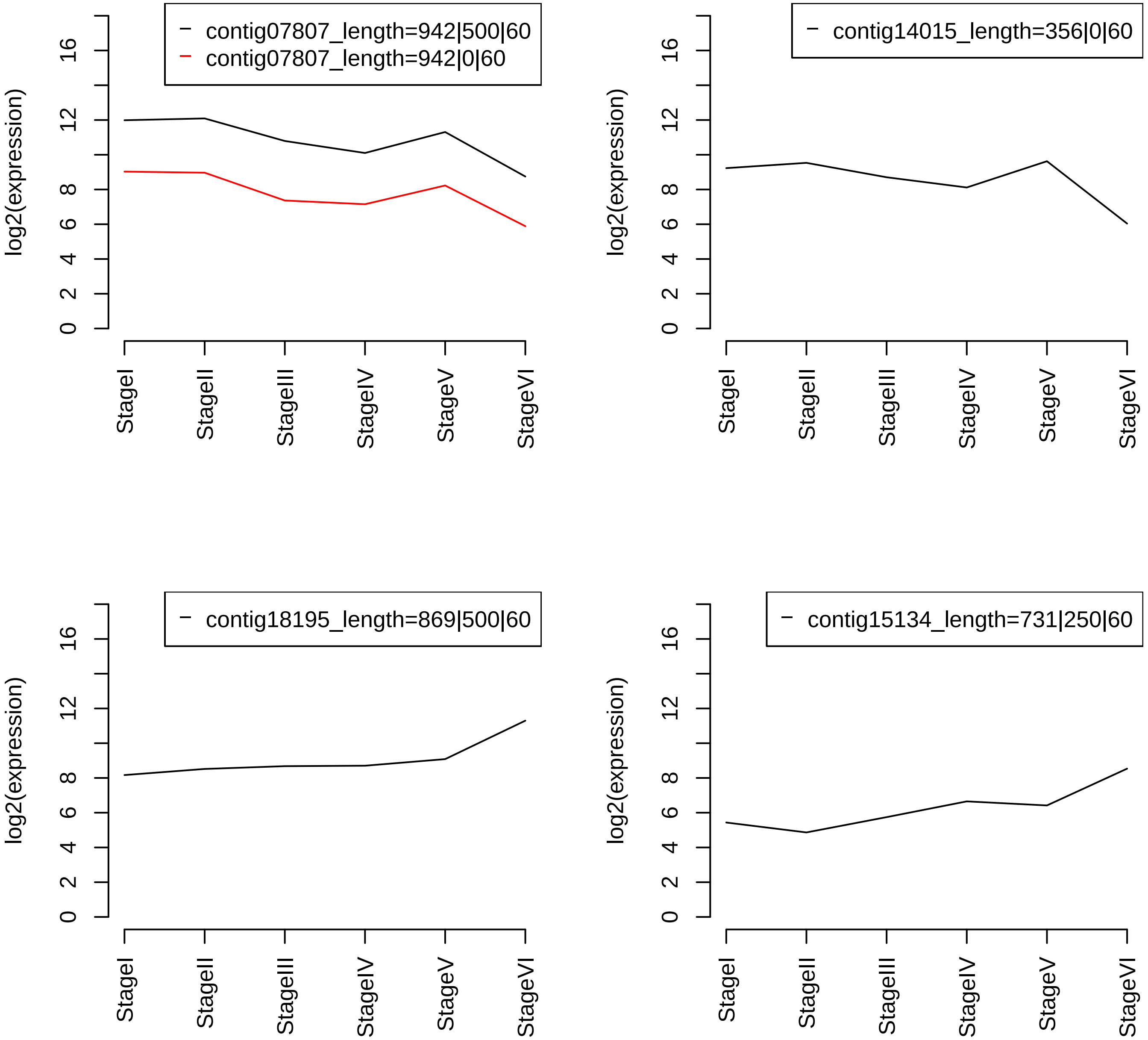

contig07807_length=942|500|60
contig07807_length=942|0|60
log2(expression)
0
2
4
6
8
12
16
StageI
StageII
StageIII
StageIV
StageV
StageVI
contig14015_length=356|0|60
contig18195_length=869|500|60
contig15134_length=731|250|60

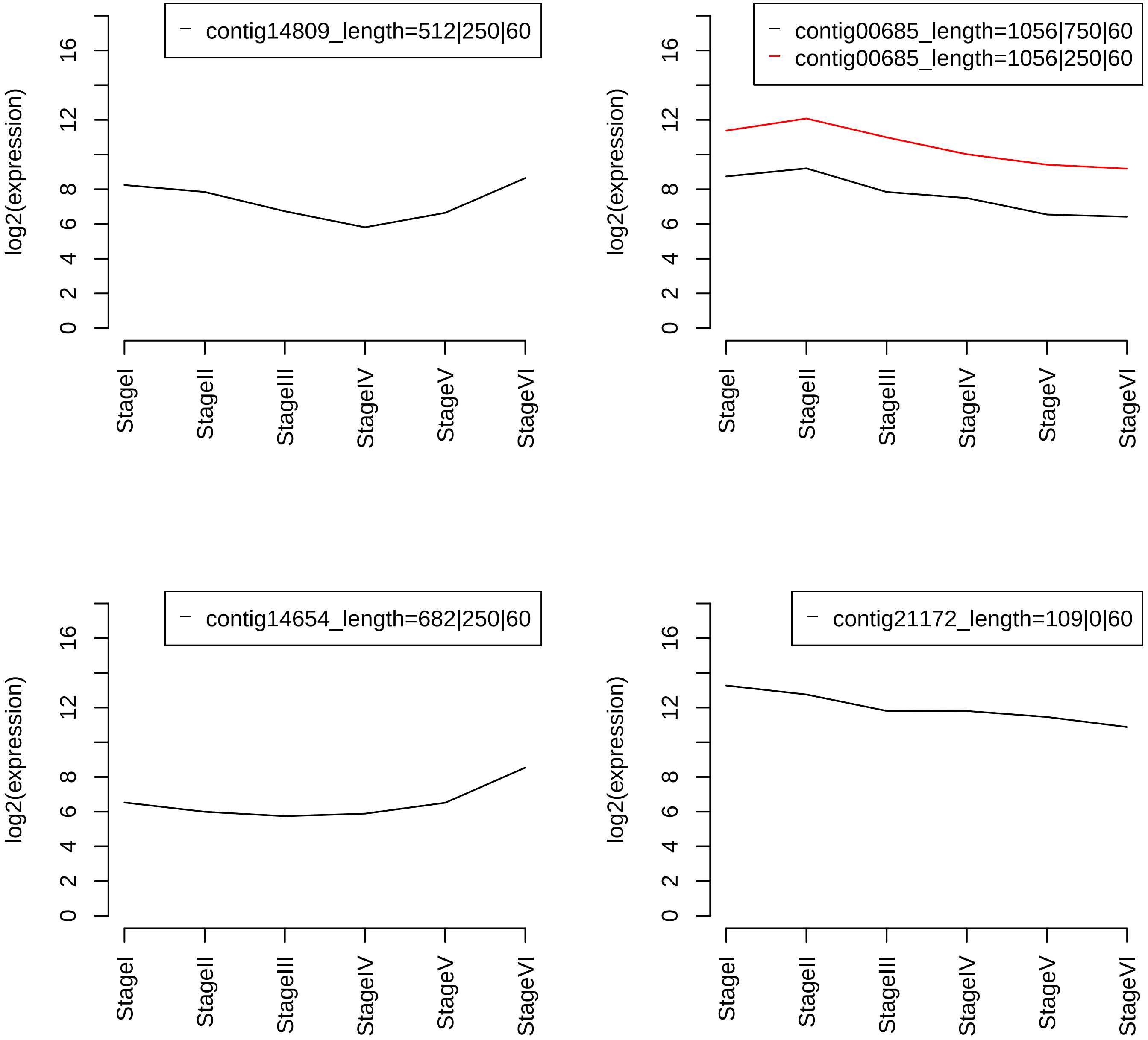
contig14809_length=512|250|60
contig00685_length=1056|750|60
contig00685_length=1056|250|60
contig14654_length=682|250|60
contig21172_length=109|0|60
log2(expression)
0
2
4
6
8
12
16
StageI
StageII
StageIII
StageIV
StageV
StageVI

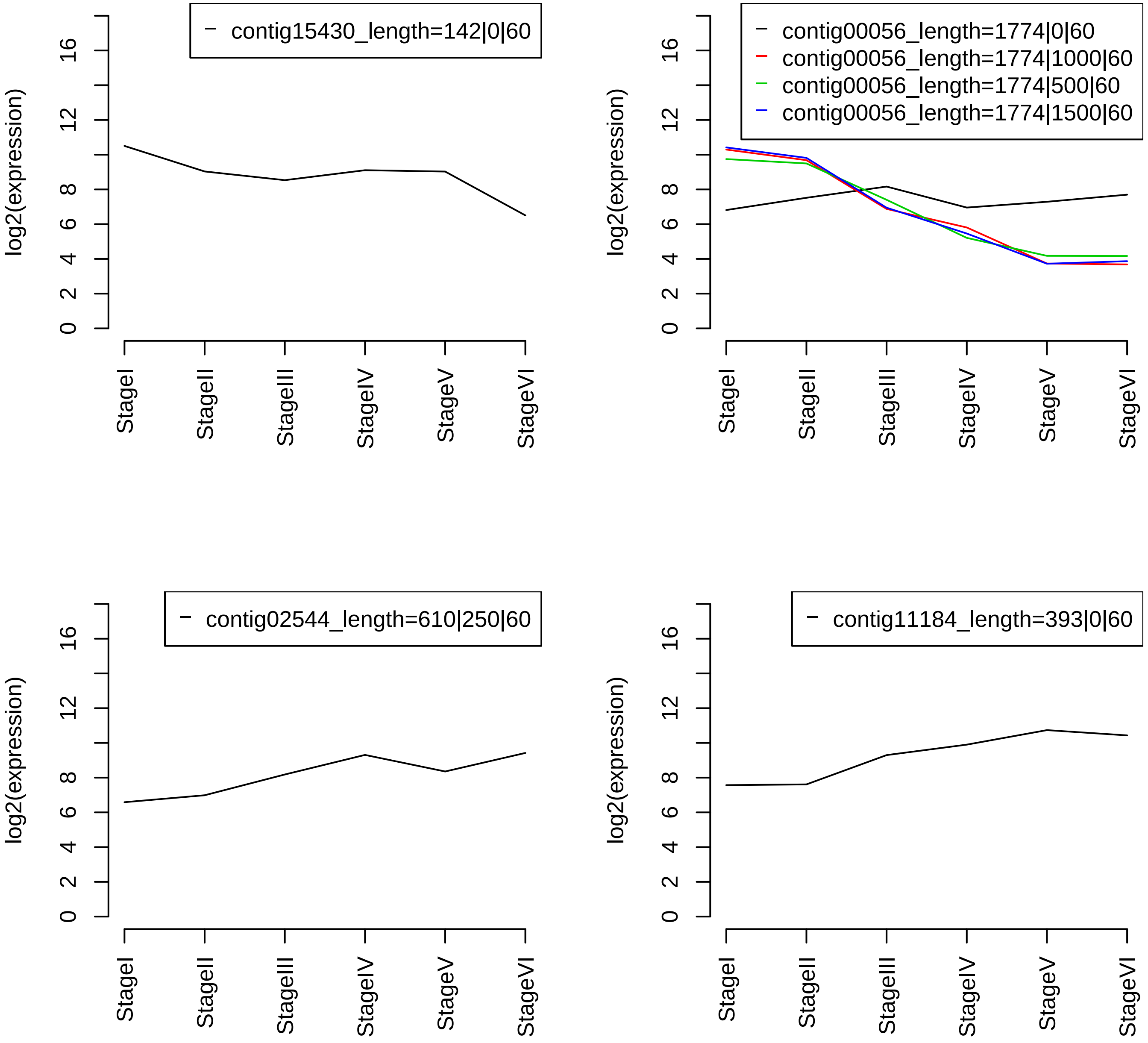

contig15430_length=142|0|60
contig00056_length=1774|0|60
contig00056_length=1774|1000|60
contig00056_length=1774|500|60
contig00056_length=1774|1500|60
contig02544_length=610|250|60
contig11184_length=393|0|60
log2(expression)
0 2 4 6 8 12 16
StageI StageII StageIII StageIV StageV StageVI

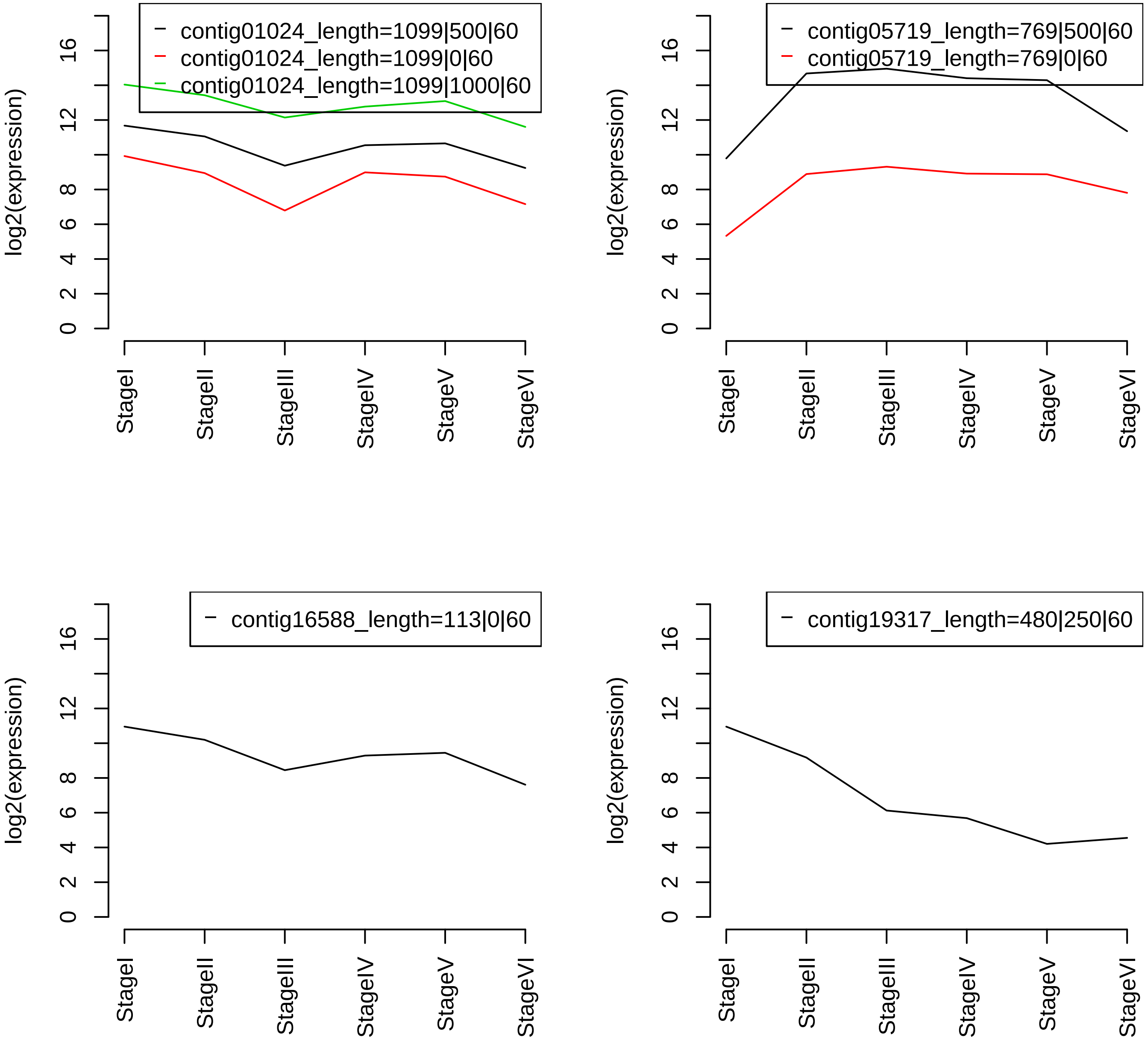
contig01024_length=1099|500|60
contig01024_length=1099|0|60
contig01024_length=1099|1000|60
contig05719_length=769|500|60
contig05719_length=769|0|60
contig16588_length=113|0|60
contig19317_length=480|250|60
log2(expression)
0
2
4
6
8
12
16
StageI
StageII
StageIII
StageIV
StageV
StageVI

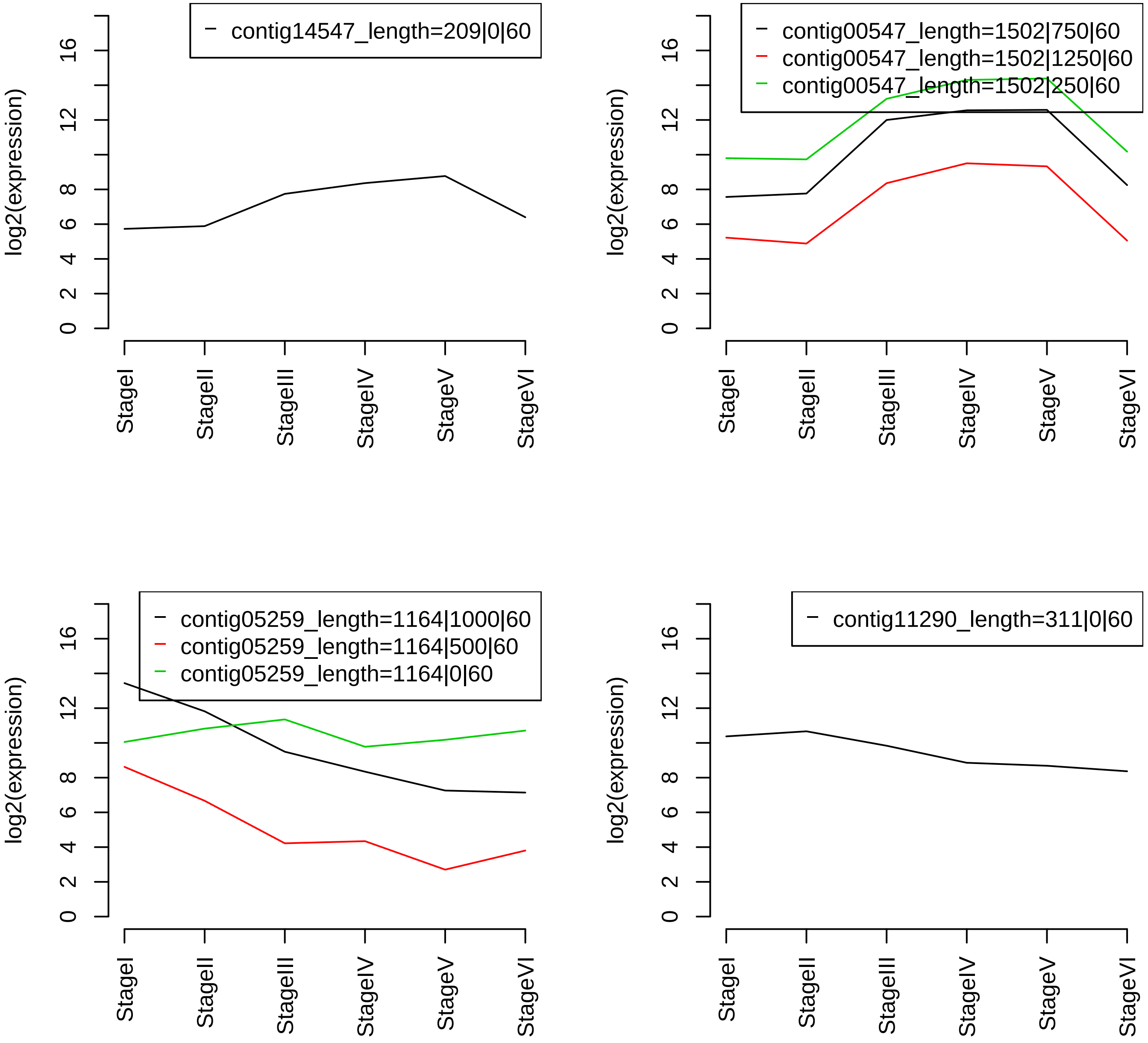
contig14547_length=209|0|60
contig00547_length=1502|750|60
contig00547_length=1502|1250|60
contig00547_length=1502|250|60
contig05259_length=1164|1000|60
contig05259_length=1164|500|60
contig05259_length=1164|0|60
contig11290_length=311|0|60
log2(expression)
0
2
4
6
8
12
16
StageI
StageII
StageIII
StageIV
StageV
StageVI

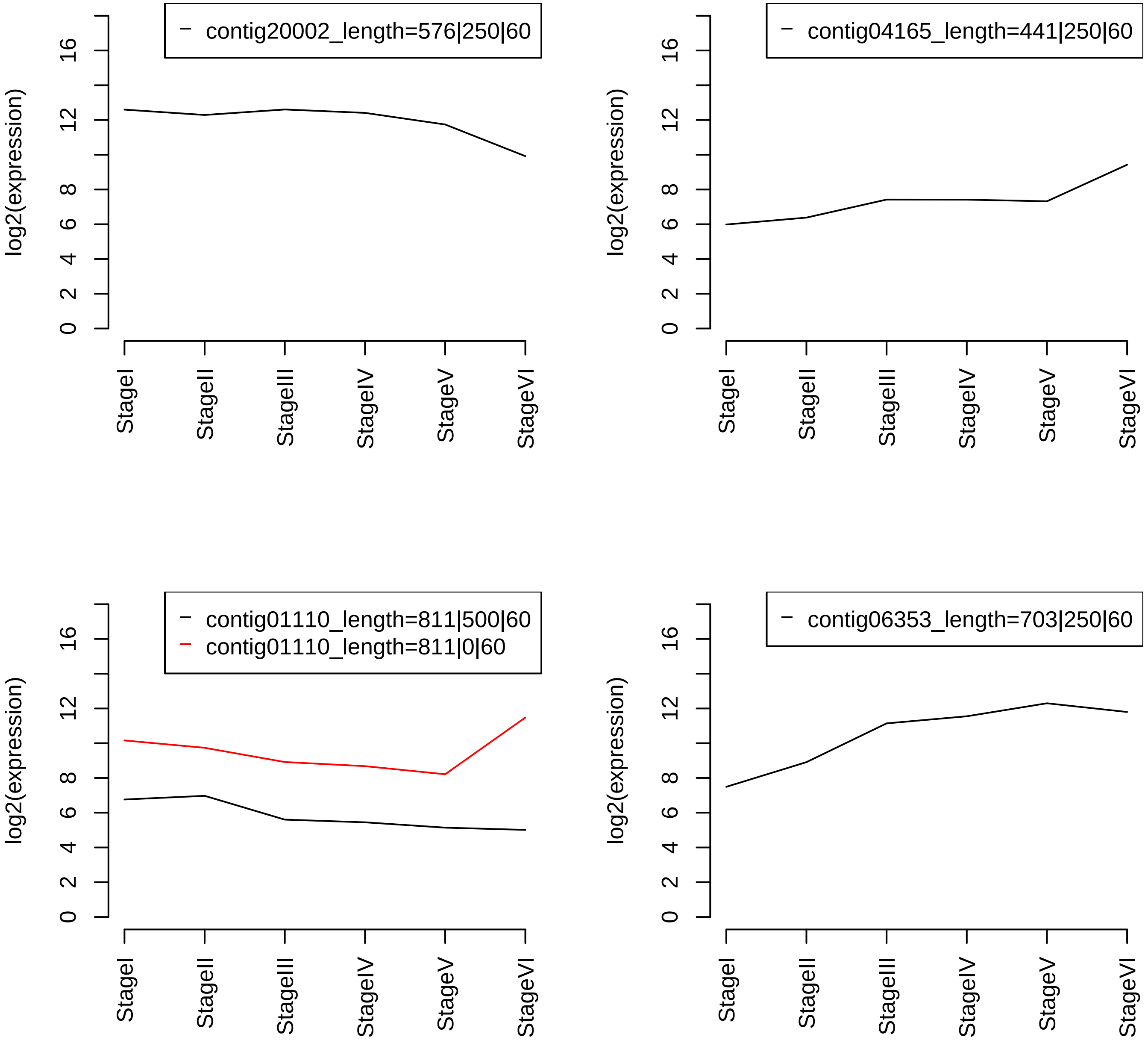

contig20002_length=576|250|60
contig04165_length=441|250|60
contig01110_length=811|500|60
contig01110_length=811|0|60
contig06353_length=703|250|60
log2(expression)
0
2
4
6
8
12
16
StageI
StageII
StageIII
StageIV
StageV
StageVI

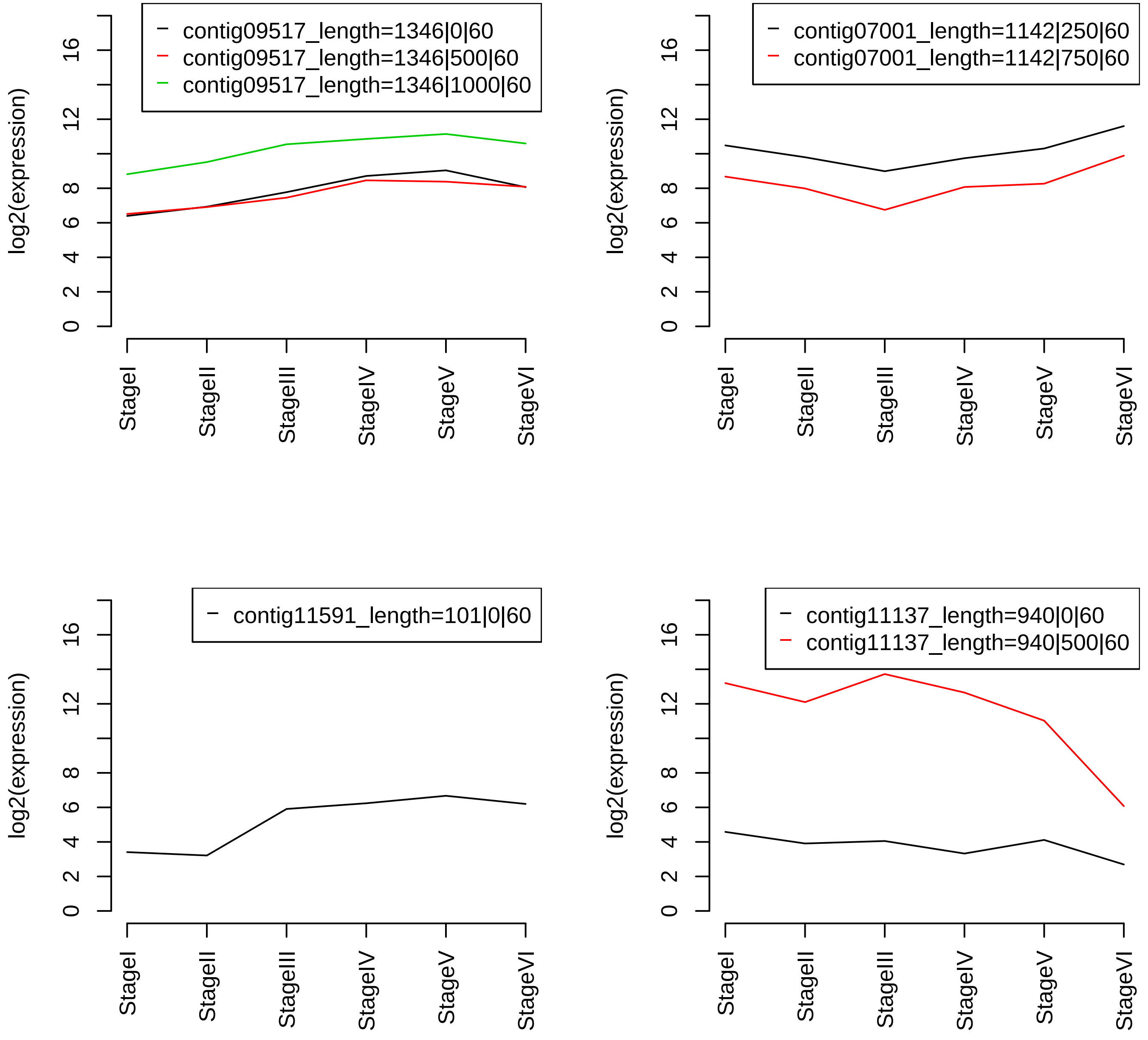

contig09517_length=1346|0|60
contig09517_length=1346|500|60
contig09517_length=1346|1000|60
contig07001_length=1142|250|60
contig07001_length=1142|750|60
contig11591_length=101|0|60
contig11137_length=940|0|60
contig11137_length=940|500|60
log2(expression)
0
2
4
6
8
12
16
StageI
StageII
StageIII
StageIV
StageV
StageVI

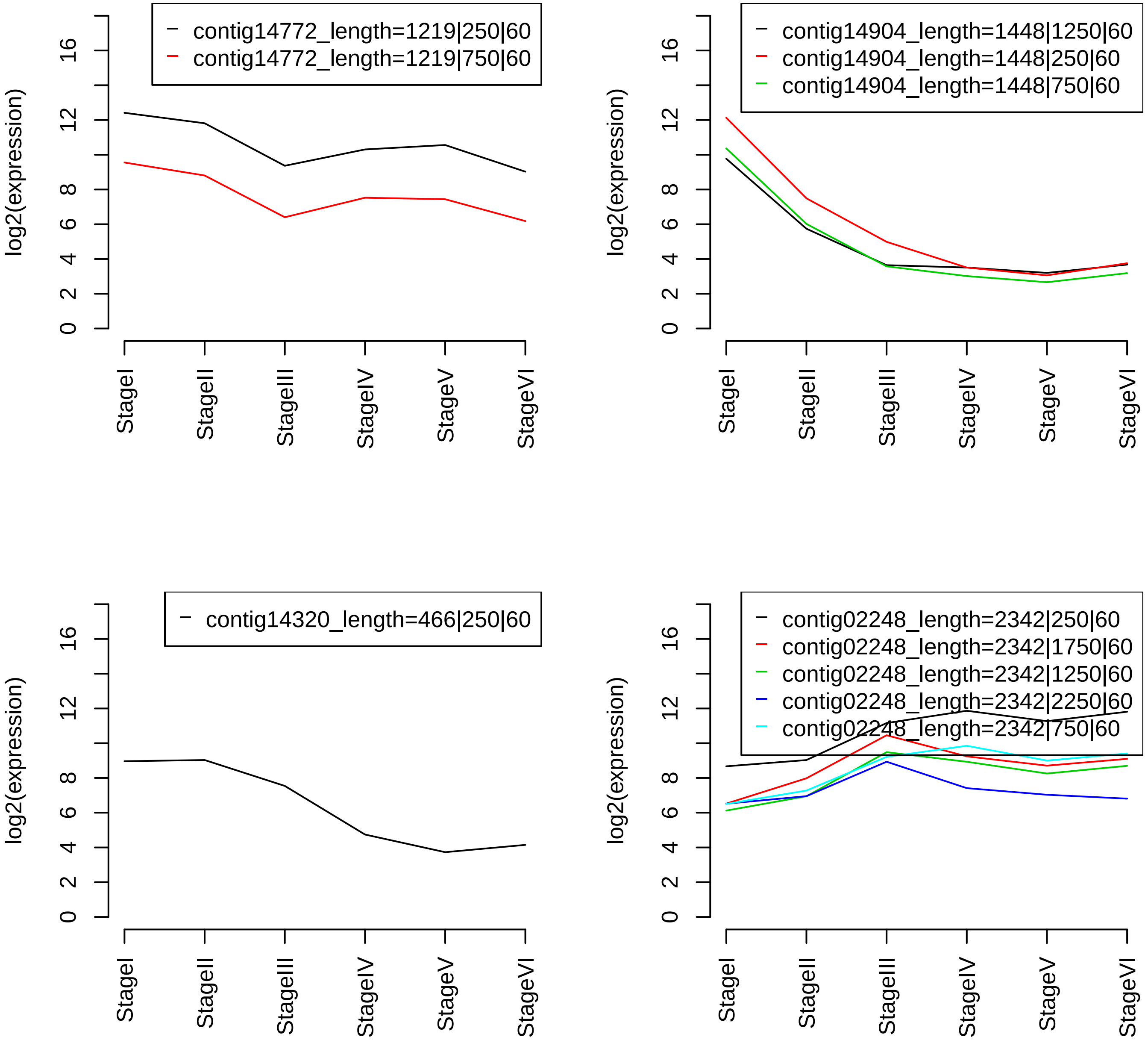

contig14772_length=1219|250|60
contig14772_length=1219|750|60
contig14904_length=1448|1250|60
contig14904_length=1448|250|60
contig14904_length=1448|750|60
contig14320_length=466|250|60
contig02248_length=2342|250|60
contig02248_length=2342|1750|60
contig02248_length=2342|1250|60
contig02248_length=2342|2250|60
contig02248_length=2342|750|60
log2(expression)
0 2 4 6 8 12 16
StageI StageII StageIII StageIV StageV StageVI

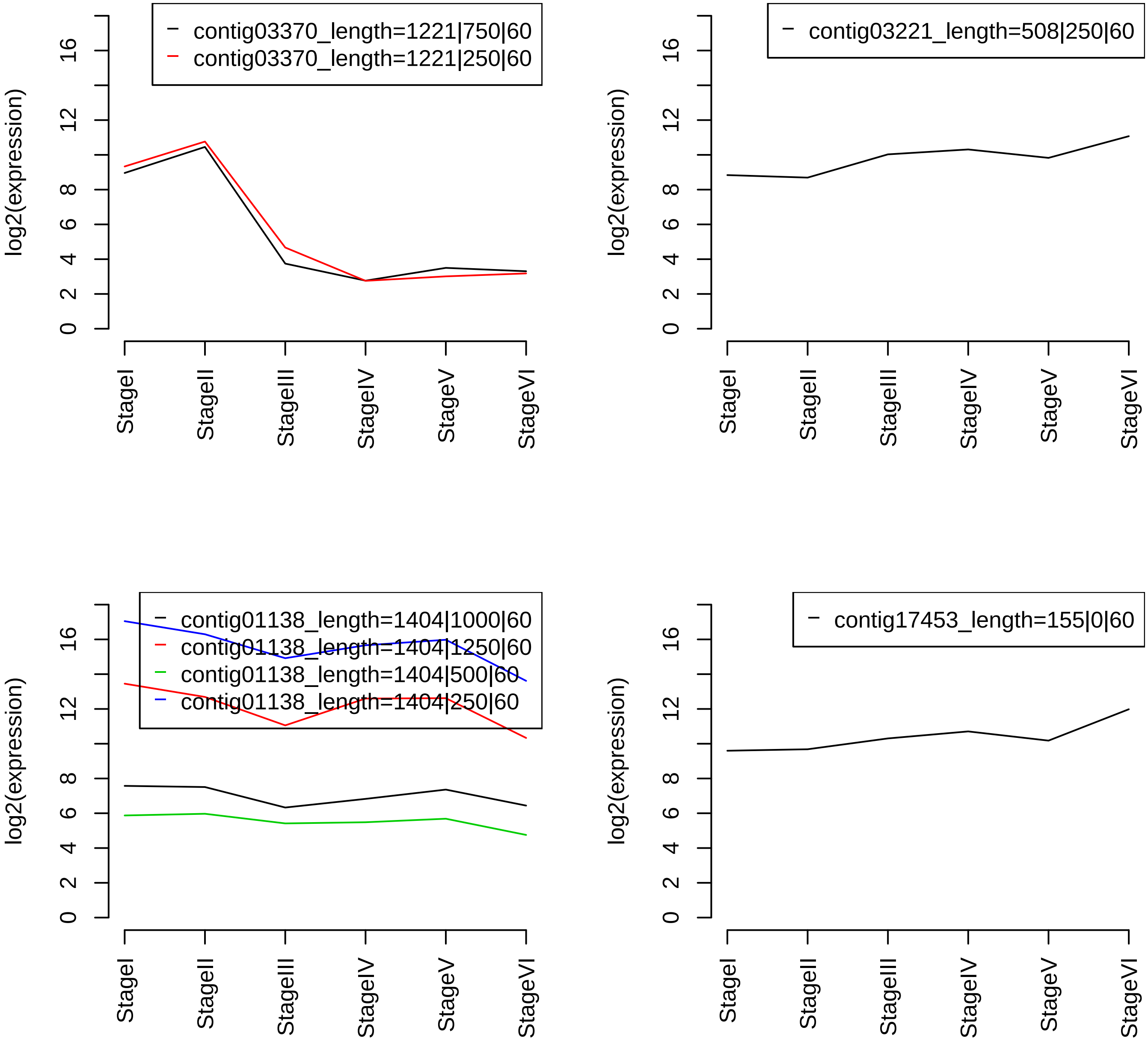

contig03370_length=1221|750|60
contig03370_length=1221|250|60
contig03221_length=508|250|60
contig01138_length=1404|1000|60
contig01138_length=1404|1250|60
contig01138_length=1404|500|60
contig01138_length=1404|250|60
contig17453_length=155|0|60
log2(expression)
0 2 4 6 8 12 16
StageI StageII StageIII StageIV StageV StageVI

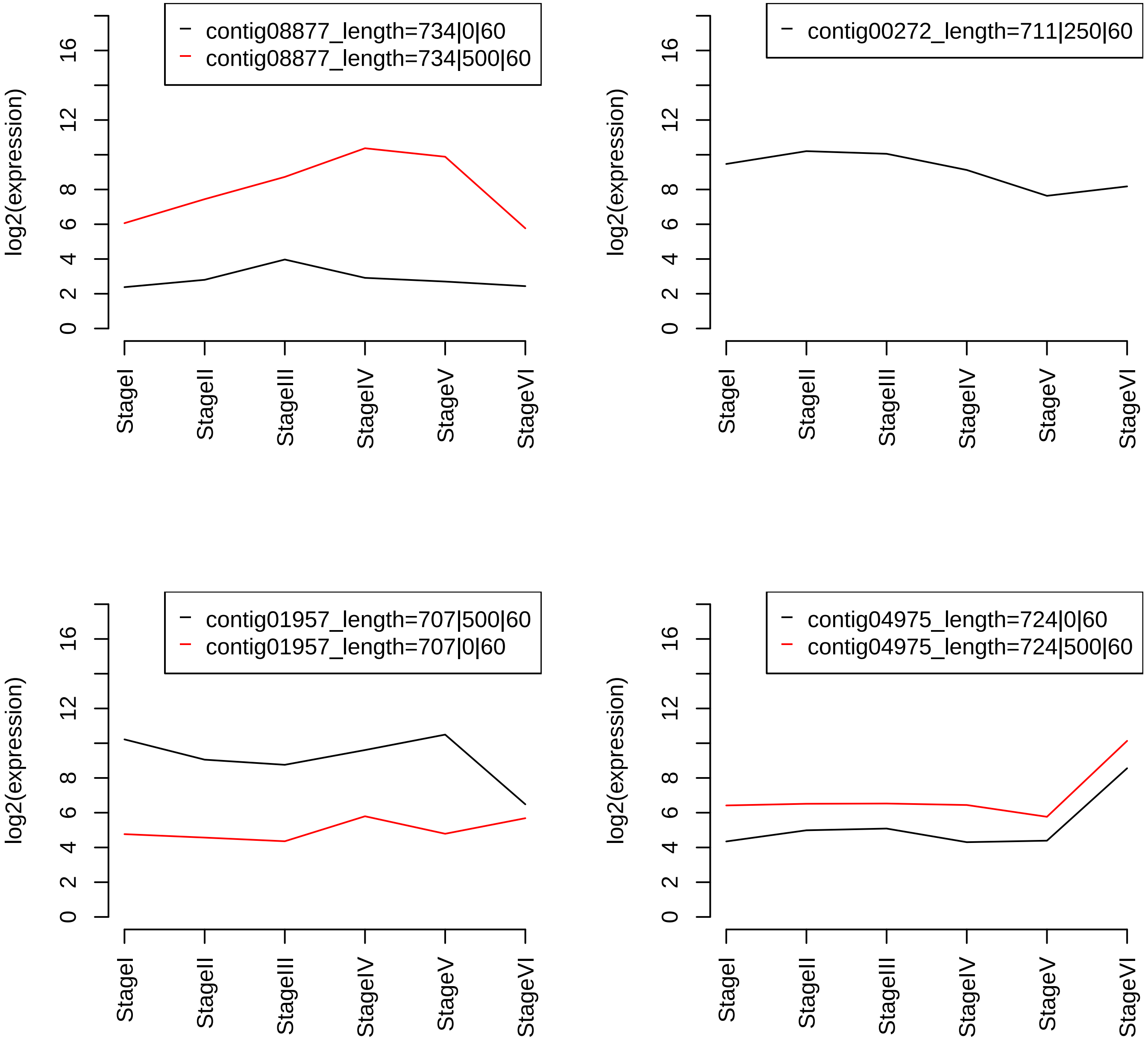
contig08877_length=734|0|60
contig08877_length=734|500|60
contig00272_length=711|250|60
contig01957_length=707|500|60
contig01957_length=707|0|60
contig04975_length=724|0|60
contig04975_length=724|500|60
log2(expression)
StageI
StageII
StageIII
StageIV
StageV
StageVI

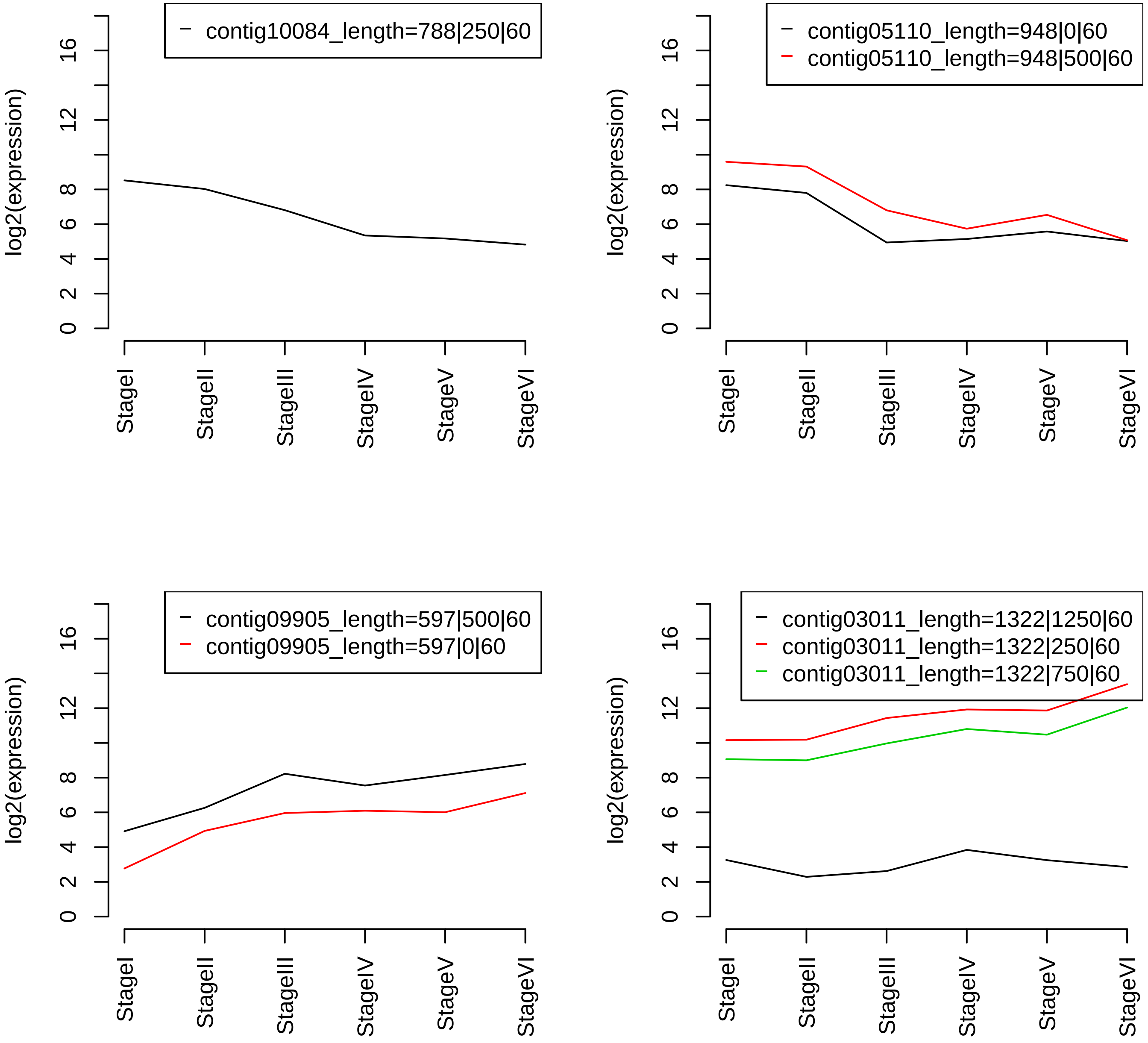

contig10084_length=788|250|60
contig05110_length=948|0|60
contig05110_length=948|500|60
contig09905_length=597|500|60
contig09905_length=597|0|60
contig03011_length=1322|1250|60
contig03011_length=1322|250|60
contig03011_length=1322|750|60
log2(expression)
0
2
4
6
8
12
16
StageI
StageII
StageIII
StageIV
StageV
StageVI

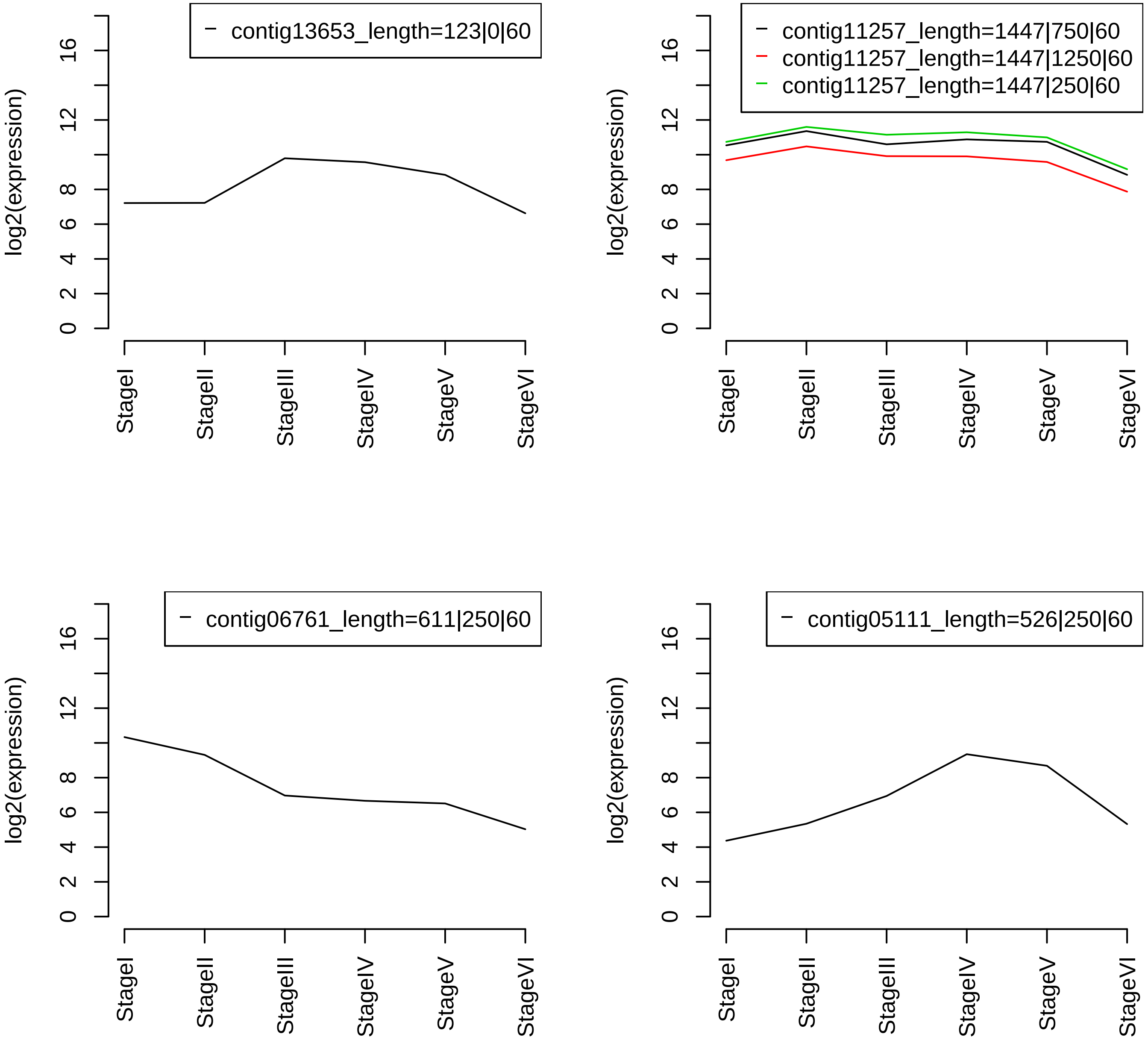
contig13653_length=123|0|60
contig11257_length=1447|750|60
contig11257_length=1447|1250|60
contig11257_length=1447|250|60
contig06761_length=611|250|60
contig05111_length=526|250|60
log2(expression)
0 2 4 6 8 12 16
StageI
StageII
StageIII
StageIV
StageV
StageVI

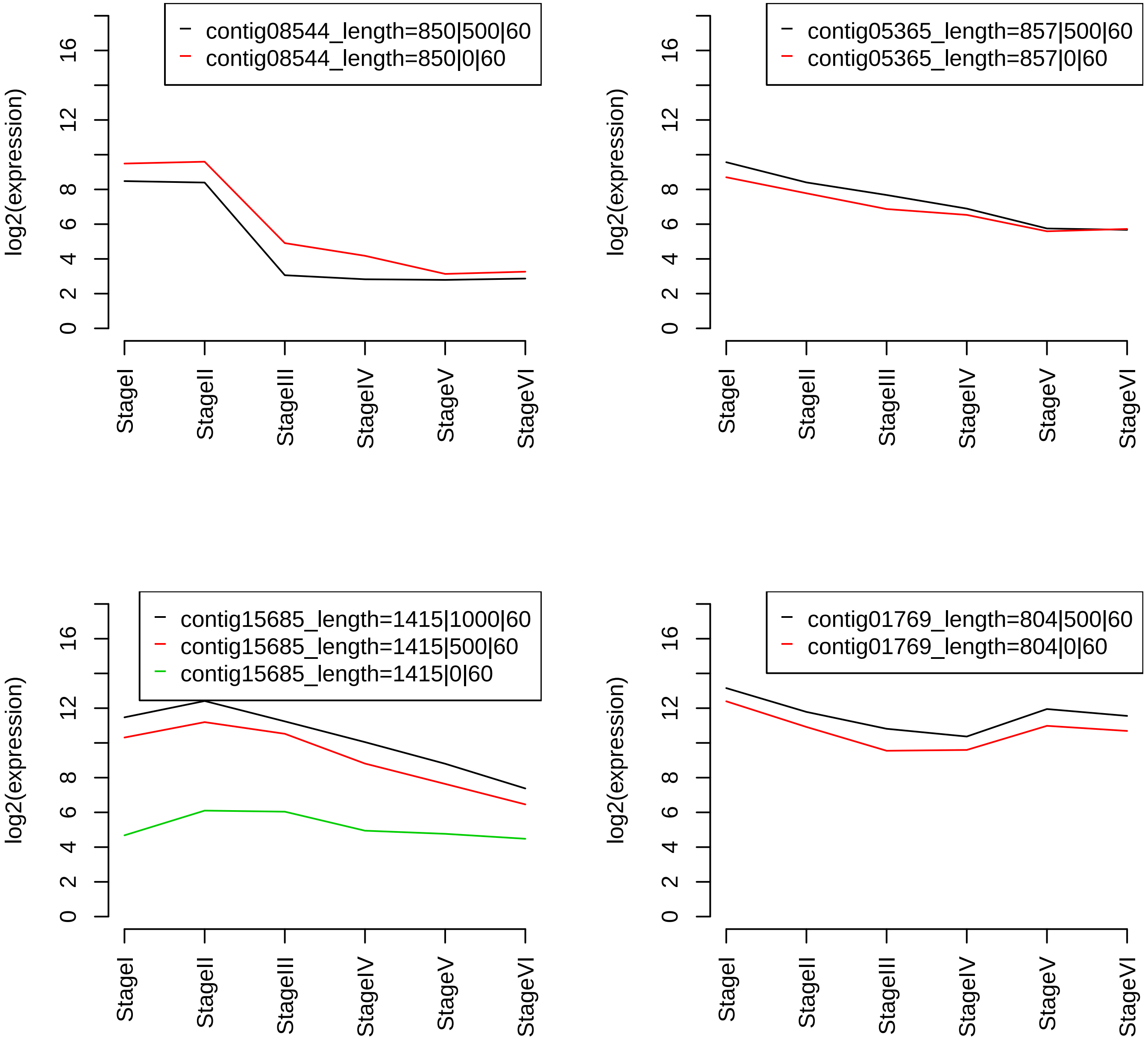
contig08544_length=850|500|60
contig08544_length=850|0|60
contig05365_length=857|500|60
contig05365_length=857|0|60
contig15685_length=1415|1000|60
contig15685_length=1415|500|60
contig15685_length=1415|0|60
contig01769_length=804|500|60
contig01769_length=804|0|60
log2(expression)
0
2
4
6
8
12
16
StageI
StageII
StageIII
StageIV
StageV
StageVI

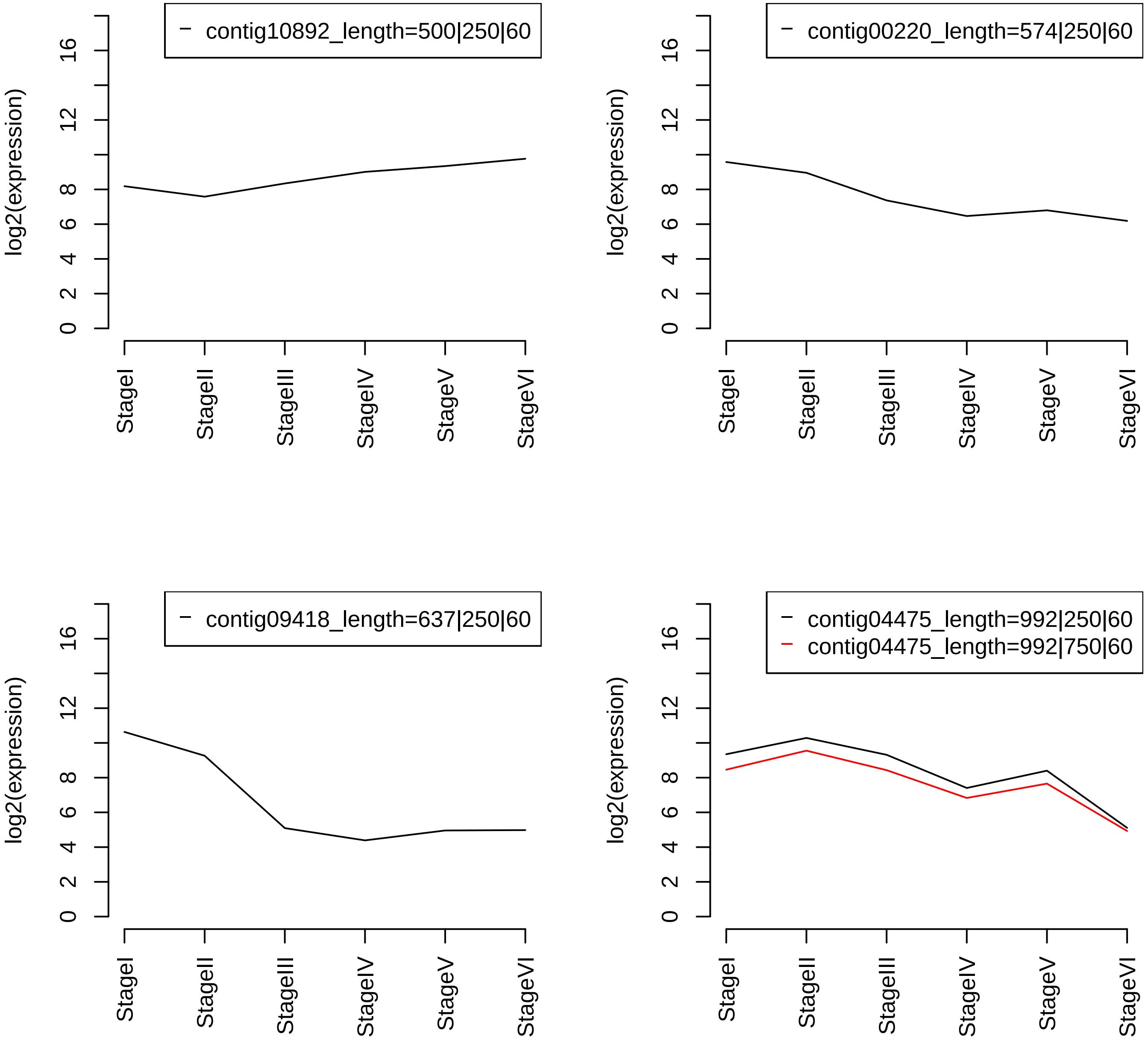

contig10892_length=500|250|60
contig00220_length=574|250|60
contig09418_length=637|250|60
contig04475_length=992|250|60
contig04475_length=992|750|60
log2(expression)
0
2
4
6
8
12
16
StageI
StageII
StageIII
StageIV
StageV
StageVI

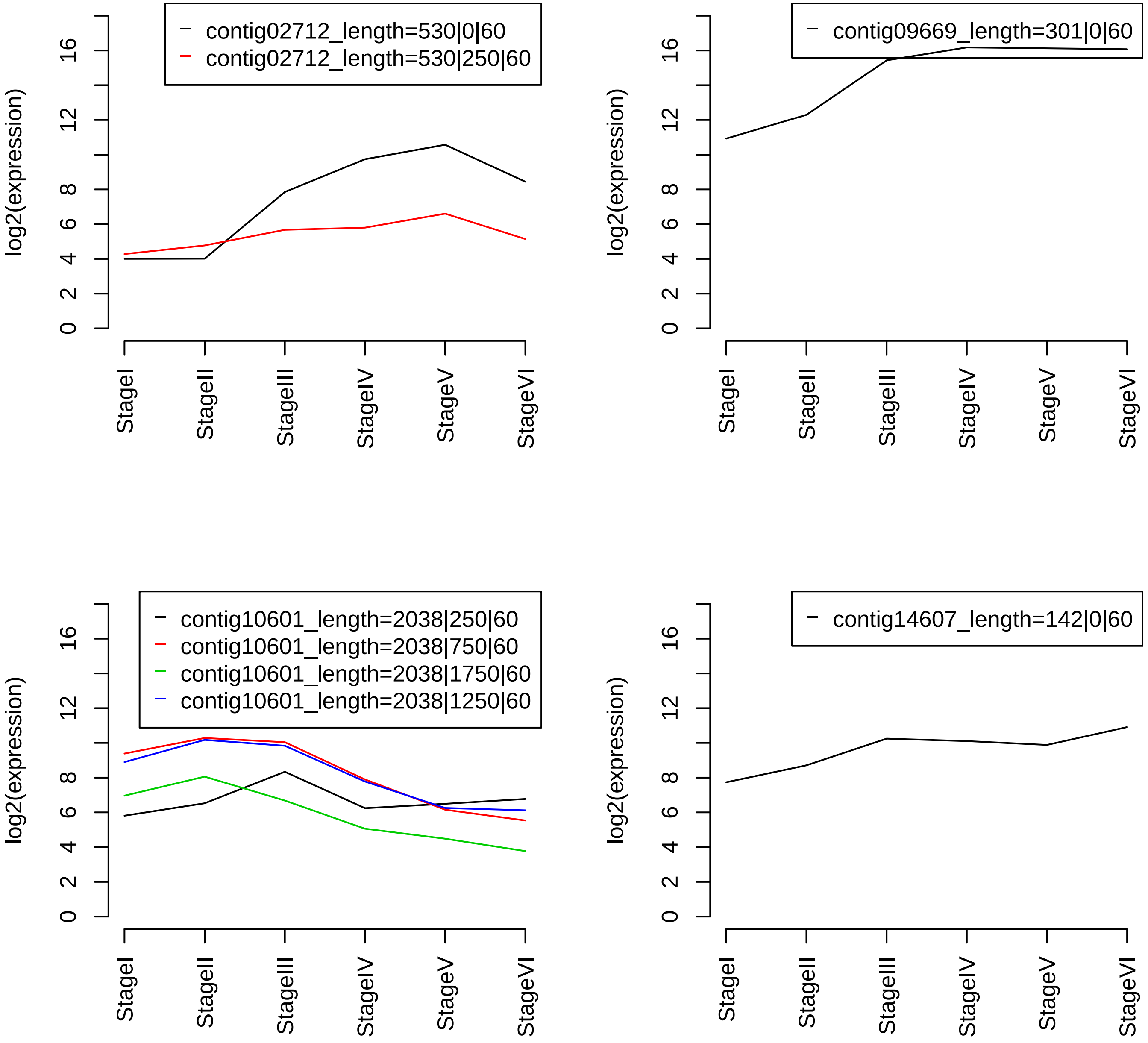
contig02712_length=530|0|60
contig02712_length=530|250|60
contig09669_length=301|0|60
contig10601_length=2038|250|60
contig10601_length=2038|750|60
contig10601_length=2038|1750|60
contig10601_length=2038|1250|60
contig14607_length=142|0|60
log2(expression)
0
2
4
6
8
12
16
StageI
StageII
StageIII
StageIV
StageV
StageVI

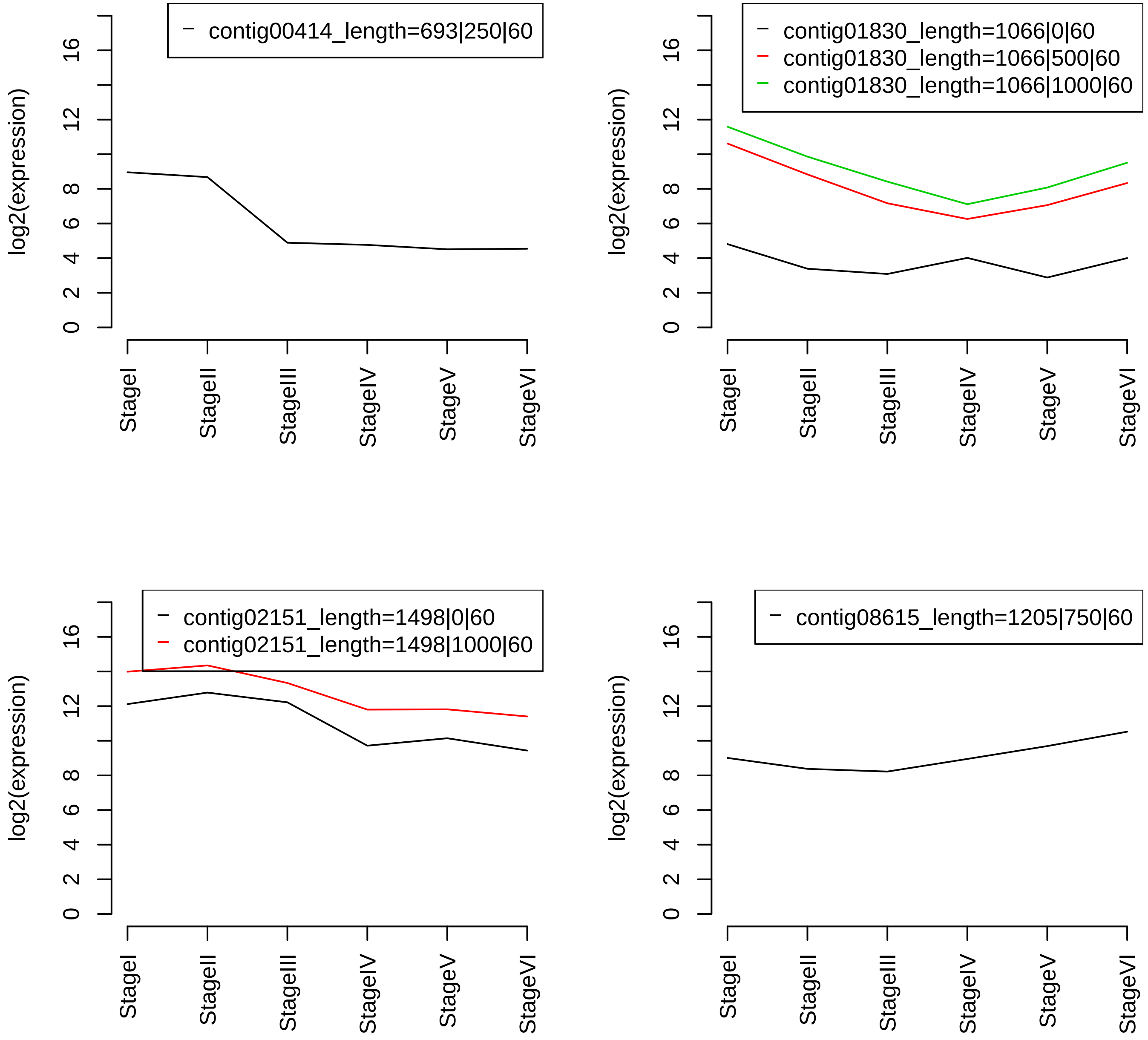
contig00414_length=693|250|60
contig01830_length=1066|0|60
contig01830_length=1066|500|60
contig01830_length=1066|1000|60
contig02151_length=1498|0|60
contig02151_length=1498|1000|60
contig08615_length=1205|750|60
log2(expression)
0
2
4
6
8
12
16
StageI
StageII
StageIII
StageIV
StageV
StageVI

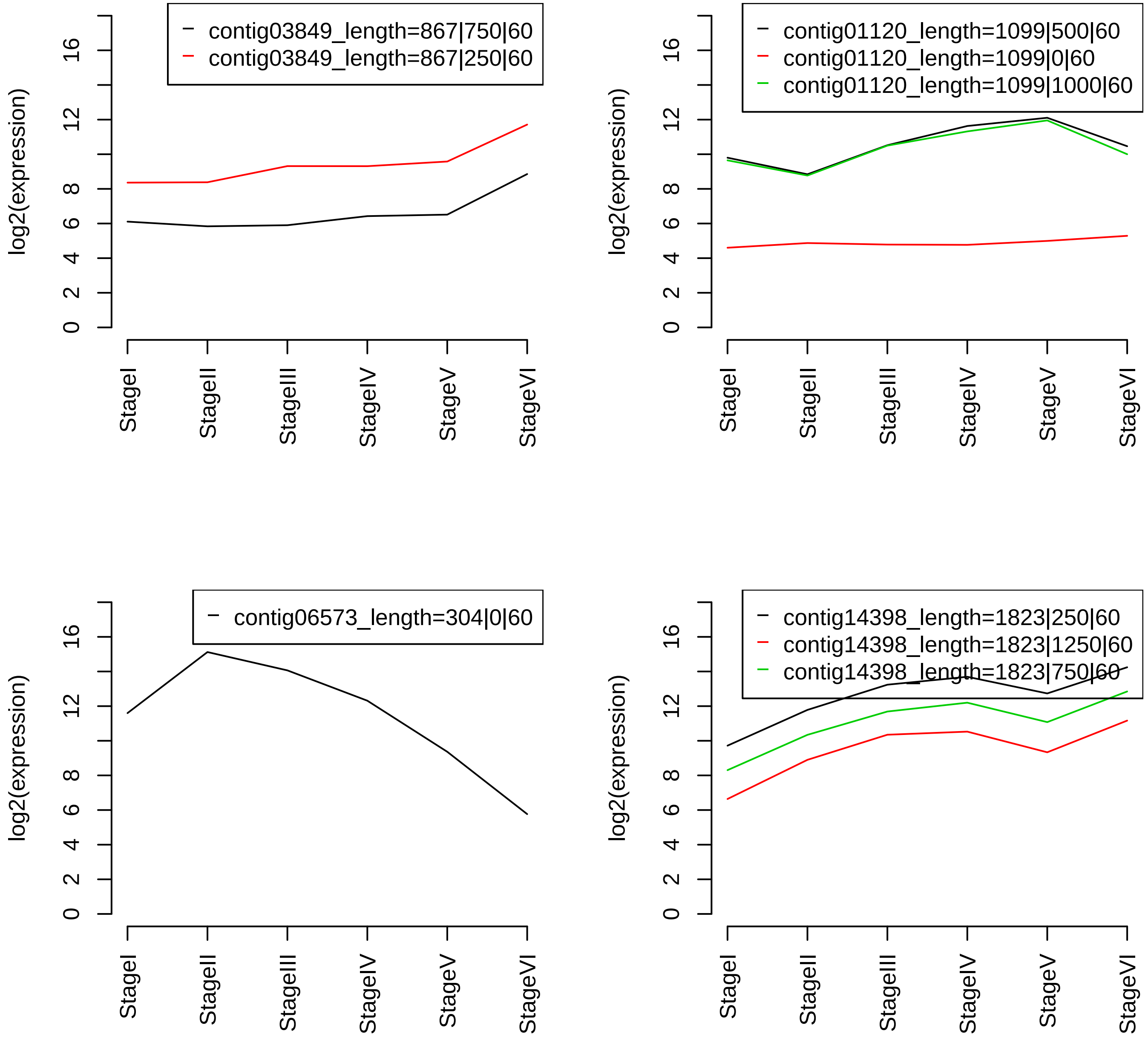
contig03849_length=867|750|60
contig03849_length=867|250|60
log2(expression)
0 2 4 6 8 12 16
StageI StageII StageIII StageIV StageV StageVI
contig01120_length=1099|500|60
contig01120_length=1099|0|60
contig01120_length=1099|1000|60
log2(expression)
0 2 4 6 8 12 16
StageI StageII StageIII StageIV StageV StageVI
contig06573_length=304|0|60
log2(expression)
0 2 4 6 8 12 16
StageI StageII StageIII StageIV StageV StageVI
contig14398_length=1823|250|60
contig14398_length=1823|1250|60
contig14398_length=1823|750|60
log2(expression)
0 2 4 6 8 12 16
StageI StageII StageIII StageIV StageV StageVI

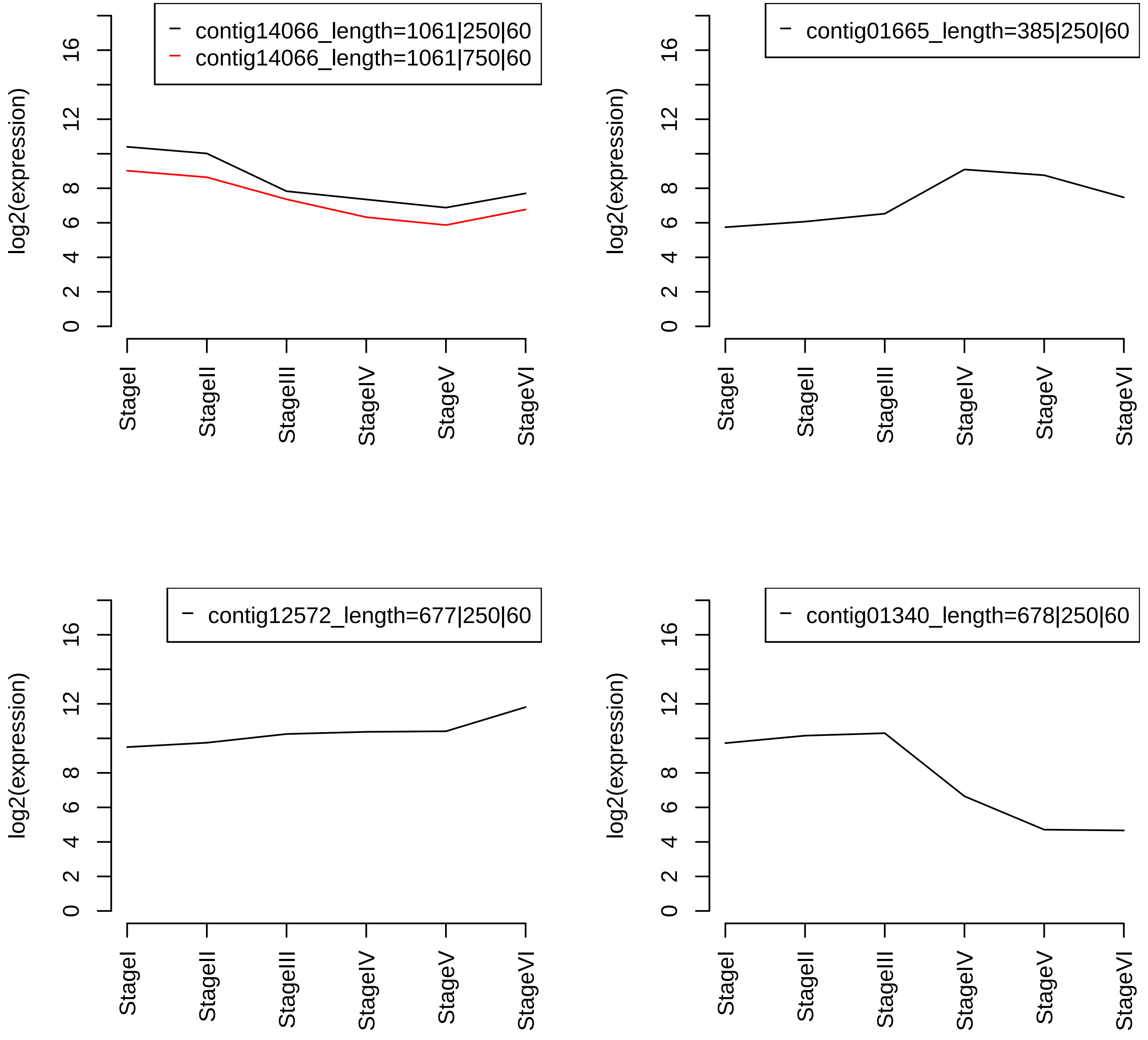
contig14066_length=1061|250|60
contig14066_length=1061|750|60
log2(expression)
0
2
4
6
8
12
16
StageI
StageII
StageIII
StageIV
StageV
StageVI
contig01665_length=385|250|60
contig12572_length=677|250|60
contig01340_length=678|250|60

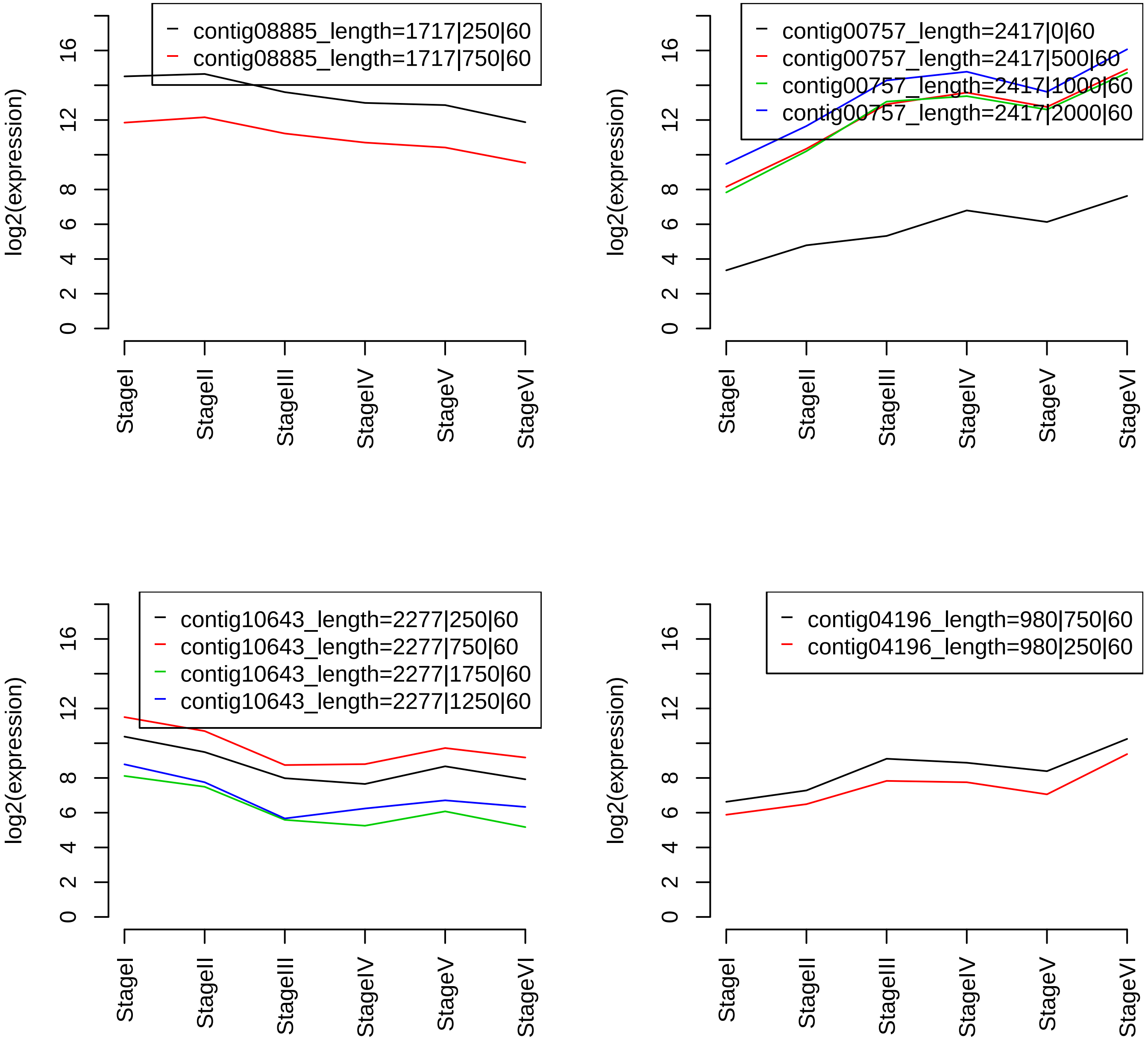
contig08885_length=1717|250|60
contig08885_length=1717|750|60
contig00757_length=2417|0|60
contig00757_length=2417|500|60
contig00757_length=2417|1000|60
contig00757_length=2417|2000|60
contig10643_length=2277|250|60
contig10643_length=2277|750|60
contig10643_length=2277|1750|60
contig10643_length=2277|1250|60
contig04196_length=980|750|60
contig04196_length=980|250|60
log2(expression)
0
2
4
6
8
12
16
StageI
StageII
StageIII
StageIV
StageV
StageVI

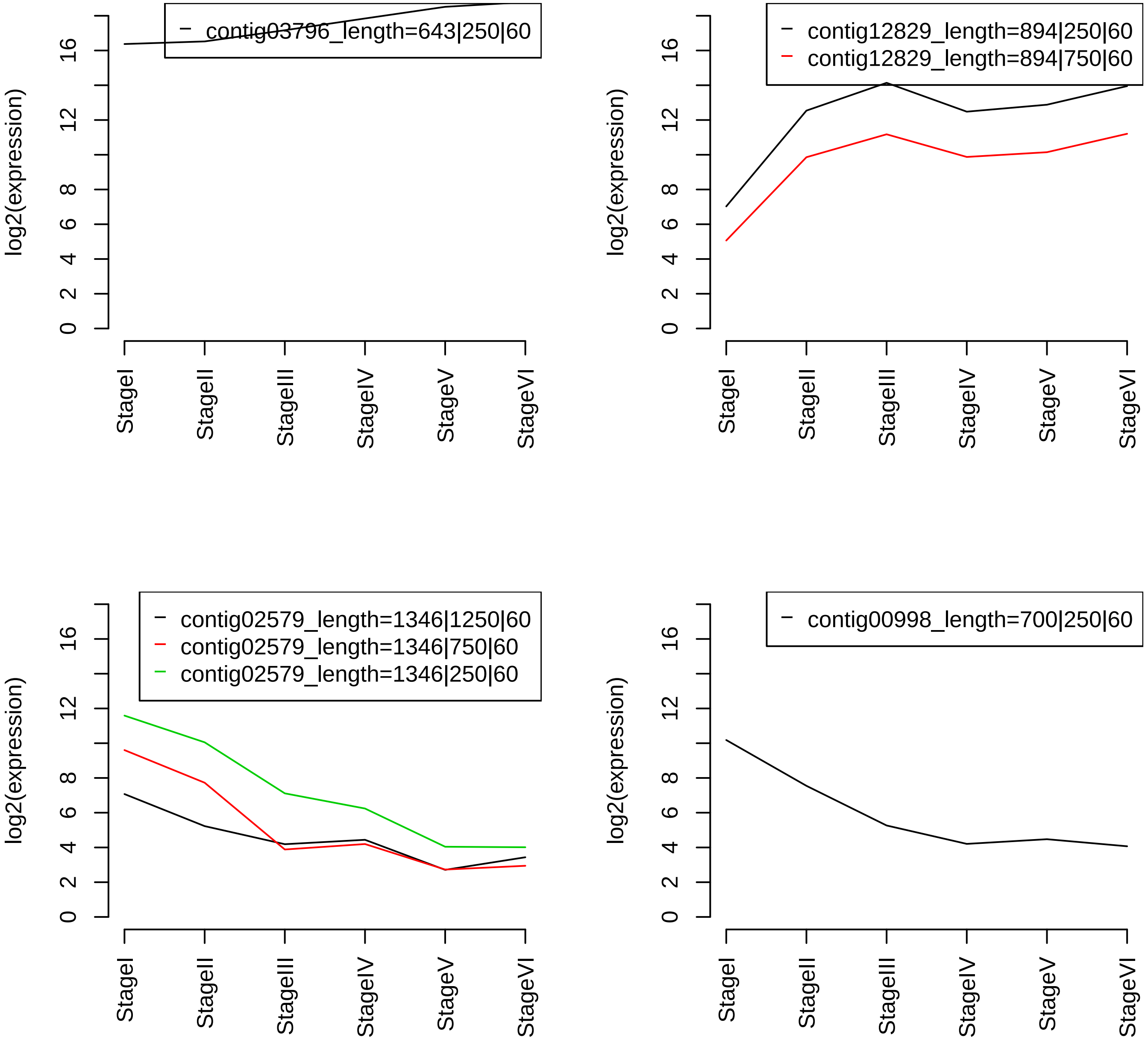
contig03796_length=643|250|60
contig12829_length=894|250|60
contig12829_length=894|750|60
contig02579_length=1346|1250|60
contig02579_length=1346|750|60
contig02579_length=1346|250|60
contig00998_length=700|250|60
log2(expression)
0 2 4 6 8 12 16
StageI StageII StageIII StageIV StageV StageVI

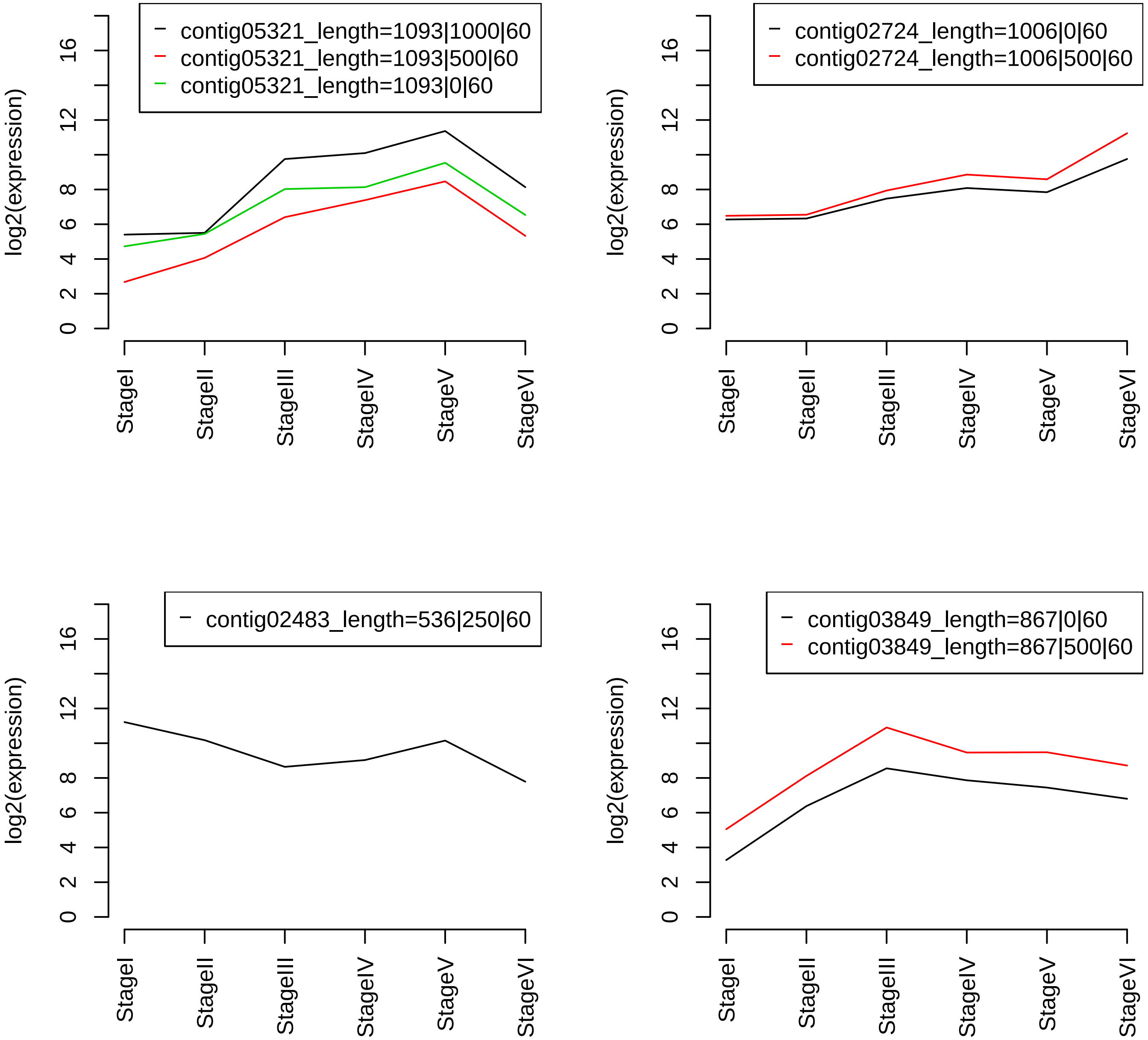

contig05321_length=1093|1000|60
contig05321_length=1093|500|60
contig05321_length=1093|0|60
contig02724_length=1006|0|60
contig02724_length=1006|500|60
contig02483_length=536|250|60
contig03849_length=867|0|60
contig03849_length=867|500|60
log2(expression)
0
2
4
6
8
12
16
StageI
StageII
StageIII
StageIV
StageV
StageVI

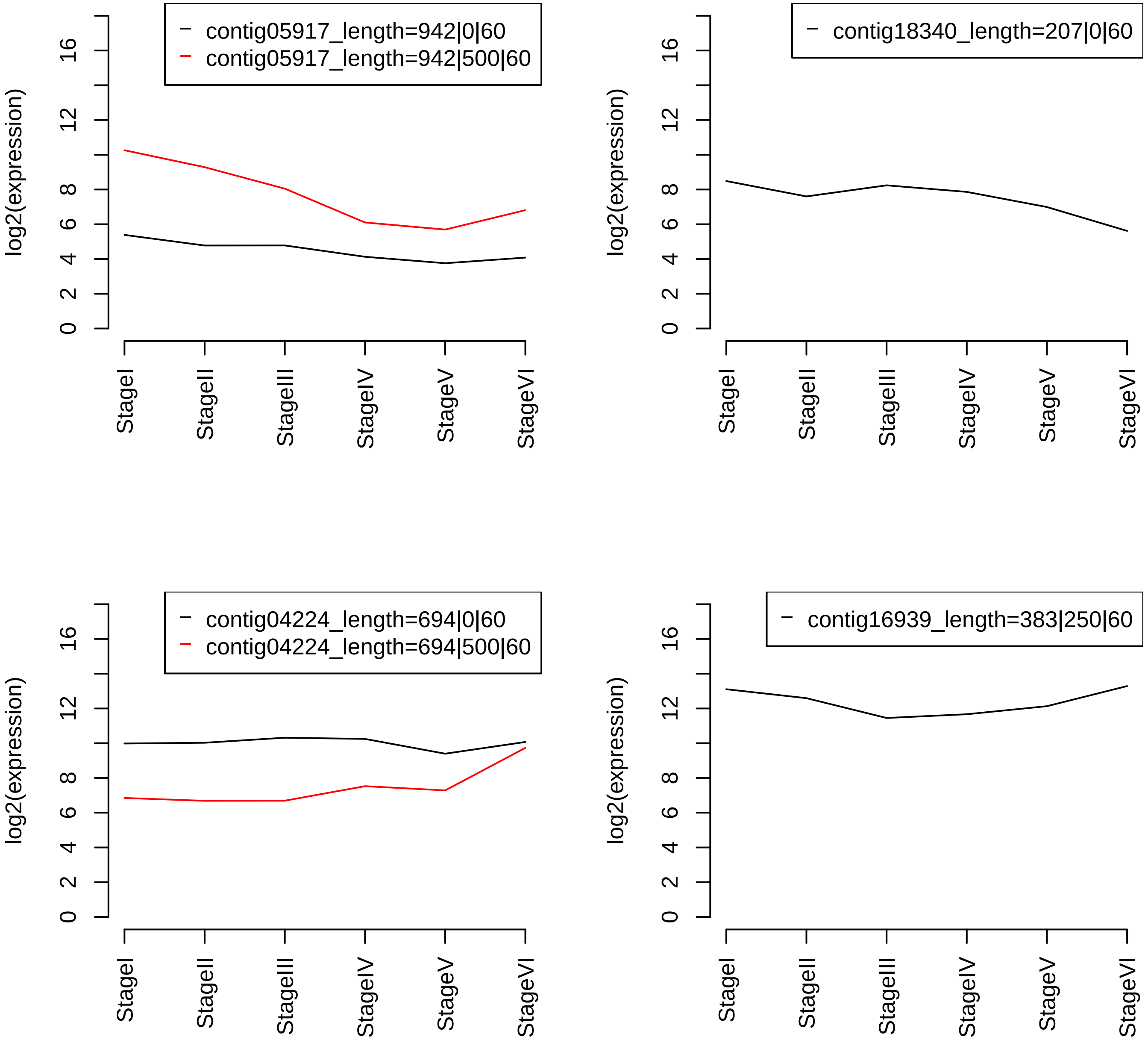

contig05917_length=942|0|60
contig05917_length=942|500|60
contig18340_length=207|0|60
contig04224_length=694|0|60
contig04224_length=694|500|60
contig16939_length=383|250|60
log2(expression)
0 2 4 6 8 12 16
StageI StageII StageIII StageIV StageV StageVI

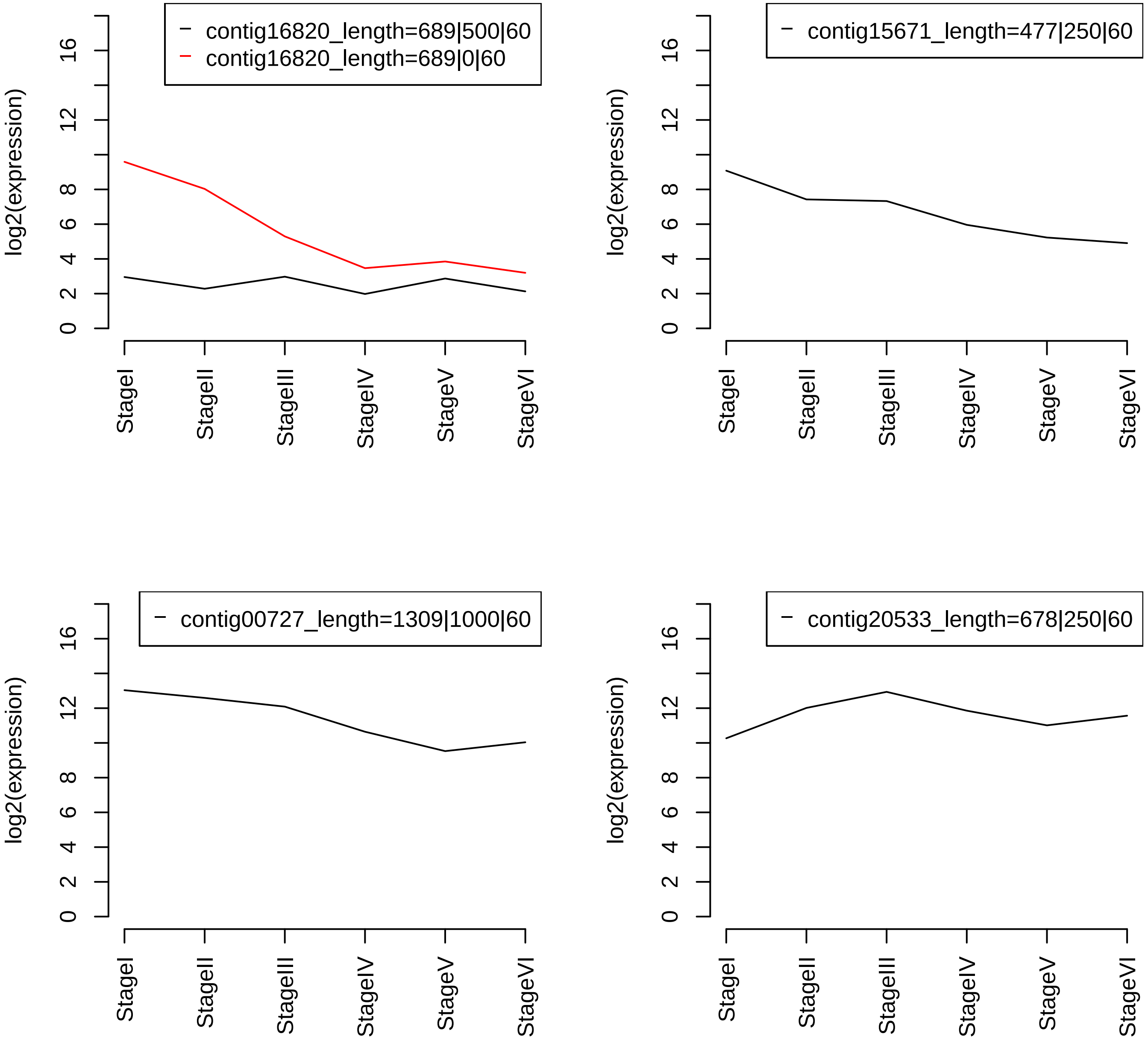
contig16820_length=689|500|60
contig16820_length=689|0|60
contig15671_length=477|250|60
contig00727_length=1309|1000|60
contig20533_length=678|250|60
log2(expression)
0
2
4
6
8
12
16
StageI
StageII
StageIII
StageIV
StageV
StageVI

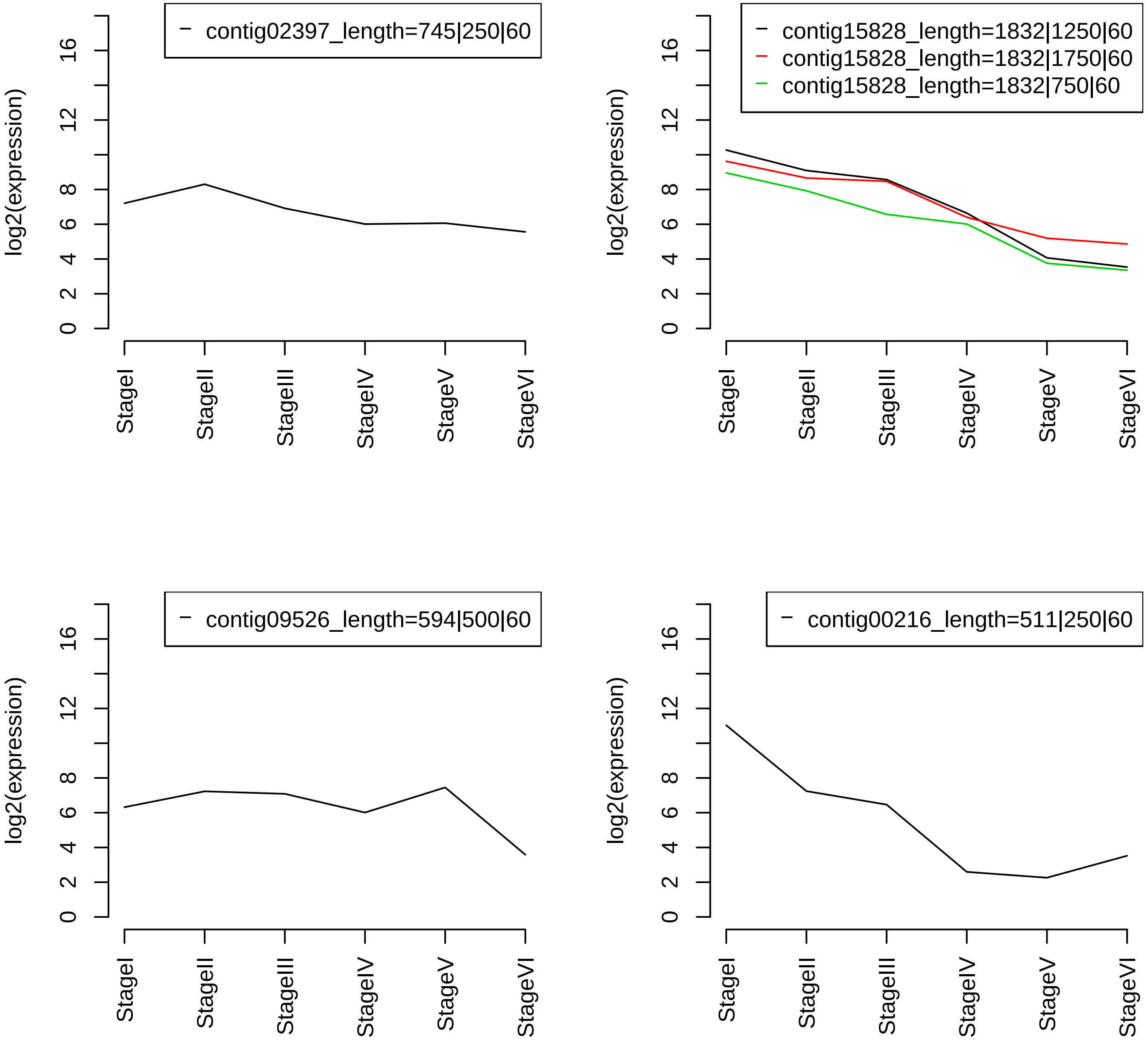

contig02397_length=745|250|60
contig15828_length=1832|1250|60
contig15828_length=1832|1750|60
contig15828_length=1832|750|60
contig09526_length=594|500|60
contig00216_length=511|250|60
log2(expression)
0
2
4
6
8
12
16
StageI
StageII
StageIII
StageIV
StageV
StageVI

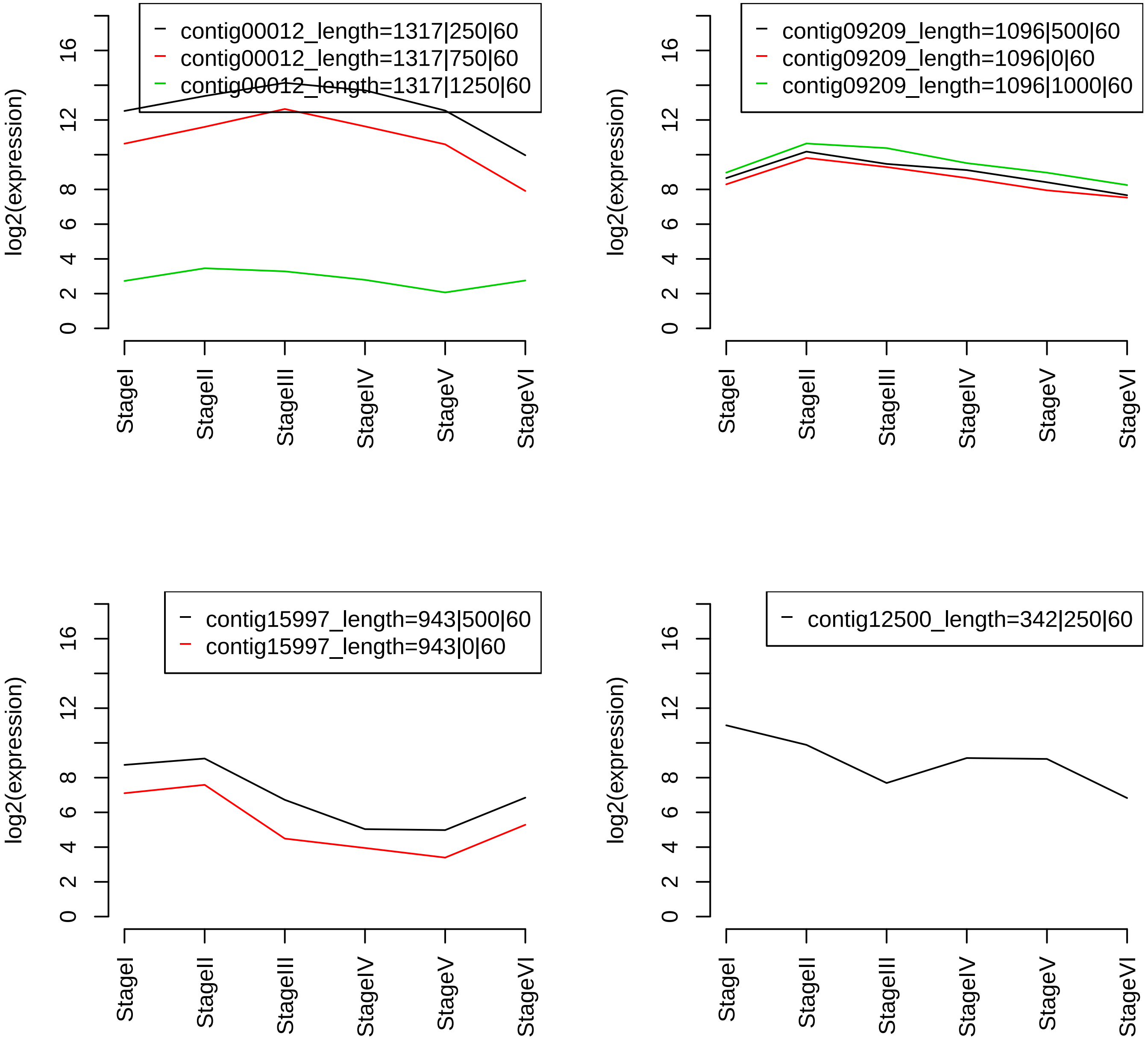

contig00012_length=1317|250|60
contig00012_length=1317|750|60
contig00012_length=1317|1250|60
contig09209_length=1096|500|60
contig09209_length=1096|0|60
contig09209_length=1096|1000|60
contig15997_length=943|500|60
contig15997_length=943|0|60
contig12500_length=342|250|60
log2(expression)
0
2
4
6
8
12
16
StageI
StageII
StageIII
StageIV
StageV
StageVI

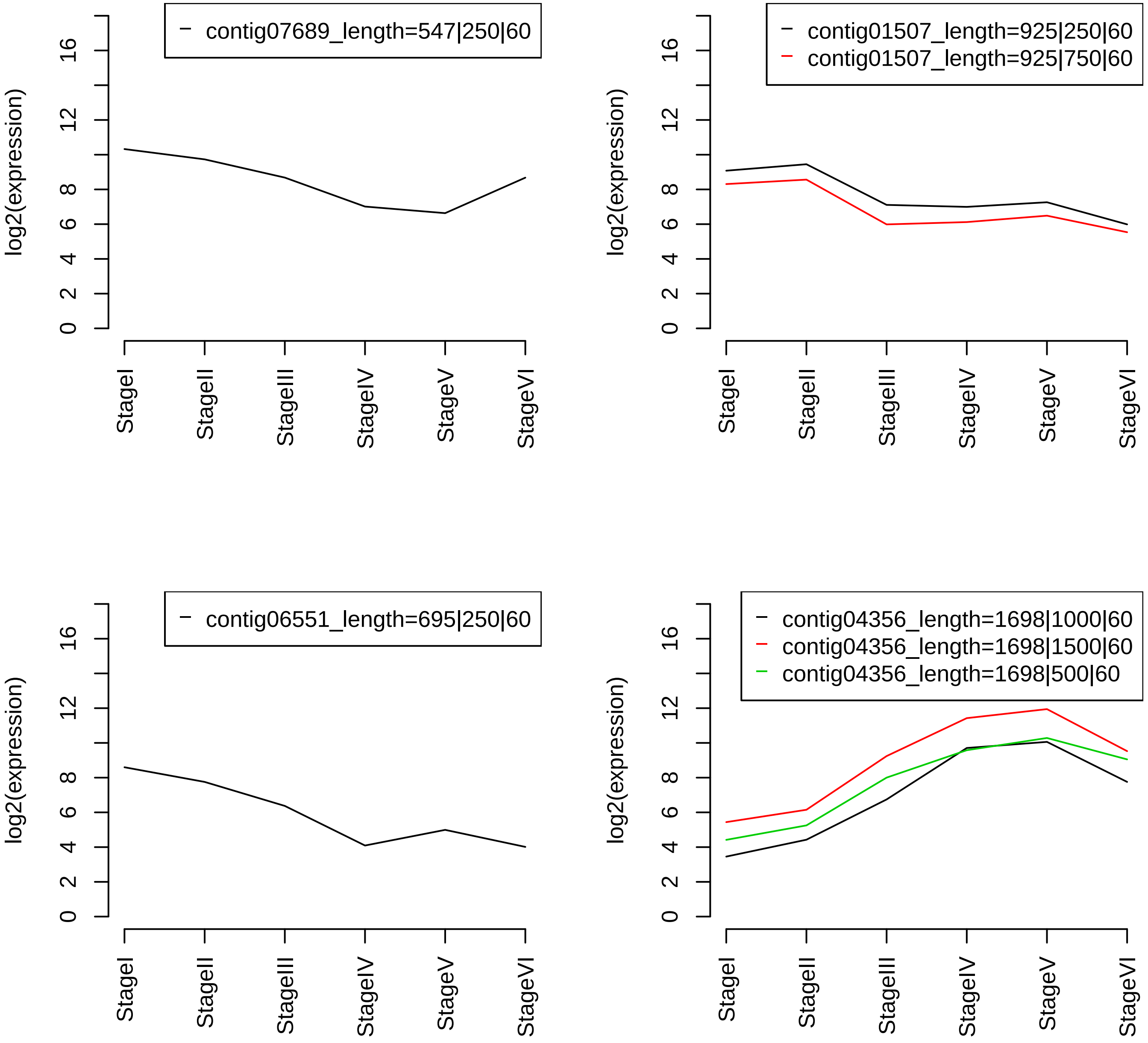

contig07689_length=547|250|60
contig01507_length=925|250|60
contig01507_length=925|750|60
contig06551_length=695|250|60
contig04356_length=1698|1000|60
contig04356_length=1698|1500|60
contig04356_length=1698|500|60
log2(expression)
0 2 4 6 8 12 16
StageI StageII StageIII StageIV StageV StageVI

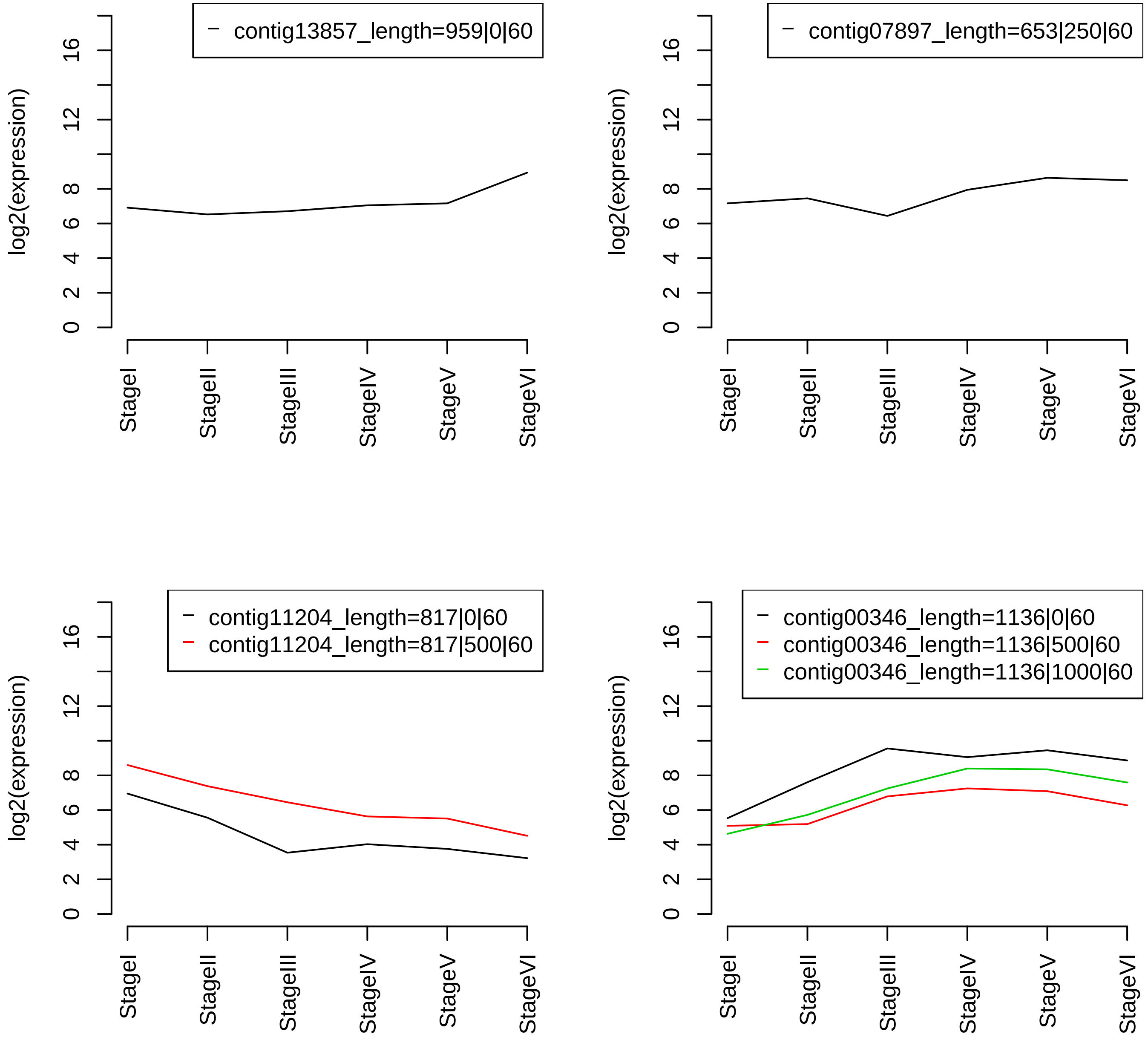

contig13857_length=959|0|60
contig07897_length=653|250|60
contig11204_length=817|0|60
contig11204_length=817|500|60
contig00346_length=1136|0|60
contig00346_length=1136|500|60
contig00346_length=1136|1000|60
log2(expression)
0
2
4
6
8
12
16
StageI
StageII
StageIII
StageIV
StageV
StageVI

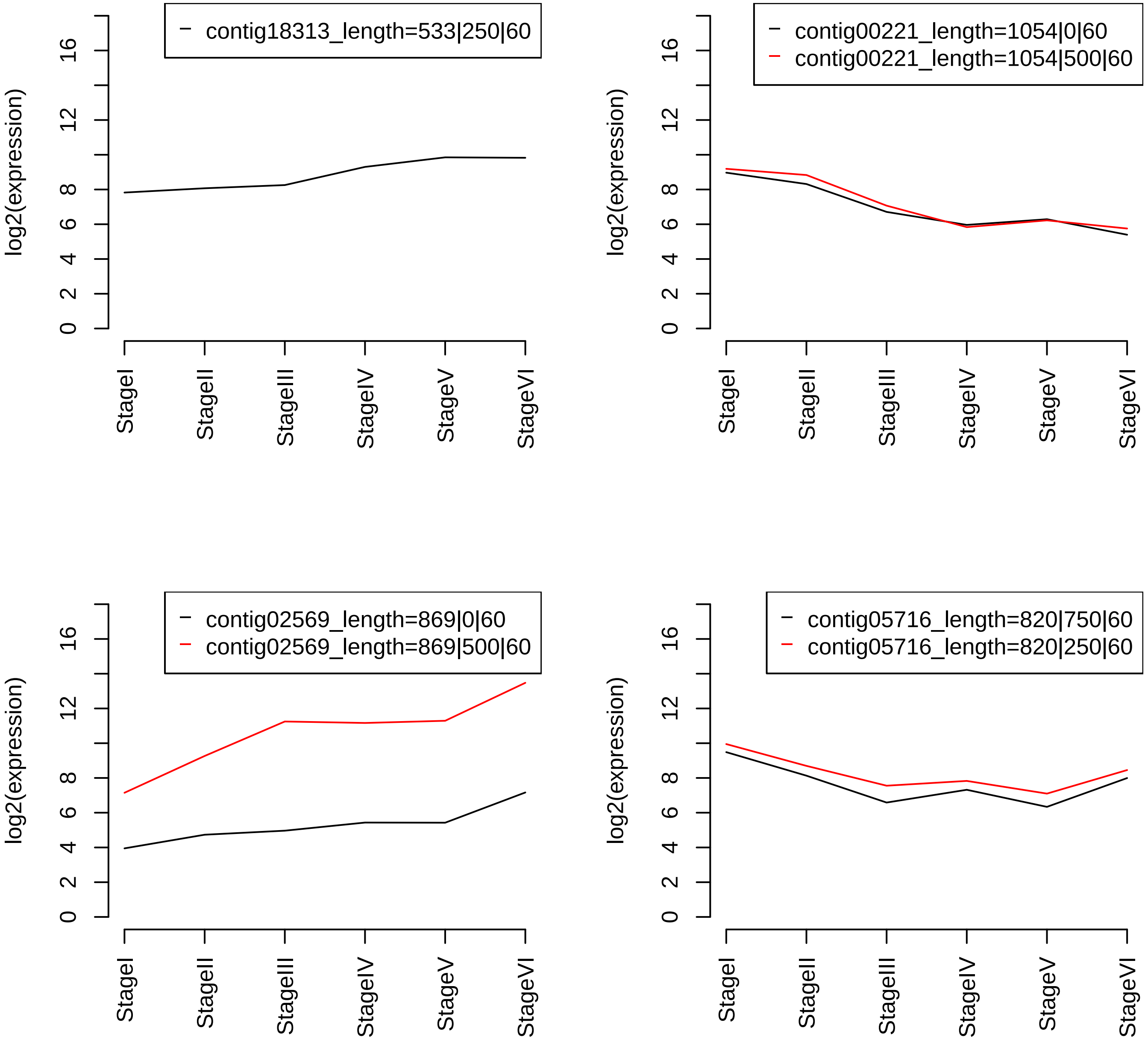
contig18313_length=533|250|60
contig00221_length=1054|0|60
contig00221_length=1054|500|60
contig02569_length=869|0|60
contig02569_length=869|500|60
contig05716_length=820|750|60
contig05716_length=820|250|60
log2(expression)
StageI
StageII
StageIII
StageIV
StageV
StageVI

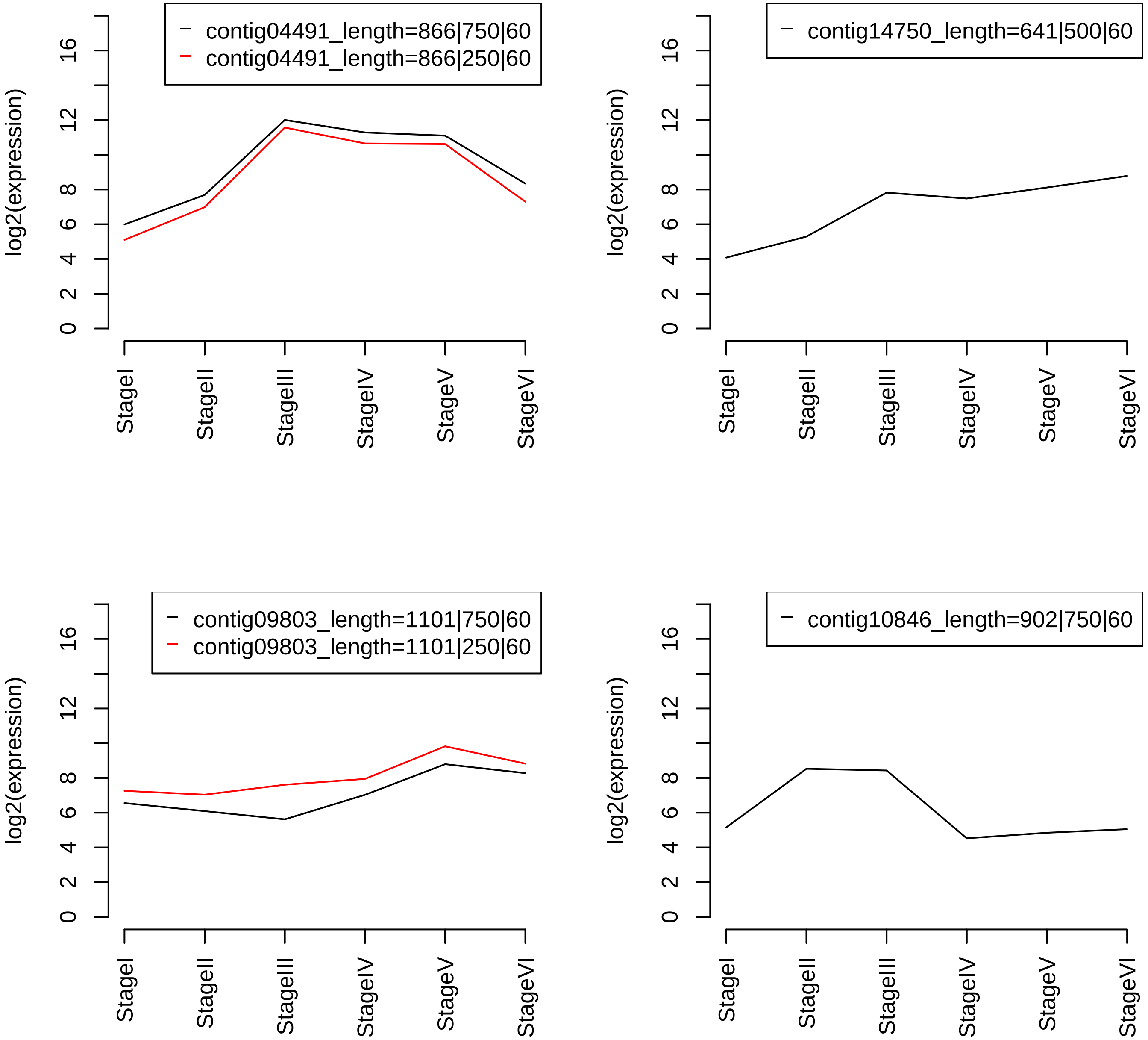
contig04491_length=866|750|60
contig04491_length=866|250|60
contig14750_length=641|500|60
contig09803_length=1101|750|60
contig09803_length=1101|250|60
contig10846_length=902|750|60
log2(expression)
0
2
4
6
8
12
16
StageI
StageII
StageIII
StageIV
StageV
StageVI

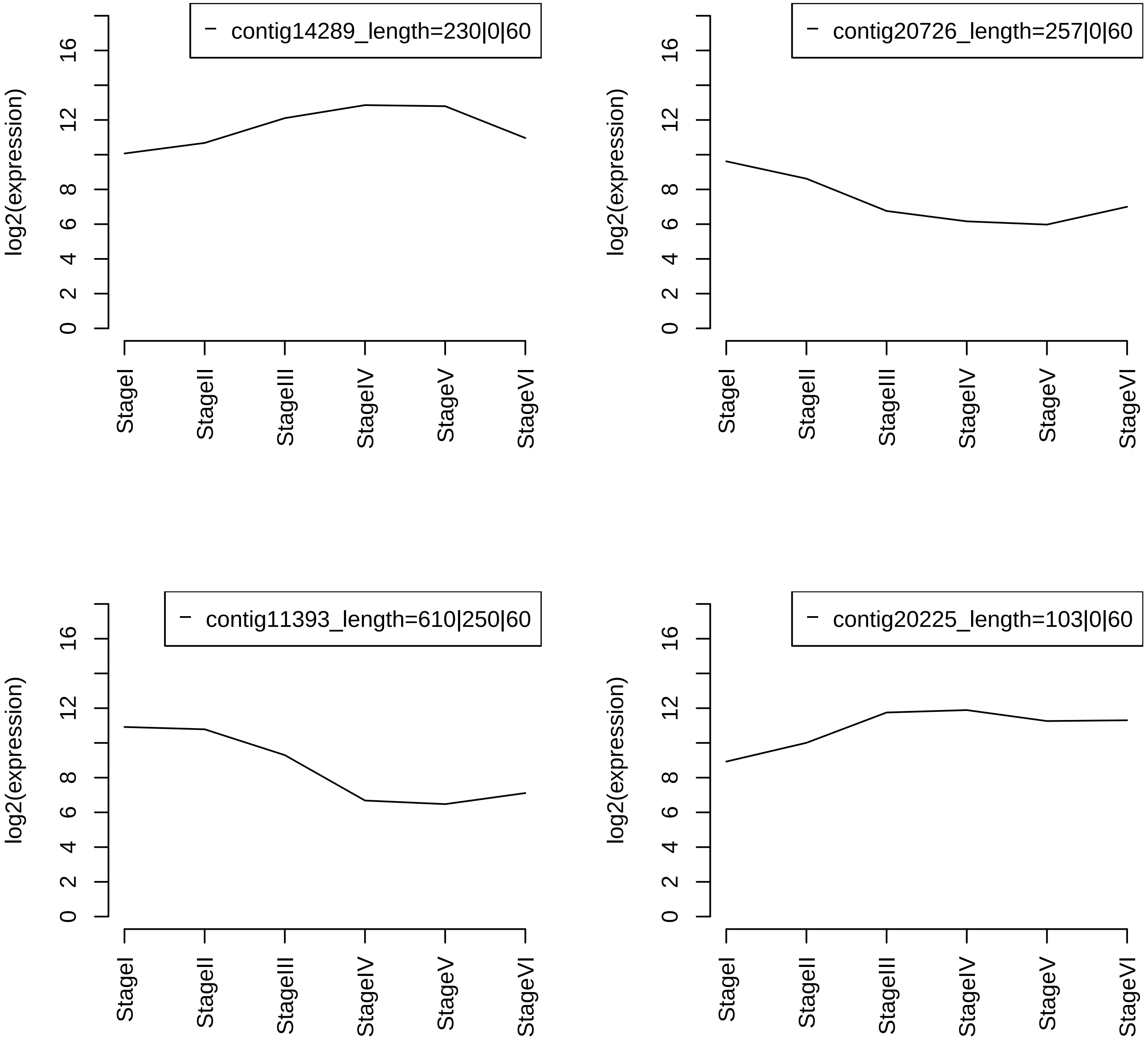
contig14289_length=230|0|60
contig20726_length=257|0|60
contig11393_length=610|250|60
contig20225_length=103|0|60
log2(expression)
0
2
4
6
8
12
16
StageI
StageII
StageIII
StageIV
StageV
StageVI

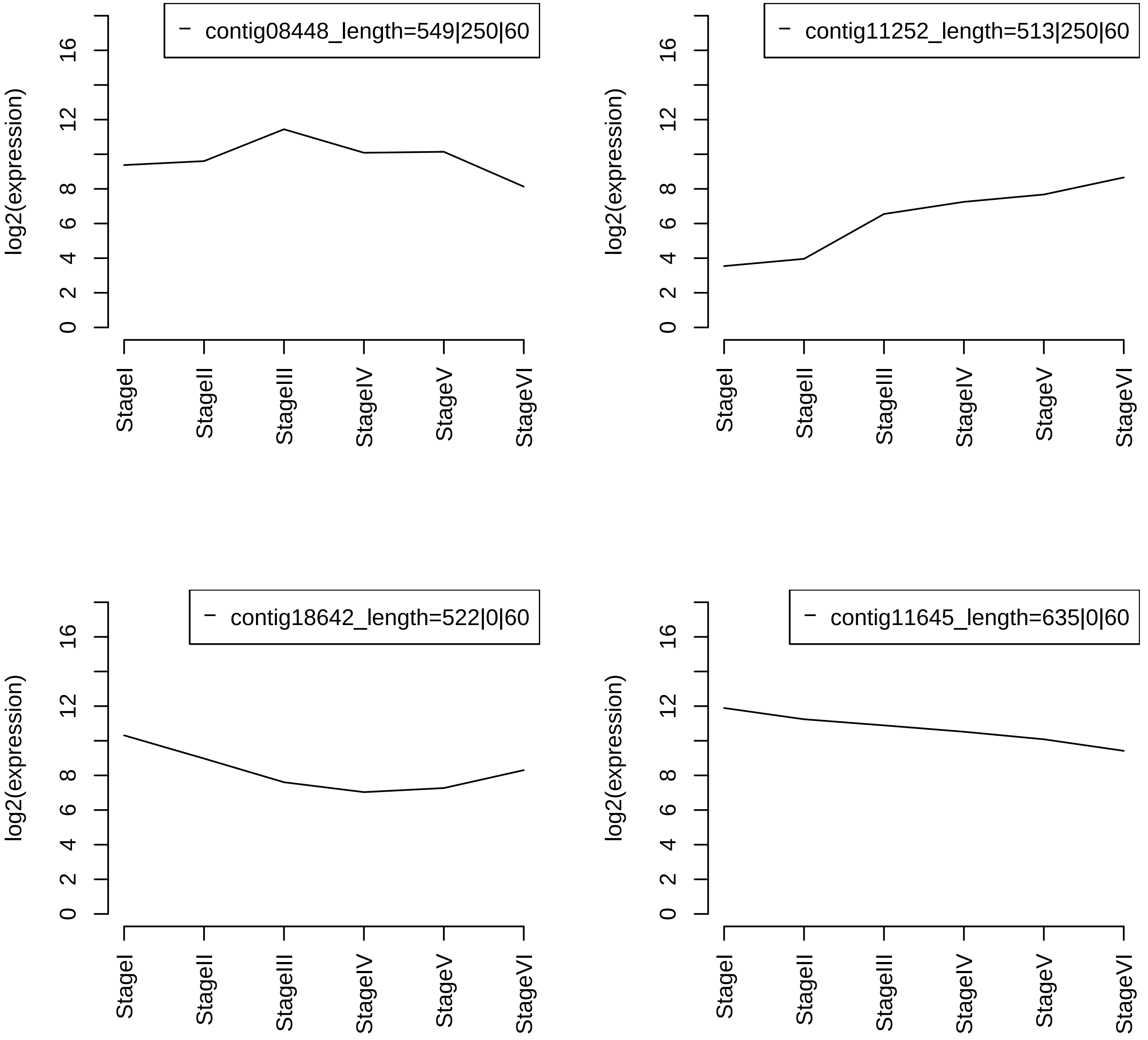
contig08448_length=549|250|60
contig11252_length=513|250|60
contig18642_length=522|0|60
contig11645_length=635|0|60
log2(expression)
0
2
4
6
8
12
16
StageI
StageII
StageIII
StageIV
StageV
StageVI

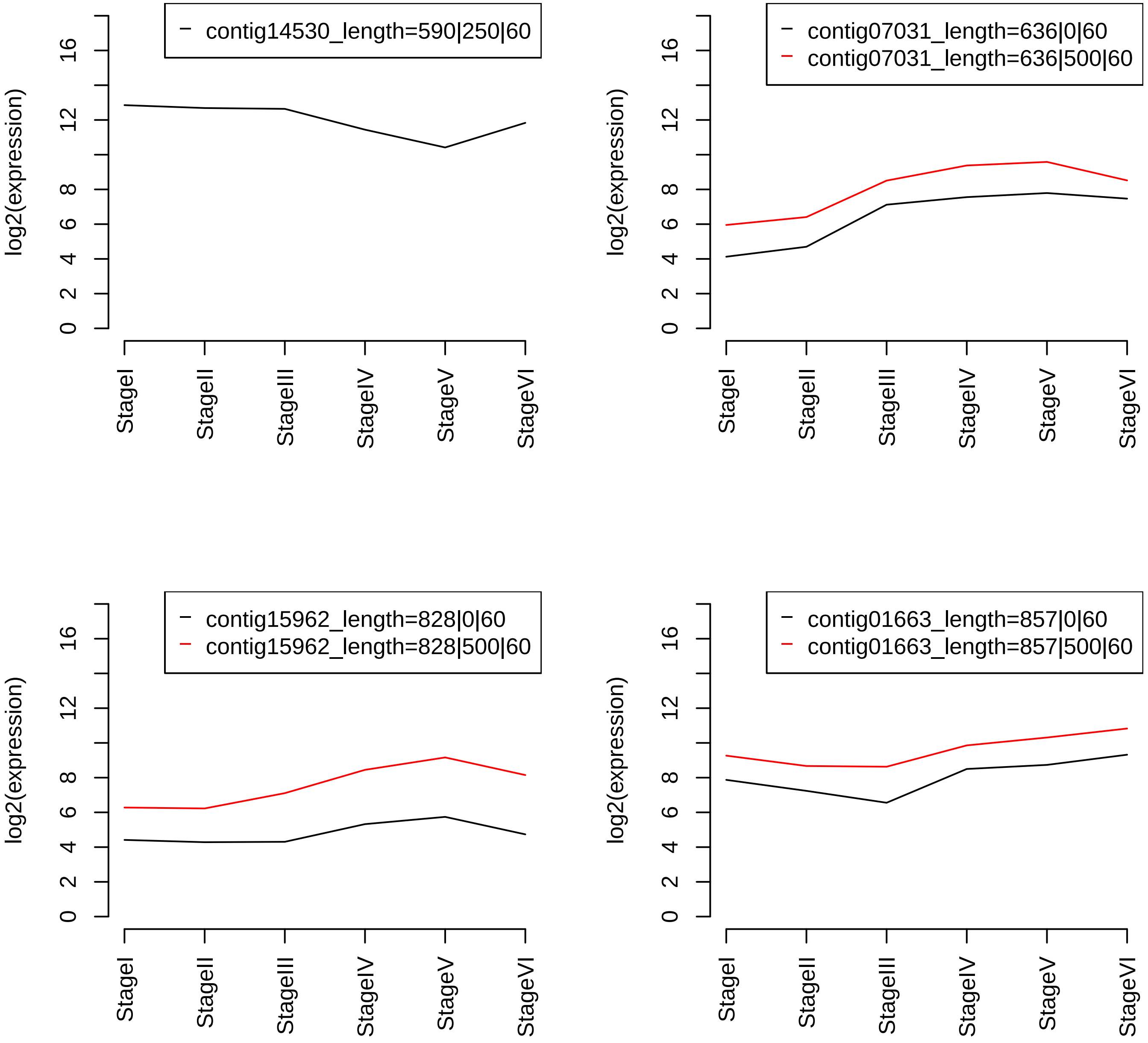

contig14530_length=590|250|60
contig07031_length=636|0|60
contig07031_length=636|500|60
contig15962_length=828|0|60
contig15962_length=828|500|60
contig01663_length=857|0|60
contig01663_length=857|500|60
log2(expression)
0 2 4 6 8 12 16
StageI StageII StageIII StageIV StageV StageVI

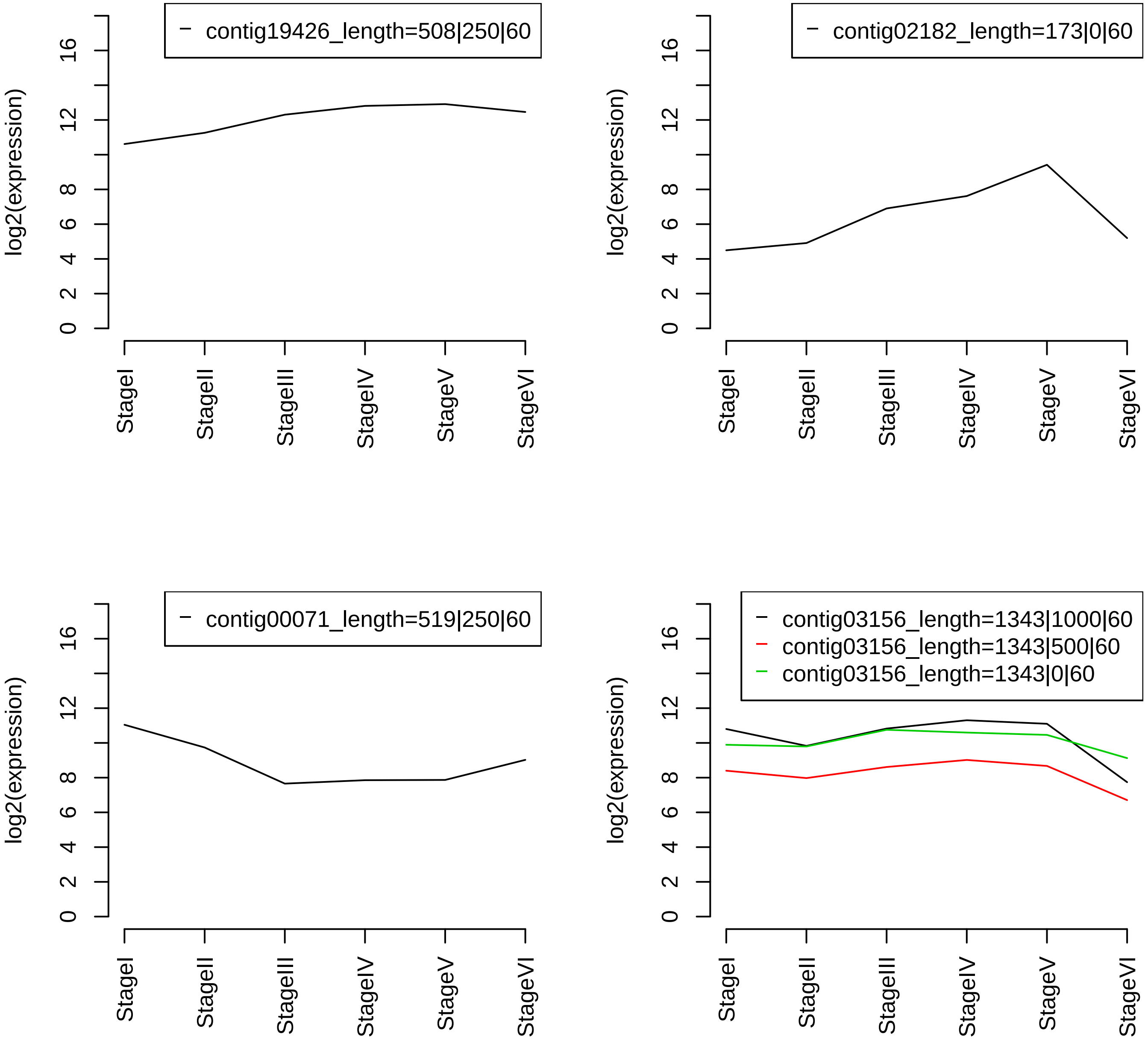
contig19426_length=508|250|60
contig02182_length=173|0|60
contig00071_length=519|250|60
contig03156_length=1343|1000|60
contig03156_length=1343|500|60
contig03156_length=1343|0|60
log2(expression)
0
2
4
6
8
12
16
StageI
StageII
StageIII
StageIV
StageV
StageVI

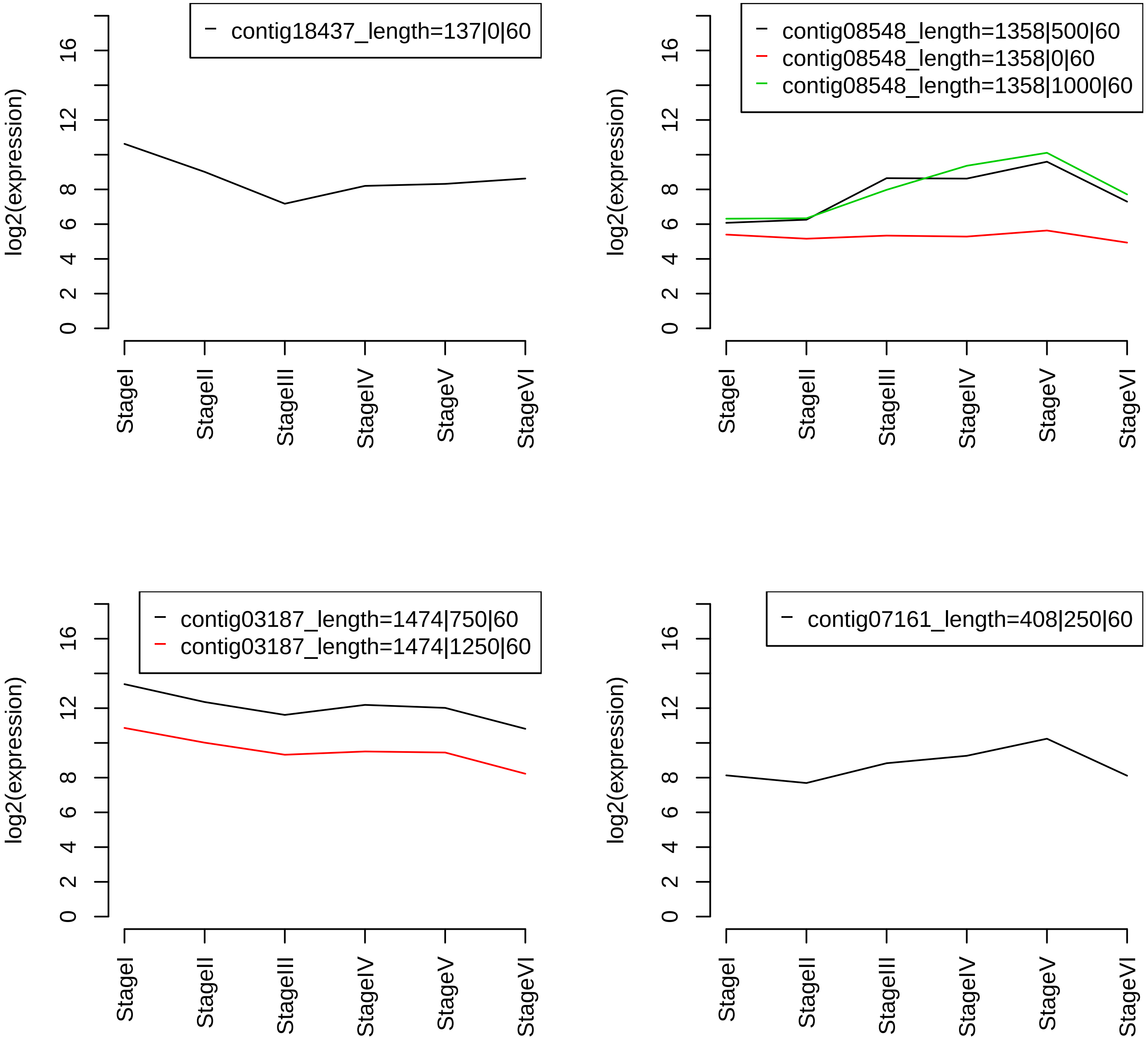
contig18437_length=137|0|60
contig08548_length=1358|500|60
contig08548_length=1358|0|60
contig08548_length=1358|1000|60
contig03187_length=1474|750|60
contig03187_length=1474|1250|60
contig07161_length=408|250|60
log2(expression)
0
2
4
6
8
12
16
StageI
StageII
StageIII
StageIV
StageV
StageVI

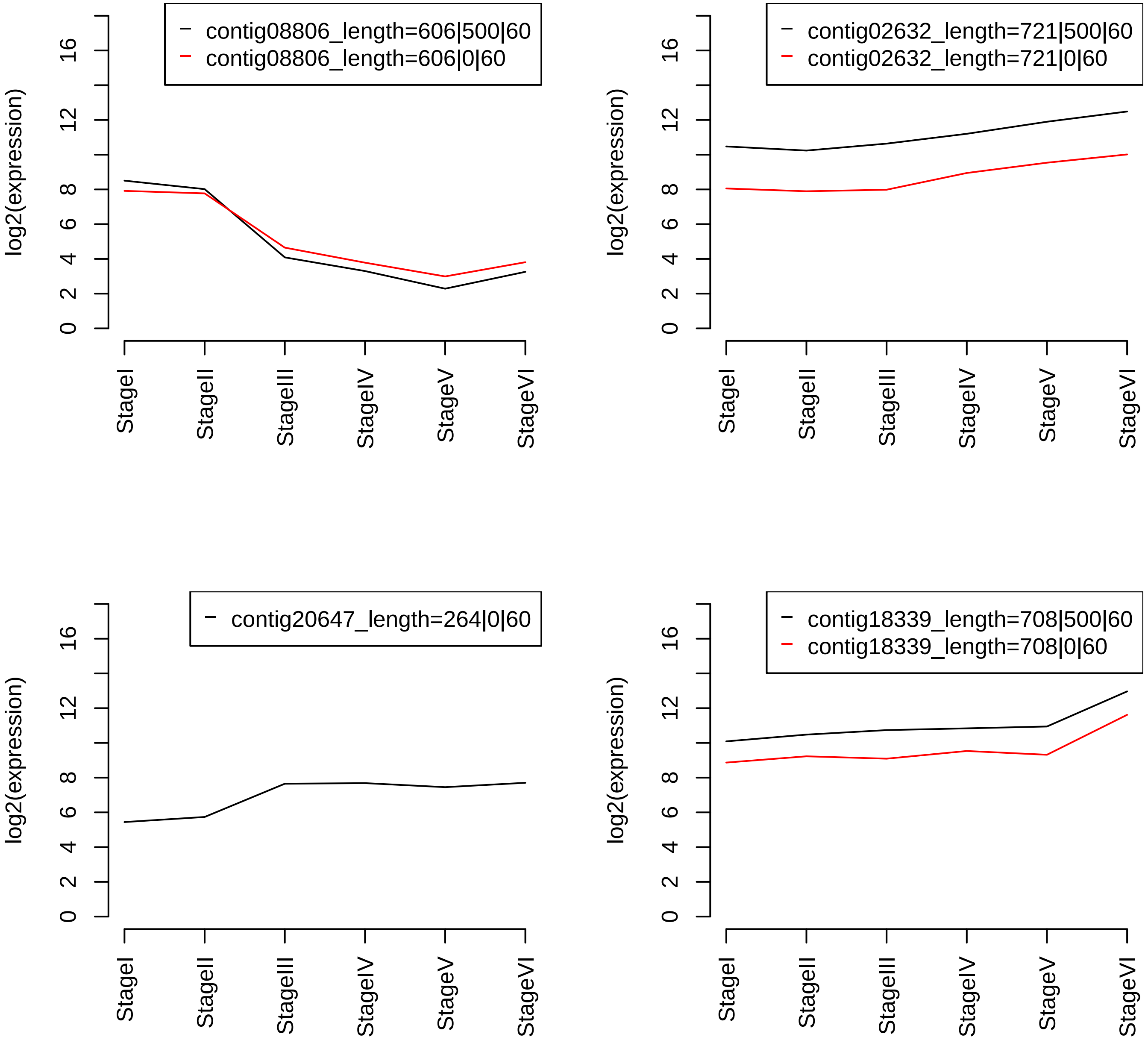

contig08806_length=606|500|60
contig08806_length=606|0|60
contig02632_length=721|500|60
contig02632_length=721|0|60
contig20647_length=264|0|60
contig18339_length=708|500|60
contig18339_length=708|0|60
log2(expression)
0 2 4 6 8 12 16
StageI StageII StageIII StageIV StageV StageVI

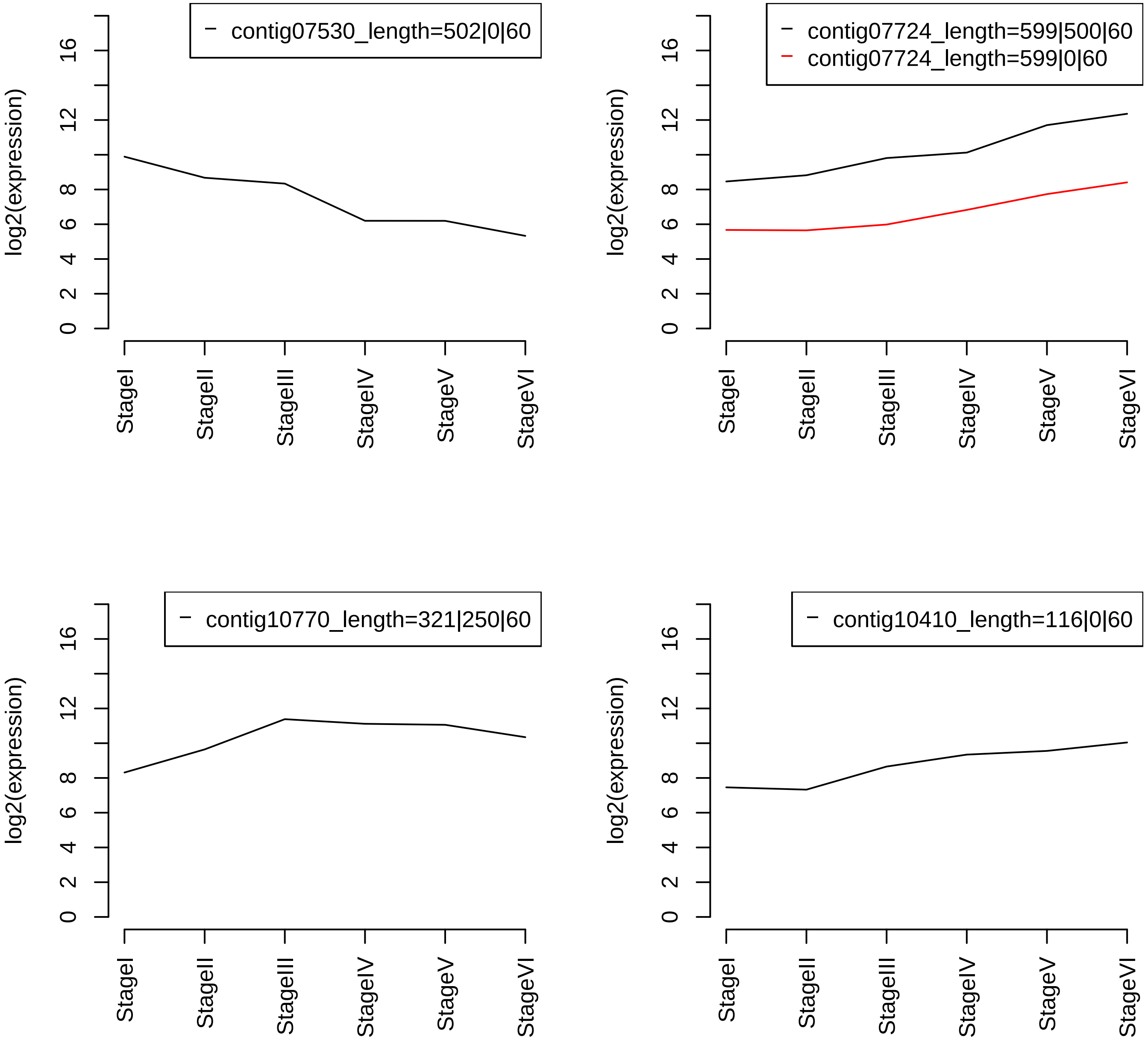
contig07530_length=502|0|60
contig07724_length=599|500|60
contig07724_length=599|0|60
contig10770_length=321|250|60
contig10410_length=116|0|60
log2(expression)
0
2
4
6
8
12
16
StageI
StageII
StageIII
StageIV
StageV
StageVI

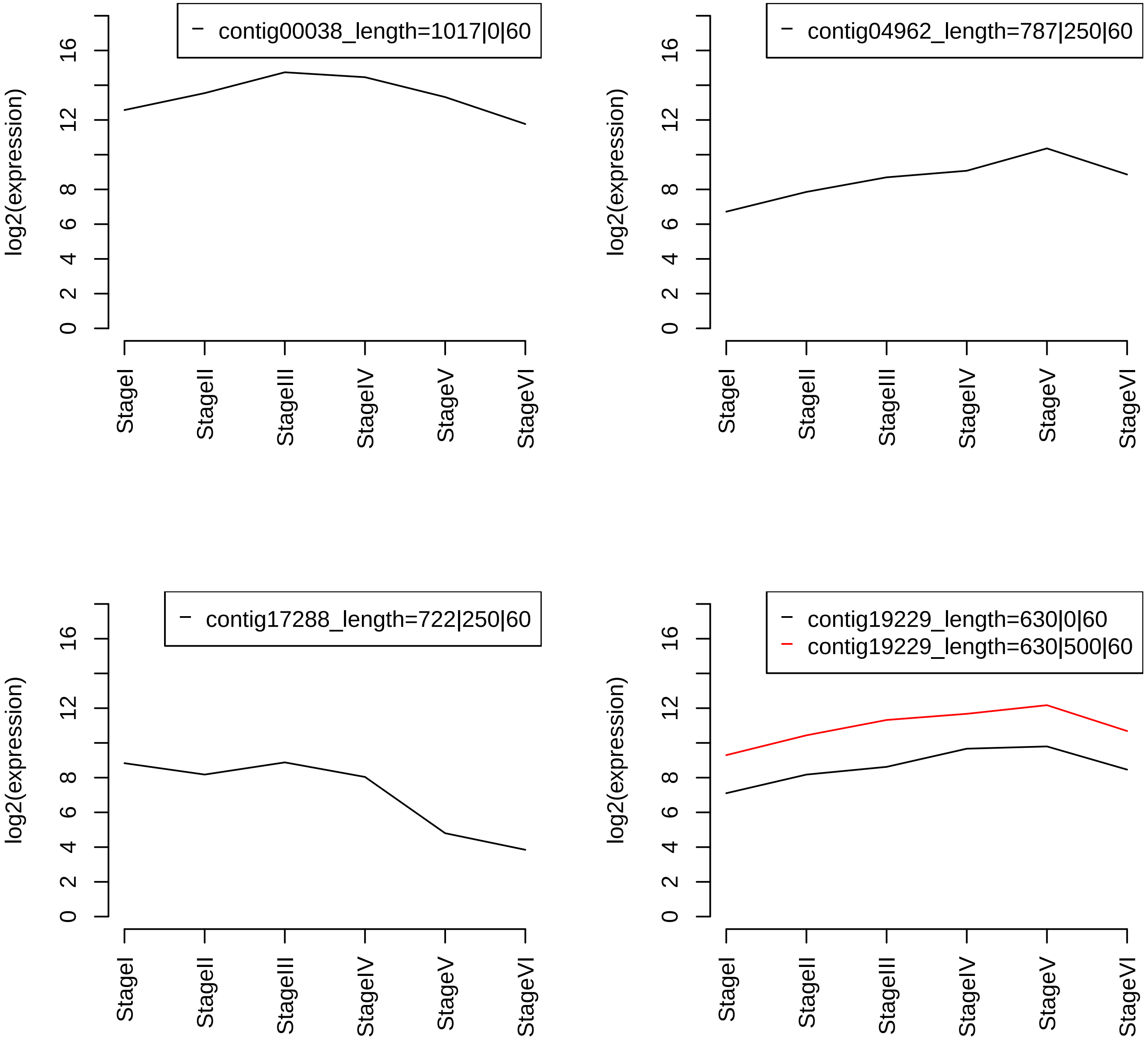
contig00038_length=1017|0|60
contig04962_length=787|250|60
contig17288_length=722|250|60
contig19229_length=630|0|60
contig19229_length=630|500|60
log2(expression)
0
2
4
6
8
12
16
StageI
StageII
StageIII
StageIV
StageV
StageVI

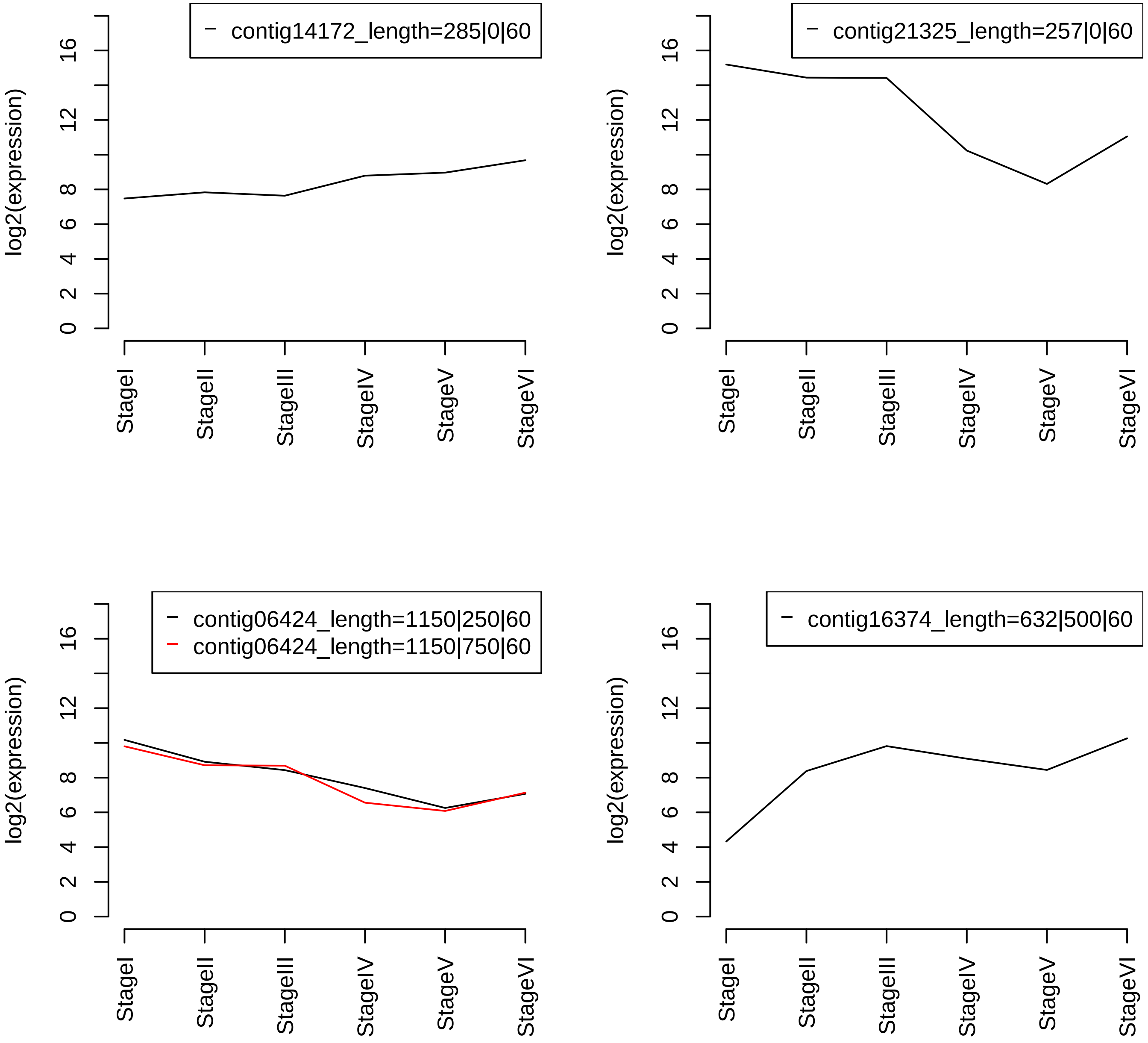

contig14172_length=285|0|60
contig21325_length=257|0|60
contig06424_length=1150|250|60
contig06424_length=1150|750|60
contig16374_length=632|500|60
log2(expression)
0
2
4
6
8
12
16
StageI
StageII
StageIII
StageIV
StageV
StageVI

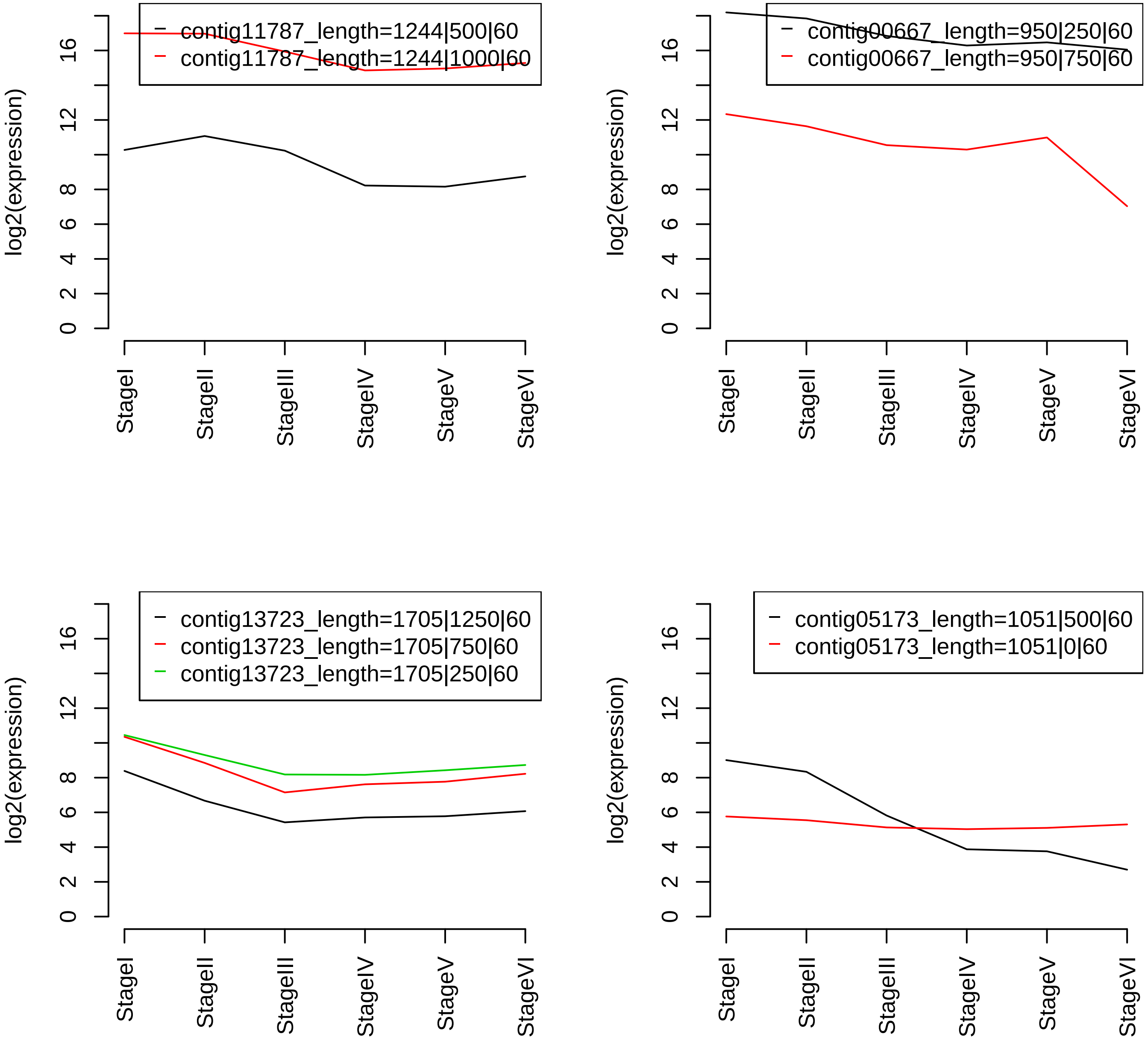
contig11787_length=1244|500|60
contig11787_length=1244|1000|60
contig00667_length=950|250|60
contig00667_length=950|750|60
contig13723_length=1705|1250|60
contig13723_length=1705|750|60
contig13723_length=1705|250|60
contig05173_length=1051|500|60
contig05173_length=1051|0|60
log2(expression)
0
2
4
6
8
12
16
StageI
StageII
StageIII
StageIV
StageV
StageVI

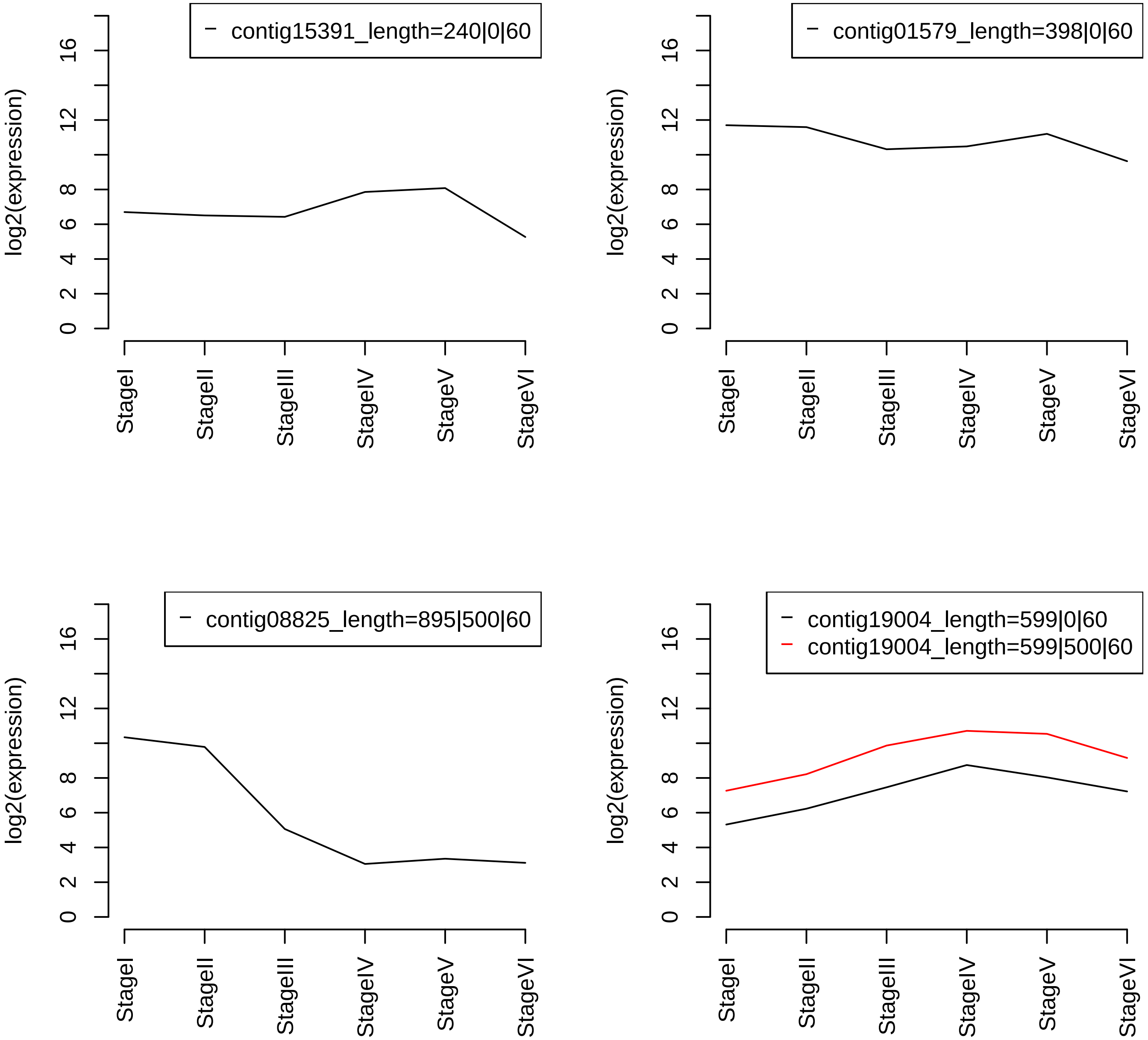
contig15391_length=240|0|60
contig01579_length=398|0|60
contig08825_length=895|500|60
contig19004_length=599|0|60
contig19004_length=599|500|60
log2(expression)
0
2
4
6
8
12
16
StageI
StageII
StageIII
StageIV
StageV
StageVI

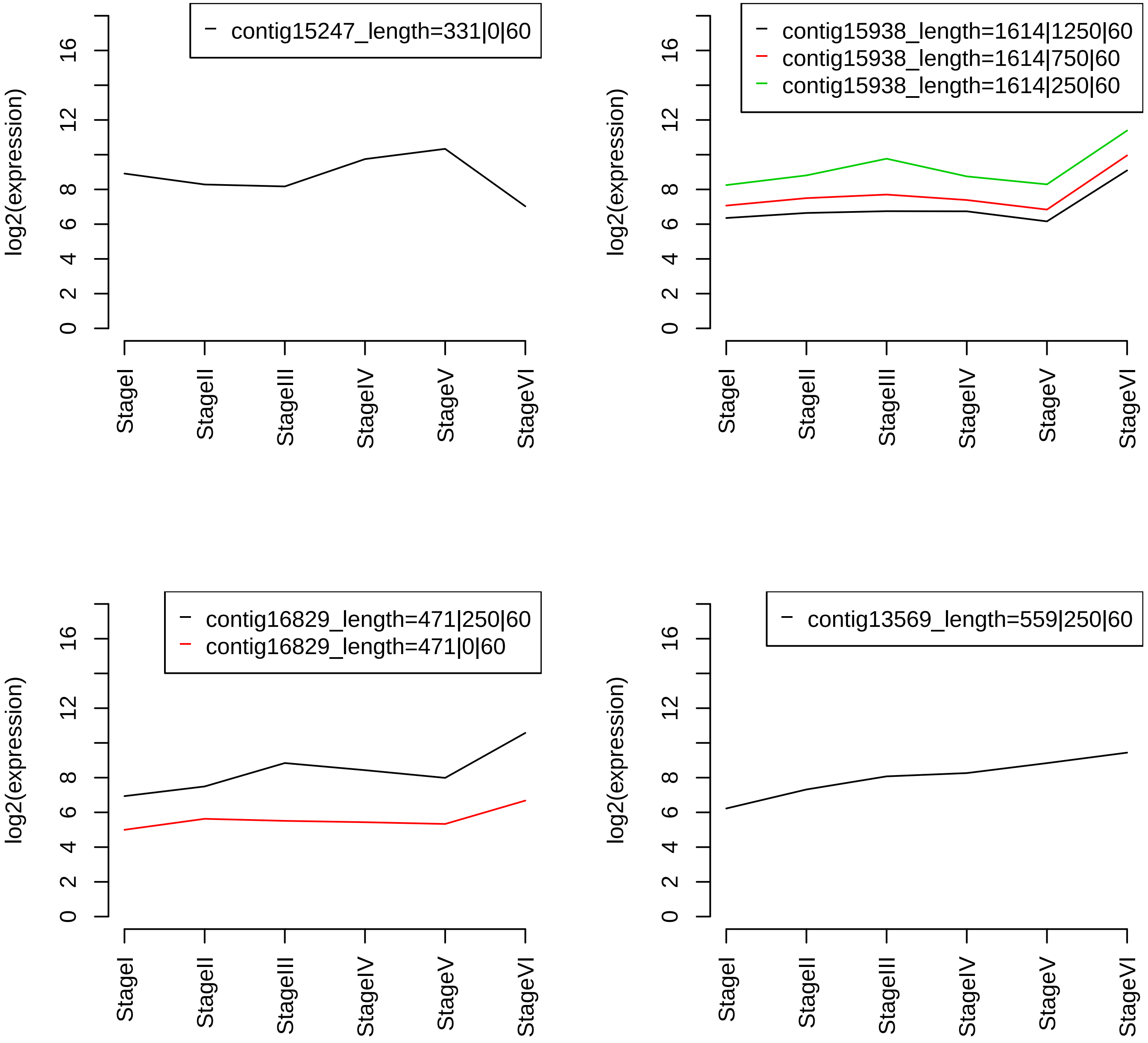
contig15247_length=331|0|60
contig15938_length=1614|1250|60
contig15938_length=1614|750|60
contig15938_length=1614|250|60
contig16829_length=471|250|60
contig16829_length=471|0|60
contig13569_length=559|250|60
log2(expression)
0 2 4 6 8 12 16
StageI StageII StageIII StageIV StageV StageVI

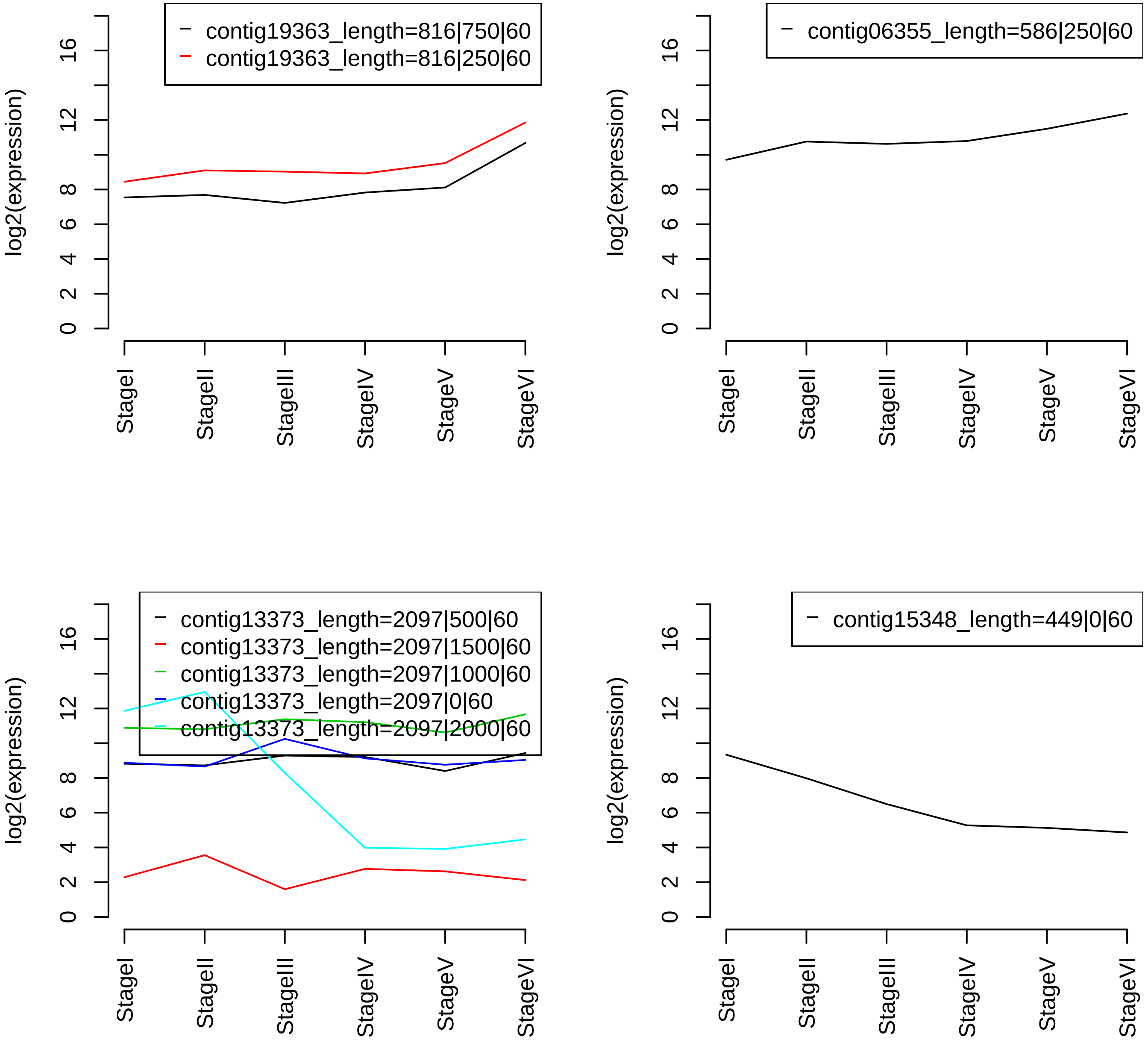
contig19363_length=816|750|60
contig19363_length=816|250|60
contig06355_length=586|250|60
contig13373_length=2097|500|60
contig13373_length=2097|1500|60
contig13373_length=2097|1000|60
contig13373_length=2097|0|60
contig13373_length=2097|2000|60
contig15348_length=449|0|60
log2(expression)
0
2
4
6
8
12
16
StageI
StageII
StageIII
StageIV
StageV
StageVI

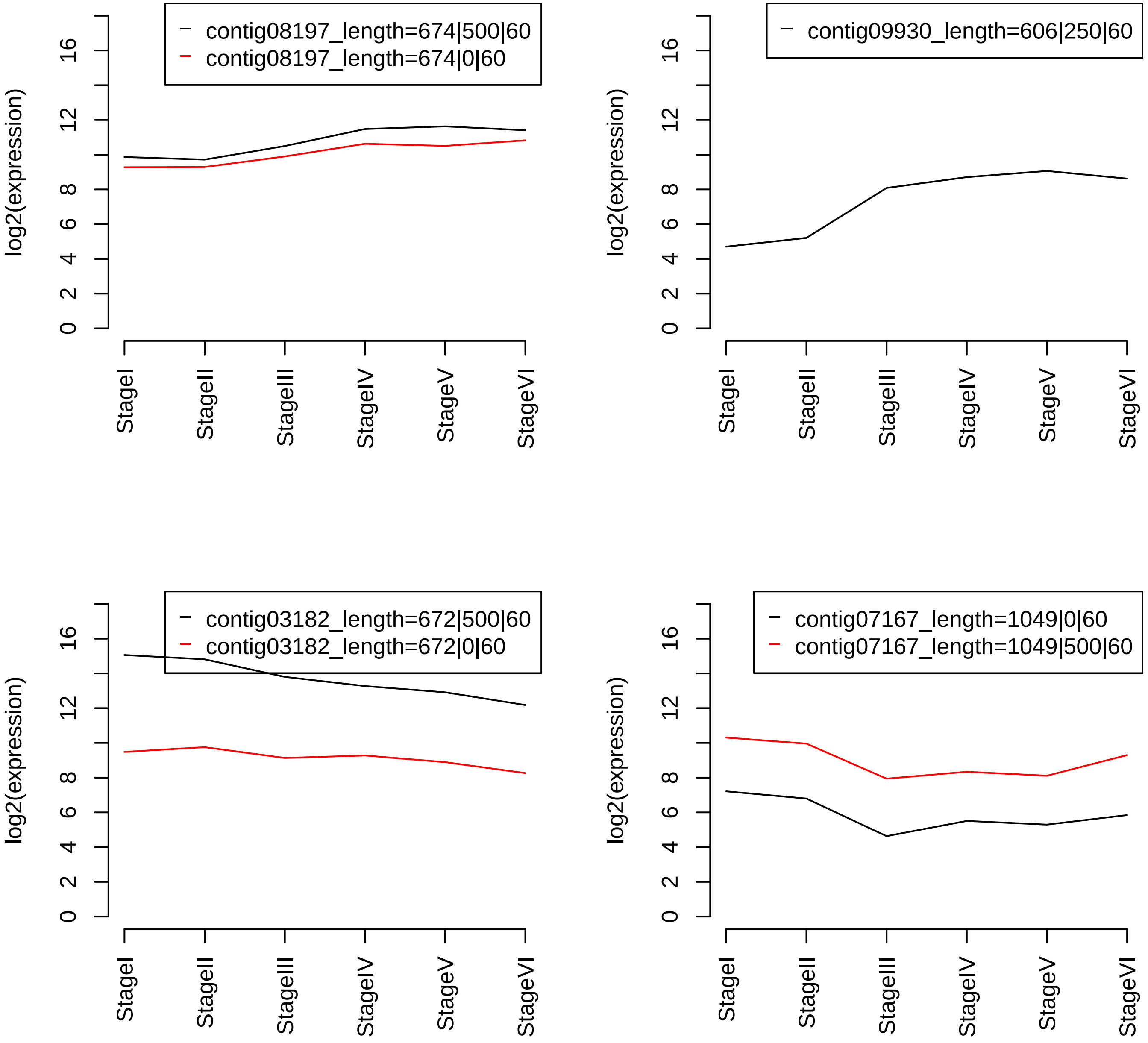

contig08197_length=674|500|60
contig08197_length=674|0|60
log2(expression)
0
2
4
6
8
12
16
StageI
StageII
StageIII
StageIV
StageV
StageVI
contig09930_length=606|250|60
contig03182_length=672|500|60
contig03182_length=672|0|60
contig07167_length=1049|0|60
contig07167_length=1049|500|60

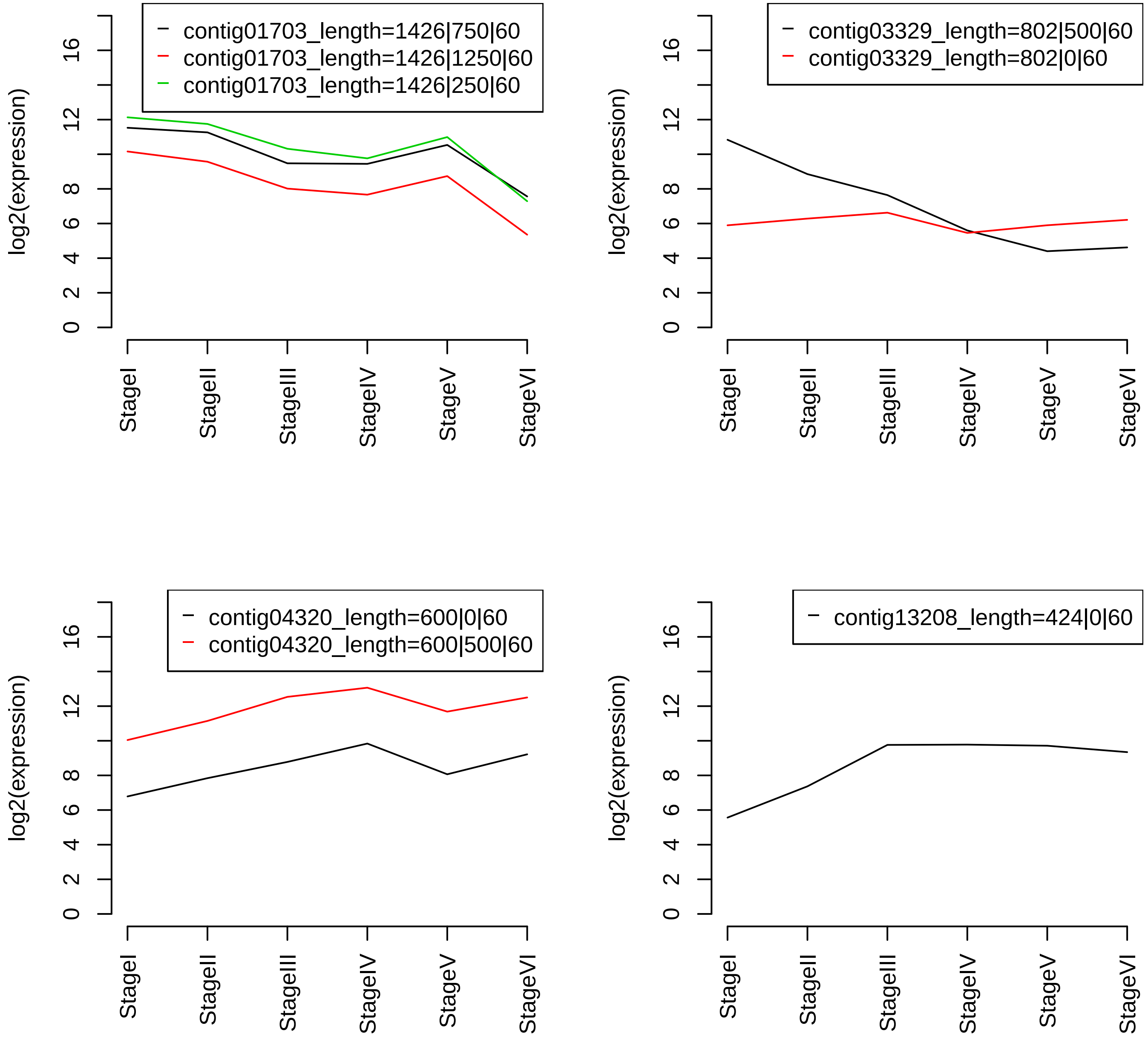

contig01703_length=1426|750|60
contig01703_length=1426|1250|60
contig01703_length=1426|250|60
contig03329_length=802|500|60
contig03329_length=802|0|60
contig04320_length=600|0|60
contig04320_length=600|500|60
contig13208_length=424|0|60
log2(expression)
0
2
4
6
8
12
16
StageI
StageII
StageIII
StageIV
StageV
StageVI

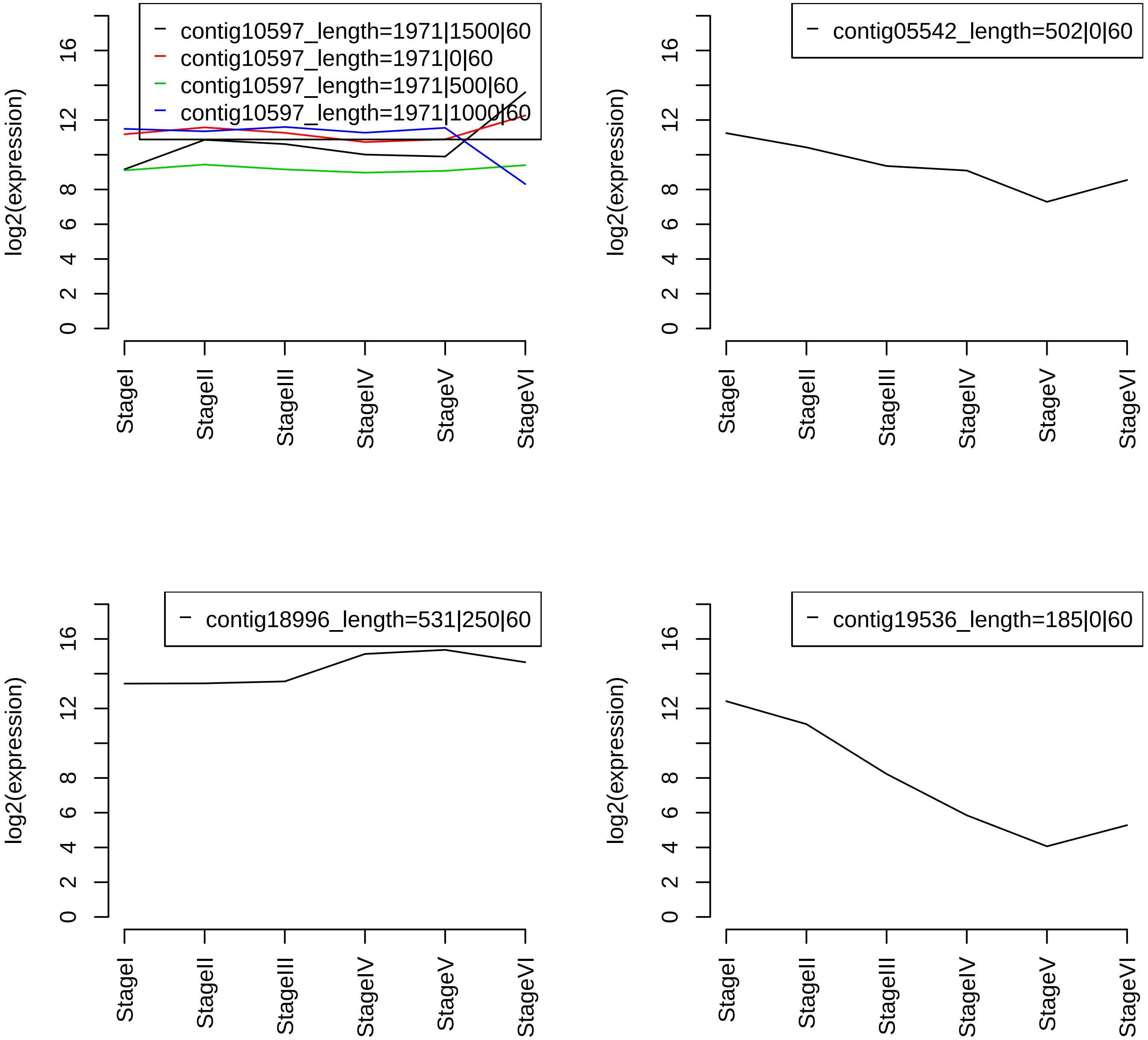

contig10597_length=1971|1500|60
contig10597_length=1971|0|60
contig10597_length=1971|500|60
contig10597_length=1971|1000|60
contig05542_length=502|0|60
contig18996_length=531|250|60
contig19536_length=185|0|60
log2(expression)
0
2
4
6
8
12
16
StageI
StageII
StageIII
StageIV
StageV
StageVI

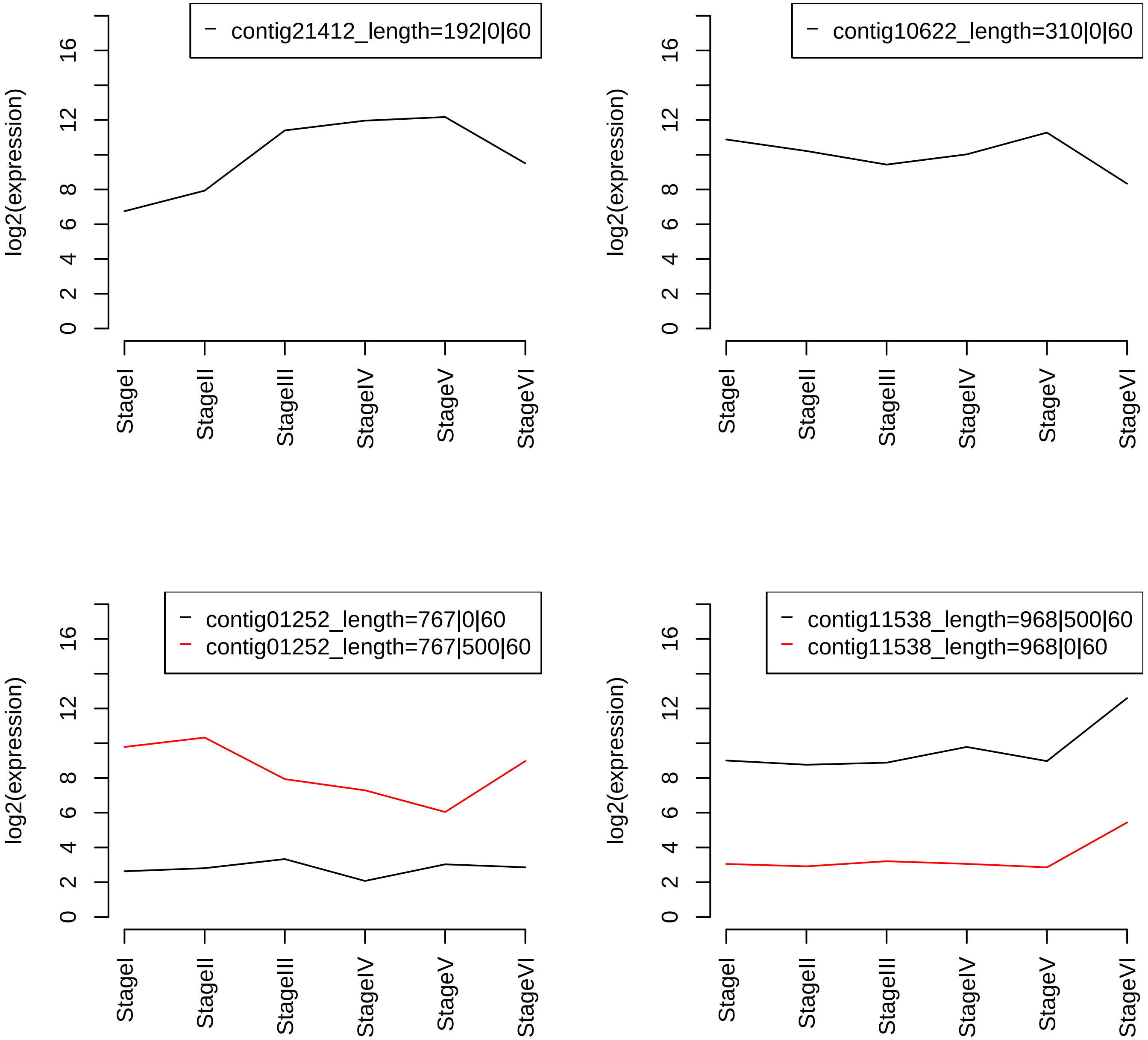

contig21412_length=192|0|60
contig10622_length=310|0|60
contig01252_length=767|0|60
contig01252_length=767|500|60
contig11538_length=968|500|60
contig11538_length=968|0|60
log2(expression)
0
2
4
6
8
12
16
StageI
StageII
StageIII
StageIV
StageV
StageVI

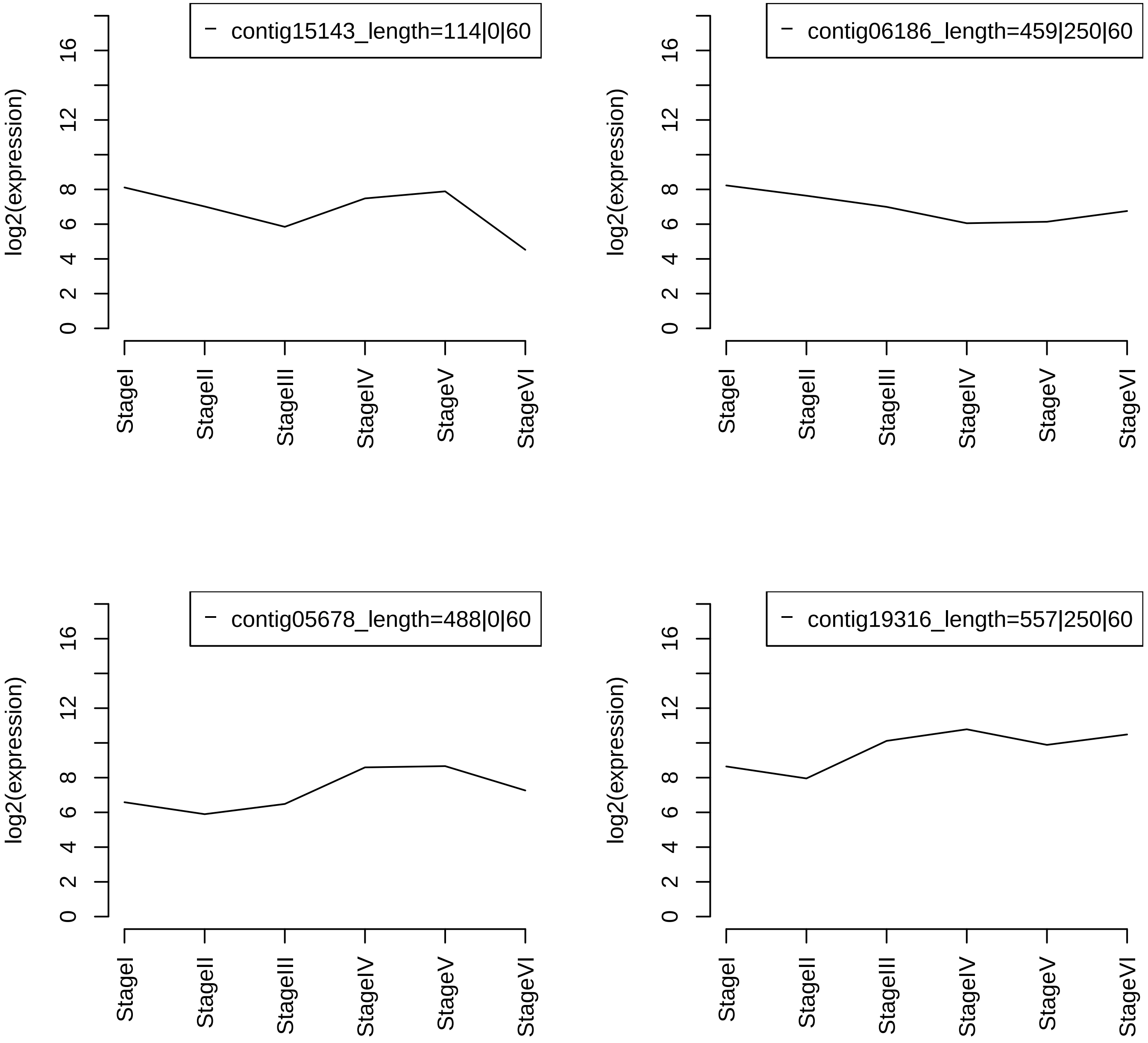
contig15143_length=114|0|60
contig06186_length=459|250|60
contig05678_length=488|0|60
contig19316_length=557|250|60
log2(expression)
0
2
4
6
8
12
16
StageI
StageII
StageIII
StageIV
StageV
StageVI

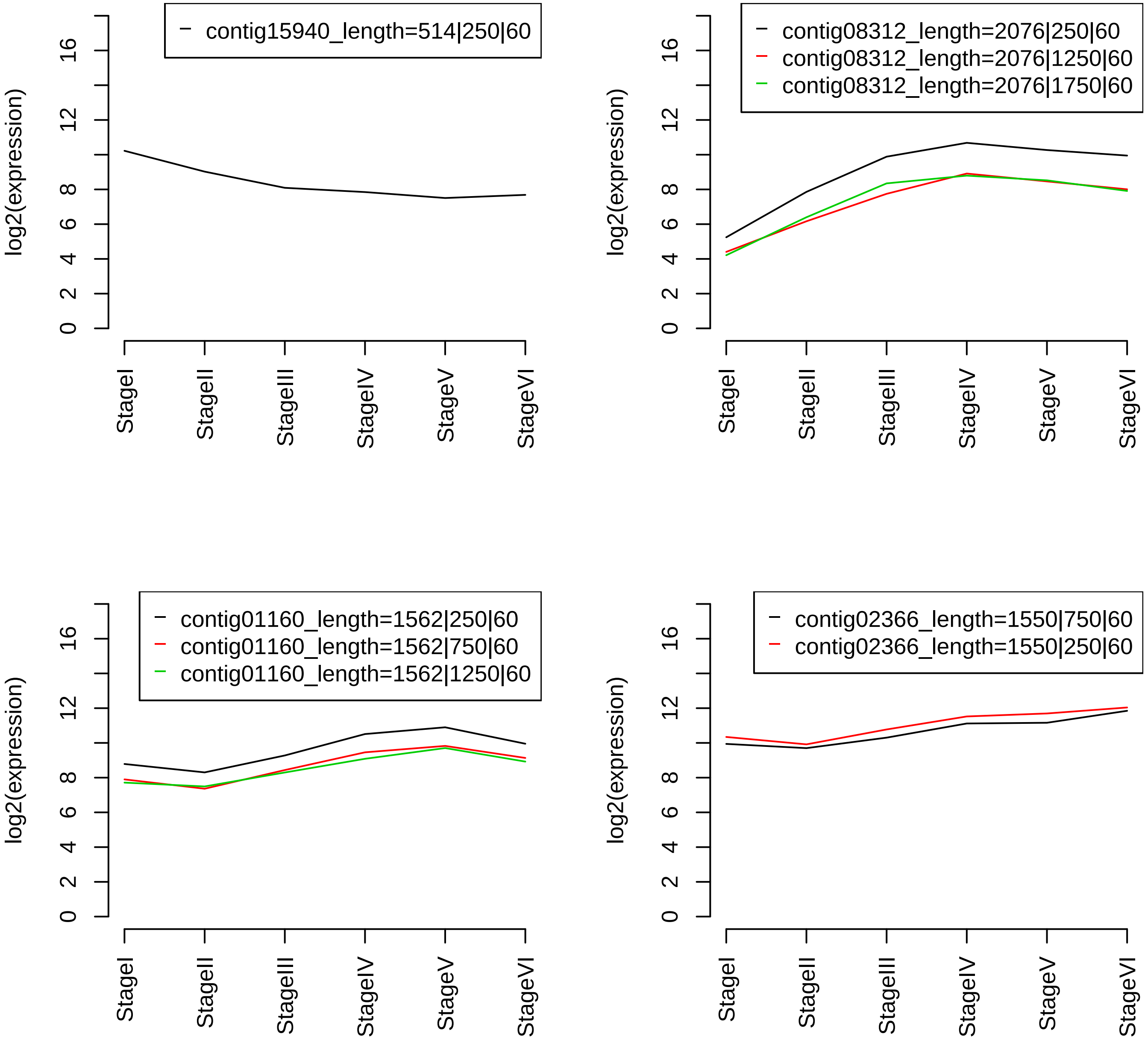
contig15940_length=514|250|60
contig08312_length=2076|250|60
contig08312_length=2076|1250|60
contig08312_length=2076|1750|60
contig01160_length=1562|250|60
contig01160_length=1562|750|60
contig01160_length=1562|1250|60
contig02366_length=1550|750|60
contig02366_length=1550|250|60
log2(expression)
0
2
4
6
8
12
16
StageI
StageII
StageIII
StageIV
StageV
StageVI

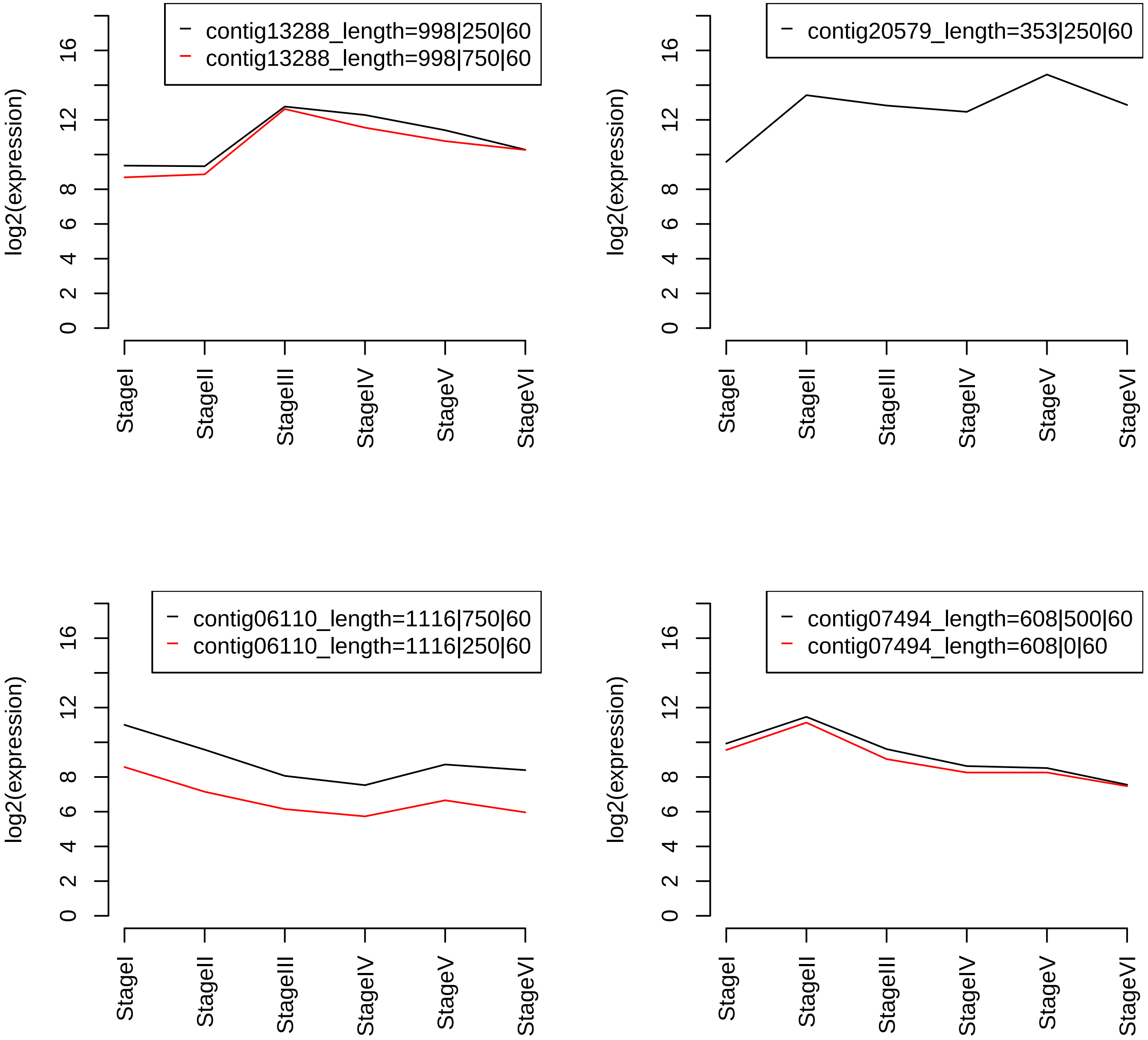

contig13288_length=998|250|60
contig13288_length=998|750|60
contig20579_length=353|250|60
contig06110_length=1116|750|60
contig06110_length=1116|250|60
contig07494_length=608|500|60
contig07494_length=608|0|60
log2(expression)
0
2
4
6
8
12
16
StageI
StageII
StageIII
StageIV
StageV
StageVI

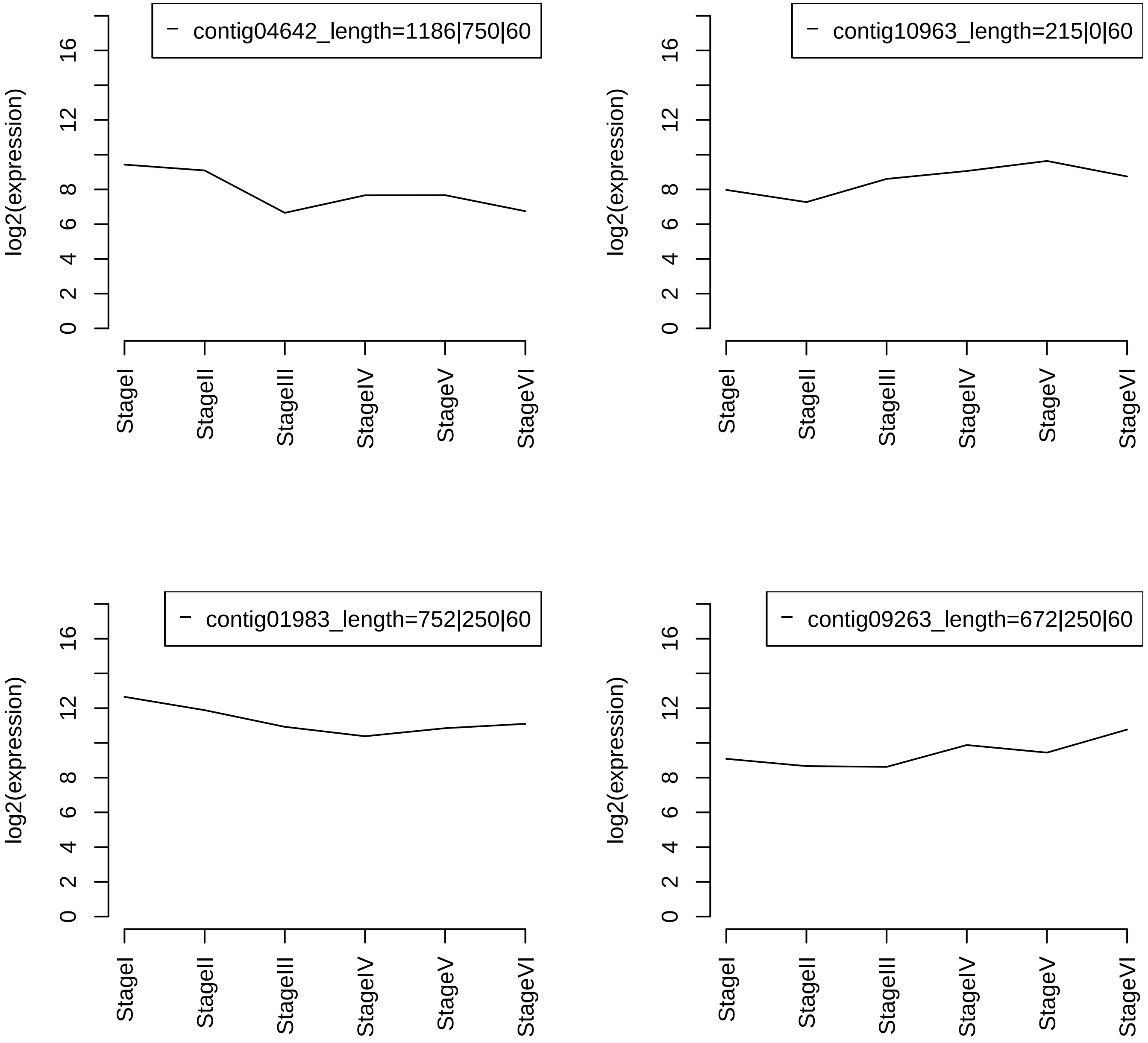
contig04642_length=1186|750|60
contig10963_length=215|0|60
contig01983_length=752|250|60
contig09263_length=672|250|60
log2(expression)
0
2
4
6
8
12
16
StageI
StageII
StageIII
StageIV
StageV
StageVI

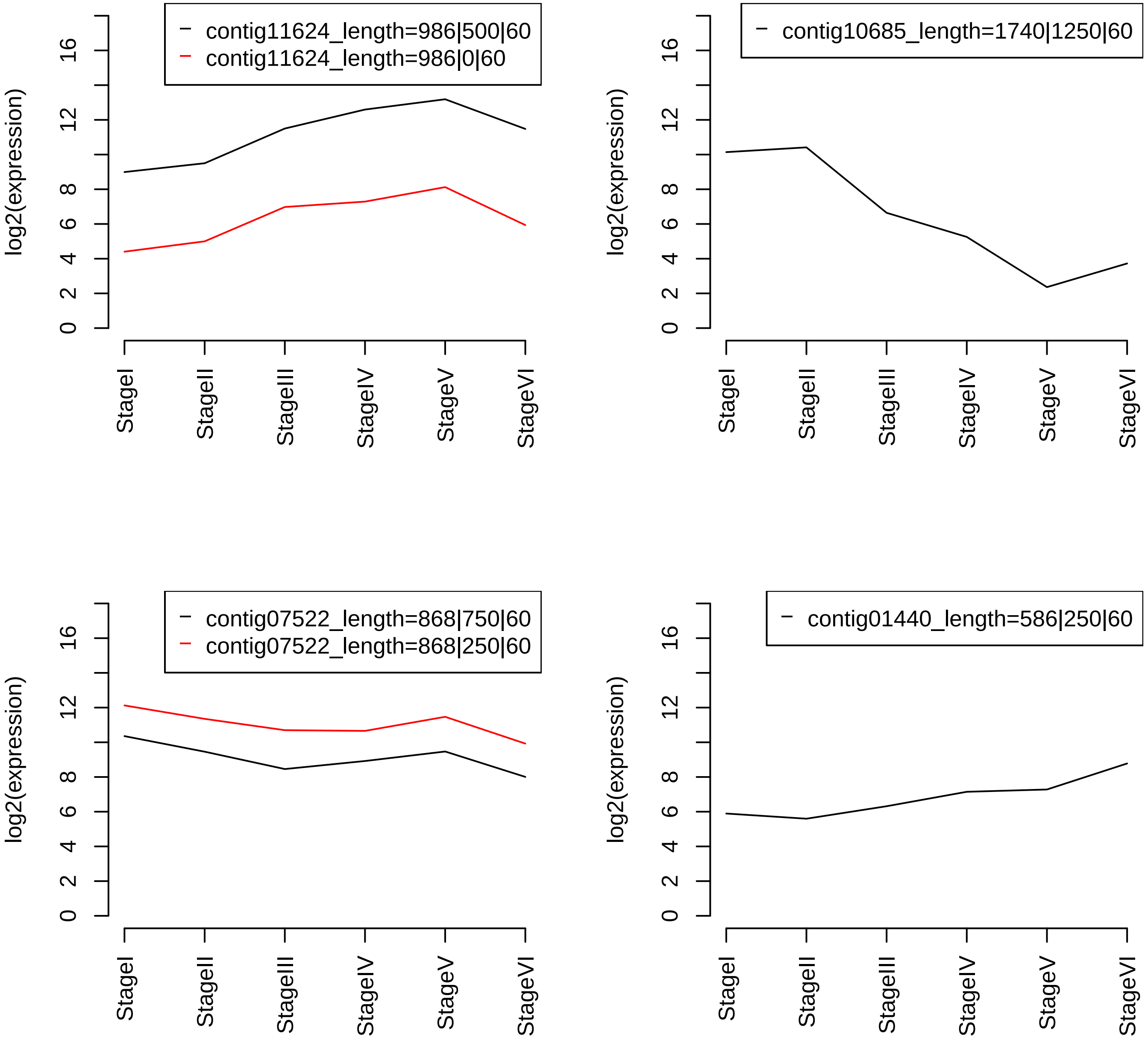
contig11624_length=986|500|60
contig11624_length=986|0|60
log2(expression)
0
2
4
6
8
12
16
StageI
StageII
StageIII
StageIV
StageV
StageVI
contig10685_length=1740|1250|60
log2(expression)
contig07522_length=868|750|60
contig07522_length=868|250|60
log2(expression)
contig01440_length=586|250|60
log2(expression)

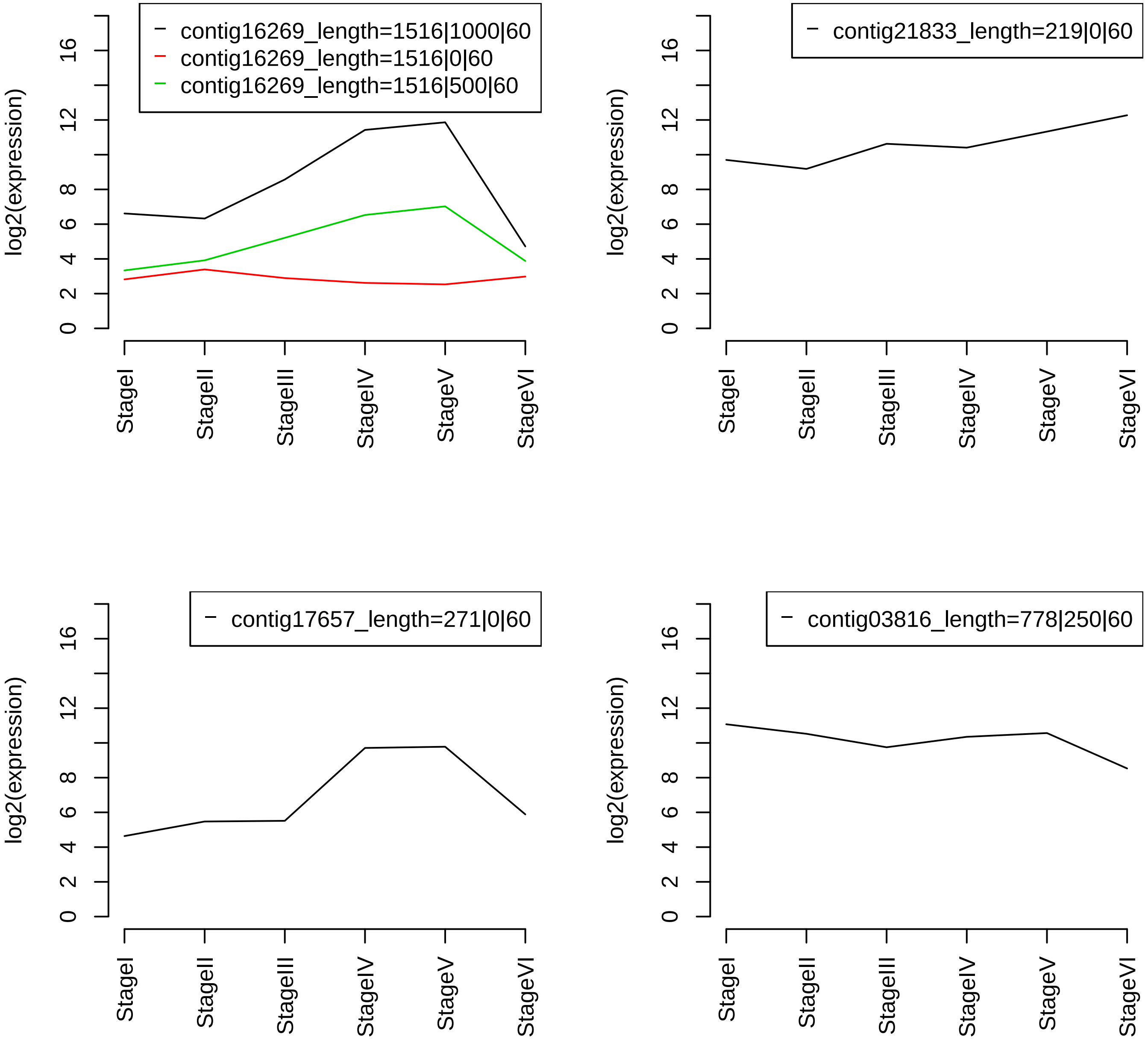
contig16269_length=1516|1000|60
contig16269_length=1516|0|60
contig16269_length=1516|500|60
contig21833_length=219|0|60
contig17657_length=271|0|60
contig03816_length=778|250|60
log2(expression)
0
2
4
6
8
12
16
StageI
StageII
StageIII
StageIV
StageV
StageVI

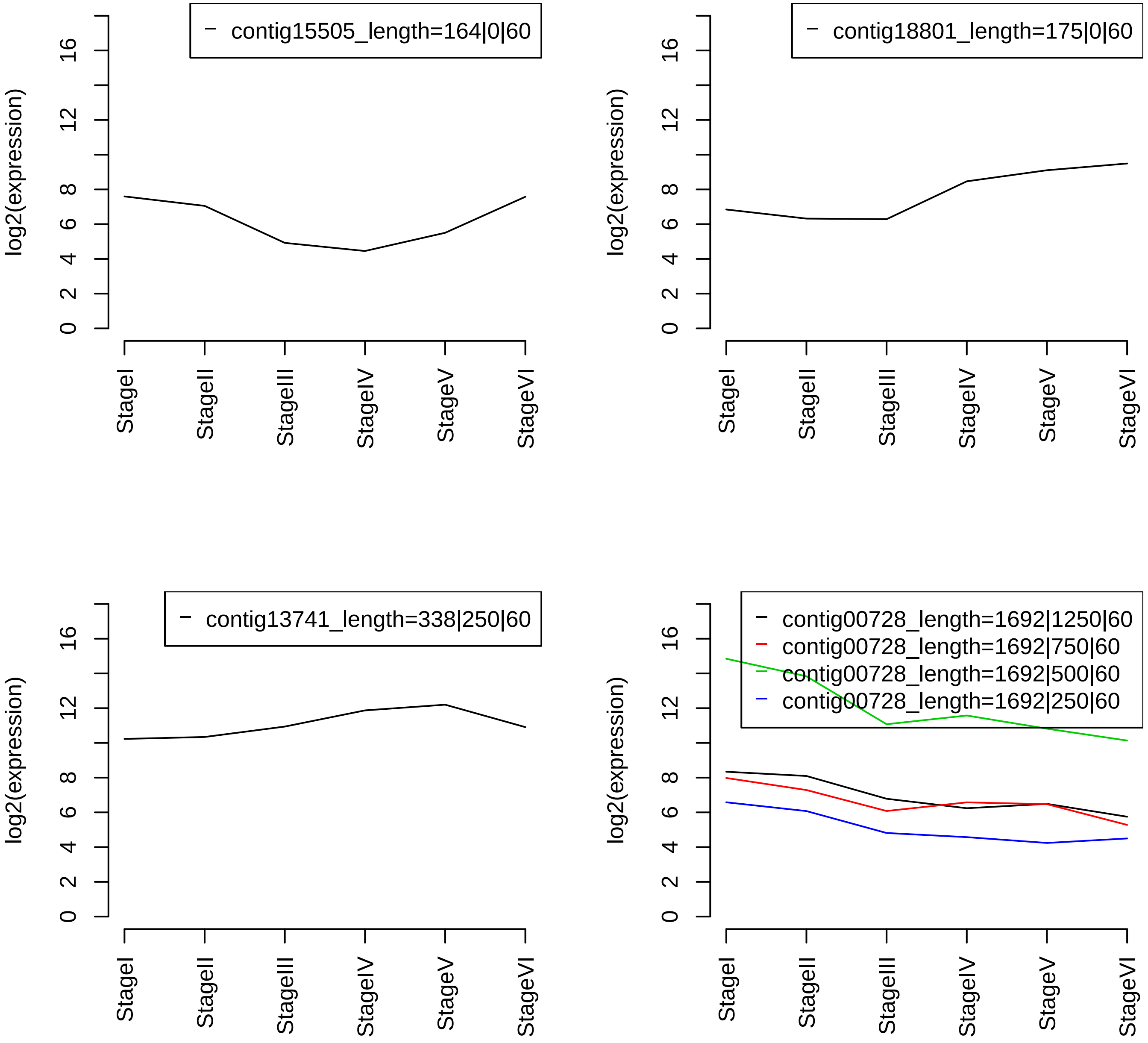

contig15505_length=164|0|60
contig18801_length=175|0|60
contig13741_length=338|250|60
contig00728_length=1692|1250|60
contig00728_length=1692|750|60
contig00728_length=1692|500|60
contig00728_length=1692|250|60
log2(expression)
0
2
4
6
8
12
16
StageI
StageII
StageIII
StageIV
StageV
StageVI

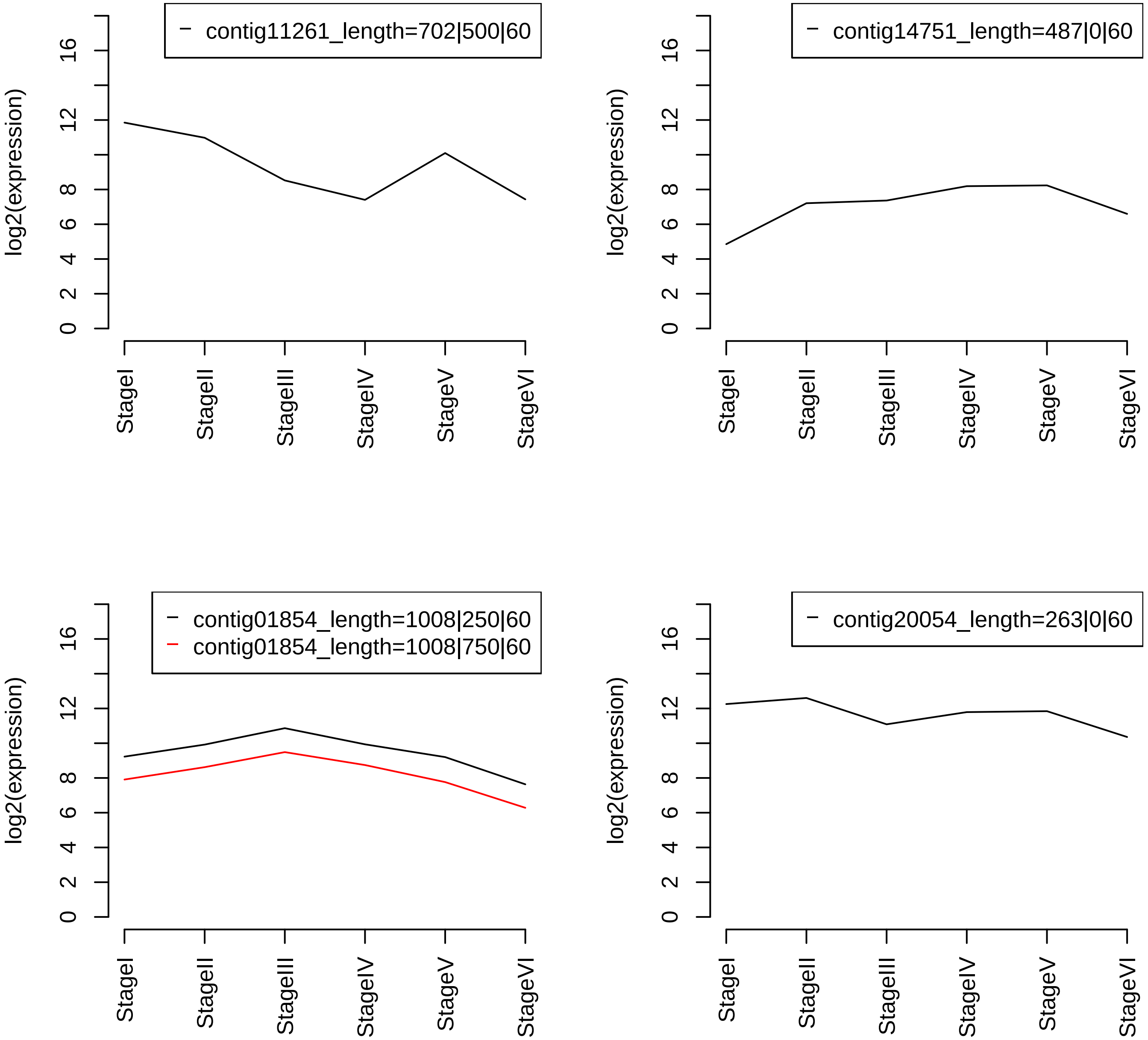

contig11261_length=702|500|60
contig14751_length=487|0|60
contig01854_length=1008|250|60
contig01854_length=1008|750|60
contig20054_length=263|0|60
log2(expression)
0
2
4
6
8
12
16
StageI
StageII
StageIII
StageIV
StageV
StageVI

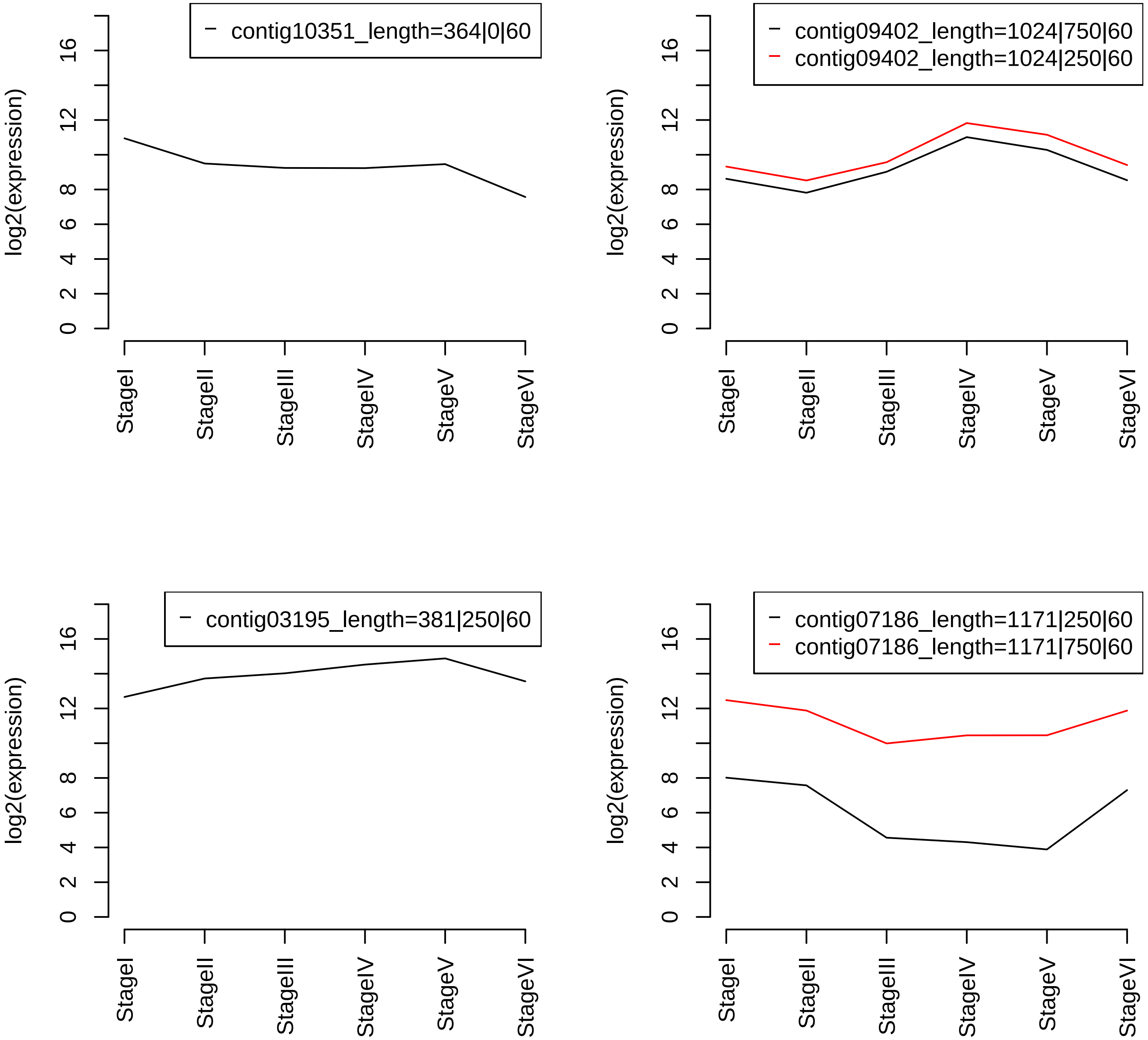
contig10351_length=364|0|60
contig09402_length=1024|750|60
contig09402_length=1024|250|60
contig03195_length=381|250|60
contig07186_length=1171|250|60
contig07186_length=1171|750|60
log2(expression)
0
2
4
6
8
12
16
StageI
StageII
StageIII
StageIV
StageV
StageVI

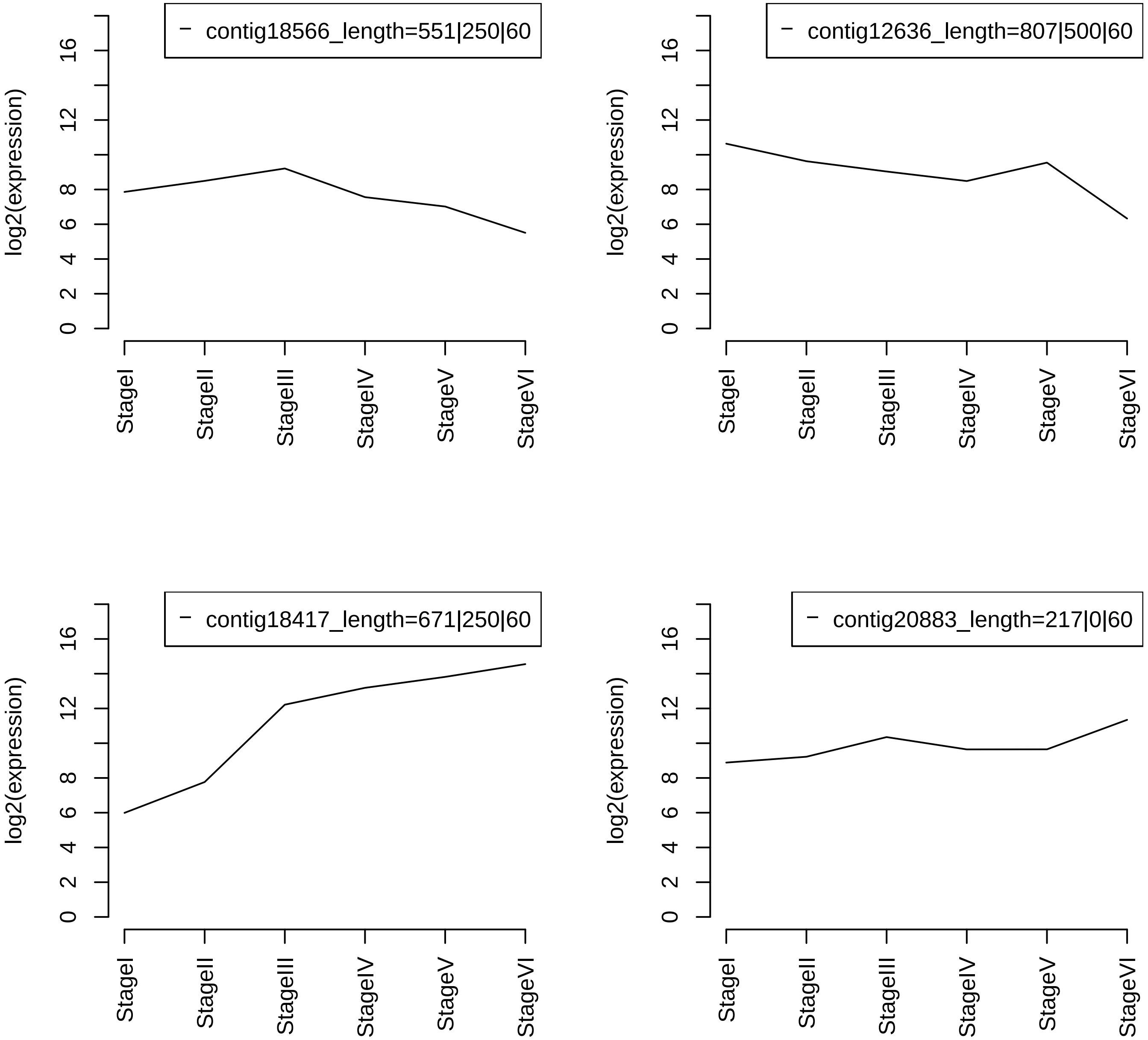

contig18566_length=551|250|60
contig12636_length=807|500|60
contig18417_length=671|250|60
contig20883_length=217|0|60
log2(expression)
0
2
4
6
8
12
16
StageI
StageII
StageIII
StageIV
StageV
StageVI

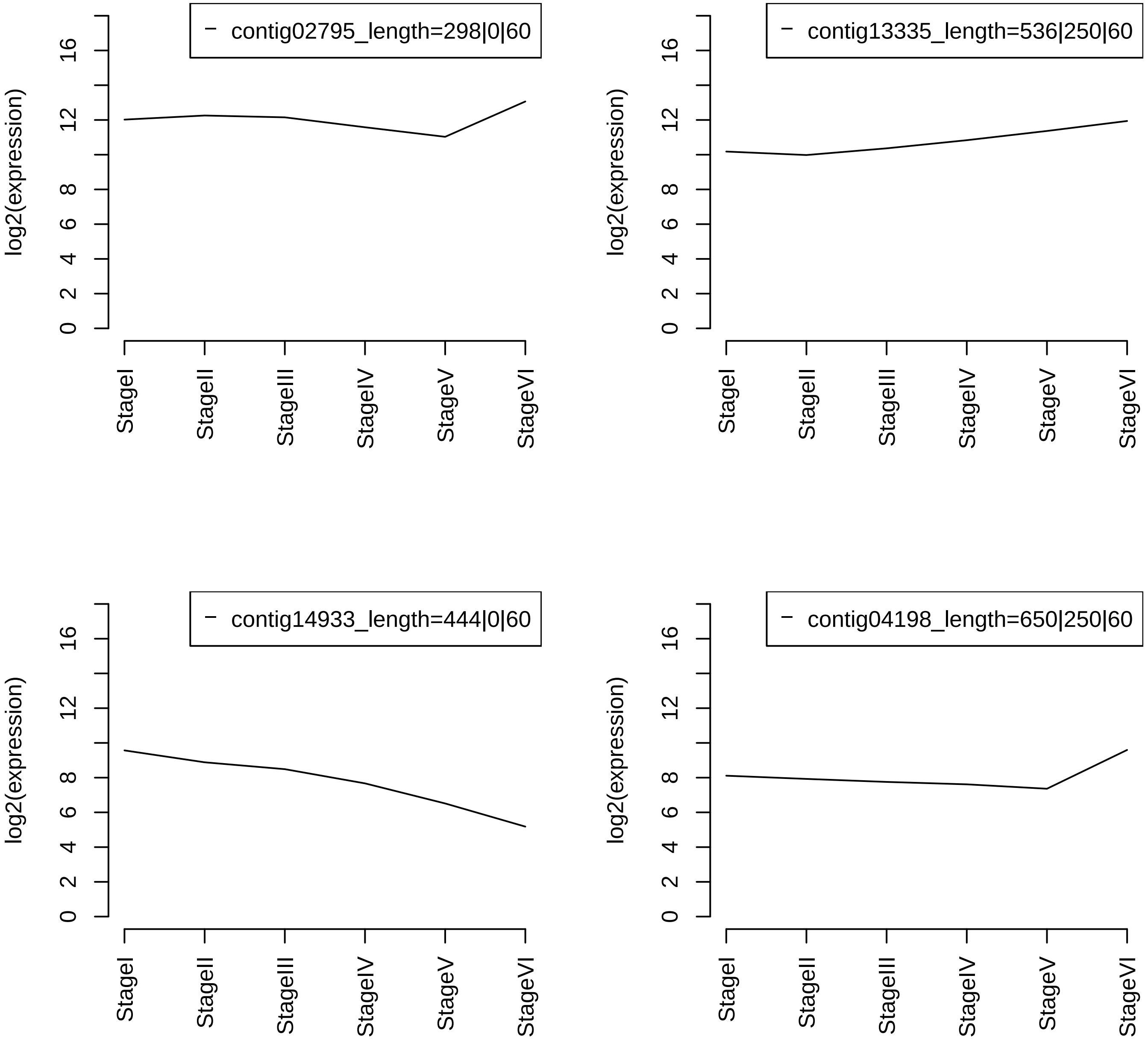

contig02795_length=298|0|60
contig13335_length=536|250|60
contig14933_length=444|0|60
contig04198_length=650|250|60
log2(expression)
0
2
4
6
8
12
16
StageI
StageII
StageIII
StageIV
StageV
StageVI

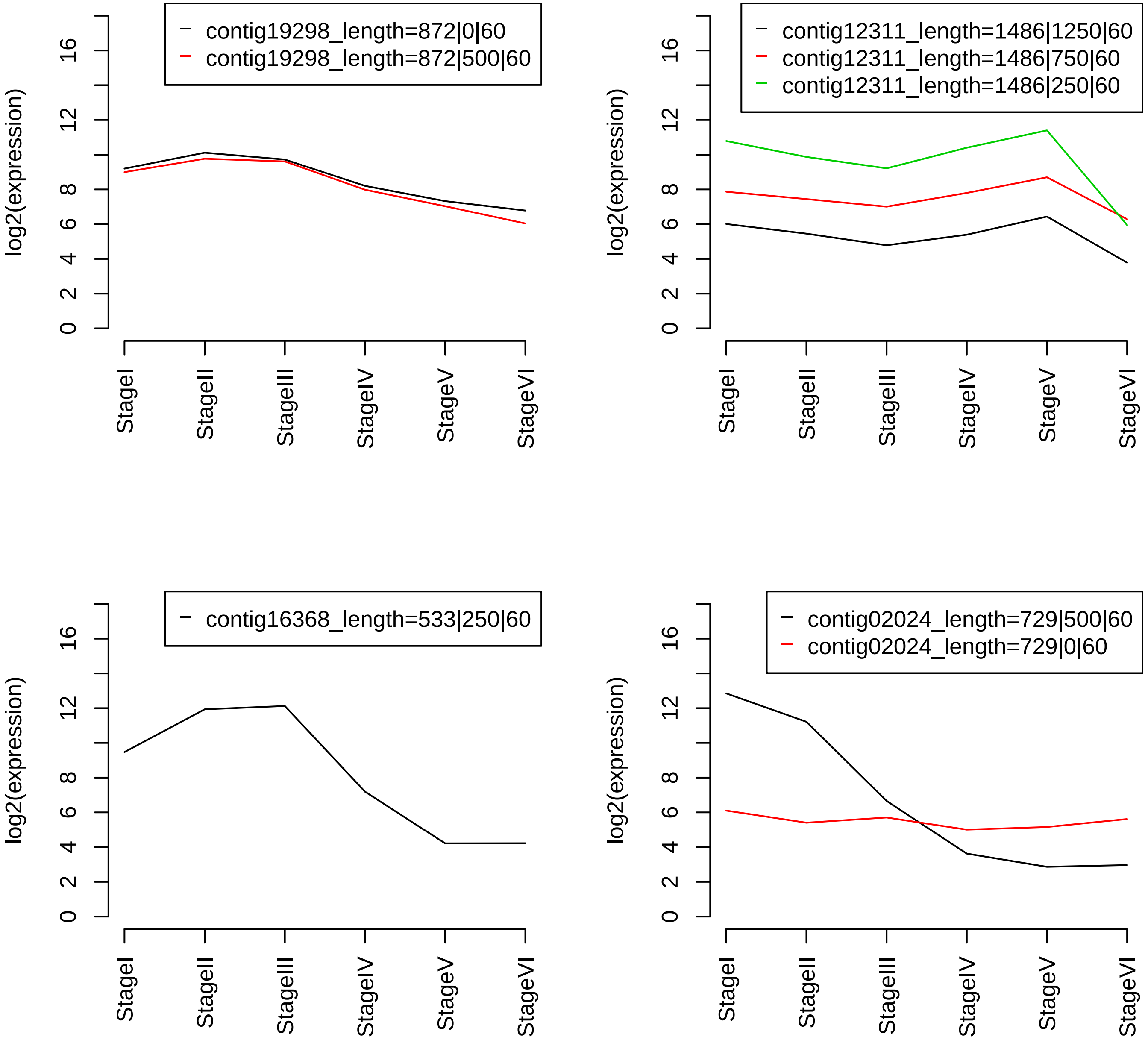
contig19298_length=872|0|60
contig19298_length=872|500|60
contig12311_length=1486|1250|60
contig12311_length=1486|750|60
contig12311_length=1486|250|60
contig16368_length=533|250|60
contig02024_length=729|500|60
contig02024_length=729|0|60
log2(expression)
StageI
StageII
StageIII
StageIV
StageV
StageVI

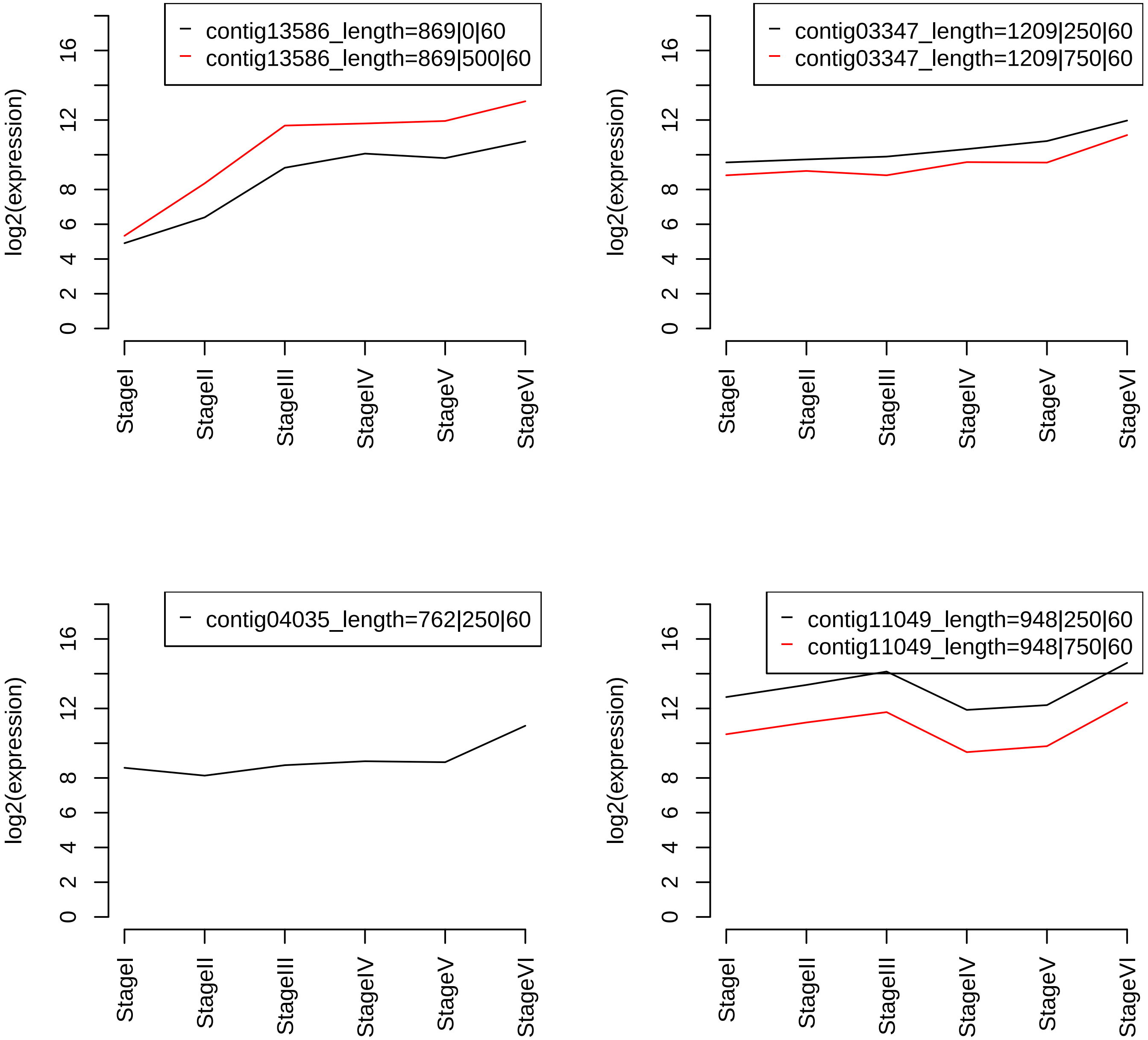
contig13586_length=869|0|60
contig13586_length=869|500|60
contig03347_length=1209|250|60
contig03347_length=1209|750|60
contig04035_length=762|250|60
contig11049_length=948|250|60
contig11049_length=948|750|60
log2(expression)
0 2 4 6 8 12 16
StageI StageII StageIII StageIV StageV StageVI

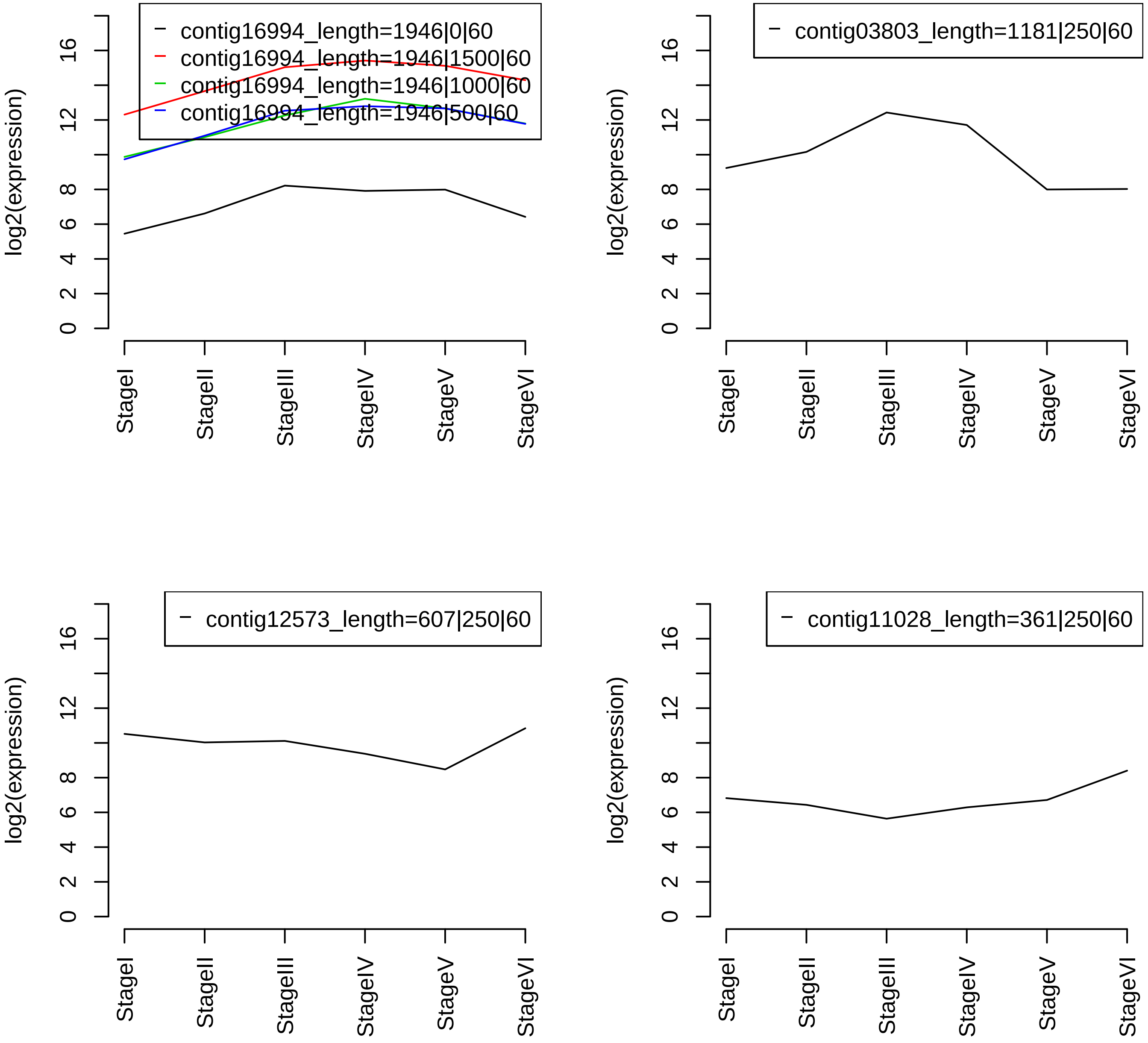

contig16994_length=1946|0|60
contig16994_length=1946|1500|60
contig16994_length=1946|1000|60
contig16994_length=1946|500|60
contig03803_length=1181|250|60
contig12573_length=607|250|60
contig11028_length=361|250|60
log2(expression)
0
2
4
6
8
12
16
StageI
StageII
StageIII
StageIV
StageV
StageVI

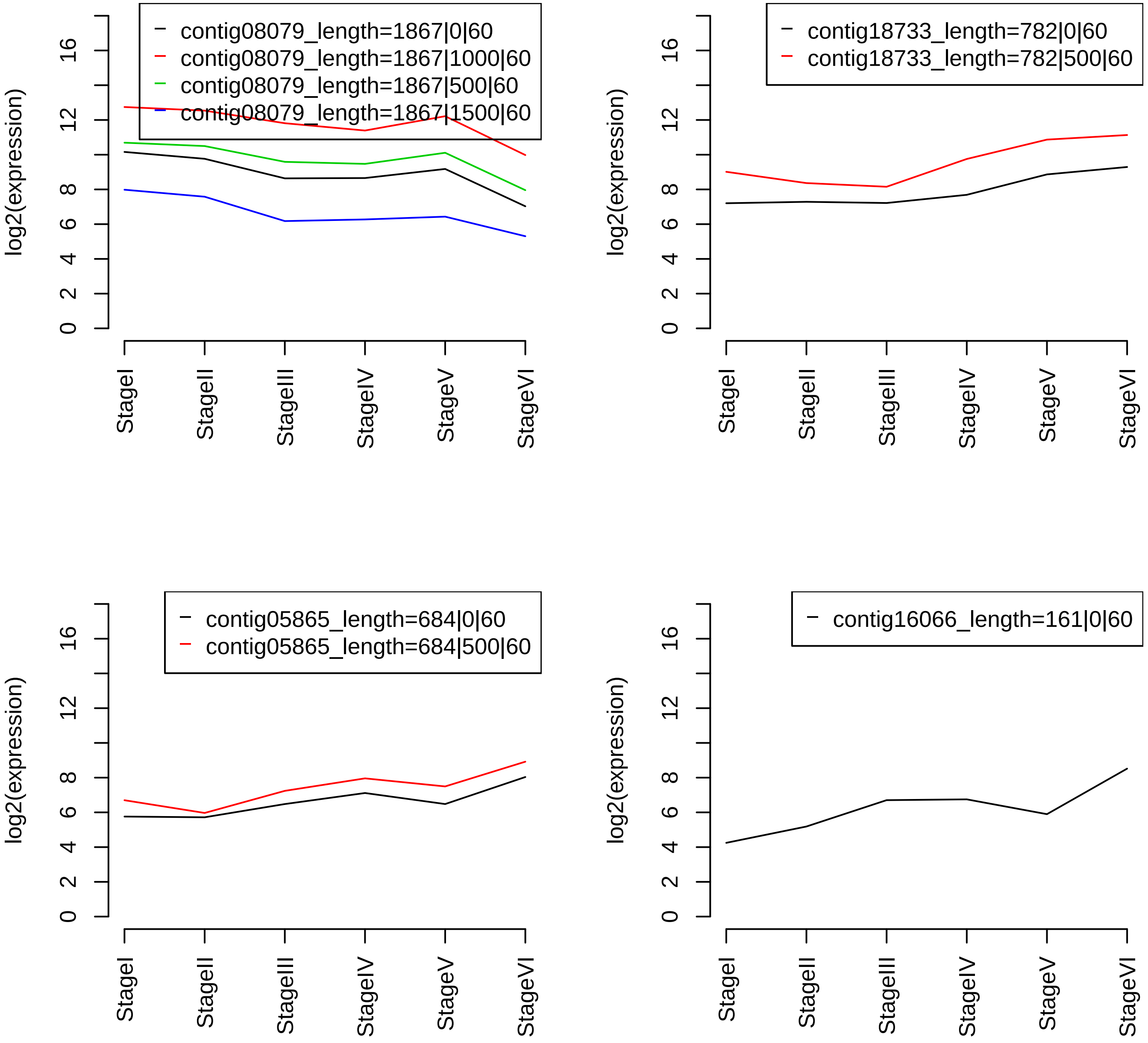

contig08079_length=1867|0|60
contig08079_length=1867|1000|60
contig08079_length=1867|500|60
contig08079_length=1867|1500|60
contig18733_length=782|0|60
contig18733_length=782|500|60
contig05865_length=684|0|60
contig05865_length=684|500|60
contig16066_length=161|0|60
log2(expression)
0 2 4 6 8 12 16
StageI StageII StageIII StageIV StageV StageVI

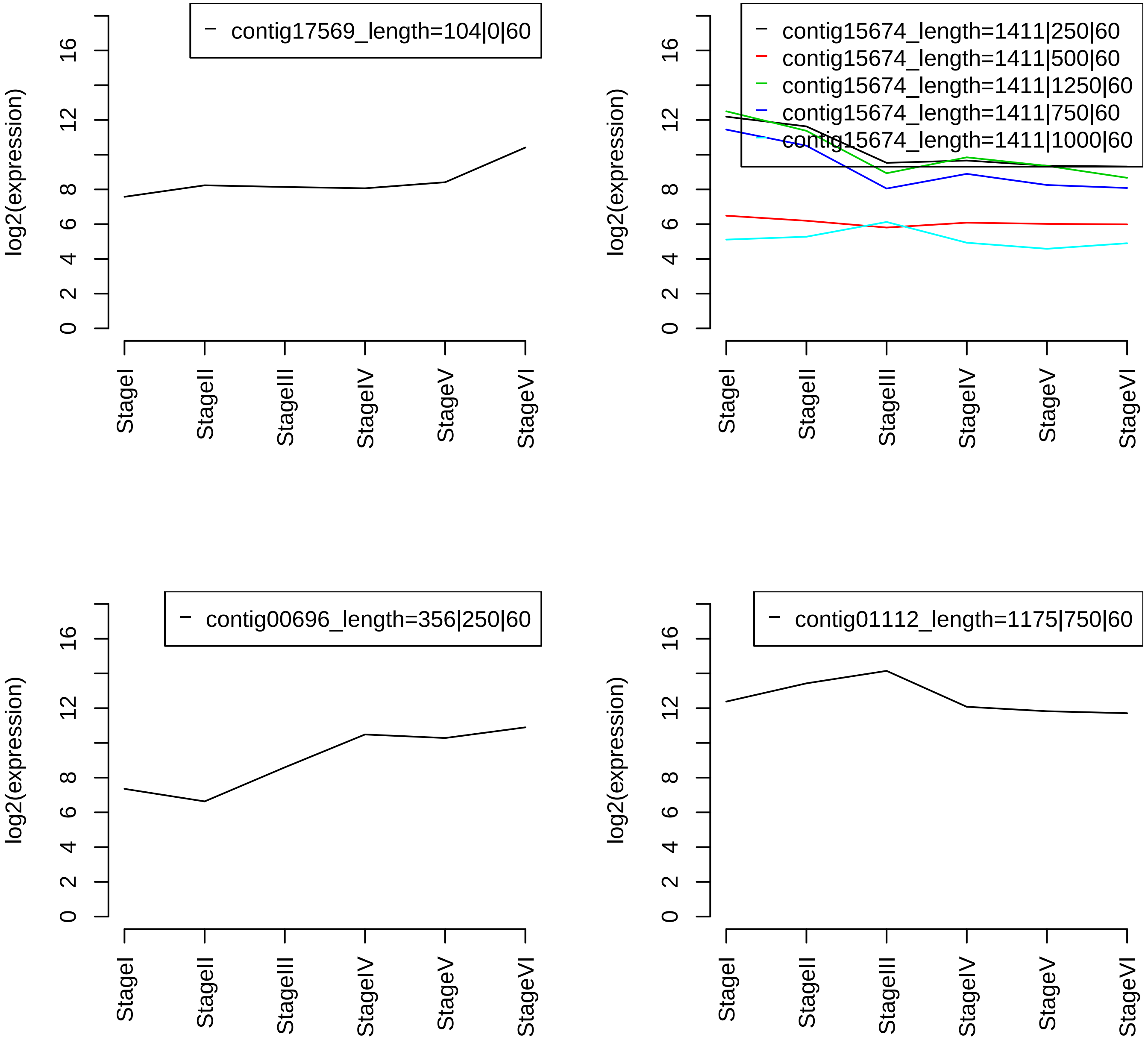
contig17569_length=104|0|60
contig15674_length=1411|250|60
contig15674_length=1411|500|60
contig15674_length=1411|1250|60
contig15674_length=1411|750|60
contig15674_length=1411|1000|60
contig00696_length=356|250|60
contig01112_length=1175|750|60
log2(expression)
0
2
4
6
8
12
16
StageI
StageII
StageIII
StageIV
StageV
StageVI

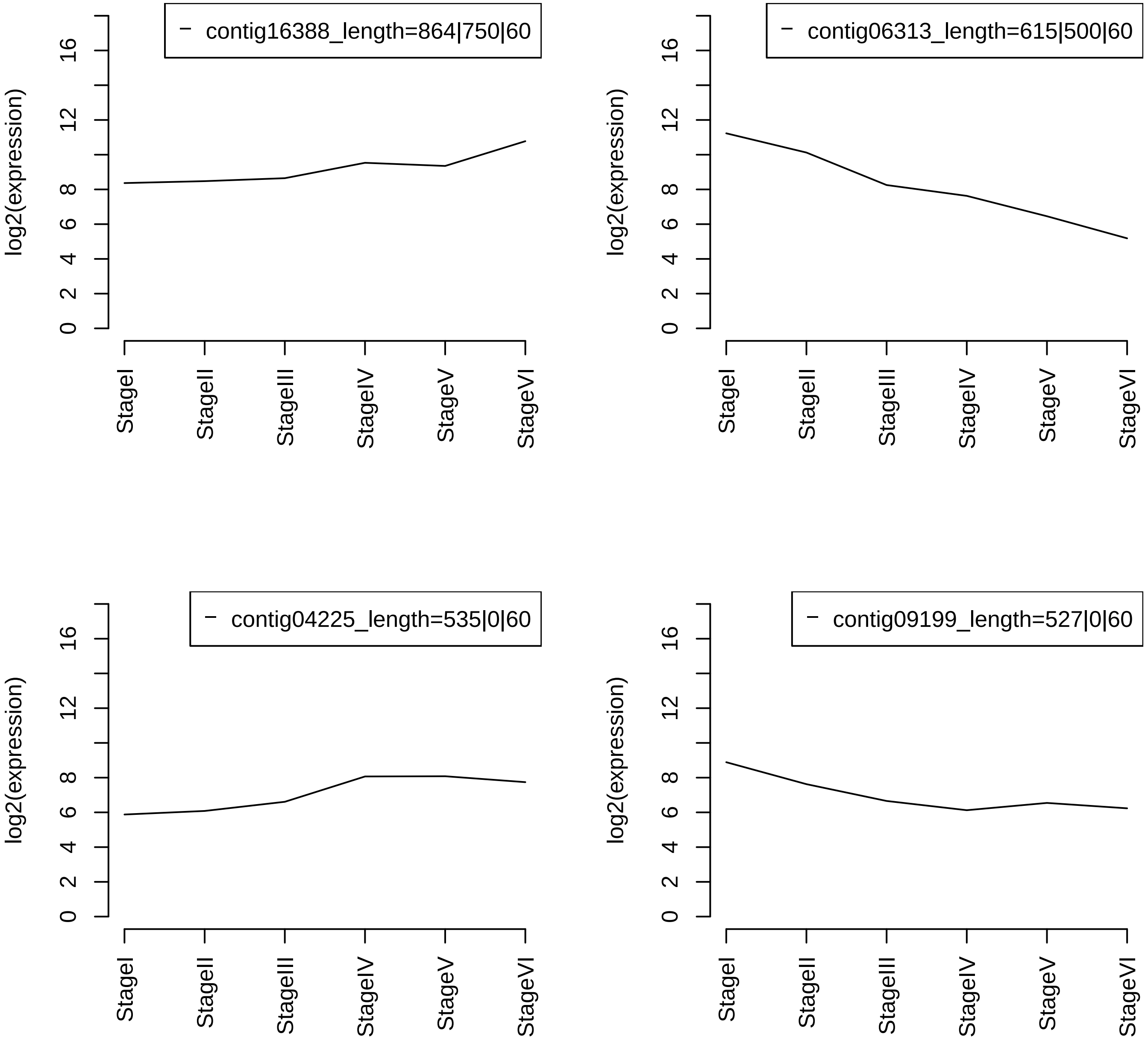
contig16388_length=864|750|60
contig06313_length=615|500|60
contig04225_length=535|0|60
contig09199_length=527|0|60
log2(expression)
0
2
4
6
8
12
16
StageI
StageII
StageIII
StageIV
StageV
StageVI

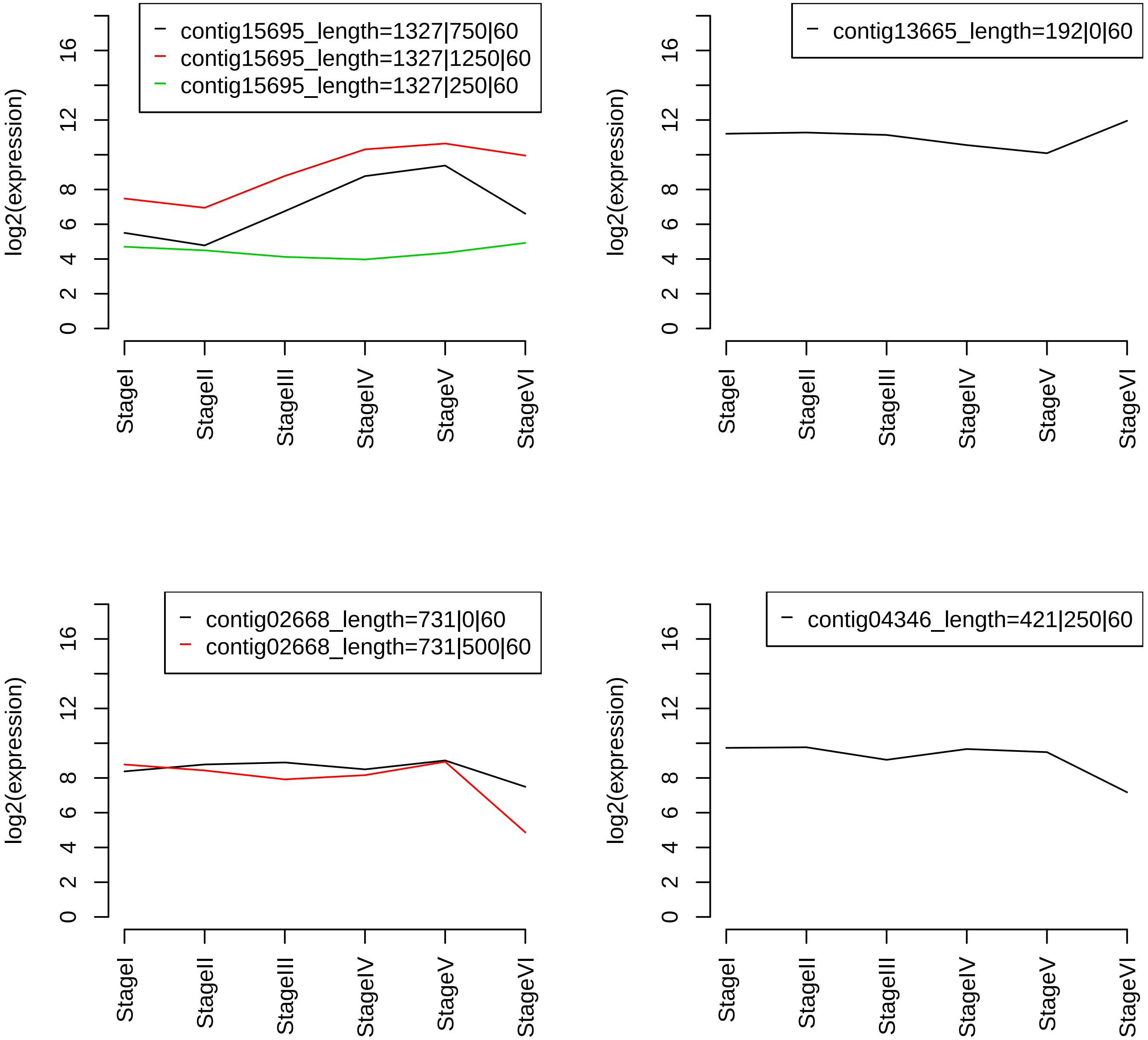
contig15695_length=1327|750|60
contig15695_length=1327|1250|60
contig15695_length=1327|250|60
contig13665_length=192|0|60
contig02668_length=731|0|60
contig02668_length=731|500|60
contig04346_length=421|250|60
log2(expression)
0 2 4 6 8 12 16
StageI StageII StageIII StageIV StageV StageVI

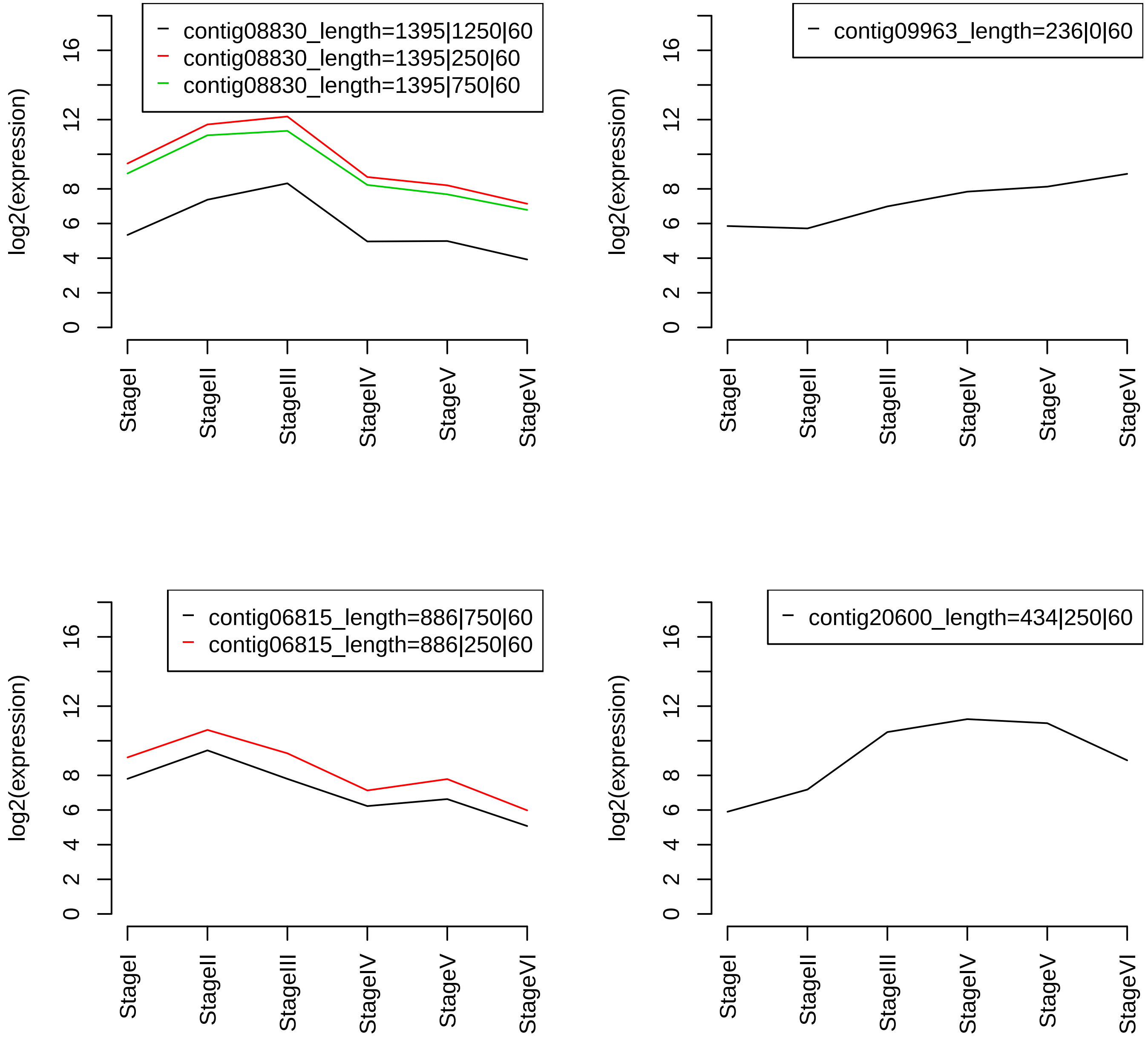

contig08830_length=1395|1250|60
contig08830_length=1395|250|60
contig08830_length=1395|750|60
contig09963_length=236|0|60
contig06815_length=886|750|60
contig06815_length=886|250|60
contig20600_length=434|250|60
log2(expression)
0
2
4
6
8
12
16
StageI
StageII
StageIII
StageIV
StageV
StageVI

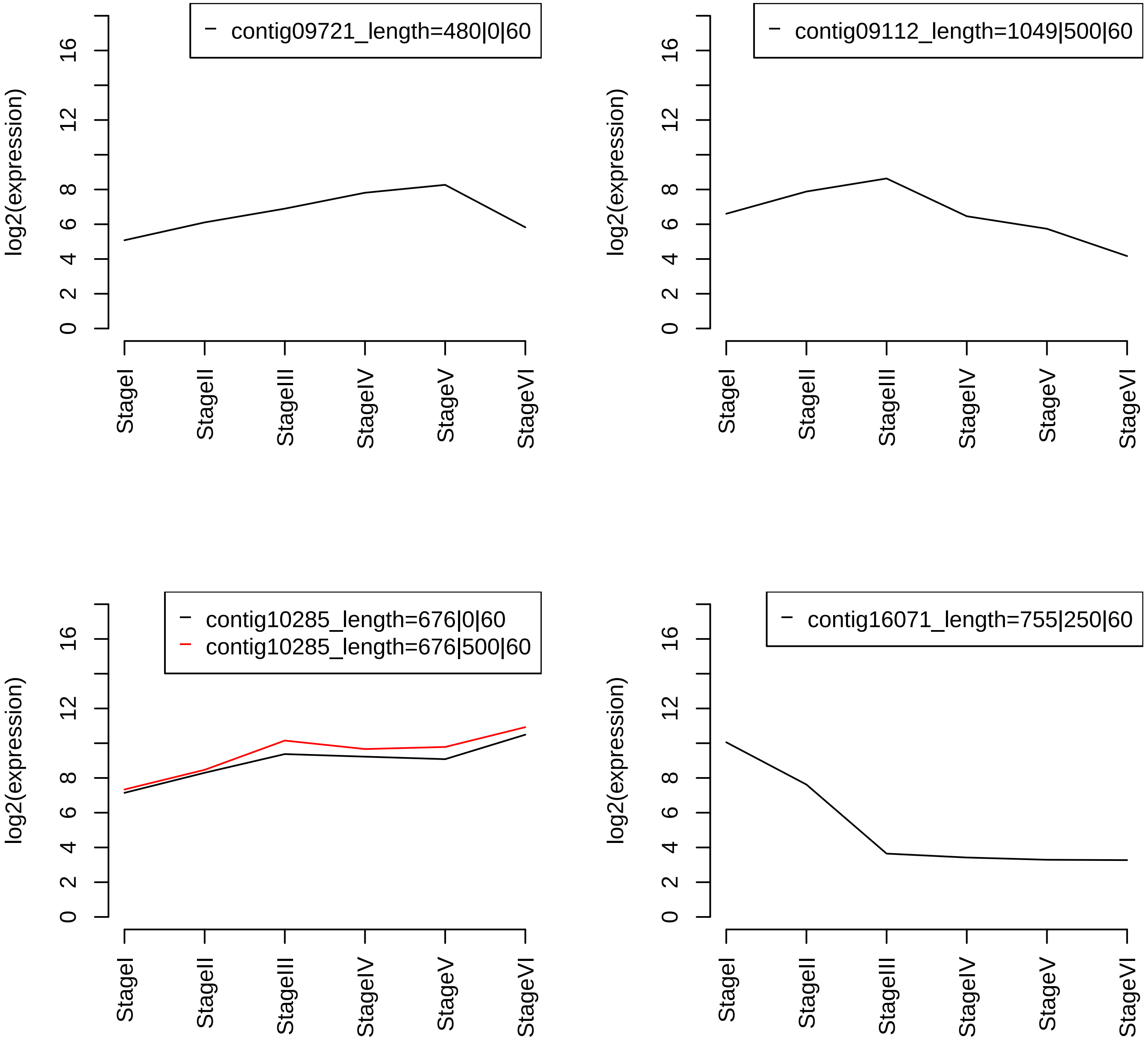

contig09721_length=480|0|60
contig09112_length=1049|500|60
contig10285_length=676|0|60
contig10285_length=676|500|60
contig16071_length=755|250|60
log2(expression)
0
2
4
6
8
12
16
StageI
StageII
StageIII
StageIV
StageV
StageVI

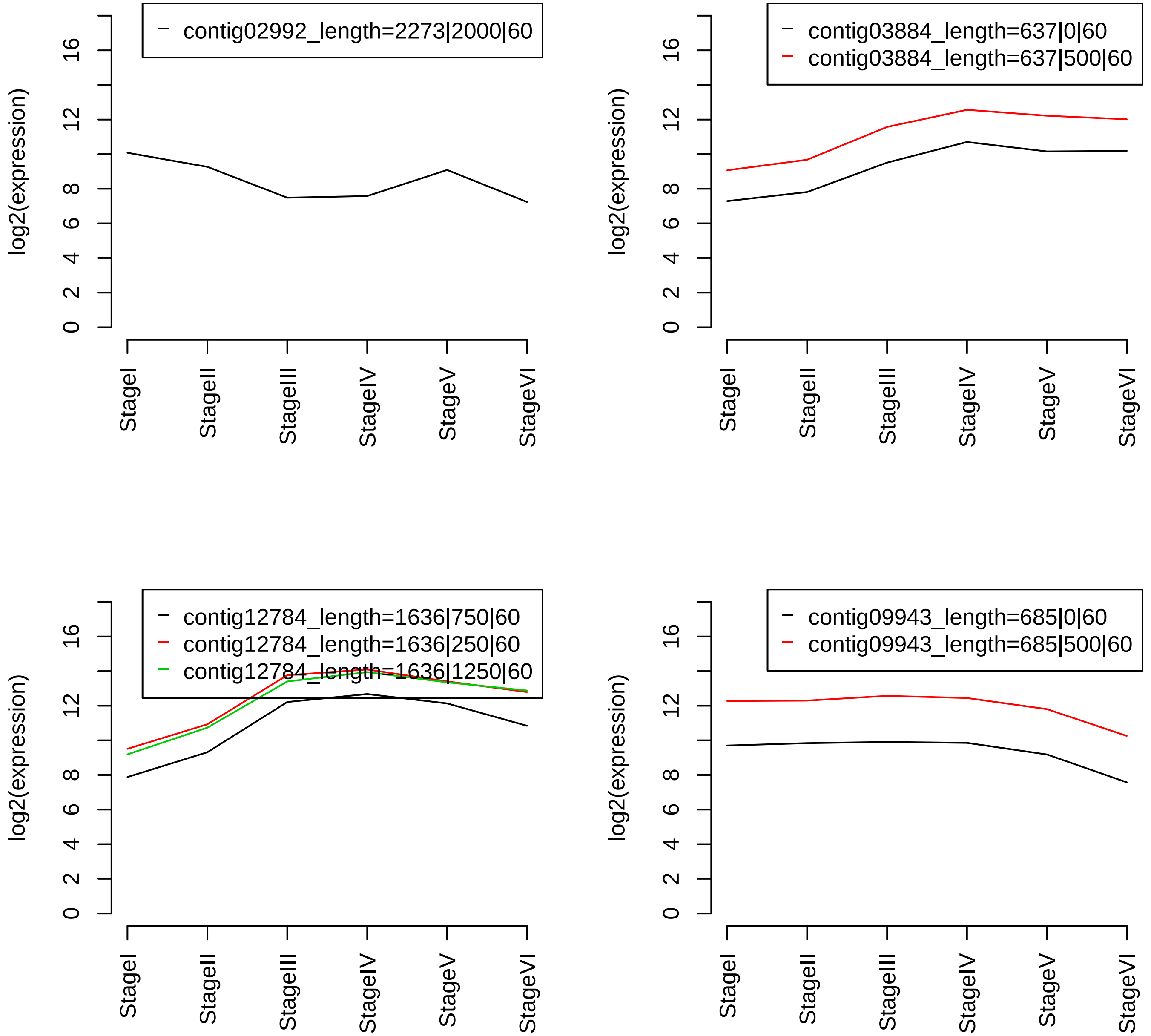
contig02992_length=2273|2000|60
contig03884_length=637|0|60
contig03884_length=637|500|60
contig12784_length=1636|750|60
contig12784_length=1636|250|60
contig12784_length=1636|1250|60
contig09943_length=685|0|60
contig09943_length=685|500|60
log2(expression)
0
2
4
6
8
12
16
StageI
StageII
StageIII
StageIV
StageV
StageVI

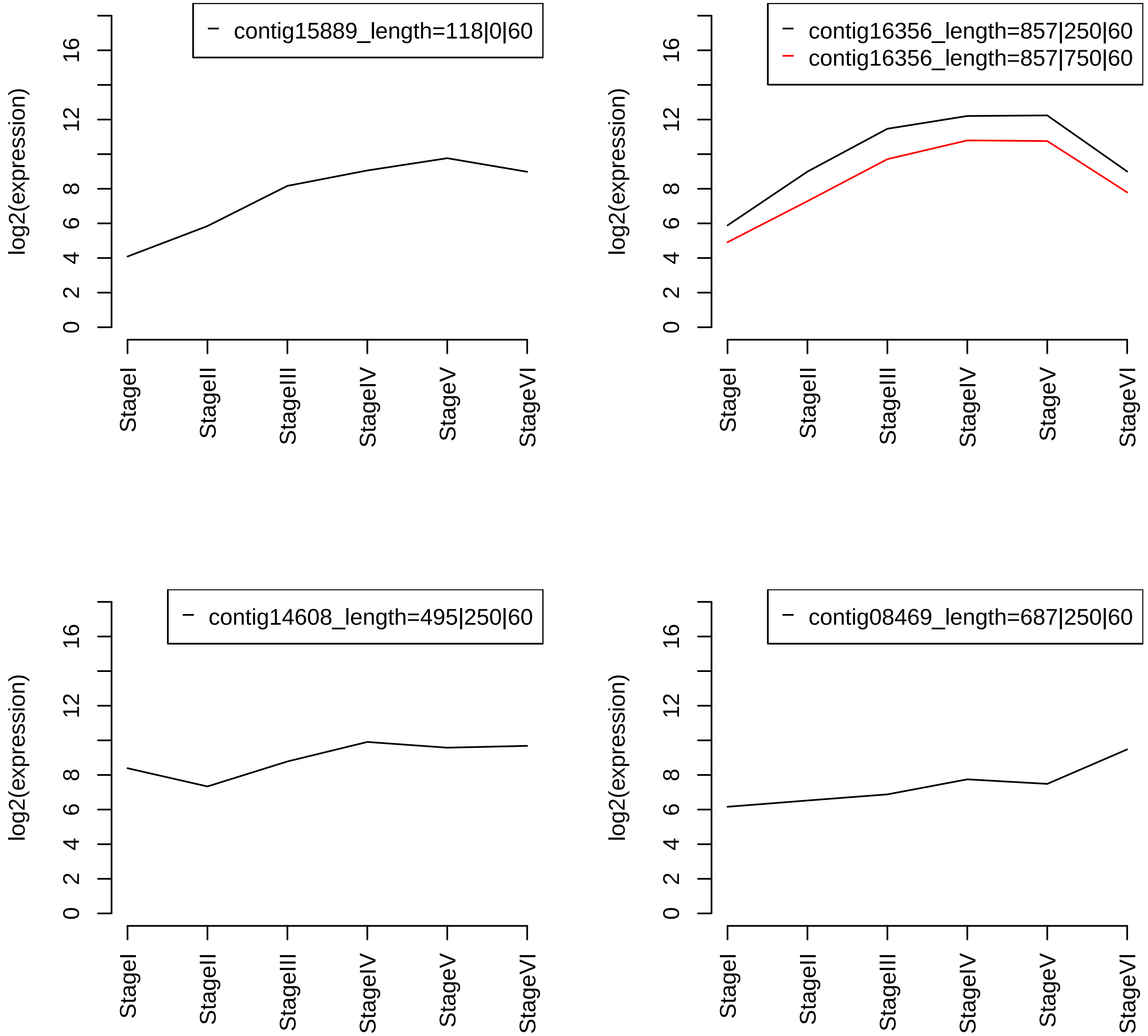
contig15889_length=118|0|60
contig16356_length=857|250|60
contig16356_length=857|750|60
contig14608_length=495|250|60
contig08469_length=687|250|60
log2(expression)
0
2
4
6
8
12
16
StageI
StageII
StageIII
StageIV
StageV
StageVI

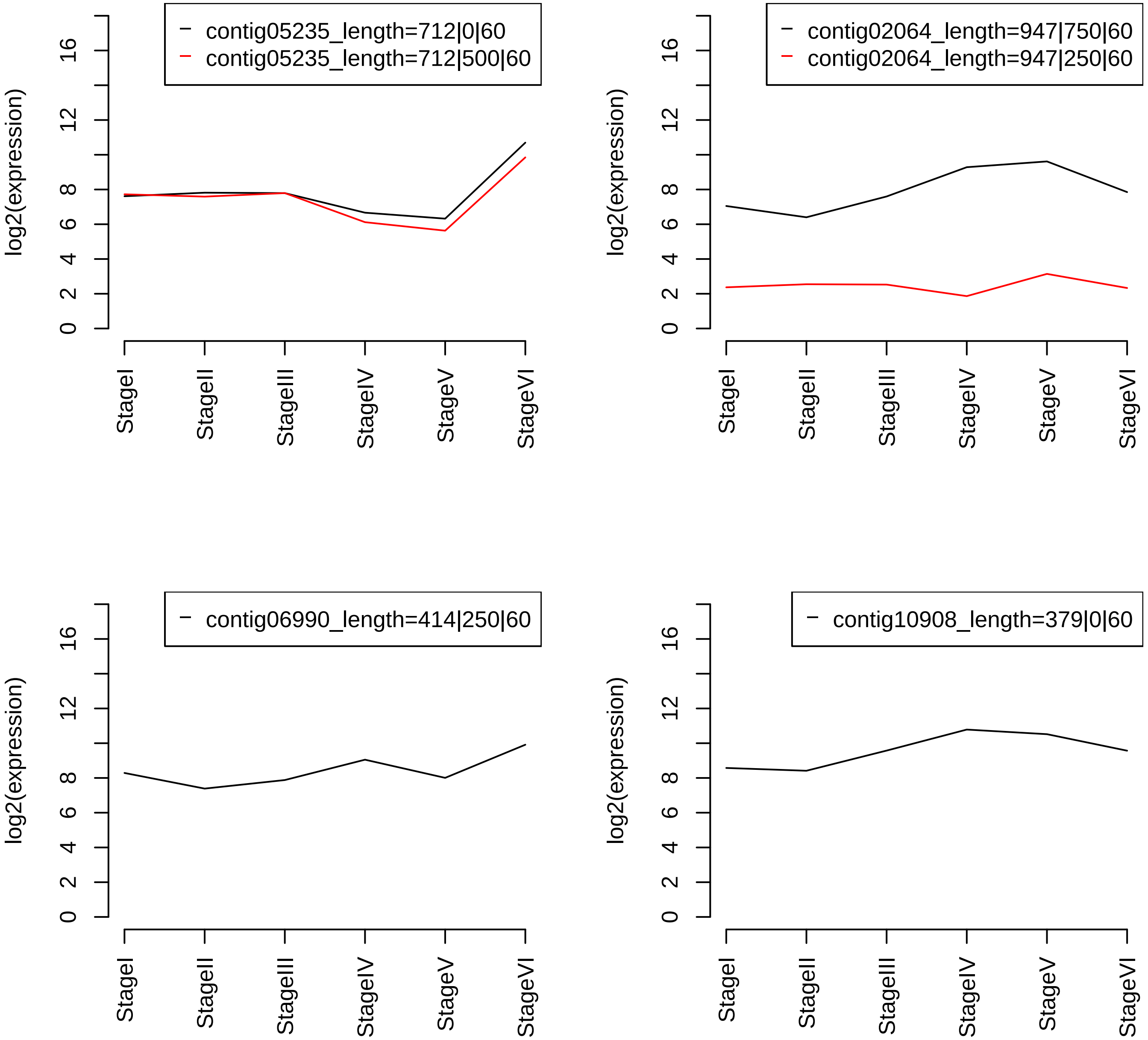
contig05235_length=712|0|60
contig05235_length=712|500|60
log2(expression)
0
2
4
6
8
12
16
StageI
StageII
StageIII
StageIV
StageV
StageVI
contig02064_length=947|750|60
contig02064_length=947|250|60
contig06990_length=414|250|60
contig10908_length=379|0|60

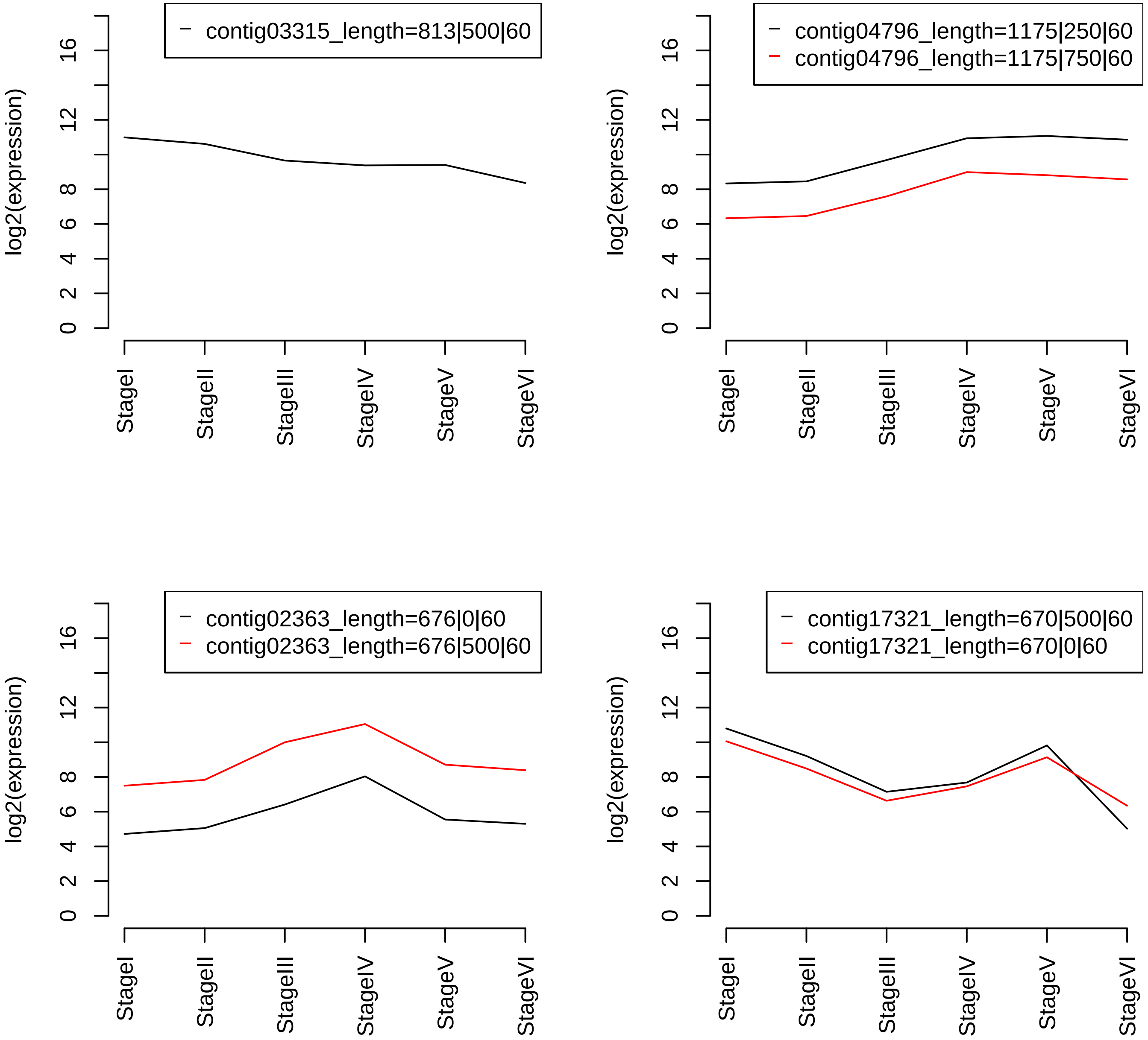
contig03315_length=813|500|60
contig04796_length=1175|250|60
contig04796_length=1175|750|60
contig02363_length=676|0|60
contig02363_length=676|500|60
contig17321_length=670|500|60
contig17321_length=670|0|60
log2(expression)
0 2 4 6 8 12 16
StageI
StageII
StageIII
StageIV
StageV
StageVI

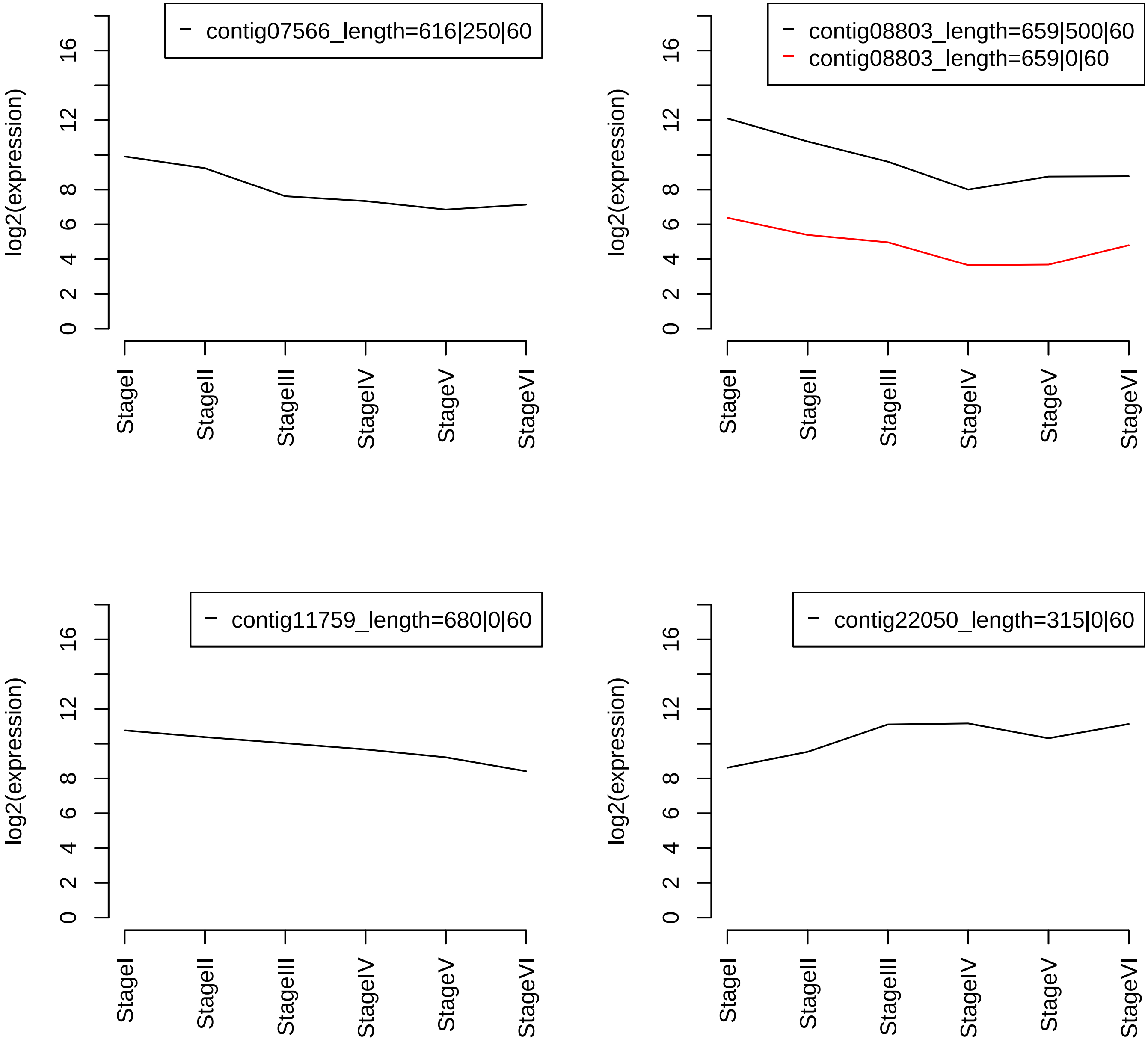
contig07566_length=616|250|60
contig08803_length=659|500|60
contig08803_length=659|0|60
contig11759_length=680|0|60
contig22050_length=315|0|60
log2(expression)
0
2
4
6
8
12
16
StageI
StageII
StageIII
StageIV
StageV
StageVI

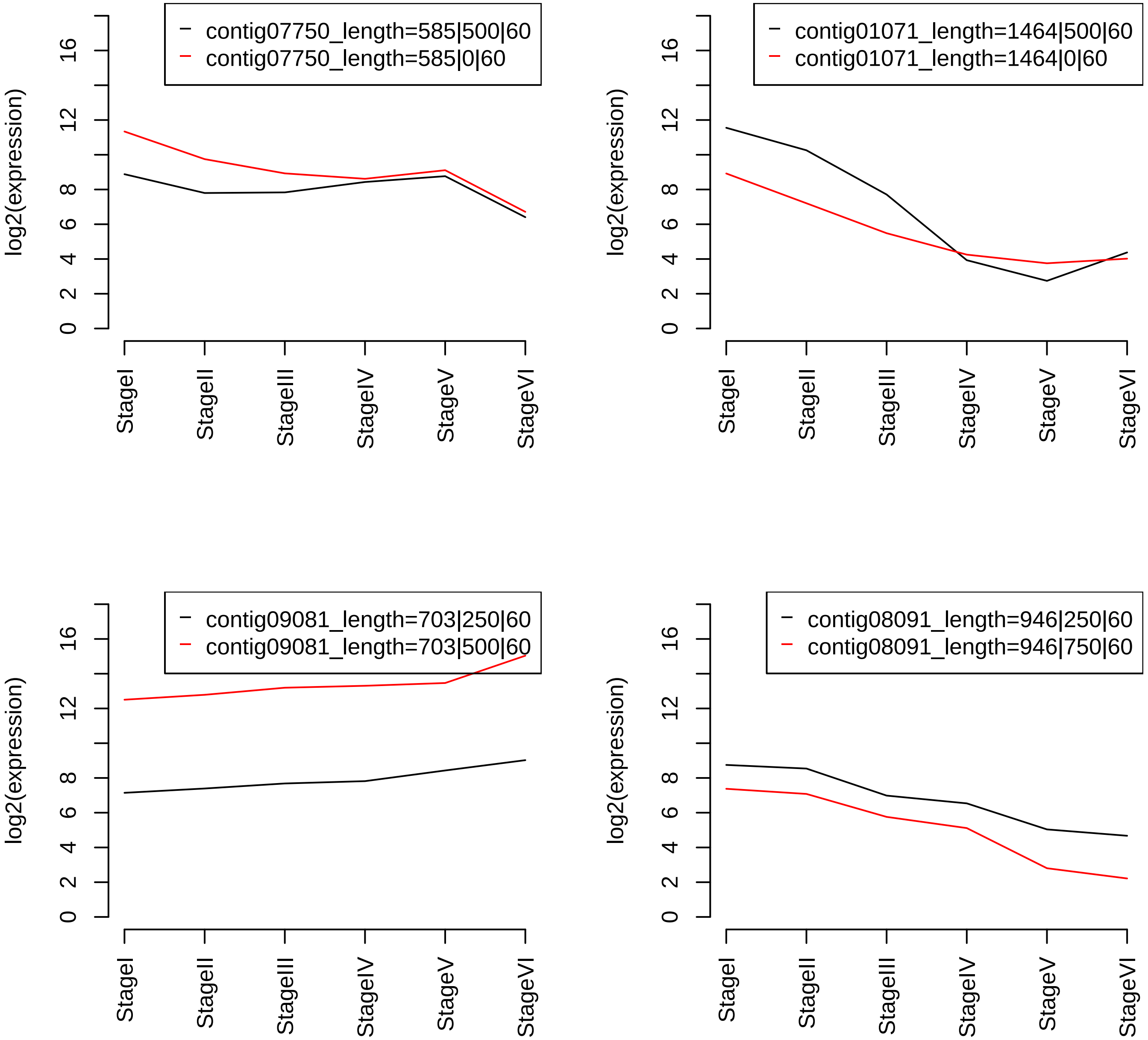

contig07750_length=585|500|60
contig07750_length=585|0|60
contig01071_length=1464|500|60
contig01071_length=1464|0|60
contig09081_length=703|250|60
contig09081_length=703|500|60
contig08091_length=946|250|60
contig08091_length=946|750|60
log2(expression)
0
2
4
6
8
12
16
StageI
StageII
StageIII
StageIV
StageV
StageVI

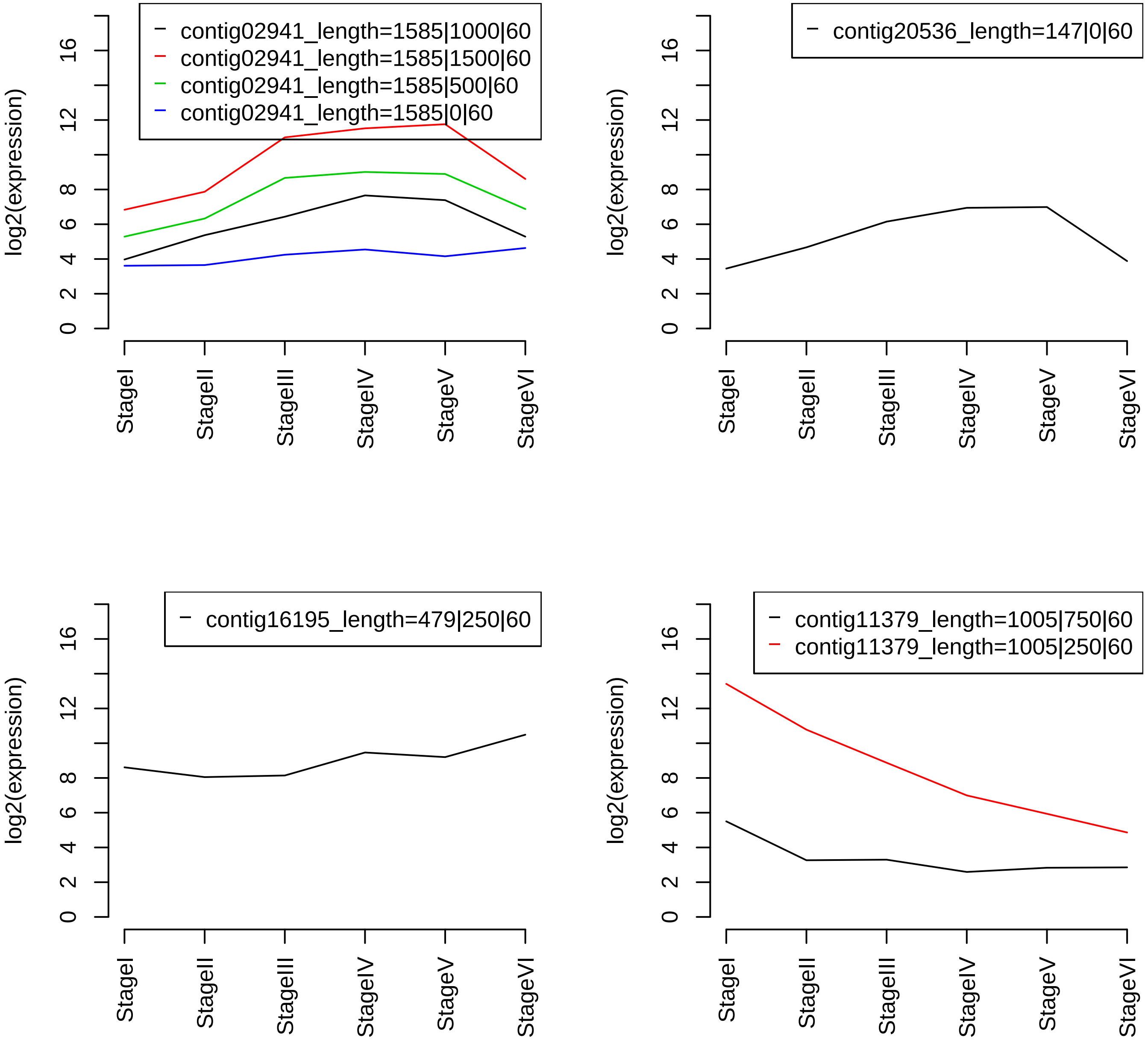

contig02941_length=1585|1000|60
contig02941_length=1585|1500|60
contig02941_length=1585|500|60
contig02941_length=1585|0|60
contig20536_length=147|0|60
contig16195_length=479|250|60
contig11379_length=1005|750|60
contig11379_length=1005|250|60
log2(expression)
0
2
4
6
8
12
16
StageI
StageII
StageIII
StageIV
StageV
StageVI

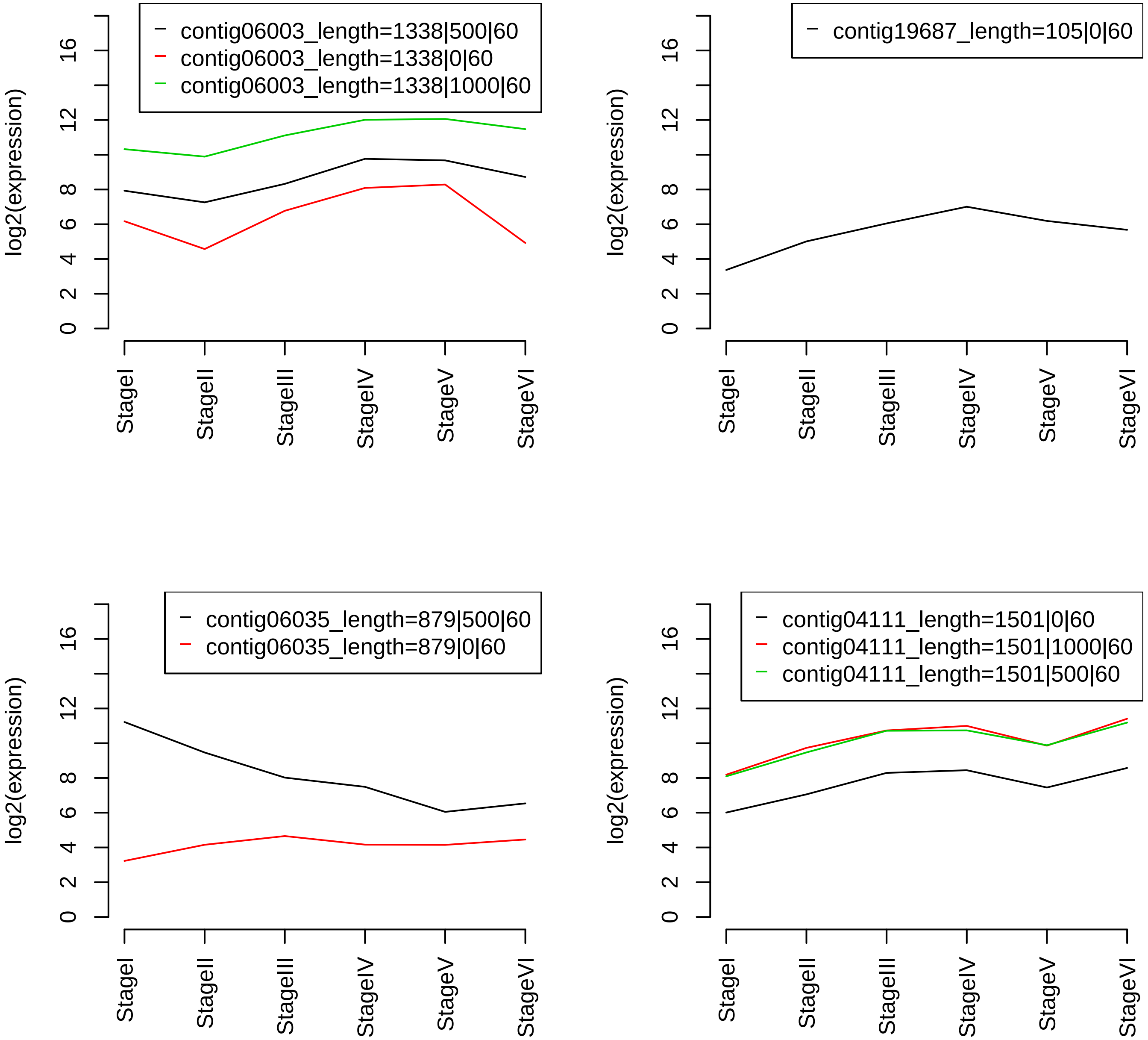
contig06003_length=1338|500|60
contig06003_length=1338|0|60
contig06003_length=1338|1000|60
contig19687_length=105|0|60
contig06035_length=879|500|60
contig06035_length=879|0|60
contig04111_length=1501|0|60
contig04111_length=1501|1000|60
contig04111_length=1501|500|60
log2(expression)
0
2
4
6
8
12
16
StageI
StageII
StageIII
StageIV
StageV
StageVI

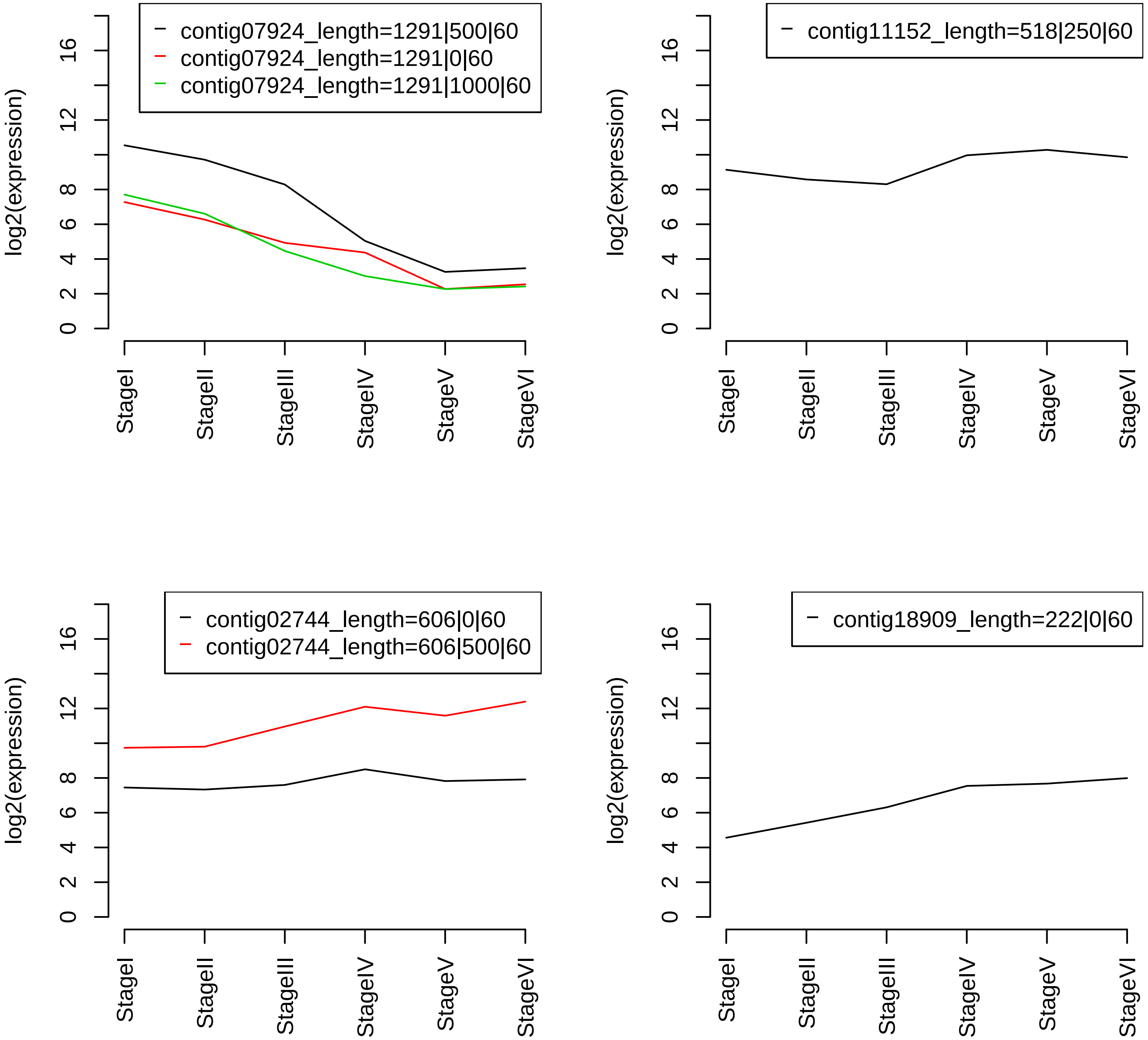
contig07924_length=1291|500|60
contig07924_length=1291|0|60
contig07924_length=1291|1000|60
contig11152_length=518|250|60
contig02744_length=606|0|60
contig02744_length=606|500|60
contig18909_length=222|0|60
log2(expression)
0
2
4
6
8
12
16
StageI
StageII
StageIII
StageIV
StageV
StageVI

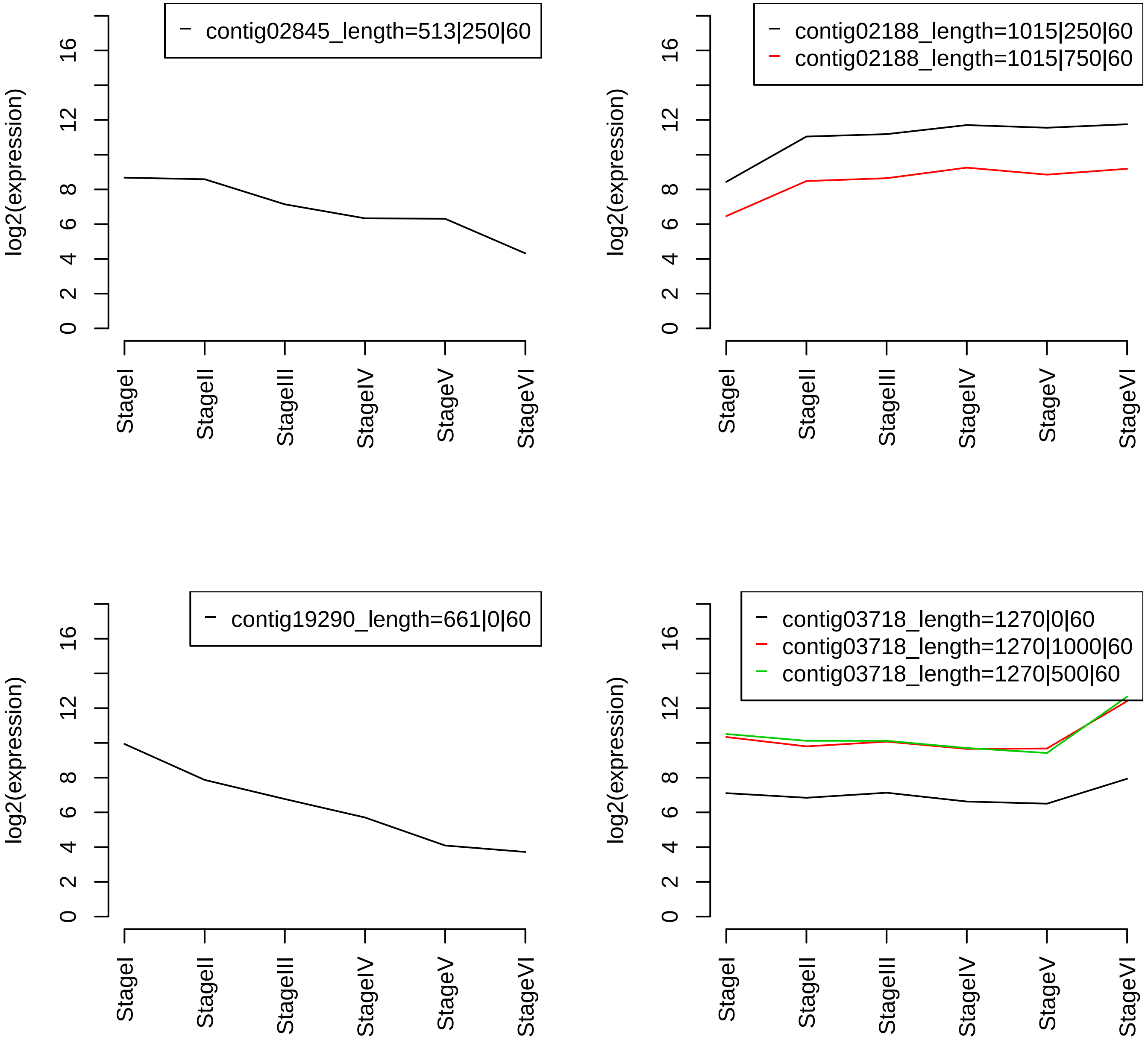
contig02845_length=513|250|60
contig02188_length=1015|250|60
contig02188_length=1015|750|60
contig19290_length=661|0|60
contig03718_length=1270|0|60
contig03718_length=1270|1000|60
contig03718_length=1270|500|60
log2(expression)
0
2
4
6
8
12
16
StageI
StageII
StageIII
StageIV
StageV
StageVI

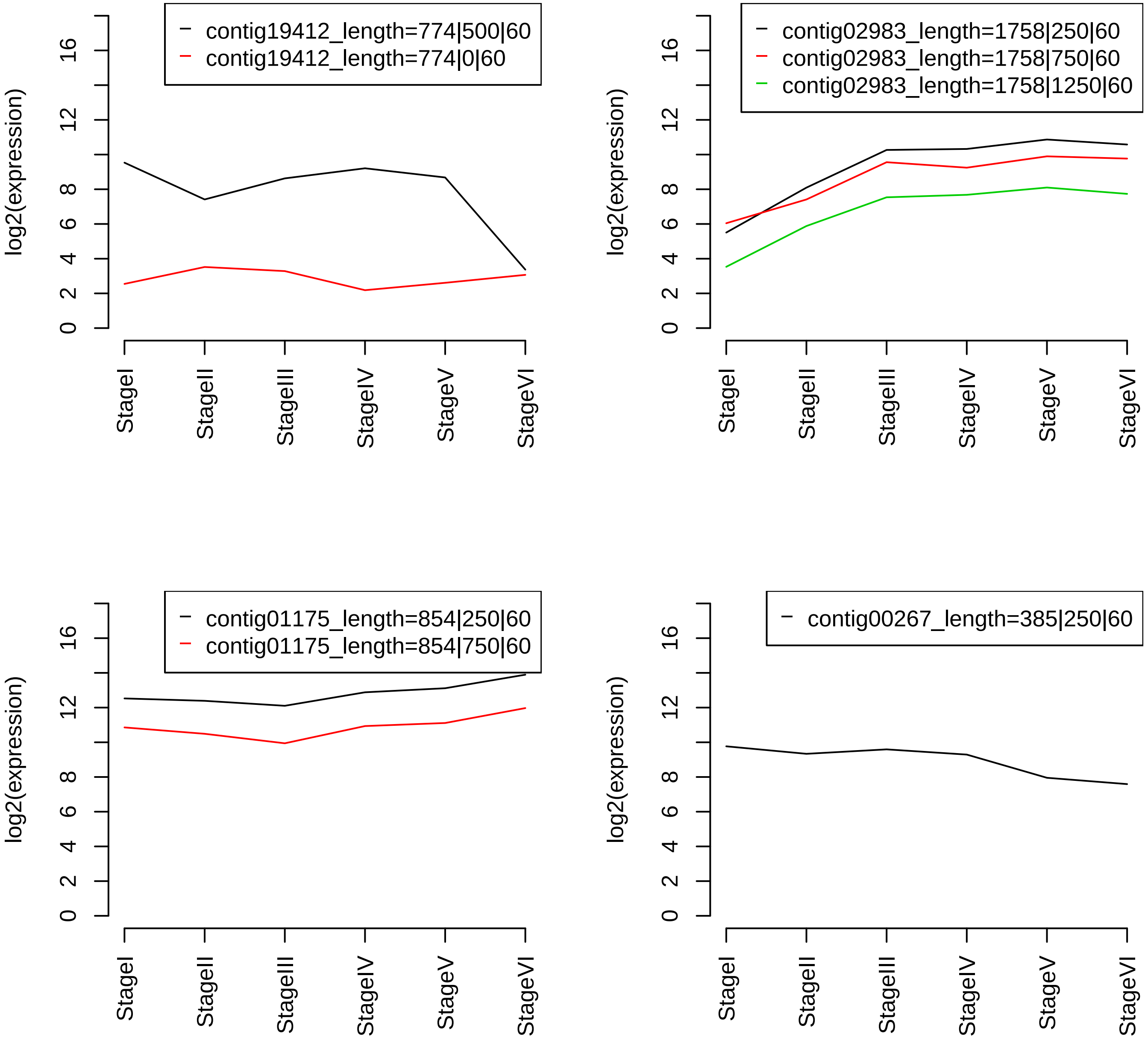

contig19412_length=774|500|60
contig19412_length=774|0|60
contig02983_length=1758|250|60
contig02983_length=1758|750|60
contig02983_length=1758|1250|60
contig01175_length=854|250|60
contig01175_length=854|750|60
contig00267_length=385|250|60
log2(expression)
0
2
4
6
8
12
16
StageI
StageII
StageIII
StageIV
StageV
StageVI

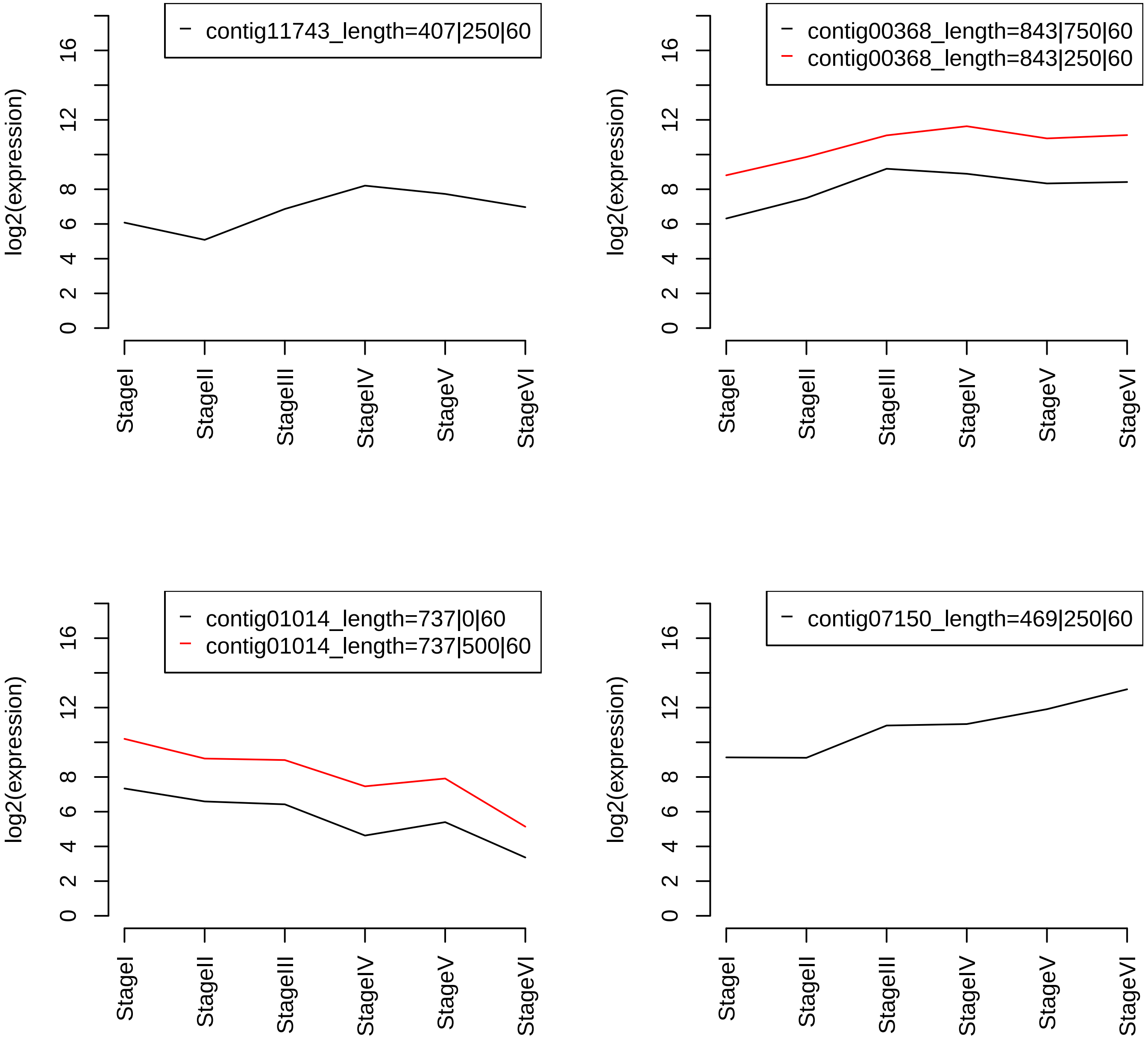
contig11743_length=407|250|60
contig00368_length=843|750|60
contig00368_length=843|250|60
contig01014_length=737|0|60
contig01014_length=737|500|60
contig07150_length=469|250|60
log2(expression)
0 2 4 6 8 12 16
StageI StageII StageIII StageIV StageV StageVI

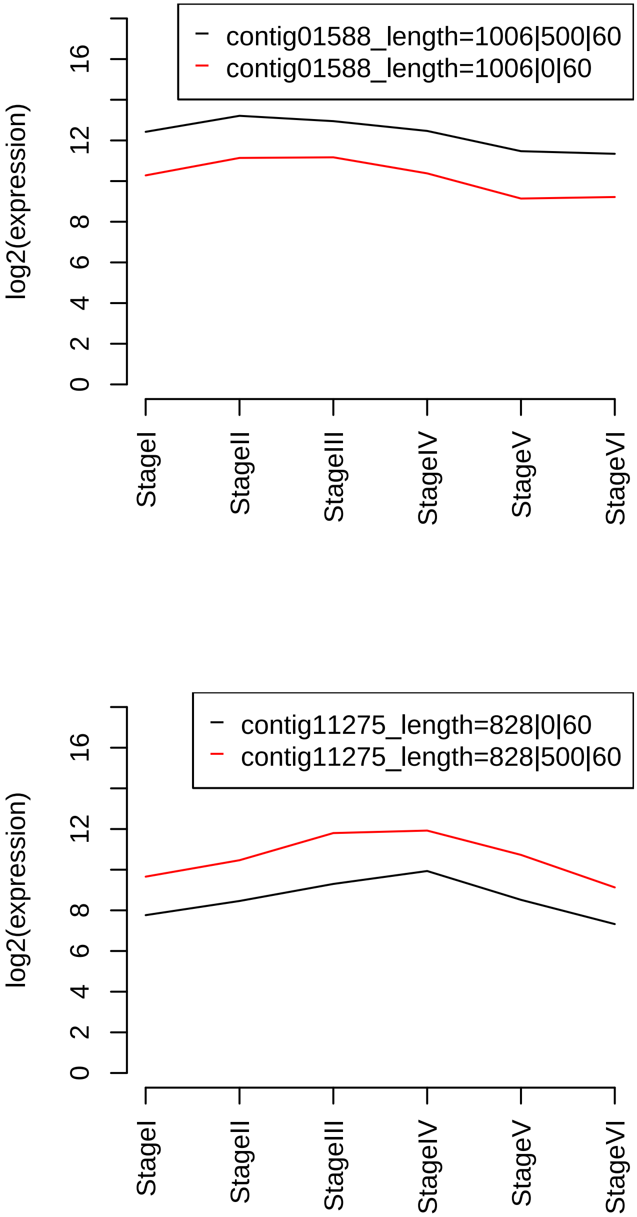
contig01588_length=1006|500|60
contig01588_length=1006|0|60
contig20846_length=535|0|60
contig11275_length=828|0|60
contig11275_length=828|500|60
contig03712_length=641|250|60
log2(expression)
0
2
4
6
8
12
16
StageI
StageII
StageIII
StageIV
StageV
StageVI

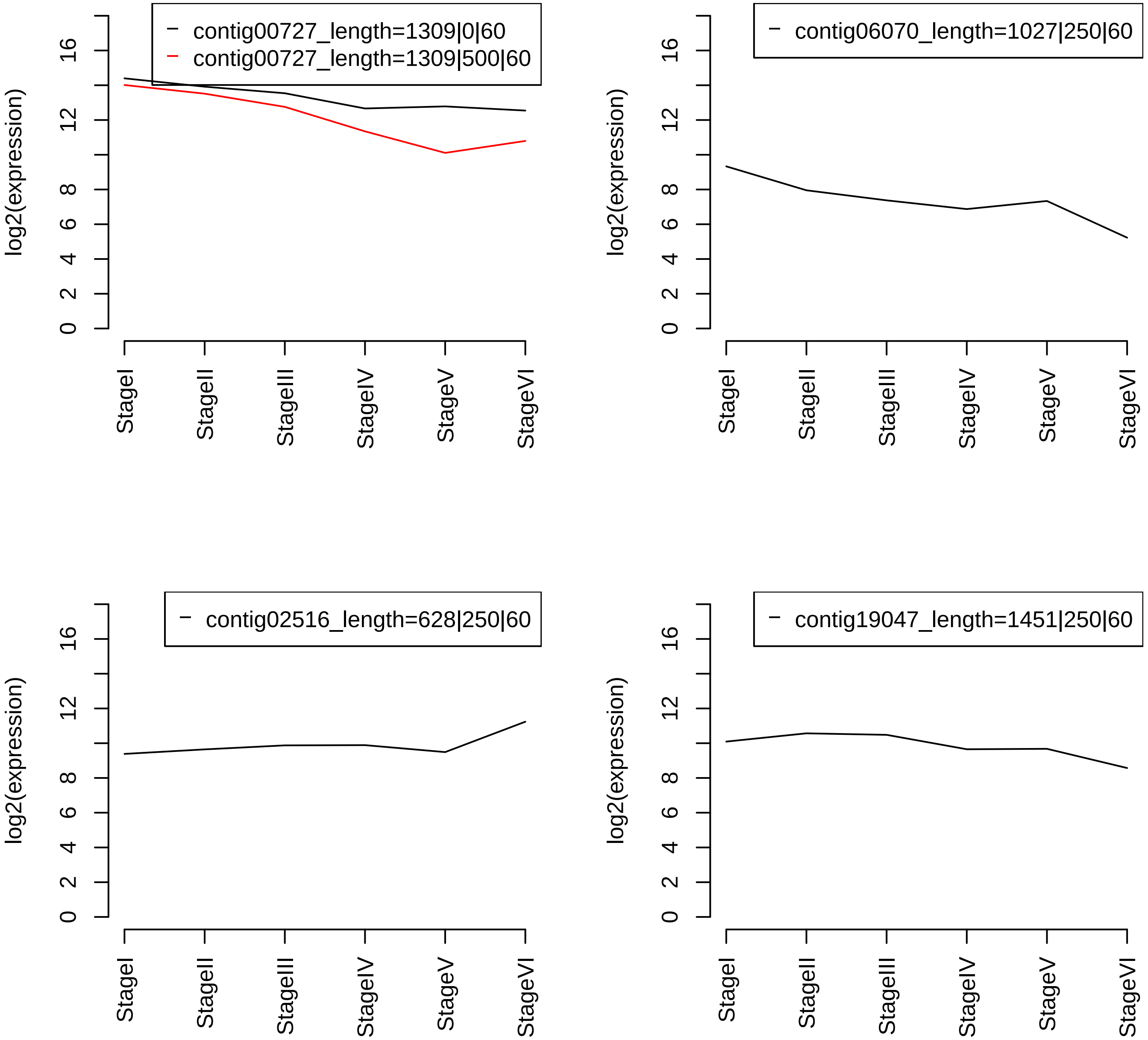

contig00727_length=1309|0|60
contig00727_length=1309|500|60
log2(expression)
0 2 4 6 8 12 16
StageI StageII StageIII StageIV StageV StageVI
contig06070_length=1027|250|60
log2(expression)
0 2 4 6 8 12 16
StageI StageII StageIII StageIV StageV StageVI
contig02516_length=628|250|60
log2(expression)
0 2 4 6 8 12 16
StageI StageII StageIII StageIV StageV StageVI
contig19047_length=1451|250|60
log2(expression)
0 2 4 6 8 12 16
StageI StageII StageIII StageIV StageV StageVI

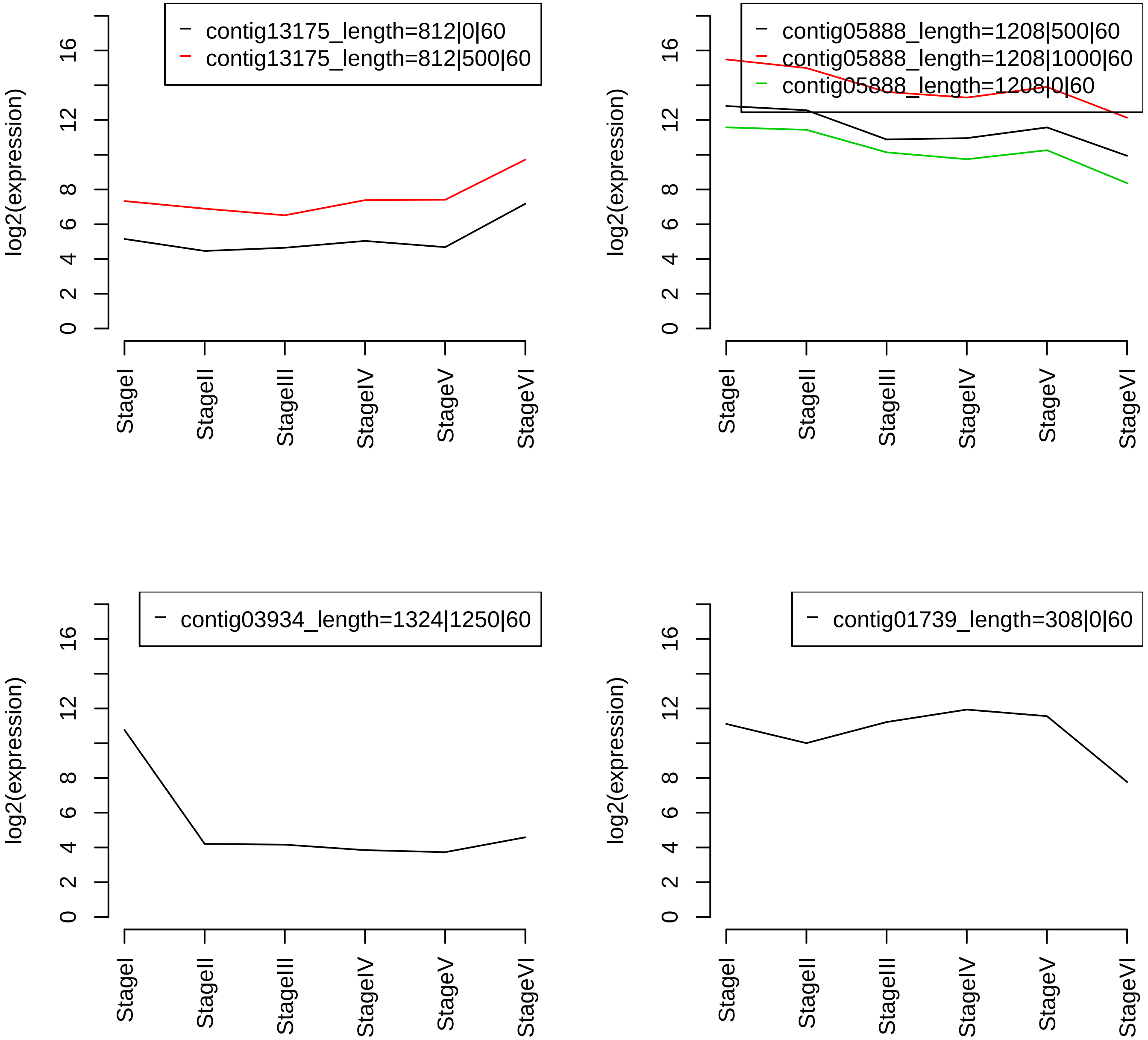

contig13175_length=812|0|60
contig13175_length=812|500|60
contig05888_length=1208|500|60
contig05888_length=1208|1000|60
contig05888_length=1208|0|60
contig03934_length=1324|1250|60
contig01739_length=308|0|60
log2(expression)
0 2 4 6 8 12 16
StageI StageII StageIII StageIV StageV StageVI

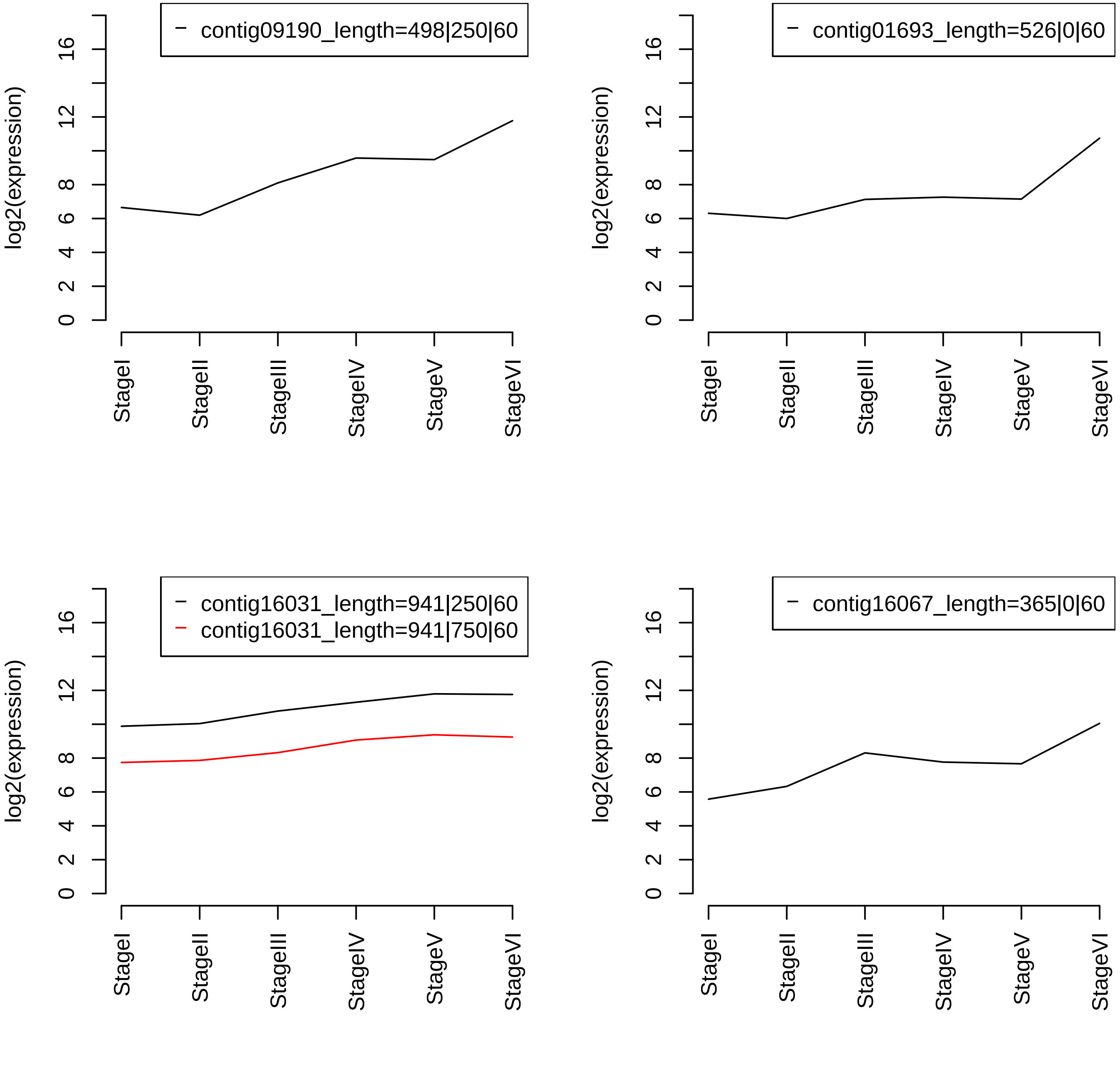

contig09190_length=498|250|60
contig01693_length=526|0|60
contig16031_length=941|250|60
contig16031_length=941|750|60
contig16067_length=365|0|60
log2(expression)
0
2
4
6
8
12
16
StageI
StageII
StageIII
StageIV
StageV
StageVI

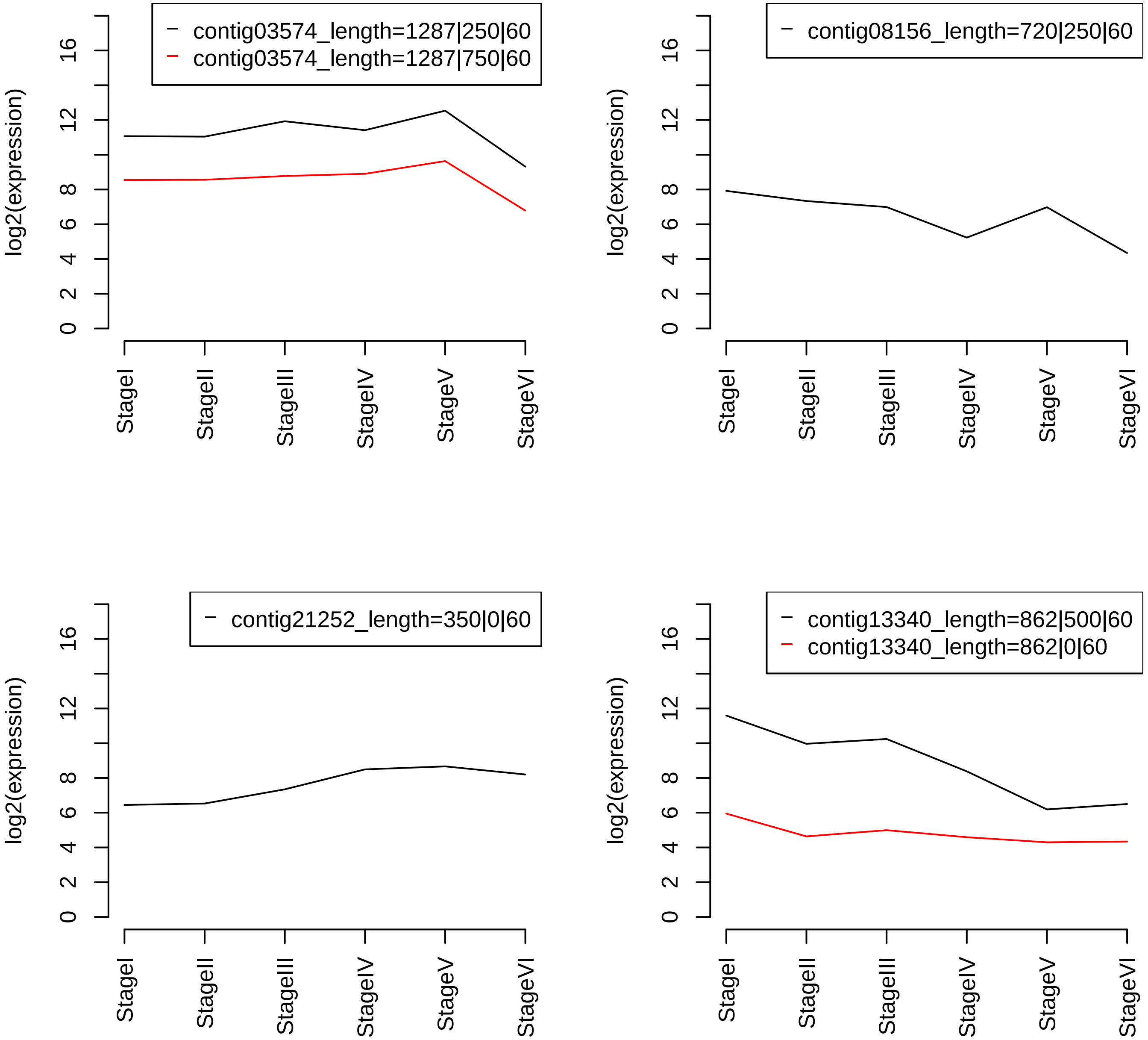
contig03574_length=1287|250|60
contig03574_length=1287|750|60
contig08156_length=720|250|60
contig21252_length=350|0|60
contig13340_length=862|500|60
contig13340_length=862|0|60
log2(expression)
0
2
4
6
8
12
16
StageI
StageII
StageIII
StageIV
StageV
StageVI

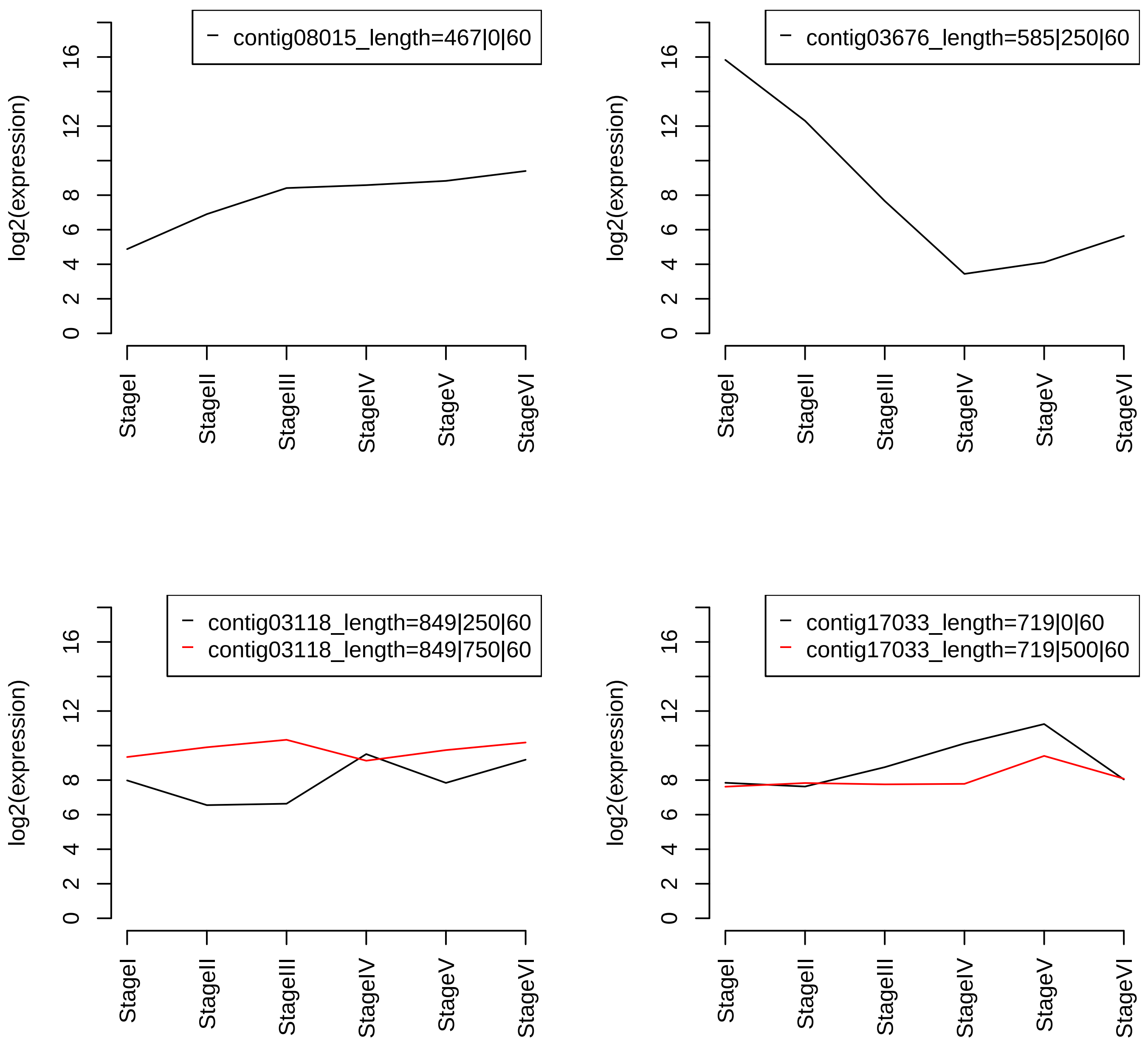
contig08015_length=467|0|60
contig03676_length=585|250|60
contig03118_length=849|250|60
contig03118_length=849|750|60
contig17033_length=719|0|60
contig17033_length=719|500|60
log2(expression)
0
2
4
6
8
12
16
StageI
StageII
StageIII
StageIV
StageV
StageVI

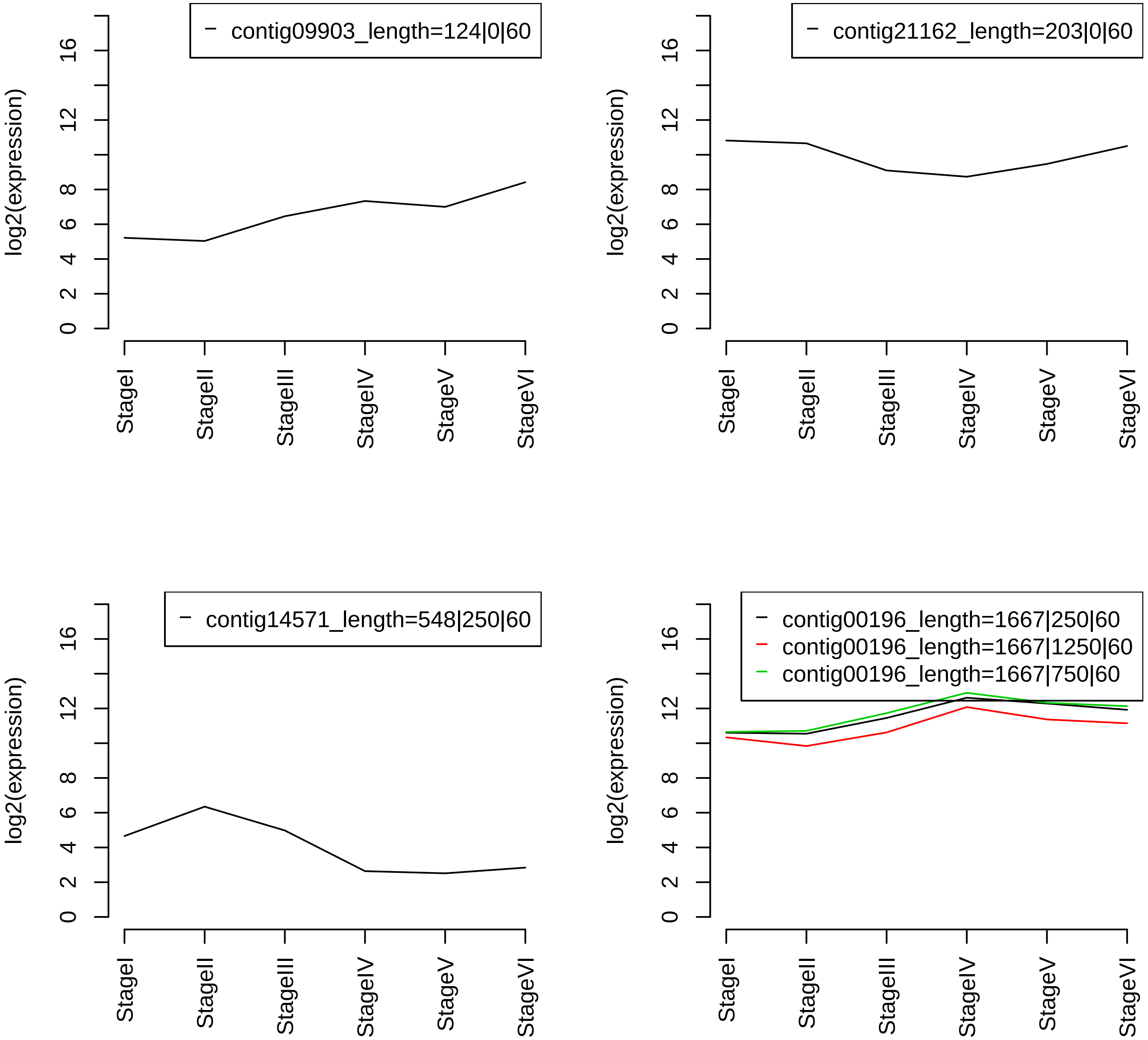
contig09903_length=124|0|60
contig21162_length=203|0|60
contig14571_length=548|250|60
contig00196_length=1667|250|60
contig00196_length=1667|1250|60
contig00196_length=1667|750|60
log2(expression)
StageI
StageII
StageIII
StageIV
StageV
StageVI

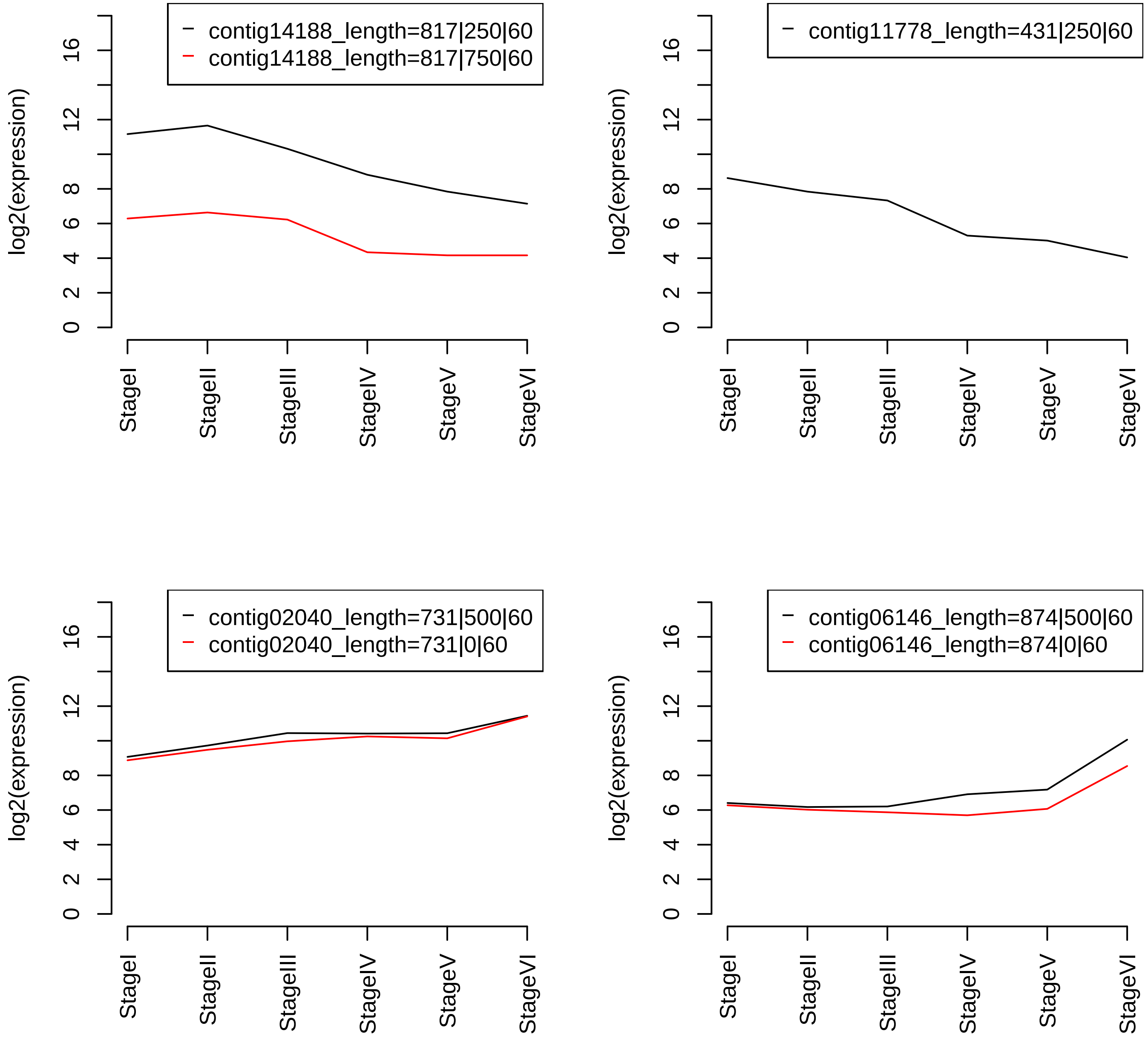

contig14188_length=817|250|60
contig14188_length=817|750|60
contig11778_length=431|250|60
contig02040_length=731|500|60
contig02040_length=731|0|60
contig06146_length=874|500|60
contig06146_length=874|0|60
log2(expression)
0 2 4 6 8 12 16
StageI StageII StageIII StageIV StageV StageVI

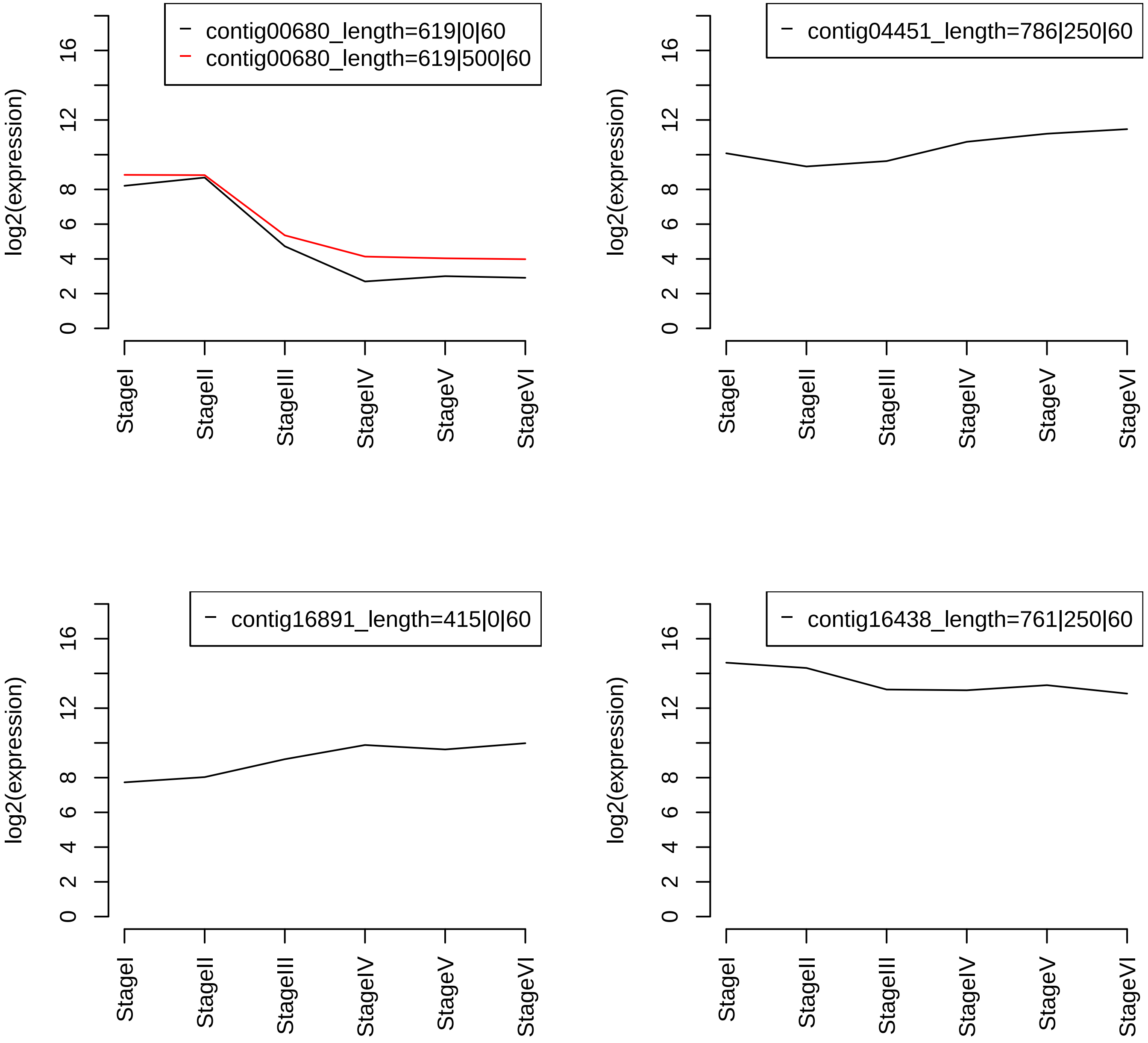
contig00680_length=619|0|60
contig00680_length=619|500|60
contig04451_length=786|250|60
contig16891_length=415|0|60
contig16438_length=761|250|60
log2(expression)
0
2
4
6
8
12
16
StageI
StageII
StageIII
StageIV
StageV
StageVI

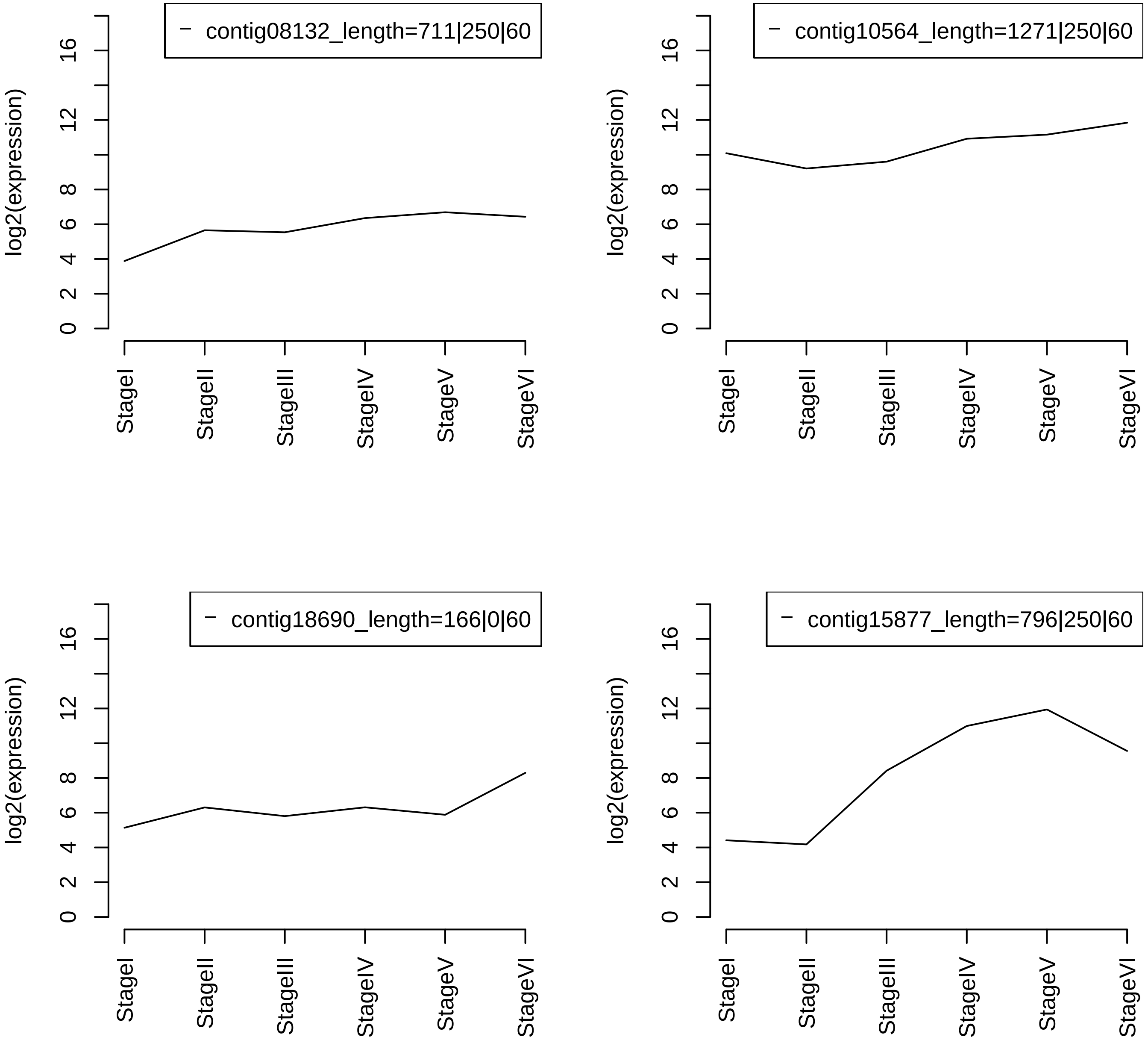
contig08132_length=711|250|60
contig10564_length=1271|250|60
contig18690_length=166|0|60
contig15877_length=796|250|60
log2(expression)
0
2
4
6
8
12
16
StageI
StageII
StageIII
StageIV
StageV
StageVI

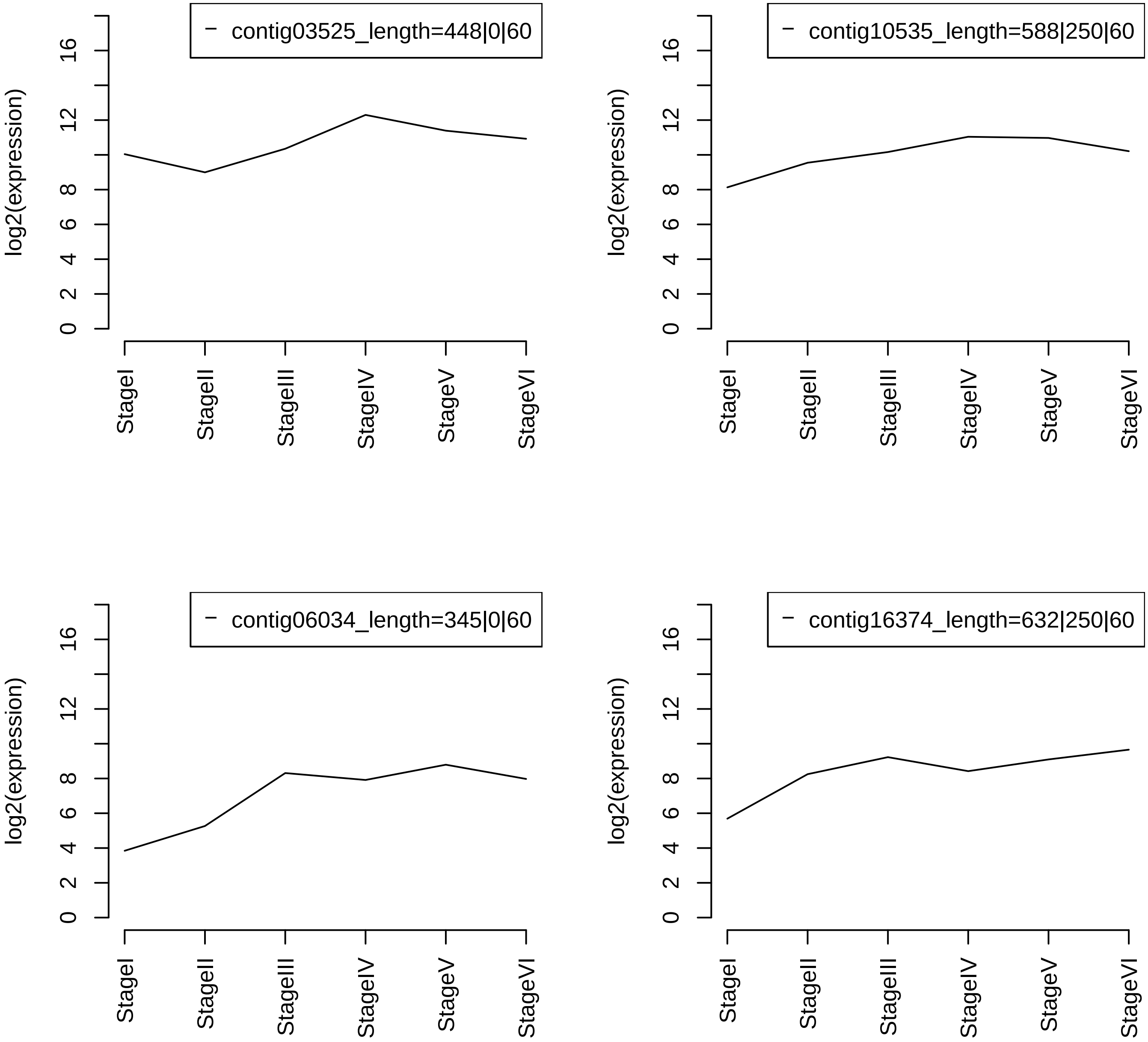

contig03525_length=448|0|60
contig10535_length=588|250|60
contig06034_length=345|0|60
contig16374_length=632|250|60
log2(expression)
0
2
4
6
8
12
16
StageI
StageII
StageIII
StageIV
StageV
StageVI

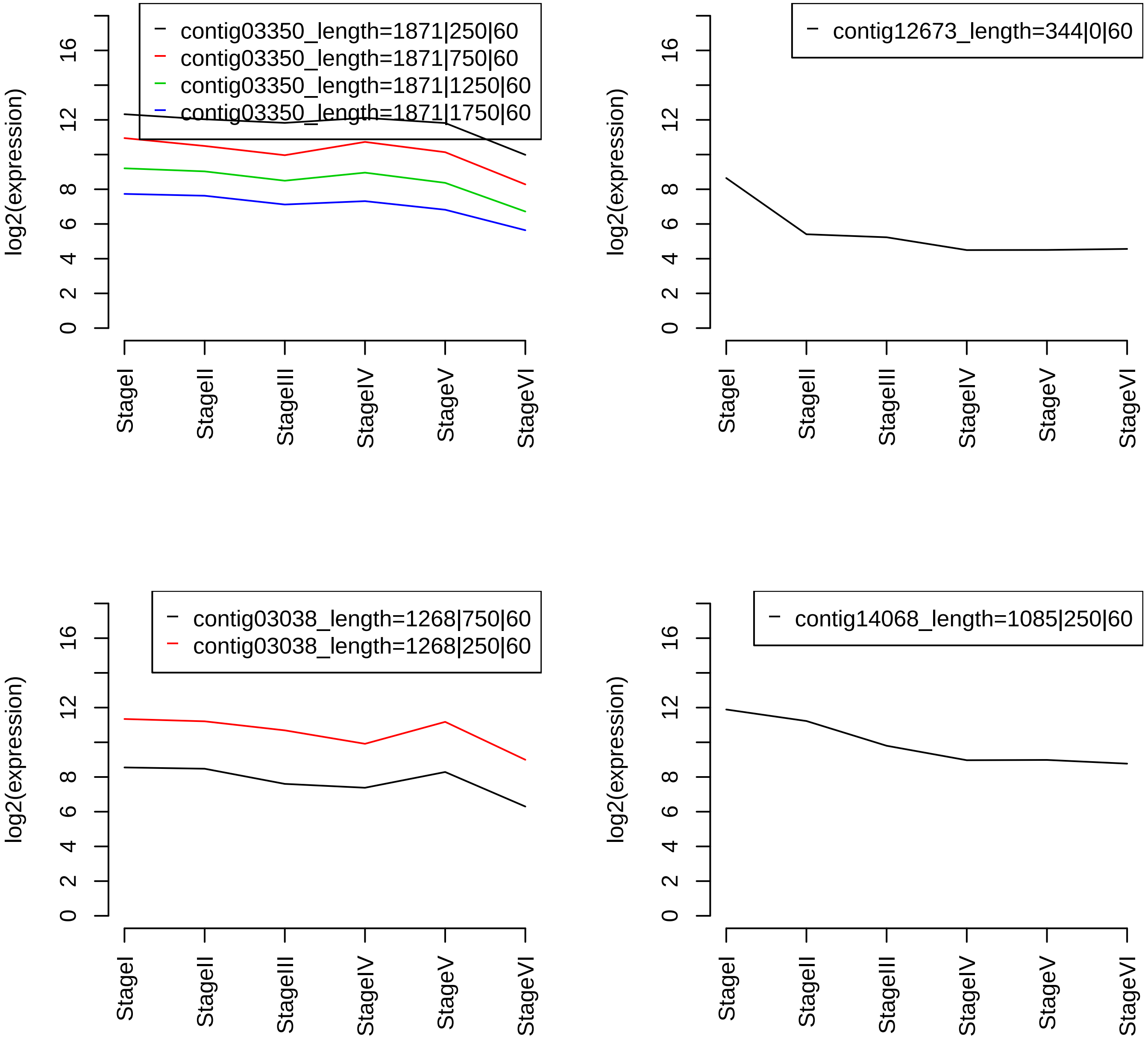
contig03350_length=1871|250|60
contig03350_length=1871|750|60
contig03350_length=1871|1250|60
contig03350_length=1871|1750|60
contig12673_length=344|0|60
contig03038_length=1268|750|60
contig03038_length=1268|250|60
contig14068_length=1085|250|60
log2(expression)
0
2
4
6
8
12
16
StageI
StageII
StageIII
StageIV
StageV
StageVI

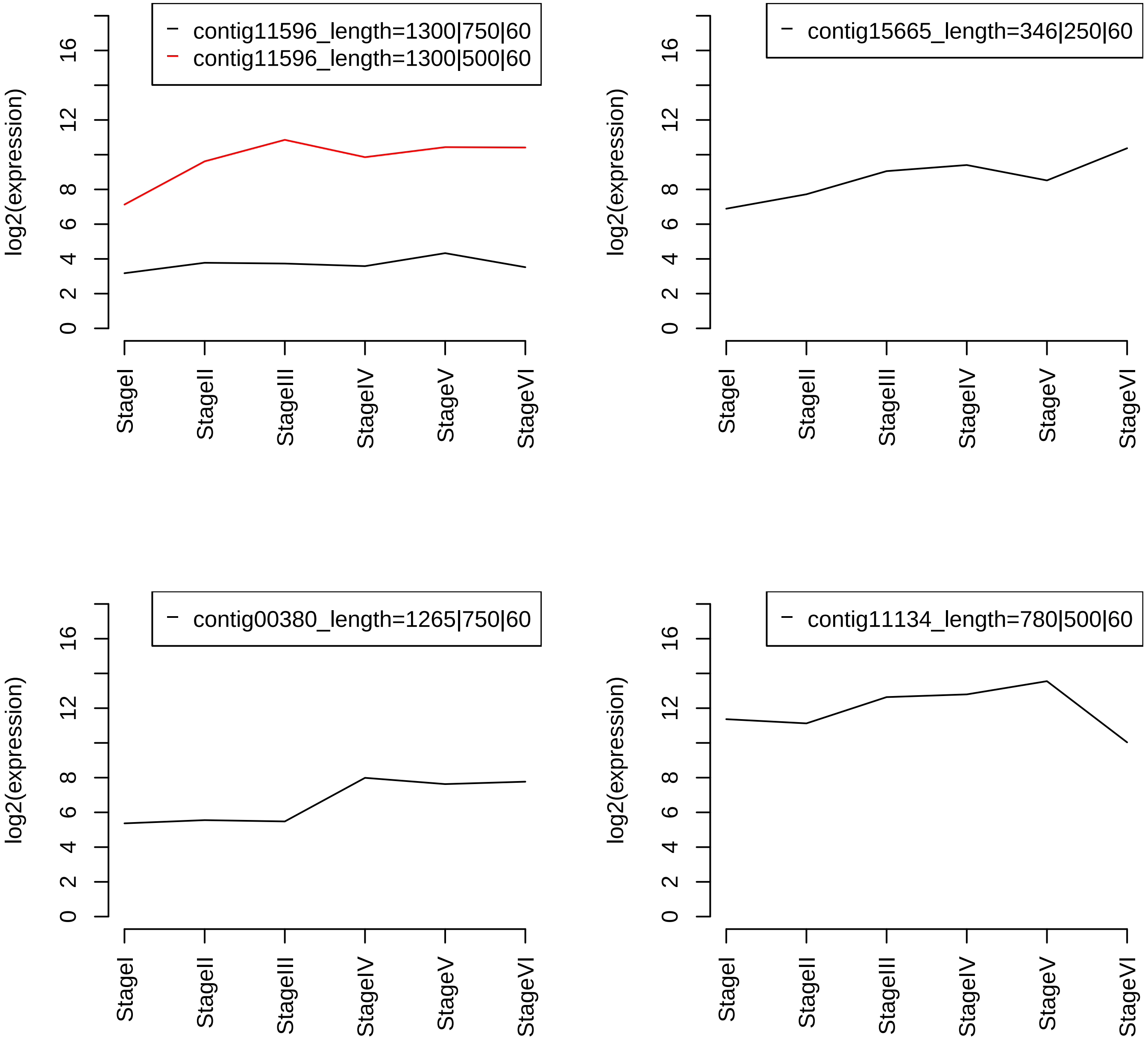

contig11596_length=1300|750|60
contig11596_length=1300|500|60
log2(expression)
0
2
4
6
8
12
16
StageI
StageII
StageIII
StageIV
StageV
StageVI
contig15665_length=346|250|60
contig00380_length=1265|750|60
contig11134_length=780|500|60

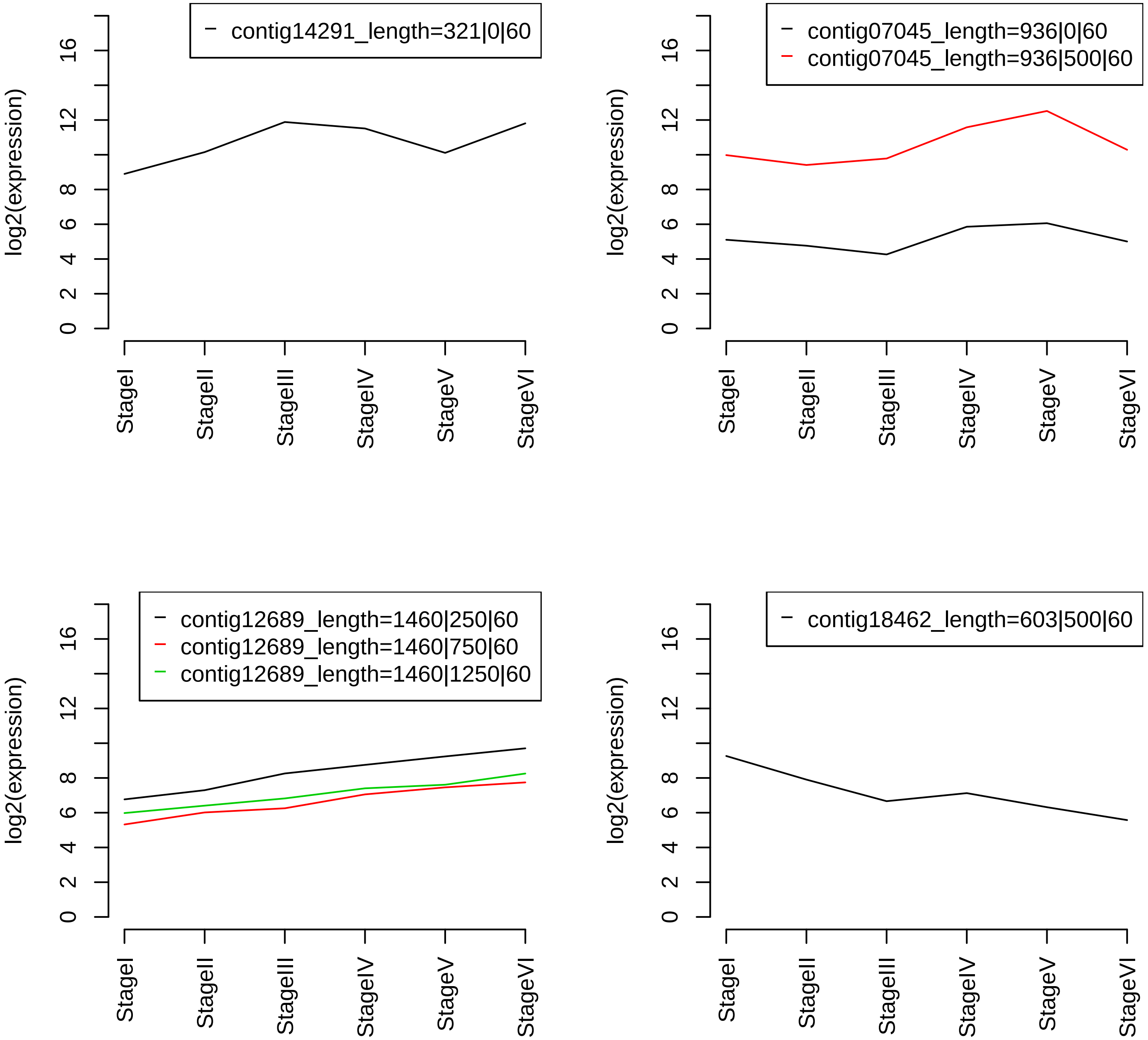

contig14291_length=321|0|60
contig07045_length=936|0|60
contig07045_length=936|500|60
contig12689_length=1460|250|60
contig12689_length=1460|750|60
contig12689_length=1460|1250|60
contig18462_length=603|500|60
log2(expression)
0 2 4 6 8 12 16
StageI StageII StageIII StageIV StageV StageVI

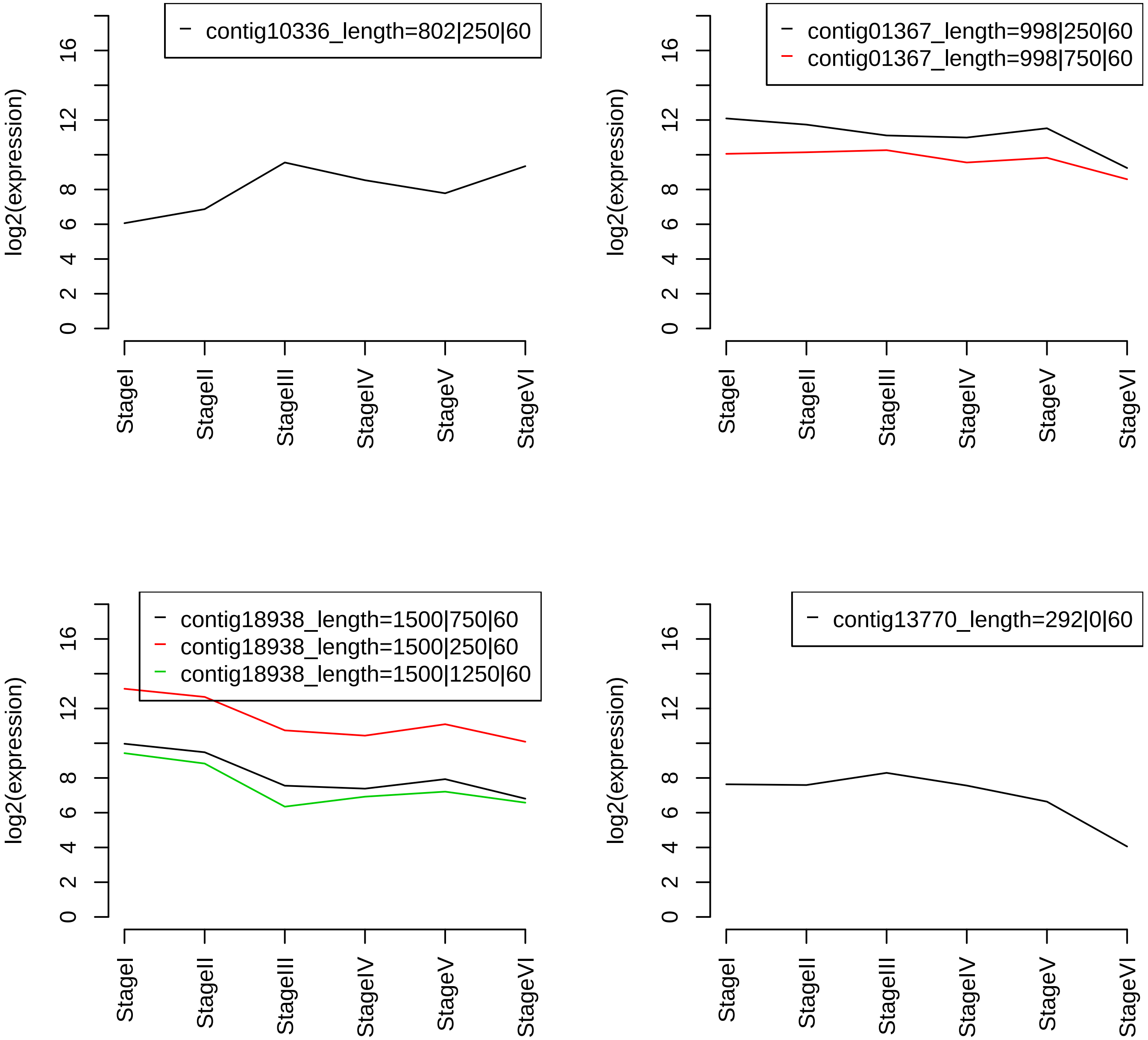
contig10336_length=802|250|60
contig01367_length=998|250|60
contig01367_length=998|750|60
contig18938_length=1500|750|60
contig18938_length=1500|250|60
contig18938_length=1500|1250|60
contig13770_length=292|0|60
log2(expression)
StageI
StageII
StageIII
StageIV
StageV
StageVI

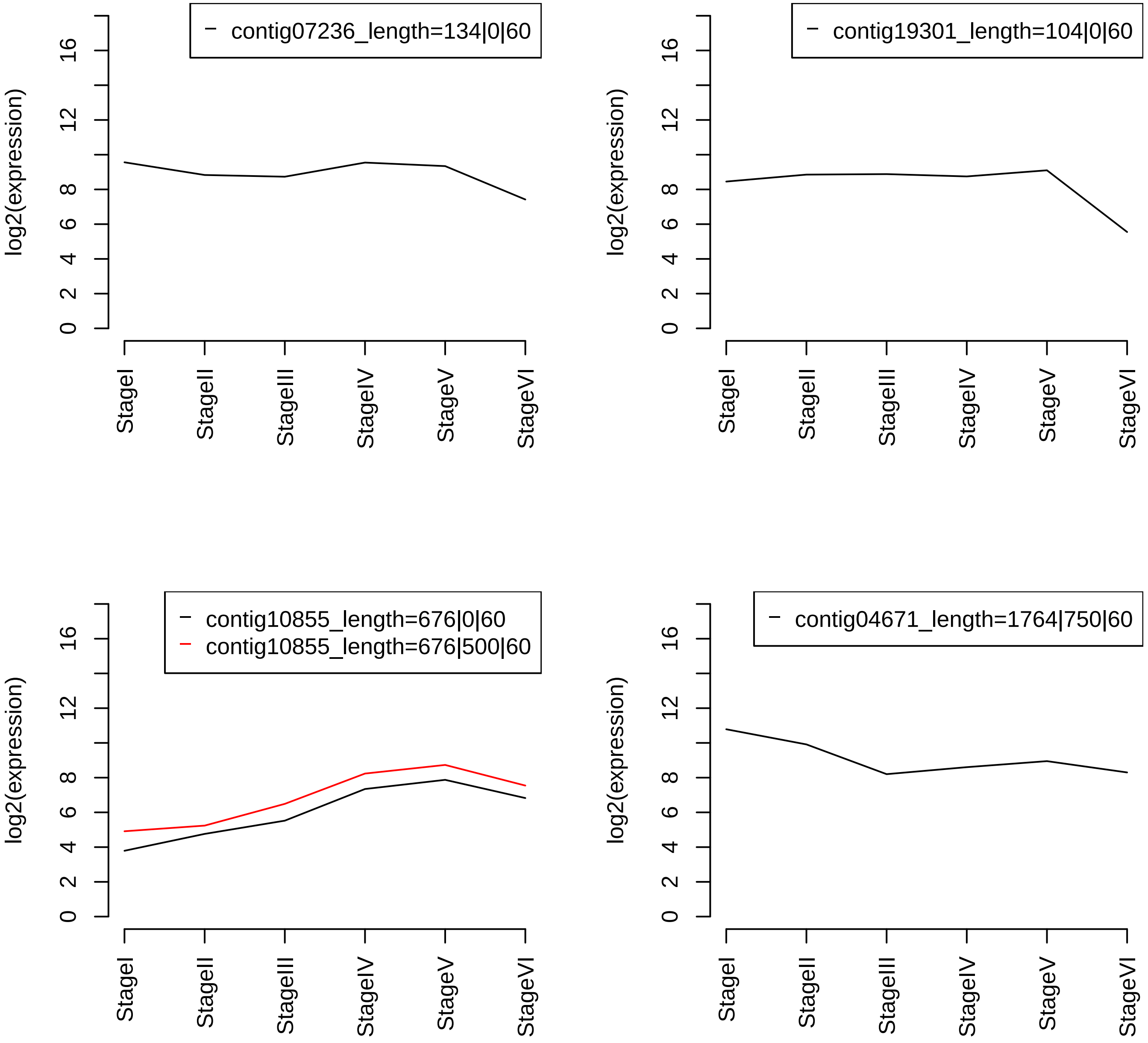

contig07236_length=134|0|60
contig19301_length=104|0|60
contig10855_length=676|0|60
contig10855_length=676|500|60
contig04671_length=1764|750|60
log2(expression)
0
2
4
6
8
12
16
StageI
StageII
StageIII
StageIV
StageV
StageVI

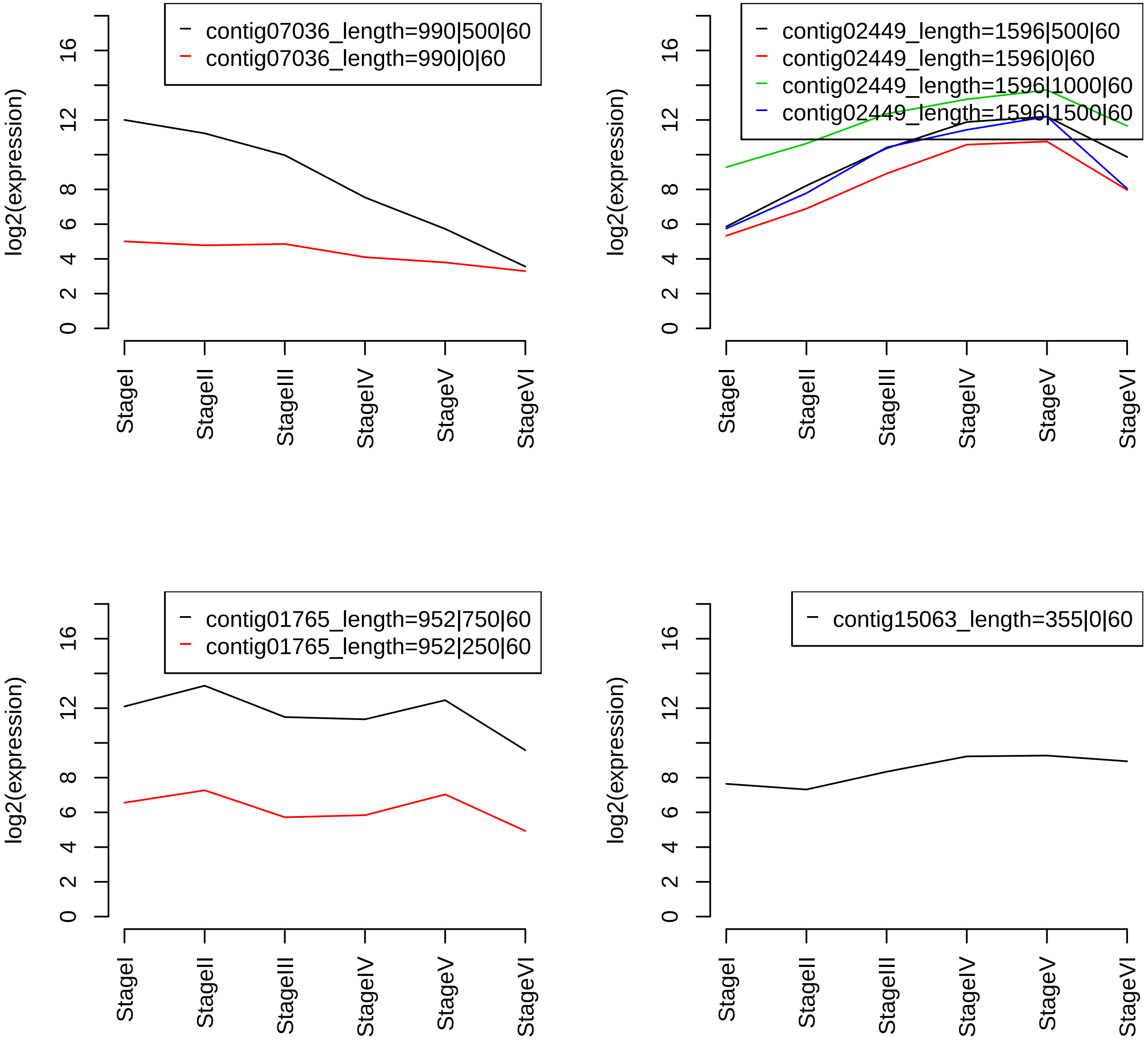
contig07036_length=990|500|60
contig07036_length=990|0|60
contig02449_length=1596|500|60
contig02449_length=1596|0|60
contig02449_length=1596|1000|60
contig02449_length=1596|1500|60
contig01765_length=952|750|60
contig01765_length=952|250|60
contig15063_length=355|0|60
log2(expression)
0 2 4 6 8 12 16
StageI StageII StageIII StageIV StageV StageVI

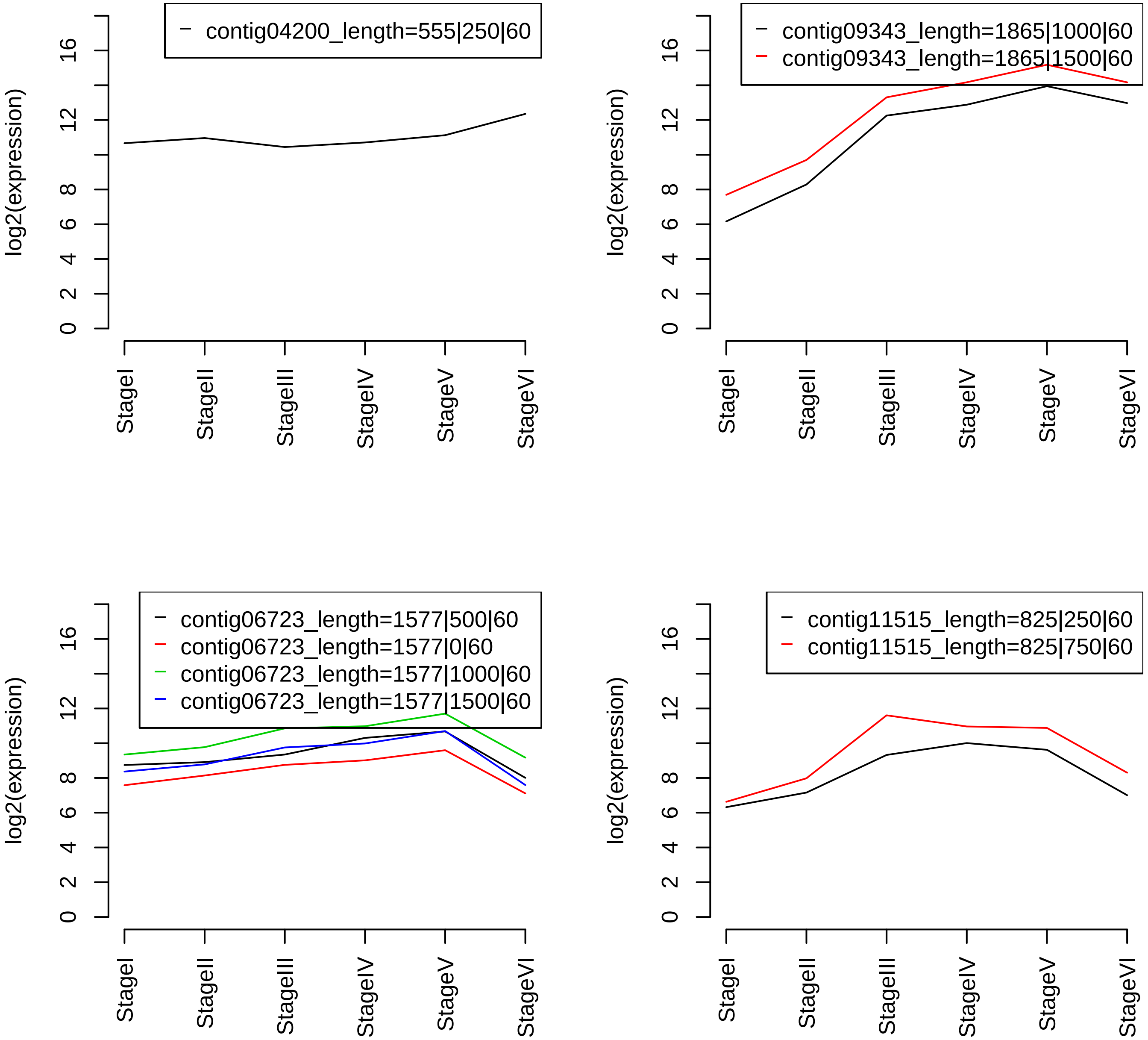

contig04200_length=555|250|60
contig09343_length=1865|1000|60
contig09343_length=1865|1500|60
contig06723_length=1577|500|60
contig06723_length=1577|0|60
contig06723_length=1577|1000|60
contig06723_length=1577|1500|60
contig11515_length=825|250|60
contig11515_length=825|750|60
log2(expression)
0 2 4 6 8 12 16
StageI StageII StageIII StageIV StageV StageVI

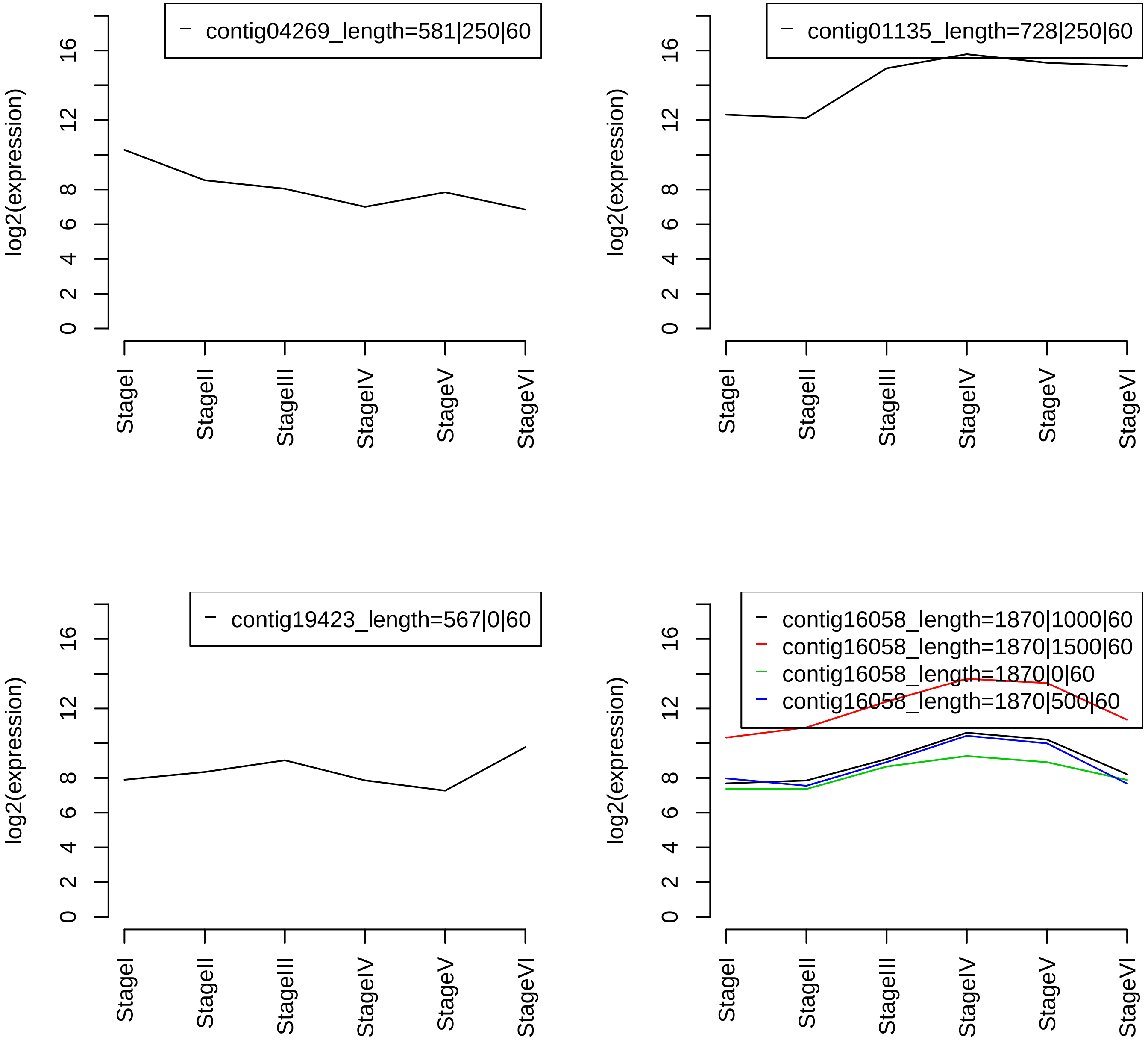
contig04269_length=581|250|60
contig01135_length=728|250|60
contig19423_length=567|0|60
contig16058_length=1870|1000|60
contig16058_length=1870|1500|60
contig16058_length=1870|0|60
contig16058_length=1870|500|60
log2(expression)
0
2
4
6
8
12
16
StageI
StageII
StageIII
StageIV
StageV
StageVI

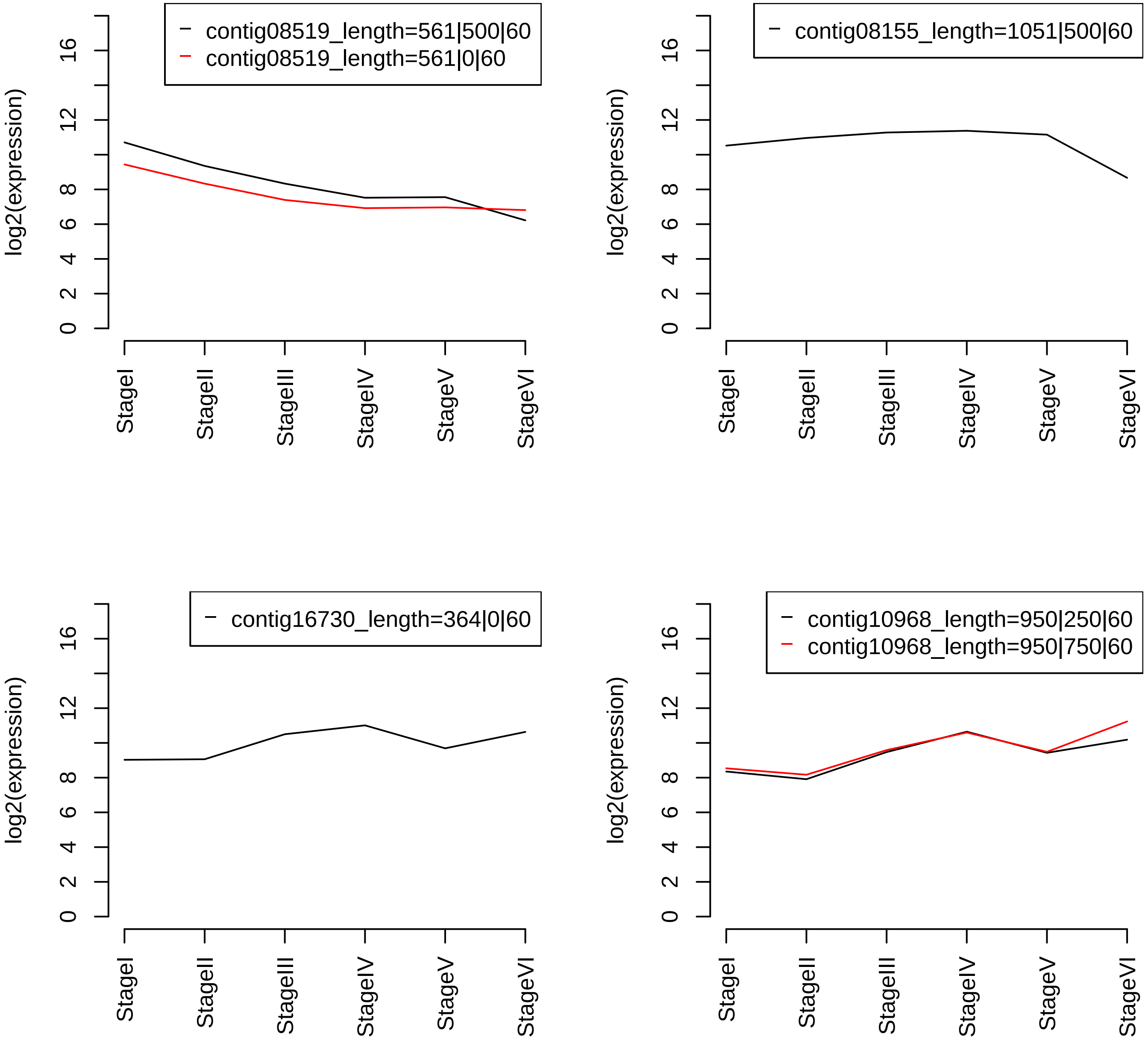

contig08519_length=561|500|60
contig08519_length=561|0|60
contig08155_length=1051|500|60
contig16730_length=364|0|60
contig10968_length=950|250|60
contig10968_length=950|750|60
log2(expression)
0 2 4 6 8 12 16
StageI StageII StageIII StageIV StageV StageVI

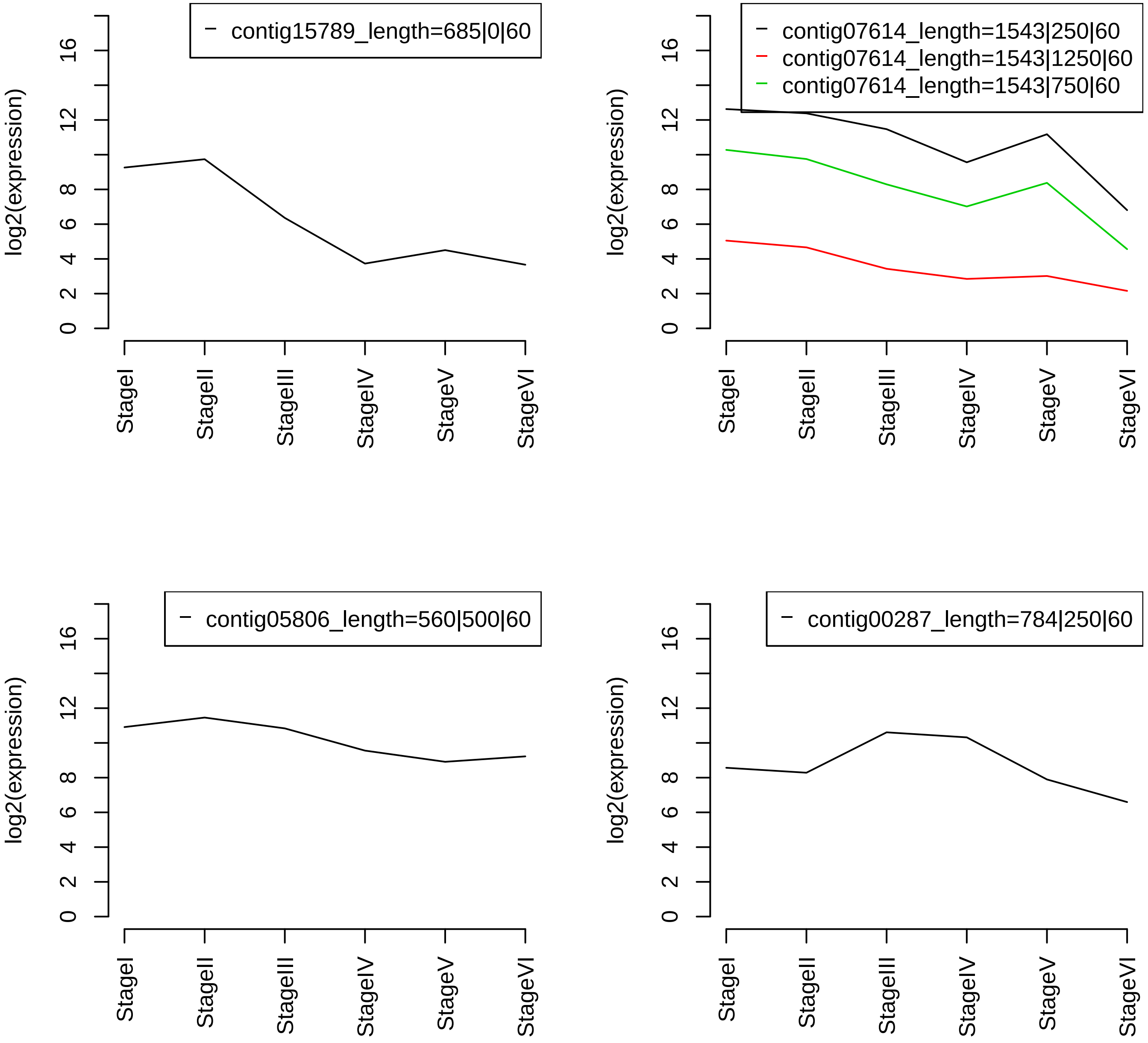
contig15789_length=685|0|60
contig07614_length=1543|250|60
contig07614_length=1543|1250|60
contig07614_length=1543|750|60
contig05806_length=560|500|60
contig00287_length=784|250|60
log2(expression)
0 2 4 6 8 12 16
StageI StageII StageIII StageIV StageV StageVI

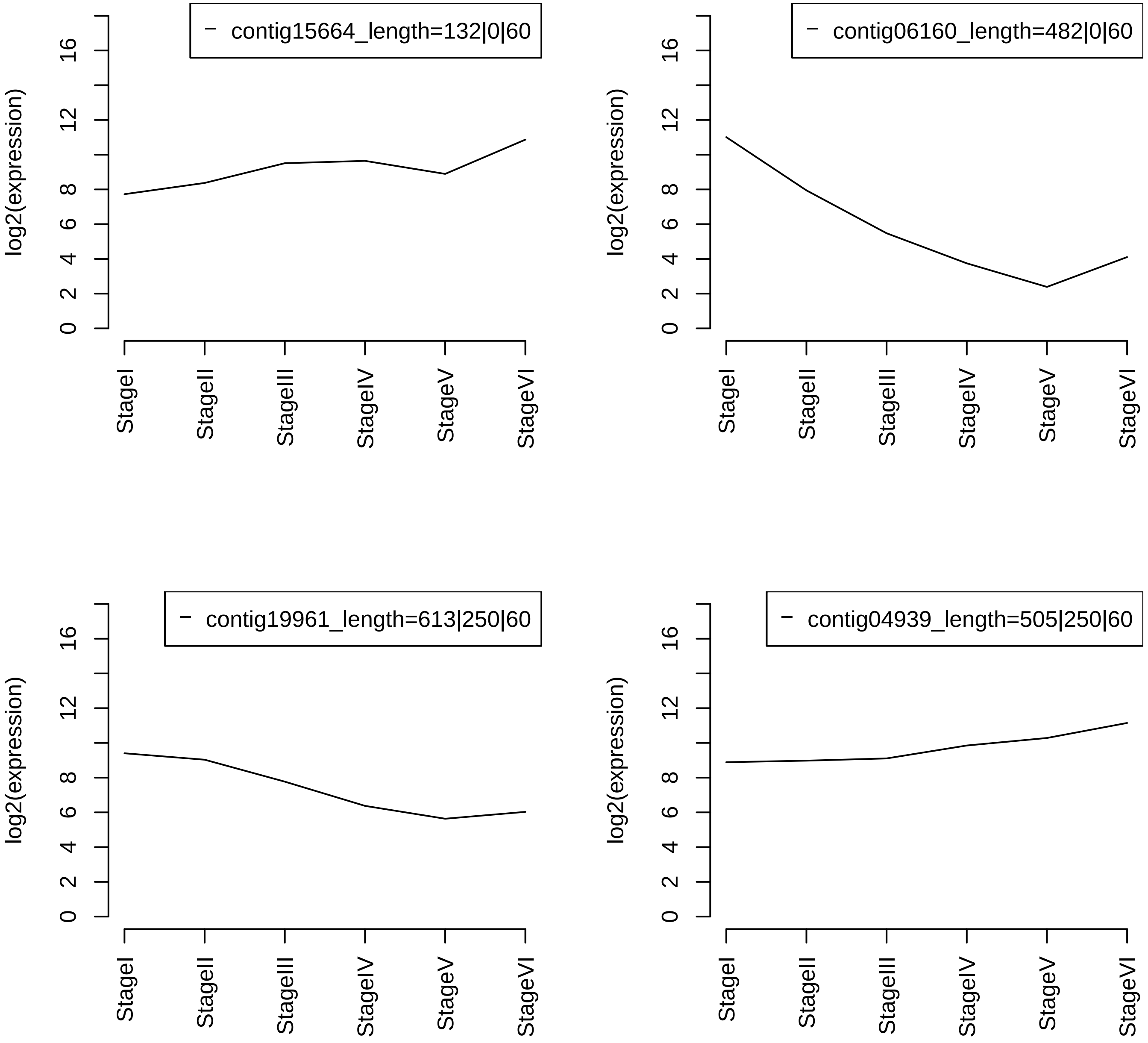
contig15664_length=132|0|60
contig06160_length=482|0|60
contig19961_length=613|250|60
contig04939_length=505|250|60
log2(expression)
0
2
4
6
8
12
16
StageI
StageII
StageIII
StageIV
StageV
StageVI

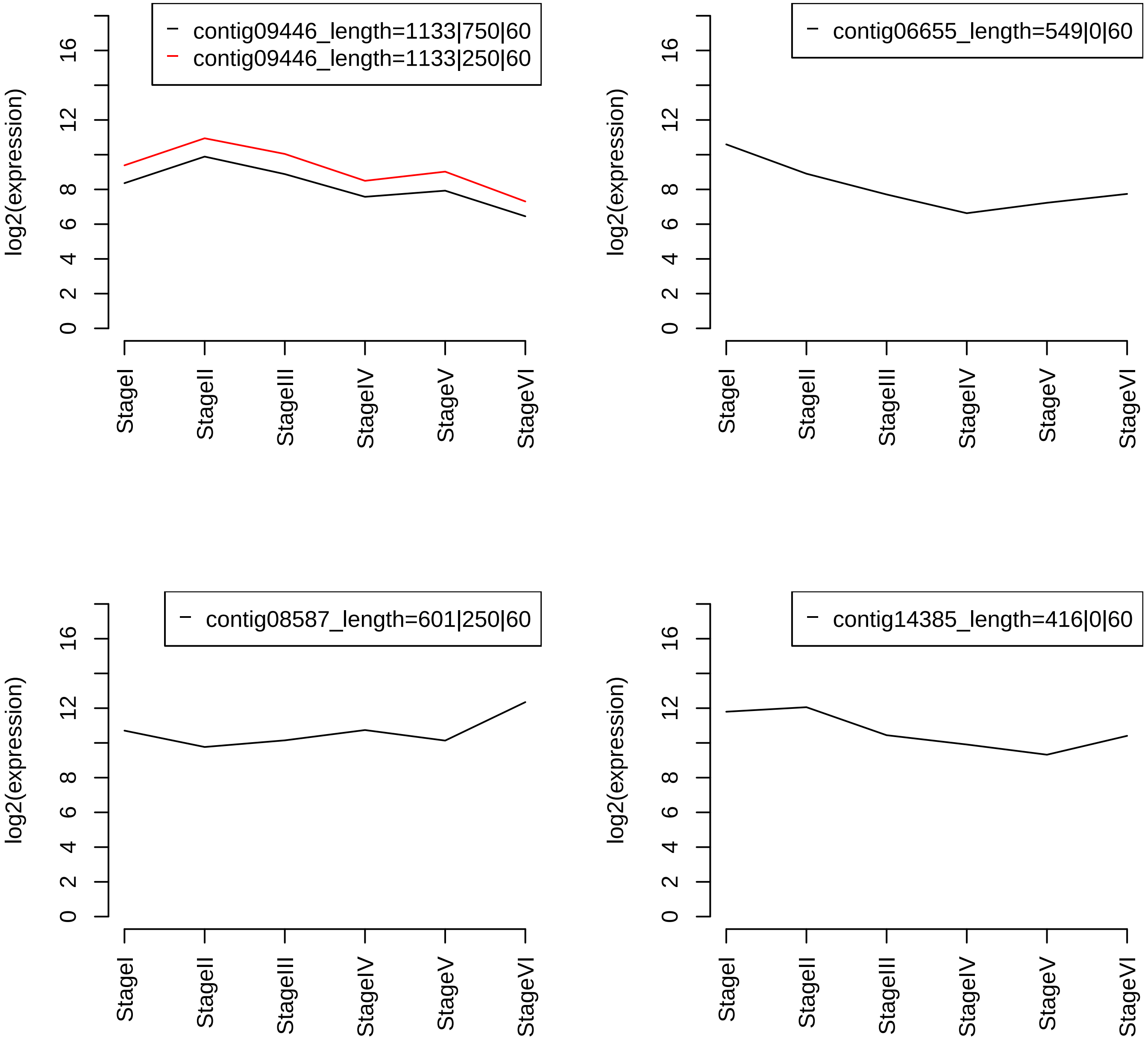
contig09446_length=1133|750|60
contig09446_length=1133|250|60
log2(expression)
0
2
4
6
8
12
16
StageI
StageII
StageIII
StageIV
StageV
StageVI
contig06655_length=549|0|60
contig08587_length=601|250|60
contig14385_length=416|0|60

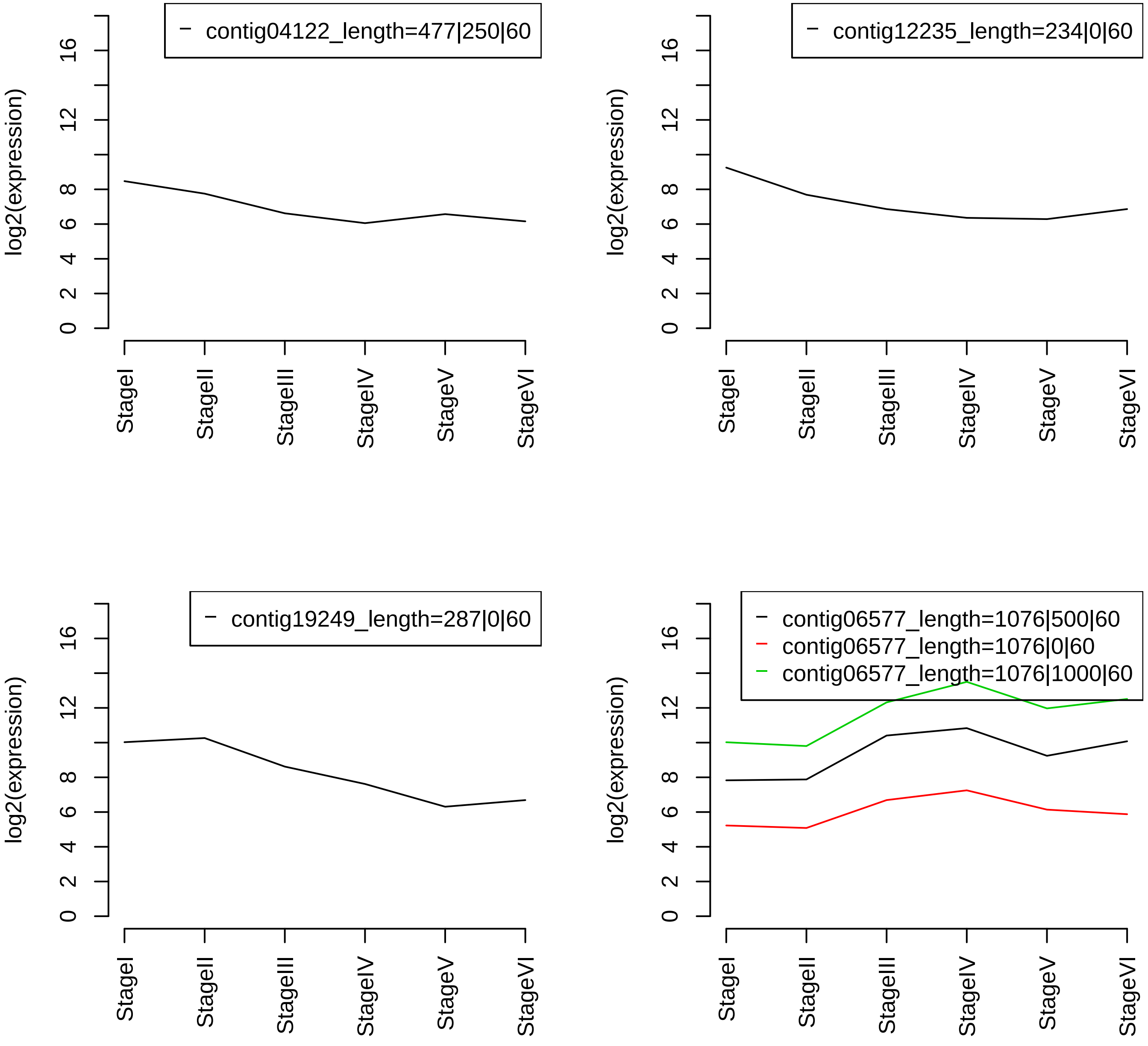
contig04122_length=477|250|60
contig12235_length=234|0|60
contig19249_length=287|0|60
contig06577_length=1076|500|60
contig06577_length=1076|0|60
contig06577_length=1076|1000|60
log2(expression)
0
2
4
6
8
12
16
StageI
StageII
StageIII
StageIV
StageV
StageVI

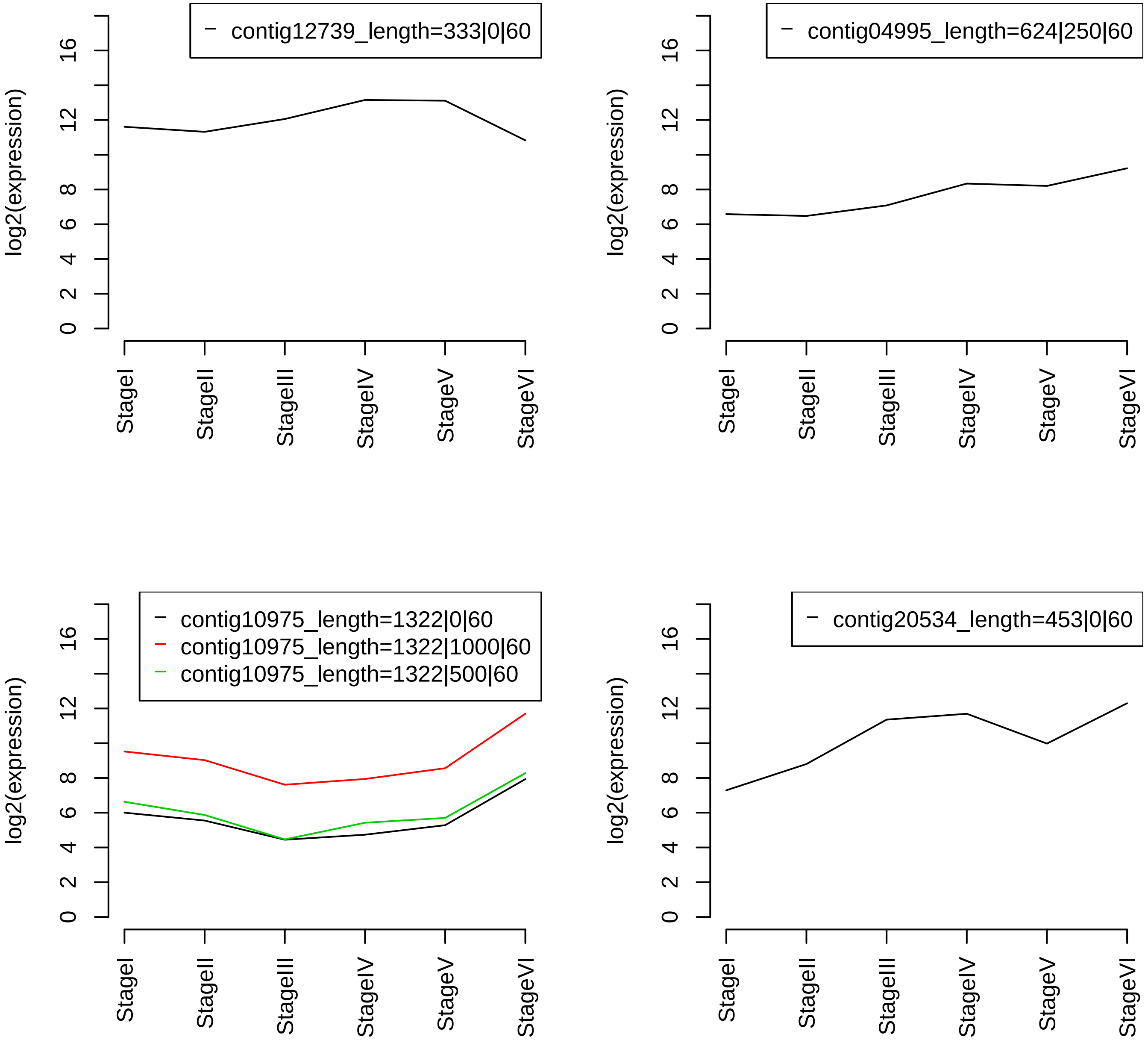

contig12739_length=333|0|60
log2(expression)
0
2
4
6
8
12
16
StageI
StageII
StageIII
StageIV
StageV
StageVI
contig04995_length=624|250|60
log2(expression)
contig10975_length=1322|0|60
contig10975_length=1322|1000|60
contig10975_length=1322|500|60
log2(expression)
contig20534_length=453|0|60
log2(expression)

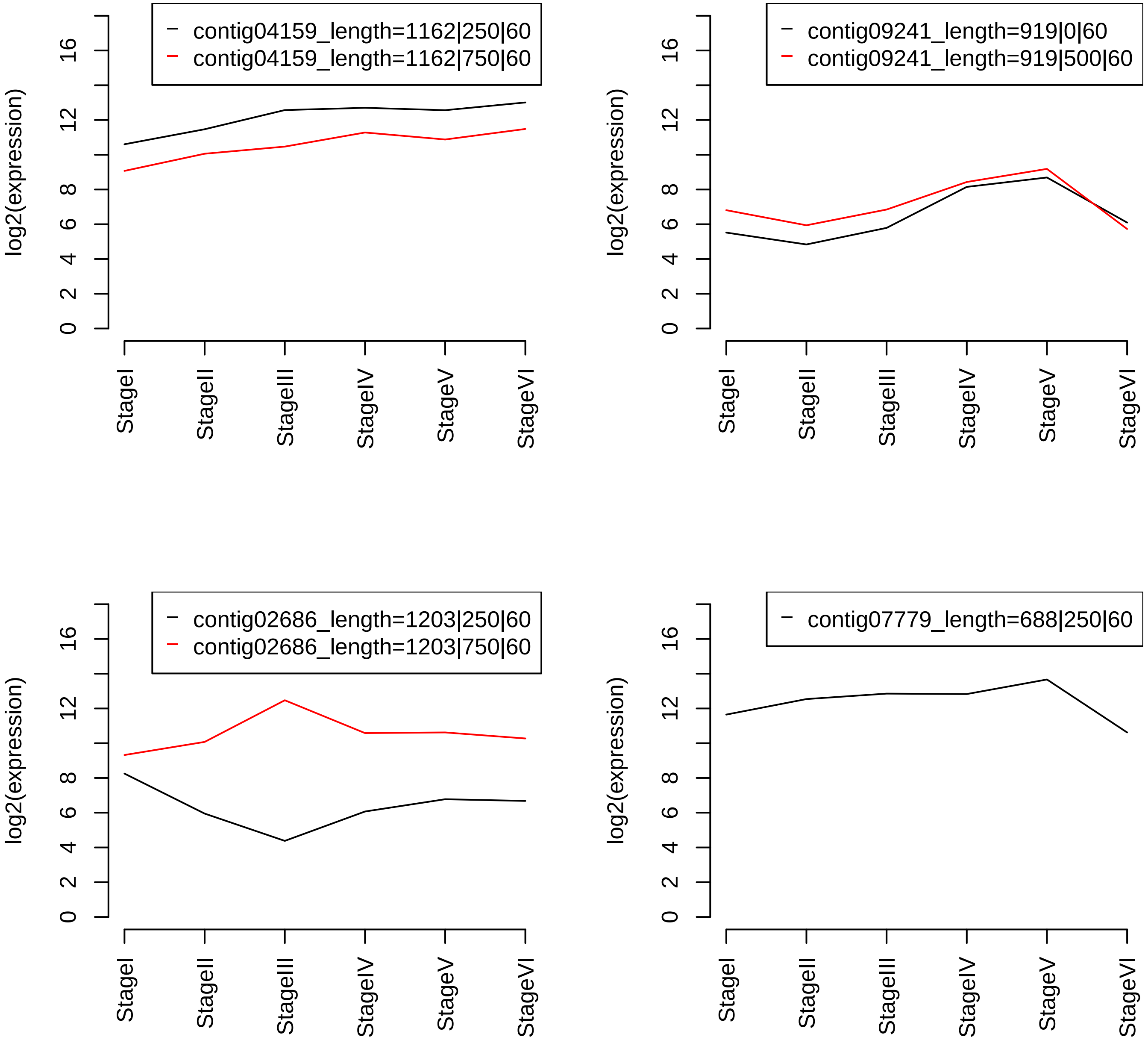
contig04159_length=1162|250|60
contig04159_length=1162|750|60
contig09241_length=919|0|60
contig09241_length=919|500|60
contig02686_length=1203|250|60
contig02686_length=1203|750|60
contig07779_length=688|250|60
log2(expression)
StageI
StageII
StageIII
StageIV
StageV
StageVI

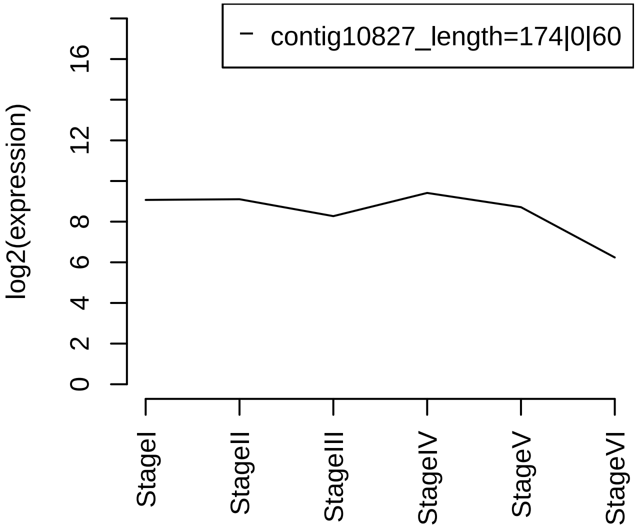
contig10827_length=174|0|60
log2(expression)
0
2
4
6
8
12
16
StageI
StageII
StageIII
StageIV
StageV
StageVI

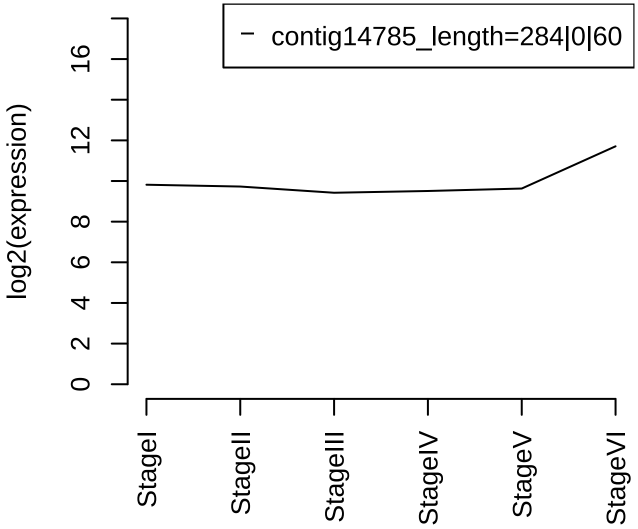
contig14785_length=284|0|60
log2(expression)
0
2
4
6
8
12
16
StageI
StageII
StageIII
StageIV
StageV
StageVI

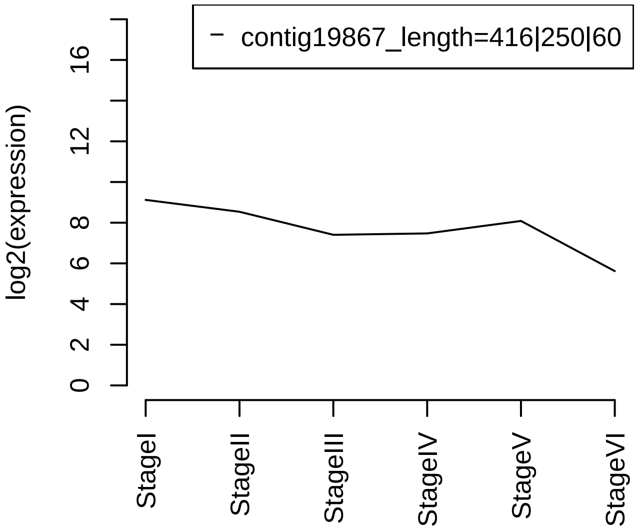
contig19867_length=416|250|60
log2(expression)
0
2
4
6
8
12
16
StageI
StageII
StageIII
StageIV
StageV
StageVI

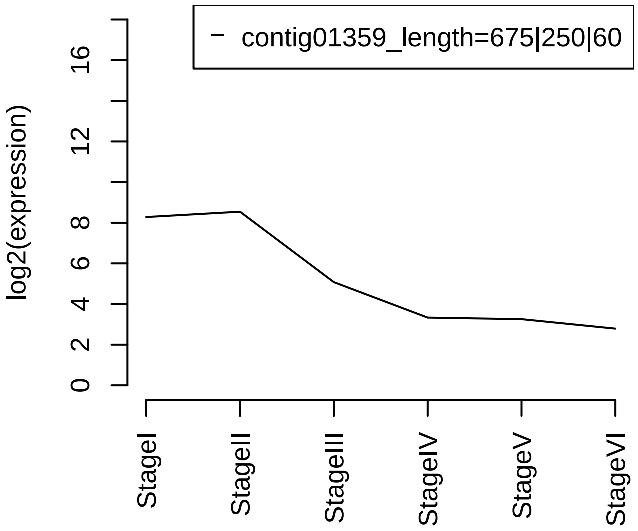
contig01359_length=675|250|60
log2(expression)
0
2
4
6
8
12
16
StageI
StageII
StageIII
StageIV
StageV
StageVI

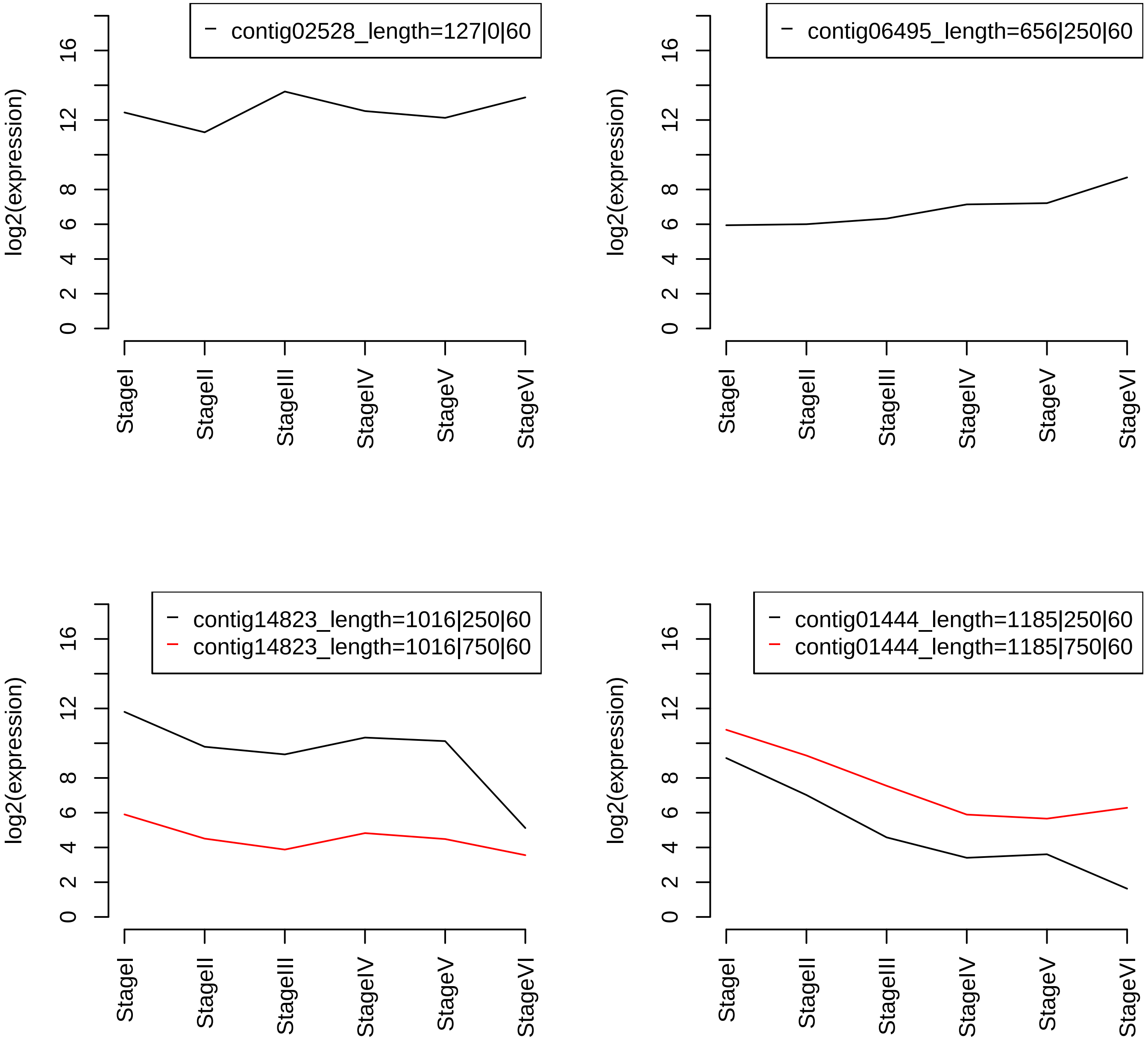
contig02528_length=127|0|60
contig06495_length=656|250|60
contig14823_length=1016|250|60
contig14823_length=1016|750|60
contig01444_length=1185|250|60
contig01444_length=1185|750|60
log2(expression)
0 2 4 6 8 12 16
StageI StageII StageIII StageIV StageV StageVI

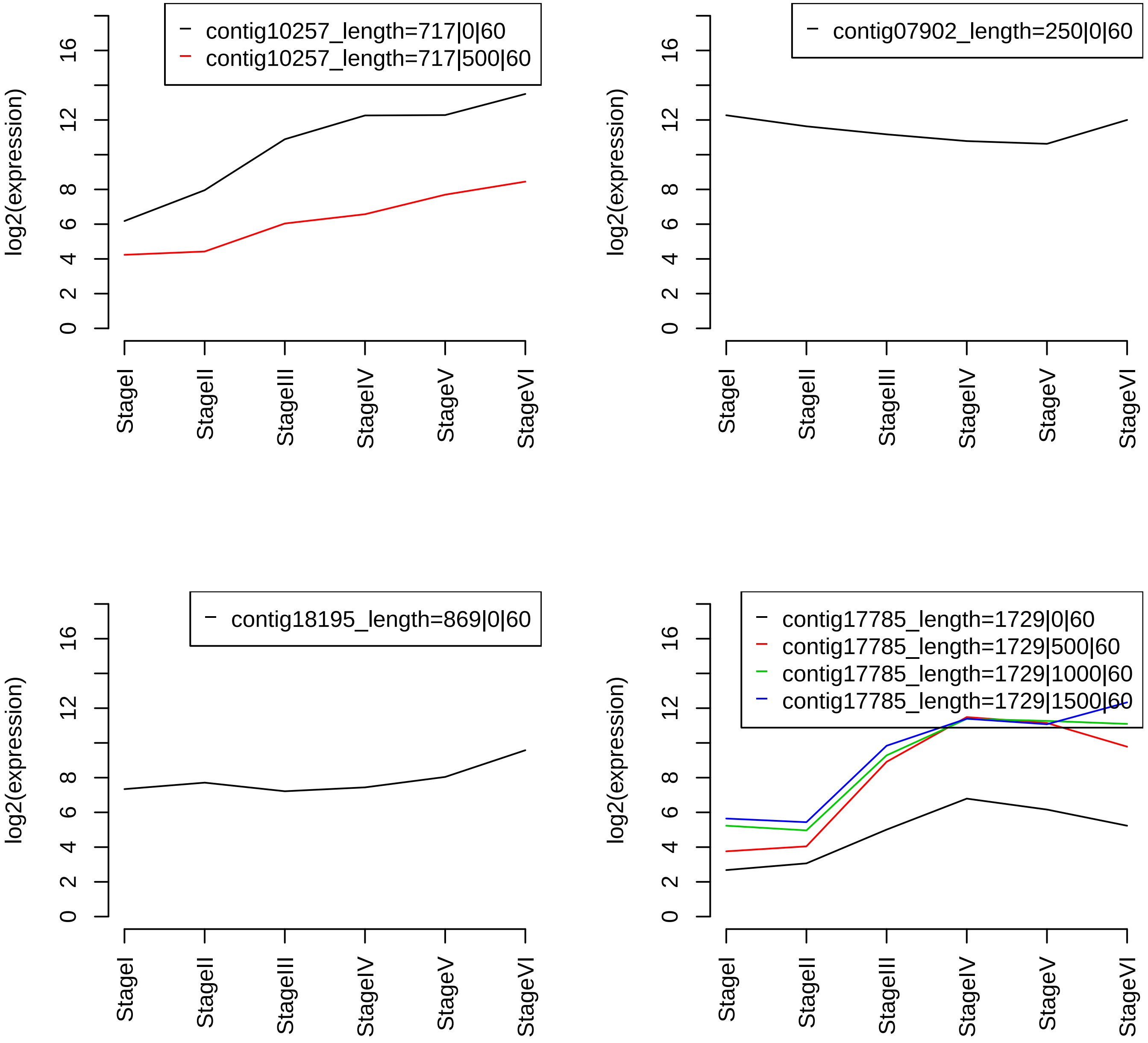

contig10257_length=717|0|60
contig10257_length=717|500|60
contig07902_length=250|0|60
contig18195_length=869|0|60
contig17785_length=1729|0|60
contig17785_length=1729|500|60
contig17785_length=1729|1000|60
contig17785_length=1729|1500|60
log2(expression)
0 2 4 6 8 12 16
StageI StageII StageIII StageIV StageV StageVI

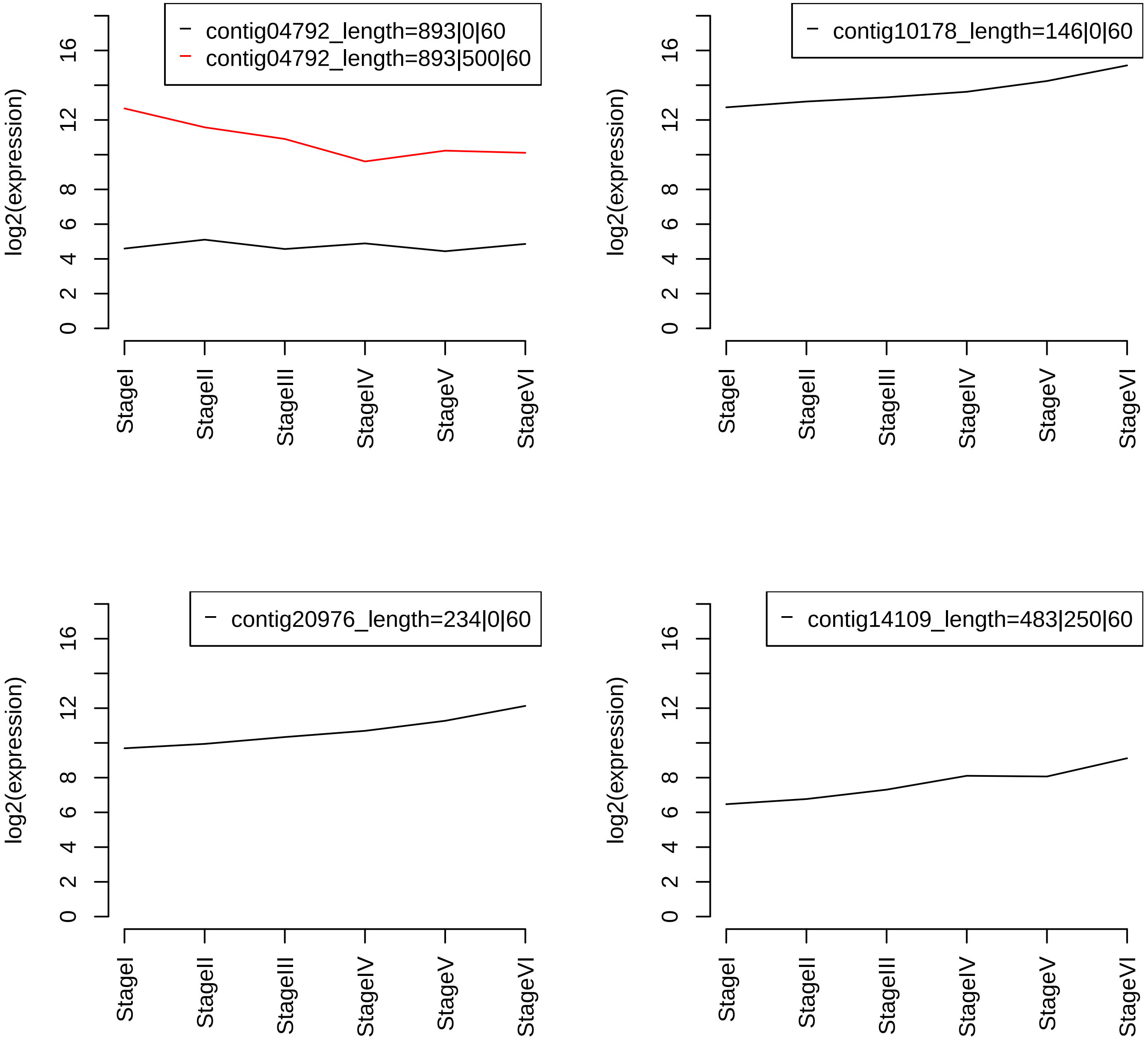
contig04792_length=893|0|60
contig04792_length=893|500|60
contig10178_length=146|0|60
contig20976_length=234|0|60
contig14109_length=483|250|60
log2(expression)
0
2
4
6
8
12
16
StageI
StageII
StageIII
StageIV
StageV
StageVI

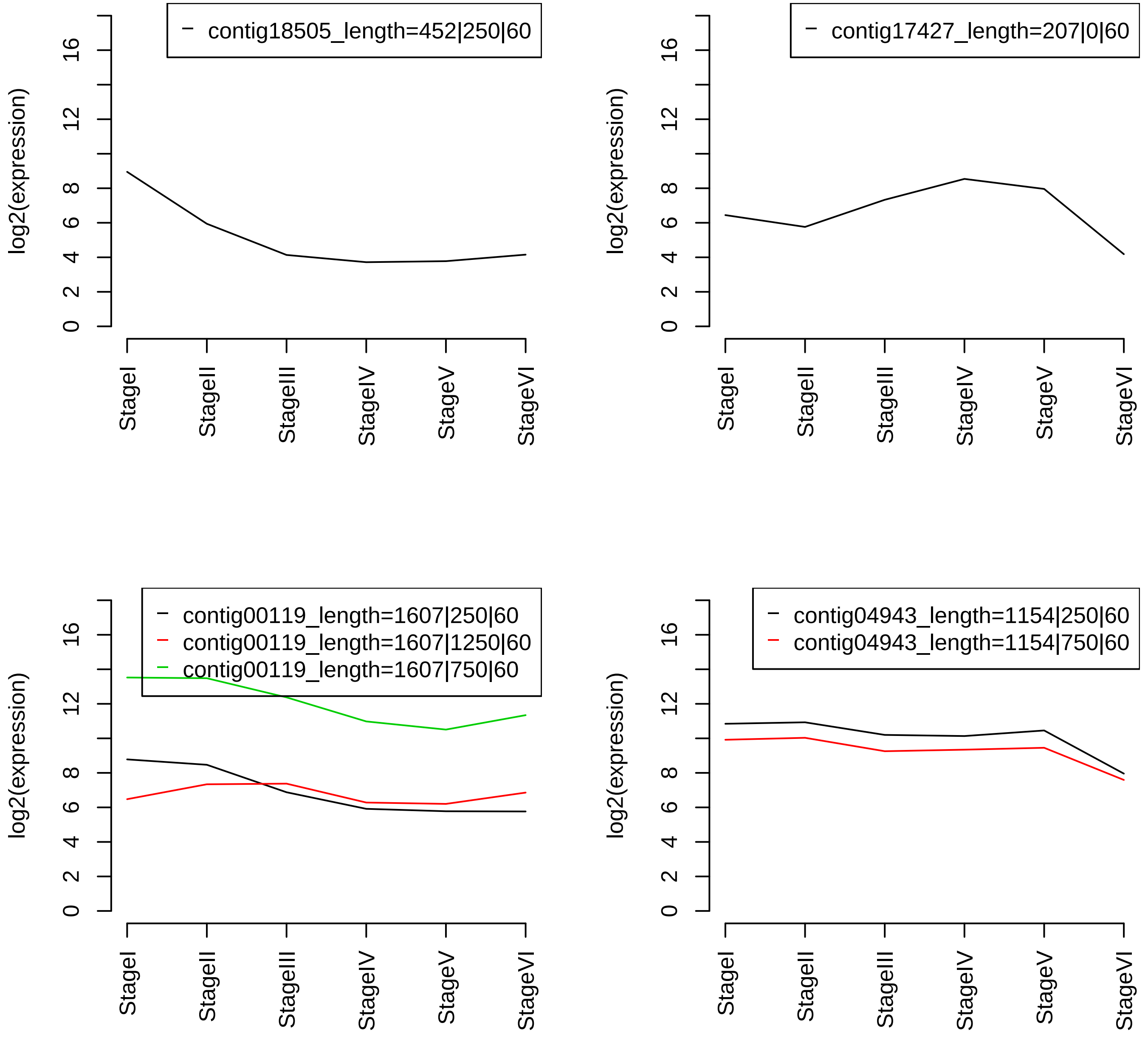

contig18505_length=452|250|60
contig17427_length=207|0|60
contig00119_length=1607|250|60
contig00119_length=1607|1250|60
contig00119_length=1607|750|60
contig04943_length=1154|250|60
contig04943_length=1154|750|60
log2(expression)
0
2
4
6
8
12
16
StageI
StageII
StageIII
StageIV
StageV
StageVI

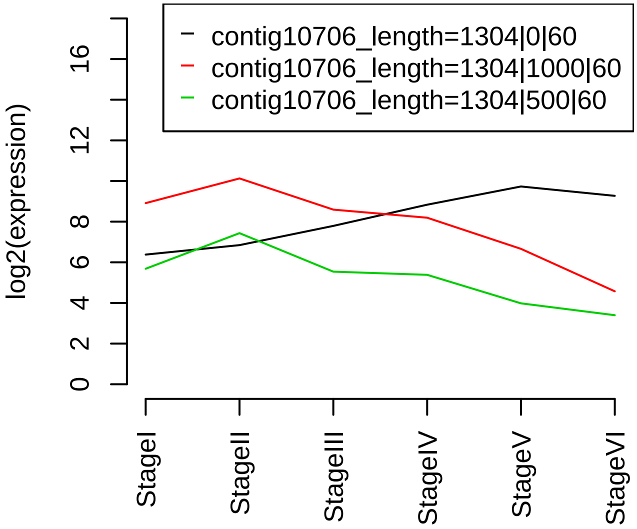
contig10706_length=1304|0|60
contig10706_length=1304|1000|60
contig10706_length=1304|500|60
log2(expression)
0
2
4
6
8
12
16
StageI
StageII
StageIII
StageIV
StageV
StageVI

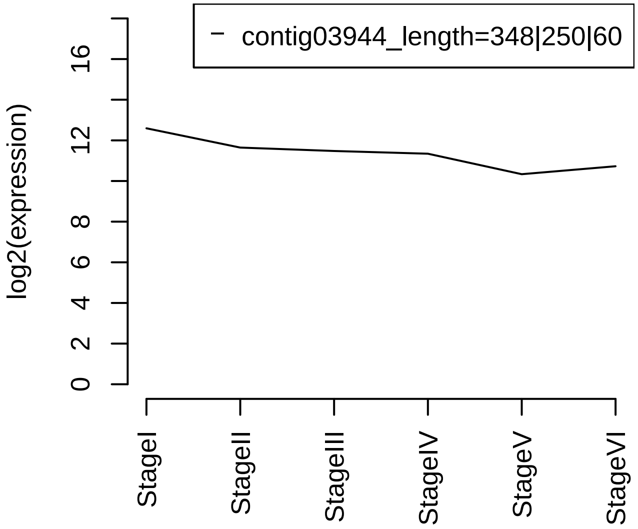
contig03944_length=348|250|60
log2(expression)
0
2
4
6
8
12
16
StageI
StageII
StageIII
StageIV
StageV
StageVI

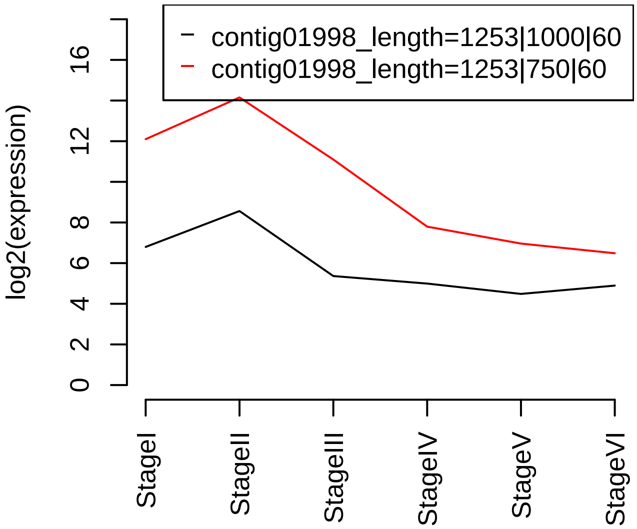
contig01998_length=1253|1000|60
contig01998_length=1253|750|60
log2(expression)
0
2
4
6
8
12
16
StageI
StageII
StageIII
StageIV
StageV
StageVI

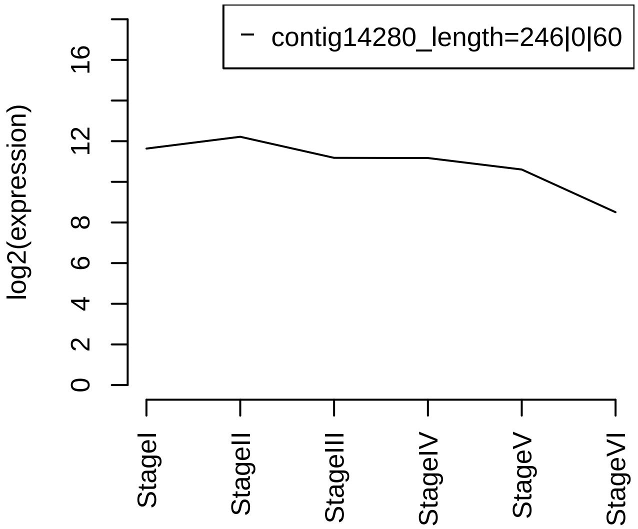
contig14280_length=246|0|60
log2(expression)
0
2
4
6
8
12
16
StageI
StageII
StageIII
StageIV
StageV
StageVI

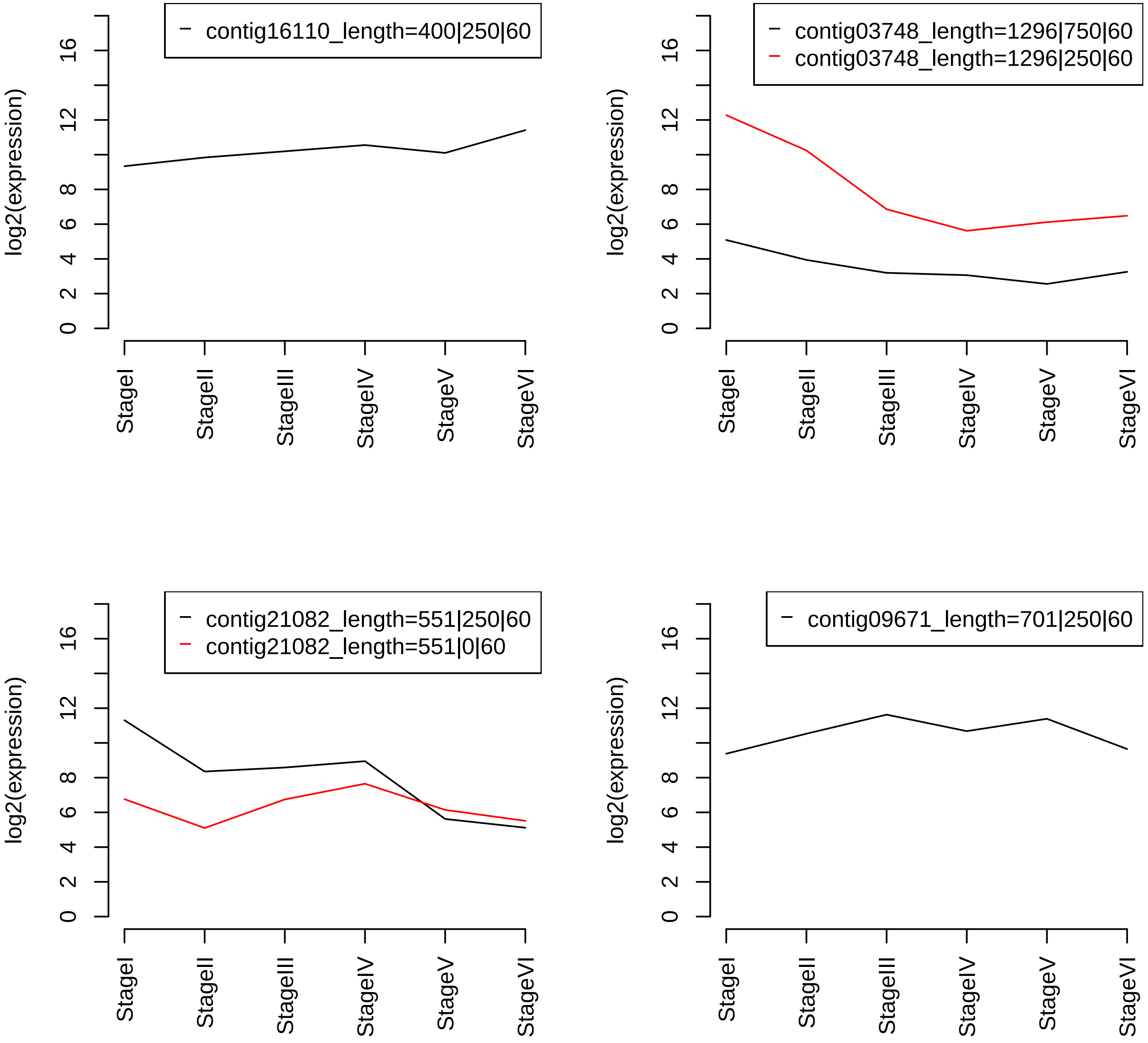

contig16110_length=400|250|60
contig03748_length=1296|750|60
contig03748_length=1296|250|60
contig21082_length=551|250|60
contig21082_length=551|0|60
contig09671_length=701|250|60
log2(expression)
0 2 4 6 8 12 16
StageI StageII StageIII StageIV StageV StageVI

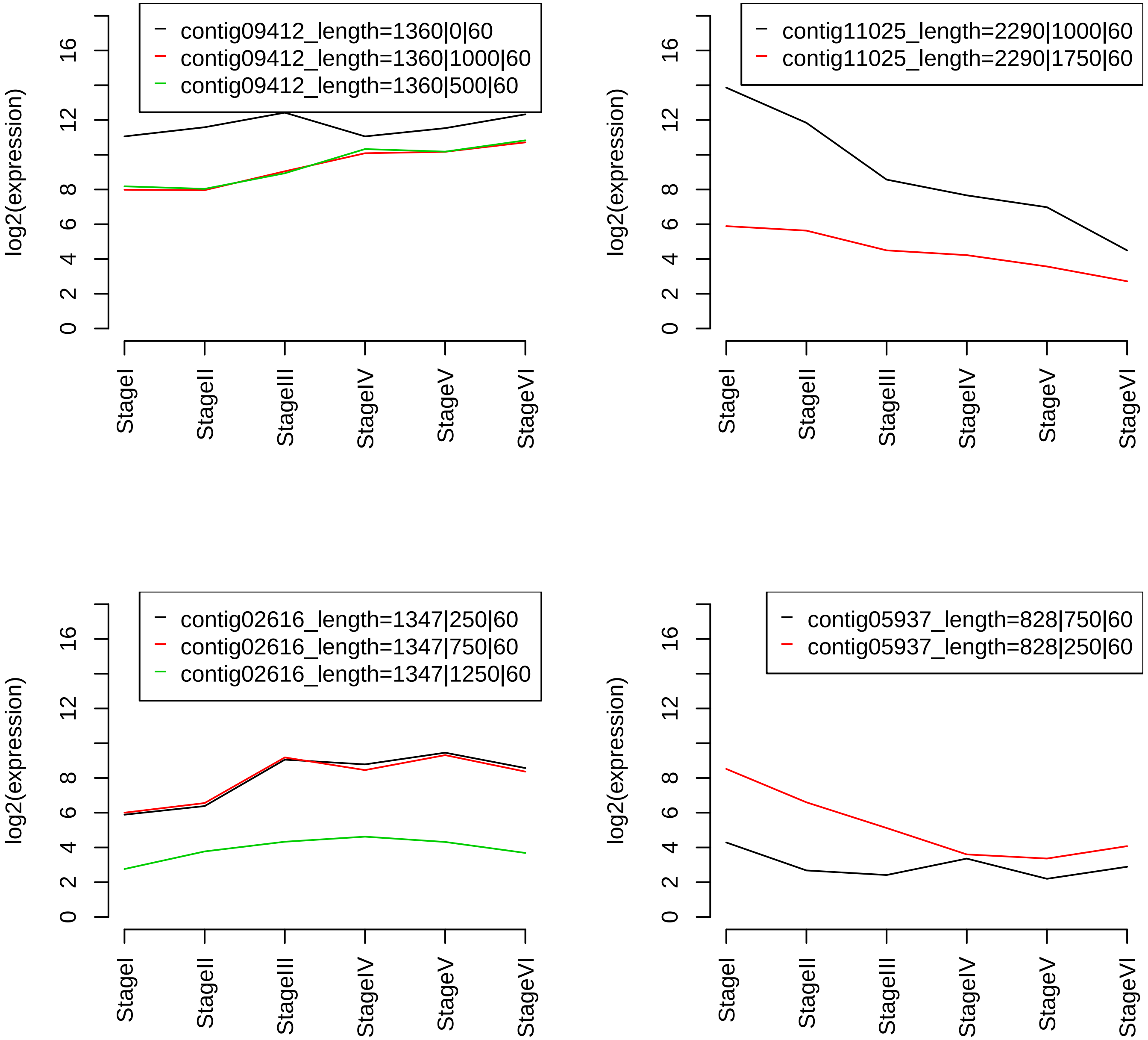

contig09412_length=1360|0|60
contig09412_length=1360|1000|60
contig09412_length=1360|500|60
contig11025_length=2290|1000|60
contig11025_length=2290|1750|60
contig02616_length=1347|250|60
contig02616_length=1347|750|60
contig02616_length=1347|1250|60
contig05937_length=828|750|60
contig05937_length=828|250|60
log2(expression)
0
2
4
6
8
12
16
StageI
StageII
StageIII
StageIV
StageV
StageVI

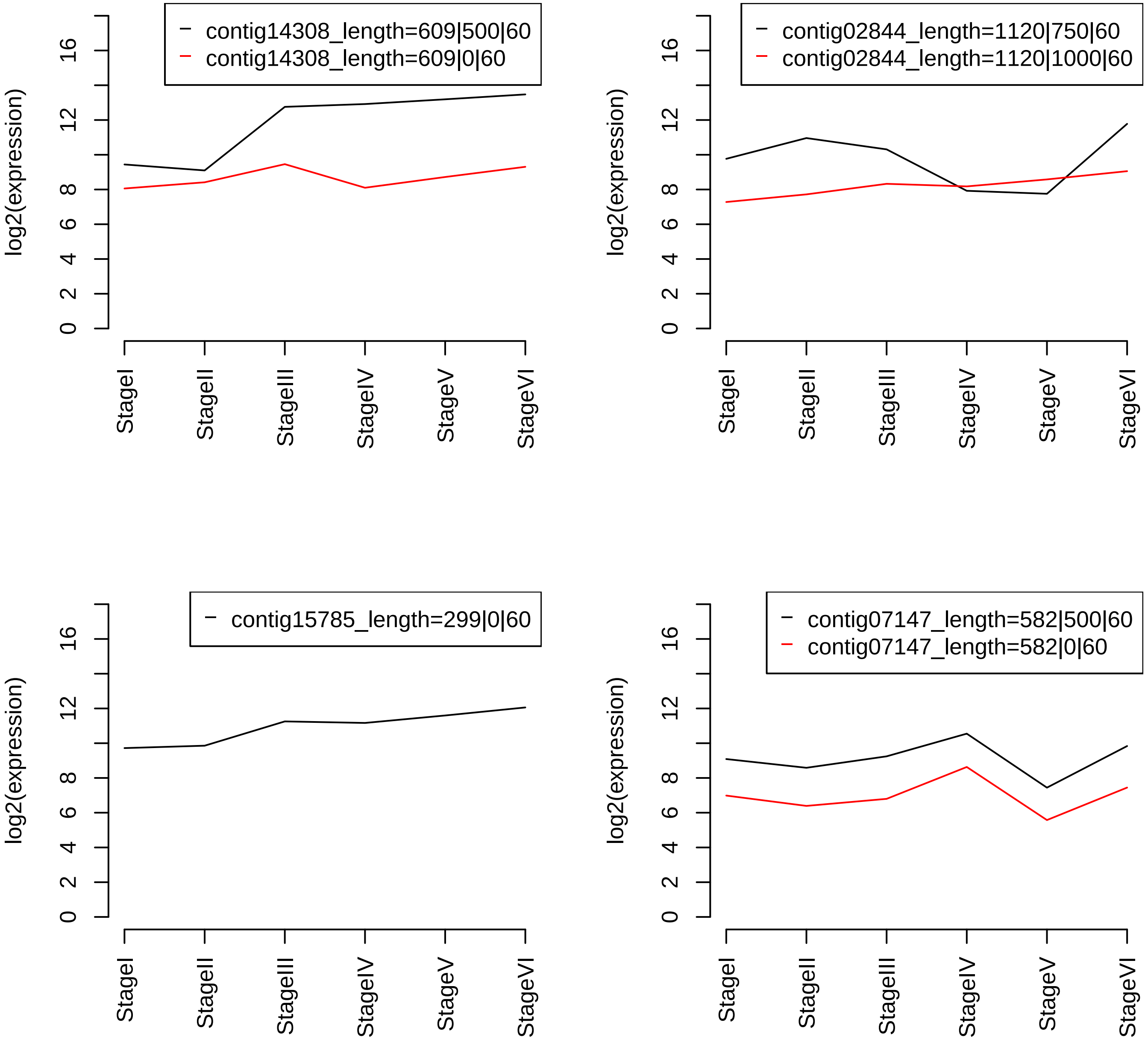
contig14308_length=609|500|60
contig14308_length=609|0|60
contig02844_length=1120|750|60
contig02844_length=1120|1000|60
contig15785_length=299|0|60
contig07147_length=582|500|60
contig07147_length=582|0|60
log2(expression)
0
2
4
6
8
12
16
StageI
StageII
StageIII
StageIV
StageV
StageVI

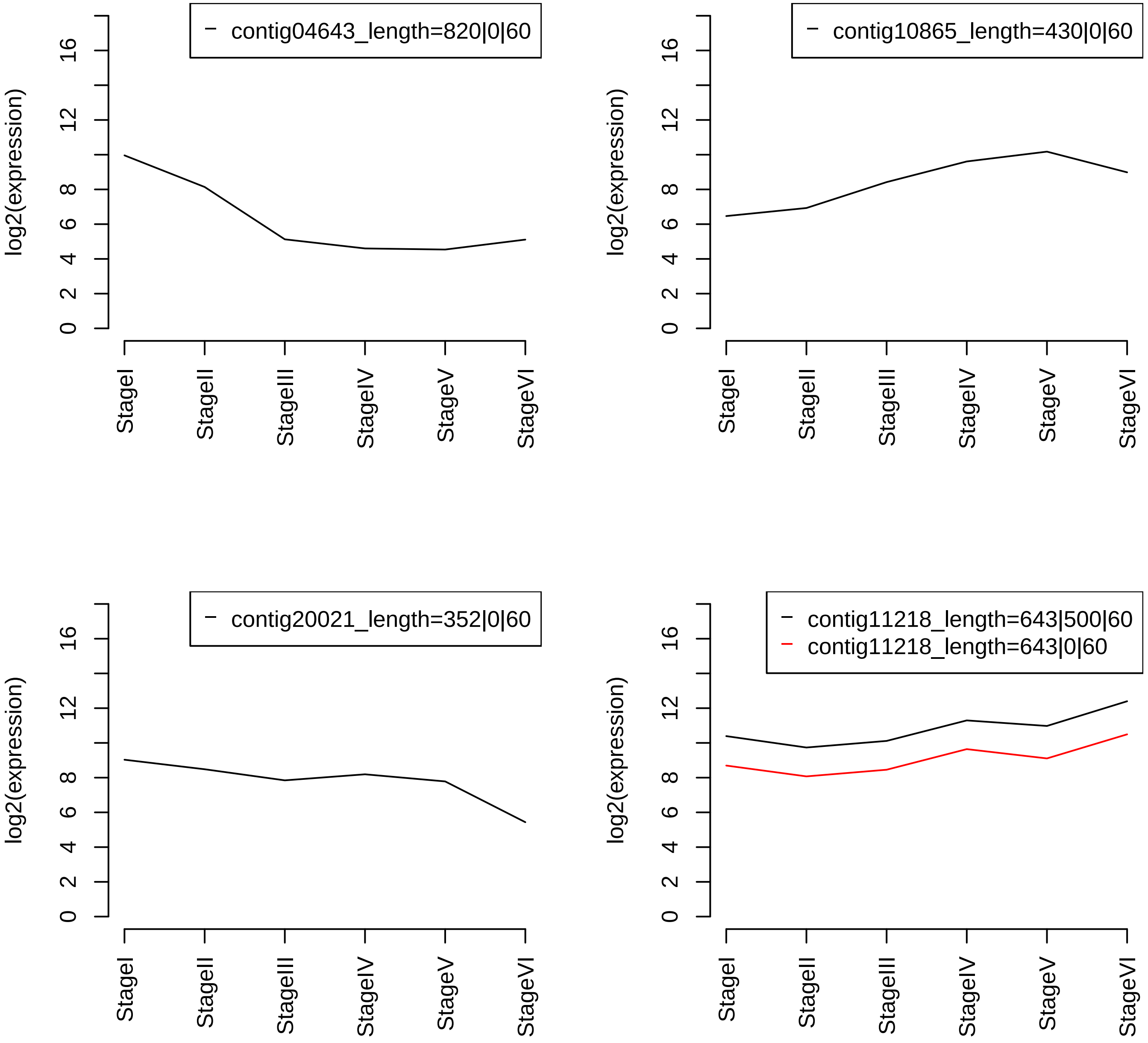

contig04643_length=820|0|60
contig10865_length=430|0|60
contig20021_length=352|0|60
contig11218_length=643|500|60
contig11218_length=643|0|60
log2(expression)
0
2
4
6
8
12
16
StageI
StageII
StageIII
StageIV
StageV
StageVI

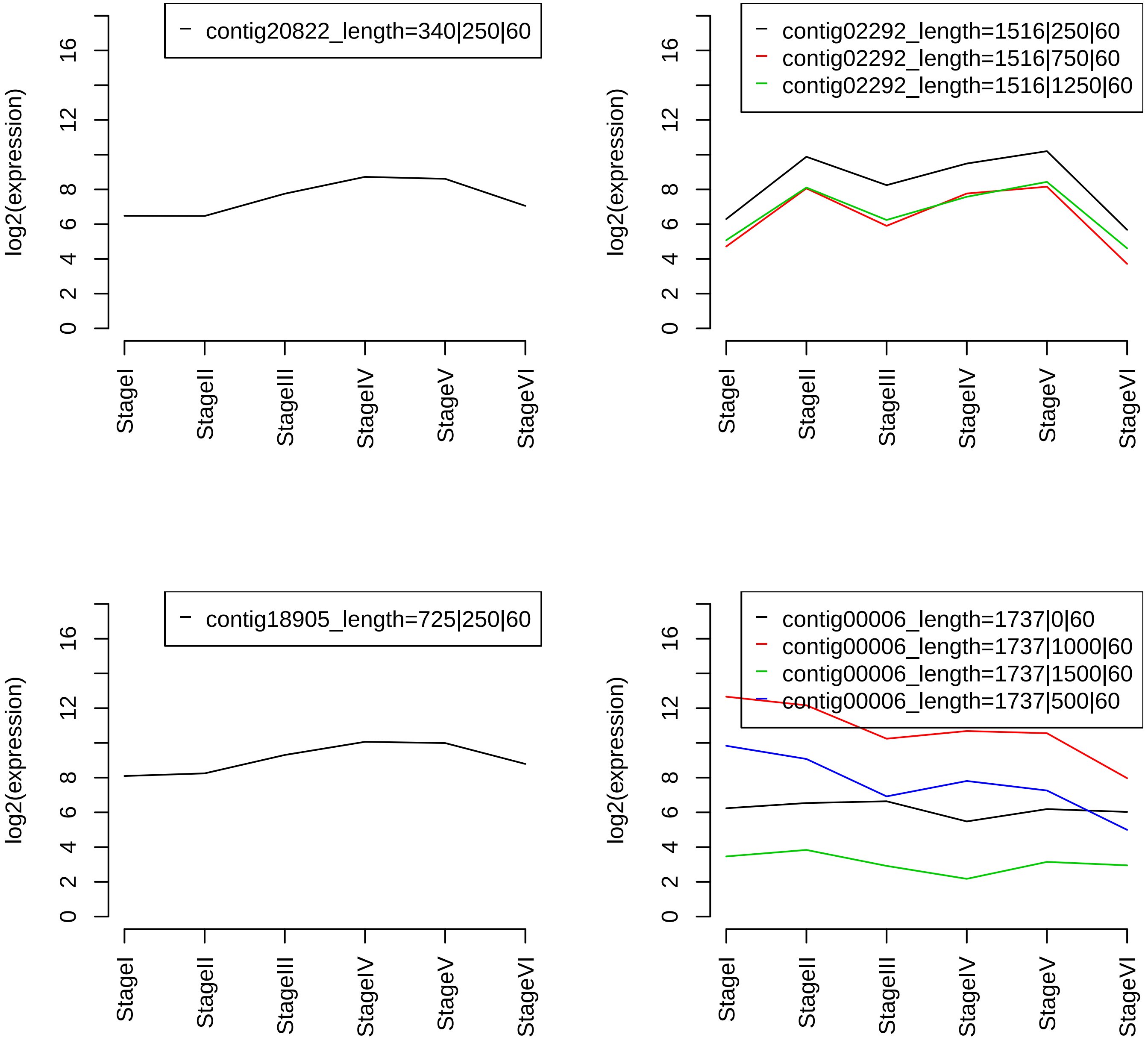
contig20822_length=340|250|60
log2(expression)
0
2
4
6
8
12
16
StageI
StageII
StageIII
StageIV
StageV
StageVI
contig02292_length=1516|250|60
contig02292_length=1516|750|60
contig02292_length=1516|1250|60
contig18905_length=725|250|60
contig00006_length=1737|0|60
contig00006_length=1737|1000|60
contig00006_length=1737|1500|60
contig00006_length=1737|500|60

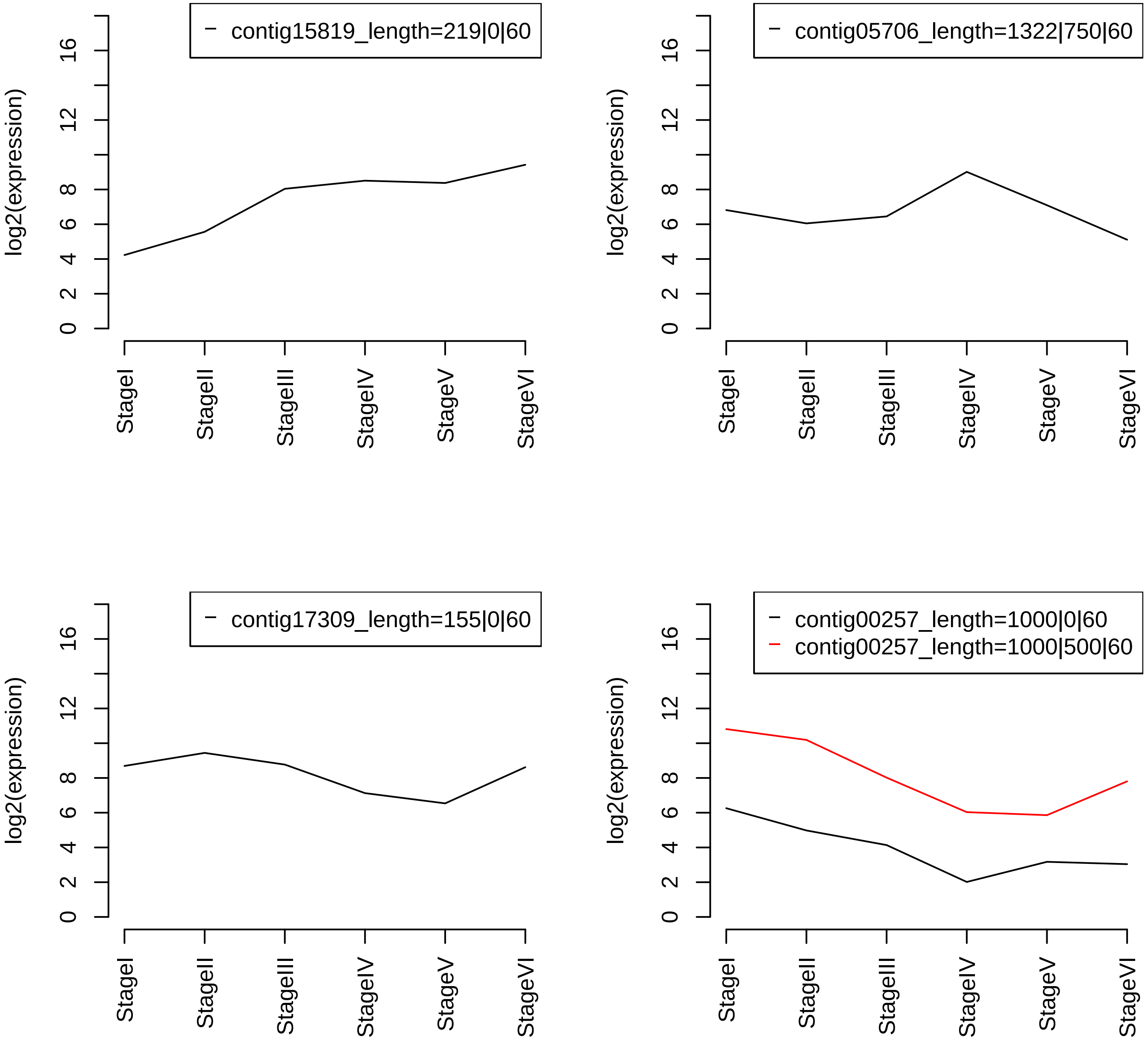
contig15819_length=219|0|60
contig05706_length=1322|750|60
contig17309_length=155|0|60
contig00257_length=1000|0|60
contig00257_length=1000|500|60
log2(expression)
0
2
4
6
8
12
16
StageI
StageII
StageIII
StageIV
StageV
StageVI

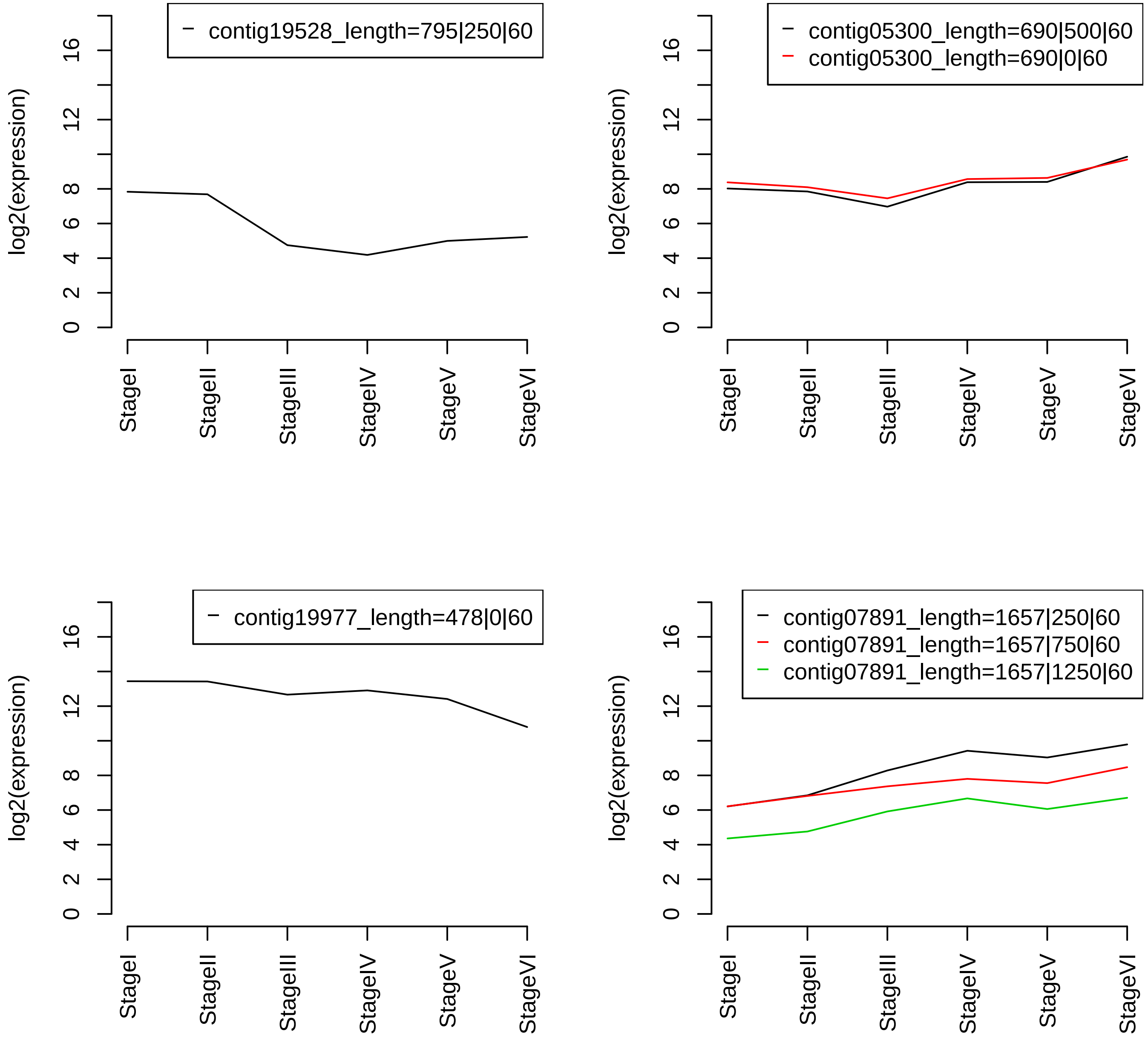
contig19528_length=795|250|60
contig05300_length=690|500|60
contig05300_length=690|0|60
contig19977_length=478|0|60
contig07891_length=1657|250|60
contig07891_length=1657|750|60
contig07891_length=1657|1250|60
log2(expression)
0
2
4
6
8
12
16
StageI
StageII
StageIII
StageIV
StageV
StageVI

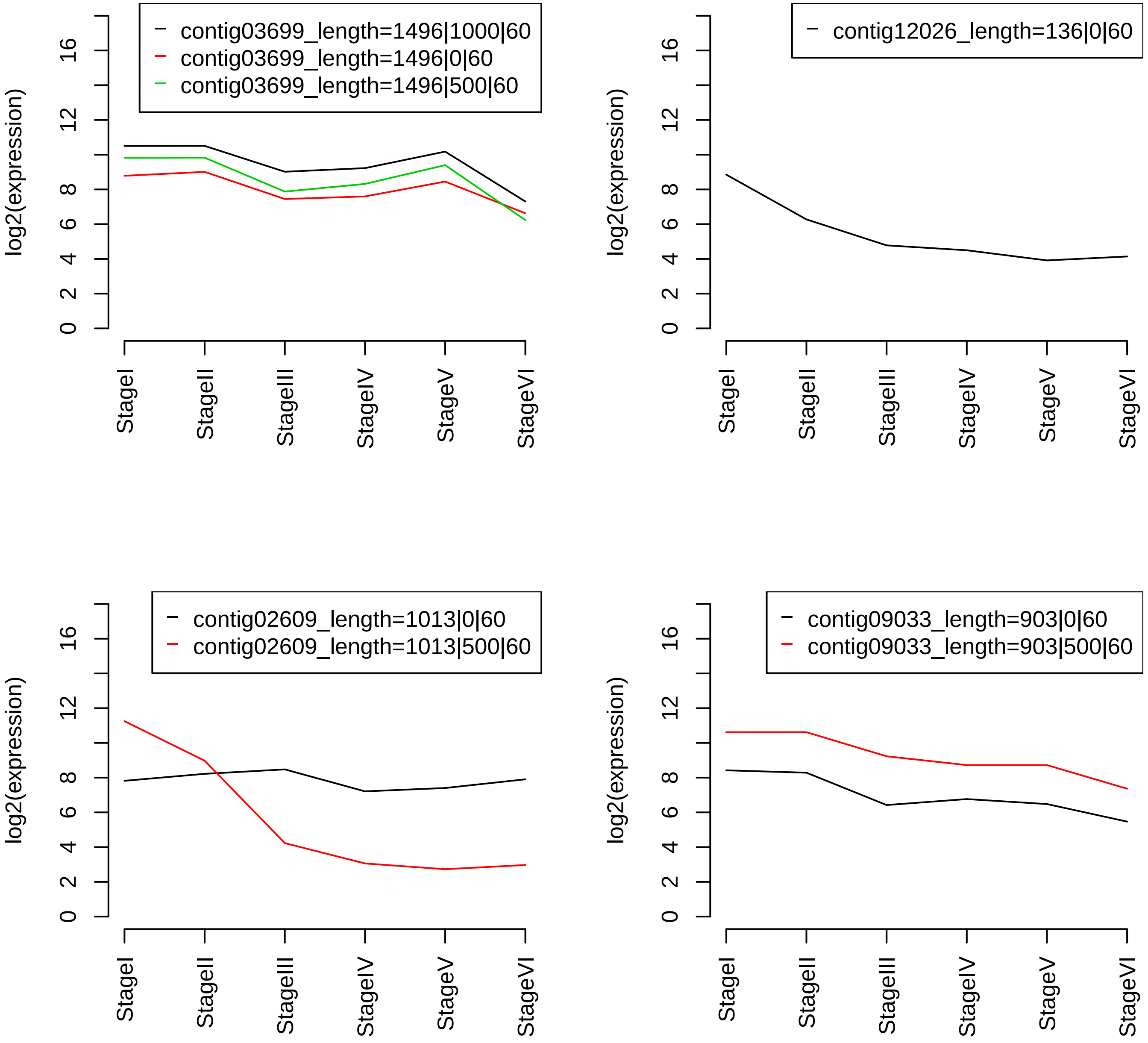
contig03699_length=1496|1000|60
contig03699_length=1496|0|60
contig03699_length=1496|500|60
contig12026_length=136|0|60
contig02609_length=1013|0|60
contig02609_length=1013|500|60
contig09033_length=903|0|60
contig09033_length=903|500|60
log2(expression)
0
2
4
6
8
12
16
StageI
StageII
StageIII
StageIV
StageV
StageVI

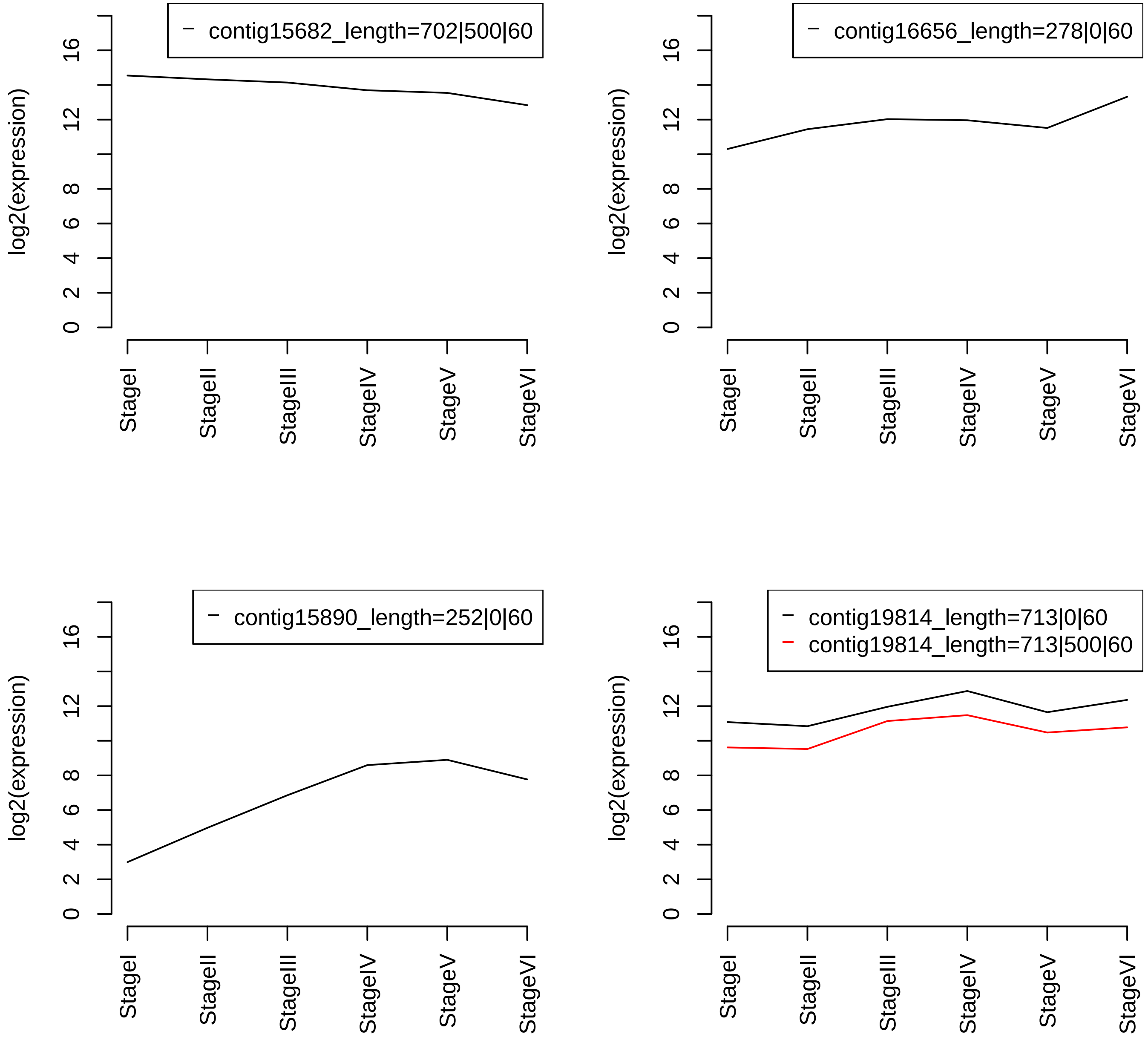
contig15682_length=702|500|60
contig16656_length=278|0|60
contig15890_length=252|0|60
contig19814_length=713|0|60
contig19814_length=713|500|60
log2(expression)
0
2
4
6
8
12
16
StageI
StageII
StageIII
StageIV
StageV
StageVI

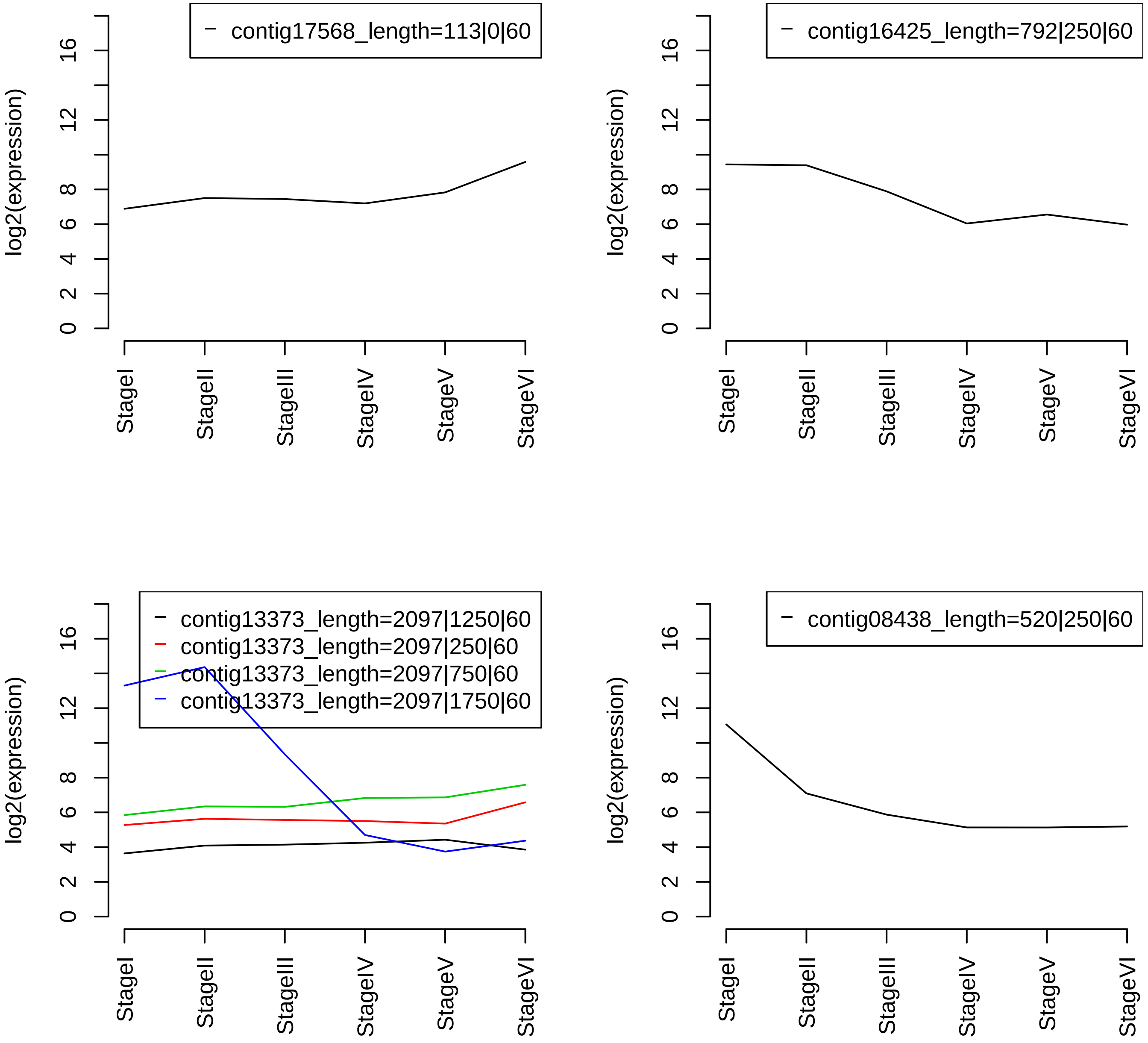
contig17568_length=113|0|60
contig16425_length=792|250|60
contig13373_length=2097|1250|60
contig13373_length=2097|250|60
contig13373_length=2097|750|60
contig13373_length=2097|1750|60
contig08438_length=520|250|60
log2(expression)
0
2
4
6
8
12
16
StageI
StageII
StageIII
StageIV
StageV
StageVI

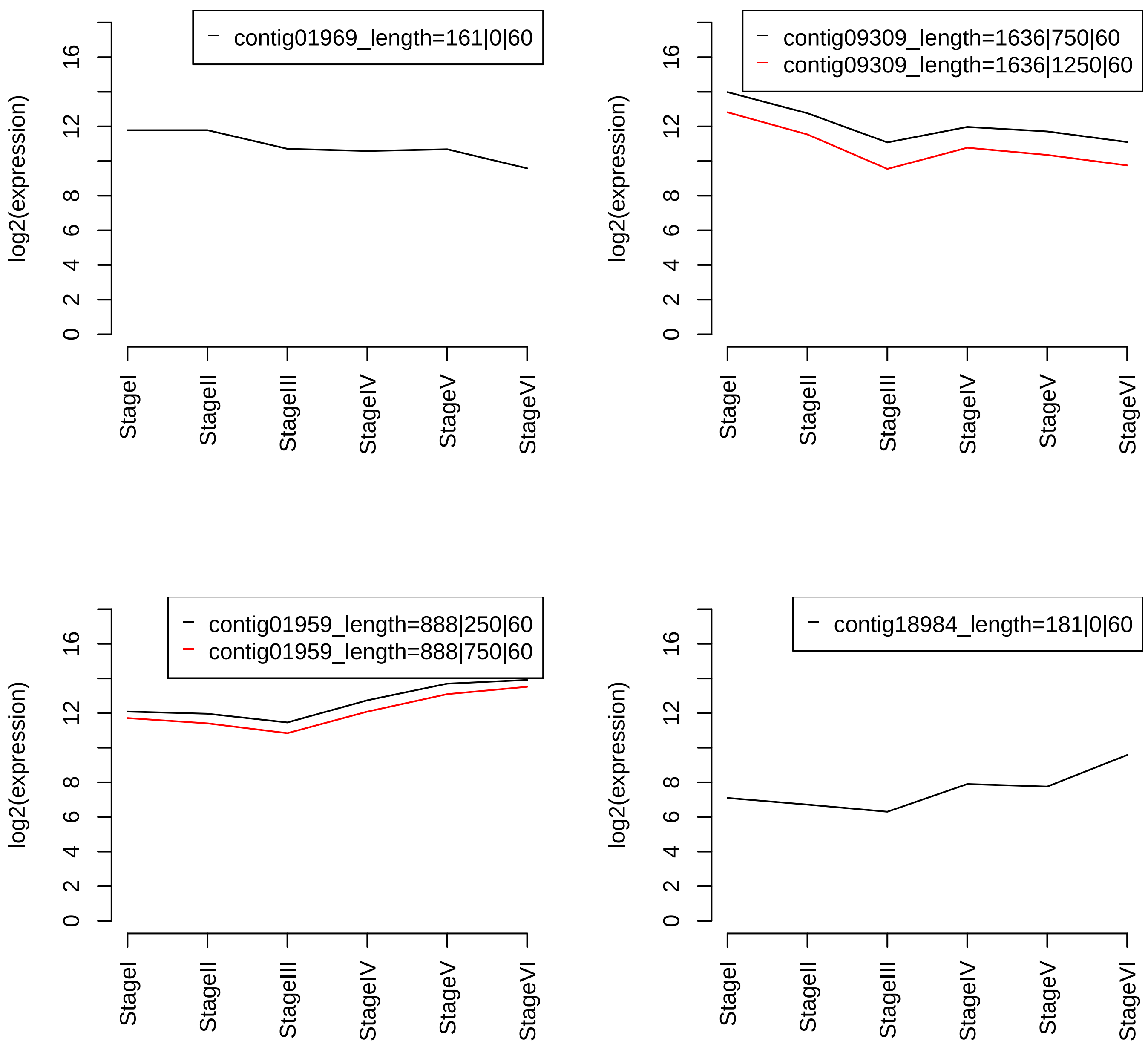
contig01969_length=161|0|60
contig09309_length=1636|750|60
contig09309_length=1636|1250|60
contig01959_length=888|250|60
contig01959_length=888|750|60
contig18984_length=181|0|60
log2(expression)
StageI
StageII
StageIII
StageIV
StageV
StageVI

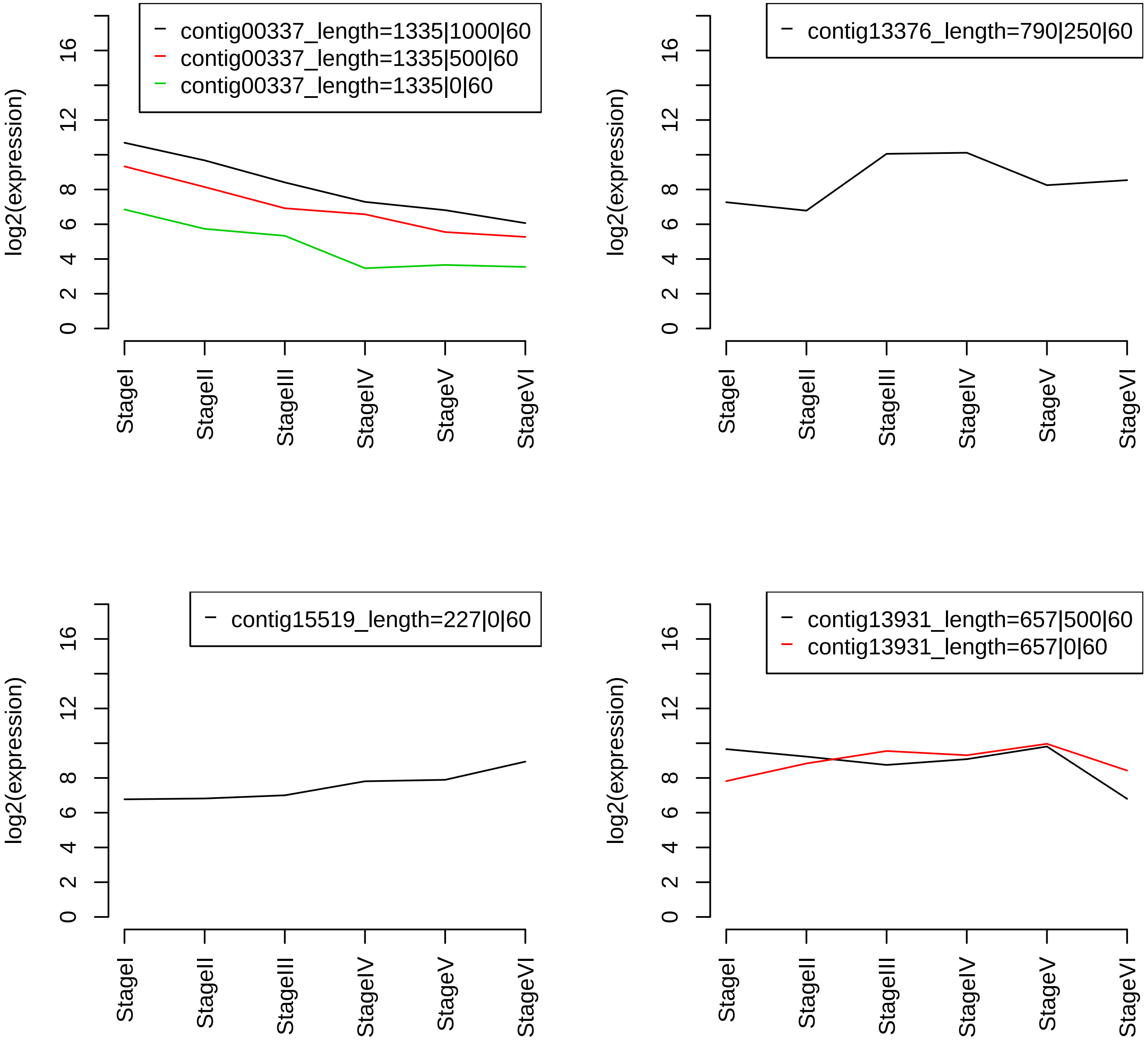
contig00337_length=1335|1000|60
contig00337_length=1335|500|60
contig00337_length=1335|0|60
contig13376_length=790|250|60
contig15519_length=227|0|60
contig13931_length=657|500|60
contig13931_length=657|0|60
log2(expression)
0
2
4
6
8
12
16
StageI
StageII
StageIII
StageIV
StageV
StageVI

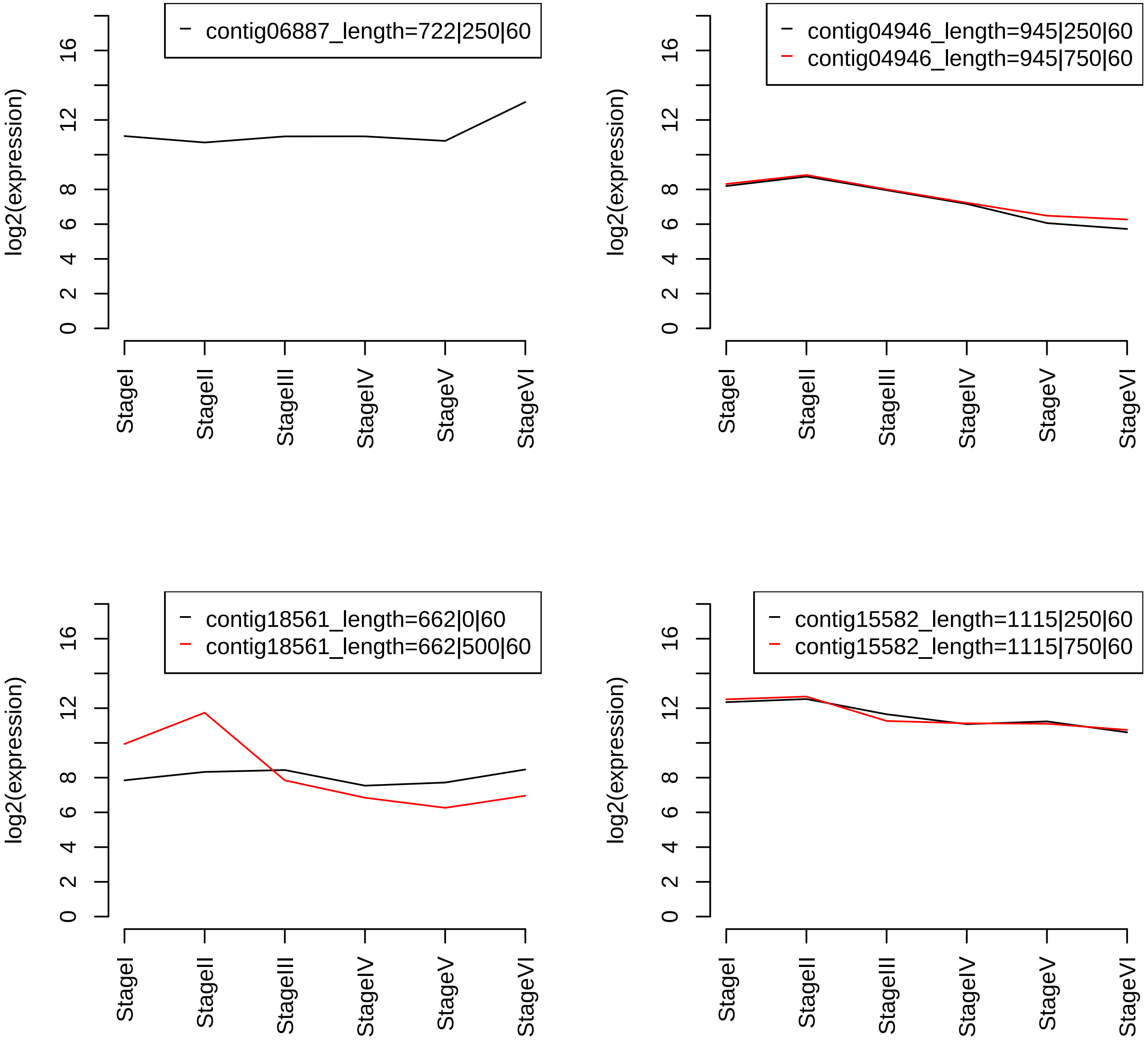
contig06887_length=722|250|60
contig04946_length=945|250|60
contig04946_length=945|750|60
contig18561_length=662|0|60
contig18561_length=662|500|60
contig15582_length=1115|250|60
contig15582_length=1115|750|60
log2(expression)
StageI
StageII
StageIII
StageIV
StageV
StageVI

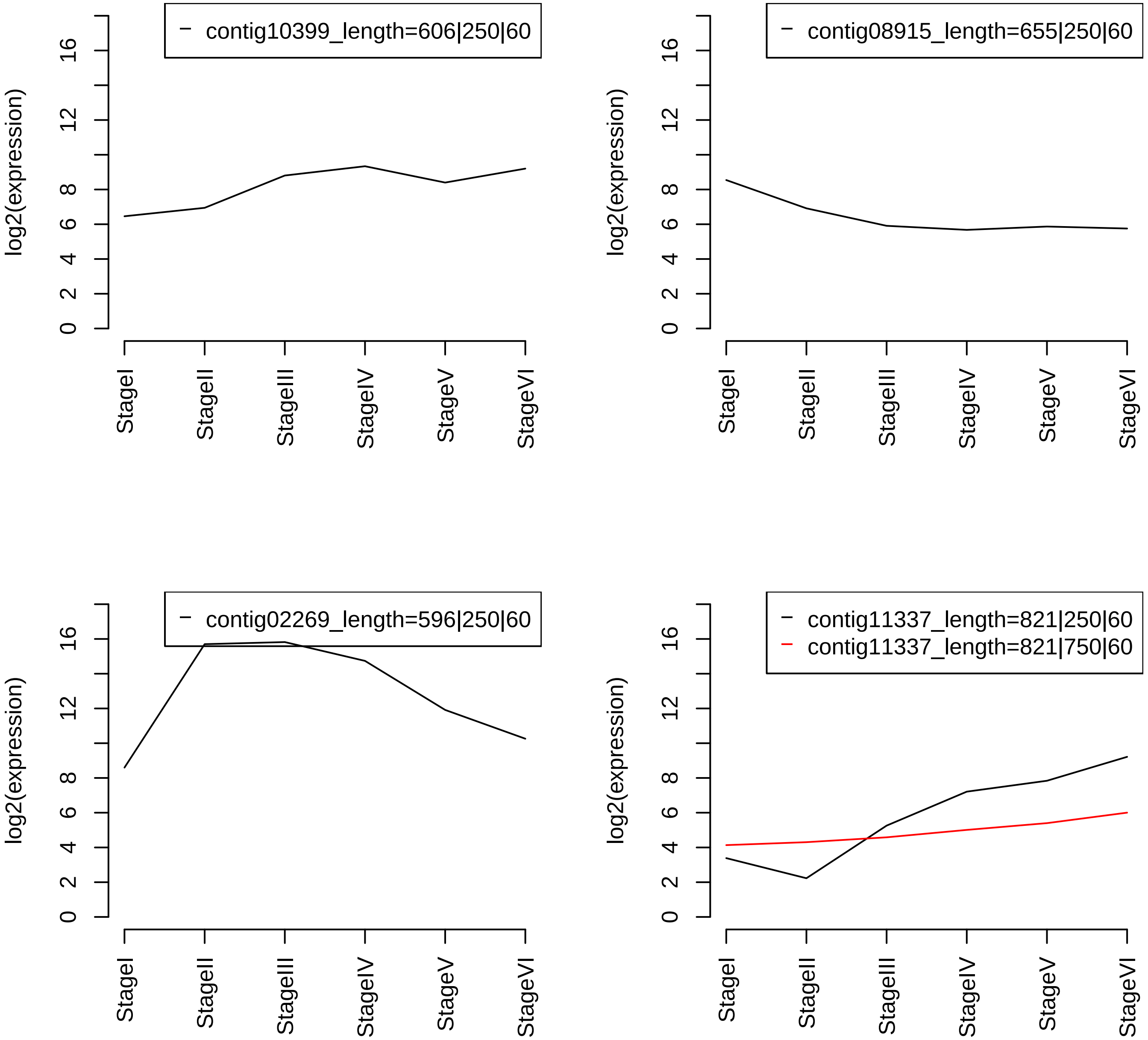

contig10399_length=606|250|60
contig08915_length=655|250|60
contig02269_length=596|250|60
contig11337_length=821|250|60
contig11337_length=821|750|60
log2(expression)
0
2
4
6
8
12
16
StageI
StageII
StageIII
StageIV
StageV
StageVI

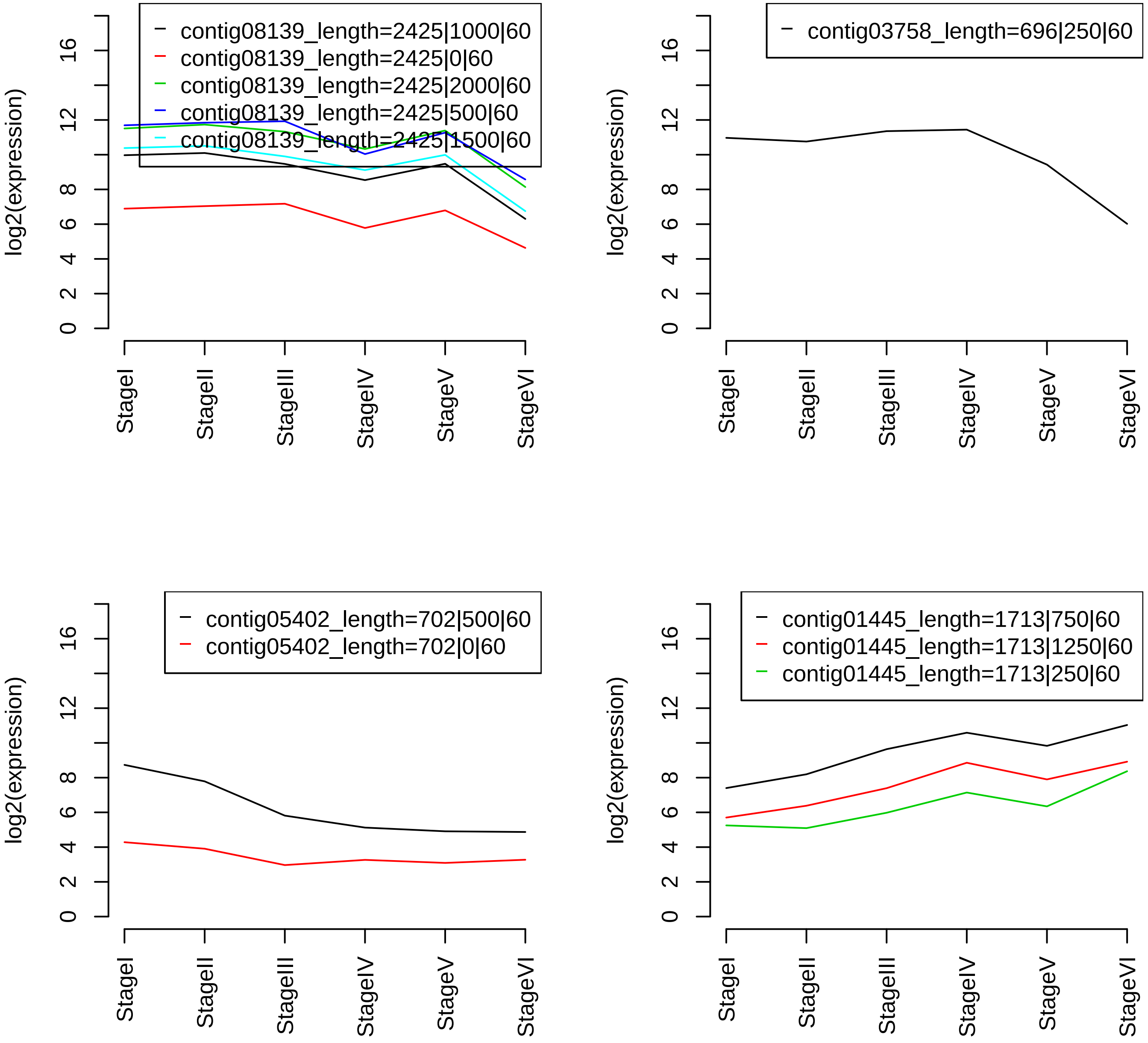
contig08139_length=2425|1000|60
contig08139_length=2425|0|60
contig08139_length=2425|2000|60
contig08139_length=2425|500|60
contig08139_length=2425|1500|60
log2(expression)
0
2
4
6
8
12
16
StageI
StageII
StageIII
StageIV
StageV
StageVI
contig03758_length=696|250|60
contig05402_length=702|500|60
contig05402_length=702|0|60
contig01445_length=1713|750|60
contig01445_length=1713|1250|60
contig01445_length=1713|250|60

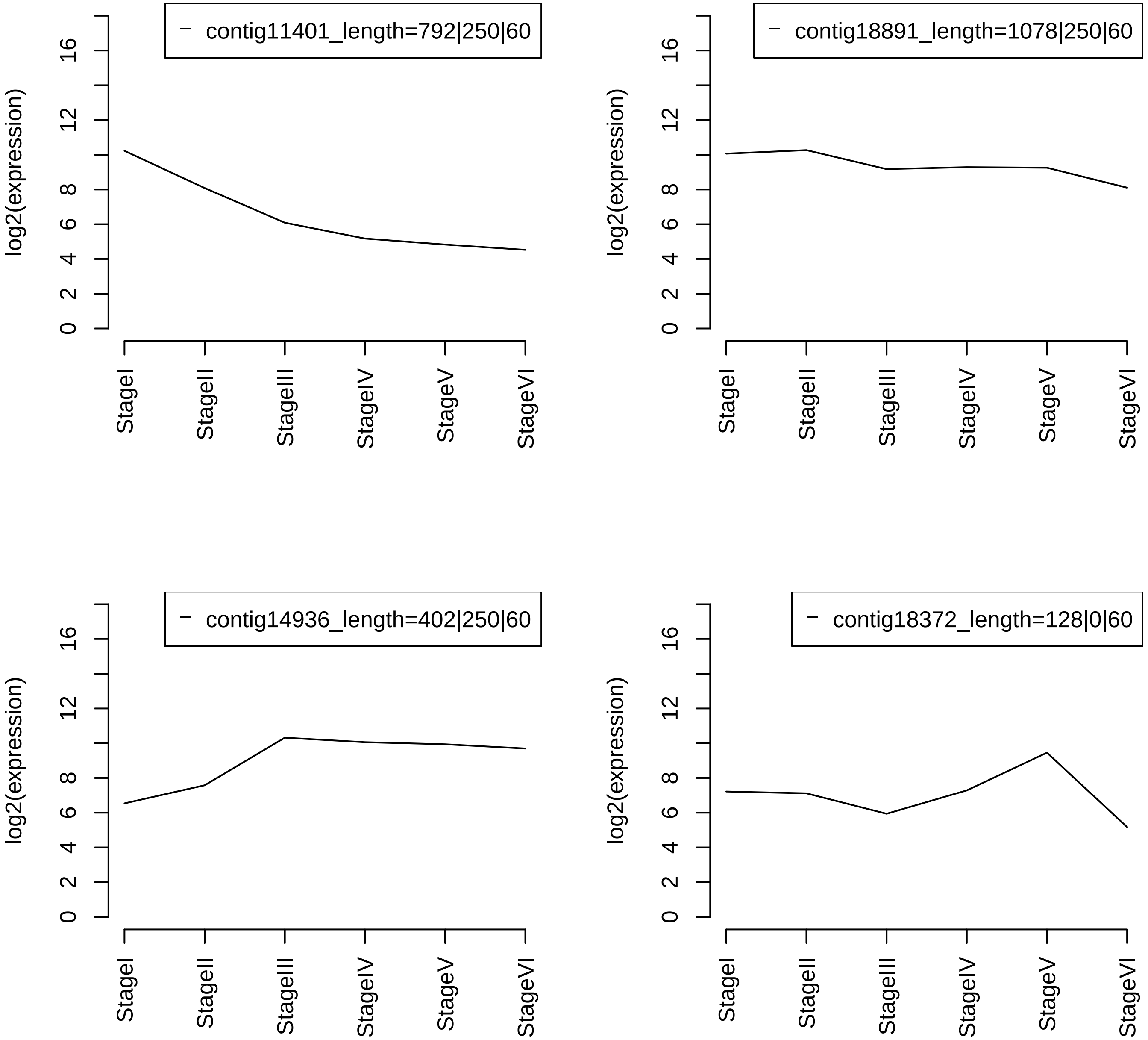

contig11401_length=792|250|60
contig18891_length=1078|250|60
contig14936_length=402|250|60
contig18372_length=128|0|60
log2(expression)
0
2
4
6
8
12
16
StageI
StageII
StageIII
StageIV
StageV
StageVI

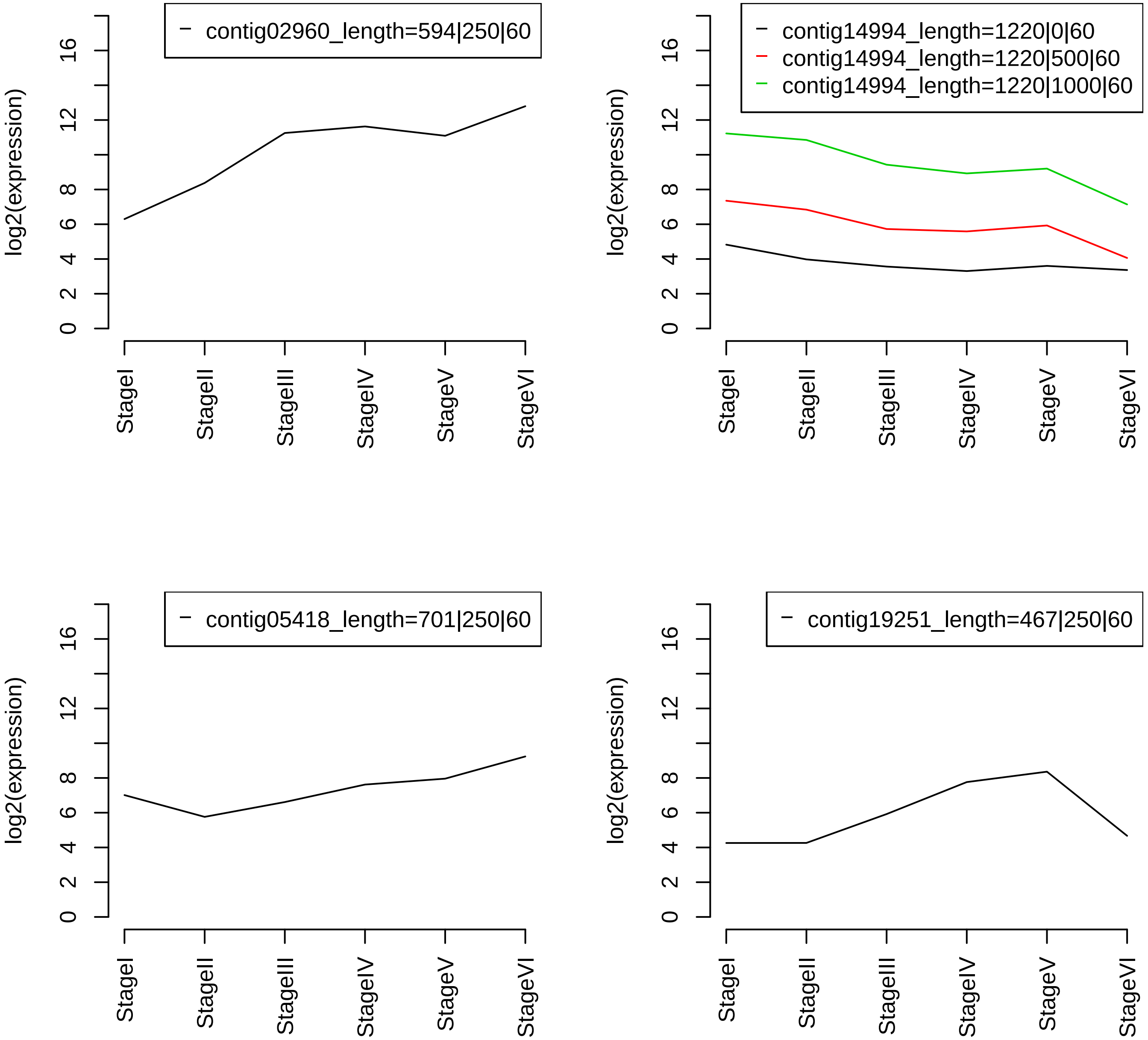

contig02960_length=594|250|60
contig14994_length=1220|0|60
contig14994_length=1220|500|60
contig14994_length=1220|1000|60
contig05418_length=701|250|60
contig19251_length=467|250|60
log2(expression)
0
2
4
6
8
12
16
StageI
StageII
StageIII
StageIV
StageV
StageVI

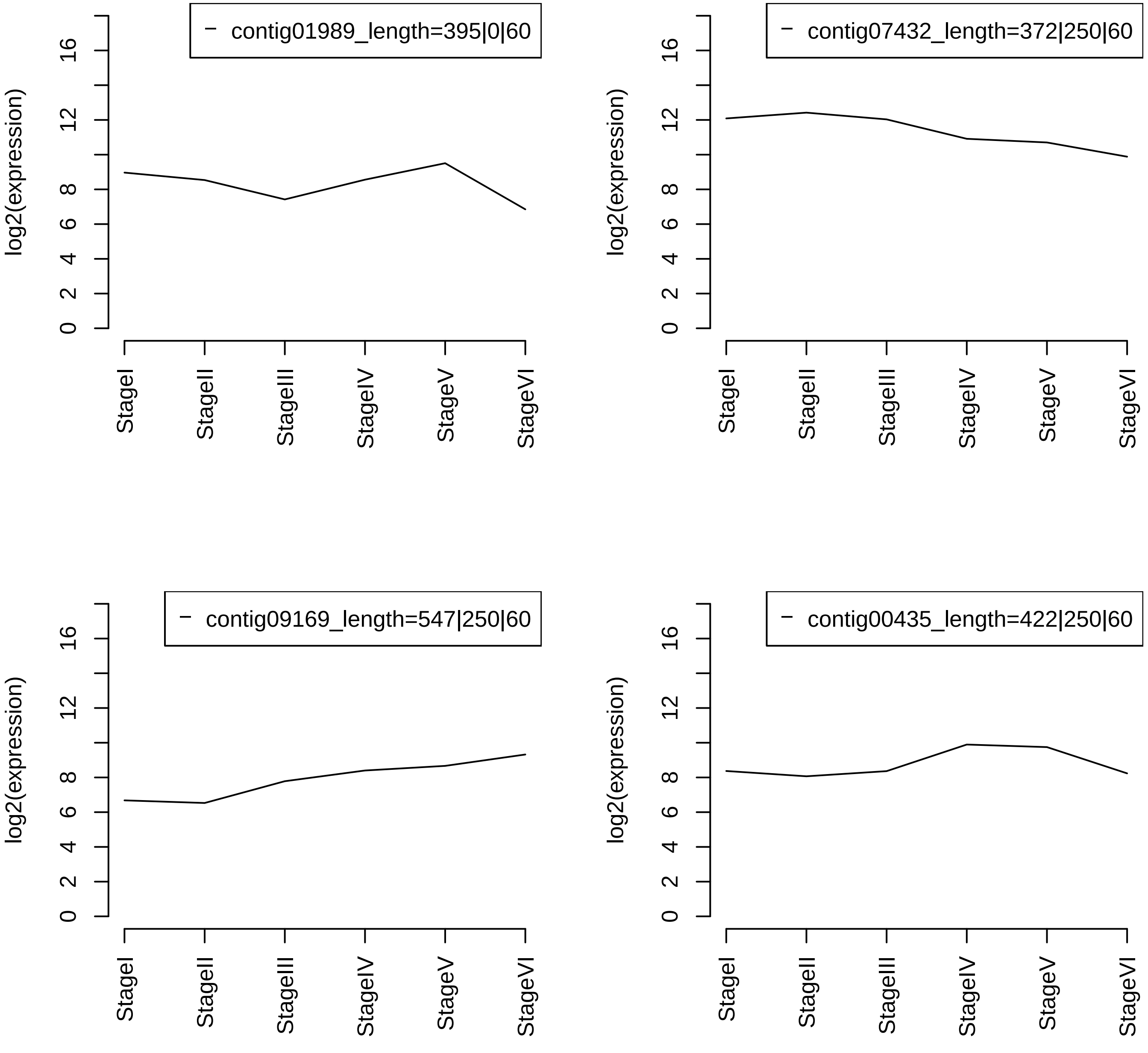

contig01989_length=395|0|60
contig07432_length=372|250|60
contig09169_length=547|250|60
contig00435_length=422|250|60
log2(expression)
0
2
4
6
8
12
16
StageI
StageII
StageIII
StageIV
StageV
StageVI

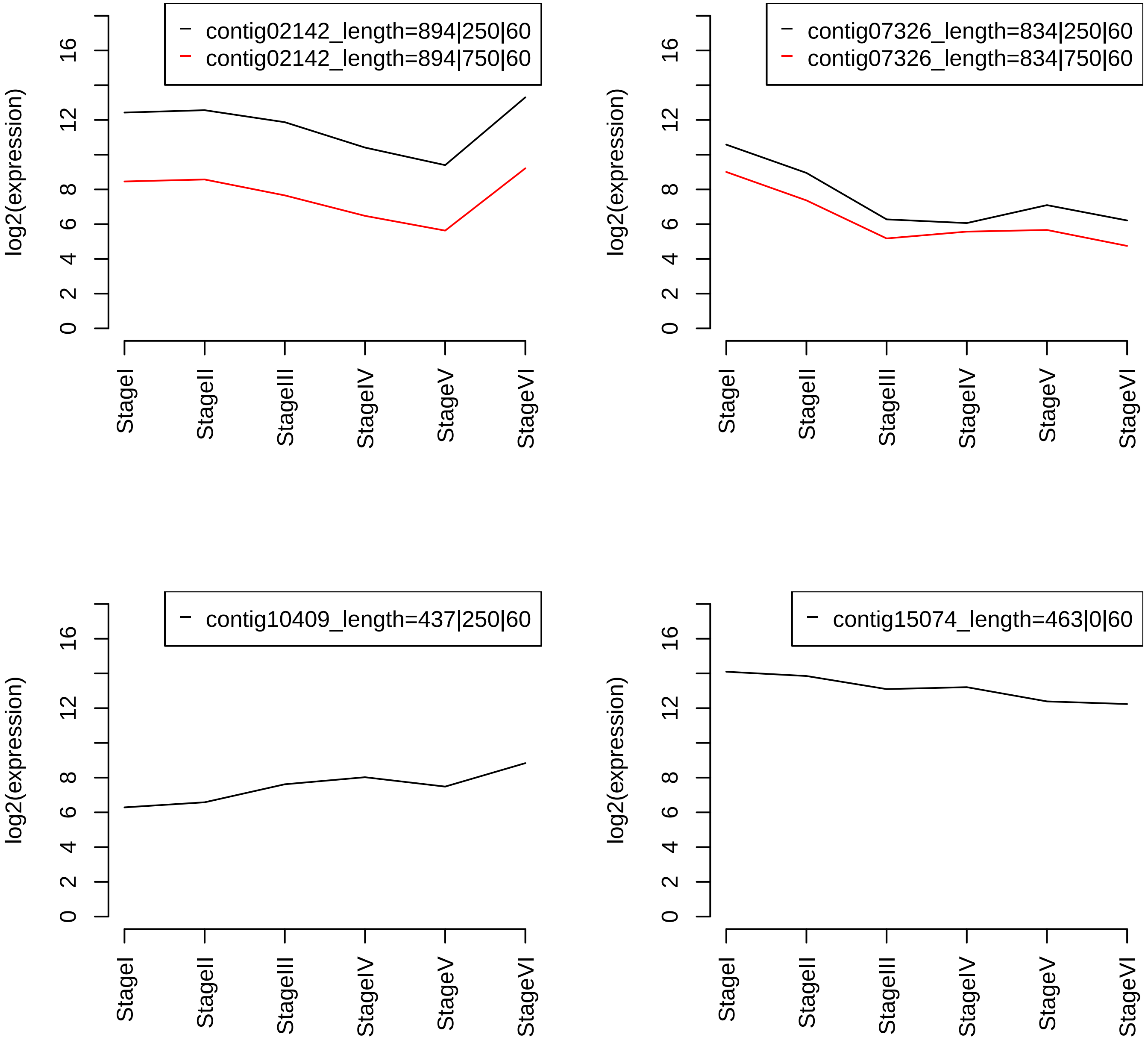

contig02142_length=894|250|60
contig02142_length=894|750|60
contig07326_length=834|250|60
contig07326_length=834|750|60
contig10409_length=437|250|60
contig15074_length=463|0|60
log2(expression)
0 2 4 6 8 12 16
StageI StageII StageIII StageIV StageV StageVI

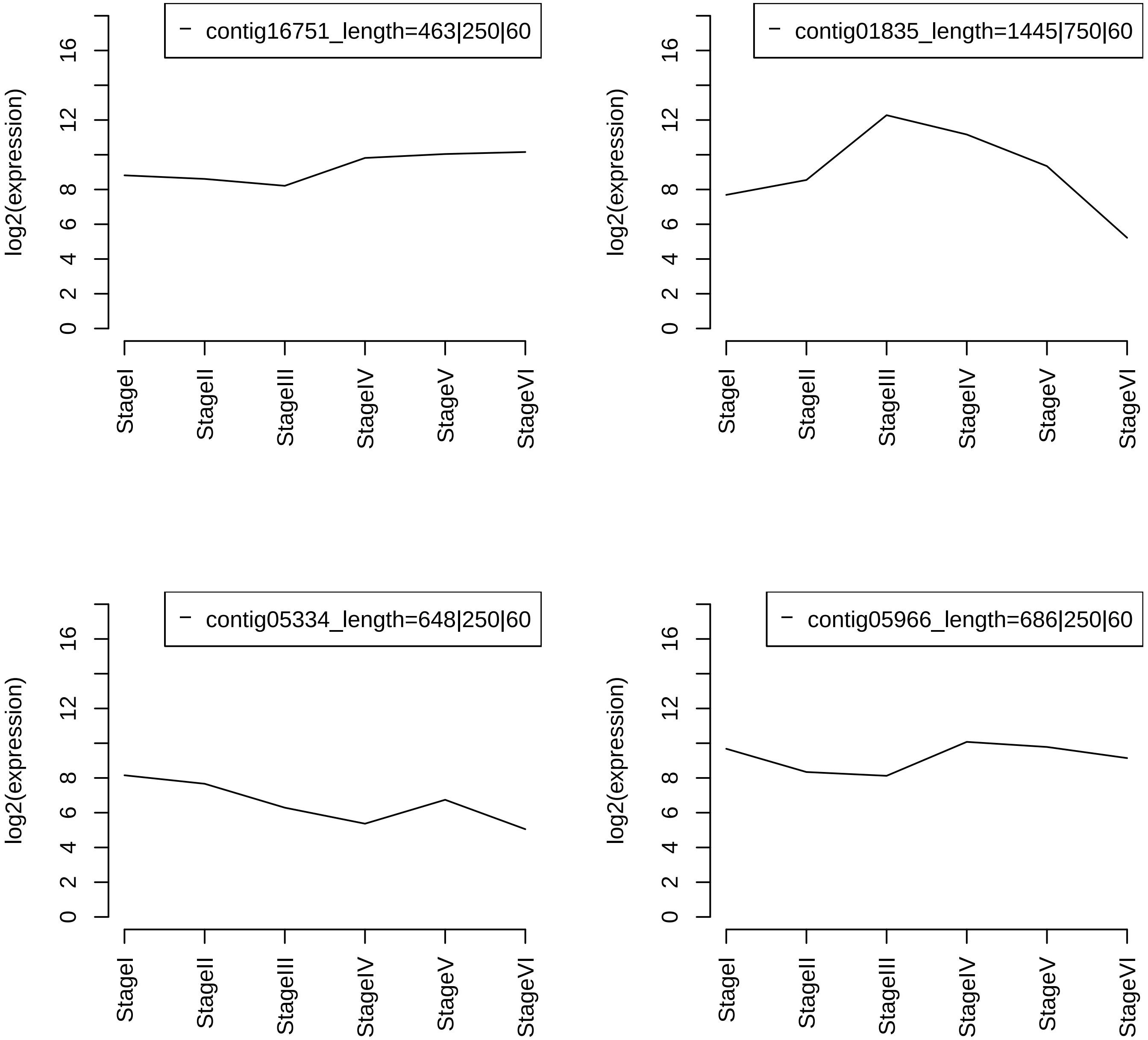
contig16751_length=463|250|60
contig01835_length=1445|750|60
contig05334_length=648|250|60
contig05966_length=686|250|60
log2(expression)
0
2
4
6
8
12
16
StageI
StageII
StageIII
StageIV
StageV
StageVI

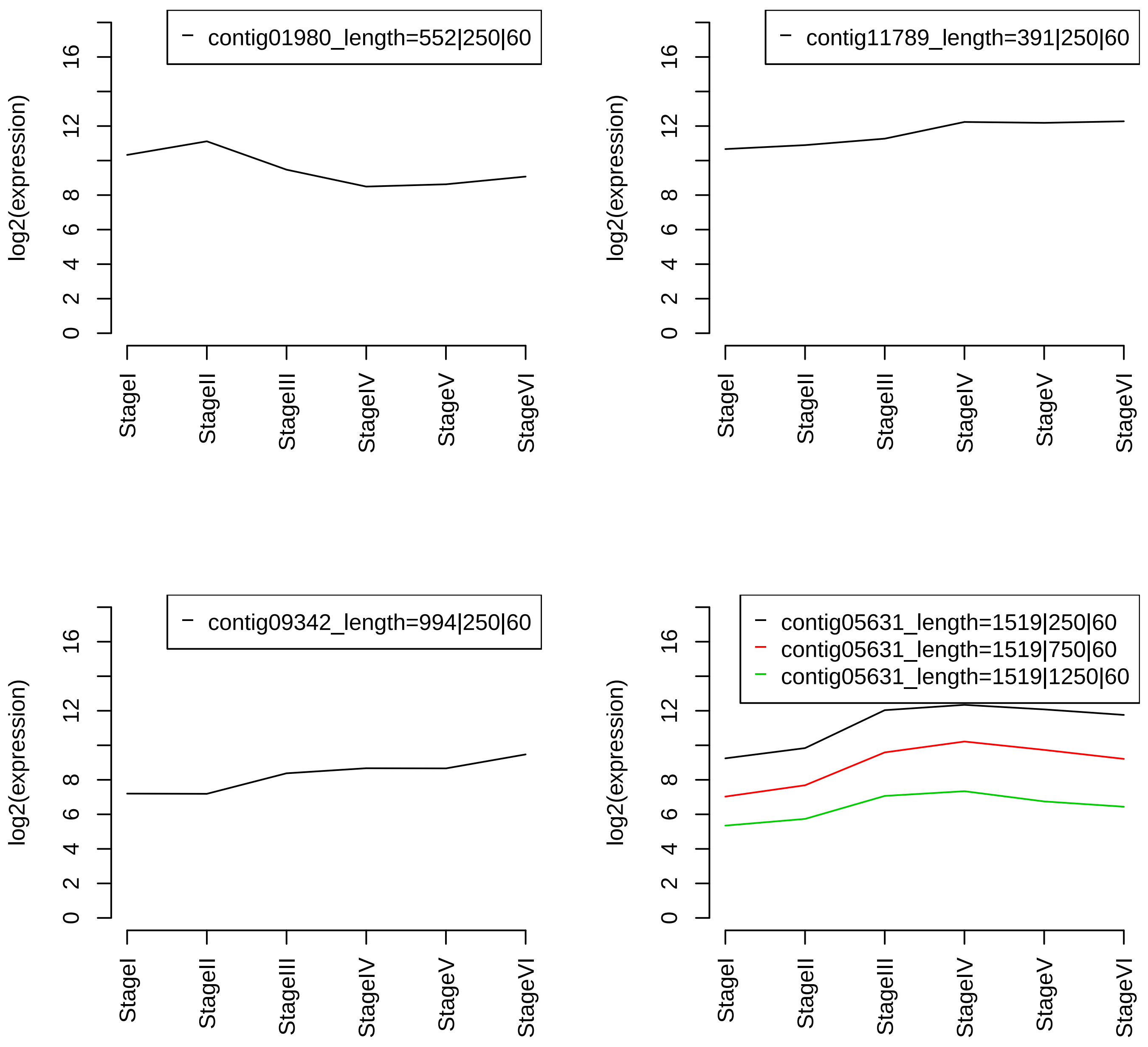
contig01980_length=552|250|60
contig11789_length=391|250|60
contig09342_length=994|250|60
contig05631_length=1519|250|60
contig05631_length=1519|750|60
contig05631_length=1519|1250|60
log2(expression)
0
2
4
6
8
12
16
StageI
StageII
StageIII
StageIV
StageV
StageVI

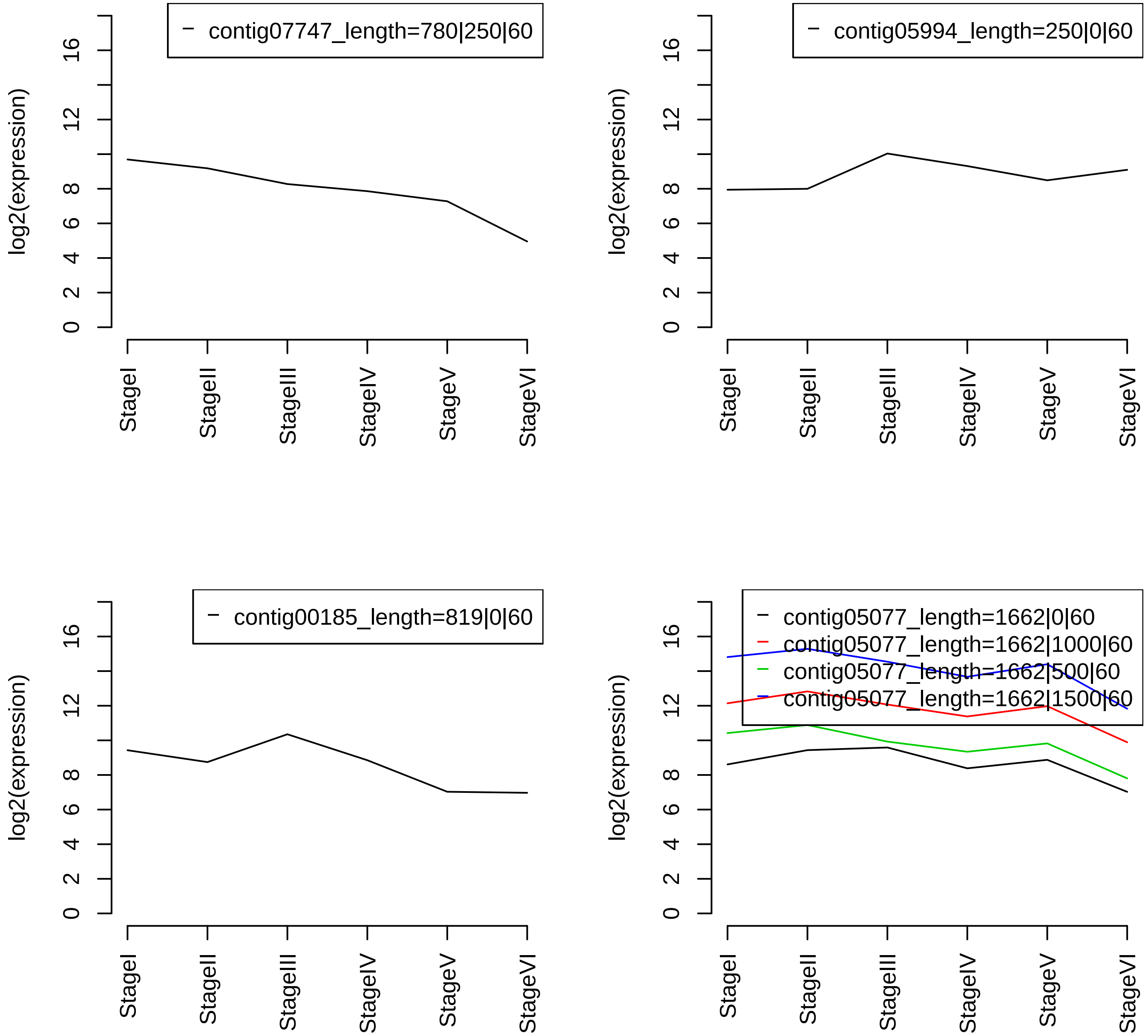
contig07747_length=780|250|60
contig05994_length=250|0|60
contig00185_length=819|0|60
contig05077_length=1662|0|60
contig05077_length=1662|1000|60
contig05077_length=1662|500|60
contig05077_length=1662|1500|60
log2(expression)
0 2 4 6 8 12 16
StageI StageII StageIII StageIV StageV StageVI

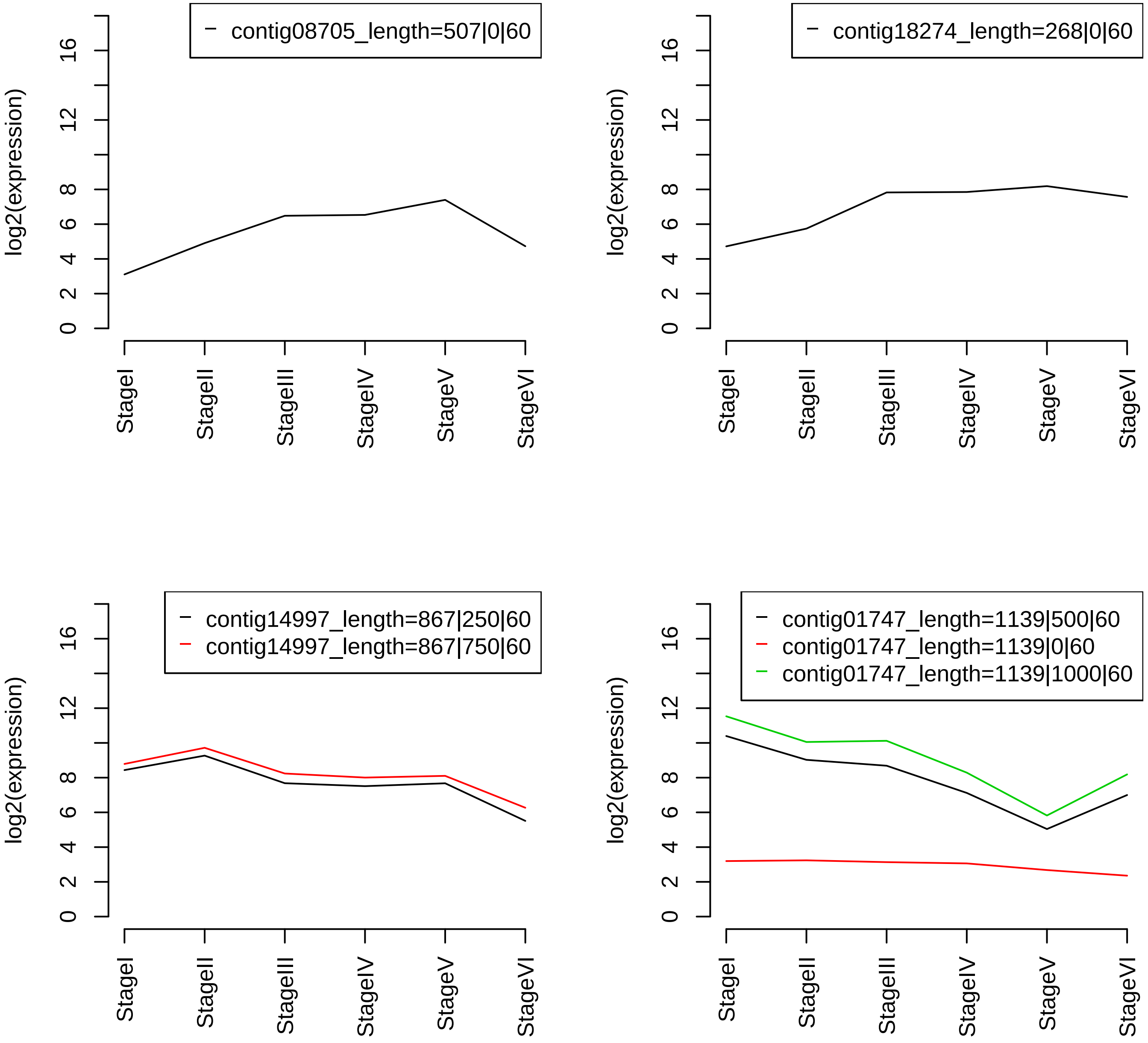
contig08705_length=507|0|60
contig18274_length=268|0|60
contig14997_length=867|250|60
contig14997_length=867|750|60
contig01747_length=1139|500|60
contig01747_length=1139|0|60
contig01747_length=1139|1000|60
log2(expression)
0 2 4 6 8 12 16
StageI
StageII
StageIII
StageIV
StageV
StageVI

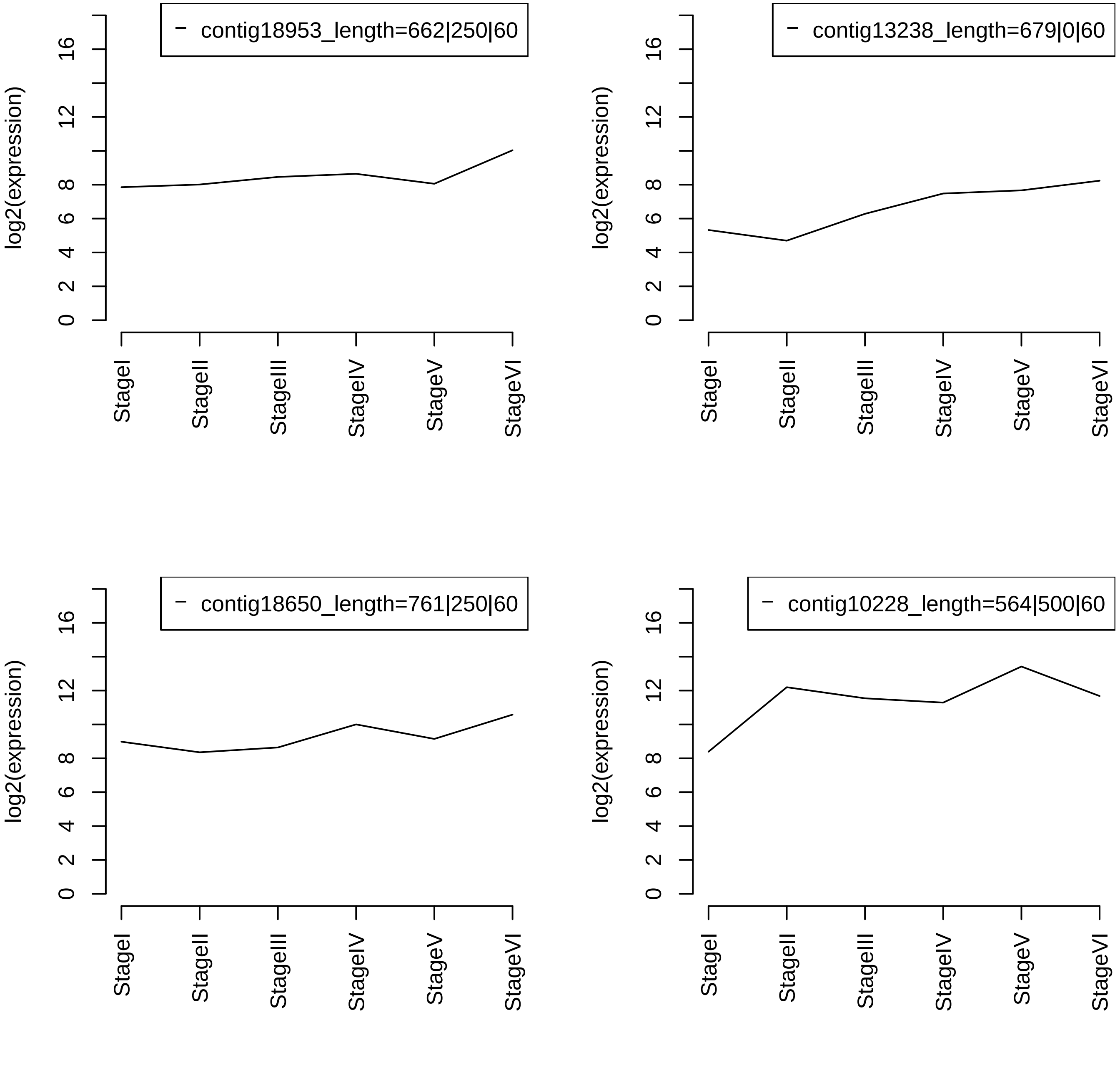
contig18953_length=662|250|60
contig13238_length=679|0|60
contig18650_length=761|250|60
contig10228_length=564|500|60
log2(expression)
0
2
4
6
8
12
16
StageI
StageII
StageIII
StageIV
StageV
StageVI

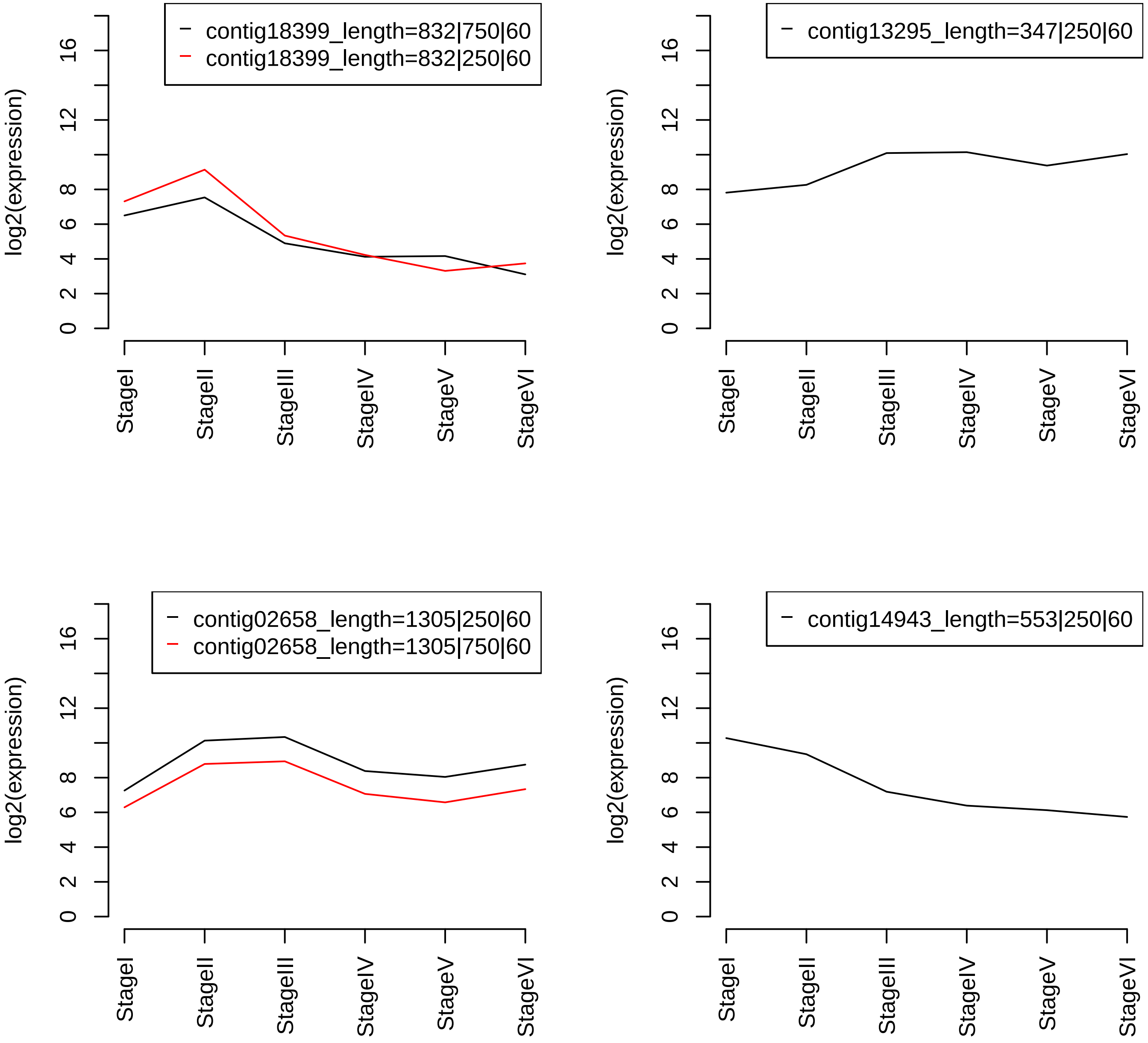

contig18399_length=832|750|60
contig18399_length=832|250|60
log2(expression)
0
2
4
6
8
12
16
StageI
StageII
StageIII
StageIV
StageV
StageVI
contig13295_length=347|250|60
contig02658_length=1305|250|60
contig02658_length=1305|750|60
contig14943_length=553|250|60

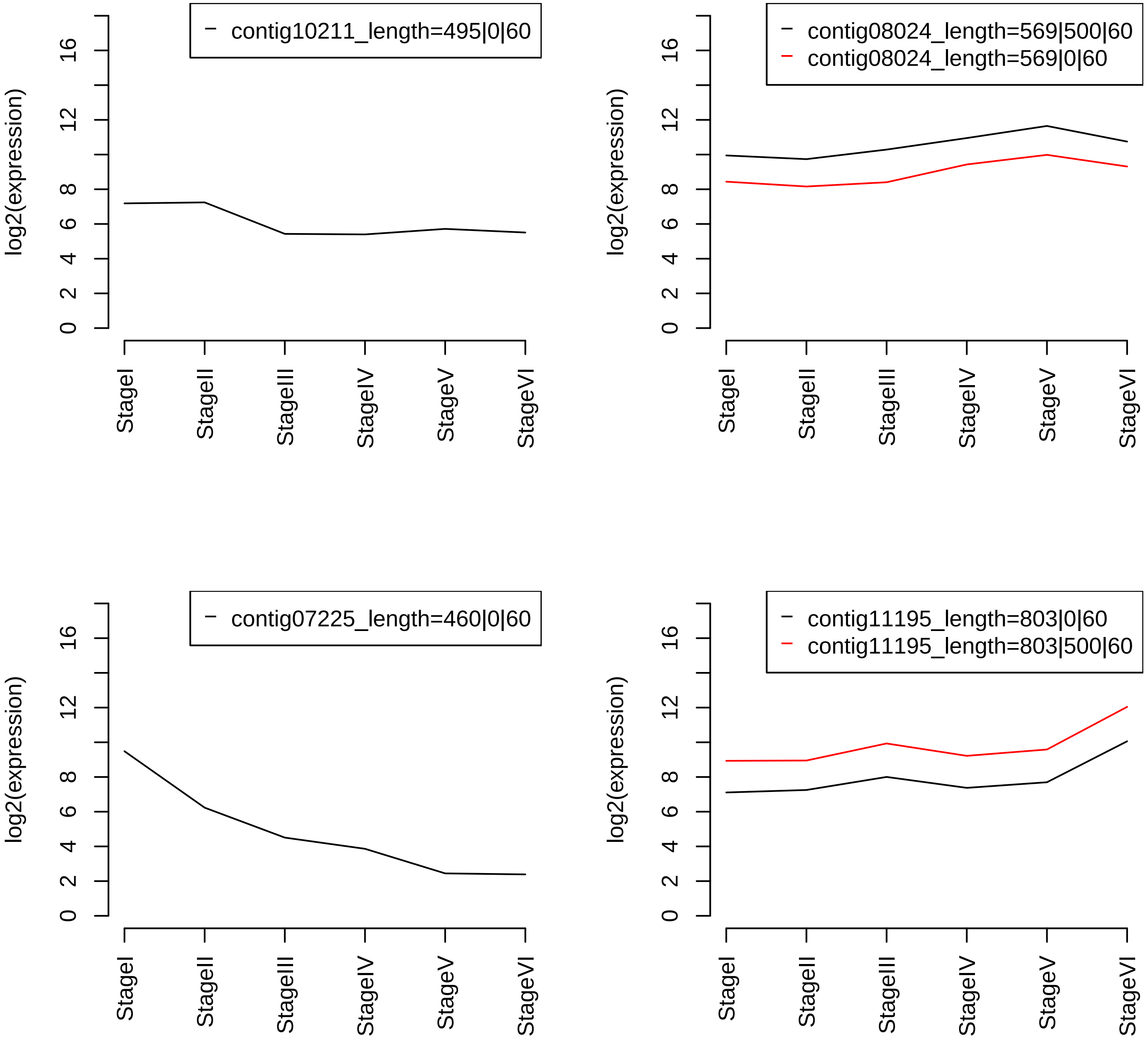
contig10211_length=495|0|60
contig08024_length=569|500|60
contig08024_length=569|0|60
contig07225_length=460|0|60
contig11195_length=803|0|60
contig11195_length=803|500|60
log2(expression)
0
2
4
6
8
12
16
StageI
StageII
StageIII
StageIV
StageV
StageVI

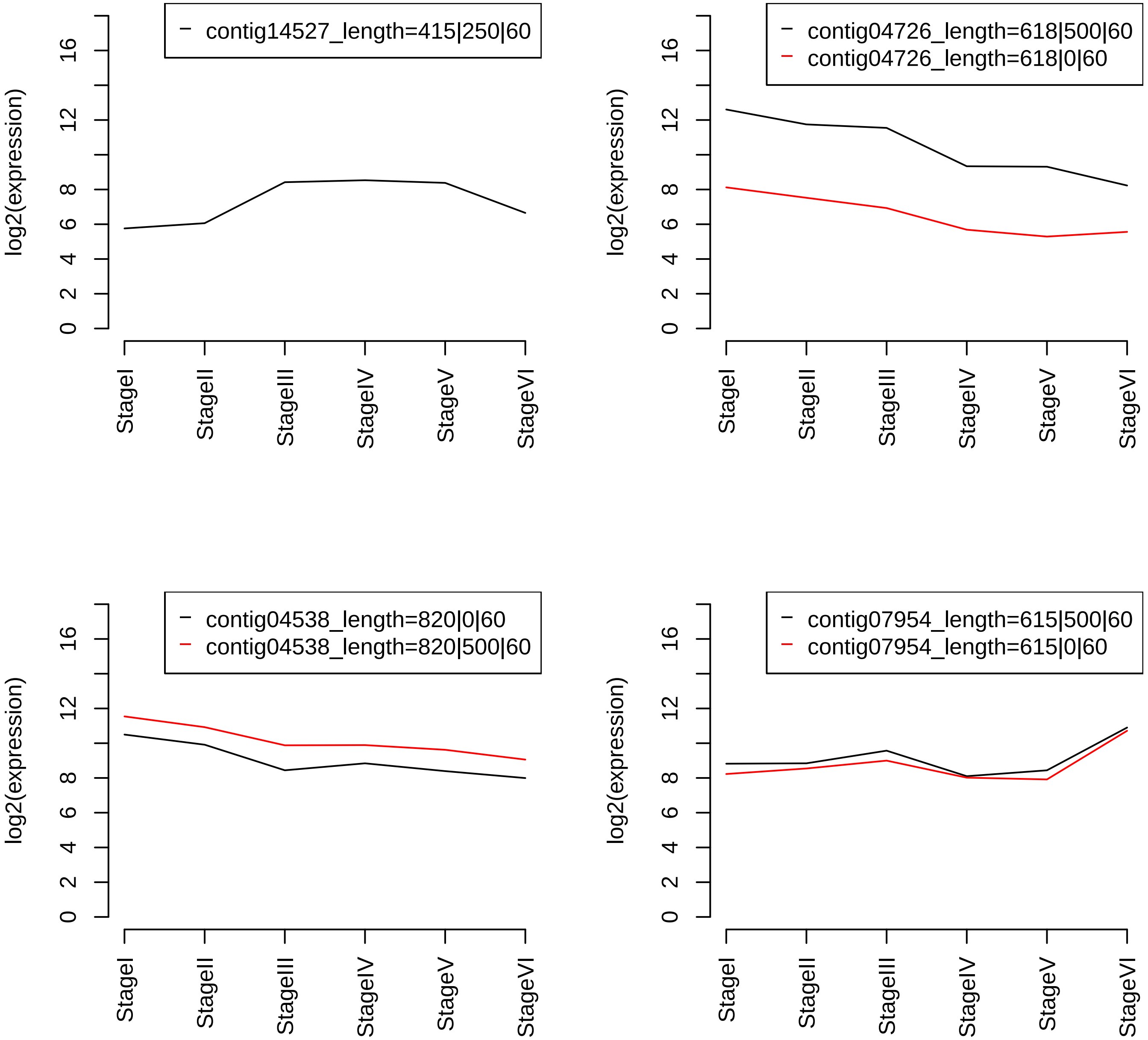

contig14527_length=415|250|60
contig04726_length=618|500|60
contig04726_length=618|0|60
contig04538_length=820|0|60
contig04538_length=820|500|60
contig07954_length=615|500|60
contig07954_length=615|0|60
log2(expression)
0
2
4
6
8
12
16
StageI
StageII
StageIII
StageIV
StageV
StageVI

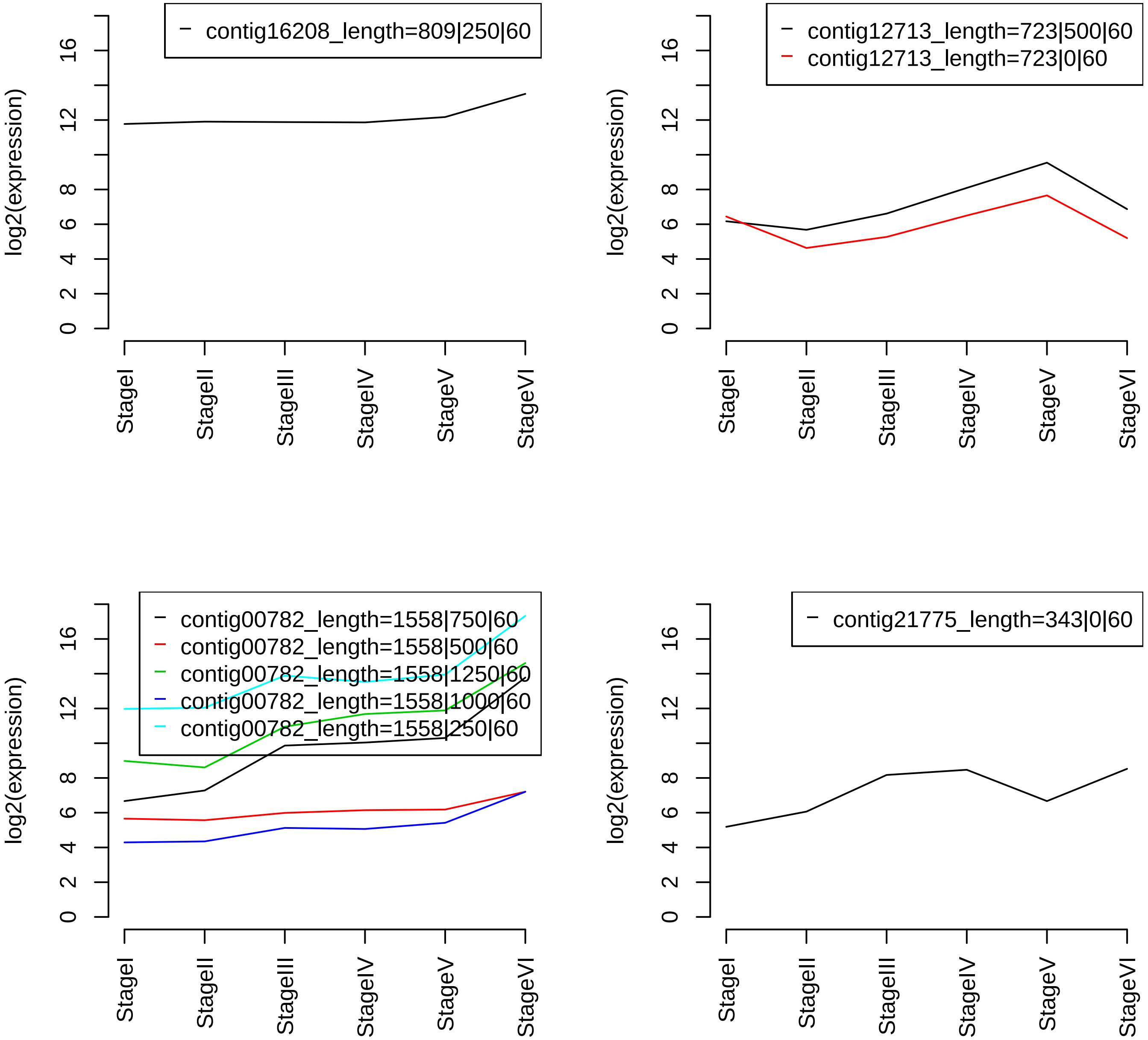
contig16208_length=809|250|60
contig12713_length=723|500|60
contig12713_length=723|0|60
contig00782_length=1558|750|60
contig00782_length=1558|500|60
contig00782_length=1558|1250|60
contig00782_length=1558|1000|60
contig00782_length=1558|250|60
contig21775_length=343|0|60
log2(expression)
StageI
StageII
StageIII
StageIV
StageV
StageVI

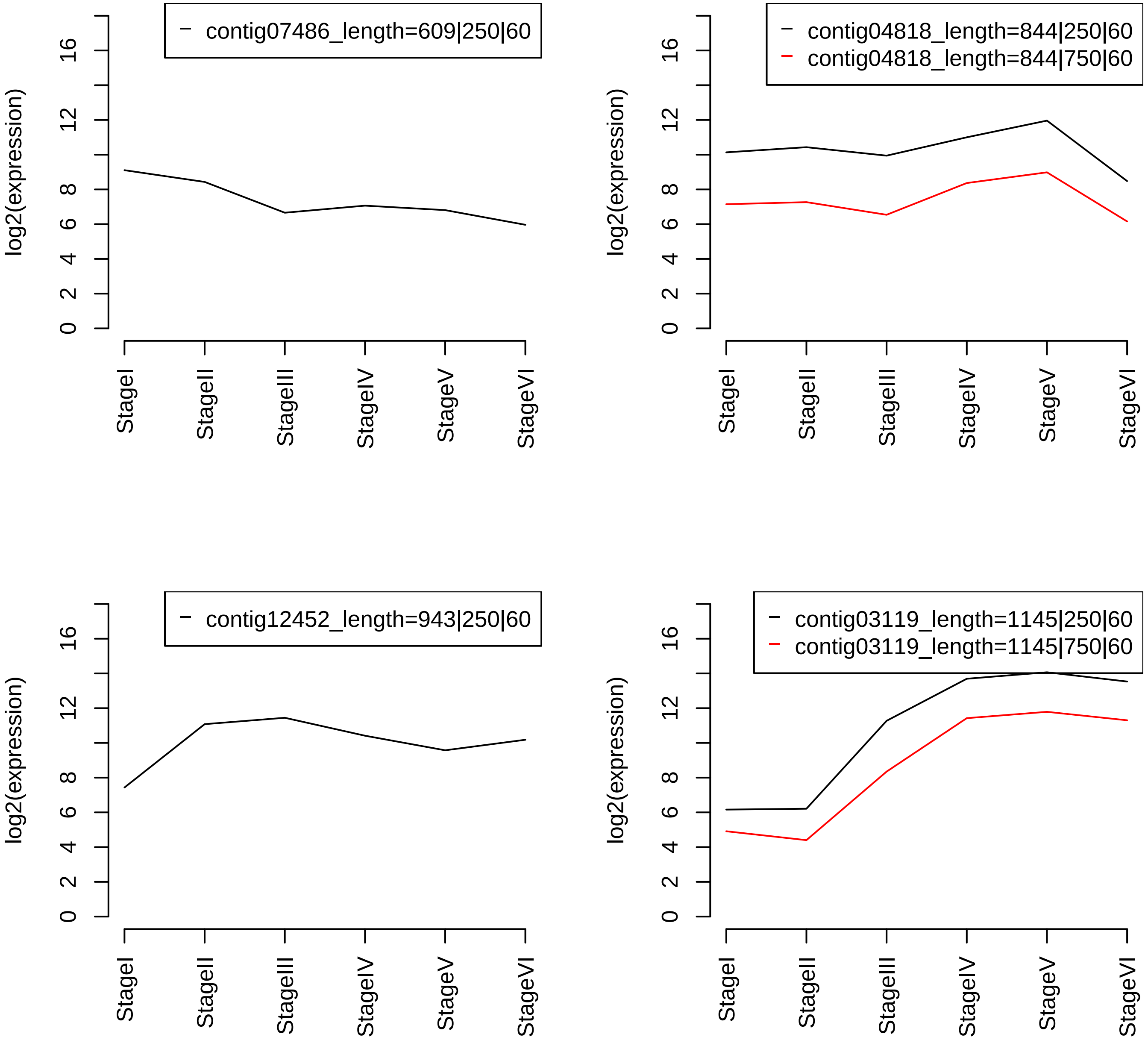
contig07486_length=609|250|60
contig04818_length=844|250|60
contig04818_length=844|750|60
contig12452_length=943|250|60
contig03119_length=1145|250|60
contig03119_length=1145|750|60
log2(expression)
0 2 4 6 8 12 16
StageI StageII StageIII StageIV StageV StageVI

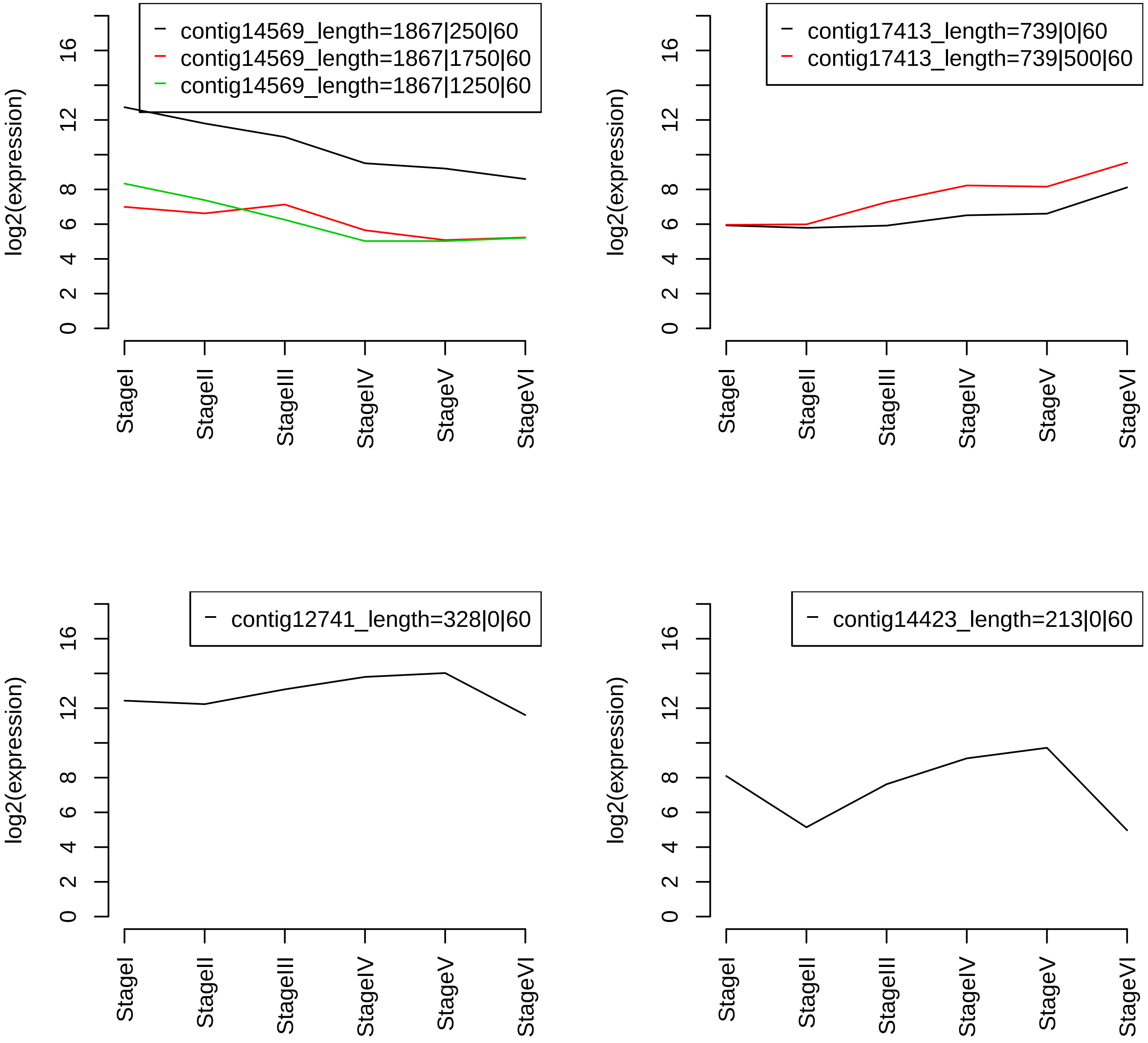

contig14569_length=1867|250|60
contig14569_length=1867|1750|60
contig14569_length=1867|1250|60
contig17413_length=739|0|60
contig17413_length=739|500|60
contig12741_length=328|0|60
contig14423_length=213|0|60
log2(expression)
0
2
4
6
8
12
16
StageI
StageII
StageIII
StageIV
StageV
StageVI

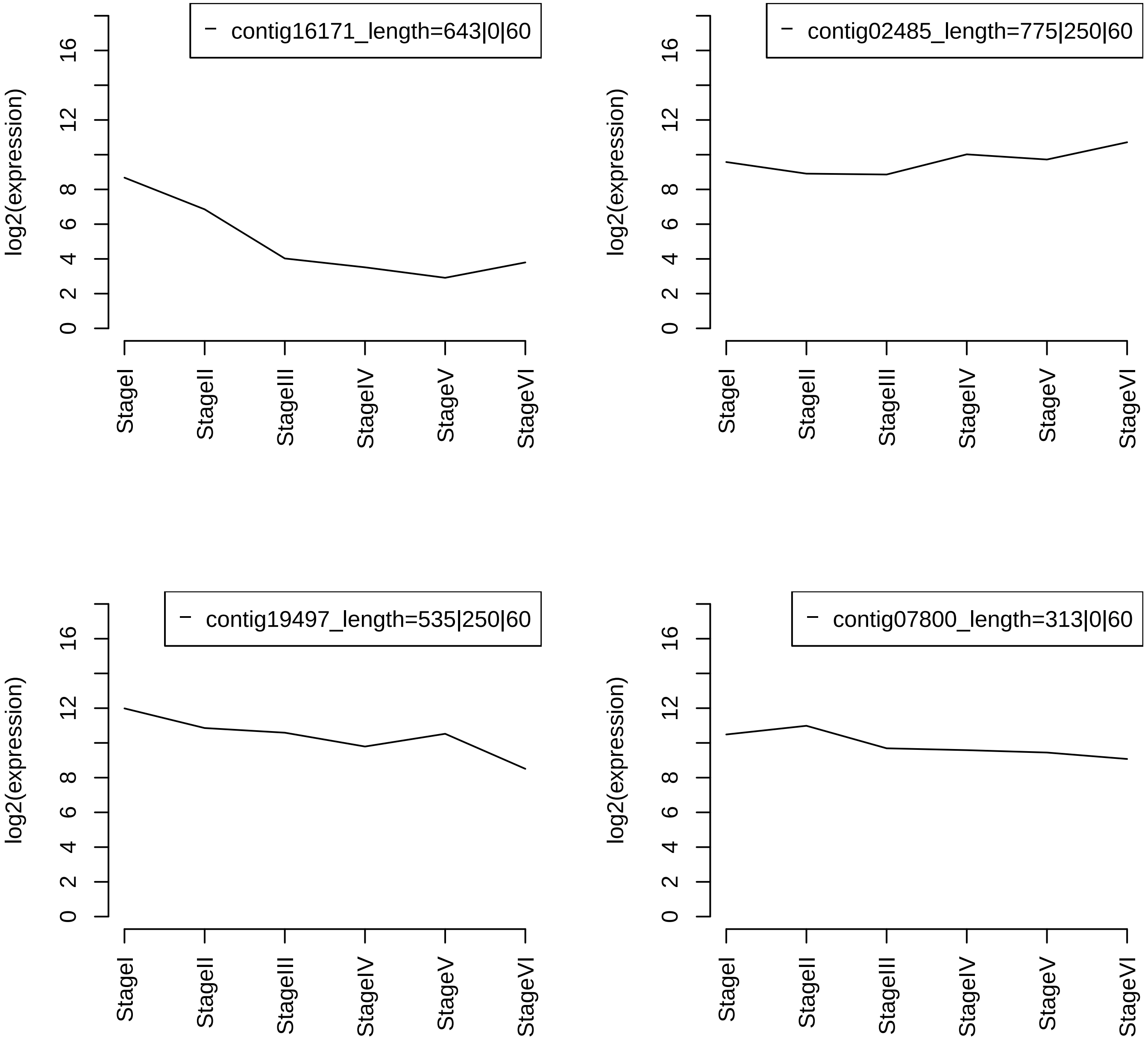
contig16171_length=643|0|60
contig02485_length=775|250|60
contig19497_length=535|250|60
contig07800_length=313|0|60
log2(expression)
0
2
4
6
8
12
16
StageI
StageII
StageIII
StageIV
StageV
StageVI

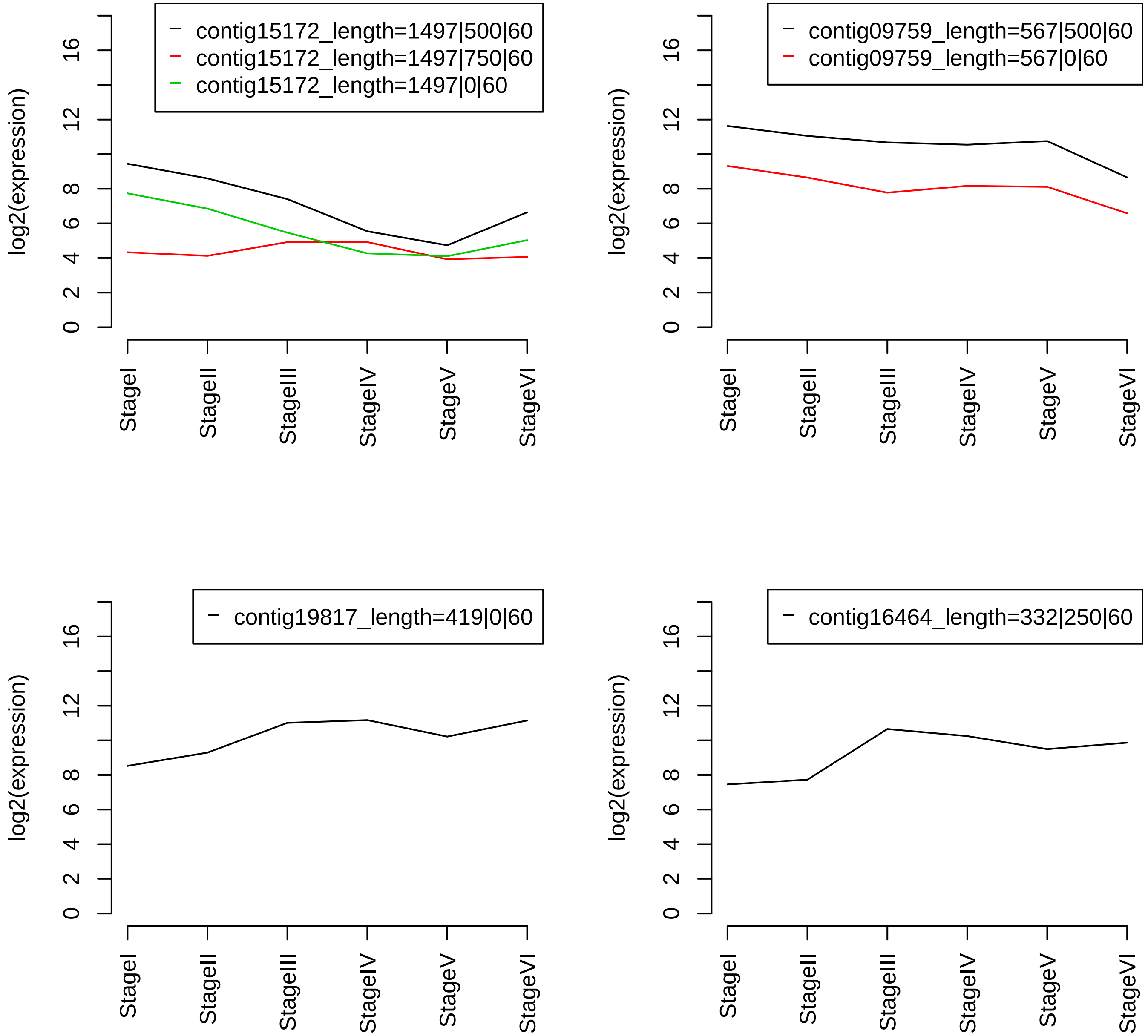
contig15172_length=1497|500|60
contig15172_length=1497|750|60
contig15172_length=1497|0|60
contig09759_length=567|500|60
contig09759_length=567|0|60
contig19817_length=419|0|60
contig16464_length=332|250|60
log2(expression)
0 2 4 6 8 12 16
StageI StageII StageIII StageIV StageV StageVI

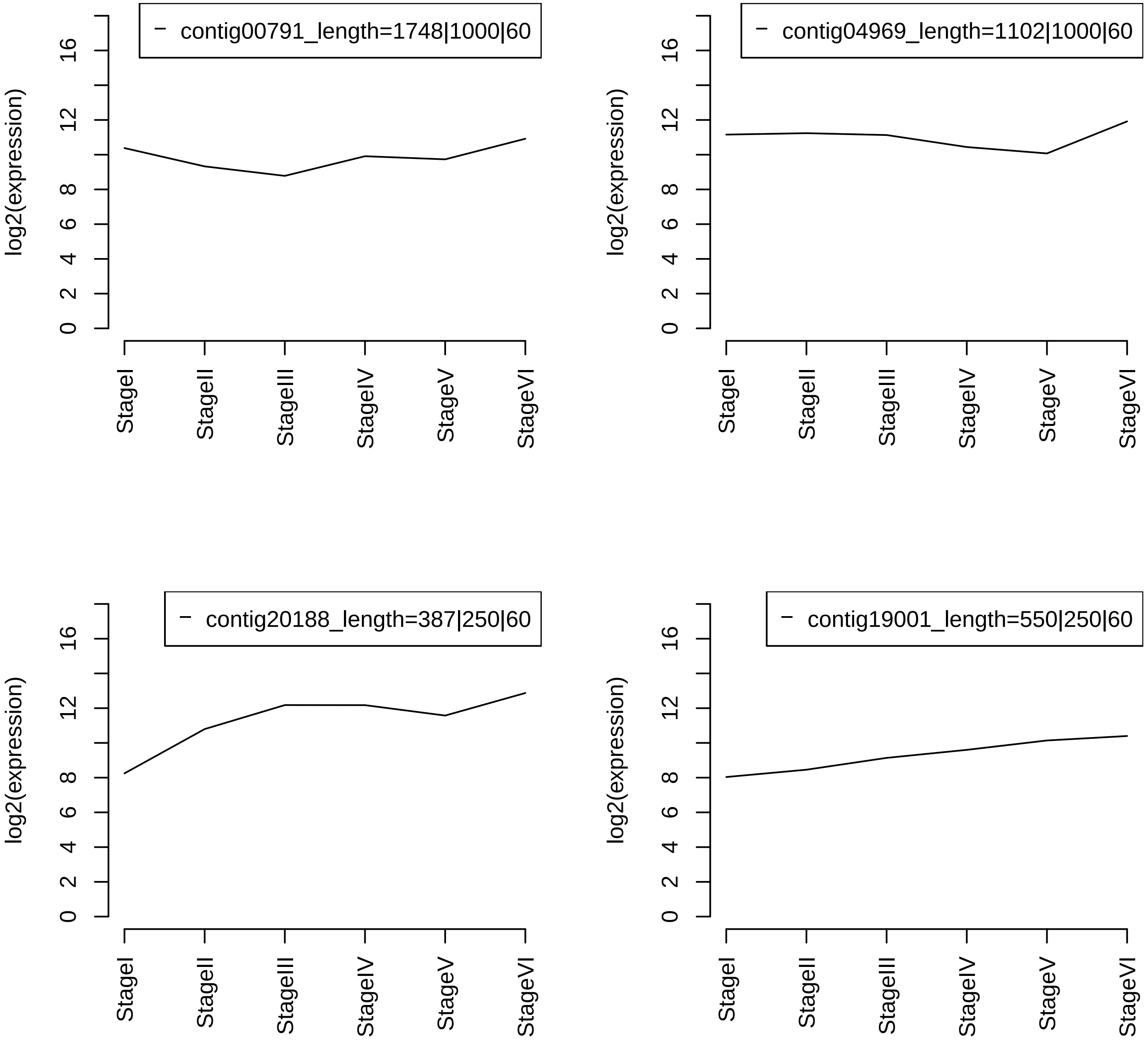

contig00791_length=1748|1000|60
contig04969_length=1102|1000|60
contig20188_length=387|250|60
contig19001_length=550|250|60
log2(expression)
0
2
4
6
8
12
16
StageI
StageII
StageIII
StageIV
StageV
StageVI

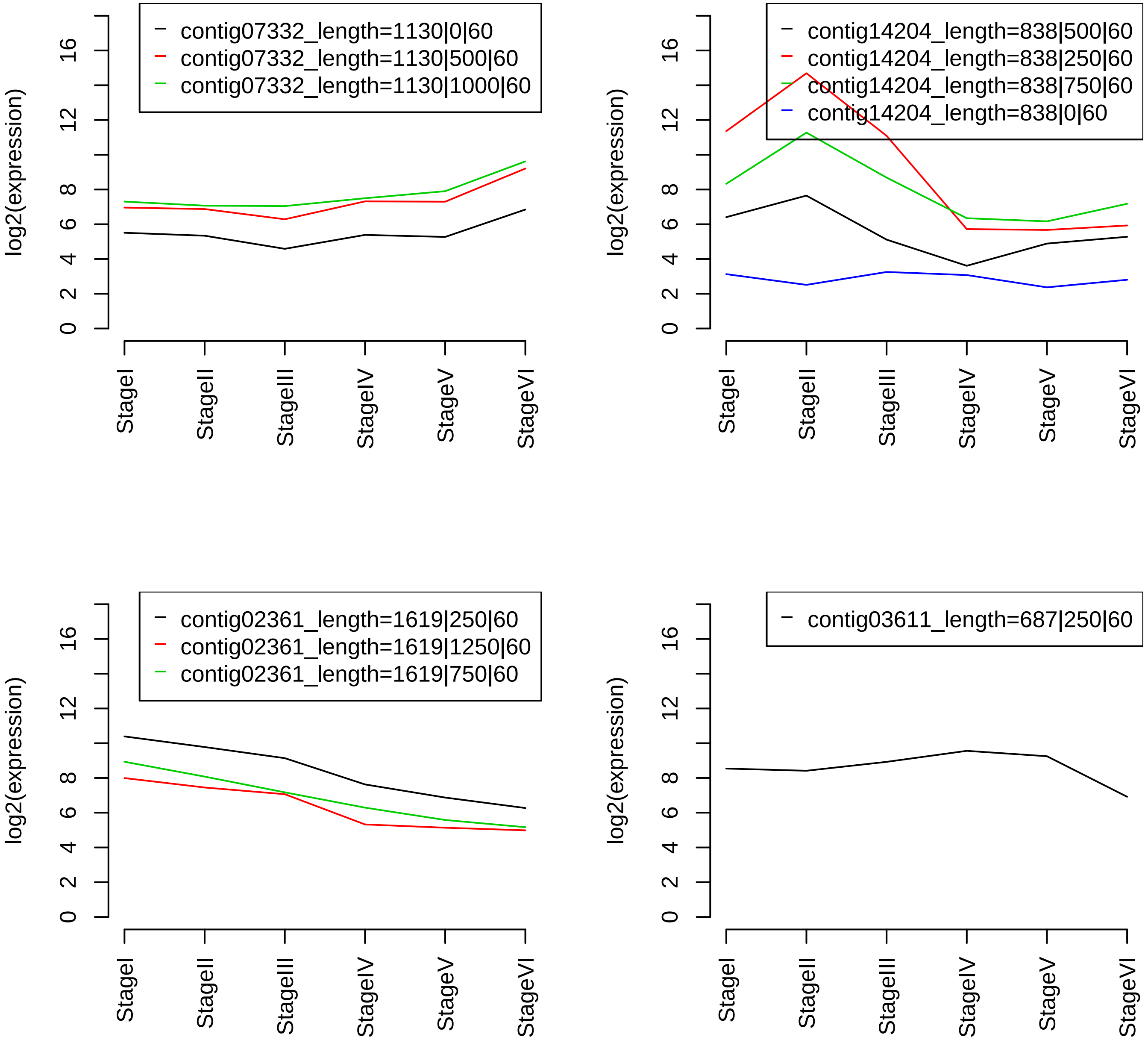
contig07332_length=1130|0|60
contig07332_length=1130|500|60
contig07332_length=1130|1000|60
contig14204_length=838|500|60
contig14204_length=838|250|60
contig14204_length=838|750|60
contig14204_length=838|0|60
contig02361_length=1619|250|60
contig02361_length=1619|1250|60
contig02361_length=1619|750|60
contig03611_length=687|250|60
log2(expression)
0
2
4
6
8
12
16
StageI
StageII
StageIII
StageIV
StageV
StageVI

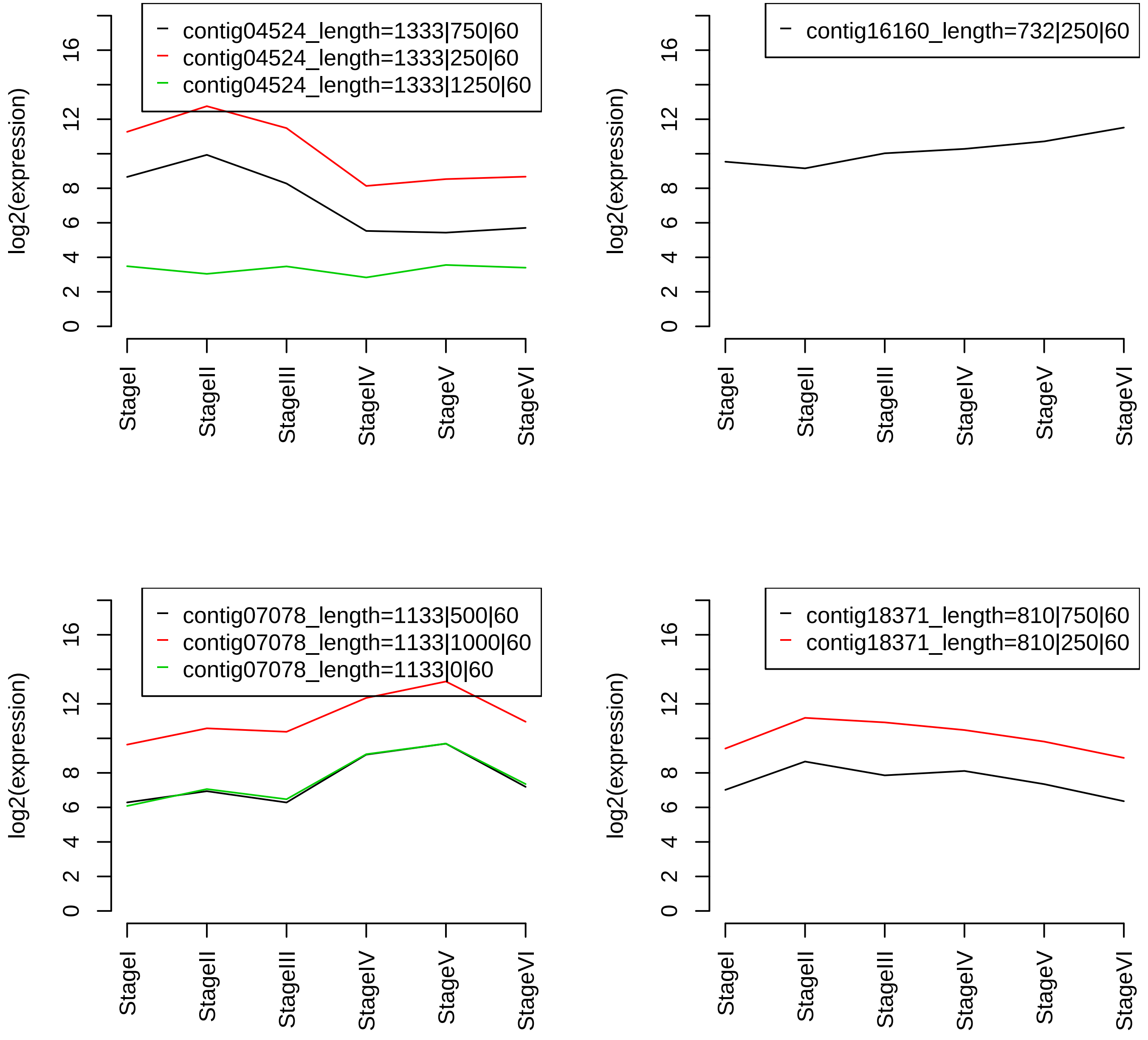

contig04524_length=1333|750|60
contig04524_length=1333|250|60
contig04524_length=1333|1250|60
contig16160_length=732|250|60
contig07078_length=1133|500|60
contig07078_length=1133|1000|60
contig07078_length=1133|0|60
contig18371_length=810|750|60
contig18371_length=810|250|60
log2(expression)
0
2
4
6
8
12
16
StageI
StageII
StageIII
StageIV
StageV
StageVI

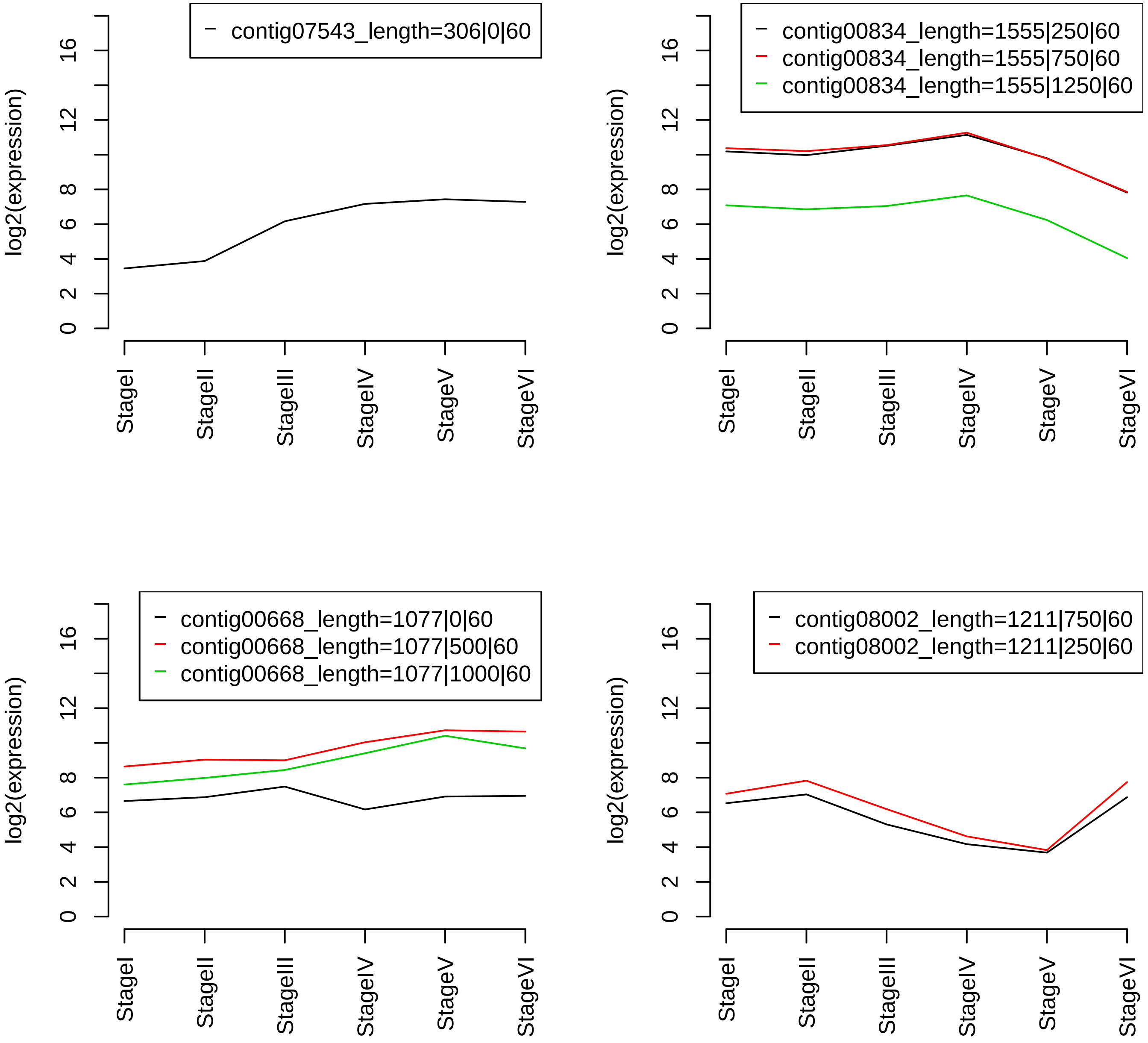
contig07543_length=306|0|60
contig00834_length=1555|250|60
contig00834_length=1555|750|60
contig00834_length=1555|1250|60
contig00668_length=1077|0|60
contig00668_length=1077|500|60
contig00668_length=1077|1000|60
contig08002_length=1211|750|60
contig08002_length=1211|250|60
log2(expression)
0
2
4
6
8
12
16
StageI
StageII
StageIII
StageIV
StageV
StageVI

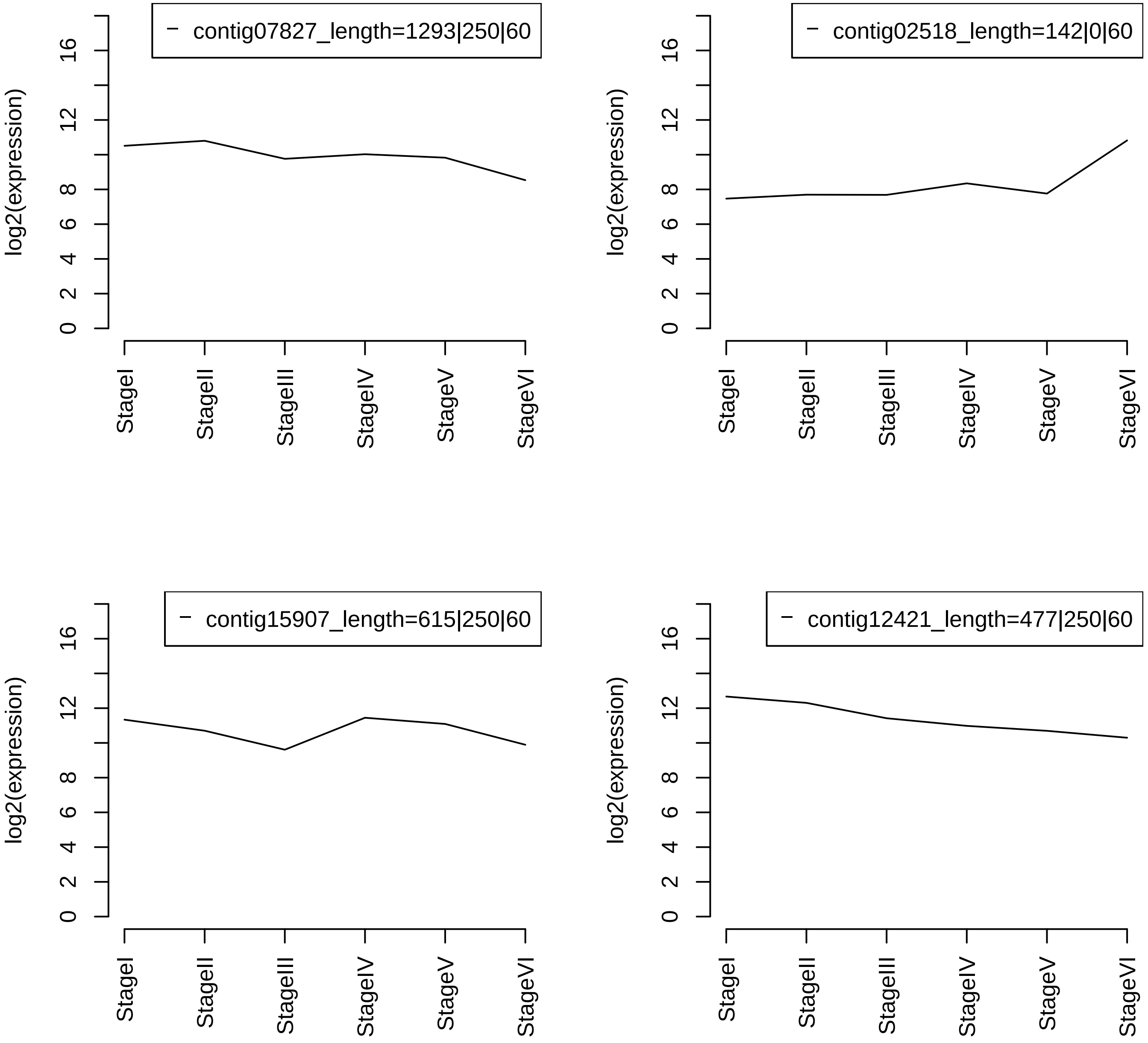
contig07827_length=1293|250|60
contig02518_length=142|0|60
contig15907_length=615|250|60
contig12421_length=477|250|60
log2(expression)
0
2
4
6
8
12
16
StageI
StageII
StageIII
StageIV
StageV
StageVI

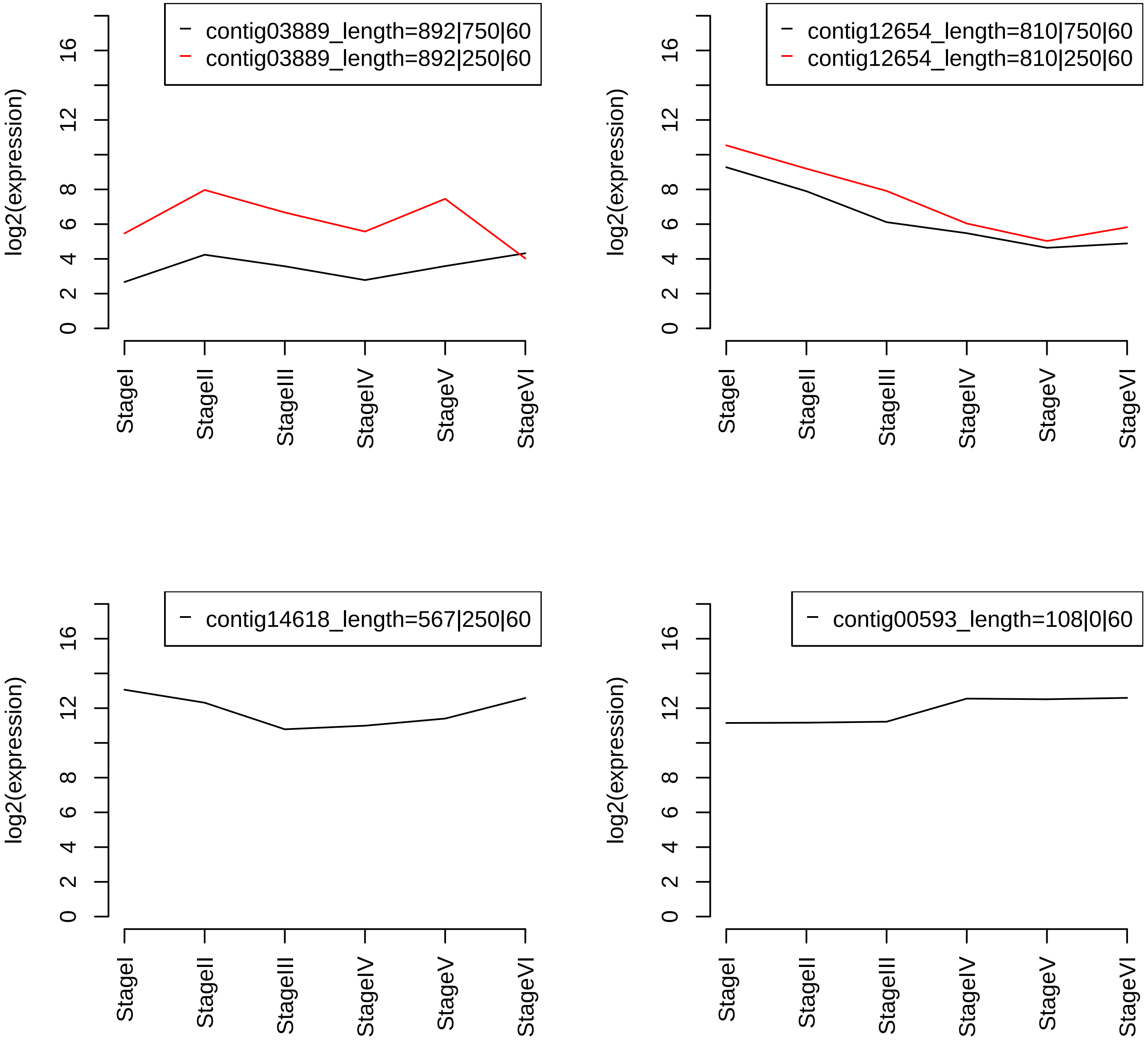
contig03889_length=892|750|60
contig03889_length=892|250|60
contig12654_length=810|750|60
contig12654_length=810|250|60
contig14618_length=567|250|60
contig00593_length=108|0|60
log2(expression)
0
2
4
6
8
12
16
StageI
StageII
StageIII
StageIV
StageV
StageVI

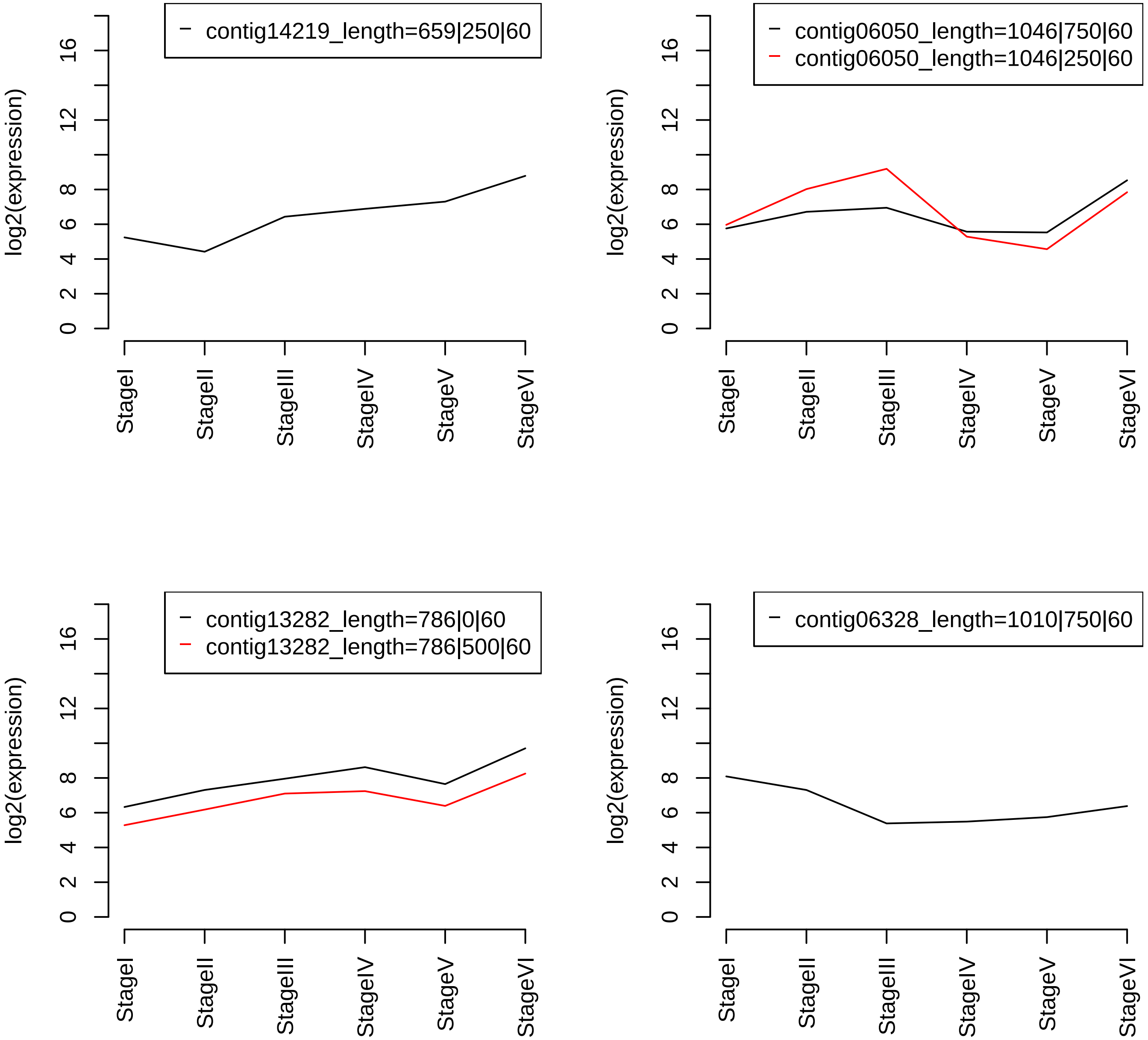

contig14219_length=659|250|60
contig06050_length=1046|750|60
contig06050_length=1046|250|60
contig13282_length=786|0|60
contig13282_length=786|500|60
contig06328_length=1010|750|60
log2(expression)
0
2
4
6
8
12
16
StageI
StageII
StageIII
StageIV
StageV
StageVI

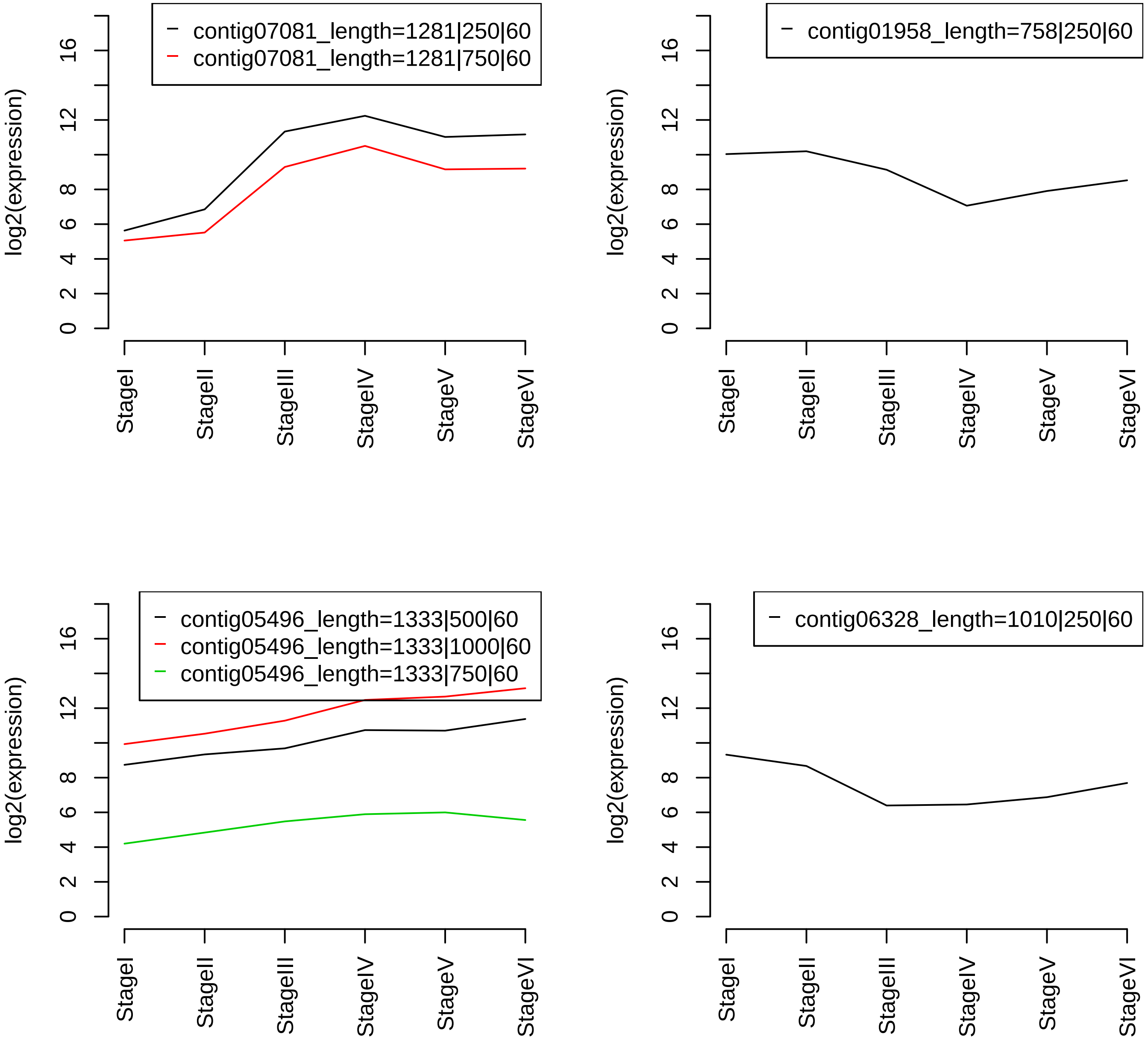
contig07081_length=1281|250|60
contig07081_length=1281|750|60
contig01958_length=758|250|60
contig05496_length=1333|500|60
contig05496_length=1333|1000|60
contig05496_length=1333|750|60
contig06328_length=1010|250|60
log2(expression)
0
2
4
6
8
12
16
StageI
StageII
StageIII
StageIV
StageV
StageVI

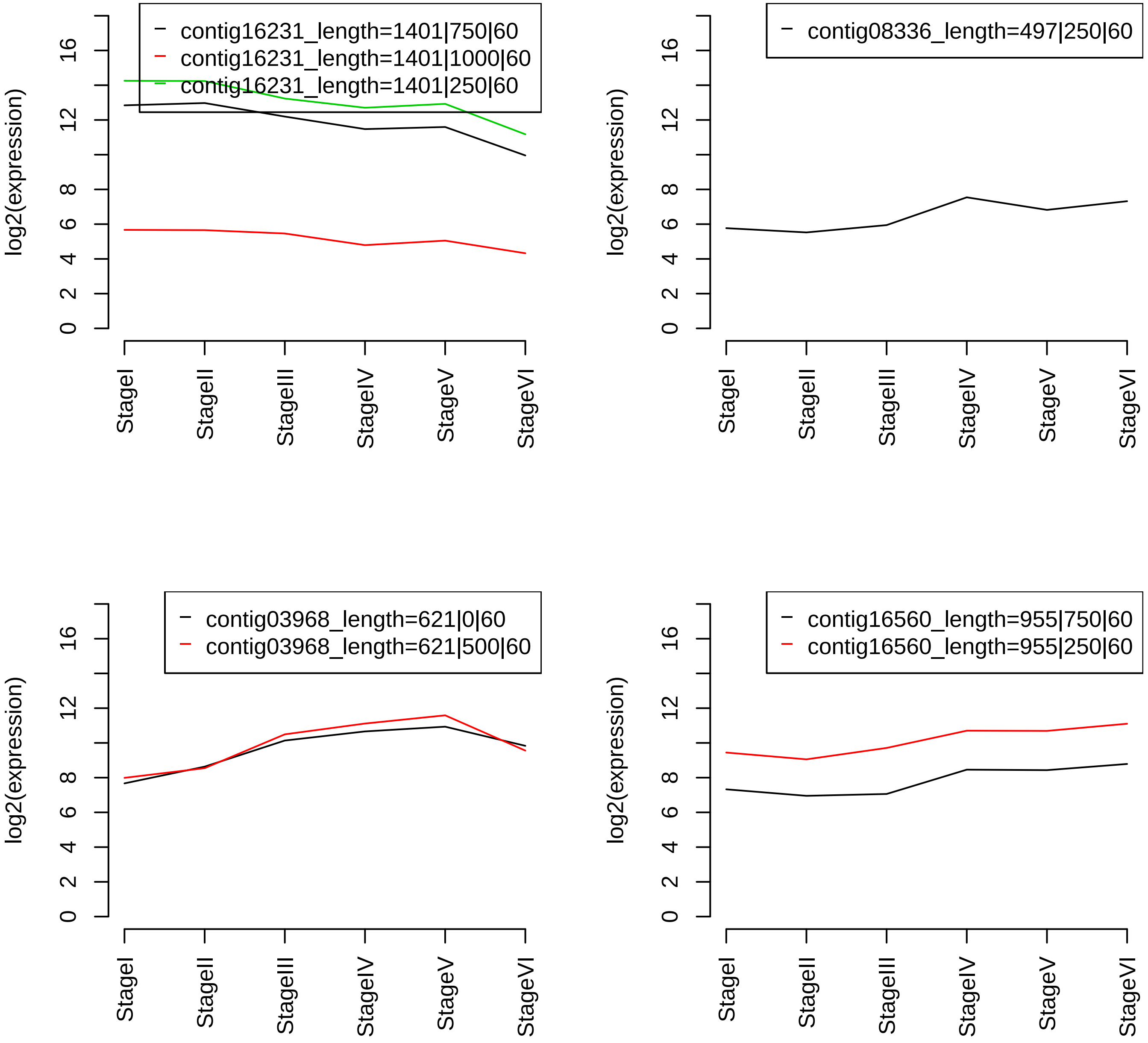
contig16231_length=1401|750|60
contig16231_length=1401|1000|60
contig16231_length=1401|250|60
log2(expression)
0
2
4
6
8
12
16
StageI
StageII
StageIII
StageIV
StageV
StageVI
contig08336_length=497|250|60
contig03968_length=621|0|60
contig03968_length=621|500|60
contig16560_length=955|750|60
contig16560_length=955|250|60

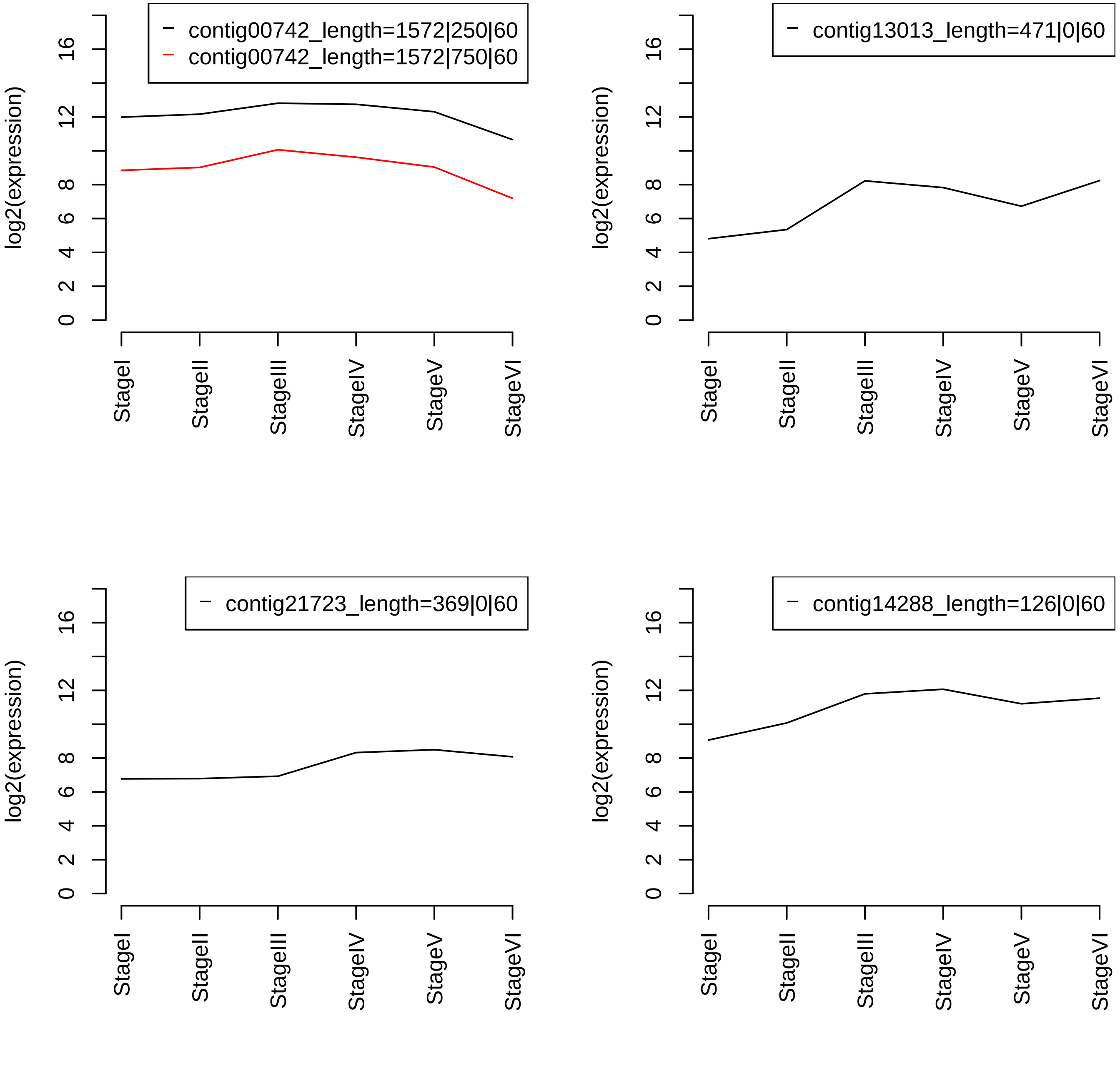

contig00742_length=1572|250|60
contig00742_length=1572|750|60
contig13013_length=471|0|60
contig21723_length=369|0|60
contig14288_length=126|0|60
log2(expression)
0
2
4
6
8
12
16
StageI
StageII
StageIII
StageIV
StageV
StageVI

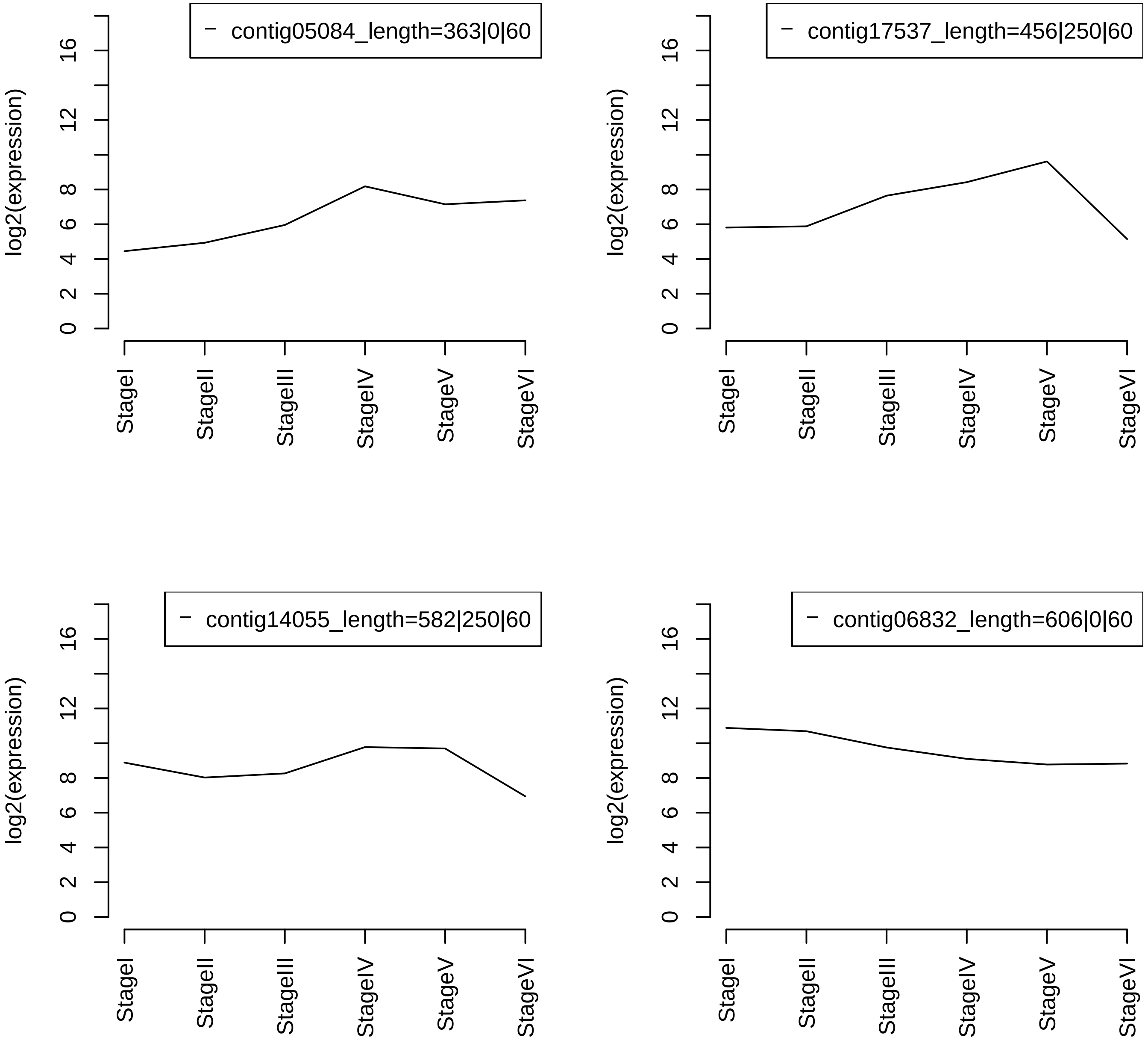

contig05084_length=363|0|60
contig17537_length=456|250|60
contig14055_length=582|250|60
contig06832_length=606|0|60
log2(expression)
0
2
4
6
8
12
16
StageI
StageII
StageIII
StageIV
StageV
StageVI

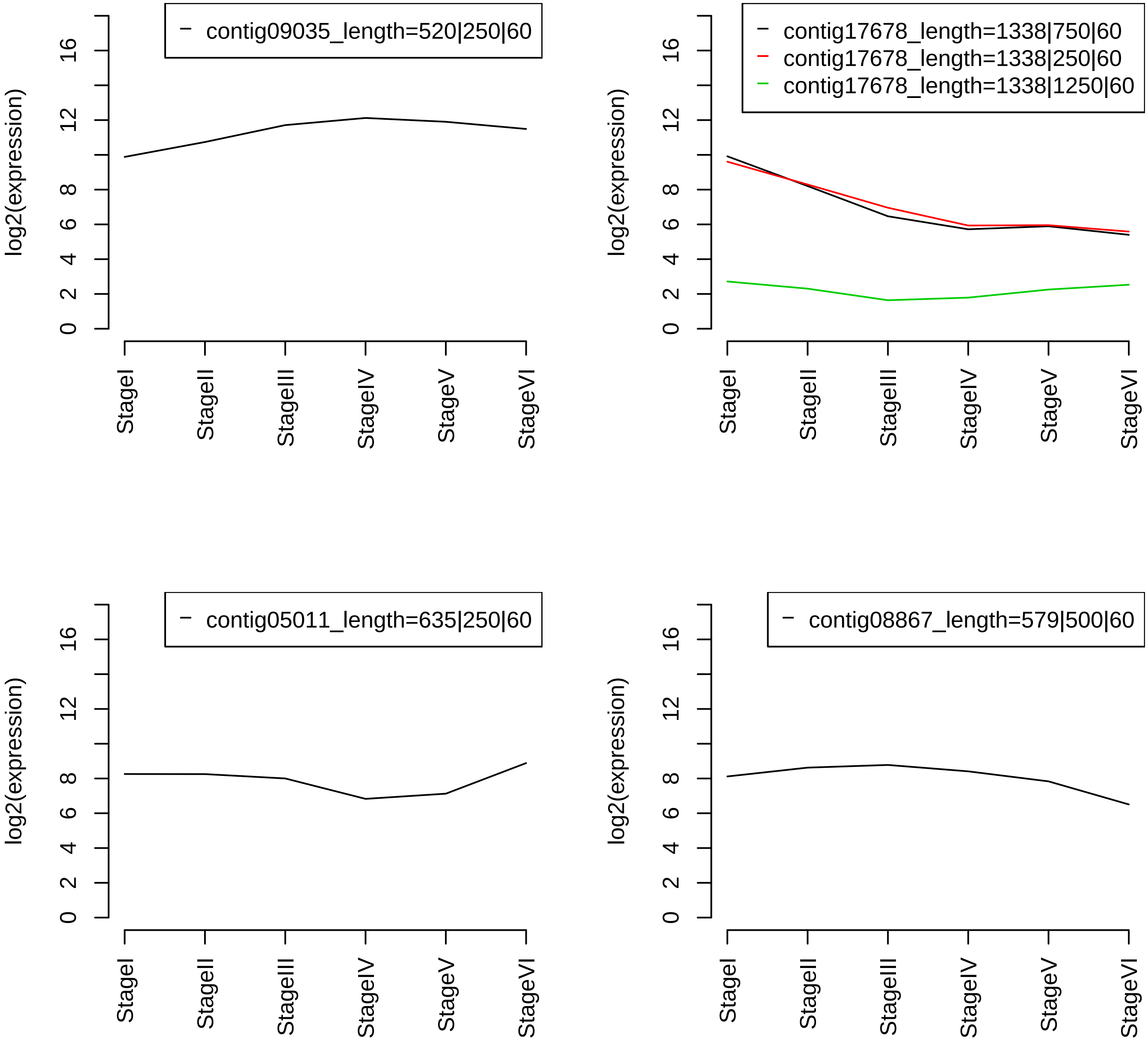
contig09035_length=520|250|60
contig17678_length=1338|750|60
contig17678_length=1338|250|60
contig17678_length=1338|1250|60
contig05011_length=635|250|60
contig08867_length=579|500|60
log2(expression)
0
2
4
6
8
12
16
StageI
StageII
StageIII
StageIV
StageV
StageVI

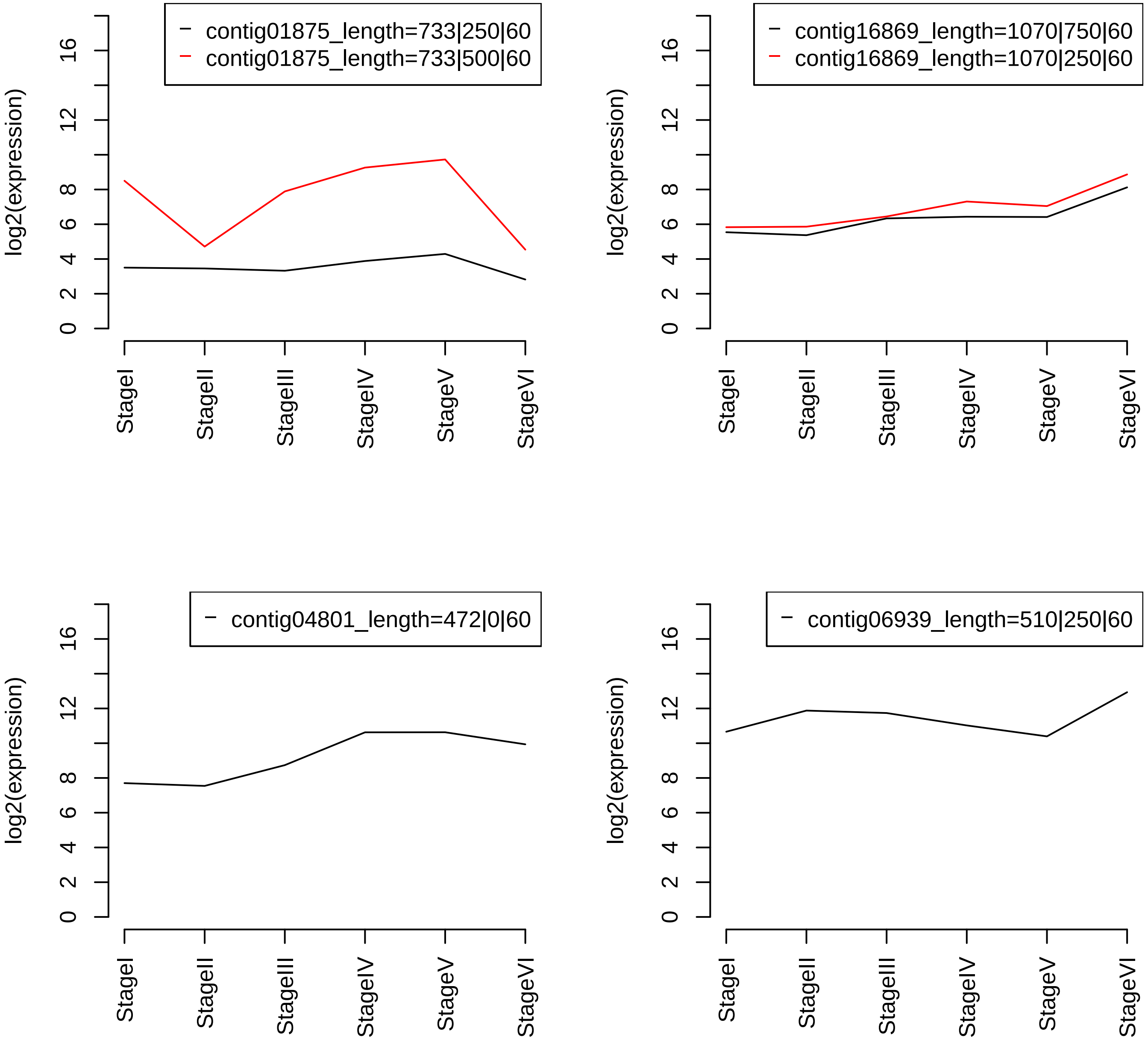

contig01875_length=733|250|60
contig01875_length=733|500|60
log2(expression)
0
2
4
6
8
12
16
StageI
StageII
StageIII
StageIV
StageV
StageVI
contig16869_length=1070|750|60
contig16869_length=1070|250|60
log2(expression)
0
2
4
6
8
12
16
StageI
StageII
StageIII
StageIV
StageV
StageVI
contig04801_length=472|0|60
log2(expression)
0
2
4
6
8
12
16
StageI
StageII
StageIII
StageIV
StageV
StageVI
contig06939_length=510|250|60
log2(expression)
0
2
4
6
8
12
16
StageI
StageII
StageIII
StageIV
StageV
StageVI

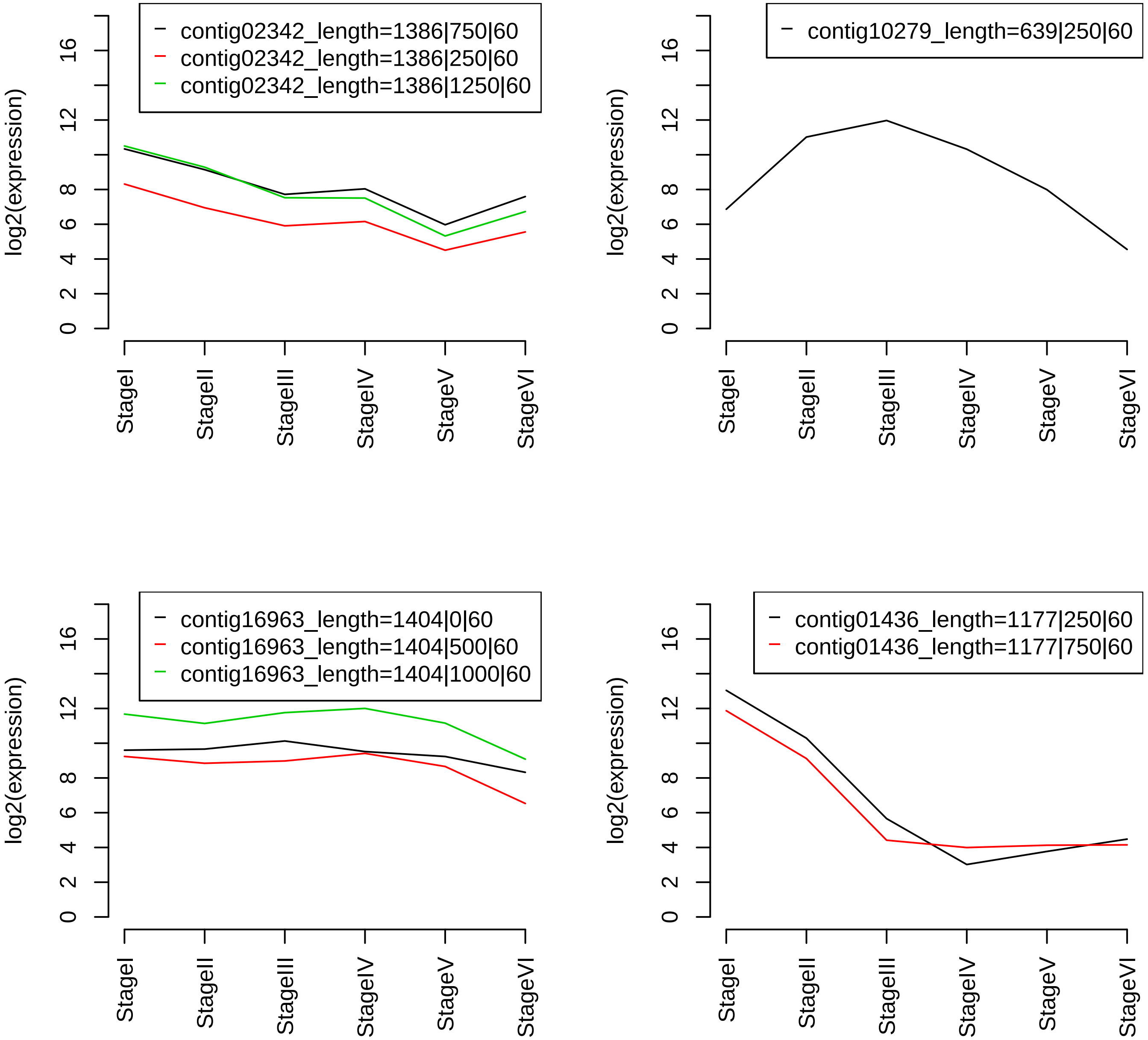

contig02342_length=1386|750|60
contig02342_length=1386|250|60
contig02342_length=1386|1250|60
log2(expression)
0
2
4
6
8
12
16
StageI
StageII
StageIII
StageIV
StageV
StageVI
contig10279_length=639|250|60
contig16963_length=1404|0|60
contig16963_length=1404|500|60
contig16963_length=1404|1000|60
contig01436_length=1177|250|60
contig01436_length=1177|750|60

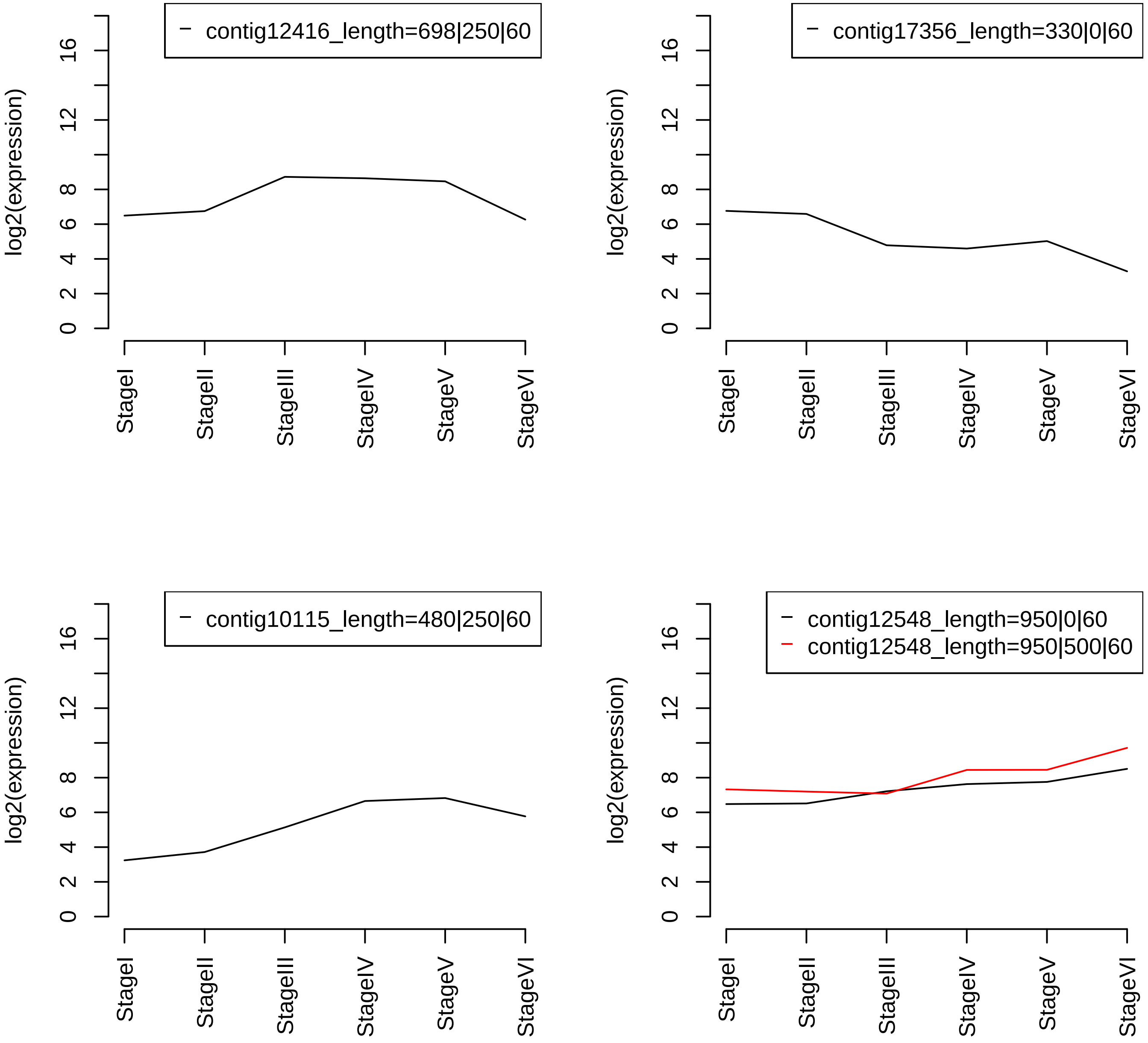
contig12416_length=698|250|60
contig17356_length=330|0|60
contig10115_length=480|250|60
contig12548_length=950|0|60
contig12548_length=950|500|60
log2(expression)
0
2
4
6
8
12
16
StageI
StageII
StageIII
StageIV
StageV
StageVI

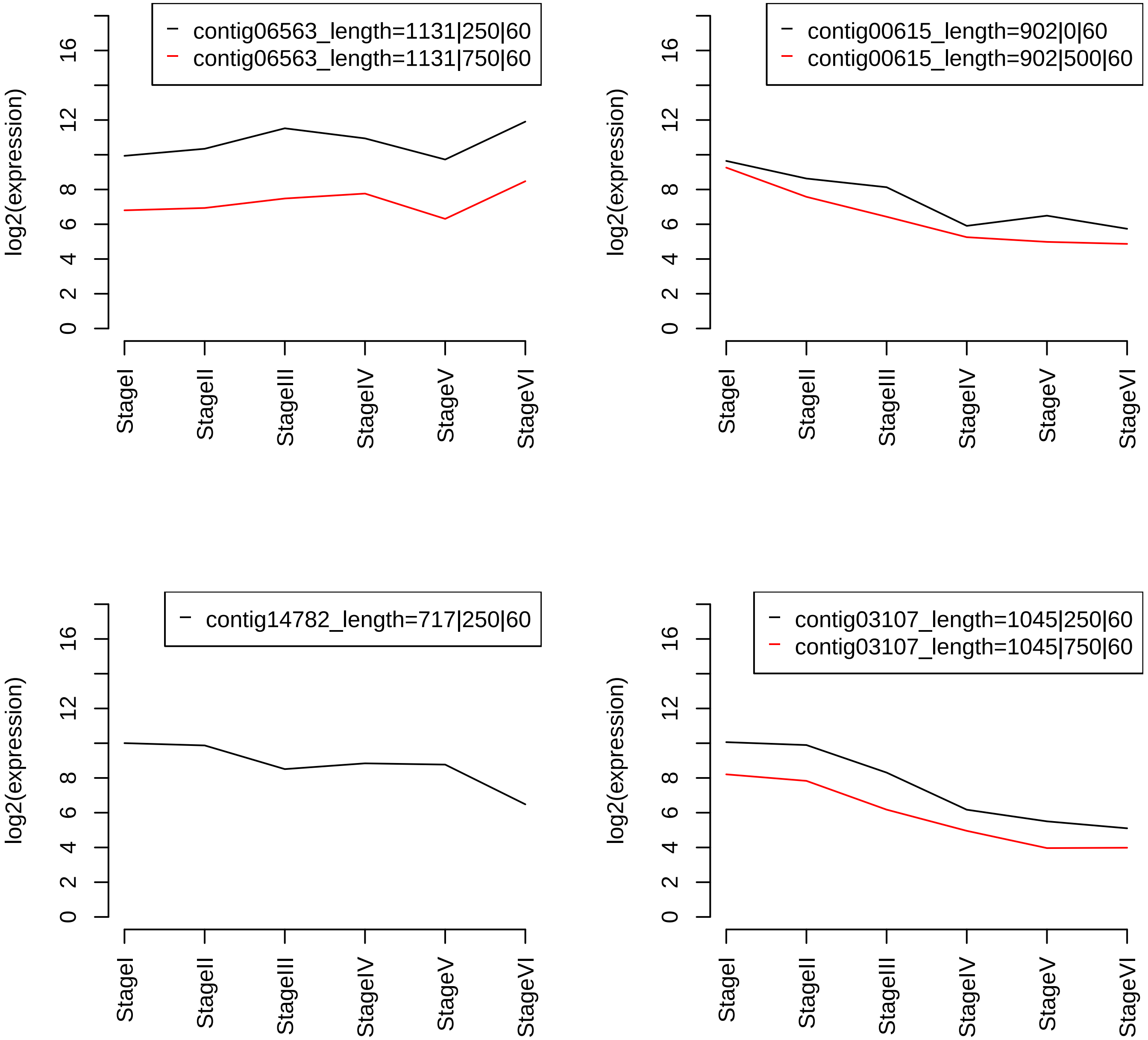
contig06563_length=1131|250|60
contig06563_length=1131|750|60
contig00615_length=902|0|60
contig00615_length=902|500|60
contig14782_length=717|250|60
contig03107_length=1045|250|60
contig03107_length=1045|750|60
log2(expression)
0
2
4
6
8
12
16
StageI
StageII
StageIII
StageIV
StageV
StageVI

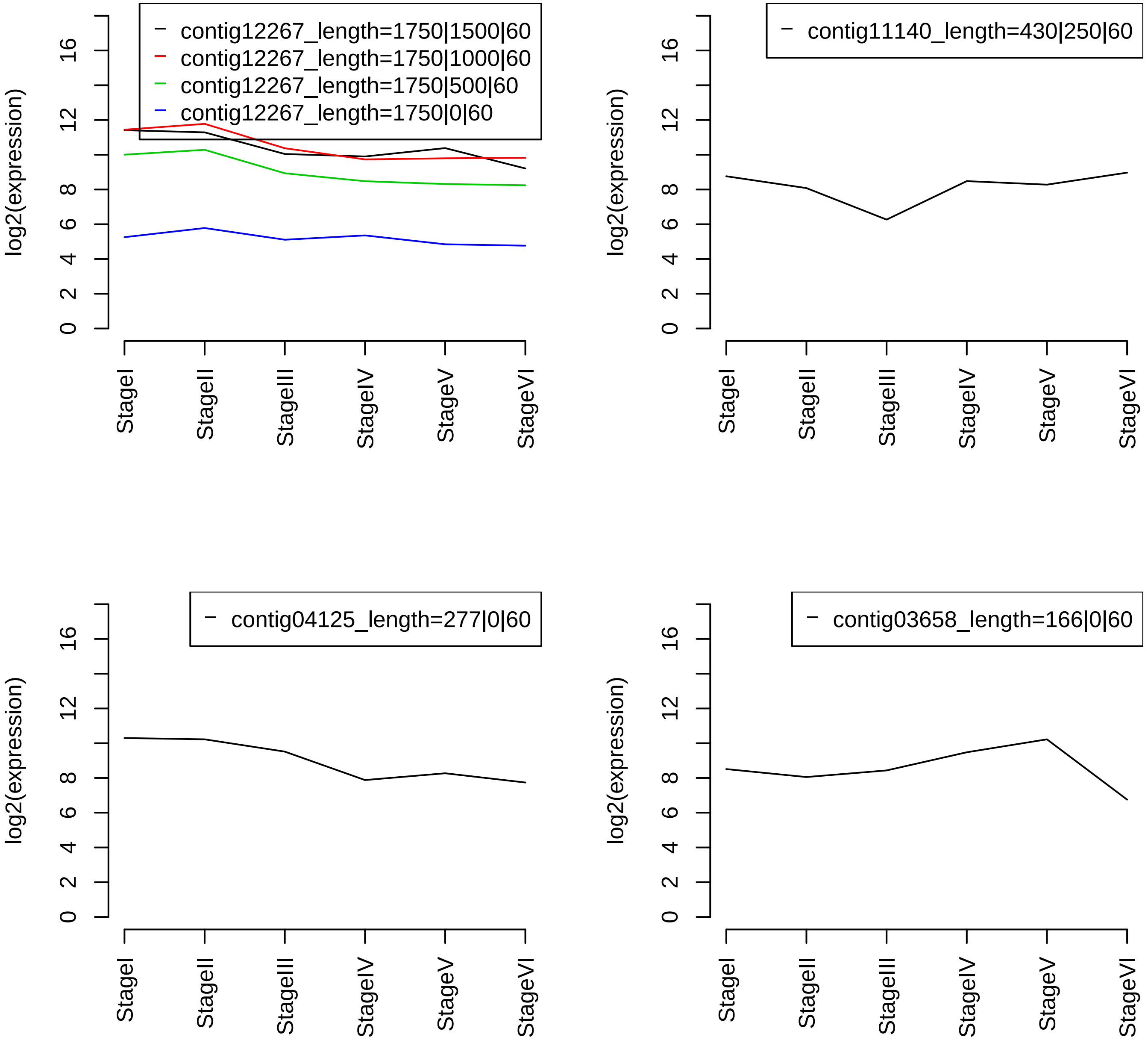

contig12267_length=1750|1500|60
contig12267_length=1750|1000|60
contig12267_length=1750|500|60
contig12267_length=1750|0|60
contig11140_length=430|250|60
contig04125_length=277|0|60
contig03658_length=166|0|60
log2(expression)
0
2
4
6
8
12
16
StageI
StageII
StageIII
StageIV
StageV
StageVI

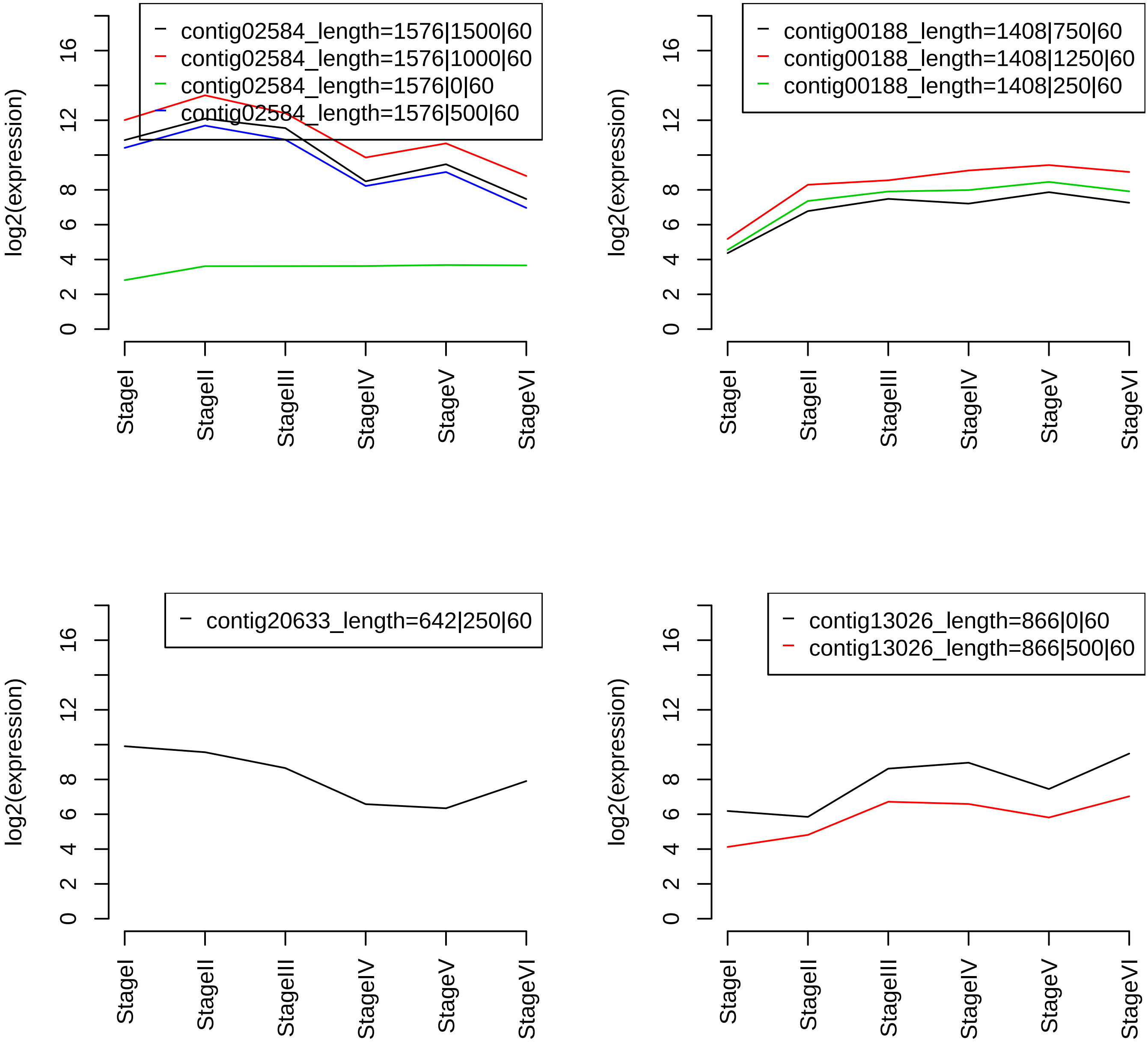
contig02584_length=1576|1500|60
contig02584_length=1576|1000|60
contig02584_length=1576|0|60
contig02584_length=1576|500|60
contig00188_length=1408|750|60
contig00188_length=1408|1250|60
contig00188_length=1408|250|60
contig20633_length=642|250|60
contig13026_length=866|0|60
contig13026_length=866|500|60
log2(expression)
0
2
4
6
8
12
16
StageI
StageII
StageIII
StageIV
StageV
StageVI

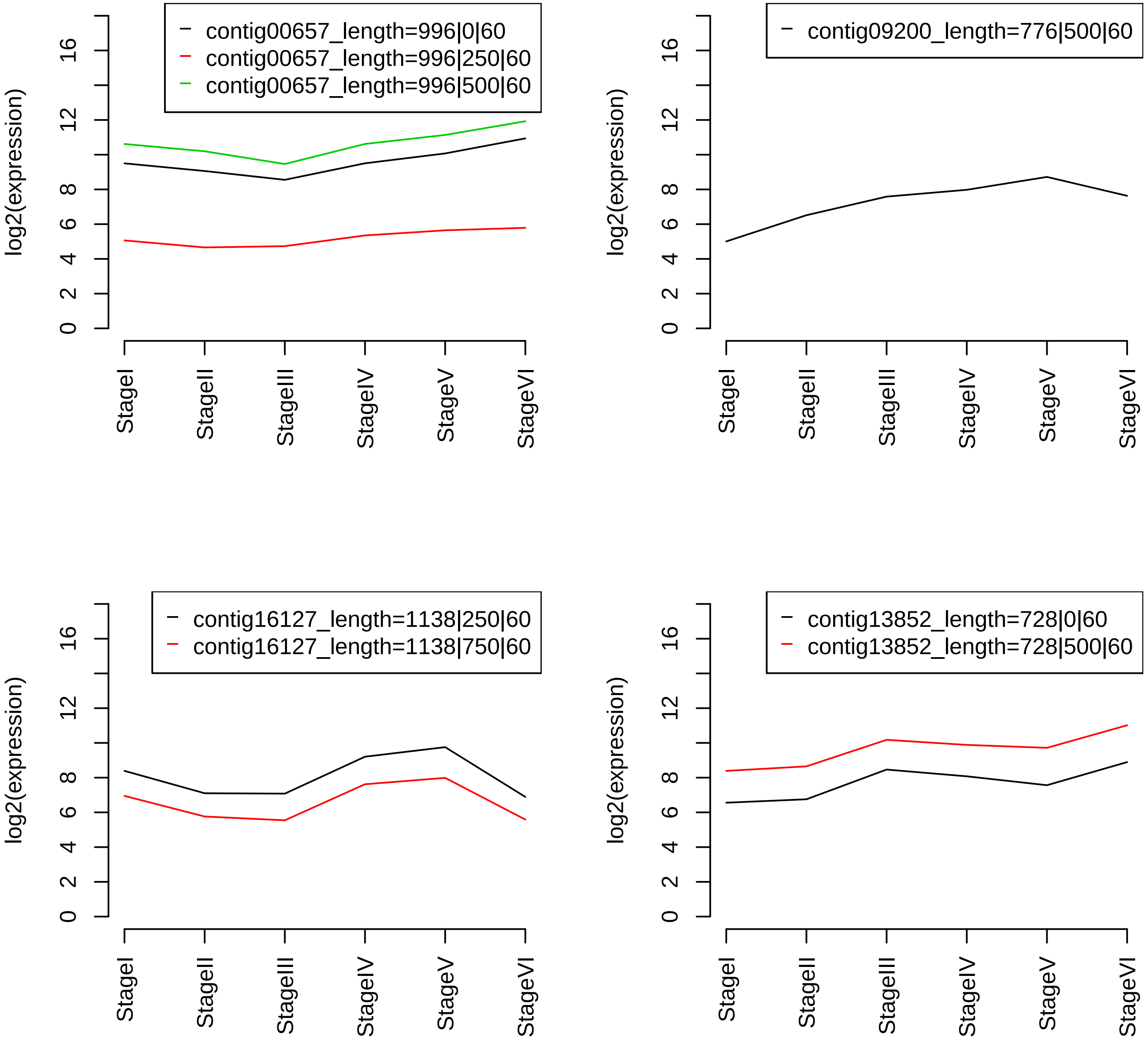

contig00657_length=996|0|60
contig00657_length=996|250|60
contig00657_length=996|500|60
contig09200_length=776|500|60
contig16127_length=1138|250|60
contig16127_length=1138|750|60
contig13852_length=728|0|60
contig13852_length=728|500|60
log2(expression)
0
2
4
6
8
12
16
StageI
StageII
StageIII
StageIV
StageV
StageVI

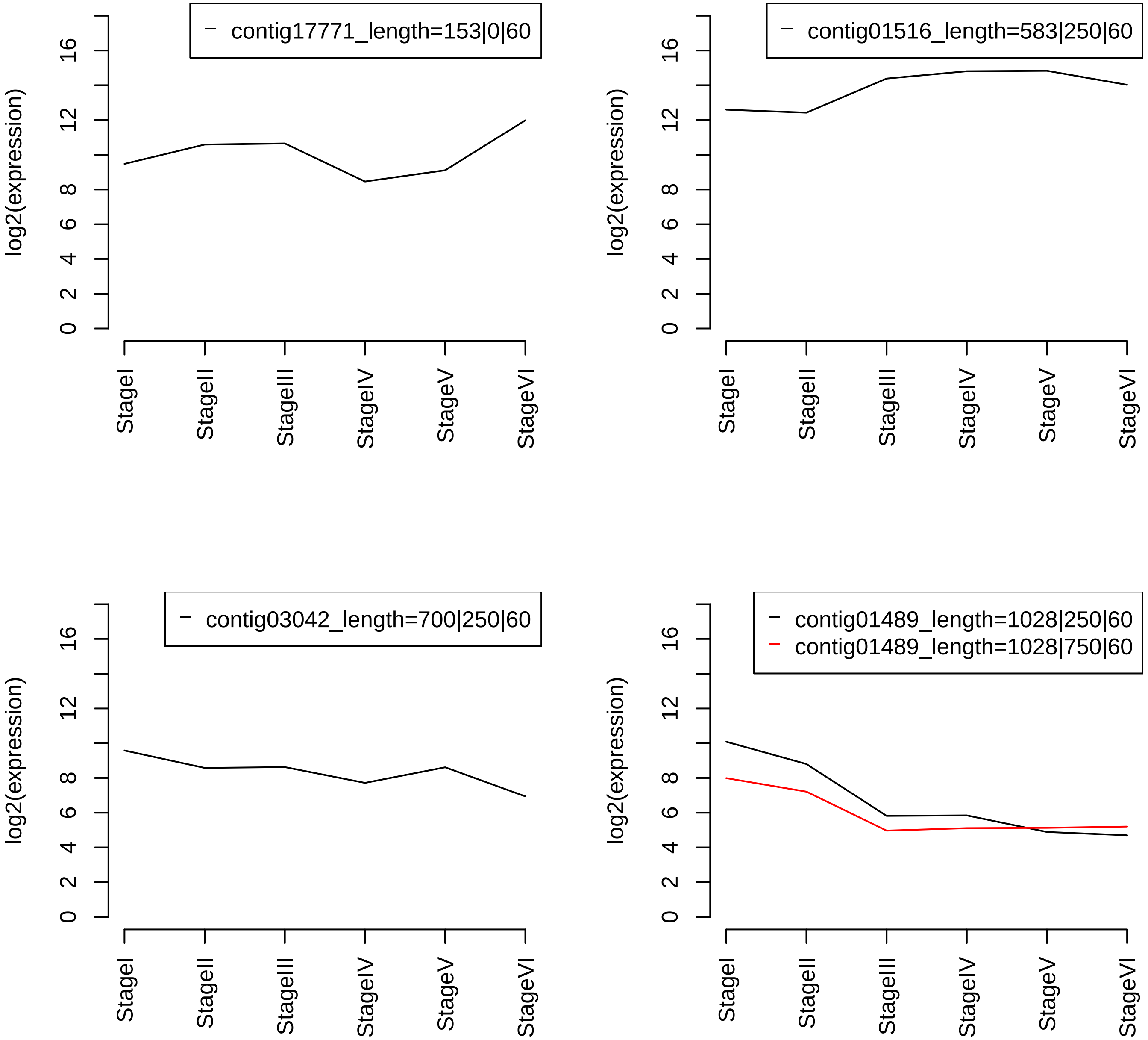

contig17771_length=153|0|60
contig01516_length=583|250|60
contig03042_length=700|250|60
contig01489_length=1028|250|60
contig01489_length=1028|750|60
log2(expression)
0
2
4
6
8
12
16
StageI
StageII
StageIII
StageIV
StageV
StageVI

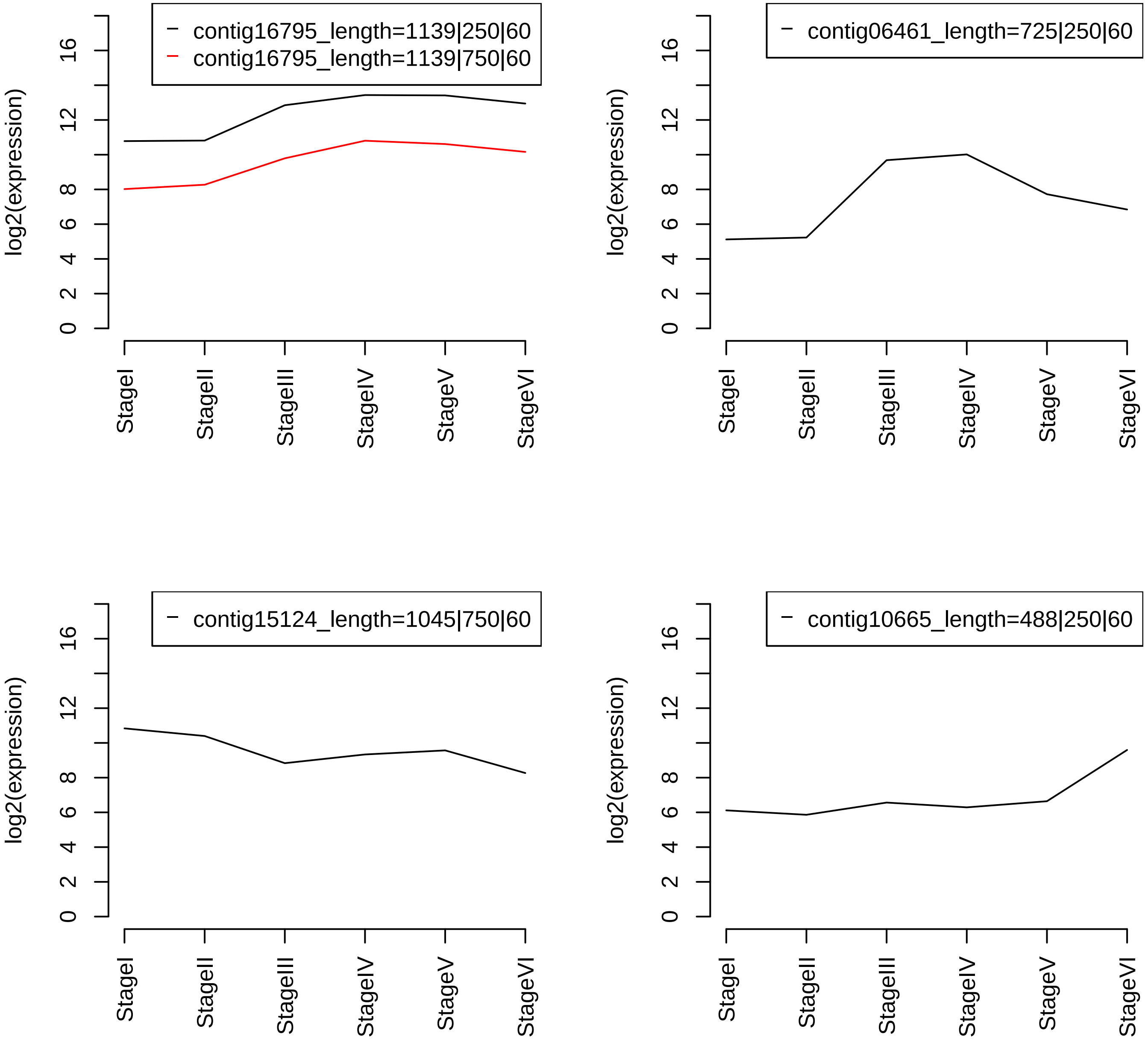
contig16795_length=1139|250|60
contig16795_length=1139|750|60
contig06461_length=725|250|60
contig15124_length=1045|750|60
contig10665_length=488|250|60
log2(expression)
0
2
4
6
8
12
16
StageI
StageII
StageIII
StageIV
StageV
StageVI

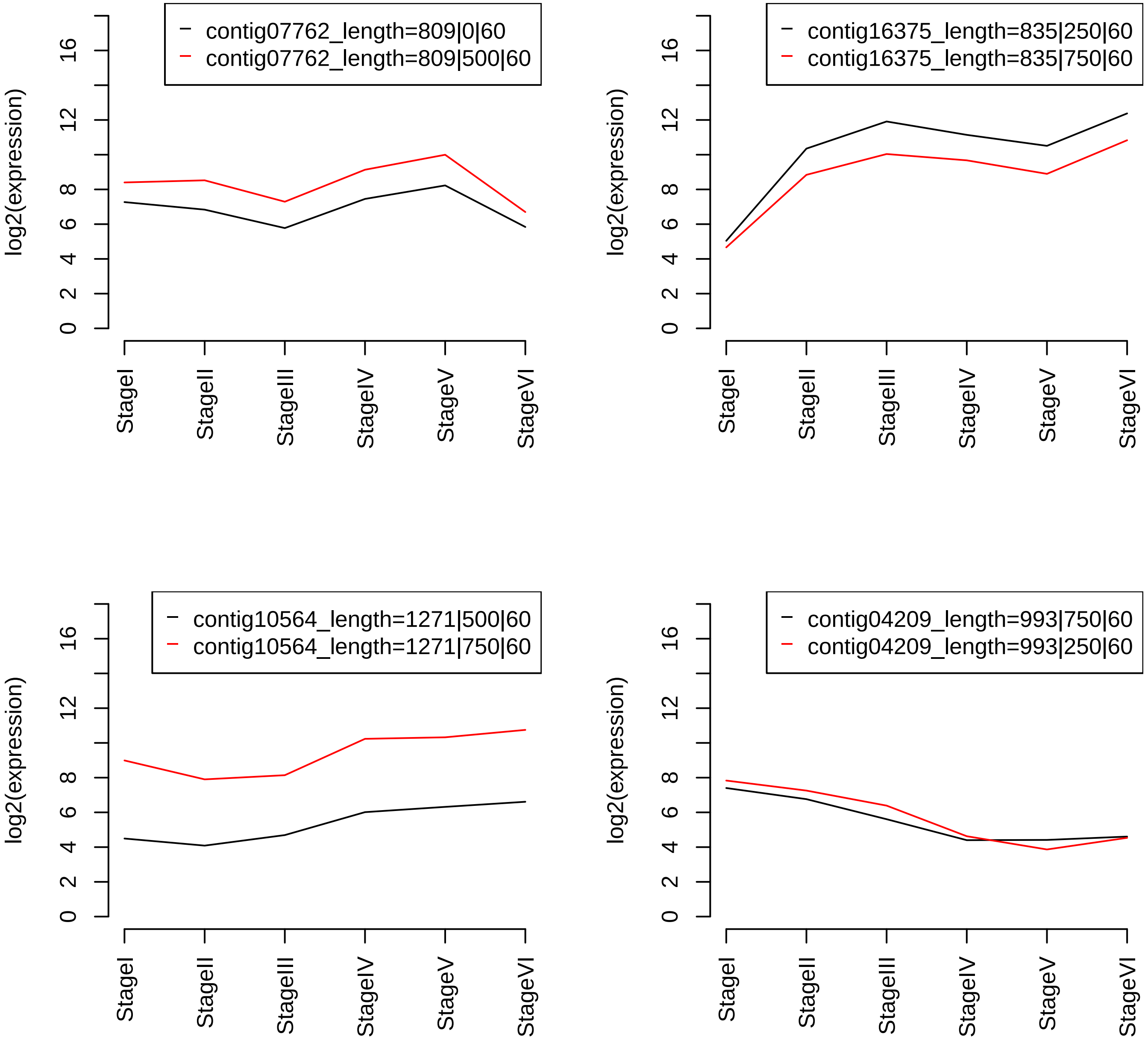

contig07762_length=809|0|60
contig07762_length=809|500|60
contig16375_length=835|250|60
contig16375_length=835|750|60
contig10564_length=1271|500|60
contig10564_length=1271|750|60
contig04209_length=993|750|60
contig04209_length=993|250|60
log2(expression)
0 2 4 6 8 12 16
StageI StageII StageIII StageIV StageV StageVI

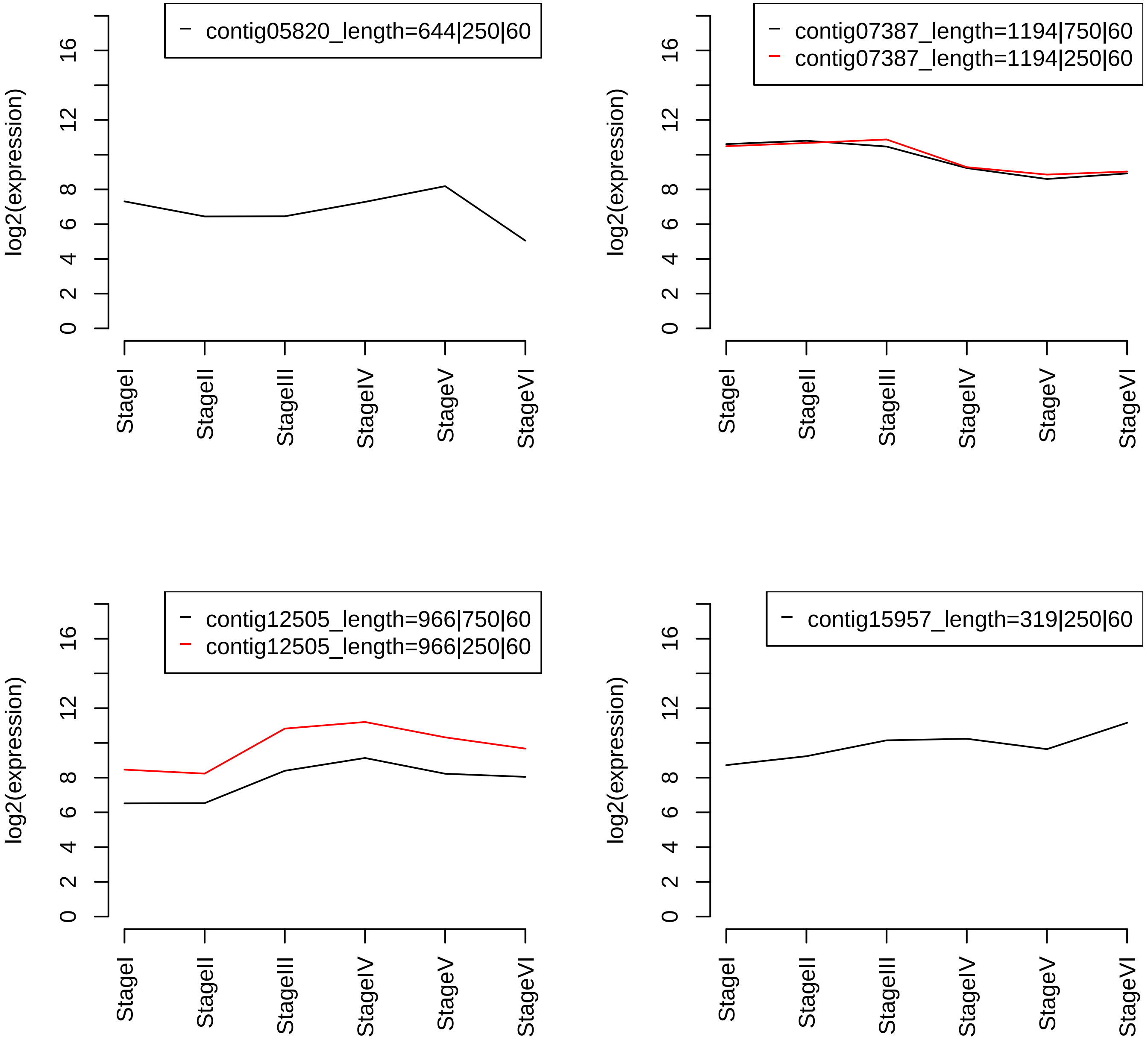

contig05820_length=644|250|60
contig07387_length=1194|750|60
contig07387_length=1194|250|60
contig12505_length=966|750|60
contig12505_length=966|250|60
contig15957_length=319|250|60
log2(expression)
0
2
4
6
8
12
16
StageI
StageII
StageIII
StageIV
StageV
StageVI

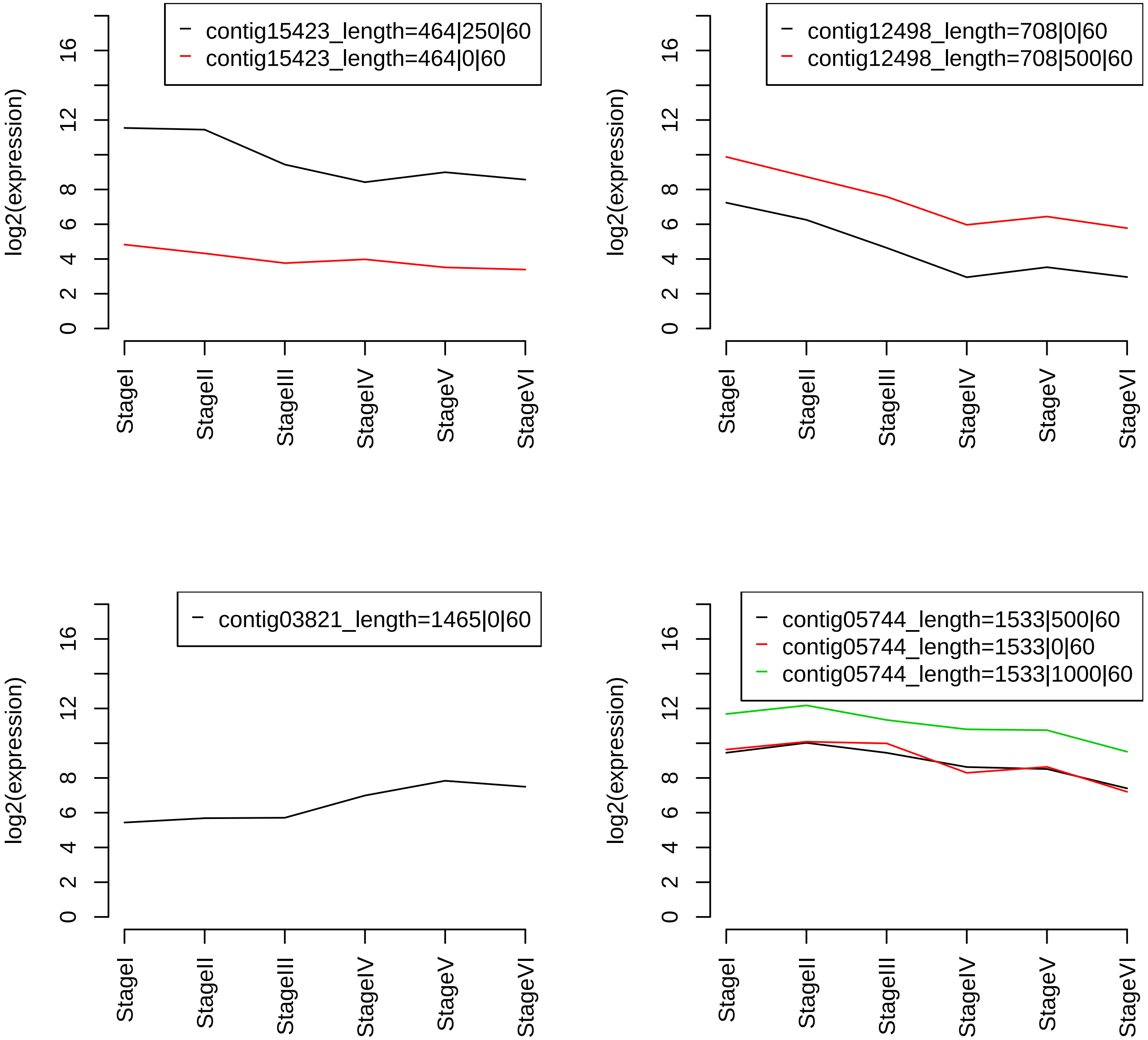
contig15423_length=464|250|60
contig15423_length=464|0|60
contig12498_length=708|0|60
contig12498_length=708|500|60
contig03821_length=1465|0|60
contig05744_length=1533|500|60
contig05744_length=1533|0|60
contig05744_length=1533|1000|60
log2(expression)
0
2
4
6
8
12
16
StageI
StageII
StageIII
StageIV
StageV
StageVI

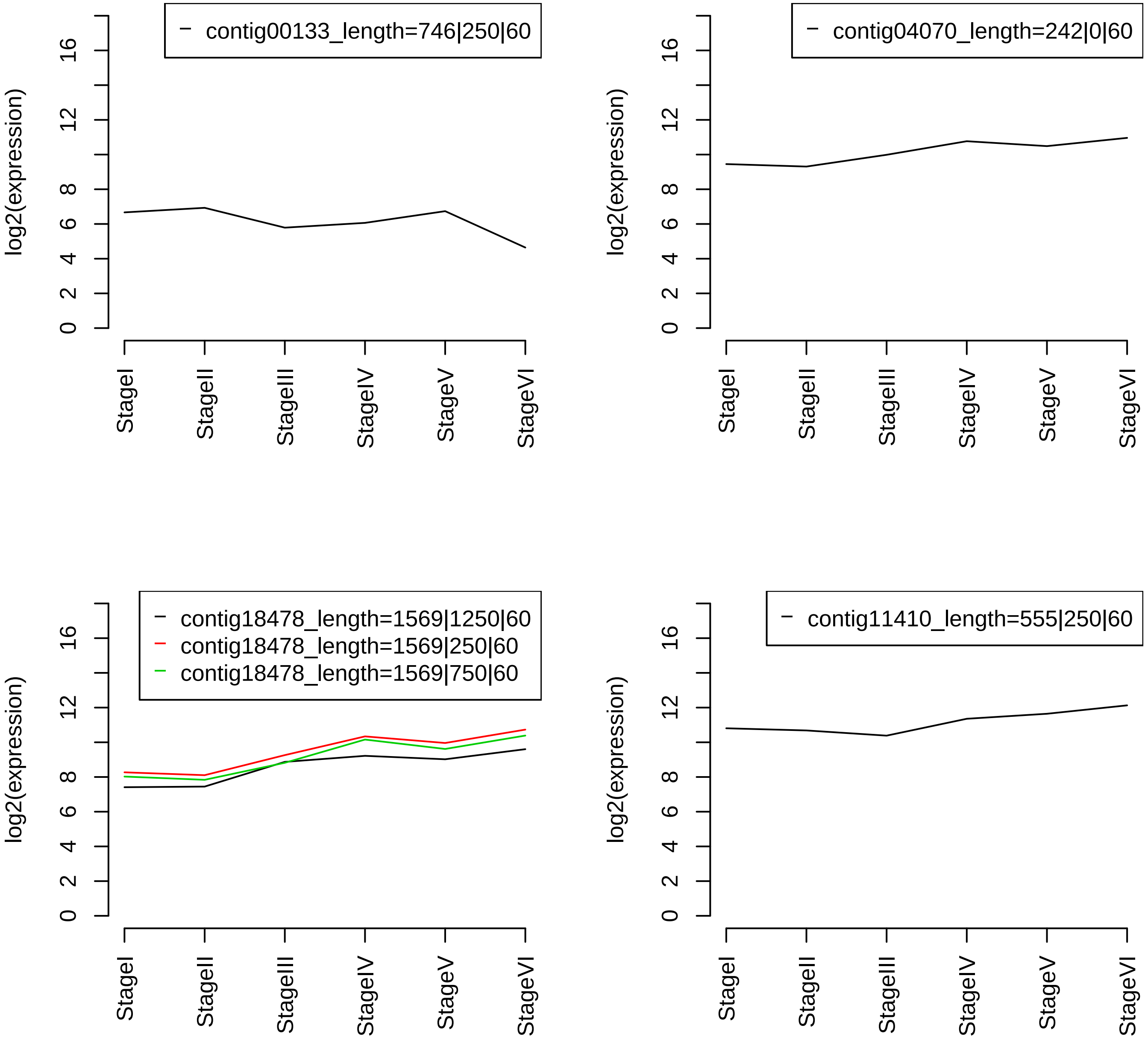

contig00133_length=746|250|60
contig04070_length=242|0|60
contig18478_length=1569|1250|60
contig18478_length=1569|250|60
contig18478_length=1569|750|60
contig11410_length=555|250|60
log2(expression)
0
2
4
6
8
12
16
StageI
StageII
StageIII
StageIV
StageV
StageVI

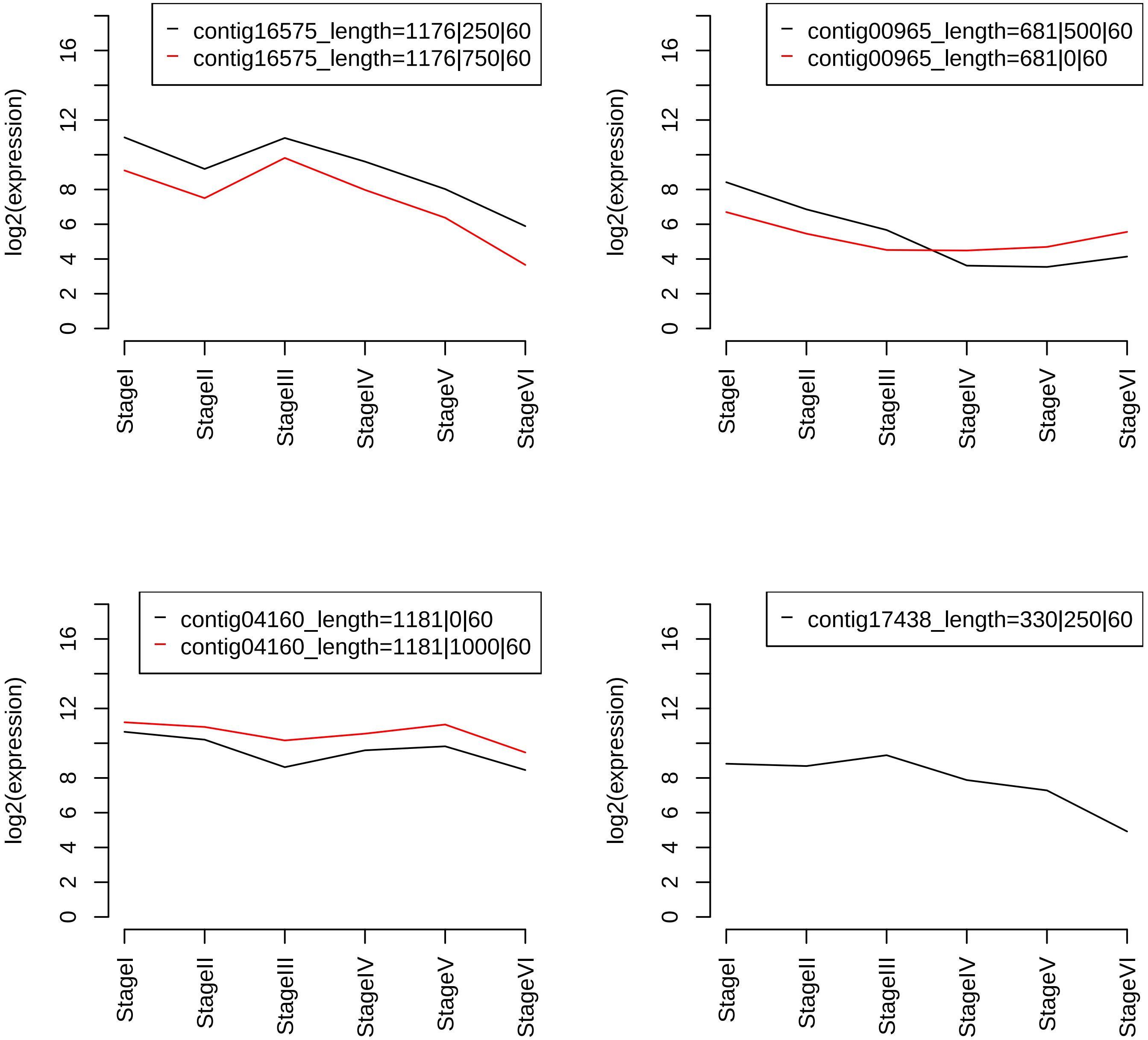
contig16575_length=1176|250|60
contig16575_length=1176|750|60
log2(expression)
0
2
4
6
8
12
16
StageI
StageII
StageIII
StageIV
StageV
StageVI
contig00965_length=681|500|60
contig00965_length=681|0|60
contig04160_length=1181|0|60
contig04160_length=1181|1000|60
contig17438_length=330|250|60

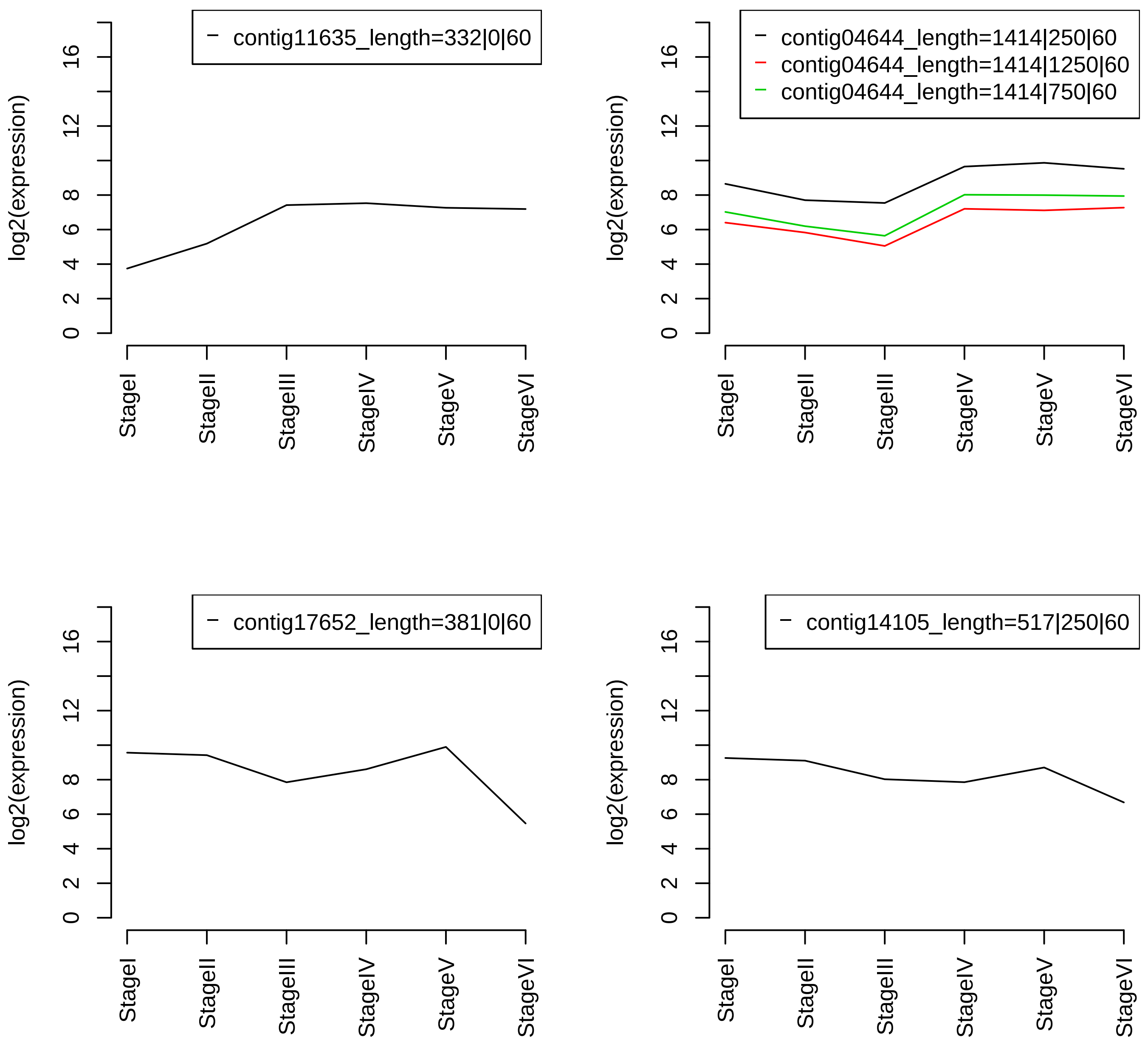

contig11635_length=332|0|60
contig04644_length=1414|250|60
contig04644_length=1414|1250|60
contig04644_length=1414|750|60
contig17652_length=381|0|60
contig14105_length=517|250|60
log2(expression)
0
2
4
6
8
12
16
StageI
StageII
StageIII
StageIV
StageV
StageVI

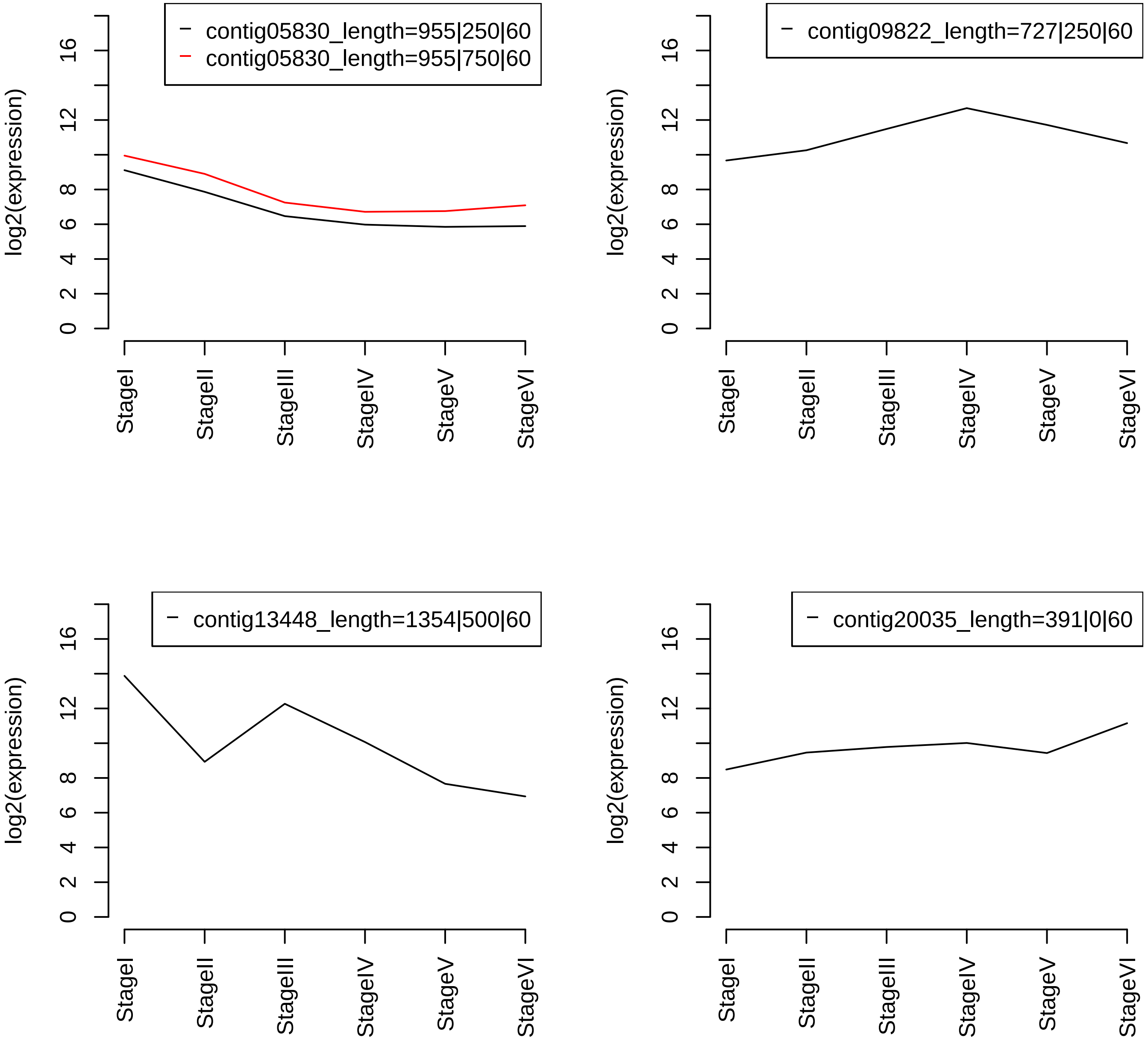

contig05830_length=955|250|60
contig05830_length=955|750|60
contig09822_length=727|250|60
contig13448_length=1354|500|60
contig20035_length=391|0|60
log2(expression)
0
2
4
6
8
12
16
StageI
StageII
StageIII
StageIV
StageV
StageVI

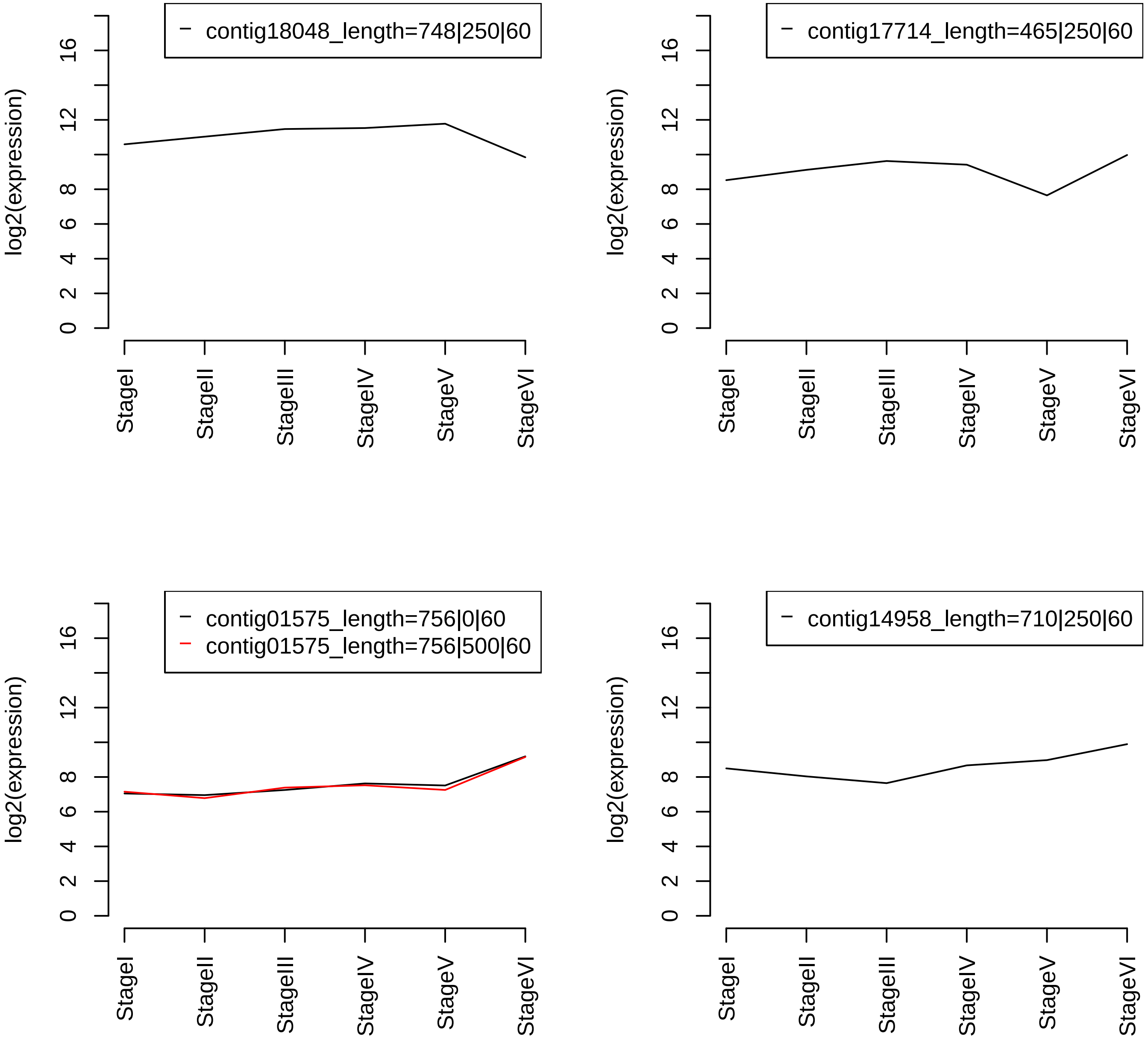

contig18048_length=748|250|60
contig17714_length=465|250|60
contig01575_length=756|0|60
contig01575_length=756|500|60
contig14958_length=710|250|60
log2(expression)
0
2
4
6
8
12
16
StageI
StageII
StageIII
StageIV
StageV
StageVI

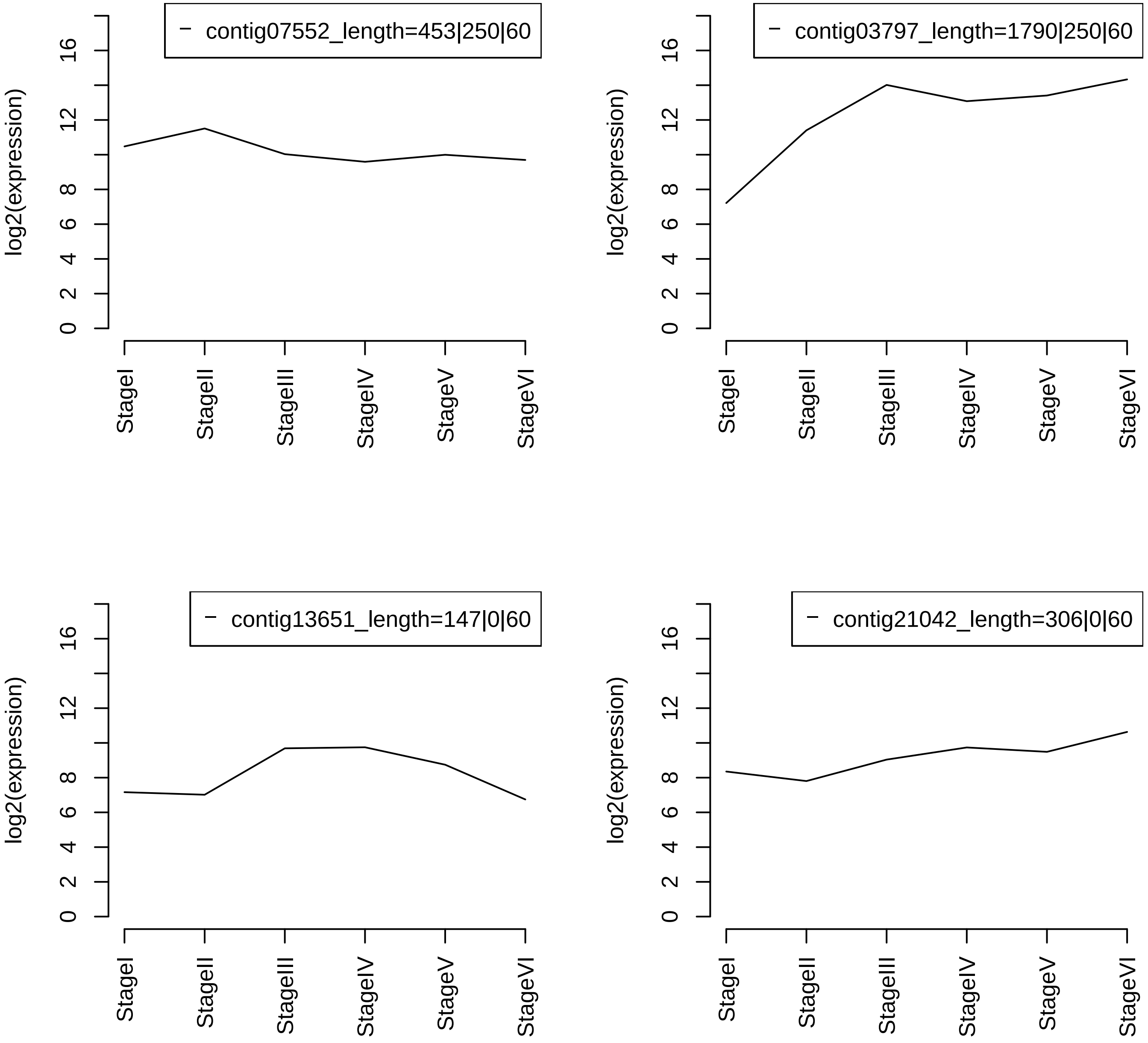
contig07552_length=453|250|60
contig03797_length=1790|250|60
contig13651_length=147|0|60
contig21042_length=306|0|60
log2(expression)
0
2
4
6
8
12
16
StageI
StageII
StageIII
StageIV
StageV
StageVI

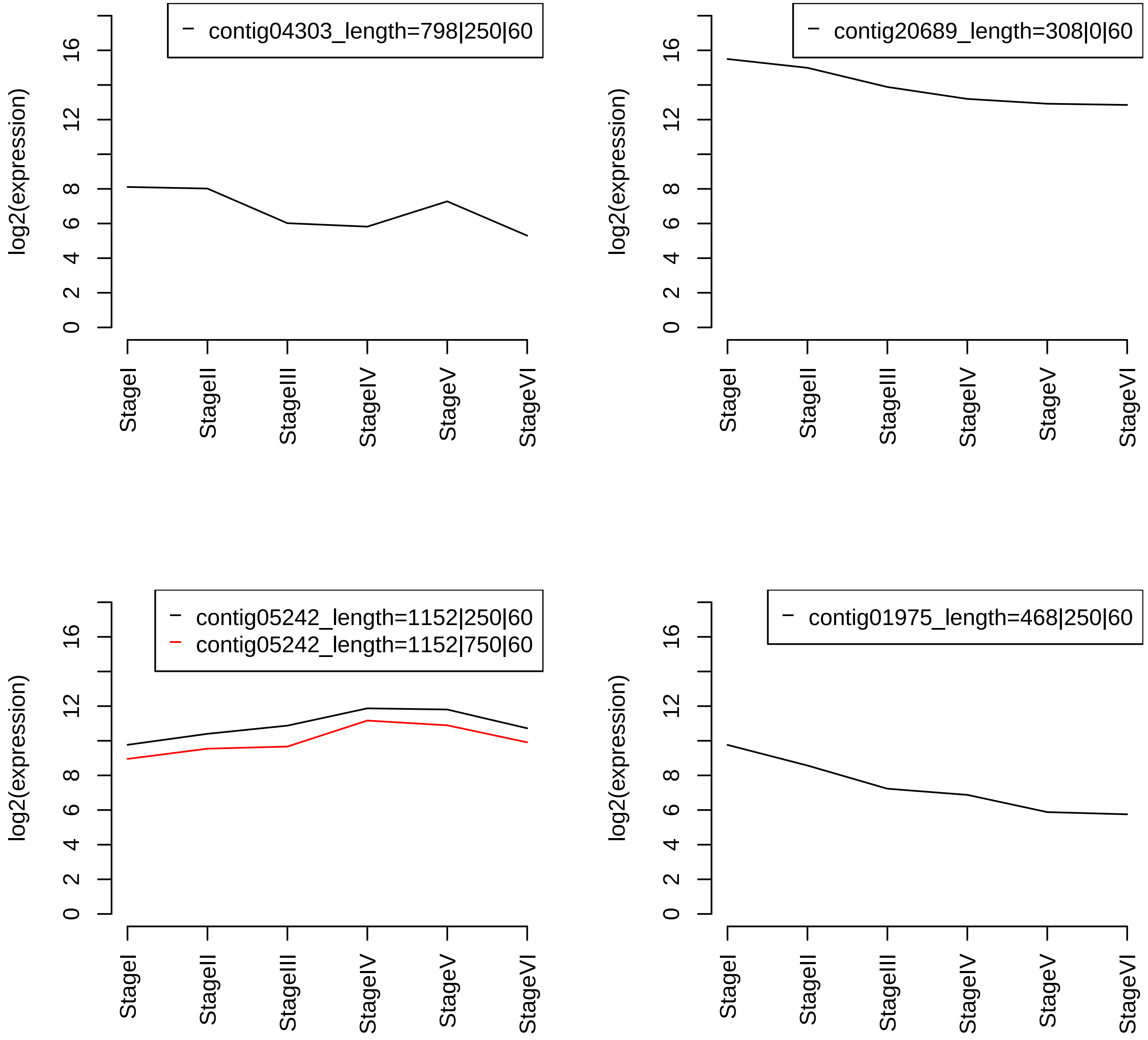
contig04303_length=798|250|60
contig20689_length=308|0|60
contig05242_length=1152|250|60
contig05242_length=1152|750|60
contig01975_length=468|250|60
log2(expression)
0
2
4
6
8
12
16
StageI
StageII
StageIII
StageIV
StageV
StageVI

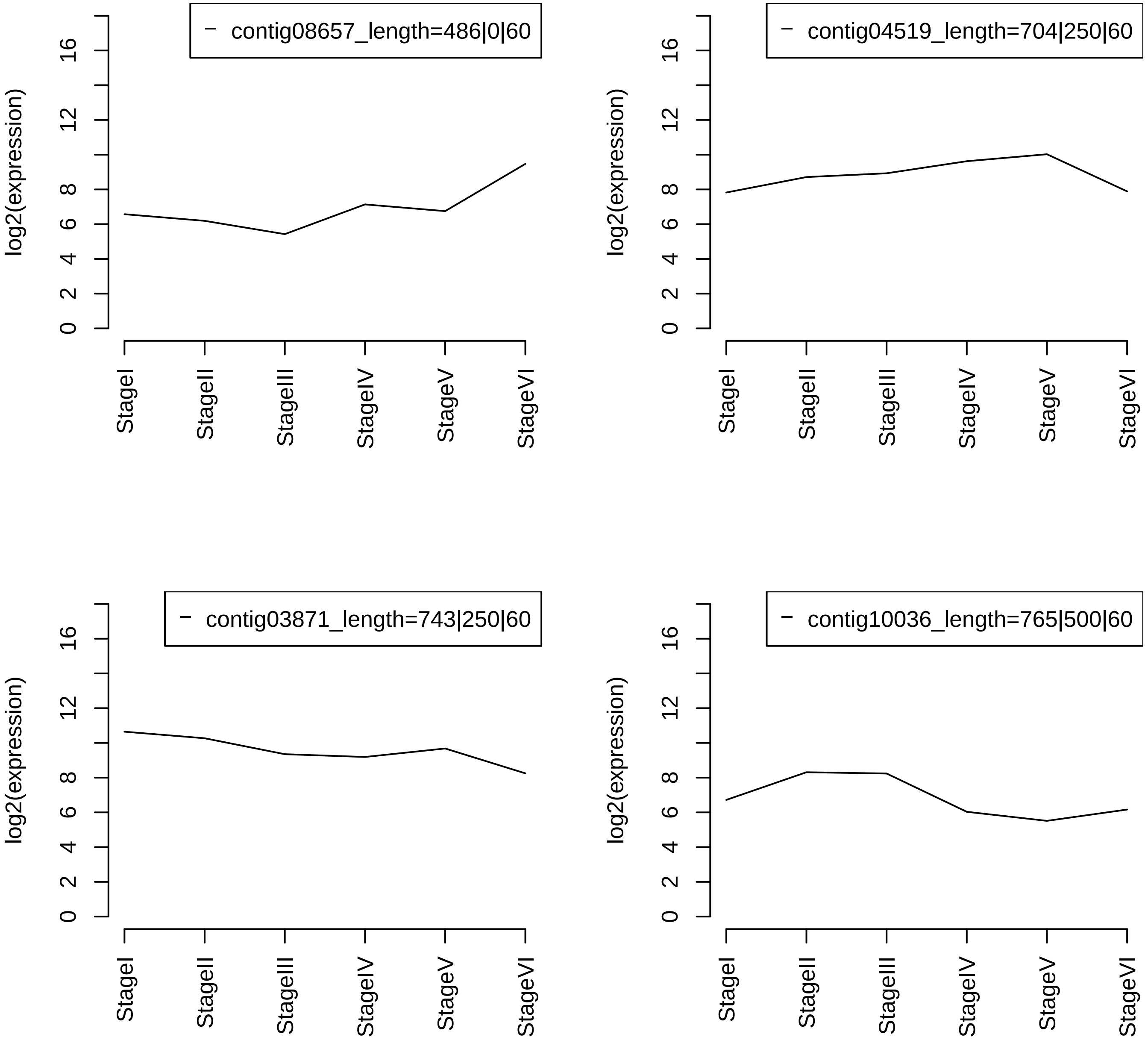
contig08657_length=486|0|60
contig04519_length=704|250|60
contig03871_length=743|250|60
contig10036_length=765|500|60
log2(expression)
0
2
4
6
8
12
16
StageI
StageII
StageIII
StageIV
StageV
StageVI

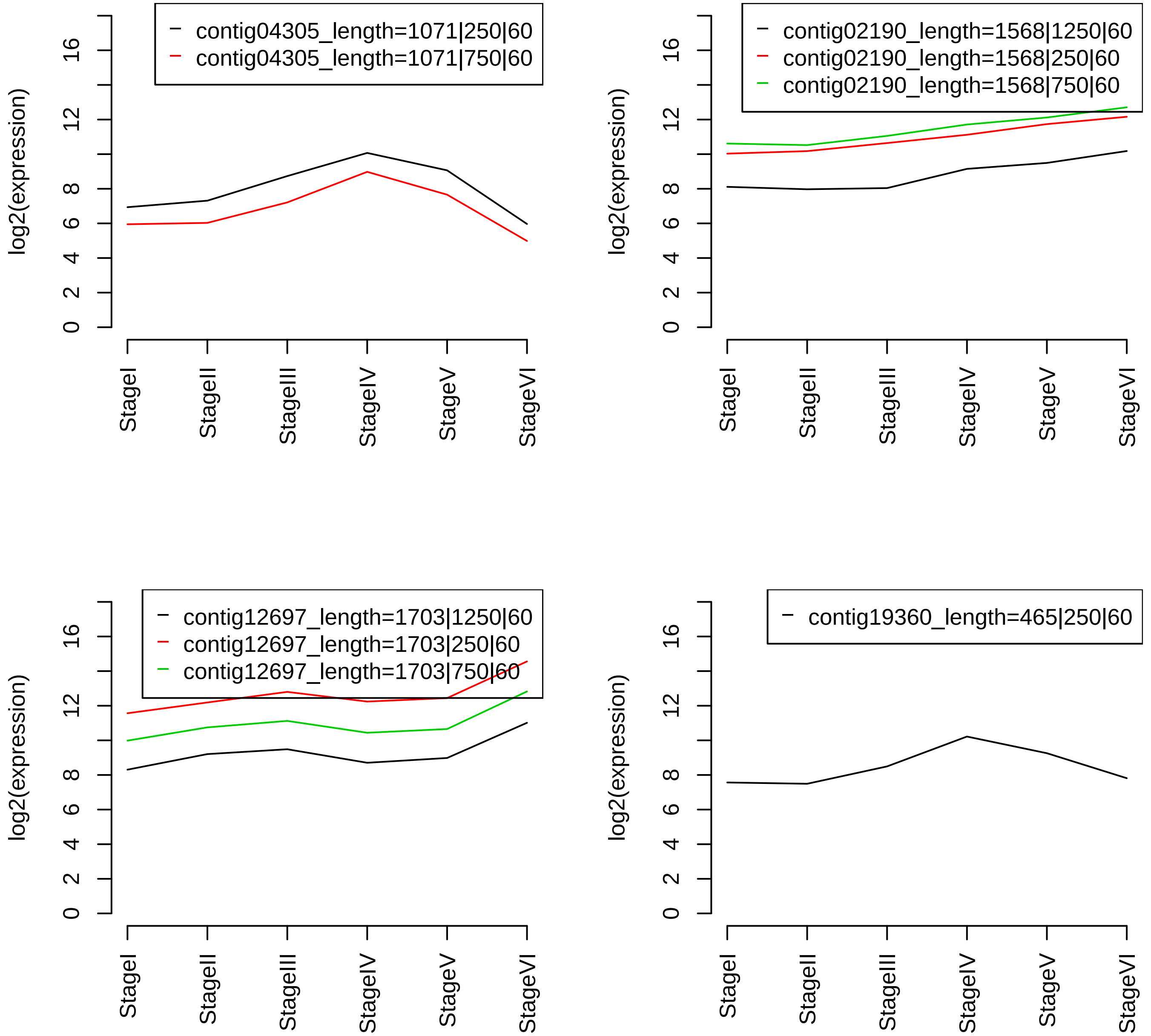
contig04305_length=1071|250|60
contig04305_length=1071|750|60
contig02190_length=1568|1250|60
contig02190_length=1568|250|60
contig02190_length=1568|750|60
contig12697_length=1703|1250|60
contig12697_length=1703|250|60
contig12697_length=1703|750|60
contig19360_length=465|250|60
log2(expression)
StageI
StageII
StageIII
StageIV
StageV
StageVI

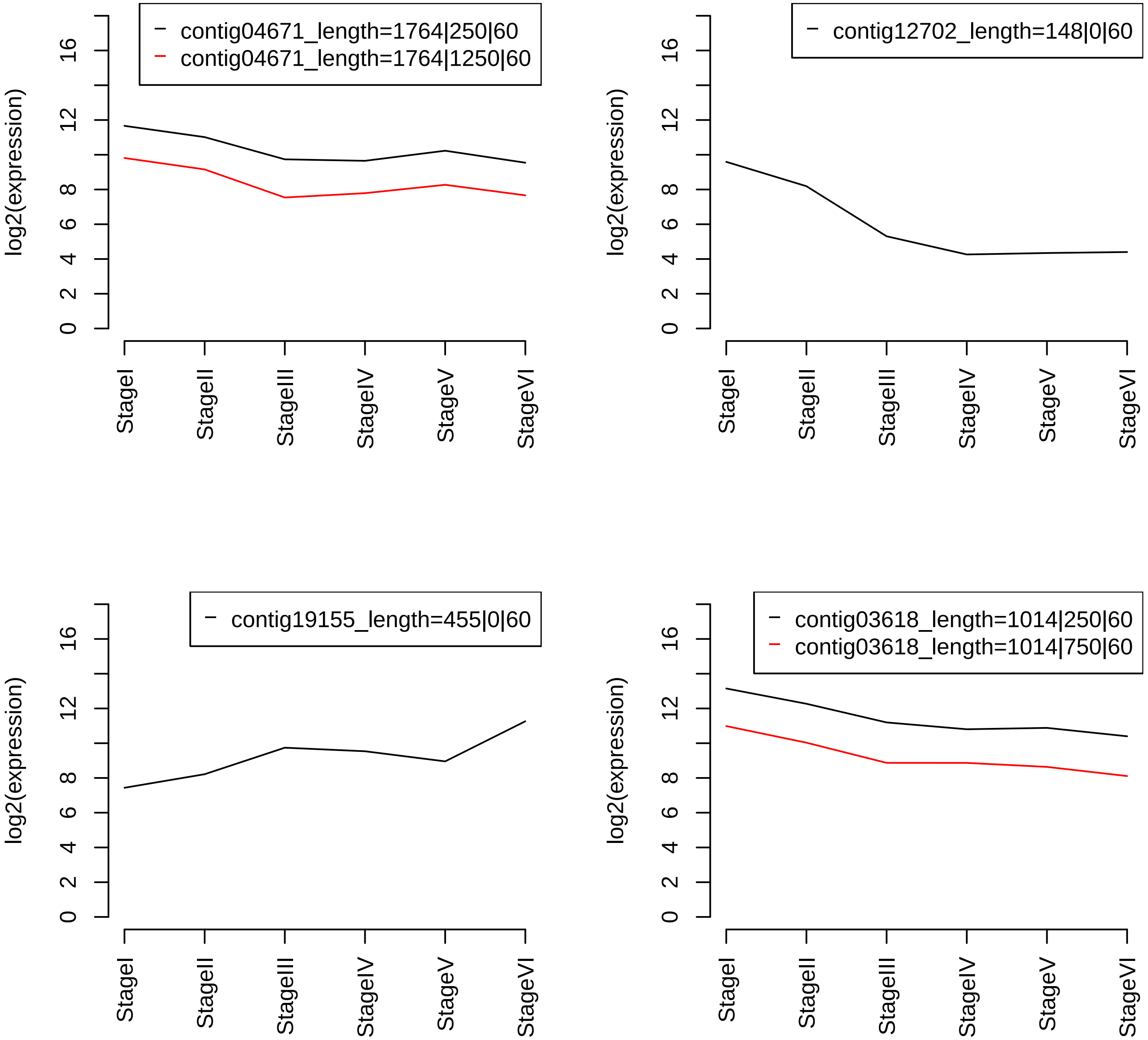
contig04671_length=1764|250|60
contig04671_length=1764|1250|60
contig12702_length=148|0|60
contig19155_length=455|0|60
contig03618_length=1014|250|60
contig03618_length=1014|750|60
log2(expression)
0
2
4
6
8
12
16
StageI
StageII
StageIII
StageIV
StageV
StageVI

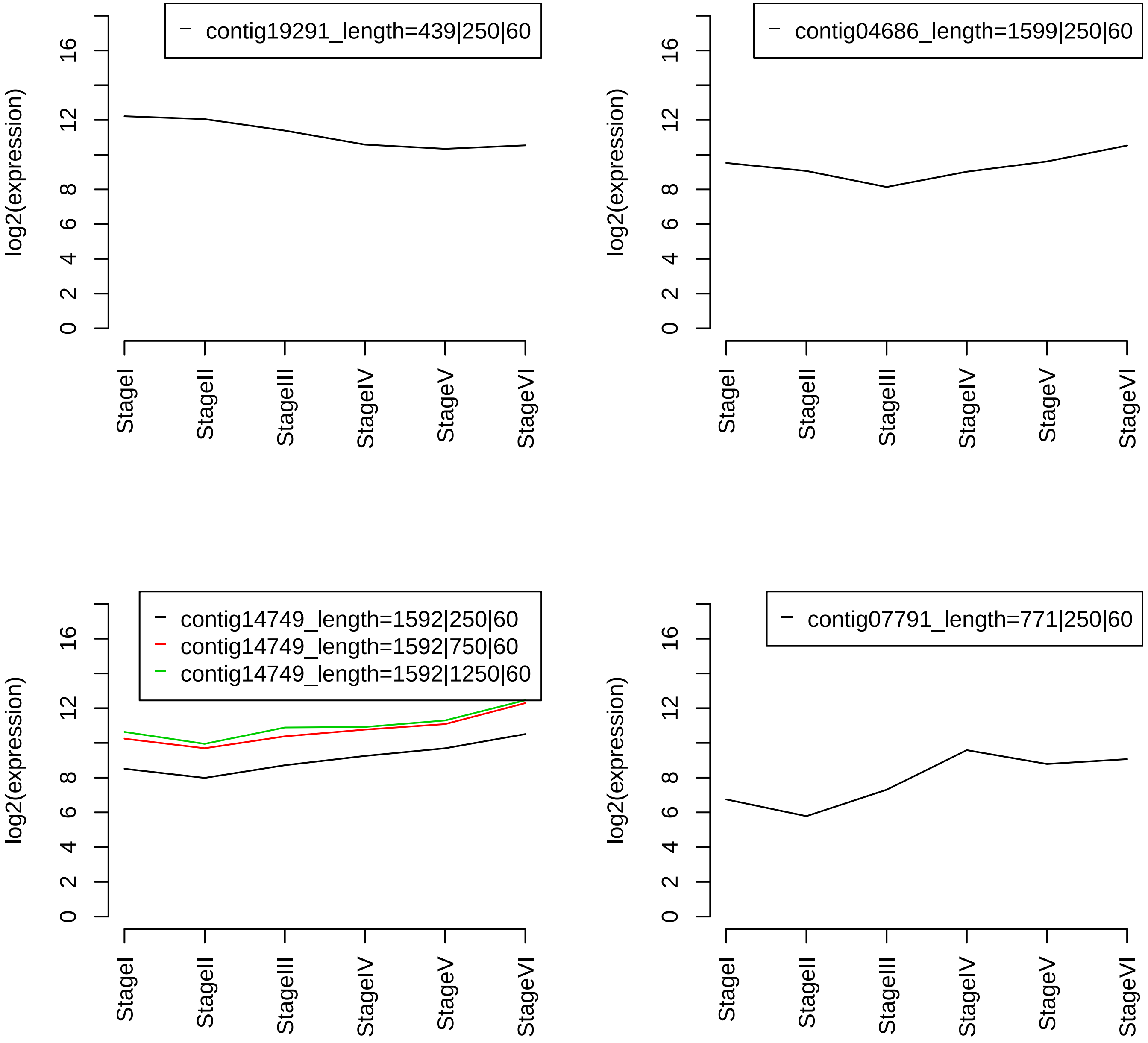
contig19291_length=439|250|60
contig04686_length=1599|250|60
contig14749_length=1592|250|60
contig14749_length=1592|750|60
contig14749_length=1592|1250|60
contig07791_length=771|250|60
log2(expression)
0
2
4
6
8
12
16
StageI
StageII
StageIII
StageIV
StageV
StageVI

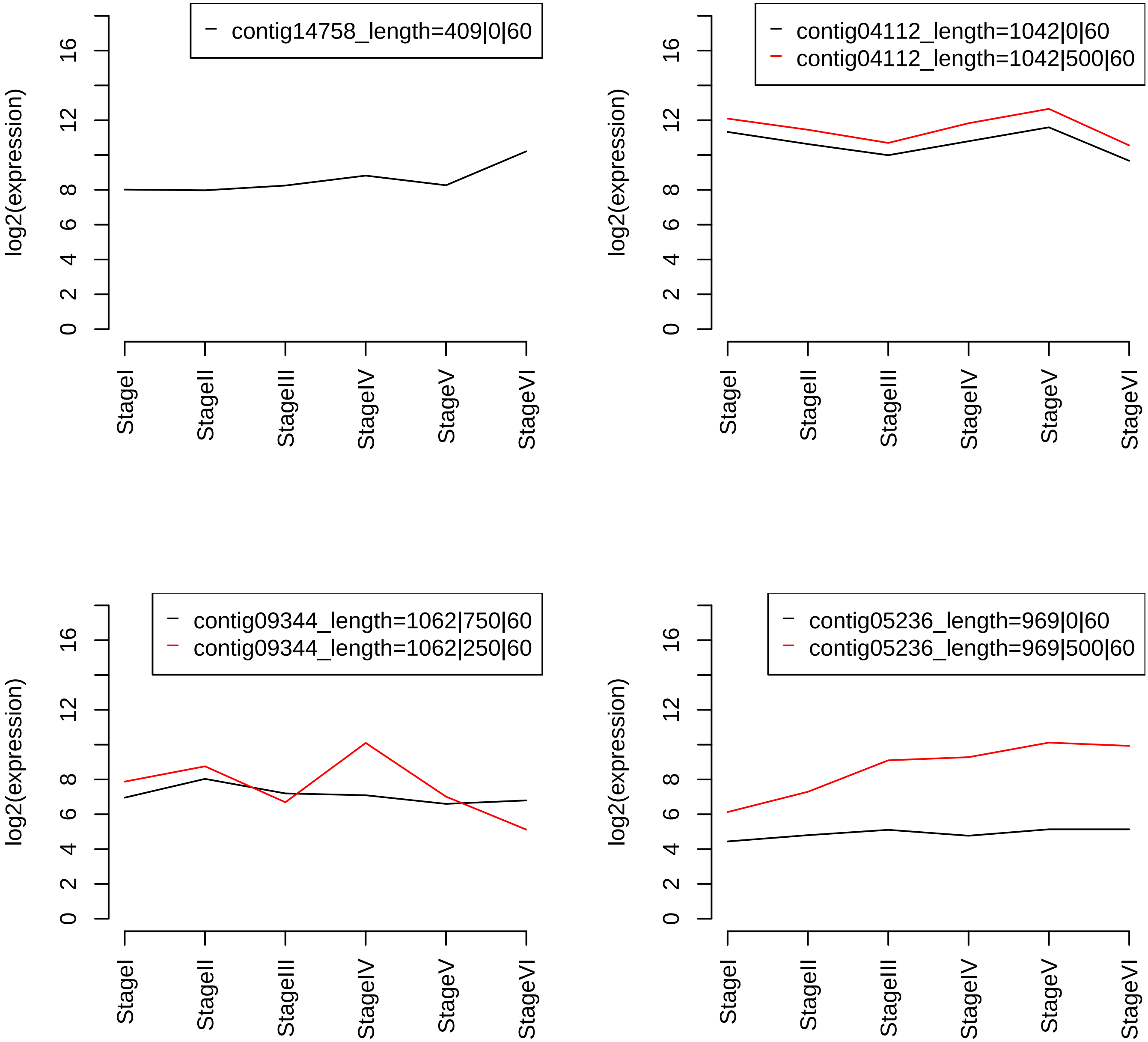

contig14758_length=409|0|60
contig04112_length=1042|0|60
contig04112_length=1042|500|60
contig09344_length=1062|750|60
contig09344_length=1062|250|60
contig05236_length=969|0|60
contig05236_length=969|500|60
log2(expression)
0
2
4
6
8
12
16
StageI
StageII
StageIII
StageIV
StageV
StageVI

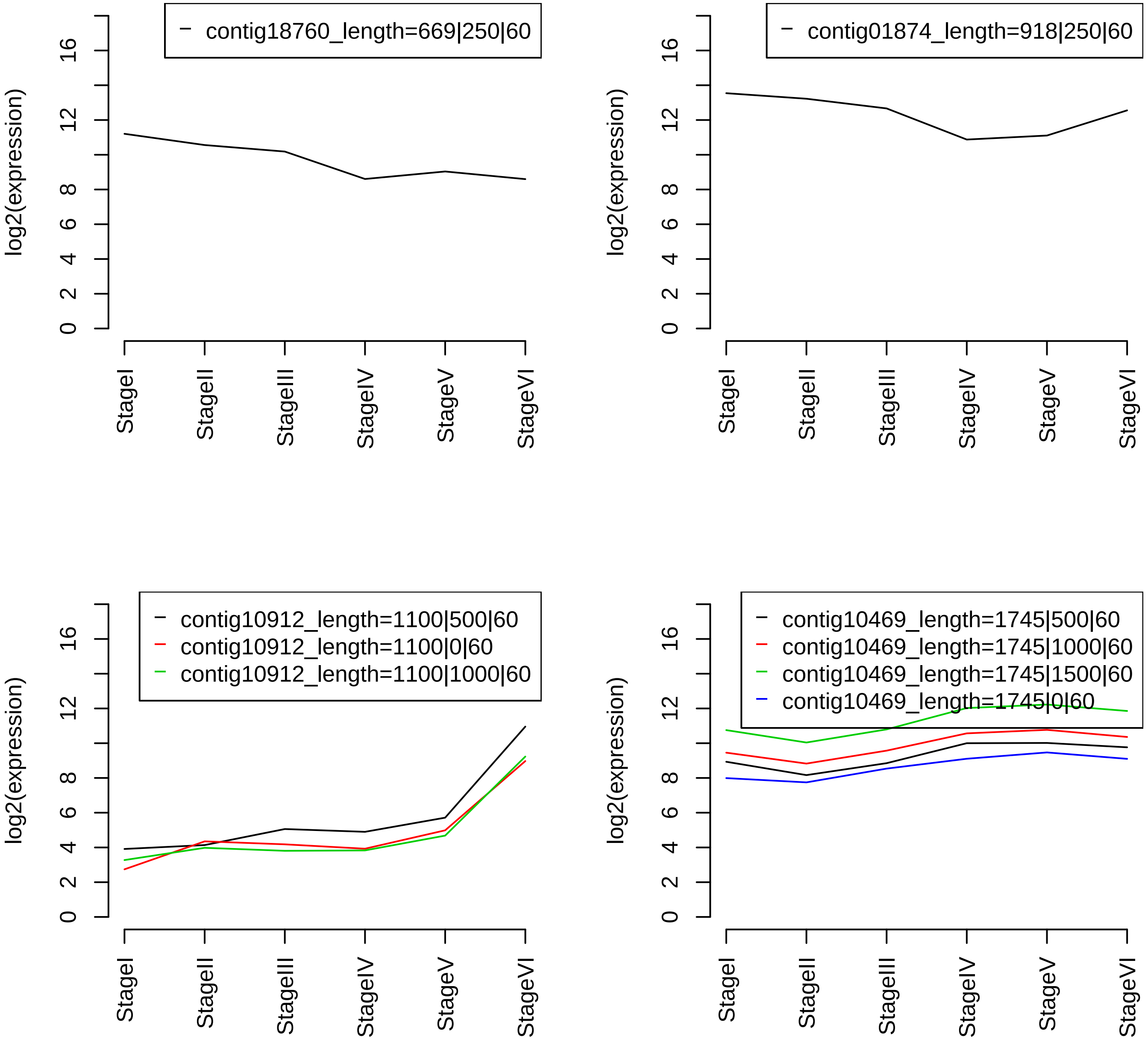

contig18760_length=669|250|60
contig01874_length=918|250|60
contig10912_length=1100|500|60
contig10912_length=1100|0|60
contig10912_length=1100|1000|60
contig10469_length=1745|500|60
contig10469_length=1745|1000|60
contig10469_length=1745|1500|60
contig10469_length=1745|0|60
log2(expression)
0
2
4
6
8
12
16
StageI
StageII
StageIII
StageIV
StageV
StageVI

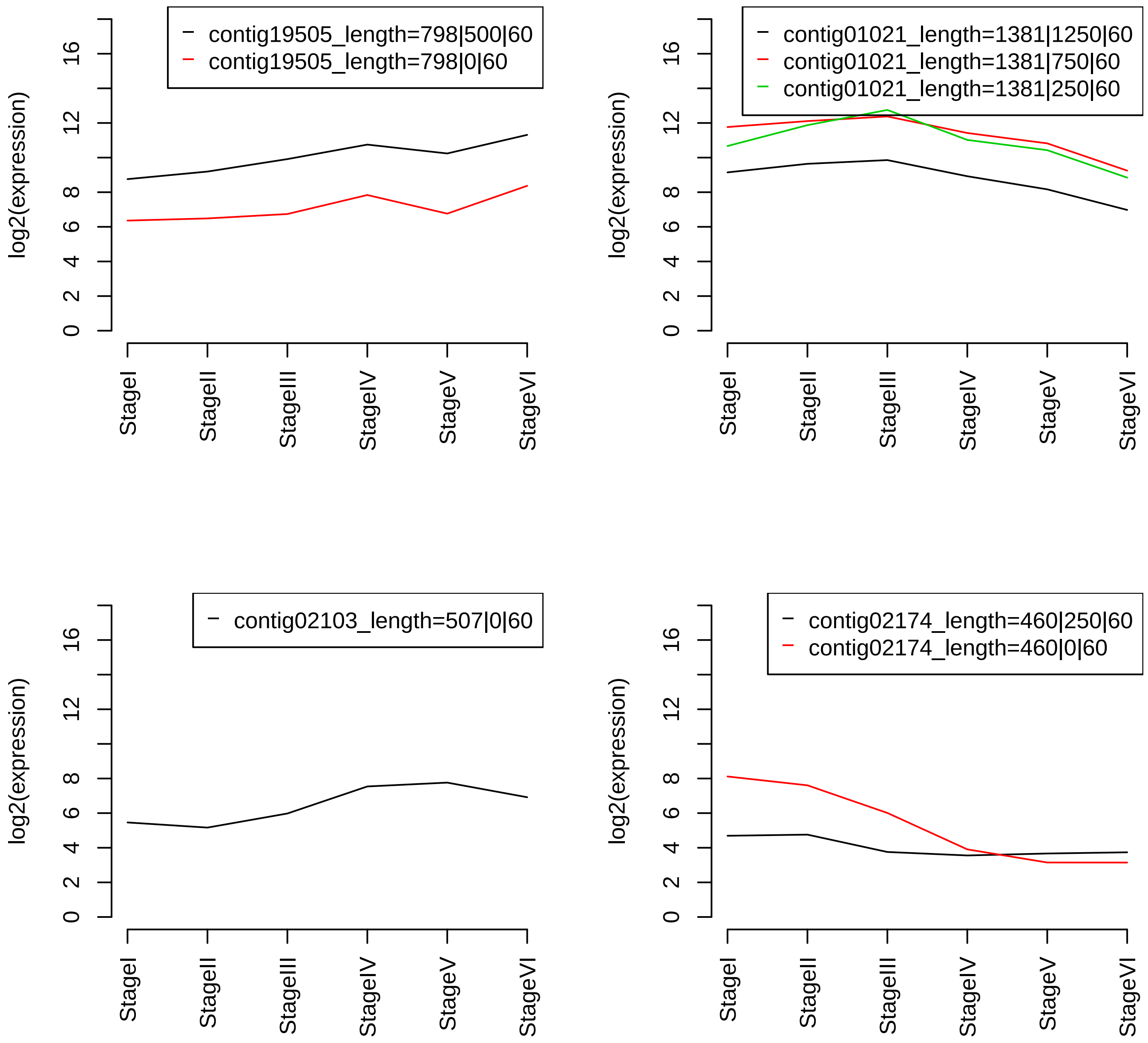

contig19505_length=798|500|60
contig19505_length=798|0|60
contig01021_length=1381|1250|60
contig01021_length=1381|750|60
contig01021_length=1381|250|60
contig02103_length=507|0|60
contig02174_length=460|250|60
contig02174_length=460|0|60
log2(expression)
0
2
4
6
8
12
16
StageI
StageII
StageIII
StageIV
StageV
StageVI

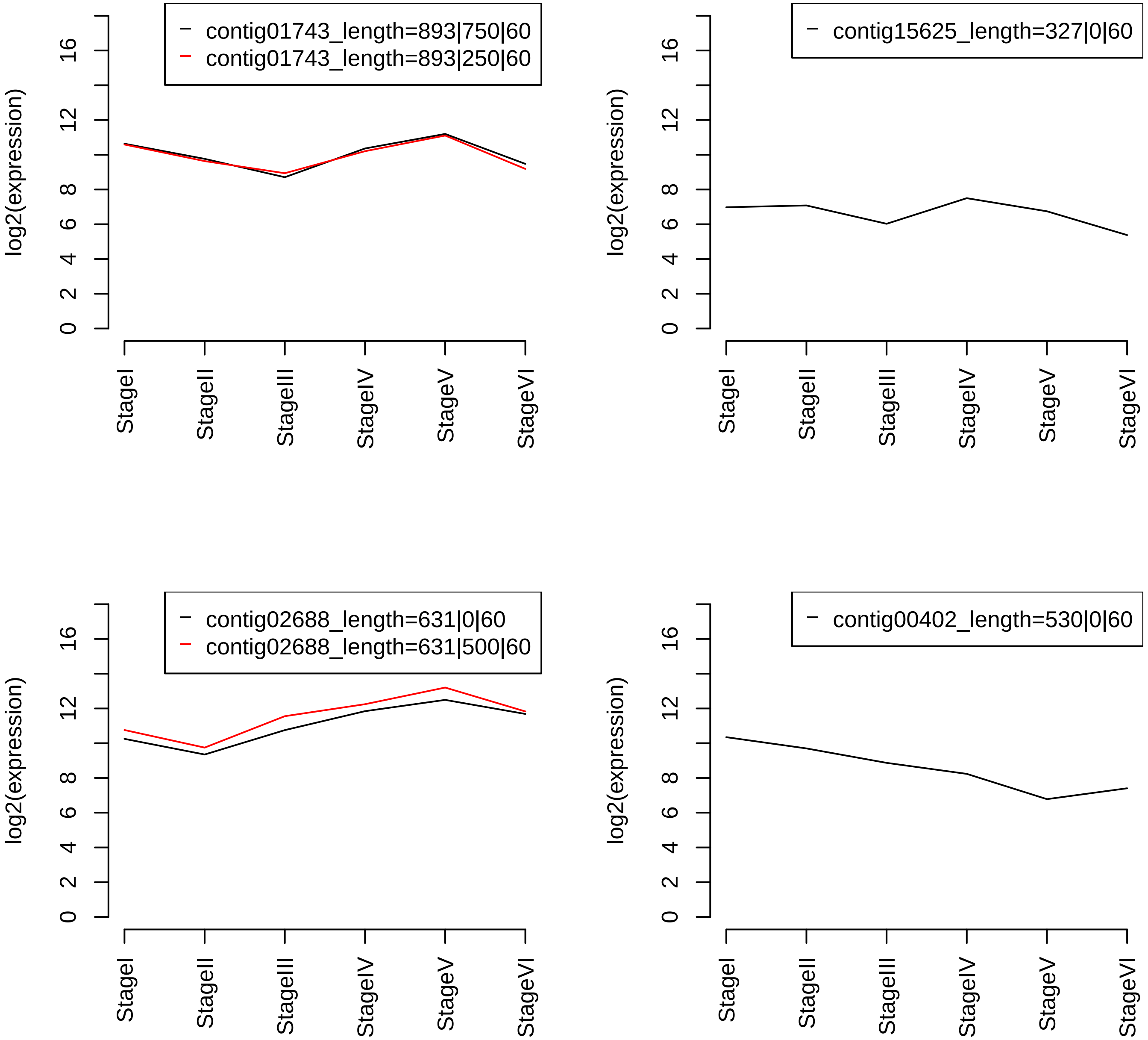
contig01743_length=893|750|60
contig01743_length=893|250|60
contig15625_length=327|0|60
contig02688_length=631|0|60
contig02688_length=631|500|60
contig00402_length=530|0|60
log2(expression)
0
2
4
6
8
12
16
StageI
StageII
StageIII
StageIV
StageV
StageVI

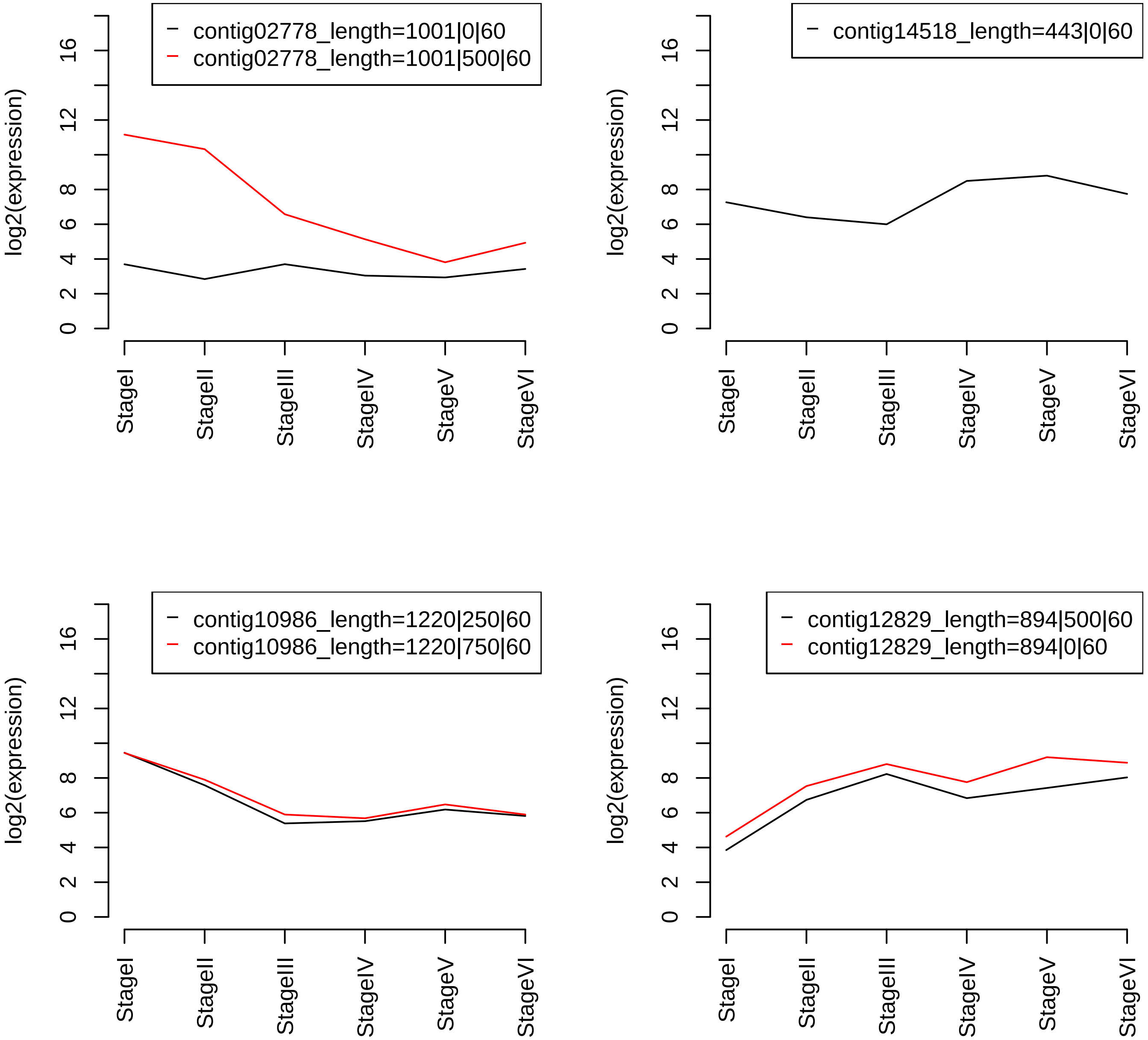
contig02778_length=1001|0|60
contig02778_length=1001|500|60
contig14518_length=443|0|60
contig10986_length=1220|250|60
contig10986_length=1220|750|60
contig12829_length=894|500|60
contig12829_length=894|0|60
log2(expression)
0
2
4
6
8
12
16
StageI
StageII
StageIII
StageIV
StageV
StageVI

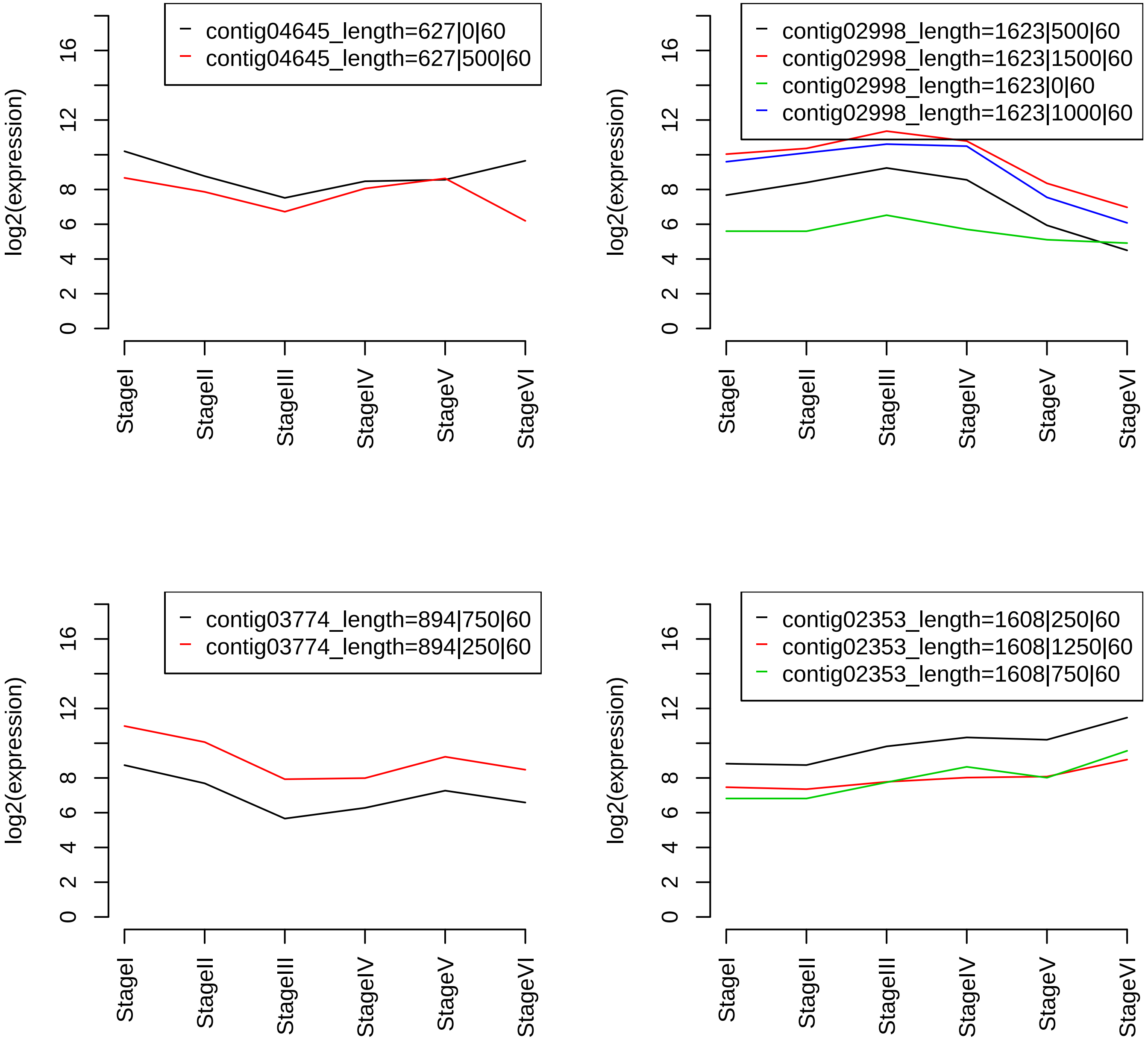
contig04645_length=627|0|60
contig04645_length=627|500|60
log2(expression)
0
2
4
6
8
12
16
StageI
StageII
StageIII
StageIV
StageV
StageVI
contig02998_length=1623|500|60
contig02998_length=1623|1500|60
contig02998_length=1623|0|60
contig02998_length=1623|1000|60
log2(expression)
0
2
4
6
8
12
16
StageI
StageII
StageIII
StageIV
StageV
StageVI
contig03774_length=894|750|60
contig03774_length=894|250|60
log2(expression)
0
2
4
6
8
12
16
StageI
StageII
StageIII
StageIV
StageV
StageVI
contig02353_length=1608|250|60
contig02353_length=1608|1250|60
contig02353_length=1608|750|60
log2(expression)
0
2
4
6
8
12
16
StageI
StageII
StageIII
StageIV
StageV
StageVI

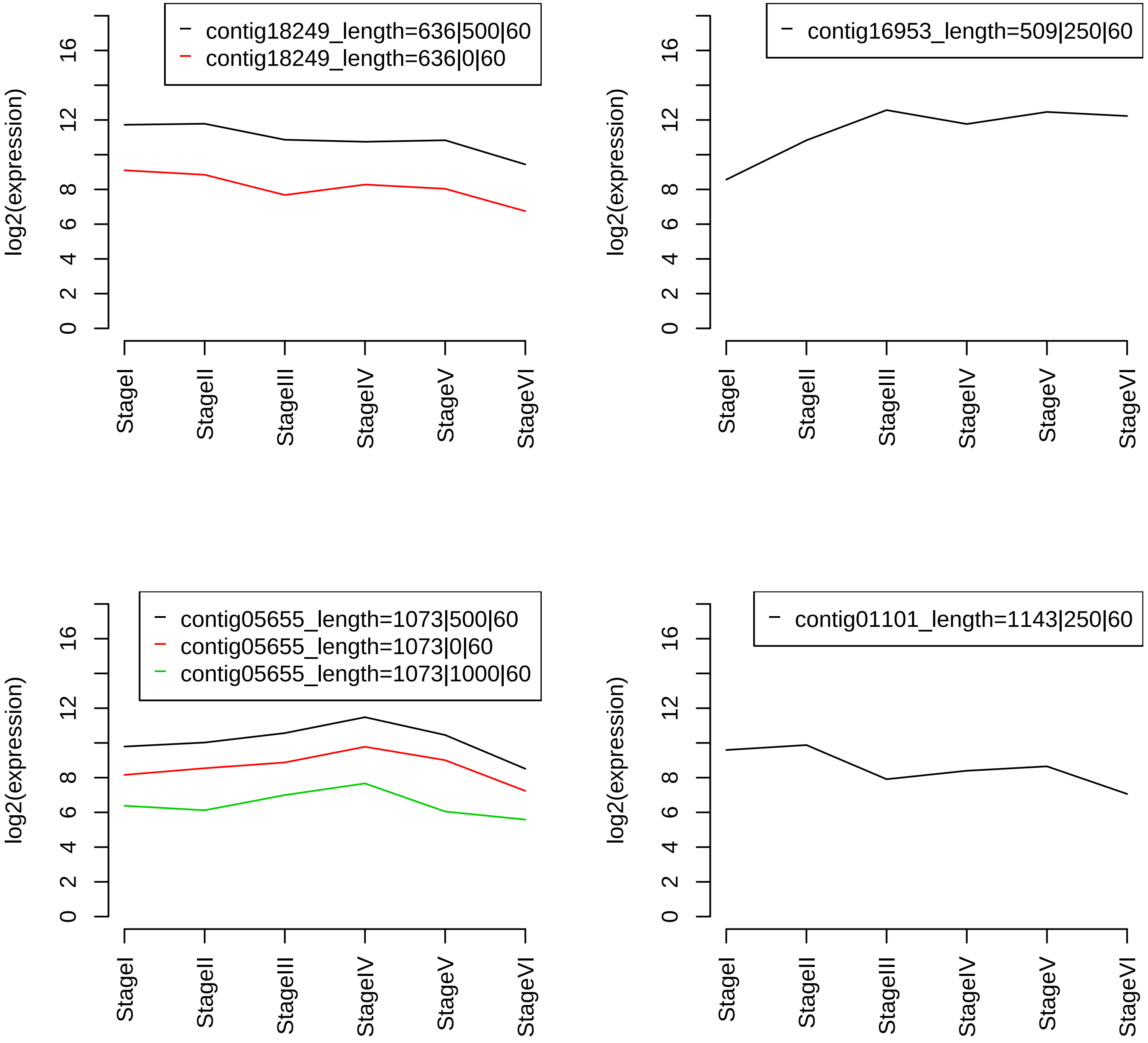

contig18249_length=636|500|60
contig18249_length=636|0|60
contig16953_length=509|250|60
contig05655_length=1073|500|60
contig05655_length=1073|0|60
contig05655_length=1073|1000|60
contig01101_length=1143|250|60
log2(expression)
0
2
4
6
8
12
16
StageI
StageII
StageIII
StageIV
StageV
StageVI

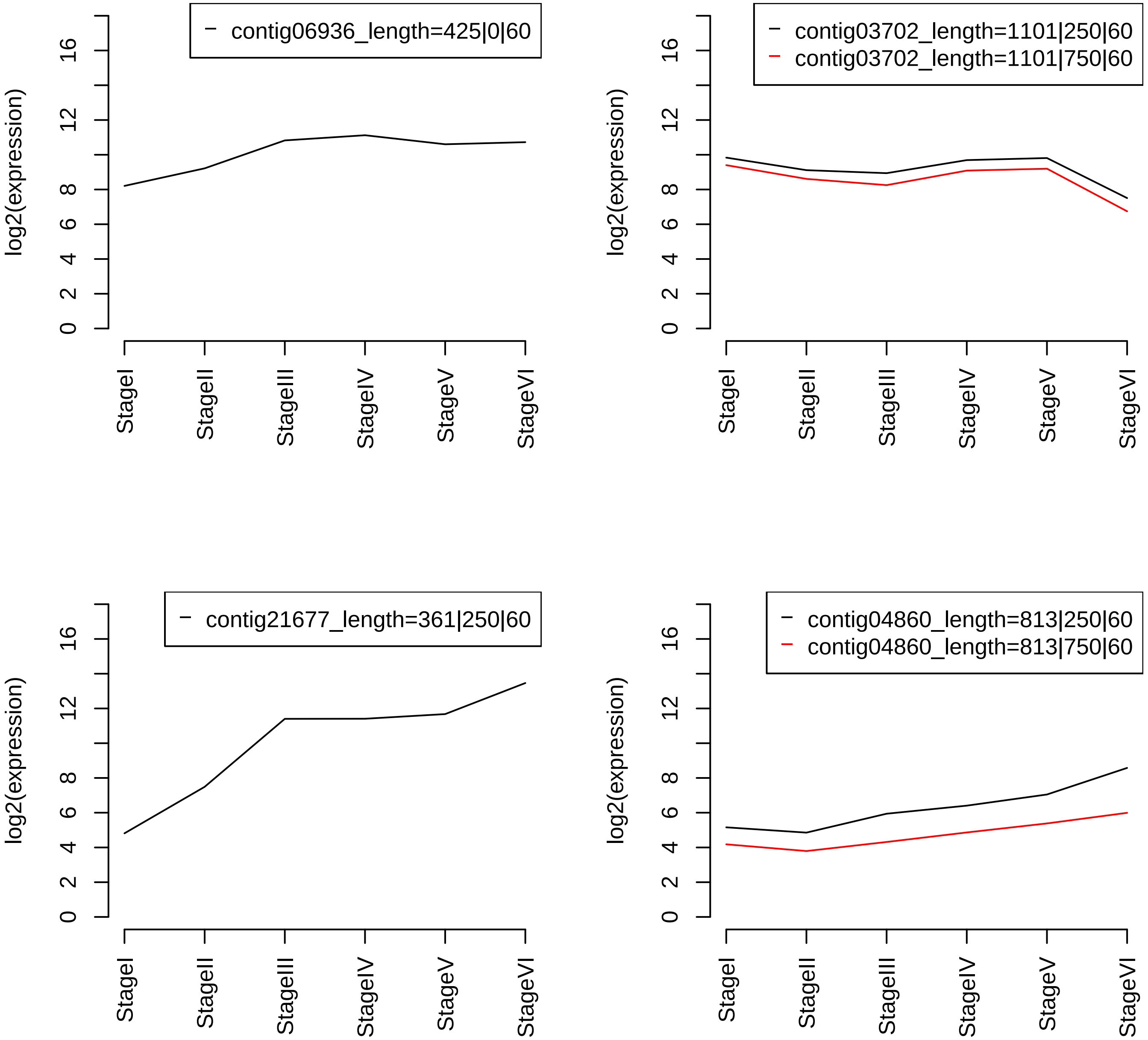

contig06936_length=425|0|60
contig03702_length=1101|250|60
contig03702_length=1101|750|60
contig21677_length=361|250|60
contig04860_length=813|250|60
contig04860_length=813|750|60
log2(expression)
0
2
4
6
8
12
16
StageI
StageII
StageIII
StageIV
StageV
StageVI

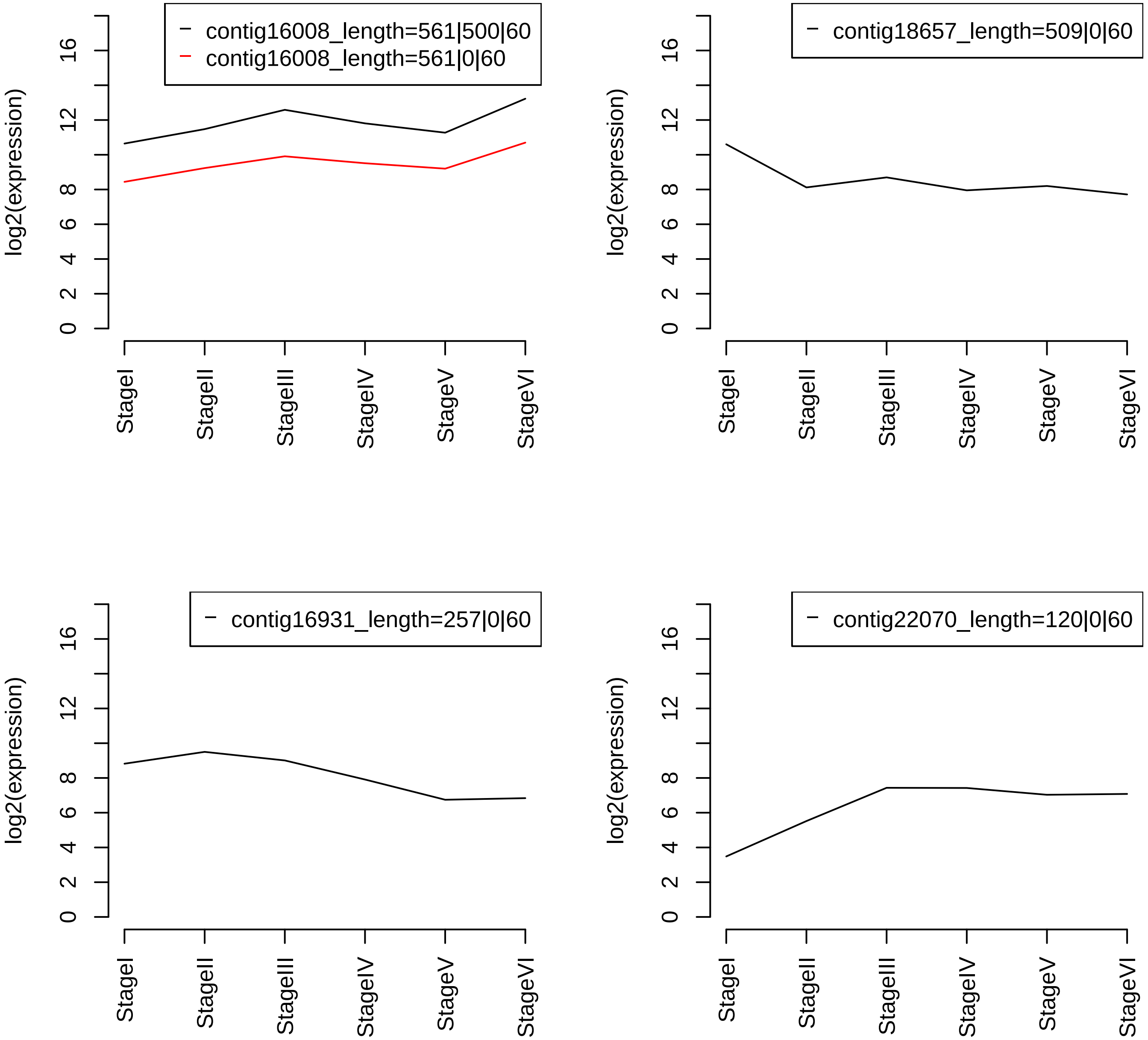

contig16008_length=561|500|60
contig16008_length=561|0|60
contig18657_length=509|0|60
contig16931_length=257|0|60
contig22070_length=120|0|60
log2(expression)
0
2
4
6
8
12
16
StageI
StageII
StageIII
StageIV
StageV
StageVI

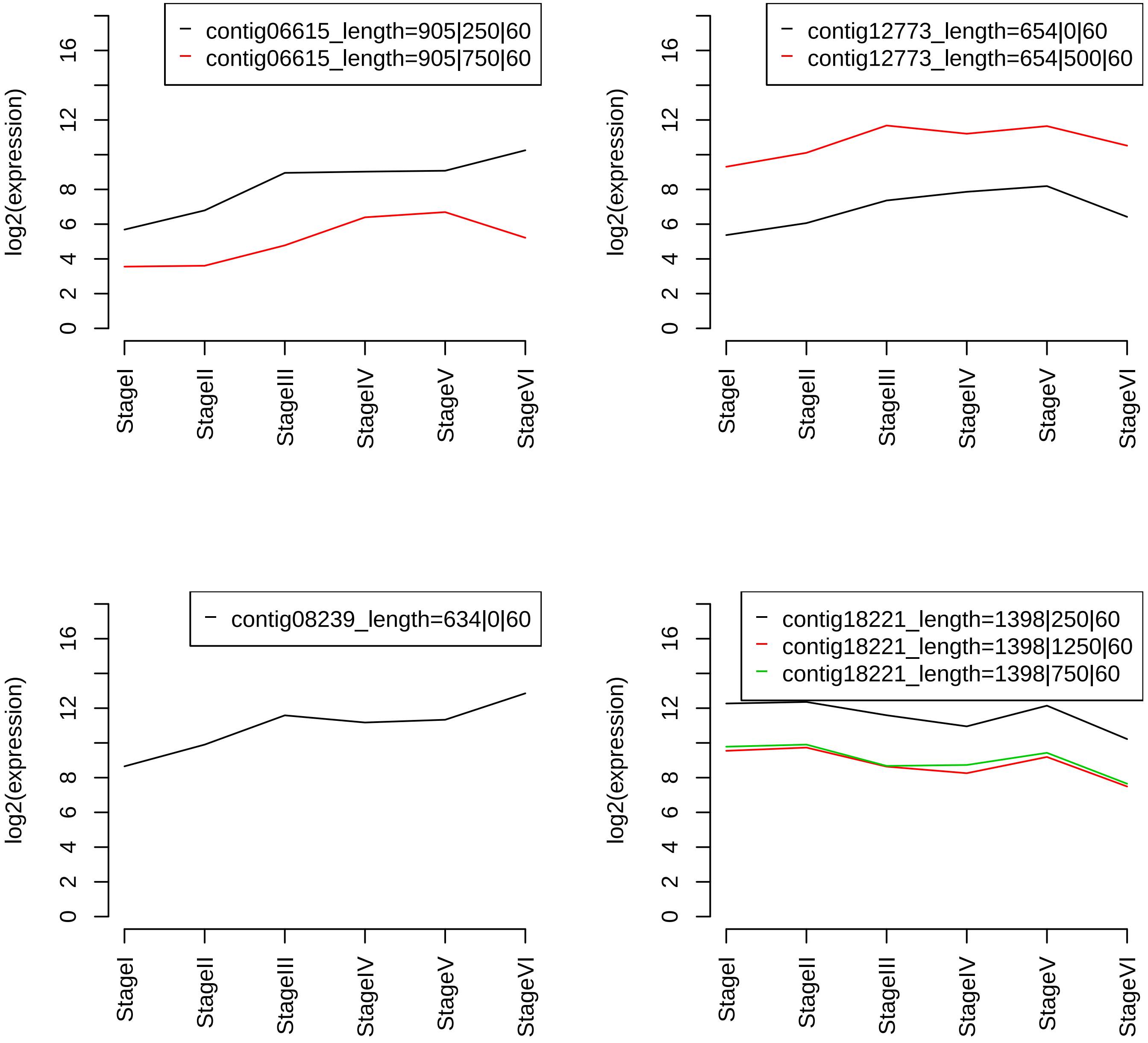
contig06615_length=905|250|60
contig06615_length=905|750|60
contig12773_length=654|0|60
contig12773_length=654|500|60
contig08239_length=634|0|60
contig18221_length=1398|250|60
contig18221_length=1398|1250|60
contig18221_length=1398|750|60
log2(expression)
0
2
4
6
8
12
16
StageI
StageII
StageIII
StageIV
StageV
StageVI

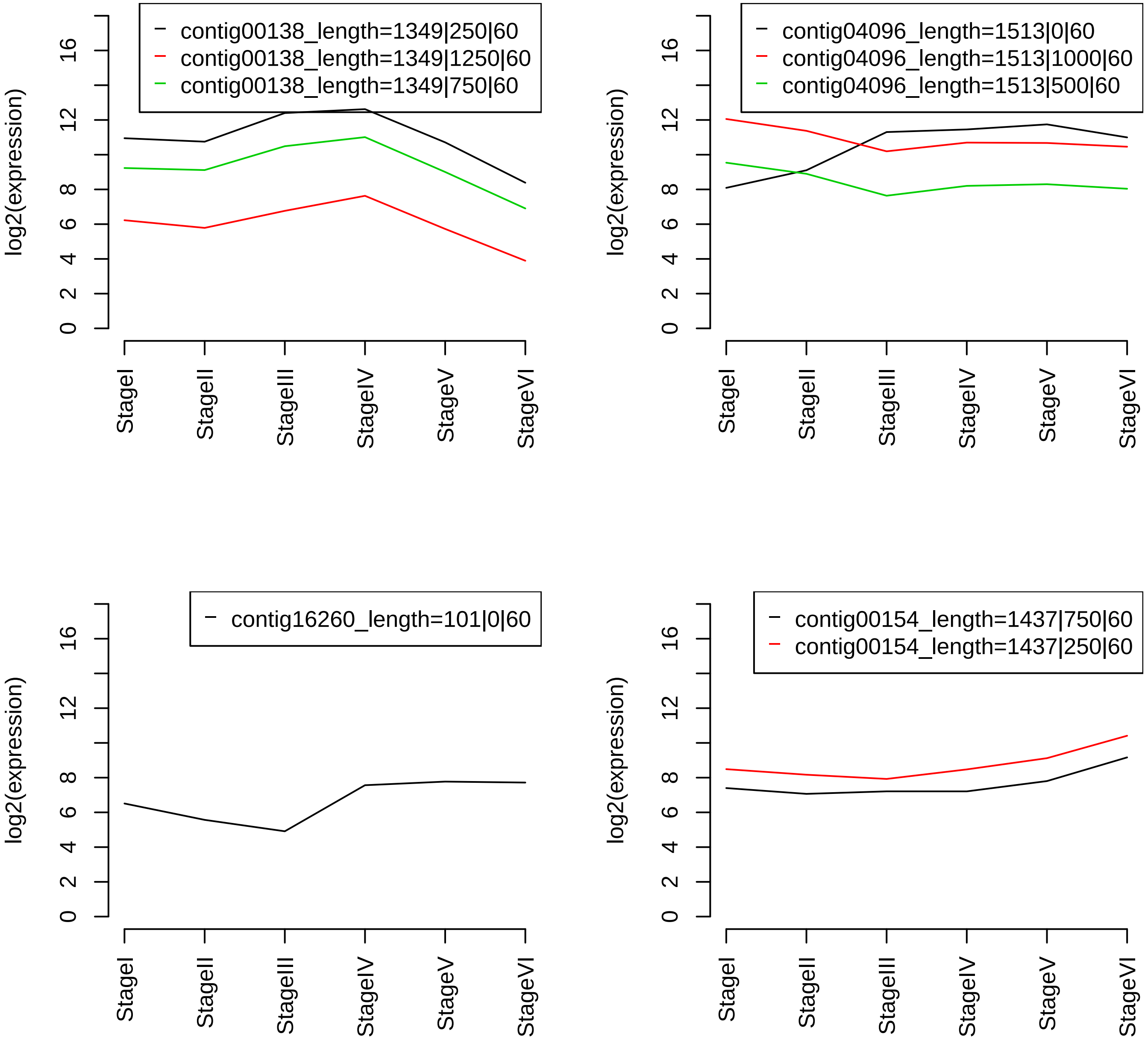

contig00138_length=1349|250|60
contig00138_length=1349|1250|60
contig00138_length=1349|750|60
contig04096_length=1513|0|60
contig04096_length=1513|1000|60
contig04096_length=1513|500|60
contig16260_length=101|0|60
contig00154_length=1437|750|60
contig00154_length=1437|250|60
log2(expression)
0
2
4
6
8
12
16
StageI
StageII
StageIII
StageIV
StageV
StageVI

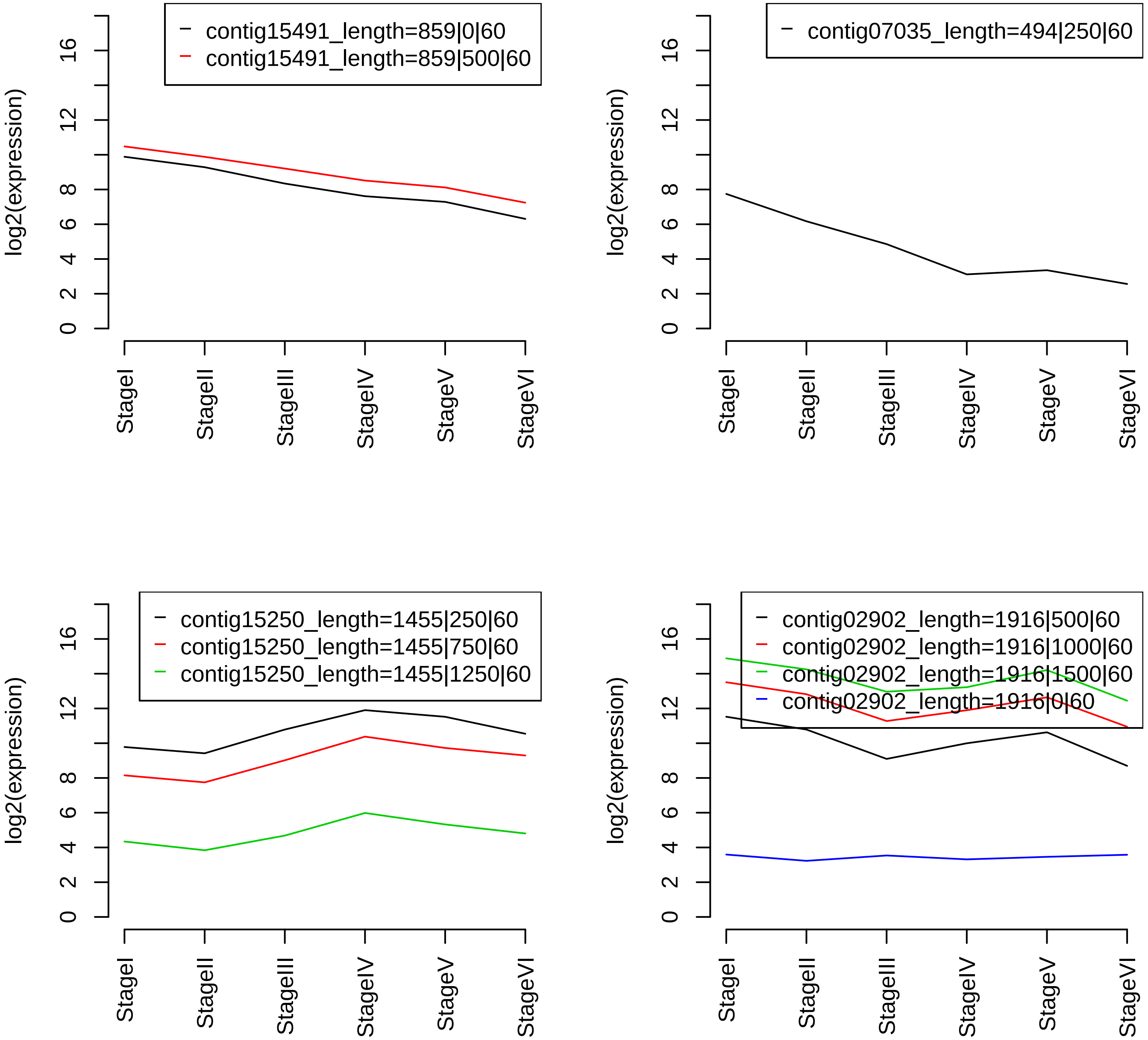

contig15491_length=859|0|60
contig15491_length=859|500|60
contig07035_length=494|250|60
contig15250_length=1455|250|60
contig15250_length=1455|750|60
contig15250_length=1455|1250|60
contig02902_length=1916|500|60
contig02902_length=1916|1000|60
contig02902_length=1916|1500|60
contig02902_length=1916|0|60
log2(expression)
0
2
4
6
8
12
16
StageI
StageII
StageIII
StageIV
StageV
StageVI

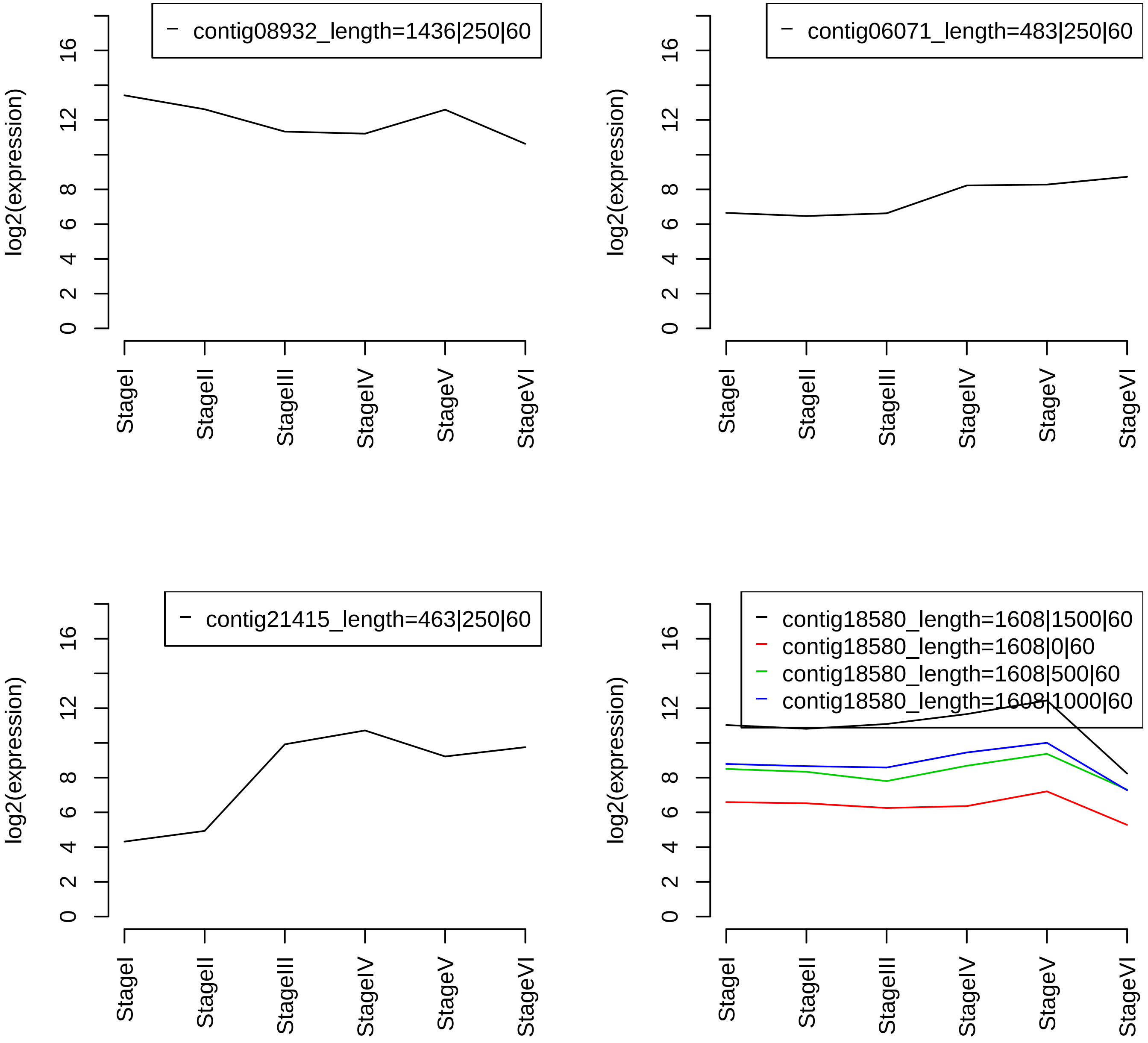

contig08932_length=1436|250|60
contig06071_length=483|250|60
contig21415_length=463|250|60
contig18580_length=1608|1500|60
contig18580_length=1608|0|60
contig18580_length=1608|500|60
contig18580_length=1608|1000|60
log2(expression)
0
2
4
6
8
12
16
StageI
StageII
StageIII
StageIV
StageV
StageVI

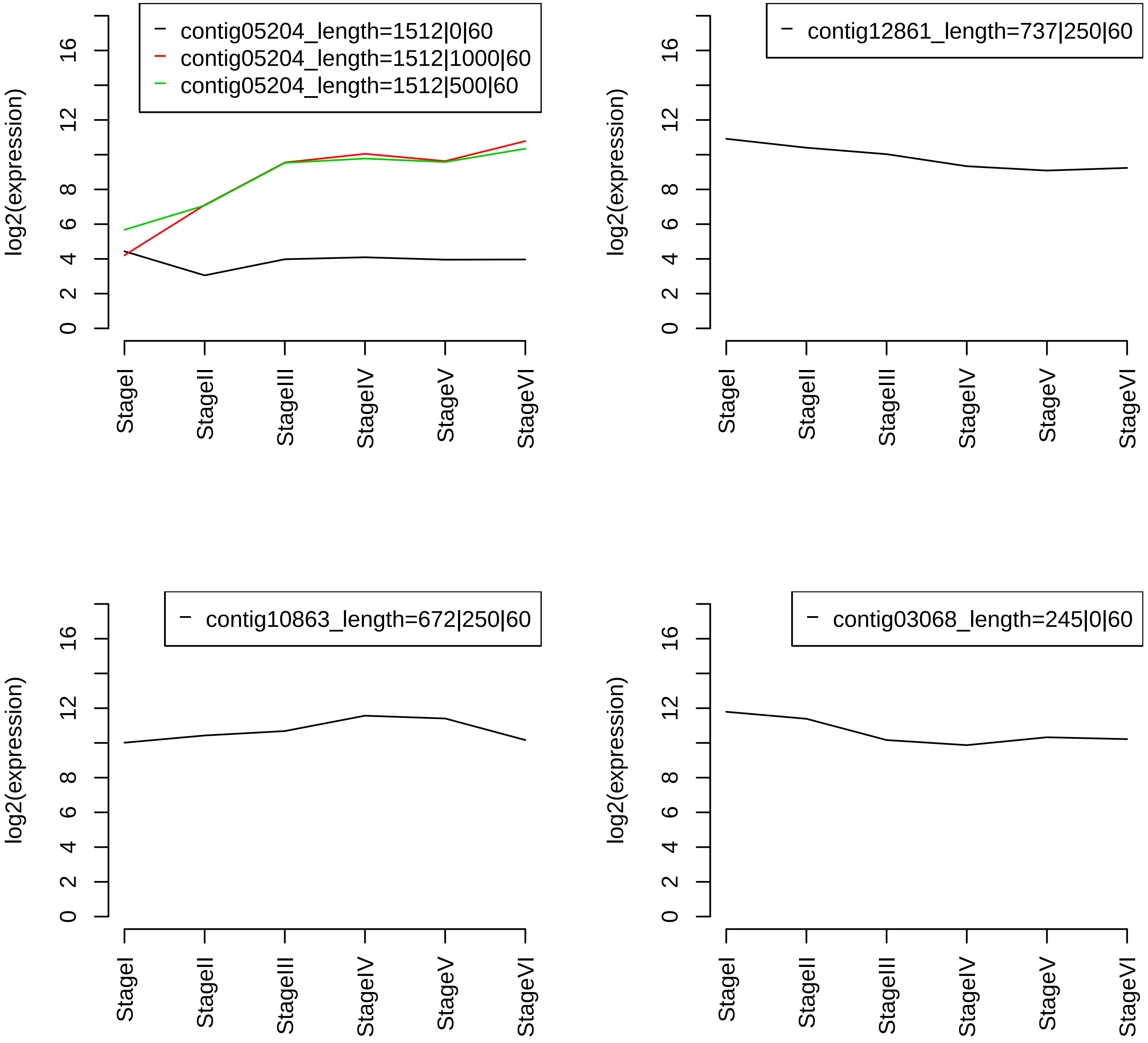

contig05204_length=1512|0|60
contig05204_length=1512|1000|60
contig05204_length=1512|500|60
contig12861_length=737|250|60
contig10863_length=672|250|60
contig03068_length=245|0|60
log2(expression)
0 2 4 6 8 12 16
StageI StageII StageIII StageIV StageV StageVI

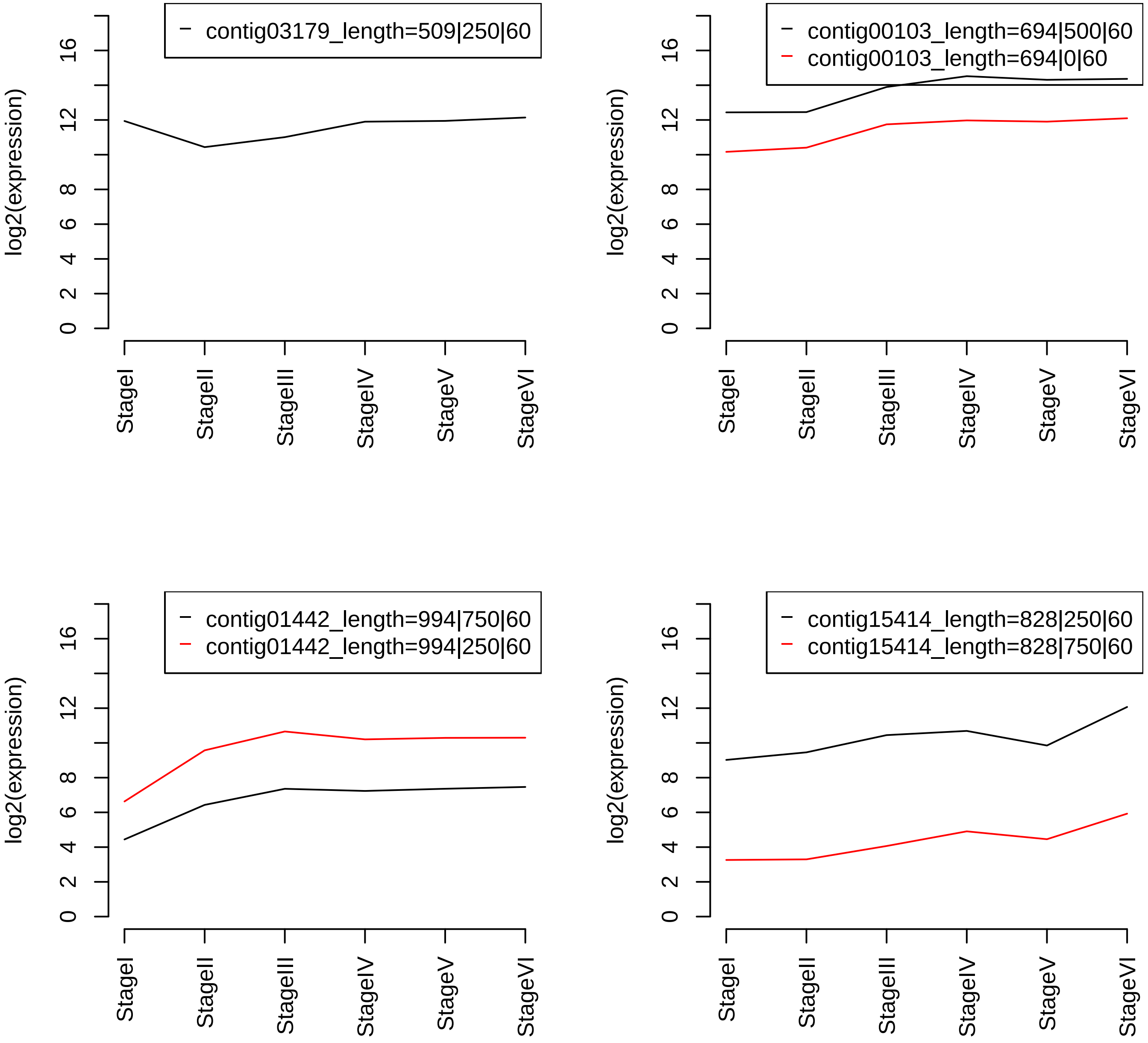

contig03179_length=509|250|60
log2(expression)
0
2
4
6
8
12
16
StageI
StageII
StageIII
StageIV
StageV
StageVI
contig00103_length=694|500|60
contig00103_length=694|0|60
contig01442_length=994|750|60
contig01442_length=994|250|60
contig15414_length=828|250|60
contig15414_length=828|750|60

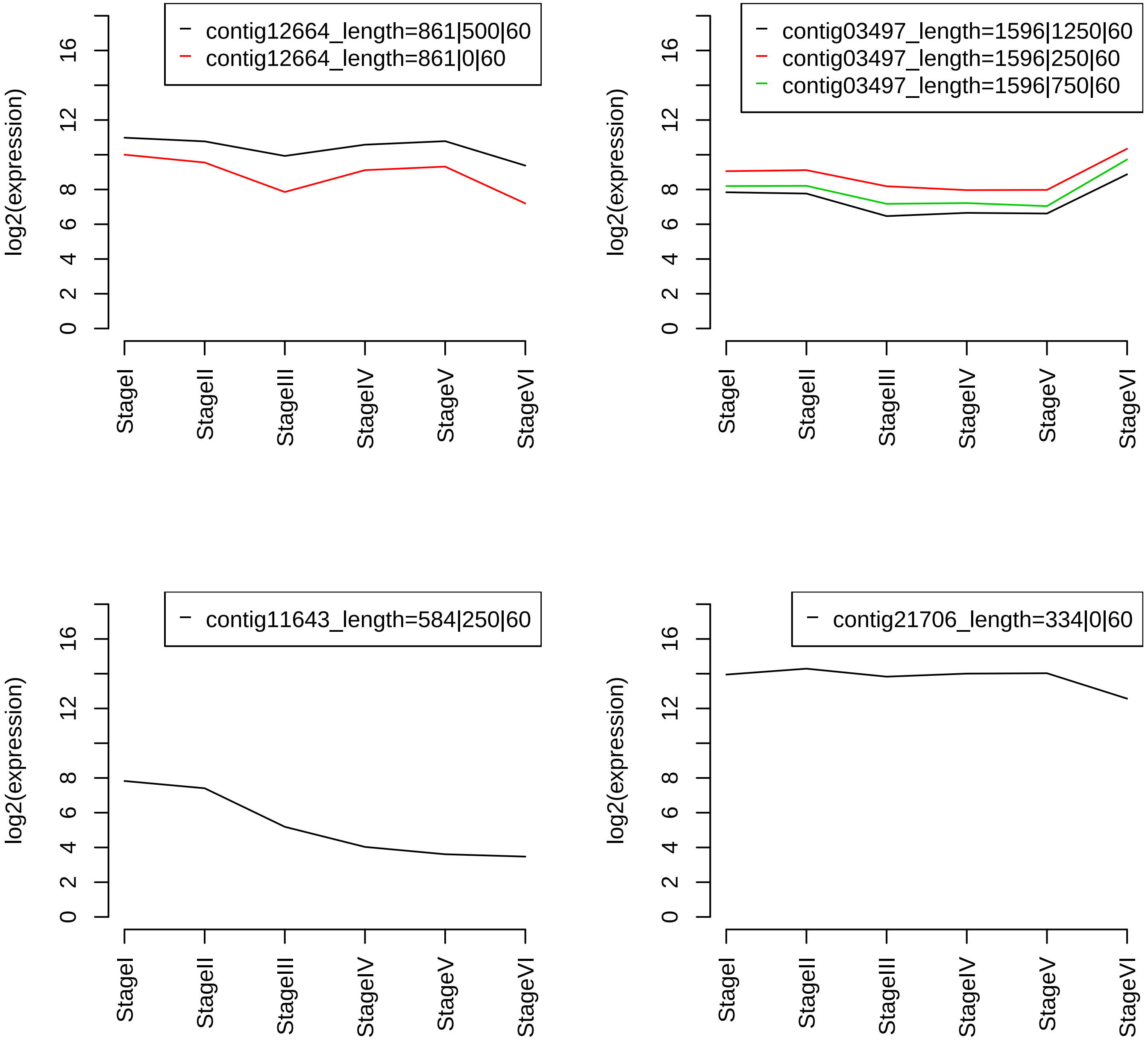
contig12664_length=861|500|60
contig12664_length=861|0|60
contig03497_length=1596|1250|60
contig03497_length=1596|250|60
contig03497_length=1596|750|60
contig11643_length=584|250|60
contig21706_length=334|0|60
log2(expression)
0 2 4 6 8 12 16
StageI StageII StageIII StageIV StageV StageVI

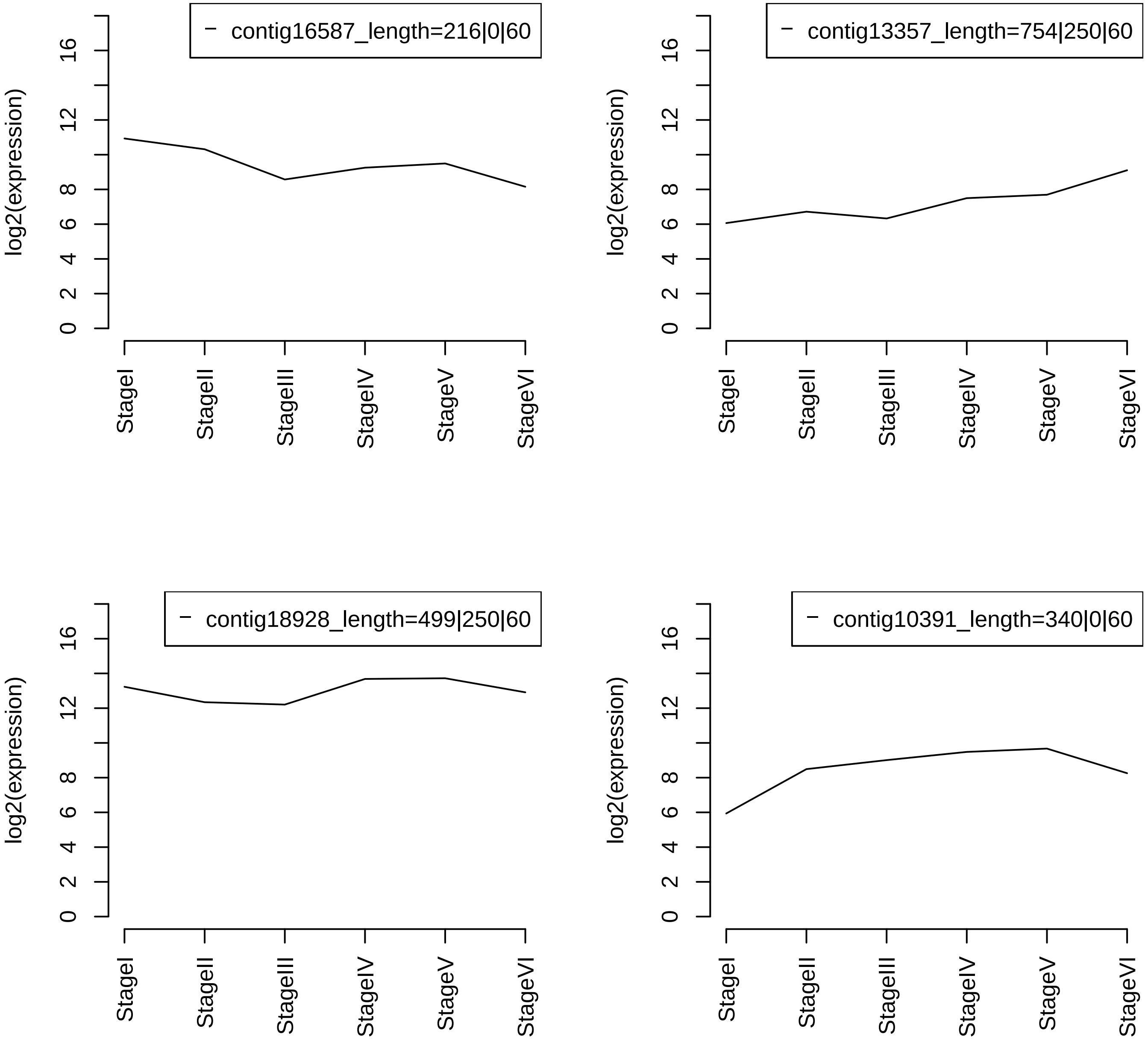
contig16587_length=216|0|60
contig13357_length=754|250|60
contig18928_length=499|250|60
contig10391_length=340|0|60
log2(expression)
0
2
4
6
8
12
16
StageI
StageII
StageIII
StageIV
StageV
StageVI

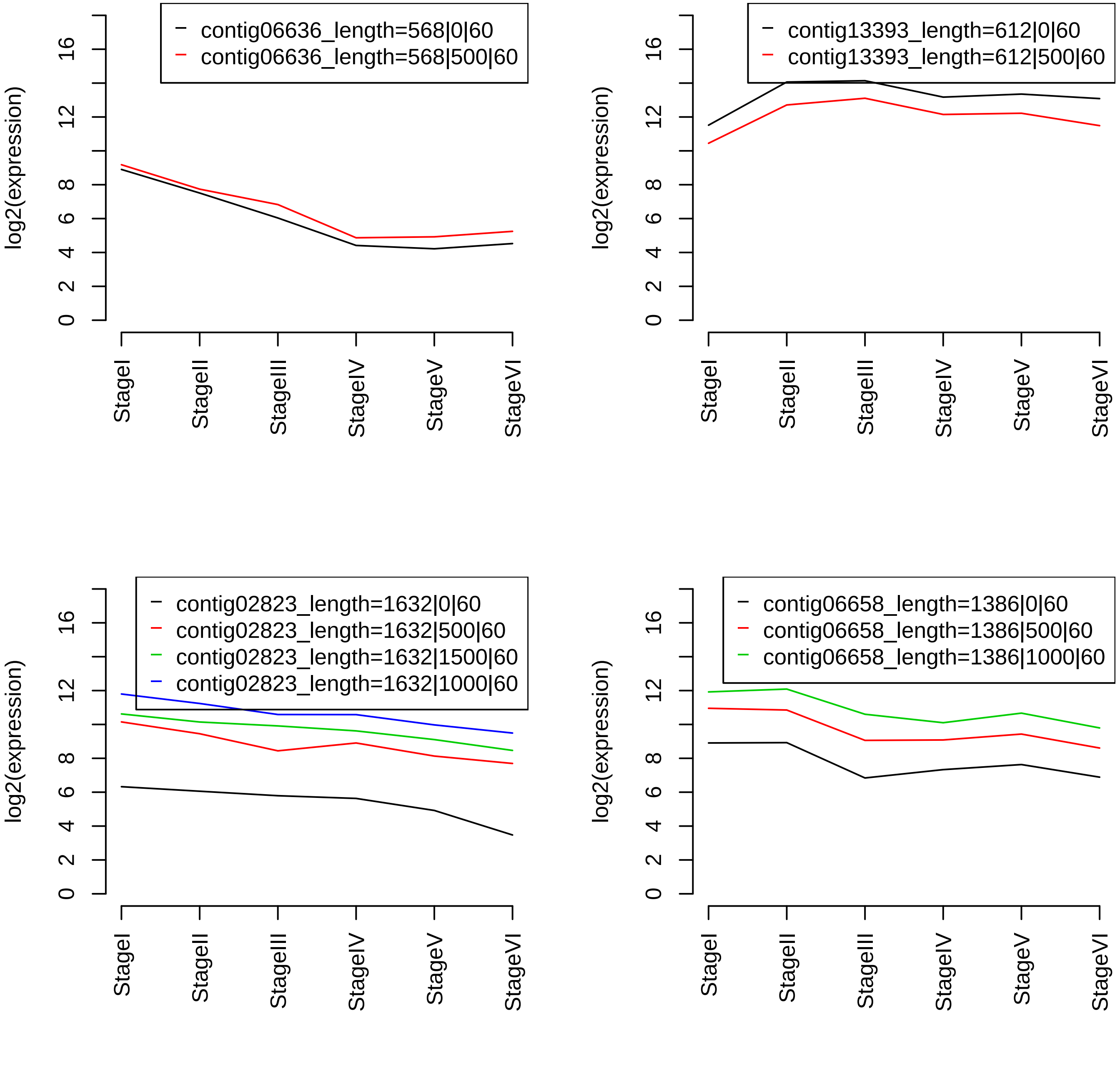
contig06636_length=568|0|60
contig06636_length=568|500|60
contig13393_length=612|0|60
contig13393_length=612|500|60
contig02823_length=1632|0|60
contig02823_length=1632|500|60
contig02823_length=1632|1500|60
contig02823_length=1632|1000|60
contig06658_length=1386|0|60
contig06658_length=1386|500|60
contig06658_length=1386|1000|60
log2(expression)
0
2
4
6
8
12
16
StageI
StageII
StageIII
StageIV
StageV
StageVI

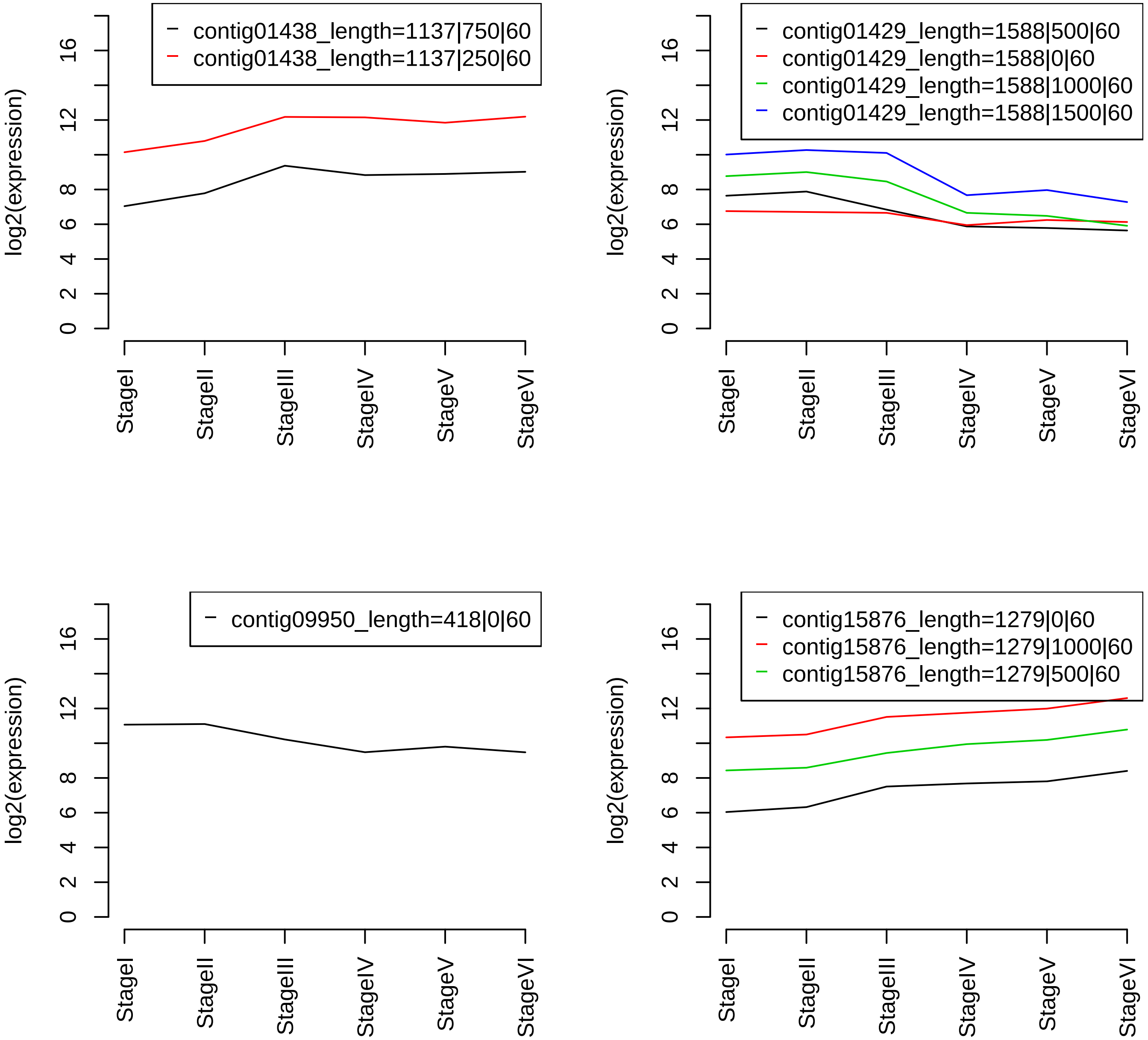

contig01438_length=1137|750|60
contig01438_length=1137|250|60
contig01429_length=1588|500|60
contig01429_length=1588|0|60
contig01429_length=1588|1000|60
contig01429_length=1588|1500|60
contig09950_length=418|0|60
contig15876_length=1279|0|60
contig15876_length=1279|1000|60
contig15876_length=1279|500|60
log2(expression)
0
2
4
6
8
12
16
StageI
StageII
StageIII
StageIV
StageV
StageVI

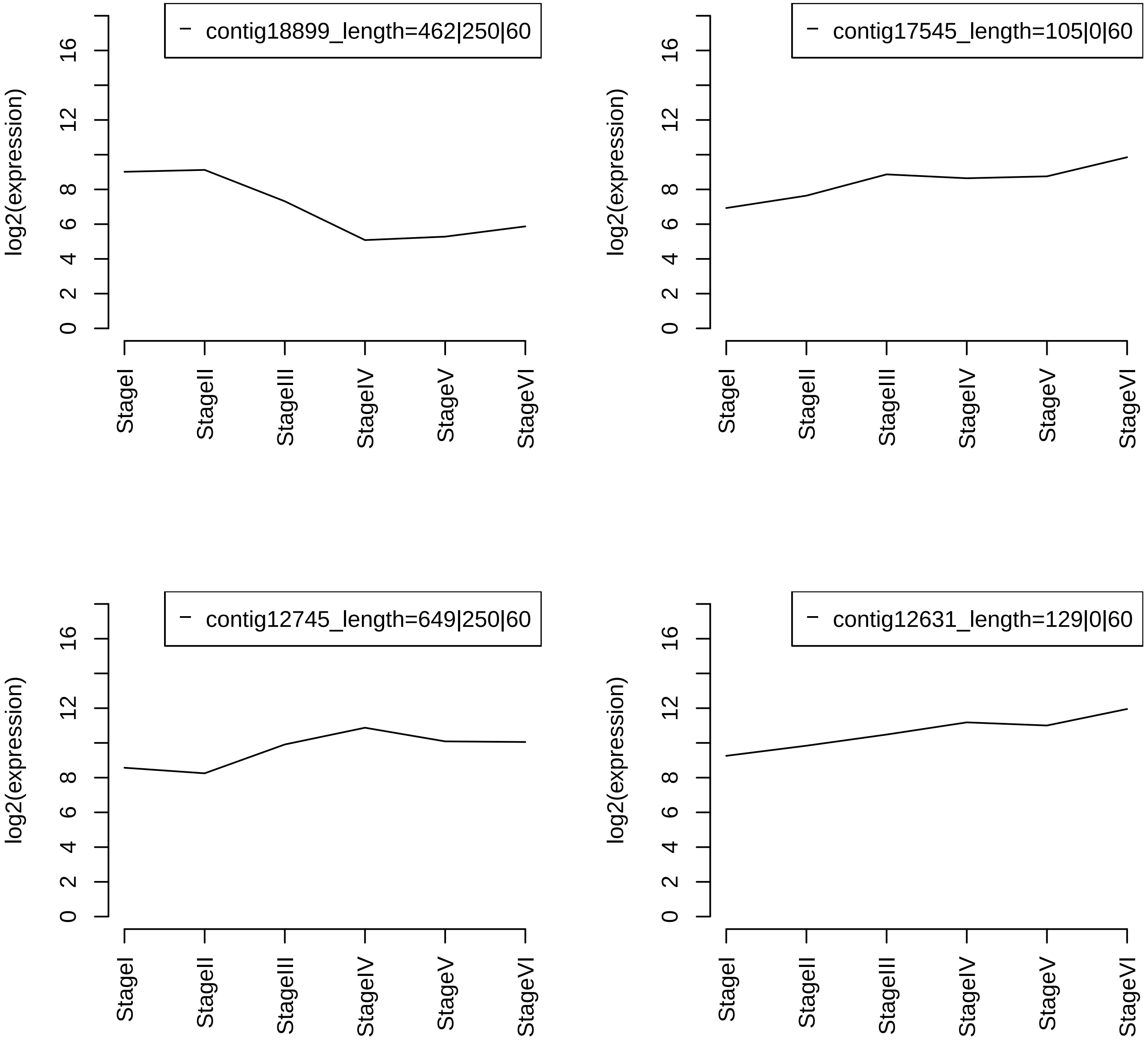
contig18899_length=462|250|60
contig17545_length=105|0|60
contig12745_length=649|250|60
contig12631_length=129|0|60
log2(expression)
0
2
4
6
8
12
16
StageI
StageII
StageIII
StageIV
StageV
StageVI

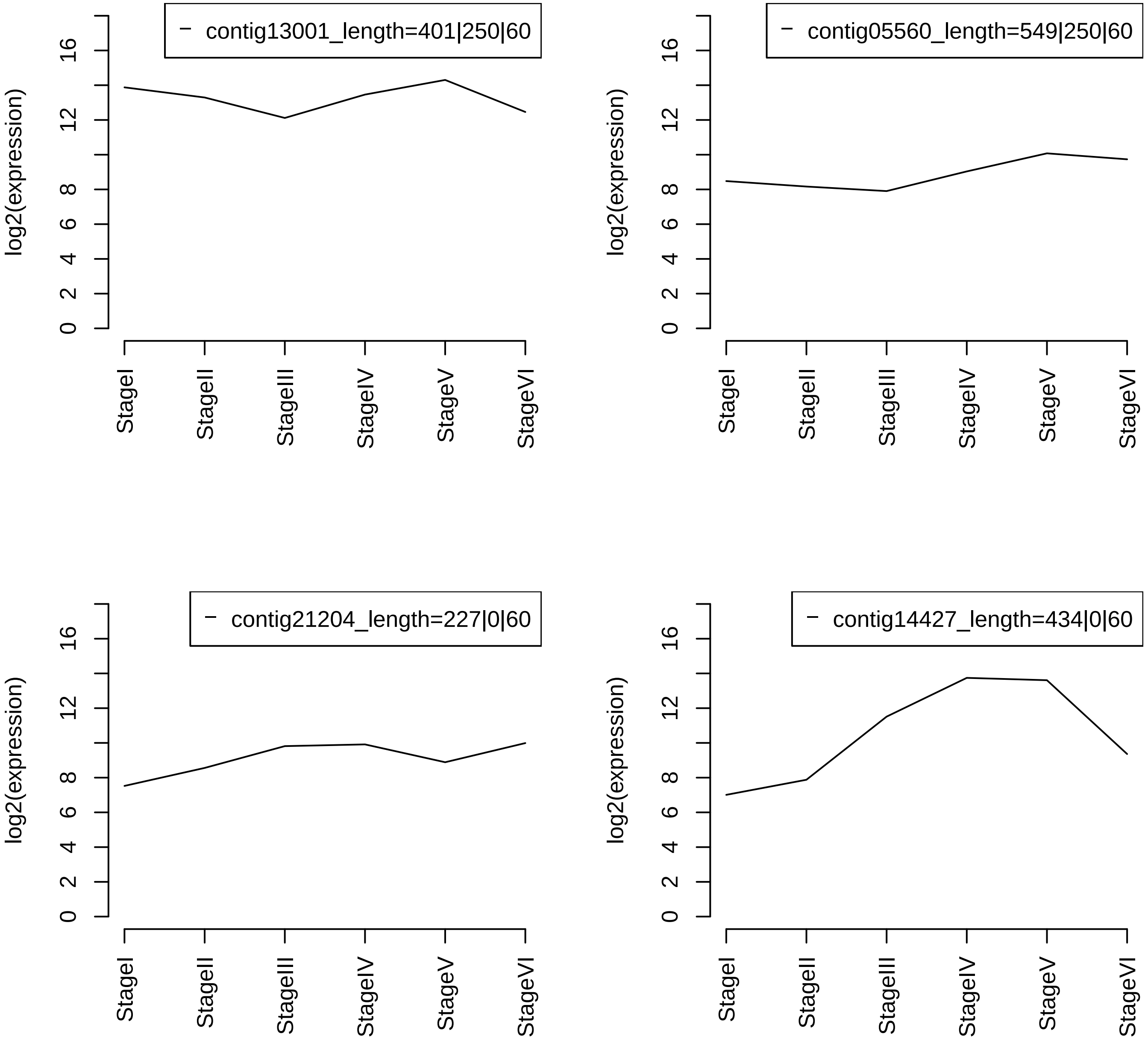
contig13001_length=401|250|60
contig05560_length=549|250|60
contig21204_length=227|0|60
contig14427_length=434|0|60
log2(expression)
0
2
4
6
8
12
16
StageI
StageII
StageIII
StageIV
StageV
StageVI

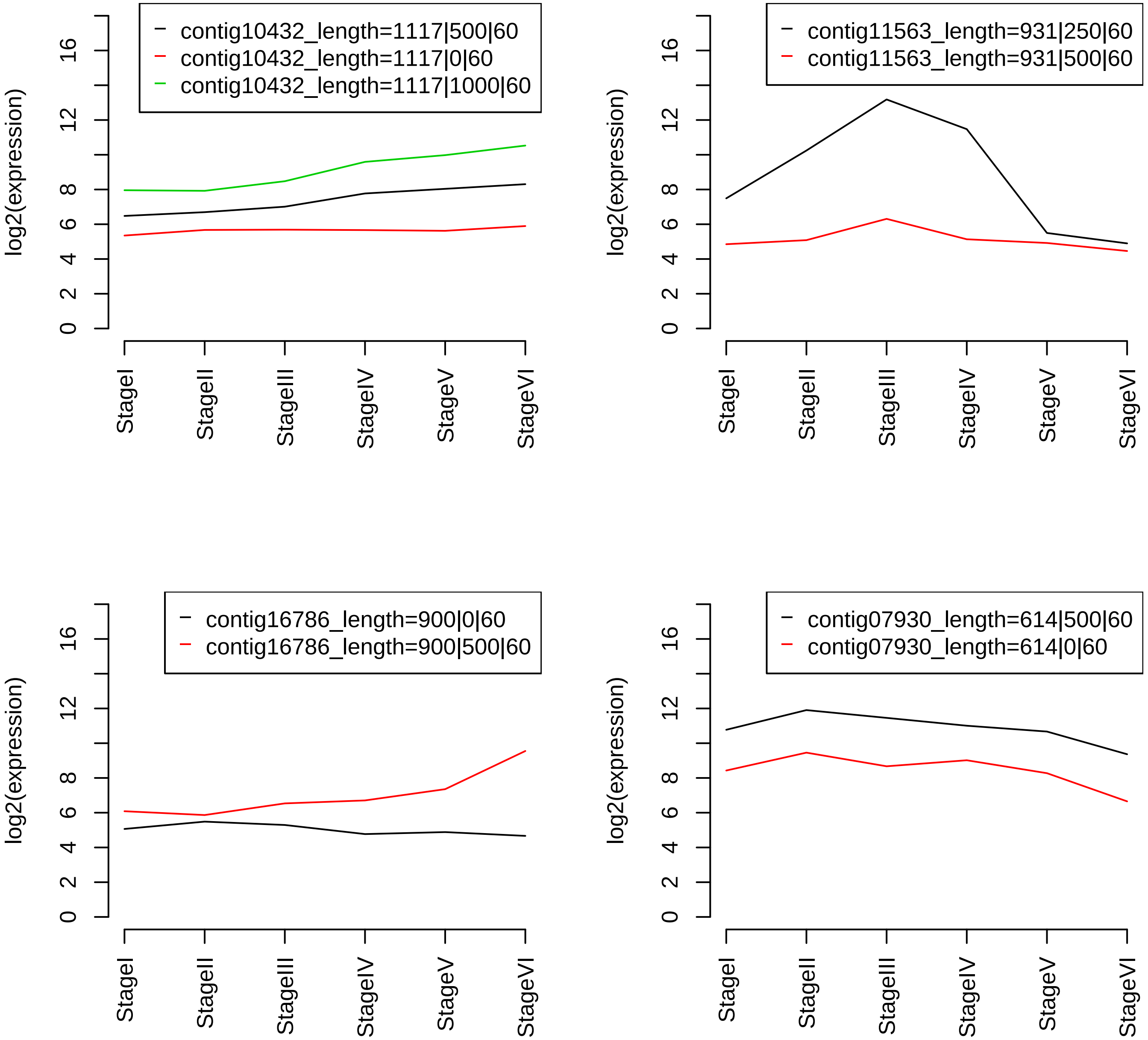

contig10432_length=1117|500|60
contig10432_length=1117|0|60
contig10432_length=1117|1000|60
contig11563_length=931|250|60
contig11563_length=931|500|60
contig16786_length=900|0|60
contig16786_length=900|500|60
contig07930_length=614|500|60
contig07930_length=614|0|60
log2(expression)
0
2
4
6
8
12
16
StageI
StageII
StageIII
StageIV
StageV
StageVI

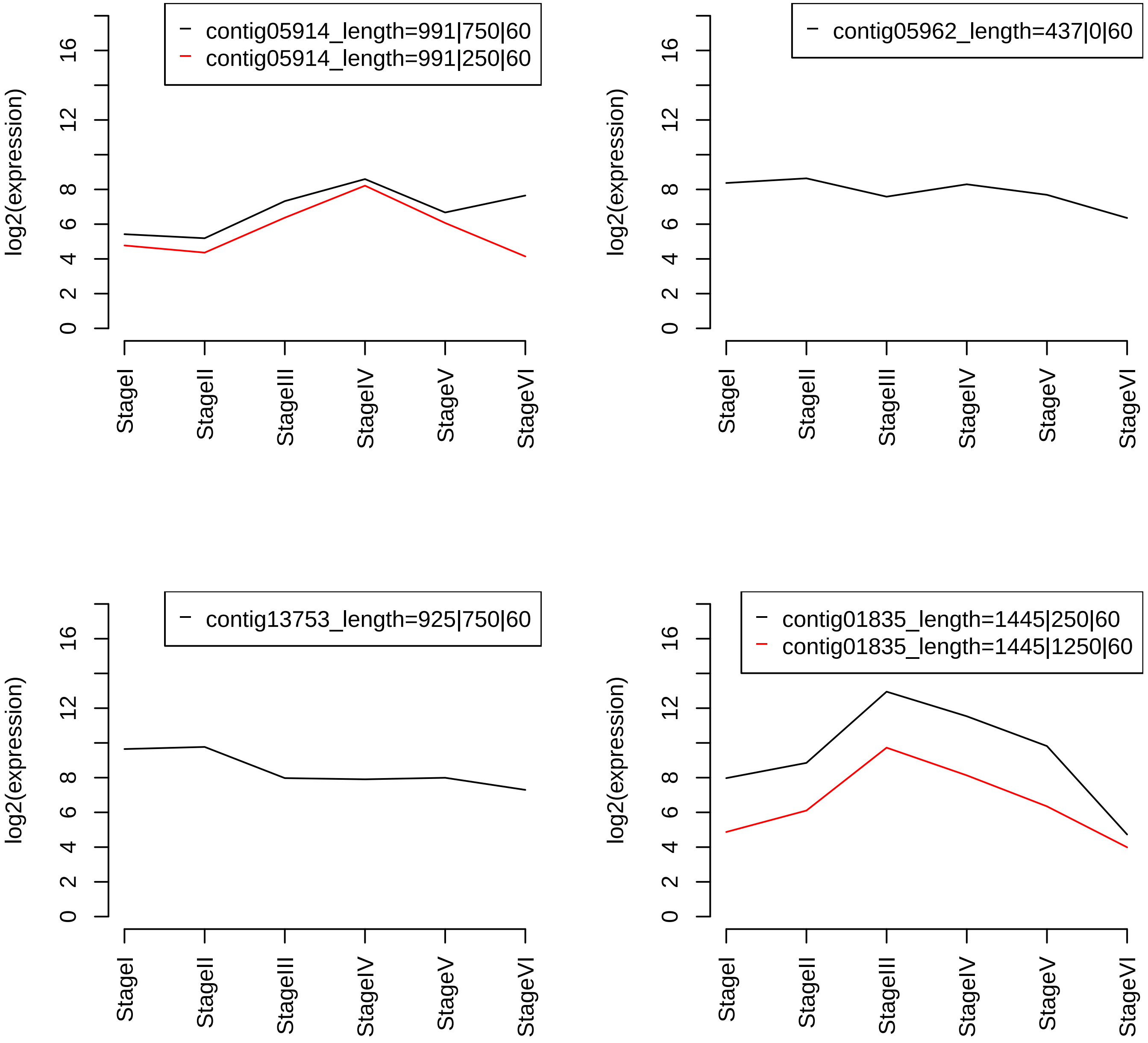
contig05914_length=991|750|60
contig05914_length=991|250|60
contig05962_length=437|0|60
contig13753_length=925|750|60
contig01835_length=1445|250|60
contig01835_length=1445|1250|60
log2(expression)
0
2
4
6
8
12
16
StageI
StageII
StageIII
StageIV
StageV
StageVI

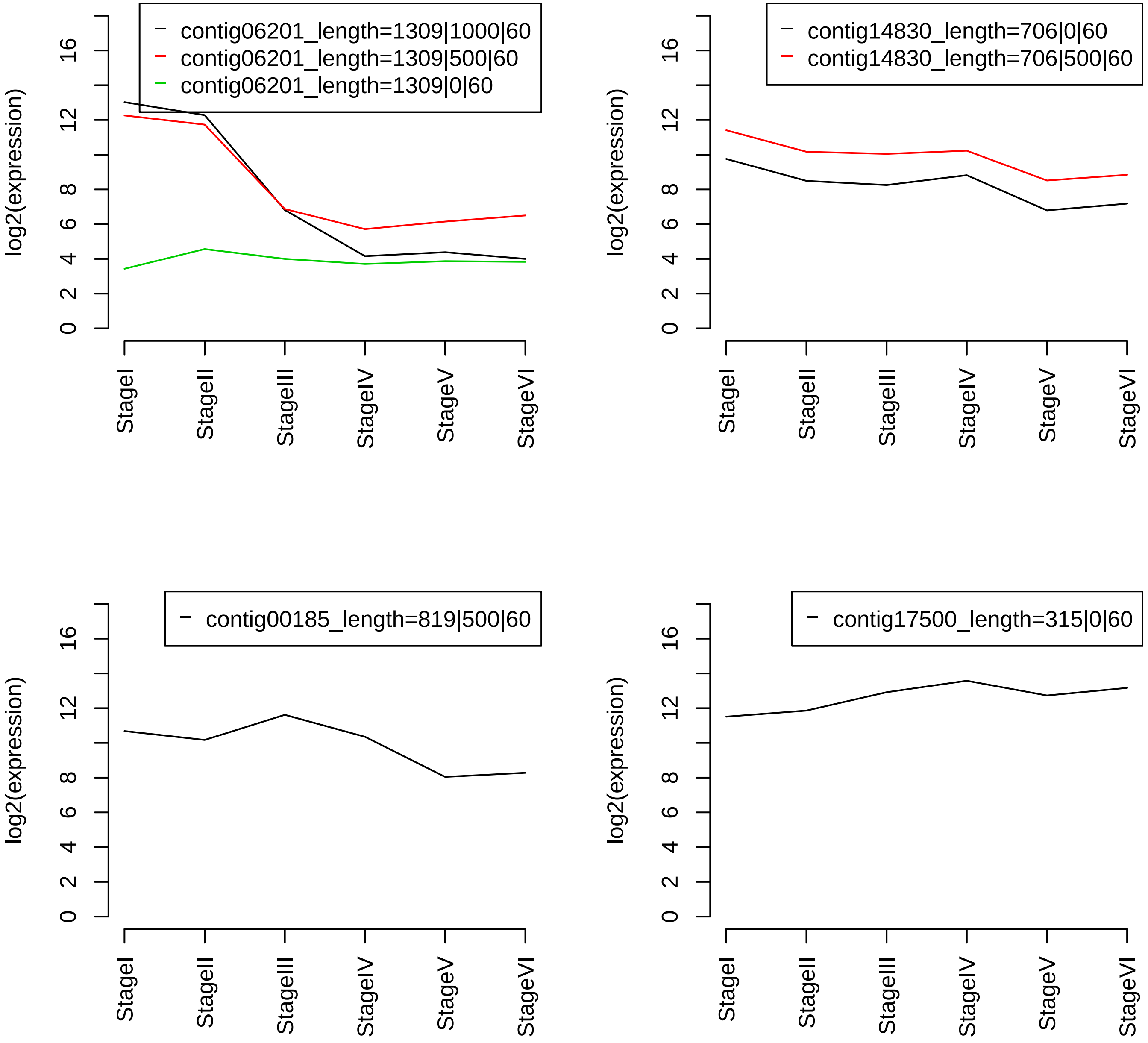
contig06201_length=1309|1000|60
contig06201_length=1309|500|60
contig06201_length=1309|0|60
contig14830_length=706|0|60
contig14830_length=706|500|60
contig00185_length=819|500|60
contig17500_length=315|0|60
log2(expression)
0
2
4
6
8
12
16
StageI
StageII
StageIII
StageIV
StageV
StageVI

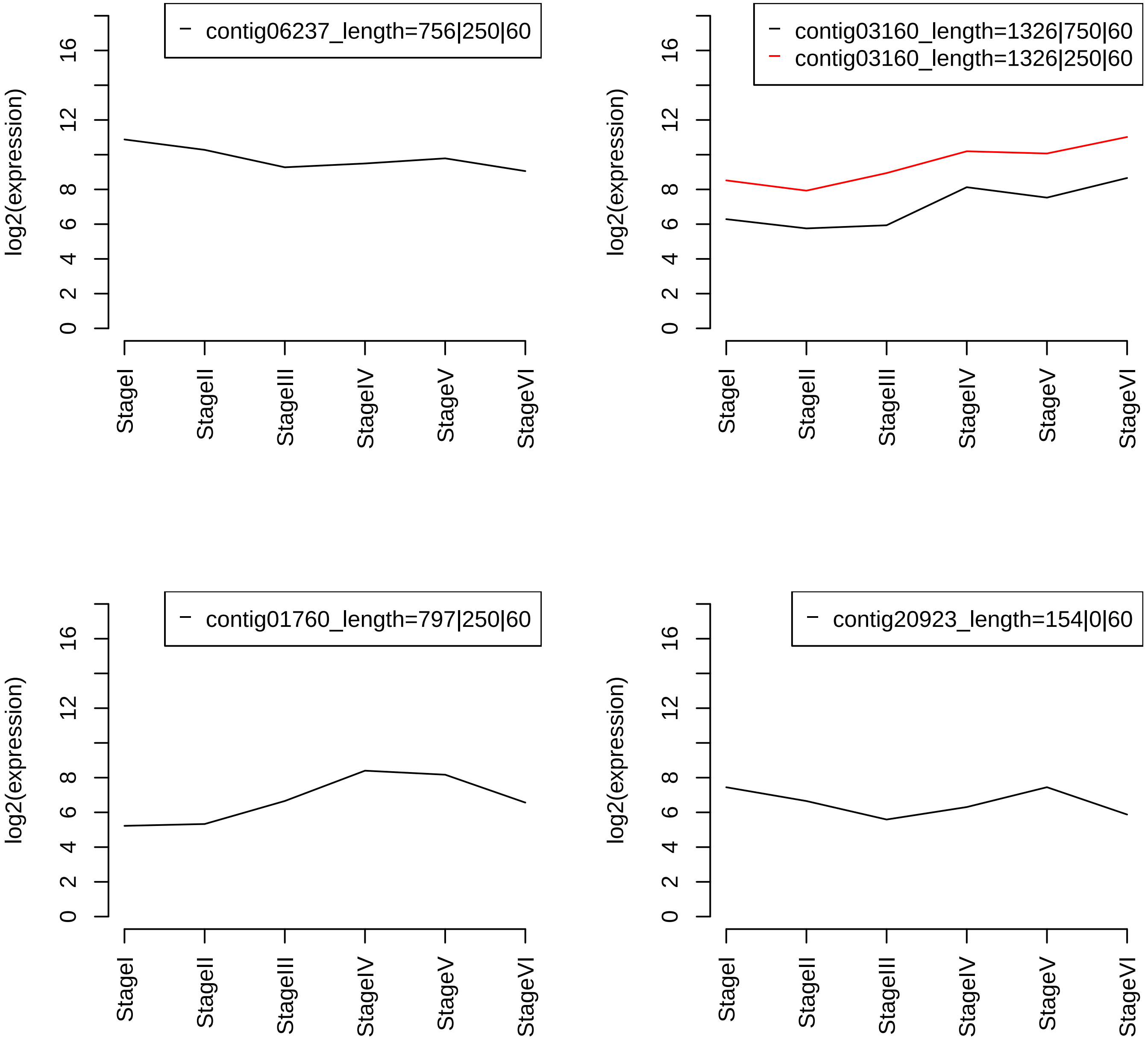

contig06237_length=756|250|60
contig03160_length=1326|750|60
contig03160_length=1326|250|60
contig01760_length=797|250|60
contig20923_length=154|0|60
log2(expression)
0
2
4
6
8
12
16
StageI
StageII
StageIII
StageIV
StageV
StageVI

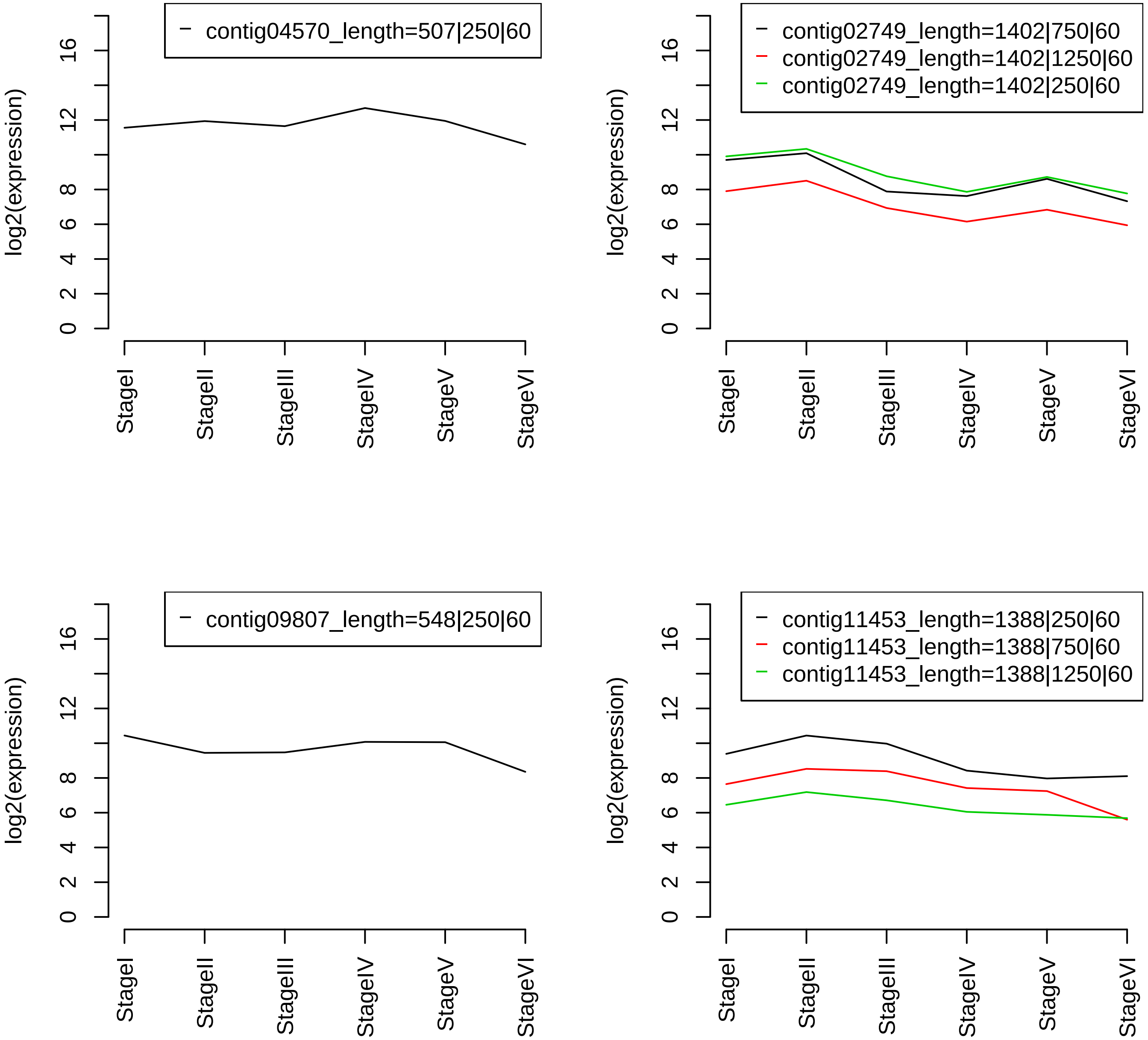

contig04570_length=507|250|60
contig02749_length=1402|750|60
contig02749_length=1402|1250|60
contig02749_length=1402|250|60
contig09807_length=548|250|60
contig11453_length=1388|250|60
contig11453_length=1388|750|60
contig11453_length=1388|1250|60
log2(expression)
0
2
4
6
8
12
16
StageI
StageII
StageIII
StageIV
StageV
StageVI

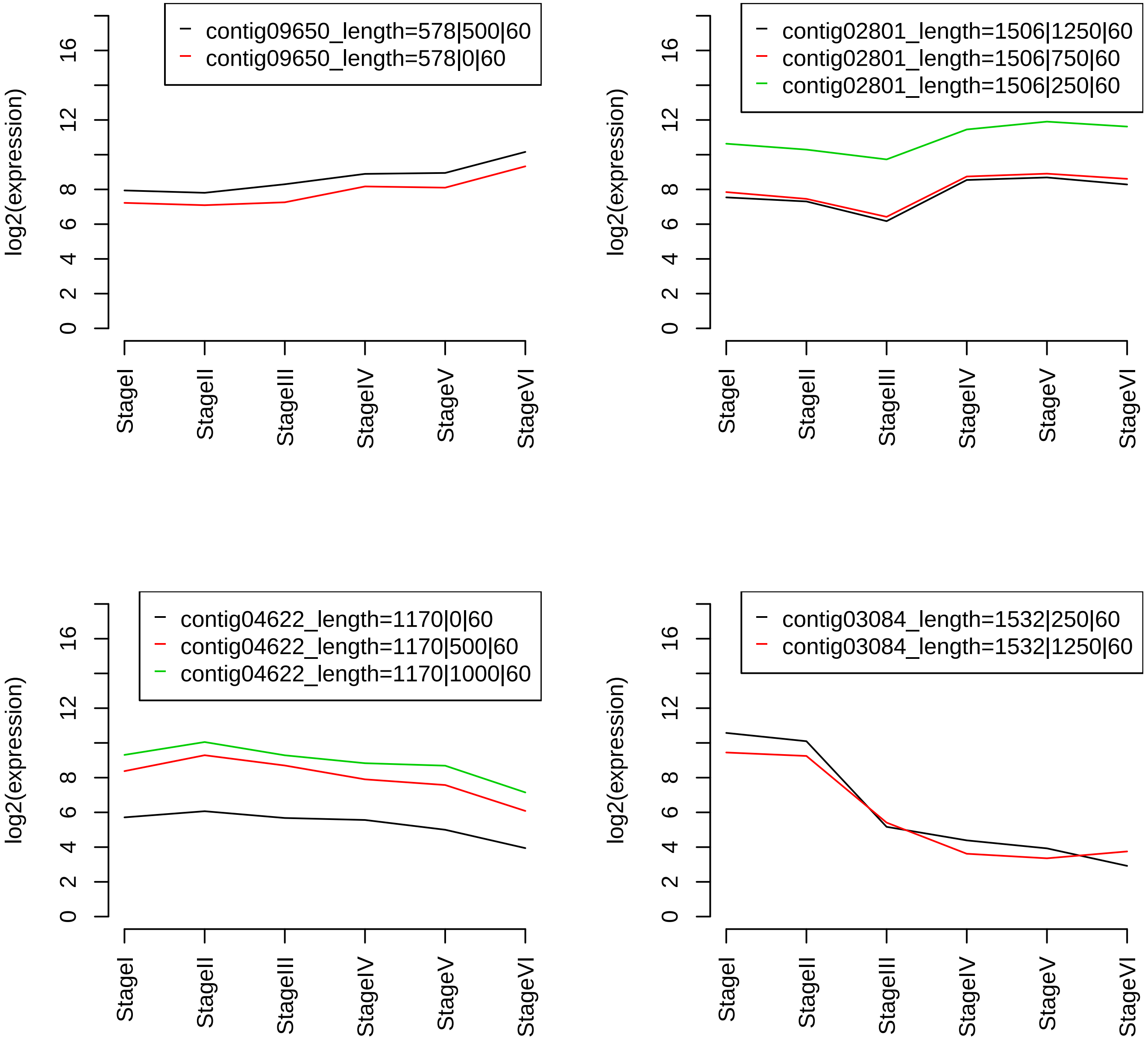
contig09650_length=578|500|60
contig09650_length=578|0|60
contig02801_length=1506|1250|60
contig02801_length=1506|750|60
contig02801_length=1506|250|60
contig04622_length=1170|0|60
contig04622_length=1170|500|60
contig04622_length=1170|1000|60
contig03084_length=1532|250|60
contig03084_length=1532|1250|60
log2(expression)
0 2 4 6 8 12 16
StageI StageII StageIII StageIV StageV StageVI

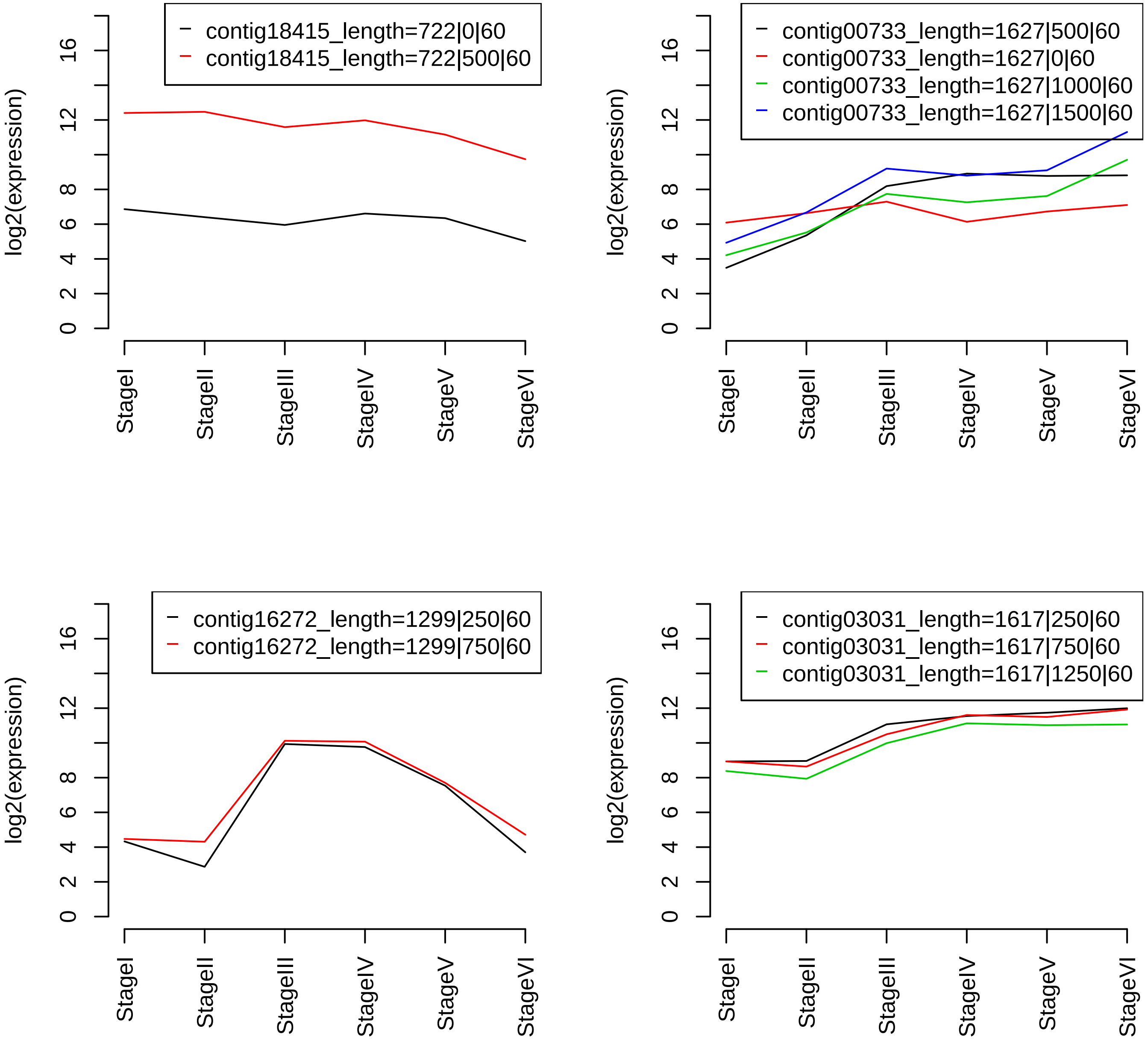
contig18415_length=722|0|60
contig18415_length=722|500|60
contig00733_length=1627|500|60
contig00733_length=1627|0|60
contig00733_length=1627|1000|60
contig00733_length=1627|1500|60
contig16272_length=1299|250|60
contig16272_length=1299|750|60
contig03031_length=1617|250|60
contig03031_length=1617|750|60
contig03031_length=1617|1250|60
log2(expression)
0
2
4
6
8
12
16
StageI
StageII
StageIII
StageIV
StageV
StageVI

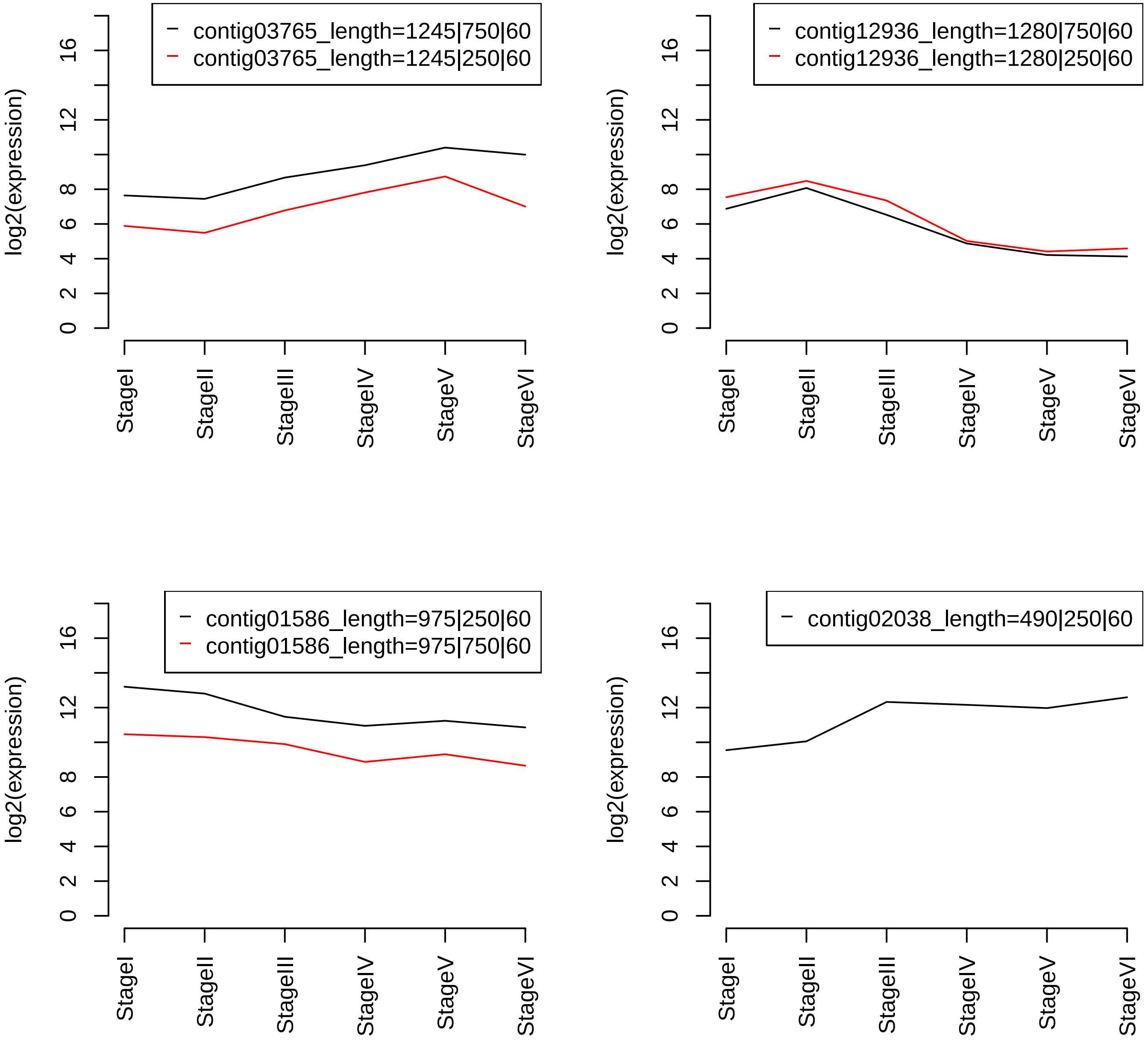
contig03765_length=1245|750|60
contig03765_length=1245|250|60
contig12936_length=1280|750|60
contig12936_length=1280|250|60
contig01586_length=975|250|60
contig01586_length=975|750|60
contig02038_length=490|250|60
log2(expression)
0
2
4
6
8
12
16
StageI
StageII
StageIII
StageIV
StageV
StageVI

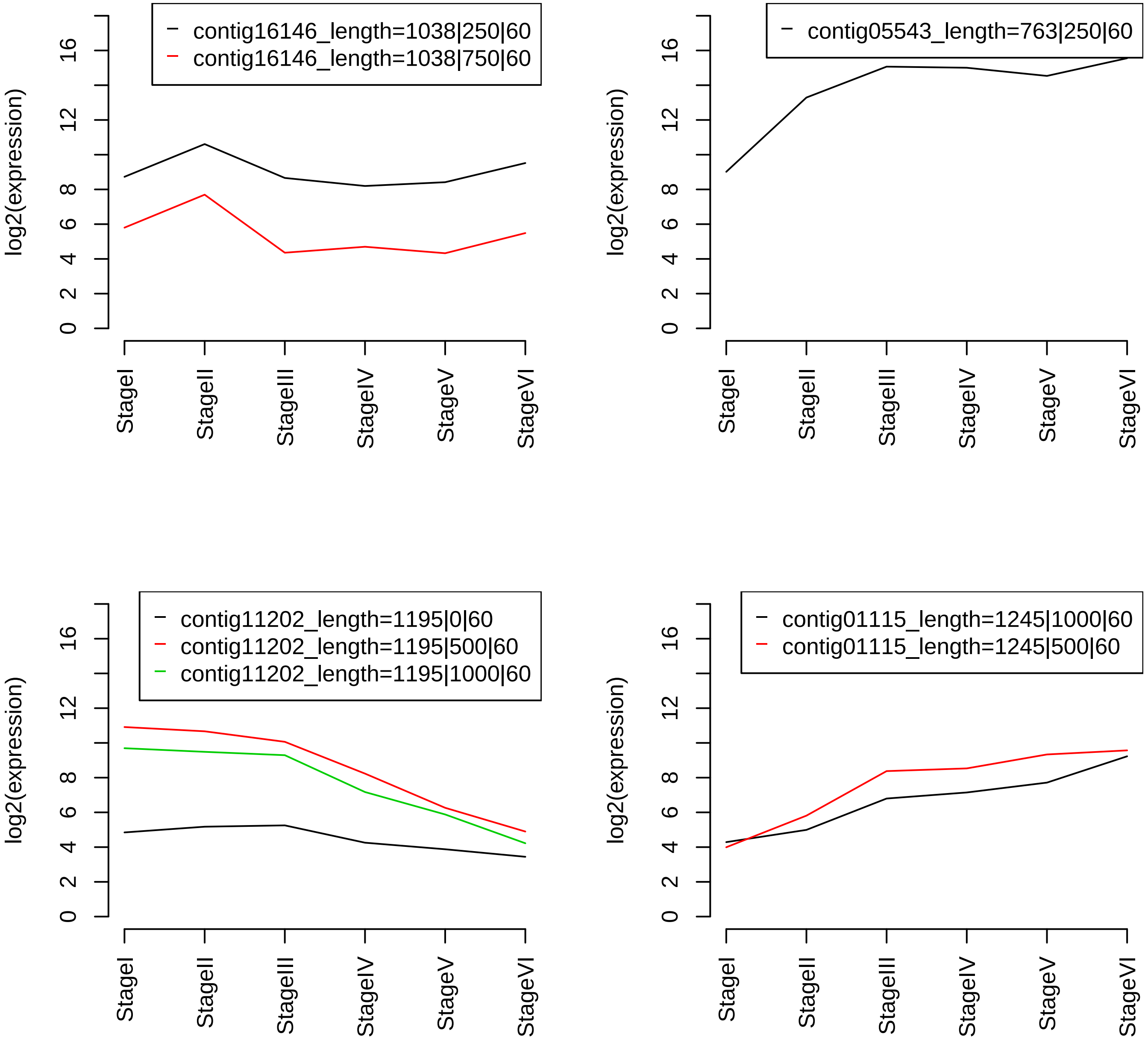
contig16146_length=1038|250|60
contig16146_length=1038|750|60
contig05543_length=763|250|60
contig11202_length=1195|0|60
contig11202_length=1195|500|60
contig11202_length=1195|1000|60
contig01115_length=1245|1000|60
contig01115_length=1245|500|60
log2(expression)
0
2
4
6
8
12
16
StageI
StageII
StageIII
StageIV
StageV
StageVI

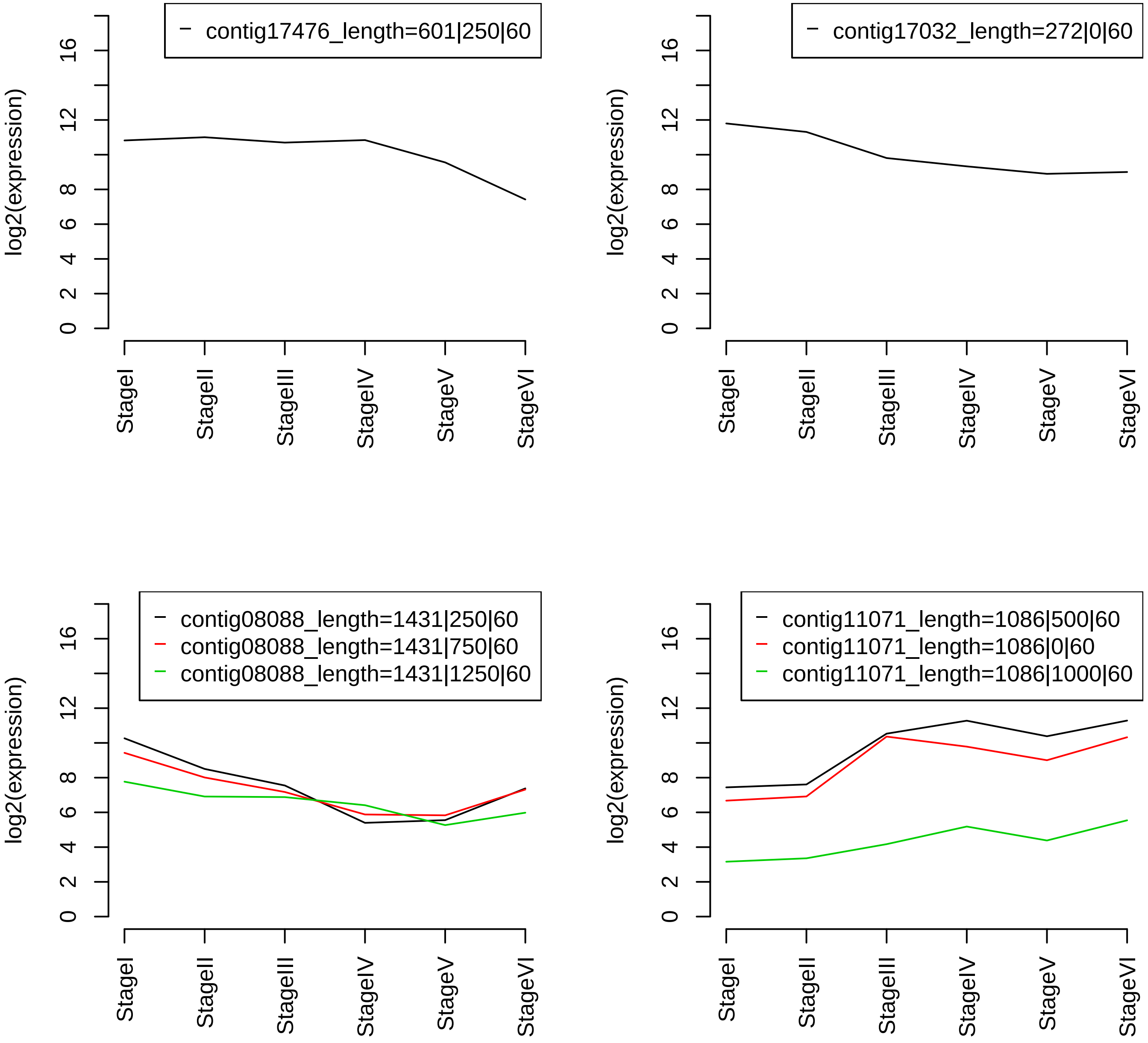
contig17476_length=601|250|60
contig17032_length=272|0|60
contig08088_length=1431|250|60
contig08088_length=1431|750|60
contig08088_length=1431|1250|60
contig11071_length=1086|500|60
contig11071_length=1086|0|60
contig11071_length=1086|1000|60
log2(expression)
0
2
4
6
8
12
16
StageI
StageII
StageIII
StageIV
StageV
StageVI

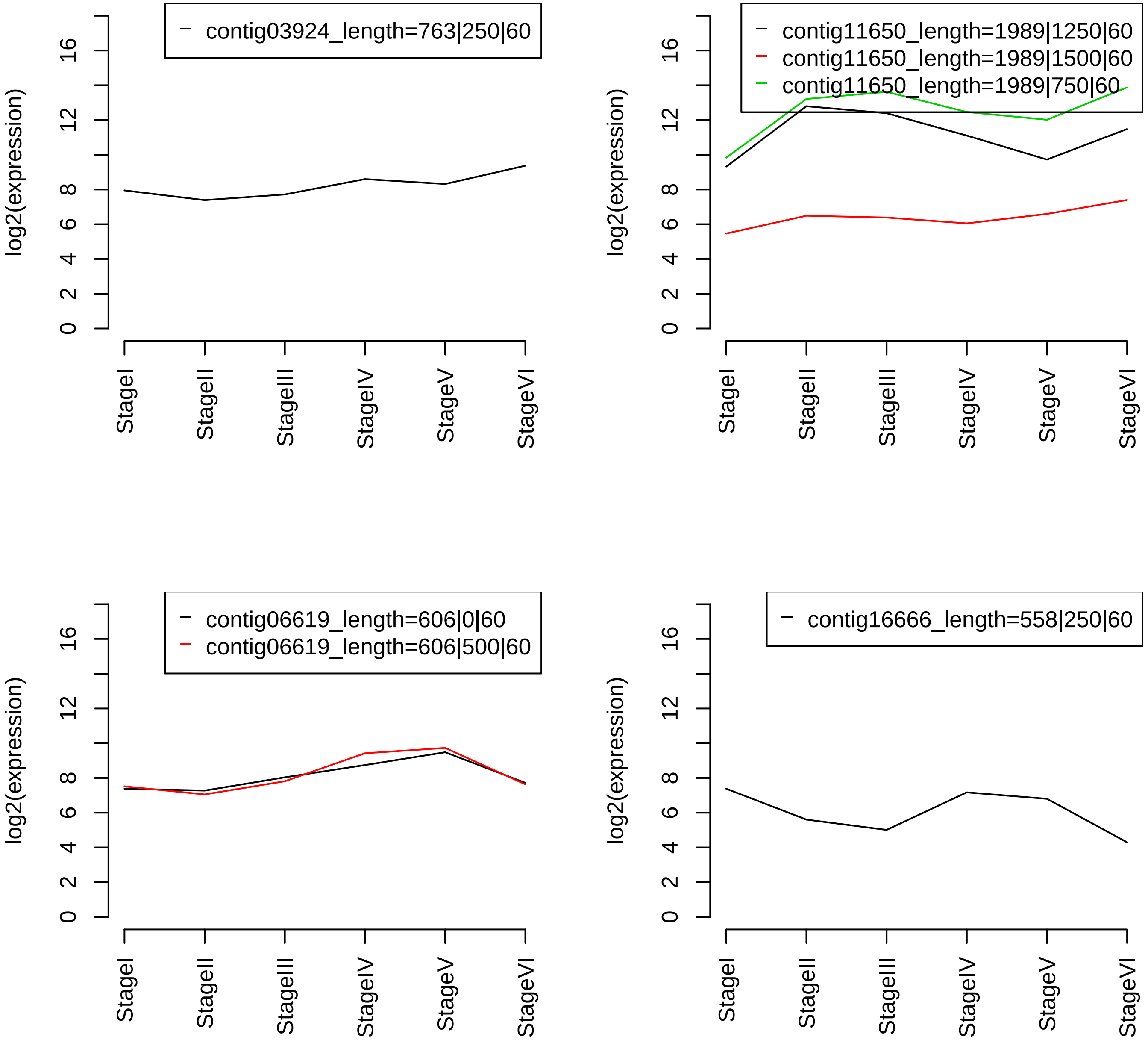
contig03924_length=763|250|60
contig11650_length=1989|1250|60
contig11650_length=1989|1500|60
contig11650_length=1989|750|60
contig06619_length=606|0|60
contig06619_length=606|500|60
contig16666_length=558|250|60
log2(expression)
0 2 4 6 8 12 16
StageI StageII StageIII StageIV StageV StageVI

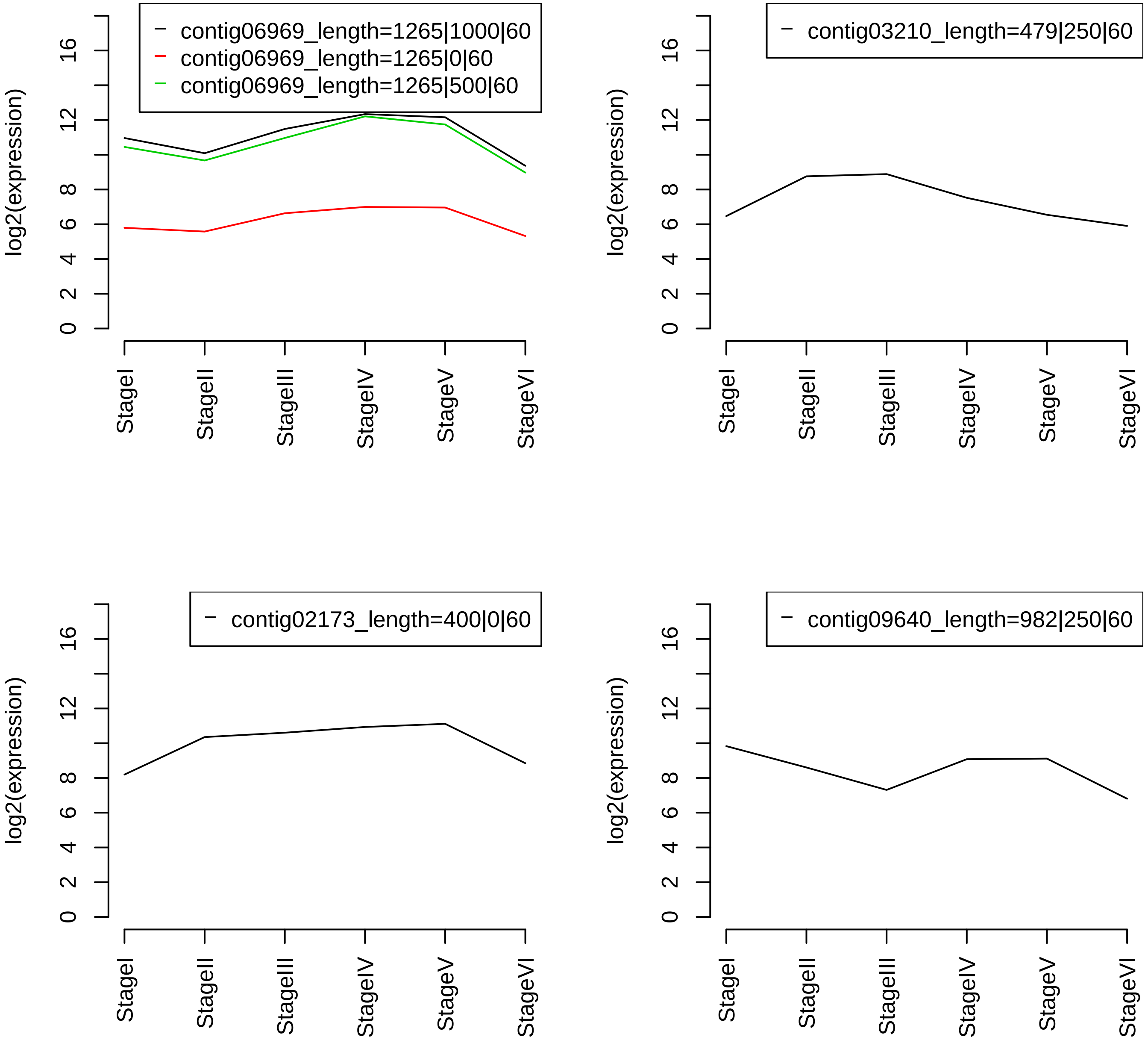

contig06969_length=1265|1000|60
contig06969_length=1265|0|60
contig06969_length=1265|500|60
contig03210_length=479|250|60
contig02173_length=400|0|60
contig09640_length=982|250|60
log2(expression)
0
2
4
6
8
12
16
StageI
StageII
StageIII
StageIV
StageV
StageVI

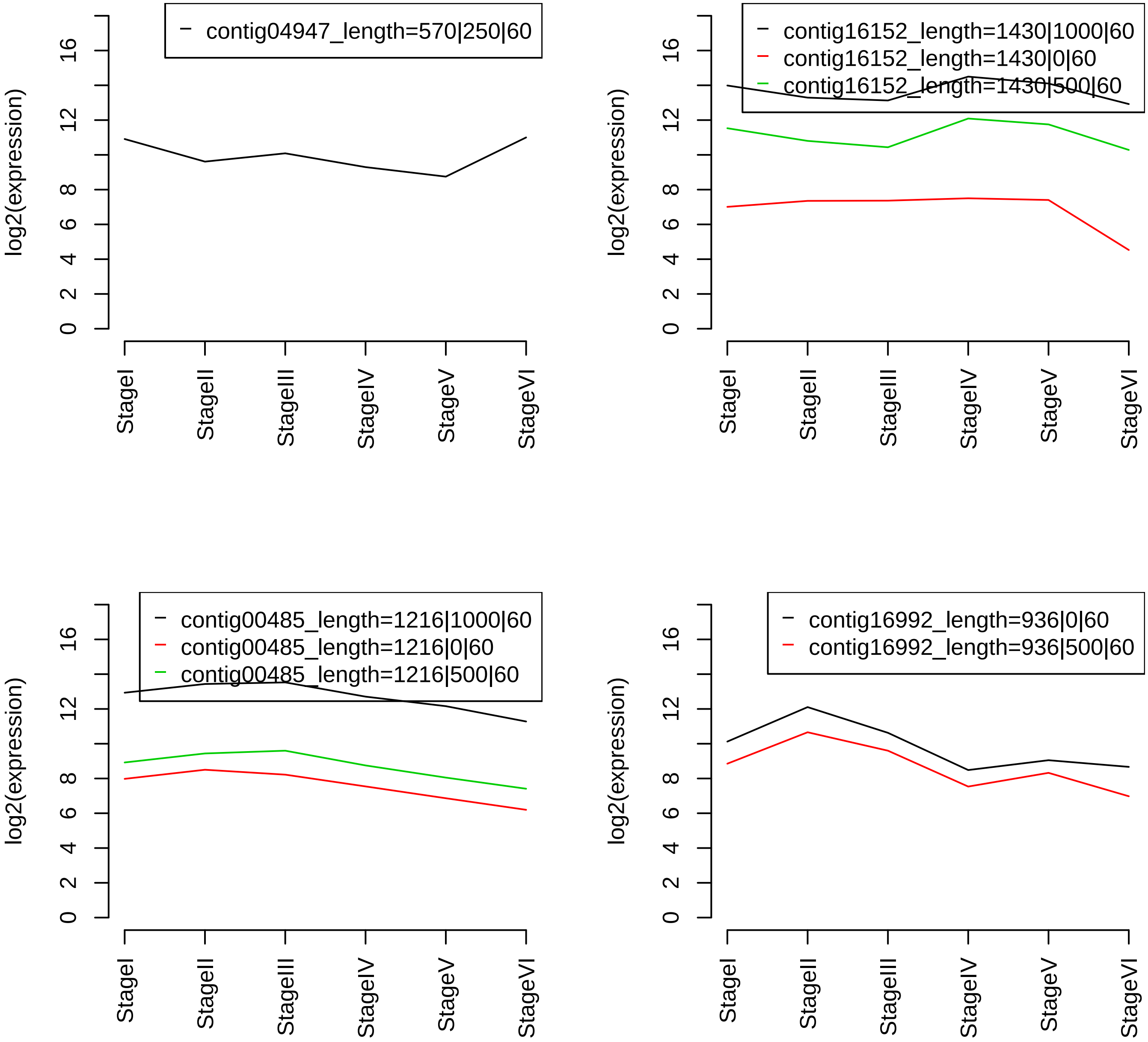
contig04947_length=570|250|60
contig16152_length=1430|1000|60
contig16152_length=1430|0|60
contig16152_length=1430|500|60
contig00485_length=1216|1000|60
contig00485_length=1216|0|60
contig00485_length=1216|500|60
contig16992_length=936|0|60
contig16992_length=936|500|60
log2(expression)
0 2 4 6 8 12 16
StageI StageII StageIII StageIV StageV StageVI

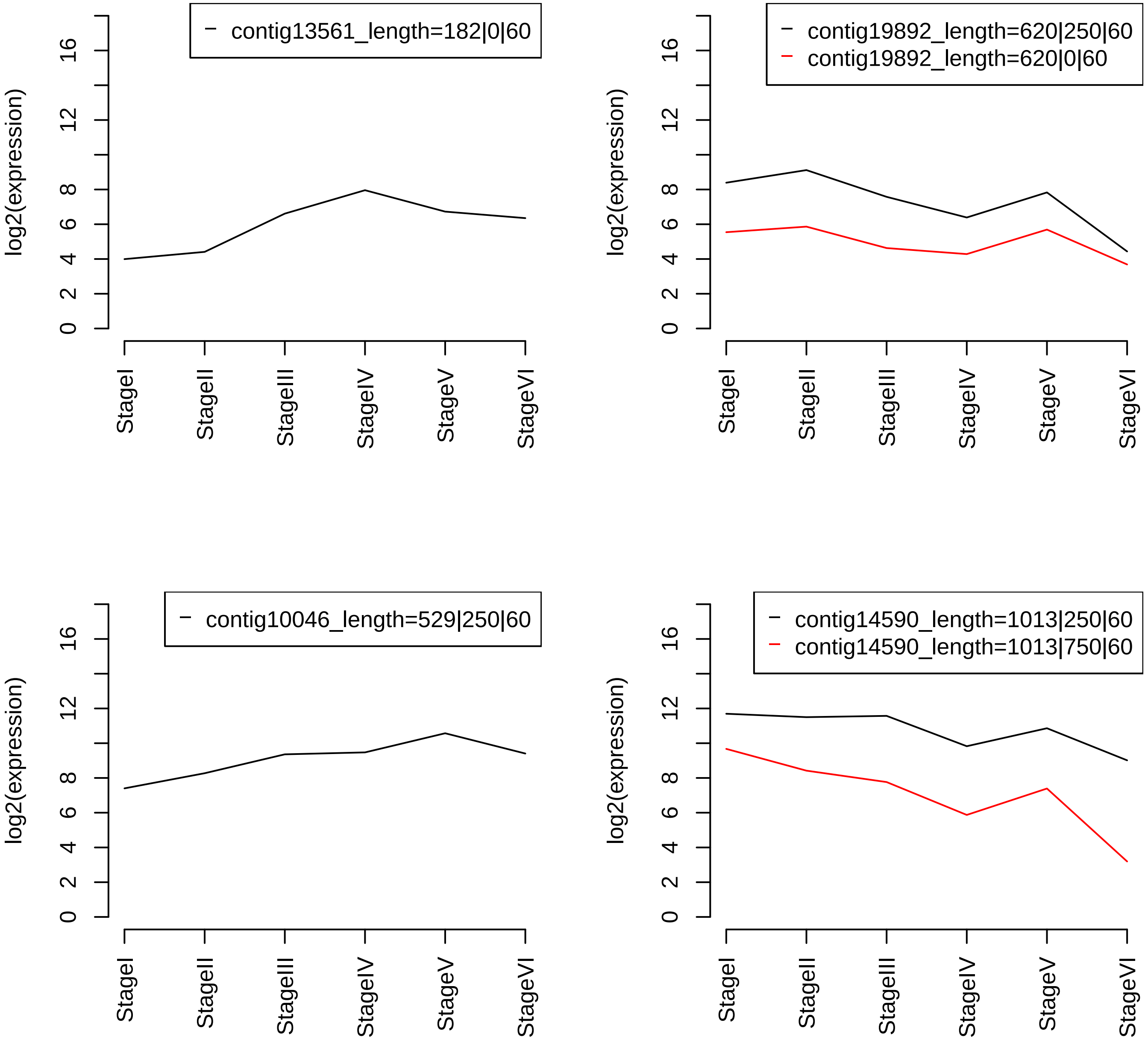
contig13561_length=182|0|60
contig19892_length=620|250|60
contig19892_length=620|0|60
contig10046_length=529|250|60
contig14590_length=1013|250|60
contig14590_length=1013|750|60
log2(expression)
StageI
StageII
StageIII
StageIV
StageV
StageVI

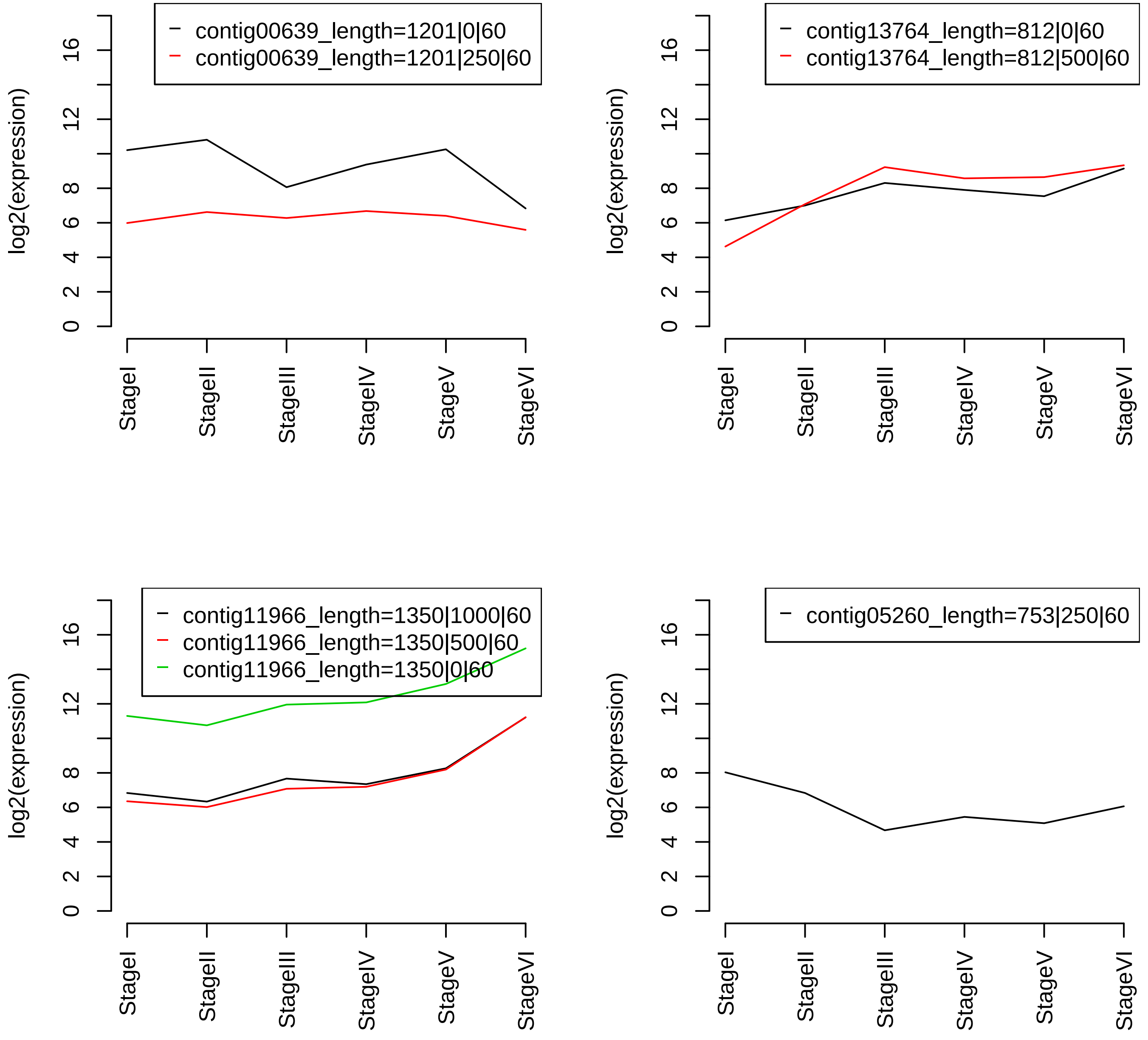

contig00639_length=1201|0|60
contig00639_length=1201|250|60
contig13764_length=812|0|60
contig13764_length=812|500|60
contig11966_length=1350|1000|60
contig11966_length=1350|500|60
contig11966_length=1350|0|60
contig05260_length=753|250|60
log2(expression)
0 2 4 6 8 12 16
StageI
StageII
StageIII
StageIV
StageV
StageVI

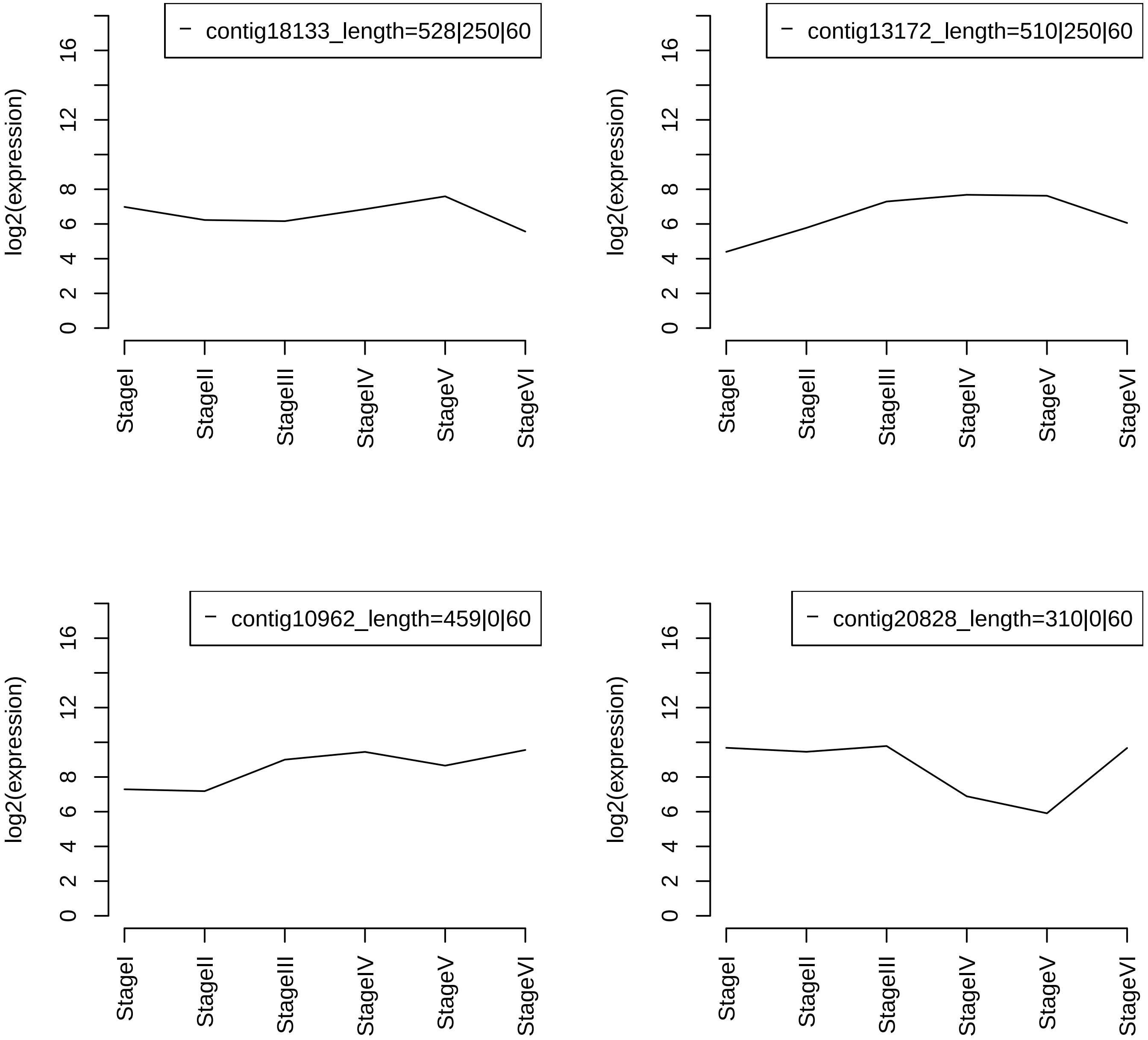
contig18133_length=528|250|60
contig13172_length=510|250|60
contig10962_length=459|0|60
contig20828_length=310|0|60
log2(expression)
0
2
4
6
8
12
16
StageI
StageII
StageIII
StageIV
StageV
StageVI

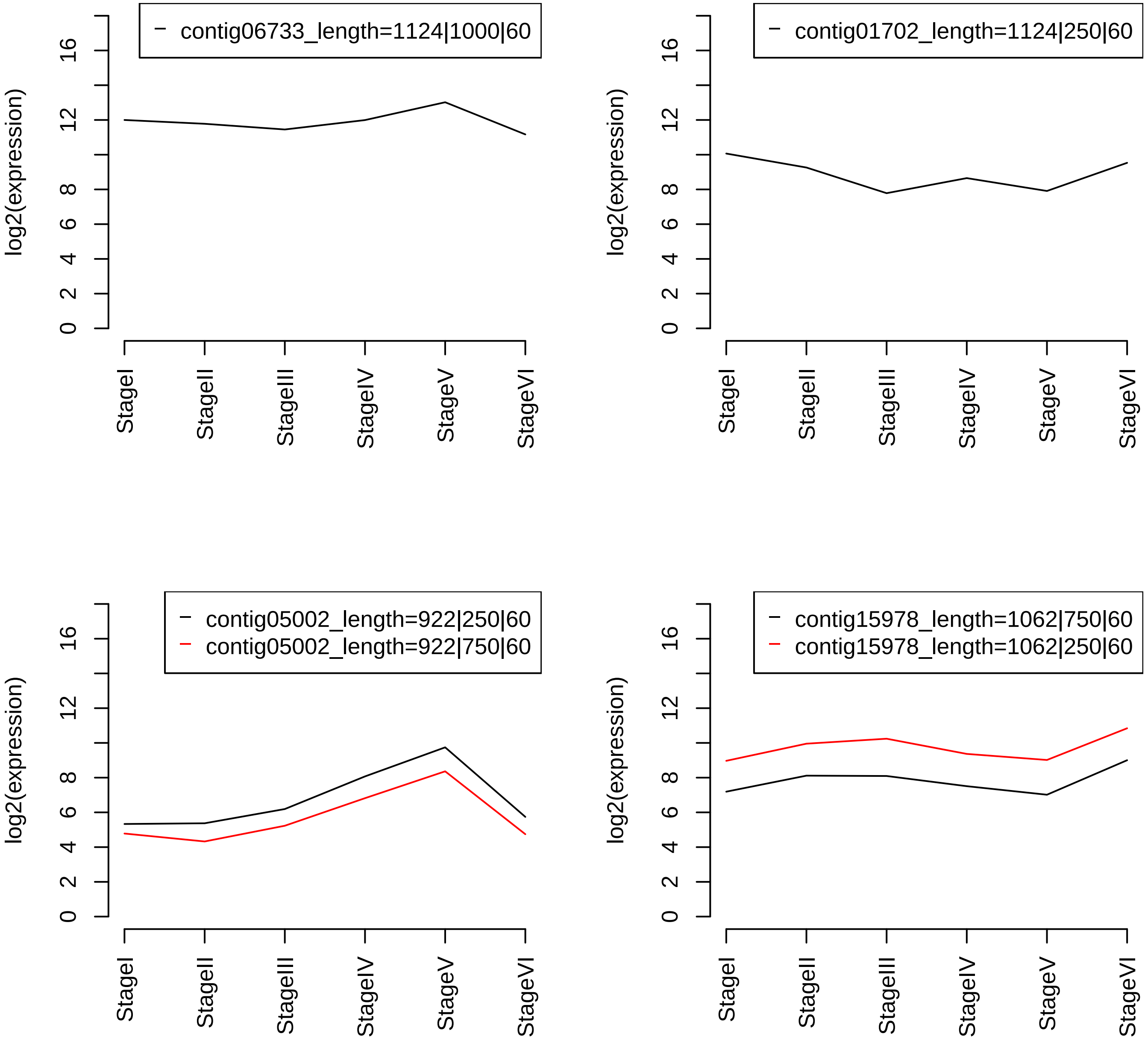

contig06733_length=1124|1000|60
contig01702_length=1124|250|60
contig05002_length=922|250|60
contig05002_length=922|750|60
contig15978_length=1062|750|60
contig15978_length=1062|250|60
log2(expression)
0
2
4
6
8
12
16
StageI
StageII
StageIII
StageIV
StageV
StageVI

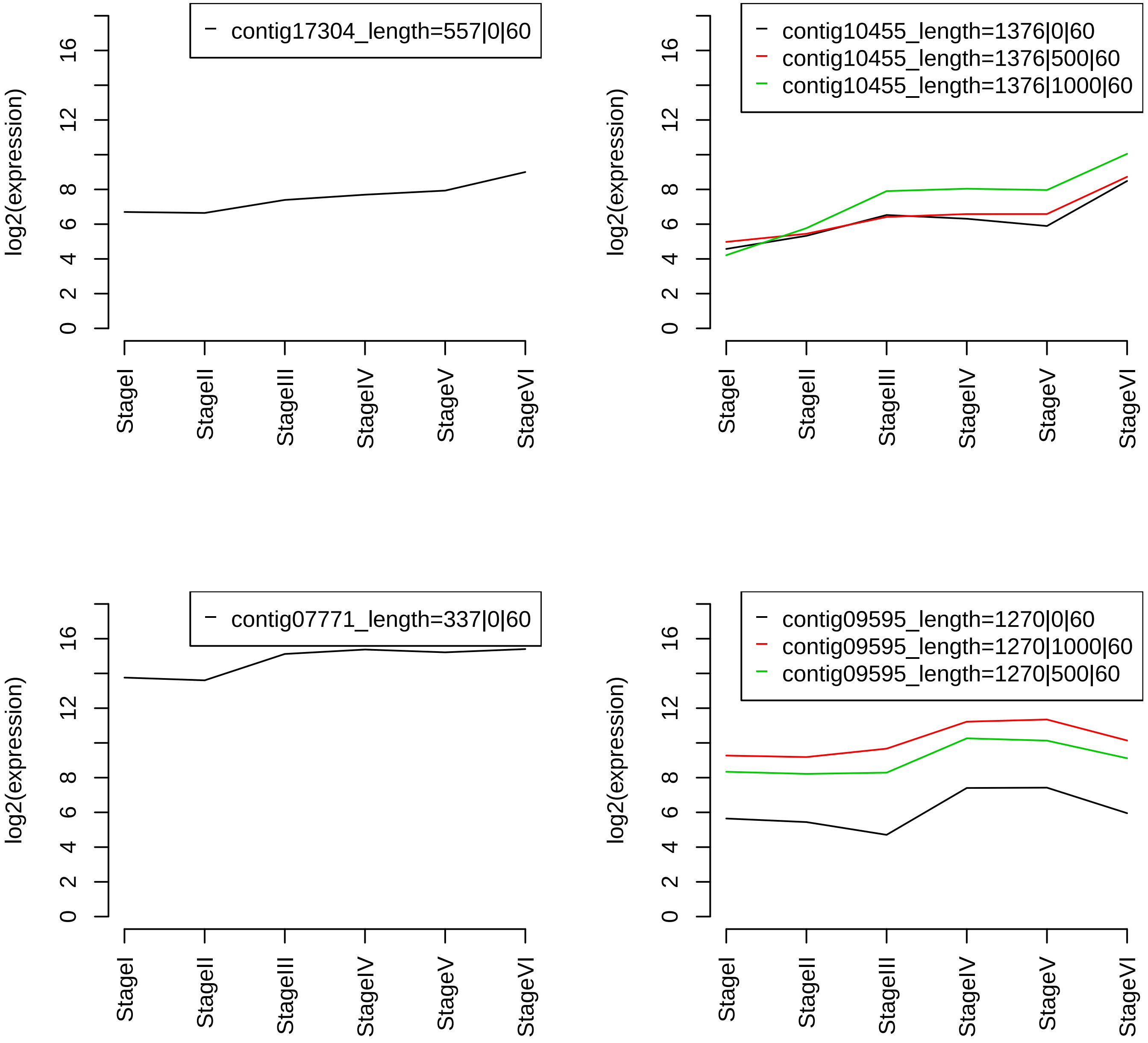

contig17304_length=557|0|60
contig10455_length=1376|0|60
contig10455_length=1376|500|60
contig10455_length=1376|1000|60
contig07771_length=337|0|60
contig09595_length=1270|0|60
contig09595_length=1270|1000|60
contig09595_length=1270|500|60
log2(expression)
0
2
4
6
8
12
16
StageI
StageII
StageIII
StageIV
StageV
StageVI

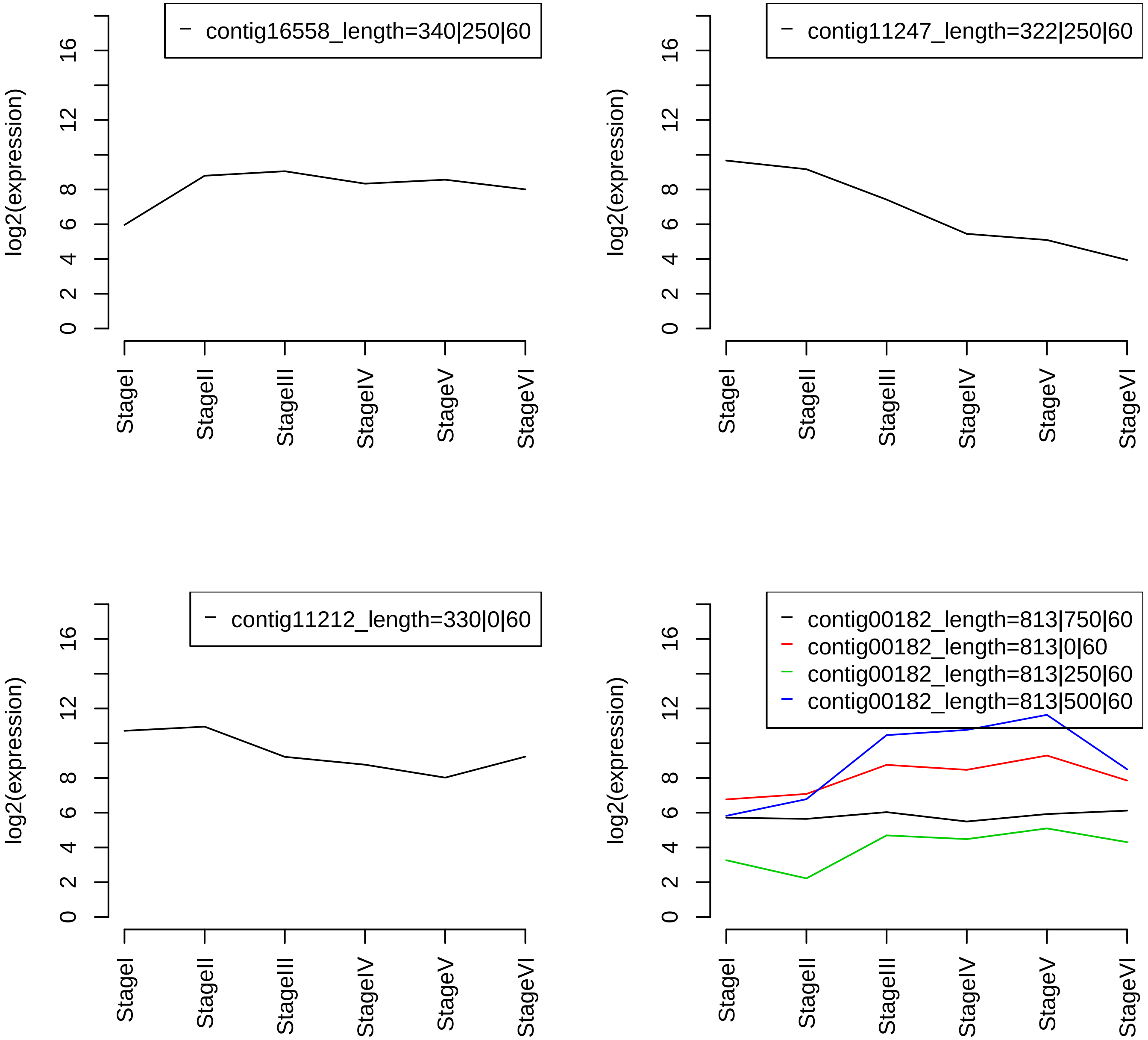

contig16558_length=340|250|60
contig11247_length=322|250|60
contig11212_length=330|0|60
contig00182_length=813|750|60
contig00182_length=813|0|60
contig00182_length=813|250|60
contig00182_length=813|500|60
log2(expression)
0
2
4
6
8
12
16
StageI
StageII
StageIII
StageIV
StageV
StageVI

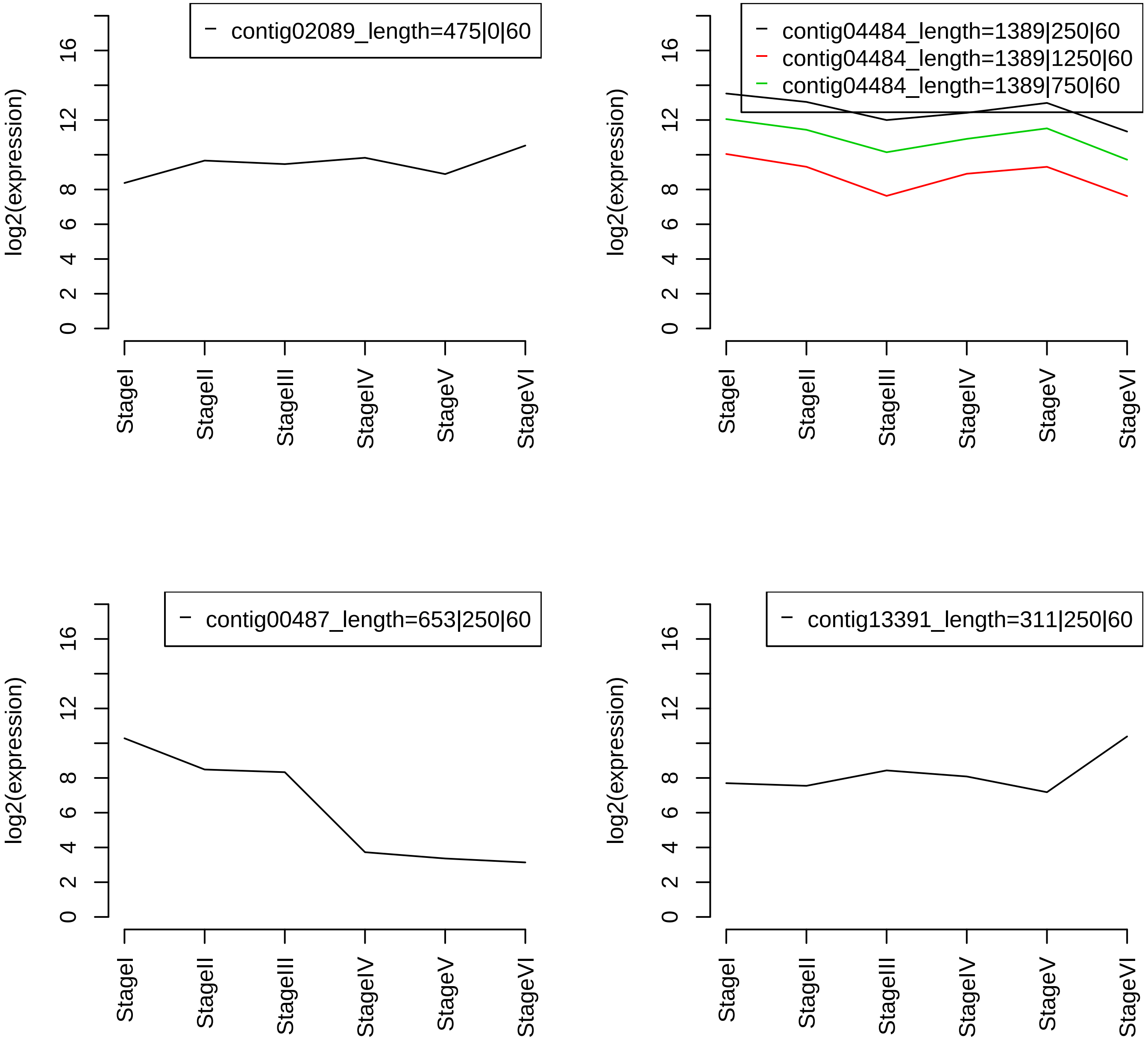
contig02089_length=475|0|60
contig04484_length=1389|250|60
contig04484_length=1389|1250|60
contig04484_length=1389|750|60
contig00487_length=653|250|60
contig13391_length=311|250|60
log2(expression)
0
2
4
6
8
12
16
StageI
StageII
StageIII
StageIV
StageV
StageVI

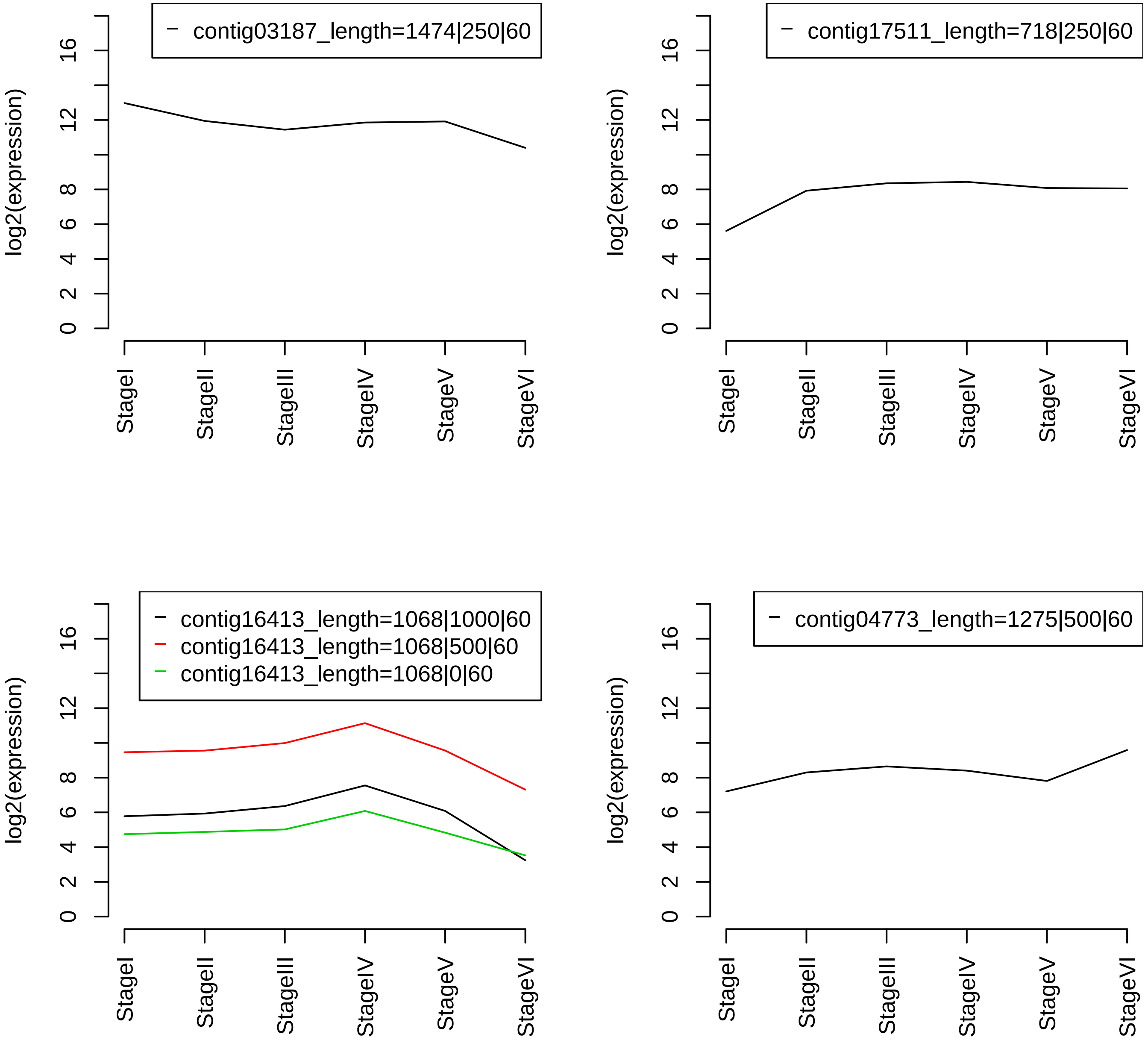

contig03187_length=1474|250|60
contig17511_length=718|250|60
contig16413_length=1068|1000|60
contig16413_length=1068|500|60
contig16413_length=1068|0|60
contig04773_length=1275|500|60
log2(expression)
StageI
StageII
StageIII
StageIV
StageV
StageVI

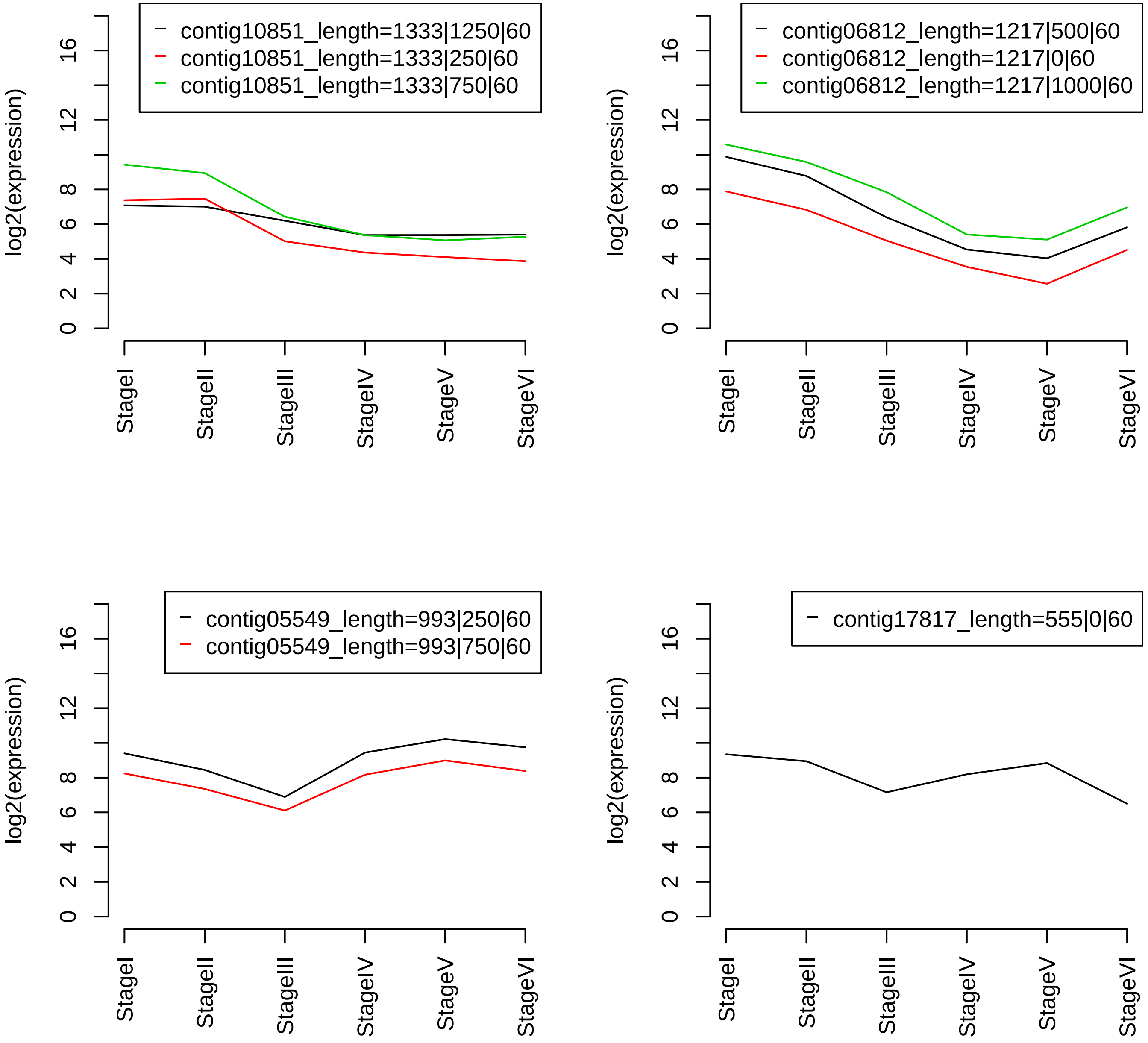

contig10851_length=1333|1250|60
contig10851_length=1333|250|60
contig10851_length=1333|750|60
contig06812_length=1217|500|60
contig06812_length=1217|0|60
contig06812_length=1217|1000|60
contig05549_length=993|250|60
contig05549_length=993|750|60
contig17817_length=555|0|60
log2(expression)
0 2 4 6 8 12 16
StageI StageII StageIII StageIV StageV StageVI

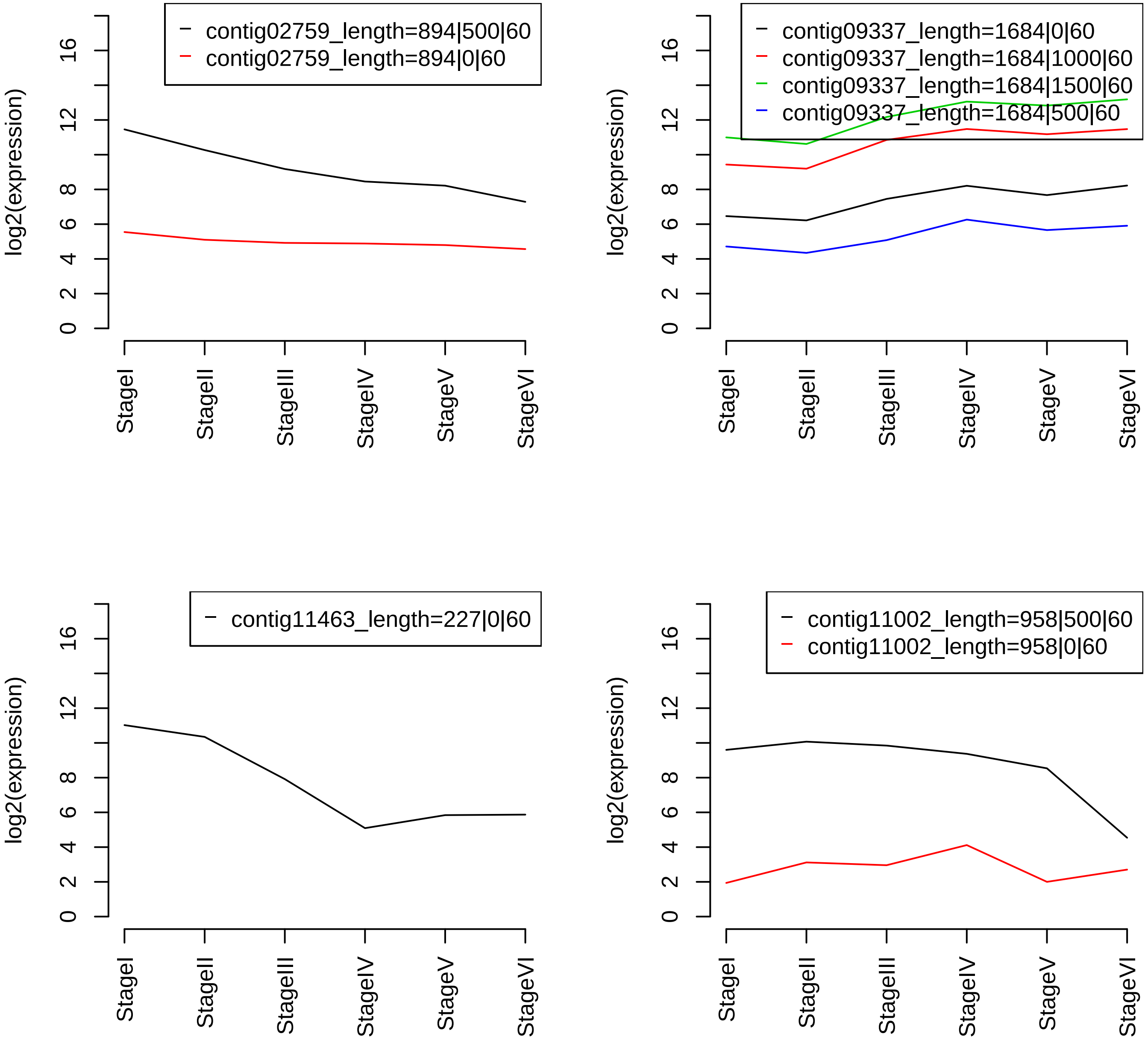
contig02759_length=894|500|60
contig02759_length=894|0|60
contig09337_length=1684|0|60
contig09337_length=1684|1000|60
contig09337_length=1684|1500|60
contig09337_length=1684|500|60
contig11463_length=227|0|60
contig11002_length=958|500|60
contig11002_length=958|0|60
log2(expression)
0
2
4
6
8
12
16
StageI
StageII
StageIII
StageIV
StageV
StageVI

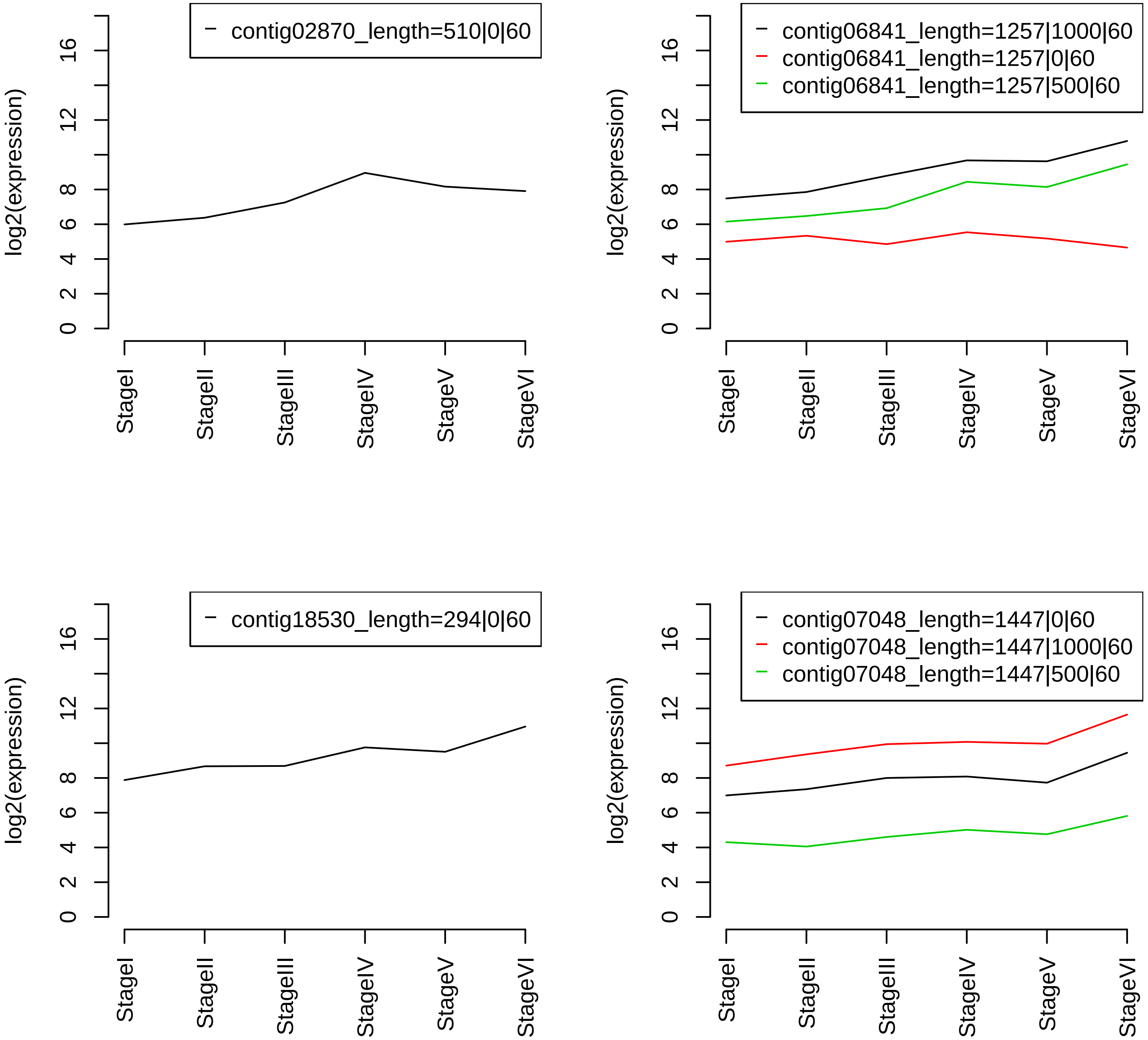

contig02870_length=510|0|60
contig06841_length=1257|1000|60
contig06841_length=1257|0|60
contig06841_length=1257|500|60
contig18530_length=294|0|60
contig07048_length=1447|0|60
contig07048_length=1447|1000|60
contig07048_length=1447|500|60
log2(expression)
0
2
4
6
8
12
16
StageI
StageII
StageIII
StageIV
StageV
StageVI

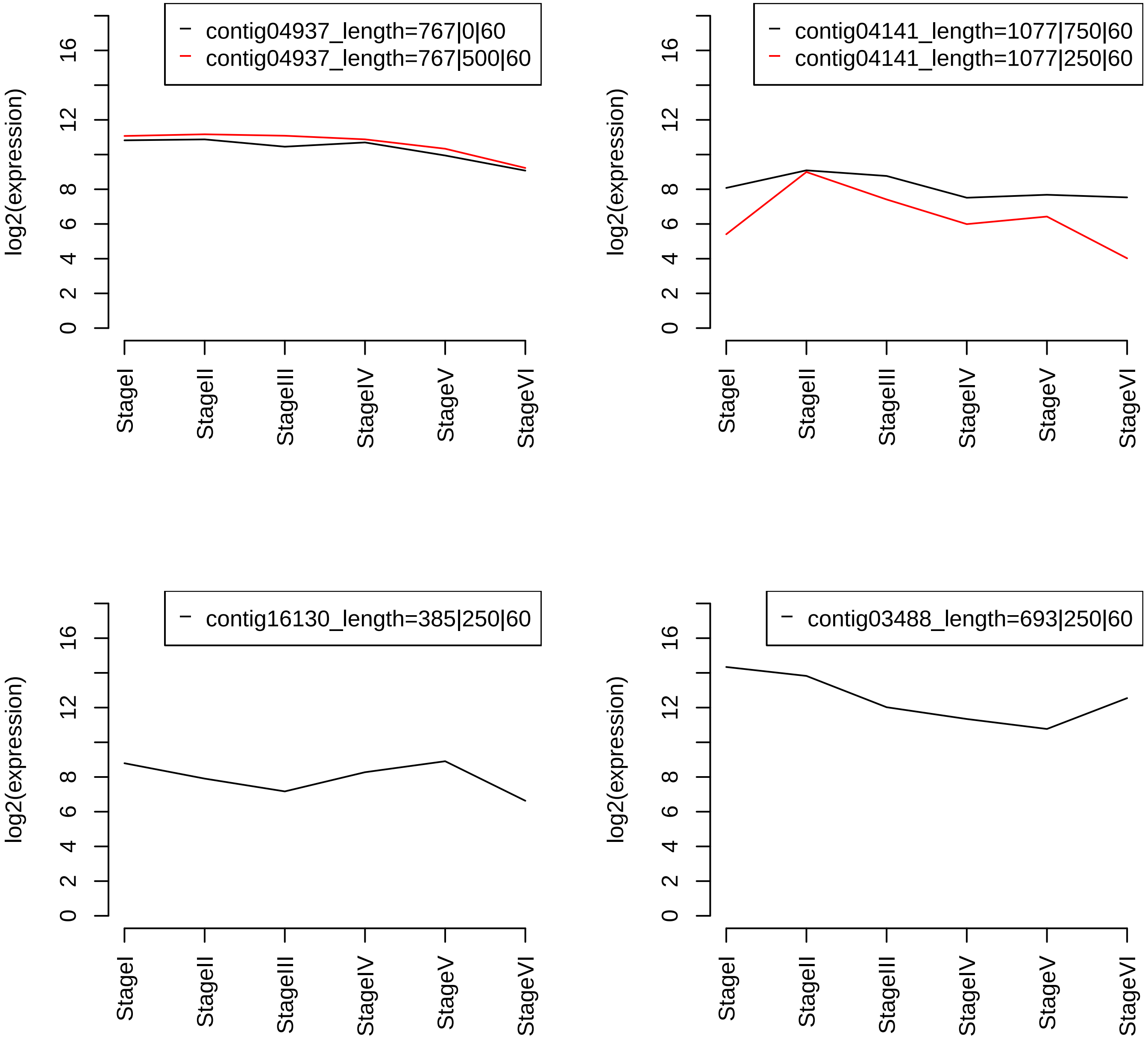
contig04937_length=767|0|60
contig04937_length=767|500|60
contig04141_length=1077|750|60
contig04141_length=1077|250|60
contig16130_length=385|250|60
contig03488_length=693|250|60
log2(expression)
0 2 4 6 8 12 16
StageI
StageII
StageIII
StageIV
StageV
StageVI

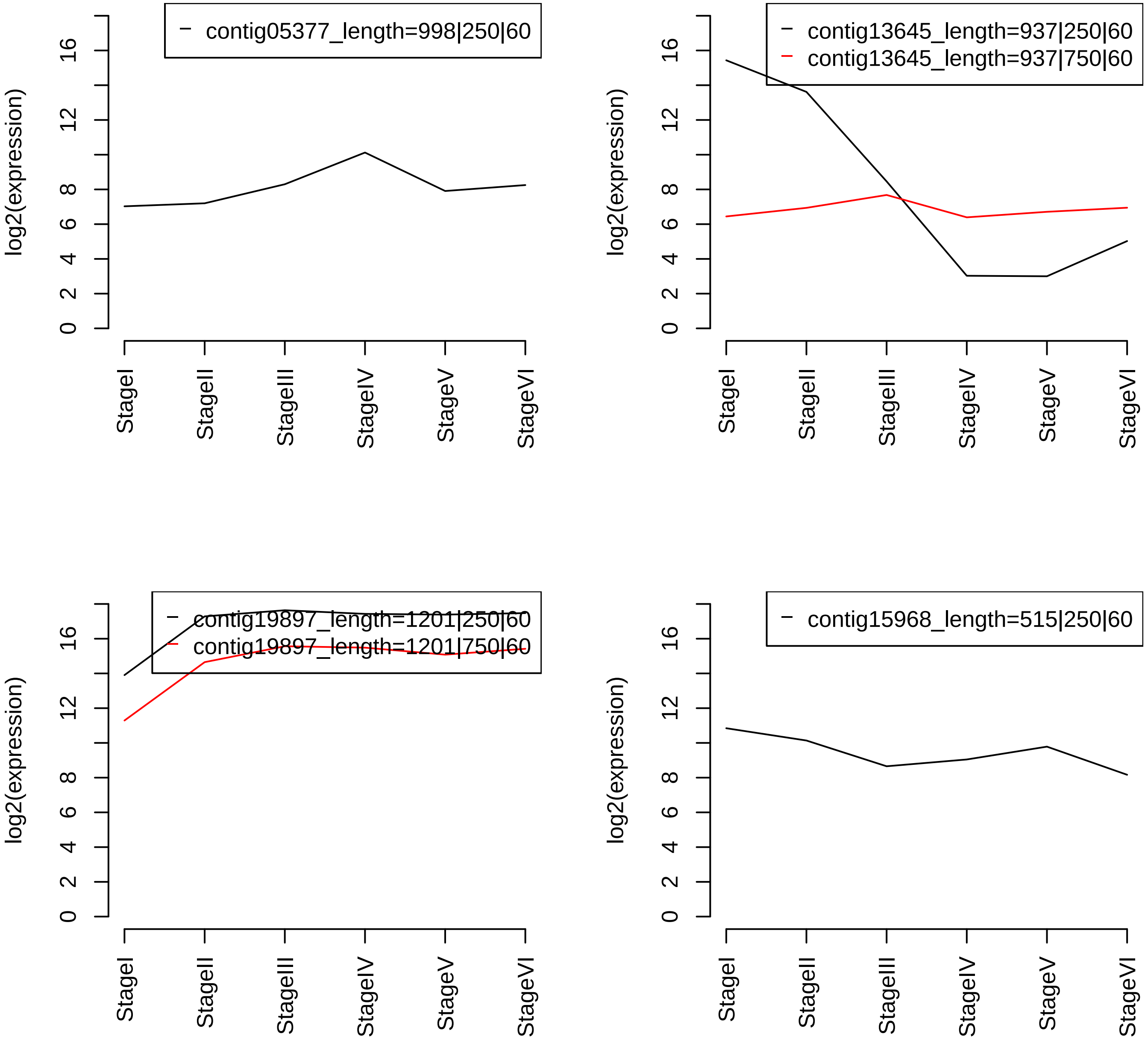
contig05377_length=998|250|60
contig13645_length=937|250|60
contig13645_length=937|750|60
contig19897_length=1201|250|60
contig19897_length=1201|750|60
contig15968_length=515|250|60
log2(expression)
StageI
StageII
StageIII
StageIV
StageV
StageVI

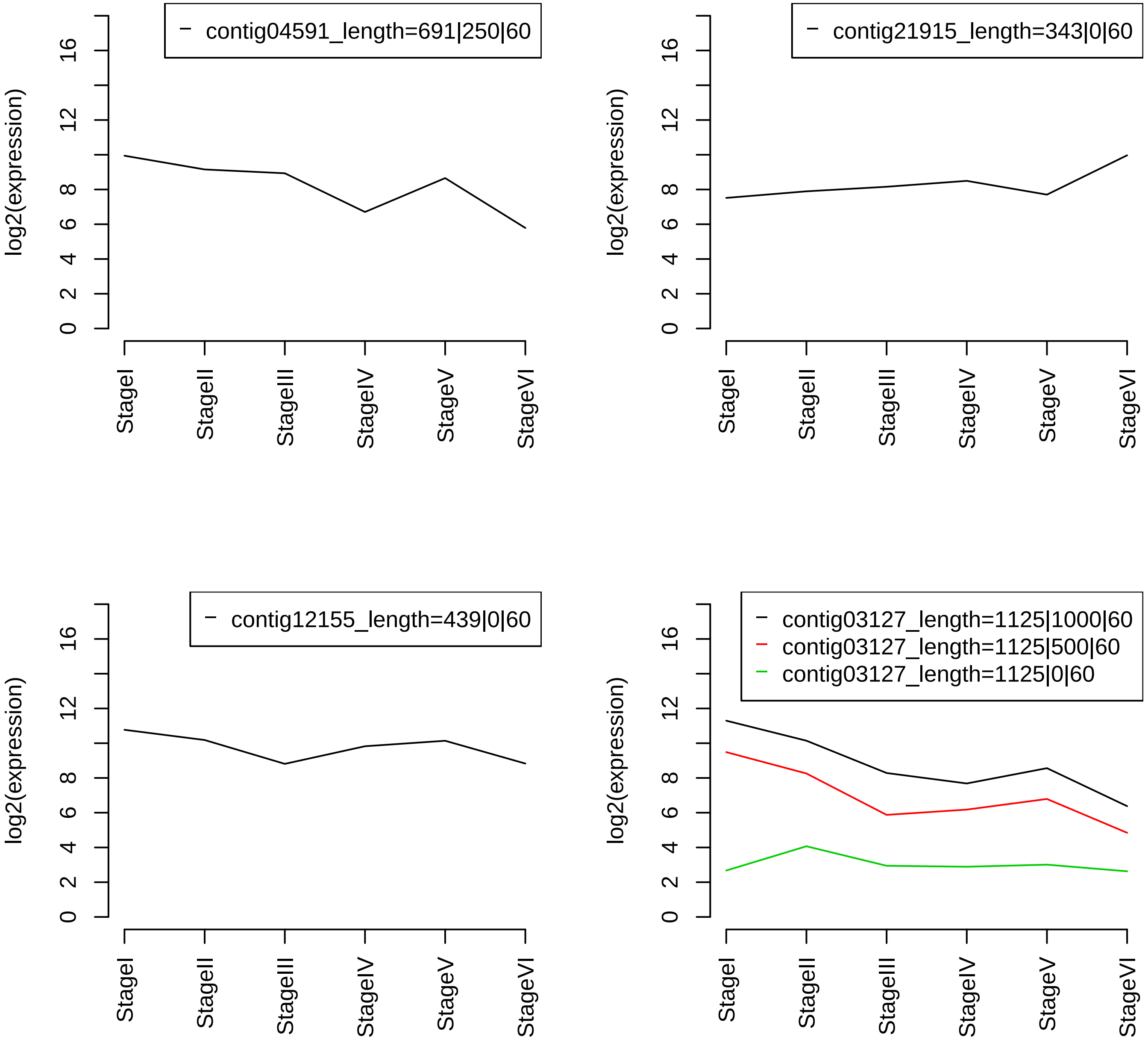
contig04591_length=691|250|60
contig21915_length=343|0|60
contig12155_length=439|0|60
contig03127_length=1125|1000|60
contig03127_length=1125|500|60
contig03127_length=1125|0|60
log2(expression)
0
2
4
6
8
12
16
StageI
StageII
StageIII
StageIV
StageV
StageVI

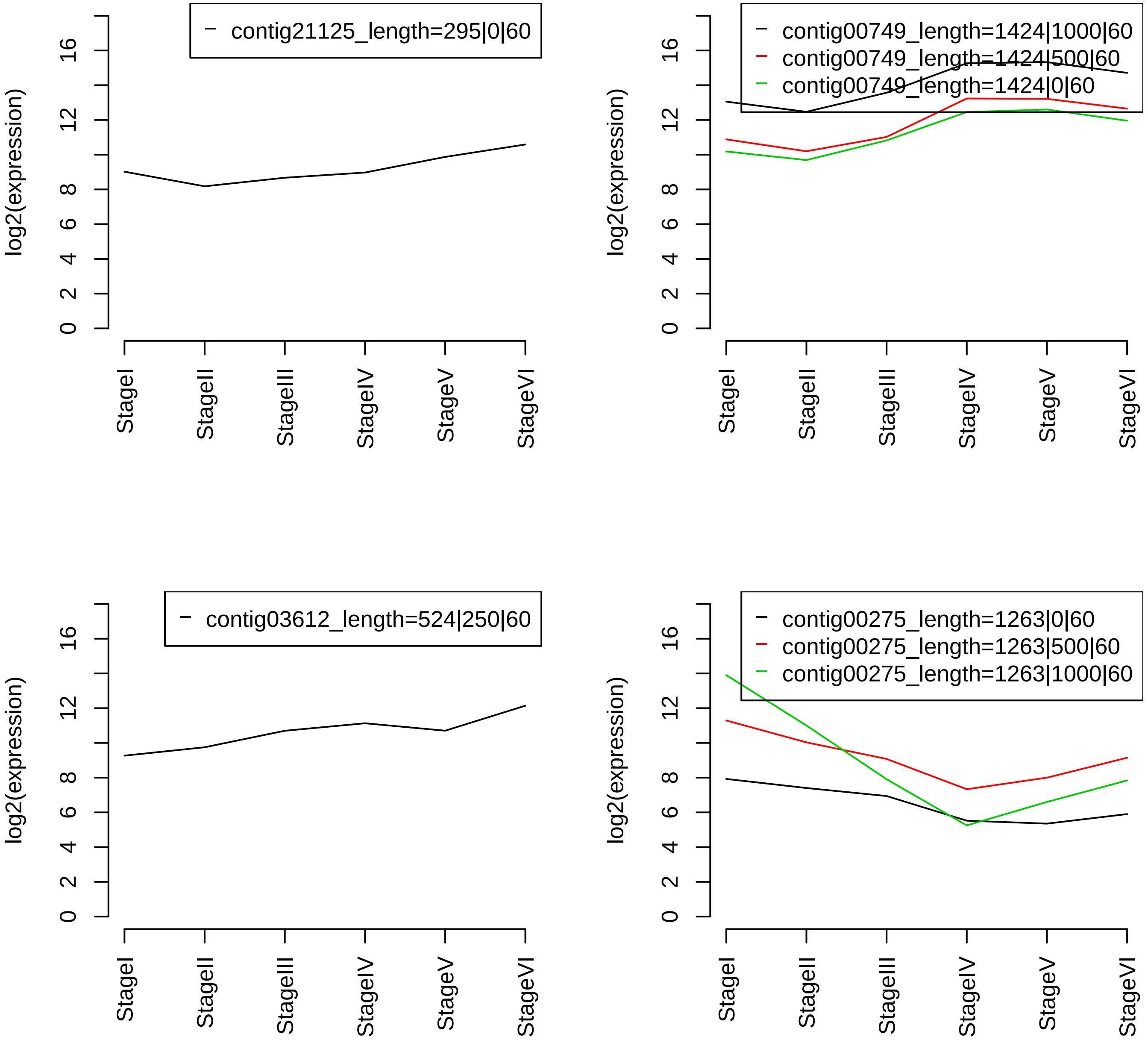

contig21125_length=295|0|60
contig00749_length=1424|1000|60
contig00749_length=1424|500|60
contig00749_length=1424|0|60
contig03612_length=524|250|60
contig00275_length=1263|0|60
contig00275_length=1263|500|60
contig00275_length=1263|1000|60
log2(expression)
0
2
4
6
8
12
16
StageI
StageII
StageIII
StageIV
StageV
StageVI

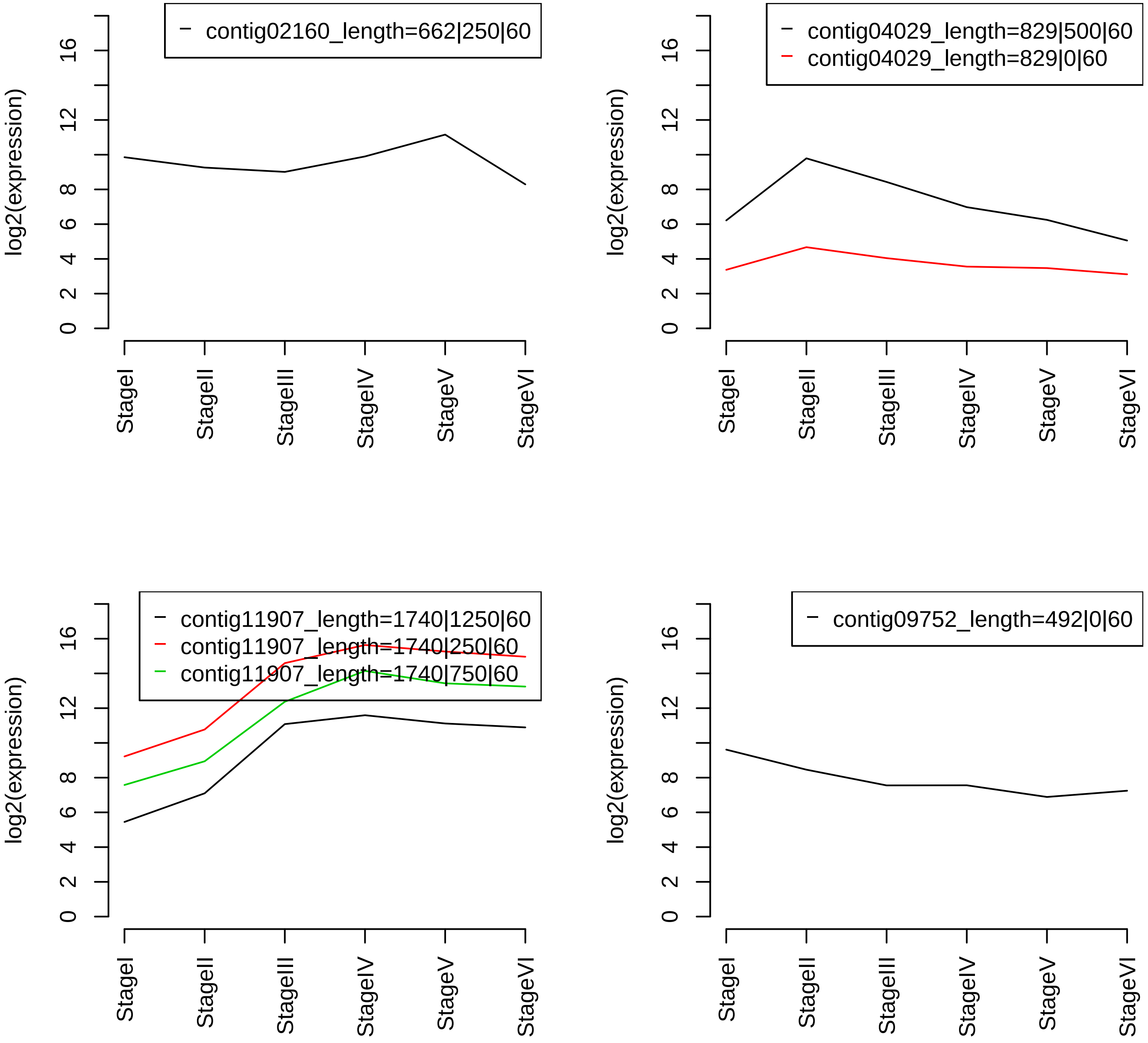
contig02160_length=662|250|60
contig04029_length=829|500|60
contig04029_length=829|0|60
contig11907_length=1740|1250|60
contig11907_length=1740|250|60
contig11907_length=1740|750|60
contig09752_length=492|0|60
log2(expression)
StageI
StageII
StageIII
StageIV
StageV
StageVI

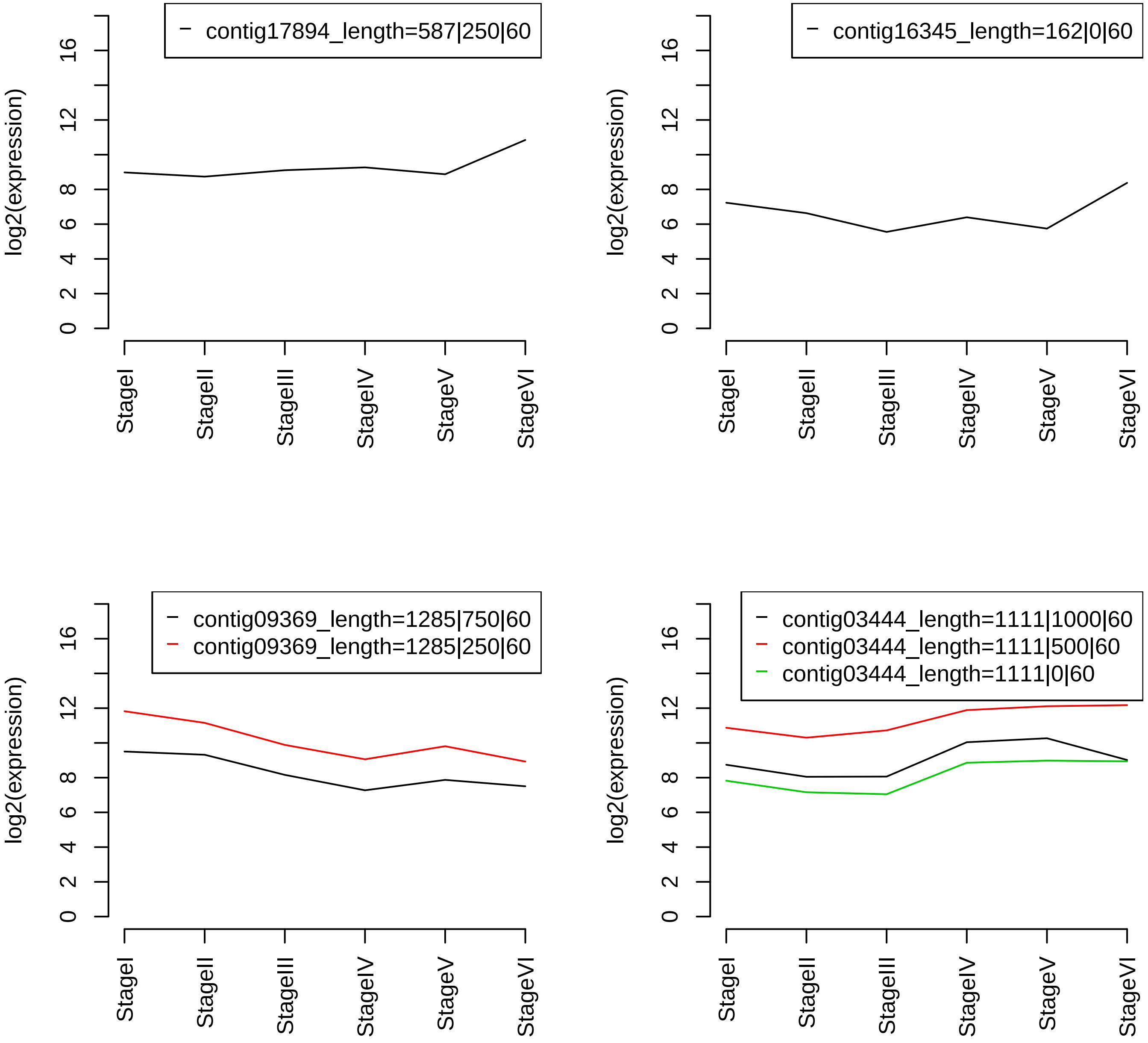

contig17894_length=587|250|60
contig16345_length=162|0|60
contig09369_length=1285|750|60
contig09369_length=1285|250|60
contig03444_length=1111|1000|60
contig03444_length=1111|500|60
contig03444_length=1111|0|60
log2(expression)
0
2
4
6
8
12
16
StageI
StageII
StageIII
StageIV
StageV
StageVI

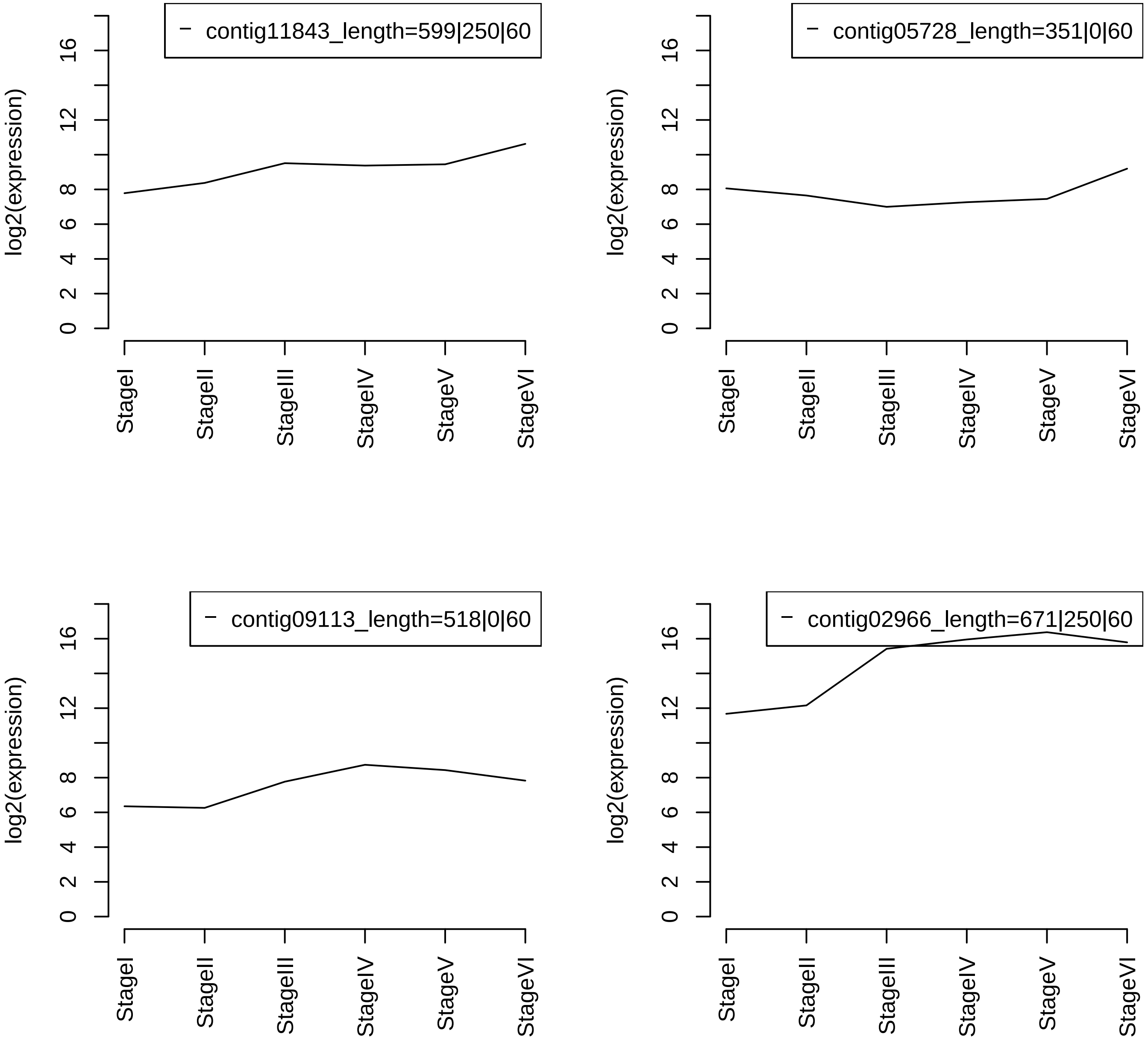

contig11843_length=599|250|60
log2(expression)
0
2
4
6
8
12
16
StageI
StageII
StageIII
StageIV
StageV
StageVI
contig05728_length=351|0|60
log2(expression)
0
2
4
6
8
12
16
StageI
StageII
StageIII
StageIV
StageV
StageVI
contig09113_length=518|0|60
log2(expression)
0
2
4
6
8
12
16
StageI
StageII
StageIII
StageIV
StageV
StageVI
contig02966_length=671|250|60
log2(expression)
0
2
4
6
8
12
16
StageI
StageII
StageIII
StageIV
StageV
StageVI

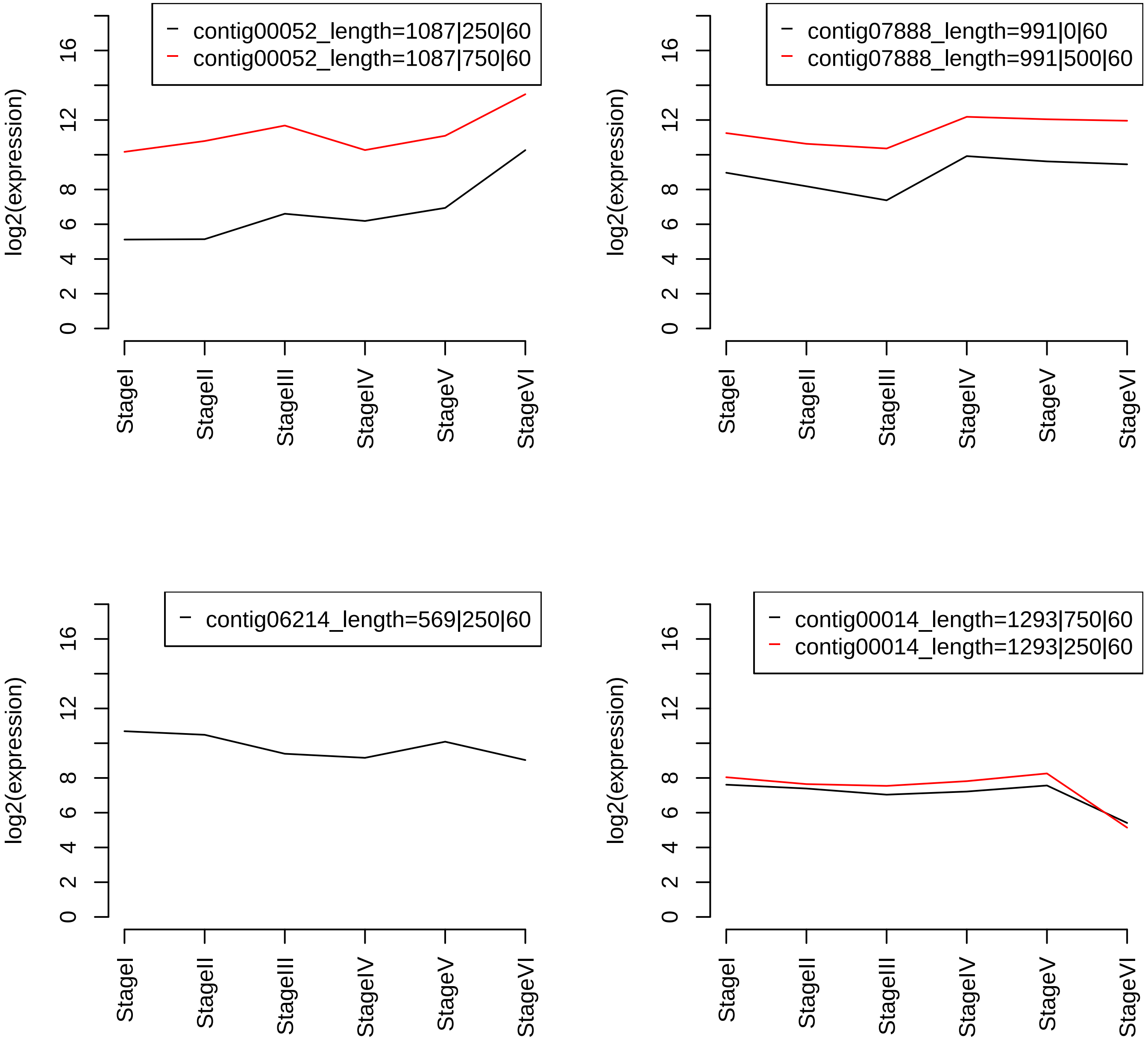

contig00052_length=1087|250|60
contig00052_length=1087|750|60
contig07888_length=991|0|60
contig07888_length=991|500|60
contig06214_length=569|250|60
contig00014_length=1293|750|60
contig00014_length=1293|250|60
log2(expression)
0 2 4 6 8 12 16
StageI StageII StageIII StageIV StageV StageVI

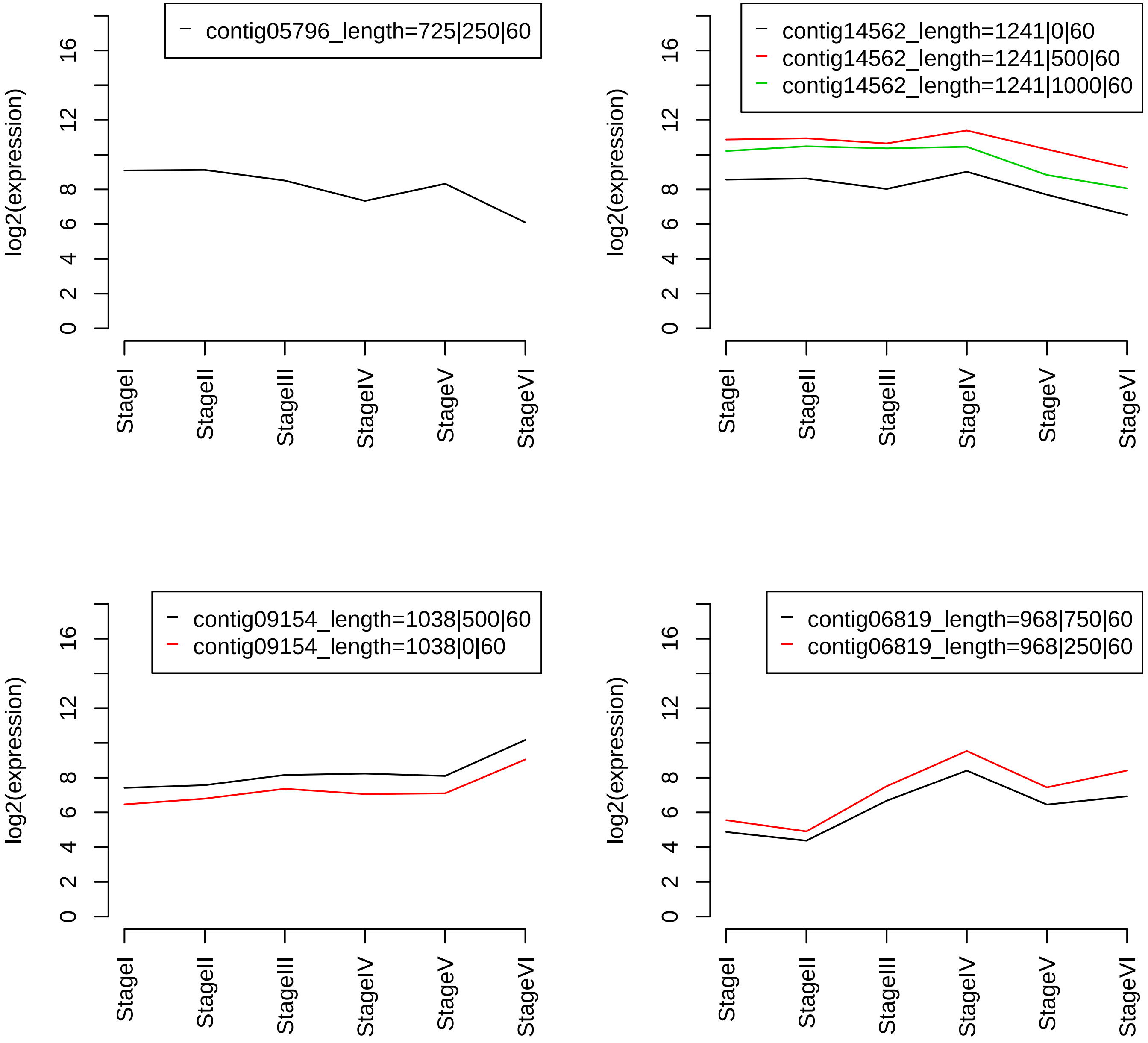

contig05796_length=725|250|60
contig14562_length=1241|0|60
contig14562_length=1241|500|60
contig14562_length=1241|1000|60
contig09154_length=1038|500|60
contig09154_length=1038|0|60
contig06819_length=968|750|60
contig06819_length=968|250|60
log2(expression)
0 2 4 6 8 12 16
StageI StageII StageIII StageIV StageV StageVI

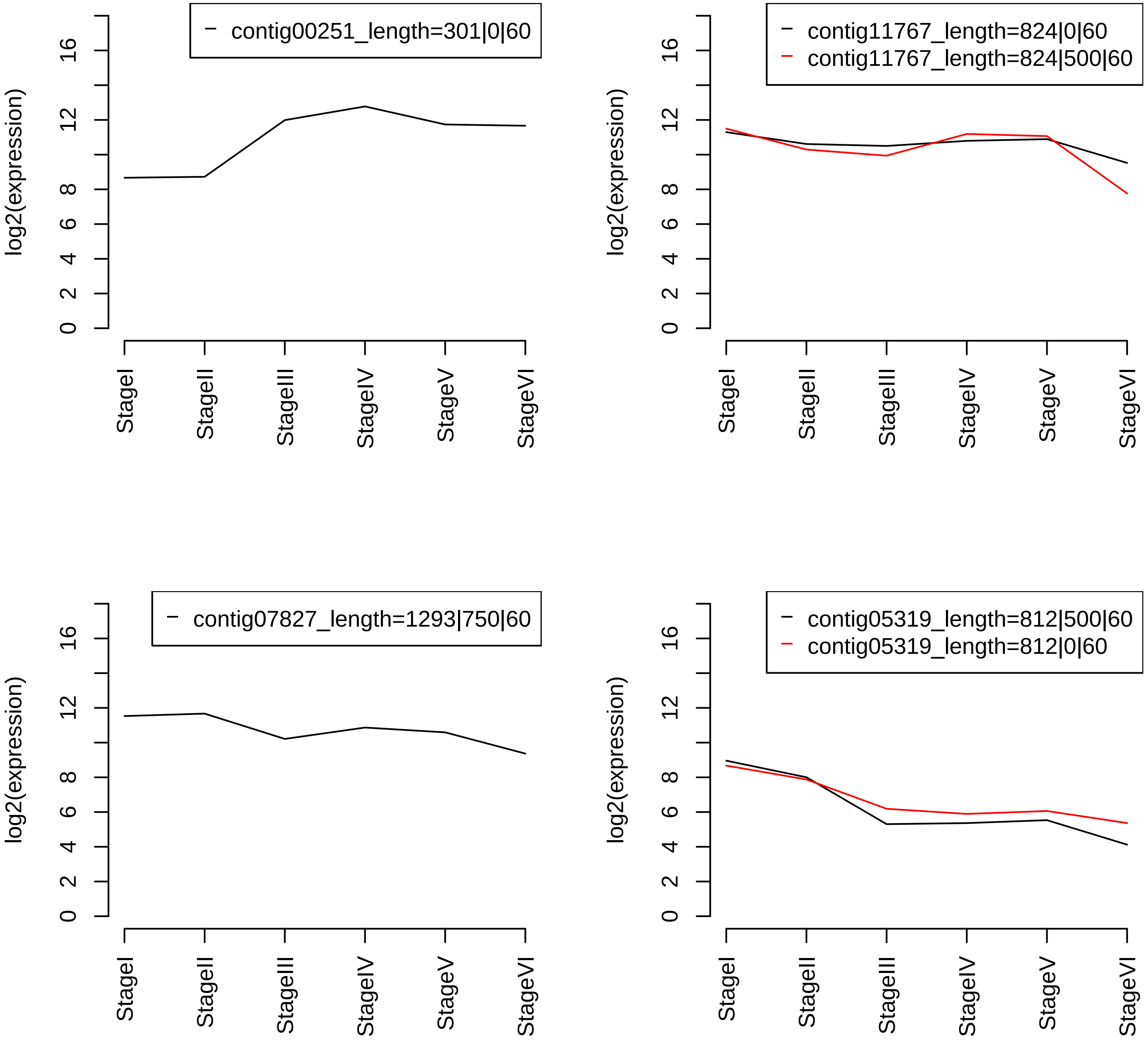
contig00251_length=301|0|60
contig11767_length=824|0|60
contig11767_length=824|500|60
contig07827_length=1293|750|60
contig05319_length=812|500|60
contig05319_length=812|0|60
log2(expression)
0
2
4
6
8
12
16
StageI
StageII
StageIII
StageIV
StageV
StageVI

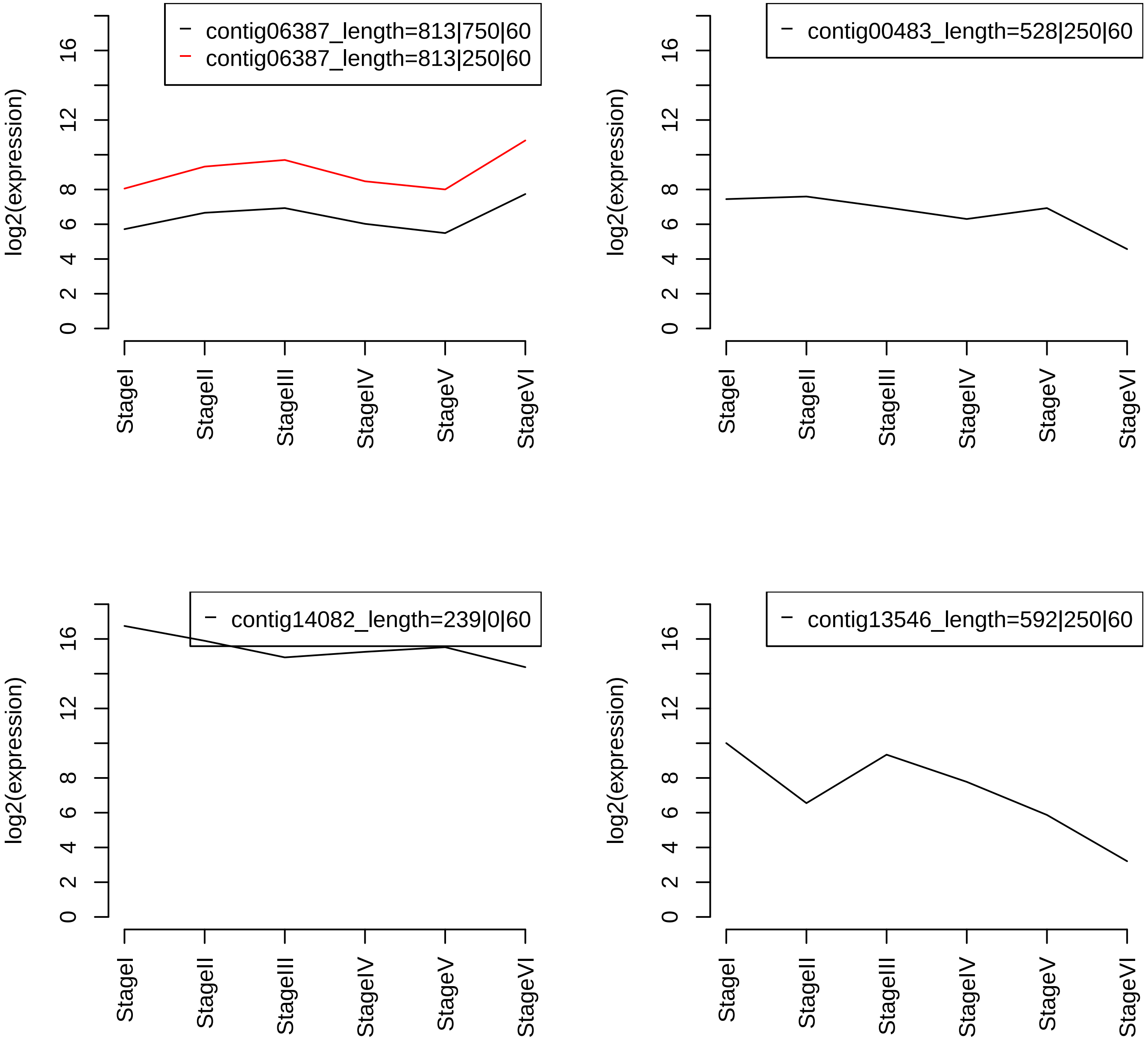

contig06387_length=813|750|60
contig06387_length=813|250|60
contig00483_length=528|250|60
contig14082_length=239|0|60
contig13546_length=592|250|60
log2(expression)
StageI
StageII
StageIII
StageIV
StageV
StageVI

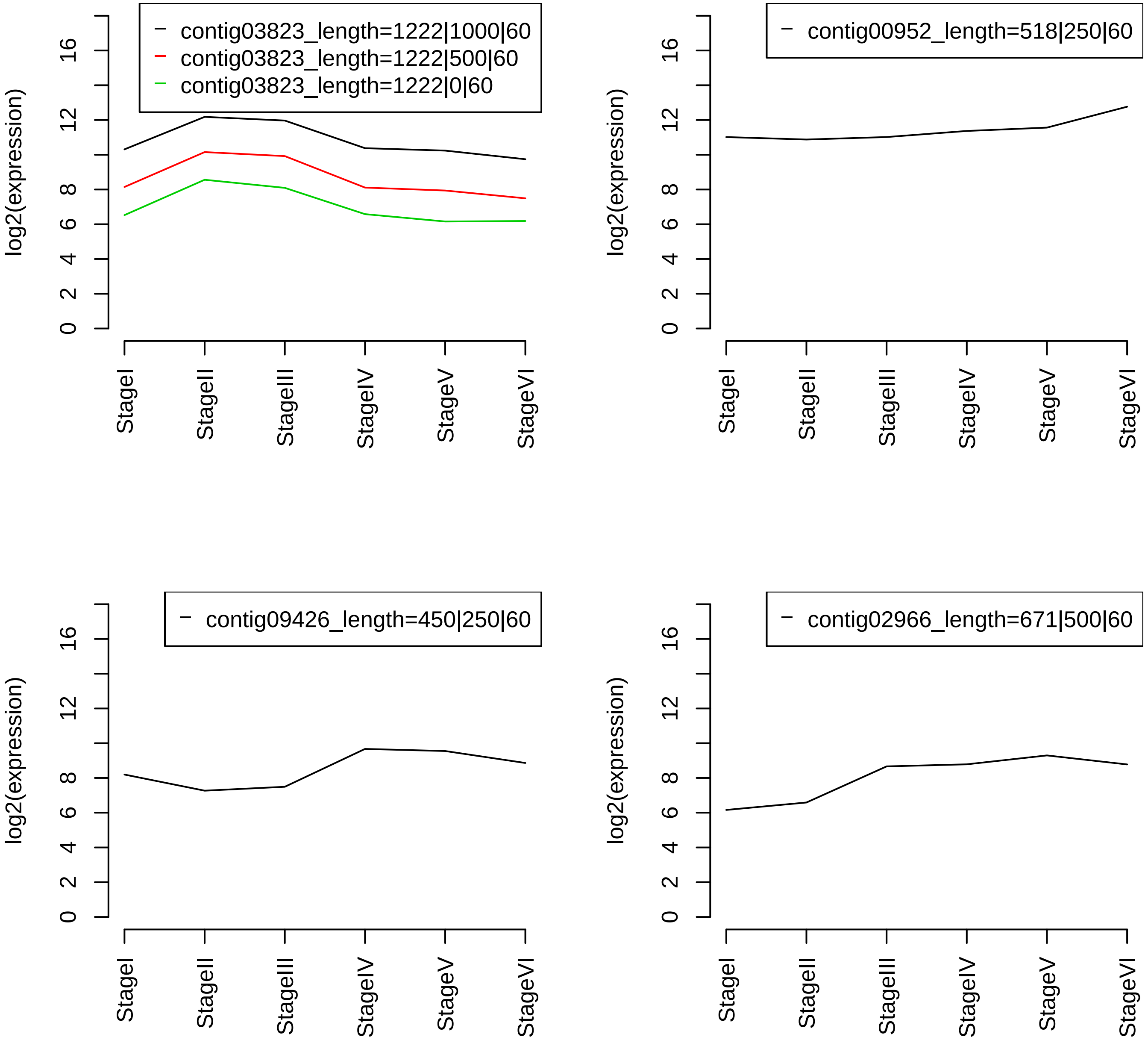

contig03823_length=1222|1000|60
contig03823_length=1222|500|60
contig03823_length=1222|0|60
log2(expression)
0
2
4
6
8
12
16
StageI
StageII
StageIII
StageIV
StageV
StageVI
contig00952_length=518|250|60
contig09426_length=450|250|60
contig02966_length=671|500|60

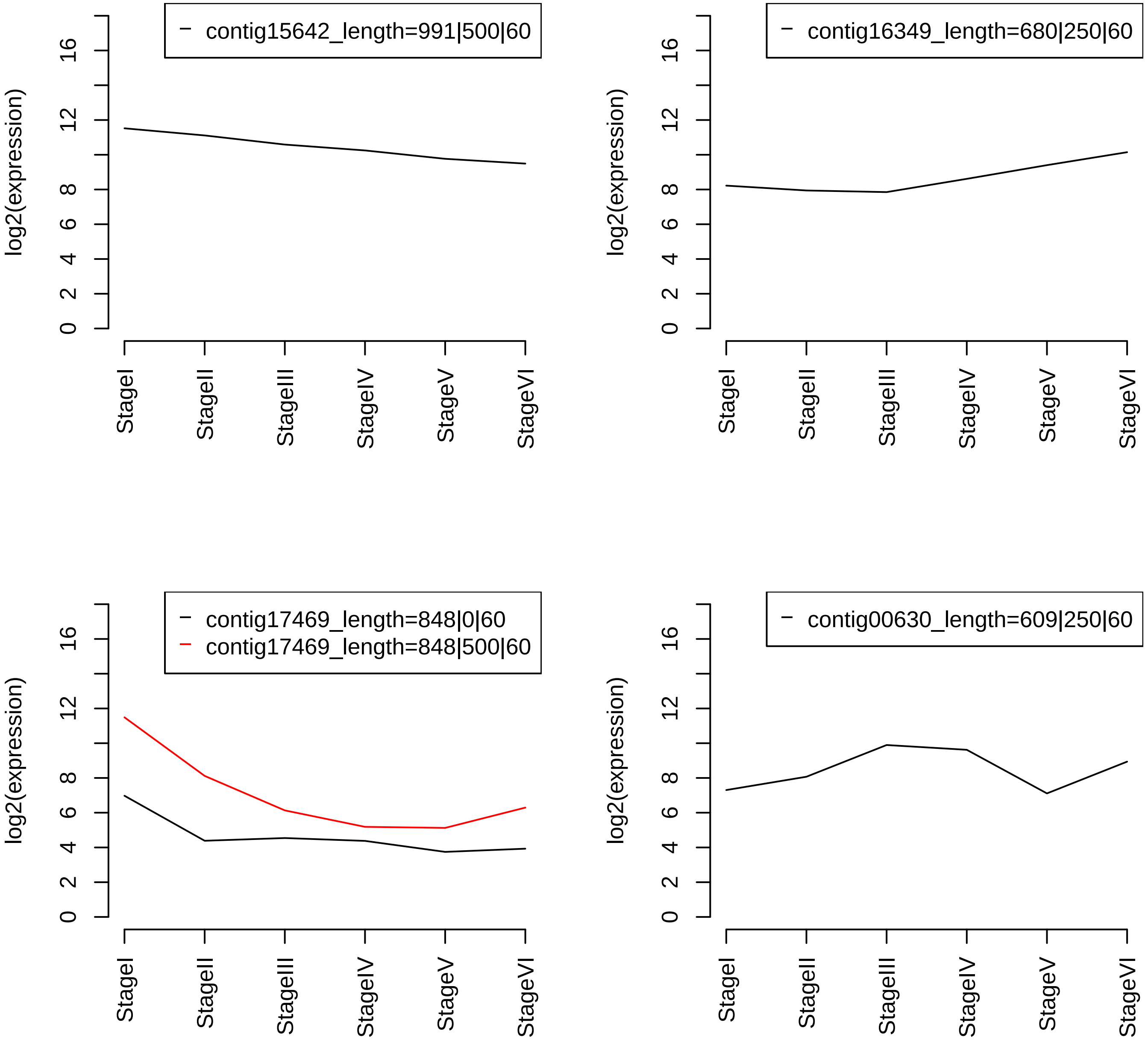

contig15642_length=991|500|60
contig16349_length=680|250|60
contig17469_length=848|0|60
contig17469_length=848|500|60
contig00630_length=609|250|60
log2(expression)
0
2
4
6
8
12
16
StageI
StageII
StageIII
StageIV
StageV
StageVI

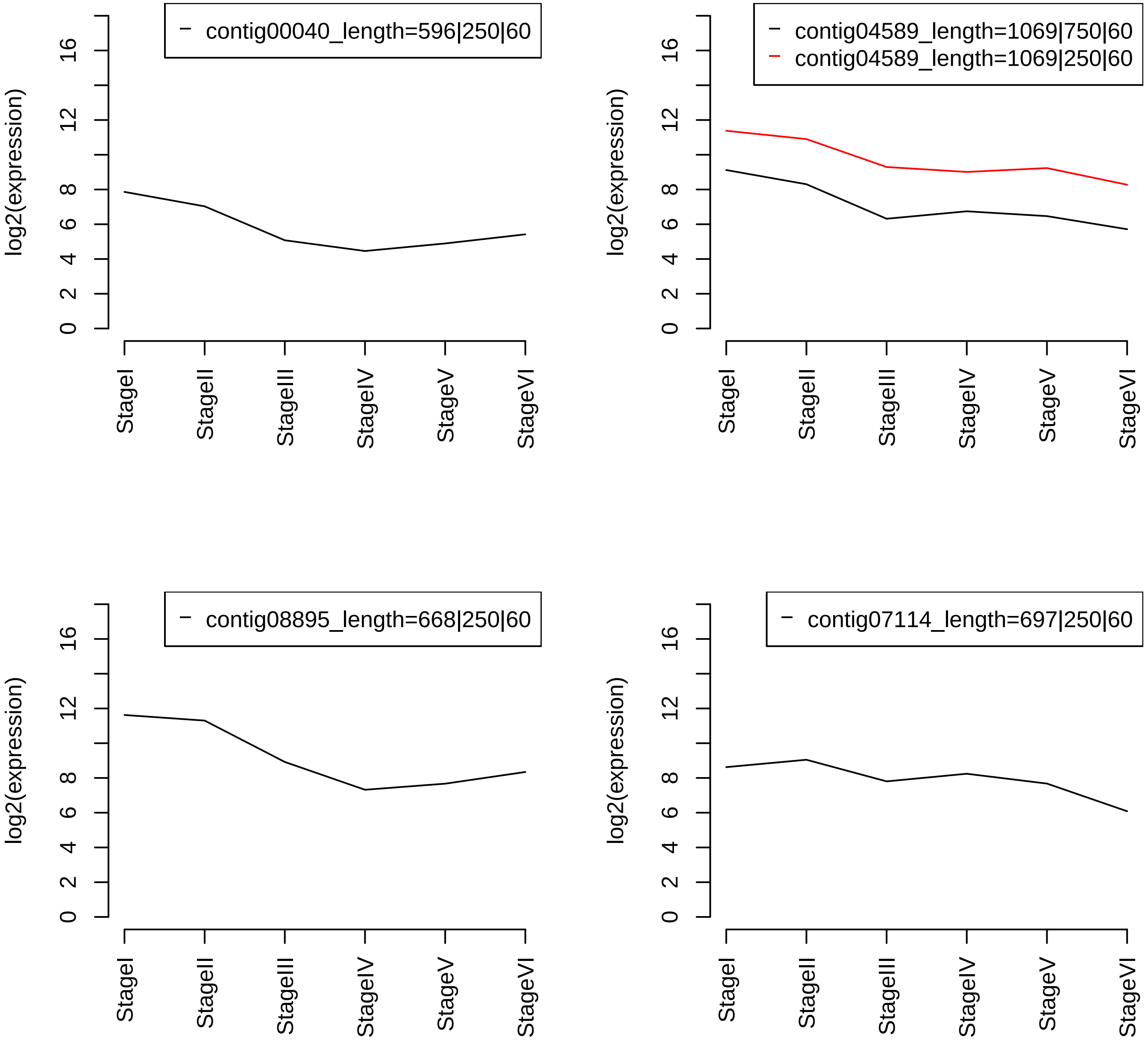
contig00040_length=596|250|60
contig04589_length=1069|750|60
contig04589_length=1069|250|60
contig08895_length=668|250|60
contig07114_length=697|250|60
log2(expression)
0
2
4
6
8
12
16
StageI
StageII
StageIII
StageIV
StageV
StageVI

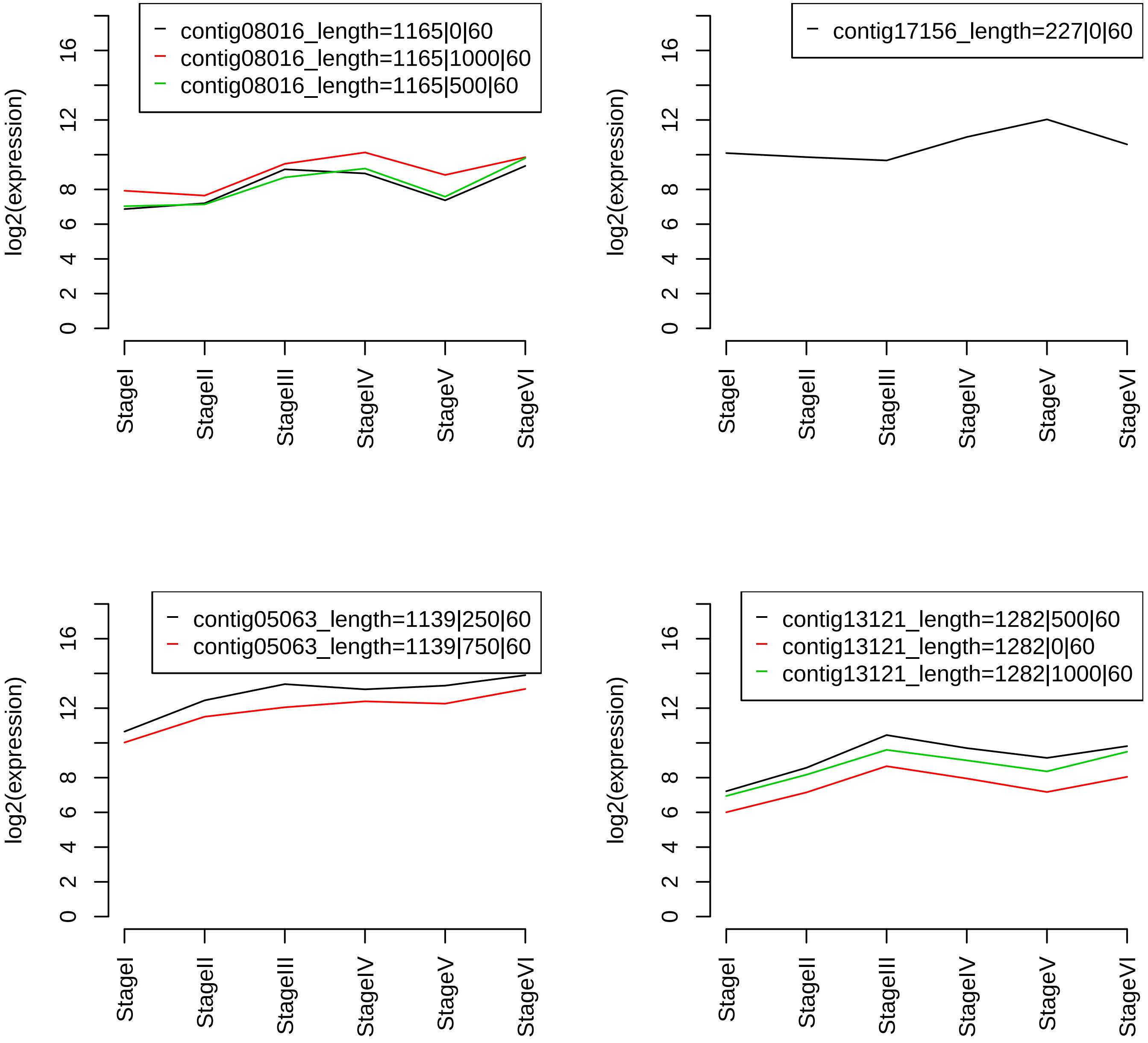
contig08016_length=1165|0|60
contig08016_length=1165|1000|60
contig08016_length=1165|500|60
contig17156_length=227|0|60
contig05063_length=1139|250|60
contig05063_length=1139|750|60
contig13121_length=1282|500|60
contig13121_length=1282|0|60
contig13121_length=1282|1000|60
log2(expression)
0
2
4
6
8
12
16
StageI
StageII
StageIII
StageIV
StageV
StageVI

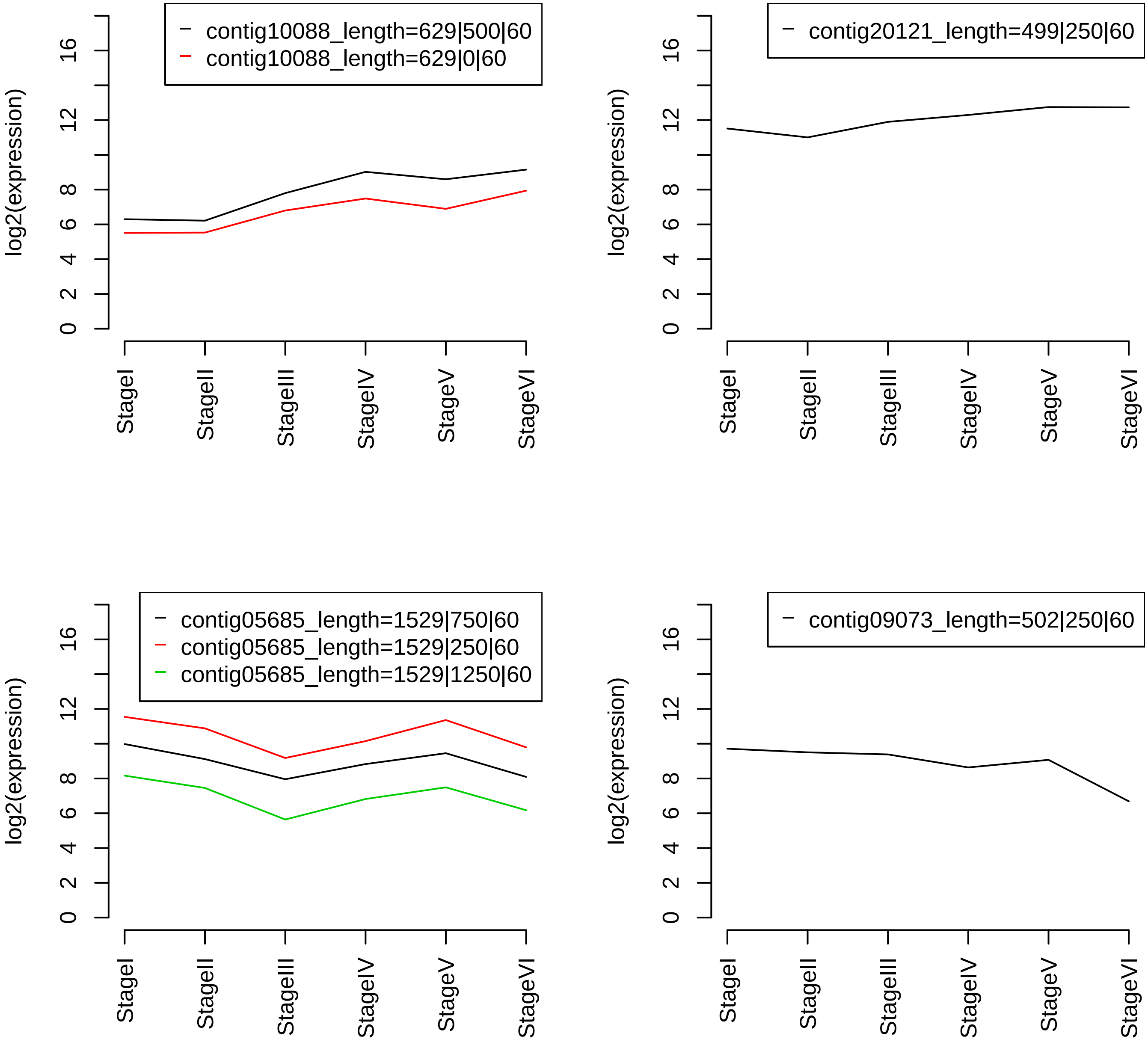

contig10088_length=629|500|60
contig10088_length=629|0|60
contig20121_length=499|250|60
contig05685_length=1529|750|60
contig05685_length=1529|250|60
contig05685_length=1529|1250|60
contig09073_length=502|250|60
log2(expression)
0
2
4
6
8
12
16
StageI
StageII
StageIII
StageIV
StageV
StageVI

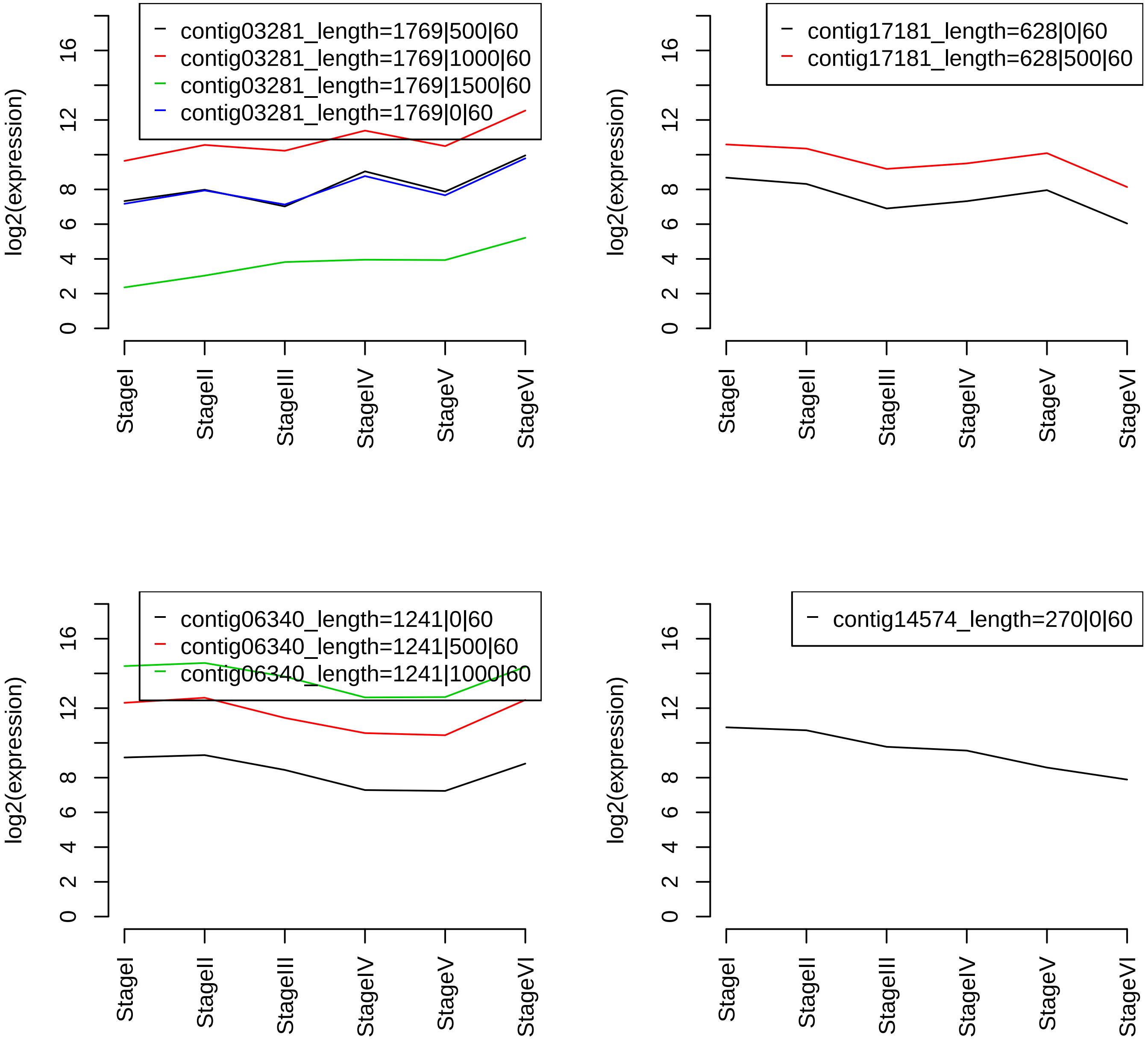
contig03281_length=1769|500|60
contig03281_length=1769|1000|60
contig03281_length=1769|1500|60
contig03281_length=1769|0|60
contig17181_length=628|0|60
contig17181_length=628|500|60
contig06340_length=1241|0|60
contig06340_length=1241|500|60
contig06340_length=1241|1000|60
contig14574_length=270|0|60
log2(expression)
StageI
StageII
StageIII
StageIV
StageV
StageVI

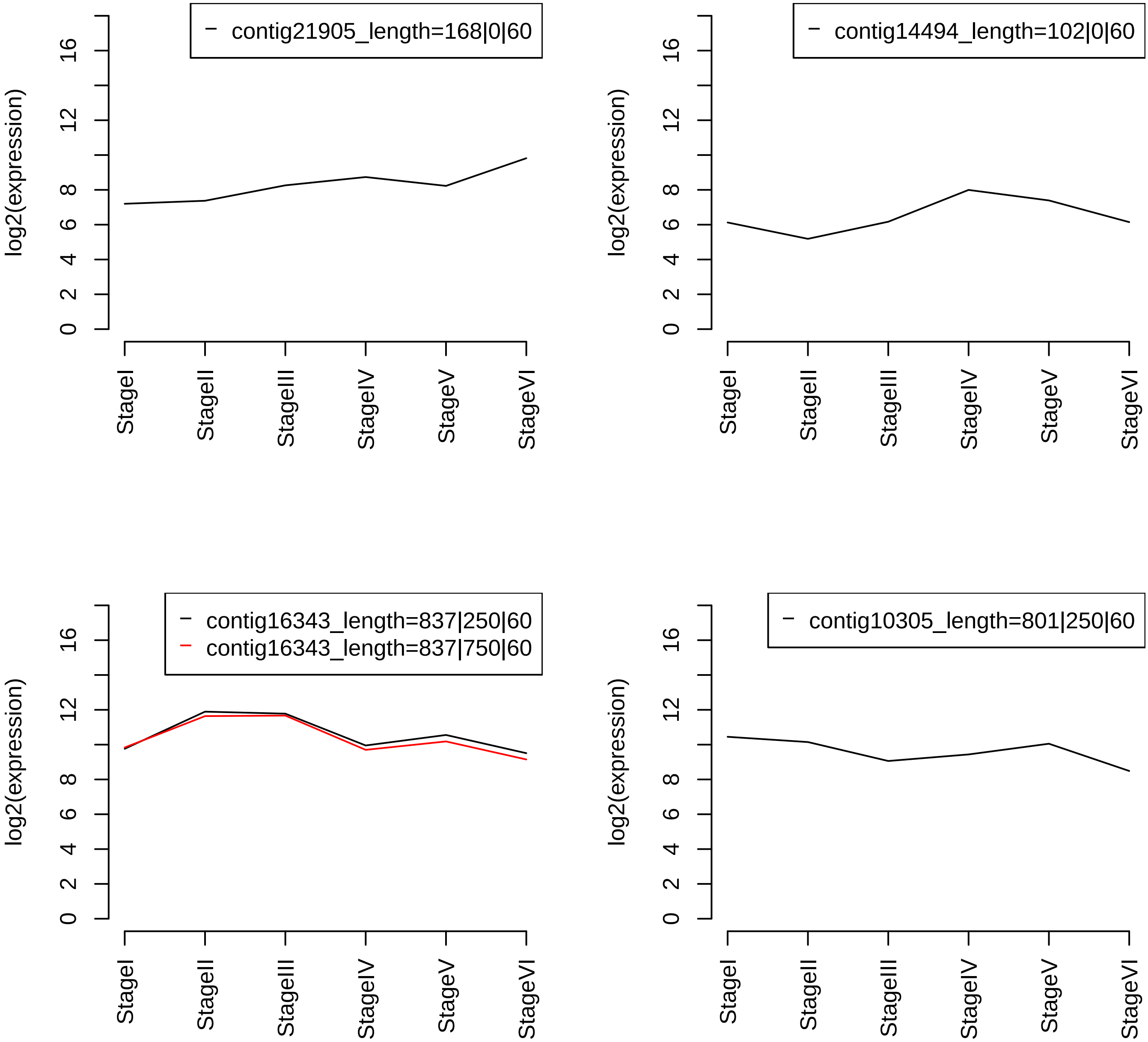

contig21905_length=168|0|60
contig14494_length=102|0|60
contig16343_length=837|250|60
contig16343_length=837|750|60
contig10305_length=801|250|60
log2(expression)
0 2 4 6 8 12 16
StageI
StageII
StageIII
StageIV
StageV
StageVI

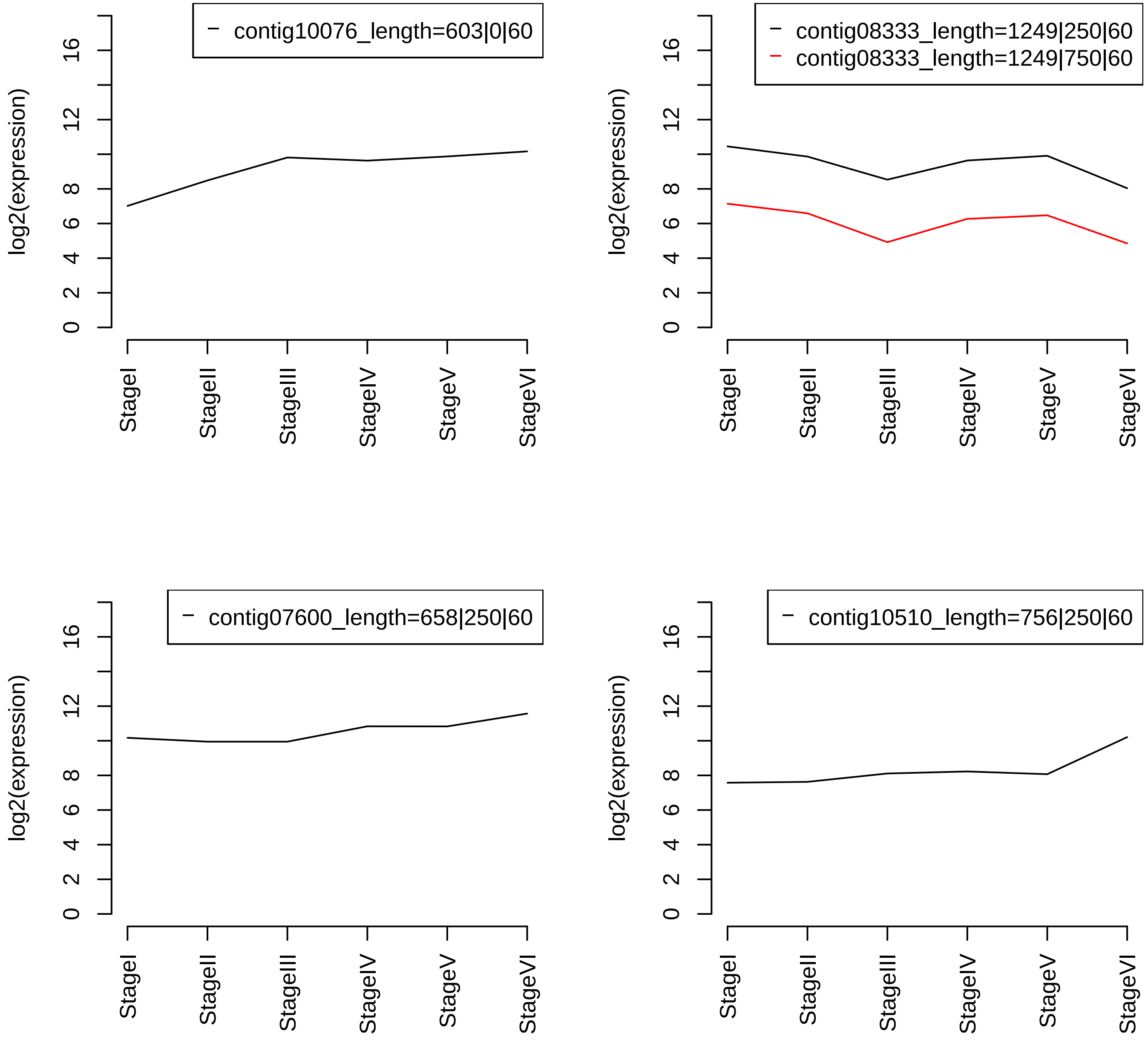

contig10076_length=603|0|60
contig08333_length=1249|250|60
contig08333_length=1249|750|60
contig07600_length=658|250|60
contig10510_length=756|250|60
log2(expression)
0
2
4
6
8
12
16
StageI
StageII
StageIII
StageIV
StageV
StageVI

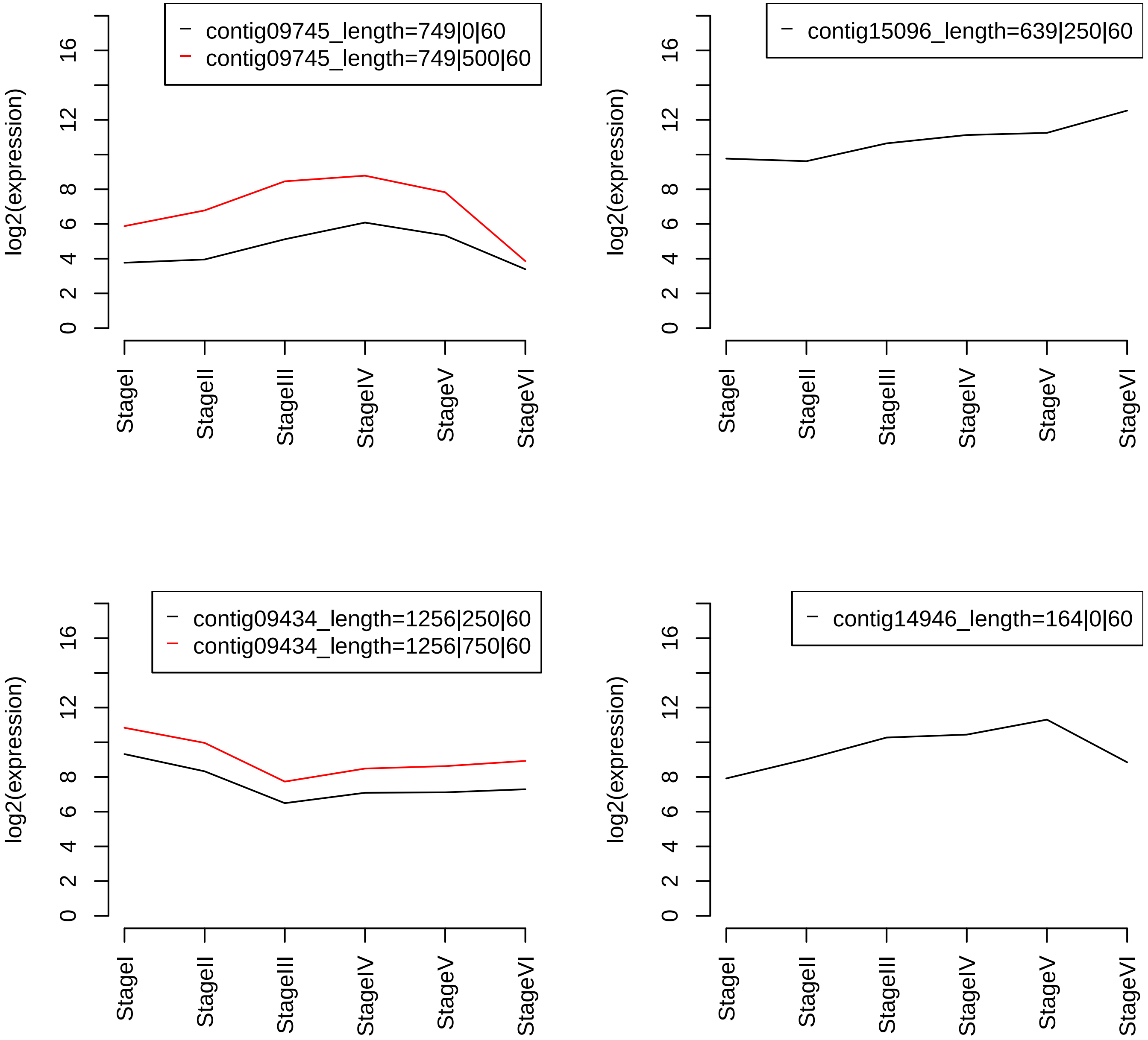
contig09745_length=749|0|60
contig09745_length=749|500|60
contig15096_length=639|250|60
contig09434_length=1256|250|60
contig09434_length=1256|750|60
contig14946_length=164|0|60
log2(expression)
0 2 4 6 8 12 16
StageI StageII StageIII StageIV StageV StageVI

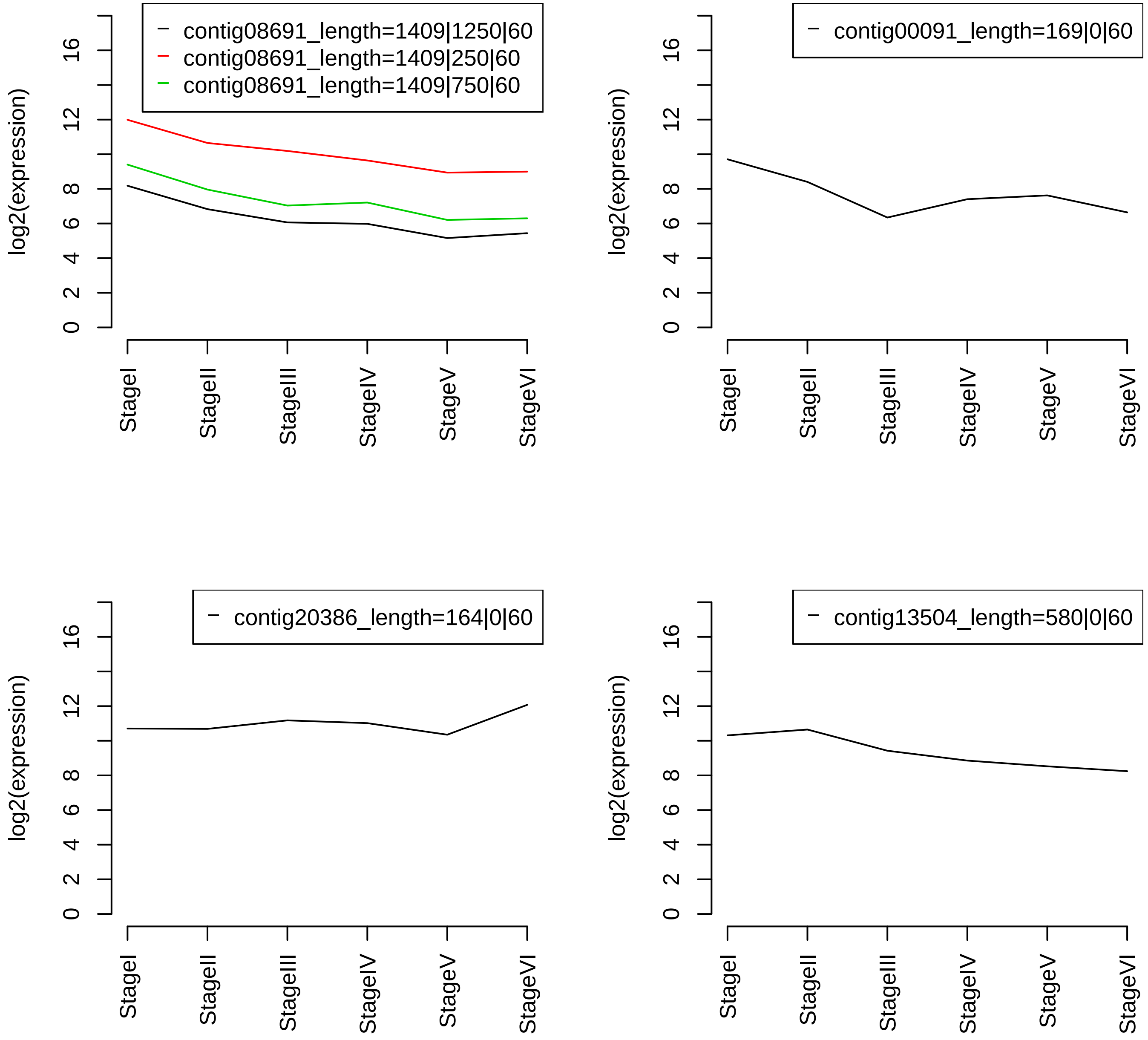

contig08691_length=1409|1250|60
contig08691_length=1409|250|60
contig08691_length=1409|750|60
log2(expression)
0
2
4
6
8
12
16
StageI
StageII
StageIII
StageIV
StageV
StageVI
contig00091_length=169|0|60
log2(expression)
0
2
4
6
8
12
16
StageI
StageII
StageIII
StageIV
StageV
StageVI
contig20386_length=164|0|60
log2(expression)
0
2
4
6
8
12
16
StageI
StageII
StageIII
StageIV
StageV
StageVI
contig13504_length=580|0|60
log2(expression)
0
2
4
6
8
12
16
StageI
StageII
StageIII
StageIV
StageV
StageVI

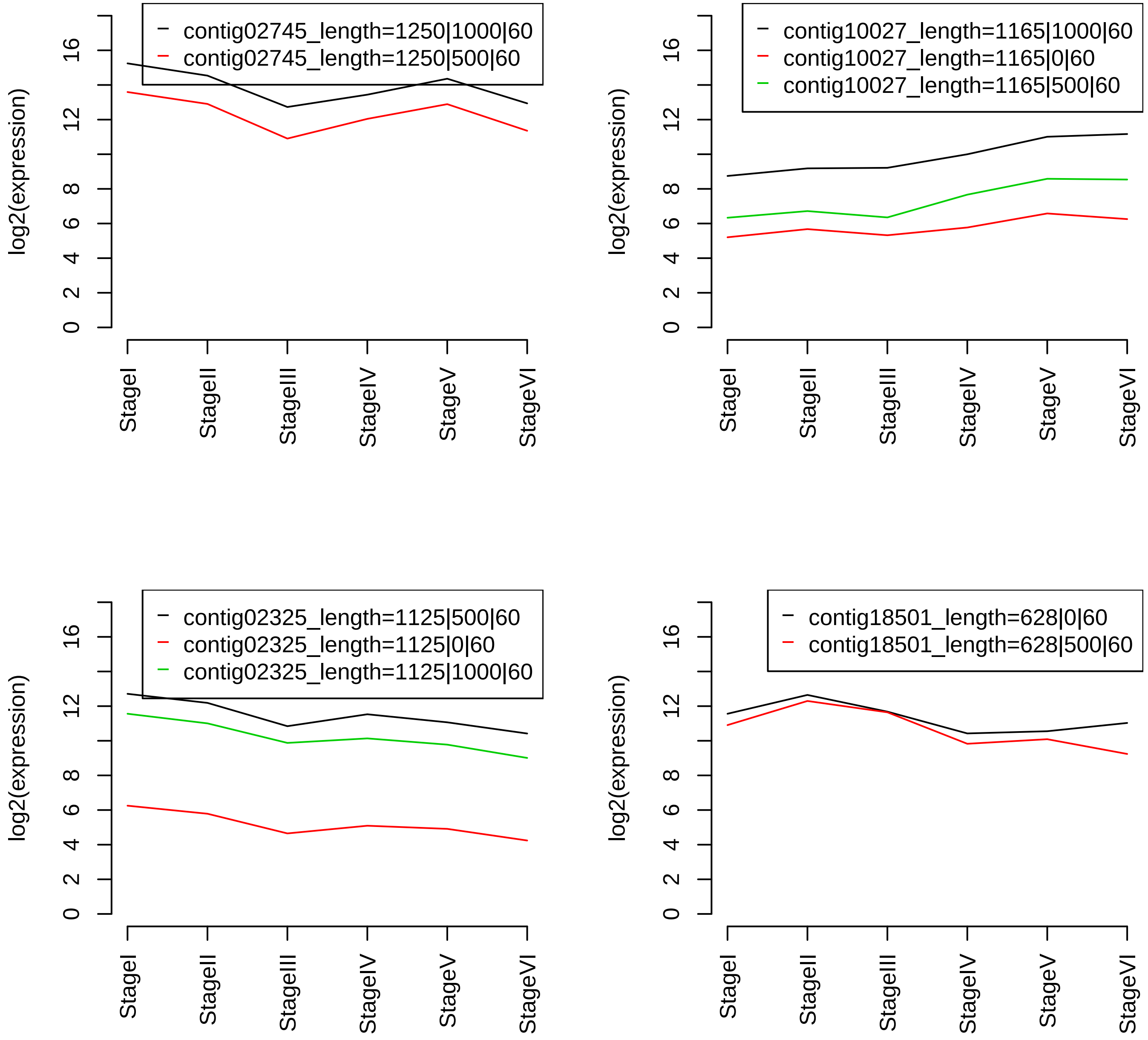

contig02745_length=1250|1000|60
contig02745_length=1250|500|60
contig10027_length=1165|1000|60
contig10027_length=1165|0|60
contig10027_length=1165|500|60
contig02325_length=1125|500|60
contig02325_length=1125|0|60
contig02325_length=1125|1000|60
contig18501_length=628|0|60
contig18501_length=628|500|60
log2(expression)
0
2
4
6
8
12
16
StageI
StageII
StageIII
StageIV
StageV
StageVI

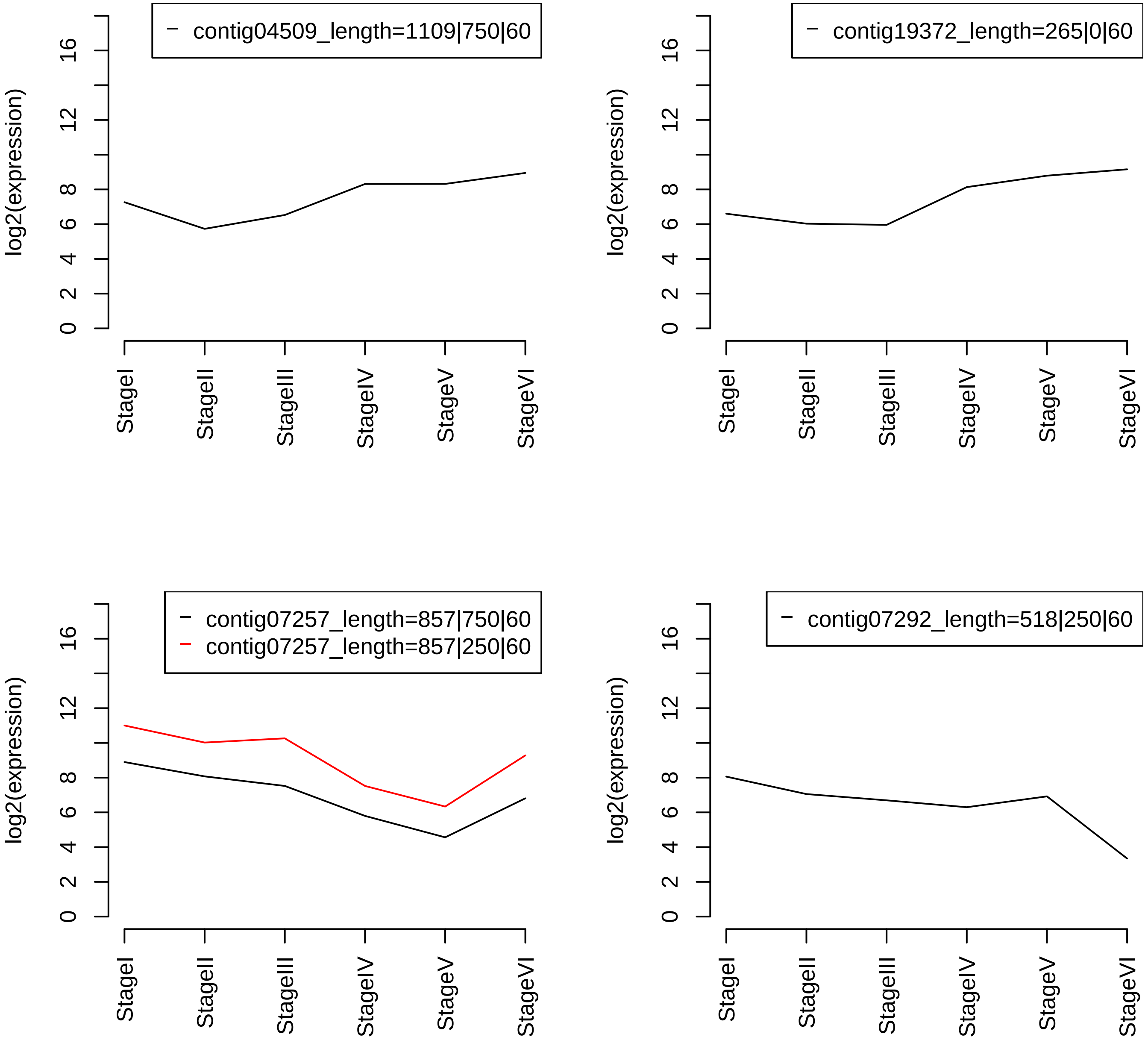

contig04509_length=1109|750|60
contig19372_length=265|0|60
contig07257_length=857|750|60
contig07257_length=857|250|60
contig07292_length=518|250|60
log2(expression)
0
2
4
6
8
12
16
StageI
StageII
StageIII
StageIV
StageV
StageVI

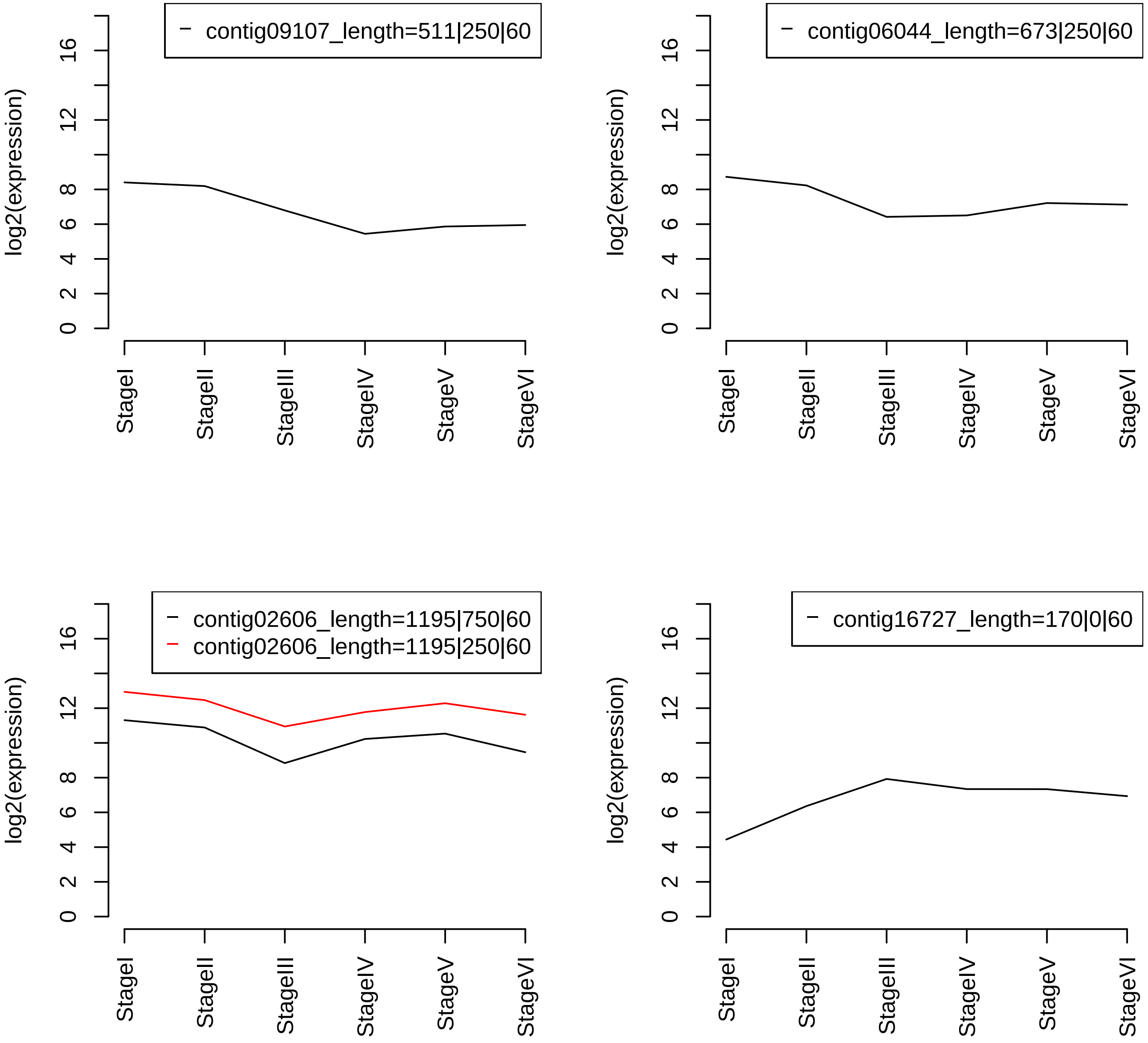

contig09107_length=511|250|60
contig06044_length=673|250|60
contig02606_length=1195|750|60
contig02606_length=1195|250|60
contig16727_length=170|0|60
log2(expression)
0
2
4
6
8
12
16
StageI
StageII
StageIII
StageIV
StageV
StageVI

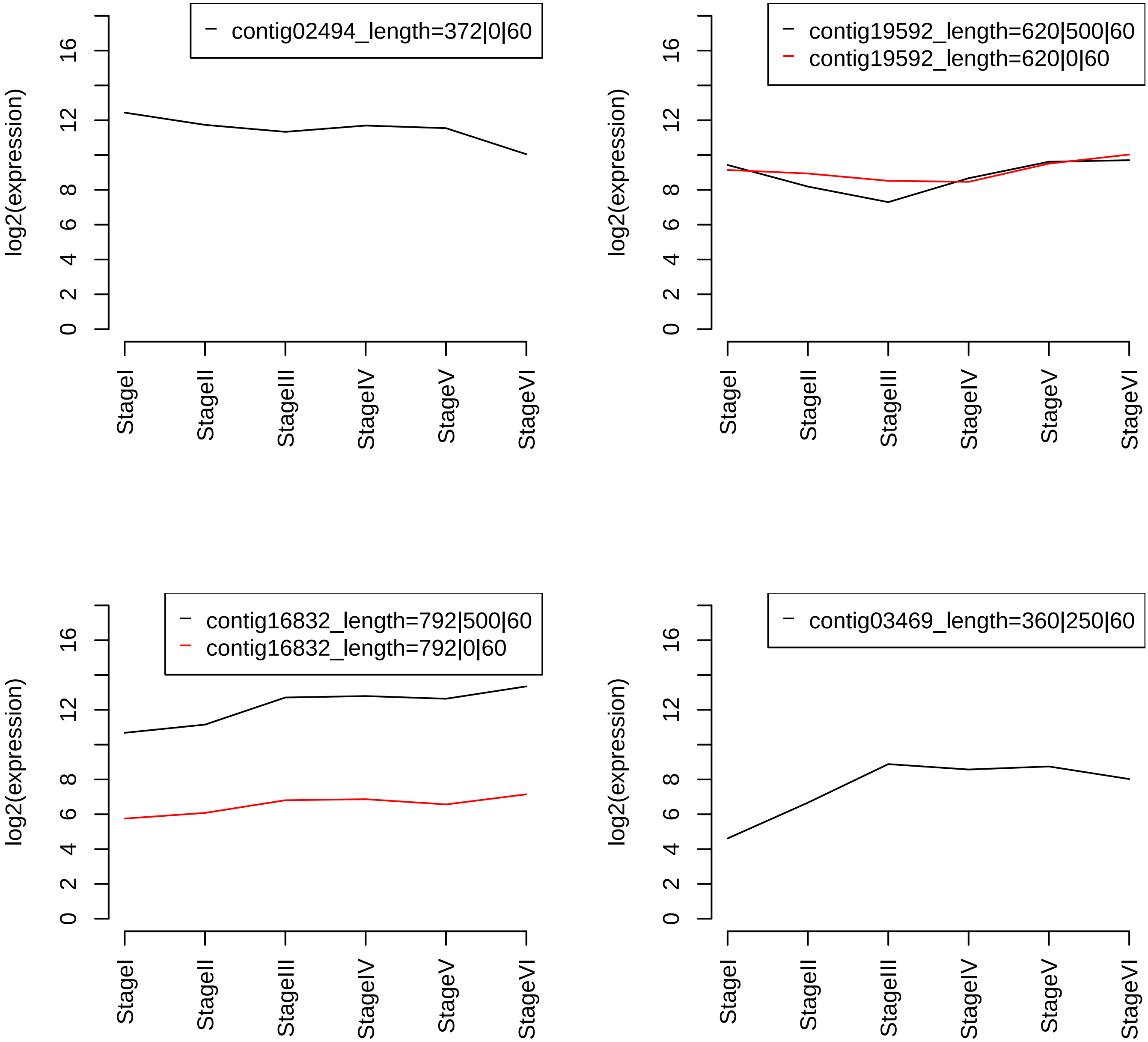

contig02494_length=372|0|60
contig19592_length=620|500|60
contig19592_length=620|0|60
contig16832_length=792|500|60
contig16832_length=792|0|60
contig03469_length=360|250|60
log2(expression)
0
2
4
6
8
12
16
StageI
StageII
StageIII
StageIV
StageV
StageVI

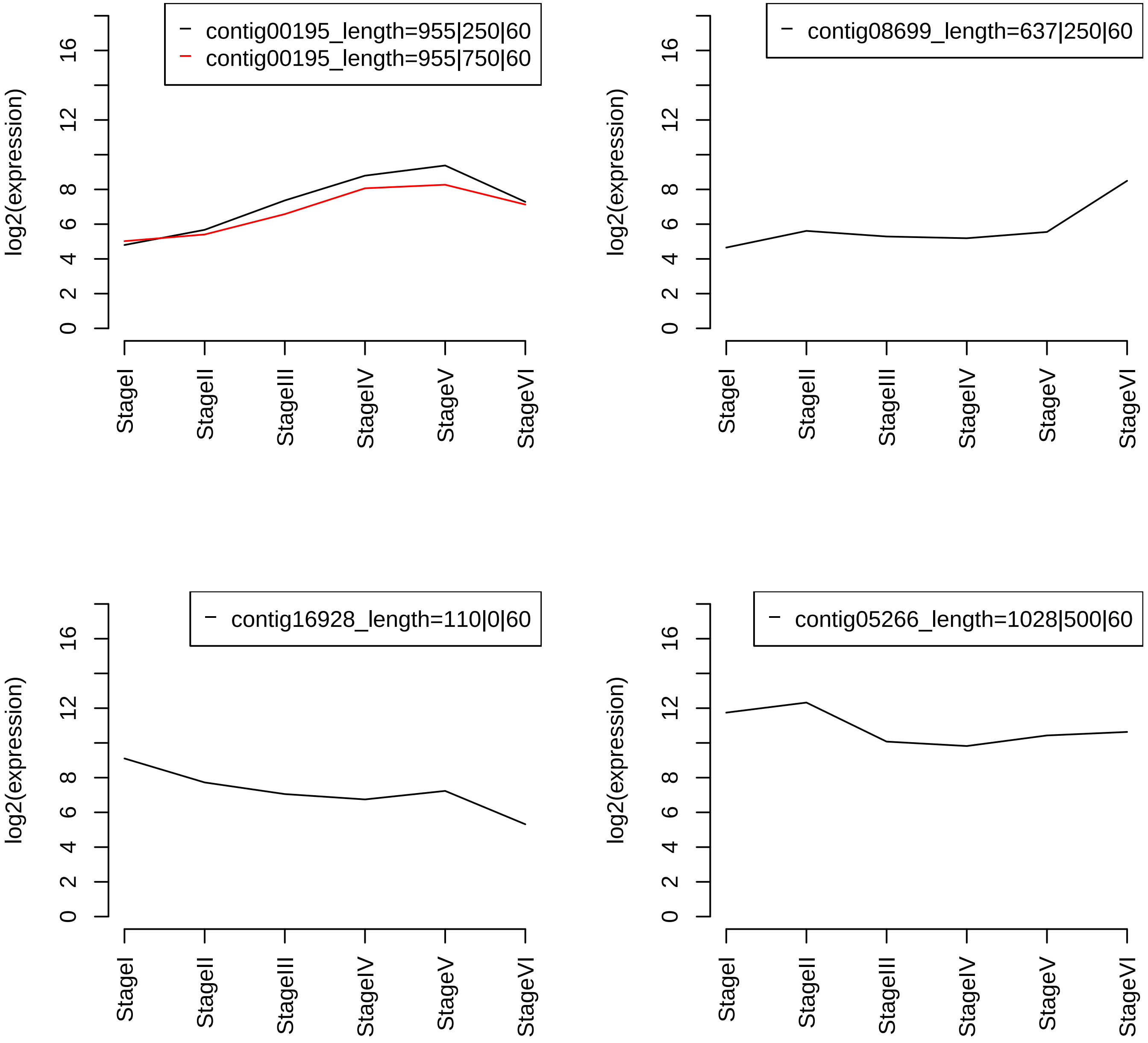
contig00195_length=955|250|60
contig00195_length=955|750|60
contig08699_length=637|250|60
contig16928_length=110|0|60
contig05266_length=1028|500|60
log2(expression)
0
2
4
6
8
12
16
StageI
StageII
StageIII
StageIV
StageV
StageVI

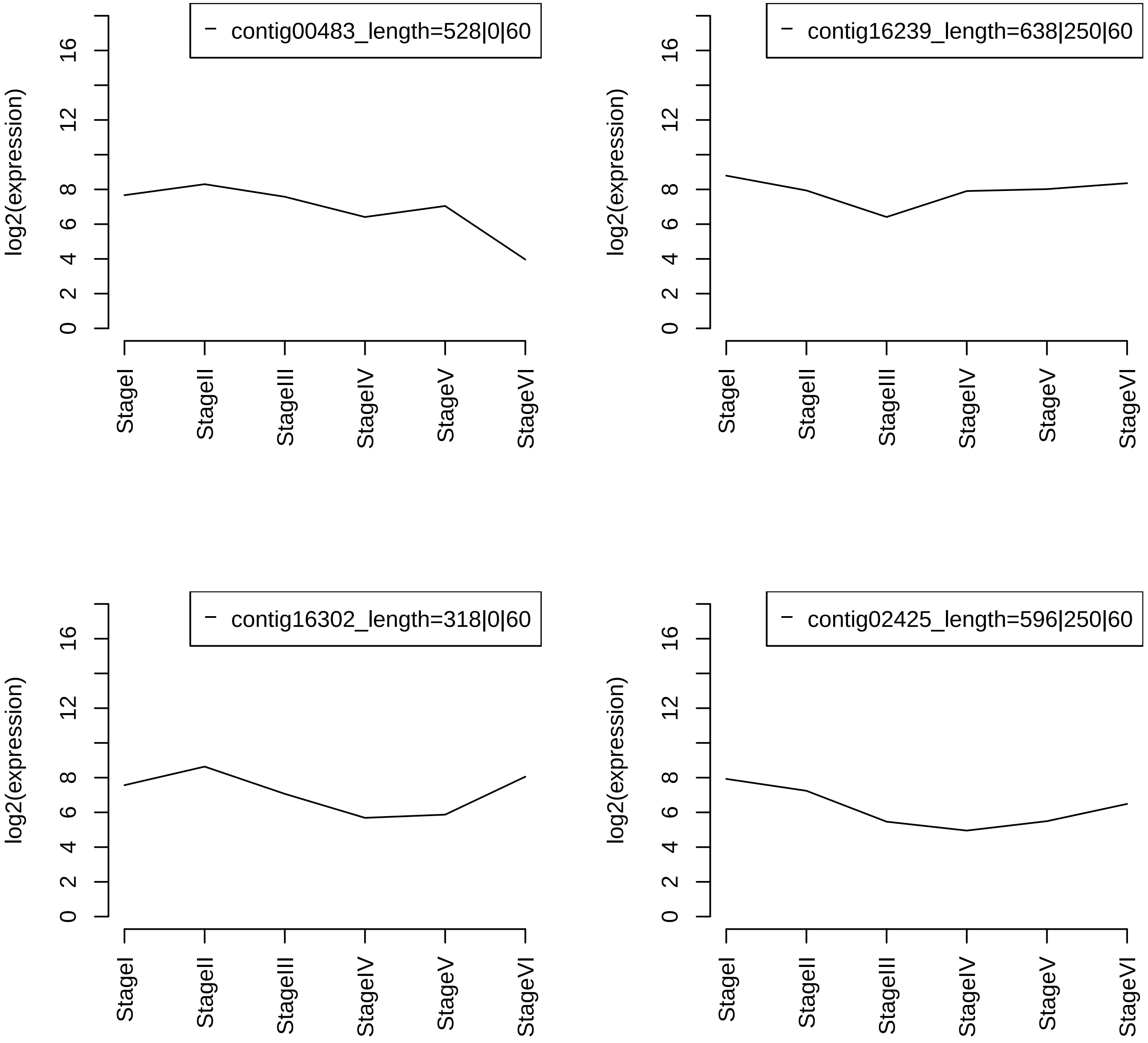
contig00483_length=528|0|60
contig16239_length=638|250|60
contig16302_length=318|0|60
contig02425_length=596|250|60
log2(expression)
0
2
4
6
8
12
16
StageI
StageII
StageIII
StageIV
StageV
StageVI

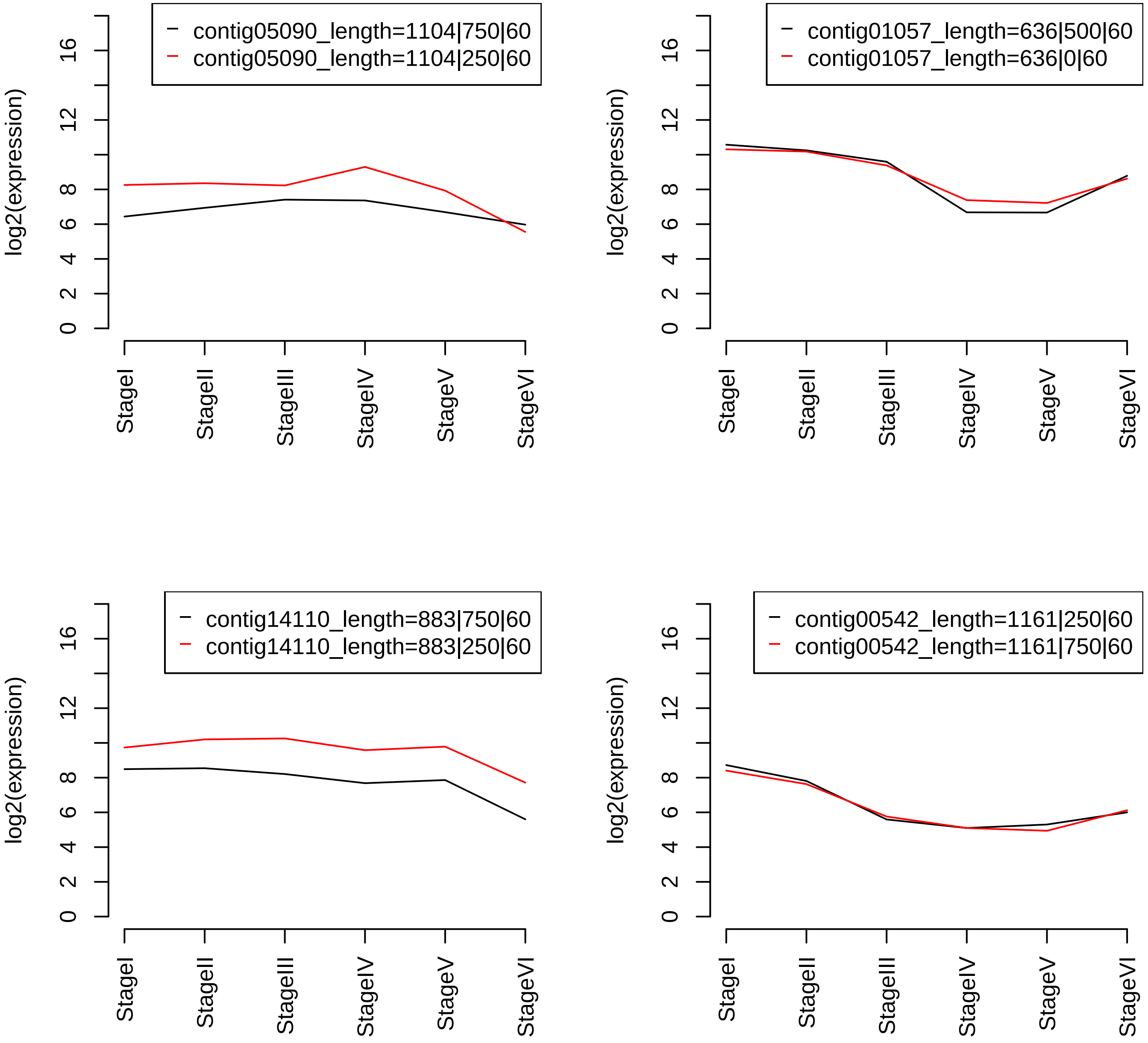

contig05090_length=1104|750|60
contig05090_length=1104|250|60
contig01057_length=636|500|60
contig01057_length=636|0|60
contig14110_length=883|750|60
contig14110_length=883|250|60
contig00542_length=1161|250|60
contig00542_length=1161|750|60
log2(expression)
0 2 4 6 8 12 16
StageI StageII StageIII StageIV StageV StageVI

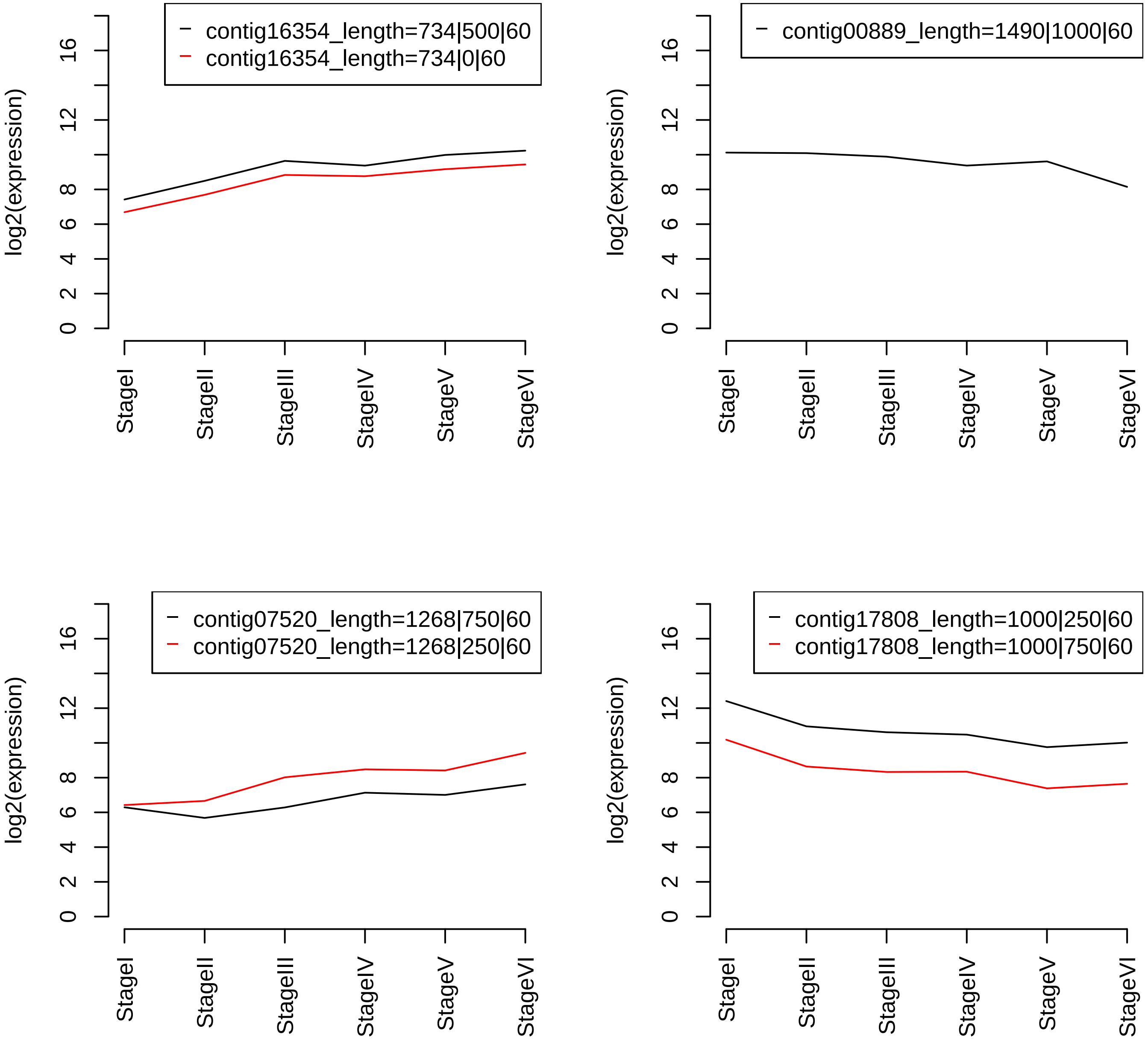
contig16354_length=734|500|60
contig16354_length=734|0|60
log2(expression)
0
2
4
6
8
12
16
StageI
StageII
StageIII
StageIV
StageV
StageVI
contig00889_length=1490|1000|60
log2(expression)
contig07520_length=1268|750|60
contig07520_length=1268|250|60
log2(expression)
contig17808_length=1000|250|60
contig17808_length=1000|750|60
log2(expression)

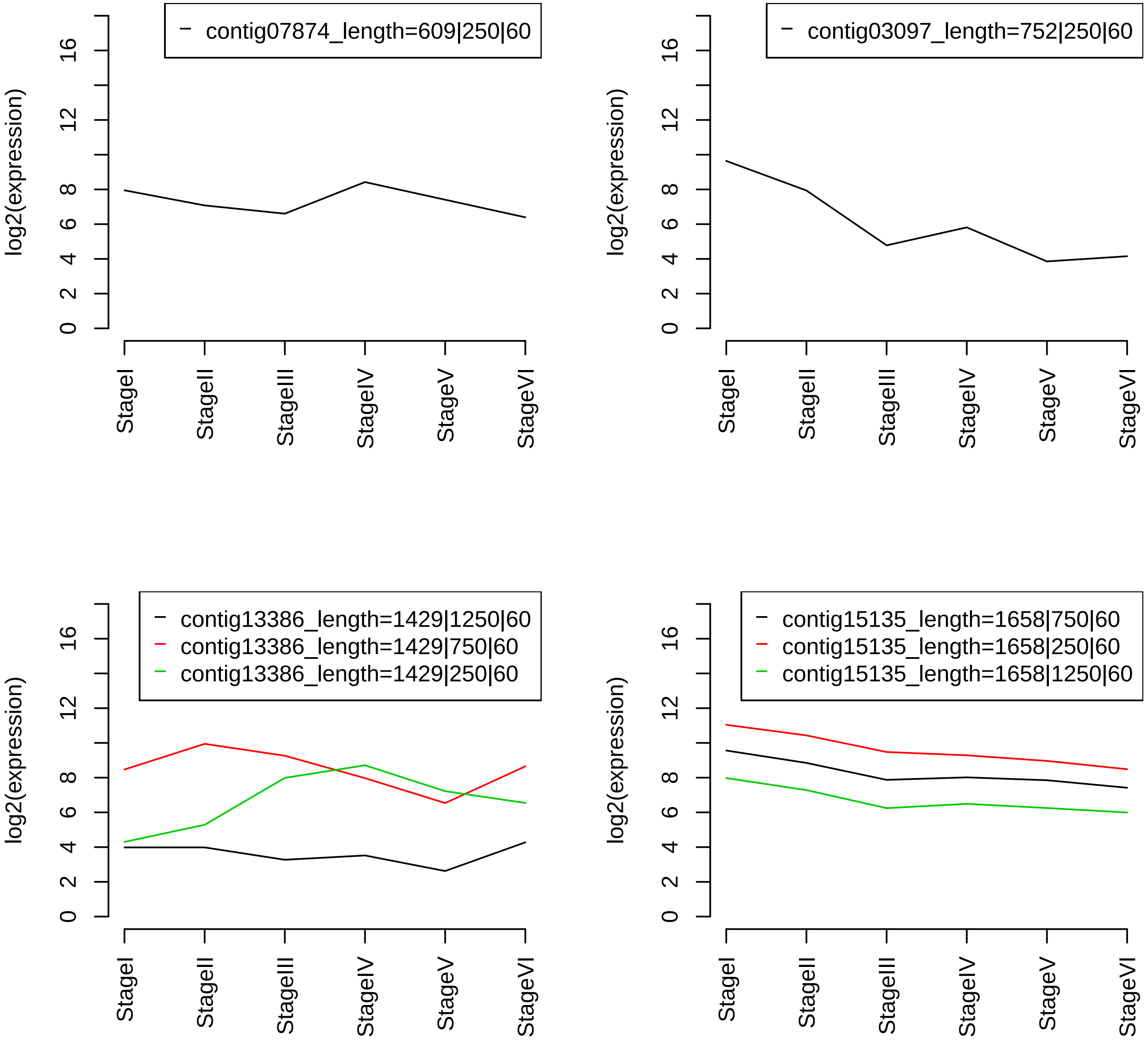

contig07874_length=609|250|60
contig03097_length=752|250|60
contig13386_length=1429|1250|60
contig13386_length=1429|750|60
contig13386_length=1429|250|60
contig15135_length=1658|750|60
contig15135_length=1658|250|60
contig15135_length=1658|1250|60
log2(expression)
0
2
4
6
8
12
16
StageI
StageII
StageIII
StageIV
StageV
StageVI

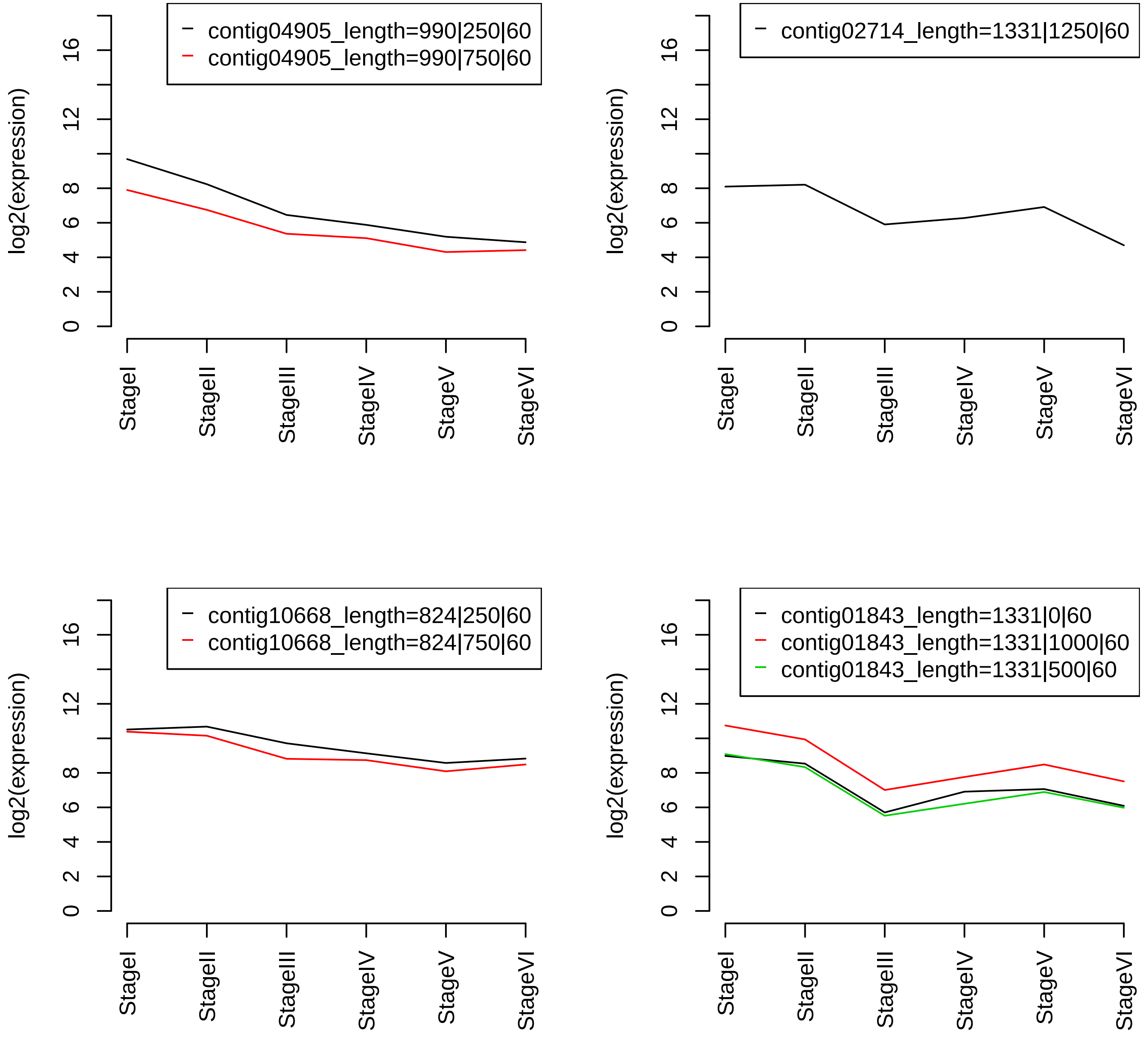
contig04905_length=990|250|60
contig04905_length=990|750|60
contig02714_length=1331|1250|60
contig10668_length=824|250|60
contig10668_length=824|750|60
contig01843_length=1331|0|60
contig01843_length=1331|1000|60
contig01843_length=1331|500|60
log2(expression)
0
2
4
6
8
12
16
StageI
StageII
StageIII
StageIV
StageV
StageVI

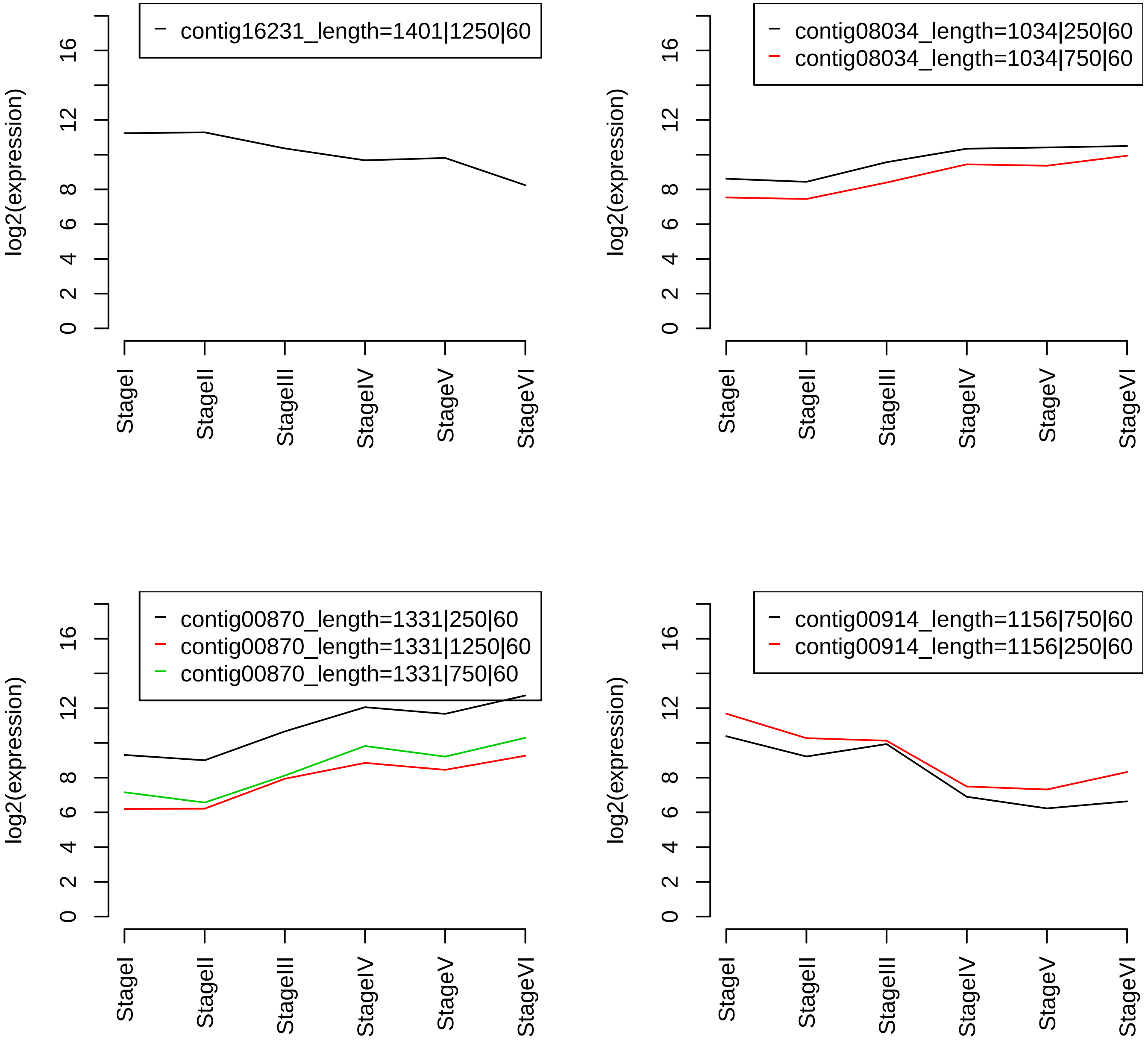

contig16231_length=1401|1250|60
contig08034_length=1034|250|60
contig08034_length=1034|750|60
contig00870_length=1331|250|60
contig00870_length=1331|1250|60
contig00870_length=1331|750|60
contig00914_length=1156|750|60
contig00914_length=1156|250|60
log2(expression)
0
2
4
6
8
12
16
StageI
StageII
StageIII
StageIV
StageV
StageVI

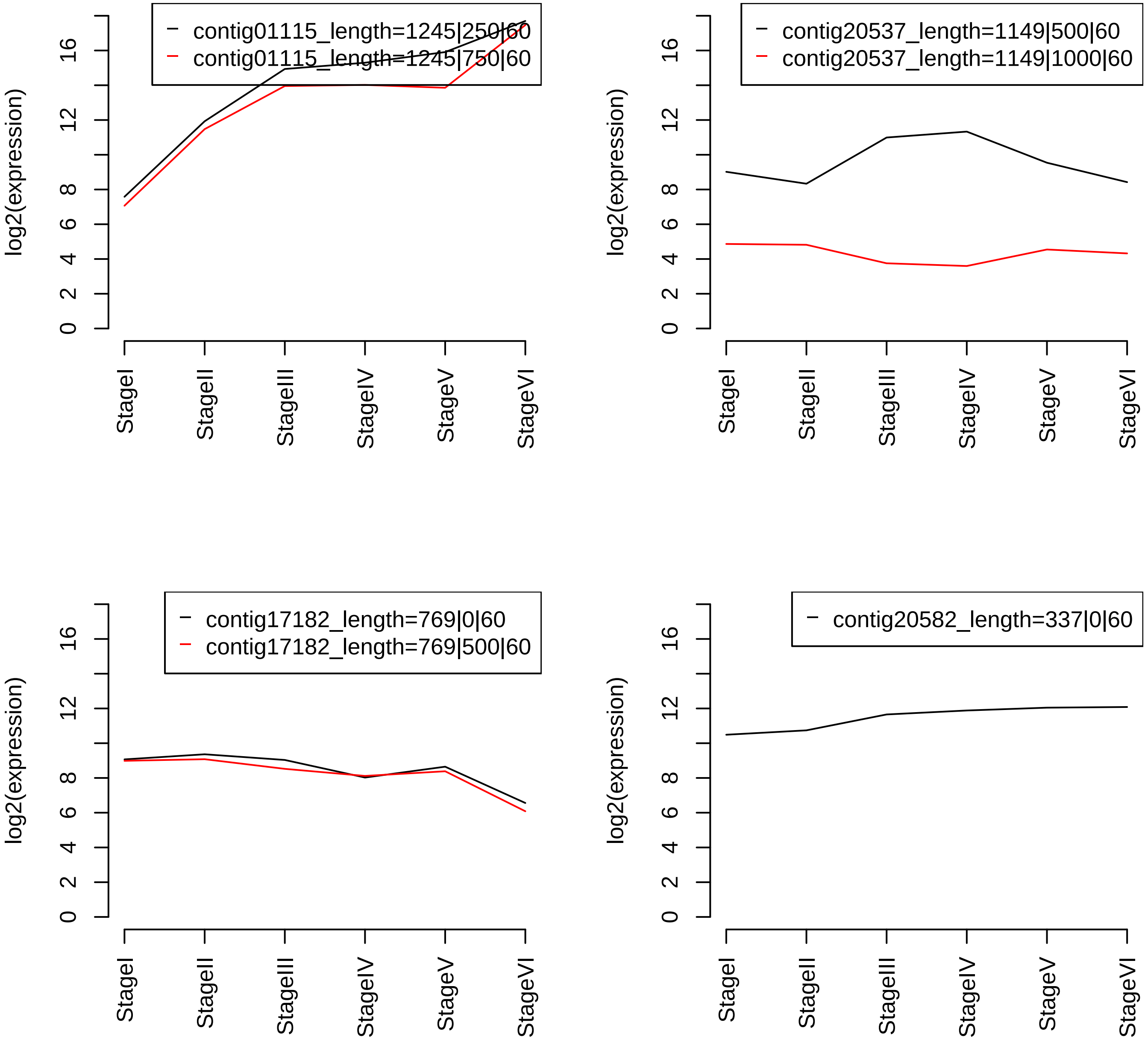
contig01115_length=1245|250|60
contig01115_length=1245|750|60
contig20537_length=1149|500|60
contig20537_length=1149|1000|60
contig17182_length=769|0|60
contig17182_length=769|500|60
contig20582_length=337|0|60
log2(expression)
0 2 4 6 8 12 16
StageI StageII StageIII StageIV StageV StageVI

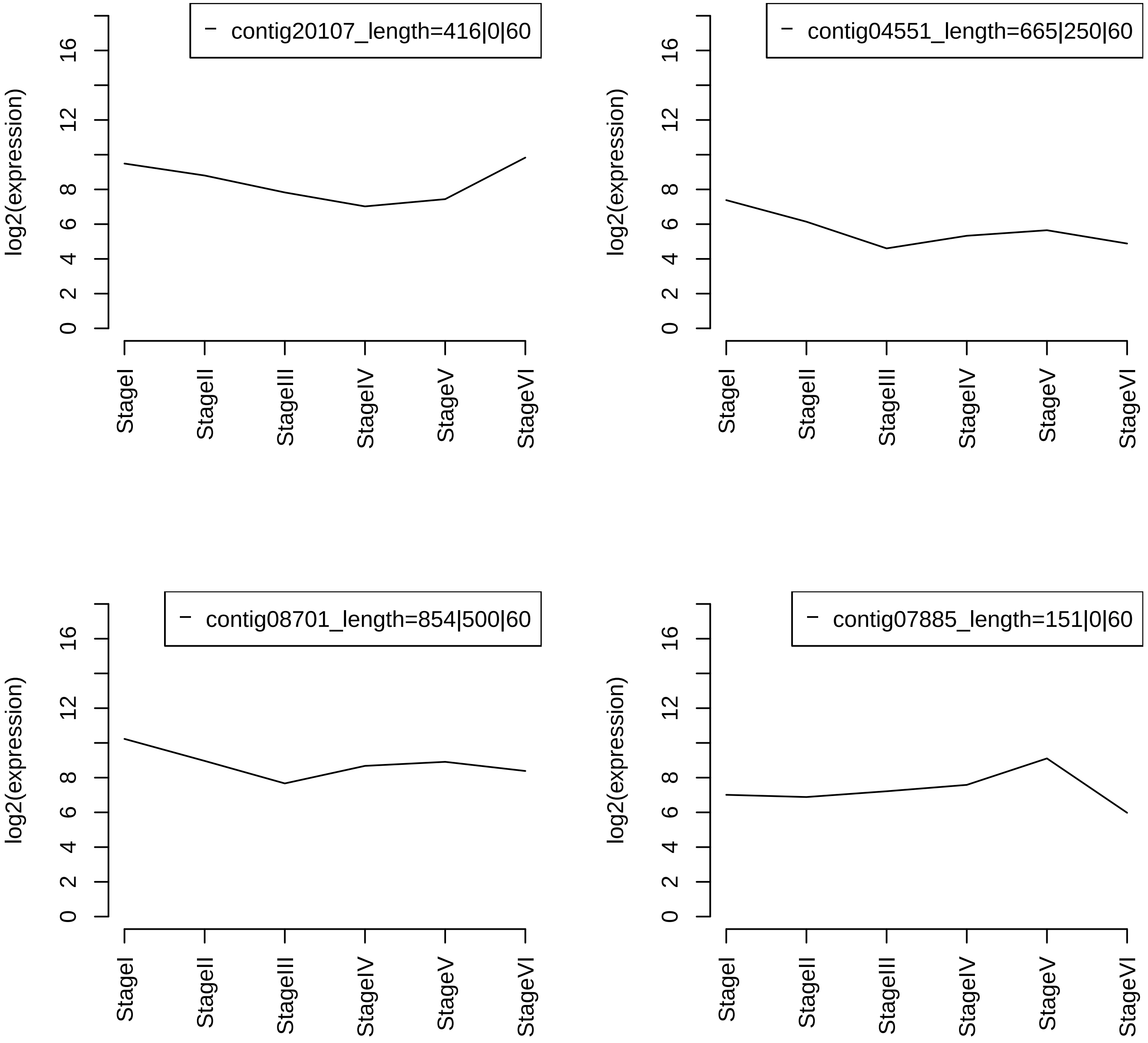
contig20107_length=416|0|60
contig04551_length=665|250|60
contig08701_length=854|500|60
contig07885_length=151|0|60
log2(expression)
0
2
4
6
8
12
16
StageI
StageII
StageIII
StageIV
StageV
StageVI

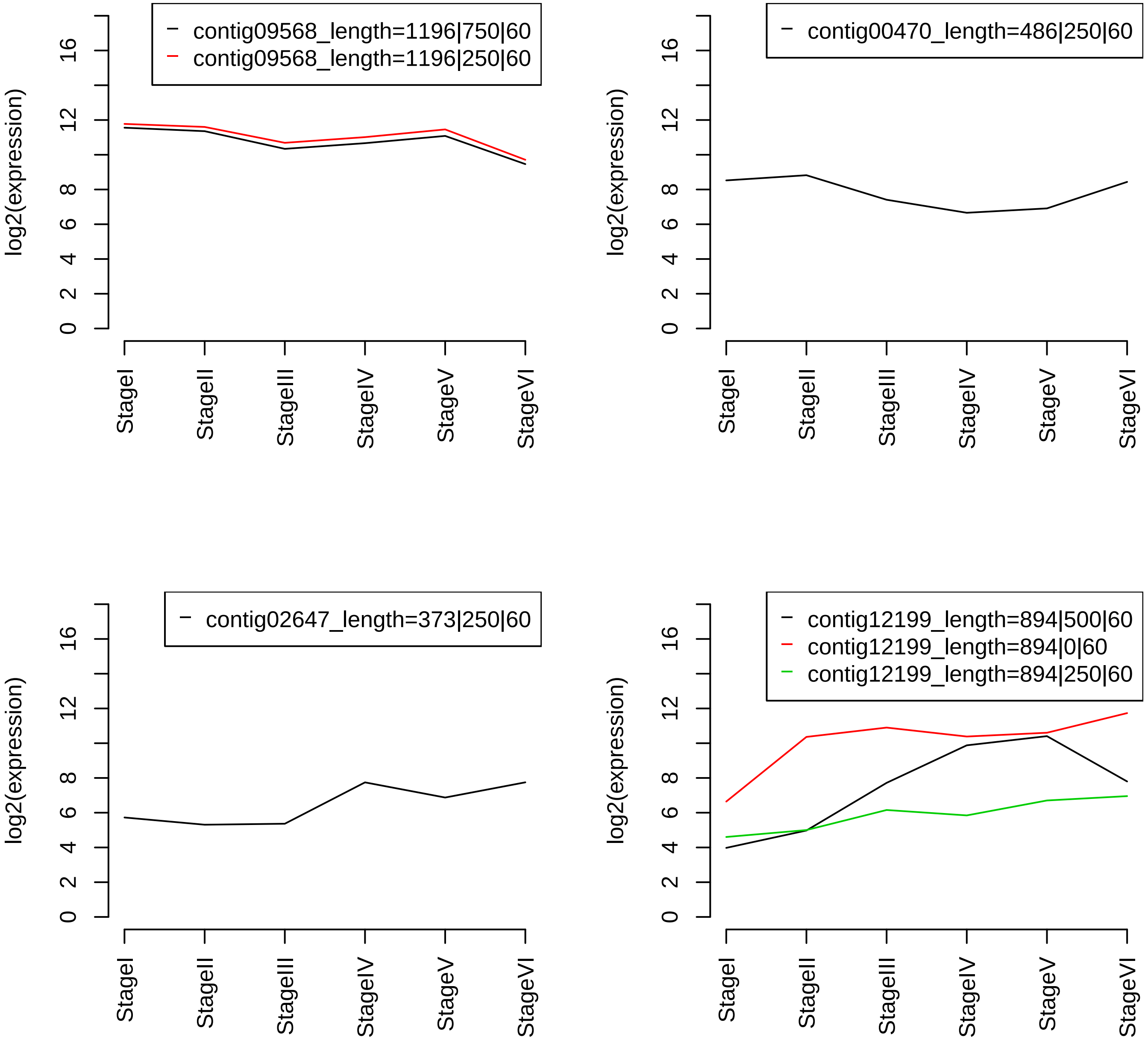

contig09568_length=1196|750|60
contig09568_length=1196|250|60
contig00470_length=486|250|60
contig02647_length=373|250|60
contig12199_length=894|500|60
contig12199_length=894|0|60
contig12199_length=894|250|60
log2(expression)
0 2 4 6 8 12 16
StageI StageII StageIII StageIV StageV StageVI

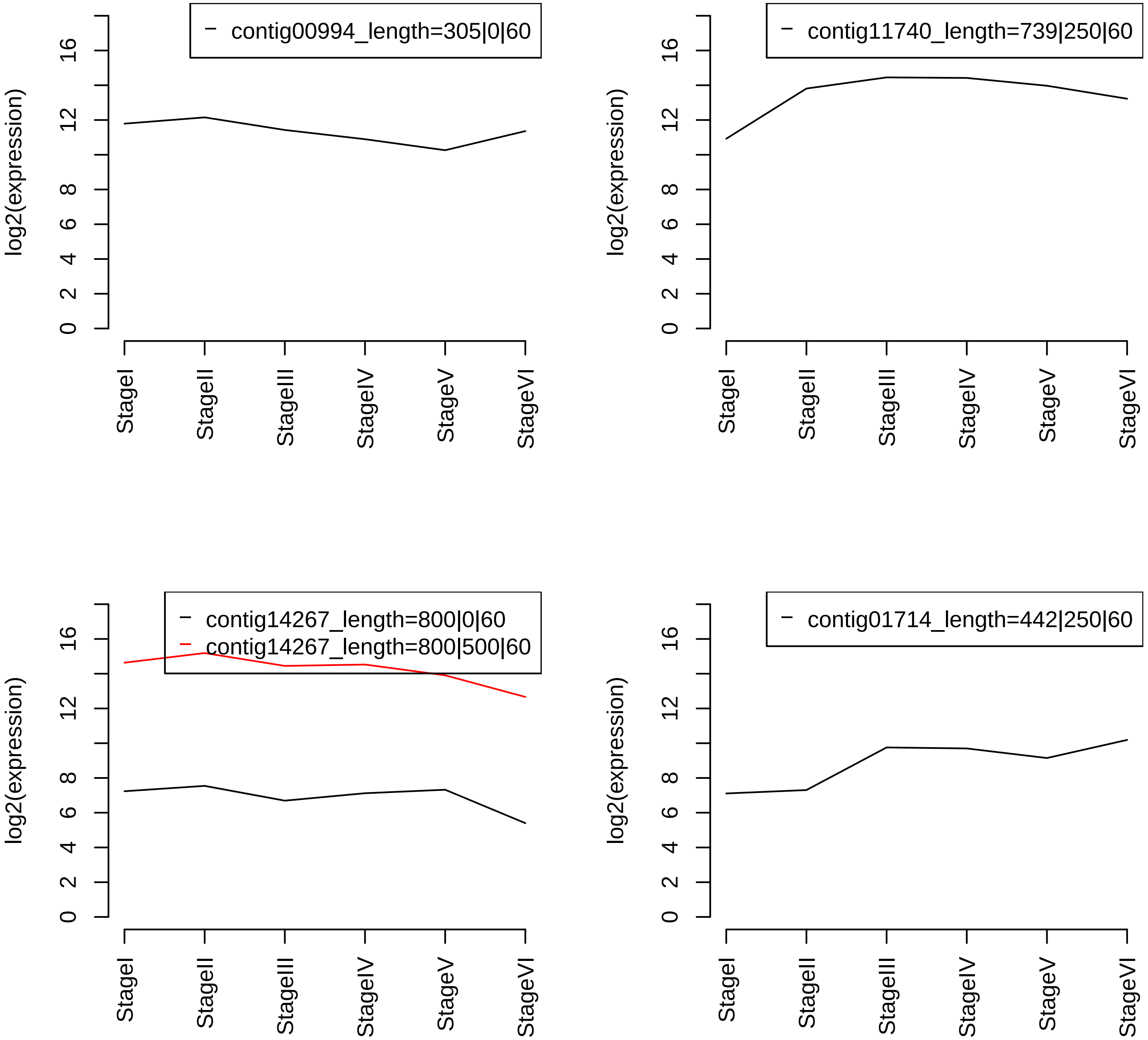

contig00994_length=305|0|60
contig11740_length=739|250|60
contig14267_length=800|0|60
contig14267_length=800|500|60
contig01714_length=442|250|60
log2(expression)
0
2
4
6
8
12
16
StageI
StageII
StageIII
StageIV
StageV
StageVI

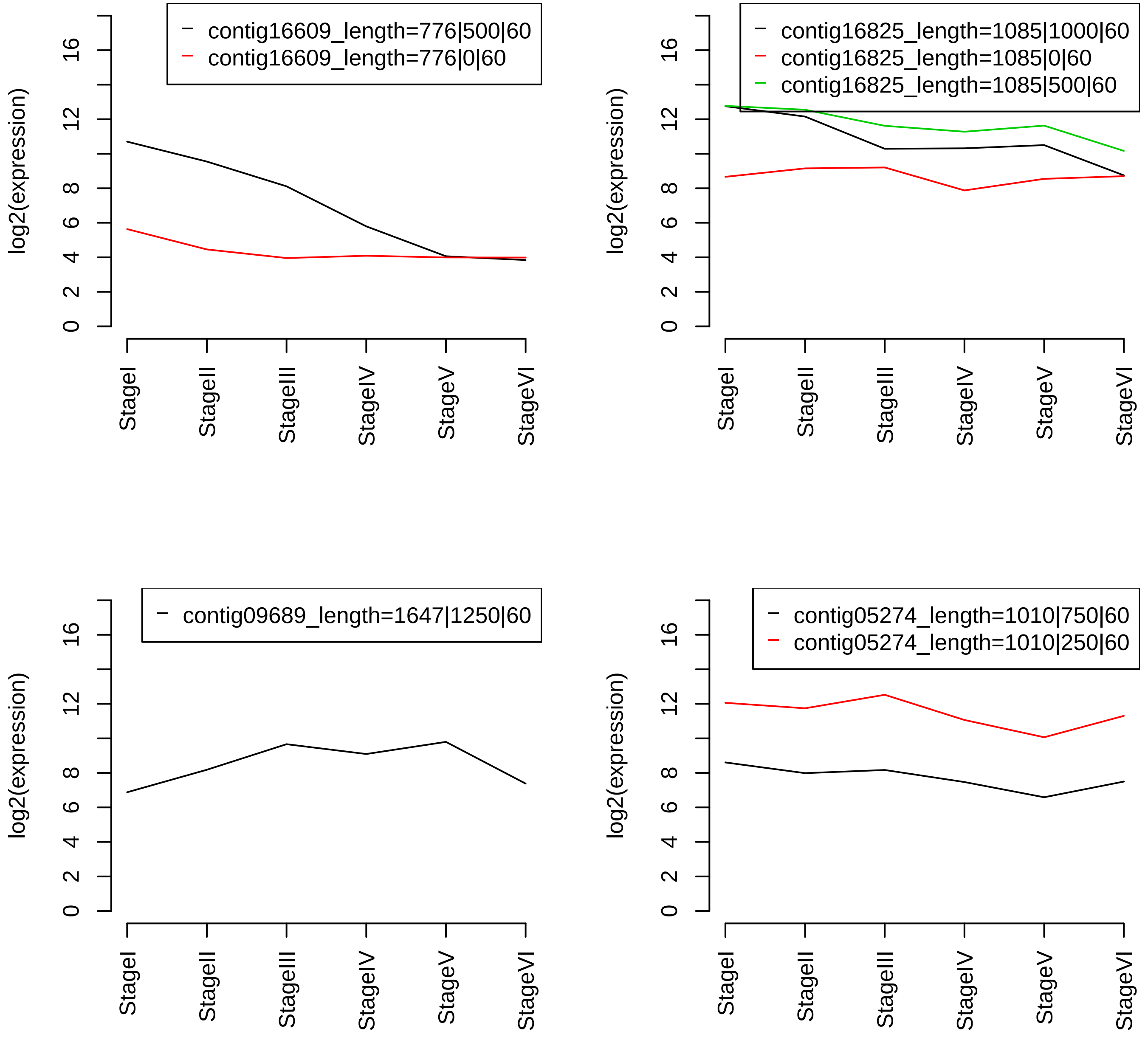

contig16609_length=776|500|60
contig16609_length=776|0|60
contig16825_length=1085|1000|60
contig16825_length=1085|0|60
contig16825_length=1085|500|60
contig09689_length=1647|1250|60
contig05274_length=1010|750|60
contig05274_length=1010|250|60
log2(expression)
0
2
4
6
8
12
16
StageI
StageII
StageIII
StageIV
StageV
StageVI

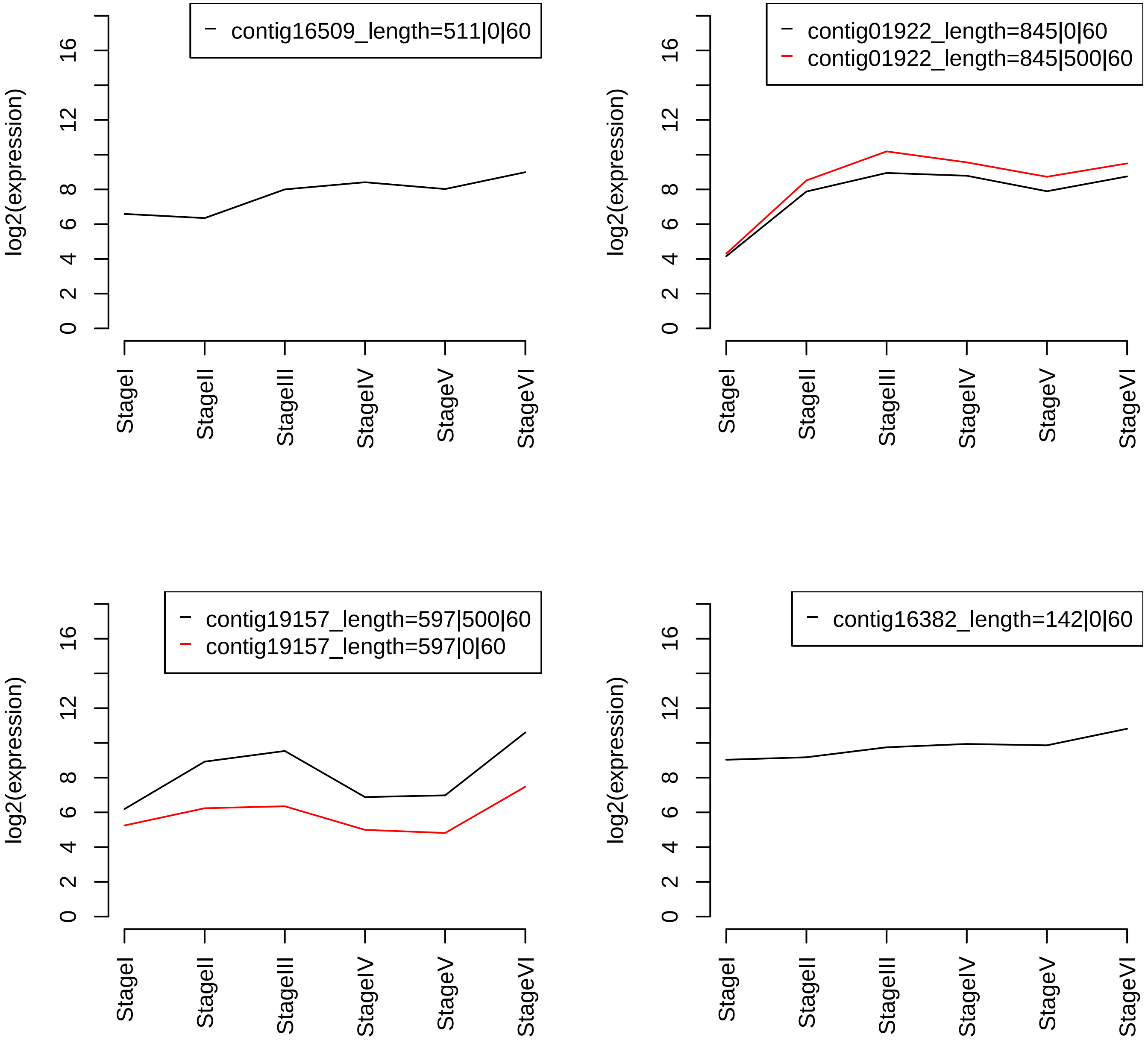
contig16509_length=511|0|60
contig01922_length=845|0|60
contig01922_length=845|500|60
contig19157_length=597|500|60
contig19157_length=597|0|60
contig16382_length=142|0|60
log2(expression)
StageI
StageII
StageIII
StageIV
StageV
StageVI

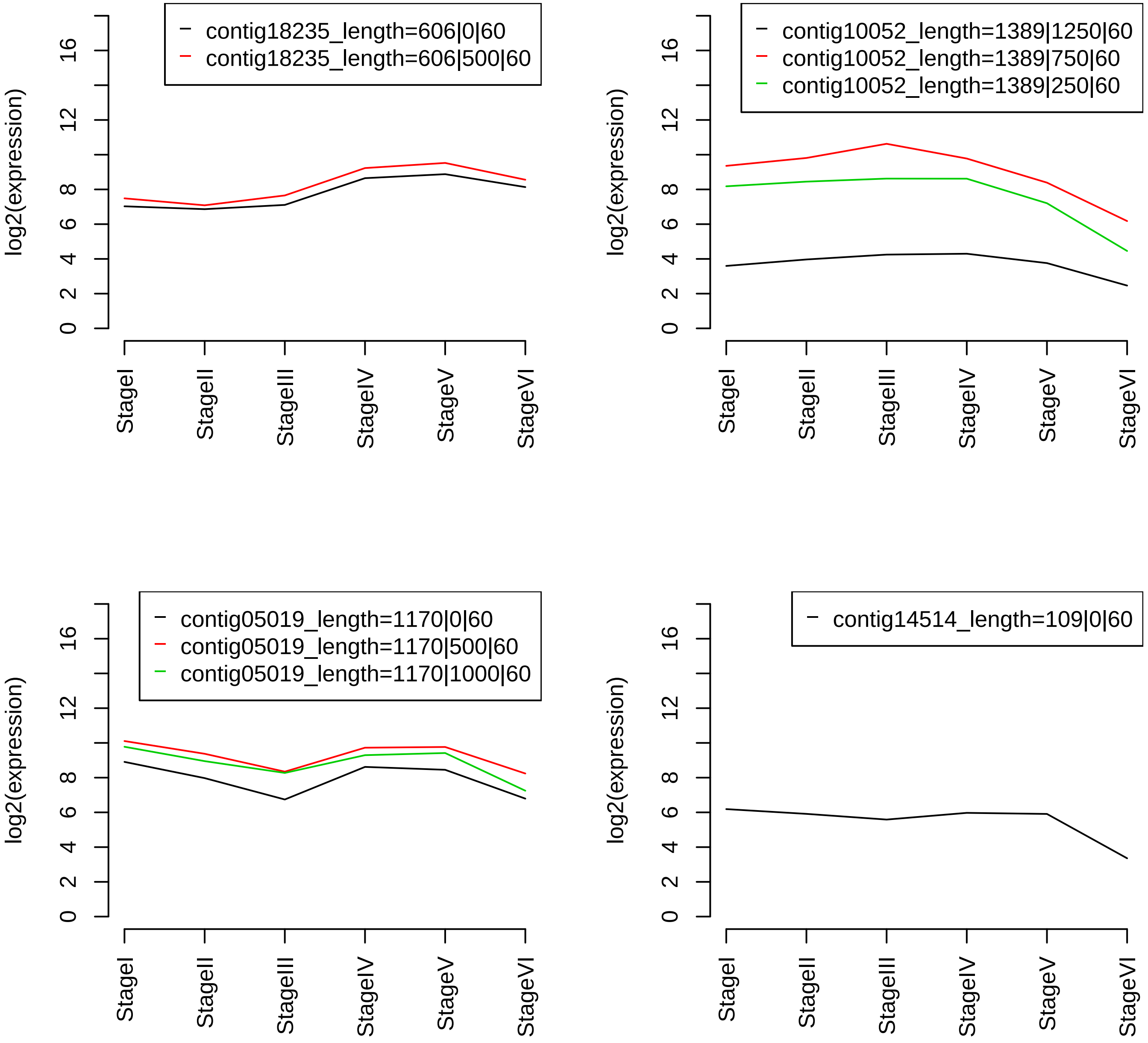
contig18235_length=606|0|60
contig18235_length=606|500|60
contig10052_length=1389|1250|60
contig10052_length=1389|750|60
contig10052_length=1389|250|60
contig05019_length=1170|0|60
contig05019_length=1170|500|60
contig05019_length=1170|1000|60
contig14514_length=109|0|60
log2(expression)
0 2 4 6 8 12 16
StageI StageII StageIII StageIV StageV StageVI

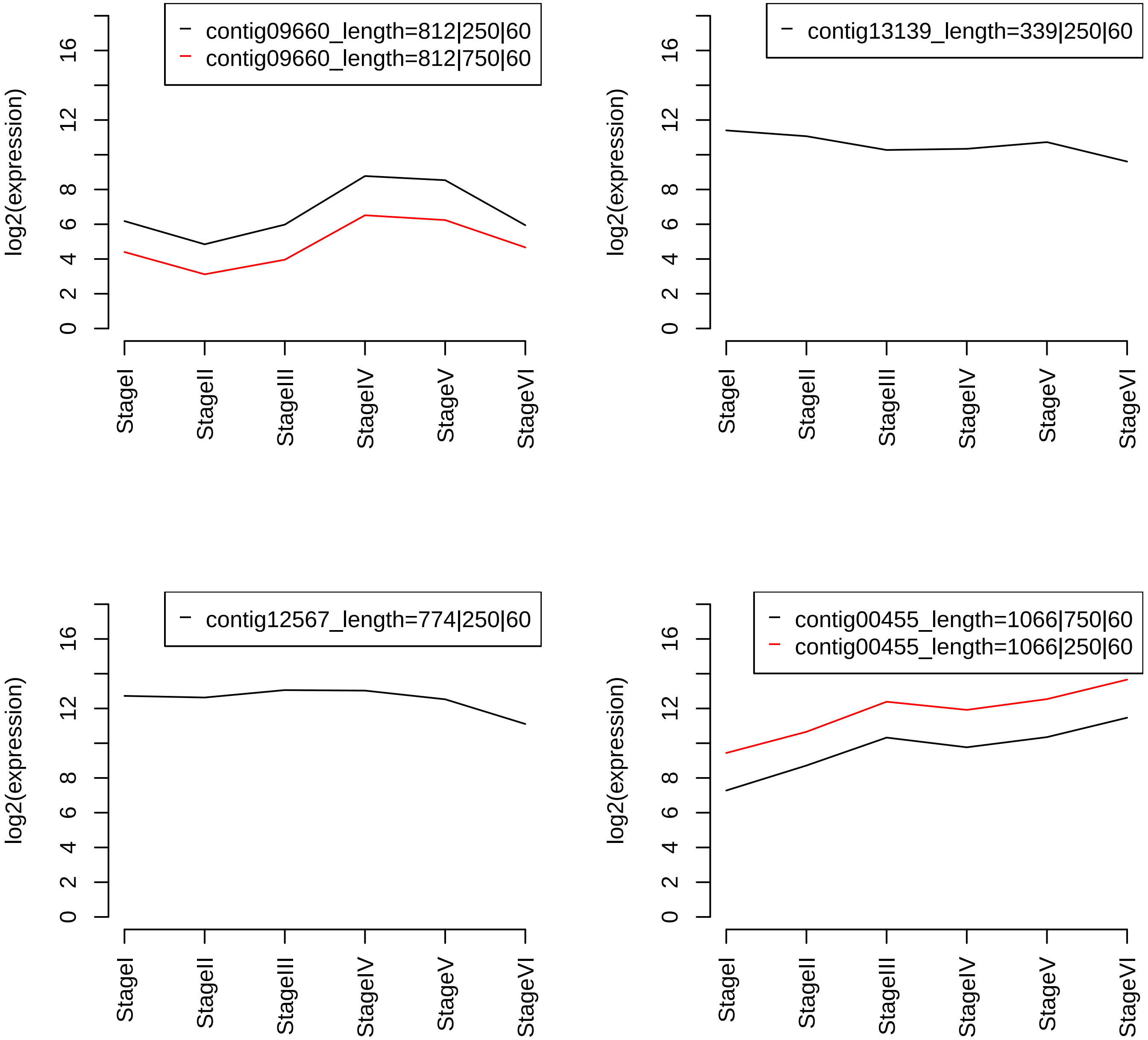

contig09660_length=812|250|60
contig09660_length=812|750|60
contig13139_length=339|250|60
contig12567_length=774|250|60
contig00455_length=1066|750|60
contig00455_length=1066|250|60
log2(expression)
0 2 4 6 8 12 16
StageI StageII StageIII StageIV StageV StageVI

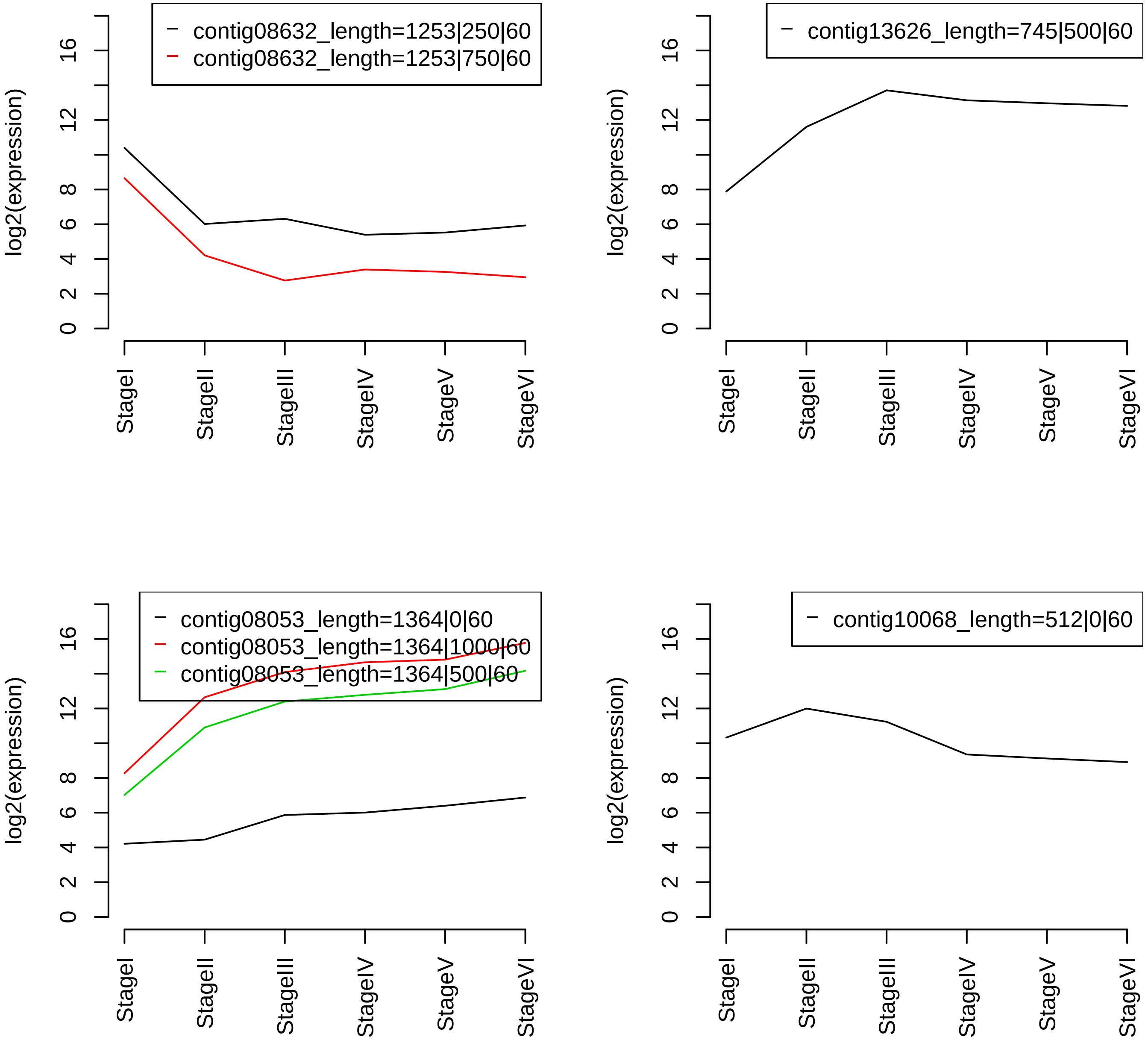
contig08632_length=1253|250|60
contig08632_length=1253|750|60
contig13626_length=745|500|60
contig08053_length=1364|0|60
contig08053_length=1364|1000|60
contig08053_length=1364|500|60
contig10068_length=512|0|60
log2(expression)
StageI
StageII
StageIII
StageIV
StageV
StageVI

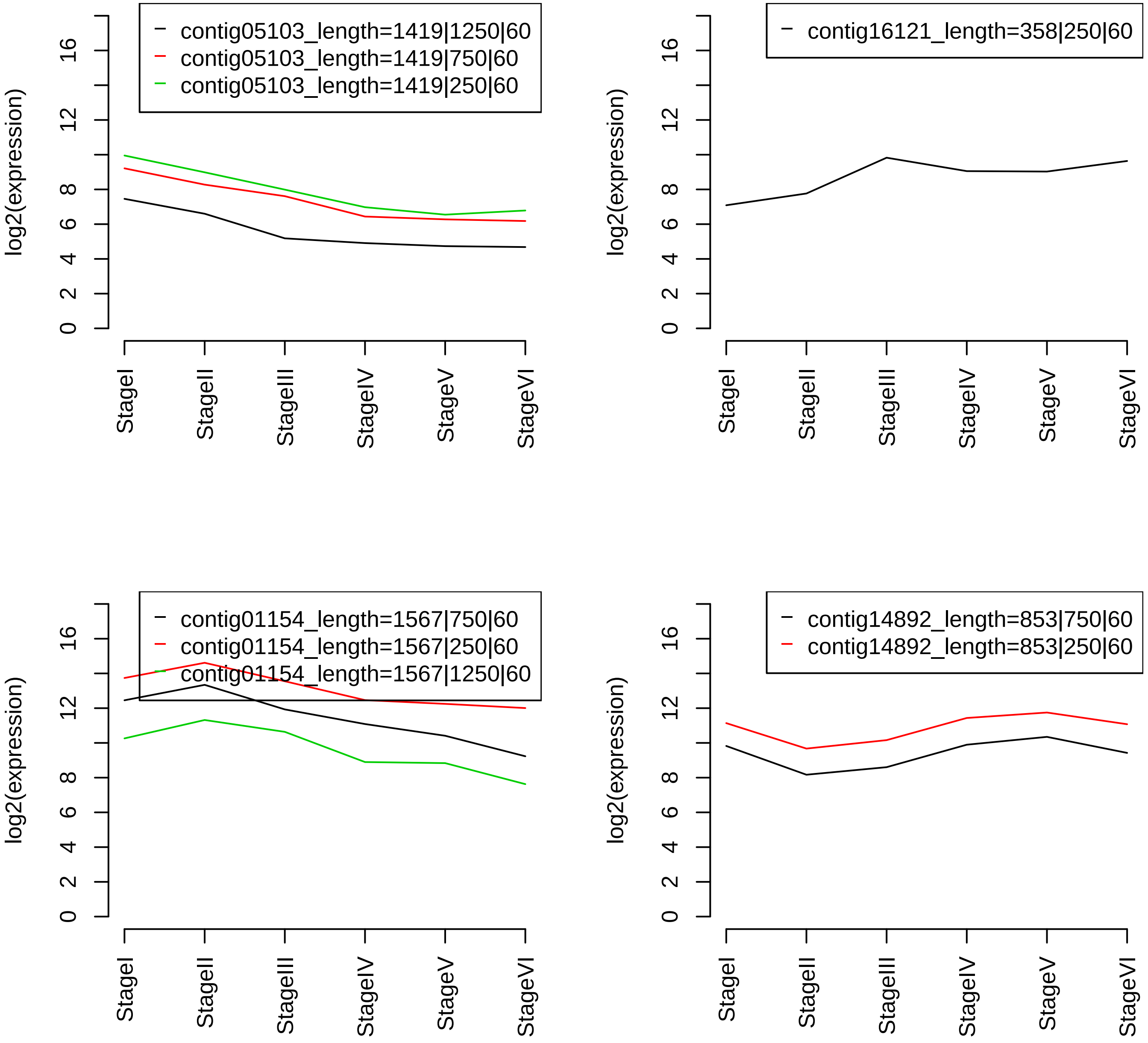

contig05103_length=1419|1250|60
contig05103_length=1419|750|60
contig05103_length=1419|250|60
contig16121_length=358|250|60
contig01154_length=1567|750|60
contig01154_length=1567|250|60
contig01154_length=1567|1250|60
contig14892_length=853|750|60
contig14892_length=853|250|60
log2(expression)
StageI
StageII
StageIII
StageIV
StageV
StageVI

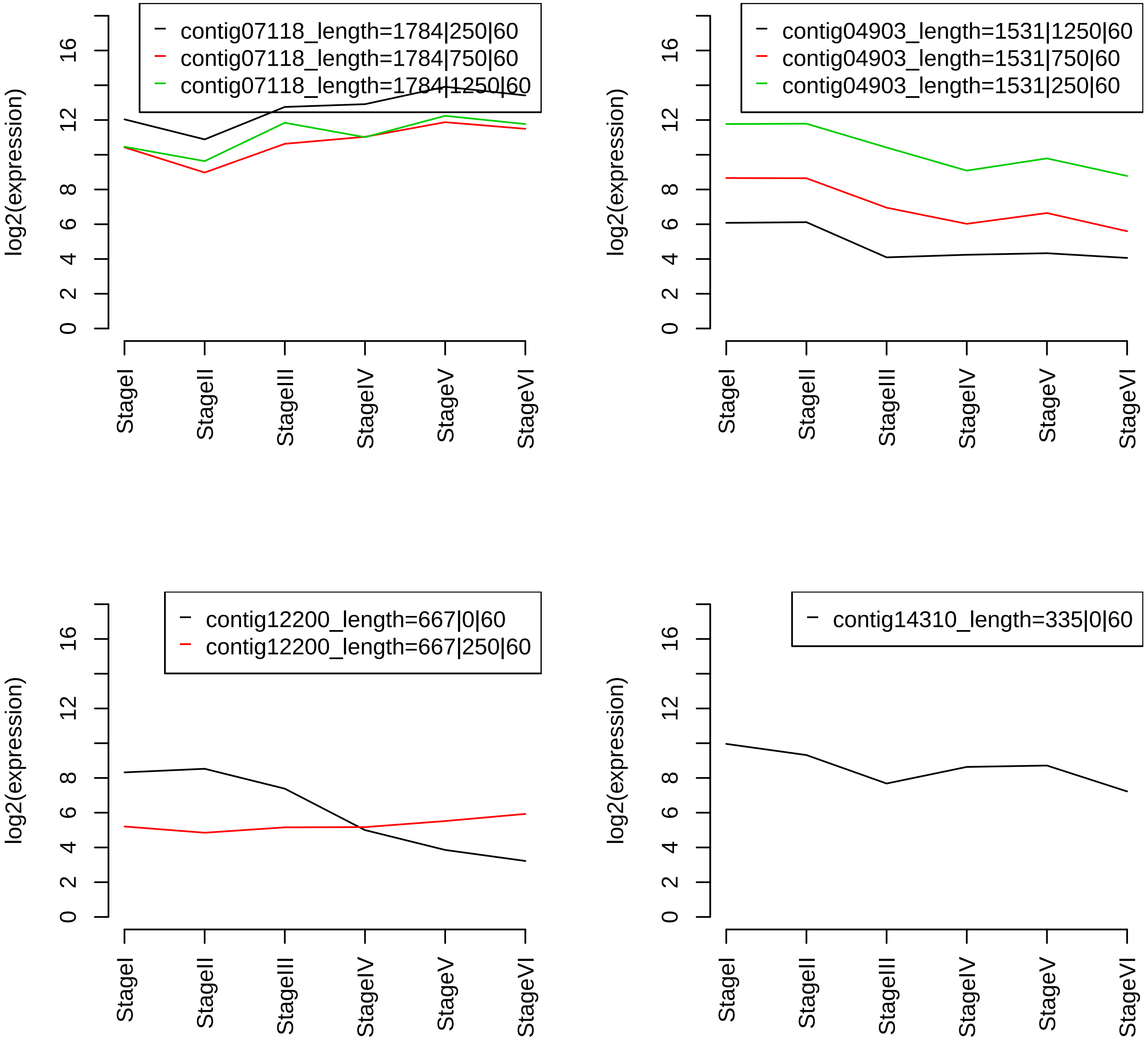

contig07118_length=1784|250|60
contig07118_length=1784|750|60
contig07118_length=1784|1250|60
contig04903_length=1531|1250|60
contig04903_length=1531|750|60
contig04903_length=1531|250|60
contig12200_length=667|0|60
contig12200_length=667|250|60
contig14310_length=335|0|60
log2(expression)
0 2 4 6 8 12 16
StageI StageII StageIII StageIV StageV StageVI

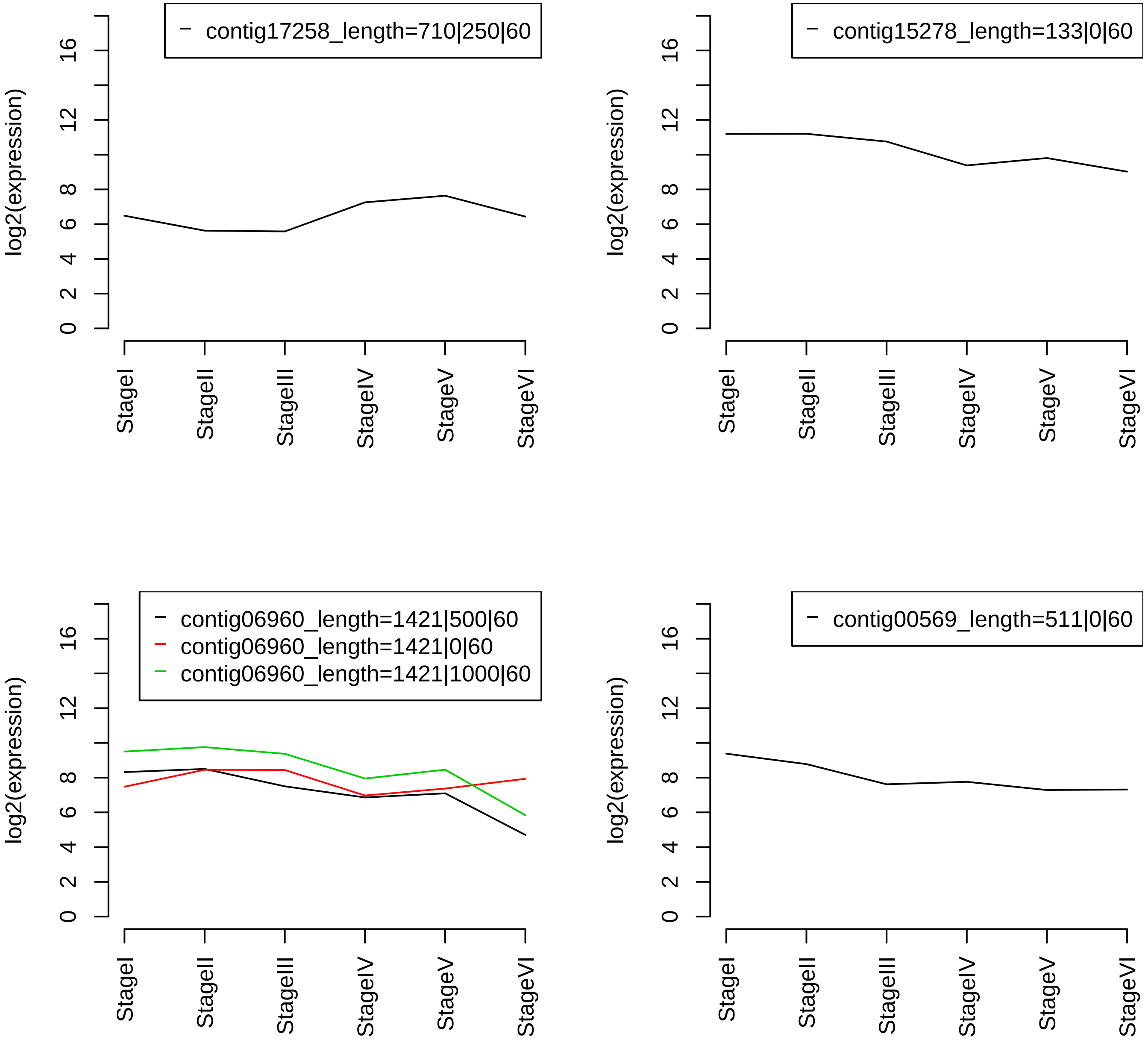

contig17258_length=710|250|60
contig15278_length=133|0|60
contig06960_length=1421|500|60
contig06960_length=1421|0|60
contig06960_length=1421|1000|60
contig00569_length=511|0|60
log2(expression)
0 2 4 6 8 12 16
StageI StageII StageIII StageIV StageV StageVI

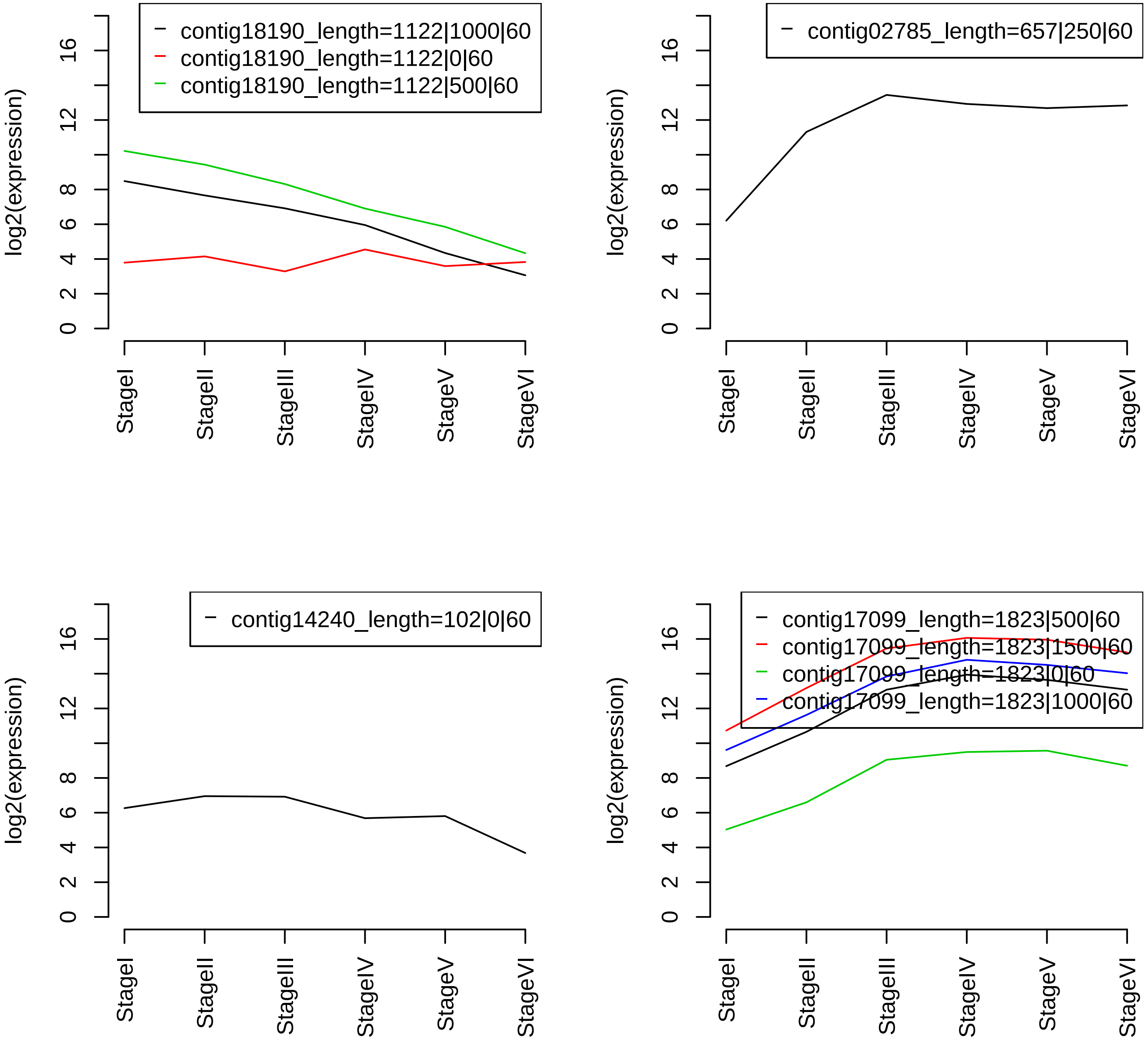

contig18190_length=1122|1000|60
contig18190_length=1122|0|60
contig18190_length=1122|500|60
contig02785_length=657|250|60
contig14240_length=102|0|60
contig17099_length=1823|500|60
contig17099_length=1823|1500|60
contig17099_length=1823|0|60
contig17099_length=1823|1000|60
log2(expression)
0
2
4
6
8
12
16
StageI
StageII
StageIII
StageIV
StageV
StageVI

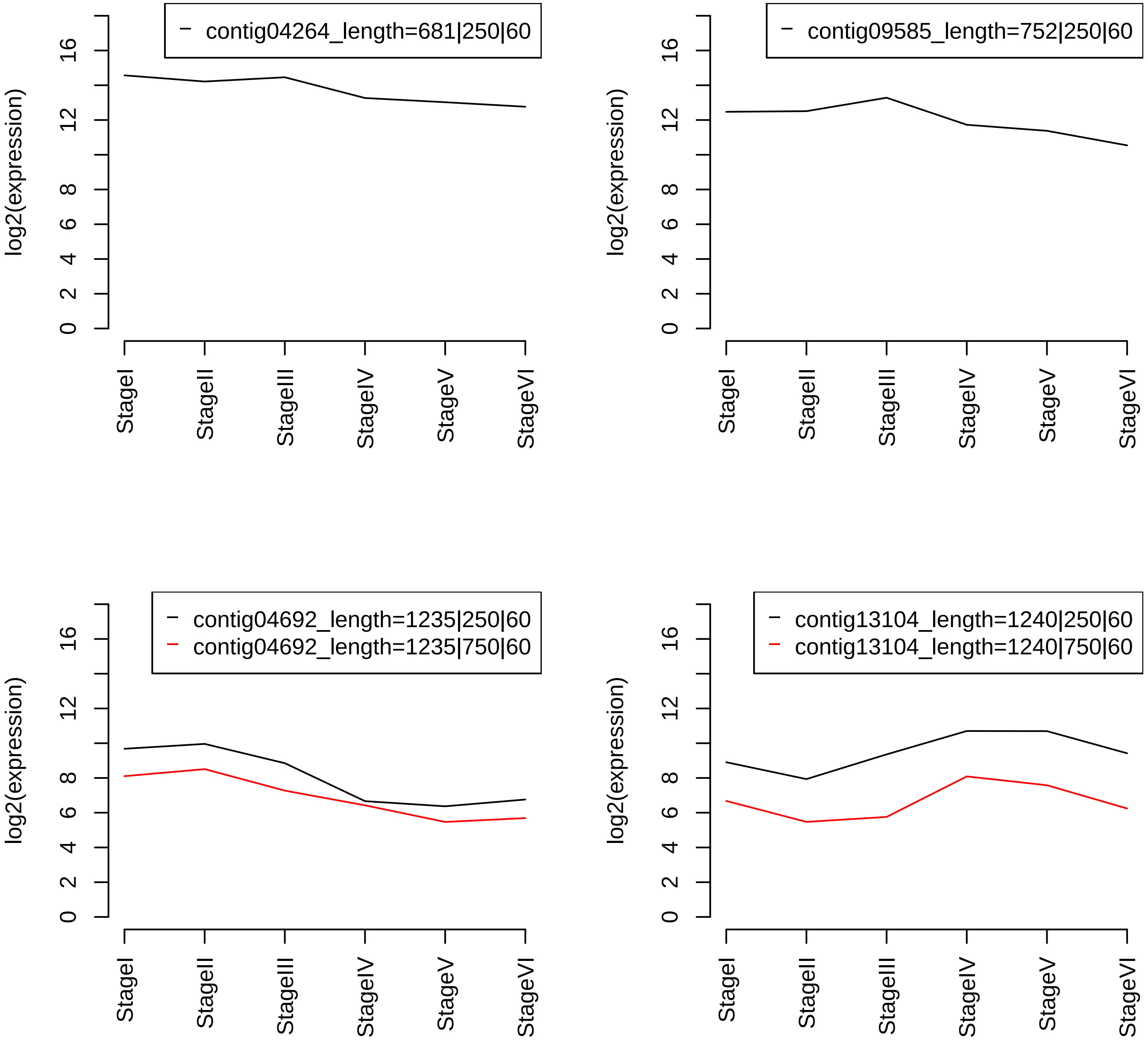

contig04264_length=681|250|60
log2(expression)
0
2
4
6
8
12
16
StageI
StageII
StageIII
StageIV
StageV
StageVI
contig09585_length=752|250|60
log2(expression)
contig04692_length=1235|250|60
contig04692_length=1235|750|60
log2(expression)
contig13104_length=1240|250|60
contig13104_length=1240|750|60
log2(expression)

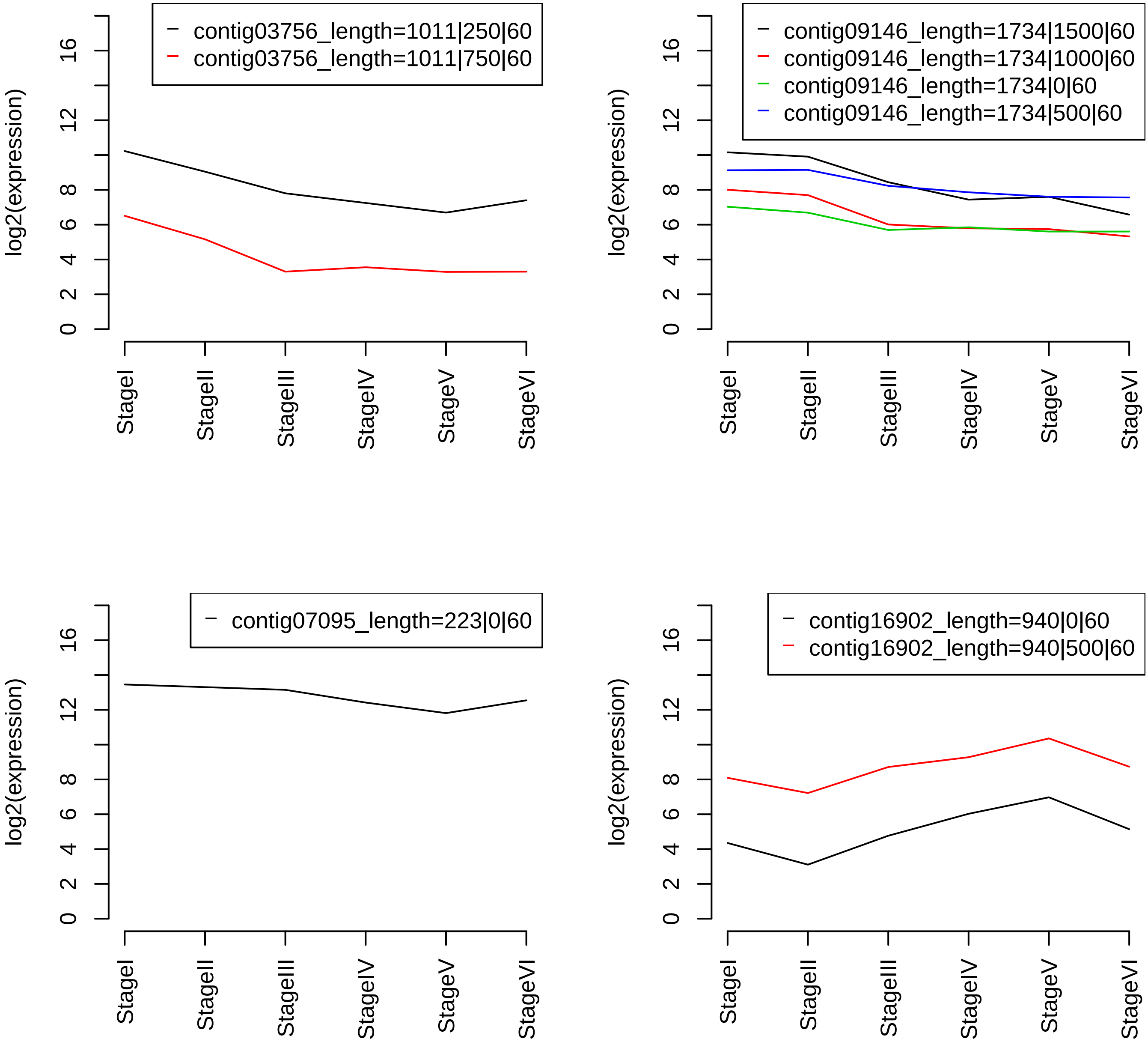

contig03756_length=1011|250|60
contig03756_length=1011|750|60
log2(expression)
0
2
4
6
8
12
16
StageI
StageII
StageIII
StageIV
StageV
StageVI
contig09146_length=1734|1500|60
contig09146_length=1734|1000|60
contig09146_length=1734|0|60
contig09146_length=1734|500|60
contig07095_length=223|0|60
contig16902_length=940|0|60
contig16902_length=940|500|60

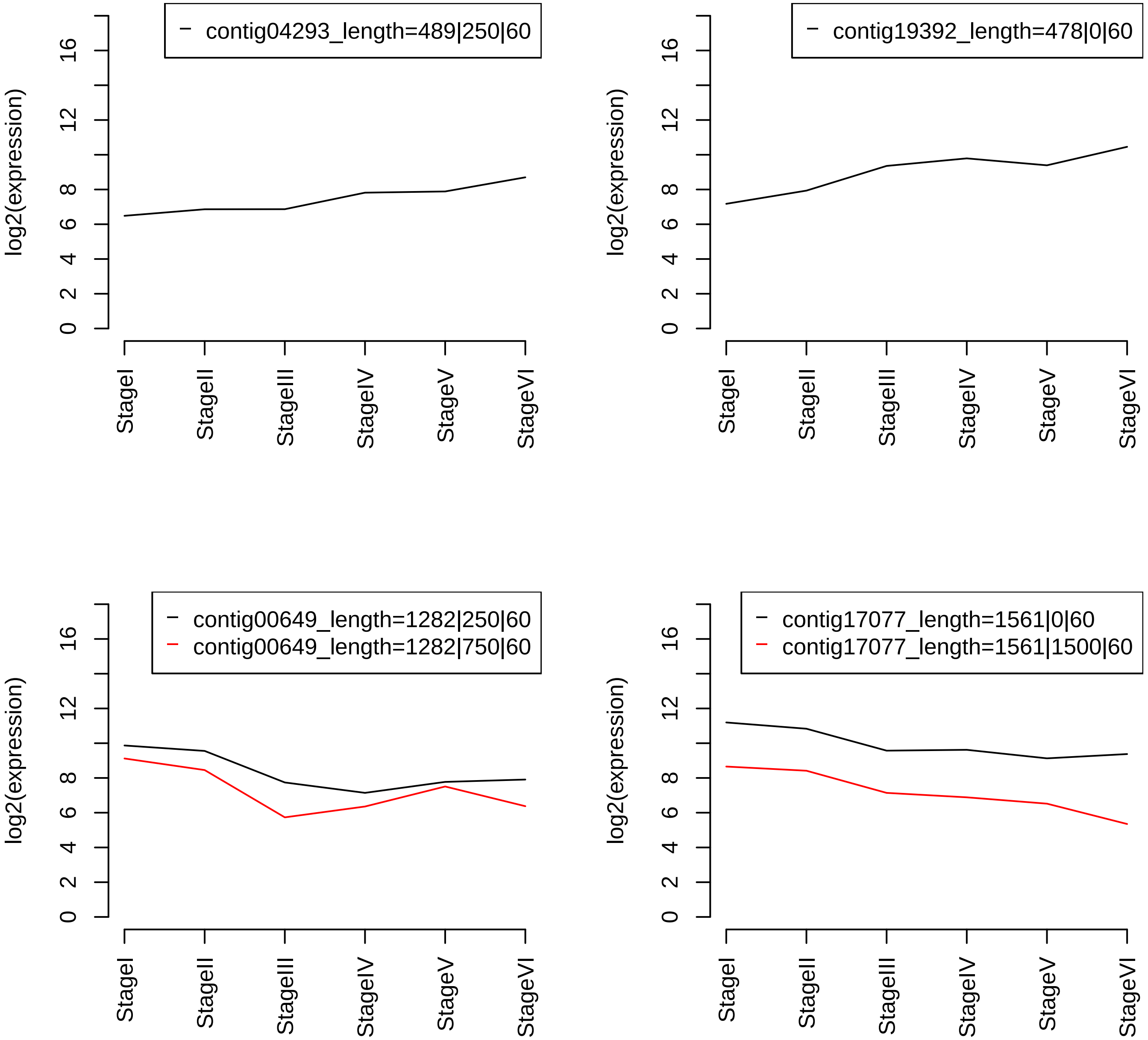
contig04293_length=489|250|60
contig19392_length=478|0|60
contig00649_length=1282|250|60
contig00649_length=1282|750|60
contig17077_length=1561|0|60
contig17077_length=1561|1500|60
log2(expression)
0 2 4 6 8 12 16
StageI
StageII
StageIII
StageIV
StageV
StageVI

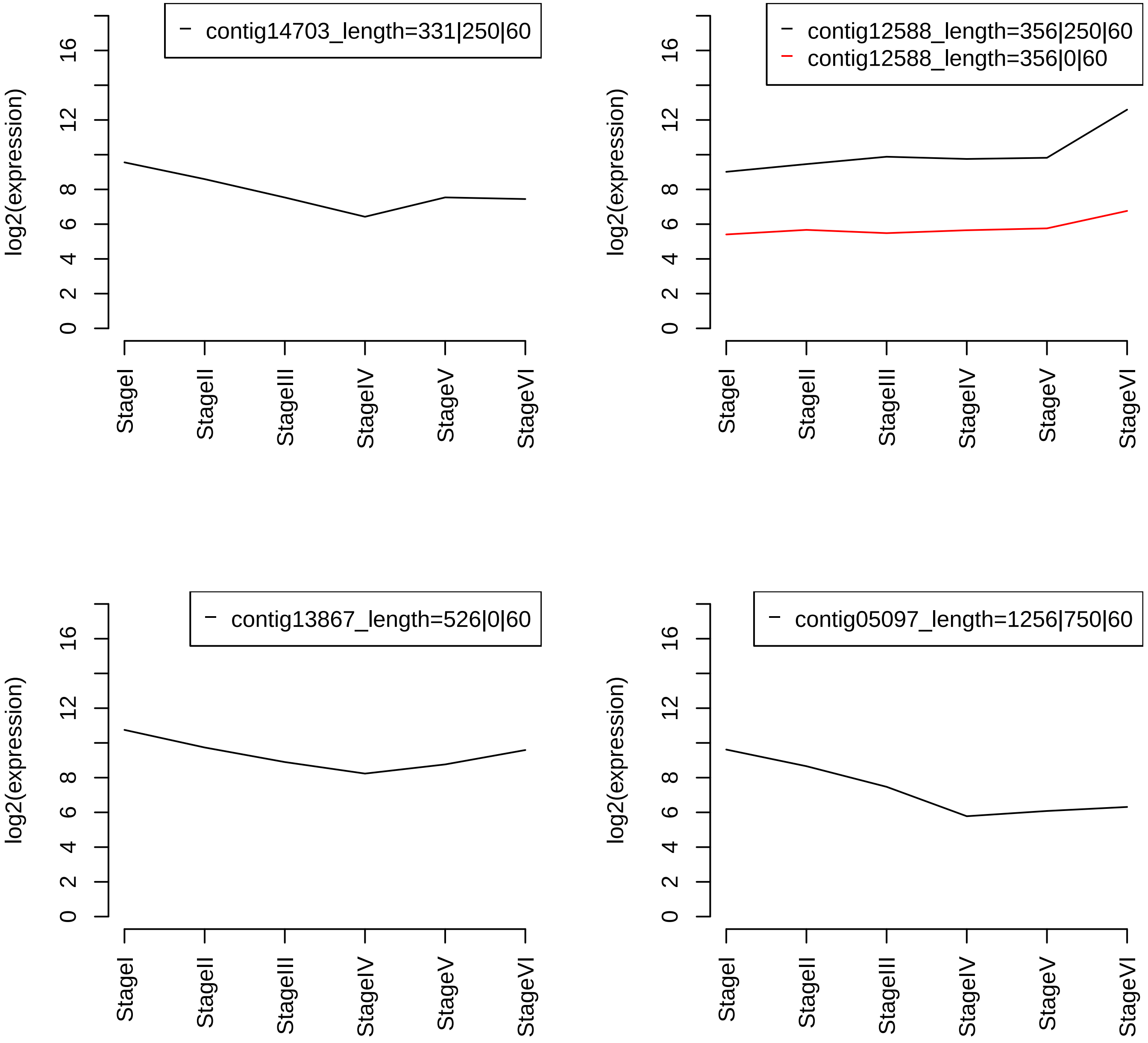

contig14703_length=331|250|60
contig12588_length=356|250|60
contig12588_length=356|0|60
contig13867_length=526|0|60
contig05097_length=1256|750|60
log2(expression)
0
2
4
6
8
12
16
StageI
StageII
StageIII
StageIV
StageV
StageVI

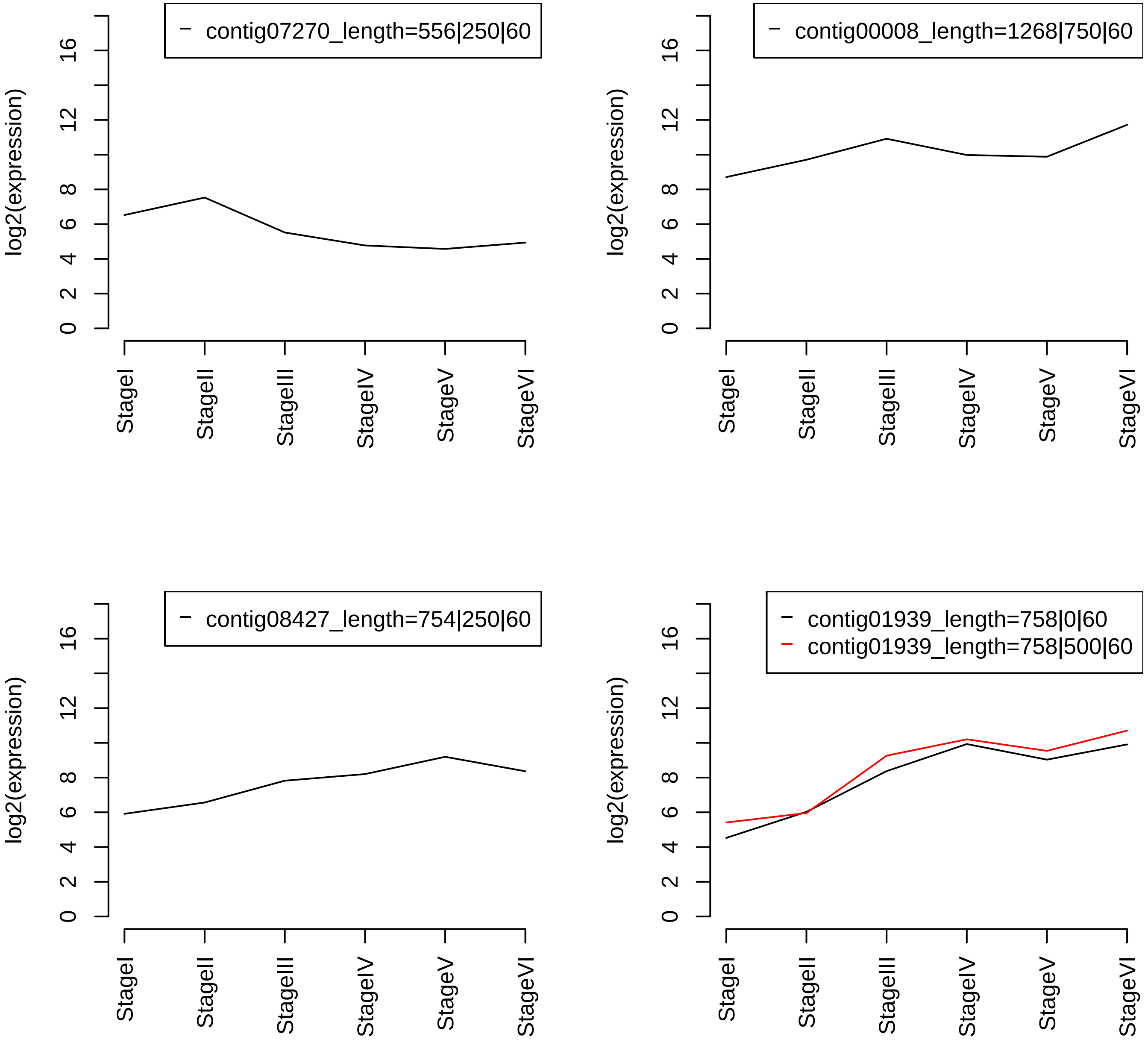

contig07270_length=556|250|60
contig00008_length=1268|750|60
contig08427_length=754|250|60
contig01939_length=758|0|60
contig01939_length=758|500|60
log2(expression)
0
2
4
6
8
12
16
StageI
StageII
StageIII
StageIV
StageV
StageVI

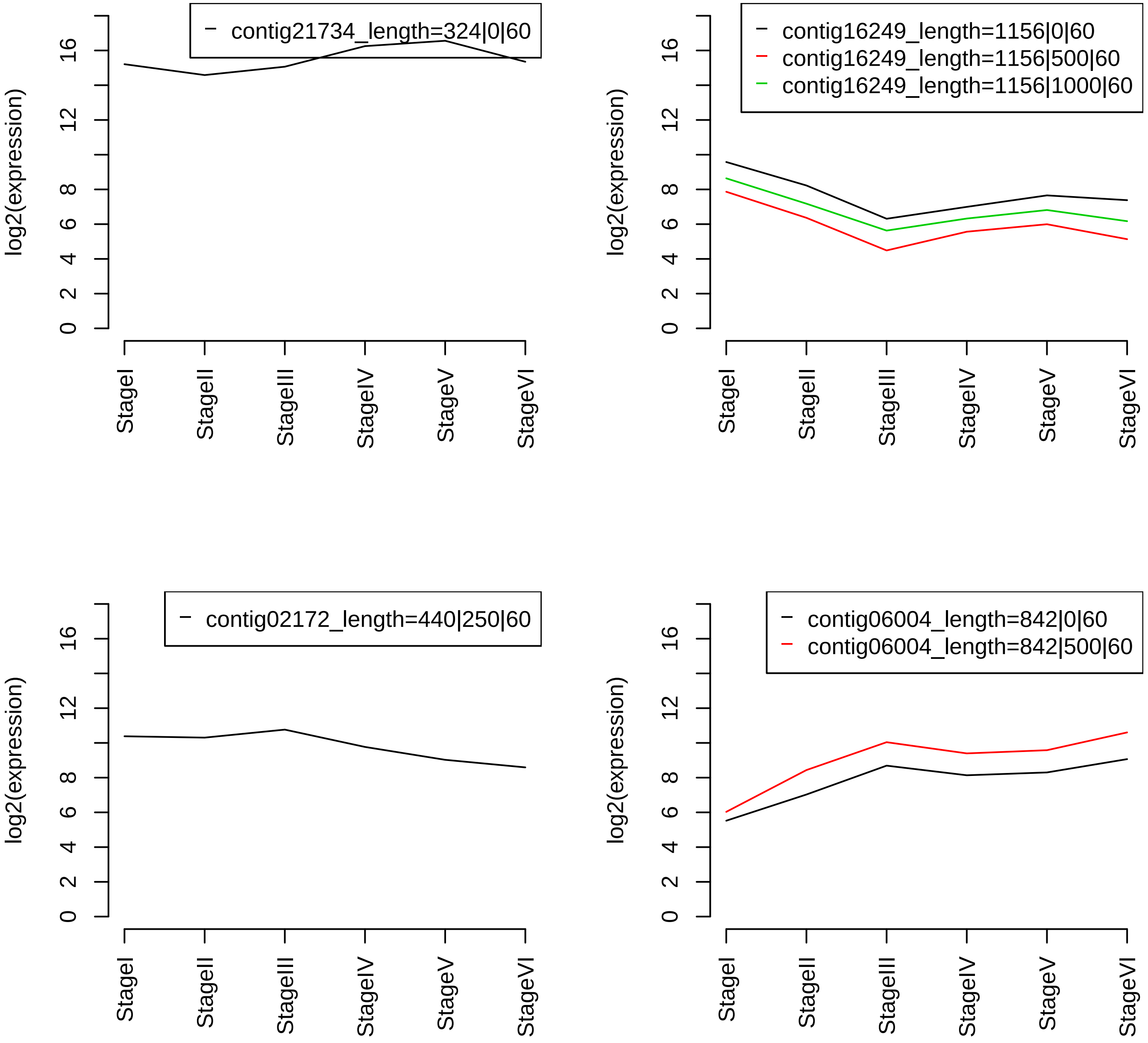
contig21734_length=324|0|60
contig16249_length=1156|0|60
contig16249_length=1156|500|60
contig16249_length=1156|1000|60
contig02172_length=440|250|60
contig06004_length=842|0|60
contig06004_length=842|500|60
log2(expression)
0 2 4 6 8 12 16
StageI
StageII
StageIII
StageIV
StageV
StageVI

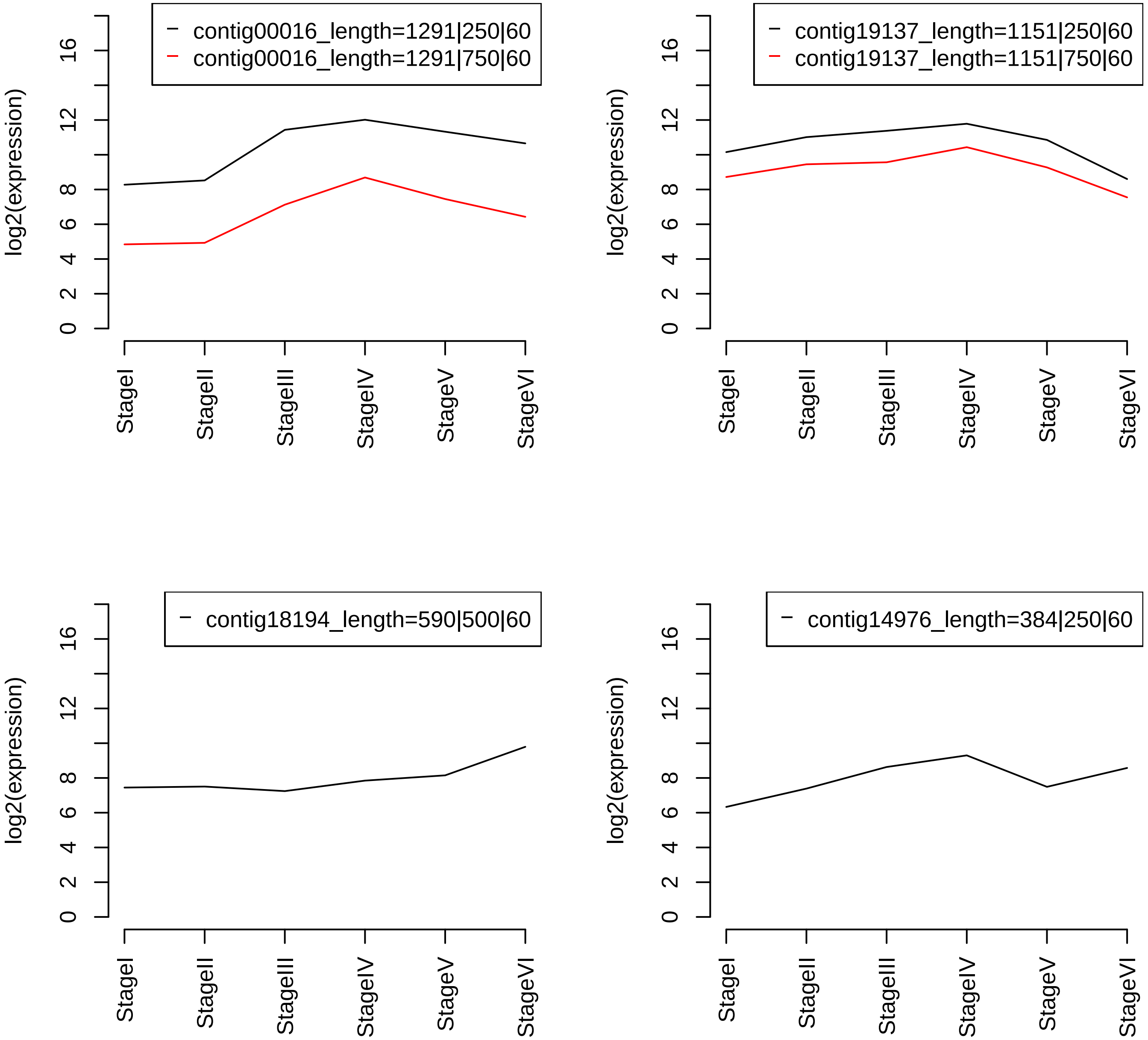

contig00016_length=1291|250|60
contig00016_length=1291|750|60
contig19137_length=1151|250|60
contig19137_length=1151|750|60
contig18194_length=590|500|60
contig14976_length=384|250|60
log2(expression)
0
2
4
6
8
12
16
StageI
StageII
StageIII
StageIV
StageV
StageVI

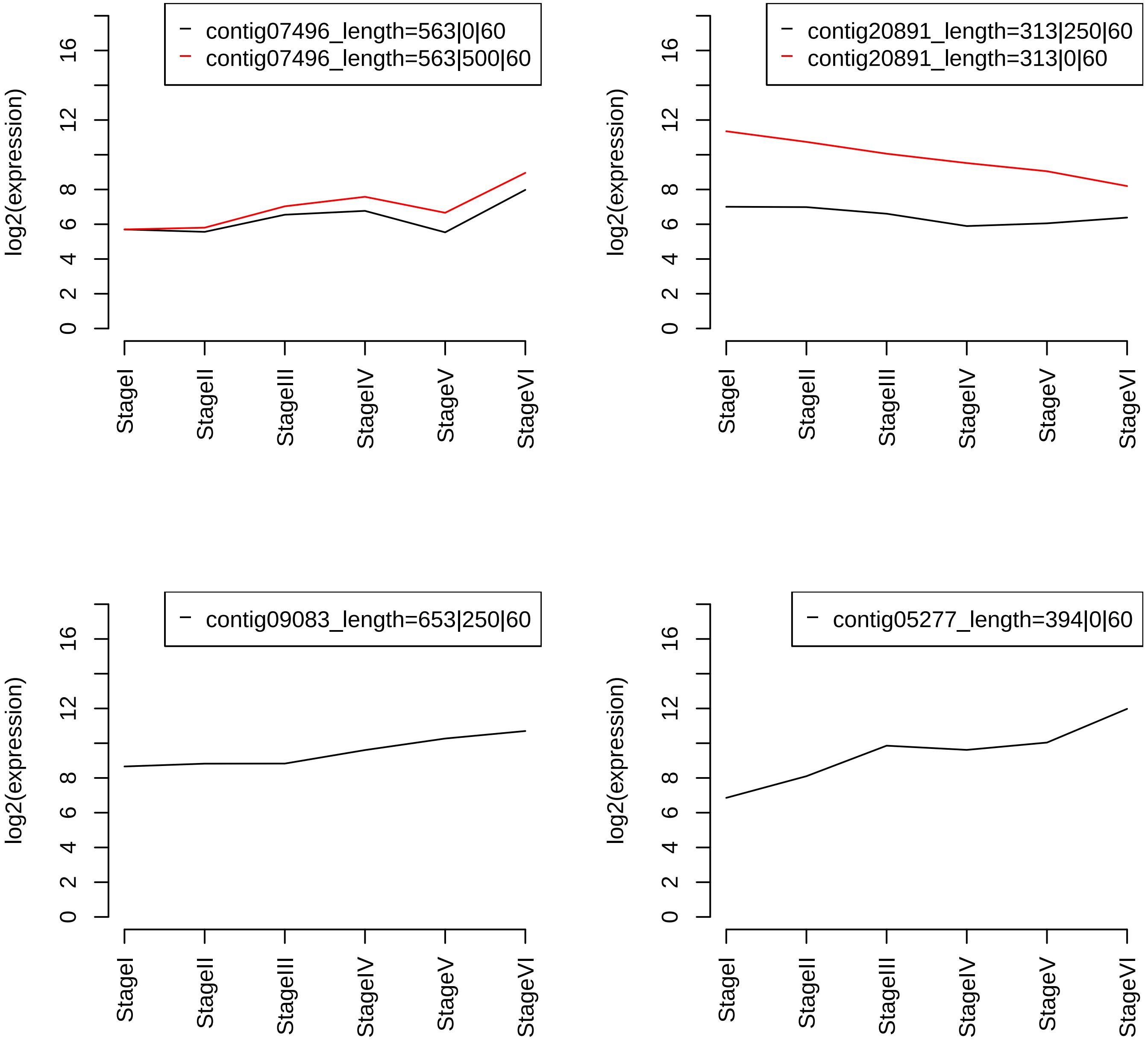

contig07496_length=563|0|60
contig07496_length=563|500|60
contig20891_length=313|250|60
contig20891_length=313|0|60
contig09083_length=653|250|60
contig05277_length=394|0|60
log2(expression)
0 2 4 6 8 12 16
StageI
StageII
StageIII
StageIV
StageV
StageVI

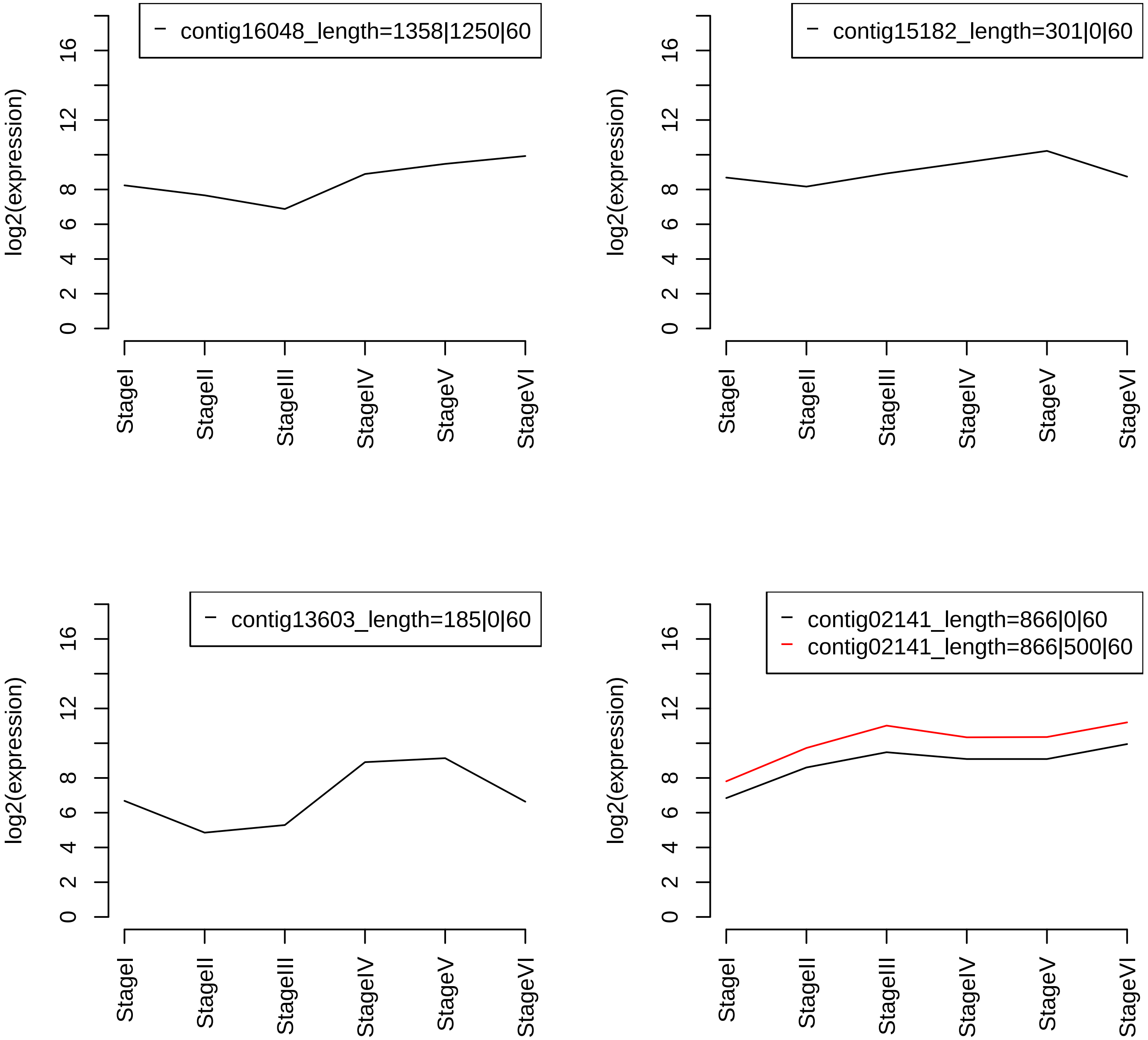

contig16048_length=1358|1250|60
contig15182_length=301|0|60
contig13603_length=185|0|60
contig02141_length=866|0|60
contig02141_length=866|500|60
log2(expression)
0
2
4
6
8
12
16
StageI
StageII
StageIII
StageIV
StageV
StageVI

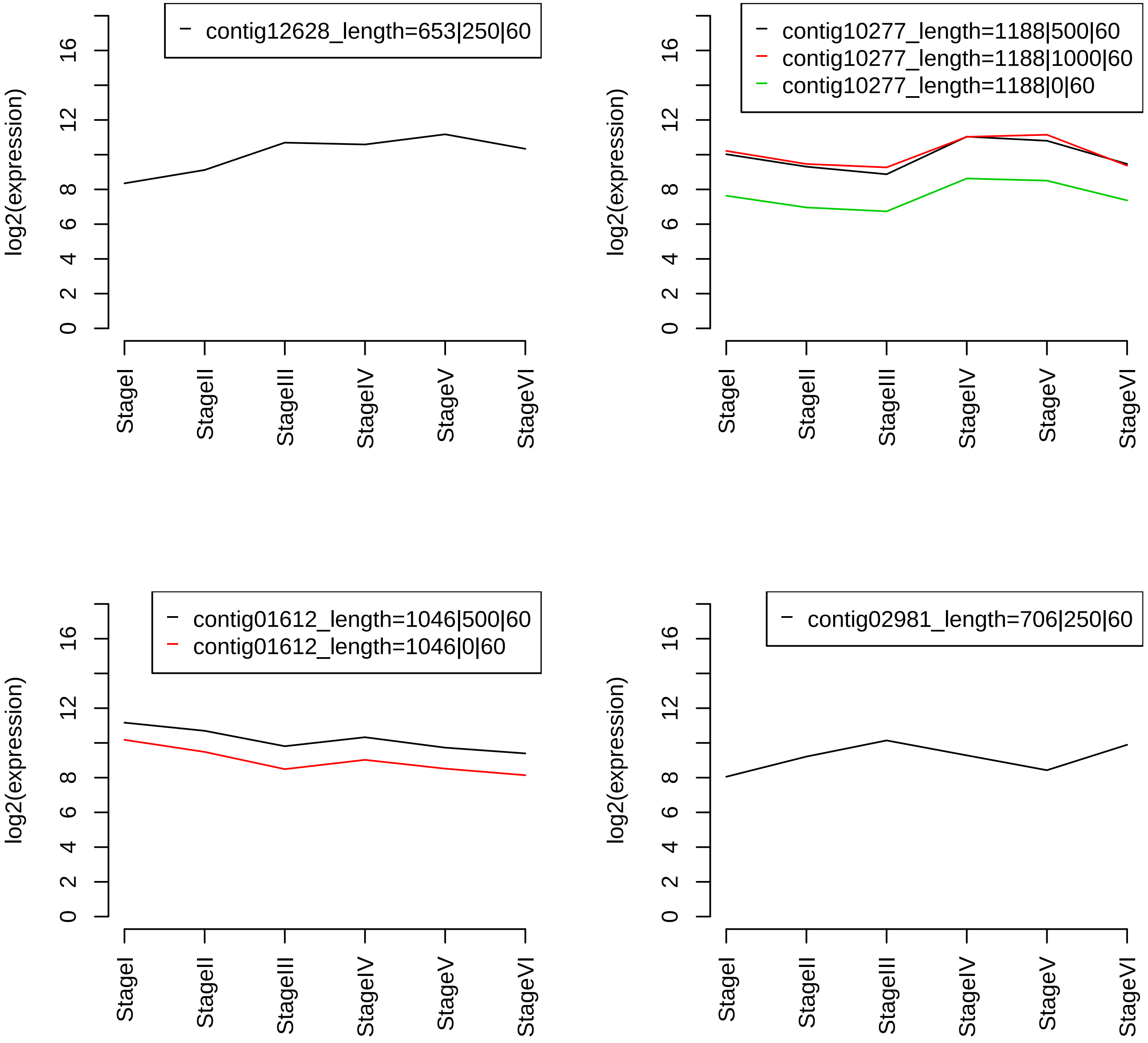
contig12628_length=653|250|60
log2(expression)
0
2
4
6
8
12
16
StageI
StageII
StageIII
StageIV
StageV
StageVI
contig10277_length=1188|500|60
contig10277_length=1188|1000|60
contig10277_length=1188|0|60
log2(expression)
0
2
4
6
8
12
16
StageI
StageII
StageIII
StageIV
StageV
StageVI
contig01612_length=1046|500|60
contig01612_length=1046|0|60
log2(expression)
0
2
4
6
8
12
16
StageI
StageII
StageIII
StageIV
StageV
StageVI
contig02981_length=706|250|60
log2(expression)
0
2
4
6
8
12
16
StageI
StageII
StageIII
StageIV
StageV
StageVI

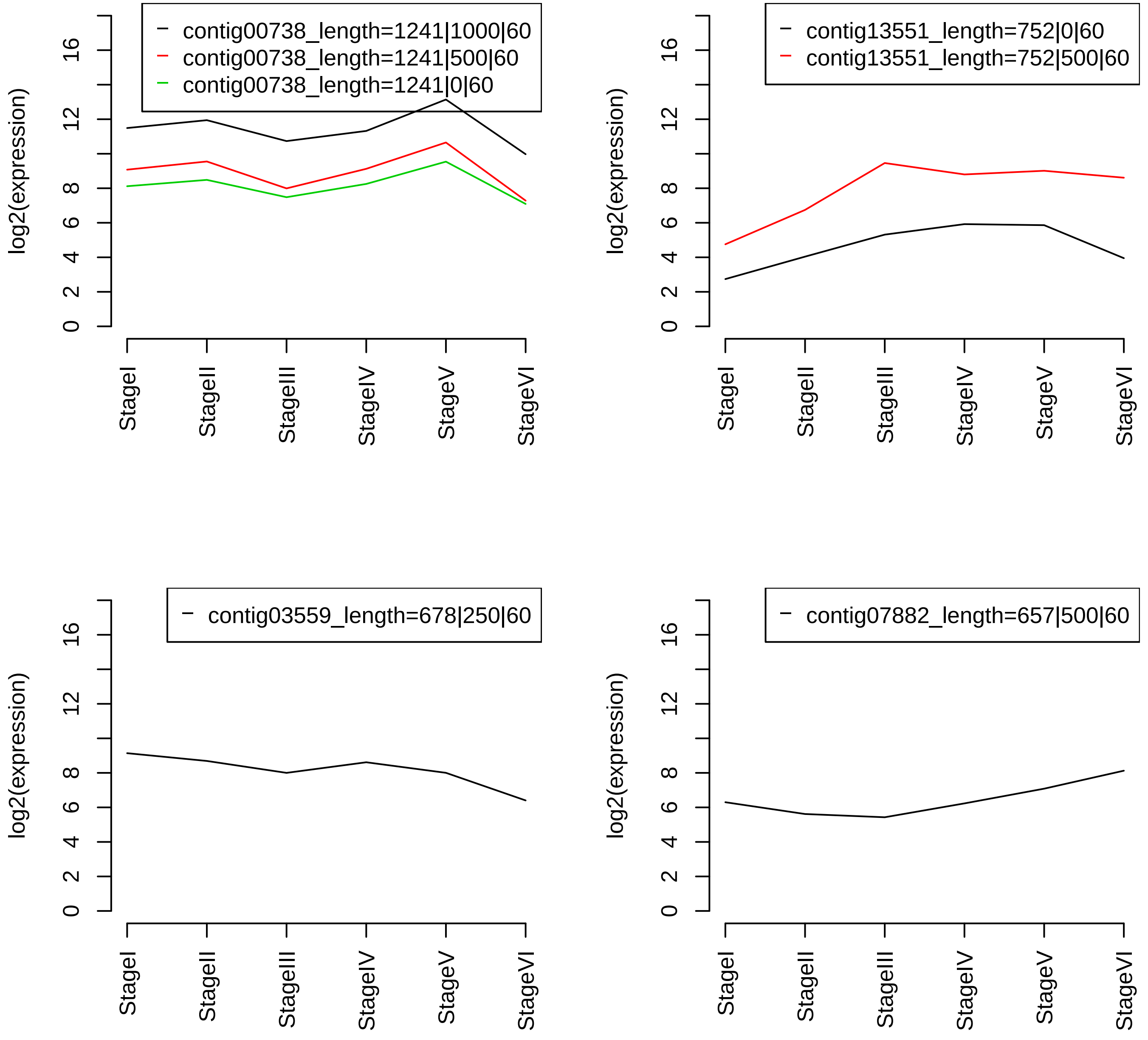
contig00738_length=1241|1000|60
contig00738_length=1241|500|60
contig00738_length=1241|0|60
contig13551_length=752|0|60
contig13551_length=752|500|60
contig03559_length=678|250|60
contig07882_length=657|500|60
log2(expression)
0 2 4 6 8 12 16
StageI StageII StageIII StageIV StageV StageVI

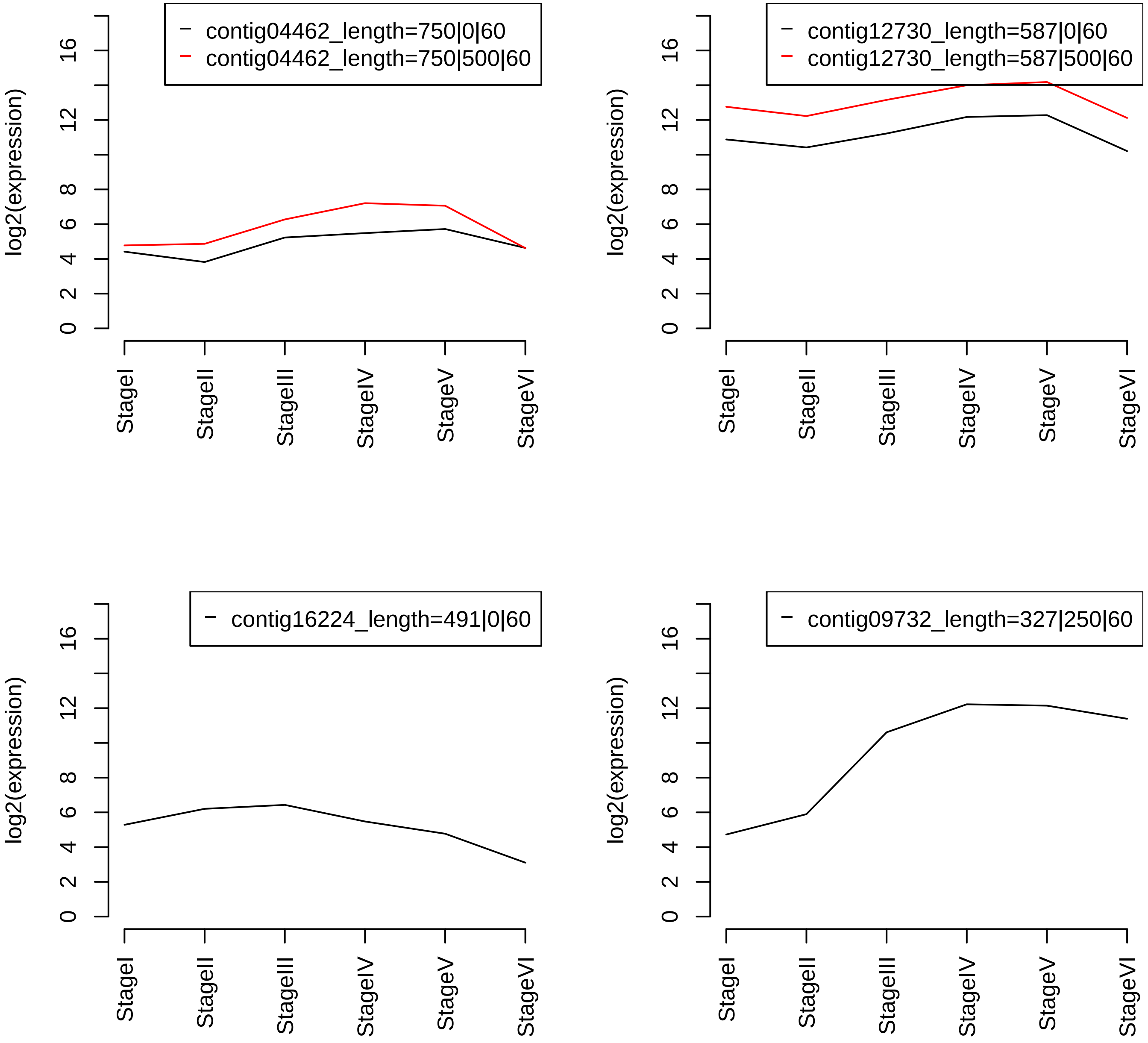
contig04462_length=750|0|60
contig04462_length=750|500|60
contig12730_length=587|0|60
contig12730_length=587|500|60
contig16224_length=491|0|60
contig09732_length=327|250|60
log2(expression)
0
2
4
6
8
12
16
StageI
StageII
StageIII
StageIV
StageV
StageVI

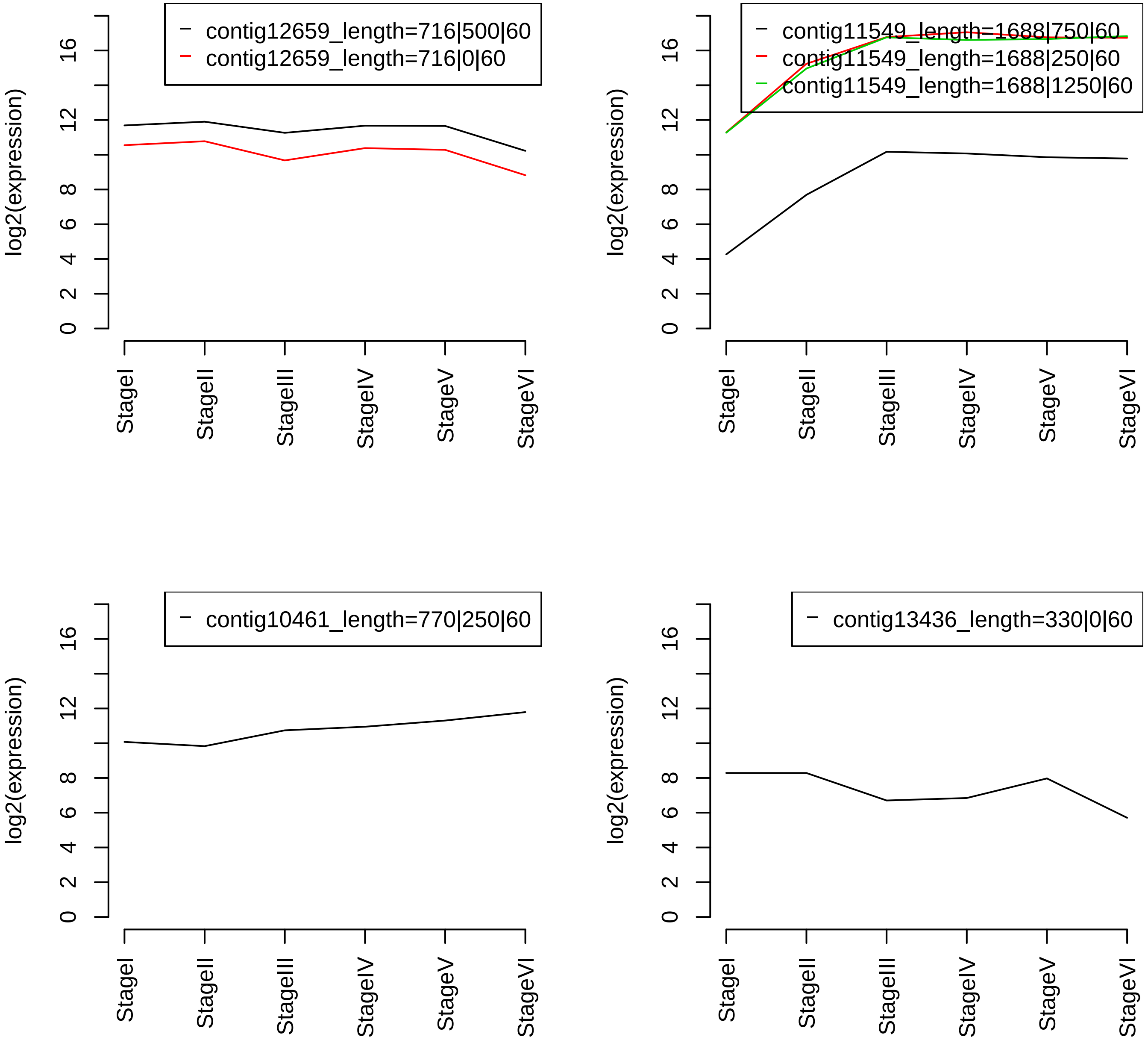
contig12659_length=716|500|60
contig12659_length=716|0|60
contig11549_length=1688|750|60
contig11549_length=1688|250|60
contig11549_length=1688|1250|60
contig10461_length=770|250|60
contig13436_length=330|0|60
log2(expression)
0 2 4 6 8 12 16
StageI StageII StageIII StageIV StageV StageVI

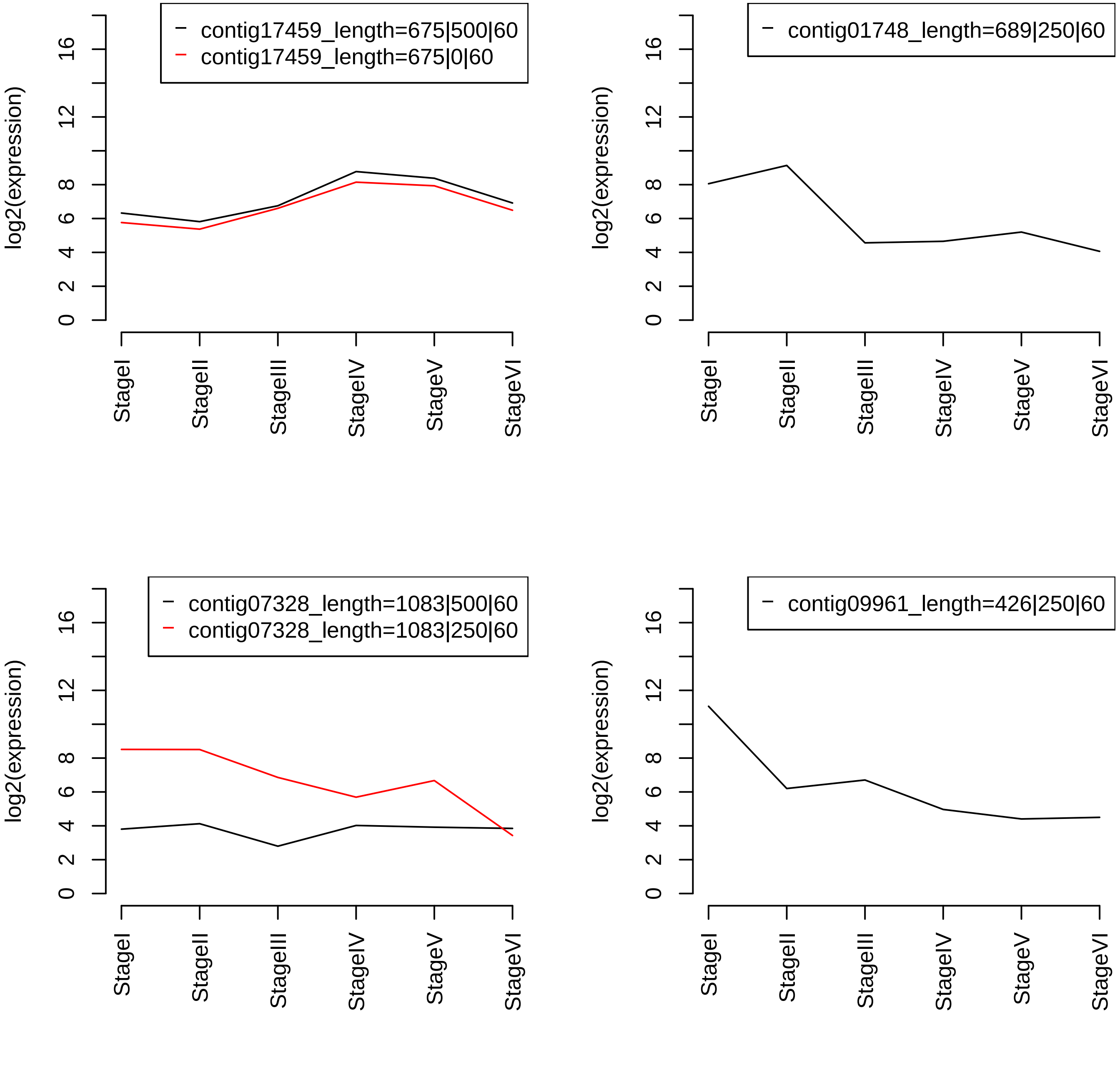
contig17459_length=675|500|60
contig17459_length=675|0|60
contig01748_length=689|250|60
contig07328_length=1083|500|60
contig07328_length=1083|250|60
contig09961_length=426|250|60
log2(expression)
0
2
4
6
8
12
16
StageI
StageII
StageIII
StageIV
StageV
StageVI

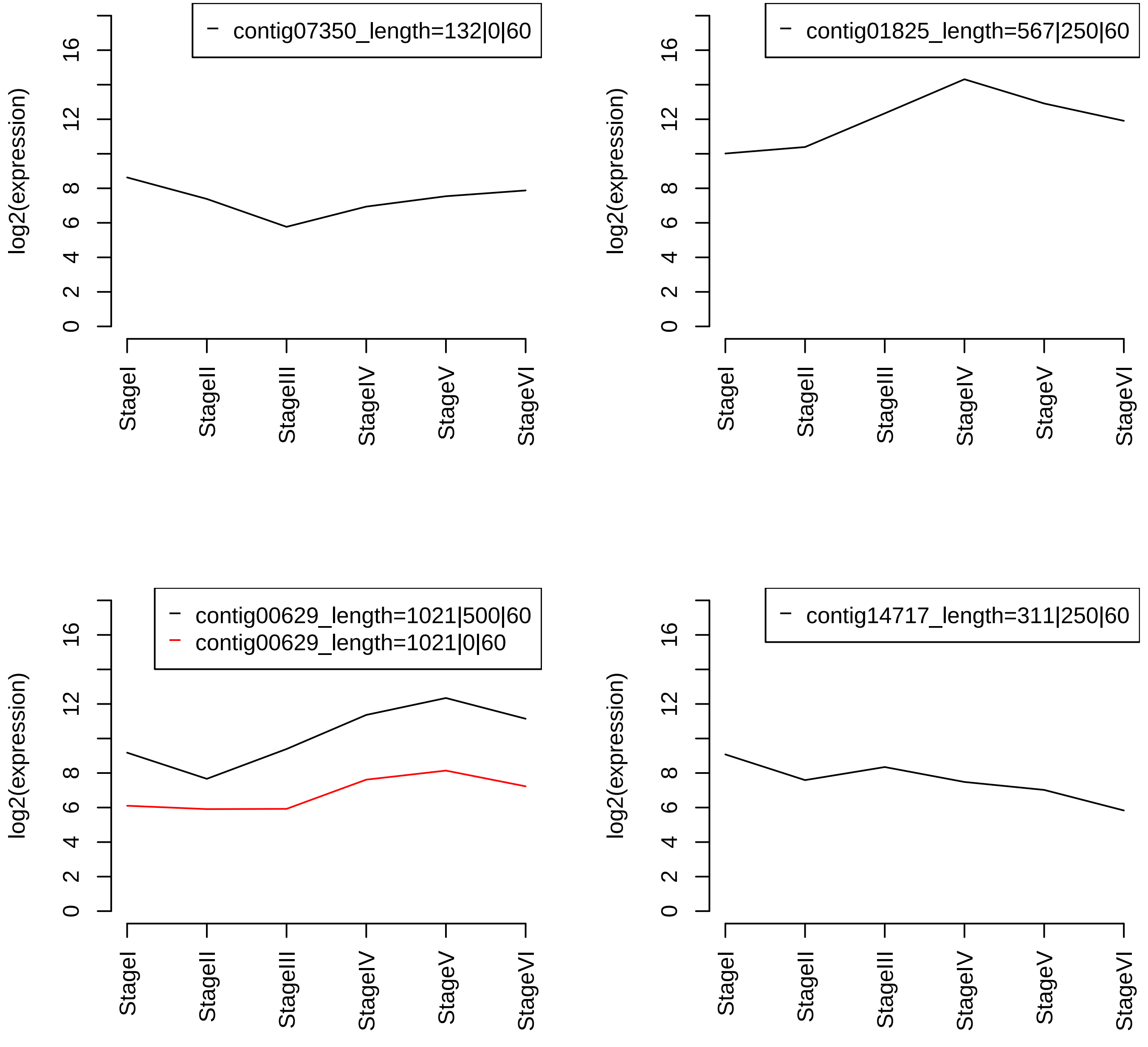

contig07350_length=132|0|60
contig01825_length=567|250|60
contig00629_length=1021|500|60
contig00629_length=1021|0|60
contig14717_length=311|250|60
log2(expression)
0
2
4
6
8
12
16
StageI
StageII
StageIII
StageIV
StageV
StageVI

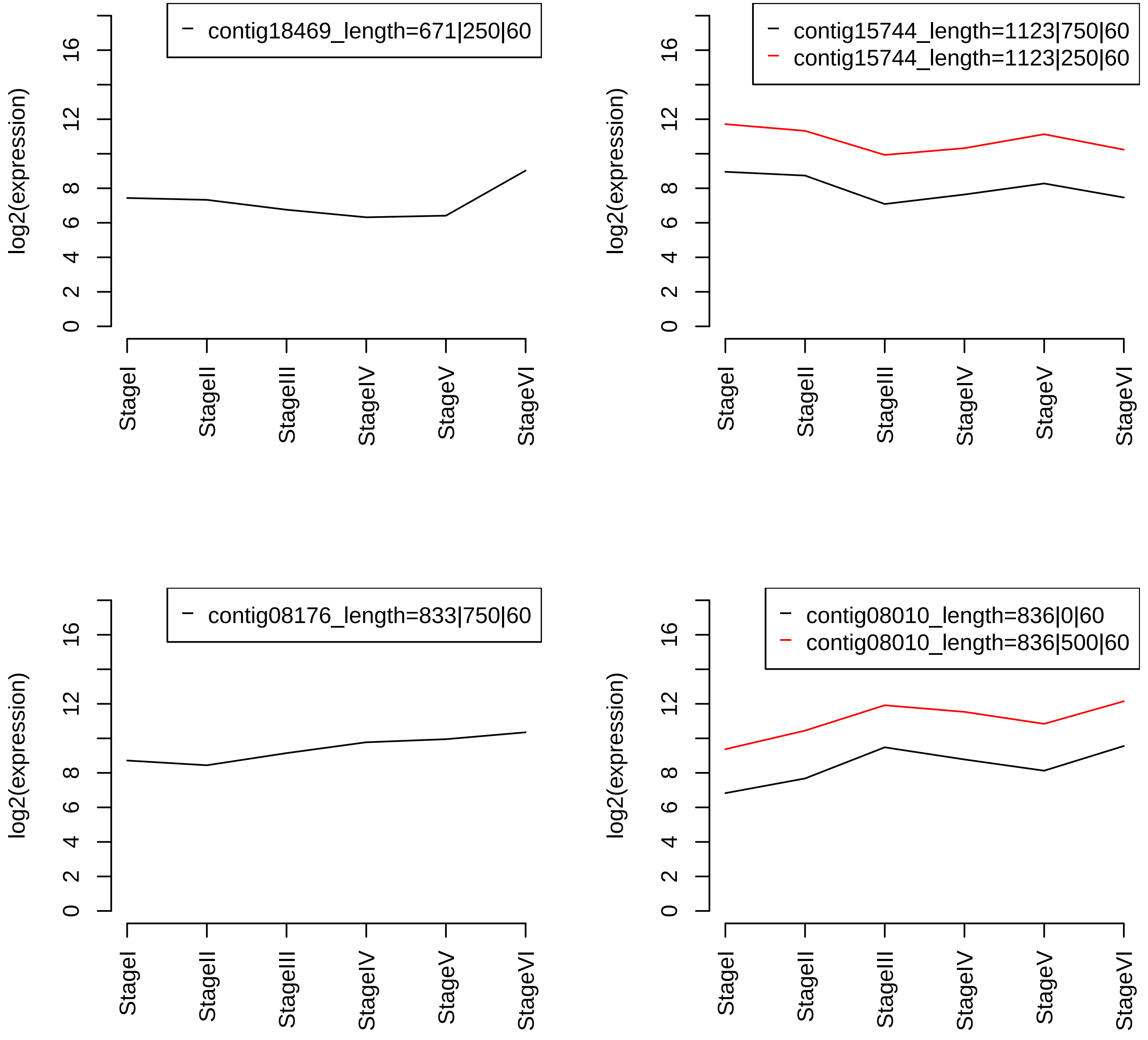
contig18469_length=671|250|60
contig15744_length=1123|750|60
contig15744_length=1123|250|60
contig08176_length=833|750|60
contig08010_length=836|0|60
contig08010_length=836|500|60
log2(expression)
0
2
4
6
8
12
16
StageI
StageII
StageIII
StageIV
StageV
StageVI

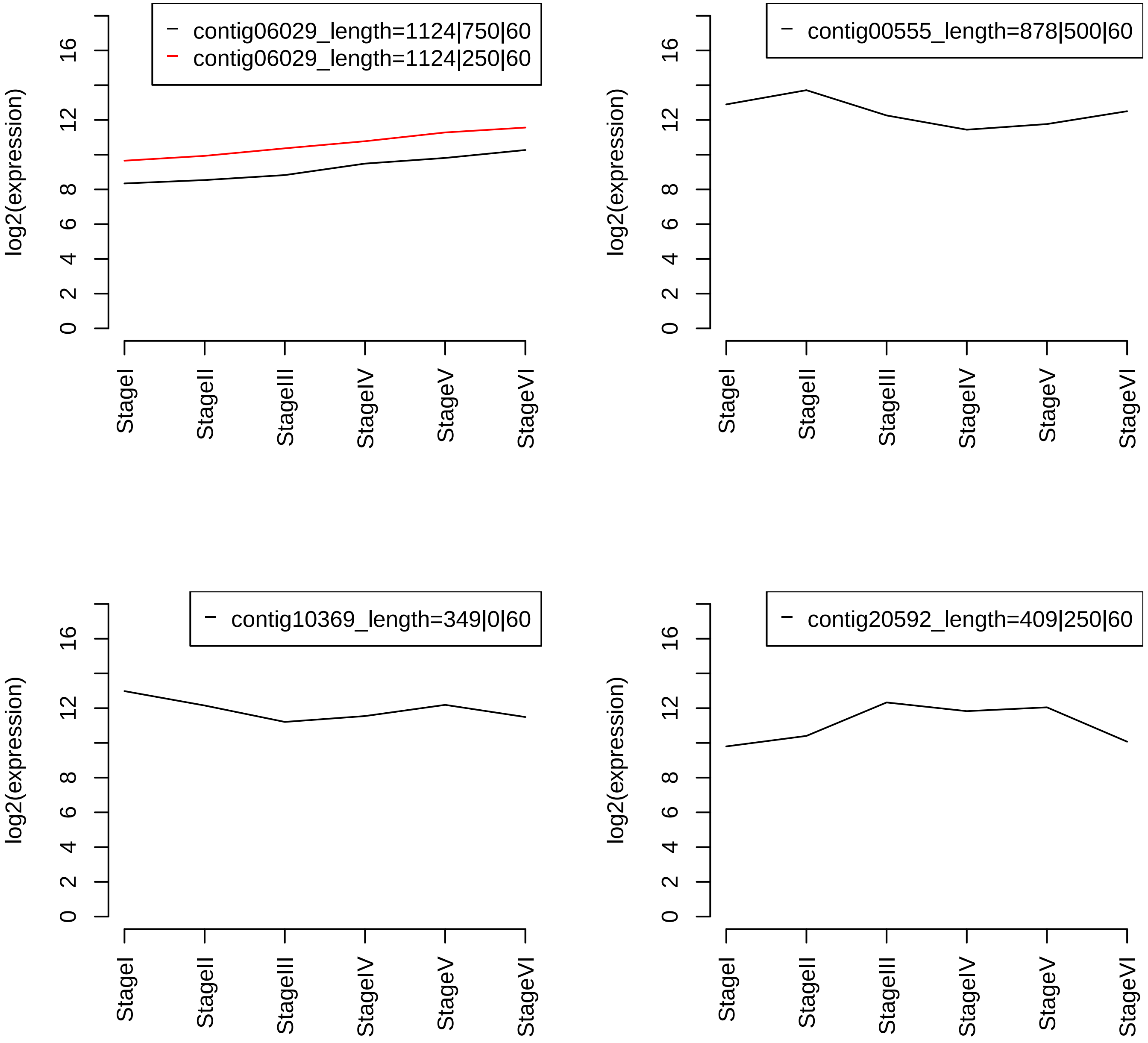
contig06029_length=1124|750|60
contig06029_length=1124|250|60
contig00555_length=878|500|60
contig10369_length=349|0|60
contig20592_length=409|250|60
log2(expression)
StageI
StageII
StageIII
StageIV
StageV
StageVI

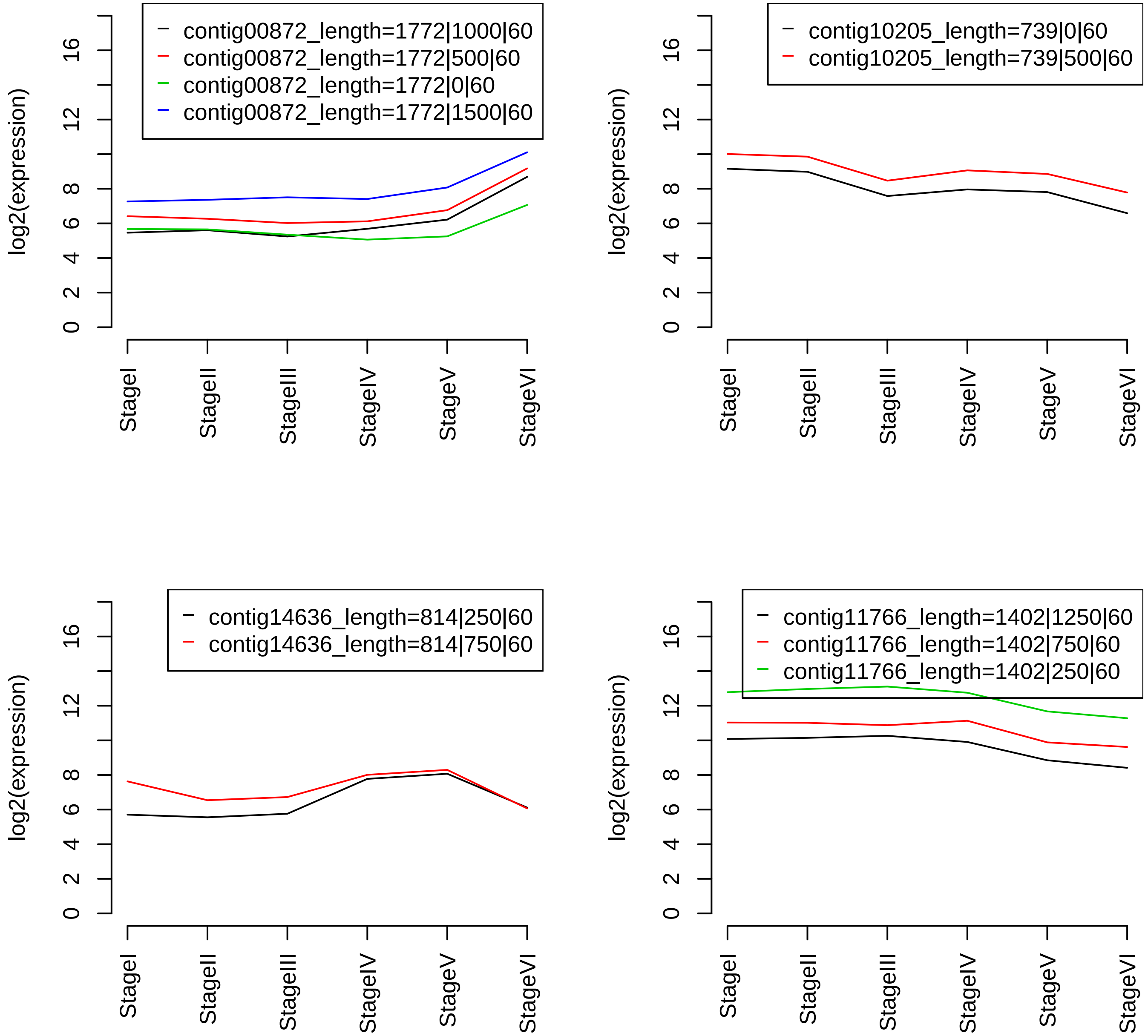
contig00872_length=1772|1000|60
contig00872_length=1772|500|60
contig00872_length=1772|0|60
contig00872_length=1772|1500|60
log2(expression)
StageI
StageII
StageIII
StageIV
StageV
StageVI
contig10205_length=739|0|60
contig10205_length=739|500|60
contig14636_length=814|250|60
contig14636_length=814|750|60
contig11766_length=1402|1250|60
contig11766_length=1402|750|60
contig11766_length=1402|250|60

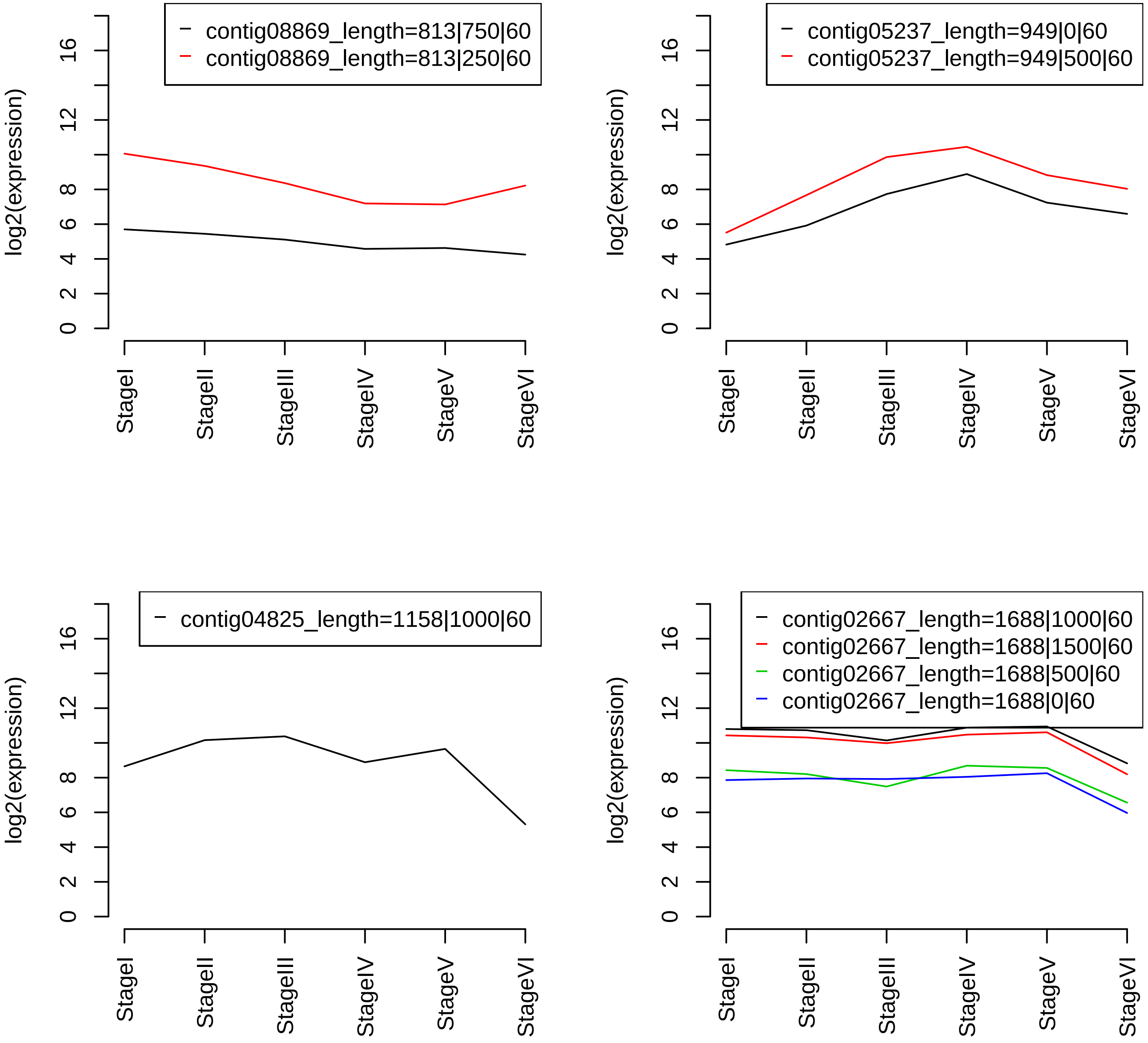

contig08869_length=813|750|60
contig08869_length=813|250|60
contig05237_length=949|0|60
contig05237_length=949|500|60
contig04825_length=1158|1000|60
contig02667_length=1688|1000|60
contig02667_length=1688|1500|60
contig02667_length=1688|500|60
contig02667_length=1688|0|60
log2(expression)
0 2 4 6 8 12 16
StageI StageII StageIII StageIV StageV StageVI

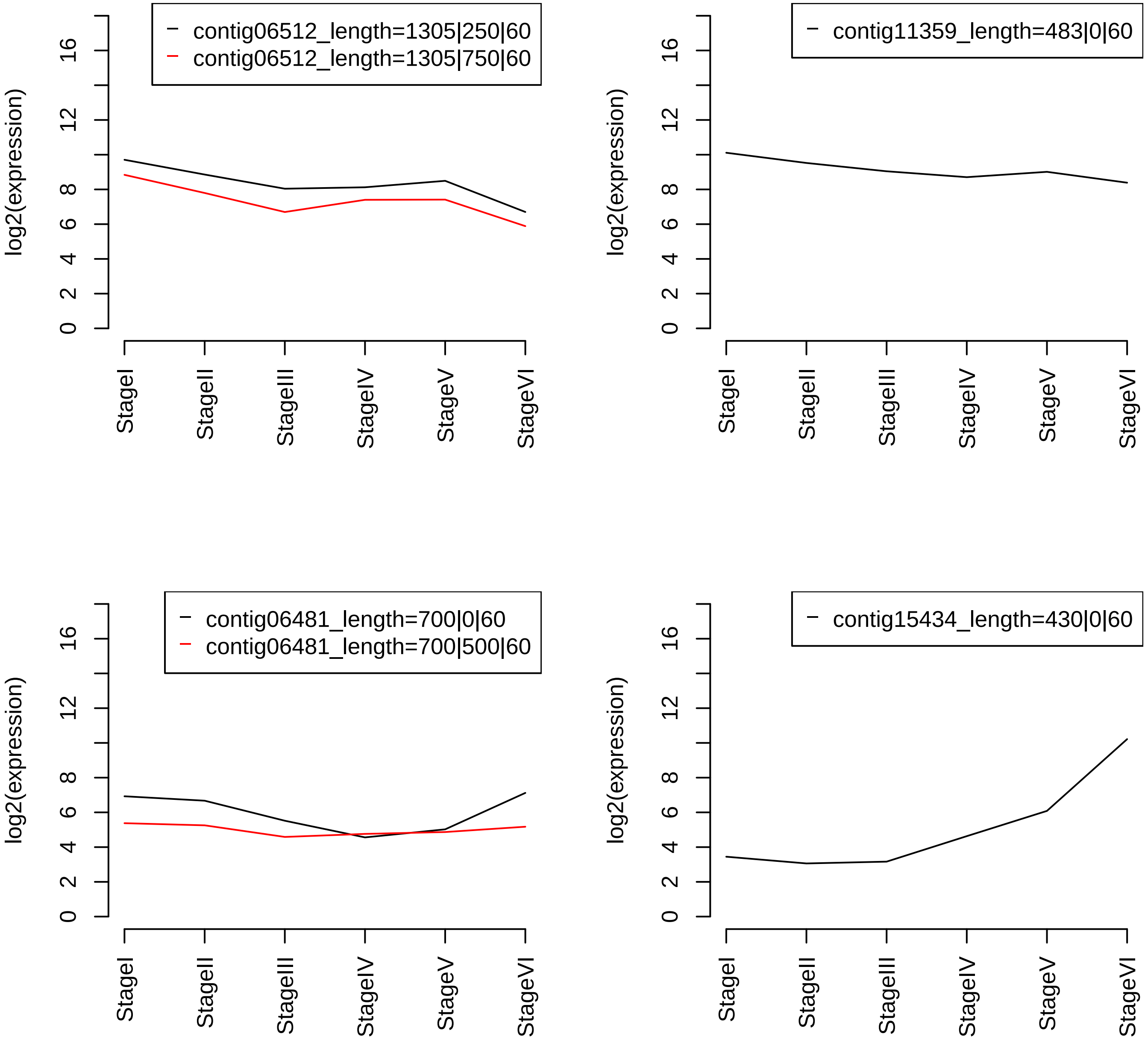
contig06512_length=1305|250|60
contig06512_length=1305|750|60
contig11359_length=483|0|60
contig06481_length=700|0|60
contig06481_length=700|500|60
contig15434_length=430|0|60
log2(expression)
0
2
4
6
8
12
16
StageI
StageII
StageIII
StageIV
StageV
StageVI

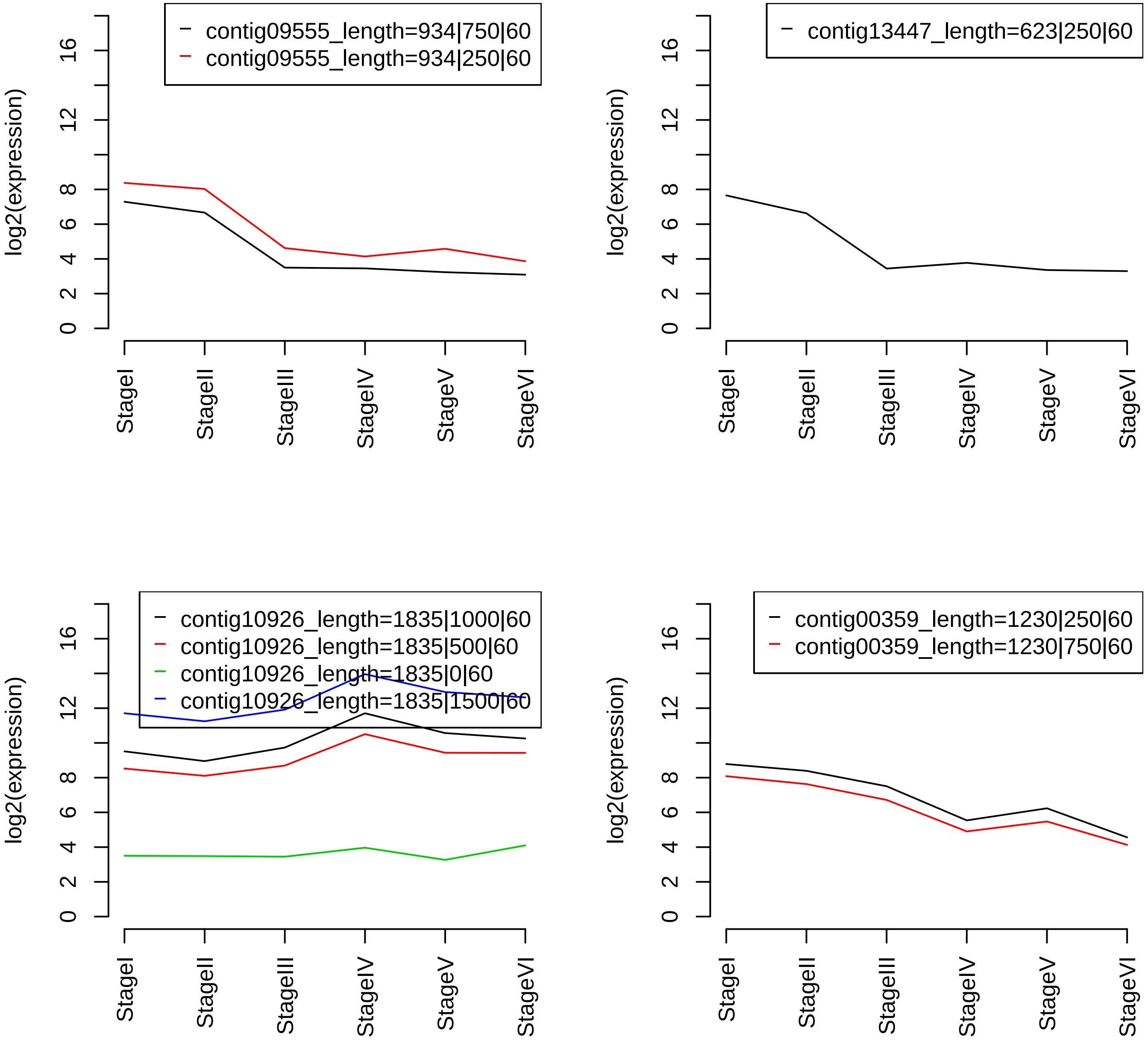

contig09555_length=934|750|60
contig09555_length=934|250|60
contig13447_length=623|250|60
contig10926_length=1835|1000|60
contig10926_length=1835|500|60
contig10926_length=1835|0|60
contig10926_length=1835|1500|60
contig00359_length=1230|250|60
contig00359_length=1230|750|60
log2(expression)
0 2 4 6 8 12 16
StageI StageII StageIII StageIV StageV StageVI

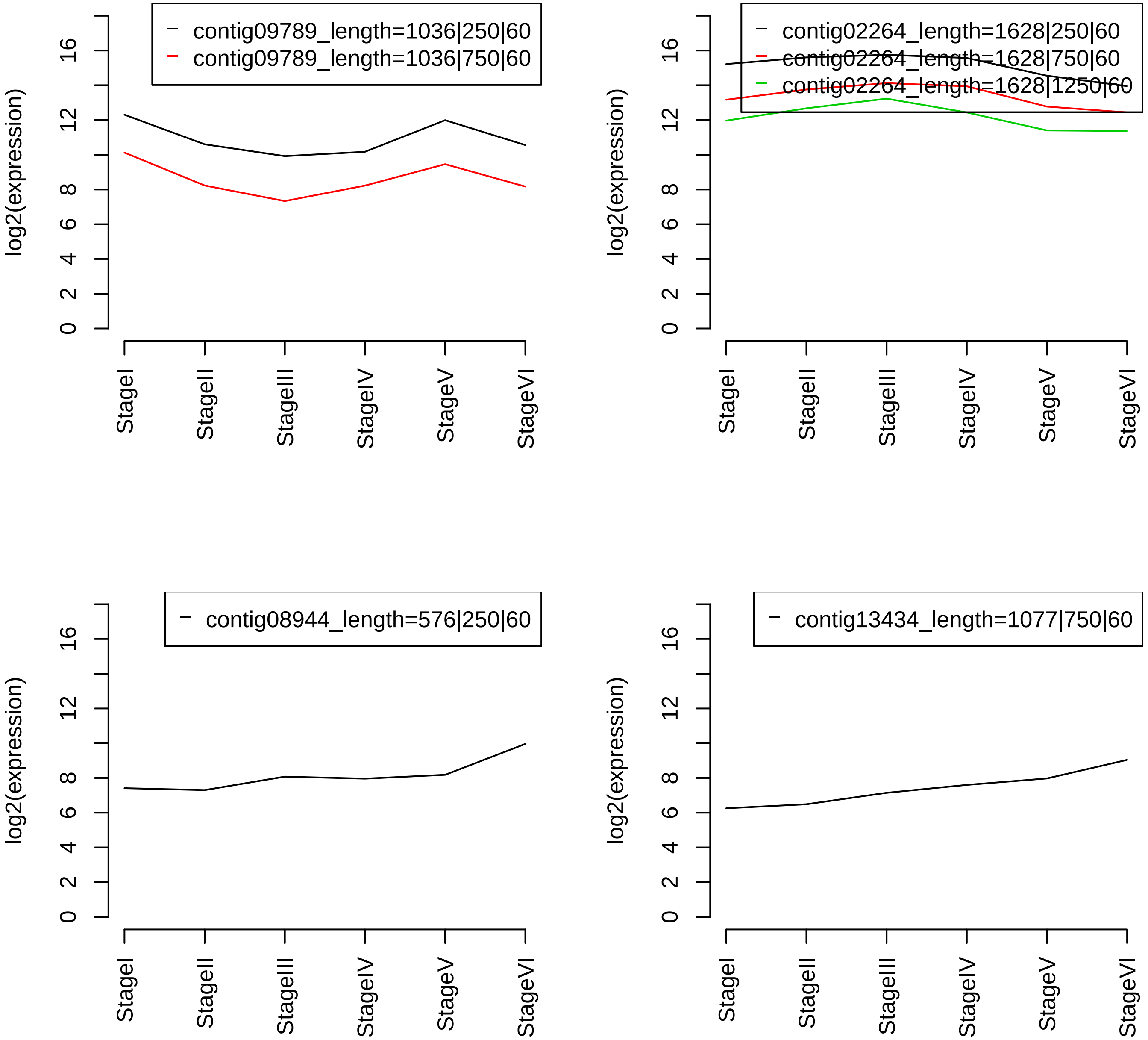

contig09789_length=1036|250|60
contig09789_length=1036|750|60
contig02264_length=1628|250|60
contig02264_length=1628|750|60
contig02264_length=1628|1250|60
contig08944_length=576|250|60
contig13434_length=1077|750|60
log2(expression)
0 2 4 6 8 12 16
StageI StageII StageIII StageIV StageV StageVI

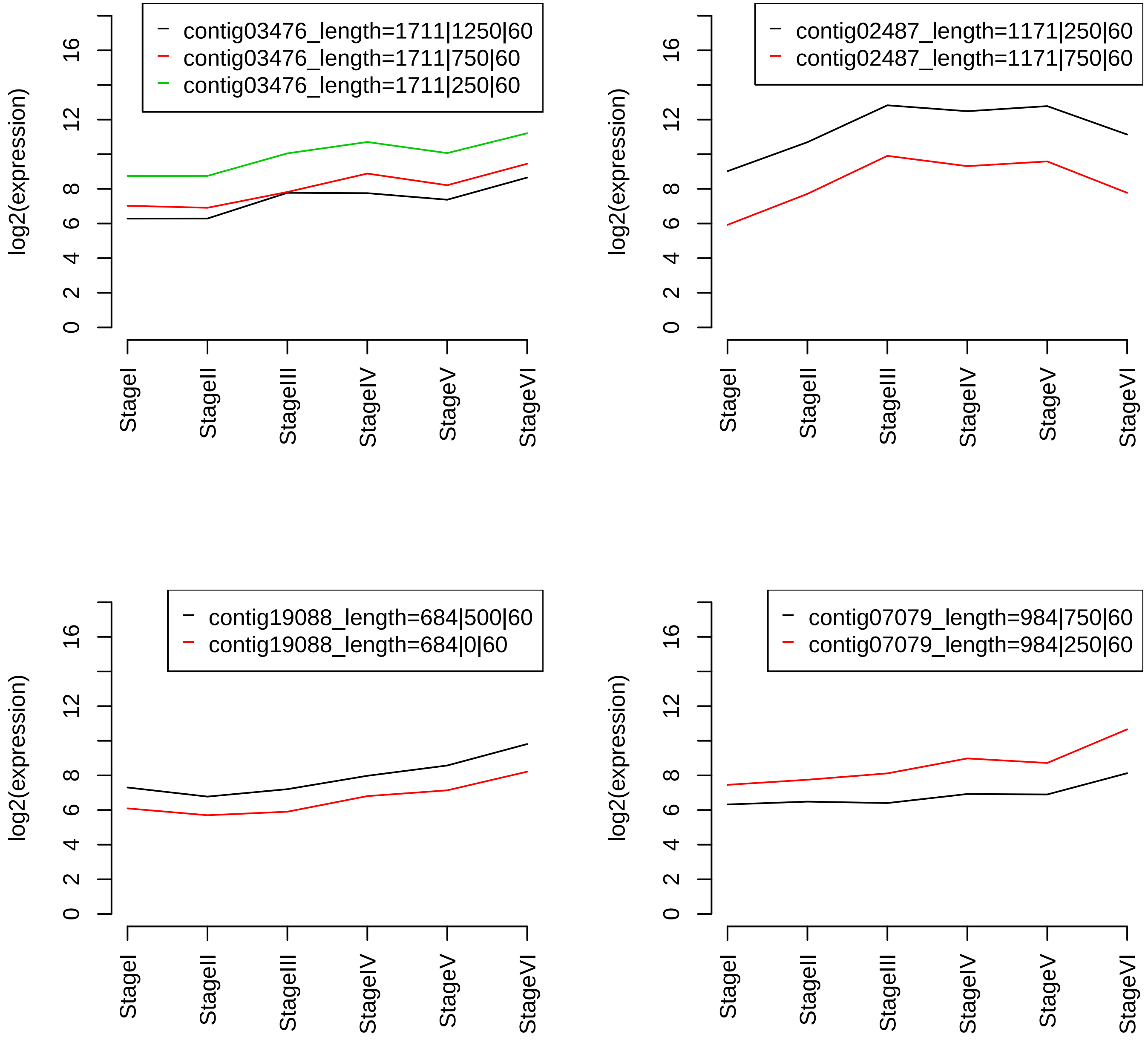
contig03476_length=1711|1250|60
contig03476_length=1711|750|60
contig03476_length=1711|250|60
contig02487_length=1171|250|60
contig02487_length=1171|750|60
contig19088_length=684|500|60
contig19088_length=684|0|60
contig07079_length=984|750|60
contig07079_length=984|250|60
log2(expression)
0
2
4
6
8
12
16
StageI
StageII
StageIII
StageIV
StageV
StageVI

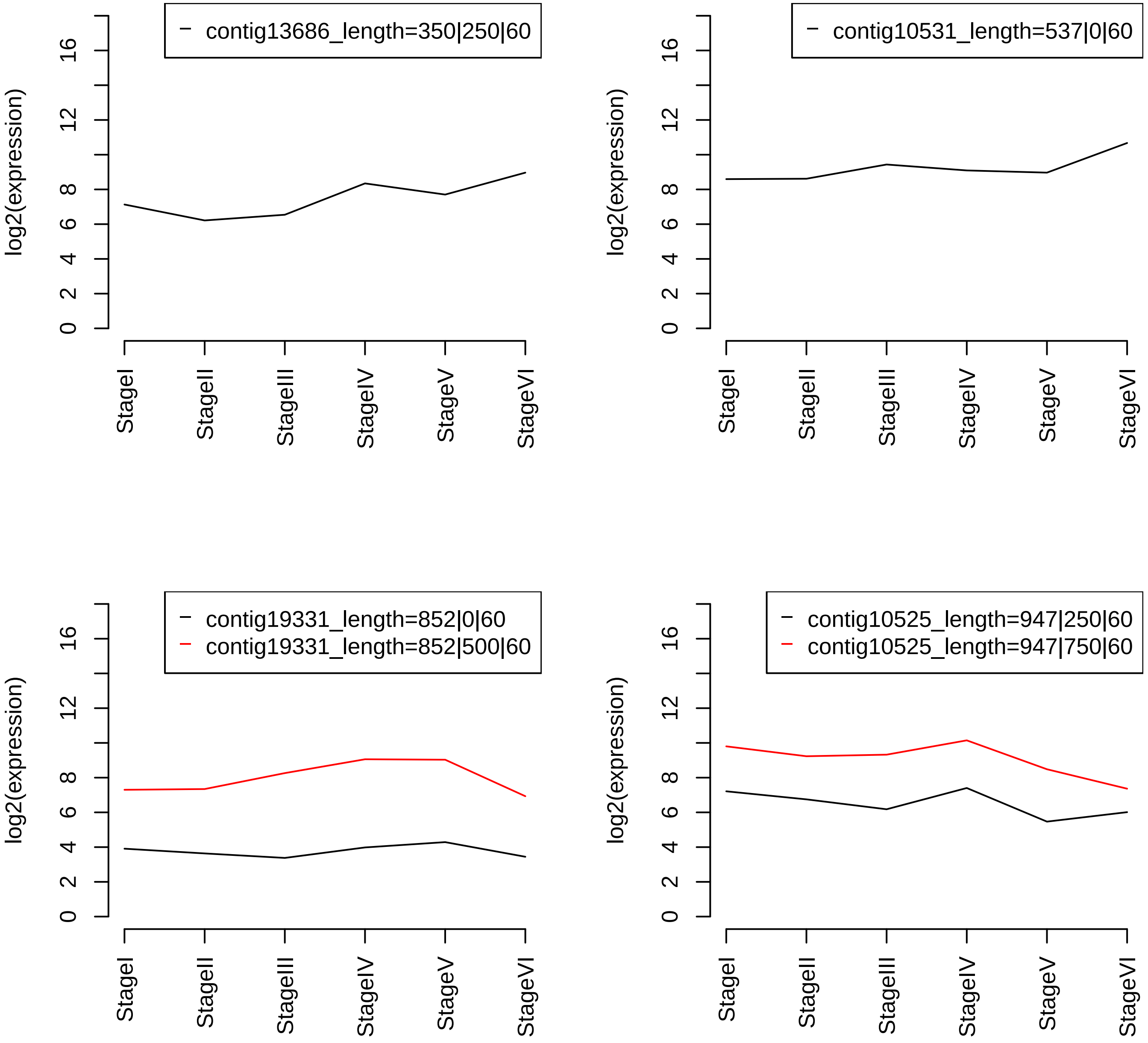

contig13686_length=350|250|60
contig10531_length=537|0|60
contig19331_length=852|0|60
contig19331_length=852|500|60
contig10525_length=947|250|60
contig10525_length=947|750|60
log2(expression)
0
2
4
6
8
12
16
StageI
StageII
StageIII
StageIV
StageV
StageVI

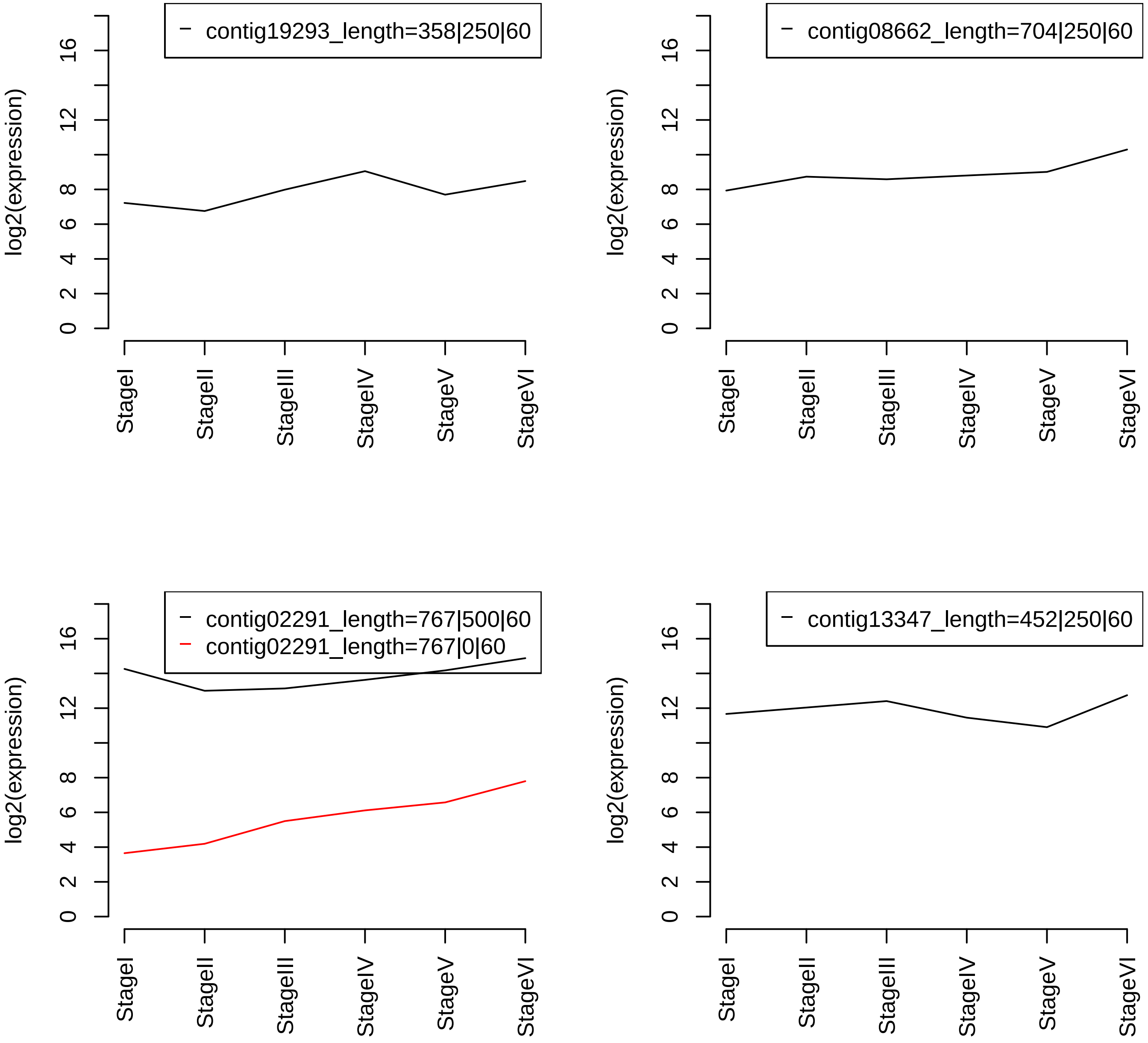

contig19293_length=358|250|60
log2(expression)
0 2 4 6 8 12 16
StageI StageII StageIII StageIV StageV StageVI
contig08662_length=704|250|60
log2(expression)
0 2 4 6 8 12 16
StageI StageII StageIII StageIV StageV StageVI
contig02291_length=767|500|60
contig02291_length=767|0|60
log2(expression)
0 2 4 6 8 12 16
StageI StageII StageIII StageIV StageV StageVI
contig13347_length=452|250|60
log2(expression)
0 2 4 6 8 12 16
StageI StageII StageIII StageIV StageV StageVI

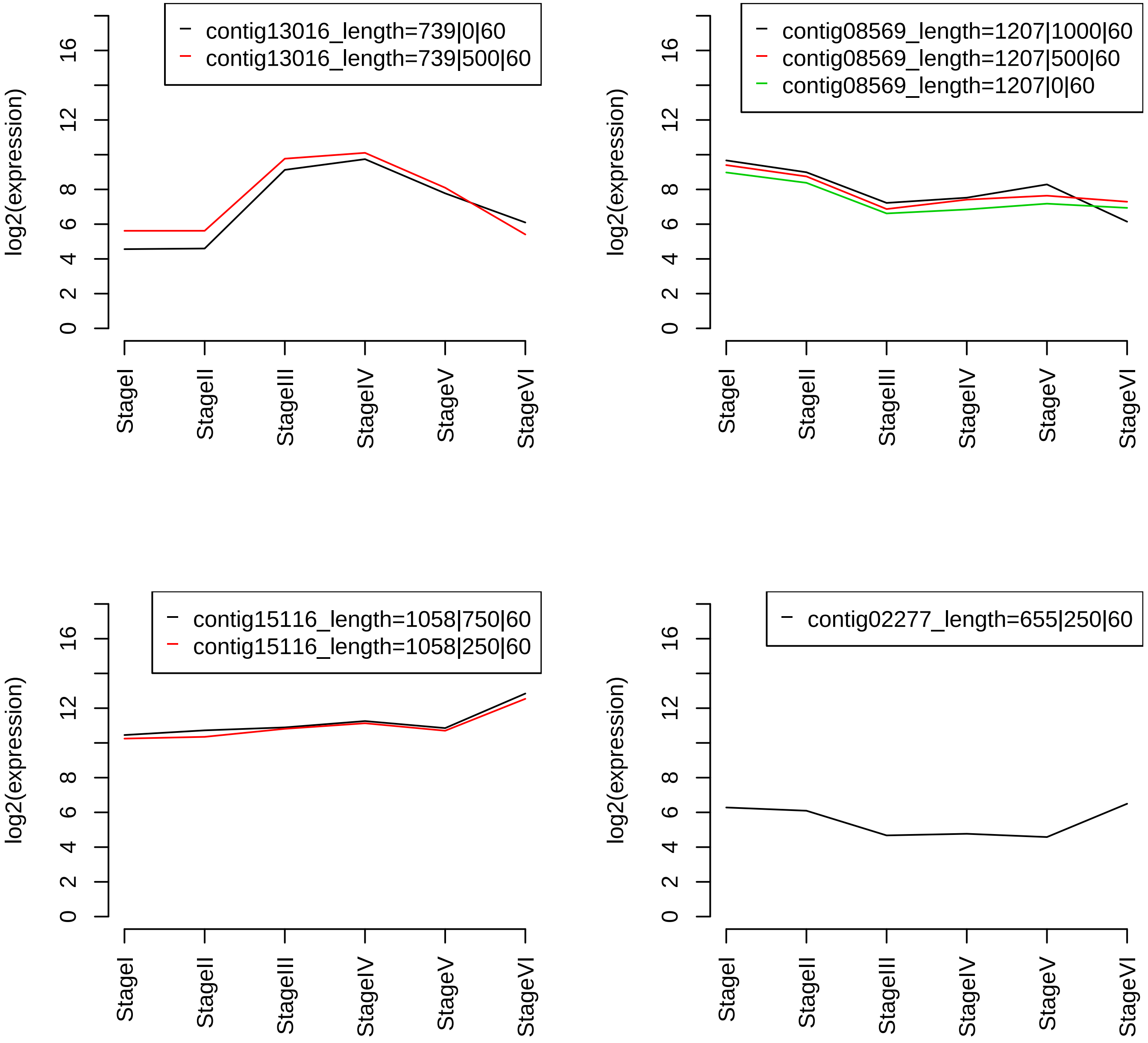

contig13016_length=739|0|60
contig13016_length=739|500|60
log2(expression)
0 2 4 6 8 12 16
StageI StageII StageIII StageIV StageV StageVI
contig08569_length=1207|1000|60
contig08569_length=1207|500|60
contig08569_length=1207|0|60
log2(expression)
0 2 4 6 8 12 16
StageI StageII StageIII StageIV StageV StageVI
contig15116_length=1058|750|60
contig15116_length=1058|250|60
log2(expression)
0 2 4 6 8 12 16
StageI StageII StageIII StageIV StageV StageVI
contig02277_length=655|250|60
log2(expression)
0 2 4 6 8 12 16
StageI StageII StageIII StageIV StageV StageVI

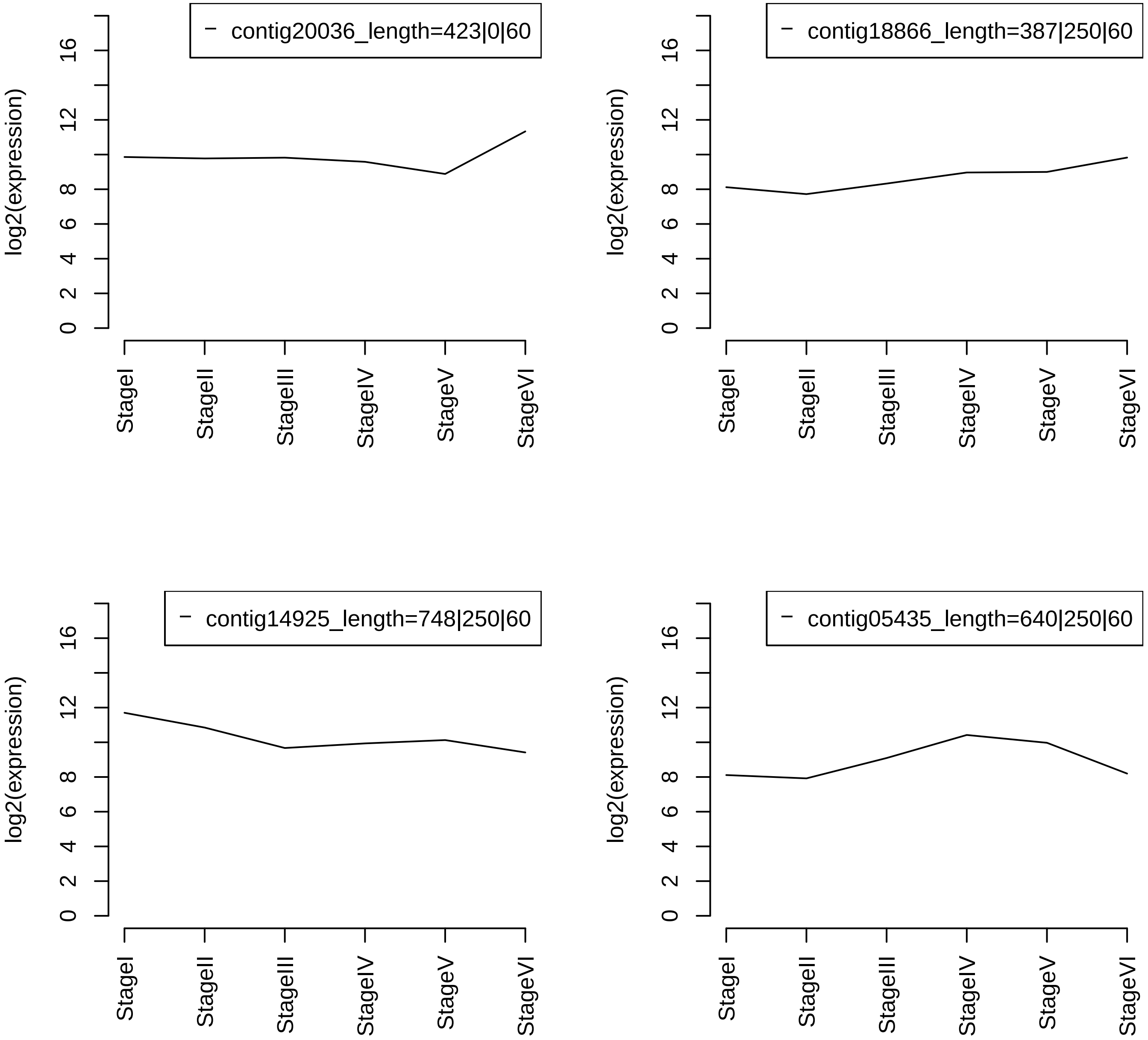
contig20036_length=423|0|60
contig18866_length=387|250|60
contig14925_length=748|250|60
contig05435_length=640|250|60
log2(expression)
0
2
4
6
8
12
16
StageI
StageII
StageIII
StageIV
StageV
StageVI

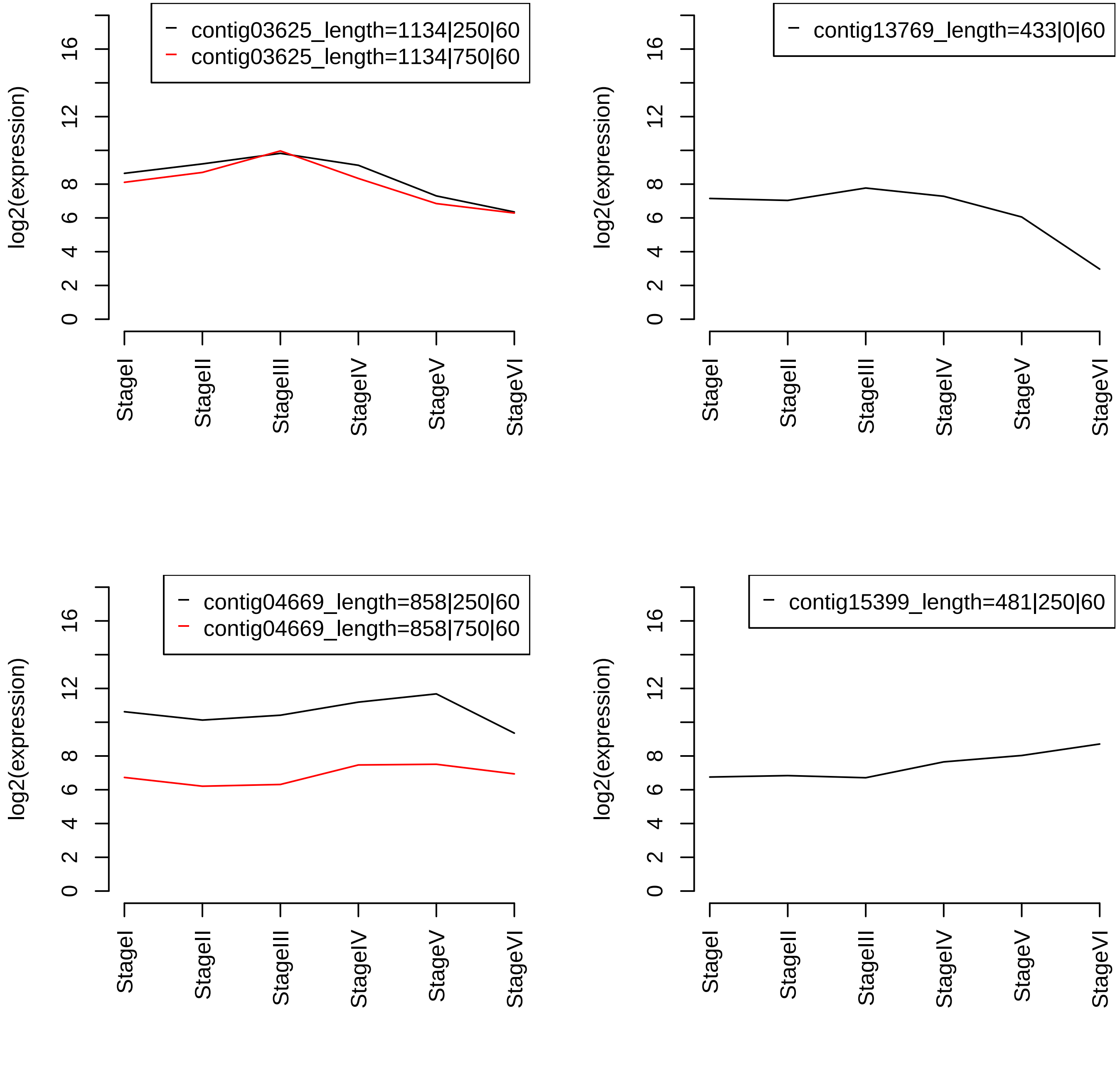

contig03625_length=1134|250|60
contig03625_length=1134|750|60
contig13769_length=433|0|60
contig04669_length=858|250|60
contig04669_length=858|750|60
contig15399_length=481|250|60
log2(expression)
0
2
4
6
8
12
16
StageI
StageII
StageIII
StageIV
StageV
StageVI

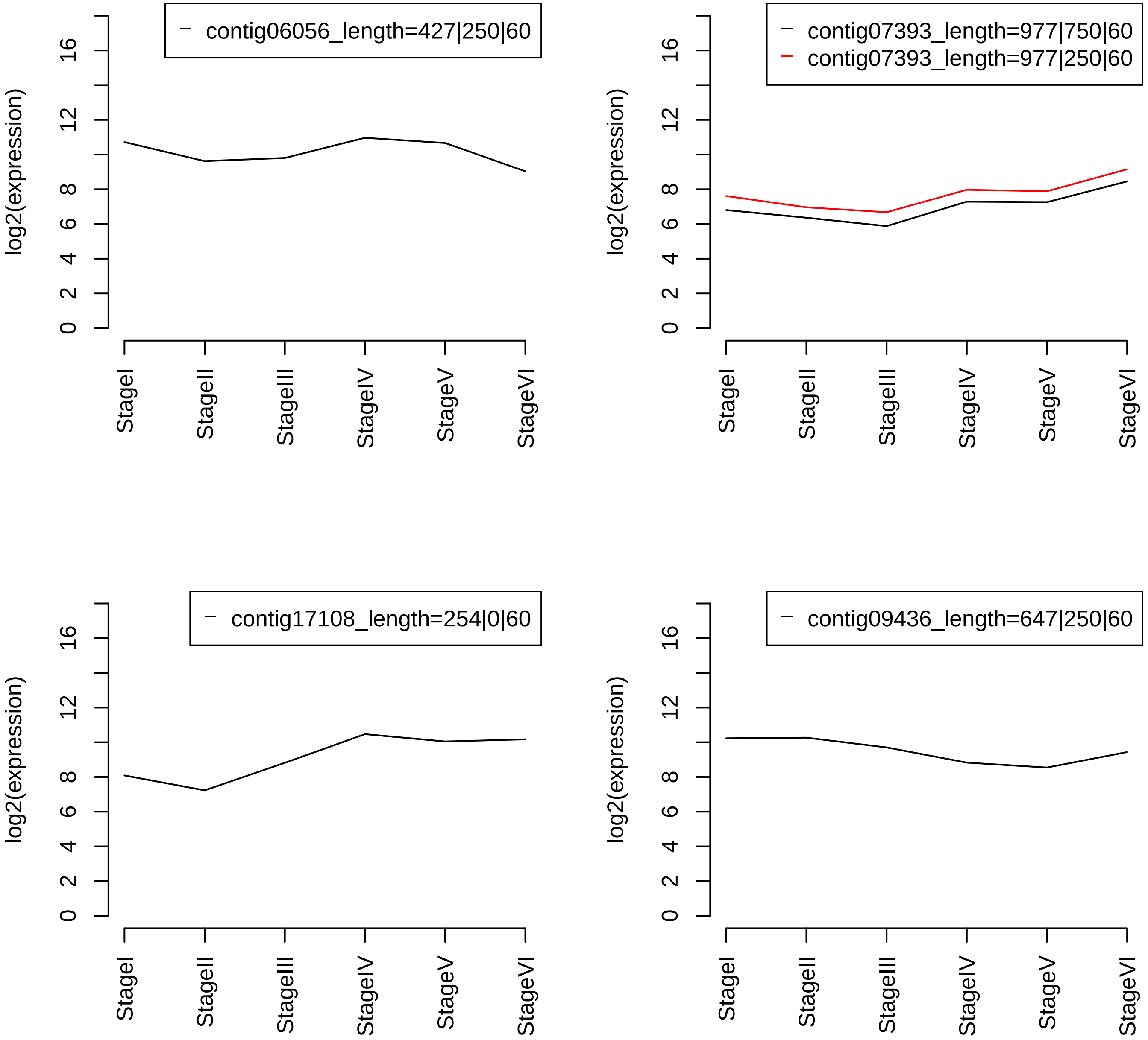
contig06056_length=427|250|60
contig07393_length=977|750|60
contig07393_length=977|250|60
contig17108_length=254|0|60
contig09436_length=647|250|60
log2(expression)
0 2 4 6 8 12 16
StageI
StageII
StageIII
StageIV
StageV
StageVI

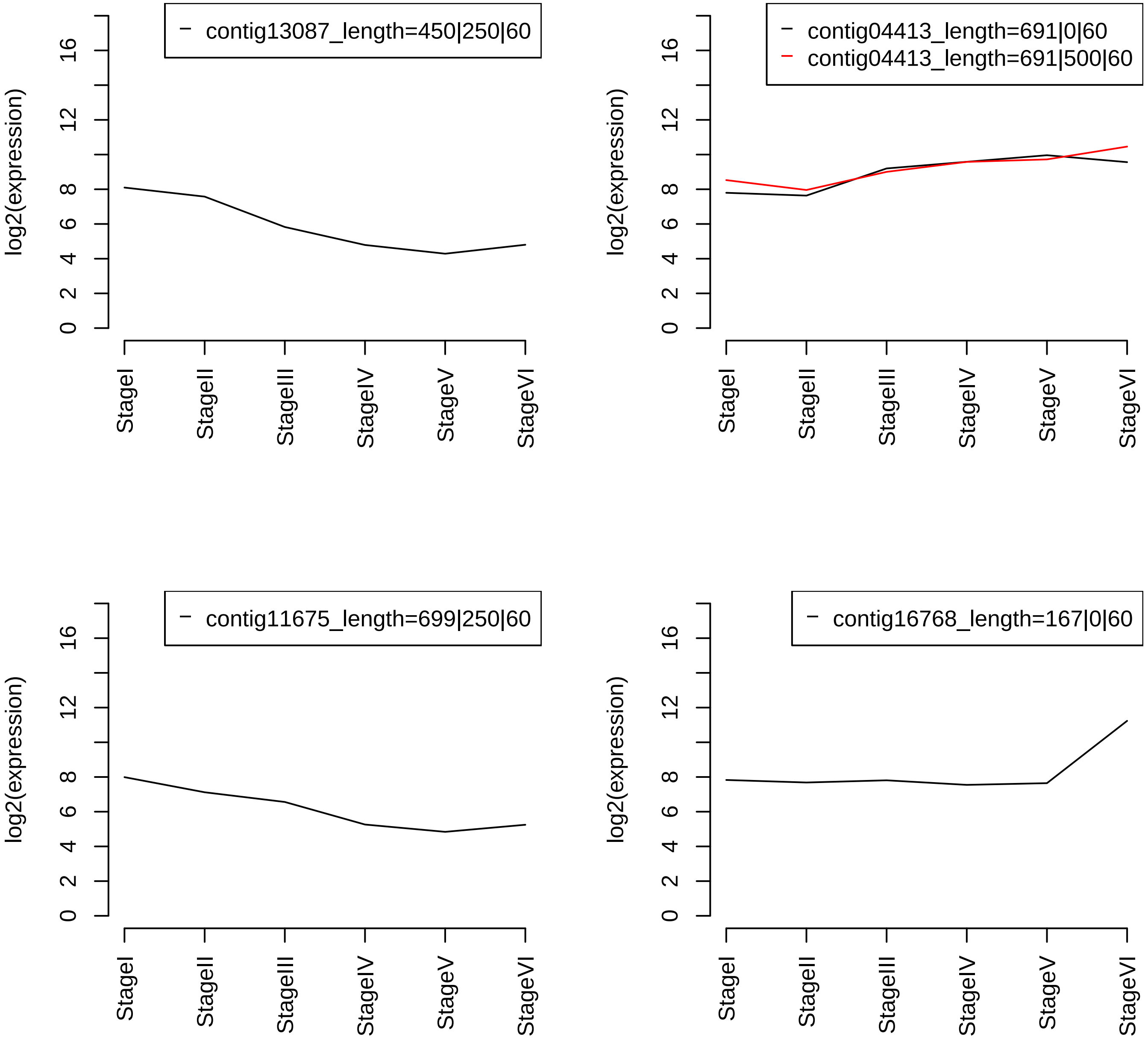
contig13087_length=450|250|60
contig04413_length=691|0|60
contig04413_length=691|500|60
contig11675_length=699|250|60
contig16768_length=167|0|60
log2(expression)
0
2
4
6
8
12
16
StageI
StageII
StageIII
StageIV
StageV
StageVI

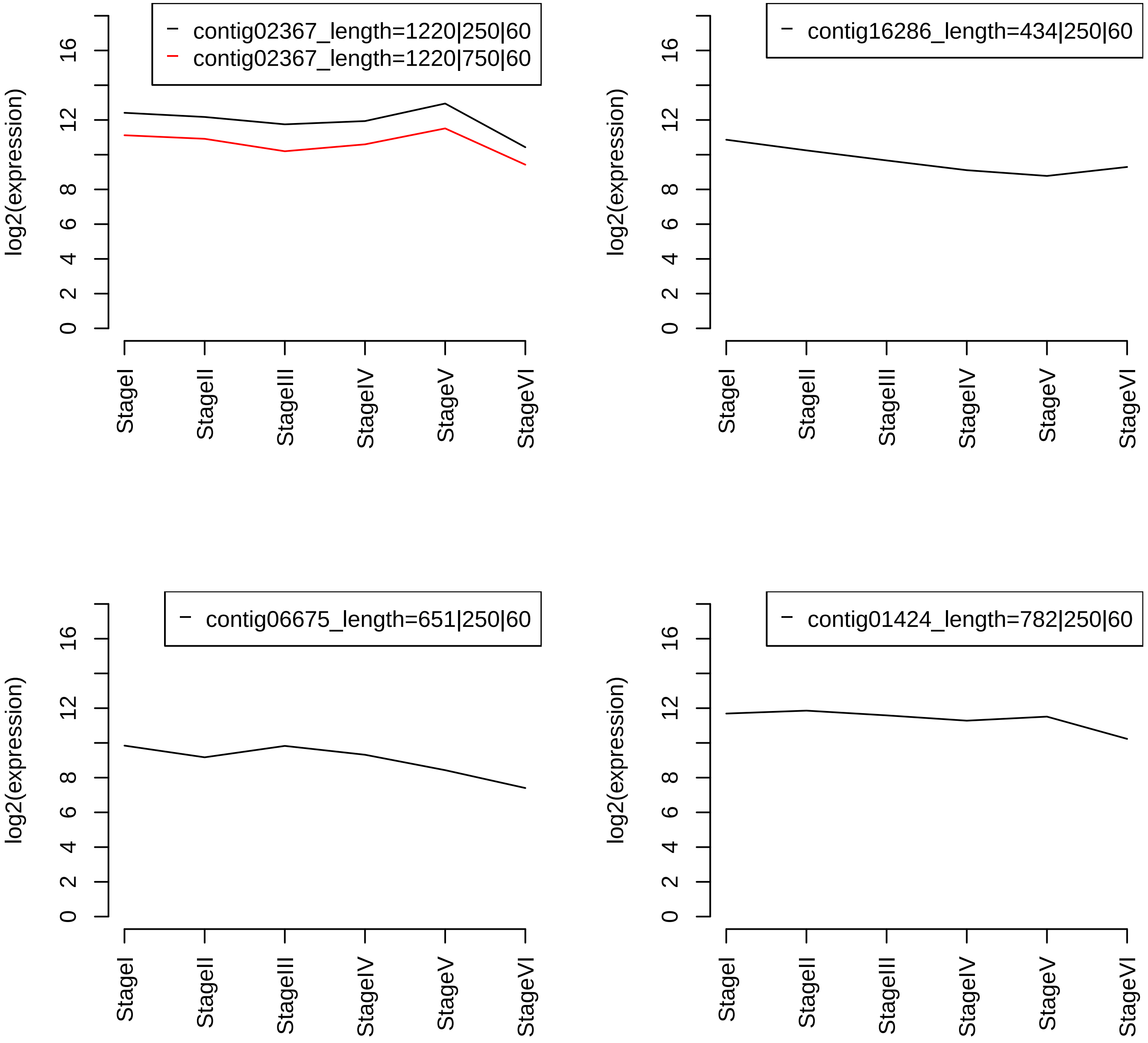

contig02367_length=1220|250|60
contig02367_length=1220|750|60
contig16286_length=434|250|60
contig06675_length=651|250|60
contig01424_length=782|250|60
log2(expression)
0
2
4
6
8
12
16
StageI
StageII
StageIII
StageIV
StageV
StageVI

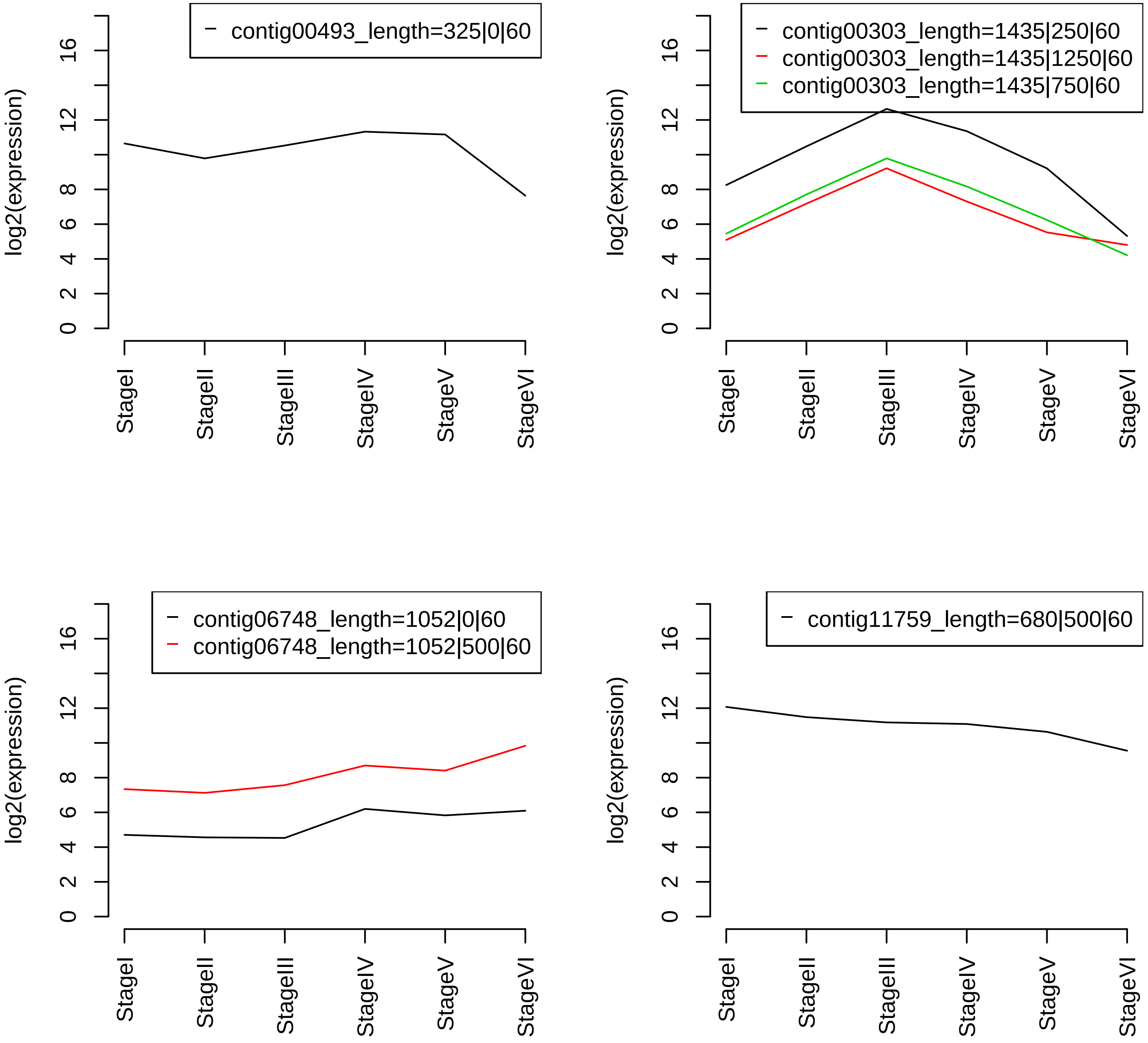
contig00493_length=325|0|60
contig00303_length=1435|250|60
contig00303_length=1435|1250|60
contig00303_length=1435|750|60
contig06748_length=1052|0|60
contig06748_length=1052|500|60
contig11759_length=680|500|60
log2(expression)
0
2
4
6
8
12
16
StageI
StageII
StageIII
StageIV
StageV
StageVI

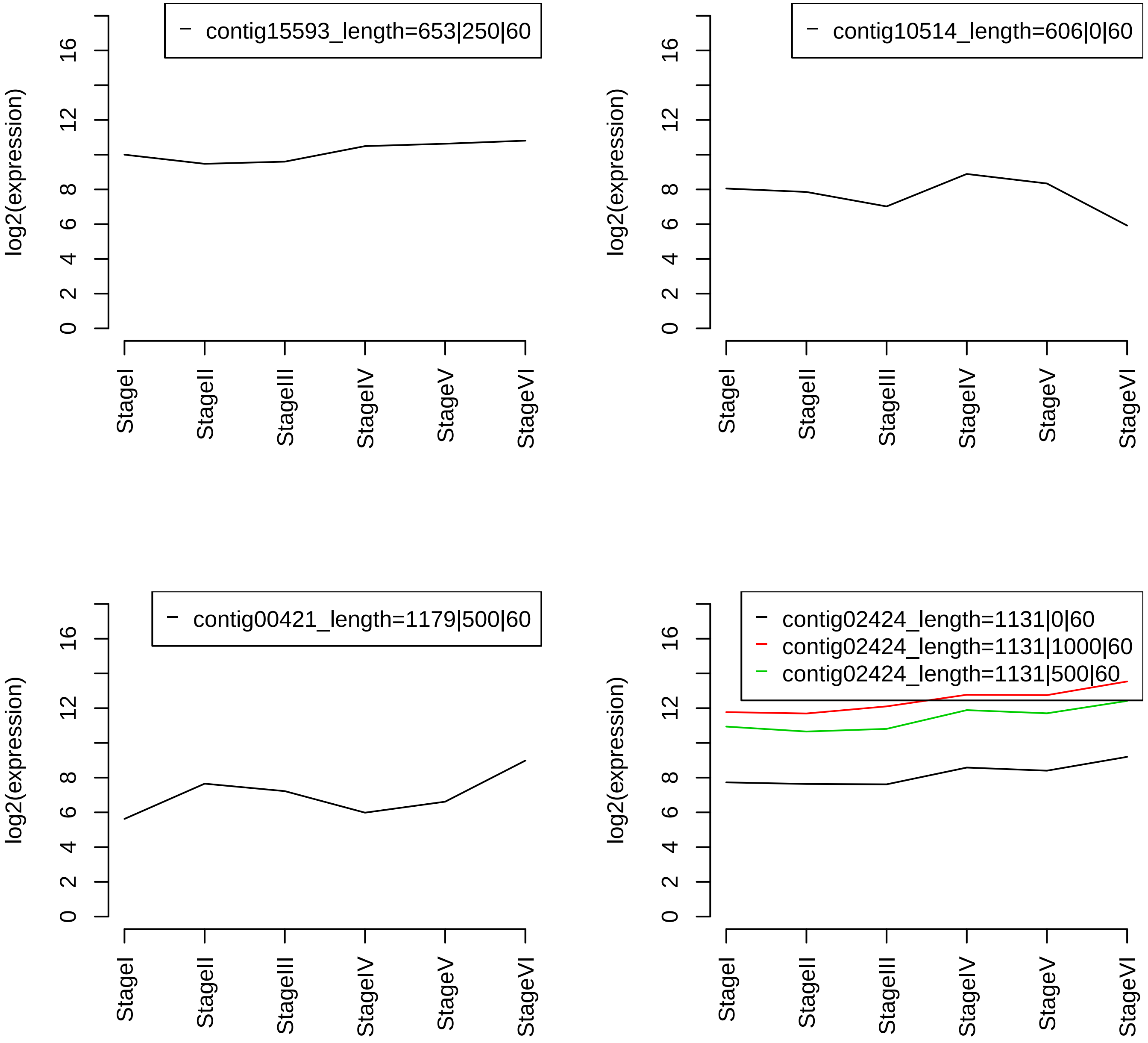
contig15593_length=653|250|60
contig10514_length=606|0|60
contig00421_length=1179|500|60
contig02424_length=1131|0|60
contig02424_length=1131|1000|60
contig02424_length=1131|500|60
log2(expression)
0 2 4 6 8 12 16
StageI
StageII
StageIII
StageIV
StageV
StageVI

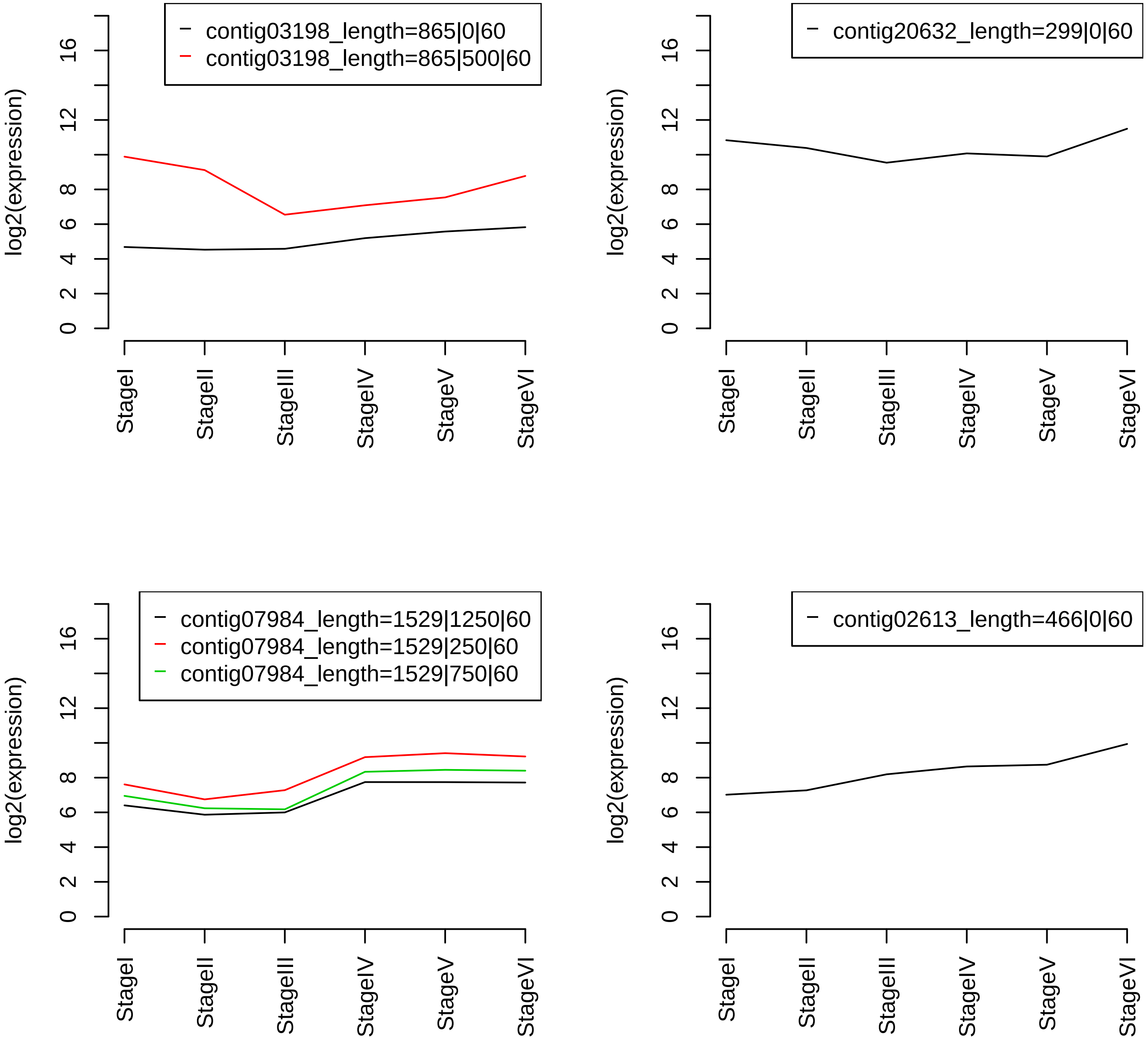
contig03198_length=865|0|60
contig03198_length=865|500|60
contig20632_length=299|0|60
contig07984_length=1529|1250|60
contig07984_length=1529|250|60
contig07984_length=1529|750|60
contig02613_length=466|0|60
log2(expression)
0
2
4
6
8
12
16
StageI
StageII
StageIII
StageIV
StageV
StageVI

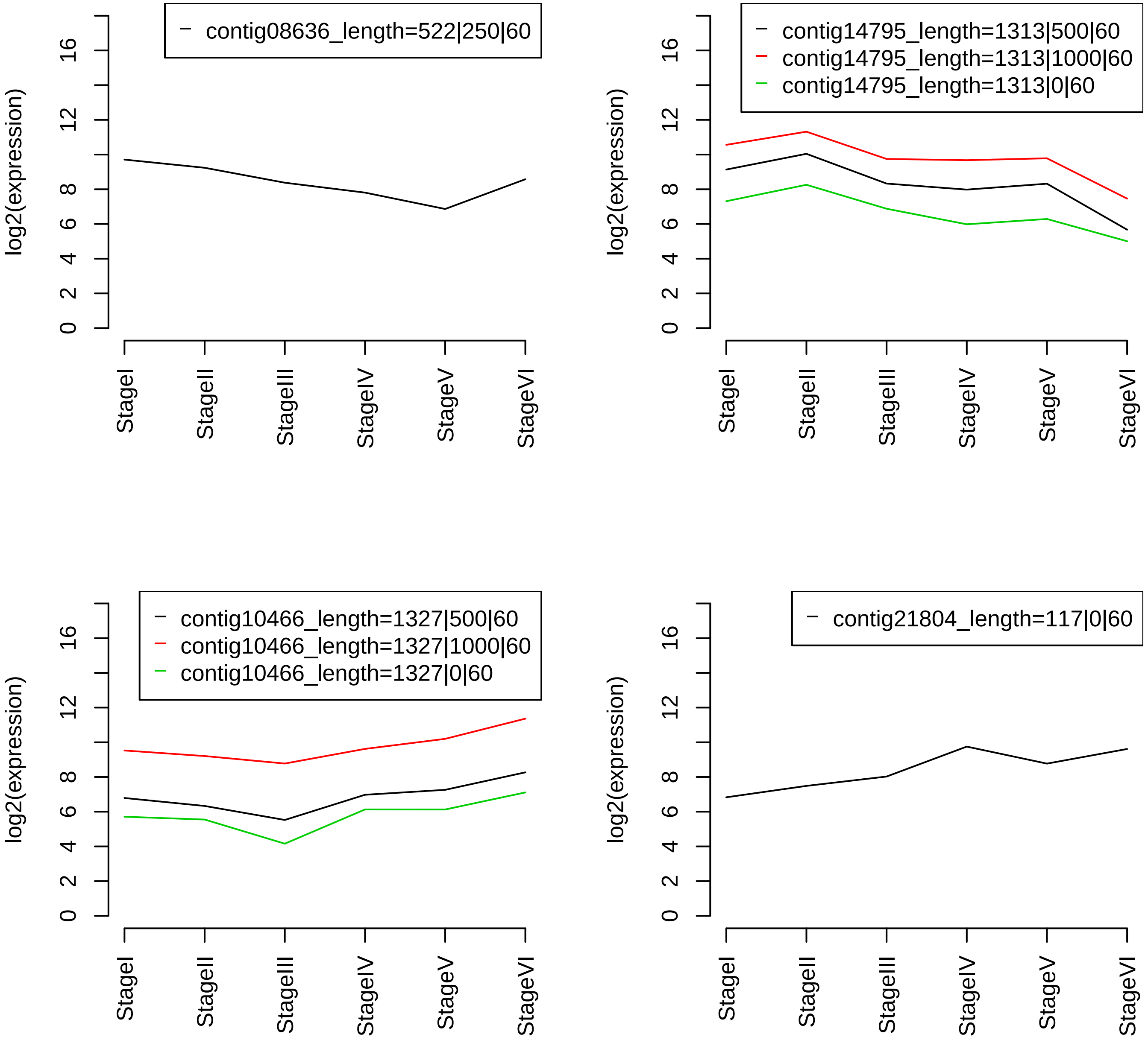
contig08636_length=522|250|60
contig14795_length=1313|500|60
contig14795_length=1313|1000|60
contig14795_length=1313|0|60
contig10466_length=1327|500|60
contig10466_length=1327|1000|60
contig10466_length=1327|0|60
contig21804_length=117|0|60
log2(expression)
0
2
4
6
8
12
16
StageI
StageII
StageIII
StageIV
StageV
StageVI

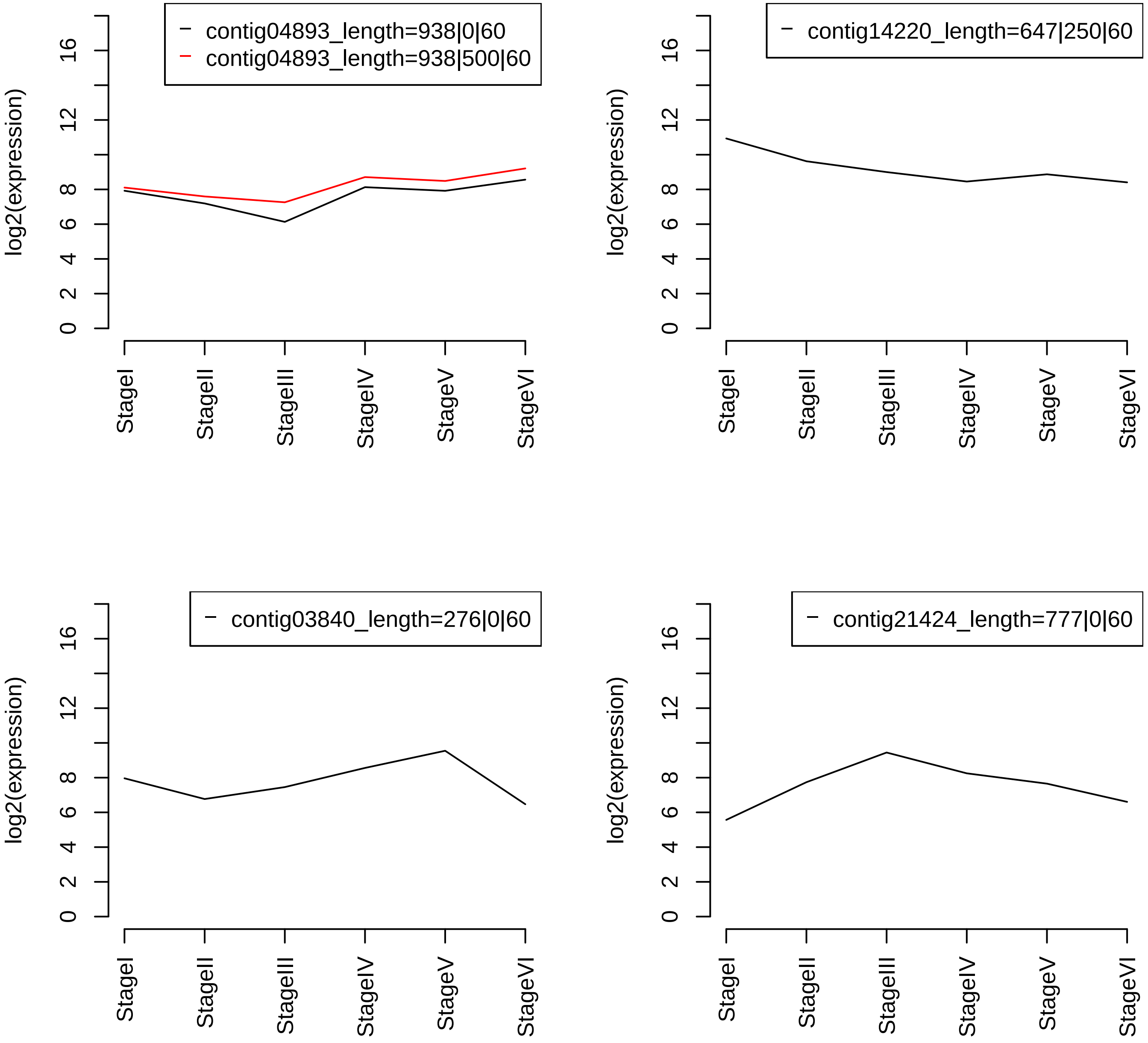

contig04893_length=938|0|60
contig04893_length=938|500|60
contig14220_length=647|250|60
contig03840_length=276|0|60
contig21424_length=777|0|60
log2(expression)
0
2
4
6
8
12
16
StageI
StageII
StageIII
StageIV
StageV
StageVI

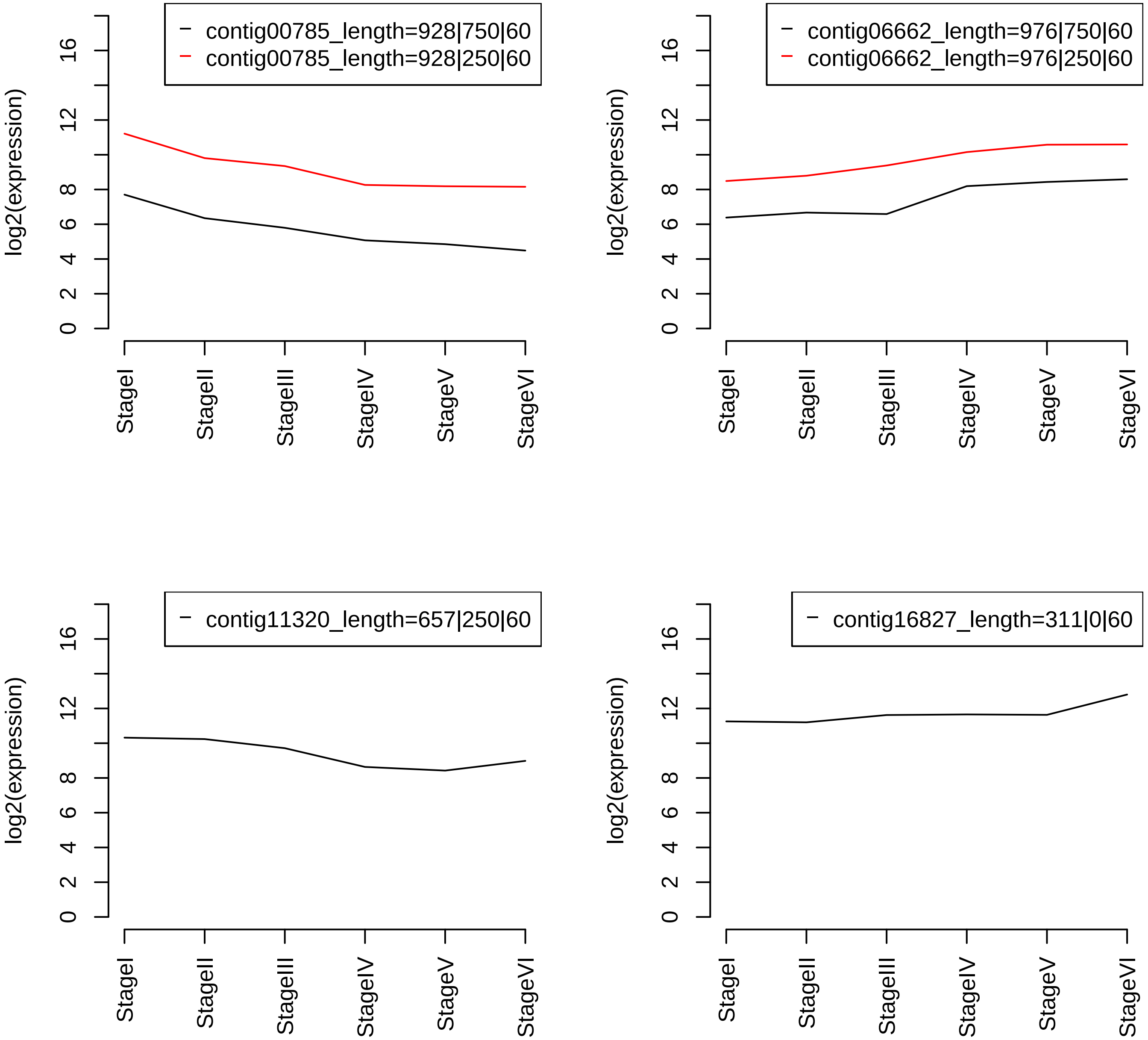

contig00785_length=928|750|60
contig00785_length=928|250|60
contig06662_length=976|750|60
contig06662_length=976|250|60
contig11320_length=657|250|60
contig16827_length=311|0|60
log2(expression)
0 2 4 6 8 12 16
StageI StageII StageIII StageIV StageV StageVI

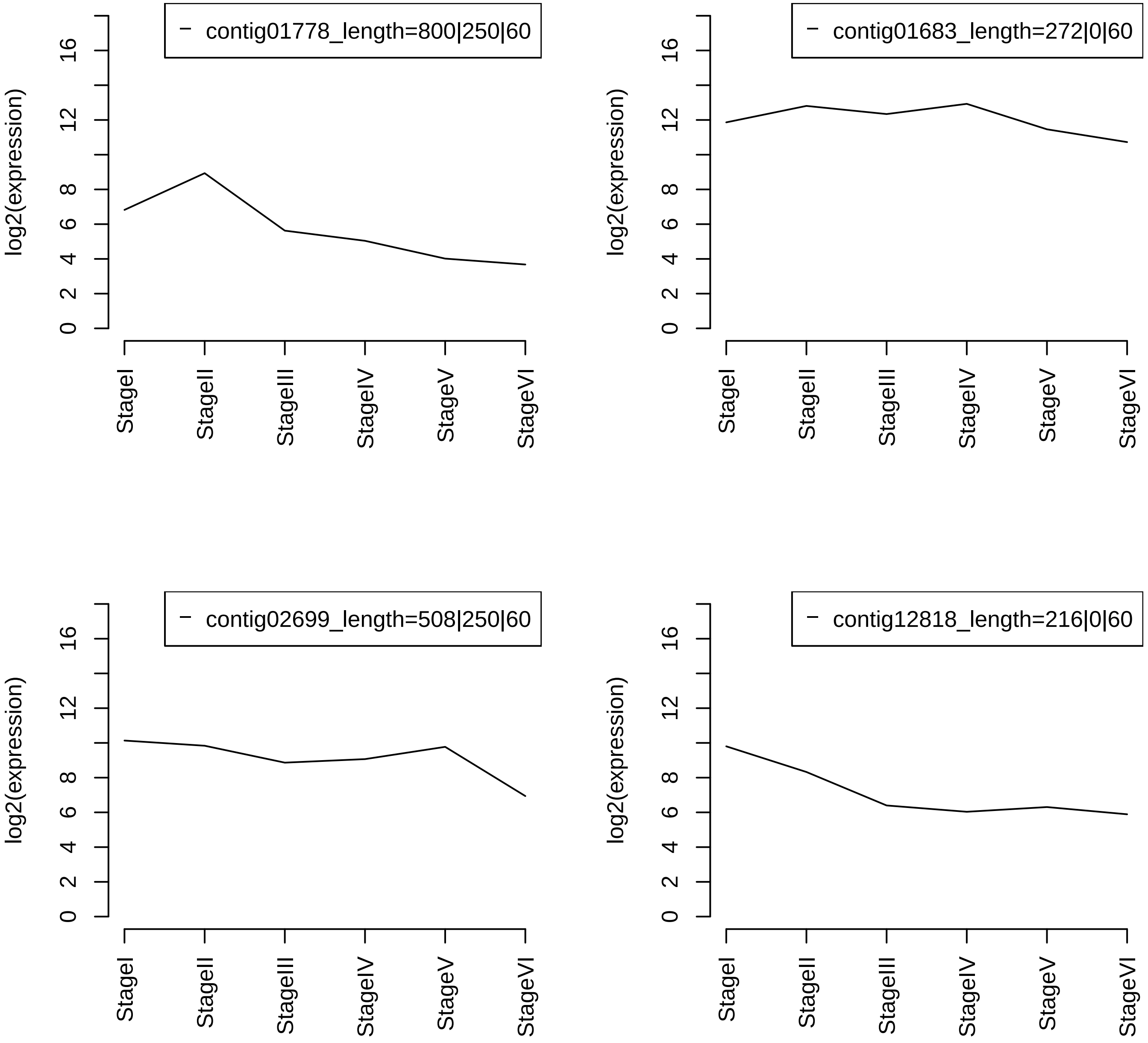

contig01778_length=800|250|60
contig01683_length=272|0|60
contig02699_length=508|250|60
contig12818_length=216|0|60
log2(expression)
0
2
4
6
8
12
16
StageI
StageII
StageIII
StageIV
StageV
StageVI

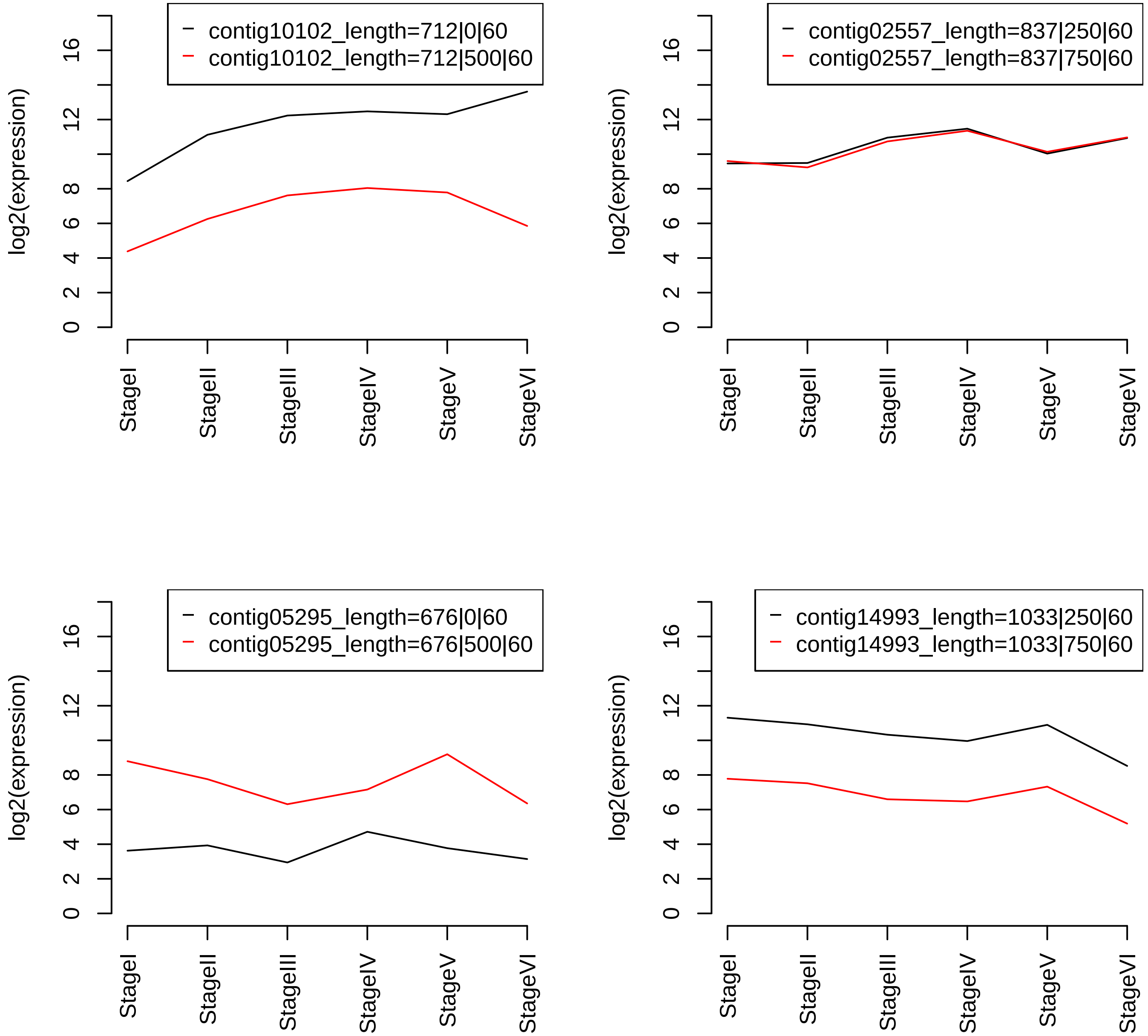
contig10102_length=712|0|60
contig10102_length=712|500|60
contig02557_length=837|250|60
contig02557_length=837|750|60
contig05295_length=676|0|60
contig05295_length=676|500|60
contig14993_length=1033|250|60
contig14993_length=1033|750|60
log2(expression)
0
2
4
6
8
12
16
StageI
StageII
StageIII
StageIV
StageV
StageVI

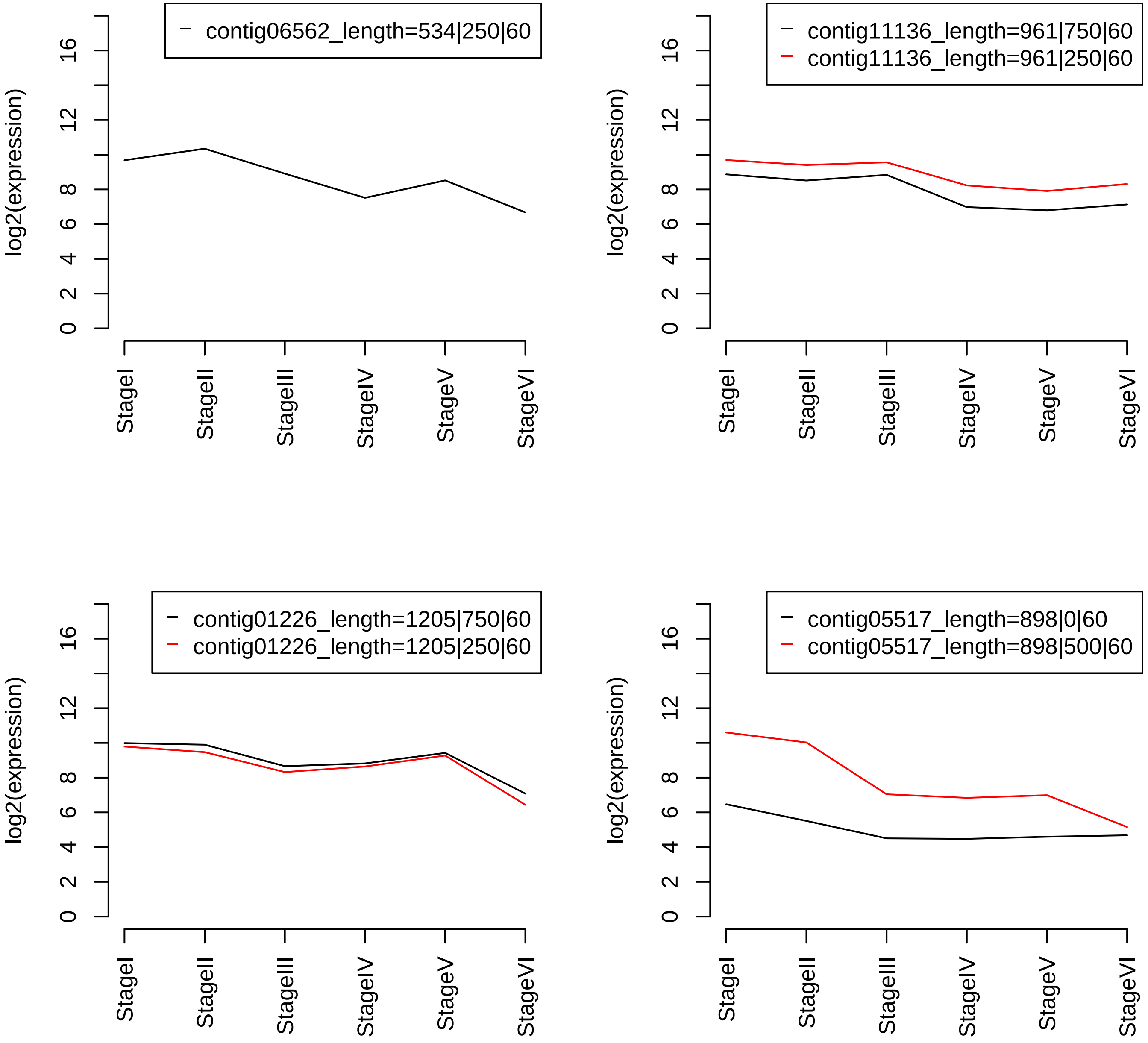

contig06562_length=534|250|60
contig11136_length=961|750|60
contig11136_length=961|250|60
contig01226_length=1205|750|60
contig01226_length=1205|250|60
contig05517_length=898|0|60
contig05517_length=898|500|60
log2(expression)
0 2 4 6 8 12 16
StageI
StageII
StageIII
StageIV
StageV
StageVI

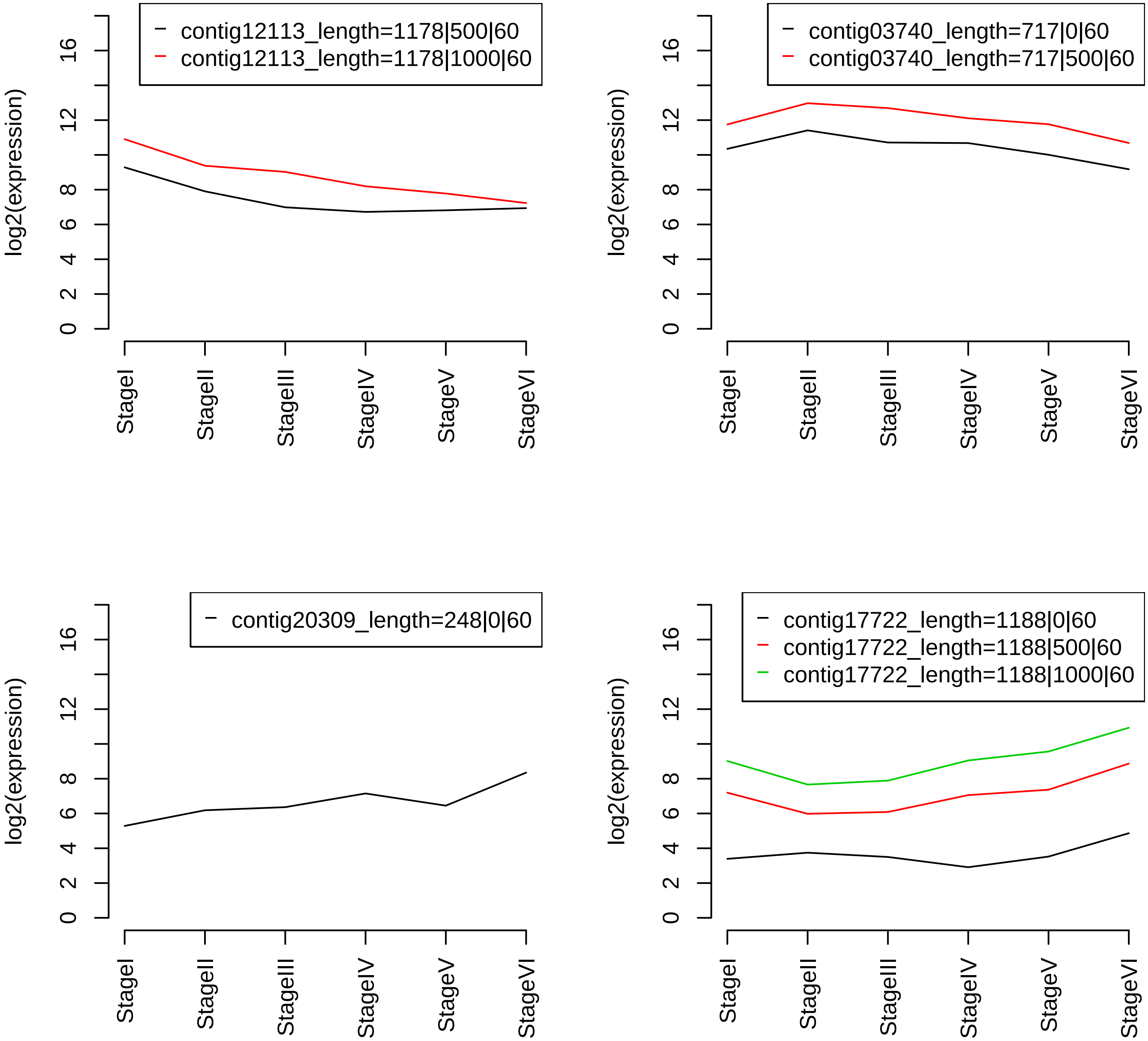
contig12113_length=1178|500|60
contig12113_length=1178|1000|60
contig03740_length=717|0|60
contig03740_length=717|500|60
contig20309_length=248|0|60
contig17722_length=1188|0|60
contig17722_length=1188|500|60
contig17722_length=1188|1000|60
log2(expression)
StageI
StageII
StageIII
StageIV
StageV
StageVI

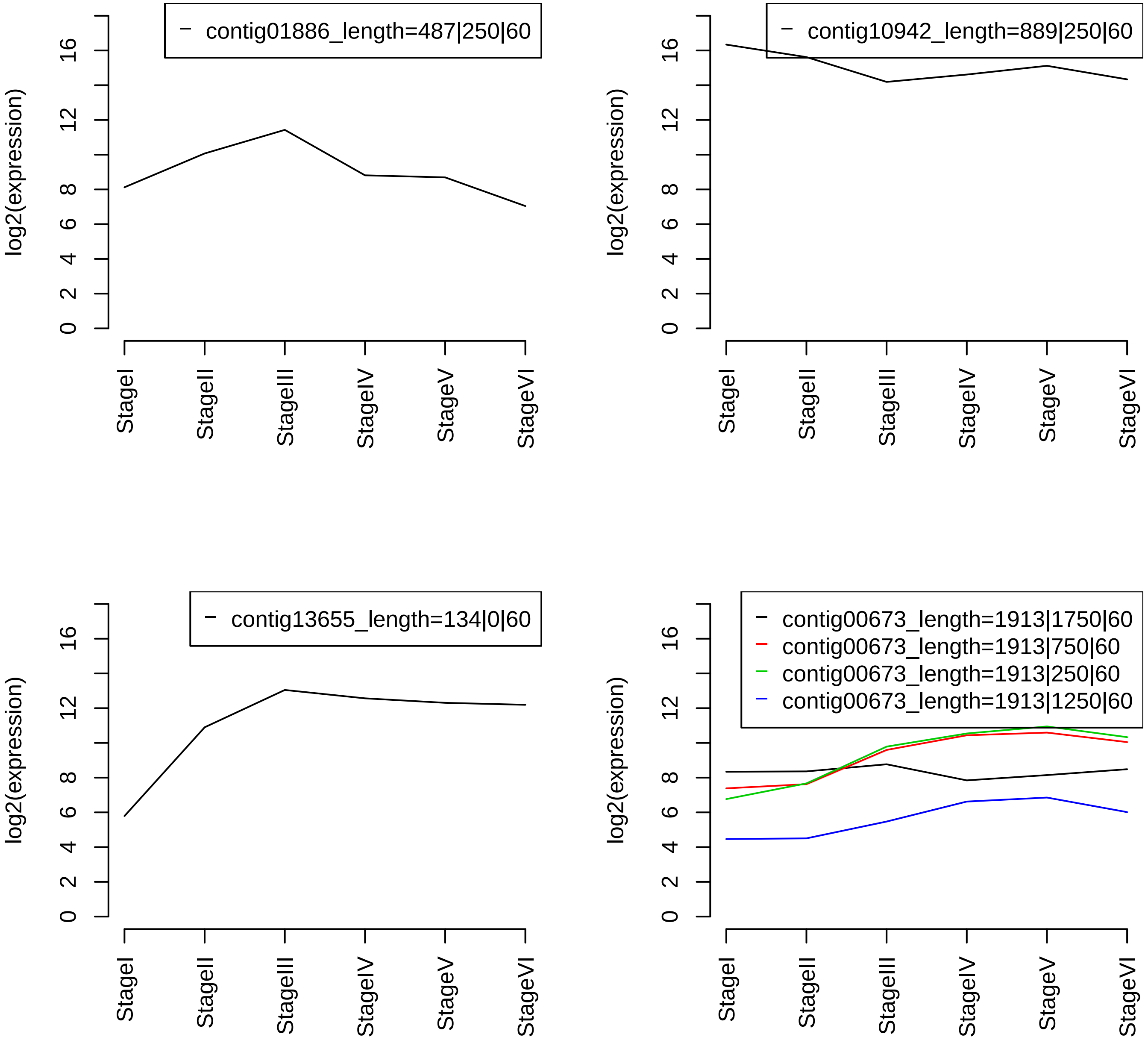

contig01886_length=487|250|60
contig10942_length=889|250|60
contig13655_length=134|0|60
contig00673_length=1913|1750|60
contig00673_length=1913|750|60
contig00673_length=1913|250|60
contig00673_length=1913|1250|60
log2(expression)
0
2
4
6
8
12
16
StageI
StageII
StageIII
StageIV
StageV
StageVI

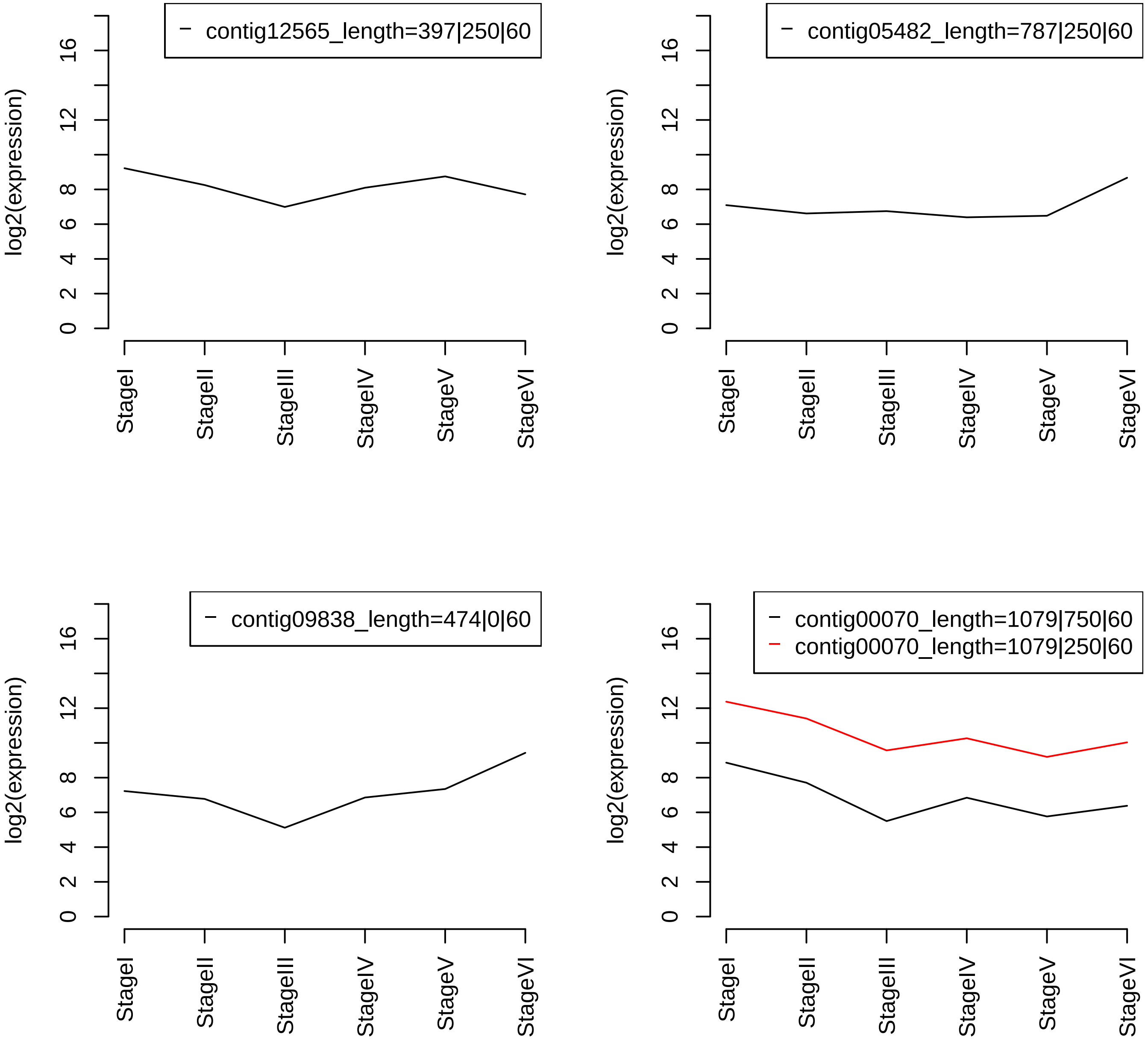

contig12565_length=397|250|60
contig05482_length=787|250|60
contig09838_length=474|0|60
contig00070_length=1079|750|60
contig00070_length=1079|250|60
log2(expression)
0
2
4
6
8
12
16
StageI
StageII
StageIII
StageIV
StageV
StageVI

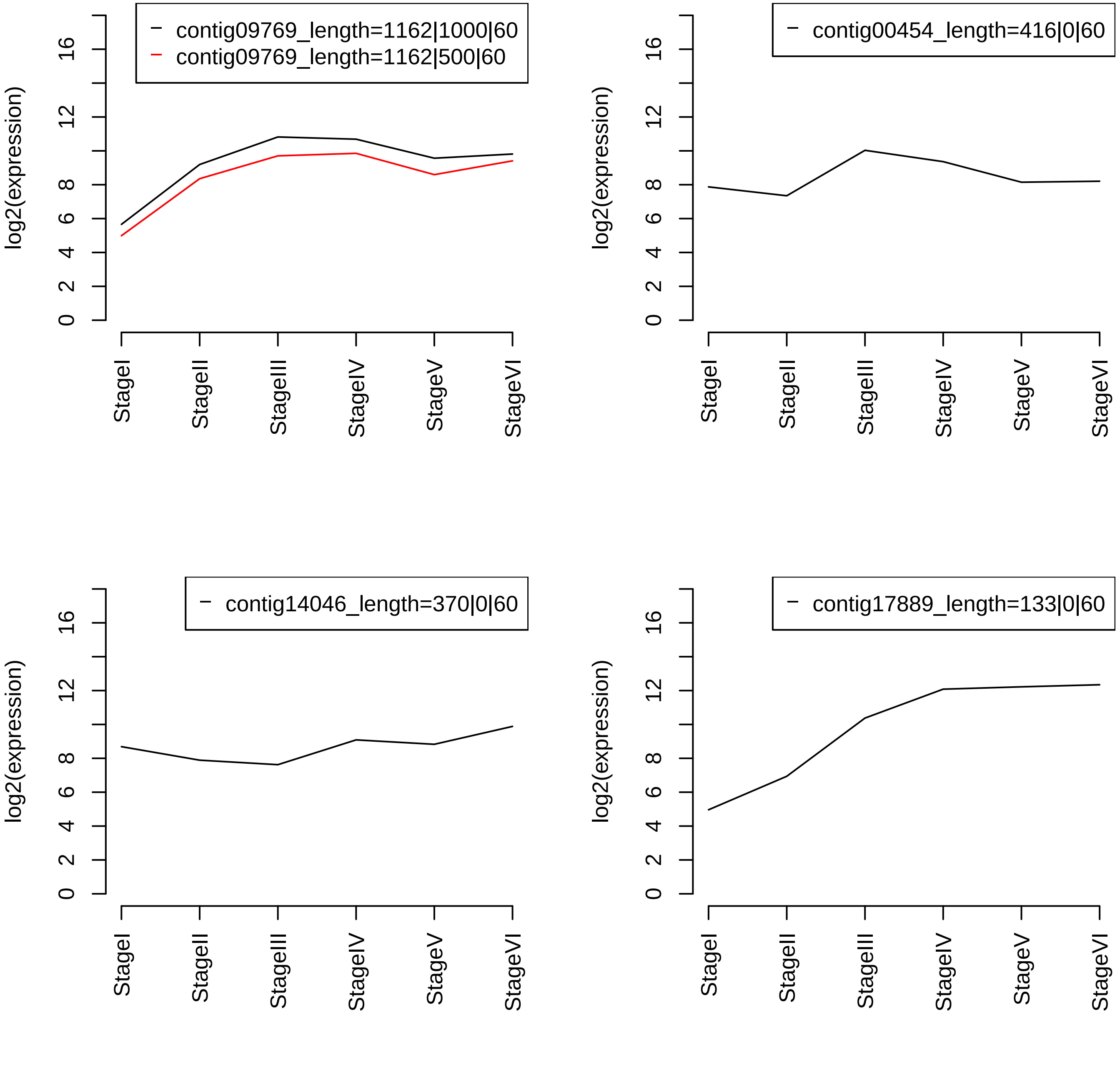

contig09769_length=1162|1000|60
contig09769_length=1162|500|60
contig00454_length=416|0|60
contig14046_length=370|0|60
contig17889_length=133|0|60
log2(expression)
0
2
4
6
8
12
16
StageI
StageII
StageIII
StageIV
StageV
StageVI

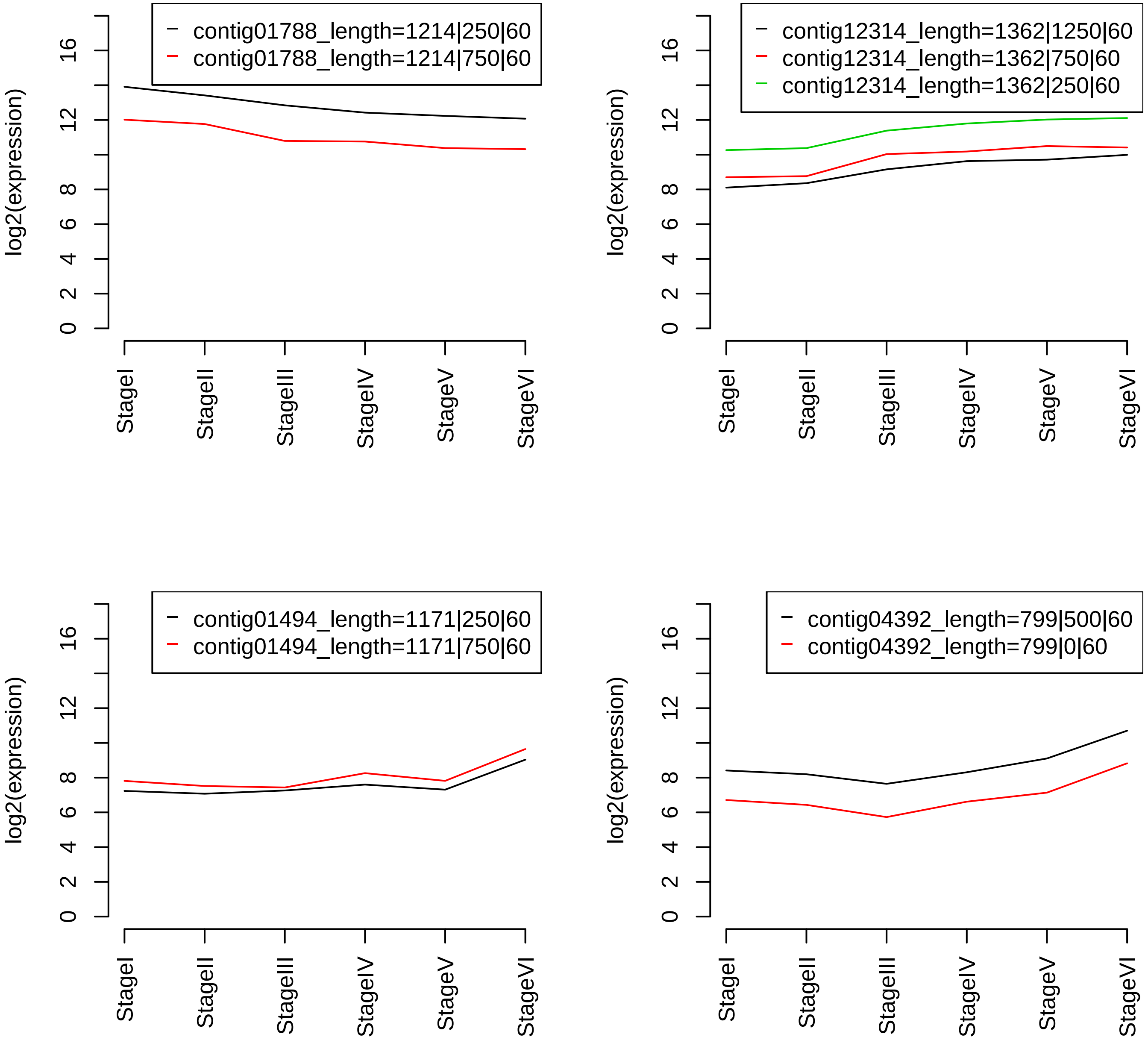

contig01788_length=1214|250|60
contig01788_length=1214|750|60
contig12314_length=1362|1250|60
contig12314_length=1362|750|60
contig12314_length=1362|250|60
contig01494_length=1171|250|60
contig01494_length=1171|750|60
contig04392_length=799|500|60
contig04392_length=799|0|60
log2(expression)
0 2 4 6 8 12 16
StageI StageII StageIII StageIV StageV StageVI

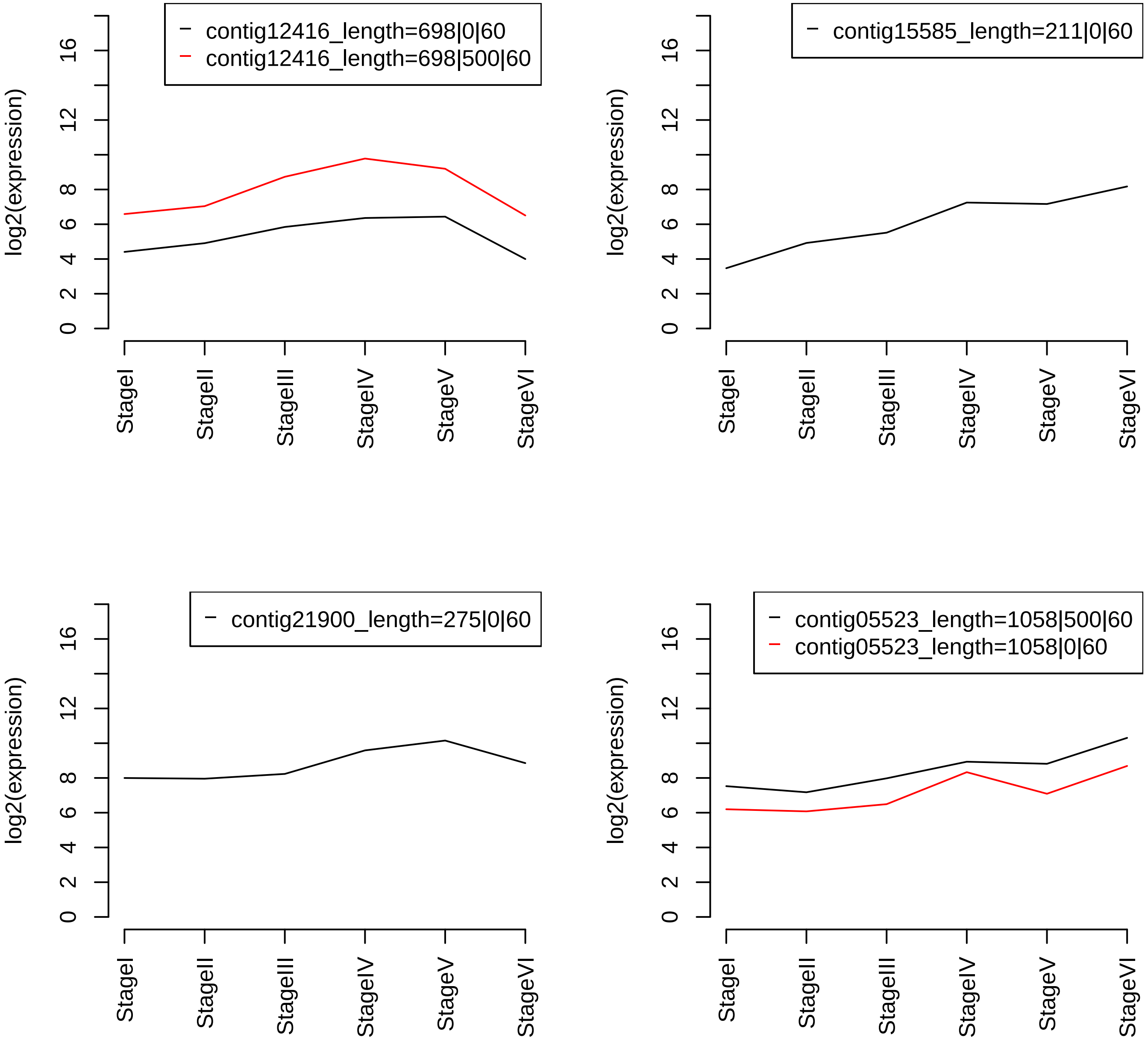
contig12416_length=698|0|60
contig12416_length=698|500|60
contig15585_length=211|0|60
contig21900_length=275|0|60
contig05523_length=1058|500|60
contig05523_length=1058|0|60
log2(expression)
0
2
4
6
8
12
16
StageI
StageII
StageIII
StageIV
StageV
StageVI

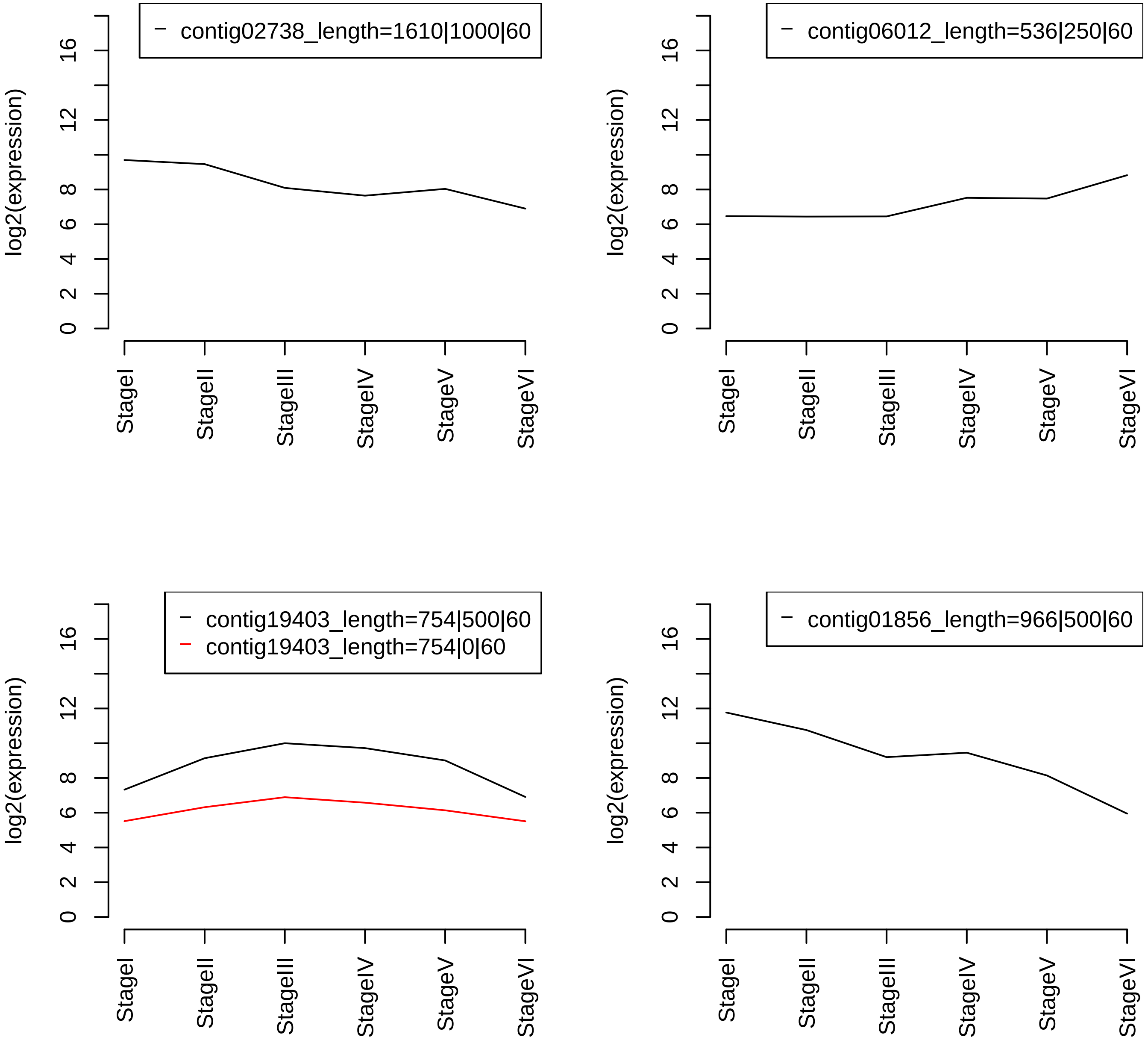

contig02738_length=1610|1000|60
contig06012_length=536|250|60
contig19403_length=754|500|60
contig19403_length=754|0|60
contig01856_length=966|500|60
log2(expression)
0
2
4
6
8
12
16
StageI
StageII
StageIII
StageIV
StageV
StageVI

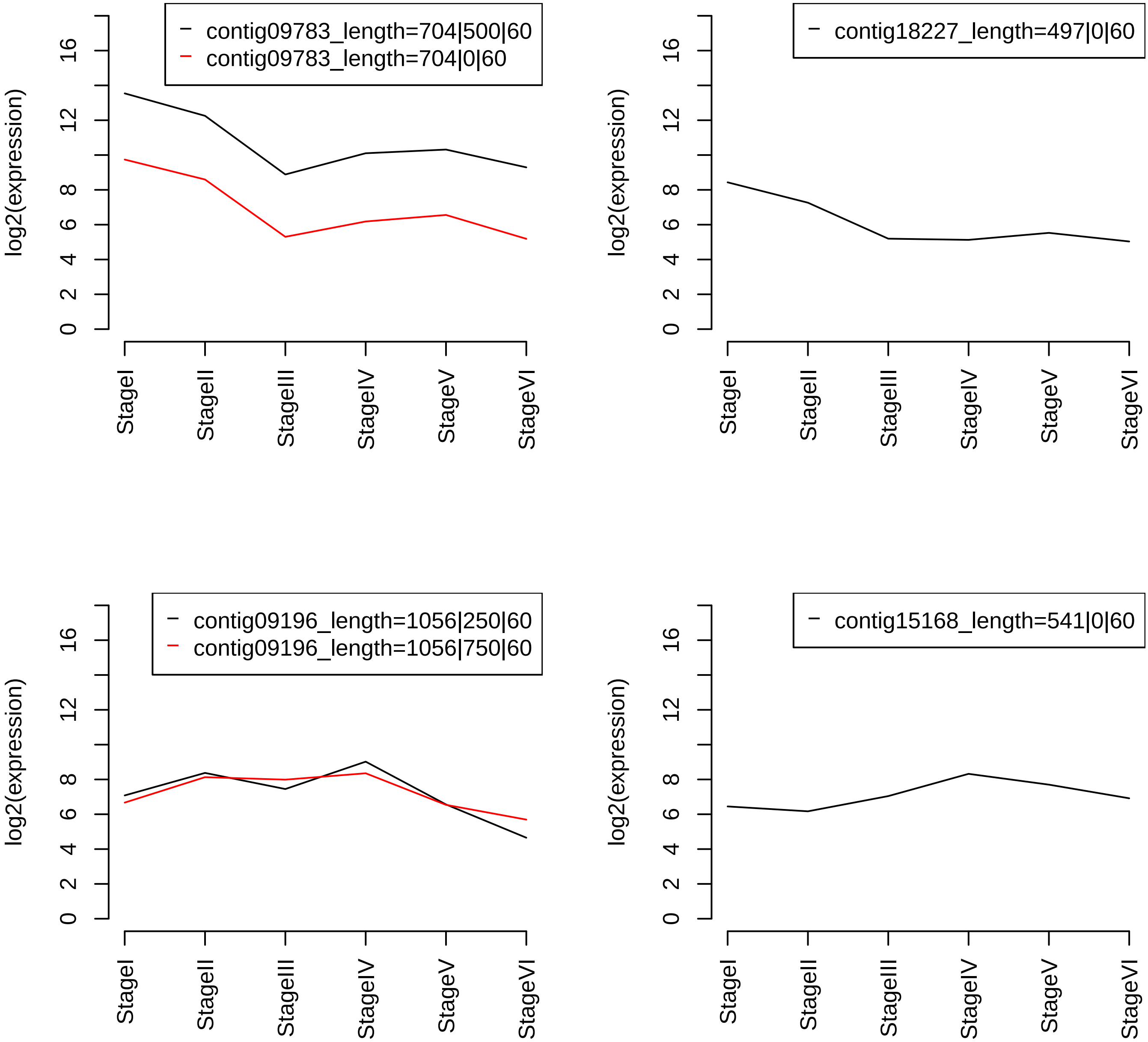

contig09783_length=704|500|60
contig09783_length=704|0|60
contig18227_length=497|0|60
contig09196_length=1056|250|60
contig09196_length=1056|750|60
contig15168_length=541|0|60
log2(expression)
0
2
4
6
8
12
16
StageI
StageII
StageIII
StageIV
StageV
StageVI

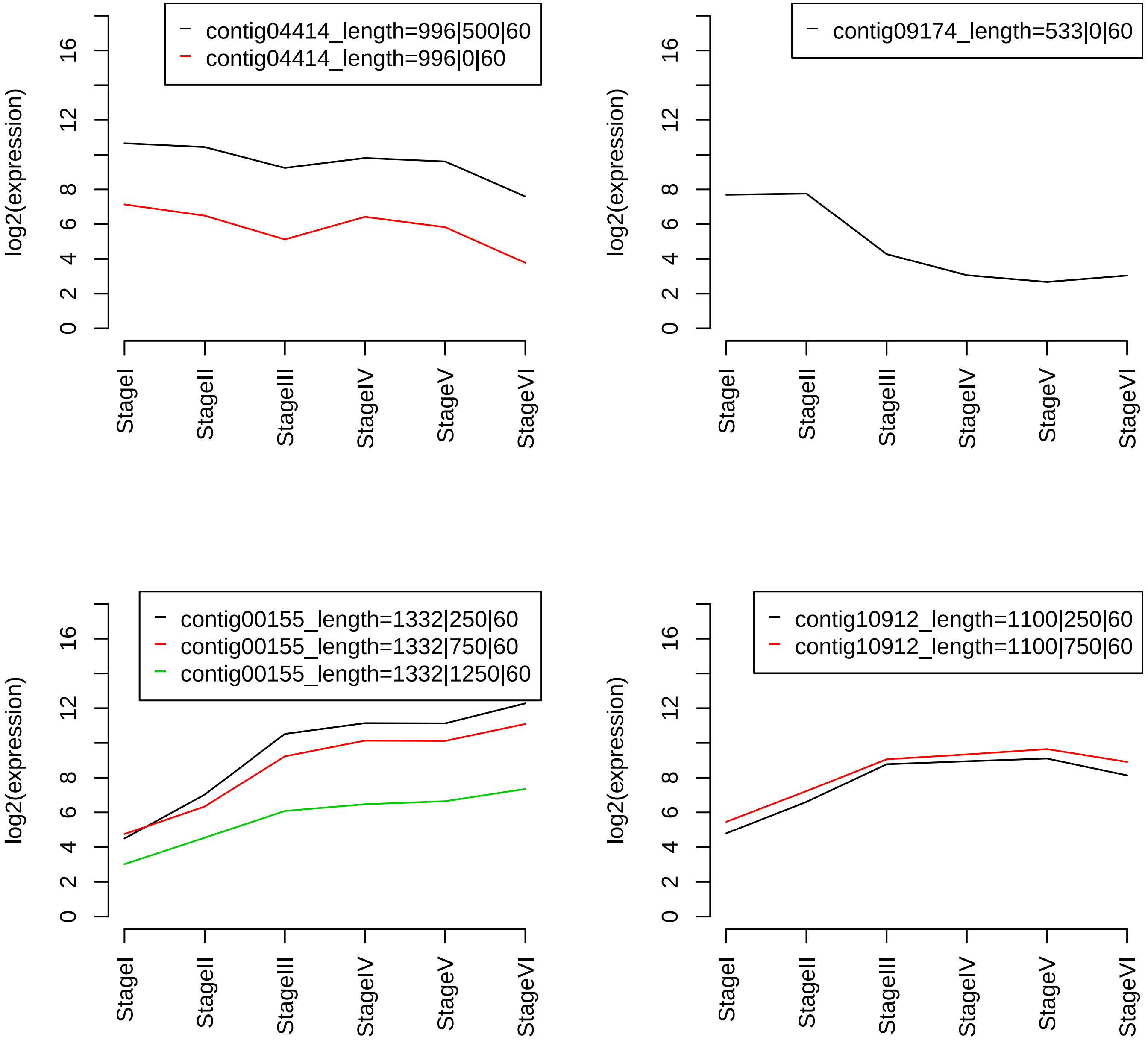
contig04414_length=996|500|60
contig04414_length=996|0|60
contig09174_length=533|0|60
contig00155_length=1332|250|60
contig00155_length=1332|750|60
contig00155_length=1332|1250|60
contig10912_length=1100|250|60
contig10912_length=1100|750|60
log2(expression)
0
2
4
6
8
12
16
StageI
StageII
StageIII
StageIV
StageV
StageVI

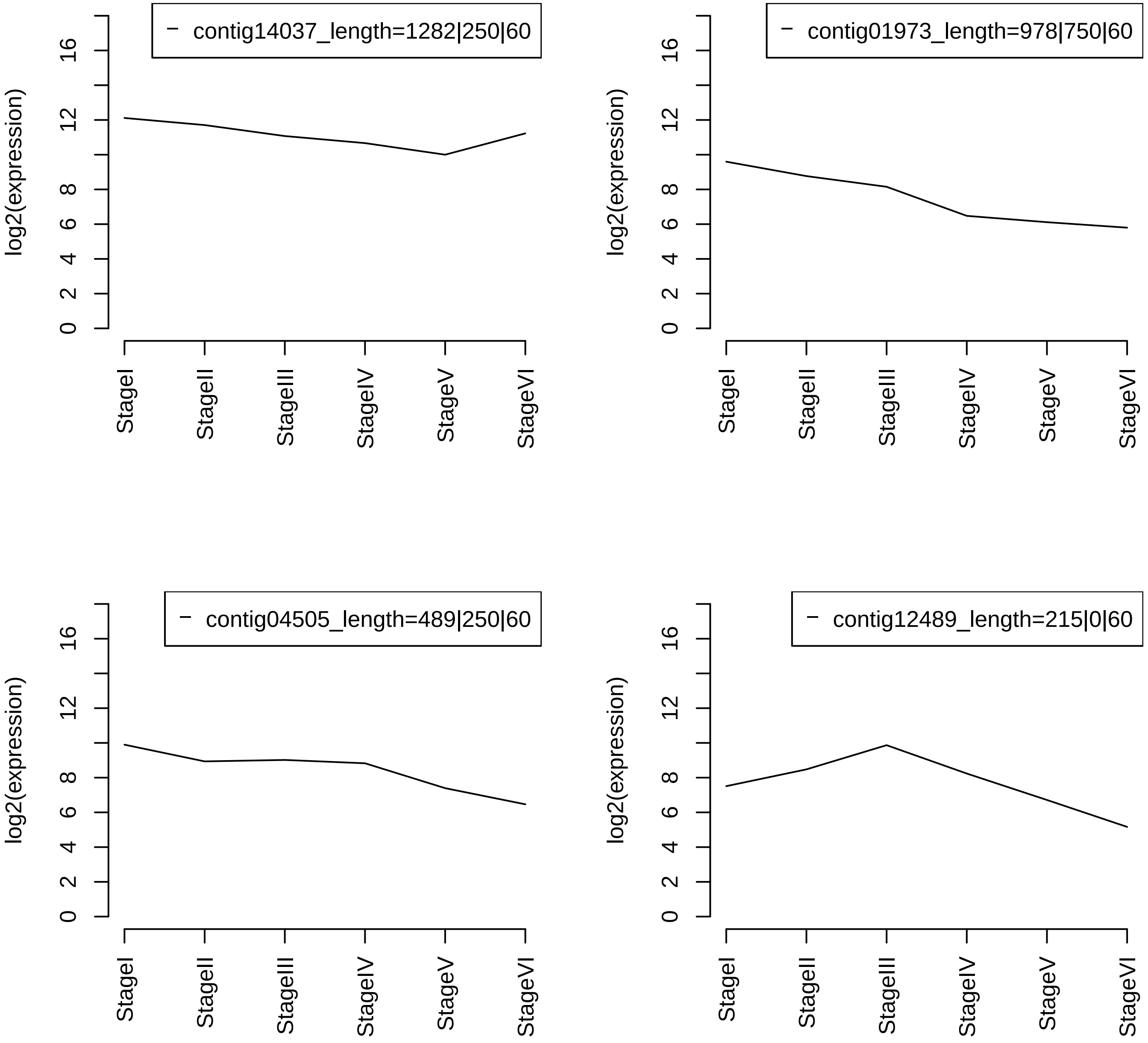
contig14037_length=1282|250|60
contig01973_length=978|750|60
contig04505_length=489|250|60
contig12489_length=215|0|60
log2(expression)
0
2
4
6
8
12
16
StageI
StageII
StageIII
StageIV
StageV
StageVI

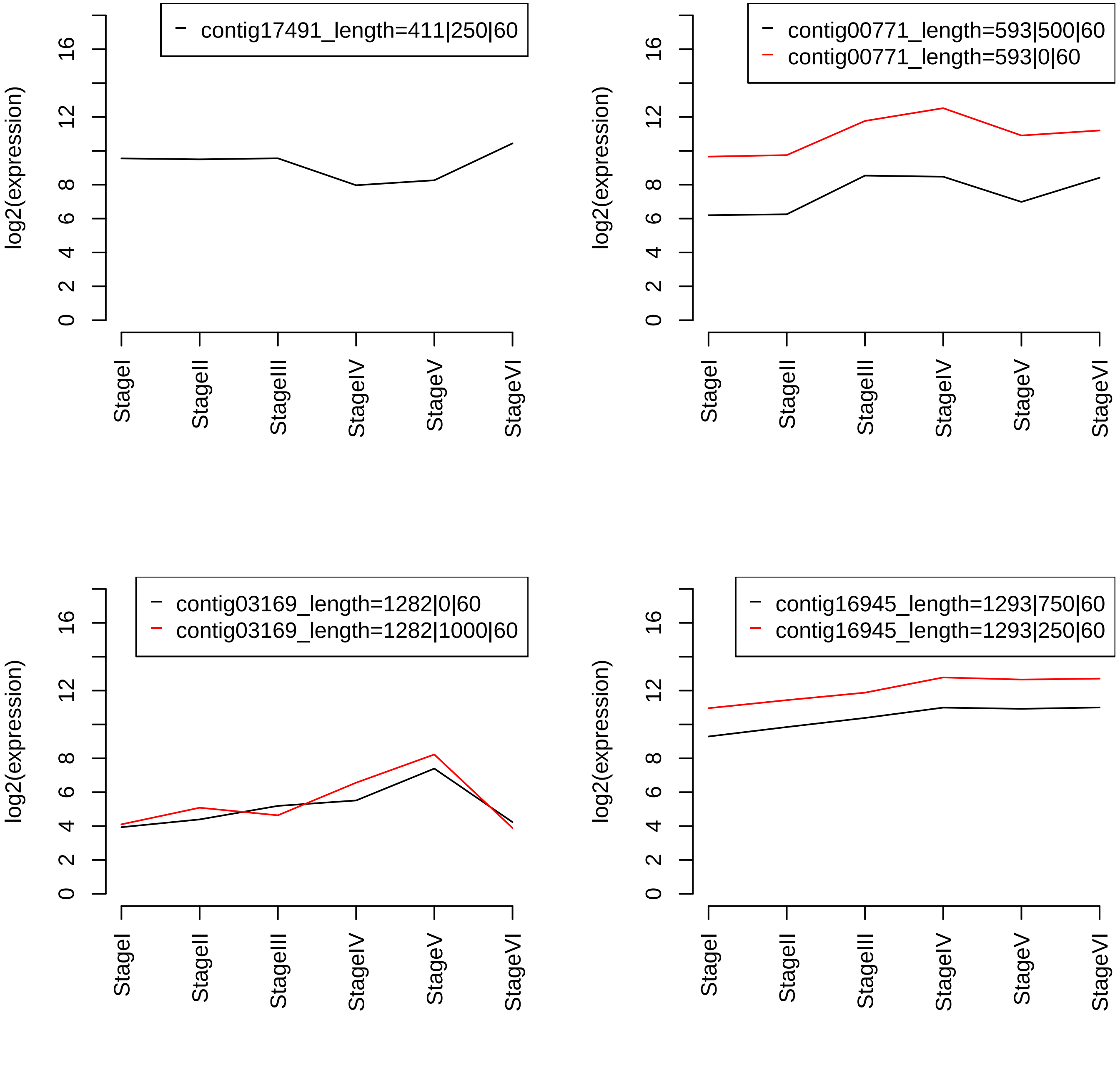

contig17491_length=411|250|60
contig00771_length=593|500|60
contig00771_length=593|0|60
contig03169_length=1282|0|60
contig03169_length=1282|1000|60
contig16945_length=1293|750|60
contig16945_length=1293|250|60
log2(expression)
0
2
4
6
8
12
16
StageI
StageII
StageIII
StageIV
StageV
StageVI

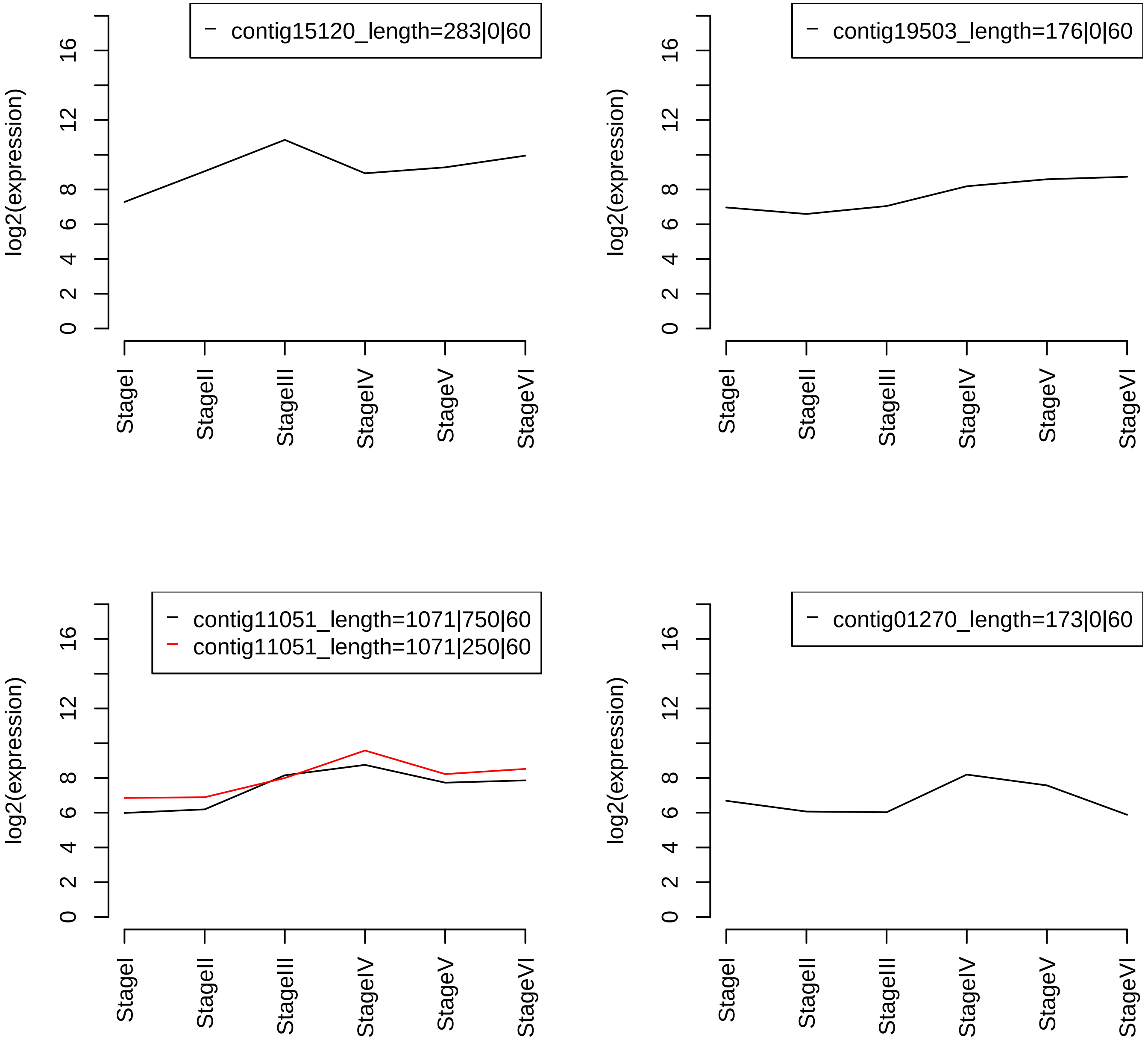
contig15120_length=283|0|60
contig19503_length=176|0|60
contig11051_length=1071|750|60
contig11051_length=1071|250|60
contig01270_length=173|0|60
log2(expression)
0
2
4
6
8
12
16
StageI
StageII
StageIII
StageIV
StageV
StageVI

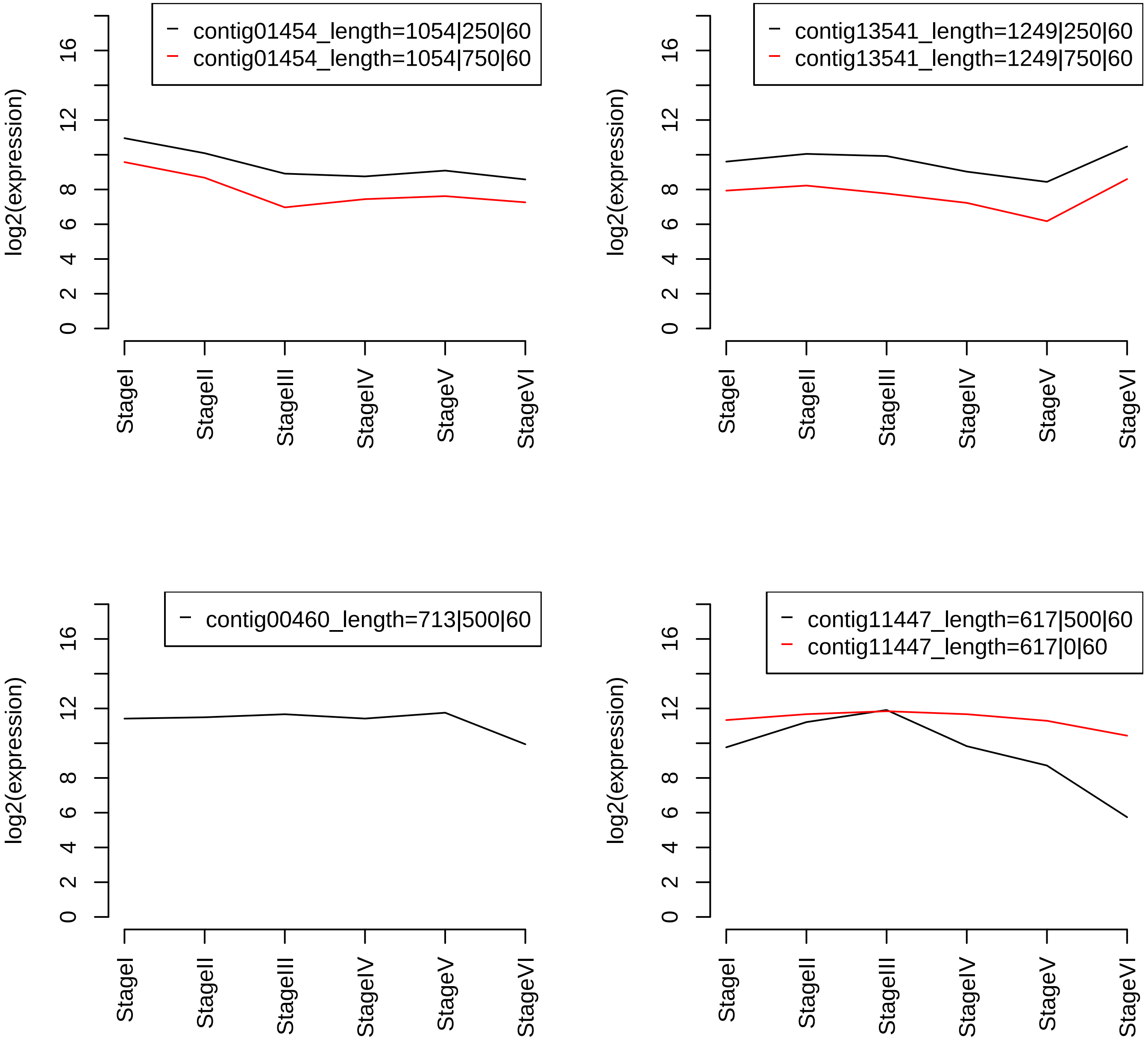

contig01454_length=1054|250|60
contig01454_length=1054|750|60
contig13541_length=1249|250|60
contig13541_length=1249|750|60
contig00460_length=713|500|60
contig11447_length=617|500|60
contig11447_length=617|0|60
log2(expression)
0 2 4 6 8 12 16
StageI StageII StageIII StageIV StageV StageVI

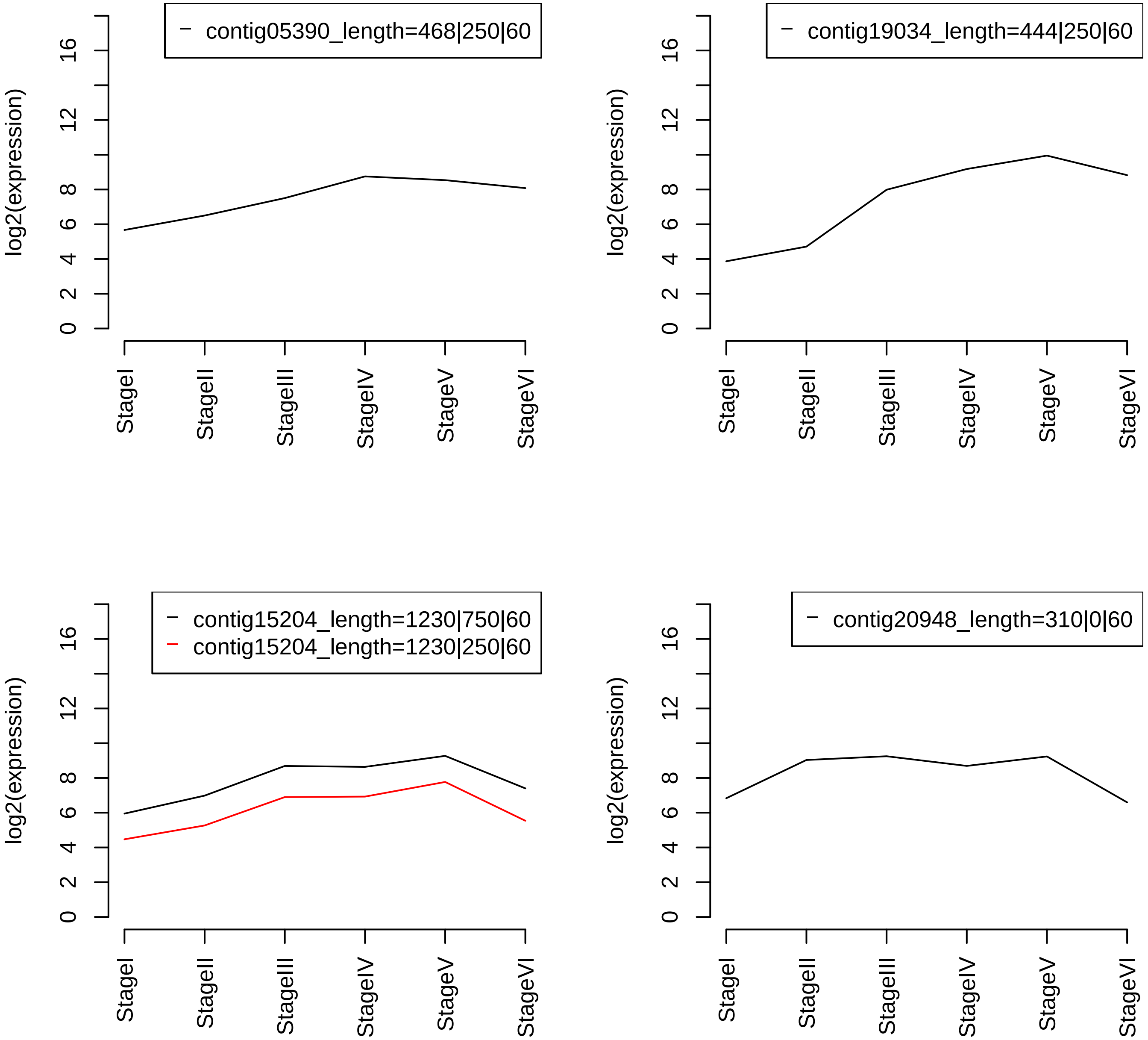
contig05390_length=468|250|60
contig19034_length=444|250|60
contig15204_length=1230|750|60
contig15204_length=1230|250|60
contig20948_length=310|0|60
log2(expression)
0
2
4
6
8
12
16
StageI
StageII
StageIII
StageIV
StageV
StageVI

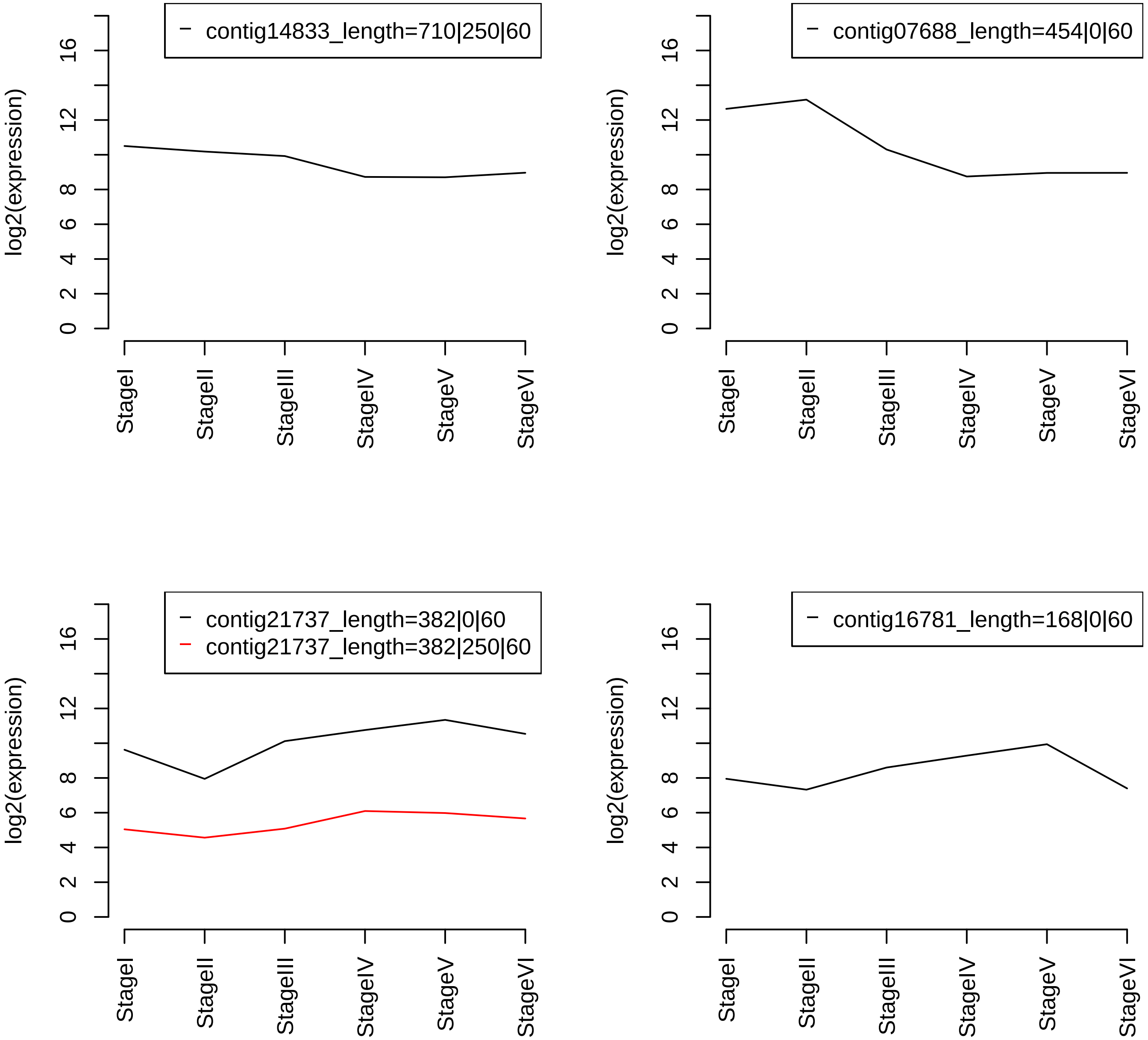
contig14833_length=710|250|60
contig07688_length=454|0|60
contig21737_length=382|0|60
contig21737_length=382|250|60
contig16781_length=168|0|60
log2(expression)
0
2
4
6
8
12
16
StageI
StageII
StageIII
StageIV
StageV
StageVI

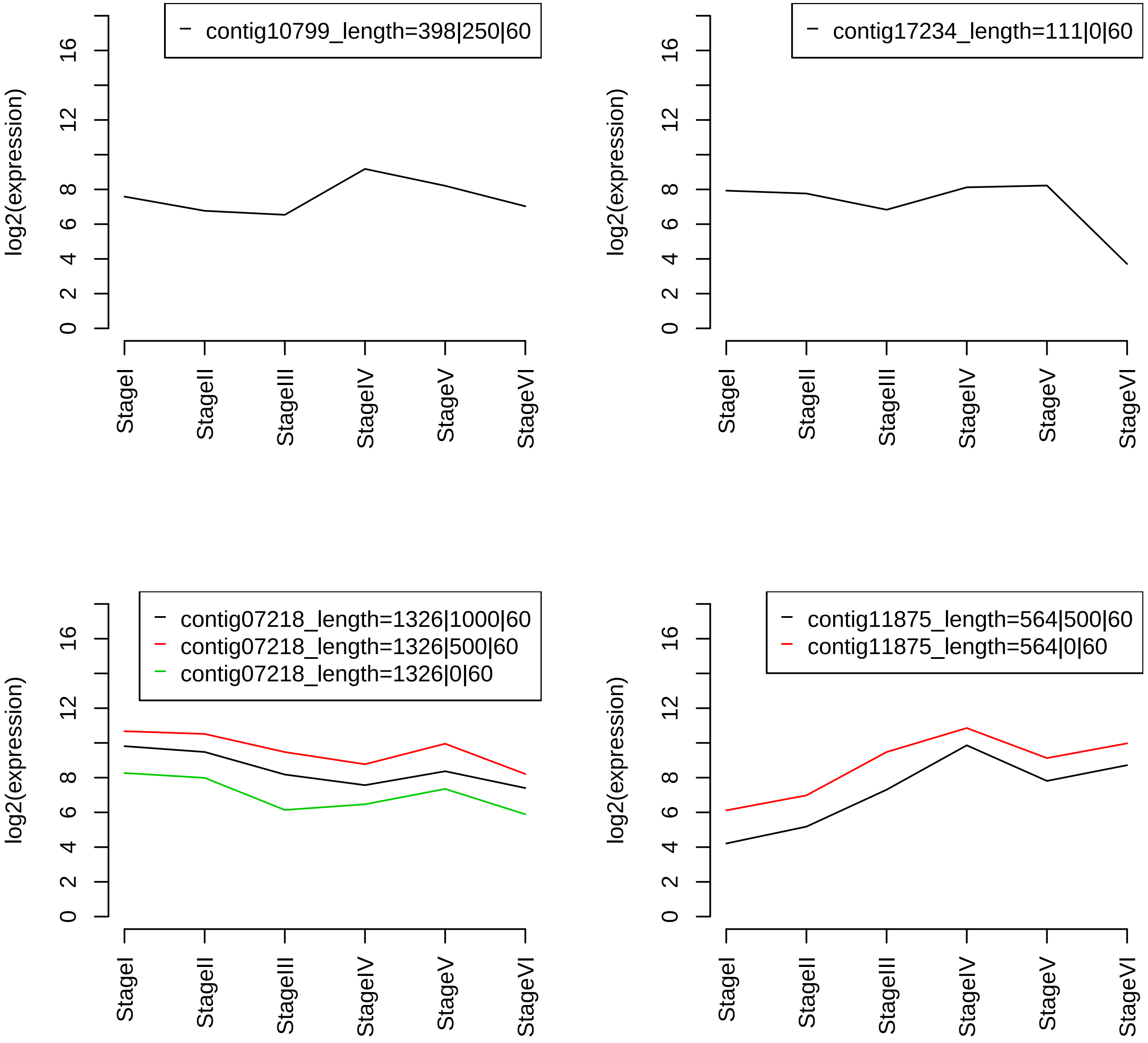
contig10799_length=398|250|60
contig17234_length=111|0|60
contig07218_length=1326|1000|60
contig07218_length=1326|500|60
contig07218_length=1326|0|60
contig11875_length=564|500|60
contig11875_length=564|0|60
log2(expression)
0
2
4
6
8
12
16
StageI
StageII
StageIII
StageIV
StageV
StageVI

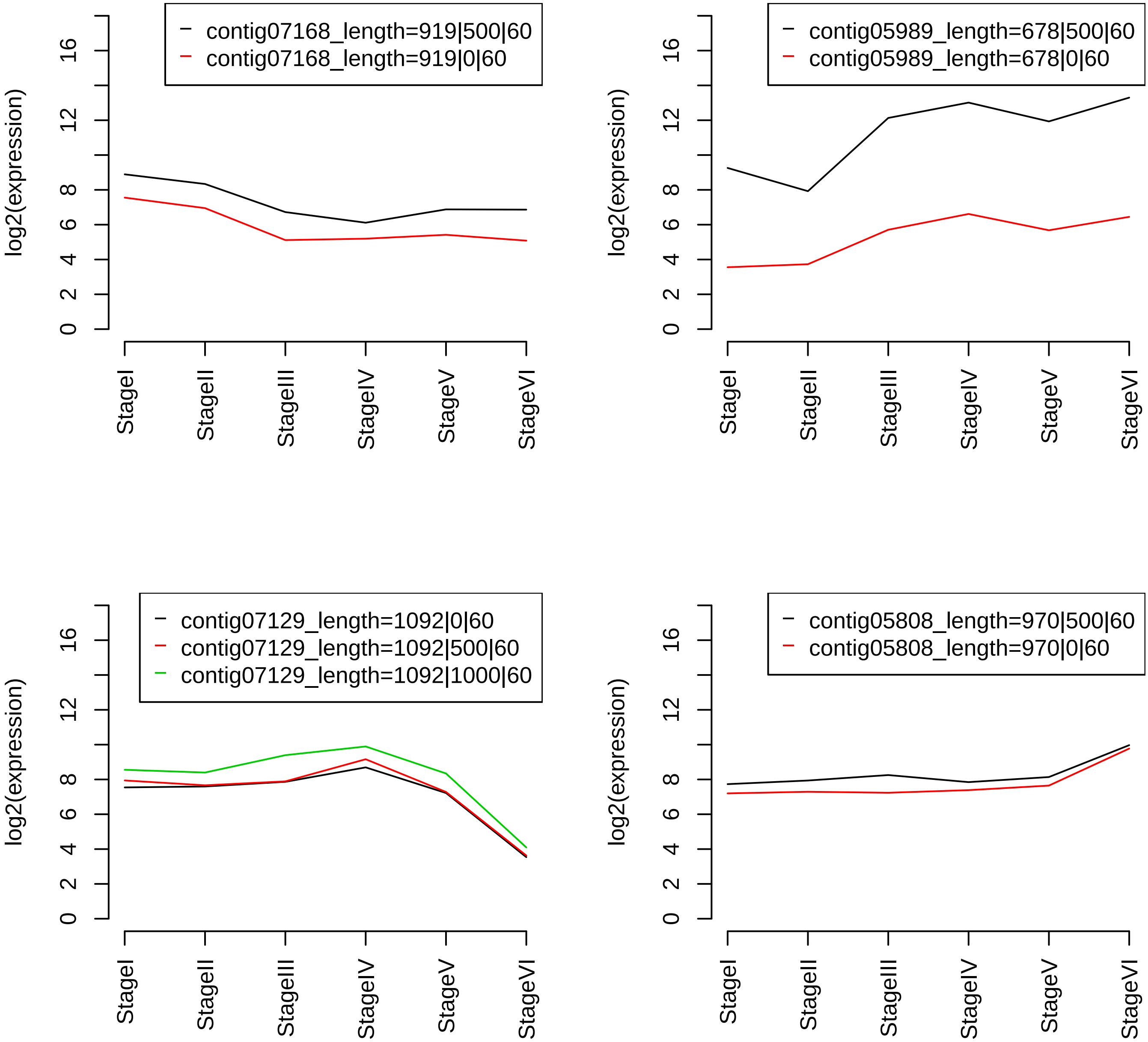

contig07168_length=919|500|60
contig07168_length=919|0|60
contig05989_length=678|500|60
contig05989_length=678|0|60
contig07129_length=1092|0|60
contig07129_length=1092|500|60
contig07129_length=1092|1000|60
contig05808_length=970|500|60
contig05808_length=970|0|60
log2(expression)
0
2
4
6
8
12
16
StageI
StageII
StageIII
StageIV
StageV
StageVI

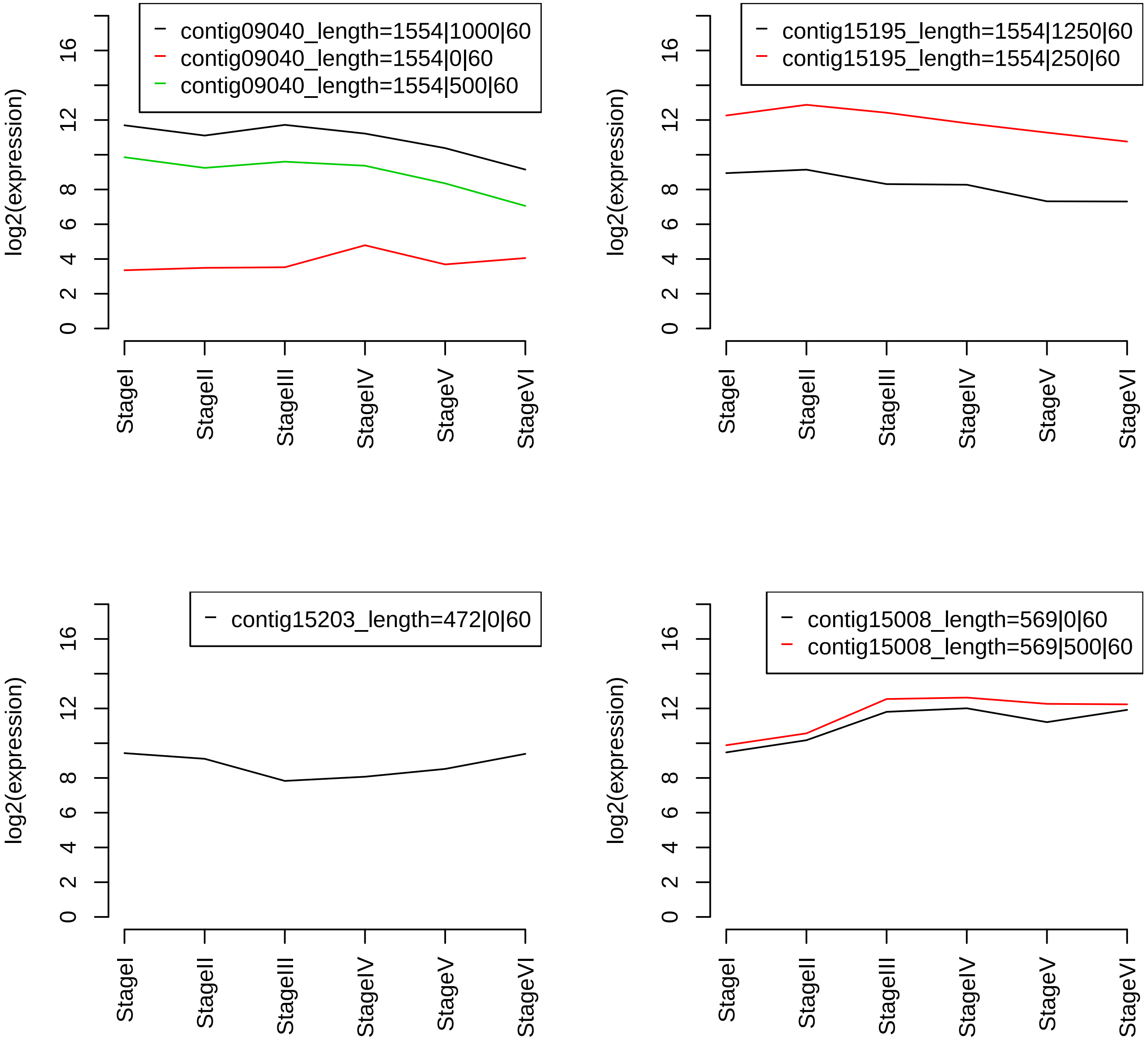

contig09040_length=1554|1000|60
contig09040_length=1554|0|60
contig09040_length=1554|500|60
log2(expression)
0
2
4
6
8
12
16
StageI
StageII
StageIII
StageIV
StageV
StageVI
contig15195_length=1554|1250|60
contig15195_length=1554|250|60
contig15203_length=472|0|60
contig15008_length=569|0|60
contig15008_length=569|500|60

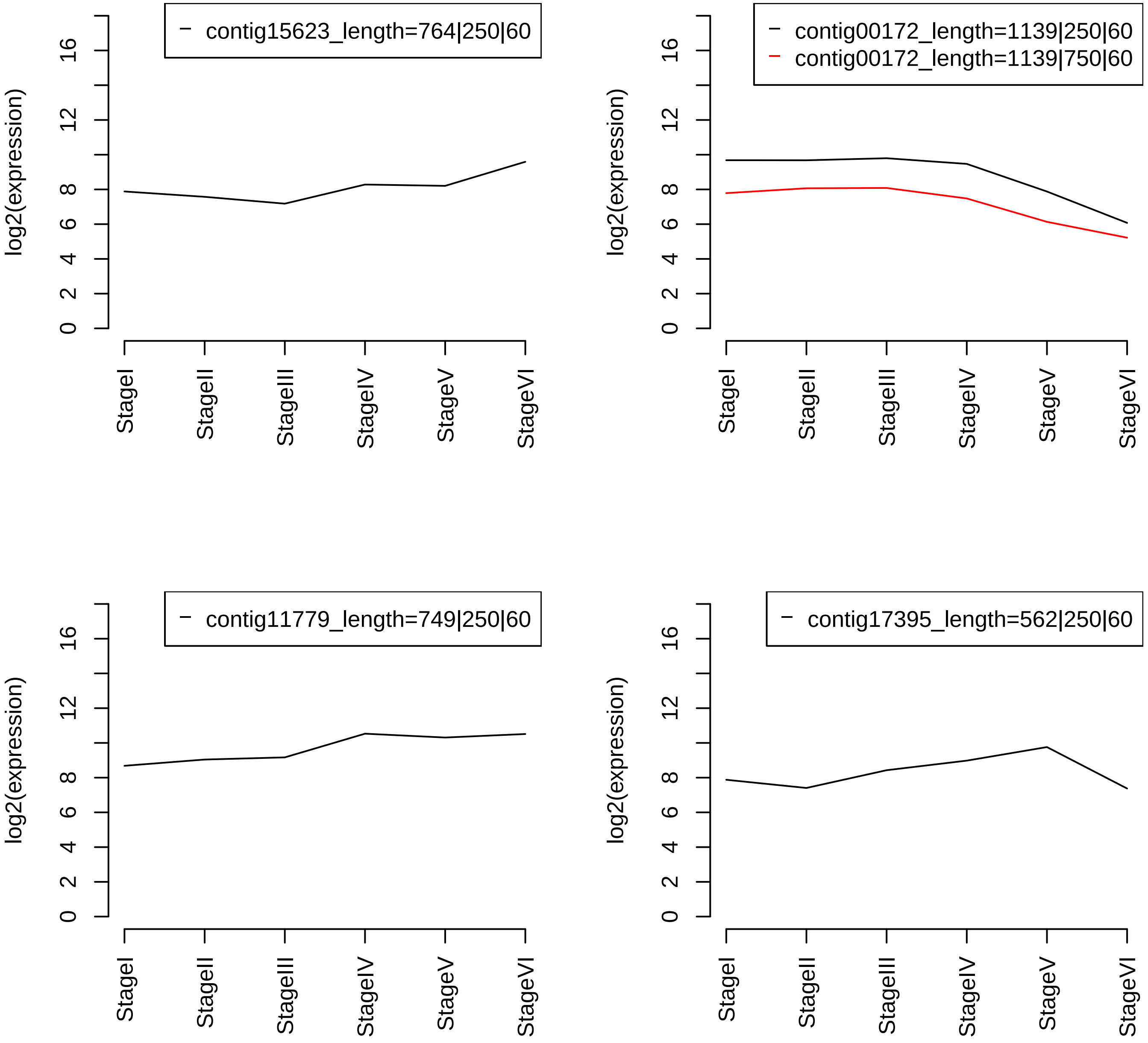

contig15623_length=764|250|60
contig00172_length=1139|250|60
contig00172_length=1139|750|60
contig11779_length=749|250|60
contig17395_length=562|250|60
log2(expression)
0
2
4
6
8
12
16
StageI
StageII
StageIII
StageIV
StageV
StageVI

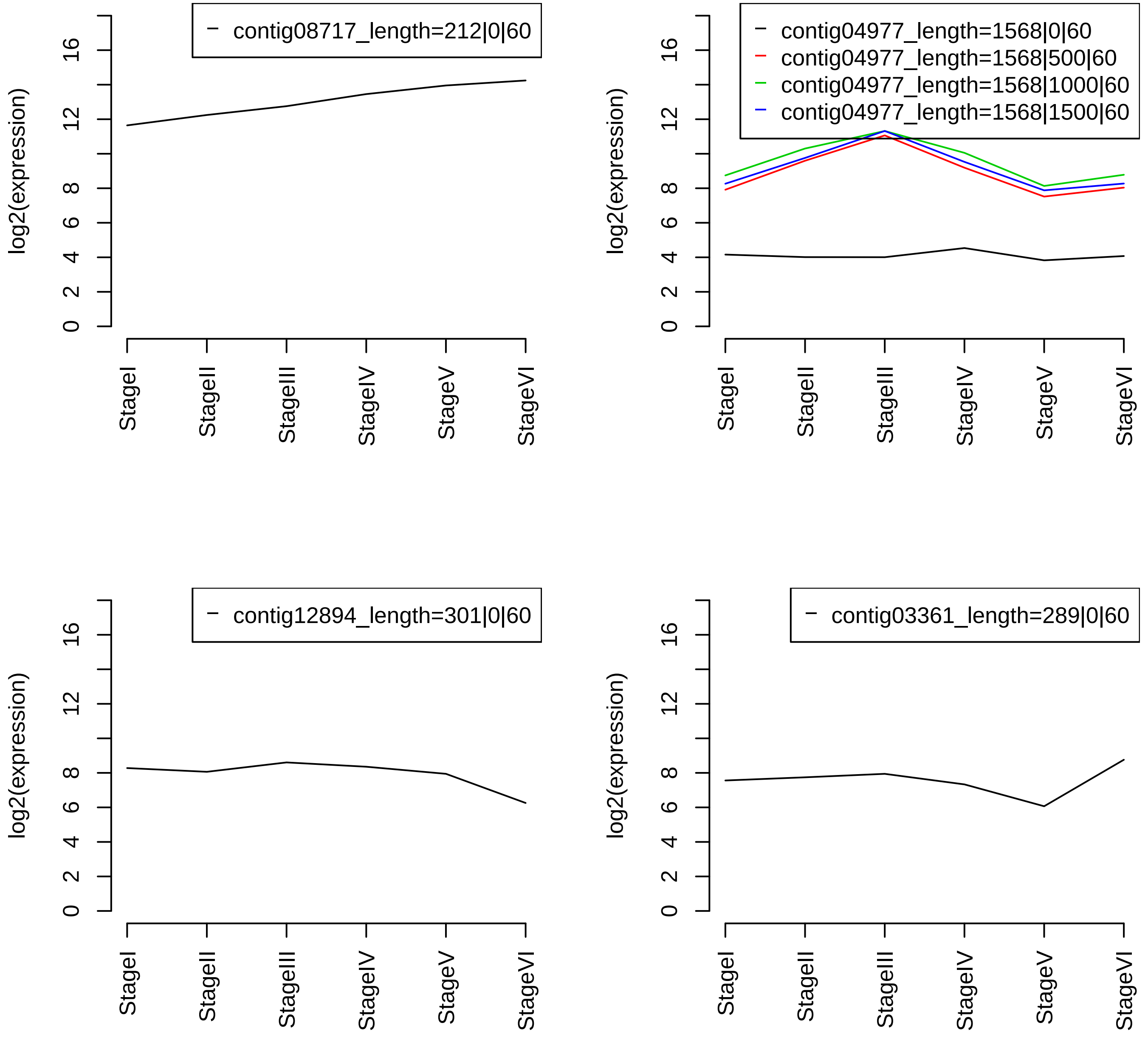

contig08717_length=212|0|60
contig04977_length=1568|0|60
contig04977_length=1568|500|60
contig04977_length=1568|1000|60
contig04977_length=1568|1500|60
contig12894_length=301|0|60
contig03361_length=289|0|60
log2(expression)
0
2
4
6
8
12
16
StageI
StageII
StageIII
StageIV
StageV
StageVI

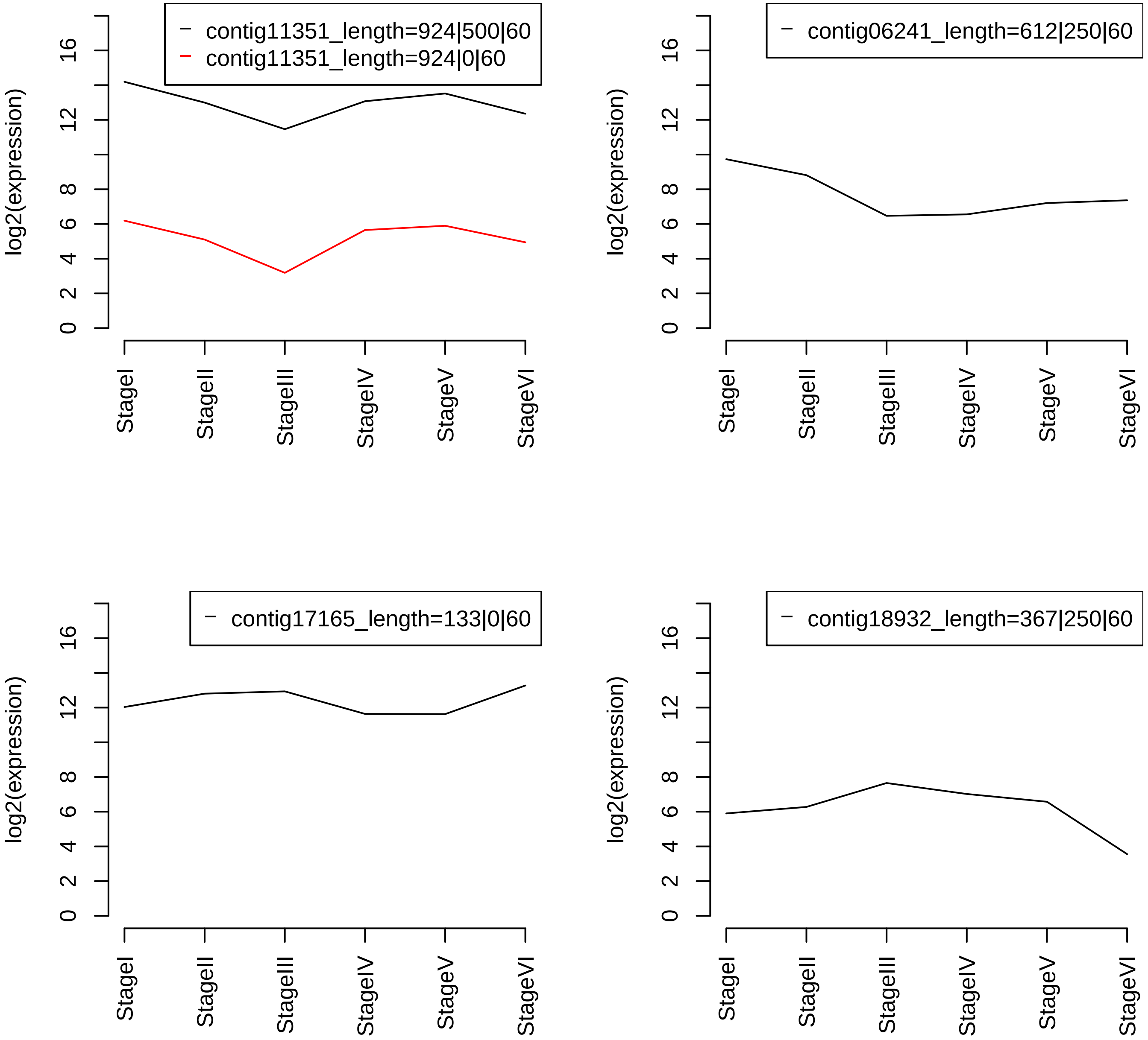
contig11351_length=924|500|60
contig11351_length=924|0|60
contig06241_length=612|250|60
contig17165_length=133|0|60
contig18932_length=367|250|60
log2(expression)
0
2
4
6
8
12
16
StageI
StageII
StageIII
StageIV
StageV
StageVI

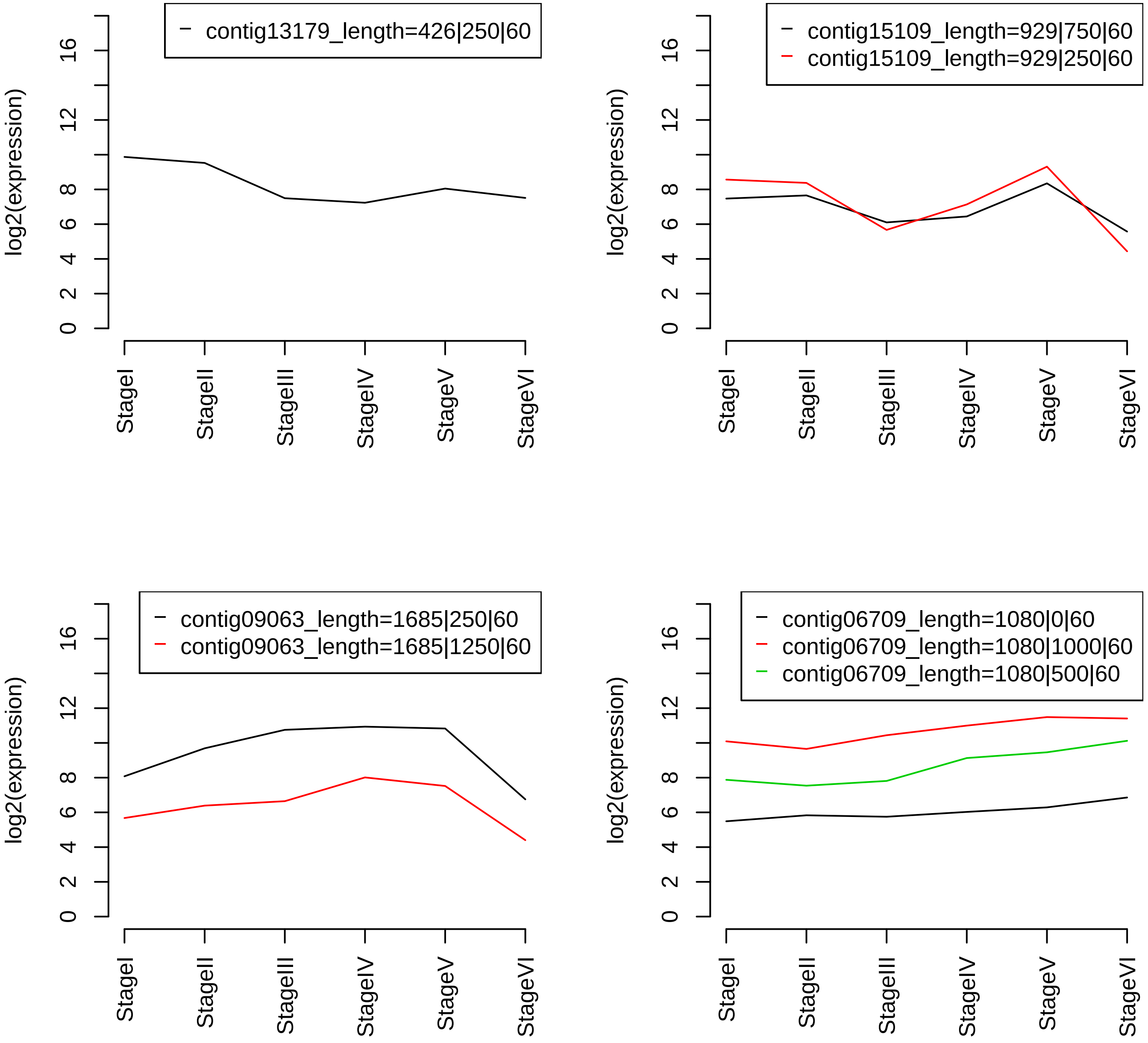

contig13179_length=426|250|60
contig15109_length=929|750|60
contig15109_length=929|250|60
contig09063_length=1685|250|60
contig09063_length=1685|1250|60
contig06709_length=1080|0|60
contig06709_length=1080|1000|60
contig06709_length=1080|500|60
log2(expression)
0
2
4
6
8
12
16
StageI
StageII
StageIII
StageIV
StageV
StageVI

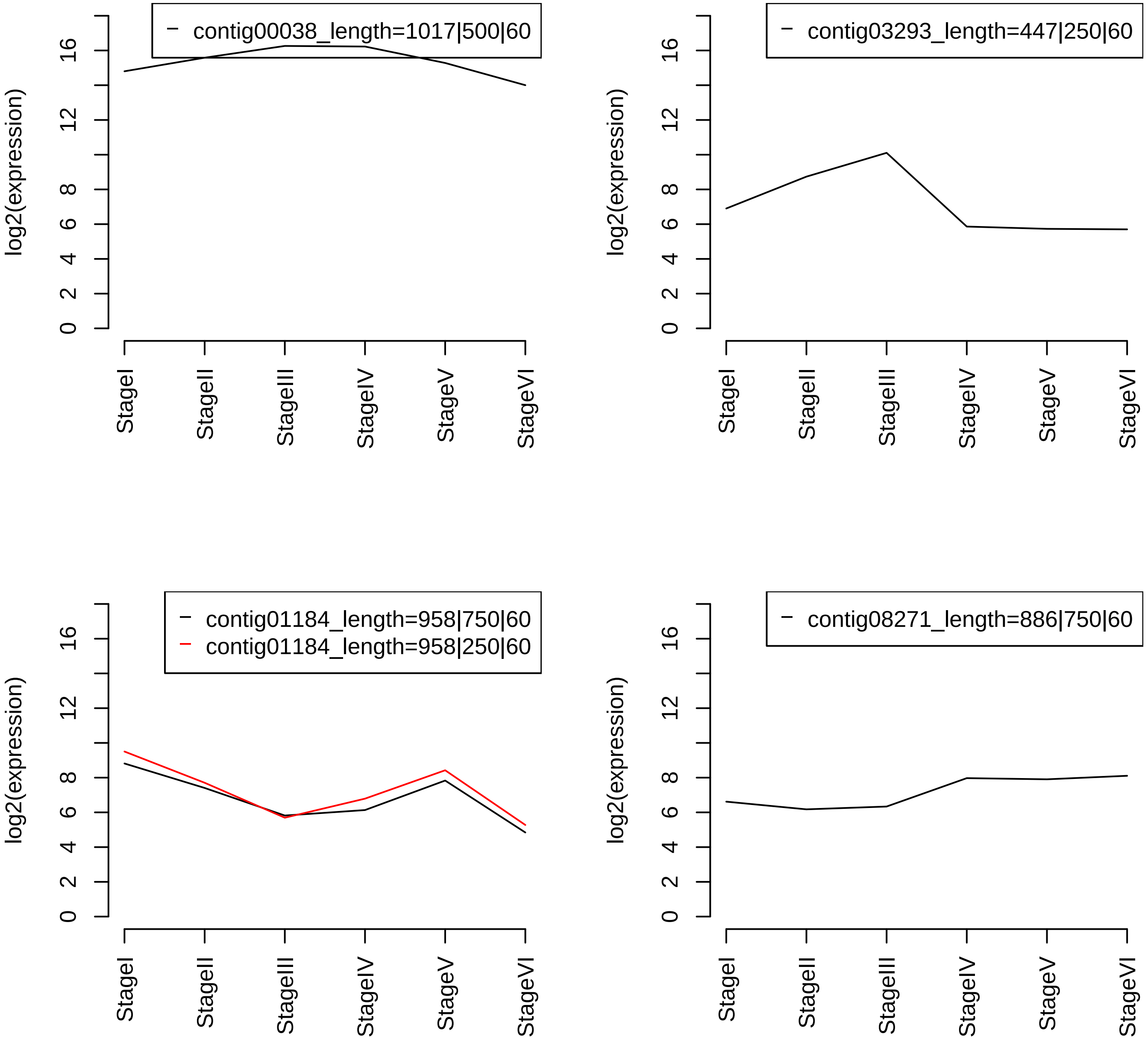
contig00038_length=1017|500|60
contig03293_length=447|250|60
contig01184_length=958|750|60
contig01184_length=958|250|60
contig08271_length=886|750|60
log2(expression)
StageI
StageII
StageIII
StageIV
StageV
StageVI

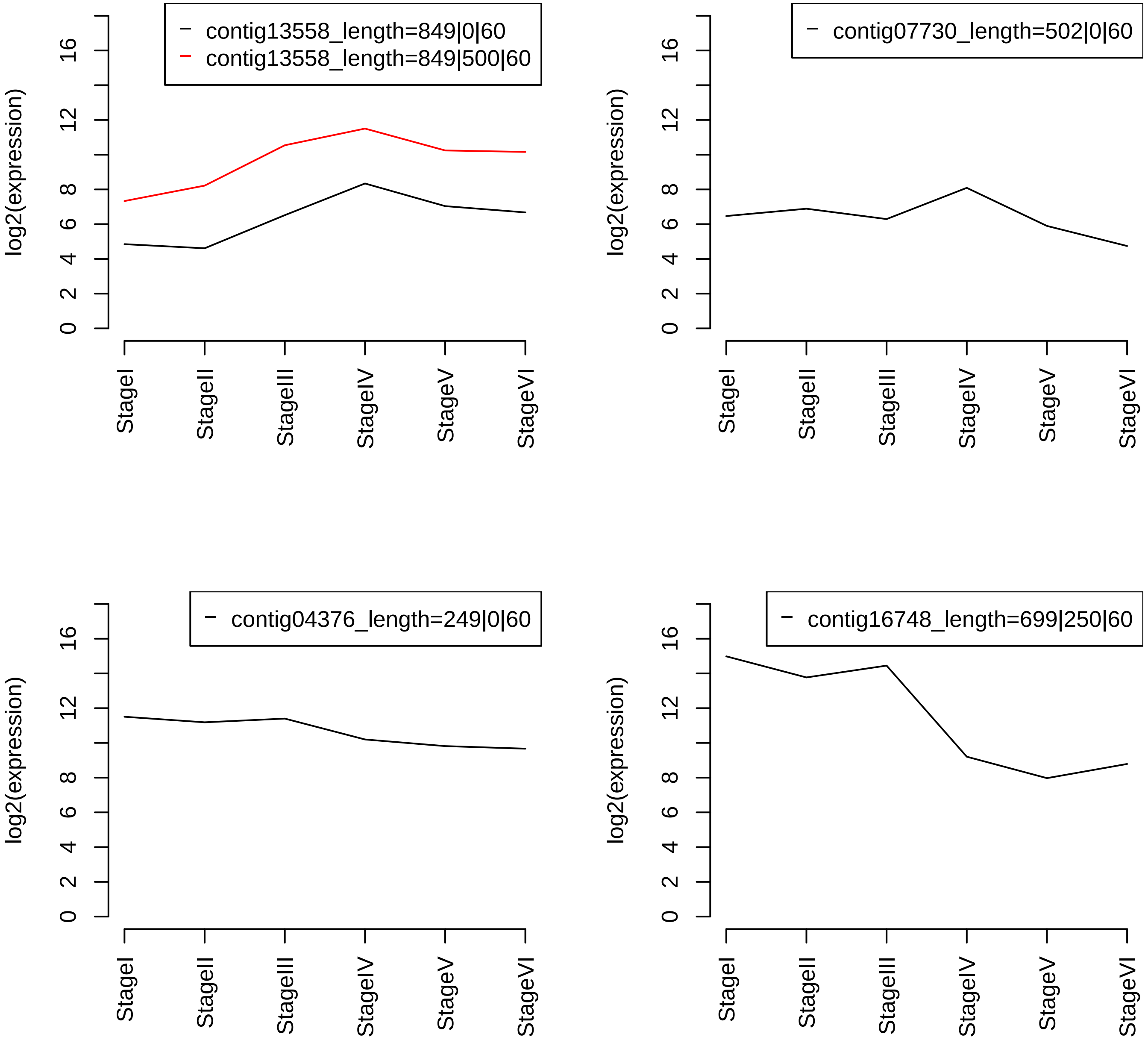
contig13558_length=849|0|60
contig13558_length=849|500|60
contig07730_length=502|0|60
contig04376_length=249|0|60
contig16748_length=699|250|60
log2(expression)
0
2
4
6
8
12
16
StageI
StageII
StageIII
StageIV
StageV
StageVI

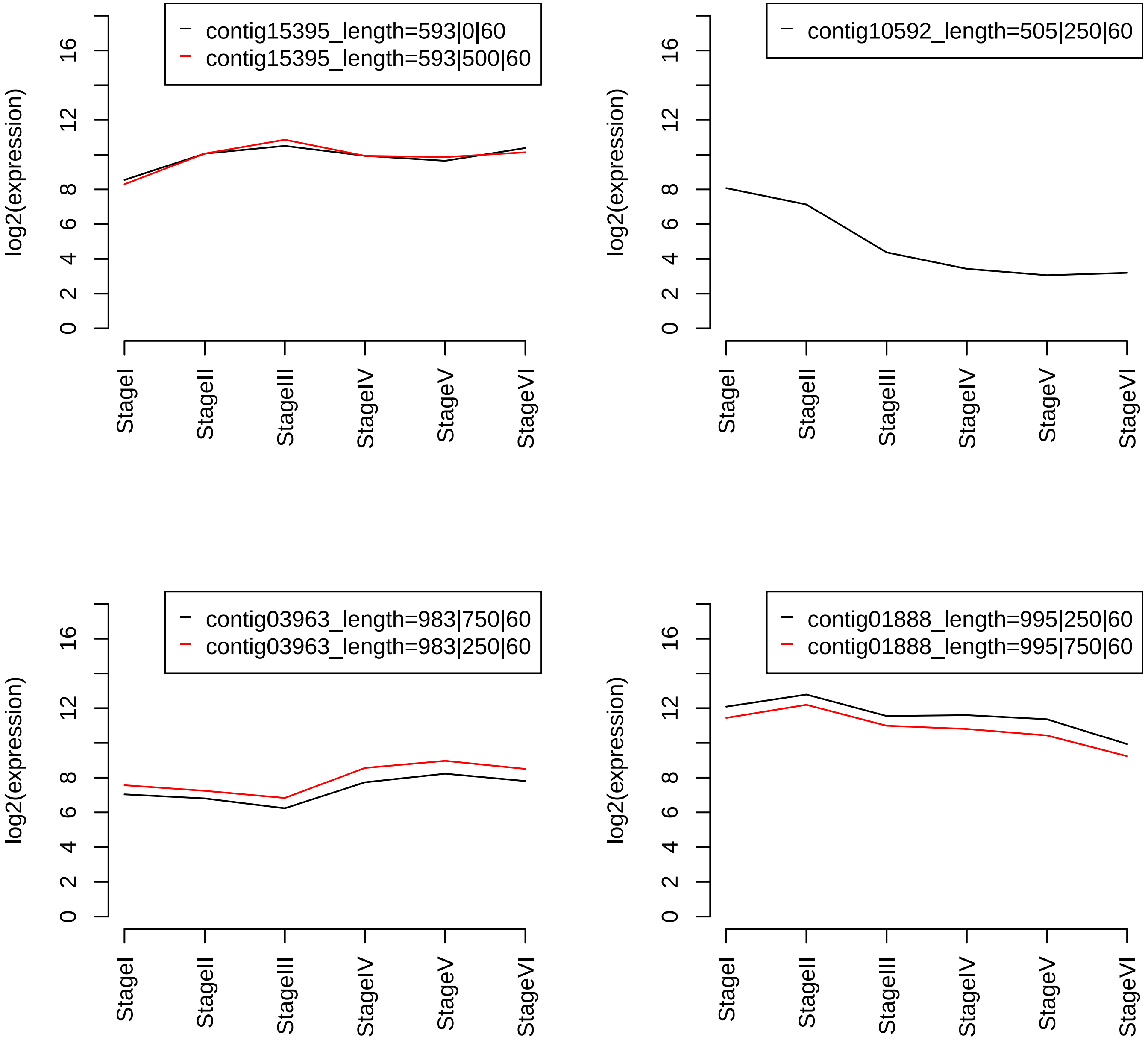

contig15395_length=593|0|60
contig15395_length=593|500|60
log2(expression)
0
2
4
6
8
12
16
StageI
StageII
StageIII
StageIV
StageV
StageVI
contig10592_length=505|250|60
log2(expression)
0
2
4
6
8
12
16
StageI
StageII
StageIII
StageIV
StageV
StageVI
contig03963_length=983|750|60
contig03963_length=983|250|60
log2(expression)
0
2
4
6
8
12
16
StageI
StageII
StageIII
StageIV
StageV
StageVI
contig01888_length=995|250|60
contig01888_length=995|750|60
log2(expression)
0
2
4
6
8
12
16
StageI
StageII
StageIII
StageIV
StageV
StageVI

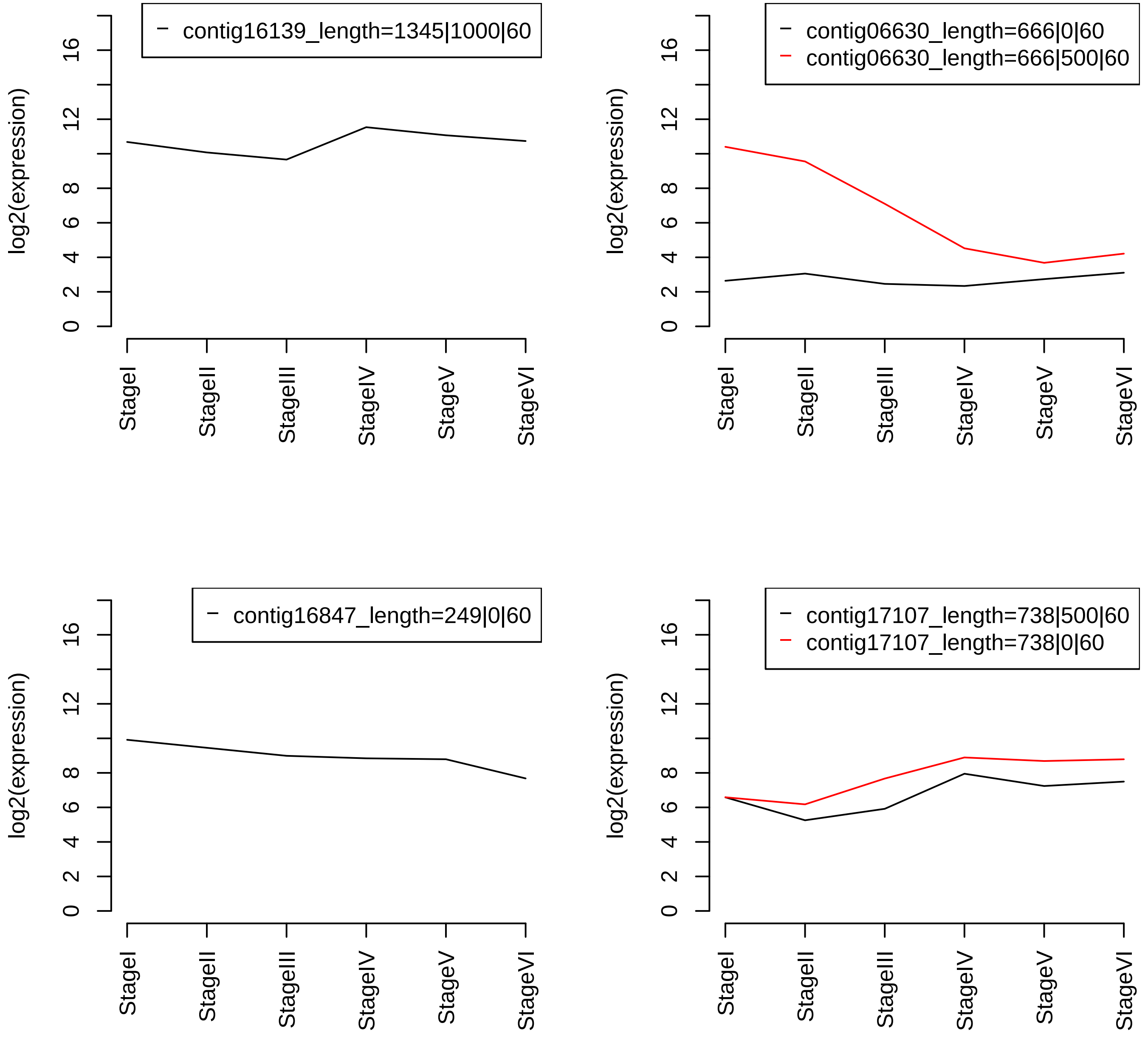

contig16139_length=1345|1000|60
contig06630_length=666|0|60
contig06630_length=666|500|60
contig16847_length=249|0|60
contig17107_length=738|500|60
contig17107_length=738|0|60
log2(expression)
0
2
4
6
8
12
16
StageI
StageII
StageIII
StageIV
StageV
StageVI

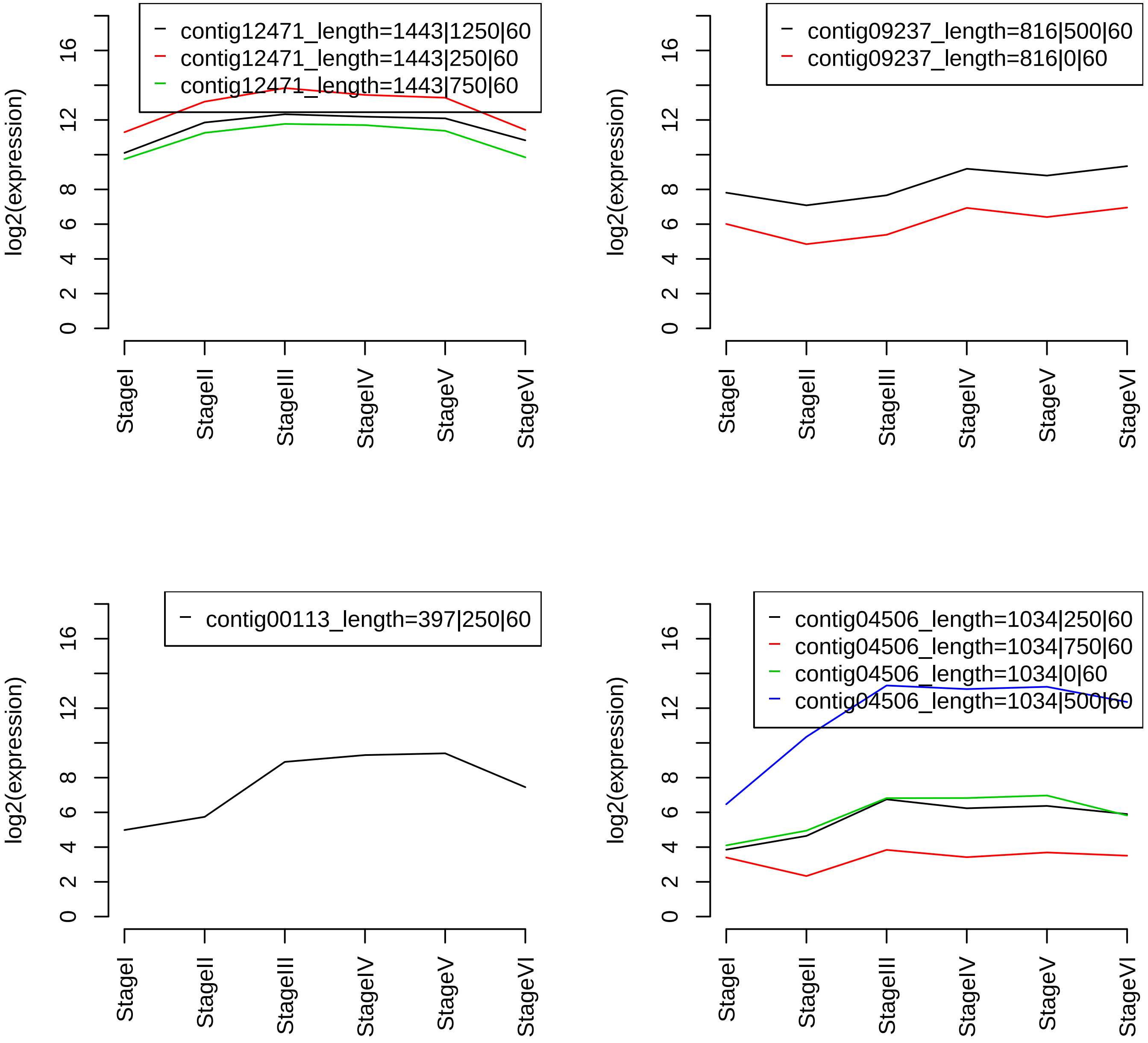
contig12471_length=1443|1250|60
contig12471_length=1443|250|60
contig12471_length=1443|750|60
contig09237_length=816|500|60
contig09237_length=816|0|60
contig00113_length=397|250|60
contig04506_length=1034|250|60
contig04506_length=1034|750|60
contig04506_length=1034|0|60
contig04506_length=1034|500|60
log2(expression)
StageI
StageII
StageIII
StageIV
StageV
StageVI

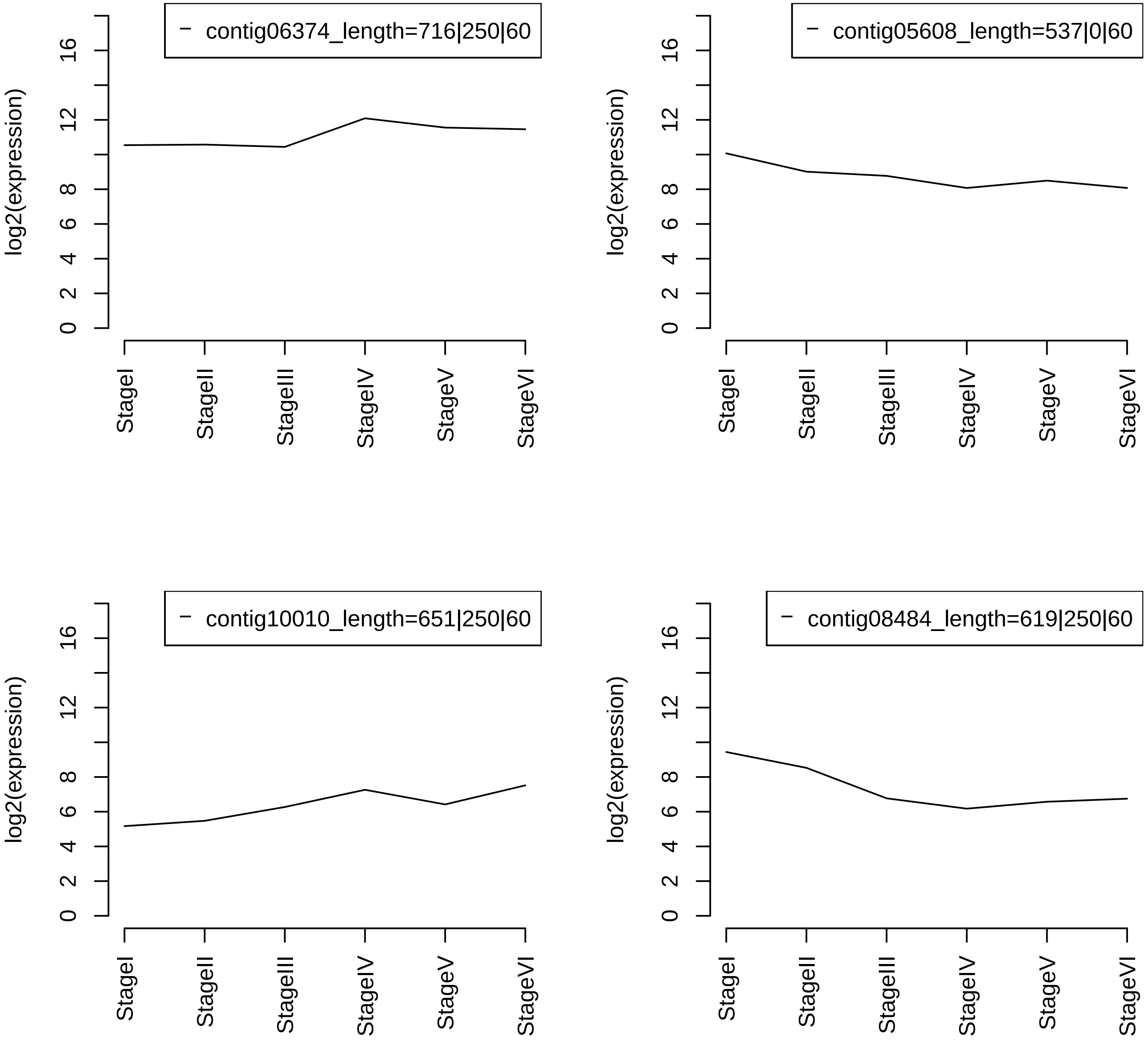
contig06374_length=716|250|60
contig05608_length=537|0|60
contig10010_length=651|250|60
contig08484_length=619|250|60
log2(expression)
0
2
4
6
8
12
16
StageI
StageII
StageIII
StageIV
StageV
StageVI

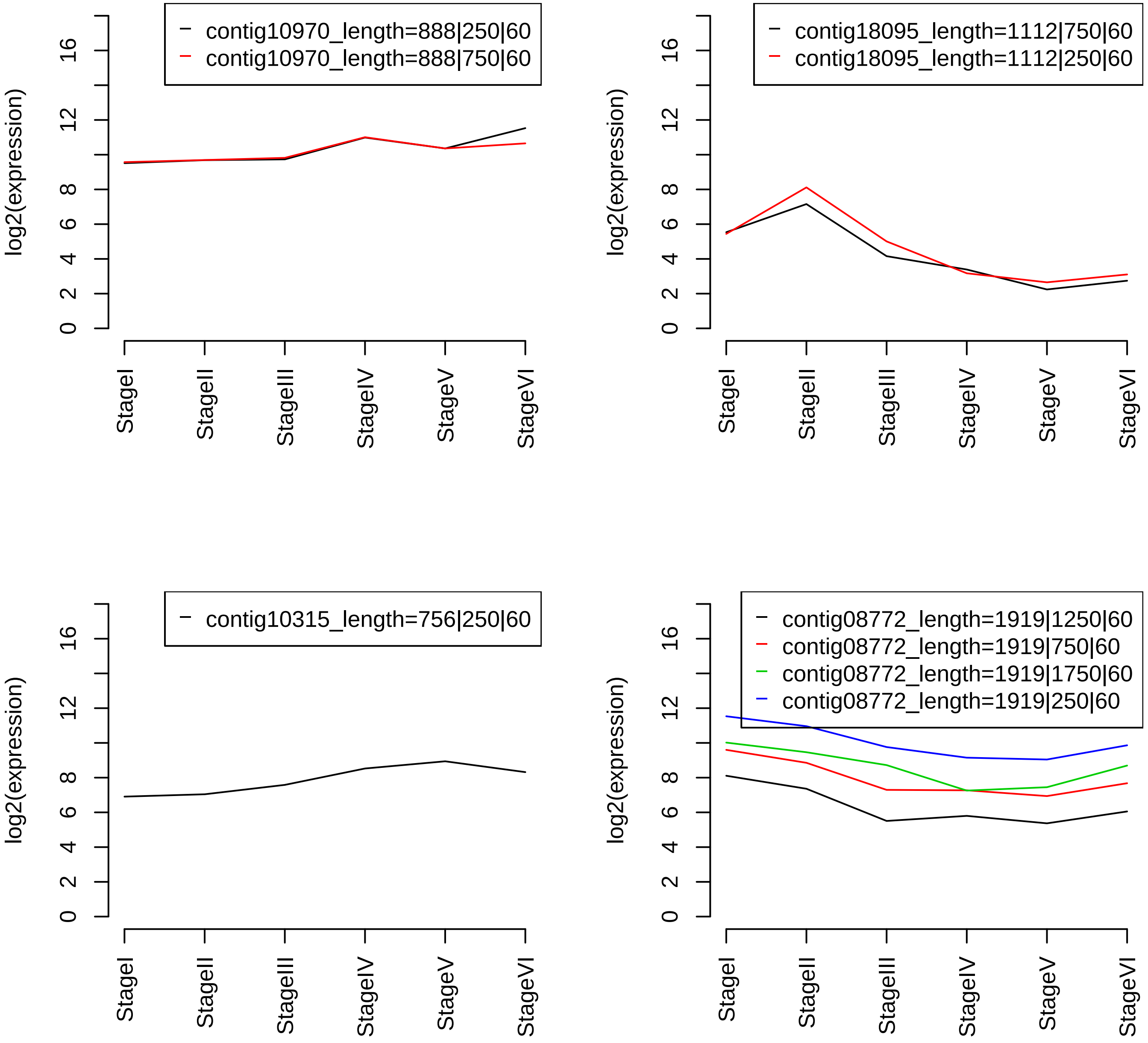
contig10970_length=888|250|60
contig10970_length=888|750|60
contig18095_length=1112|750|60
contig18095_length=1112|250|60
contig10315_length=756|250|60
contig08772_length=1919|1250|60
contig08772_length=1919|750|60
contig08772_length=1919|1750|60
contig08772_length=1919|250|60
log2(expression)
0
2
4
6
8
12
16
StageI
StageII
StageIII
StageIV
StageV
StageVI

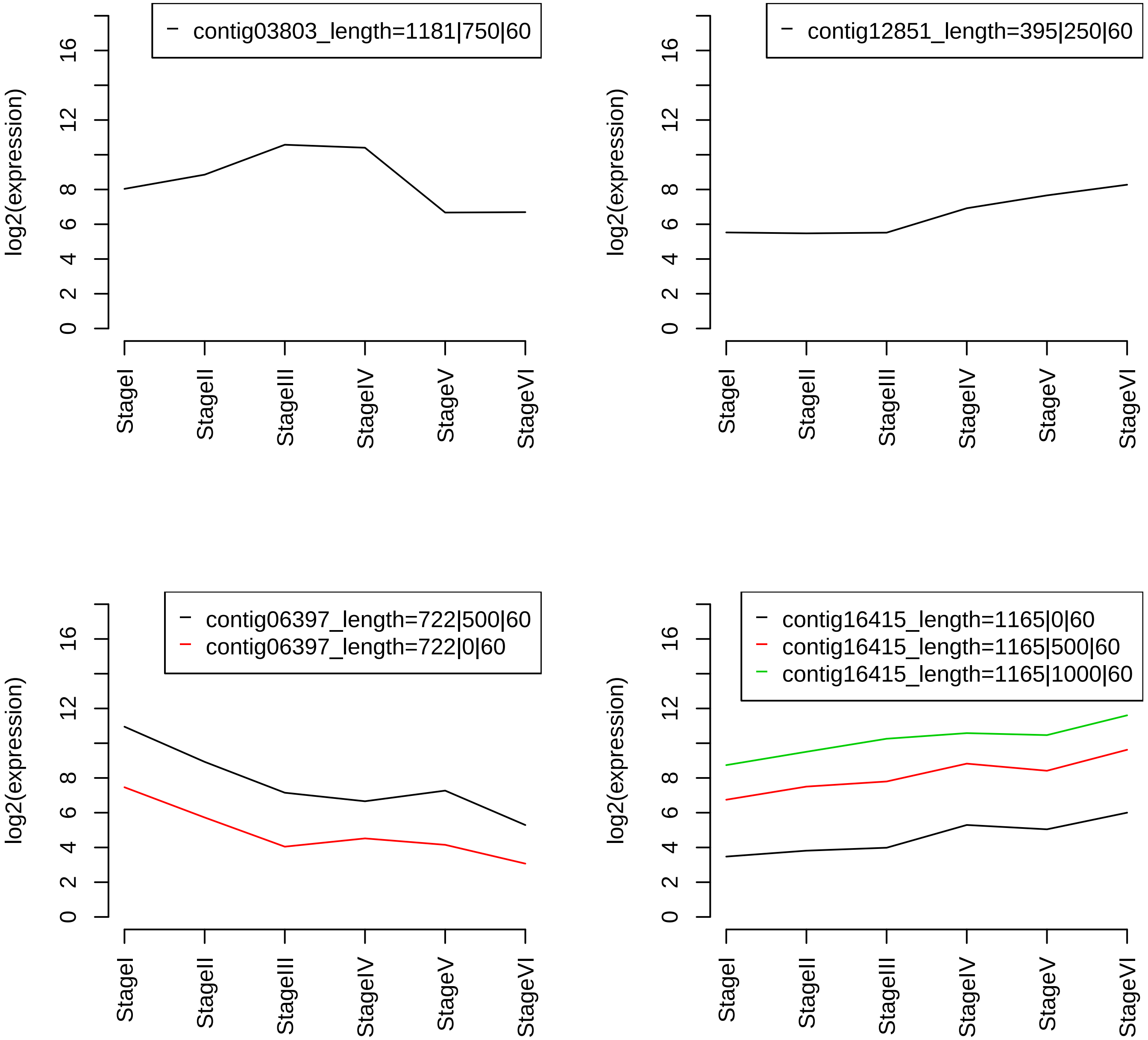

contig03803_length=1181|750|60
contig12851_length=395|250|60
contig06397_length=722|500|60
contig06397_length=722|0|60
contig16415_length=1165|0|60
contig16415_length=1165|500|60
contig16415_length=1165|1000|60
log2(expression)
0
2
4
6
8
12
16
StageI
StageII
StageIII
StageIV
StageV
StageVI

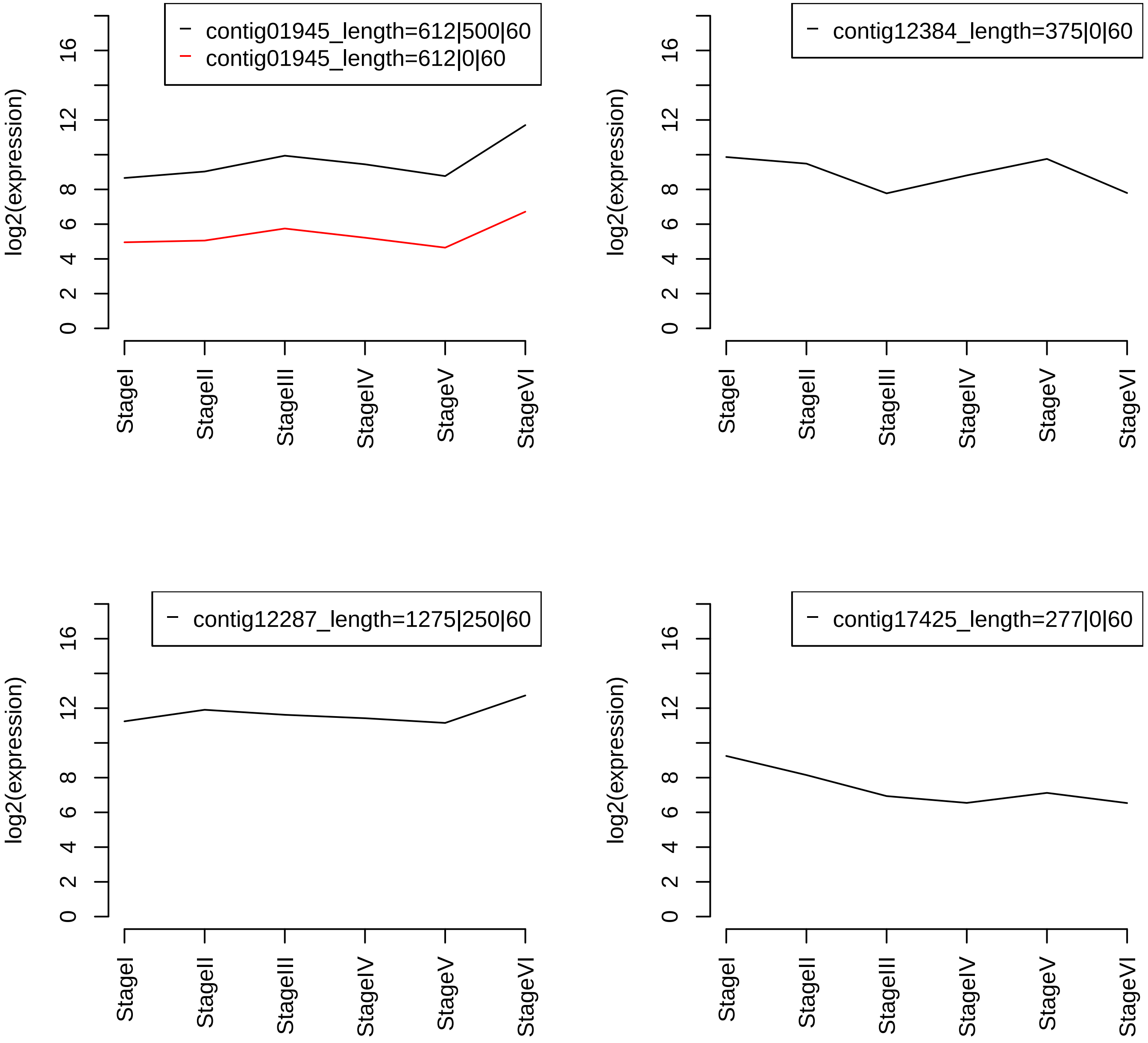
contig01945_length=612|500|60
contig01945_length=612|0|60
contig12384_length=375|0|60
contig12287_length=1275|250|60
contig17425_length=277|0|60
log2(expression)
0
2
4
6
8
12
16
StageI
StageII
StageIII
StageIV
StageV
StageVI

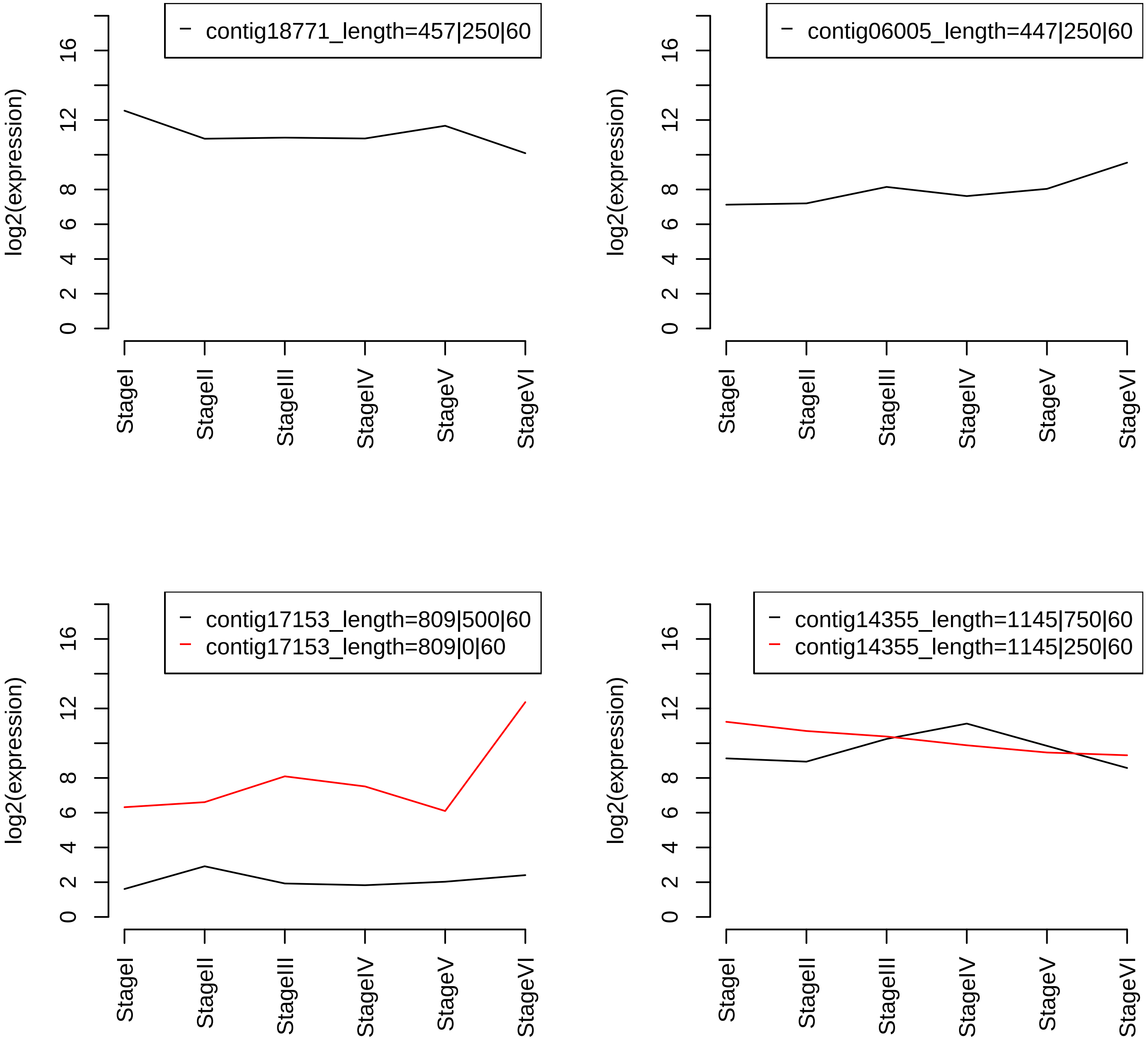
contig18771_length=457|250|60
contig06005_length=447|250|60
contig17153_length=809|500|60
contig17153_length=809|0|60
contig14355_length=1145|750|60
contig14355_length=1145|250|60
log2(expression)
0
2
4
6
8
12
16
StageI
StageII
StageIII
StageIV
StageV
StageVI

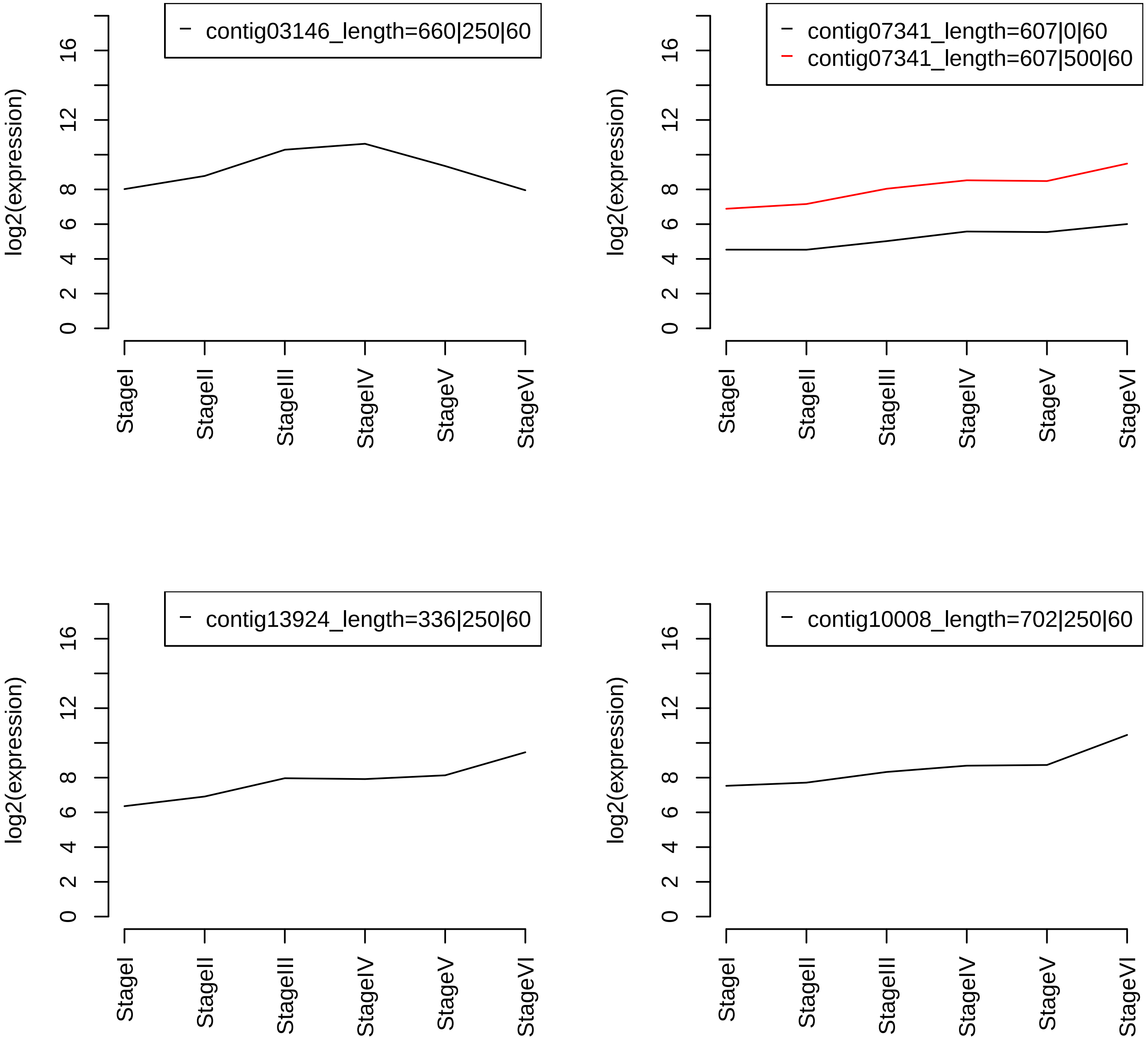

contig03146_length=660|250|60
contig07341_length=607|0|60
contig07341_length=607|500|60
contig13924_length=336|250|60
contig10008_length=702|250|60
log2(expression)
0
2
4
6
8
12
16
StageI
StageII
StageIII
StageIV
StageV
StageVI

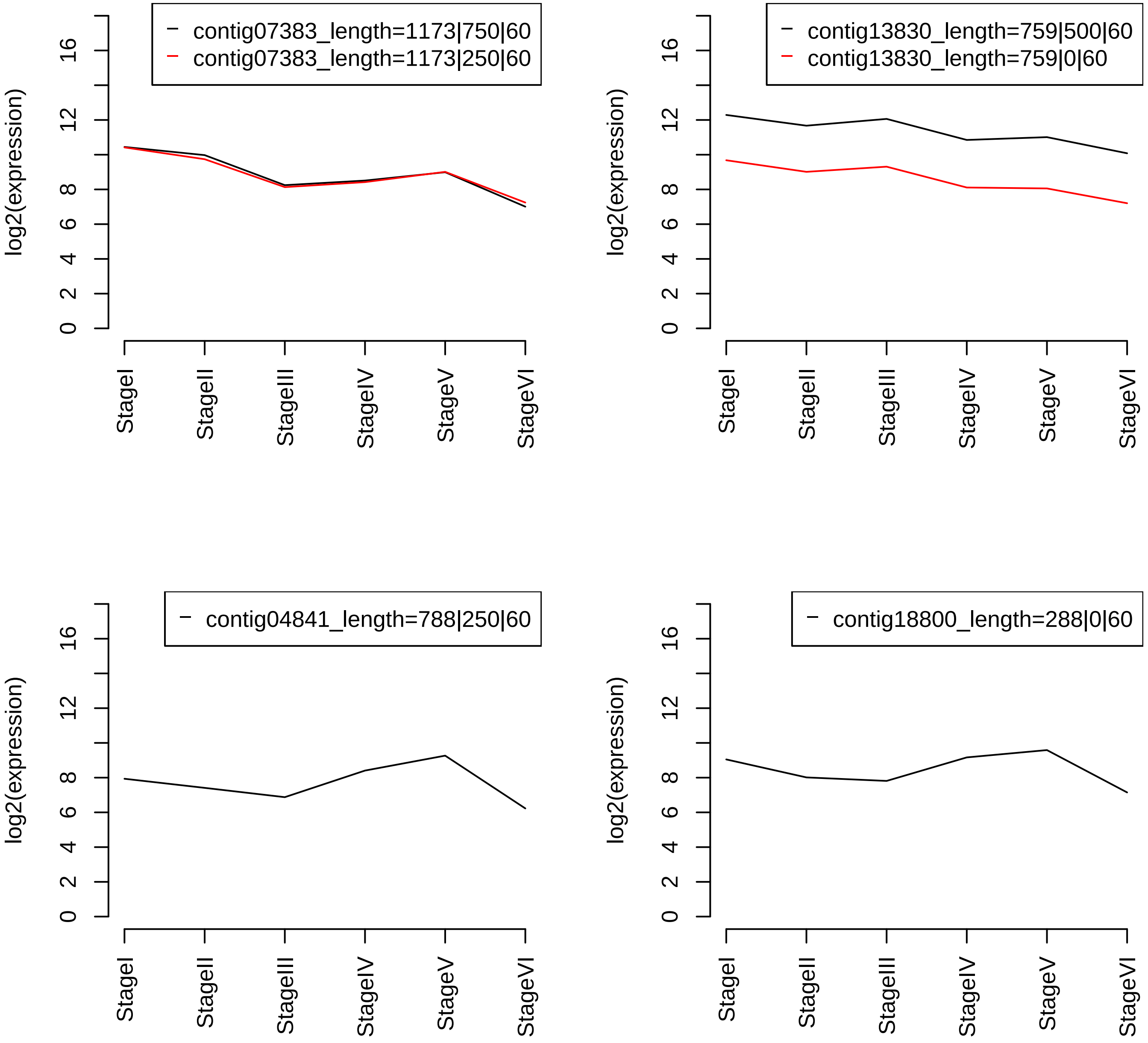
contig07383_length=1173|750|60
contig07383_length=1173|250|60
contig13830_length=759|500|60
contig13830_length=759|0|60
contig04841_length=788|250|60
contig18800_length=288|0|60
log2(expression)
0
2
4
6
8
12
16
StageI
StageII
StageIII
StageIV
StageV
StageVI

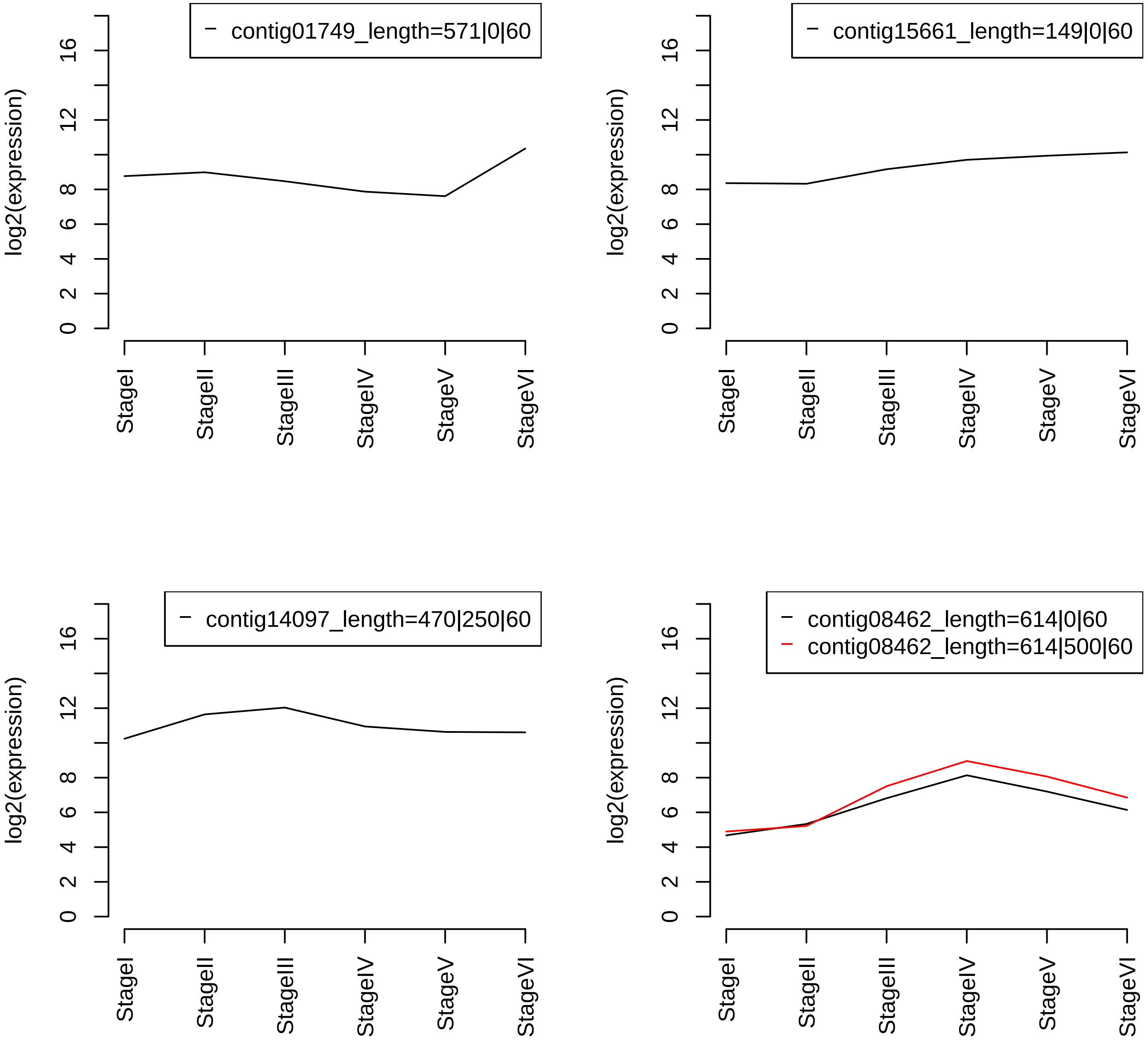
contig01749_length=571|0|60
contig15661_length=149|0|60
contig14097_length=470|250|60
contig08462_length=614|0|60
contig08462_length=614|500|60
log2(expression)
0
2
4
6
8
12
16
StageI
StageII
StageIII
StageIV
StageV
StageVI

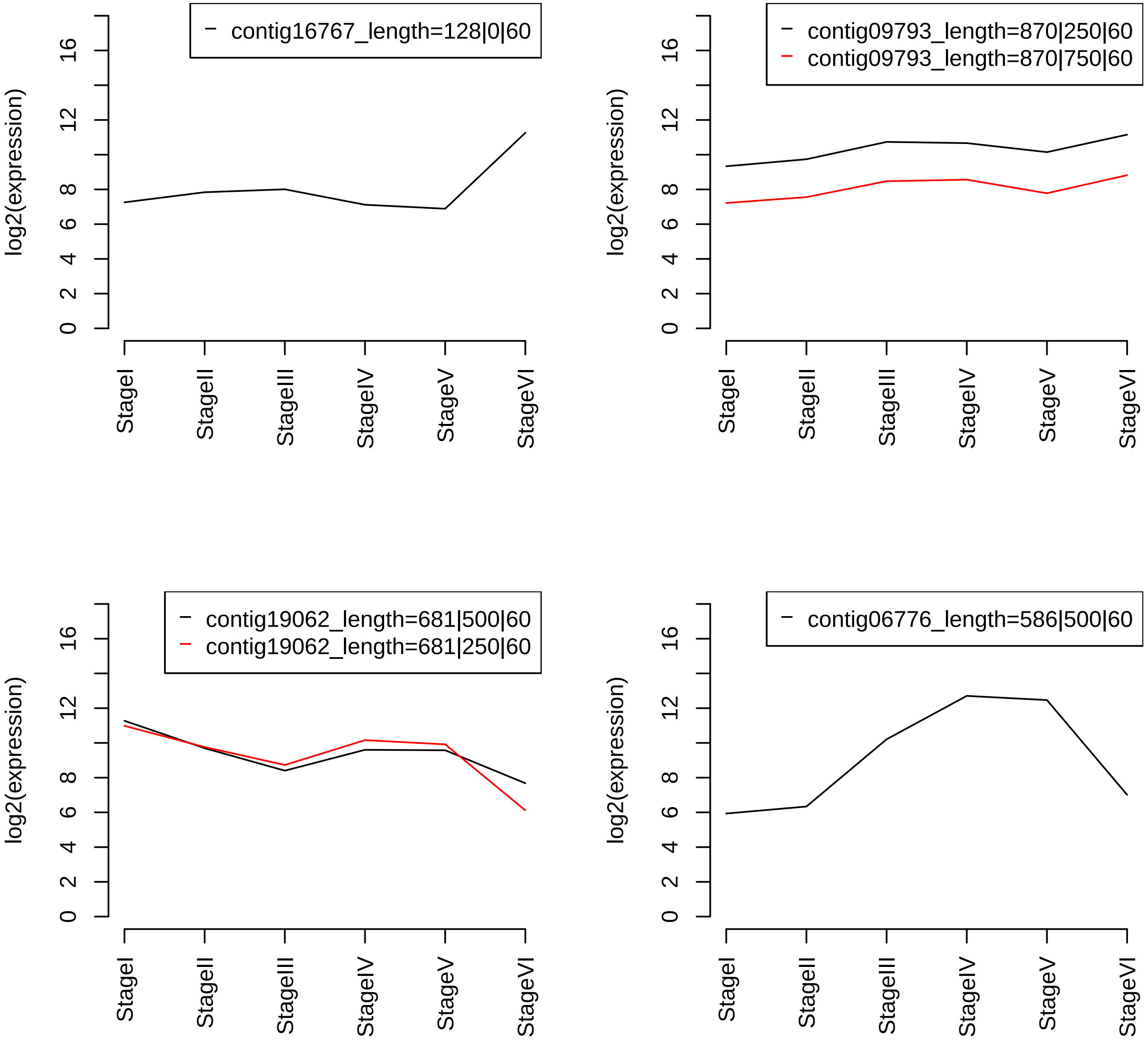

contig16767_length=128|0|60
contig09793_length=870|250|60
contig09793_length=870|750|60
contig19062_length=681|500|60
contig19062_length=681|250|60
contig06776_length=586|500|60
log2(expression)
0
2
4
6
8
12
16
StageI
StageII
StageIII
StageIV
StageV
StageVI

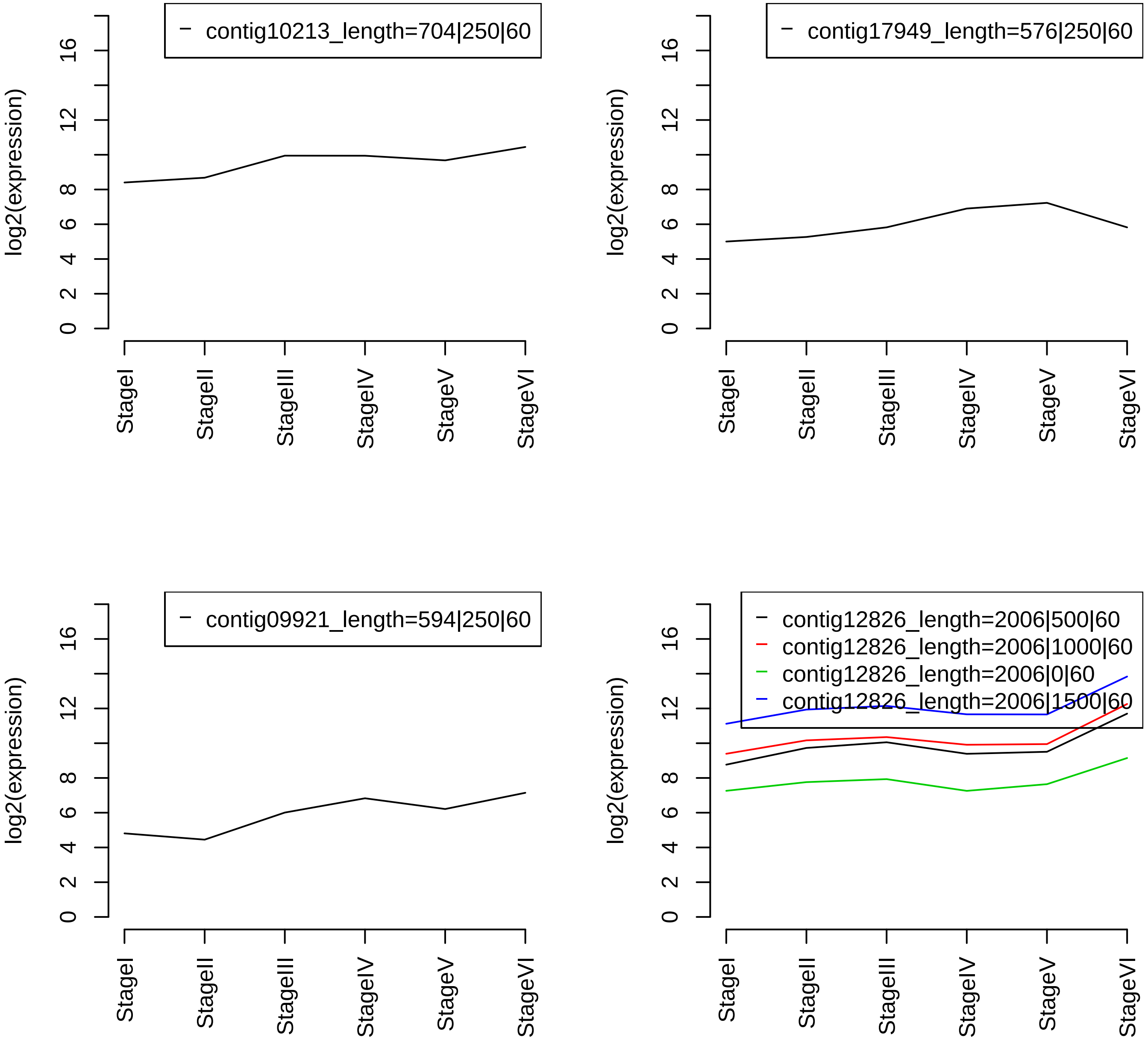

contig10213_length=704|250|60
contig17949_length=576|250|60
contig09921_length=594|250|60
contig12826_length=2006|500|60
contig12826_length=2006|1000|60
contig12826_length=2006|0|60
contig12826_length=2006|1500|60
log2(expression)
0 2 4 6 8 12 16
StageI StageII StageIII StageIV StageV StageVI

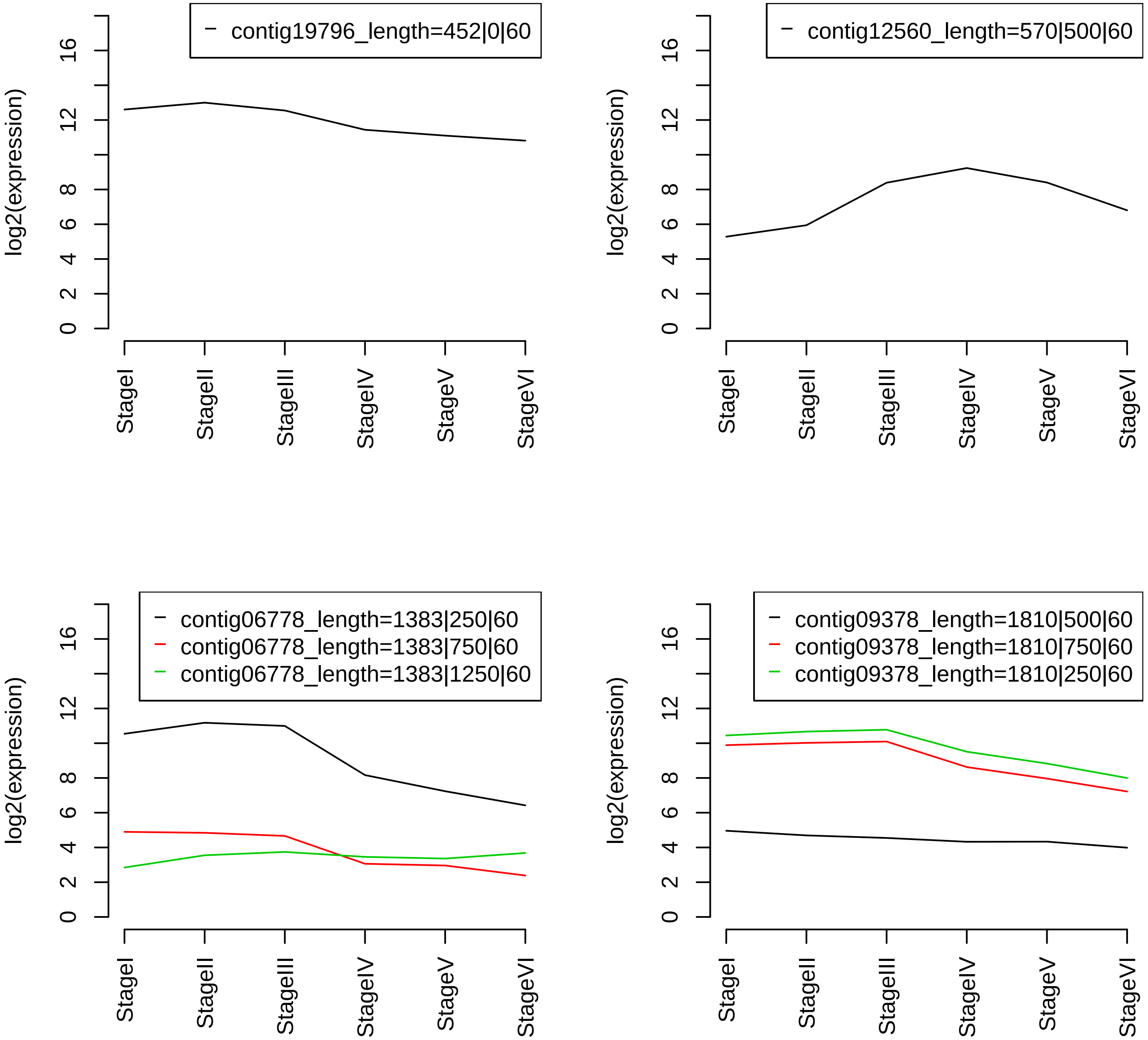
contig19796_length=452|0|60
contig12560_length=570|500|60
contig06778_length=1383|250|60
contig06778_length=1383|750|60
contig06778_length=1383|1250|60
contig09378_length=1810|500|60
contig09378_length=1810|750|60
contig09378_length=1810|250|60
log2(expression)
0
2
4
6
8
12
16
StageI
StageII
StageIII
StageIV
StageV
StageVI

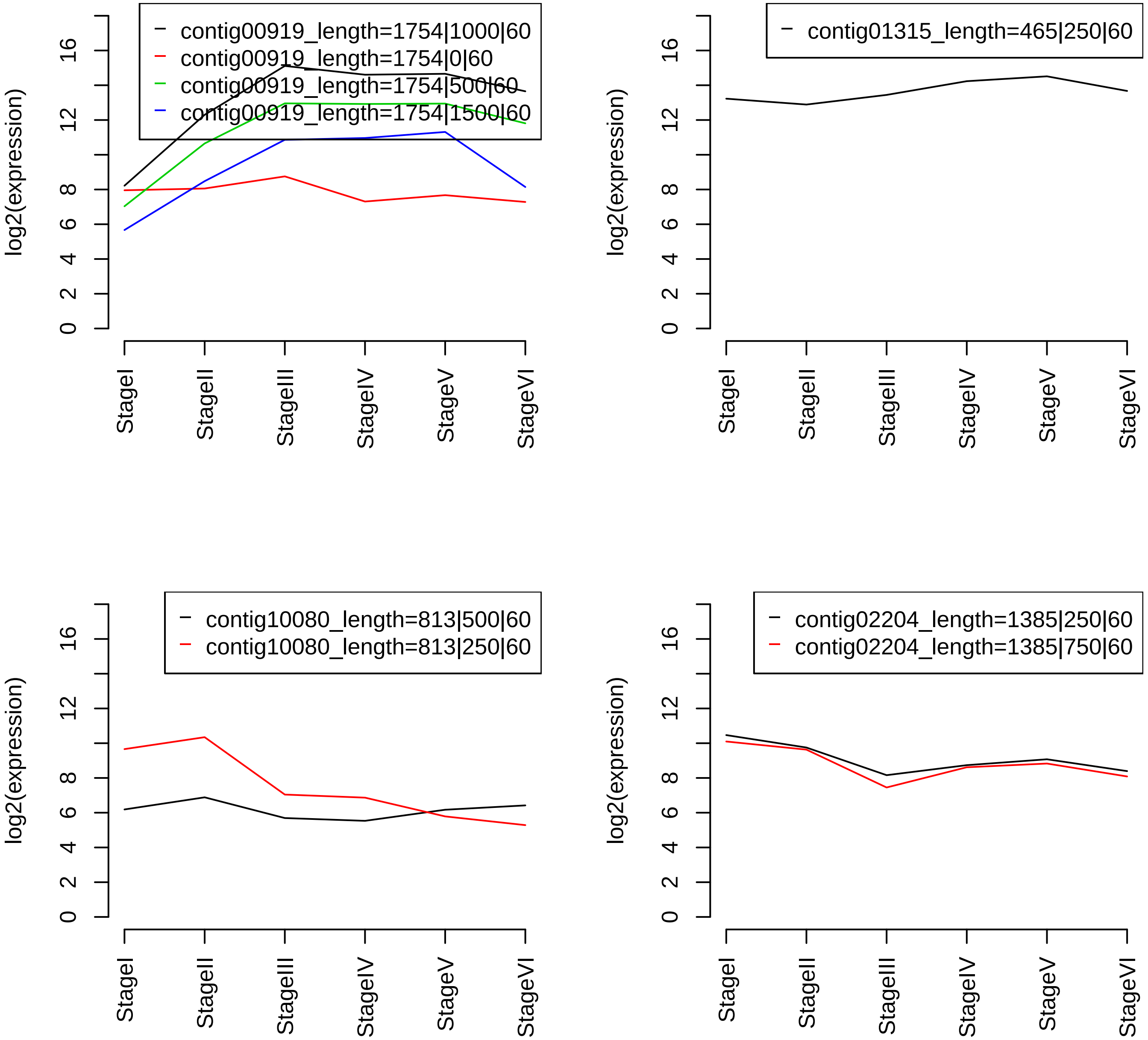

contig00919_length=1754|1000|60
contig00919_length=1754|0|60
contig00919_length=1754|500|60
contig00919_length=1754|1500|60
contig01315_length=465|250|60
contig10080_length=813|500|60
contig10080_length=813|250|60
contig02204_length=1385|250|60
contig02204_length=1385|750|60
log2(expression)
0
2
4
6
8
12
16
StageI
StageII
StageIII
StageIV
StageV
StageVI

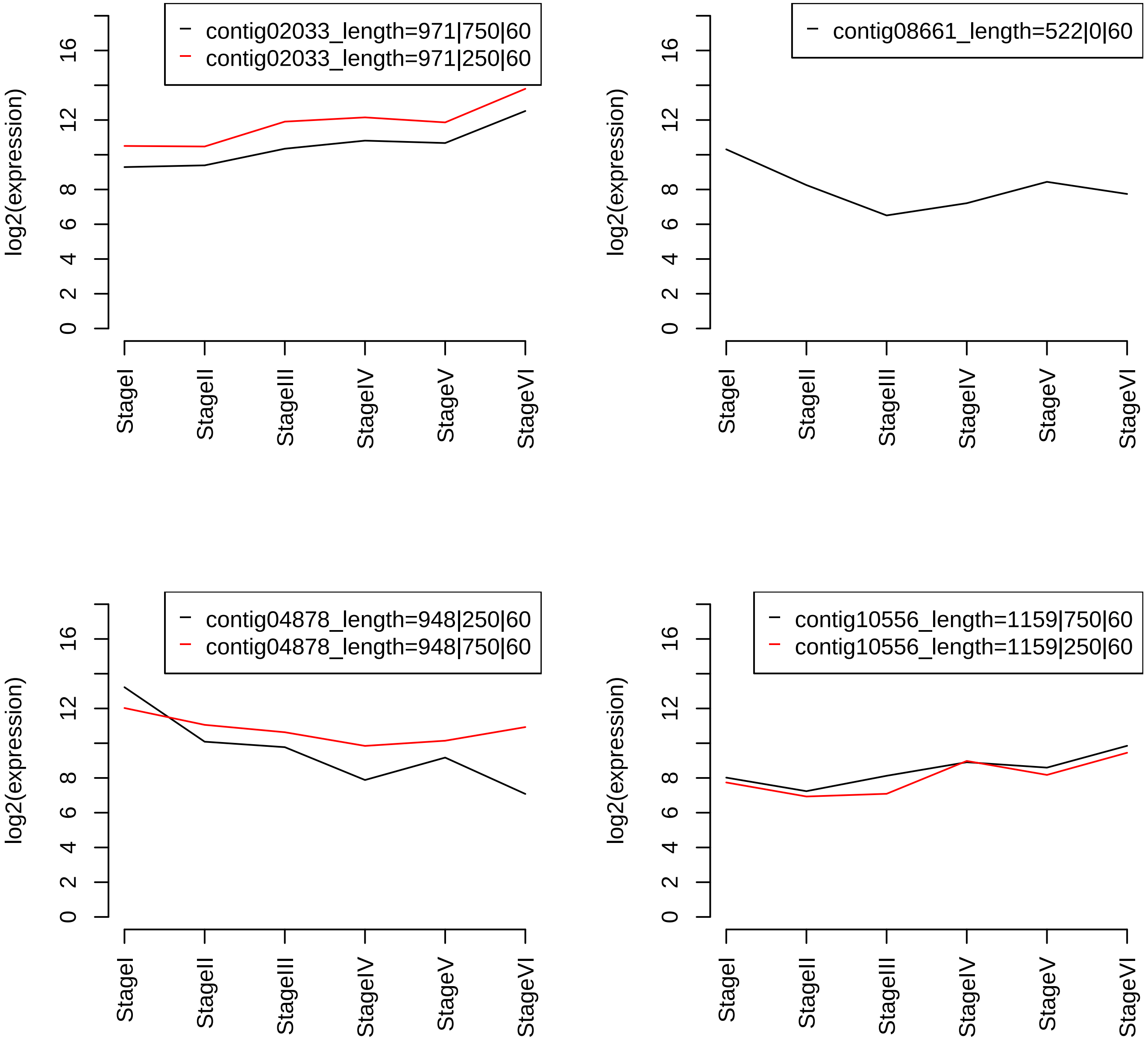
contig02033_length=971|750|60
contig02033_length=971|250|60
contig08661_length=522|0|60
contig04878_length=948|250|60
contig04878_length=948|750|60
contig10556_length=1159|750|60
contig10556_length=1159|250|60
log2(expression)
0
2
4
6
8
12
16
StageI
StageII
StageIII
StageIV
StageV
StageVI

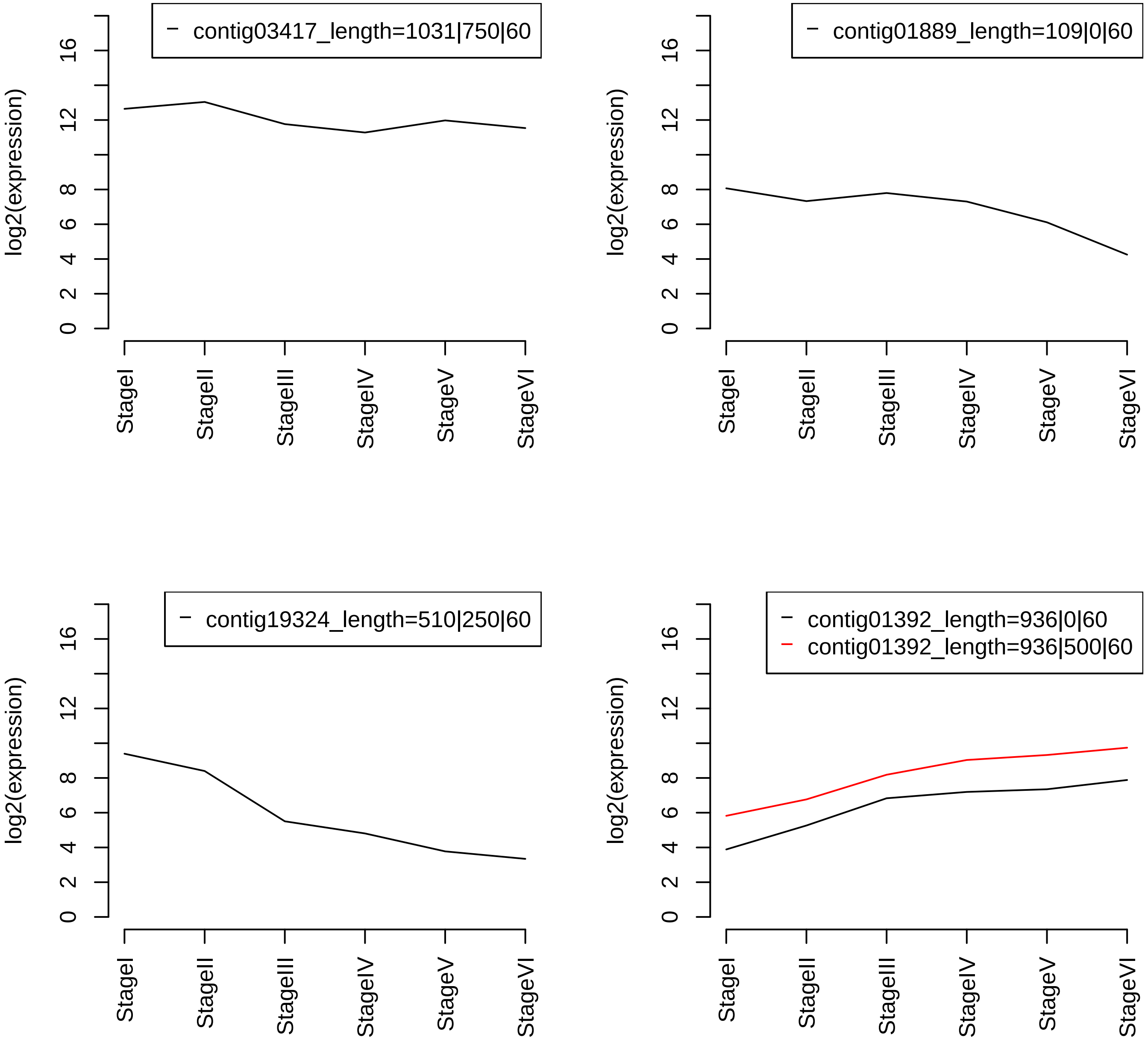
contig03417_length=1031|750|60
contig01889_length=109|0|60
contig19324_length=510|250|60
contig01392_length=936|0|60
contig01392_length=936|500|60
log2(expression)
0
2
4
6
8
12
16
StageI
StageII
StageIII
StageIV
StageV
StageVI

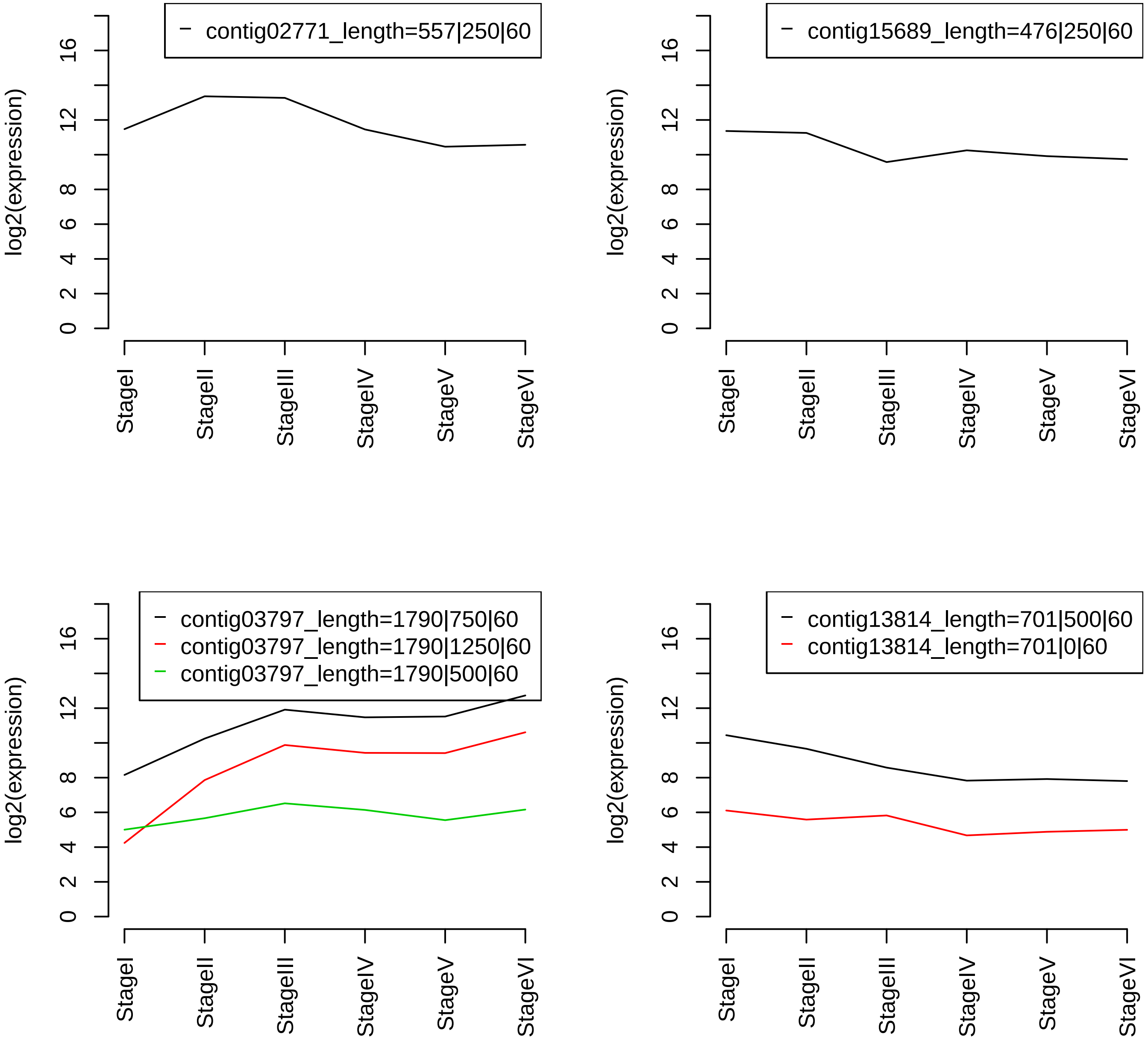

contig02771_length=557|250|60
contig15689_length=476|250|60
contig03797_length=1790|750|60
contig03797_length=1790|1250|60
contig03797_length=1790|500|60
contig13814_length=701|500|60
contig13814_length=701|0|60
log2(expression)
0
2
4
6
8
12
16
StageI
StageII
StageIII
StageIV
StageV
StageVI

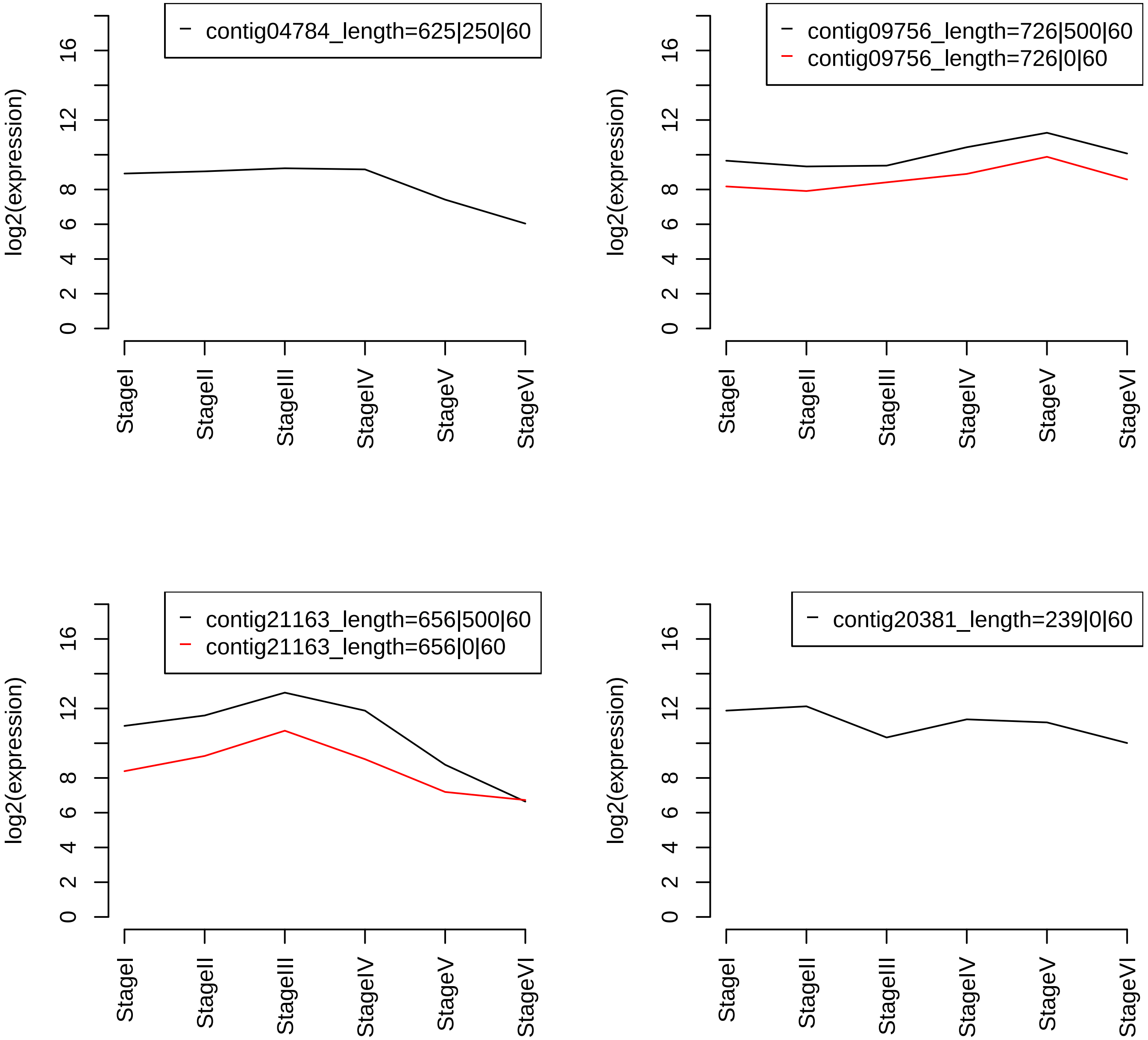

contig04784_length=625|250|60
contig09756_length=726|500|60
contig09756_length=726|0|60
contig21163_length=656|500|60
contig21163_length=656|0|60
contig20381_length=239|0|60
log2(expression)
0
2
4
6
8
12
16
StageI
StageII
StageIII
StageIV
StageV
StageVI

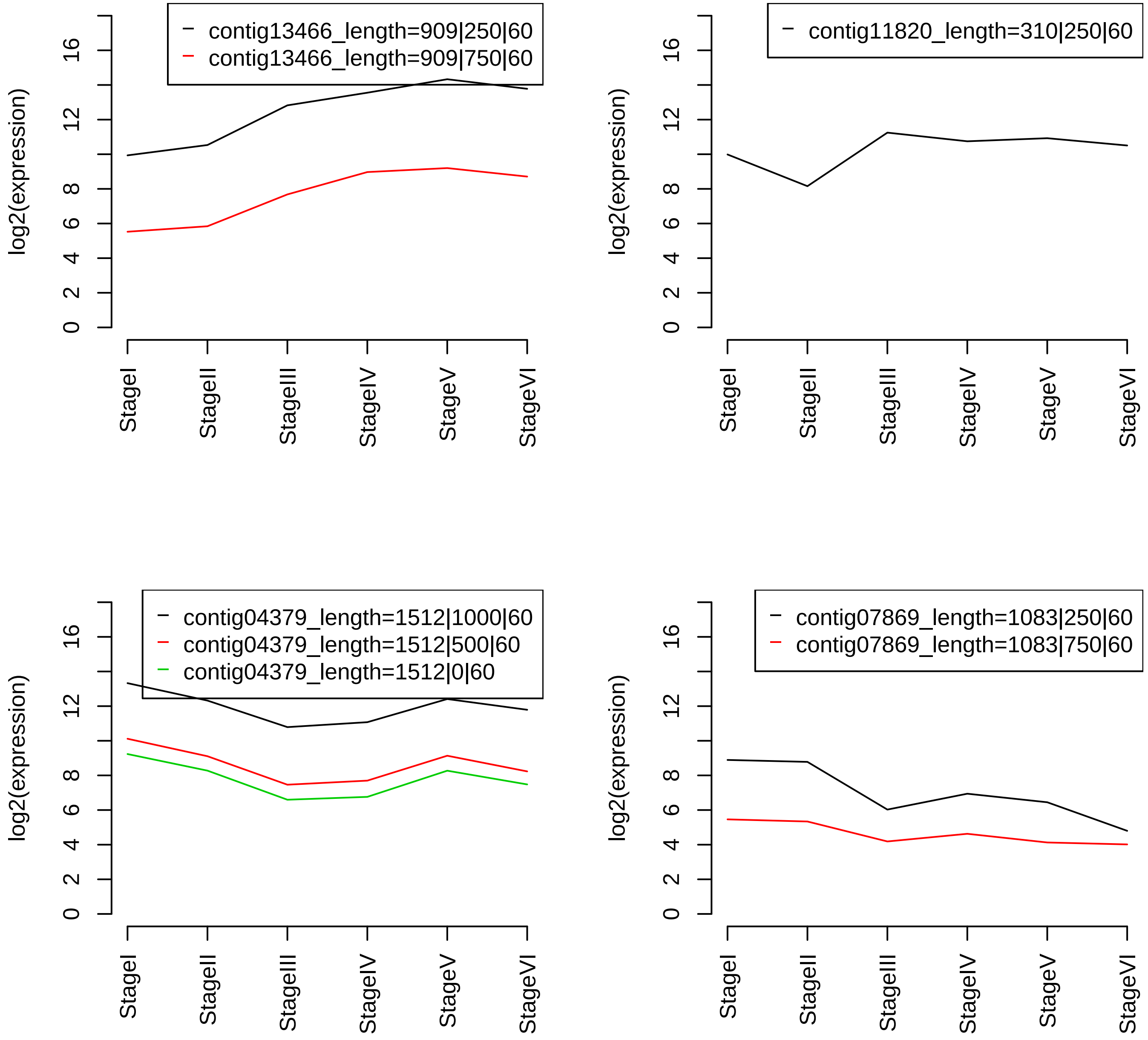

contig13466_length=909|250|60
contig13466_length=909|750|60
contig11820_length=310|250|60
contig04379_length=1512|1000|60
contig04379_length=1512|500|60
contig04379_length=1512|0|60
contig07869_length=1083|250|60
contig07869_length=1083|750|60
log2(expression)
StageI
StageII
StageIII
StageIV
StageV
StageVI

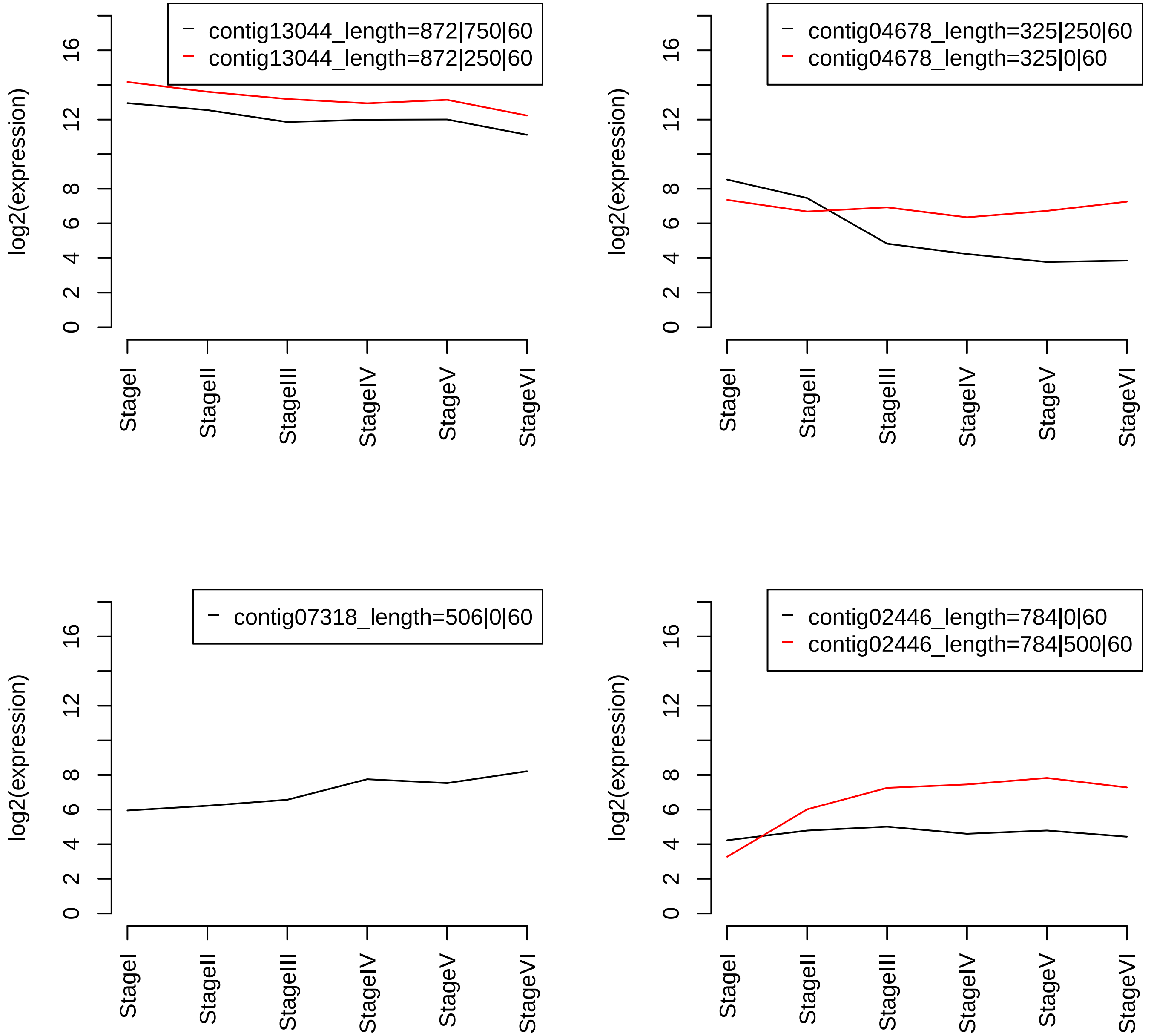
contig13044_length=872|750|60
contig13044_length=872|250|60
contig04678_length=325|250|60
contig04678_length=325|0|60
contig07318_length=506|0|60
contig02446_length=784|0|60
contig02446_length=784|500|60
log2(expression)
0
2
4
6
8
12
16
StageI
StageII
StageIII
StageIV
StageV
StageVI

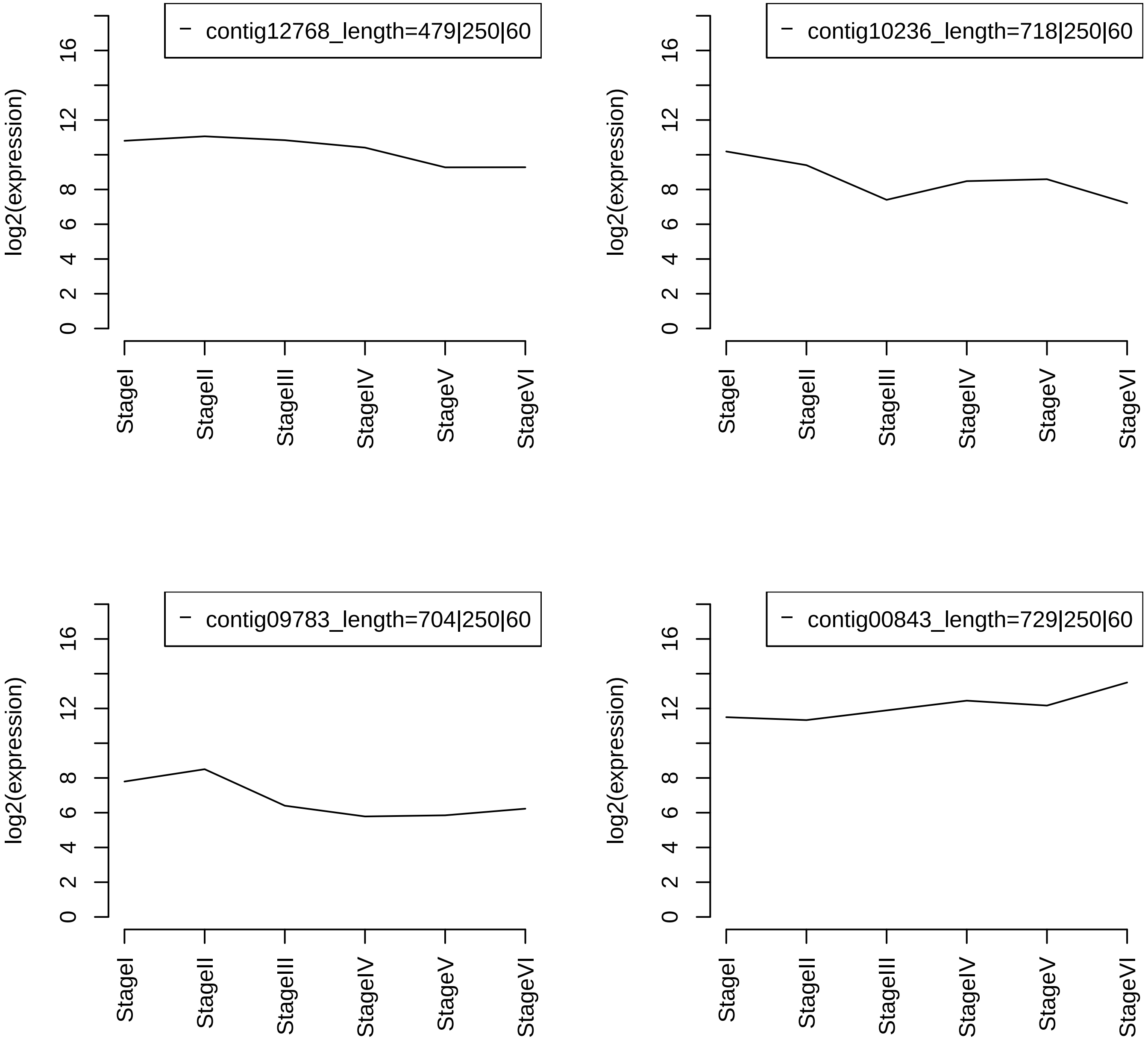
contig12768_length=479|250|60
contig10236_length=718|250|60
contig09783_length=704|250|60
contig00843_length=729|250|60
log2(expression)
0
2
4
6
8
12
16
StageI
StageII
StageIII
StageIV
StageV
StageVI

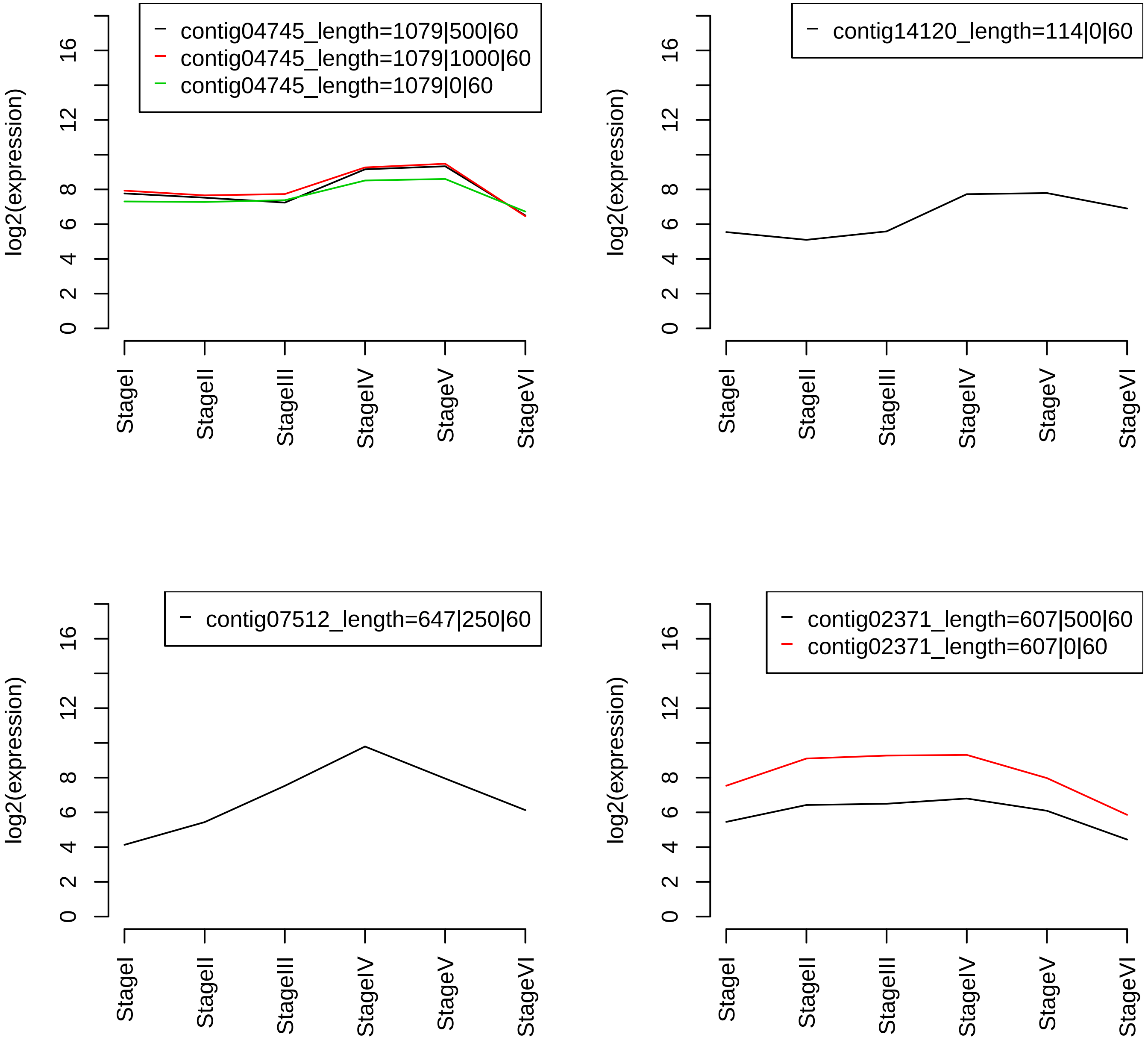

contig04745_length=1079|500|60
contig04745_length=1079|1000|60
contig04745_length=1079|0|60
contig14120_length=114|0|60
contig07512_length=647|250|60
contig02371_length=607|500|60
contig02371_length=607|0|60
log2(expression)
0
2
4
6
8
12
16
StageI
StageII
StageIII
StageIV
StageV
StageVI

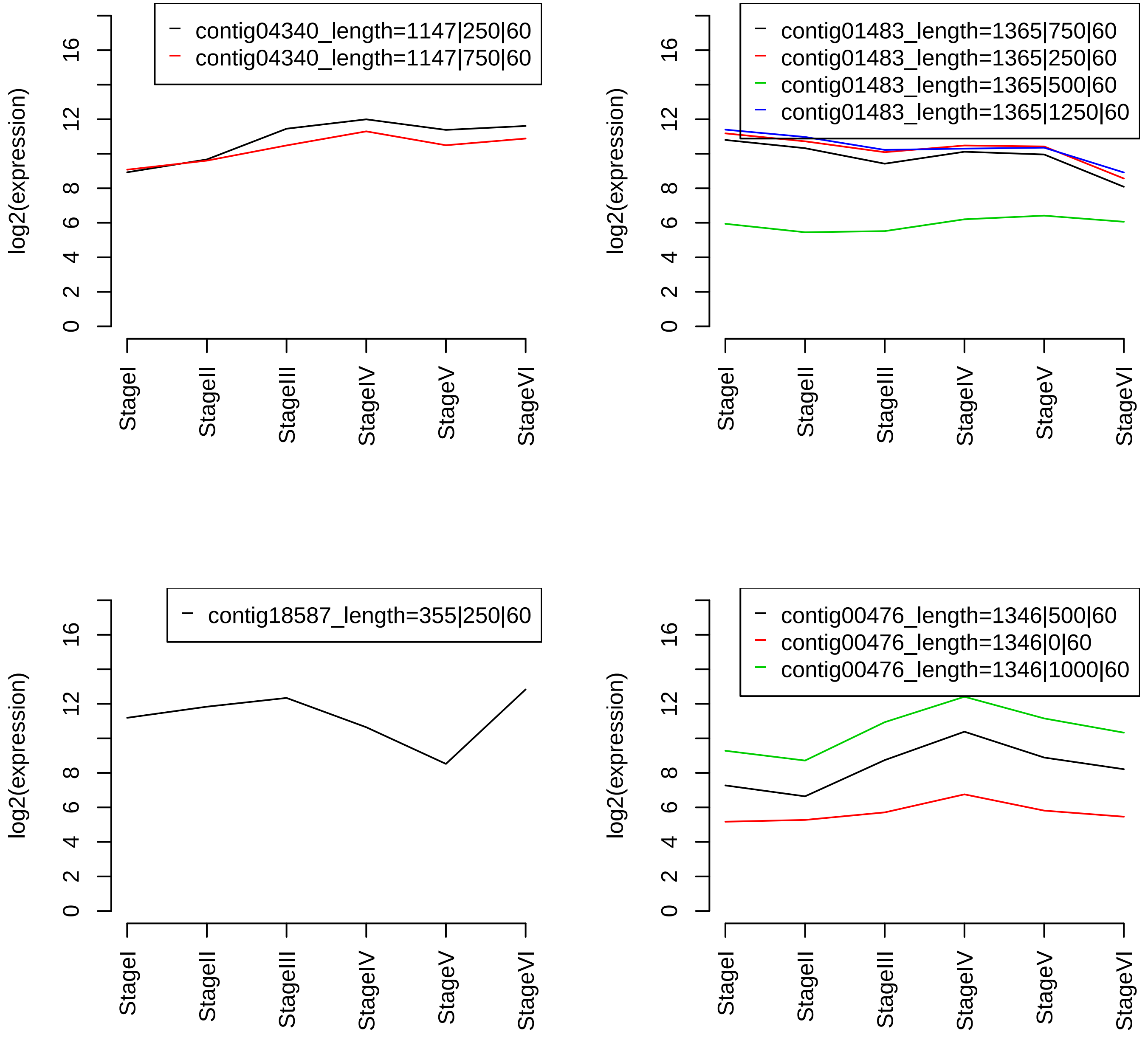

contig04340_length=1147|250|60
contig04340_length=1147|750|60
log2(expression)
0 2 4 6 8 12 16
StageI StageII StageIII StageIV StageV StageVI
contig01483_length=1365|750|60
contig01483_length=1365|250|60
contig01483_length=1365|500|60
contig01483_length=1365|1250|60
log2(expression)
0 2 4 6 8 12 16
StageI StageII StageIII StageIV StageV StageVI
contig18587_length=355|250|60
log2(expression)
0 2 4 6 8 12 16
StageI StageII StageIII StageIV StageV StageVI
contig00476_length=1346|500|60
contig00476_length=1346|0|60
contig00476_length=1346|1000|60
log2(expression)
0 2 4 6 8 12 16
StageI StageII StageIII StageIV StageV StageVI

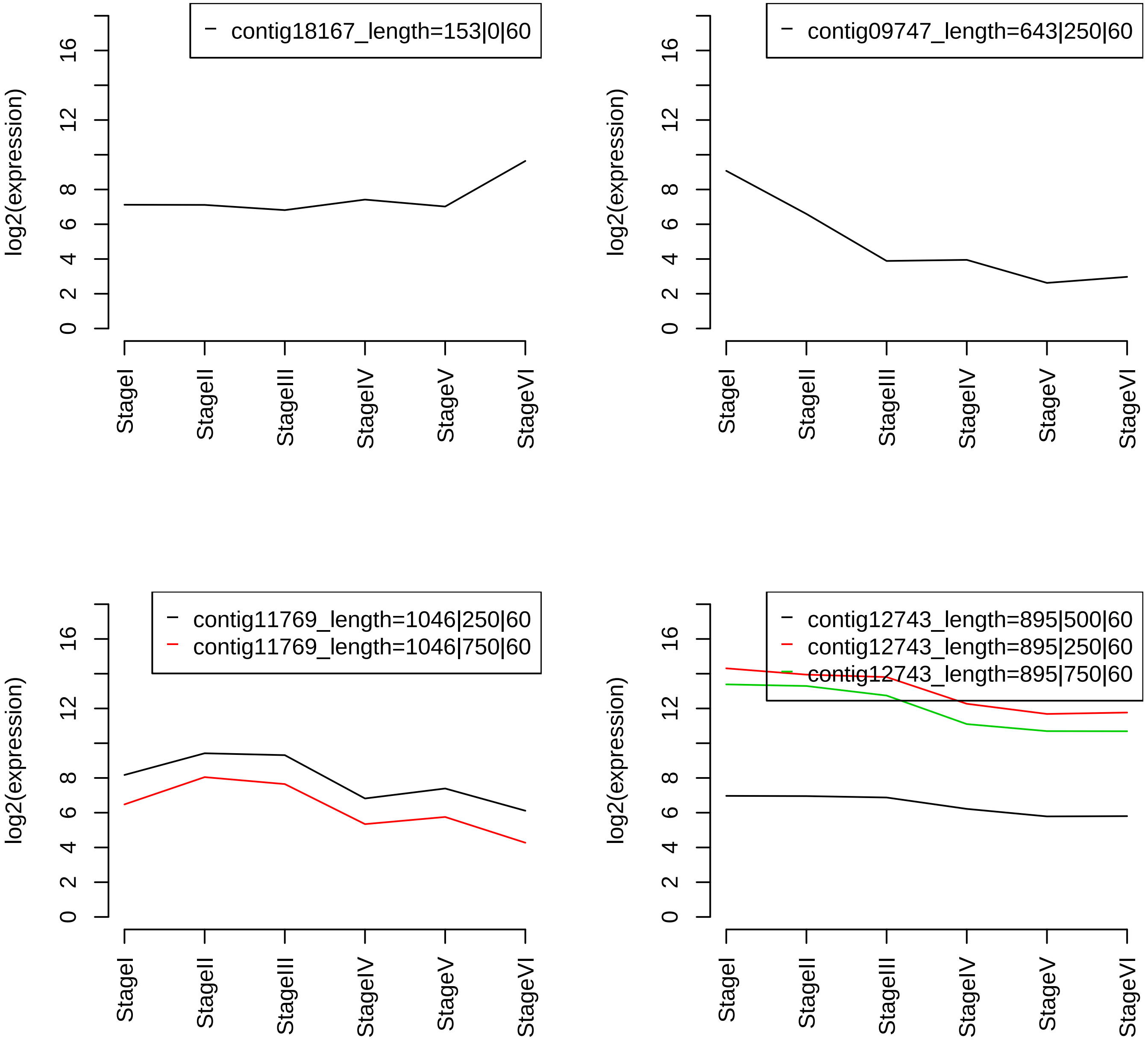
contig18167_length=153|0|60
contig09747_length=643|250|60
contig11769_length=1046|250|60
contig11769_length=1046|750|60
contig12743_length=895|500|60
contig12743_length=895|250|60
contig12743_length=895|750|60
log2(expression)
0
2
4
6
8
12
16
StageI
StageII
StageIII
StageIV
StageV
StageVI

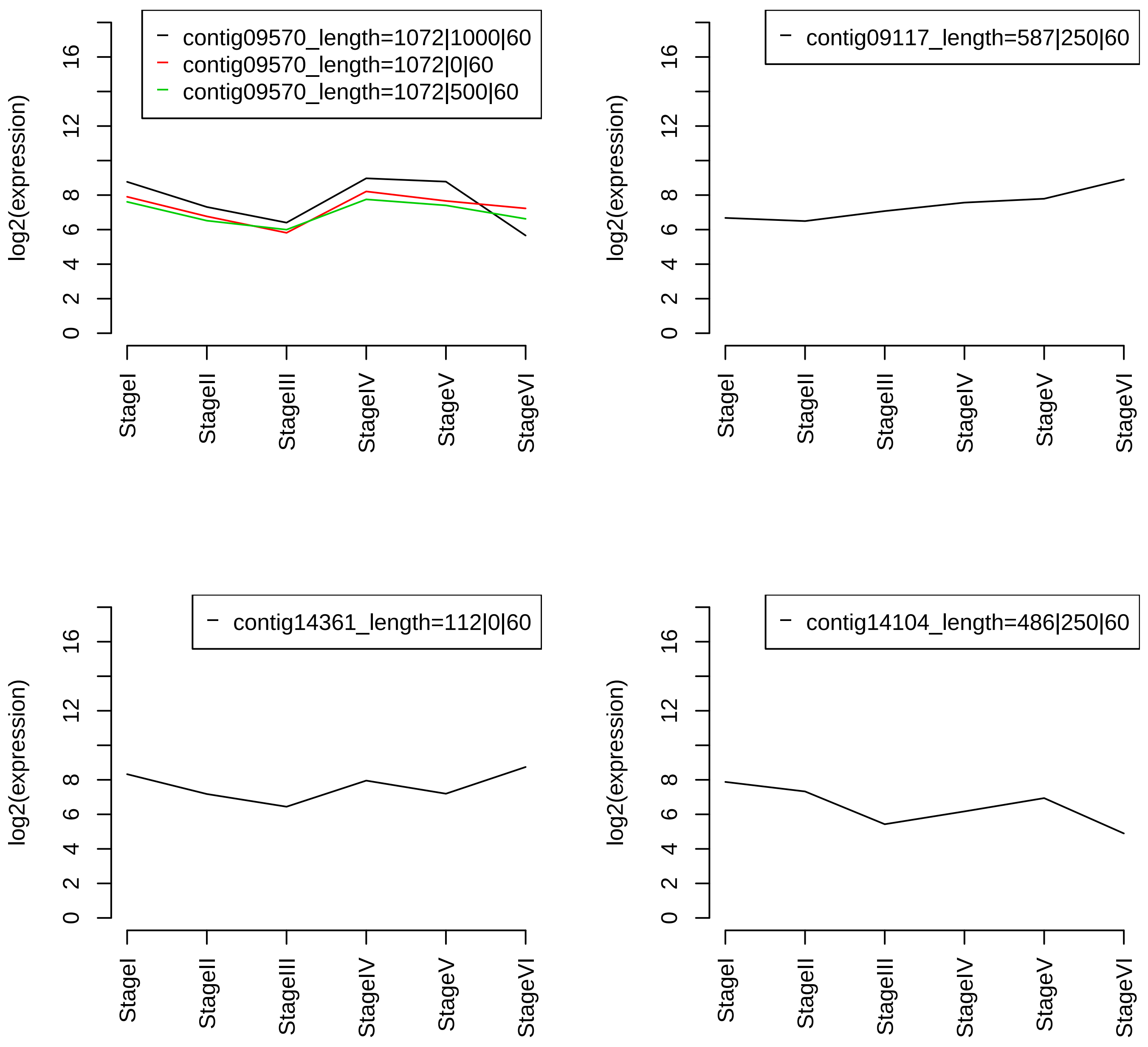
contig09570_length=1072|1000|60
contig09570_length=1072|0|60
contig09570_length=1072|500|60
contig09117_length=587|250|60
contig14361_length=112|0|60
contig14104_length=486|250|60
log2(expression)
0
2
4
6
8
12
16
StageI
StageII
StageIII
StageIV
StageV
StageVI

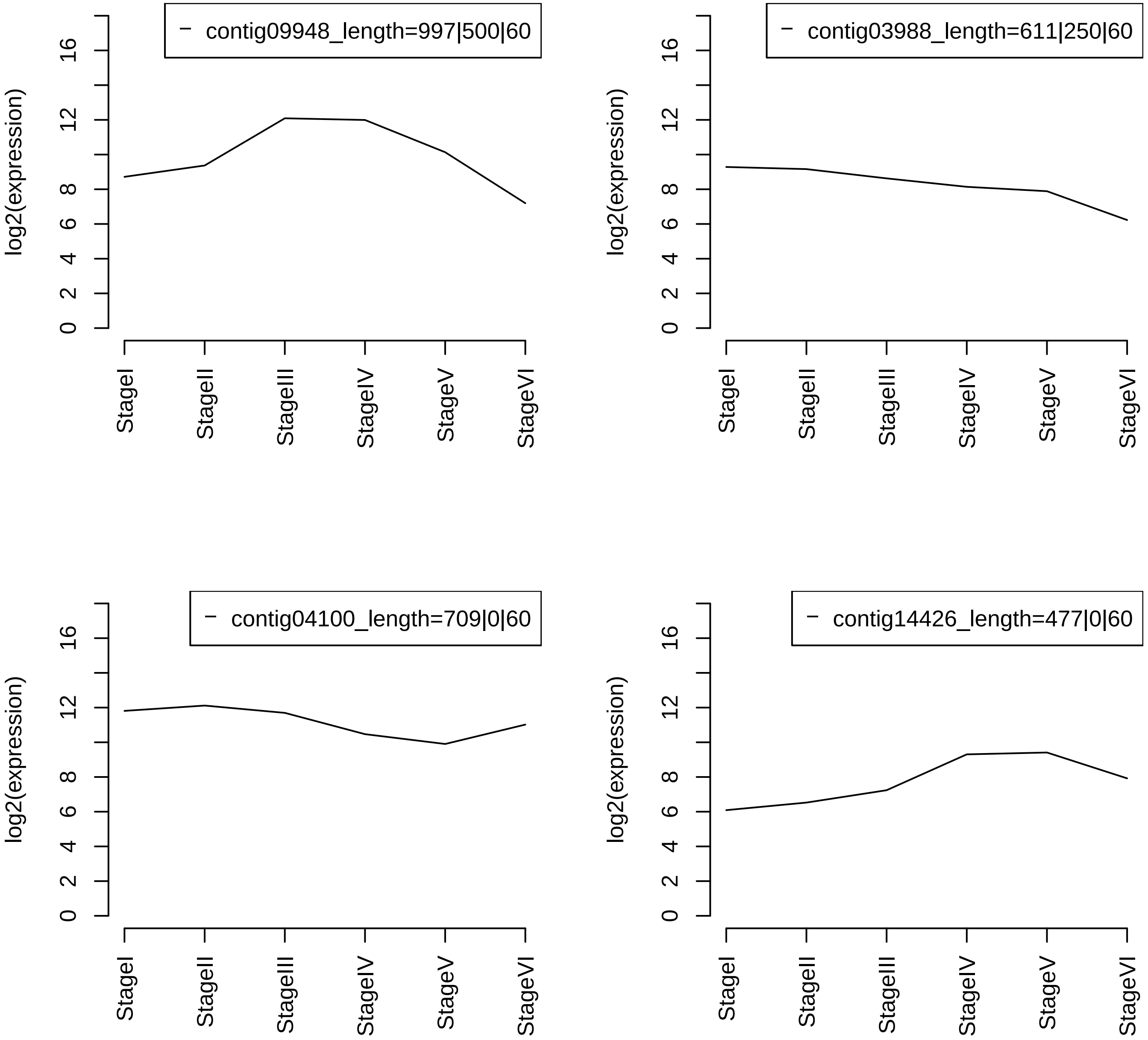
contig09948_length=997|500|60
contig03988_length=611|250|60
contig04100_length=709|0|60
contig14426_length=477|0|60
log2(expression)
0
2
4
6
8
12
16
StageI
StageII
StageIII
StageIV
StageV
StageVI

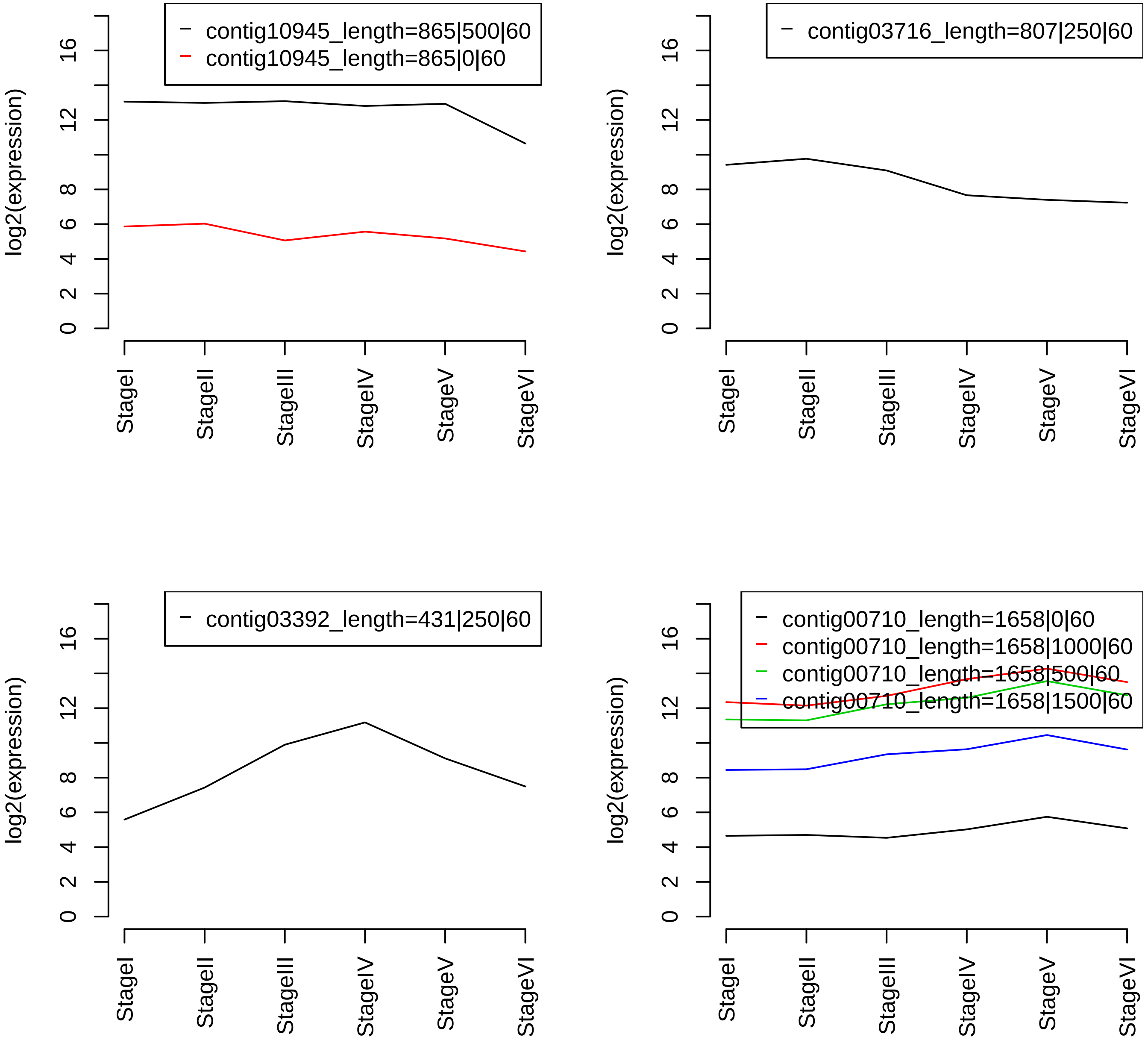

contig10945_length=865|500|60
contig10945_length=865|0|60
contig03716_length=807|250|60
contig03392_length=431|250|60
contig00710_length=1658|0|60
contig00710_length=1658|1000|60
contig00710_length=1658|500|60
contig00710_length=1658|1500|60
log2(expression)
StageI
StageII
StageIII
StageIV
StageV
StageVI

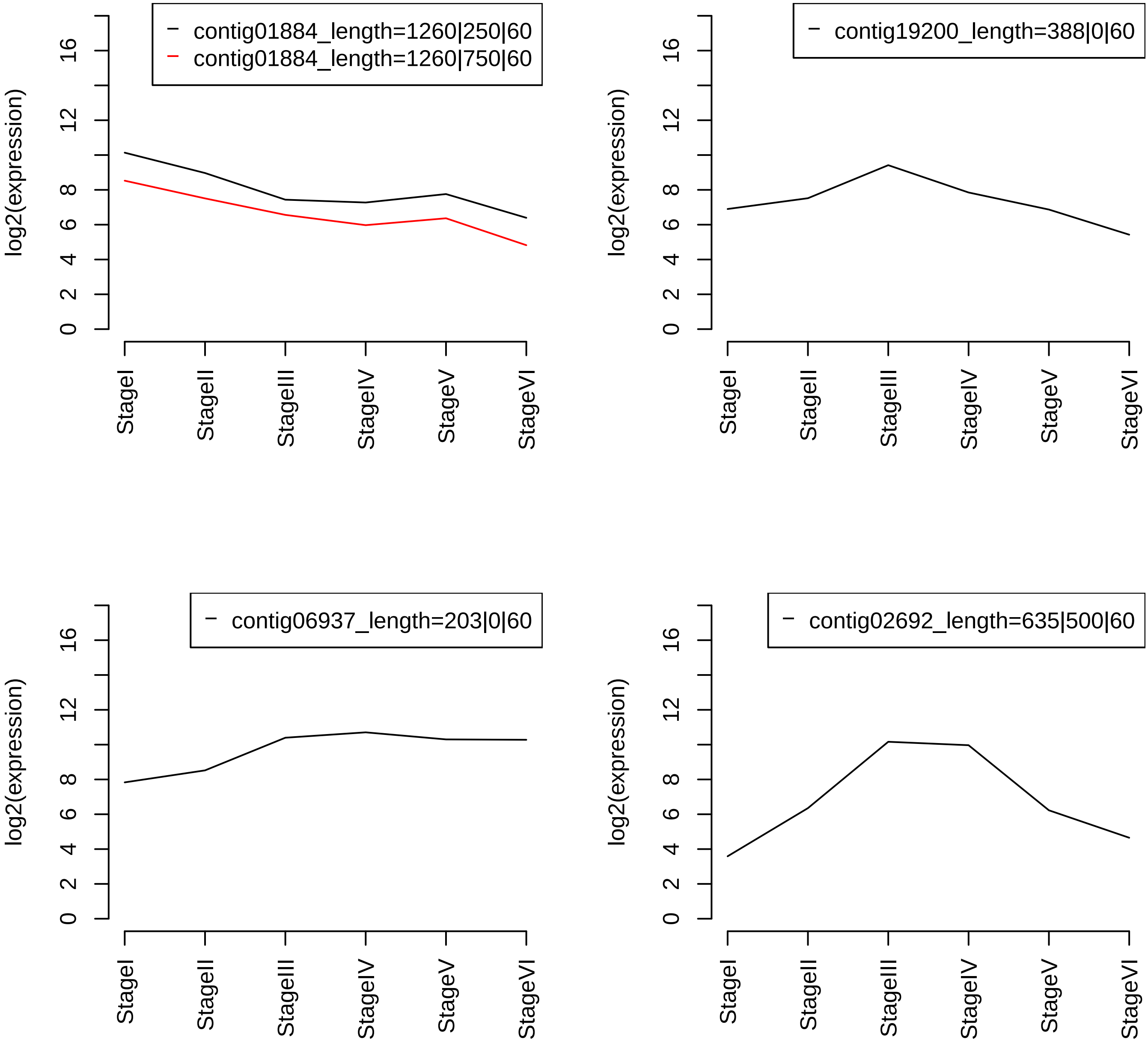
contig01884_length=1260|250|60
contig01884_length=1260|750|60
contig19200_length=388|0|60
contig06937_length=203|0|60
contig02692_length=635|500|60
log2(expression)
0
2
4
6
8
12
16
StageI
StageII
StageIII
StageIV
StageV
StageVI

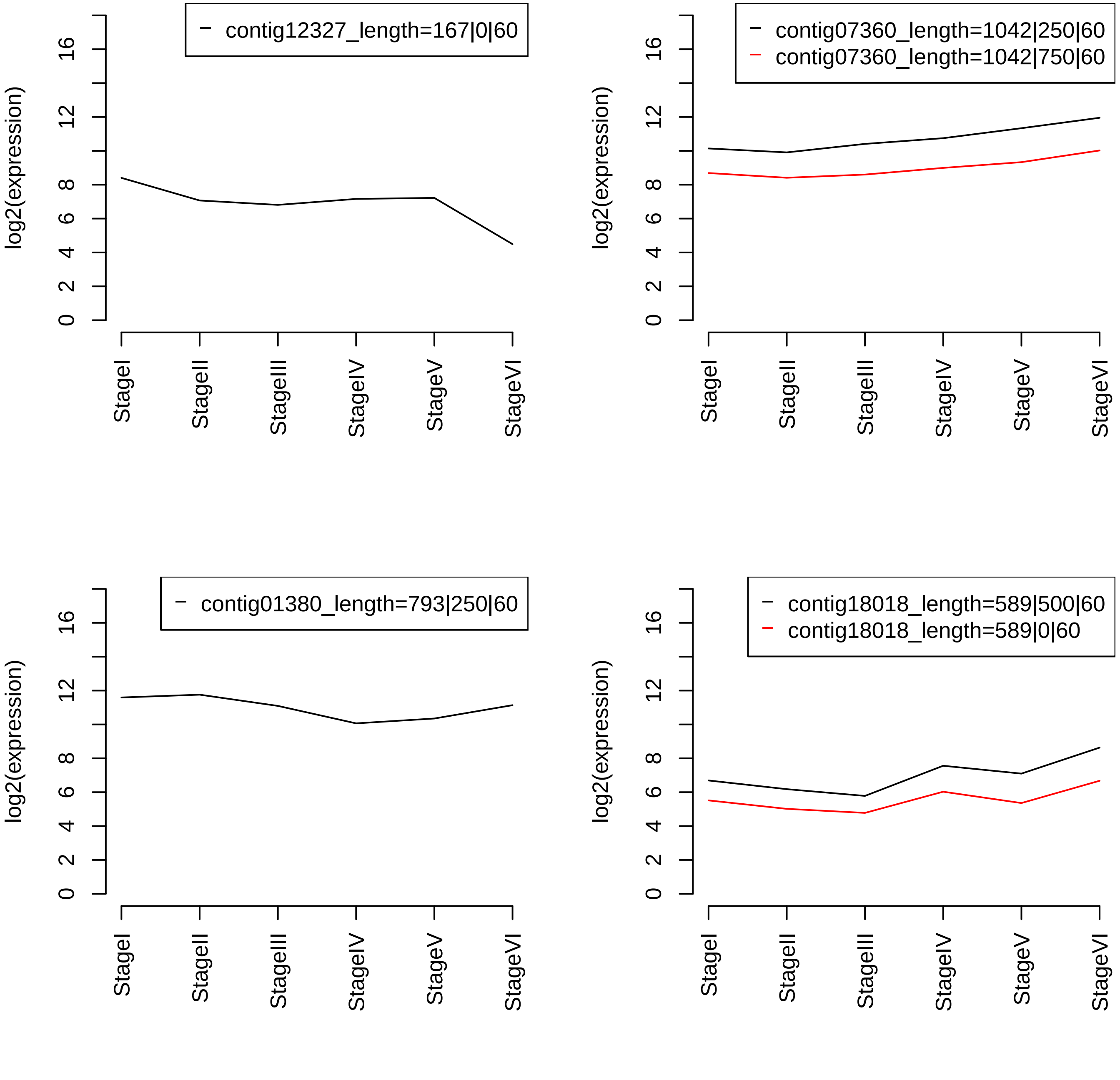
contig12327_length=167|0|60
contig07360_length=1042|250|60
contig07360_length=1042|750|60
contig01380_length=793|250|60
contig18018_length=589|500|60
contig18018_length=589|0|60
log2(expression)
0 2 4 6 8 12 16
StageI StageII StageIII StageIV StageV StageVI

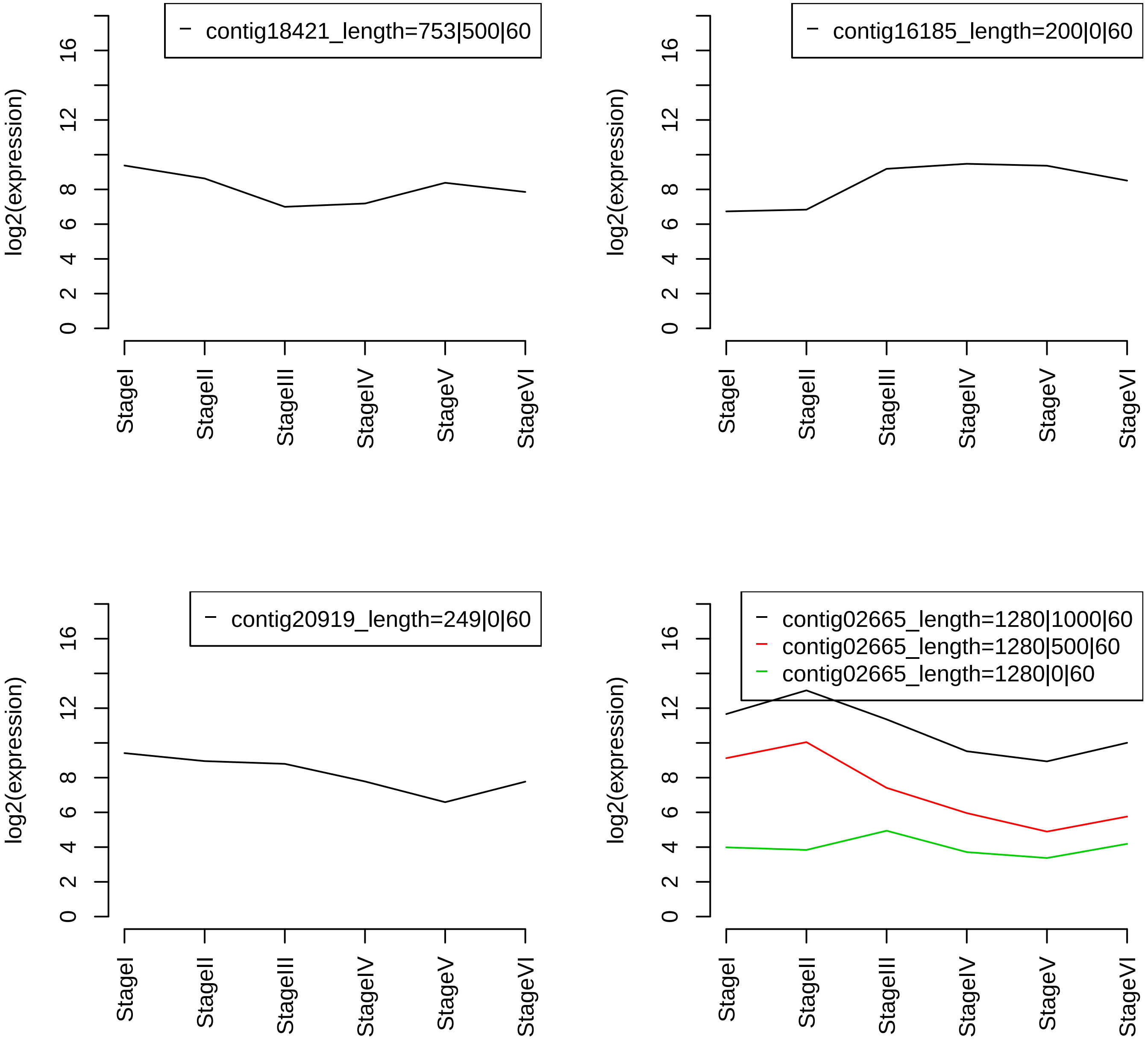

contig18421_length=753|500|60
contig16185_length=200|0|60
contig20919_length=249|0|60
contig02665_length=1280|1000|60
contig02665_length=1280|500|60
contig02665_length=1280|0|60
log2(expression)
0
2
4
6
8
12
16
StageI
StageII
StageIII
StageIV
StageV
StageVI

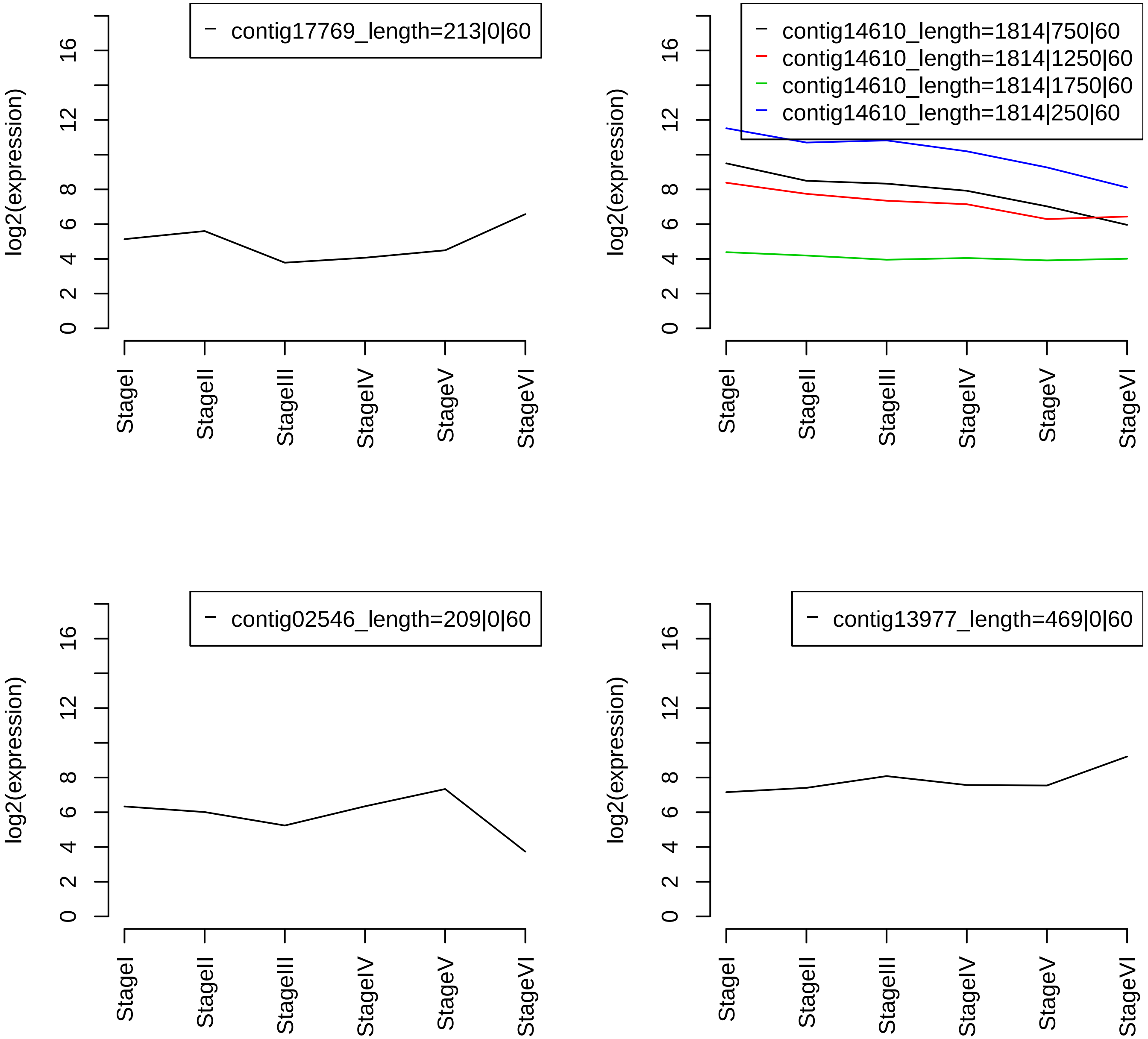

contig17769_length=213|0|60
contig14610_length=1814|750|60
contig14610_length=1814|1250|60
contig14610_length=1814|1750|60
contig14610_length=1814|250|60
contig02546_length=209|0|60
contig13977_length=469|0|60
log2(expression)
0
2
4
6
8
12
16
StageI
StageII
StageIII
StageIV
StageV
StageVI

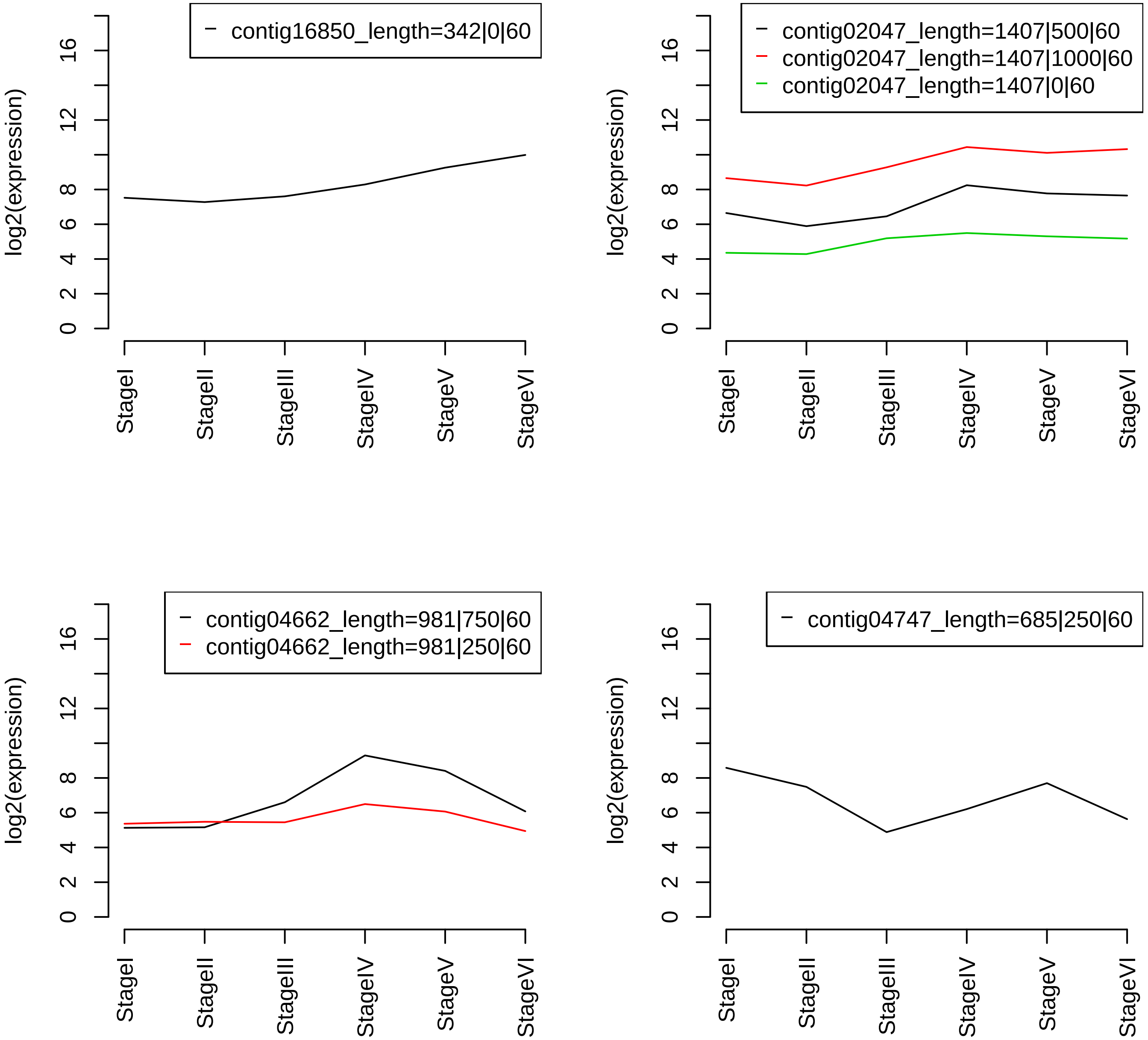

contig16850_length=342|0|60
contig02047_length=1407|500|60
contig02047_length=1407|1000|60
contig02047_length=1407|0|60
contig04662_length=981|750|60
contig04662_length=981|250|60
contig04747_length=685|250|60
log2(expression)
0
2
4
6
8
12
16
StageI
StageII
StageIII
StageIV
StageV
StageVI

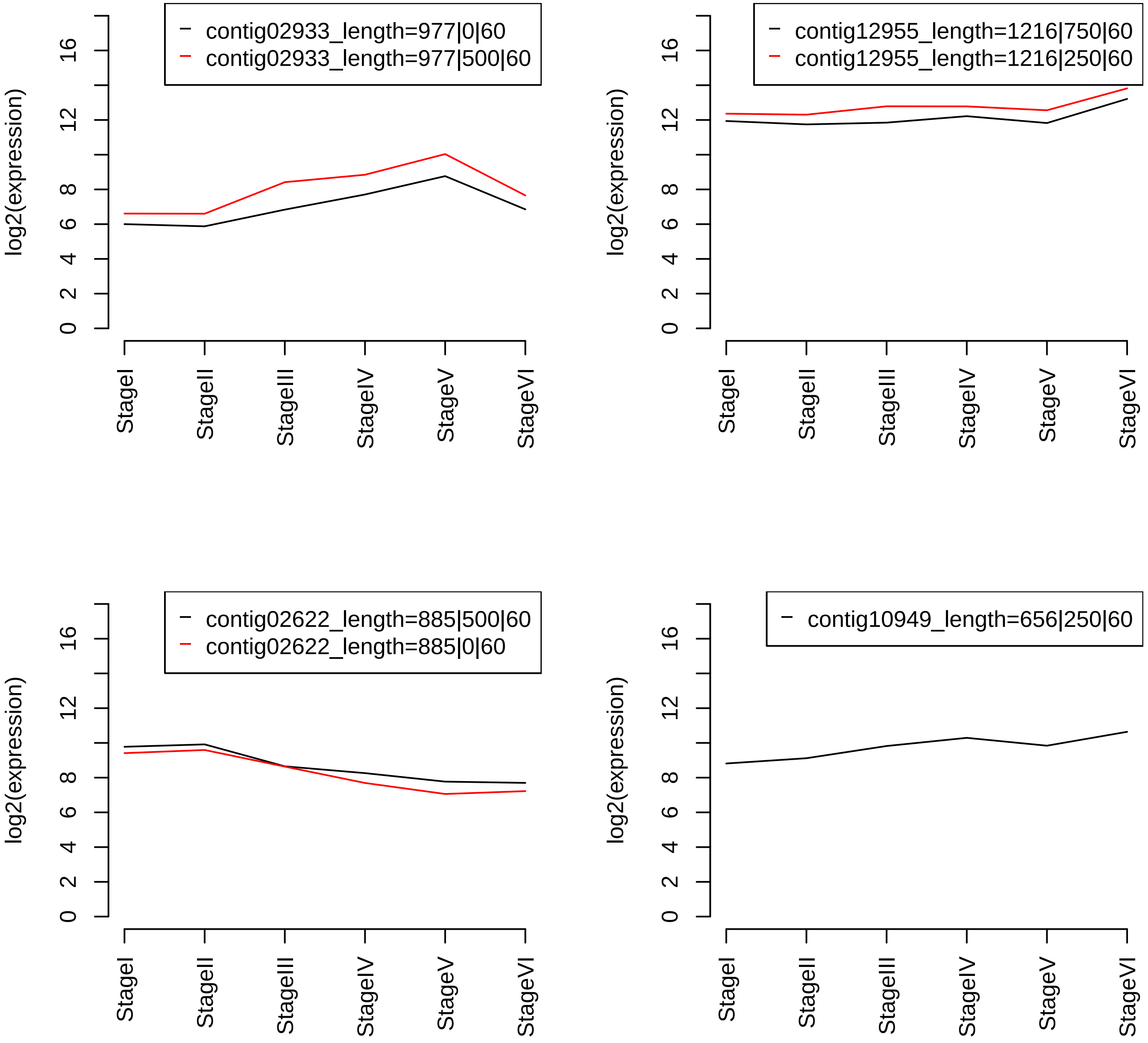

contig02933_length=977|0|60
contig02933_length=977|500|60
log2(expression)
0
2
4
6
8
12
16
StageI
StageII
StageIII
StageIV
StageV
StageVI
contig12955_length=1216|750|60
contig12955_length=1216|250|60
contig02622_length=885|500|60
contig02622_length=885|0|60
contig10949_length=656|250|60

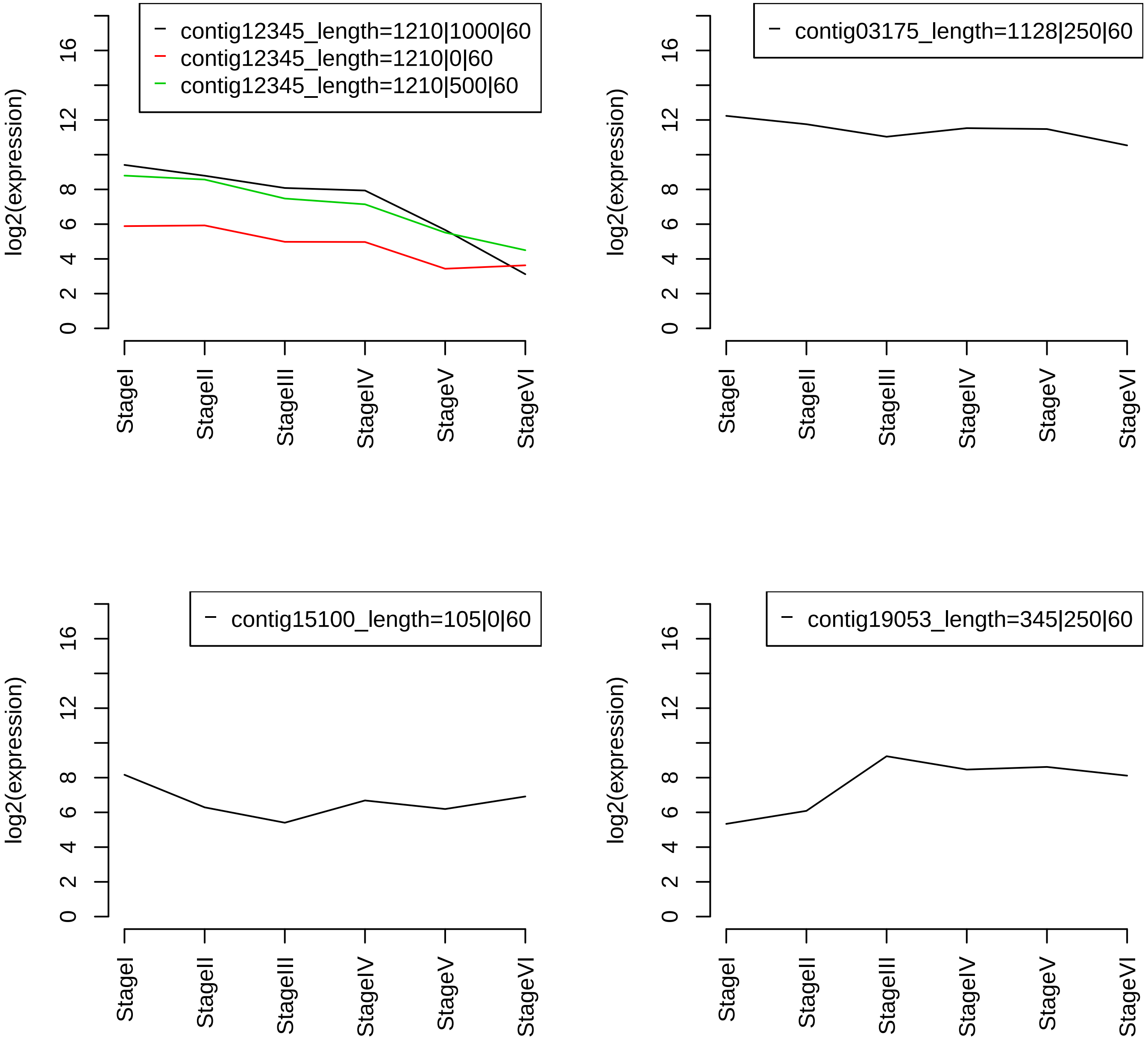
contig12345_length=1210|1000|60
contig12345_length=1210|0|60
contig12345_length=1210|500|60
contig03175_length=1128|250|60
contig15100_length=105|0|60
contig19053_length=345|250|60
log2(expression)
0
2
4
6
8
12
16
StageI
StageII
StageIII
StageIV
StageV
StageVI

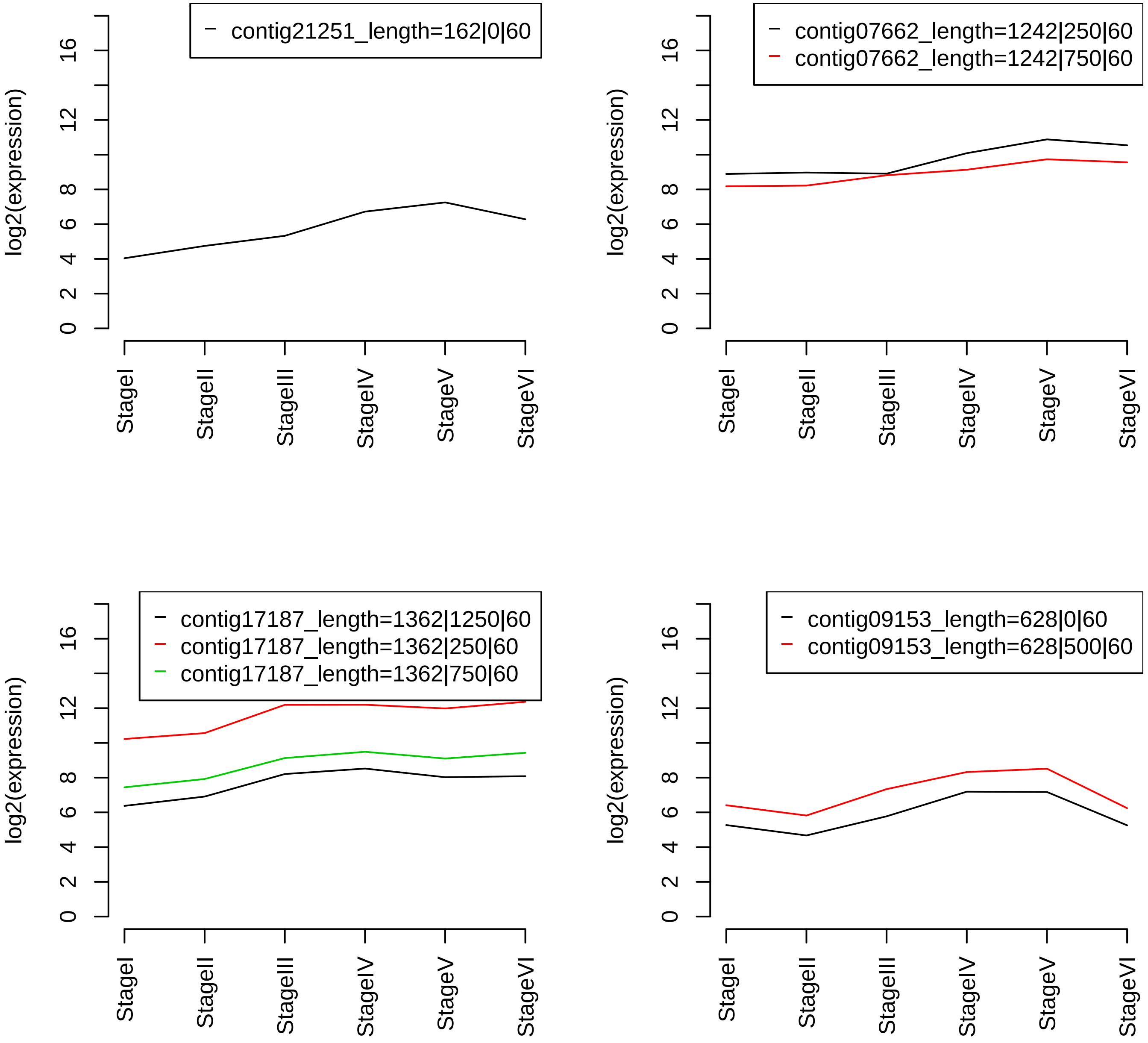

contig21251_length=162|0|60
contig07662_length=1242|250|60
contig07662_length=1242|750|60
contig17187_length=1362|1250|60
contig17187_length=1362|250|60
contig17187_length=1362|750|60
contig09153_length=628|0|60
contig09153_length=628|500|60
log2(expression)
0
2
4
6
8
12
16
StageI
StageII
StageIII
StageIV
StageV
StageVI

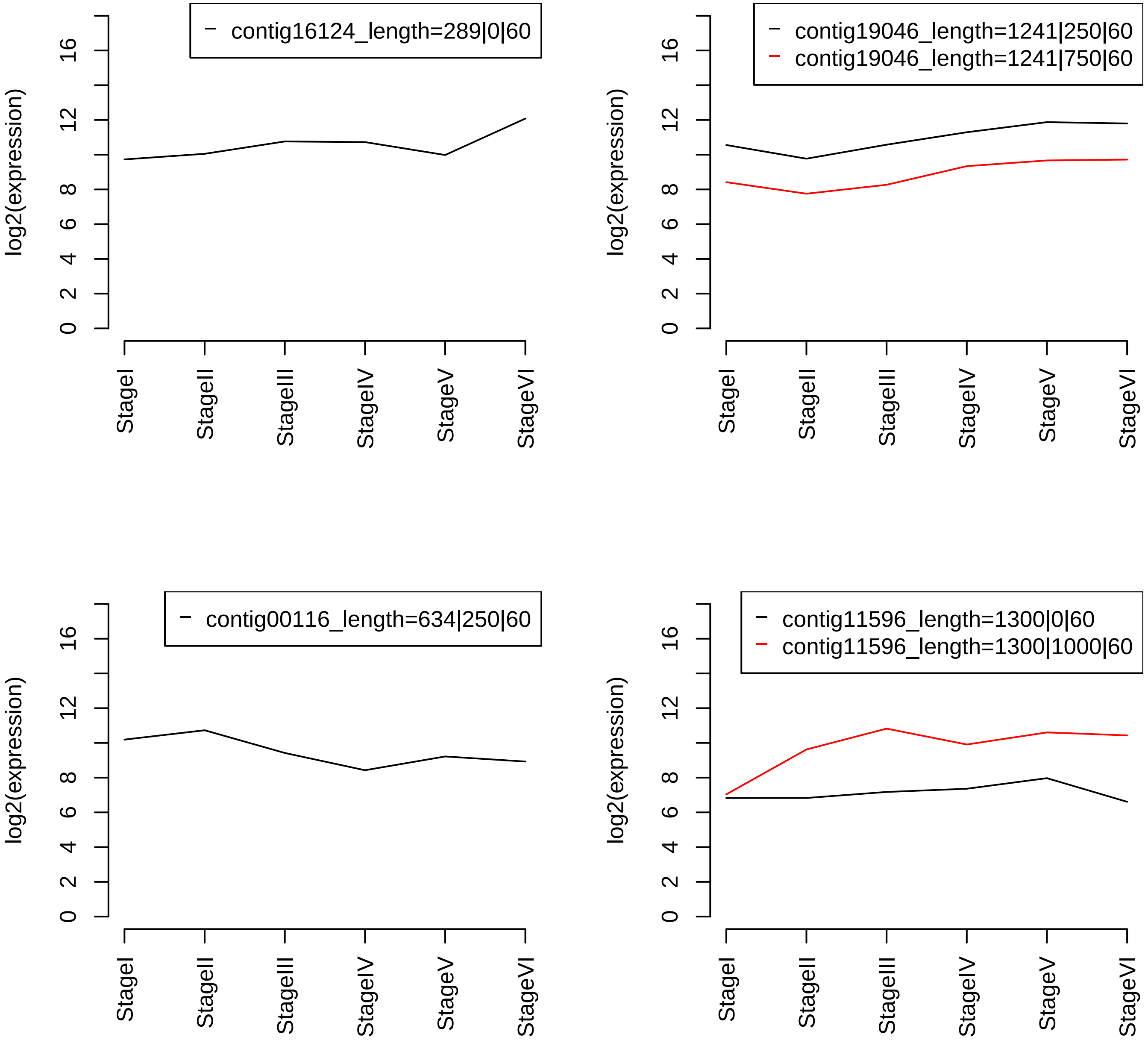

contig16124_length=289|0|60
contig19046_length=1241|250|60
contig19046_length=1241|750|60
contig00116_length=634|250|60
contig11596_length=1300|0|60
contig11596_length=1300|1000|60
log2(expression)
0
2
4
6
8
12
16
StageI
StageII
StageIII
StageIV
StageV
StageVI

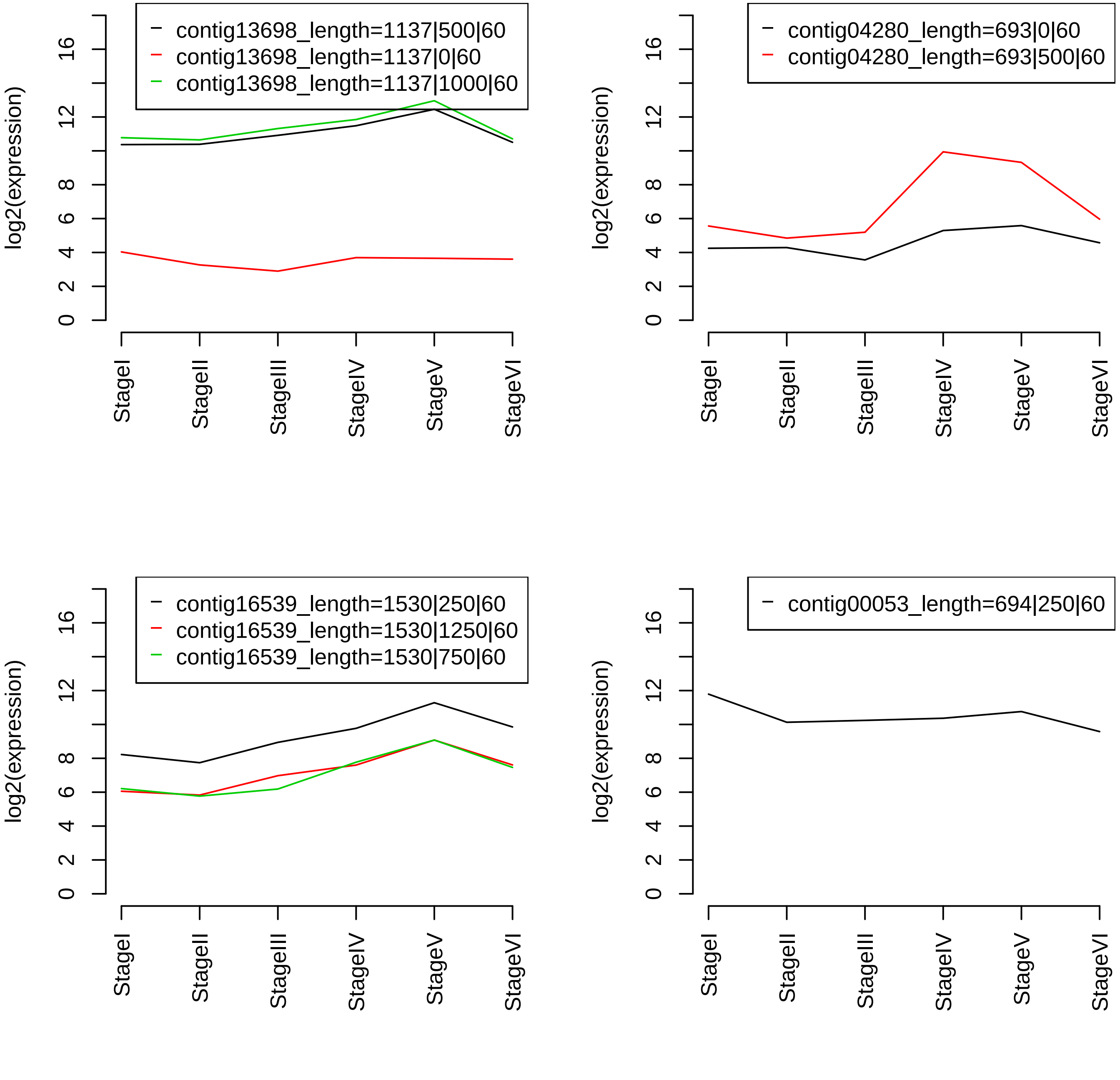
contig13698_length=1137|500|60
contig13698_length=1137|0|60
contig13698_length=1137|1000|60
log2(expression)
0
2
4
6
8
12
16
StageI
StageII
StageIII
StageIV
StageV
StageVI
contig04280_length=693|0|60
contig04280_length=693|500|60
contig16539_length=1530|250|60
contig16539_length=1530|1250|60
contig16539_length=1530|750|60
contig00053_length=694|250|60

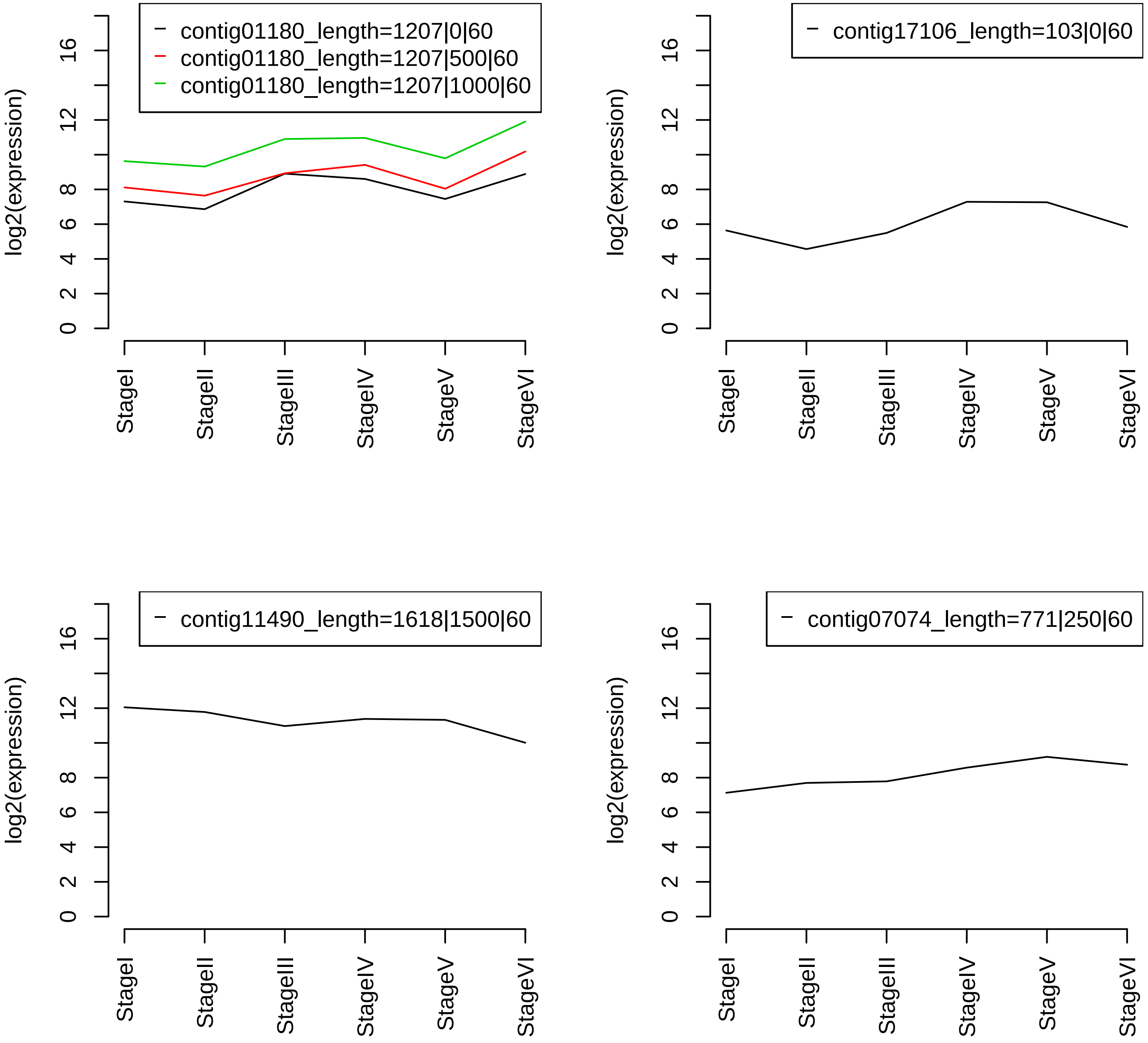
contig01180_length=1207|0|60
contig01180_length=1207|500|60
contig01180_length=1207|1000|60
log2(expression)
0
2
4
6
8
12
16
StageI
StageII
StageIII
StageIV
StageV
StageVI
contig17106_length=103|0|60
contig11490_length=1618|1500|60
contig07074_length=771|250|60

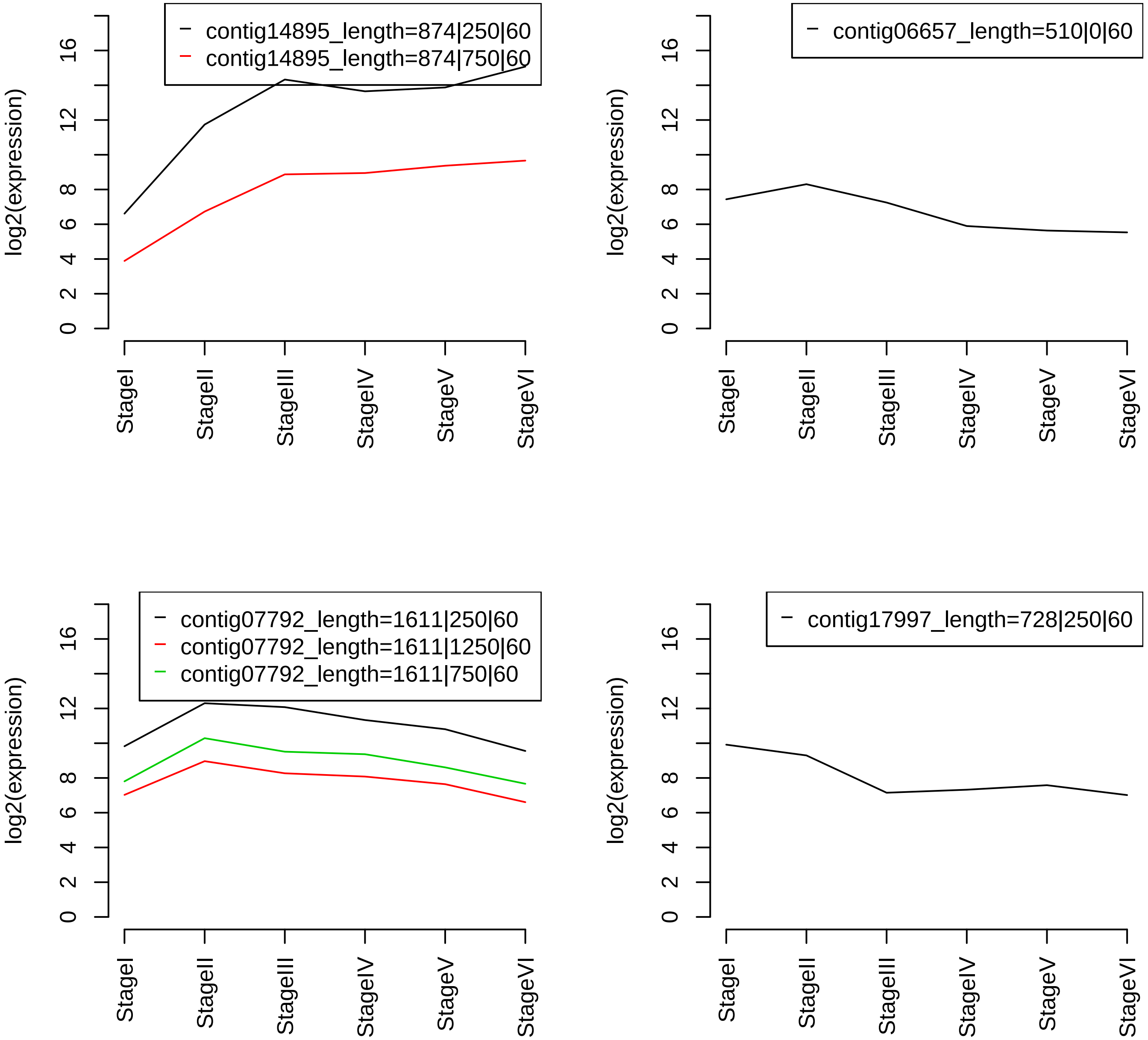

contig14895_length=874|250|60
contig14895_length=874|750|60
log2(expression)
0
2
4
6
8
12
16
StageI
StageII
StageIII
StageIV
StageV
StageVI
contig06657_length=510|0|60
log2(expression)
0
2
4
6
8
12
16
StageI
StageII
StageIII
StageIV
StageV
StageVI
contig07792_length=1611|250|60
contig07792_length=1611|1250|60
contig07792_length=1611|750|60
log2(expression)
0
2
4
6
8
12
16
StageI
StageII
StageIII
StageIV
StageV
StageVI
contig17997_length=728|250|60
log2(expression)
0
2
4
6
8
12
16
StageI
StageII
StageIII
StageIV
StageV
StageVI

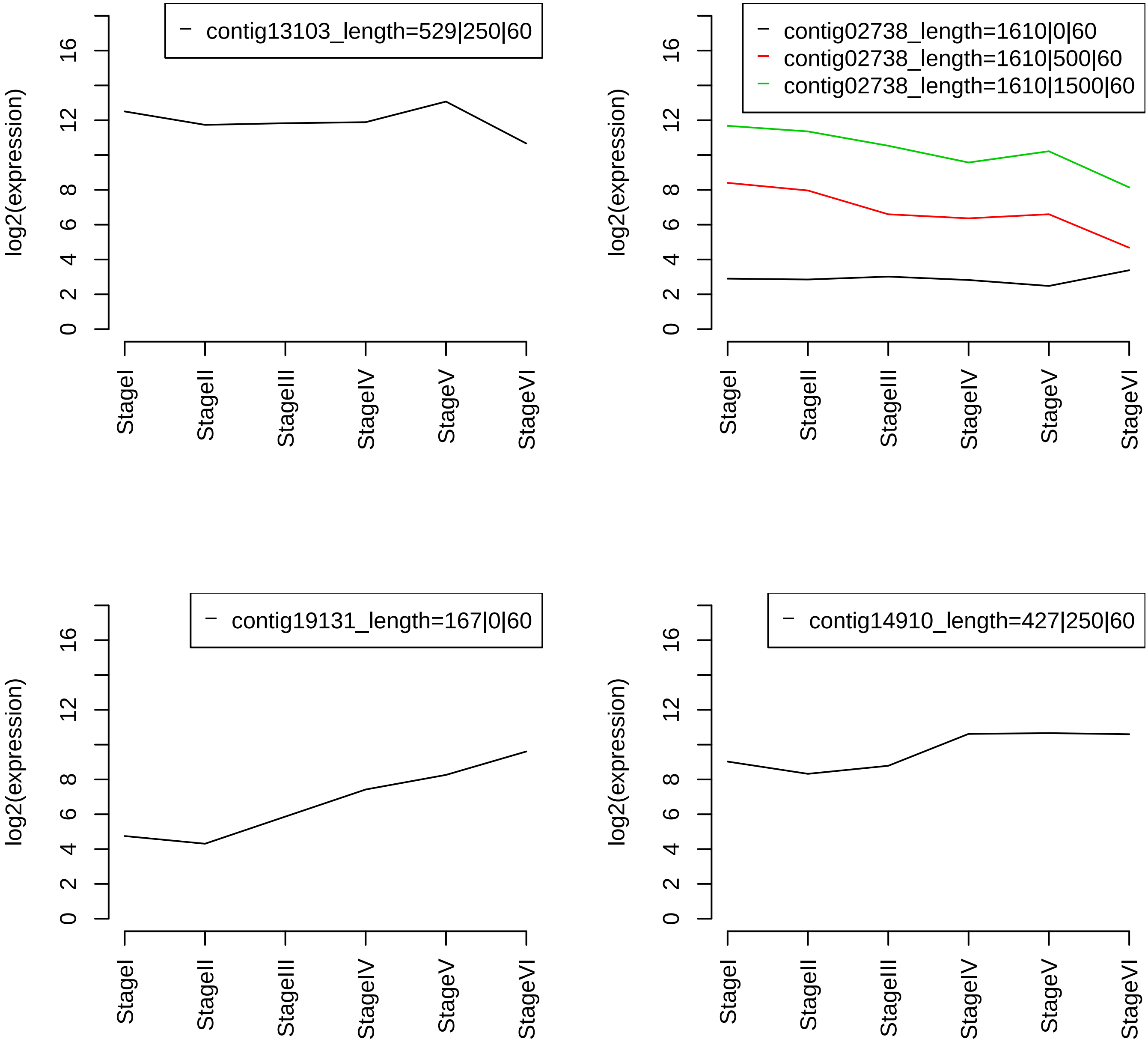
contig13103_length=529|250|60
contig02738_length=1610|0|60
contig02738_length=1610|500|60
contig02738_length=1610|1500|60
contig19131_length=167|0|60
contig14910_length=427|250|60
log2(expression)
0
2
4
6
8
12
16
StageI
StageII
StageIII
StageIV
StageV
StageVI

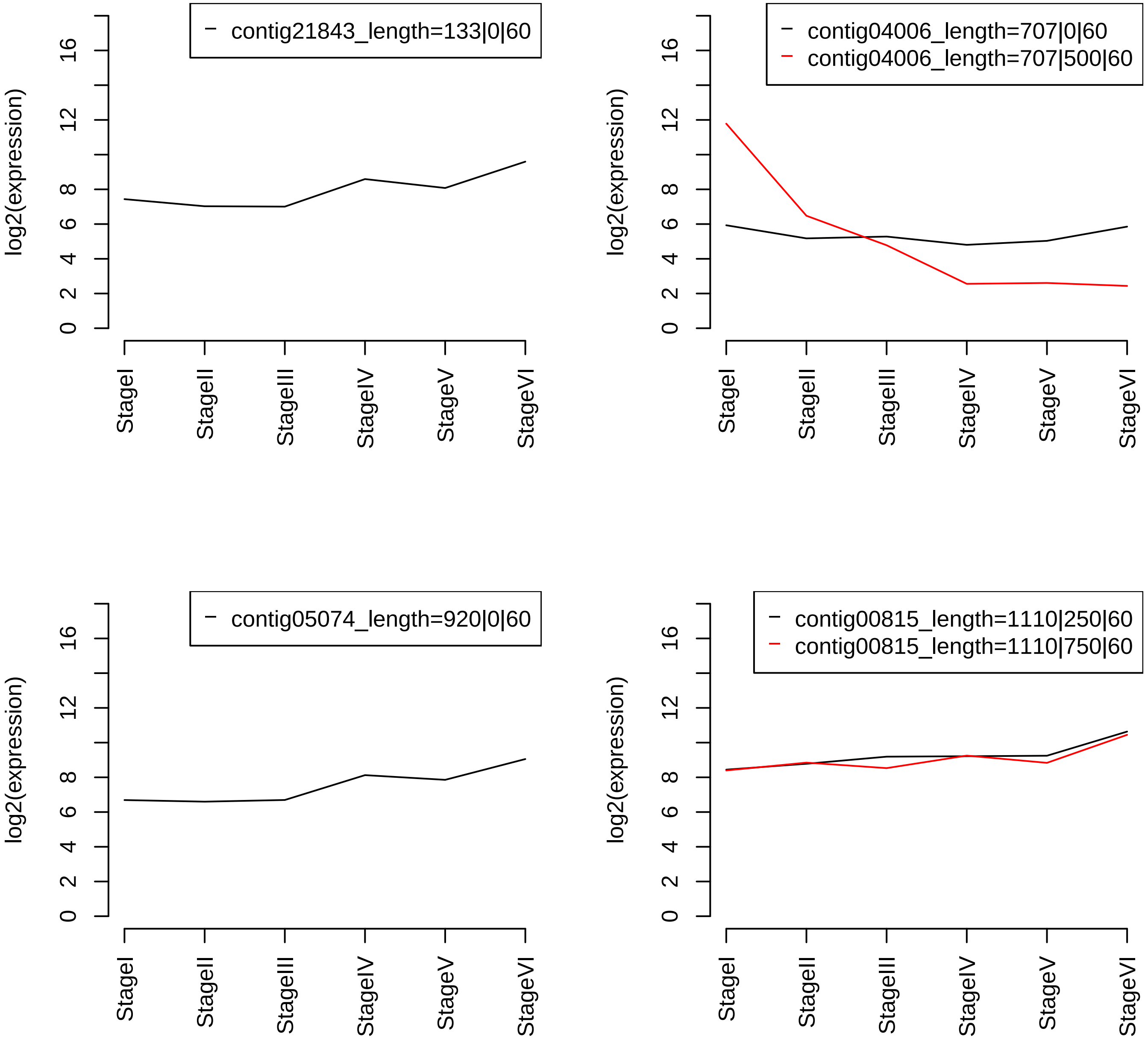

contig21843_length=133|0|60
contig04006_length=707|0|60
contig04006_length=707|500|60
contig05074_length=920|0|60
contig00815_length=1110|250|60
contig00815_length=1110|750|60
log2(expression)
0
2
4
6
8
12
16
StageI
StageII
StageIII
StageIV
StageV
StageVI

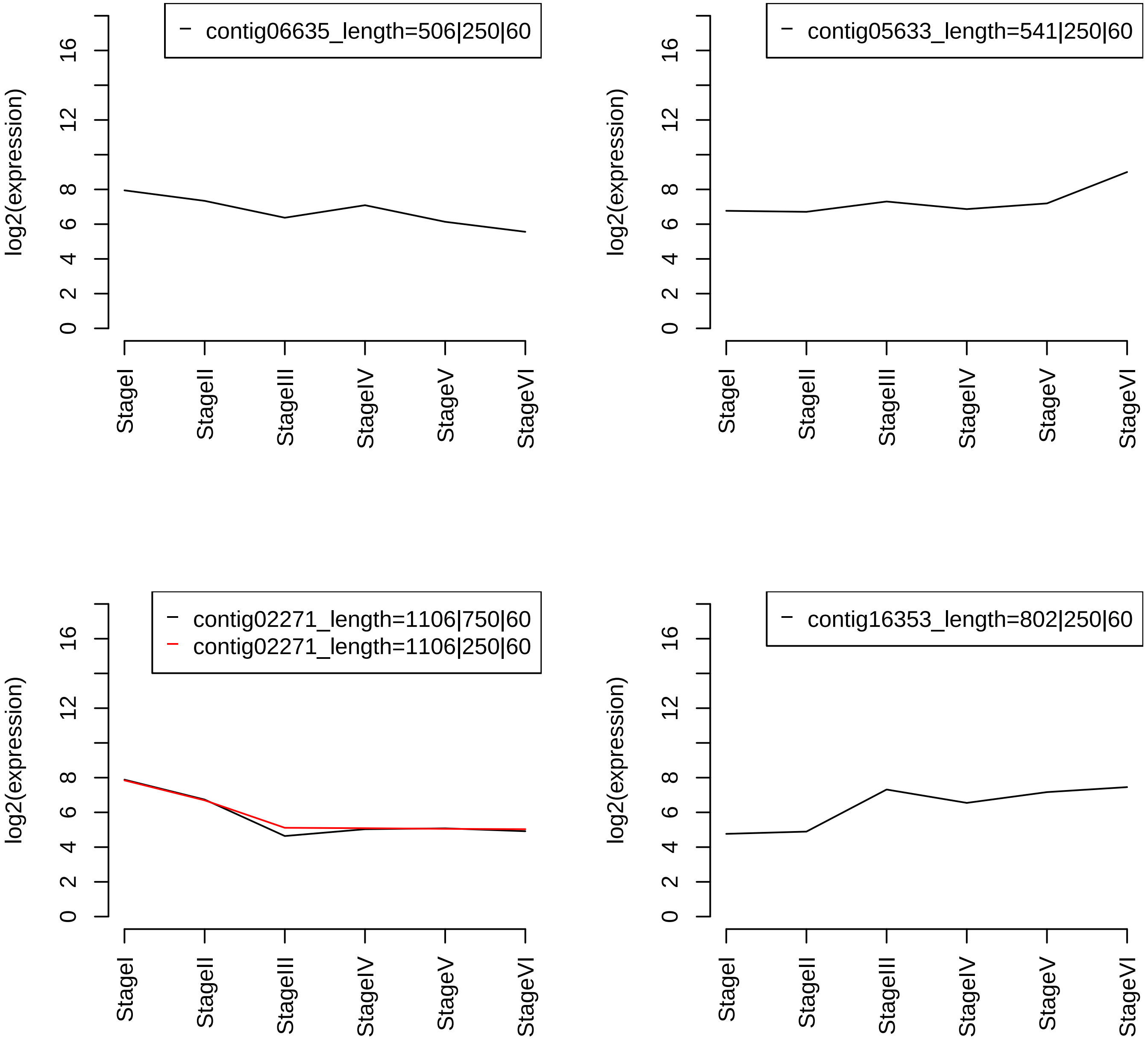

contig06635_length=506|250|60
contig05633_length=541|250|60
contig02271_length=1106|750|60
contig02271_length=1106|250|60
contig16353_length=802|250|60
log2(expression)
0 2 4 6 8 12 16
StageI
StageII
StageIII
StageIV
StageV
StageVI

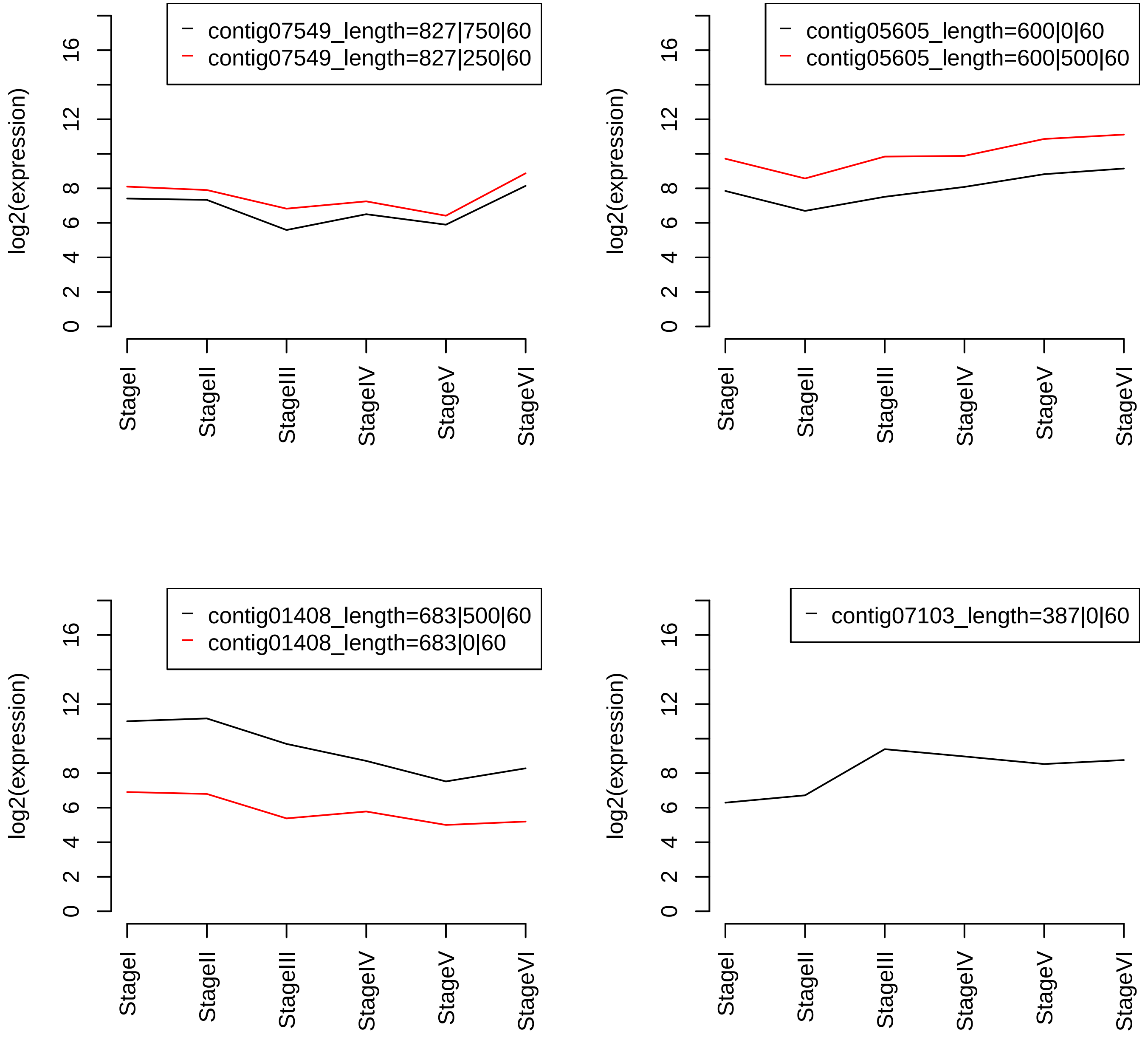

contig07549_length=827|750|60
contig07549_length=827|250|60
log2(expression)
0
2
4
6
8
12
16
StageI
StageII
StageIII
StageIV
StageV
StageVI
contig05605_length=600|0|60
contig05605_length=600|500|60
contig01408_length=683|500|60
contig01408_length=683|0|60
contig07103_length=387|0|60

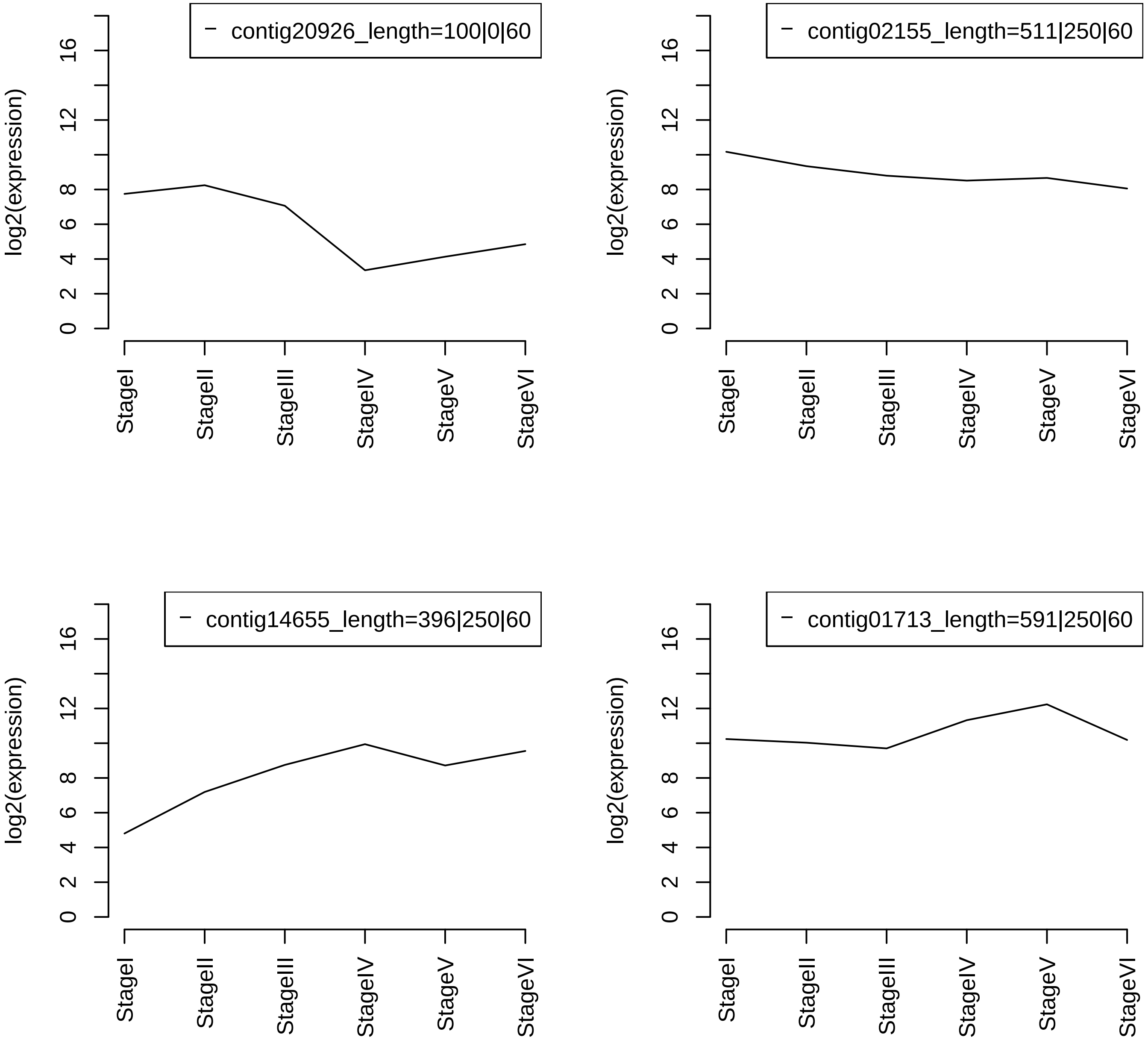
contig20926_length=100|0|60
contig02155_length=511|250|60
contig14655_length=396|250|60
contig01713_length=591|250|60
log2(expression)
0
2
4
6
8
12
16
StageI
StageII
StageIII
StageIV
StageV
StageVI

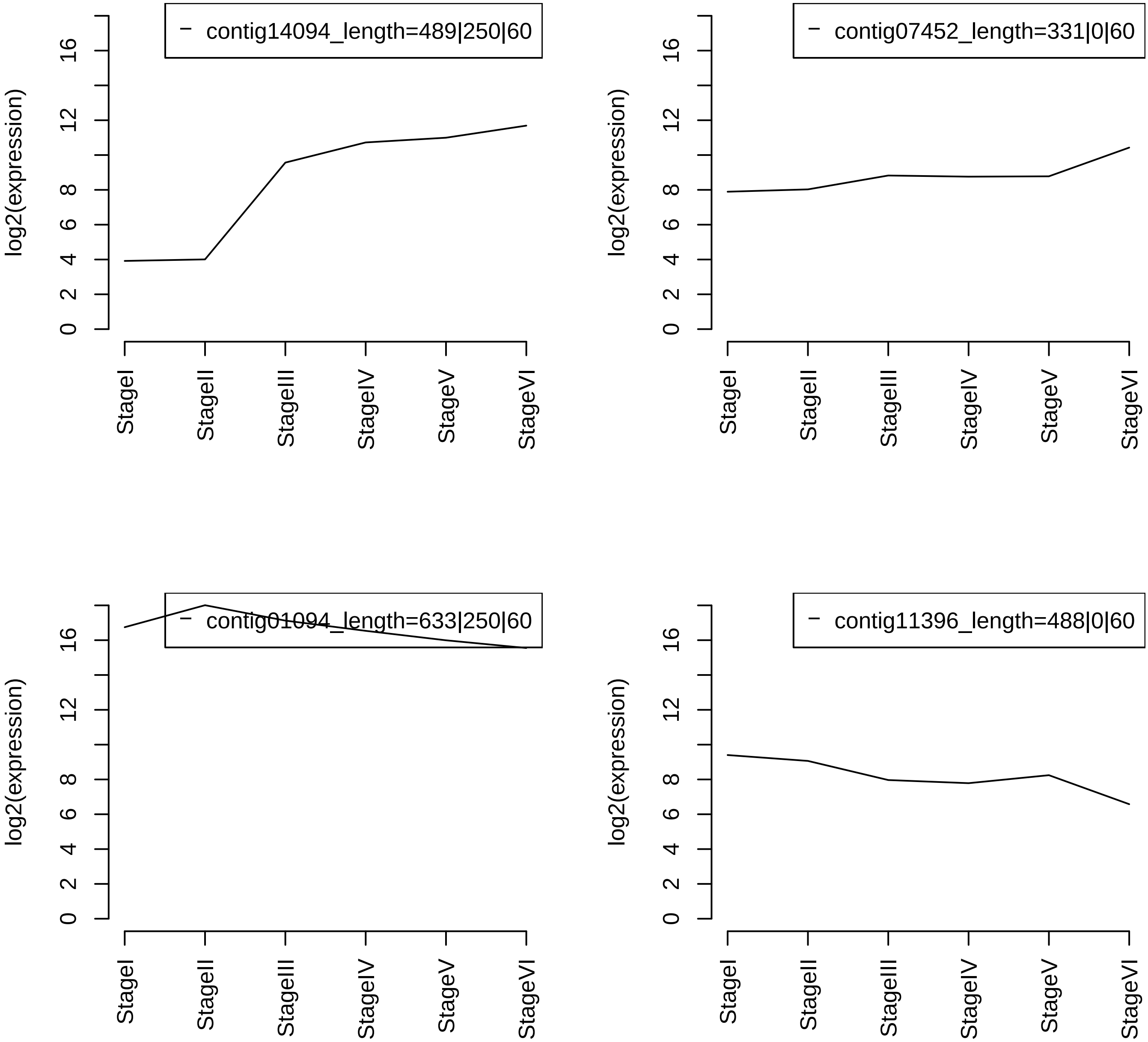

contig14094_length=489|250|60
contig07452_length=331|0|60
contig01094_length=633|250|60
contig11396_length=488|0|60
log2(expression)
0
2
4
6
8
12
16
StageI
StageII
StageIII
StageIV
StageV
StageVI

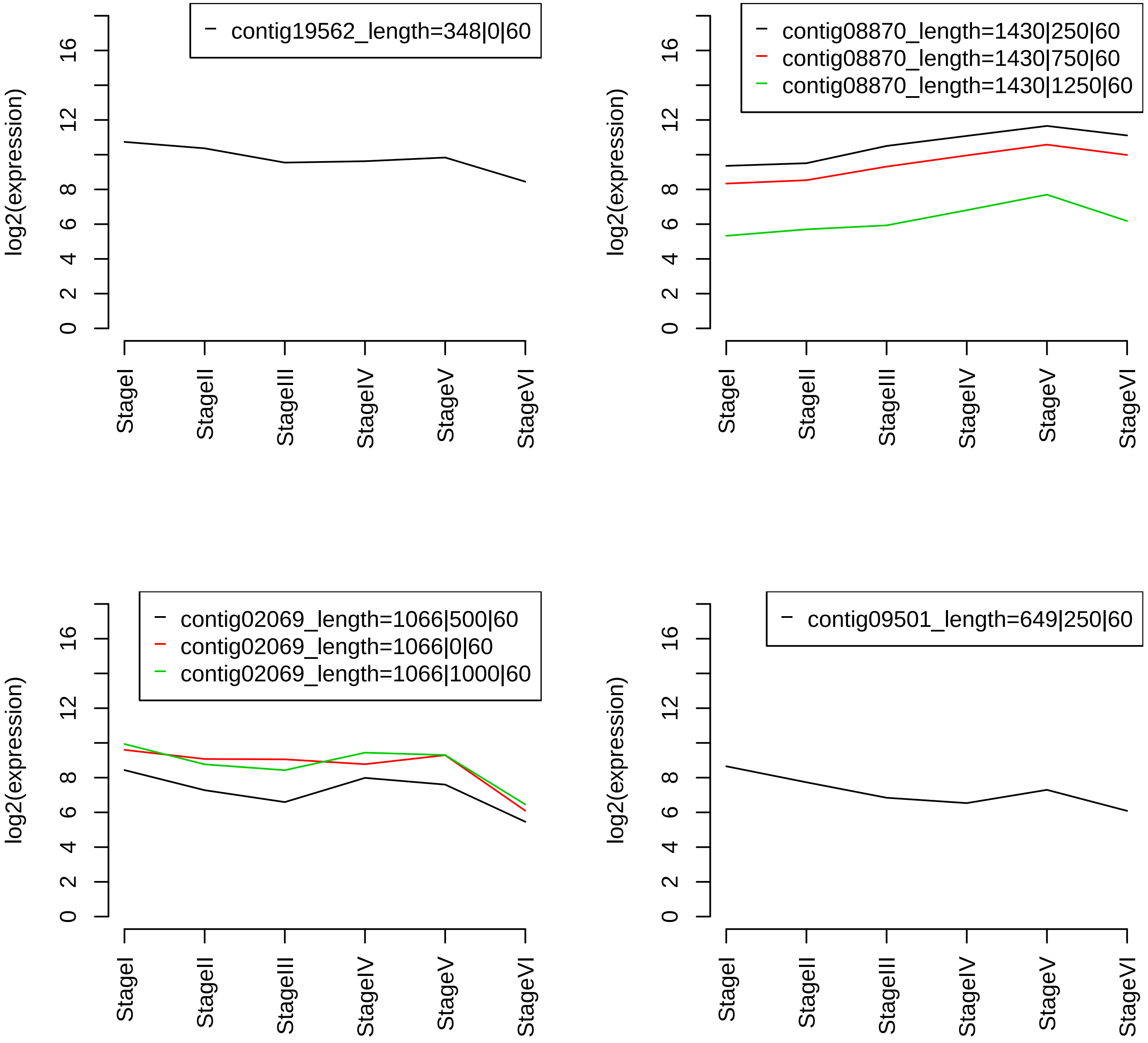

contig19562_length=348|0|60
contig08870_length=1430|250|60
contig08870_length=1430|750|60
contig08870_length=1430|1250|60
contig02069_length=1066|500|60
contig02069_length=1066|0|60
contig02069_length=1066|1000|60
contig09501_length=649|250|60
log2(expression)
0
2
4
6
8
12
16
StageI
StageII
StageIII
StageIV
StageV
StageVI

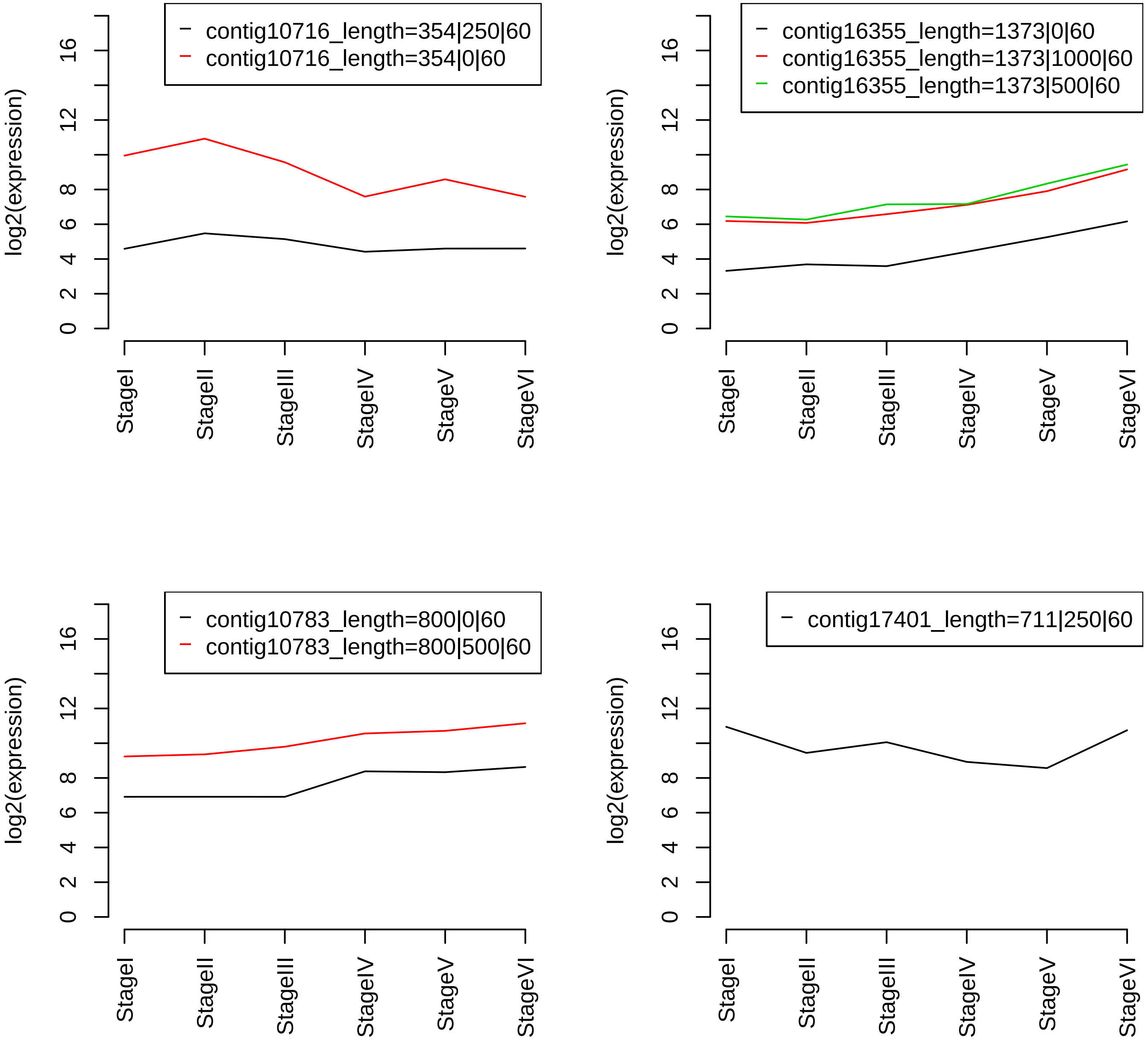

contig10716_length=354|250|60
contig10716_length=354|0|60
contig16355_length=1373|0|60
contig16355_length=1373|1000|60
contig16355_length=1373|500|60
contig10783_length=800|0|60
contig10783_length=800|500|60
contig17401_length=711|250|60
log2(expression)
StageI
StageII
StageIII
StageIV
StageV
StageVI

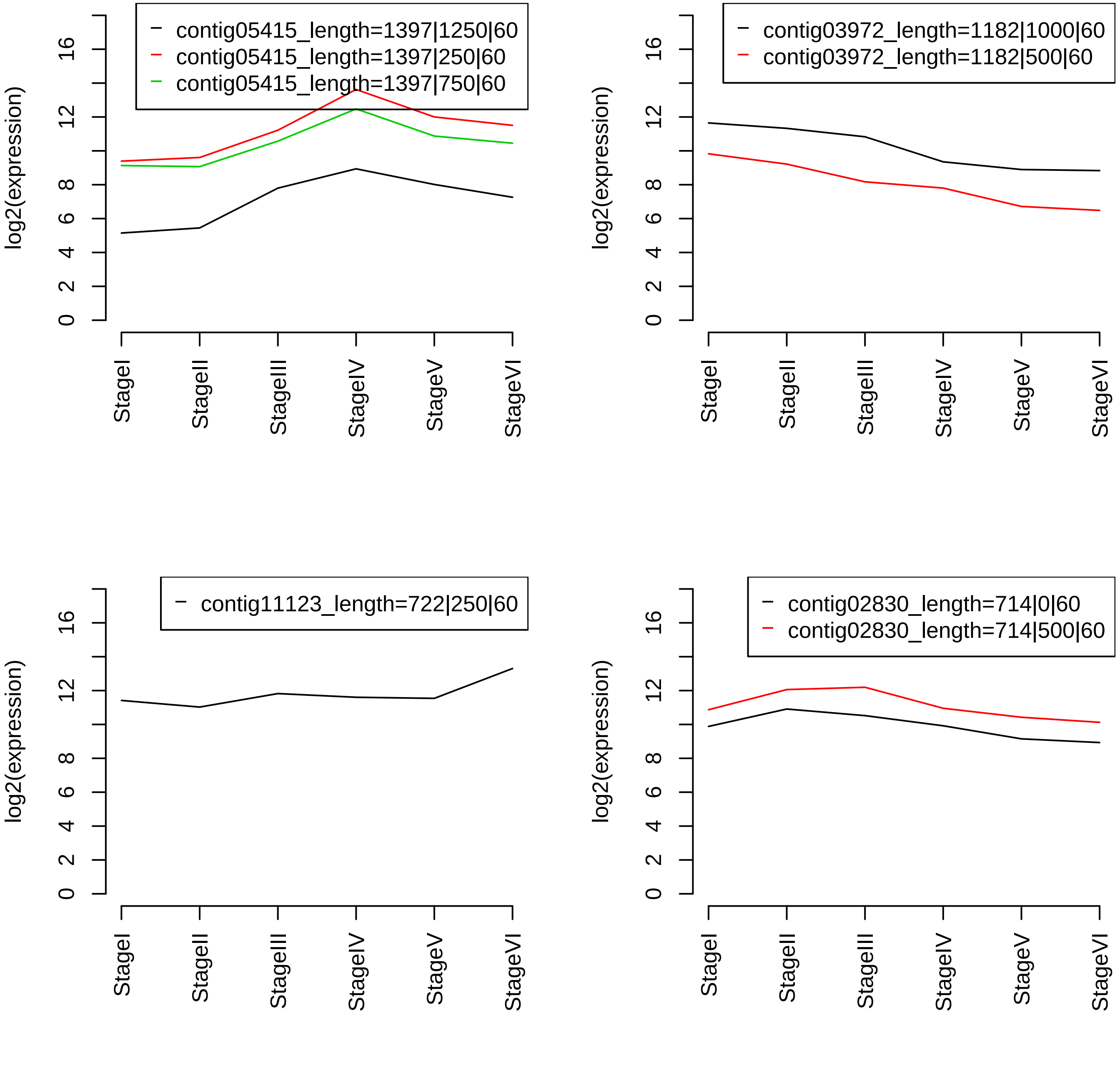

contig05415_length=1397|1250|60
contig05415_length=1397|250|60
contig05415_length=1397|750|60
log2(expression)
0
2
4
6
8
12
16
StageI
StageII
StageIII
StageIV
StageV
StageVI
contig03972_length=1182|1000|60
contig03972_length=1182|500|60
contig11123_length=722|250|60
contig02830_length=714|0|60
contig02830_length=714|500|60

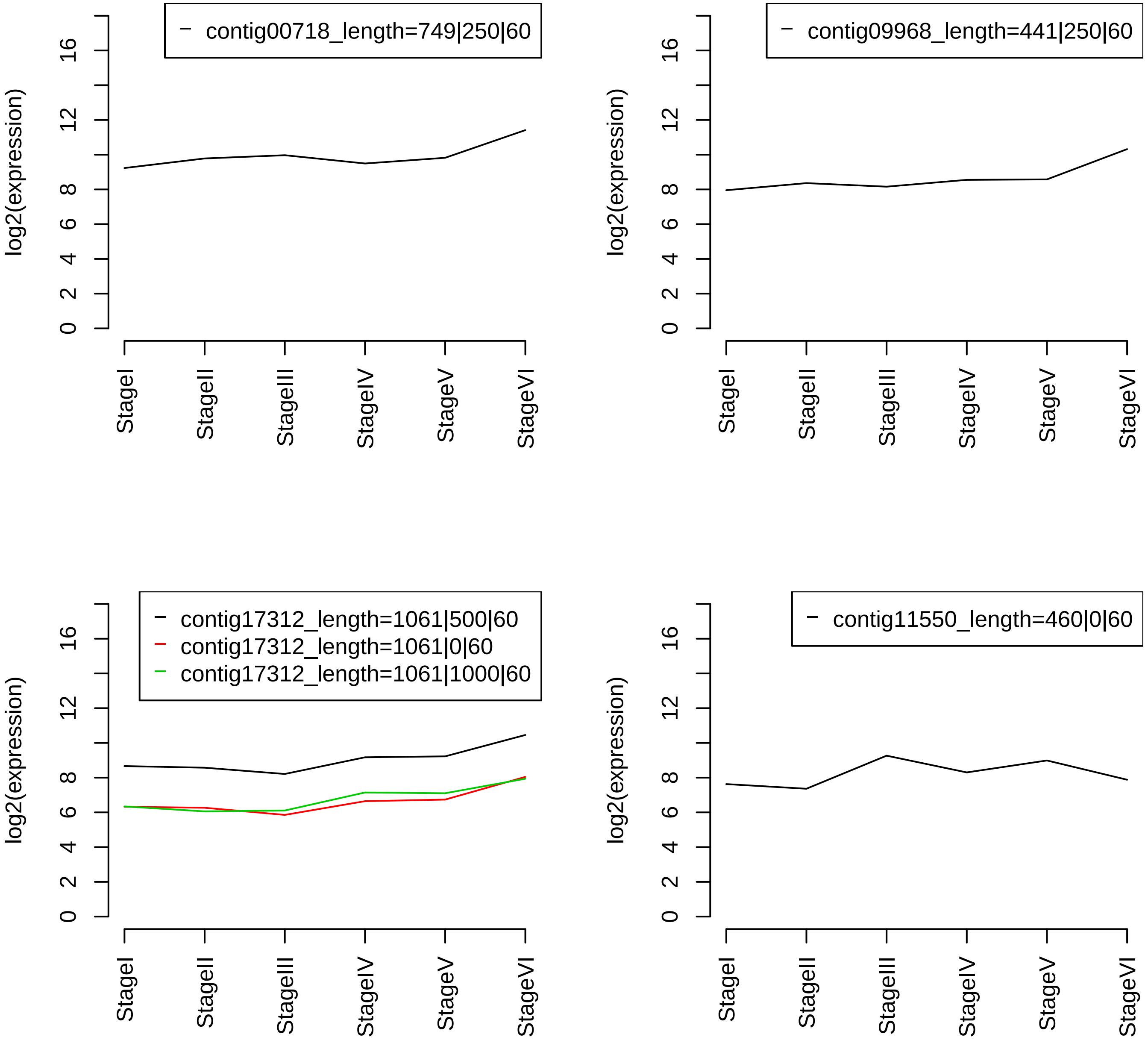
contig00718_length=749|250|60
log2(expression)
0
2
4
6
8
12
16
StageI
StageII
StageIII
StageIV
StageV
StageVI
contig09968_length=441|250|60
contig17312_length=1061|500|60
contig17312_length=1061|0|60
contig17312_length=1061|1000|60
contig11550_length=460|0|60

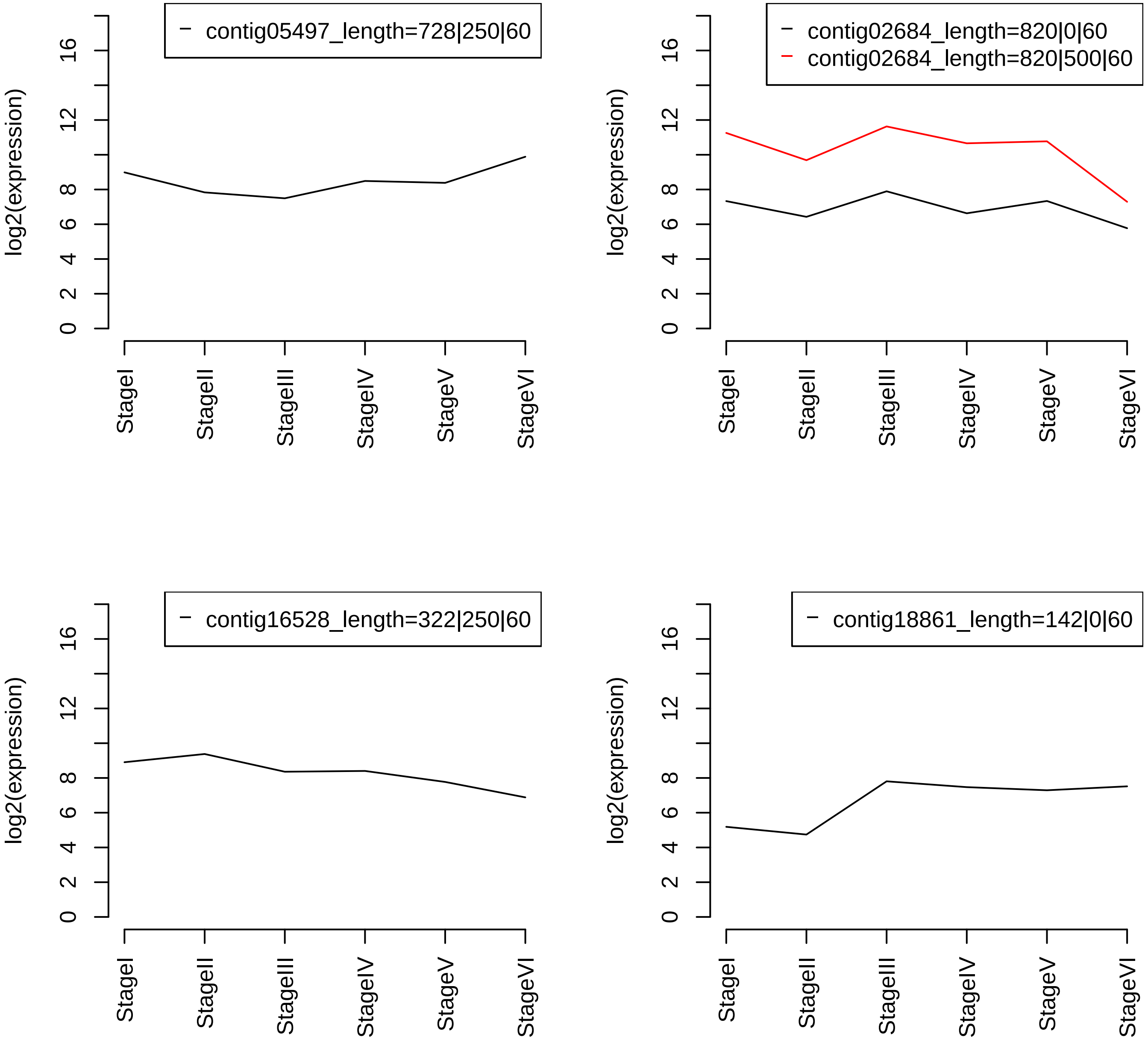

contig05497_length=728|250|60
log2(expression)
0
2
4
6
8
12
16
StageI
StageII
StageIII
StageIV
StageV
StageVI
contig02684_length=820|0|60
contig02684_length=820|500|60
log2(expression)
0
2
4
6
8
12
16
StageI
StageII
StageIII
StageIV
StageV
StageVI
contig16528_length=322|250|60
log2(expression)
0
2
4
6
8
12
16
StageI
StageII
StageIII
StageIV
StageV
StageVI
contig18861_length=142|0|60
log2(expression)
0
2
4
6
8
12
16
StageI
StageII
StageIII
StageIV
StageV
StageVI

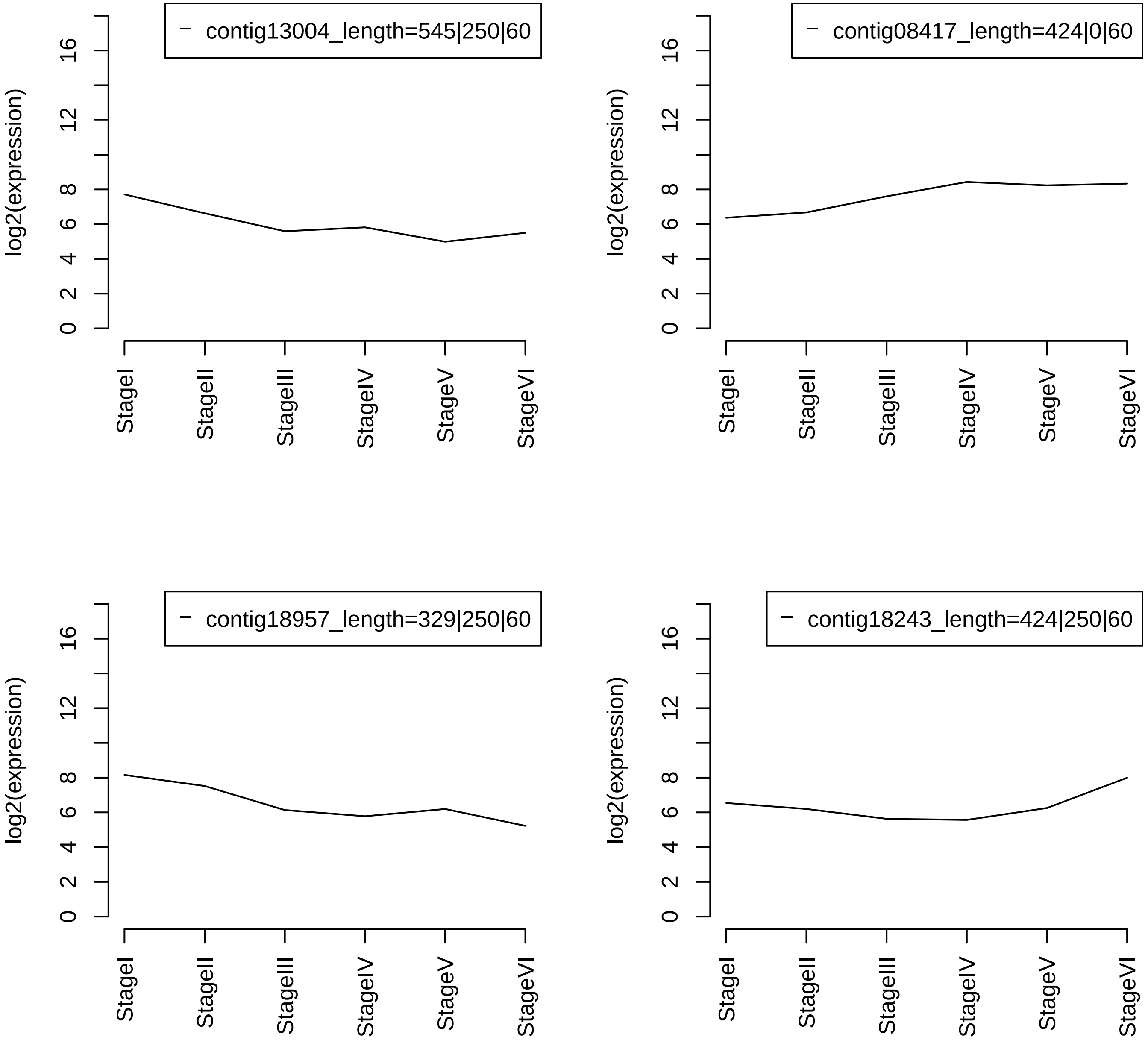
contig13004_length=545|250|60
contig08417_length=424|0|60
contig18957_length=329|250|60
contig18243_length=424|250|60
log2(expression)
0
2
4
6
8
12
16
StageI
StageII
StageIII
StageIV
StageV
StageVI

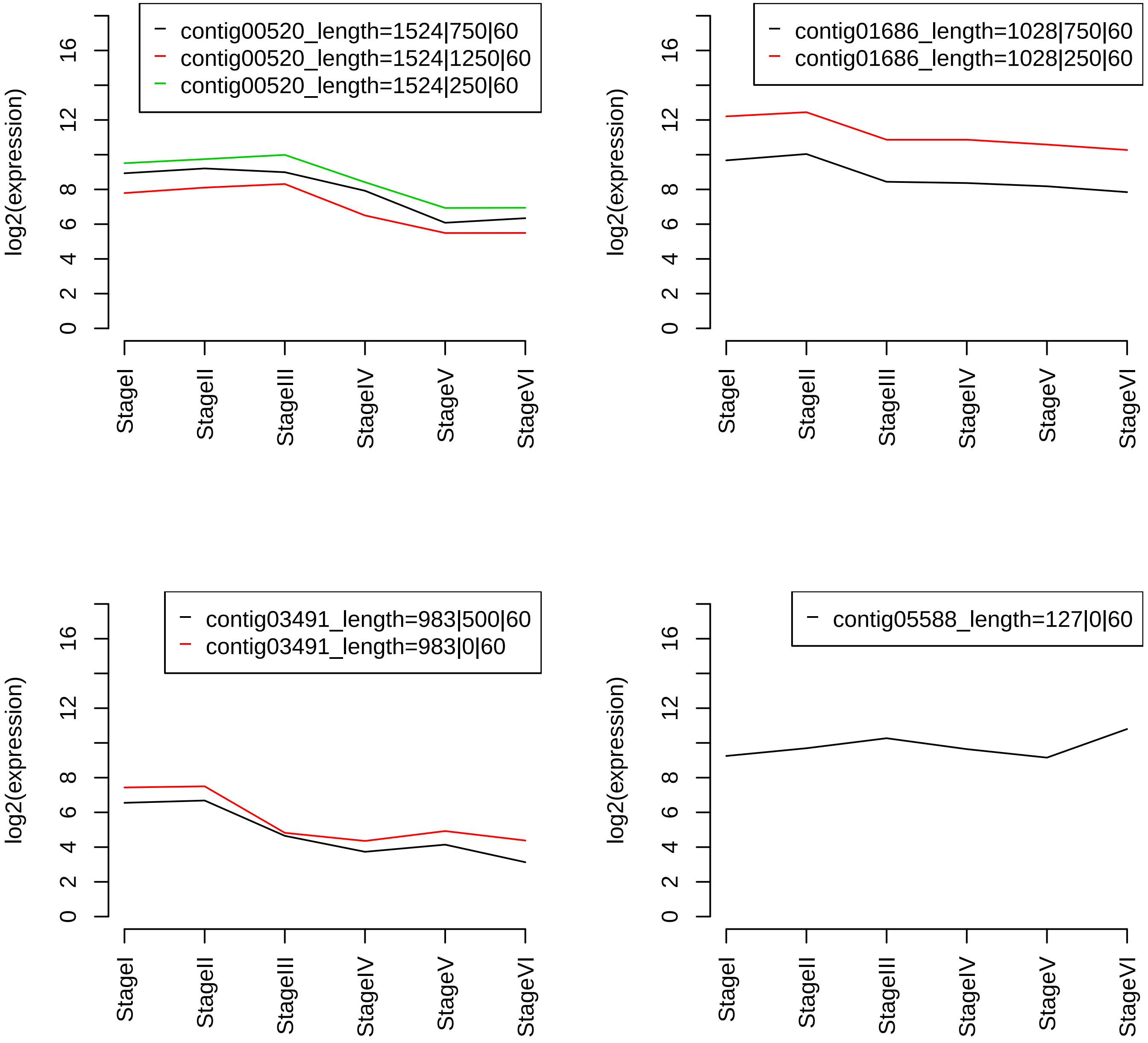

contig00520_length=1524|750|60
contig00520_length=1524|1250|60
contig00520_length=1524|250|60
contig01686_length=1028|750|60
contig01686_length=1028|250|60
contig03491_length=983|500|60
contig03491_length=983|0|60
contig05588_length=127|0|60
log2(expression)
0
2
4
6
8
12
16
StageI
StageII
StageIII
StageIV
StageV
StageVI

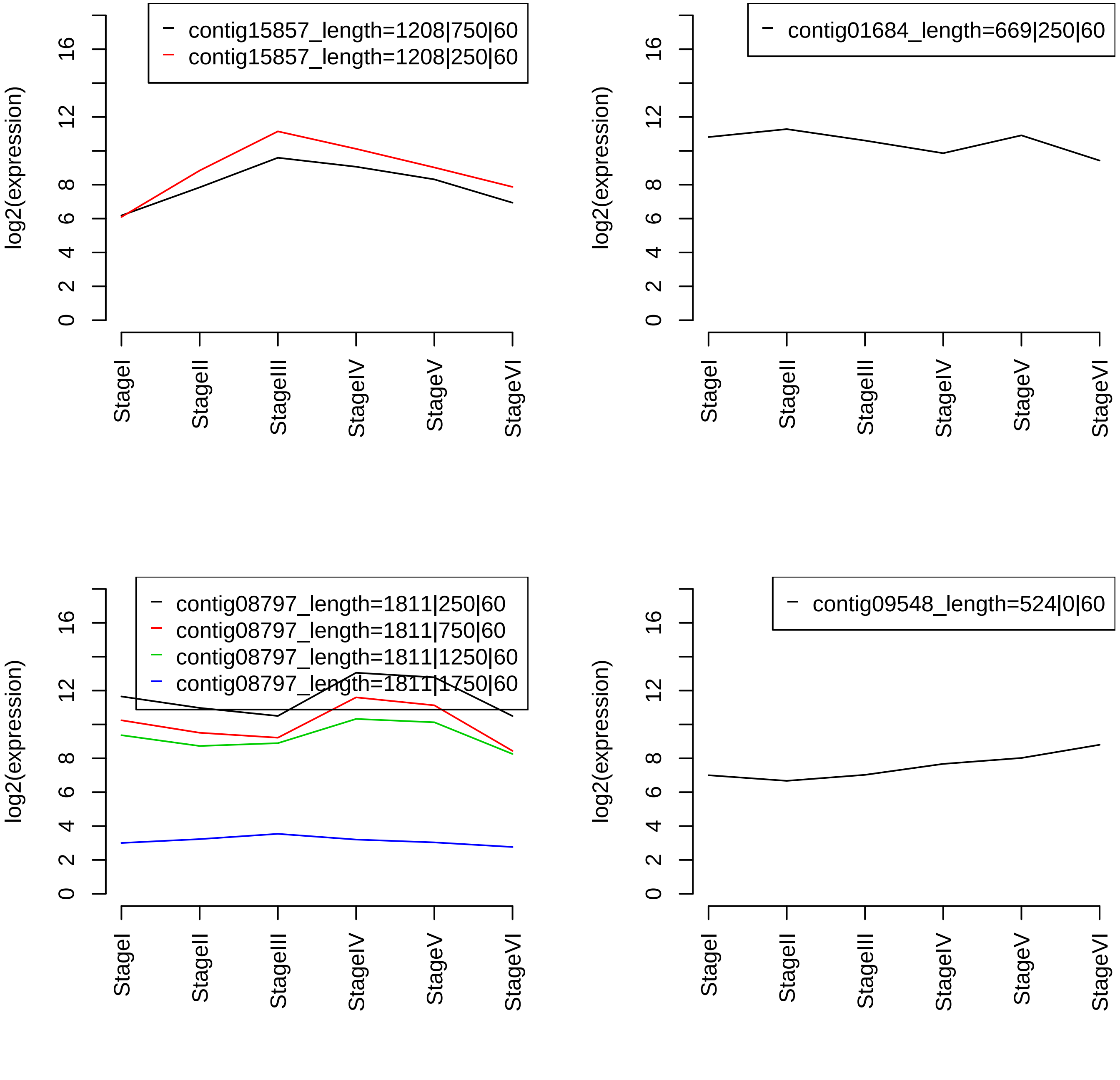
contig15857_length=1208|750|60
contig15857_length=1208|250|60
contig01684_length=669|250|60
contig08797_length=1811|250|60
contig08797_length=1811|750|60
contig08797_length=1811|1250|60
contig08797_length=1811|1750|60
contig09548_length=524|0|60
log2(expression)
0
2
4
6
8
12
16
StageI
StageII
StageIII
StageIV
StageV
StageVI

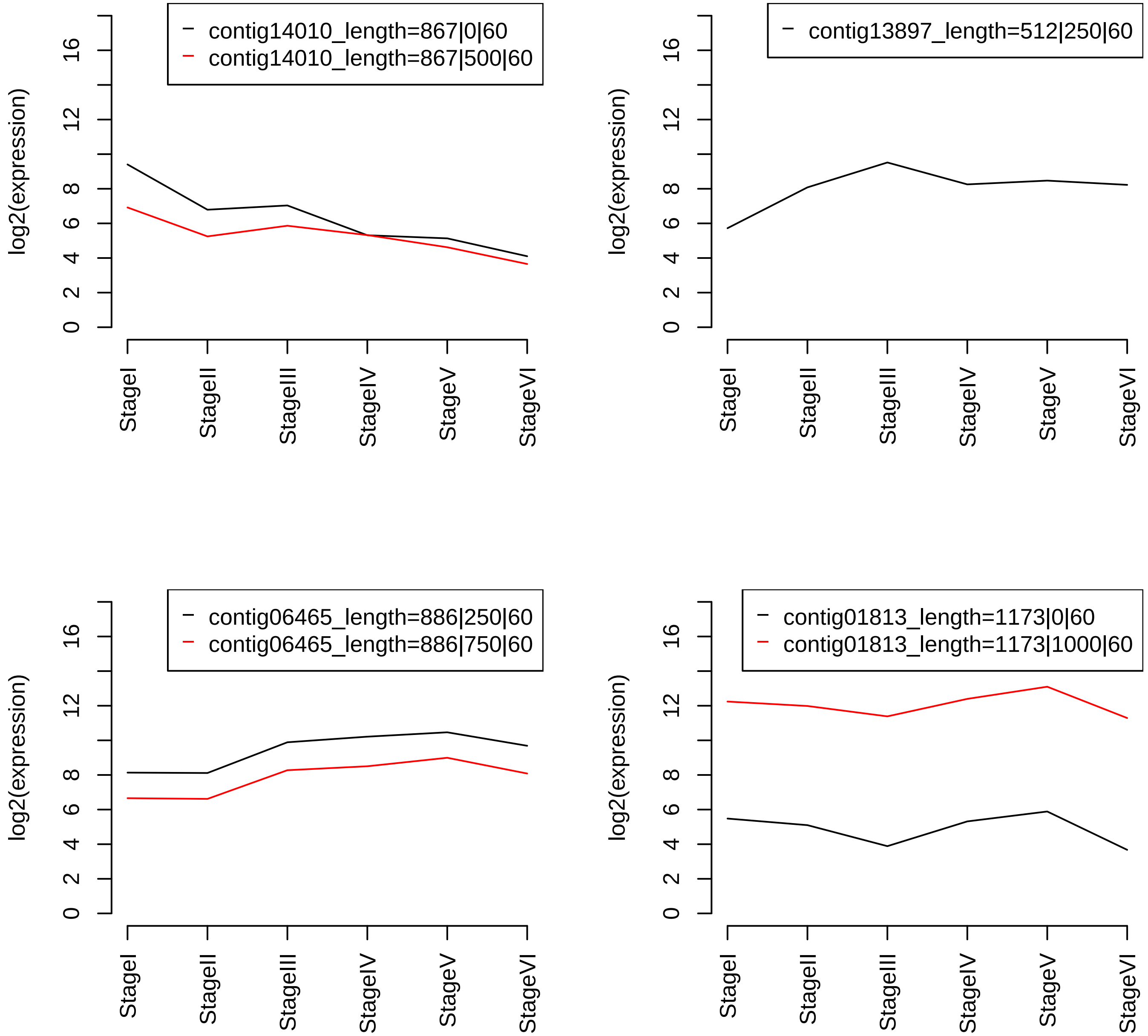
contig14010_length=867|0|60
contig14010_length=867|500|60
contig13897_length=512|250|60
contig06465_length=886|250|60
contig06465_length=886|750|60
contig01813_length=1173|0|60
contig01813_length=1173|1000|60
log2(expression)
0
2
4
6
8
12
16
StageI
StageII
StageIII
StageIV
StageV
StageVI

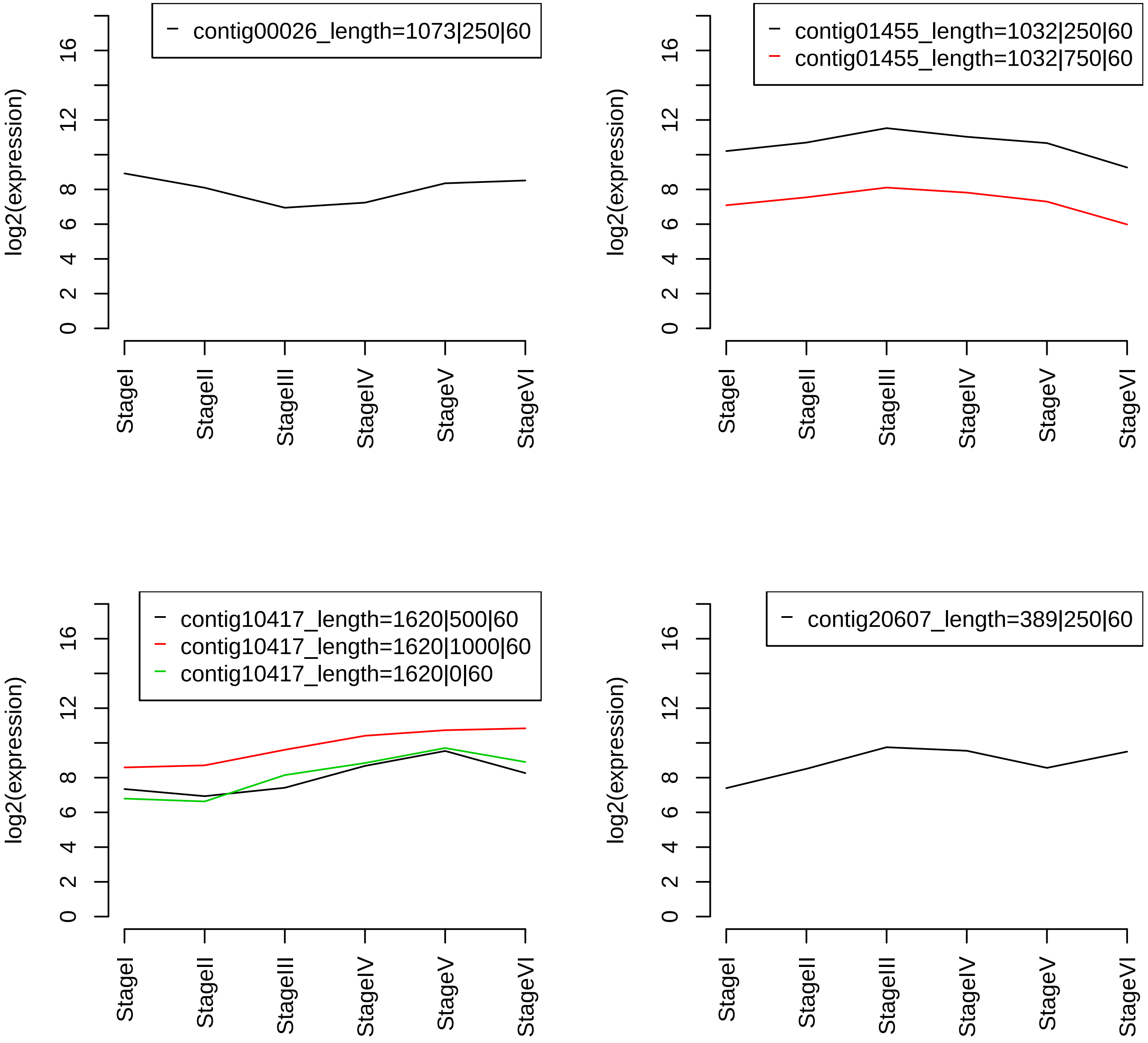
contig00026_length=1073|250|60
contig01455_length=1032|250|60
contig01455_length=1032|750|60
contig10417_length=1620|500|60
contig10417_length=1620|1000|60
contig10417_length=1620|0|60
contig20607_length=389|250|60
log2(expression)
0
2
4
6
8
12
16
StageI
StageII
StageIII
StageIV
StageV
StageVI

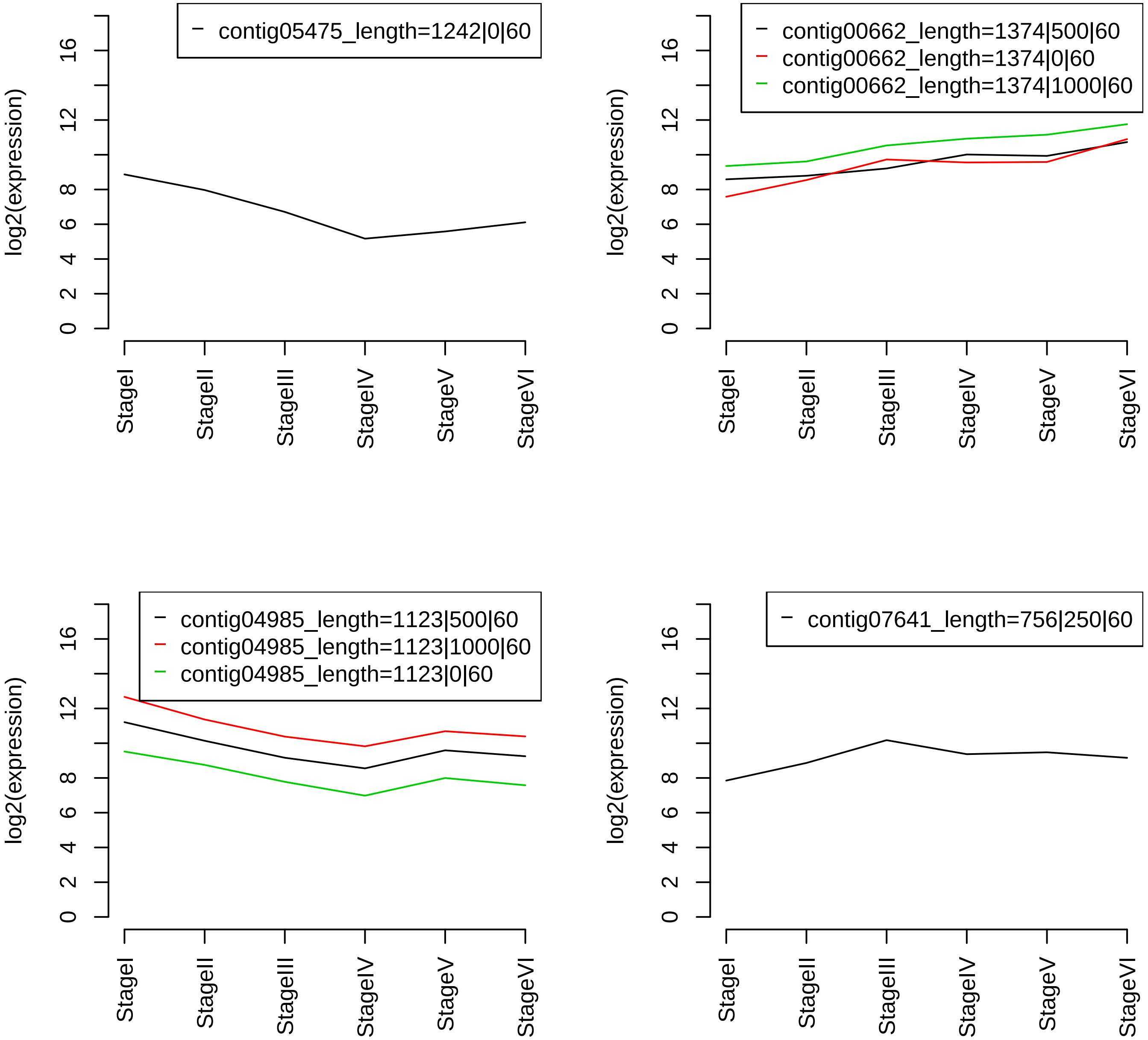
contig05475_length=1242|0|60
contig00662_length=1374|500|60
contig00662_length=1374|0|60
contig00662_length=1374|1000|60
contig04985_length=1123|500|60
contig04985_length=1123|1000|60
contig04985_length=1123|0|60
contig07641_length=756|250|60
log2(expression)
0
2
4
6
8
12
16
StageI
StageII
StageIII
StageIV
StageV
StageVI

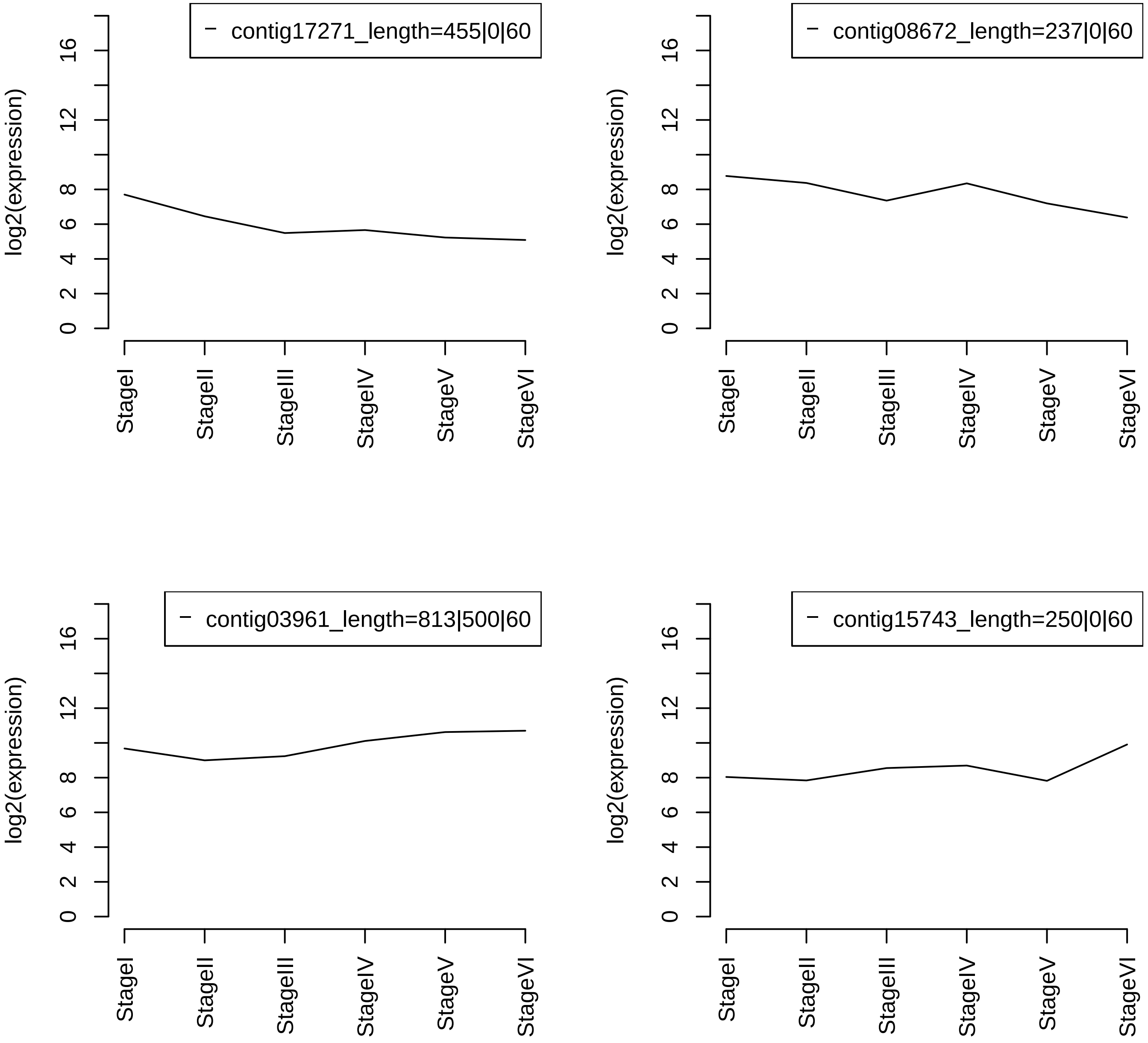
contig17271_length=455|0|60
contig08672_length=237|0|60
contig03961_length=813|500|60
contig15743_length=250|0|60
log2(expression)
0
2
4
6
8
12
16
StageI
StageII
StageIII
StageIV
StageV
StageVI

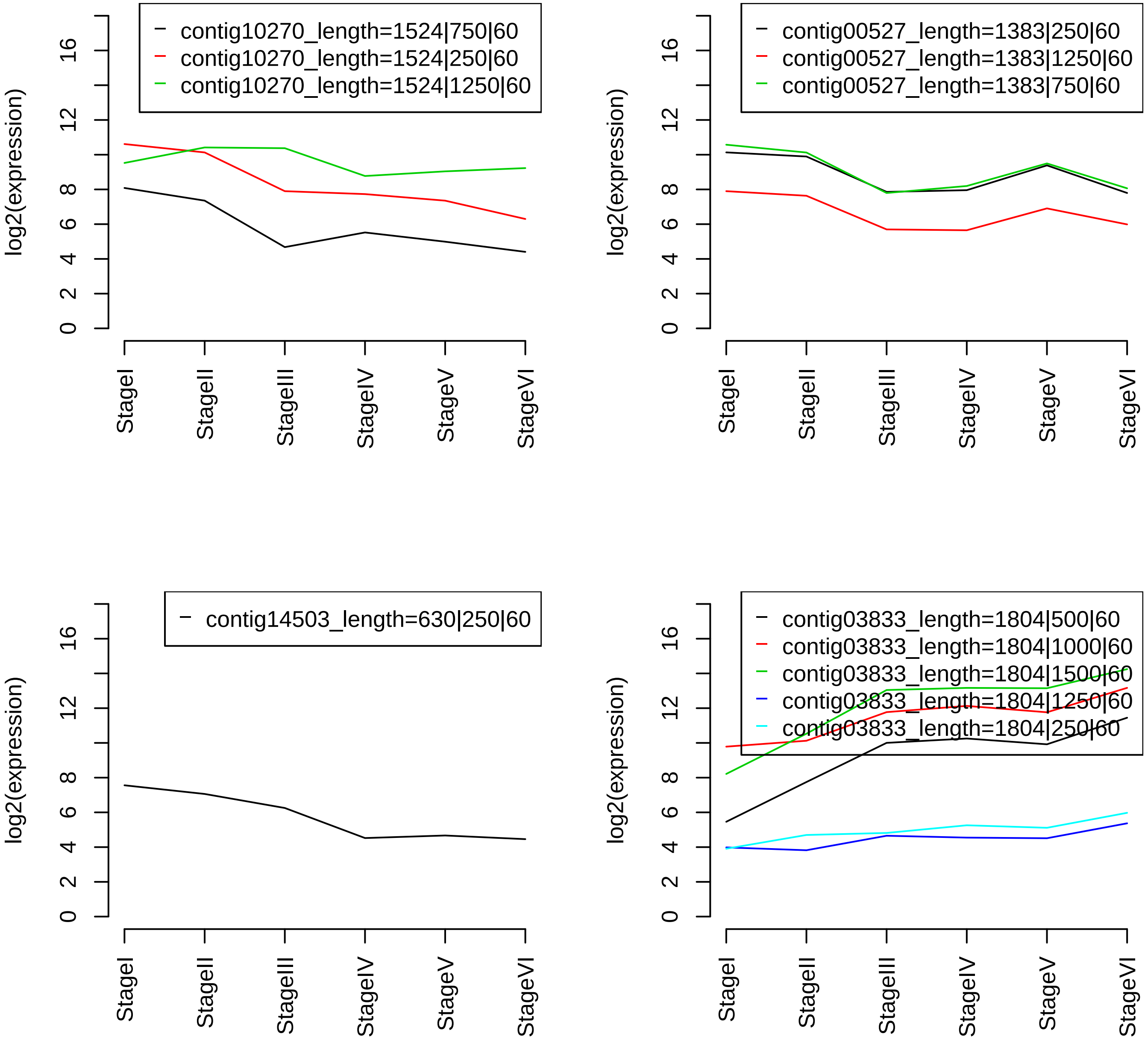

contig10270_length=1524|750|60
contig10270_length=1524|250|60
contig10270_length=1524|1250|60
log2(expression)
0 2 4 6 8 12 16
StageI StageII StageIII StageIV StageV StageVI
contig00527_length=1383|250|60
contig00527_length=1383|1250|60
contig00527_length=1383|750|60
log2(expression)
0 2 4 6 8 12 16
StageI StageII StageIII StageIV StageV StageVI
contig14503_length=630|250|60
log2(expression)
0 2 4 6 8 12 16
StageI StageII StageIII StageIV StageV StageVI
contig03833_length=1804|500|60
contig03833_length=1804|1000|60
contig03833_length=1804|1500|60
contig03833_length=1804|1250|60
contig03833_length=1804|250|60
log2(expression)
0 2 4 6 8 12 16
StageI StageII StageIII StageIV StageV StageVI

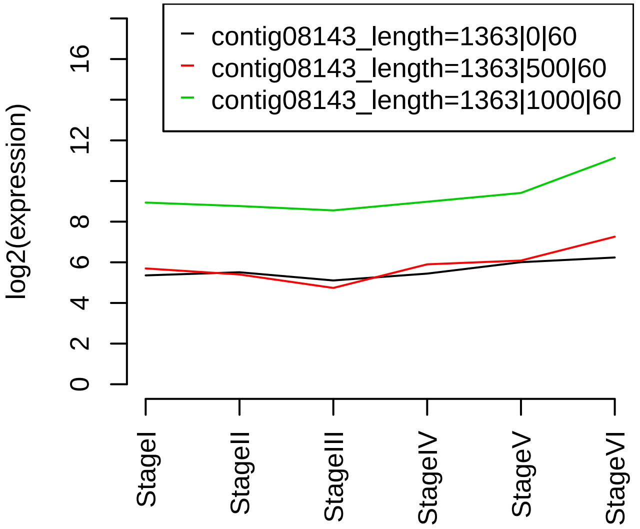
contig08143_length=1363|0|60
contig08143_length=1363|500|60
contig08143_length=1363|1000|60
log2(expression)
0
2
4
6
8
12
16
StageI
StageII
StageIII
StageIV
StageV
StageVI

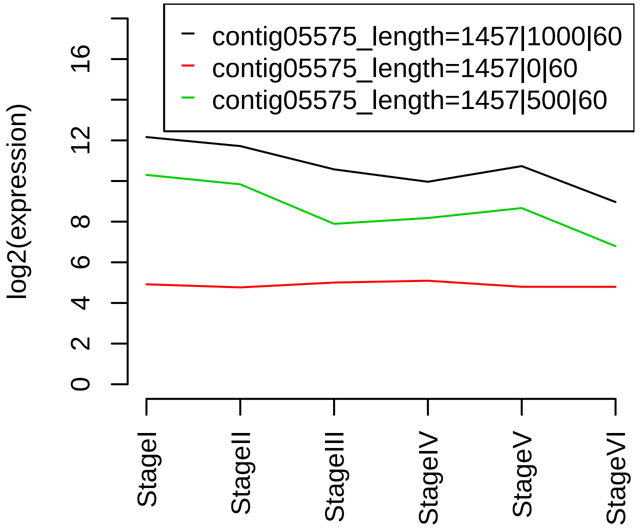
contig05575_length=1457|1000|60
contig05575_length=1457|0|60
contig05575_length=1457|500|60
log2(expression)
0
2
4
6
8
12
16
StageI
StageII
StageIII
StageIV
StageV
StageVI

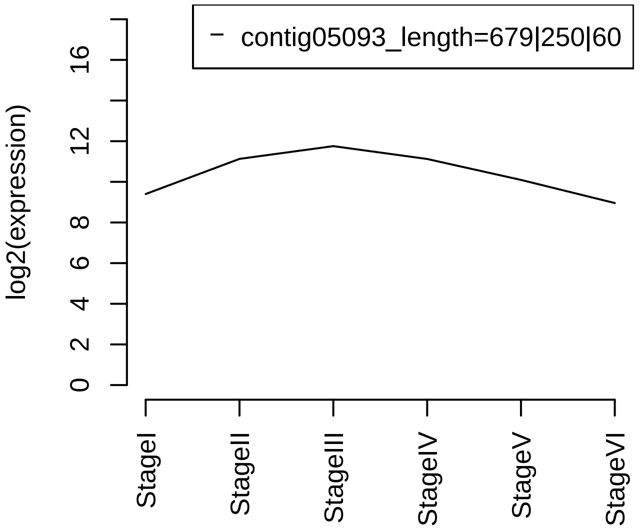
contig05093_length=679|250|60
log2(expression)
0
2
4
6
8
12
16
StageI
StageII
StageIII
StageIV
StageV
StageVI

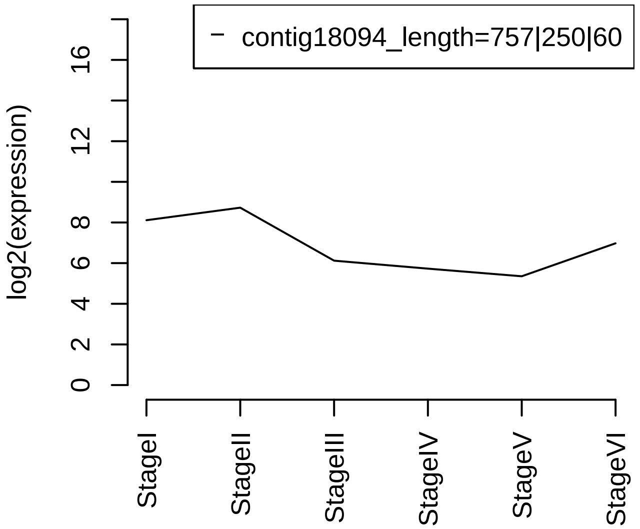
contig18094_length=757|250|60
log2(expression)
0
2
4
6
8
12
16
StageI
StageII
StageIII
StageIV
StageV
StageVI

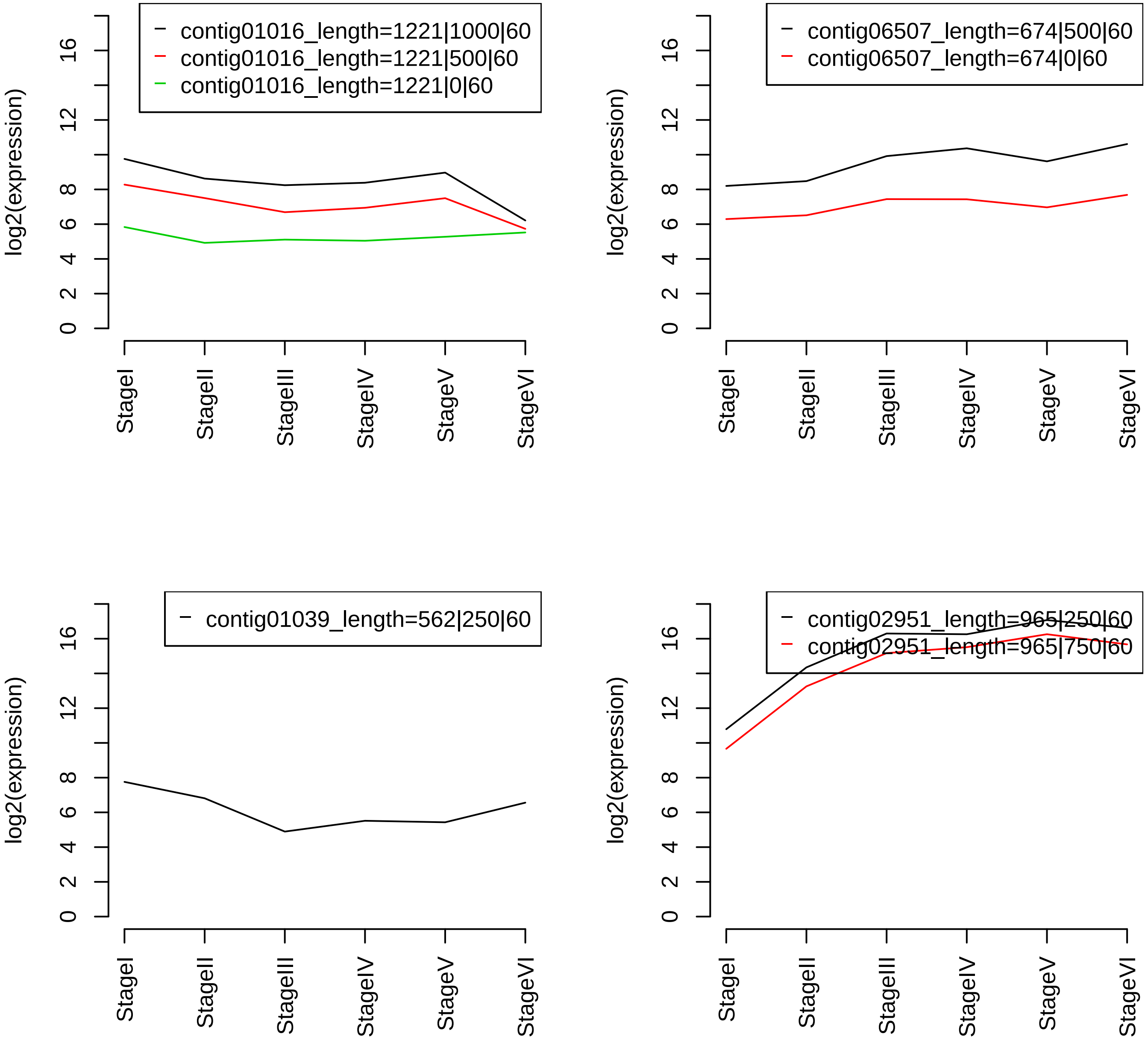
contig01016_length=1221|1000|60
contig01016_length=1221|500|60
contig01016_length=1221|0|60
log2(expression)
0
2
4
6
8
12
16
StageI
StageII
StageIII
StageIV
StageV
StageVI
contig06507_length=674|500|60
contig06507_length=674|0|60
contig01039_length=562|250|60
contig02951_length=965|250|60
contig02951_length=965|750|60

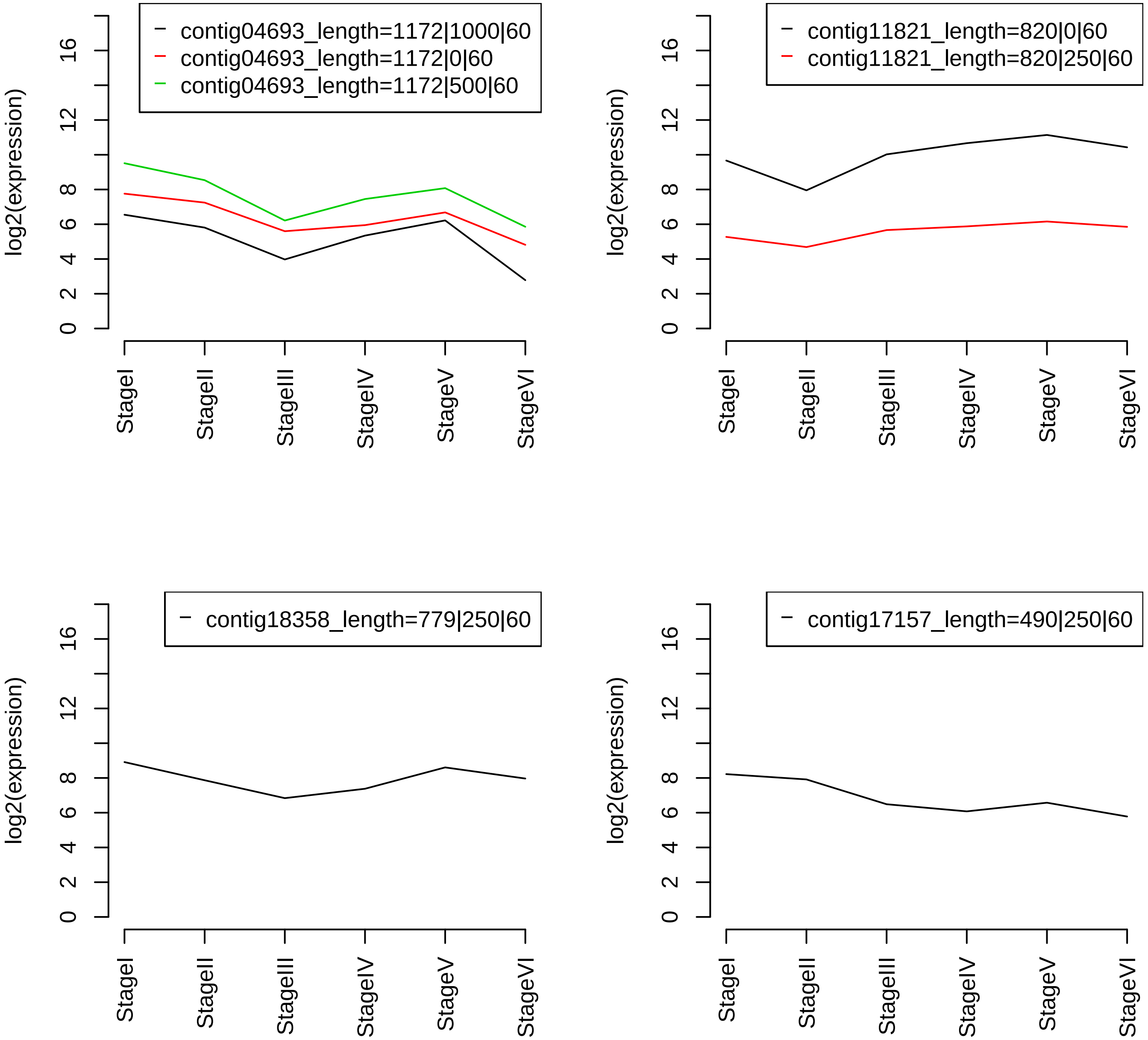

contig04693_length=1172|1000|60
contig04693_length=1172|0|60
contig04693_length=1172|500|60
contig11821_length=820|0|60
contig11821_length=820|250|60
contig18358_length=779|250|60
contig17157_length=490|250|60
log2(expression)
0
2
4
6
8
12
16
StageI
StageII
StageIII
StageIV
StageV
StageVI

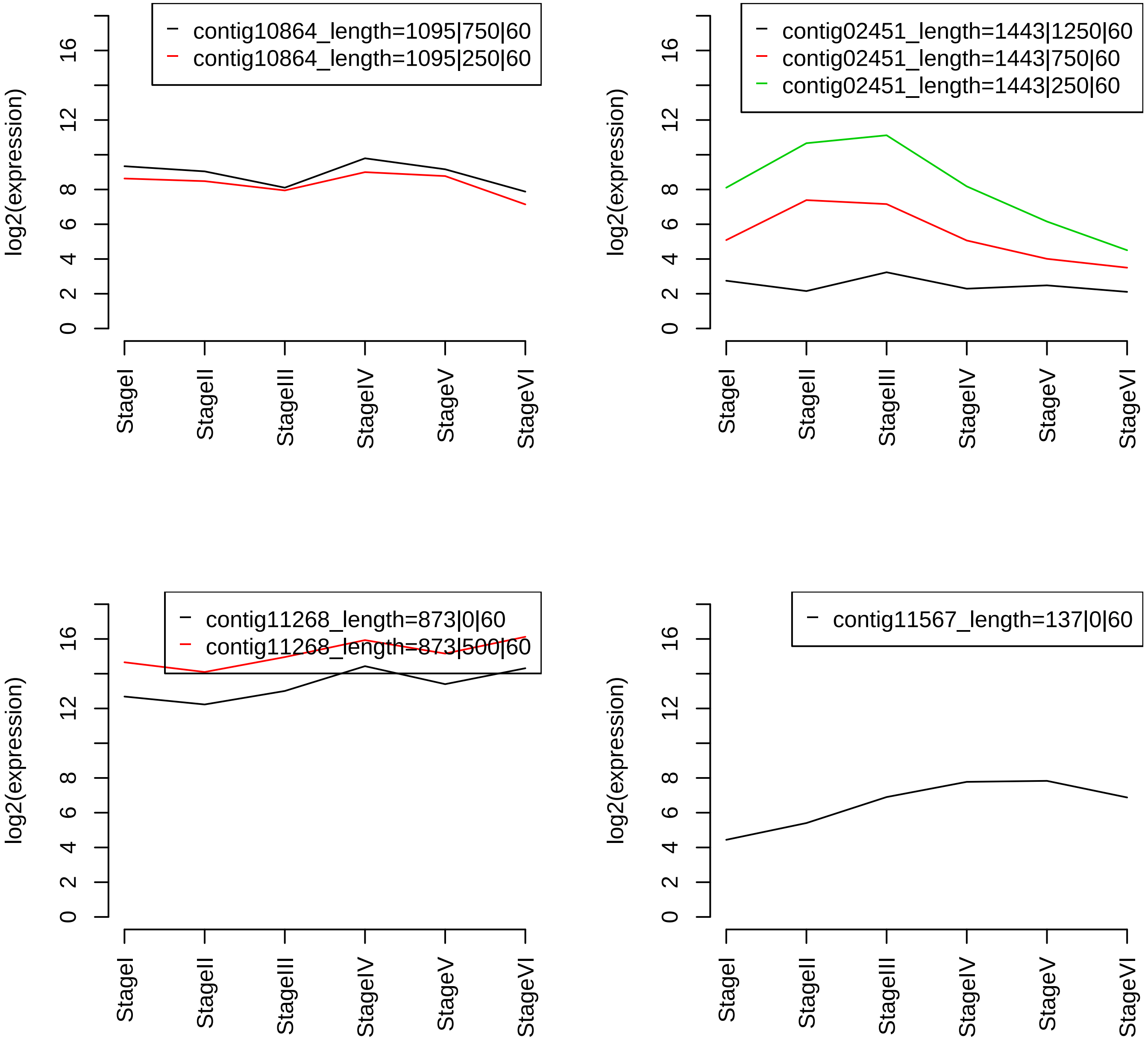

contig10864_length=1095|750|60
contig10864_length=1095|250|60
contig02451_length=1443|1250|60
contig02451_length=1443|750|60
contig02451_length=1443|250|60
contig11268_length=873|0|60
contig11268_length=873|500|60
contig11567_length=137|0|60
log2(expression)
StageI
StageII
StageIII
StageIV
StageV
StageVI

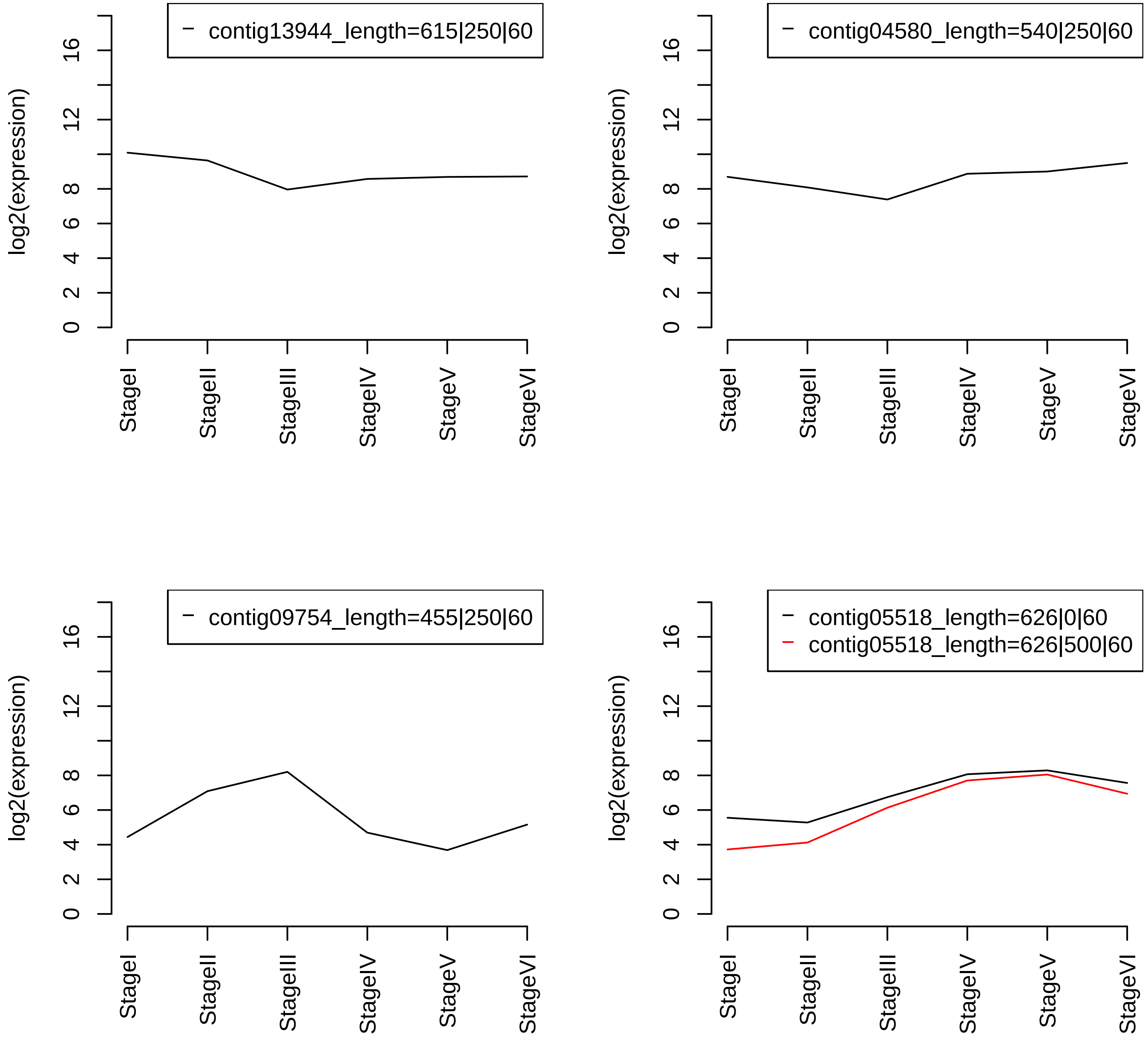

contig13944_length=615|250|60
contig04580_length=540|250|60
contig09754_length=455|250|60
contig05518_length=626|0|60
contig05518_length=626|500|60
log2(expression)
0 2 4 6 8 12 16
StageI StageII StageIII StageIV StageV StageVI

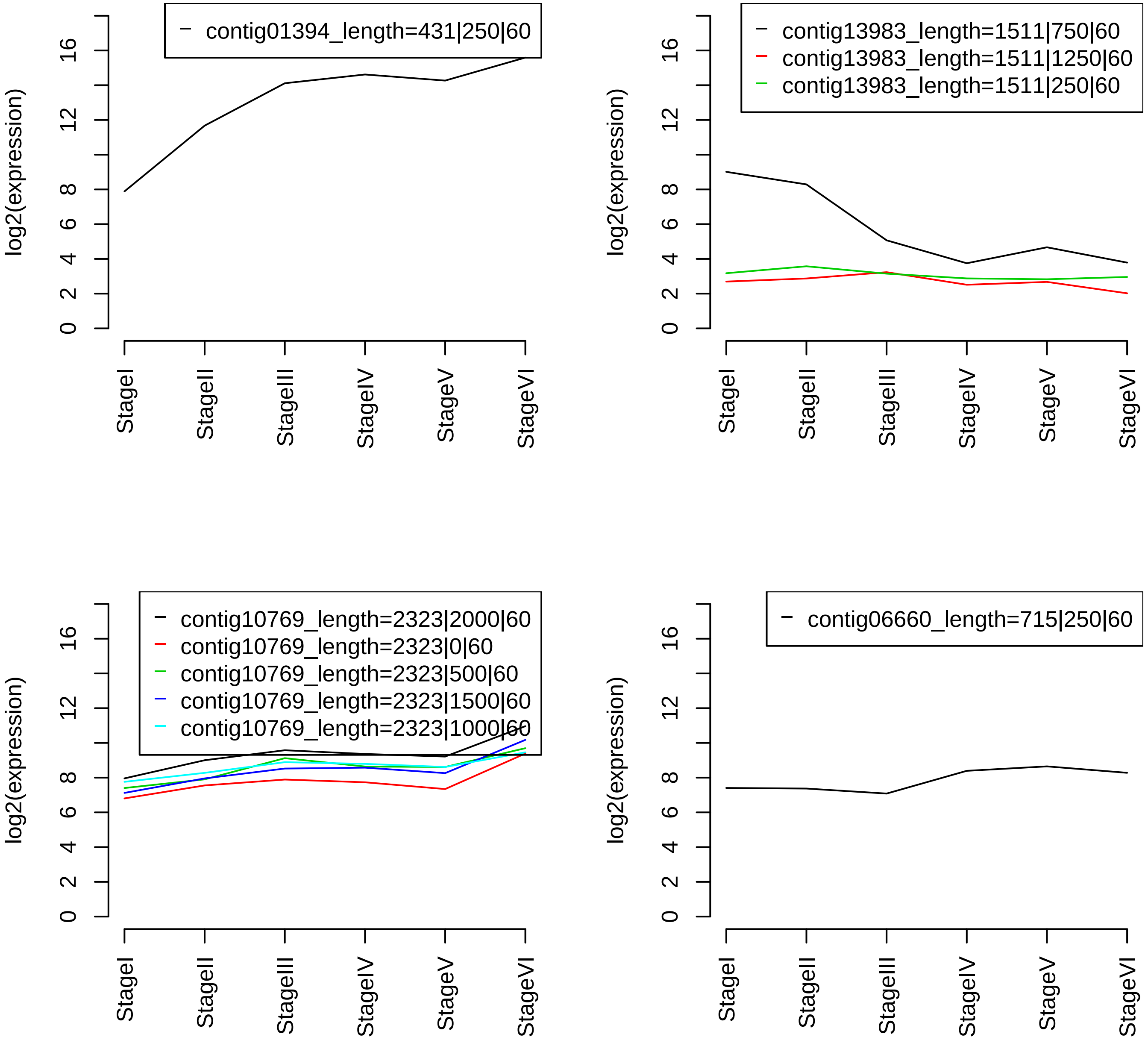

contig01394_length=431|250|60
contig13983_length=1511|750|60
contig13983_length=1511|1250|60
contig13983_length=1511|250|60
contig10769_length=2323|2000|60
contig10769_length=2323|0|60
contig10769_length=2323|500|60
contig10769_length=2323|1500|60
contig10769_length=2323|1000|60
contig06660_length=715|250|60
log2(expression)
0 2 4 6 8 12 16
StageI StageII StageIII StageIV StageV StageVI

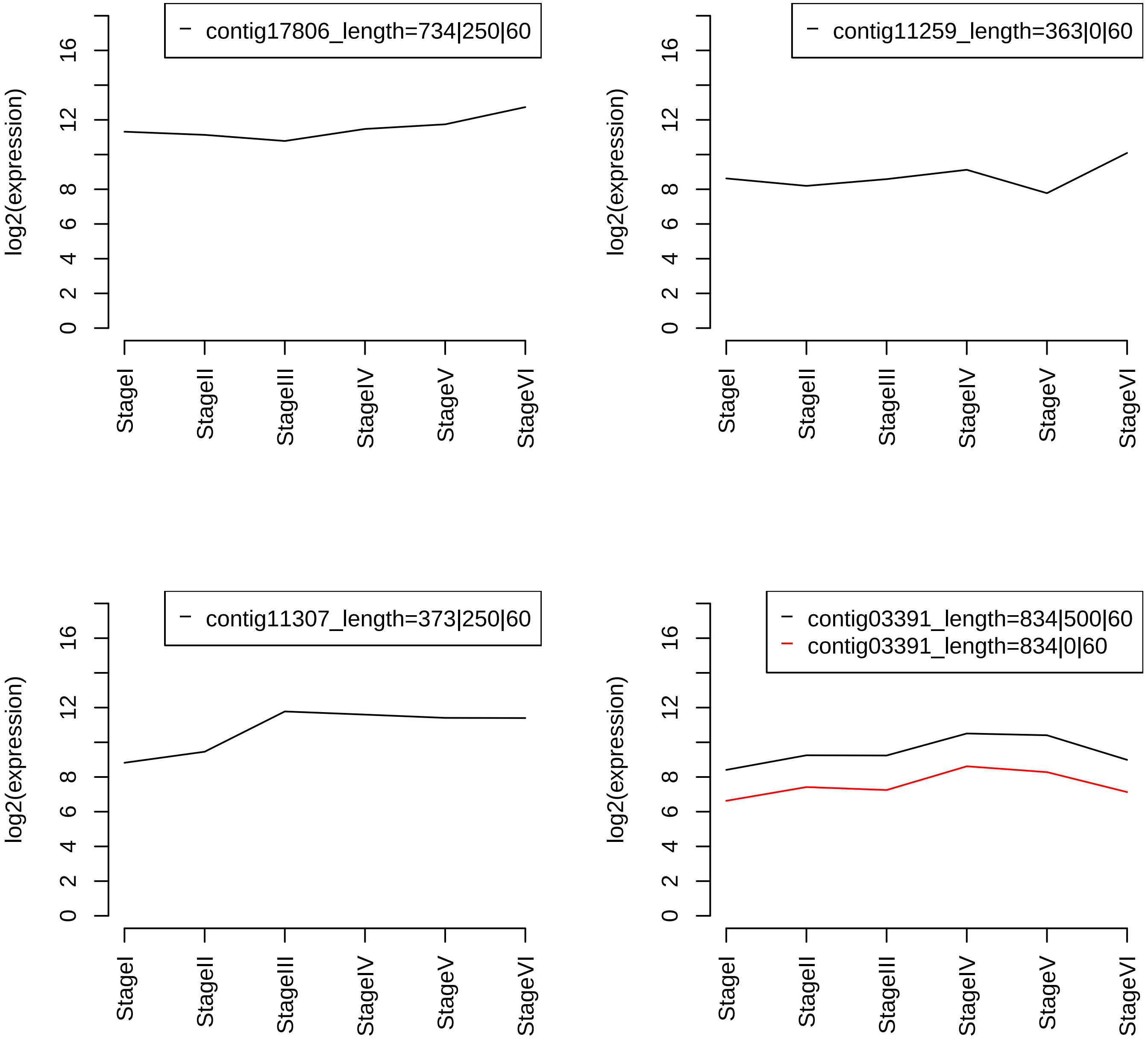
contig17806_length=734|250|60
contig11259_length=363|0|60
contig11307_length=373|250|60
contig03391_length=834|500|60
contig03391_length=834|0|60
log2(expression)
0
2
4
6
8
12
16
StageI
StageII
StageIII
StageIV
StageV
StageVI

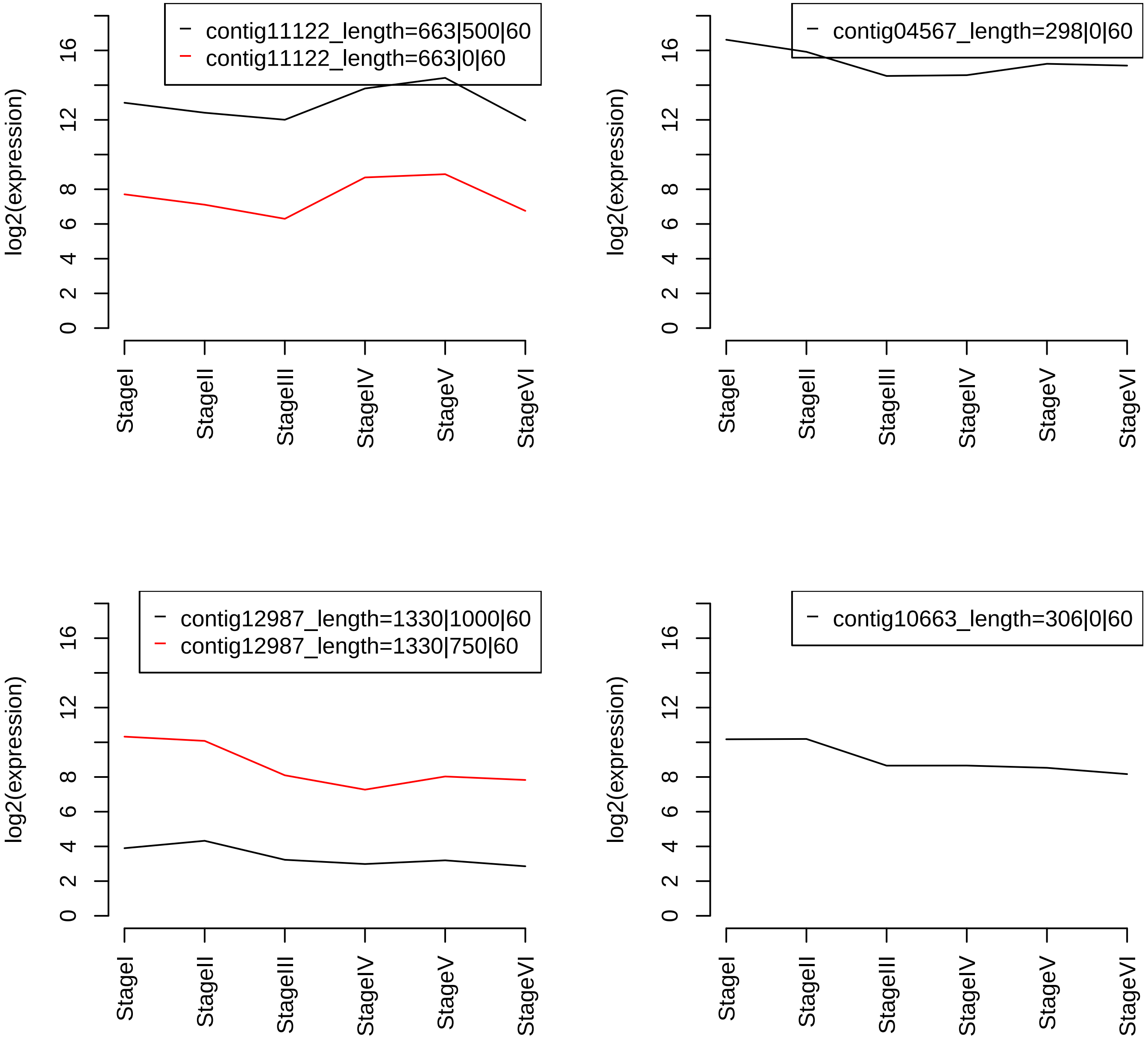
contig11122_length=663|500|60
contig11122_length=663|0|60
contig04567_length=298|0|60
contig12987_length=1330|1000|60
contig12987_length=1330|750|60
contig10663_length=306|0|60
log2(expression)
0
2
4
6
8
12
16
StageI
StageII
StageIII
StageIV
StageV
StageVI

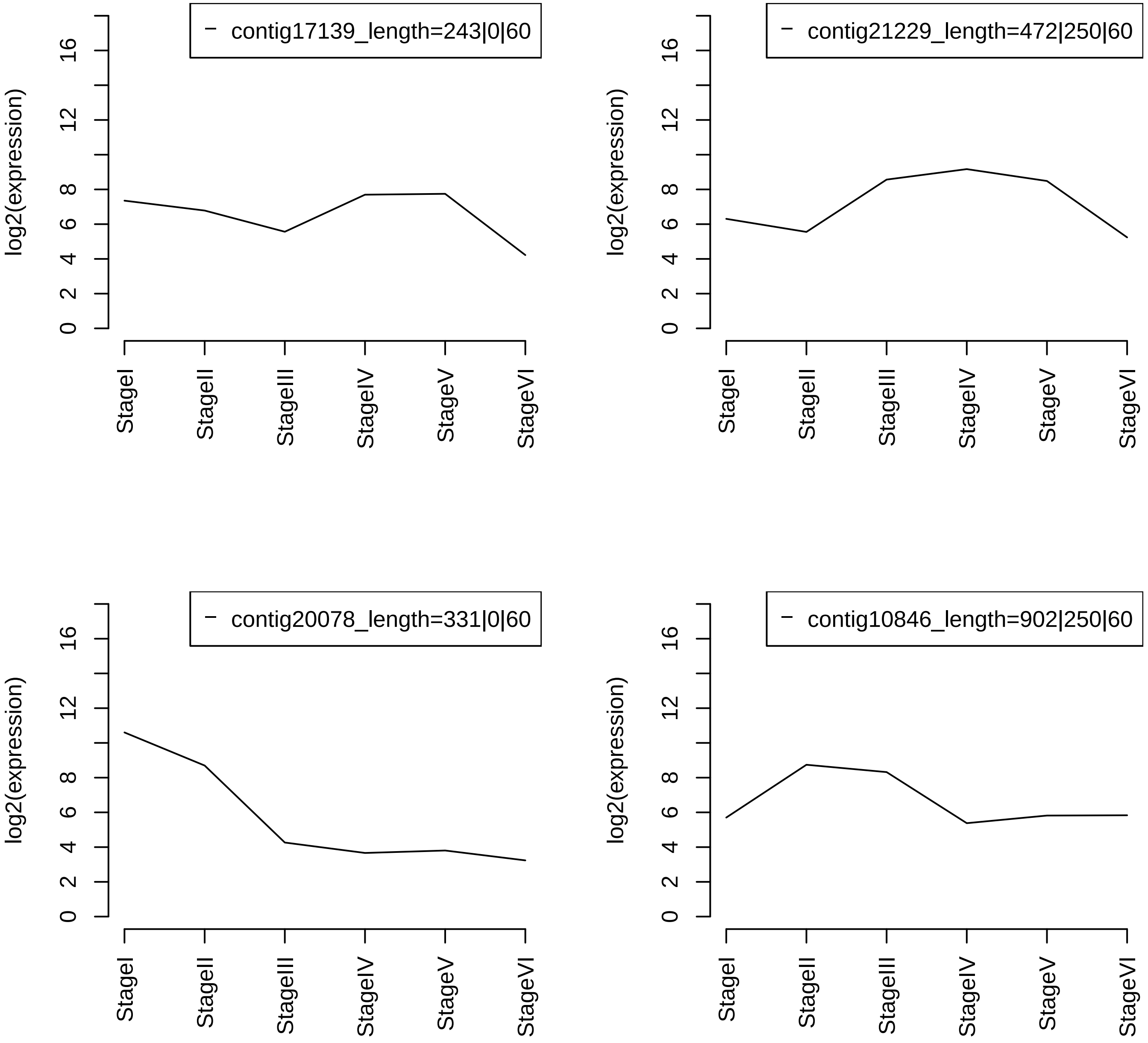
contig17139_length=243|0|60
contig21229_length=472|250|60
contig20078_length=331|0|60
contig10846_length=902|250|60
log2(expression)
0
2
4
6
8
12
16
StageI
StageII
StageIII
StageIV
StageV
StageVI

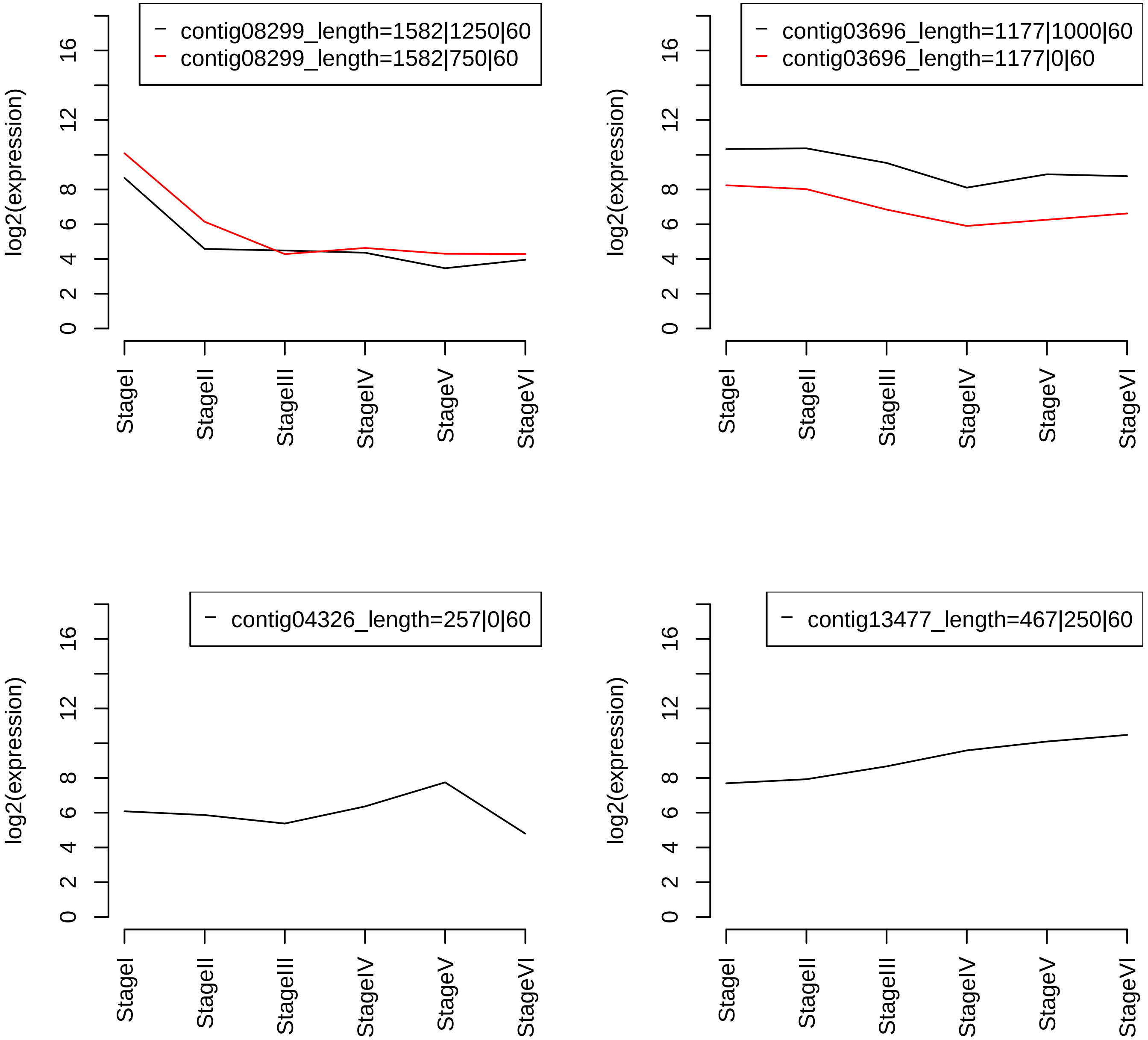
contig08299_length=1582|1250|60
contig08299_length=1582|750|60
contig03696_length=1177|1000|60
contig03696_length=1177|0|60
contig04326_length=257|0|60
contig13477_length=467|250|60
log2(expression)
0
2
4
6
8
12
16
StageI
StageII
StageIII
StageIV
StageV
StageVI

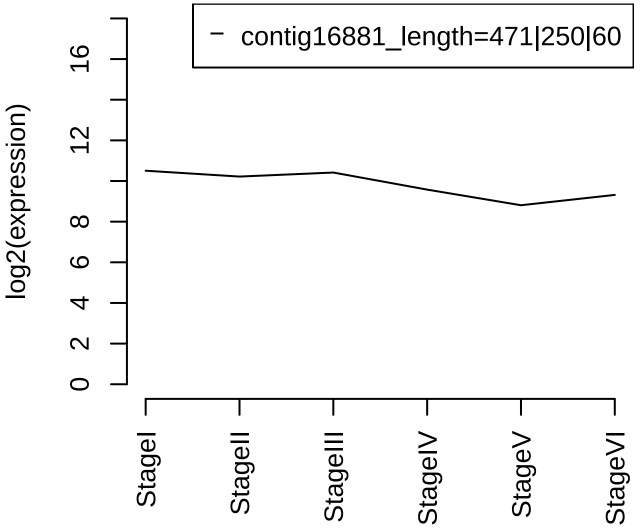
contig16881_length=471|250|60
log2(expression)
0
2
4
6
8
12
16
StageI
StageII
StageIII
StageIV
StageV
StageVI
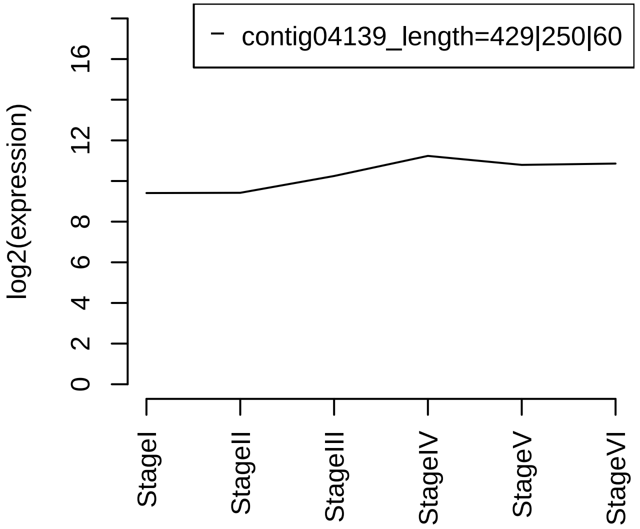
contig04139_length=429|250|60
log2(expression)
0
2
4
6
8
12
16
StageI
StageII
StageIII
StageIV
StageV
StageVI
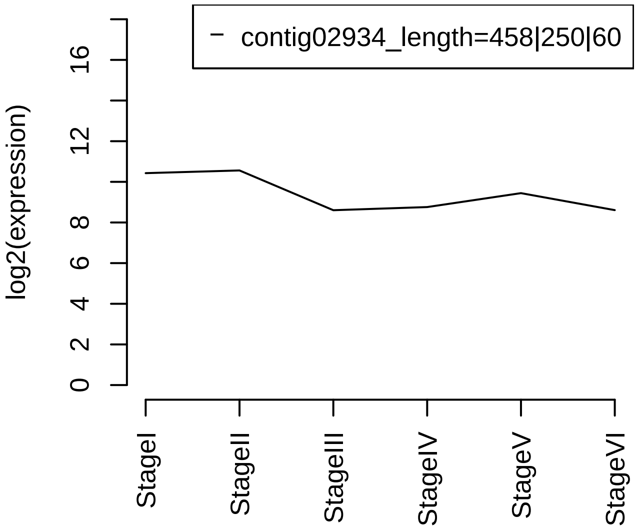
contig02934_length=458|250|60
log2(expression)
0
2
4
6
8
12
16
StageI
StageII
StageIII
StageIV
StageV
StageVI
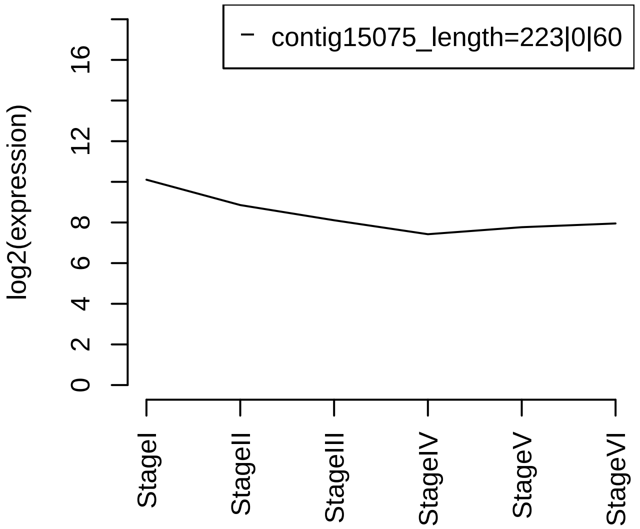
contig15075_length=223|0|60
log2(expression)
0
2
4
6
8
12
16
StageI
StageII
StageIII
StageIV
StageV
StageVI

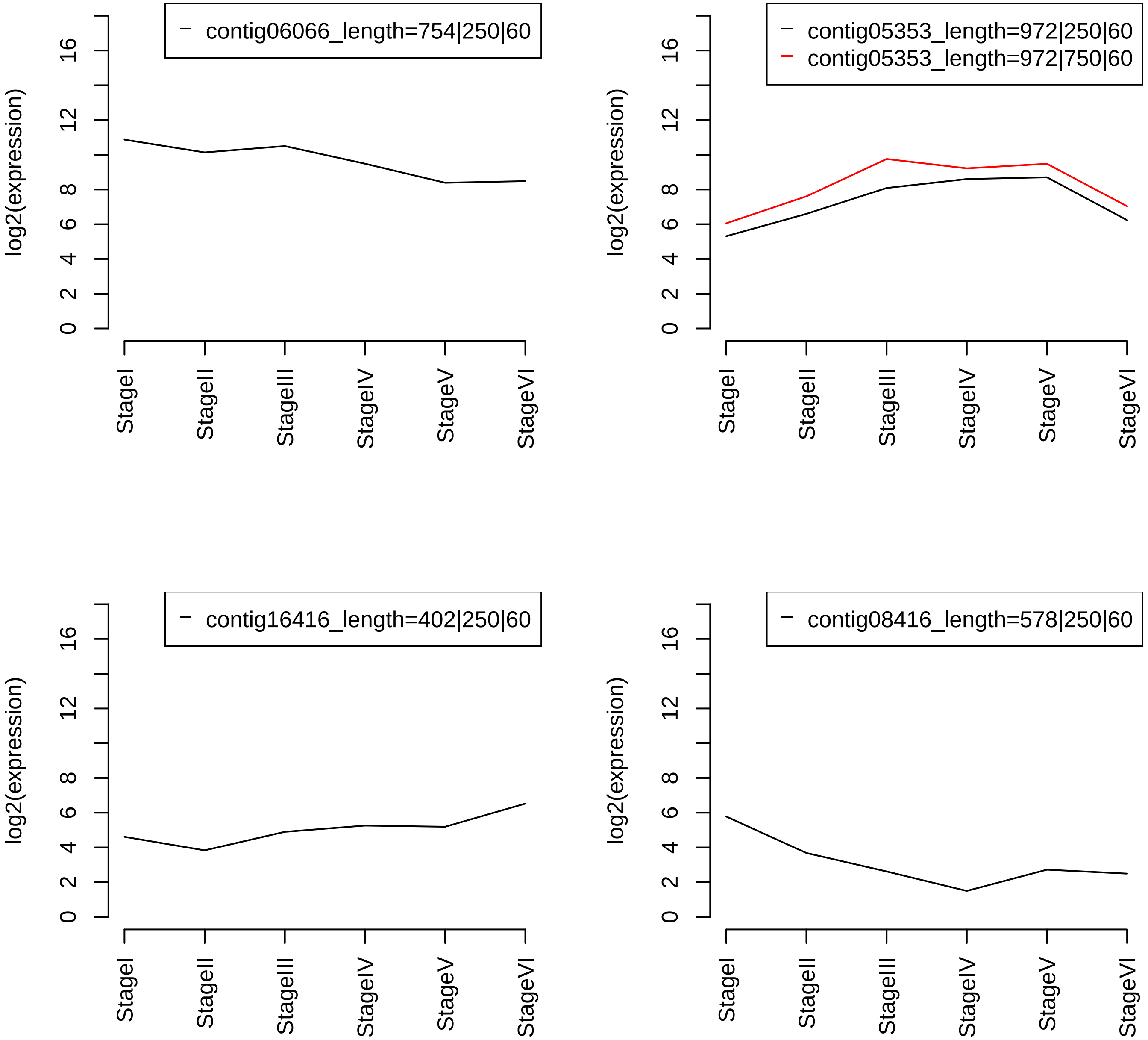
contig06066_length=754|250|60
contig05353_length=972|250|60
contig05353_length=972|750|60
contig16416_length=402|250|60
contig08416_length=578|250|60
log2(expression)
0
2
4
6
8
12
16
StageI
StageII
StageIII
StageIV
StageV
StageVI

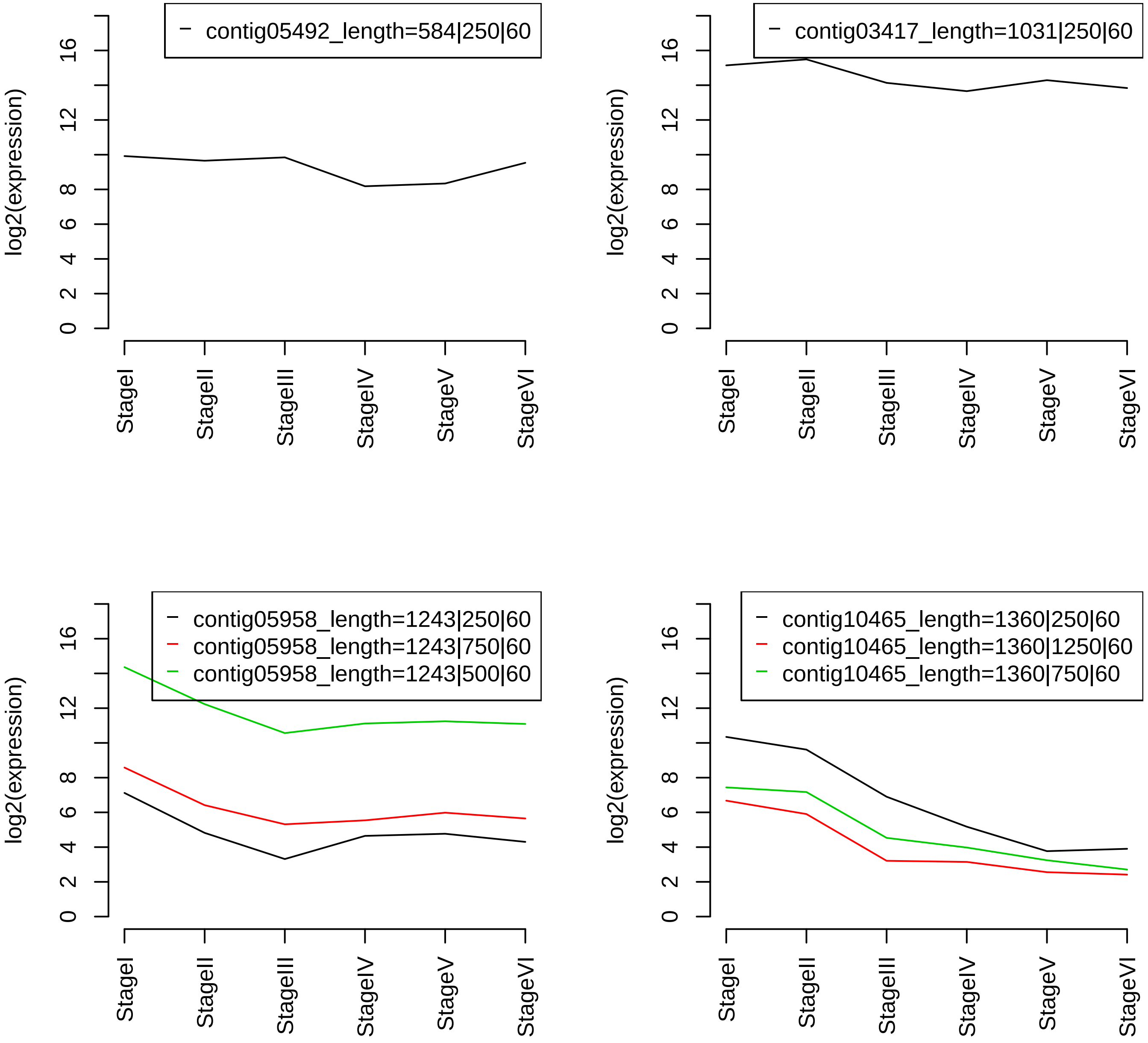
contig05492_length=584|250|60
contig03417_length=1031|250|60
contig05958_length=1243|250|60
contig05958_length=1243|750|60
contig05958_length=1243|500|60
contig10465_length=1360|250|60
contig10465_length=1360|1250|60
contig10465_length=1360|750|60
log2(expression)
0 2 4 6 8 12 16
StageI StageII StageIII StageIV StageV StageVI

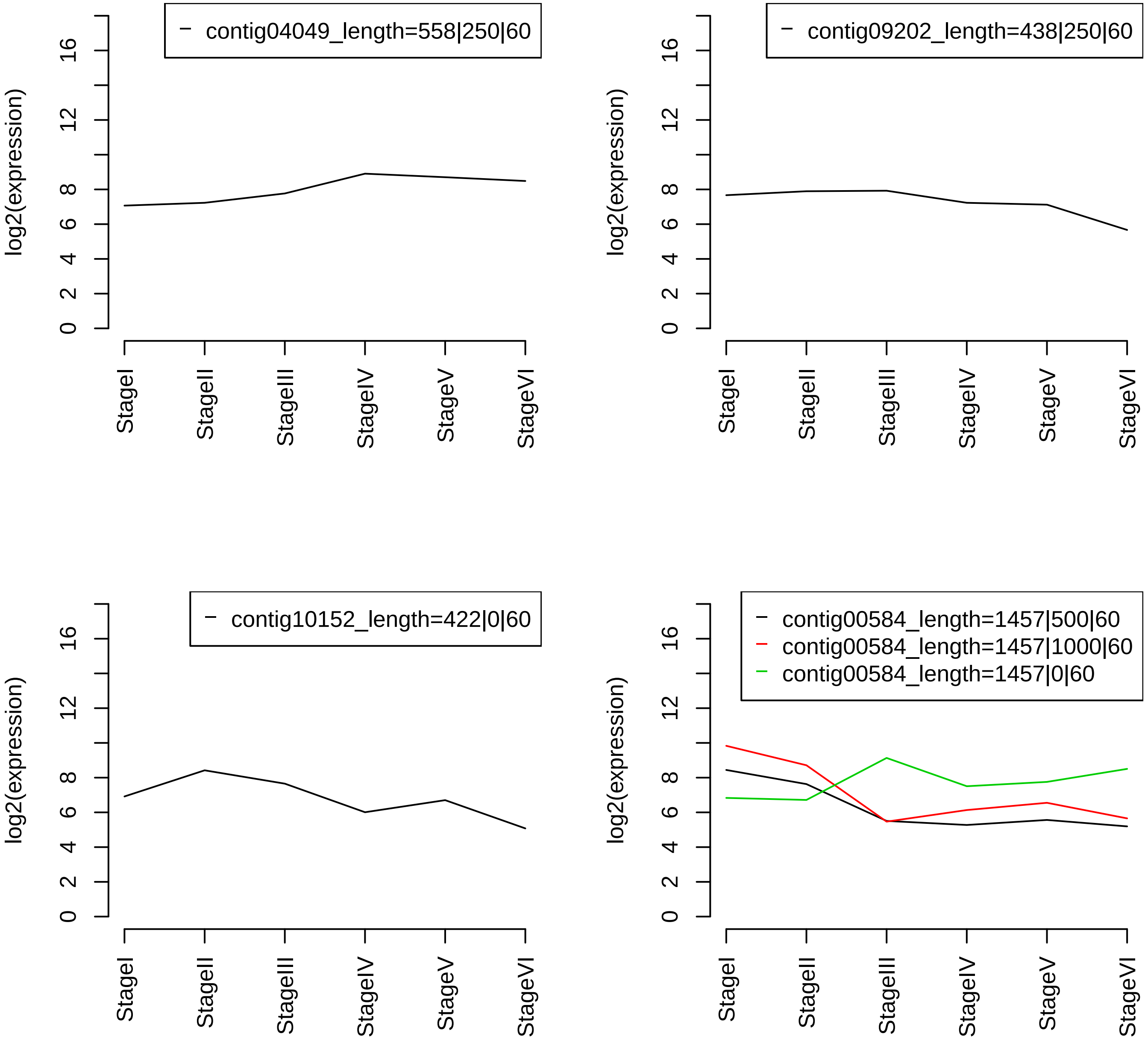
contig04049_length=558|250|60
contig09202_length=438|250|60
contig10152_length=422|0|60
contig00584_length=1457|500|60
contig00584_length=1457|1000|60
contig00584_length=1457|0|60
log2(expression)
0
2
4
6
8
12
16
StageI
StageII
StageIII
StageIV
StageV
StageVI

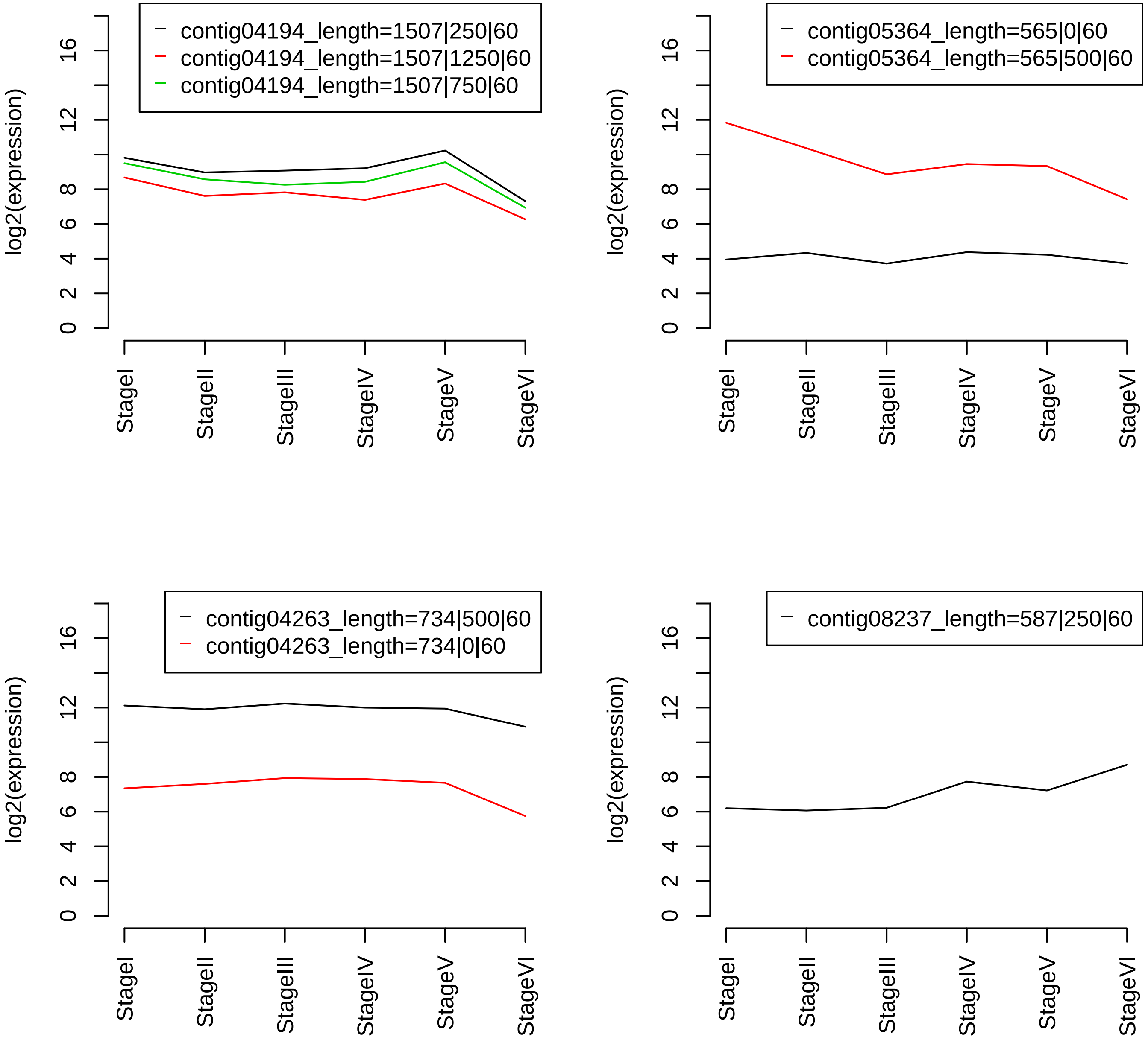
contig04194_length=1507|250|60
contig04194_length=1507|1250|60
contig04194_length=1507|750|60
log2(expression)
0
2
4
6
8
12
16
StageI
StageII
StageIII
StageIV
StageV
StageVI
contig05364_length=565|0|60
contig05364_length=565|500|60
contig04263_length=734|500|60
contig04263_length=734|0|60
contig08237_length=587|250|60

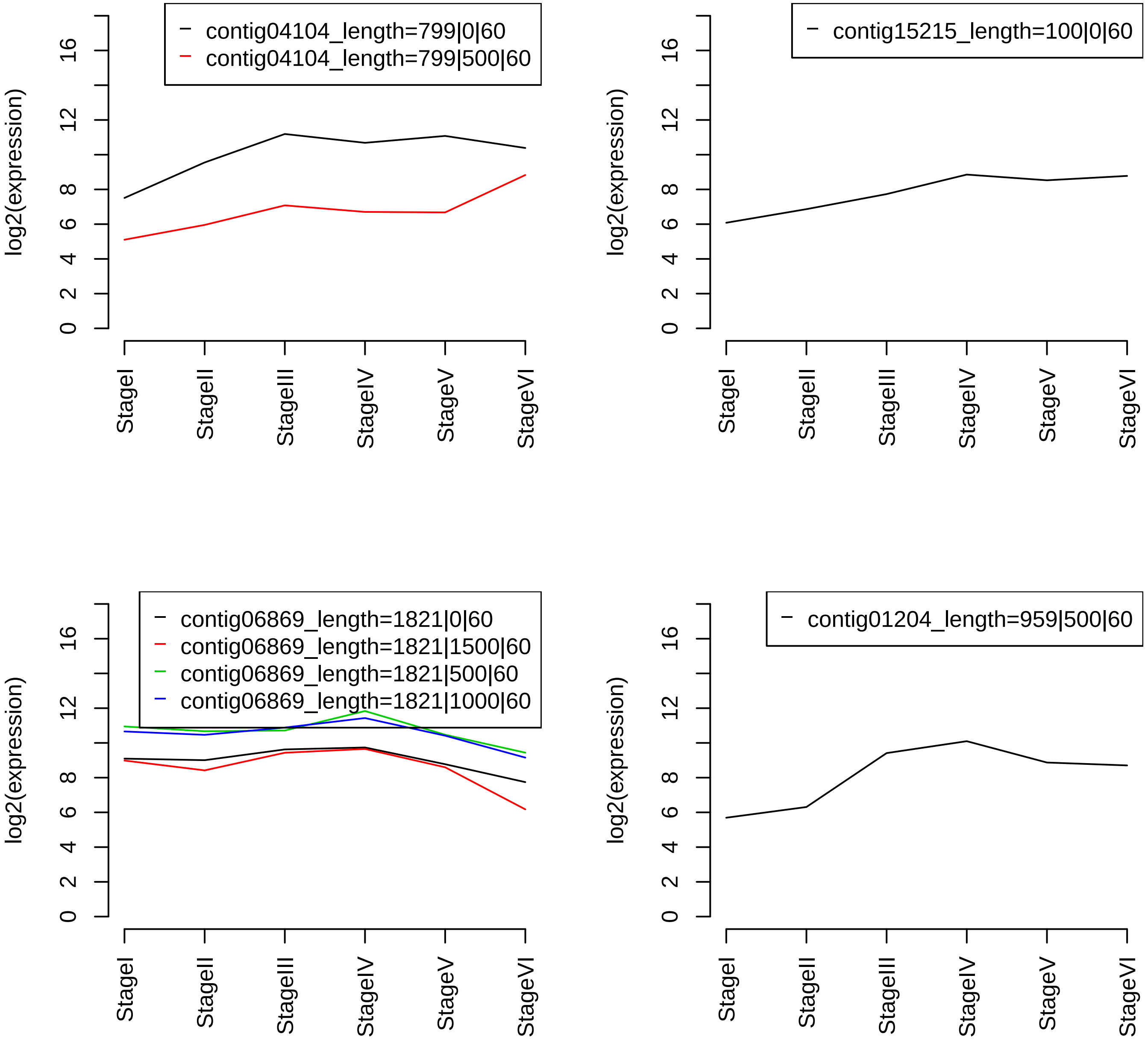
contig04104_length=799|0|60
contig04104_length=799|500|60
log2(expression)
0 2 4 6 8 12 16
StageI StageII StageIII StageIV StageV StageVI
contig15215_length=100|0|60
log2(expression)
0 2 4 6 8 12 16
StageI StageII StageIII StageIV StageV StageVI
contig06869_length=1821|0|60
contig06869_length=1821|1500|60
contig06869_length=1821|500|60
contig06869_length=1821|1000|60
log2(expression)
0 2 4 6 8 12 16
StageI StageII StageIII StageIV StageV StageVI
contig01204_length=959|500|60
log2(expression)
0 2 4 6 8 12 16
StageI StageII StageIII StageIV StageV StageVI

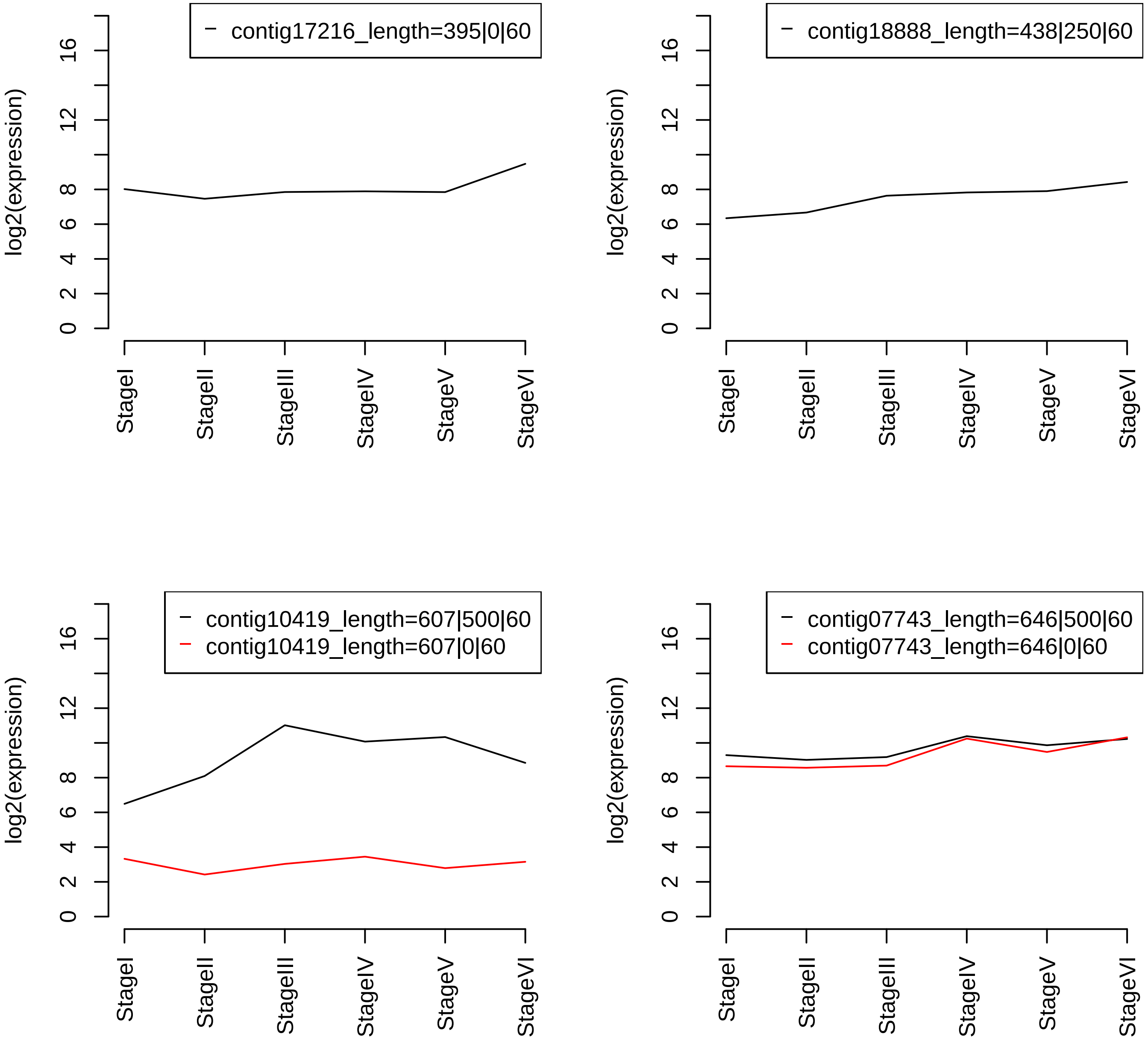
contig17216_length=395|0|60
contig18888_length=438|250|60
contig10419_length=607|500|60
contig10419_length=607|0|60
contig07743_length=646|500|60
contig07743_length=646|0|60
log2(expression)
0
2
4
6
8
12
16
StageI
StageII
StageIII
StageIV
StageV
StageVI

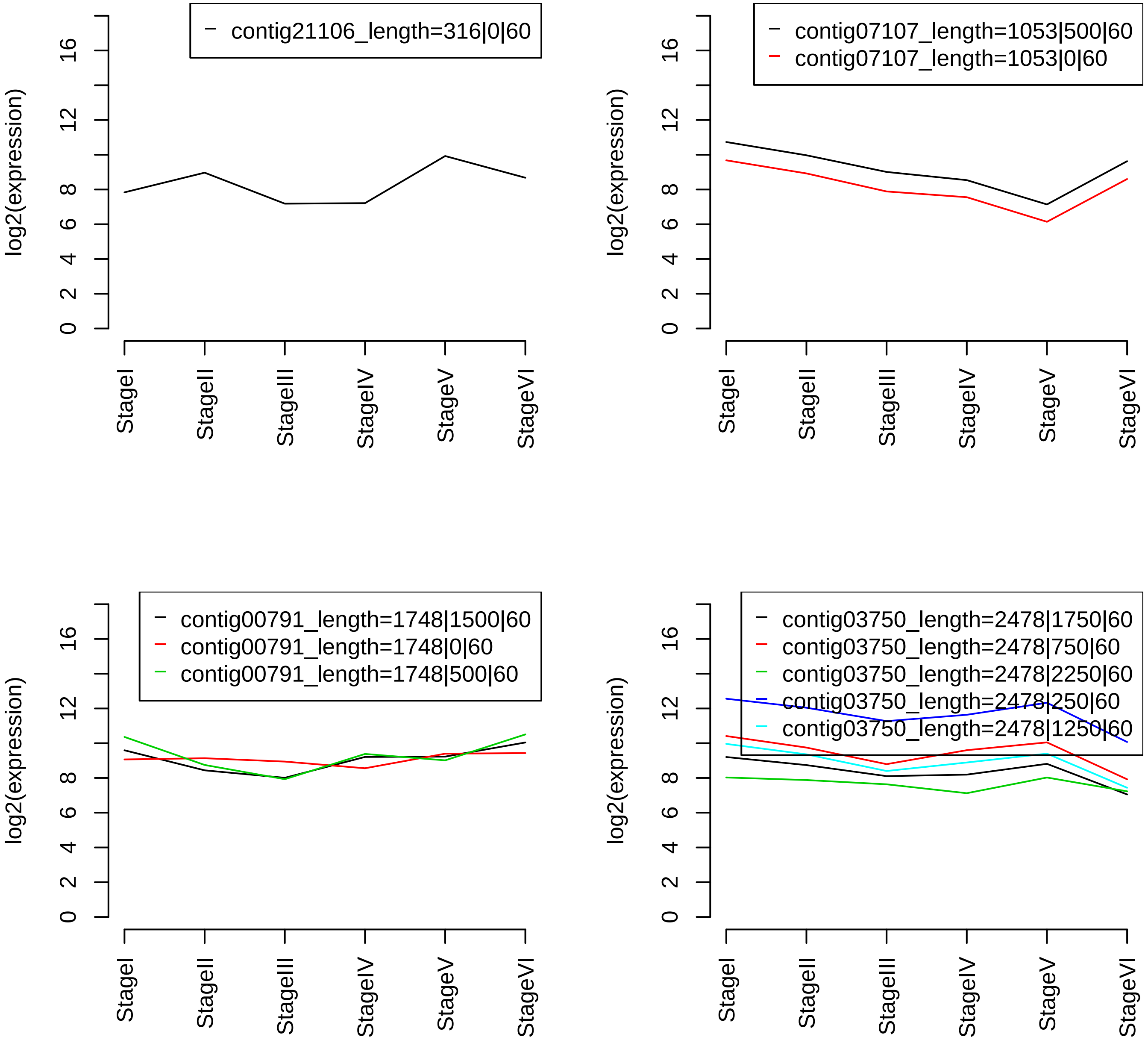

contig21106_length=316|0|60
contig07107_length=1053|500|60
contig07107_length=1053|0|60
contig00791_length=1748|1500|60
contig00791_length=1748|0|60
contig00791_length=1748|500|60
contig03750_length=2478|1750|60
contig03750_length=2478|750|60
contig03750_length=2478|2250|60
contig03750_length=2478|250|60
contig03750_length=2478|1250|60
log2(expression)
0
2
4
6
8
12
16
StageI
StageII
StageIII
StageIV
StageV
StageVI

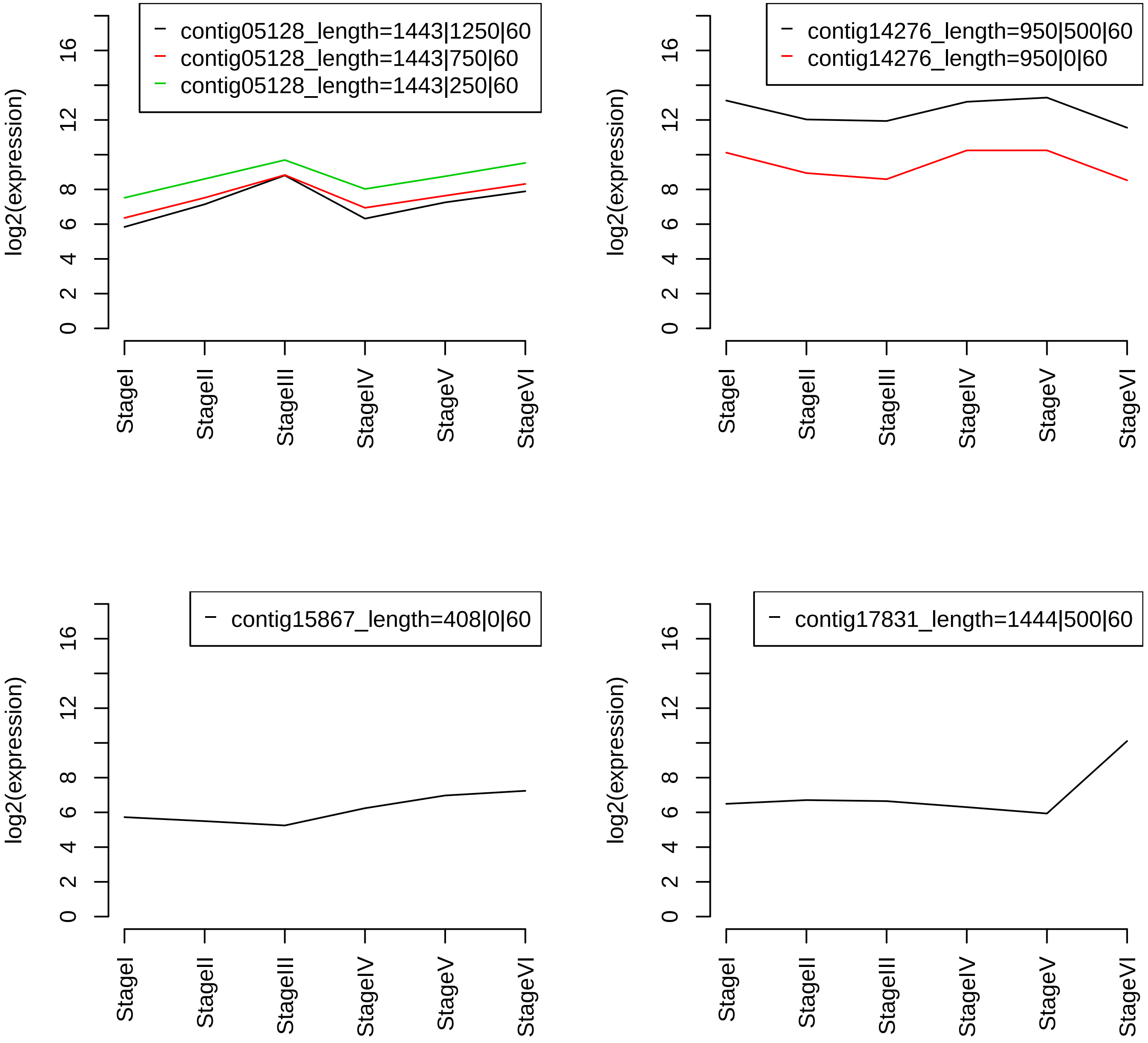

contig05128_length=1443|1250|60
contig05128_length=1443|750|60
contig05128_length=1443|250|60
contig14276_length=950|500|60
contig14276_length=950|0|60
contig15867_length=408|0|60
contig17831_length=1444|500|60
log2(expression)
0 2 4 6 8 12 16
StageI
StageII
StageIII
StageIV
StageV
StageVI

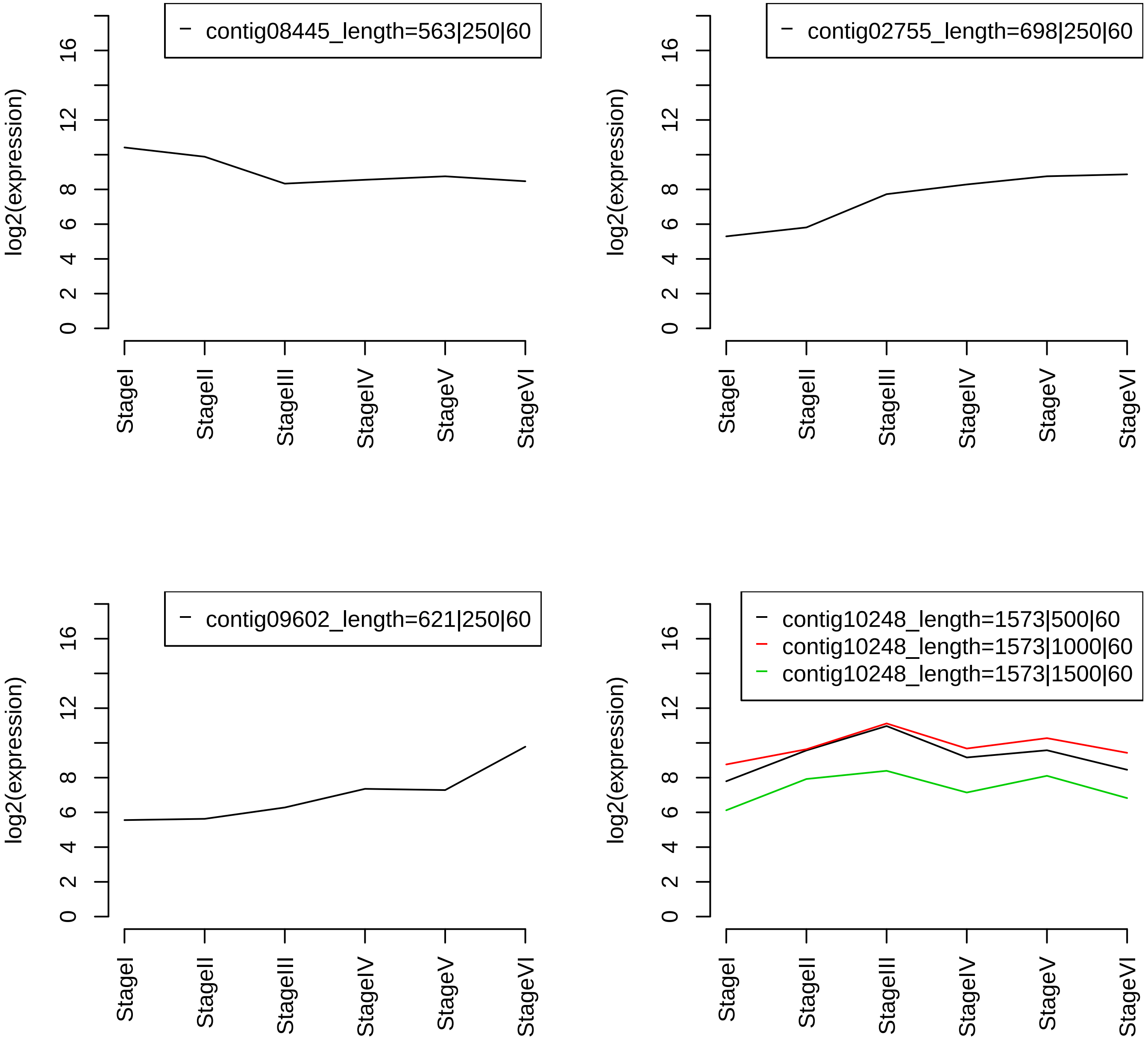
contig08445_length=563|250|60
contig02755_length=698|250|60
contig09602_length=621|250|60
contig10248_length=1573|500|60
contig10248_length=1573|1000|60
contig10248_length=1573|1500|60
log2(expression)
0
2
4
6
8
12
16
StageI
StageII
StageIII
StageIV
StageV
StageVI

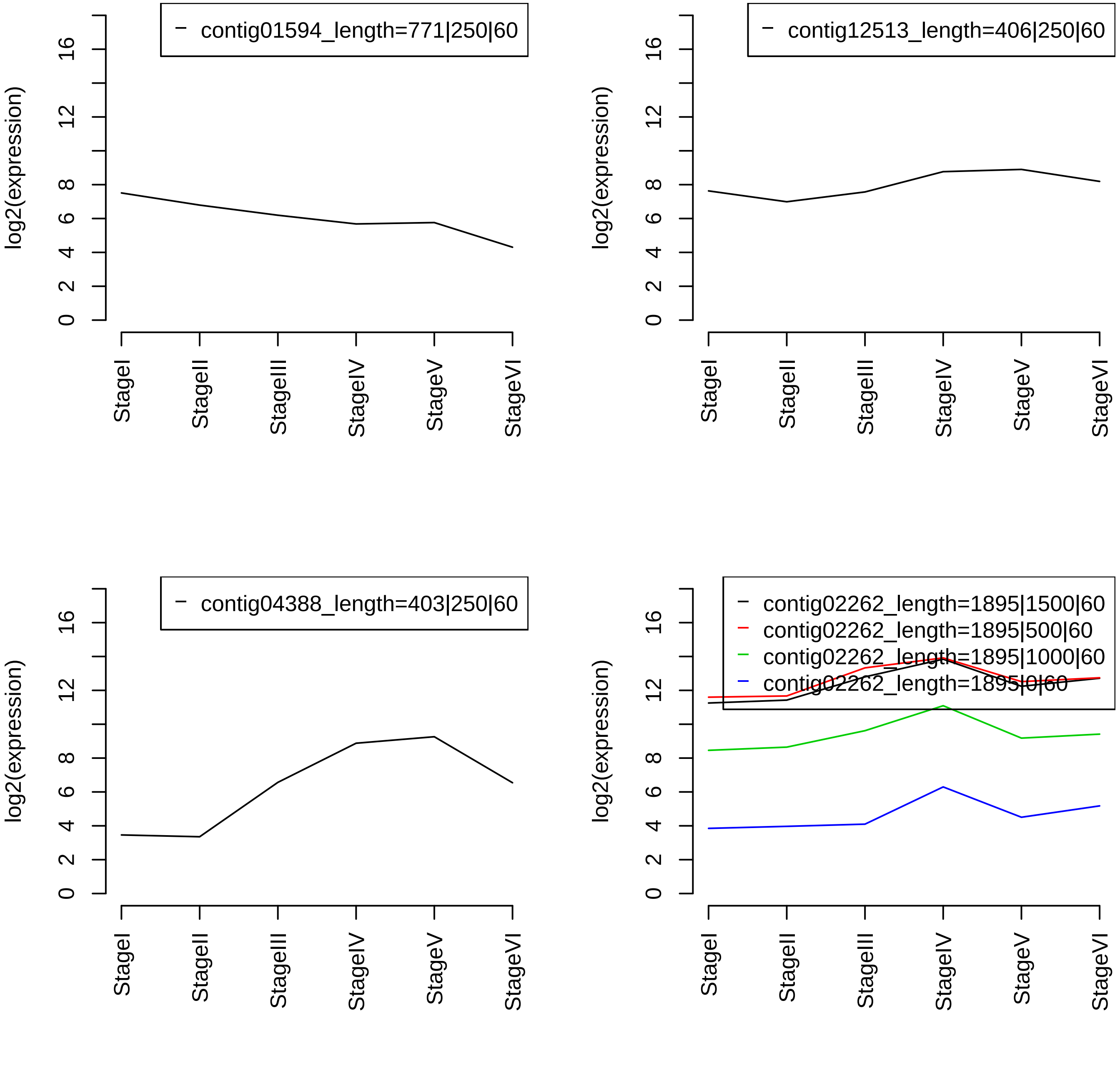
contig01594_length=771|250|60
contig12513_length=406|250|60
contig04388_length=403|250|60
contig02262_length=1895|1500|60
contig02262_length=1895|500|60
contig02262_length=1895|1000|60
contig02262_length=1895|0|60
log2(expression)
0
2
4
6
8
12
16
StageI
StageII
StageIII
StageIV
StageV
StageVI

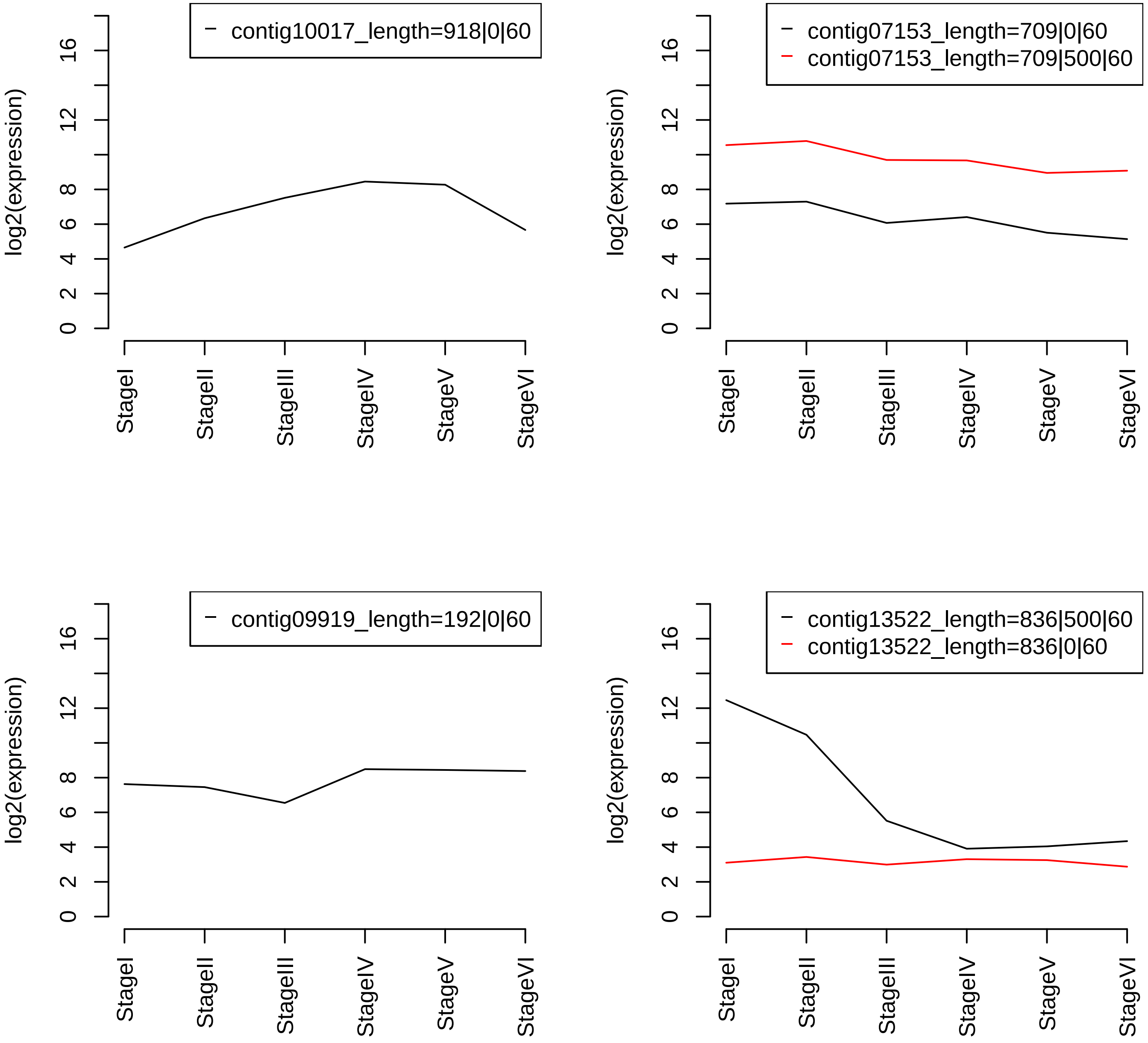

contig10017_length=918|0|60
contig07153_length=709|0|60
contig07153_length=709|500|60
contig09919_length=192|0|60
contig13522_length=836|500|60
contig13522_length=836|0|60
log2(expression)
0
2
4
6
8
12
16
StageI
StageII
StageIII
StageIV
StageV
StageVI

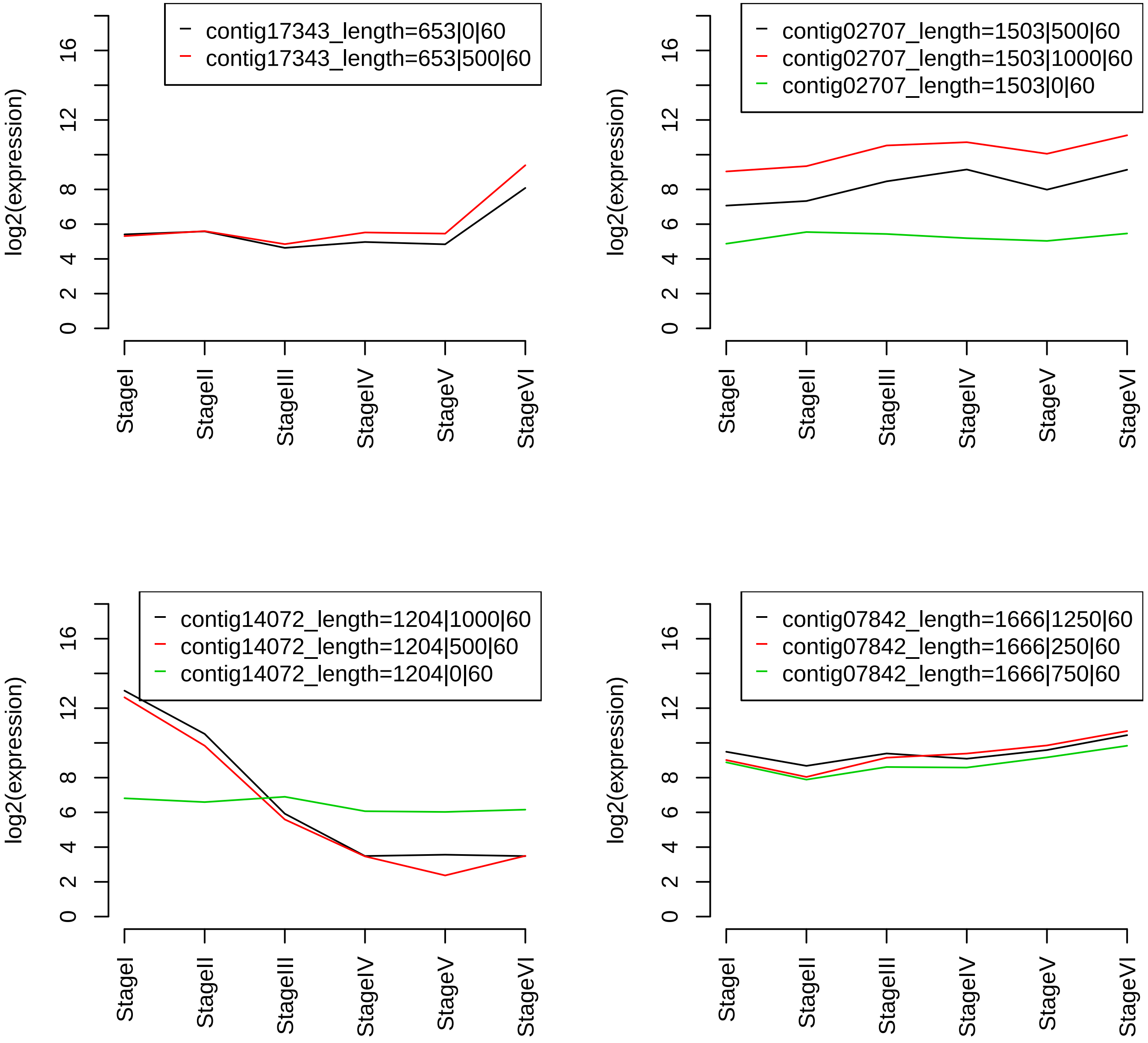

contig17343_length=653|0|60
contig17343_length=653|500|60
contig02707_length=1503|500|60
contig02707_length=1503|1000|60
contig02707_length=1503|0|60
contig14072_length=1204|1000|60
contig14072_length=1204|500|60
contig14072_length=1204|0|60
contig07842_length=1666|1250|60
contig07842_length=1666|250|60
contig07842_length=1666|750|60
log2(expression)
0
2
4
6
8
12
16
StageI
StageII
StageIII
StageIV
StageV
StageVI

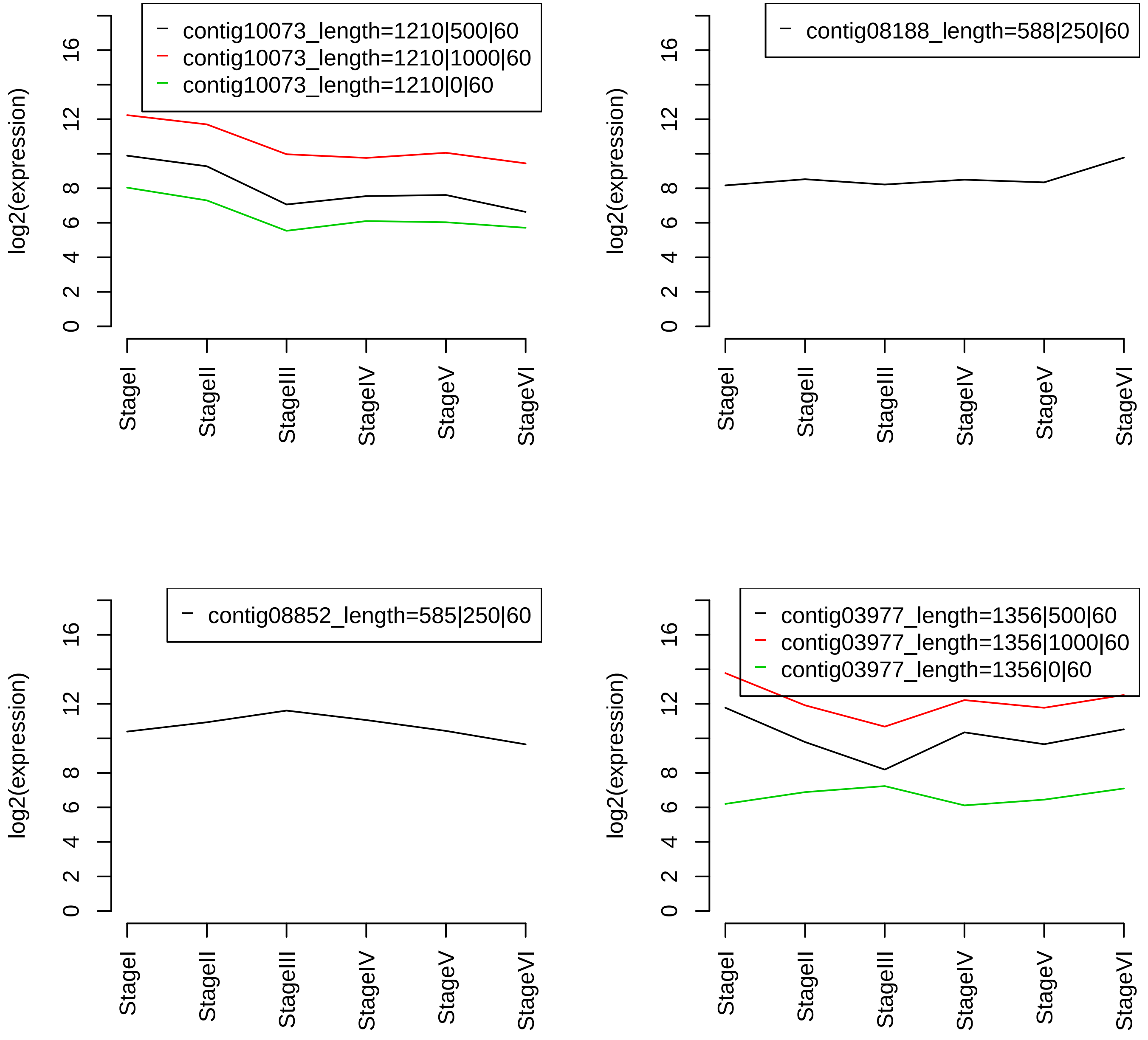
contig10073_length=1210|500|60
contig10073_length=1210|1000|60
contig10073_length=1210|0|60
log2(expression)
0
2
4
6
8
12
16
StageI
StageII
StageIII
StageIV
StageV
StageVI
contig08188_length=588|250|60
contig08852_length=585|250|60
contig03977_length=1356|500|60
contig03977_length=1356|1000|60
contig03977_length=1356|0|60

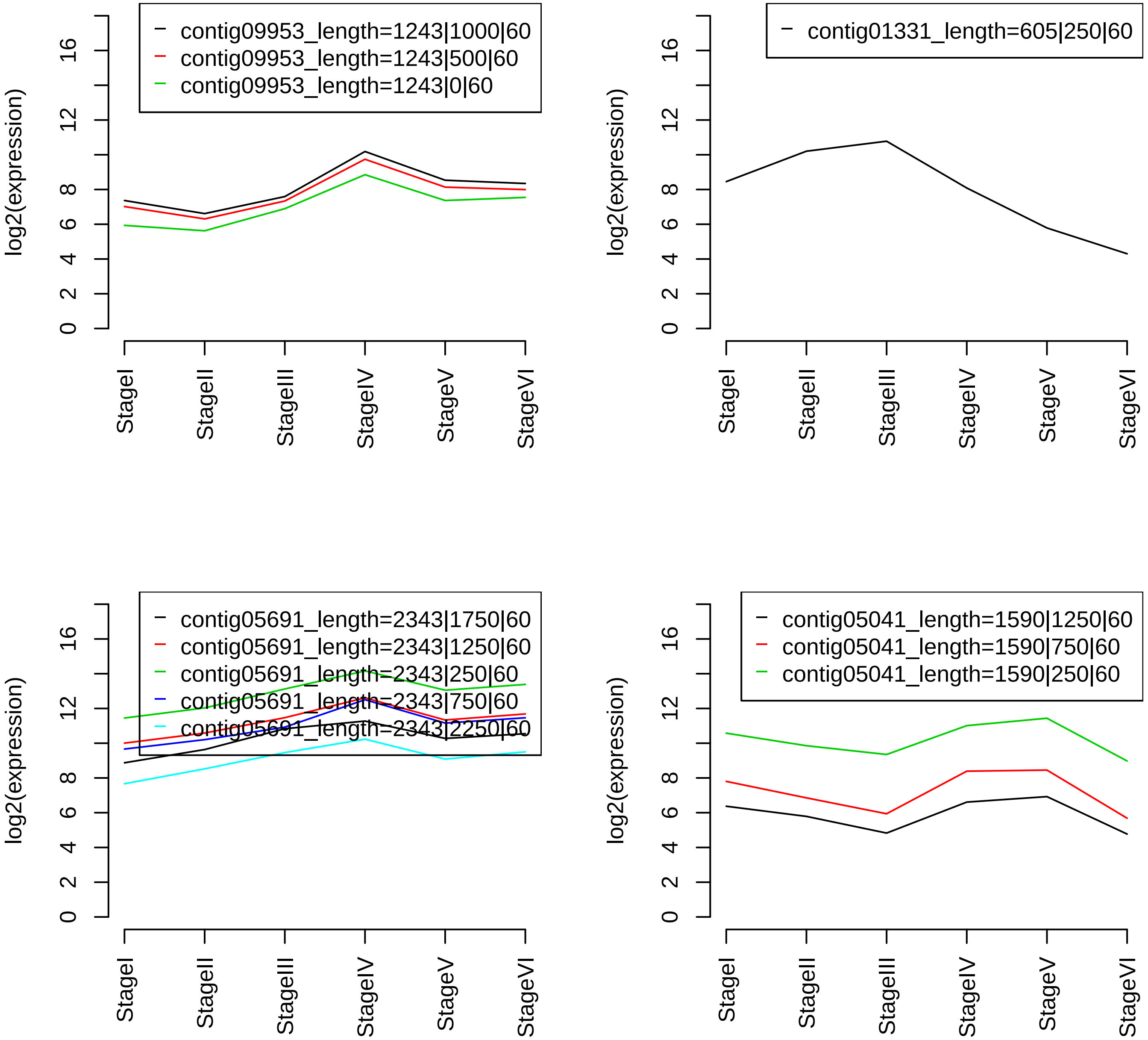

contig09953_length=1243|1000|60
contig09953_length=1243|500|60
contig09953_length=1243|0|60
contig01331_length=605|250|60
contig05691_length=2343|1750|60
contig05691_length=2343|1250|60
contig05691_length=2343|250|60
contig05691_length=2343|750|60
contig05691_length=2343|2250|60
contig05041_length=1590|1250|60
contig05041_length=1590|750|60
contig05041_length=1590|250|60
log2(expression)
0 2 4 6 8 12 16
StageI StageII StageIII StageIV StageV StageVI

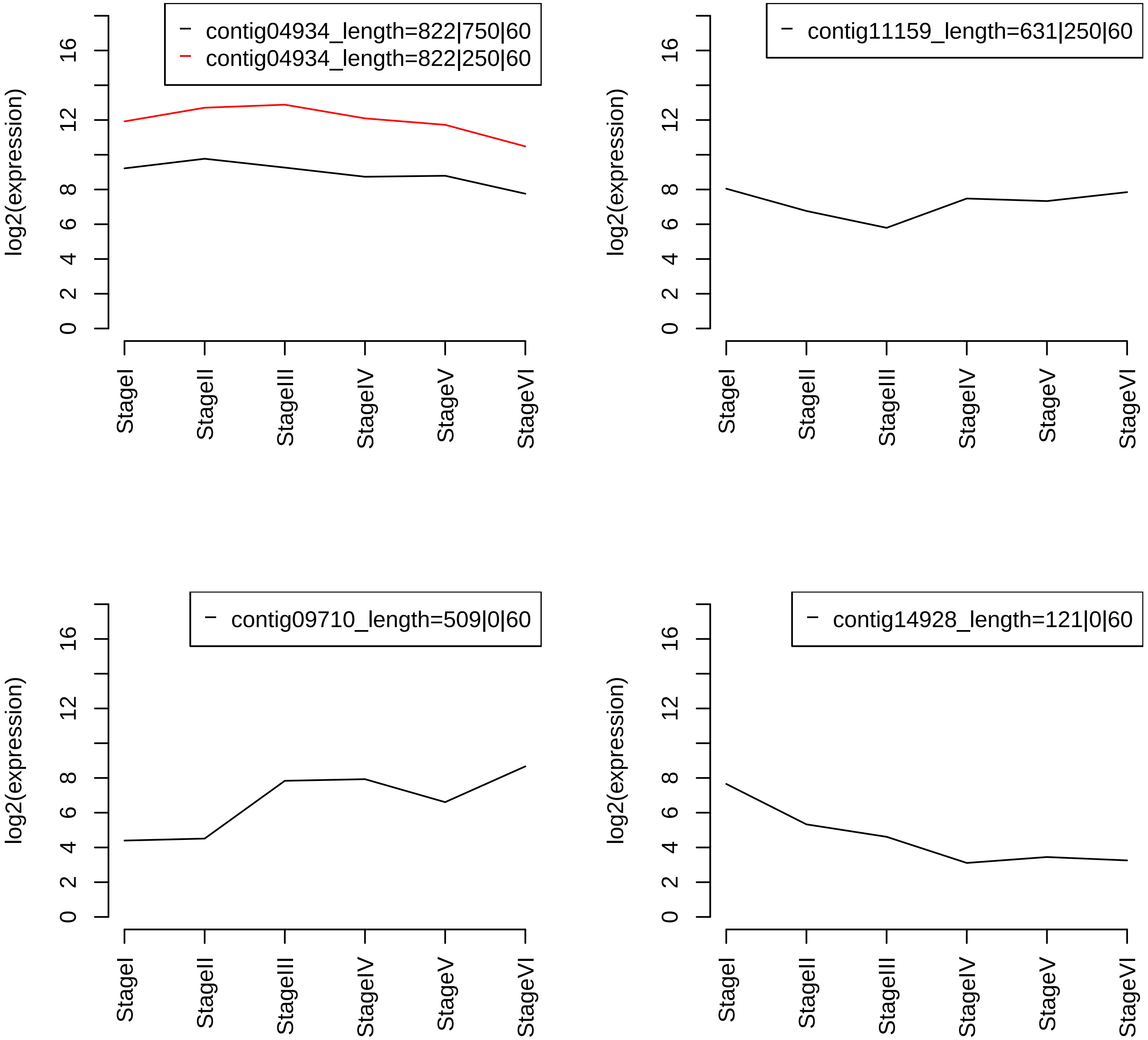

contig04934_length=822|750|60
contig04934_length=822|250|60
contig11159_length=631|250|60
contig09710_length=509|0|60
contig14928_length=121|0|60
log2(expression)
0
2
4
6
8
12
16
StageI
StageII
StageIII
StageIV
StageV
StageVI

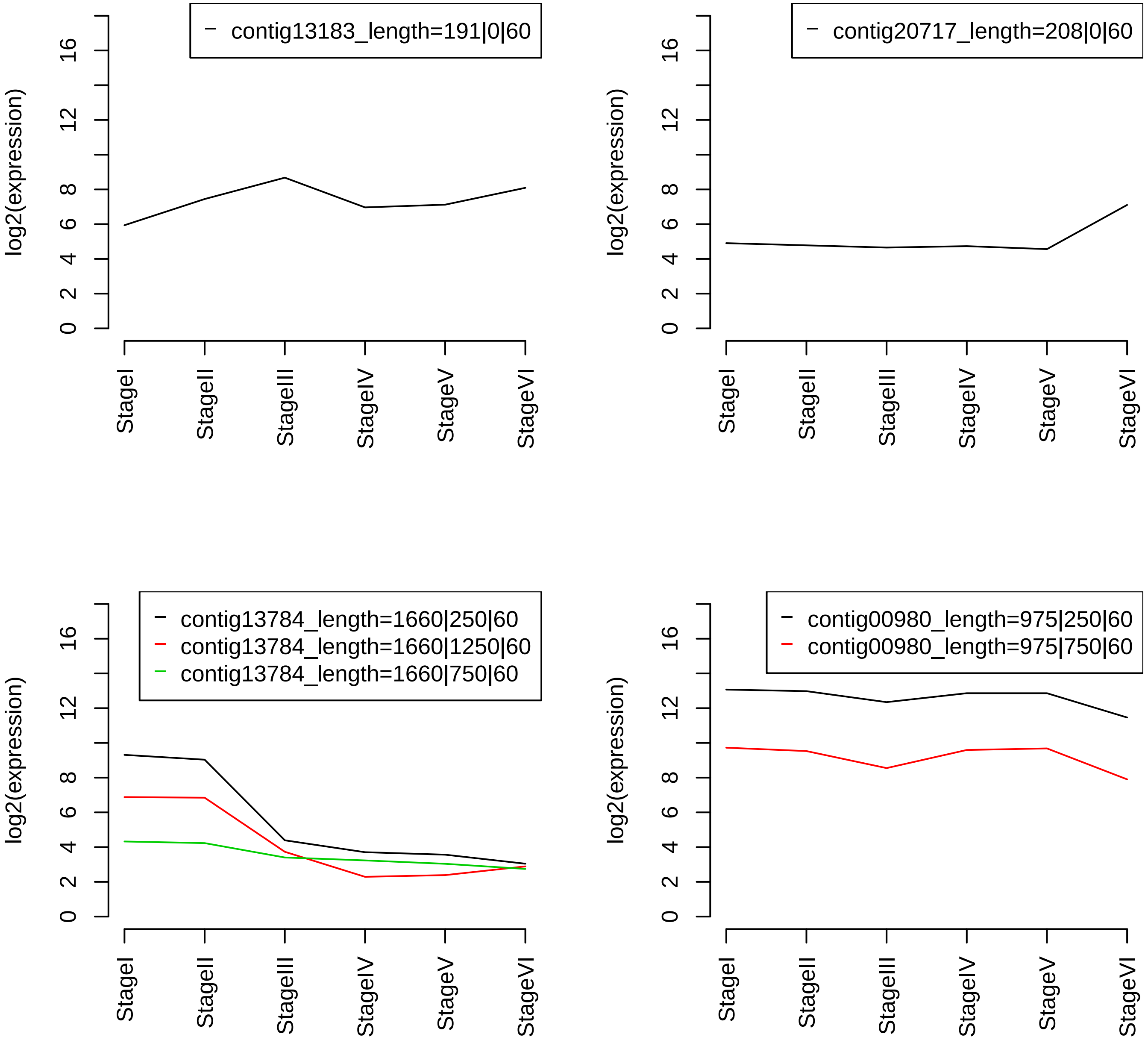
contig13183_length=191|0|60
contig20717_length=208|0|60
contig13784_length=1660|250|60
contig13784_length=1660|1250|60
contig13784_length=1660|750|60
contig00980_length=975|250|60
contig00980_length=975|750|60
log2(expression)
0
2
4
6
8
12
16
StageI
StageII
StageIII
StageIV
StageV
StageVI

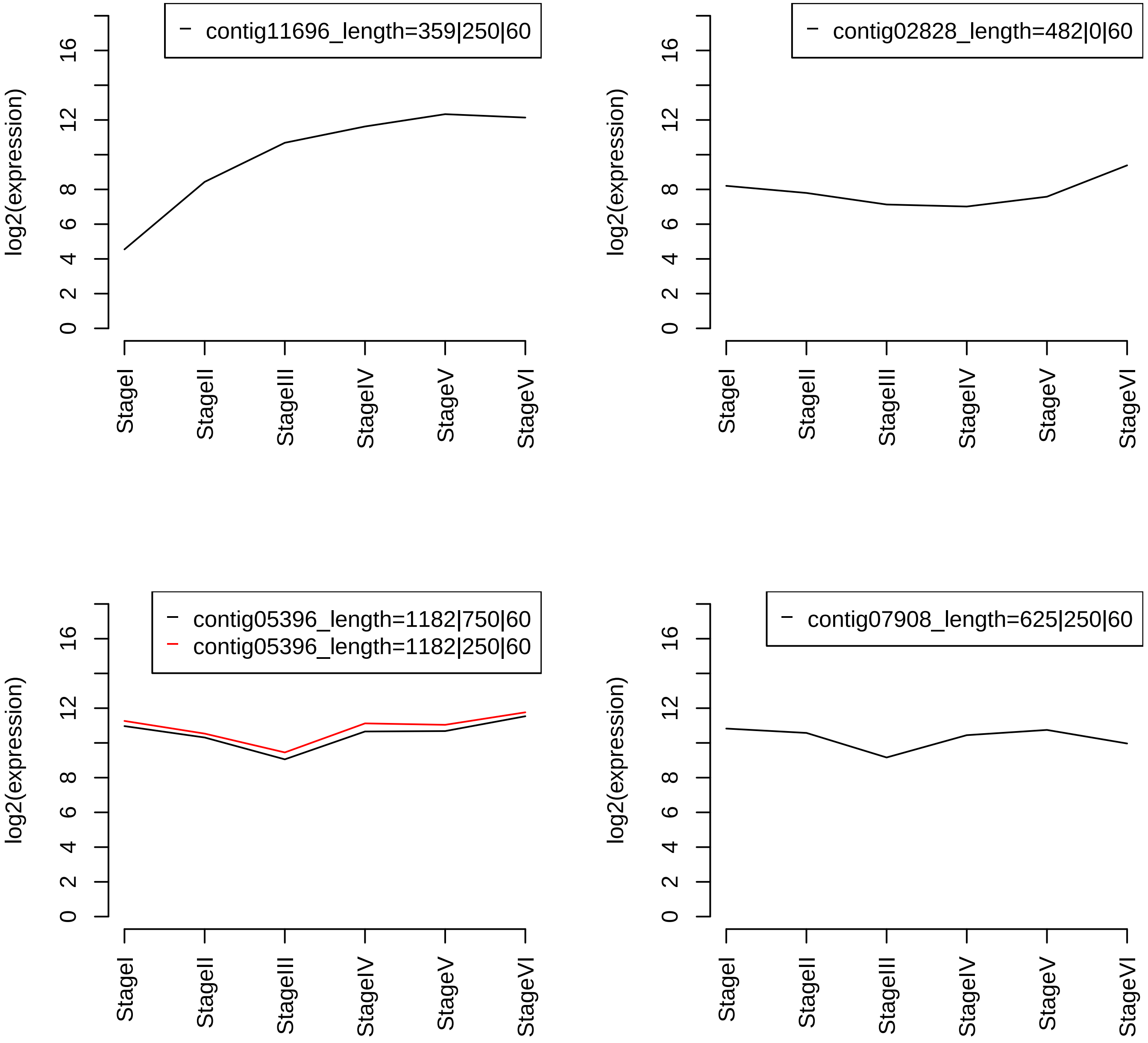
contig11696_length=359|250|60
contig02828_length=482|0|60
contig05396_length=1182|750|60
contig05396_length=1182|250|60
contig07908_length=625|250|60
log2(expression)
0
2
4
6
8
12
16
StageI
StageII
StageIII
StageIV
StageV
StageVI

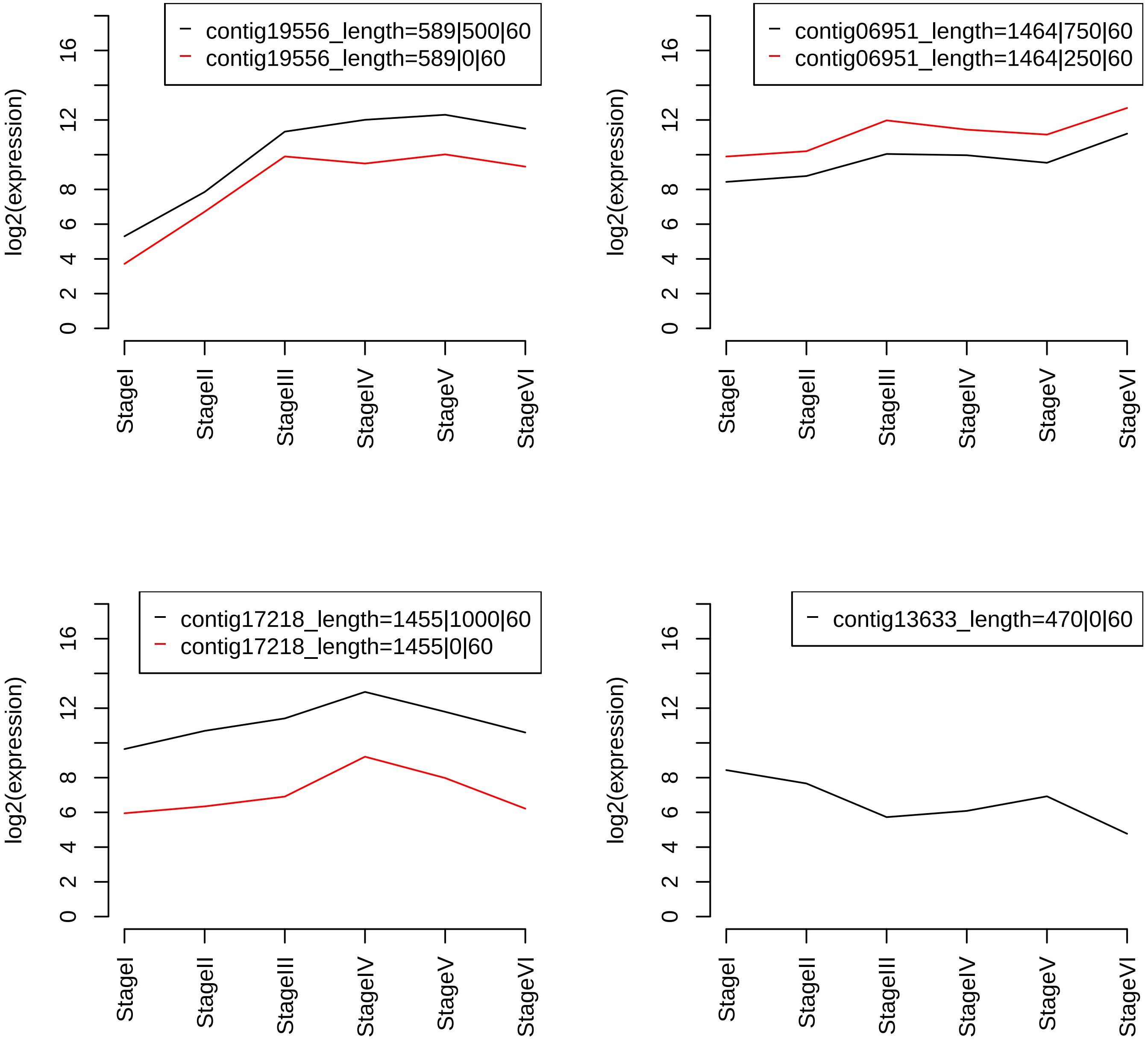
contig19556_length=589|500|60
contig19556_length=589|0|60
contig06951_length=1464|750|60
contig06951_length=1464|250|60
contig17218_length=1455|1000|60
contig17218_length=1455|0|60
contig13633_length=470|0|60
log2(expression)
0
2
4
6
8
12
16
StageI
StageII
StageIII
StageIV
StageV
StageVI

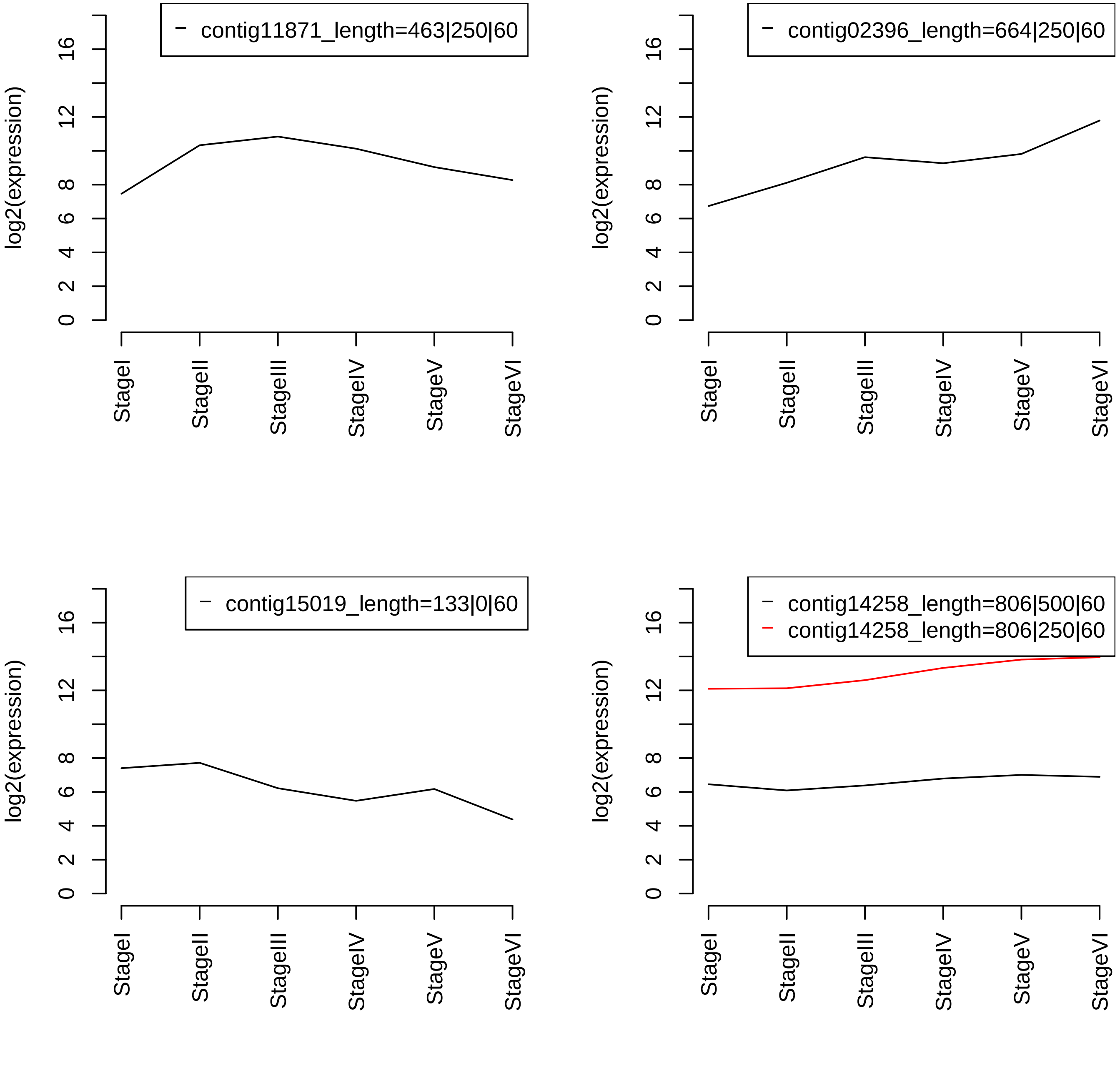
contig11871_length=463|250|60
contig02396_length=664|250|60
contig15019_length=133|0|60
contig14258_length=806|500|60
contig14258_length=806|250|60
log2(expression)
0 2 4 6 8 12 16
StageI
StageII
StageIII
StageIV
StageV
StageVI

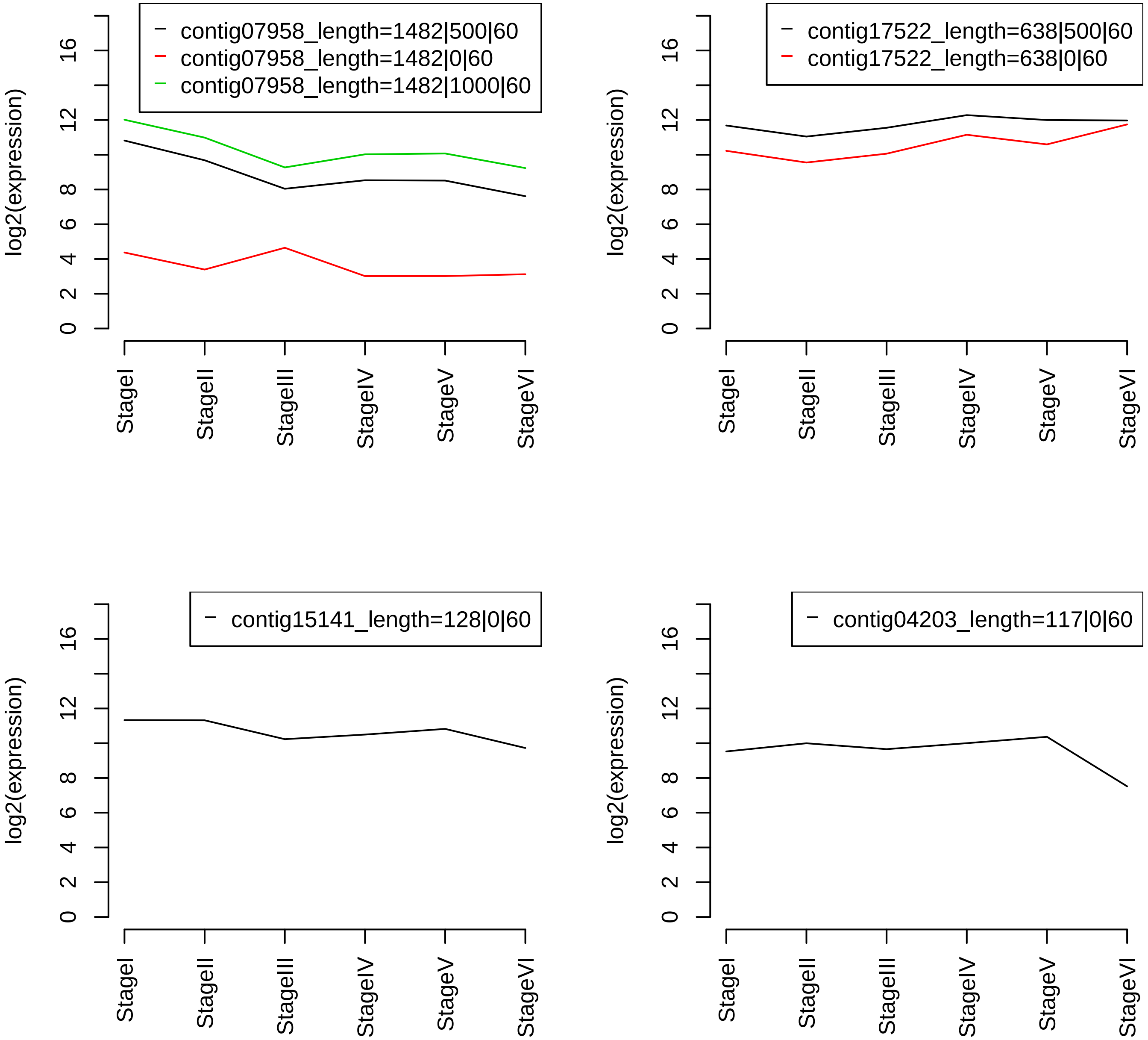

contig07958_length=1482|500|60
contig07958_length=1482|0|60
contig07958_length=1482|1000|60
contig17522_length=638|500|60
contig17522_length=638|0|60
contig15141_length=128|0|60
contig04203_length=117|0|60
log2(expression)
0
2
4
6
8
12
16
StageI
StageII
StageIII
StageIV
StageV
StageVI

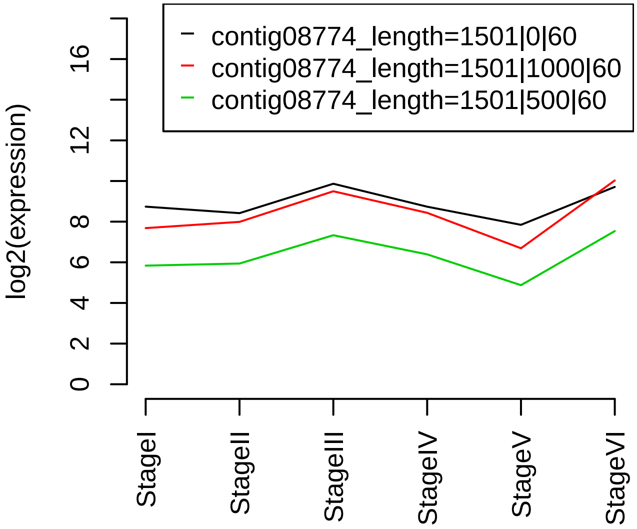
contig08774_length=1501|0|60
contig08774_length=1501|1000|60
contig08774_length=1501|500|60
log2(expression)
0
2
4
6
8
12
16
StageI
StageII
StageIII
StageIV
StageV
StageVI

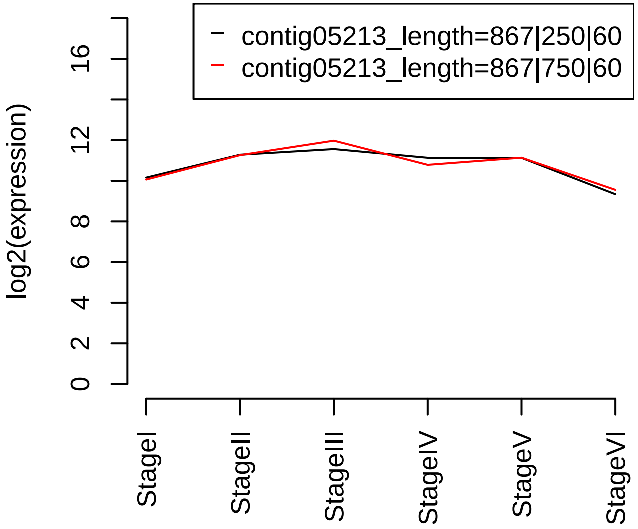
contig05213_length=867|250|60
contig05213_length=867|750|60
log2(expression)
0
2
4
6
8
12
16
StageI
StageII
StageIII
StageIV
StageV
StageVI

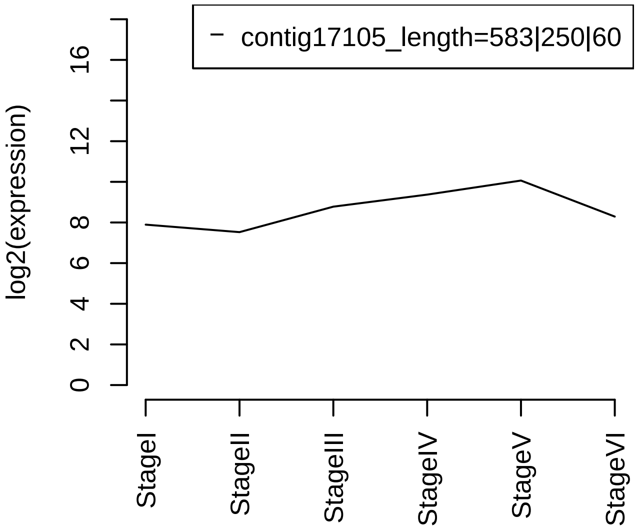
contig17105_length=583|250|60
log2(expression)
0
2
4
6
8
12
16
StageI
StageII
StageIII
StageIV
StageV
StageVI

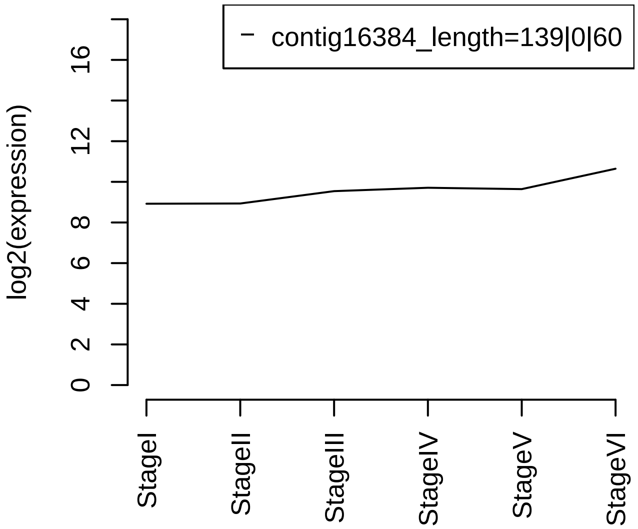
contig16384_length=139|0|60
log2(expression)
0
2
4
6
8
12
16
StageI
StageII
StageIII
StageIV
StageV
StageVI

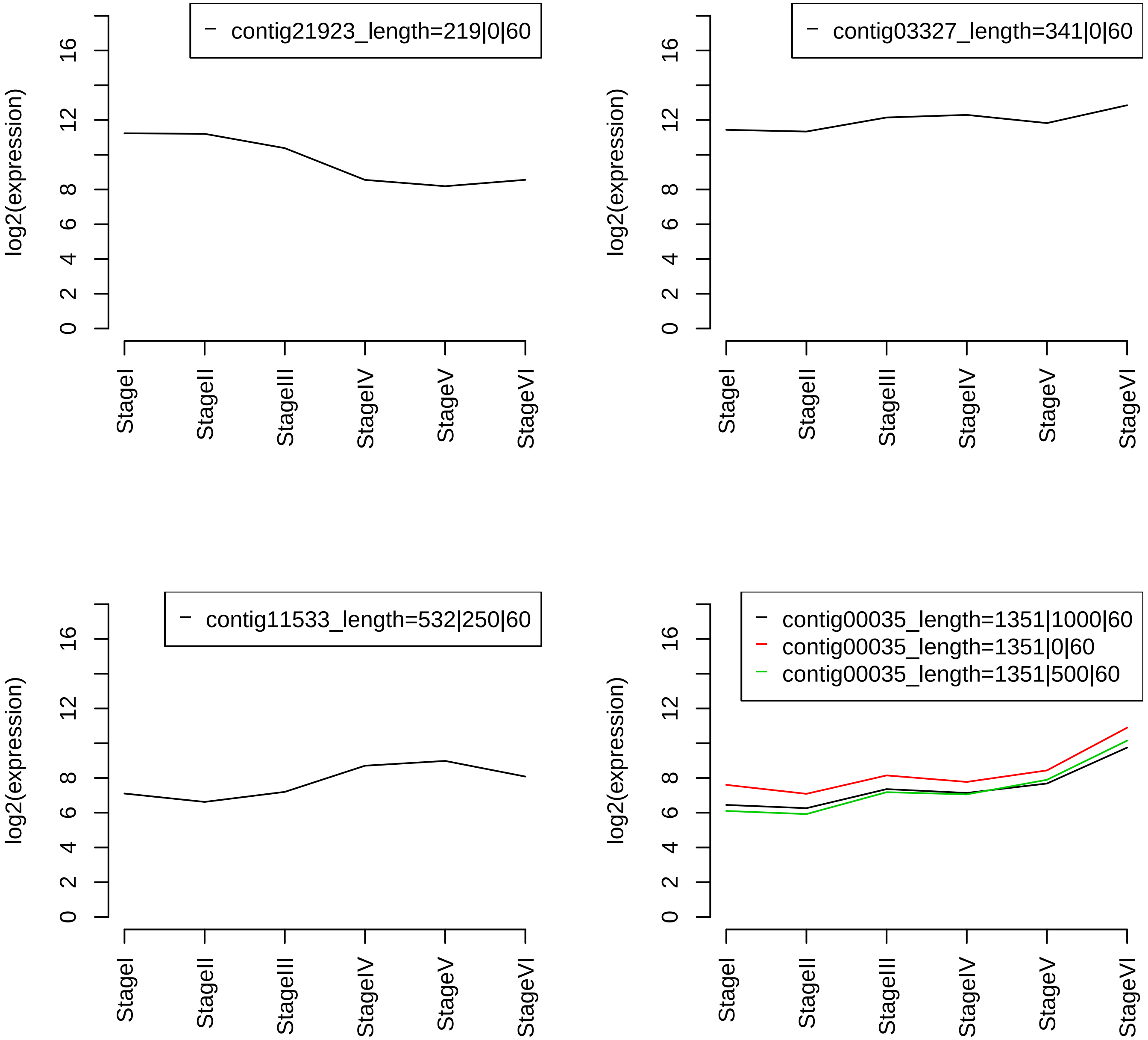
contig21923_length=219|0|60
contig03327_length=341|0|60
contig11533_length=532|250|60
contig00035_length=1351|1000|60
contig00035_length=1351|0|60
contig00035_length=1351|500|60
log2(expression)
0 2 4 6 8 12 16
StageI StageII StageIII StageIV StageV StageVI

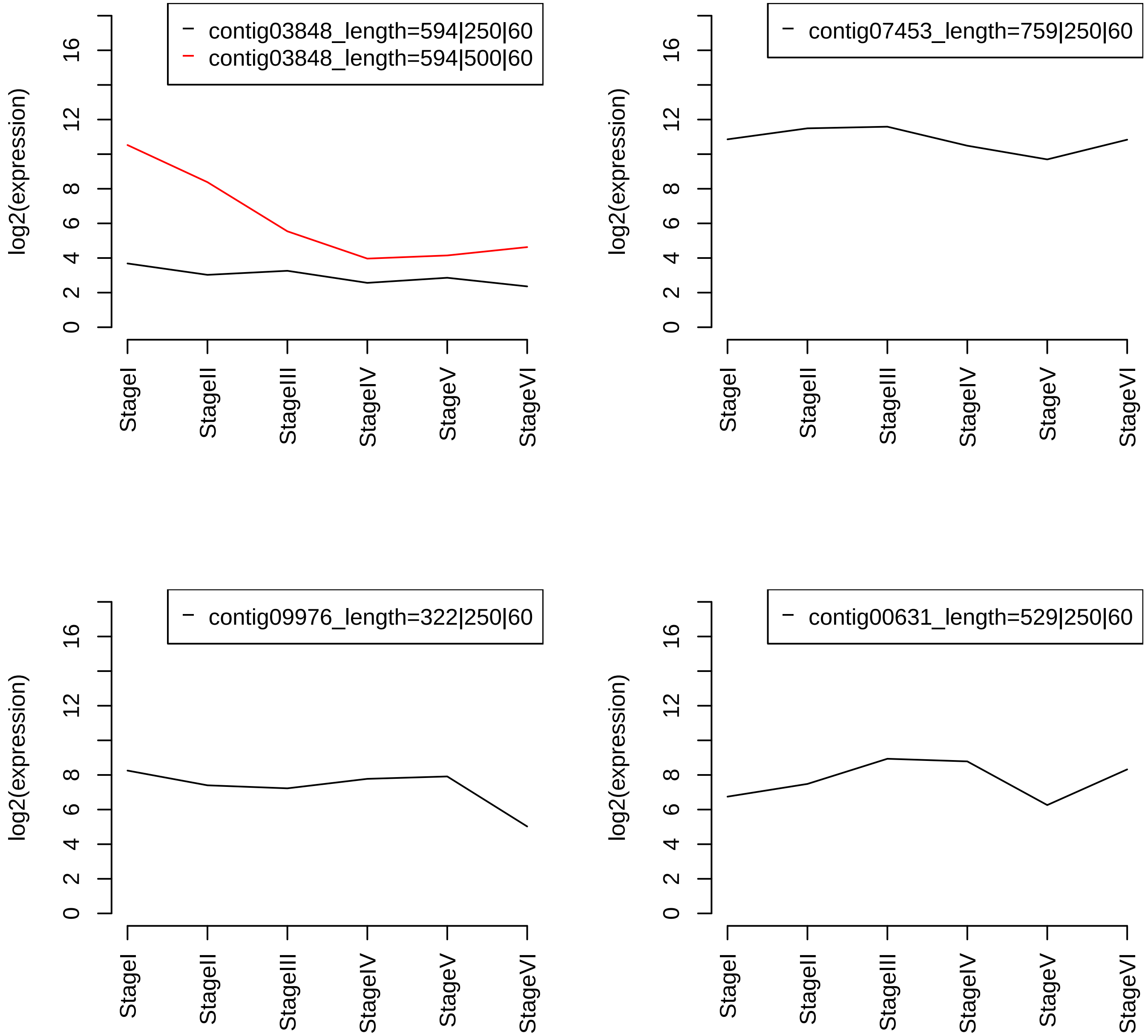

contig03848_length=594|250|60
contig03848_length=594|500|60
contig07453_length=759|250|60
contig09976_length=322|250|60
contig00631_length=529|250|60
log2(expression)
0
2
4
6
8
12
16
StageI
StageII
StageIII
StageIV
StageV
StageVI

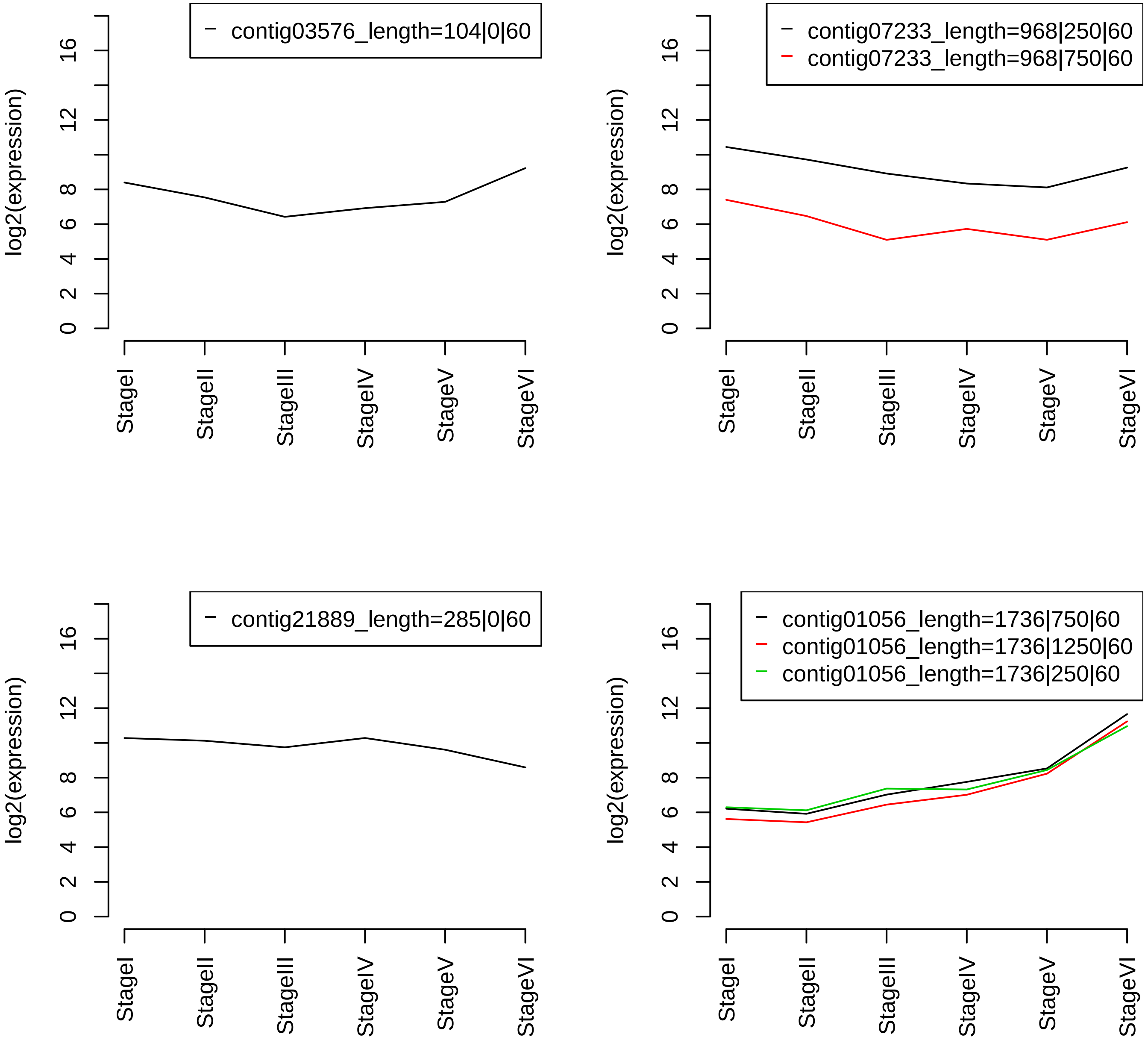
contig03576_length=104|0|60
contig07233_length=968|250|60
contig07233_length=968|750|60
contig21889_length=285|0|60
contig01056_length=1736|750|60
contig01056_length=1736|1250|60
contig01056_length=1736|250|60
log2(expression)
0 2 4 6 8 12 16
StageI StageII StageIII StageIV StageV StageVI

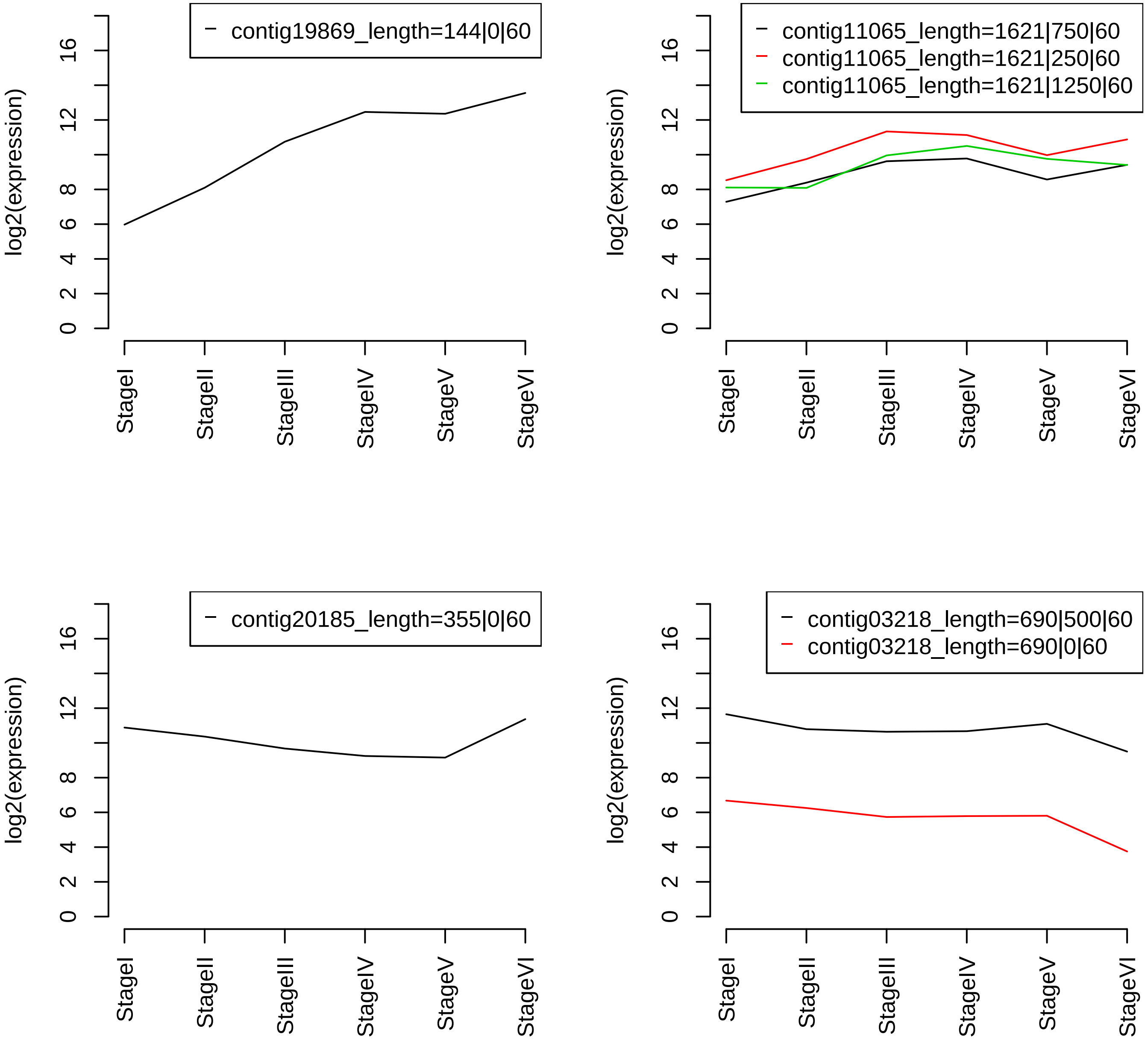

contig19869_length=144|0|60
contig11065_length=1621|750|60
contig11065_length=1621|250|60
contig11065_length=1621|1250|60
contig20185_length=355|0|60
contig03218_length=690|500|60
contig03218_length=690|0|60
log2(expression)
0
2
4
6
8
12
16
StageI
StageII
StageIII
StageIV
StageV
StageVI

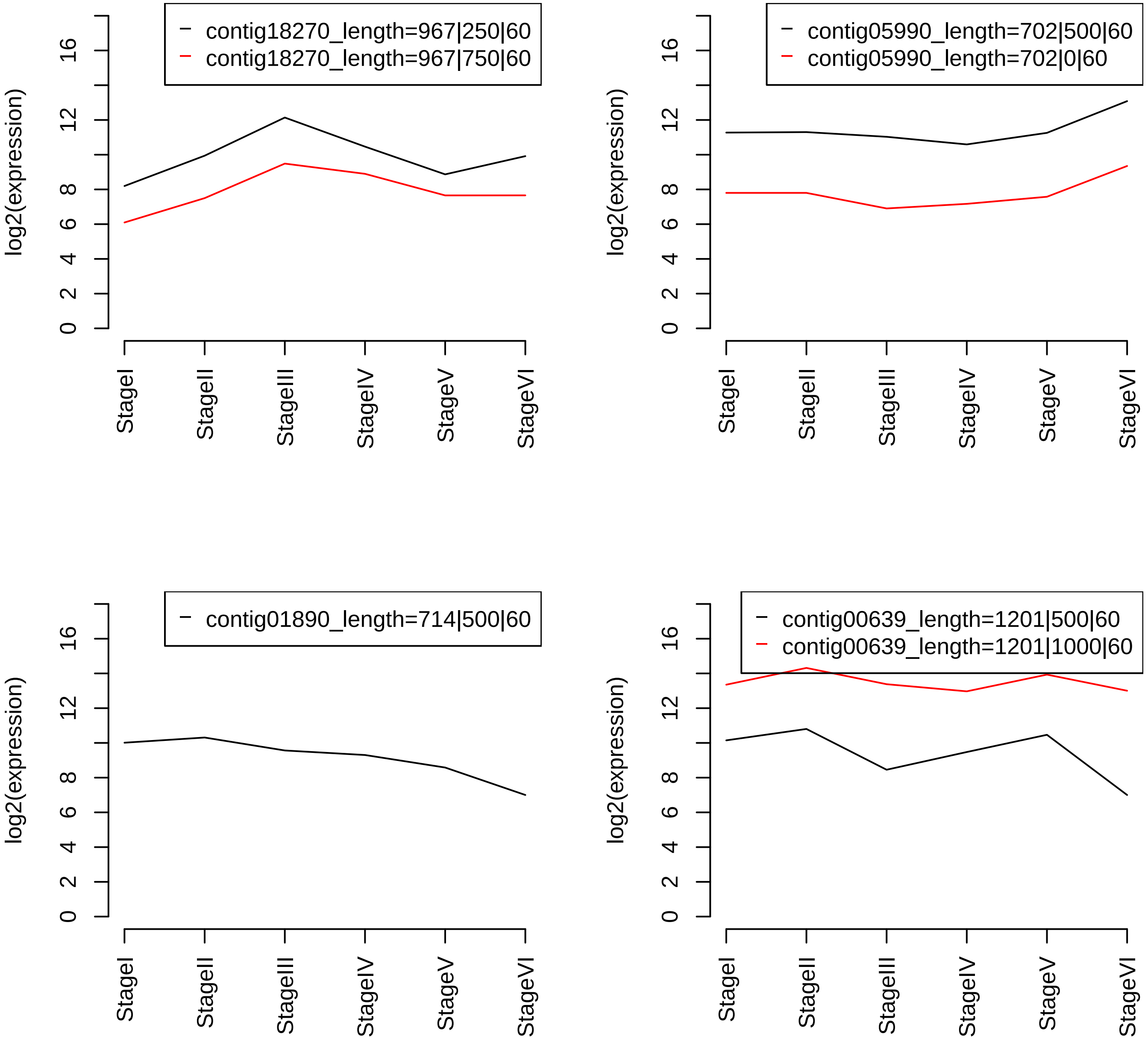
contig18270_length=967|250|60
contig18270_length=967|750|60
contig05990_length=702|500|60
contig05990_length=702|0|60
contig01890_length=714|500|60
contig00639_length=1201|500|60
contig00639_length=1201|1000|60
log2(expression)
0 2 4 6 8 12 16
StageI StageII StageIII StageIV StageV StageVI

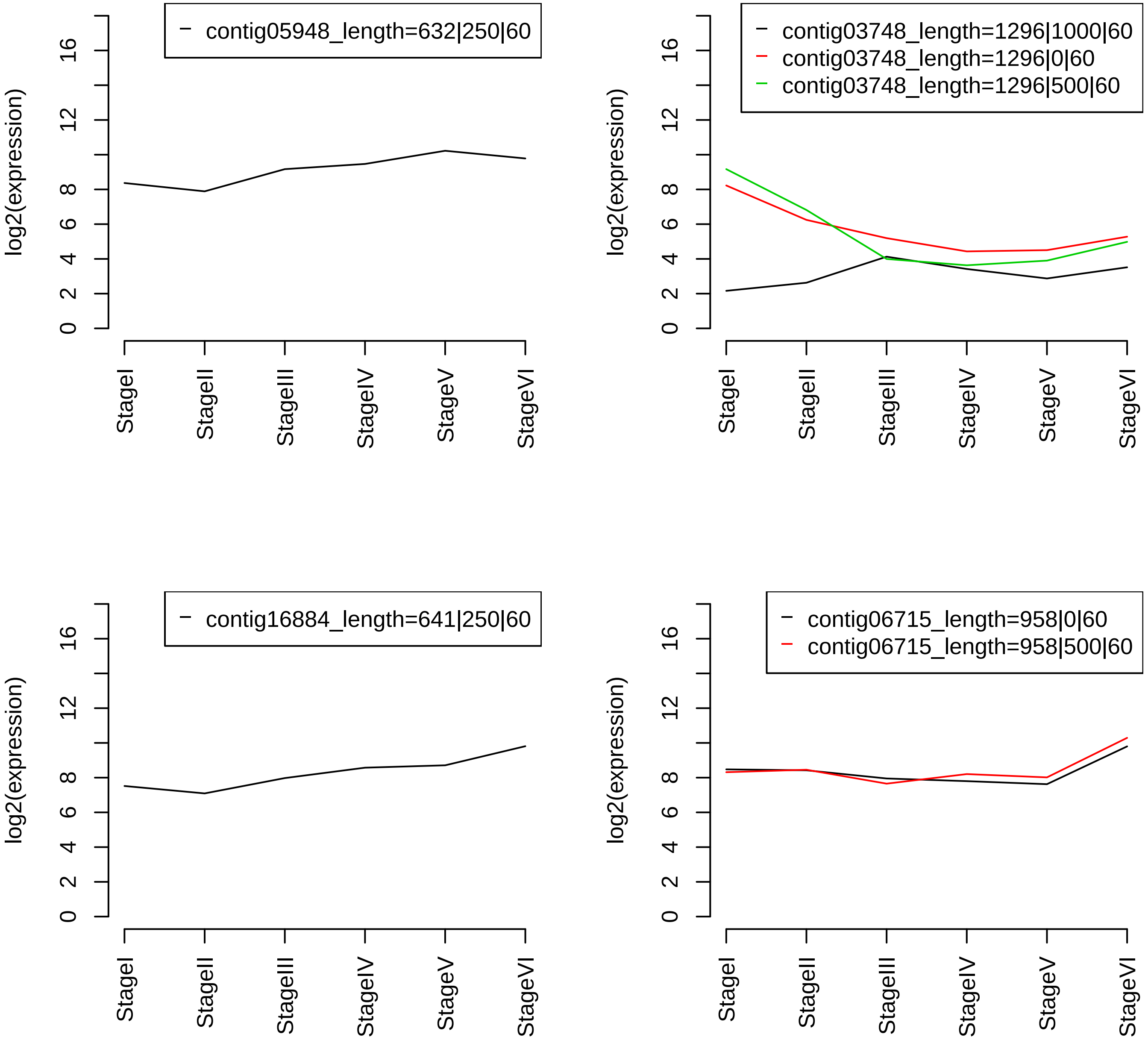
contig05948_length=632|250|60
contig03748_length=1296|1000|60
contig03748_length=1296|0|60
contig03748_length=1296|500|60
contig16884_length=641|250|60
contig06715_length=958|0|60
contig06715_length=958|500|60
log2(expression)
0
2
4
6
8
12
16
StageI
StageII
StageIII
StageIV
StageV
StageVI

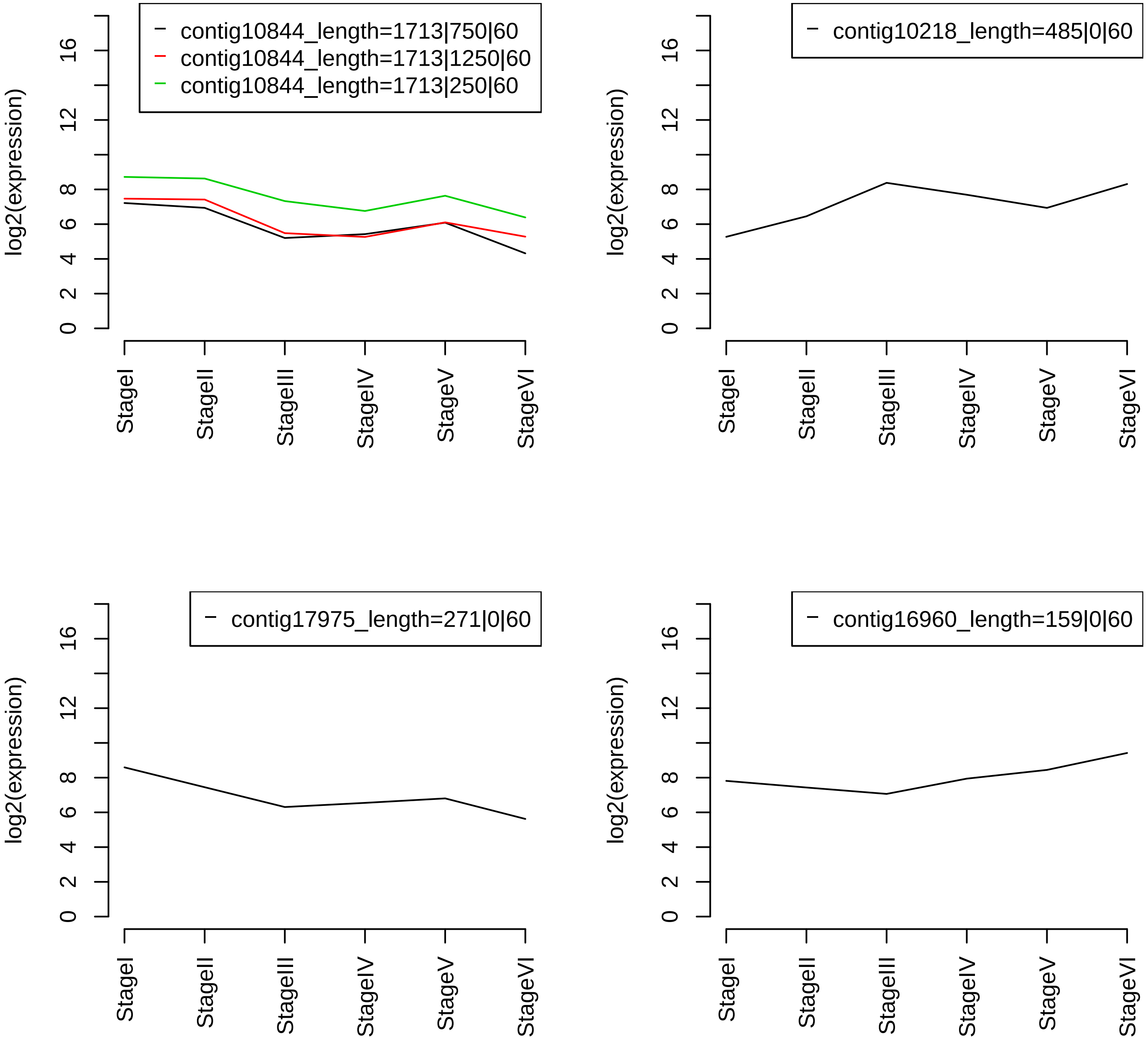
contig10844_length=1713|750|60
contig10844_length=1713|1250|60
contig10844_length=1713|250|60
contig10218_length=485|0|60
contig17975_length=271|0|60
contig16960_length=159|0|60
log2(expression)
0
2
4
6
8
12
16
StageI
StageII
StageIII
StageIV
StageV
StageVI

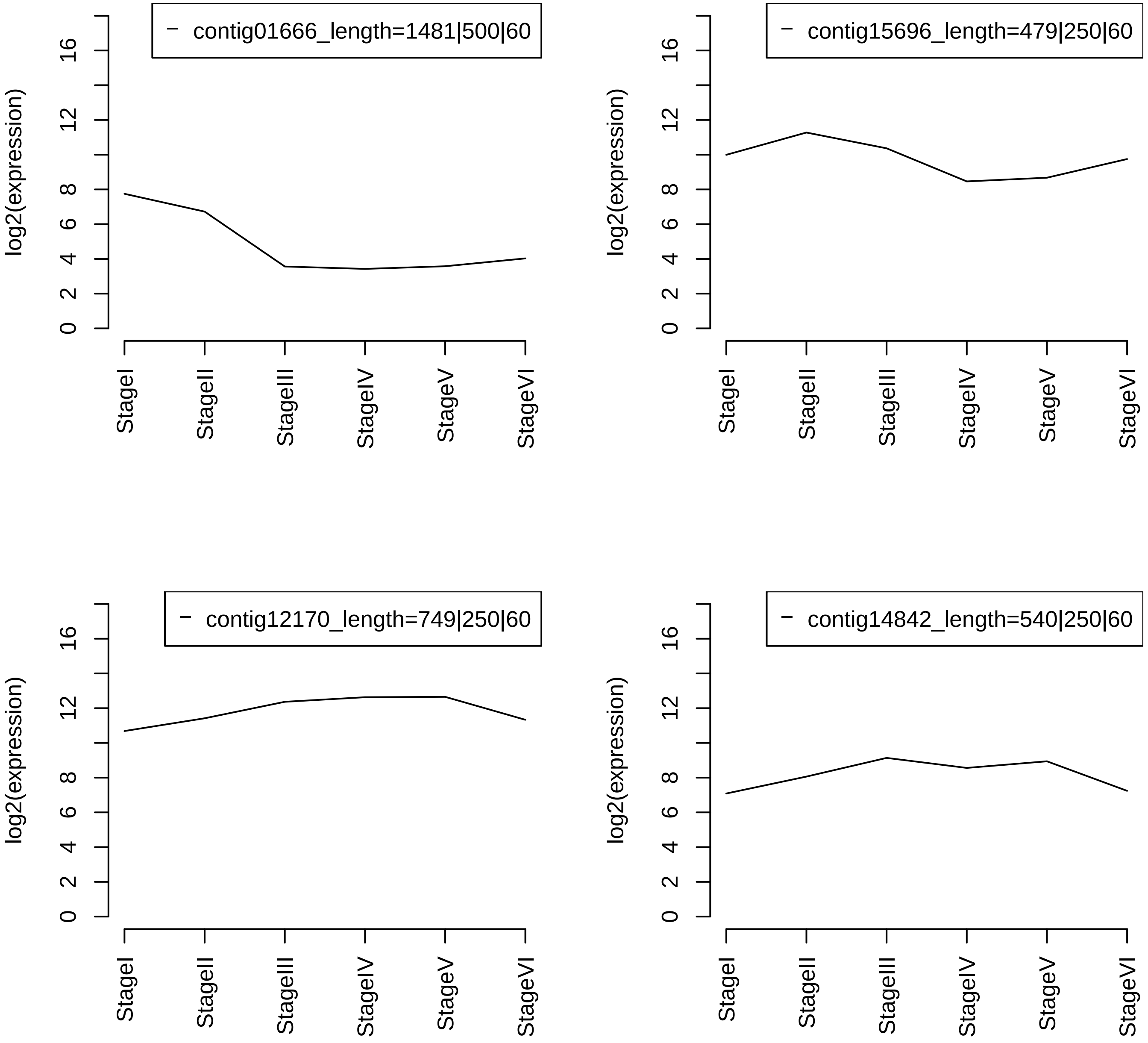
contig01666_length=1481|500|60
contig15696_length=479|250|60
contig12170_length=749|250|60
contig14842_length=540|250|60
log2(expression)
0
2
4
6
8
12
16
StageI
StageII
StageIII
StageIV
StageV
StageVI

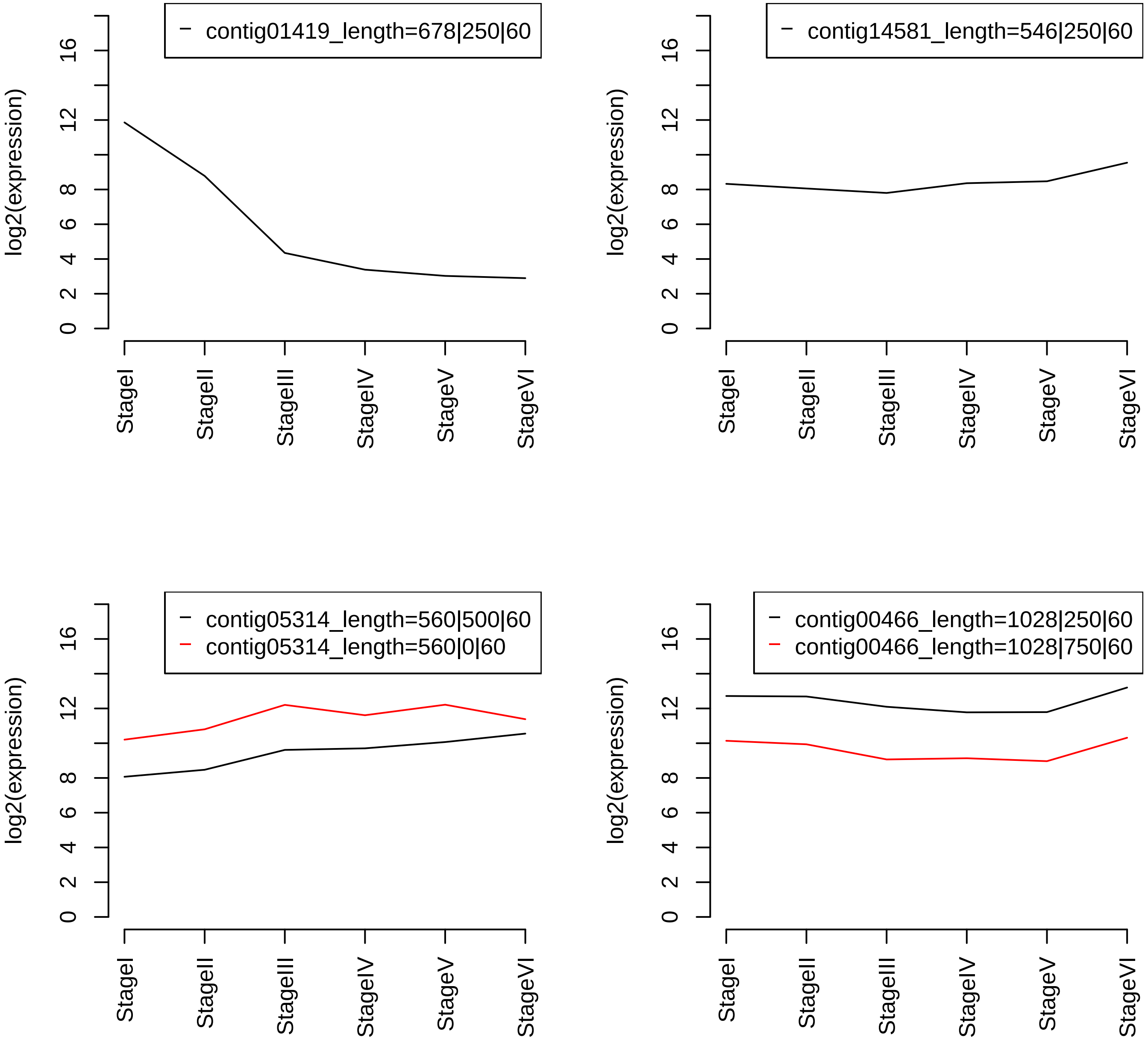
contig01419_length=678|250|60
contig14581_length=546|250|60
contig05314_length=560|500|60
contig05314_length=560|0|60
contig00466_length=1028|250|60
contig00466_length=1028|750|60
log2(expression)
0
2
4
6
8
12
16
StageI
StageII
StageIII
StageIV
StageV
StageVI

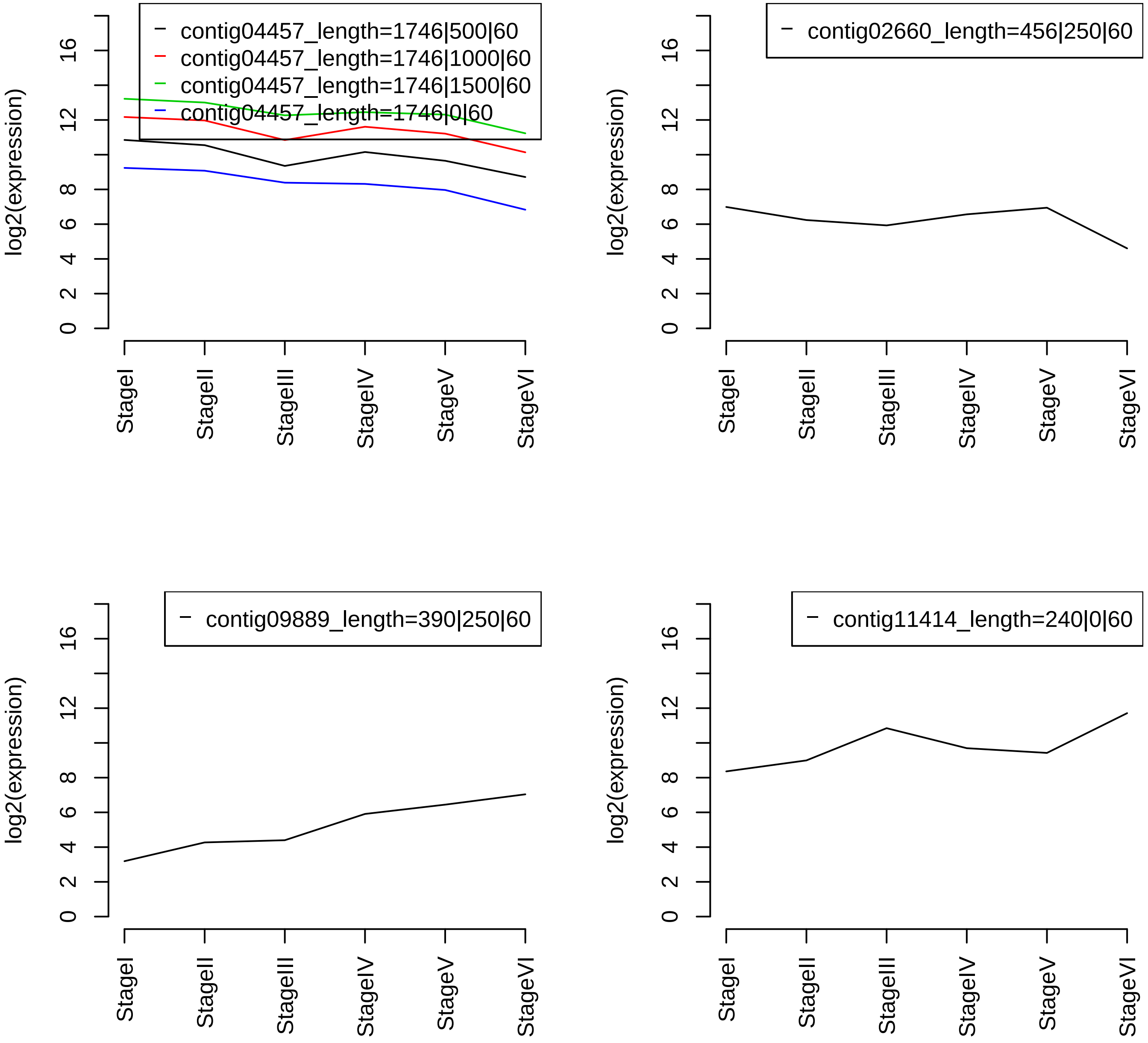

contig04457_length=1746|500|60
contig04457_length=1746|1000|60
contig04457_length=1746|1500|60
contig04457_length=1746|0|60
contig02660_length=456|250|60
contig09889_length=390|250|60
contig11414_length=240|0|60
log2(expression)
0
2
4
6
8
12
16
StageI
StageII
StageIII
StageIV
StageV
StageVI

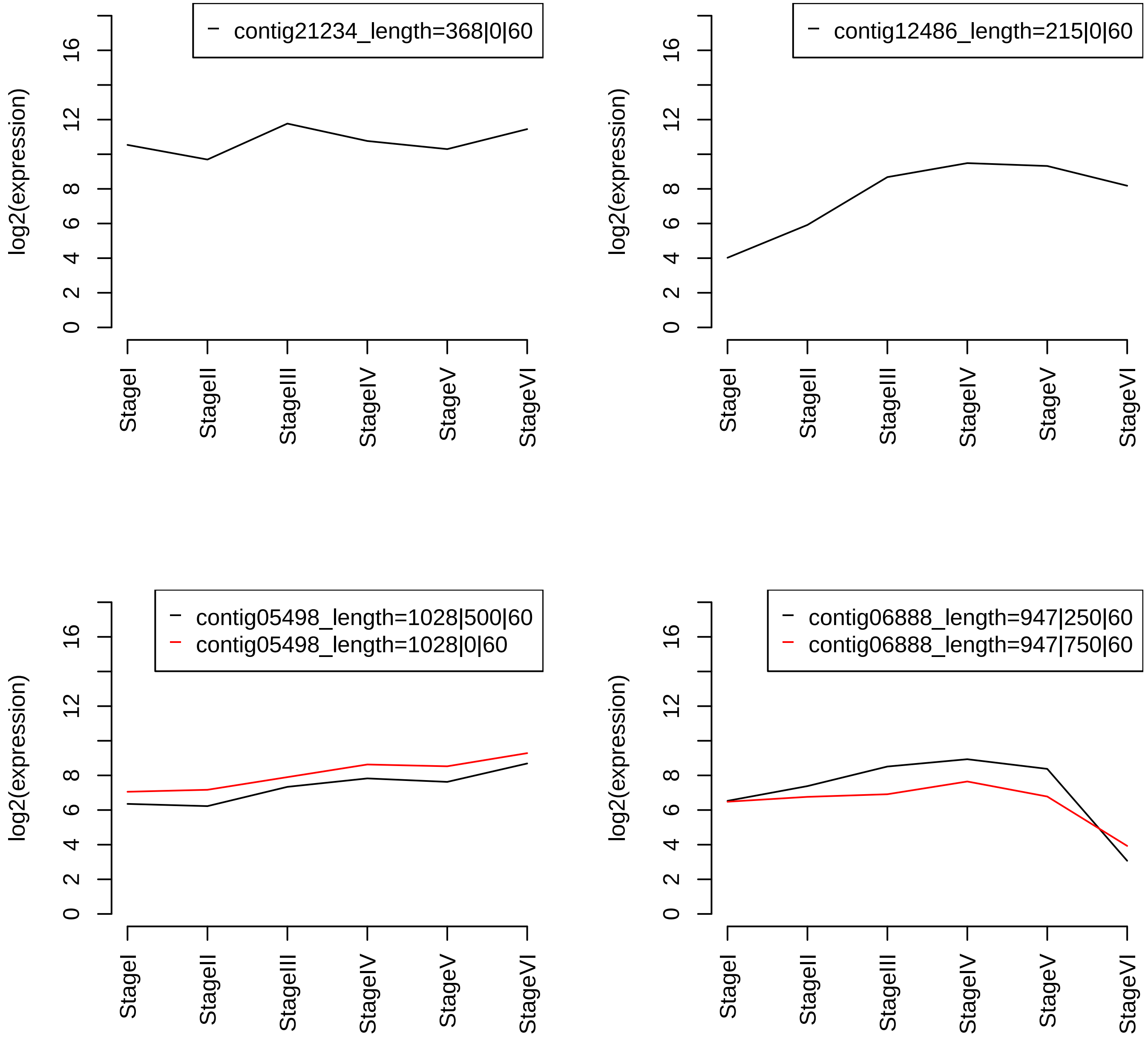
contig21234_length=368|0|60
contig12486_length=215|0|60
contig05498_length=1028|500|60
contig05498_length=1028|0|60
contig06888_length=947|250|60
contig06888_length=947|750|60
log2(expression)
0
2
4
6
8
12
16
StageI
StageII
StageIII
StageIV
StageV
StageVI

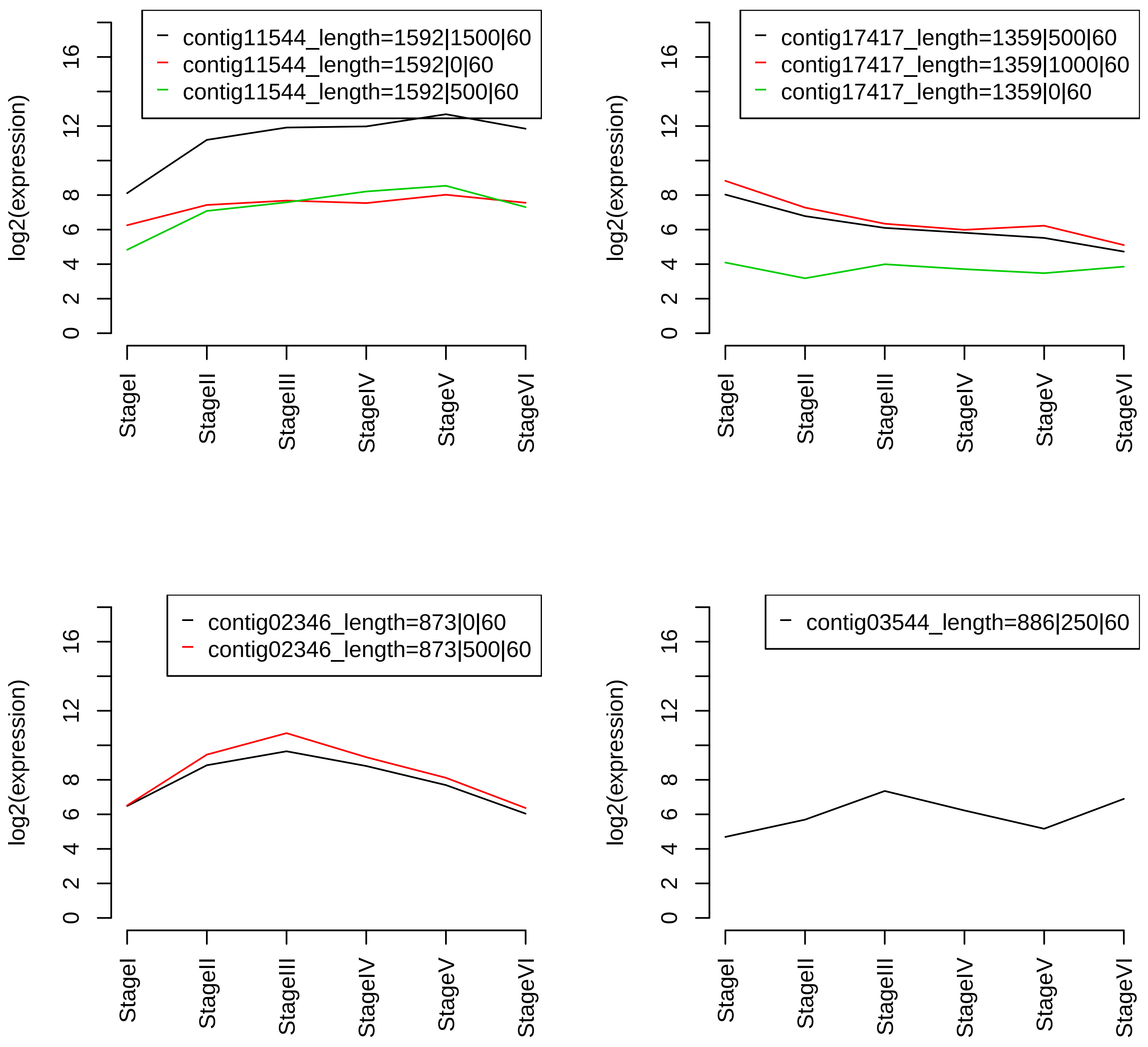
contig11544_length=1592|1500|60
contig11544_length=1592|0|60
contig11544_length=1592|500|60
log2(expression)
0
2
4
6
8
12
16
StageI
StageII
StageIII
StageIV
StageV
StageVI
contig17417_length=1359|500|60
contig17417_length=1359|1000|60
contig17417_length=1359|0|60
contig02346_length=873|0|60
contig02346_length=873|500|60
contig03544_length=886|250|60

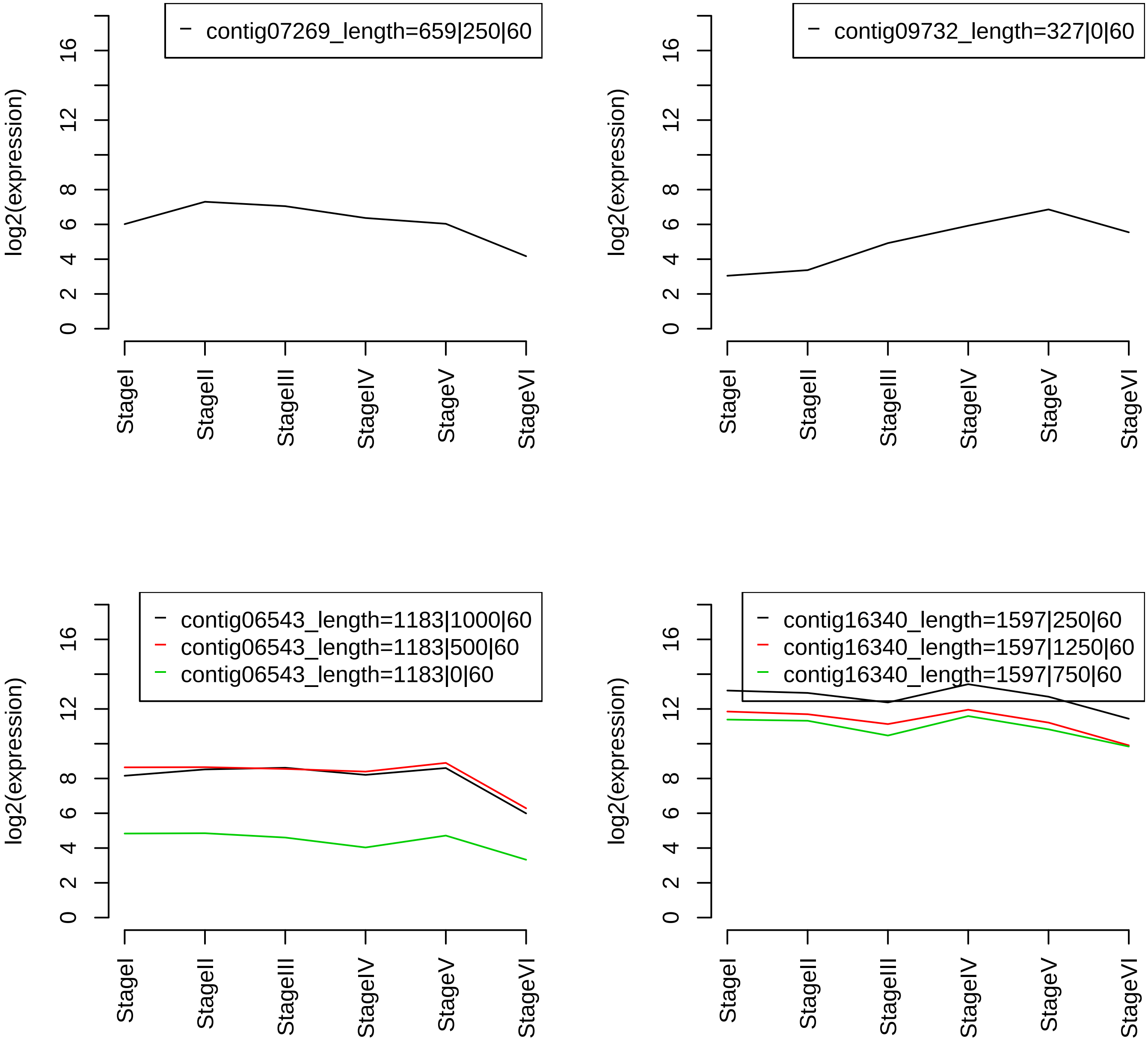
contig07269_length=659|250|60
contig09732_length=327|0|60
contig06543_length=1183|1000|60
contig06543_length=1183|500|60
contig06543_length=1183|0|60
contig16340_length=1597|250|60
contig16340_length=1597|1250|60
contig16340_length=1597|750|60
log2(expression)
0
2
4
6
8
12
16
StageI
StageII
StageIII
StageIV
StageV
StageVI

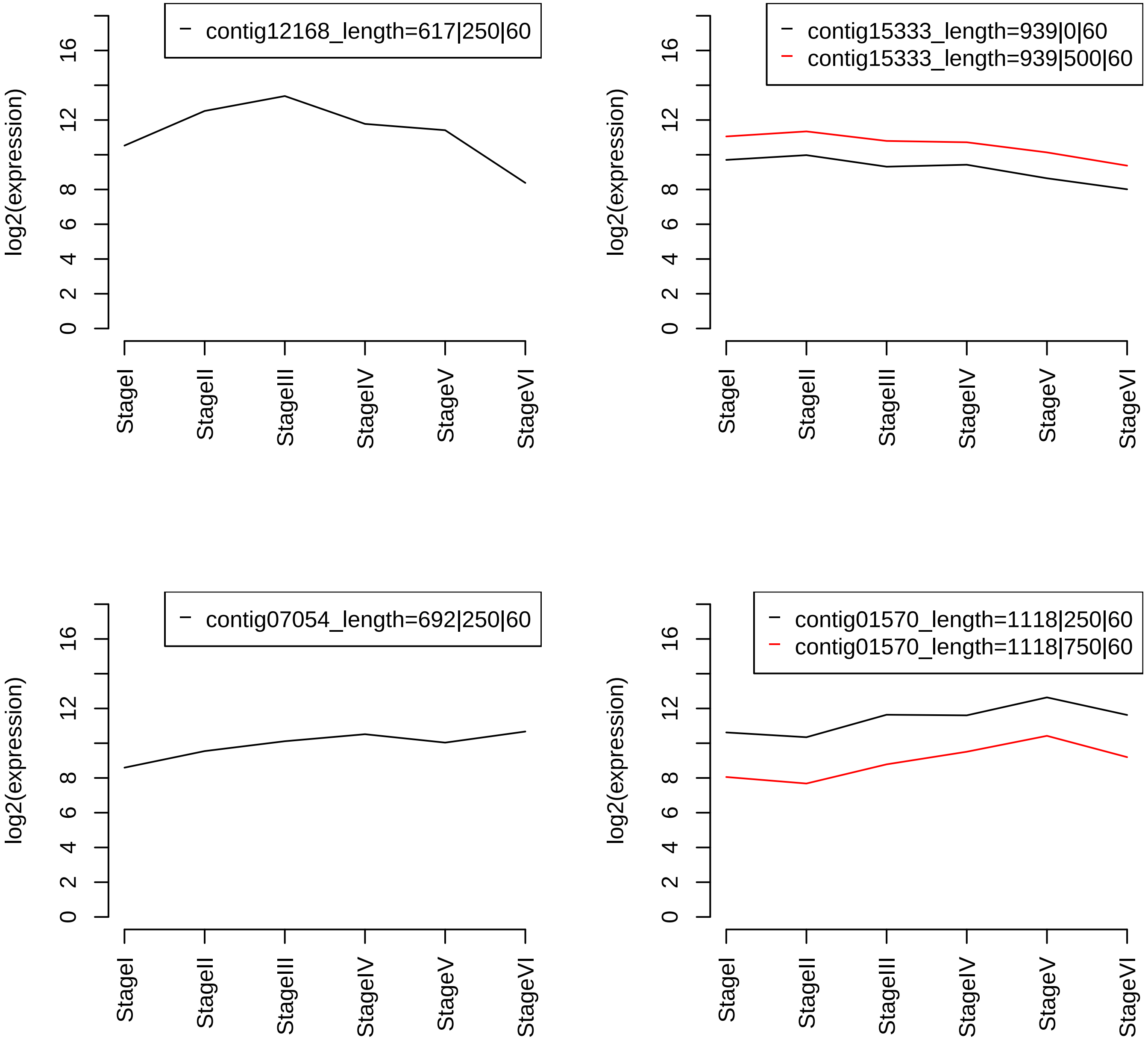
contig12168_length=617|250|60
log2(expression)
0 2 4 6 8 12 16
StageI StageII StageIII StageIV StageV StageVI
contig15333_length=939|0|60
contig15333_length=939|500|60
log2(expression)
0 2 4 6 8 12 16
StageI StageII StageIII StageIV StageV StageVI
contig07054_length=692|250|60
log2(expression)
0 2 4 6 8 12 16
StageI StageII StageIII StageIV StageV StageVI
contig01570_length=1118|250|60
contig01570_length=1118|750|60
log2(expression)
0 2 4 6 8 12 16
StageI StageII StageIII StageIV StageV StageVI

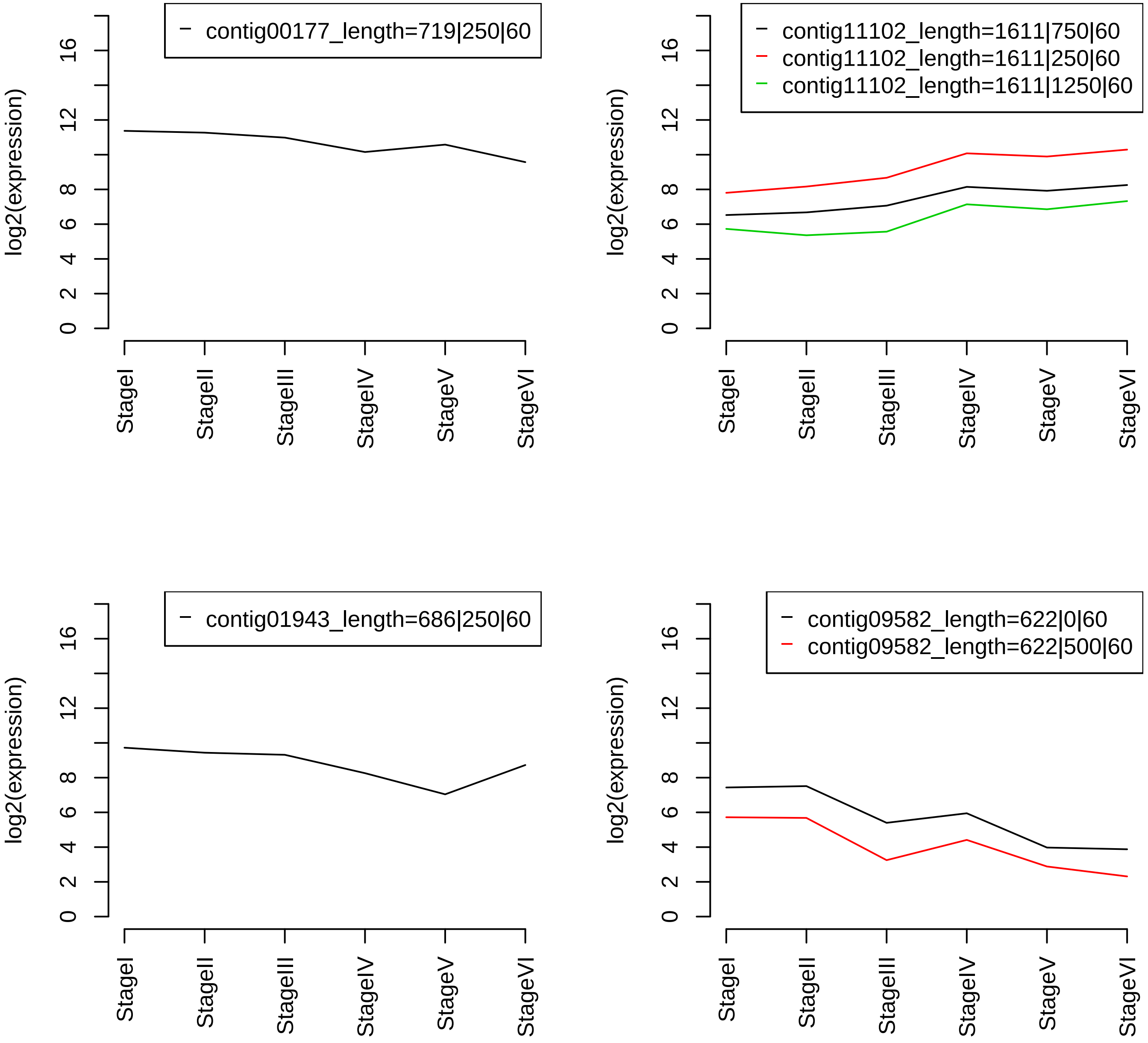

contig00177_length=719|250|60
contig11102_length=1611|750|60
contig11102_length=1611|250|60
contig11102_length=1611|1250|60
contig01943_length=686|250|60
contig09582_length=622|0|60
contig09582_length=622|500|60
log2(expression)
0 2 4 6 8 12 16
StageI StageII StageIII StageIV StageV StageVI

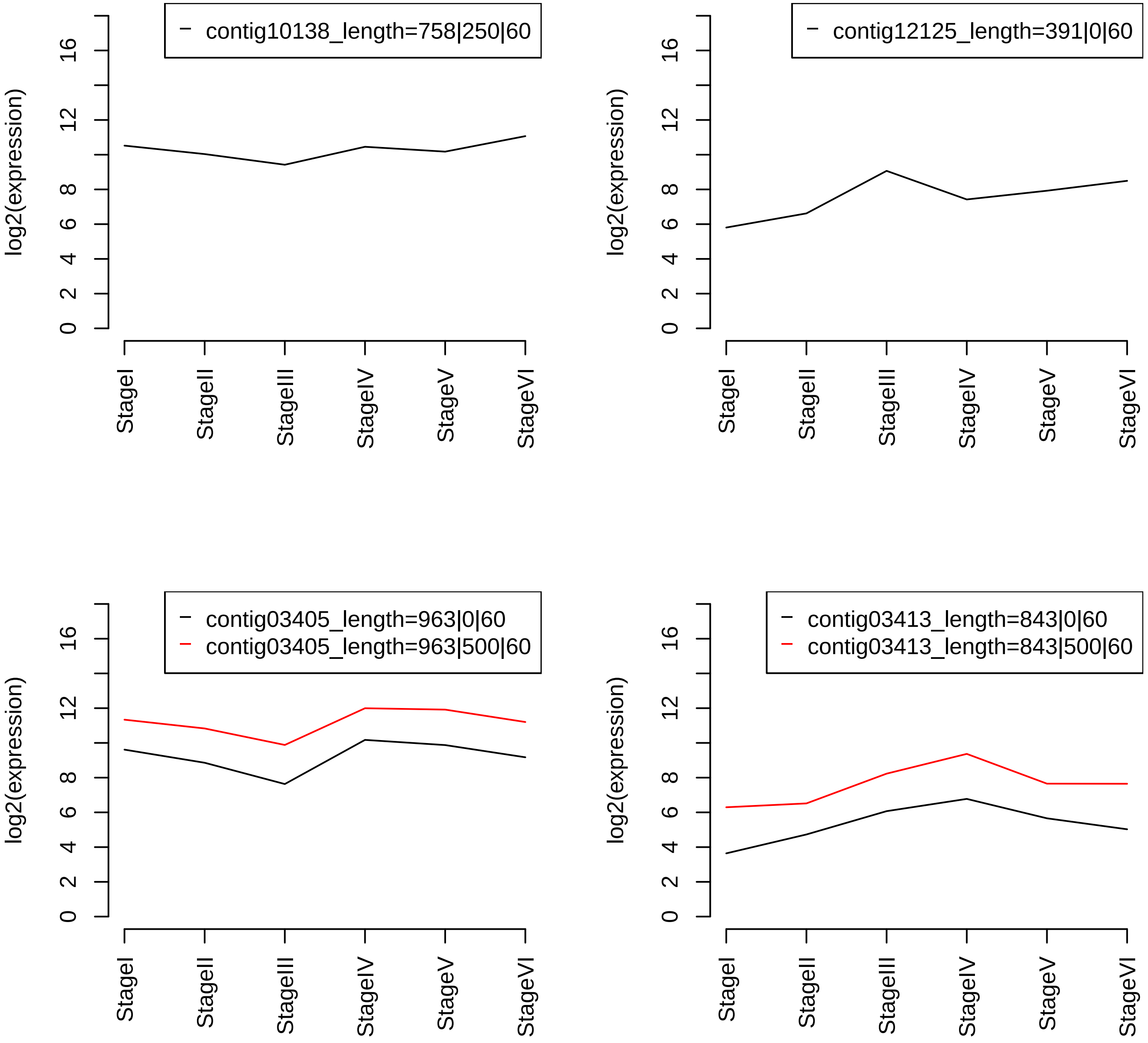

contig10138_length=758|250|60
contig12125_length=391|0|60
contig03405_length=963|0|60
contig03405_length=963|500|60
contig03413_length=843|0|60
contig03413_length=843|500|60
log2(expression)
0
2
4
6
8
12
16
StageI
StageII
StageIII
StageIV
StageV
StageVI

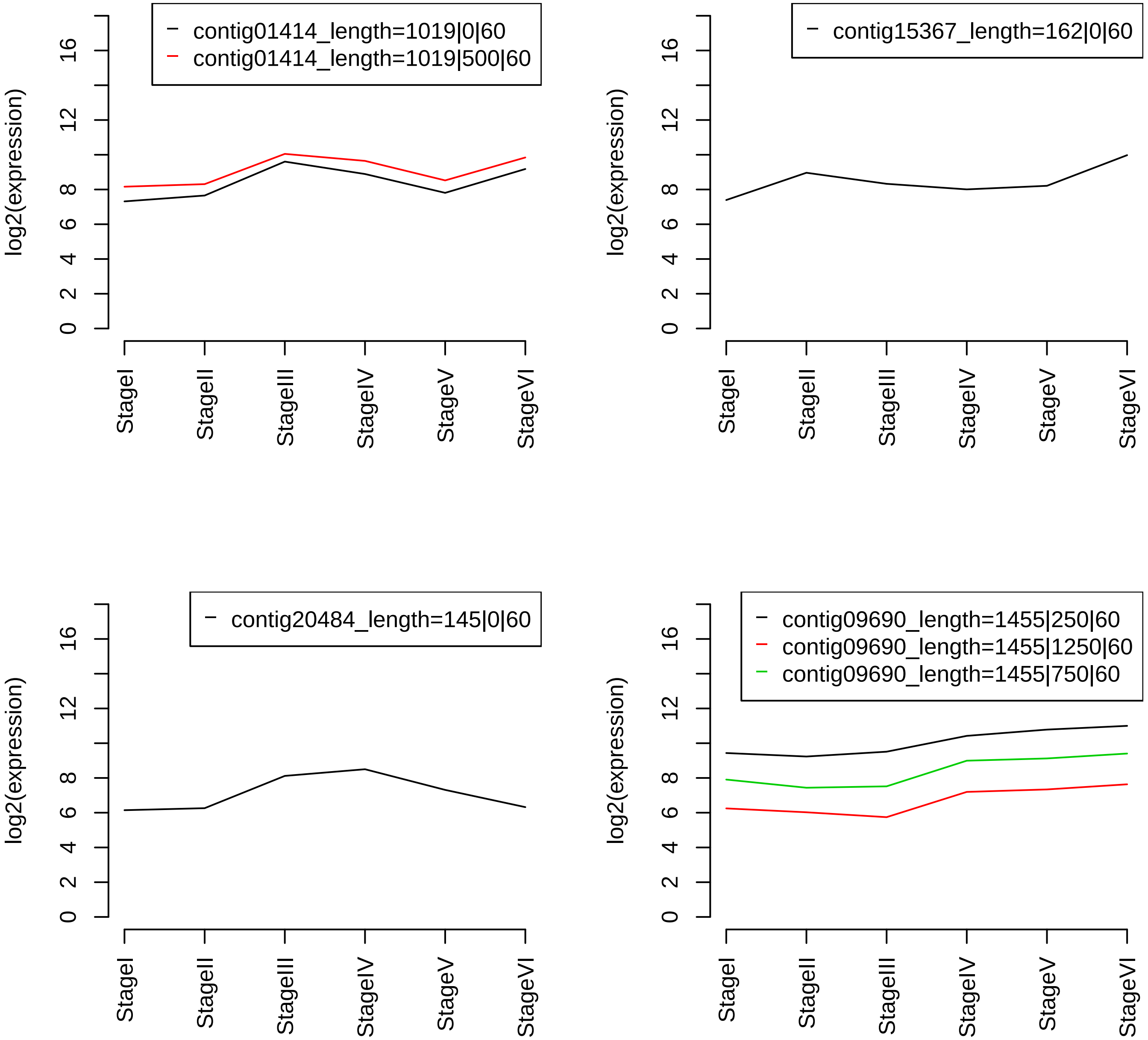
contig01414_length=1019|0|60
contig01414_length=1019|500|60
log2(expression)
0
2
4
6
8
12
16
StageI
StageII
StageIII
StageIV
StageV
StageVI
contig15367_length=162|0|60
log2(expression)
StageI
StageII
StageIII
StageIV
StageV
StageVI
contig20484_length=145|0|60
log2(expression)
StageI
StageII
StageIII
StageIV
StageV
StageVI
contig09690_length=1455|250|60
contig09690_length=1455|1250|60
contig09690_length=1455|750|60
log2(expression)
StageI
StageII
StageIII
StageIV
StageV
StageVI

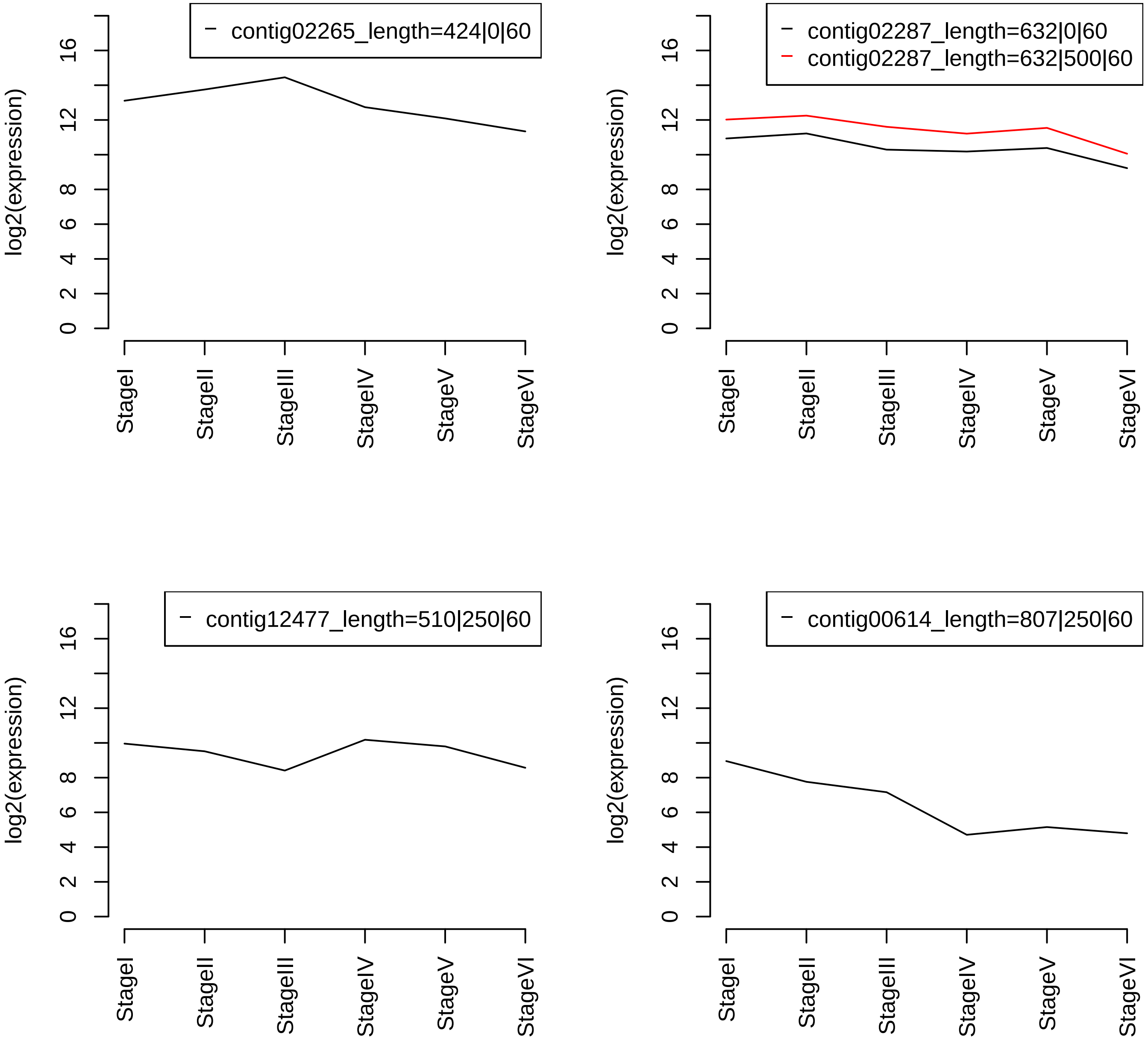

contig02265_length=424|0|60
contig02287_length=632|0|60
contig02287_length=632|500|60
contig12477_length=510|250|60
contig00614_length=807|250|60
log2(expression)
0
2
4
6
8
12
16
StageI
StageII
StageIII
StageIV
StageV
StageVI

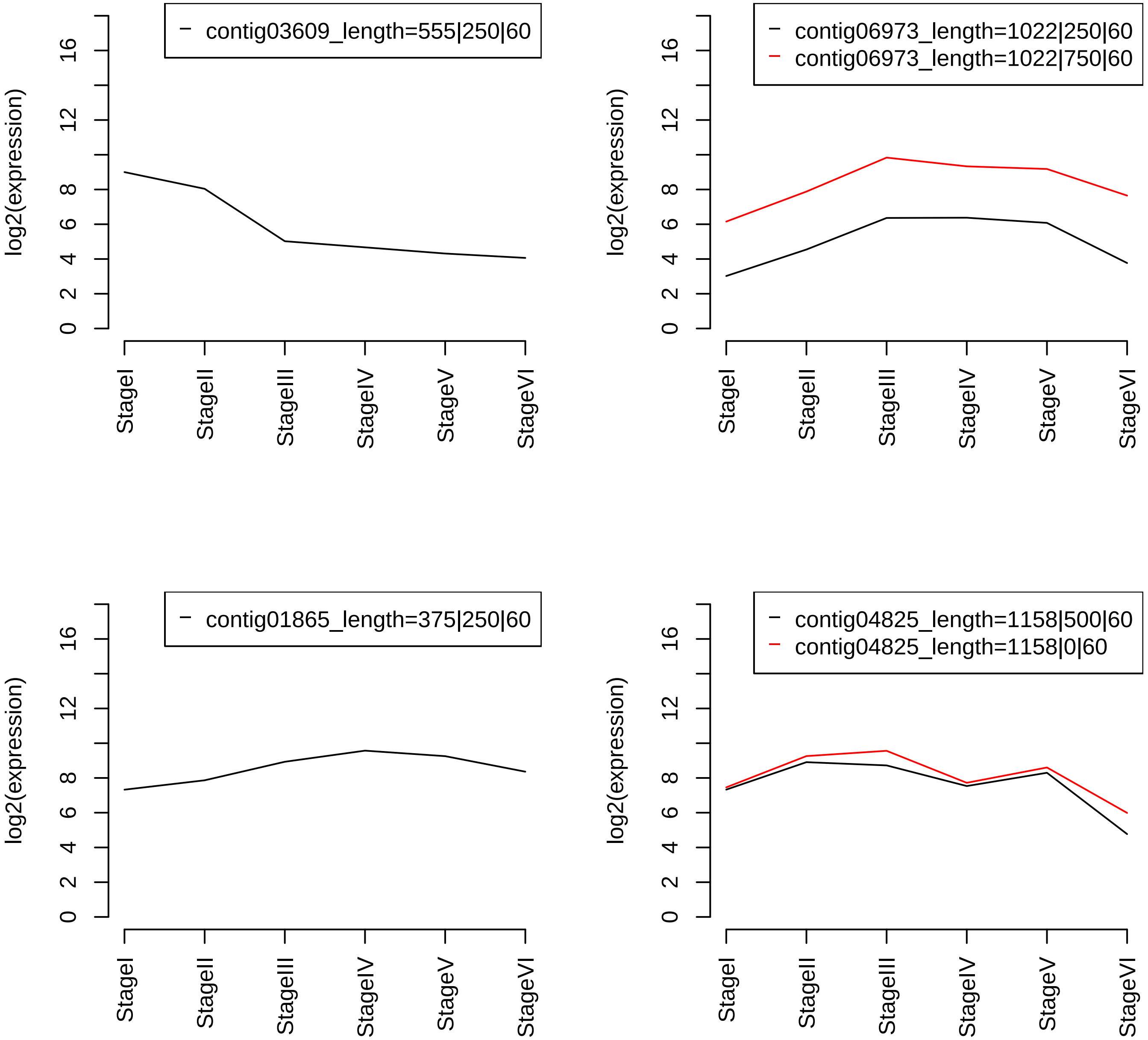

contig03609_length=555|250|60
contig06973_length=1022|250|60
contig06973_length=1022|750|60
contig01865_length=375|250|60
contig04825_length=1158|500|60
contig04825_length=1158|0|60
log2(expression)
0
2
4
6
8
12
16
StageI
StageII
StageIII
StageIV
StageV
StageVI

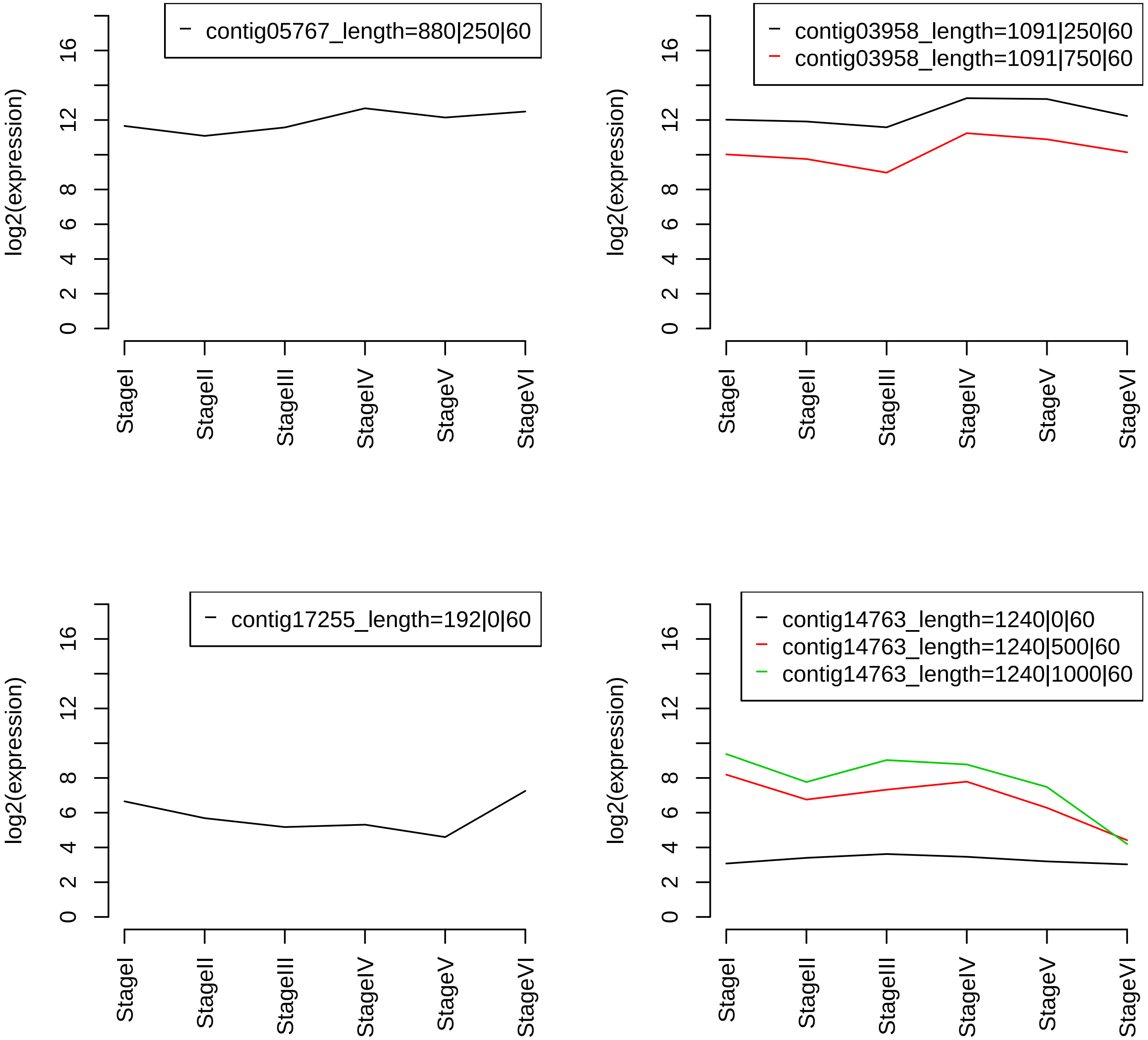

contig05767_length=880|250|60
contig03958_length=1091|250|60
contig03958_length=1091|750|60
contig17255_length=192|0|60
contig14763_length=1240|0|60
contig14763_length=1240|500|60
contig14763_length=1240|1000|60
log2(expression)
StageI
StageII
StageIII
StageIV
StageV
StageVI

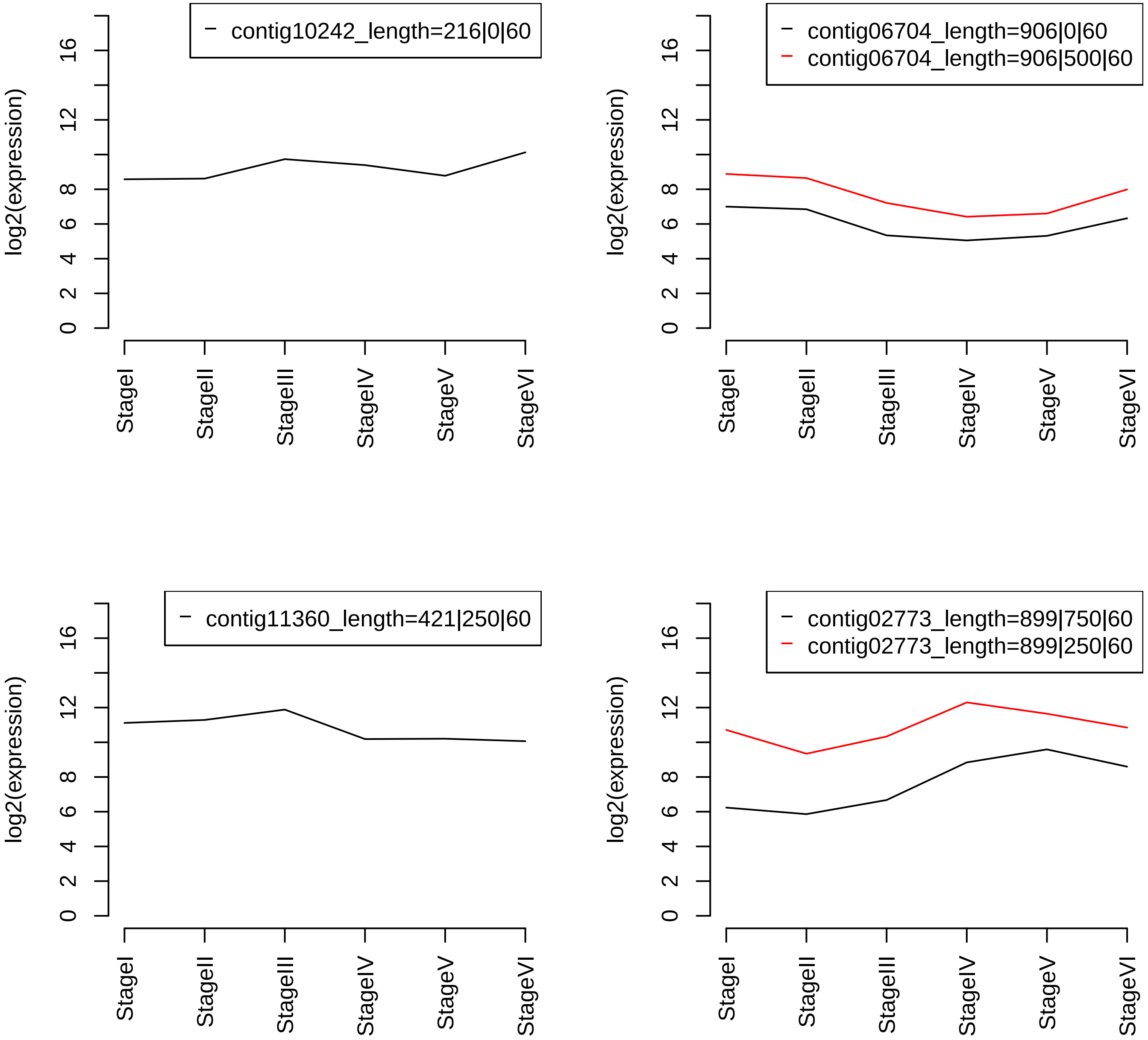
contig10242_length=216|0|60
contig06704_length=906|0|60
contig06704_length=906|500|60
contig11360_length=421|250|60
contig02773_length=899|750|60
contig02773_length=899|250|60
log2(expression)
0
2
4
6
8
12
16
StageI
StageII
StageIII
StageIV
StageV
StageVI

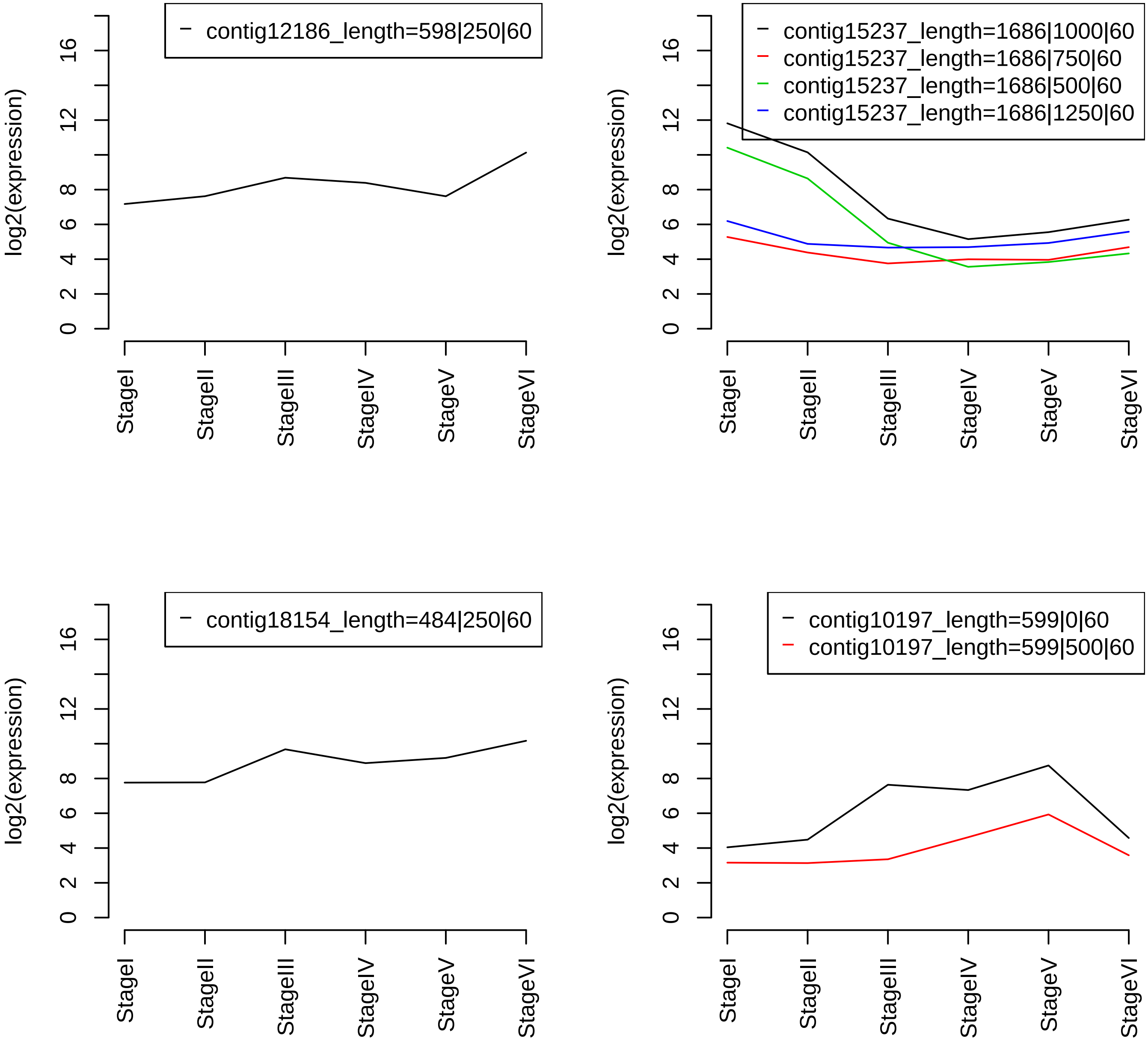
contig12186_length=598|250|60
contig15237_length=1686|1000|60
contig15237_length=1686|750|60
contig15237_length=1686|500|60
contig15237_length=1686|1250|60
contig18154_length=484|250|60
contig10197_length=599|0|60
contig10197_length=599|500|60
log2(expression)
0
2
4
6
8
12
16
StageI
StageII
StageIII
StageIV
StageV
StageVI

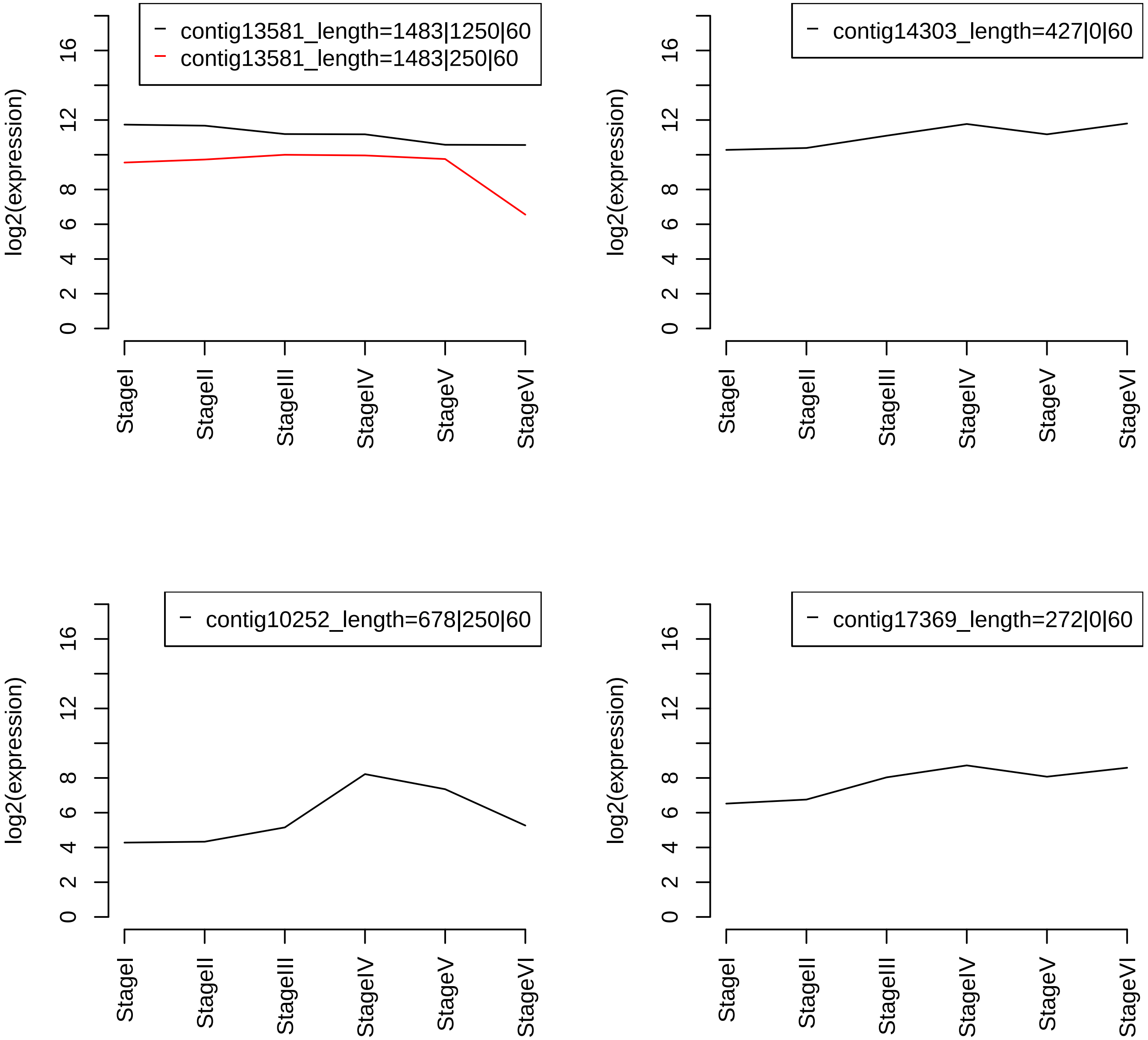

contig13581_length=1483|1250|60
contig13581_length=1483|250|60
contig14303_length=427|0|60
contig10252_length=678|250|60
contig17369_length=272|0|60
log2(expression)
0
2
4
6
8
12
16
StageI
StageII
StageIII
StageIV
StageV
StageVI

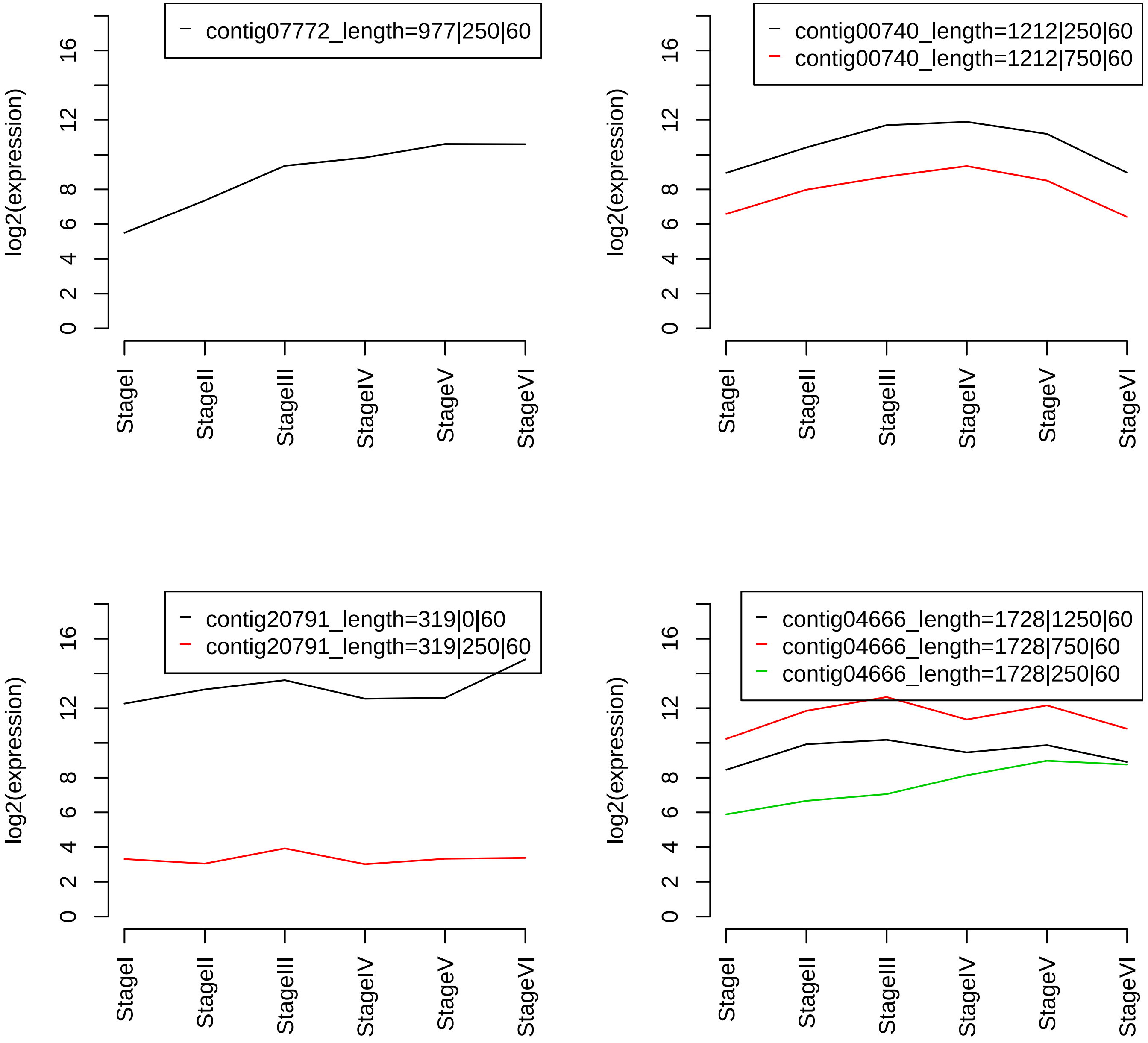
contig07772_length=977|250|60
contig00740_length=1212|250|60
contig00740_length=1212|750|60
contig20791_length=319|0|60
contig20791_length=319|250|60
contig04666_length=1728|1250|60
contig04666_length=1728|750|60
contig04666_length=1728|250|60
log2(expression)
0 2 4 6 8 12 16
StageI StageII StageIII StageIV StageV StageVI

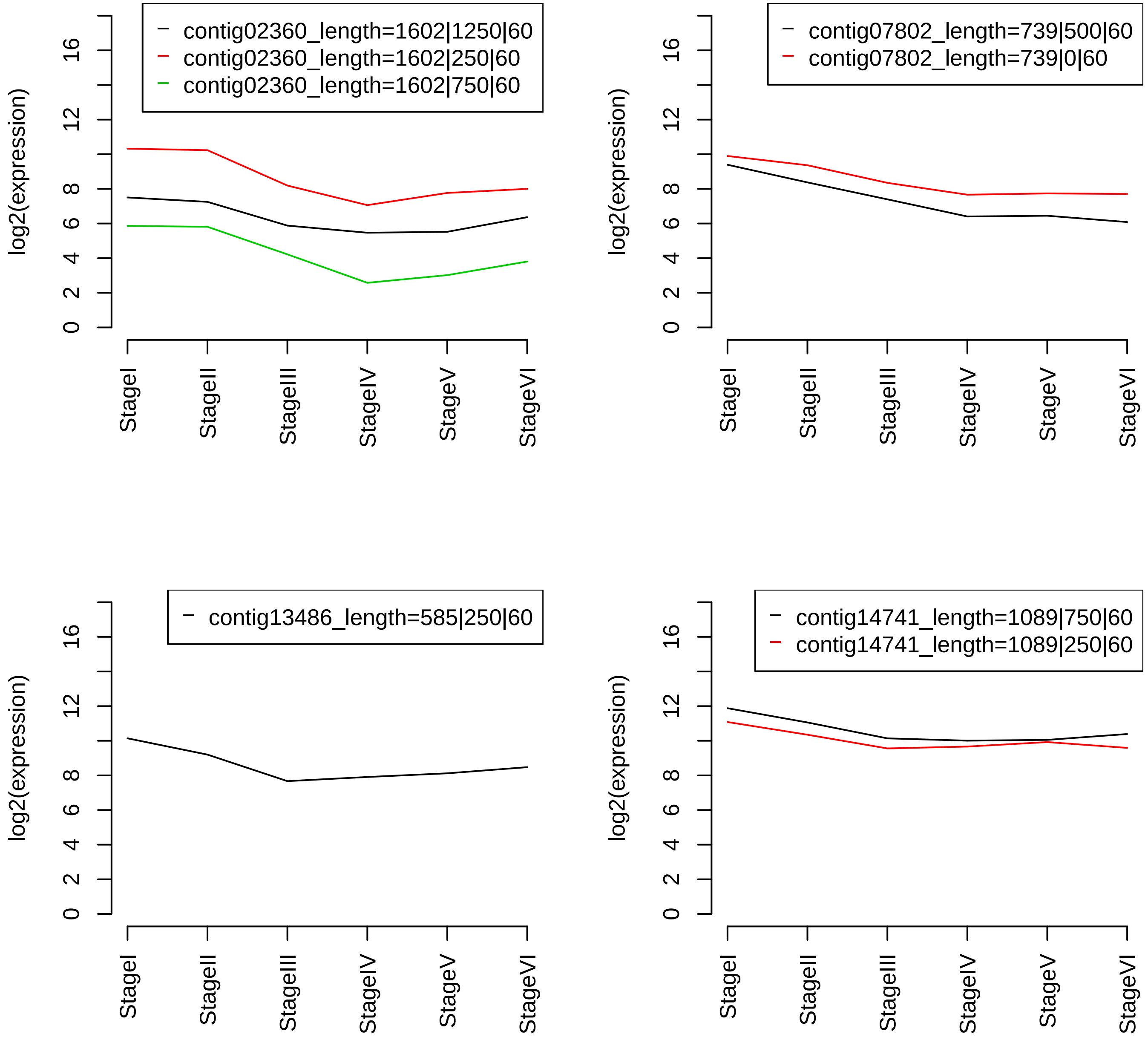
contig02360_length=1602|1250|60
contig02360_length=1602|250|60
contig02360_length=1602|750|60
contig07802_length=739|500|60
contig07802_length=739|0|60
contig13486_length=585|250|60
contig14741_length=1089|750|60
contig14741_length=1089|250|60
log2(expression)
StageI
StageII
StageIII
StageIV
StageV
StageVI

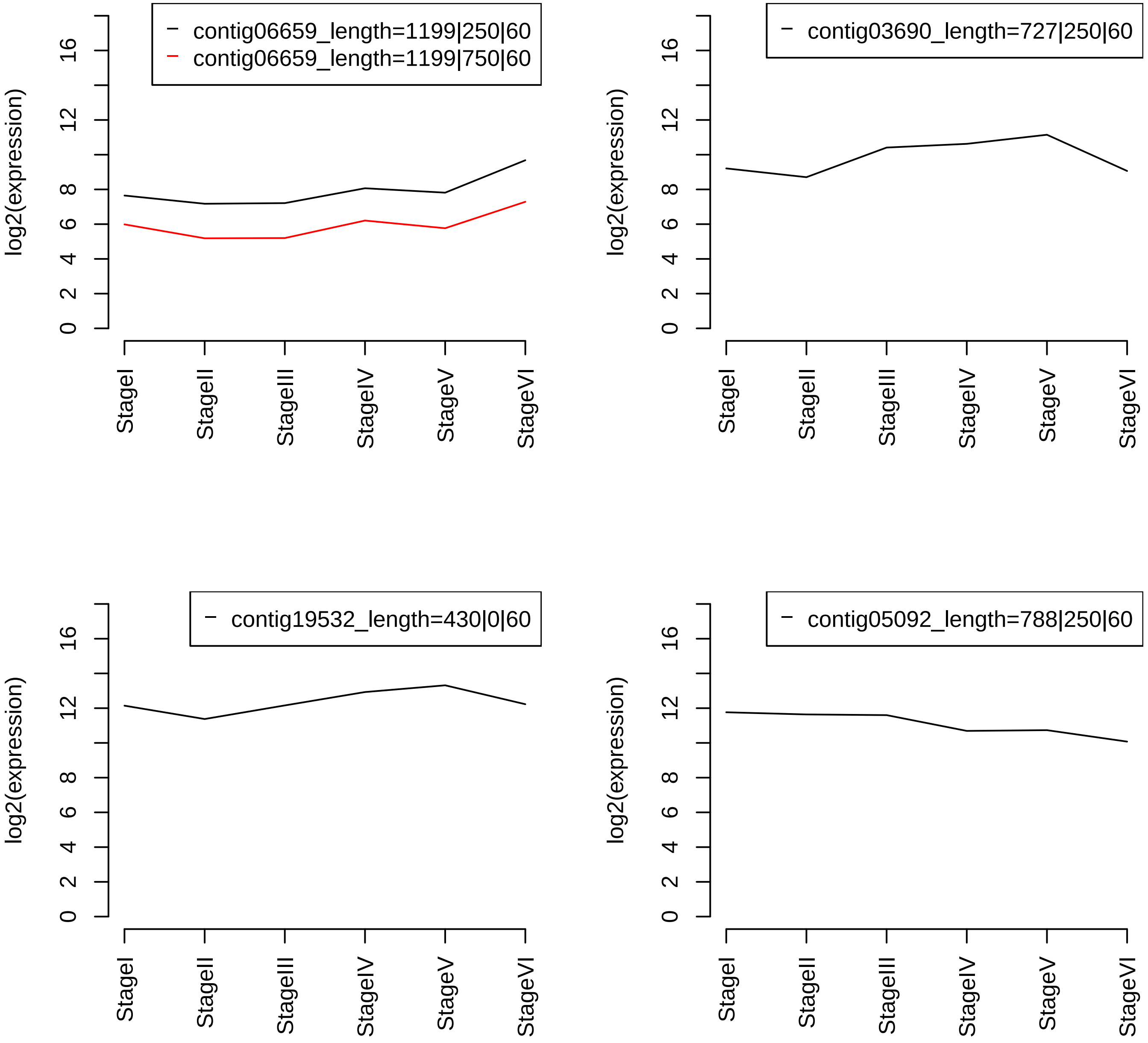

contig06659_length=1199|250|60
contig06659_length=1199|750|60
contig03690_length=727|250|60
contig19532_length=430|0|60
contig05092_length=788|250|60
log2(expression)
0
2
4
6
8
12
16
StageI
StageII
StageIII
StageIV
StageV
StageVI

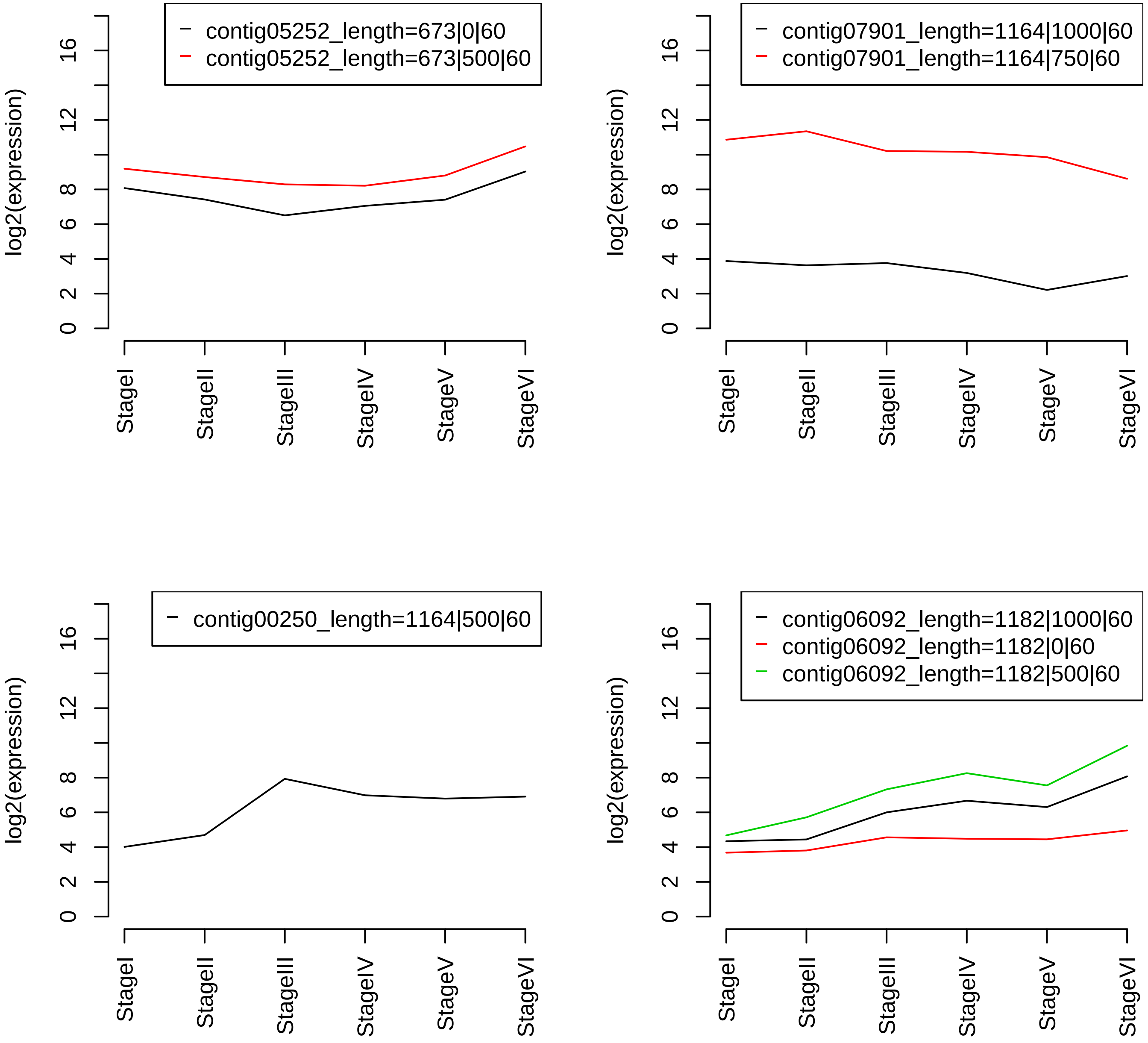
contig05252_length=673|0|60
contig05252_length=673|500|60
log2(expression)
0
2
4
6
8
12
16
StageI
StageII
StageIII
StageIV
StageV
StageVI
contig07901_length=1164|1000|60
contig07901_length=1164|750|60
contig00250_length=1164|500|60
contig06092_length=1182|1000|60
contig06092_length=1182|0|60
contig06092_length=1182|500|60

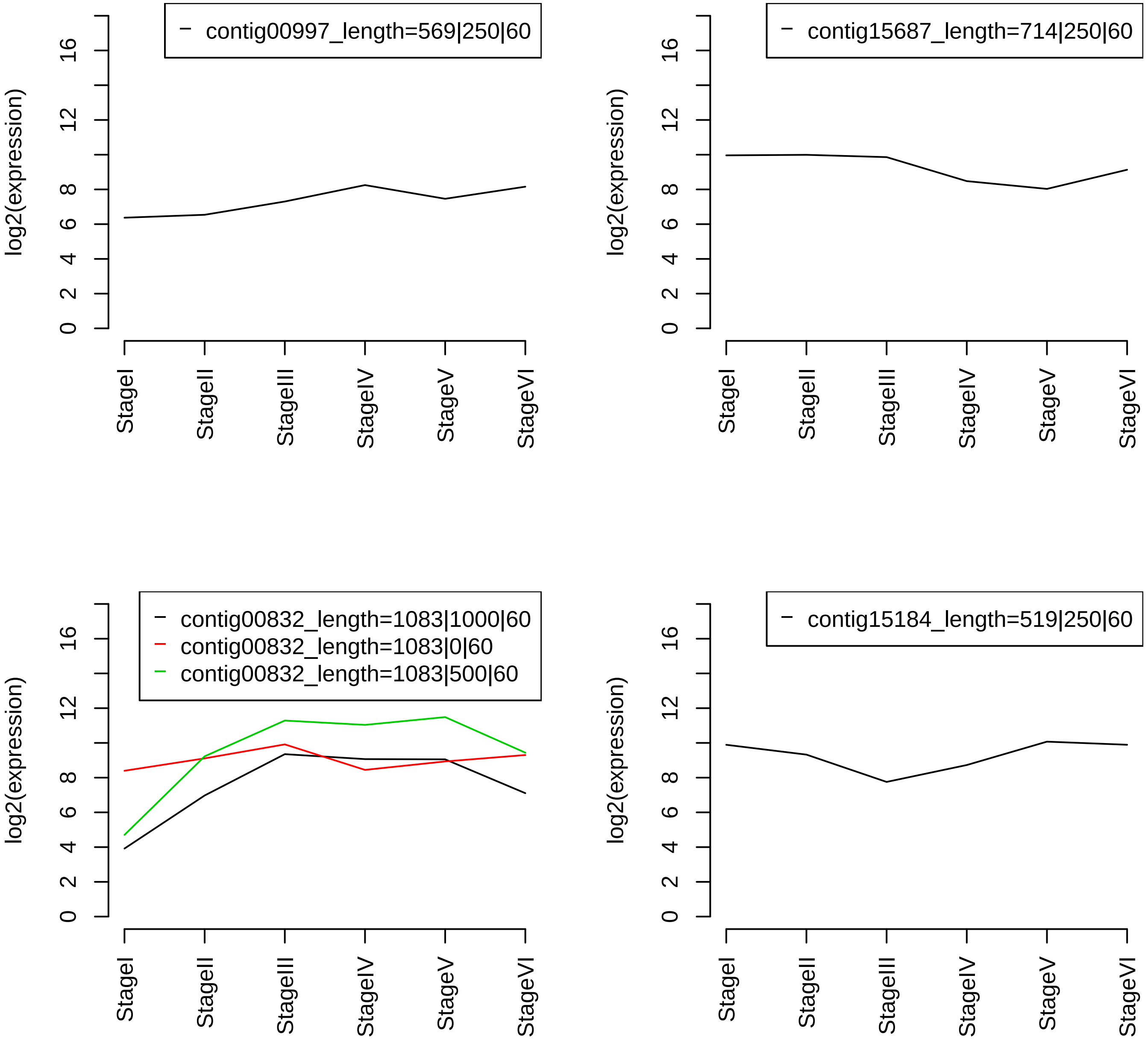
contig00997_length=569|250|60
contig15687_length=714|250|60
contig00832_length=1083|1000|60
contig00832_length=1083|0|60
contig00832_length=1083|500|60
contig15184_length=519|250|60
log2(expression)
0 2 4 6 8 12 16
StageI StageII StageIII StageIV StageV StageVI

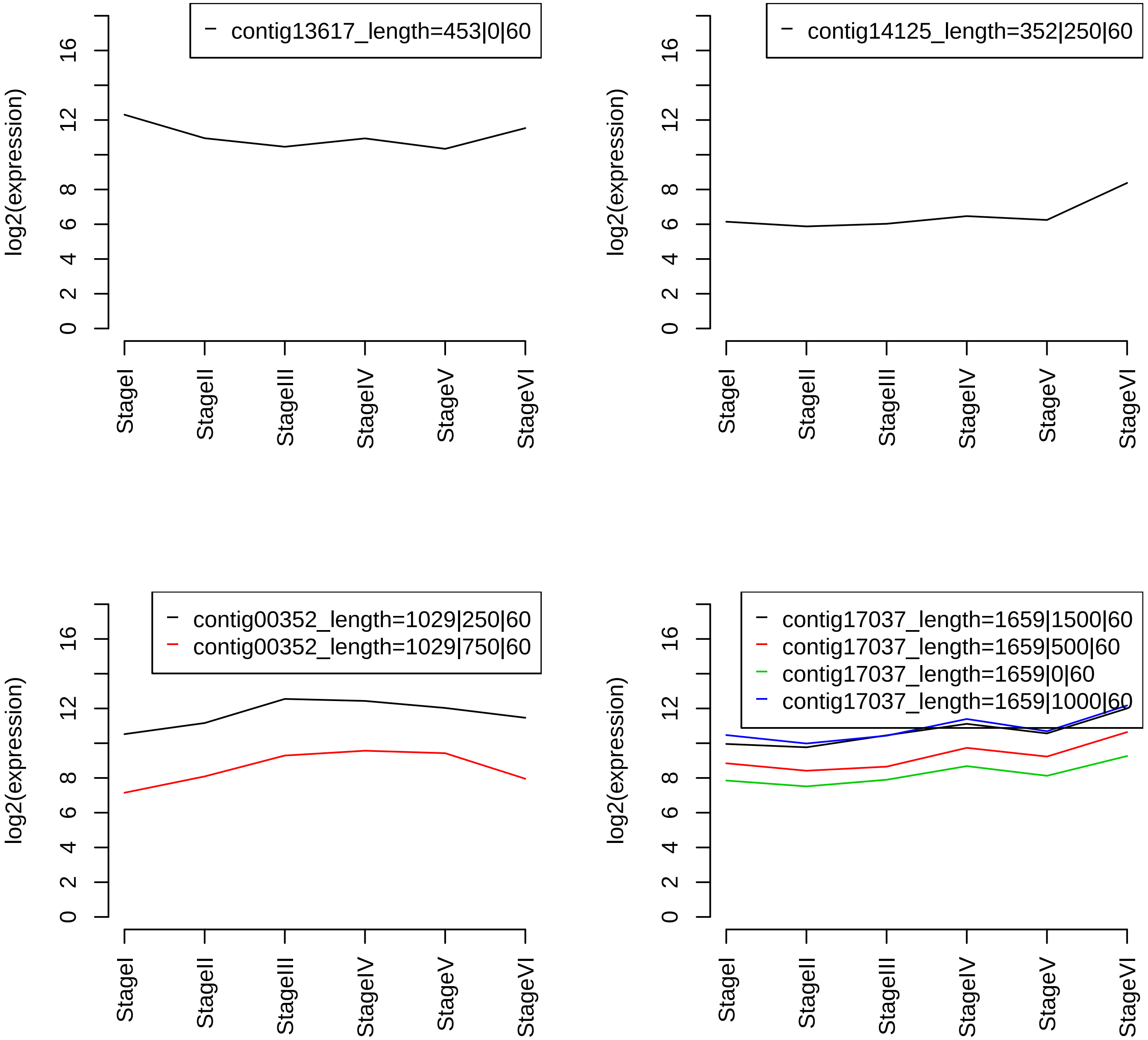

contig13617_length=453|0|60
contig14125_length=352|250|60
contig00352_length=1029|250|60
contig00352_length=1029|750|60
contig17037_length=1659|1500|60
contig17037_length=1659|500|60
contig17037_length=1659|0|60
contig17037_length=1659|1000|60
log2(expression)
0 2 4 6 8 12 16
StageI StageII StageIII StageIV StageV StageVI

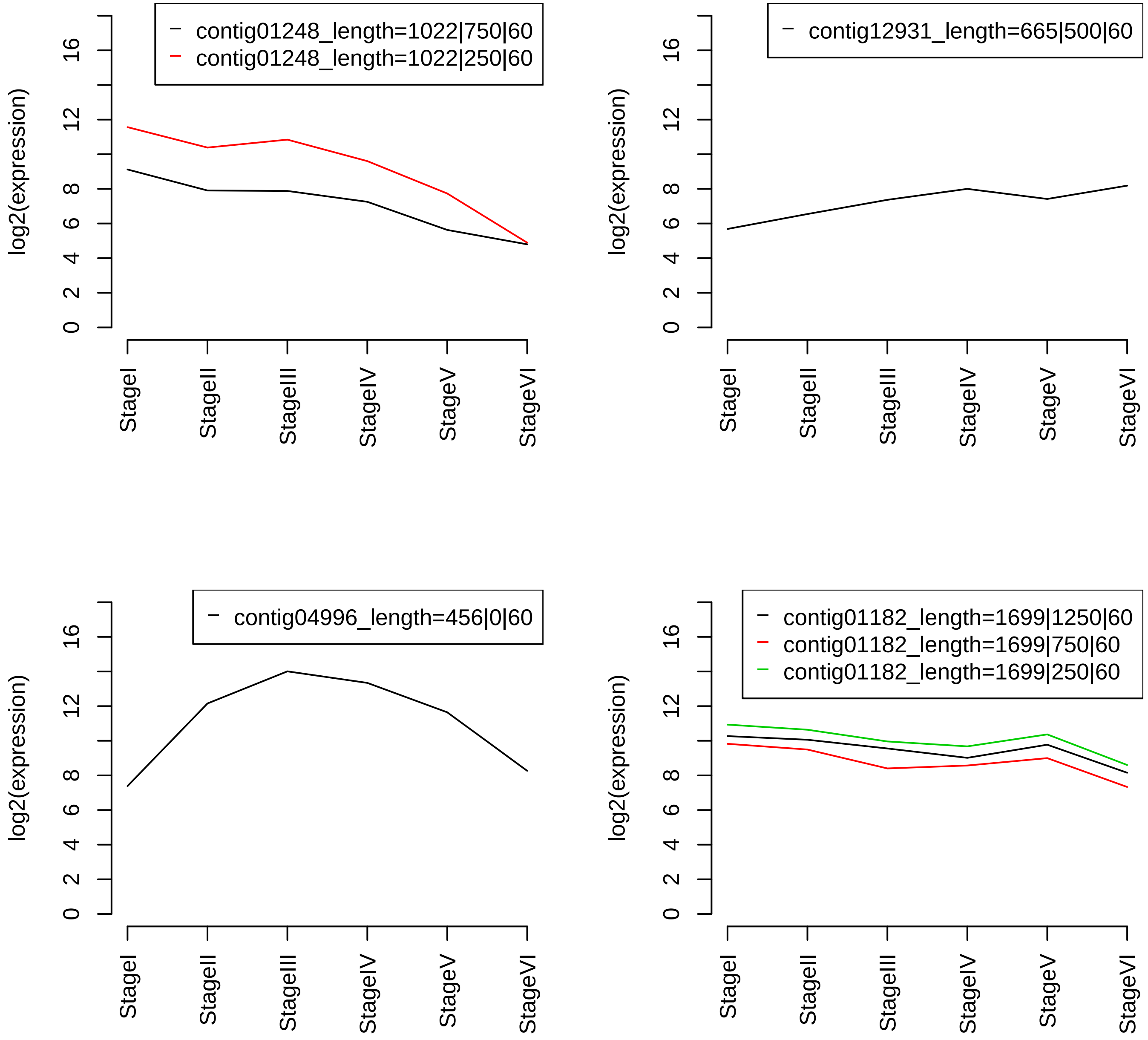
contig01248_length=1022|750|60
contig01248_length=1022|250|60
contig12931_length=665|500|60
contig04996_length=456|0|60
contig01182_length=1699|1250|60
contig01182_length=1699|750|60
contig01182_length=1699|250|60
log2(expression)
0
2
4
6
8
12
16
StageI
StageII
StageIII
StageIV
StageV
StageVI

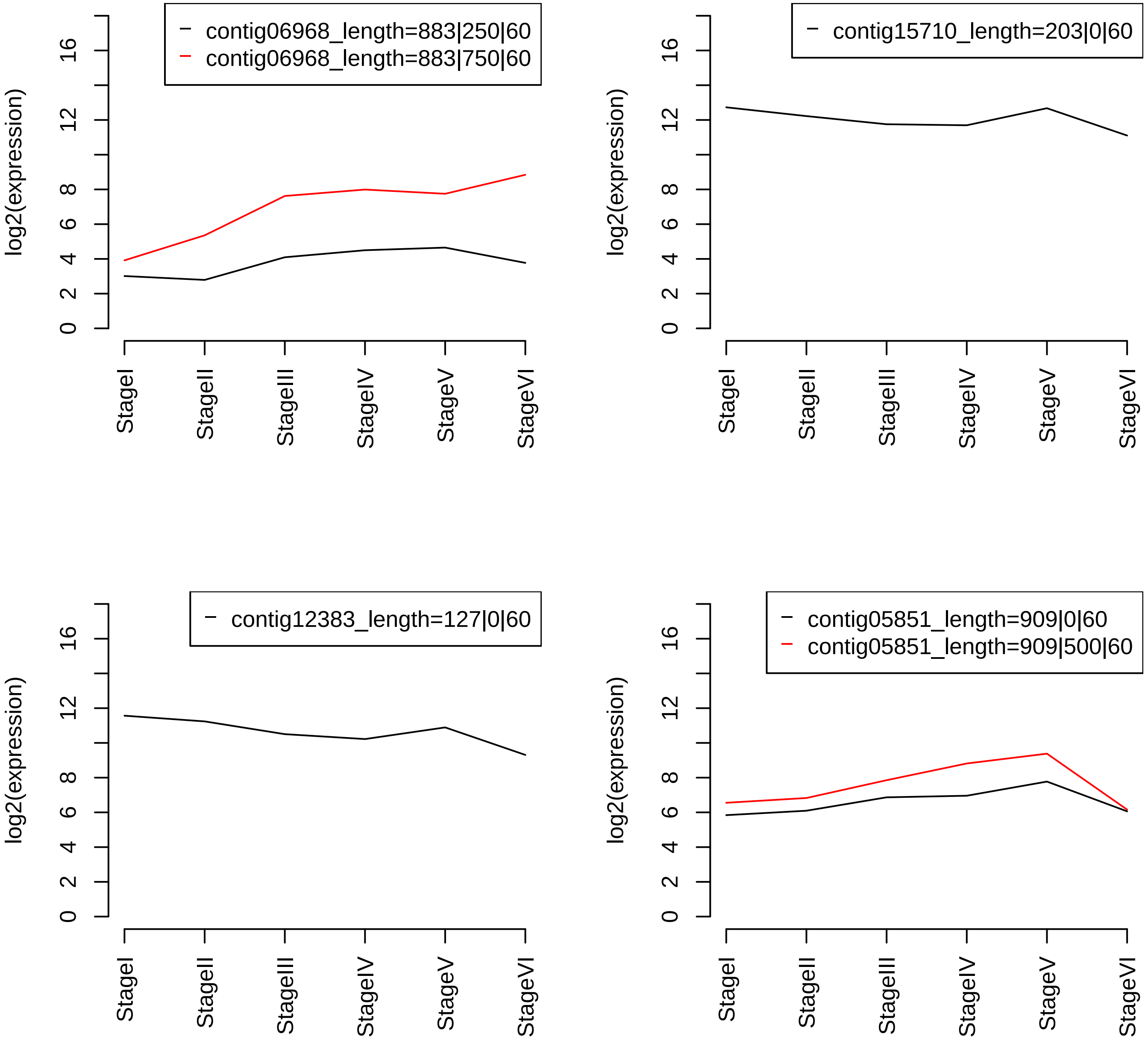
contig06968_length=883|250|60
contig06968_length=883|750|60
contig15710_length=203|0|60
contig12383_length=127|0|60
contig05851_length=909|0|60
contig05851_length=909|500|60
log2(expression)
0
2
4
6
8
12
16
StageI
StageII
StageIII
StageIV
StageV
StageVI

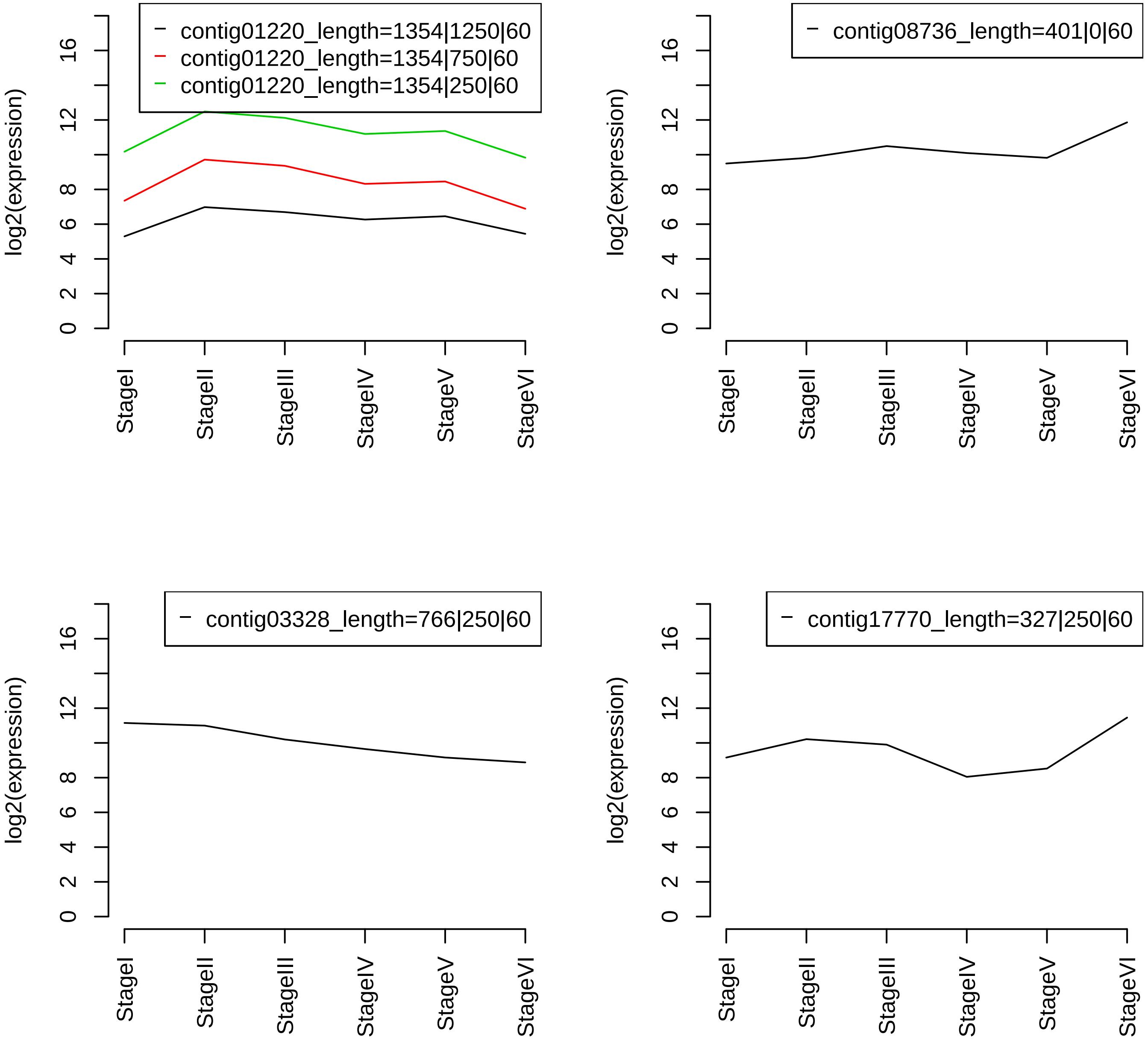

contig01220_length=1354|1250|60
contig01220_length=1354|750|60
contig01220_length=1354|250|60
contig08736_length=401|0|60
contig03328_length=766|250|60
contig17770_length=327|250|60
log2(expression)
0
2
4
6
8
12
16
StageI
StageII
StageIII
StageIV
StageV
StageVI

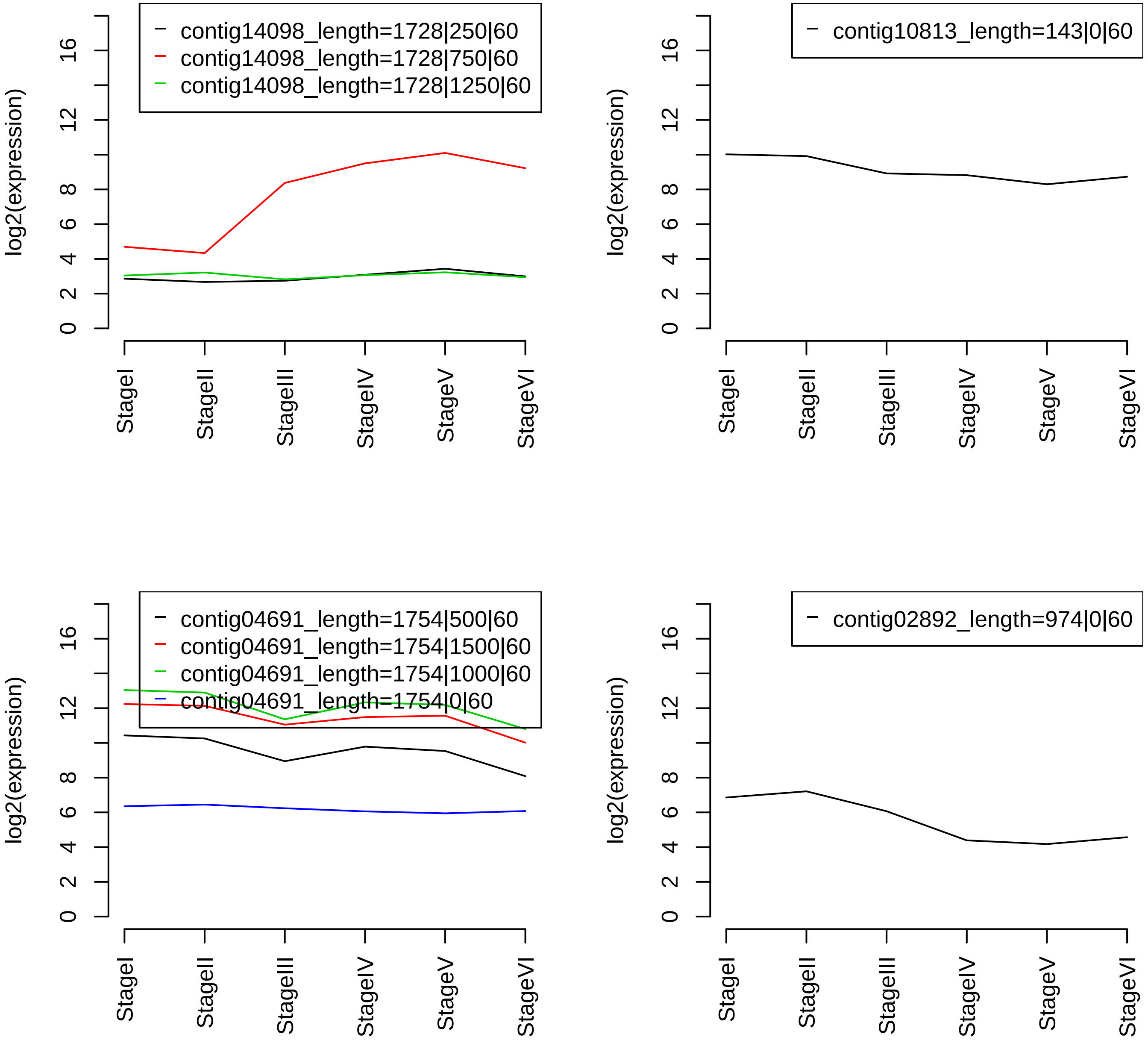
contig14098_length=1728|250|60
contig14098_length=1728|750|60
contig14098_length=1728|1250|60
contig10813_length=143|0|60
contig04691_length=1754|500|60
contig04691_length=1754|1500|60
contig04691_length=1754|1000|60
contig04691_length=1754|0|60
contig02892_length=974|0|60
log2(expression)
0 2 4 6 8 12 16
StageI StageII StageIII StageIV StageV StageVI

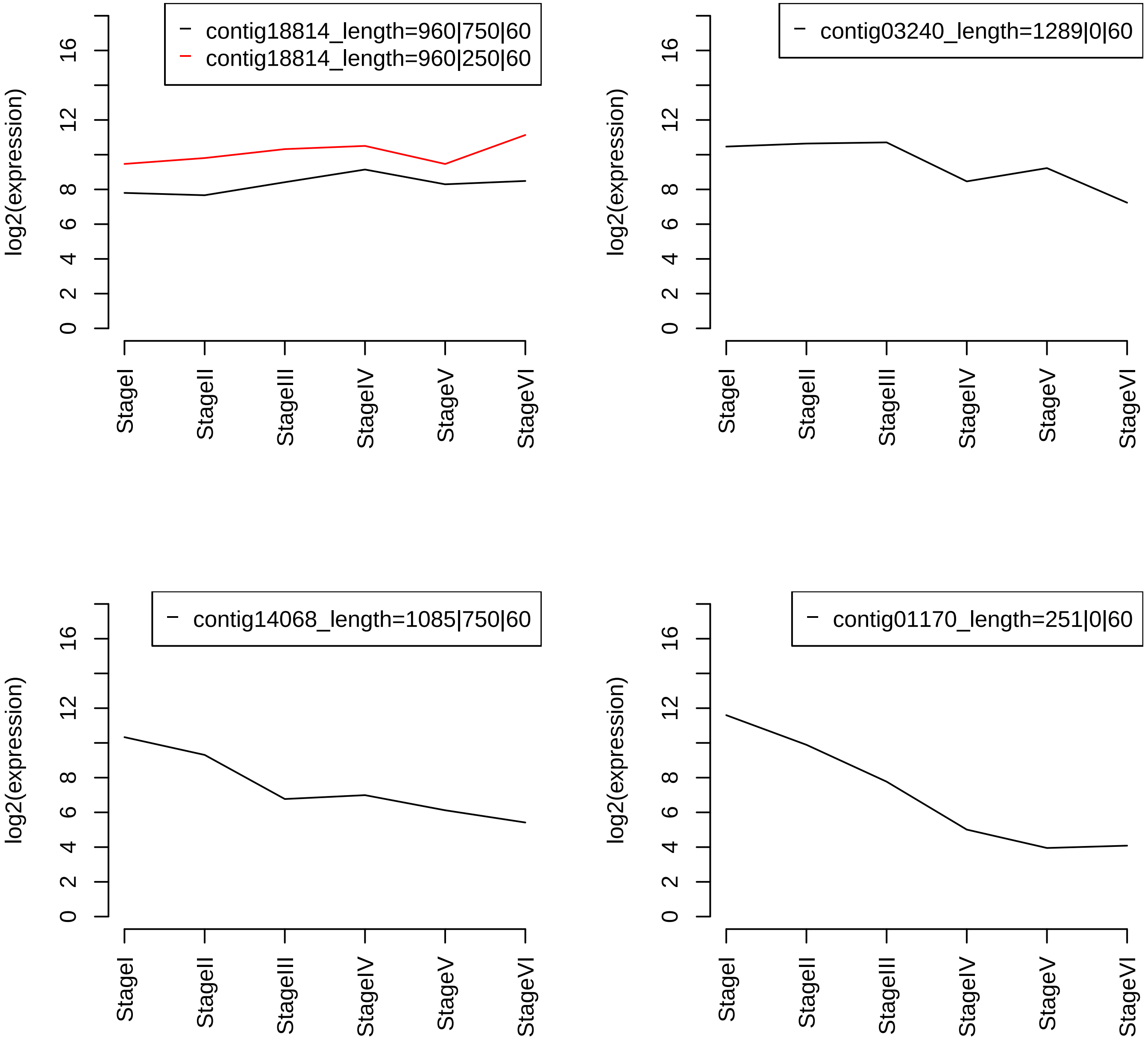
contig18814_length=960|750|60
contig18814_length=960|250|60
contig03240_length=1289|0|60
contig14068_length=1085|750|60
contig01170_length=251|0|60
log2(expression)
0
2
4
6
8
12
16
StageI
StageII
StageIII
StageIV
StageV
StageVI

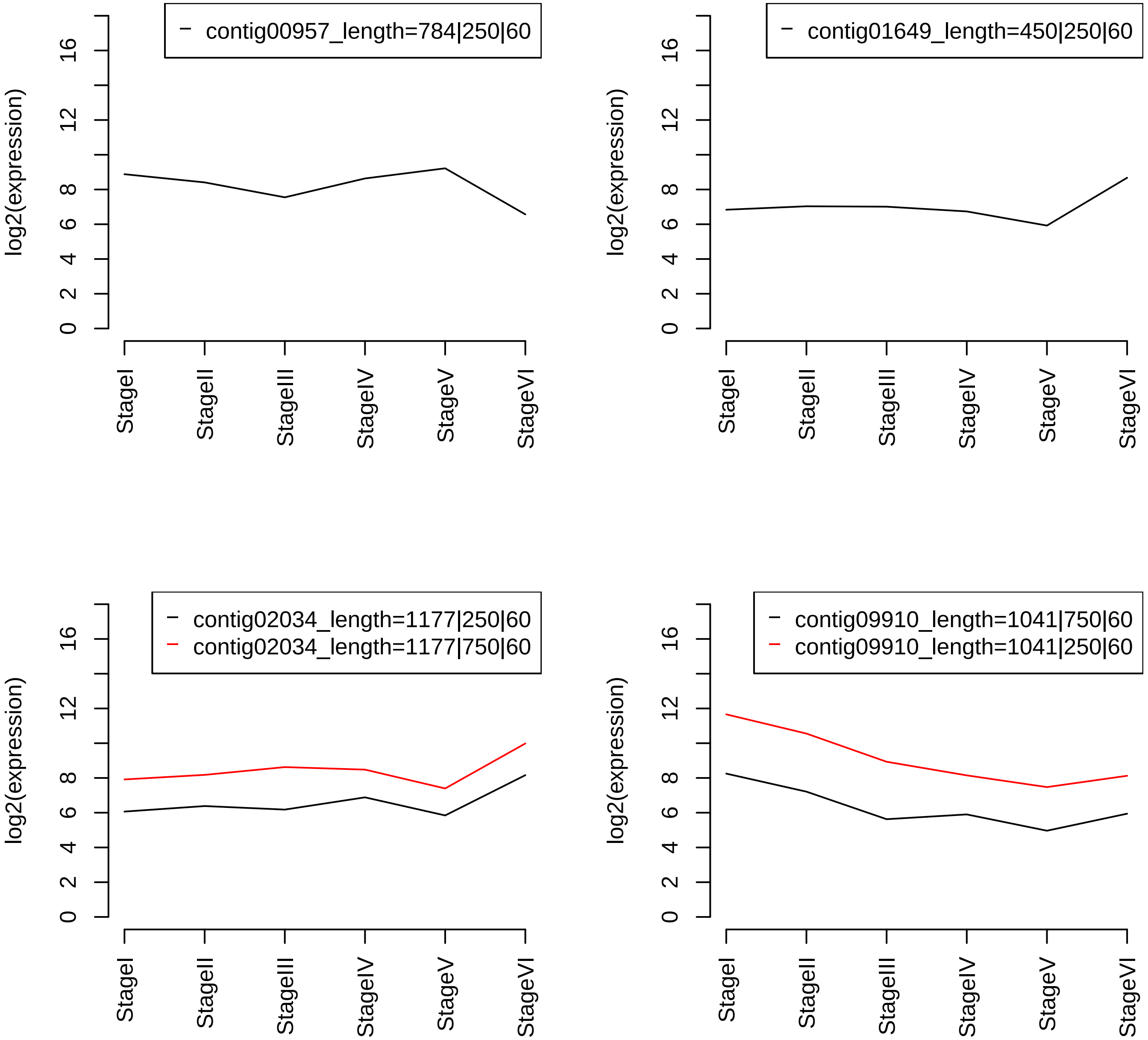

contig00957_length=784|250|60
contig01649_length=450|250|60
contig02034_length=1177|250|60
contig02034_length=1177|750|60
contig09910_length=1041|750|60
contig09910_length=1041|250|60
log2(expression)
0
2
4
6
8
12
16
StageI
StageII
StageIII
StageIV
StageV
StageVI

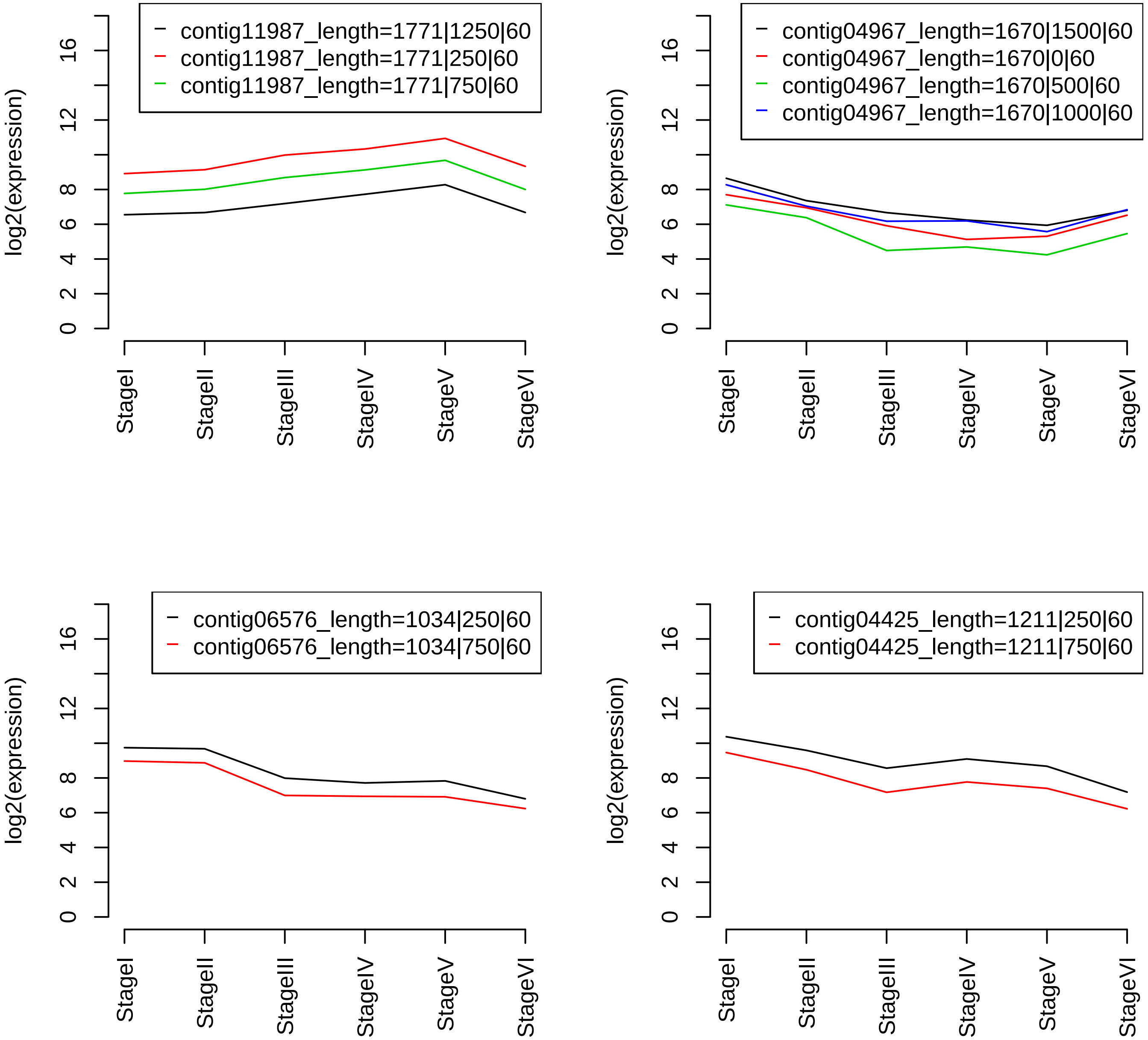
contig11987_length=1771|1250|60
contig11987_length=1771|250|60
contig11987_length=1771|750|60
contig04967_length=1670|1500|60
contig04967_length=1670|0|60
contig04967_length=1670|500|60
contig04967_length=1670|1000|60
contig06576_length=1034|250|60
contig06576_length=1034|750|60
contig04425_length=1211|250|60
contig04425_length=1211|750|60
log2(expression)
StageI
StageII
StageIII
StageIV
StageV
StageVI

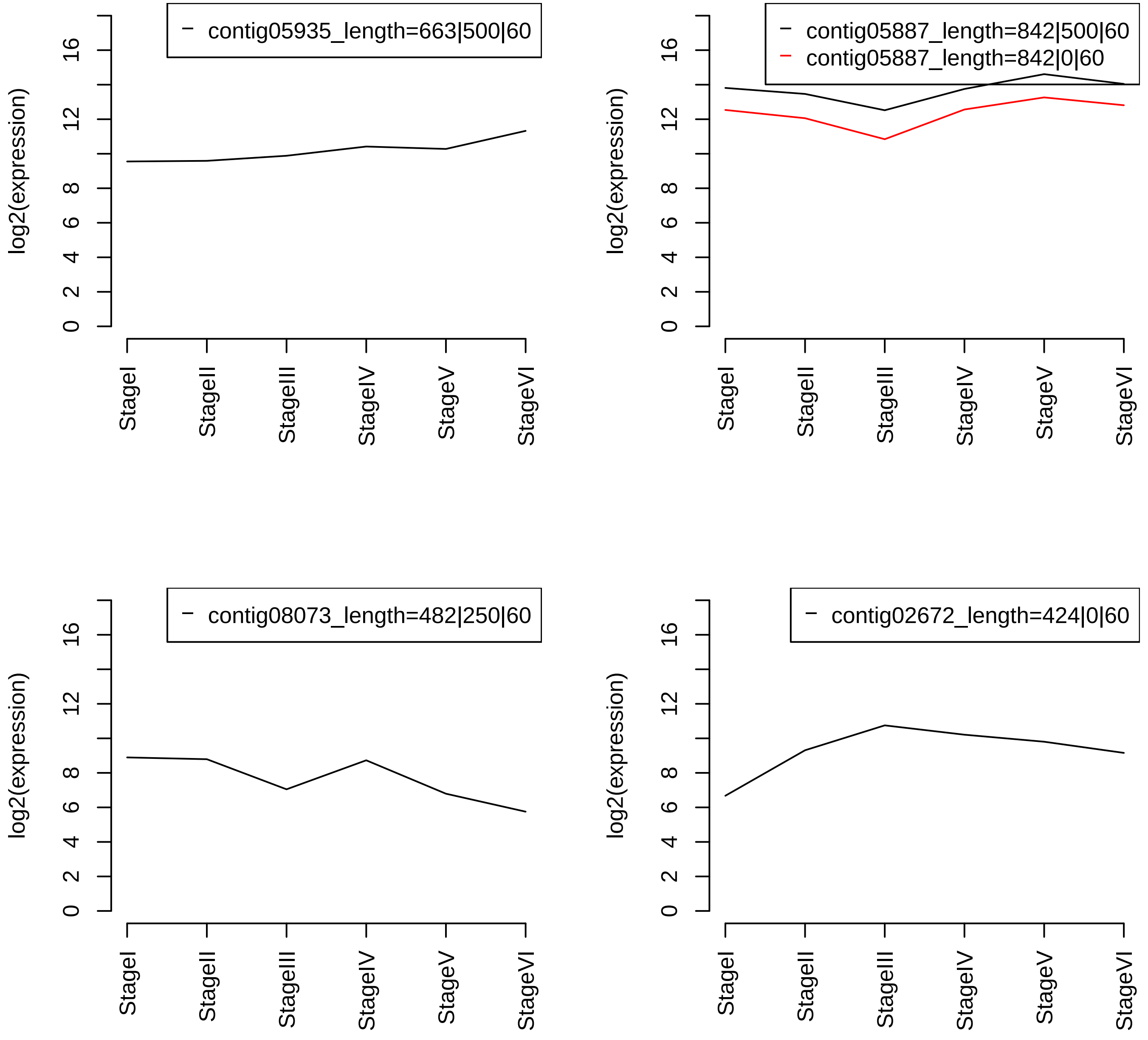

contig05935_length=663|500|60
contig05887_length=842|500|60
contig05887_length=842|0|60
contig08073_length=482|250|60
contig02672_length=424|0|60
log2(expression)
StageI
StageII
StageIII
StageIV
StageV
StageVI
0
2
4
6
8
12
16

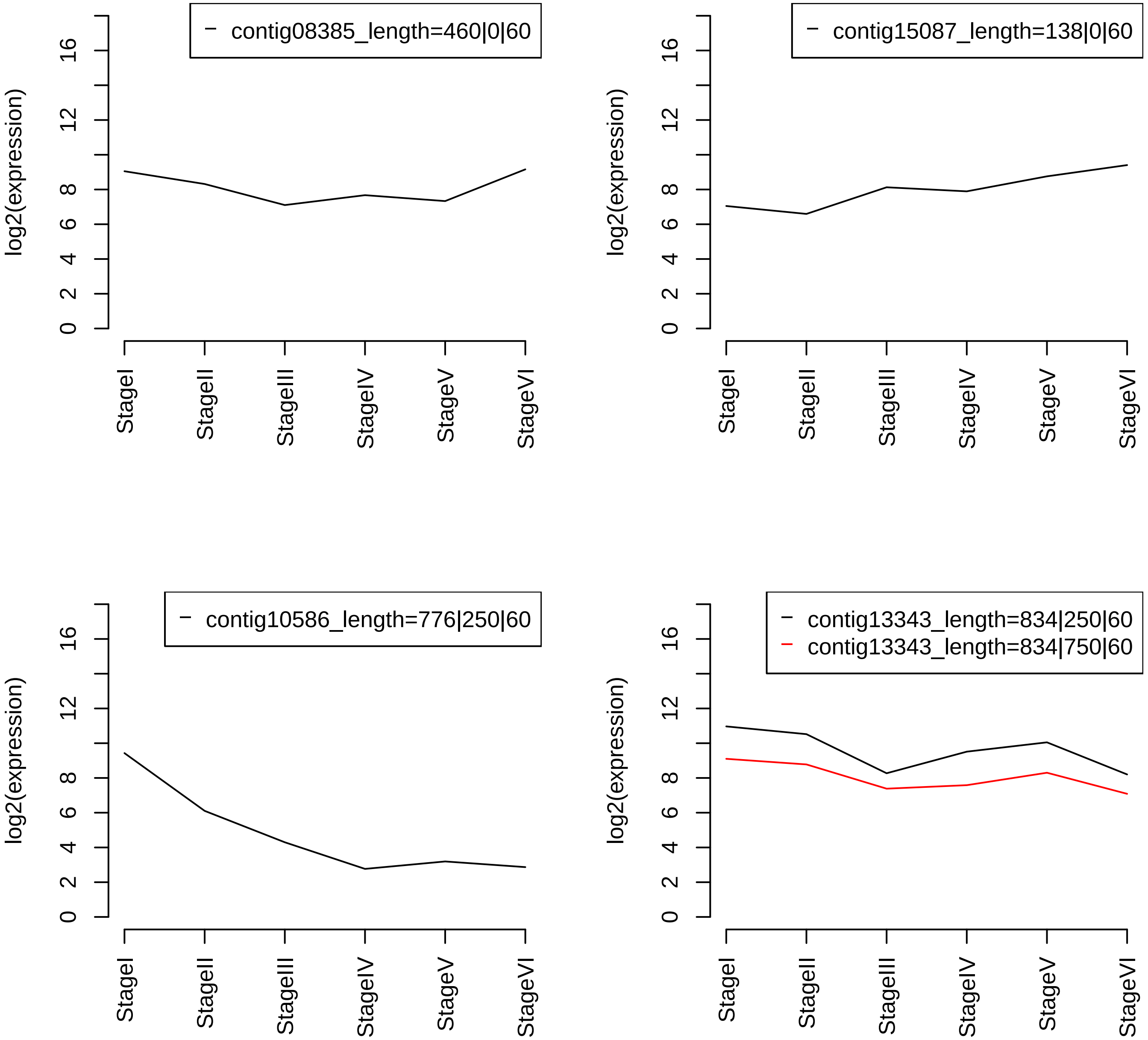
contig08385_length=460|0|60
contig15087_length=138|0|60
contig10586_length=776|250|60
contig13343_length=834|250|60
contig13343_length=834|750|60
log2(expression)
0
2
4
6
8
12
16
StageI
StageII
StageIII
StageIV
StageV
StageVI

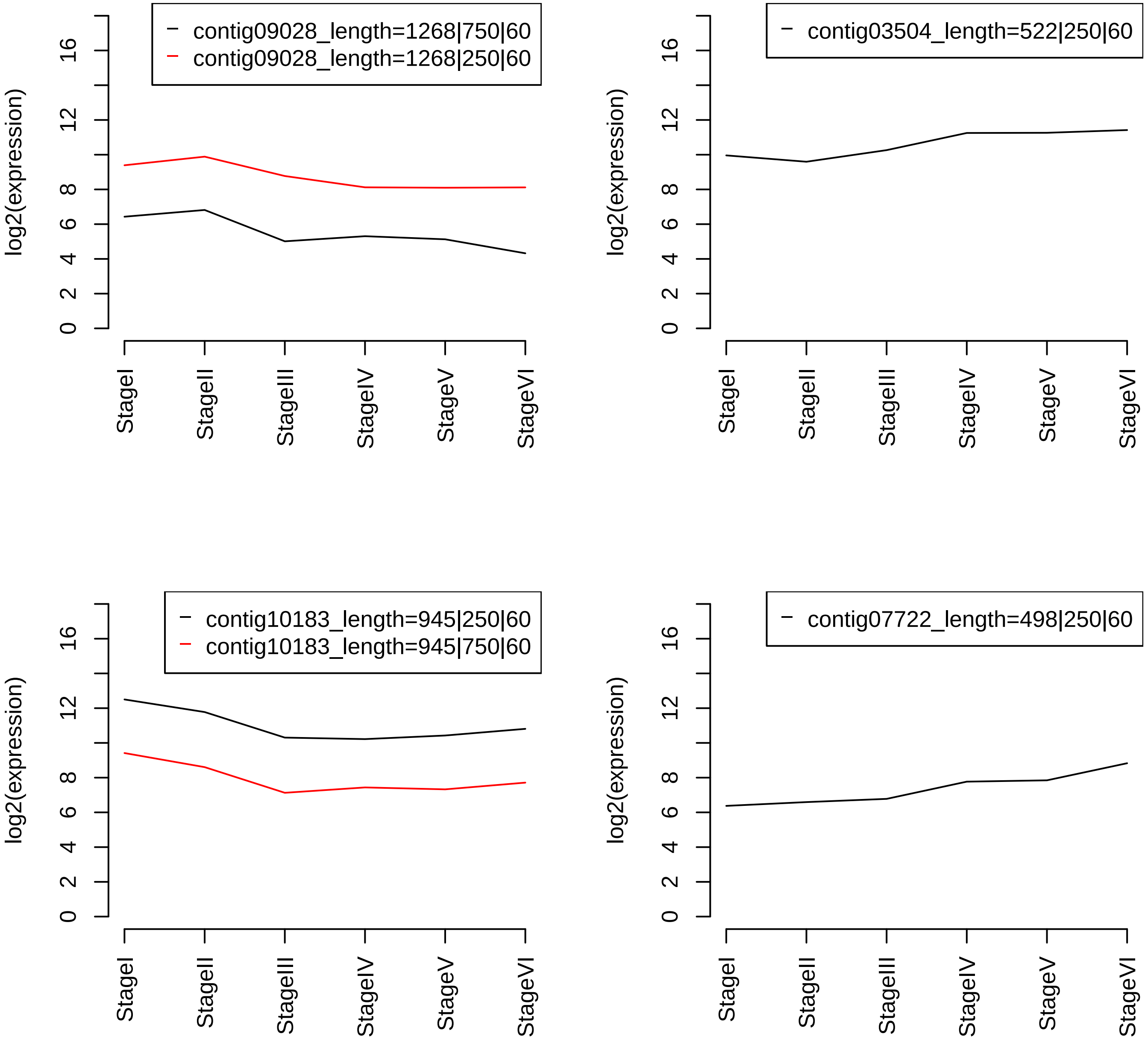
contig09028_length=1268|750|60
contig09028_length=1268|250|60
log2(expression)
0
2
4
6
8
12
16
StageI
StageII
StageIII
StageIV
StageV
StageVI
contig03504_length=522|250|60
log2(expression)
0
2
4
6
8
12
16
StageI
StageII
StageIII
StageIV
StageV
StageVI
contig10183_length=945|250|60
contig10183_length=945|750|60
log2(expression)
0
2
4
6
8
12
16
StageI
StageII
StageIII
StageIV
StageV
StageVI
contig07722_length=498|250|60
log2(expression)
0
2
4
6
8
12
16
StageI
StageII
StageIII
StageIV
StageV
StageVI

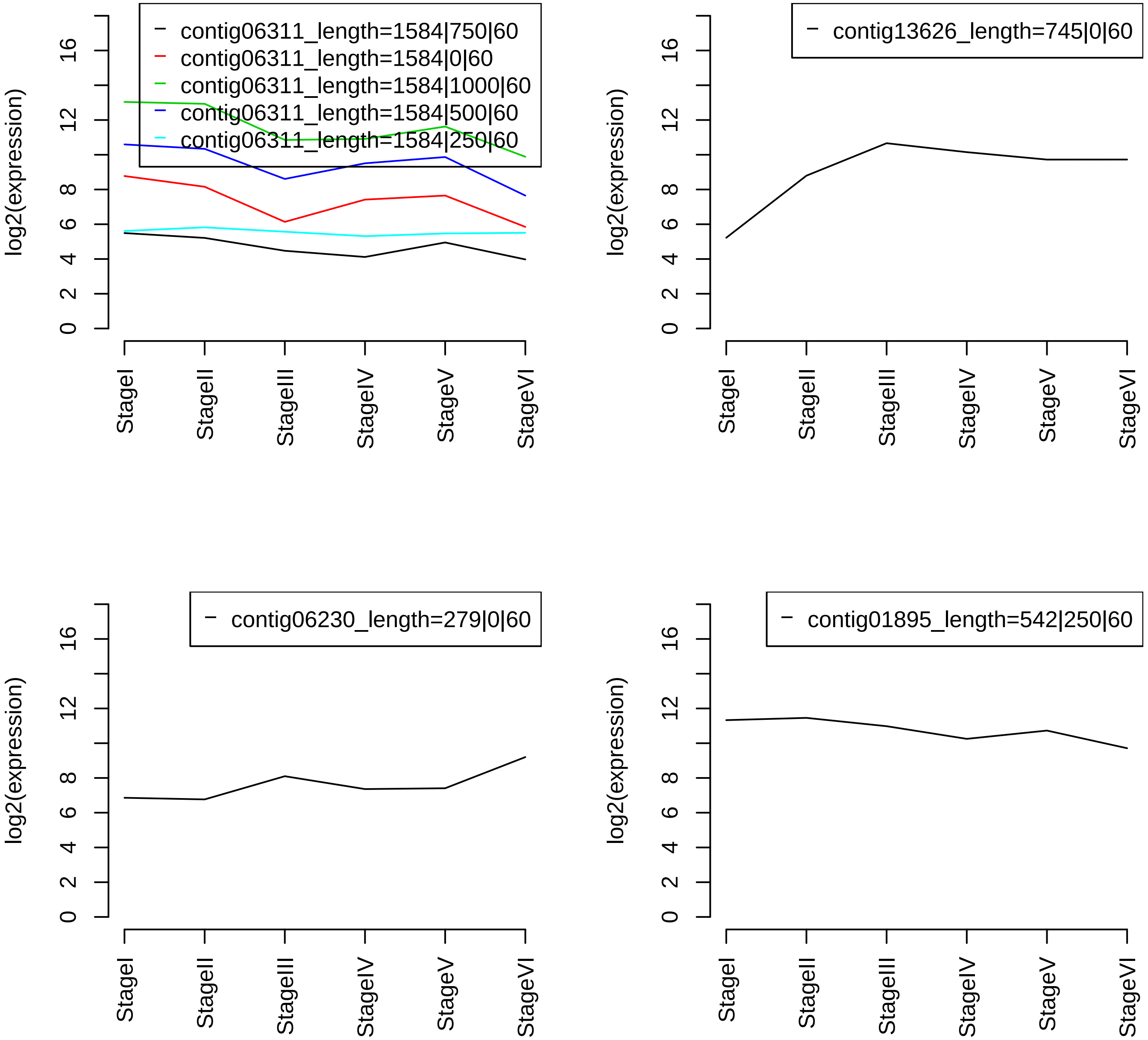
contig06311_length=1584|750|60
contig06311_length=1584|0|60
contig06311_length=1584|1000|60
contig06311_length=1584|500|60
contig06311_length=1584|250|60
log2(expression)
0 2 4 6 8 12 16
StageI StageII StageIII StageIV StageV StageVI
contig13626_length=745|0|60
log2(expression)
0 2 4 6 8 12 16
StageI StageII StageIII StageIV StageV StageVI
contig06230_length=279|0|60
log2(expression)
0 2 4 6 8 12 16
StageI StageII StageIII StageIV StageV StageVI
contig01895_length=542|250|60
log2(expression)
0 2 4 6 8 12 16
StageI StageII StageIII StageIV StageV StageVI

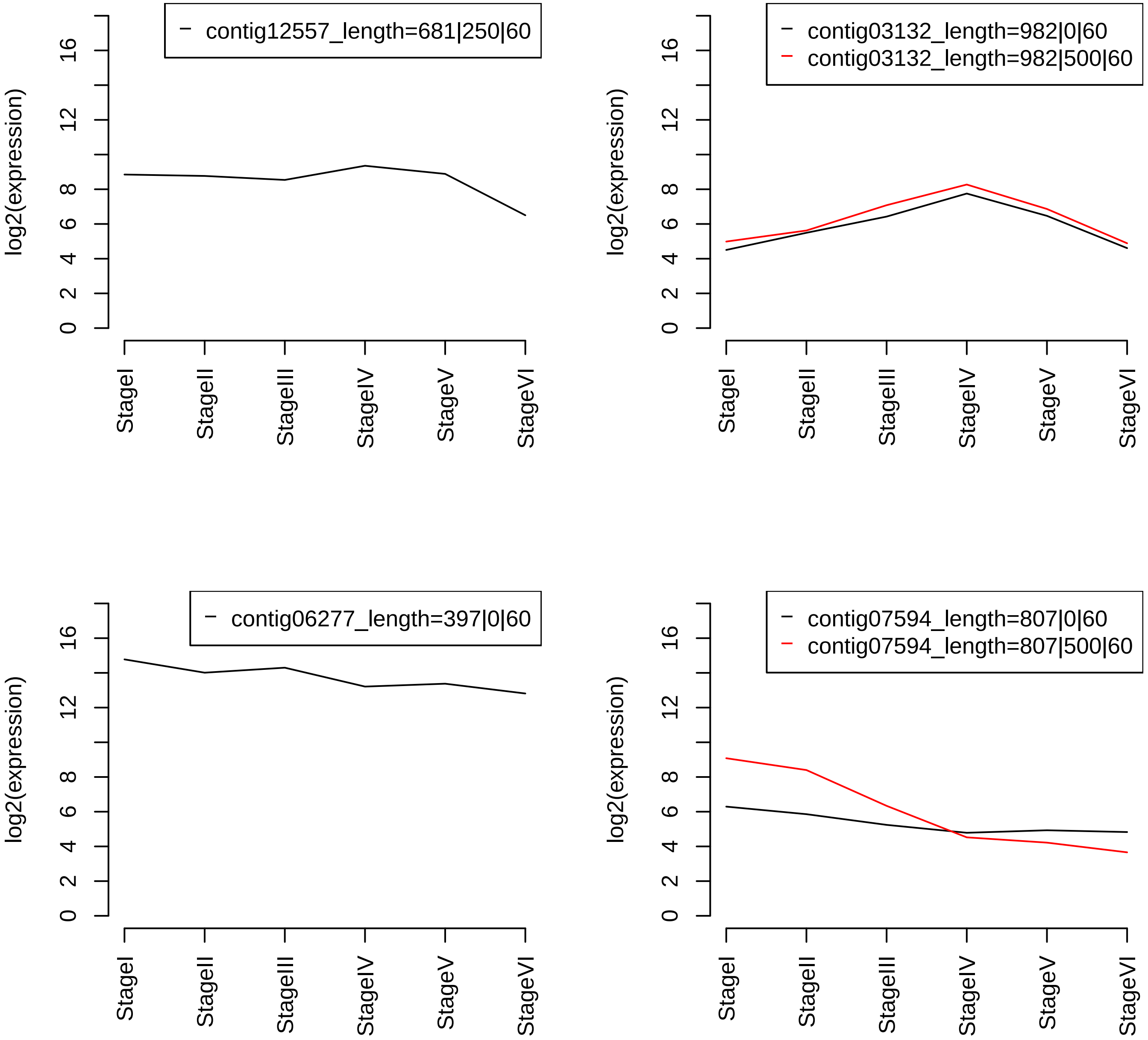
contig12557_length=681|250|60
contig03132_length=982|0|60
contig03132_length=982|500|60
contig06277_length=397|0|60
contig07594_length=807|0|60
contig07594_length=807|500|60
log2(expression)
0 2 4 6 8 12 16
StageI StageII StageIII StageIV StageV StageVI

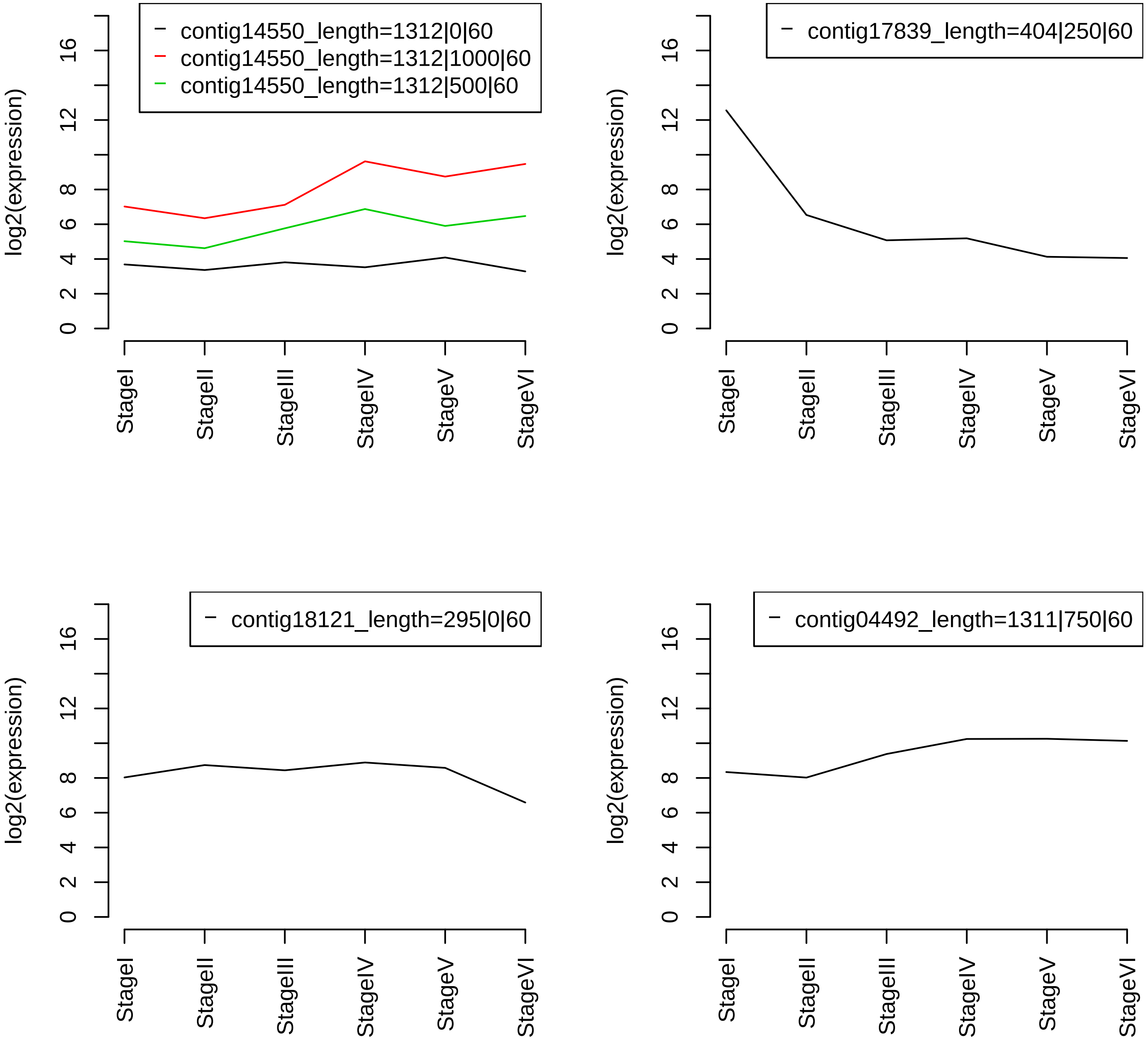

contig14550_length=1312|0|60
contig14550_length=1312|1000|60
contig14550_length=1312|500|60
log2(expression)
0
2
4
6
8
12
16
StageI
StageII
StageIII
StageIV
StageV
StageVI
contig17839_length=404|250|60
contig18121_length=295|0|60
contig04492_length=1311|750|60

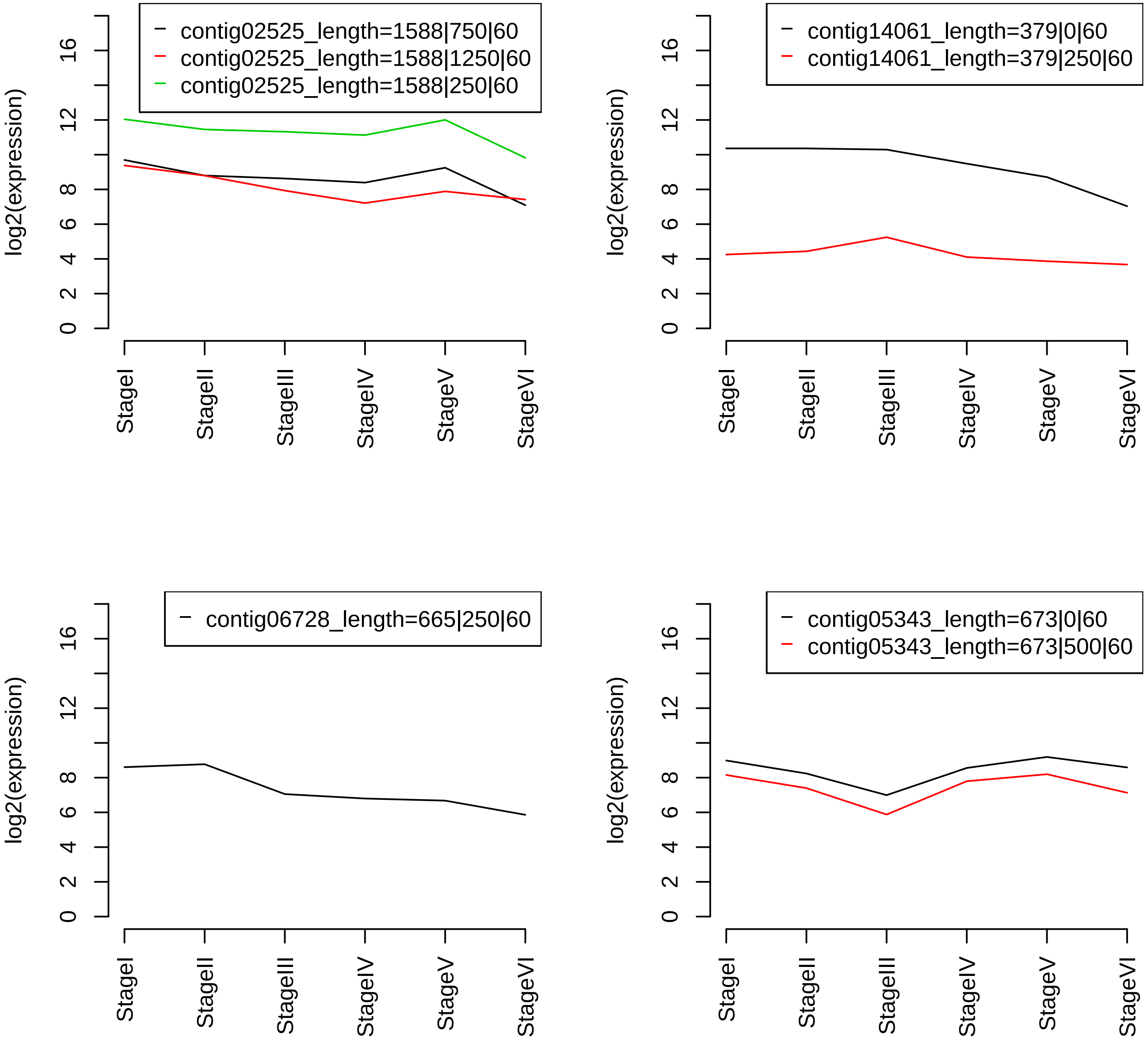
contig02525_length=1588|750|60
contig02525_length=1588|1250|60
contig02525_length=1588|250|60
contig14061_length=379|0|60
contig14061_length=379|250|60
contig06728_length=665|250|60
contig05343_length=673|0|60
contig05343_length=673|500|60
log2(expression)
0 2 4 6 8 12 16
StageI StageII StageIII StageIV StageV StageVI

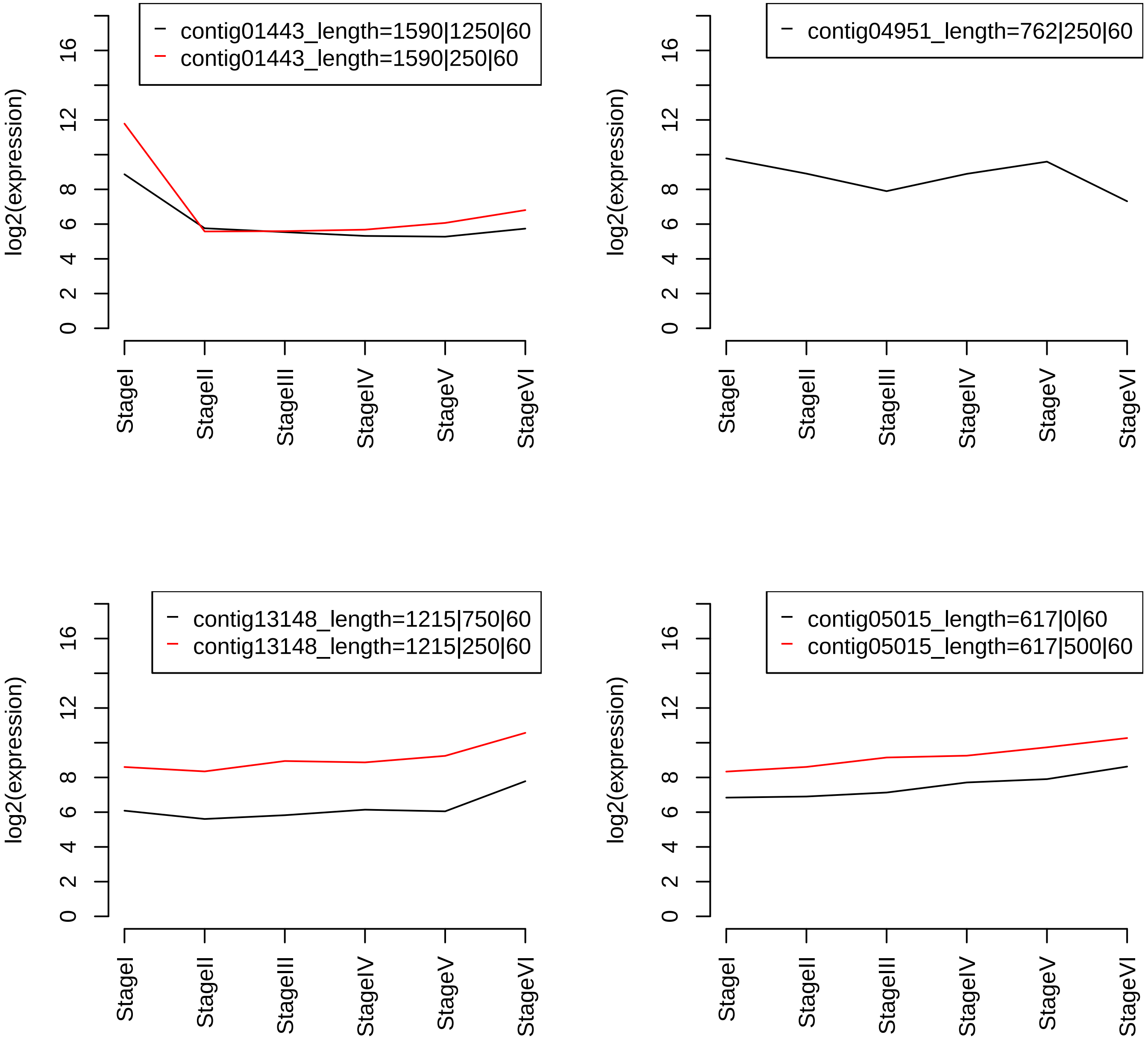
contig01443_length=1590|1250|60
contig01443_length=1590|250|60
log2(expression)
0
2
4
6
8
12
16
StageI
StageII
StageIII
StageIV
StageV
StageVI
contig04951_length=762|250|60
contig13148_length=1215|750|60
contig13148_length=1215|250|60
contig05015_length=617|0|60
contig05015_length=617|500|60

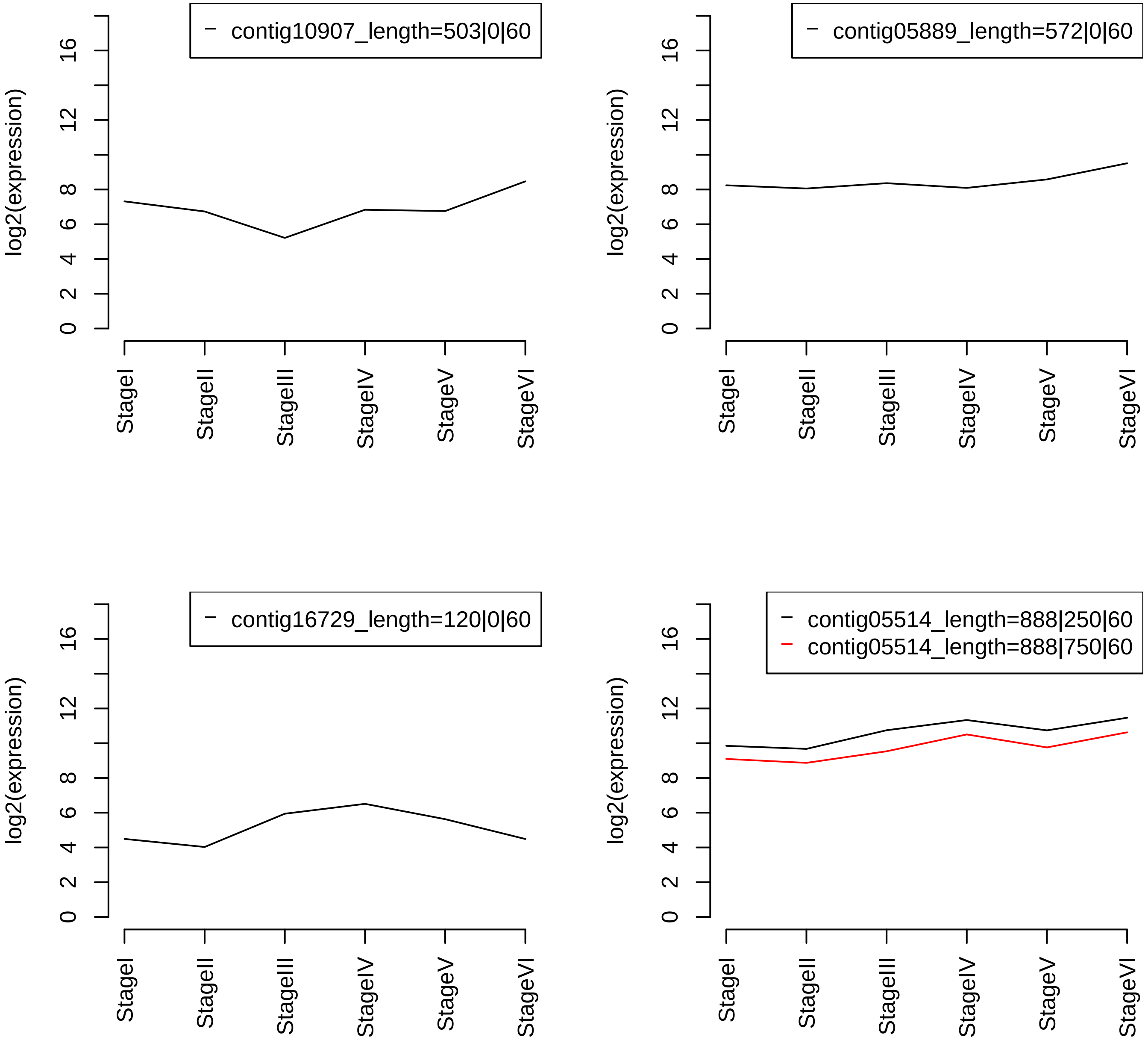

contig10907_length=503|0|60
contig05889_length=572|0|60
contig16729_length=120|0|60
contig05514_length=888|250|60
contig05514_length=888|750|60
log2(expression)
0
2
4
6
8
12
16
StageI
StageII
StageIII
StageIV
StageV
StageVI

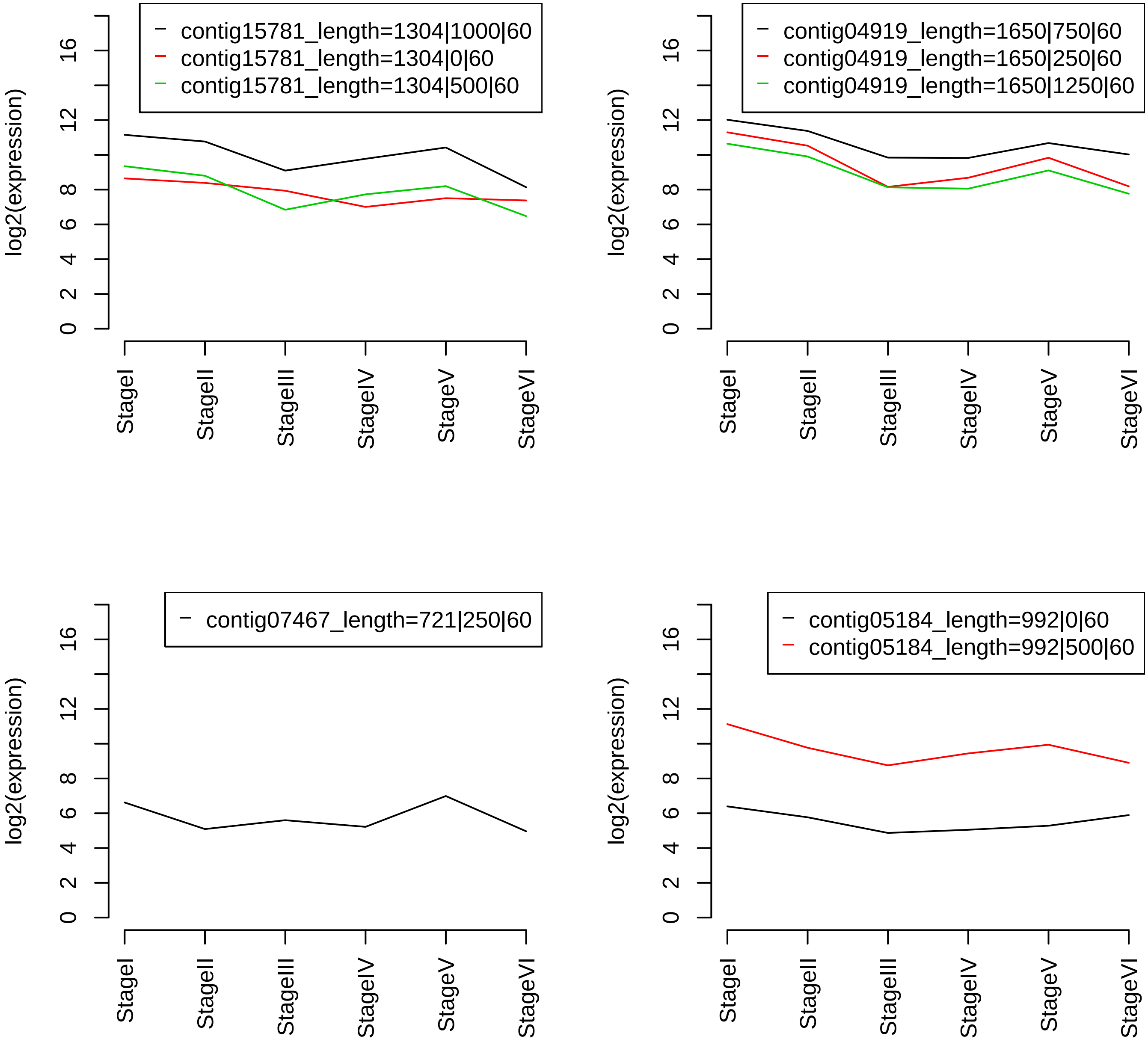

contig15781_length=1304|1000|60
contig15781_length=1304|0|60
contig15781_length=1304|500|60
contig04919_length=1650|750|60
contig04919_length=1650|250|60
contig04919_length=1650|1250|60
contig07467_length=721|250|60
contig05184_length=992|0|60
contig05184_length=992|500|60
log2(expression)
0
2
4
6
8
12
16
StageI
StageII
StageIII
StageIV
StageV
StageVI

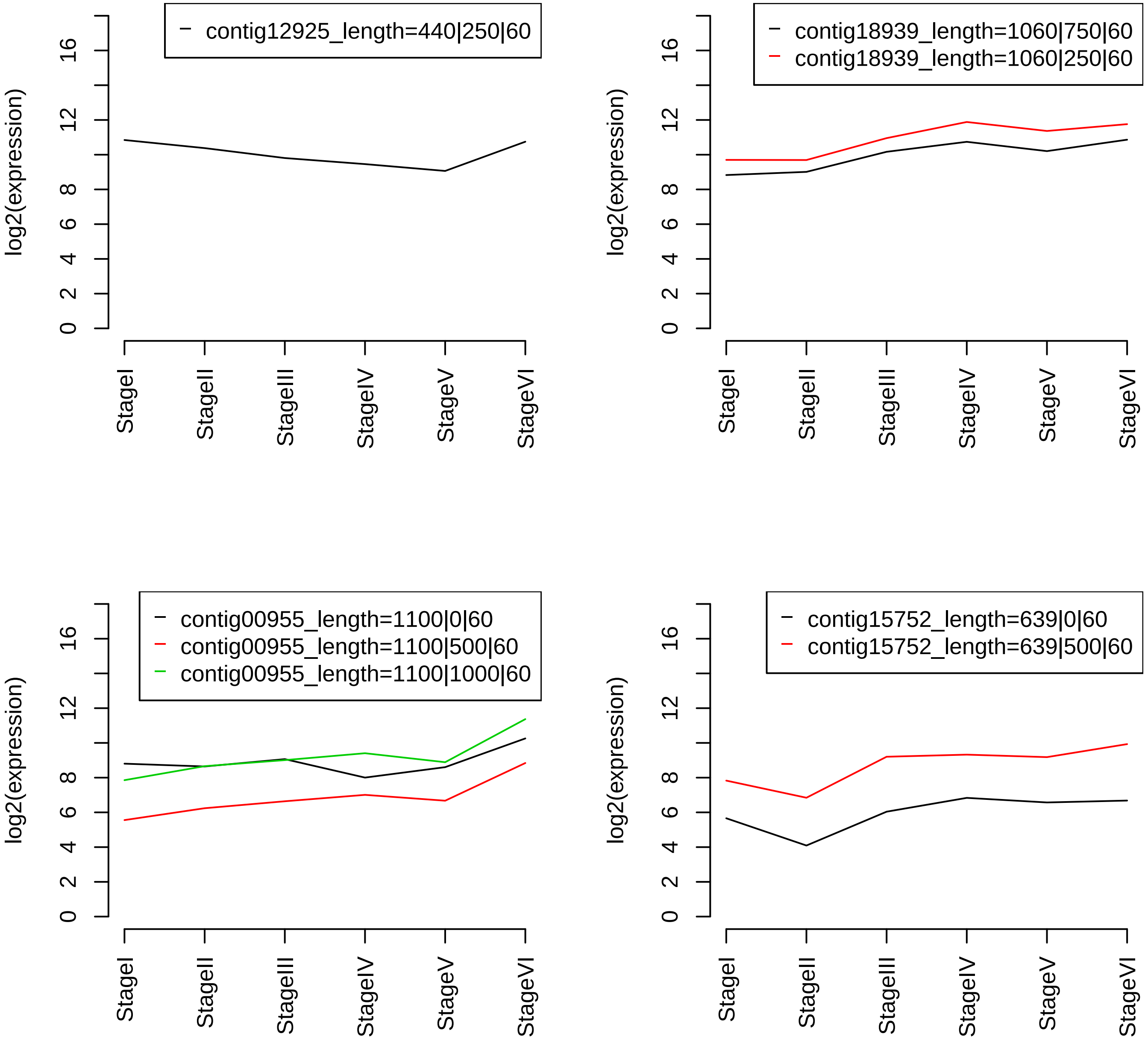
contig12925_length=440|250|60
contig18939_length=1060|750|60
contig18939_length=1060|250|60
contig00955_length=1100|0|60
contig00955_length=1100|500|60
contig00955_length=1100|1000|60
contig15752_length=639|0|60
contig15752_length=639|500|60
log2(expression)
0
2
4
6
8
12
16
StageI
StageII
StageIII
StageIV
StageV
StageVI

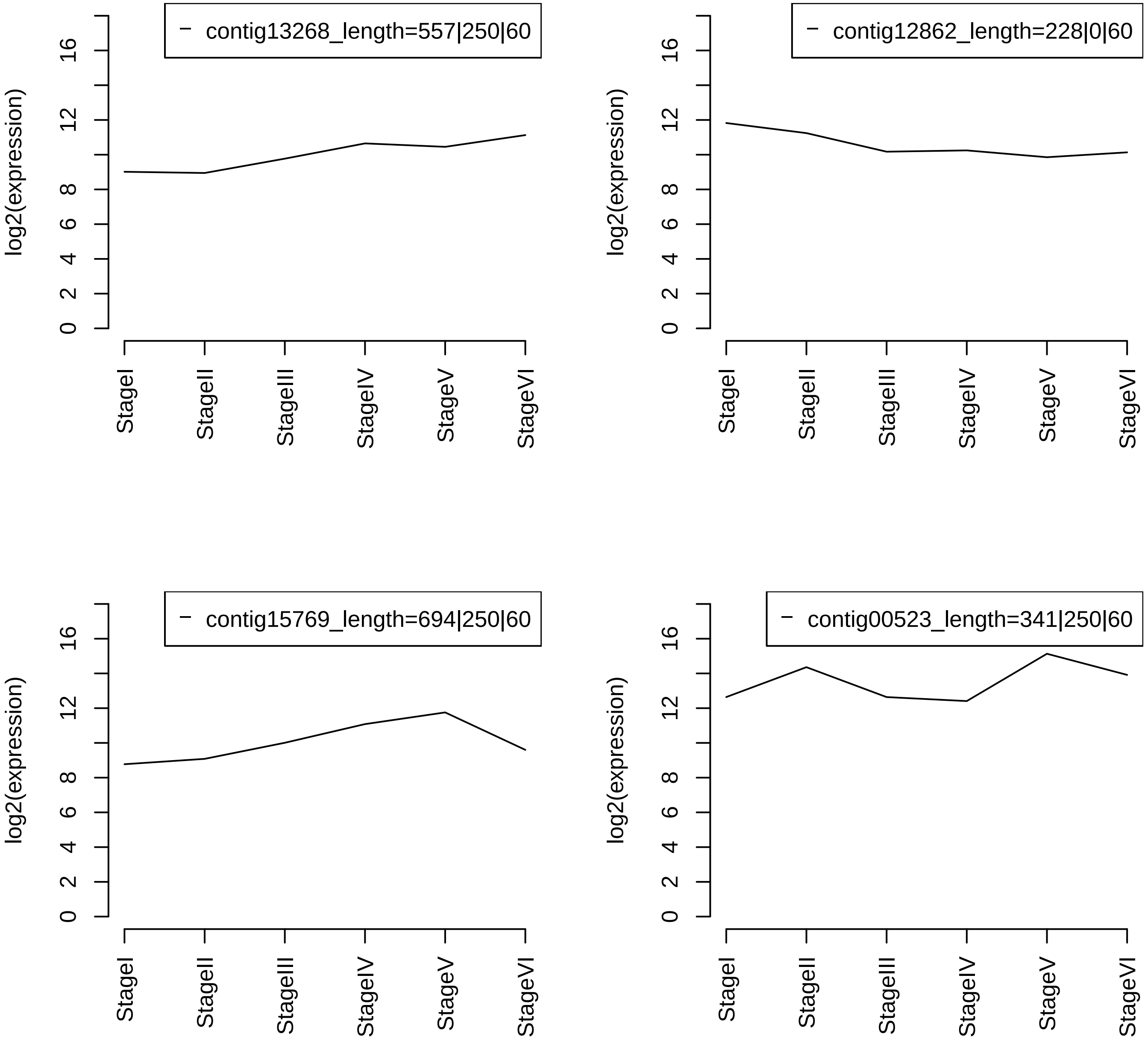

contig13268_length=557|250|60
contig12862_length=228|0|60
contig15769_length=694|250|60
contig00523_length=341|250|60
log2(expression)
0
2
4
6
8
12
16
StageI
StageII
StageIII
StageIV
StageV
StageVI

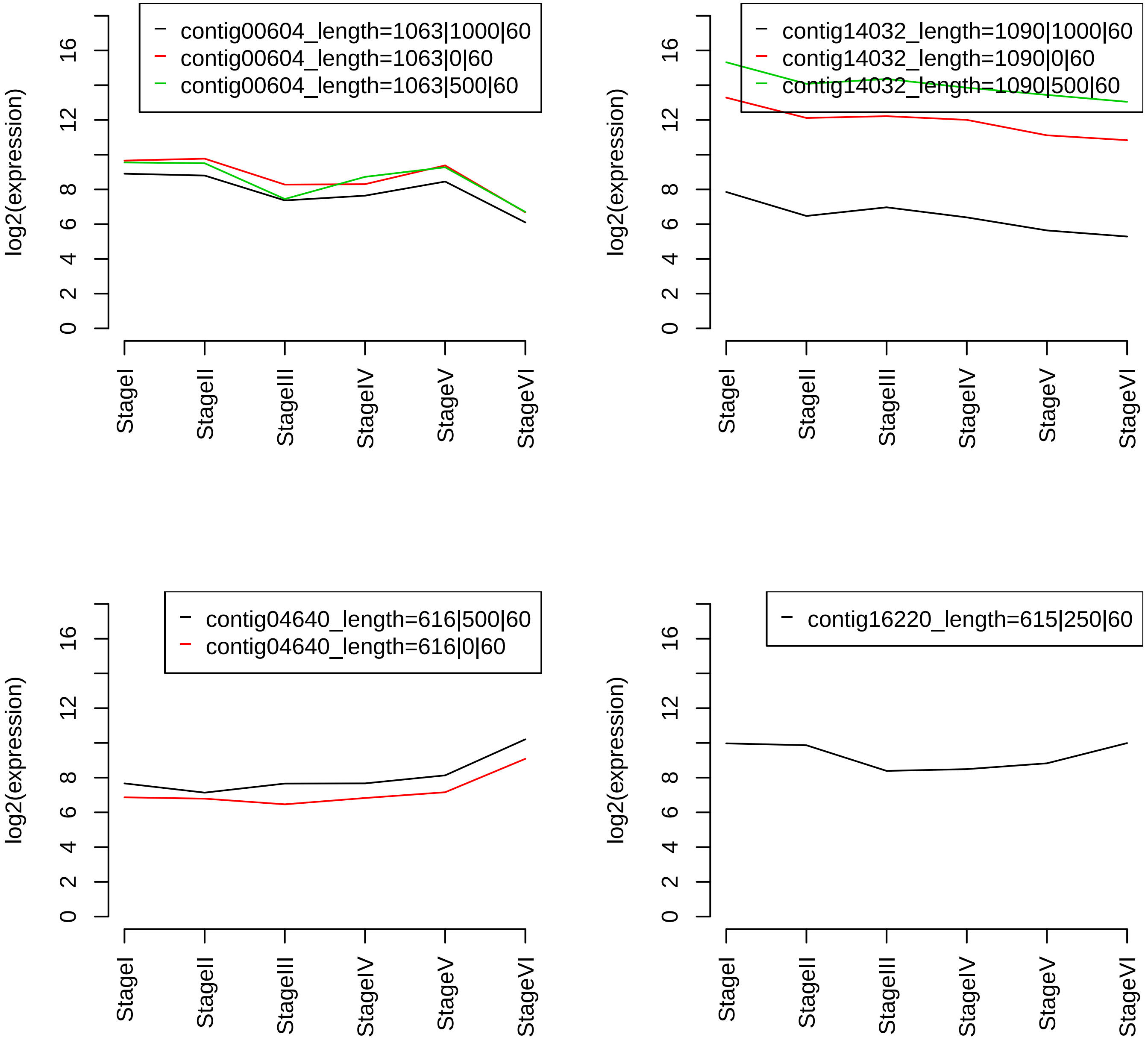

contig00604_length=1063|1000|60
contig00604_length=1063|0|60
contig00604_length=1063|500|60
contig14032_length=1090|1000|60
contig14032_length=1090|0|60
contig14032_length=1090|500|60
contig04640_length=616|500|60
contig04640_length=616|0|60
contig16220_length=615|250|60
log2(expression)
0
2
4
6
8
12
16
StageI
StageII
StageIII
StageIV
StageV
StageVI

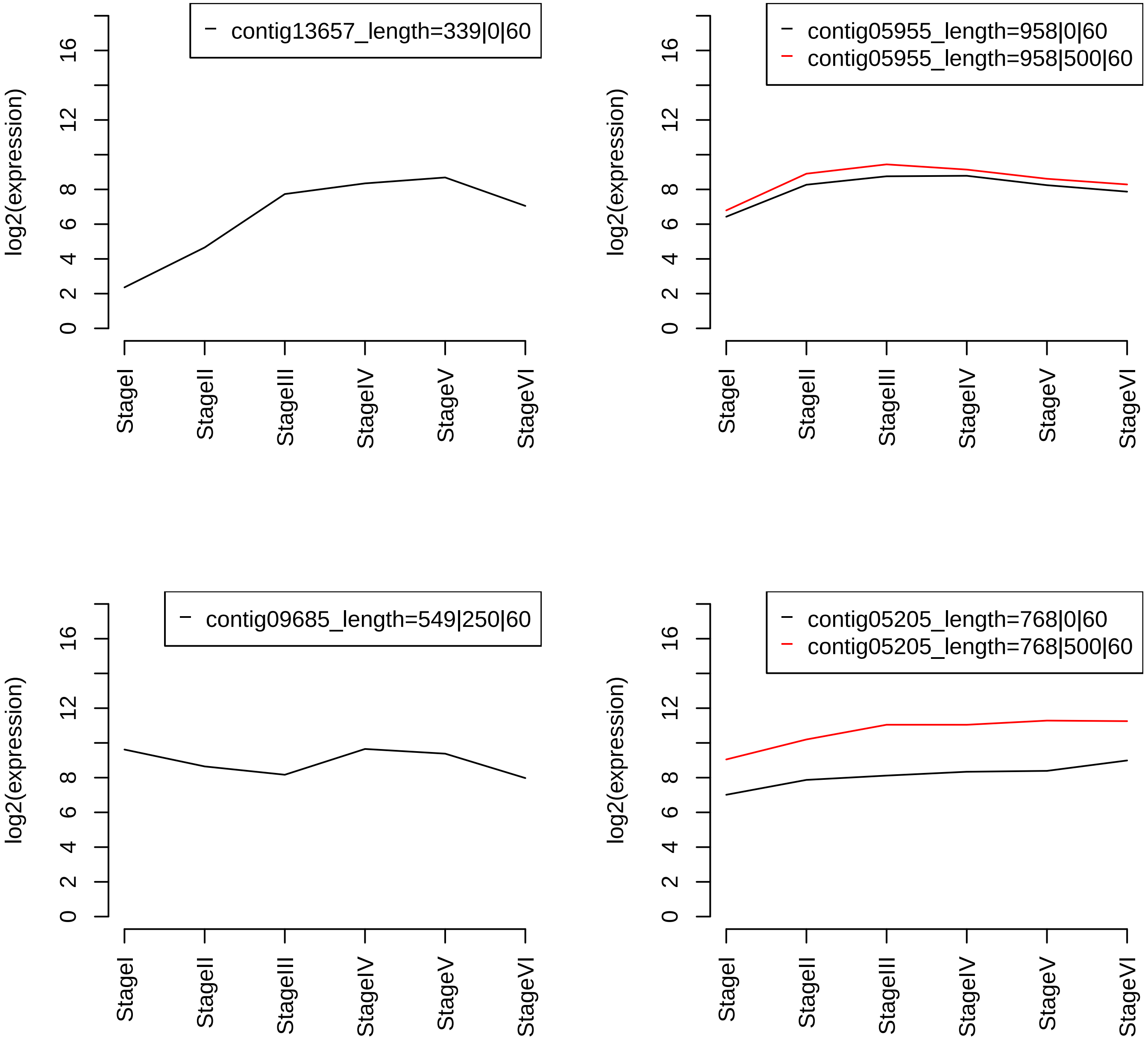
contig13657_length=339|0|60
contig05955_length=958|0|60
contig05955_length=958|500|60
contig09685_length=549|250|60
contig05205_length=768|0|60
contig05205_length=768|500|60
log2(expression)
0
2
4
6
8
12
16
StageI
StageII
StageIII
StageIV
StageV
StageVI

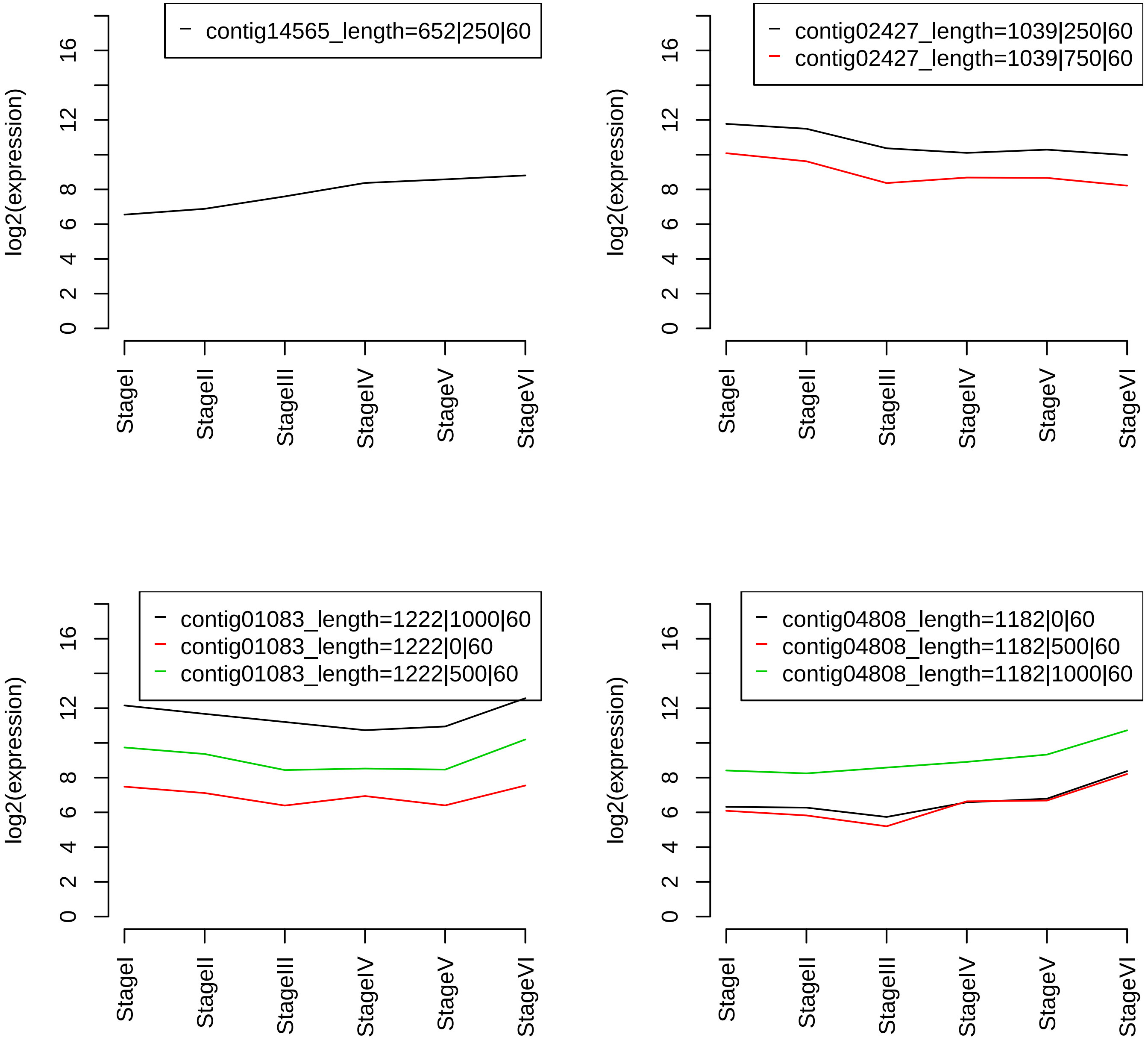

contig14565_length=652|250|60
contig02427_length=1039|250|60
contig02427_length=1039|750|60
contig01083_length=1222|1000|60
contig01083_length=1222|0|60
contig01083_length=1222|500|60
contig04808_length=1182|0|60
contig04808_length=1182|500|60
contig04808_length=1182|1000|60
log2(expression)
0
2
4
6
8
12
16
StageI
StageII
StageIII
StageIV
StageV
StageVI

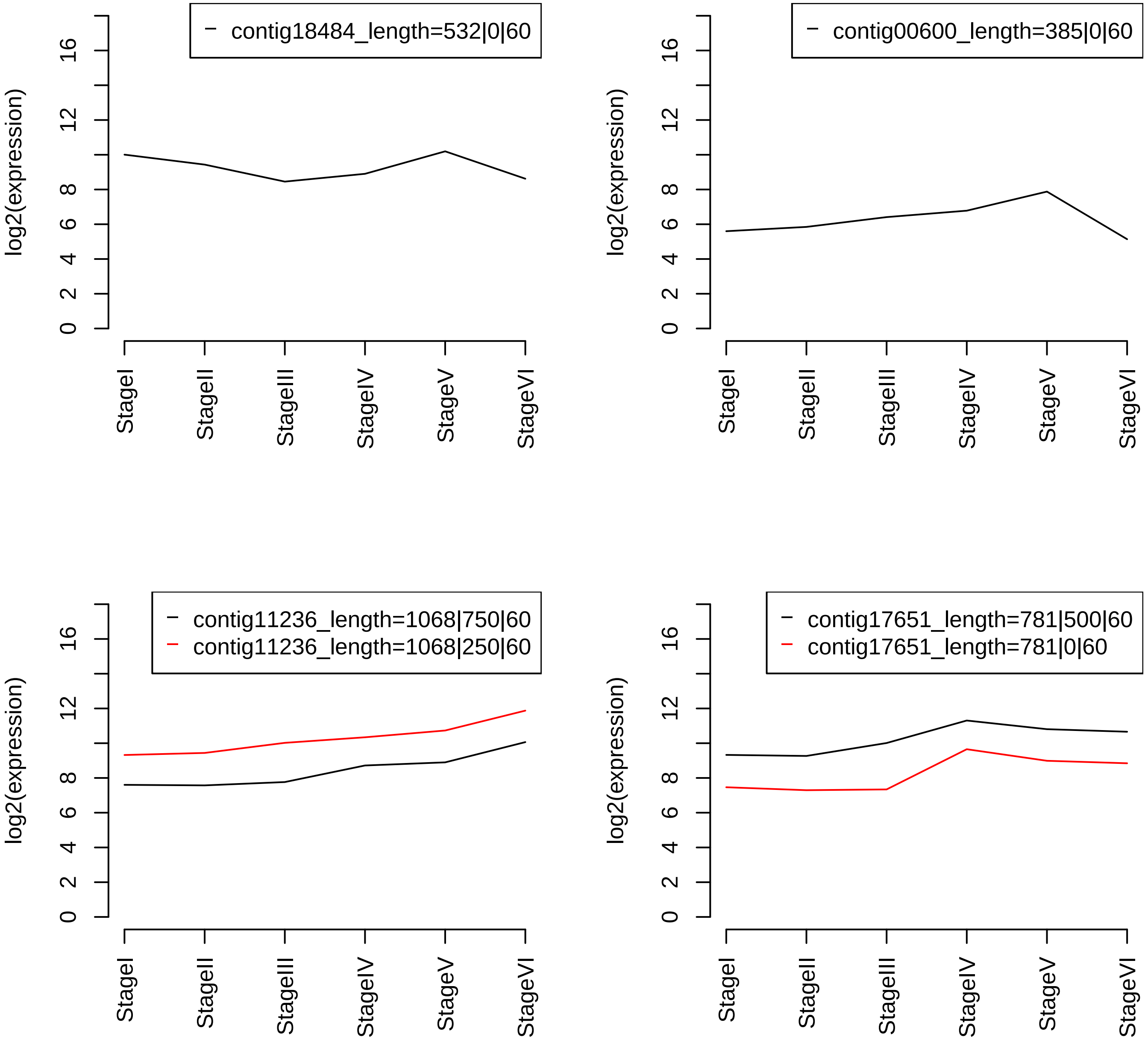
contig18484_length=532|0|60
contig00600_length=385|0|60
contig11236_length=1068|750|60
contig11236_length=1068|250|60
contig17651_length=781|500|60
contig17651_length=781|0|60
log2(expression)
0
2
4
6
8
12
16
StageI
StageII
StageIII
StageIV
StageV
StageVI

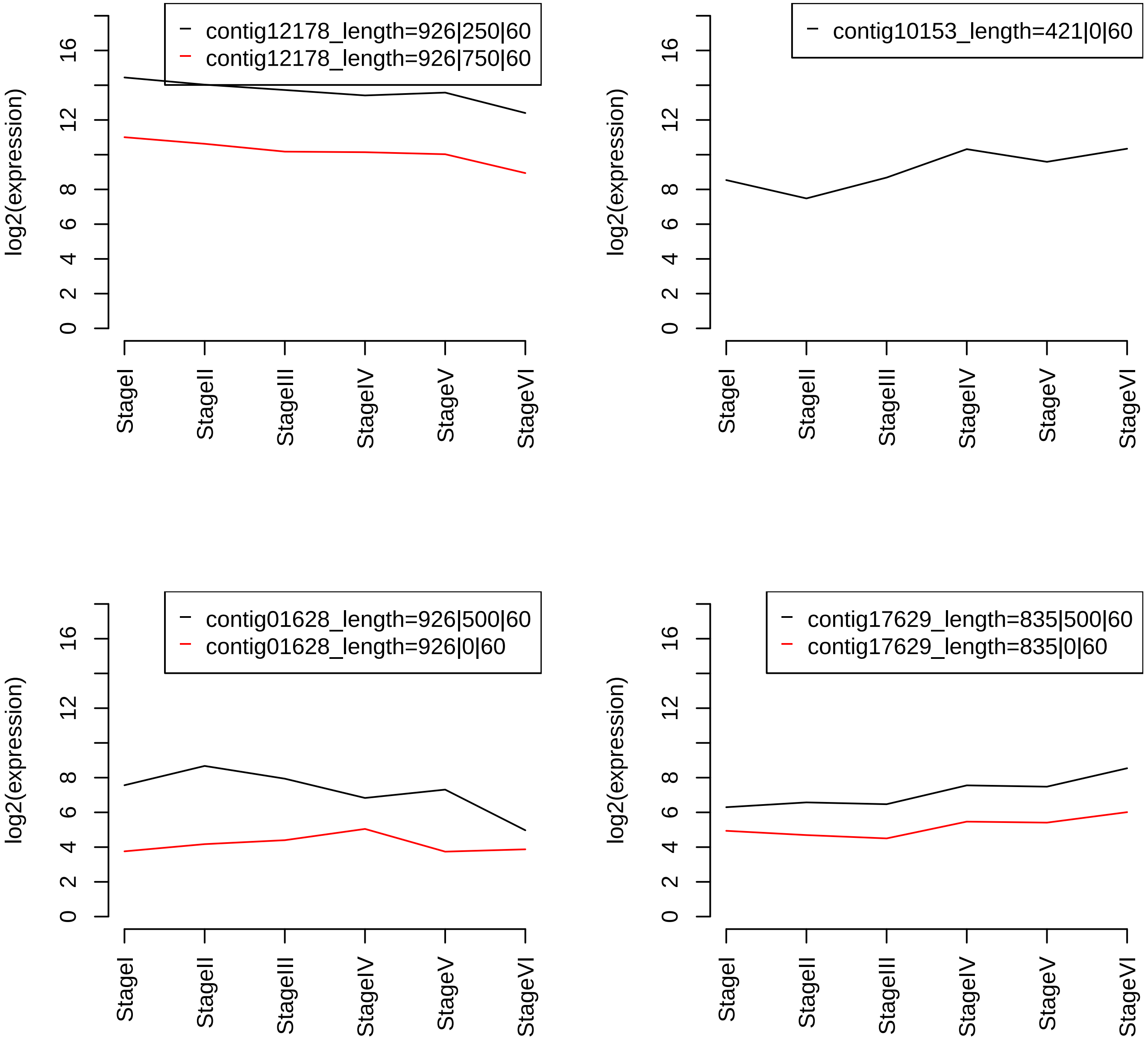
contig12178_length=926|250|60
contig12178_length=926|750|60
contig10153_length=421|0|60
contig01628_length=926|500|60
contig01628_length=926|0|60
contig17629_length=835|500|60
contig17629_length=835|0|60
log2(expression)
0
2
4
6
8
12
16
StageI
StageII
StageIII
StageIV
StageV
StageVI

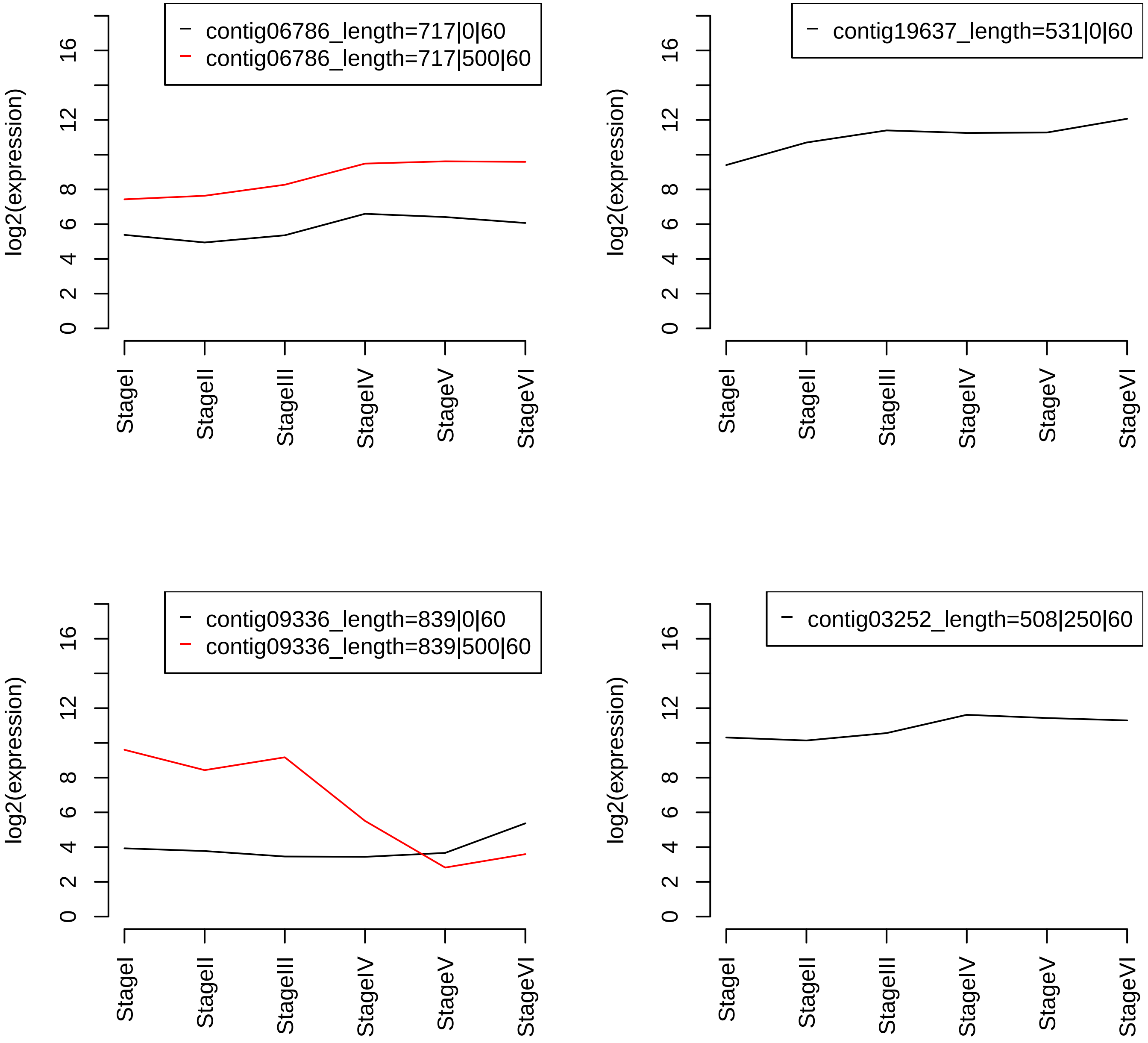
contig06786_length=717|0|60
contig06786_length=717|500|60
contig19637_length=531|0|60
contig09336_length=839|0|60
contig09336_length=839|500|60
contig03252_length=508|250|60
log2(expression)
0
2
4
6
8
12
16
StageI
StageII
StageIII
StageIV
StageV
StageVI

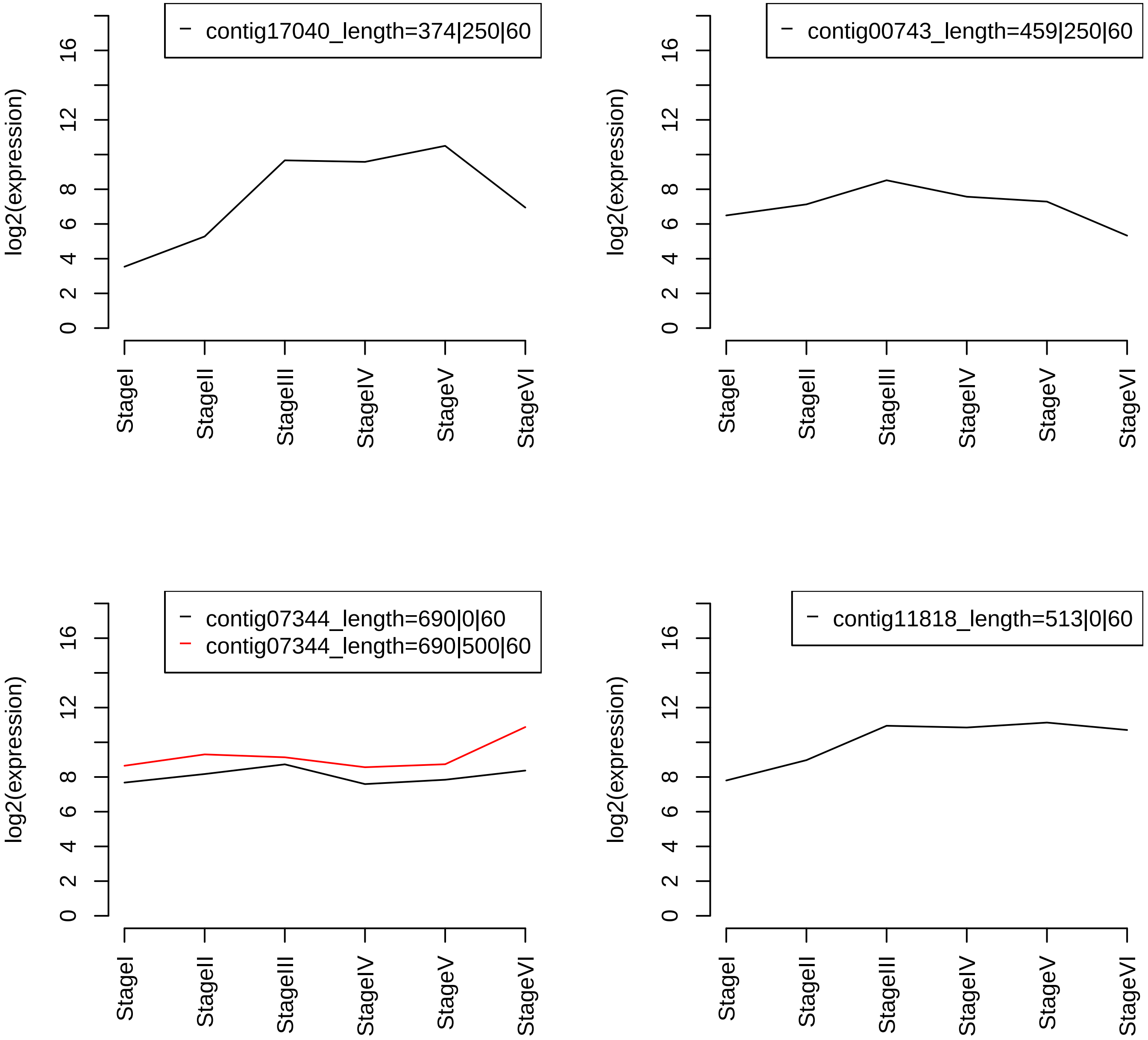
contig17040_length=374|250|60
contig00743_length=459|250|60
contig07344_length=690|0|60
contig07344_length=690|500|60
contig11818_length=513|0|60
log2(expression)
0
2
4
6
8
12
16
StageI
StageII
StageIII
StageIV
StageV
StageVI

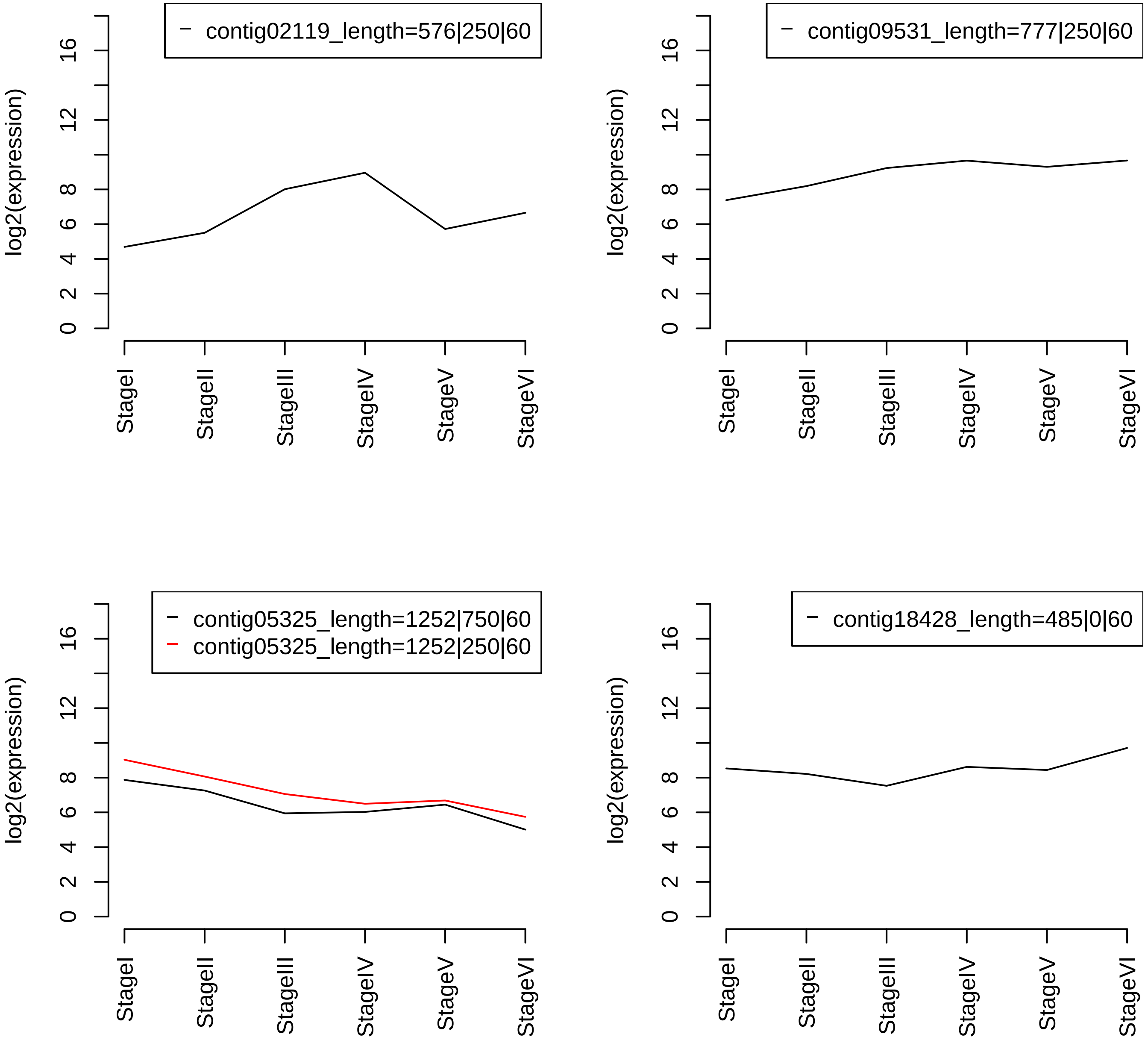
contig02119_length=576|250|60
contig09531_length=777|250|60
contig05325_length=1252|750|60
contig05325_length=1252|250|60
contig18428_length=485|0|60
log2(expression)
0
2
4
6
8
12
16
StageI
StageII
StageIII
StageIV
StageV
StageVI

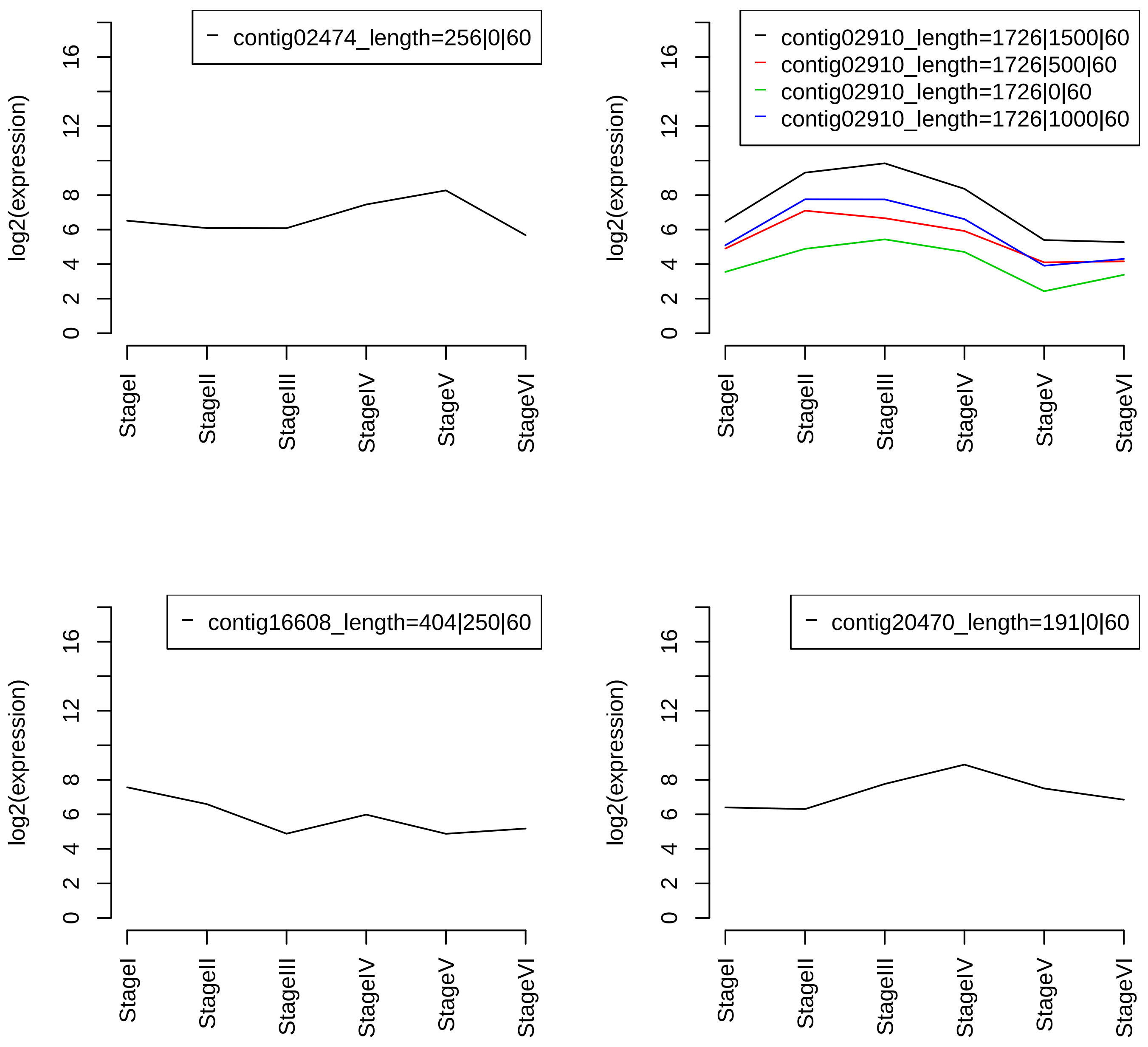
contig02474_length=256|0|60
contig02910_length=1726|1500|60
contig02910_length=1726|500|60
contig02910_length=1726|0|60
contig02910_length=1726|1000|60
contig16608_length=404|250|60
contig20470_length=191|0|60
log2(expression)
0
2
4
6
8
12
16
StageI
StageII
StageIII
StageIV
StageV
StageVI

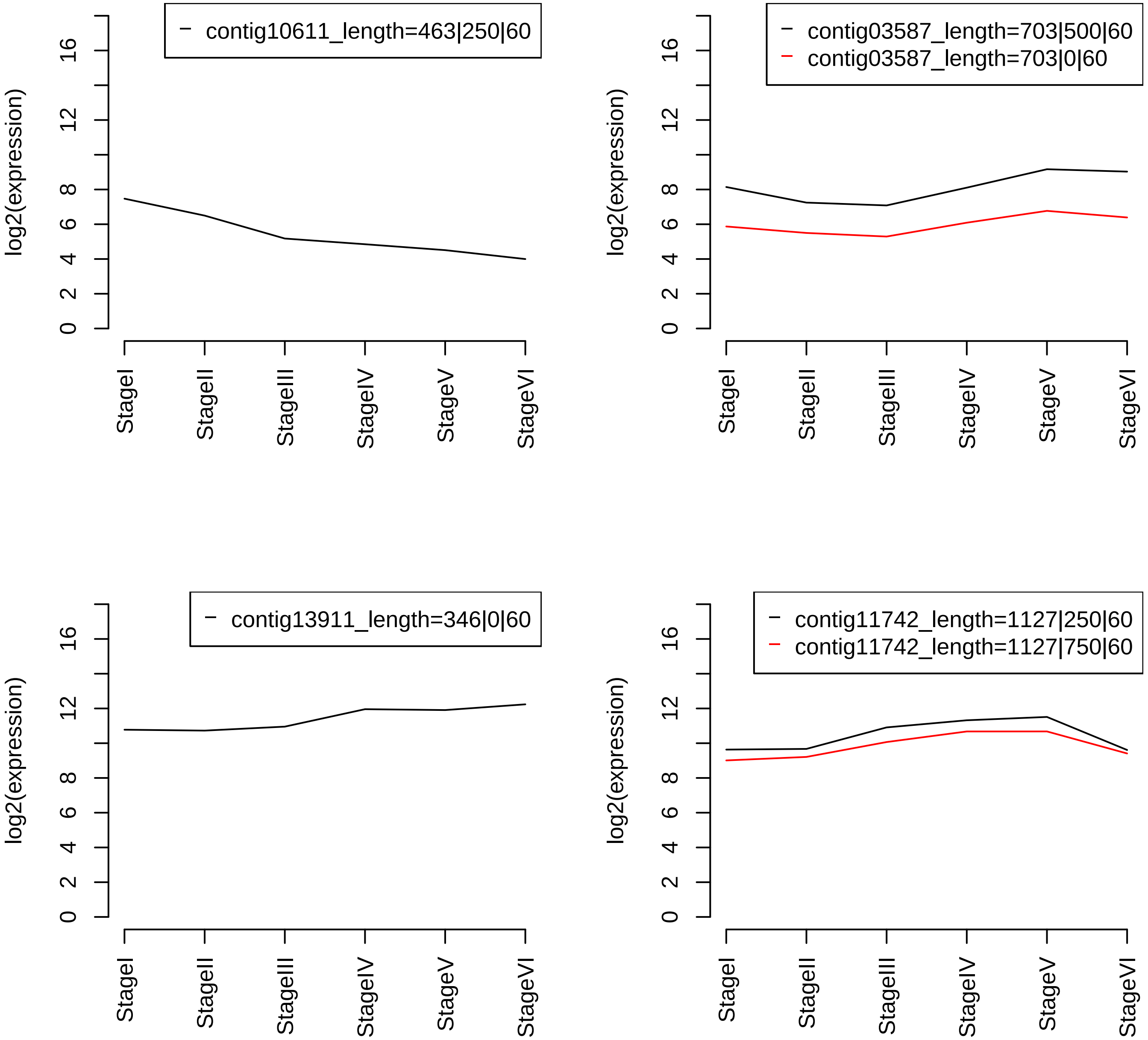
contig10611_length=463|250|60
contig03587_length=703|500|60
contig03587_length=703|0|60
contig13911_length=346|0|60
contig11742_length=1127|250|60
contig11742_length=1127|750|60
log2(expression)
0
2
4
6
8
12
16
StageI
StageII
StageIII
StageIV
StageV
StageVI

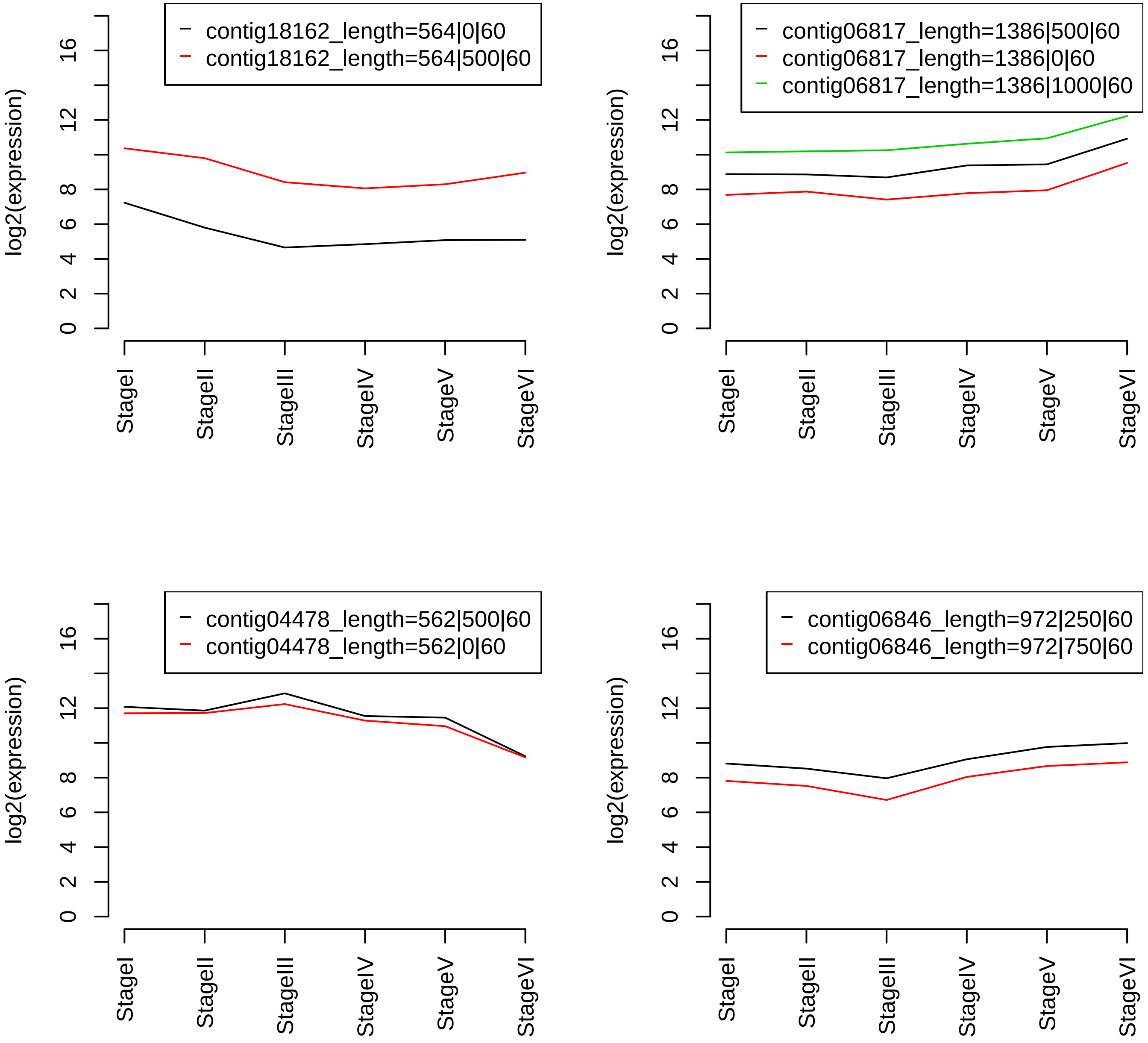

contig18162_length=564|0|60
contig18162_length=564|500|60
contig06817_length=1386|500|60
contig06817_length=1386|0|60
contig06817_length=1386|1000|60
contig04478_length=562|500|60
contig04478_length=562|0|60
contig06846_length=972|250|60
contig06846_length=972|750|60
log2(expression)
0 2 4 6 8 12 16
StageI StageII StageIII StageIV StageV StageVI

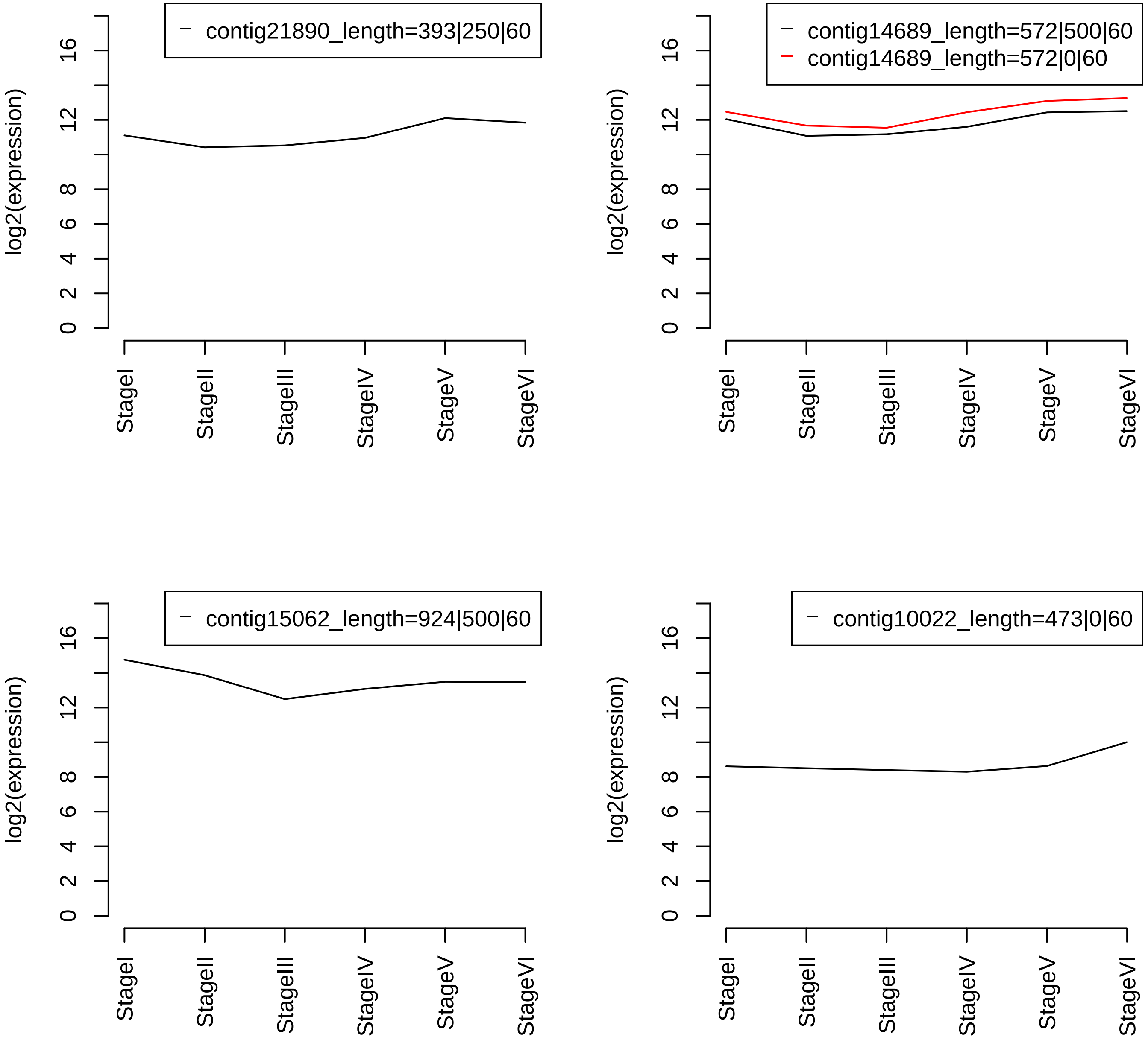

contig21890_length=393|250|60
log2(expression)
0
2
4
6
8
12
16
StageI
StageII
StageIII
StageIV
StageV
StageVI
contig14689_length=572|500|60
contig14689_length=572|0|60
log2(expression)
0
2
4
6
8
12
16
StageI
StageII
StageIII
StageIV
StageV
StageVI
contig15062_length=924|500|60
log2(expression)
0
2
4
6
8
12
16
StageI
StageII
StageIII
StageIV
StageV
StageVI
contig10022_length=473|0|60
log2(expression)
0
2
4
6
8
12
16
StageI
StageII
StageIII
StageIV
StageV
StageVI

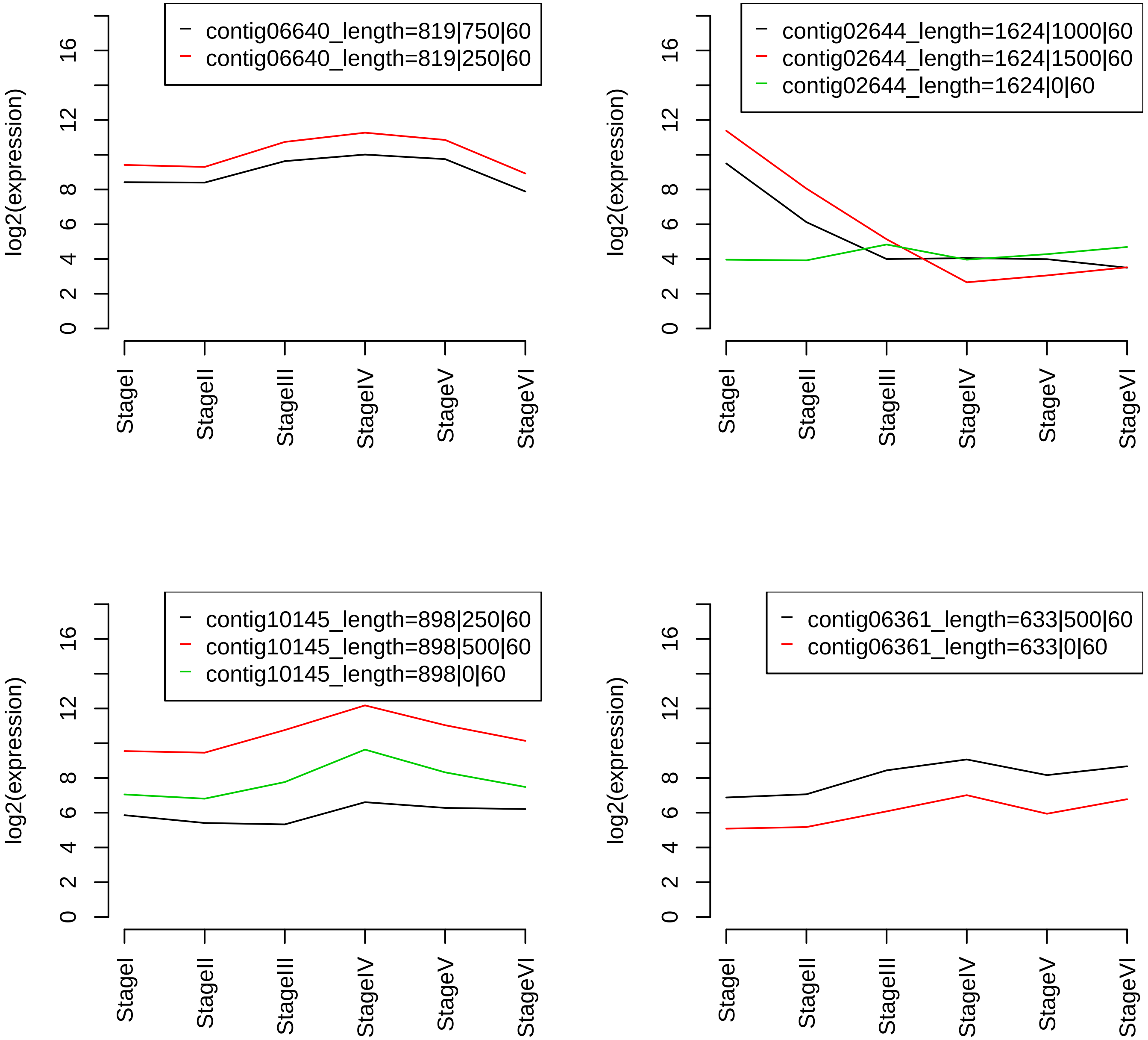

contig06640_length=819|750|60
contig06640_length=819|250|60
contig02644_length=1624|1000|60
contig02644_length=1624|1500|60
contig02644_length=1624|0|60
contig10145_length=898|250|60
contig10145_length=898|500|60
contig10145_length=898|0|60
contig06361_length=633|500|60
contig06361_length=633|0|60
log2(expression)
0 2 4 6 8 12 16
StageI StageII StageIII StageIV StageV StageVI

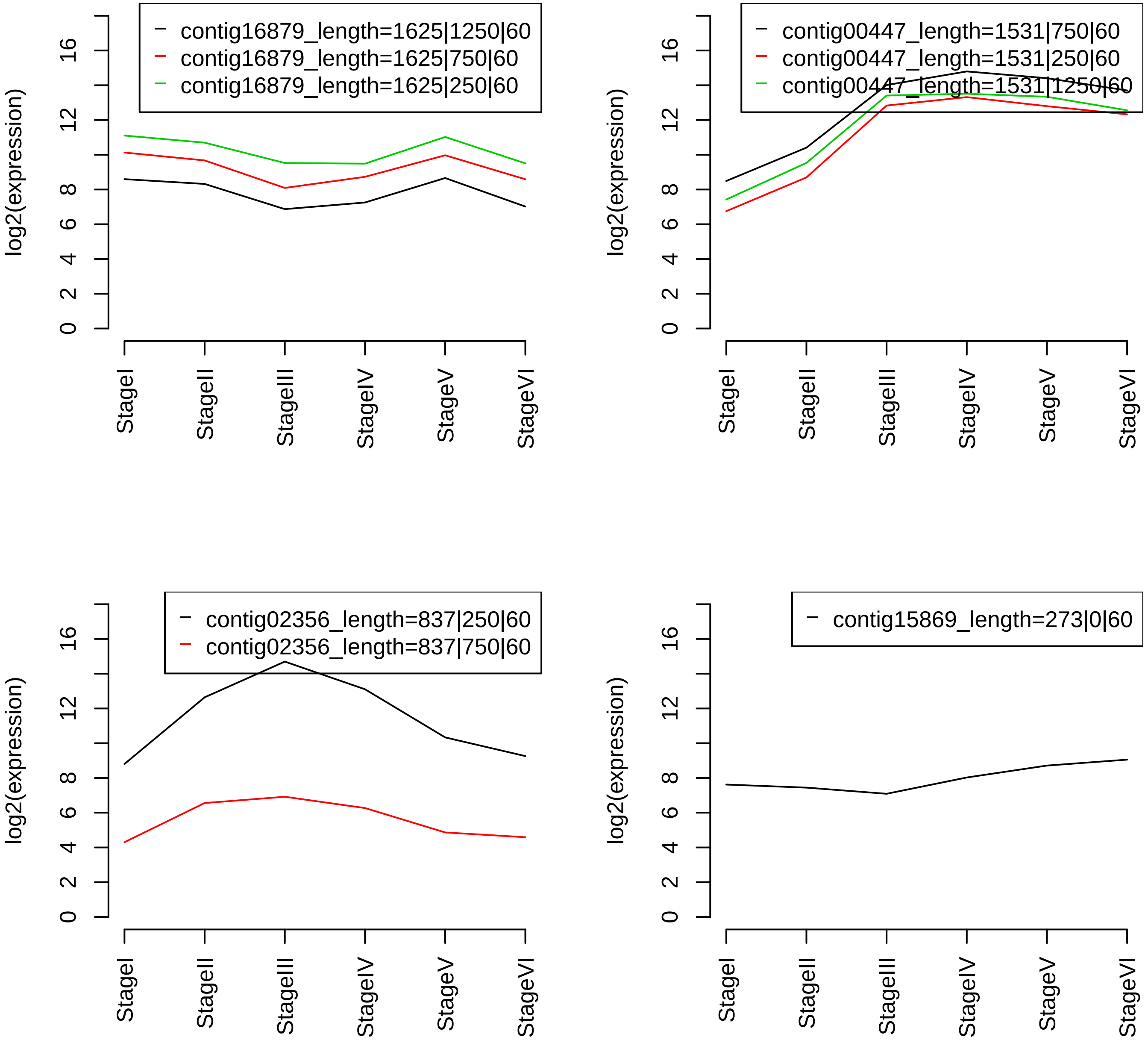
contig16879_length=1625|1250|60
contig16879_length=1625|750|60
contig16879_length=1625|250|60
log2(expression)
0
2
4
6
8
12
16
StageI
StageII
StageIII
StageIV
StageV
StageVI
contig00447_length=1531|750|60
contig00447_length=1531|250|60
contig00447_length=1531|1250|60
contig02356_length=837|250|60
contig02356_length=837|750|60
contig15869_length=273|0|60

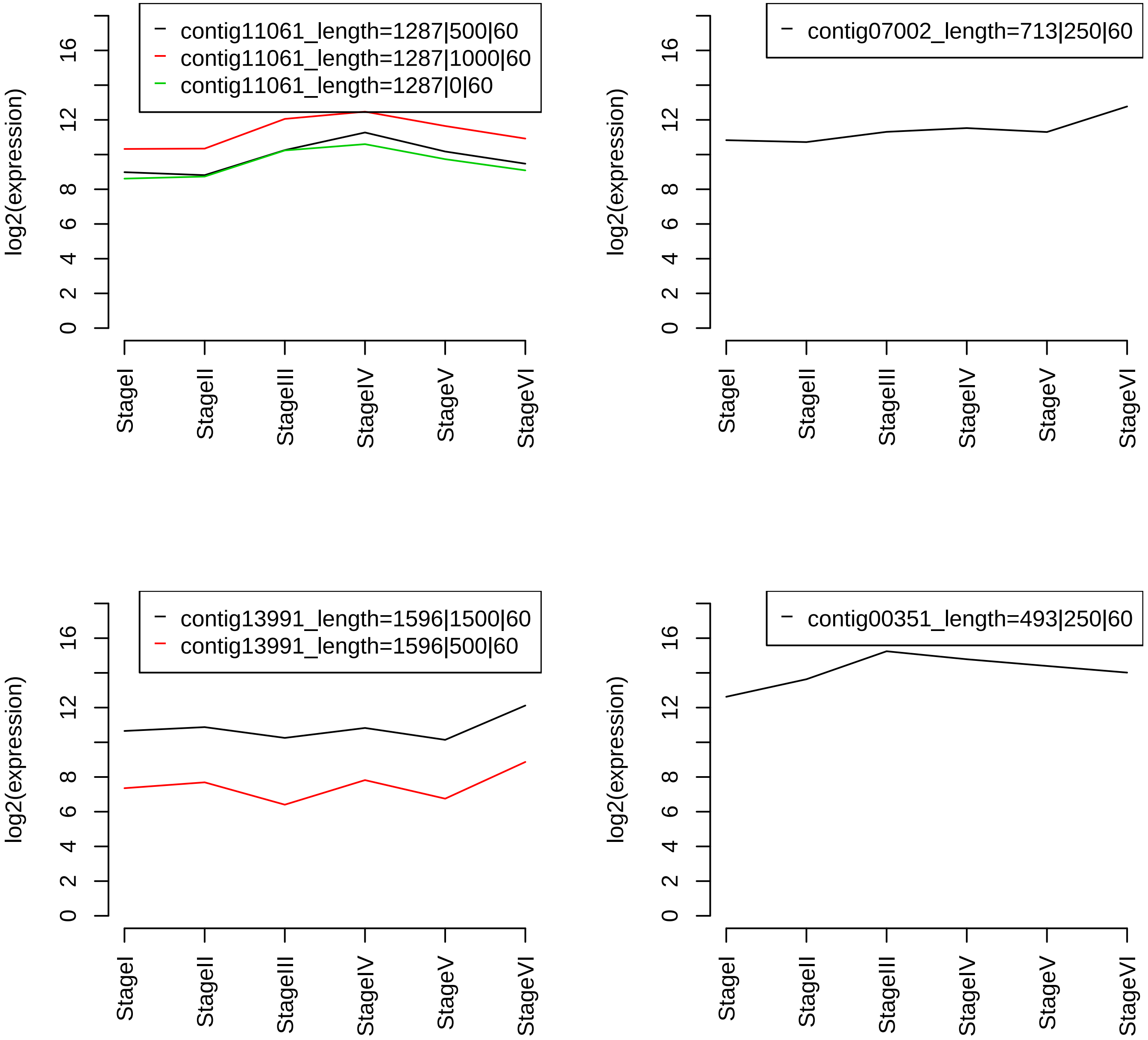
contig11061_length=1287|500|60
contig11061_length=1287|1000|60
contig11061_length=1287|0|60
log2(expression)
0 2 4 6 8 12 16
StageI StageII StageIII StageIV StageV StageVI
contig07002_length=713|250|60
log2(expression)
0 2 4 6 8 12 16
StageI StageII StageIII StageIV StageV StageVI
contig13991_length=1596|1500|60
contig13991_length=1596|500|60
log2(expression)
0 2 4 6 8 12 16
StageI StageII StageIII StageIV StageV StageVI
contig00351_length=493|250|60
log2(expression)
0 2 4 6 8 12 16
StageI StageII StageIII StageIV StageV StageVI

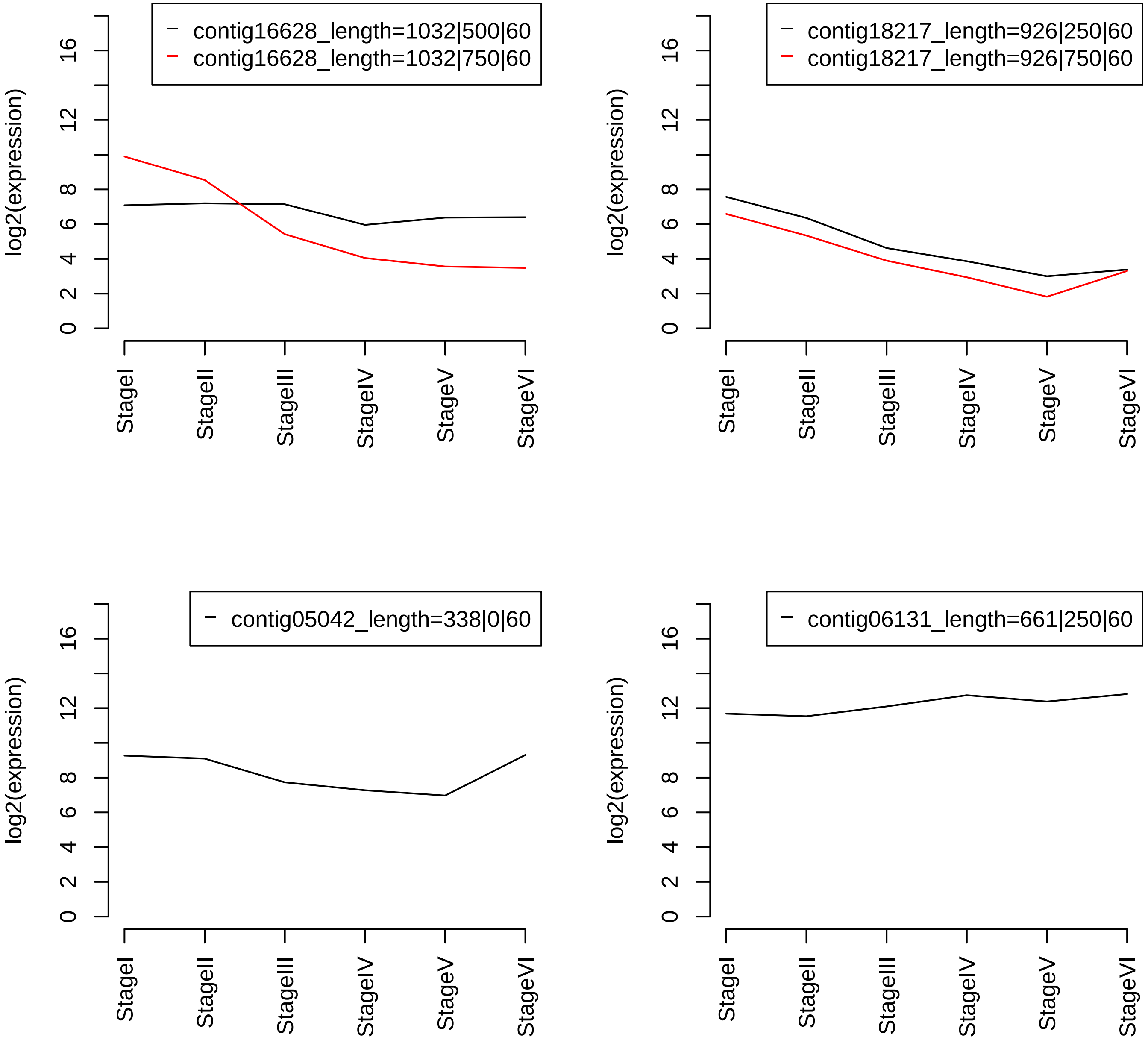
contig16628_length=1032|500|60
contig16628_length=1032|750|60
contig18217_length=926|250|60
contig18217_length=926|750|60
contig05042_length=338|0|60
contig06131_length=661|250|60
log2(expression)
StageI
StageII
StageIII
StageIV
StageV
StageVI

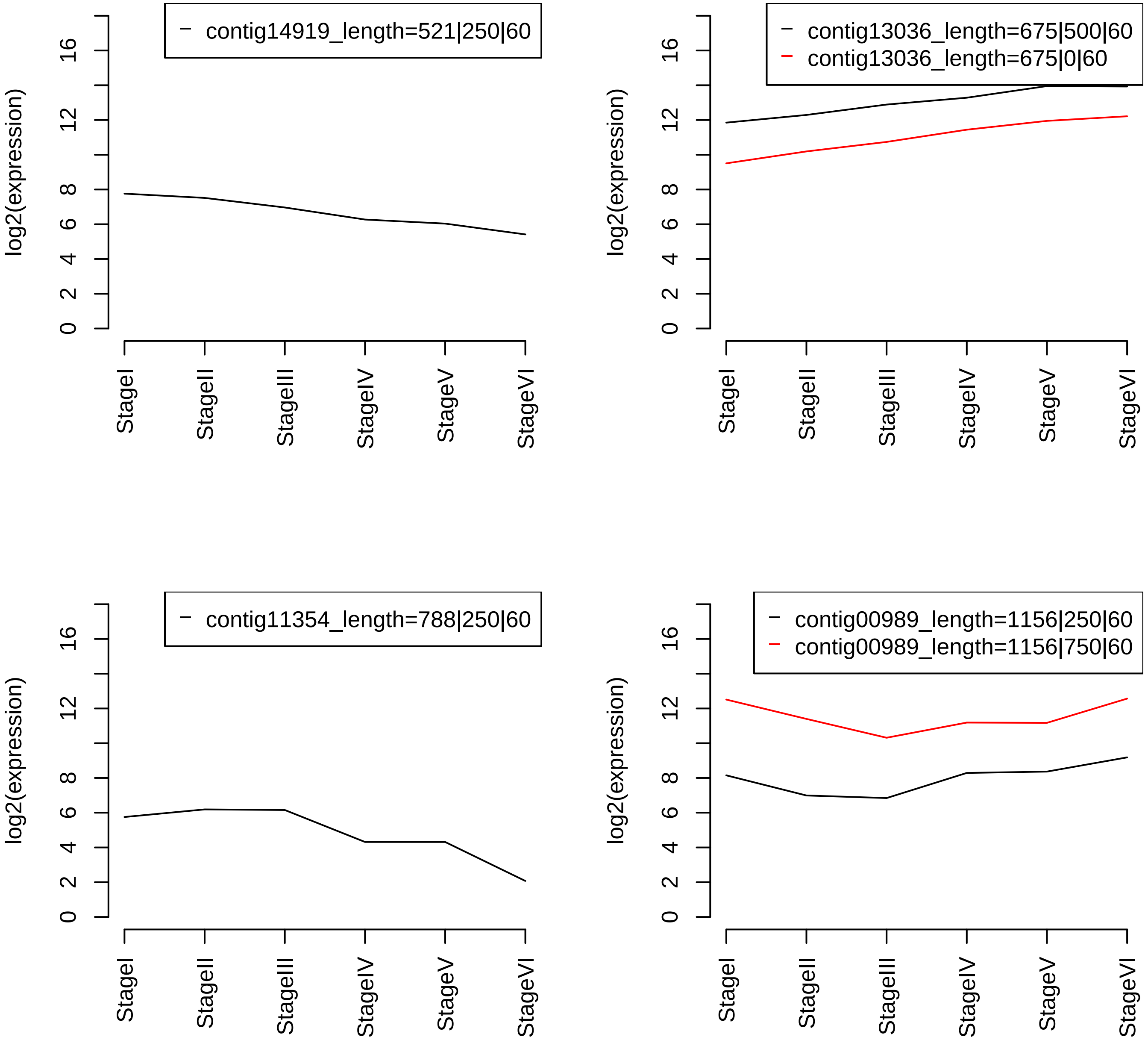
contig14919_length=521|250|60
contig13036_length=675|500|60
contig13036_length=675|0|60
contig11354_length=788|250|60
contig00989_length=1156|250|60
contig00989_length=1156|750|60
log2(expression)
0
2
4
6
8
12
16
StageI
StageII
StageIII
StageIV
StageV
StageVI

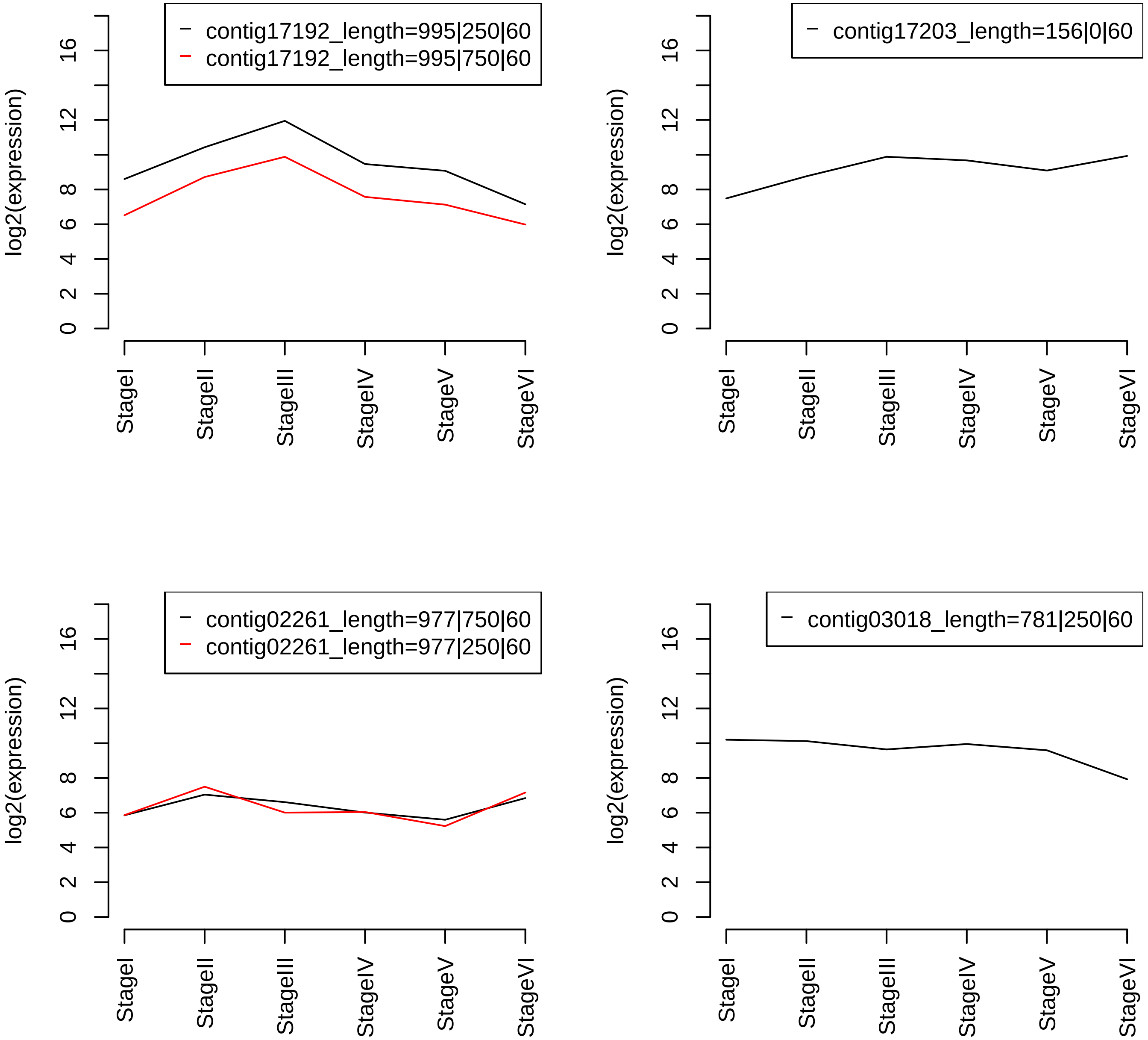
contig17192_length=995|250|60
contig17192_length=995|750|60
contig17203_length=156|0|60
contig02261_length=977|750|60
contig02261_length=977|250|60
contig03018_length=781|250|60
log2(expression)
0
2
4
6
8
12
16
StageI
StageII
StageIII
StageIV
StageV
StageVI

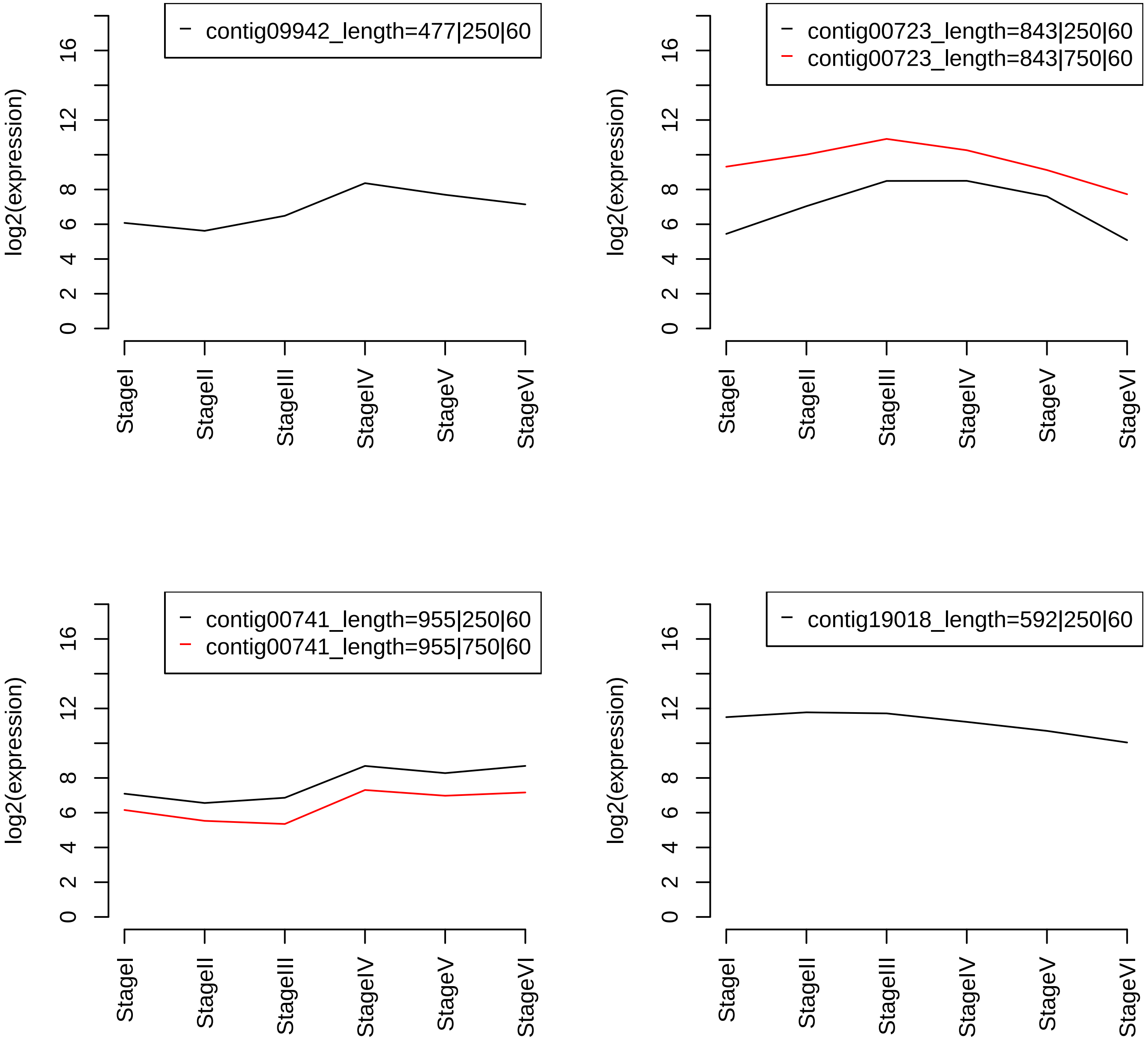

contig09942_length=477|250|60
contig00723_length=843|250|60
contig00723_length=843|750|60
contig00741_length=955|250|60
contig00741_length=955|750|60
contig19018_length=592|250|60
log2(expression)
0 2 4 6 8 12 16
StageI
StageII
StageIII
StageIV
StageV
StageVI

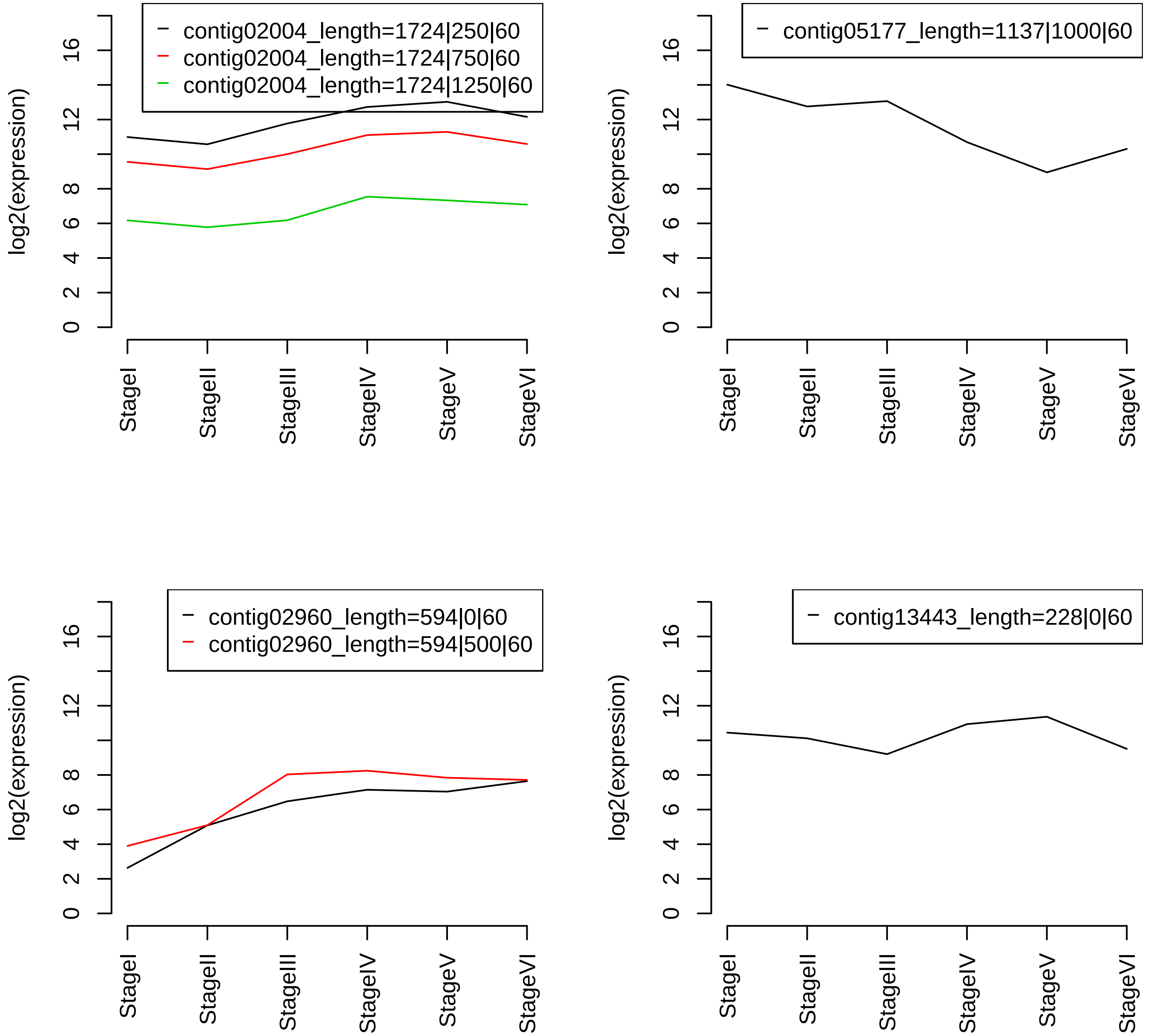

contig02004_length=1724|250|60
contig02004_length=1724|750|60
contig02004_length=1724|1250|60
log2(expression)
0
2
4
6
8
12
16
StageI
StageII
StageIII
StageIV
StageV
StageVI
contig05177_length=1137|1000|60
contig02960_length=594|0|60
contig02960_length=594|500|60
contig13443_length=228|0|60

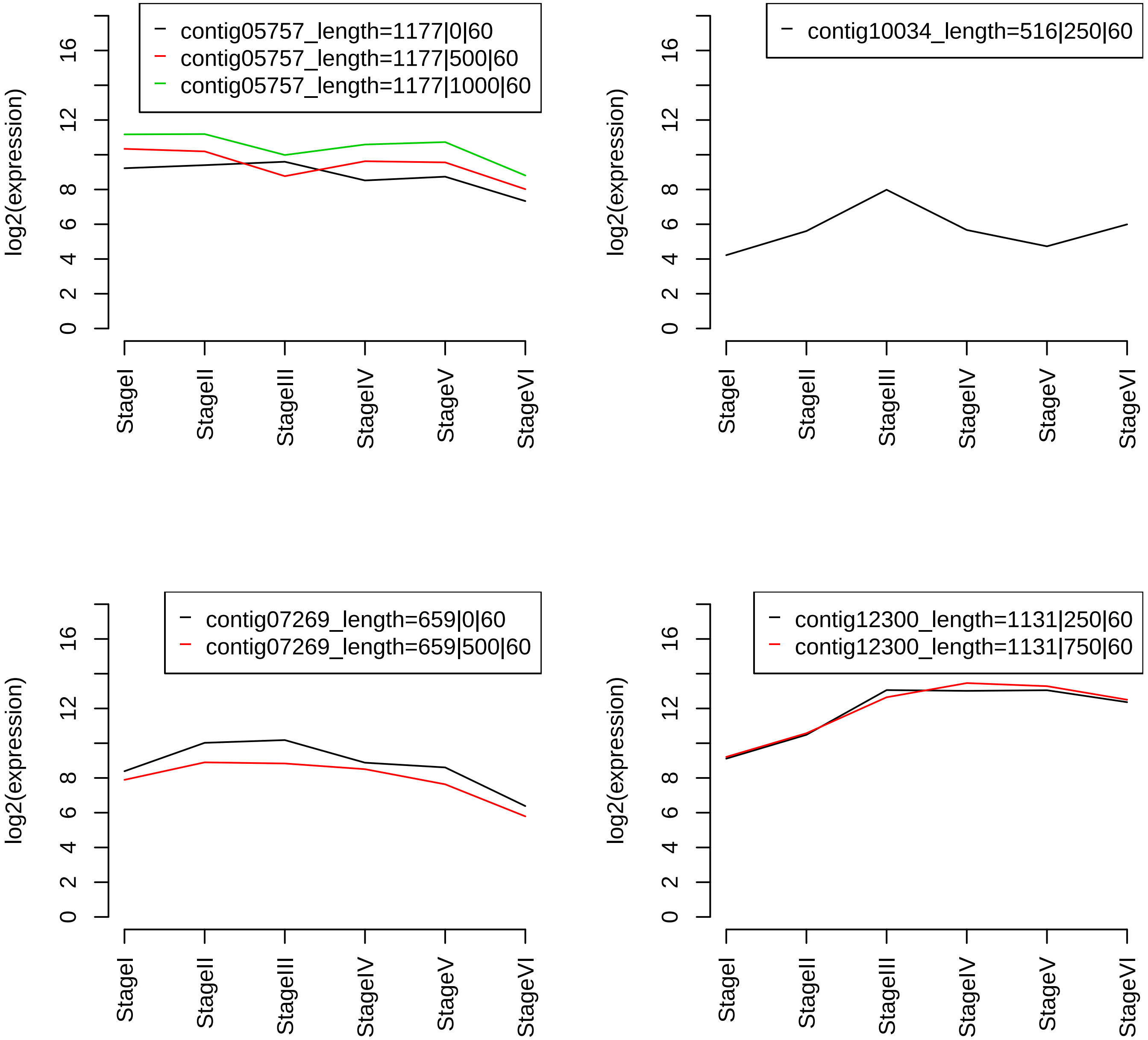
contig05757_length=1177|0|60
contig05757_length=1177|500|60
contig05757_length=1177|1000|60
contig10034_length=516|250|60
contig07269_length=659|0|60
contig07269_length=659|500|60
contig12300_length=1131|250|60
contig12300_length=1131|750|60
log2(expression)
StageI
StageII
StageIII
StageIV
StageV
StageVI

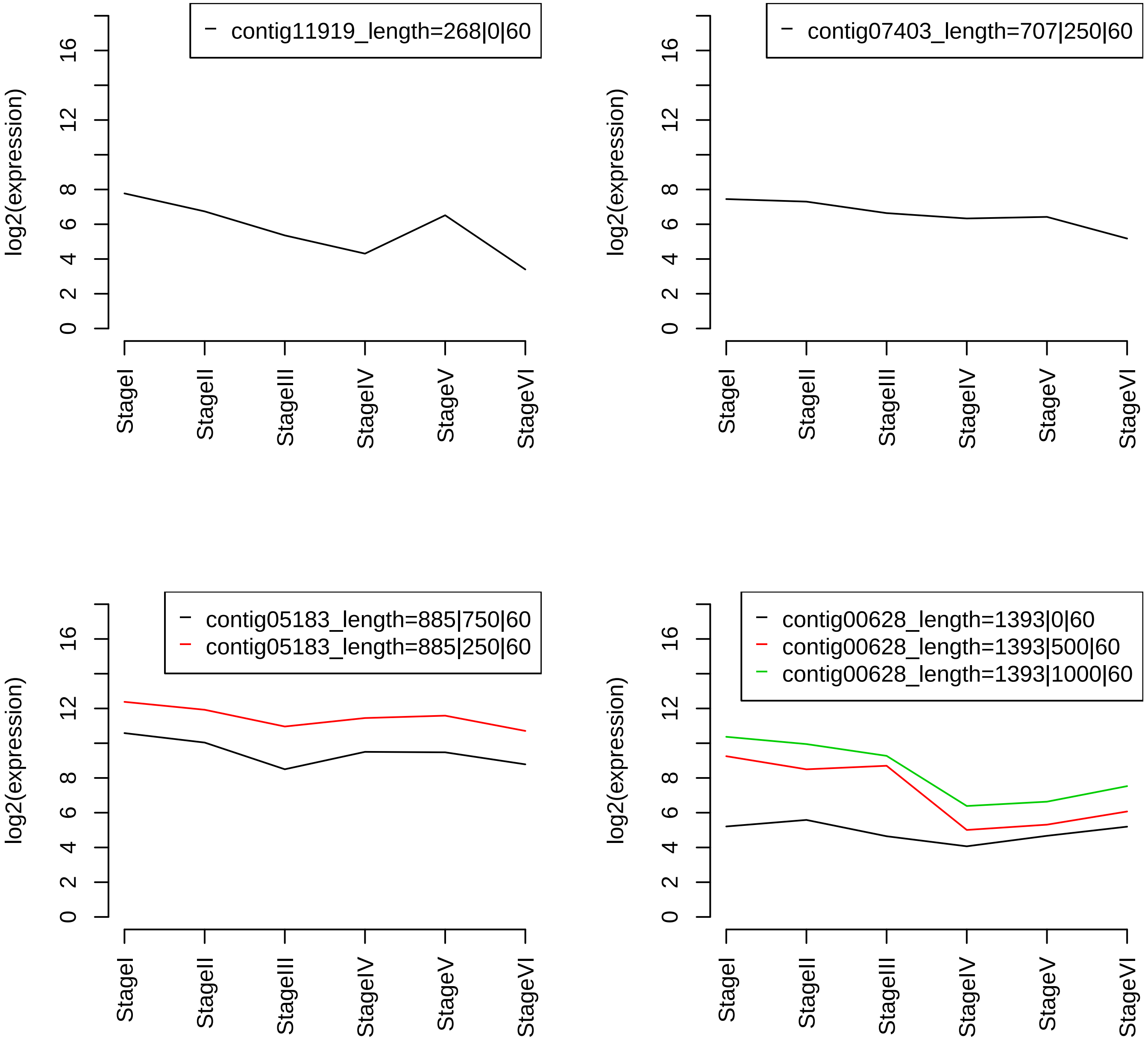
contig11919_length=268|0|60
contig07403_length=707|250|60
contig05183_length=885|750|60
contig05183_length=885|250|60
contig00628_length=1393|0|60
contig00628_length=1393|500|60
contig00628_length=1393|1000|60
log2(expression)
0
2
4
6
8
12
16
StageI
StageII
StageIII
StageIV
StageV
StageVI

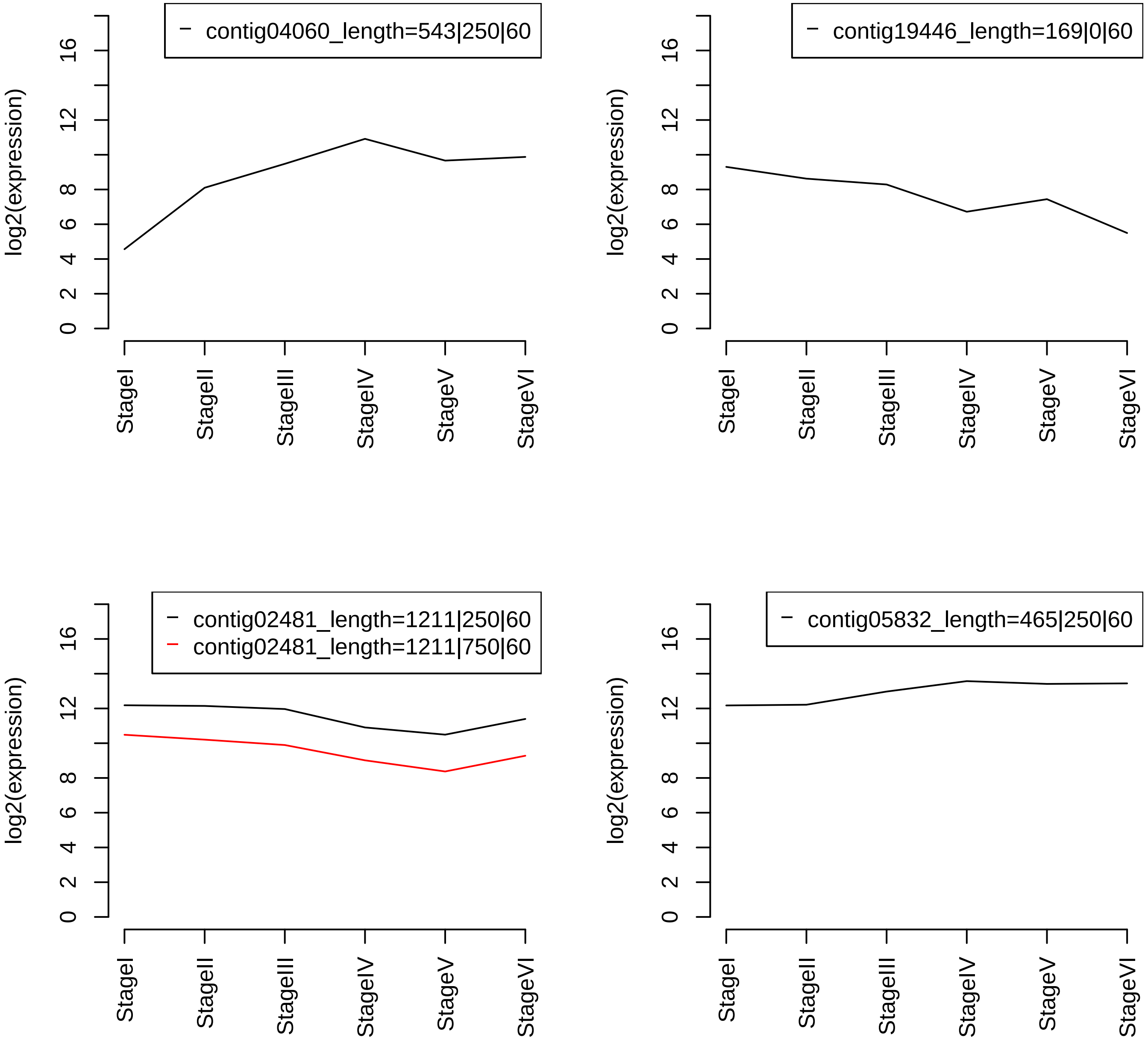

contig04060_length=543|250|60
contig19446_length=169|0|60
contig02481_length=1211|250|60
contig02481_length=1211|750|60
contig05832_length=465|250|60
log2(expression)
0
2
4
6
8
12
16
StageI
StageII
StageIII
StageIV
StageV
StageVI

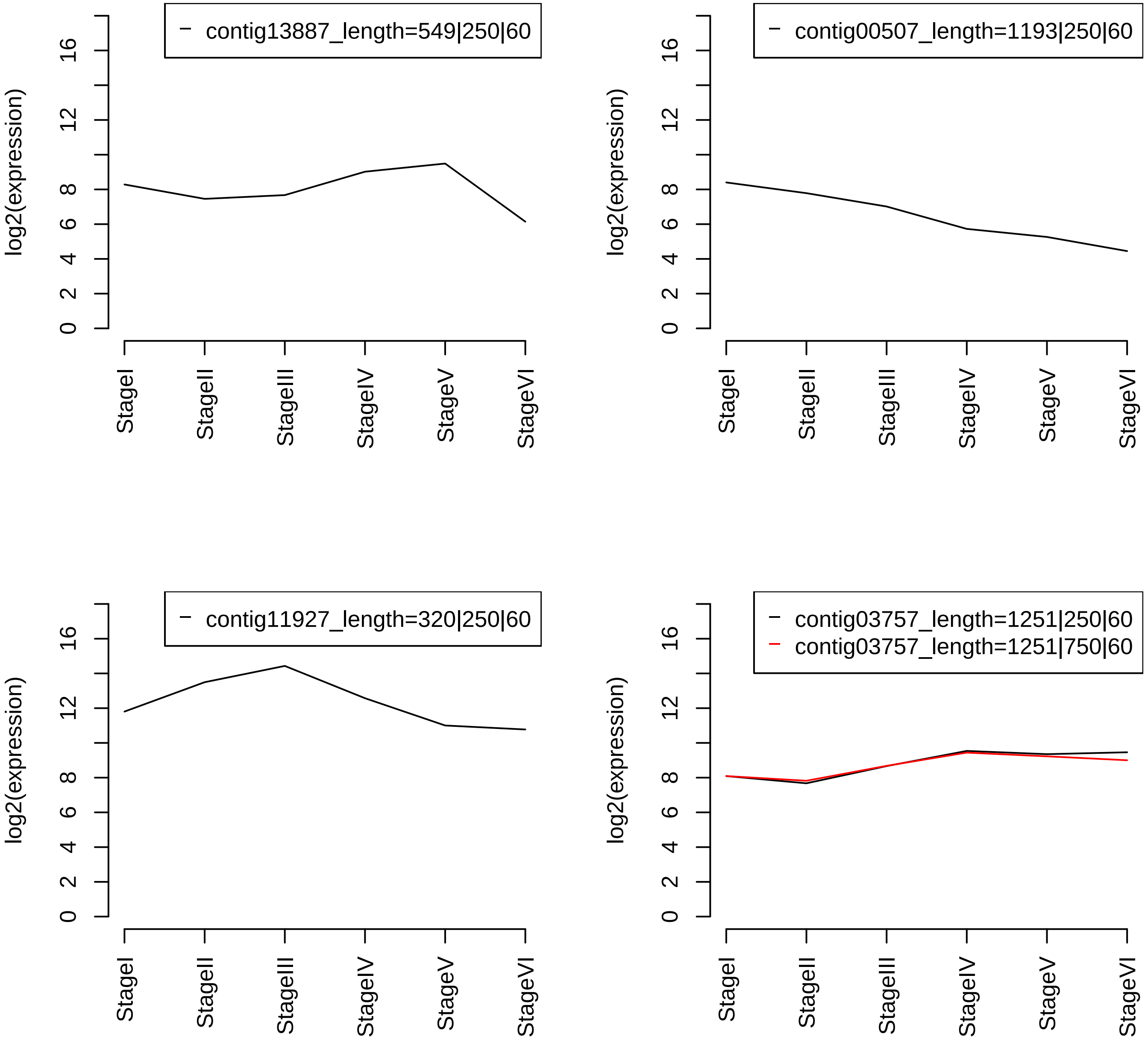

contig13887_length=549|250|60
contig00507_length=1193|250|60
contig11927_length=320|250|60
contig03757_length=1251|250|60
contig03757_length=1251|750|60
log2(expression)
0
2
4
6
8
12
16
StageI
StageII
StageIII
StageIV
StageV
StageVI

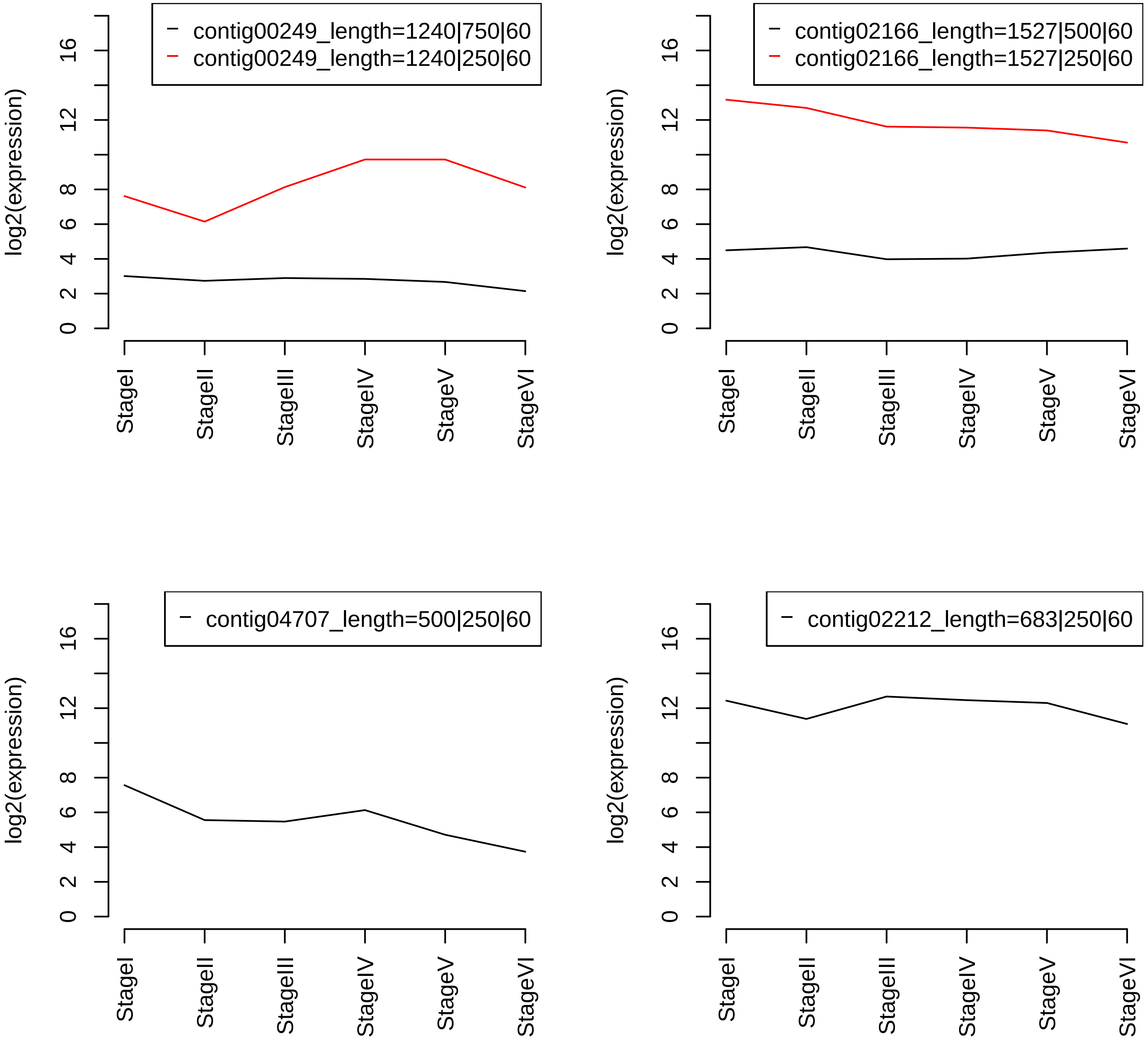
contig00249_length=1240|750|60
contig00249_length=1240|250|60
contig02166_length=1527|500|60
contig02166_length=1527|250|60
contig04707_length=500|250|60
contig02212_length=683|250|60
log2(expression)
0
2
4
6
8
12
16
StageI
StageII
StageIII
StageIV
StageV
StageVI

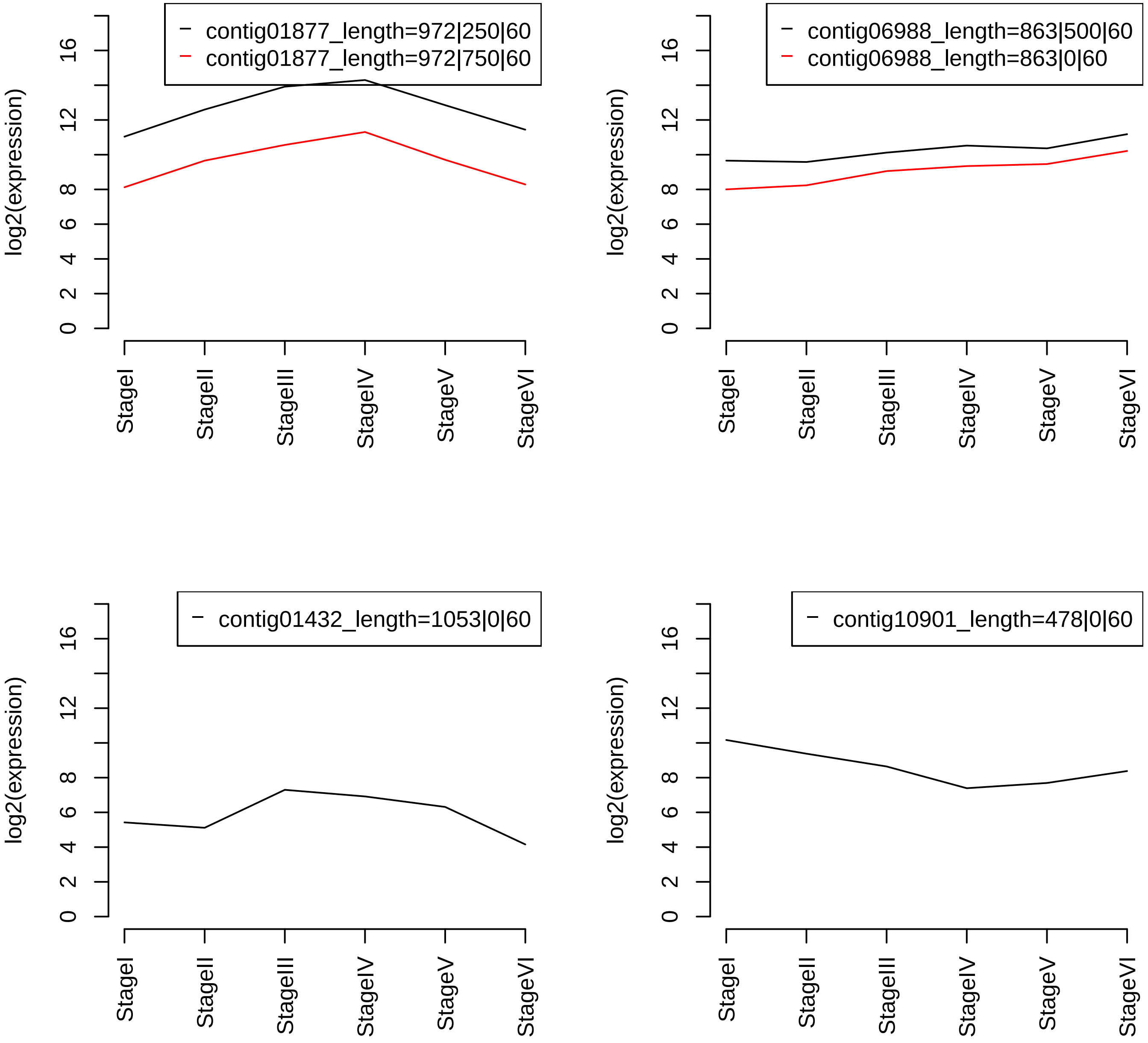
contig01877_length=972|250|60
contig01877_length=972|750|60
log2(expression)
0
2
4
6
8
12
16
StageI
StageII
StageIII
StageIV
StageV
StageVI
contig06988_length=863|500|60
contig06988_length=863|0|60
contig01432_length=1053|0|60
contig10901_length=478|0|60

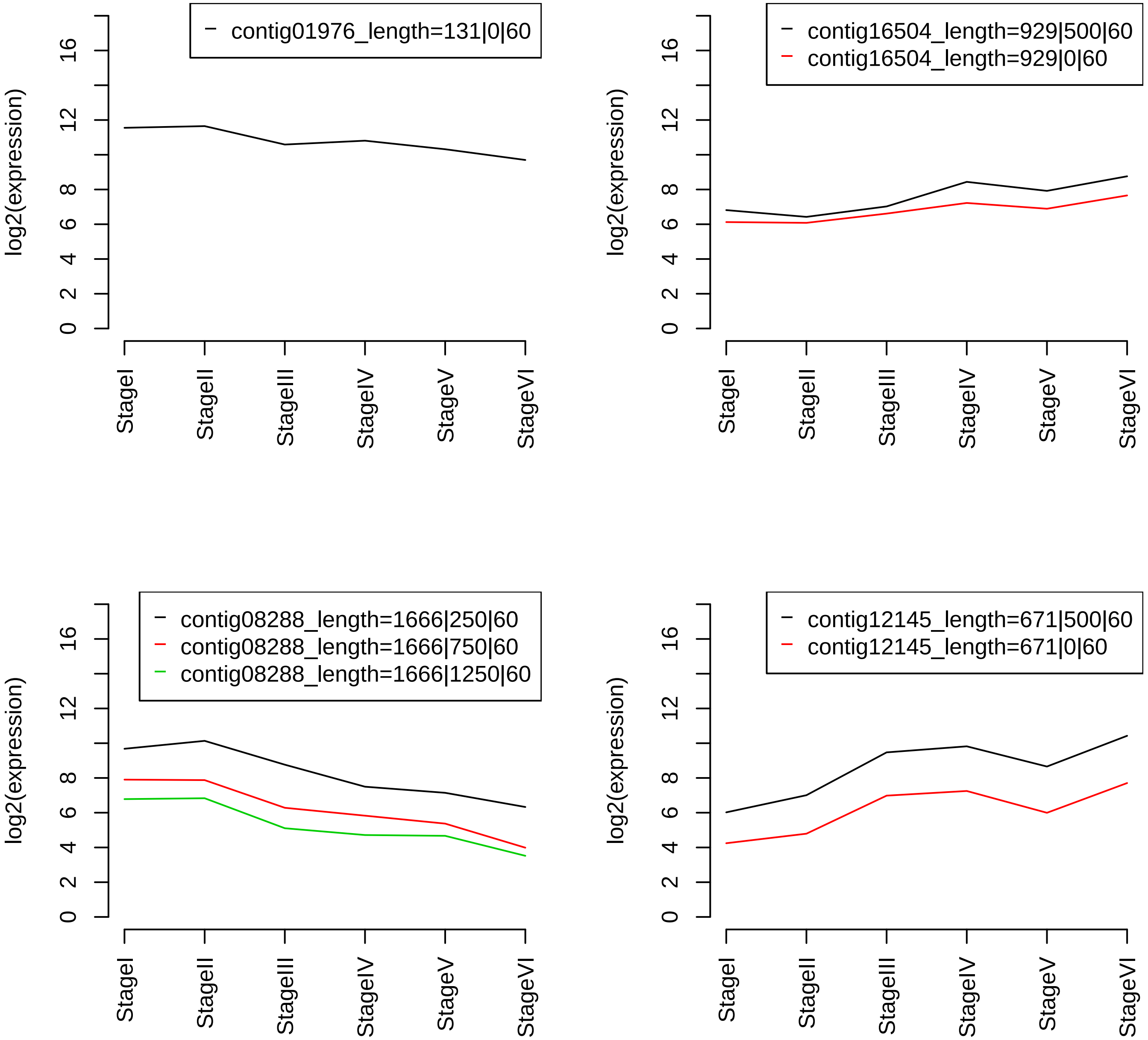

contig01976_length=131|0|60
log2(expression)
0
2
4
6
8
12
16
StageI
StageII
StageIII
StageIV
StageV
StageVI
contig16504_length=929|500|60
contig16504_length=929|0|60
contig08288_length=1666|250|60
contig08288_length=1666|750|60
contig08288_length=1666|1250|60
contig12145_length=671|500|60
contig12145_length=671|0|60

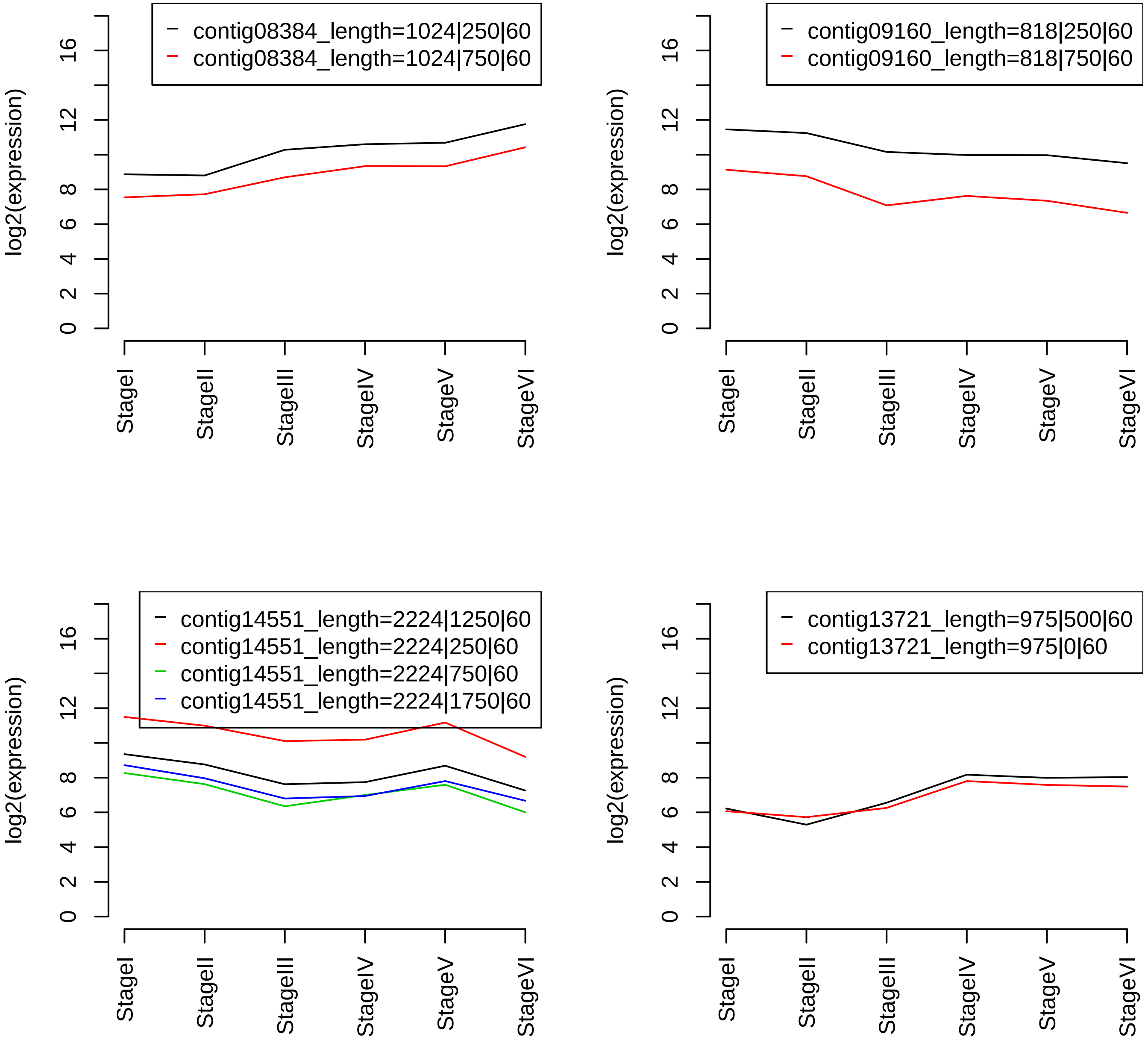
contig08384_length=1024|250|60
contig08384_length=1024|750|60
log2(expression)
0
2
4
6
8
12
16
StageI
StageII
StageIII
StageIV
StageV
StageVI
contig09160_length=818|250|60
contig09160_length=818|750|60
log2(expression)
StageI
StageII
StageIII
StageIV
StageV
StageVI
contig14551_length=2224|1250|60
contig14551_length=2224|250|60
contig14551_length=2224|750|60
contig14551_length=2224|1750|60
log2(expression)
StageI
StageII
StageIII
StageIV
StageV
StageVI
contig13721_length=975|500|60
contig13721_length=975|0|60
log2(expression)
StageI
StageII
StageIII
StageIV
StageV
StageVI

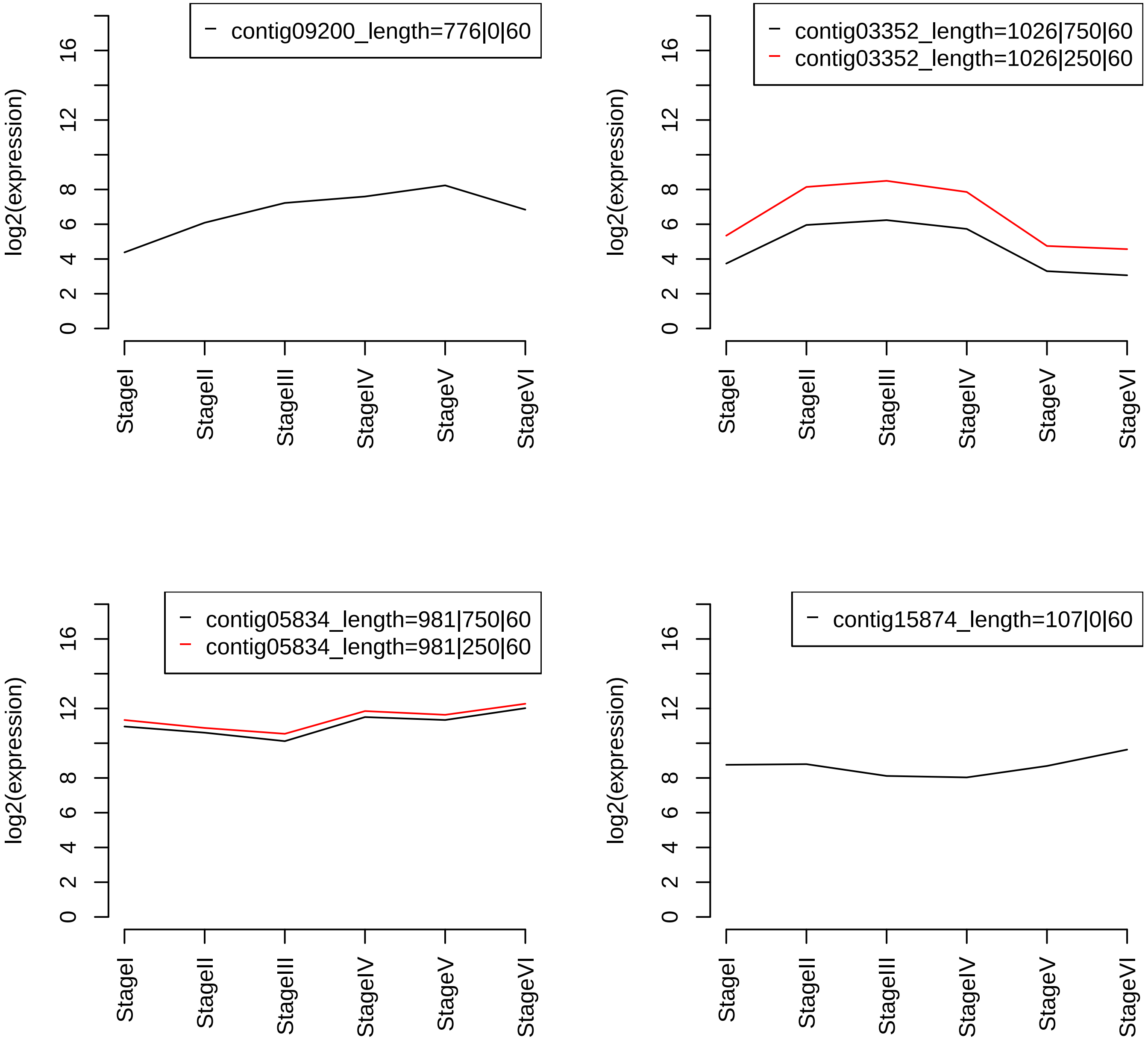

contig09200_length=776|0|60
contig03352_length=1026|750|60
contig03352_length=1026|250|60
contig05834_length=981|750|60
contig05834_length=981|250|60
contig15874_length=107|0|60
log2(expression)
0
2
4
6
8
12
16
StageI
StageII
StageIII
StageIV
StageV
StageVI

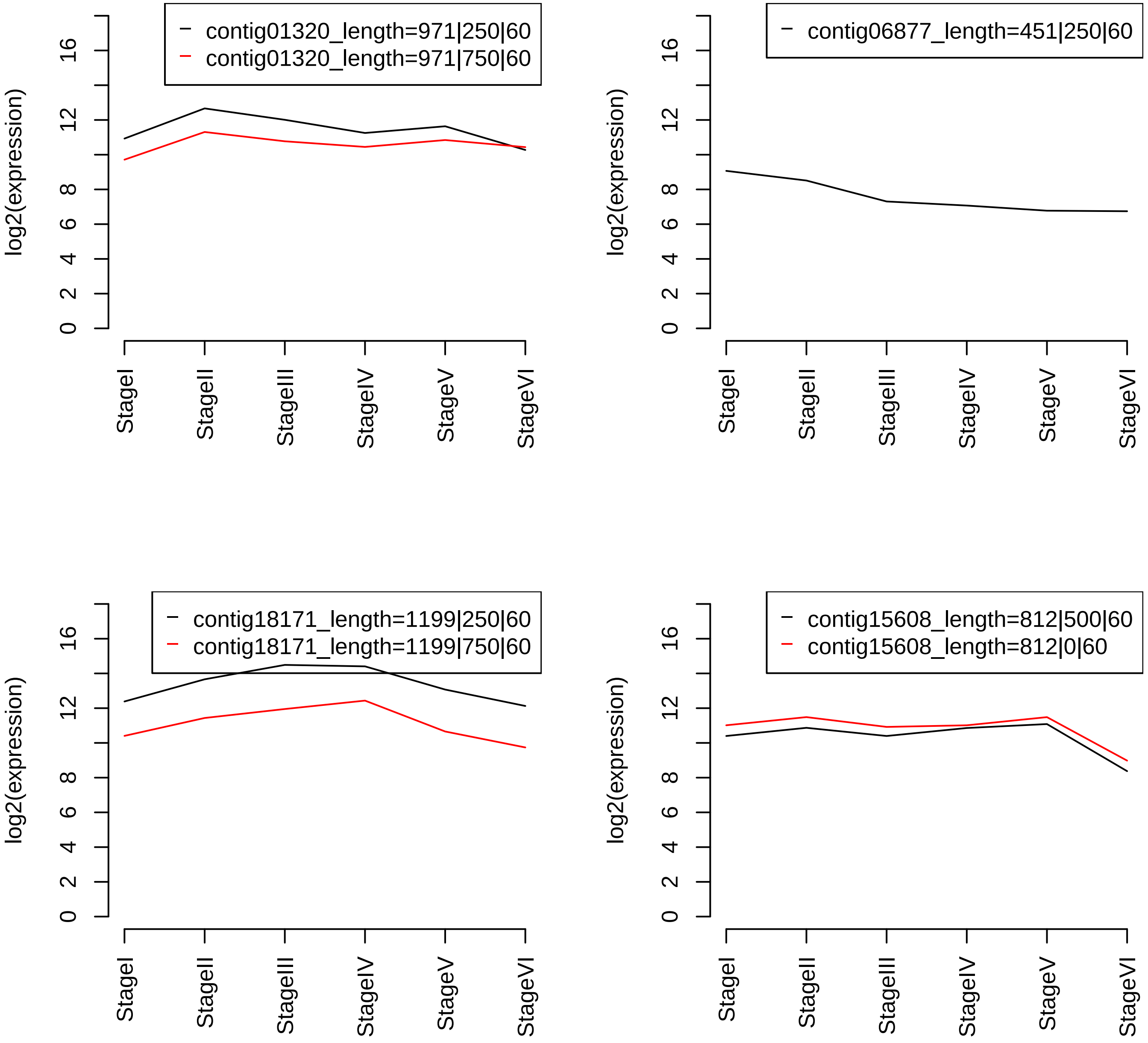
contig01320_length=971|250|60
contig01320_length=971|750|60
contig06877_length=451|250|60
contig18171_length=1199|250|60
contig18171_length=1199|750|60
contig15608_length=812|500|60
contig15608_length=812|0|60
log2(expression)
StageI
StageII
StageIII
StageIV
StageV
StageVI

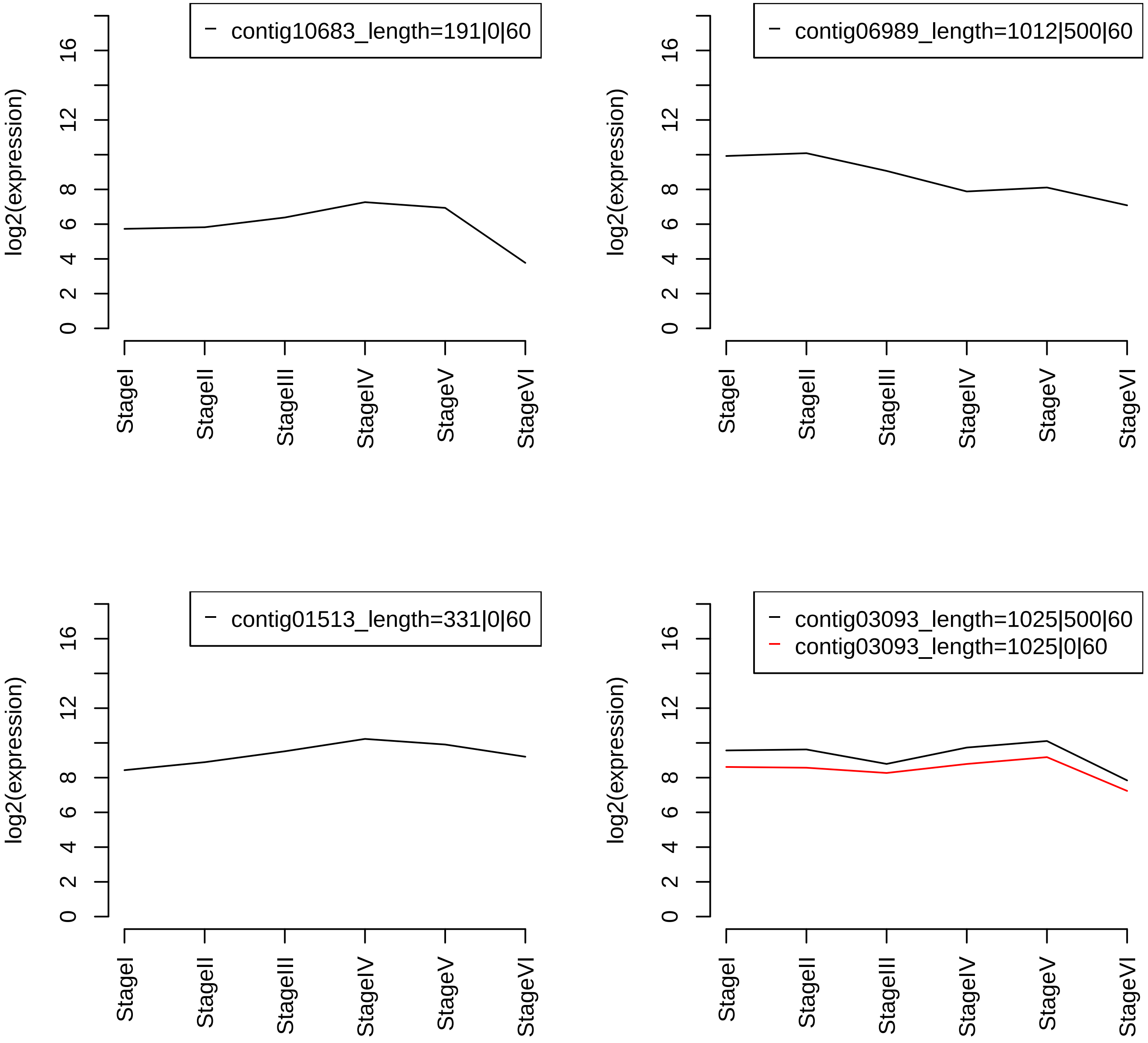

contig10683_length=191|0|60
contig06989_length=1012|500|60
contig01513_length=331|0|60
contig03093_length=1025|500|60
contig03093_length=1025|0|60
log2(expression)
0
2
4
6
8
12
16
StageI
StageII
StageIII
StageIV
StageV
StageVI

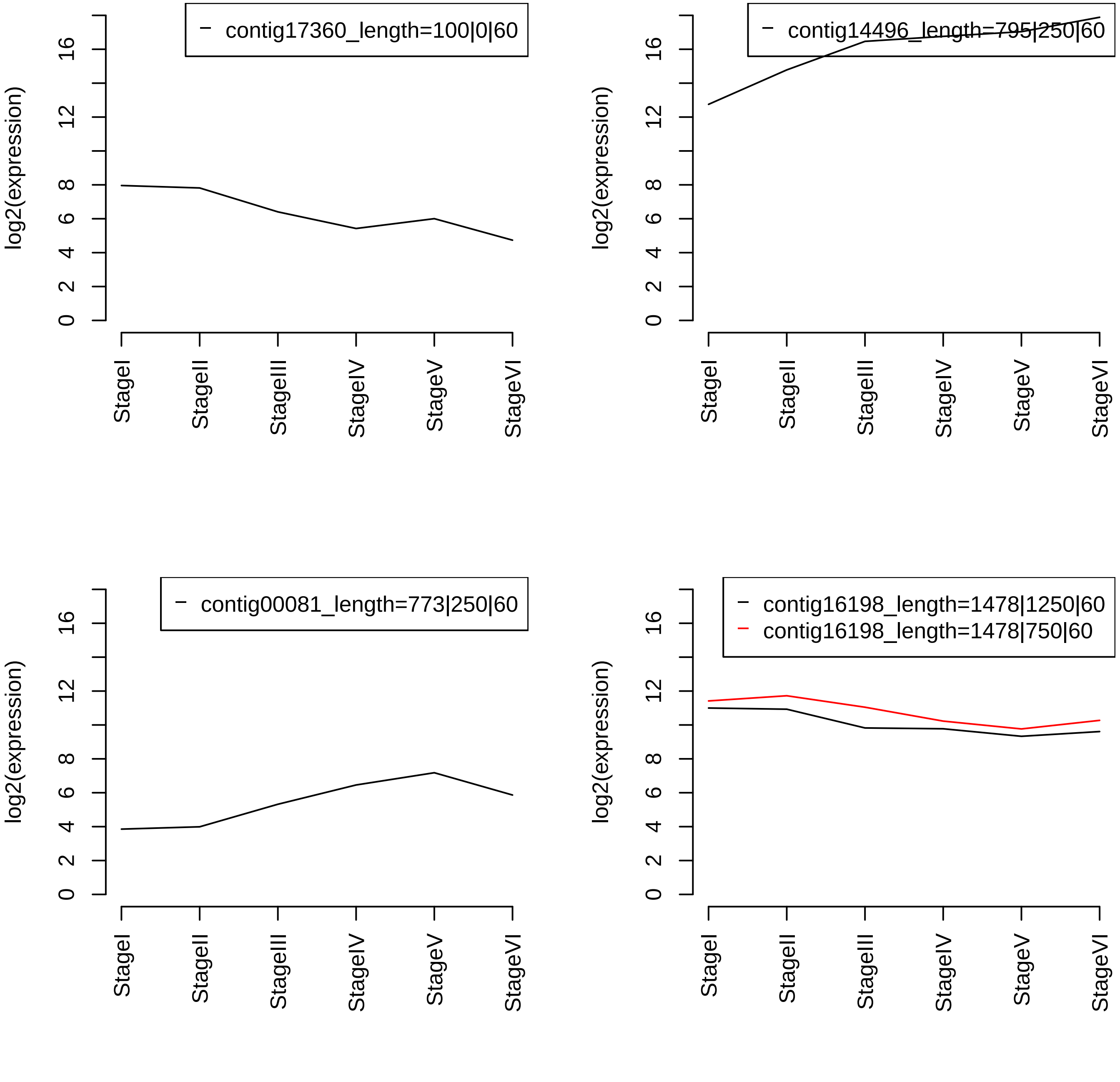

contig17360_length=100|0|60
contig14496_length=795|250|60
contig00081_length=773|250|60
contig16198_length=1478|1250|60
contig16198_length=1478|750|60
log2(expression)
0
2
4
6
8
12
16
StageI
StageII
StageIII
StageIV
StageV
StageVI

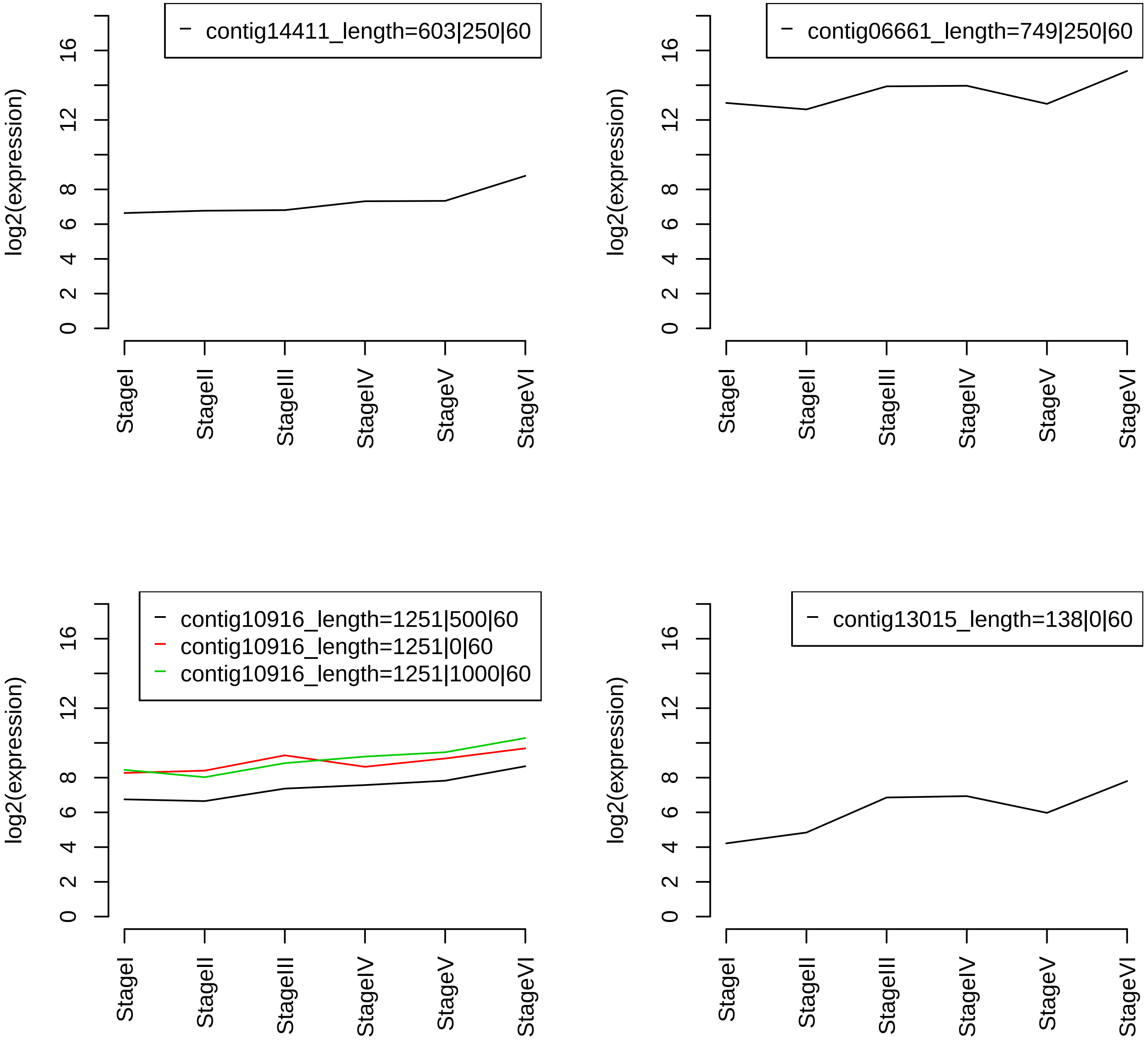

contig14411_length=603|250|60
contig06661_length=749|250|60
contig10916_length=1251|500|60
contig10916_length=1251|0|60
contig10916_length=1251|1000|60
contig13015_length=138|0|60
log2(expression)
0
2
4
6
8
12
16
StageI
StageII
StageIII
StageIV
StageV
StageVI

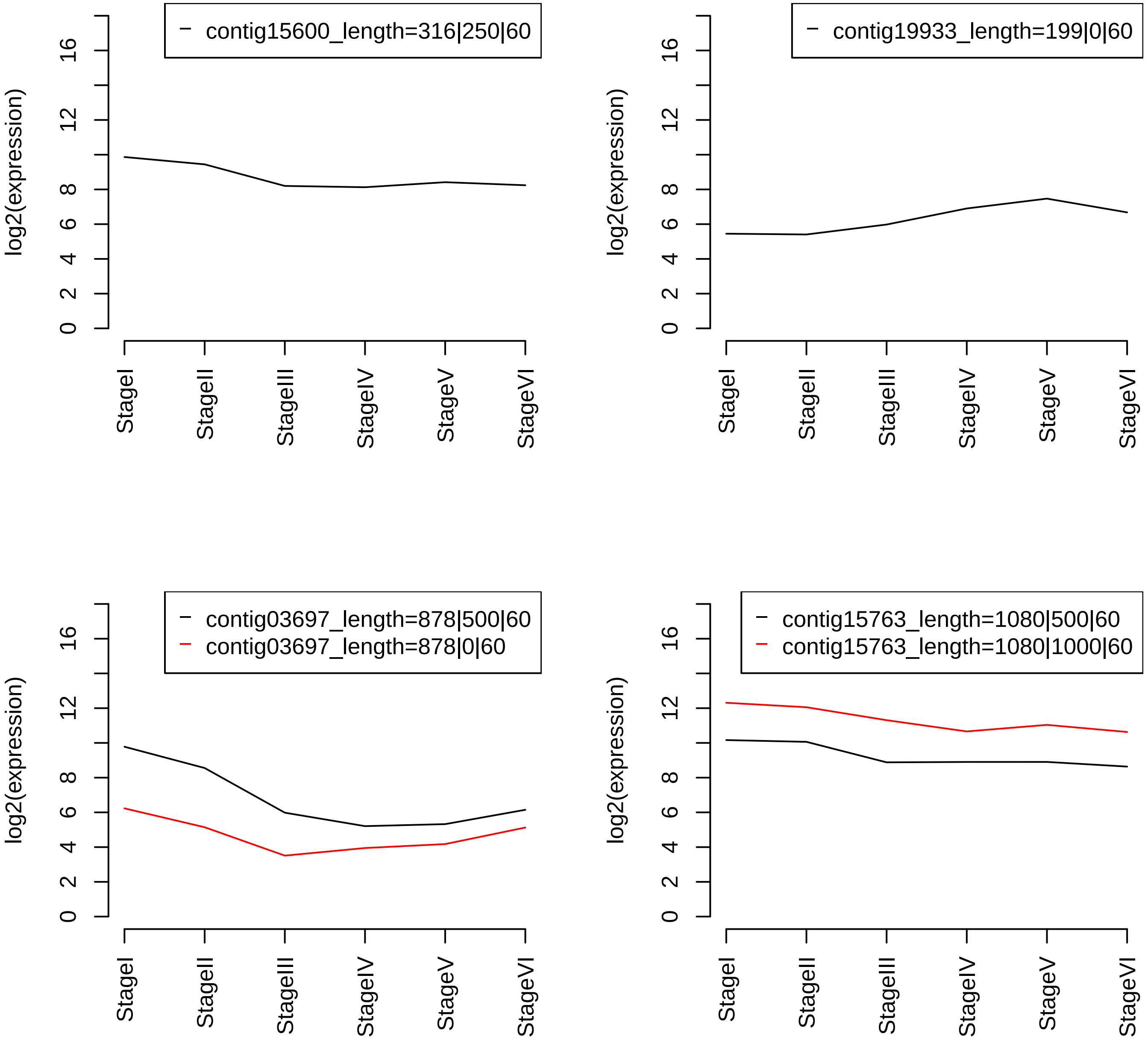
contig15600_length=316|250|60
contig19933_length=199|0|60
contig03697_length=878|500|60
contig03697_length=878|0|60
contig15763_length=1080|500|60
contig15763_length=1080|1000|60
log2(expression)
0
2
4
6
8
12
16
StageI
StageII
StageIII
StageIV
StageV
StageVI

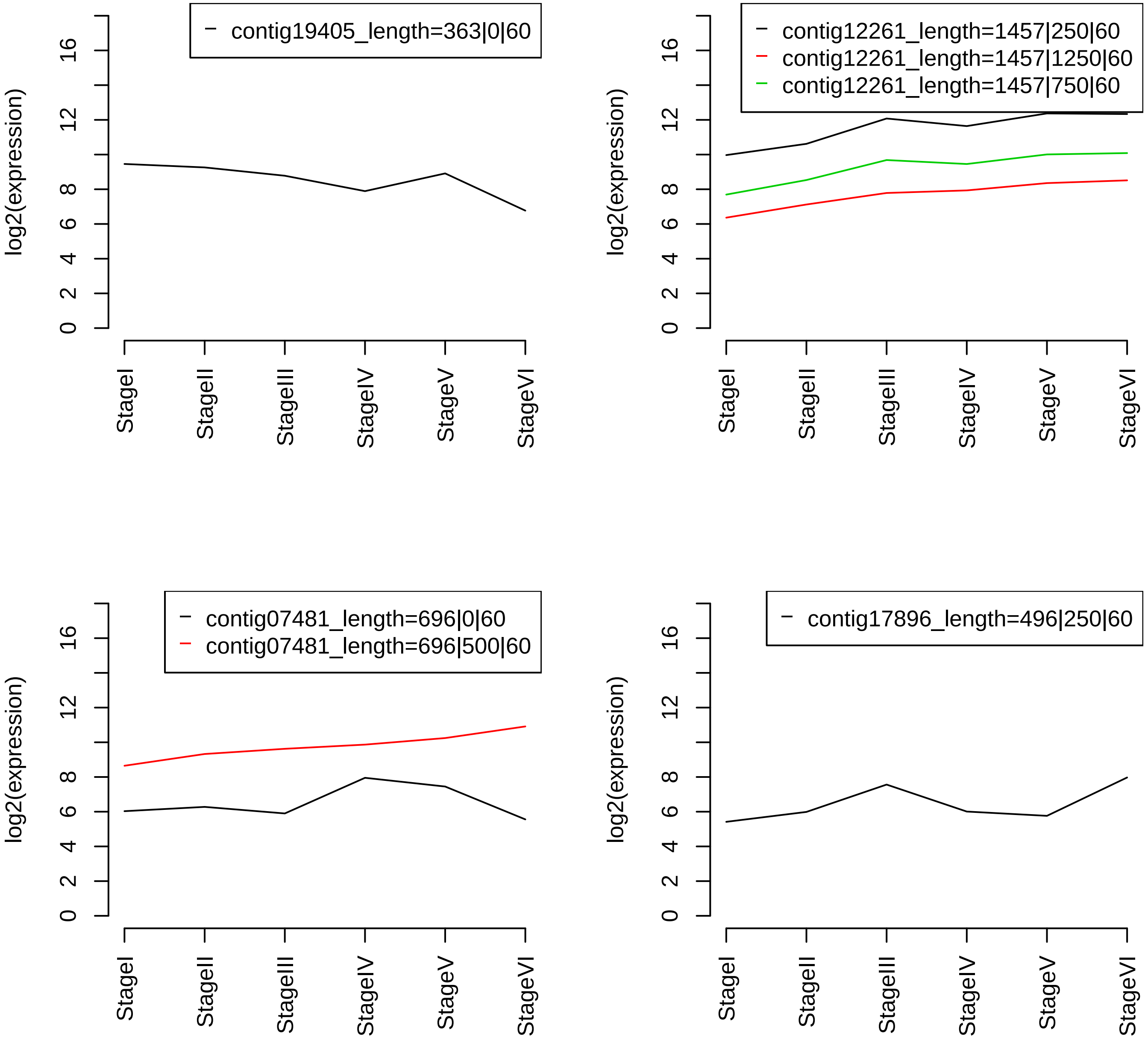
contig19405_length=363|0|60
contig12261_length=1457|250|60
contig12261_length=1457|1250|60
contig12261_length=1457|750|60
contig07481_length=696|0|60
contig07481_length=696|500|60
contig17896_length=496|250|60
log2(expression)
0 2 4 6 8 12 16
StageI StageII StageIII StageIV StageV StageVI

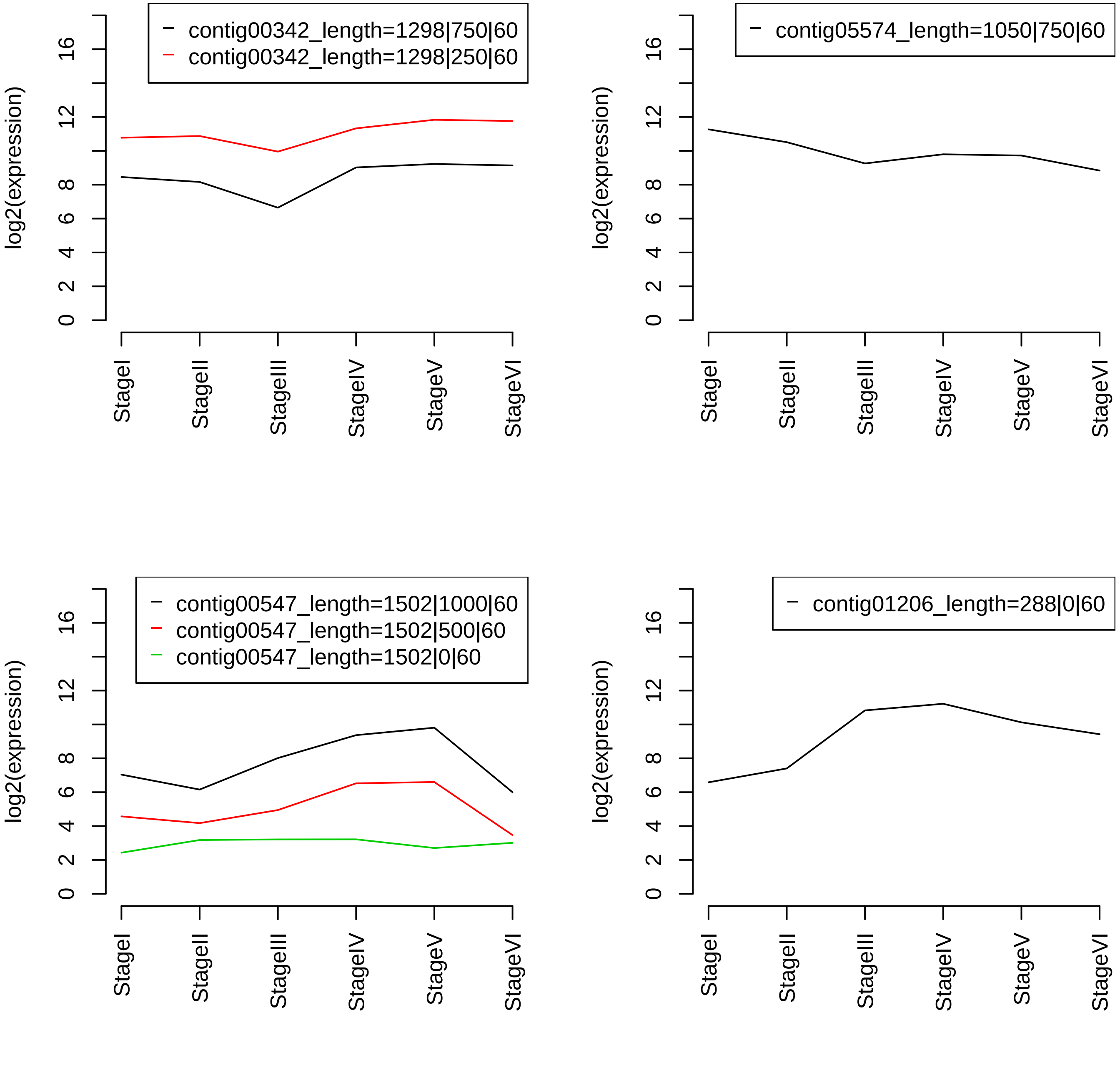

contig00342_length=1298|750|60
contig00342_length=1298|250|60
log2(expression)
0
2
4
6
8
12
16
StageI
StageII
StageIII
StageIV
StageV
StageVI
contig05574_length=1050|750|60
log2(expression)
contig00547_length=1502|1000|60
contig00547_length=1502|500|60
contig00547_length=1502|0|60
log2(expression)
contig01206_length=288|0|60
log2(expression)

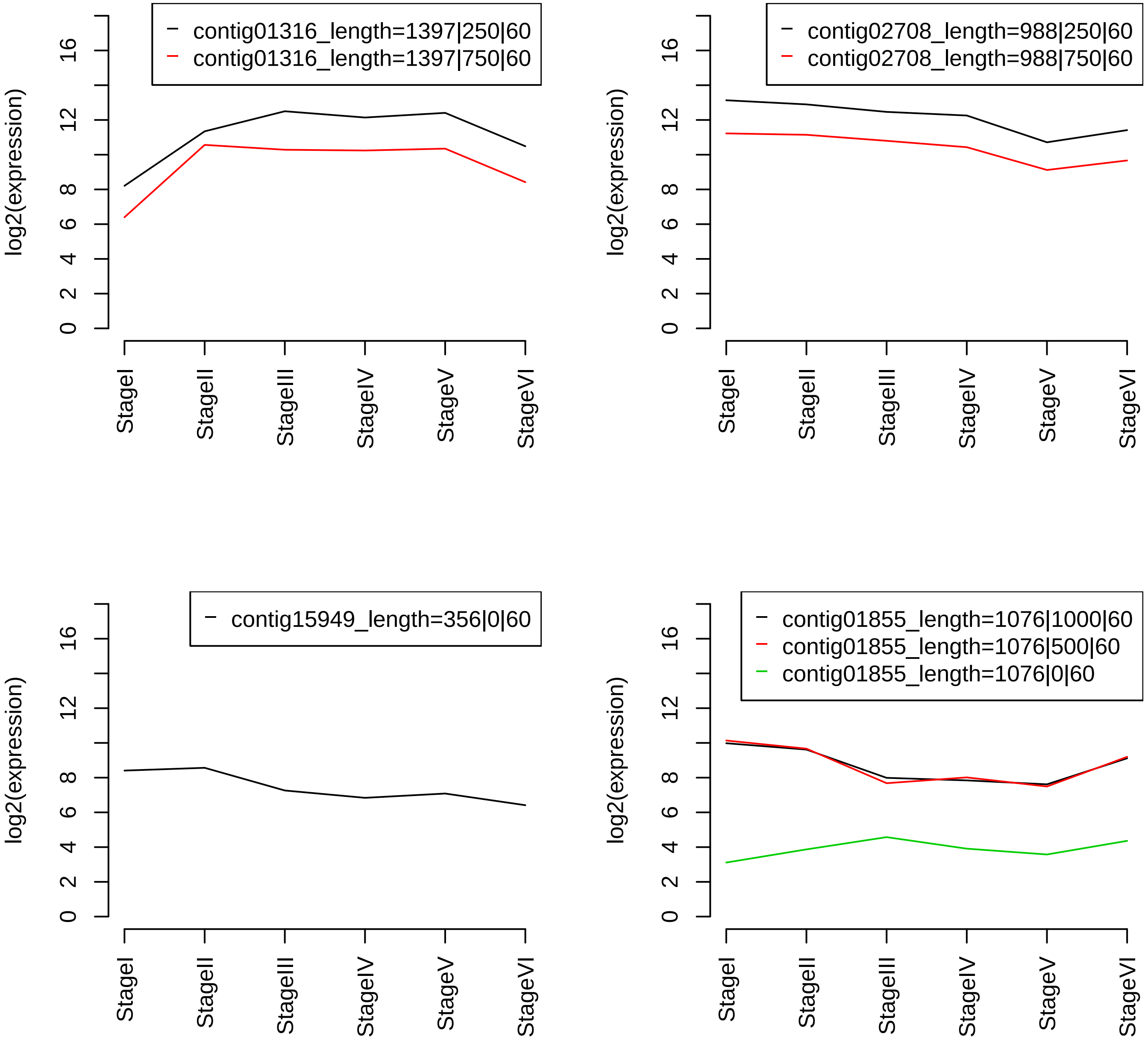
contig01316_length=1397|250|60
contig01316_length=1397|750|60
contig02708_length=988|250|60
contig02708_length=988|750|60
contig15949_length=356|0|60
contig01855_length=1076|1000|60
contig01855_length=1076|500|60
contig01855_length=1076|0|60
log2(expression)
0
2
4
6
8
12
16
StageI
StageII
StageIII
StageIV
StageV
StageVI

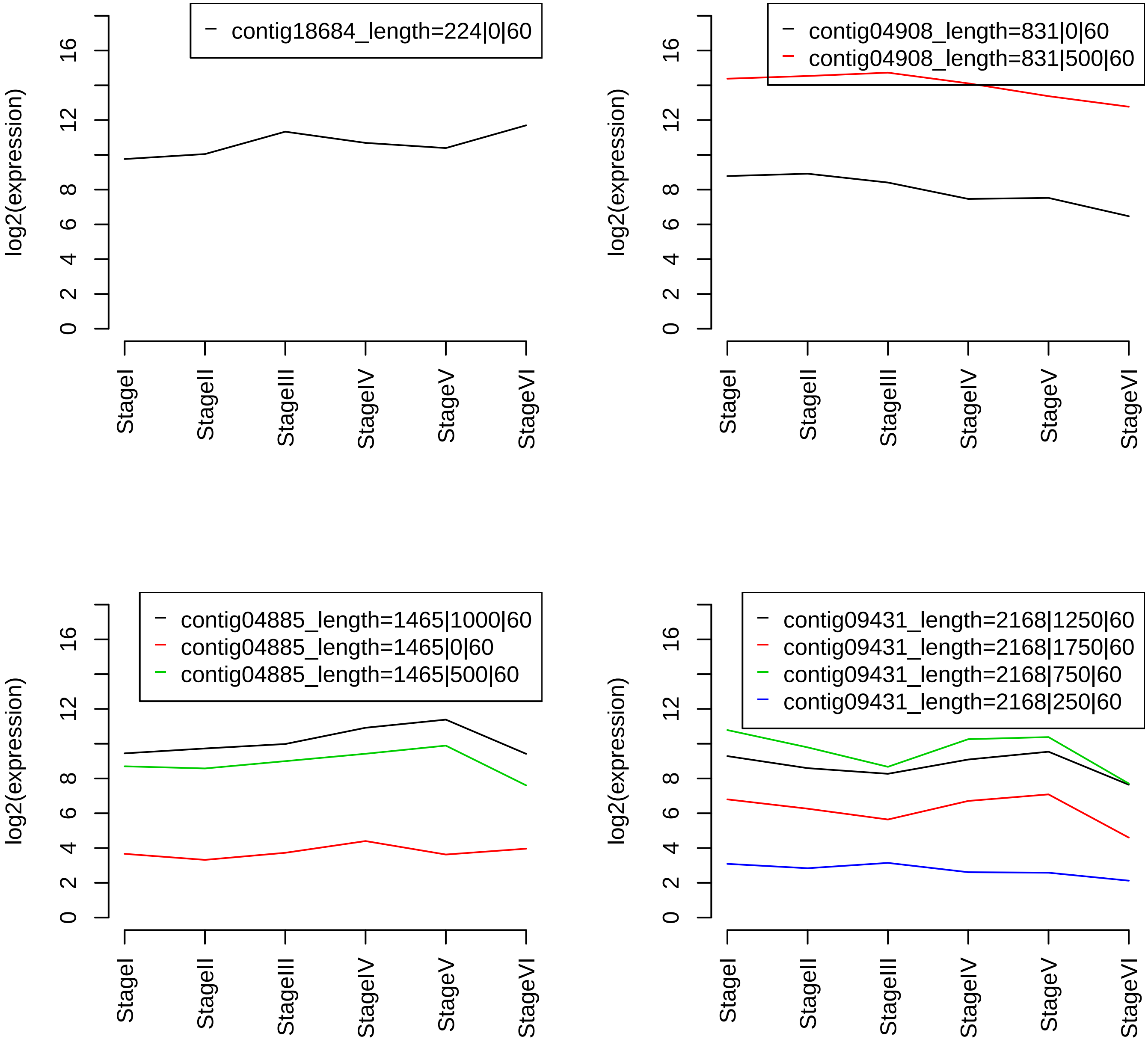

contig18684_length=224|0|60
contig04908_length=831|0|60
contig04908_length=831|500|60
contig04885_length=1465|1000|60
contig04885_length=1465|0|60
contig04885_length=1465|500|60
contig09431_length=2168|1250|60
contig09431_length=2168|1750|60
contig09431_length=2168|750|60
contig09431_length=2168|250|60
log2(expression)
0 2 4 6 8 12 16
StageI
StageII
StageIII
StageIV
StageV
StageVI

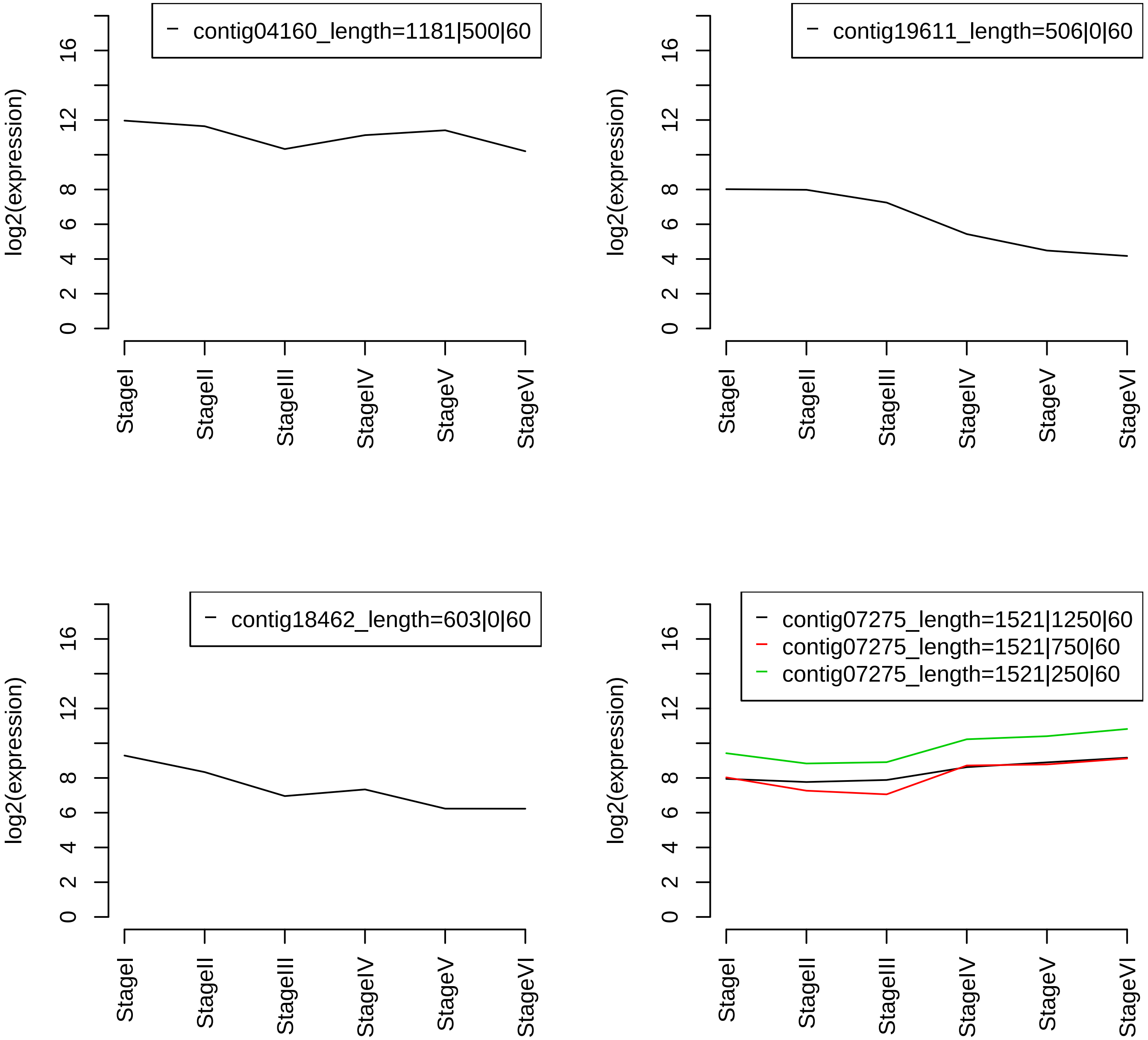
contig04160_length=1181|500|60
contig19611_length=506|0|60
contig18462_length=603|0|60
contig07275_length=1521|1250|60
contig07275_length=1521|750|60
contig07275_length=1521|250|60
log2(expression)
0 2 4 6 8 12 16
StageI
StageII
StageIII
StageIV
StageV
StageVI

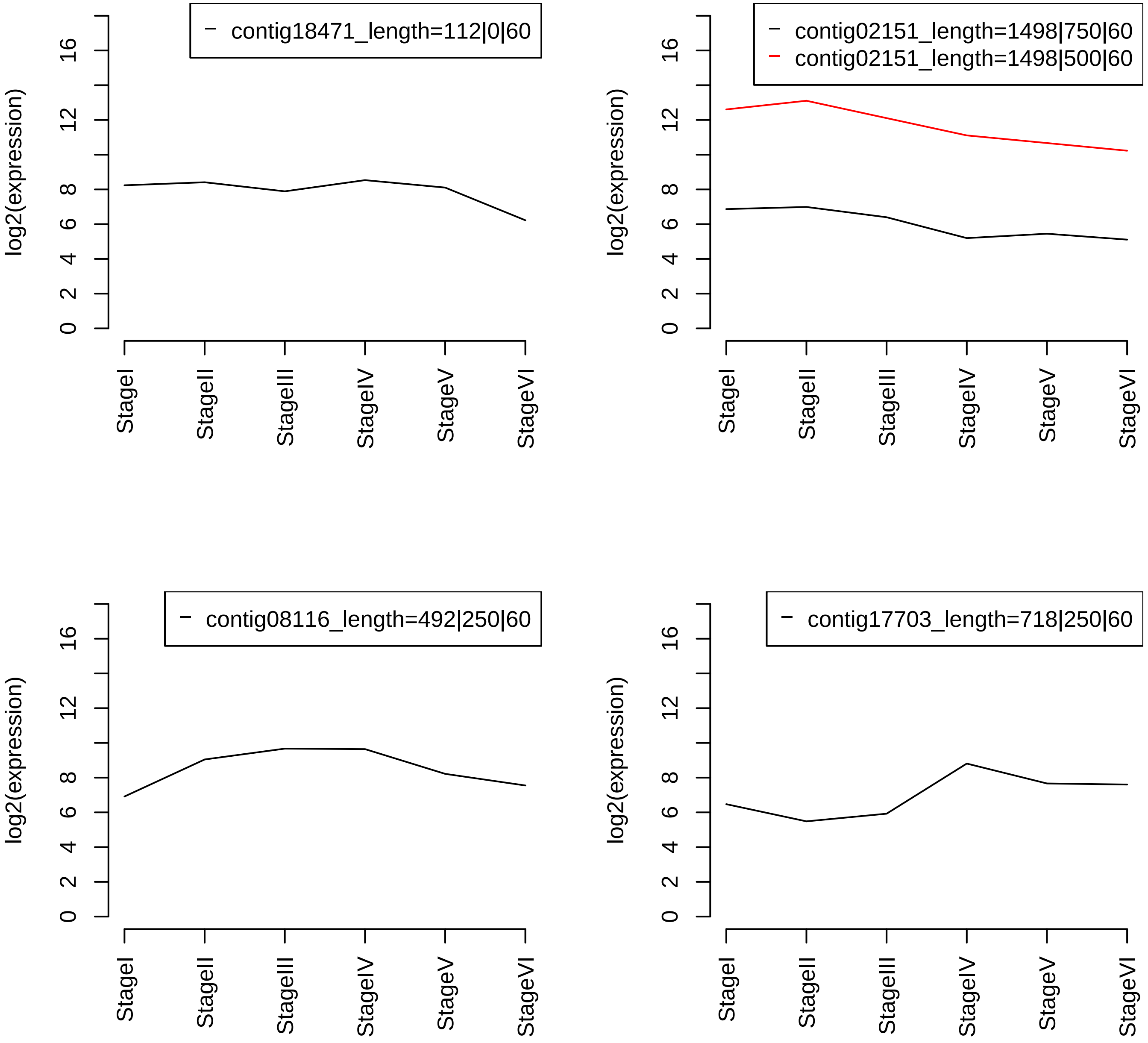
contig18471_length=112|0|60
contig02151_length=1498|750|60
contig02151_length=1498|500|60
contig08116_length=492|250|60
contig17703_length=718|250|60
log2(expression)
0 2 4 6 8 12 16
StageI StageII StageIII StageIV StageV StageVI

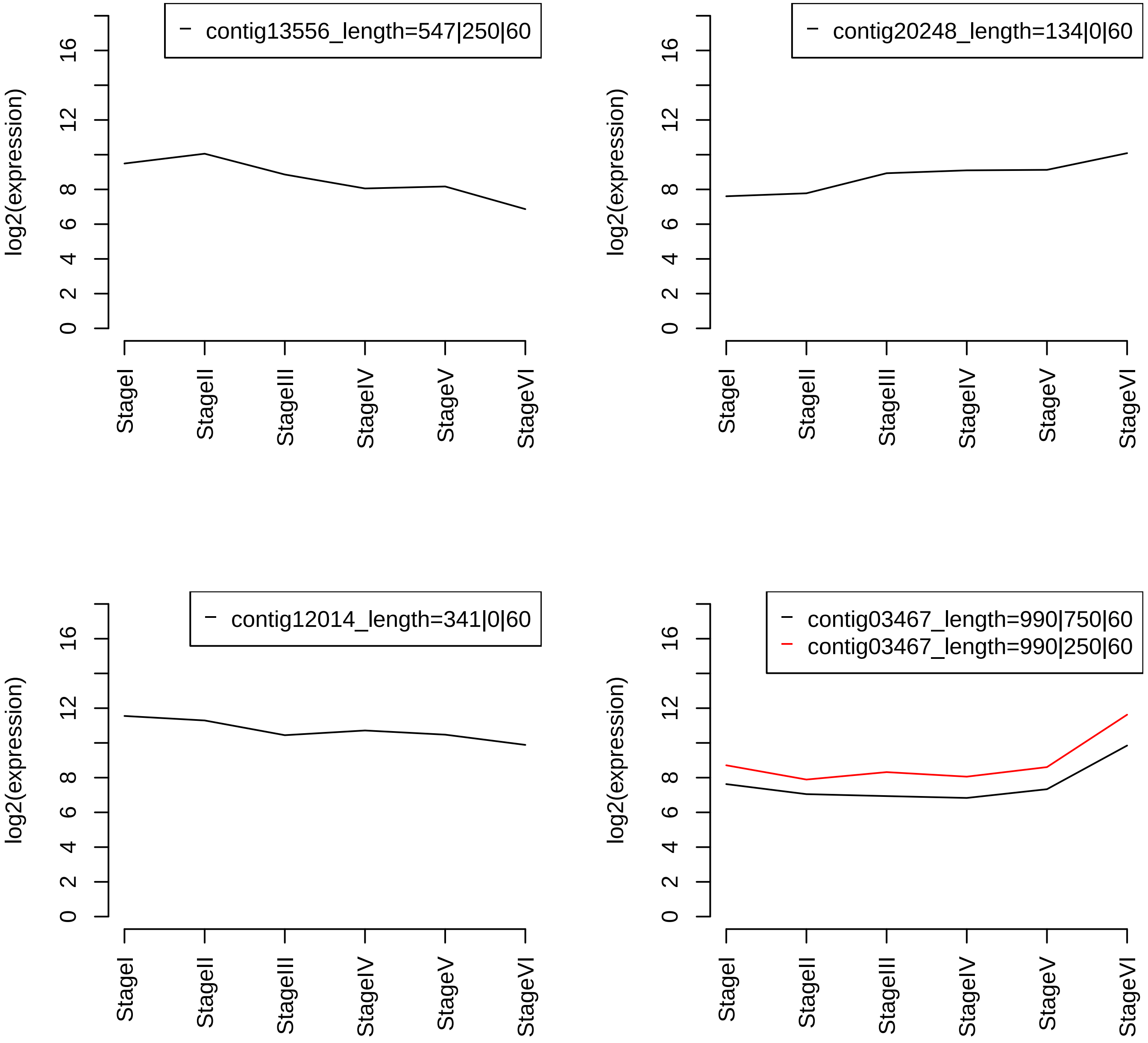

contig13556_length=547|250|60
contig20248_length=134|0|60
contig12014_length=341|0|60
contig03467_length=990|750|60
contig03467_length=990|250|60
log2(expression)
0 2 4 6 8 12 16
StageI StageII StageIII StageIV StageV StageVI

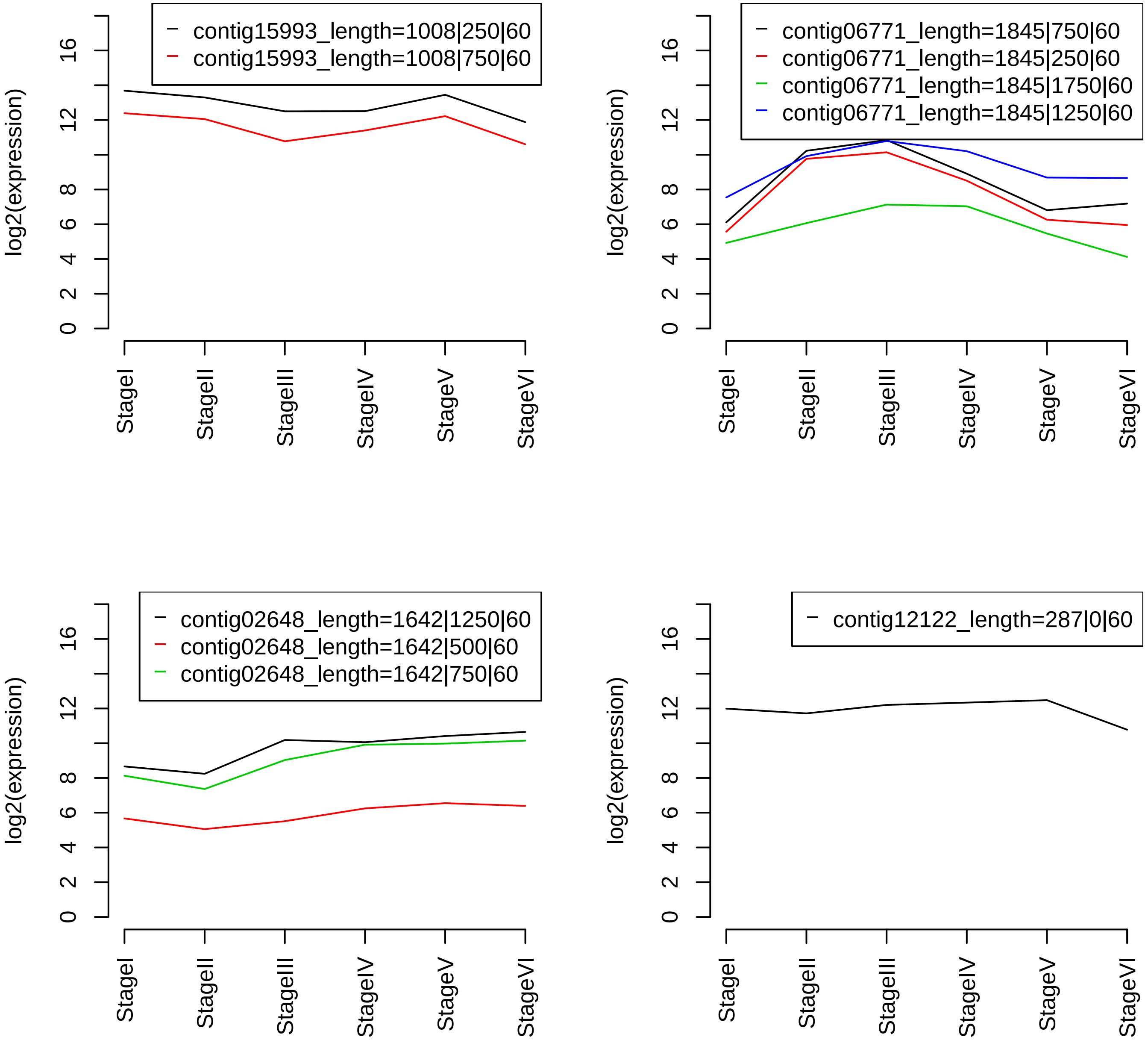

contig15993_length=1008|250|60
contig15993_length=1008|750|60
contig06771_length=1845|750|60
contig06771_length=1845|250|60
contig06771_length=1845|1750|60
contig06771_length=1845|1250|60
contig02648_length=1642|1250|60
contig02648_length=1642|500|60
contig02648_length=1642|750|60
contig12122_length=287|0|60
log2(expression)
0
2
4
6
8
12
16
StageI
StageII
StageIII
StageIV
StageV
StageVI

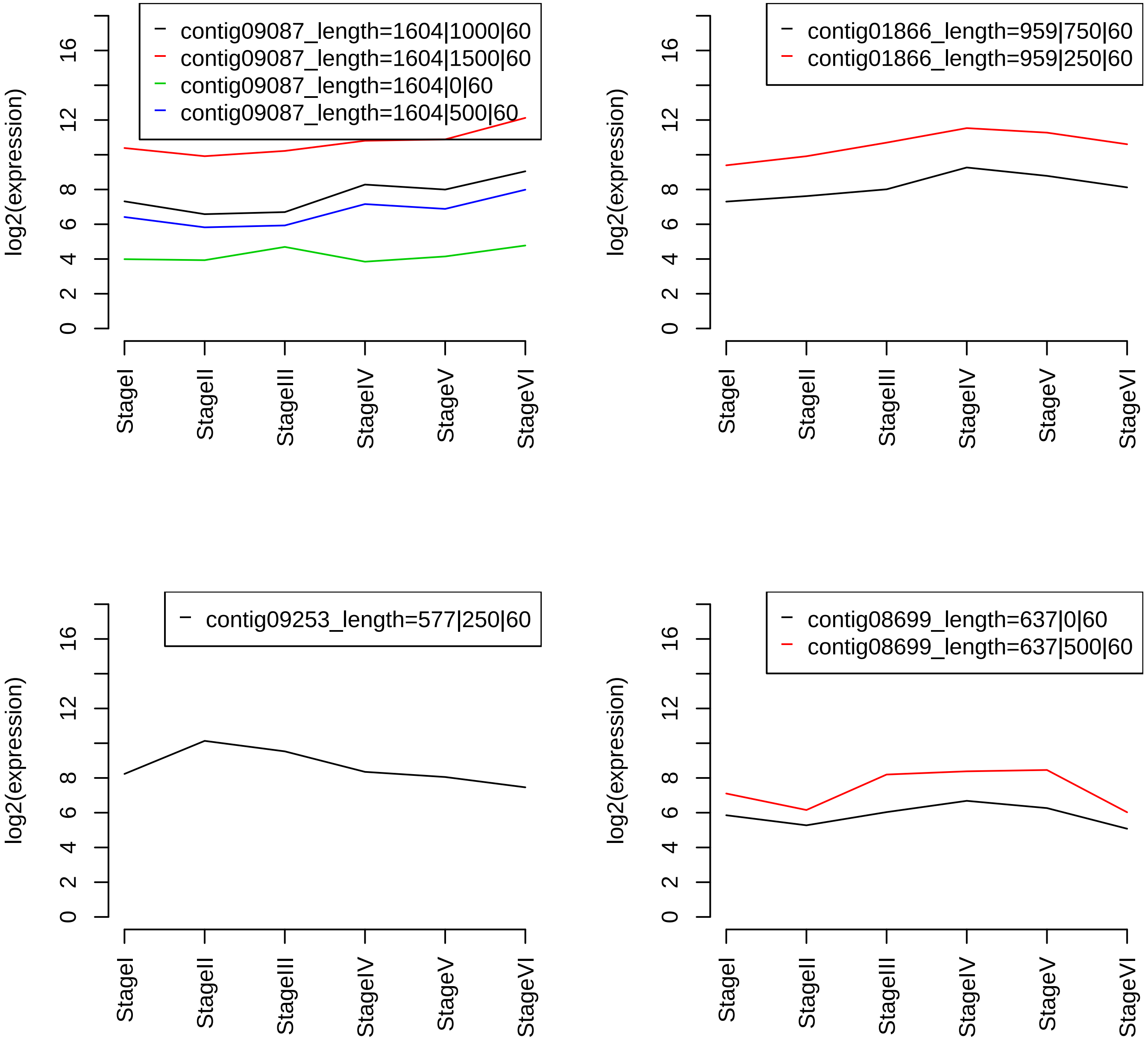

contig09087_length=1604|1000|60
contig09087_length=1604|1500|60
contig09087_length=1604|0|60
contig09087_length=1604|500|60
contig01866_length=959|750|60
contig01866_length=959|250|60
contig09253_length=577|250|60
contig08699_length=637|0|60
contig08699_length=637|500|60
log2(expression)
0
2
4
6
8
12
16
StageI
StageII
StageIII
StageIV
StageV
StageVI

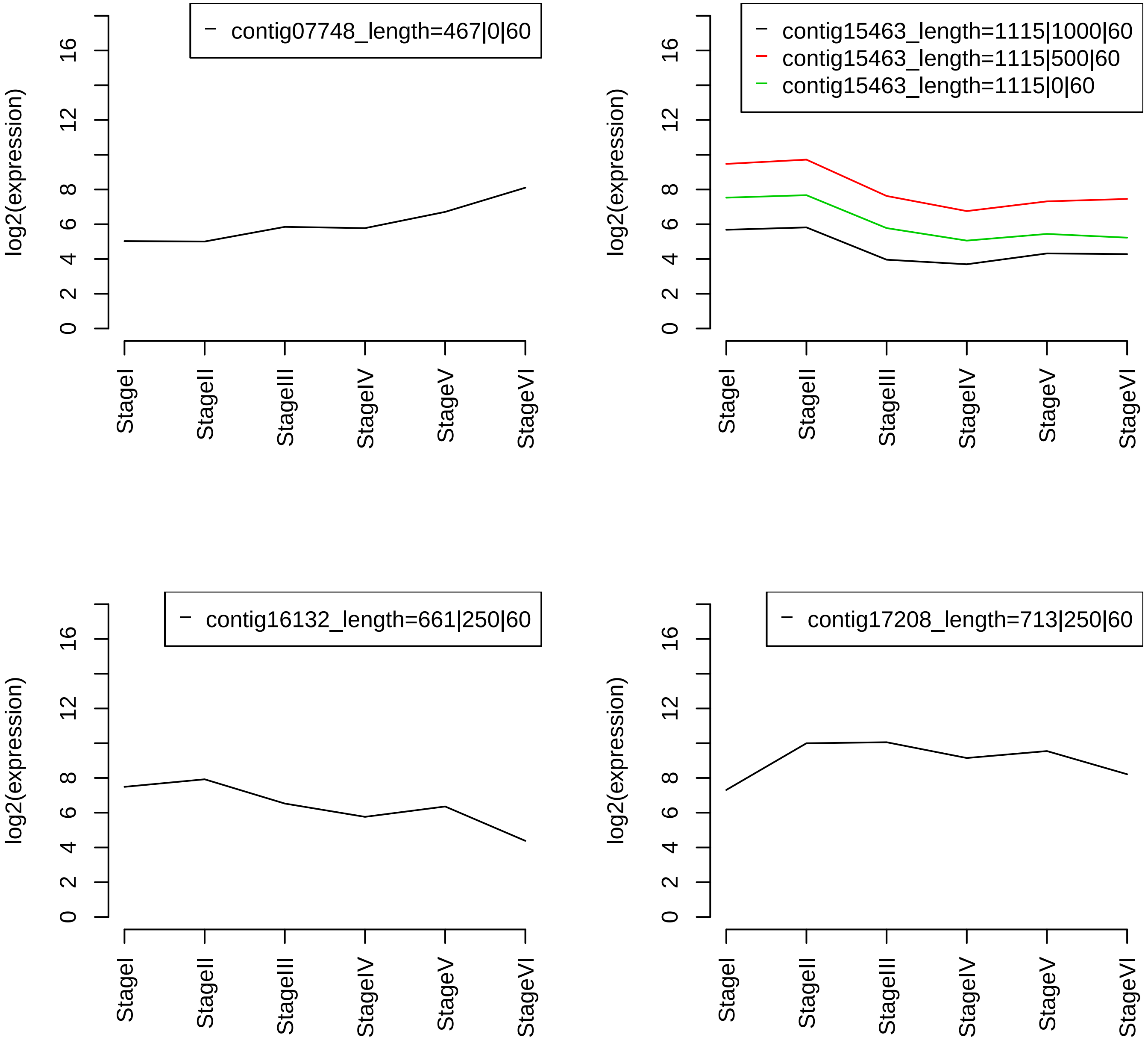
contig07748_length=467|0|60
contig15463_length=1115|1000|60
contig15463_length=1115|500|60
contig15463_length=1115|0|60
contig16132_length=661|250|60
contig17208_length=713|250|60
log2(expression)
0 2 4 6 8 12 16
StageI StageII StageIII StageIV StageV StageVI

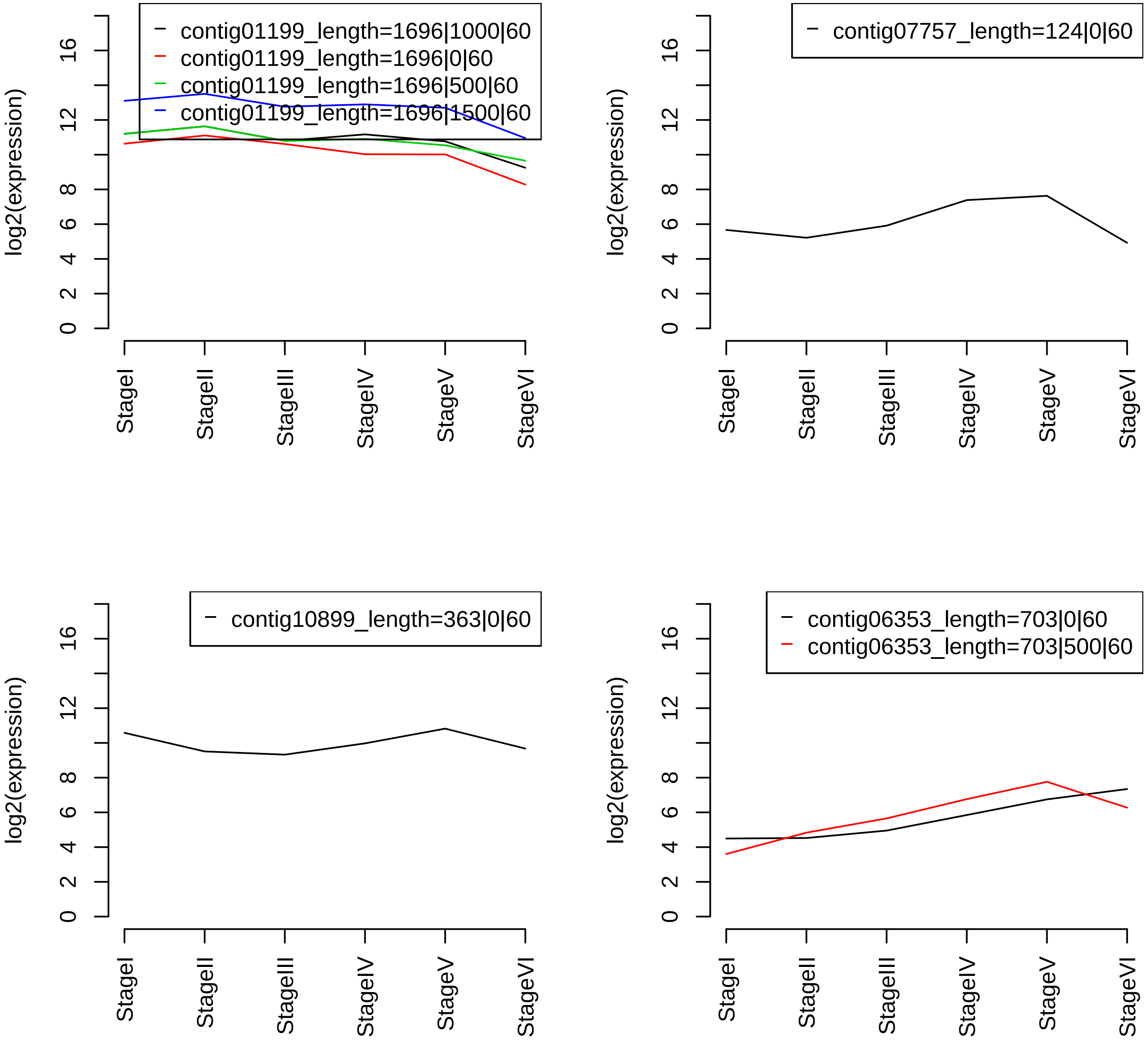

contig01199_length=1696|1000|60
contig01199_length=1696|0|60
contig01199_length=1696|500|60
contig01199_length=1696|1500|60
log2(expression)
0 2 4 6 8 12 16
StageI StageII StageIII StageIV StageV StageVI
contig07757_length=124|0|60
log2(expression)
0 2 4 6 8 12 16
StageI StageII StageIII StageIV StageV StageVI
contig10899_length=363|0|60
log2(expression)
0 2 4 6 8 12 16
StageI StageII StageIII StageIV StageV StageVI
contig06353_length=703|0|60
contig06353_length=703|500|60
log2(expression)
0 2 4 6 8 12 16
StageI StageII StageIII StageIV StageV StageVI

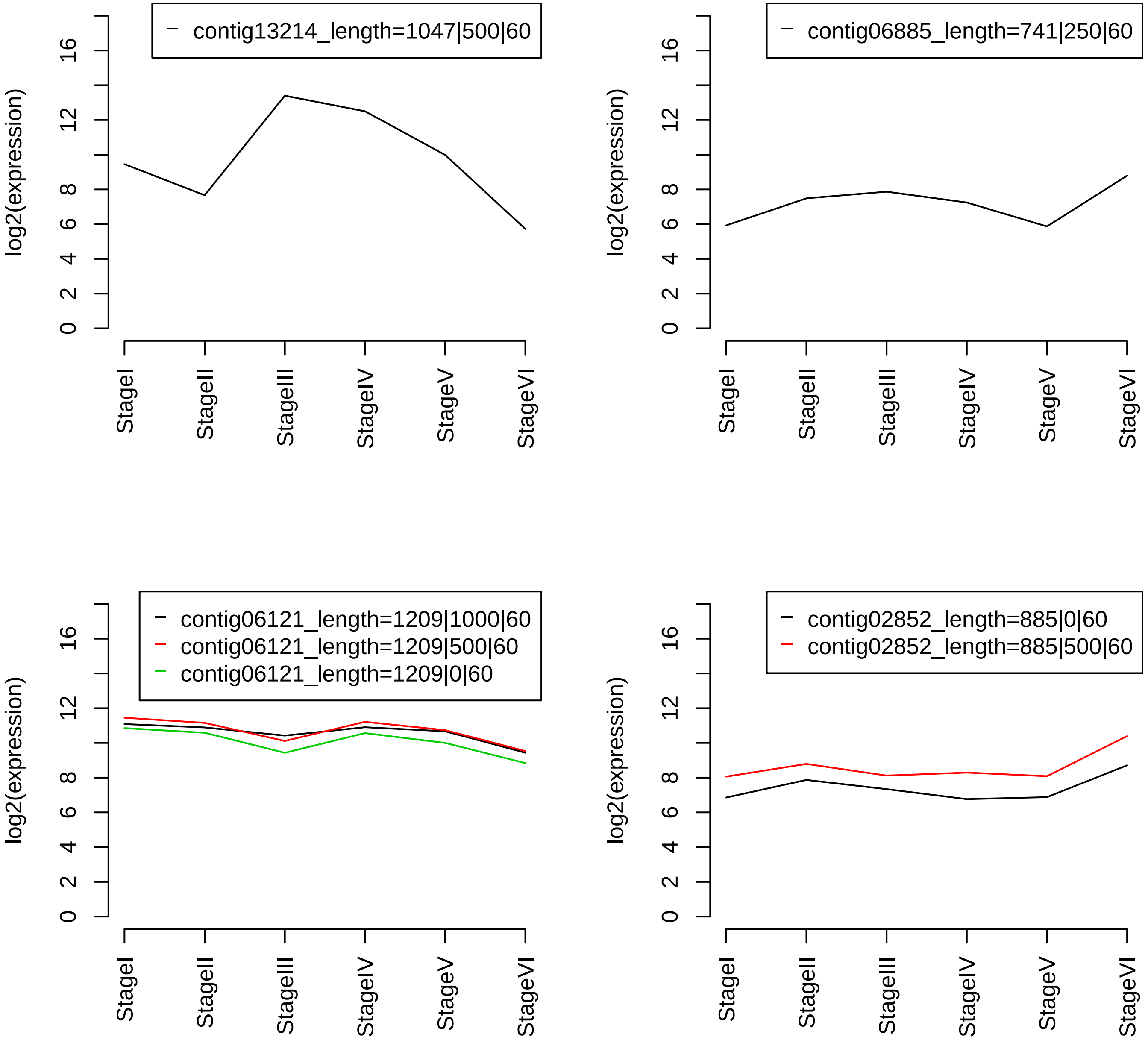

contig13214_length=1047|500|60
contig06885_length=741|250|60
contig06121_length=1209|1000|60
contig06121_length=1209|500|60
contig06121_length=1209|0|60
contig02852_length=885|0|60
contig02852_length=885|500|60
log2(expression)
0
2
4
6
8
12
16
StageI
StageII
StageIII
StageIV
StageV
StageVI

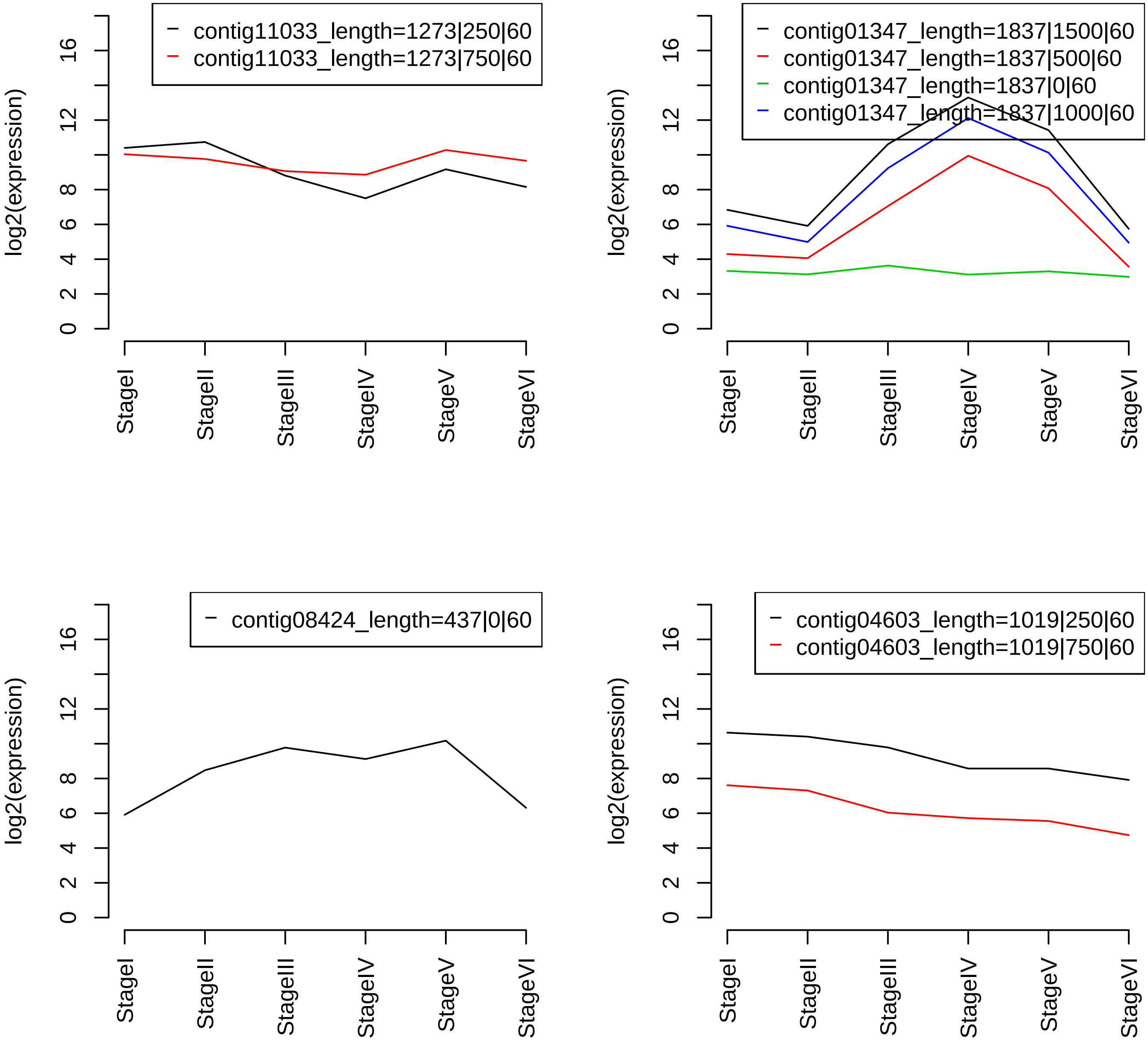
contig11033_length=1273|250|60
contig11033_length=1273|750|60
contig01347_length=1837|1500|60
contig01347_length=1837|500|60
contig01347_length=1837|0|60
contig01347_length=1837|1000|60
contig08424_length=437|0|60
contig04603_length=1019|250|60
contig04603_length=1019|750|60
log2(expression)
0 2 4 6 8 12 16
StageI
StageII
StageIII
StageIV
StageV
StageVI

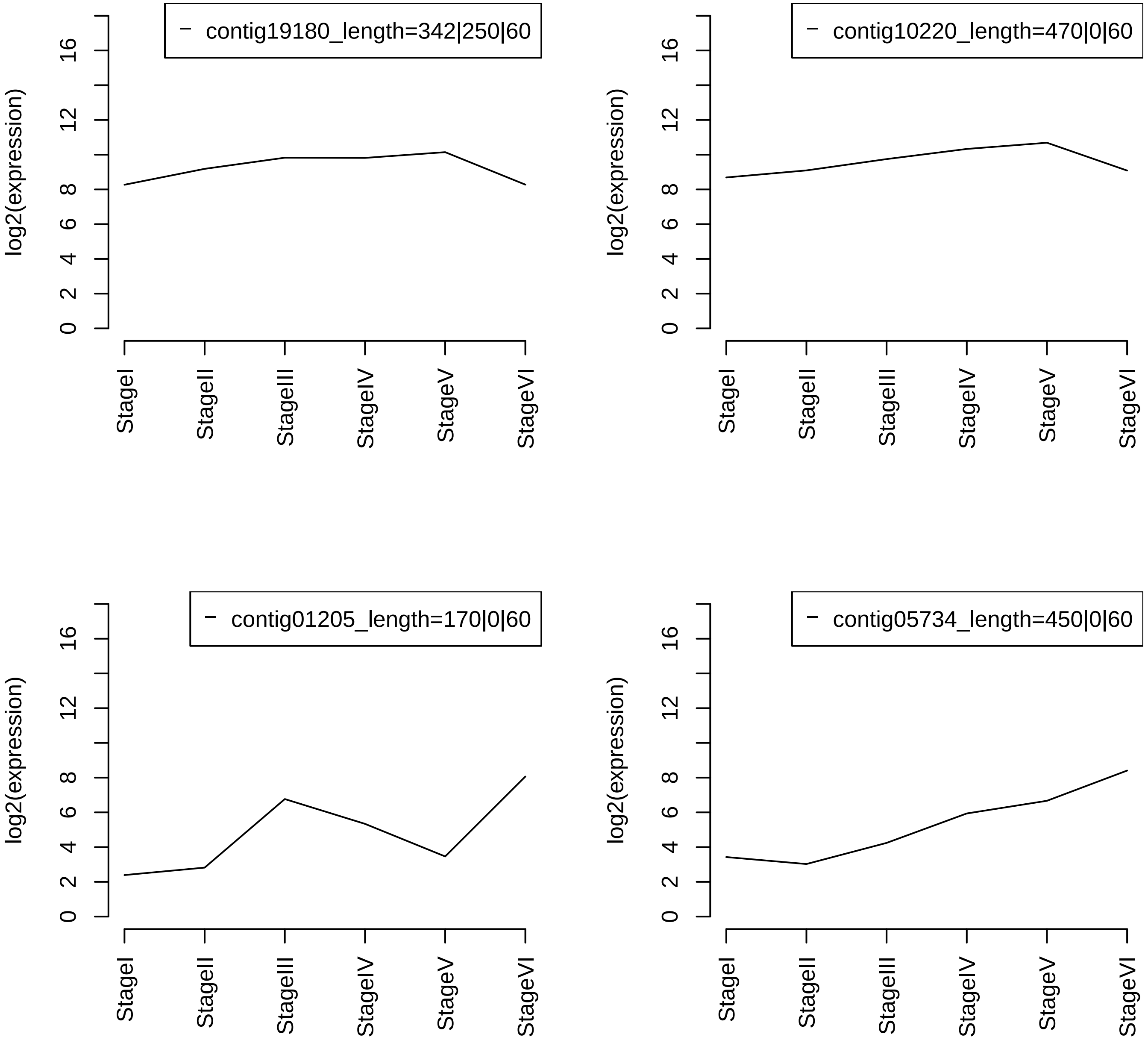

contig19180_length=342|250|60
contig10220_length=470|0|60
contig01205_length=170|0|60
contig05734_length=450|0|60
log2(expression)
0
2
4
6
8
12
16
StageI
StageII
StageIII
StageIV
StageV
StageVI

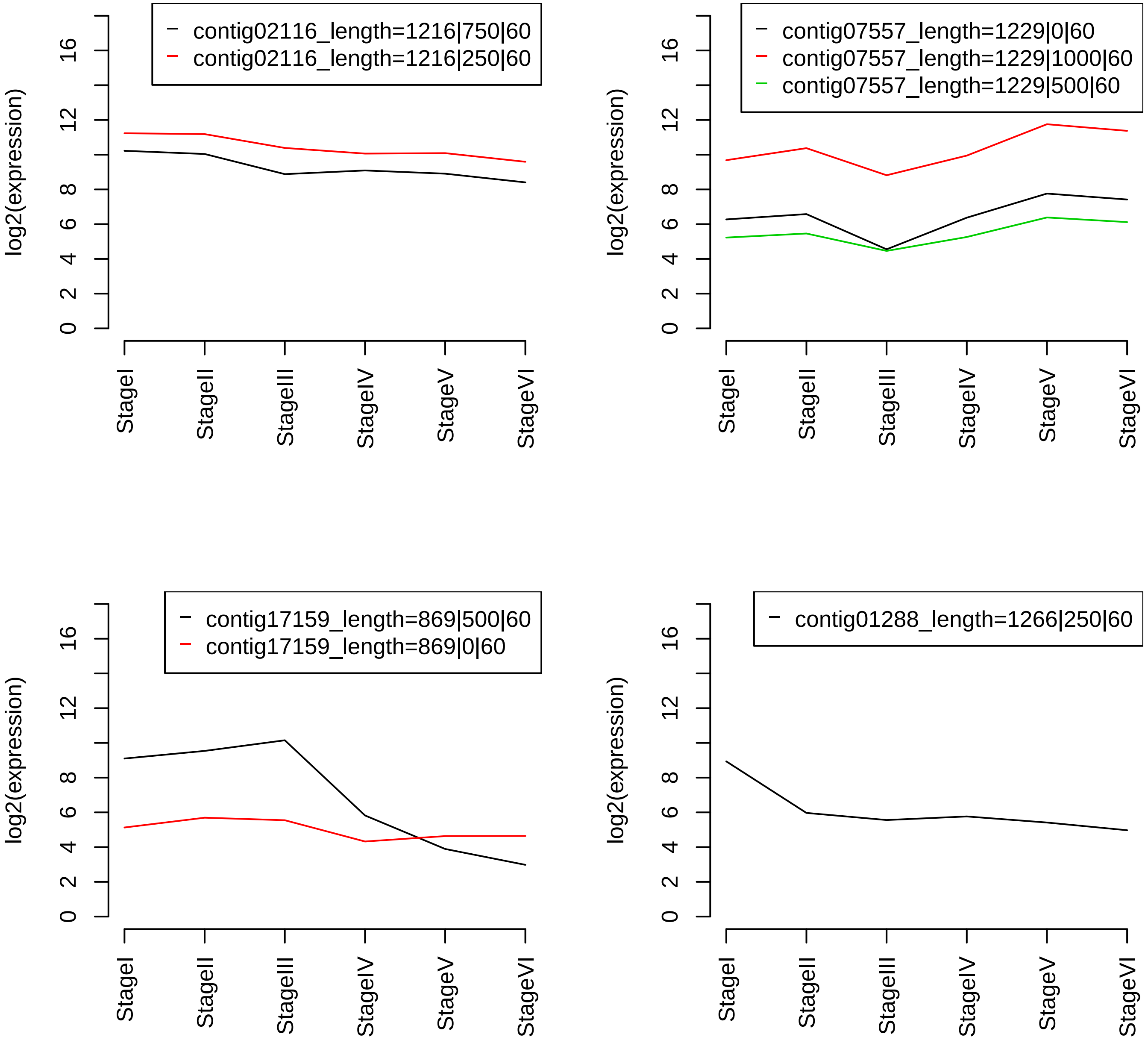

contig02116_length=1216|750|60
contig02116_length=1216|250|60
contig07557_length=1229|0|60
contig07557_length=1229|1000|60
contig07557_length=1229|500|60
contig17159_length=869|500|60
contig17159_length=869|0|60
contig01288_length=1266|250|60
log2(expression)
0
2
4
6
8
12
16
StageI
StageII
StageIII
StageIV
StageV
StageVI

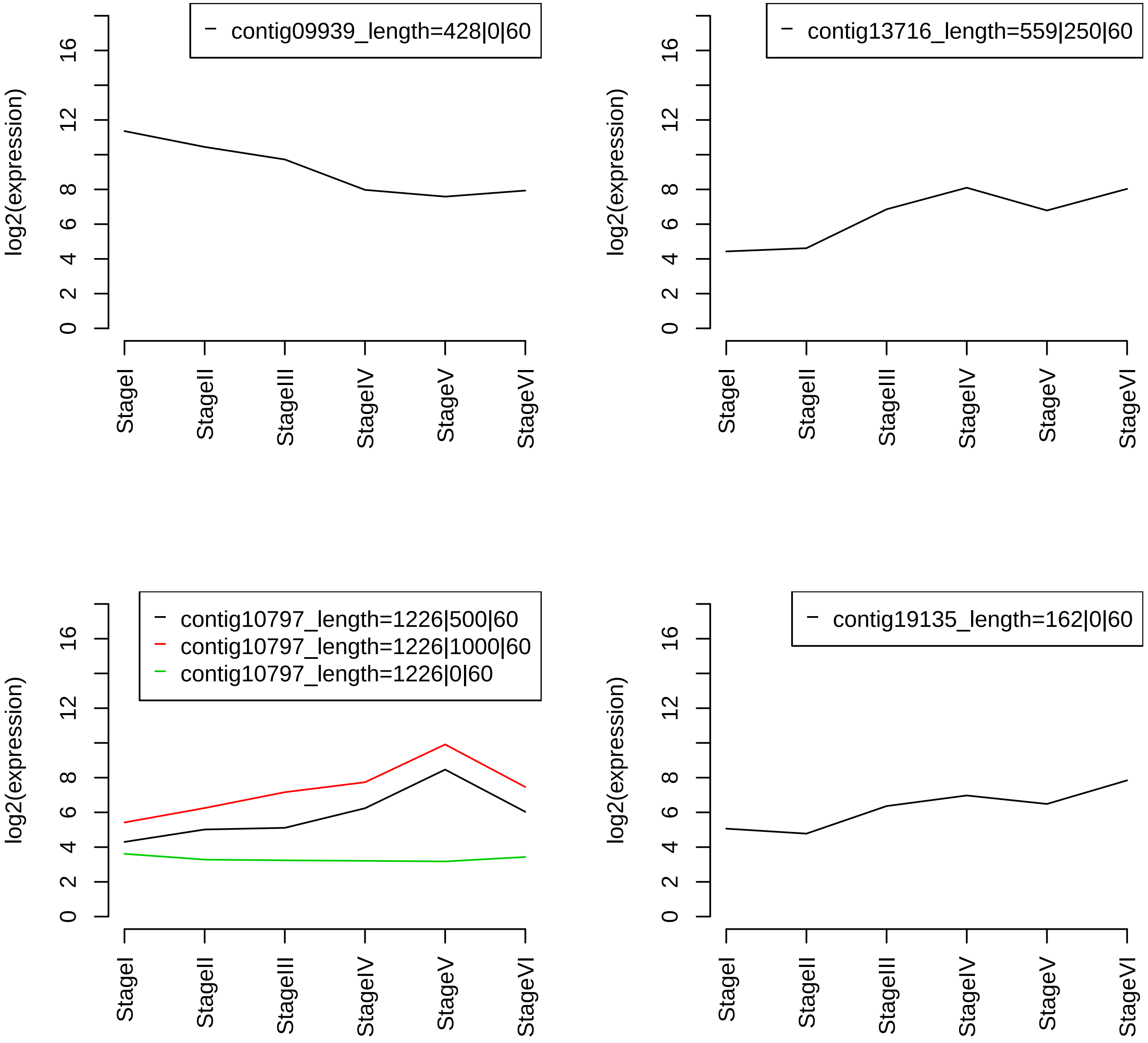
contig09939_length=428|0|60
contig13716_length=559|250|60
contig10797_length=1226|500|60
contig10797_length=1226|1000|60
contig10797_length=1226|0|60
contig19135_length=162|0|60
log2(expression)
0 2 4 6 8 12 16
StageI
StageII
StageIII
StageIV
StageV
StageVI

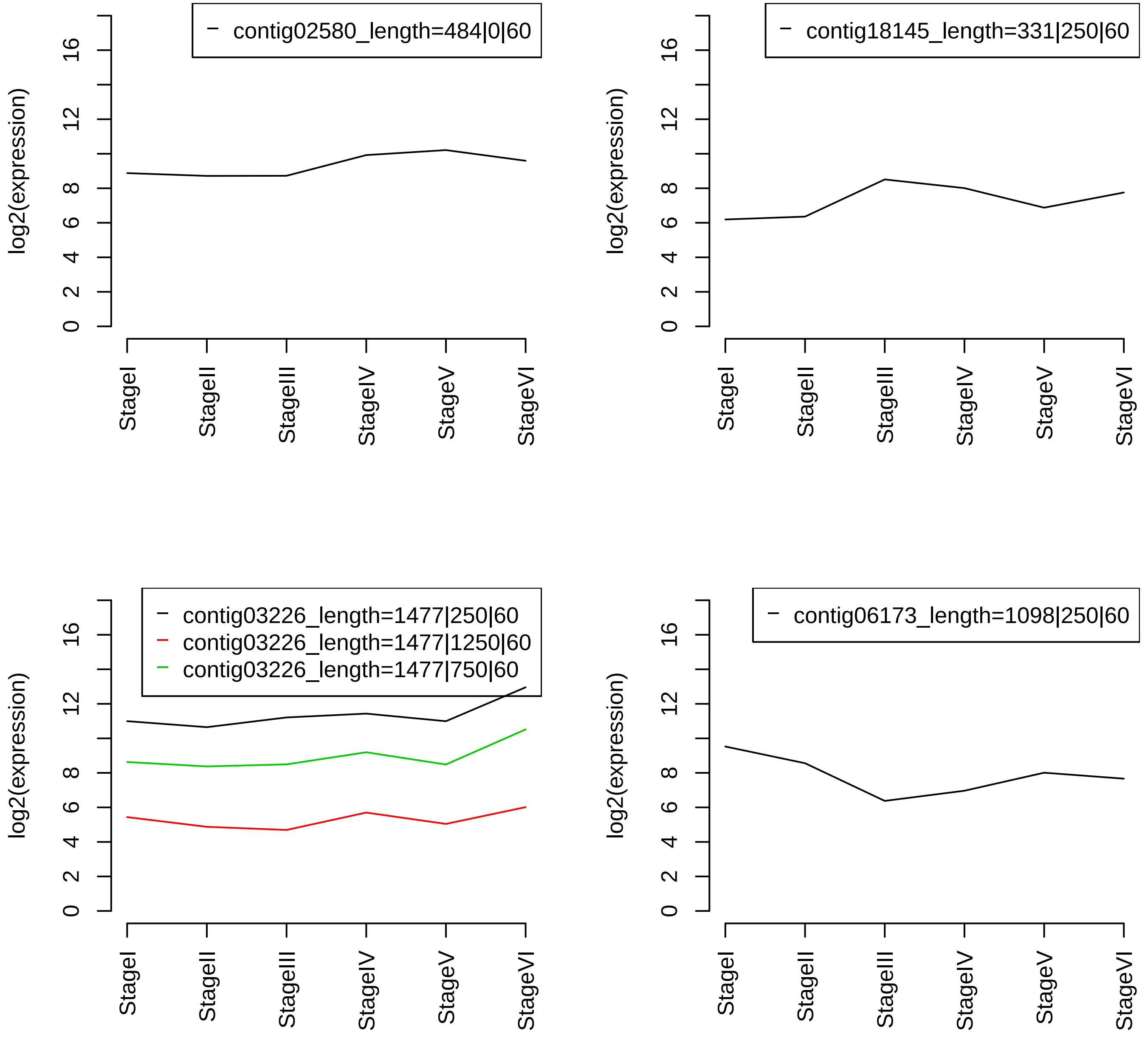

contig02580_length=484|0|60
contig18145_length=331|250|60
contig03226_length=1477|250|60
contig03226_length=1477|1250|60
contig03226_length=1477|750|60
contig06173_length=1098|250|60
log2(expression)
0
2
4
6
8
12
16
StageI
StageII
StageIII
StageIV
StageV
StageVI

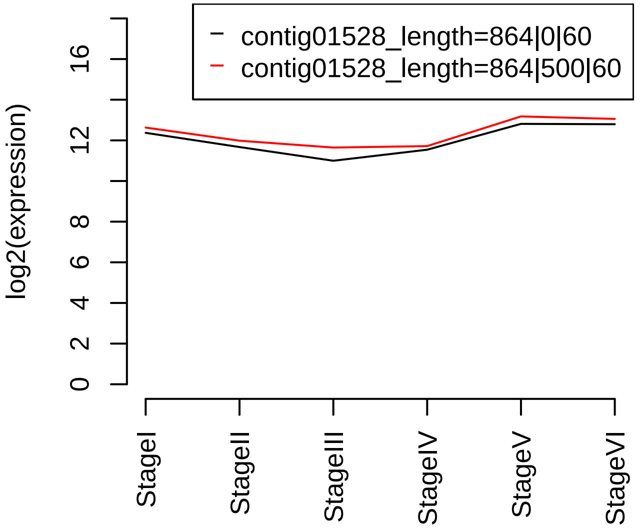

contig01528_length=864|0|60
contig01528_length=864|500|60
log2(expression)
0
2
4
6
8
12
16
StageI
StageII
StageIII
StageIV
StageV
StageVI

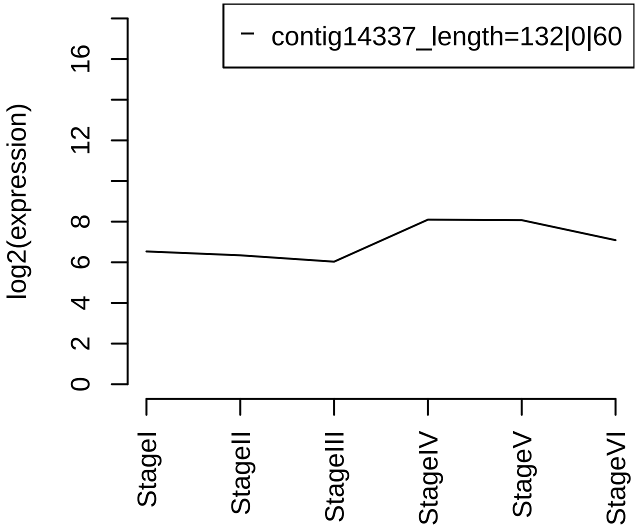

contig14337_length=132|0|60
log2(expression)
0
2
4
6
8
12
16
StageI
StageII
StageIII
StageIV
StageV
StageVI

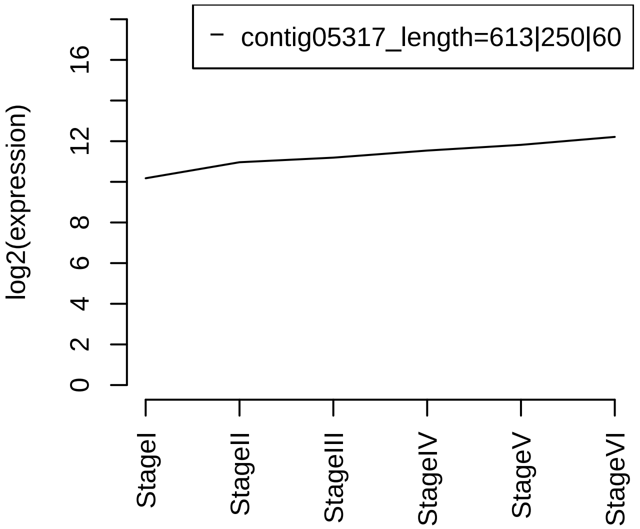

contig05317_length=613|250|60
log2(expression)
0
2
4
6
8
12
16
StageI
StageII
StageIII
StageIV
StageV
StageVI

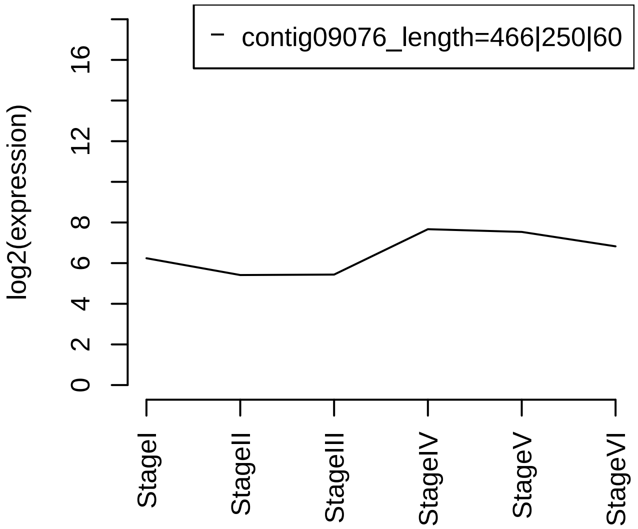

contig09076_length=466|250|60
log2(expression)
0
2
4
6
8
12
16
StageI
StageII
StageIII
StageIV
StageV
StageVI

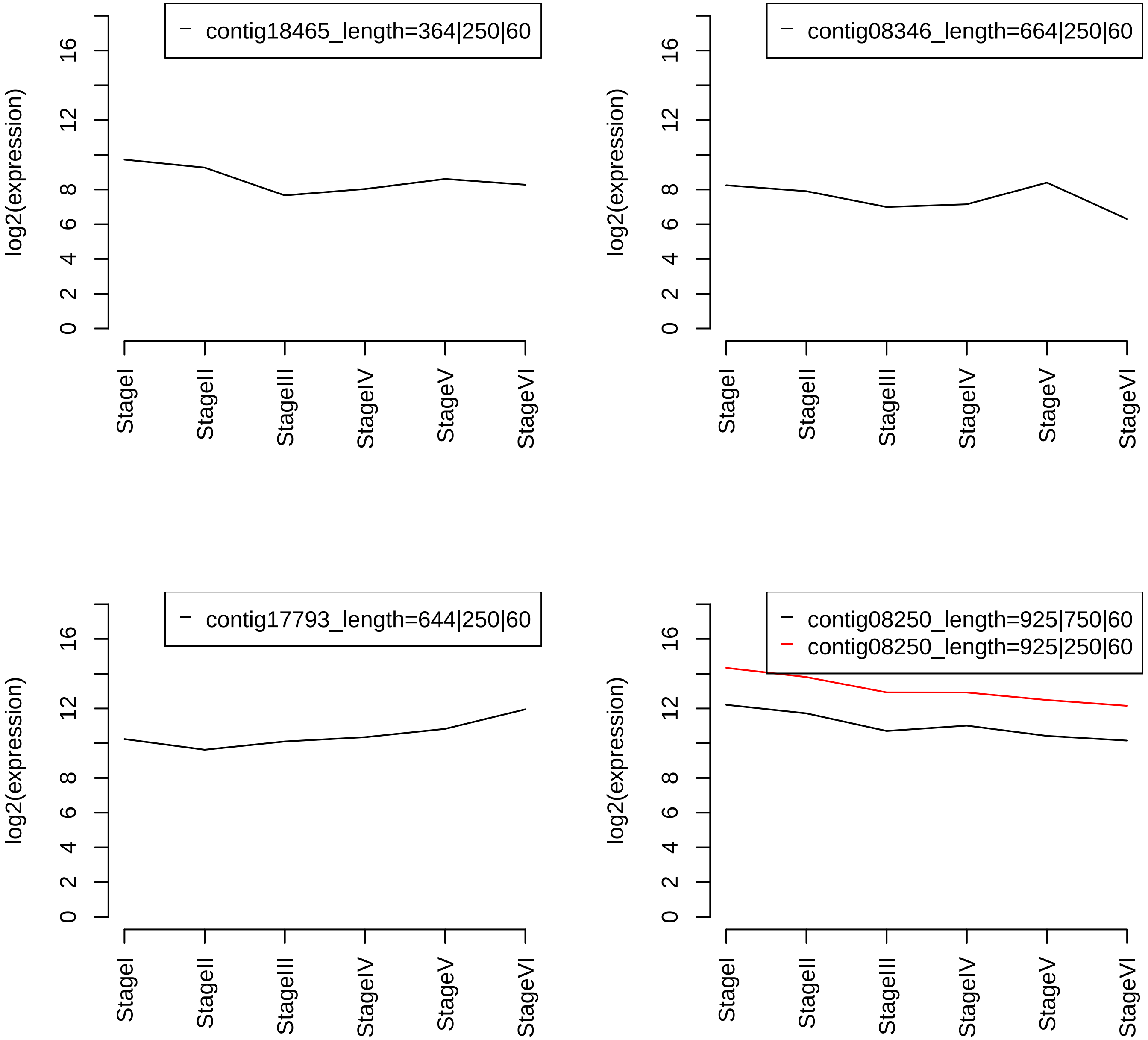
contig18465_length=364|250|60
log2(expression)
0 2 4 6 8 12 16
StageI StageII StageIII StageIV StageV StageVI
contig08346_length=664|250|60
log2(expression)
0 2 4 6 8 12 16
StageI StageII StageIII StageIV StageV StageVI
contig17793_length=644|250|60
log2(expression)
0 2 4 6 8 12 16
StageI StageII StageIII StageIV StageV StageVI
contig08250_length=925|750|60
contig08250_length=925|250|60
log2(expression)
0 2 4 6 8 12 16
StageI StageII StageIII StageIV StageV StageVI

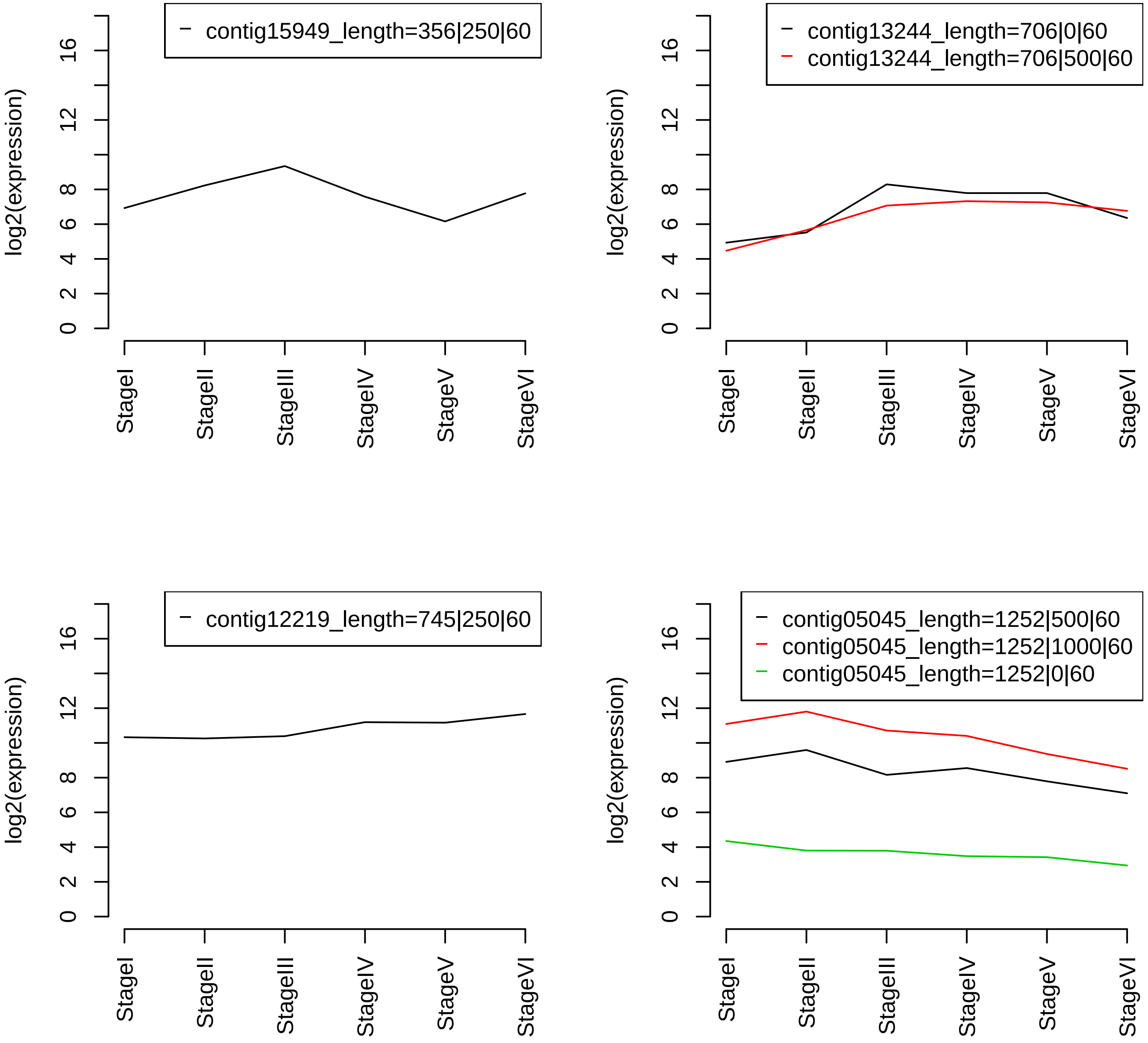

contig15949_length=356|250|60
contig13244_length=706|0|60
contig13244_length=706|500|60
contig12219_length=745|250|60
contig05045_length=1252|500|60
contig05045_length=1252|1000|60
contig05045_length=1252|0|60
log2(expression)
0
2
4
6
8
12
16
StageI
StageII
StageIII
StageIV
StageV
StageVI

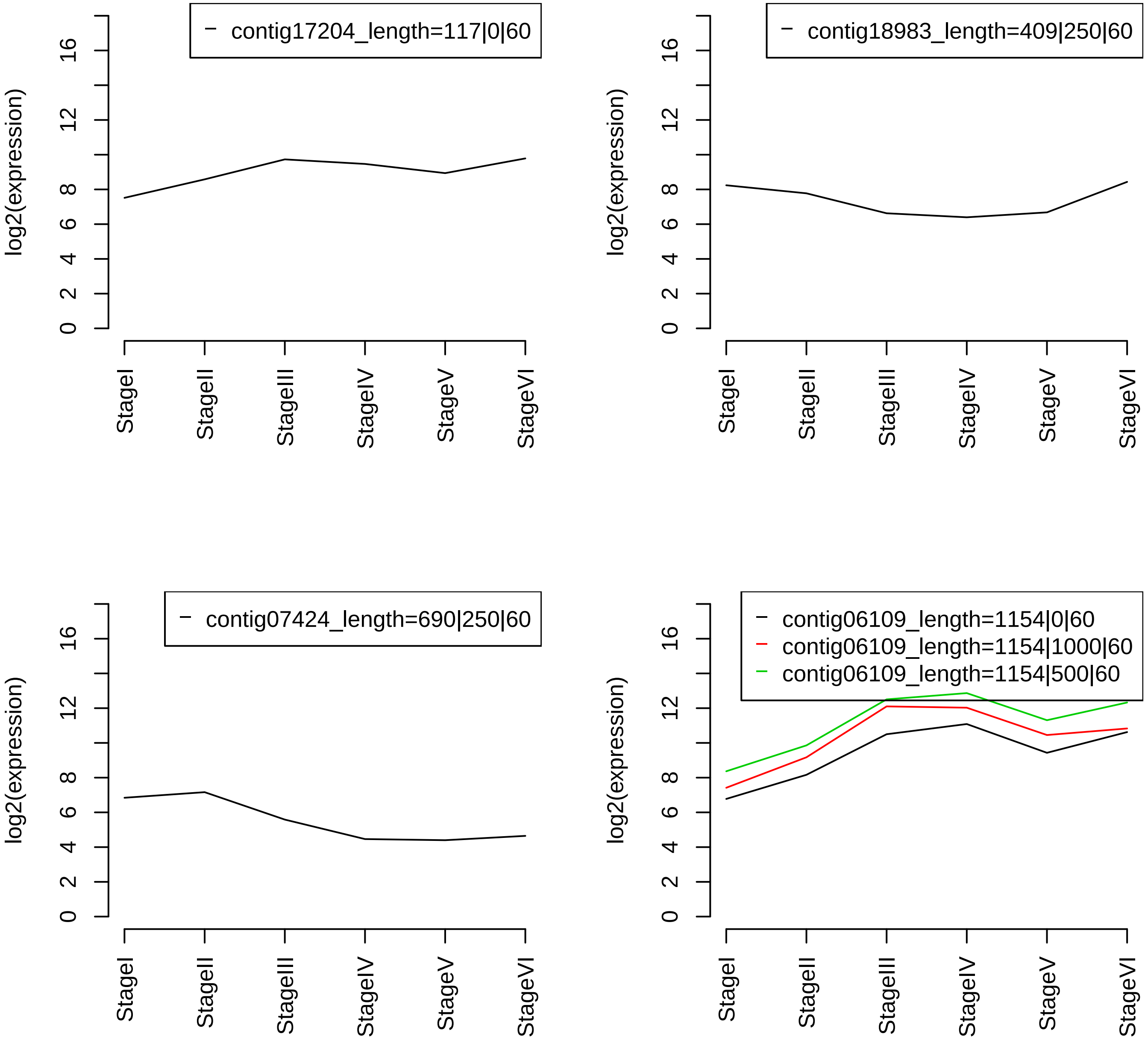

contig17204_length=117|0|60
log2(expression)
0
2
4
6
8
12
16
StageI
StageII
StageIII
StageIV
StageV
StageVI
contig18983_length=409|250|60
log2(expression)
0
2
4
6
8
12
16
StageI
StageII
StageIII
StageIV
StageV
StageVI
contig07424_length=690|250|60
log2(expression)
0
2
4
6
8
12
16
StageI
StageII
StageIII
StageIV
StageV
StageVI
contig06109_length=1154|0|60
contig06109_length=1154|1000|60
contig06109_length=1154|500|60
log2(expression)
0
2
4
6
8
12
16
StageI
StageII
StageIII
StageIV
StageV
StageVI

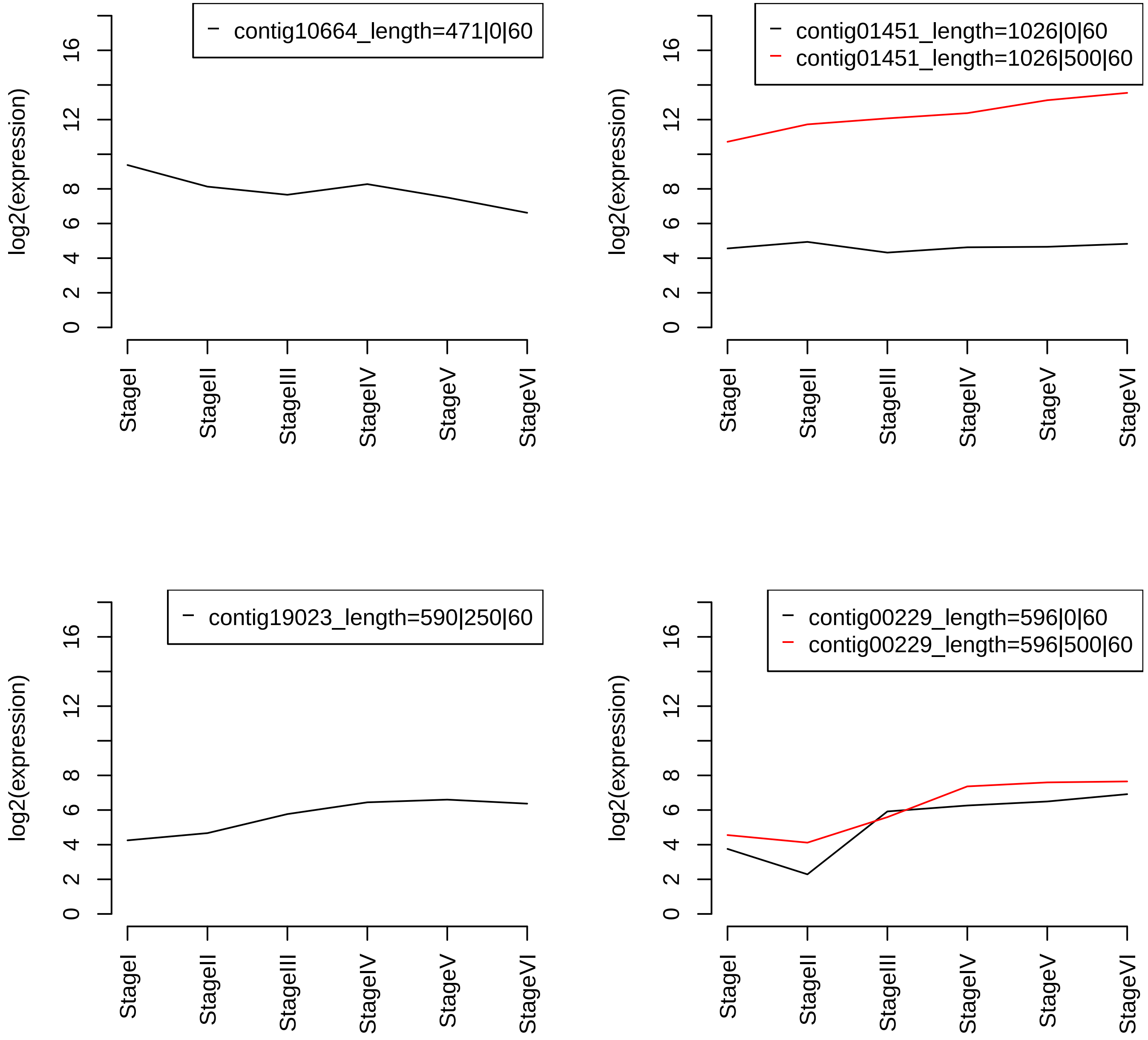
contig10664_length=471|0|60
contig01451_length=1026|0|60
contig01451_length=1026|500|60
contig19023_length=590|250|60
contig00229_length=596|0|60
contig00229_length=596|500|60
log2(expression)
0
2
4
6
8
12
16
StageI
StageII
StageIII
StageIV
StageV
StageVI

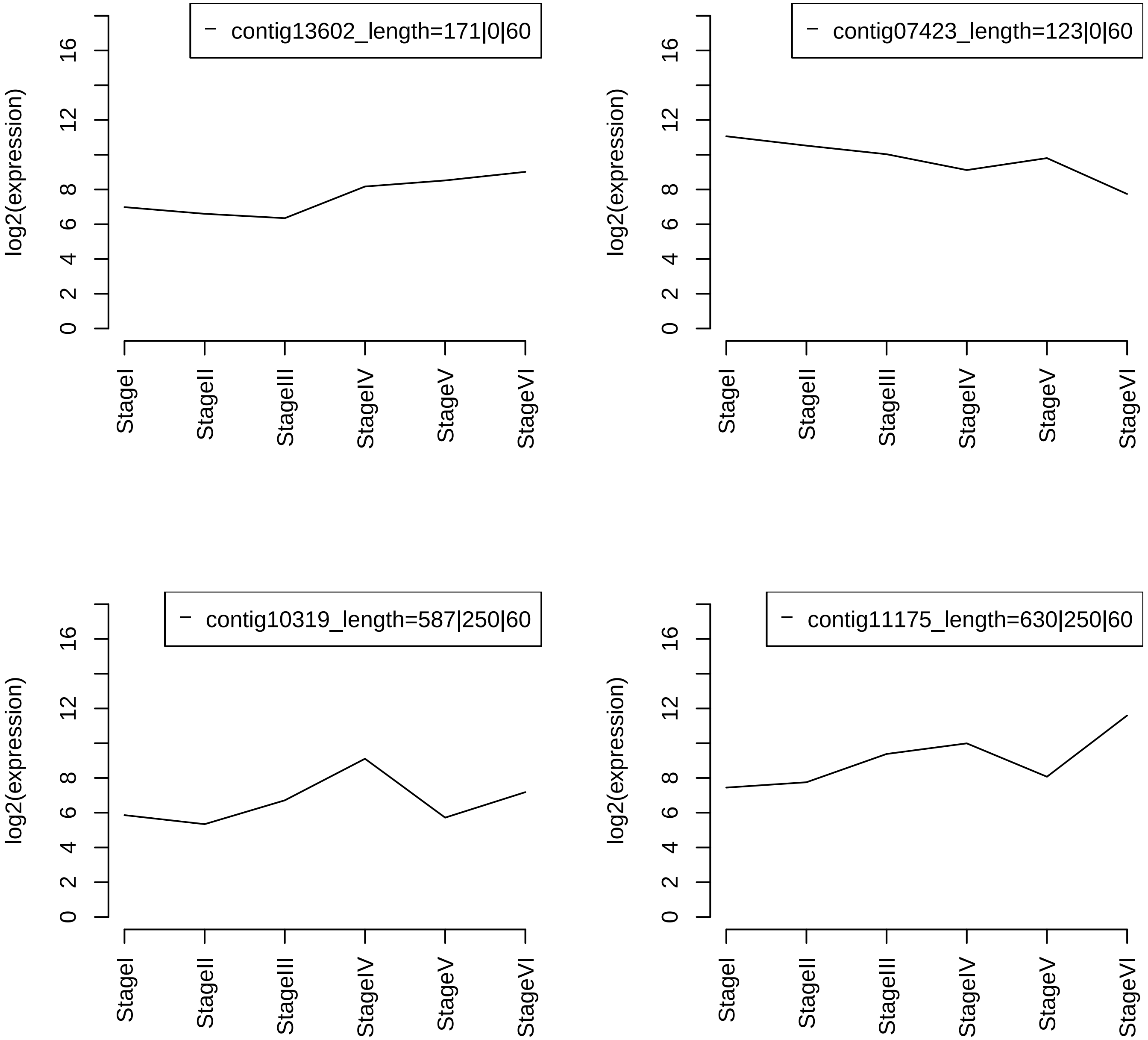
contig13602_length=171|0|60
contig07423_length=123|0|60
contig10319_length=587|250|60
contig11175_length=630|250|60
log2(expression)
0
2
4
6
8
12
16
StageI
StageII
StageIII
StageIV
StageV
StageVI

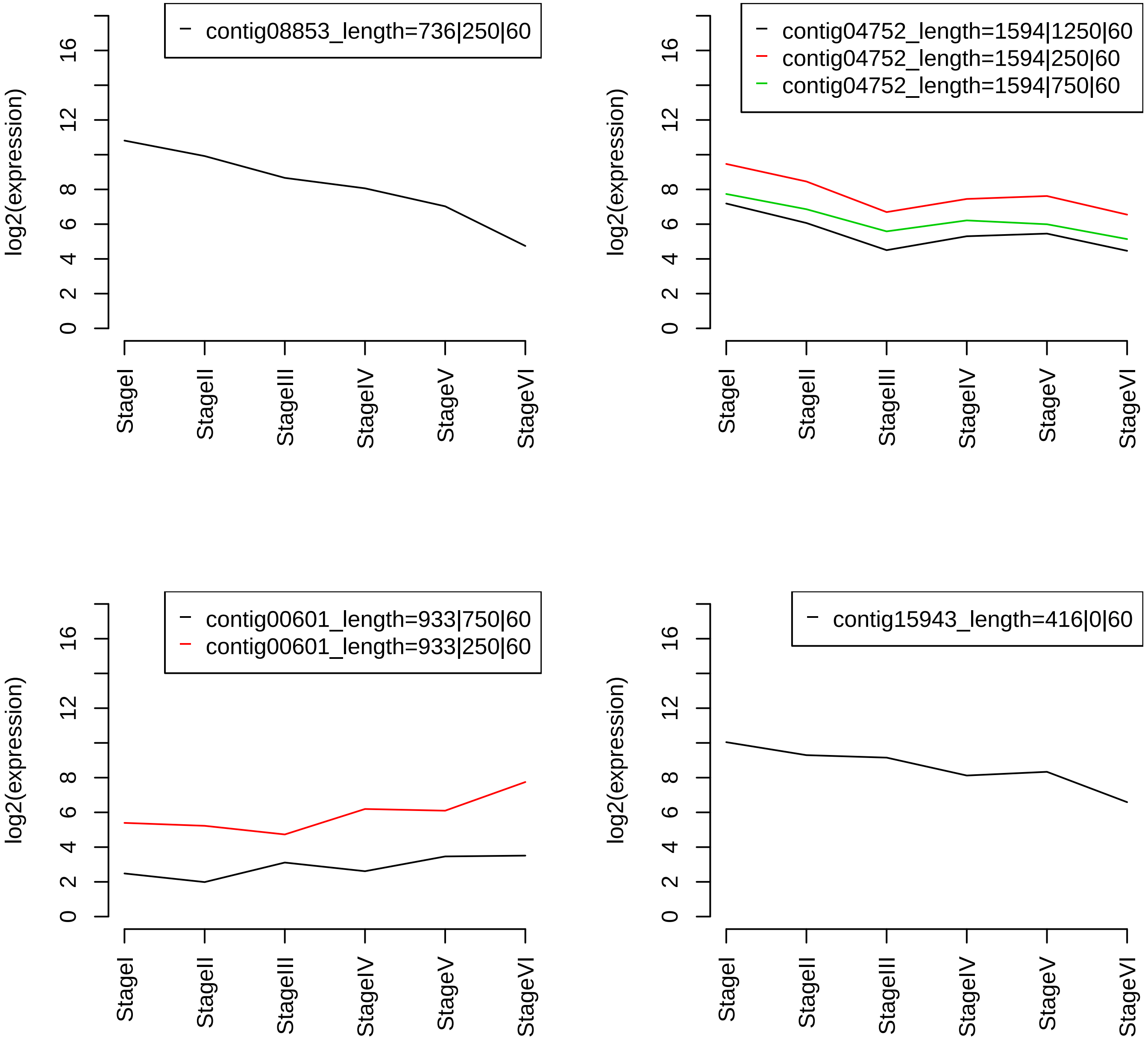

contig08853_length=736|250|60
contig04752_length=1594|1250|60
contig04752_length=1594|250|60
contig04752_length=1594|750|60
contig00601_length=933|750|60
contig00601_length=933|250|60
contig15943_length=416|0|60
log2(expression)
StageI
StageII
StageIII
StageIV
StageV
StageVI

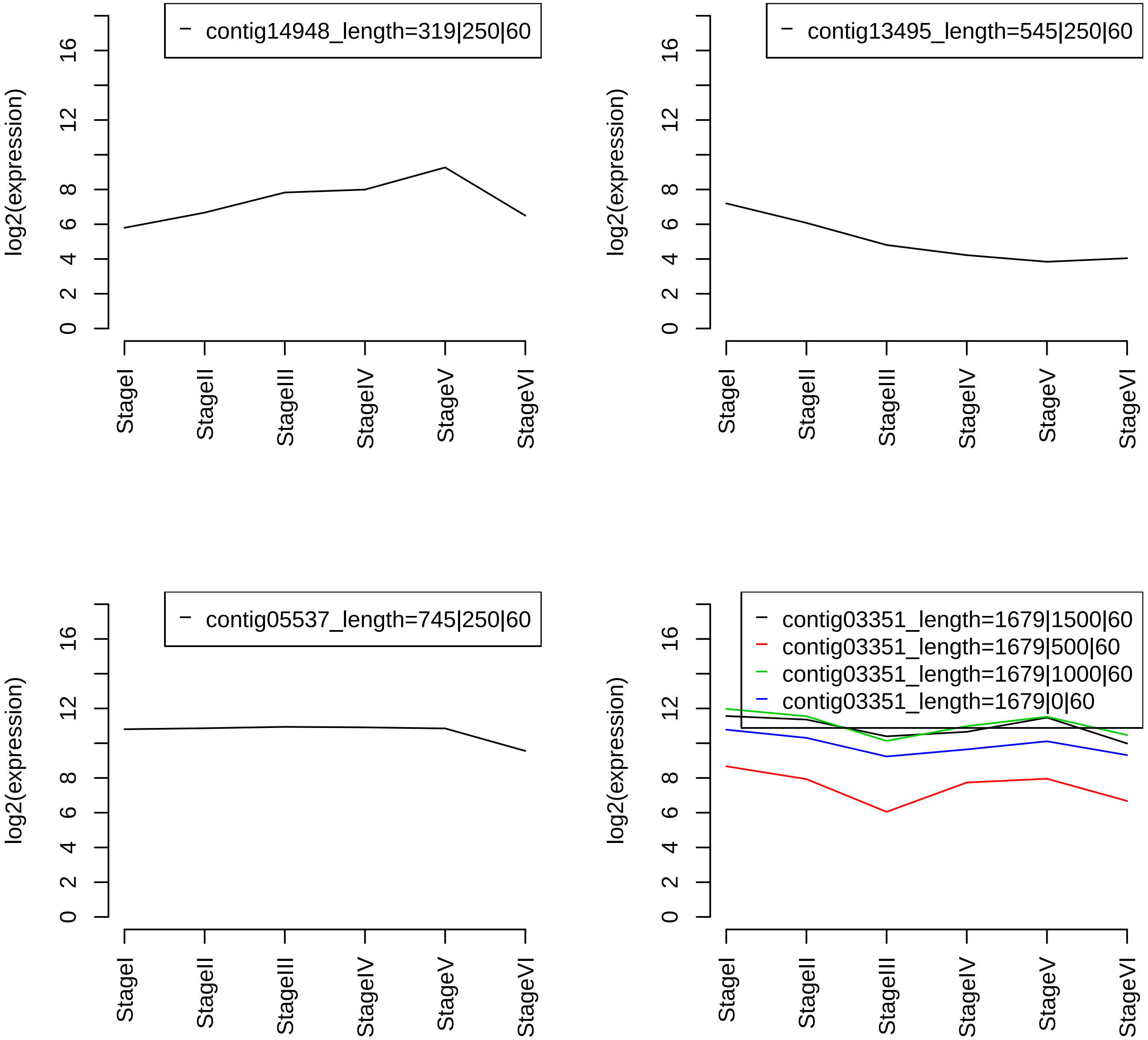

contig14948_length=319|250|60
contig13495_length=545|250|60
contig05537_length=745|250|60
contig03351_length=1679|1500|60
contig03351_length=1679|500|60
contig03351_length=1679|1000|60
contig03351_length=1679|0|60
log2(expression)
0
2
4
6
8
12
16
StageI
StageII
StageIII
StageIV
StageV
StageVI

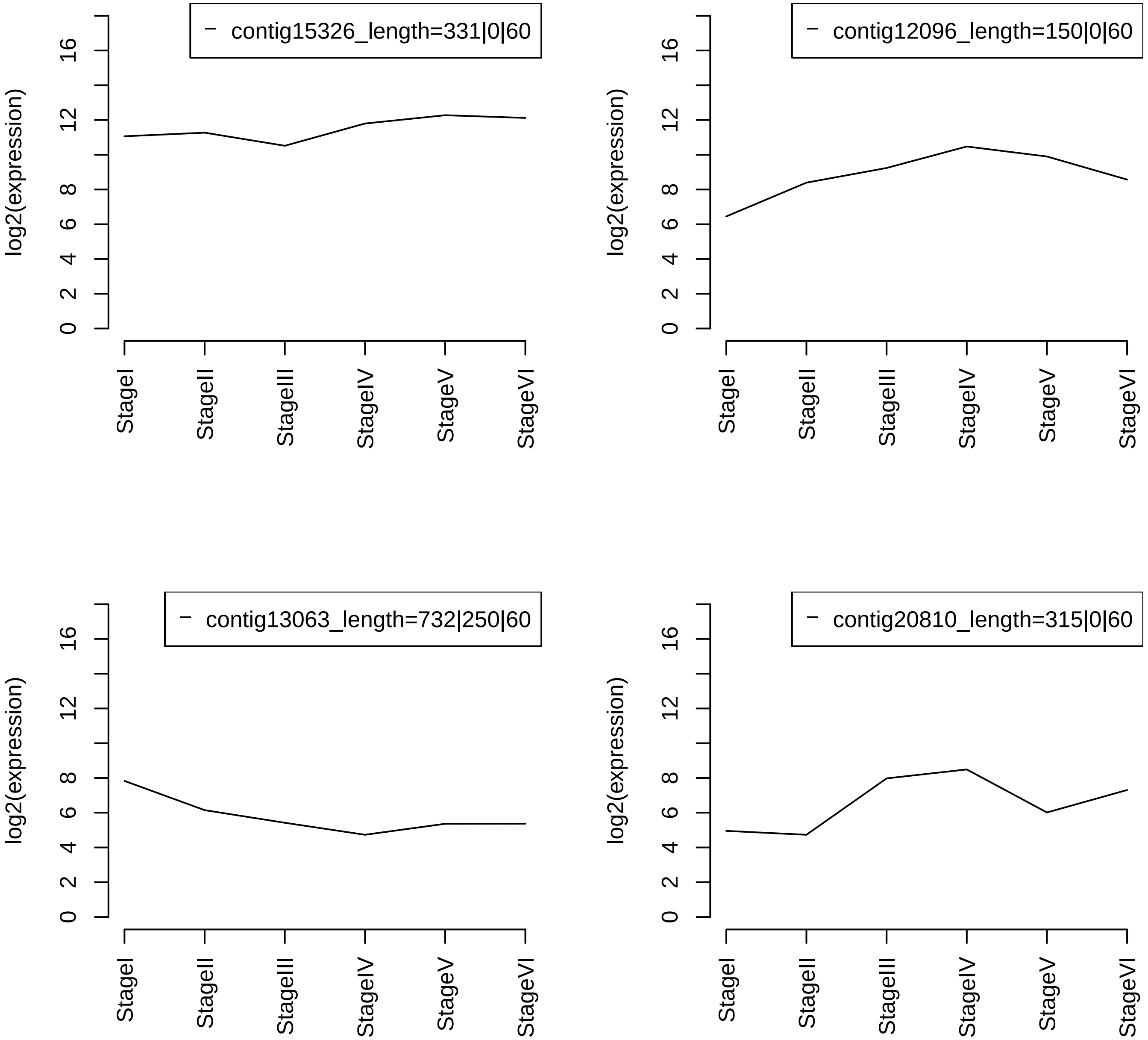

contig15326_length=331|0|60
contig12096_length=150|0|60
contig13063_length=732|250|60
contig20810_length=315|0|60
log2(expression)
0
2
4
6
8
12
16
StageI
StageII
StageIII
StageIV
StageV
StageVI

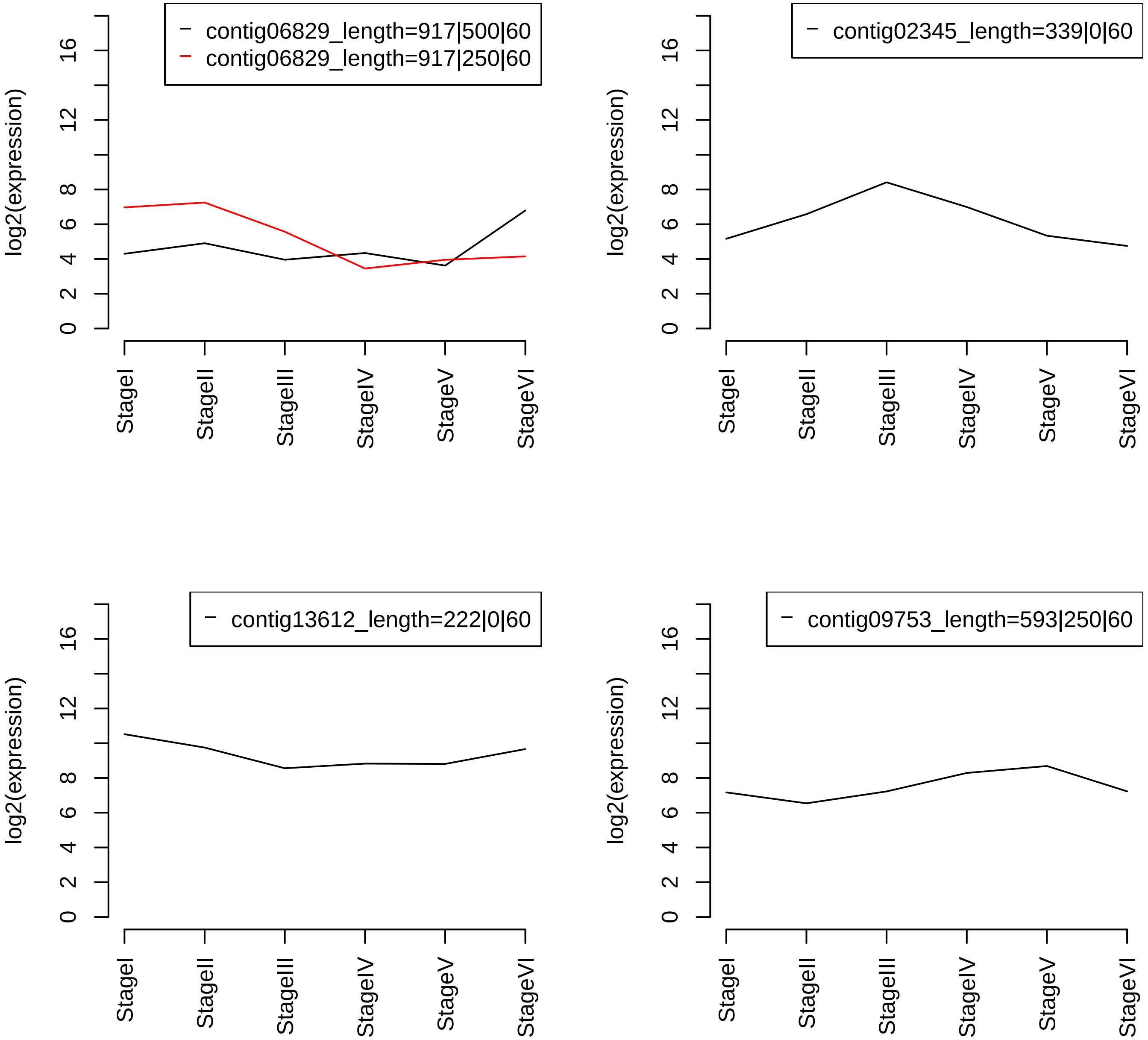
contig06829_length=917|500|60
contig06829_length=917|250|60
contig02345_length=339|0|60
contig13612_length=222|0|60
contig09753_length=593|250|60
log2(expression)
StageI
StageII
StageIII
StageIV
StageV
StageVI

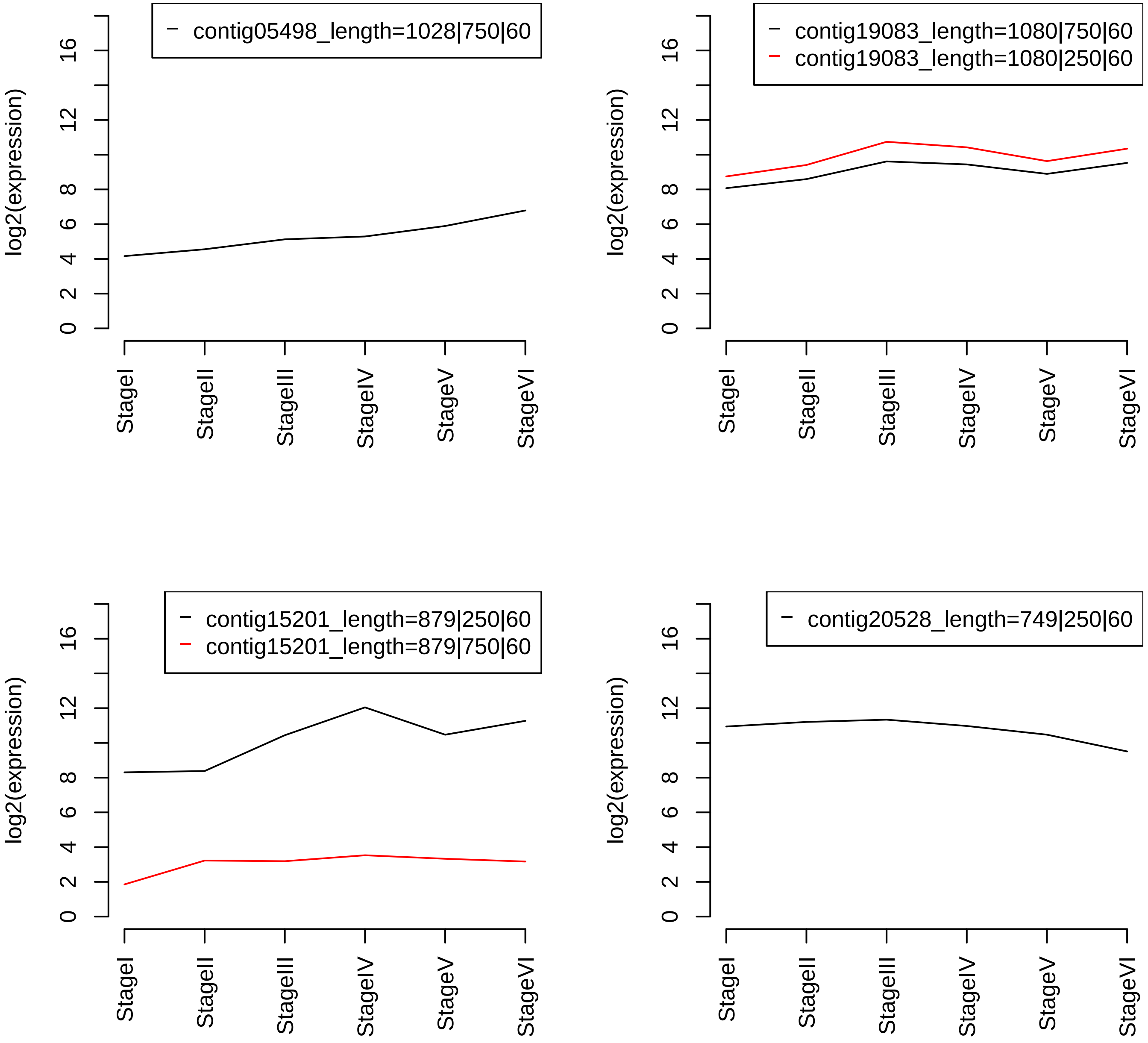

contig05498_length=1028|750|60
contig19083_length=1080|750|60
contig19083_length=1080|250|60
contig15201_length=879|250|60
contig15201_length=879|750|60
contig20528_length=749|250|60
log2(expression)
0
2
4
6
8
12
16
StageI
StageII
StageIII
StageIV
StageV
StageVI

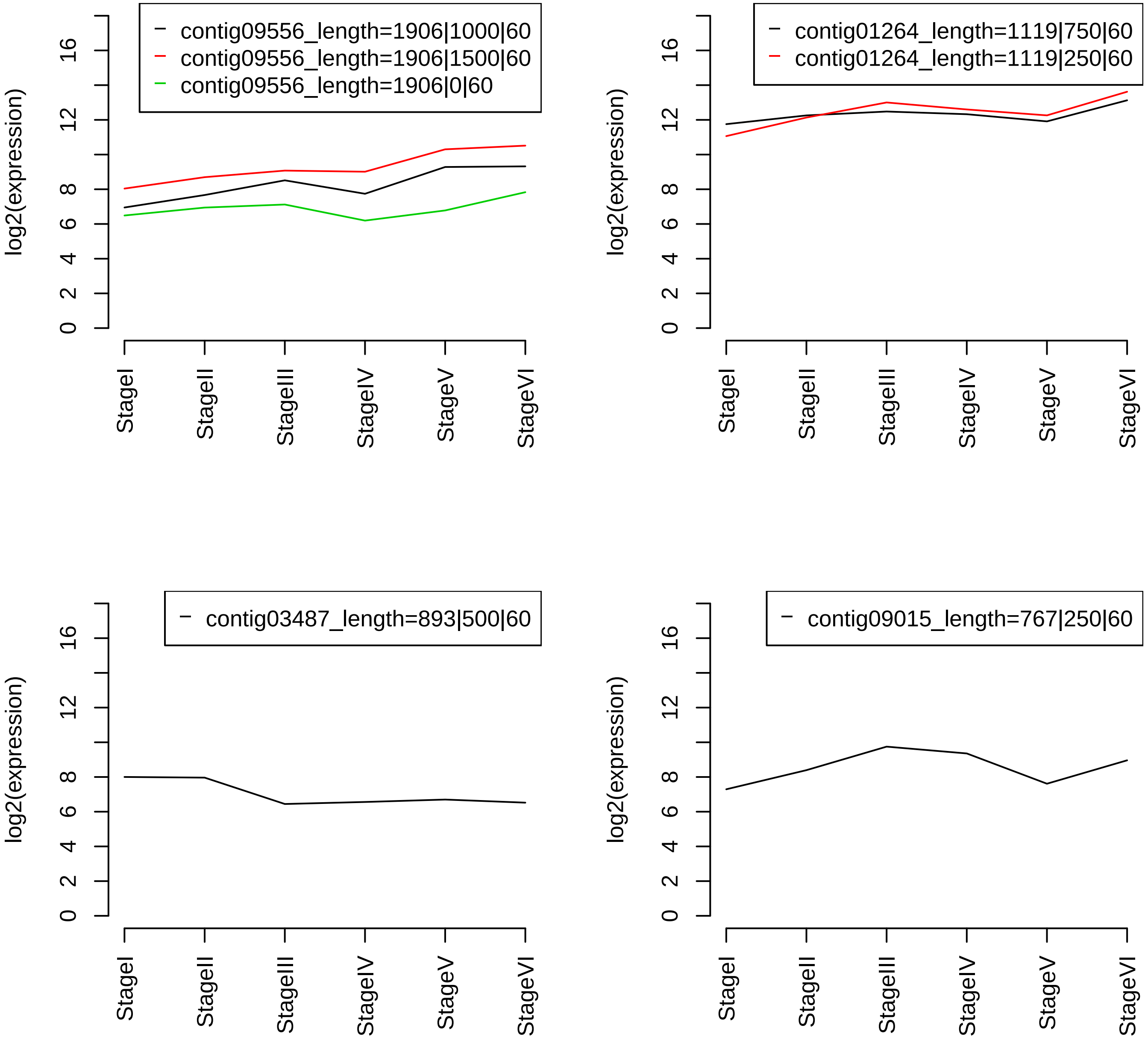
contig09556_length=1906|1000|60
contig09556_length=1906|1500|60
contig09556_length=1906|0|60
contig01264_length=1119|750|60
contig01264_length=1119|250|60
contig03487_length=893|500|60
contig09015_length=767|250|60
log2(expression)
0
2
4
6
8
12
16
StageI
StageII
StageIII
StageIV
StageV
StageVI

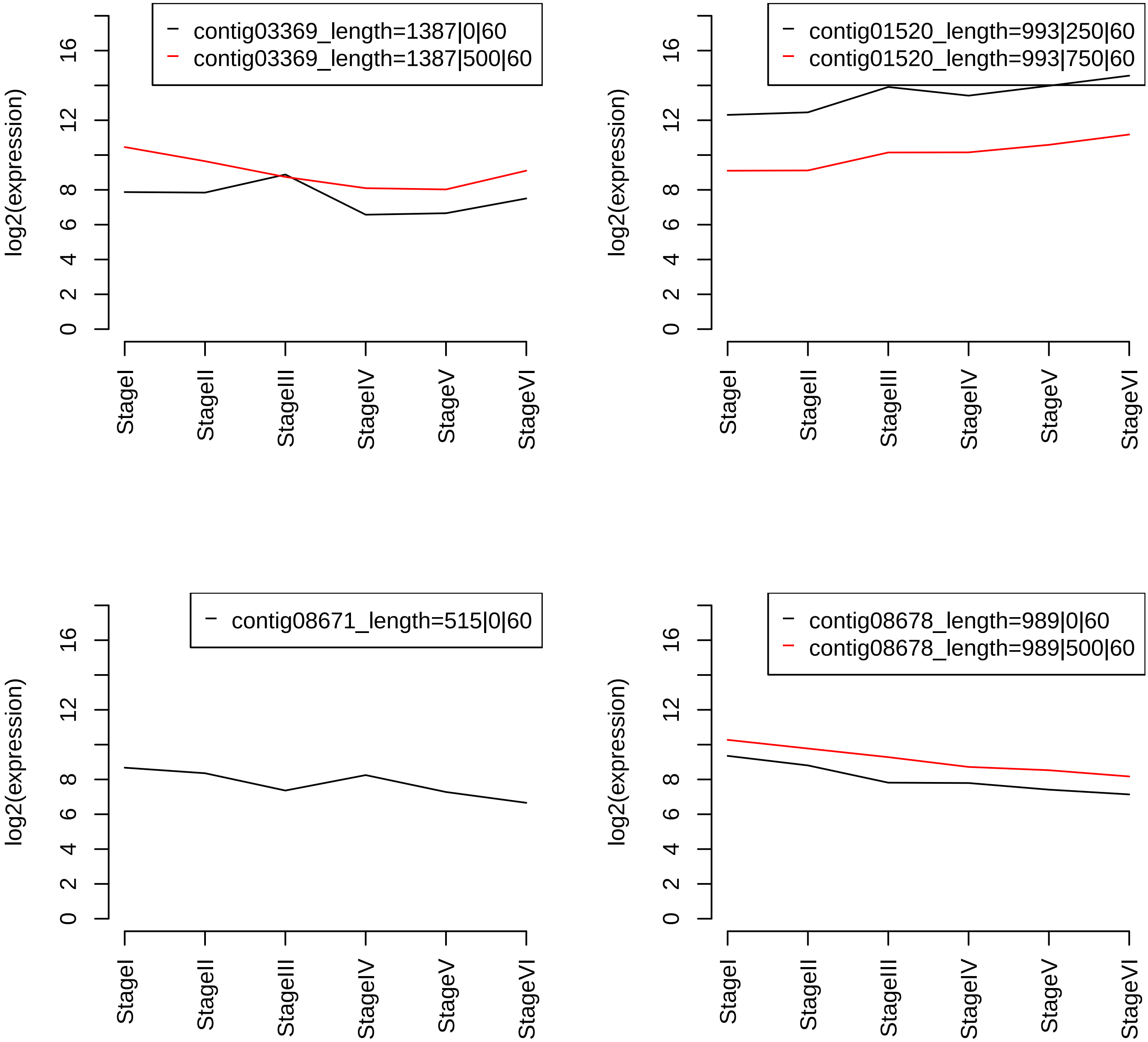

contig03369_length=1387|0|60
contig03369_length=1387|500|60
contig01520_length=993|250|60
contig01520_length=993|750|60
contig08671_length=515|0|60
contig08678_length=989|0|60
contig08678_length=989|500|60
log2(expression)
0
2
4
6
8
12
16
StageI
StageII
StageIII
StageIV
StageV
StageVI

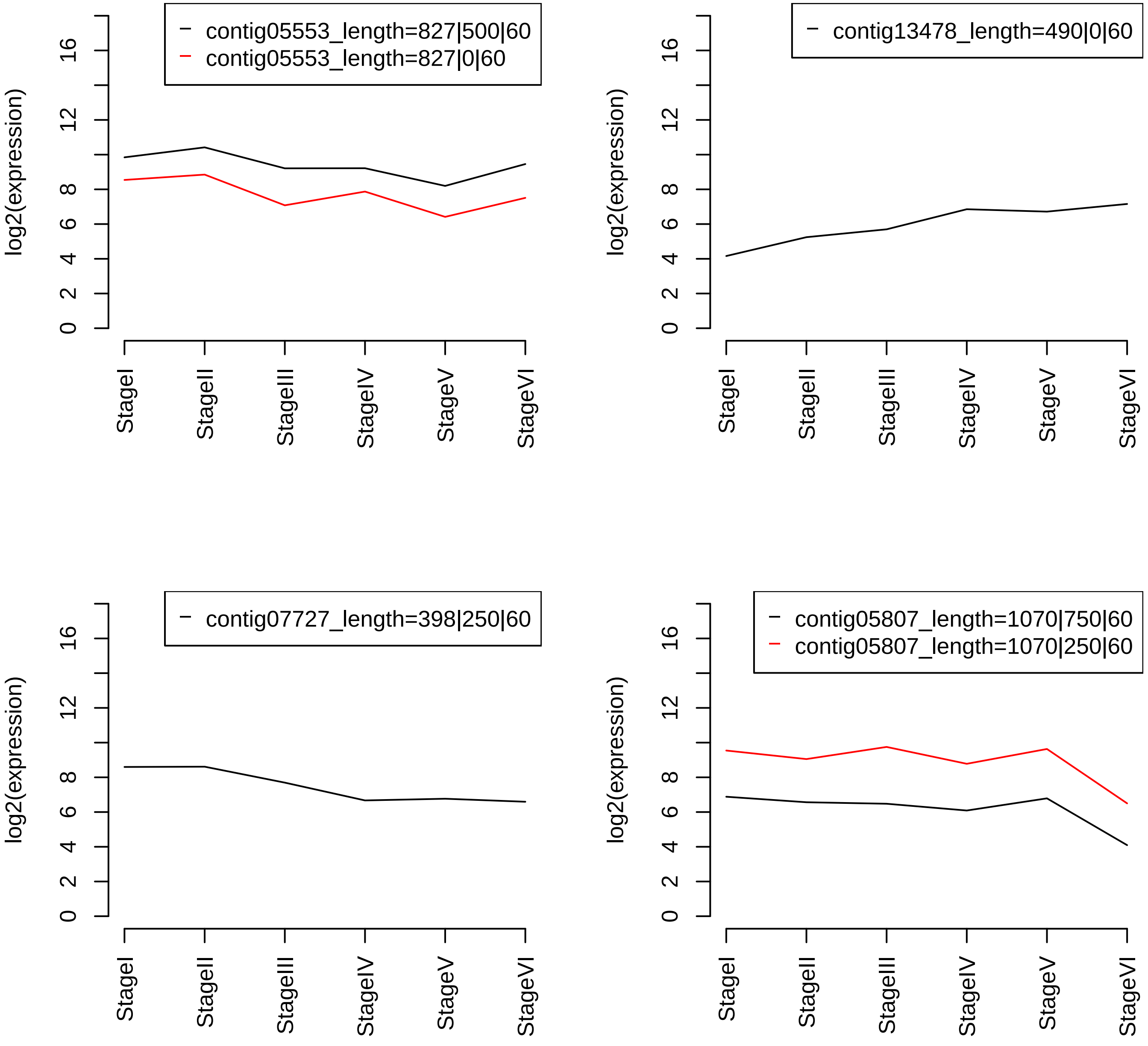

contig05553_length=827|500|60
contig05553_length=827|0|60
contig13478_length=490|0|60
contig07727_length=398|250|60
contig05807_length=1070|750|60
contig05807_length=1070|250|60
log2(expression)
0
2
4
6
8
12
16
StageI
StageII
StageIII
StageIV
StageV
StageVI

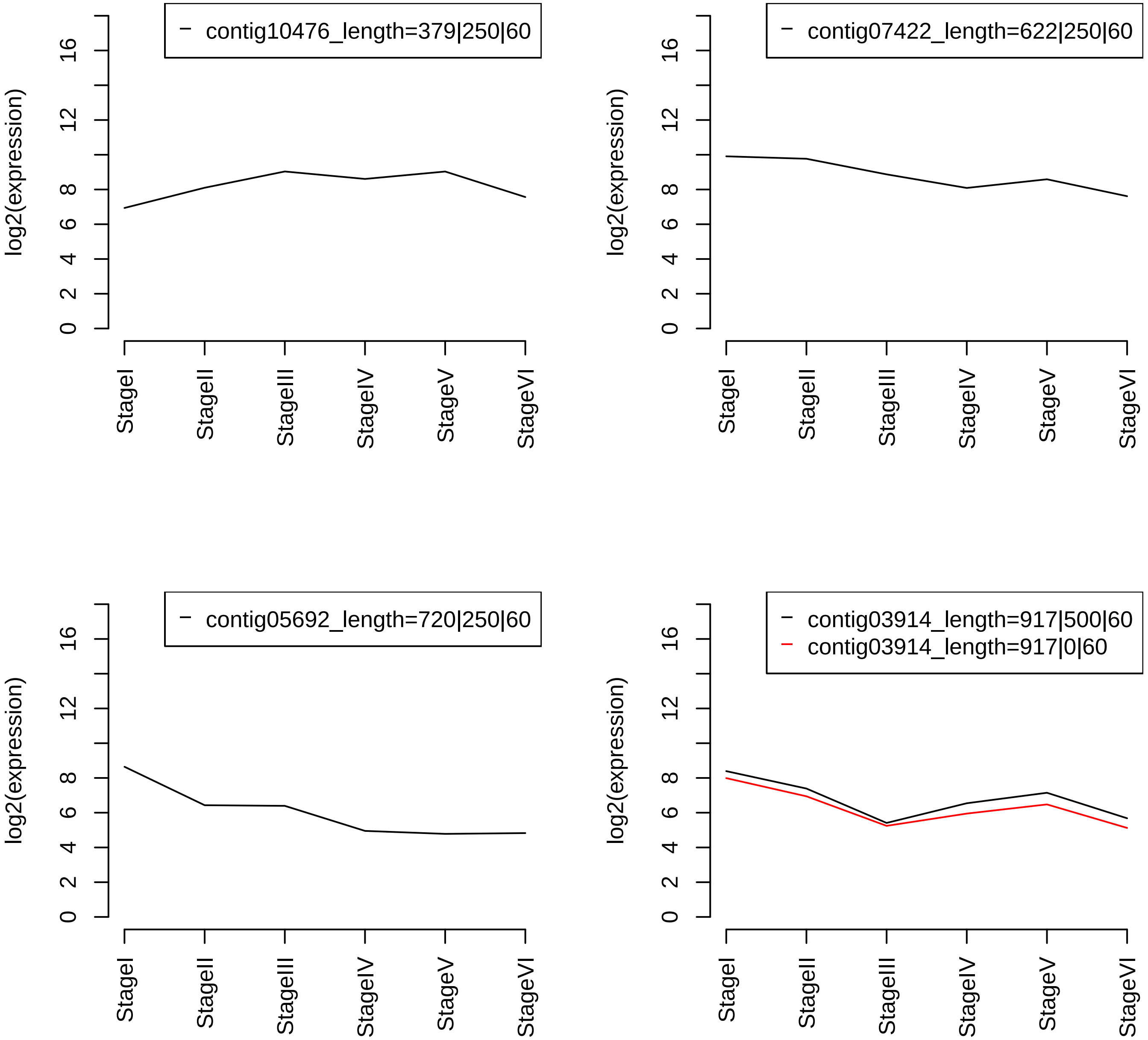
contig10476_length=379|250|60
contig07422_length=622|250|60
contig05692_length=720|250|60
contig03914_length=917|500|60
contig03914_length=917|0|60
log2(expression)
0
2
4
6
8
12
16
StageI
StageII
StageIII
StageIV
StageV
StageVI

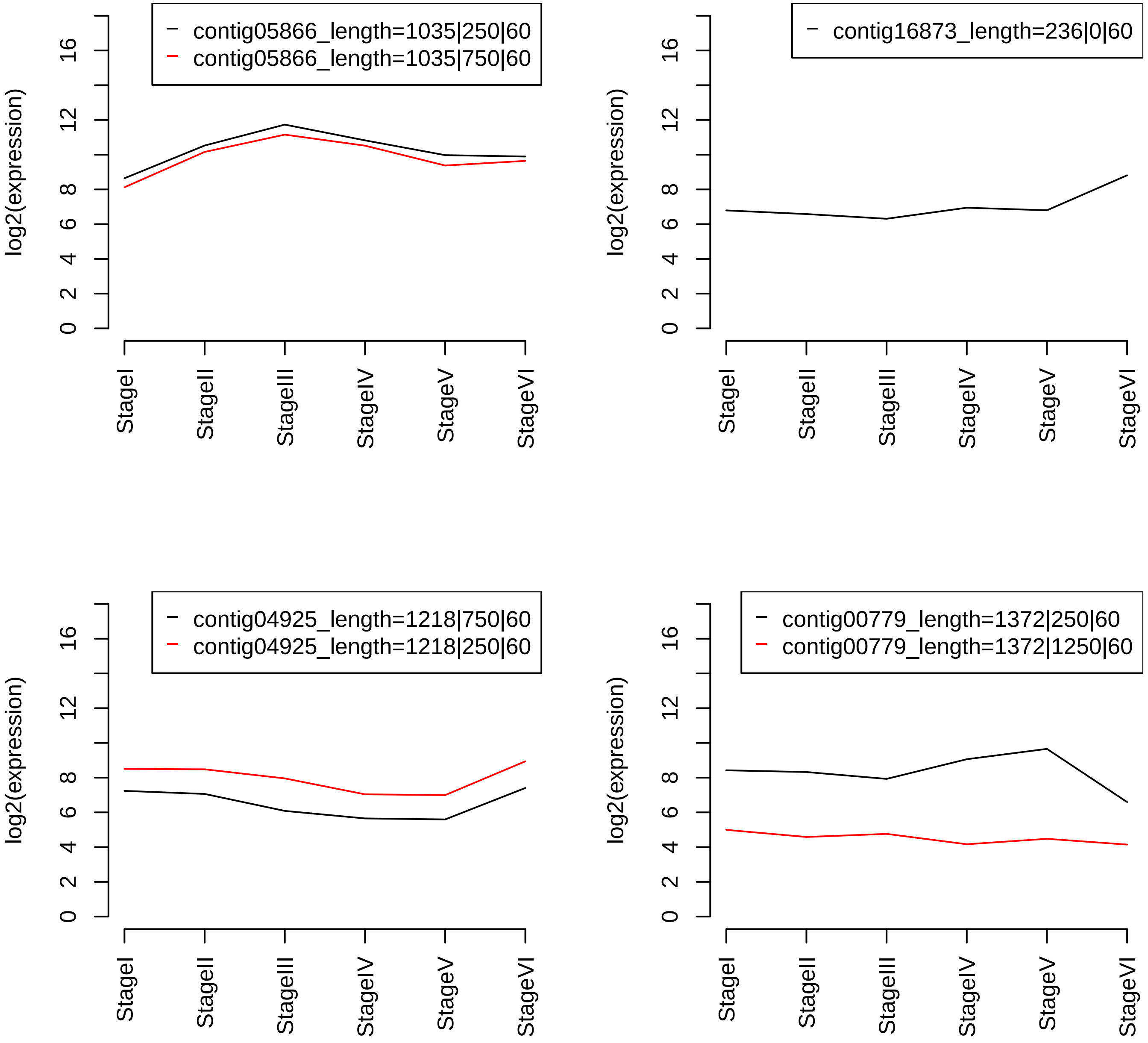
contig05866_length=1035|250|60
contig05866_length=1035|750|60
contig16873_length=236|0|60
contig04925_length=1218|750|60
contig04925_length=1218|250|60
contig00779_length=1372|250|60
contig00779_length=1372|1250|60
log2(expression)
0 2 4 6 8 12 16
StageI StageII StageIII StageIV StageV StageVI

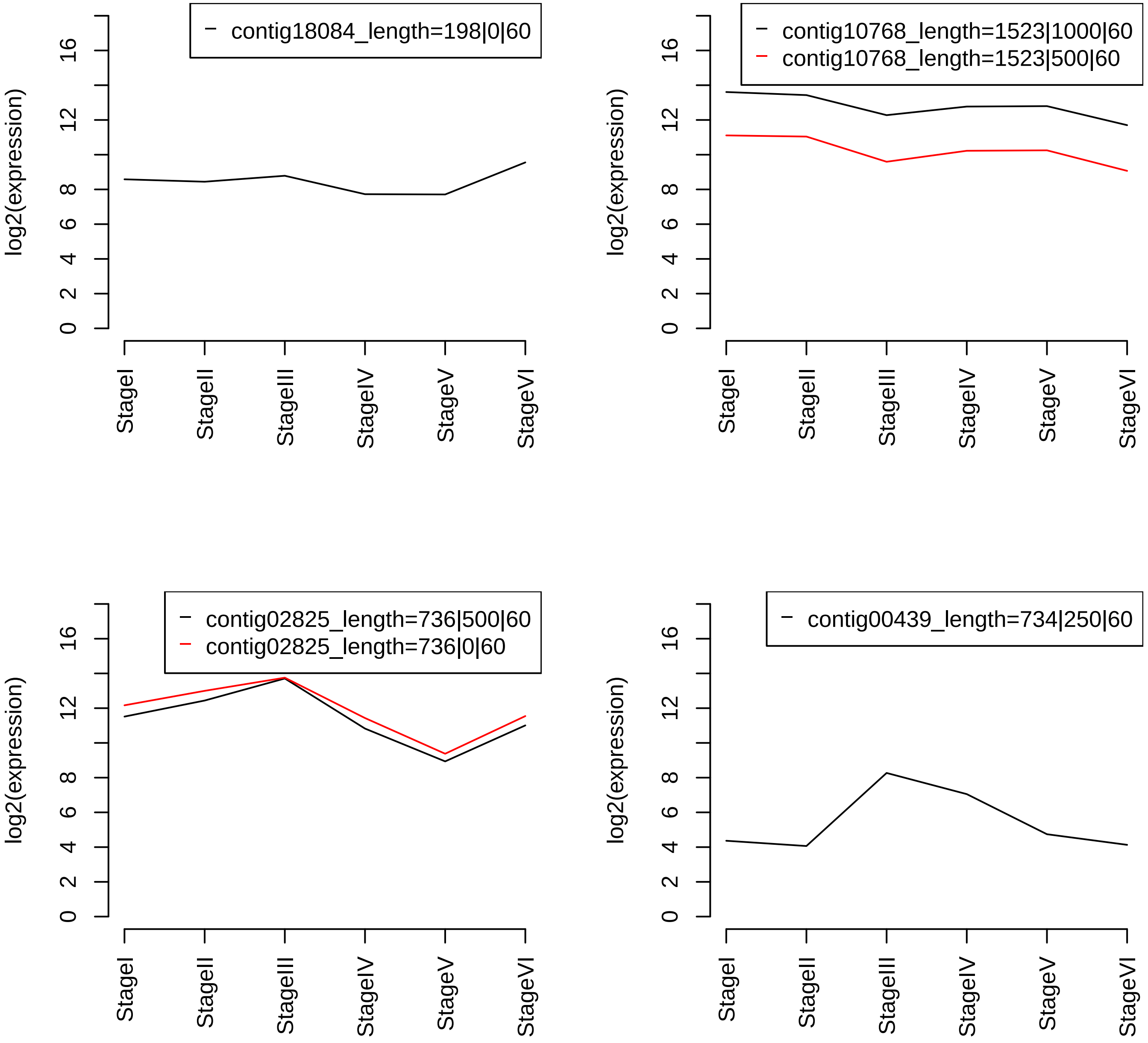

contig18084_length=198|0|60
contig10768_length=1523|1000|60
contig10768_length=1523|500|60
contig02825_length=736|500|60
contig02825_length=736|0|60
contig00439_length=734|250|60
log2(expression)
0 2 4 6 8 12 16
StageI
StageII
StageIII
StageIV
StageV
StageVI

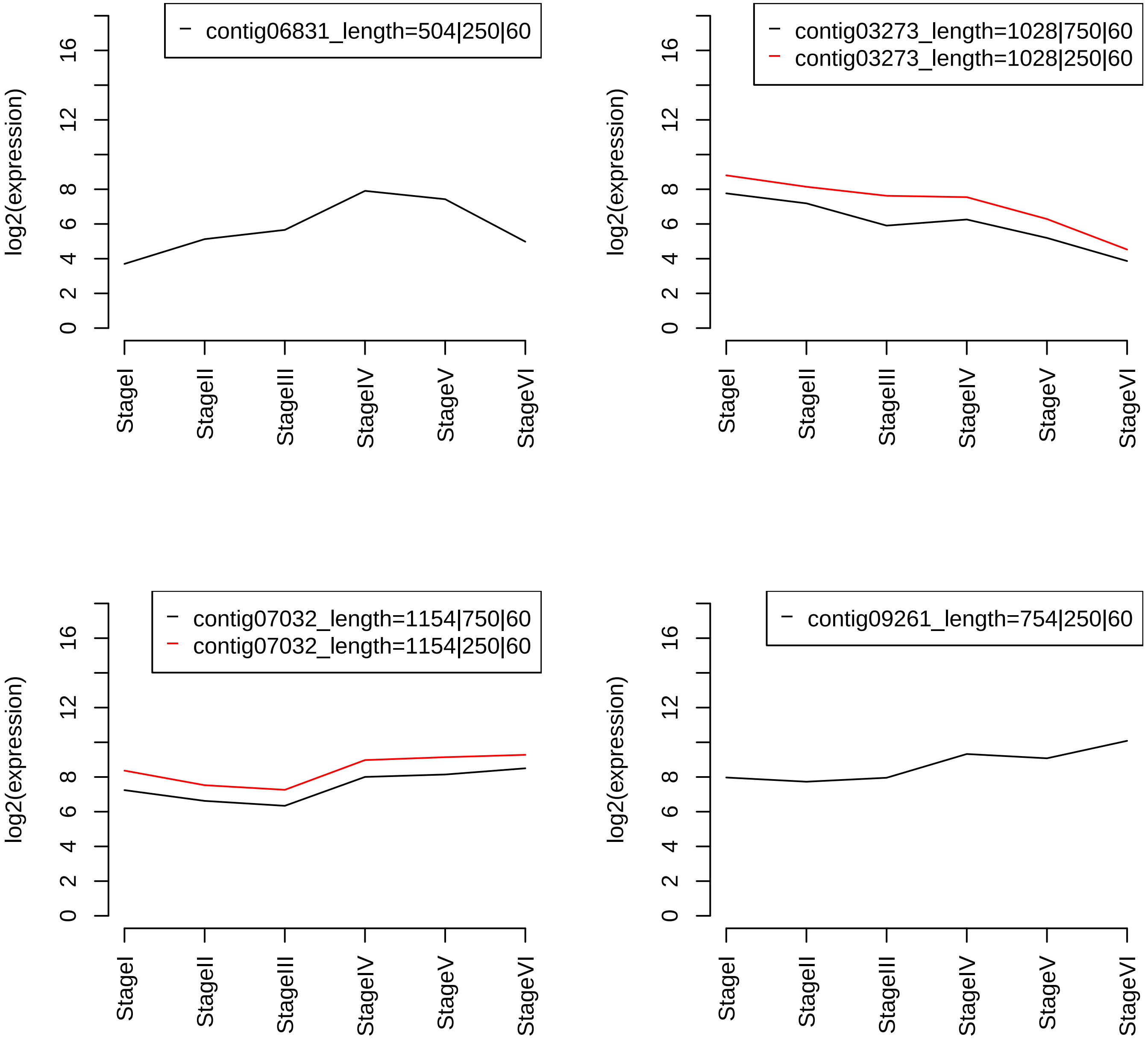

contig06831_length=504|250|60
contig03273_length=1028|750|60
contig03273_length=1028|250|60
contig07032_length=1154|750|60
contig07032_length=1154|250|60
contig09261_length=754|250|60
log2(expression)
0
2
4
6
8
12
16
StageI
StageII
StageIII
StageIV
StageV
StageVI

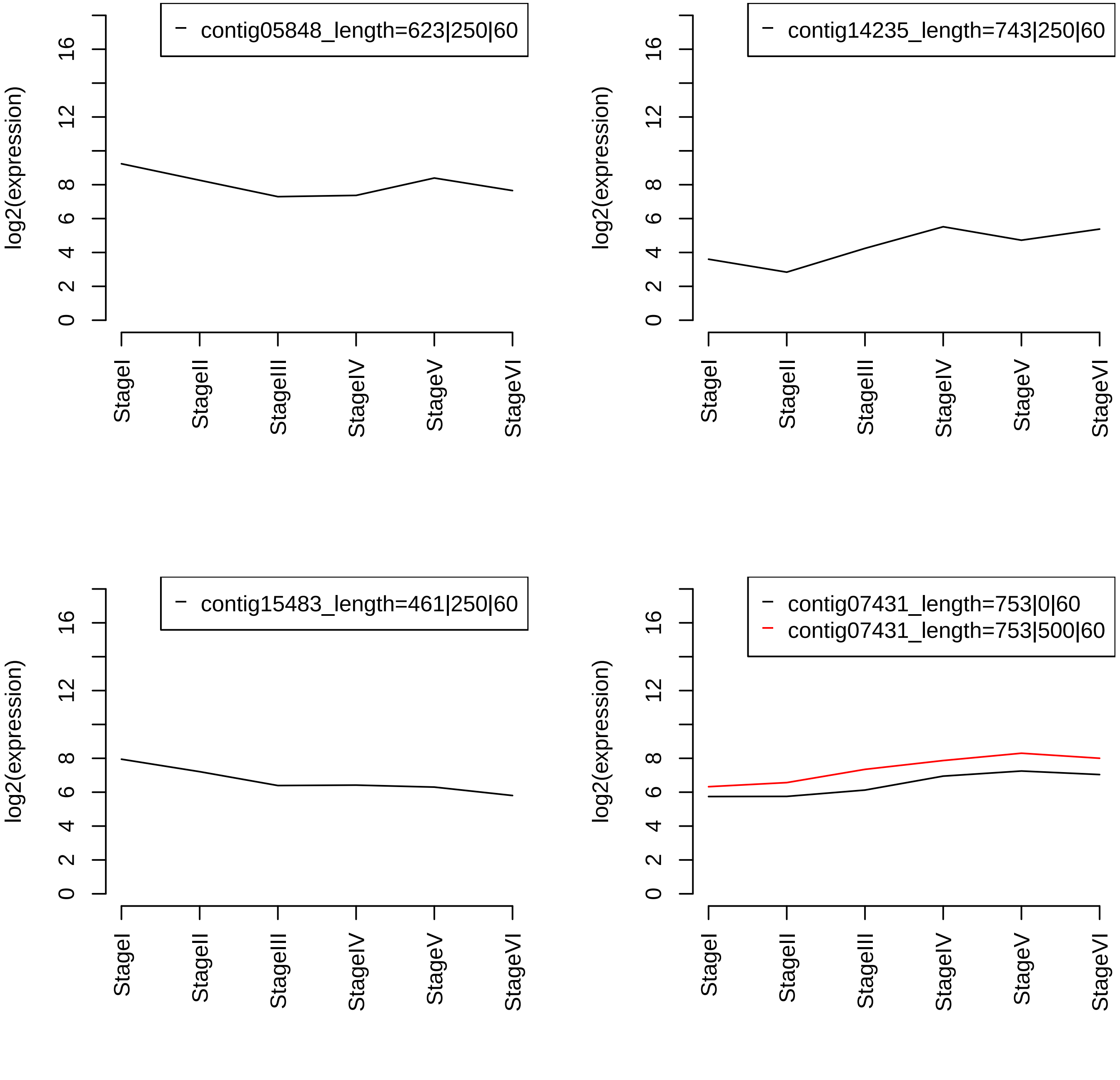
contig05848_length=623|250|60
contig14235_length=743|250|60
contig15483_length=461|250|60
contig07431_length=753|0|60
contig07431_length=753|500|60
log2(expression)
0
2
4
6
8
12
16
StageI
StageII
StageIII
StageIV
StageV
StageVI

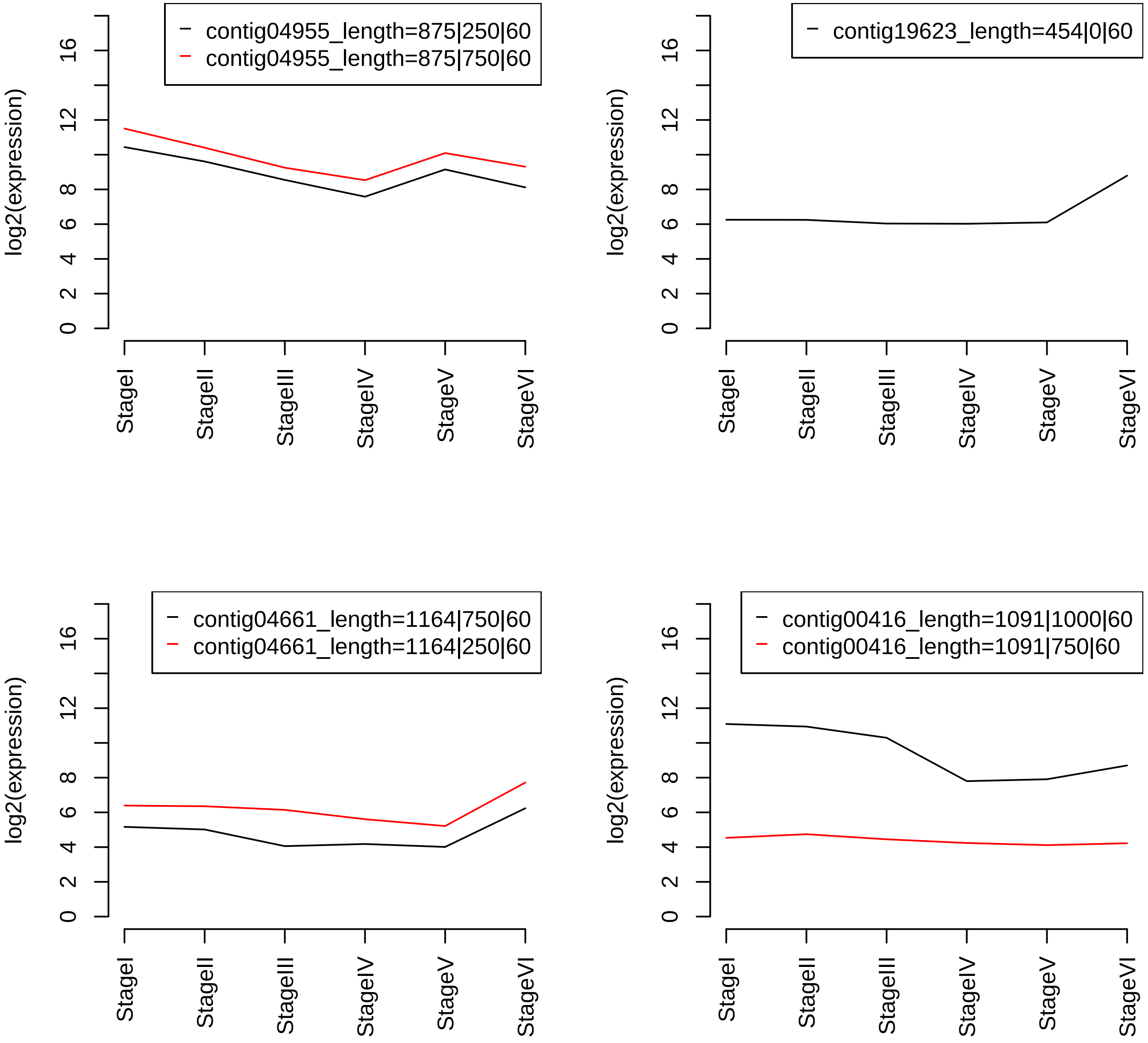

contig04955_length=875|250|60
contig04955_length=875|750|60
log2(expression)
0
2
4
6
8
12
16
StageI
StageII
StageIII
StageIV
StageV
StageVI
contig19623_length=454|0|60
log2(expression)
0
2
4
6
8
12
16
StageI
StageII
StageIII
StageIV
StageV
StageVI
contig04661_length=1164|750|60
contig04661_length=1164|250|60
log2(expression)
0
2
4
6
8
12
16
StageI
StageII
StageIII
StageIV
StageV
StageVI
contig00416_length=1091|1000|60
contig00416_length=1091|750|60
log2(expression)
0
2
4
6
8
12
16
StageI
StageII
StageIII
StageIV
StageV
StageVI

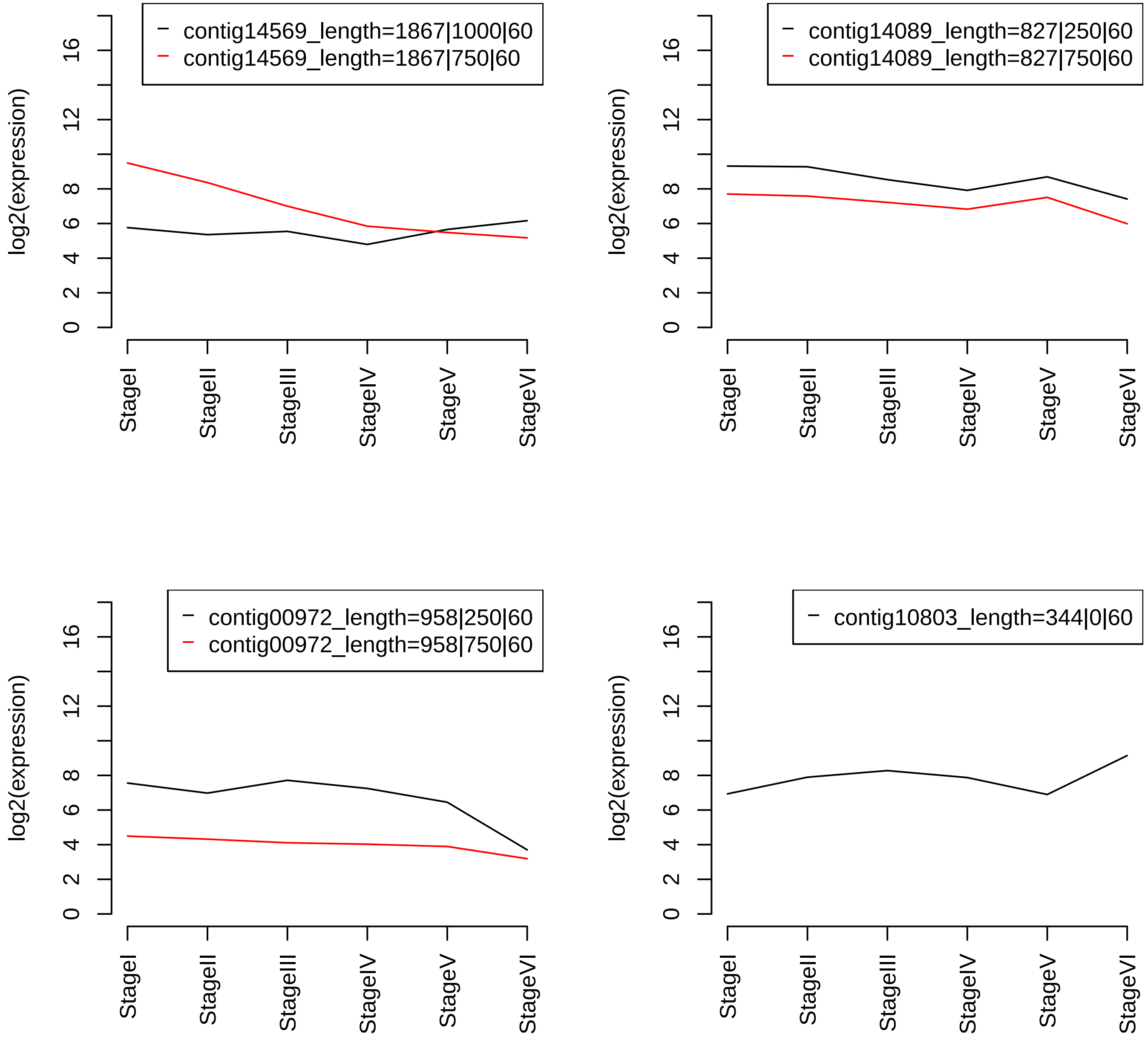
contig14569_length=1867|1000|60
contig14569_length=1867|750|60
contig14089_length=827|250|60
contig14089_length=827|750|60
contig00972_length=958|250|60
contig00972_length=958|750|60
contig10803_length=344|0|60
log2(expression)
0
2
4
6
8
12
16
StageI
StageII
StageIII
StageIV
StageV
StageVI

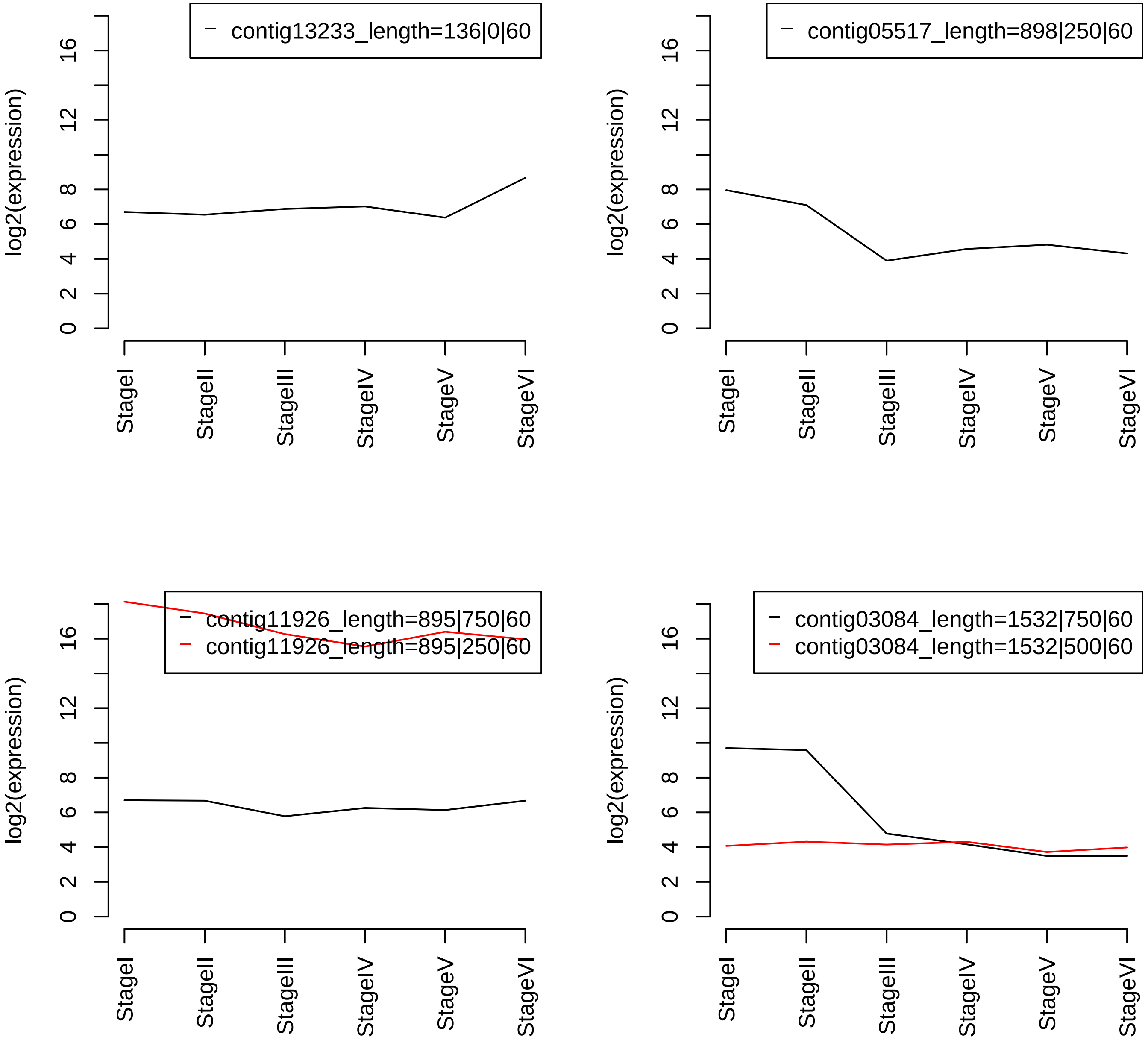
contig13233_length=136|0|60
contig05517_length=898|250|60
contig11926_length=895|750|60
contig11926_length=895|250|60
contig03084_length=1532|750|60
contig03084_length=1532|500|60
log2(expression)
0
2
4
6
8
12
16
StageI
StageII
StageIII
StageIV
StageV
StageVI

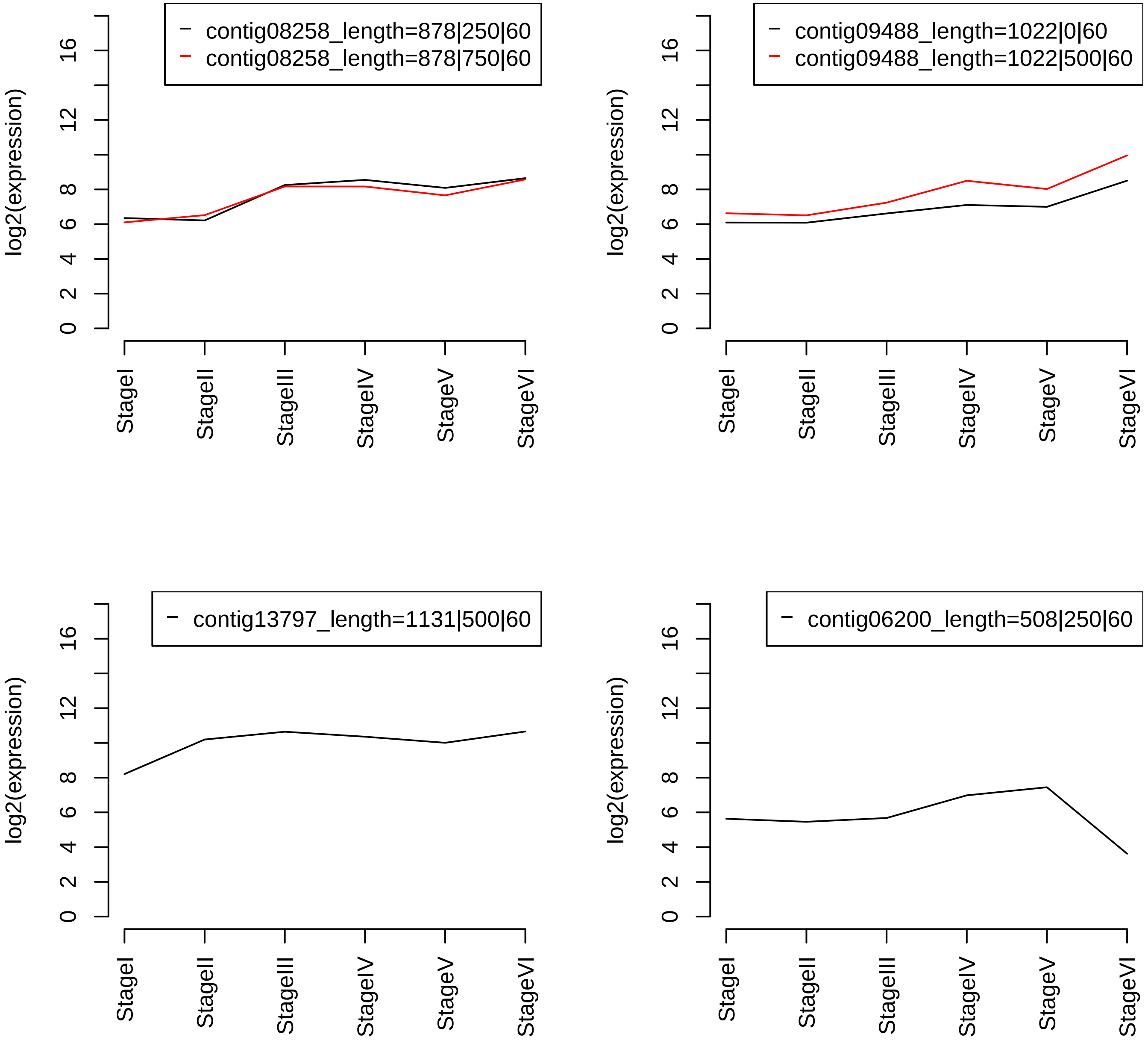
contig08258_length=878|250|60
contig08258_length=878|750|60
contig09488_length=1022|0|60
contig09488_length=1022|500|60
contig13797_length=1131|500|60
contig06200_length=508|250|60
log2(expression)
0
2
4
6
8
12
16
StageI
StageII
StageIII
StageIV
StageV
StageVI

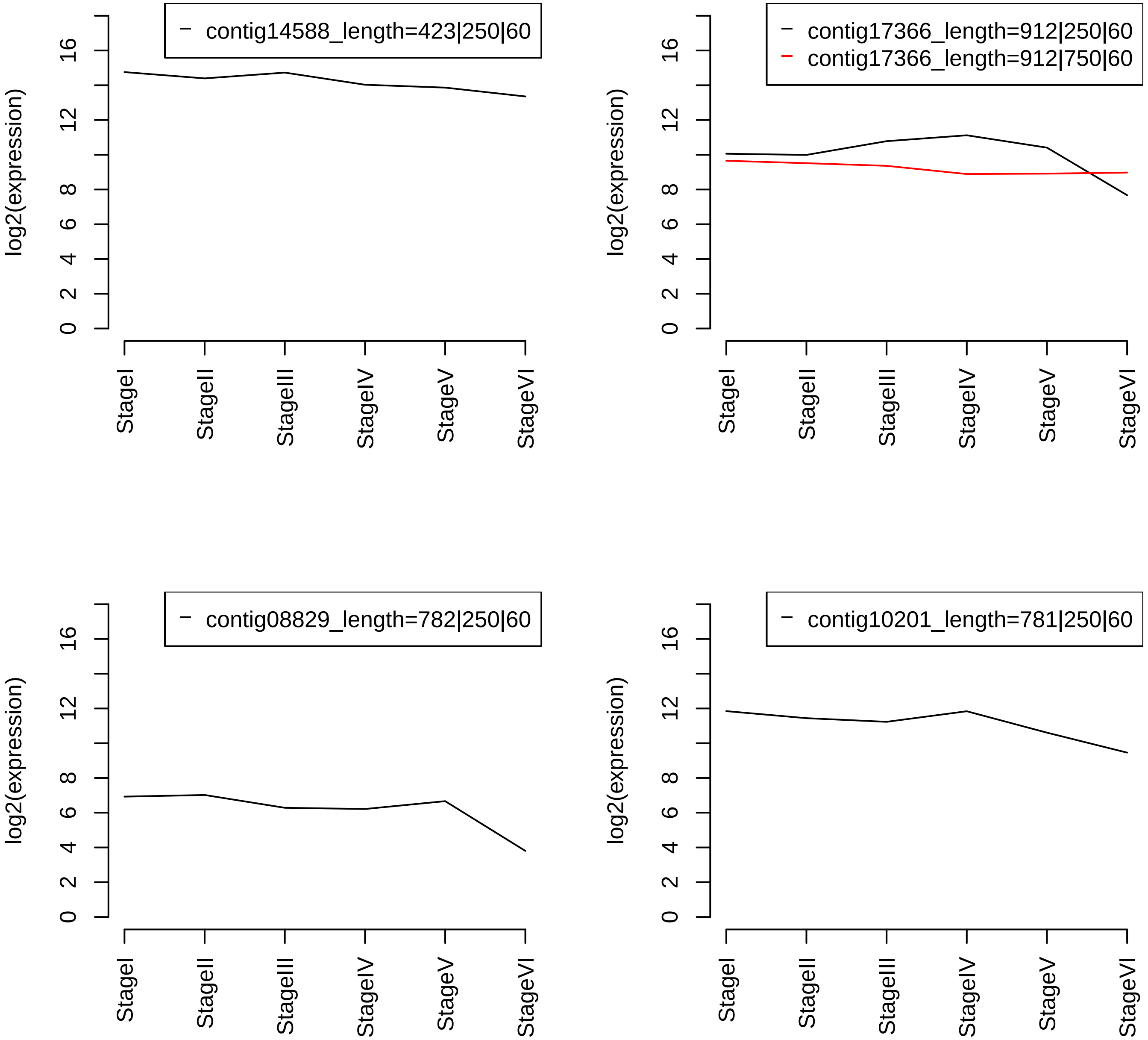

contig14588_length=423|250|60
contig17366_length=912|250|60
contig17366_length=912|750|60
contig08829_length=782|250|60
contig10201_length=781|250|60
log2(expression)
0
2
4
6
8
12
16
StageI
StageII
StageIII
StageIV
StageV
StageVI

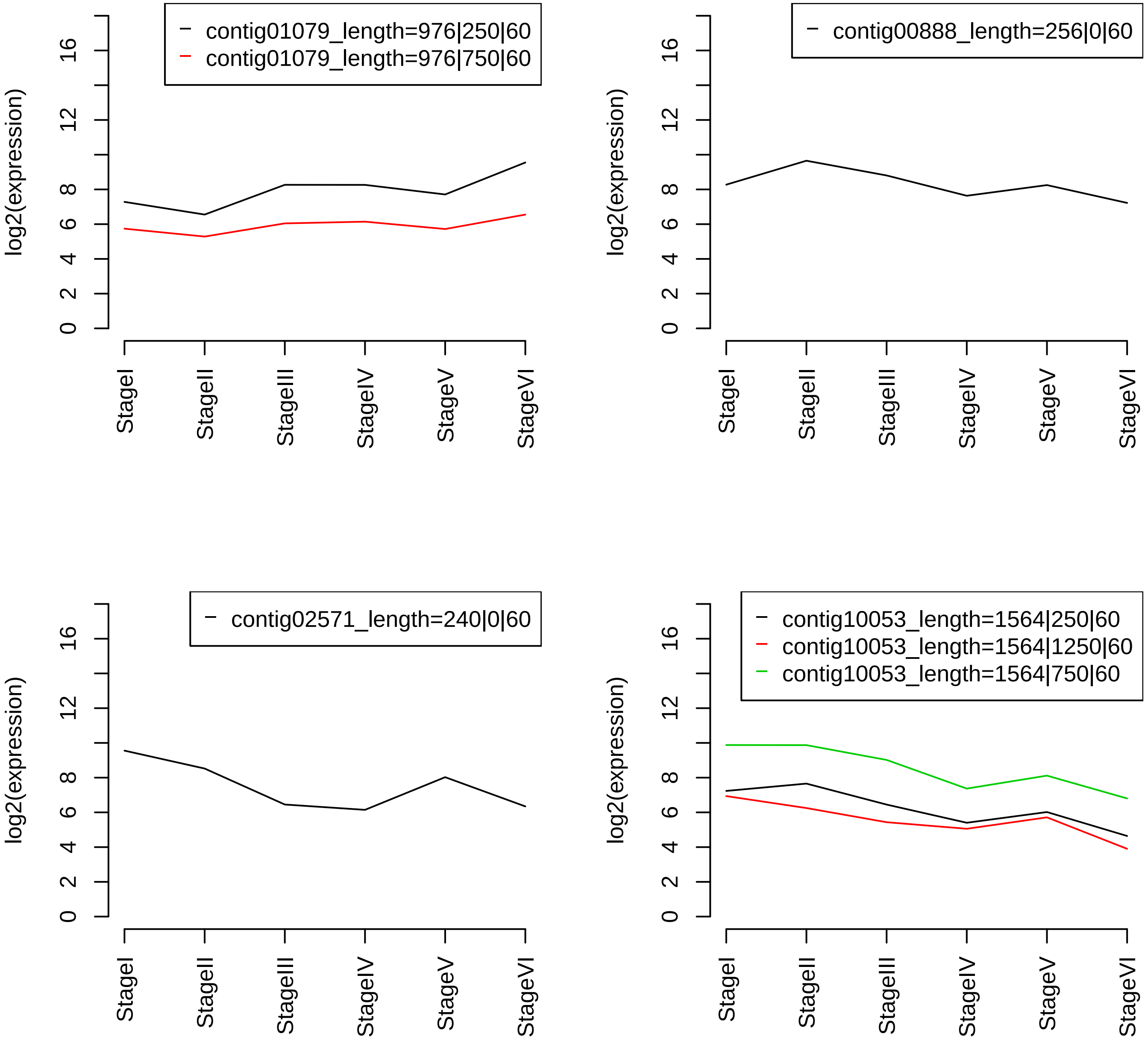
contig01079_length=976|250|60
contig01079_length=976|750|60
log2(expression)
0
2
4
6
8
12
16
StageI
StageII
StageIII
StageIV
StageV
StageVI
contig00888_length=256|0|60
log2(expression)
0
2
4
6
8
12
16
StageI
StageII
StageIII
StageIV
StageV
StageVI
contig02571_length=240|0|60
log2(expression)
0
2
4
6
8
12
16
StageI
StageII
StageIII
StageIV
StageV
StageVI
contig10053_length=1564|250|60
contig10053_length=1564|1250|60
contig10053_length=1564|750|60
log2(expression)
0
2
4
6
8
12
16
StageI
StageII
StageIII
StageIV
StageV
StageVI

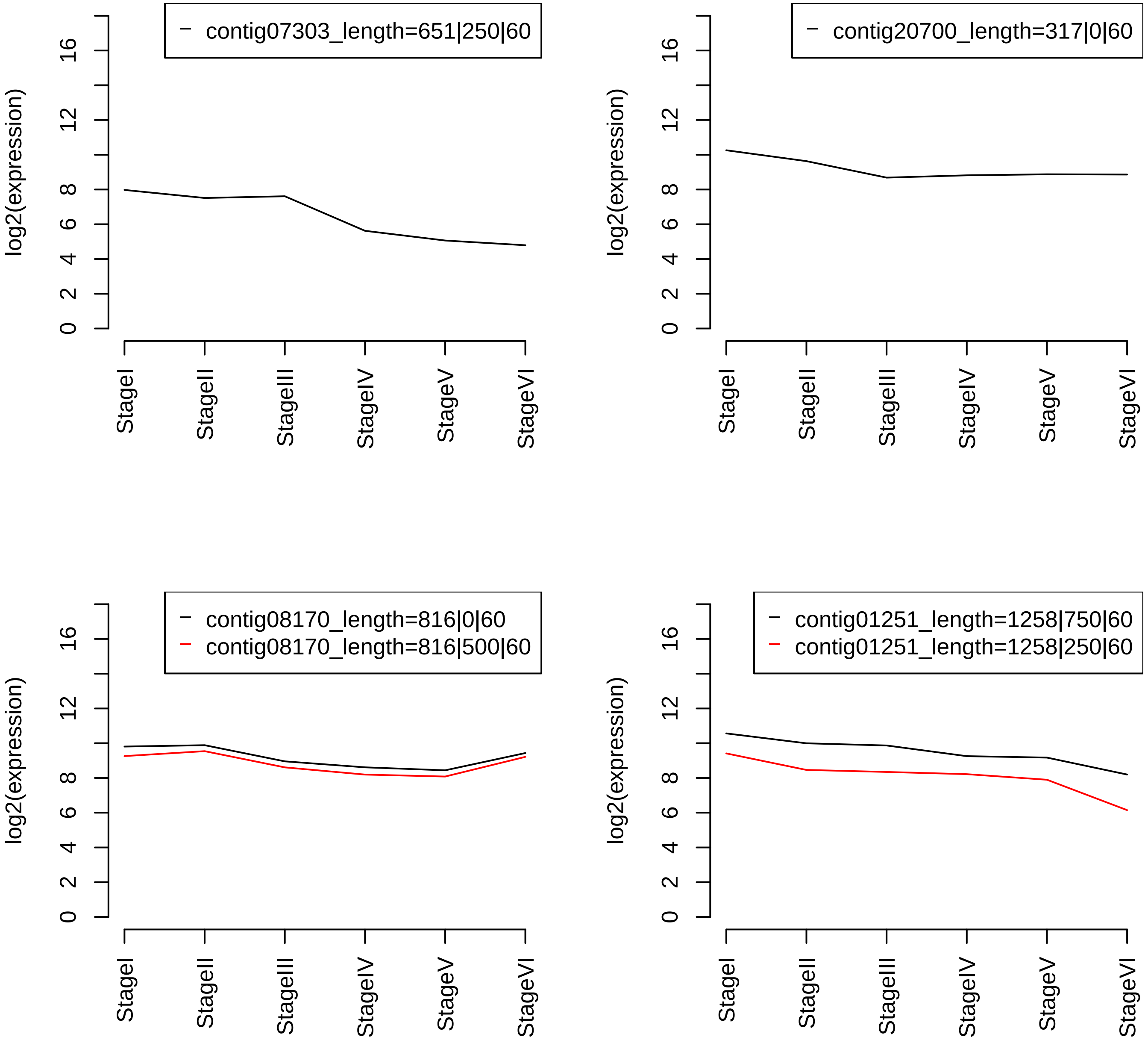
contig07303_length=651|250|60
contig20700_length=317|0|60
contig08170_length=816|0|60
contig08170_length=816|500|60
contig01251_length=1258|750|60
contig01251_length=1258|250|60
log2(expression)
0
2
4
6
8
12
16
StageI
StageII
StageIII
StageIV
StageV
StageVI

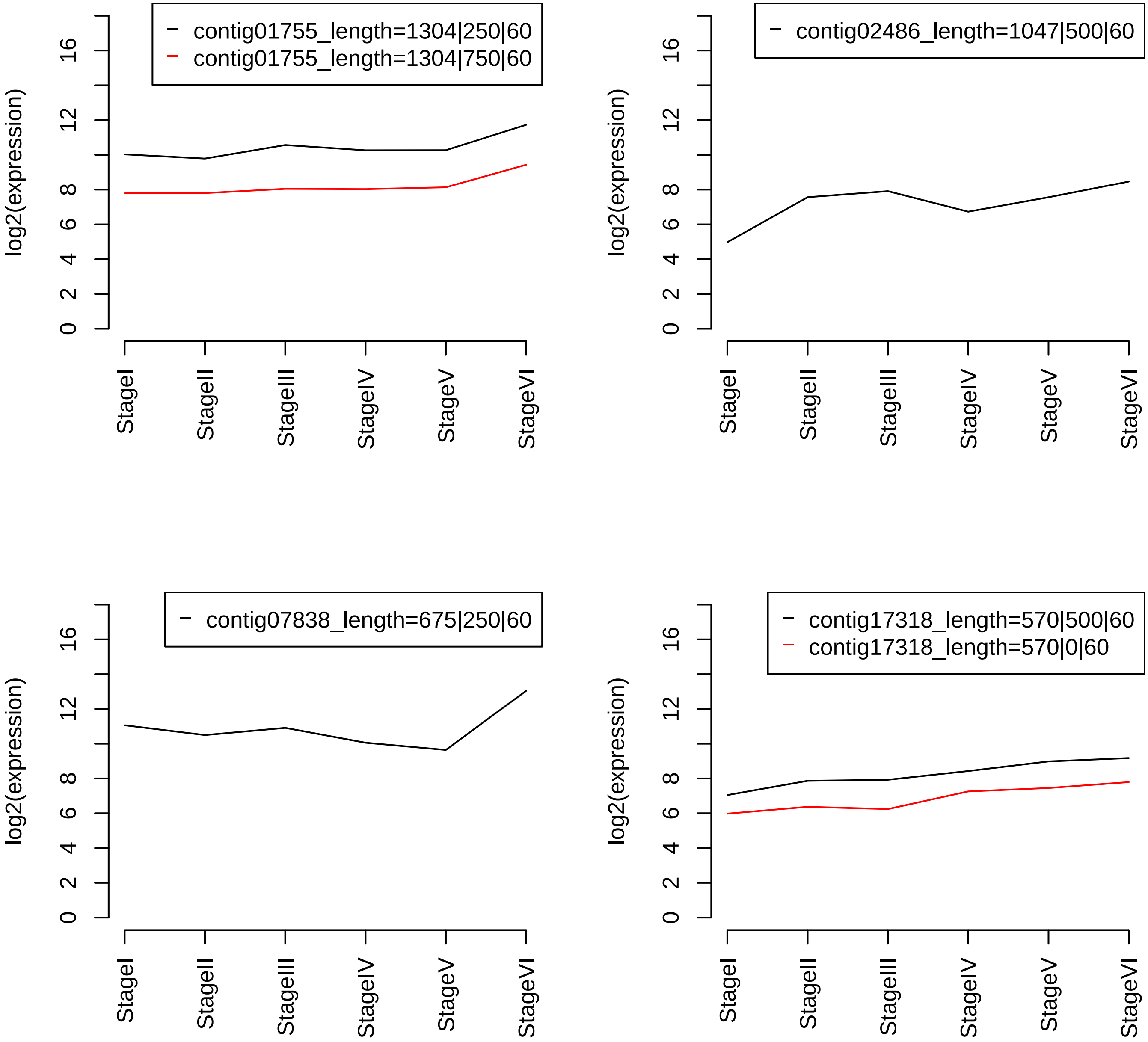
contig01755_length=1304|250|60
contig01755_length=1304|750|60
contig02486_length=1047|500|60
contig07838_length=675|250|60
contig17318_length=570|500|60
contig17318_length=570|0|60
log2(expression)
0
2
4
6
8
12
16
StageI
StageII
StageIII
StageIV
StageV
StageVI

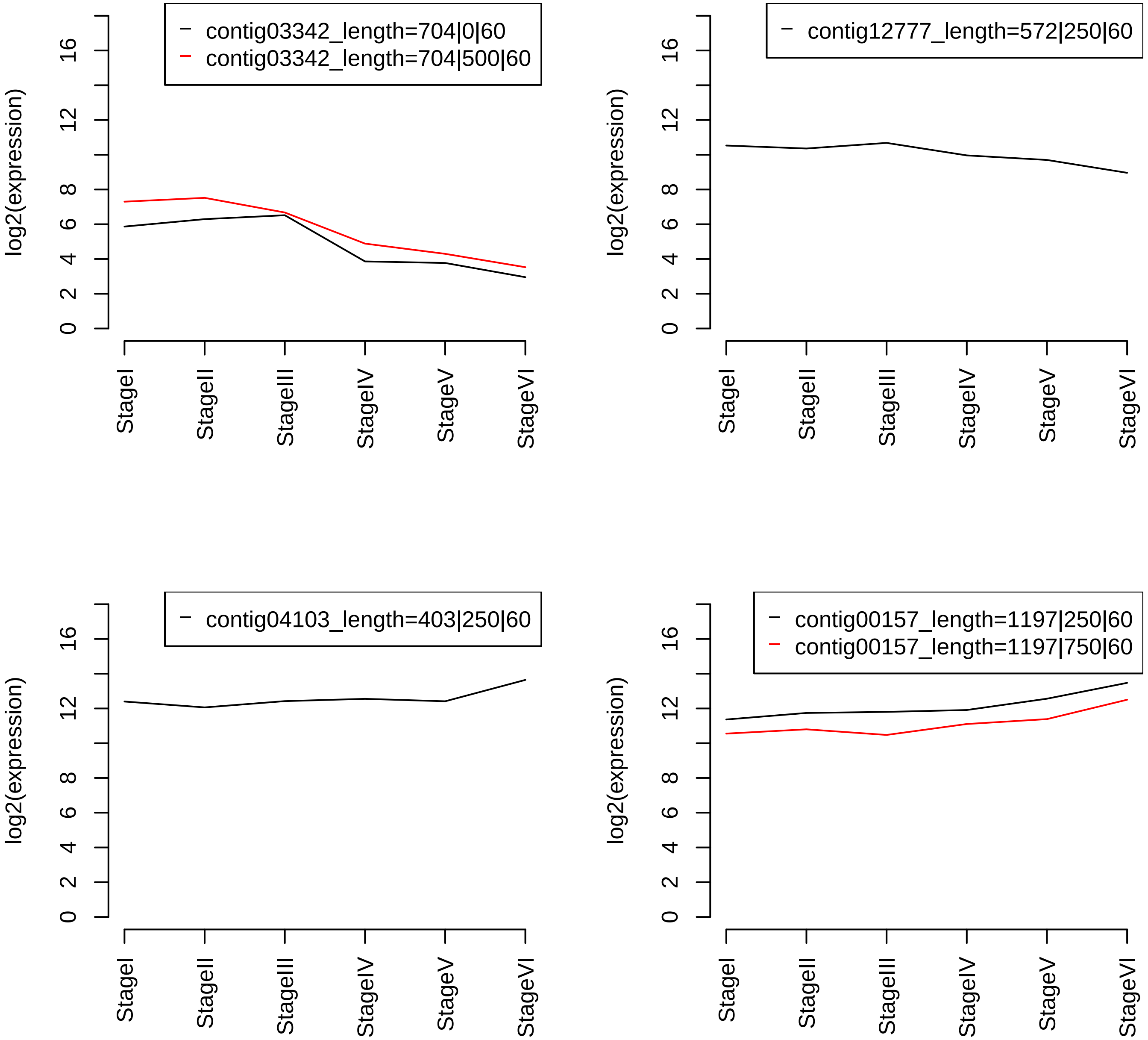
contig03342_length=704|0|60
contig03342_length=704|500|60
contig12777_length=572|250|60
contig04103_length=403|250|60
contig00157_length=1197|250|60
contig00157_length=1197|750|60
log2(expression)
0
2
4
6
8
12
16
StageI
StageII
StageIII
StageIV
StageV
StageVI

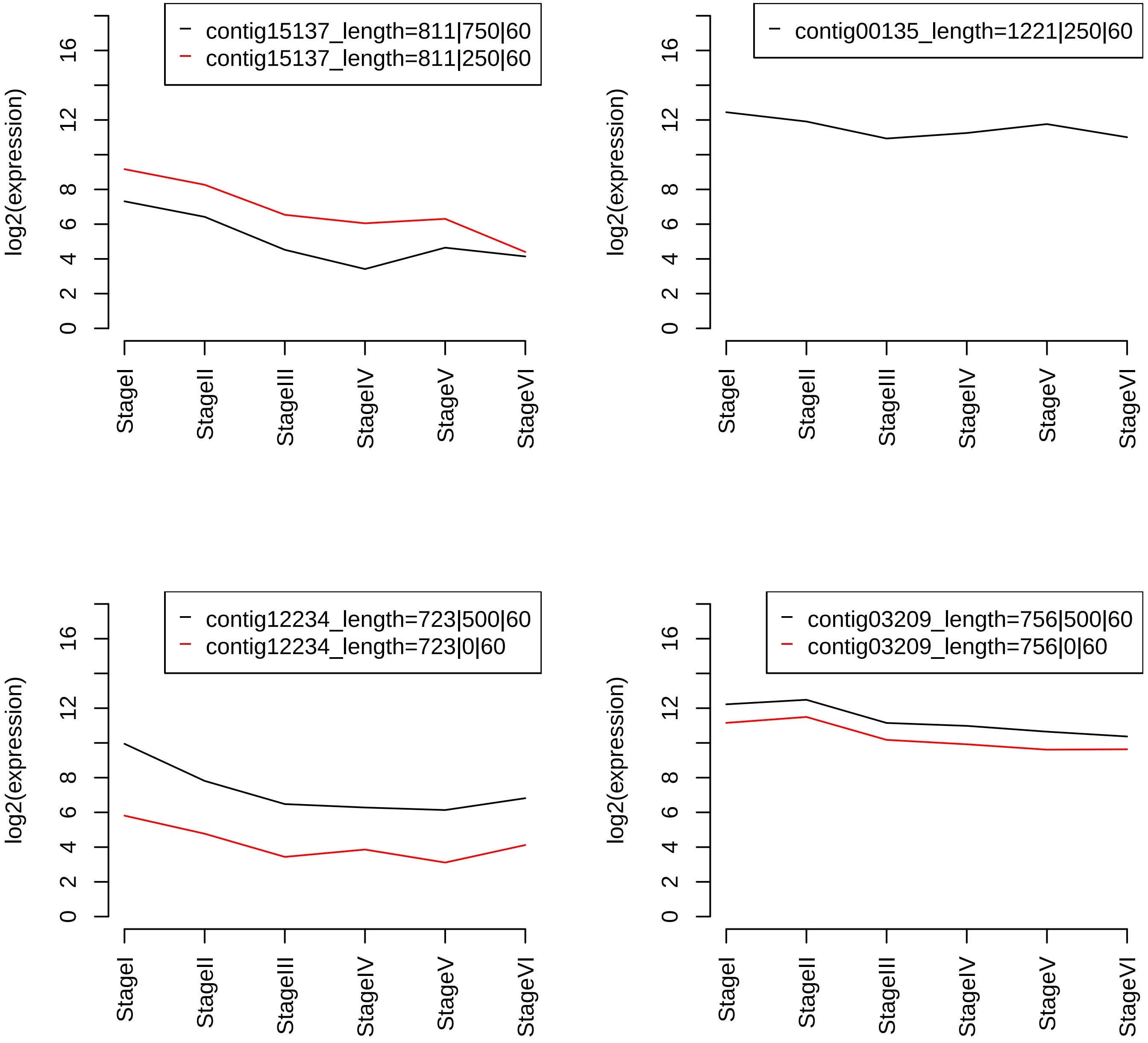
contig15137_length=811|750|60
contig15137_length=811|250|60
contig00135_length=1221|250|60
contig12234_length=723|500|60
contig12234_length=723|0|60
contig03209_length=756|500|60
contig03209_length=756|0|60
log2(expression)
0
2
4
6
8
12
16
StageI
StageII
StageIII
StageIV
StageV
StageVI

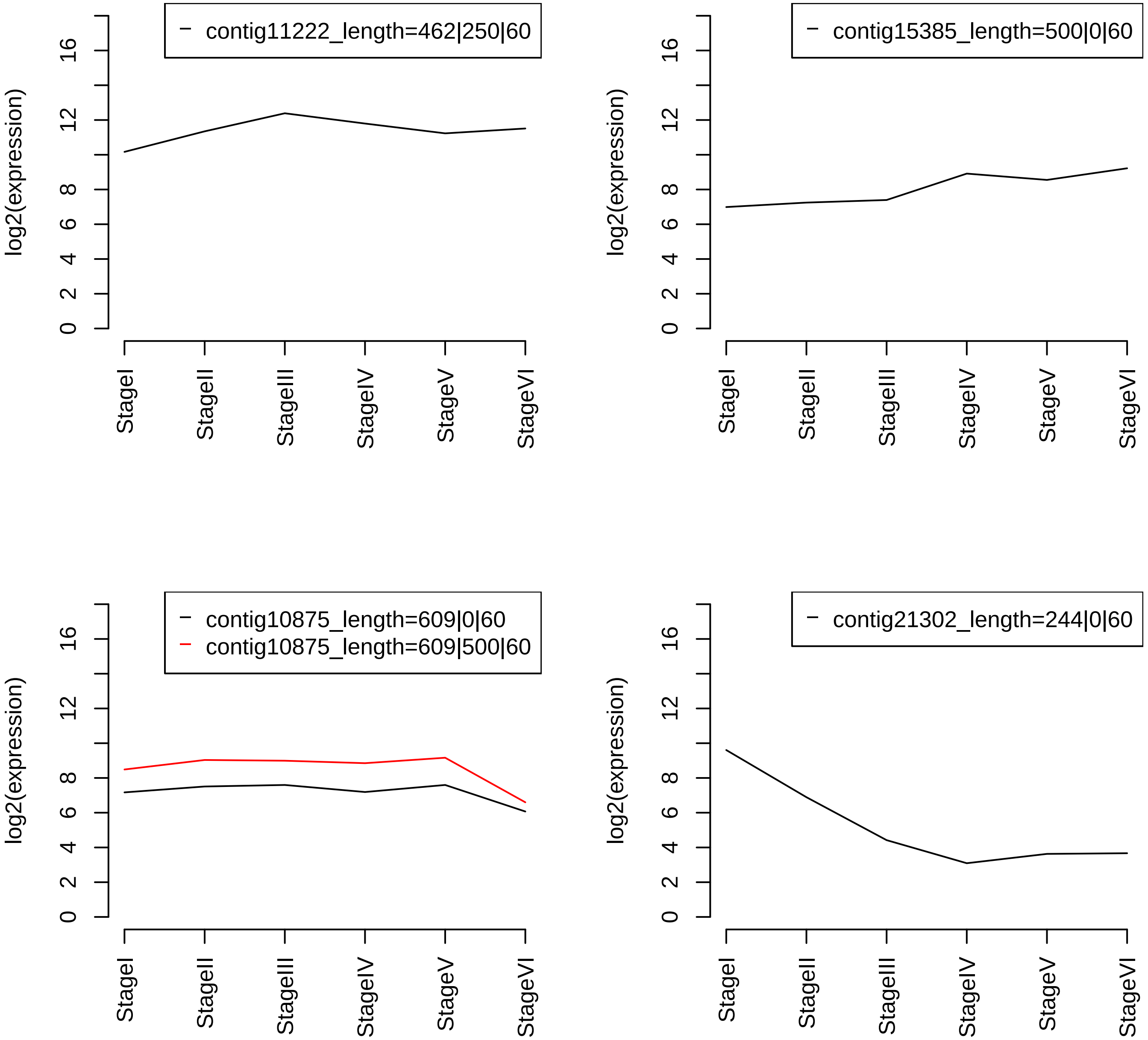
contig11222_length=462|250|60
contig15385_length=500|0|60
contig10875_length=609|0|60
contig10875_length=609|500|60
contig21302_length=244|0|60
log2(expression)
0
2
4
6
8
12
16
StageI
StageII
StageIII
StageIV
StageV
StageVI

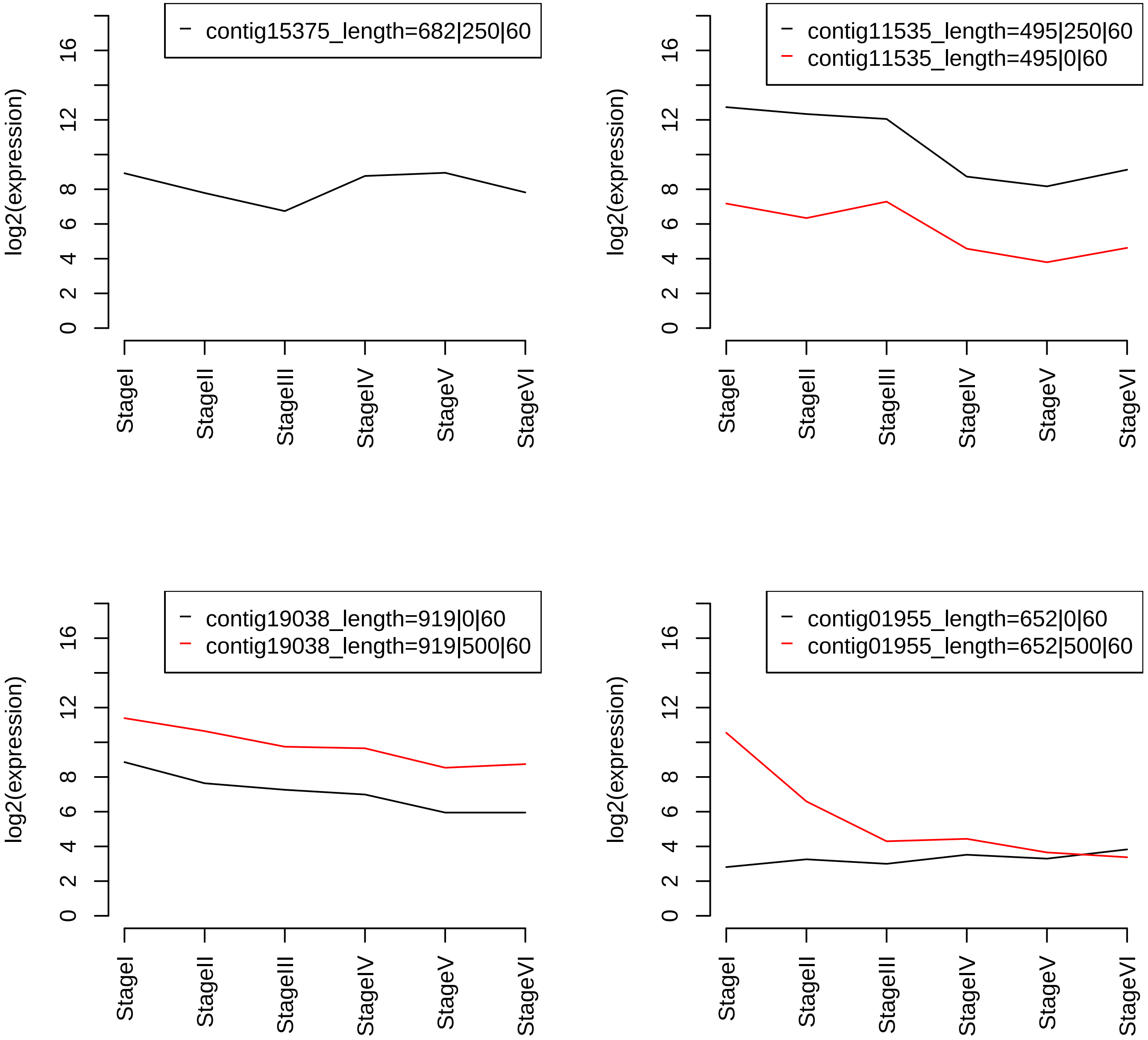

contig15375_length=682|250|60
contig11535_length=495|250|60
contig11535_length=495|0|60
contig19038_length=919|0|60
contig19038_length=919|500|60
contig01955_length=652|0|60
contig01955_length=652|500|60
log2(expression)
0 2 4 6 8 12 16
StageI StageII StageIII StageIV StageV StageVI

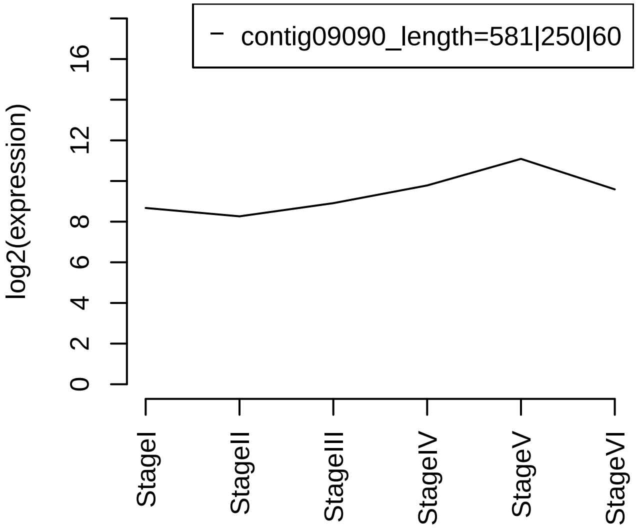
contig09090_length=581|250|60
log2(expression)
0
2
4
6
8
12
16
StageI
StageII
StageIII
StageIV
StageV
StageVI

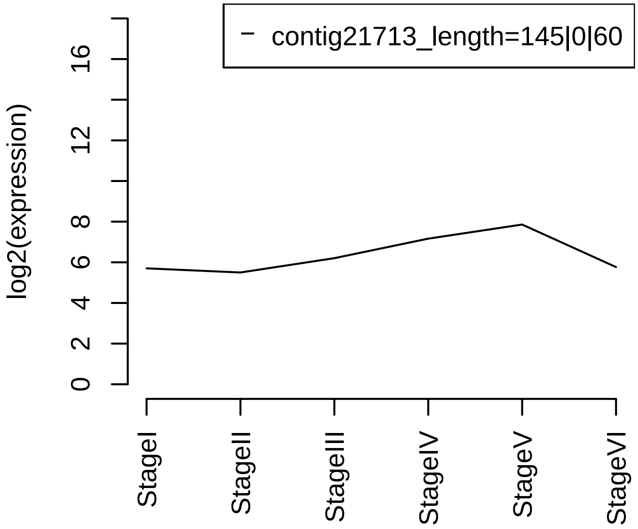
contig21713_length=145|0|60
log2(expression)
0
2
4
6
8
12
16
StageI
StageII
StageIII
StageIV
StageV
StageVI

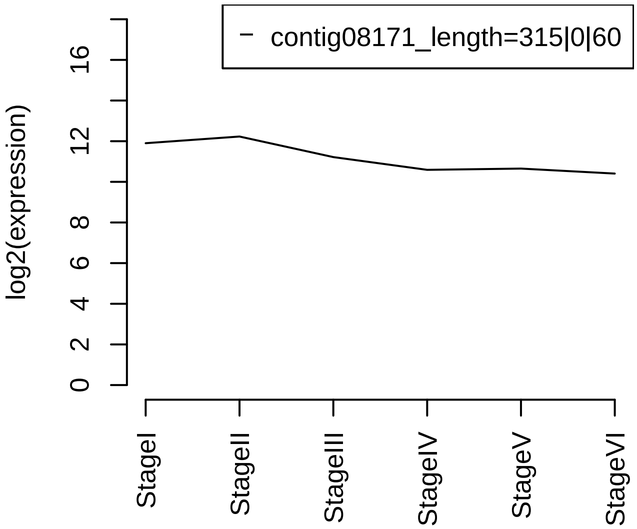
contig08171_length=315|0|60
log2(expression)
0
2
4
6
8
12
16
StageI
StageII
StageIII
StageIV
StageV
StageVI

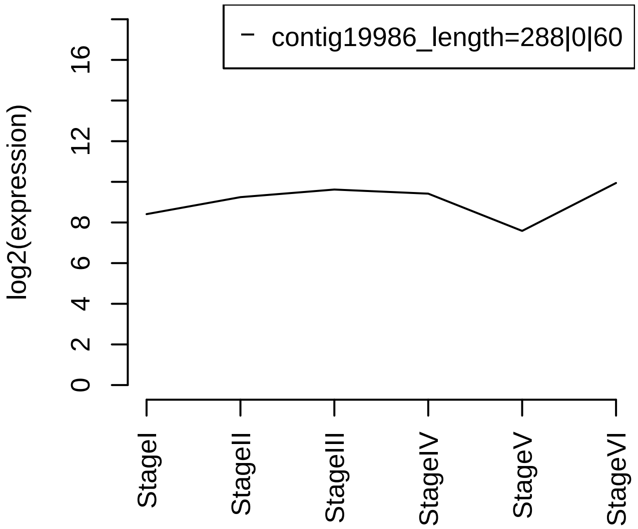
contig19986_length=288|0|60
log2(expression)
0
2
4
6
8
12
16
StageI
StageII
StageIII
StageIV
StageV
StageVI

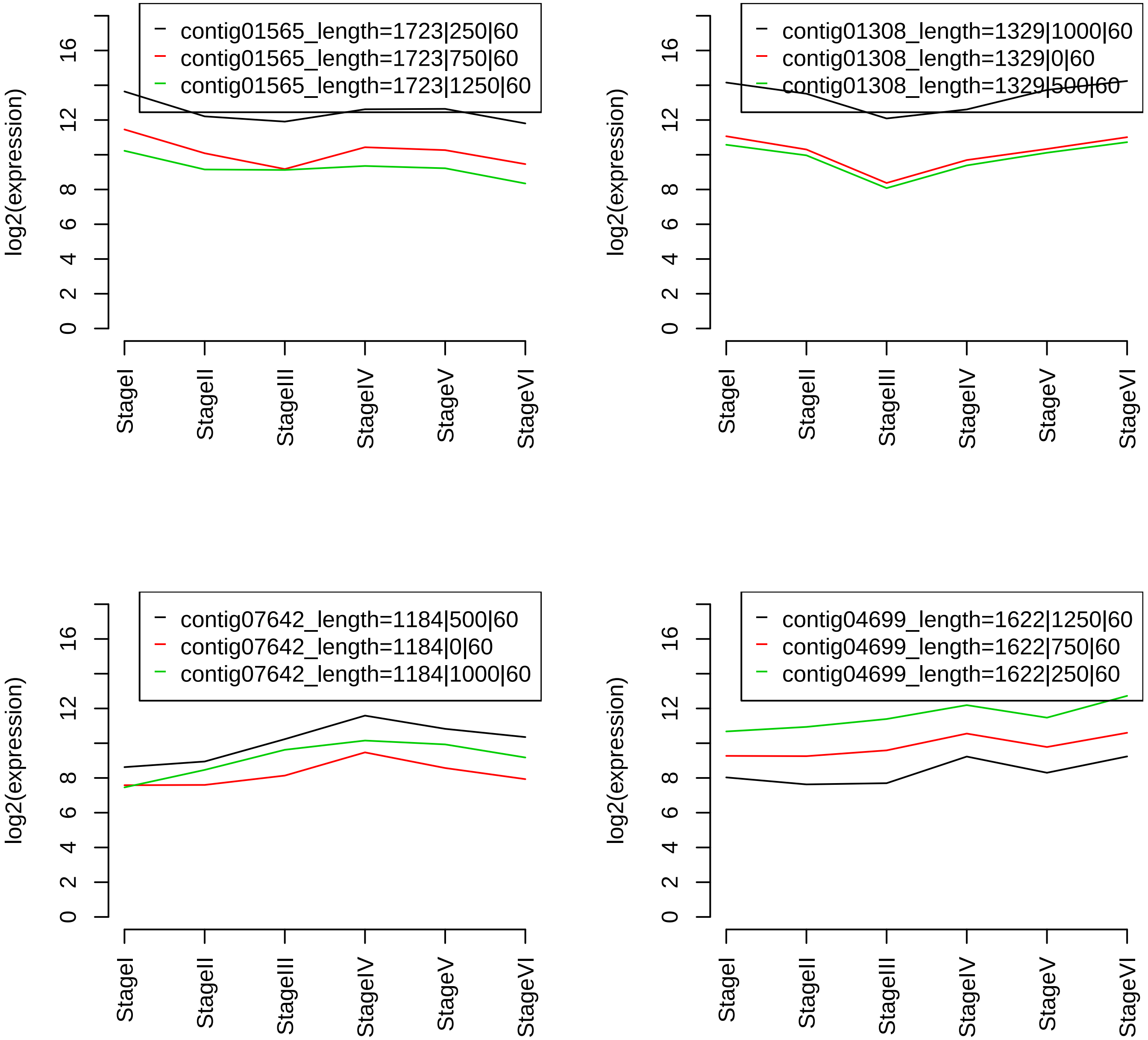

contig01565_length=1723|250|60
contig01565_length=1723|750|60
contig01565_length=1723|1250|60
contig01308_length=1329|1000|60
contig01308_length=1329|0|60
contig01308_length=1329|500|60
contig07642_length=1184|500|60
contig07642_length=1184|0|60
contig07642_length=1184|1000|60
contig04699_length=1622|1250|60
contig04699_length=1622|750|60
contig04699_length=1622|250|60
log2(expression)
0
2
4
6
8
12
16
StageI
StageII
StageIII
StageIV
StageV
StageVI

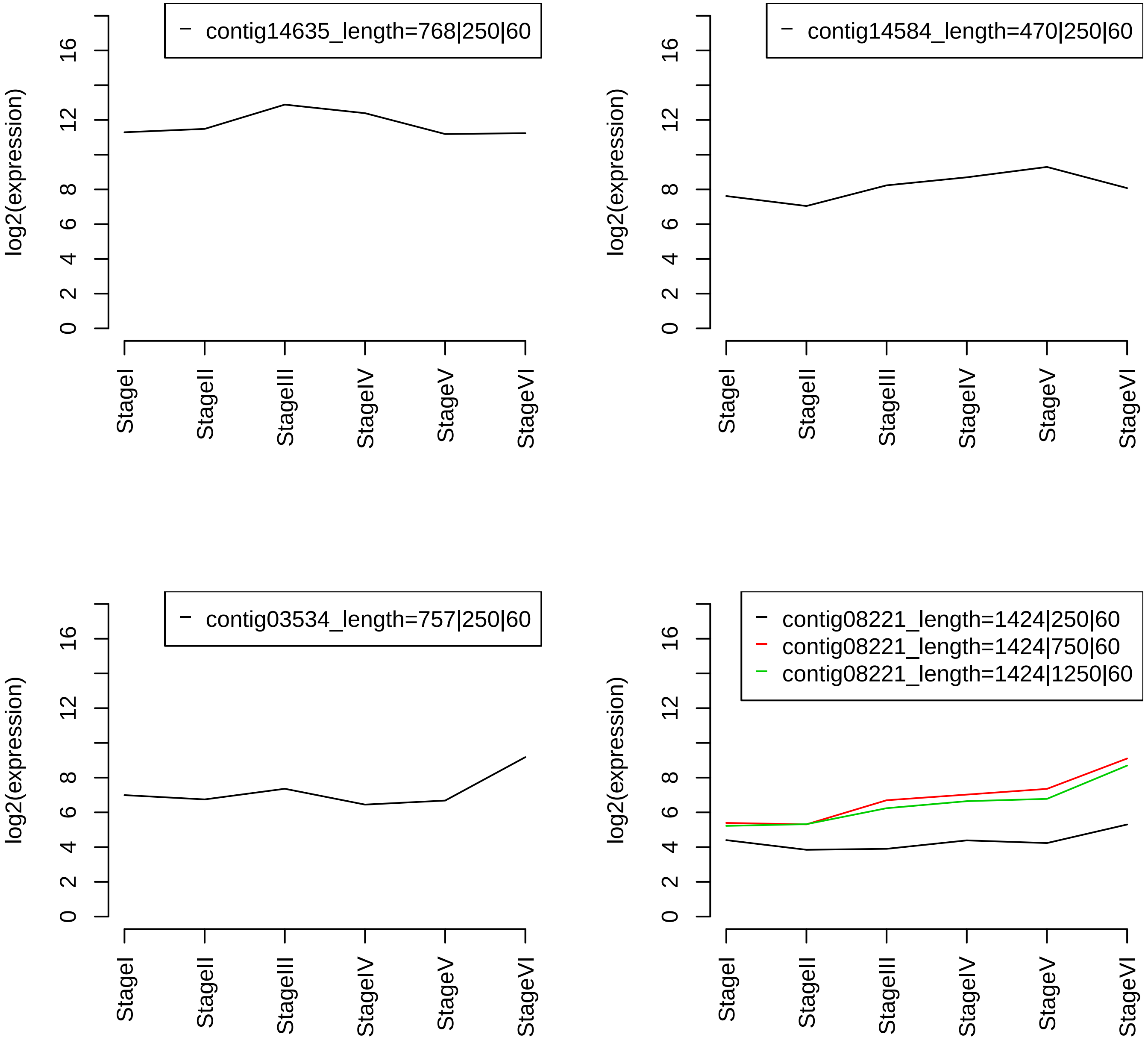
contig14635_length=768|250|60
contig14584_length=470|250|60
contig03534_length=757|250|60
contig08221_length=1424|250|60
contig08221_length=1424|750|60
contig08221_length=1424|1250|60
log2(expression)
0
2
4
6
8
12
16
StageI
StageII
StageIII
StageIV
StageV
StageVI

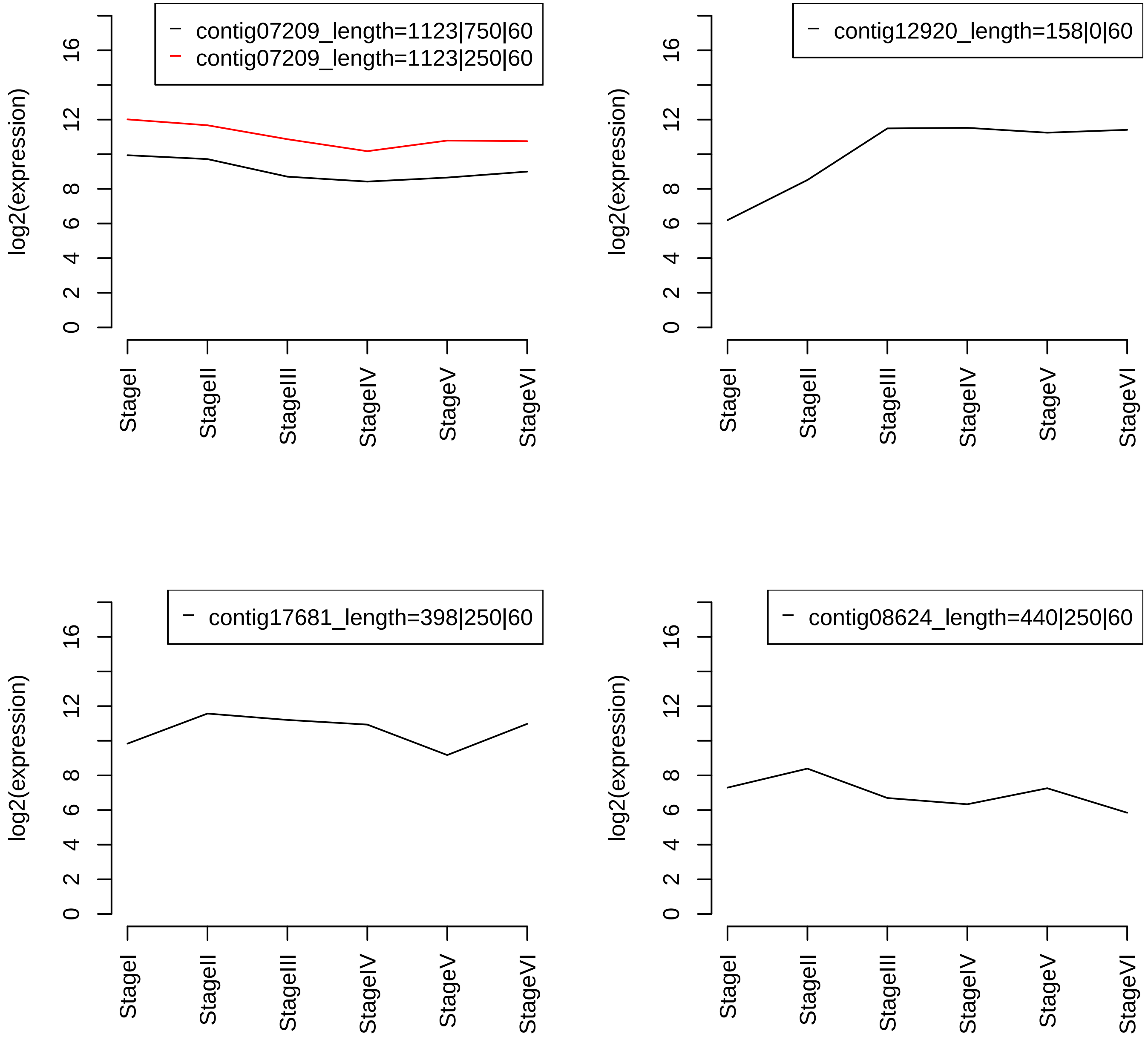

contig07209_length=1123|750|60
contig07209_length=1123|250|60
contig12920_length=158|0|60
contig17681_length=398|250|60
contig08624_length=440|250|60
log2(expression)
0 2 4 6 8 12 16
StageI StageII StageIII StageIV StageV StageVI

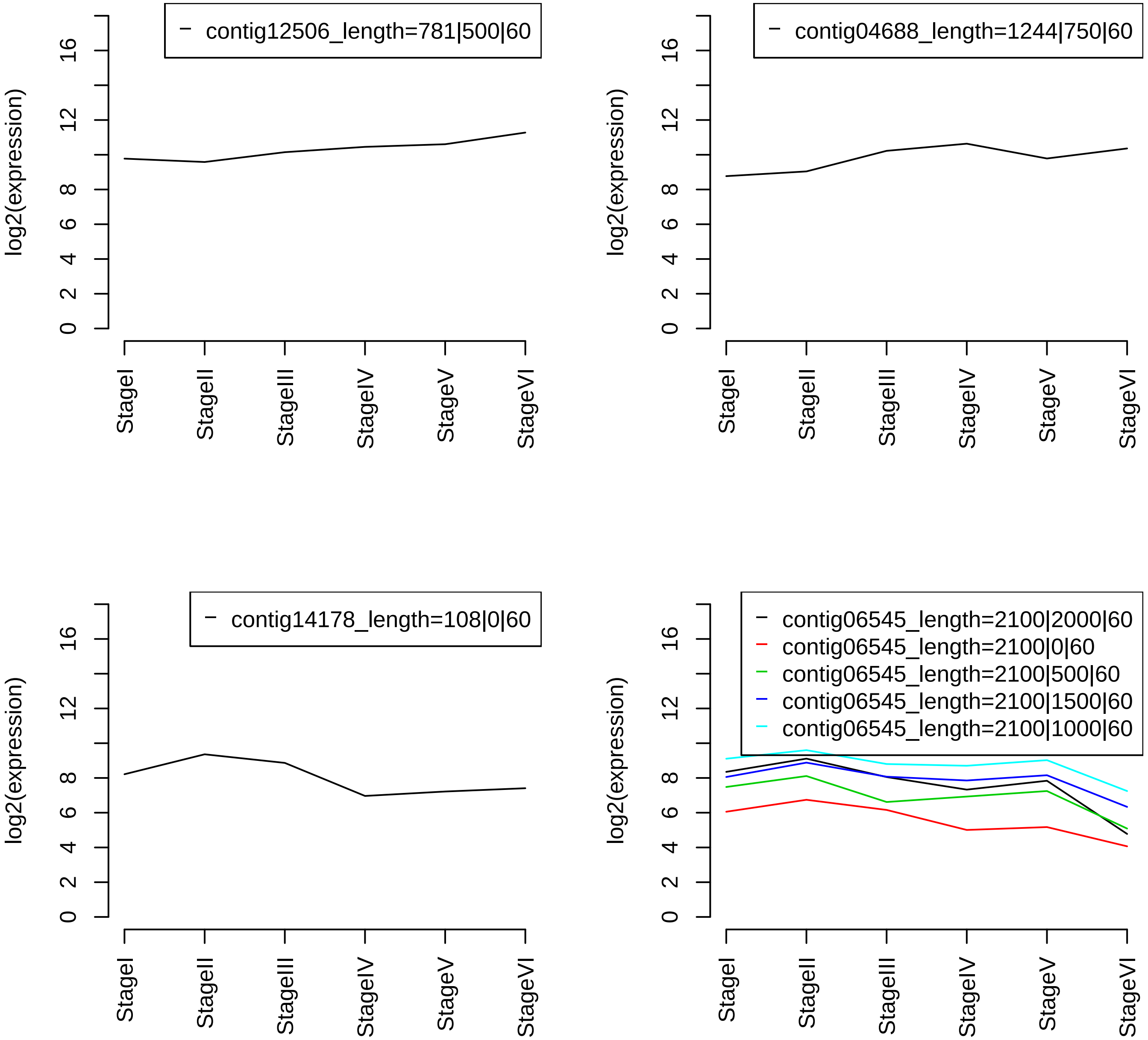
contig12506_length=781|500|60
contig04688_length=1244|750|60
contig14178_length=108|0|60
contig06545_length=2100|2000|60
contig06545_length=2100|0|60
contig06545_length=2100|500|60
contig06545_length=2100|1500|60
contig06545_length=2100|1000|60
log2(expression)
0
2
4
6
8
12
16
StageI
StageII
StageIII
StageIV
StageV
StageVI

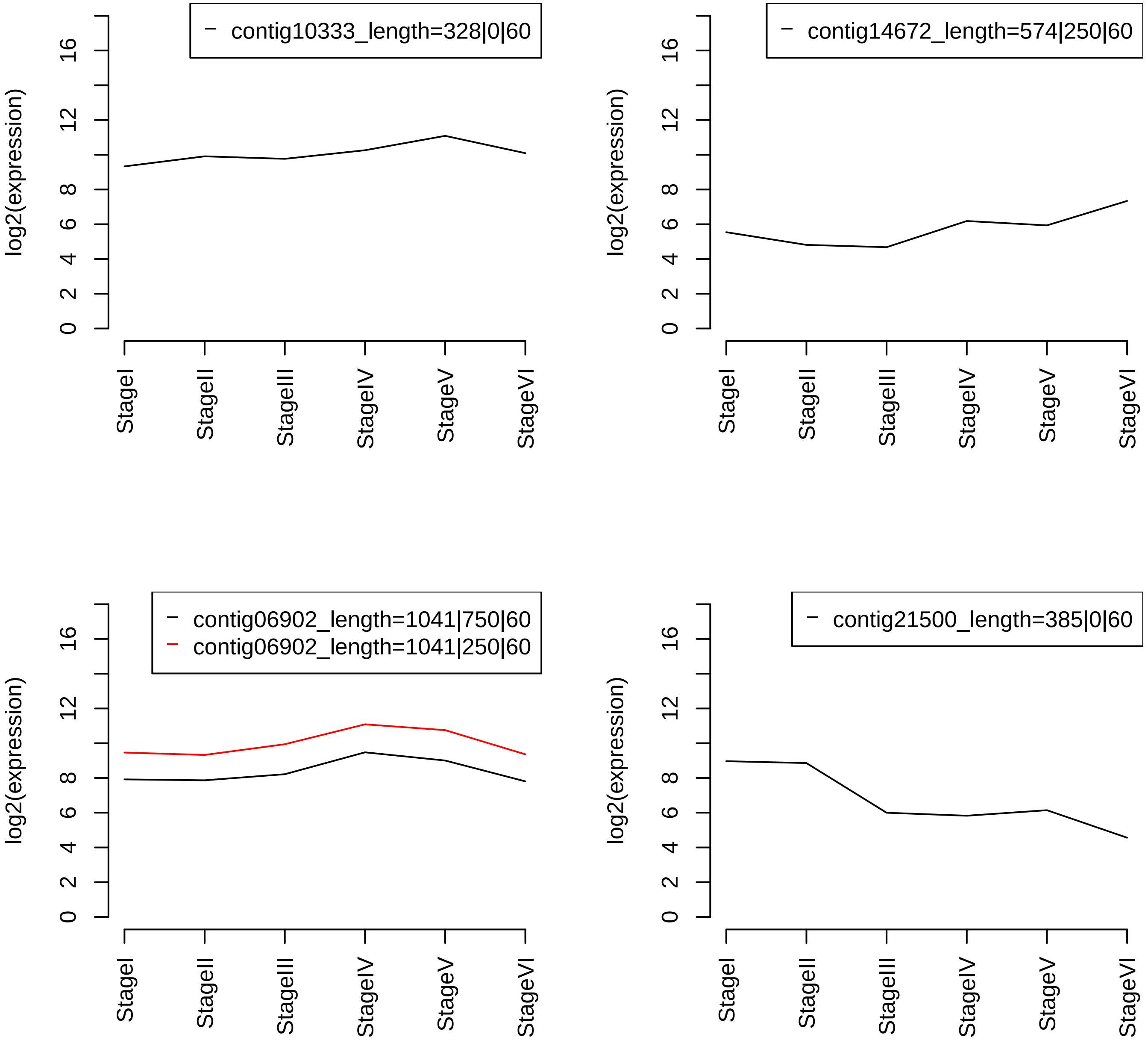
contig10333_length=328|0|60
contig14672_length=574|250|60
contig06902_length=1041|750|60
contig06902_length=1041|250|60
contig21500_length=385|0|60
log2(expression)
0 2 4 6 8 12 16
StageI
StageII
StageIII
StageIV
StageV
StageVI

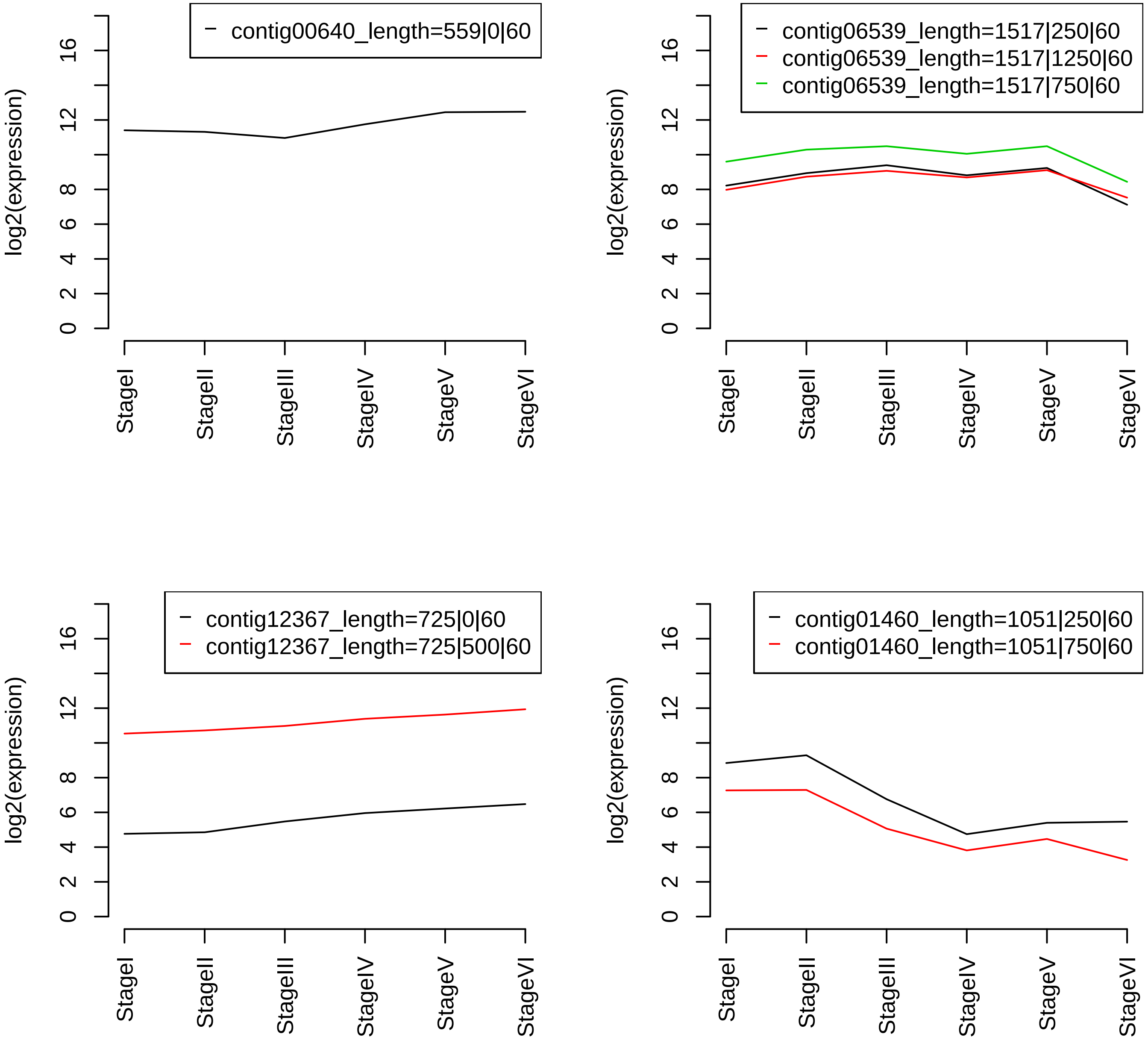

contig00640_length=559|0|60
contig06539_length=1517|250|60
contig06539_length=1517|1250|60
contig06539_length=1517|750|60
contig12367_length=725|0|60
contig12367_length=725|500|60
contig01460_length=1051|250|60
contig01460_length=1051|750|60
log2(expression)
0
2
4
6
8
12
16
StageI
StageII
StageIII
StageIV
StageV
StageVI

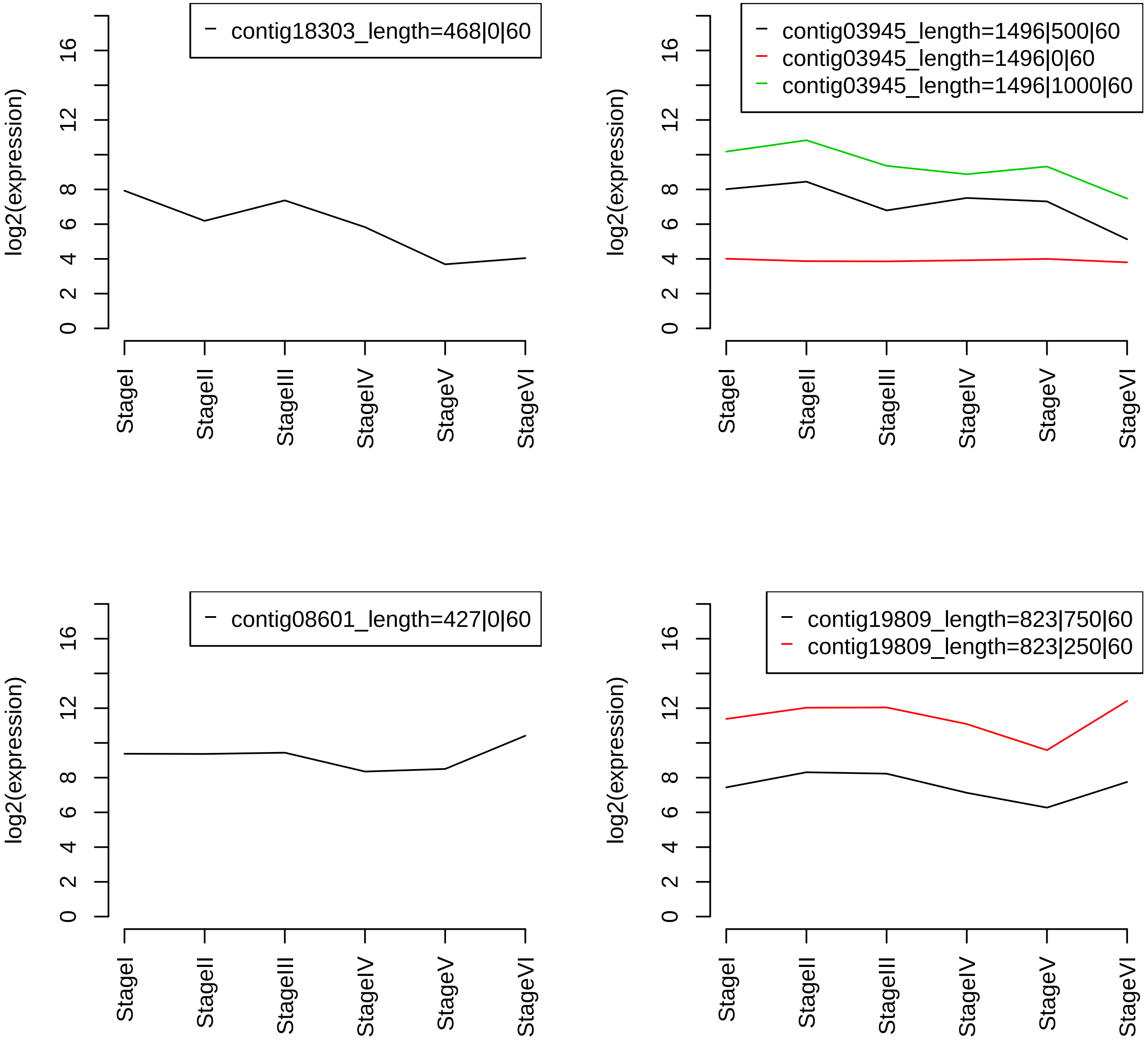

contig18303_length=468|0|60
contig03945_length=1496|500|60
contig03945_length=1496|0|60
contig03945_length=1496|1000|60
contig08601_length=427|0|60
contig19809_length=823|750|60
contig19809_length=823|250|60
log2(expression)
0
2
4
6
8
12
16
StageI
StageII
StageIII
StageIV
StageV
StageVI

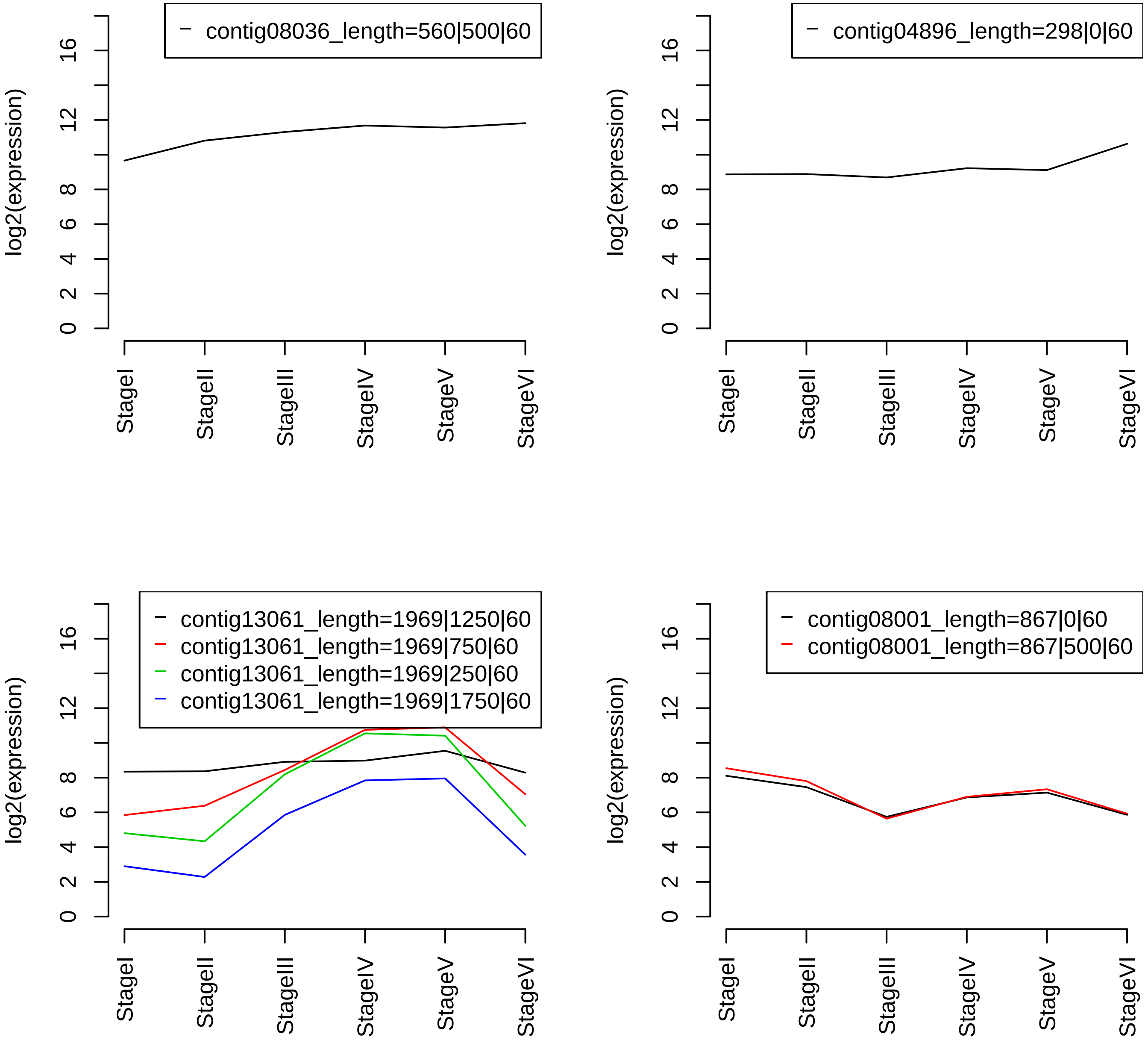
contig08036_length=560|500|60
contig04896_length=298|0|60
contig13061_length=1969|1250|60
contig13061_length=1969|750|60
contig13061_length=1969|250|60
contig13061_length=1969|1750|60
contig08001_length=867|0|60
contig08001_length=867|500|60
log2(expression)
0
2
4
6
8
12
16
StageI
StageII
StageIII
StageIV
StageV
StageVI

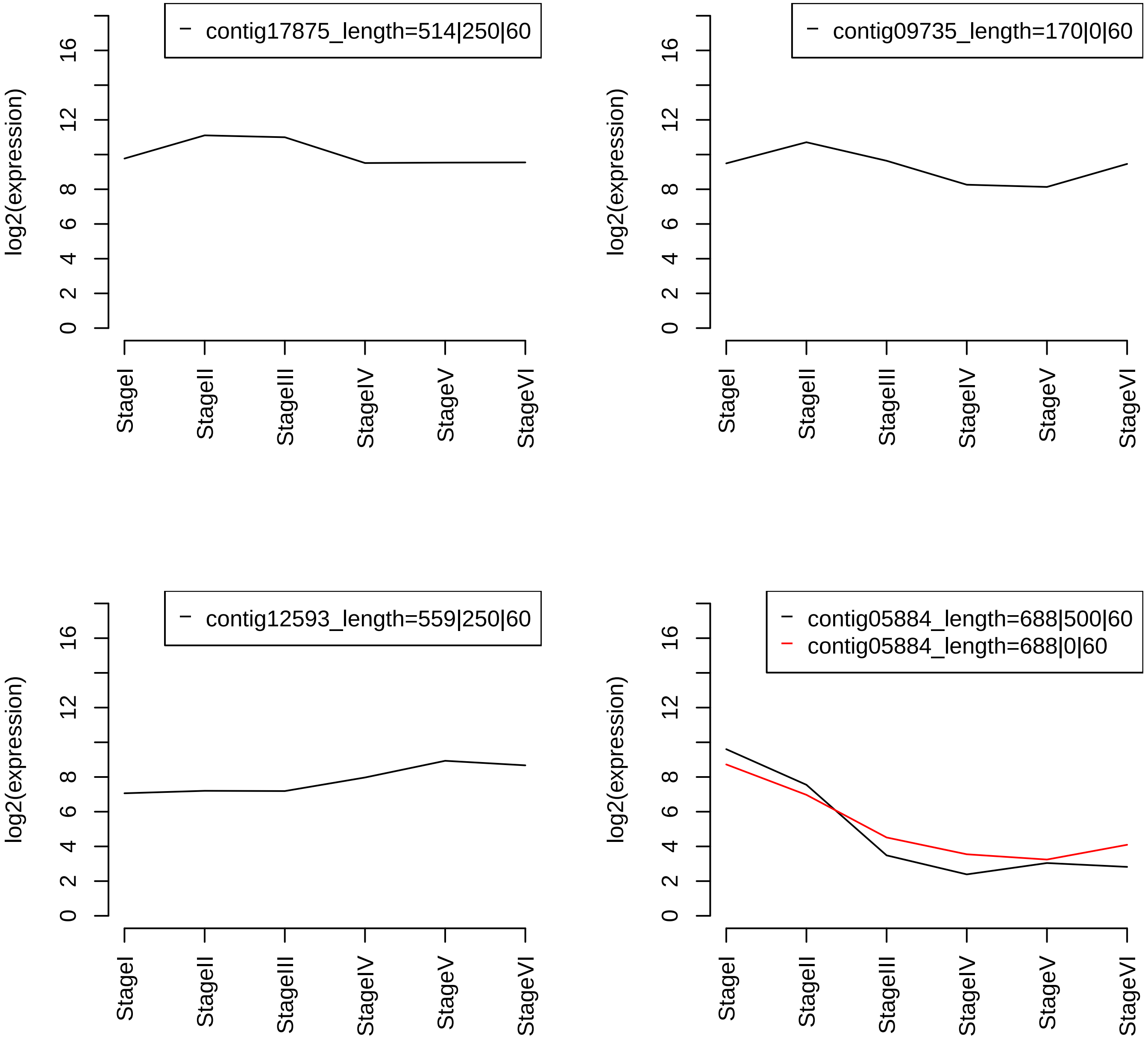
contig17875_length=514|250|60
contig09735_length=170|0|60
contig12593_length=559|250|60
contig05884_length=688|500|60
contig05884_length=688|0|60
log2(expression)
0
2
4
6
8
12
16
StageI
StageII
StageIII
StageIV
StageV
StageVI

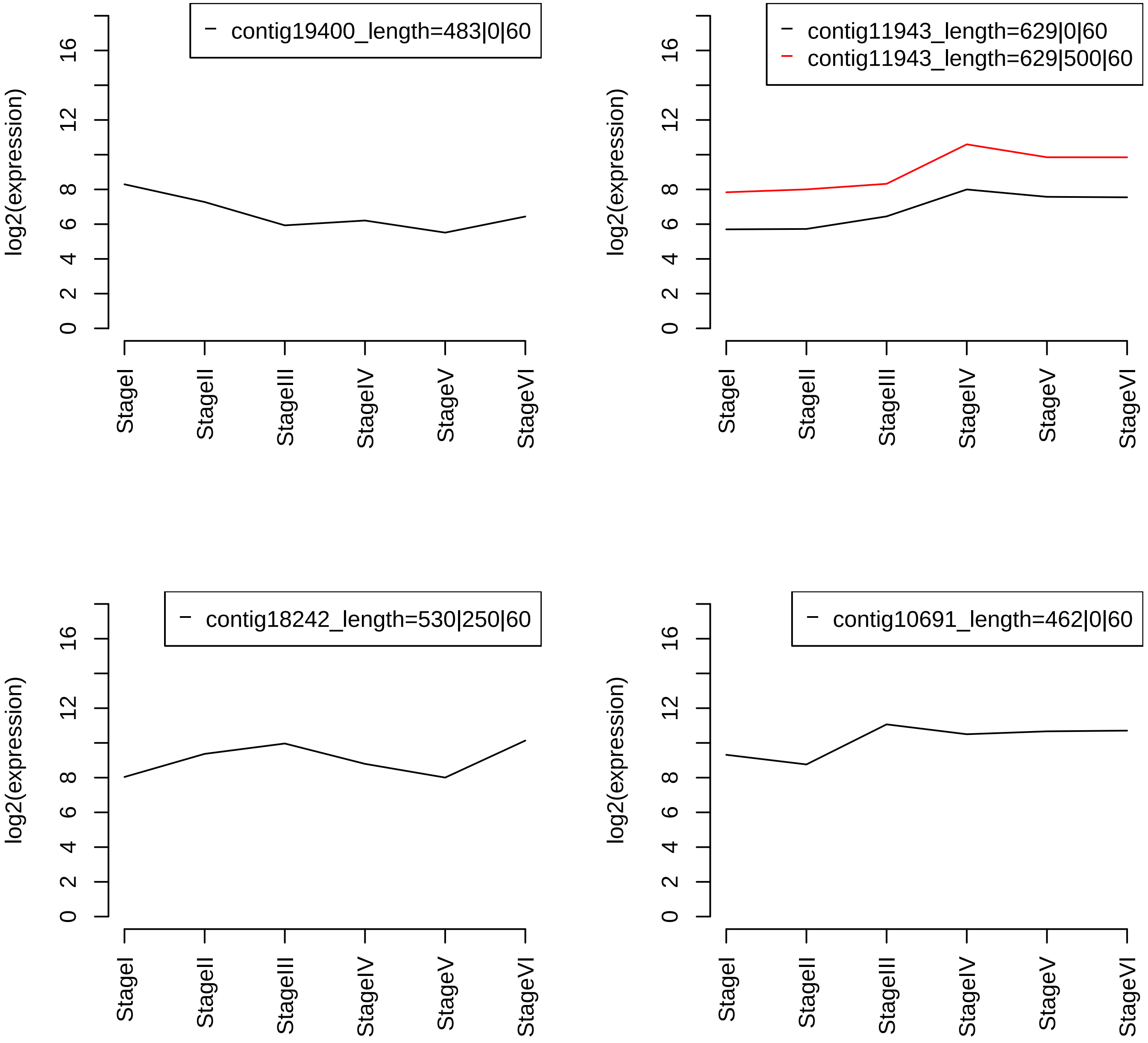
contig19400_length=483|0|60
contig11943_length=629|0|60
contig11943_length=629|500|60
contig18242_length=530|250|60
contig10691_length=462|0|60
log2(expression)
0 2 4 6 8 12 16
StageI StageII StageIII StageIV StageV StageVI

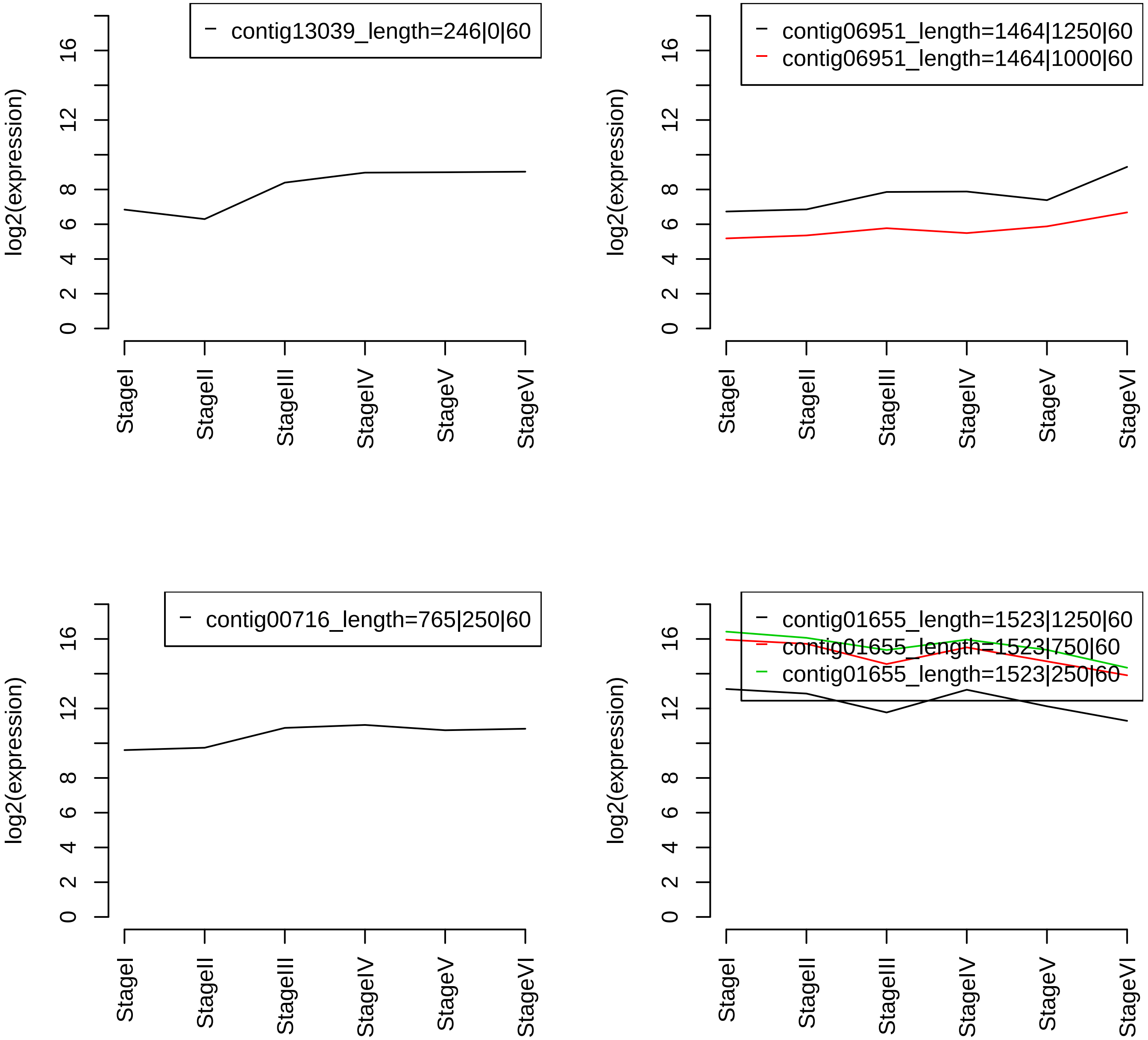

contig13039_length=246|0|60
contig06951_length=1464|1250|60
contig06951_length=1464|1000|60
contig00716_length=765|250|60
contig01655_length=1523|1250|60
contig01655_length=1523|750|60
contig01655_length=1523|250|60
log2(expression)
0 2 4 6 8 12 16
StageI StageII StageIII StageIV StageV StageVI

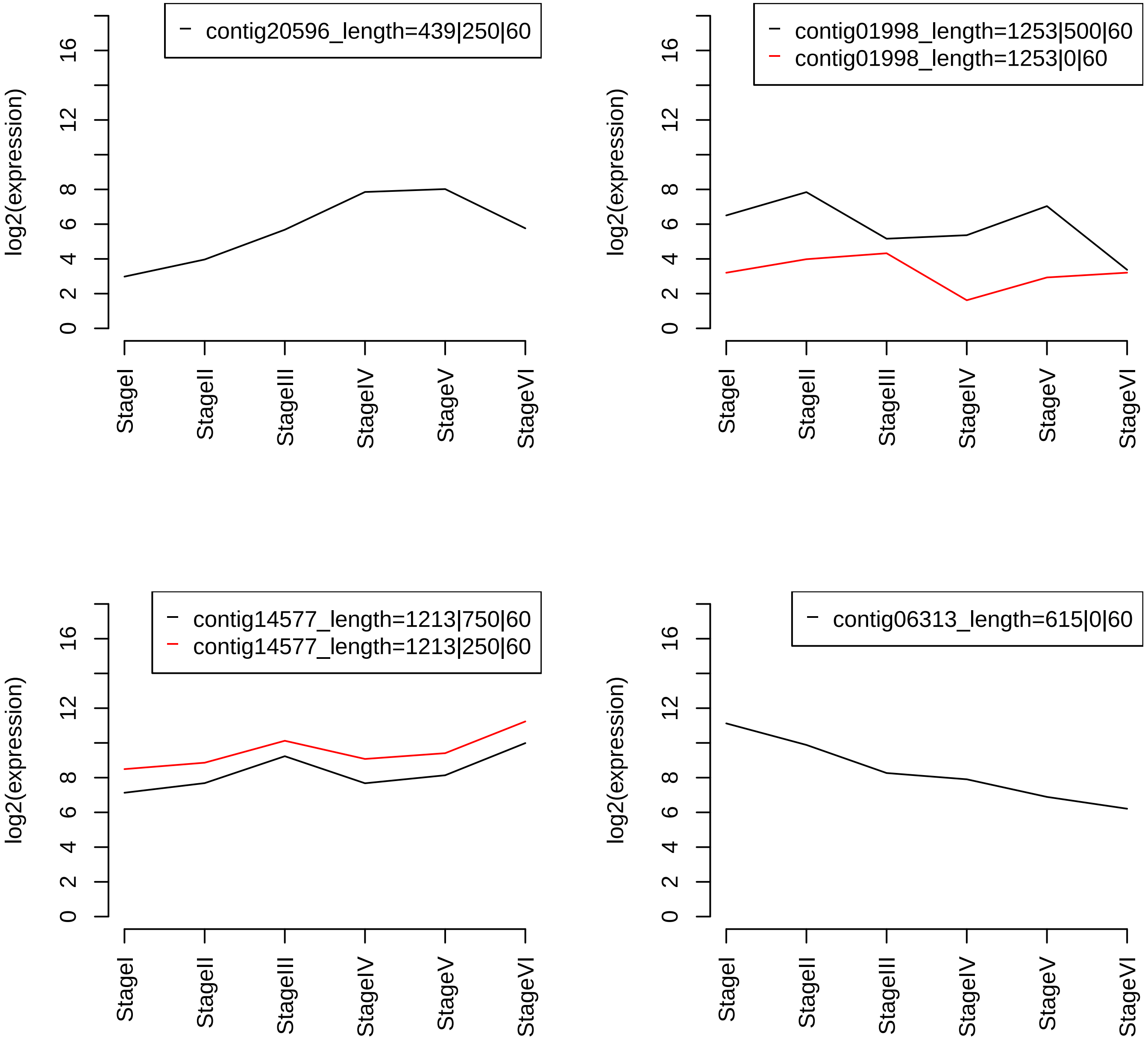

contig20596_length=439|250|60
contig01998_length=1253|500|60
contig01998_length=1253|0|60
contig14577_length=1213|750|60
contig14577_length=1213|250|60
contig06313_length=615|0|60
log2(expression)
0
2
4
6
8
12
16
StageI
StageII
StageIII
StageIV
StageV
StageVI

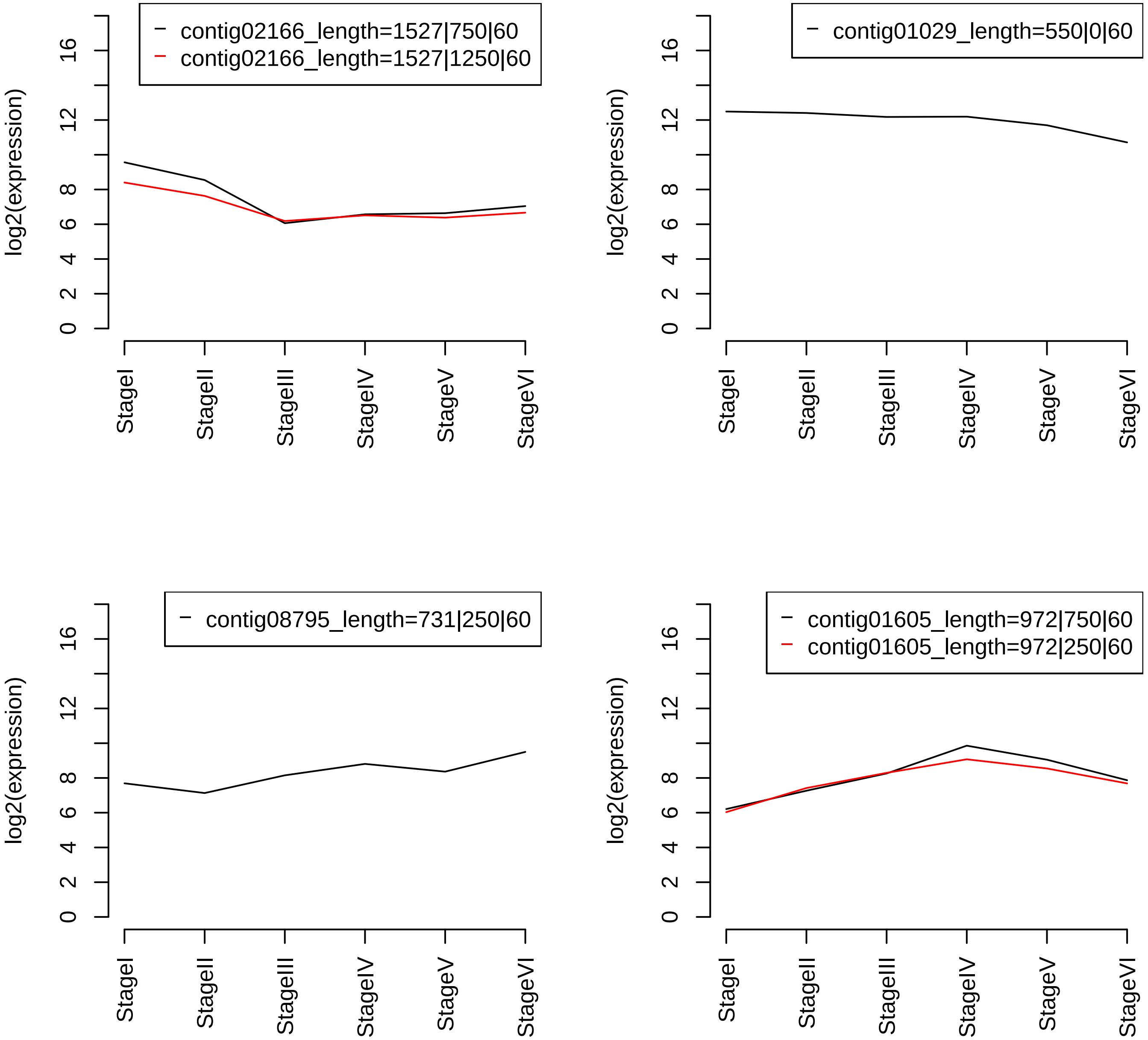

contig02166_length=1527|750|60
contig02166_length=1527|1250|60
contig01029_length=550|0|60
contig08795_length=731|250|60
contig01605_length=972|750|60
contig01605_length=972|250|60
log2(expression)
0
2
4
6
8
12
16
StageI
StageII
StageIII
StageIV
StageV
StageVI

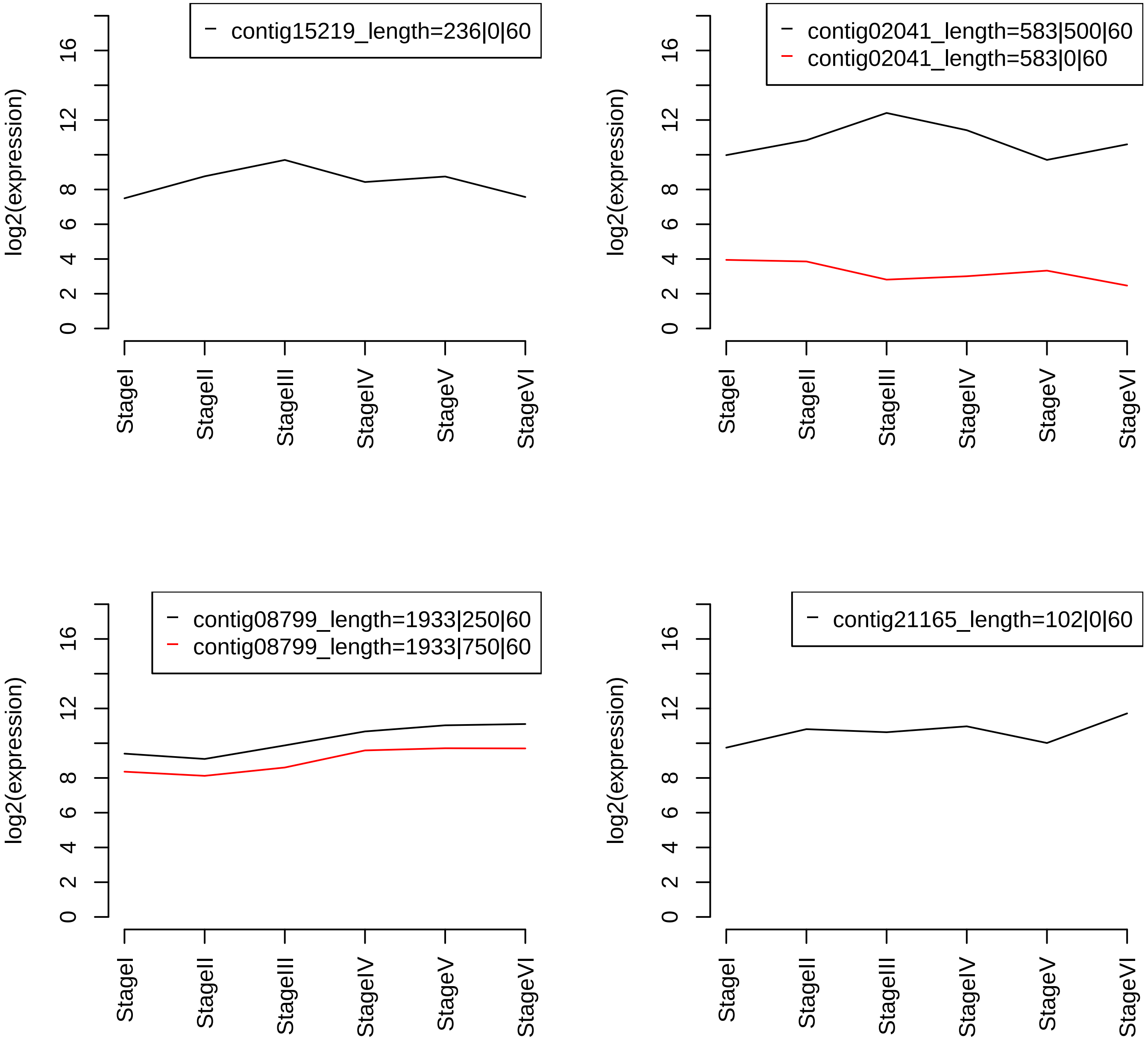
contig15219_length=236|0|60
contig02041_length=583|500|60
contig02041_length=583|0|60
contig08799_length=1933|250|60
contig08799_length=1933|750|60
contig21165_length=102|0|60
log2(expression)
0 2 4 6 8 12 16
StageI StageII StageIII StageIV StageV StageVI

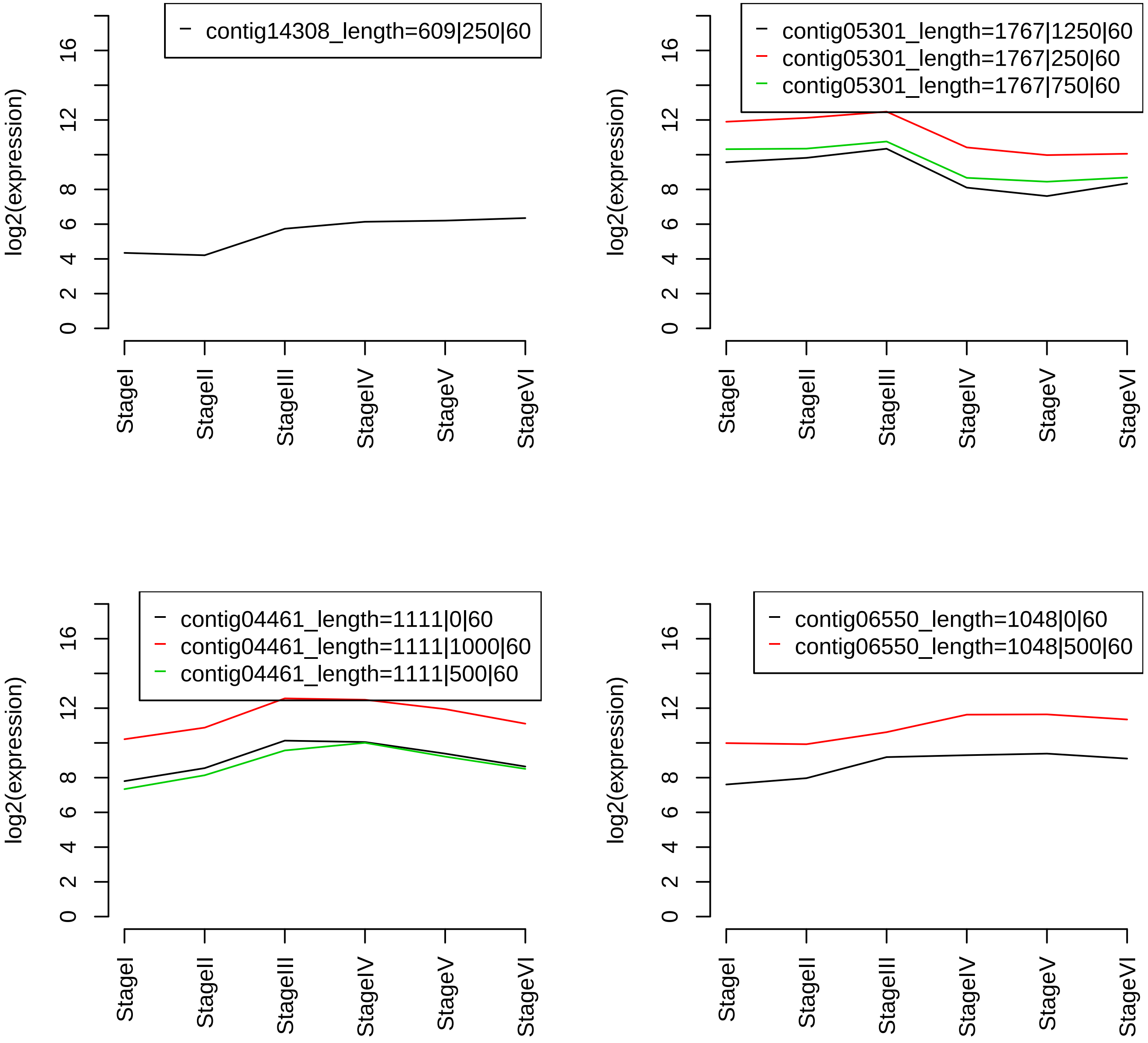

contig14308_length=609|250|60
contig05301_length=1767|1250|60
contig05301_length=1767|250|60
contig05301_length=1767|750|60
contig04461_length=1111|0|60
contig04461_length=1111|1000|60
contig04461_length=1111|500|60
contig06550_length=1048|0|60
contig06550_length=1048|500|60
log2(expression)
0
2
4
6
8
12
16
StageI
StageII
StageIII
StageIV
StageV
StageVI

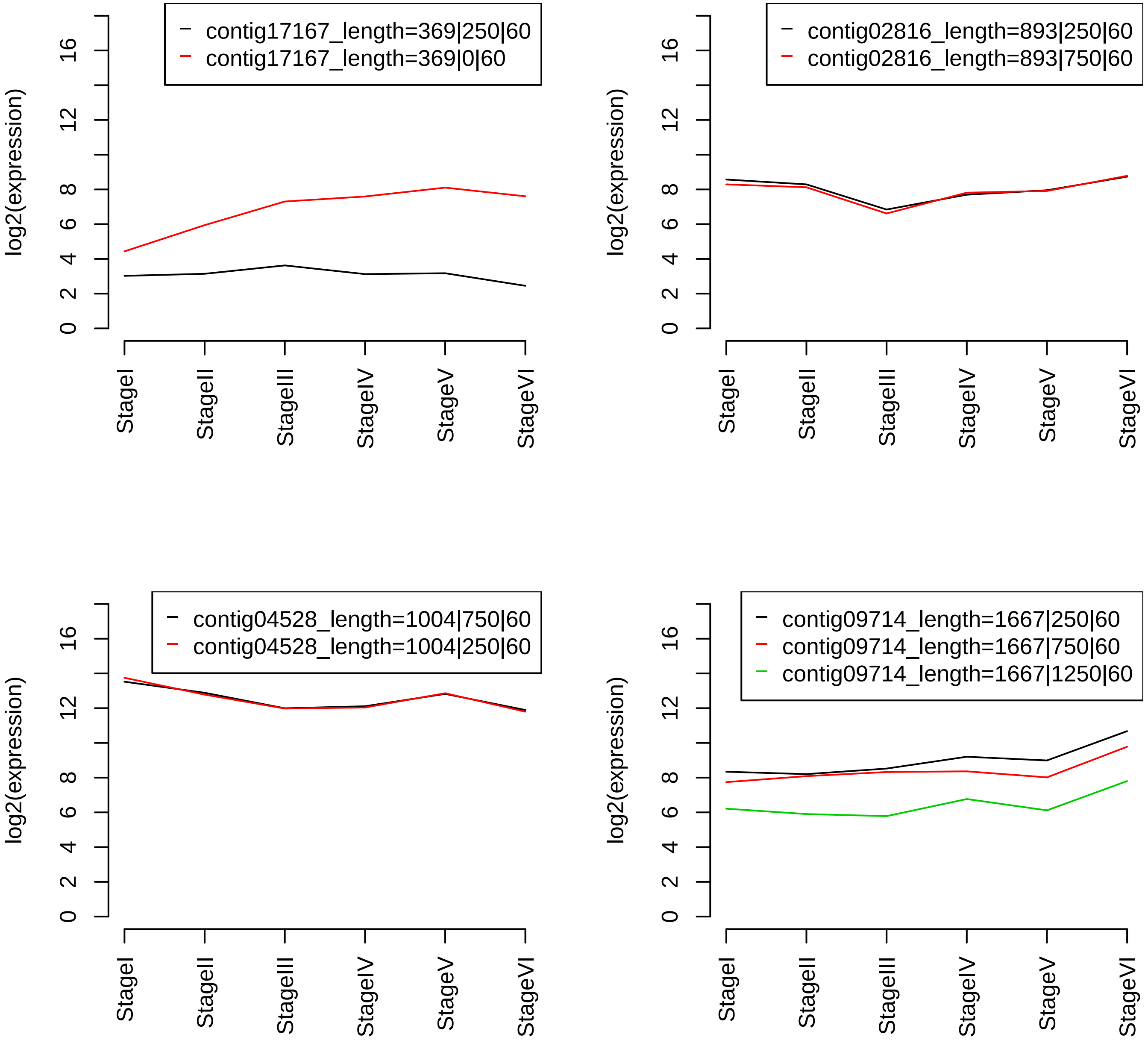
contig17167_length=369|250|60
contig17167_length=369|0|60
contig02816_length=893|250|60
contig02816_length=893|750|60
contig04528_length=1004|750|60
contig04528_length=1004|250|60
contig09714_length=1667|250|60
contig09714_length=1667|750|60
contig09714_length=1667|1250|60
log2(expression)
0 2 4 6 8 12 16
StageI StageII StageIII StageIV StageV StageVI

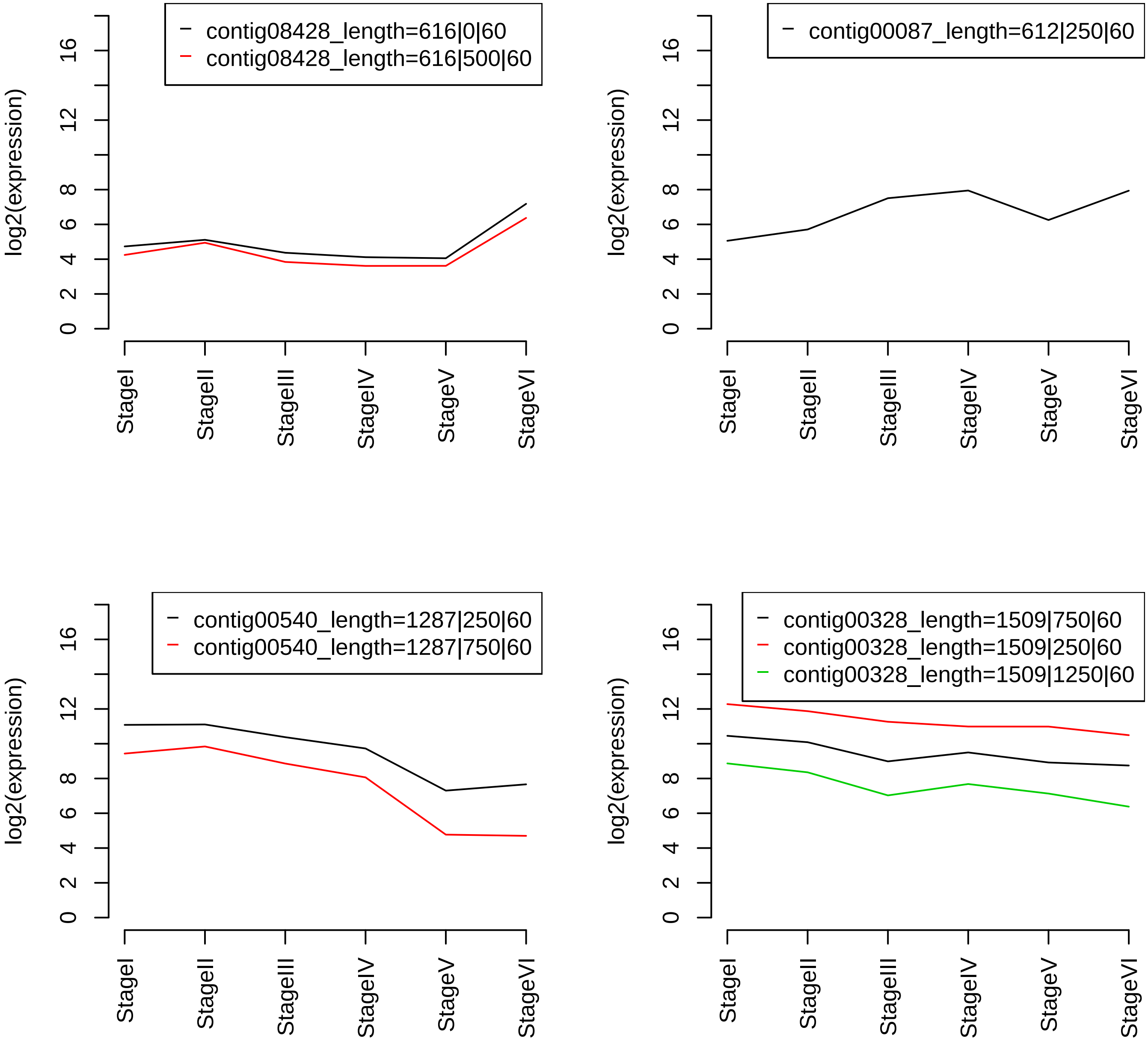

contig08428_length=616|0|60
contig08428_length=616|500|60
contig00087_length=612|250|60
contig00540_length=1287|250|60
contig00540_length=1287|750|60
contig00328_length=1509|750|60
contig00328_length=1509|250|60
contig00328_length=1509|1250|60
log2(expression)
0
2
4
6
8
12
16
StageI
StageII
StageIII
StageIV
StageV
StageVI

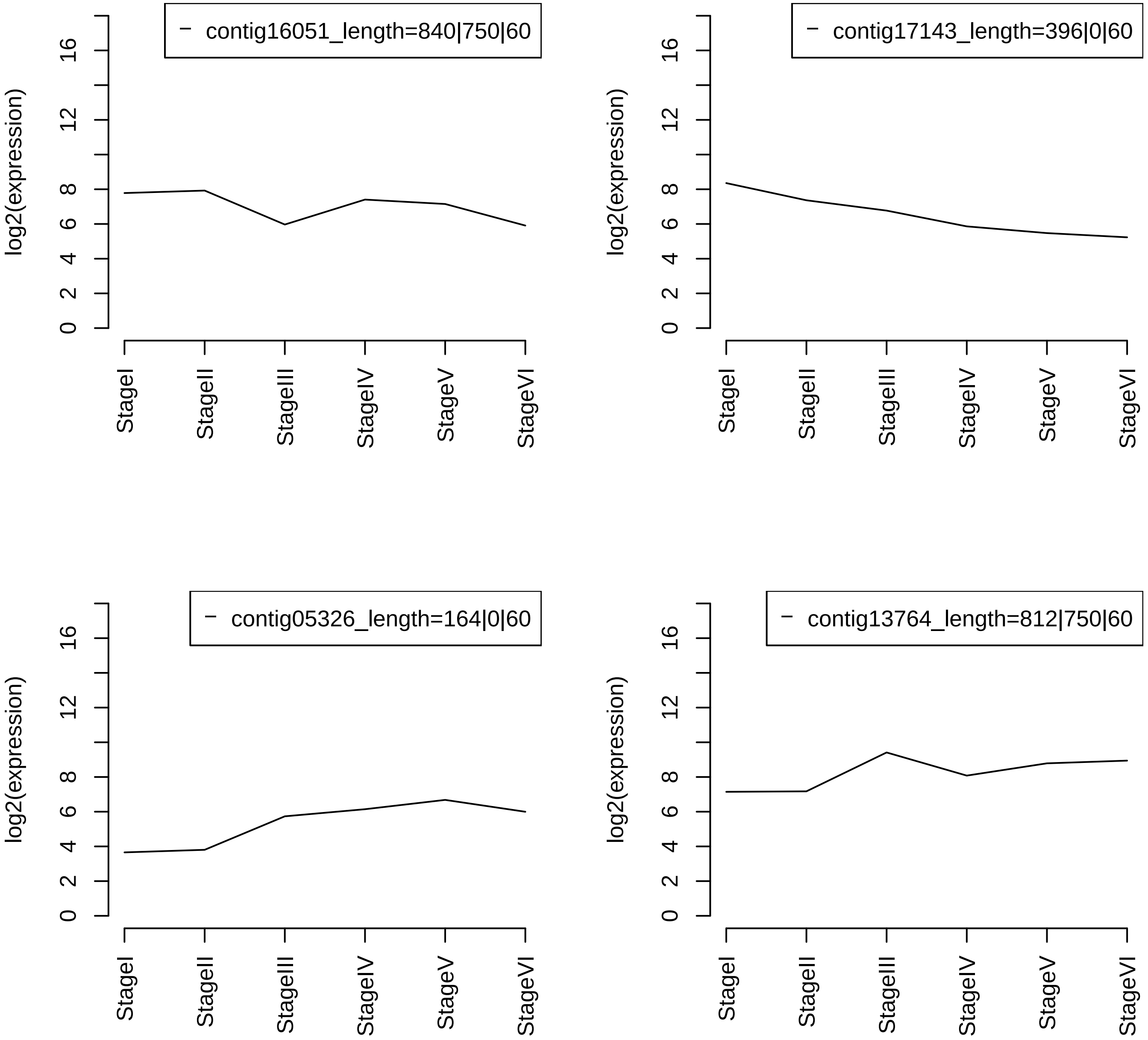

contig16051_length=840|750|60
contig17143_length=396|0|60
contig05326_length=164|0|60
contig13764_length=812|750|60
log2(expression)
0
2
4
6
8
12
16
StageI
StageII
StageIII
StageIV
StageV
StageVI

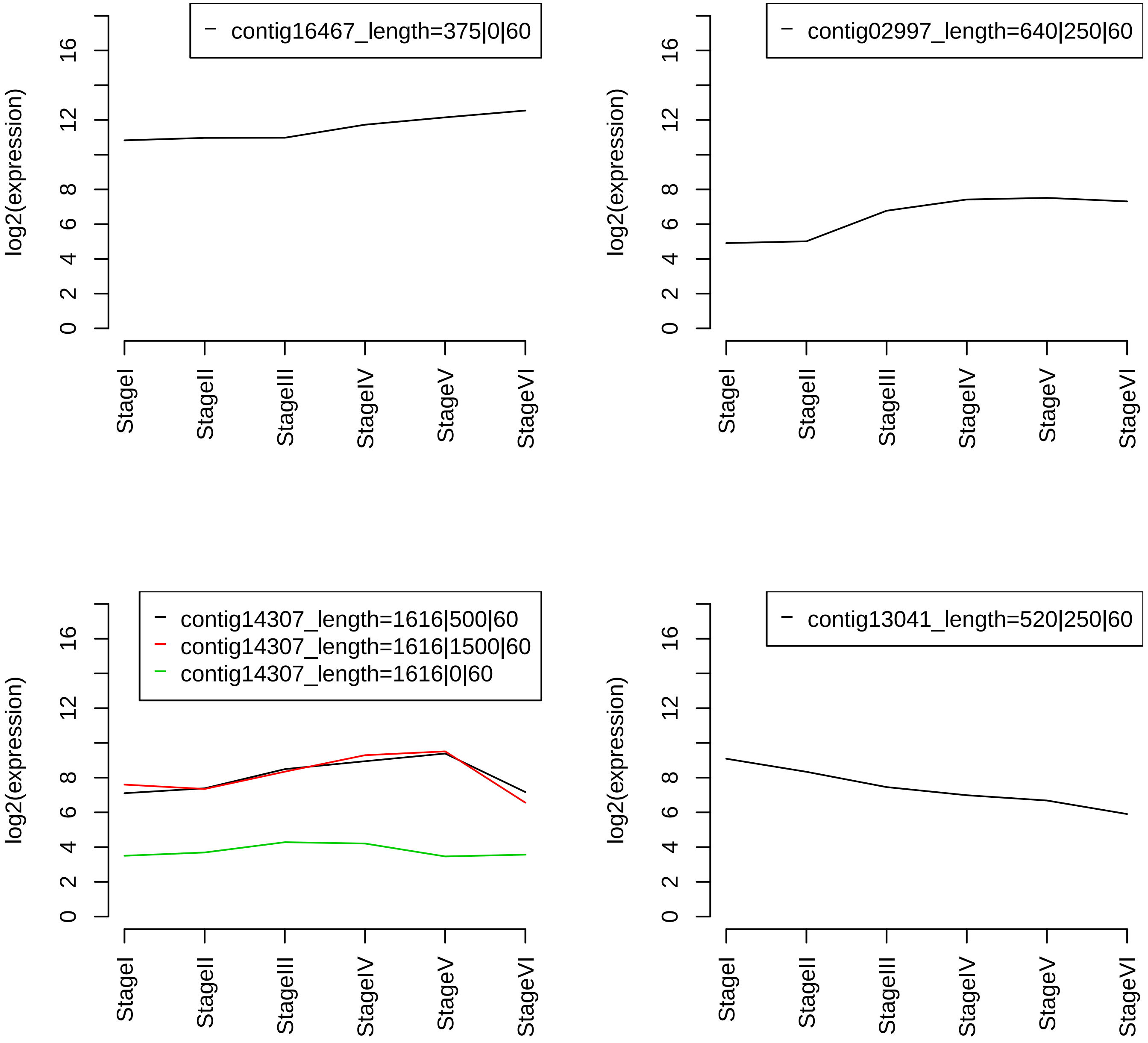
contig16467_length=375|0|60
contig02997_length=640|250|60
contig14307_length=1616|500|60
contig14307_length=1616|1500|60
contig14307_length=1616|0|60
contig13041_length=520|250|60
log2(expression)
0
2
4
6
8
12
16
StageI
StageII
StageIII
StageIV
StageV
StageVI

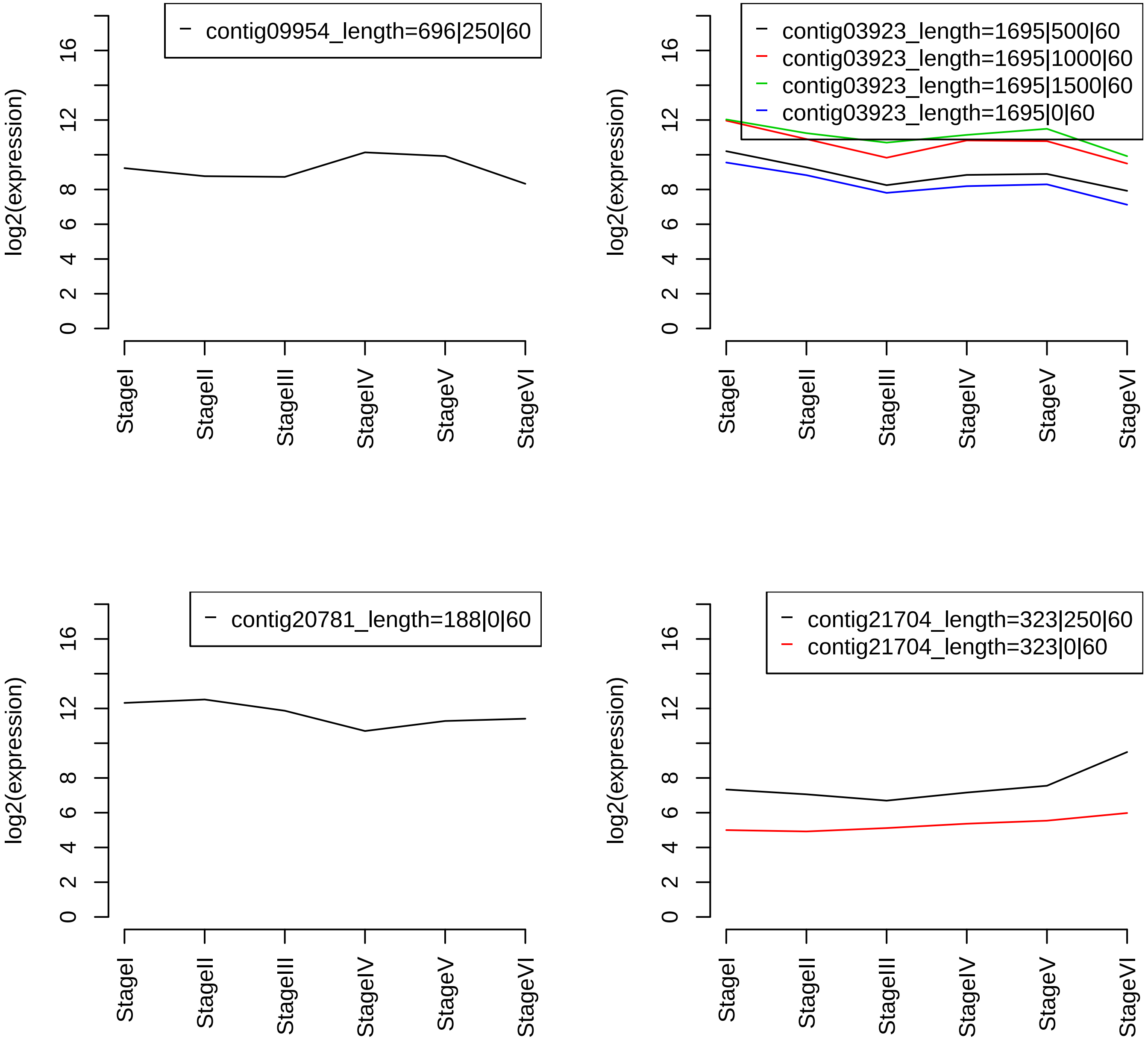

contig09954_length=696|250|60
log2(expression)
0 2 4 6 8 12 16
StageI StageII StageIII StageIV StageV StageVI
contig03923_length=1695|500|60
contig03923_length=1695|1000|60
contig03923_length=1695|1500|60
contig03923_length=1695|0|60
log2(expression)
0 2 4 6 8 12 16
StageI StageII StageIII StageIV StageV StageVI
contig20781_length=188|0|60
log2(expression)
0 2 4 6 8 12 16
StageI StageII StageIII StageIV StageV StageVI
contig21704_length=323|250|60
contig21704_length=323|0|60
log2(expression)
0 2 4 6 8 12 16
StageI StageII StageIII StageIV StageV StageVI

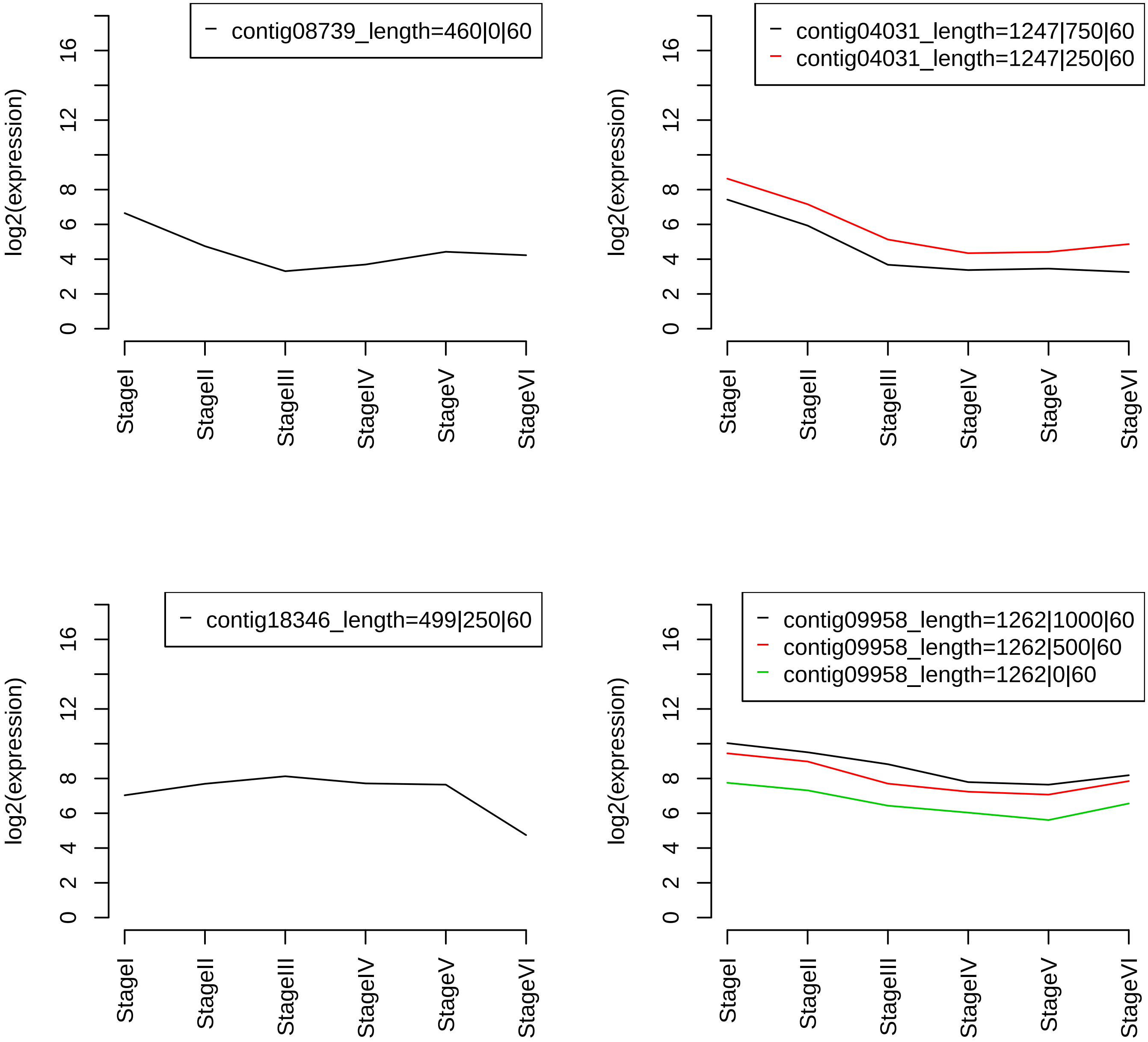
contig08739_length=460|0|60
contig04031_length=1247|750|60
contig04031_length=1247|250|60
contig18346_length=499|250|60
contig09958_length=1262|1000|60
contig09958_length=1262|500|60
contig09958_length=1262|0|60
log2(expression)
0
2
4
6
8
12
16
StageI
StageII
StageIII
StageIV
StageV
StageVI

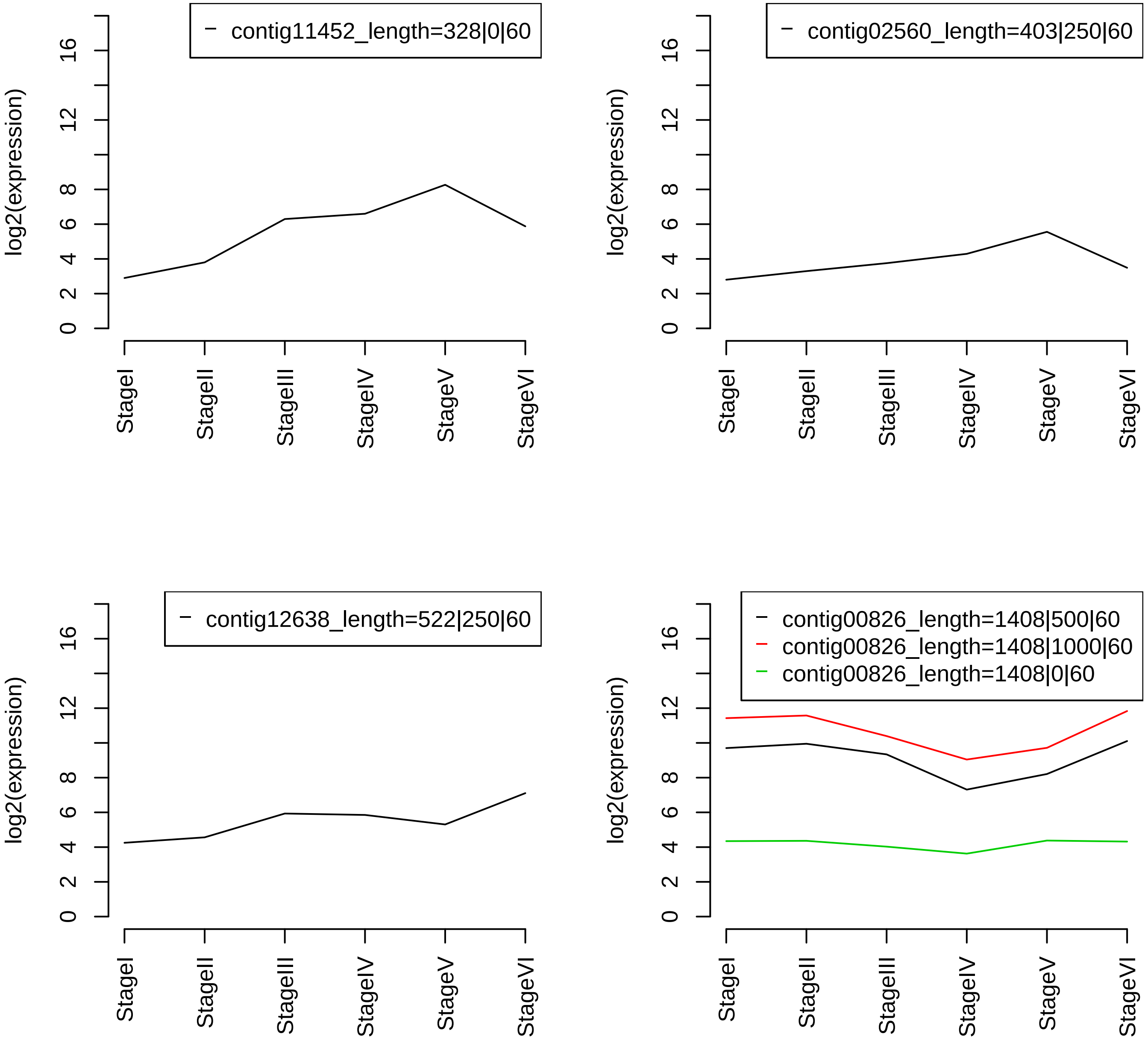

contig11452_length=328|0|60
log2(expression)
0
2
4
6
8
12
16
StageI
StageII
StageIII
StageIV
StageV
StageVI
contig02560_length=403|250|60
log2(expression)
contig12638_length=522|250|60
log2(expression)
contig00826_length=1408|500|60
contig00826_length=1408|1000|60
contig00826_length=1408|0|60
log2(expression)

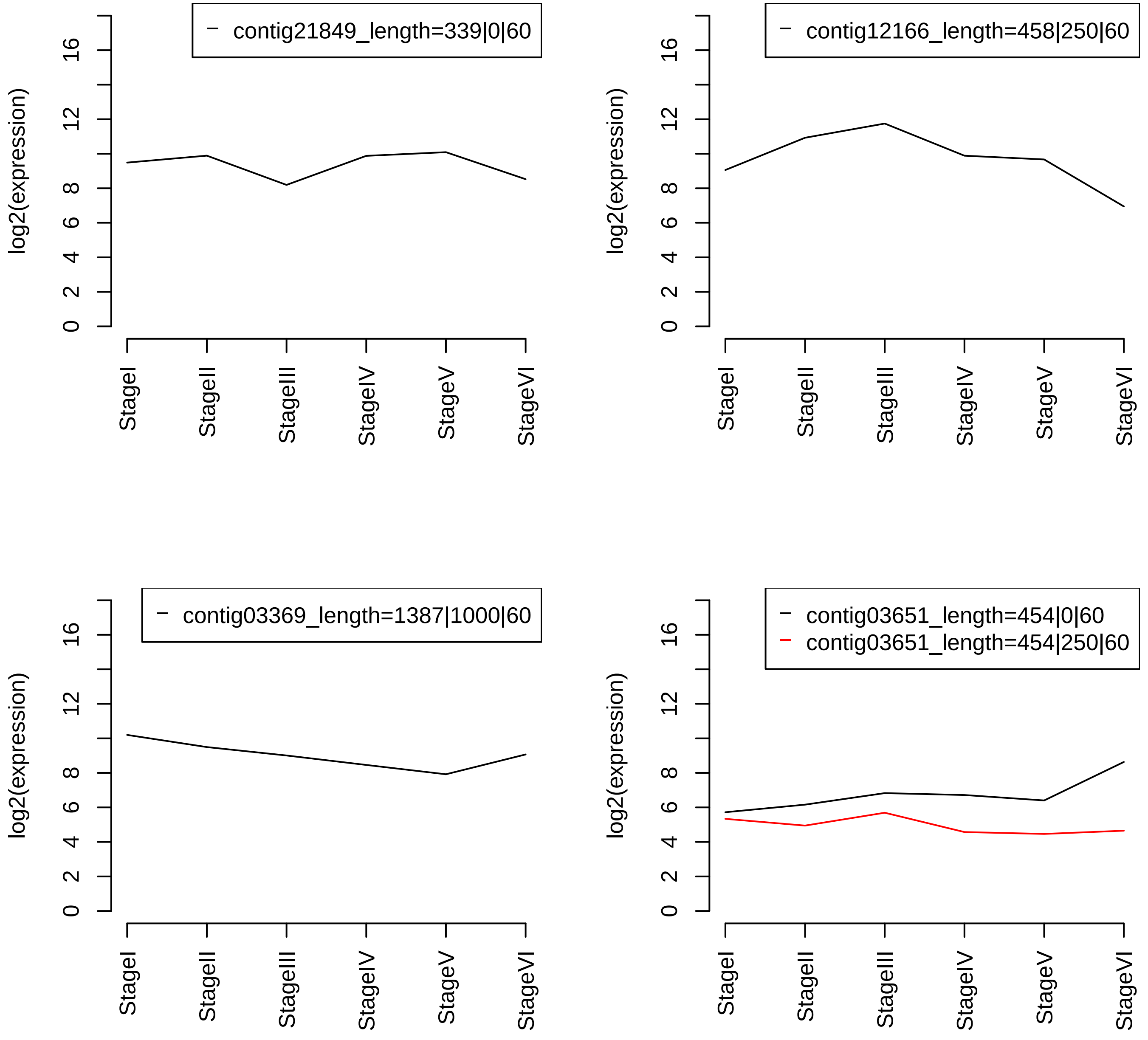
contig21849_length=339|0|60
contig12166_length=458|250|60
contig03369_length=1387|1000|60
contig03651_length=454|0|60
contig03651_length=454|250|60
log2(expression)
0
2
4
6
8
12
16
StageI
StageII
StageIII
StageIV
StageV
StageVI

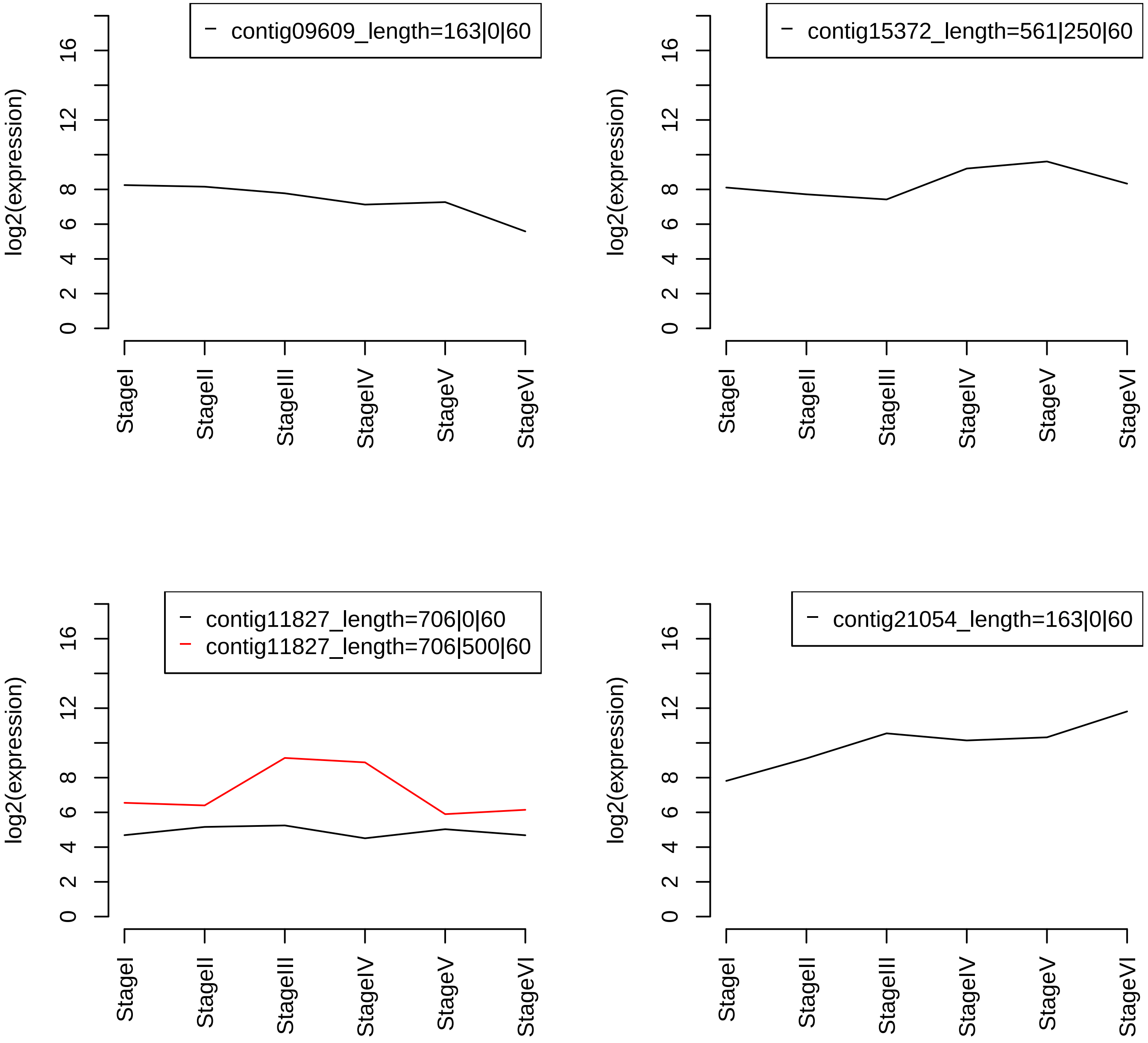
contig09609_length=163|0|60
contig15372_length=561|250|60
contig11827_length=706|0|60
contig11827_length=706|500|60
contig21054_length=163|0|60
log2(expression)
0
2
4
6
8
12
16
StageI
StageII
StageIII
StageIV
StageV
StageVI

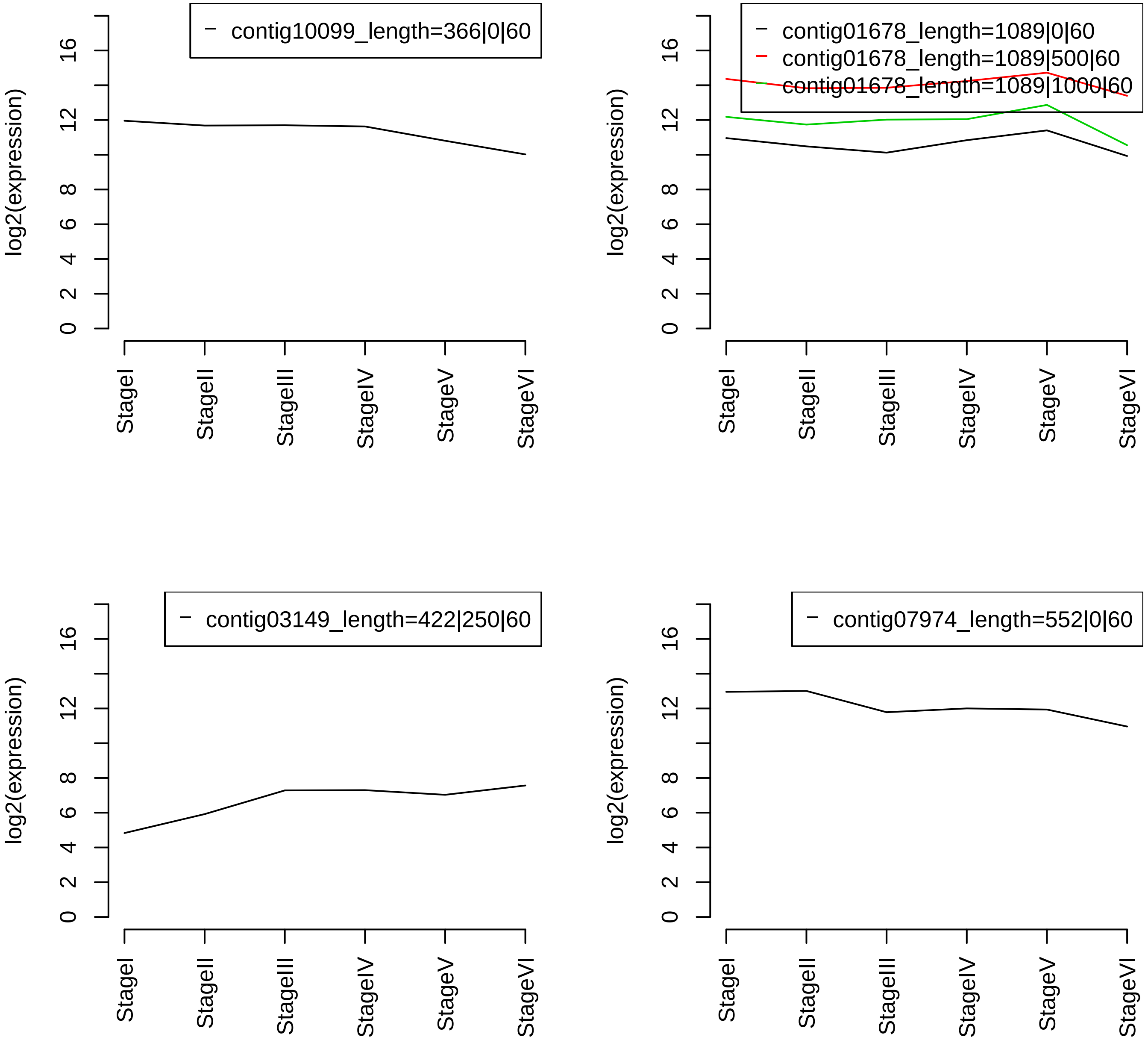

contig10099_length=366|0|60
contig01678_length=1089|0|60
contig01678_length=1089|500|60
contig01678_length=1089|1000|60
contig03149_length=422|250|60
contig07974_length=552|0|60
log2(expression)
0
2
4
6
8
12
16
StageI
StageII
StageIII
StageIV
StageV
StageVI

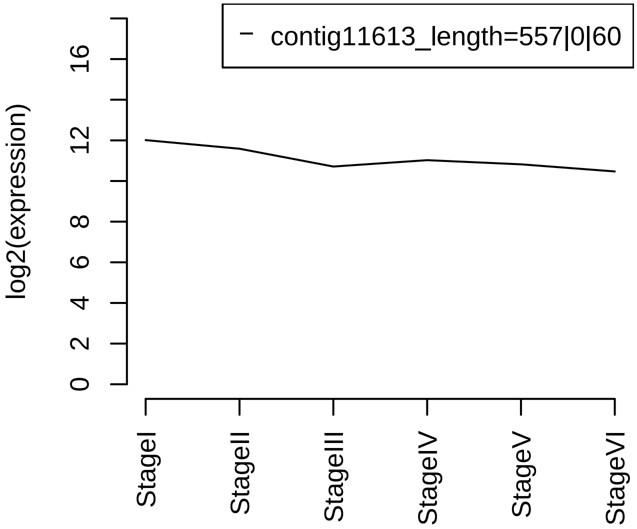

contig11613_length=557|0|60
log2(expression)
0
2
4
6
8
12
16
StageI
StageII
StageIII
StageIV
StageV
StageVI

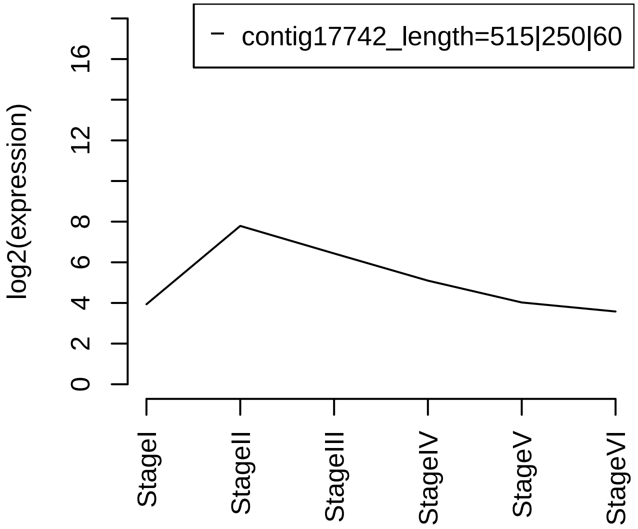

contig17742_length=515|250|60
log2(expression)
0
2
4
6
8
12
16
StageI
StageII
StageIII
StageIV
StageV
StageVI

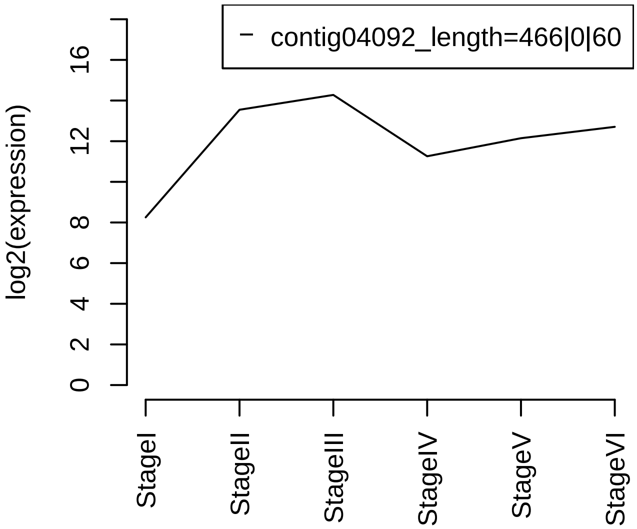

contig04092_length=466|0|60
log2(expression)
0
2
4
6
8
12
16
StageI
StageII
StageIII
StageIV
StageV
StageVI

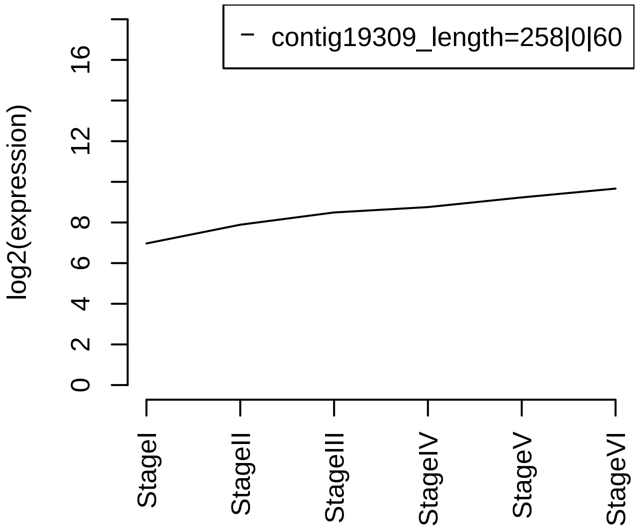

contig19309_length=258|0|60
log2(expression)
0
2
4
6
8
12
16
StageI
StageII
StageIII
StageIV
StageV
StageVI

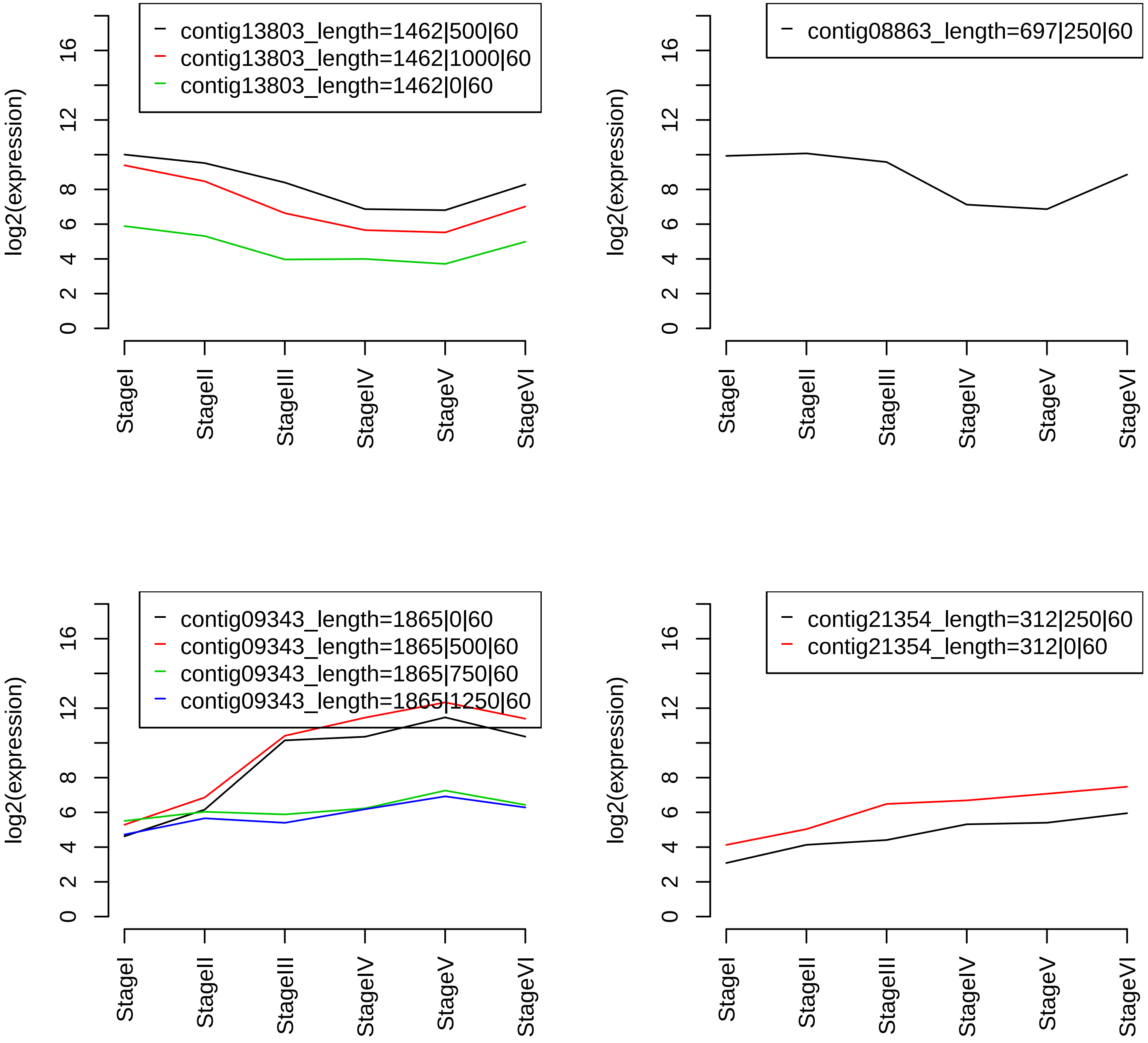

contig13803_length=1462|500|60
contig13803_length=1462|1000|60
contig13803_length=1462|0|60
contig08863_length=697|250|60
contig09343_length=1865|0|60
contig09343_length=1865|500|60
contig09343_length=1865|750|60
contig09343_length=1865|1250|60
contig21354_length=312|250|60
contig21354_length=312|0|60
log2(expression)
0
2
4
6
8
12
16
StageI
StageII
StageIII
StageIV
StageV
StageVI

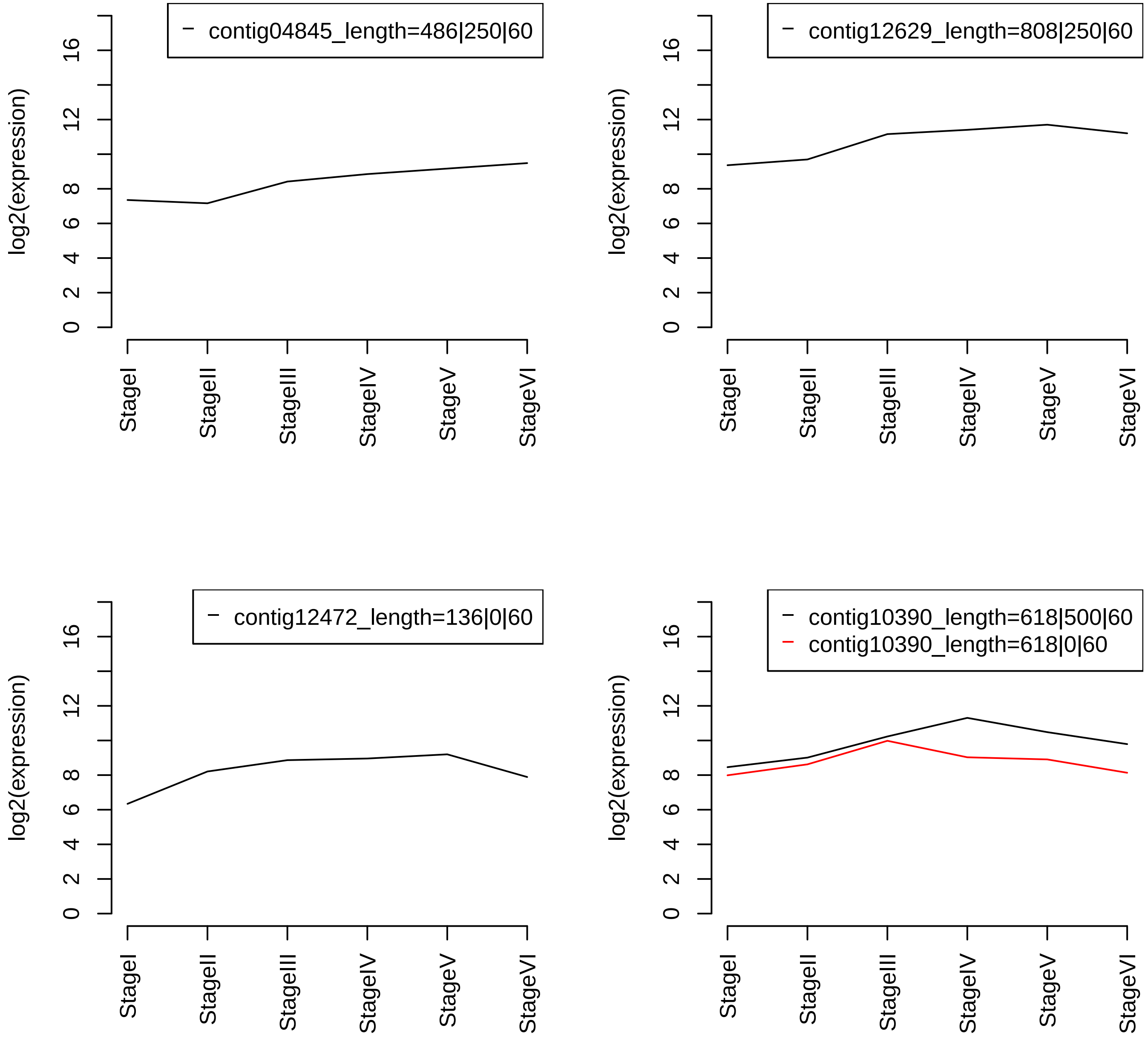
contig04845_length=486|250|60
contig12629_length=808|250|60
contig12472_length=136|0|60
contig10390_length=618|500|60
contig10390_length=618|0|60
log2(expression)
0
2
4
6
8
12
16
StageI
StageII
StageIII
StageIV
StageV
StageVI

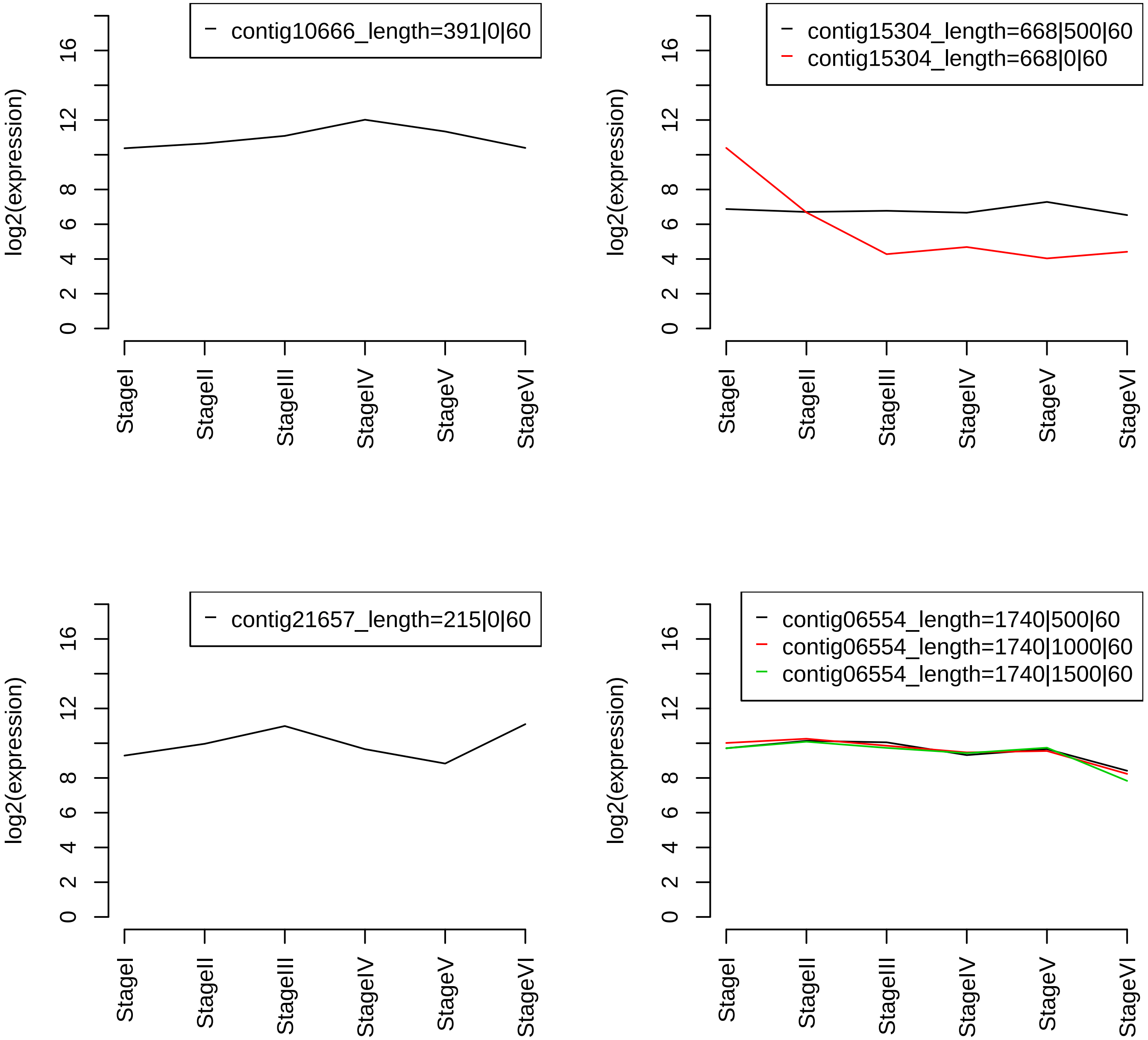
contig10666_length=391|0|60
contig15304_length=668|500|60
contig15304_length=668|0|60
contig21657_length=215|0|60
contig06554_length=1740|500|60
contig06554_length=1740|1000|60
contig06554_length=1740|1500|60
log2(expression)
0
2
4
6
8
12
16
StageI
StageII
StageIII
StageIV
StageV
StageVI

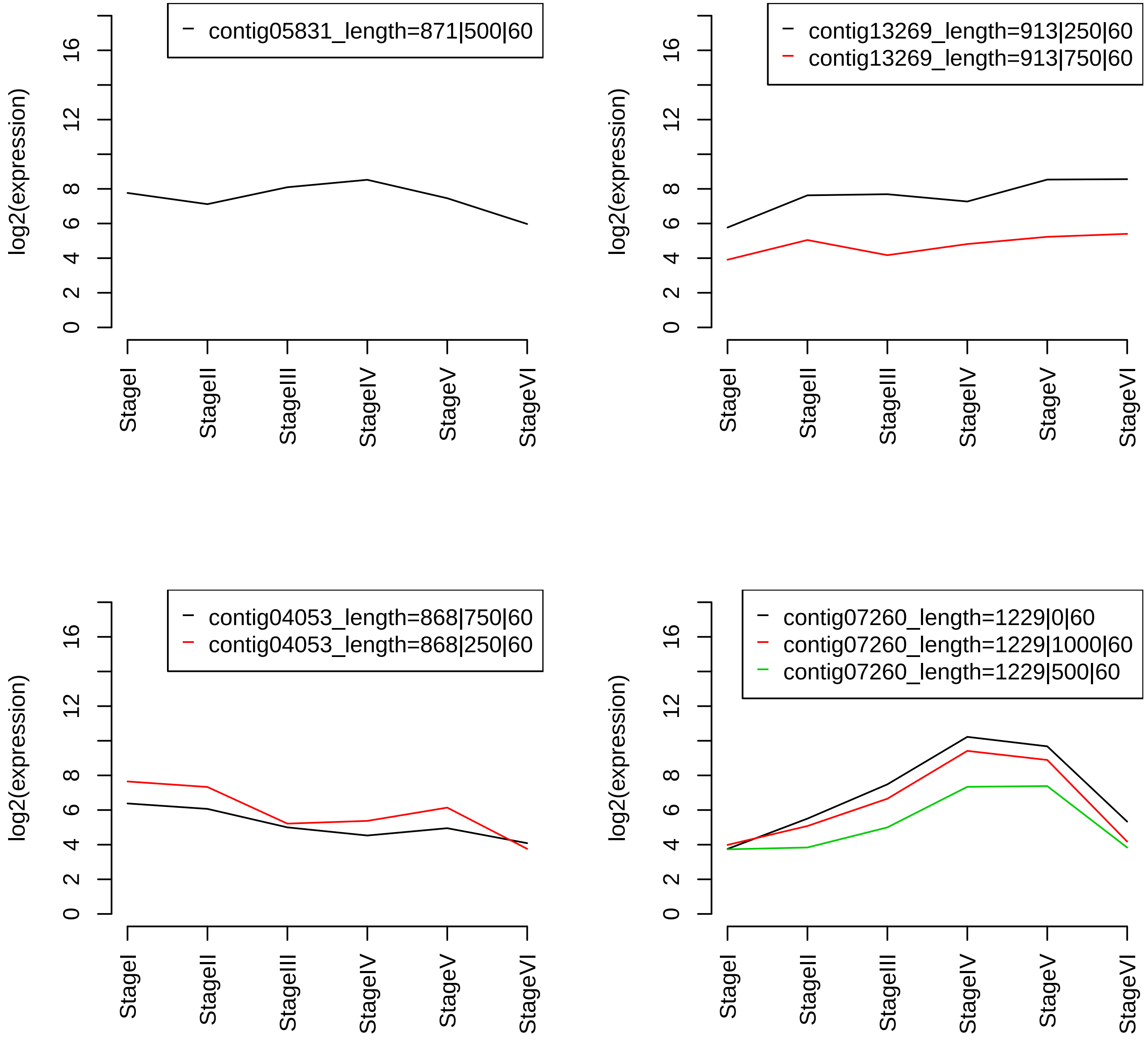

contig05831_length=871|500|60
contig13269_length=913|250|60
contig13269_length=913|750|60
contig04053_length=868|750|60
contig04053_length=868|250|60
contig07260_length=1229|0|60
contig07260_length=1229|1000|60
contig07260_length=1229|500|60
log2(expression)
0
2
4
6
8
12
16
StageI
StageII
StageIII
StageIV
StageV
StageVI

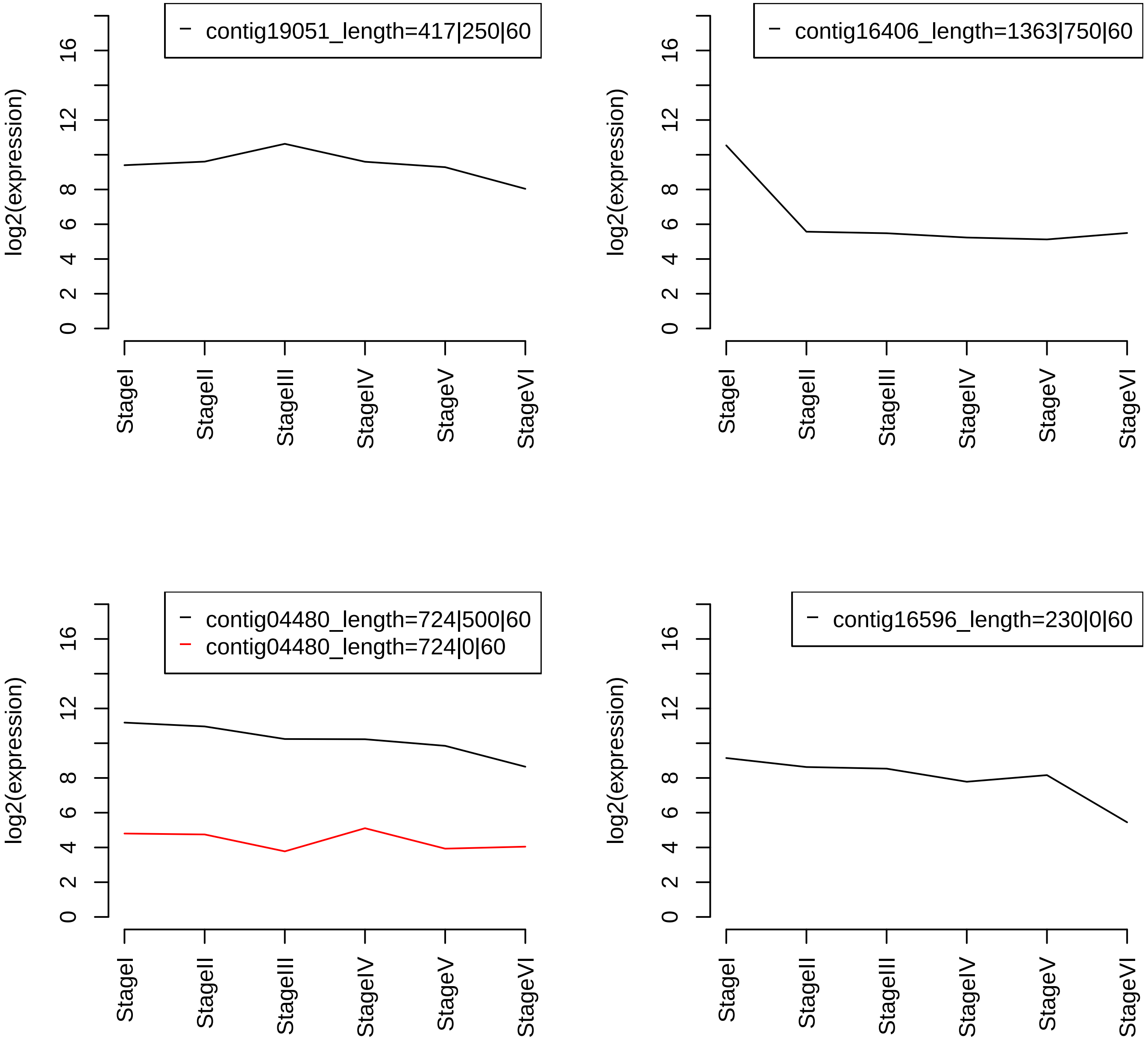

contig19051_length=417|250|60
contig16406_length=1363|750|60
contig04480_length=724|500|60
contig04480_length=724|0|60
contig16596_length=230|0|60
log2(expression)
0
2
4
6
8
12
16
StageI
StageII
StageIII
StageIV
StageV
StageVI

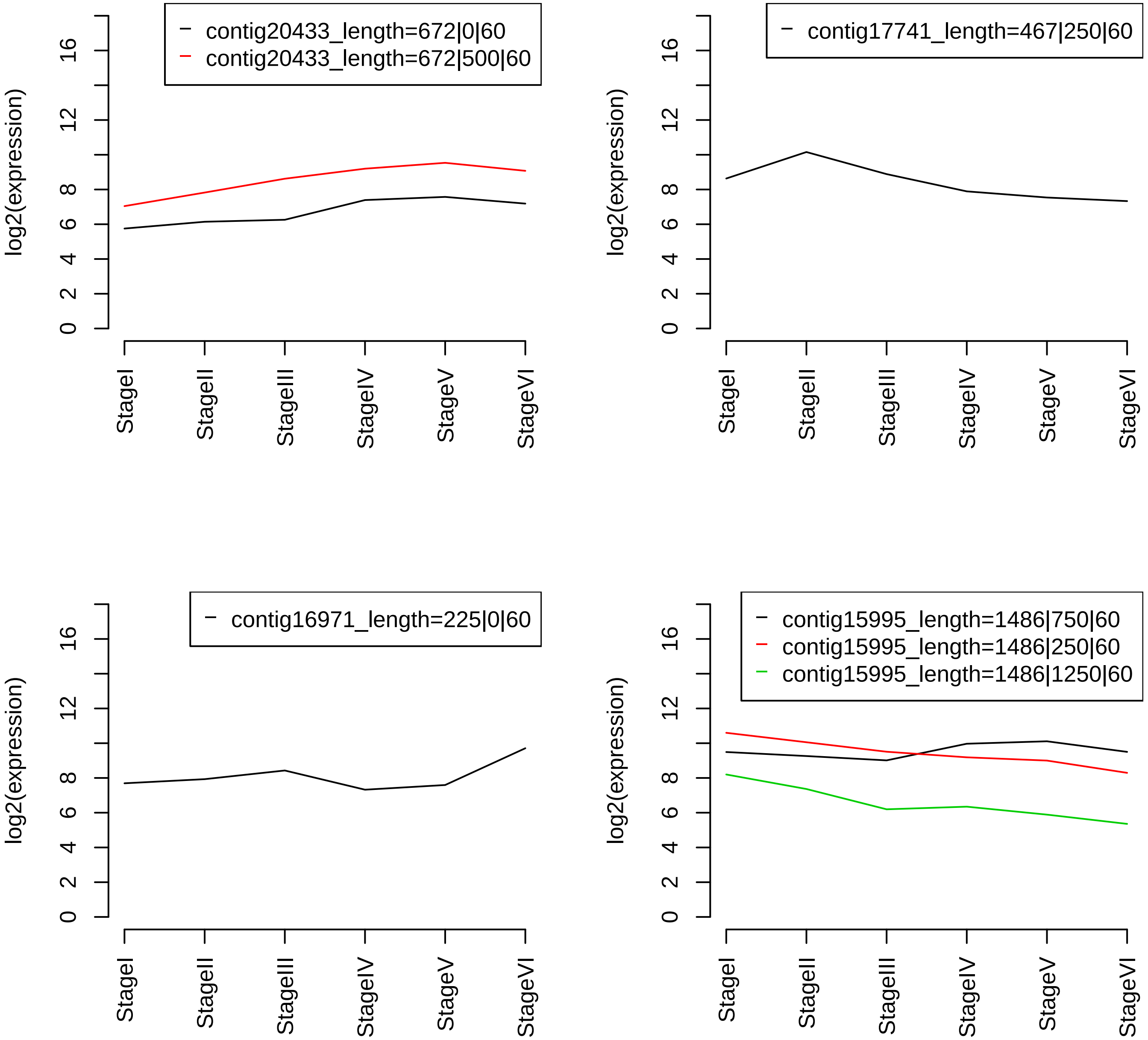
contig20433_length=672|0|60
contig20433_length=672|500|60
contig17741_length=467|250|60
contig16971_length=225|0|60
contig15995_length=1486|750|60
contig15995_length=1486|250|60
contig15995_length=1486|1250|60
log2(expression)
0
2
4
6
8
12
16
StageI
StageII
StageIII
StageIV
StageV
StageVI

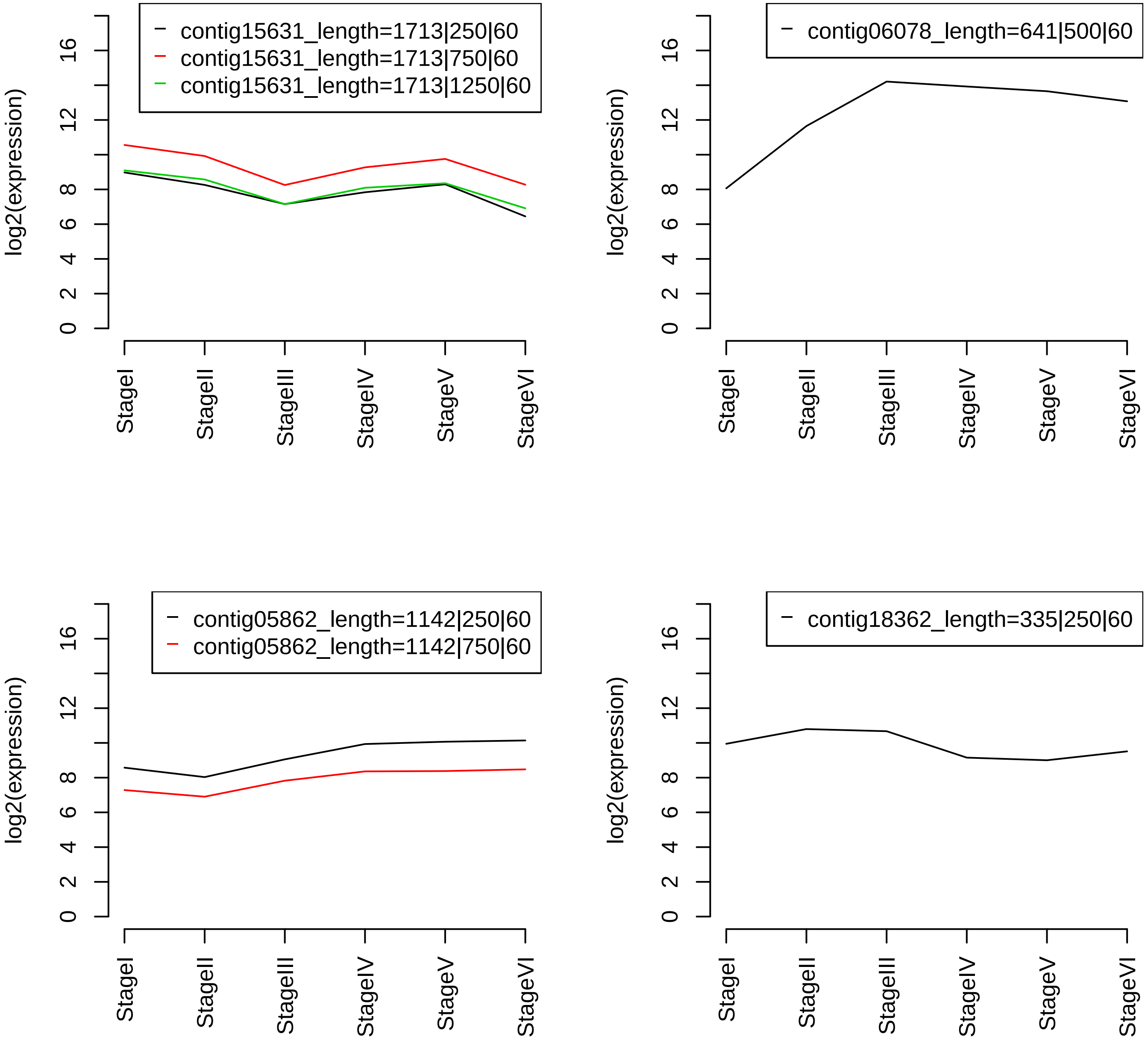

contig15631_length=1713|250|60
contig15631_length=1713|750|60
contig15631_length=1713|1250|60
contig06078_length=641|500|60
contig05862_length=1142|250|60
contig05862_length=1142|750|60
contig18362_length=335|250|60
log2(expression)
StageI
StageII
StageIII
StageIV
StageV
StageVI

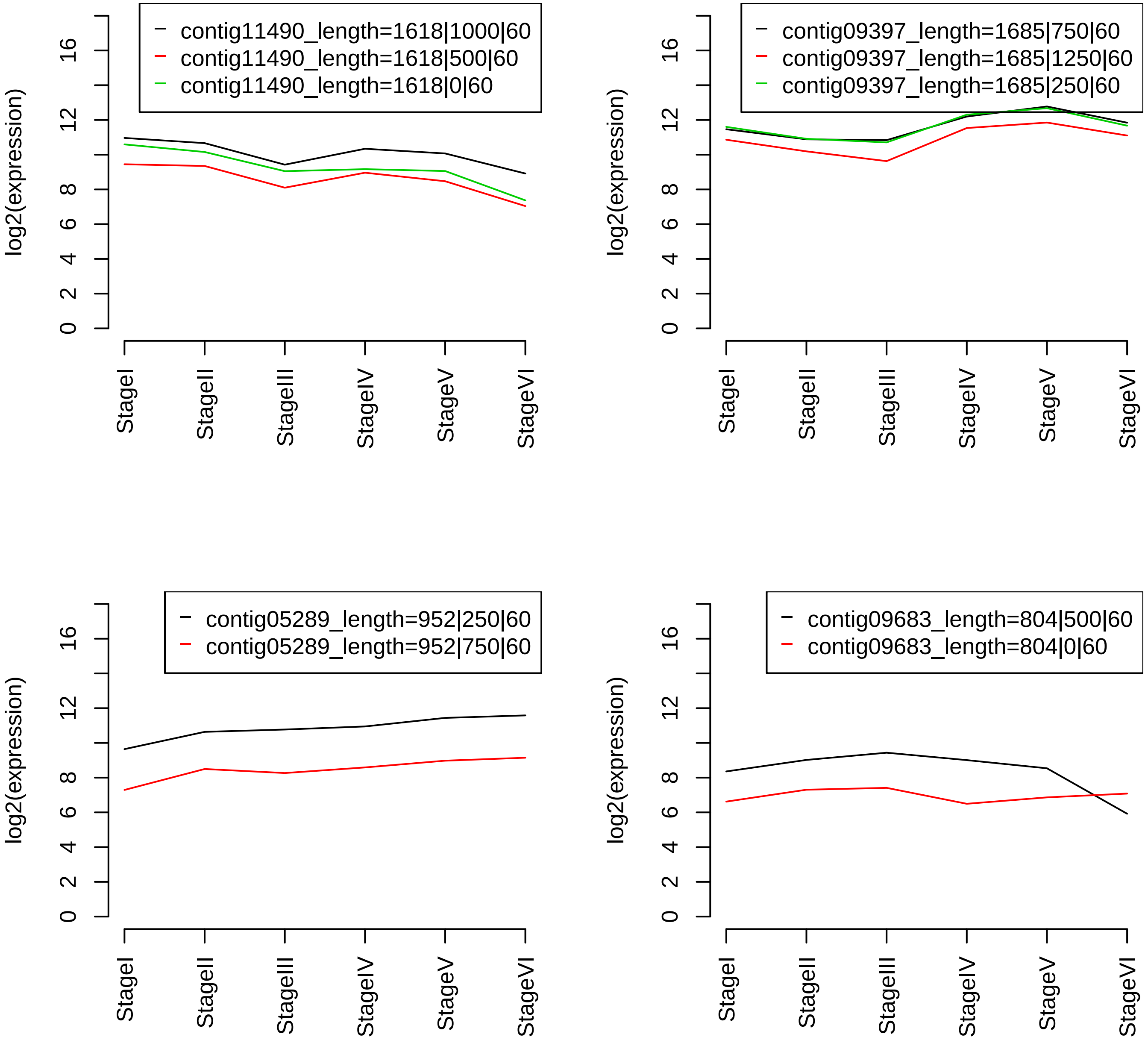
contig11490_length=1618|1000|60
contig11490_length=1618|500|60
contig11490_length=1618|0|60
contig09397_length=1685|750|60
contig09397_length=1685|1250|60
contig09397_length=1685|250|60
contig05289_length=952|250|60
contig05289_length=952|750|60
contig09683_length=804|500|60
contig09683_length=804|0|60
log2(expression)
0
2
4
6
8
12
16
StageI
StageII
StageIII
StageIV
StageV
StageVI

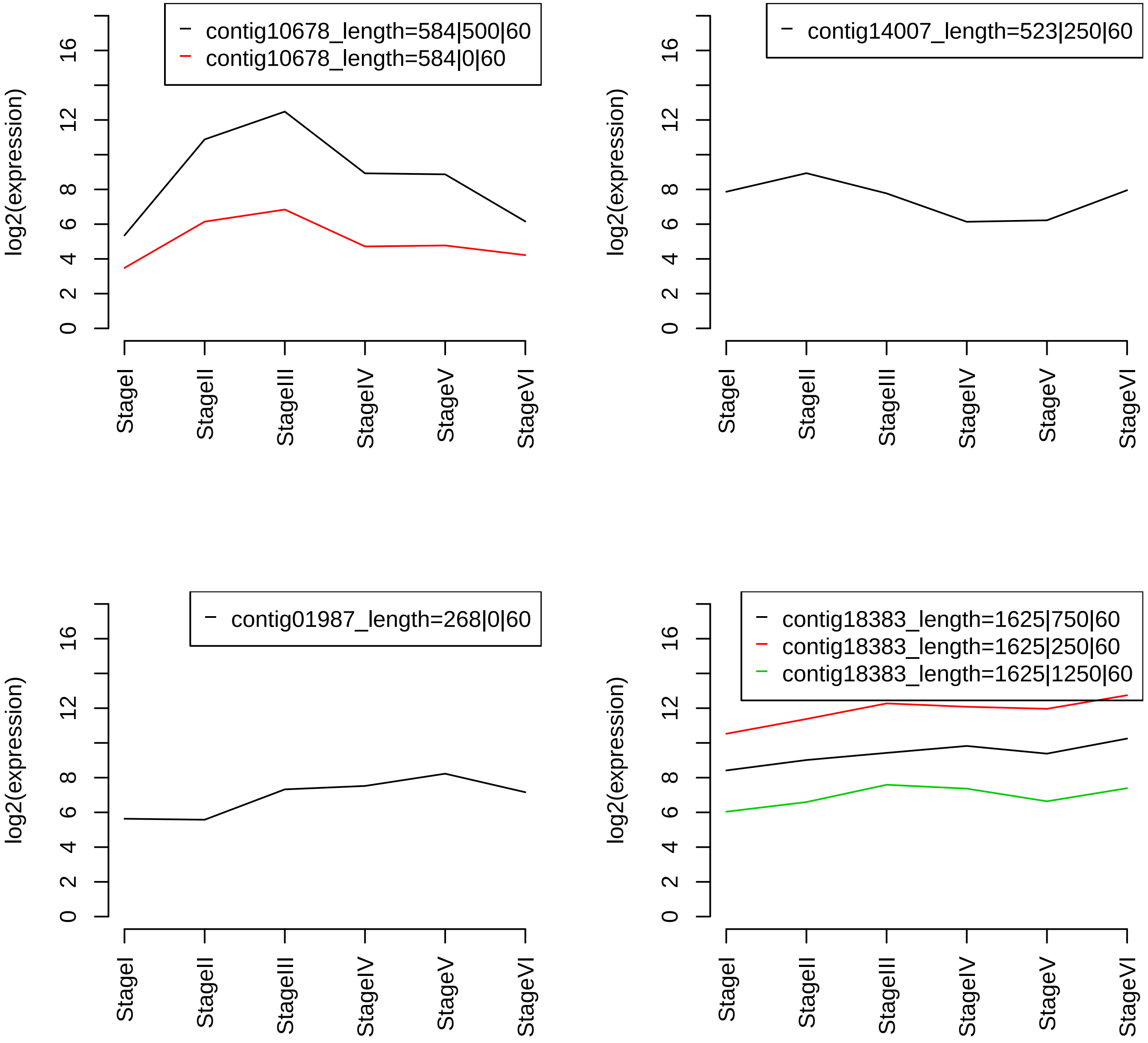

contig10678_length=584|500|60
contig10678_length=584|0|60
contig14007_length=523|250|60
contig01987_length=268|0|60
contig18383_length=1625|750|60
contig18383_length=1625|250|60
contig18383_length=1625|1250|60
log2(expression)
0
2
4
6
8
12
16
StageI
StageII
StageIII
StageIV
StageV
StageVI

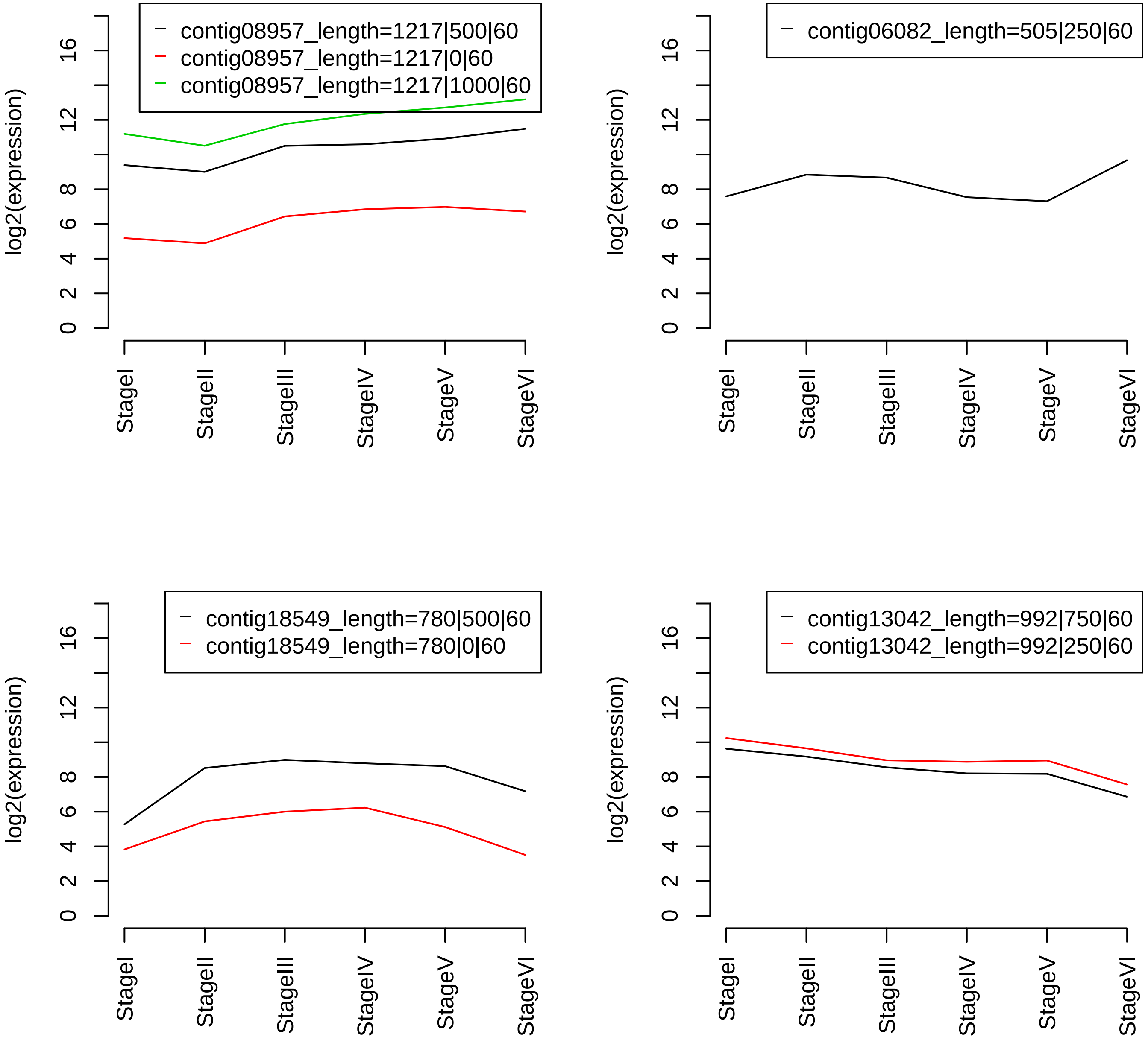
contig08957_length=1217|500|60
contig08957_length=1217|0|60
contig08957_length=1217|1000|60
contig06082_length=505|250|60
contig18549_length=780|500|60
contig18549_length=780|0|60
contig13042_length=992|750|60
contig13042_length=992|250|60
log2(expression)
0
2
4
6
8
12
16
StageI
StageII
StageIII
StageIV
StageV
StageVI

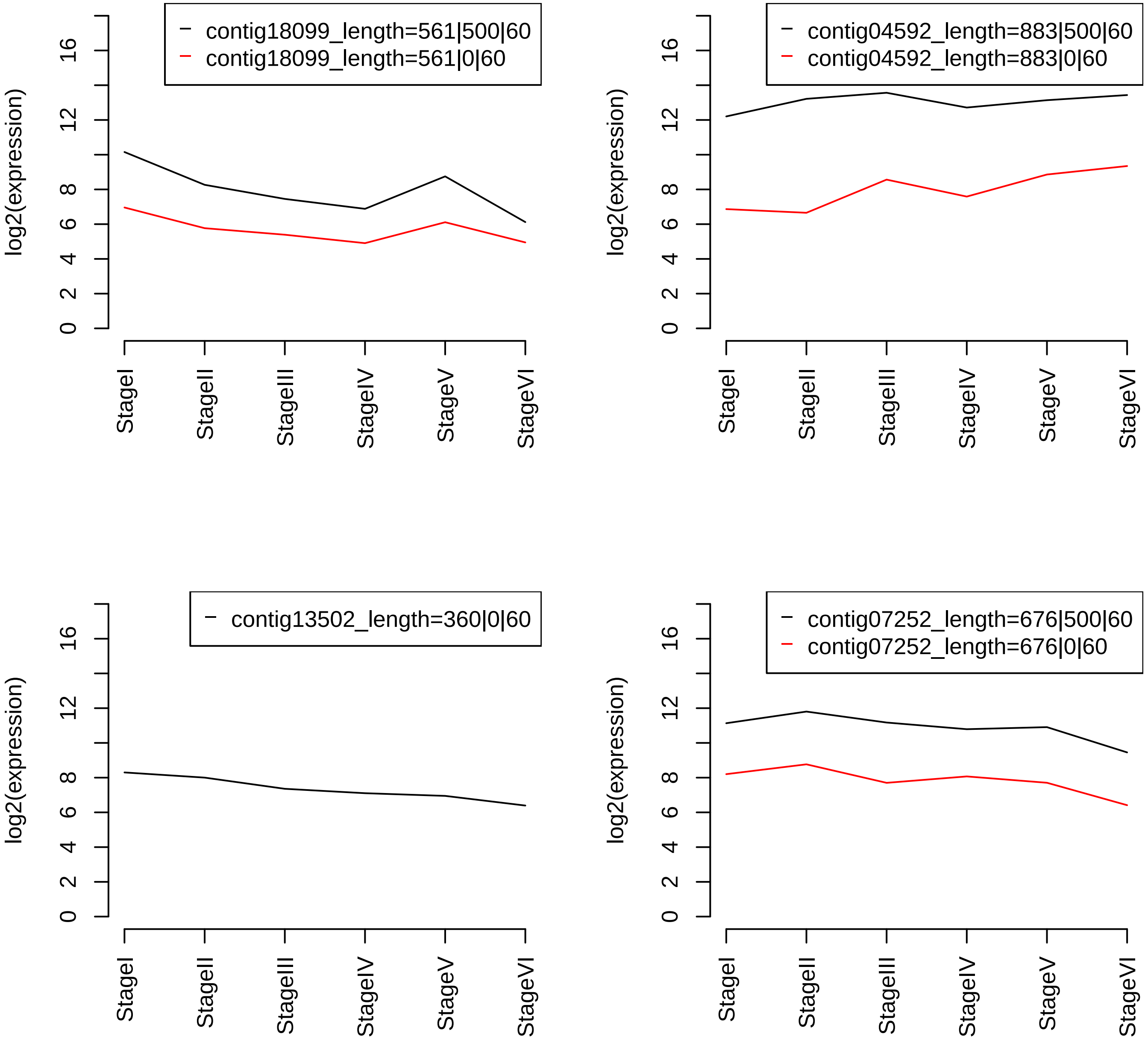
contig18099_length=561|500|60
contig18099_length=561|0|60
contig04592_length=883|500|60
contig04592_length=883|0|60
contig13502_length=360|0|60
contig07252_length=676|500|60
contig07252_length=676|0|60
log2(expression)
0
2
4
6
8
12
16
StageI
StageII
StageIII
StageIV
StageV
StageVI

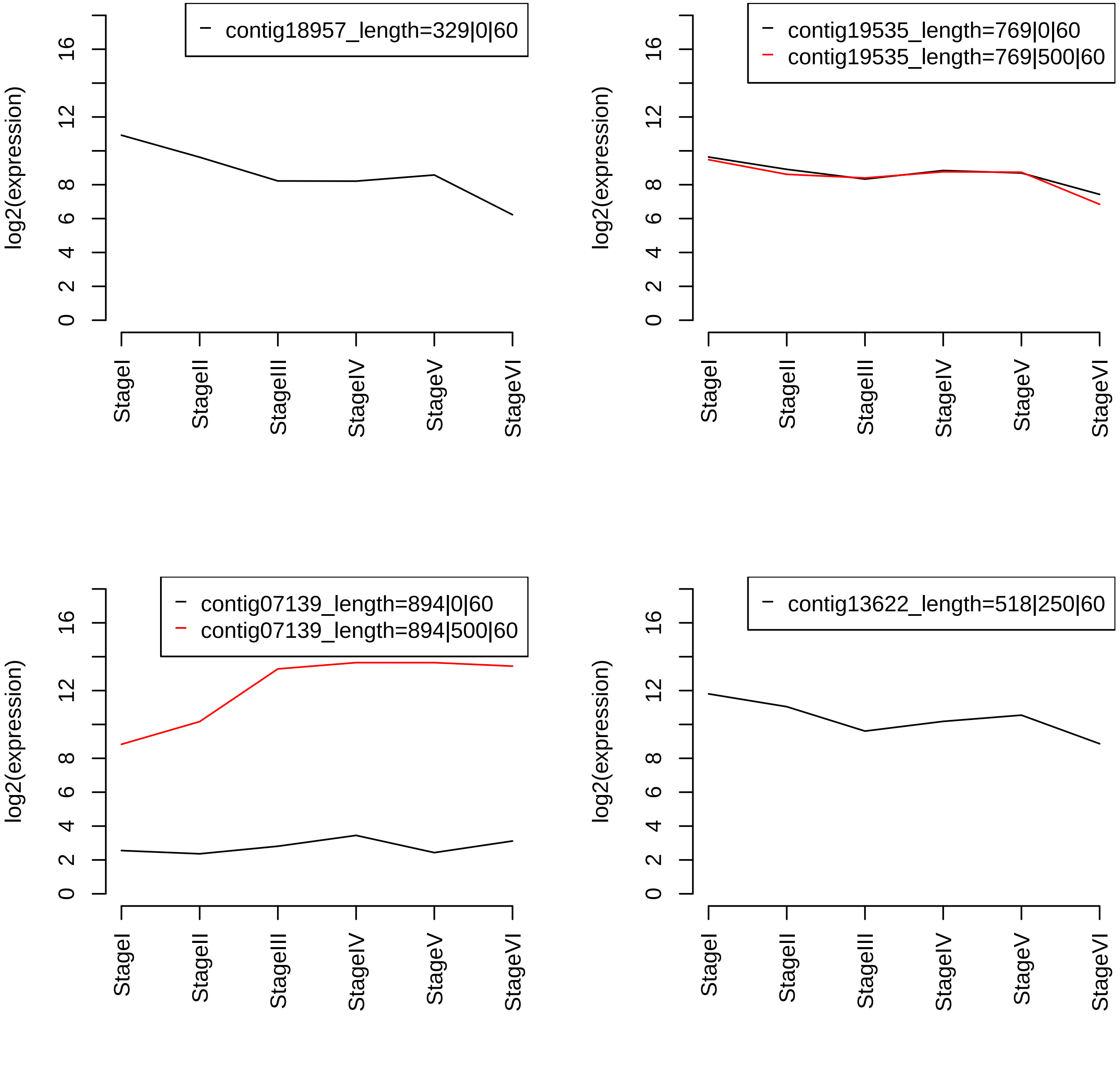

contig18957_length=329|0|60
contig19535_length=769|0|60
contig19535_length=769|500|60
contig07139_length=894|0|60
contig07139_length=894|500|60
contig13622_length=518|250|60
log2(expression)
0 2 4 6 8 12 16
StageI
StageII
StageIII
StageIV
StageV
StageVI

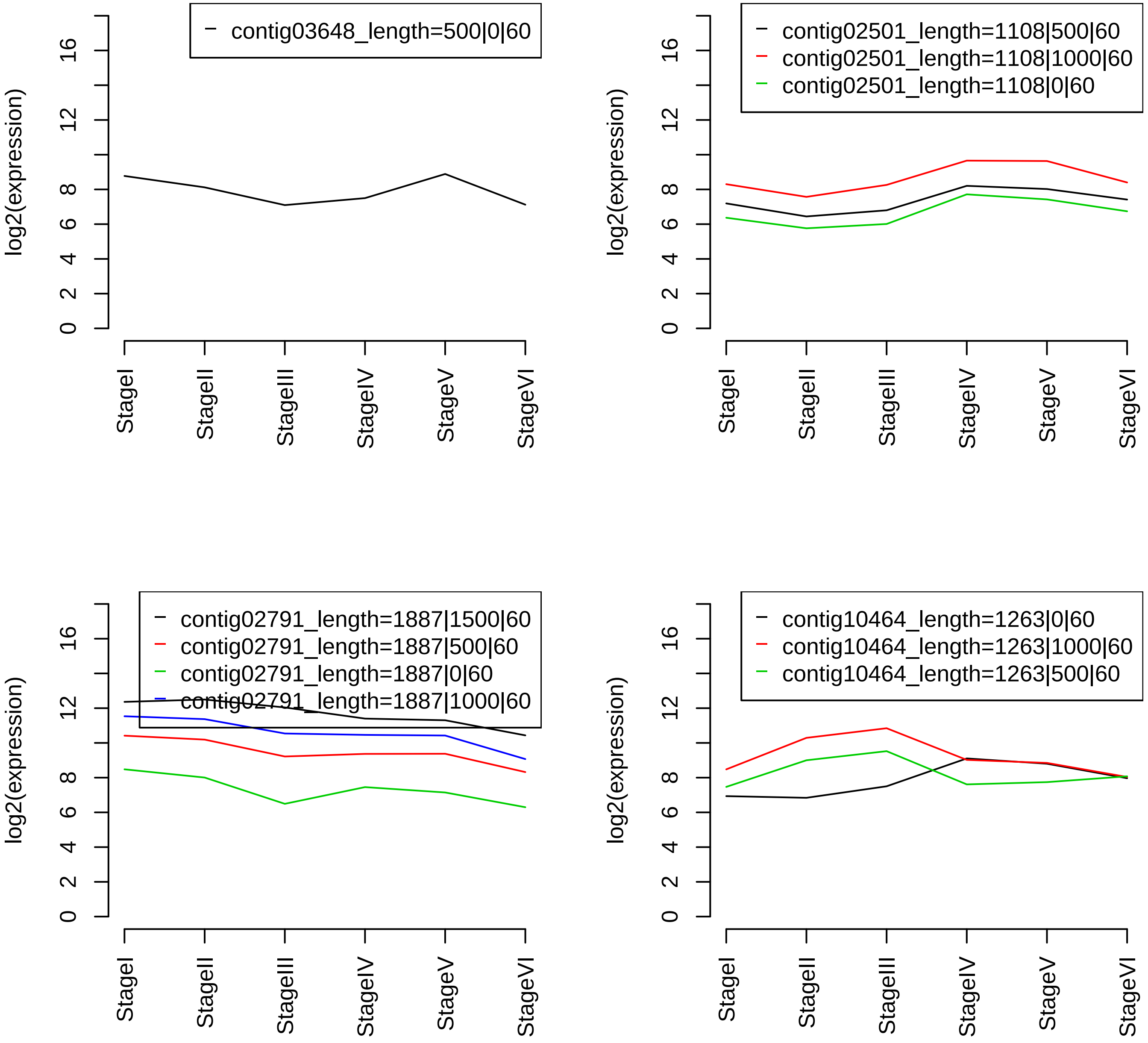
contig03648_length=500|0|60
log2(expression)
0
2
4
6
8
12
16
StageI
StageII
StageIII
StageIV
StageV
StageVI
contig02501_length=1108|500|60
contig02501_length=1108|1000|60
contig02501_length=1108|0|60
contig02791_length=1887|1500|60
contig02791_length=1887|500|60
contig02791_length=1887|0|60
contig02791_length=1887|1000|60
contig10464_length=1263|0|60
contig10464_length=1263|1000|60
contig10464_length=1263|500|60

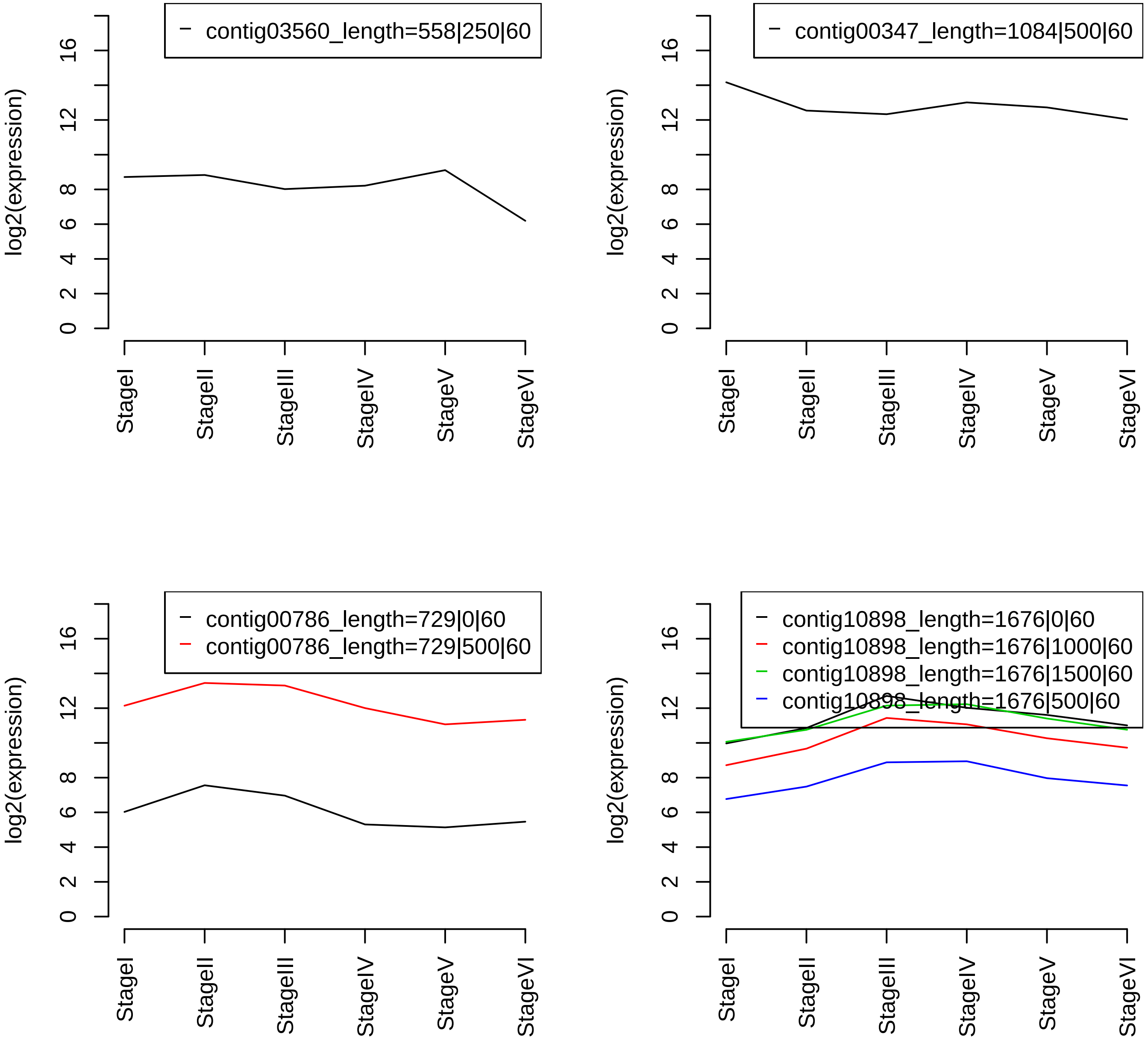

contig03560_length=558|250|60
contig00347_length=1084|500|60
contig00786_length=729|0|60
contig00786_length=729|500|60
contig10898_length=1676|0|60
contig10898_length=1676|1000|60
contig10898_length=1676|1500|60
contig10898_length=1676|500|60
log2(expression)
StageI
StageII
StageIII
StageIV
StageV
StageVI

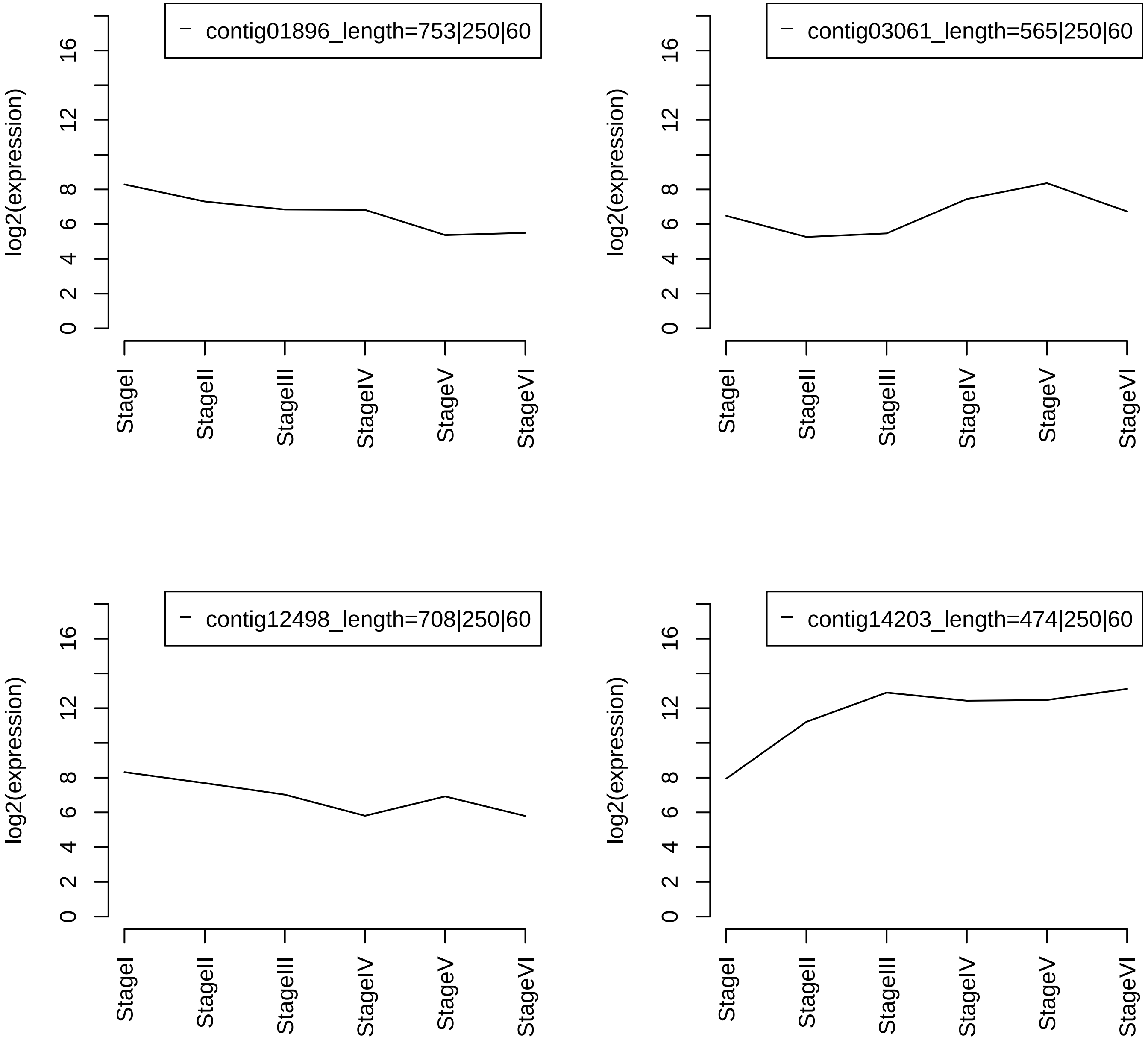

contig01896_length=753|250|60
contig03061_length=565|250|60
contig12498_length=708|250|60
contig14203_length=474|250|60
log2(expression)
0
2
4
6
8
12
16
StageI
StageII
StageIII
StageIV
StageV
StageVI

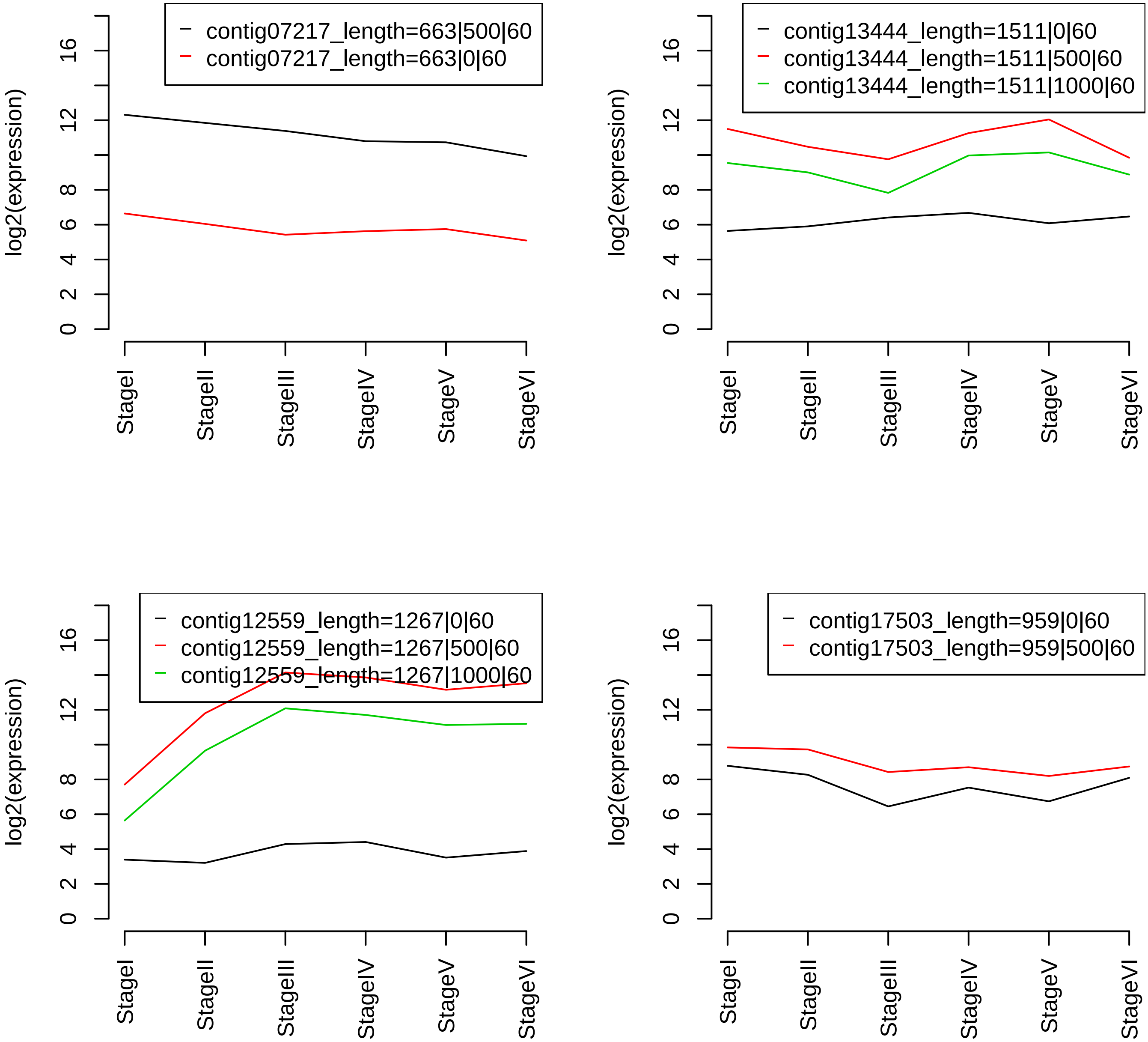
contig07217_length=663|500|60
contig07217_length=663|0|60
contig13444_length=1511|0|60
contig13444_length=1511|500|60
contig13444_length=1511|1000|60
contig12559_length=1267|0|60
contig12559_length=1267|500|60
contig12559_length=1267|1000|60
contig17503_length=959|0|60
contig17503_length=959|500|60
log2(expression)
0 2 4 6 8 12 16
StageI StageII StageIII StageIV StageV StageVI

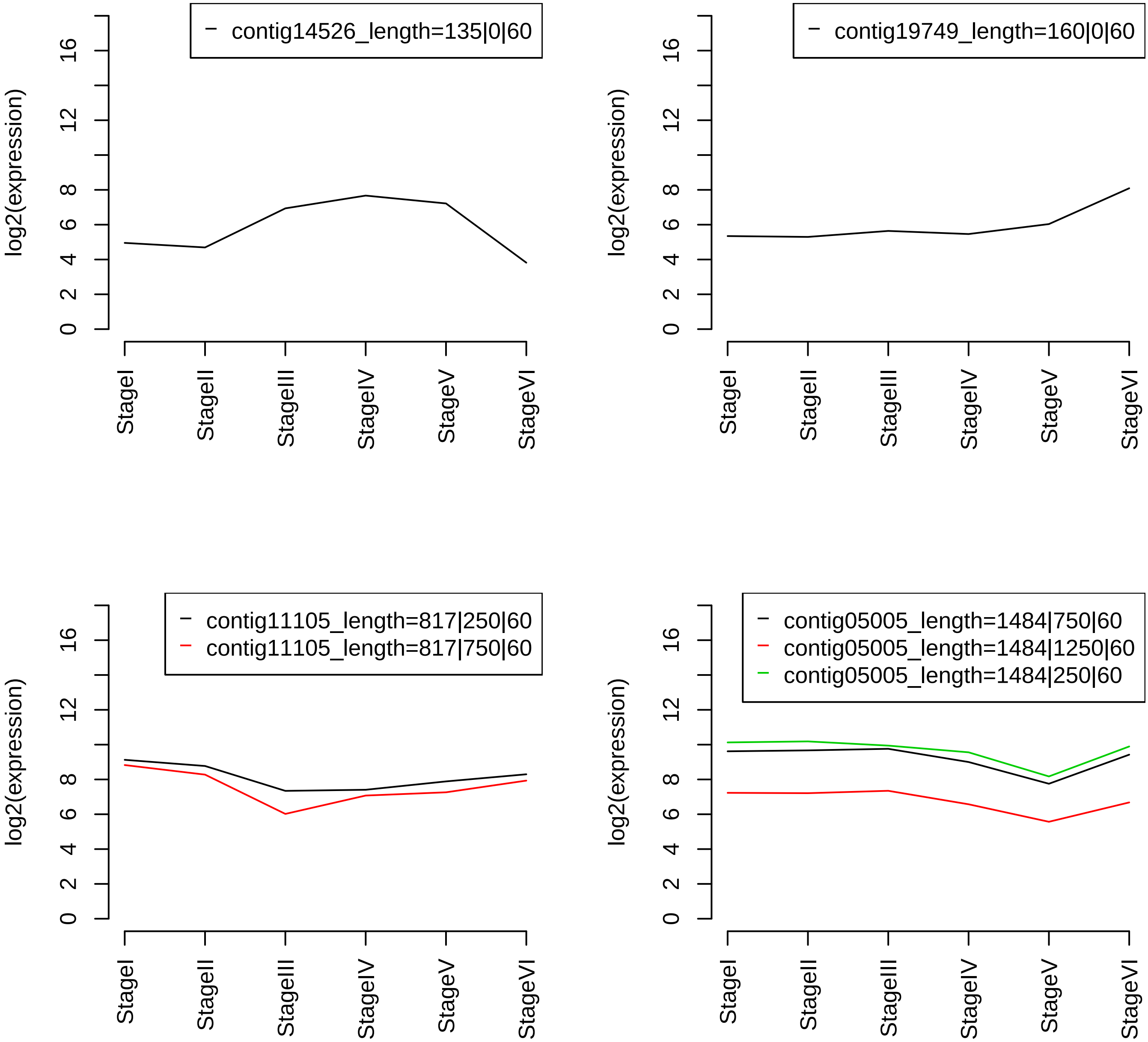
contig14526_length=135|0|60
contig19749_length=160|0|60
contig11105_length=817|250|60
contig11105_length=817|750|60
contig05005_length=1484|750|60
contig05005_length=1484|1250|60
contig05005_length=1484|250|60
log2(expression)
0
2
4
6
8
12
16
StageI
StageII
StageIII
StageIV
StageV
StageVI

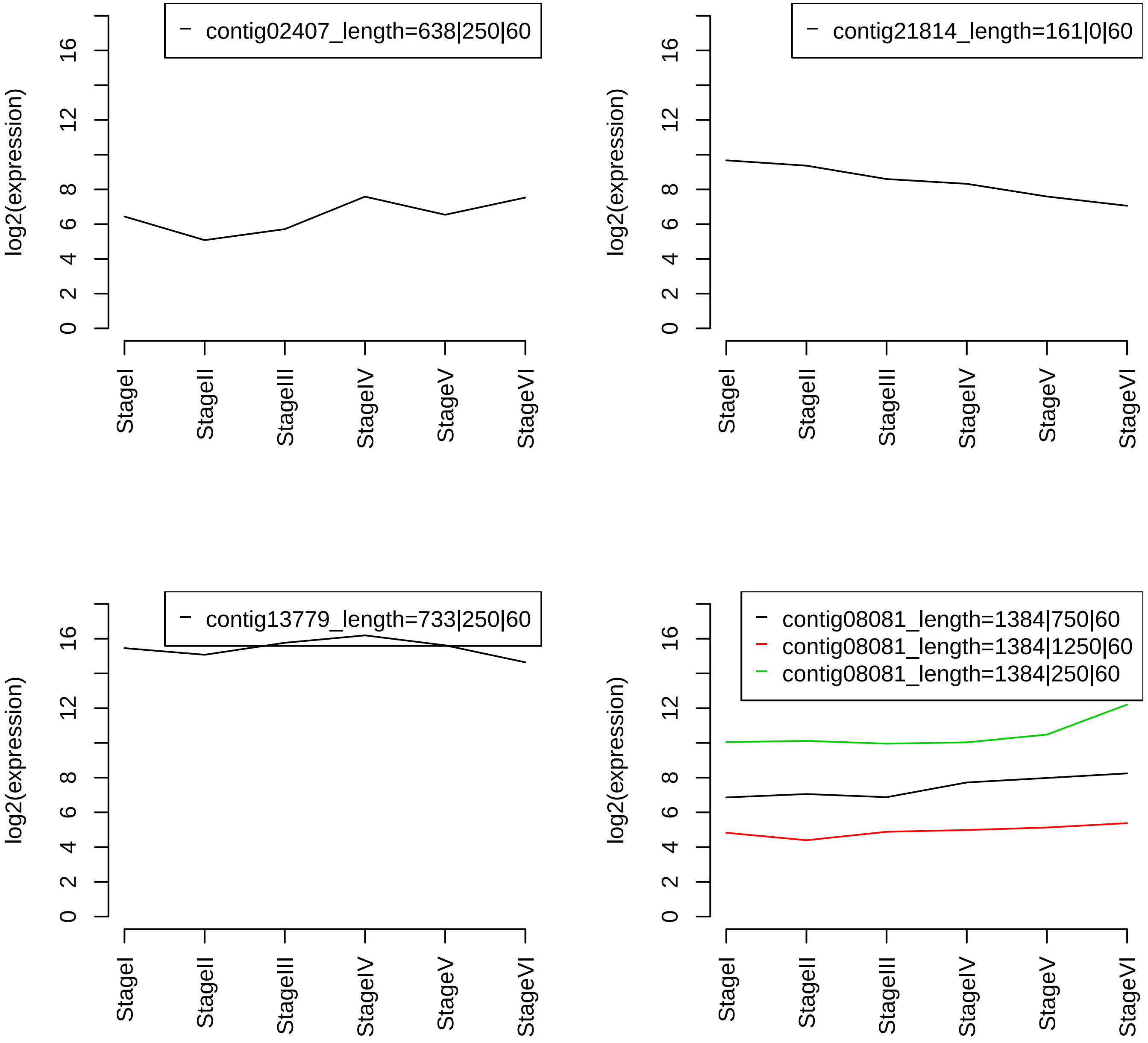
contig02407_length=638|250|60
contig21814_length=161|0|60
contig13779_length=733|250|60
contig08081_length=1384|750|60
contig08081_length=1384|1250|60
contig08081_length=1384|250|60
log2(expression)
0
2
4
6
8
12
16
StageI
StageII
StageIII
StageIV
StageV
StageVI

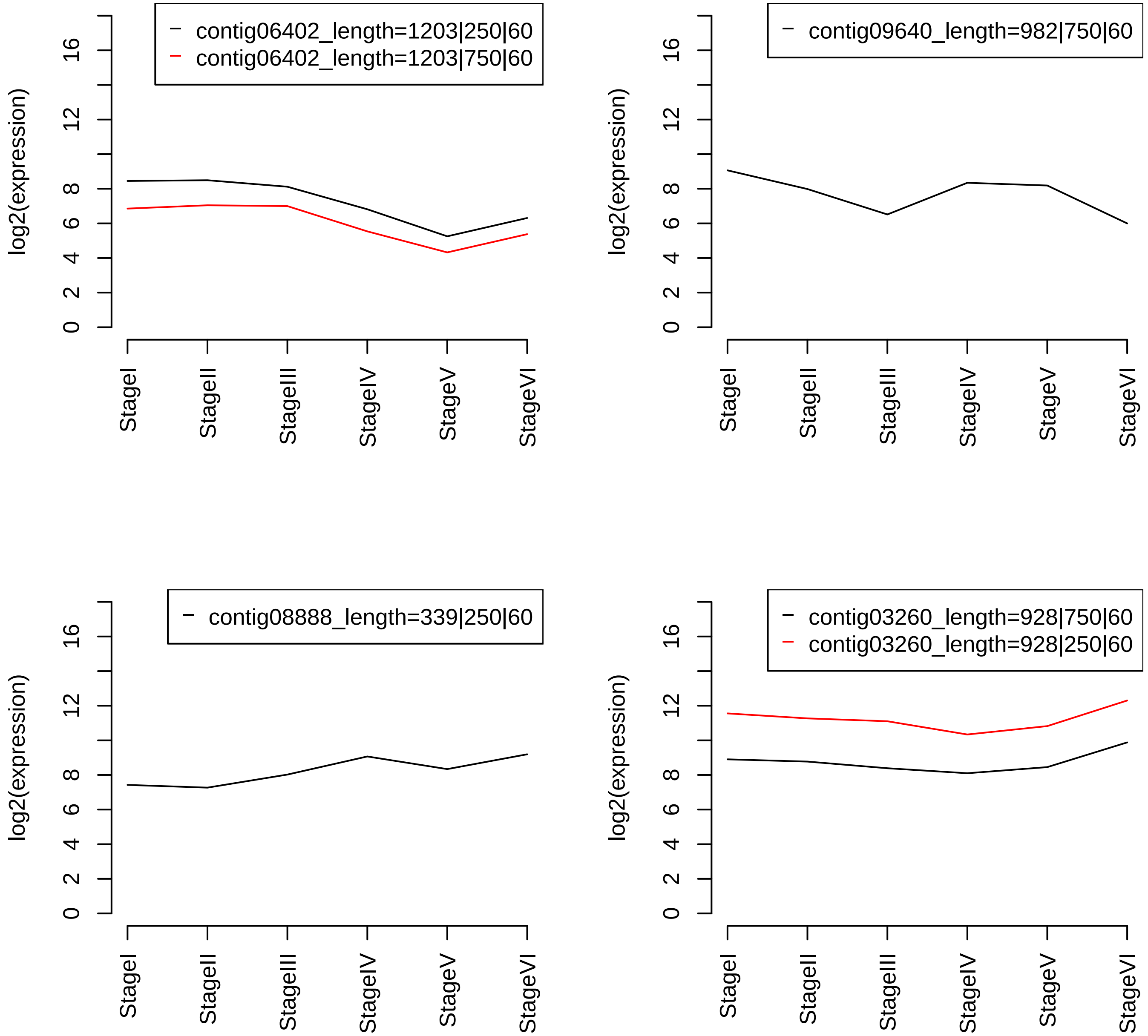
contig06402_length=1203|250|60
contig06402_length=1203|750|60
contig09640_length=982|750|60
contig08888_length=339|250|60
contig03260_length=928|750|60
contig03260_length=928|250|60
log2(expression)
0
2
4
6
8
12
16
StageI
StageII
StageIII
StageIV
StageV
StageVI

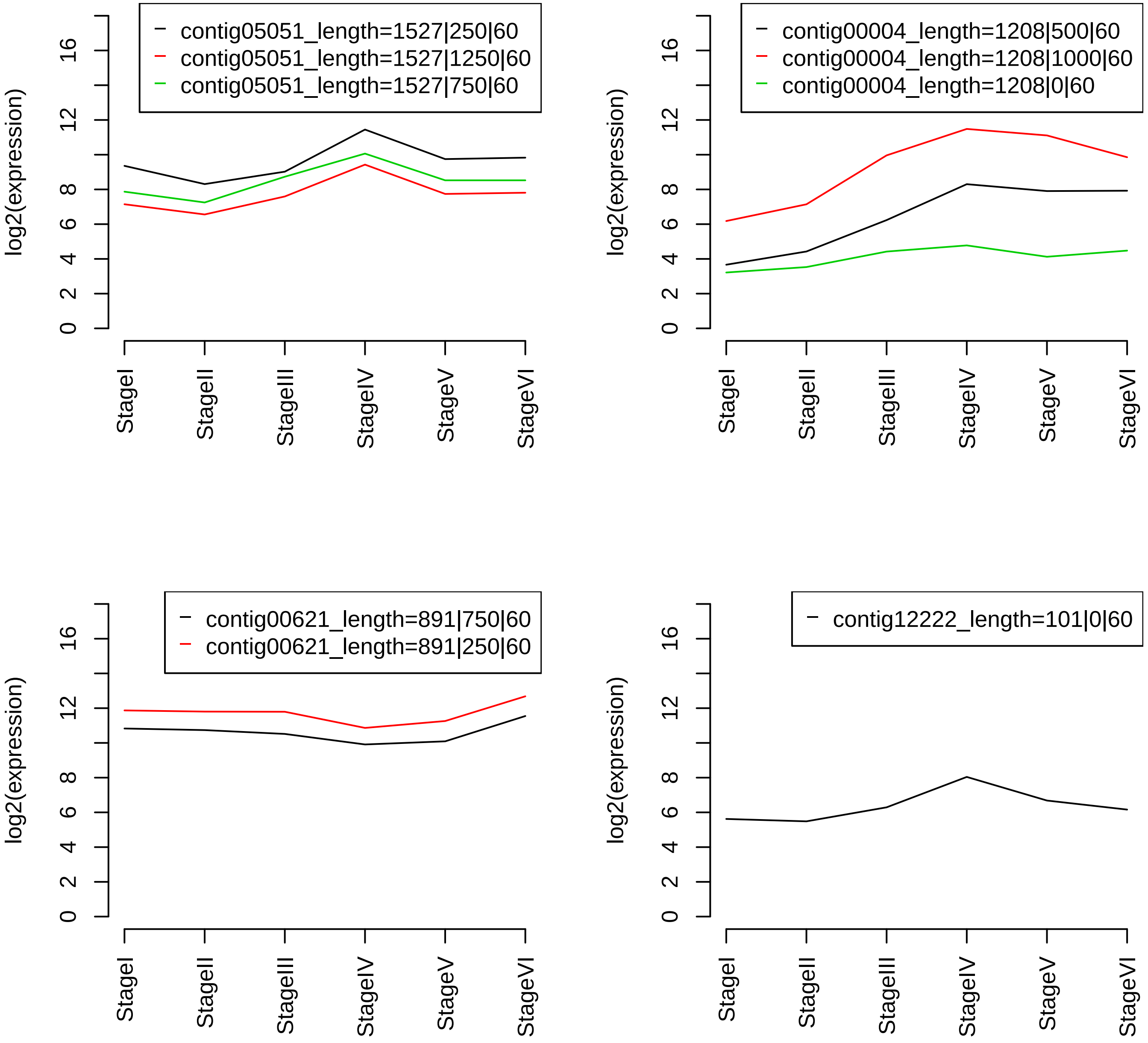
contig05051_length=1527|250|60
contig05051_length=1527|1250|60
contig05051_length=1527|750|60
contig00004_length=1208|500|60
contig00004_length=1208|1000|60
contig00004_length=1208|0|60
contig00621_length=891|750|60
contig00621_length=891|250|60
contig12222_length=101|0|60
log2(expression)
0 2 4 6 8 12 16
StageI StageII StageIII StageIV StageV StageVI

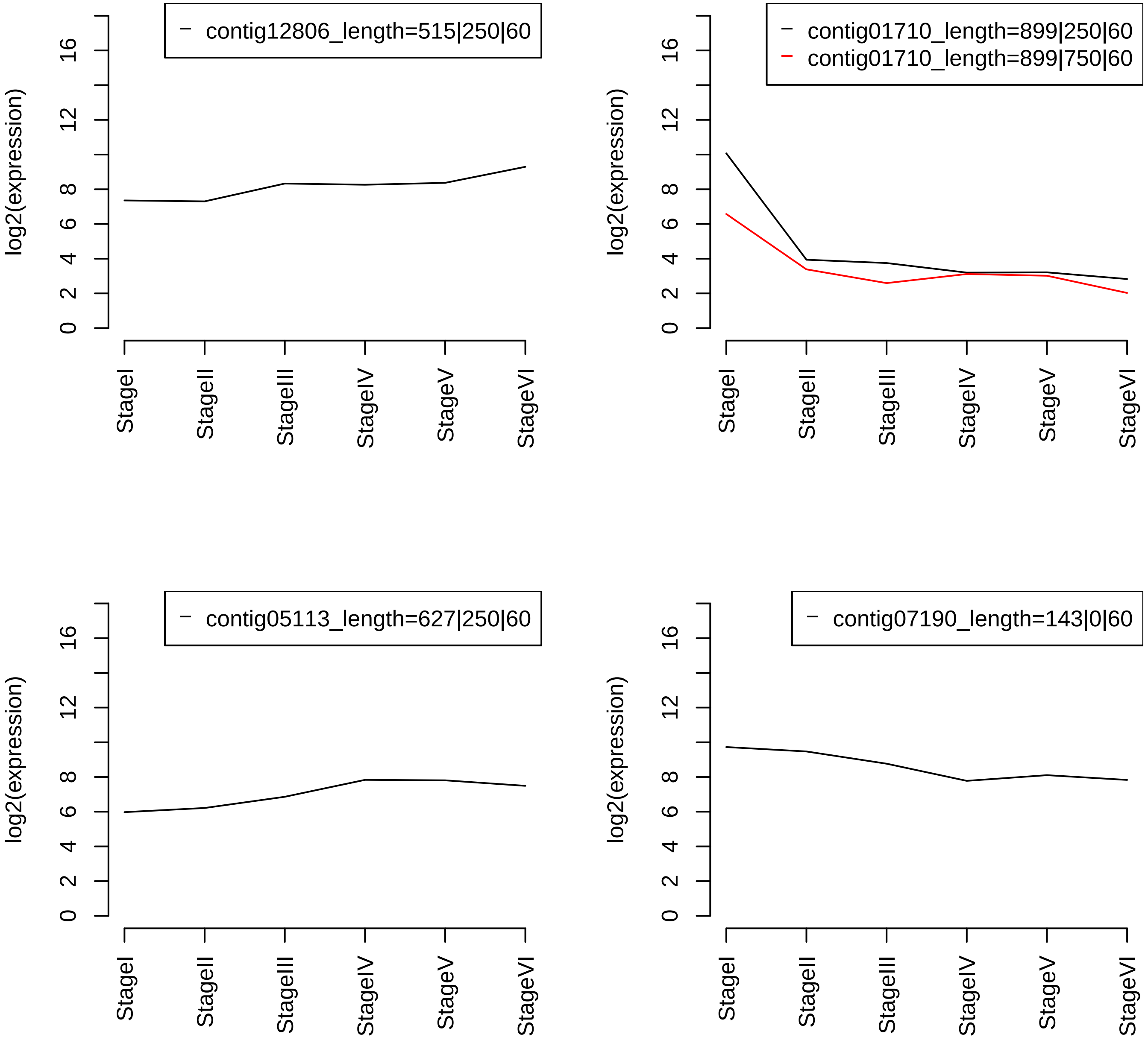

contig12806_length=515|250|60
contig01710_length=899|250|60
contig01710_length=899|750|60
contig05113_length=627|250|60
contig07190_length=143|0|60
log2(expression)
0
2
4
6
8
12
16
StageI
StageII
StageIII
StageIV
StageV
StageVI

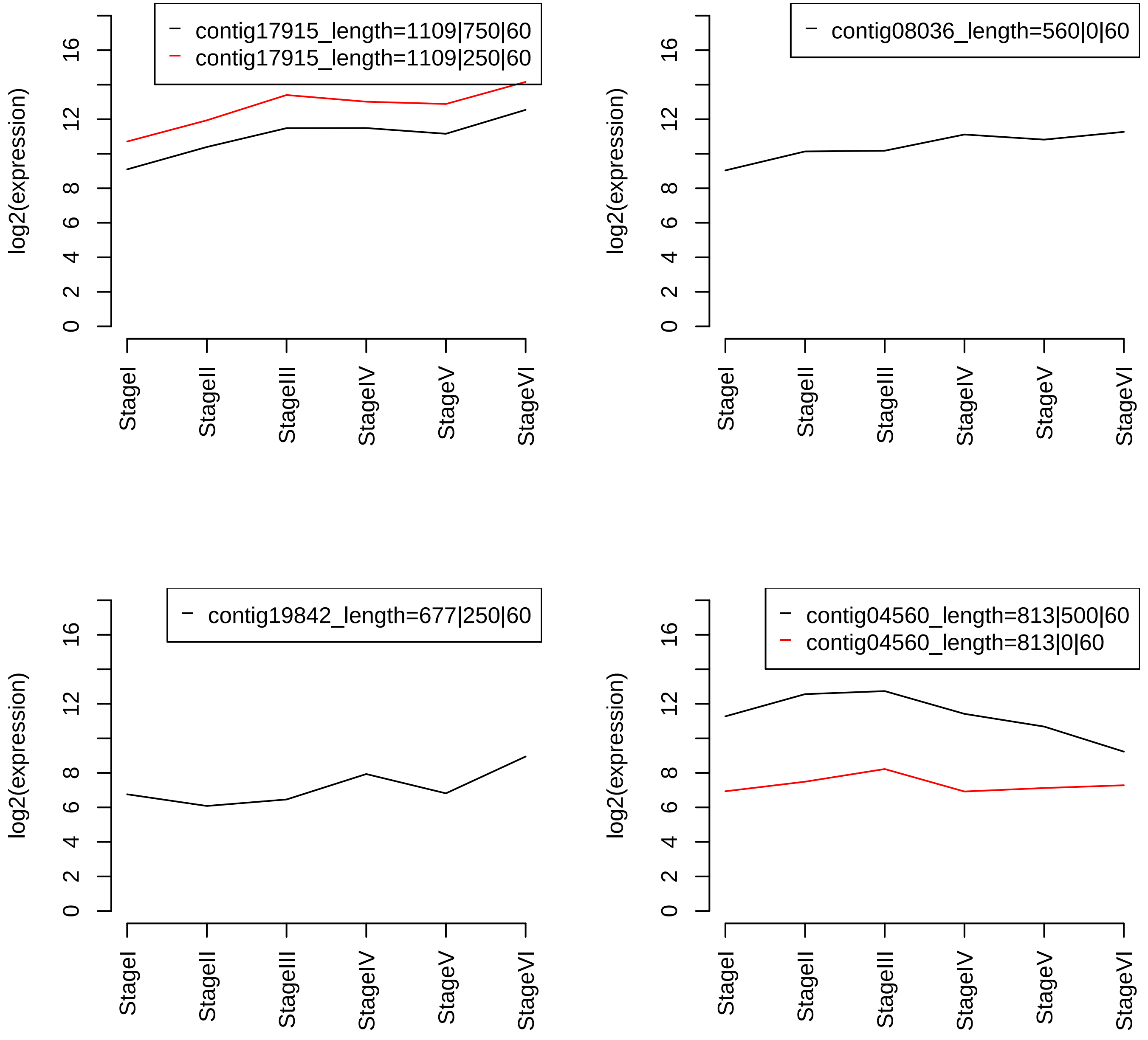

contig17915_length=1109|750|60
contig17915_length=1109|250|60
contig08036_length=560|0|60
contig19842_length=677|250|60
contig04560_length=813|500|60
contig04560_length=813|0|60
log2(expression)
0
2
4
6
8
12
16
StageI
StageII
StageIII
StageIV
StageV
StageVI

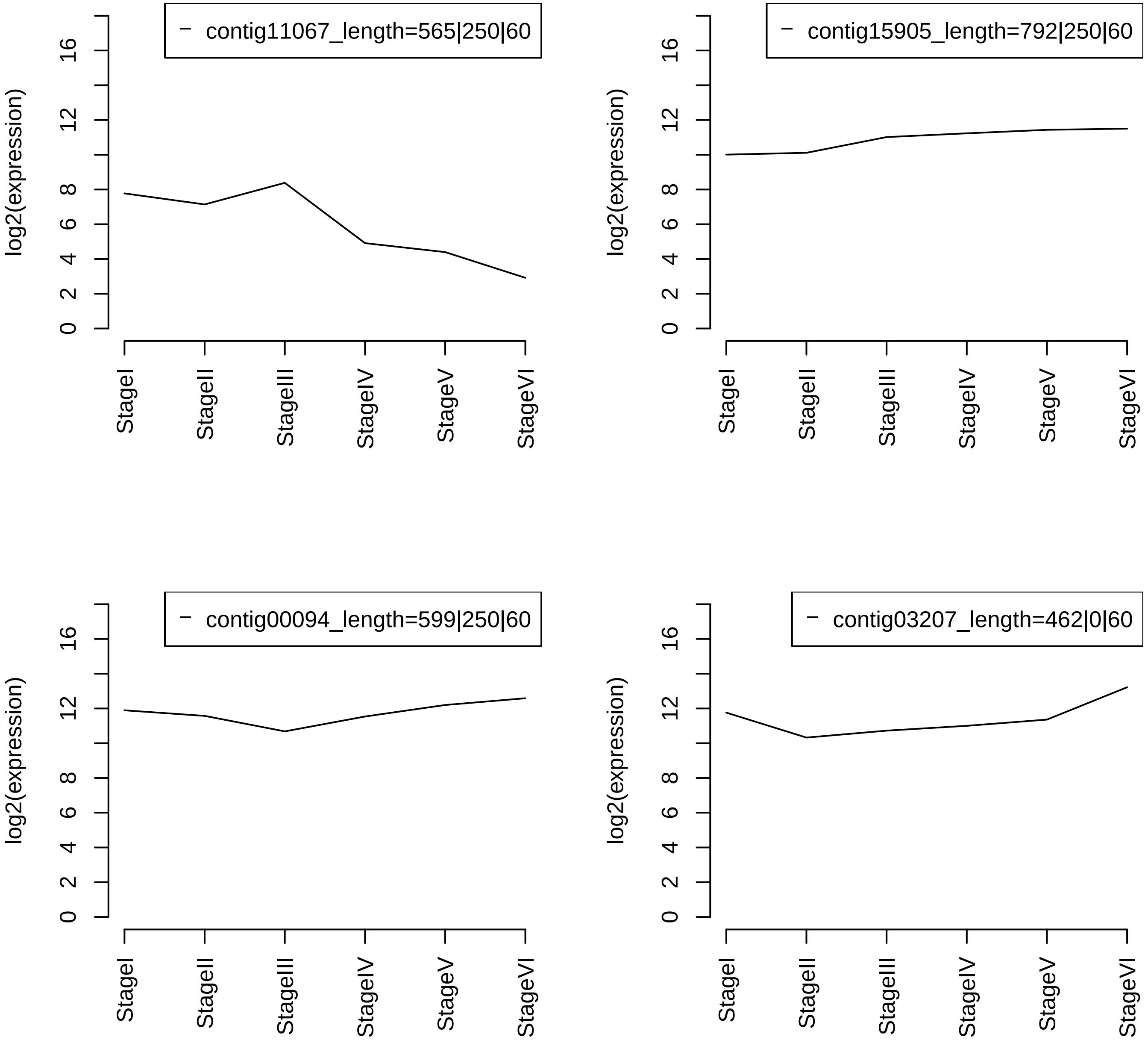

contig11067_length=565|250|60
contig15905_length=792|250|60
contig00094_length=599|250|60
contig03207_length=462|0|60
log2(expression)
0
2
4
6
8
12
16
StageI
StageII
StageIII
StageIV
StageV
StageVI

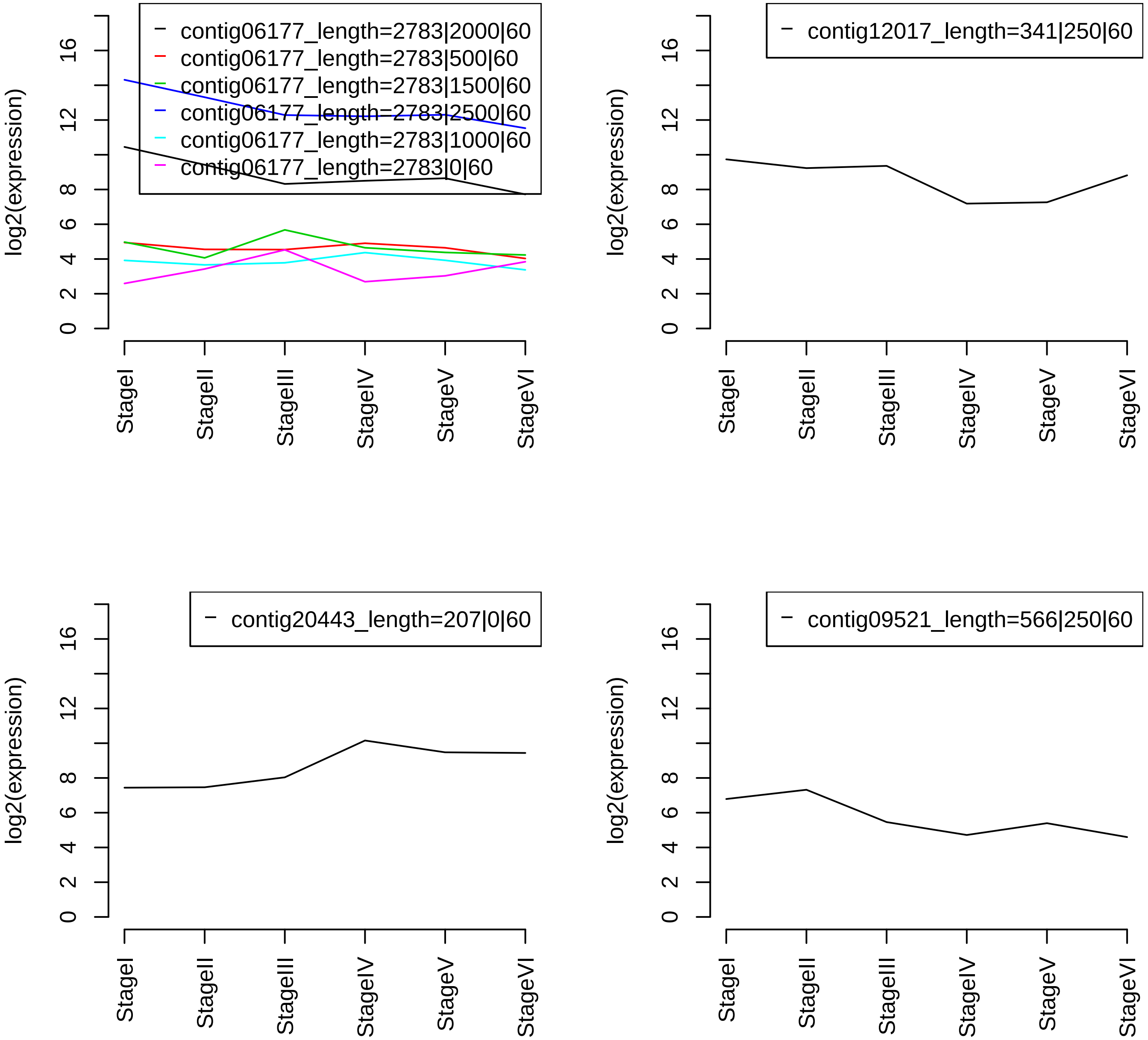
contig06177_length=2783|2000|60
contig06177_length=2783|500|60
contig06177_length=2783|1500|60
contig06177_length=2783|2500|60
contig06177_length=2783|1000|60
contig06177_length=2783|0|60
contig12017_length=341|250|60
contig20443_length=207|0|60
contig09521_length=566|250|60
log2(expression)
0
2
4
6
8
12
16
StageI
StageII
StageIII
StageIV
StageV
StageVI

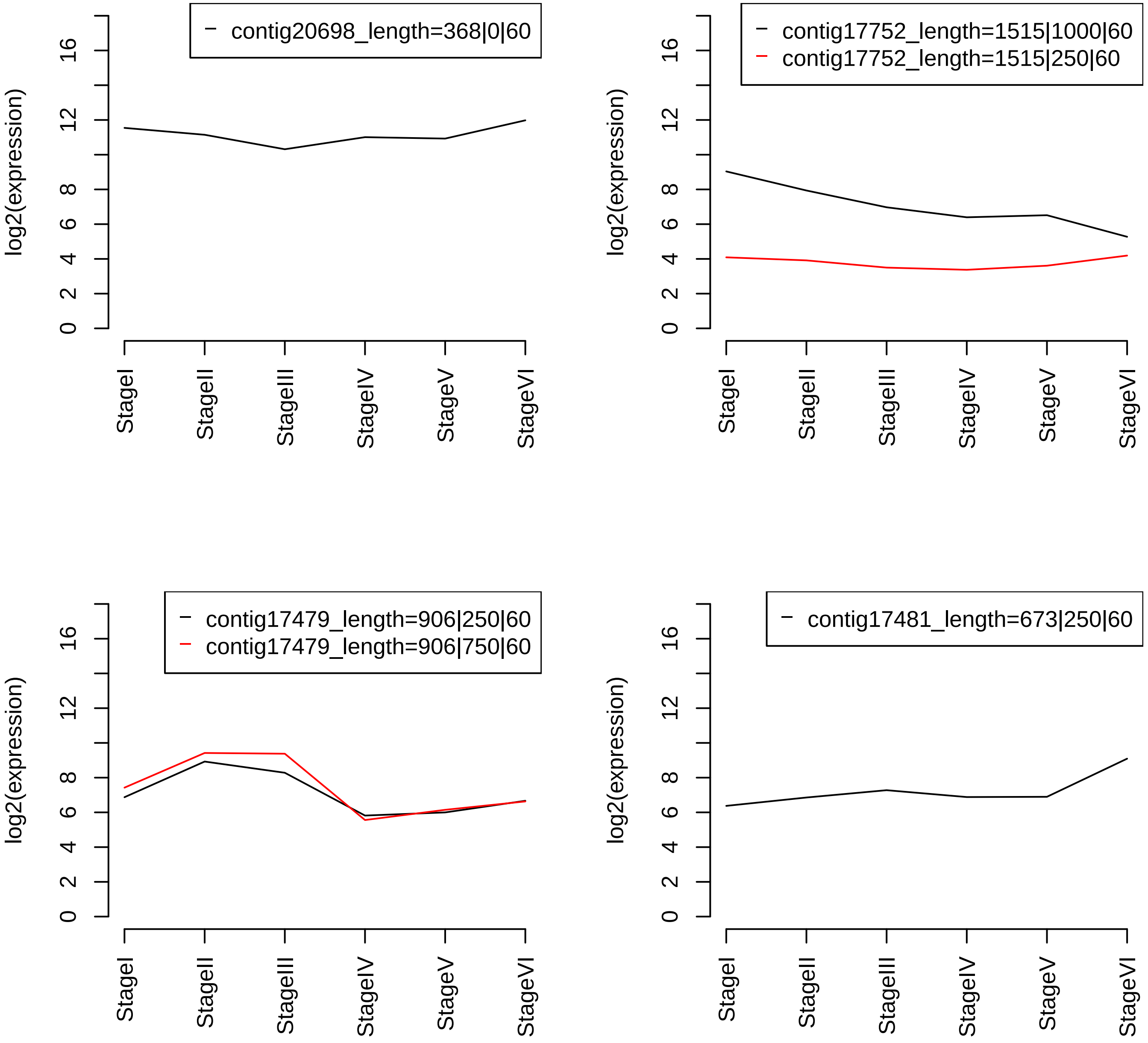

contig20698_length=368|0|60
contig17752_length=1515|1000|60
contig17752_length=1515|250|60
contig17479_length=906|250|60
contig17479_length=906|750|60
contig17481_length=673|250|60
log2(expression)
0
2
4
6
8
12
16
StageI
StageII
StageIII
StageIV
StageV
StageVI

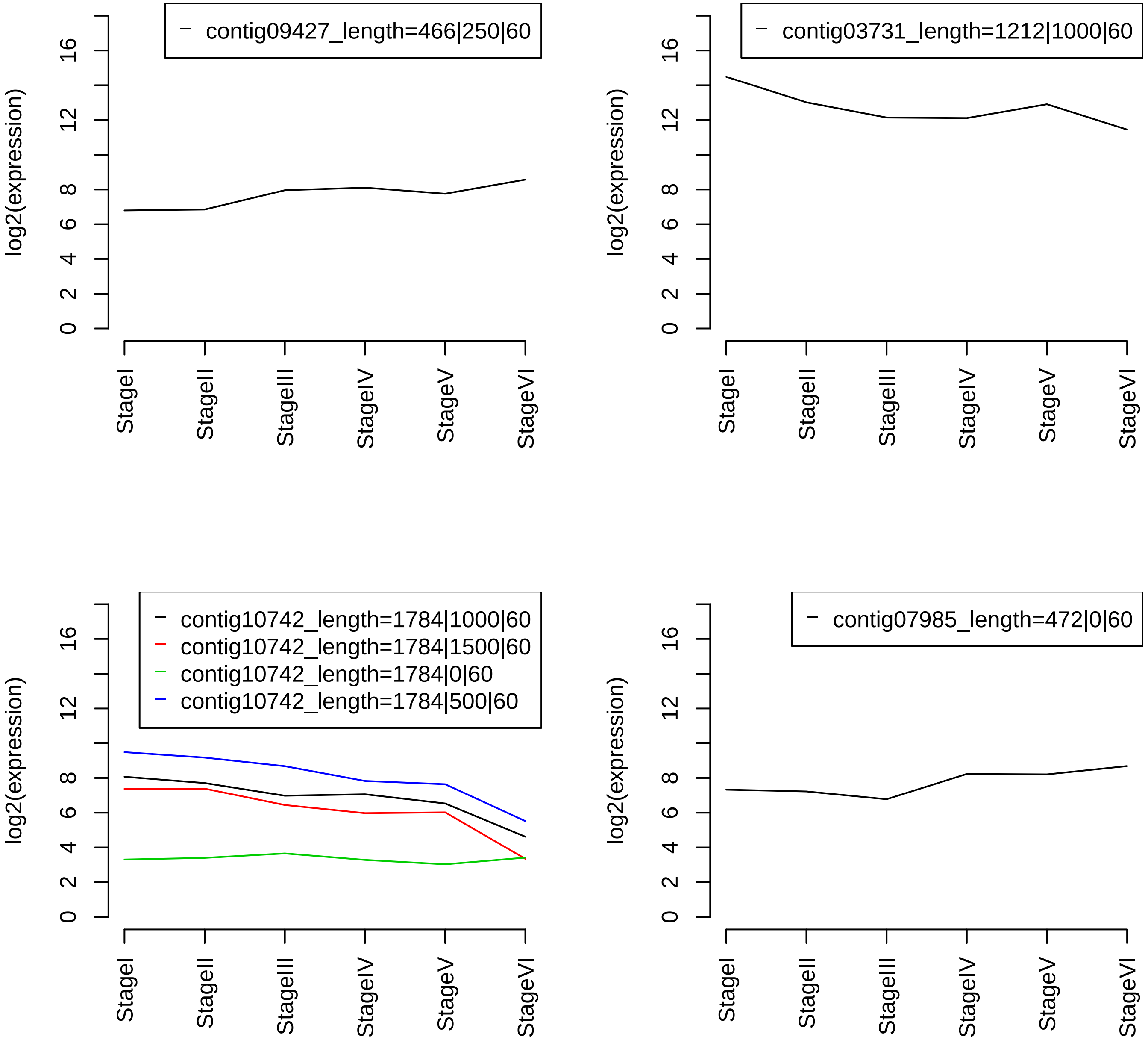
contig09427_length=466|250|60
log2(expression)
0
2
4
6
8
12
16
StageI
StageII
StageIII
StageIV
StageV
StageVI
contig03731_length=1212|1000|60
log2(expression)
0
2
4
6
8
12
16
StageI
StageII
StageIII
StageIV
StageV
StageVI
contig10742_length=1784|1000|60
contig10742_length=1784|1500|60
contig10742_length=1784|0|60
contig10742_length=1784|500|60
log2(expression)
0
2
4
6
8
12
16
StageI
StageII
StageIII
StageIV
StageV
StageVI
contig07985_length=472|0|60
log2(expression)
0
2
4
6
8
12
16
StageI
StageII
StageIII
StageIV
StageV
StageVI

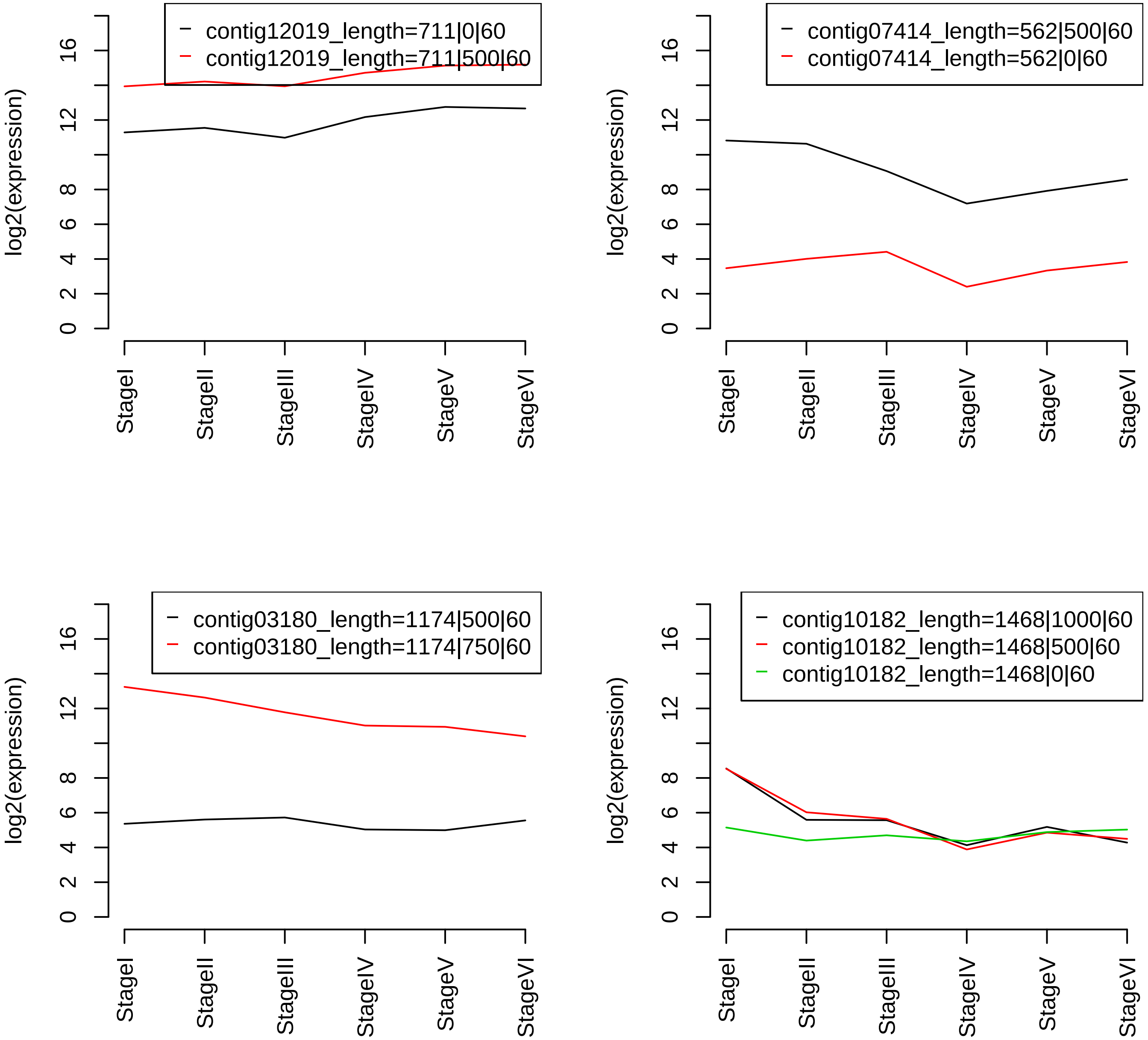
contig12019_length=711|0|60
contig12019_length=711|500|60
contig07414_length=562|500|60
contig07414_length=562|0|60
contig03180_length=1174|500|60
contig03180_length=1174|750|60
contig10182_length=1468|1000|60
contig10182_length=1468|500|60
contig10182_length=1468|0|60
log2(expression)
0 2 4 6 8 12 16
StageI StageII StageIII StageIV StageV StageVI

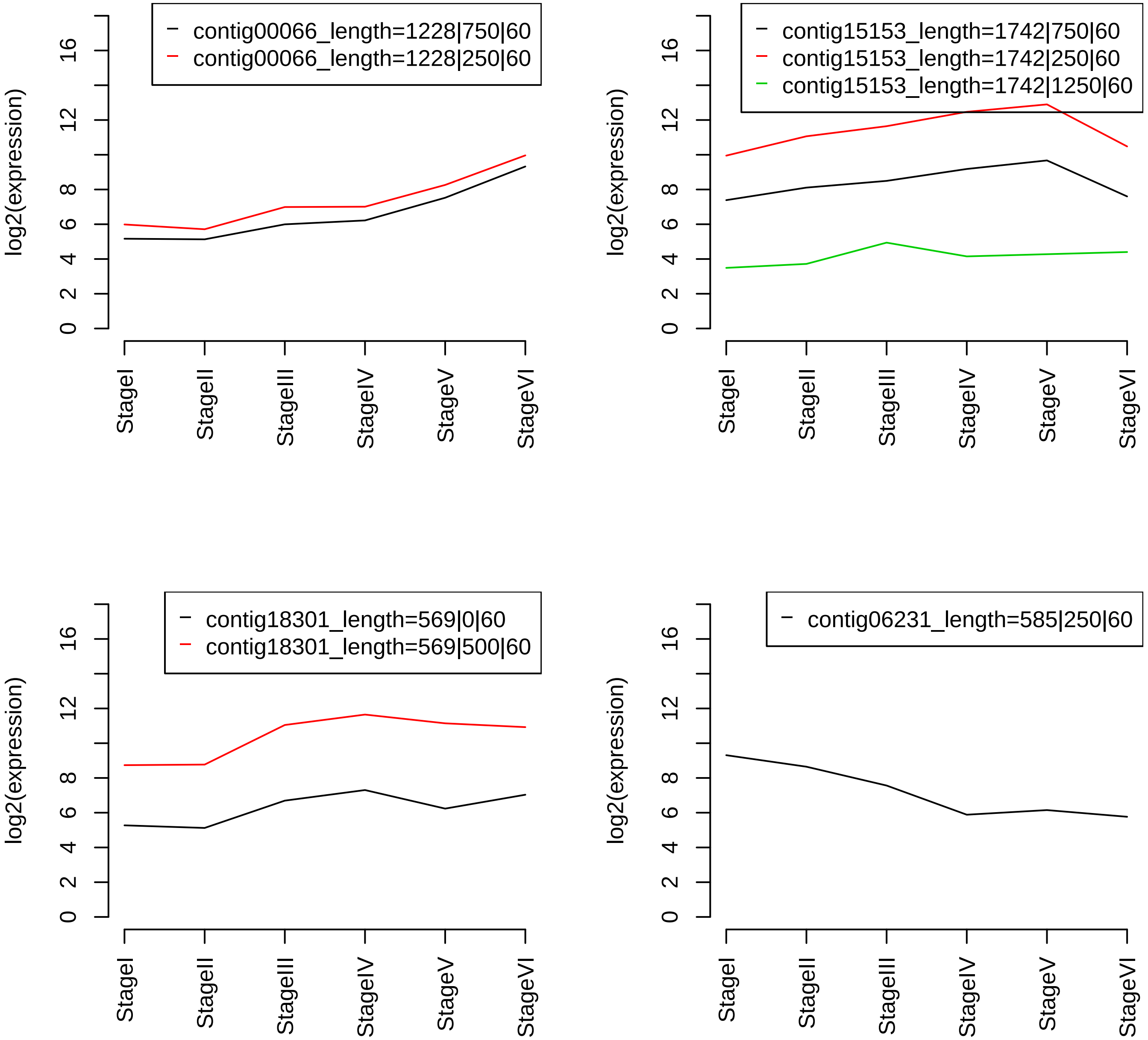

contig00066_length=1228|750|60
contig00066_length=1228|250|60
contig15153_length=1742|750|60
contig15153_length=1742|250|60
contig15153_length=1742|1250|60
contig18301_length=569|0|60
contig18301_length=569|500|60
contig06231_length=585|250|60
log2(expression)
0
2
4
6
8
12
16
StageI
StageII
StageIII
StageIV
StageV
StageVI

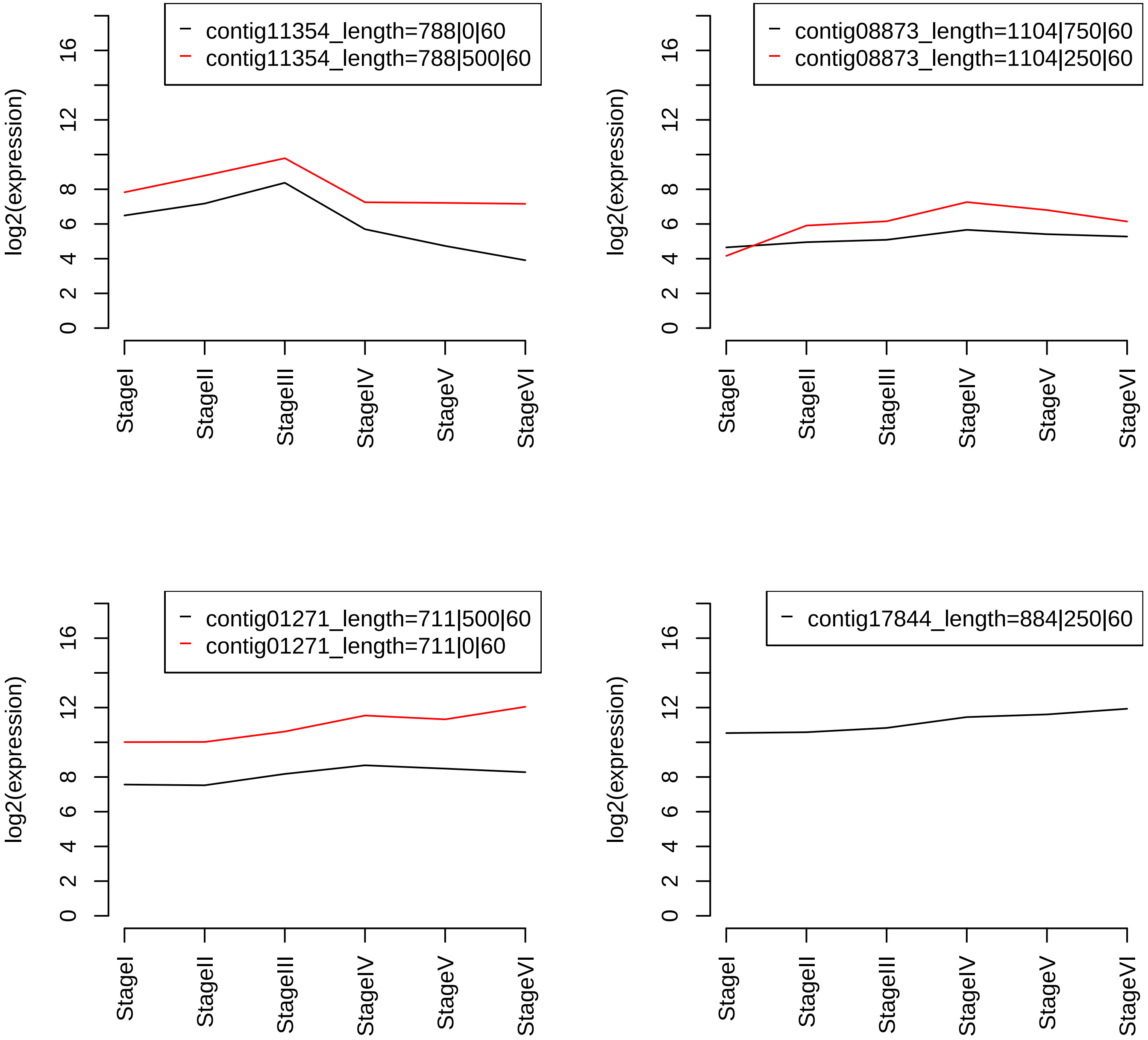
contig11354_length=788|0|60
contig11354_length=788|500|60
contig08873_length=1104|750|60
contig08873_length=1104|250|60
contig01271_length=711|500|60
contig01271_length=711|0|60
contig17844_length=884|250|60
log2(expression)
0 2 4 6 8 12 16
StageI
StageII
StageIII
StageIV
StageV
StageVI

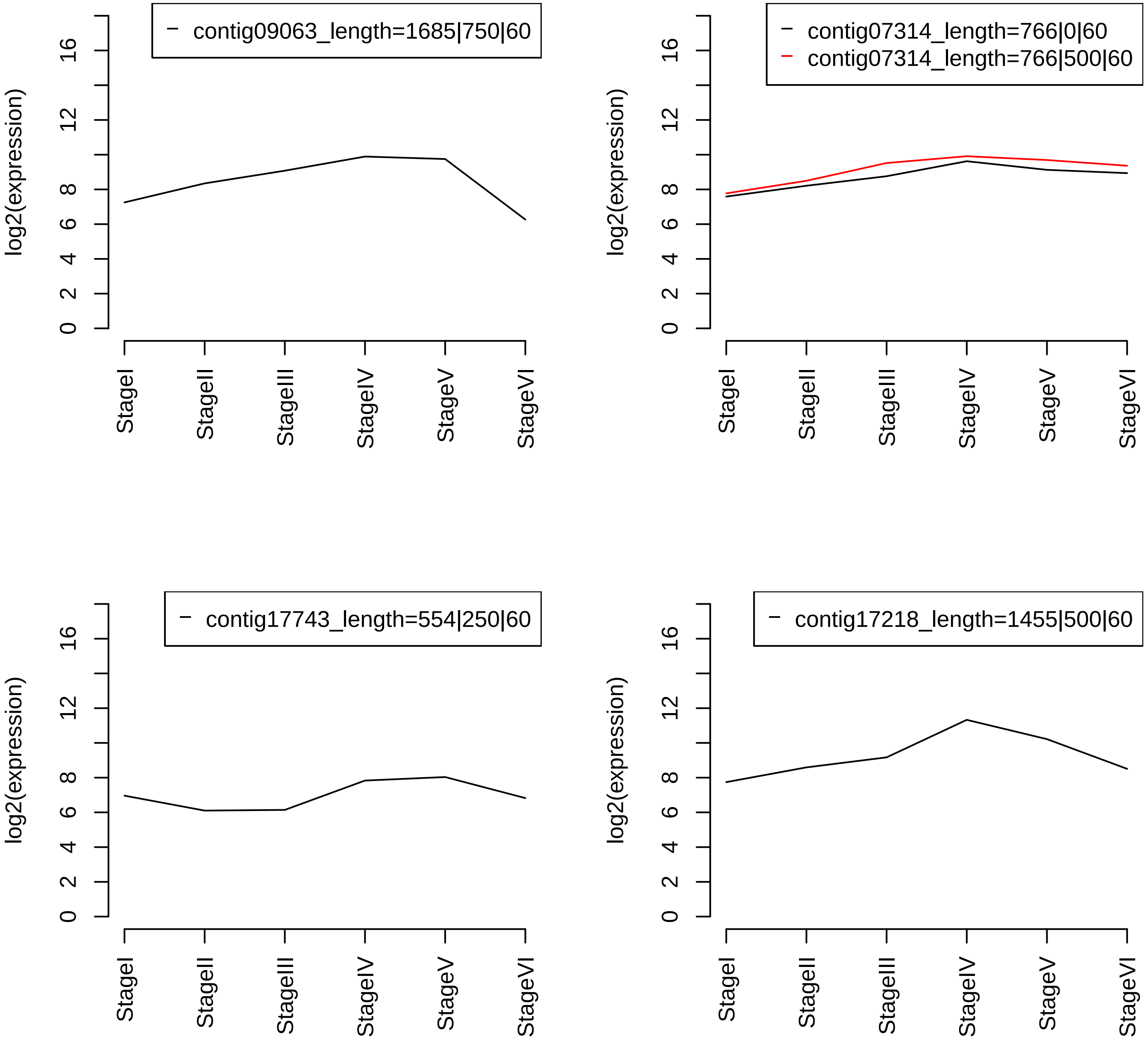
contig09063_length=1685|750|60
contig07314_length=766|0|60
contig07314_length=766|500|60
contig17743_length=554|250|60
contig17218_length=1455|500|60
log2(expression)
0
2
4
6
8
12
16
StageI
StageII
StageIII
StageIV
StageV
StageVI

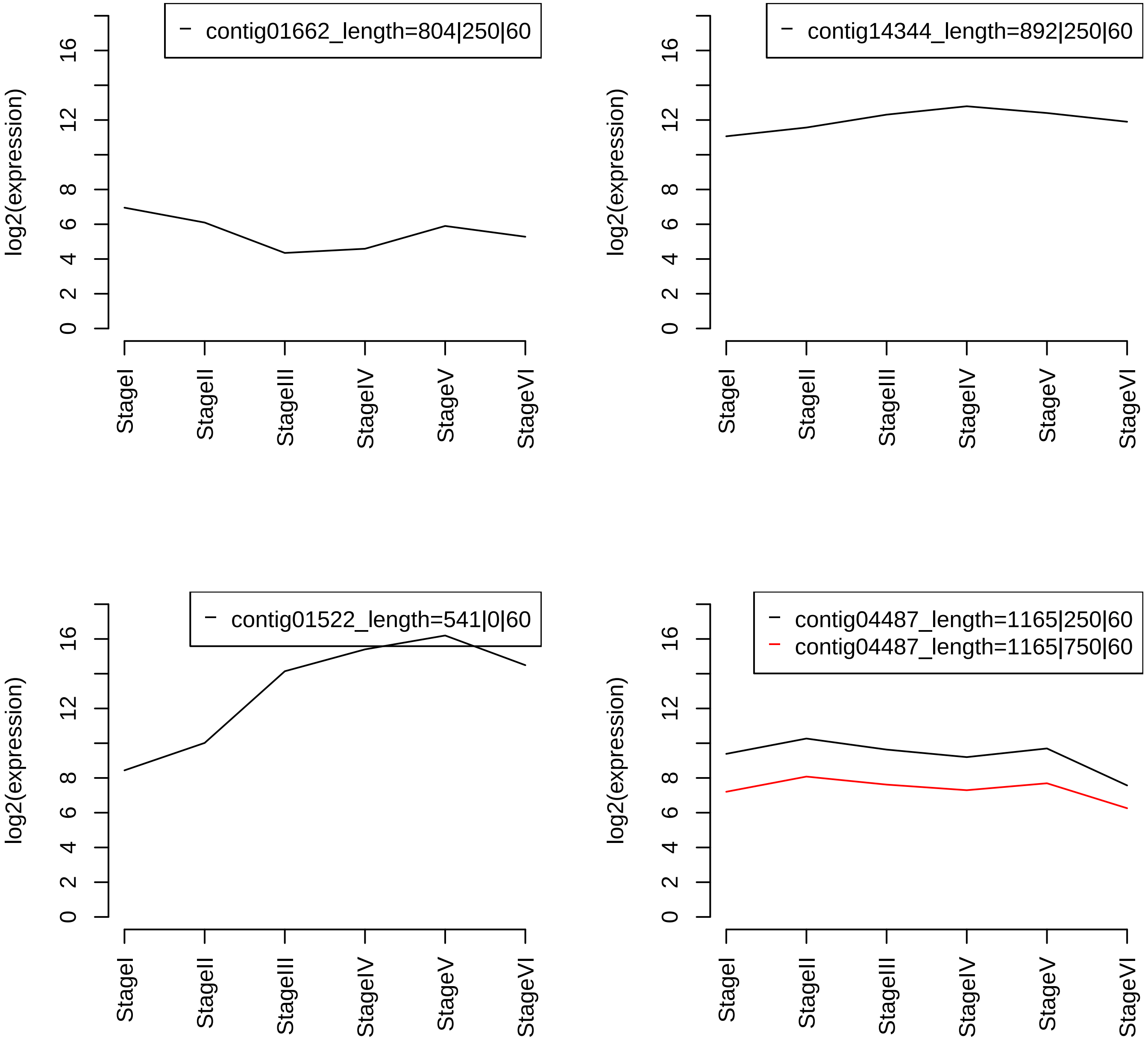
contig01662_length=804|250|60
contig14344_length=892|250|60
contig01522_length=541|0|60
contig04487_length=1165|250|60
contig04487_length=1165|750|60
log2(expression)
0
2
4
6
8
12
16
StageI
StageII
StageIII
StageIV
StageV
StageVI

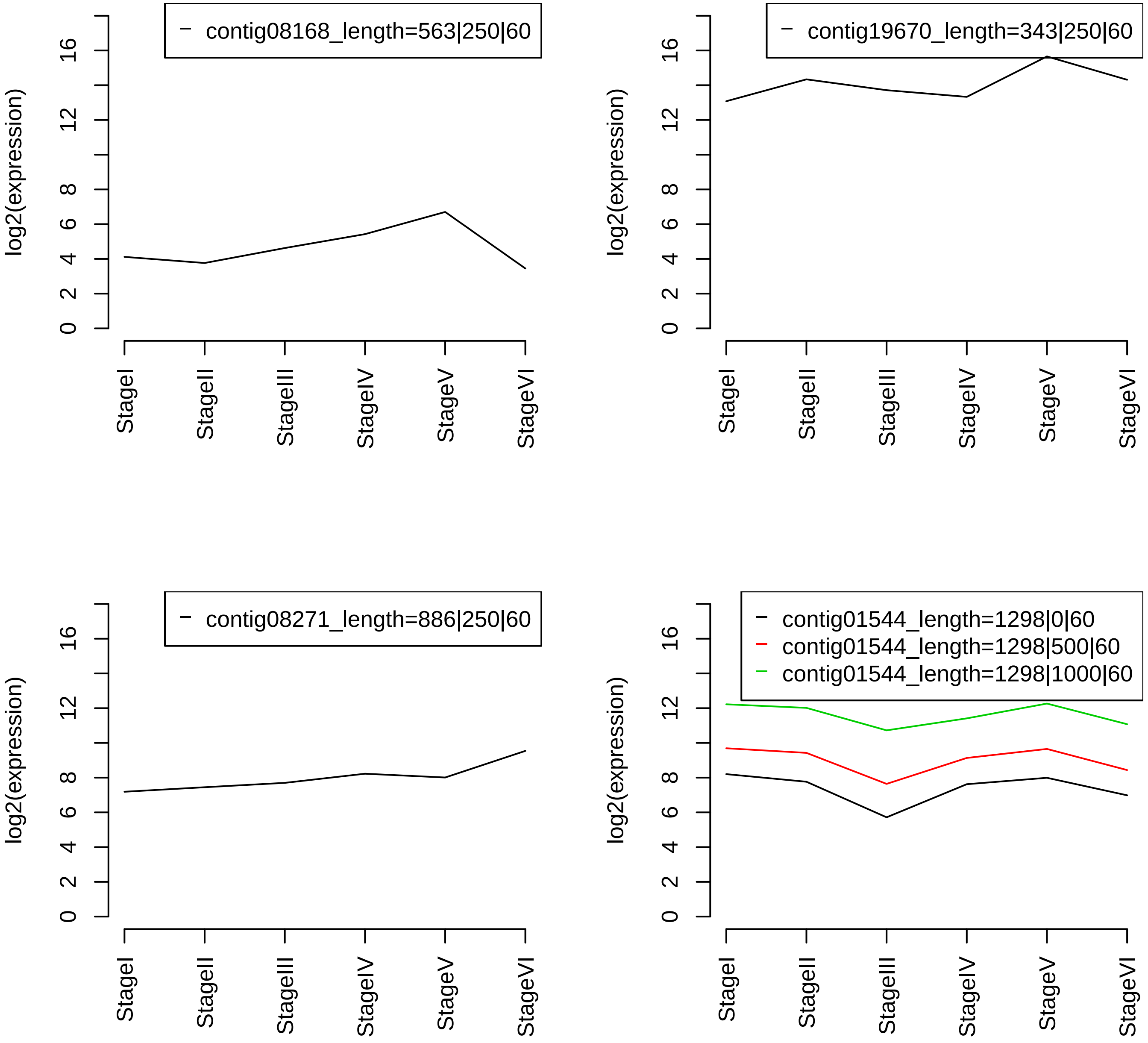
contig08168_length=563|250|60
contig19670_length=343|250|60
contig08271_length=886|250|60
contig01544_length=1298|0|60
contig01544_length=1298|500|60
contig01544_length=1298|1000|60
log2(expression)
0
2
4
6
8
12
16
StageI
StageII
StageIII
StageIV
StageV
StageVI

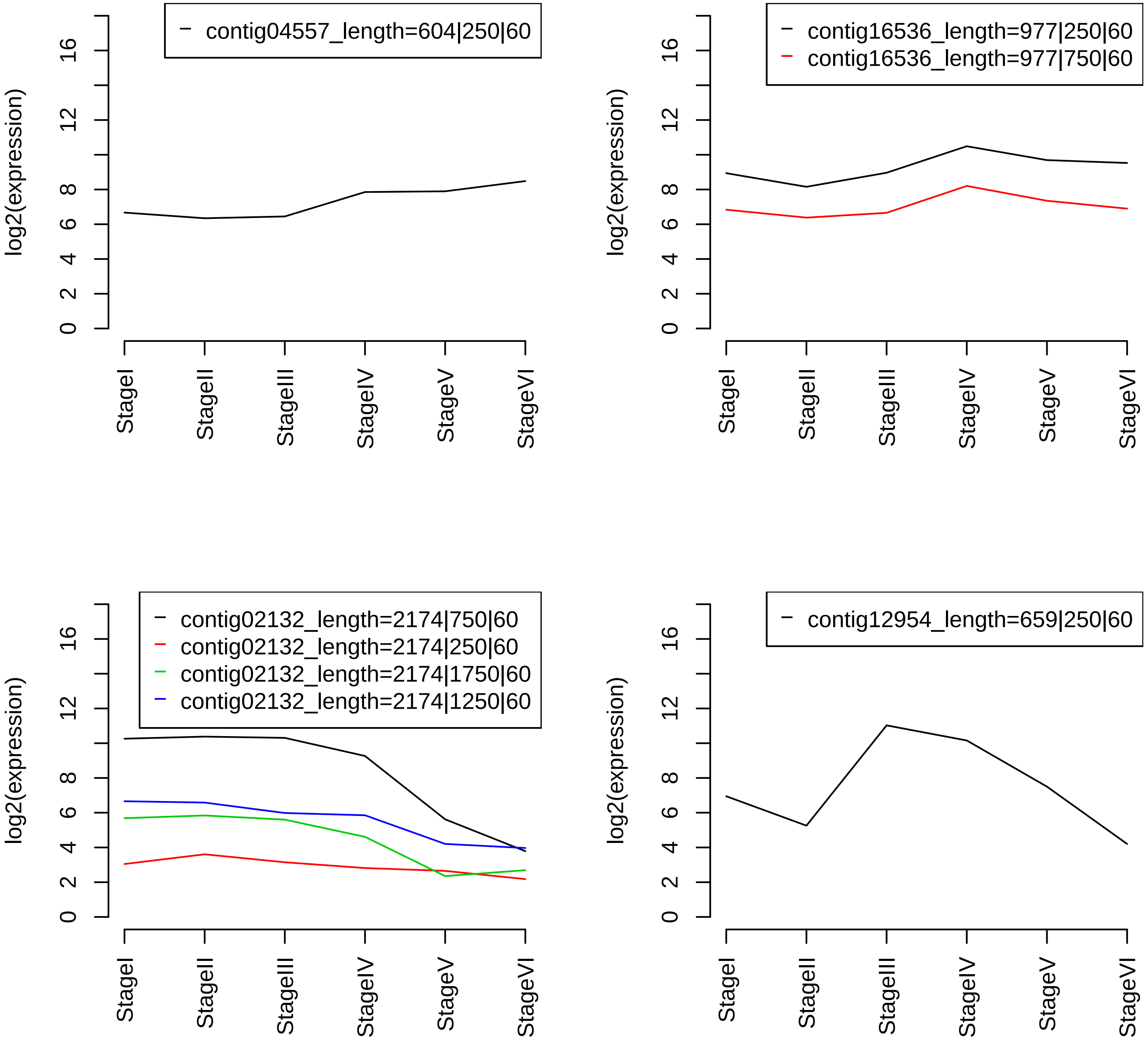

contig04557_length=604|250|60
contig16536_length=977|250|60
contig16536_length=977|750|60
contig02132_length=2174|750|60
contig02132_length=2174|250|60
contig02132_length=2174|1750|60
contig02132_length=2174|1250|60
contig12954_length=659|250|60
log2(expression)
0
2
4
6
8
12
16
StageI
StageII
StageIII
StageIV
StageV
StageVI

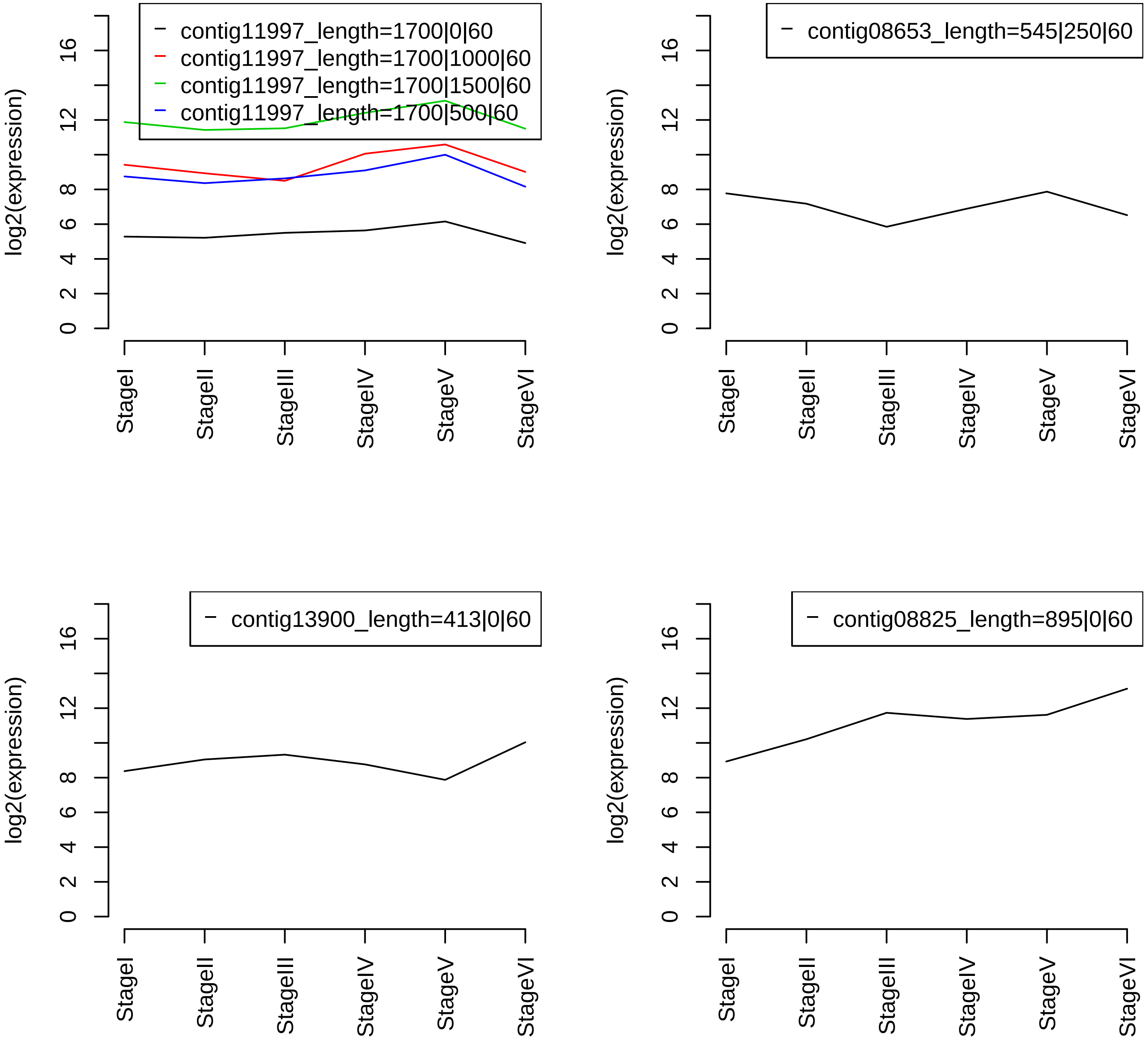

contig11997_length=1700|0|60
contig11997_length=1700|1000|60
contig11997_length=1700|1500|60
contig11997_length=1700|500|60
contig08653_length=545|250|60
contig13900_length=413|0|60
contig08825_length=895|0|60
log2(expression)
0
2
4
6
8
12
16
StageI
StageII
StageIII
StageIV
StageV
StageVI

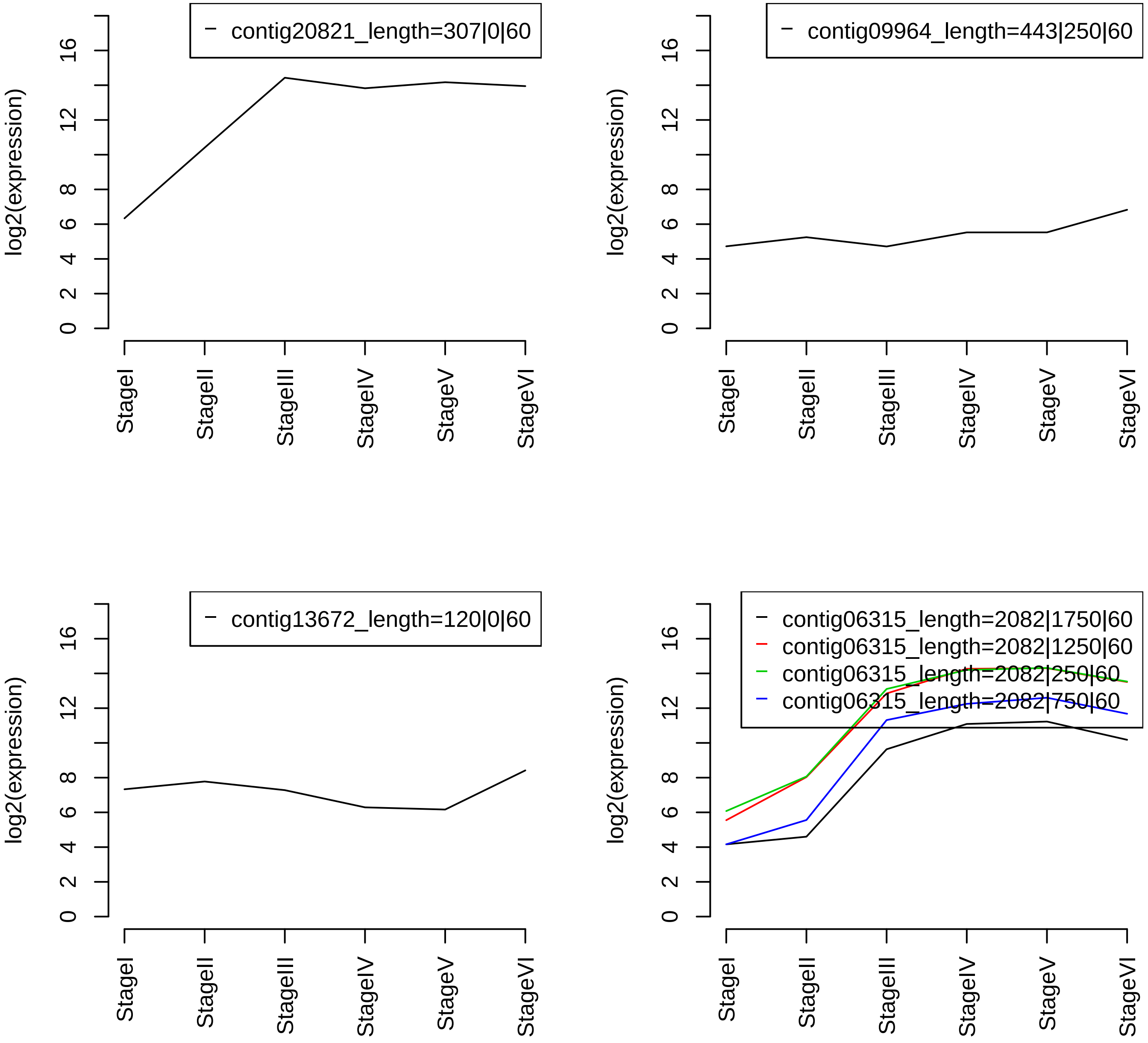

contig20821_length=307|0|60
contig09964_length=443|250|60
contig13672_length=120|0|60
contig06315_length=2082|1750|60
contig06315_length=2082|1250|60
contig06315_length=2082|250|60
contig06315_length=2082|750|60
log2(expression)
0 2 4 6 8 12 16
StageI StageII StageIII StageIV StageV StageVI

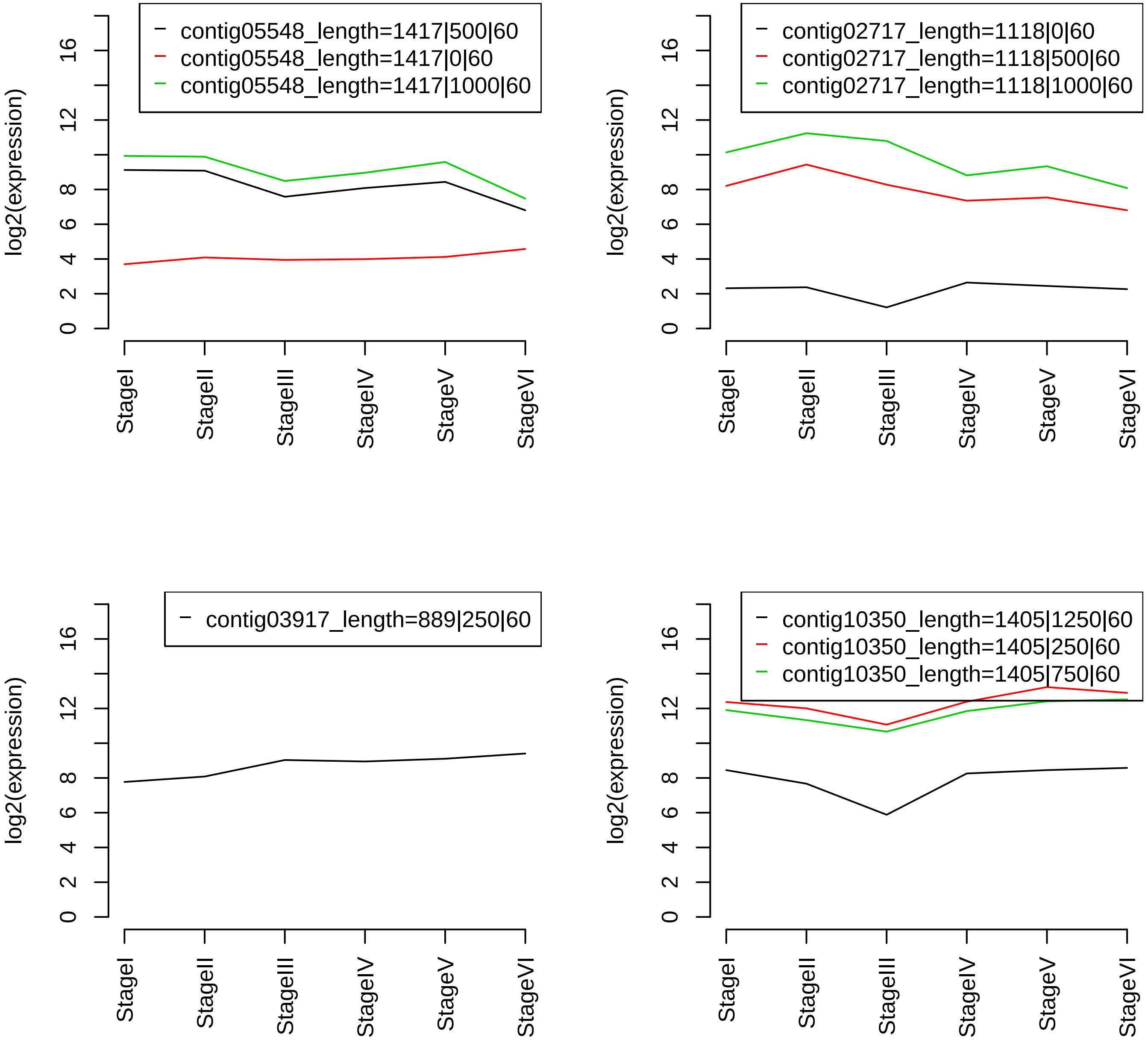
contig05548_length=1417|500|60
contig05548_length=1417|0|60
contig05548_length=1417|1000|60
contig02717_length=1118|0|60
contig02717_length=1118|500|60
contig02717_length=1118|1000|60
contig03917_length=889|250|60
contig10350_length=1405|1250|60
contig10350_length=1405|250|60
contig10350_length=1405|750|60
log2(expression)
0 2 4 6 8 12 16
StageI StageII StageIII StageIV StageV StageVI

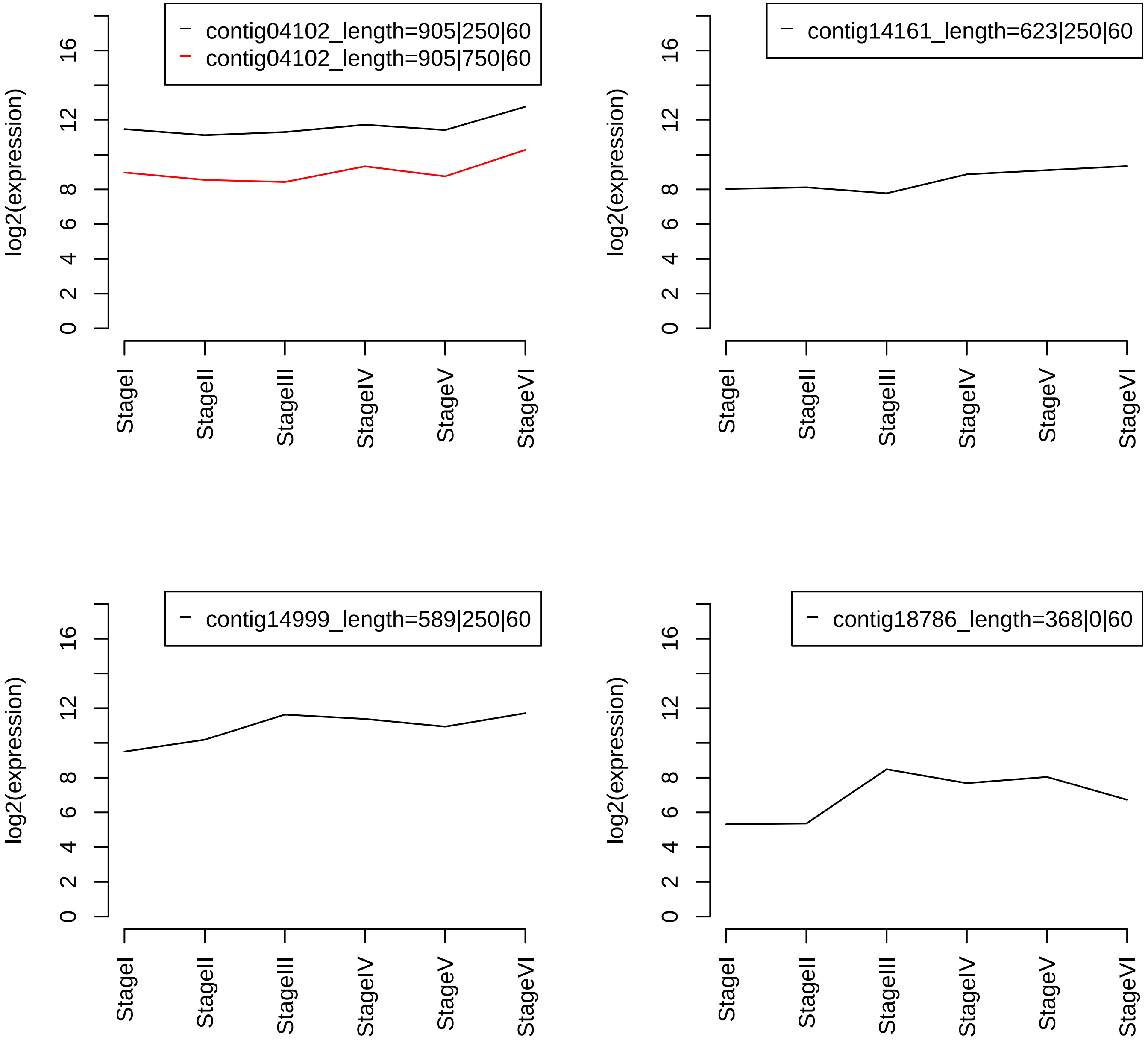
contig04102_length=905|250|60
contig04102_length=905|750|60
contig14161_length=623|250|60
contig14999_length=589|250|60
contig18786_length=368|0|60
log2(expression)
0
2
4
6
8
12
16
StageI
StageII
StageIII
StageIV
StageV
StageVI

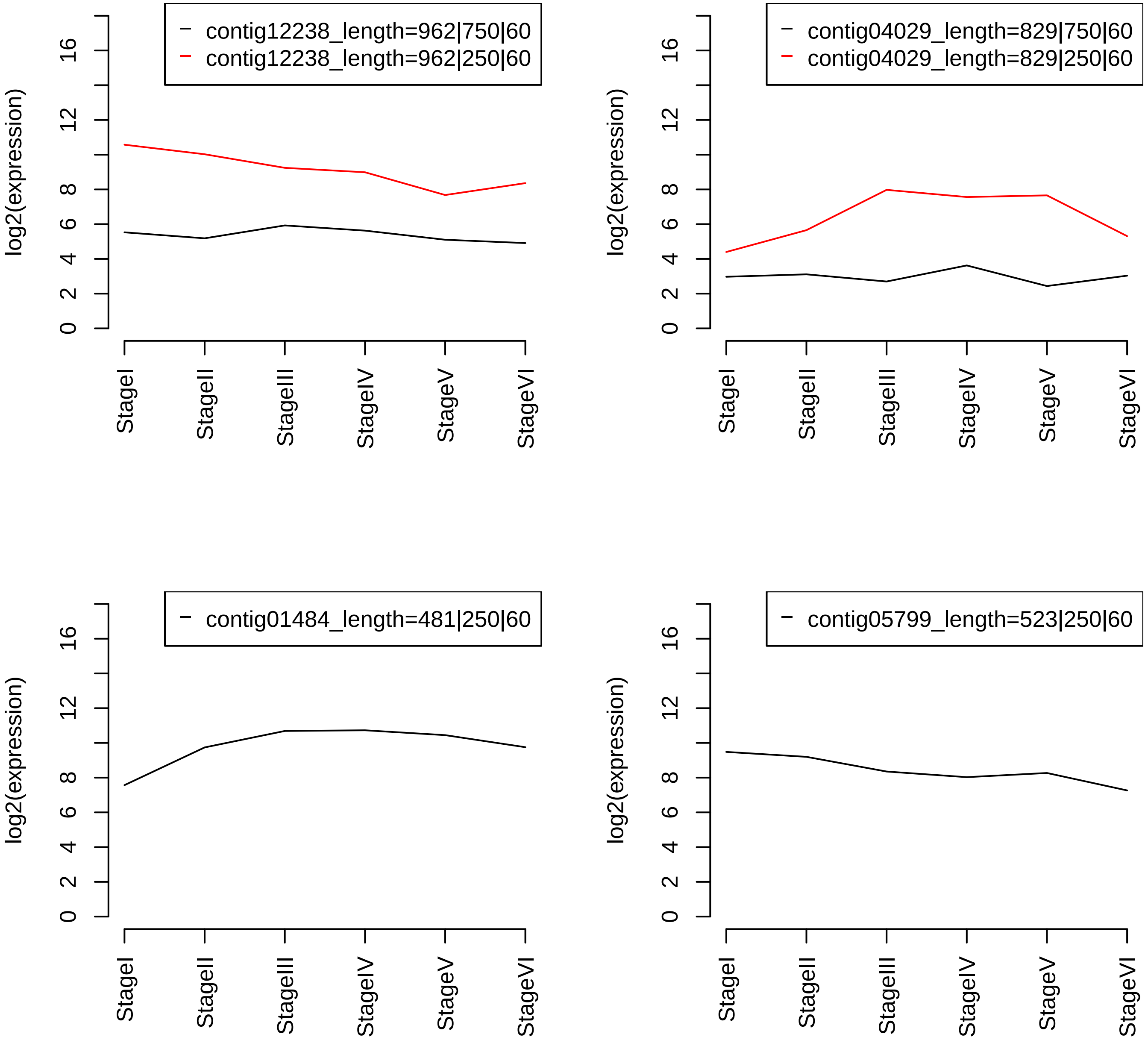
contig12238_length=962|750|60
contig12238_length=962|250|60
contig04029_length=829|750|60
contig04029_length=829|250|60
contig01484_length=481|250|60
contig05799_length=523|250|60
log2(expression)
0
2
4
6
8
12
16
StageI
StageII
StageIII
StageIV
StageV
StageVI

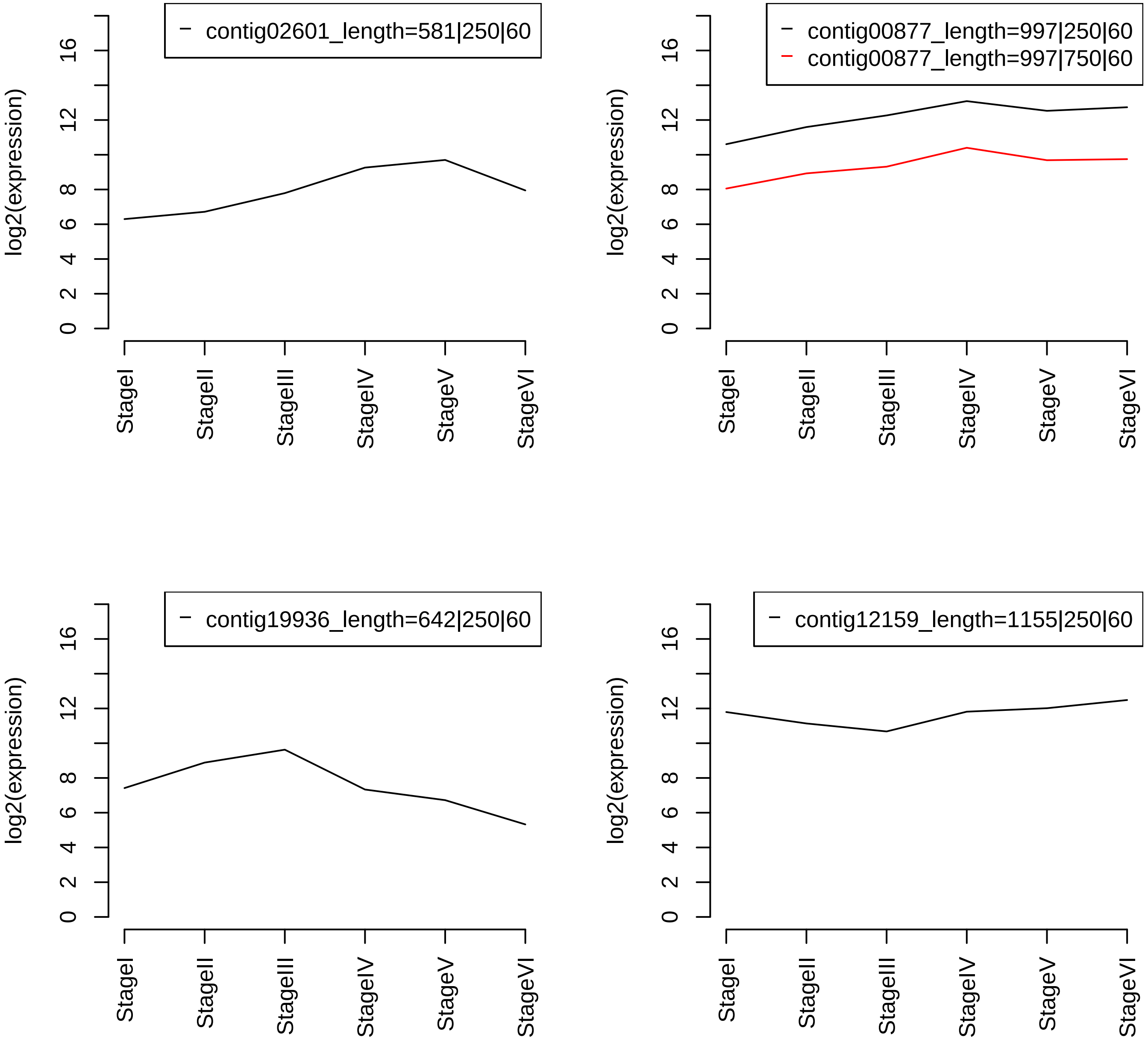
contig02601_length=581|250|60
contig00877_length=997|250|60
contig00877_length=997|750|60
contig19936_length=642|250|60
contig12159_length=1155|250|60
log2(expression)
0
2
4
6
8
12
16
StageI
StageII
StageIII
StageIV
StageV
StageVI

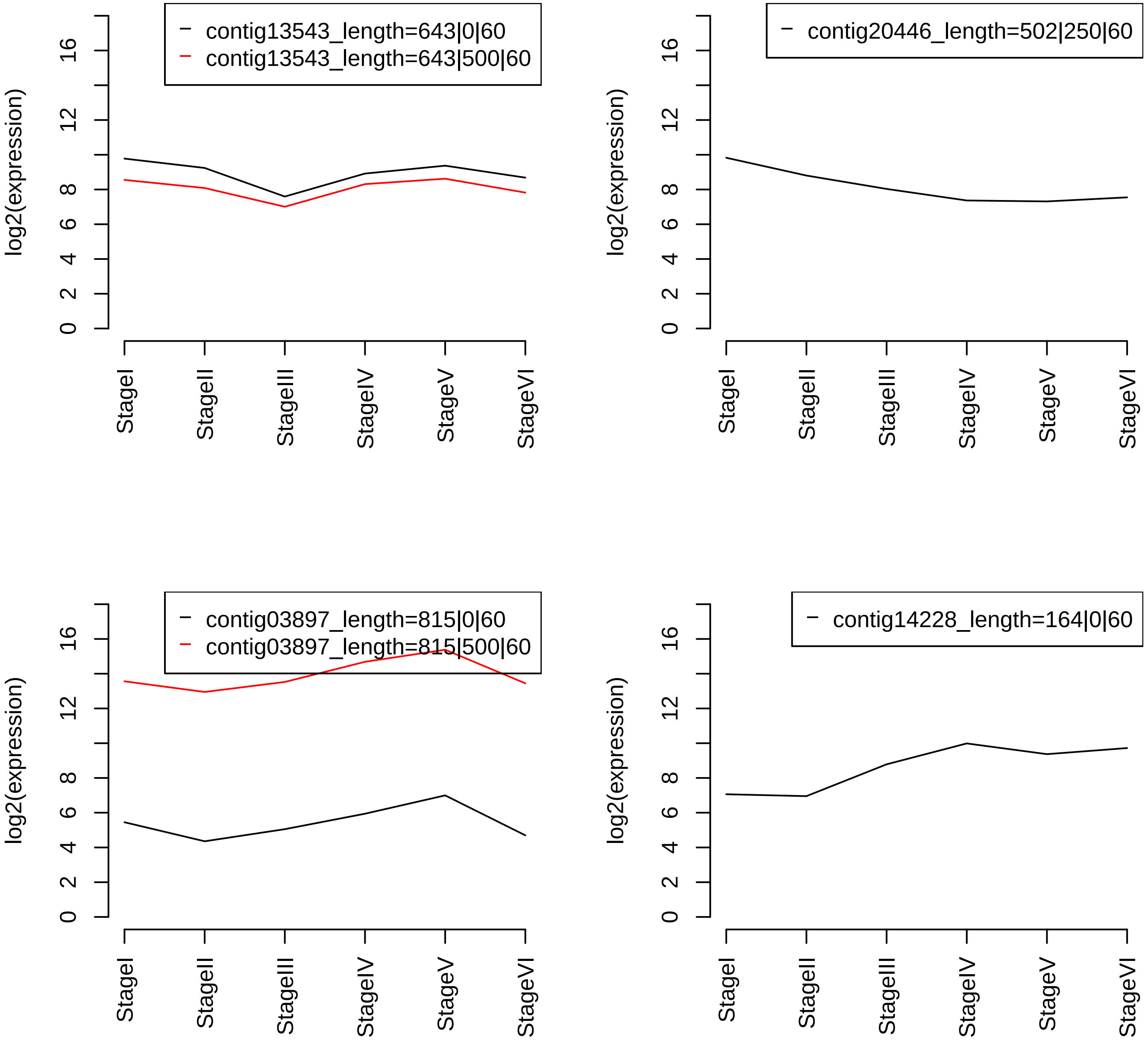
contig13543_length=643|0|60
contig13543_length=643|500|60
contig20446_length=502|250|60
contig03897_length=815|0|60
contig03897_length=815|500|60
contig14228_length=164|0|60
log2(expression)
0 2 4 6 8 12 16
StageI StageII StageIII StageIV StageV StageVI

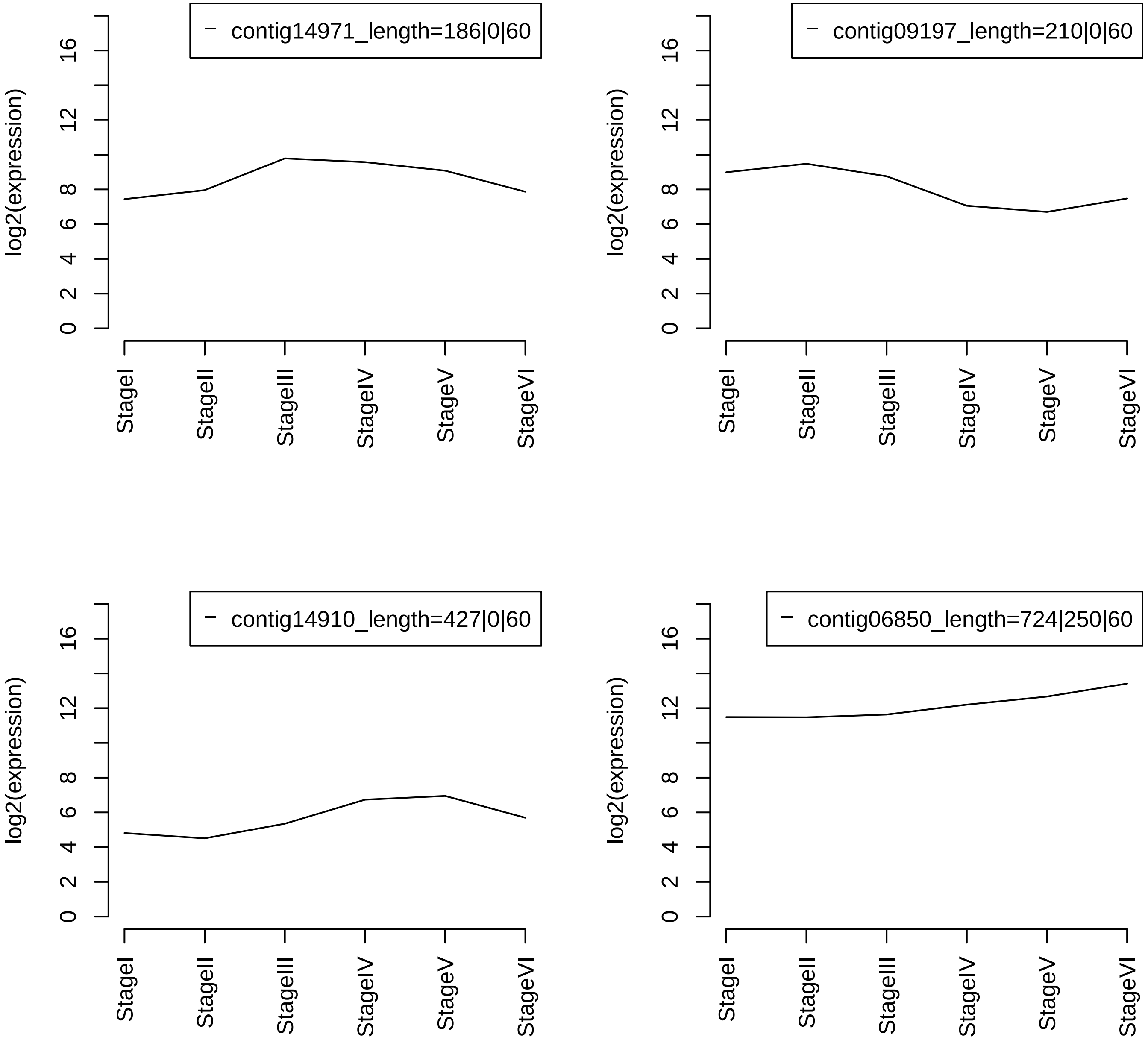
contig14971_length=186|0|60
contig09197_length=210|0|60
contig14910_length=427|0|60
contig06850_length=724|250|60
log2(expression)
0
2
4
6
8
12
16
StageI
StageII
StageIII
StageIV
StageV
StageVI

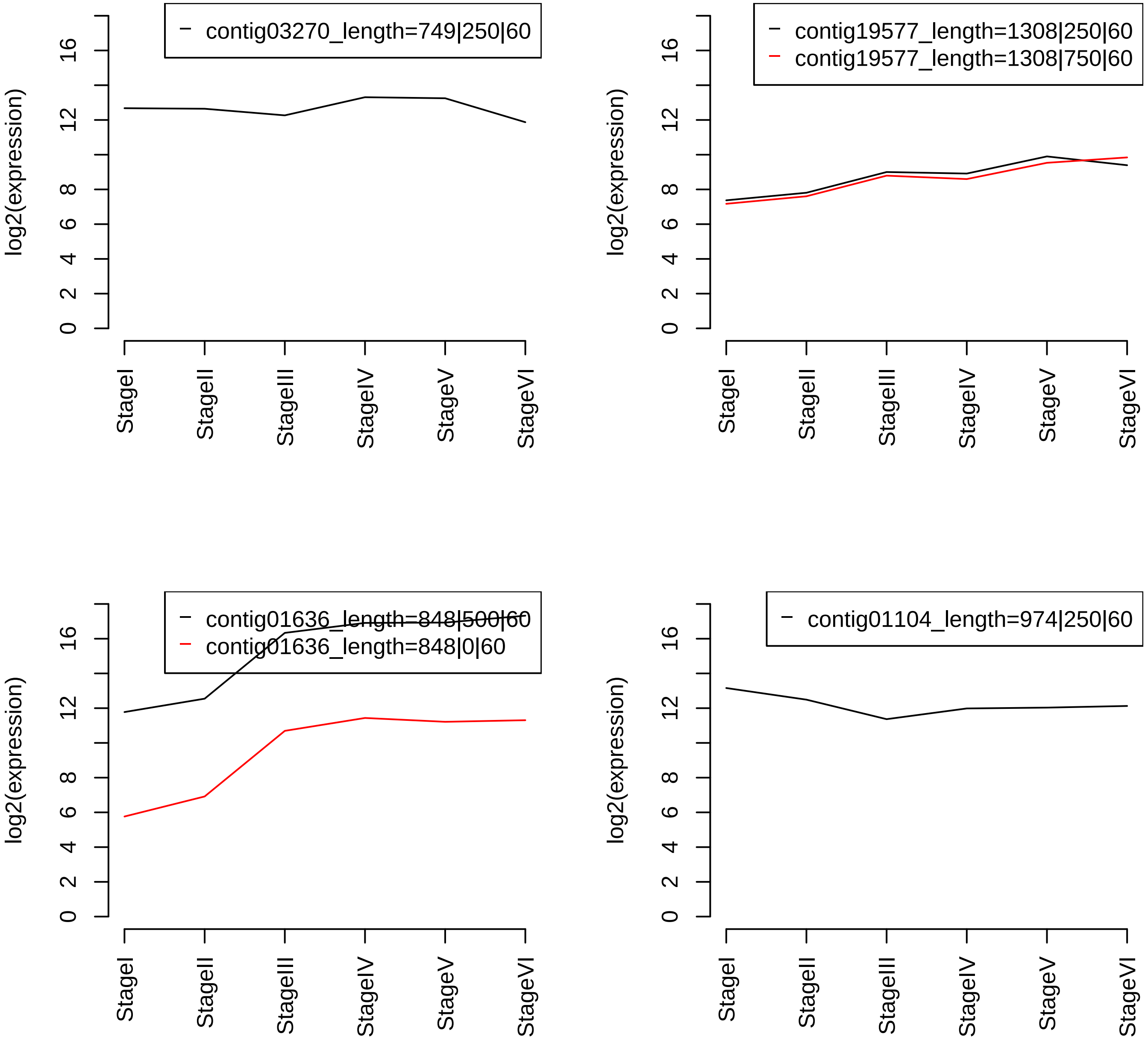
contig03270_length=749|250|60
contig19577_length=1308|250|60
contig19577_length=1308|750|60
contig01636_length=848|500|60
contig01636_length=848|0|60
contig01104_length=974|250|60
log2(expression)
0
2
4
6
8
12
16
StageI
StageII
StageIII
StageIV
StageV
StageVI

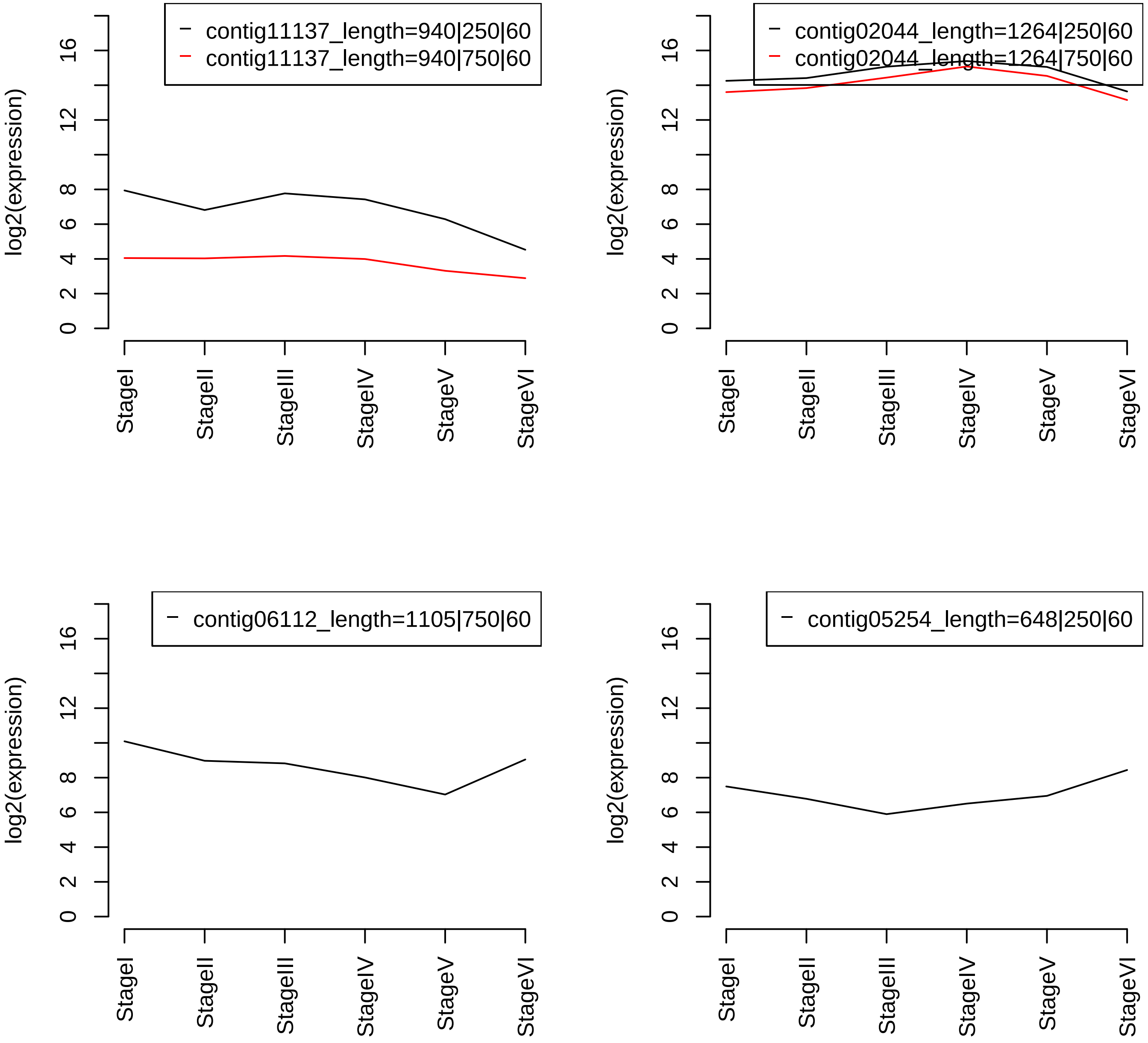
contig11137_length=940|250|60
contig11137_length=940|750|60
contig02044_length=1264|250|60
contig02044_length=1264|750|60
contig06112_length=1105|750|60
contig05254_length=648|250|60
log2(expression)
0 2 4 6 8 12 16
StageI StageII StageIII StageIV StageV StageVI

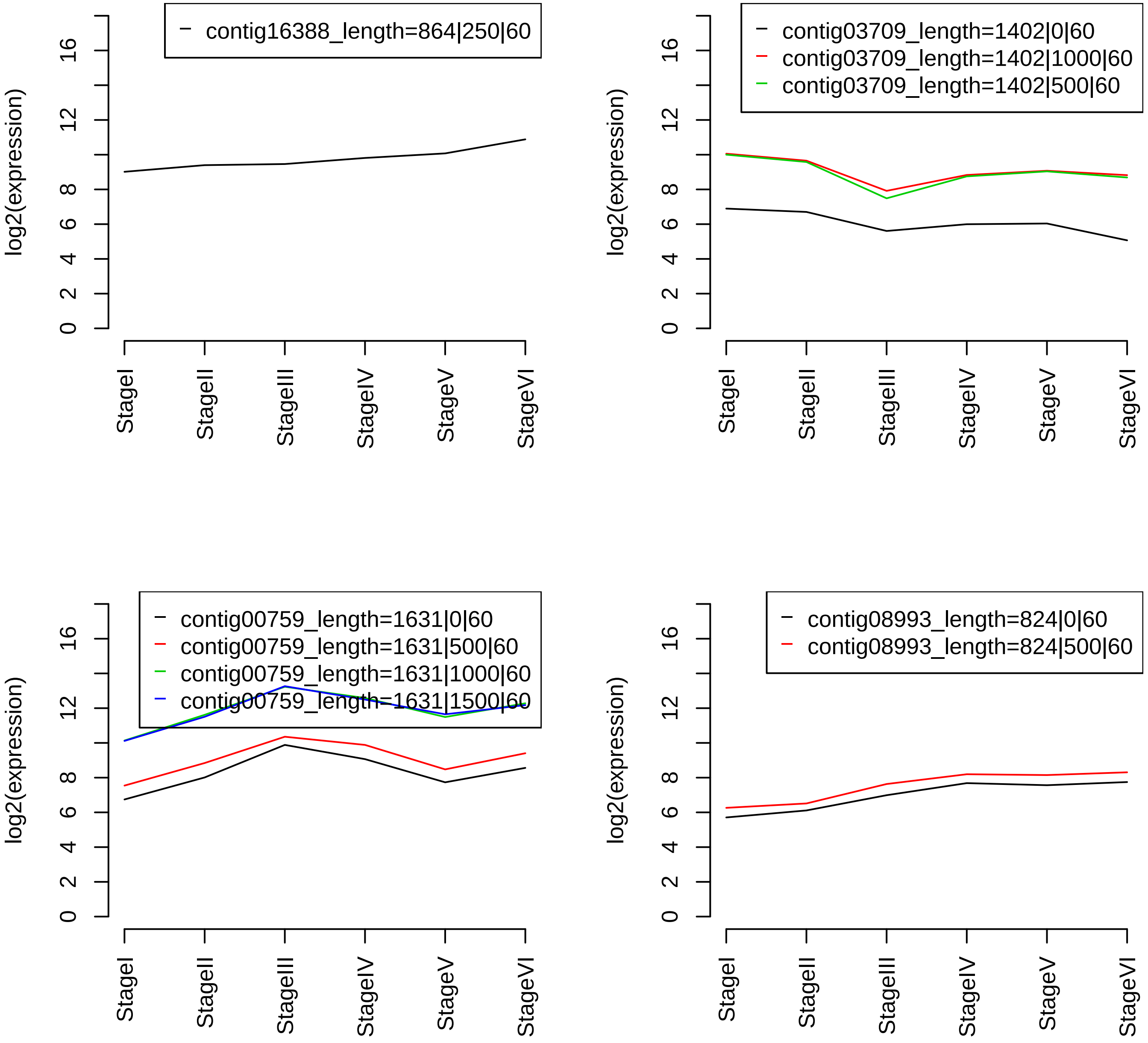

contig16388_length=864|250|60
contig03709_length=1402|0|60
contig03709_length=1402|1000|60
contig03709_length=1402|500|60
contig00759_length=1631|0|60
contig00759_length=1631|500|60
contig00759_length=1631|1000|60
contig00759_length=1631|1500|60
contig08993_length=824|0|60
contig08993_length=824|500|60
log2(expression)
0 2 4 6 8 12 16
StageI StageII StageIII StageIV StageV StageVI

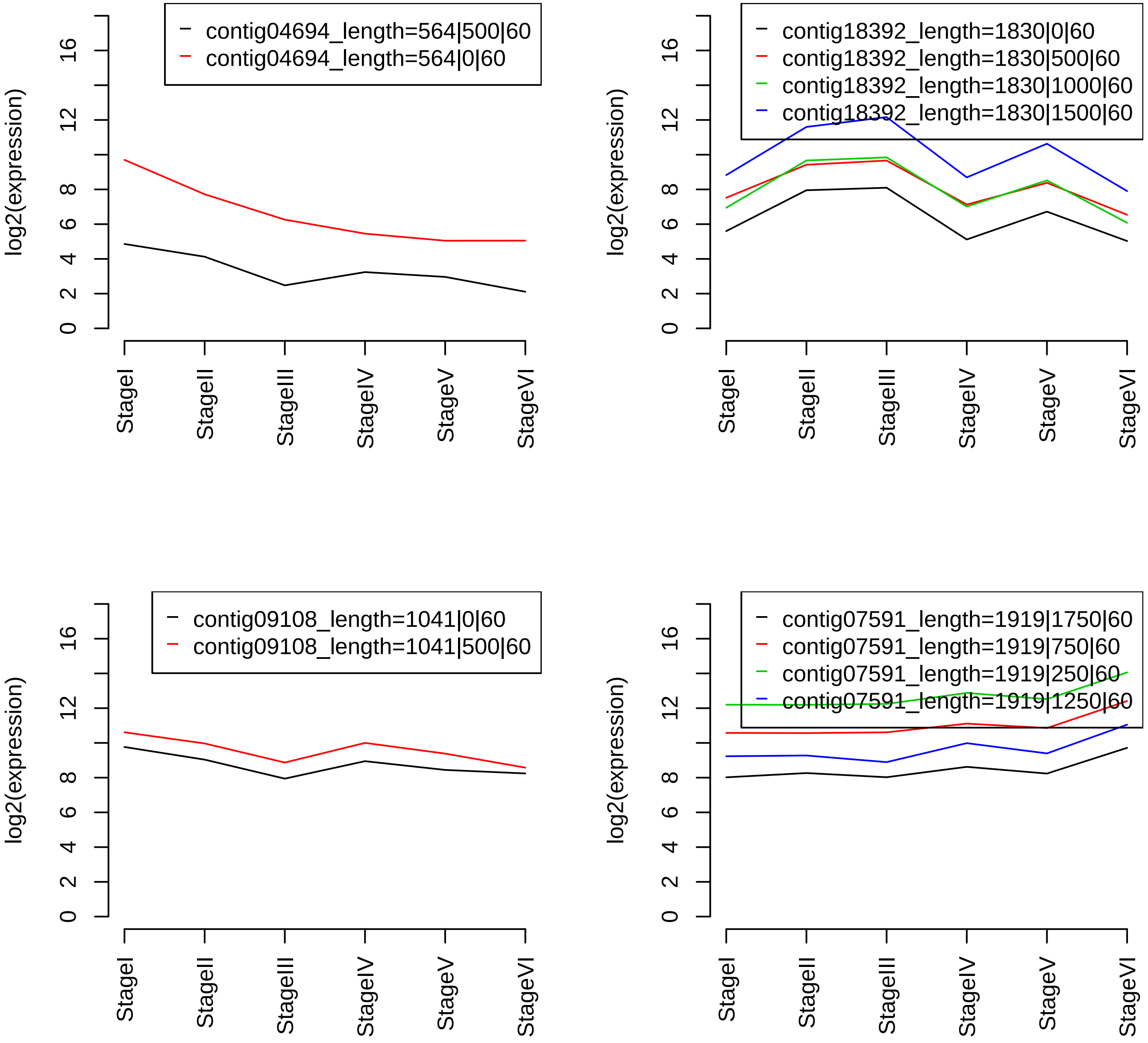
contig04694_length=564|500|60
contig04694_length=564|0|60
contig18392_length=1830|0|60
contig18392_length=1830|500|60
contig18392_length=1830|1000|60
contig18392_length=1830|1500|60
contig09108_length=1041|0|60
contig09108_length=1041|500|60
contig07591_length=1919|1750|60
contig07591_length=1919|750|60
contig07591_length=1919|250|60
contig07591_length=1919|1250|60
log2(expression)
0
2
4
6
8
12
16
StageI
StageII
StageIII
StageIV
StageV
StageVI

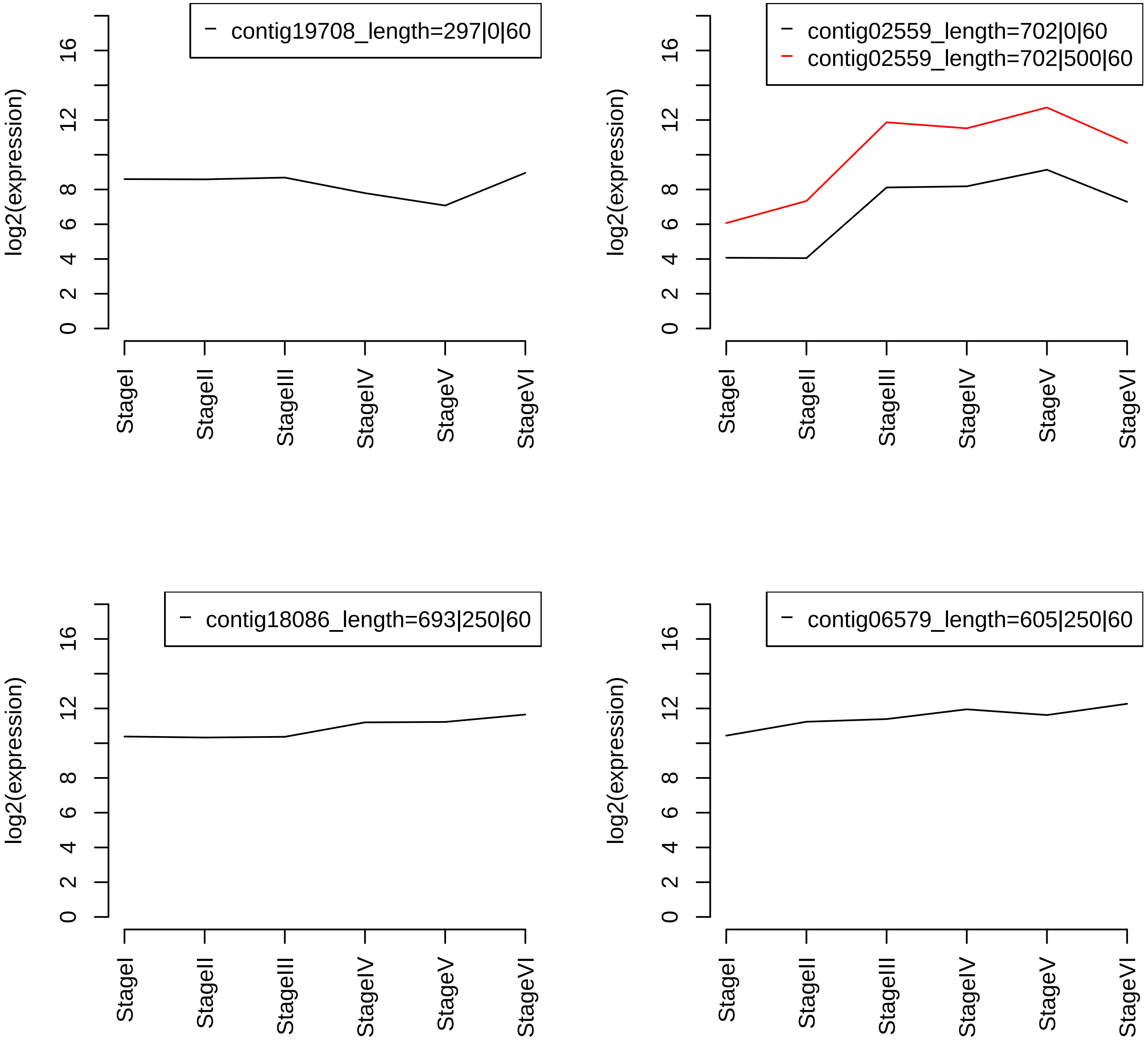

contig19708_length=297|0|60
contig02559_length=702|0|60
contig02559_length=702|500|60
contig18086_length=693|250|60
contig06579_length=605|250|60
log2(expression)
0
2
4
6
8
12
16
StageI
StageII
StageIII
StageIV
StageV
StageVI

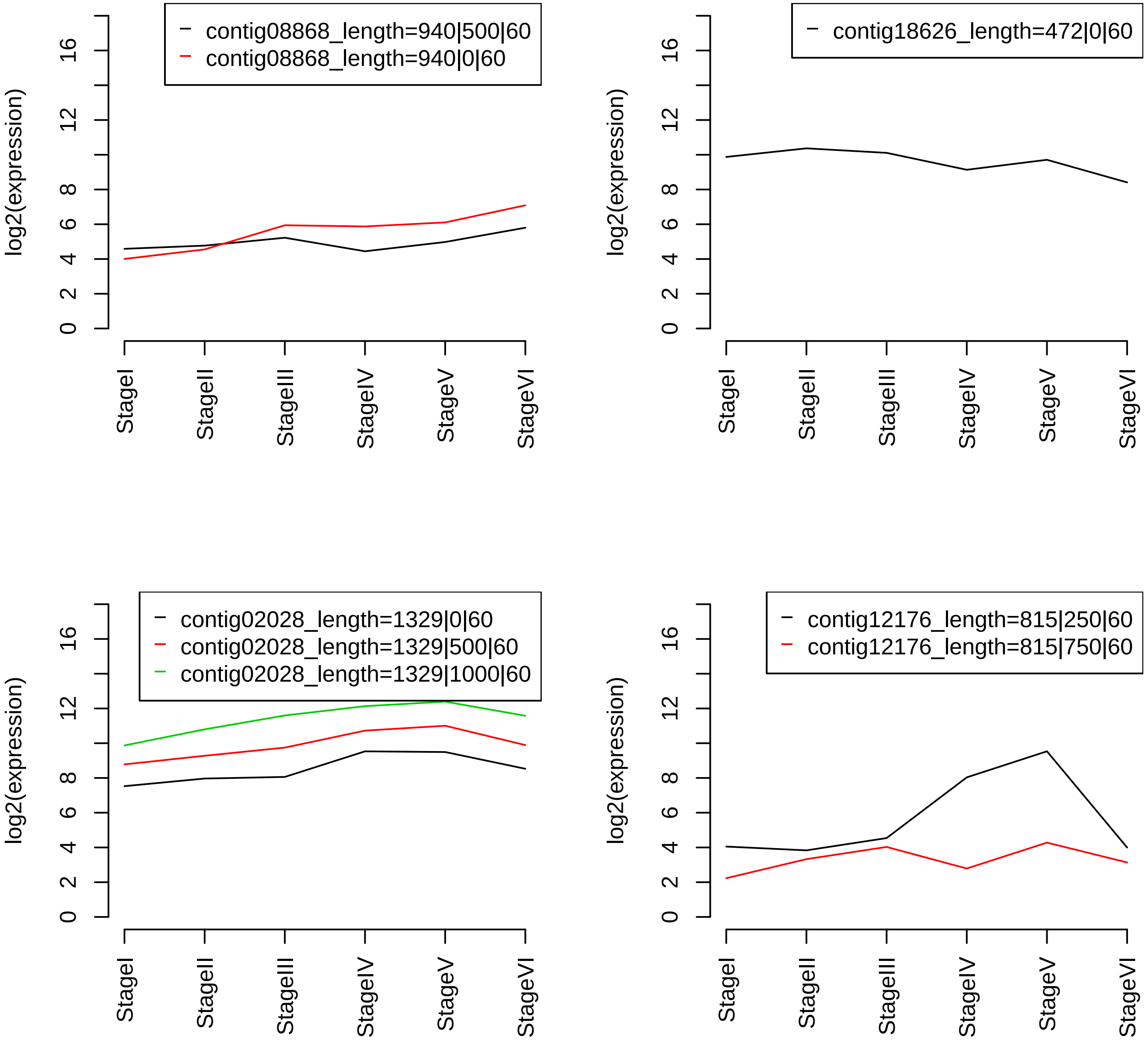

contig08868_length=940|500|60
contig08868_length=940|0|60
contig18626_length=472|0|60
contig02028_length=1329|0|60
contig02028_length=1329|500|60
contig02028_length=1329|1000|60
contig12176_length=815|250|60
contig12176_length=815|750|60
log2(expression)
0
2
4
6
8
12
16
StageI
StageII
StageIII
StageIV
StageV
StageVI

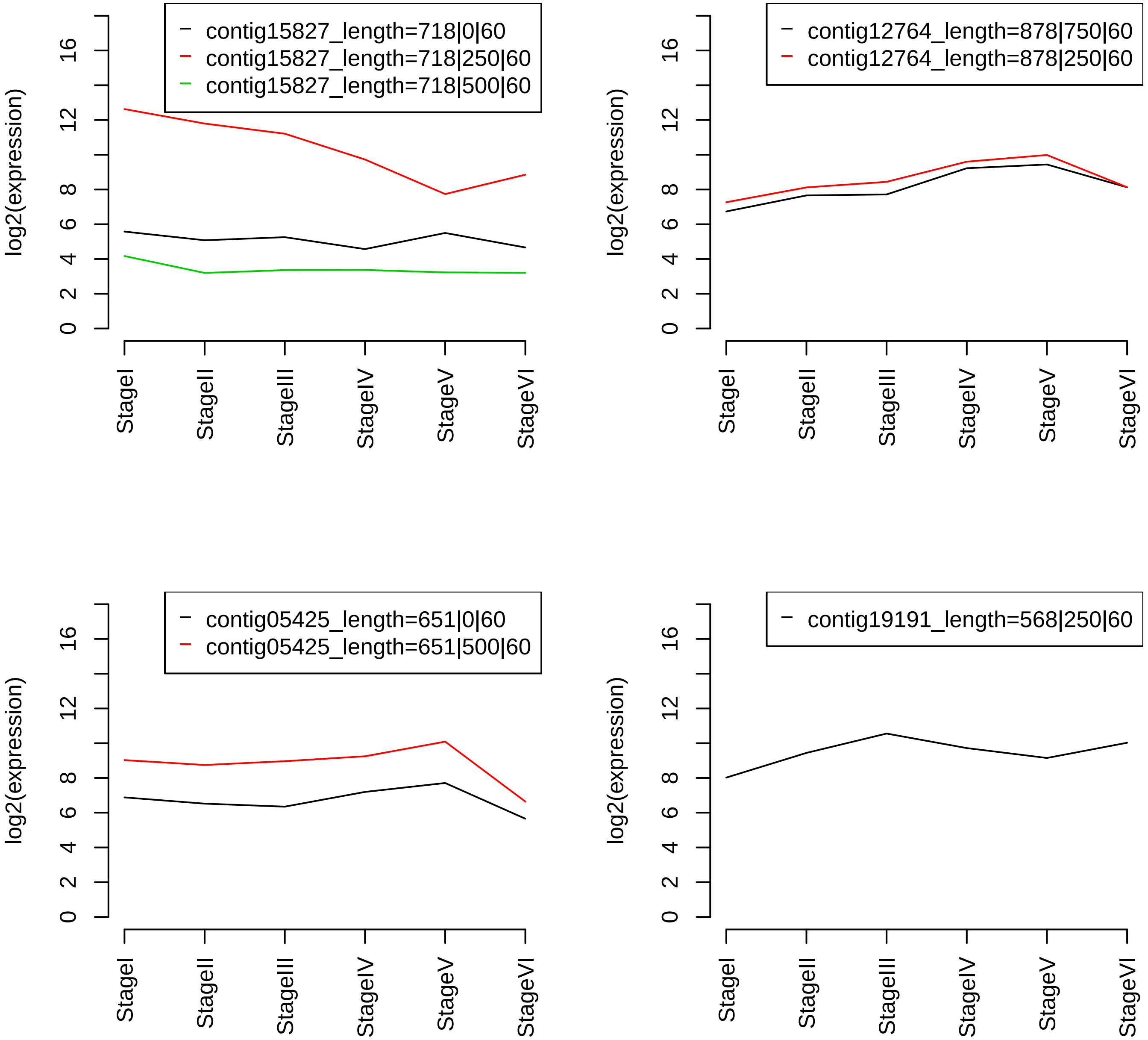

contig15827_length=718|0|60
contig15827_length=718|250|60
contig15827_length=718|500|60
log2(expression)
0
2
4
6
8
12
16
StageI
StageII
StageIII
StageIV
StageV
StageVI
contig12764_length=878|750|60
contig12764_length=878|250|60
contig05425_length=651|0|60
contig05425_length=651|500|60
contig19191_length=568|250|60

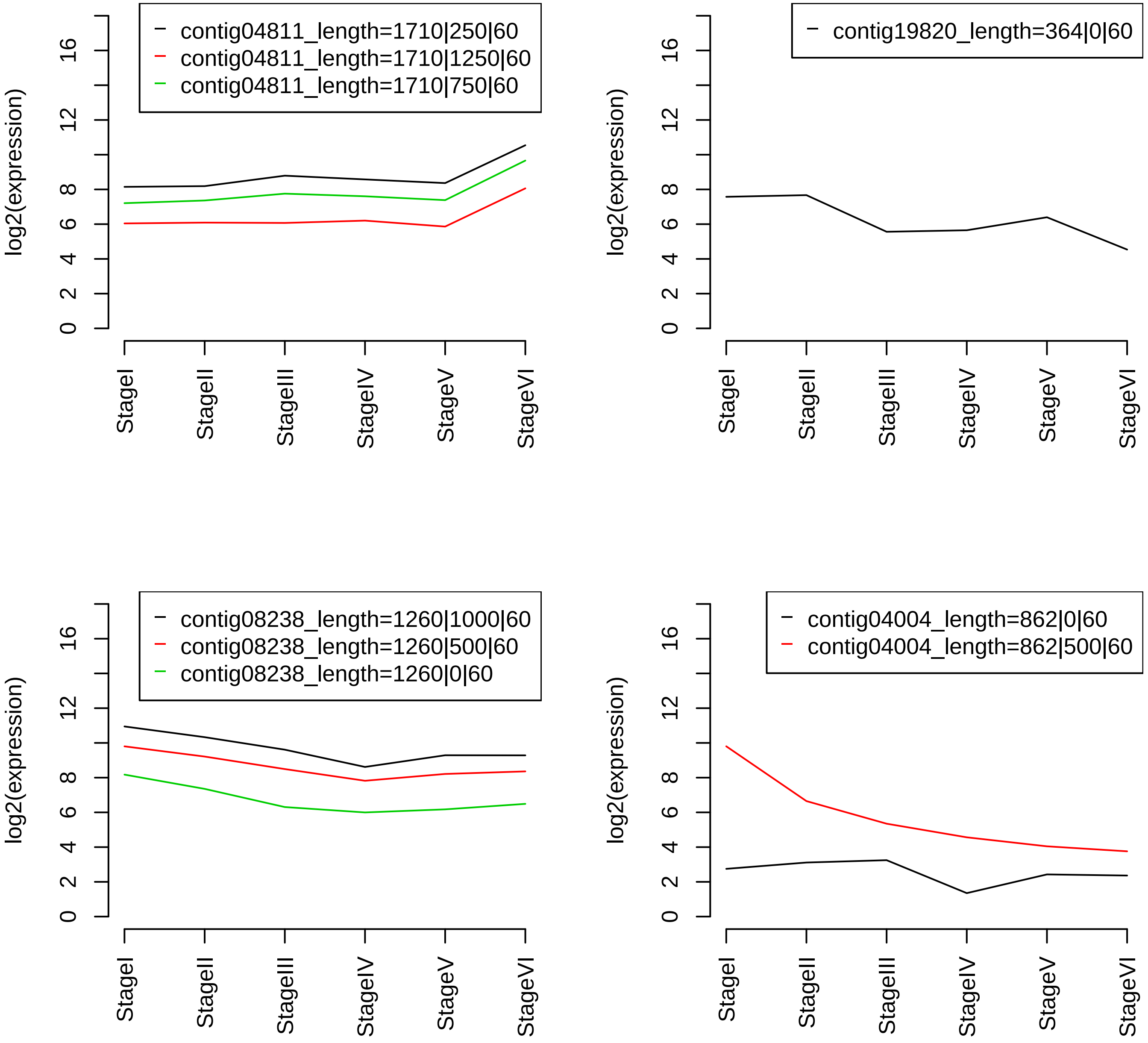

contig04811_length=1710|250|60
contig04811_length=1710|1250|60
contig04811_length=1710|750|60
contig19820_length=364|0|60
contig08238_length=1260|1000|60
contig08238_length=1260|500|60
contig08238_length=1260|0|60
contig04004_length=862|0|60
contig04004_length=862|500|60
log2(expression)
0
2
4
6
8
12
16
StageI
StageII
StageIII
StageIV
StageV
StageVI

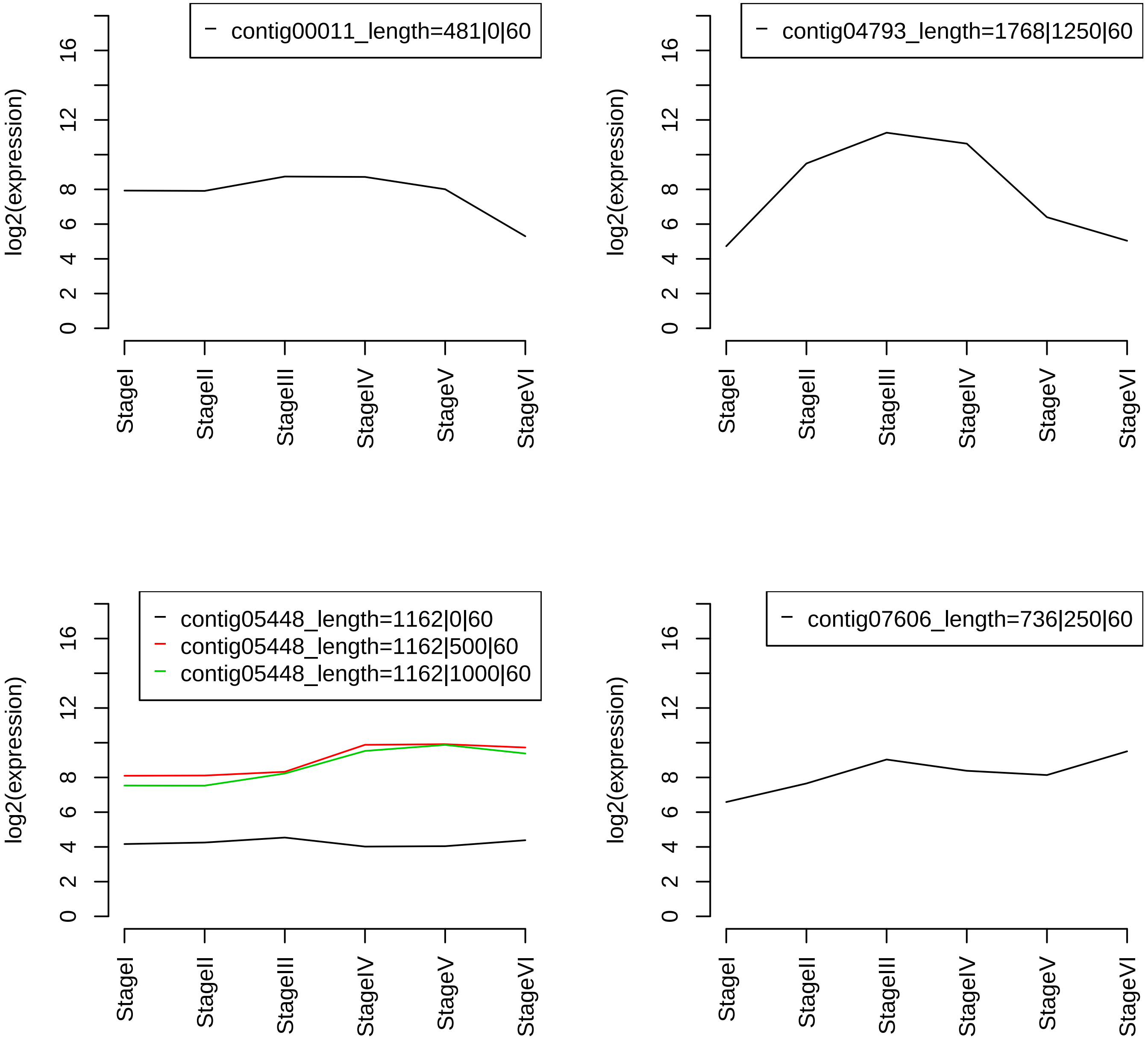
contig00011_length=481|0|60
contig04793_length=1768|1250|60
contig05448_length=1162|0|60
contig05448_length=1162|500|60
contig05448_length=1162|1000|60
contig07606_length=736|250|60
log2(expression)
0
2
4
6
8
12
16
StageI
StageII
StageIII
StageIV
StageV
StageVI

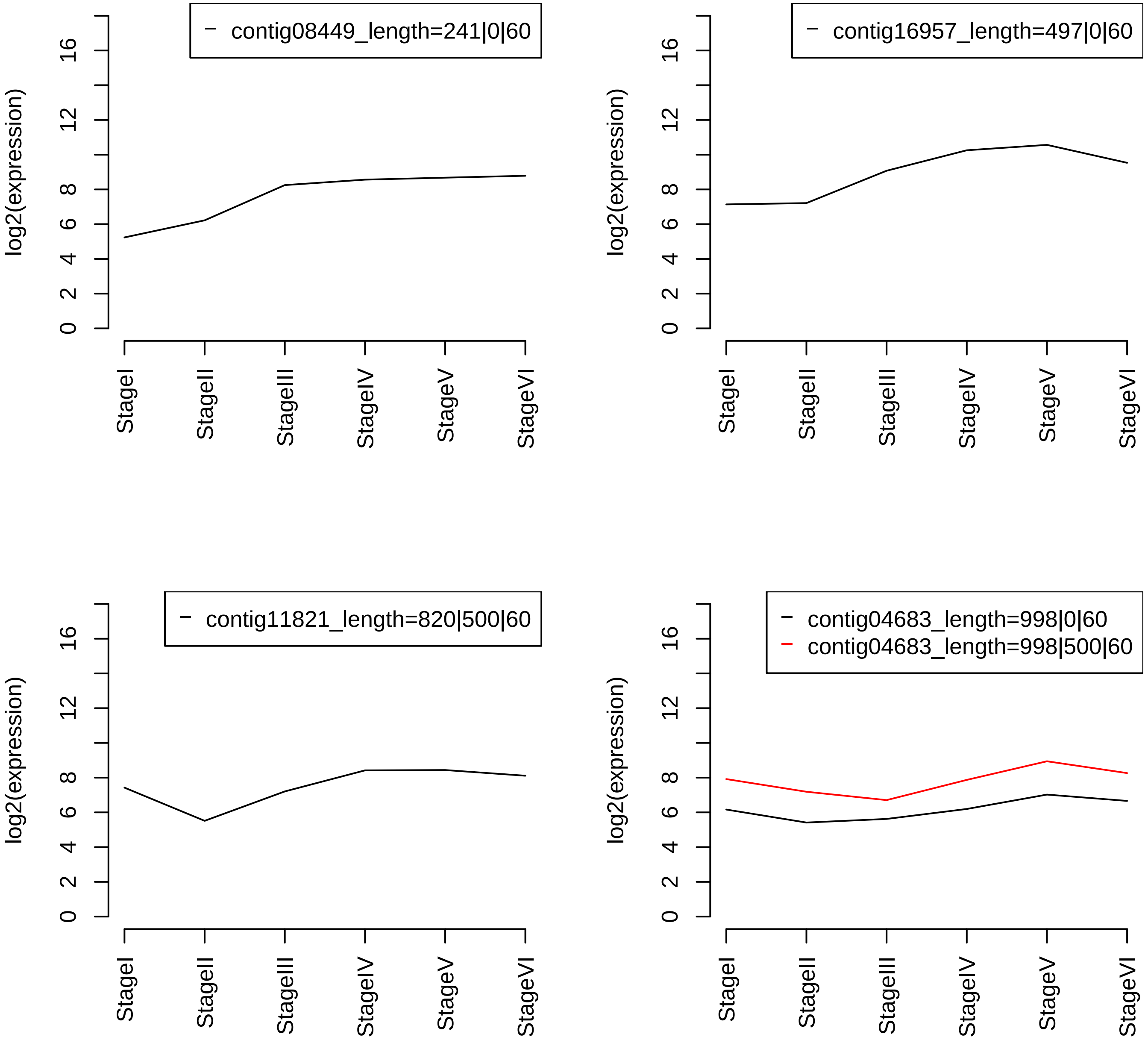

contig08449_length=241|0|60
contig16957_length=497|0|60
contig11821_length=820|500|60
contig04683_length=998|0|60
contig04683_length=998|500|60
log2(expression)
0
2
4
6
8
12
16
StageI
StageII
StageIII
StageIV
StageV
StageVI

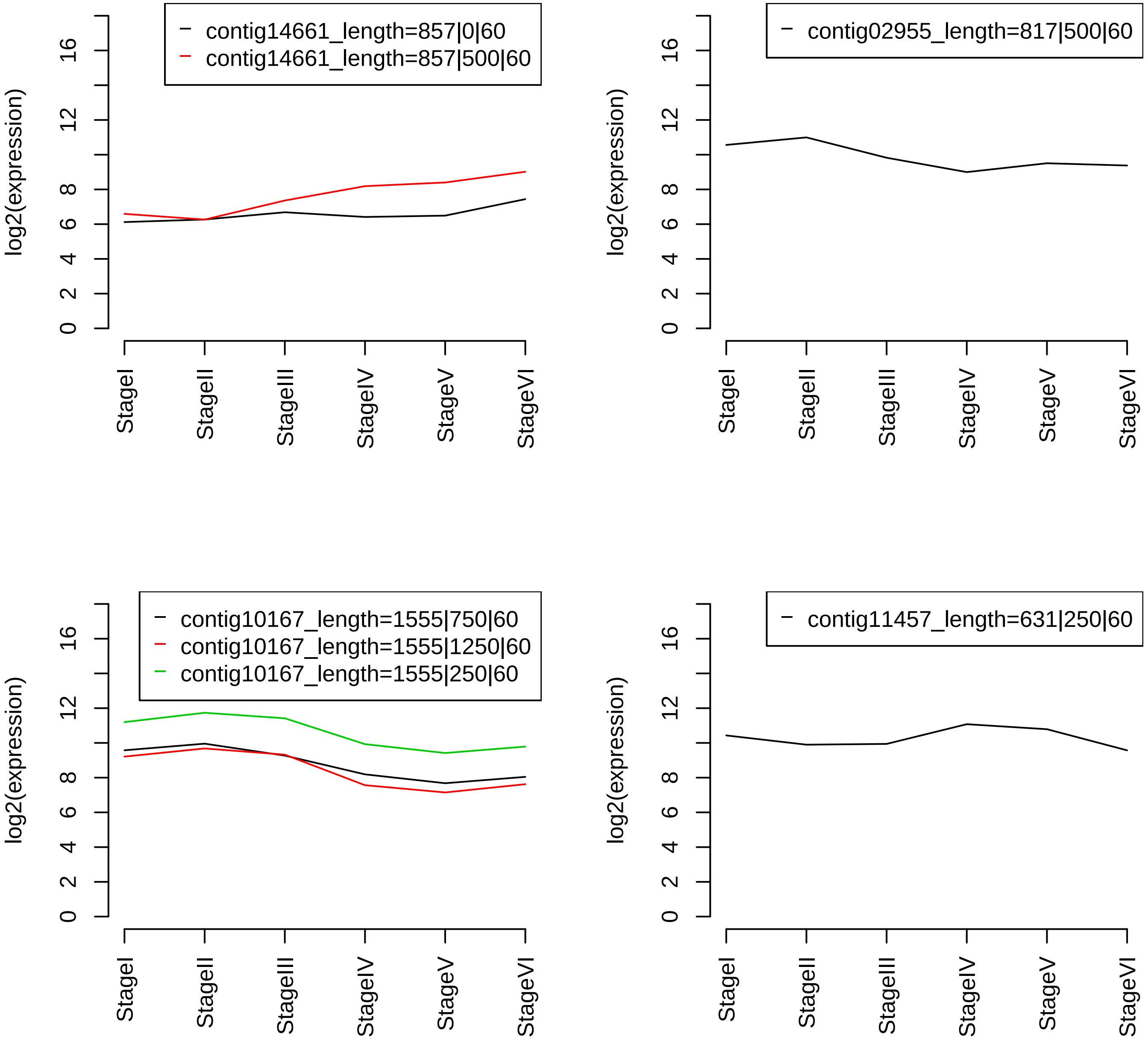

contig14661_length=857|0|60
contig14661_length=857|500|60
contig02955_length=817|500|60
contig10167_length=1555|750|60
contig10167_length=1555|1250|60
contig10167_length=1555|250|60
contig11457_length=631|250|60
log2(expression)
0
2
4
6
8
12
16
StageI
StageII
StageIII
StageIV
StageV
StageVI

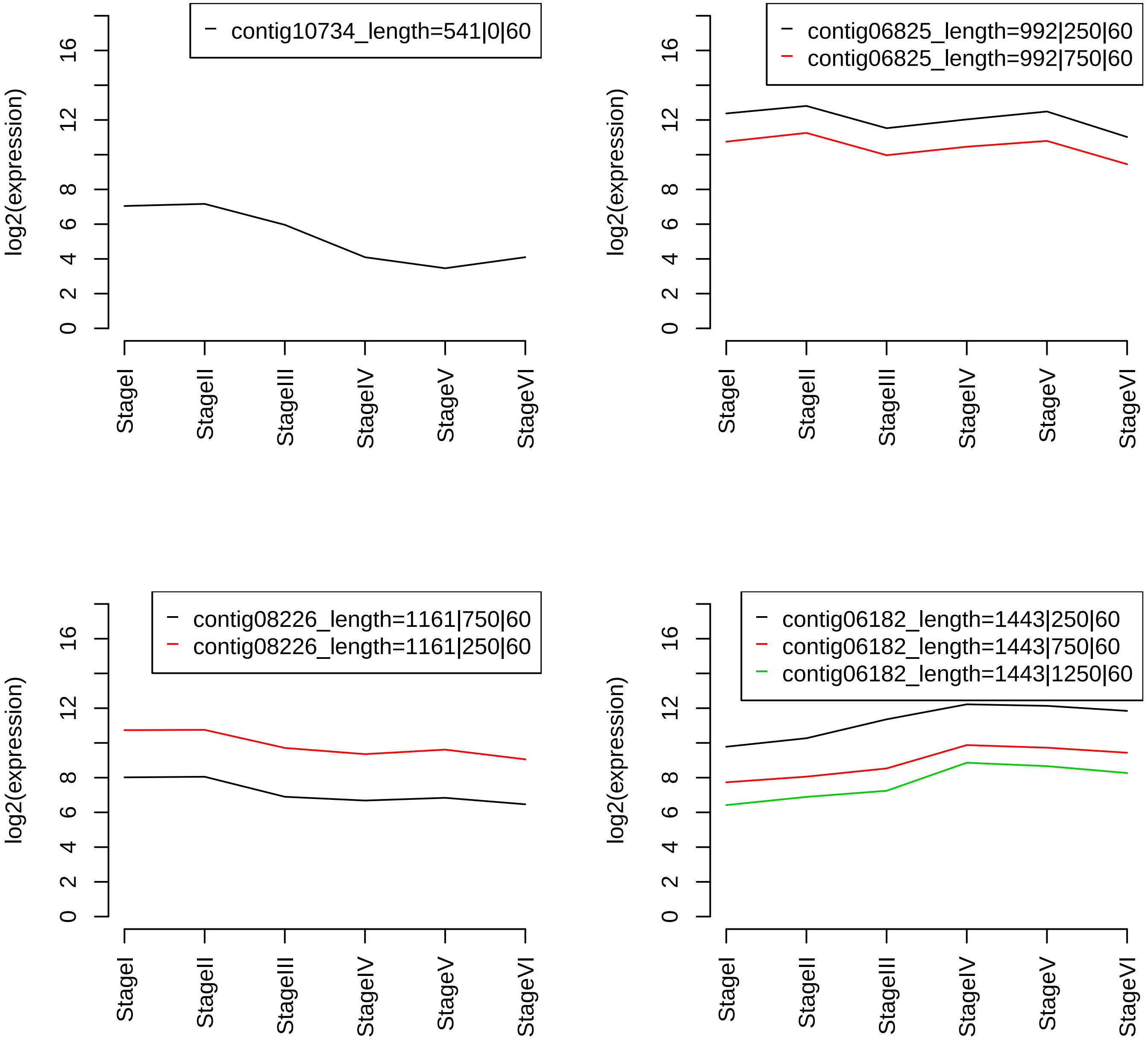

contig10734_length=541|0|60
contig06825_length=992|250|60
contig06825_length=992|750|60
contig08226_length=1161|750|60
contig08226_length=1161|250|60
contig06182_length=1443|250|60
contig06182_length=1443|750|60
contig06182_length=1443|1250|60
log2(expression)
0 2 4 6 8 12 16
StageI StageII StageIII StageIV StageV StageVI

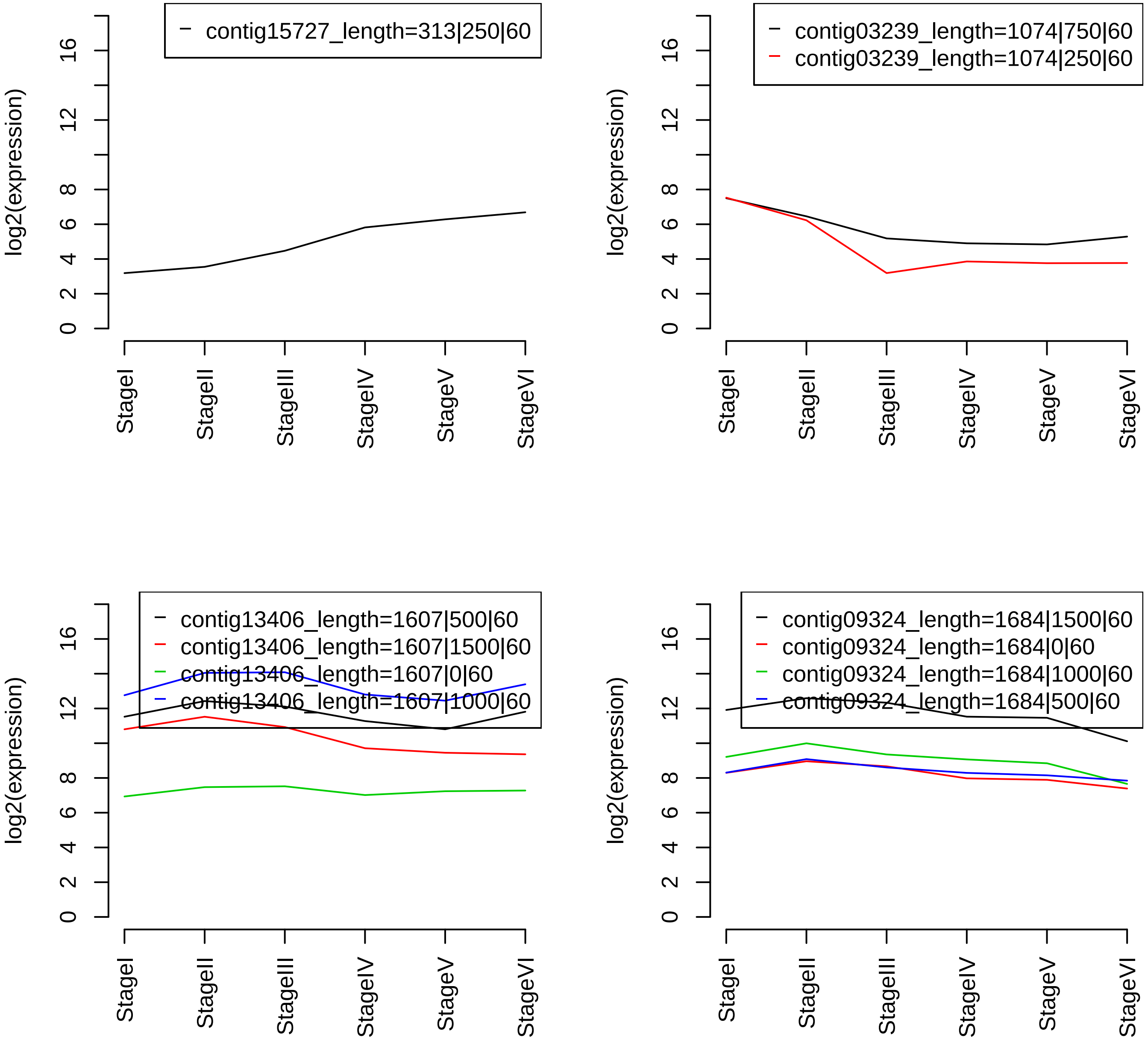
contig15727_length=313|250|60
contig03239_length=1074|750|60
contig03239_length=1074|250|60
contig13406_length=1607|500|60
contig13406_length=1607|1500|60
contig13406_length=1607|0|60
contig13406_length=1607|1000|60
contig09324_length=1684|1500|60
contig09324_length=1684|0|60
contig09324_length=1684|1000|60
contig09324_length=1684|500|60
log2(expression)
0 2 4 6 8 12 16
StageI StageII StageIII StageIV StageV StageVI

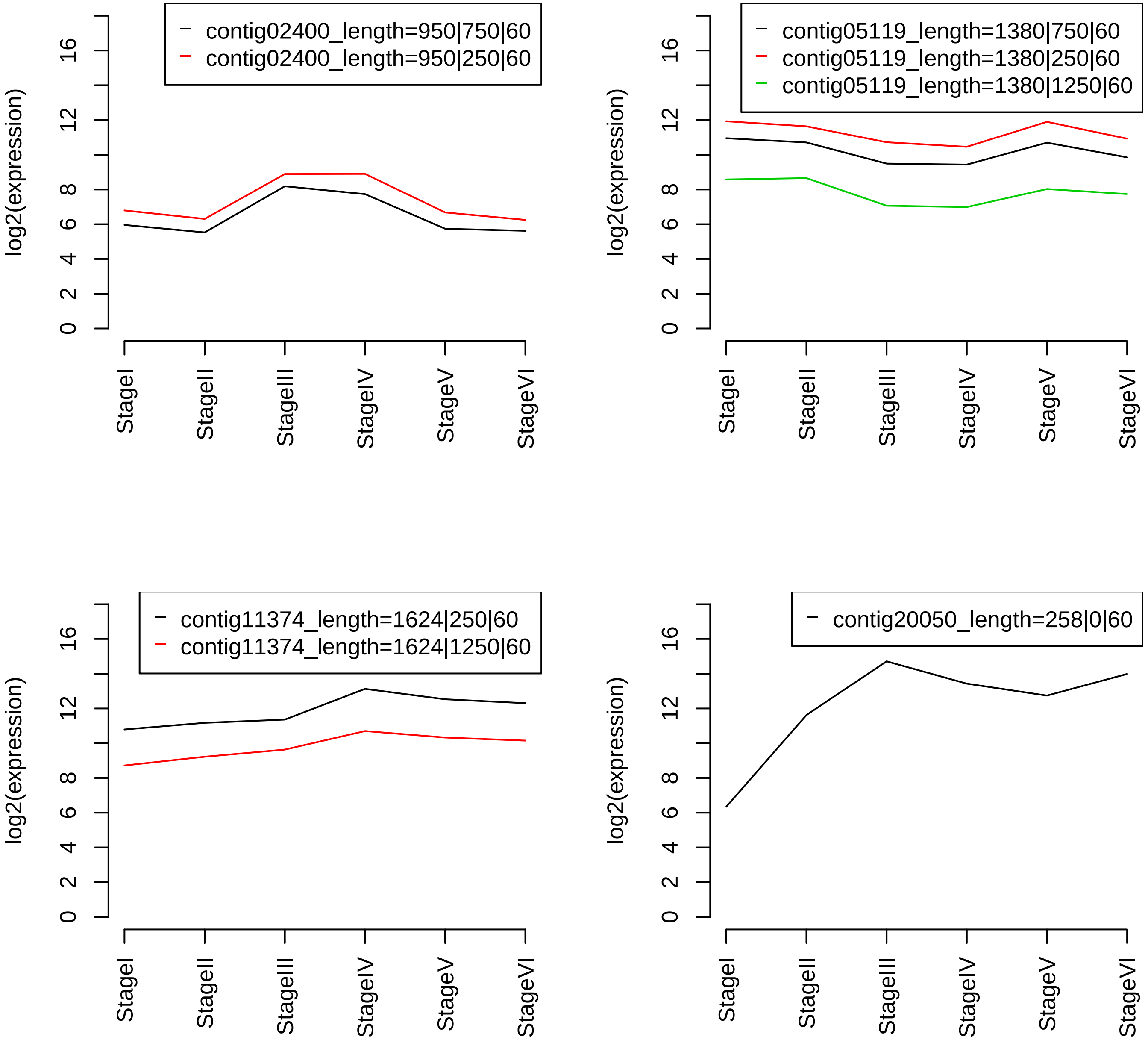

contig02400_length=950|750|60
contig02400_length=950|250|60
log2(expression)
0
2
4
6
8
12
16
StageI
StageII
StageIII
StageIV
StageV
StageVI
contig05119_length=1380|750|60
contig05119_length=1380|250|60
contig05119_length=1380|1250|60
contig11374_length=1624|250|60
contig11374_length=1624|1250|60
contig20050_length=258|0|60

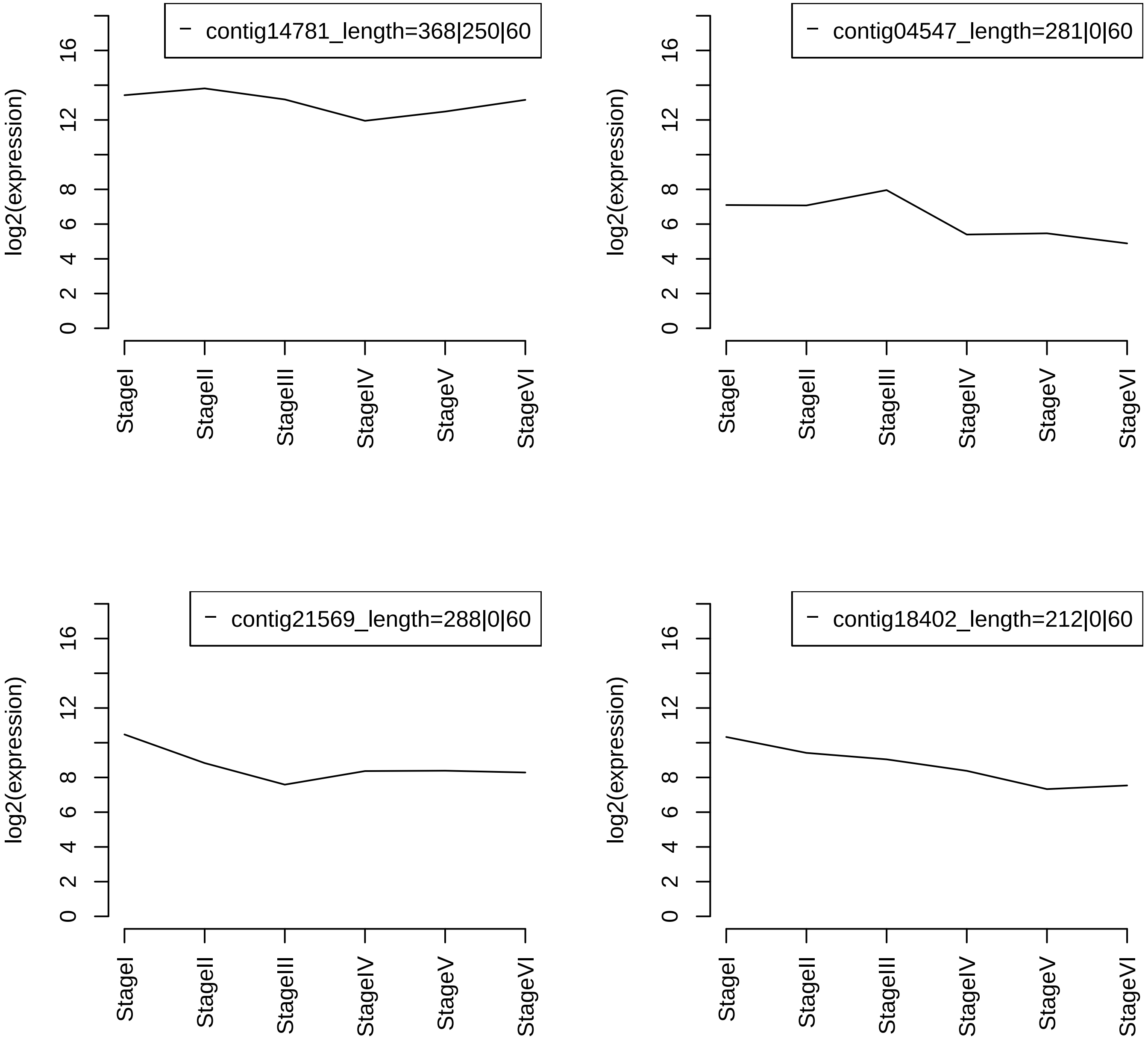
contig14781_length=368|250|60
contig04547_length=281|0|60
contig21569_length=288|0|60
contig18402_length=212|0|60
log2(expression)
0
2
4
6
8
12
16
StageI
StageII
StageIII
StageIV
StageV
StageVI

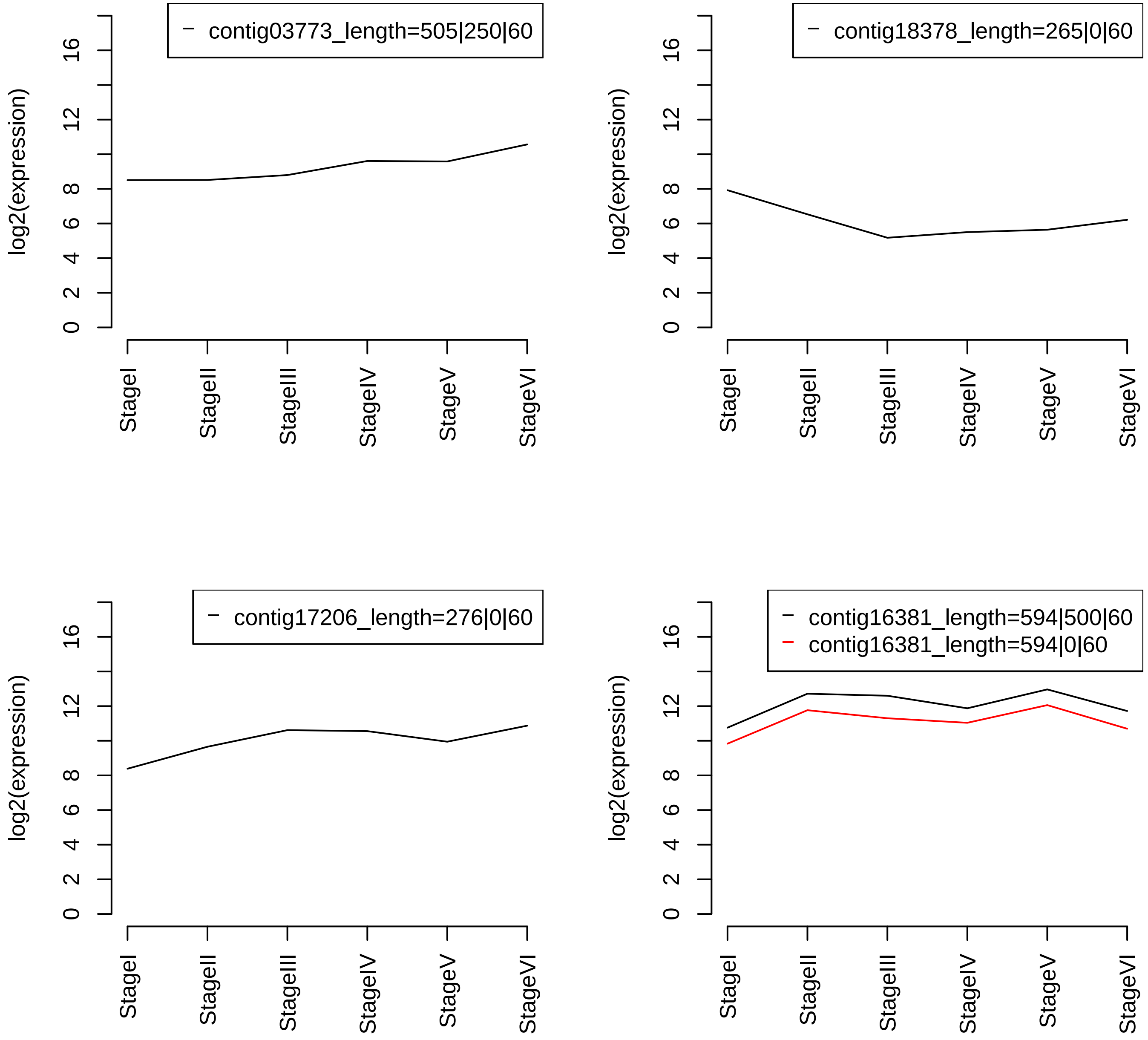
contig03773_length=505|250|60
contig18378_length=265|0|60
contig17206_length=276|0|60
contig16381_length=594|500|60
contig16381_length=594|0|60
log2(expression)
0 2 4 6 8 12 16
StageI
StageII
StageIII
StageIV
StageV
StageVI

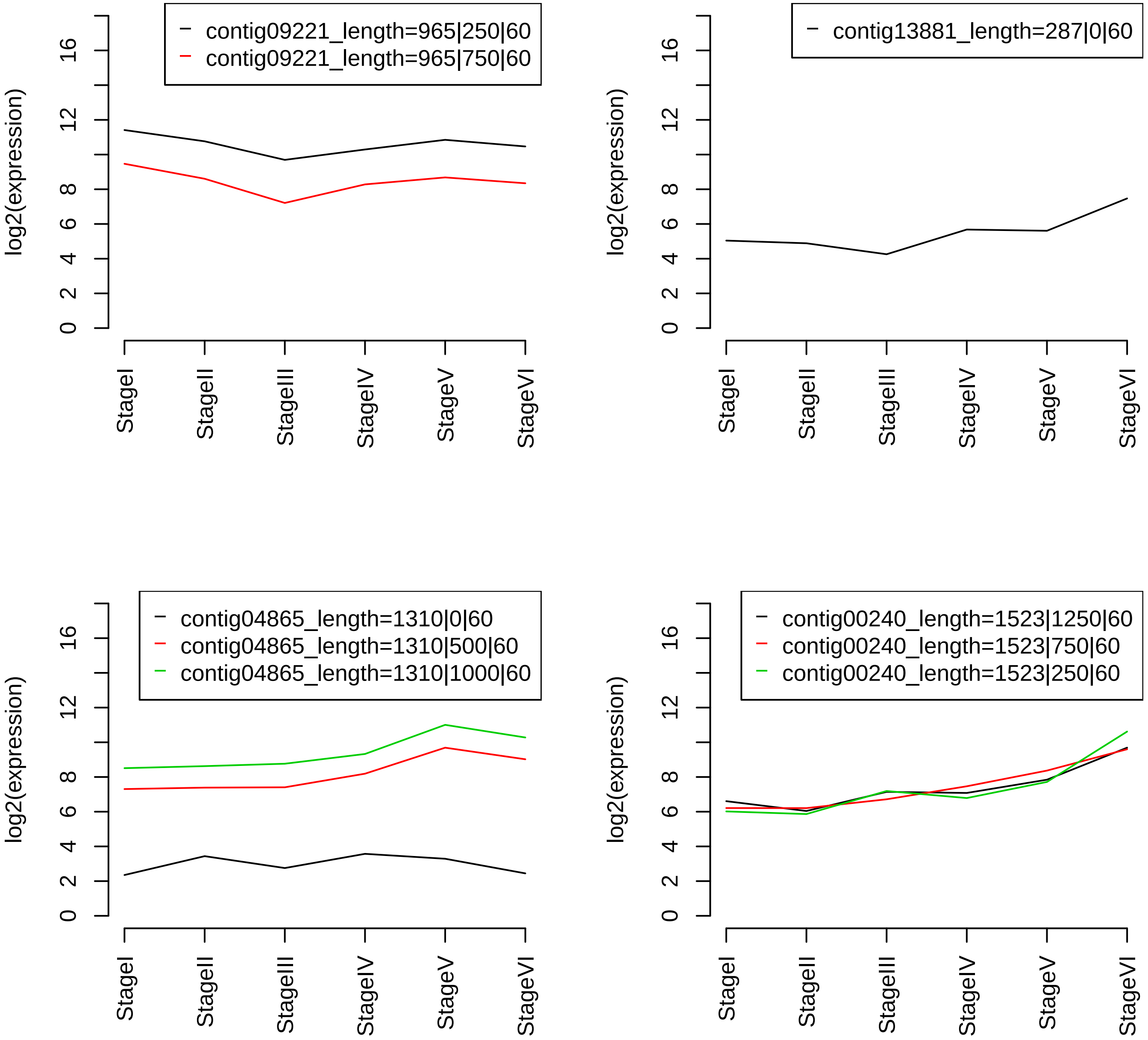
contig09221_length=965|250|60
contig09221_length=965|750|60
contig13881_length=287|0|60
contig04865_length=1310|0|60
contig04865_length=1310|500|60
contig04865_length=1310|1000|60
contig00240_length=1523|1250|60
contig00240_length=1523|750|60
contig00240_length=1523|250|60
log2(expression)
0
2
4
6
8
12
16
StageI
StageII
StageIII
StageIV
StageV
StageVI

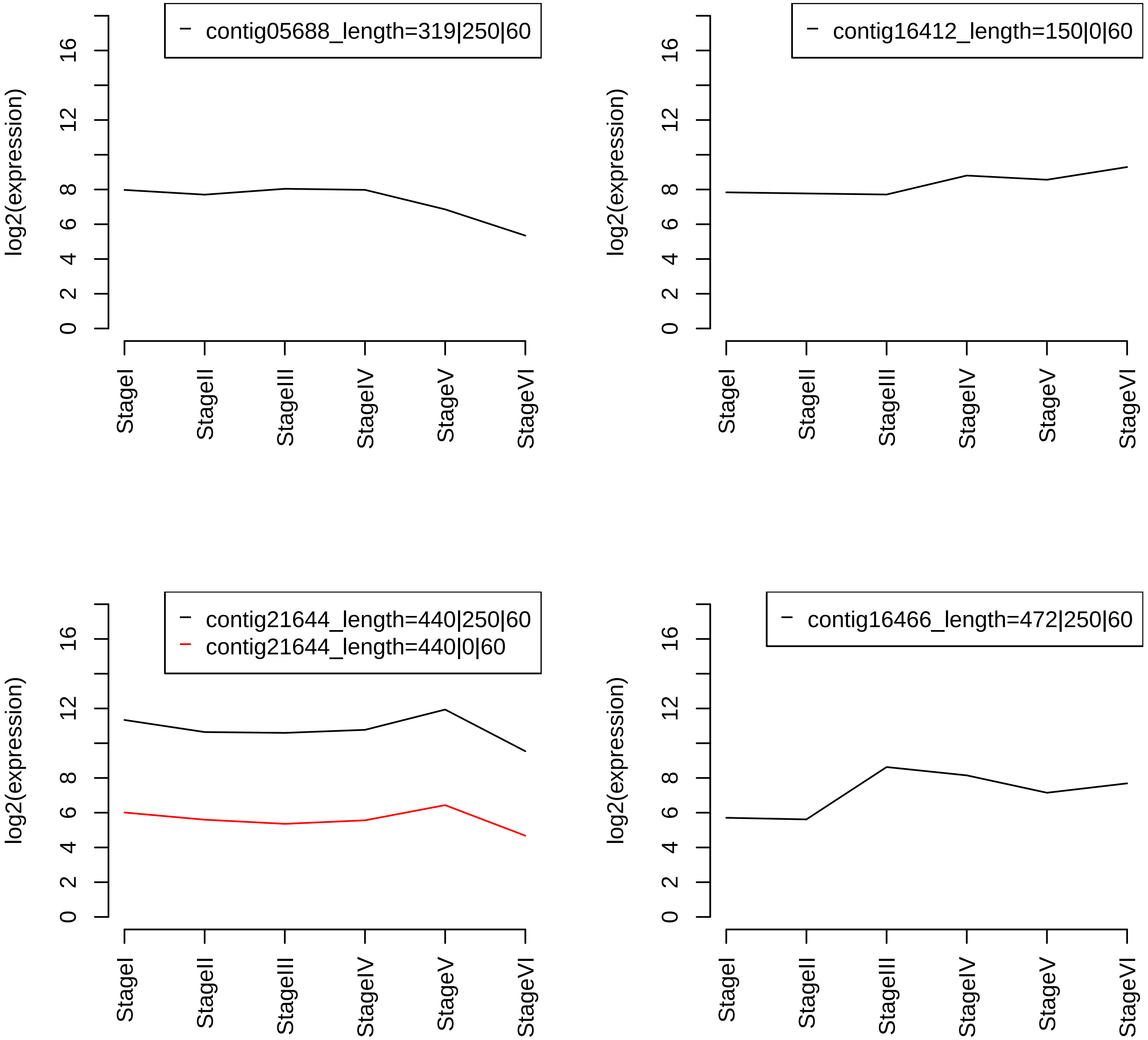

contig05688_length=319|250|60
contig16412_length=150|0|60
contig21644_length=440|250|60
contig21644_length=440|0|60
contig16466_length=472|250|60
log2(expression)
0
2
4
6
8
12
16
StageI
StageII
StageIII
StageIV
StageV
StageVI

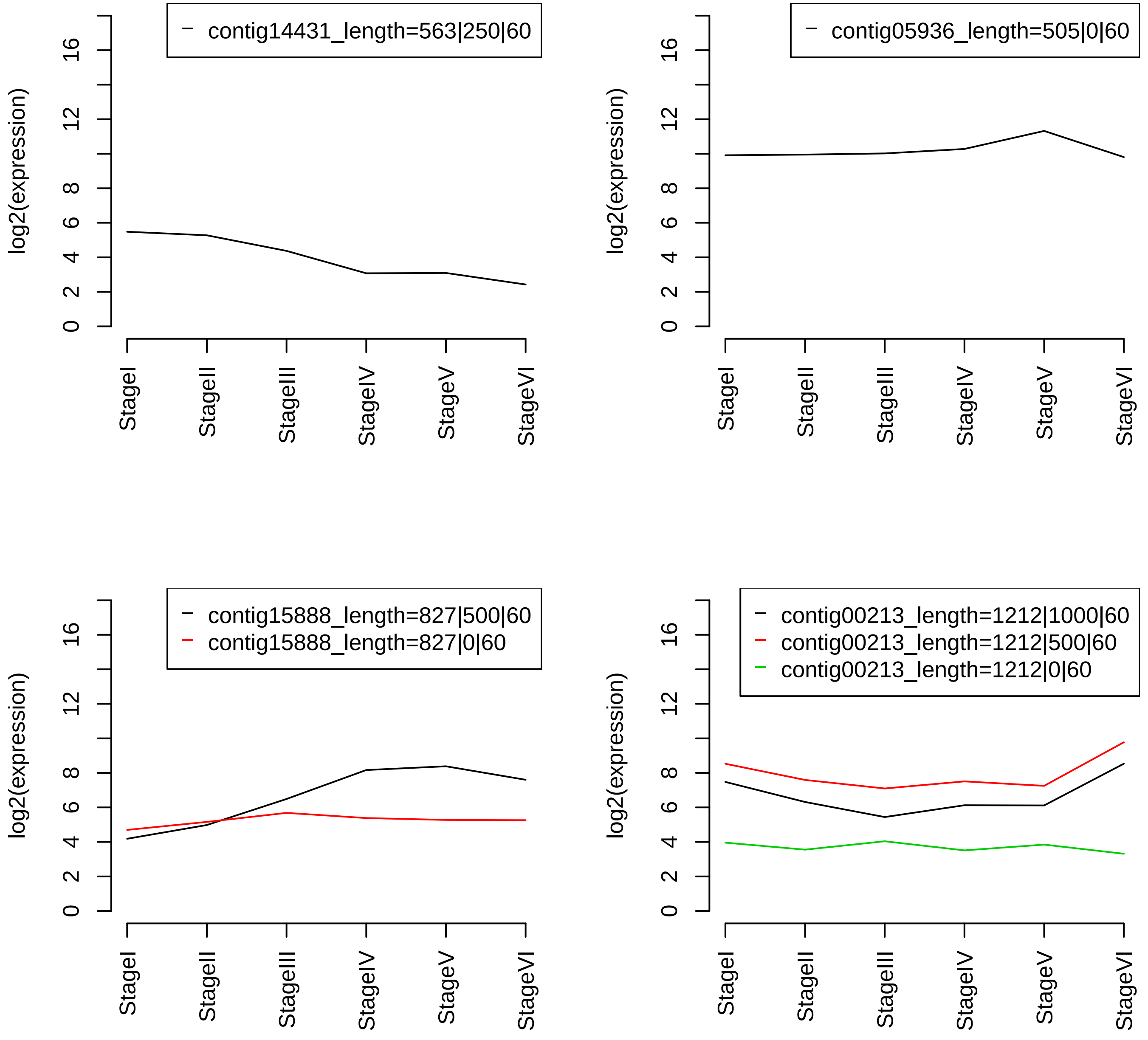

contig14431_length=563|250|60
contig05936_length=505|0|60
contig15888_length=827|500|60
contig15888_length=827|0|60
contig00213_length=1212|1000|60
contig00213_length=1212|500|60
contig00213_length=1212|0|60
log2(expression)
0
2
4
6
8
12
16
StageI
StageII
StageIII
StageIV
StageV
StageVI

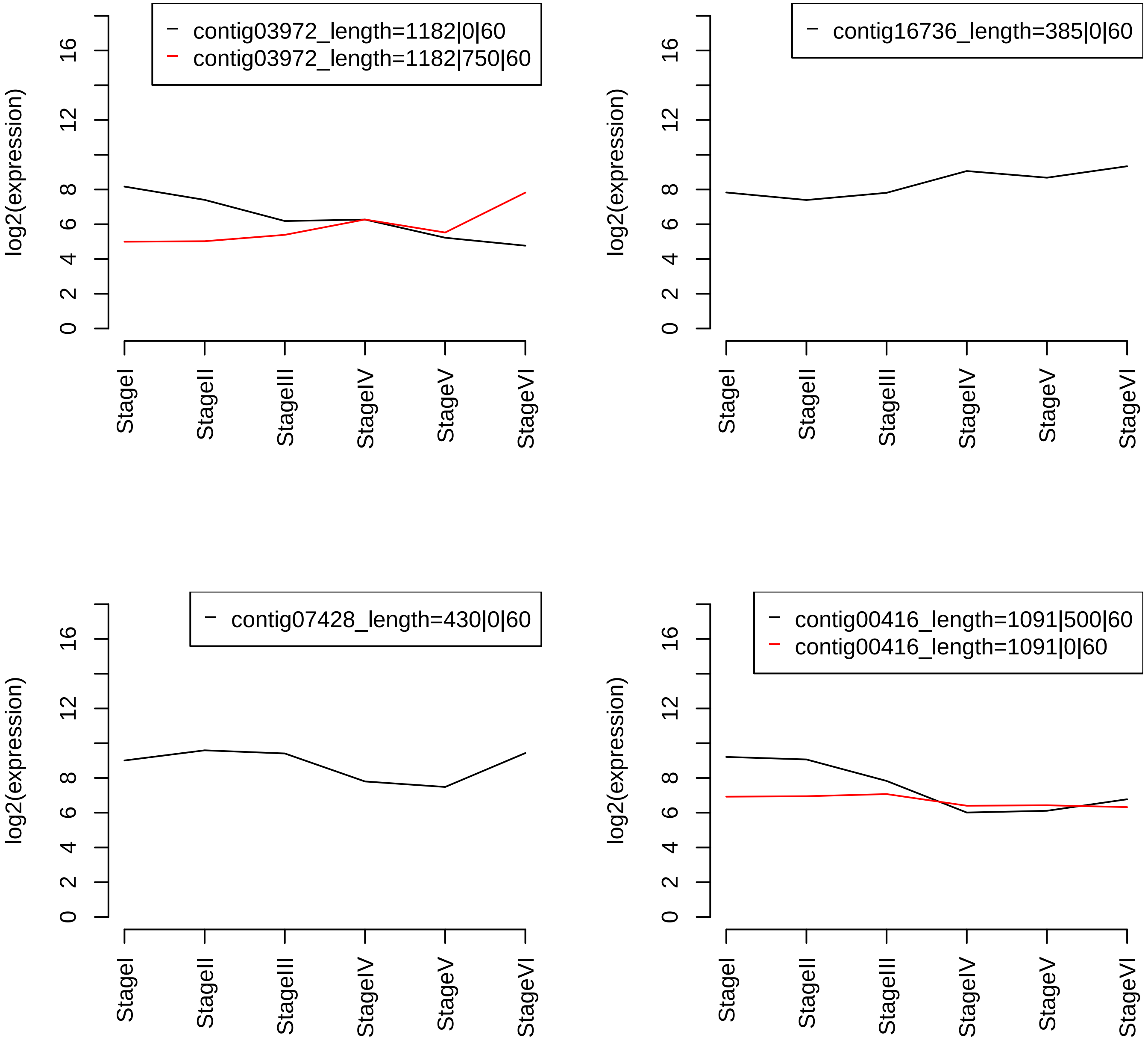

contig03972_length=1182|0|60
contig03972_length=1182|750|60
contig16736_length=385|0|60
contig07428_length=430|0|60
contig00416_length=1091|500|60
contig00416_length=1091|0|60
log2(expression)
0
2
4
6
8
12
16
StageI
StageII
StageIII
StageIV
StageV
StageVI

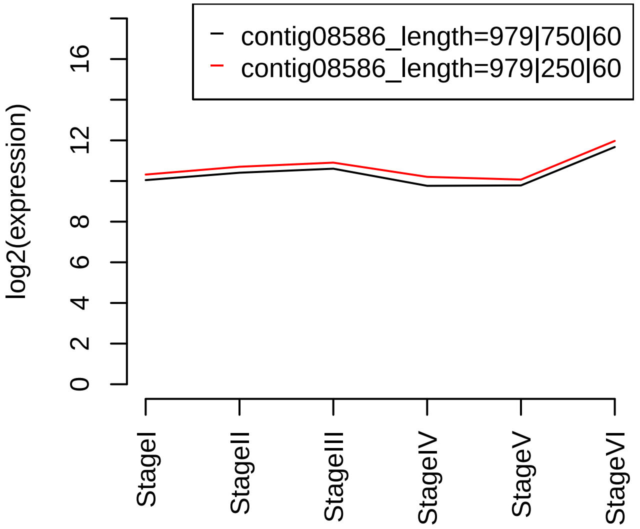

contig08586_length=979|750|60
contig08586_length=979|250|60
log2(expression)
0
2
4
6
8
12
16
StageI
StageII
StageIII
StageIV
StageV
StageVI

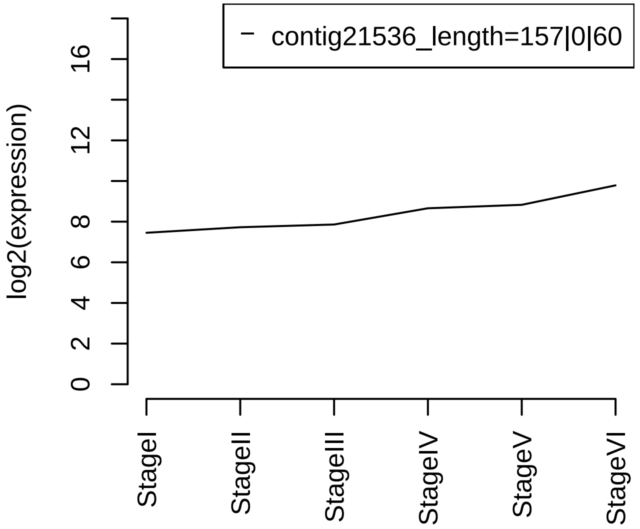

contig21536_length=157|0|60
log2(expression)
0
2
4
6
8
12
16
StageI
StageII
StageIII
StageIV
StageV
StageVI

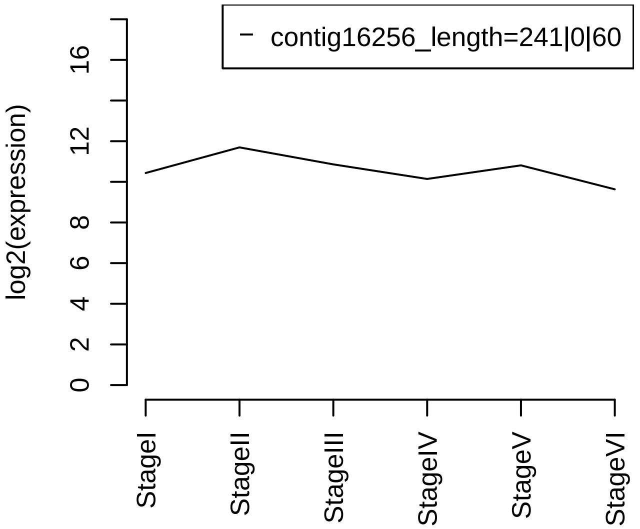

contig16256_length=241|0|60
log2(expression)
0
2
4
6
8
12
16
StageI
StageII
StageIII
StageIV
StageV
StageVI

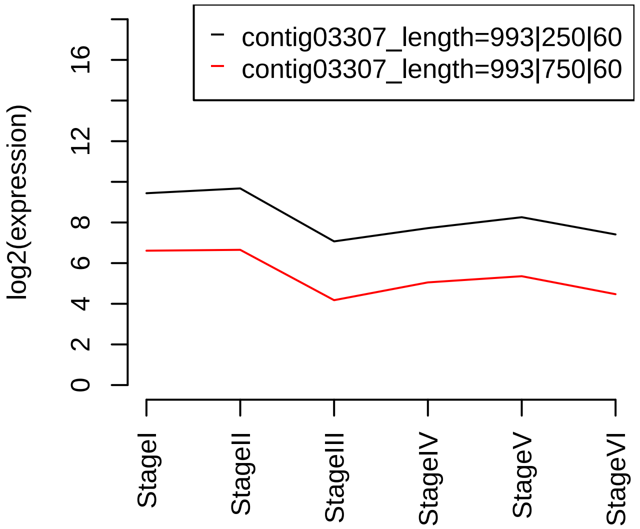

contig03307_length=993|250|60
contig03307_length=993|750|60
log2(expression)
0
2
4
6
8
12
16
StageI
StageII
StageIII
StageIV
StageV
StageVI

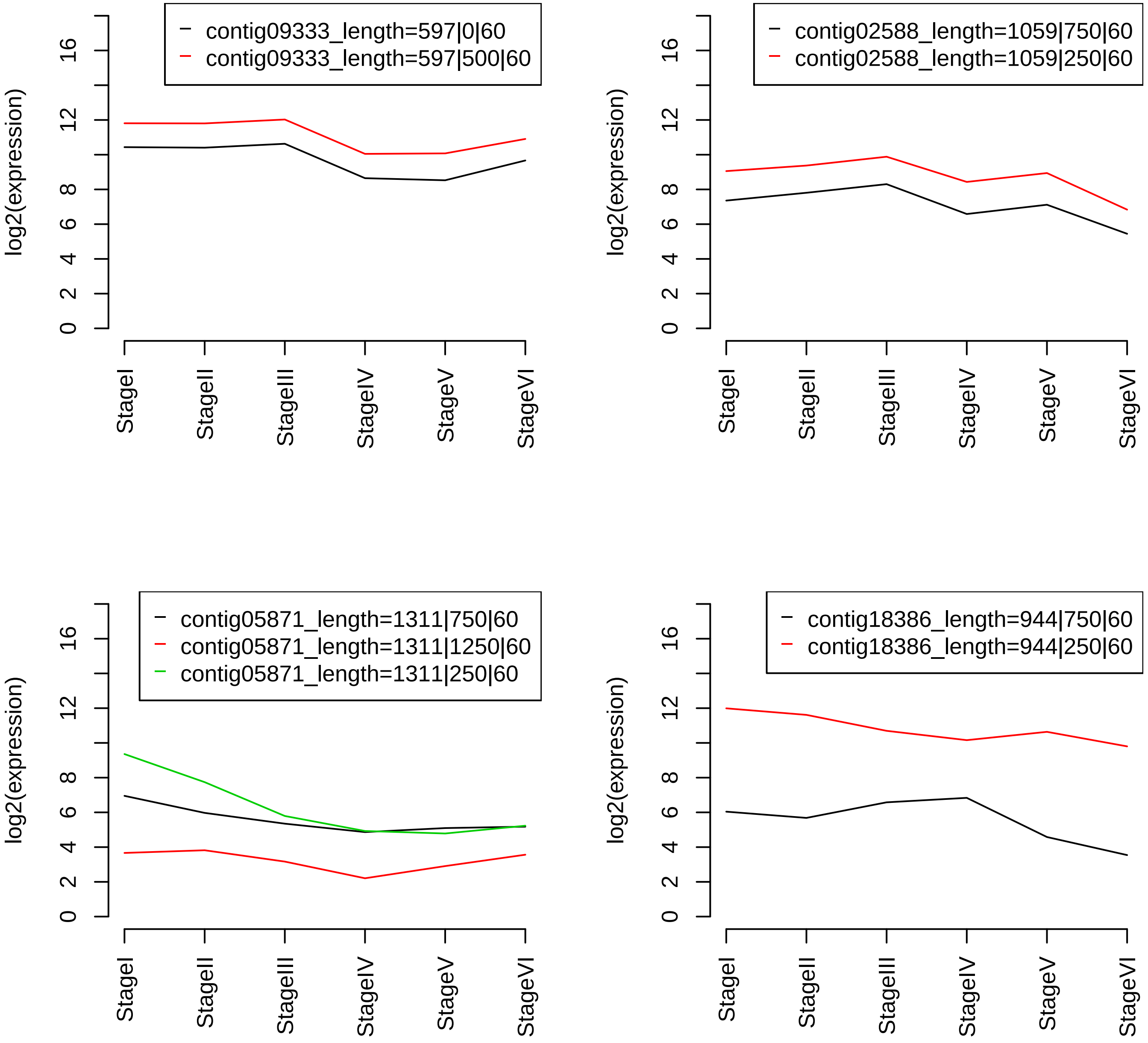
contig09333_length=597|0|60
contig09333_length=597|500|60
contig02588_length=1059|750|60
contig02588_length=1059|250|60
contig05871_length=1311|750|60
contig05871_length=1311|1250|60
contig05871_length=1311|250|60
contig18386_length=944|750|60
contig18386_length=944|250|60
log2(expression)
0 2 4 6 8 12 16
StageI StageII StageIII StageIV StageV StageVI

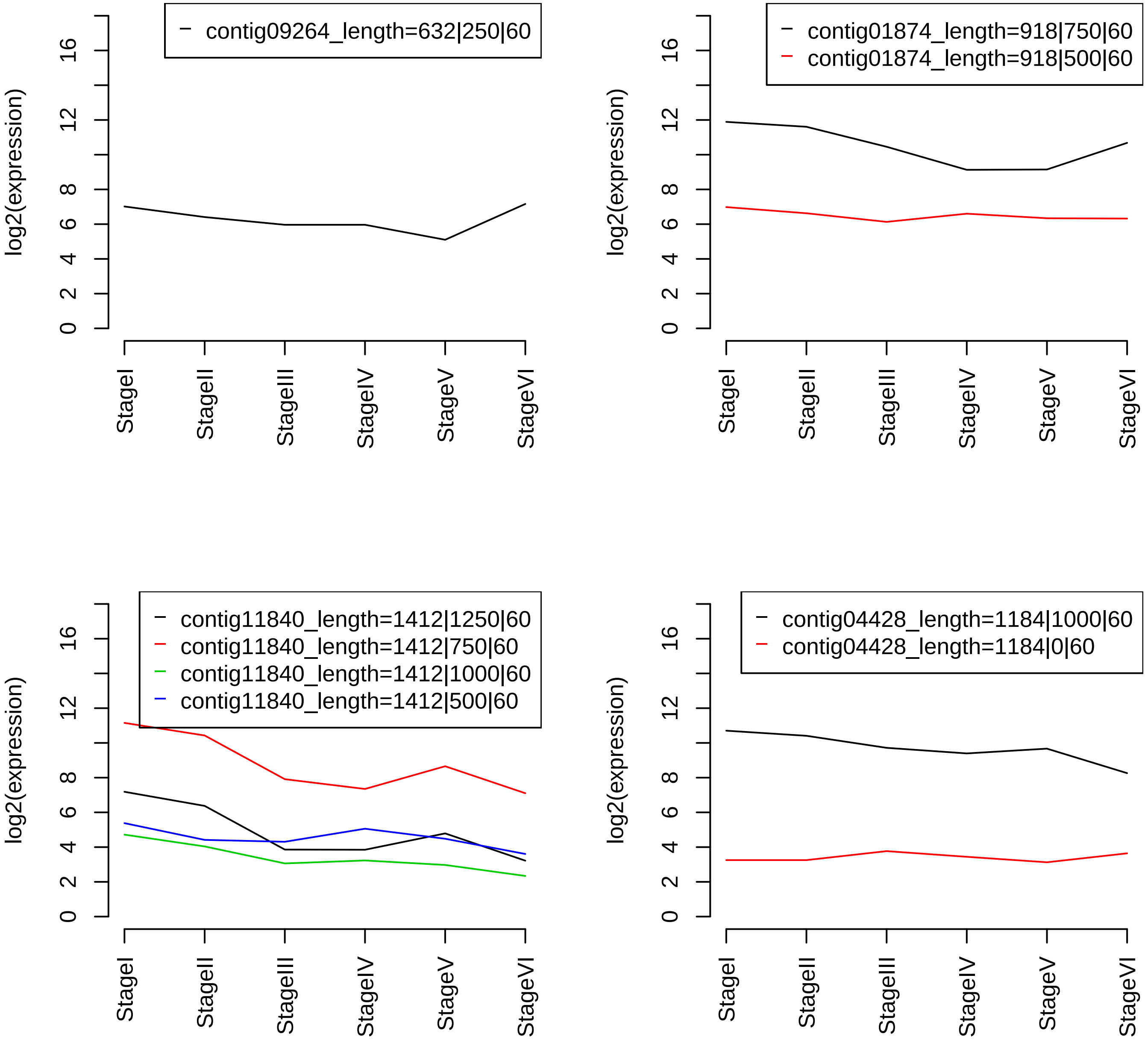
contig09264_length=632|250|60
contig01874_length=918|750|60
contig01874_length=918|500|60
contig11840_length=1412|1250|60
contig11840_length=1412|750|60
contig11840_length=1412|1000|60
contig11840_length=1412|500|60
contig04428_length=1184|1000|60
contig04428_length=1184|0|60
log2(expression)
StageI
StageII
StageIII
StageIV
StageV
StageVI

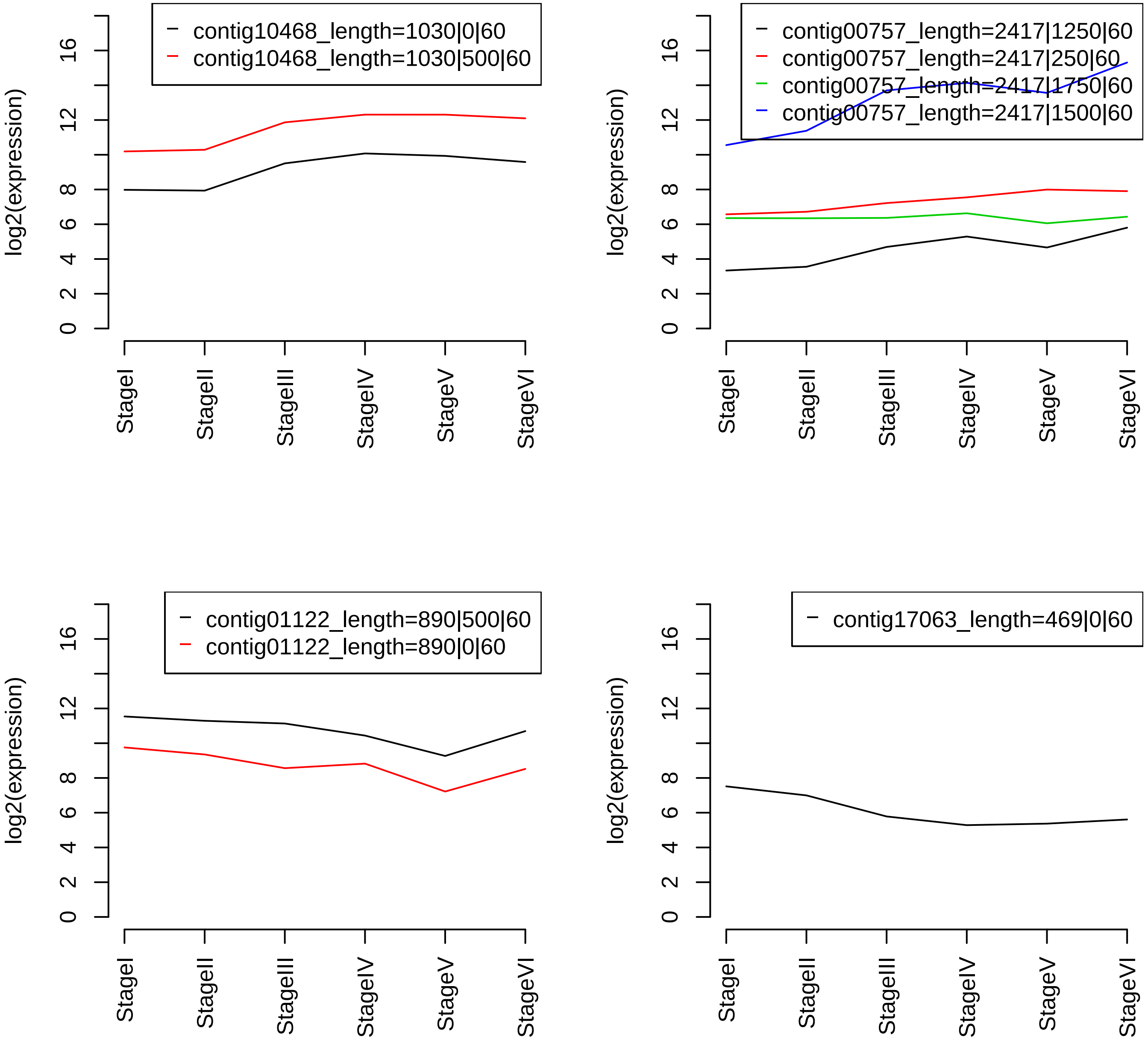

contig10468_length=1030|0|60
contig10468_length=1030|500|60
contig00757_length=2417|1250|60
contig00757_length=2417|250|60
contig00757_length=2417|1750|60
contig00757_length=2417|1500|60
contig01122_length=890|500|60
contig01122_length=890|0|60
contig17063_length=469|0|60
log2(expression)
0
2
4
6
8
12
16
StageI
StageII
StageIII
StageIV
StageV
StageVI

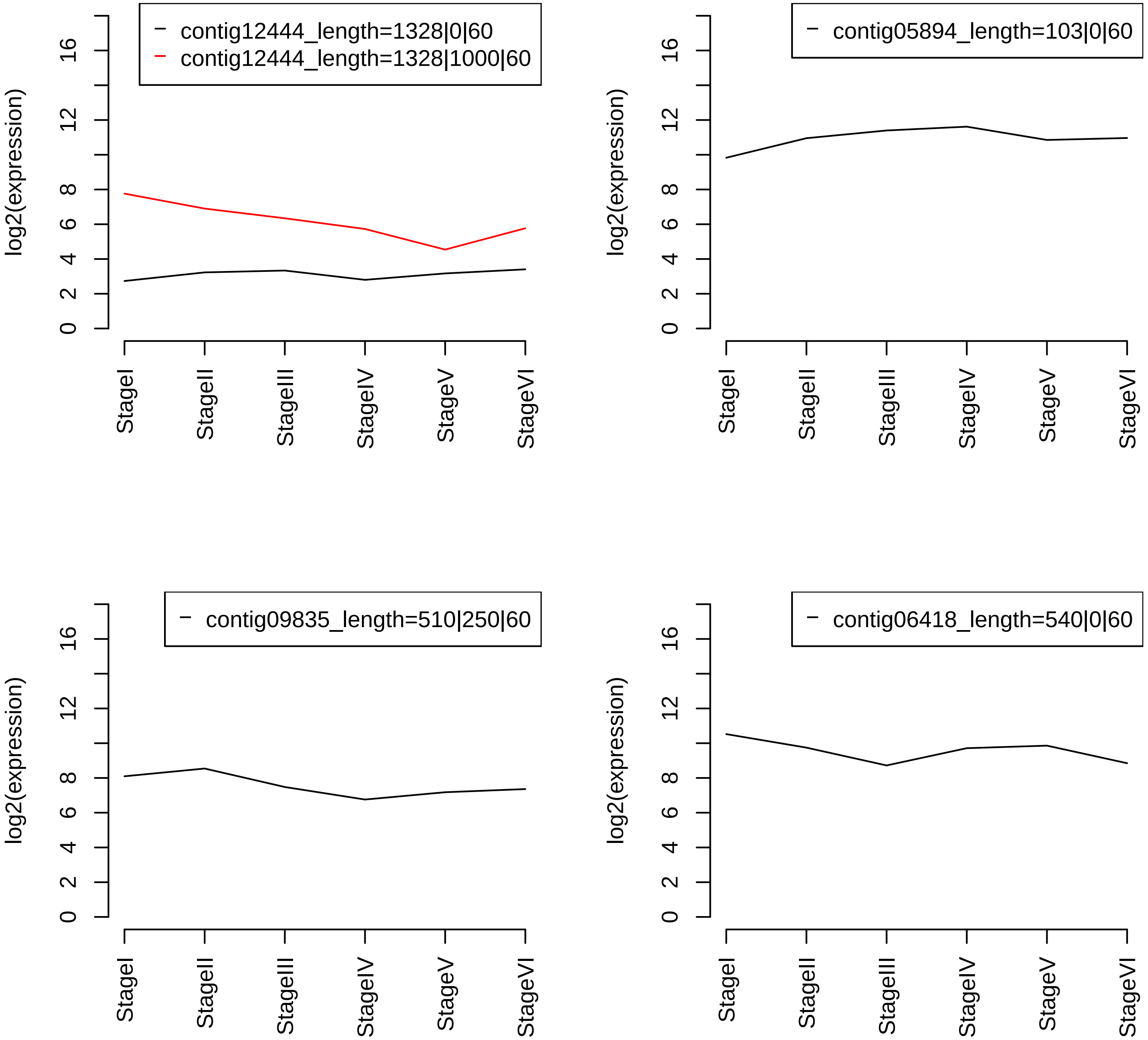

contig12444_length=1328|0|60
contig12444_length=1328|1000|60
contig05894_length=103|0|60
contig09835_length=510|250|60
contig06418_length=540|0|60
log2(expression)
0
2
4
6
8
12
16
StageI
StageII
StageIII
StageIV
StageV
StageVI

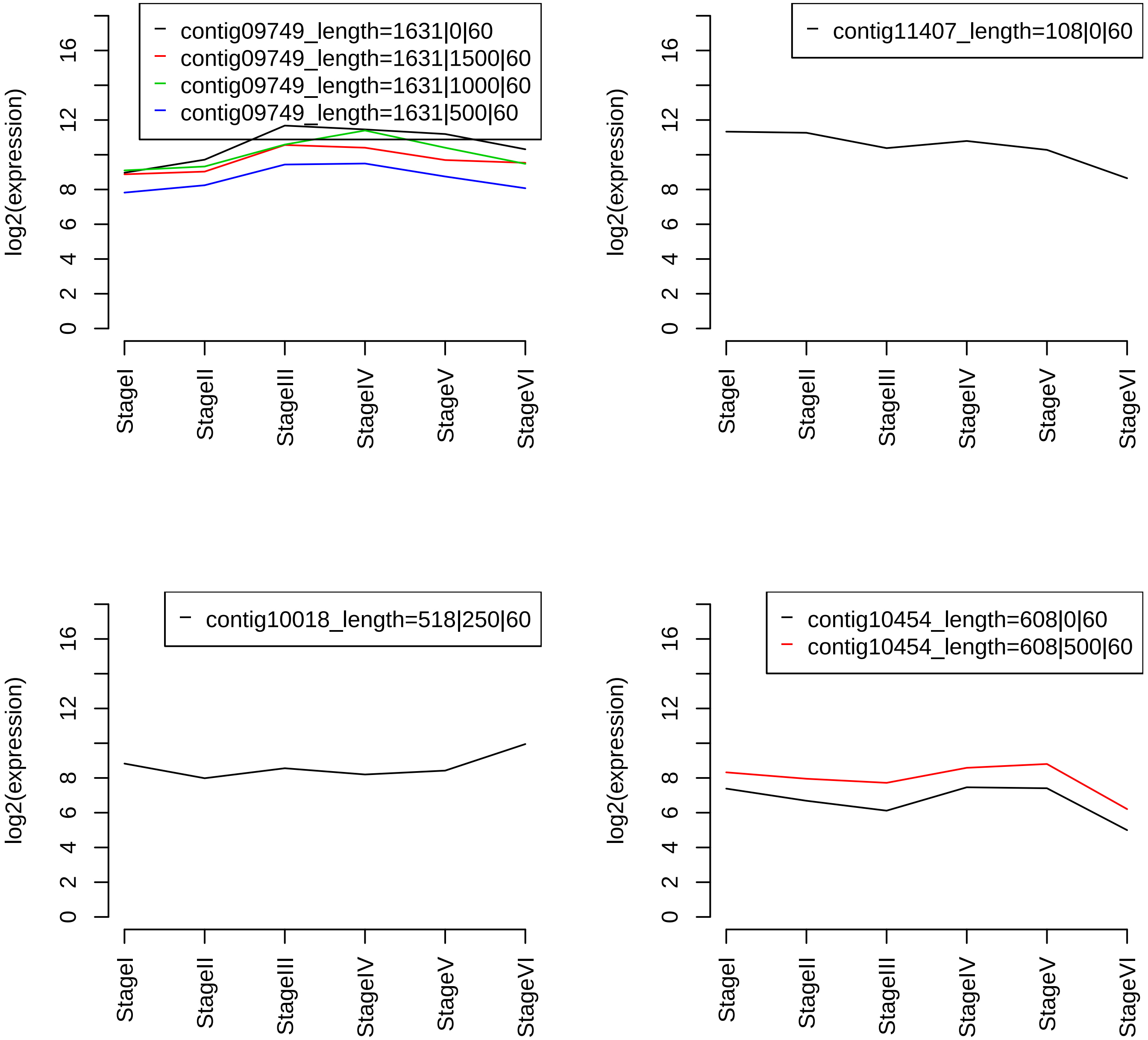
contig09749_length=1631|0|60
contig09749_length=1631|1500|60
contig09749_length=1631|1000|60
contig09749_length=1631|500|60
contig11407_length=108|0|60
contig10018_length=518|250|60
contig10454_length=608|0|60
contig10454_length=608|500|60
log2(expression)
0
2
4
6
8
12
16
StageI
StageII
StageIII
StageIV
StageV
StageVI

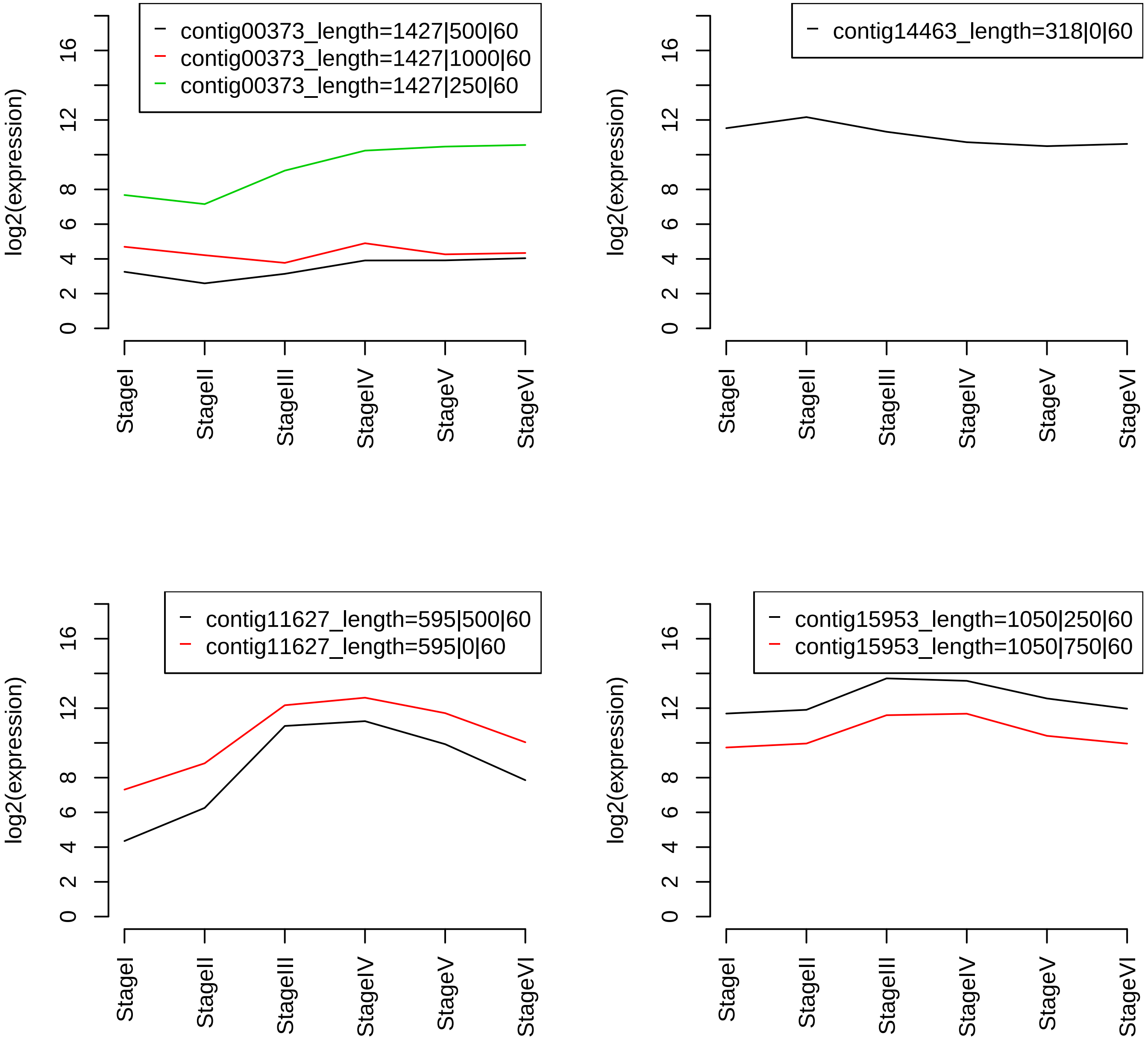
contig00373_length=1427|500|60
contig00373_length=1427|1000|60
contig00373_length=1427|250|60
contig14463_length=318|0|60
contig11627_length=595|500|60
contig11627_length=595|0|60
contig15953_length=1050|250|60
contig15953_length=1050|750|60
log2(expression)
0
2
4
6
8
12
16
StageI
StageII
StageIII
StageIV
StageV
StageVI

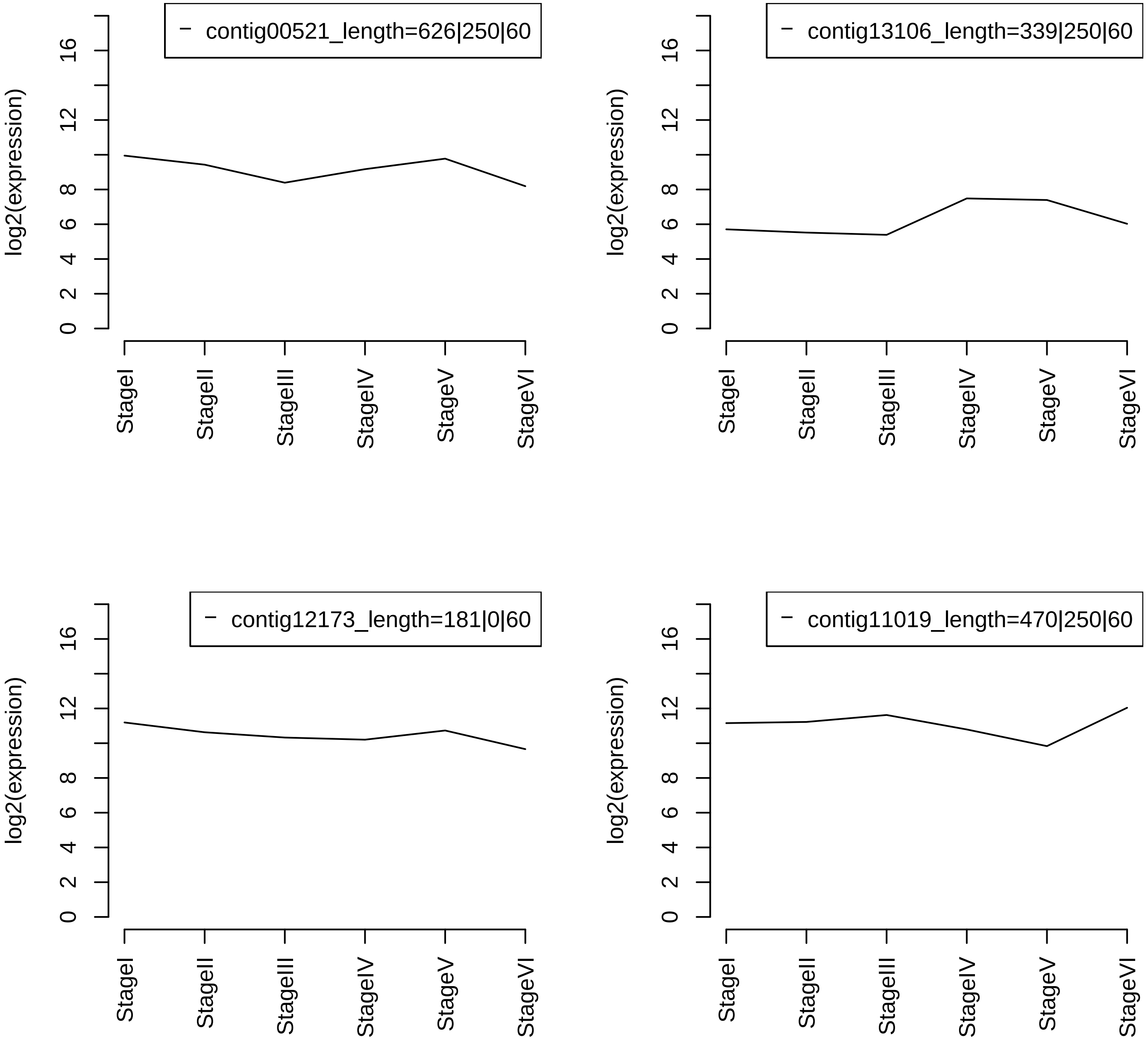

contig00521_length=626|250|60
contig13106_length=339|250|60
contig12173_length=181|0|60
contig11019_length=470|250|60
log2(expression)
0
2
4
6
8
12
16
StageI
StageII
StageIII
StageIV
StageV
StageVI

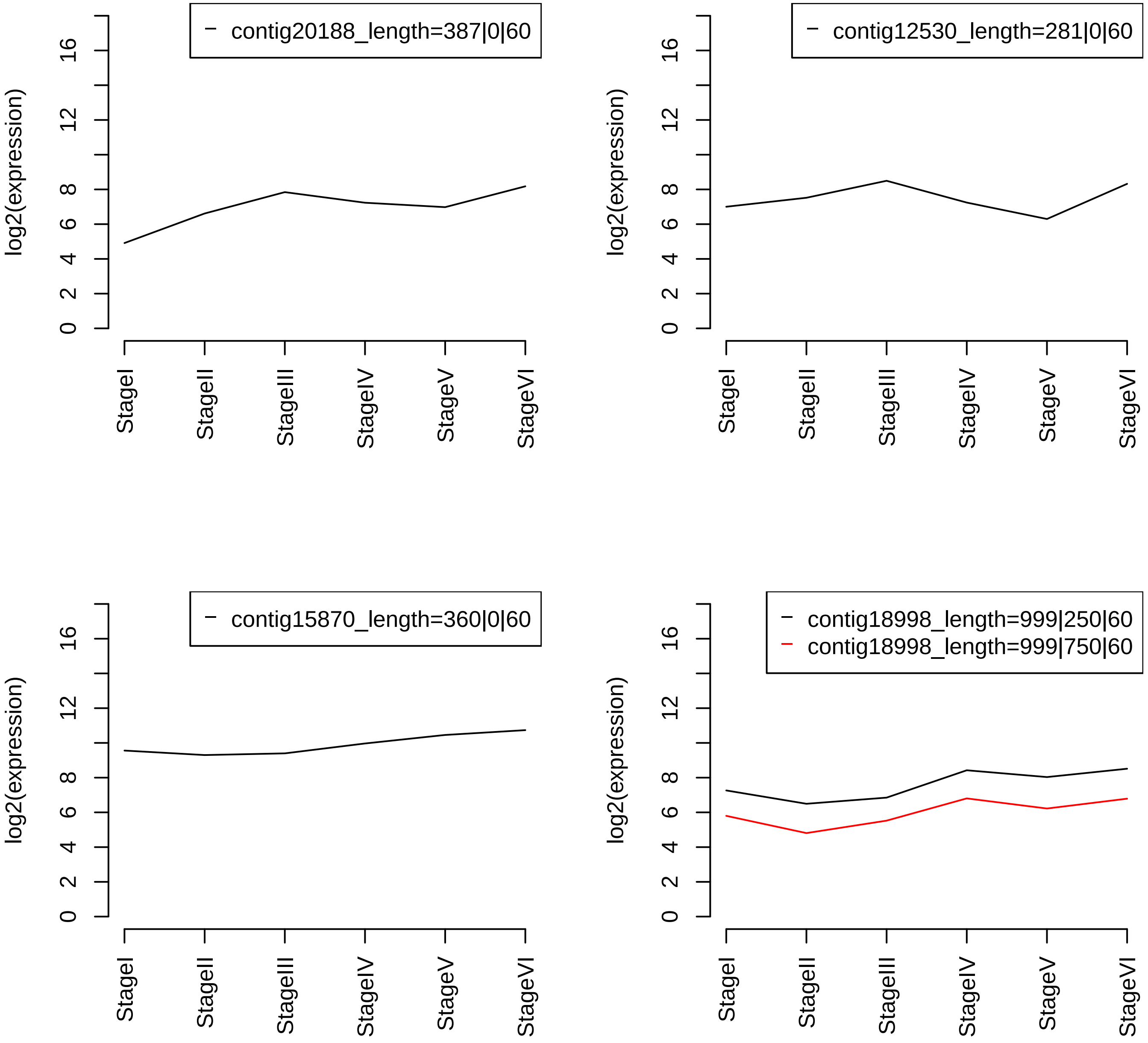

contig20188_length=387|0|60
log2(expression)
0
2
4
6
8
12
16
StageI
StageII
StageIII
StageIV
StageV
StageVI
contig12530_length=281|0|60
log2(expression)
contig15870_length=360|0|60
log2(expression)
contig18998_length=999|250|60
contig18998_length=999|750|60
log2(expression)

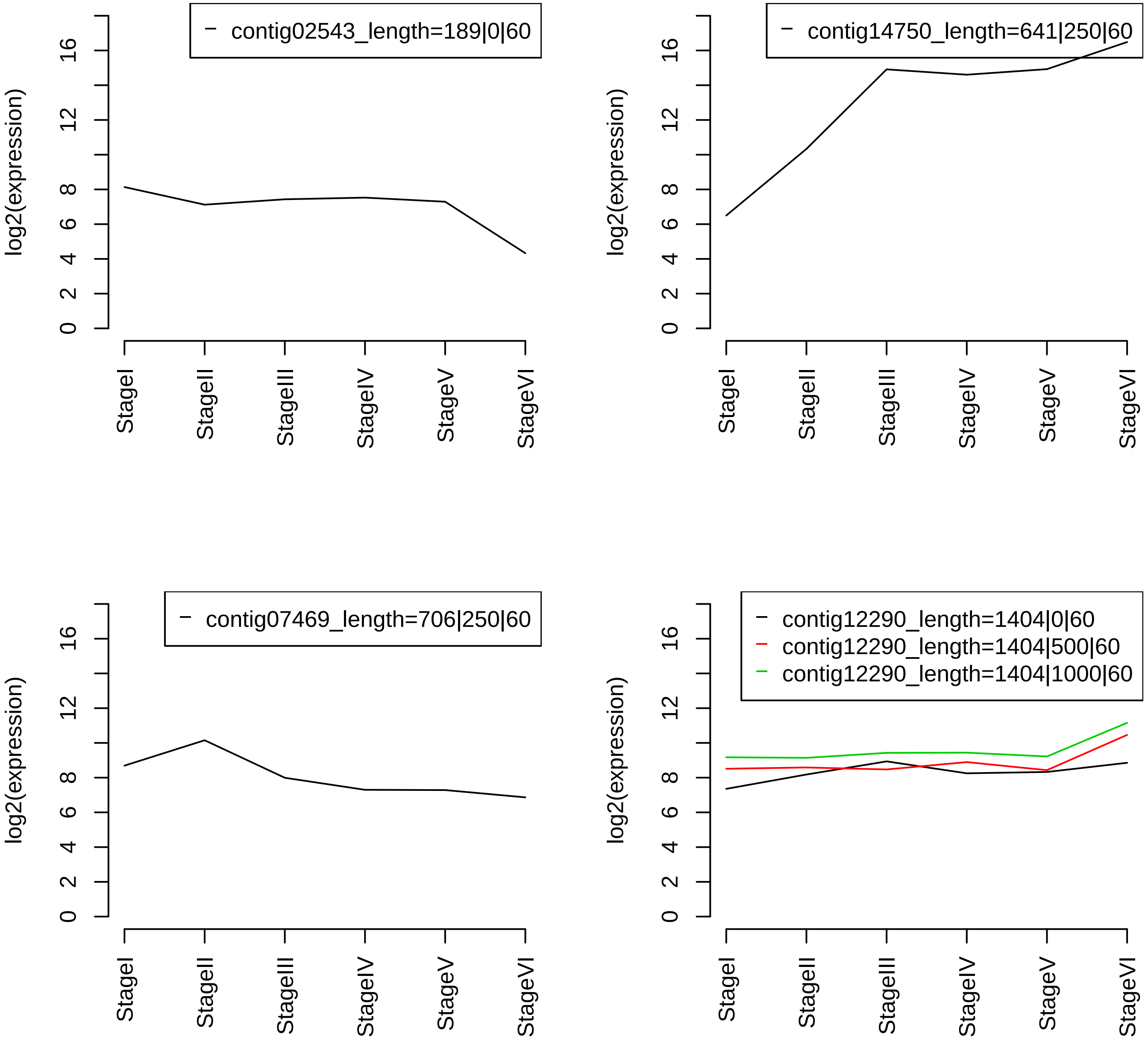

contig02543_length=189|0|60
contig14750_length=641|250|60
contig07469_length=706|250|60
contig12290_length=1404|0|60
contig12290_length=1404|500|60
contig12290_length=1404|1000|60
log2(expression)
0
2
4
6
8
12
16
StageI
StageII
StageIII
StageIV
StageV
StageVI

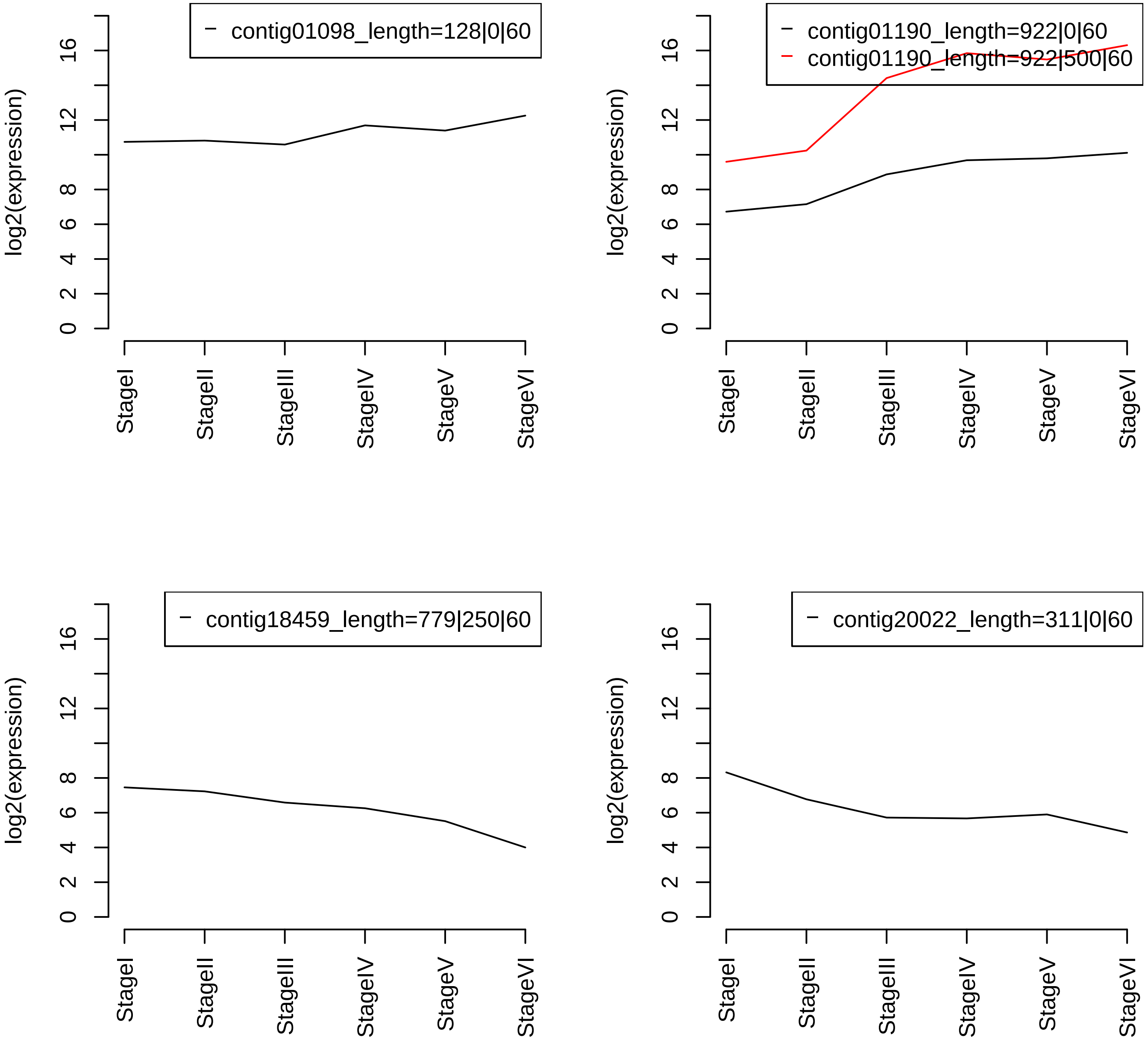

contig01098_length=128|0|60
contig01190_length=922|0|60
contig01190_length=922|500|60
contig18459_length=779|250|60
contig20022_length=311|0|60
log2(expression)
0
2
4
6
8
12
16
StageI
StageII
StageIII
StageIV
StageV
StageVI

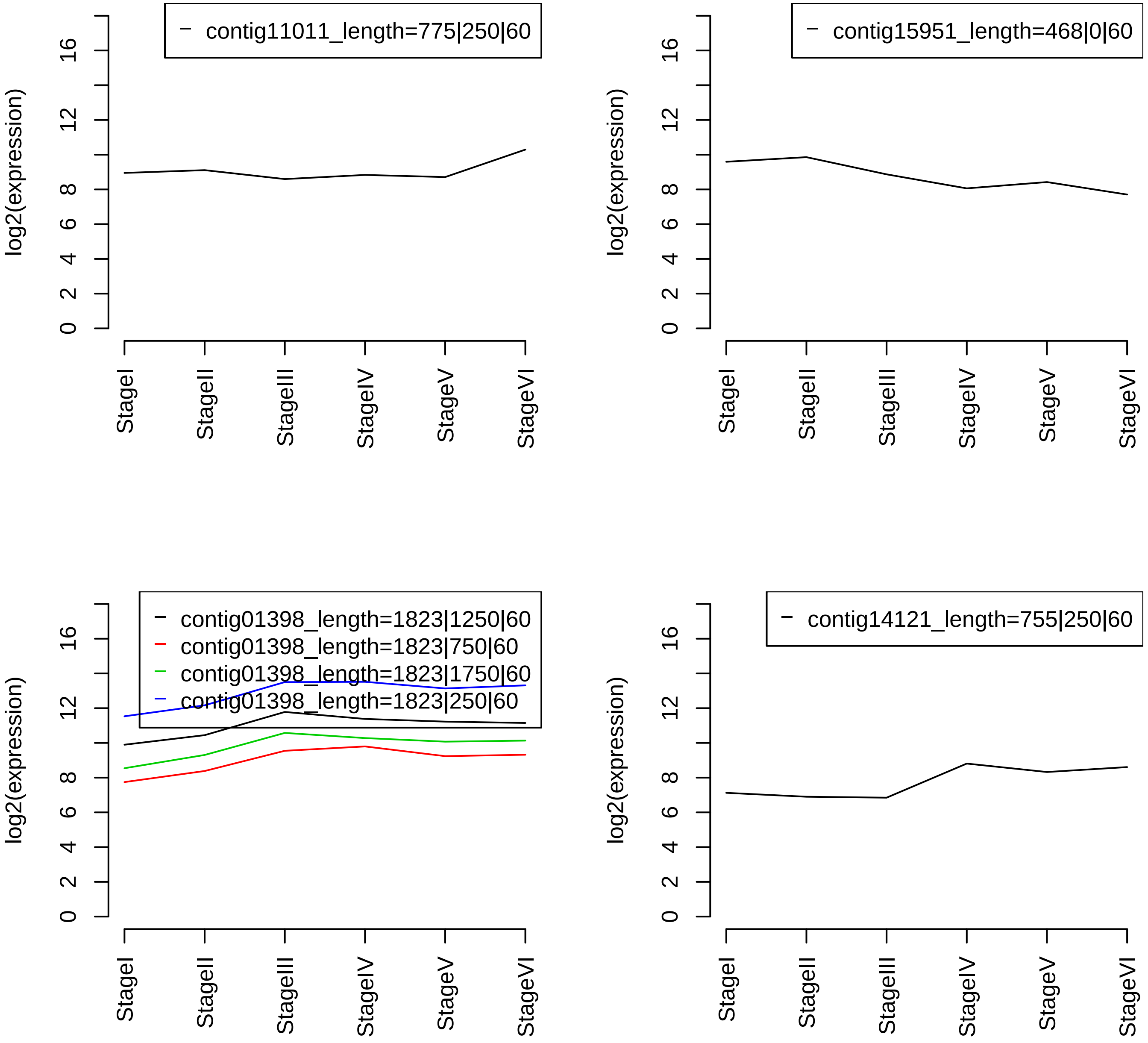
contig11011_length=775|250|60
contig15951_length=468|0|60
contig01398_length=1823|1250|60
contig01398_length=1823|750|60
contig01398_length=1823|1750|60
contig01398_length=1823|250|60
contig14121_length=755|250|60
log2(expression)
0
2
4
6
8
12
16
StageI
StageII
StageIII
StageIV
StageV
StageVI

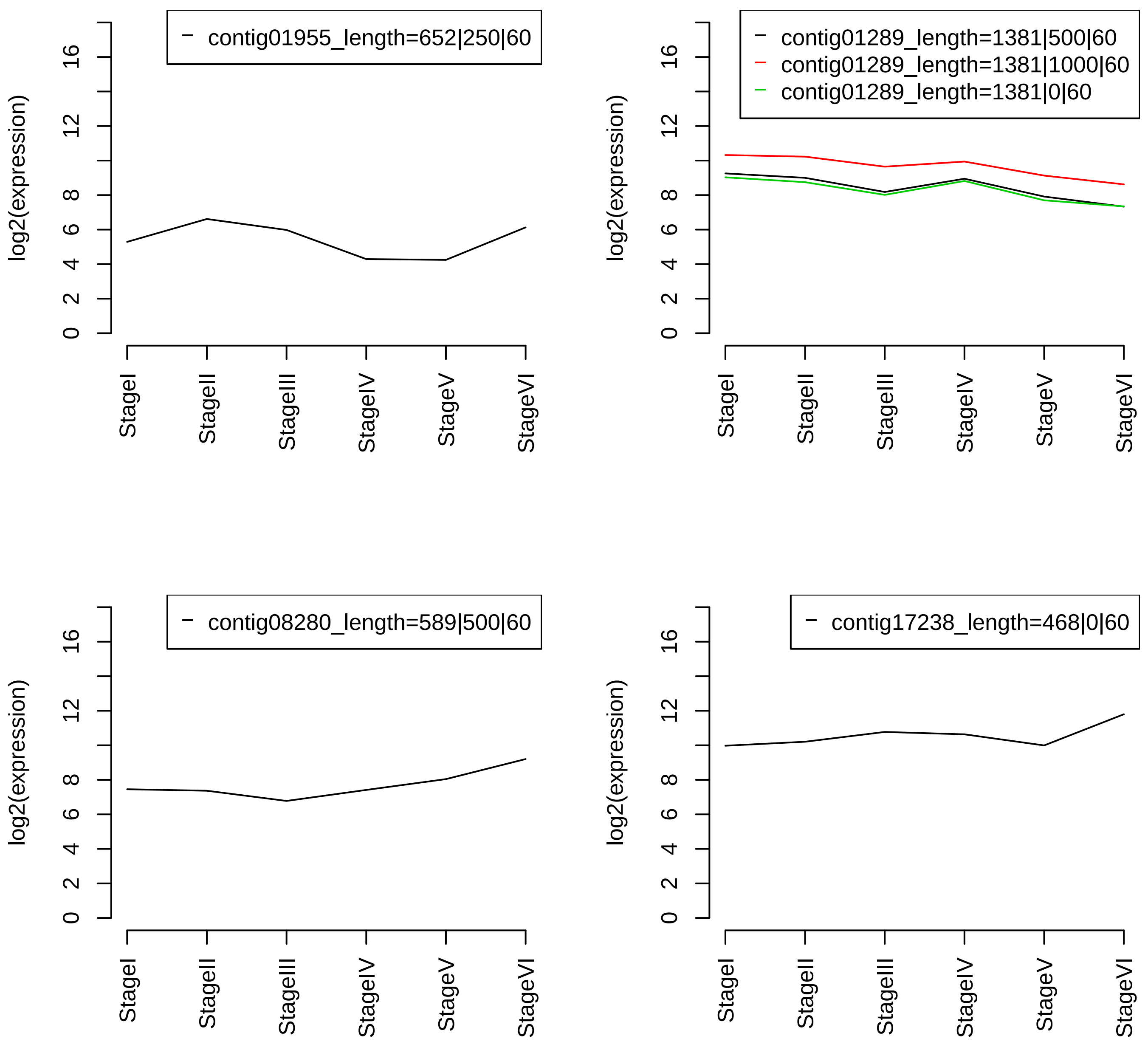
contig01955_length=652|250|60
contig01289_length=1381|500|60
contig01289_length=1381|1000|60
contig01289_length=1381|0|60
contig08280_length=589|500|60
contig17238_length=468|0|60
log2(expression)
0
2
4
6
8
12
16
StageI
StageII
StageIII
StageIV
StageV
StageVI

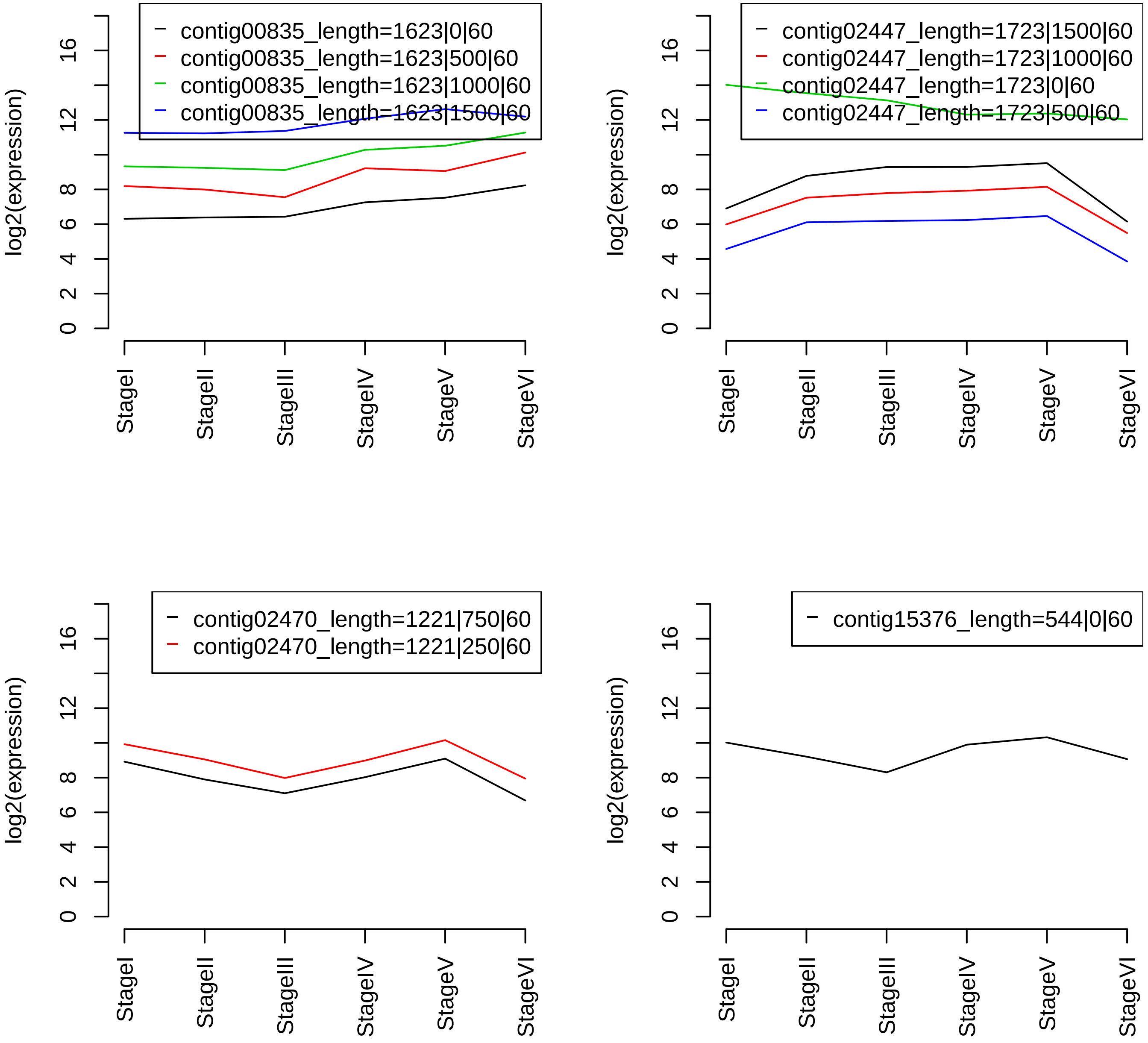

contig00835_length=1623|0|60
contig00835_length=1623|500|60
contig00835_length=1623|1000|60
contig00835_length=1623|1500|60
contig02447_length=1723|1500|60
contig02447_length=1723|1000|60
contig02447_length=1723|0|60
contig02447_length=1723|500|60
contig02470_length=1221|750|60
contig02470_length=1221|250|60
contig15376_length=544|0|60
log2(expression)
0
2
4
6
8
12
16
StageI
StageII
StageIII
StageIV
StageV
StageVI

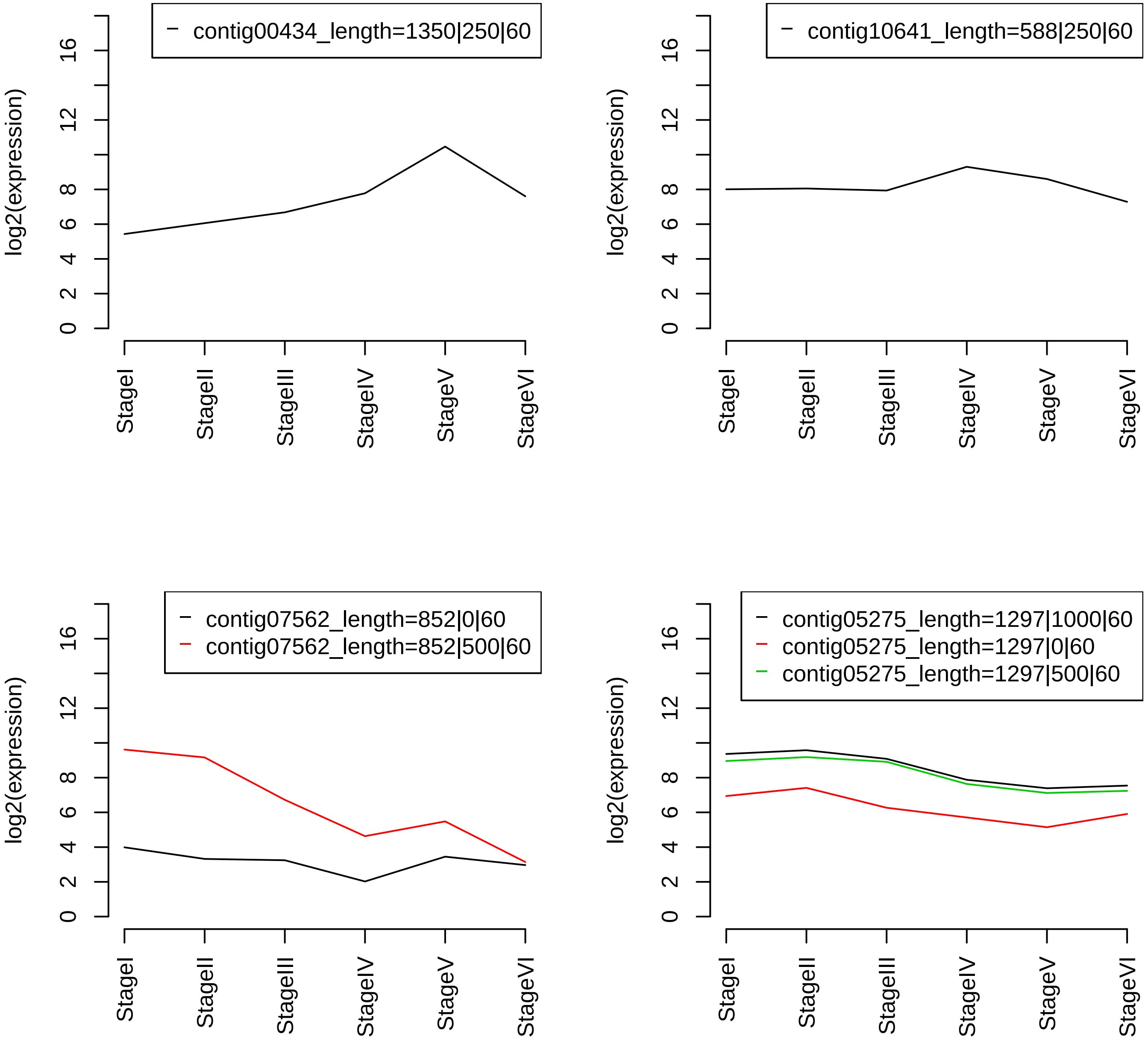

contig00434_length=1350|250|60
contig10641_length=588|250|60
contig07562_length=852|0|60
contig07562_length=852|500|60
contig05275_length=1297|1000|60
contig05275_length=1297|0|60
contig05275_length=1297|500|60
log2(expression)
0 2 4 6 8 12 16
StageI StageII StageIII StageIV StageV StageVI

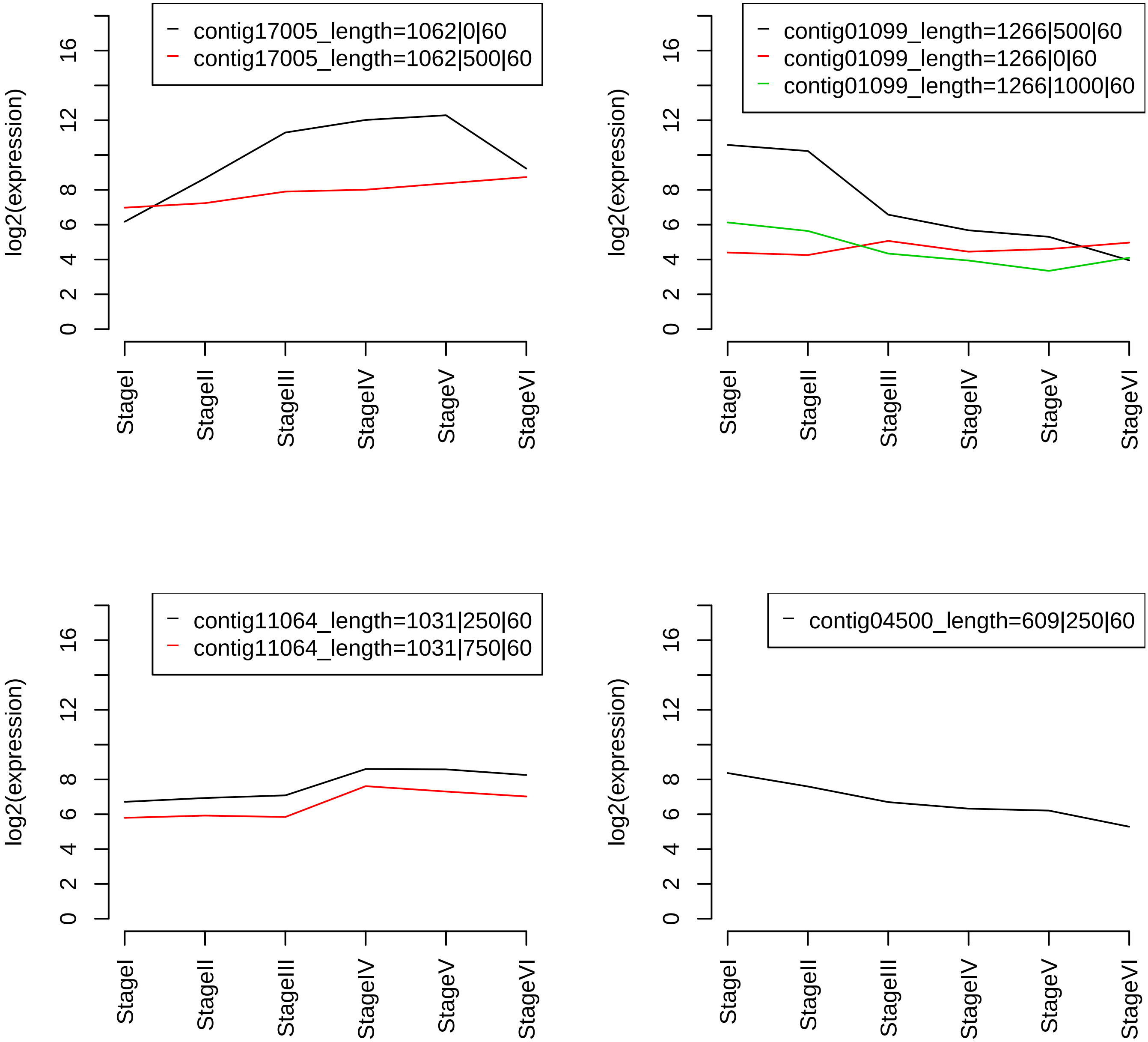
contig17005_length=1062|0|60
contig17005_length=1062|500|60
log2(expression)
0
2
4
6
8
12
16
StageI
StageII
StageIII
StageIV
StageV
StageVI
contig01099_length=1266|500|60
contig01099_length=1266|0|60
contig01099_length=1266|1000|60
log2(expression)
contig11064_length=1031|250|60
contig11064_length=1031|750|60
log2(expression)
contig04500_length=609|250|60
log2(expression)

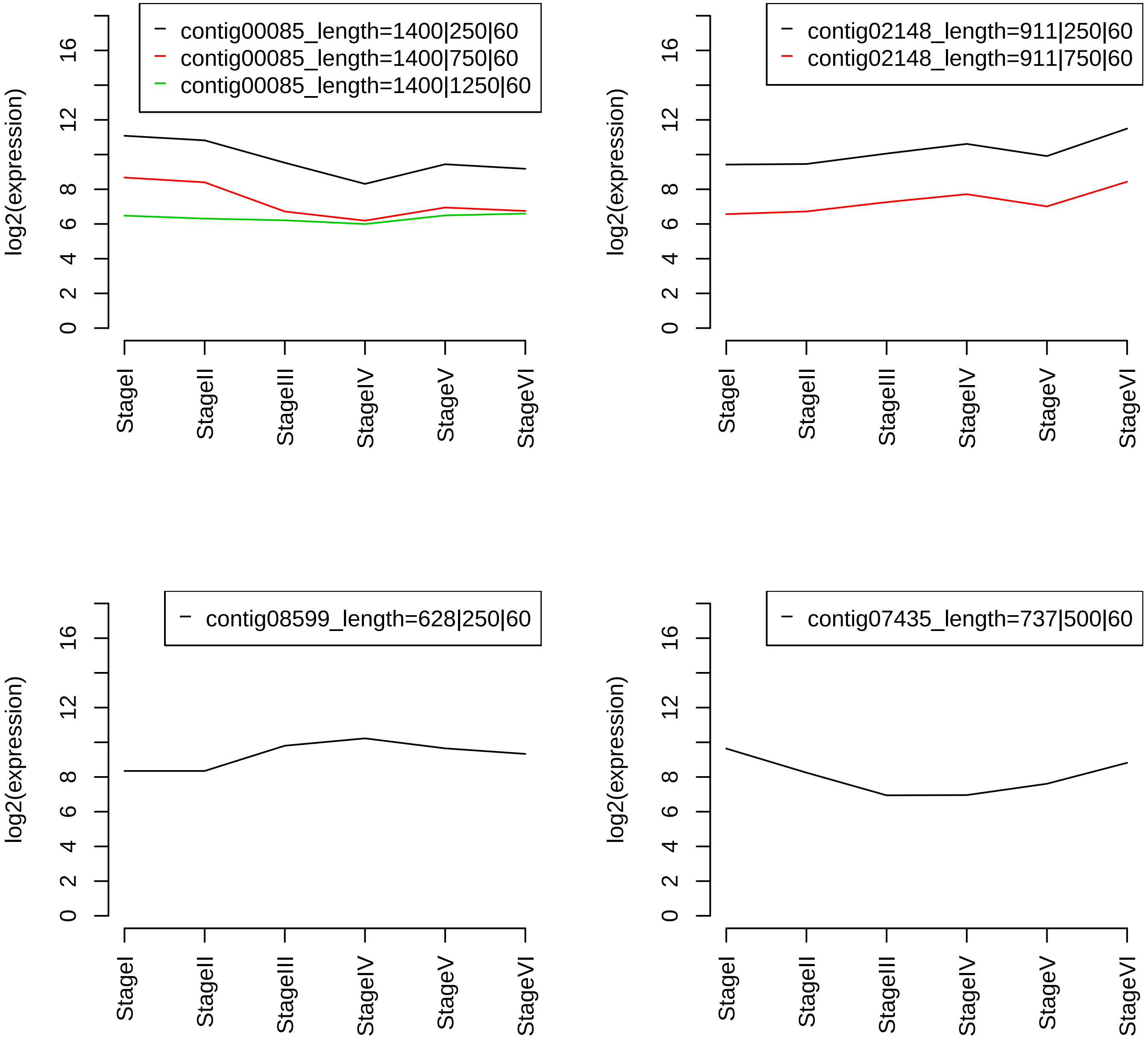
contig00085_length=1400|250|60
contig00085_length=1400|750|60
contig00085_length=1400|1250|60
contig02148_length=911|250|60
contig02148_length=911|750|60
contig08599_length=628|250|60
contig07435_length=737|500|60
log2(expression)
0
2
4
6
8
12
16
StageI
StageII
StageIII
StageIV
StageV
StageVI

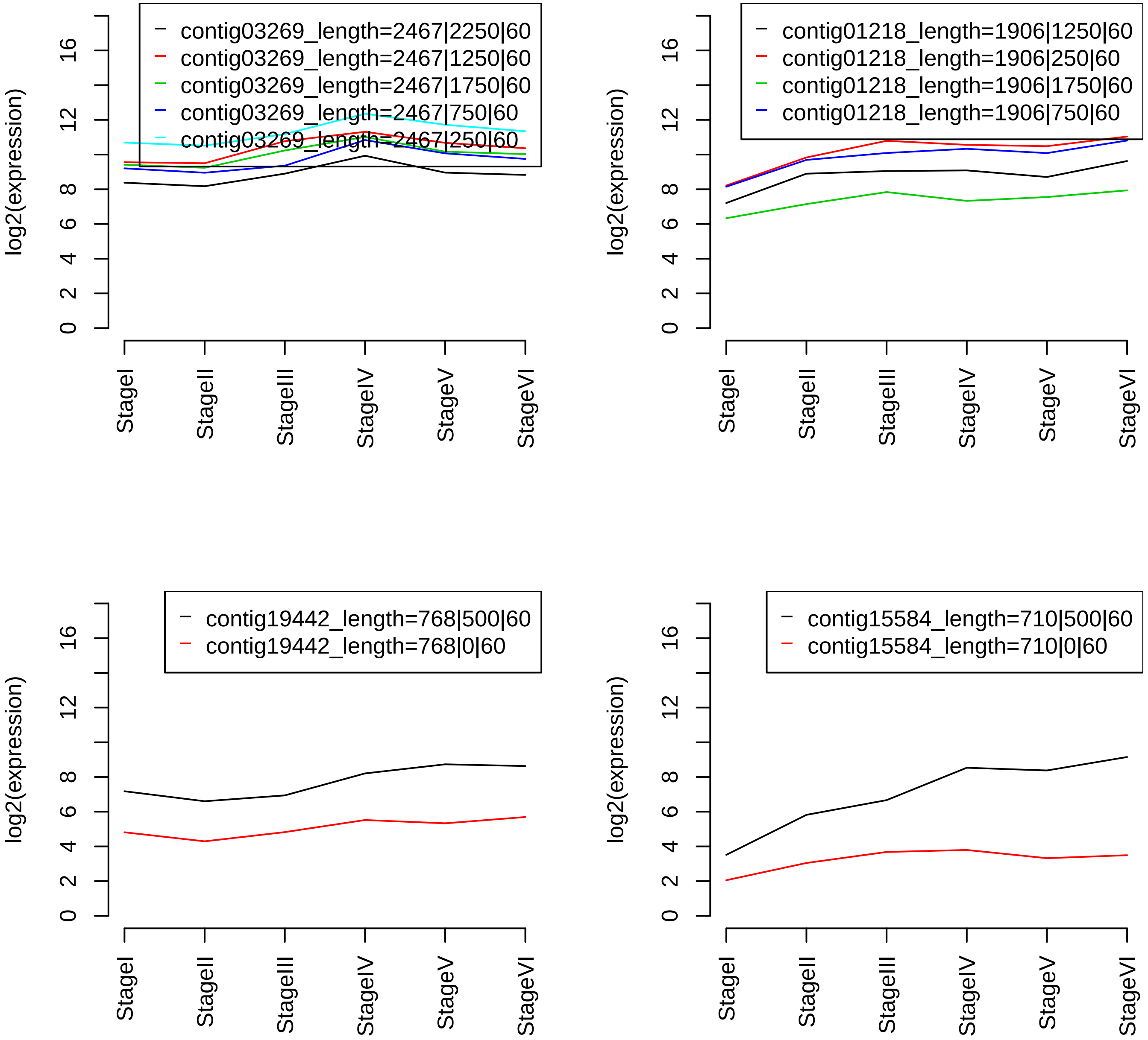
contig03269_length=2467|2250|60
contig03269_length=2467|1250|60
contig03269_length=2467|1750|60
contig03269_length=2467|750|60
contig03269_length=2467|250|60
contig01218_length=1906|1250|60
contig01218_length=1906|250|60
contig01218_length=1906|1750|60
contig01218_length=1906|750|60
contig19442_length=768|500|60
contig19442_length=768|0|60
contig15584_length=710|500|60
contig15584_length=710|0|60
log2(expression)
0
2
4
6
8
12
16
StageI
StageII
StageIII
StageIV
StageV
StageVI

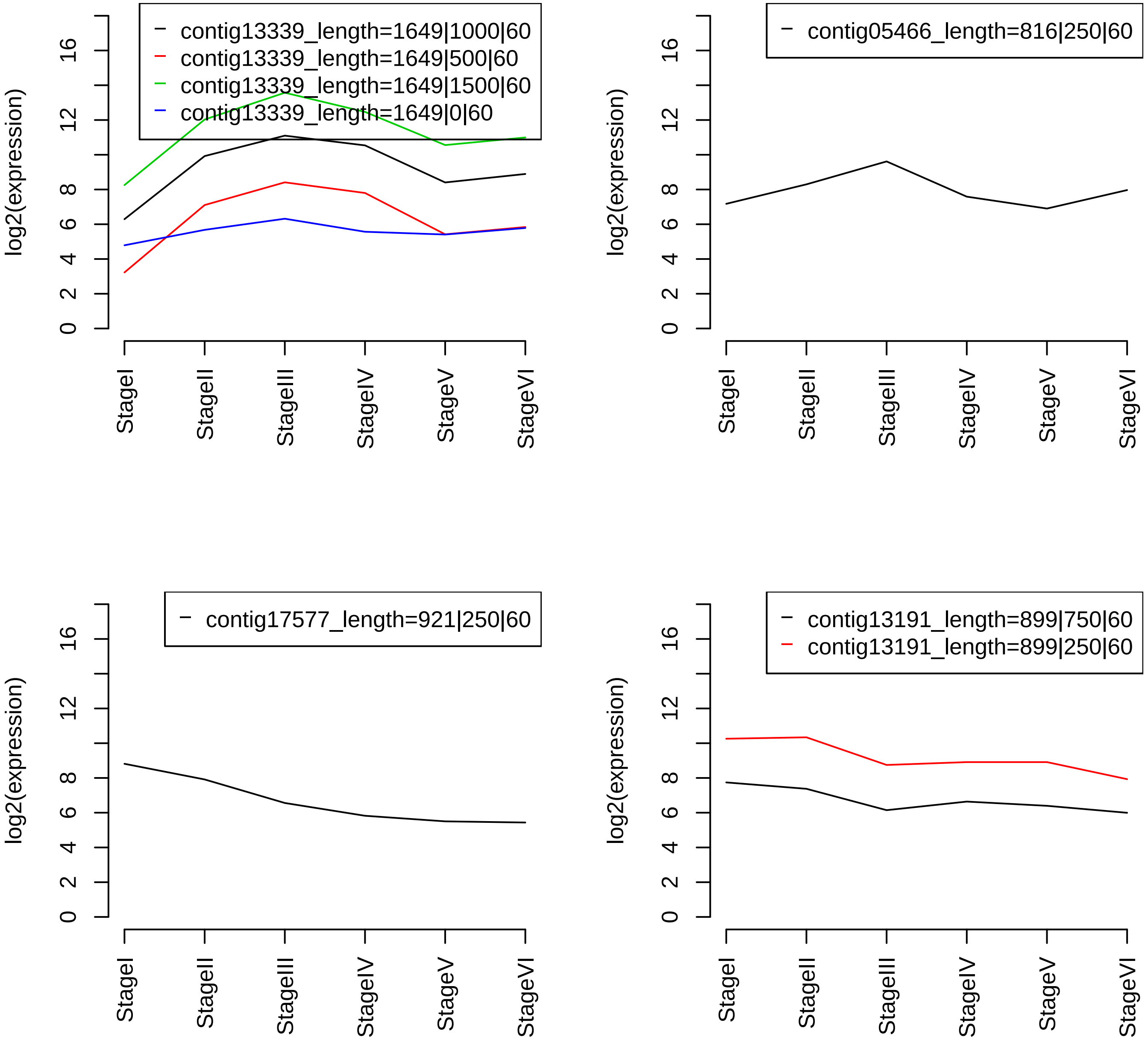
contig13339_length=1649|1000|60
contig13339_length=1649|500|60
contig13339_length=1649|1500|60
contig13339_length=1649|0|60
log2(expression)
0
2
4
6
8
12
16
StageI
StageII
StageIII
StageIV
StageV
StageVI
contig05466_length=816|250|60
contig17577_length=921|250|60
contig13191_length=899|750|60
contig13191_length=899|250|60

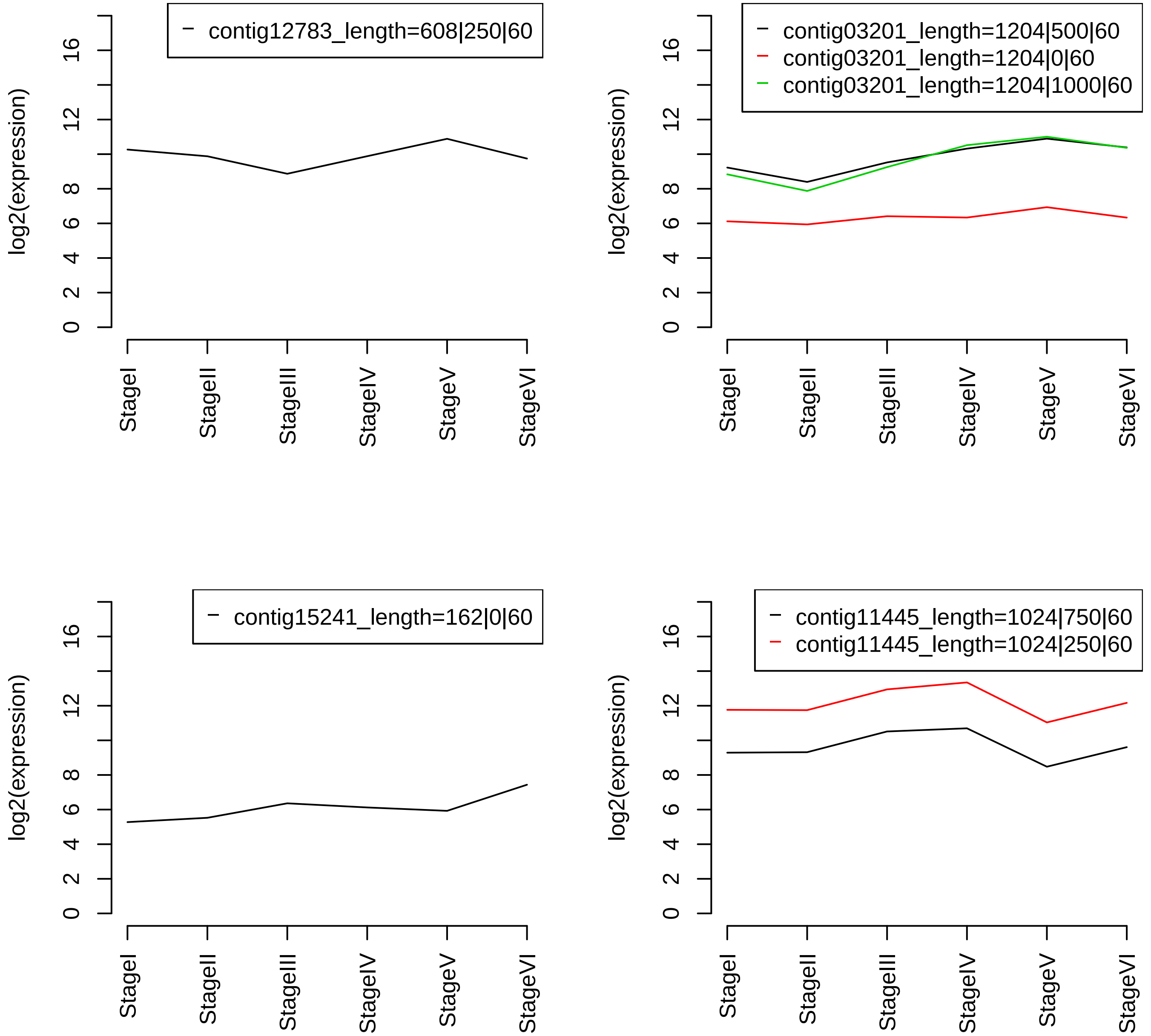
contig12783_length=608|250|60
log2(expression)
0
2
4
6
8
12
16
StageI
StageII
StageIII
StageIV
StageV
StageVI
contig03201_length=1204|500|60
contig03201_length=1204|0|60
contig03201_length=1204|1000|60
log2(expression)
contig15241_length=162|0|60
log2(expression)
contig11445_length=1024|750|60
contig11445_length=1024|250|60
log2(expression)

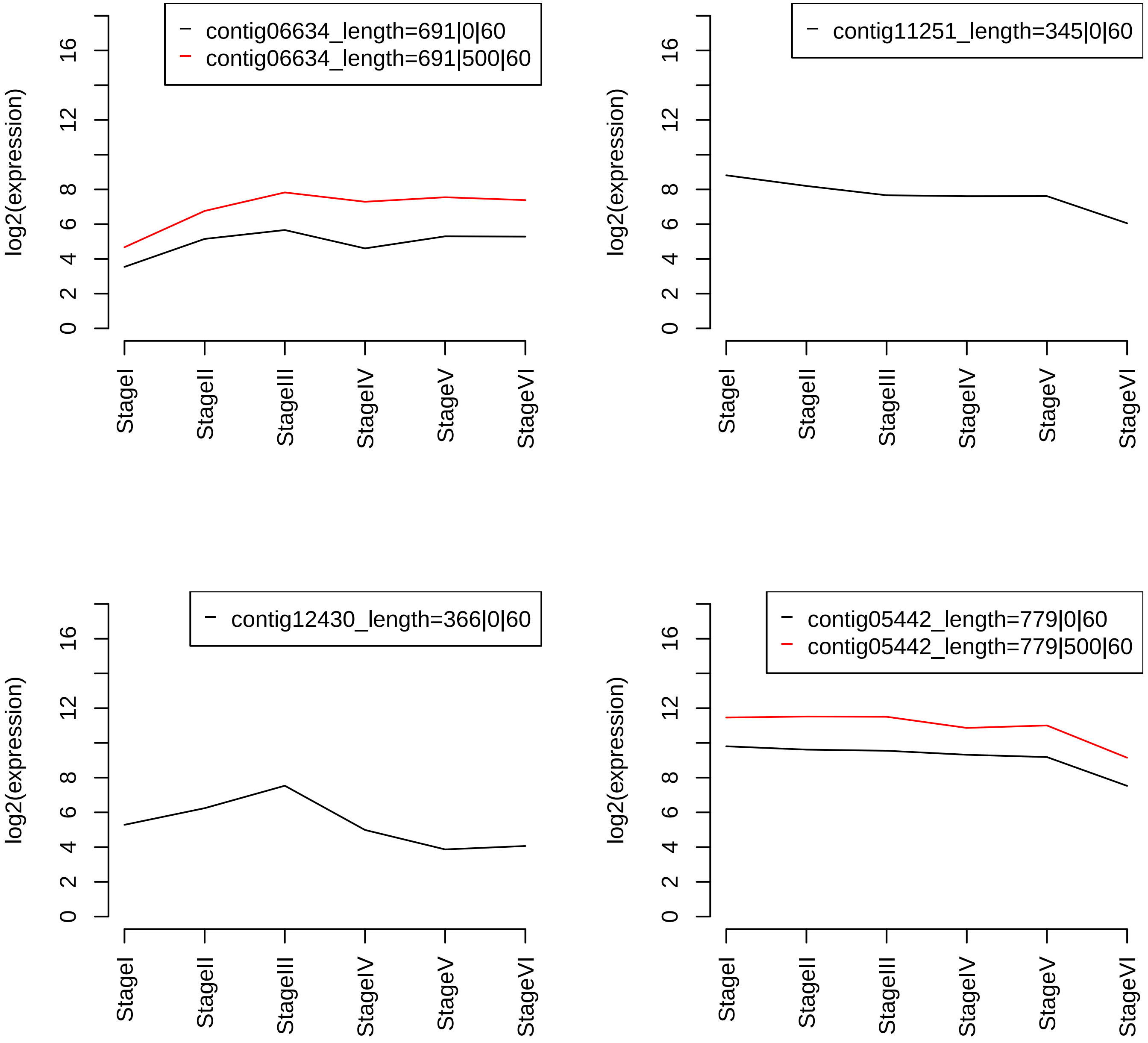
contig06634_length=691|0|60
contig06634_length=691|500|60
contig11251_length=345|0|60
contig12430_length=366|0|60
contig05442_length=779|0|60
contig05442_length=779|500|60
log2(expression)
StageI
StageII
StageIII
StageIV
StageV
StageVI

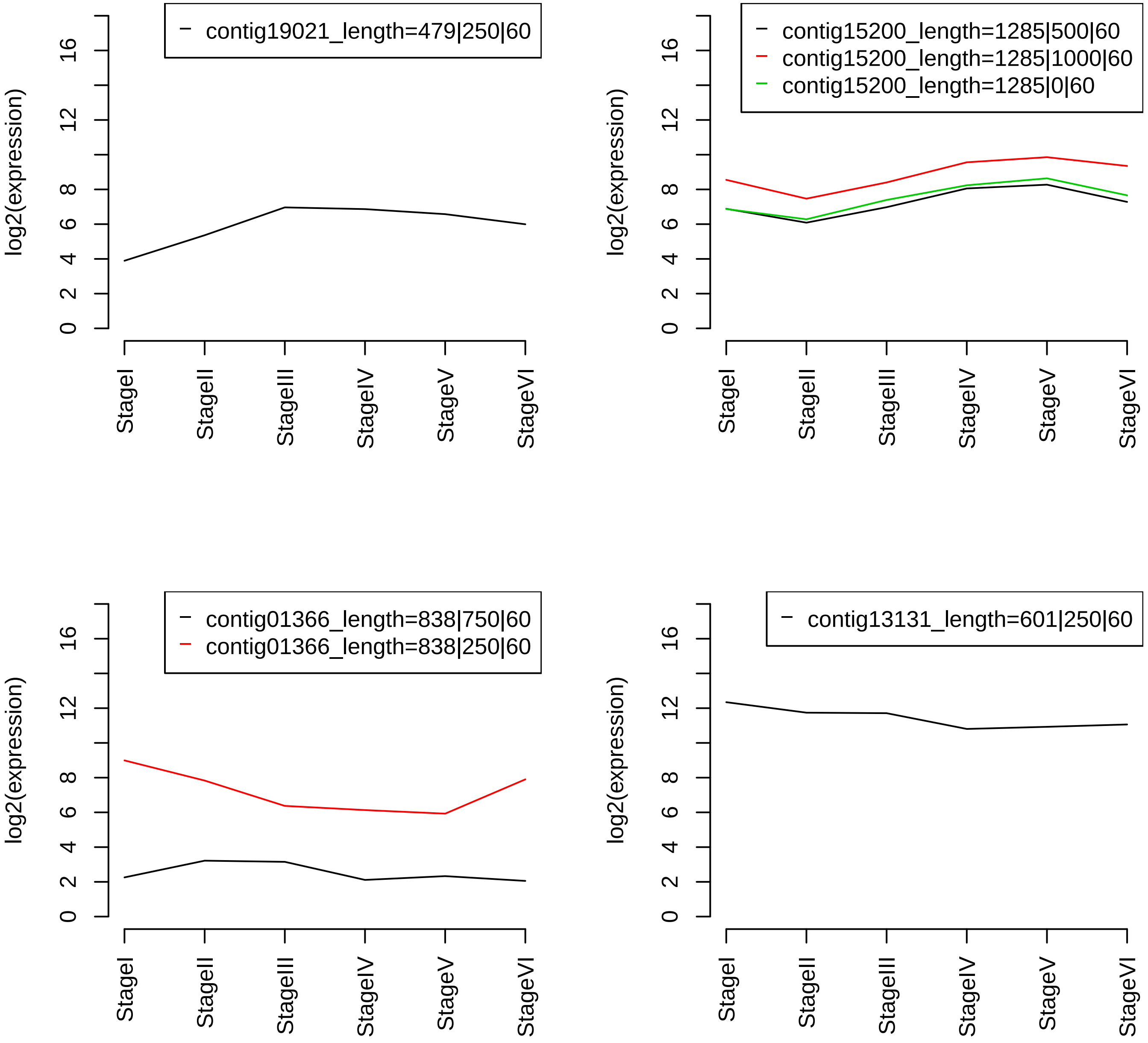

contig19021_length=479|250|60
contig15200_length=1285|500|60
contig15200_length=1285|1000|60
contig15200_length=1285|0|60
contig01366_length=838|750|60
contig01366_length=838|250|60
contig13131_length=601|250|60
log2(expression)
0
2
4
6
8
12
16
StageI
StageII
StageIII
StageIV
StageV
StageVI

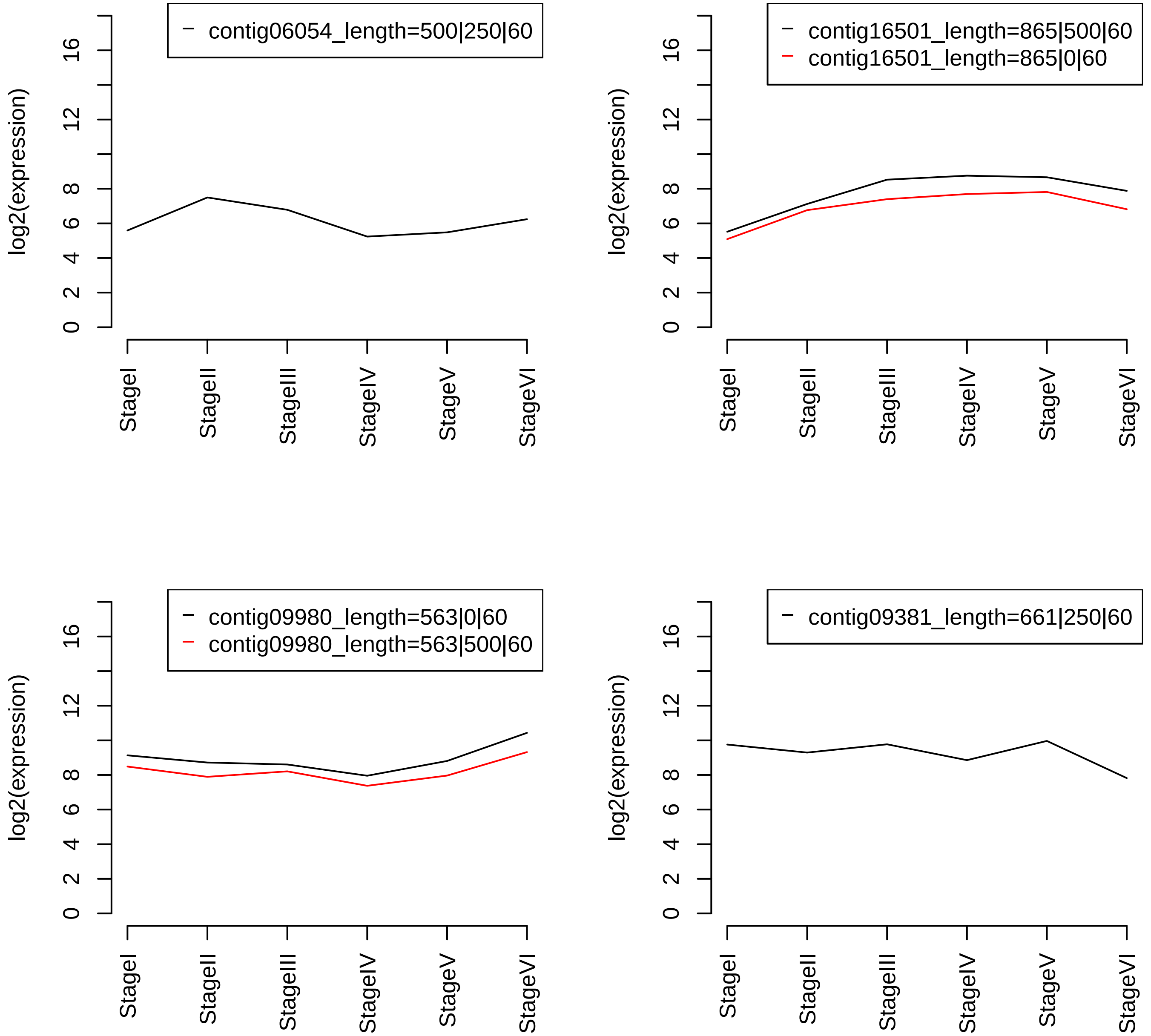

contig06054_length=500|250|60
contig16501_length=865|500|60
contig16501_length=865|0|60
contig09980_length=563|0|60
contig09980_length=563|500|60
contig09381_length=661|250|60
log2(expression)
0
2
4
6
8
12
16
StageI
StageII
StageIII
StageIV
StageV
StageVI

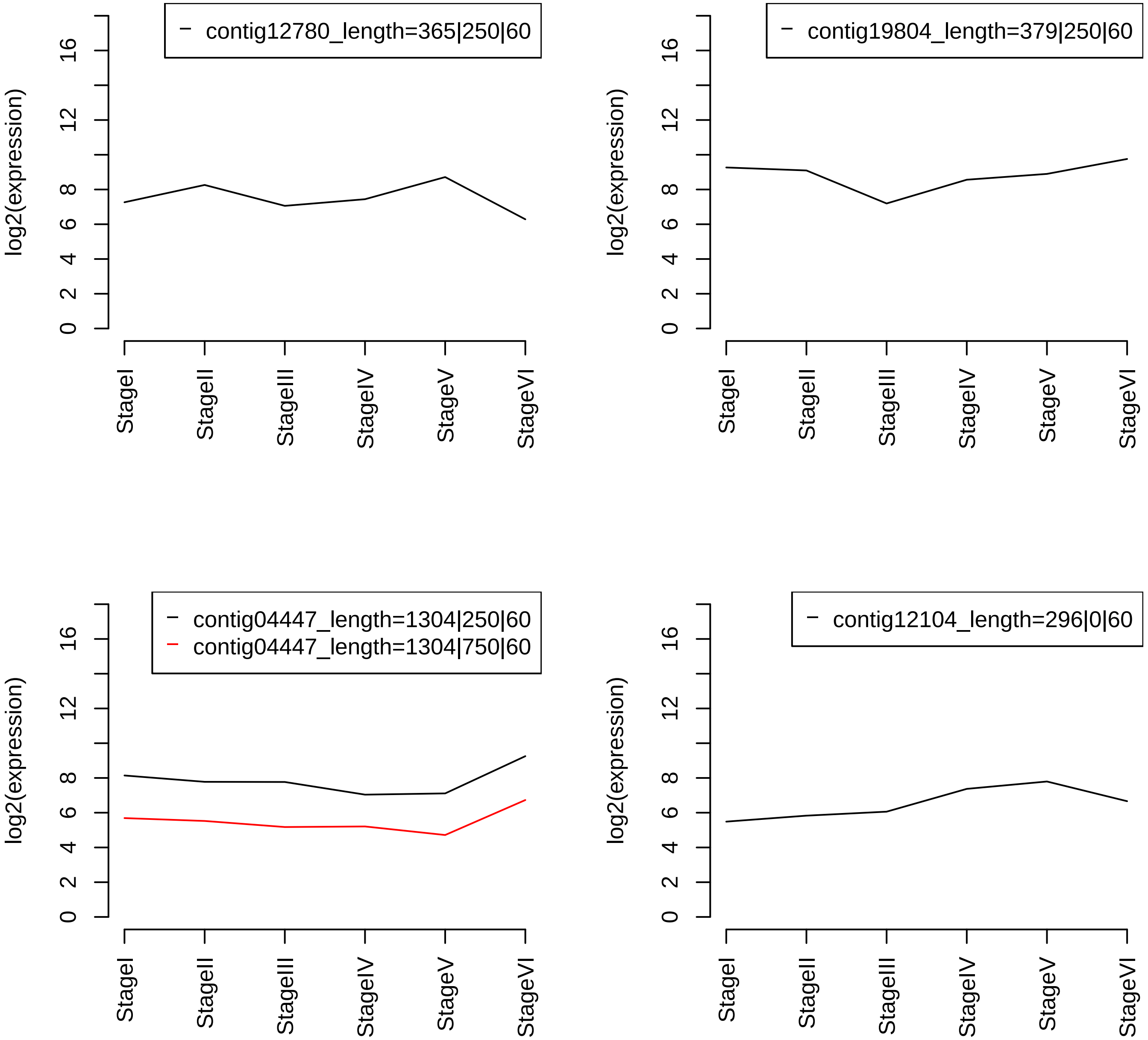
contig12780_length=365|250|60
contig19804_length=379|250|60
contig04447_length=1304|250|60
contig04447_length=1304|750|60
contig12104_length=296|0|60
log2(expression)
0
2
4
6
8
12
16
StageI
StageII
StageIII
StageIV
StageV
StageVI

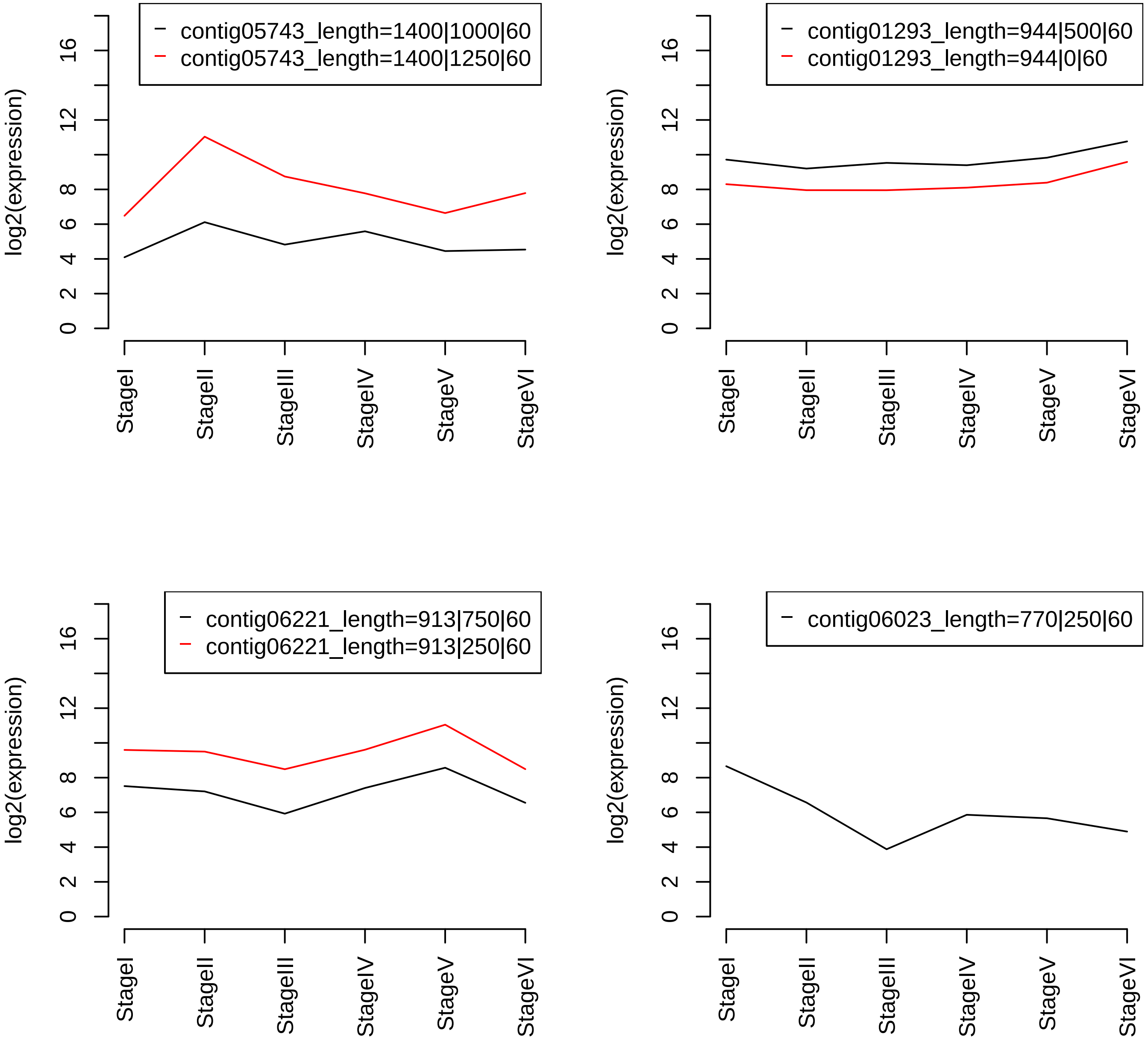

contig05743_length=1400|1000|60
contig05743_length=1400|1250|60
contig01293_length=944|500|60
contig01293_length=944|0|60
contig06221_length=913|750|60
contig06221_length=913|250|60
contig06023_length=770|250|60
log2(expression)
0
2
4
6
8
12
16
StageI
StageII
StageIII
StageIV
StageV
StageVI

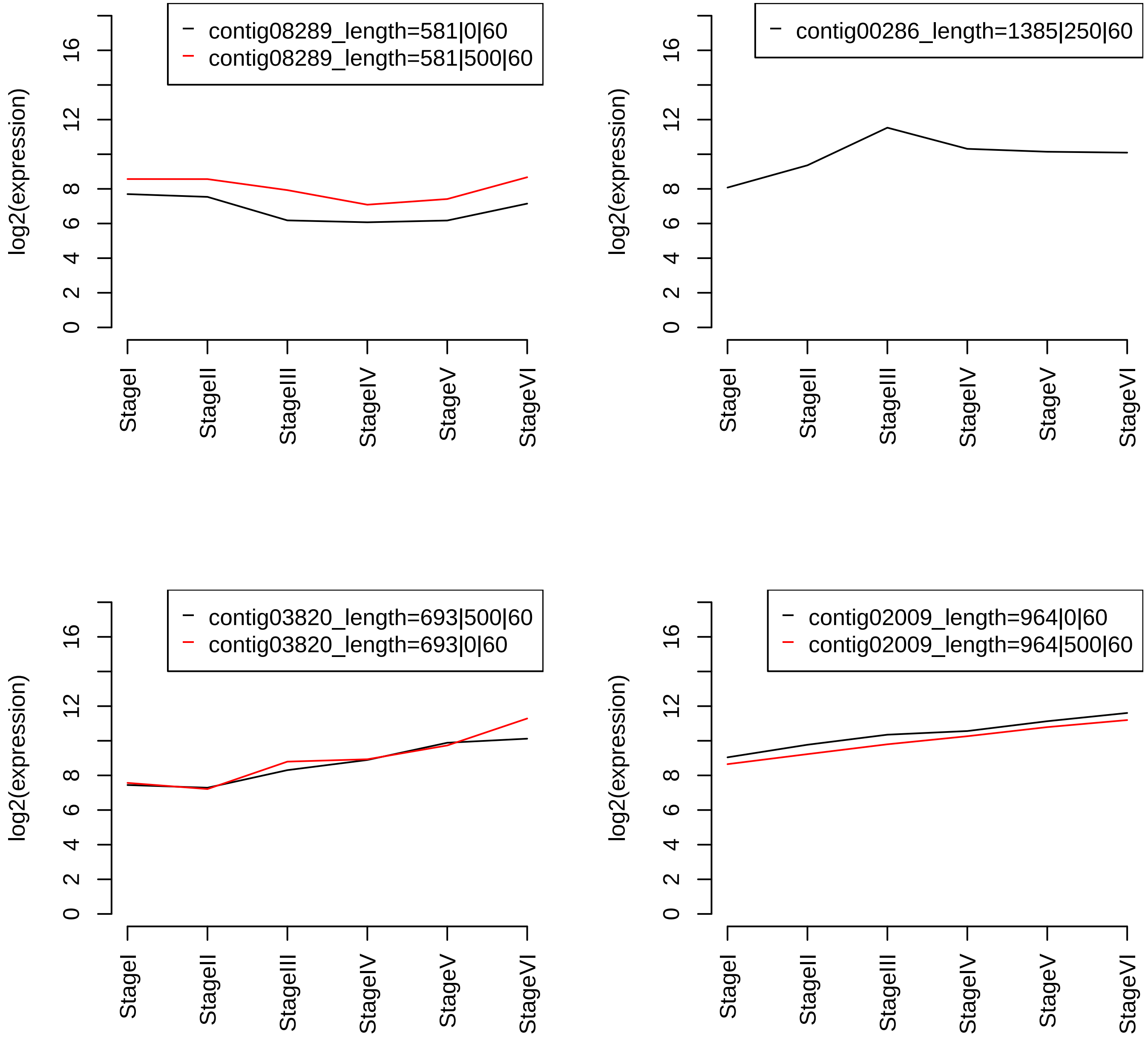
contig08289_length=581|0|60
contig08289_length=581|500|60
log2(expression)
0
2
4
6
8
12
16
StageI
StageII
StageIII
StageIV
StageV
StageVI
contig00286_length=1385|250|60
contig03820_length=693|500|60
contig03820_length=693|0|60
contig02009_length=964|0|60
contig02009_length=964|500|60

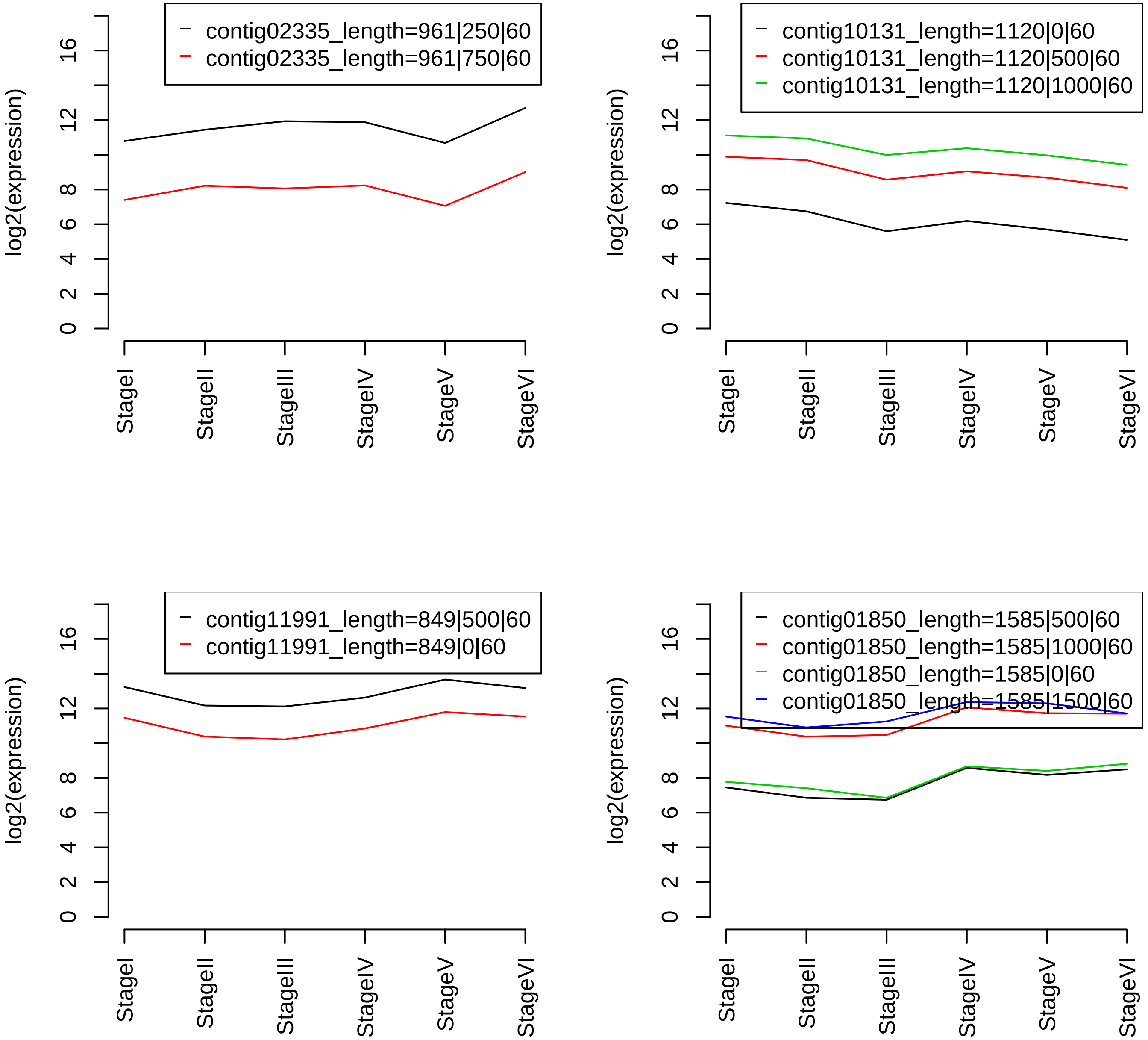

contig02335_length=961|250|60
contig02335_length=961|750|60
contig10131_length=1120|0|60
contig10131_length=1120|500|60
contig10131_length=1120|1000|60
contig11991_length=849|500|60
contig11991_length=849|0|60
contig01850_length=1585|500|60
contig01850_length=1585|1000|60
contig01850_length=1585|0|60
contig01850_length=1585|1500|60
log2(expression)
0 2 4 6 8 12 16
StageI StageII StageIII StageIV StageV StageVI

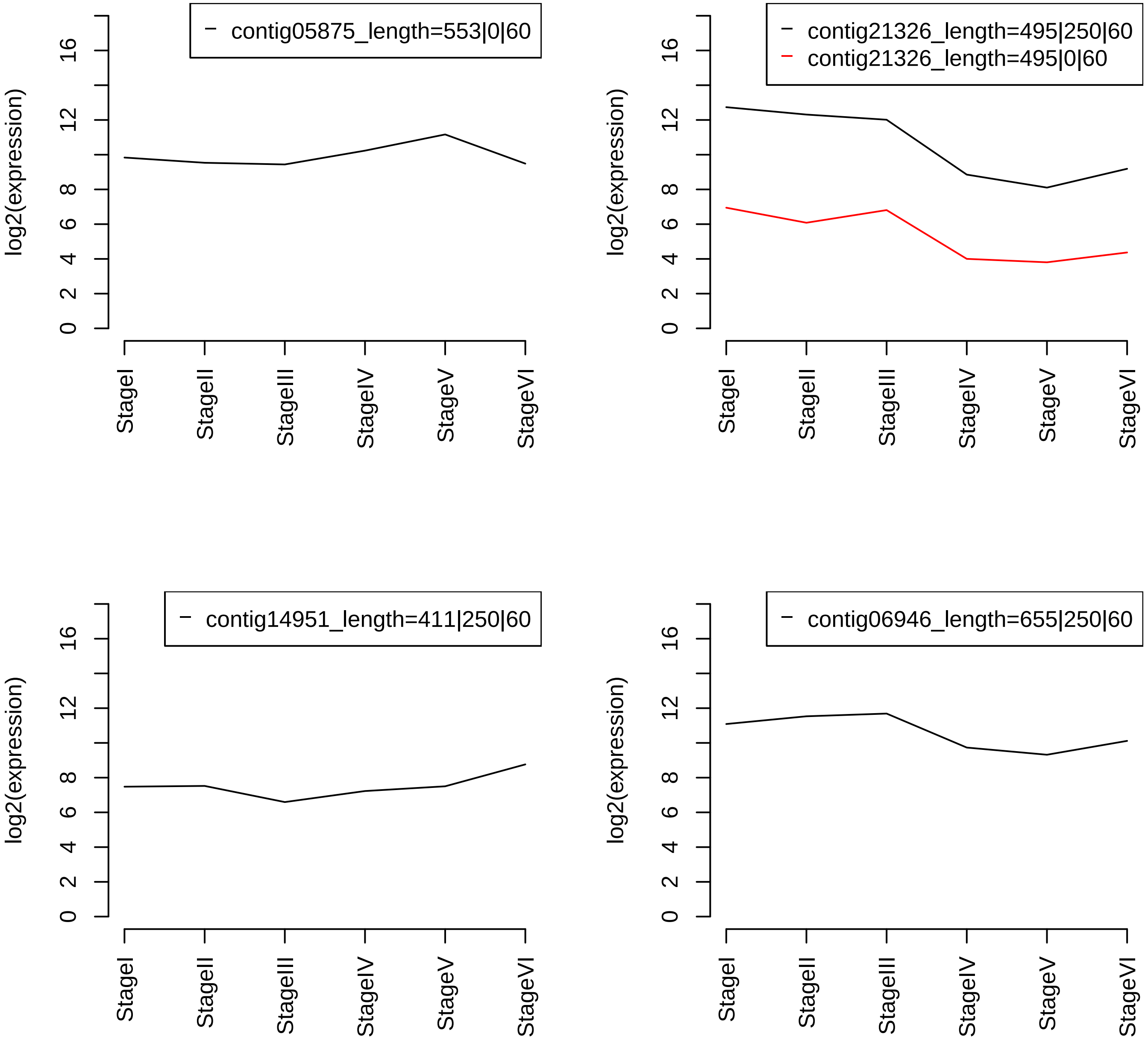
contig05875_length=553|0|60
contig21326_length=495|250|60
contig21326_length=495|0|60
contig14951_length=411|250|60
contig06946_length=655|250|60
log2(expression)
0
2
4
6
8
12
16
StageI
StageII
StageIII
StageIV
StageV
StageVI

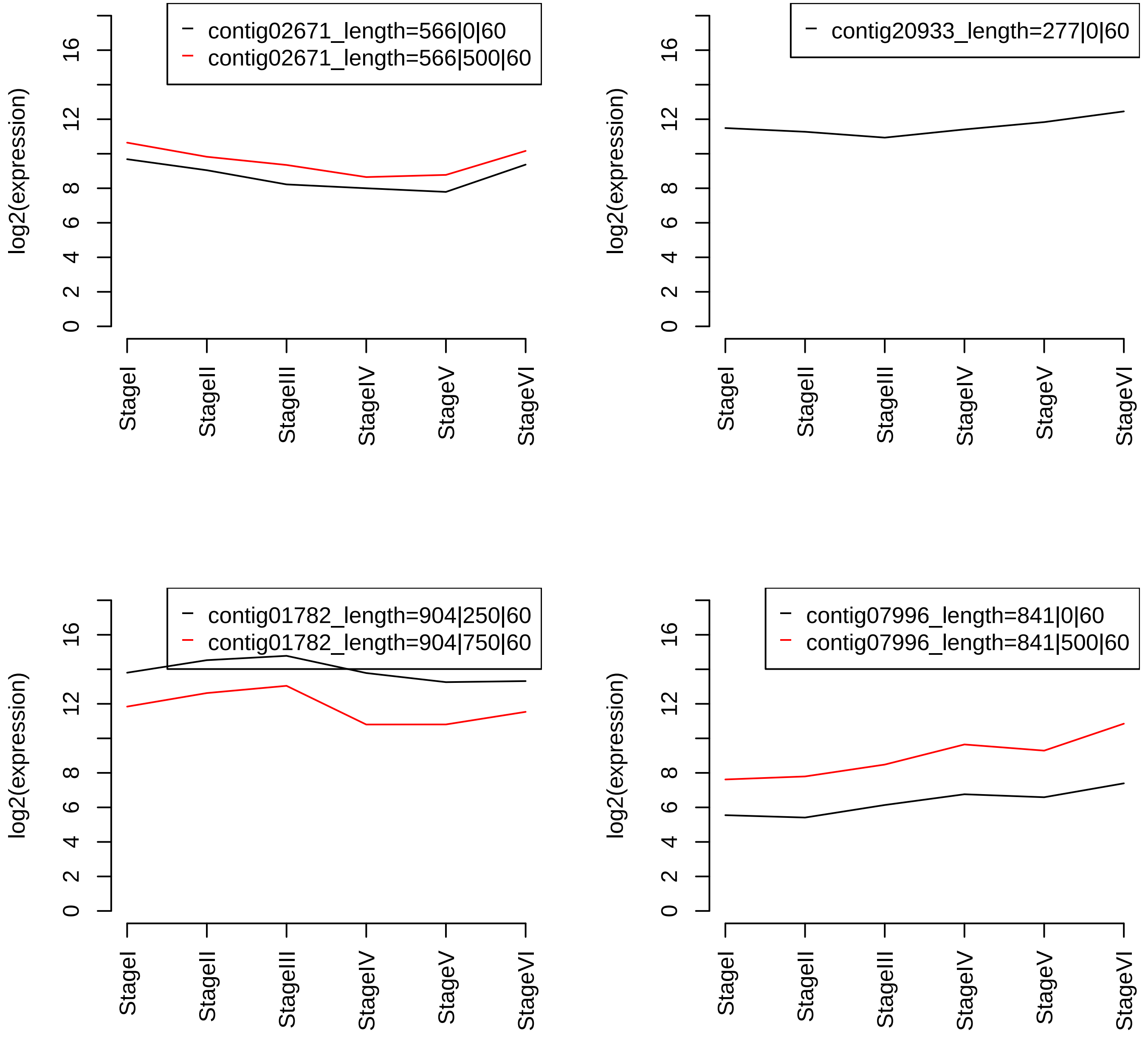
contig02671_length=566|0|60
contig02671_length=566|500|60
contig20933_length=277|0|60
contig01782_length=904|250|60
contig01782_length=904|750|60
contig07996_length=841|0|60
contig07996_length=841|500|60
log2(expression)
0
2
4
6
8
12
16
StageI
StageII
StageIII
StageIV
StageV
StageVI

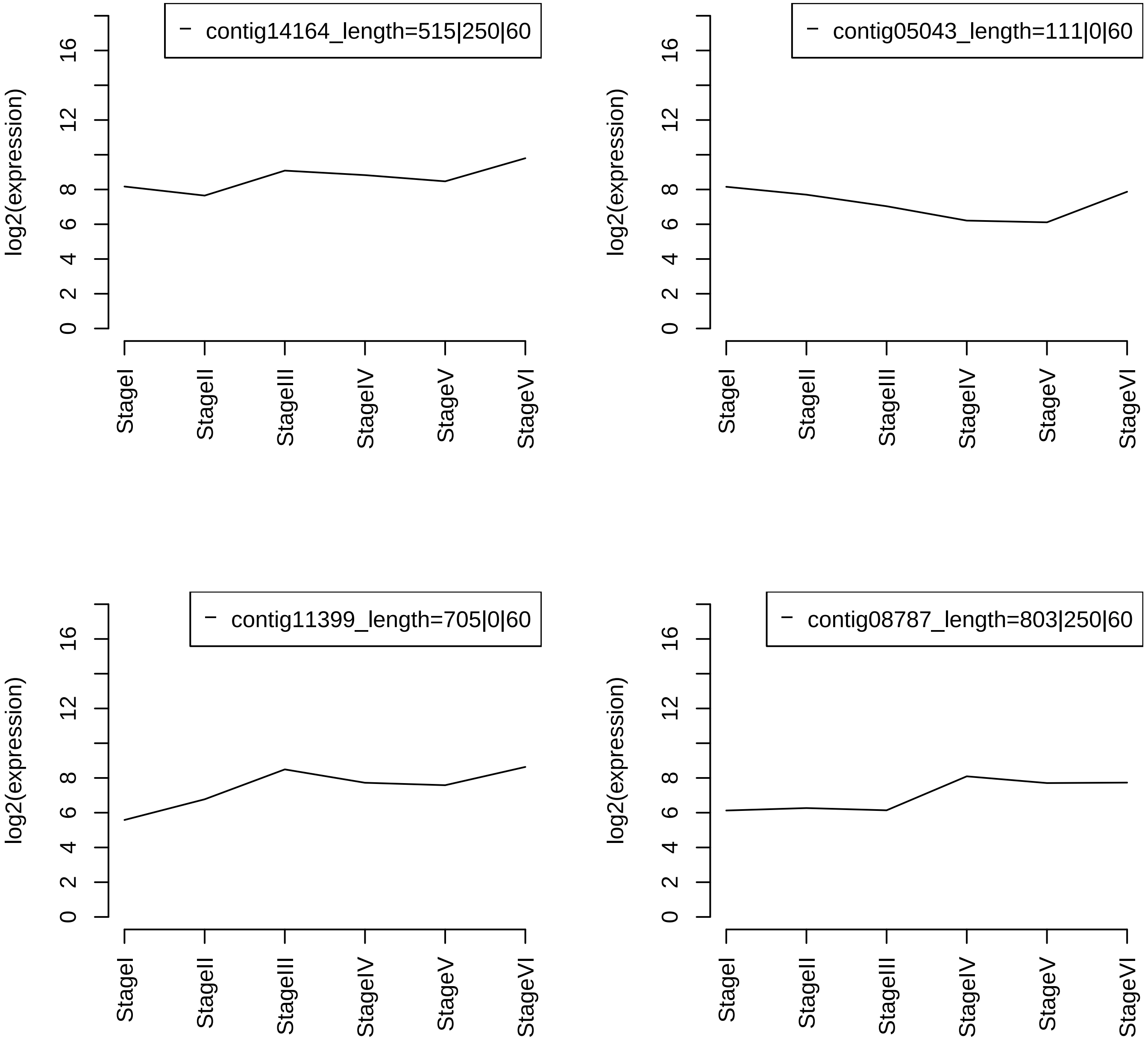

contig14164_length=515|250|60
contig05043_length=111|0|60
contig11399_length=705|0|60
contig08787_length=803|250|60
log2(expression)
0
2
4
6
8
12
16
StageI
StageII
StageIII
StageIV
StageV
StageVI

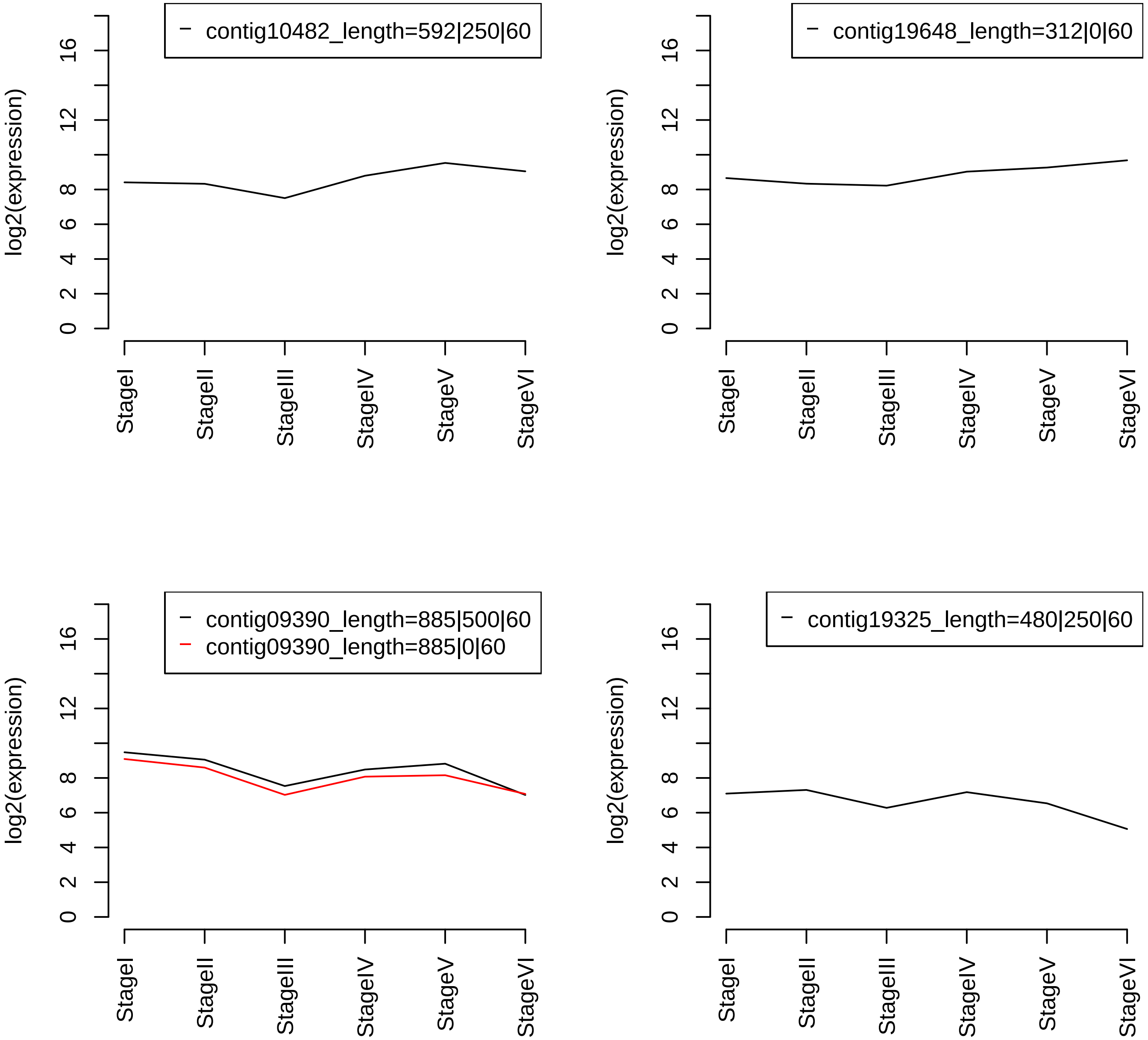
contig10482_length=592|250|60
contig19648_length=312|0|60
contig09390_length=885|500|60
contig09390_length=885|0|60
contig19325_length=480|250|60
log2(expression)
0
2
4
6
8
12
16
StageI
StageII
StageIII
StageIV
StageV
StageVI

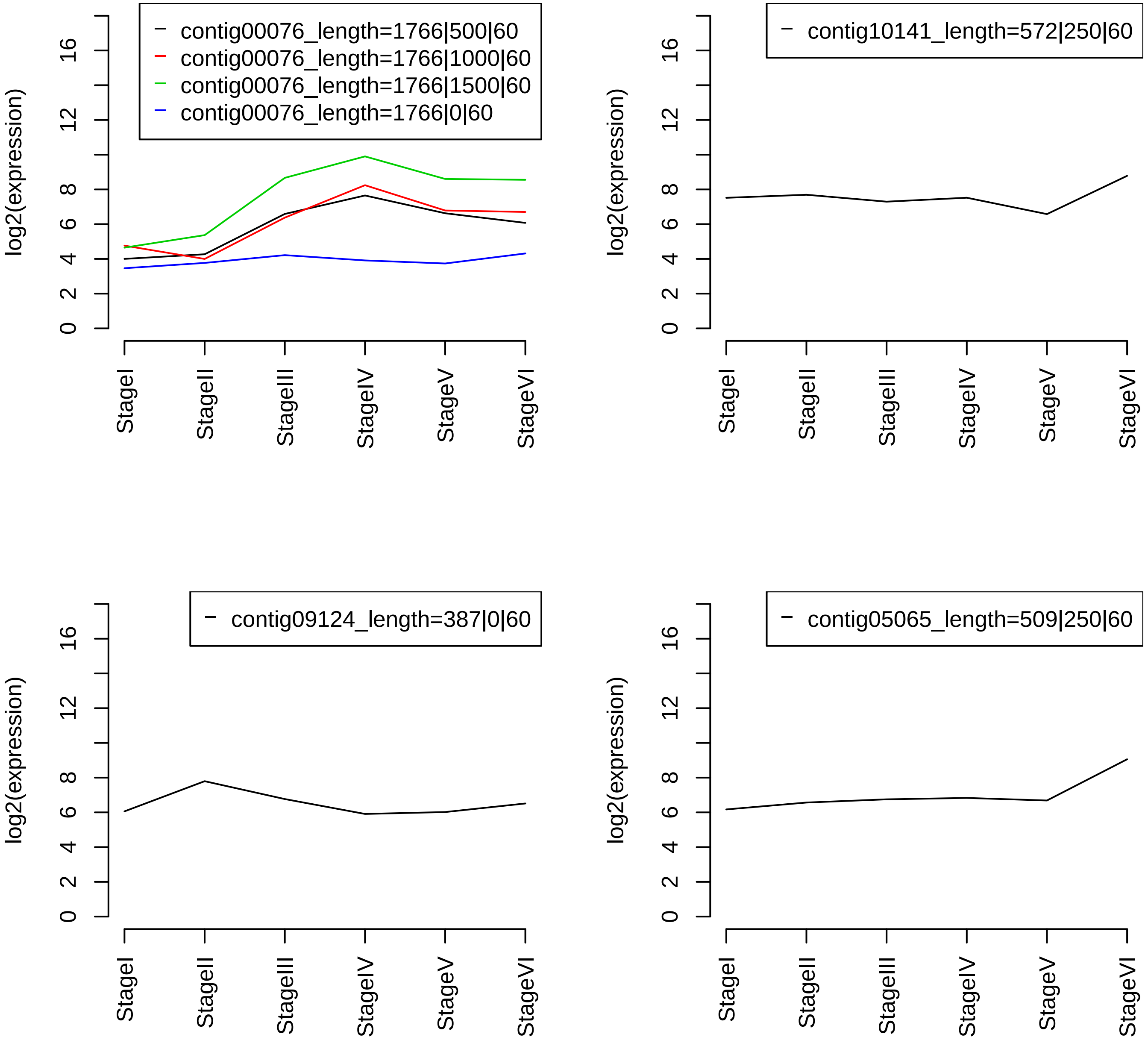
contig00076_length=1766|500|60
contig00076_length=1766|1000|60
contig00076_length=1766|1500|60
contig00076_length=1766|0|60
contig10141_length=572|250|60
contig09124_length=387|0|60
contig05065_length=509|250|60
log2(expression)
0
2
4
6
8
12
16
StageI
StageII
StageIII
StageIV
StageV
StageVI

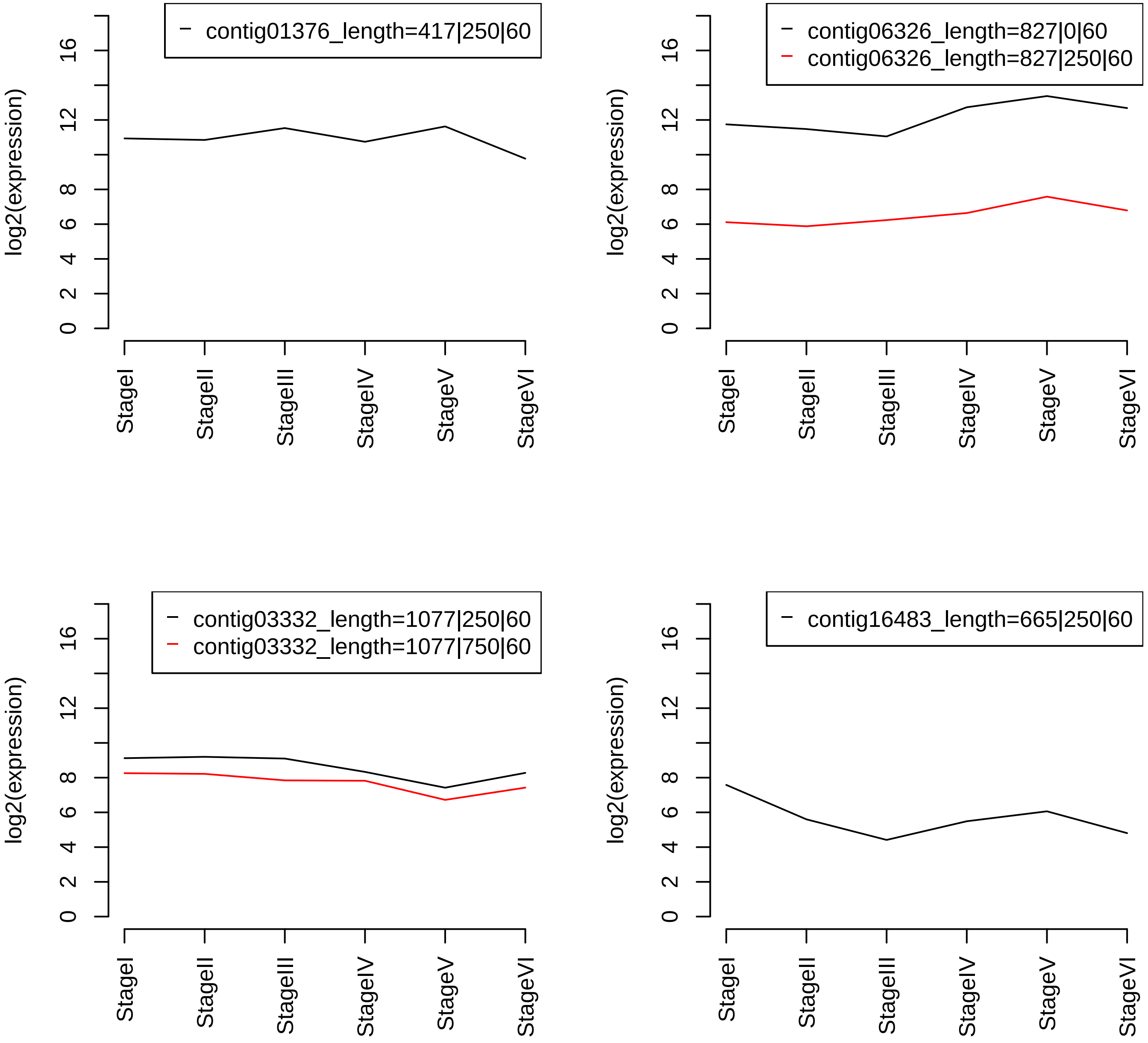
contig01376_length=417|250|60
contig06326_length=827|0|60
contig06326_length=827|250|60
contig03332_length=1077|250|60
contig03332_length=1077|750|60
contig16483_length=665|250|60
log2(expression)
0
2
4
6
8
12
16
StageI
StageII
StageIII
StageIV
StageV
StageVI

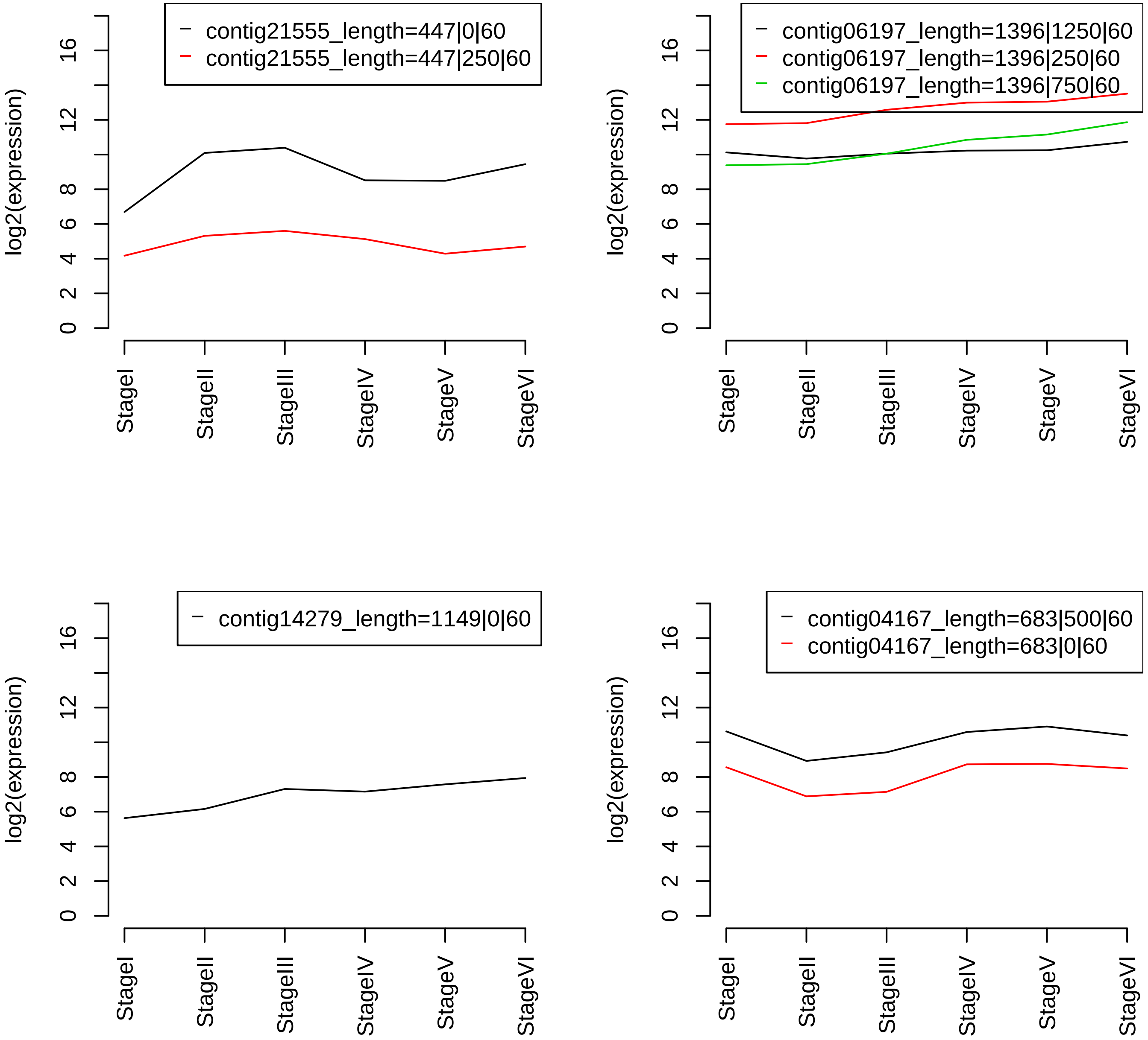
contig21555_length=447|0|60
contig21555_length=447|250|60
log2(expression)
0
2
4
6
8
12
16
StageI
StageII
StageIII
StageIV
StageV
StageVI
contig06197_length=1396|1250|60
contig06197_length=1396|250|60
contig06197_length=1396|750|60
log2(expression)
0
2
4
6
8
12
16
StageI
StageII
StageIII
StageIV
StageV
StageVI
contig14279_length=1149|0|60
log2(expression)
0
2
4
6
8
12
16
StageI
StageII
StageIII
StageIV
StageV
StageVI
contig04167_length=683|500|60
contig04167_length=683|0|60
log2(expression)
0
2
4
6
8
12
16
StageI
StageII
StageIII
StageIV
StageV
StageVI

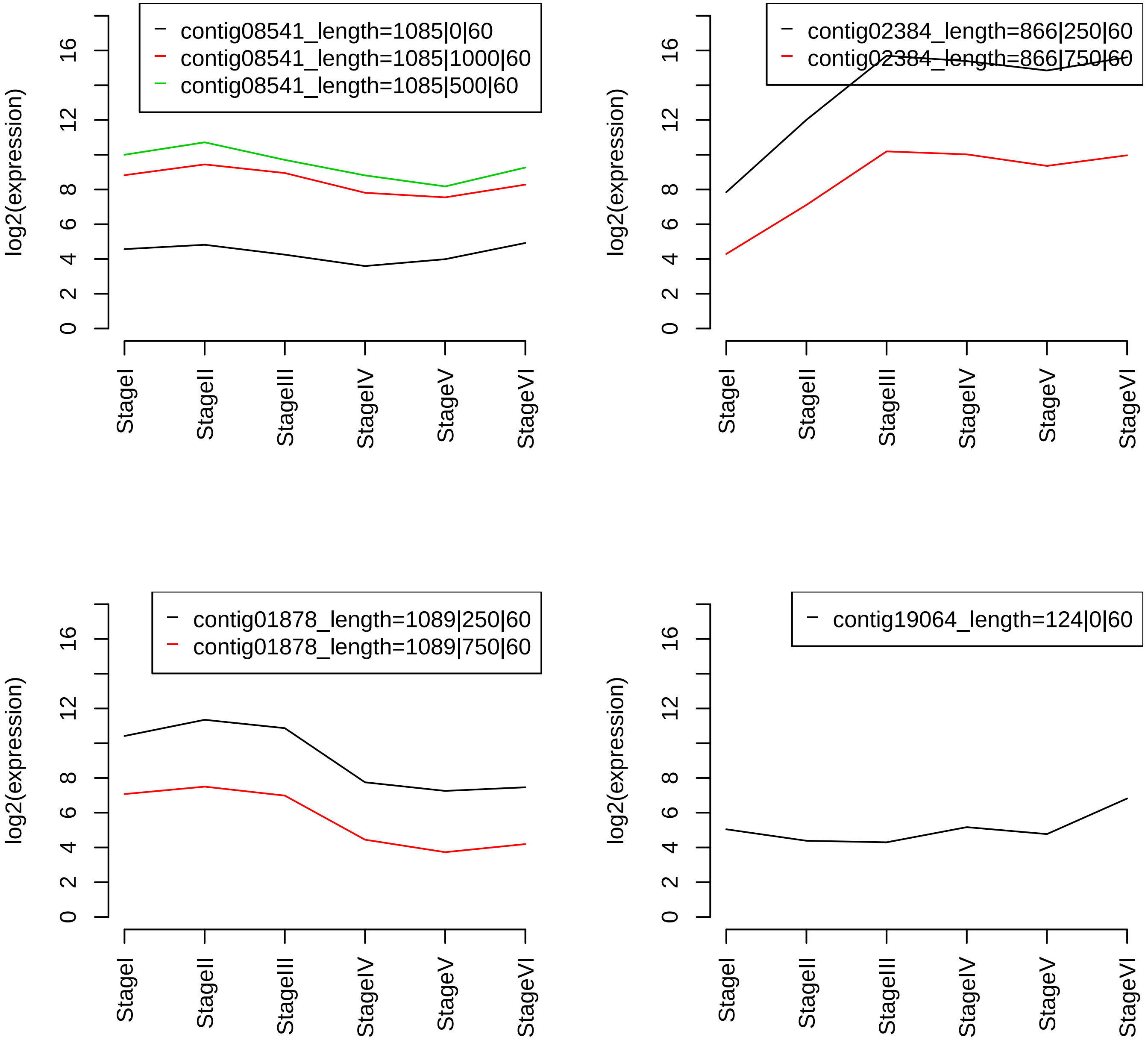
contig08541_length=1085|0|60
contig08541_length=1085|1000|60
contig08541_length=1085|500|60
log2(expression)
StageI
StageII
StageIII
StageIV
StageV
StageVI
contig02384_length=866|250|60
contig02384_length=866|750|60
contig01878_length=1089|250|60
contig01878_length=1089|750|60
contig19064_length=124|0|60

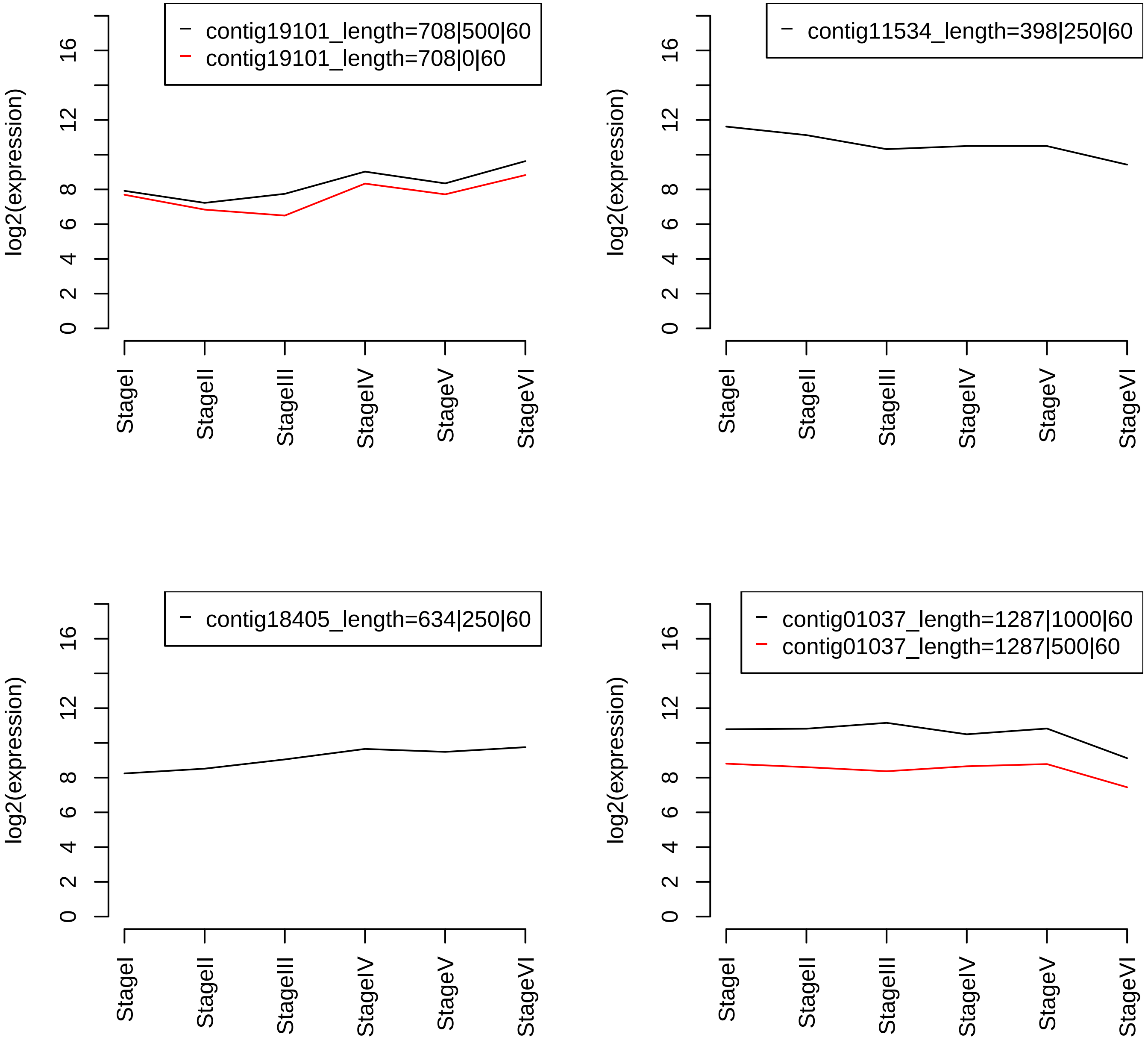

contig19101_length=708|500|60
contig19101_length=708|0|60
contig11534_length=398|250|60
contig18405_length=634|250|60
contig01037_length=1287|1000|60
contig01037_length=1287|500|60
log2(expression)
0
2
4
6
8
12
16
StageI
StageII
StageIII
StageIV
StageV
StageVI

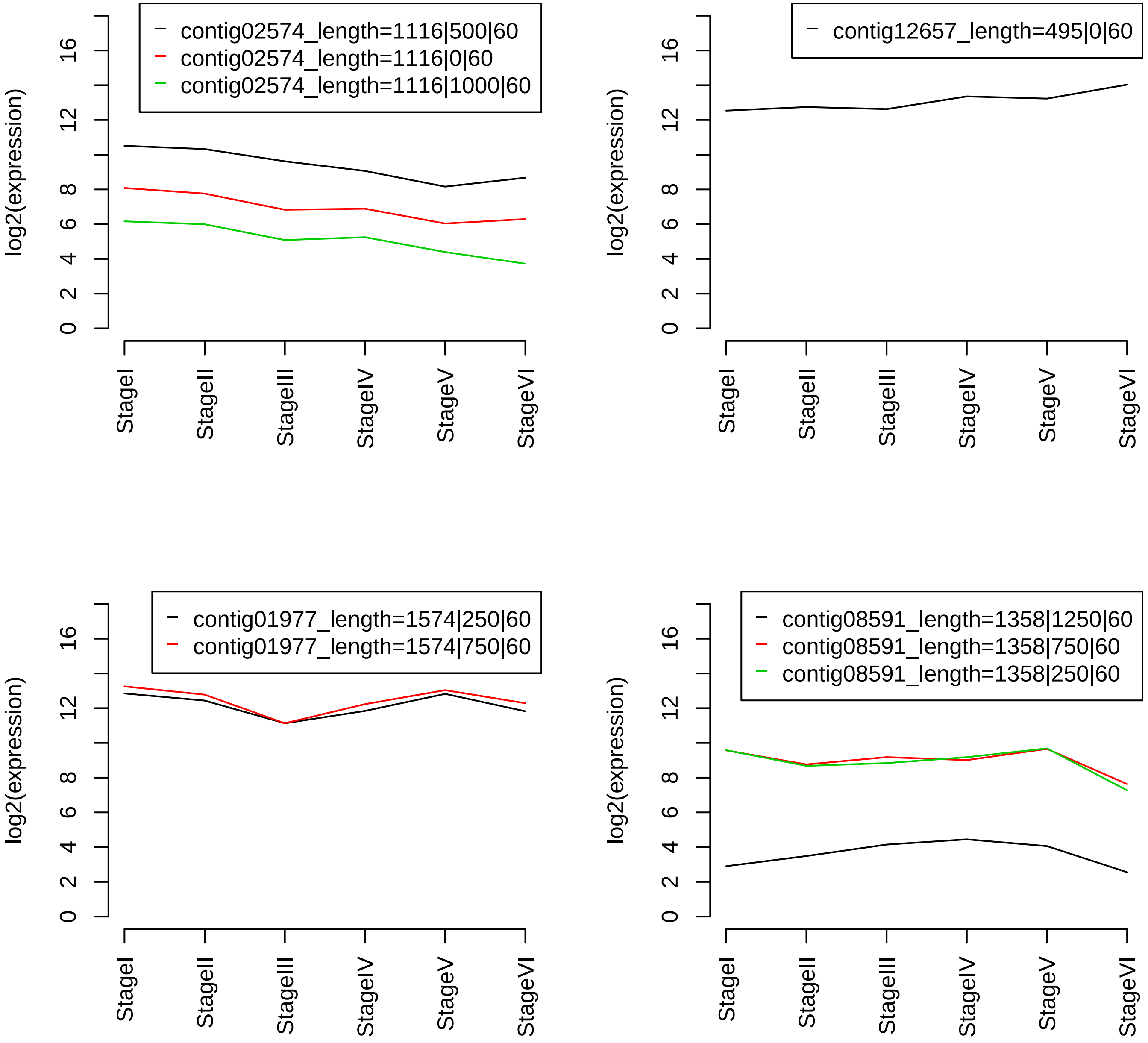

contig02574_length=1116|500|60
contig02574_length=1116|0|60
contig02574_length=1116|1000|60
contig12657_length=495|0|60
contig01977_length=1574|250|60
contig01977_length=1574|750|60
contig08591_length=1358|1250|60
contig08591_length=1358|750|60
contig08591_length=1358|250|60
log2(expression)
0
2
4
6
8
12
16
StageI
StageII
StageIII
StageIV
StageV
StageVI

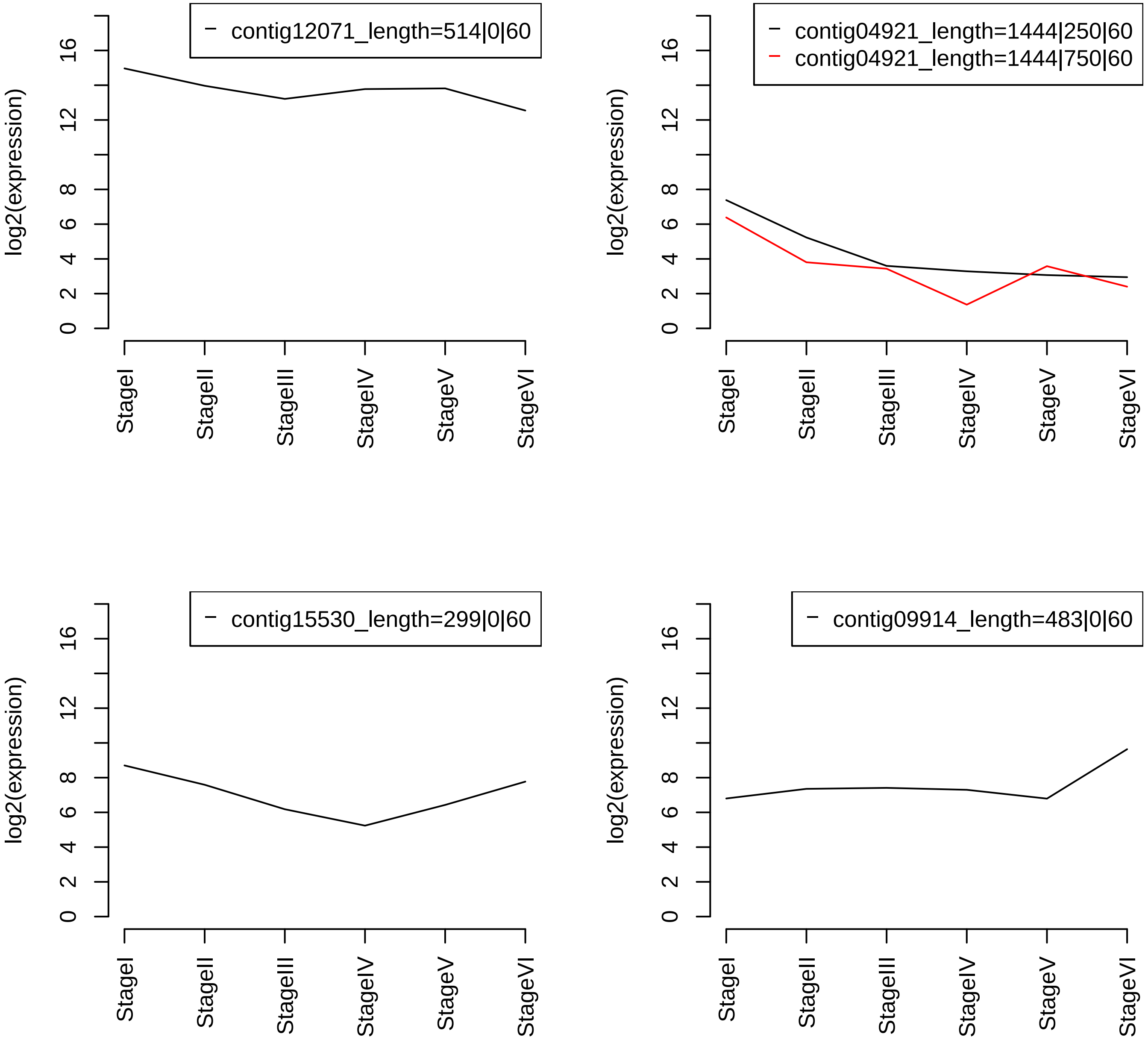

contig12071_length=514|0|60
contig04921_length=1444|250|60
contig04921_length=1444|750|60
contig15530_length=299|0|60
contig09914_length=483|0|60
log2(expression)
0
2
4
6
8
12
16
StageI
StageII
StageIII
StageIV
StageV
StageVI

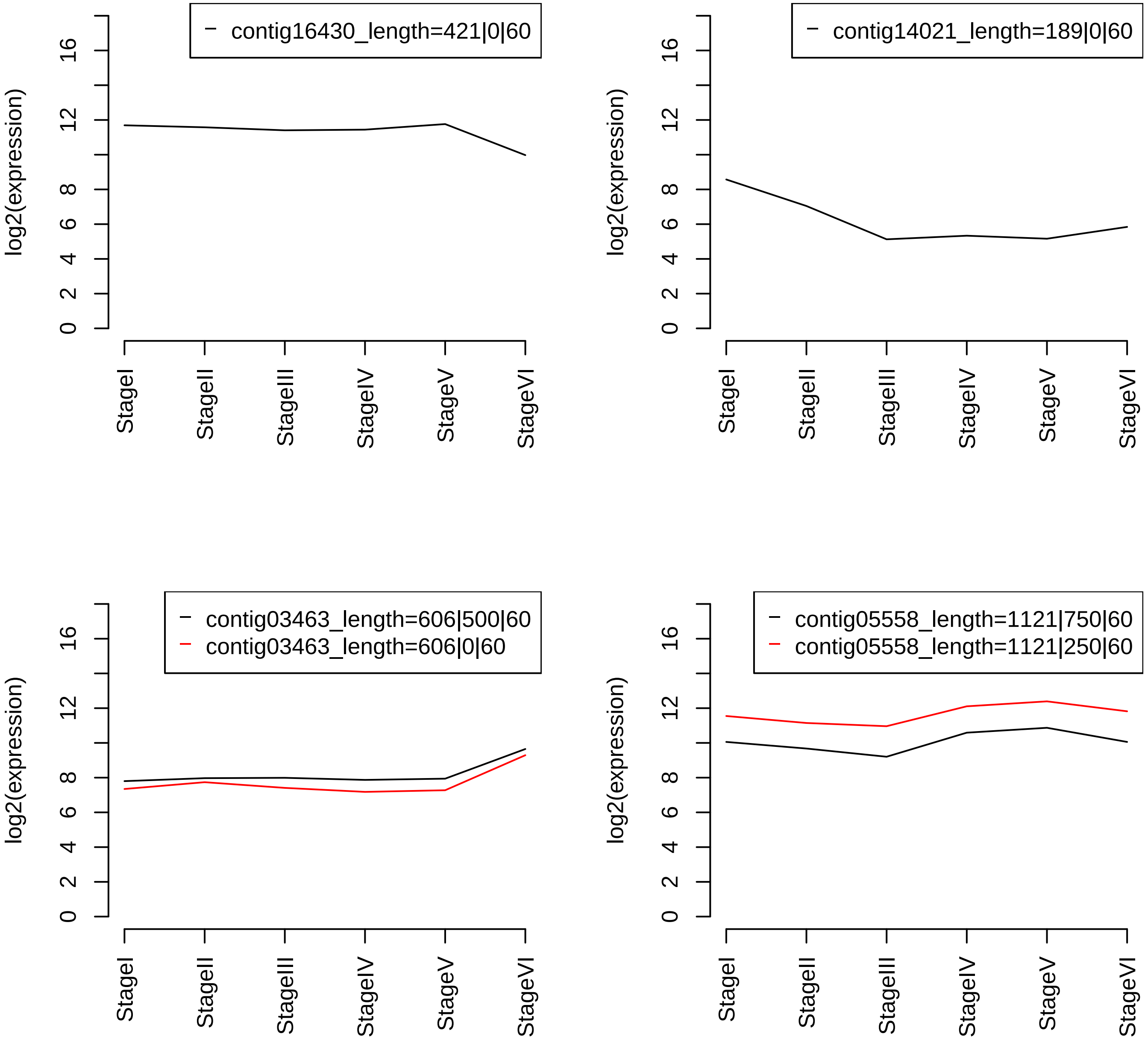
contig16430_length=421|0|60
contig14021_length=189|0|60
contig03463_length=606|500|60
contig03463_length=606|0|60
contig05558_length=1121|750|60
contig05558_length=1121|250|60
log2(expression)
0
2
4
6
8
12
16
StageI
StageII
StageIII
StageIV
StageV
StageVI

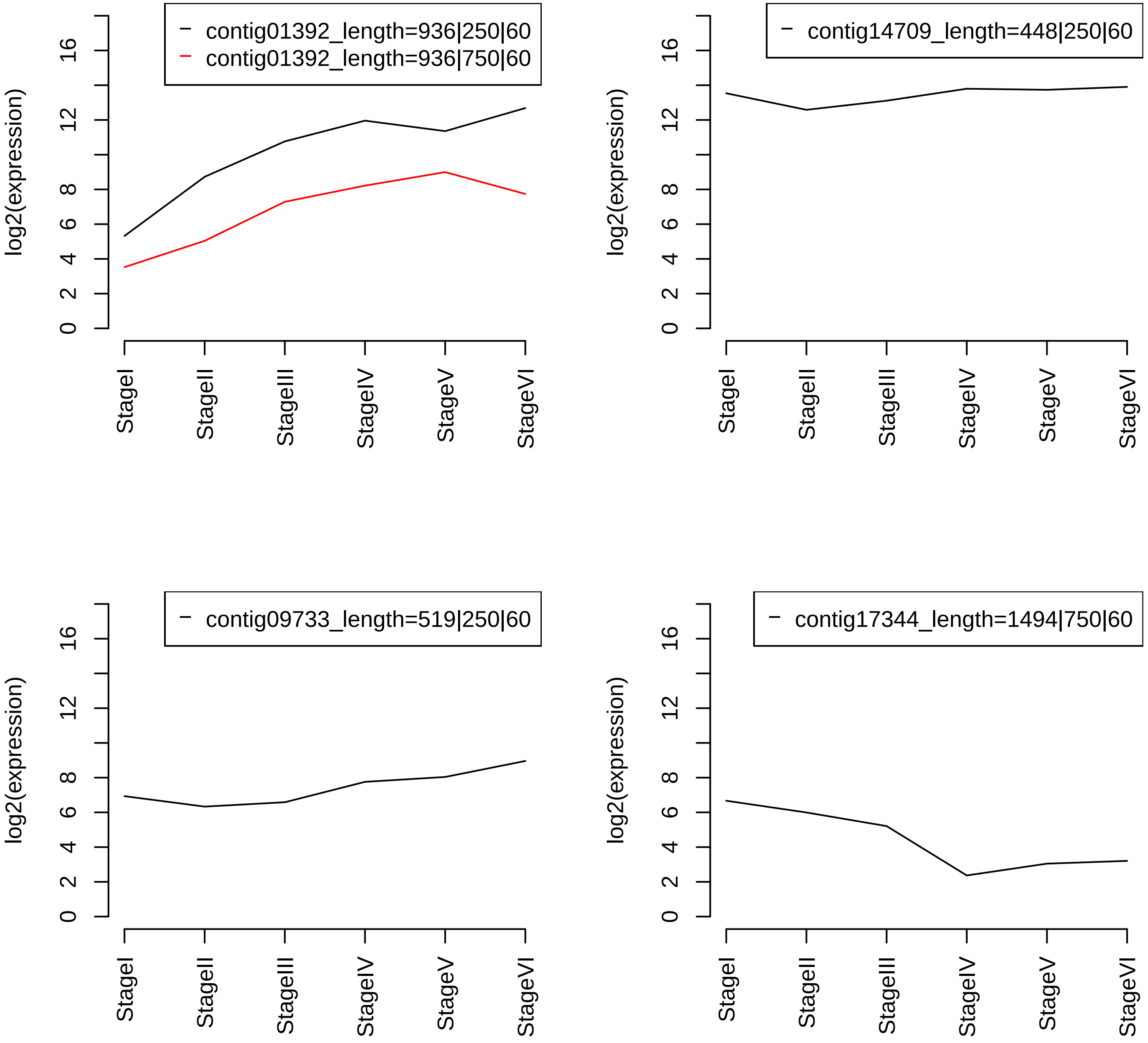
contig01392_length=936|250|60
contig01392_length=936|750|60
contig14709_length=448|250|60
contig09733_length=519|250|60
contig17344_length=1494|750|60
log2(expression)
0
2
4
6
8
12
16
StageI
StageII
StageIII
StageIV
StageV
StageVI

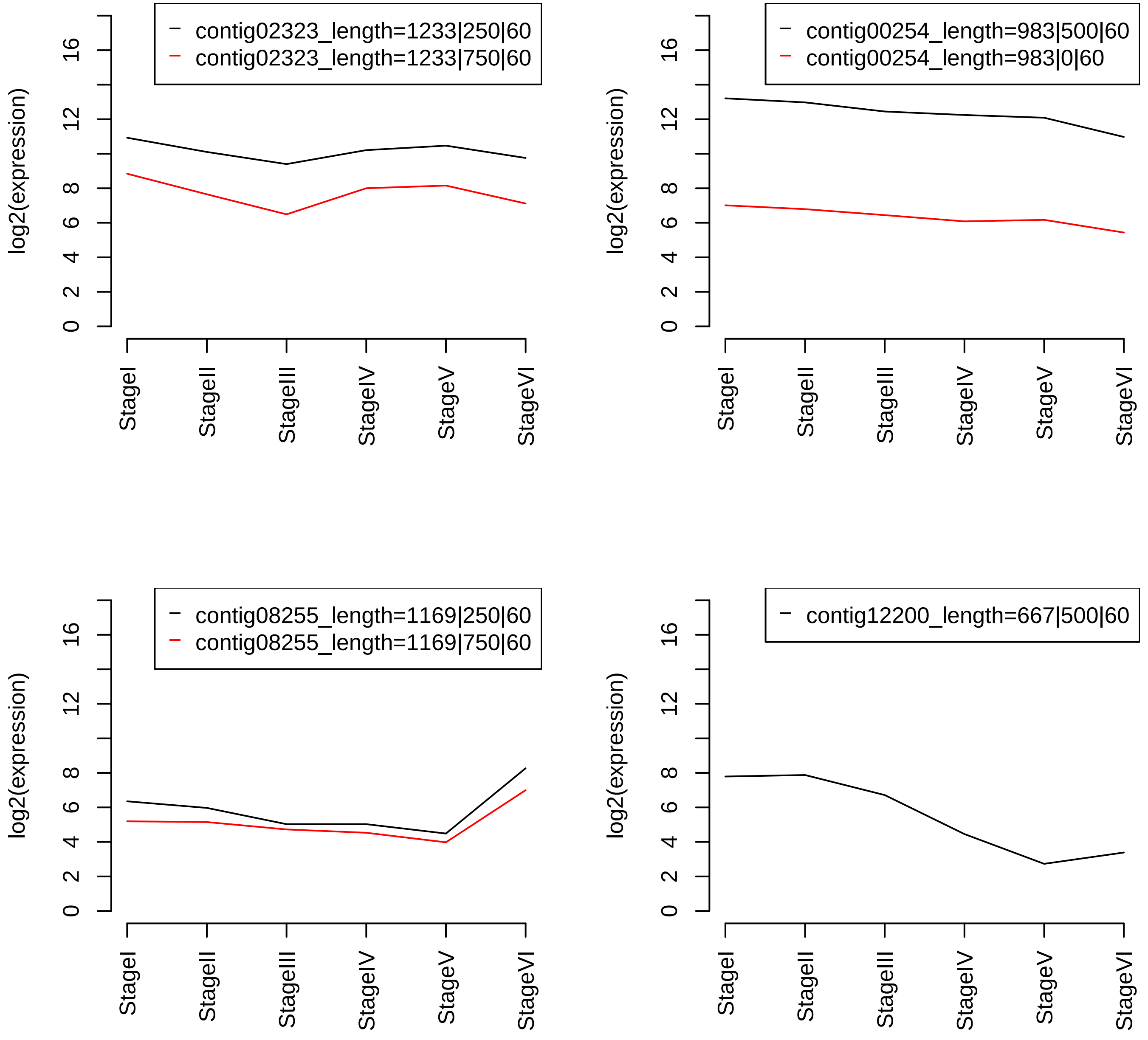

contig02323_length=1233|250|60
contig02323_length=1233|750|60
contig00254_length=983|500|60
contig00254_length=983|0|60
contig08255_length=1169|250|60
contig08255_length=1169|750|60
contig12200_length=667|500|60
log2(expression)
0
2
4
6
8
12
16
StageI
StageII
StageIII
StageIV
StageV
StageVI

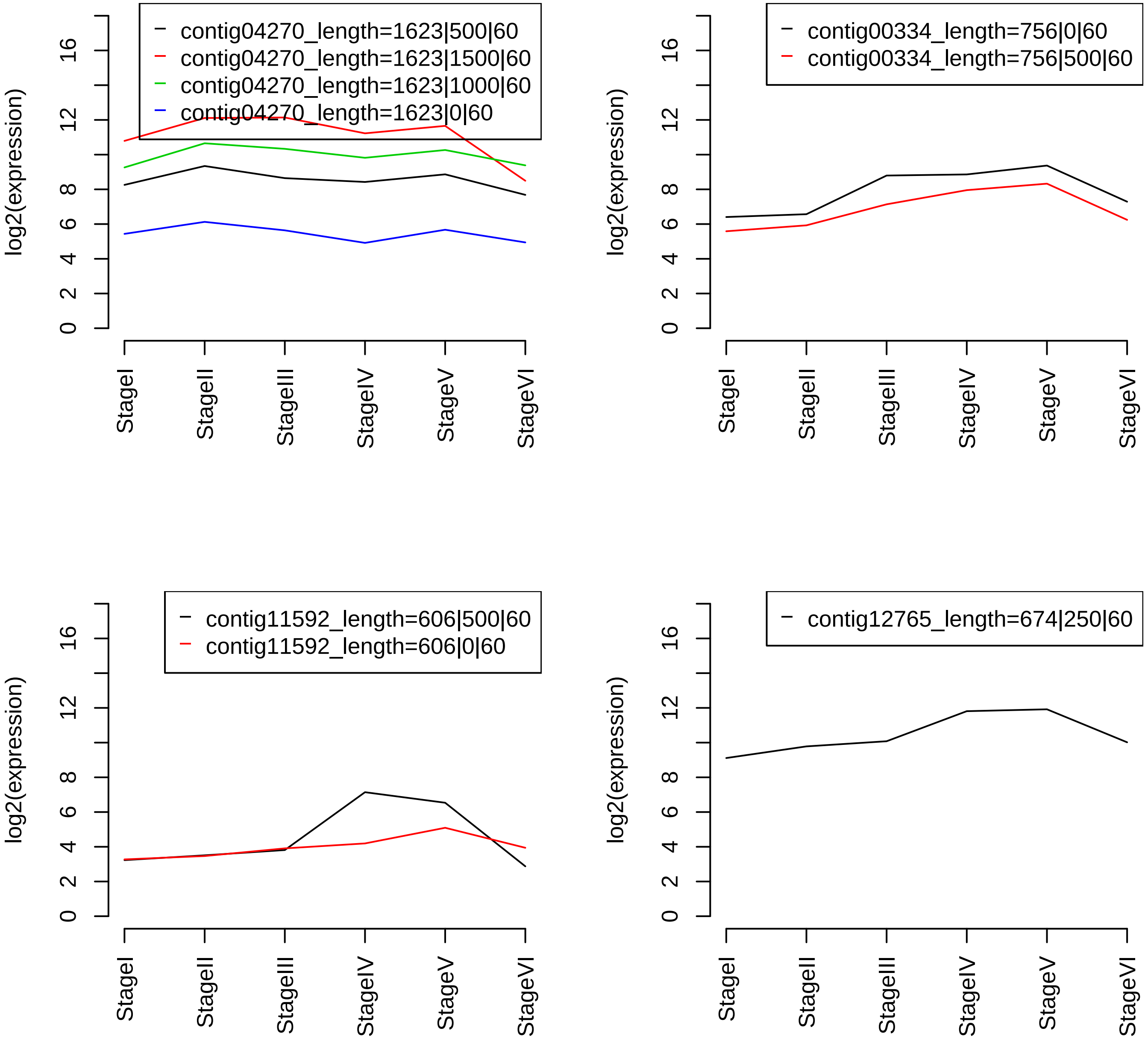

contig04270_length=1623|500|60
contig04270_length=1623|1500|60
contig04270_length=1623|1000|60
contig04270_length=1623|0|60
contig00334_length=756|0|60
contig00334_length=756|500|60
contig11592_length=606|500|60
contig11592_length=606|0|60
contig12765_length=674|250|60
log2(expression)
0
2
4
6
8
12
16
StageI
StageII
StageIII
StageIV
StageV
StageVI

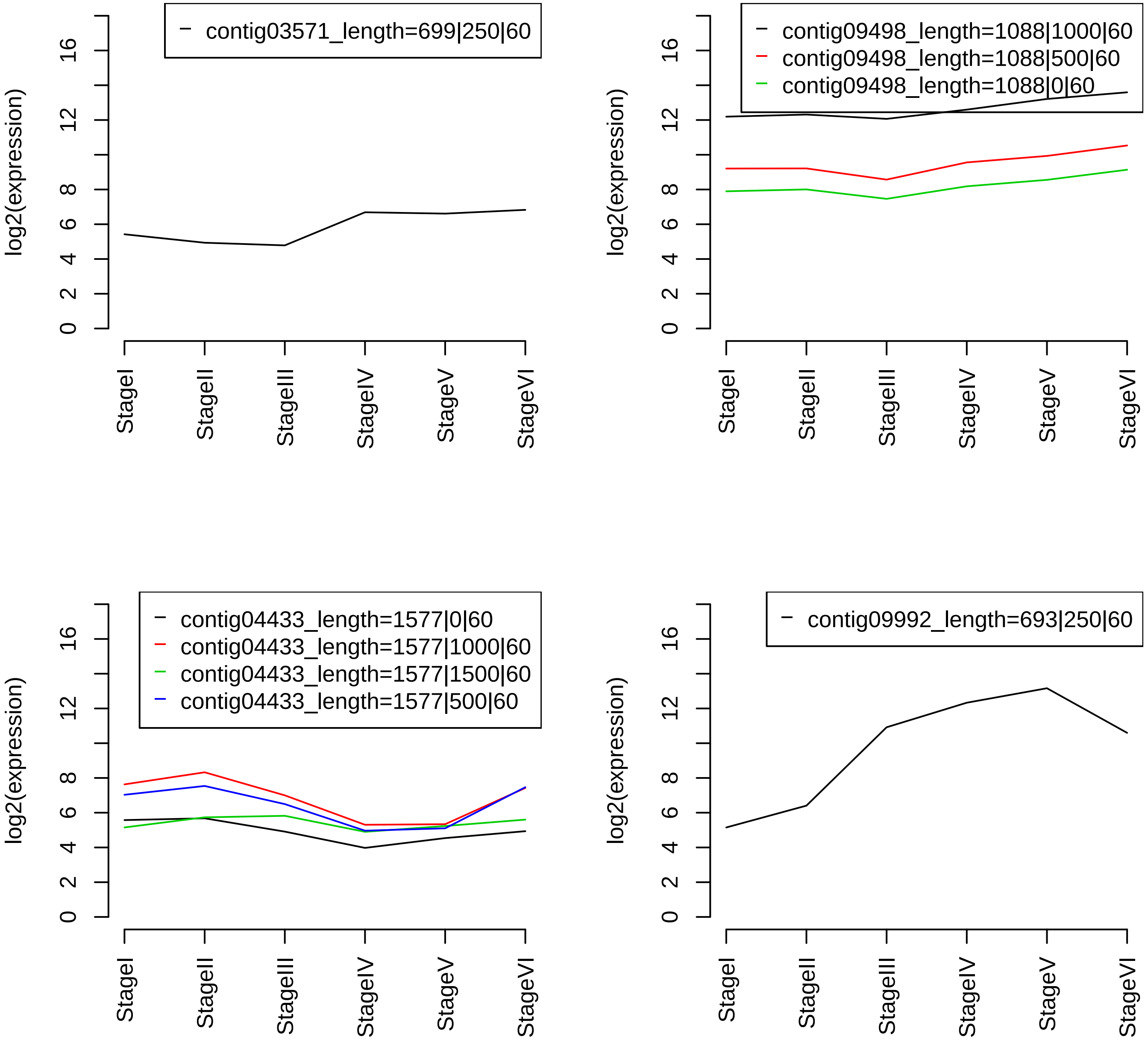
contig03571_length=699|250|60
contig09498_length=1088|1000|60
contig09498_length=1088|500|60
contig09498_length=1088|0|60
contig04433_length=1577|0|60
contig04433_length=1577|1000|60
contig04433_length=1577|1500|60
contig04433_length=1577|500|60
contig09992_length=693|250|60
log2(expression)
0
2
4
6
8
12
16
StageI
StageII
StageIII
StageIV
StageV
StageVI

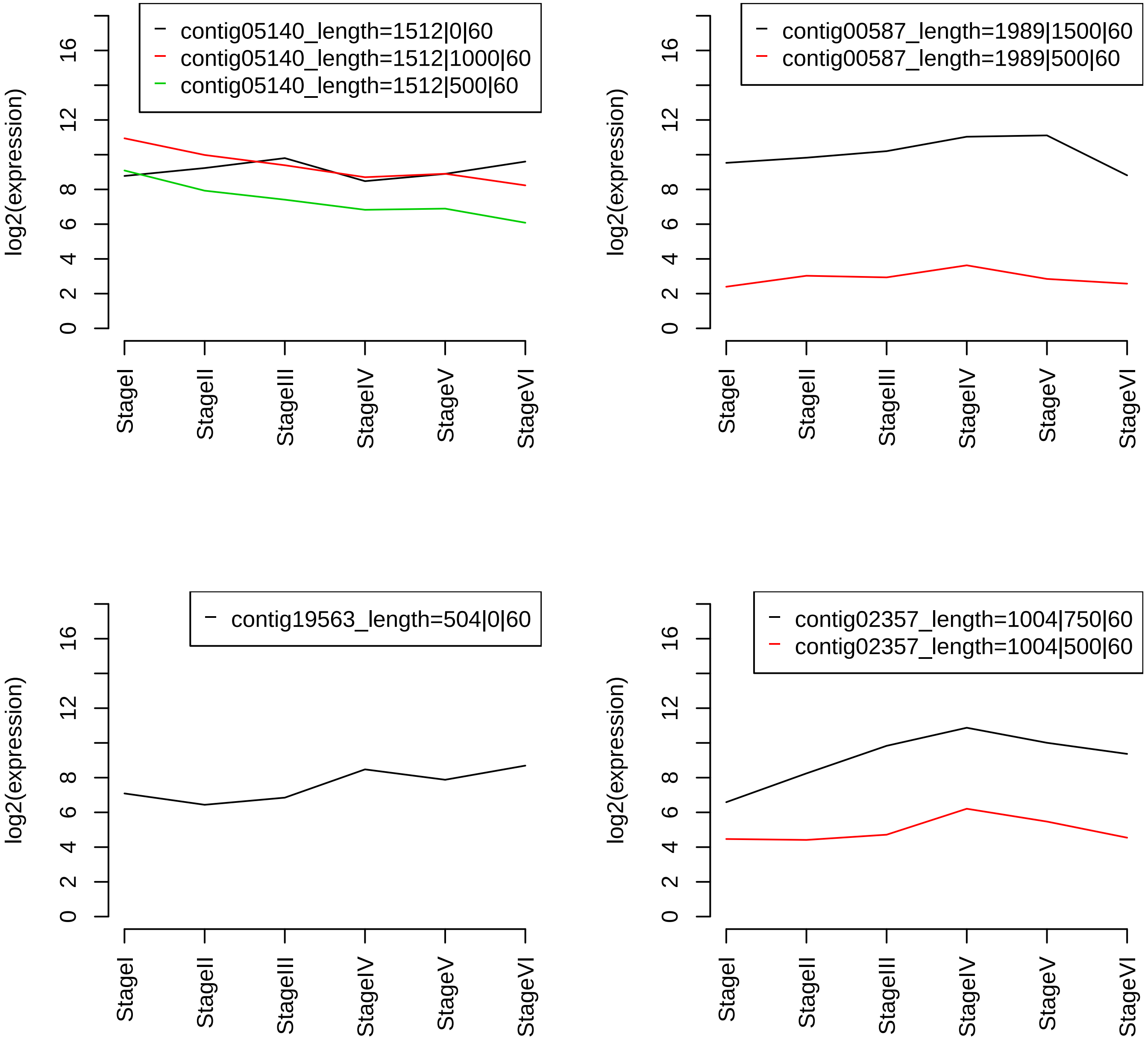
contig05140_length=1512|0|60
contig05140_length=1512|1000|60
contig05140_length=1512|500|60
contig00587_length=1989|1500|60
contig00587_length=1989|500|60
contig19563_length=504|0|60
contig02357_length=1004|750|60
contig02357_length=1004|500|60
log2(expression)
0
2
4
6
8
12
16
StageI
StageII
StageIII
StageIV
StageV
StageVI

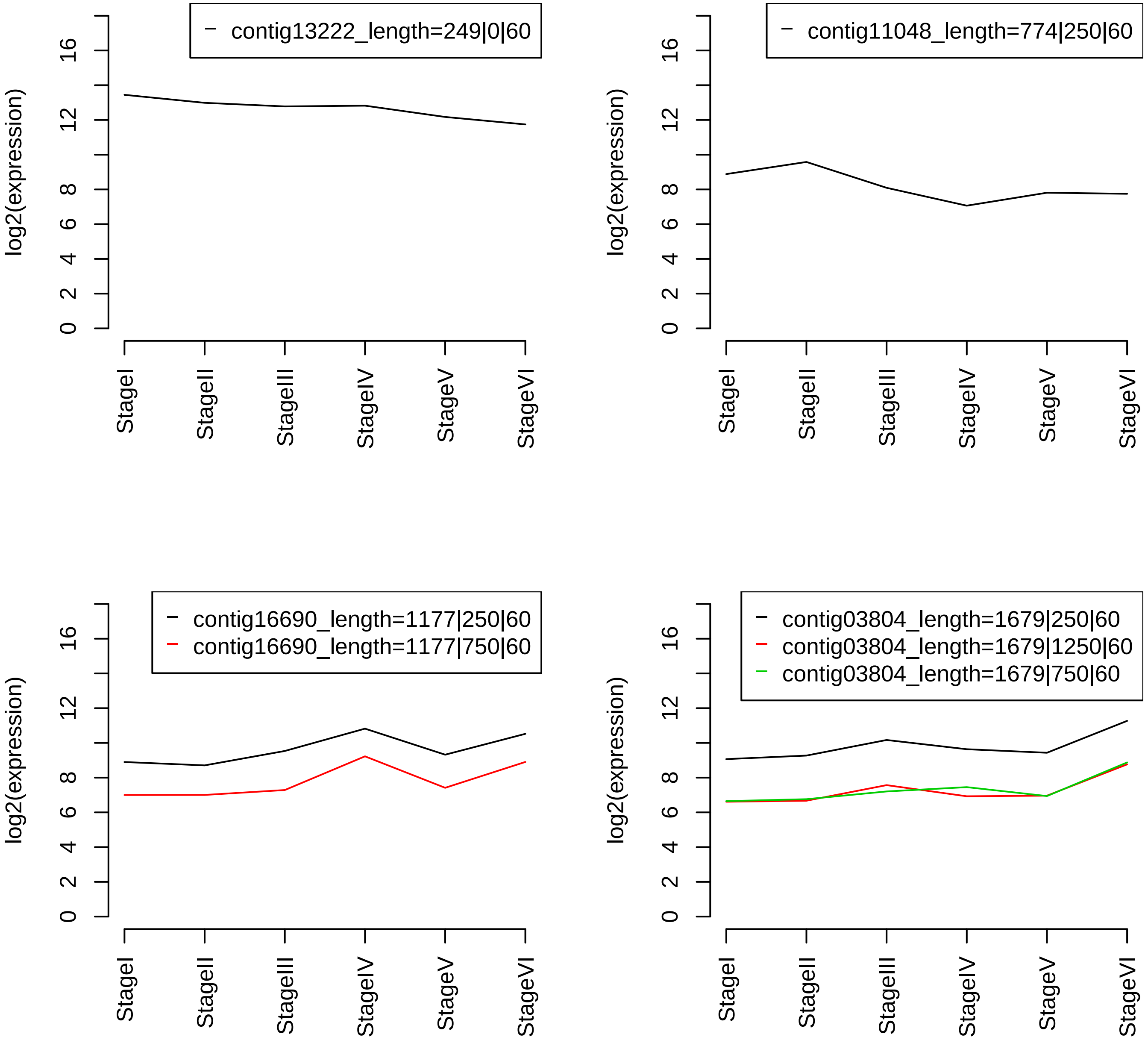

contig13222_length=249|0|60
contig11048_length=774|250|60
contig16690_length=1177|250|60
contig16690_length=1177|750|60
contig03804_length=1679|250|60
contig03804_length=1679|1250|60
contig03804_length=1679|750|60
log2(expression)
0
2
4
6
8
12
16
StageI
StageII
StageIII
StageIV
StageV
StageVI

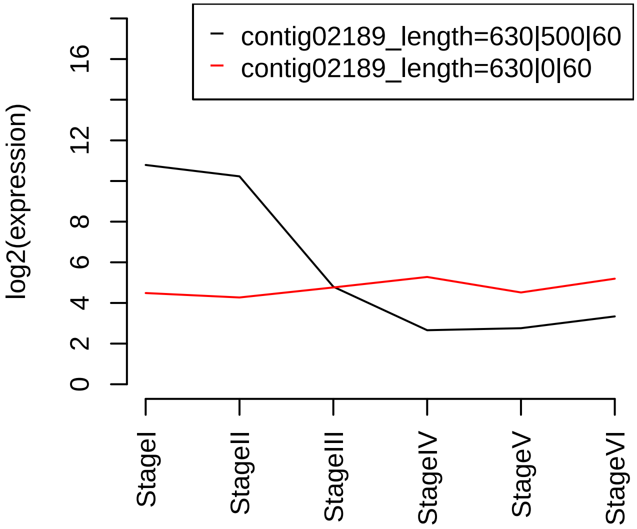

contig02189_length=630|500|60
contig02189_length=630|0|60
log2(expression)
0
2
4
6
8
12
16
StageI
StageII
StageIII
StageIV
StageV
StageVI

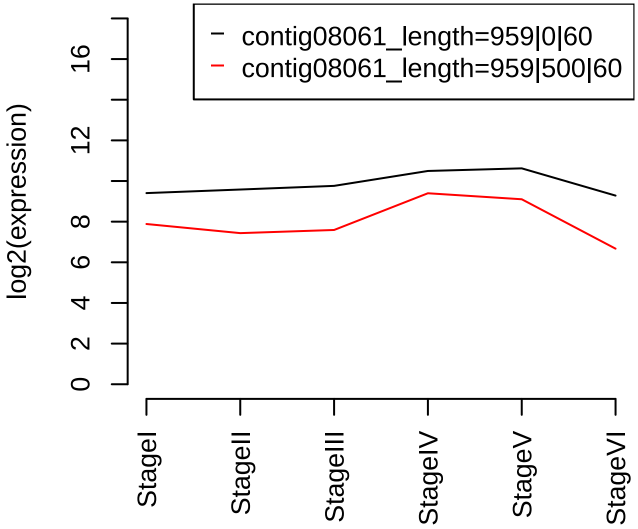

contig08061_length=959|0|60
contig08061_length=959|500|60
log2(expression)
0
2
4
6
8
12
16
StageI
StageII
StageIII
StageIV
StageV
StageVI

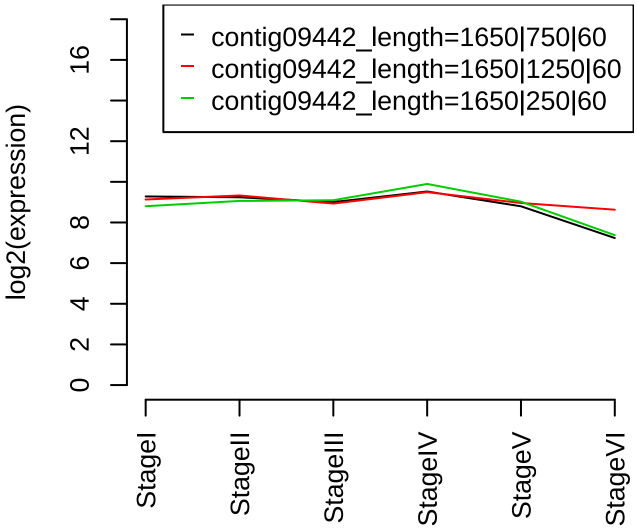

contig09442_length=1650|750|60
contig09442_length=1650|1250|60
contig09442_length=1650|250|60
log2(expression)
0
2
4
6
8
12
16
StageI
StageII
StageIII
StageIV
StageV
StageVI

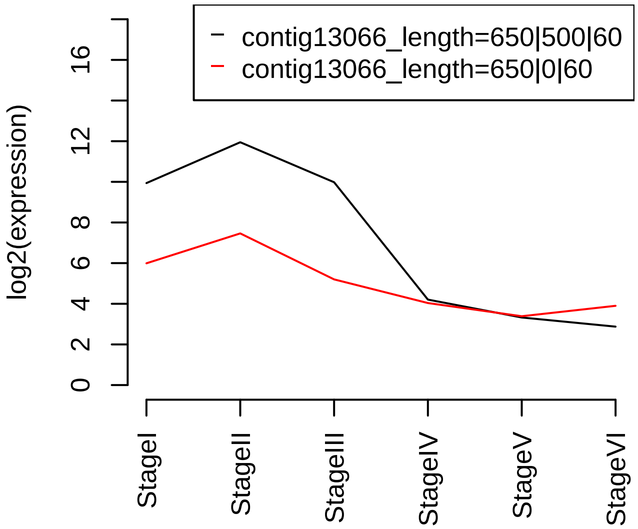

contig13066_length=650|500|60
contig13066_length=650|0|60
log2(expression)
0
2
4
6
8
12
16
StageI
StageII
StageIII
StageIV
StageV
StageVI

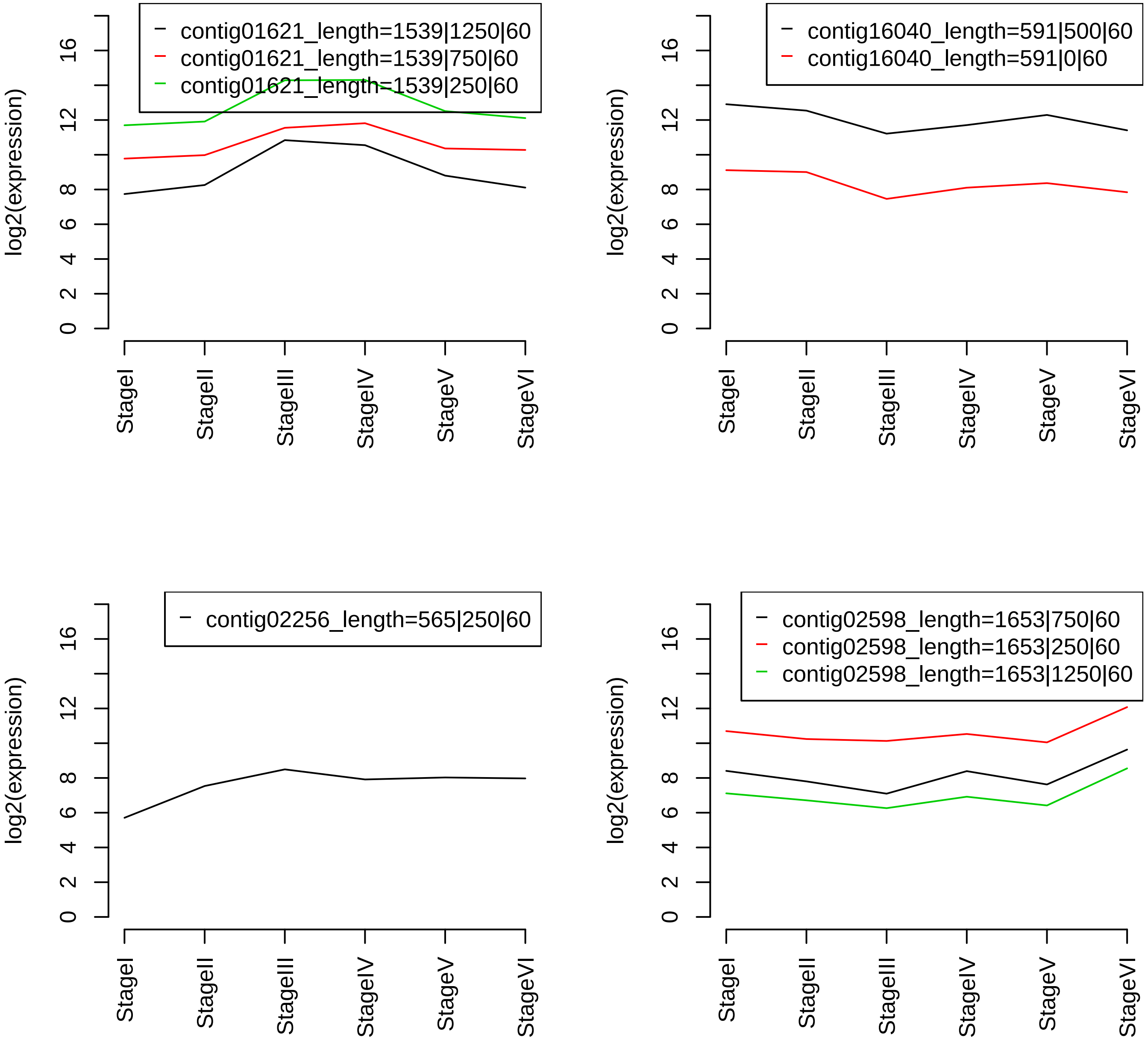

contig01621_length=1539|1250|60
contig01621_length=1539|750|60
contig01621_length=1539|250|60
log2(expression)
StageI
StageII
StageIII
StageIV
StageV
StageVI
contig16040_length=591|500|60
contig16040_length=591|0|60
contig02256_length=565|250|60
contig02598_length=1653|750|60
contig02598_length=1653|250|60
contig02598_length=1653|1250|60

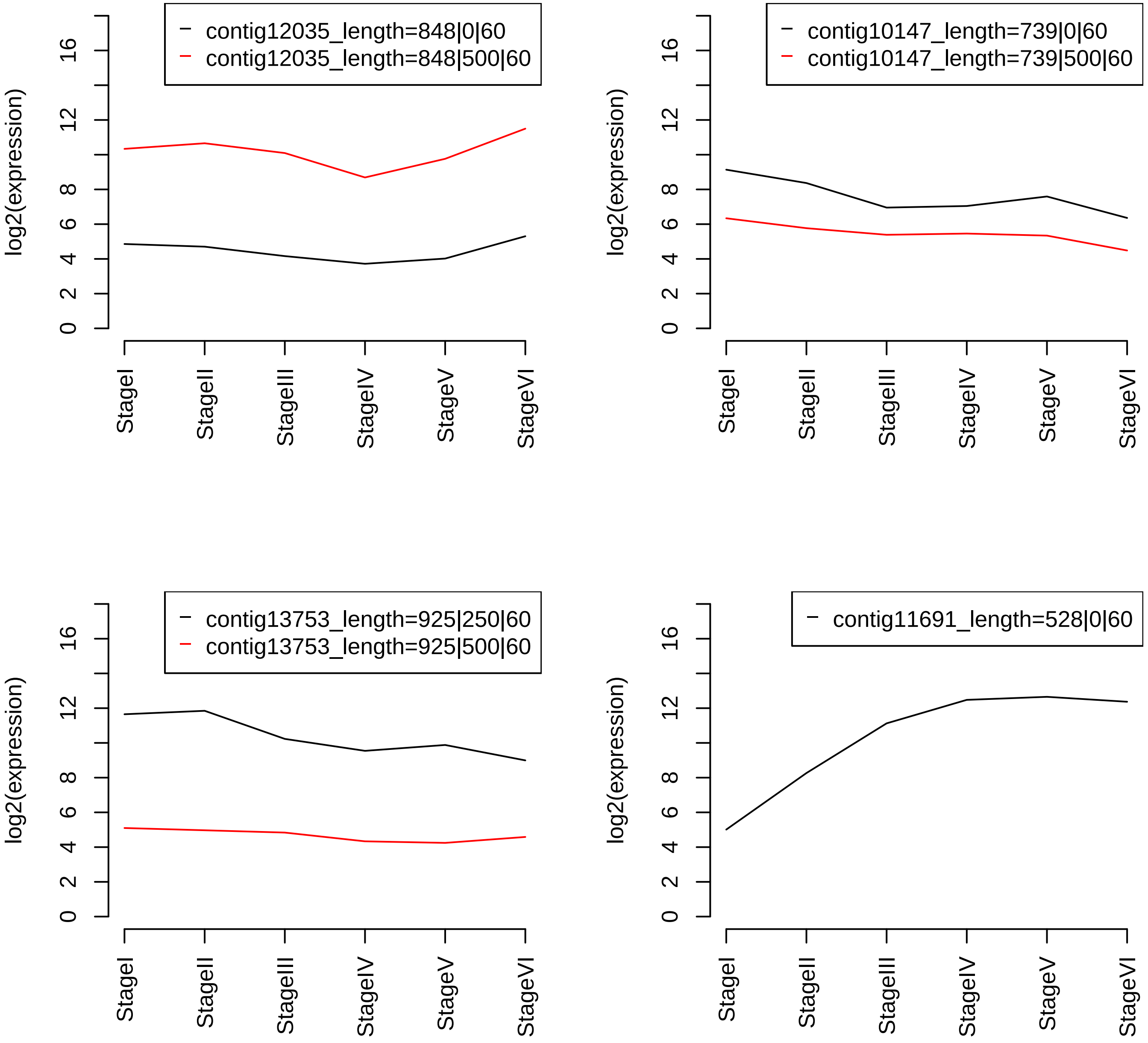
contig12035_length=848|0|60
contig12035_length=848|500|60
contig10147_length=739|0|60
contig10147_length=739|500|60
contig13753_length=925|250|60
contig13753_length=925|500|60
contig11691_length=528|0|60
log2(expression)
0 2 4 6 8 12 16
StageI StageII StageIII StageIV StageV StageVI

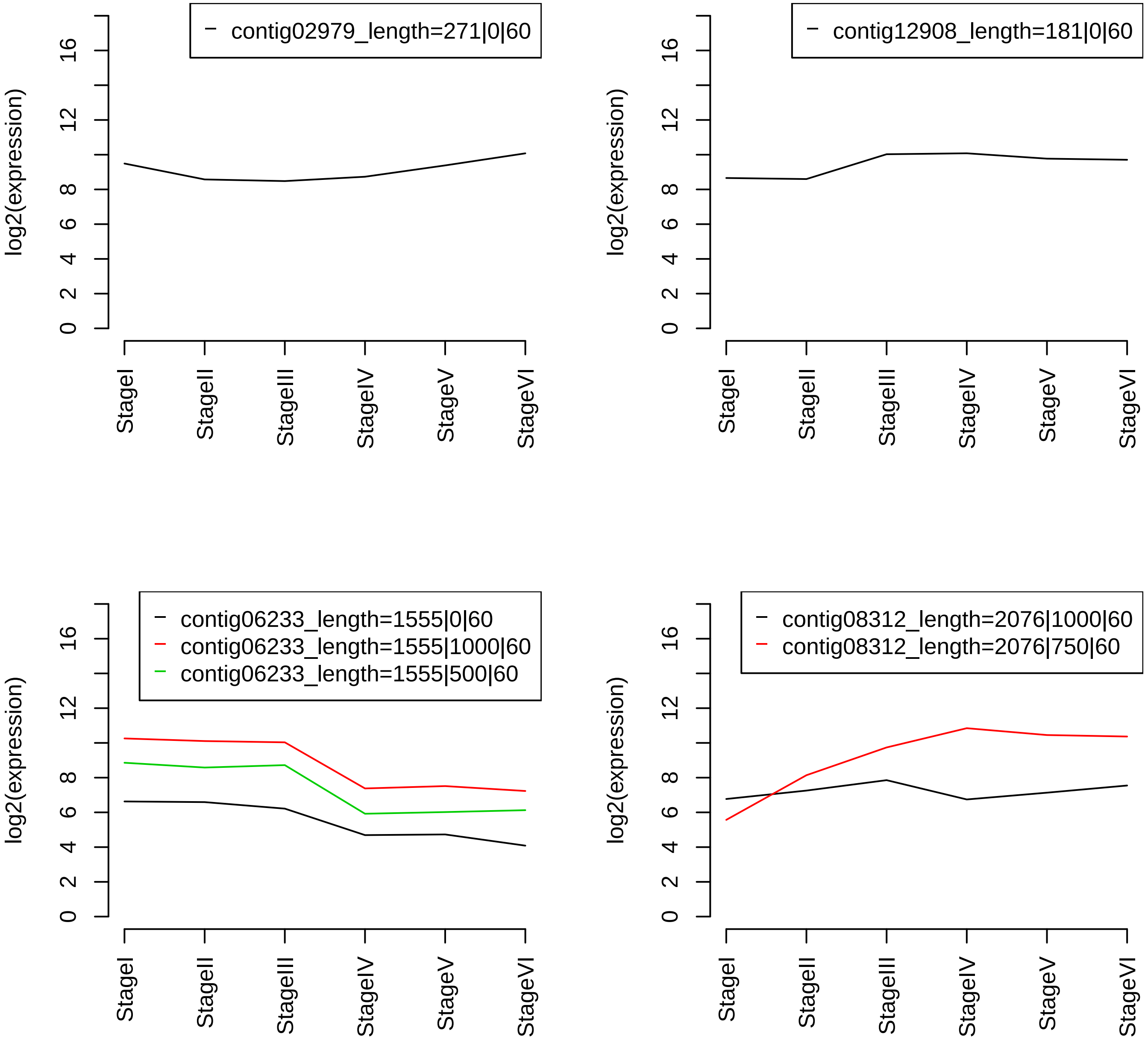
contig02979_length=271|0|60
contig12908_length=181|0|60
contig06233_length=1555|0|60
contig06233_length=1555|1000|60
contig06233_length=1555|500|60
contig08312_length=2076|1000|60
contig08312_length=2076|750|60
log2(expression)
0
2
4
6
8
12
16
StageI
StageII
StageIII
StageIV
StageV
StageVI

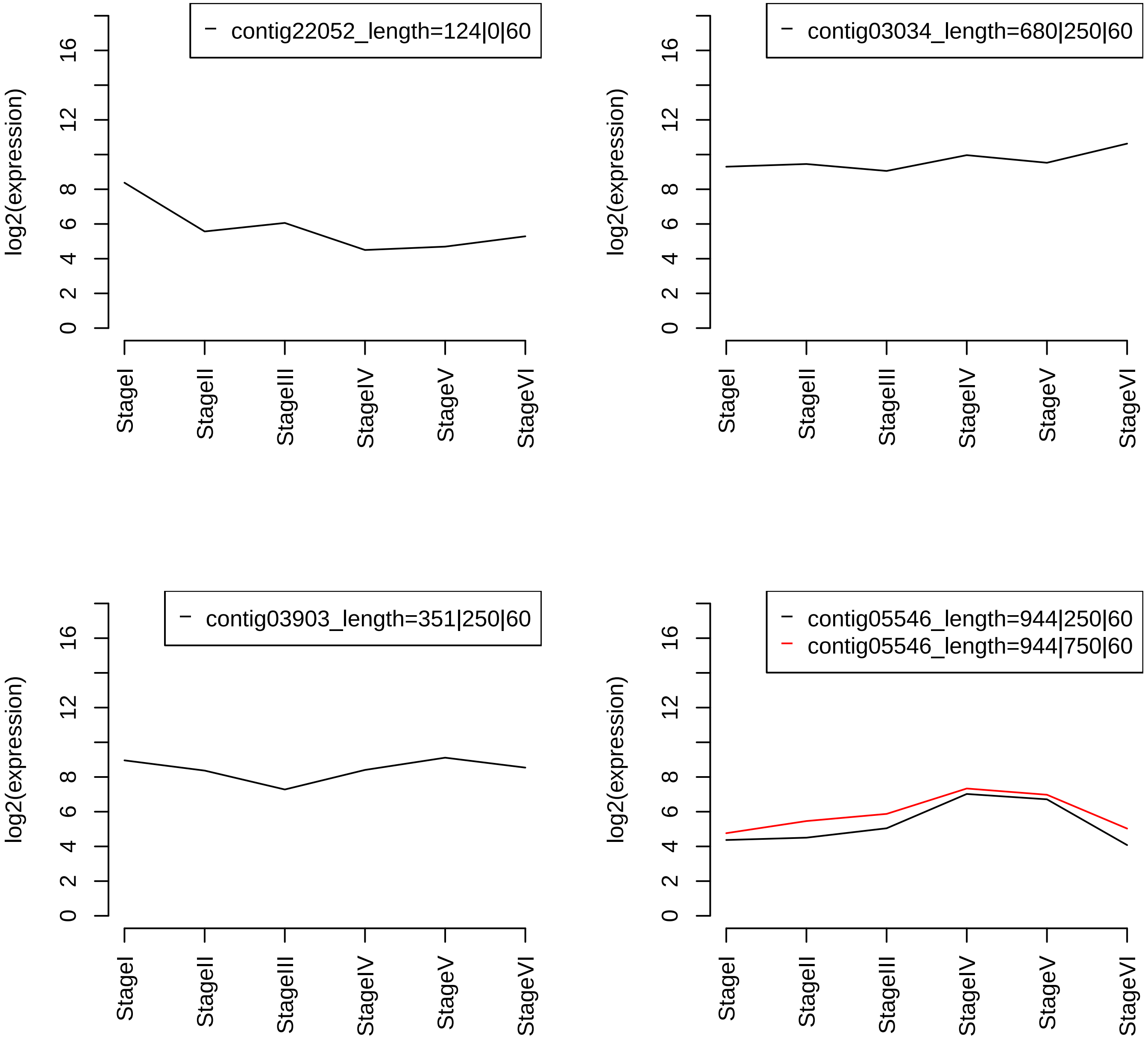
contig22052_length=124|0|60
contig03034_length=680|250|60
contig03903_length=351|250|60
contig05546_length=944|250|60
contig05546_length=944|750|60
log2(expression)
0
2
4
6
8
12
16
StageI
StageII
StageIII
StageIV
StageV
StageVI

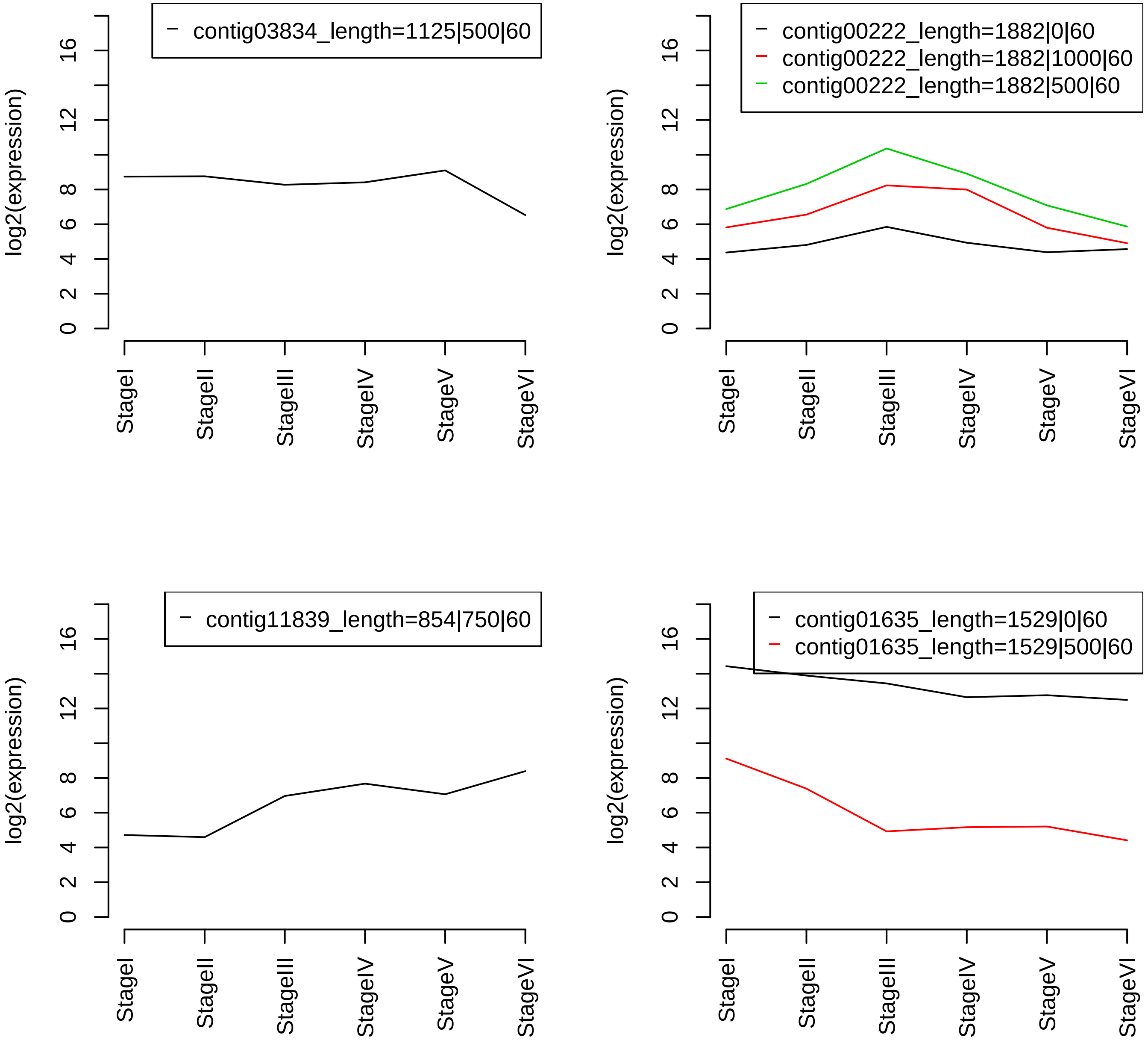
contig03834_length=1125|500|60
contig00222_length=1882|0|60
contig00222_length=1882|1000|60
contig00222_length=1882|500|60
contig11839_length=854|750|60
contig01635_length=1529|0|60
contig01635_length=1529|500|60
log2(expression)
0
2
4
6
8
12
16
StageI
StageII
StageIII
StageIV
StageV
StageVI

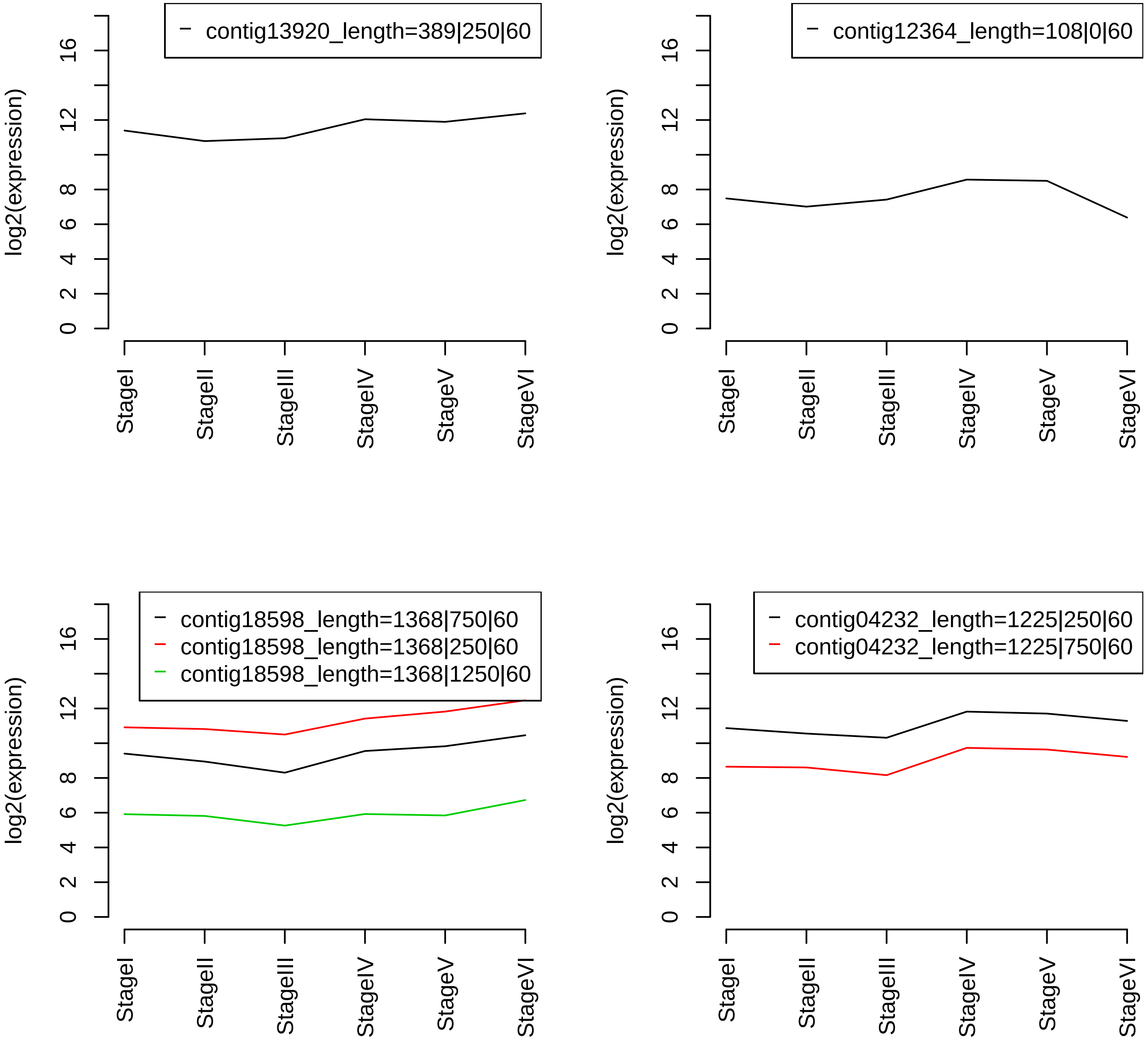

contig13920_length=389|250|60
contig12364_length=108|0|60
contig18598_length=1368|750|60
contig18598_length=1368|250|60
contig18598_length=1368|1250|60
contig04232_length=1225|250|60
contig04232_length=1225|750|60
log2(expression)
0
2
4
6
8
12
16
StageI
StageII
StageIII
StageIV
StageV
StageVI

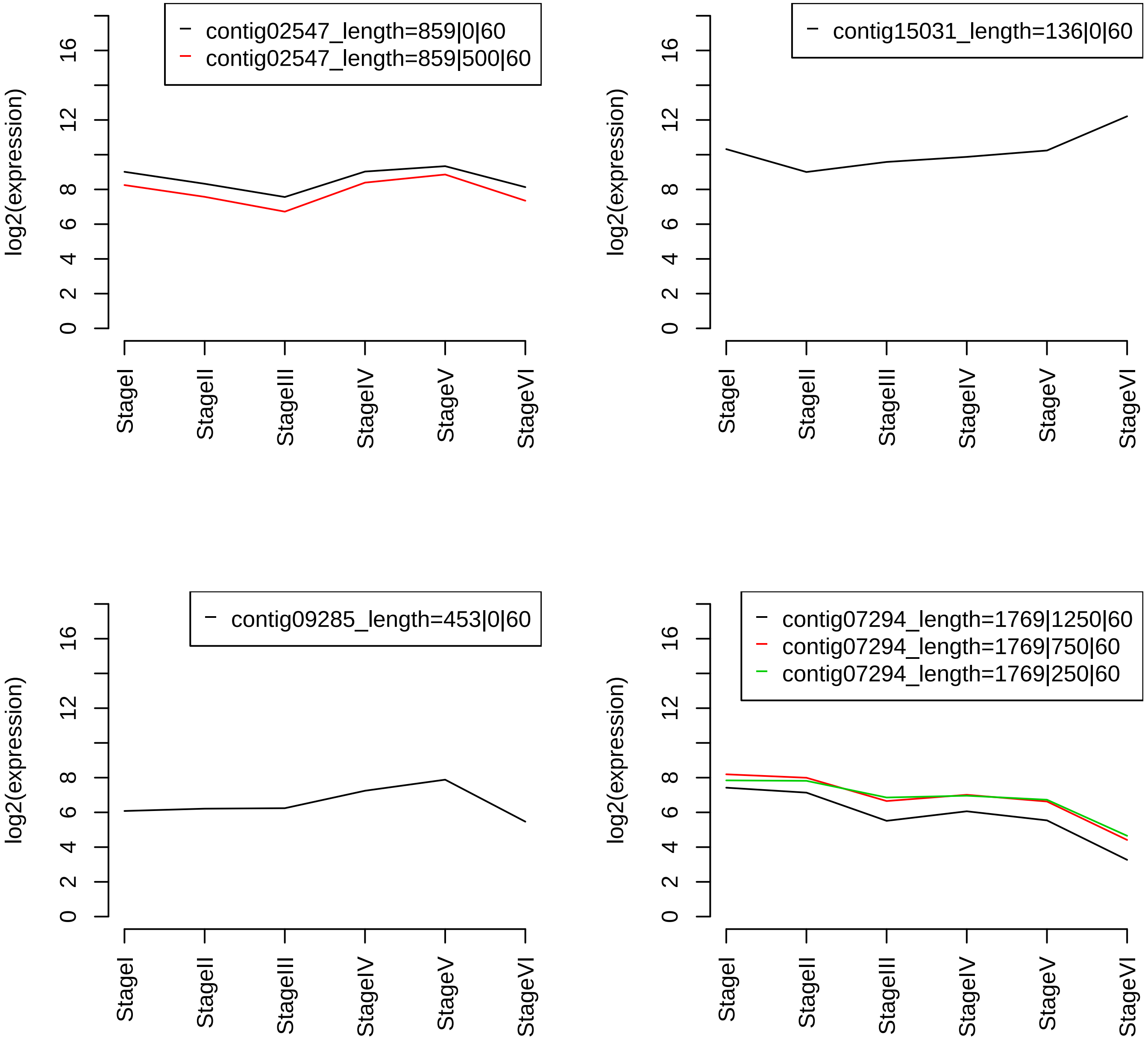

contig02547_length=859|0|60
contig02547_length=859|500|60
contig15031_length=136|0|60
contig09285_length=453|0|60
contig07294_length=1769|1250|60
contig07294_length=1769|750|60
contig07294_length=1769|250|60
log2(expression)
0
2
4
6
8
12
16
StageI
StageII
StageIII
StageIV
StageV
StageVI

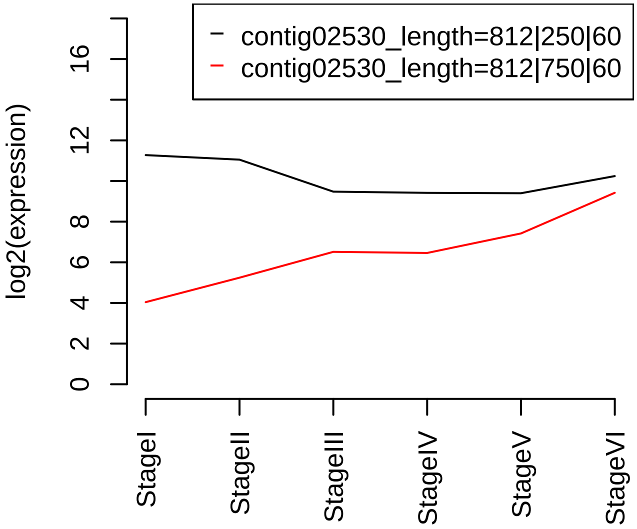

contig02530_length=812|250|60
contig02530_length=812|750|60
log2(expression)
0
2
4
6
8
12
16
StageI
StageII
StageIII
StageIV
StageV
StageVI

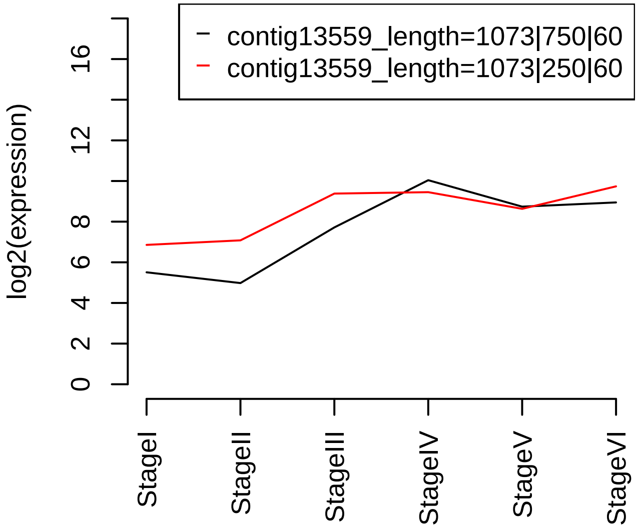

contig13559_length=1073|750|60
contig13559_length=1073|250|60
log2(expression)
0
2
4
6
8
12
16
StageI
StageII
StageIII
StageIV
StageV
StageVI

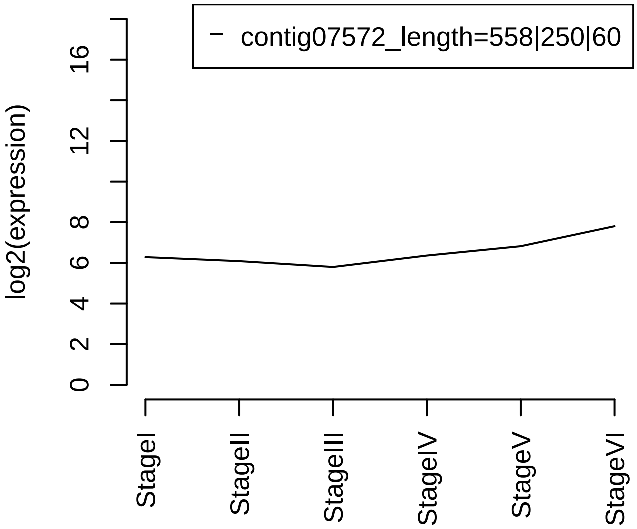

contig07572_length=558|250|60
log2(expression)
0
2
4
6
8
12
16
StageI
StageII
StageIII
StageIV
StageV
StageVI

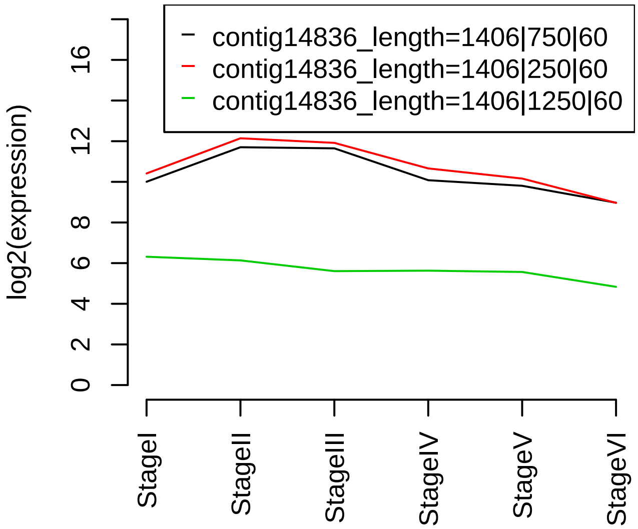

contig14836_length=1406|750|60
contig14836_length=1406|250|60
contig14836_length=1406|1250|60
log2(expression)
0
2
4
6
8
12
16
StageI
StageII
StageIII
StageIV
StageV
StageVI

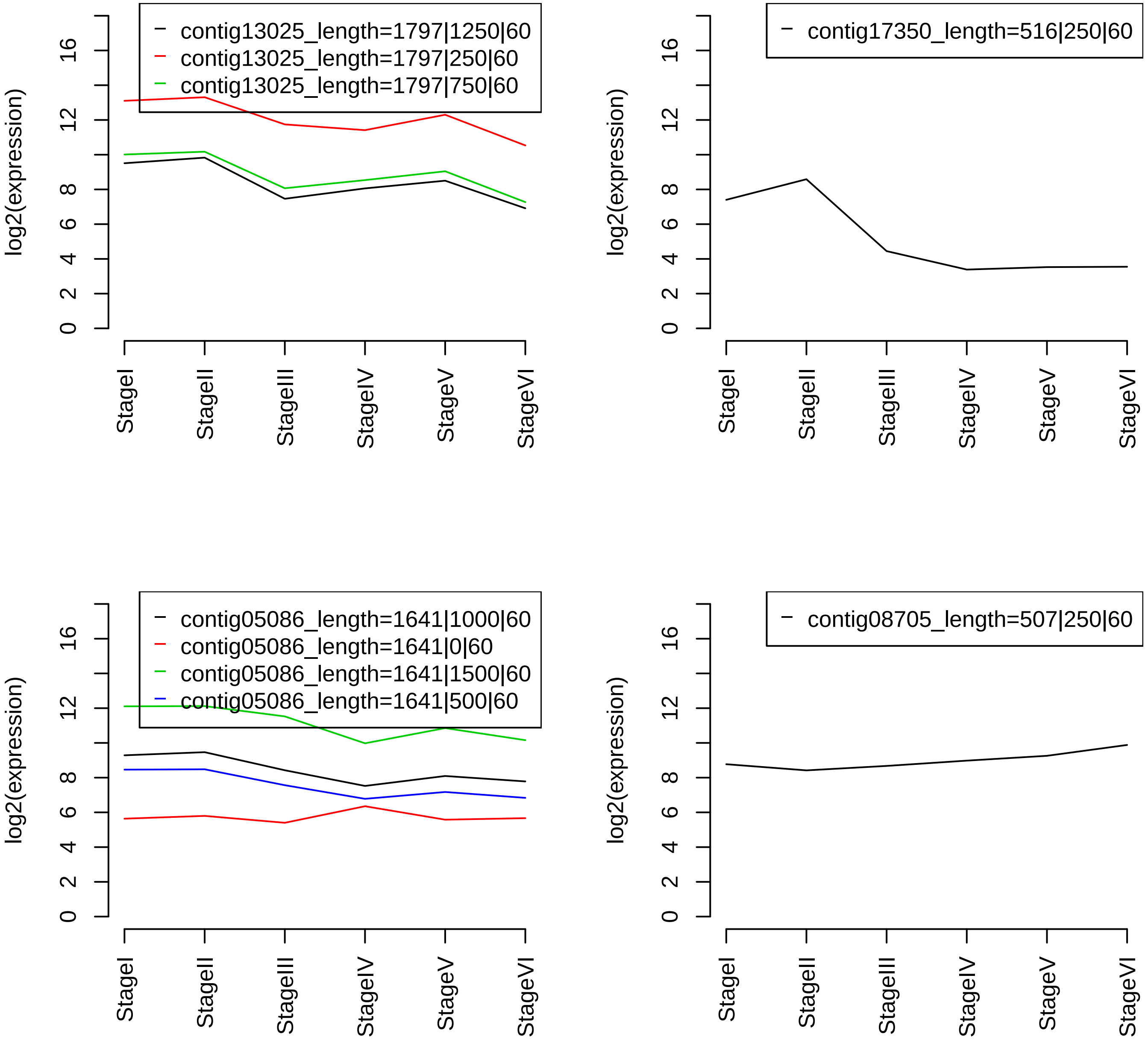

contig13025_length=1797|1250|60
contig13025_length=1797|250|60
contig13025_length=1797|750|60
contig17350_length=516|250|60
contig05086_length=1641|1000|60
contig05086_length=1641|0|60
contig05086_length=1641|1500|60
contig05086_length=1641|500|60
contig08705_length=507|250|60
log2(expression)
0
2
4
6
8
12
16
StageI
StageII
StageIII
StageIV
StageV
StageVI

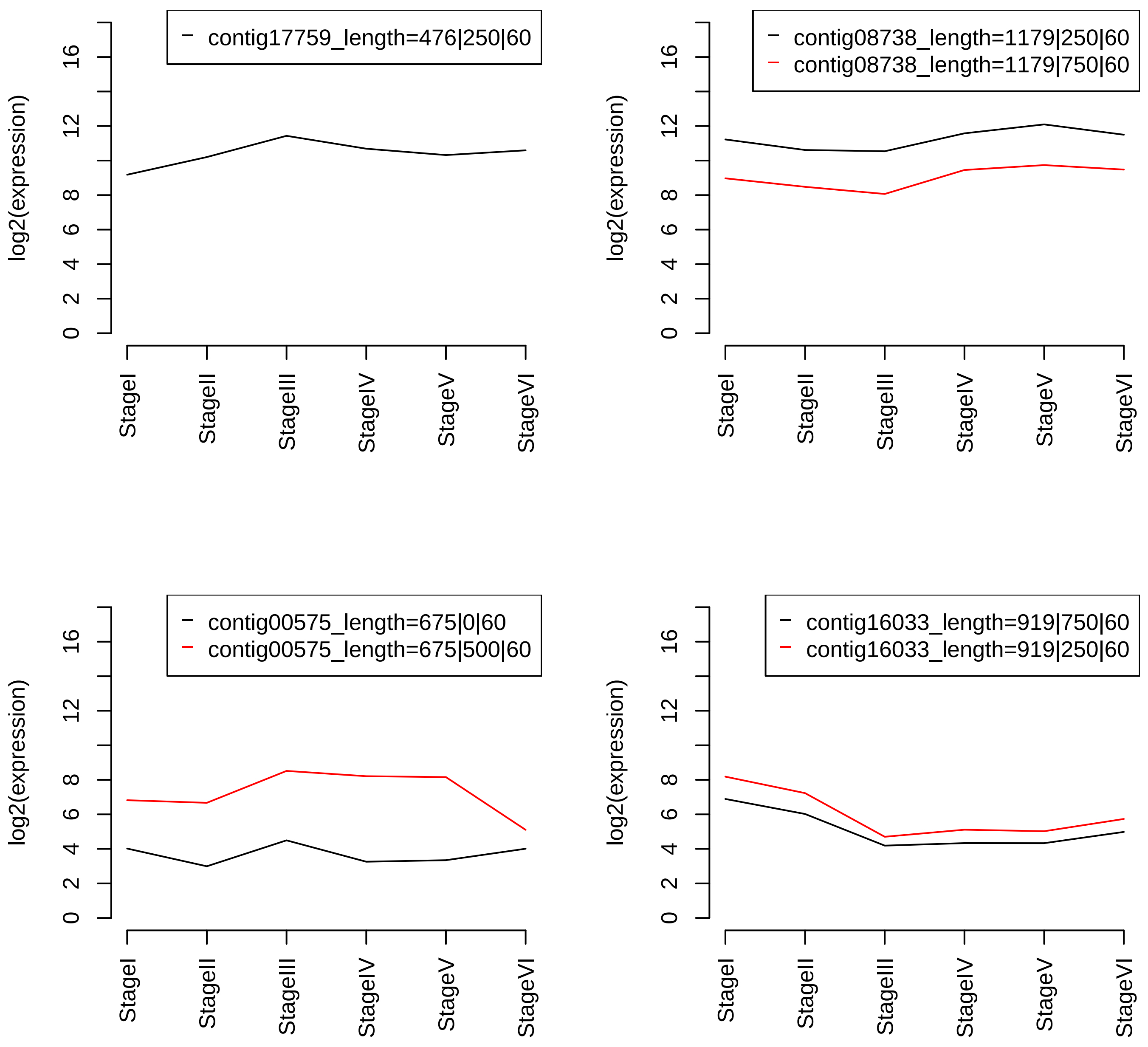

contig17759_length=476|250|60
contig08738_length=1179|250|60
contig08738_length=1179|750|60
contig00575_length=675|0|60
contig00575_length=675|500|60
contig16033_length=919|750|60
contig16033_length=919|250|60
log2(expression)
0 2 4 6 8 12 16
StageI StageII StageIII StageIV StageV StageVI

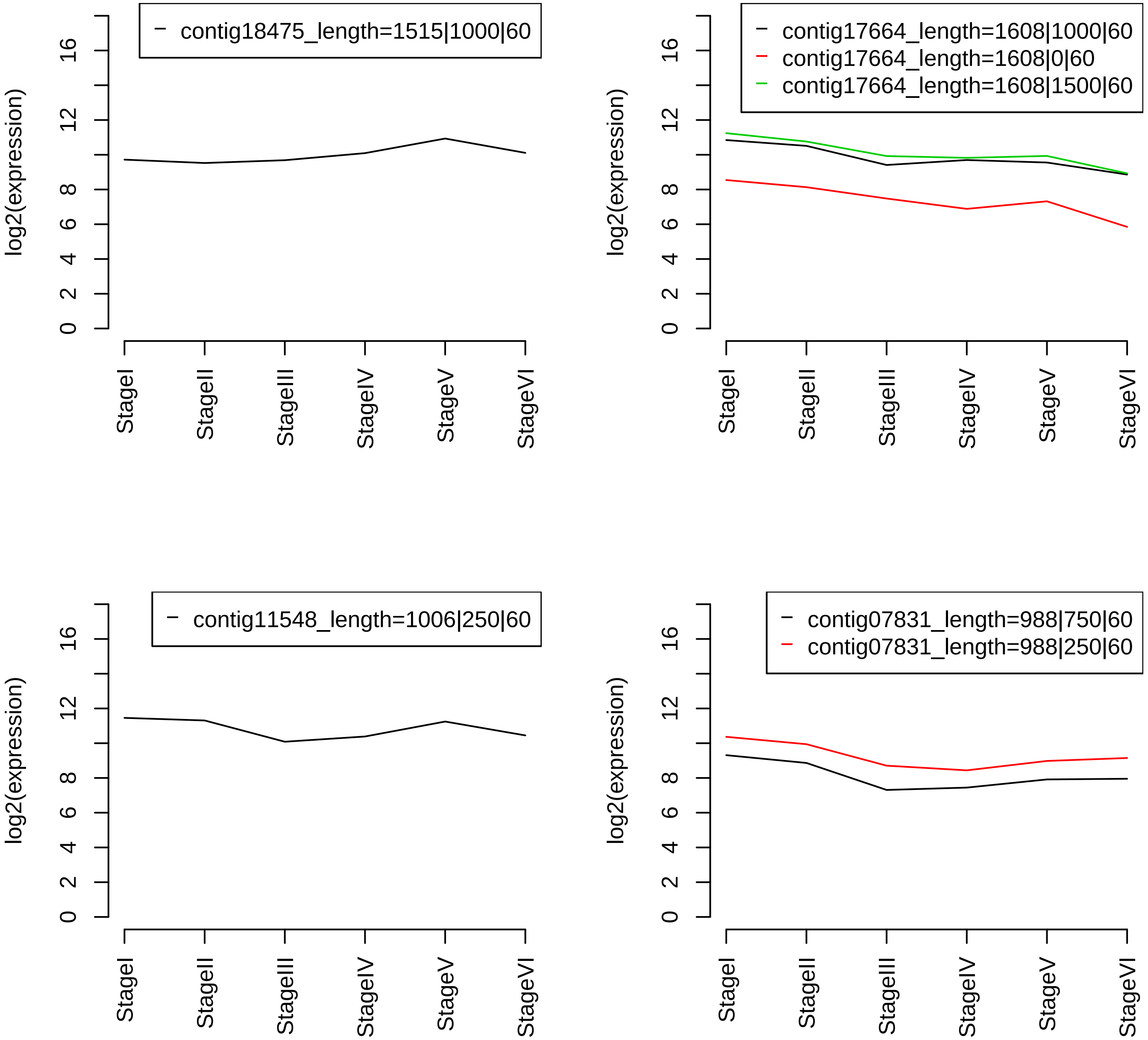
contig18475_length=1515|1000|60
contig17664_length=1608|1000|60
contig17664_length=1608|0|60
contig17664_length=1608|1500|60
contig11548_length=1006|250|60
contig07831_length=988|750|60
contig07831_length=988|250|60
log2(expression)
0
2
4
6
8
12
16
StageI
StageII
StageIII
StageIV
StageV
StageVI

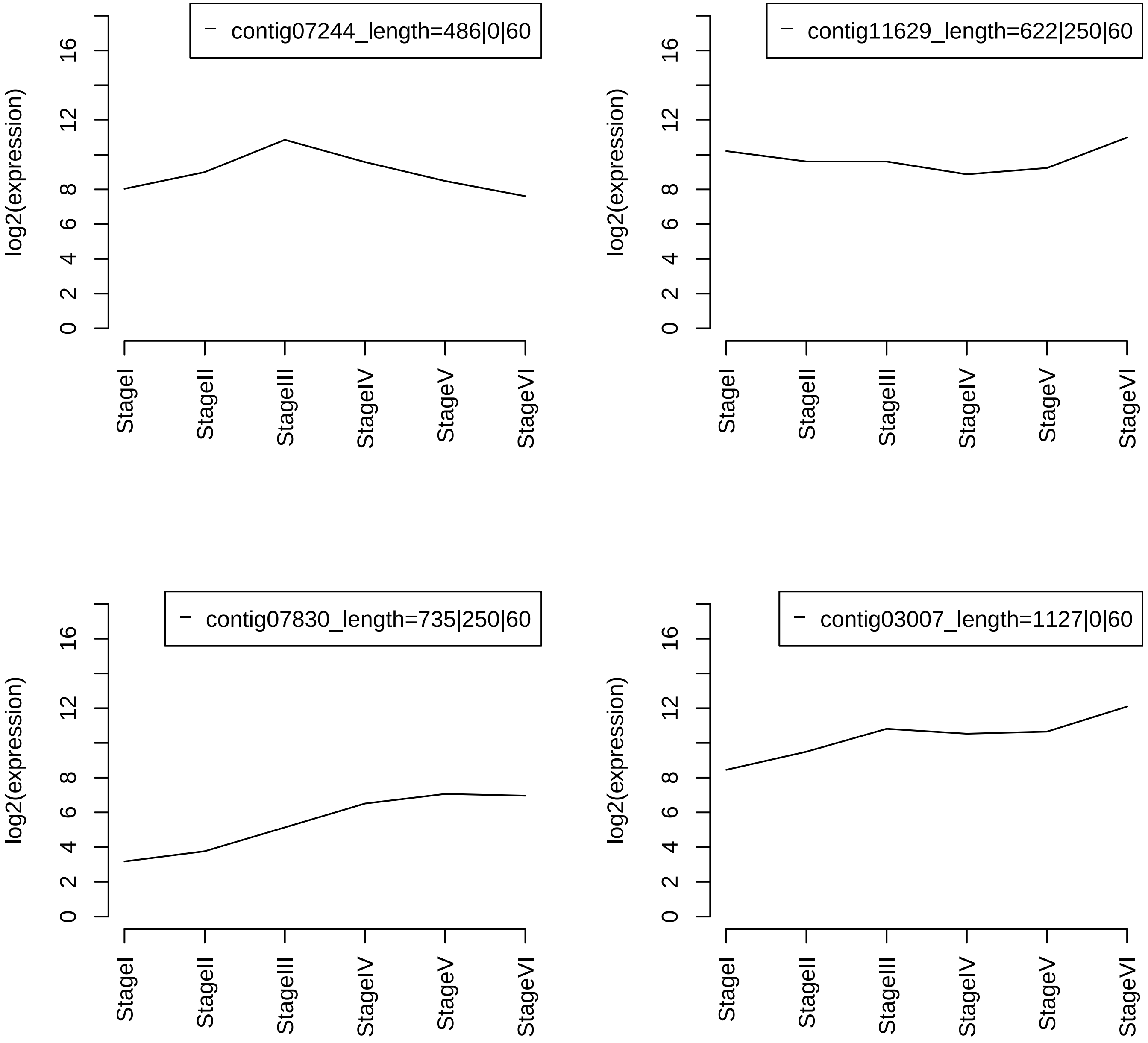
contig07244_length=486|0|60
contig11629_length=622|250|60
contig07830_length=735|250|60
contig03007_length=1127|0|60
log2(expression)
0
2
4
6
8
12
16
StageI
StageII
StageIII
StageIV
StageV
StageVI

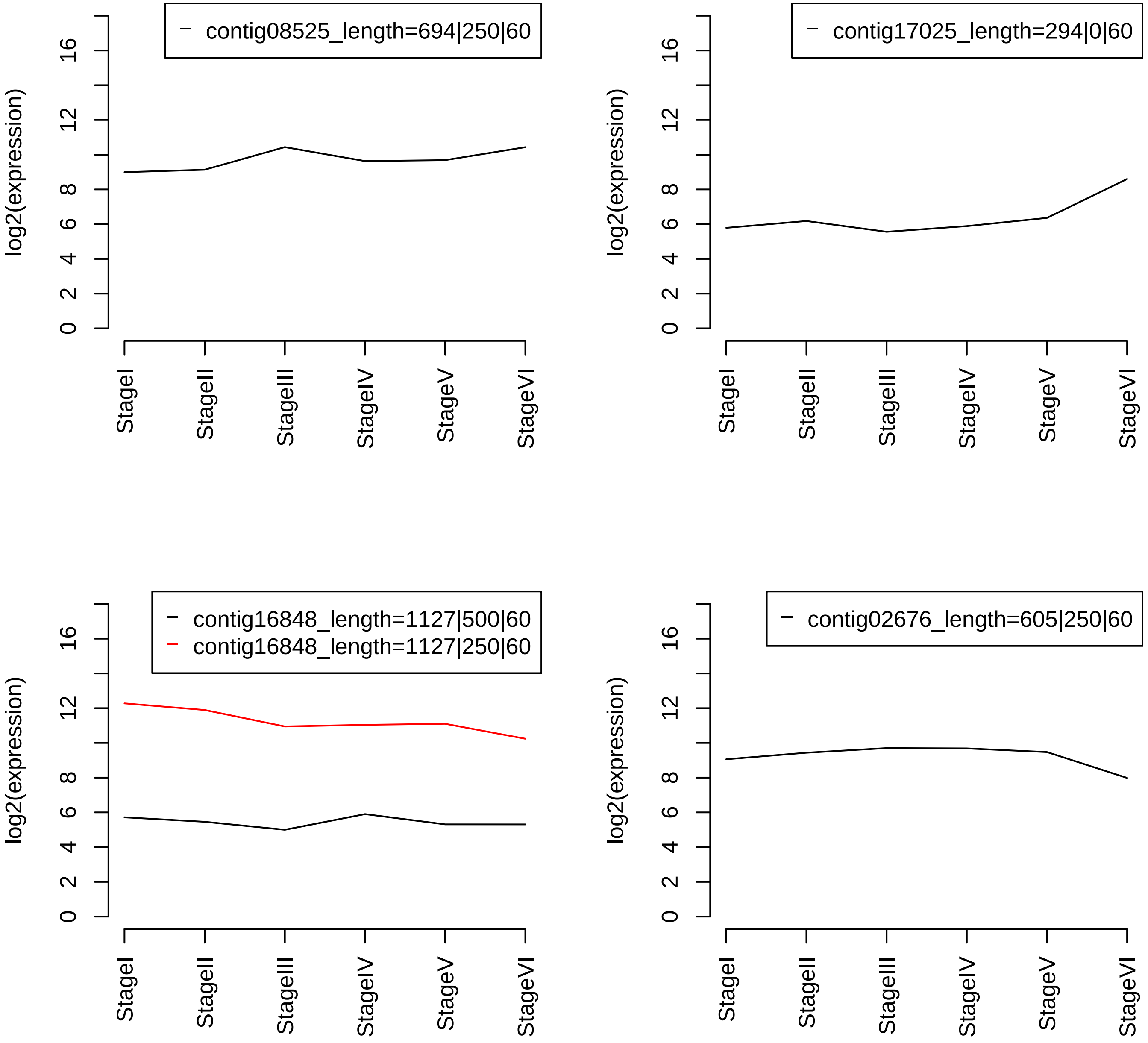
contig08525_length=694|250|60
contig17025_length=294|0|60
contig16848_length=1127|500|60
contig16848_length=1127|250|60
contig02676_length=605|250|60
log2(expression)
0
2
4
6
8
12
16
StageI
StageII
StageIII
StageIV
StageV
StageVI

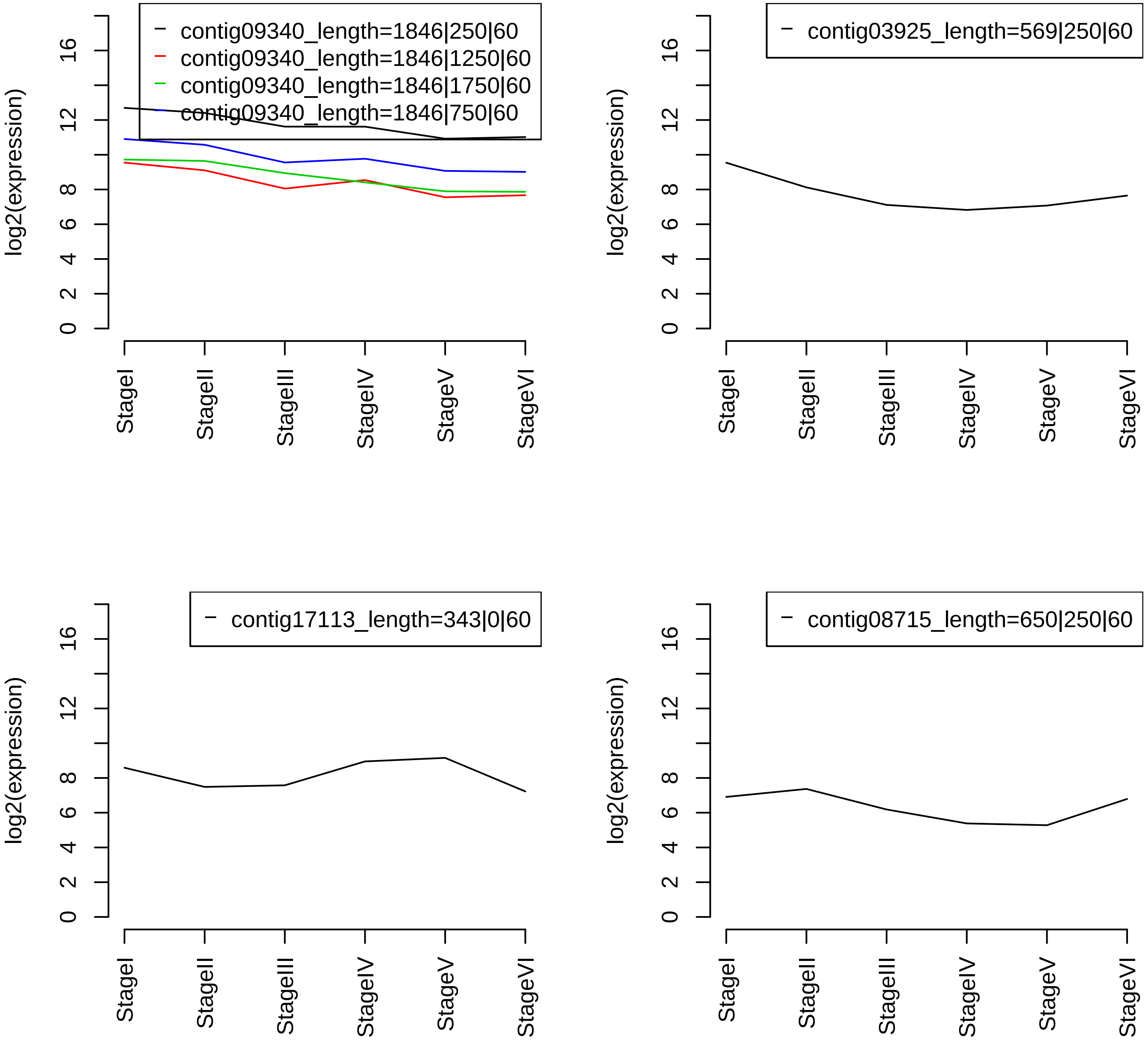
contig09340_length=1846|250|60
contig09340_length=1846|1250|60
contig09340_length=1846|1750|60
contig09340_length=1846|750|60
contig03925_length=569|250|60
contig17113_length=343|0|60
contig08715_length=650|250|60
log2(expression)
0
2
4
6
8
12
16
StageI
StageII
StageIII
StageIV
StageV
StageVI

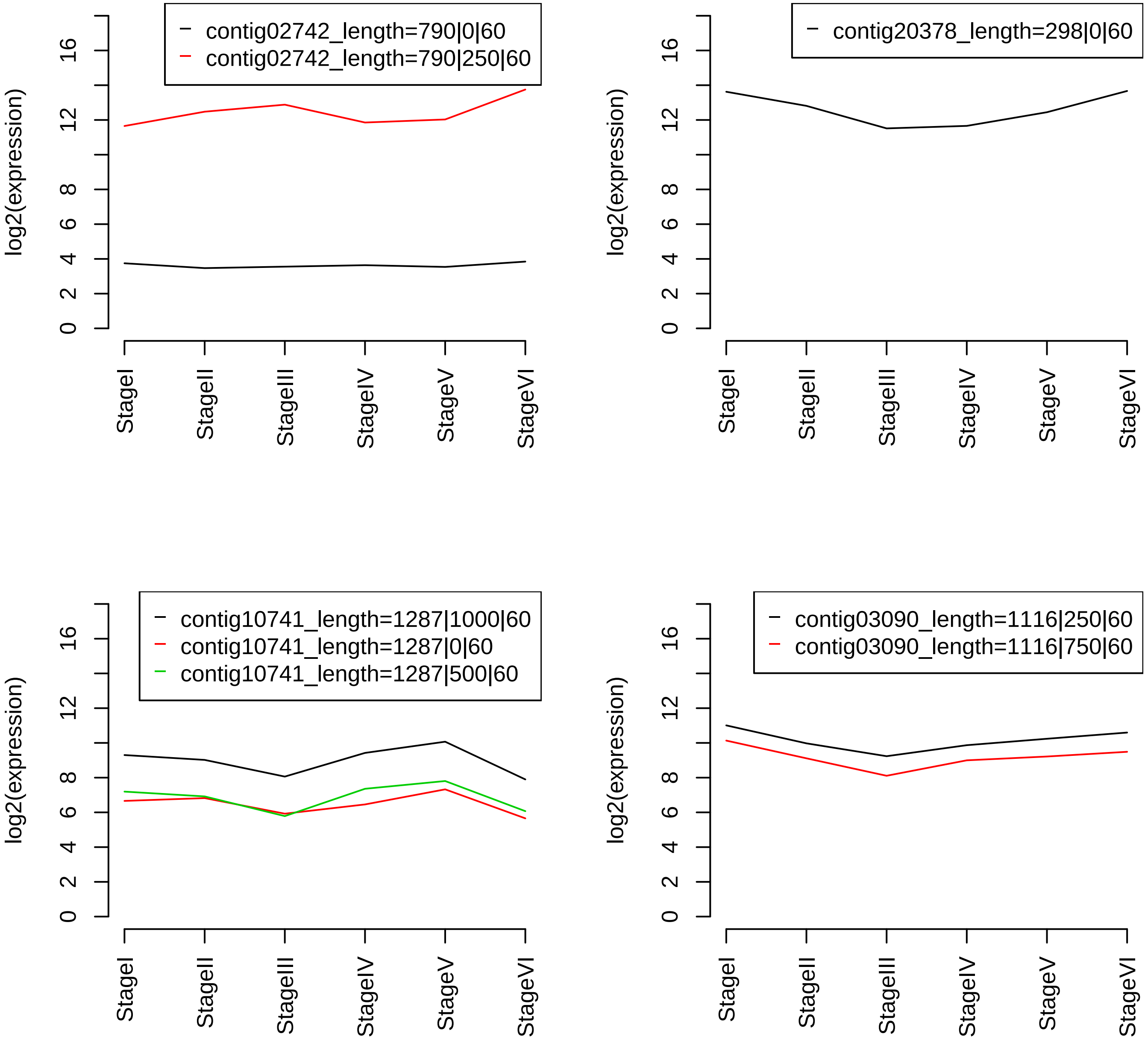

contig02742_length=790|0|60
contig02742_length=790|250|60
contig20378_length=298|0|60
contig10741_length=1287|1000|60
contig10741_length=1287|0|60
contig10741_length=1287|500|60
contig03090_length=1116|250|60
contig03090_length=1116|750|60
log2(expression)
0
2
4
6
8
12
16
StageI
StageII
StageIII
StageIV
StageV
StageVI

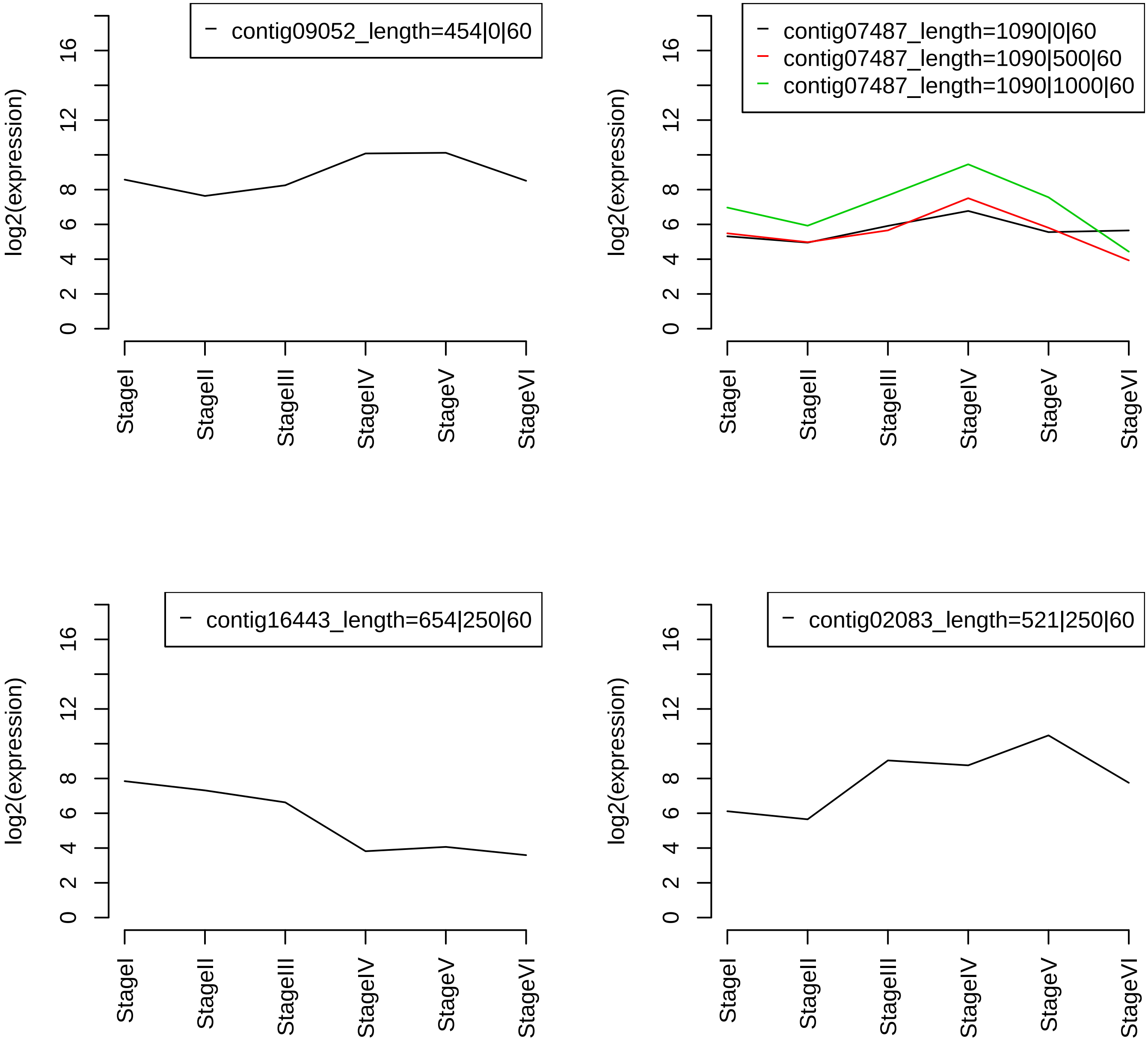
contig09052_length=454|0|60
contig07487_length=1090|0|60
contig07487_length=1090|500|60
contig07487_length=1090|1000|60
contig16443_length=654|250|60
contig02083_length=521|250|60
log2(expression)
0
2
4
6
8
12
16
StageI
StageII
StageIII
StageIV
StageV
StageVI

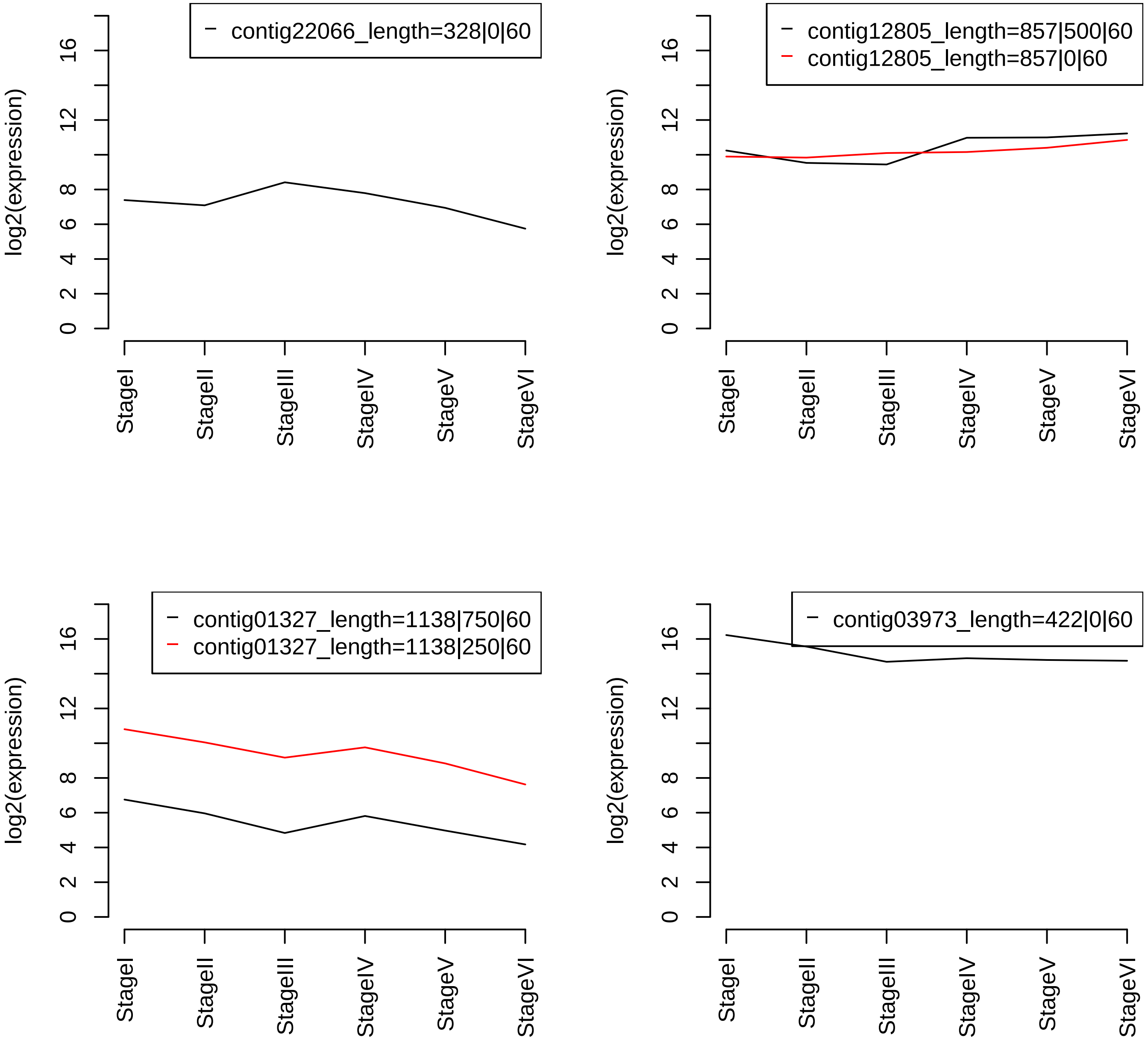

contig22066_length=328|0|60
contig12805_length=857|500|60
contig12805_length=857|0|60
contig01327_length=1138|750|60
contig01327_length=1138|250|60
contig03973_length=422|0|60
log2(expression)
0
2
4
6
8
12
16
StageI
StageII
StageIII
StageIV
StageV
StageVI

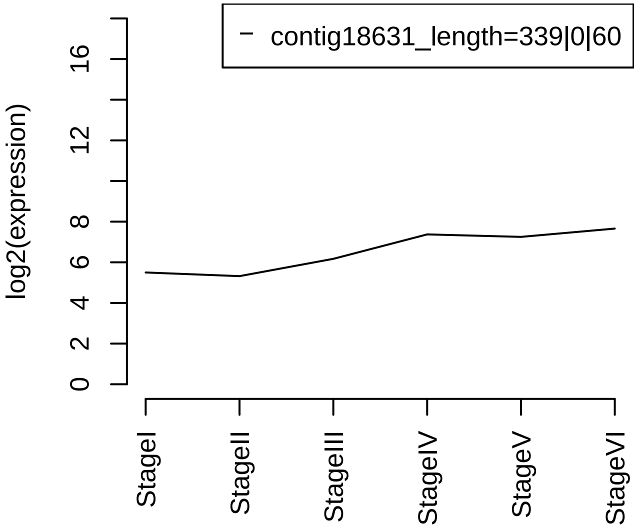
contig18631_length=339|0|60
log2(expression)
0
2
4
6
8
12
16
StageI
StageII
StageIII
StageIV
StageV
StageVI

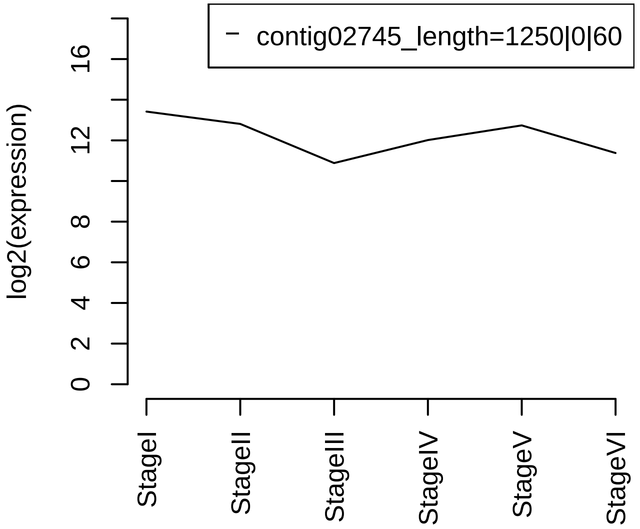
contig02745_length=1250|0|60
log2(expression)
0
2
4
6
8
12
16
StageI
StageII
StageIII
StageIV
StageV
StageVI

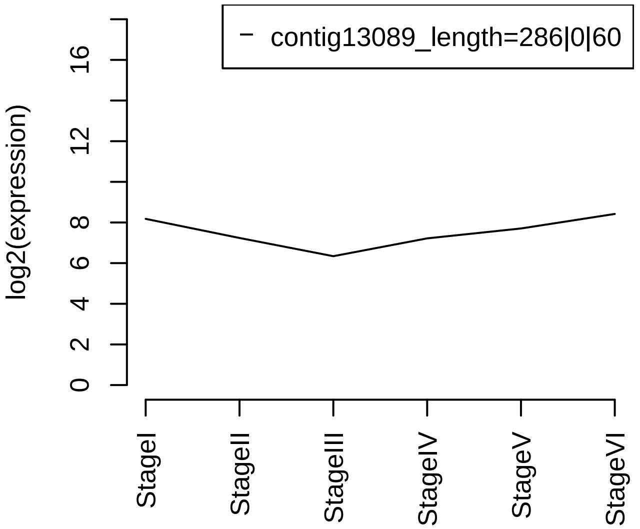
contig13089_length=286|0|60
log2(expression)
0
2
4
6
8
12
16
StageI
StageII
StageIII
StageIV
StageV
StageVI

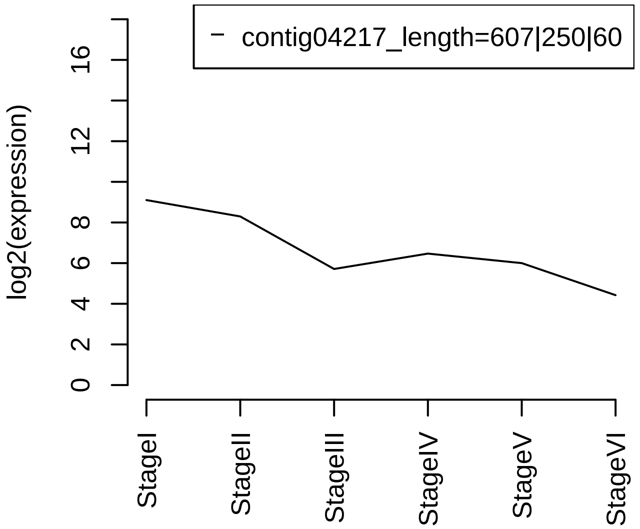
contig04217_length=607|250|60
log2(expression)
0
2
4
6
8
12
16
StageI
StageII
StageIII
StageIV
StageV
StageVI

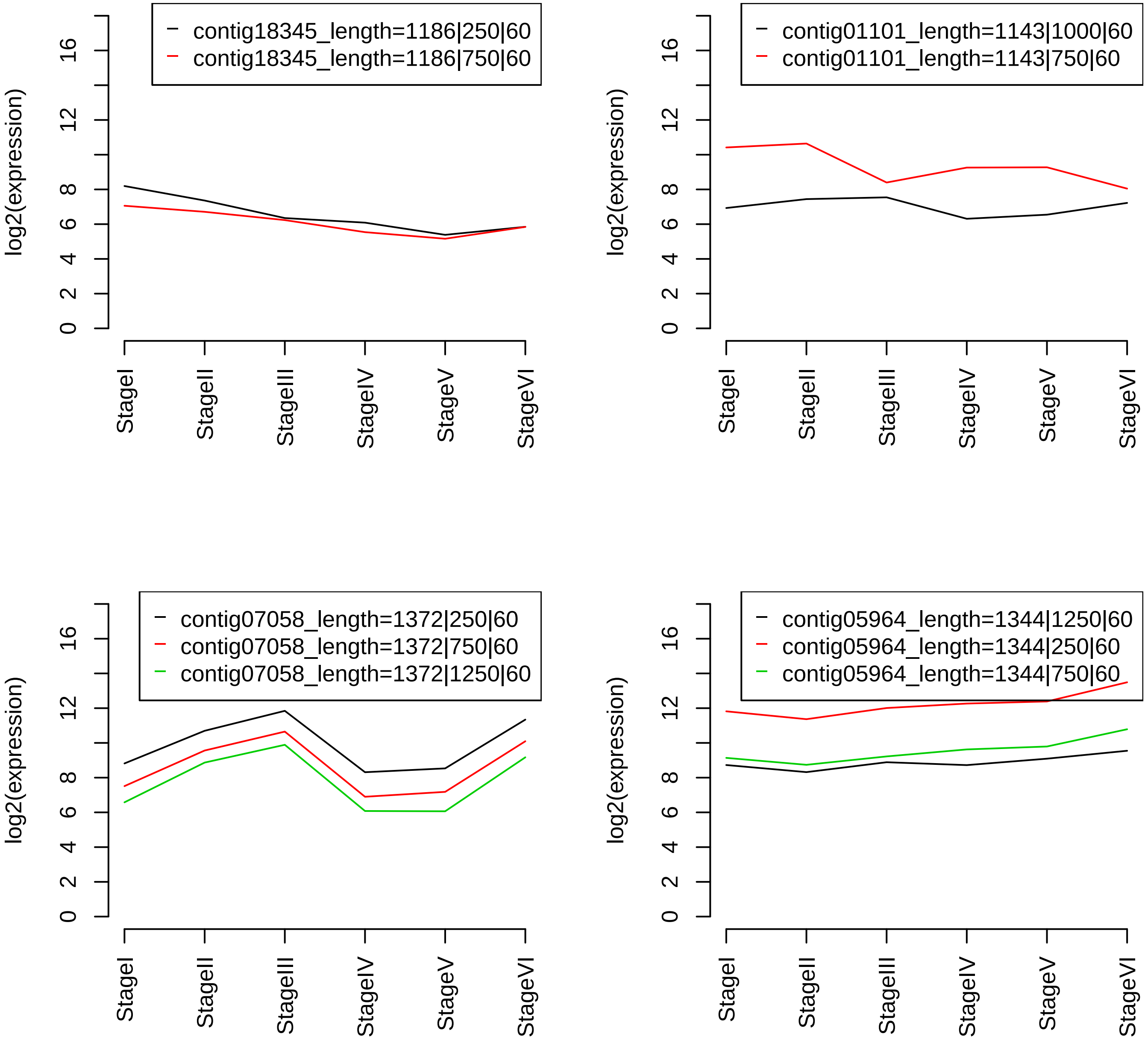
contig18345_length=1186|250|60
contig18345_length=1186|750|60
contig01101_length=1143|1000|60
contig01101_length=1143|750|60
contig07058_length=1372|250|60
contig07058_length=1372|750|60
contig07058_length=1372|1250|60
contig05964_length=1344|1250|60
contig05964_length=1344|250|60
contig05964_length=1344|750|60
log2(expression)
0
2
4
6
8
12
16
StageI
StageII
StageIII
StageIV
StageV
StageVI

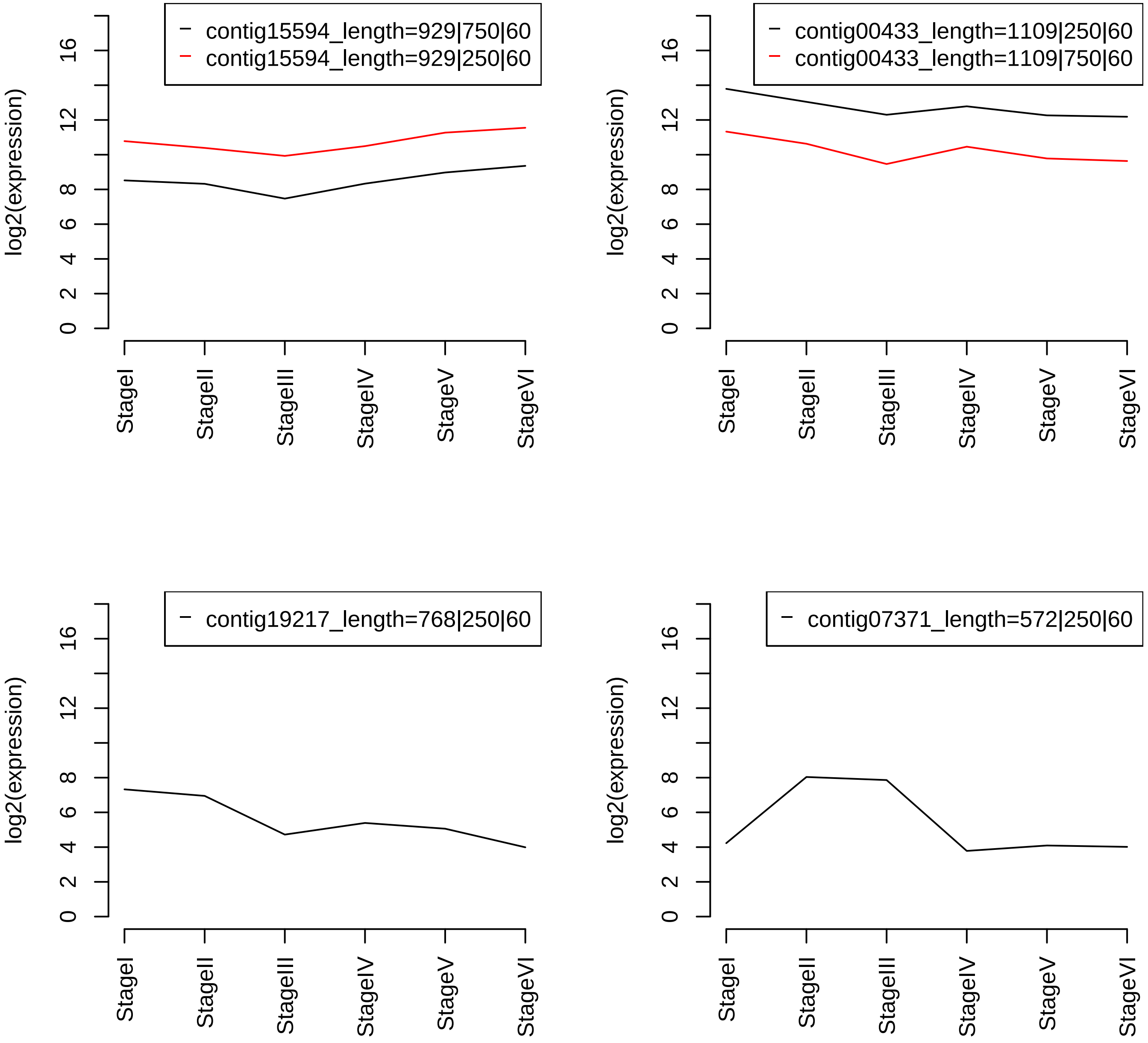

contig15594_length=929|750|60
contig15594_length=929|250|60
log2(expression)
0
2
4
6
8
12
16
StageI
StageII
StageIII
StageIV
StageV
StageVI
contig00433_length=1109|250|60
contig00433_length=1109|750|60
contig19217_length=768|250|60
contig07371_length=572|250|60

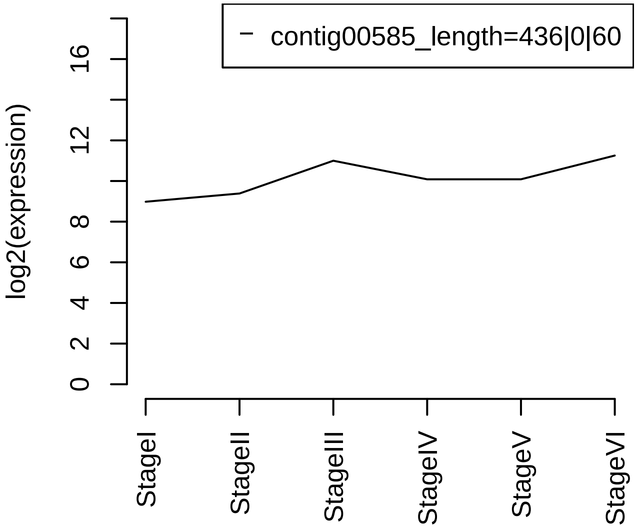
contig00585_length=436|0|60
log2(expression)
0
2
4
6
8
12
16
StageI
StageII
StageIII
StageIV
StageV
StageVI

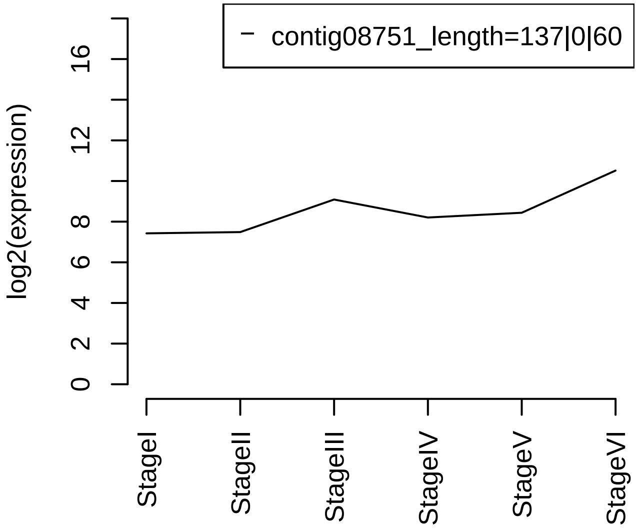
contig08751_length=137|0|60
log2(expression)
0
2
4
6
8
12
16
StageI
StageII
StageIII
StageIV
StageV
StageVI

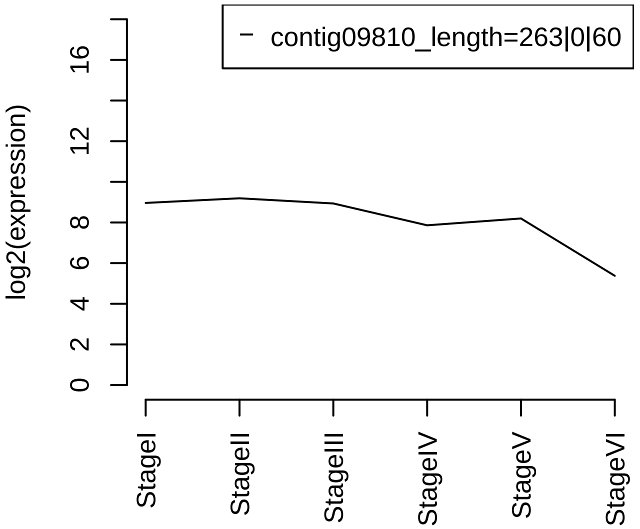
contig09810_length=263|0|60
log2(expression)
0
2
4
6
8
12
16
StageI
StageII
StageIII
StageIV
StageV
StageVI

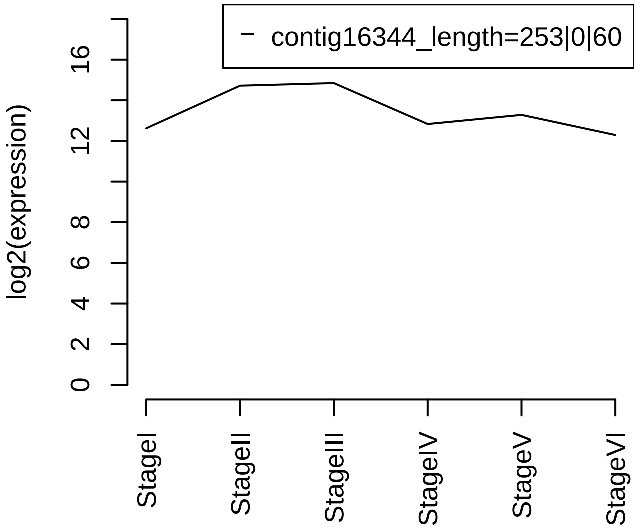
contig16344_length=253|0|60
log2(expression)
0
2
4
6
8
12
16
StageI
StageII
StageIII
StageIV
StageV
StageVI

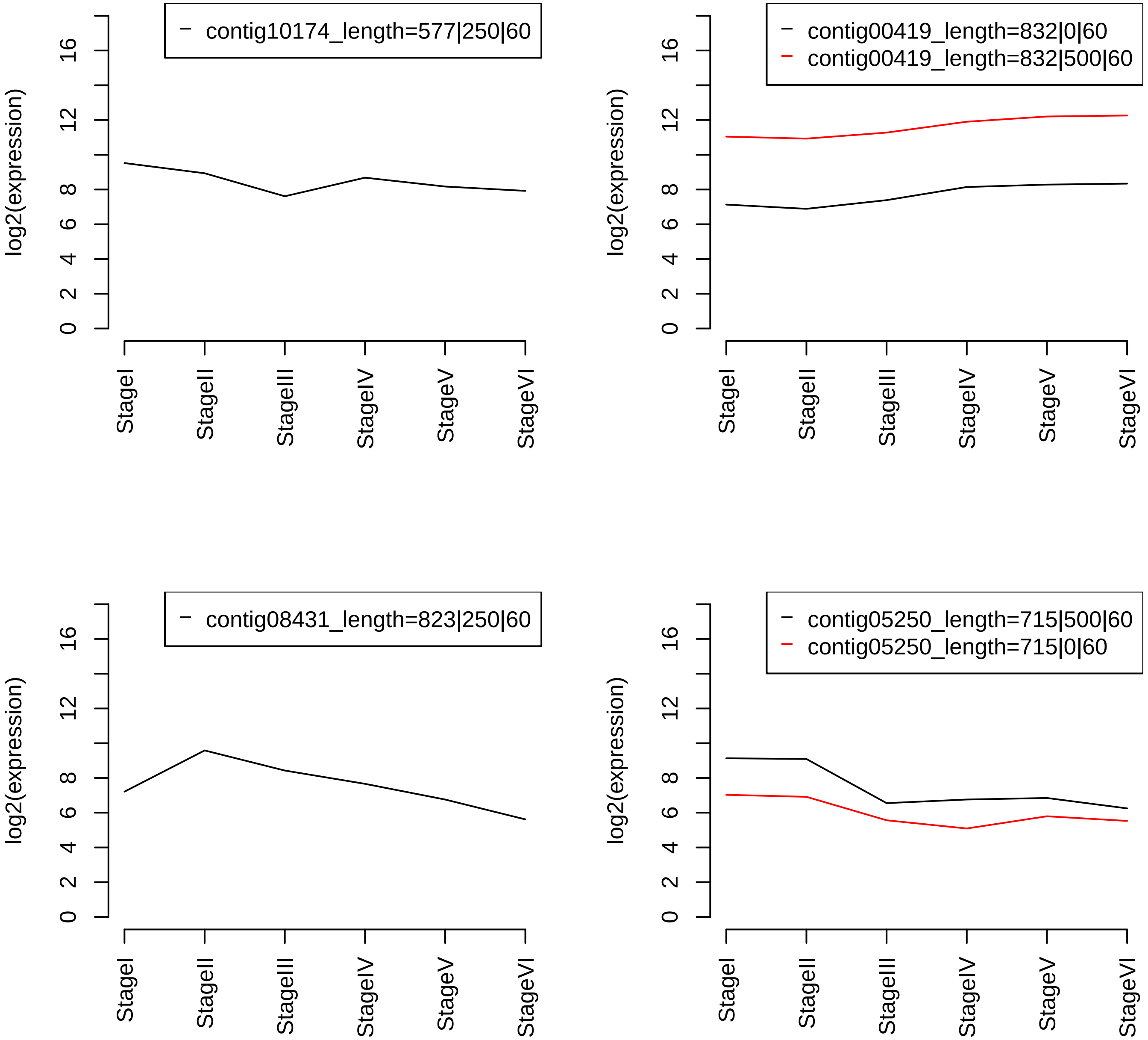
contig10174_length=577|250|60
contig00419_length=832|0|60
contig00419_length=832|500|60
contig08431_length=823|250|60
contig05250_length=715|500|60
contig05250_length=715|0|60
log2(expression)
0
2
4
6
8
12
16
StageI
StageII
StageIII
StageIV
StageV
StageVI

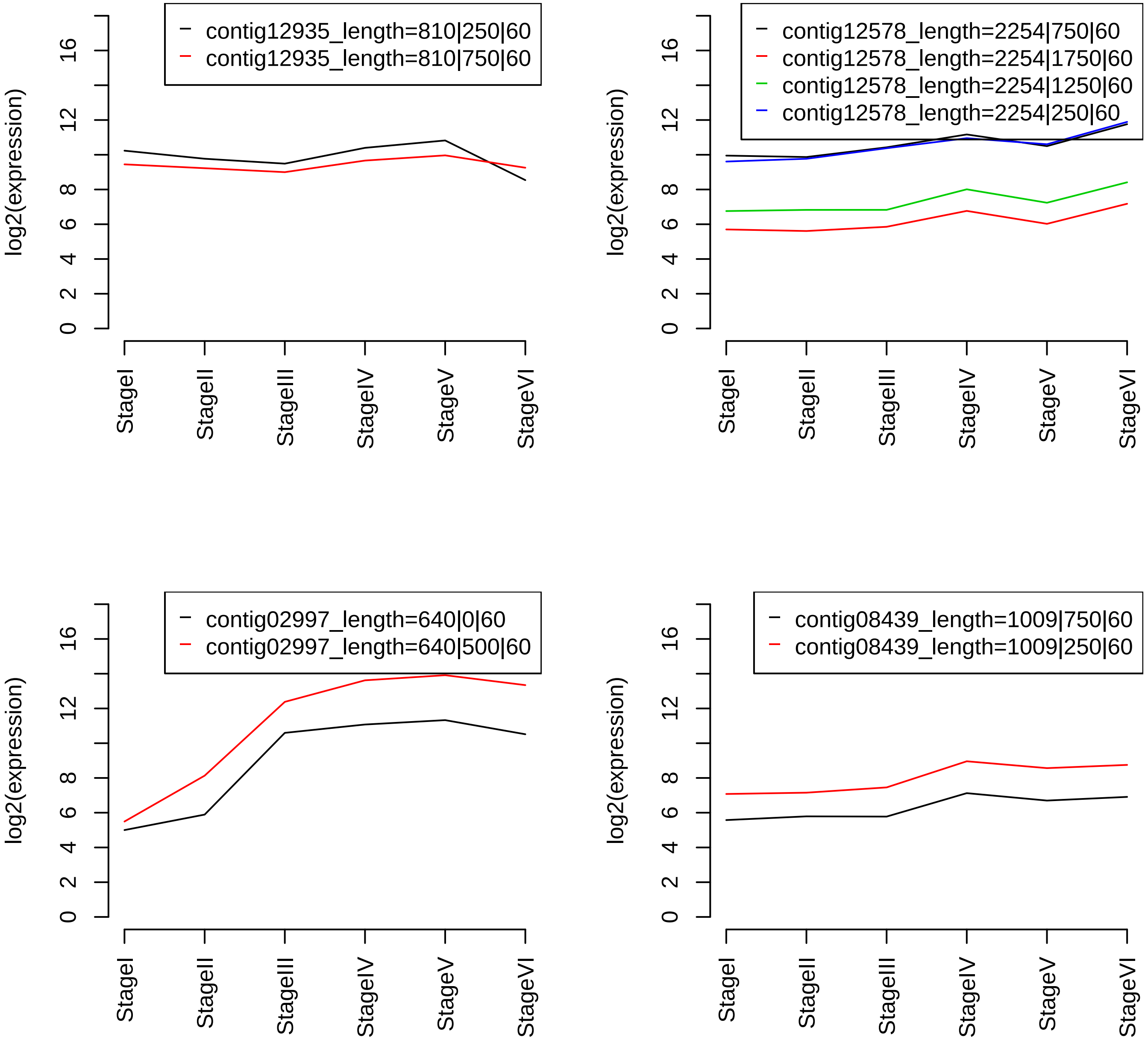
contig12935_length=810|250|60
contig12935_length=810|750|60
contig12578_length=2254|750|60
contig12578_length=2254|1750|60
contig12578_length=2254|1250|60
contig12578_length=2254|250|60
contig02997_length=640|0|60
contig02997_length=640|500|60
contig08439_length=1009|750|60
contig08439_length=1009|250|60
log2(expression)
0 2 4 6 8 12 16
StageI StageII StageIII StageIV StageV StageVI

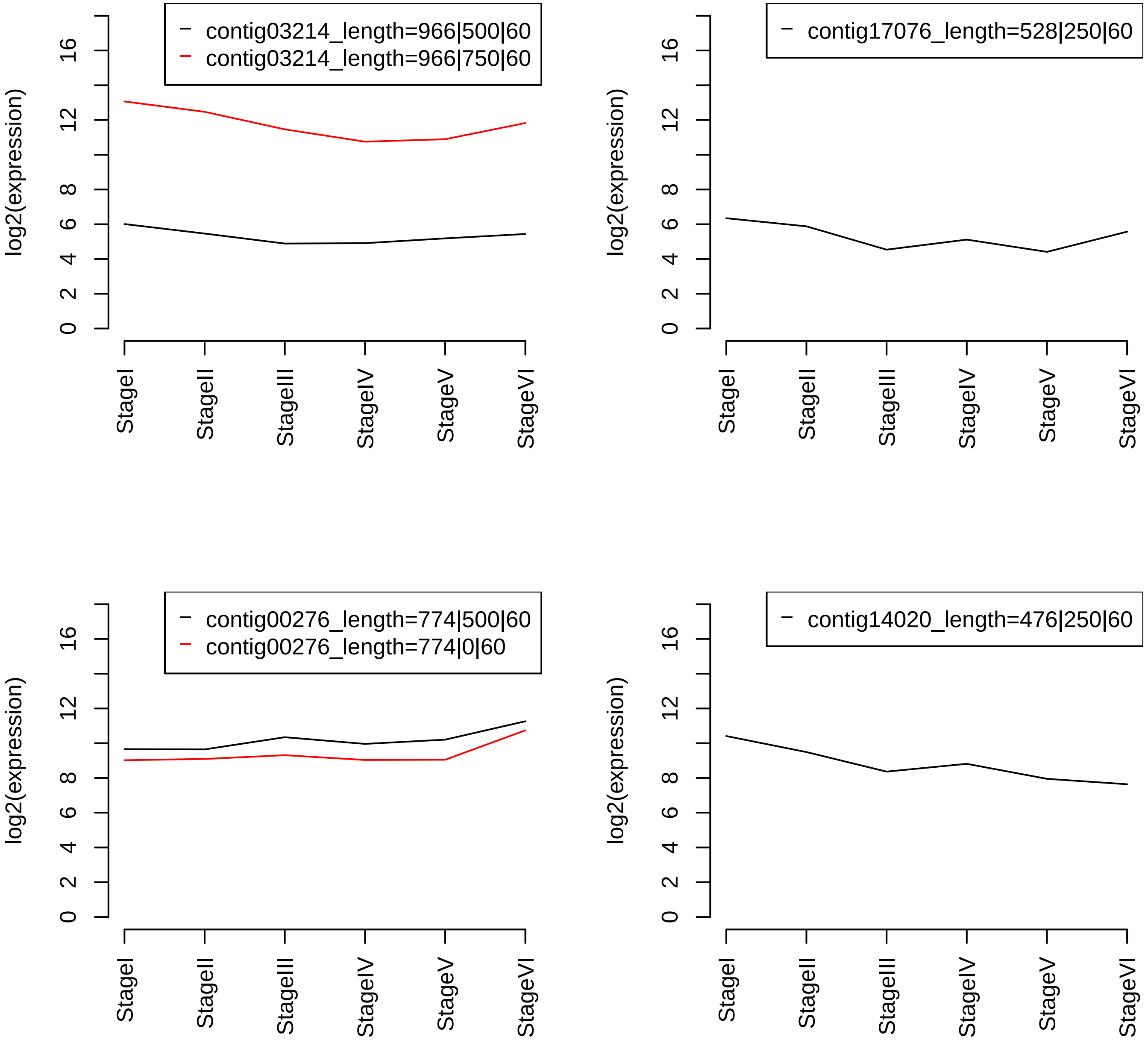
contig03214_length=966|500|60
contig03214_length=966|750|60
log2(expression)
0
2
4
6
8
12
16
StageI
StageII
StageIII
StageIV
StageV
StageVI
contig17076_length=528|250|60
contig00276_length=774|500|60
contig00276_length=774|0|60
contig14020_length=476|250|60

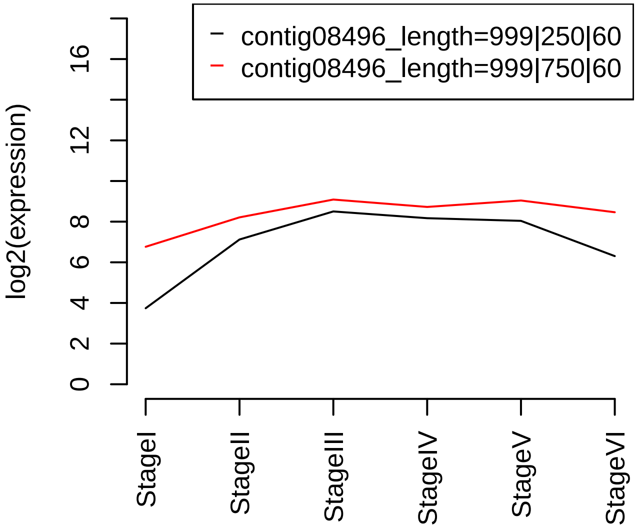

contig08496_length=999|250|60
contig08496_length=999|750|60
log2(expression)
0
2
4
6
8
12
16
StageI
StageII
StageIII
StageIV
StageV
StageVI

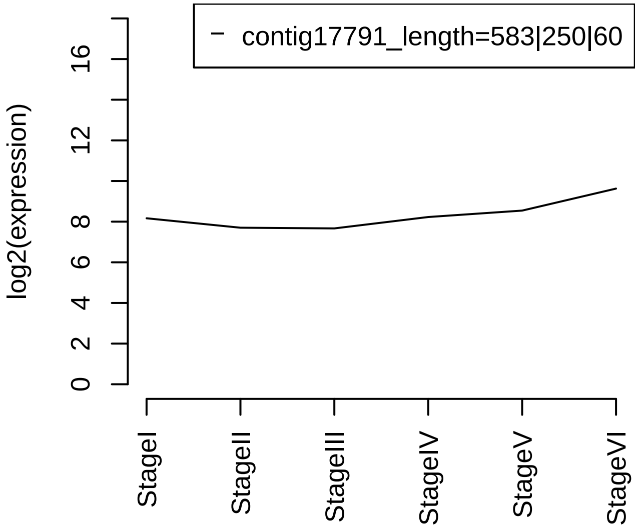

contig17791_length=583|250|60
log2(expression)
0
2
4
6
8
12
16
StageI
StageII
StageIII
StageIV
StageV
StageVI

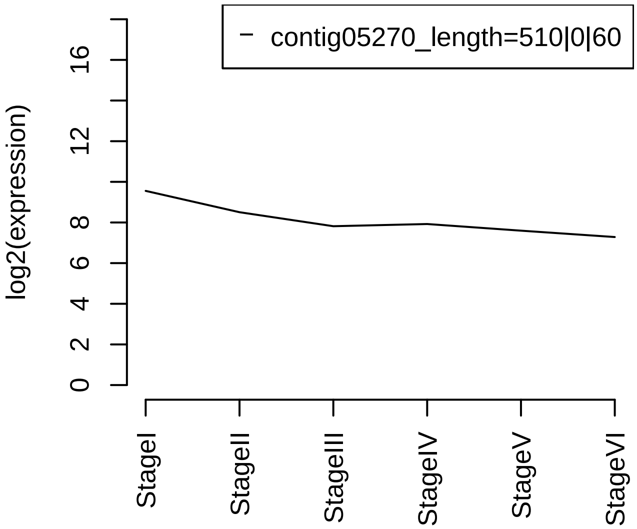

contig05270_length=510|0|60
log2(expression)
0
2
4
6
8
12
16
StageI
StageII
StageIII
StageIV
StageV
StageVI

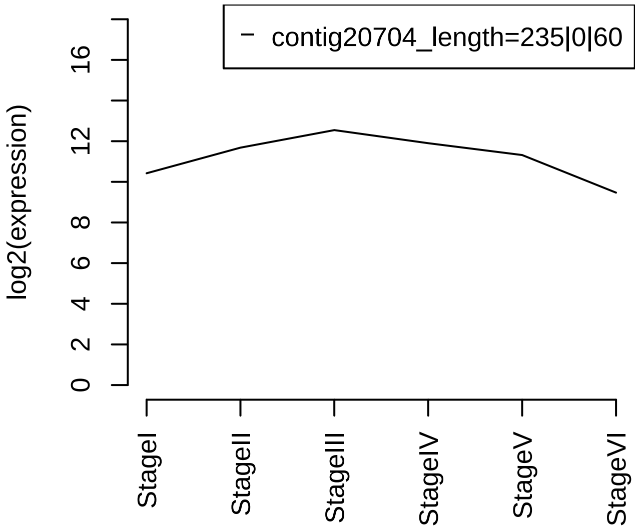

contig20704_length=235|0|60
log2(expression)
0
2
4
6
8
12
16
StageI
StageII
StageIII
StageIV
StageV
StageVI

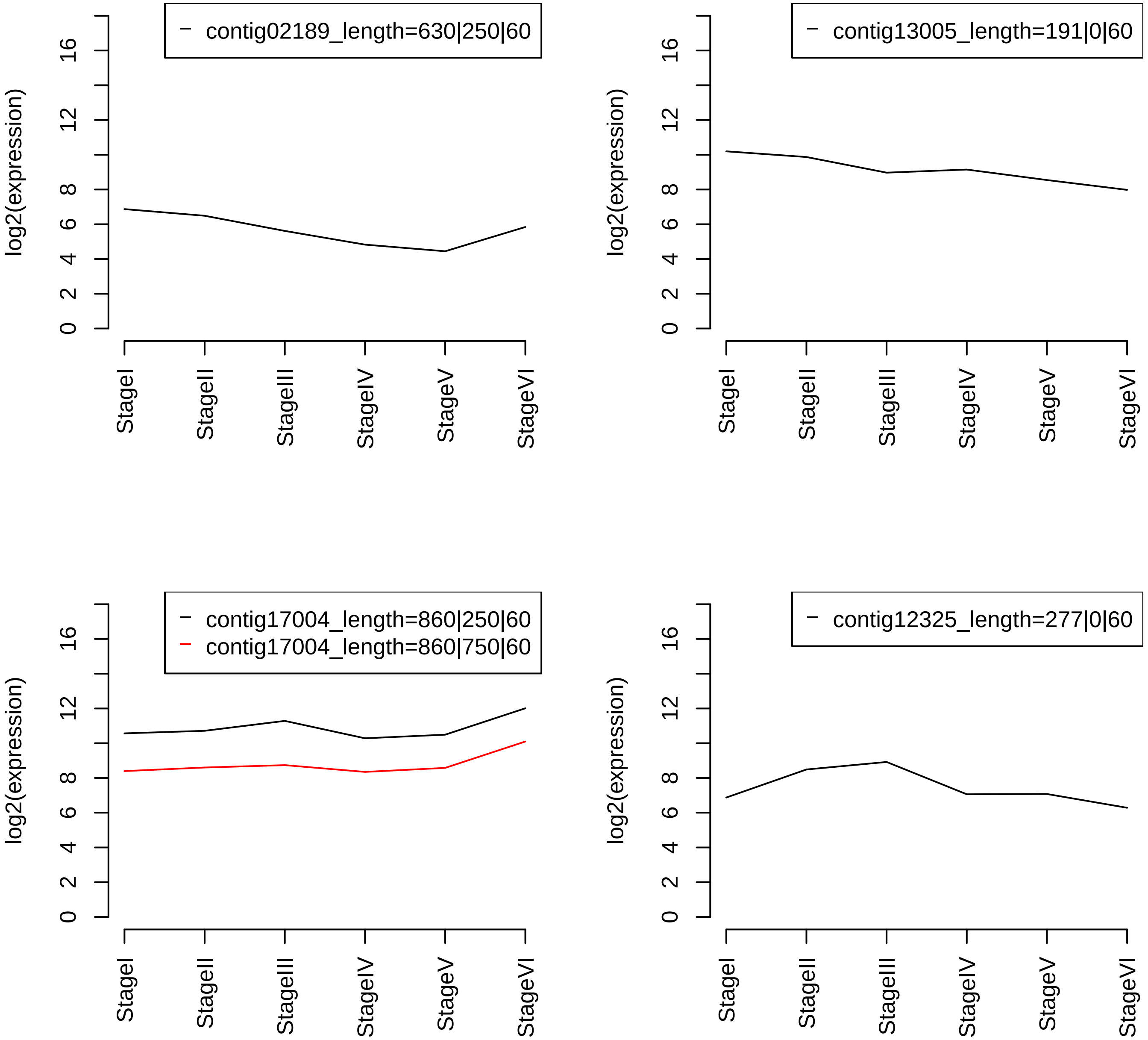
contig02189_length=630|250|60
contig13005_length=191|0|60
contig17004_length=860|250|60
contig17004_length=860|750|60
contig12325_length=277|0|60
log2(expression)
0
2
4
6
8
12
16
StageI
StageII
StageIII
StageIV
StageV
StageVI

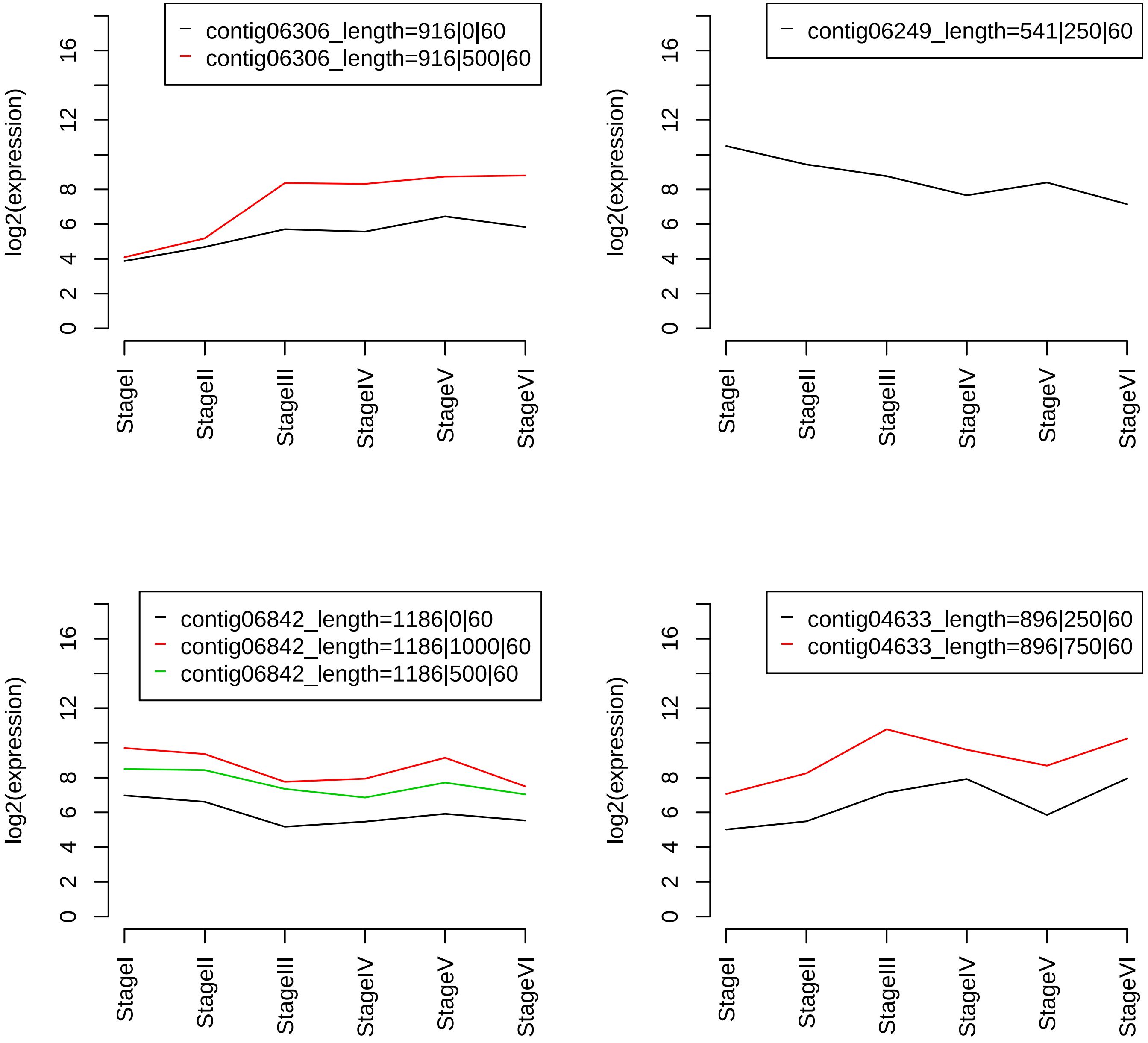

contig06306_length=916|0|60
contig06306_length=916|500|60
contig06249_length=541|250|60
contig06842_length=1186|0|60
contig06842_length=1186|1000|60
contig06842_length=1186|500|60
contig04633_length=896|250|60
contig04633_length=896|750|60
log2(expression)
0
2
4
6
8
12
16
StageI
StageII
StageIII
StageIV
StageV
StageVI

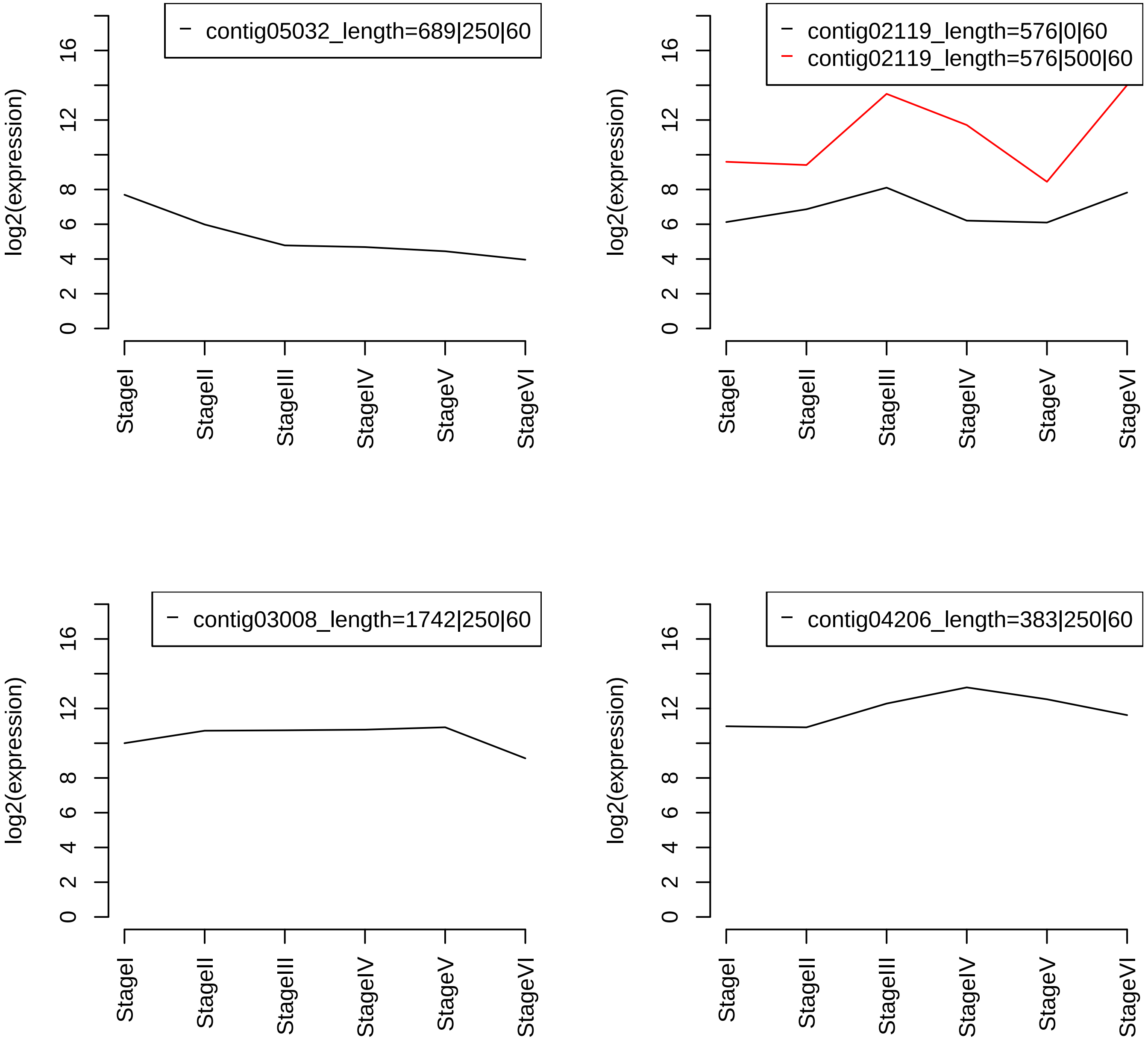
contig05032_length=689|250|60
contig02119_length=576|0|60
contig02119_length=576|500|60
contig03008_length=1742|250|60
contig04206_length=383|250|60
log2(expression)
0
2
4
6
8
12
16
StageI
StageII
StageIII
StageIV
StageV
StageVI

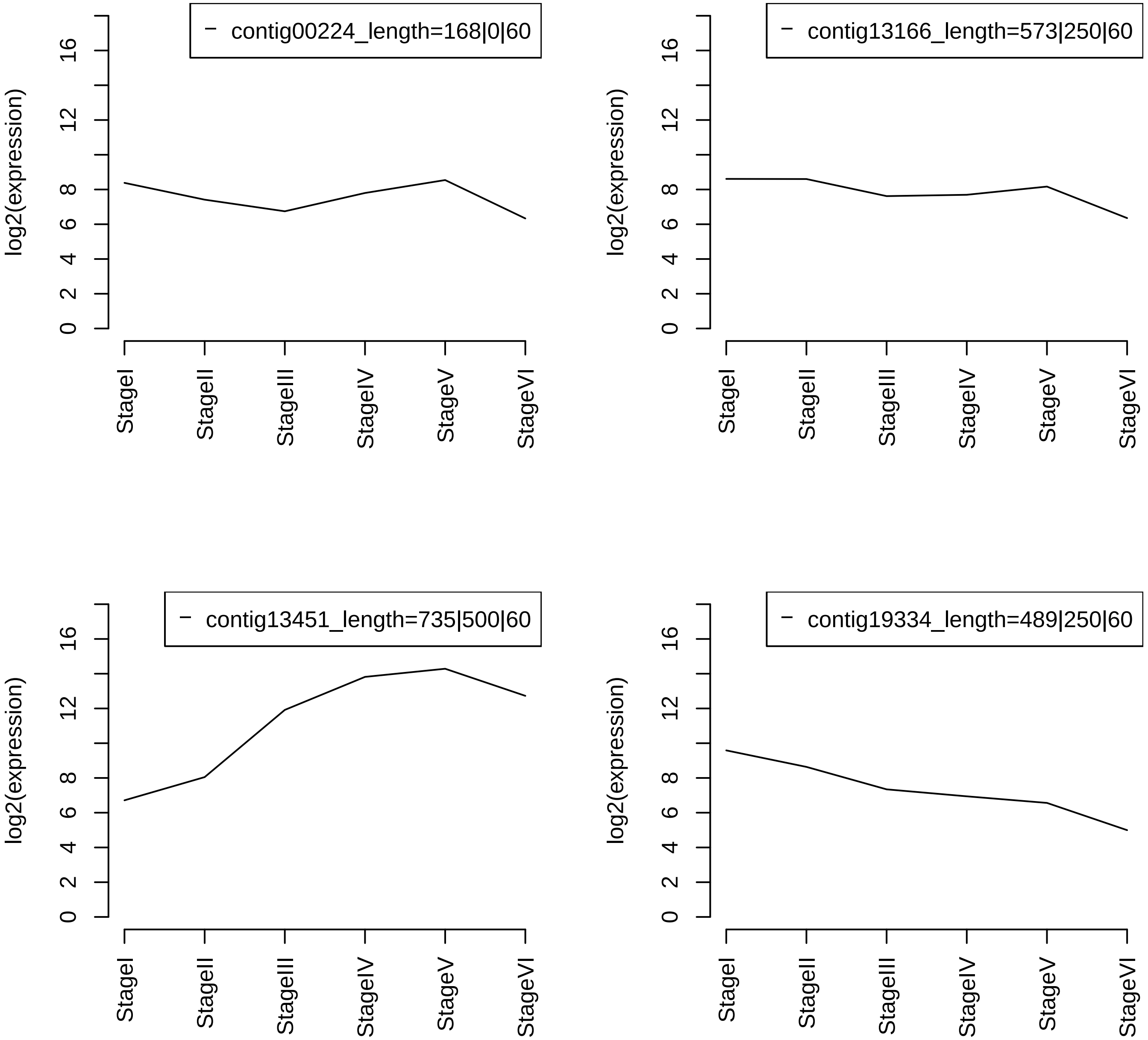
contig00224_length=168|0|60
contig13166_length=573|250|60
contig13451_length=735|500|60
contig19334_length=489|250|60
log2(expression)
0
2
4
6
8
12
16
StageI
StageII
StageIII
StageIV
StageV
StageVI

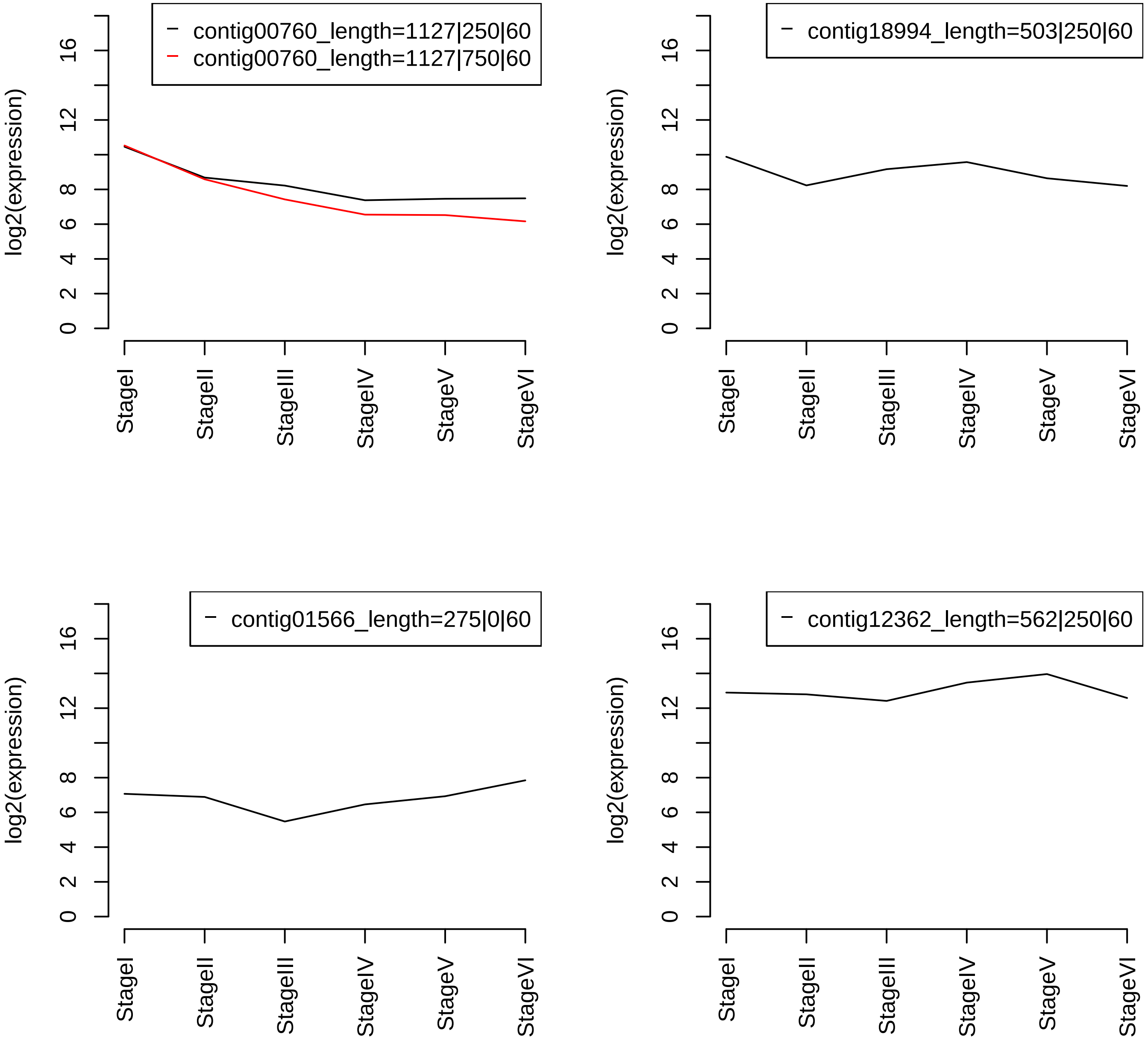
contig00760_length=1127|250|60
contig00760_length=1127|750|60
contig18994_length=503|250|60
contig01566_length=275|0|60
contig12362_length=562|250|60
log2(expression)
StageI
StageII
StageIII
StageIV
StageV
StageVI

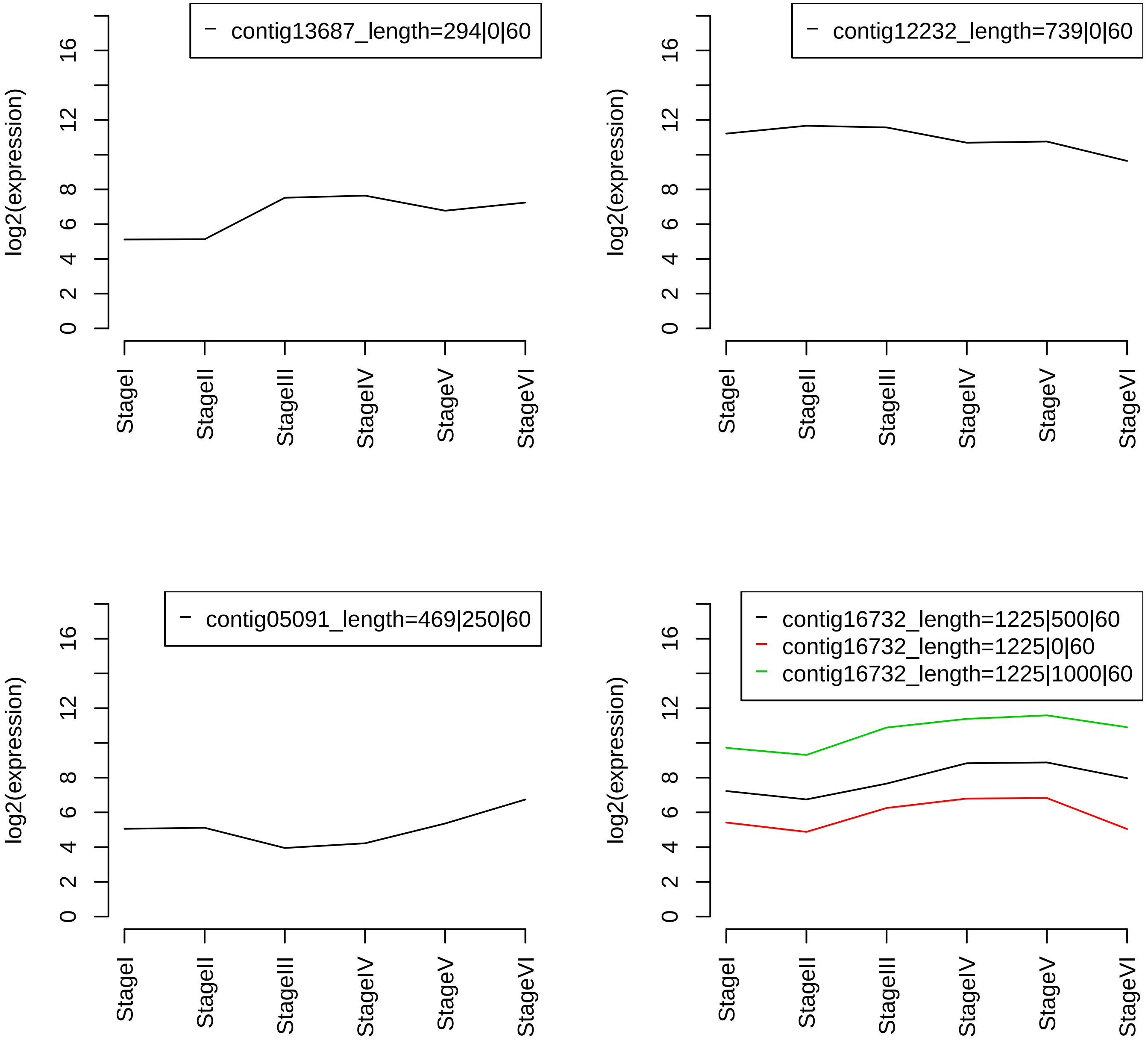
contig13687_length=294|0|60
contig12232_length=739|0|60
contig05091_length=469|250|60
contig16732_length=1225|500|60
contig16732_length=1225|0|60
contig16732_length=1225|1000|60
log2(expression)
0
2
4
6
8
12
16
StageI
StageII
StageIII
StageIV
StageV
StageVI

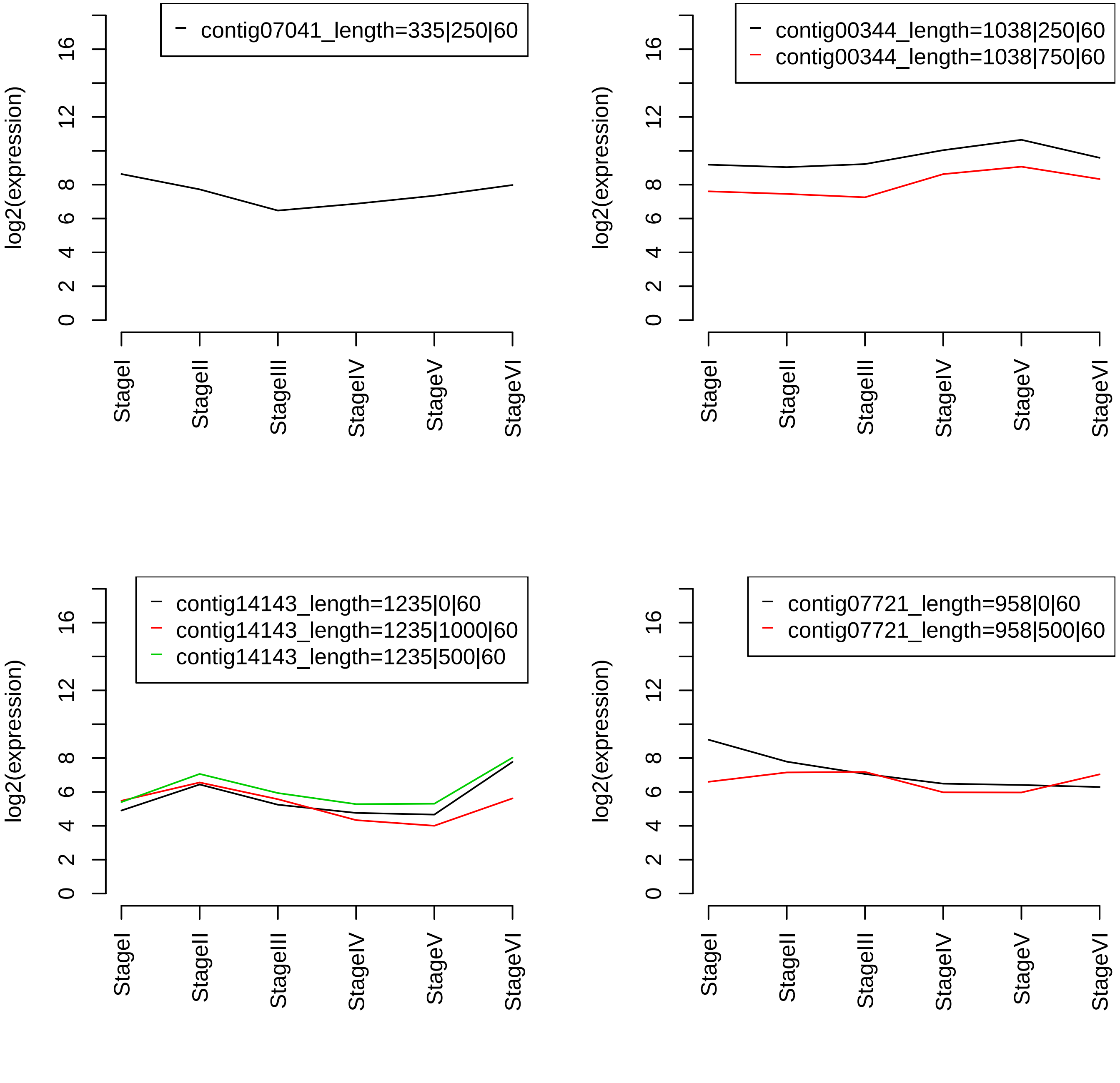

contig07041_length=335|250|60
contig00344_length=1038|250|60
contig00344_length=1038|750|60
contig14143_length=1235|0|60
contig14143_length=1235|1000|60
contig14143_length=1235|500|60
contig07721_length=958|0|60
contig07721_length=958|500|60
log2(expression)
0
2
4
6
8
12
16
StageI
StageII
StageIII
StageIV
StageV
StageVI

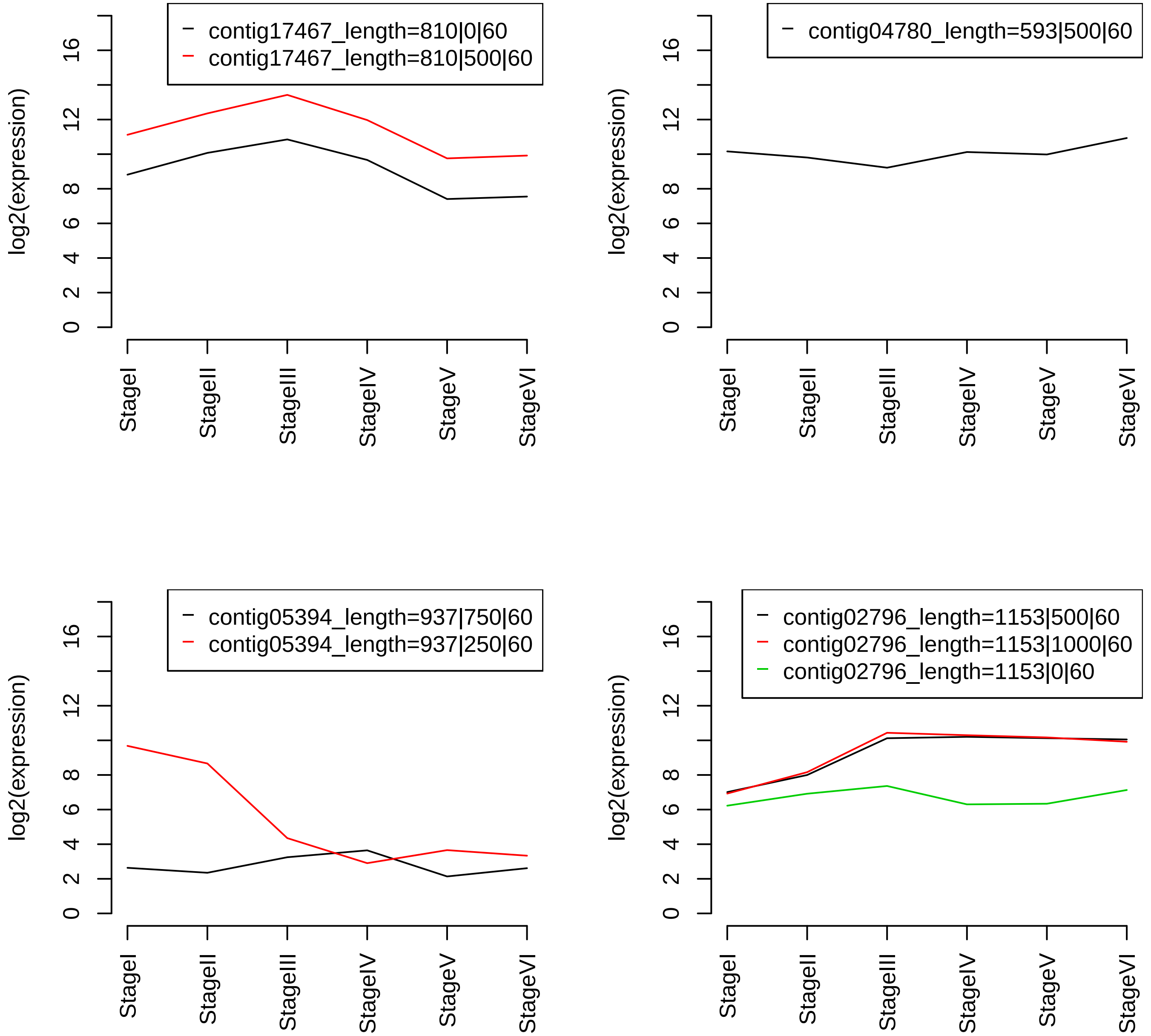
contig17467_length=810|0|60
contig17467_length=810|500|60
log2(expression)
0
2
4
6
8
12
16
StageI
StageII
StageIII
StageIV
StageV
StageVI
contig04780_length=593|500|60
log2(expression)
0
2
4
6
8
12
16
StageI
StageII
StageIII
StageIV
StageV
StageVI
contig05394_length=937|750|60
contig05394_length=937|250|60
log2(expression)
0
2
4
6
8
12
16
StageI
StageII
StageIII
StageIV
StageV
StageVI
contig02796_length=1153|500|60
contig02796_length=1153|1000|60
contig02796_length=1153|0|60
log2(expression)
0
2
4
6
8
12
16
StageI
StageII
StageIII
StageIV
StageV
StageVI

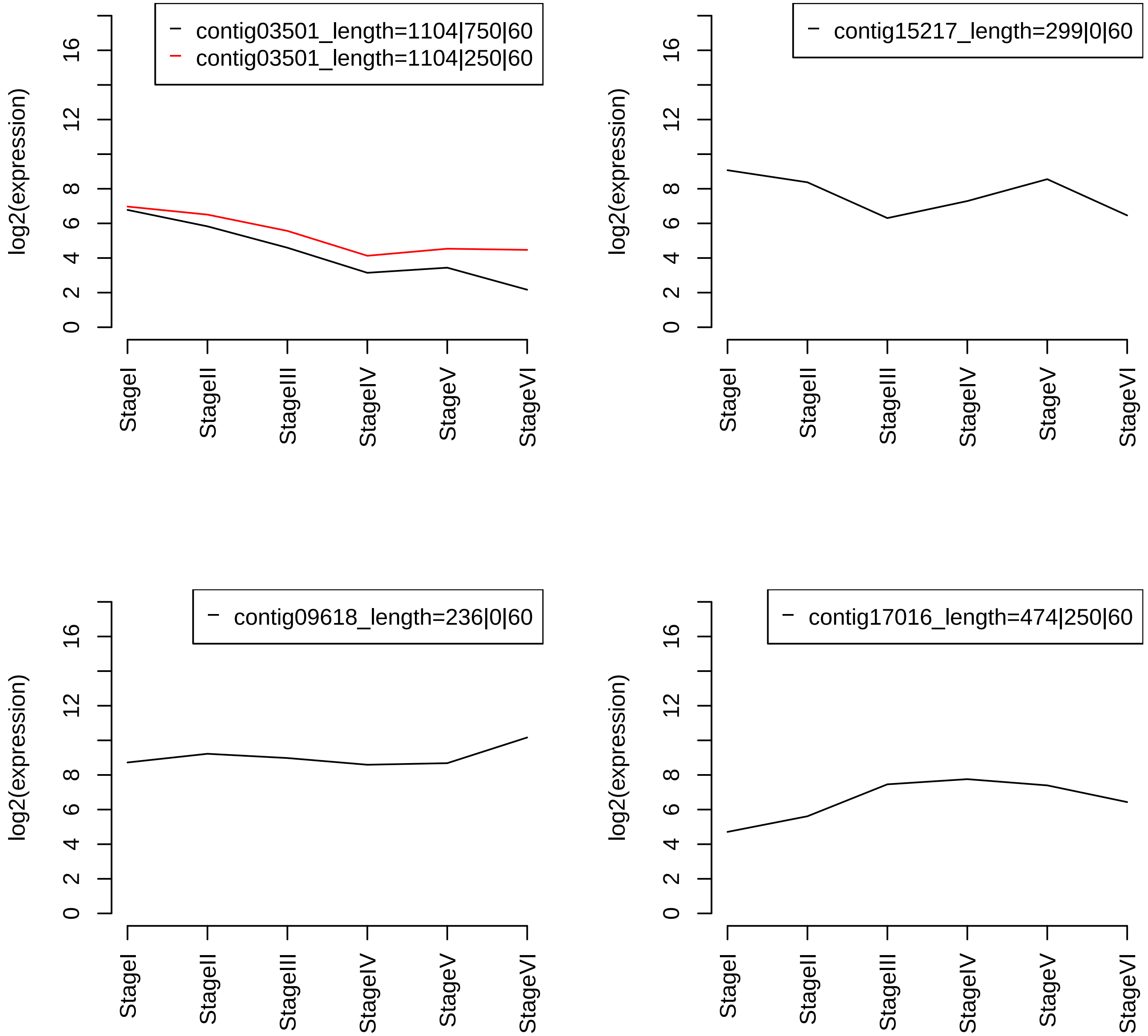
contig03501_length=1104|750|60
contig03501_length=1104|250|60
contig15217_length=299|0|60
contig09618_length=236|0|60
contig17016_length=474|250|60
log2(expression)
0
2
4
6
8
12
16
StageI
StageII
StageIII
StageIV
StageV
StageVI

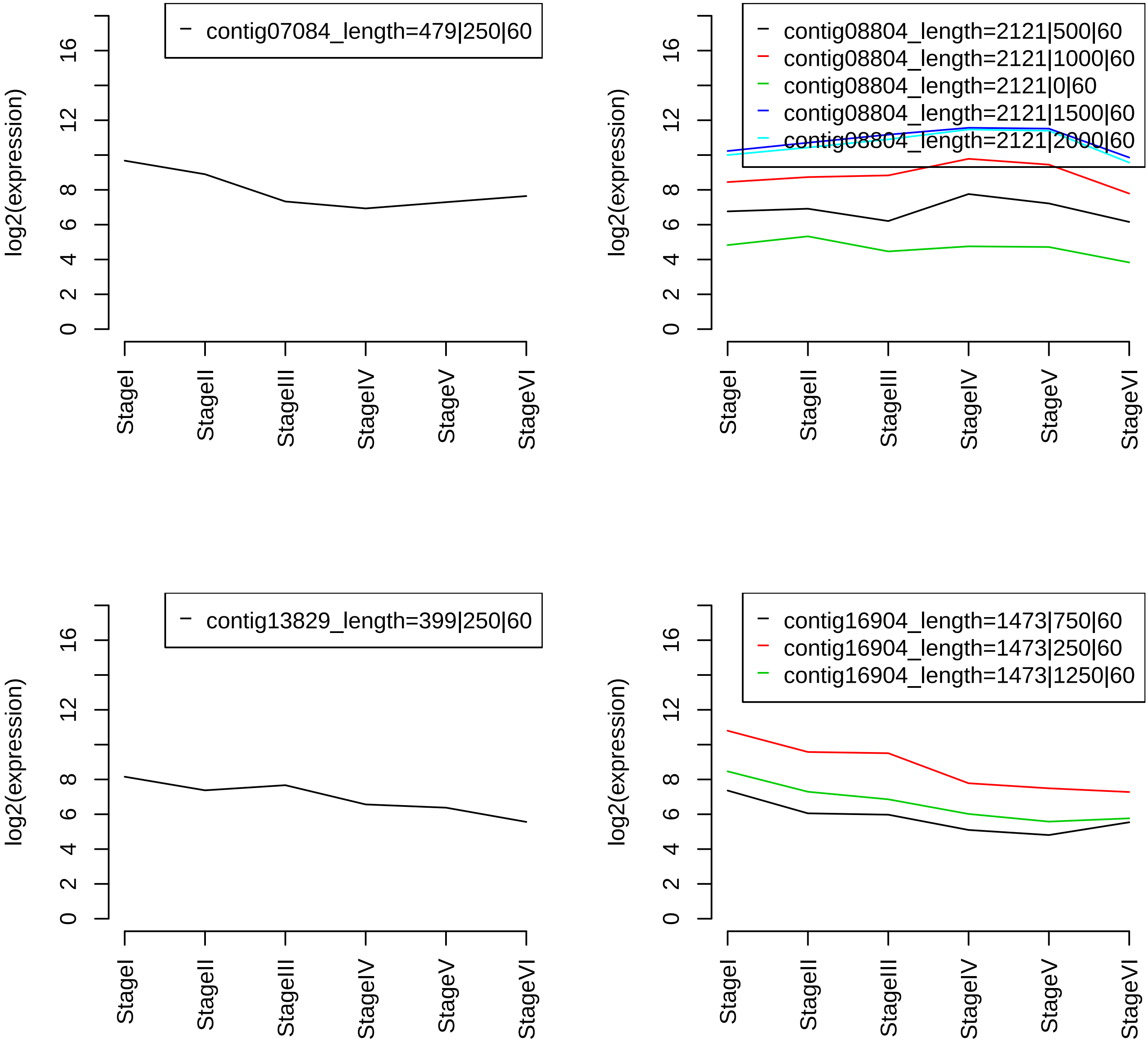
contig07084_length=479|250|60
log2(expression)
0
2
4
6
8
12
16
StageI
StageII
StageIII
StageIV
StageV
StageVI
contig08804_length=2121|500|60
contig08804_length=2121|1000|60
contig08804_length=2121|0|60
contig08804_length=2121|1500|60
contig08804_length=2121|2000|60
contig13829_length=399|250|60
contig16904_length=1473|750|60
contig16904_length=1473|250|60
contig16904_length=1473|1250|60

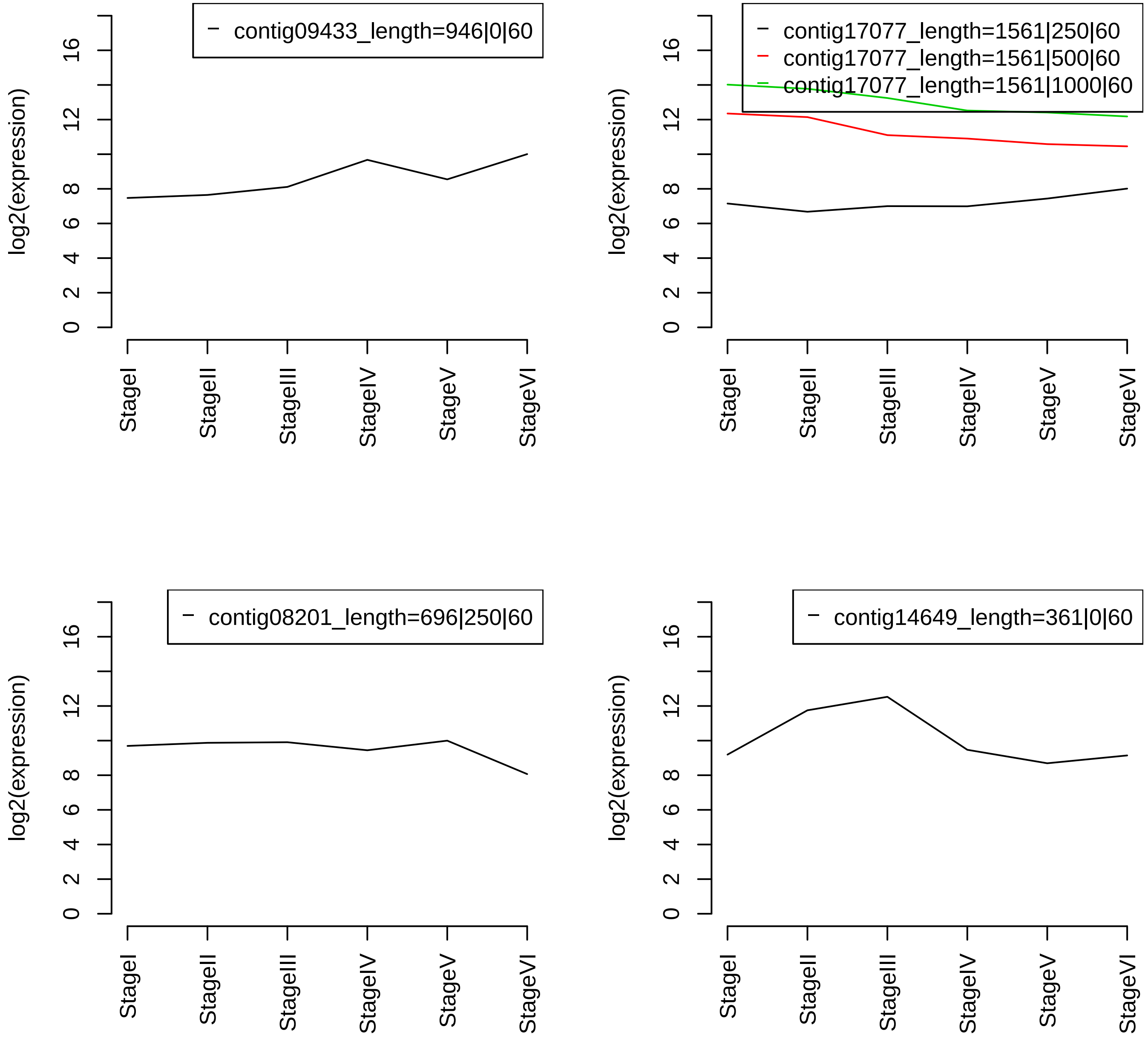

contig09433_length=946|0|60
contig17077_length=1561|250|60
contig17077_length=1561|500|60
contig17077_length=1561|1000|60
contig08201_length=696|250|60
contig14649_length=361|0|60
log2(expression)
0
2
4
6
8
12
16
StageI
StageII
StageIII
StageIV
StageV
StageVI

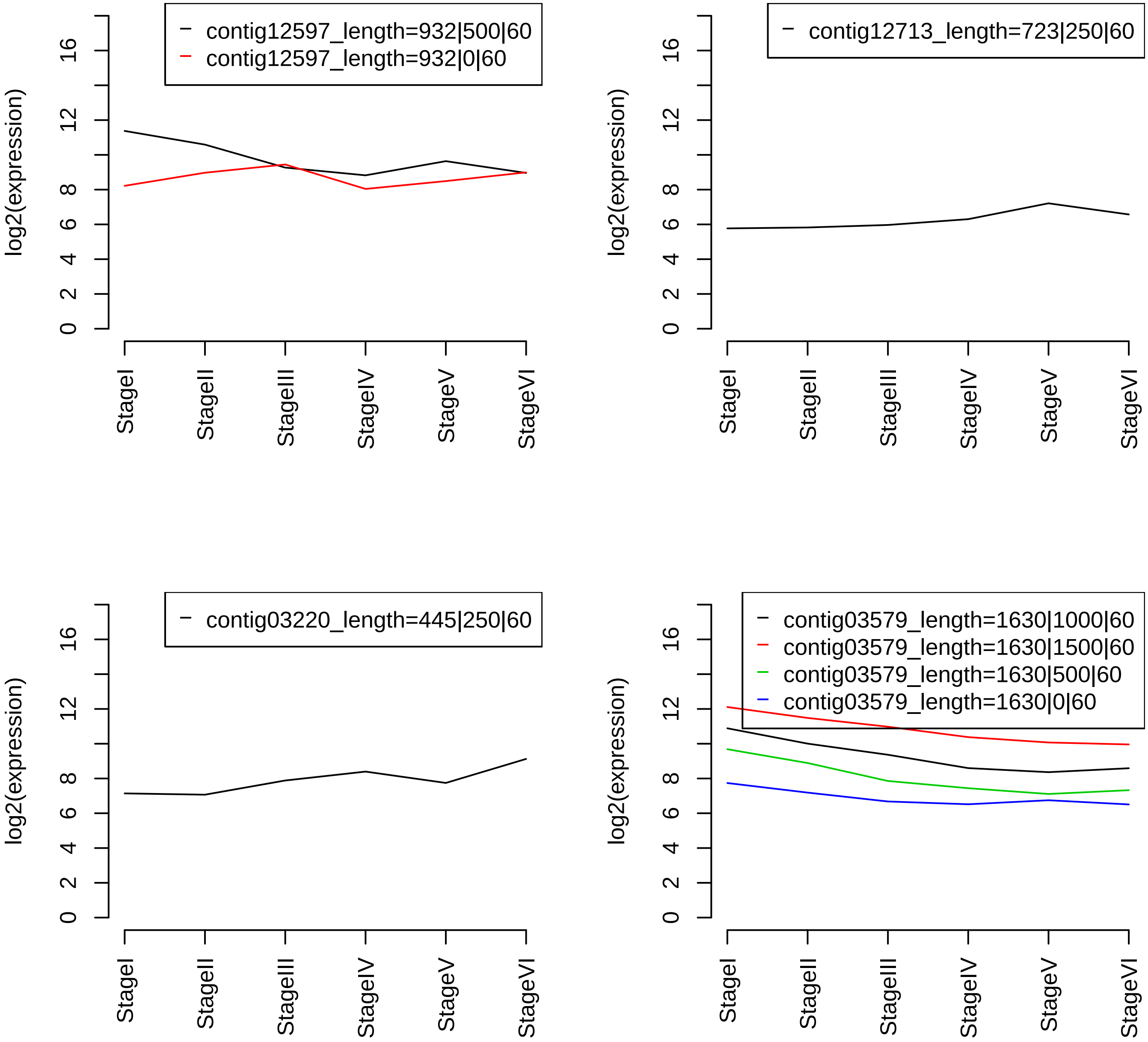
contig12597_length=932|500|60
contig12597_length=932|0|60
contig12713_length=723|250|60
contig03220_length=445|250|60
contig03579_length=1630|1000|60
contig03579_length=1630|1500|60
contig03579_length=1630|500|60
contig03579_length=1630|0|60
log2(expression)
0 2 4 6 8 12 16
StageI StageII StageIII StageIV StageV StageVI

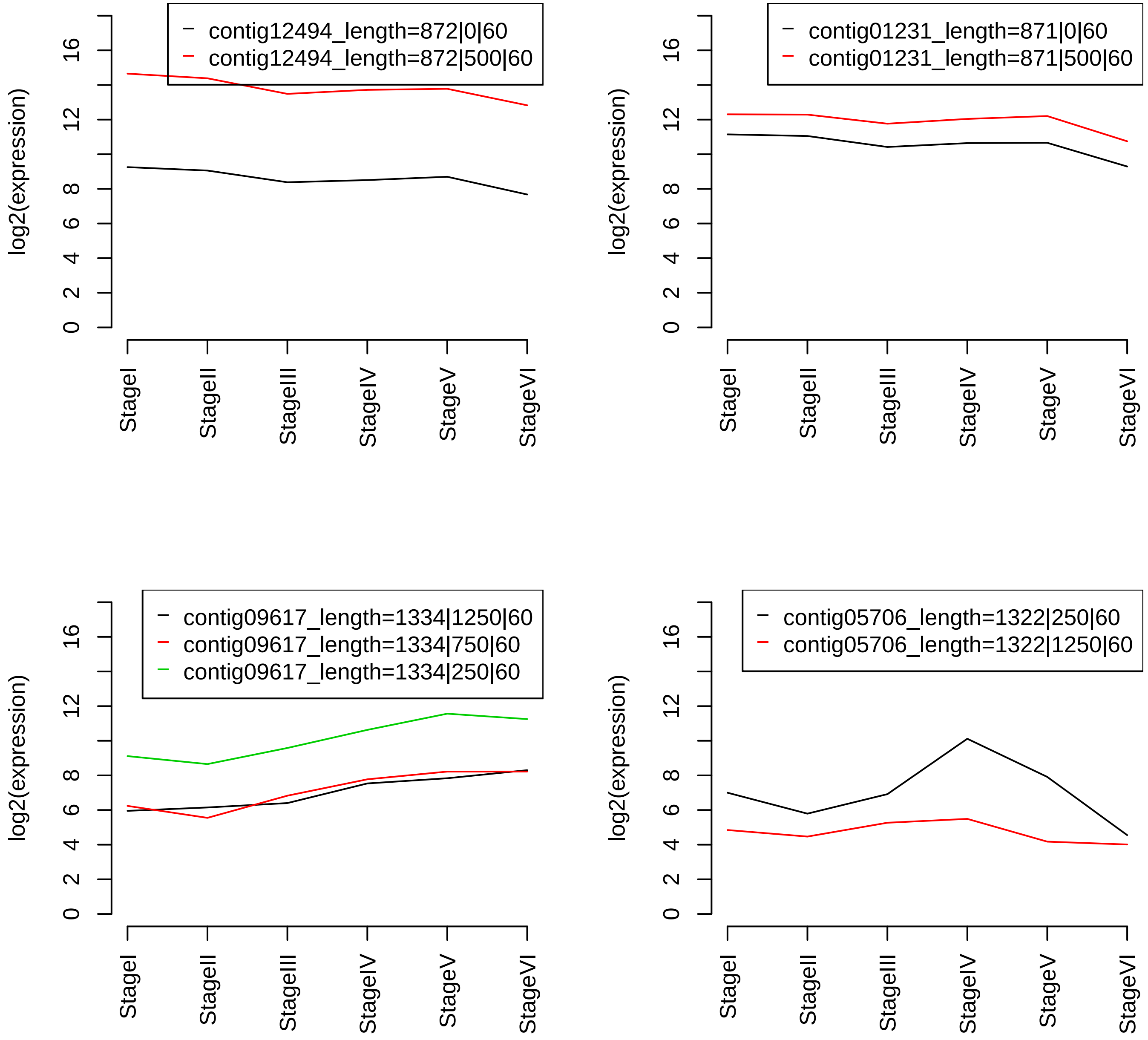
contig12494_length=872|0|60
contig12494_length=872|500|60
log2(expression)
0
2
4
6
8
12
16
StageI
StageII
StageIII
StageIV
StageV
StageVI
contig01231_length=871|0|60
contig01231_length=871|500|60
log2(expression)
StageI
StageII
StageIII
StageIV
StageV
StageVI
contig09617_length=1334|1250|60
contig09617_length=1334|750|60
contig09617_length=1334|250|60
log2(expression)
StageI
StageII
StageIII
StageIV
StageV
StageVI
contig05706_length=1322|250|60
contig05706_length=1322|1250|60
log2(expression)
StageI
StageII
StageIII
StageIV
StageV
StageVI

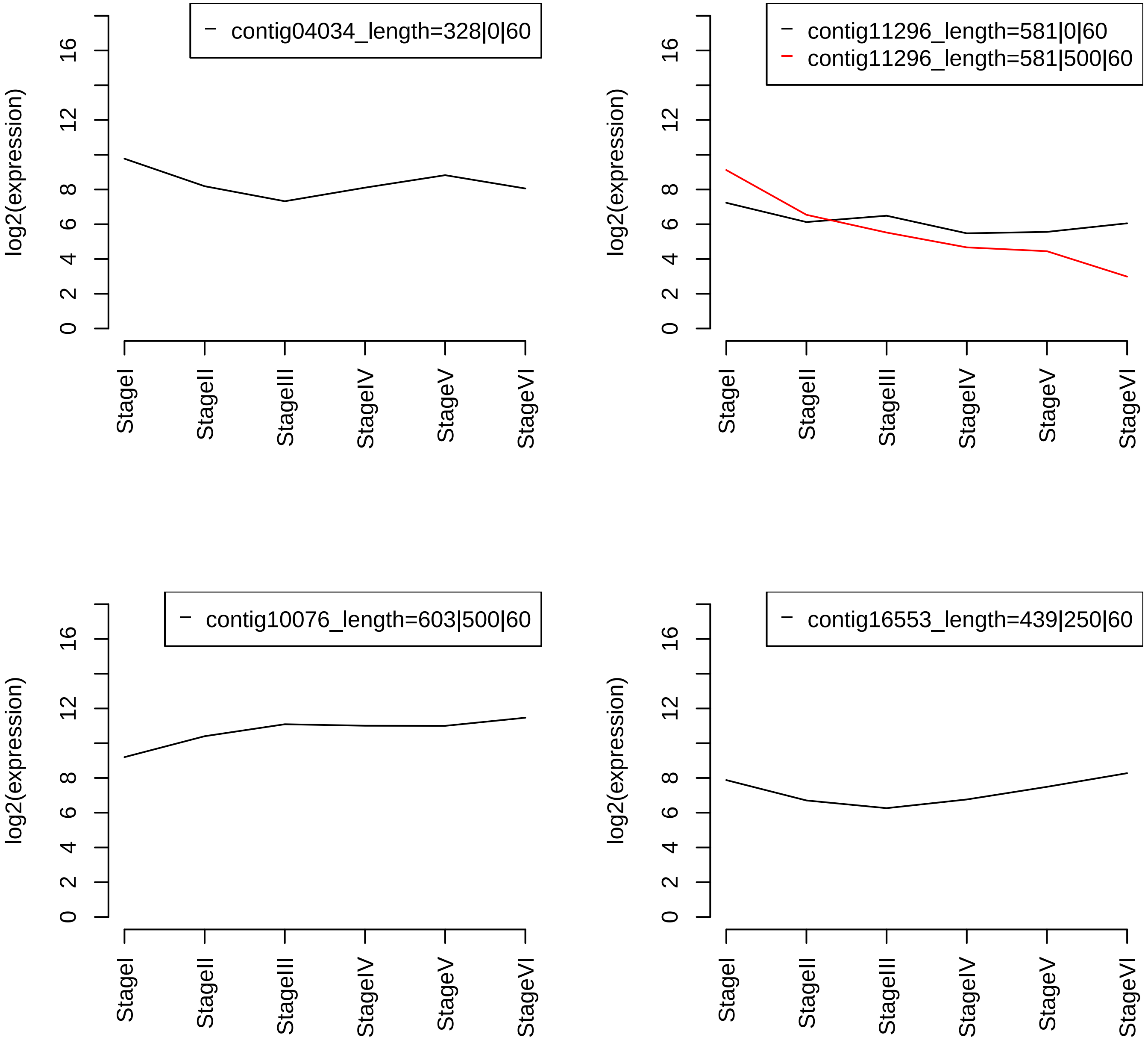

contig04034_length=328|0|60
contig11296_length=581|0|60
contig11296_length=581|500|60
contig10076_length=603|500|60
contig16553_length=439|250|60
log2(expression)
0
2
4
6
8
12
16
StageI
StageII
StageIII
StageIV
StageV
StageVI

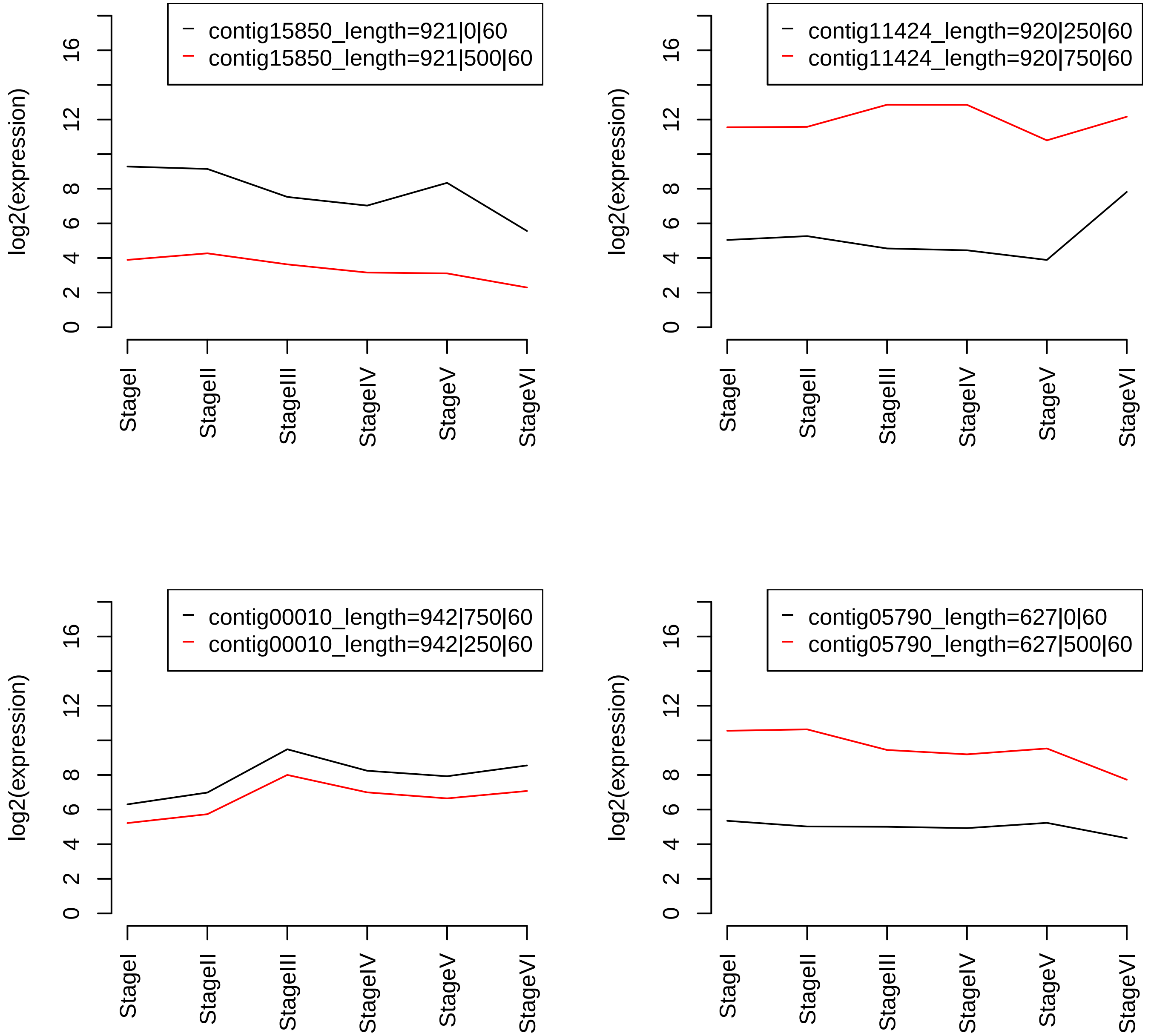
contig15850_length=921|0|60
contig15850_length=921|500|60
contig11424_length=920|250|60
contig11424_length=920|750|60
contig00010_length=942|750|60
contig00010_length=942|250|60
contig05790_length=627|0|60
contig05790_length=627|500|60
log2(expression)
0
2
4
6
8
12
16
StageI
StageII
StageIII
StageIV
StageV
StageVI

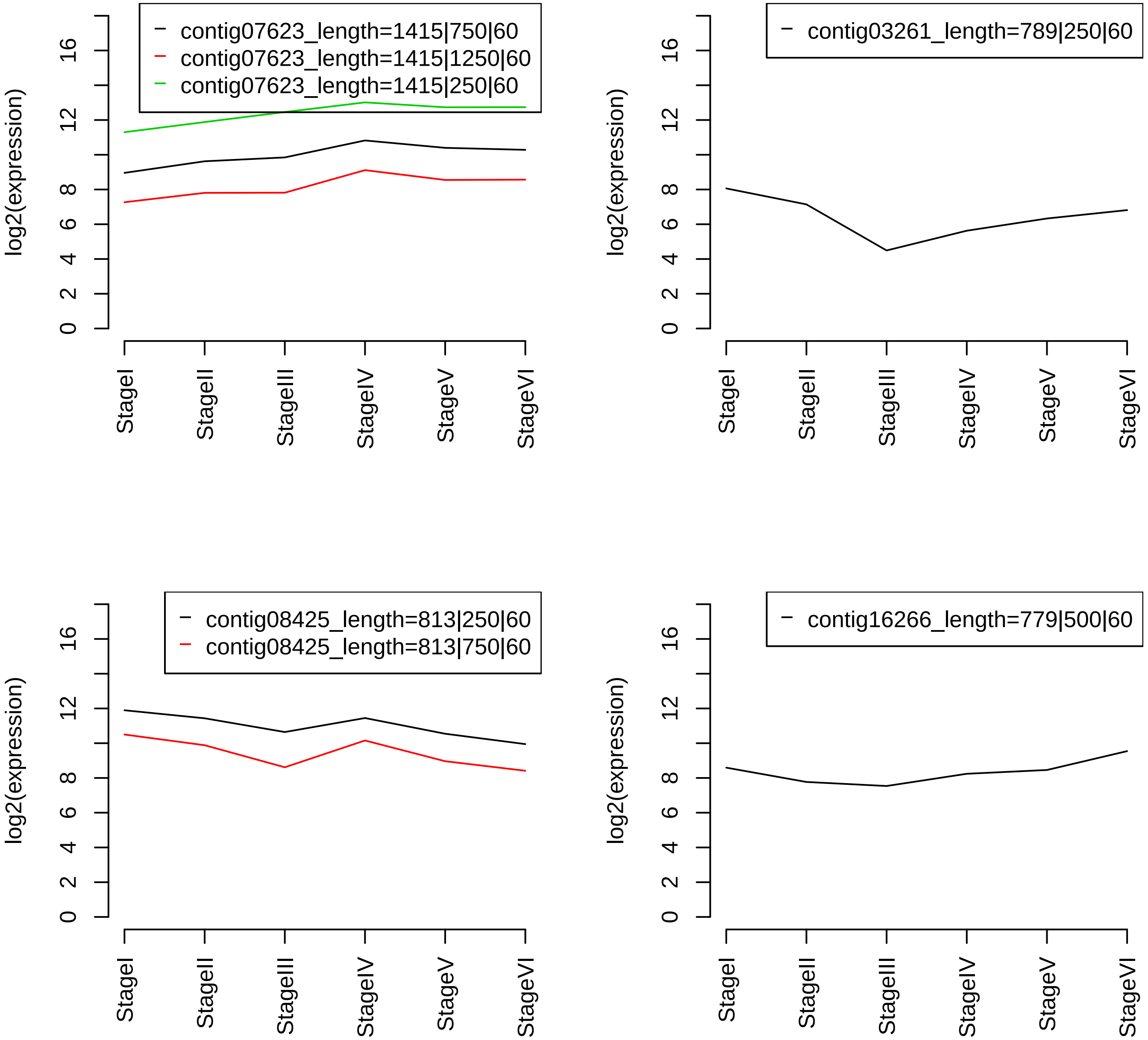
contig07623_length=1415|750|60
contig07623_length=1415|1250|60
contig07623_length=1415|250|60
contig03261_length=789|250|60
contig08425_length=813|250|60
contig08425_length=813|750|60
contig16266_length=779|500|60
log2(expression)
0
2
4
6
8
12
16
StageI
StageII
StageIII
StageIV
StageV
StageVI

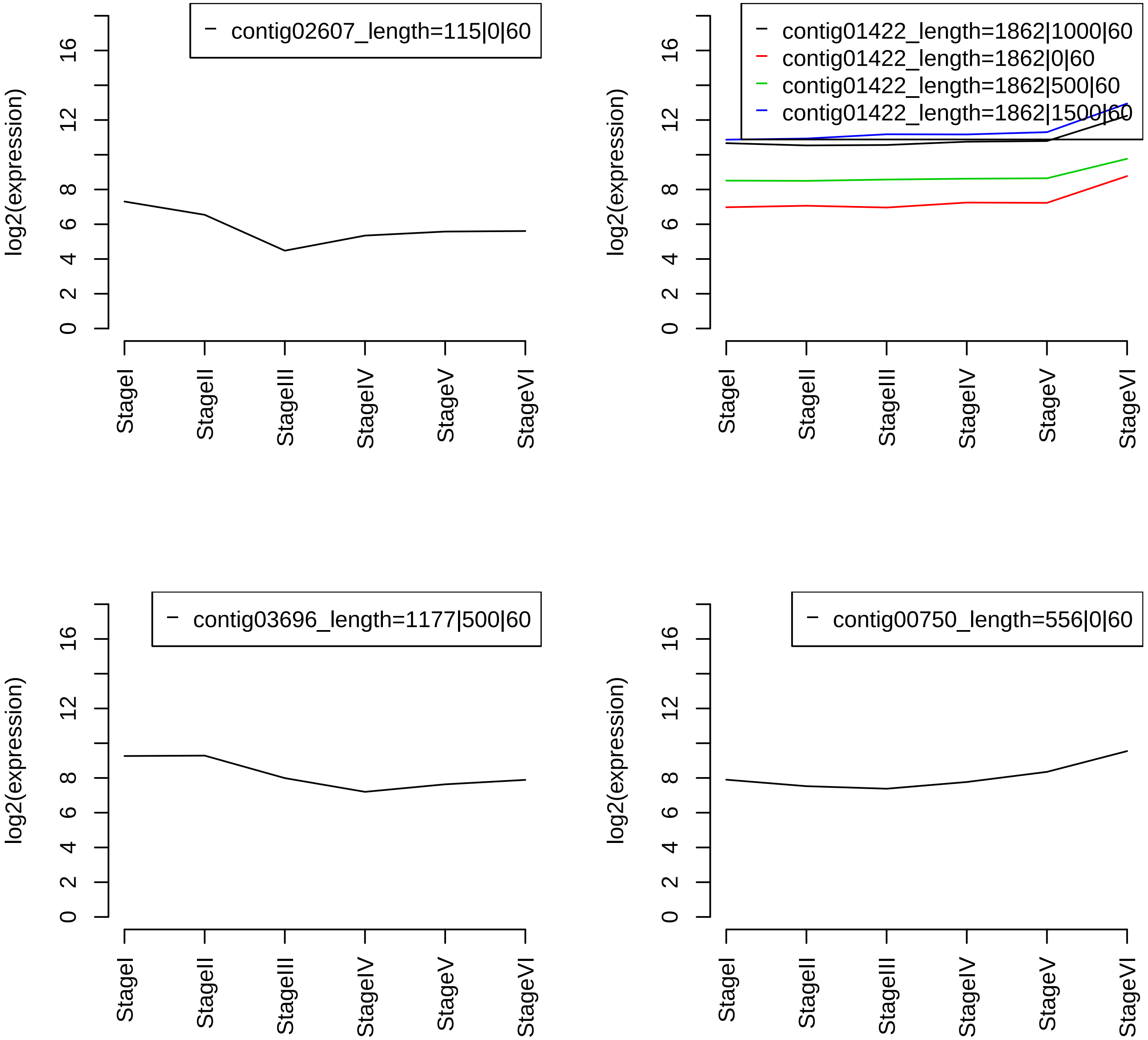

contig02607_length=115|0|60
contig01422_length=1862|1000|60
contig01422_length=1862|0|60
contig01422_length=1862|500|60
contig01422_length=1862|1500|60
contig03696_length=1177|500|60
contig00750_length=556|0|60
log2(expression)
0 2 4 6 8 12 16
StageI StageII StageIII StageIV StageV StageVI

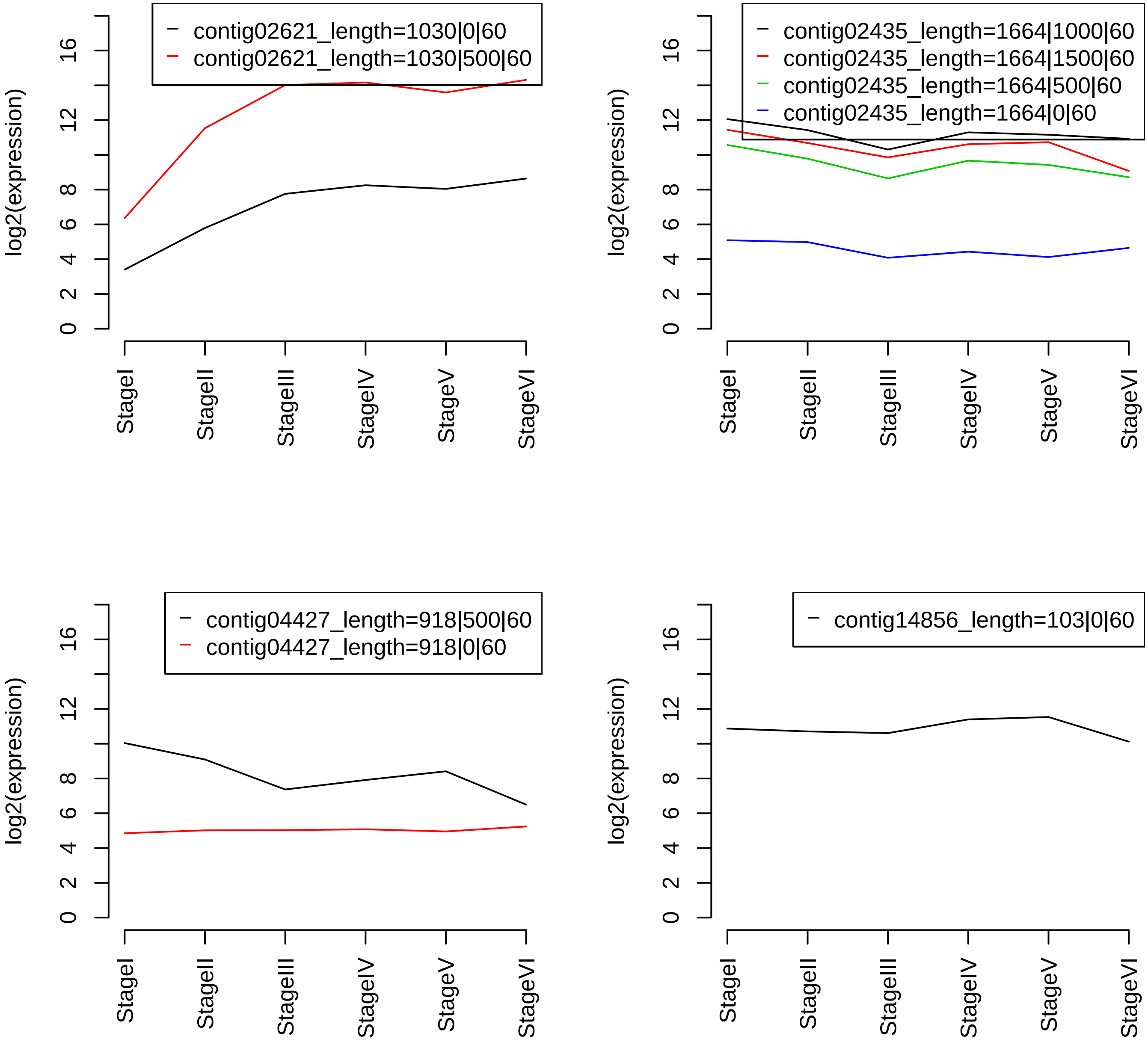
contig02621_length=1030|0|60
contig02621_length=1030|500|60
contig02435_length=1664|1000|60
contig02435_length=1664|1500|60
contig02435_length=1664|500|60
contig02435_length=1664|0|60
contig04427_length=918|500|60
contig04427_length=918|0|60
contig14856_length=103|0|60
log2(expression)
0
2
4
6
8
12
16
StageI
StageII
StageIII
StageIV
StageV
StageVI

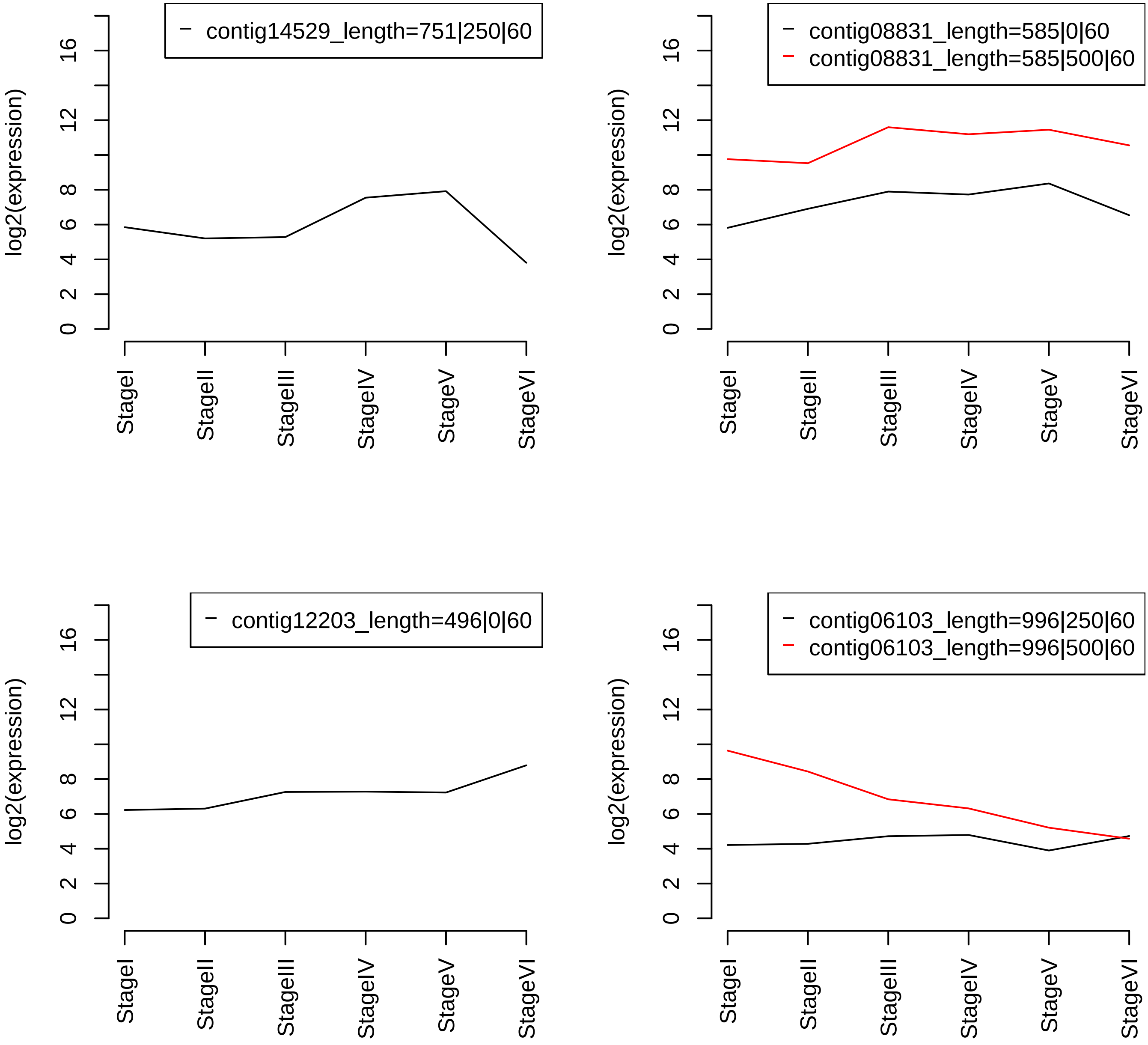
contig14529_length=751|250|60
contig08831_length=585|0|60
contig08831_length=585|500|60
contig12203_length=496|0|60
contig06103_length=996|250|60
contig06103_length=996|500|60
log2(expression)
0
2
4
6
8
12
16
StageI
StageII
StageIII
StageIV
StageV
StageVI

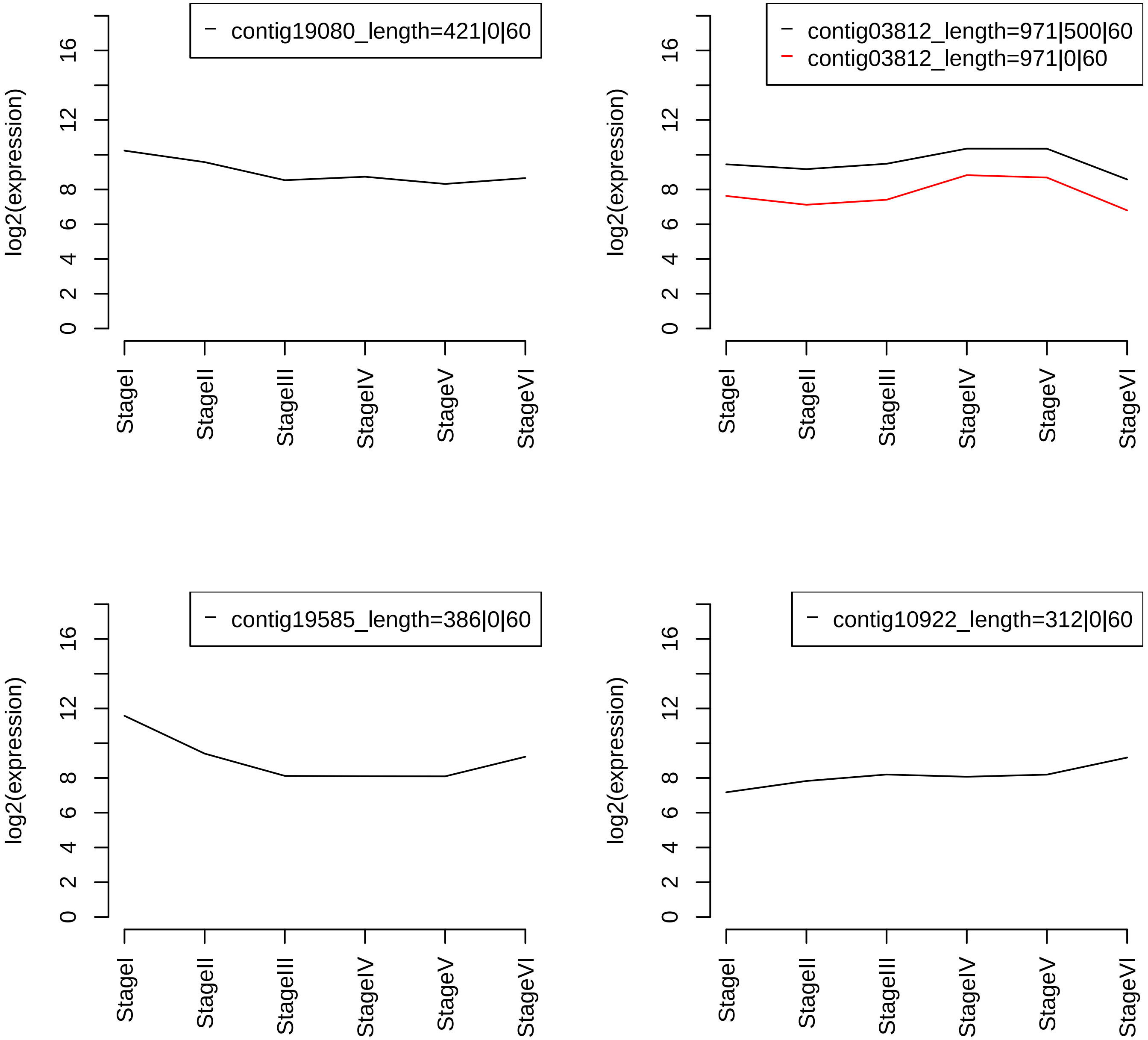

contig19080_length=421|0|60
contig03812_length=971|500|60
contig03812_length=971|0|60
contig19585_length=386|0|60
contig10922_length=312|0|60
log2(expression)
0
2
4
6
8
12
16
StageI
StageII
StageIII
StageIV
StageV
StageVI

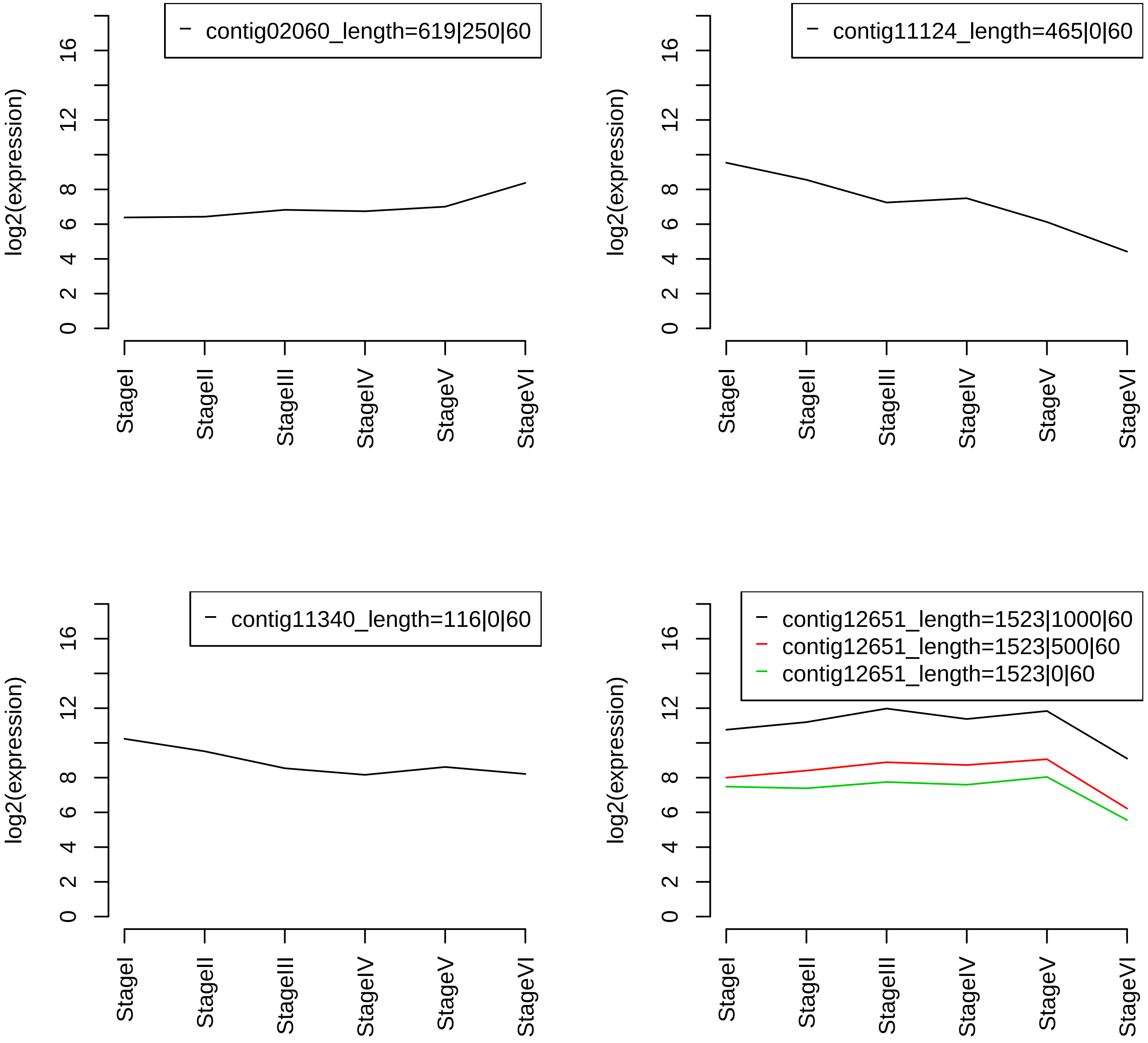

contig02060_length=619|250|60
contig11124_length=465|0|60
contig11340_length=116|0|60
contig12651_length=1523|1000|60
contig12651_length=1523|500|60
contig12651_length=1523|0|60
log2(expression)
0
2
4
6
8
12
16
StageI
StageII
StageIII
StageIV
StageV
StageVI

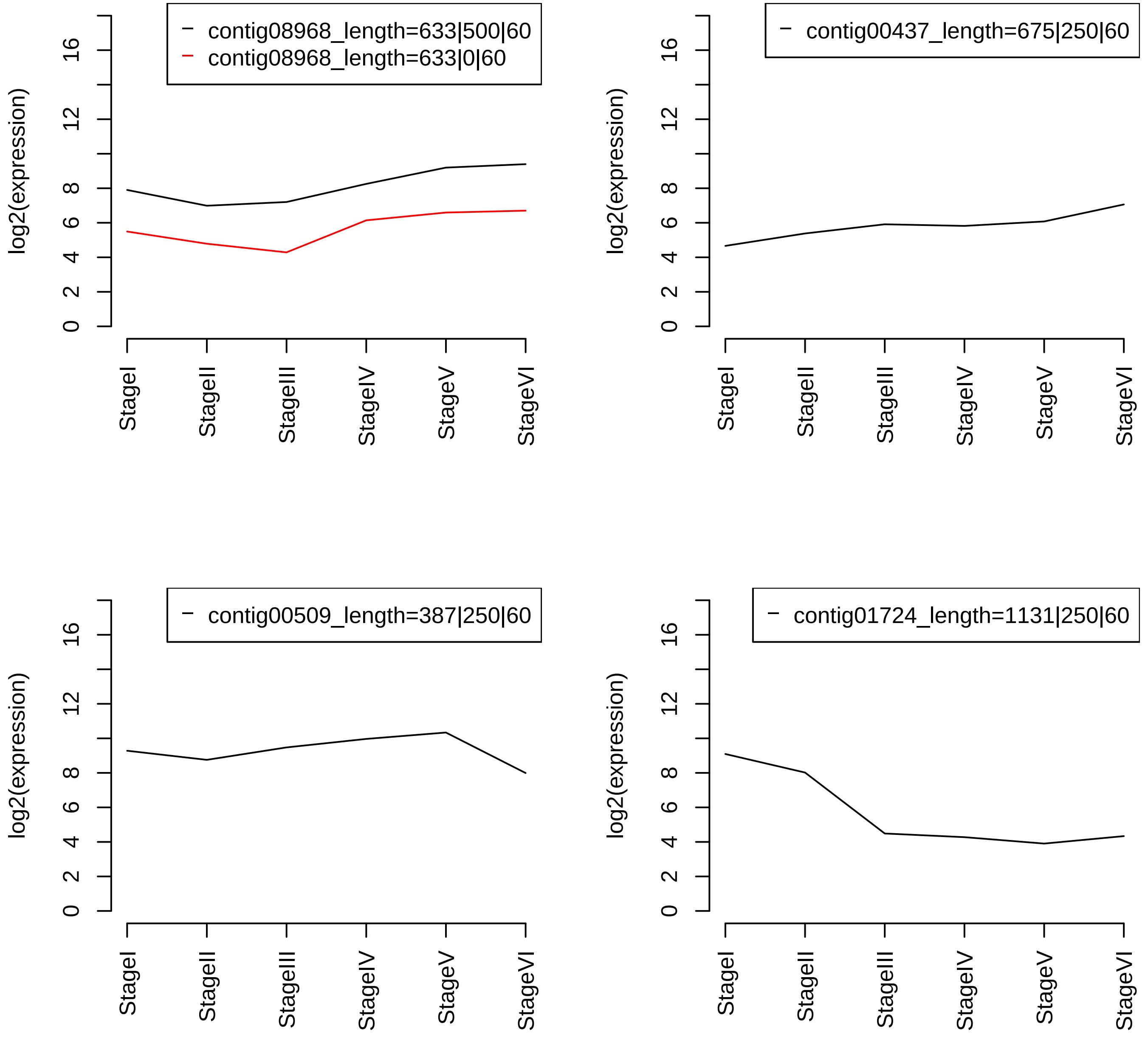
contig08968_length=633|500|60
contig08968_length=633|0|60
contig00437_length=675|250|60
contig00509_length=387|250|60
contig01724_length=1131|250|60
log2(expression)
0
2
4
6
8
12
16
StageI
StageII
StageIII
StageIV
StageV
StageVI

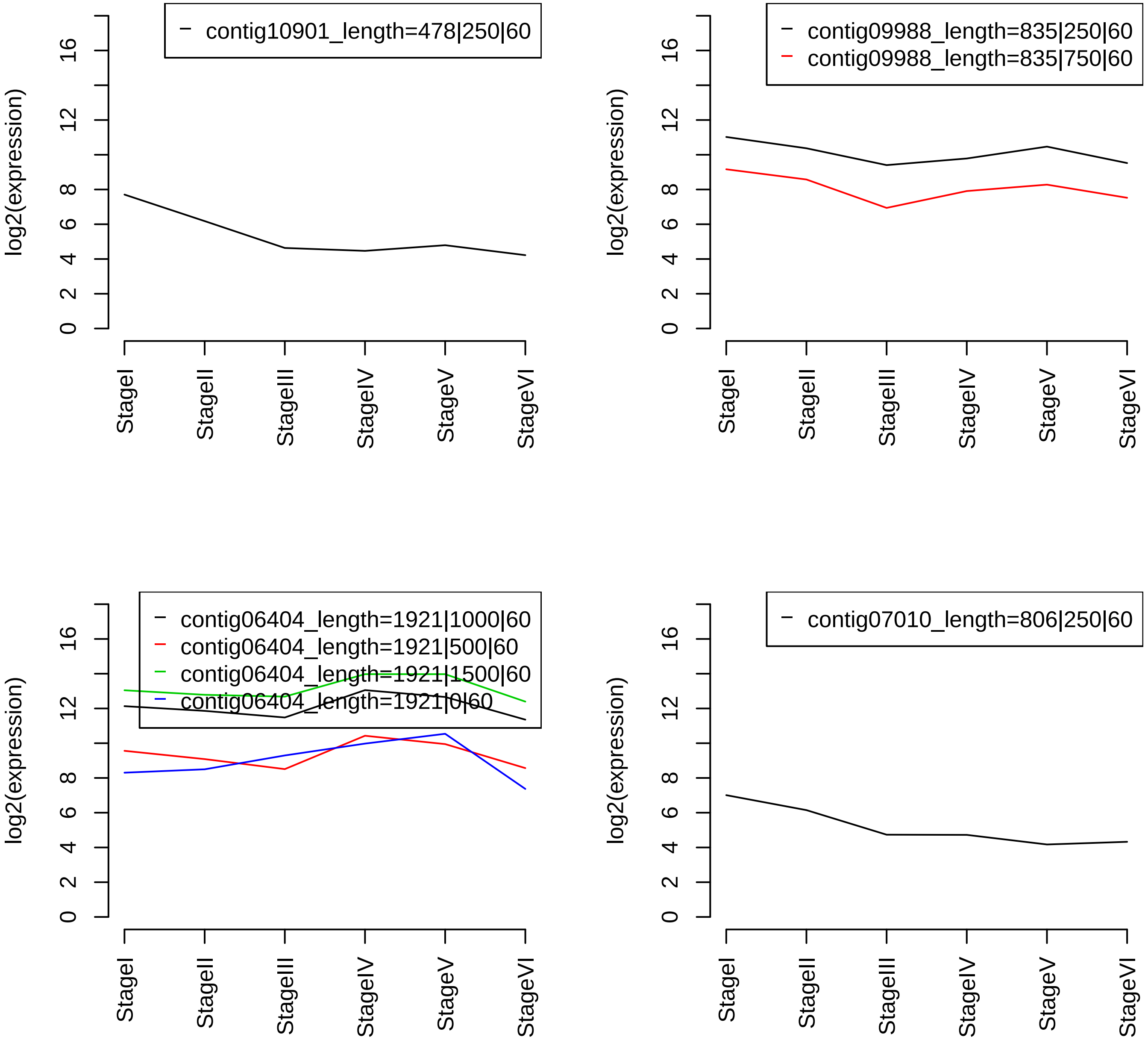

contig10901_length=478|250|60
contig09988_length=835|250|60
contig09988_length=835|750|60
contig06404_length=1921|1000|60
contig06404_length=1921|500|60
contig06404_length=1921|1500|60
contig06404_length=1921|0|60
contig07010_length=806|250|60
log2(expression)
0
2
4
6
8
12
16
StageI
StageII
StageIII
StageIV
StageV
StageVI

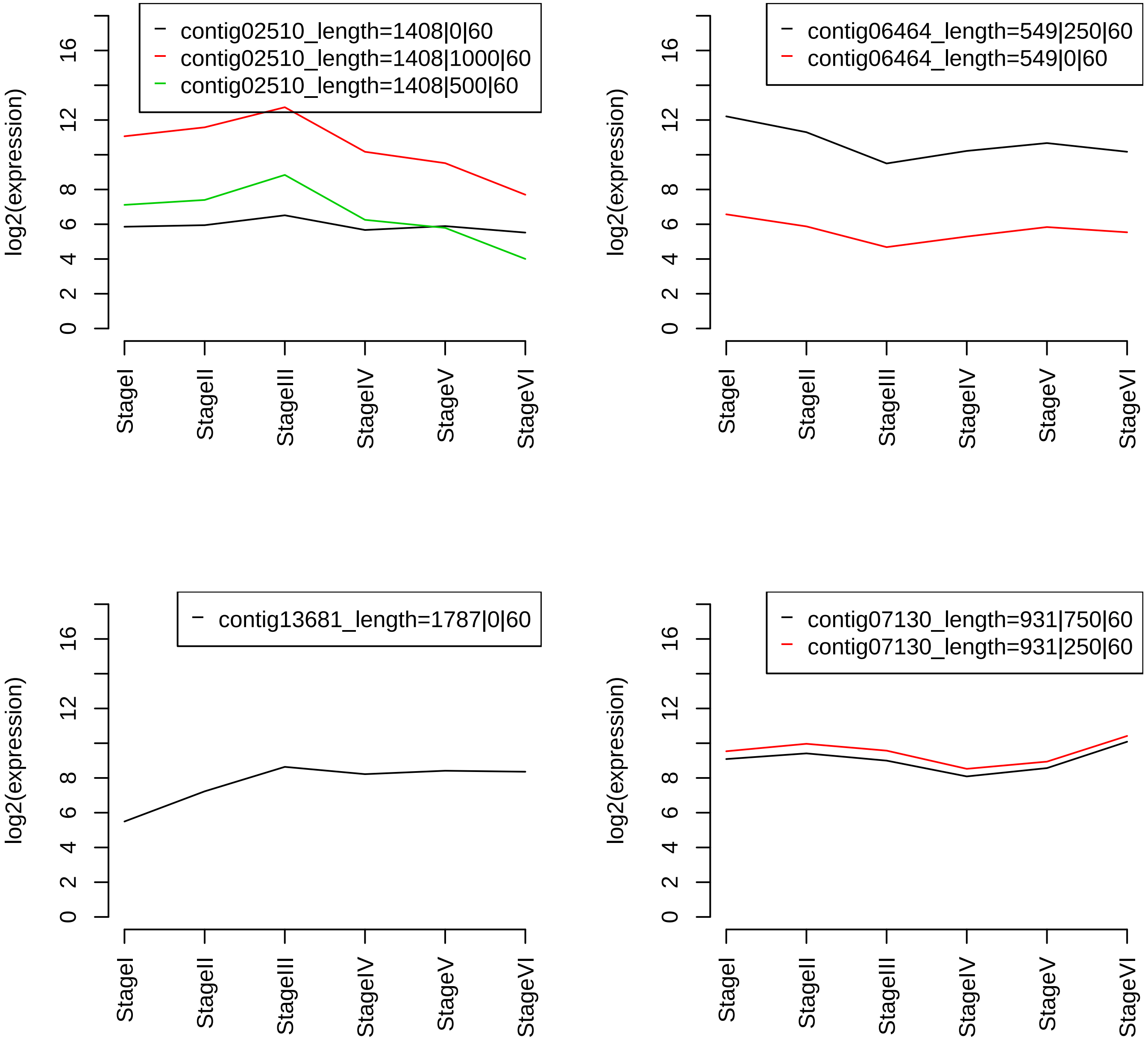

contig02510_length=1408|0|60
contig02510_length=1408|1000|60
contig02510_length=1408|500|60
log2(expression)
0
2
4
6
8
12
16
StageI
StageII
StageIII
StageIV
StageV
StageVI
contig06464_length=549|250|60
contig06464_length=549|0|60
contig13681_length=1787|0|60
contig07130_length=931|750|60
contig07130_length=931|250|60

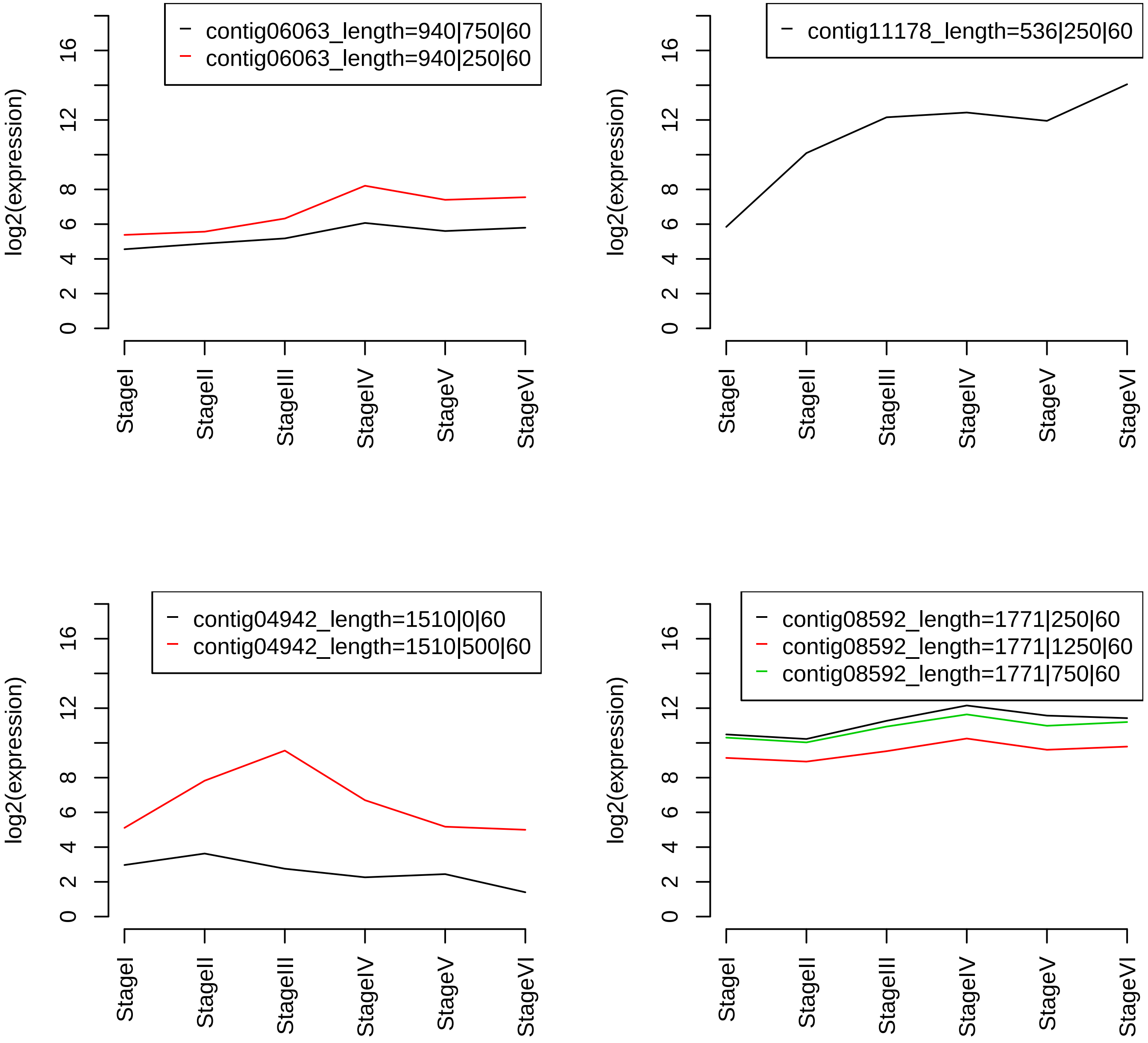

contig06063_length=940|750|60
contig06063_length=940|250|60
contig11178_length=536|250|60
contig04942_length=1510|0|60
contig04942_length=1510|500|60
contig08592_length=1771|250|60
contig08592_length=1771|1250|60
contig08592_length=1771|750|60
log2(expression)
0
2
4
6
8
12
16
StageI
StageII
StageIII
StageIV
StageV
StageVI

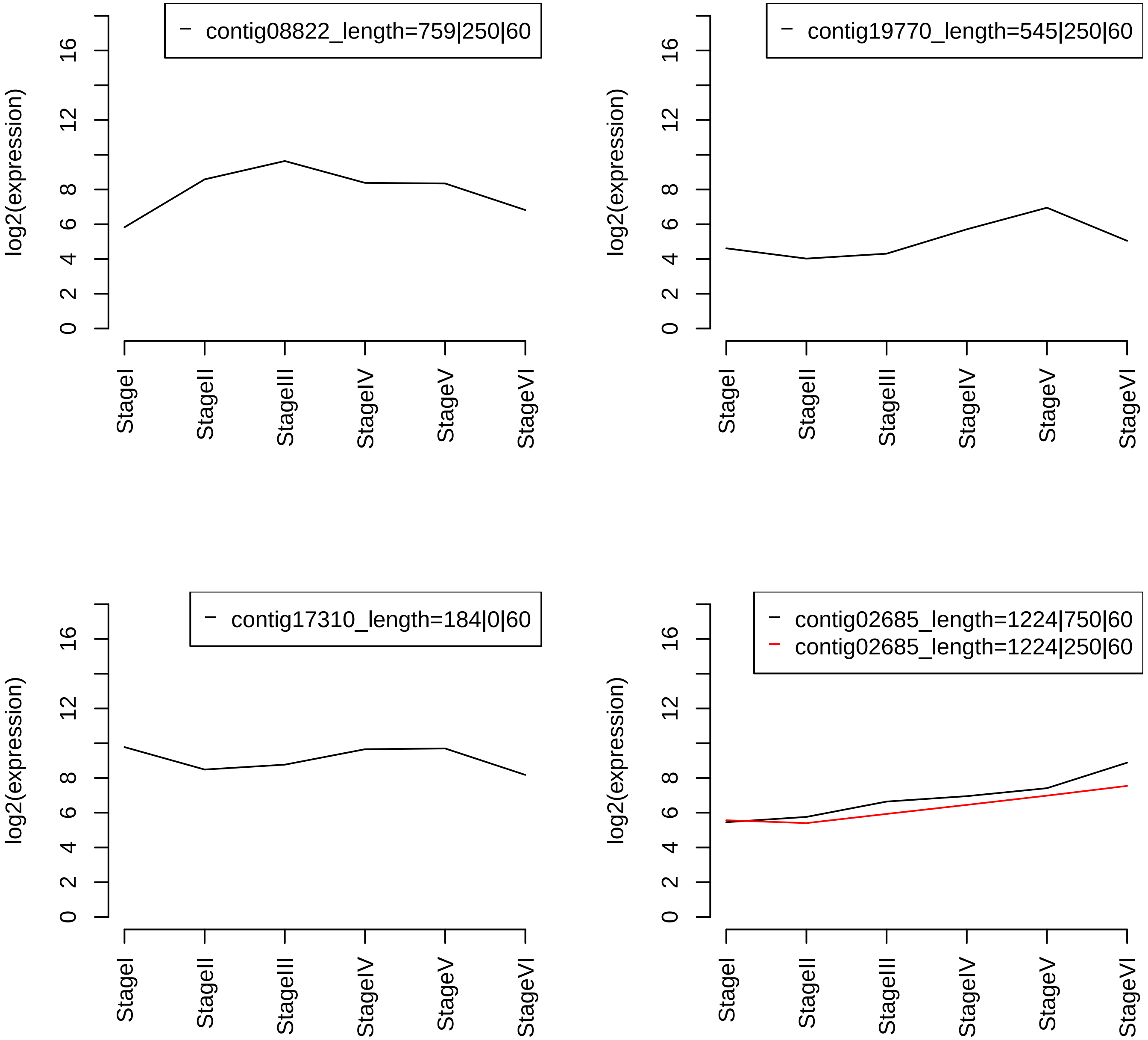
contig08822_length=759|250|60
contig19770_length=545|250|60
contig17310_length=184|0|60
contig02685_length=1224|750|60
contig02685_length=1224|250|60
log2(expression)
0
2
4
6
8
12
16
StageI
StageII
StageIII
StageIV
StageV
StageVI

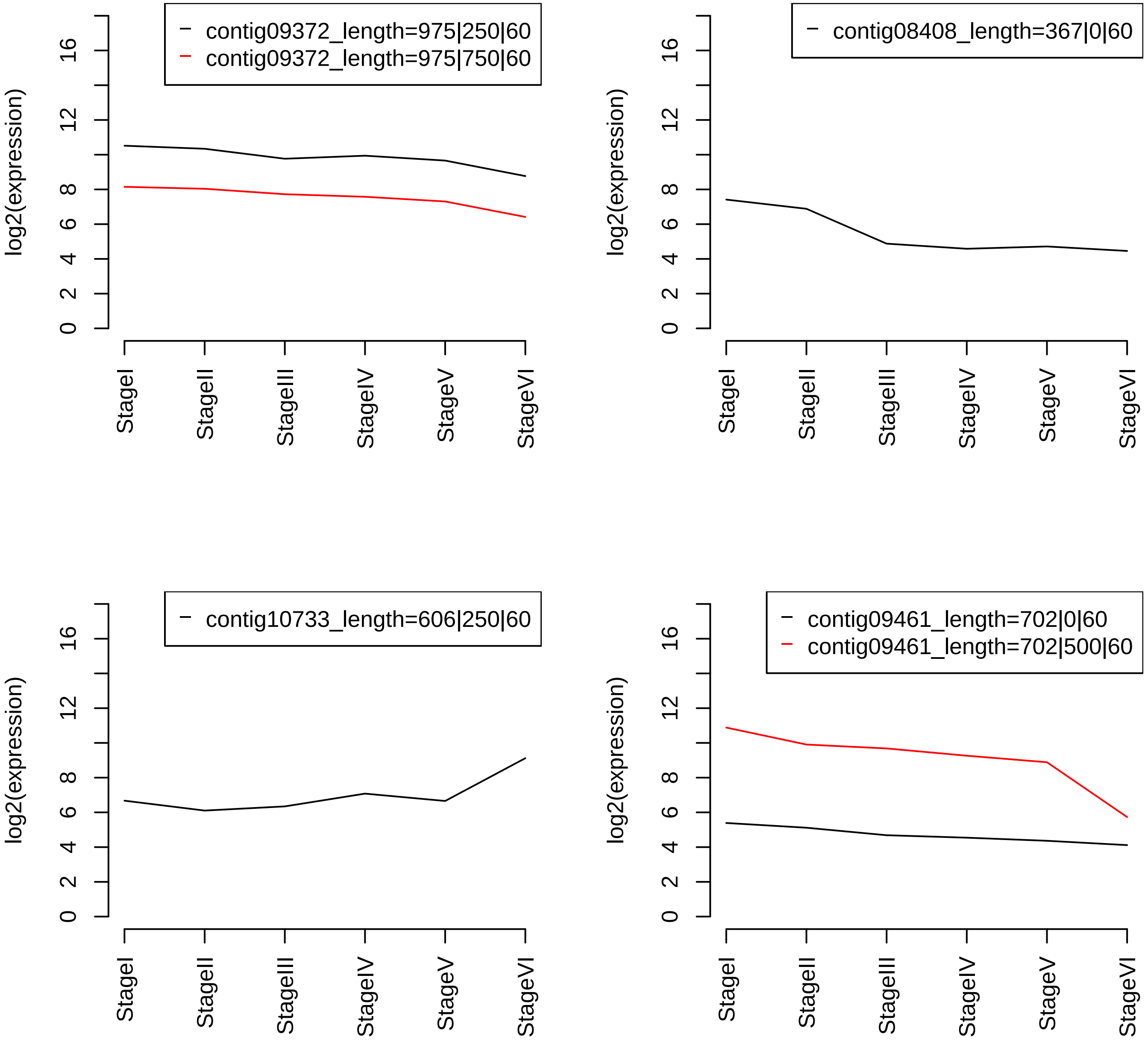
contig09372_length=975|250|60
contig09372_length=975|750|60
contig08408_length=367|0|60
contig10733_length=606|250|60
contig09461_length=702|0|60
contig09461_length=702|500|60
log2(expression)
0
2
4
6
8
12
16
StageI
StageII
StageIII
StageIV
StageV
StageVI

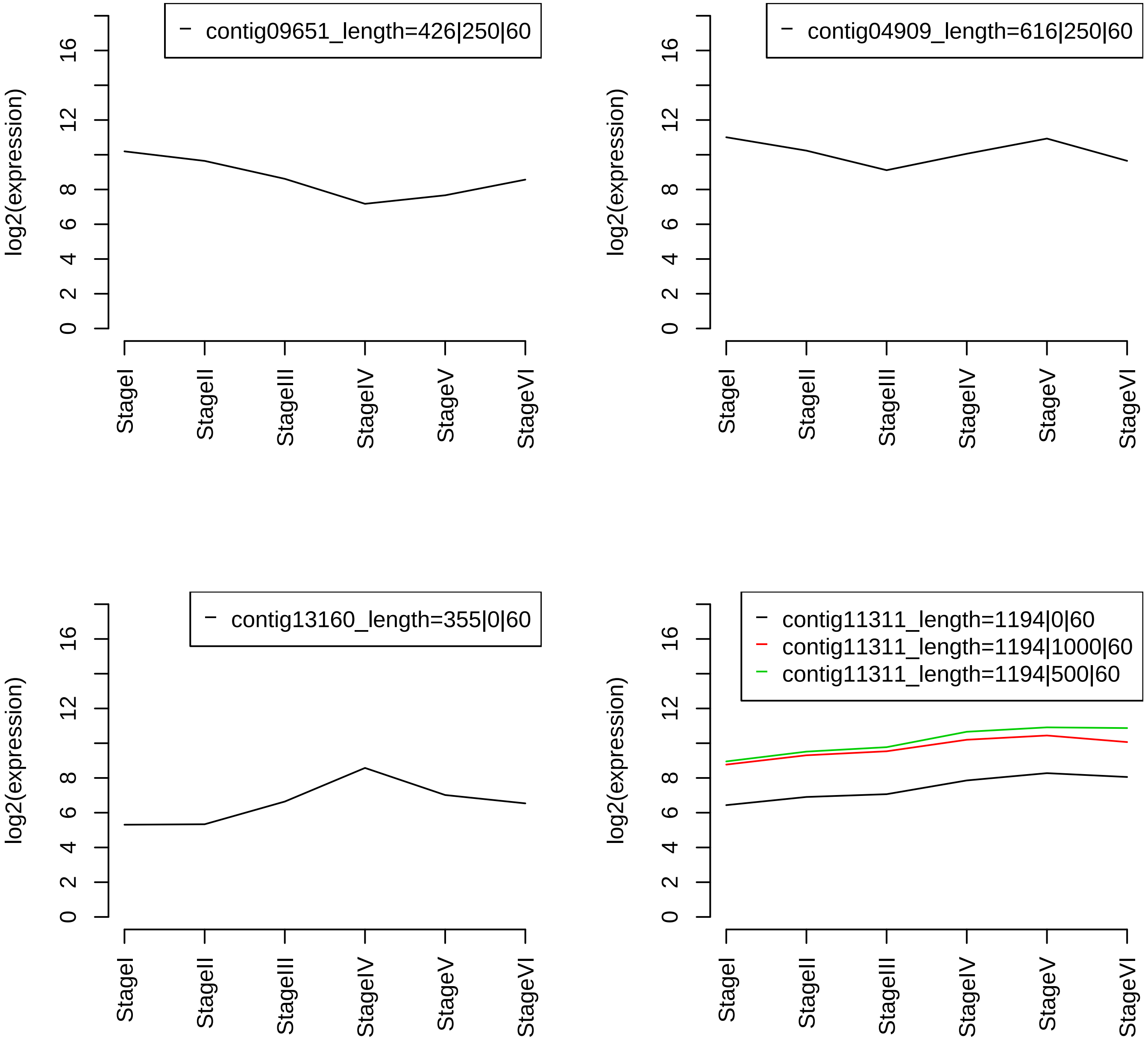
contig09651_length=426|250|60
contig04909_length=616|250|60
contig13160_length=355|0|60
contig11311_length=1194|0|60
contig11311_length=1194|1000|60
contig11311_length=1194|500|60
log2(expression)
0
2
4
6
8
12
16
StageI
StageII
StageIII
StageIV
StageV
StageVI

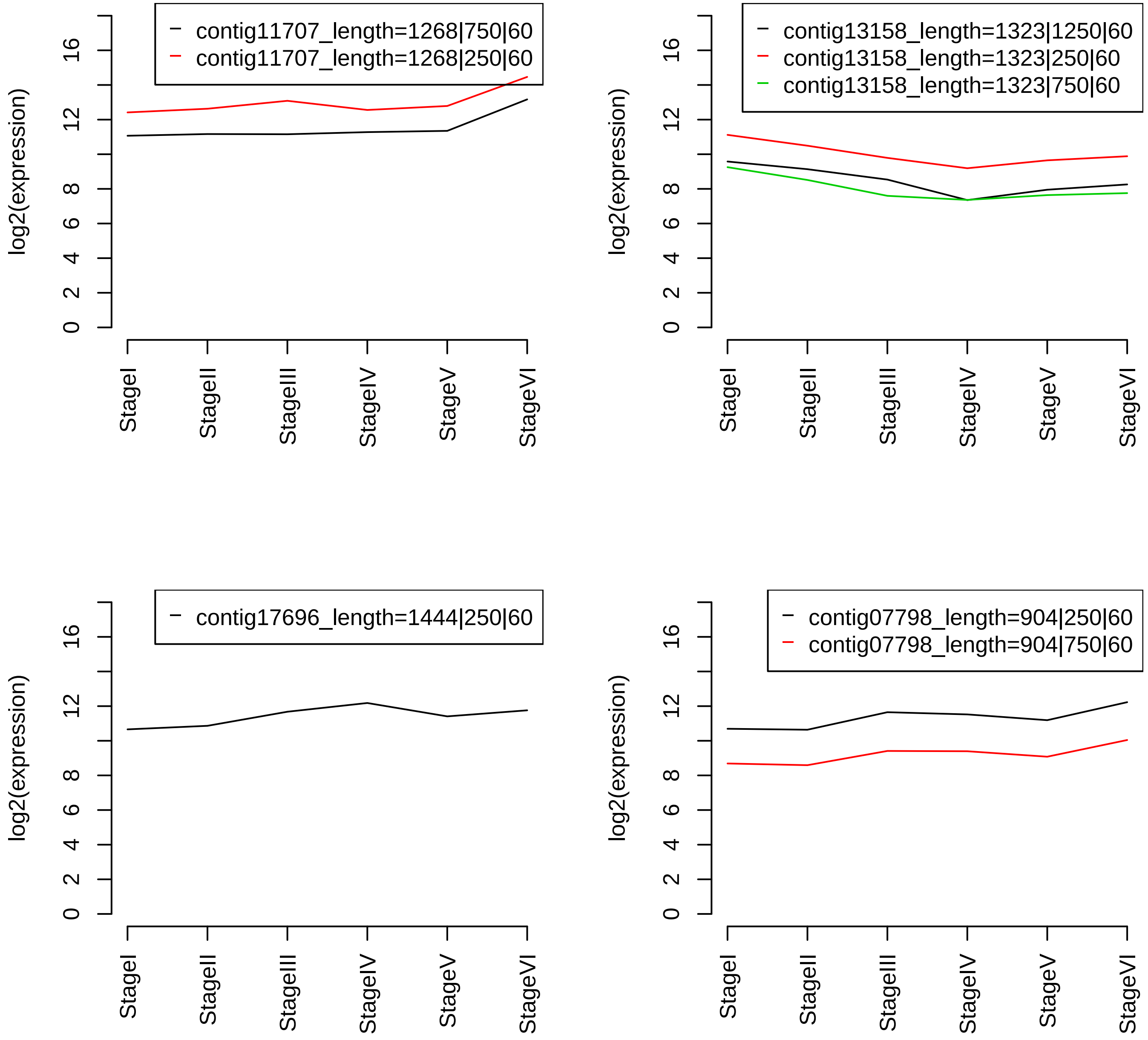

contig11707_length=1268|750|60
contig11707_length=1268|250|60
contig13158_length=1323|1250|60
contig13158_length=1323|250|60
contig13158_length=1323|750|60
contig17696_length=1444|250|60
contig07798_length=904|250|60
contig07798_length=904|750|60
log2(expression)
0 2 4 6 8 12 16
StageI StageII StageIII StageIV StageV StageVI

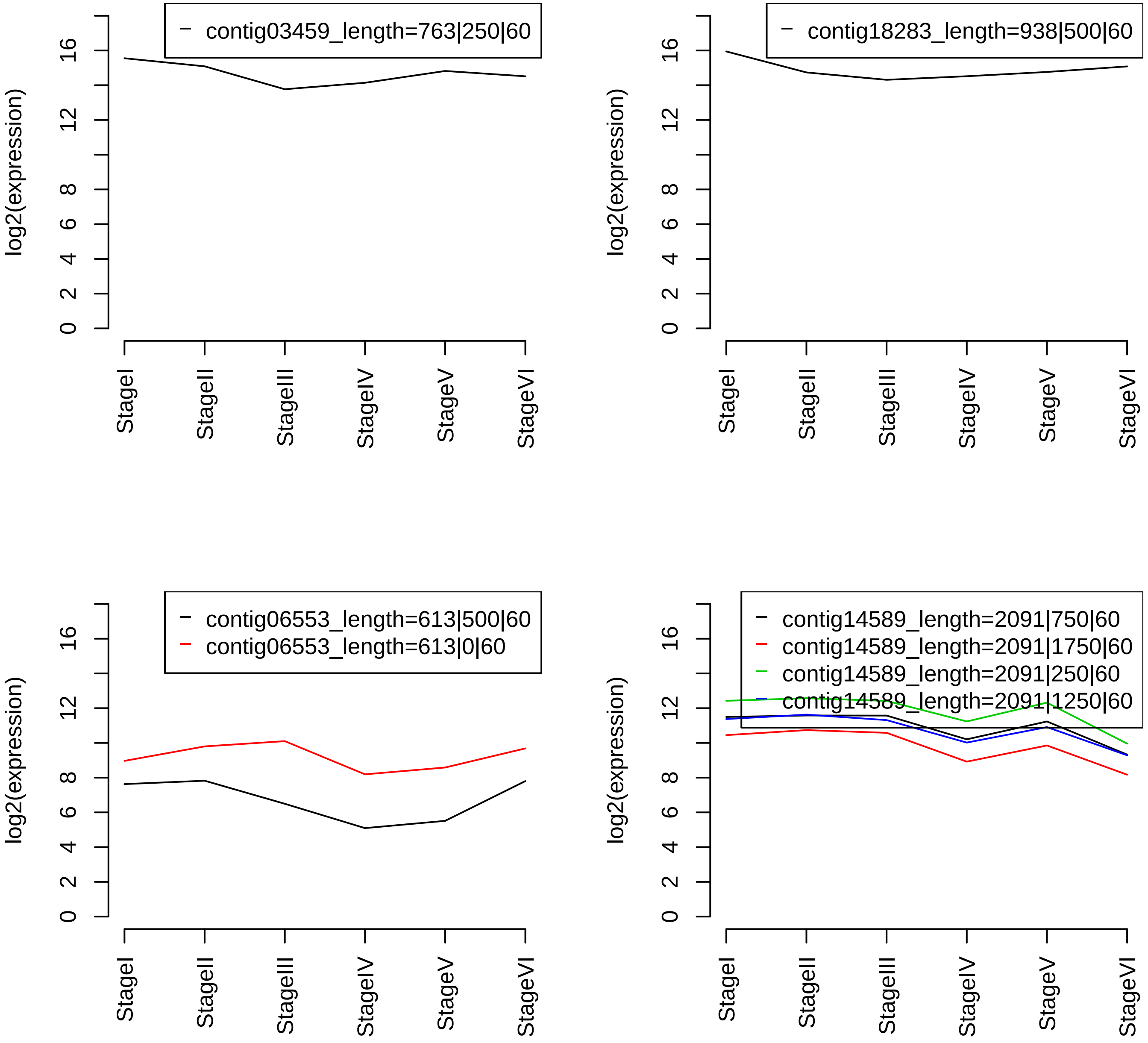
contig03459_length=763|250|60
contig18283_length=938|500|60
contig06553_length=613|500|60
contig06553_length=613|0|60
contig14589_length=2091|750|60
contig14589_length=2091|1750|60
contig14589_length=2091|250|60
contig14589_length=2091|1250|60
log2(expression)
0
2
4
6
8
12
16
StageI
StageII
StageIII
StageIV
StageV
StageVI

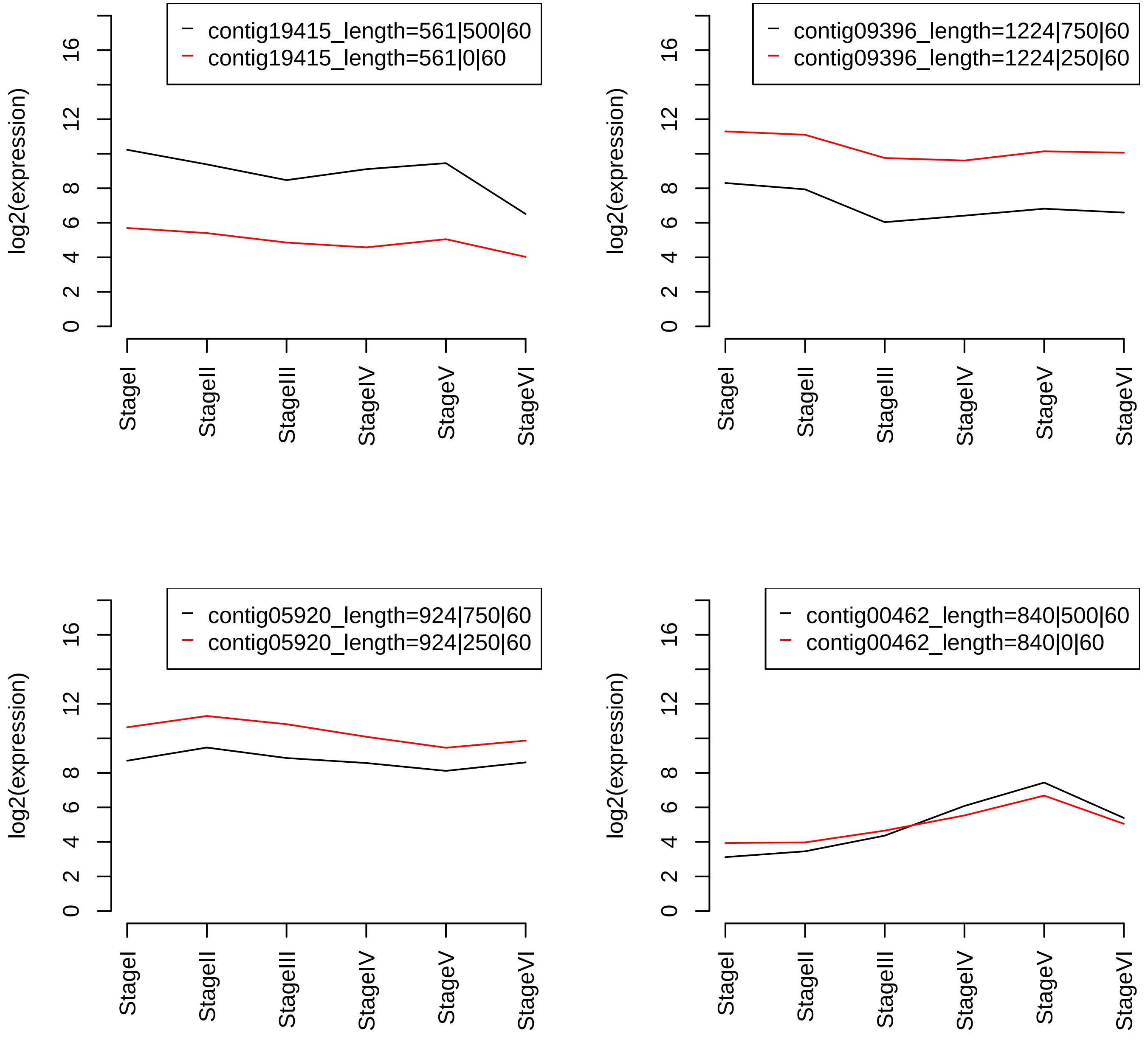

contig19415_length=561|500|60
contig19415_length=561|0|60
contig09396_length=1224|750|60
contig09396_length=1224|250|60
contig05920_length=924|750|60
contig05920_length=924|250|60
contig00462_length=840|500|60
contig00462_length=840|0|60
log2(expression)
0 2 4 6 8 12 16
StageI StageII StageIII StageIV StageV StageVI

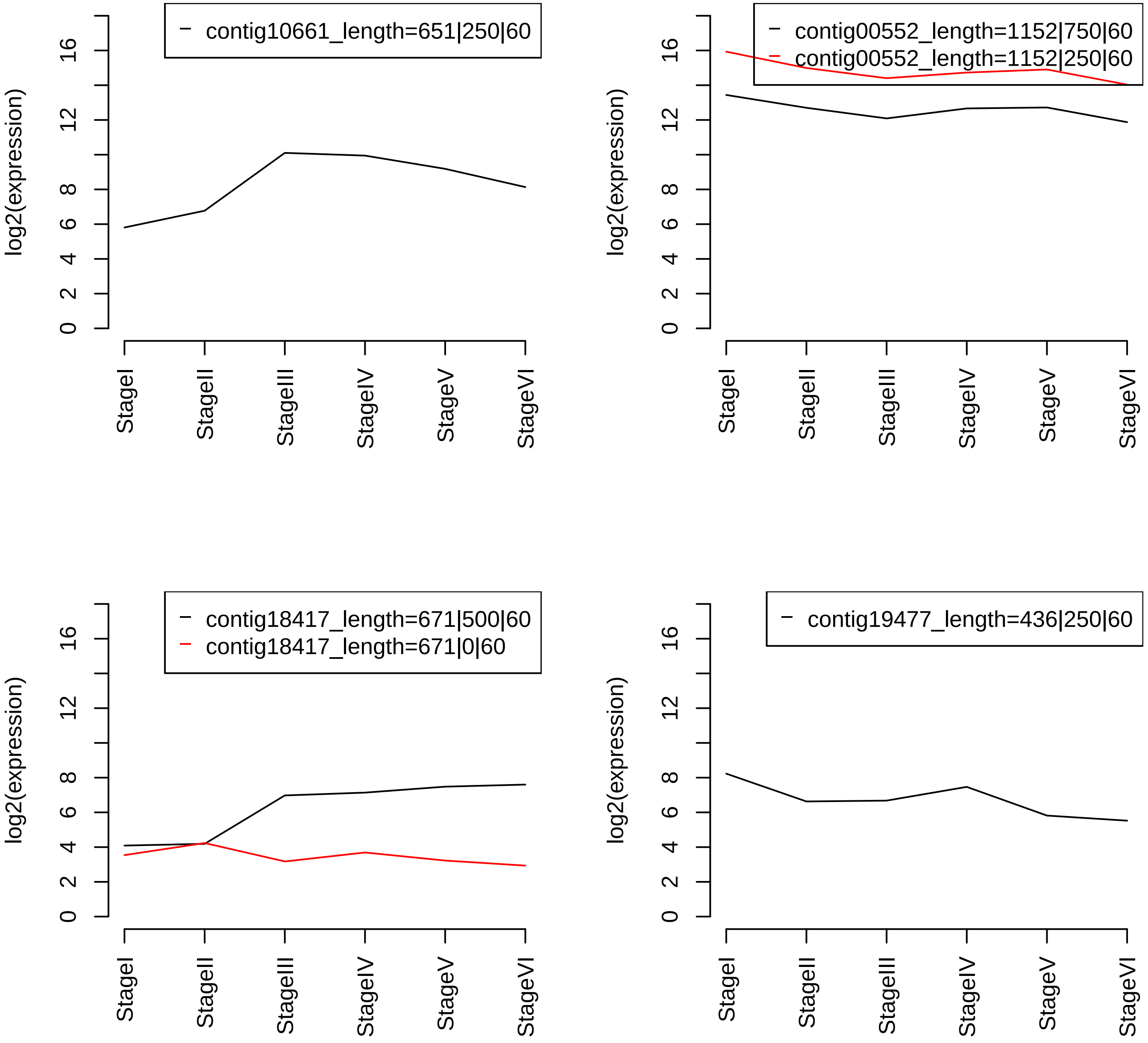
contig10661_length=651|250|60
contig00552_length=1152|750|60
contig00552_length=1152|250|60
contig18417_length=671|500|60
contig18417_length=671|0|60
contig19477_length=436|250|60
log2(expression)
0
2
4
6
8
12
16
StageI
StageII
StageIII
StageIV
StageV
StageVI

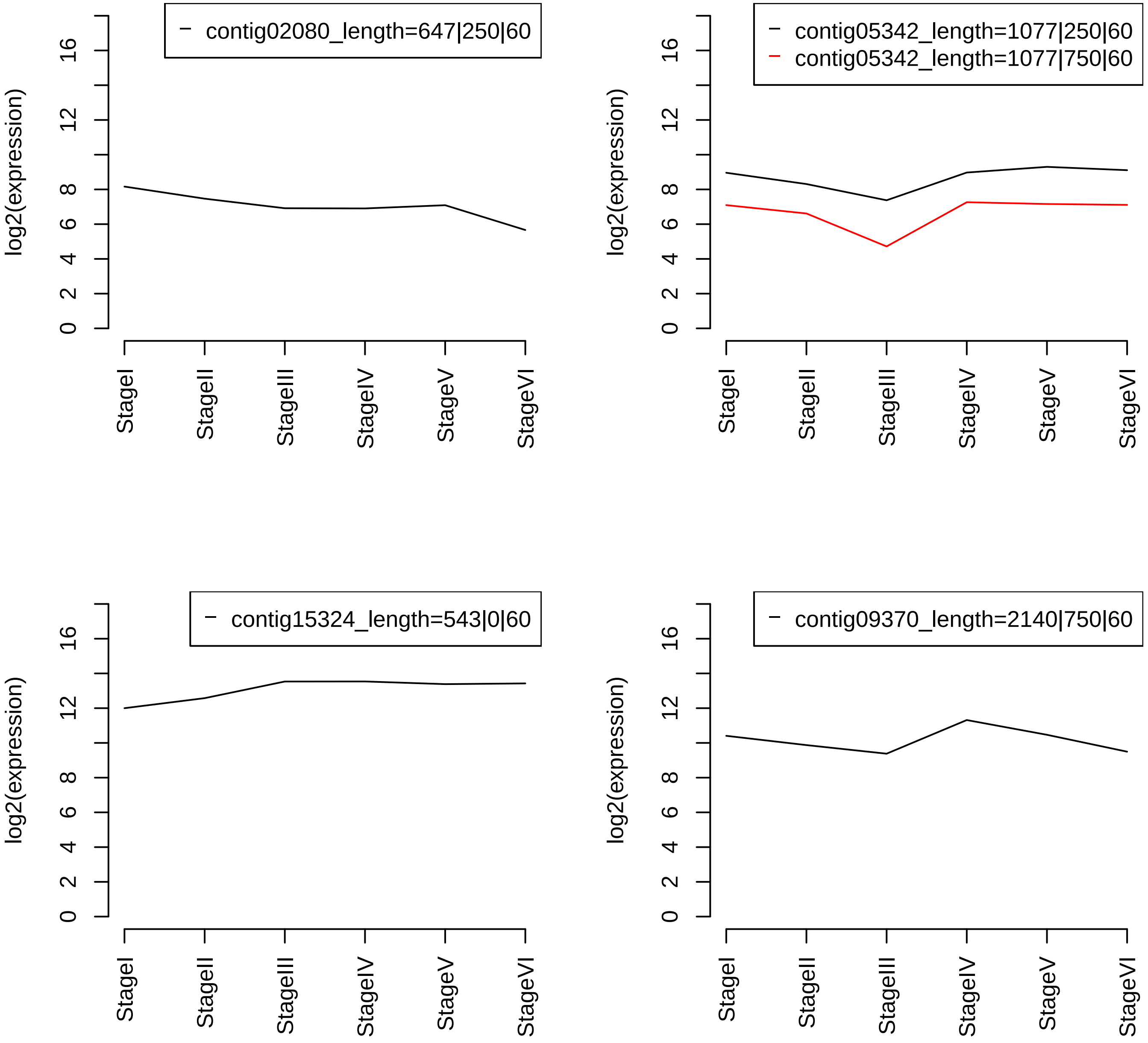
contig02080_length=647|250|60
contig05342_length=1077|250|60
contig05342_length=1077|750|60
contig15324_length=543|0|60
contig09370_length=2140|750|60
log2(expression)
0
2
4
6
8
12
16
StageI
StageII
StageIII
StageIV
StageV
StageVI

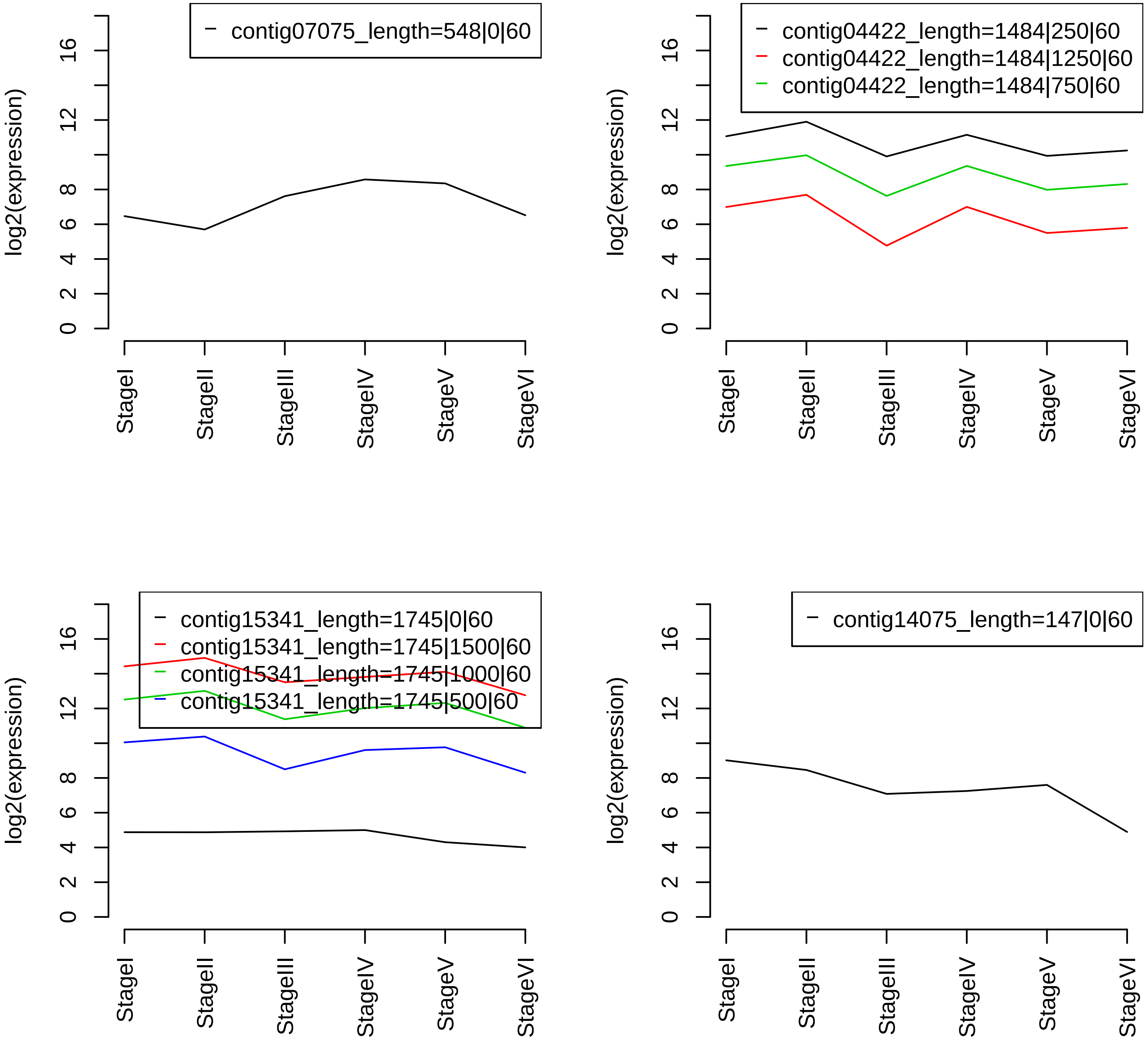
contig07075_length=548|0|60
contig04422_length=1484|250|60
contig04422_length=1484|1250|60
contig04422_length=1484|750|60
contig15341_length=1745|0|60
contig15341_length=1745|1500|60
contig15341_length=1745|1000|60
contig15341_length=1745|500|60
contig14075_length=147|0|60
log2(expression)
0
2
4
6
8
12
16
StageI
StageII
StageIII
StageIV
StageV
StageVI

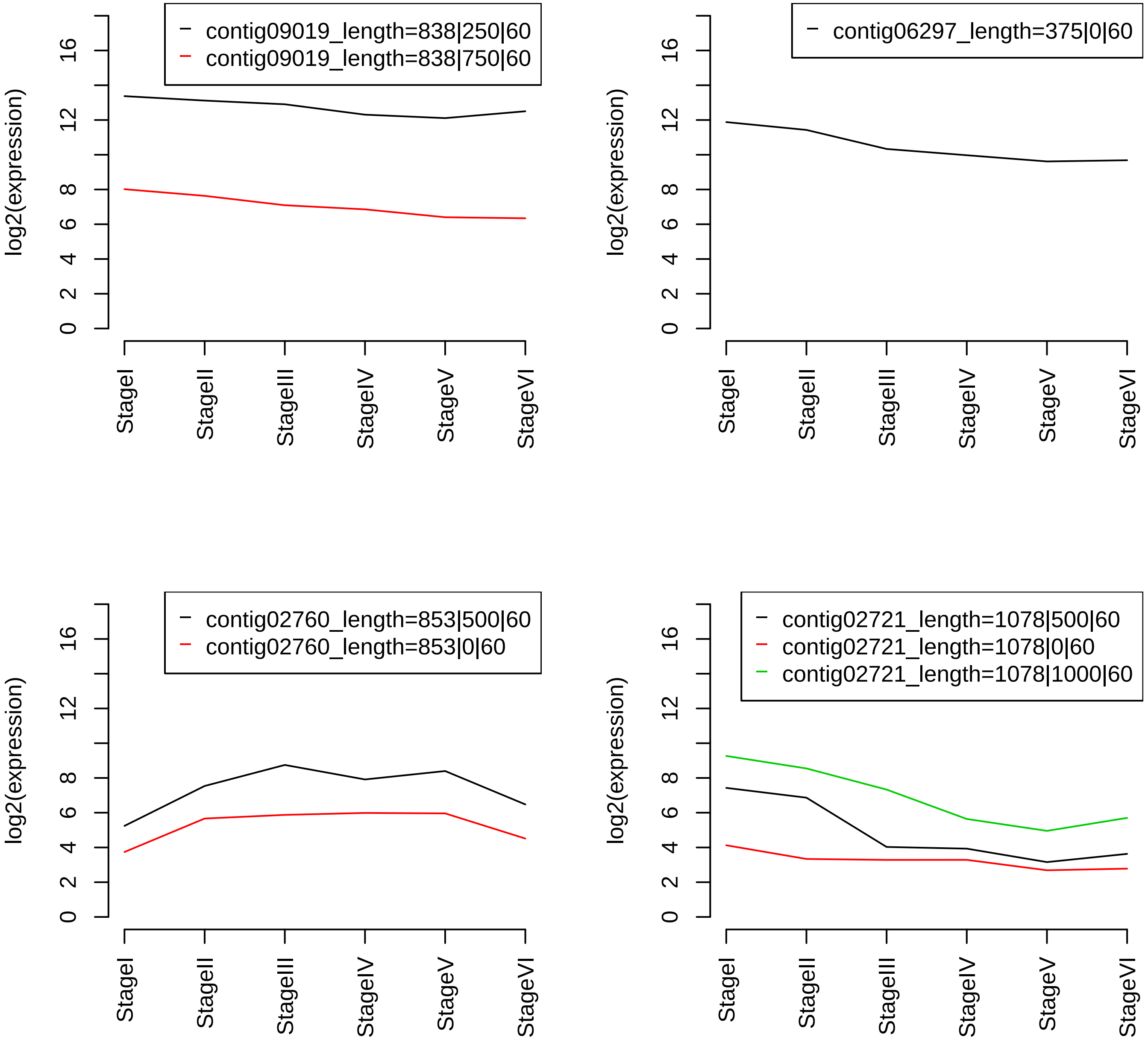
contig09019_length=838|250|60
contig09019_length=838|750|60
contig06297_length=375|0|60
contig02760_length=853|500|60
contig02760_length=853|0|60
contig02721_length=1078|500|60
contig02721_length=1078|0|60
contig02721_length=1078|1000|60
log2(expression)
0
2
4
6
8
12
16
StageI
StageII
StageIII
StageIV
StageV
StageVI

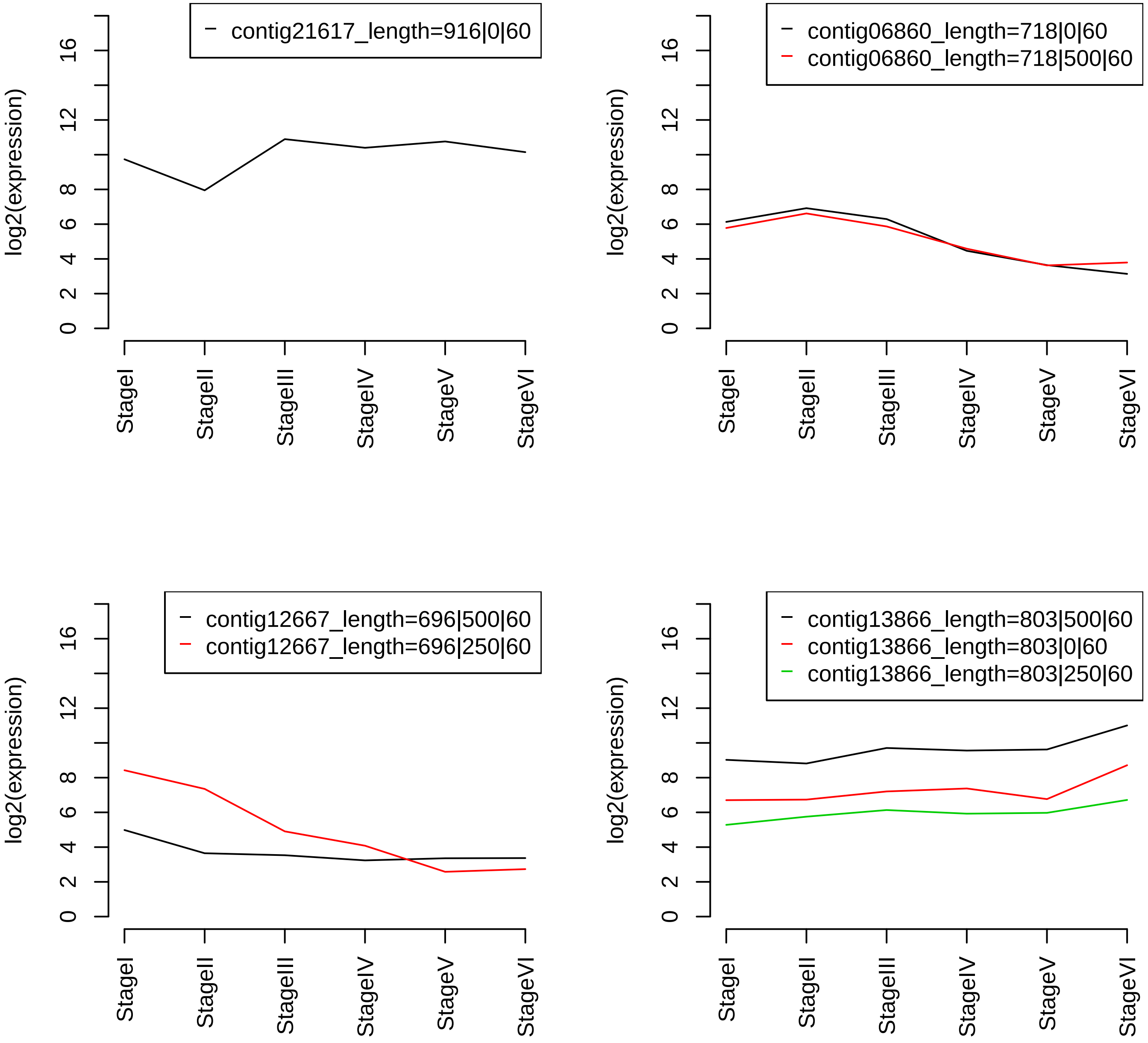

contig21617_length=916|0|60
contig06860_length=718|0|60
contig06860_length=718|500|60
contig12667_length=696|500|60
contig12667_length=696|250|60
contig13866_length=803|500|60
contig13866_length=803|0|60
contig13866_length=803|250|60
log2(expression)
0
2
4
6
8
12
16
StageI
StageII
StageIII
StageIV
StageV
StageVI

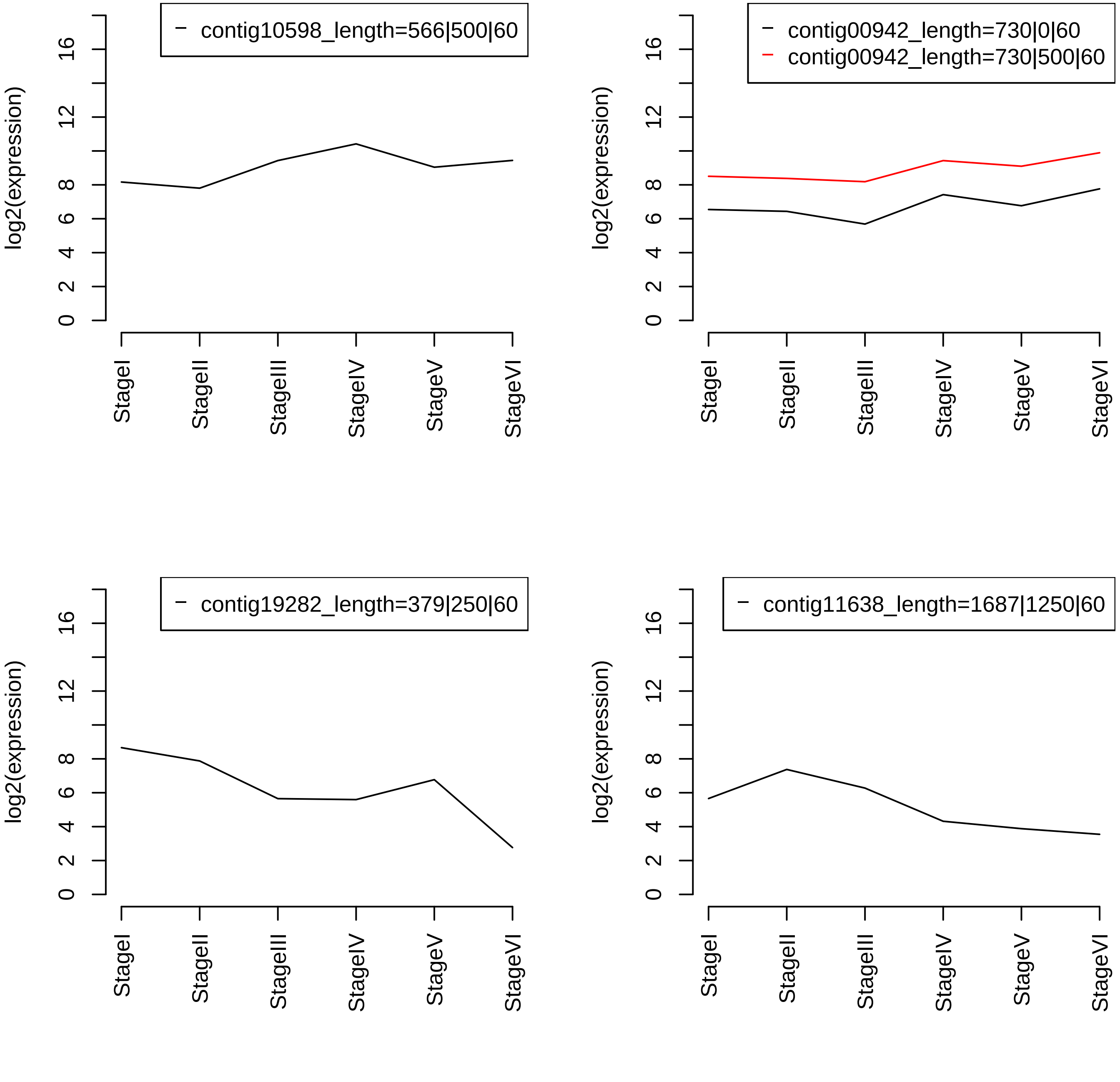

contig10598_length=566|500|60
contig00942_length=730|0|60
contig00942_length=730|500|60
contig19282_length=379|250|60
contig11638_length=1687|1250|60
log2(expression)
0
2
4
6
8
12
16
StageI
StageII
StageIII
StageIV
StageV
StageVI

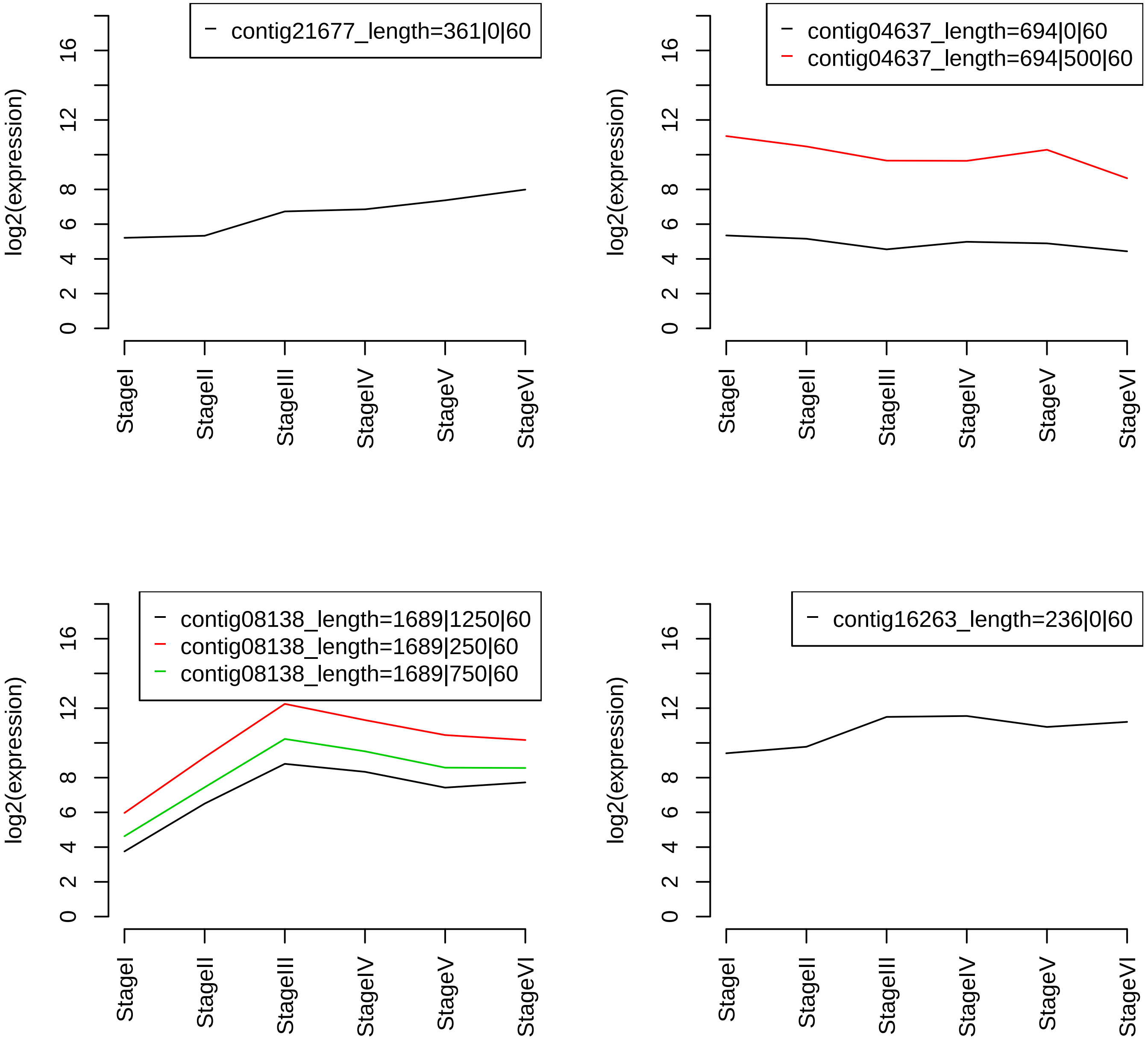

contig21677_length=361|0|60
contig04637_length=694|0|60
contig04637_length=694|500|60
contig08138_length=1689|1250|60
contig08138_length=1689|250|60
contig08138_length=1689|750|60
contig16263_length=236|0|60
log2(expression)
0
2
4
6
8
12
16
StageI
StageII
StageIII
StageIV
StageV
StageVI

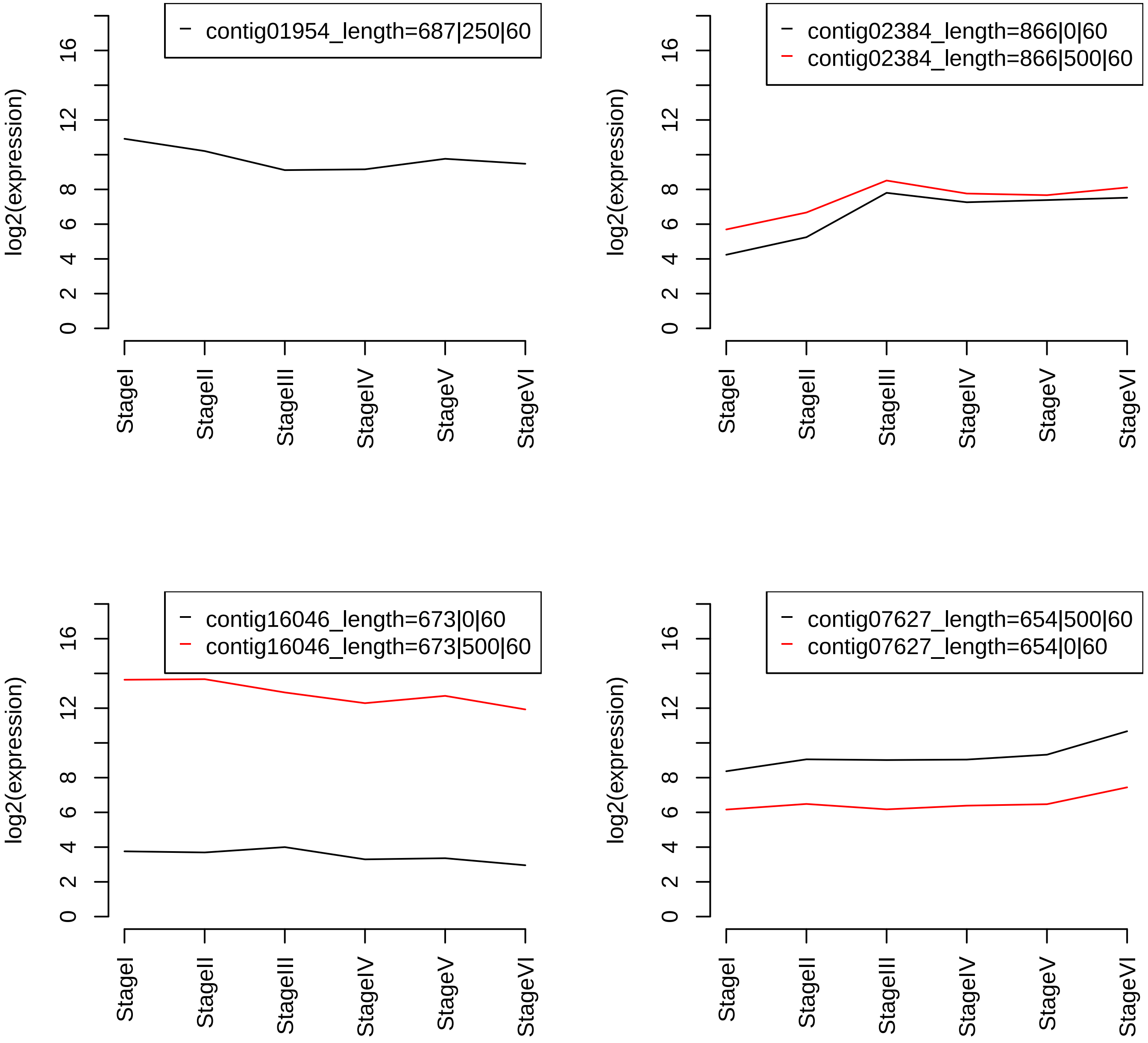
contig01954_length=687|250|60
contig02384_length=866|0|60
contig02384_length=866|500|60
contig16046_length=673|0|60
contig16046_length=673|500|60
contig07627_length=654|500|60
contig07627_length=654|0|60
log2(expression)
0 2 4 6 8 12 16
StageI
StageII
StageIII
StageIV
StageV
StageVI

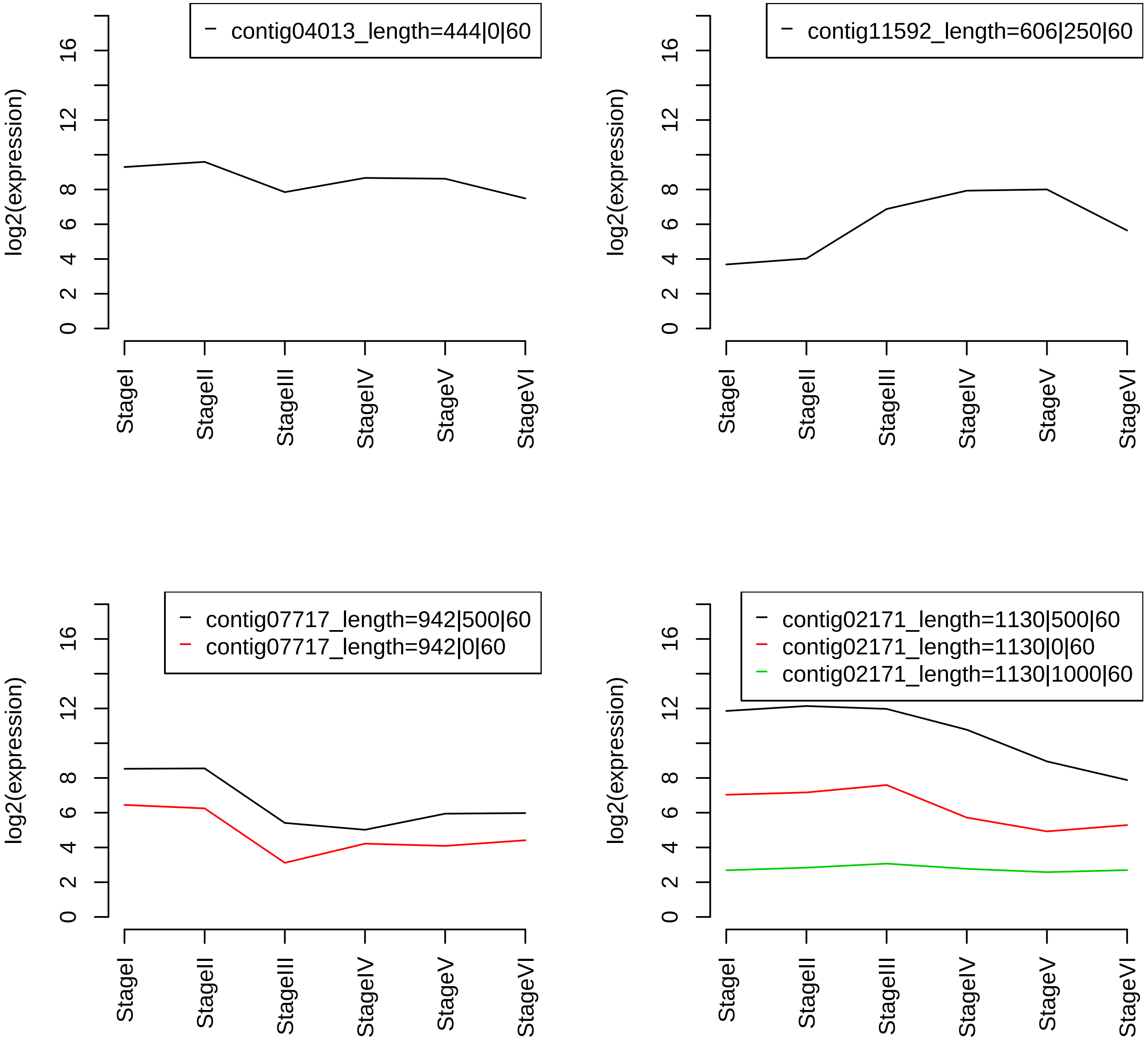
contig04013_length=444|0|60
contig11592_length=606|250|60
contig07717_length=942|500|60
contig07717_length=942|0|60
contig02171_length=1130|500|60
contig02171_length=1130|0|60
contig02171_length=1130|1000|60
log2(expression)
0
2
4
6
8
12
16
StageI
StageII
StageIII
StageIV
StageV
StageVI

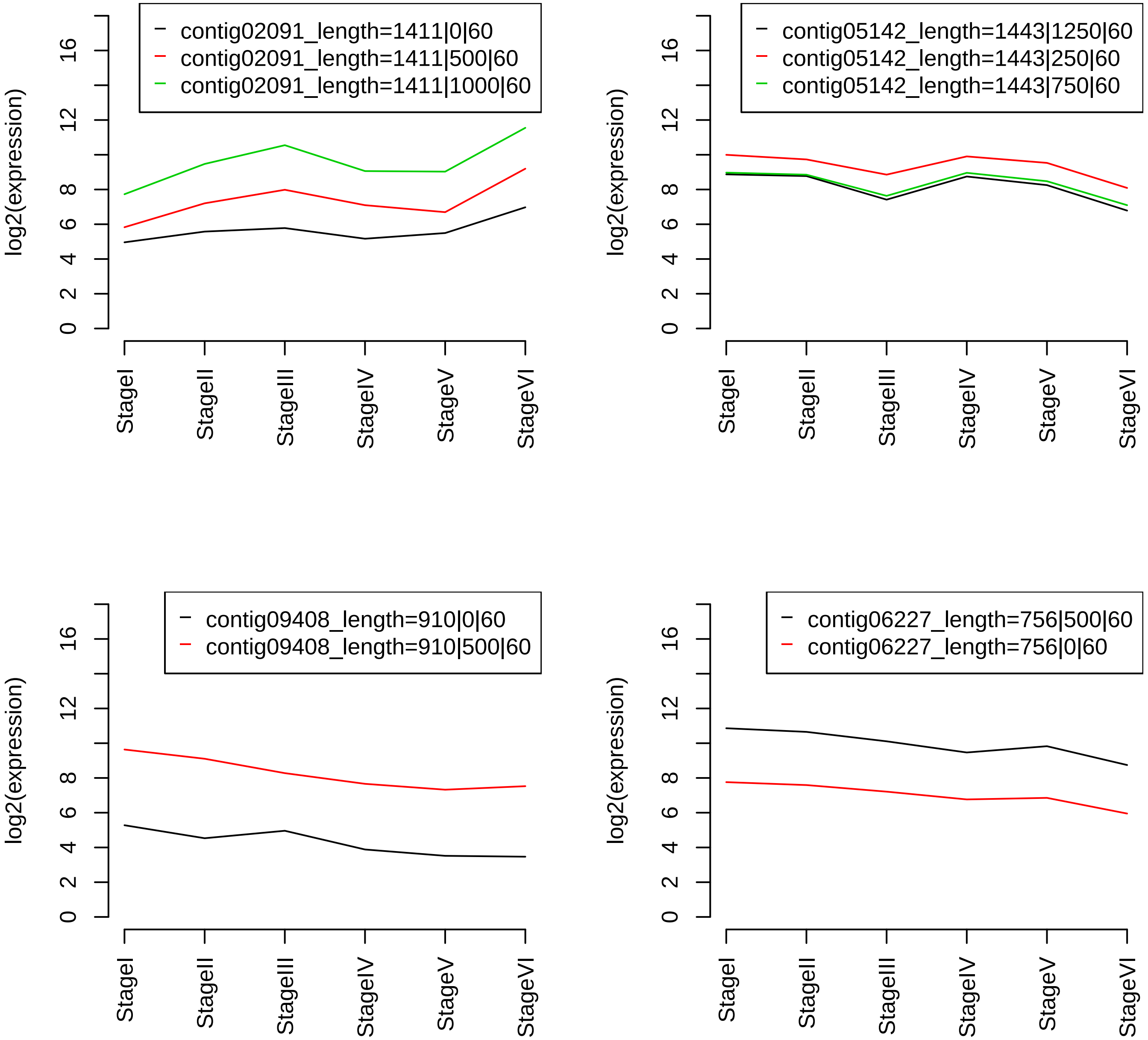

contig02091_length=1411|0|60
contig02091_length=1411|500|60
contig02091_length=1411|1000|60
contig05142_length=1443|1250|60
contig05142_length=1443|250|60
contig05142_length=1443|750|60
contig09408_length=910|0|60
contig09408_length=910|500|60
contig06227_length=756|500|60
contig06227_length=756|0|60
log2(expression)
0
2
4
6
8
12
16
StageI
StageII
StageIII
StageIV
StageV
StageVI

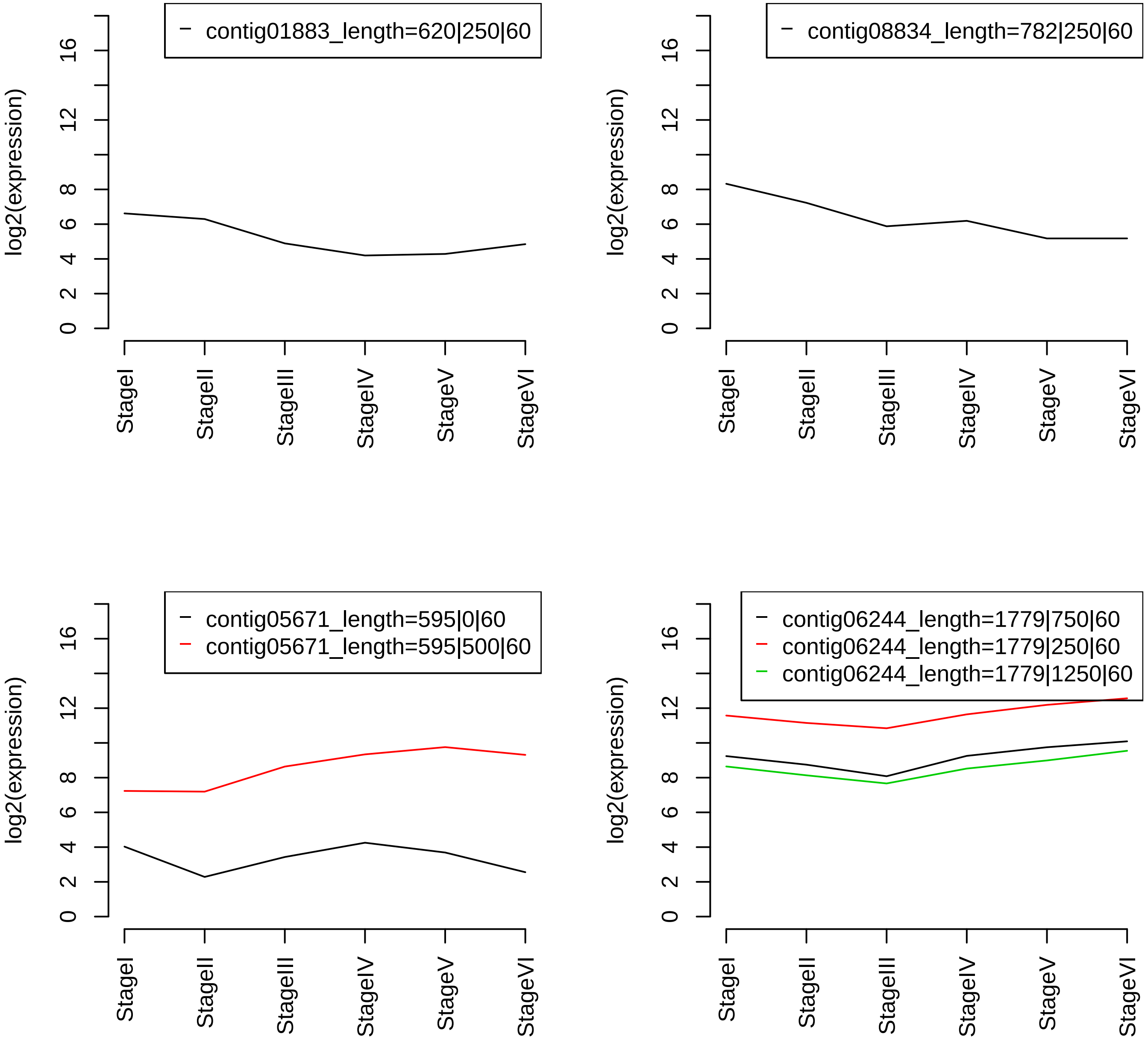

contig01883_length=620|250|60
contig08834_length=782|250|60
contig05671_length=595|0|60
contig05671_length=595|500|60
contig06244_length=1779|750|60
contig06244_length=1779|250|60
contig06244_length=1779|1250|60
log2(expression)
0
2
4
6
8
12
16
StageI
StageII
StageIII
StageIV
StageV
StageVI

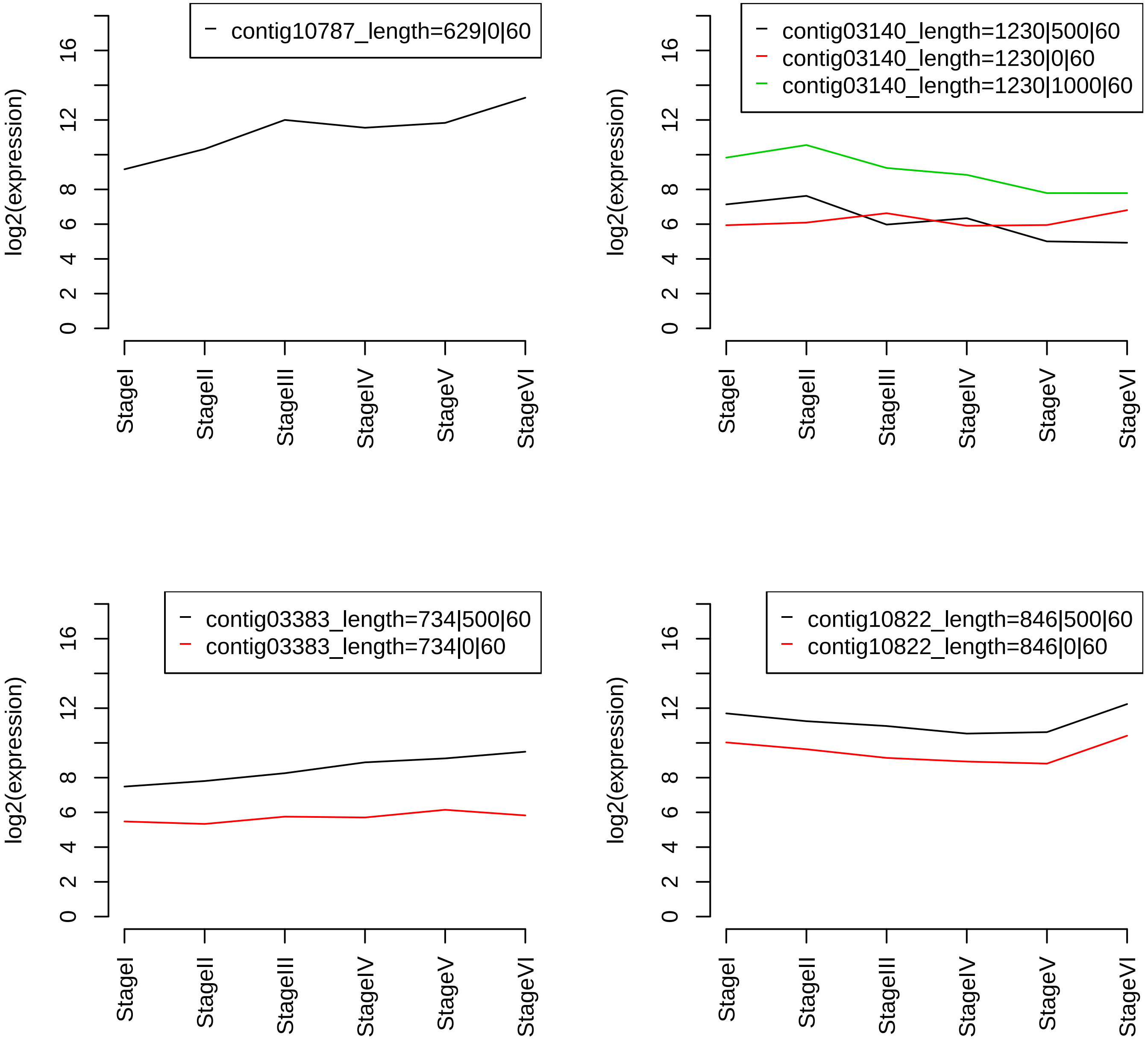
contig10787_length=629|0|60
contig03140_length=1230|500|60
contig03140_length=1230|0|60
contig03140_length=1230|1000|60
contig03383_length=734|500|60
contig03383_length=734|0|60
contig10822_length=846|500|60
contig10822_length=846|0|60
log2(expression)
0 2 4 6 8 12 16
StageI StageII StageIII StageIV StageV StageVI

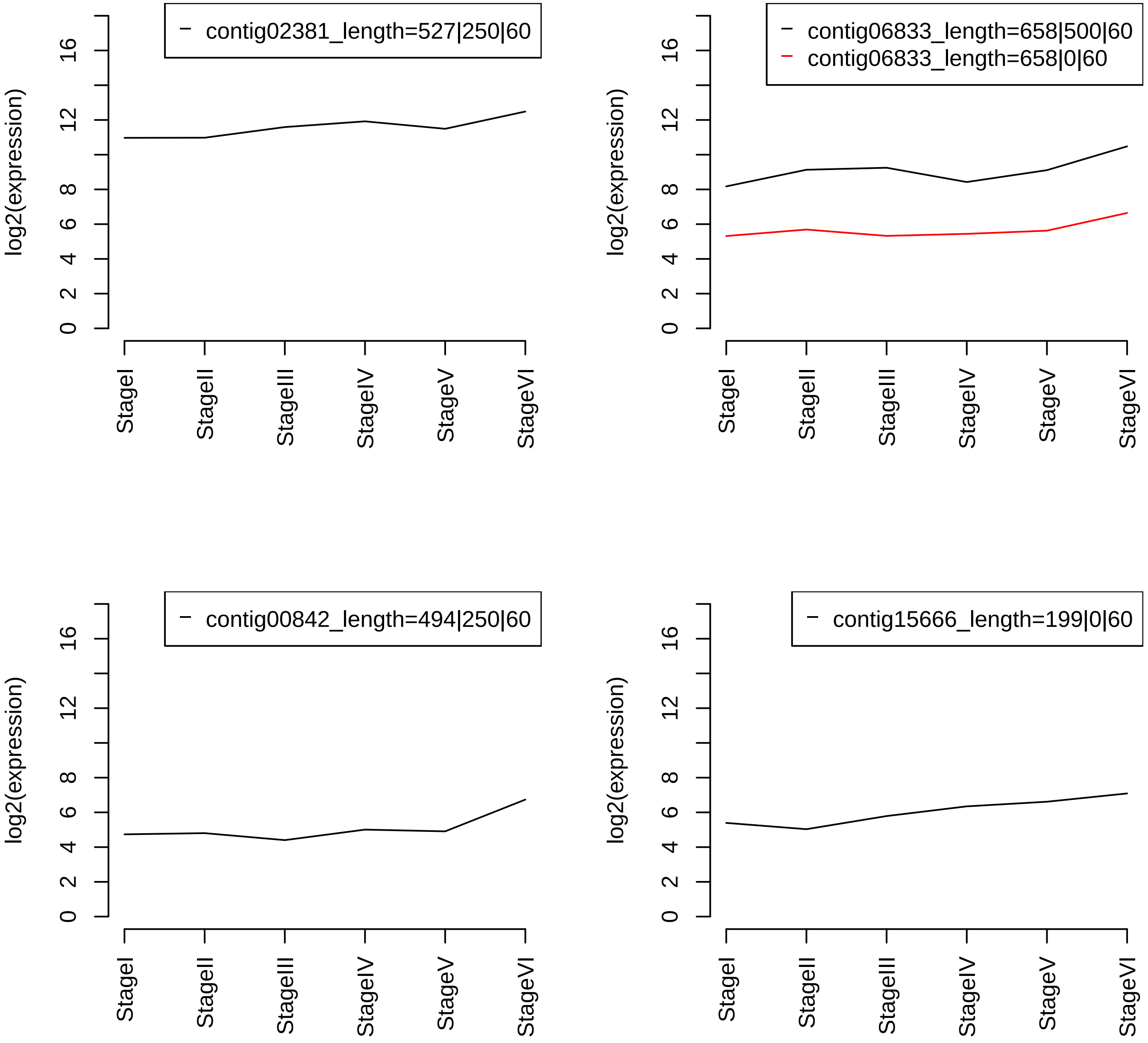

contig02381_length=527|250|60
contig06833_length=658|500|60
contig06833_length=658|0|60
contig00842_length=494|250|60
contig15666_length=199|0|60
log2(expression)
0
2
4
6
8
12
16
StageI
StageII
StageIII
StageIV
StageV
StageVI

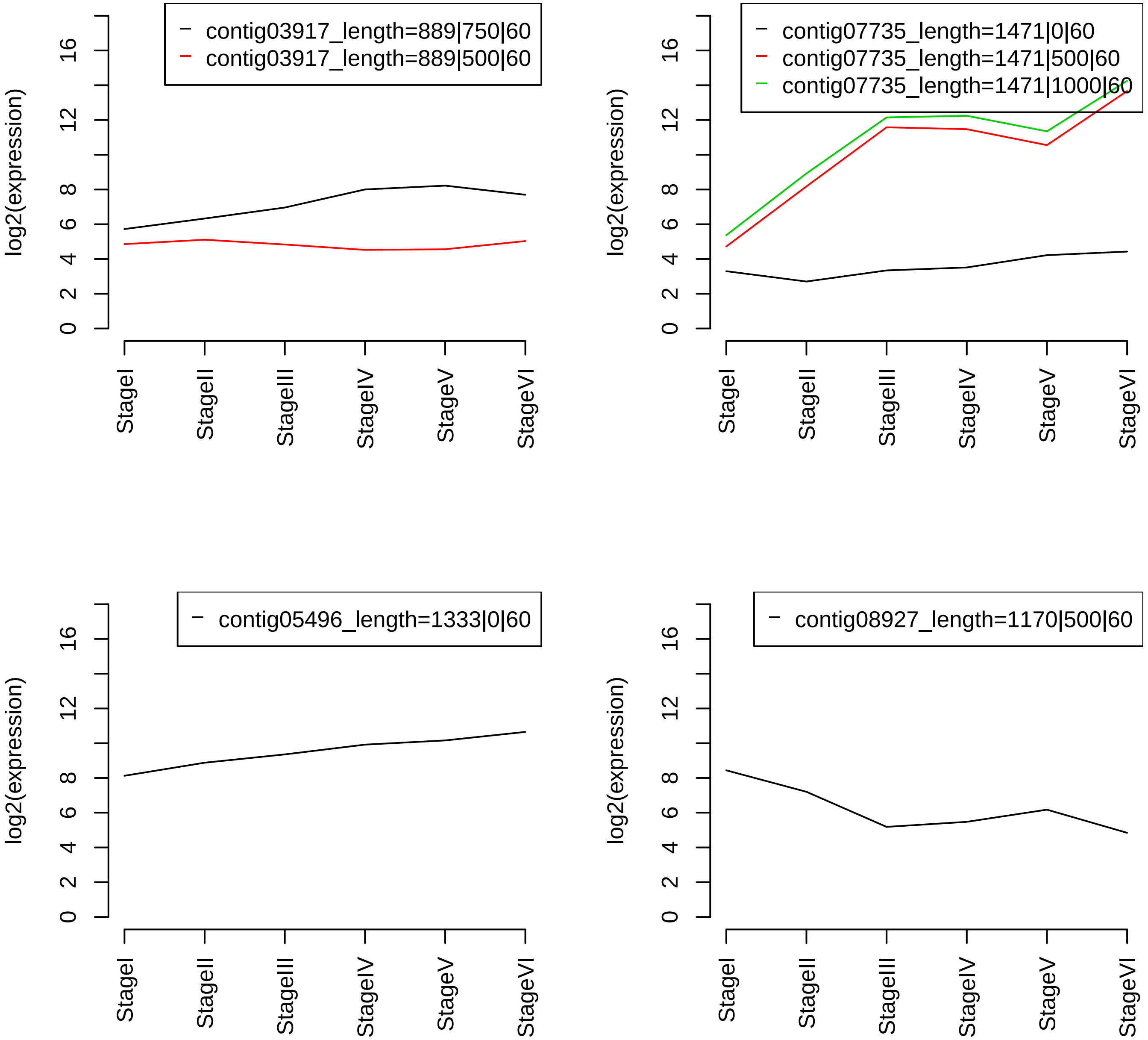

contig03917_length=889|750|60
contig03917_length=889|500|60
contig07735_length=1471|0|60
contig07735_length=1471|500|60
contig07735_length=1471|1000|60
contig05496_length=1333|0|60
contig08927_length=1170|500|60
log2(expression)
0
2
4
6
8
12
16
StageI
StageII
StageIII
StageIV
StageV
StageVI

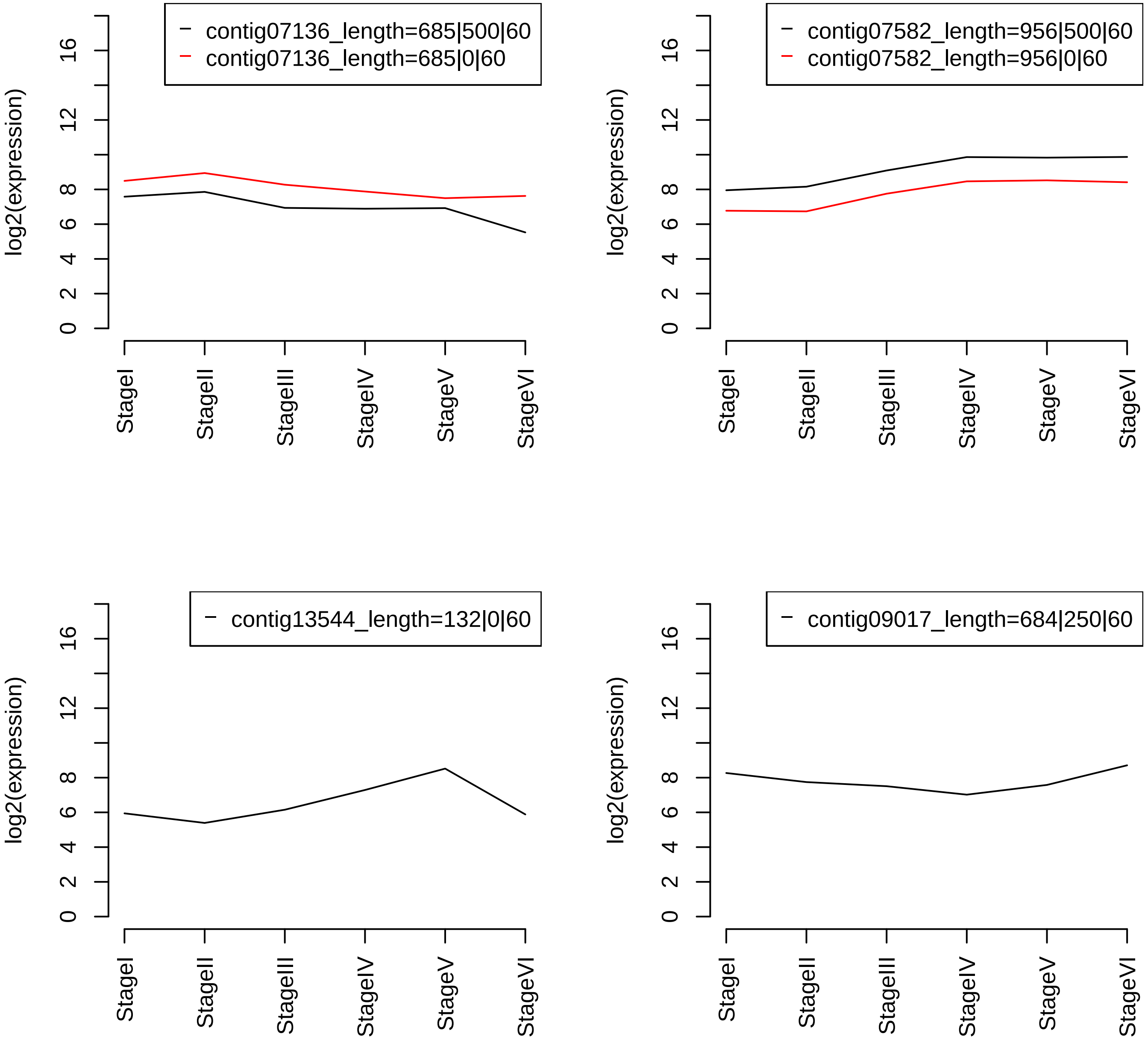

contig07136_length=685|500|60
contig07136_length=685|0|60
contig07582_length=956|500|60
contig07582_length=956|0|60
contig13544_length=132|0|60
contig09017_length=684|250|60
log2(expression)
0
2
4
6
8
12
16
StageI
StageII
StageIII
StageIV
StageV
StageVI

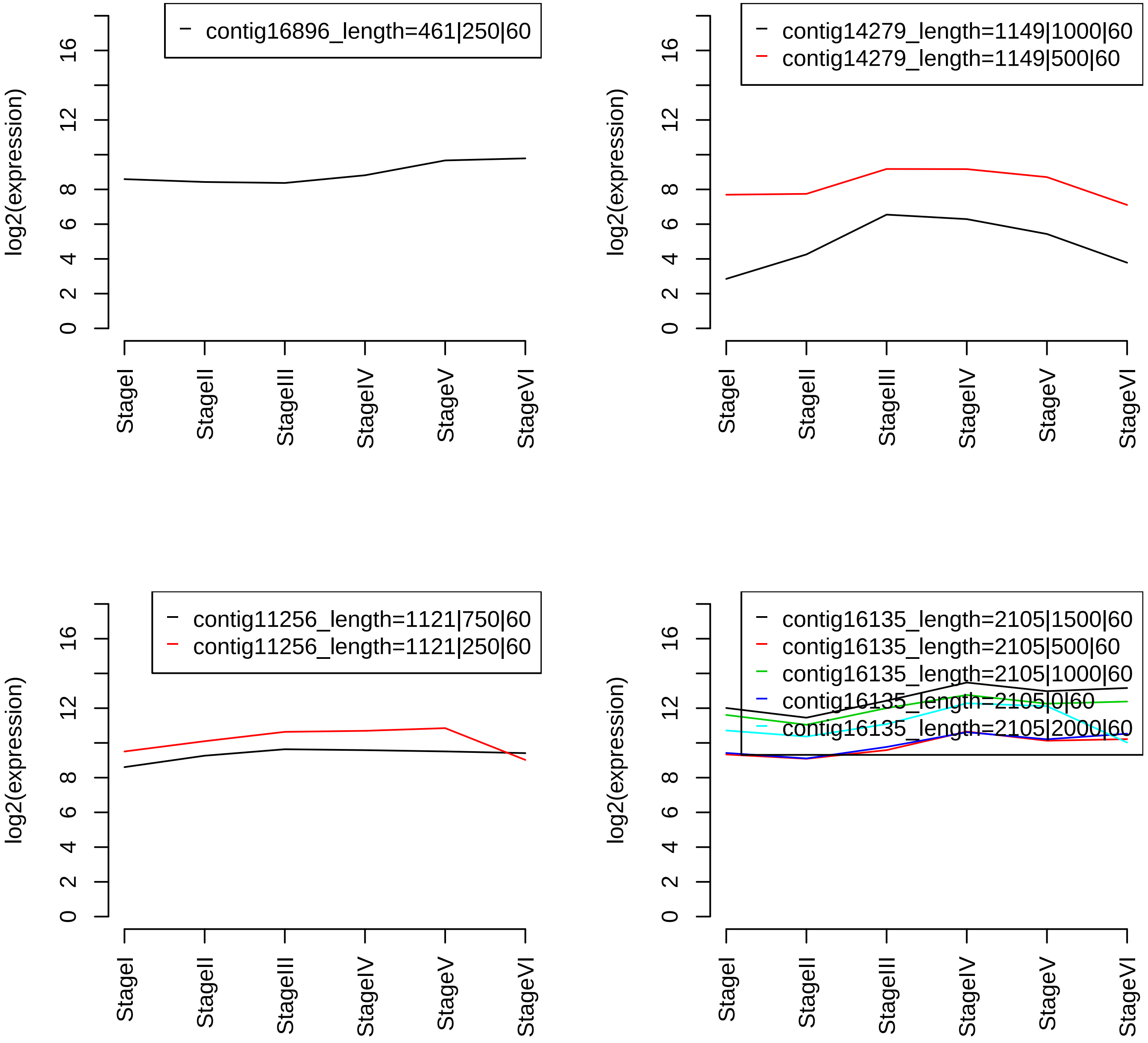

contig16896_length=461|250|60
contig14279_length=1149|1000|60
contig14279_length=1149|500|60
contig11256_length=1121|750|60
contig11256_length=1121|250|60
contig16135_length=2105|1500|60
contig16135_length=2105|500|60
contig16135_length=2105|1000|60
contig16135_length=2105|0|60
contig16135_length=2105|2000|60
log2(expression)
0
2
4
6
8
12
16
StageI
StageII
StageIII
StageIV
StageV
StageVI

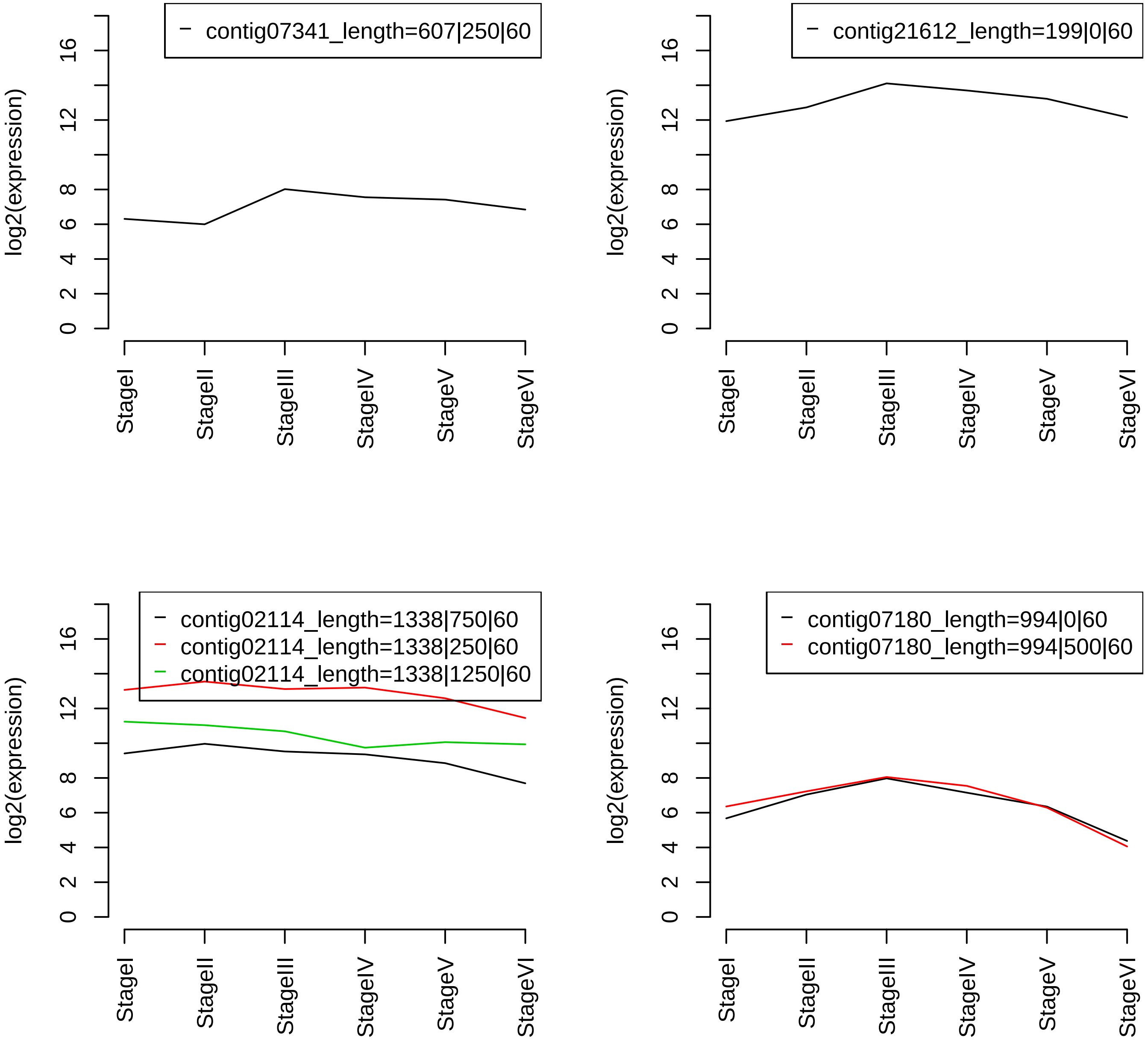
contig07341_length=607|250|60
contig21612_length=199|0|60
contig02114_length=1338|750|60
contig02114_length=1338|250|60
contig02114_length=1338|1250|60
contig07180_length=994|0|60
contig07180_length=994|500|60
log2(expression)
0
2
4
6
8
12
16
StageI
StageII
StageIII
StageIV
StageV
StageVI

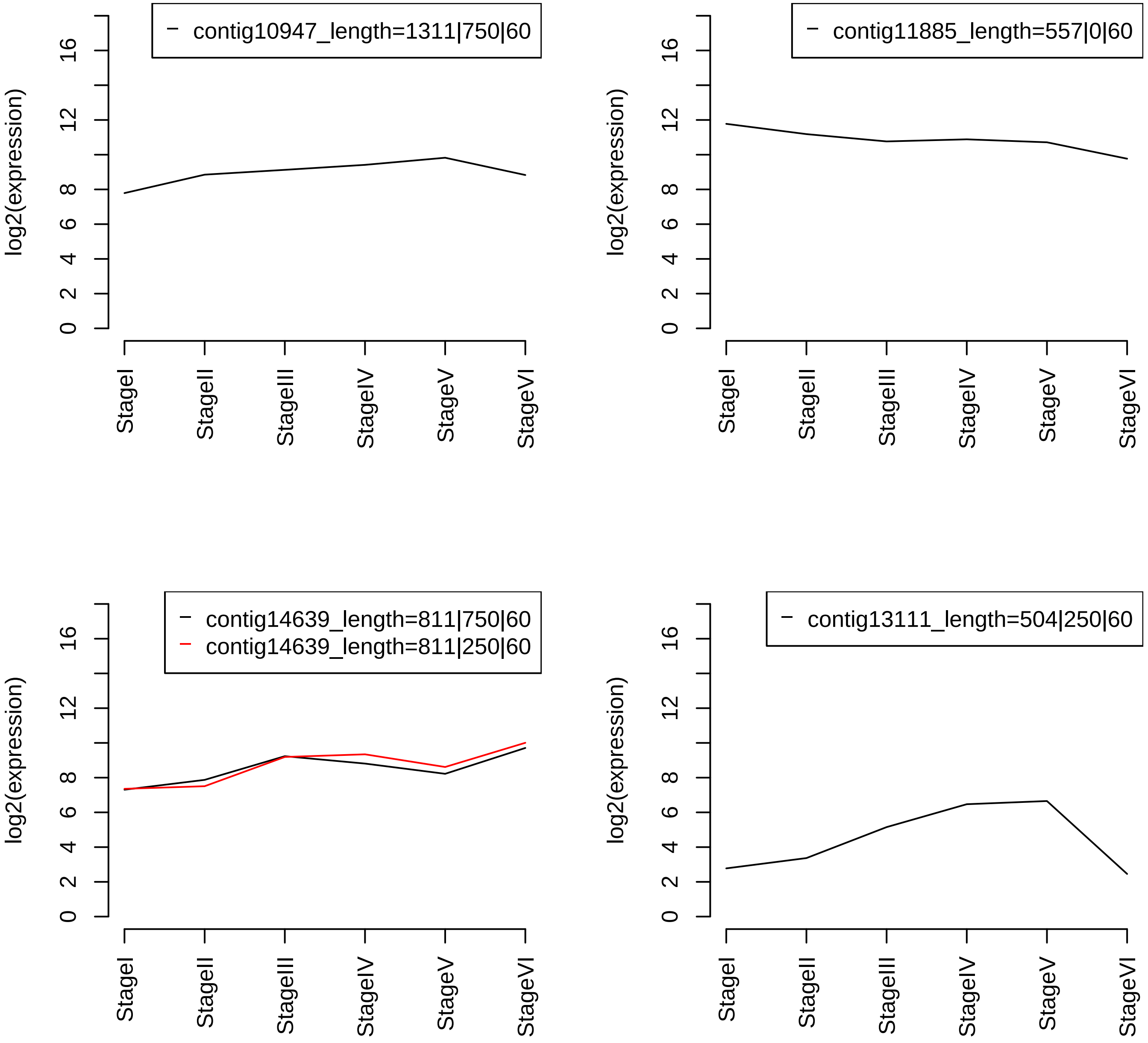

contig10947_length=1311|750|60
contig11885_length=557|0|60
contig14639_length=811|750|60
contig14639_length=811|250|60
contig13111_length=504|250|60
log2(expression)
0
2
4
6
8
12
16
StageI
StageII
StageIII
StageIV
StageV
StageVI

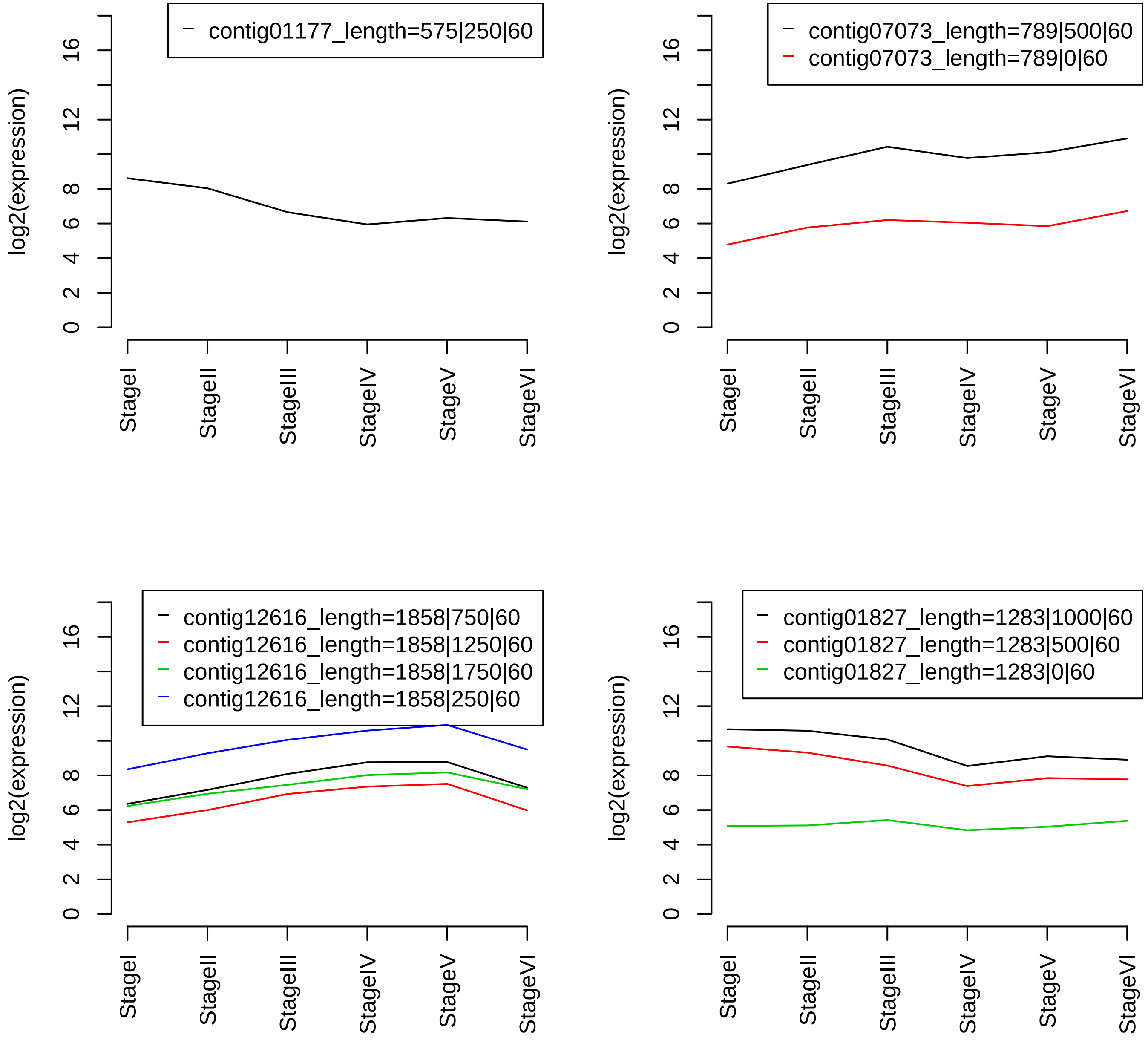
contig01177_length=575|250|60
contig07073_length=789|500|60
contig07073_length=789|0|60
contig12616_length=1858|750|60
contig12616_length=1858|1250|60
contig12616_length=1858|1750|60
contig12616_length=1858|250|60
contig01827_length=1283|1000|60
contig01827_length=1283|500|60
contig01827_length=1283|0|60
log2(expression)
0
2
4
6
8
12
16
StageI
StageII
StageIII
StageIV
StageV
StageVI

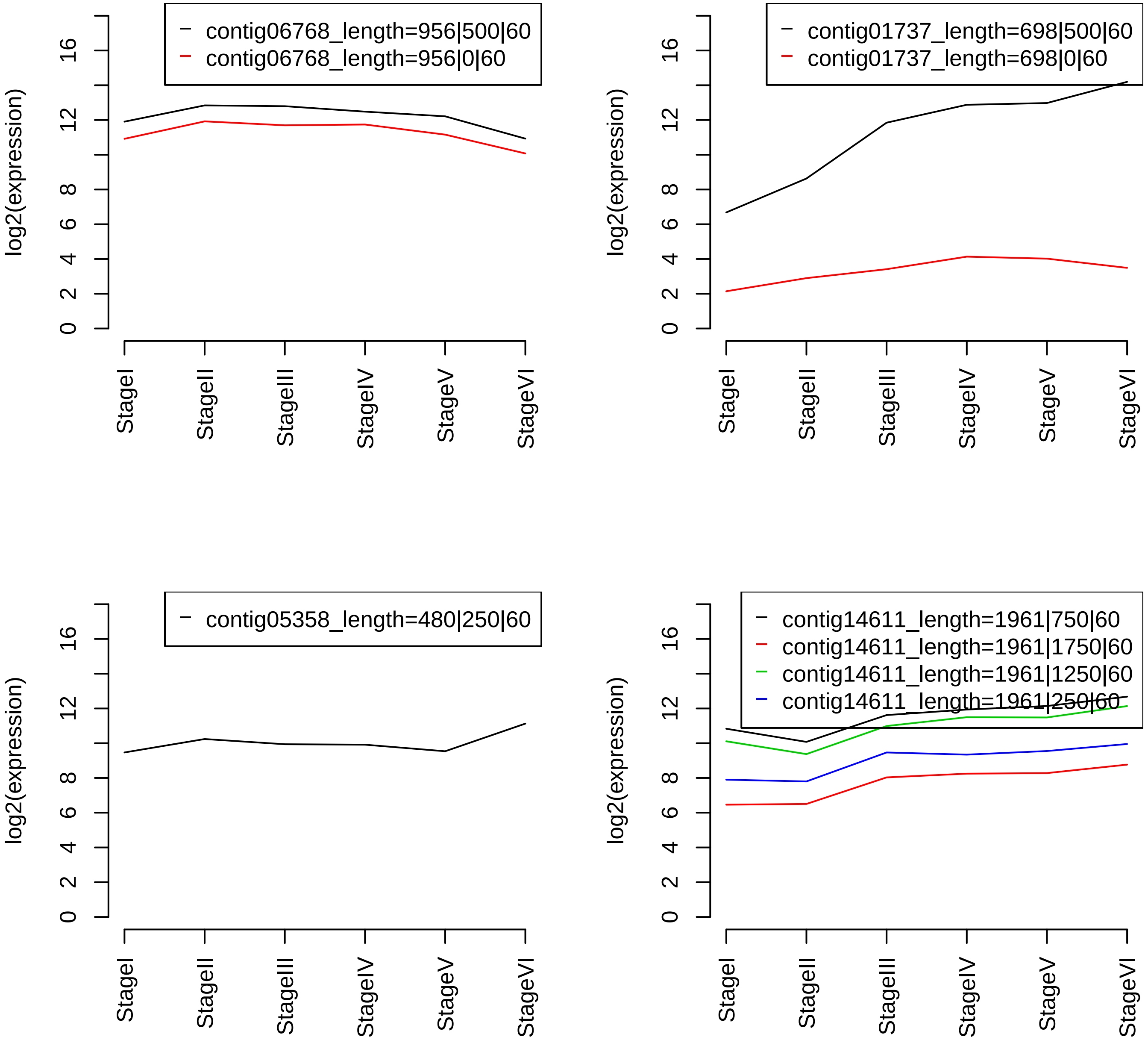

contig06768_length=956|500|60
contig06768_length=956|0|60
contig01737_length=698|500|60
contig01737_length=698|0|60
contig05358_length=480|250|60
contig14611_length=1961|750|60
contig14611_length=1961|1750|60
contig14611_length=1961|1250|60
contig14611_length=1961|250|60
log2(expression)
0 2 4 6 8 12 16
StageI
StageII
StageIII
StageIV
StageV
StageVI

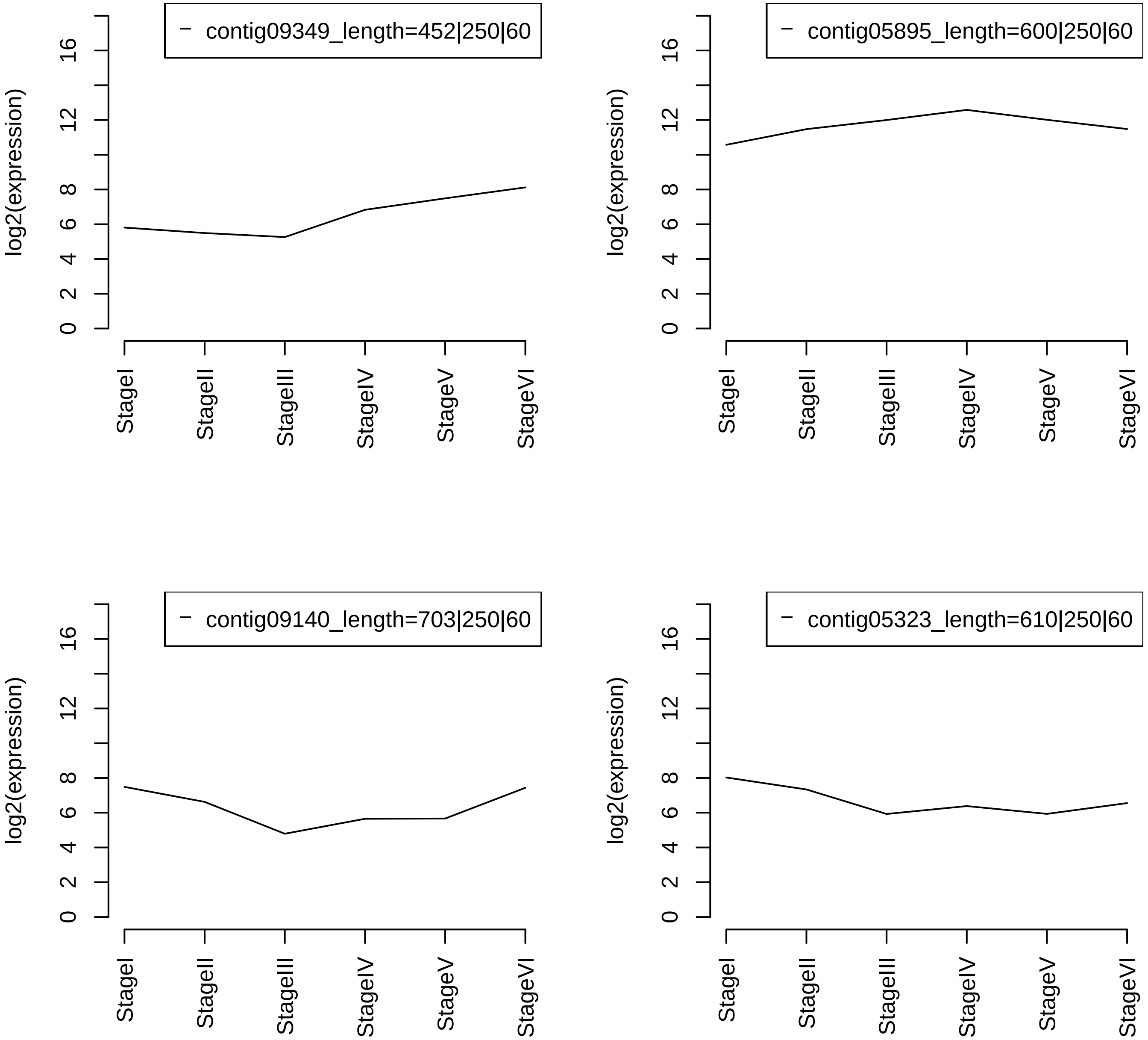
contig09349_length=452|250|60
contig05895_length=600|250|60
contig09140_length=703|250|60
contig05323_length=610|250|60
log2(expression)
0
2
4
6
8
12
16
StageI
StageII
StageIII
StageIV
StageV
StageVI

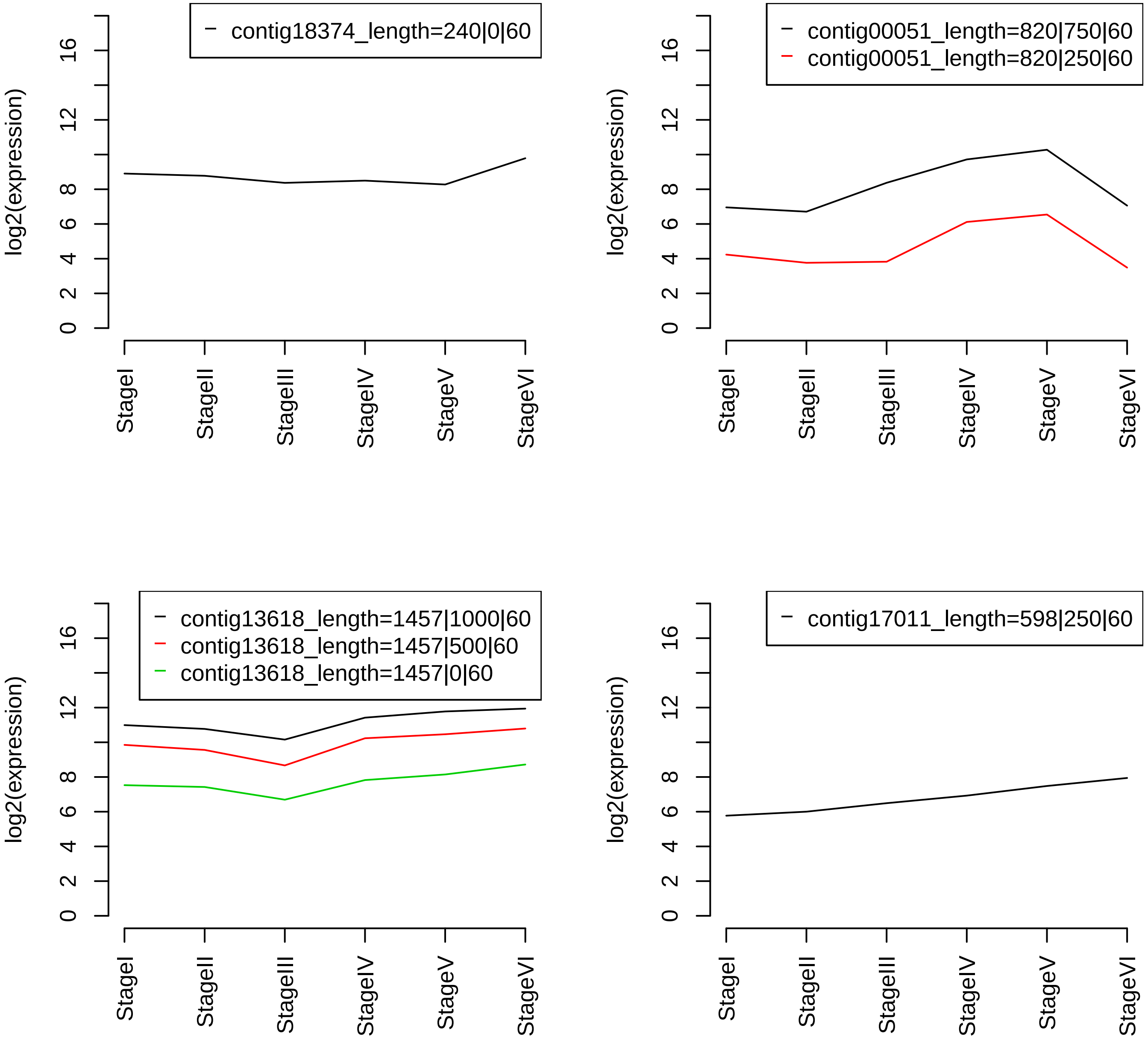
contig18374_length=240|0|60
contig00051_length=820|750|60
contig00051_length=820|250|60
contig13618_length=1457|1000|60
contig13618_length=1457|500|60
contig13618_length=1457|0|60
contig17011_length=598|250|60
log2(expression)
0
2
4
6
8
12
16
StageI
StageII
StageIII
StageIV
StageV
StageVI

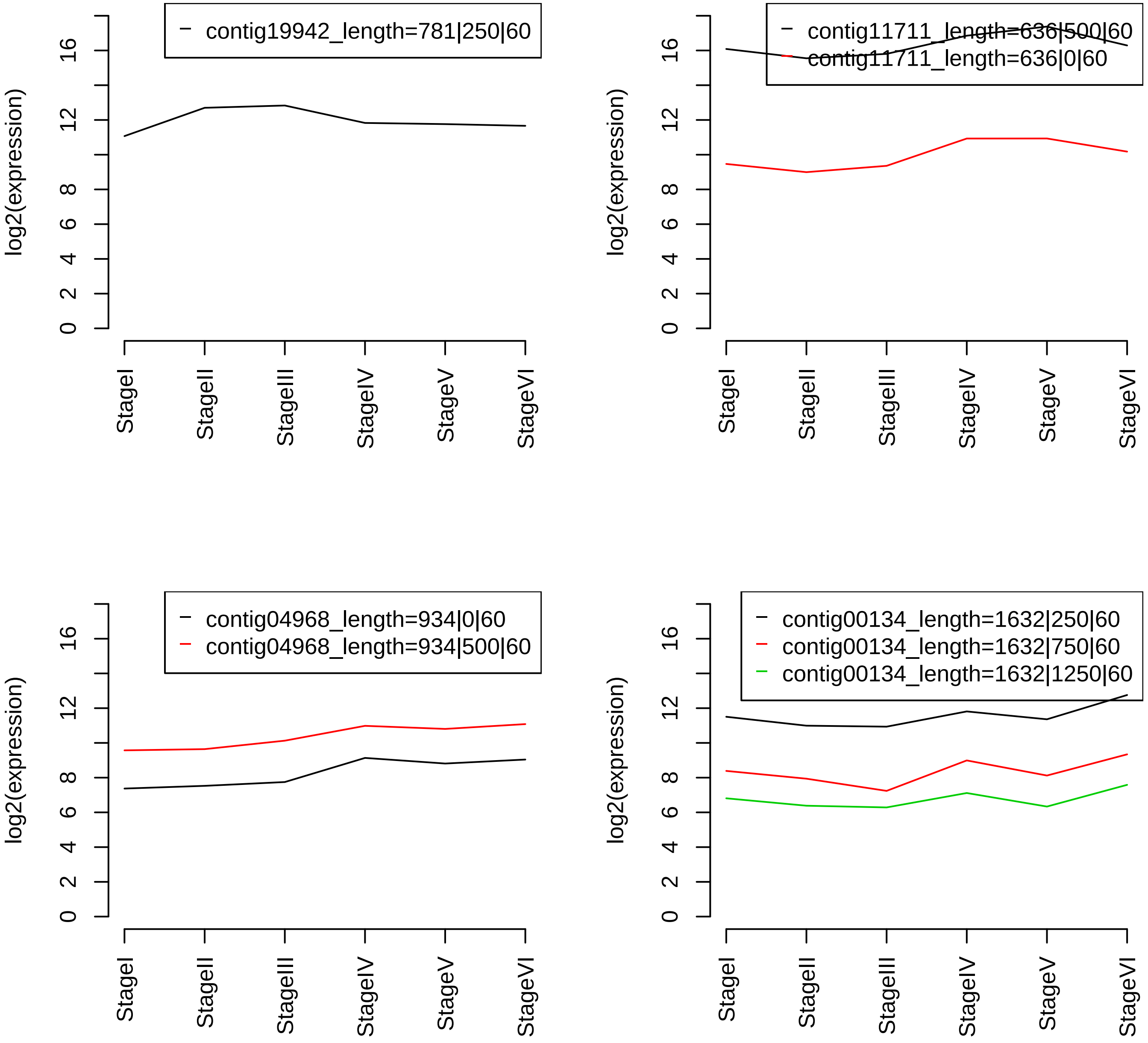
contig19942_length=781|250|60
contig11711_length=636|500|60
contig11711_length=636|0|60
contig04968_length=934|0|60
contig04968_length=934|500|60
contig00134_length=1632|250|60
contig00134_length=1632|750|60
contig00134_length=1632|1250|60
log2(expression)
0
2
4
6
8
12
16
StageI
StageII
StageIII
StageIV
StageV
StageVI

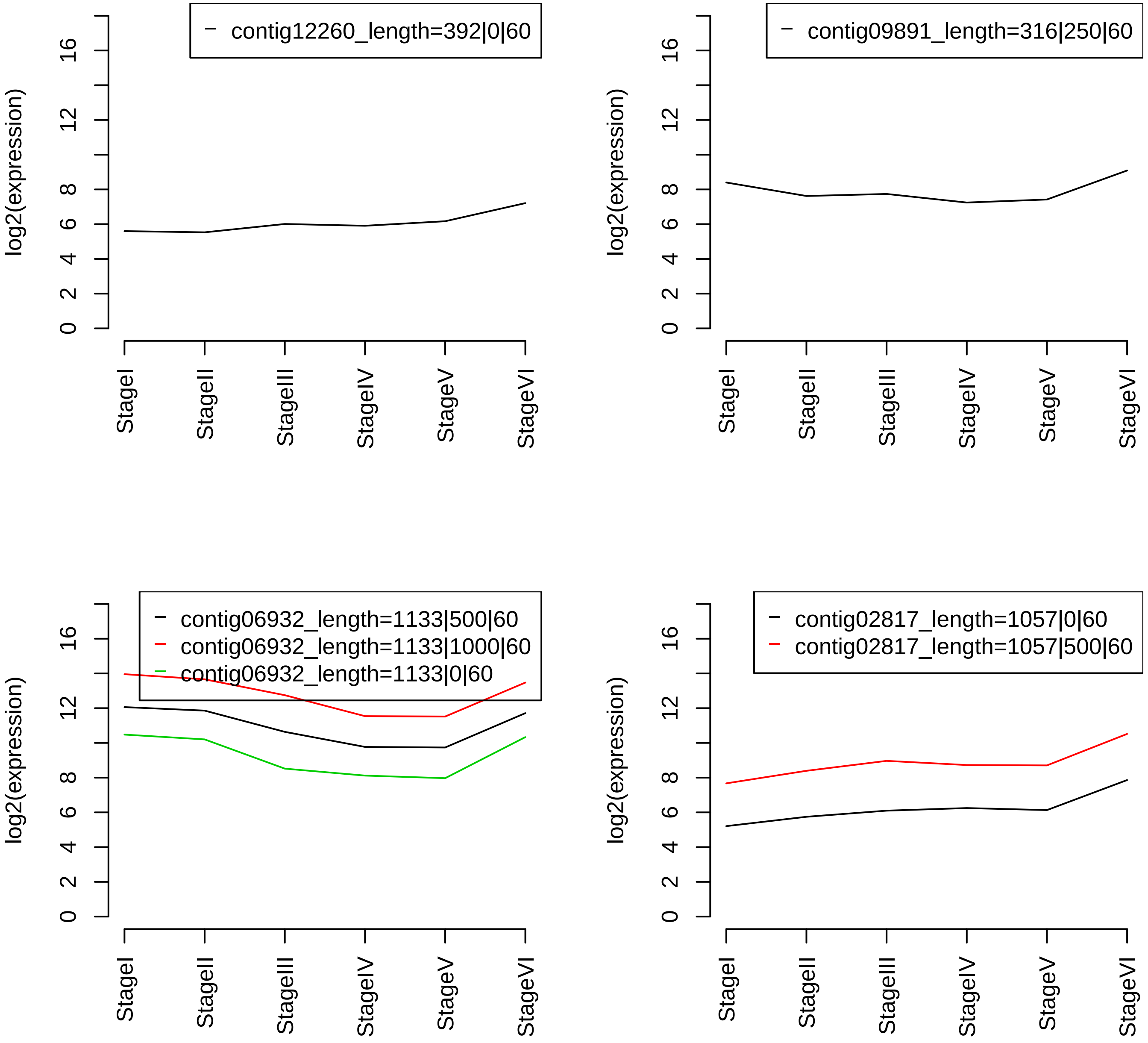

contig12260_length=392|0|60
contig09891_length=316|250|60
contig06932_length=1133|500|60
contig06932_length=1133|1000|60
contig06932_length=1133|0|60
contig02817_length=1057|0|60
contig02817_length=1057|500|60
log2(expression)
0
2
4
6
8
12
16
StageI
StageII
StageIII
StageIV
StageV
StageVI

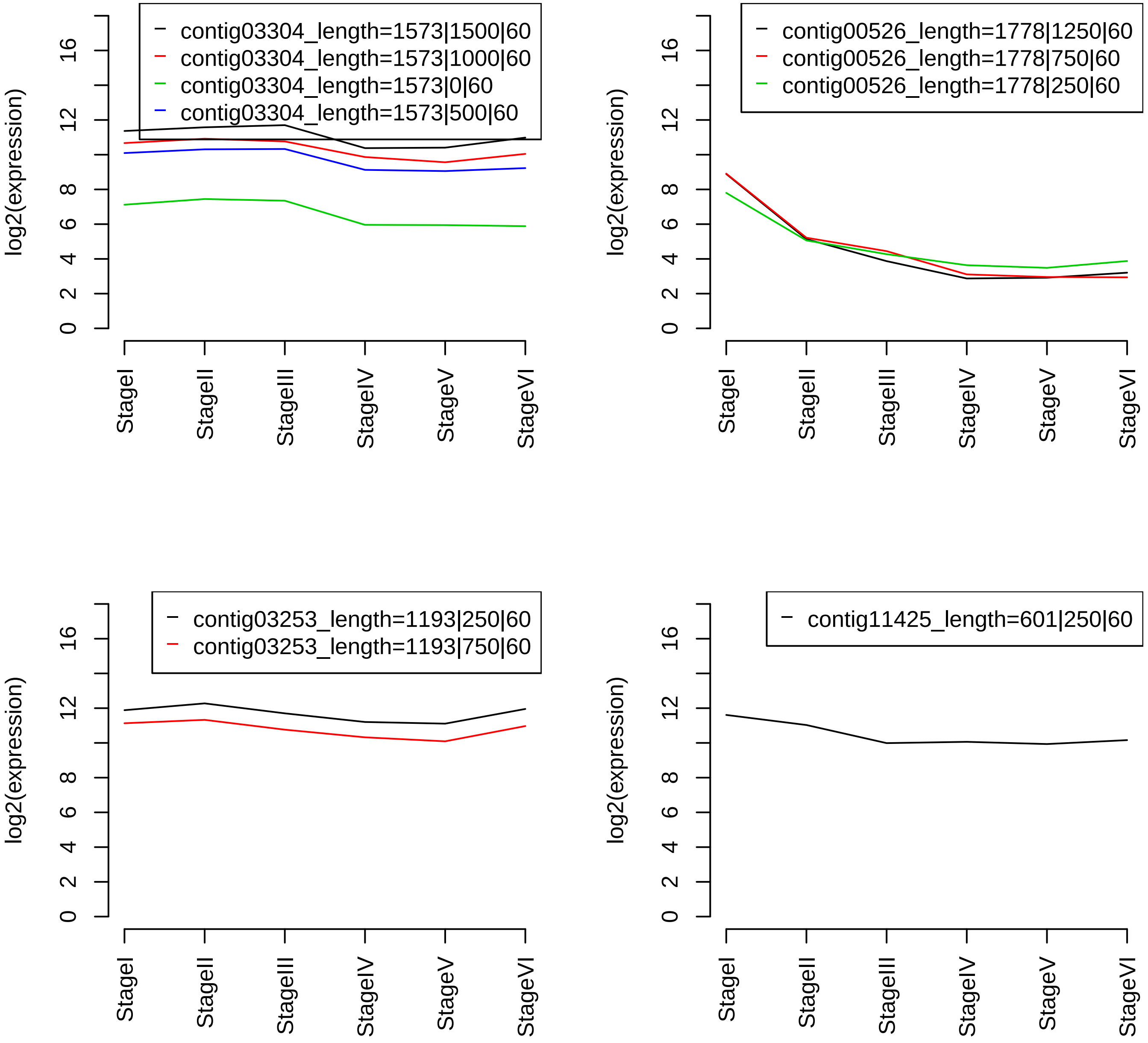

contig03304_length=1573|1500|60
contig03304_length=1573|1000|60
contig03304_length=1573|0|60
contig03304_length=1573|500|60
contig00526_length=1778|1250|60
contig00526_length=1778|750|60
contig00526_length=1778|250|60
contig03253_length=1193|250|60
contig03253_length=1193|750|60
contig11425_length=601|250|60
log2(expression)
0 2 4 6 8 12 16
StageI StageII StageIII StageIV StageV StageVI

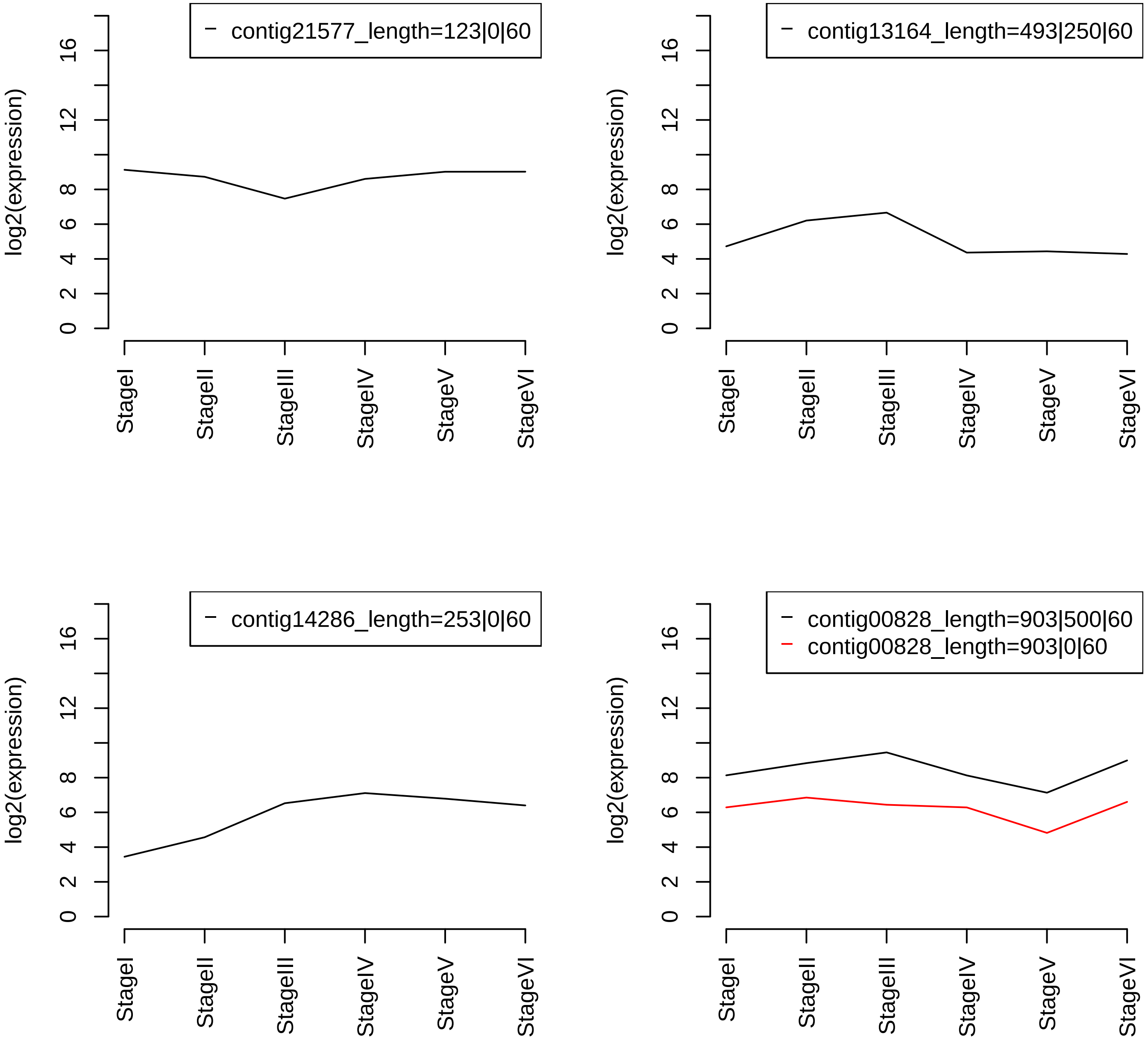

contig21577_length=123|0|60
contig13164_length=493|250|60
contig14286_length=253|0|60
contig00828_length=903|500|60
contig00828_length=903|0|60
log2(expression)
0
2
4
6
8
12
16
StageI
StageII
StageIII
StageIV
StageV
StageVI

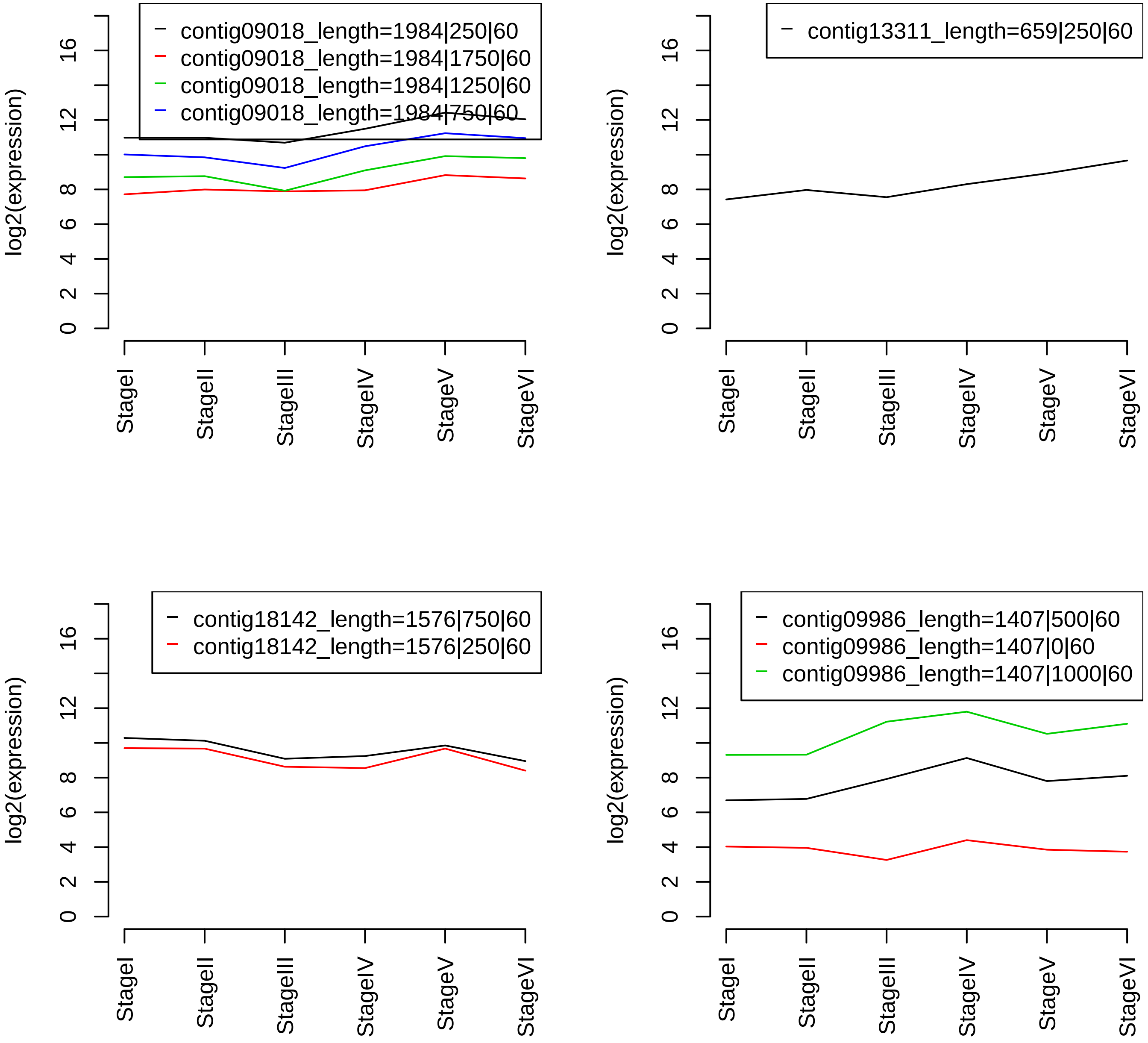

contig09018_length=1984|250|60
contig09018_length=1984|1750|60
contig09018_length=1984|1250|60
contig09018_length=1984|750|60
log2(expression)
0
2
4
6
8
12
16
StageI
StageII
StageIII
StageIV
StageV
StageVI
contig13311_length=659|250|60
contig18142_length=1576|750|60
contig18142_length=1576|250|60
contig09986_length=1407|500|60
contig09986_length=1407|0|60
contig09986_length=1407|1000|60

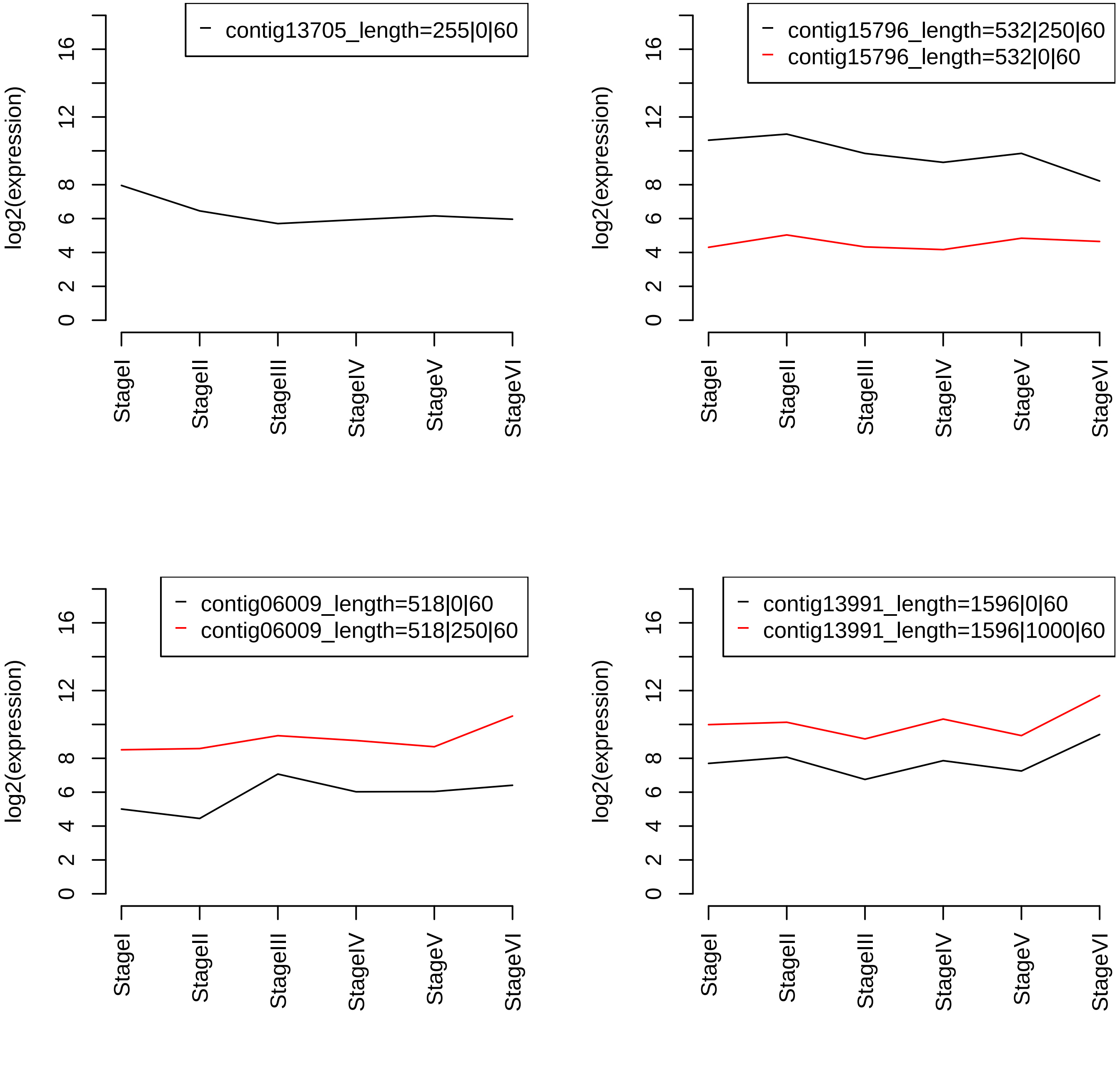
contig13705_length=255|0|60
contig15796_length=532|250|60
contig15796_length=532|0|60
contig06009_length=518|0|60
contig06009_length=518|250|60
contig13991_length=1596|0|60
contig13991_length=1596|1000|60
log2(expression)
0
2
4
6
8
12
16
StageI
StageII
StageIII
StageIV
StageV
StageVI

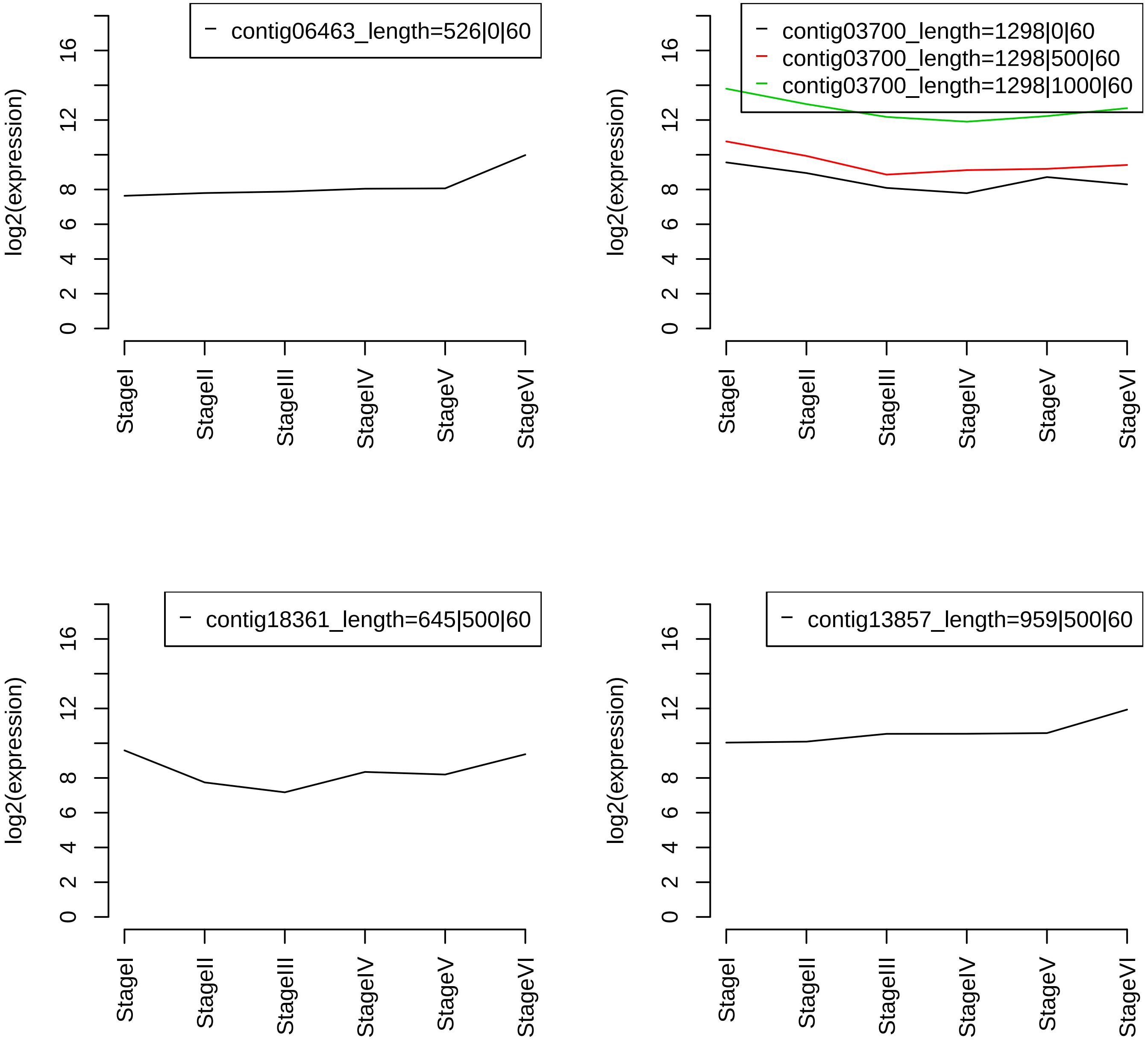
contig06463_length=526|0|60
contig03700_length=1298|0|60
contig03700_length=1298|500|60
contig03700_length=1298|1000|60
contig18361_length=645|500|60
contig13857_length=959|500|60
log2(expression)
0
2
4
6
8
12
16
StageI
StageII
StageIII
StageIV
StageV
StageVI

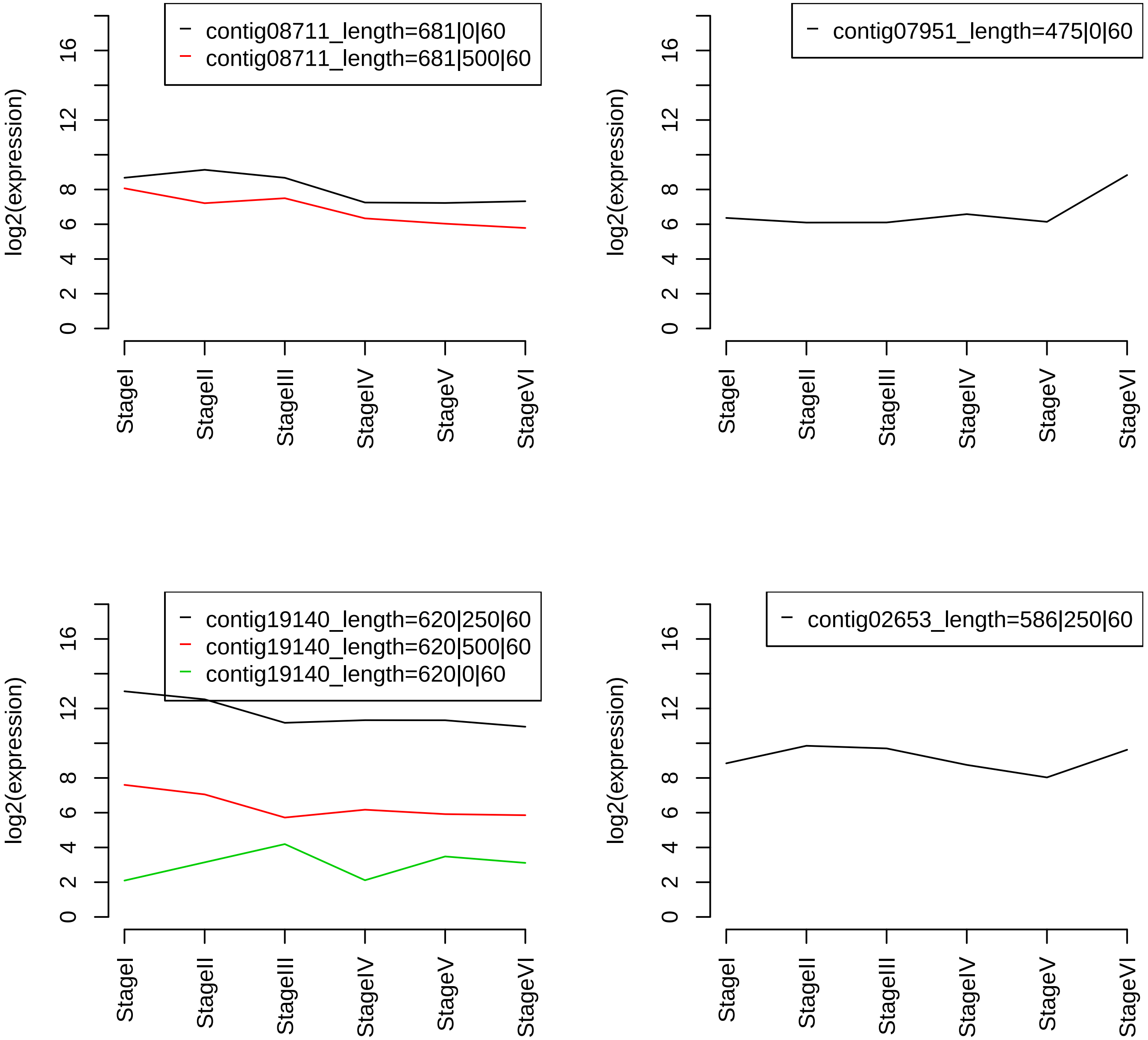
contig08711_length=681|0|60
contig08711_length=681|500|60
contig07951_length=475|0|60
contig19140_length=620|250|60
contig19140_length=620|500|60
contig19140_length=620|0|60
contig02653_length=586|250|60
log2(expression)
StageI
StageII
StageIII
StageIV
StageV
StageVI

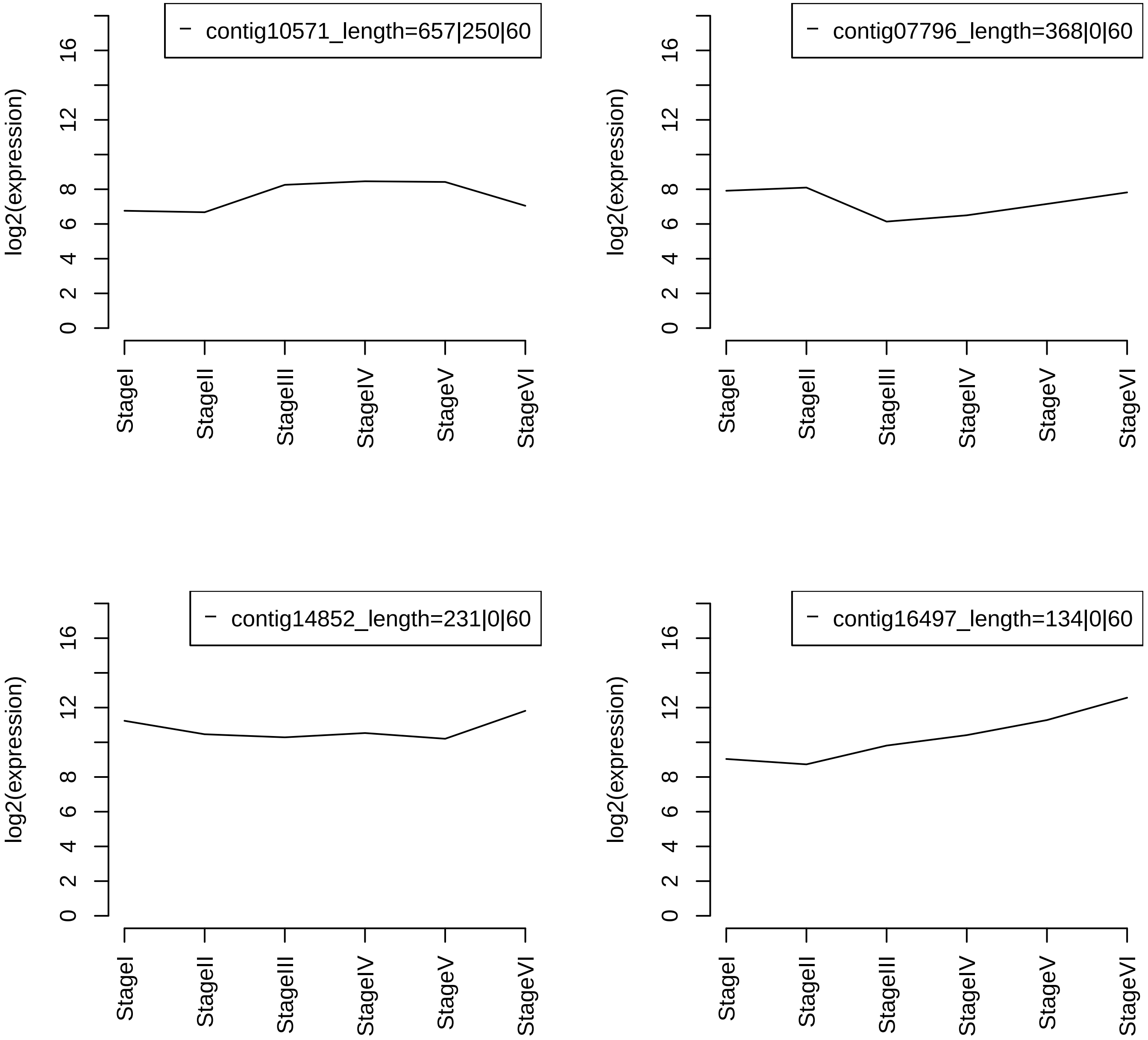
contig10571_length=657|250|60
contig07796_length=368|0|60
contig14852_length=231|0|60
contig16497_length=134|0|60
log2(expression)
0
2
4
6
8
12
16
StageI
StageII
StageIII
StageIV
StageV
StageVI

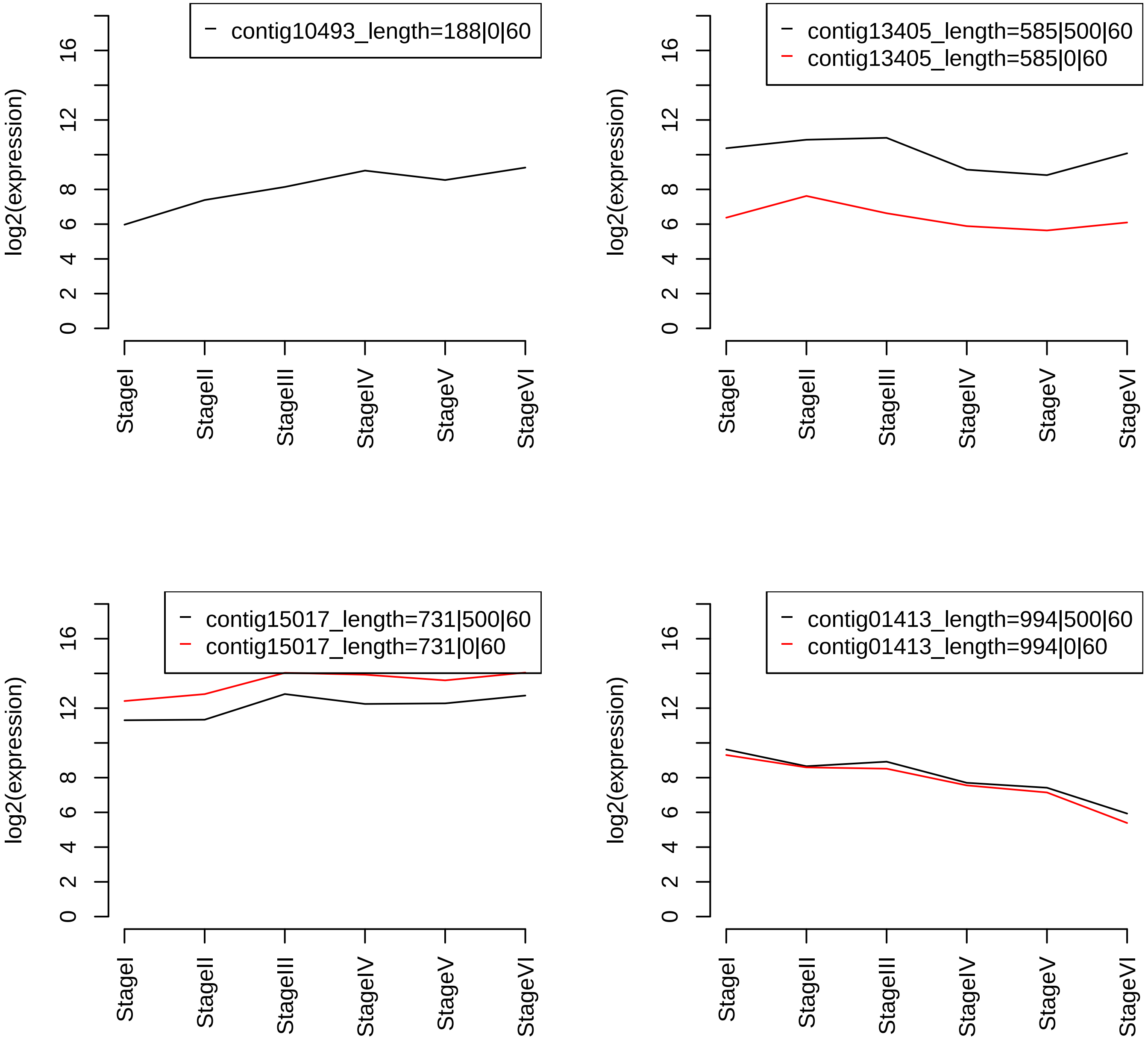

contig10493_length=188|0|60
contig13405_length=585|500|60
contig13405_length=585|0|60
contig15017_length=731|500|60
contig15017_length=731|0|60
contig01413_length=994|500|60
contig01413_length=994|0|60
log2(expression)
0
2
4
6
8
12
16
StageI
StageII
StageIII
StageIV
StageV
StageVI

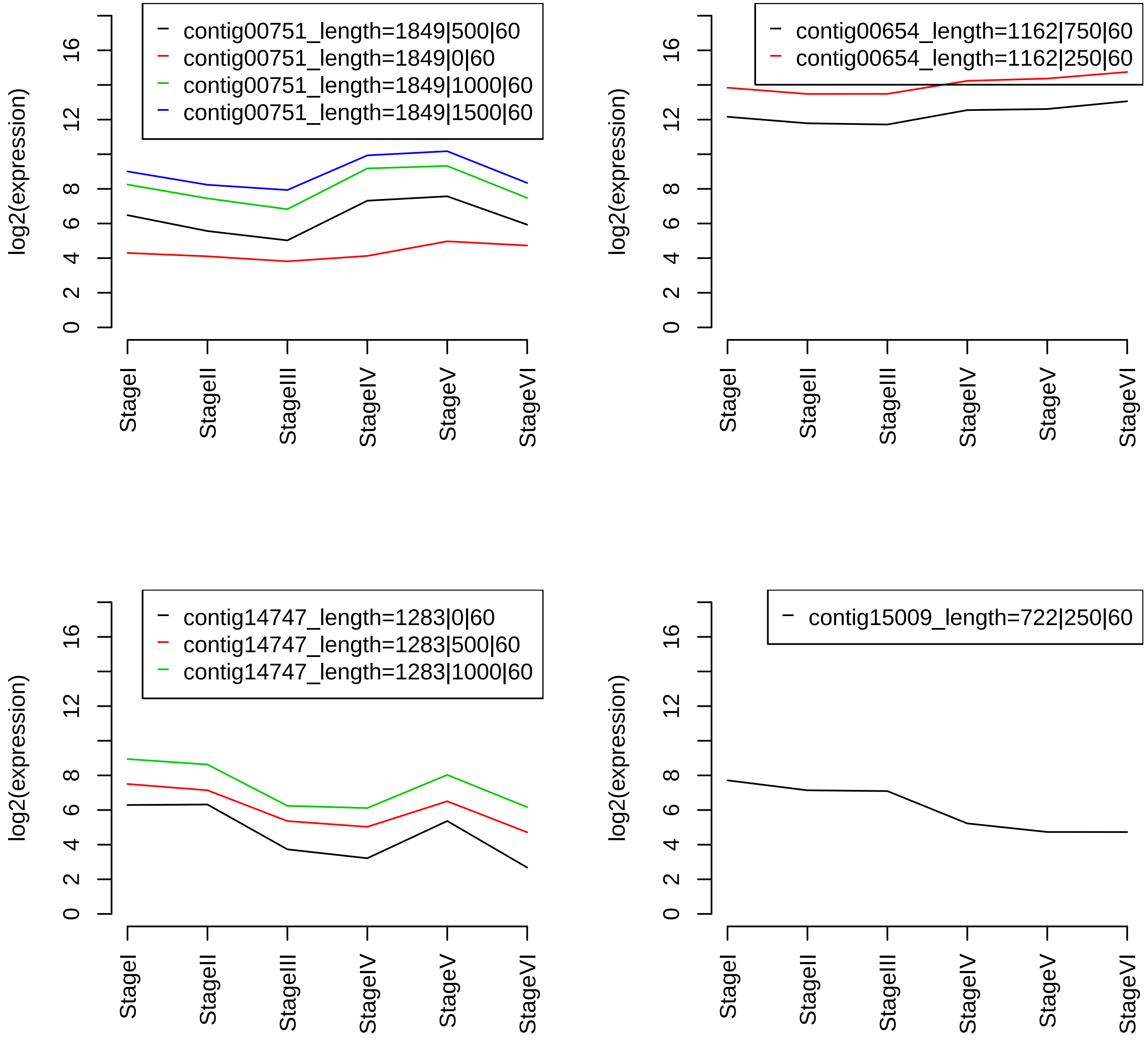
contig00751_length=1849|500|60
contig00751_length=1849|0|60
contig00751_length=1849|1000|60
contig00751_length=1849|1500|60
contig00654_length=1162|750|60
contig00654_length=1162|250|60
contig14747_length=1283|0|60
contig14747_length=1283|500|60
contig14747_length=1283|1000|60
contig15009_length=722|250|60
log2(expression)
0
2
4
6
8
12
16
StageI
StageII
StageIII
StageIV
StageV
StageVI

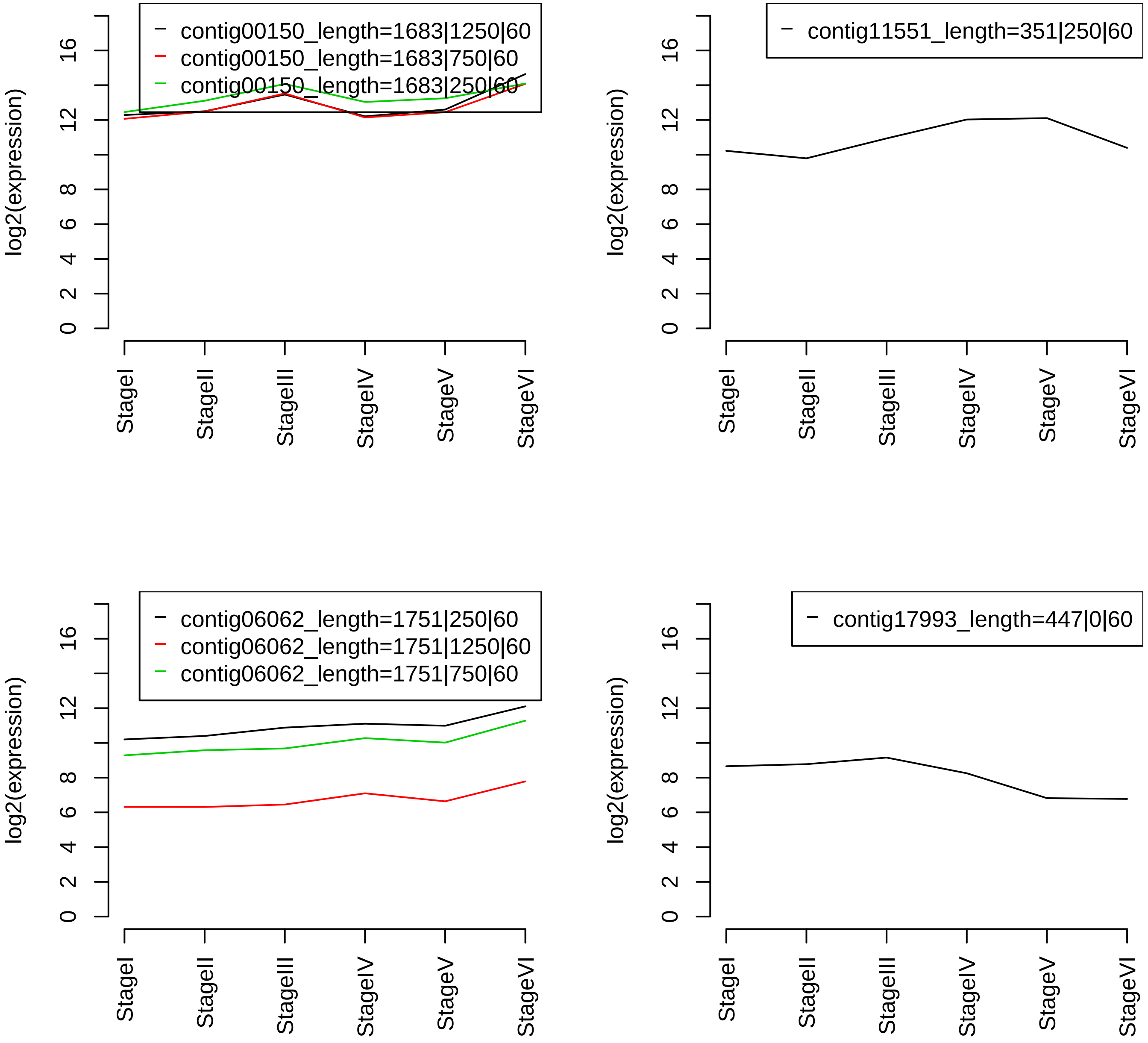

contig00150_length=1683|1250|60
contig00150_length=1683|750|60
contig00150_length=1683|250|60
contig11551_length=351|250|60
contig06062_length=1751|250|60
contig06062_length=1751|1250|60
contig06062_length=1751|750|60
contig17993_length=447|0|60
log2(expression)
0
2
4
6
8
12
16
StageI
StageII
StageIII
StageIV
StageV
StageVI

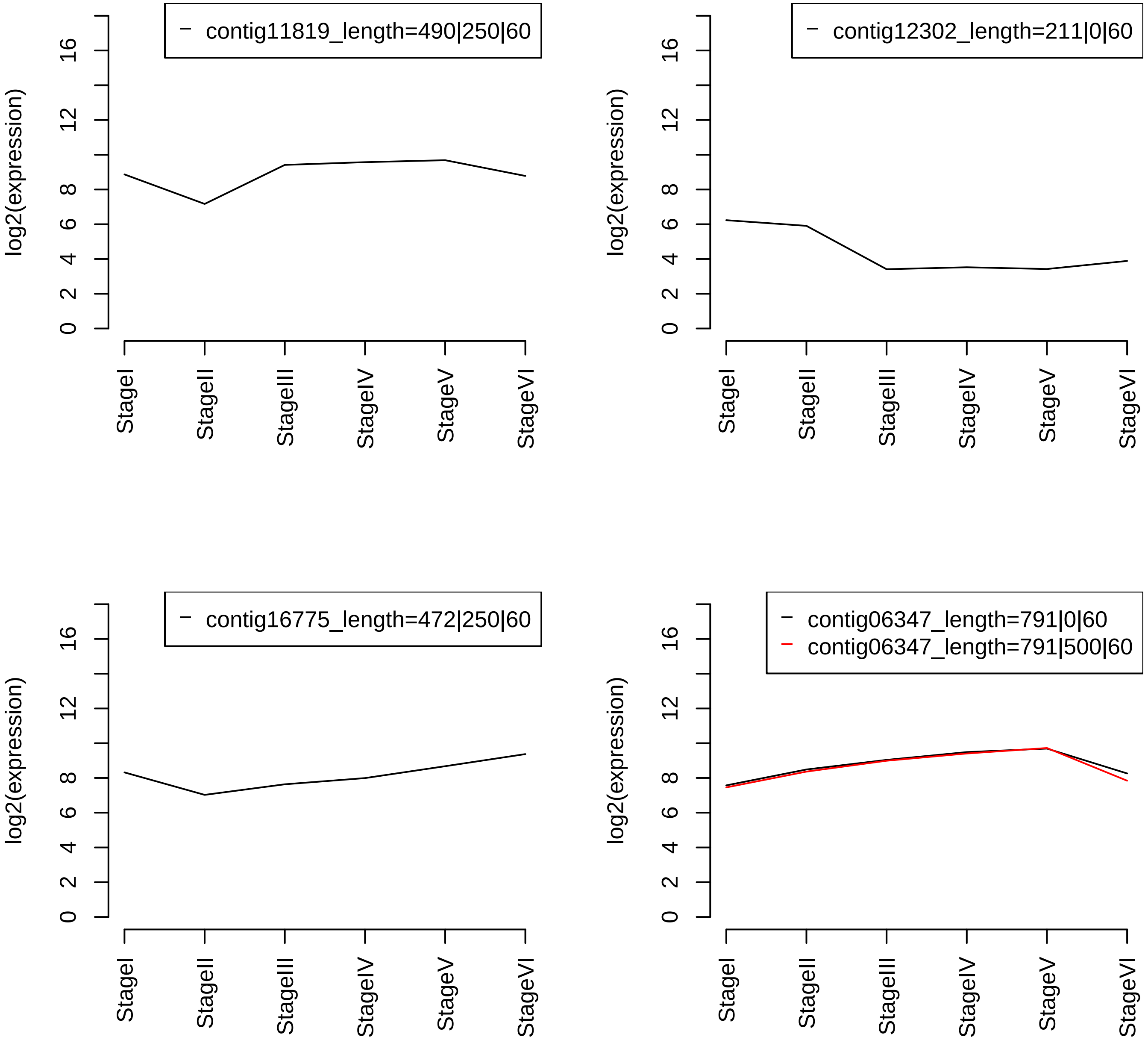
contig11819_length=490|250|60
contig12302_length=211|0|60
contig16775_length=472|250|60
contig06347_length=791|0|60
contig06347_length=791|500|60
log2(expression)
0
2
4
6
8
12
16
StageI
StageII
StageIII
StageIV
StageV
StageVI

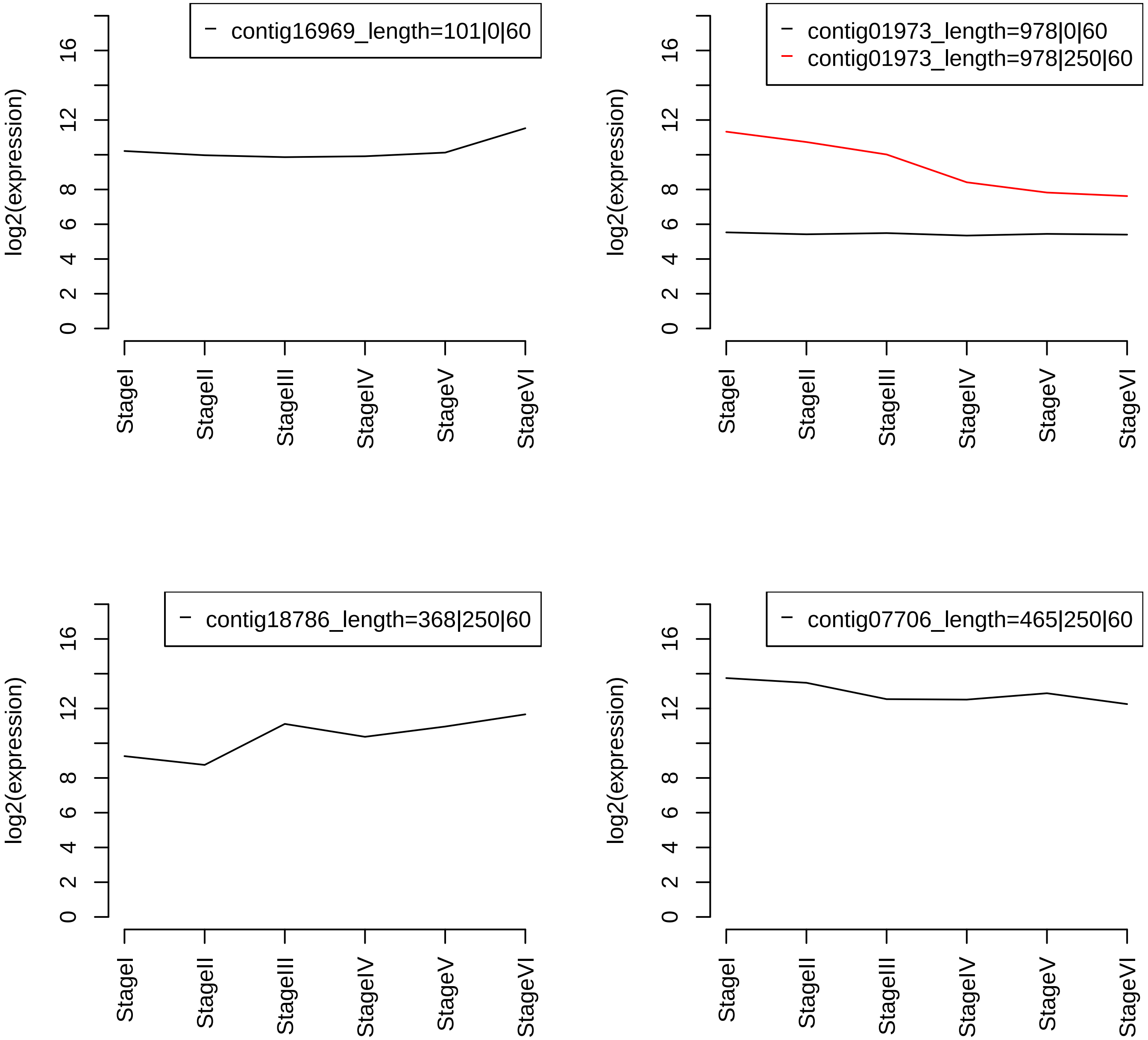

contig16969_length=101|0|60
contig01973_length=978|0|60
contig01973_length=978|250|60
contig18786_length=368|250|60
contig07706_length=465|250|60
log2(expression)
0
2
4
6
8
12
16
StageI
StageII
StageIII
StageIV
StageV
StageVI

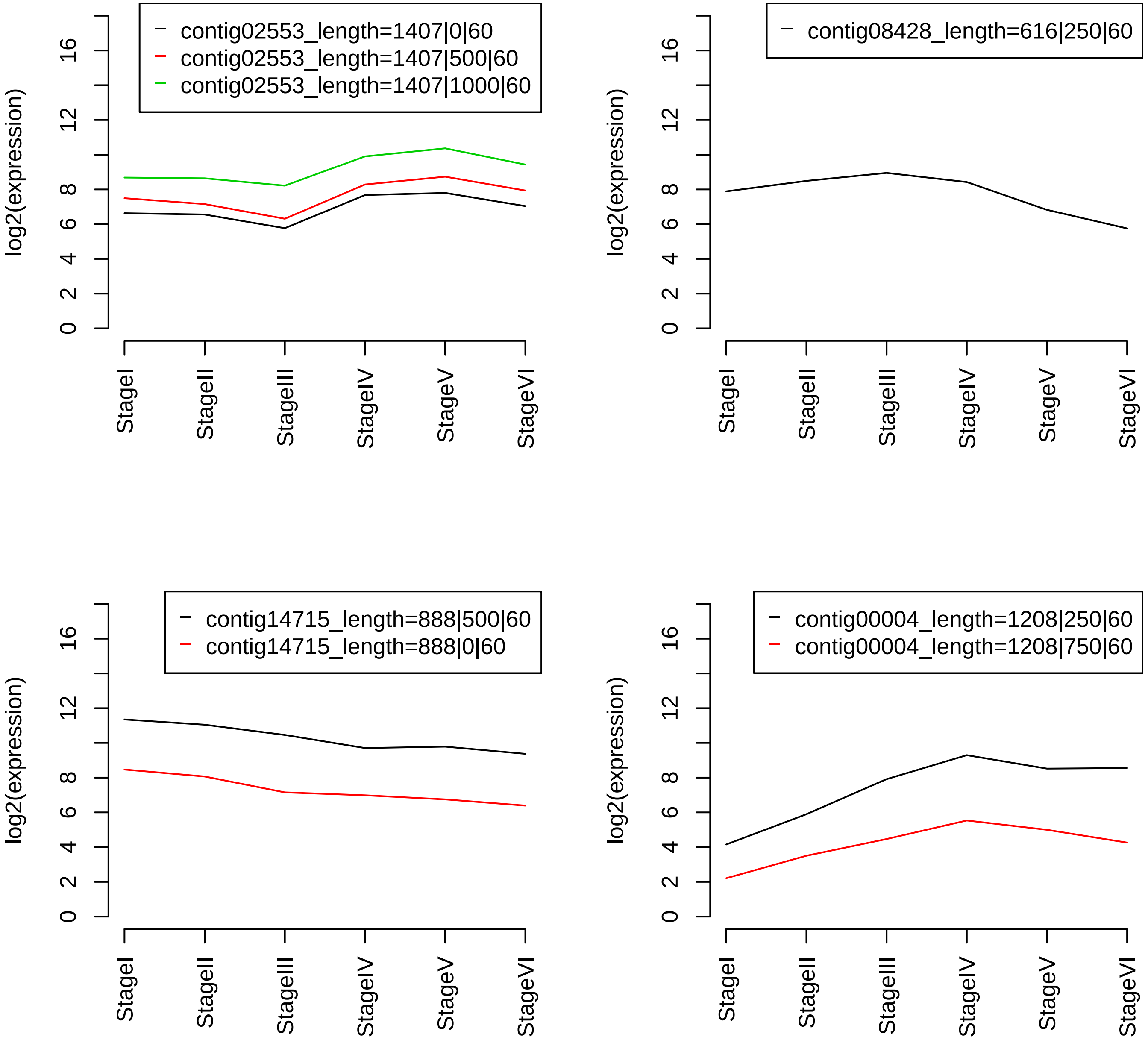

contig02553_length=1407|0|60
contig02553_length=1407|500|60
contig02553_length=1407|1000|60
contig08428_length=616|250|60
contig14715_length=888|500|60
contig14715_length=888|0|60
contig00004_length=1208|250|60
contig00004_length=1208|750|60
log2(expression)
0 2 4 6 8 12 16
StageI StageII StageIII StageIV StageV StageVI

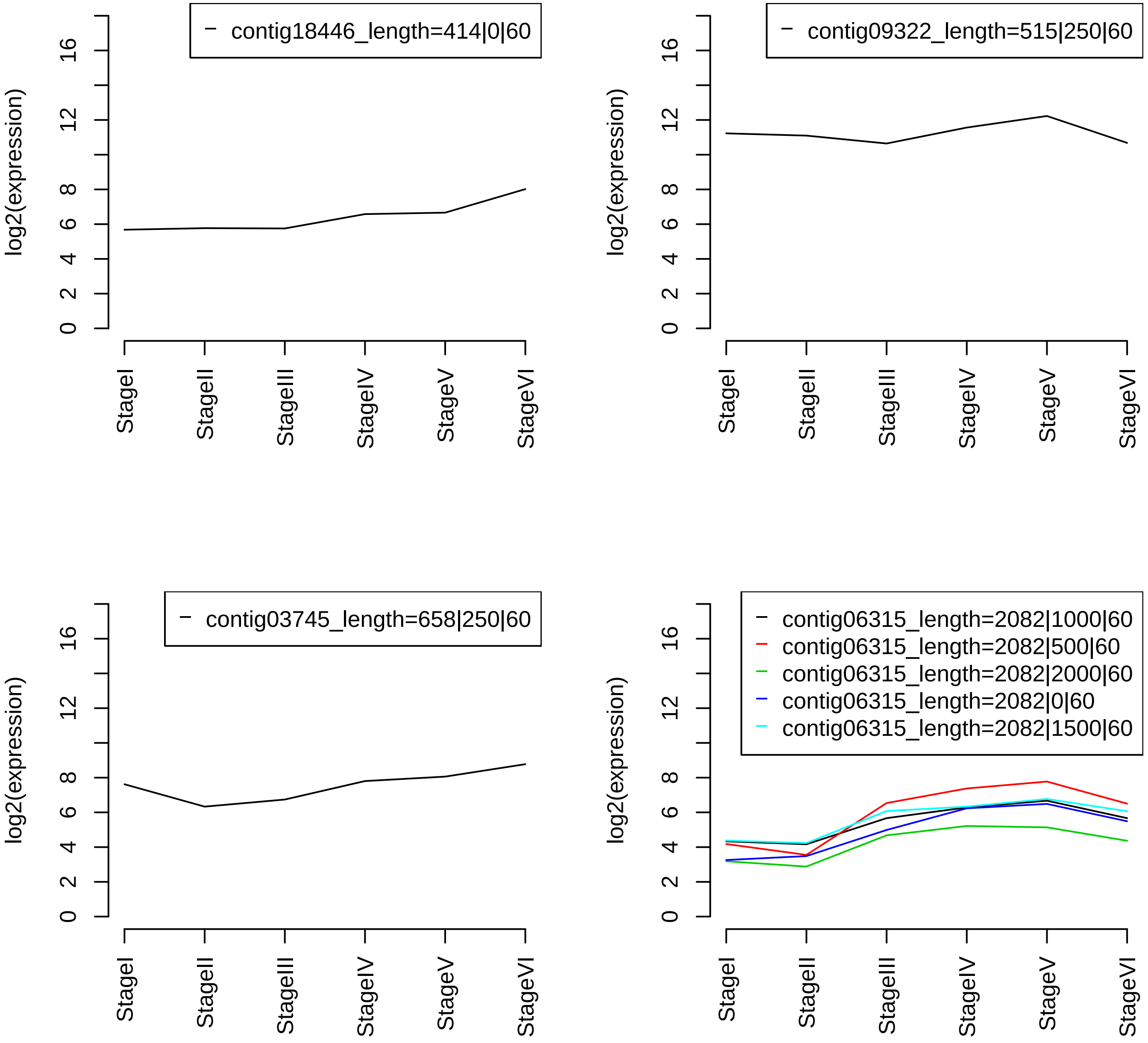

contig18446_length=414|0|60
contig09322_length=515|250|60
contig03745_length=658|250|60
contig06315_length=2082|1000|60
contig06315_length=2082|500|60
contig06315_length=2082|2000|60
contig06315_length=2082|0|60
contig06315_length=2082|1500|60
log2(expression)
0
2
4
6
8
12
16
StageI
StageII
StageIII
StageIV
StageV
StageVI

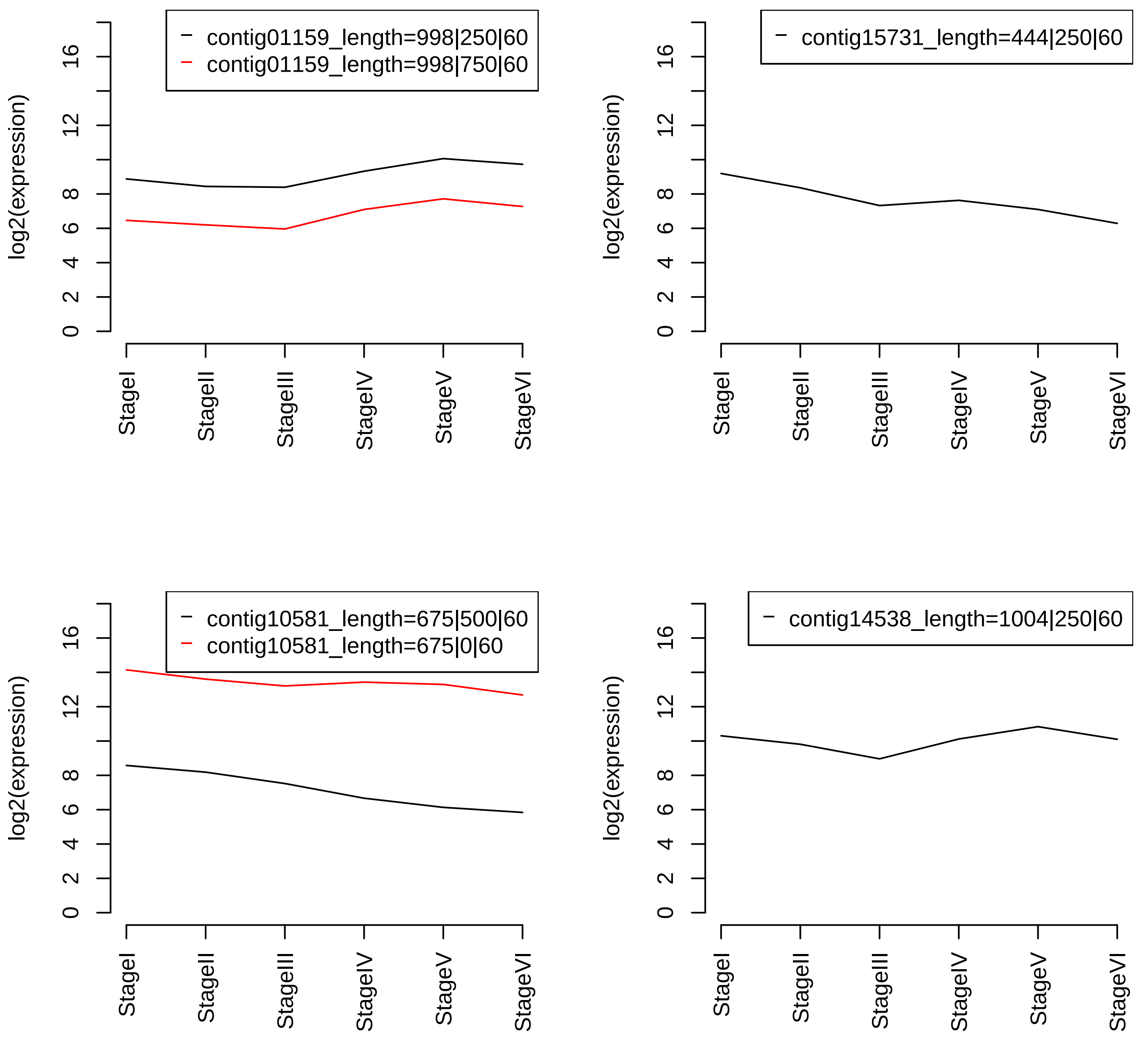

contig01159_length=998|250|60
contig01159_length=998|750|60
contig15731_length=444|250|60
contig10581_length=675|500|60
contig10581_length=675|0|60
contig14538_length=1004|250|60
log2(expression)
0
2
4
6
8
12
16
StageI
StageII
StageIII
StageIV
StageV
StageVI

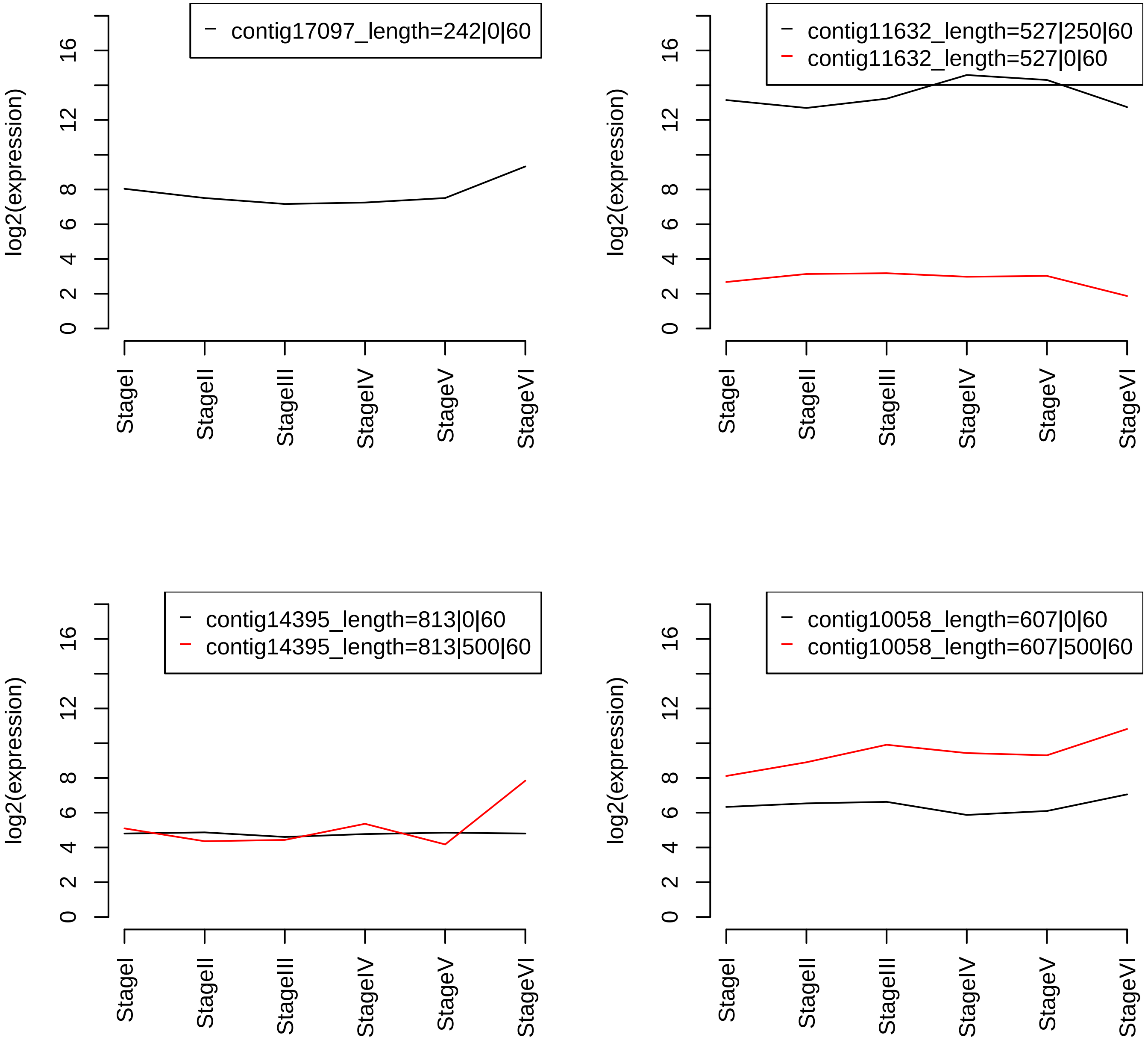

contig17097_length=242|0|60
contig11632_length=527|250|60
contig11632_length=527|0|60
contig14395_length=813|0|60
contig14395_length=813|500|60
contig10058_length=607|0|60
contig10058_length=607|500|60
log2(expression)
0
2
4
6
8
12
16
StageI
StageII
StageIII
StageIV
StageV
StageVI

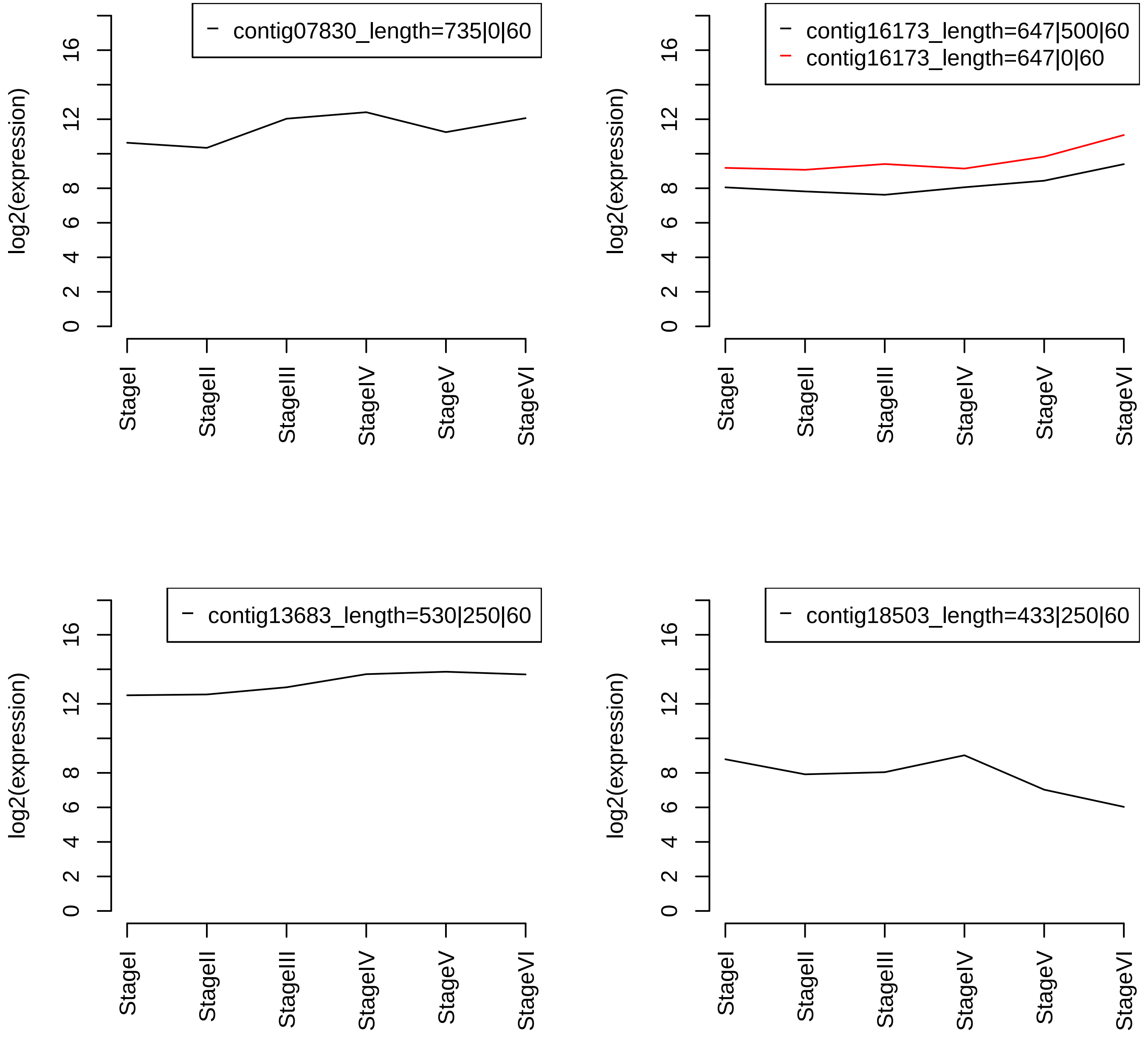
contig07830_length=735|0|60
contig16173_length=647|500|60
contig16173_length=647|0|60
contig13683_length=530|250|60
contig18503_length=433|250|60
log2(expression)
0
2
4
6
8
12
16
StageI
StageII
StageIII
StageIV
StageV
StageVI

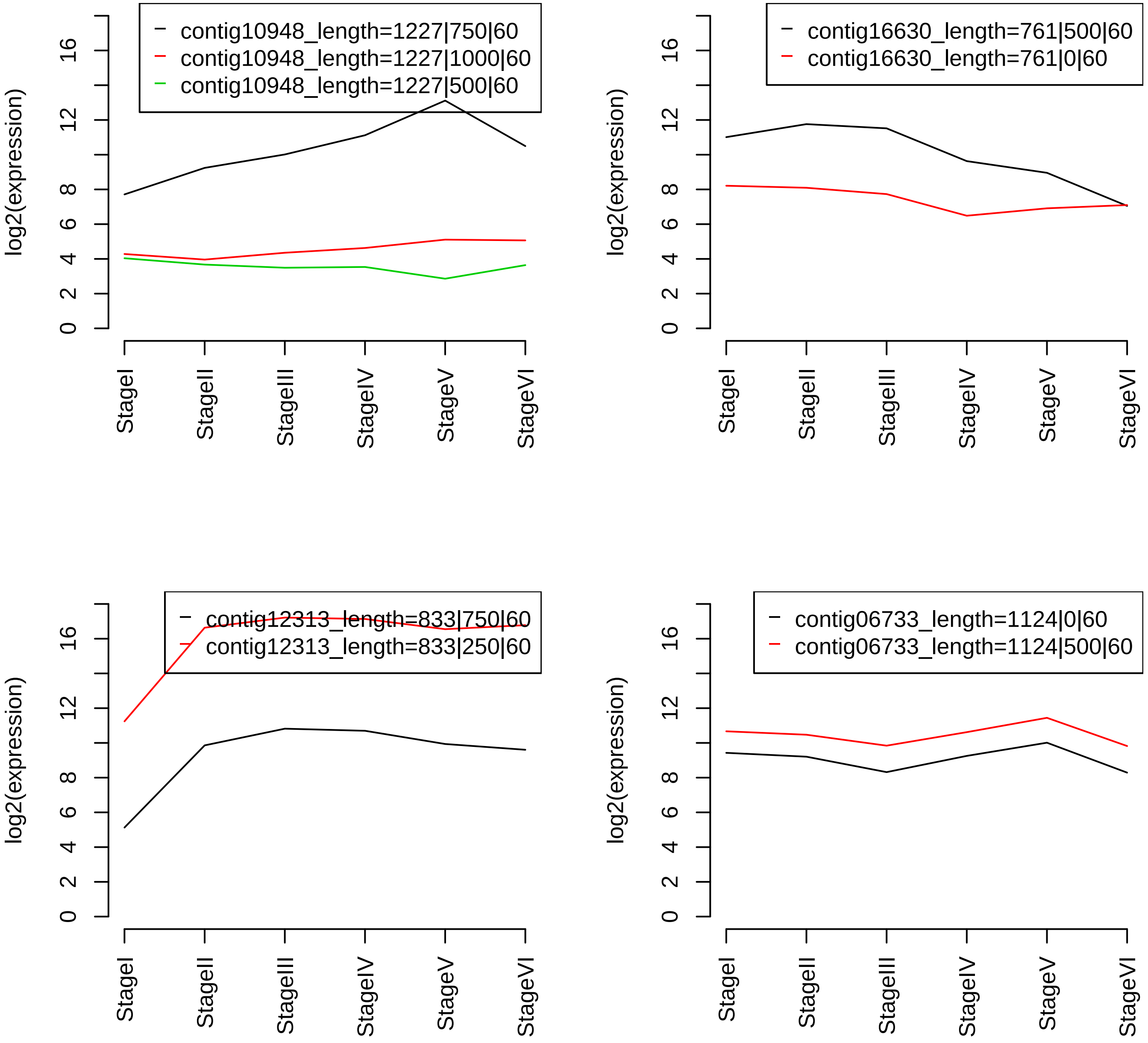
contig10948_length=1227|750|60
contig10948_length=1227|1000|60
contig10948_length=1227|500|60
log2(expression)
0
2
4
6
8
12
16
StageI
StageII
StageIII
StageIV
StageV
StageVI
contig16630_length=761|500|60
contig16630_length=761|0|60
contig12313_length=833|750|60
contig12313_length=833|250|60
contig06733_length=1124|0|60
contig06733_length=1124|500|60

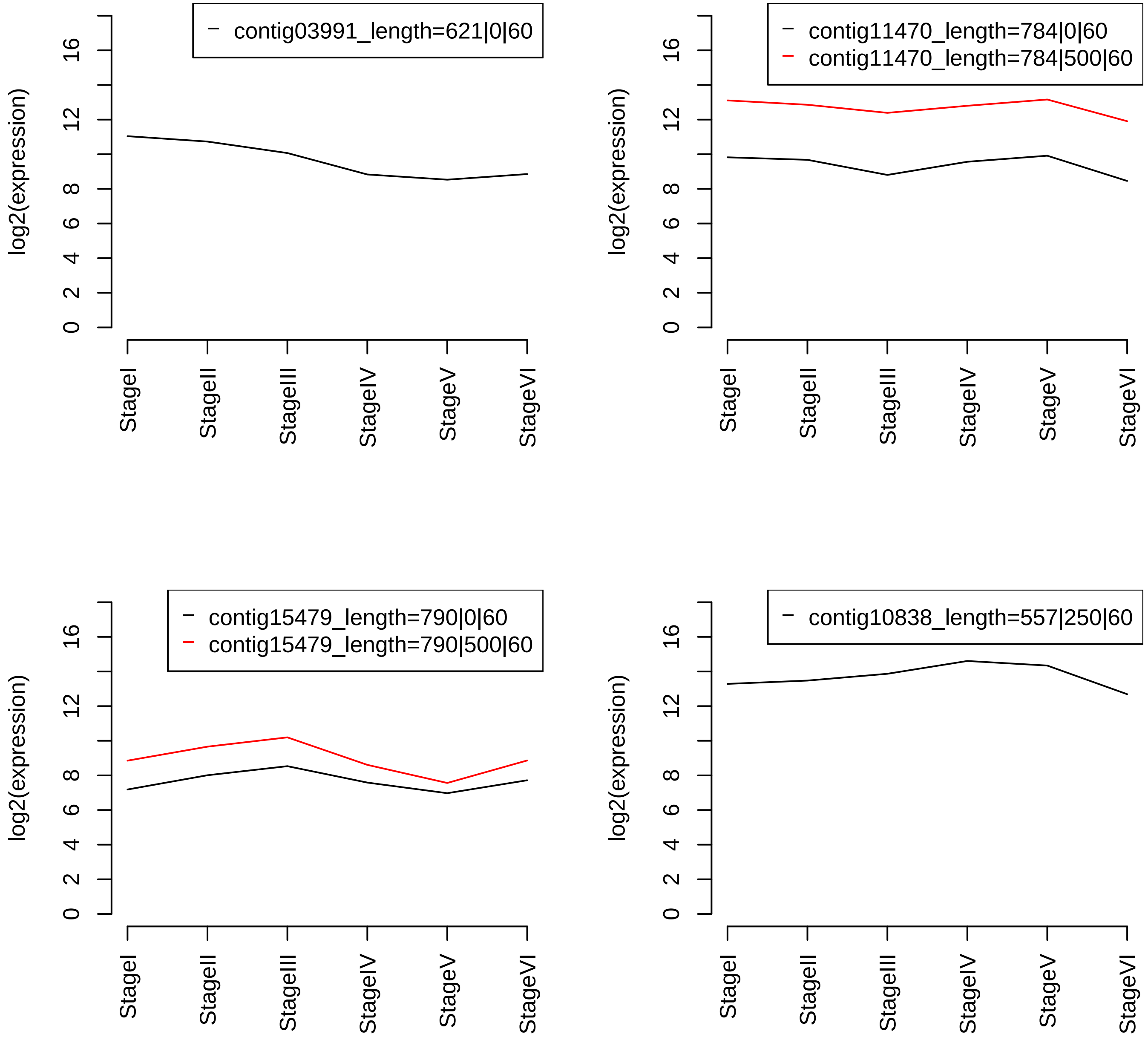

contig03991_length=621|0|60
contig11470_length=784|0|60
contig11470_length=784|500|60
contig15479_length=790|0|60
contig15479_length=790|500|60
contig10838_length=557|250|60
log2(expression)
0
2
4
6
8
12
16
StageI
StageII
StageIII
StageIV
StageV
StageVI

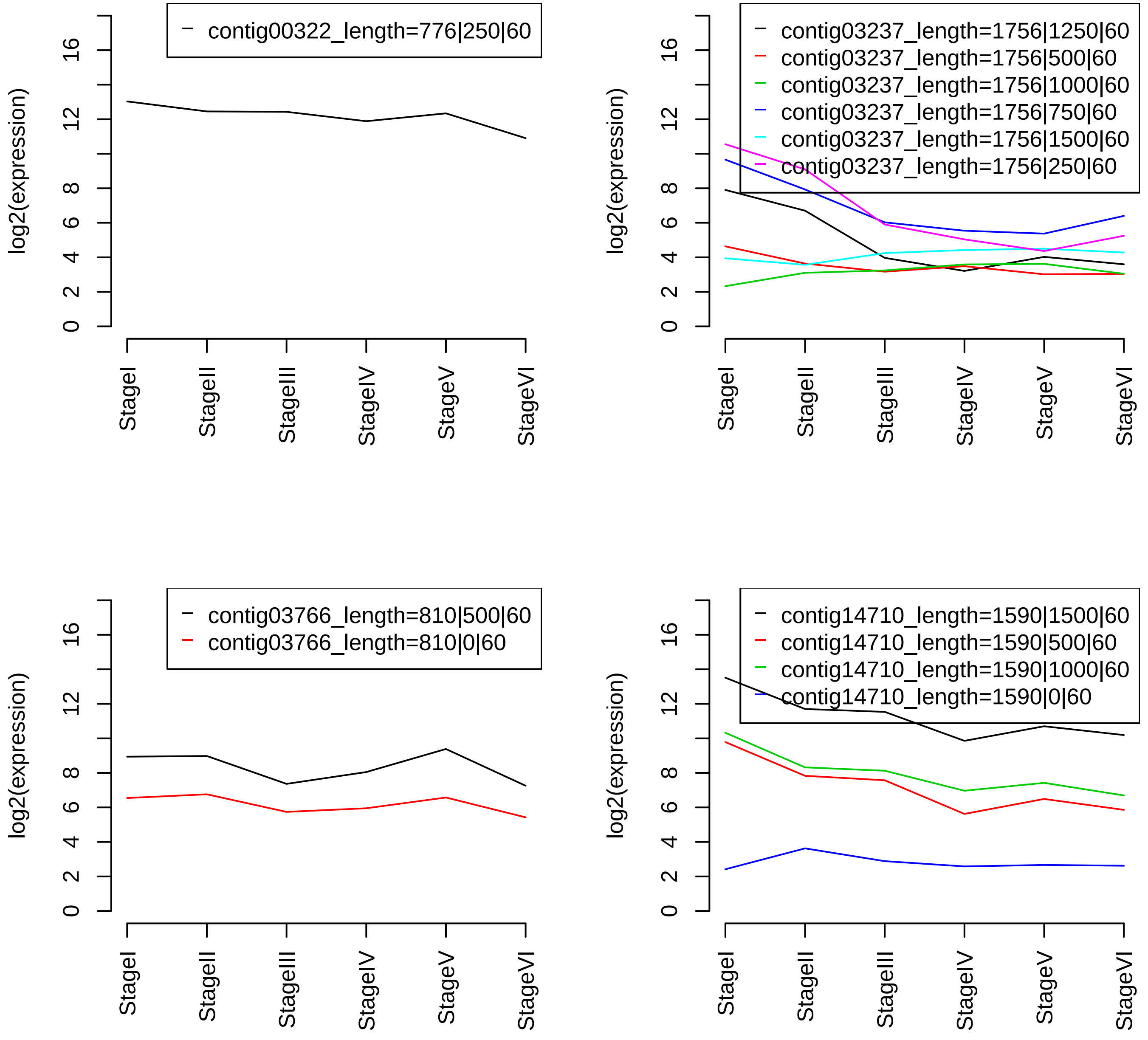

contig00322_length=776|250|60
contig03237_length=1756|1250|60
contig03237_length=1756|500|60
contig03237_length=1756|1000|60
contig03237_length=1756|750|60
contig03237_length=1756|1500|60
contig03237_length=1756|250|60
contig03766_length=810|500|60
contig03766_length=810|0|60
contig14710_length=1590|1500|60
contig14710_length=1590|500|60
contig14710_length=1590|1000|60
contig14710_length=1590|0|60
log2(expression)
0
2
4
6
8
12
16
StageI
StageII
StageIII
StageIV
StageV
StageVI

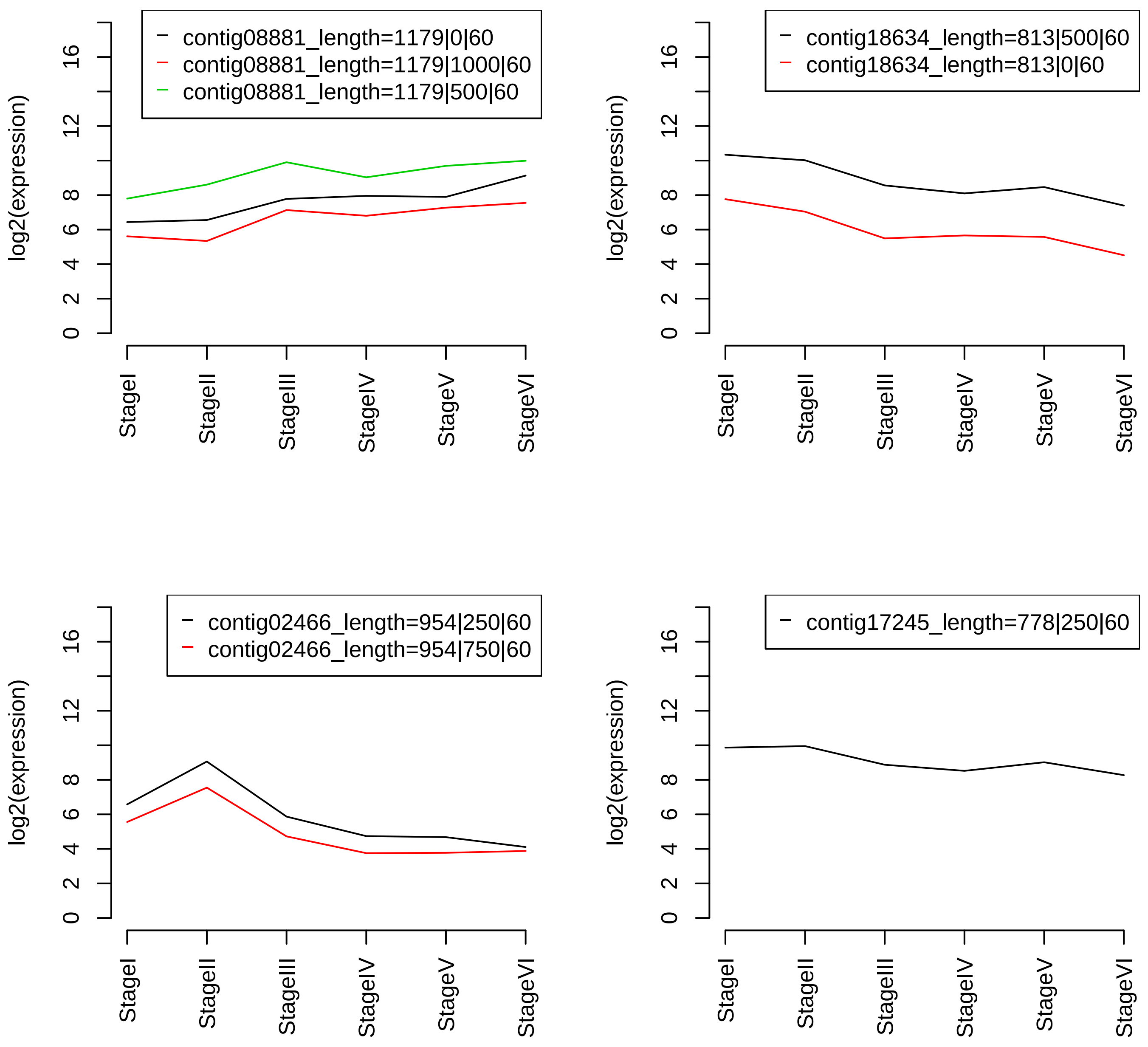
contig08881_length=1179|0|60
contig08881_length=1179|1000|60
contig08881_length=1179|500|60
log2(expression)
0
2
4
6
8
12
16
StageI
StageII
StageIII
StageIV
StageV
StageVI
contig18634_length=813|500|60
contig18634_length=813|0|60
contig02466_length=954|250|60
contig02466_length=954|750|60
contig17245_length=778|250|60

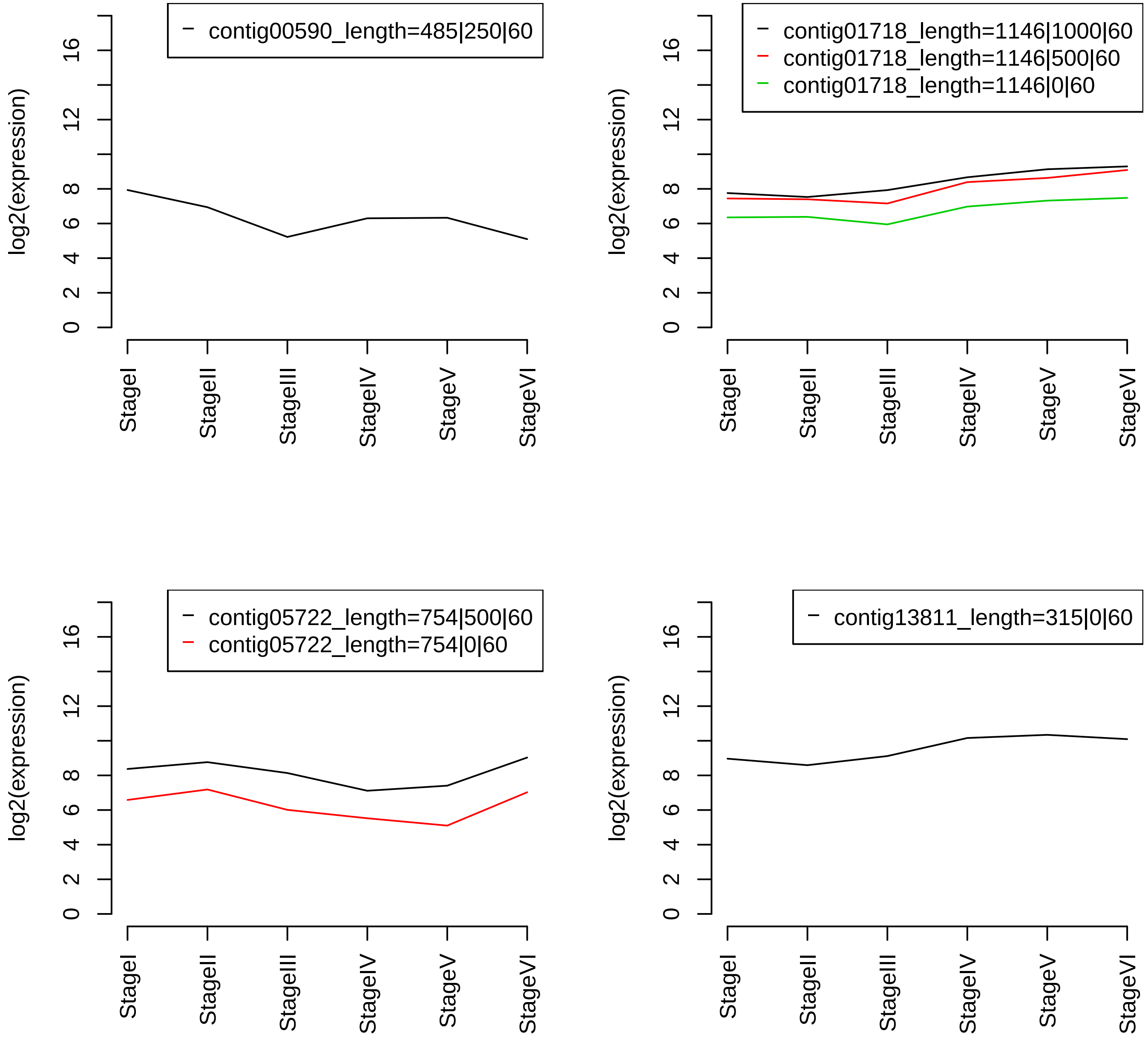

contig00590_length=485|250|60
contig01718_length=1146|1000|60
contig01718_length=1146|500|60
contig01718_length=1146|0|60
contig05722_length=754|500|60
contig05722_length=754|0|60
contig13811_length=315|0|60
log2(expression)
0 2 4 6 8 12 16
StageI StageII StageIII StageIV StageV StageVI

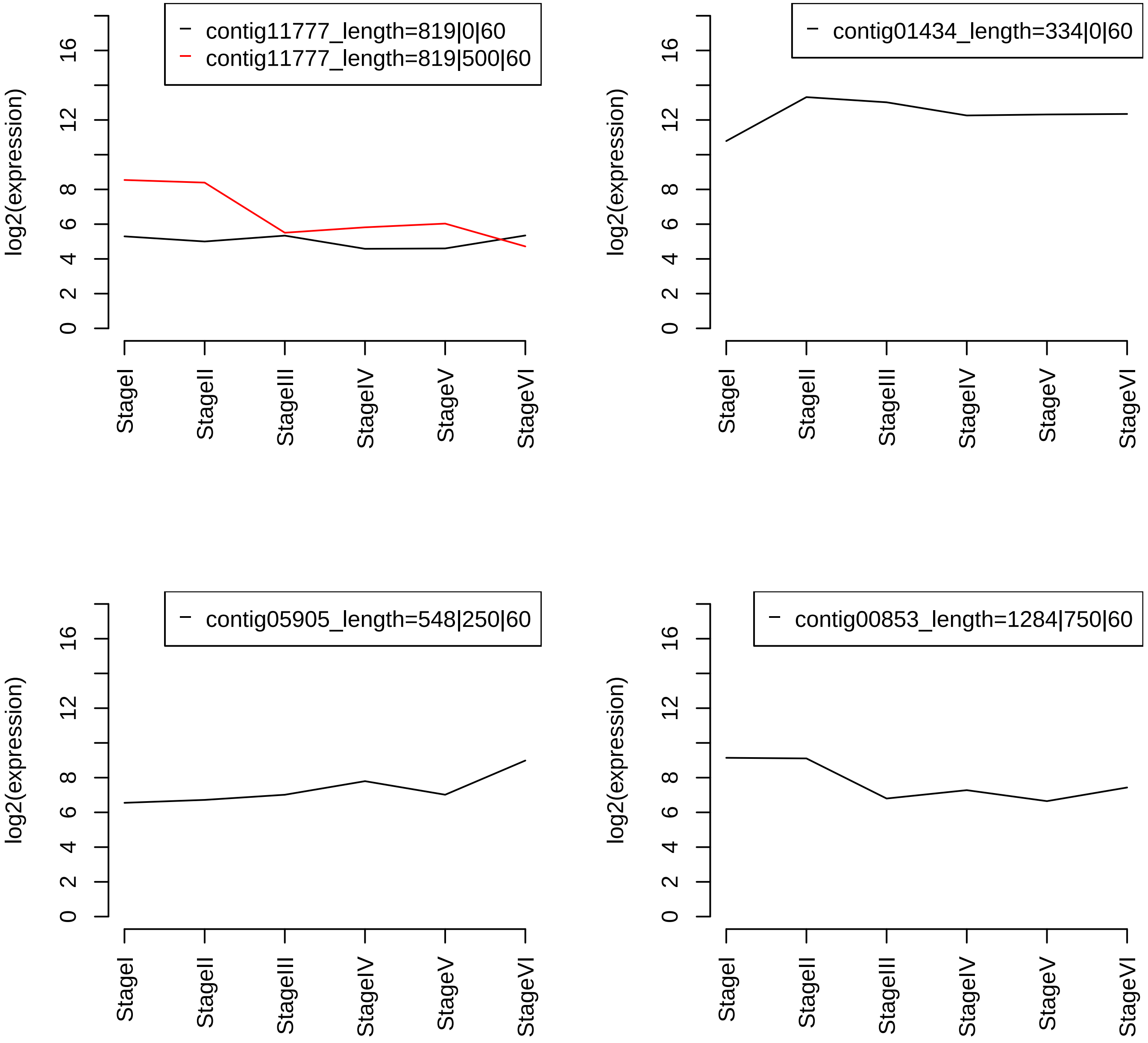

contig11777_length=819|0|60
contig11777_length=819|500|60
contig01434_length=334|0|60
contig05905_length=548|250|60
contig00853_length=1284|750|60
log2(expression)
0
2
4
6
8
12
16
StageI
StageII
StageIII
StageIV
StageV
StageVI

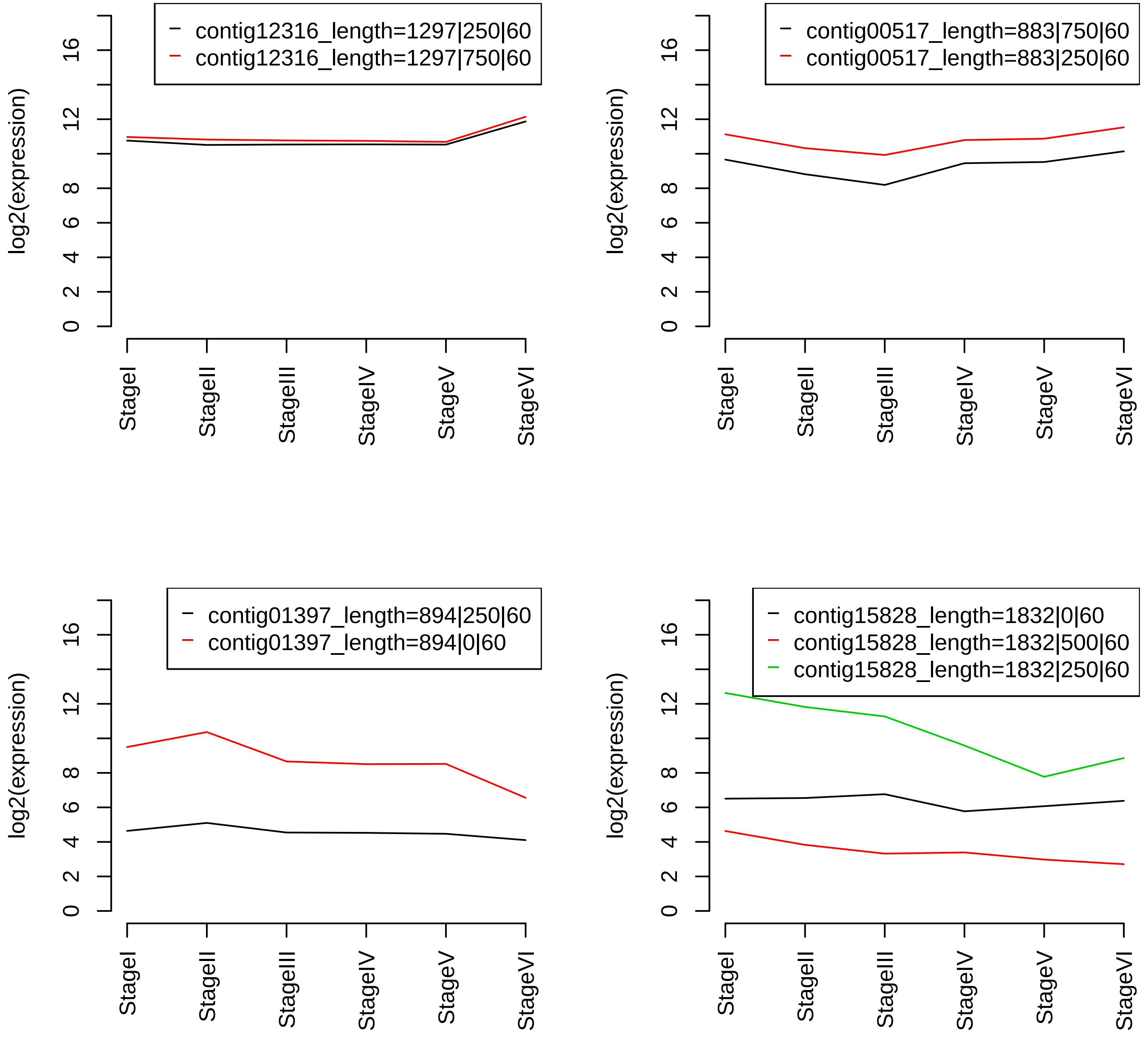

contig12316_length=1297|250|60
contig12316_length=1297|750|60
log2(expression)
0
2
4
6
8
12
16
StageI
StageII
StageIII
StageIV
StageV
StageVI
contig00517_length=883|750|60
contig00517_length=883|250|60
log2(expression)
0
2
4
6
8
12
16
StageI
StageII
StageIII
StageIV
StageV
StageVI
contig01397_length=894|250|60
contig01397_length=894|0|60
log2(expression)
0
2
4
6
8
12
16
StageI
StageII
StageIII
StageIV
StageV
StageVI
contig15828_length=1832|0|60
contig15828_length=1832|500|60
contig15828_length=1832|250|60
log2(expression)
0
2
4
6
8
12
16
StageI
StageII
StageIII
StageIV
StageV
StageVI

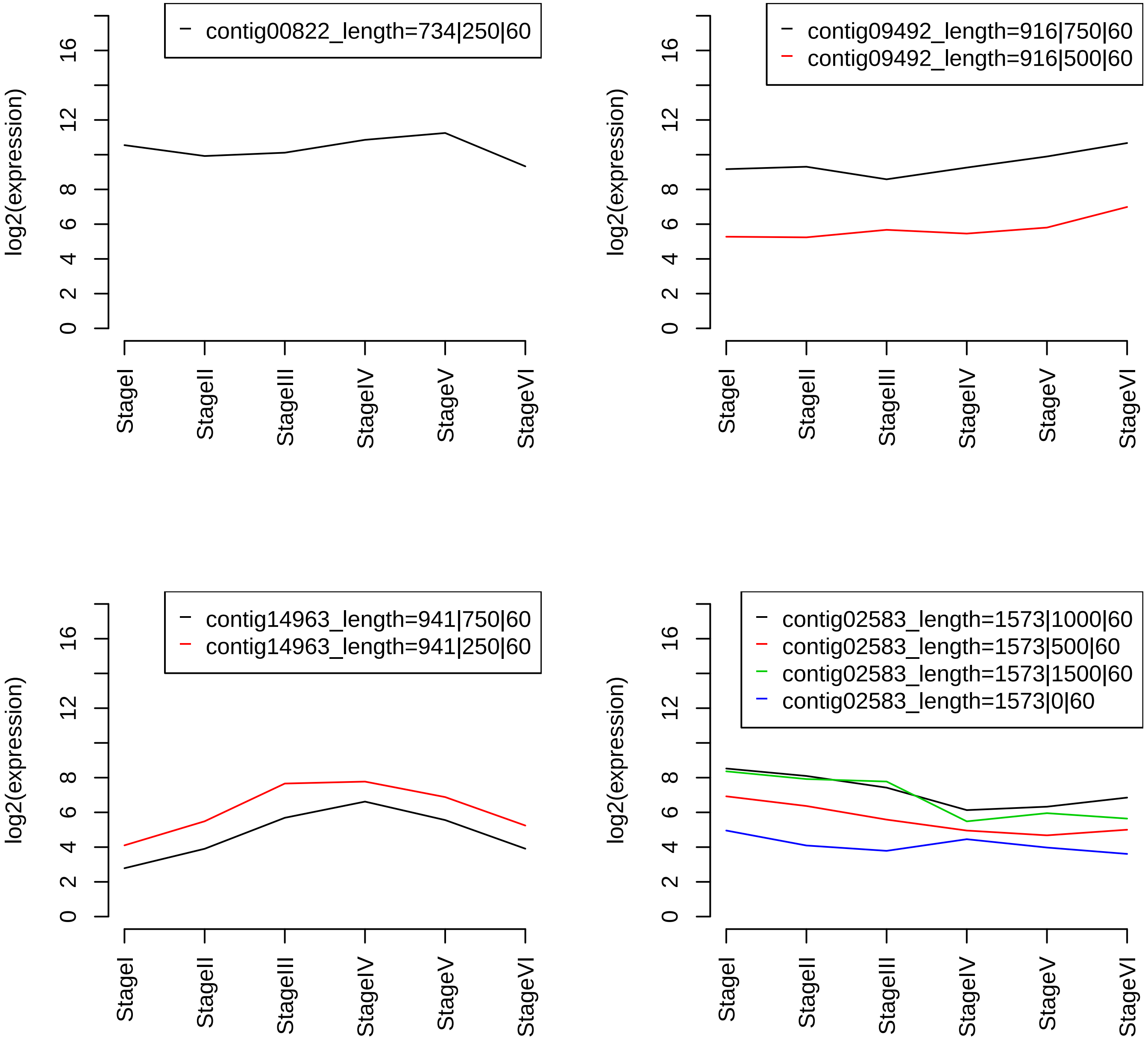

contig00822_length=734|250|60
contig09492_length=916|750|60
contig09492_length=916|500|60
contig14963_length=941|750|60
contig14963_length=941|250|60
contig02583_length=1573|1000|60
contig02583_length=1573|500|60
contig02583_length=1573|1500|60
contig02583_length=1573|0|60
log2(expression)
0
2
4
6
8
12
16
StageI
StageII
StageIII
StageIV
StageV
StageVI

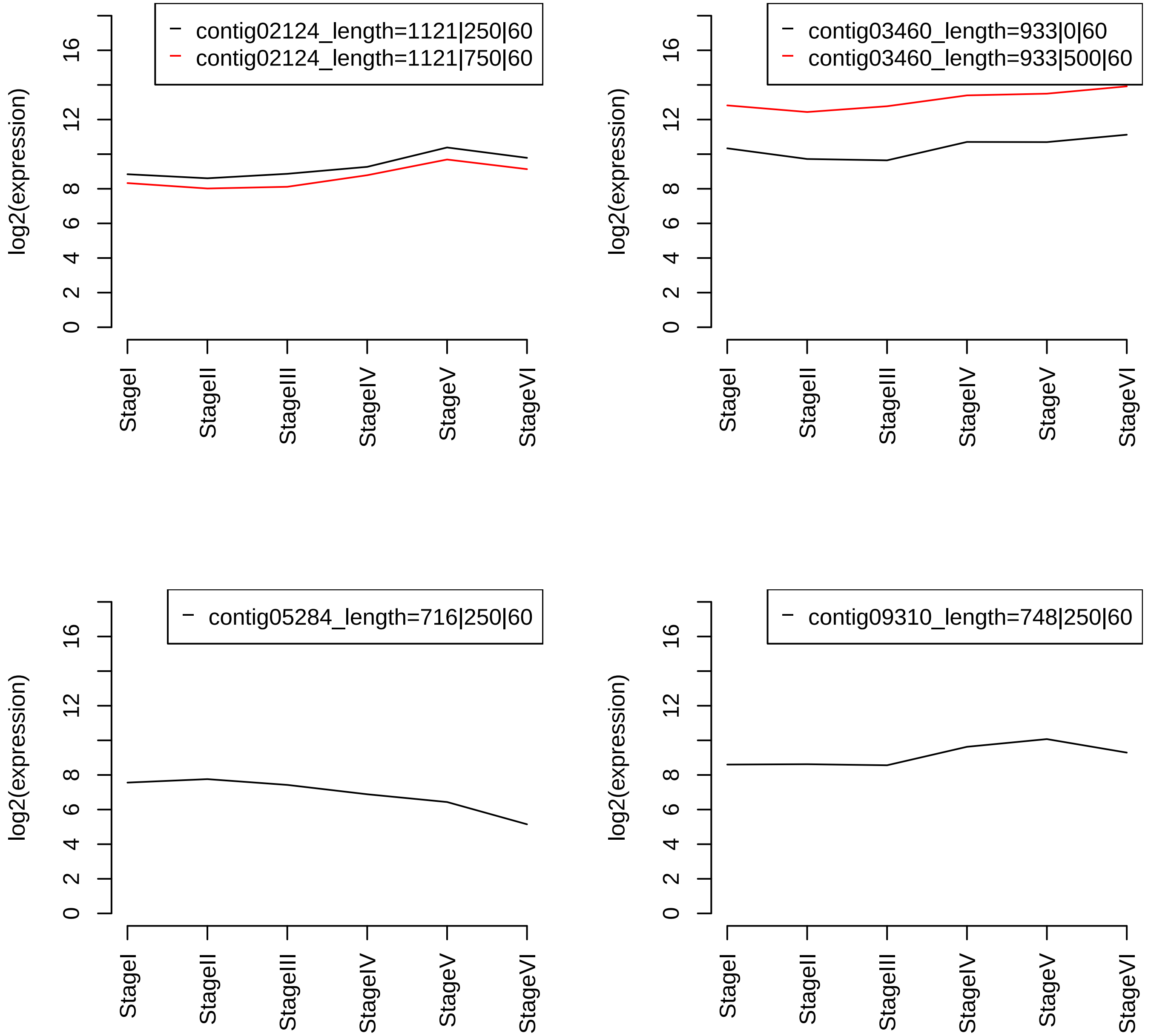

contig02124_length=1121|250|60
contig02124_length=1121|750|60
contig03460_length=933|0|60
contig03460_length=933|500|60
contig05284_length=716|250|60
contig09310_length=748|250|60
log2(expression)
0
2
4
6
8
12
16
StageI
StageII
StageIII
StageIV
StageV
StageVI

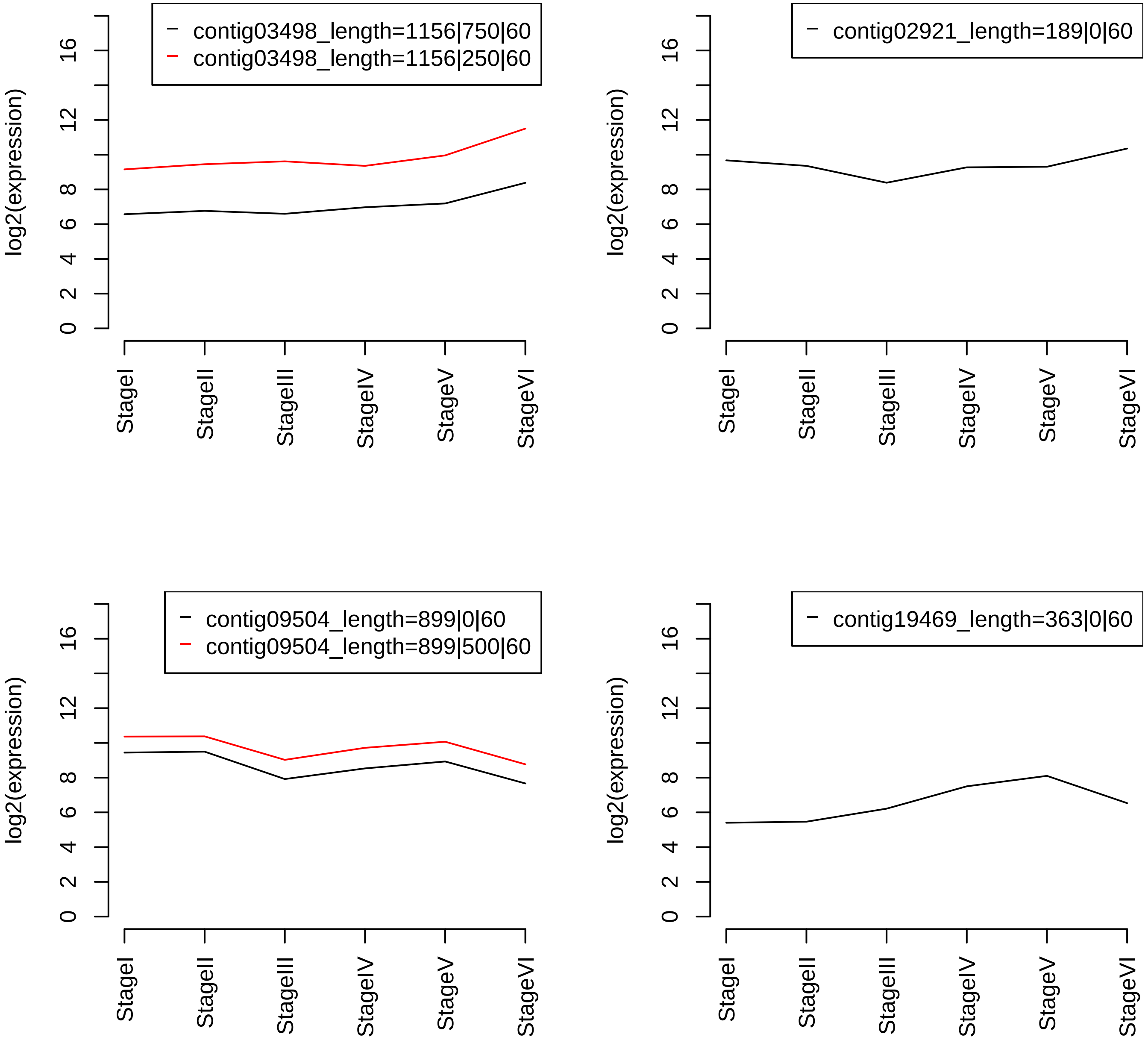

contig03498_length=1156|750|60
contig03498_length=1156|250|60
contig02921_length=189|0|60
contig09504_length=899|0|60
contig09504_length=899|500|60
contig19469_length=363|0|60
log2(expression)
0
2
4
6
8
12
16
StageI
StageII
StageIII
StageIV
StageV
StageVI

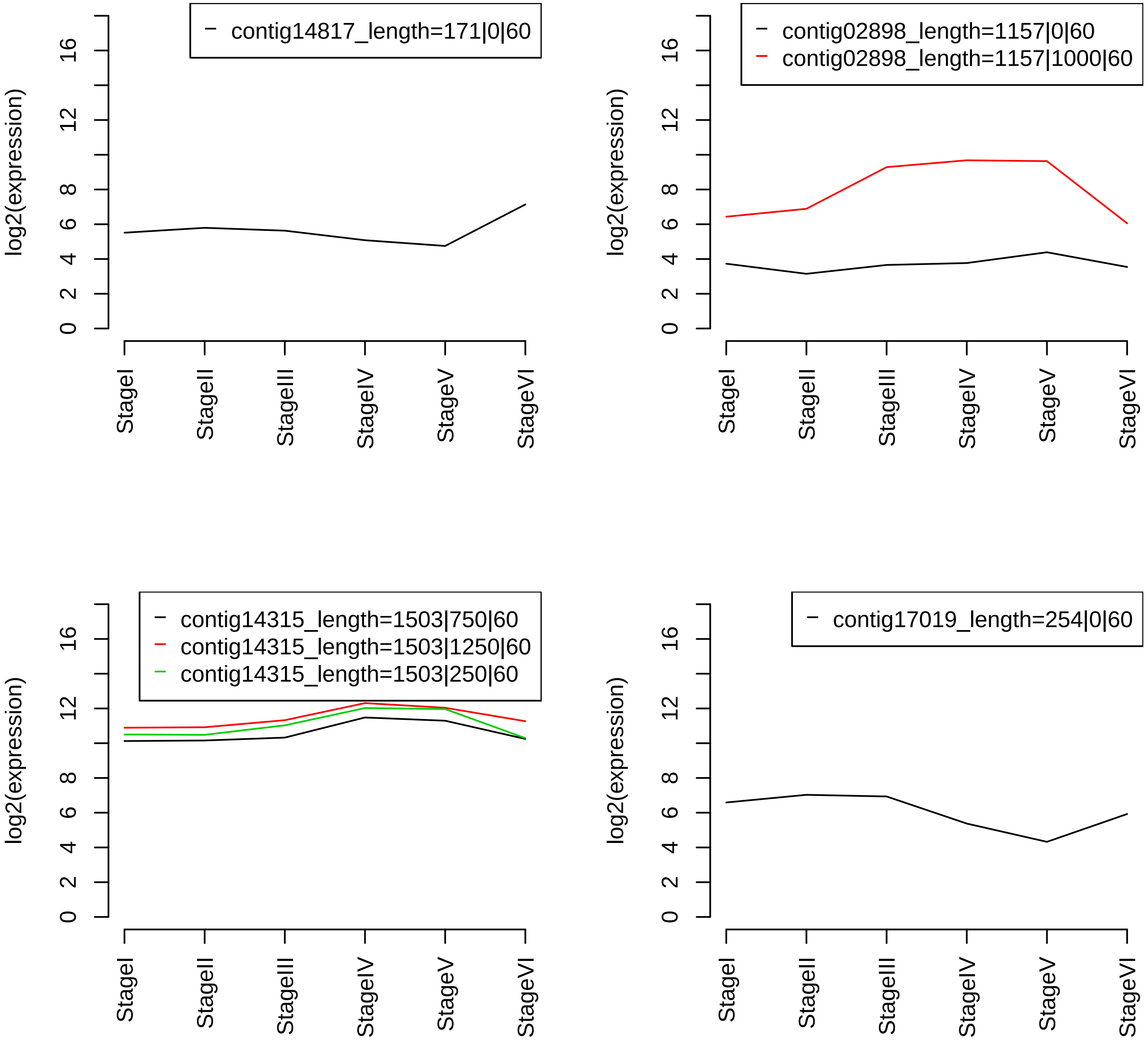
contig14817_length=171|0|60
contig02898_length=1157|0|60
contig02898_length=1157|1000|60
contig14315_length=1503|750|60
contig14315_length=1503|1250|60
contig14315_length=1503|250|60
contig17019_length=254|0|60
log2(expression)
0
2
4
6
8
12
16
StageI
StageII
StageIII
StageIV
StageV
StageVI

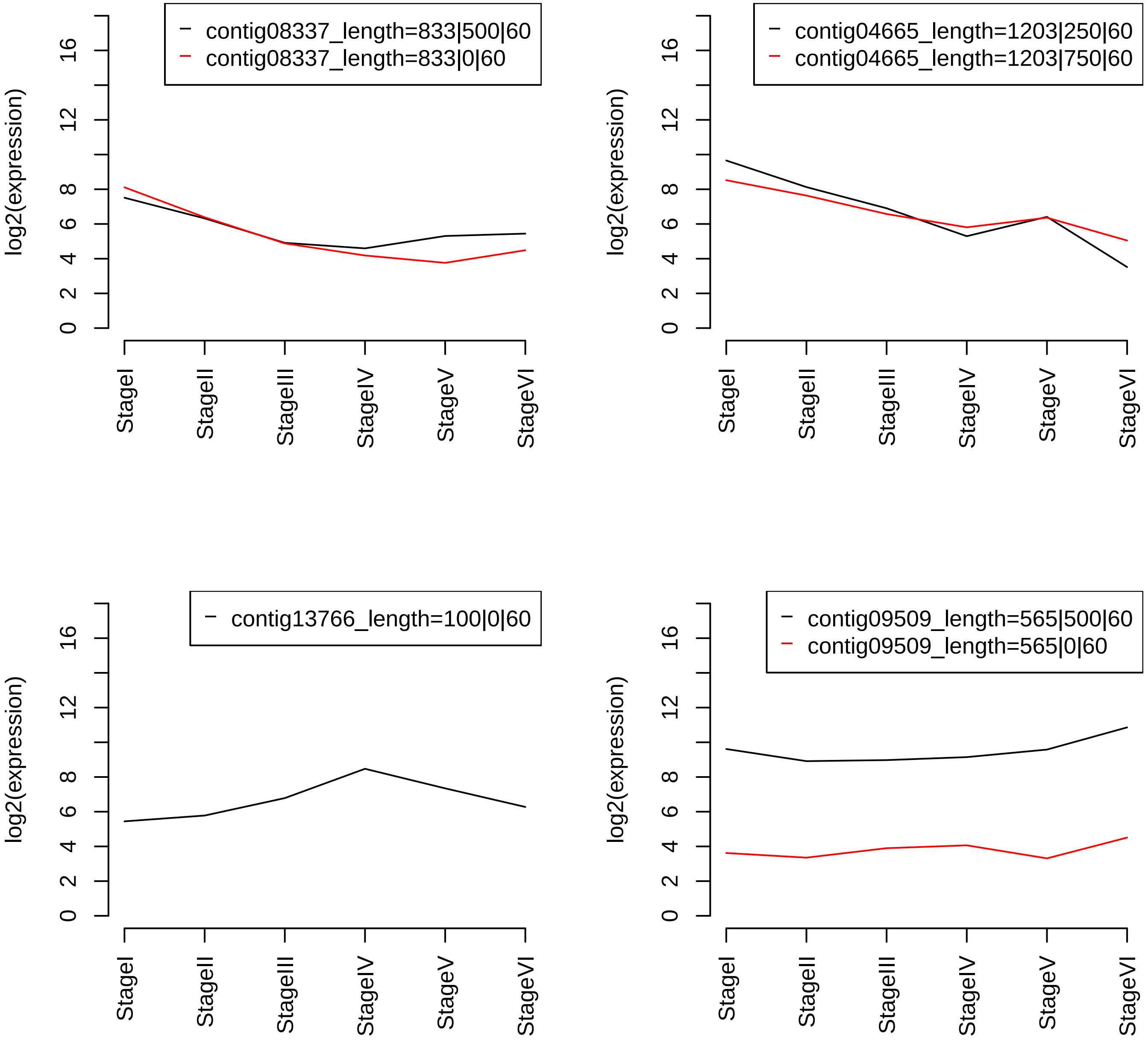

contig08337_length=833|500|60
contig08337_length=833|0|60
contig04665_length=1203|250|60
contig04665_length=1203|750|60
contig13766_length=100|0|60
contig09509_length=565|500|60
contig09509_length=565|0|60
log2(expression)
0
2
4
6
8
12
16
StageI
StageII
StageIII
StageIV
StageV
StageVI

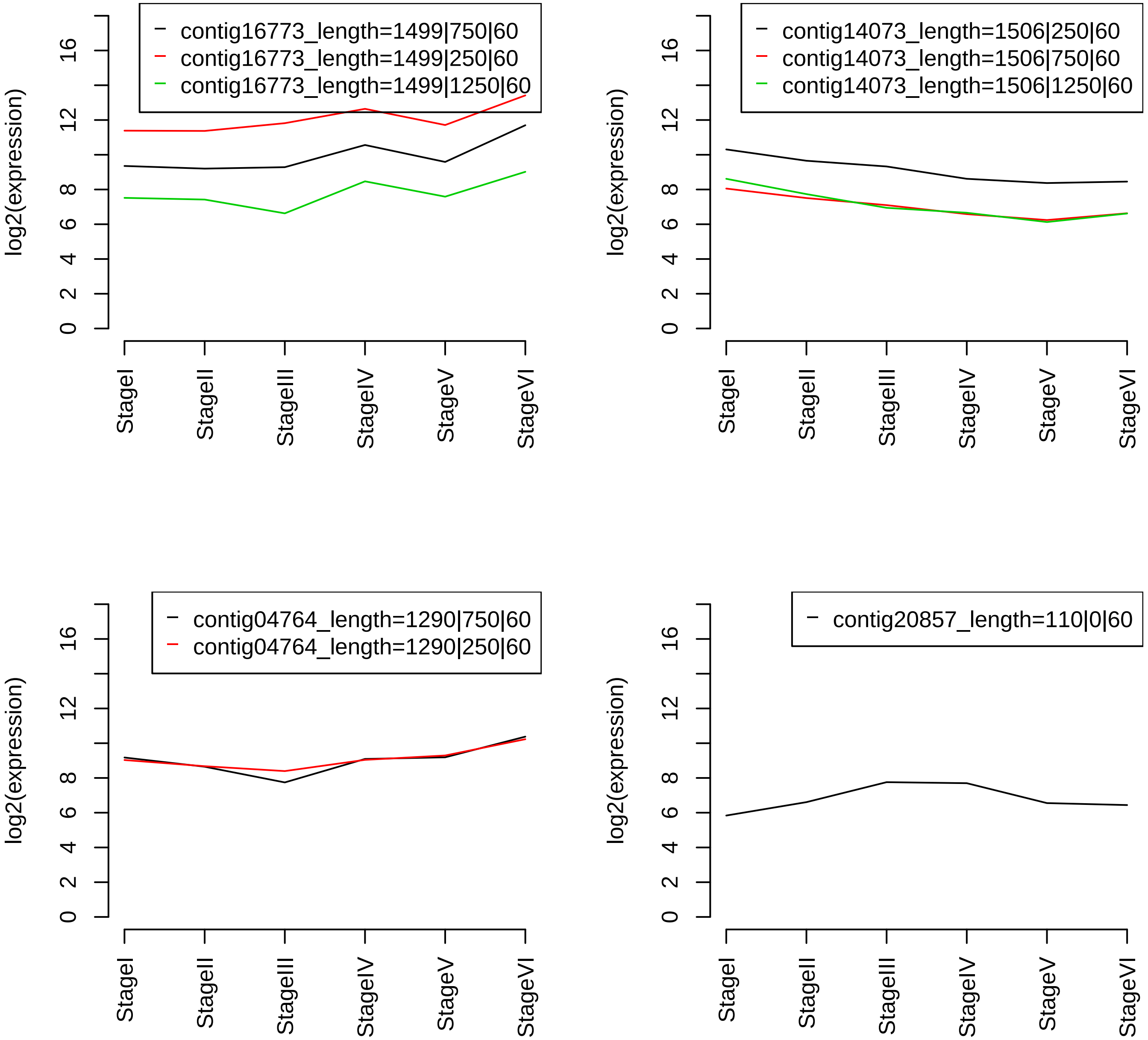
contig16773_length=1499|750|60
contig16773_length=1499|250|60
contig16773_length=1499|1250|60
contig14073_length=1506|250|60
contig14073_length=1506|750|60
contig14073_length=1506|1250|60
contig04764_length=1290|750|60
contig04764_length=1290|250|60
contig20857_length=110|0|60
log2(expression)
0
2
4
6
8
12
16
StageI
StageII
StageIII
StageIV
StageV
StageVI

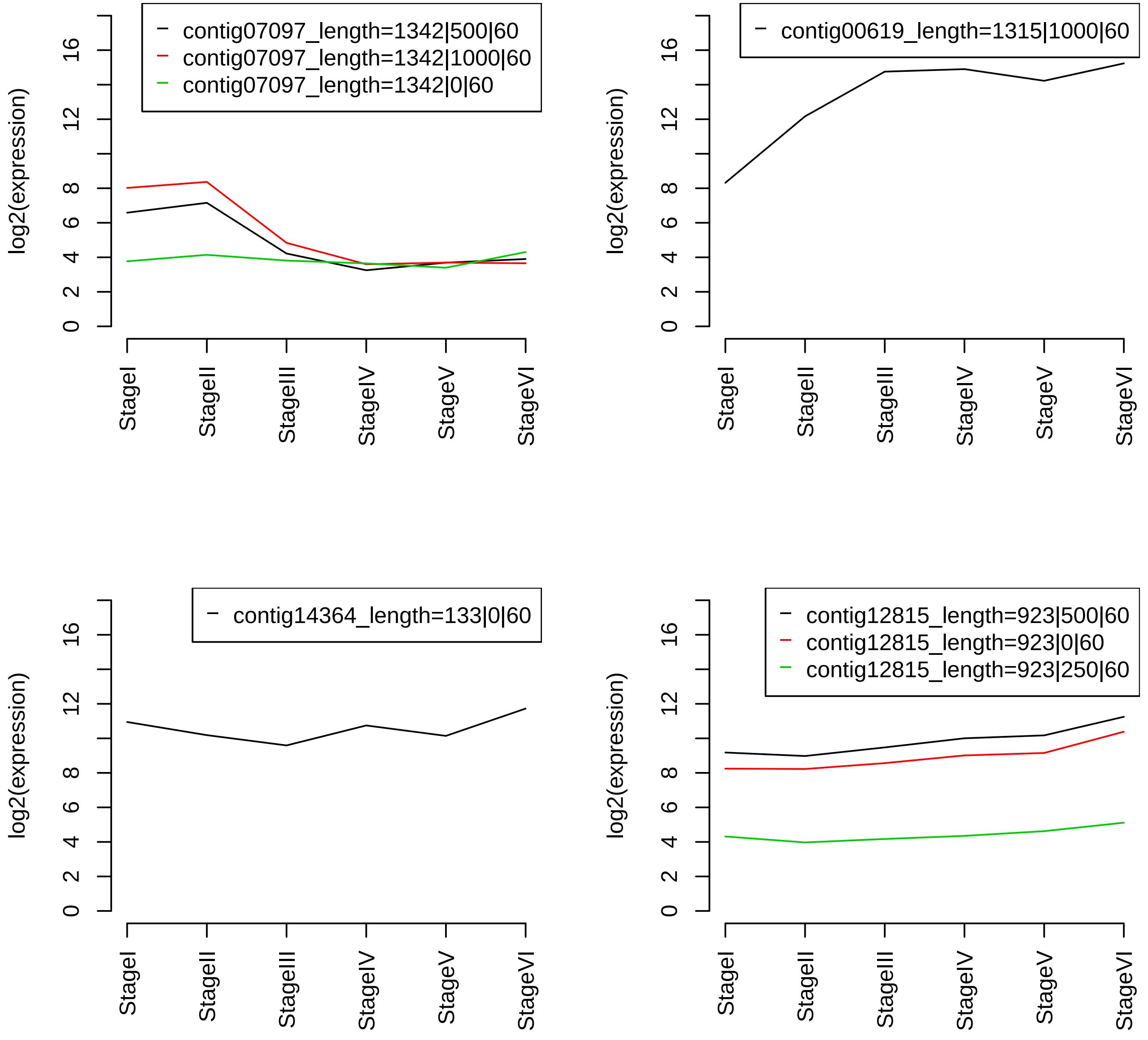

contig07097_length=1342|500|60
contig07097_length=1342|1000|60
contig07097_length=1342|0|60
contig00619_length=1315|1000|60
contig14364_length=133|0|60
contig12815_length=923|500|60
contig12815_length=923|0|60
contig12815_length=923|250|60
log2(expression)
0
2
4
6
8
12
16
StageI
StageII
StageIII
StageIV
StageV
StageVI

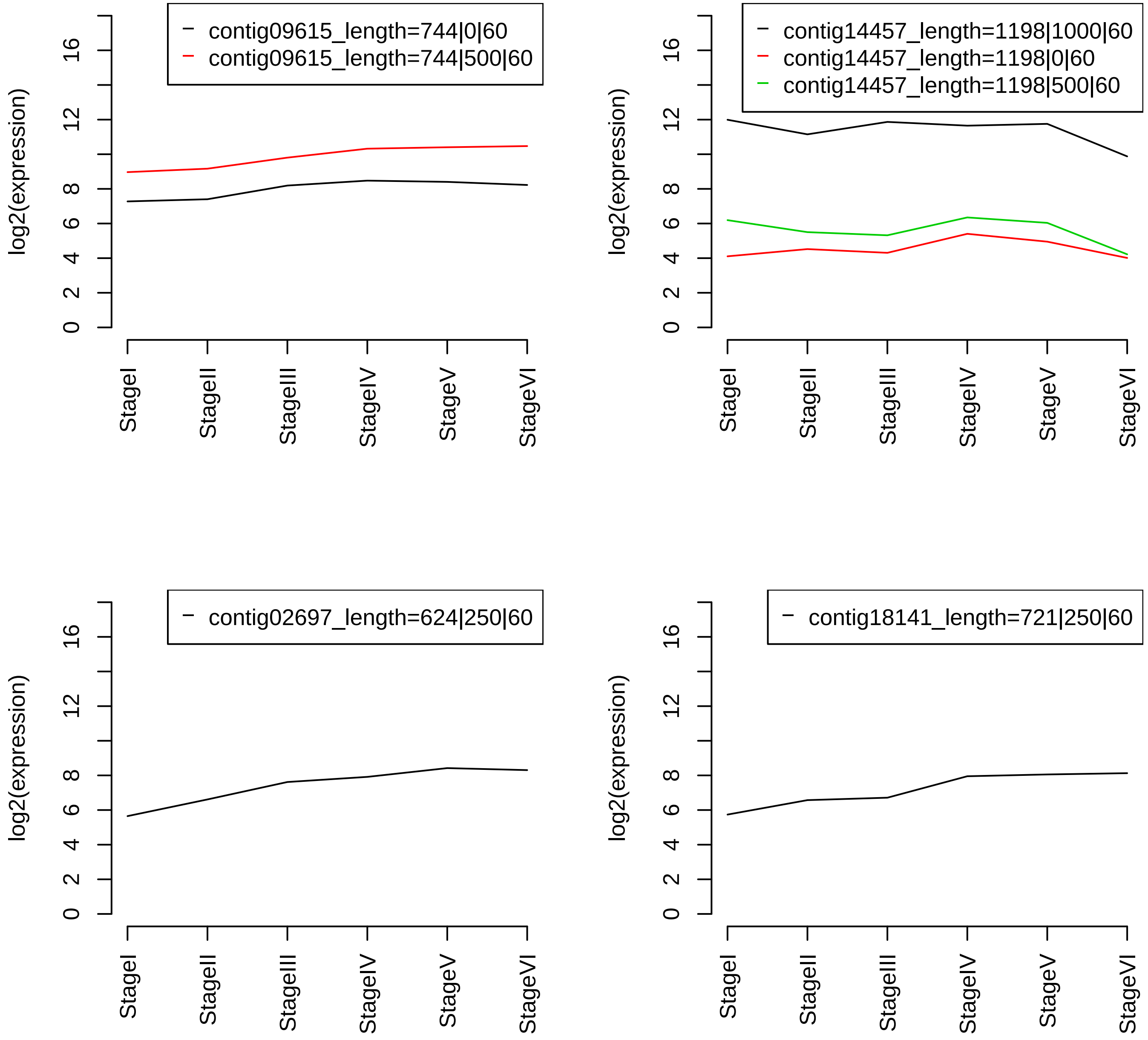
contig09615_length=744|0|60
contig09615_length=744|500|60
contig14457_length=1198|1000|60
contig14457_length=1198|0|60
contig14457_length=1198|500|60
contig02697_length=624|250|60
contig18141_length=721|250|60
log2(expression)
0
2
4
6
8
12
16
StageI
StageII
StageIII
StageIV
StageV
StageVI

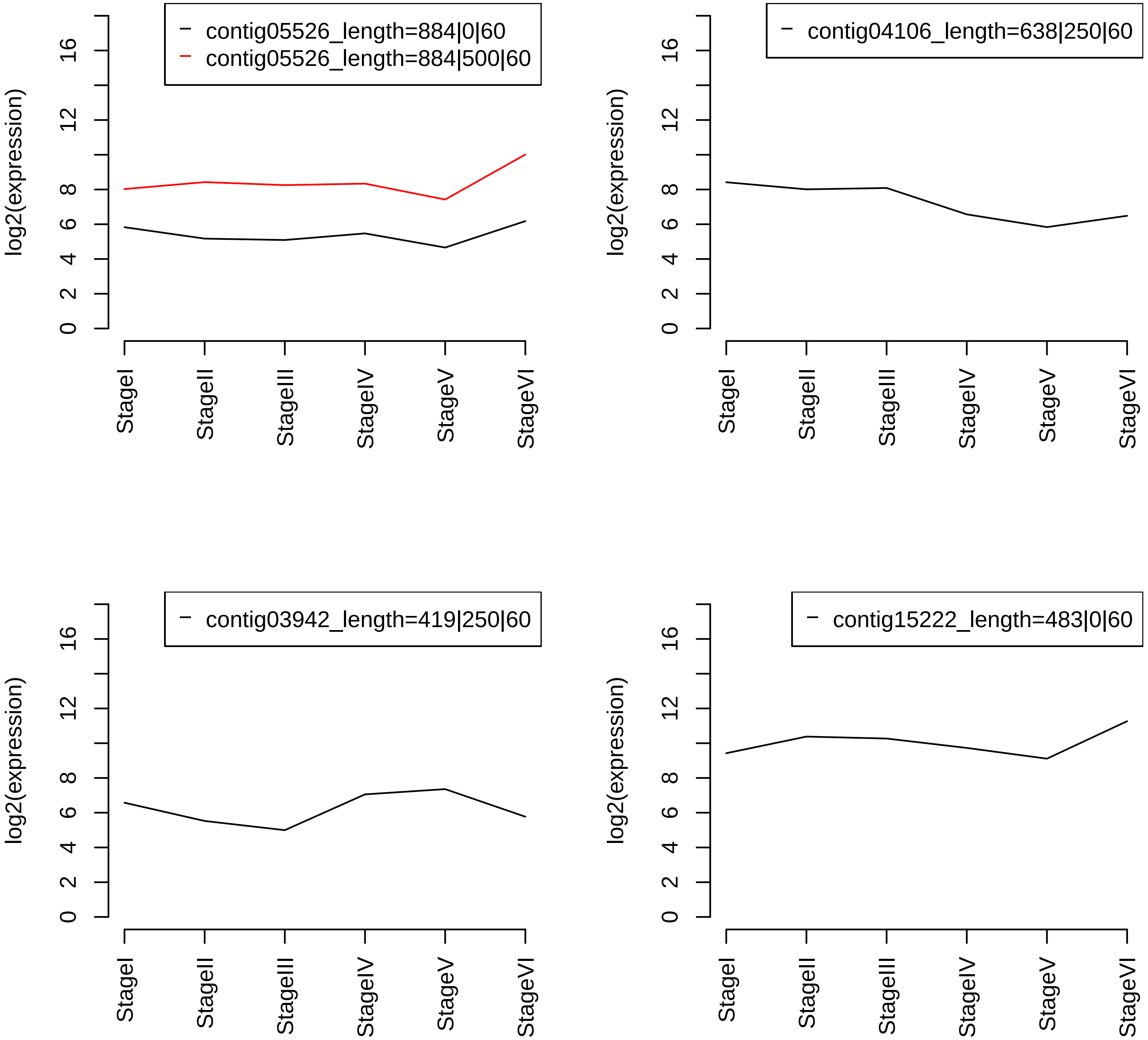

contig05526_length=884|0|60
contig05526_length=884|500|60
contig04106_length=638|250|60
contig03942_length=419|250|60
contig15222_length=483|0|60
log2(expression)
0
2
4
6
8
12
16
StageI
StageII
StageIII
StageIV
StageV
StageVI

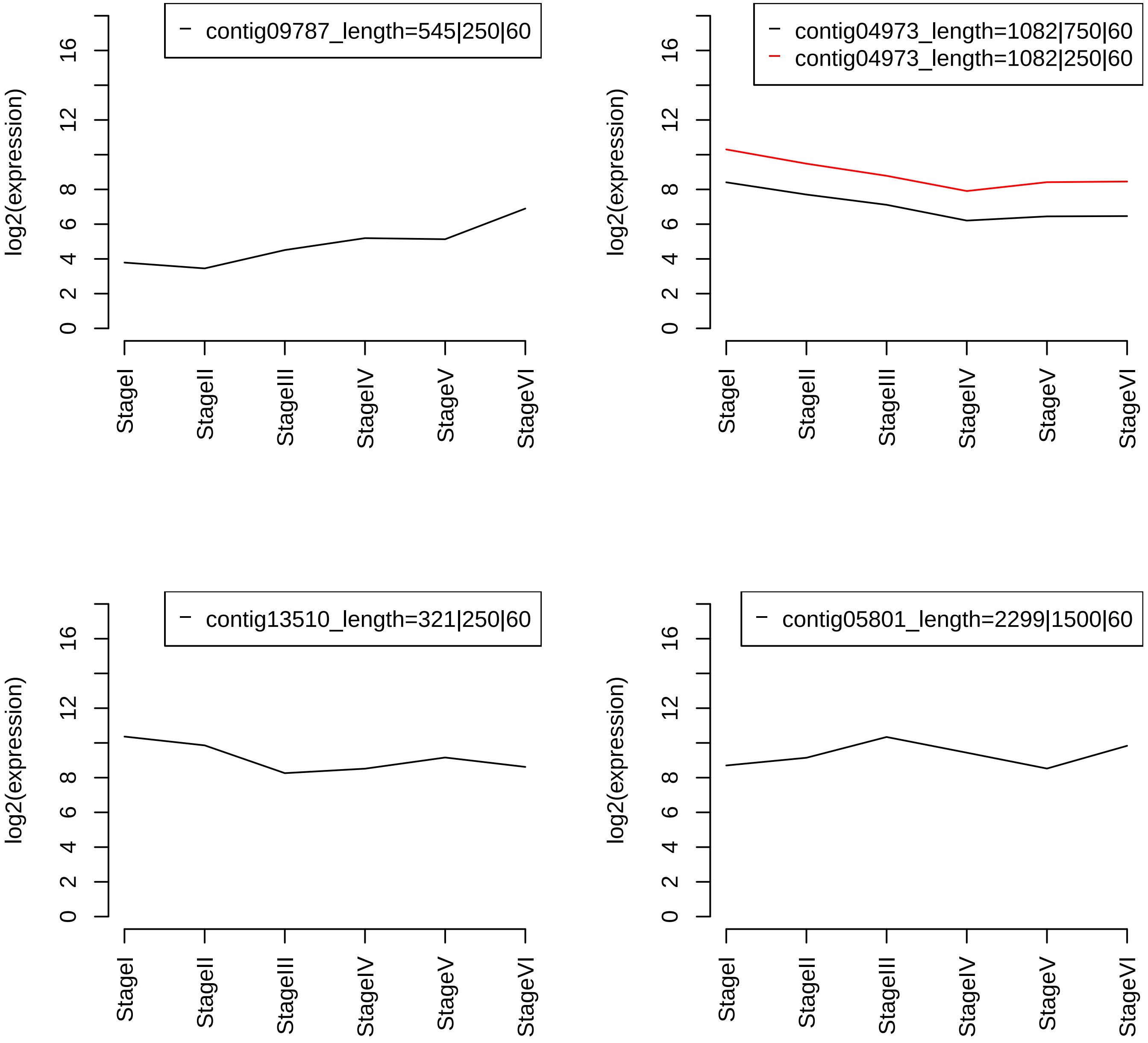

contig09787_length=545|250|60
contig04973_length=1082|750|60
contig04973_length=1082|250|60
contig13510_length=321|250|60
contig05801_length=2299|1500|60
log2(expression)
0
2
4
6
8
12
16
StageI
StageII
StageIII
StageIV
StageV
StageVI

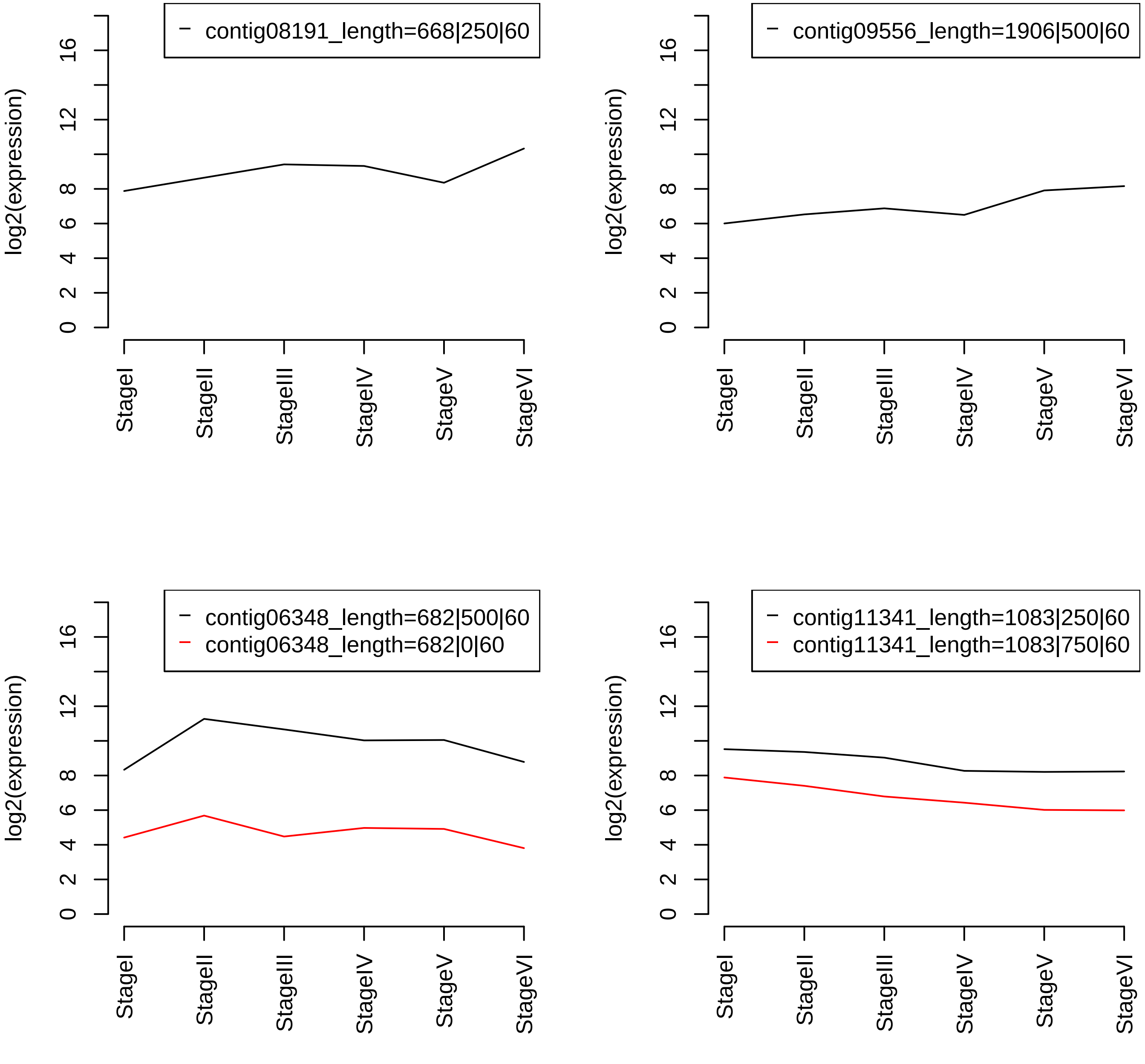

contig08191_length=668|250|60
contig09556_length=1906|500|60
contig06348_length=682|500|60
contig06348_length=682|0|60
contig11341_length=1083|250|60
contig11341_length=1083|750|60
log2(expression)
0 2 4 6 8 12 16
StageI
StageII
StageIII
StageIV
StageV
StageVI

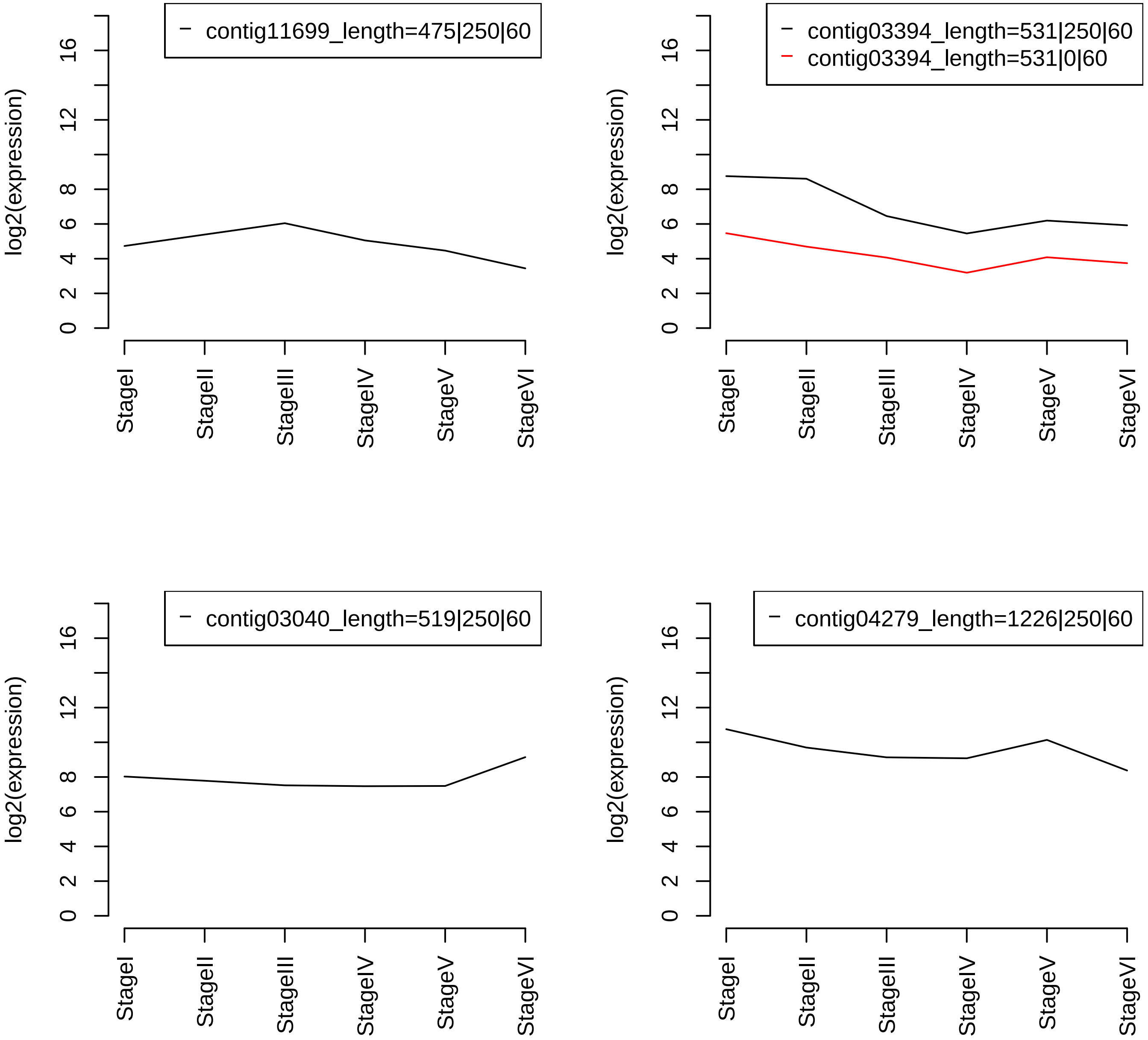
contig11699_length=475|250|60
contig03394_length=531|250|60
contig03394_length=531|0|60
contig03040_length=519|250|60
contig04279_length=1226|250|60
log2(expression)
0
2
4
6
8
12
16
StageI
StageII
StageIII
StageIV
StageV
StageVI

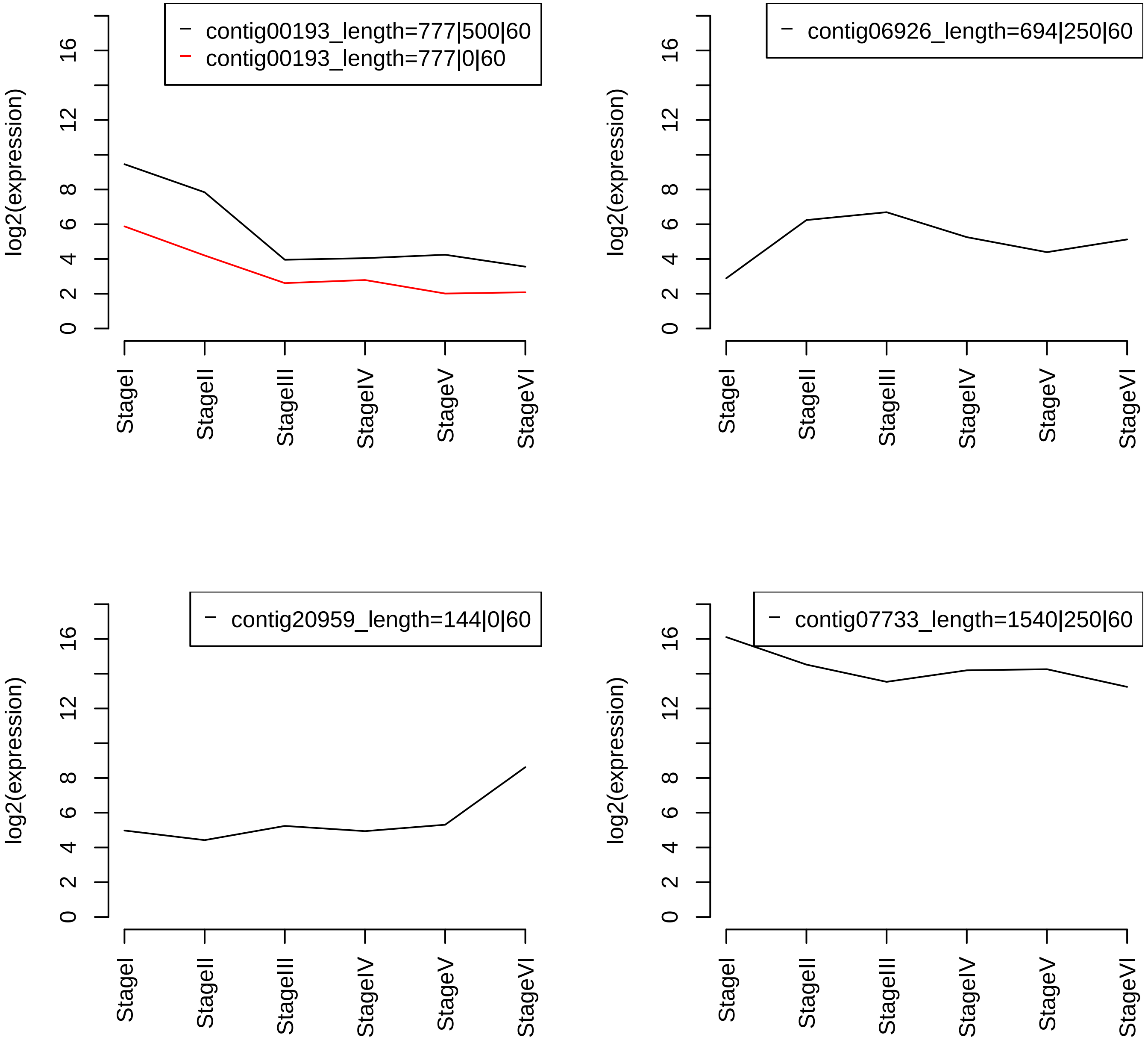

contig00193_length=777|500|60
contig00193_length=777|0|60
contig06926_length=694|250|60
contig20959_length=144|0|60
contig07733_length=1540|250|60
log2(expression)
0
2
4
6
8
12
16
StageI
StageII
StageIII
StageIV
StageV
StageVI

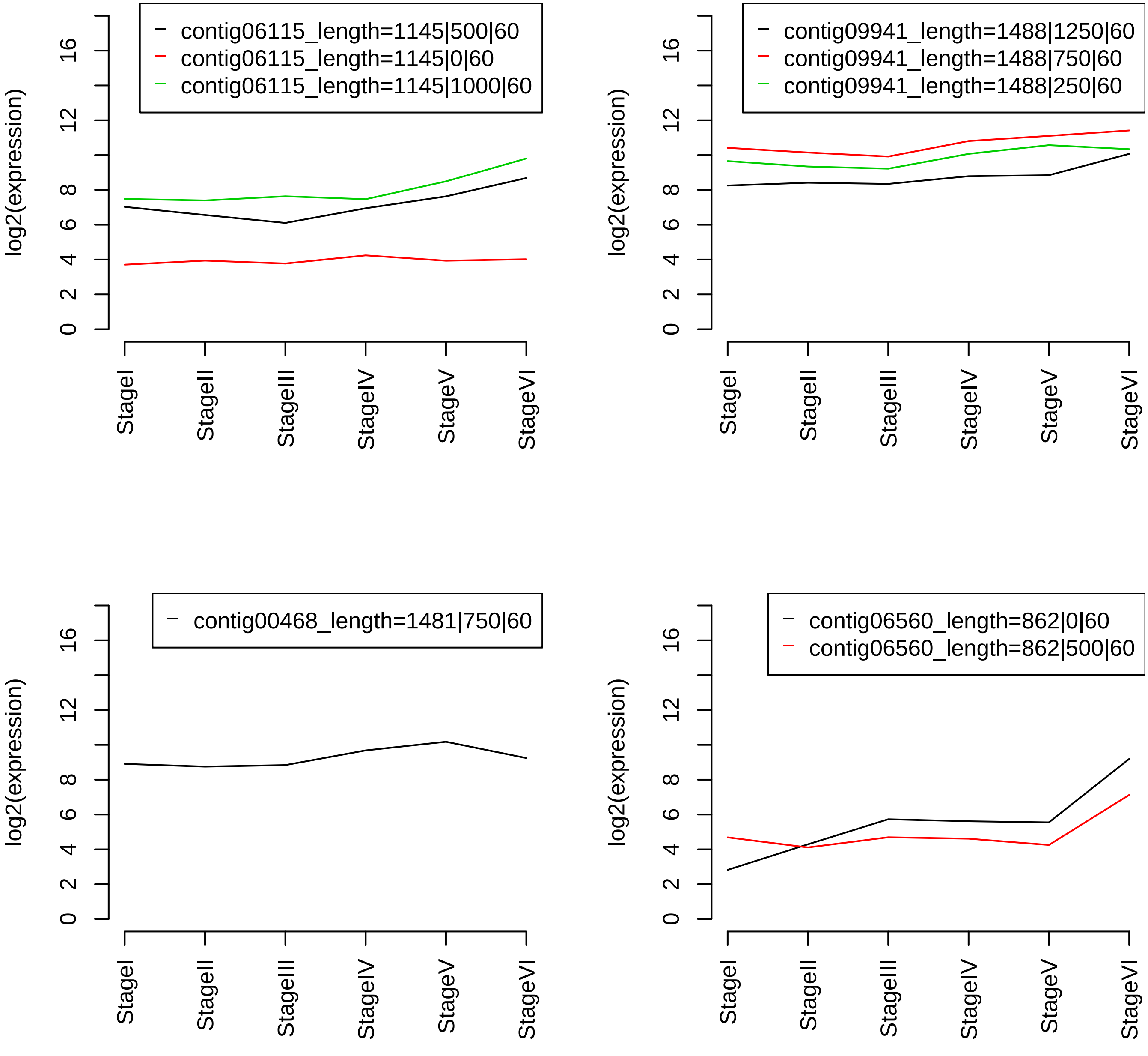

contig06115_length=1145|500|60
contig06115_length=1145|0|60
contig06115_length=1145|1000|60
contig09941_length=1488|1250|60
contig09941_length=1488|750|60
contig09941_length=1488|250|60
contig00468_length=1481|750|60
contig06560_length=862|0|60
contig06560_length=862|500|60
log2(expression)
0
2
4
6
8
12
16
StageI
StageII
StageIII
StageIV
StageV
StageVI

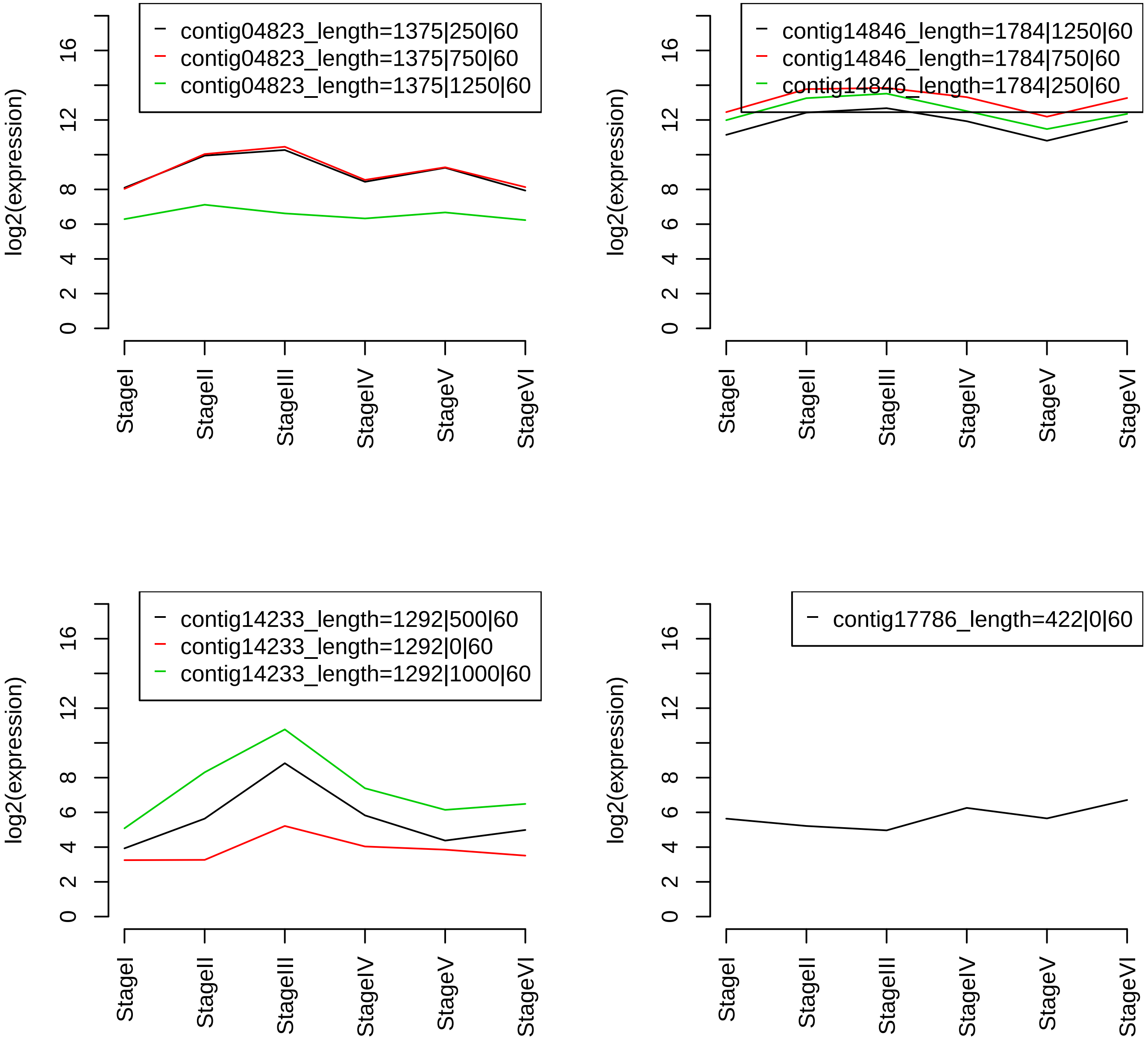

contig04823_length=1375|250|60
contig04823_length=1375|750|60
contig04823_length=1375|1250|60
contig14846_length=1784|1250|60
contig14846_length=1784|750|60
contig14846_length=1784|250|60
contig14233_length=1292|500|60
contig14233_length=1292|0|60
contig14233_length=1292|1000|60
contig17786_length=422|0|60
log2(expression)
StageI
StageII
StageIII
StageIV
StageV
StageVI

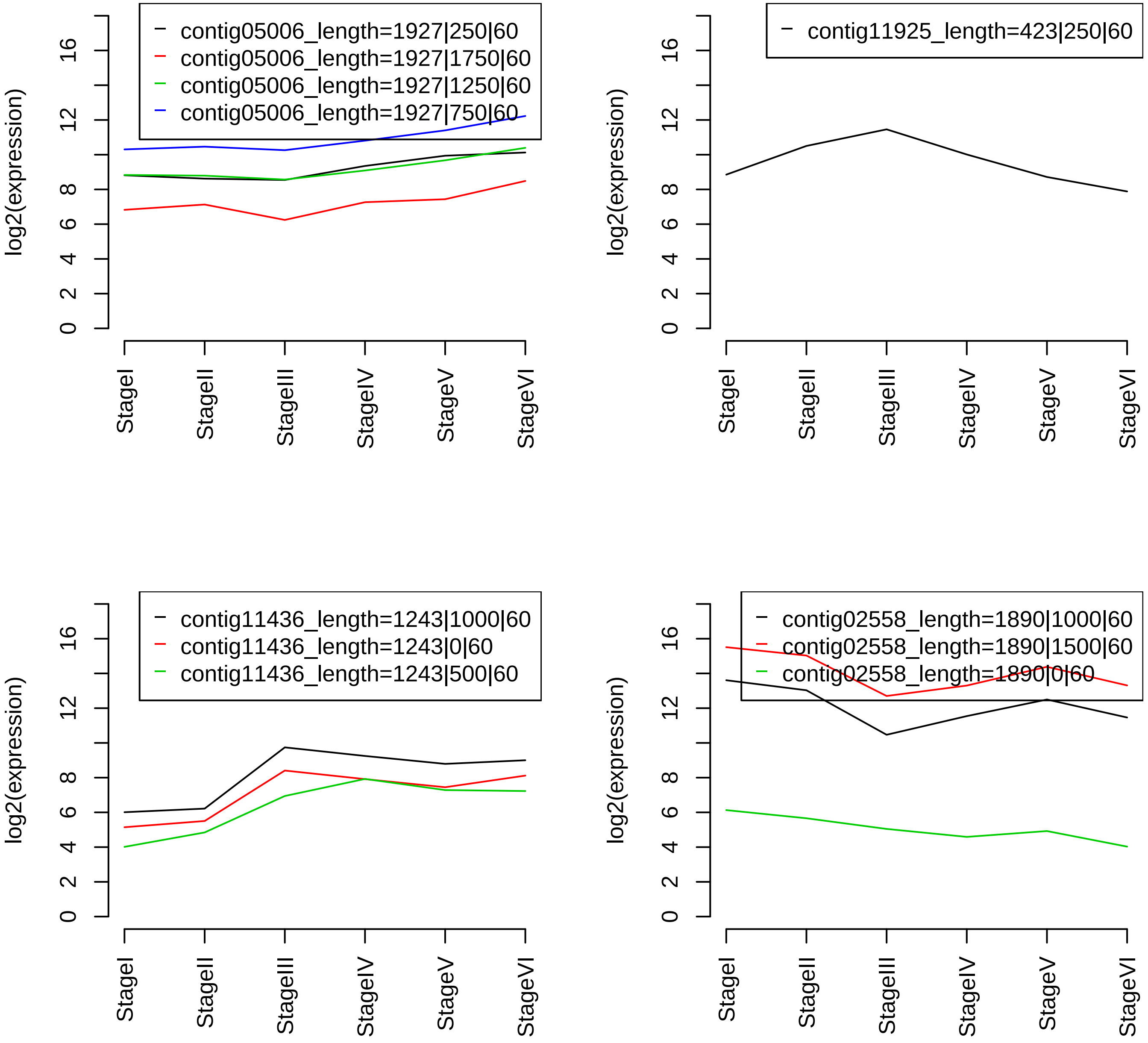
contig05006_length=1927|250|60
contig05006_length=1927|1750|60
contig05006_length=1927|1250|60
contig05006_length=1927|750|60
contig11925_length=423|250|60
contig11436_length=1243|1000|60
contig11436_length=1243|0|60
contig11436_length=1243|500|60
contig02558_length=1890|1000|60
contig02558_length=1890|1500|60
contig02558_length=1890|0|60
log2(expression)
0
2
4
6
8
12
16
StageI
StageII
StageIII
StageIV
StageV
StageVI

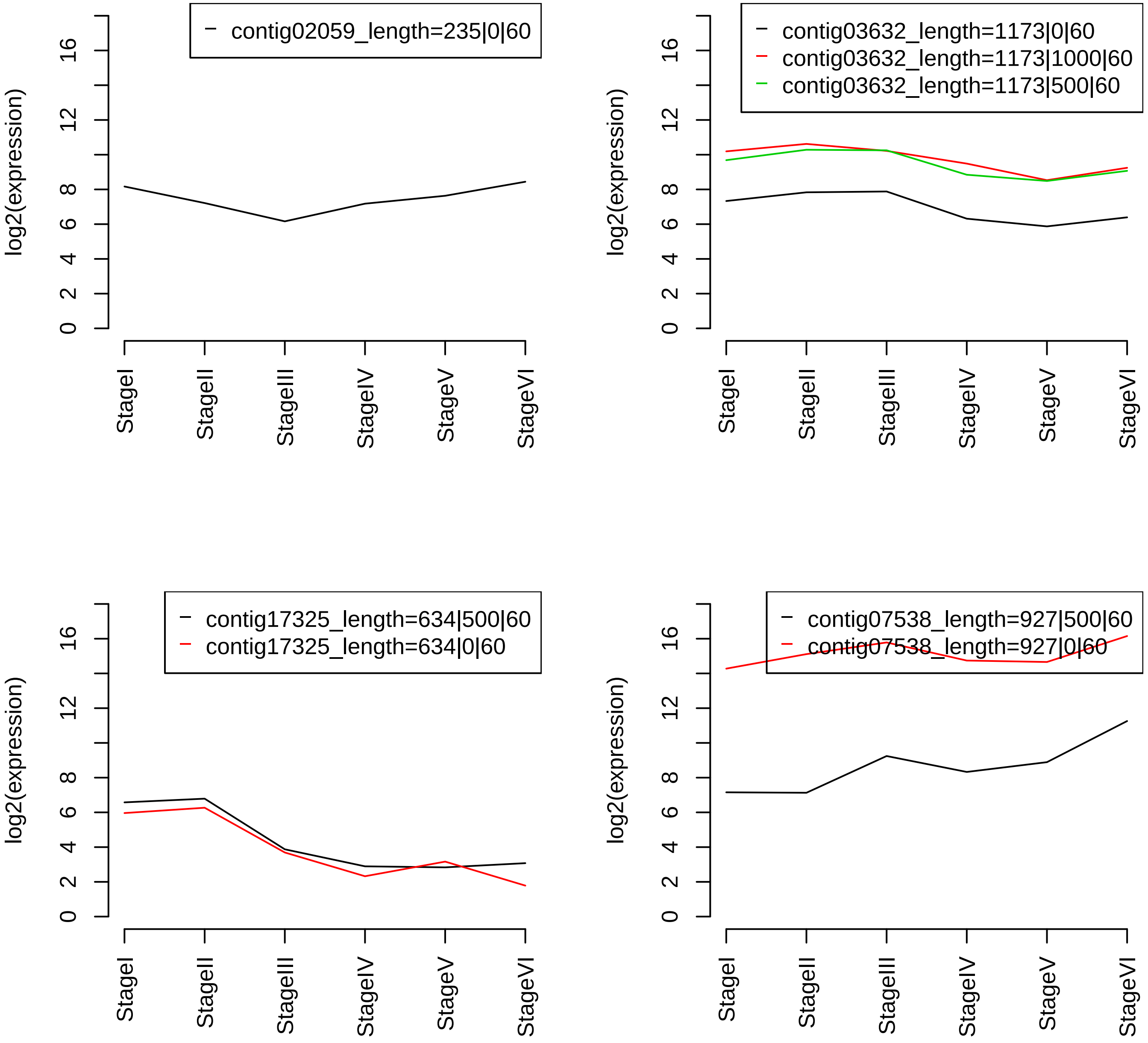

contig02059_length=235|0|60
contig03632_length=1173|0|60
contig03632_length=1173|1000|60
contig03632_length=1173|500|60
contig17325_length=634|500|60
contig17325_length=634|0|60
contig07538_length=927|500|60
contig07538_length=927|0|60
log2(expression)
0
2
4
6
8
12
16
StageI
StageII
StageIII
StageIV
StageV
StageVI

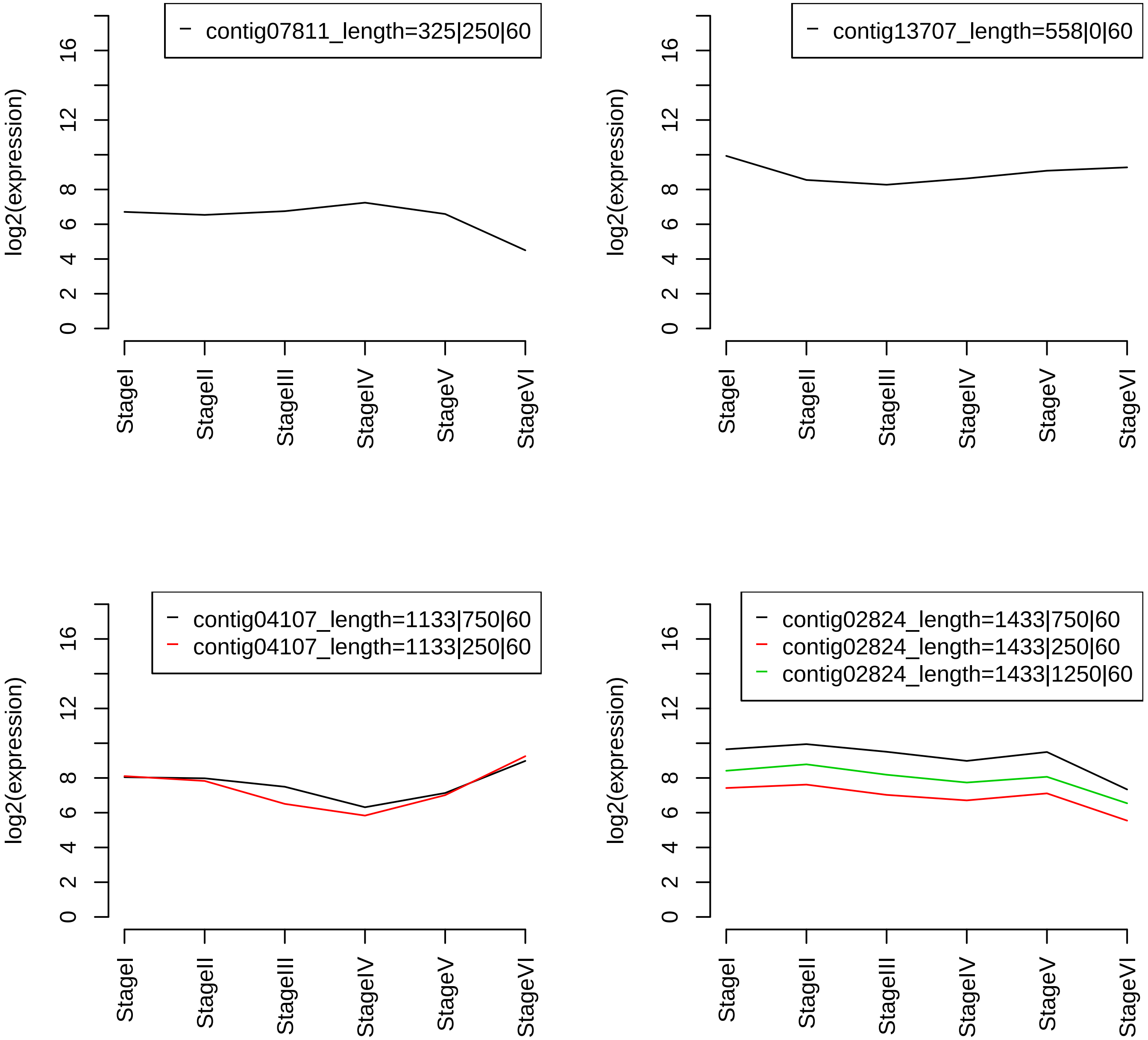

contig07811_length=325|250|60
contig13707_length=558|0|60
contig04107_length=1133|750|60
contig04107_length=1133|250|60
contig02824_length=1433|750|60
contig02824_length=1433|250|60
contig02824_length=1433|1250|60
log2(expression)
0
2
4
6
8
12
16
StageI
StageII
StageIII
StageIV
StageV
StageVI

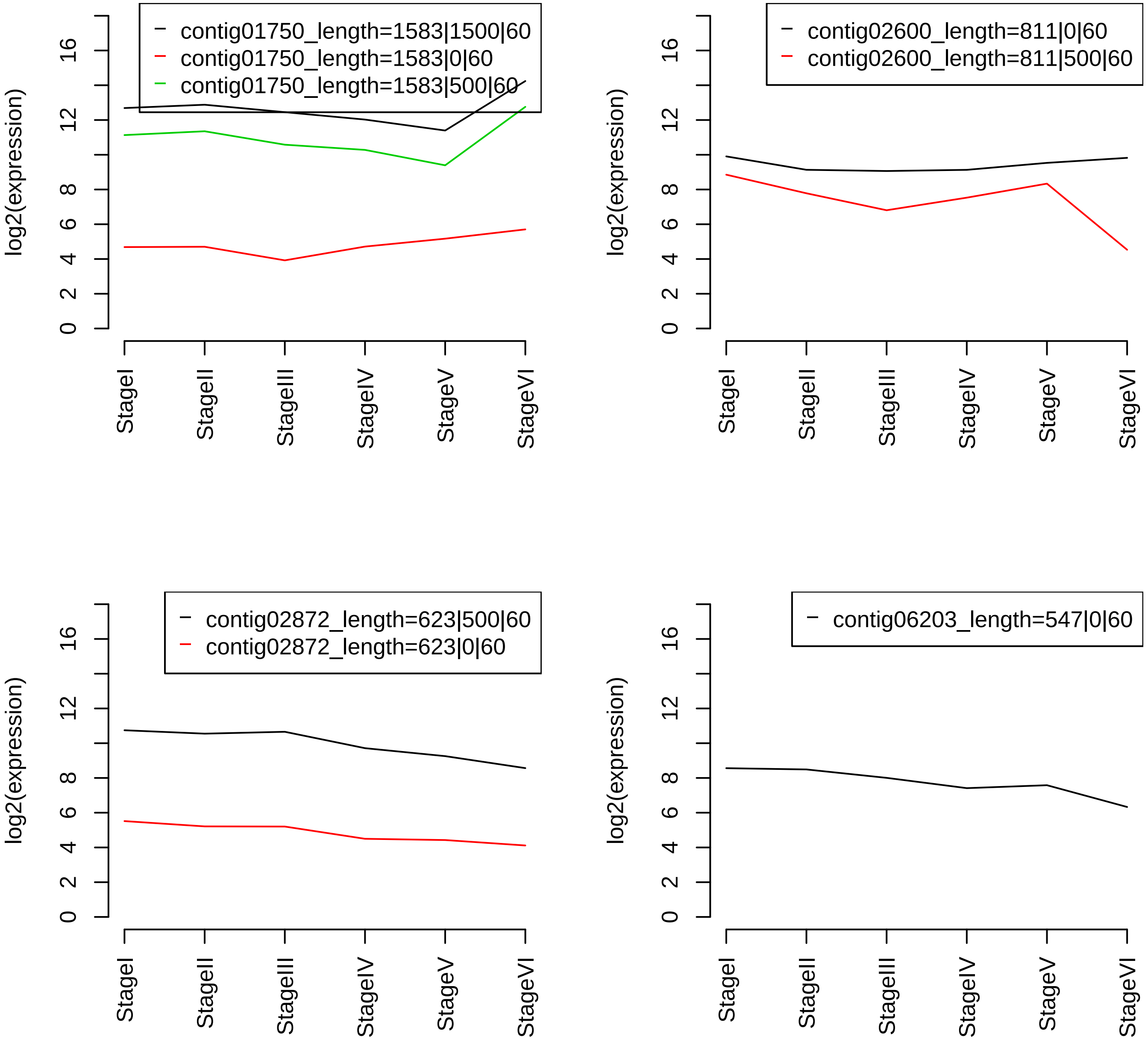

contig01750_length=1583|1500|60
contig01750_length=1583|0|60
contig01750_length=1583|500|60
contig02600_length=811|0|60
contig02600_length=811|500|60
contig02872_length=623|500|60
contig02872_length=623|0|60
contig06203_length=547|0|60
log2(expression)
0
2
4
6
8
12
16
StageI
StageII
StageIII
StageIV
StageV
StageVI

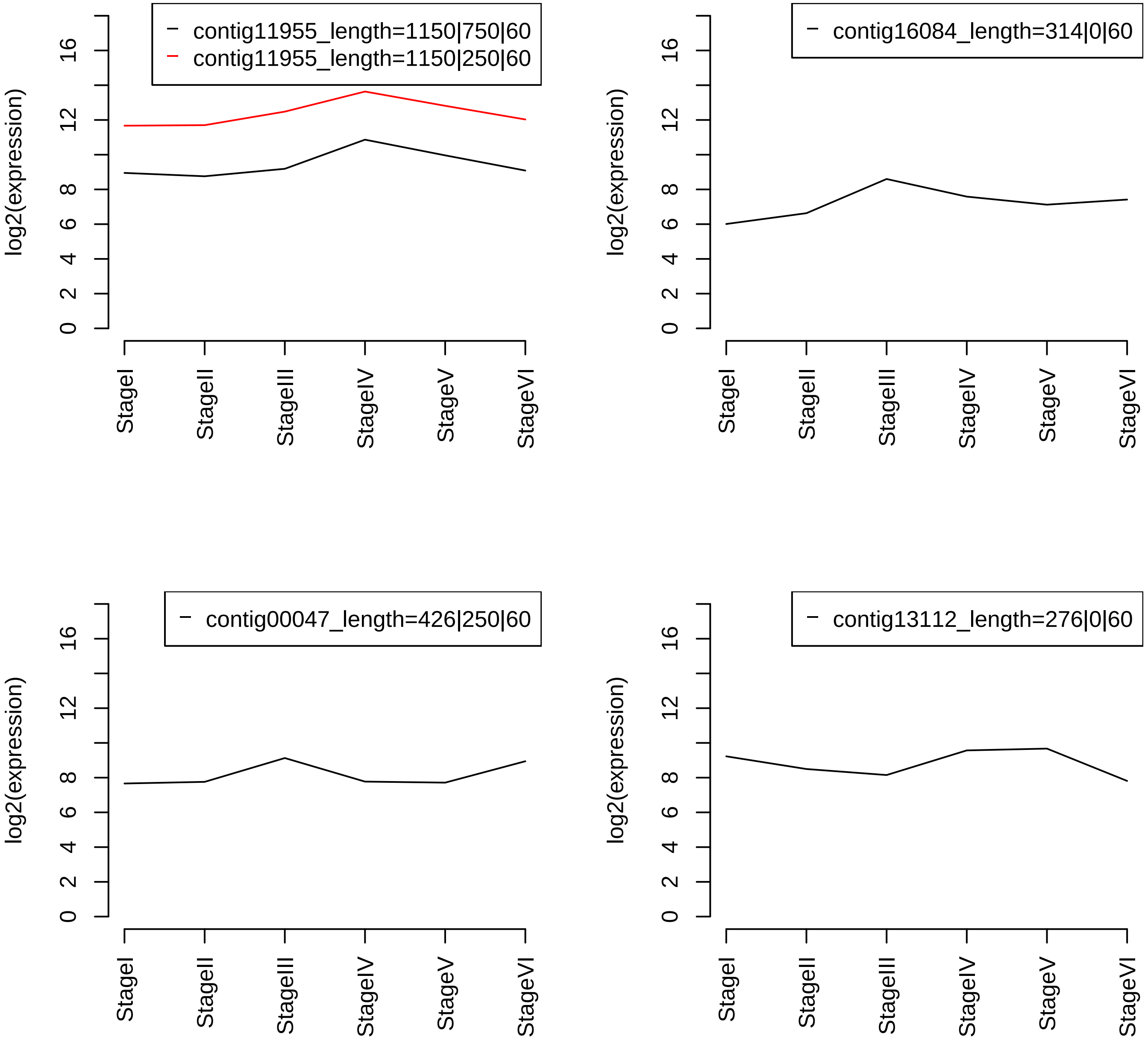
contig11955_length=1150|750|60
contig11955_length=1150|250|60
contig16084_length=314|0|60
contig00047_length=426|250|60
contig13112_length=276|0|60
log2(expression)
0
2
4
6
8
12
16
StageI
StageII
StageIII
StageIV
StageV
StageVI

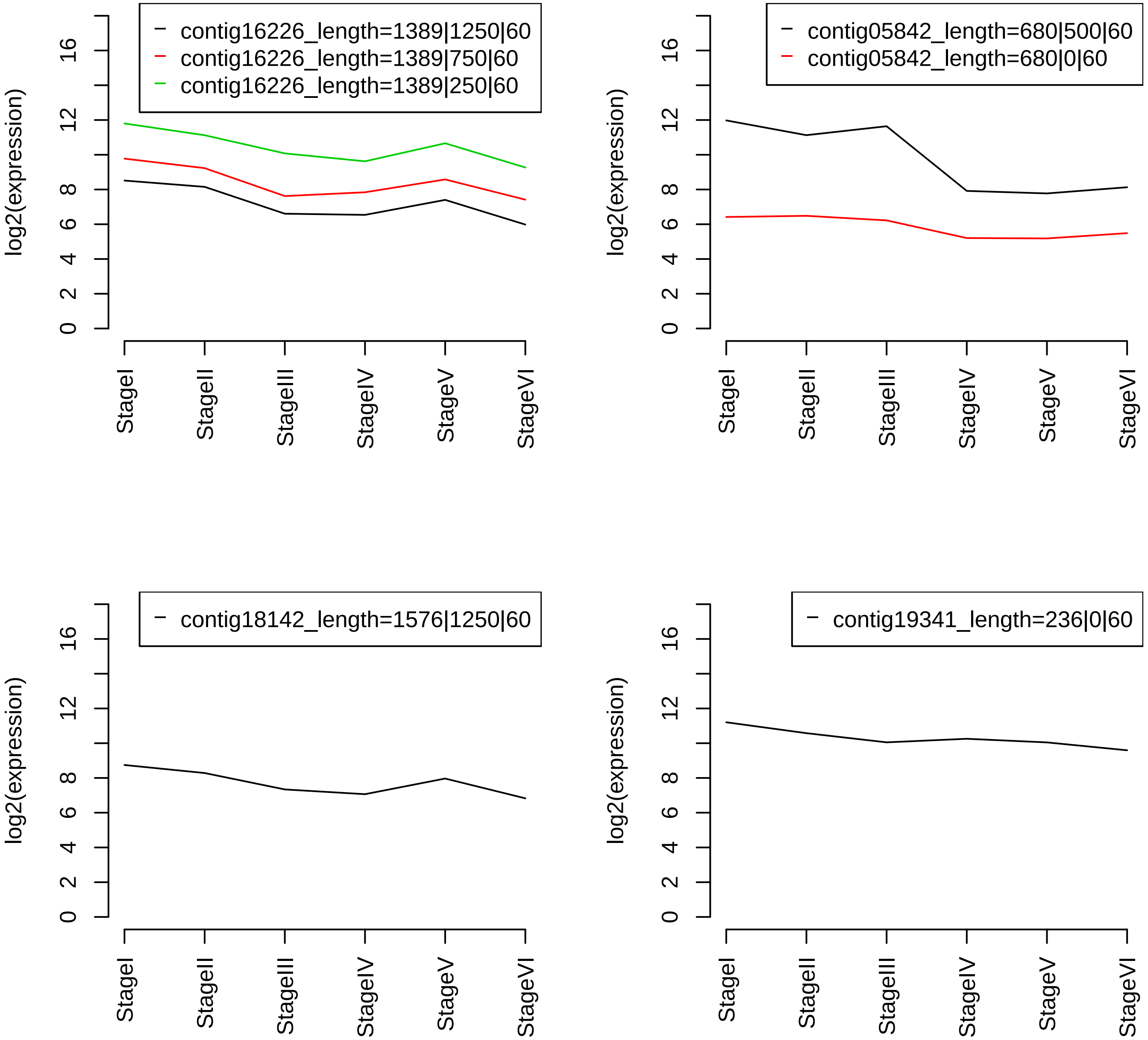

contig16226_length=1389|1250|60
contig16226_length=1389|750|60
contig16226_length=1389|250|60
contig05842_length=680|500|60
contig05842_length=680|0|60
contig18142_length=1576|1250|60
contig19341_length=236|0|60
log2(expression)
0
2
4
6
8
12
16
StageI
StageII
StageIII
StageIV
StageV
StageVI

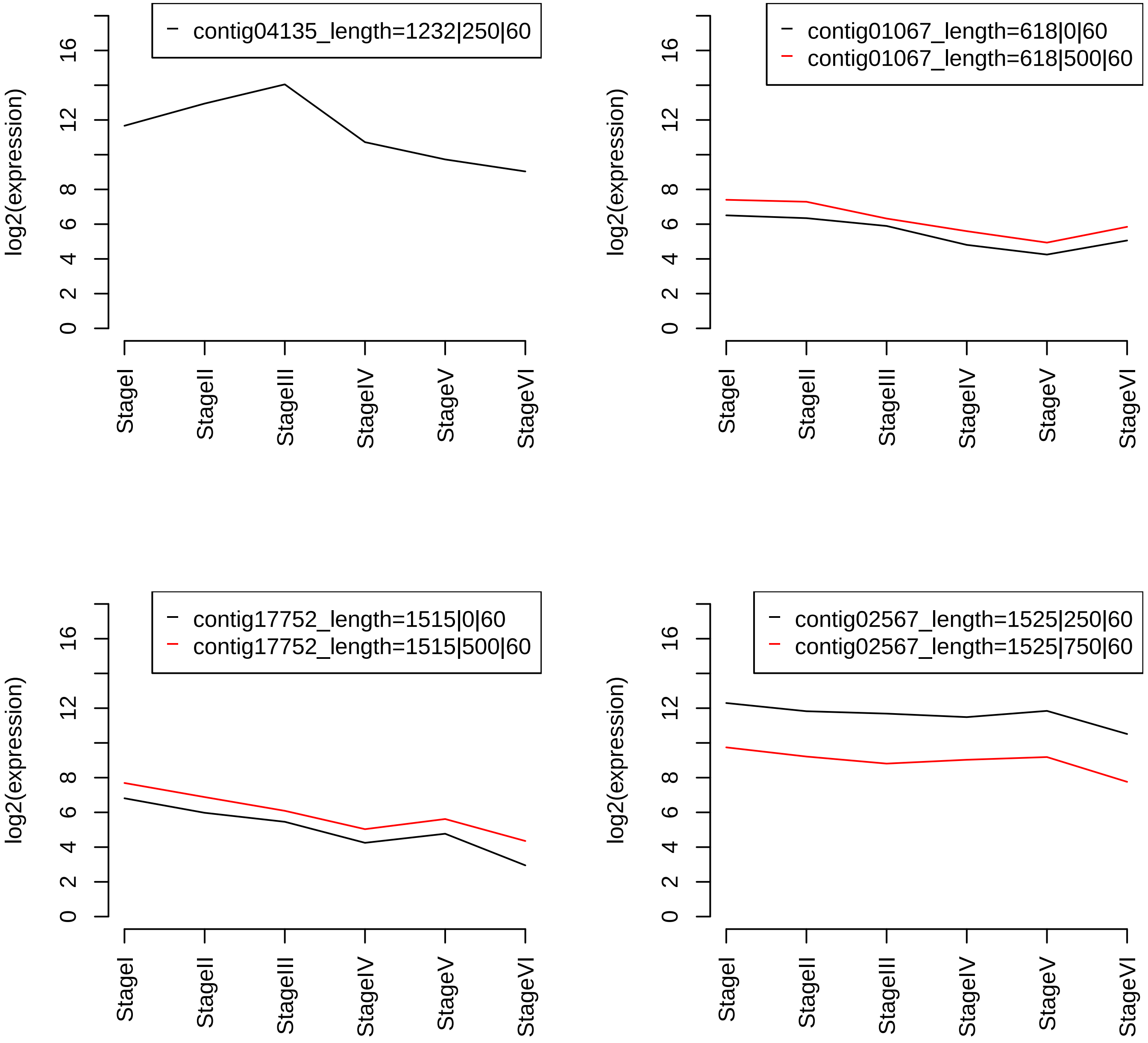
contig04135_length=1232|250|60
contig01067_length=618|0|60
contig01067_length=618|500|60
contig17752_length=1515|0|60
contig17752_length=1515|500|60
contig02567_length=1525|250|60
contig02567_length=1525|750|60
log2(expression)
0
2
4
6
8
12
16
StageI
StageII
StageIII
StageIV
StageV
StageVI

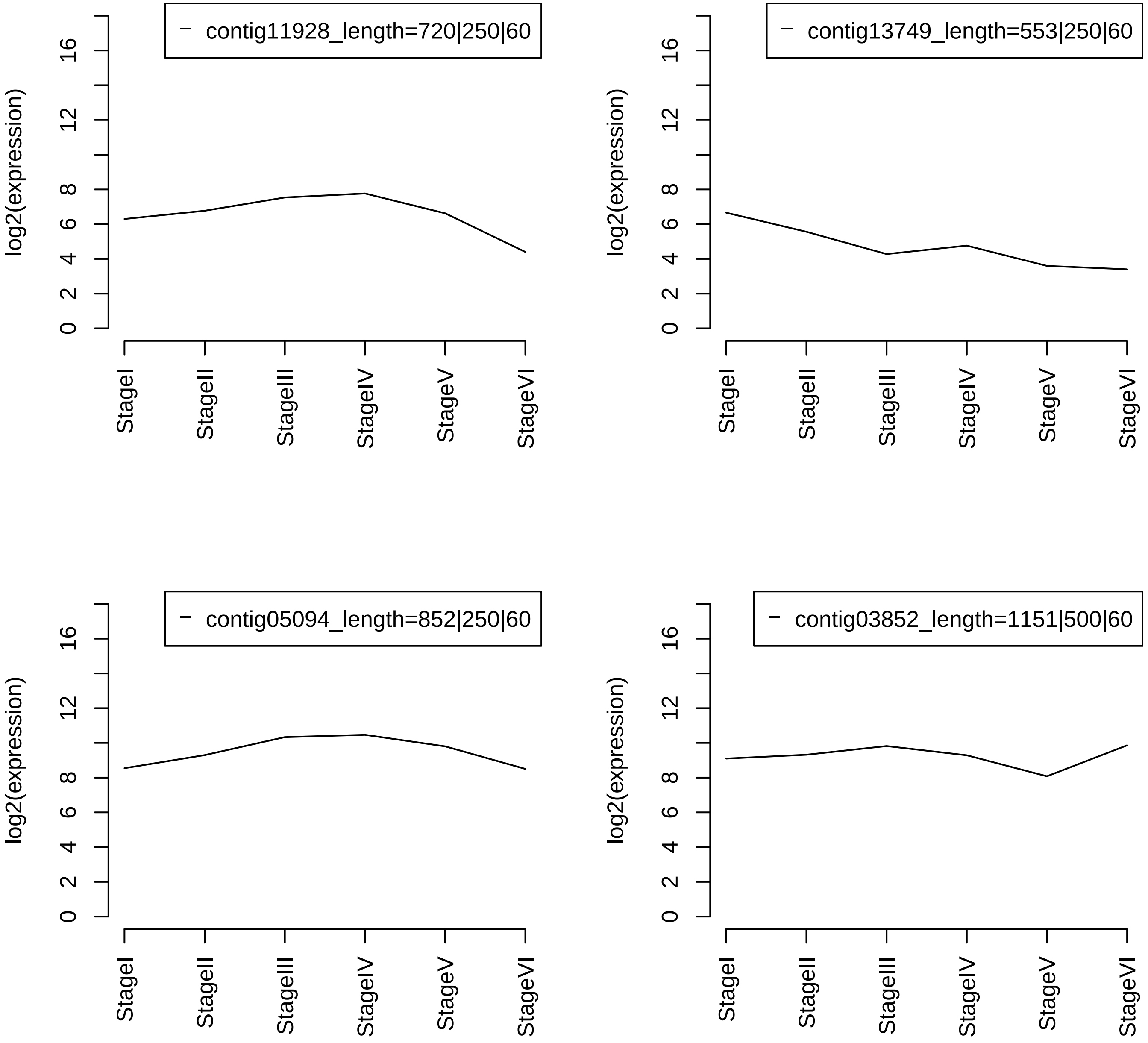

contig11928_length=720|250|60
contig13749_length=553|250|60
contig05094_length=852|250|60
contig03852_length=1151|500|60
log2(expression)
0
2
4
6
8
12
16
StageI
StageII
StageIII
StageIV
StageV
StageVI

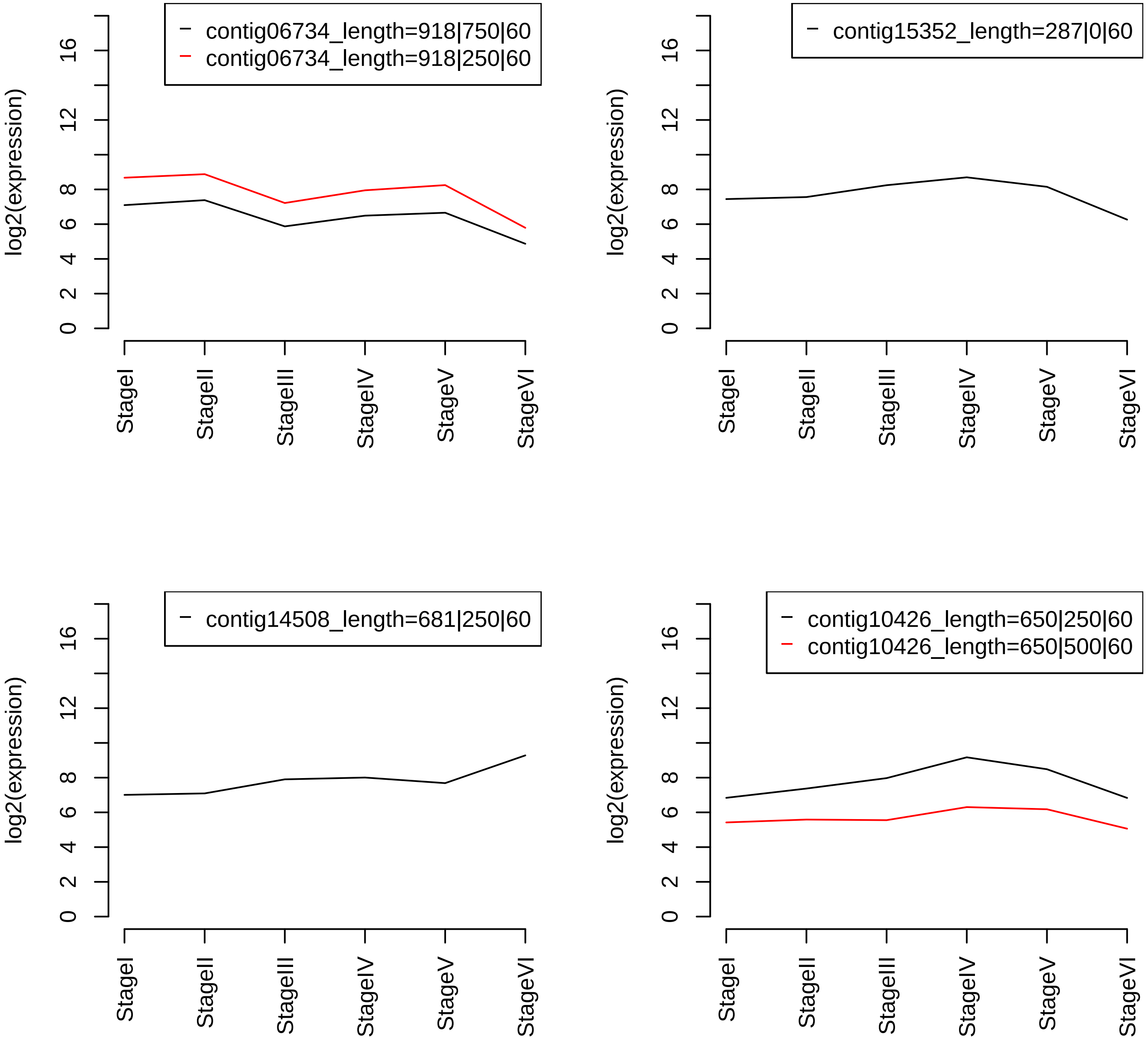
contig06734_length=918|750|60
contig06734_length=918|250|60
contig15352_length=287|0|60
contig14508_length=681|250|60
contig10426_length=650|250|60
contig10426_length=650|500|60
log2(expression)
0
2
4
6
8
12
16
StageI
StageII
StageIII
StageIV
StageV
StageVI

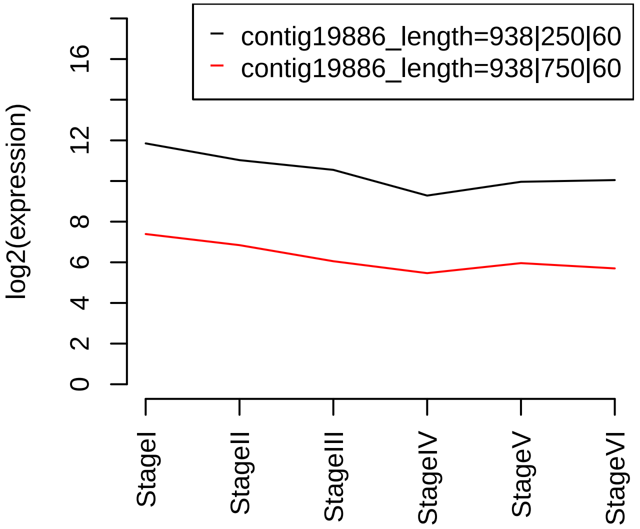

contig19886_length=938|250|60
contig19886_length=938|750|60
log2(expression)
0
2
4
6
8
12
16
StageI
StageII
StageIII
StageIV
StageV
StageVI

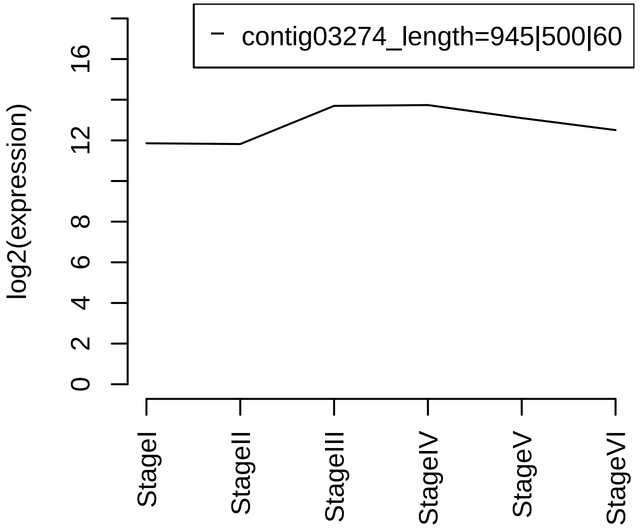

contig03274_length=945|500|60
log2(expression)
0
2
4
6
8
12
16
StageI
StageII
StageIII
StageIV
StageV
StageVI

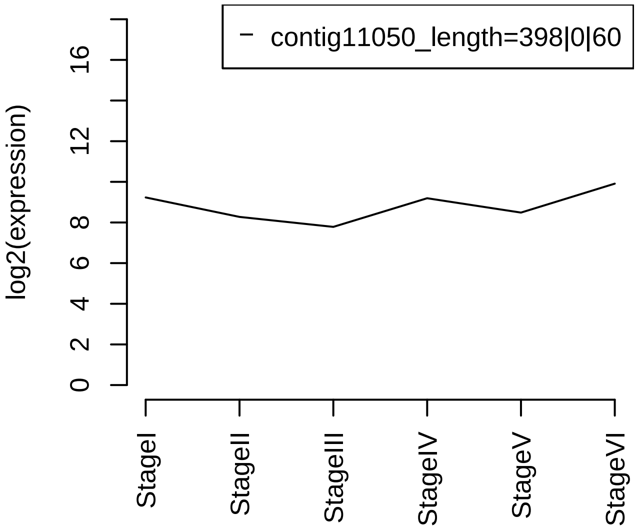

contig11050_length=398|0|60
log2(expression)
0
2
4
6
8
12
16
StageI
StageII
StageIII
StageIV
StageV
StageVI

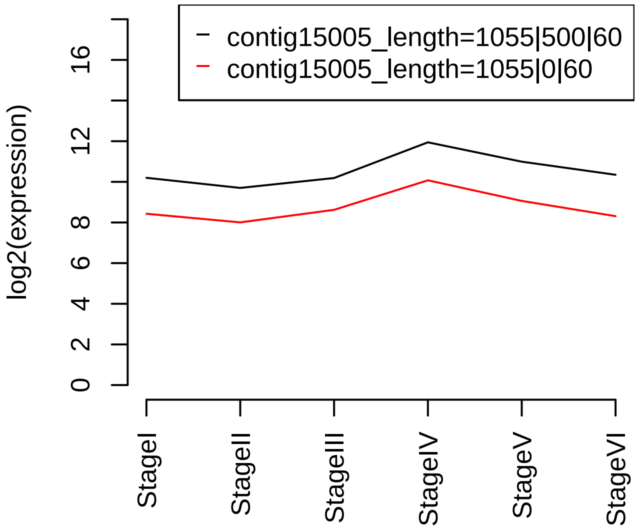

contig15005_length=1055|500|60
contig15005_length=1055|0|60
log2(expression)
0
2
4
6
8
12
16
StageI
StageII
StageIII
StageIV
StageV
StageVI

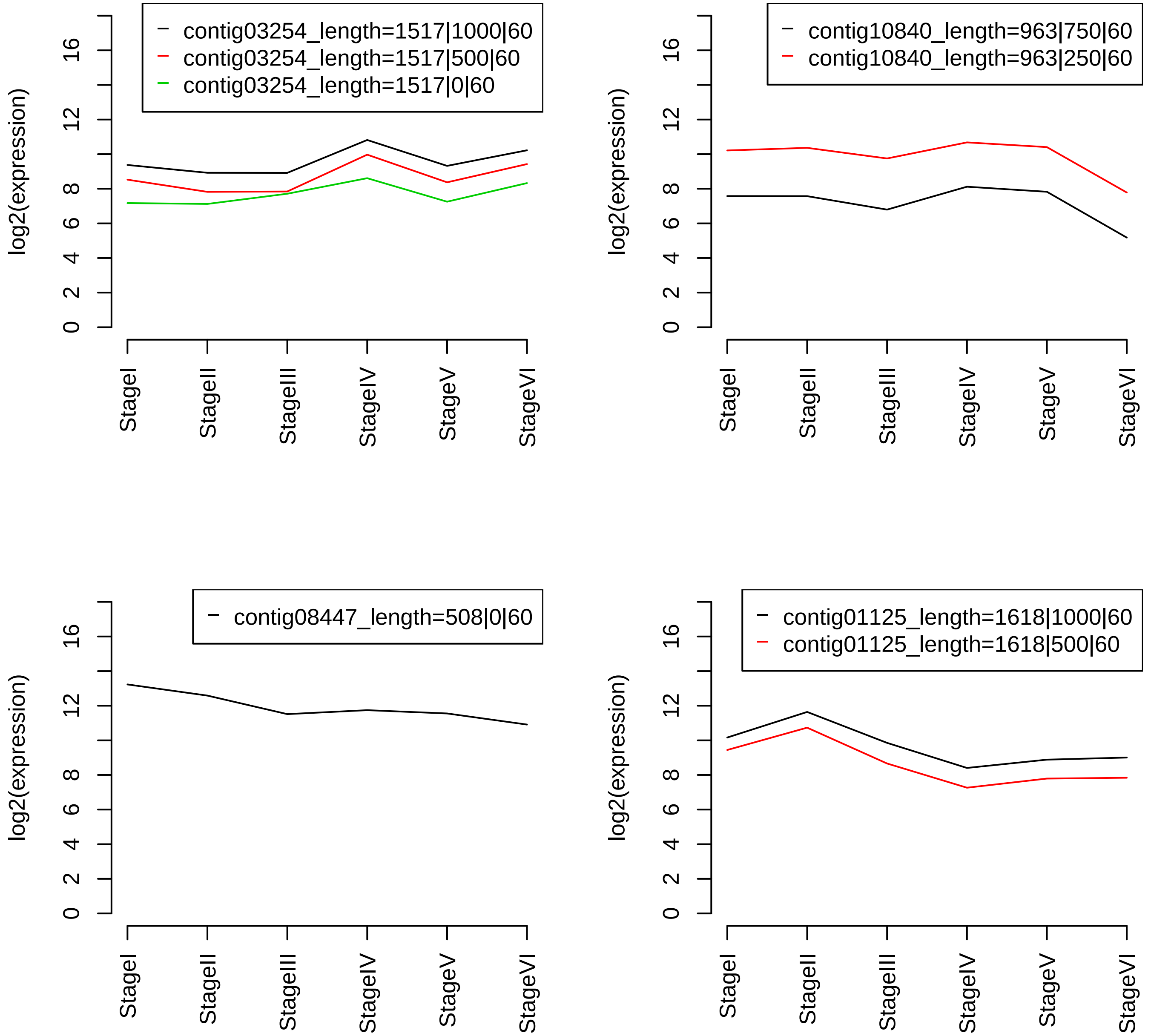

contig03254_length=1517|1000|60
contig03254_length=1517|500|60
contig03254_length=1517|0|60
contig10840_length=963|750|60
contig10840_length=963|250|60
contig08447_length=508|0|60
contig01125_length=1618|1000|60
contig01125_length=1618|500|60
log2(expression)
0 2 4 6 8 12 16
StageI StageII StageIII StageIV StageV StageVI

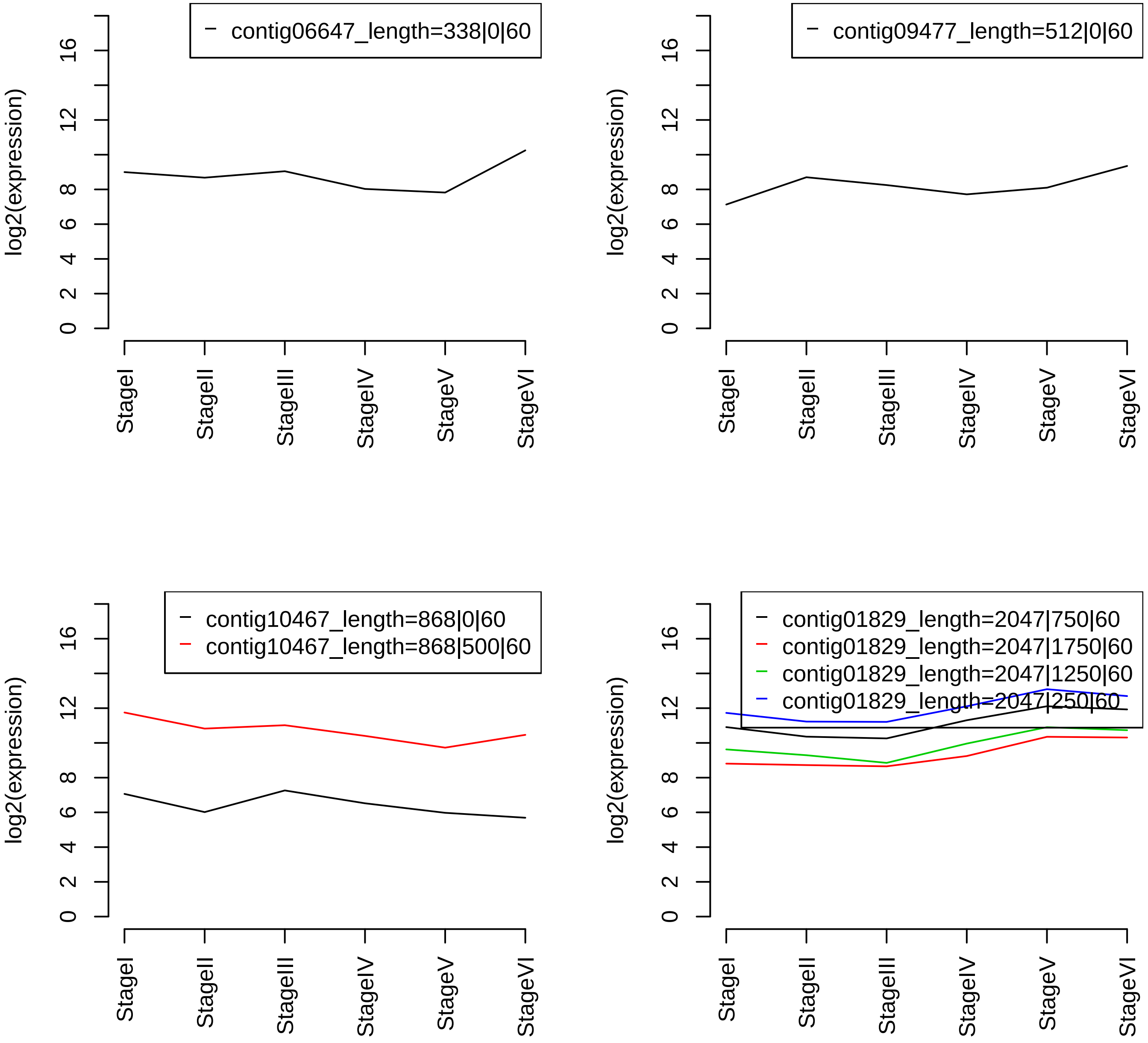
contig06647_length=338|0|60
contig09477_length=512|0|60
contig10467_length=868|0|60
contig10467_length=868|500|60
contig01829_length=2047|750|60
contig01829_length=2047|1750|60
contig01829_length=2047|1250|60
contig01829_length=2047|250|60
log2(expression)
0
2
4
6
8
12
16
StageI
StageII
StageIII
StageIV
StageV
StageVI

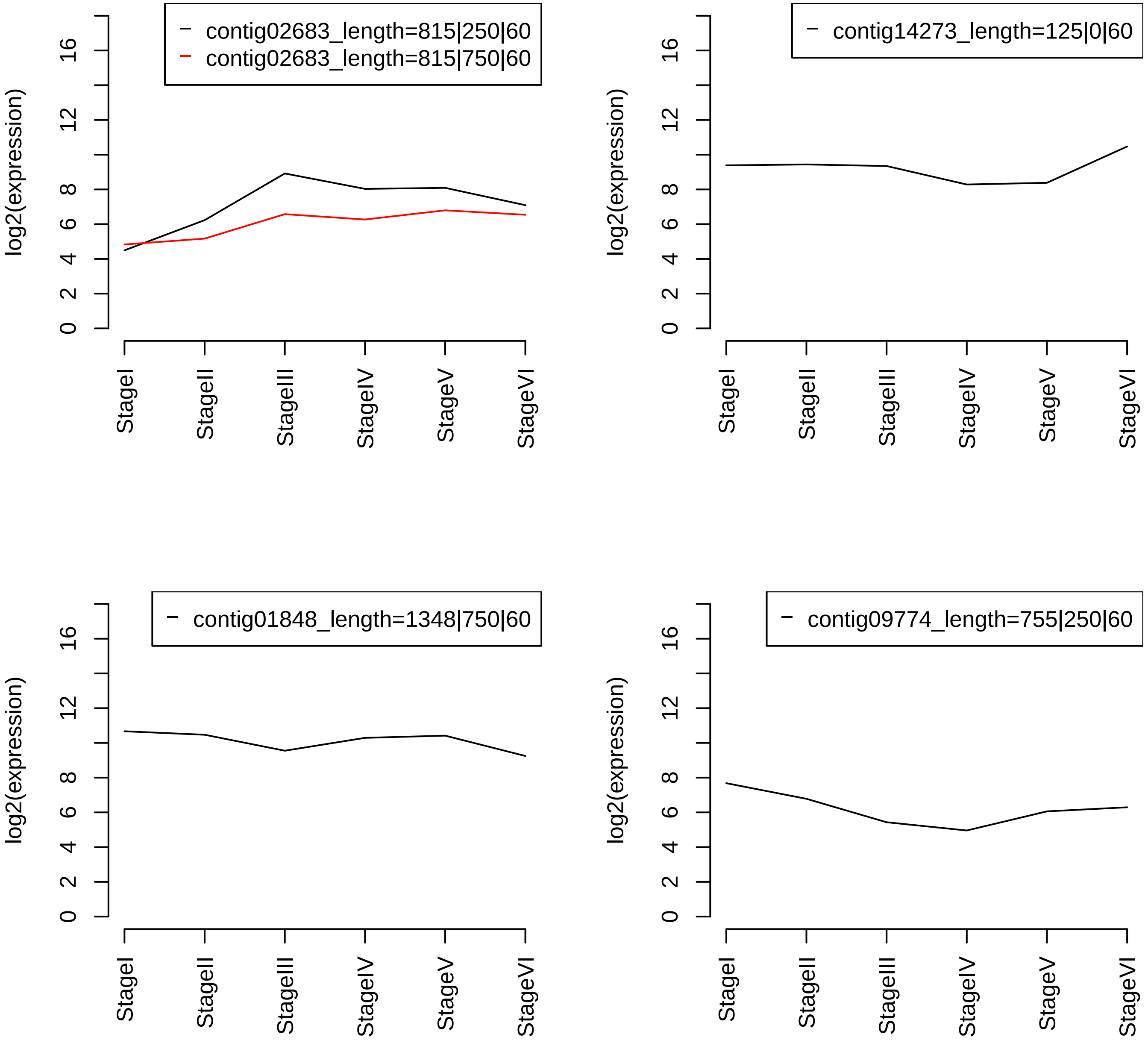
contig02683_length=815|250|60
contig02683_length=815|750|60
contig14273_length=125|0|60
contig01848_length=1348|750|60
contig09774_length=755|250|60
log2(expression)
0
2
4
6
8
12
16
StageI
StageII
StageIII
StageIV
StageV
StageVI

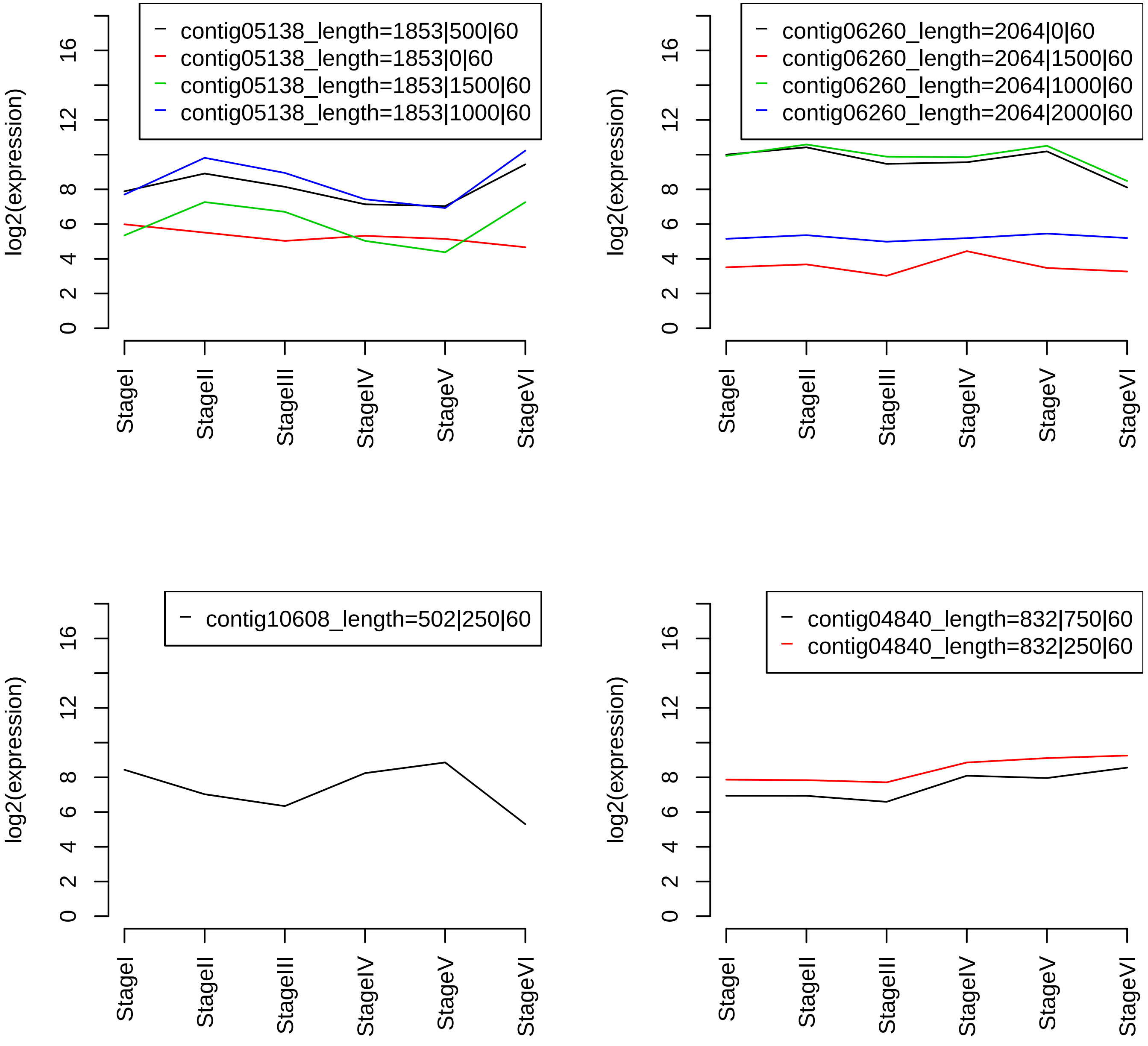

contig05138_length=1853|500|60
contig05138_length=1853|0|60
contig05138_length=1853|1500|60
contig05138_length=1853|1000|60
contig06260_length=2064|0|60
contig06260_length=2064|1500|60
contig06260_length=2064|1000|60
contig06260_length=2064|2000|60
contig10608_length=502|250|60
contig04840_length=832|750|60
contig04840_length=832|250|60
log2(expression)
0
2
4
6
8
12
16
StageI
StageII
StageIII
StageIV
StageV
StageVI

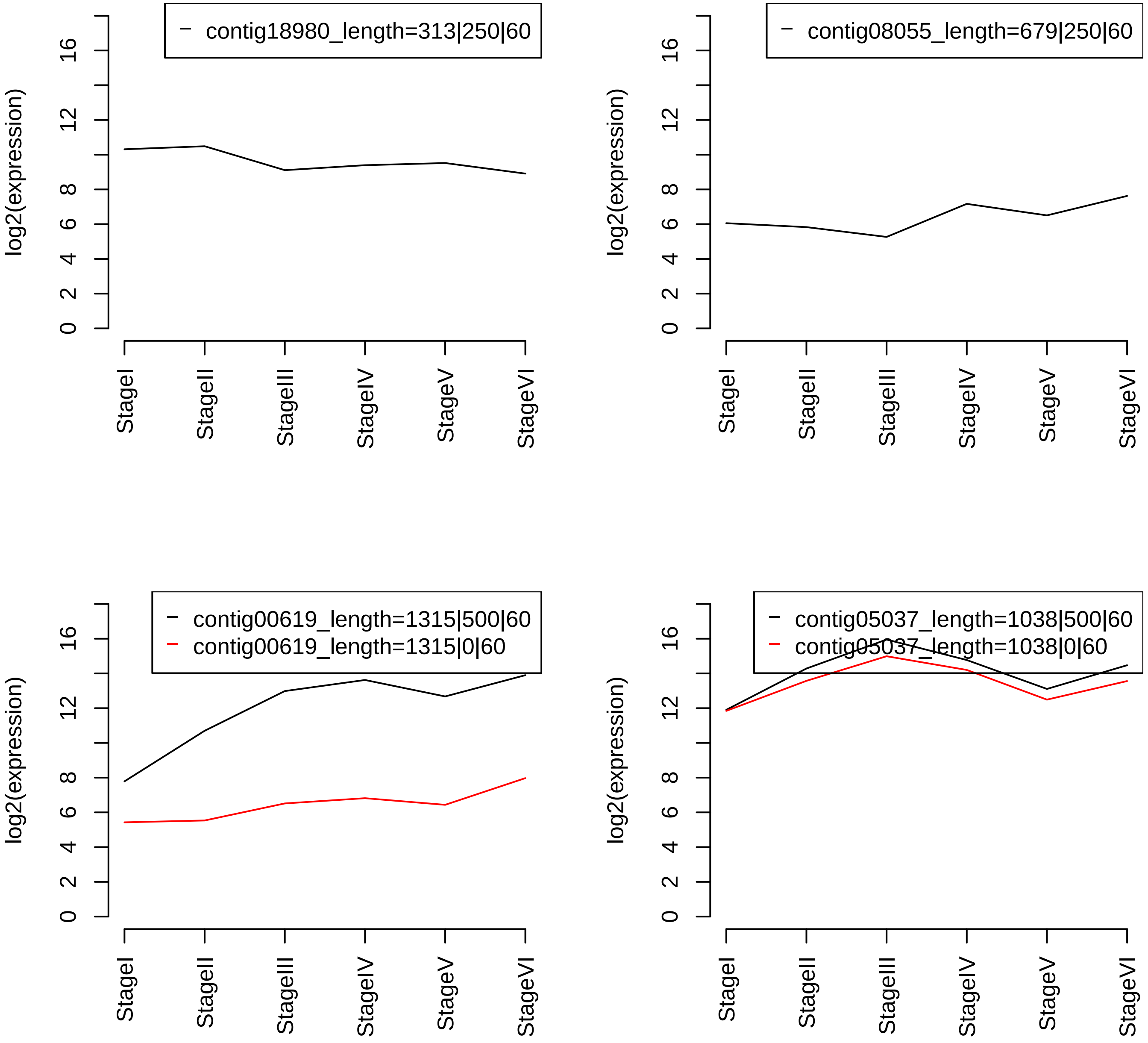

contig18980_length=313|250|60
contig08055_length=679|250|60
contig00619_length=1315|500|60
contig00619_length=1315|0|60
contig05037_length=1038|500|60
contig05037_length=1038|0|60
log2(expression)
0
2
4
6
8
12
16
StageI
StageII
StageIII
StageIV
StageV
StageVI

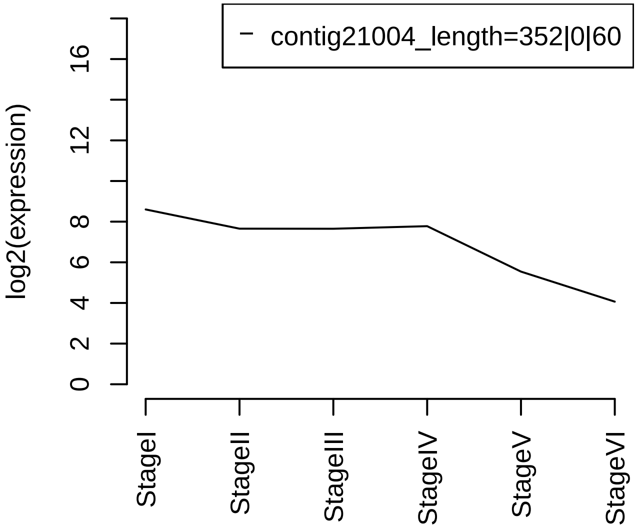
contig21004_length=352|0|60
log2(expression)
0
2
4
6
8
12
16
StageI
StageII
StageIII
StageIV
StageV
StageVI

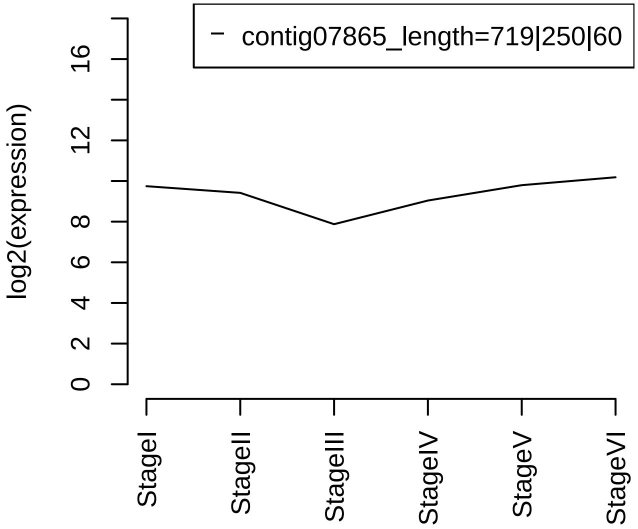
contig07865_length=719|250|60
log2(expression)
0
2
4
6
8
12
16
StageI
StageII
StageIII
StageIV
StageV
StageVI

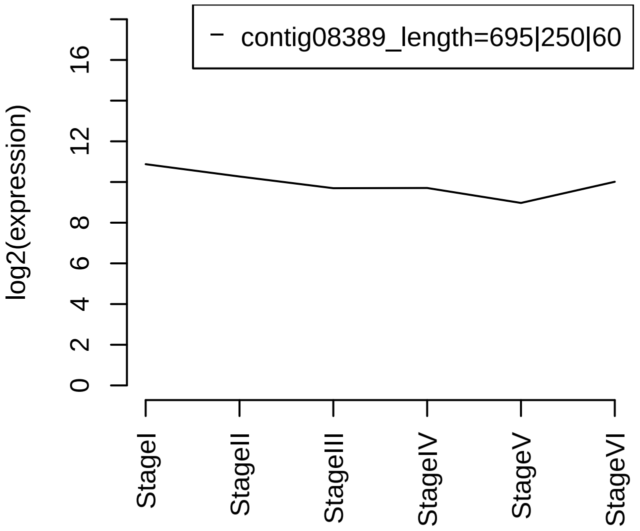
contig08389_length=695|250|60
log2(expression)
0
2
4
6
8
12
16
StageI
StageII
StageIII
StageIV
StageV
StageVI

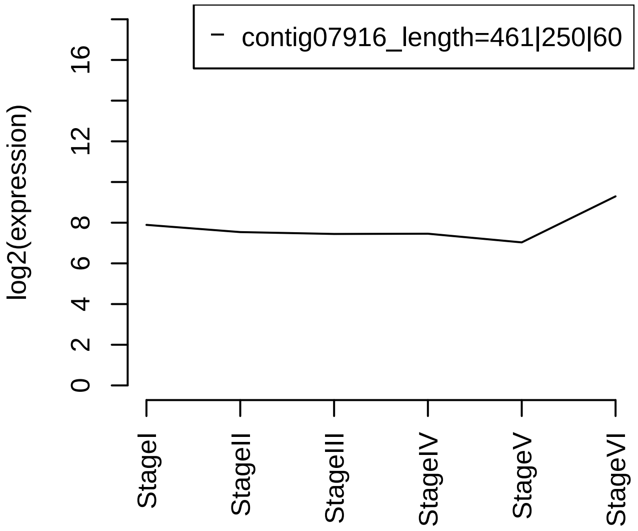
contig07916_length=461|250|60
log2(expression)
0
2
4
6
8
12
16
StageI
StageII
StageIII
StageIV
StageV
StageVI

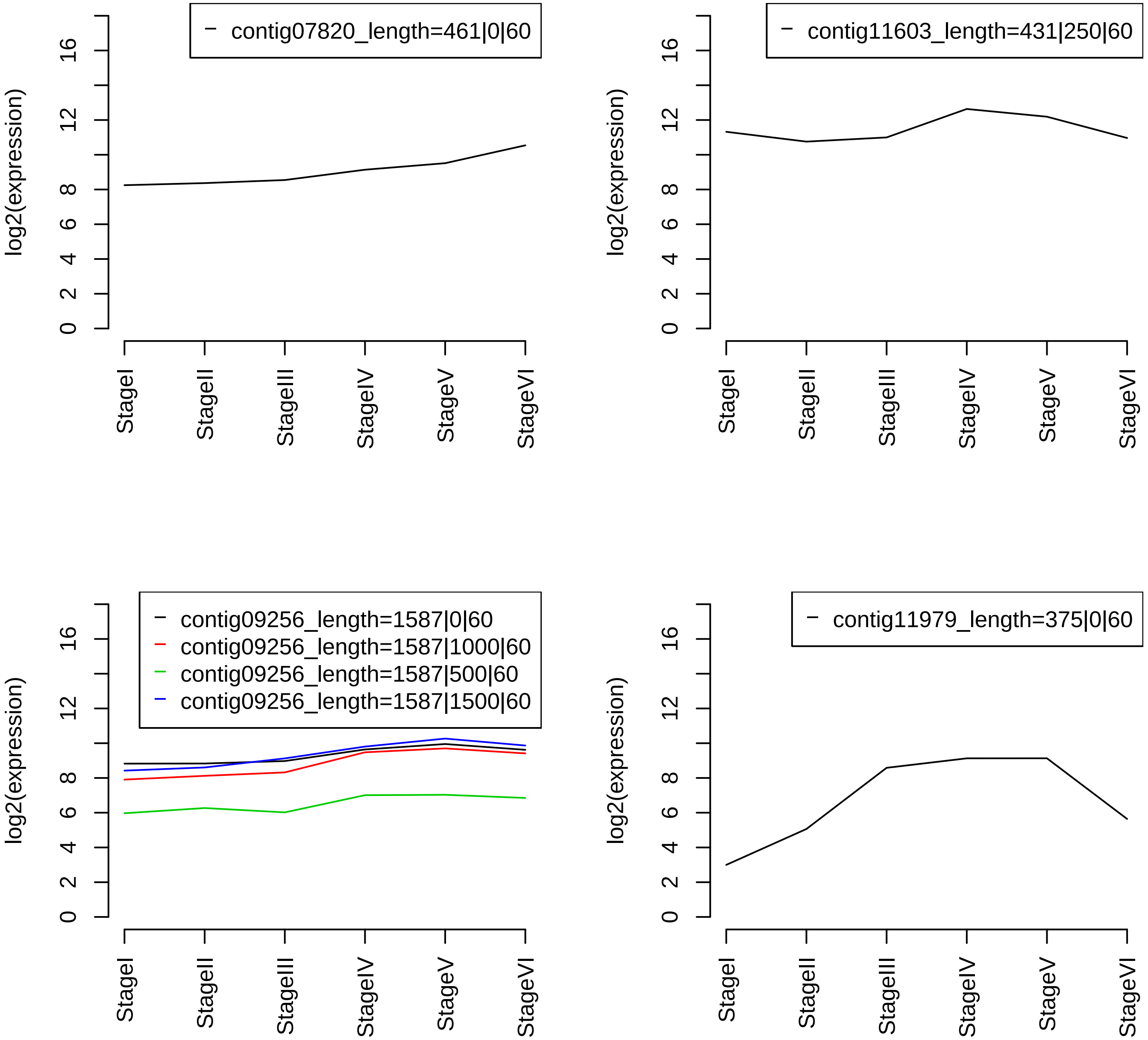
contig07820_length=461|0|60
contig11603_length=431|250|60
contig09256_length=1587|0|60
contig09256_length=1587|1000|60
contig09256_length=1587|500|60
contig09256_length=1587|1500|60
contig11979_length=375|0|60
log2(expression)
0
2
4
6
8
12
16
StageI
StageII
StageIII
StageIV
StageV
StageVI

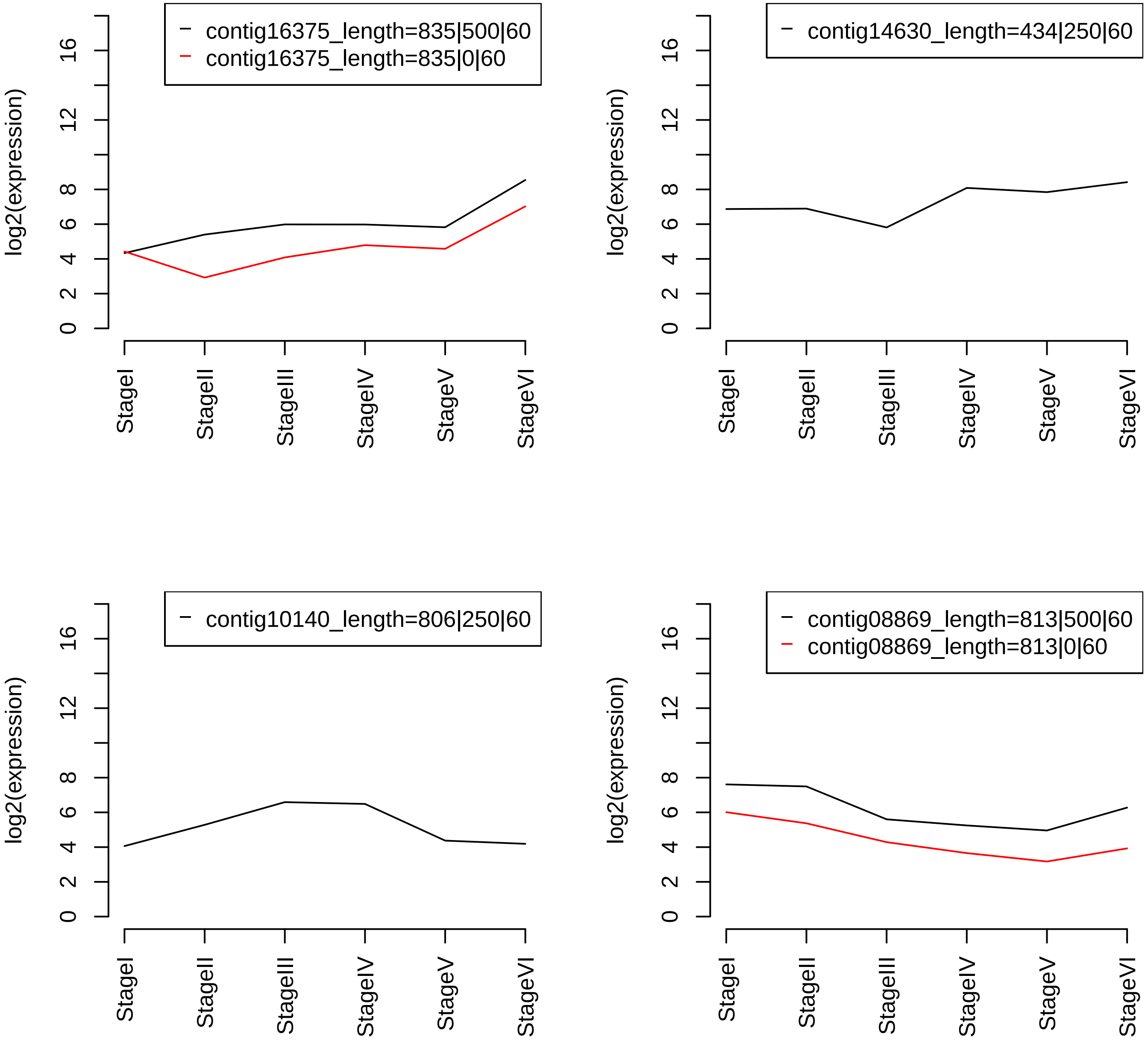
contig16375_length=835|500|60
contig16375_length=835|0|60
contig14630_length=434|250|60
contig10140_length=806|250|60
contig08869_length=813|500|60
contig08869_length=813|0|60
log2(expression)
0
2
4
6
8
12
16
StageI
StageII
StageIII
StageIV
StageV
StageVI

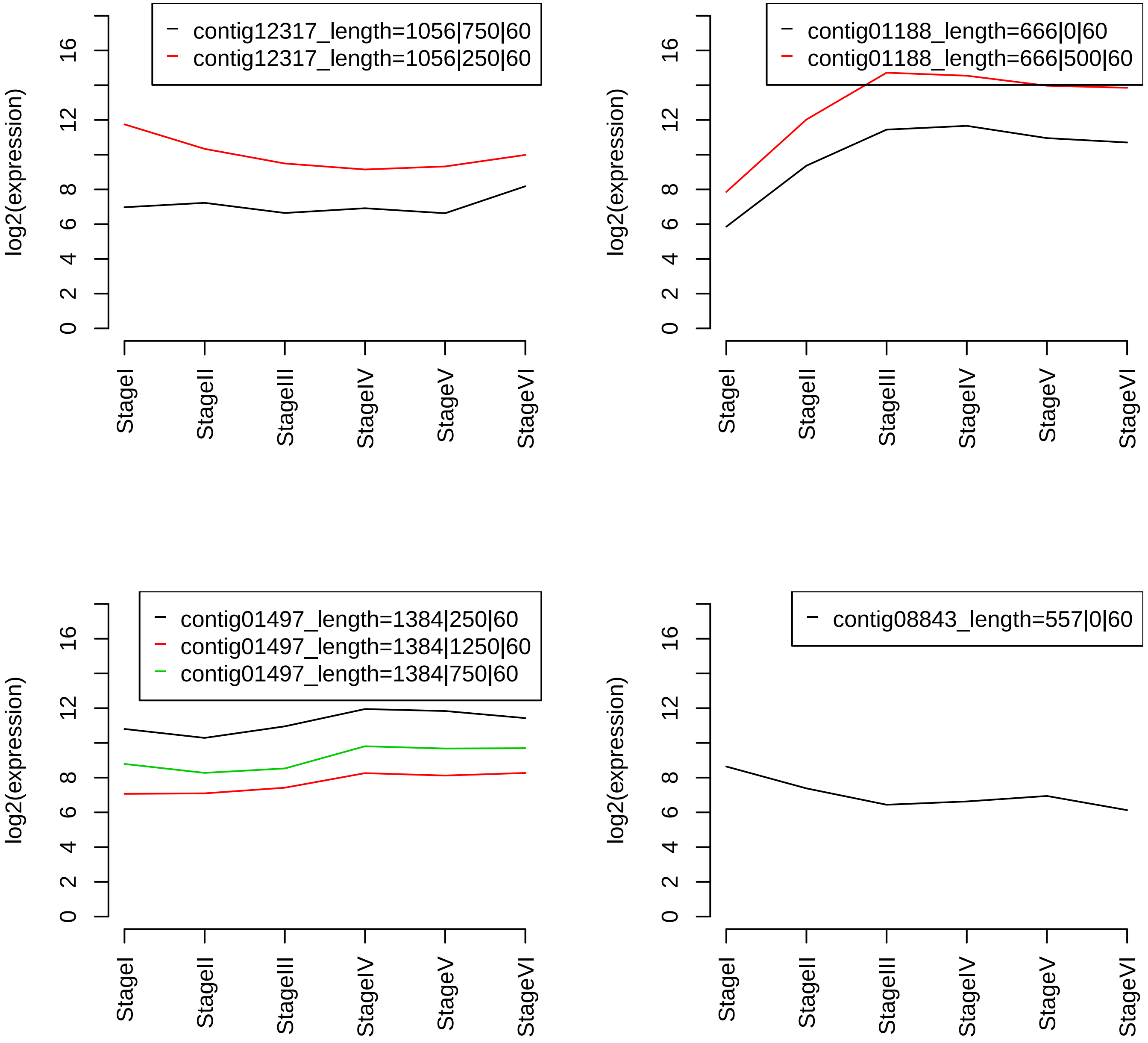

contig12317_length=1056|750|60
contig12317_length=1056|250|60
contig01188_length=666|0|60
contig01188_length=666|500|60
contig01497_length=1384|250|60
contig01497_length=1384|1250|60
contig01497_length=1384|750|60
contig08843_length=557|0|60
log2(expression)
0
2
4
6
8
12
16
StageI
StageII
StageIII
StageIV
StageV
StageVI

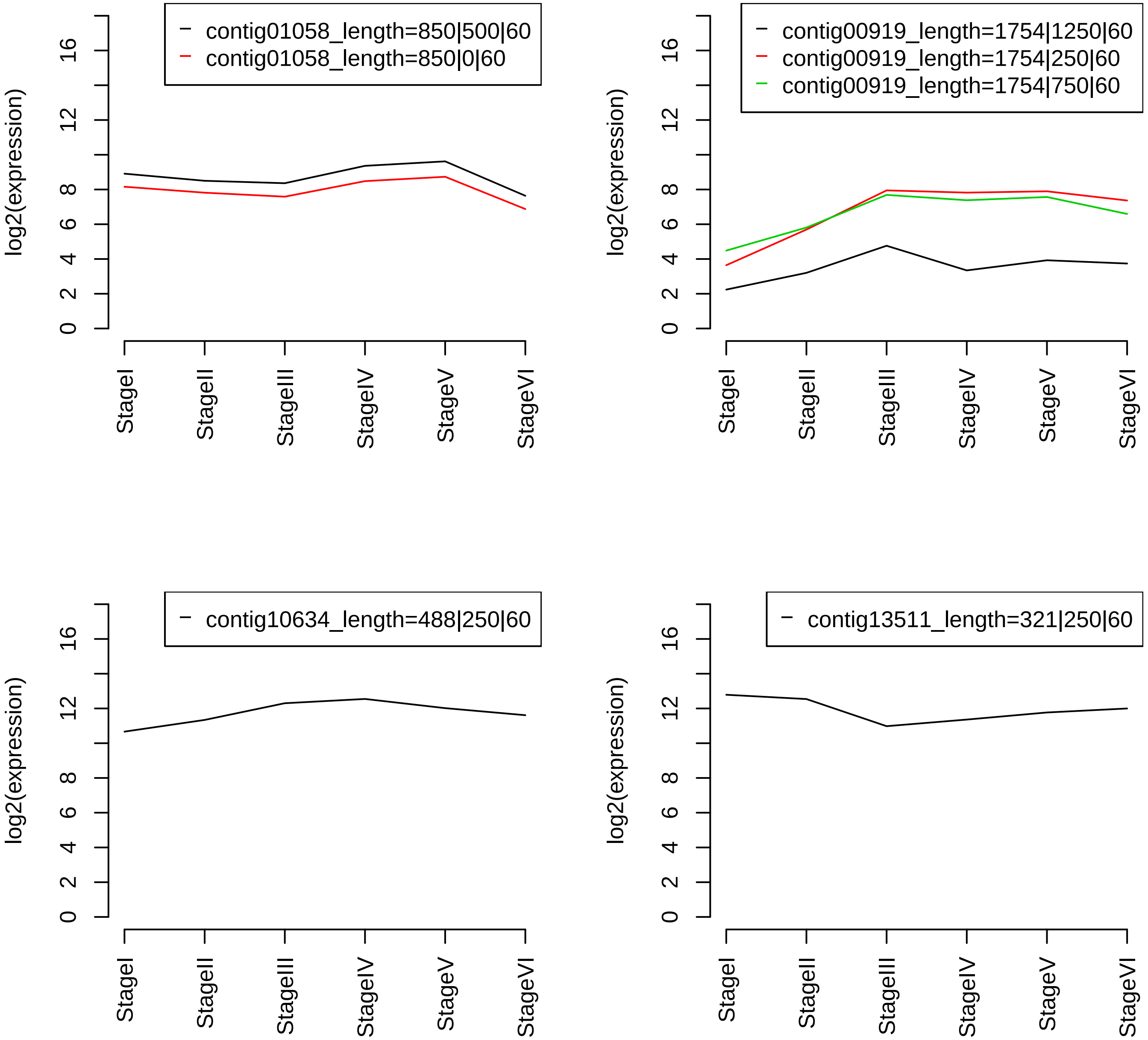
contig01058_length=850|500|60
contig01058_length=850|0|60
contig00919_length=1754|1250|60
contig00919_length=1754|250|60
contig00919_length=1754|750|60
contig10634_length=488|250|60
contig13511_length=321|250|60
log2(expression)
0
2
4
6
8
12
16
StageI
StageII
StageIII
StageIV
StageV
StageVI

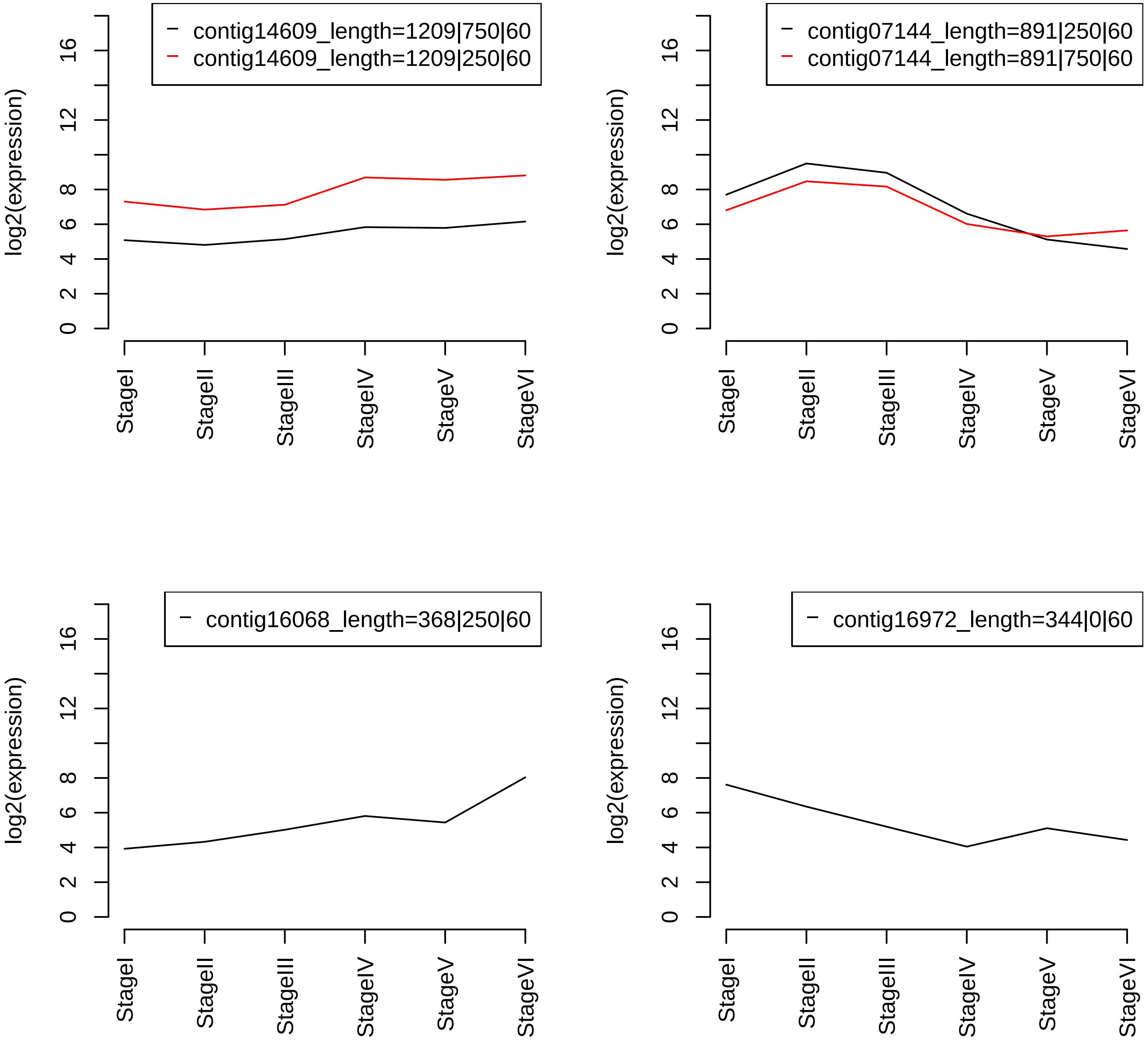
contig14609_length=1209|750|60
contig14609_length=1209|250|60
contig07144_length=891|250|60
contig07144_length=891|750|60
contig16068_length=368|250|60
contig16972_length=344|0|60
log2(expression)
0
2
4
6
8
12
16
StageI
StageII
StageIII
StageIV
StageV
StageVI

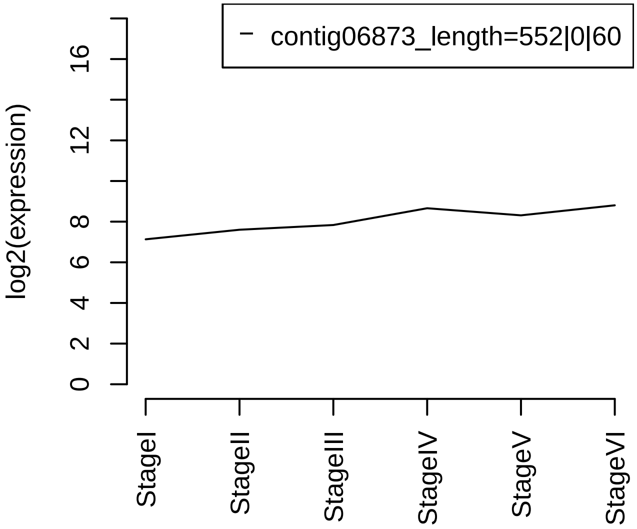
contig06873_length=552|0|60
log2(expression)
0
2
4
6
8
12
16
StageI
StageII
StageIII
StageIV
StageV
StageVI

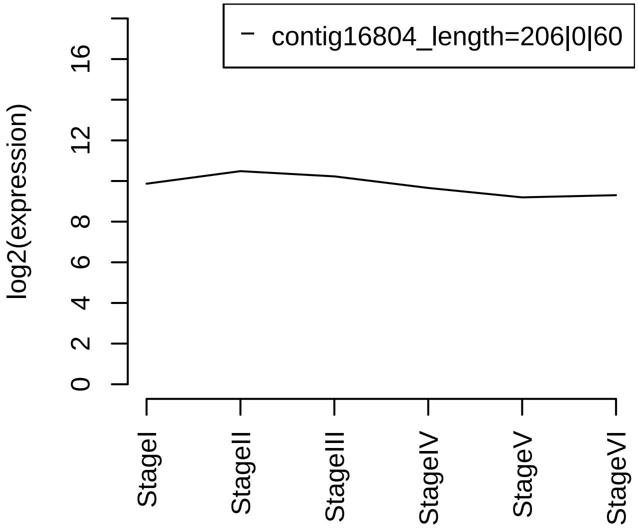
contig16804_length=206|0|60
log2(expression)
0
2
4
6
8
12
16
StageI
StageII
StageIII
StageIV
StageV
StageVI

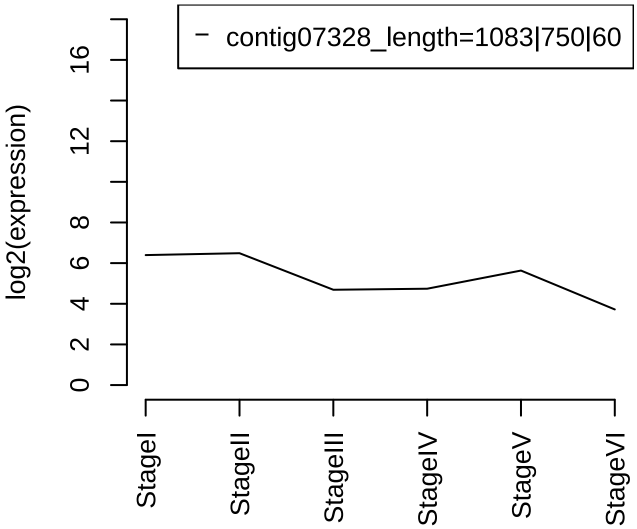
contig07328_length=1083|750|60
log2(expression)
0
2
4
6
8
12
16
StageI
StageII
StageIII
StageIV
StageV
StageVI

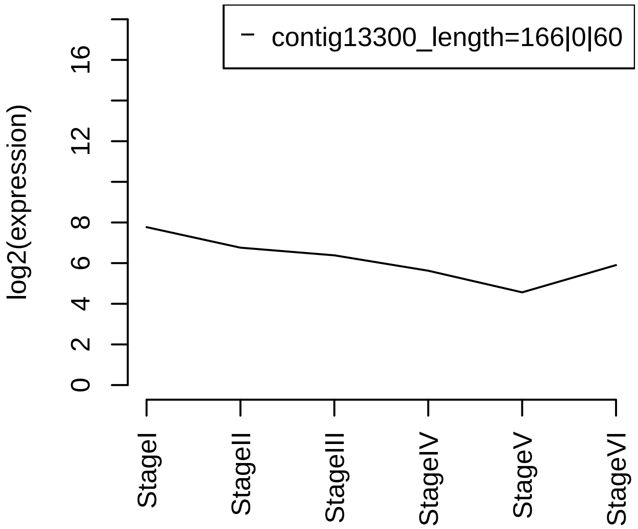
contig13300_length=166|0|60
log2(expression)
0
2
4
6
8
12
16
StageI
StageII
StageIII
StageIV
StageV
StageVI

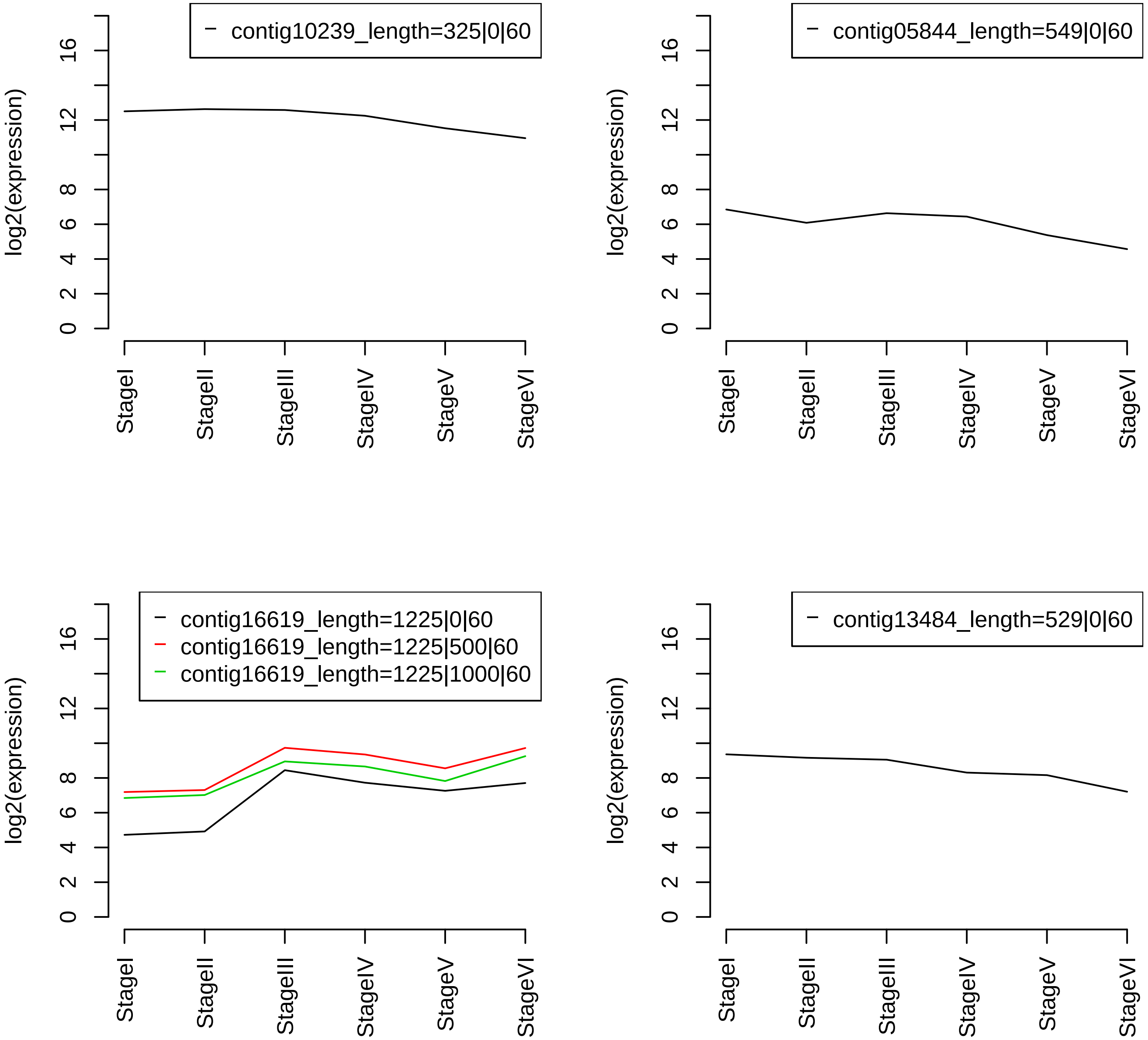
contig10239_length=325|0|60
contig05844_length=549|0|60
contig16619_length=1225|0|60
contig16619_length=1225|500|60
contig16619_length=1225|1000|60
contig13484_length=529|0|60
log2(expression)
0
2
4
6
8
12
16
StageI
StageII
StageIII
StageIV
StageV
StageVI

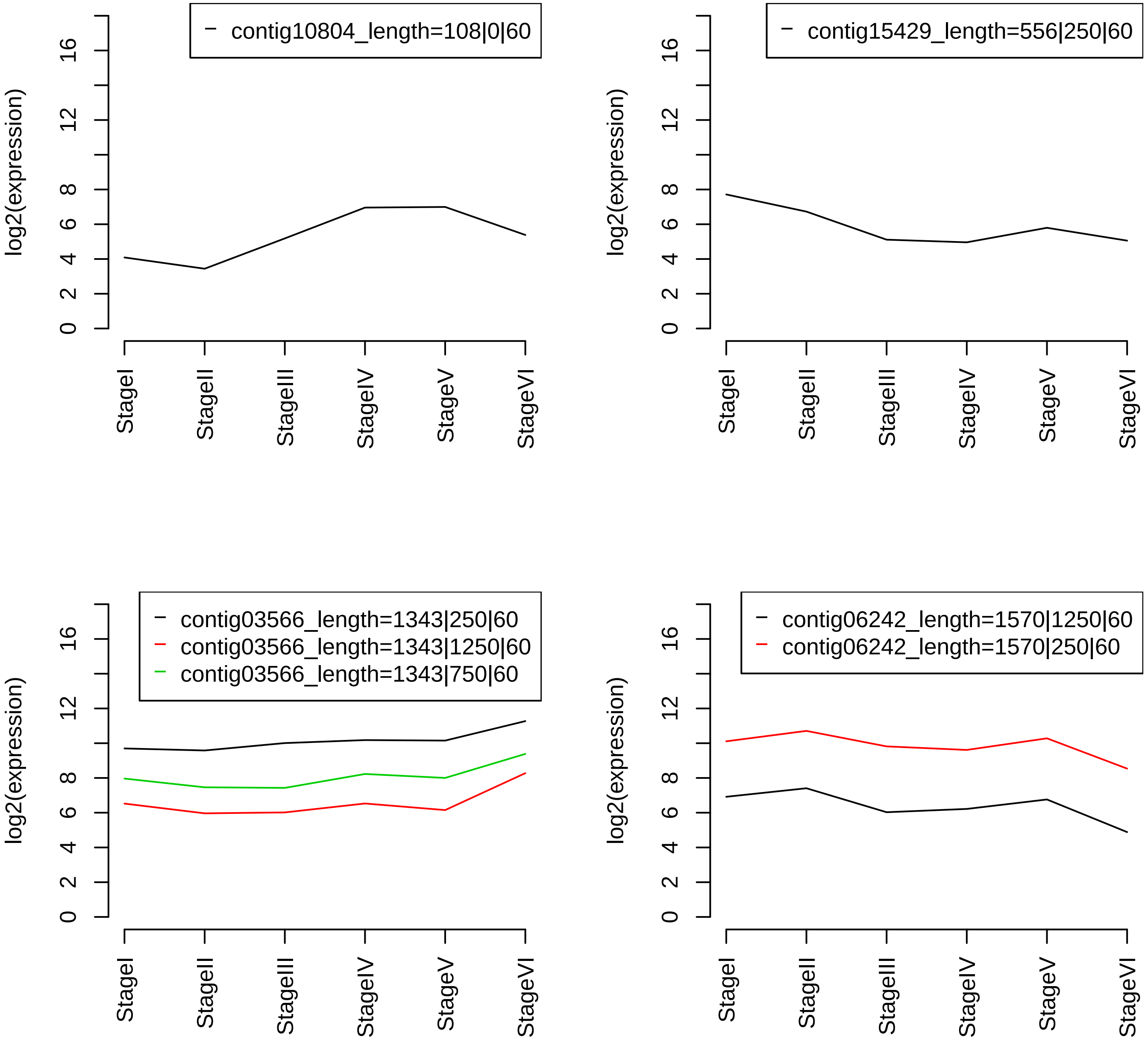
contig10804_length=108|0|60
contig15429_length=556|250|60
contig03566_length=1343|250|60
contig03566_length=1343|1250|60
contig03566_length=1343|750|60
contig06242_length=1570|1250|60
contig06242_length=1570|250|60
log2(expression)
StageI
StageII
StageIII
StageIV
StageV
StageVI

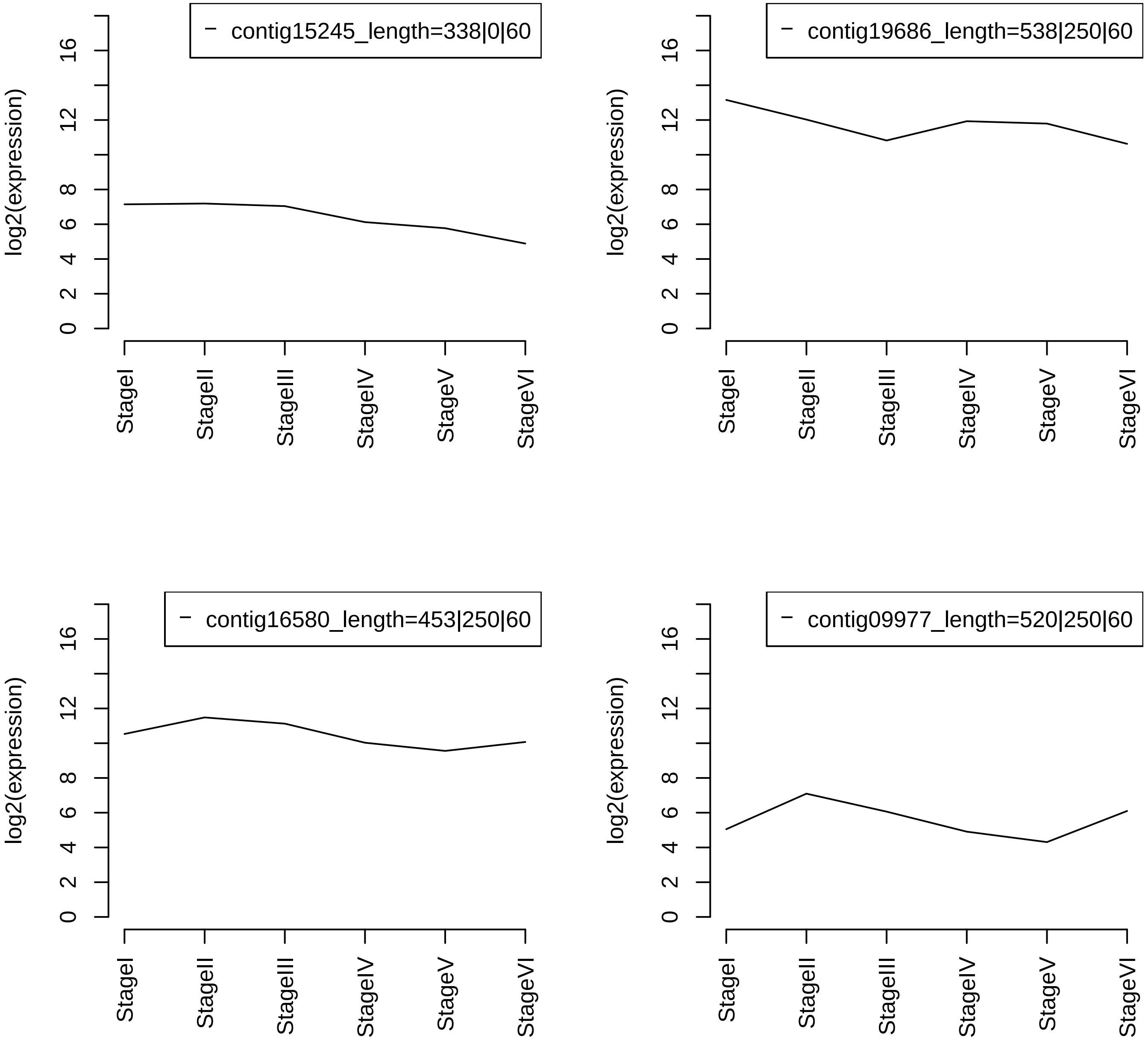
contig15245_length=338|0|60
contig19686_length=538|250|60
contig16580_length=453|250|60
contig09977_length=520|250|60
log2(expression)
0
2
4
6
8
12
16
StageI
StageII
StageIII
StageIV
StageV
StageVI

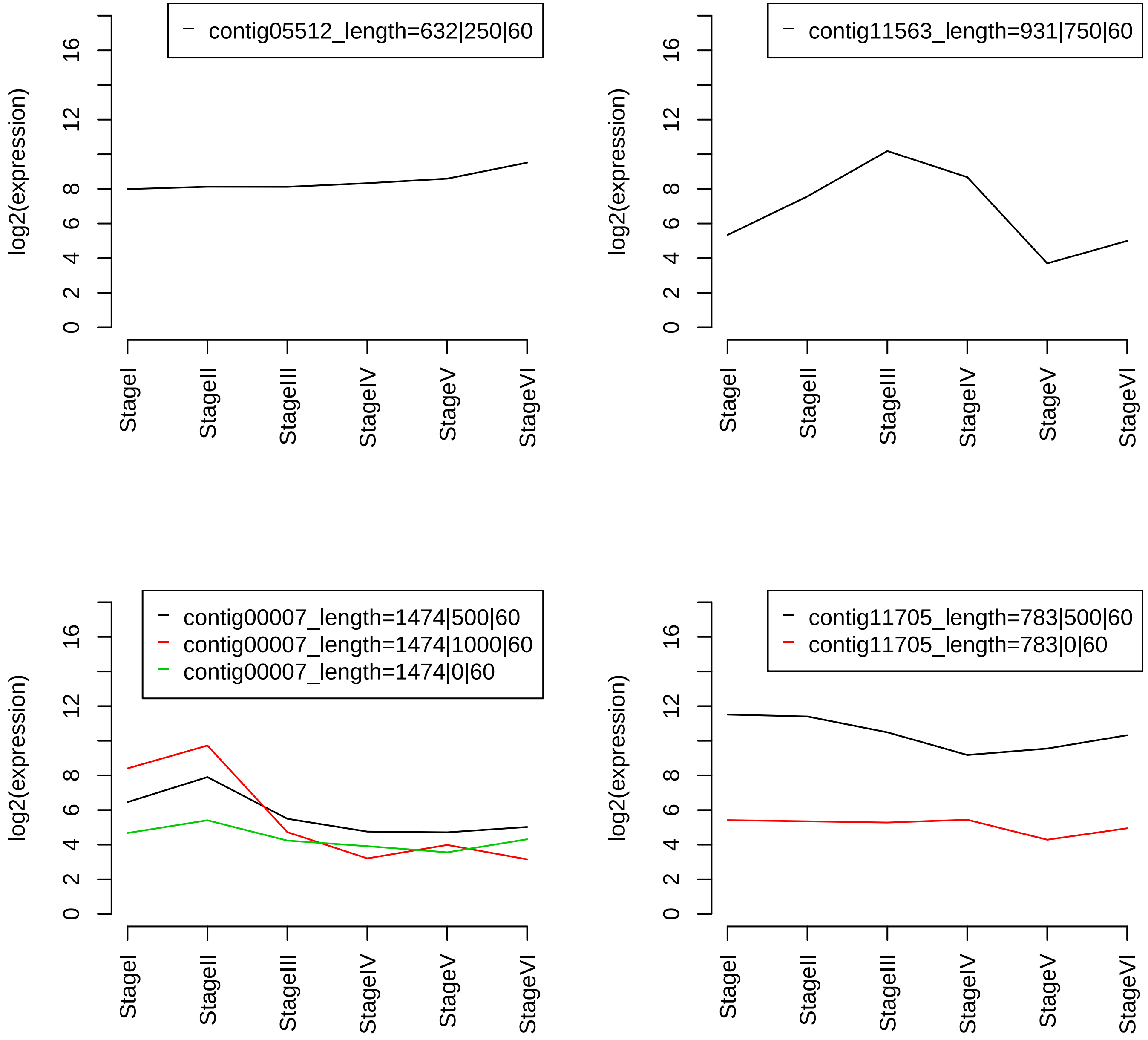

contig05512_length=632|250|60
contig11563_length=931|750|60
contig00007_length=1474|500|60
contig00007_length=1474|1000|60
contig00007_length=1474|0|60
contig11705_length=783|500|60
contig11705_length=783|0|60
log2(expression)
0
2
4
6
8
12
16
StageI
StageII
StageIII
StageIV
StageV
StageVI

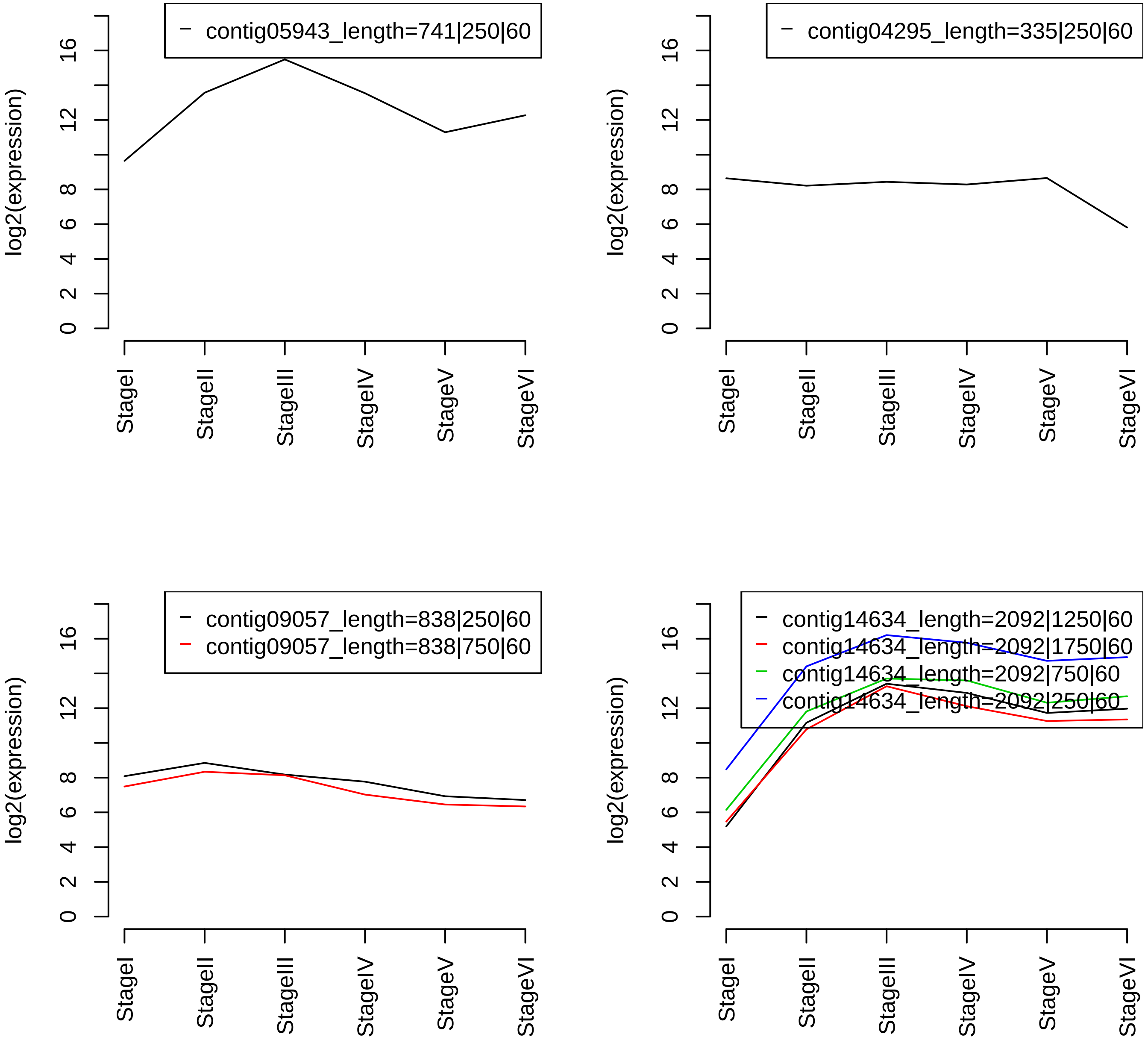
contig05943_length=741|250|60
contig04295_length=335|250|60
contig09057_length=838|250|60
contig09057_length=838|750|60
contig14634_length=2092|1250|60
contig14634_length=2092|1750|60
contig14634_length=2092|750|60
contig14634_length=2092|250|60
log2(expression)
0
2
4
6
8
12
16
StageI
StageII
StageIII
StageIV
StageV
StageVI

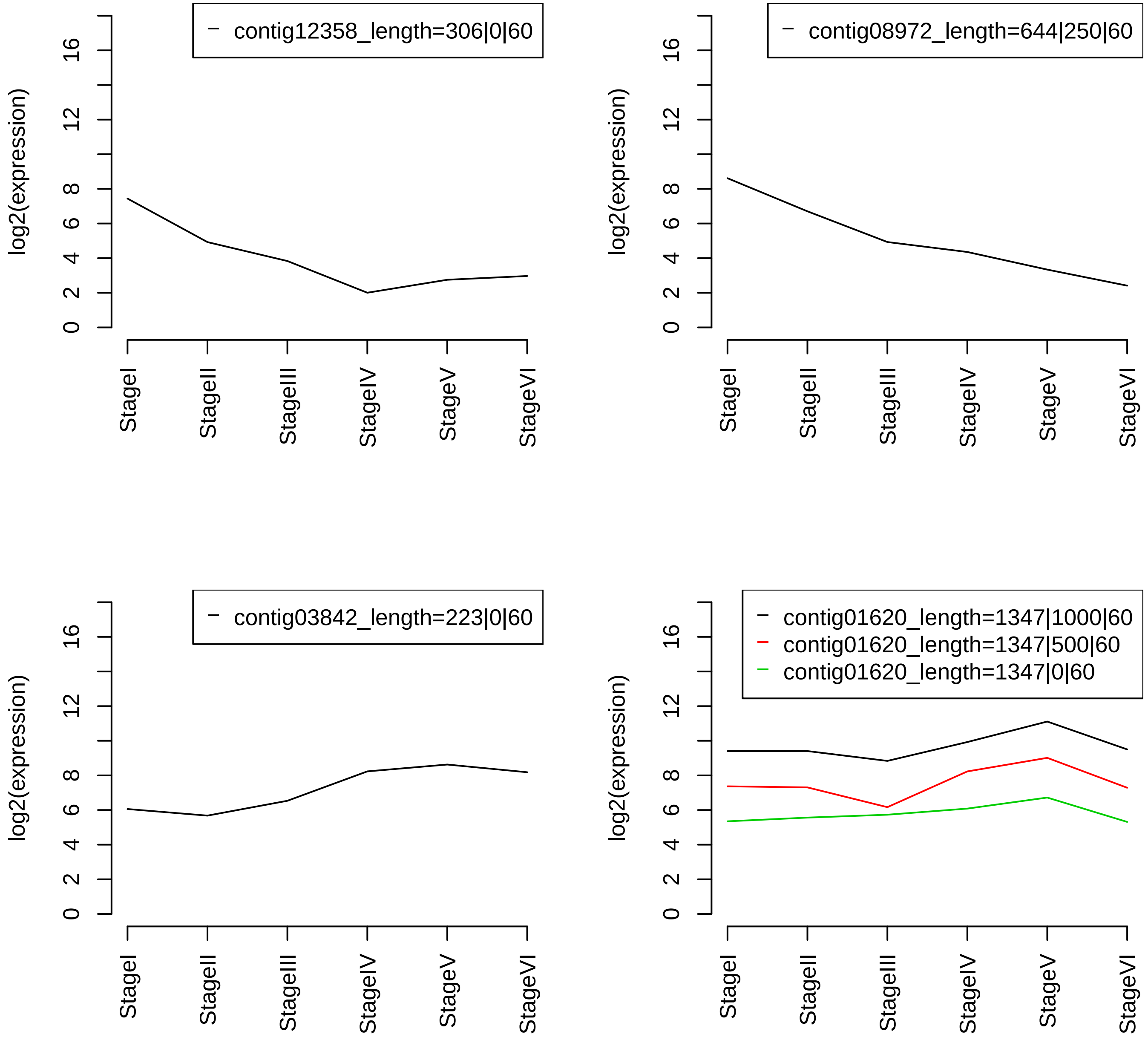

contig12358_length=306|0|60
contig08972_length=644|250|60
contig03842_length=223|0|60
contig01620_length=1347|1000|60
contig01620_length=1347|500|60
contig01620_length=1347|0|60
log2(expression)
0
2
4
6
8
12
16
StageI
StageII
StageIII
StageIV
StageV
StageVI

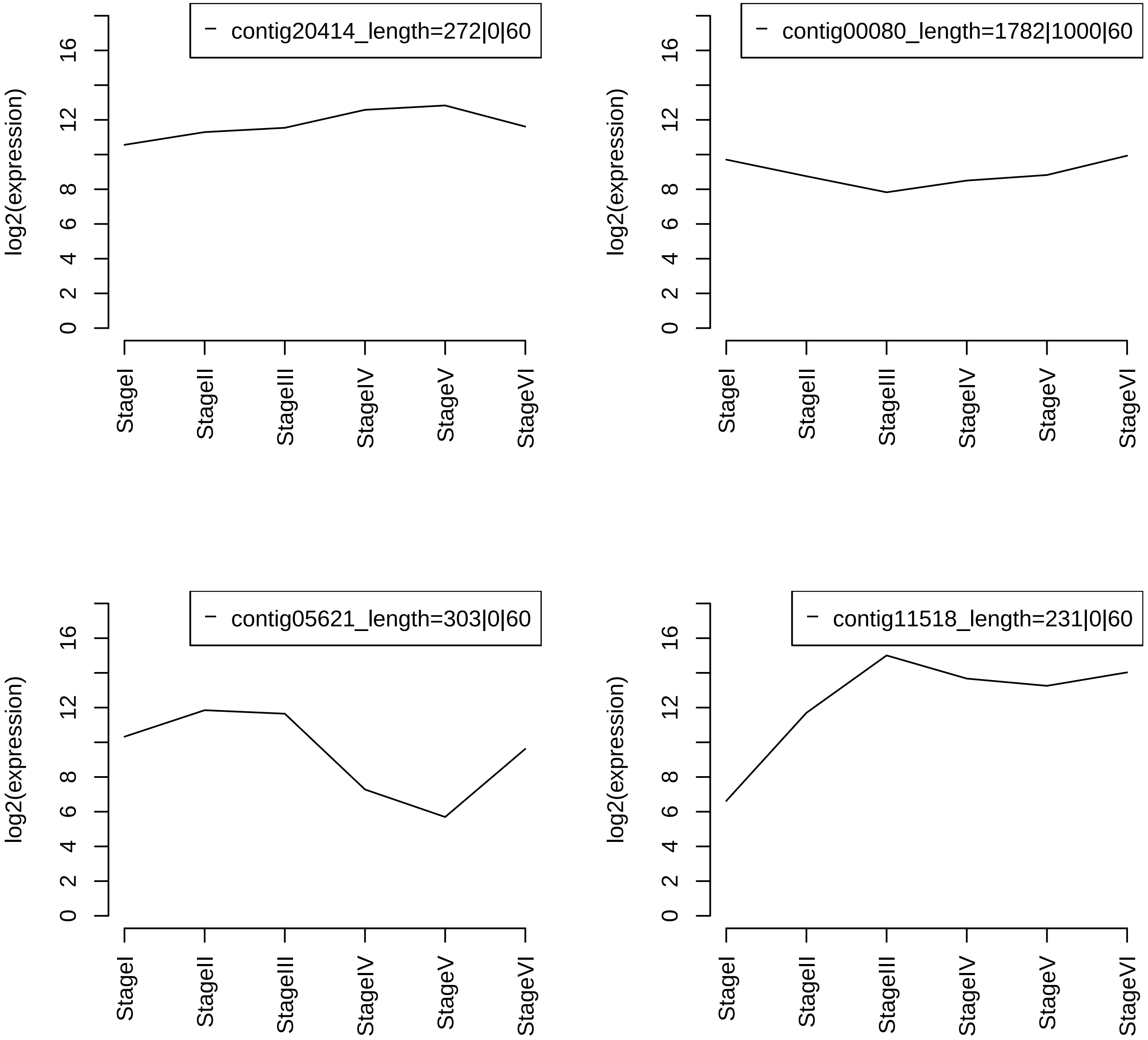

contig20414_length=272|0|60
contig00080_length=1782|1000|60
contig05621_length=303|0|60
contig11518_length=231|0|60
log2(expression)
0
2
4
6
8
12
16
StageI
StageII
StageIII
StageIV
StageV
StageVI

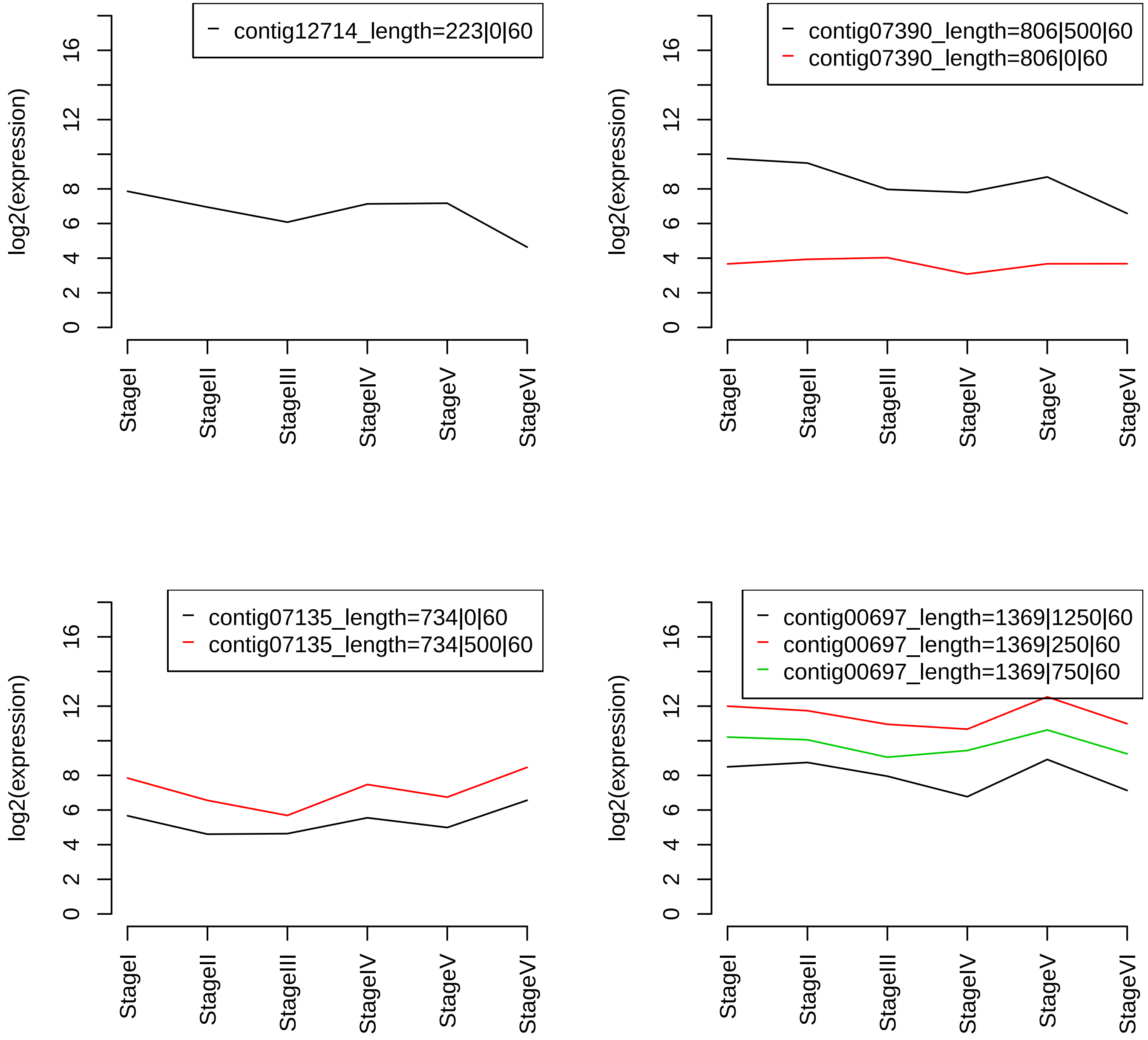
contig12714_length=223|0|60
contig07390_length=806|500|60
contig07390_length=806|0|60
contig07135_length=734|0|60
contig07135_length=734|500|60
contig00697_length=1369|1250|60
contig00697_length=1369|250|60
contig00697_length=1369|750|60
log2(expression)
StageI
StageII
StageIII
StageIV
StageV
StageVI

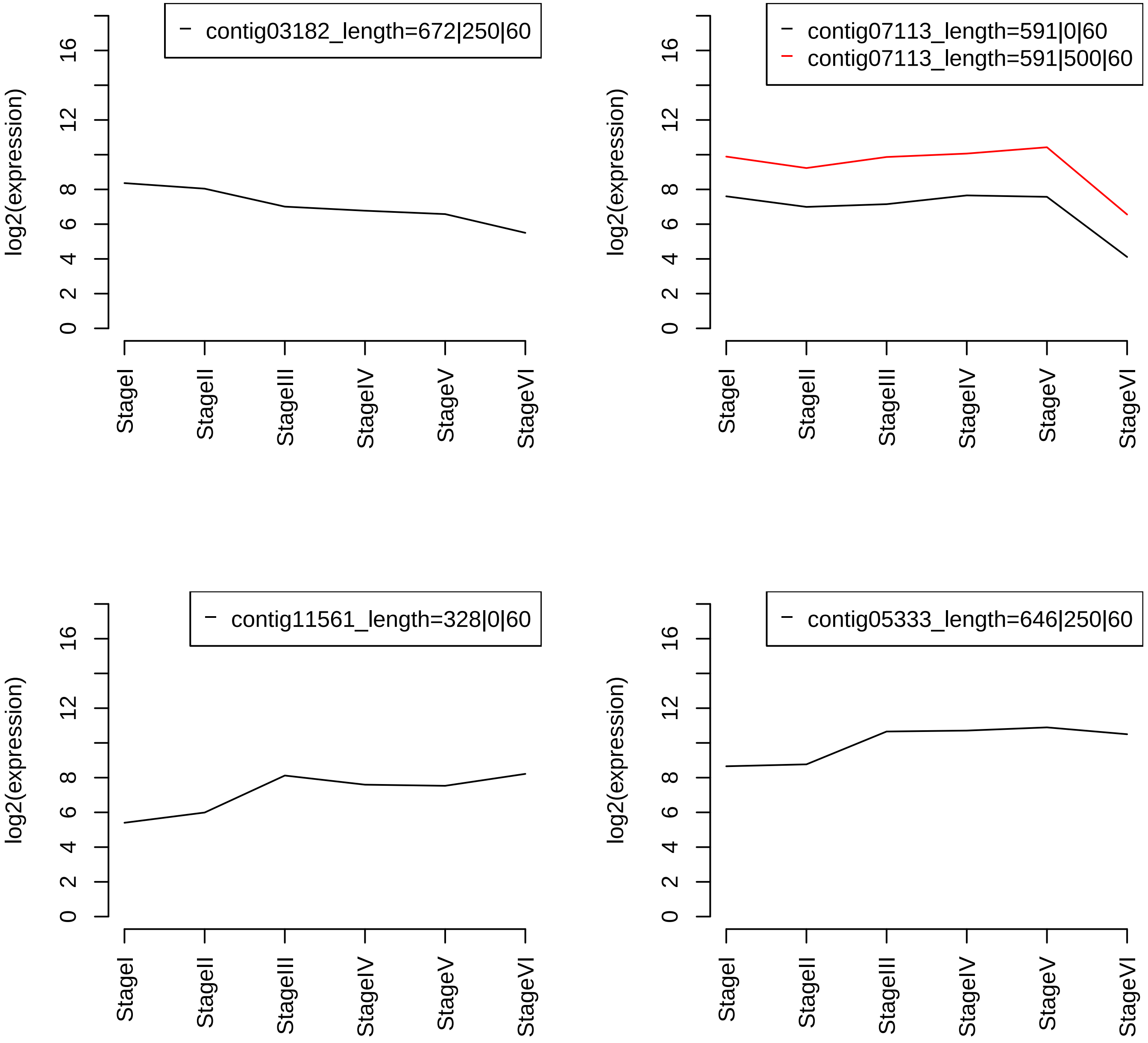
contig03182_length=672|250|60
contig07113_length=591|0|60
contig07113_length=591|500|60
contig11561_length=328|0|60
contig05333_length=646|250|60
log2(expression)
0
2
4
6
8
12
16
StageI
StageII
StageIII
StageIV
StageV
StageVI

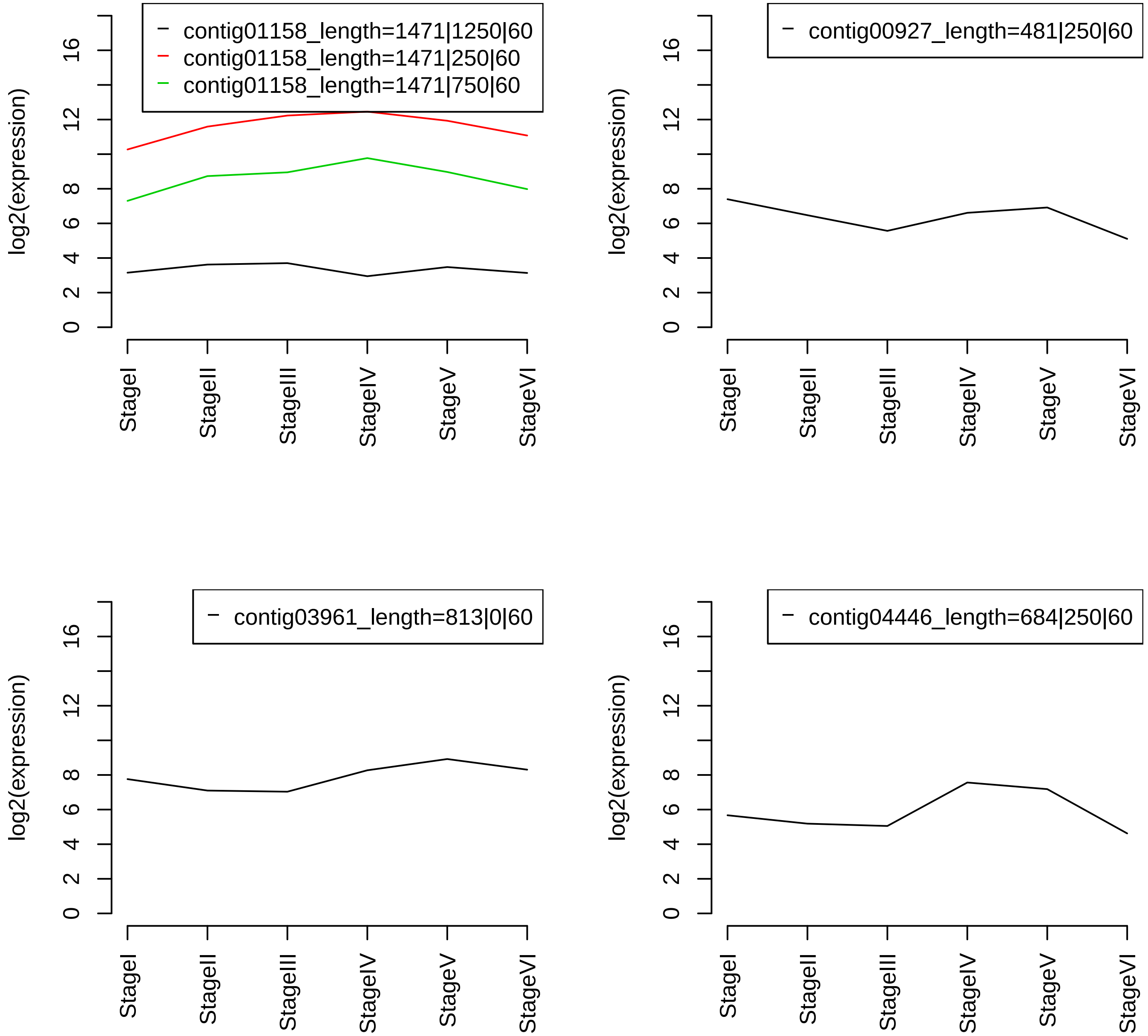
contig01158_length=1471|1250|60
contig01158_length=1471|250|60
contig01158_length=1471|750|60
contig00927_length=481|250|60
contig03961_length=813|0|60
contig04446_length=684|250|60
log2(expression)
0
2
4
6
8
12
16
StageI
StageII
StageIII
StageIV
StageV
StageVI

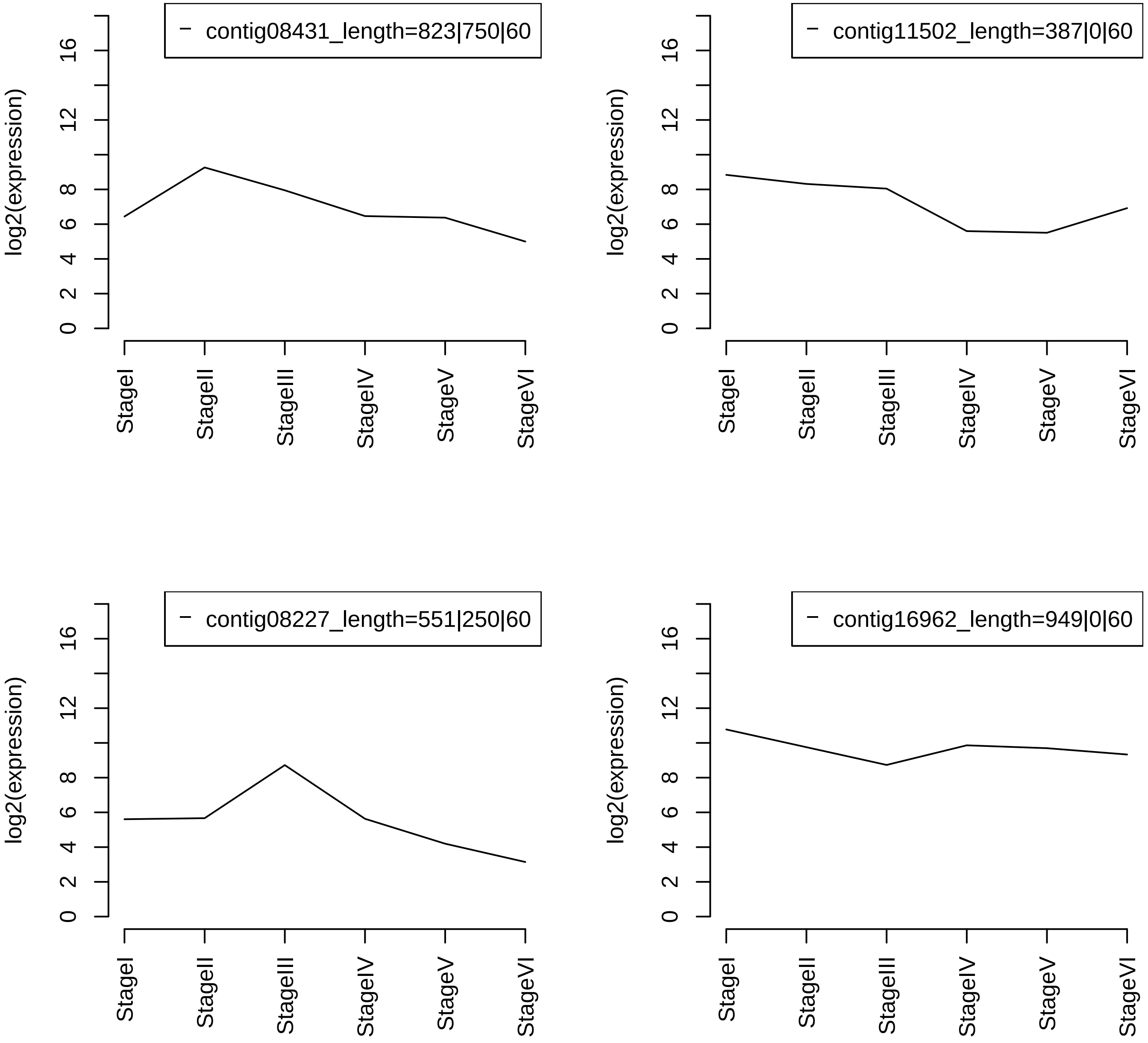
contig08431_length=823|750|60
contig11502_length=387|0|60
contig08227_length=551|250|60
contig16962_length=949|0|60
log2(expression)
0
2
4
6
8
12
16
StageI
StageII
StageIII
StageIV
StageV
StageVI

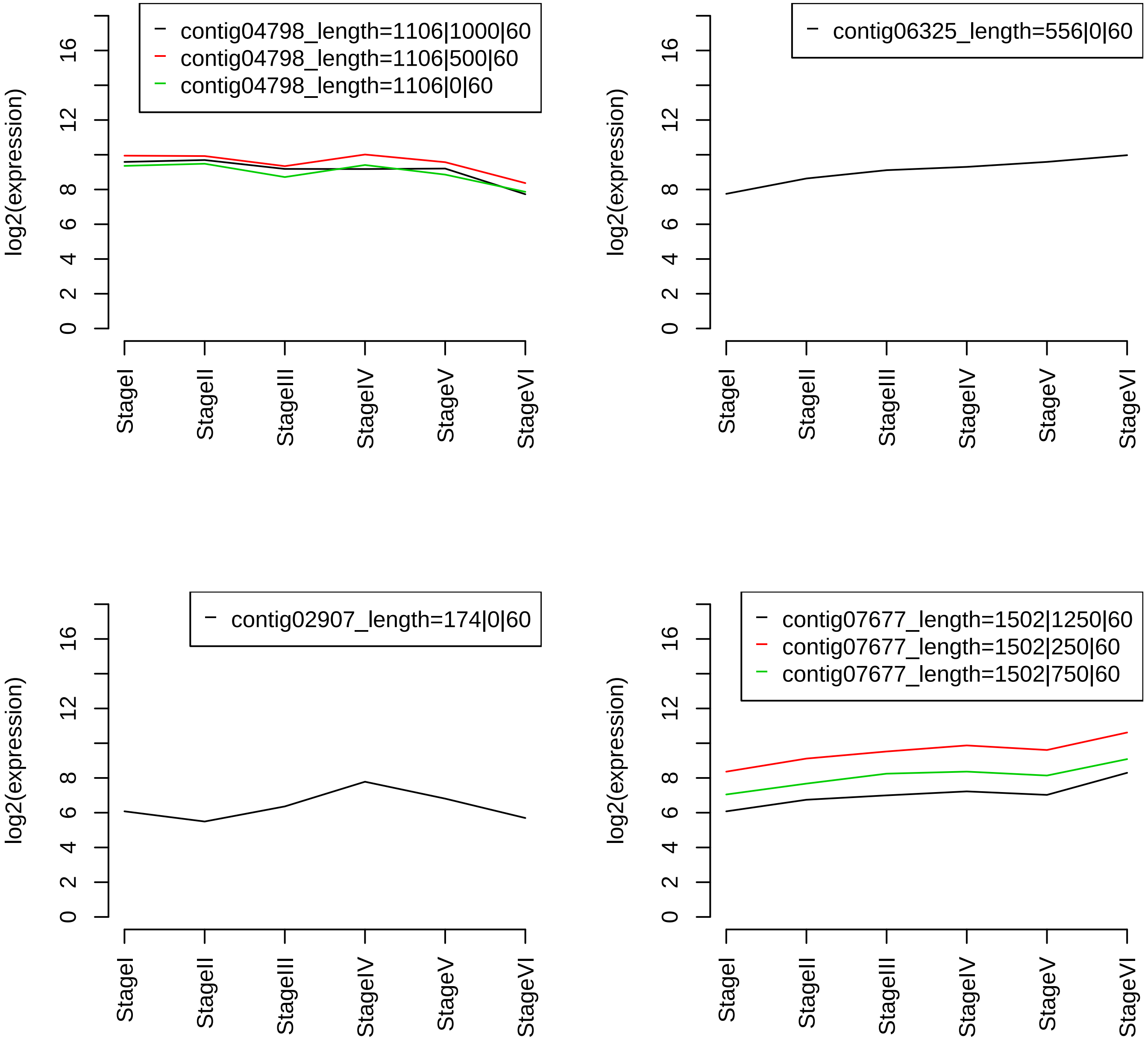
contig04798_length=1106|1000|60
contig04798_length=1106|500|60
contig04798_length=1106|0|60
contig06325_length=556|0|60
contig02907_length=174|0|60
contig07677_length=1502|1250|60
contig07677_length=1502|250|60
contig07677_length=1502|750|60
log2(expression)
0
2
4
6
8
12
16
StageI
StageII
StageIII
StageIV
StageV
StageVI

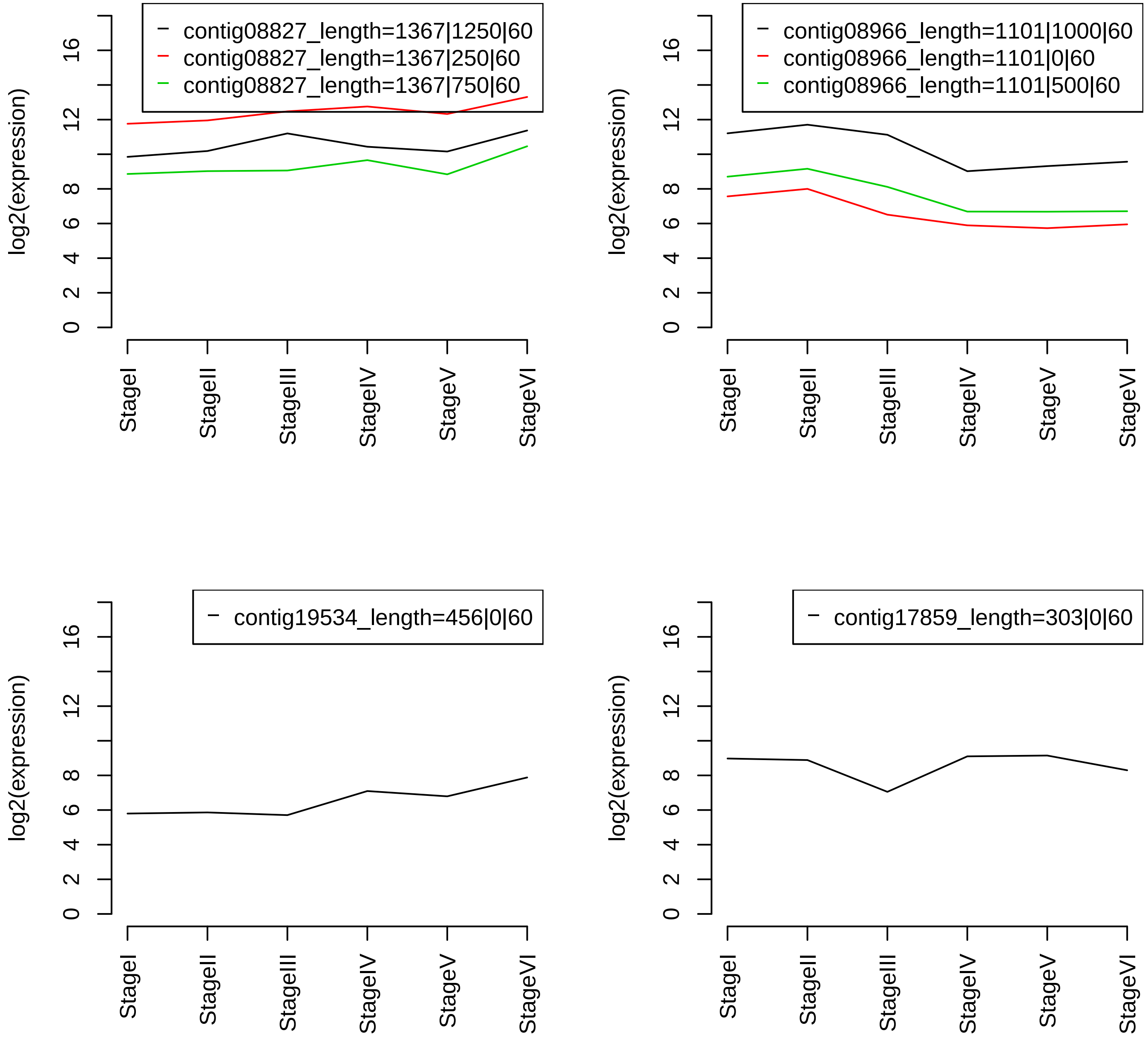
contig08827_length=1367|1250|60
contig08827_length=1367|250|60
contig08827_length=1367|750|60
log2(expression)
0
2
4
6
8
12
16
StageI
StageII
StageIII
StageIV
StageV
StageVI
contig08966_length=1101|1000|60
contig08966_length=1101|0|60
contig08966_length=1101|500|60
contig19534_length=456|0|60
contig17859_length=303|0|60

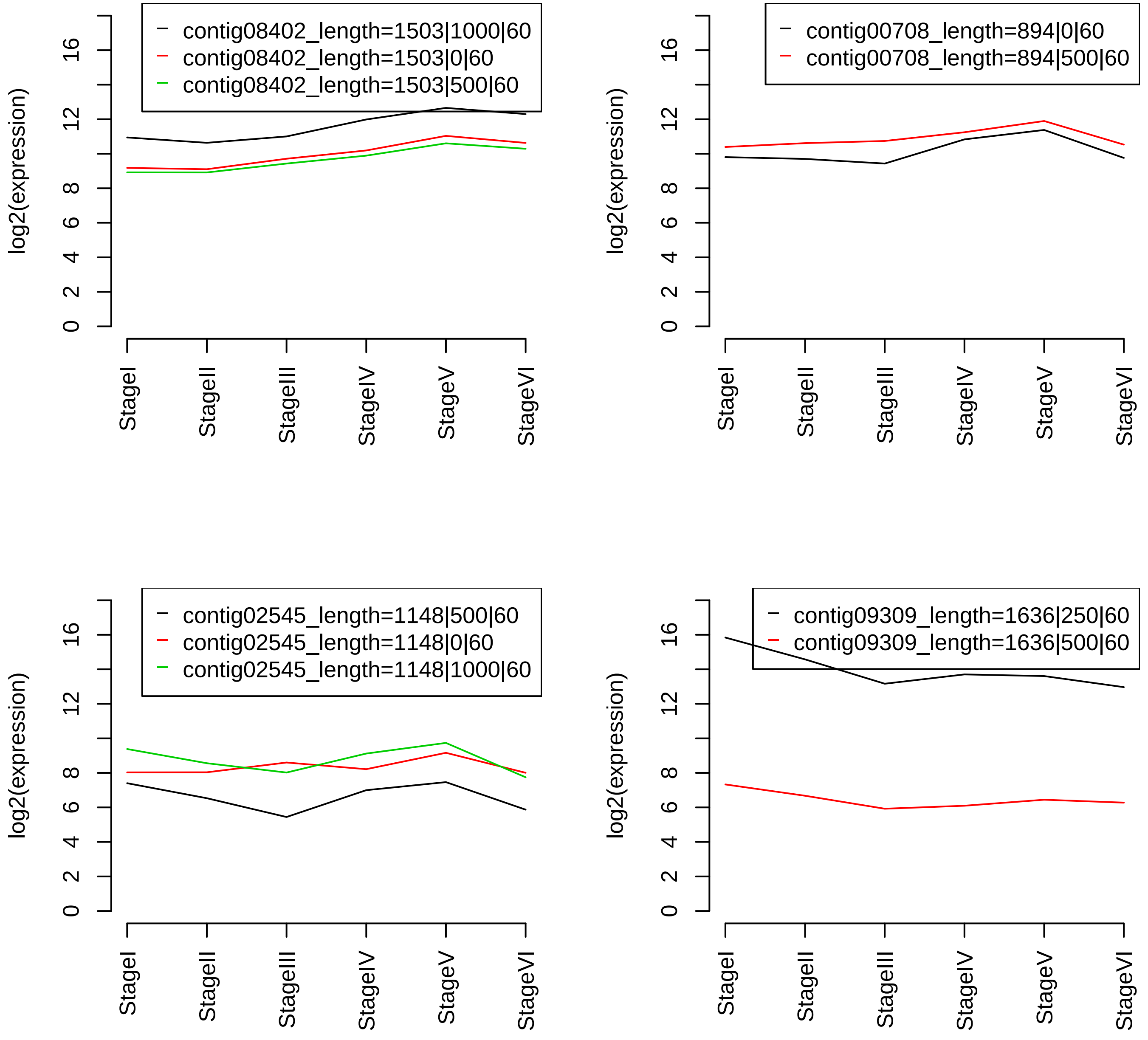

contig08402_length=1503|1000|60
contig08402_length=1503|0|60
contig08402_length=1503|500|60
contig00708_length=894|0|60
contig00708_length=894|500|60
contig02545_length=1148|500|60
contig02545_length=1148|0|60
contig02545_length=1148|1000|60
contig09309_length=1636|250|60
contig09309_length=1636|500|60
log2(expression)
0 2 4 6 8 12 16
StageI StageII StageIII StageIV StageV StageVI

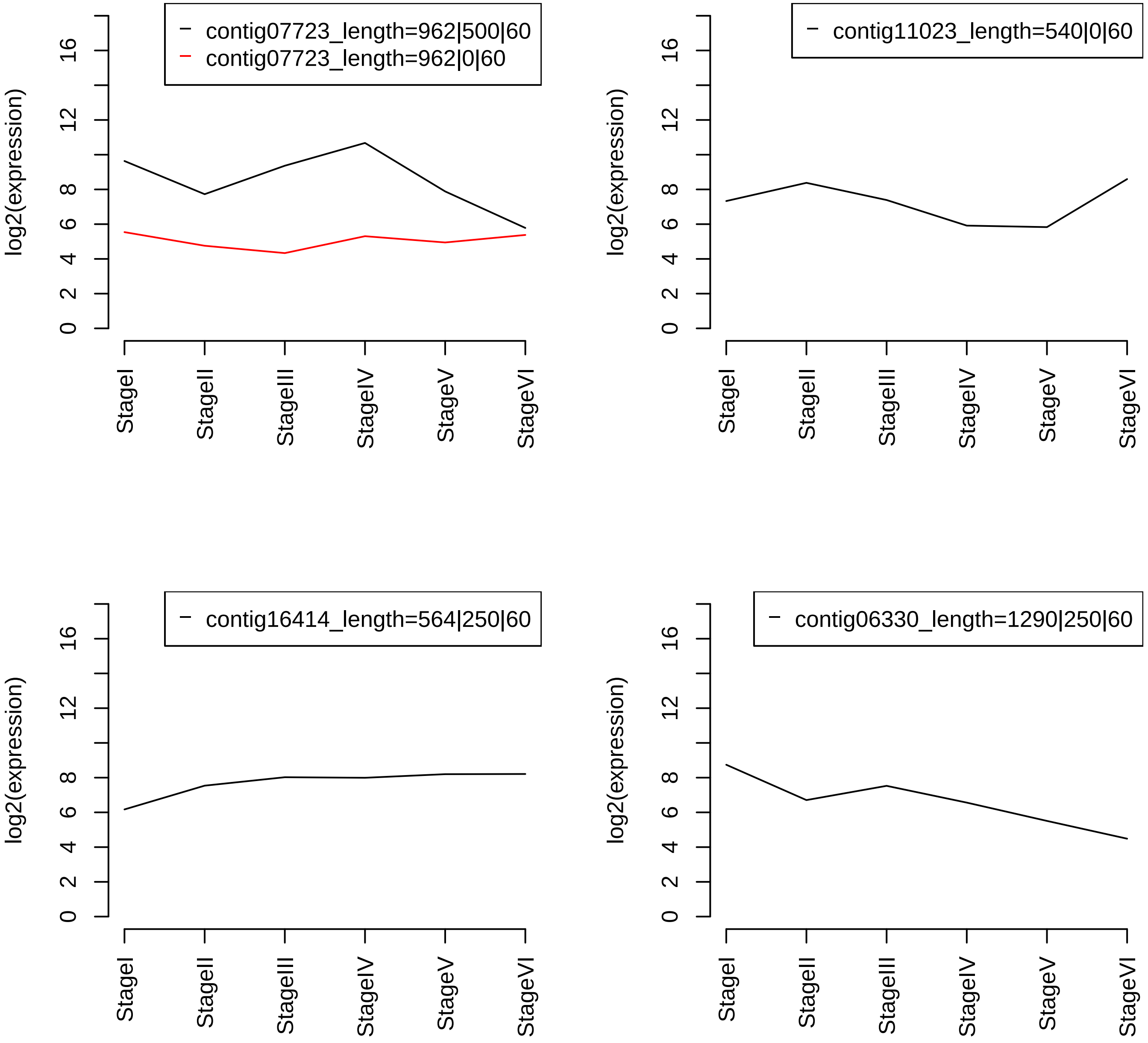
contig07723_length=962|500|60
contig07723_length=962|0|60
contig11023_length=540|0|60
contig16414_length=564|250|60
contig06330_length=1290|250|60
log2(expression)
StageI
StageII
StageIII
StageIV
StageV
StageVI

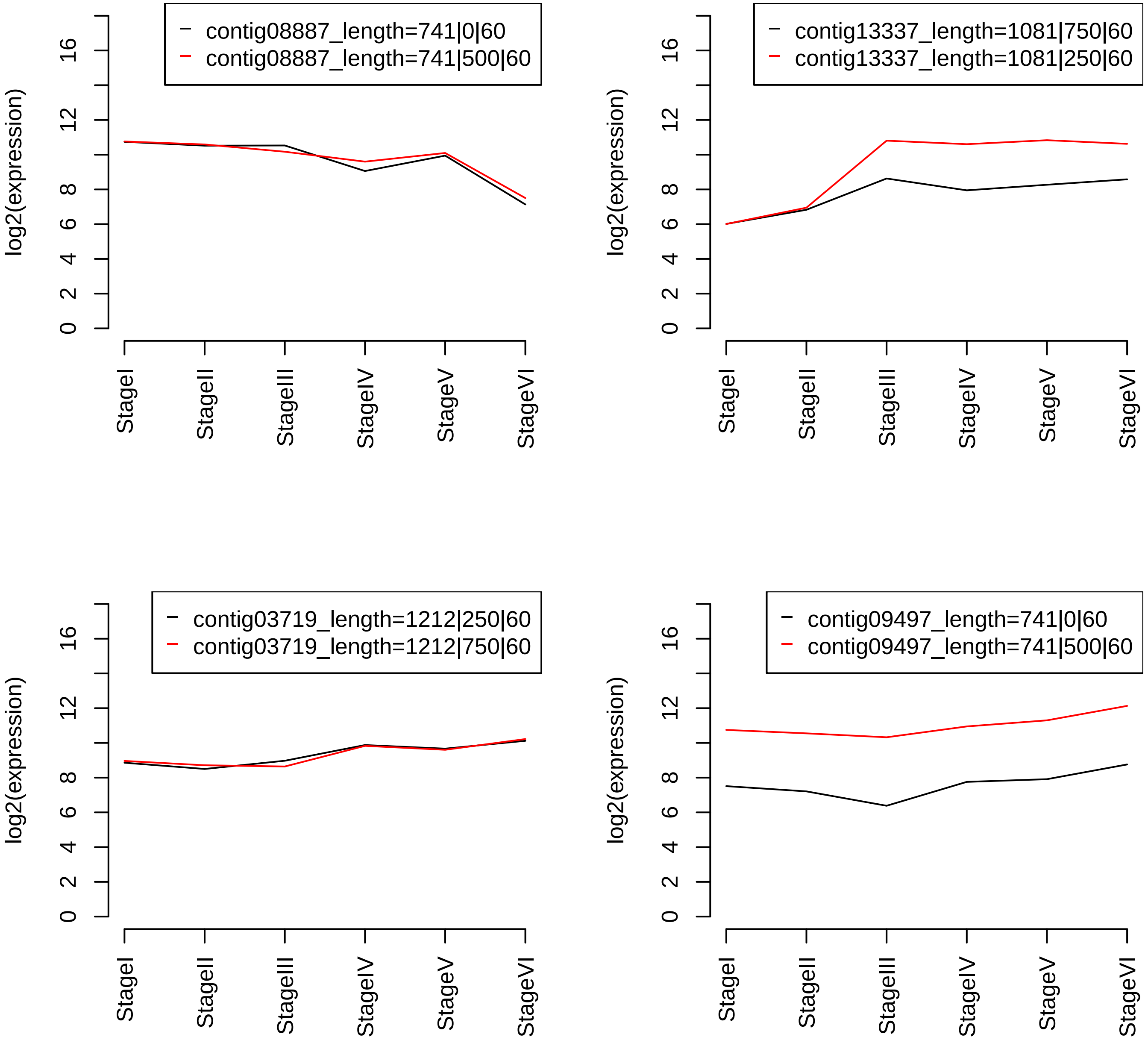

contig08887_length=741|0|60
contig08887_length=741|500|60
contig13337_length=1081|750|60
contig13337_length=1081|250|60
contig03719_length=1212|250|60
contig03719_length=1212|750|60
contig09497_length=741|0|60
contig09497_length=741|500|60
log2(expression)
0 2 4 6 8 12 16
StageI
StageII
StageIII
StageIV
StageV
StageVI

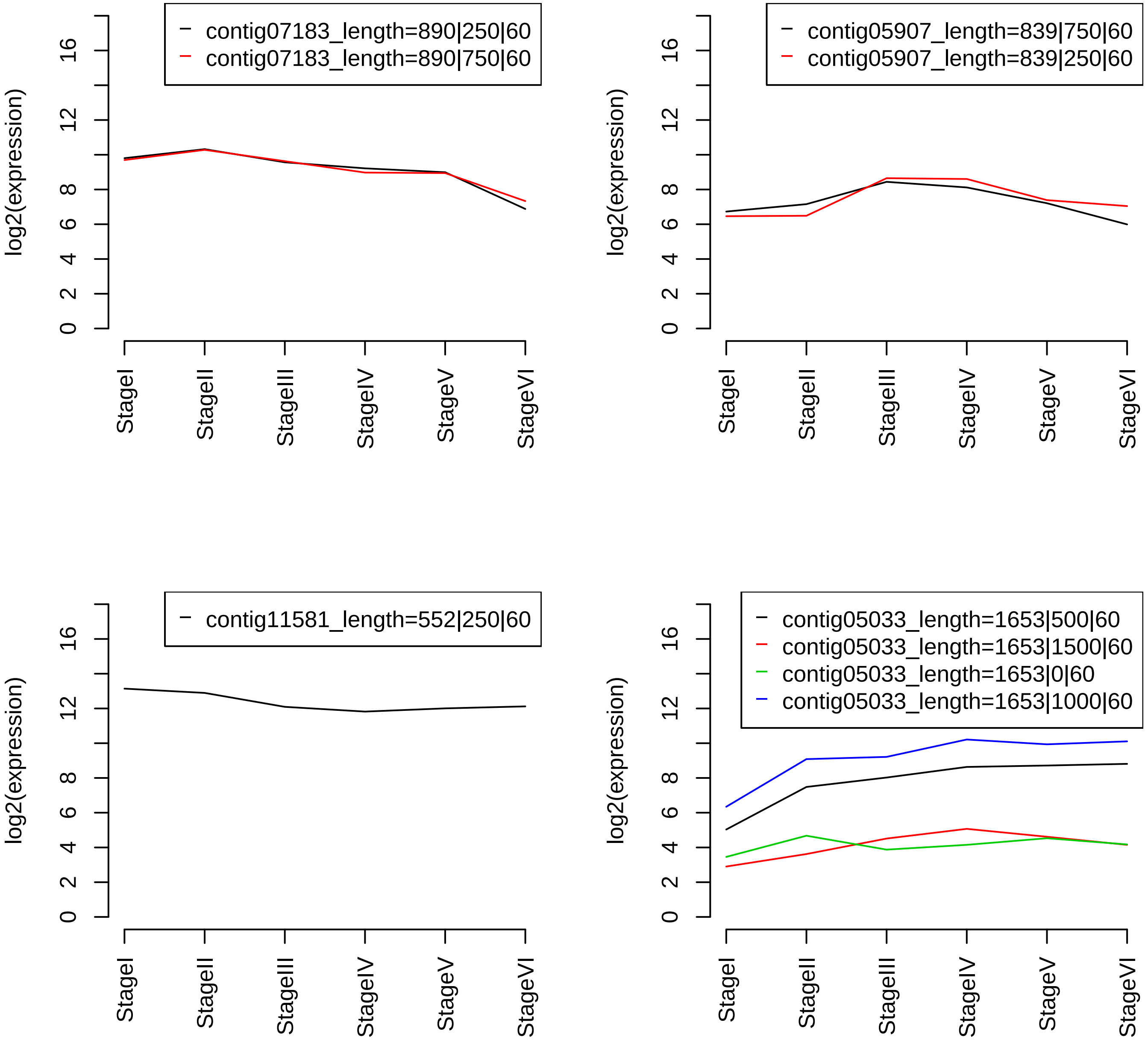

contig07183_length=890|250|60
contig07183_length=890|750|60
contig05907_length=839|750|60
contig05907_length=839|250|60
contig11581_length=552|250|60
contig05033_length=1653|500|60
contig05033_length=1653|1500|60
contig05033_length=1653|0|60
contig05033_length=1653|1000|60
log2(expression)
0 2 4 6 8 12 16
StageI
StageII
StageIII
StageIV
StageV
StageVI

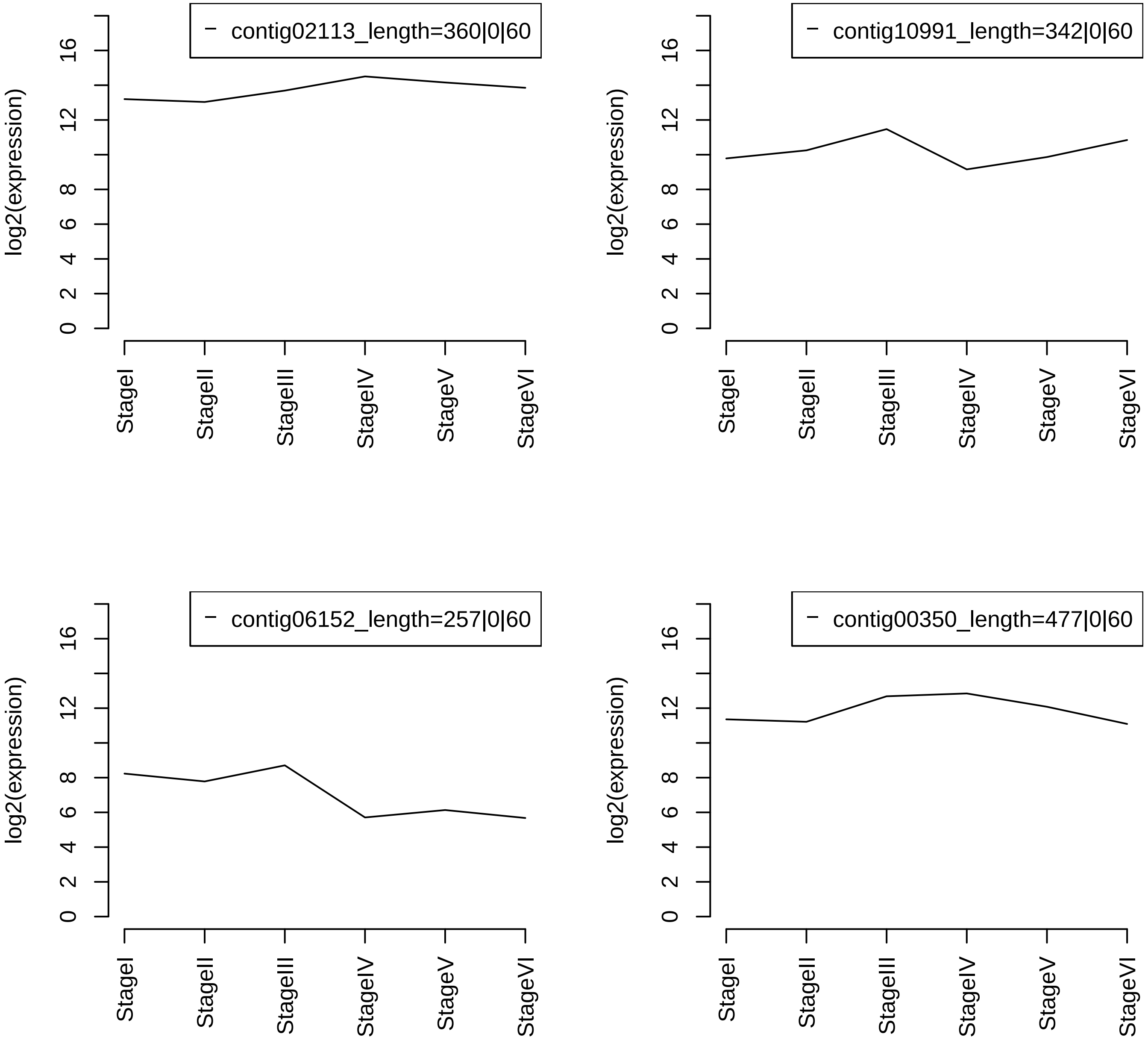
contig02113_length=360|0|60
contig10991_length=342|0|60
contig06152_length=257|0|60
contig00350_length=477|0|60
log2(expression)
0
2
4
6
8
12
16
StageI
StageII
StageIII
StageIV
StageV
StageVI

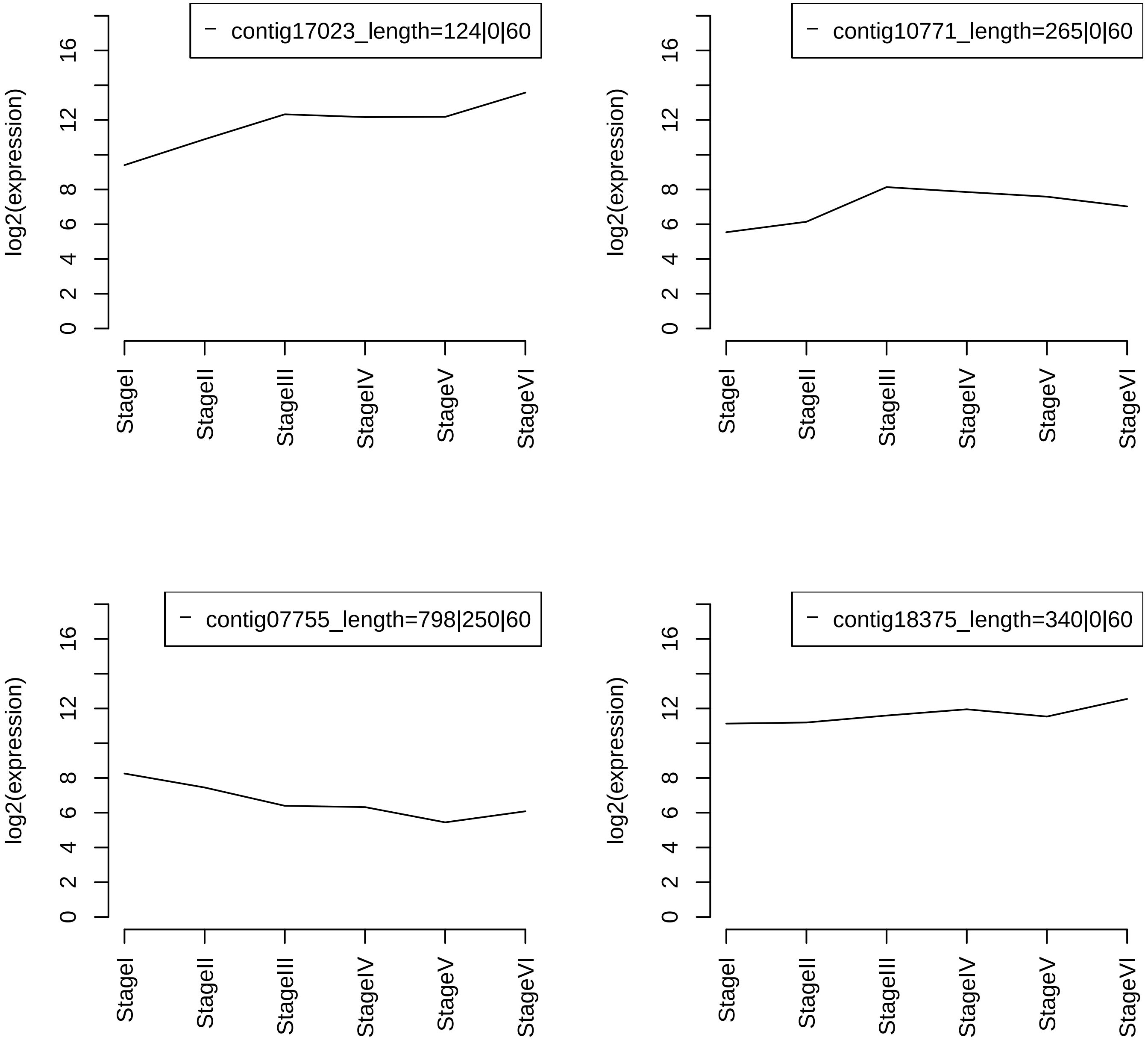
contig17023_length=124|0|60
contig10771_length=265|0|60
contig07755_length=798|250|60
contig18375_length=340|0|60
log2(expression)
0
2
4
6
8
12
16
StageI
StageII
StageIII
StageIV
StageV
StageVI

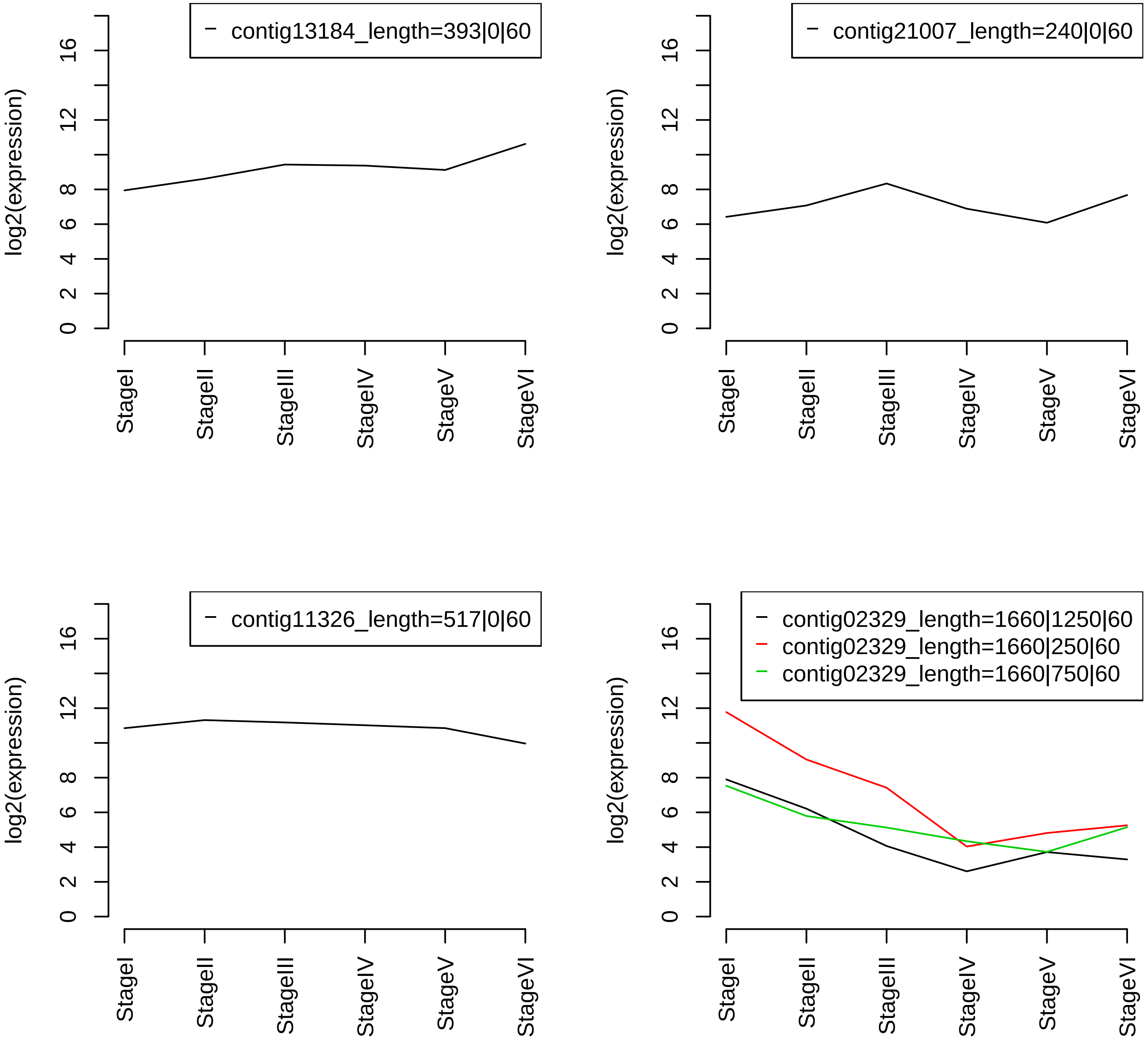
contig13184_length=393|0|60
contig21007_length=240|0|60
contig11326_length=517|0|60
contig02329_length=1660|1250|60
contig02329_length=1660|250|60
contig02329_length=1660|750|60
log2(expression)
0
2
4
6
8
12
16
StageI
StageII
StageIII
StageIV
StageV
StageVI

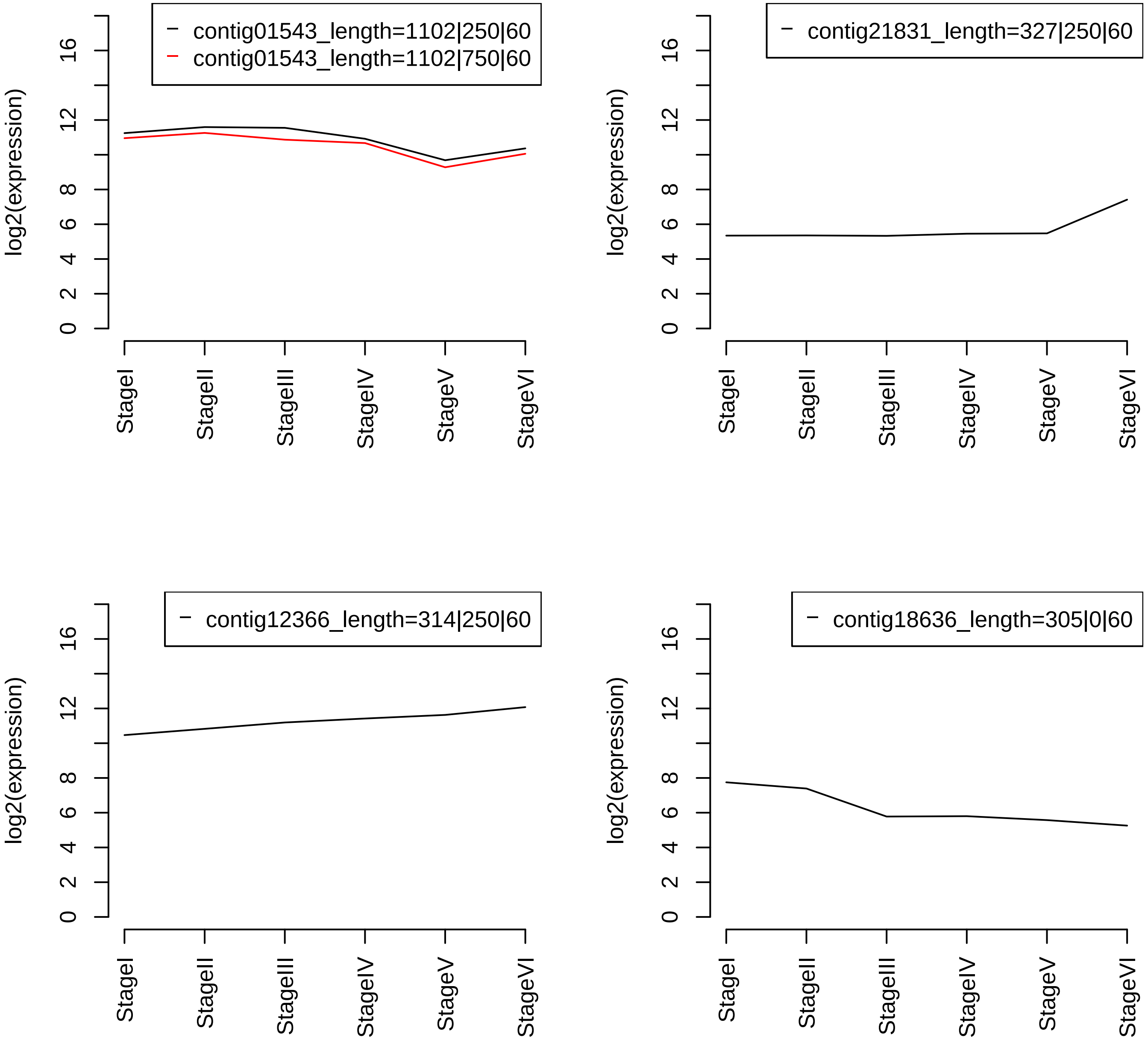

contig01543_length=1102|250|60
contig01543_length=1102|750|60
contig21831_length=327|250|60
contig12366_length=314|250|60
contig18636_length=305|0|60
log2(expression)
0
2
4
6
8
12
16
StageI
StageII
StageIII
StageIV
StageV
StageVI

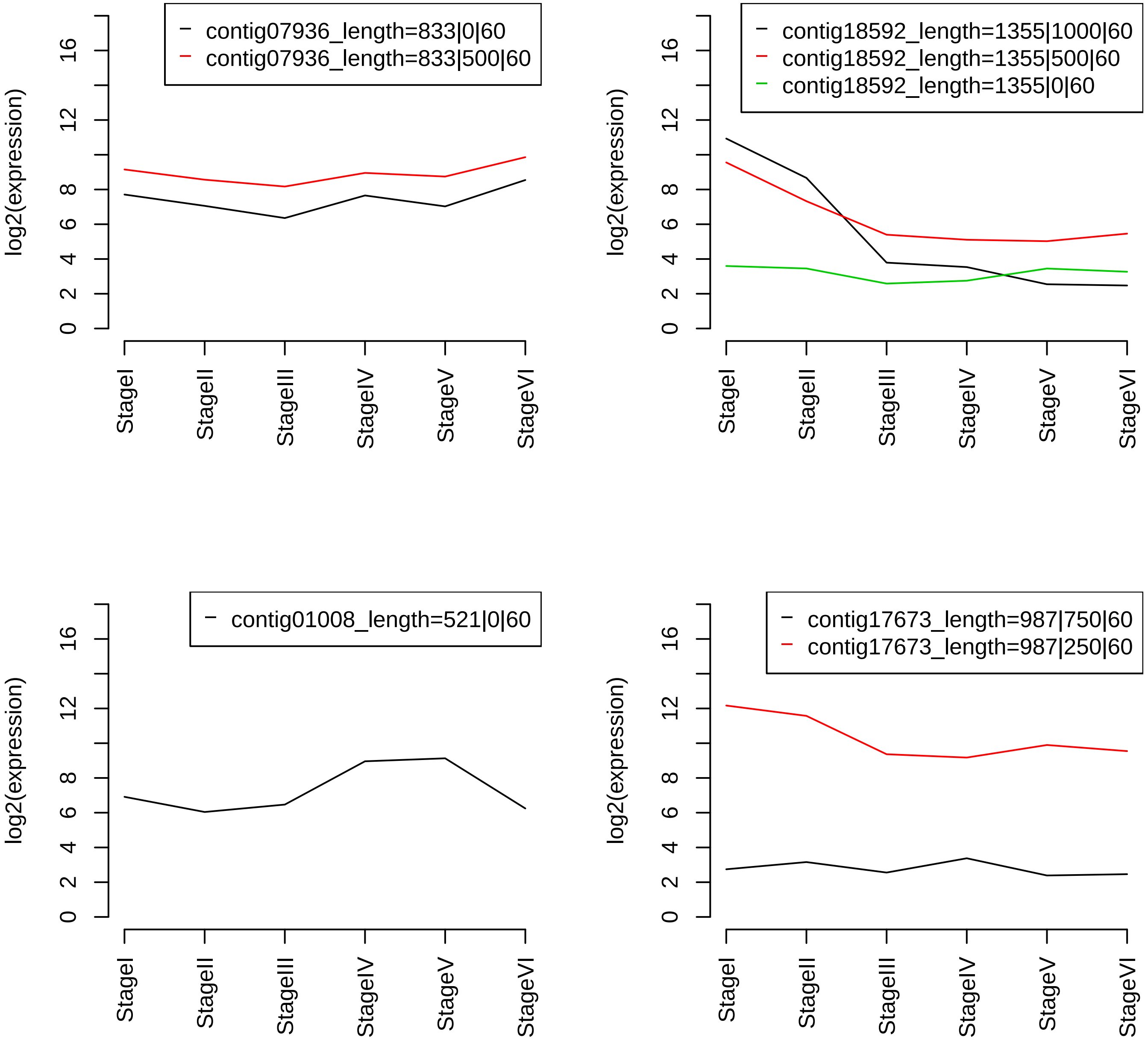

contig07936_length=833|0|60
contig07936_length=833|500|60
contig18592_length=1355|1000|60
contig18592_length=1355|500|60
contig18592_length=1355|0|60
contig01008_length=521|0|60
contig17673_length=987|750|60
contig17673_length=987|250|60
log2(expression)
0
2
4
6
8
12
16
StageI
StageII
StageIII
StageIV
StageV
StageVI

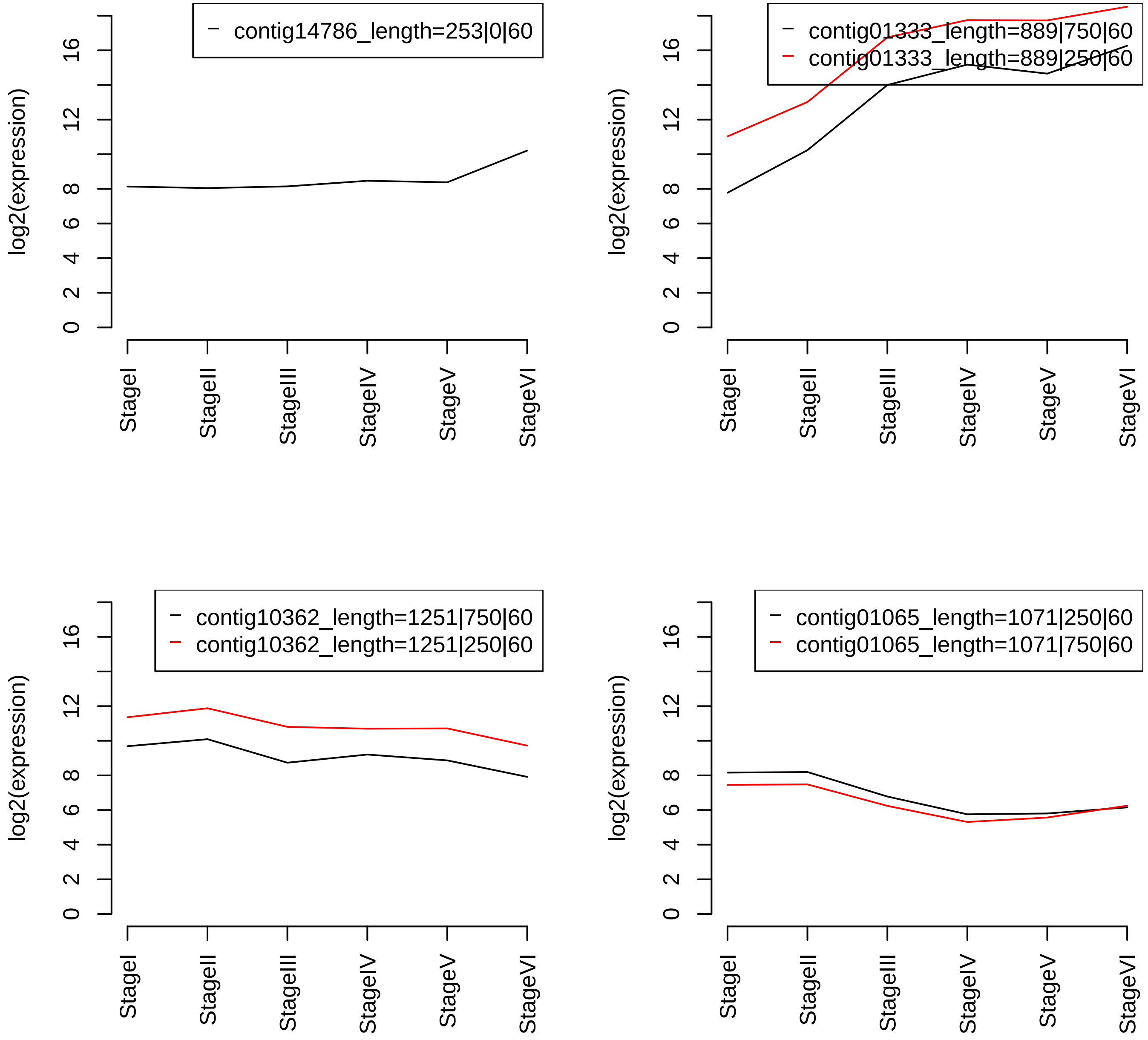
contig14786_length=253|0|60
contig01333_length=889|750|60
contig01333_length=889|250|60
contig10362_length=1251|750|60
contig10362_length=1251|250|60
contig01065_length=1071|250|60
contig01065_length=1071|750|60
log2(expression)
0
2
4
6
8
12
16
StageI
StageII
StageIII
StageIV
StageV
StageVI

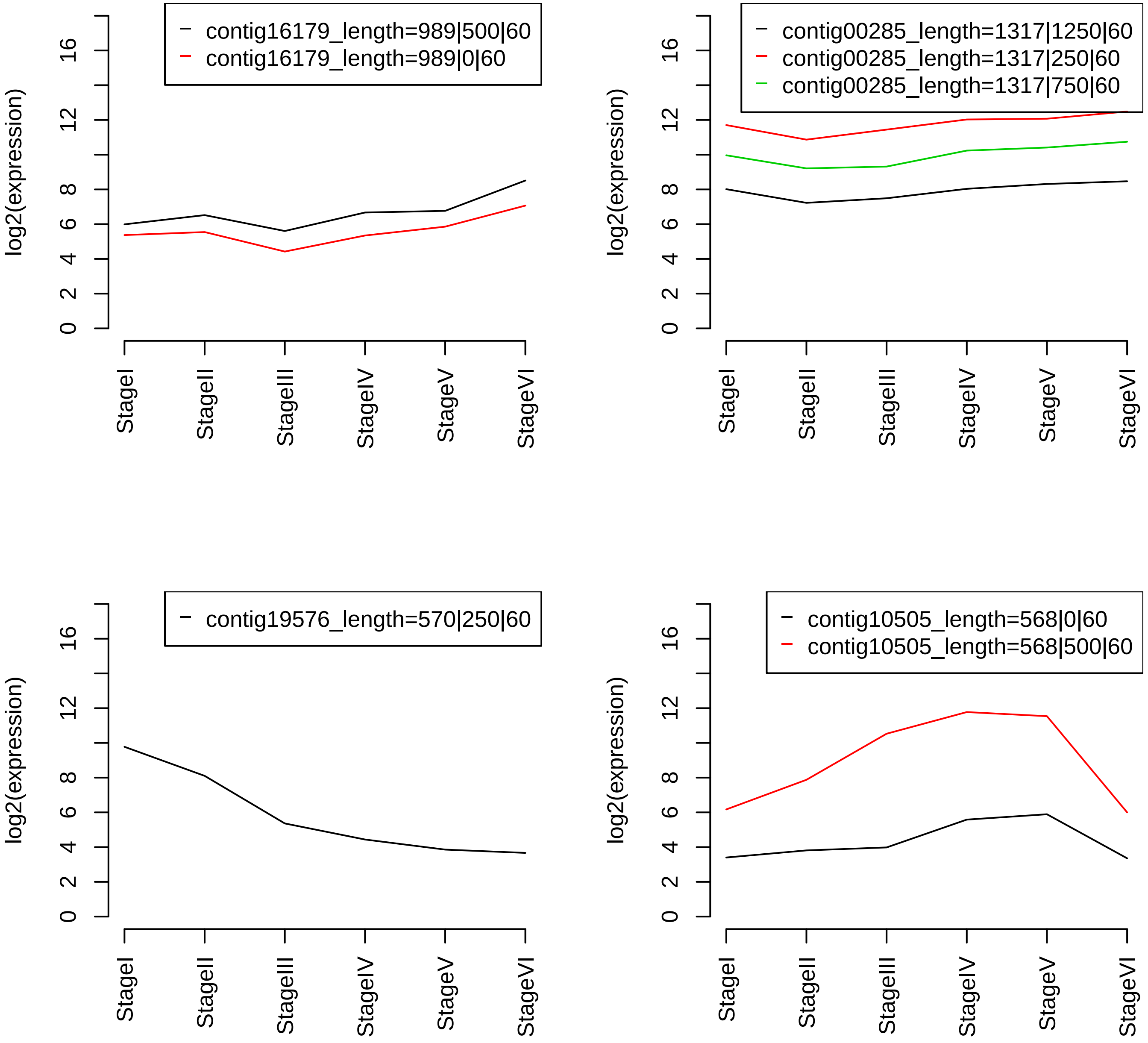
contig16179_length=989|500|60
contig16179_length=989|0|60
contig00285_length=1317|1250|60
contig00285_length=1317|250|60
contig00285_length=1317|750|60
contig19576_length=570|250|60
contig10505_length=568|0|60
contig10505_length=568|500|60
log2(expression)
0 2 4 6 8 12 16
StageI StageII StageIII StageIV StageV StageVI

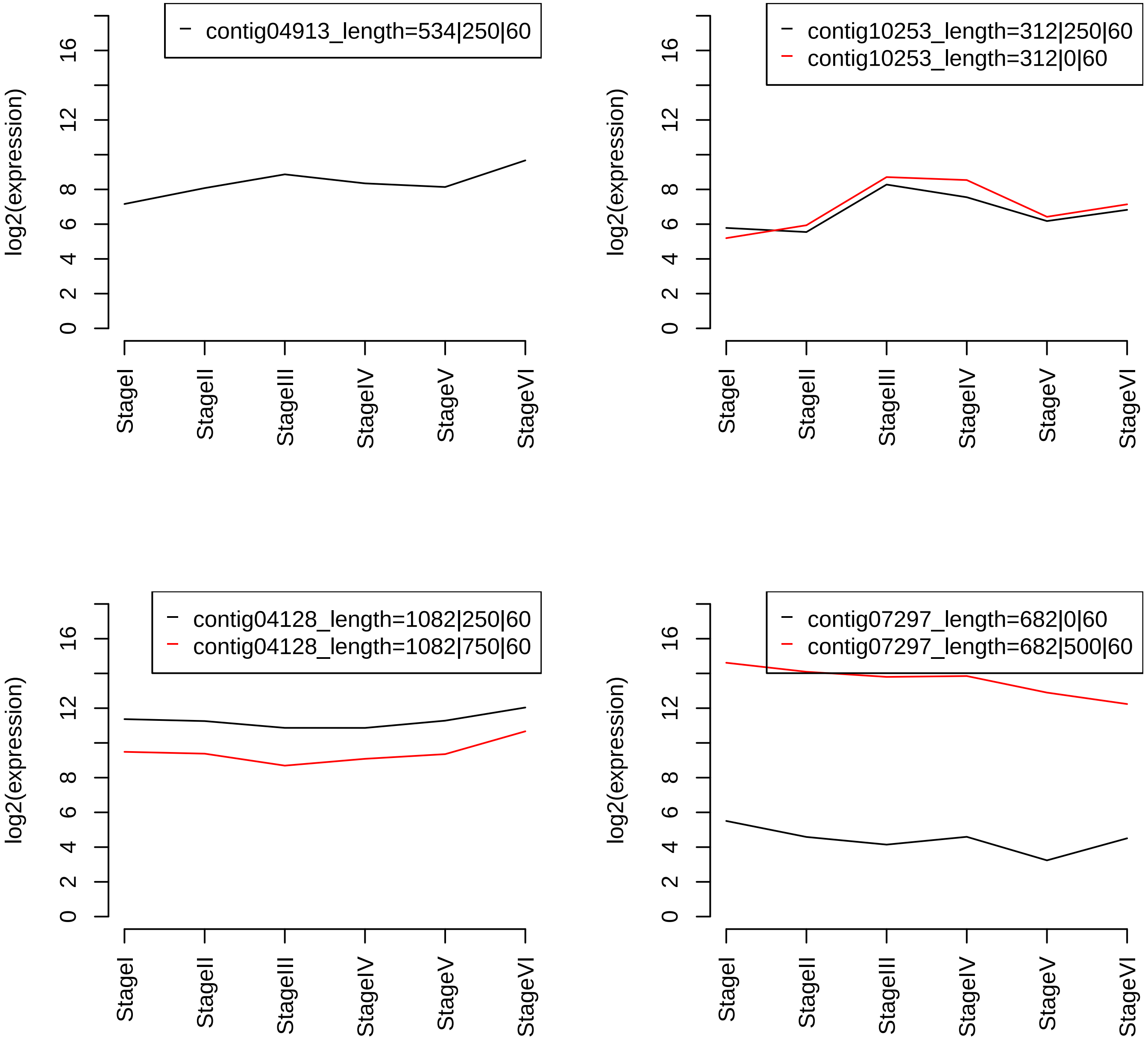
contig04913_length=534|250|60
contig10253_length=312|250|60
contig10253_length=312|0|60
contig04128_length=1082|250|60
contig04128_length=1082|750|60
contig07297_length=682|0|60
contig07297_length=682|500|60
log2(expression)
0
2
4
6
8
12
16
StageI
StageII
StageIII
StageIV
StageV
StageVI

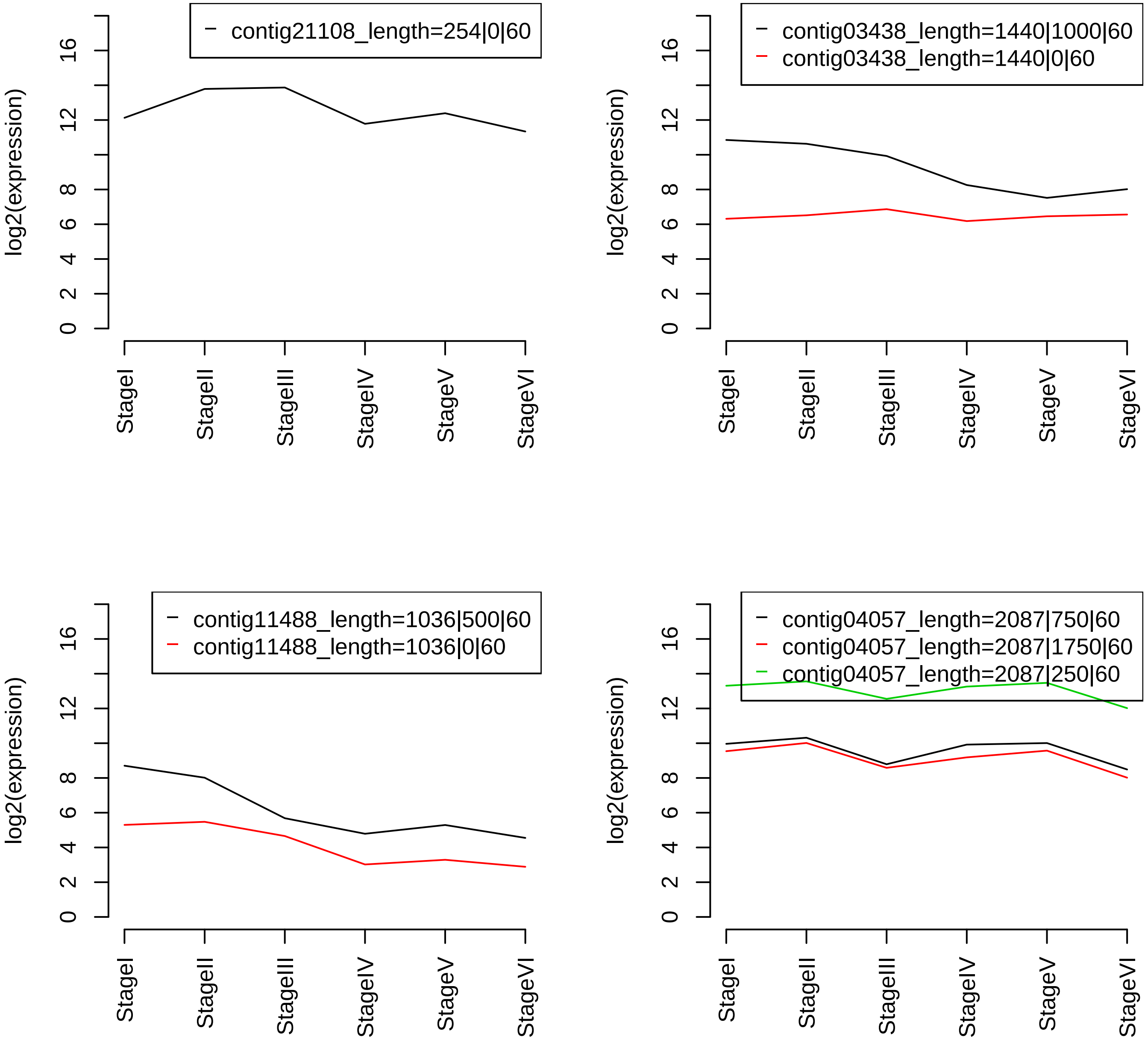

contig21108_length=254|0|60
contig03438_length=1440|1000|60
contig03438_length=1440|0|60
contig11488_length=1036|500|60
contig11488_length=1036|0|60
contig04057_length=2087|750|60
contig04057_length=2087|1750|60
contig04057_length=2087|250|60
log2(expression)
0
2
4
6
8
12
16
StageI
StageII
StageIII
StageIV
StageV
StageVI

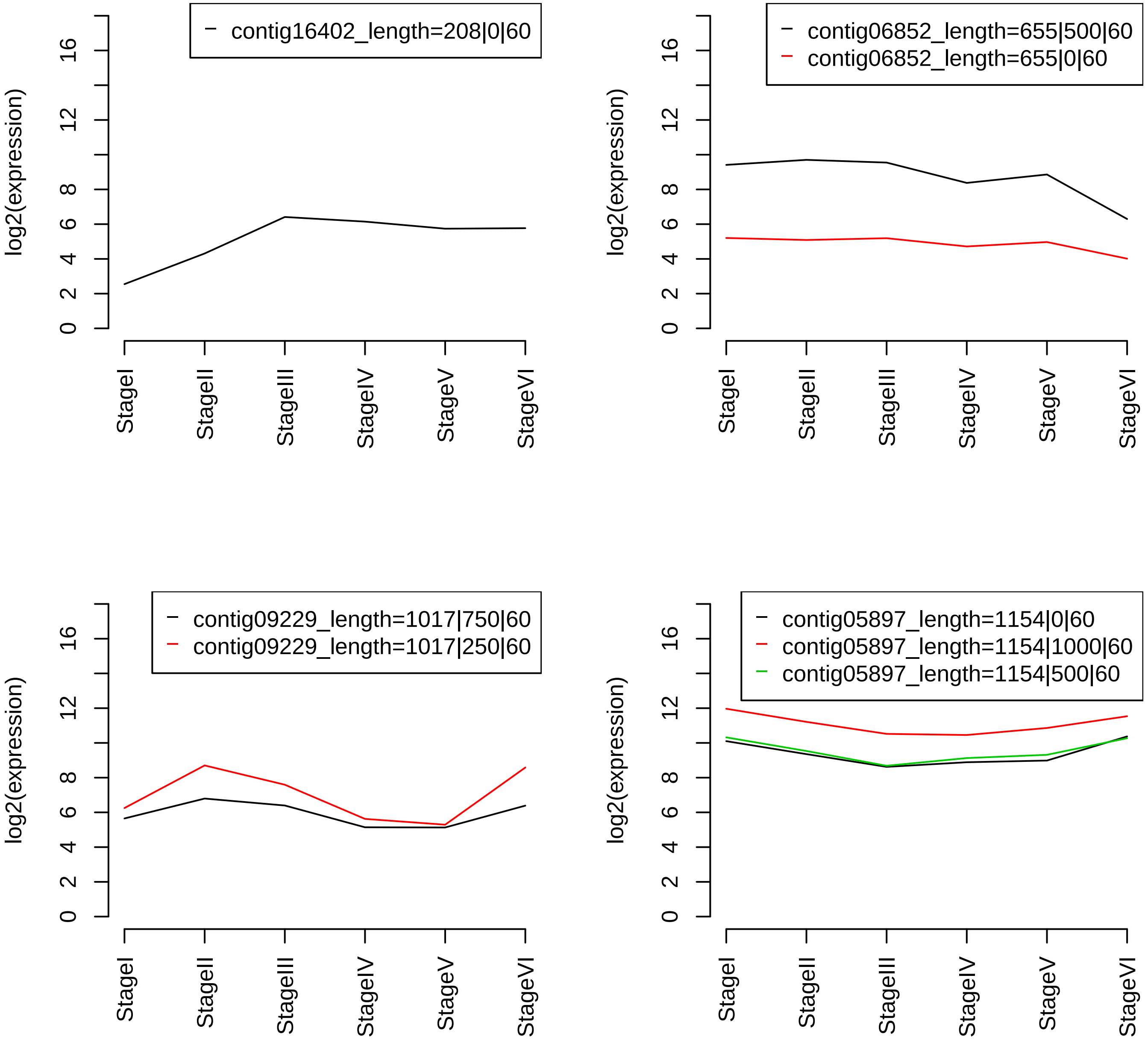

contig16402_length=208|0|60
log2(expression)
0
2
4
6
8
12
16
StageI
StageII
StageIII
StageIV
StageV
StageVI
contig06852_length=655|500|60
contig06852_length=655|0|60
log2(expression)
0
2
4
6
8
12
16
StageI
StageII
StageIII
StageIV
StageV
StageVI
contig09229_length=1017|750|60
contig09229_length=1017|250|60
log2(expression)
0
2
4
6
8
12
16
StageI
StageII
StageIII
StageIV
StageV
StageVI
contig05897_length=1154|0|60
contig05897_length=1154|1000|60
contig05897_length=1154|500|60
log2(expression)
0
2
4
6
8
12
16
StageI
StageII
StageIII
StageIV
StageV
StageVI

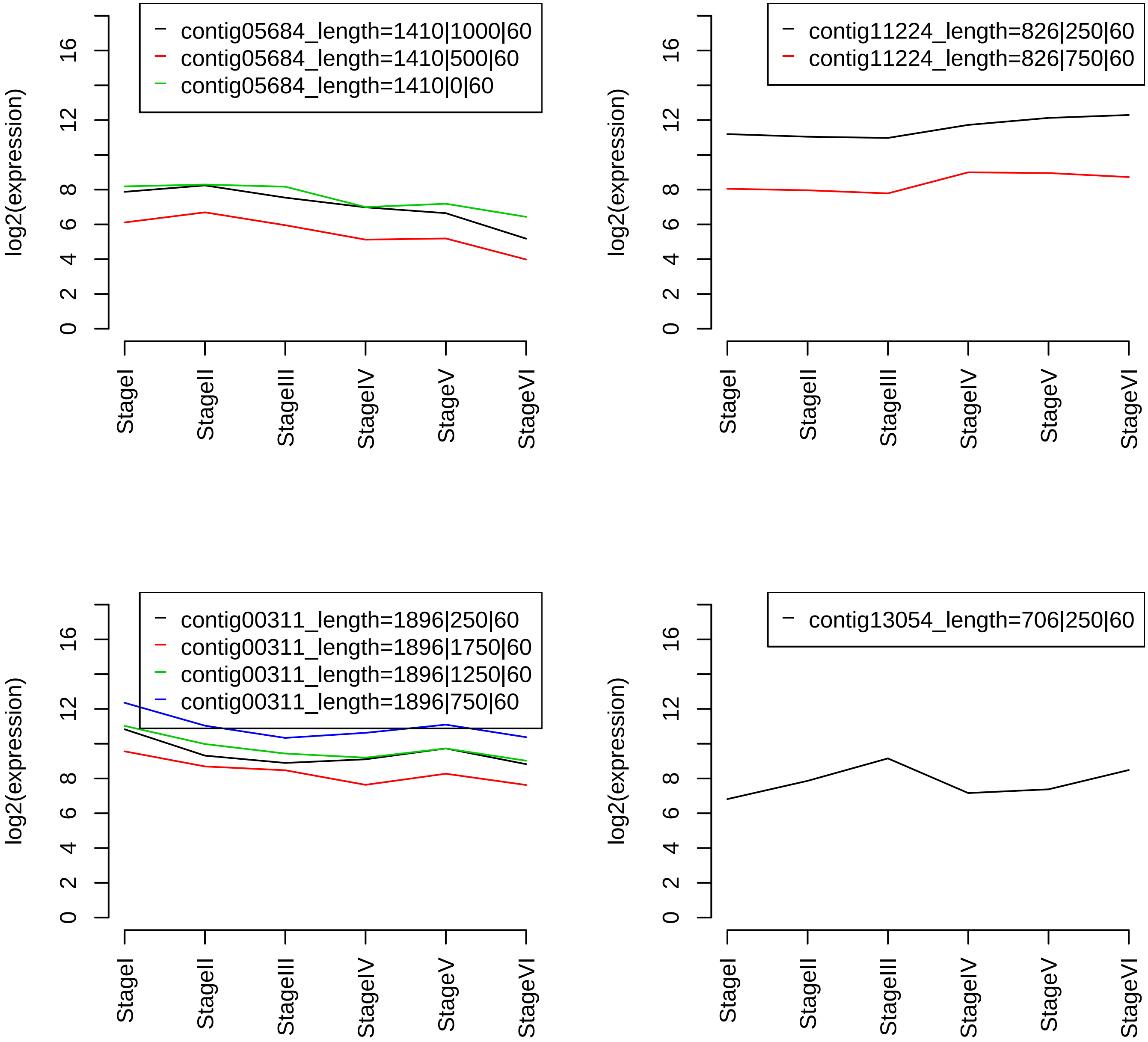

contig05684_length=1410|1000|60
contig05684_length=1410|500|60
contig05684_length=1410|0|60
contig11224_length=826|250|60
contig11224_length=826|750|60
contig00311_length=1896|250|60
contig00311_length=1896|1750|60
contig00311_length=1896|1250|60
contig00311_length=1896|750|60
contig13054_length=706|250|60
log2(expression)
0 2 4 6 8 12 16
StageI StageII StageIII StageIV StageV StageVI

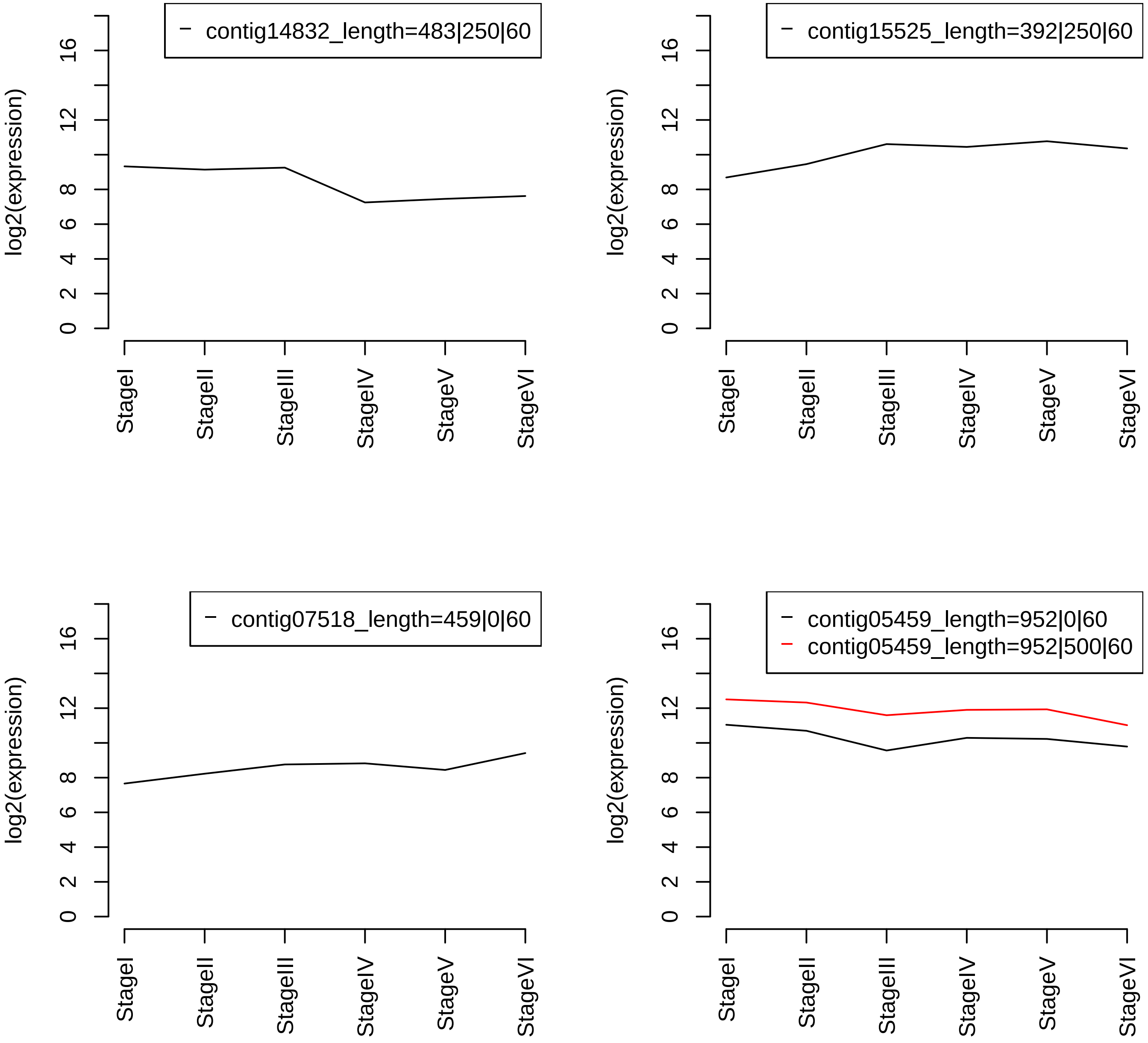

contig14832_length=483|250|60
contig15525_length=392|250|60
contig07518_length=459|0|60
contig05459_length=952|0|60
contig05459_length=952|500|60
log2(expression)
0
2
4
6
8
12
16
StageI
StageII
StageIII
StageIV
StageV
StageVI

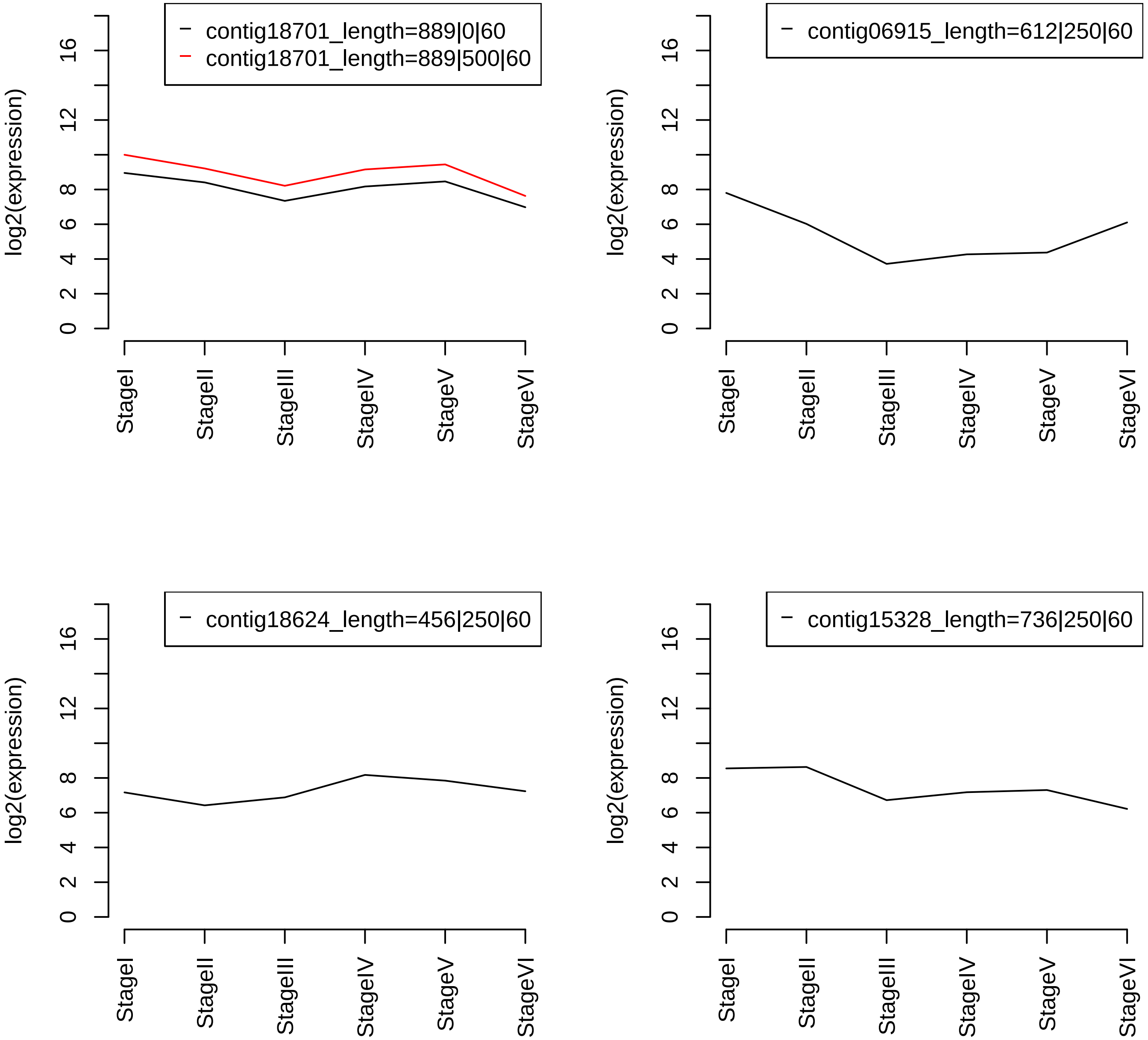
contig18701_length=889|0|60
contig18701_length=889|500|60
contig06915_length=612|250|60
contig18624_length=456|250|60
contig15328_length=736|250|60
log2(expression)
0
2
4
6
8
12
16
StageI
StageII
StageIII
StageIV
StageV
StageVI

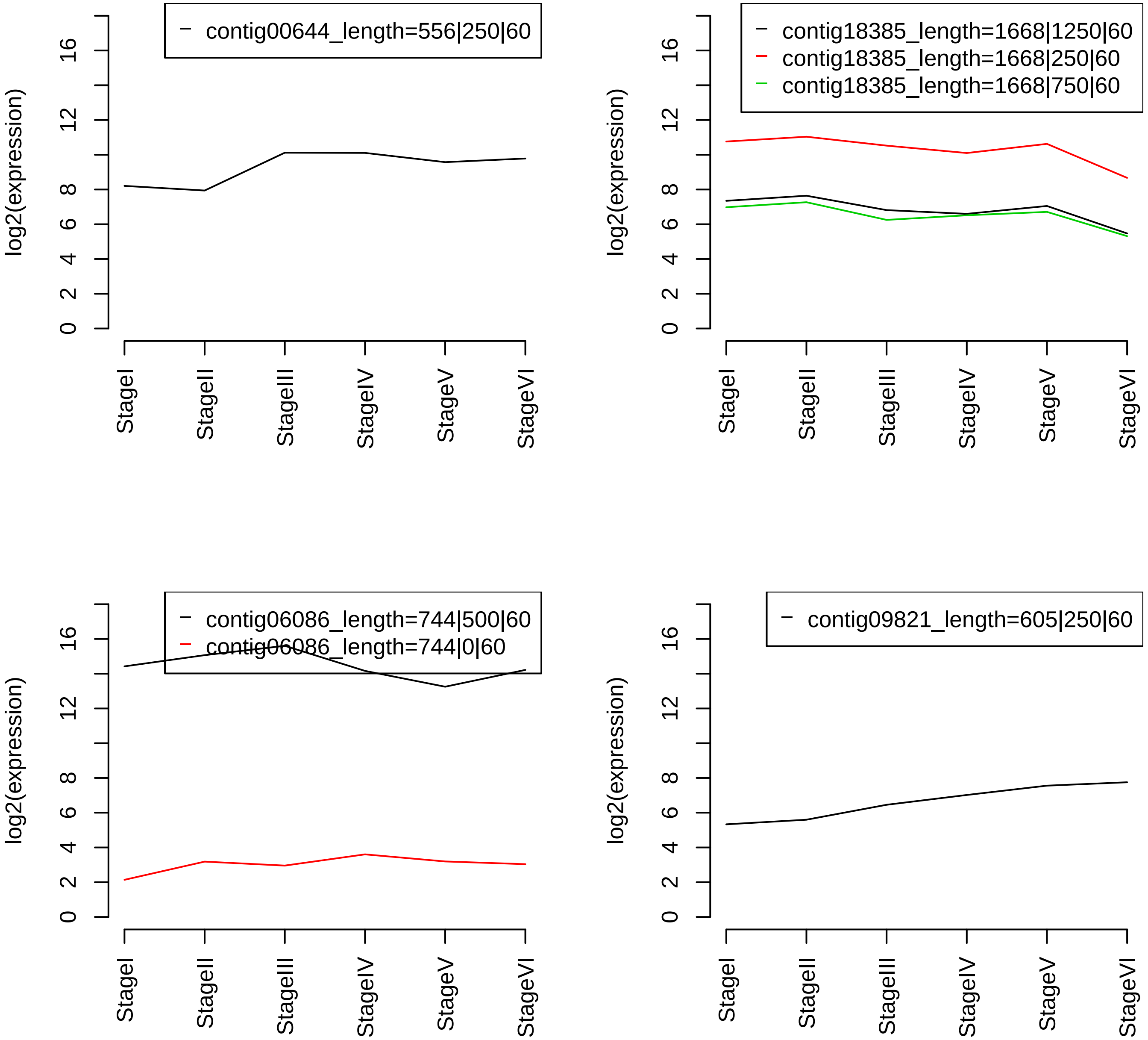
contig00644_length=556|250|60
contig18385_length=1668|1250|60
contig18385_length=1668|250|60
contig18385_length=1668|750|60
contig06086_length=744|500|60
contig06086_length=744|0|60
contig09821_length=605|250|60
log2(expression)
0
2
4
6
8
12
16
StageI
StageII
StageIII
StageIV
StageV
StageVI

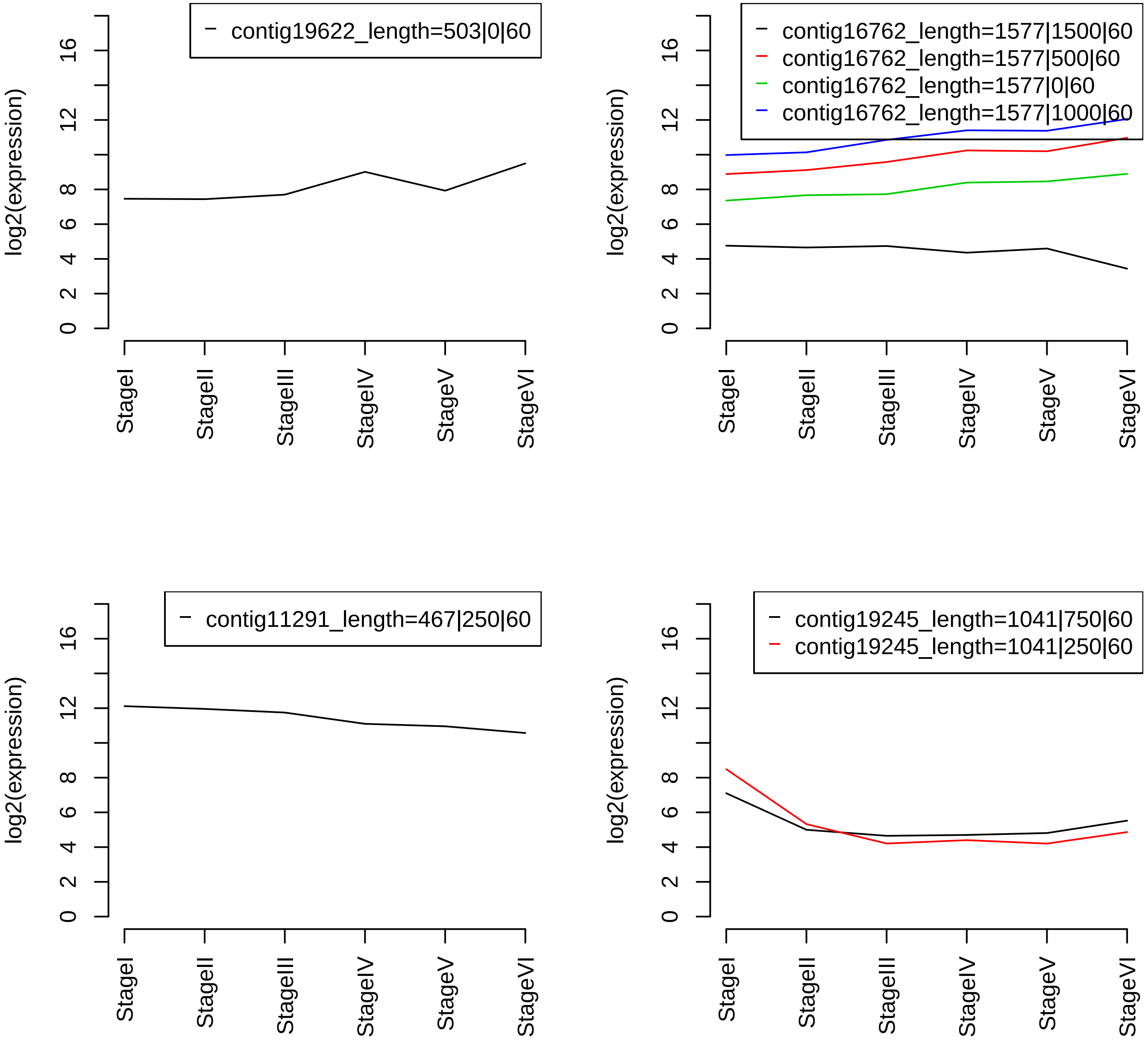
contig19622_length=503|0|60
contig16762_length=1577|1500|60
contig16762_length=1577|500|60
contig16762_length=1577|0|60
contig16762_length=1577|1000|60
contig11291_length=467|250|60
contig19245_length=1041|750|60
contig19245_length=1041|250|60
log2(expression)
0
2
4
6
8
12
16
StageI
StageII
StageIII
StageIV
StageV
StageVI

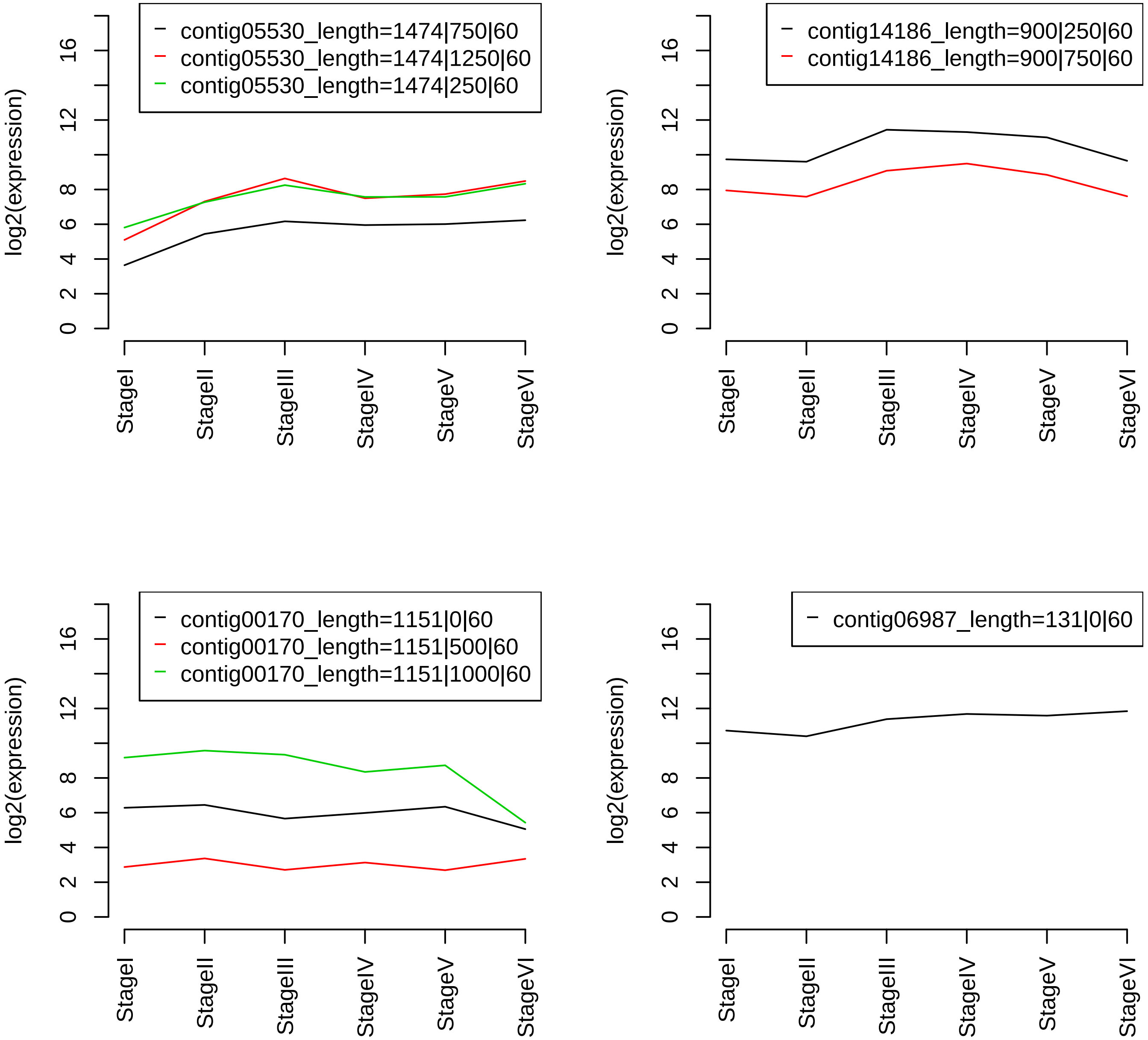
contig05530_length=1474|750|60
contig05530_length=1474|1250|60
contig05530_length=1474|250|60
contig14186_length=900|250|60
contig14186_length=900|750|60
contig00170_length=1151|0|60
contig00170_length=1151|500|60
contig00170_length=1151|1000|60
contig06987_length=131|0|60
log2(expression)
0
2
4
6
8
12
16
StageI
StageII
StageIII
StageIV
StageV
StageVI

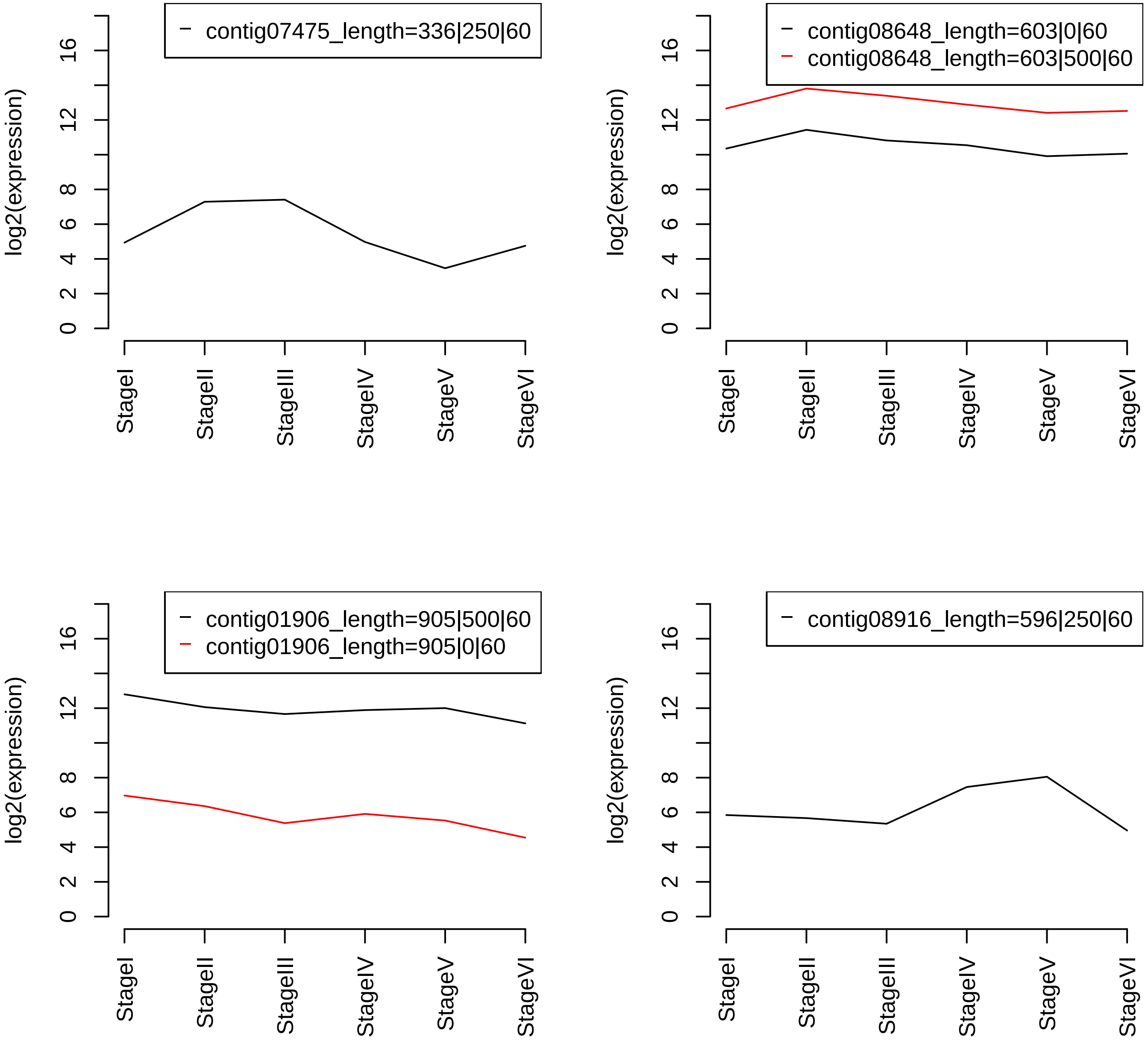

contig07475_length=336|250|60
contig08648_length=603|0|60
contig08648_length=603|500|60
contig01906_length=905|500|60
contig01906_length=905|0|60
contig08916_length=596|250|60
log2(expression)
0
2
4
6
8
12
16
StageI
StageII
StageIII
StageIV
StageV
StageVI

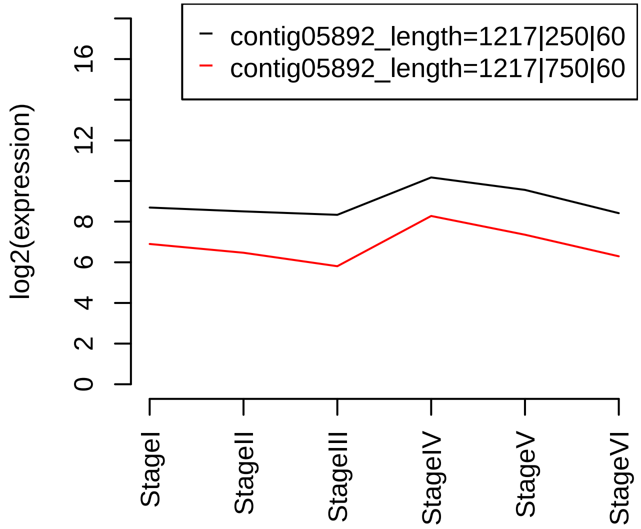
contig05892_length=1217|250|60
contig05892_length=1217|750|60
log2(expression)
0
2
4
6
8
12
16
StageI
StageII
StageIII
StageIV
StageV
StageVI

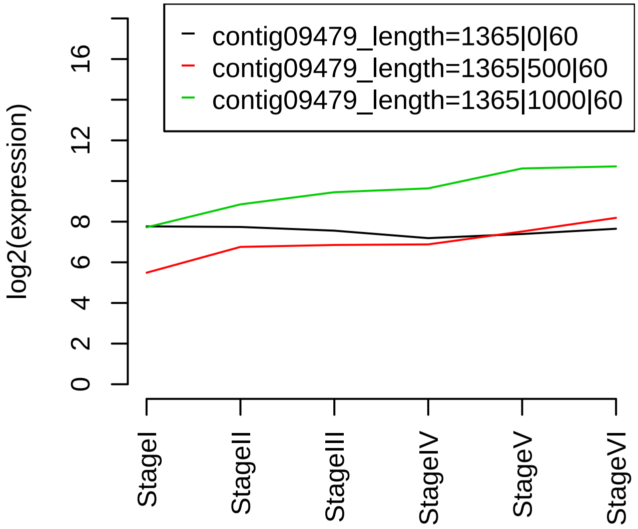
contig09479_length=1365|0|60
contig09479_length=1365|500|60
contig09479_length=1365|1000|60
log2(expression)
0
2
4
6
8
12
16
StageI
StageII
StageIII
StageIV
StageV
StageVI

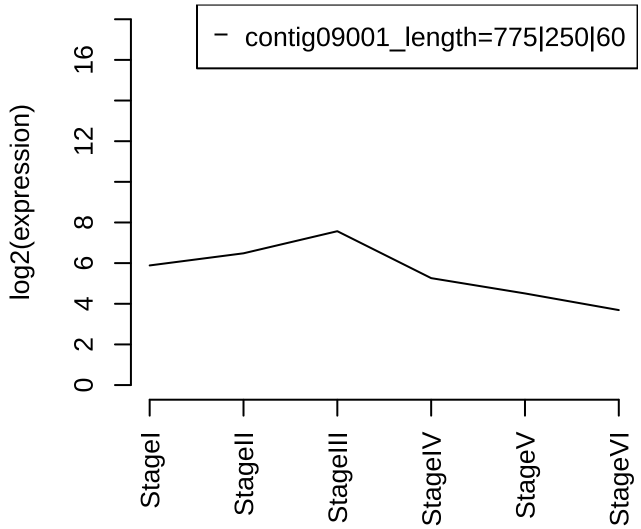
contig09001_length=775|250|60
log2(expression)
0
2
4
6
8
12
16
StageI
StageII
StageIII
StageIV
StageV
StageVI

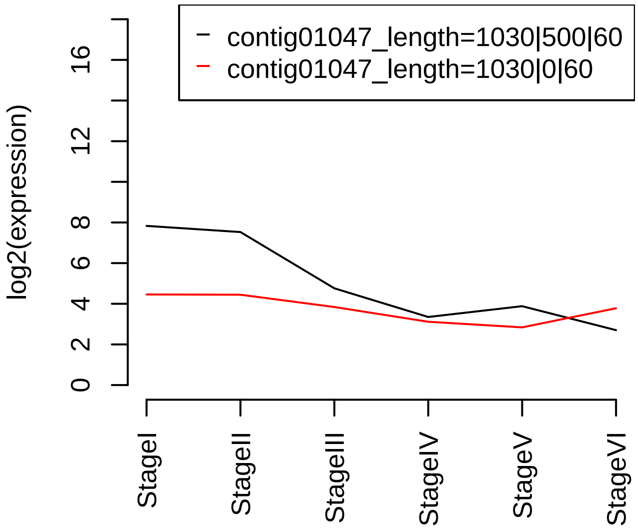
contig01047_length=1030|500|60
contig01047_length=1030|0|60
log2(expression)
0
2
4
6
8
12
16
StageI
StageII
StageIII
StageIV
StageV
StageVI

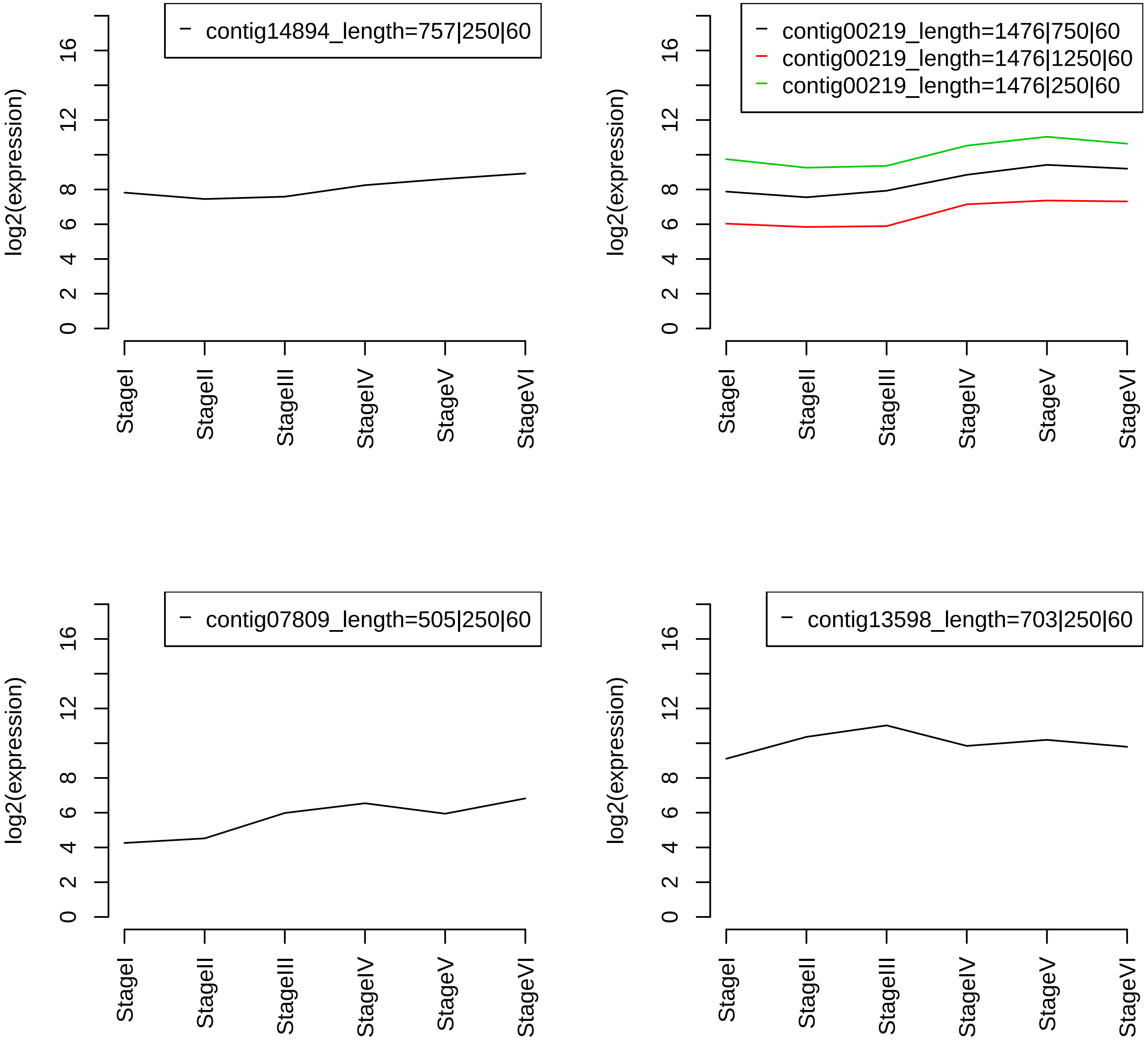
contig14894_length=757|250|60
contig00219_length=1476|750|60
contig00219_length=1476|1250|60
contig00219_length=1476|250|60
contig07809_length=505|250|60
contig13598_length=703|250|60
log2(expression)
0
2
4
6
8
12
16
StageI
StageII
StageIII
StageIV
StageV
StageVI

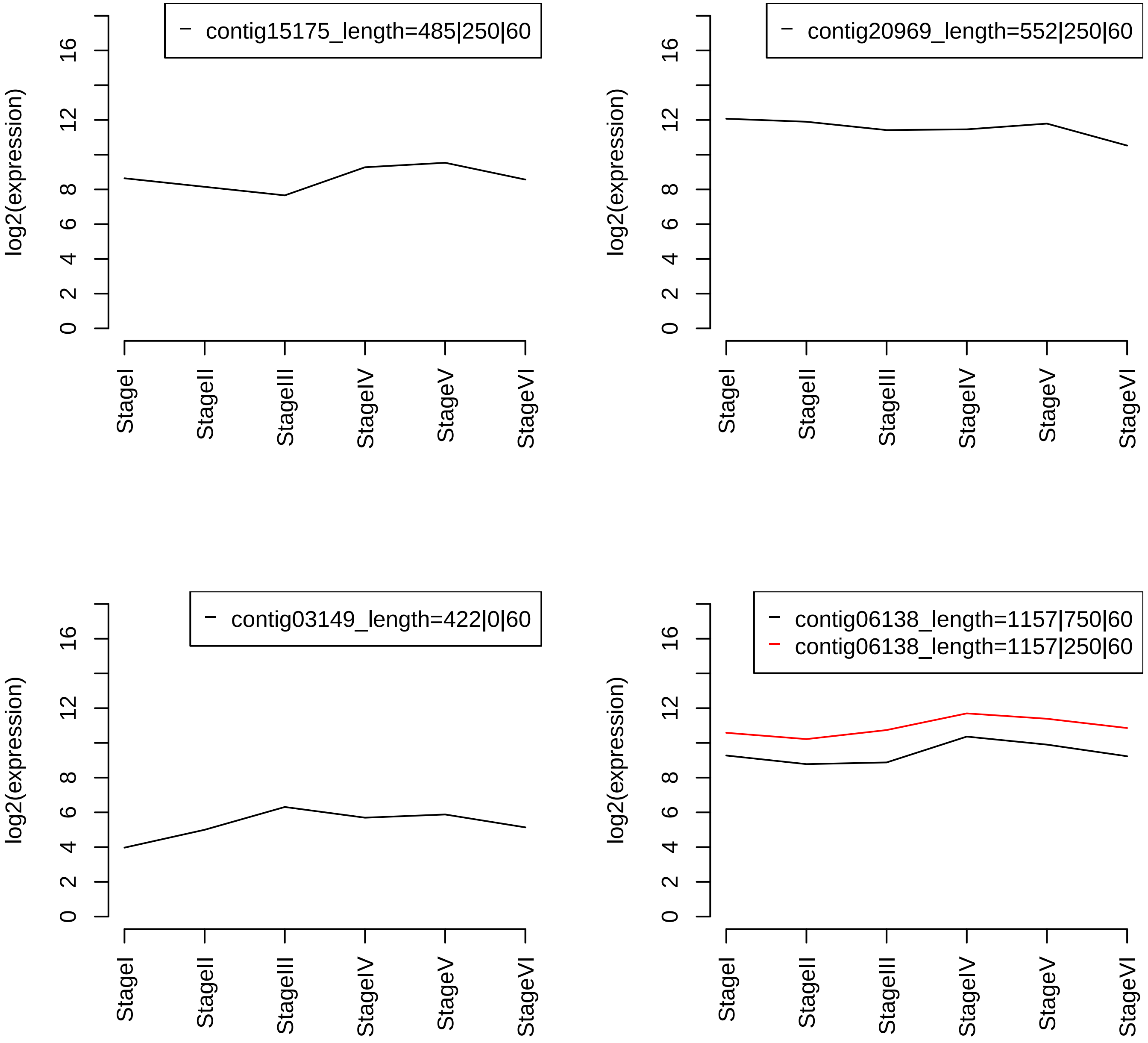
contig15175_length=485|250|60
contig20969_length=552|250|60
contig03149_length=422|0|60
contig06138_length=1157|750|60
contig06138_length=1157|250|60
log2(expression)
0
2
4
6
8
12
16
StageI
StageII
StageIII
StageIV
StageV
StageVI

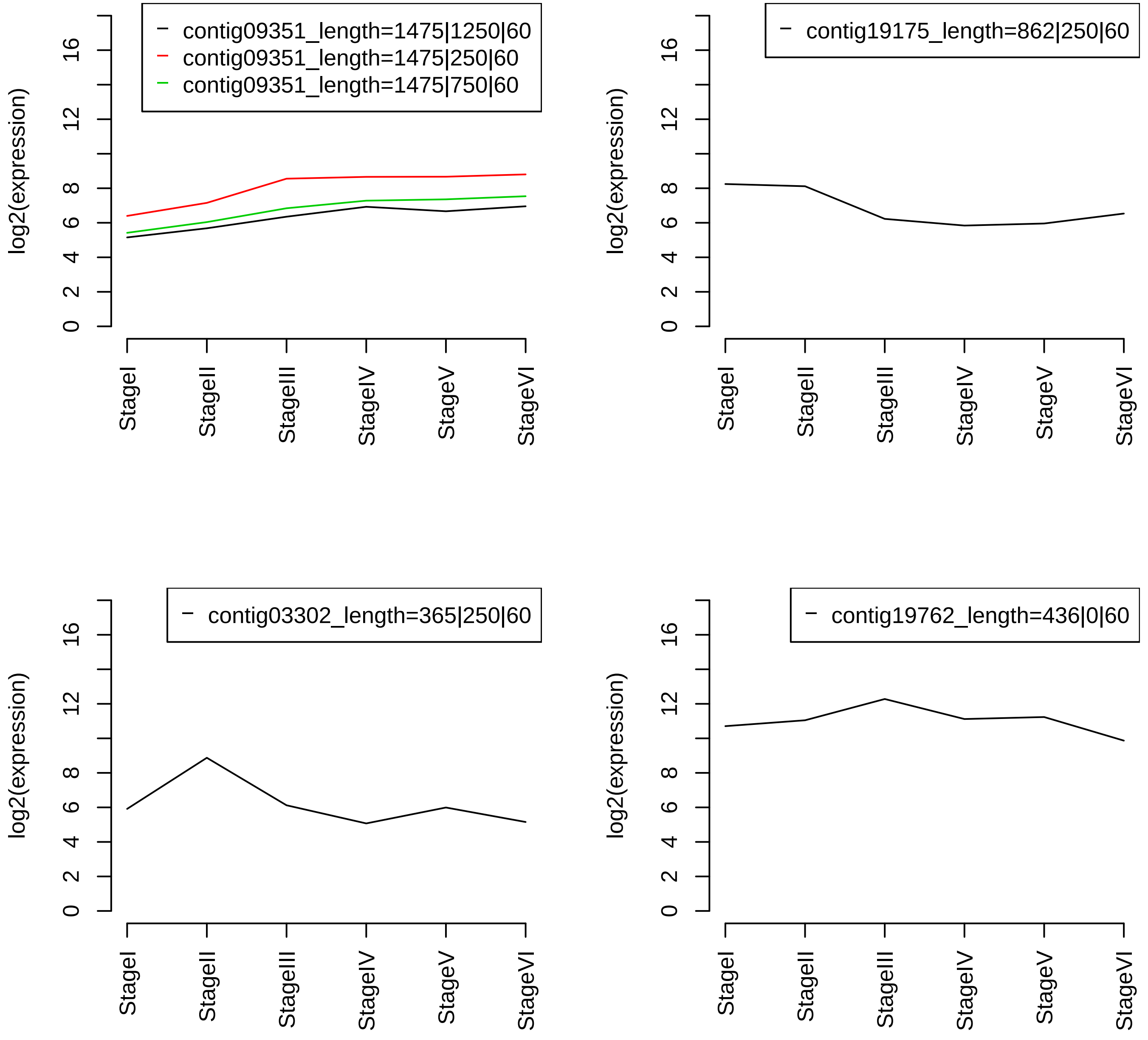

contig09351_length=1475|1250|60
contig09351_length=1475|250|60
contig09351_length=1475|750|60
contig19175_length=862|250|60
contig03302_length=365|250|60
contig19762_length=436|0|60
log2(expression)
0
2
4
6
8
12
16
StageI
StageII
StageIII
StageIV
StageV
StageVI

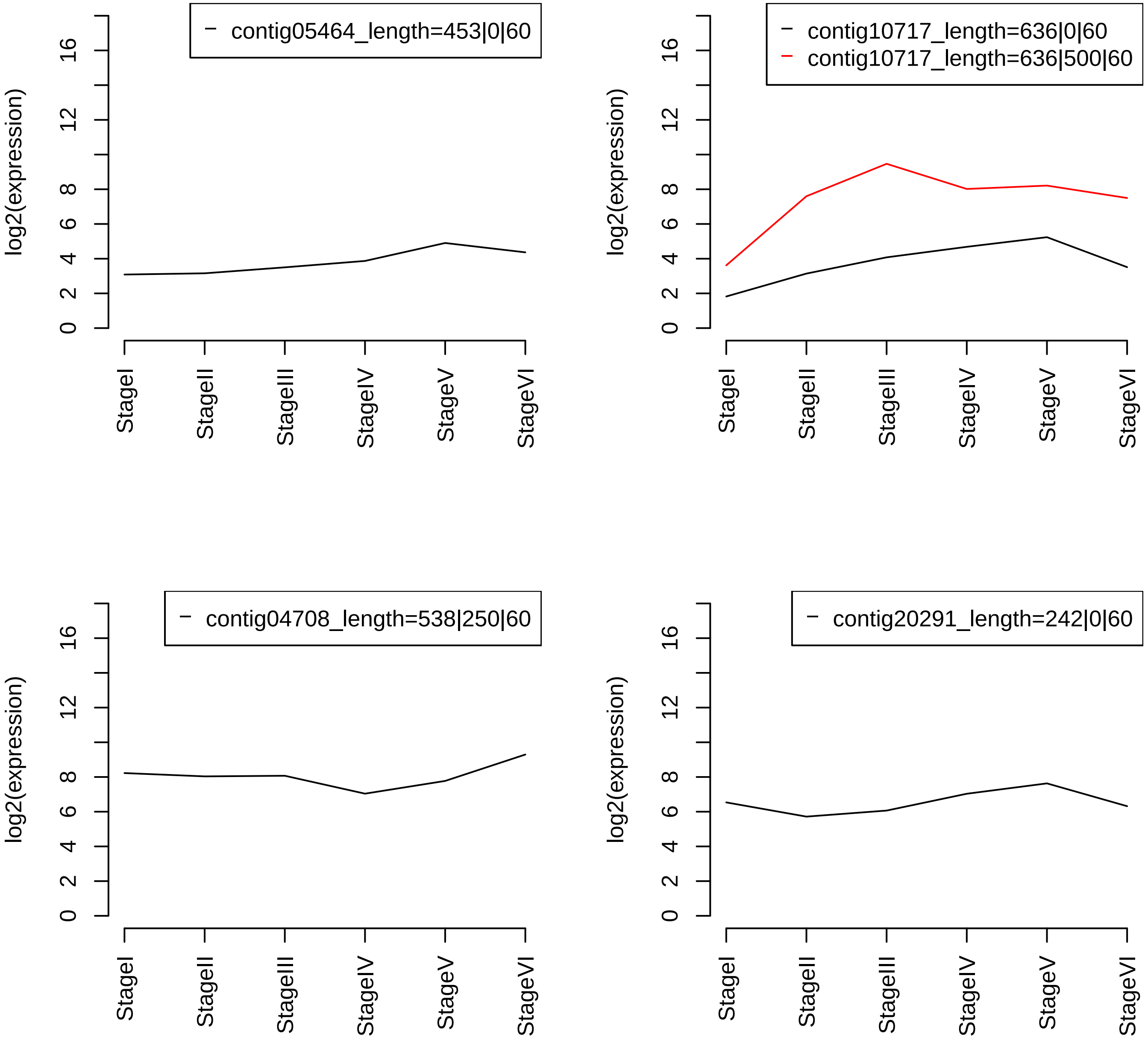
contig05464_length=453|0|60
contig10717_length=636|0|60
contig10717_length=636|500|60
contig04708_length=538|250|60
contig20291_length=242|0|60
log2(expression)
0
2
4
6
8
12
16
StageI
StageII
StageIII
StageIV
StageV
StageVI

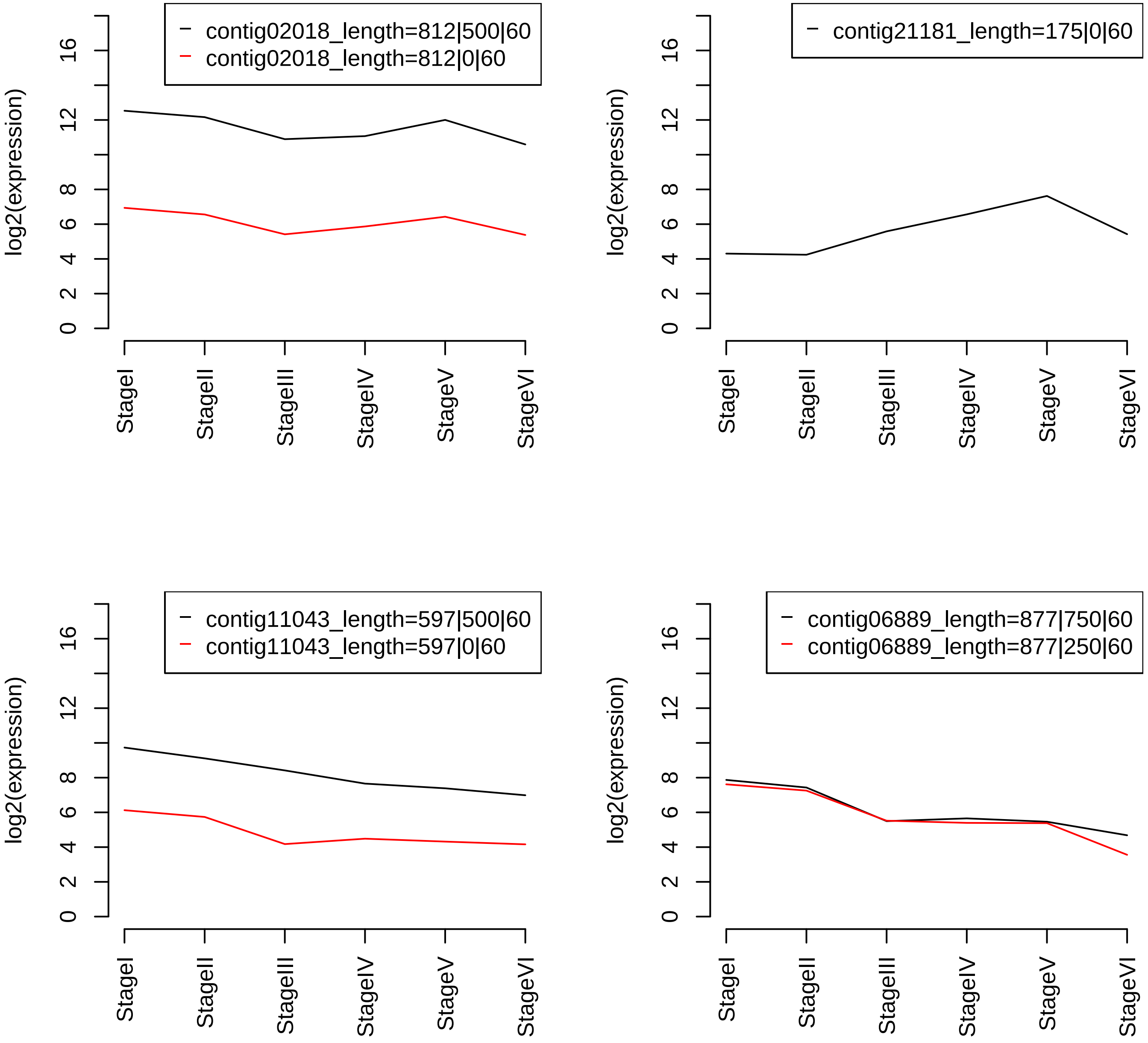

contig02018_length=812|500|60
contig02018_length=812|0|60
contig21181_length=175|0|60
contig11043_length=597|500|60
contig11043_length=597|0|60
contig06889_length=877|750|60
contig06889_length=877|250|60
log2(expression)
0
2
4
6
8
12
16
StageI
StageII
StageIII
StageIV
StageV
StageVI

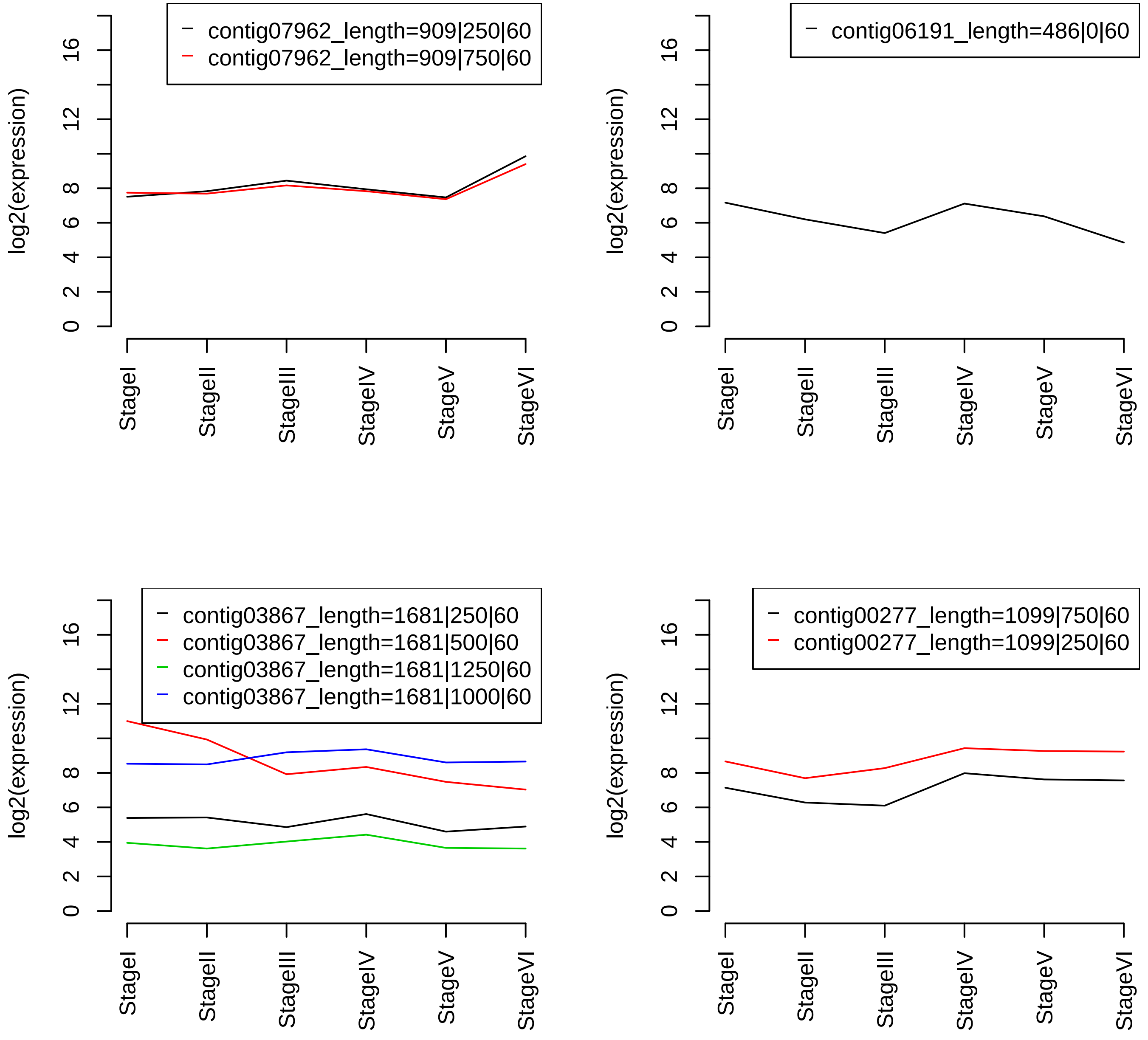

contig07962_length=909|250|60
contig07962_length=909|750|60
contig06191_length=486|0|60
contig03867_length=1681|250|60
contig03867_length=1681|500|60
contig03867_length=1681|1250|60
contig03867_length=1681|1000|60
contig00277_length=1099|750|60
contig00277_length=1099|250|60
log2(expression)
0
2
4
6
8
12
16
StageI
StageII
StageIII
StageIV
StageV
StageVI

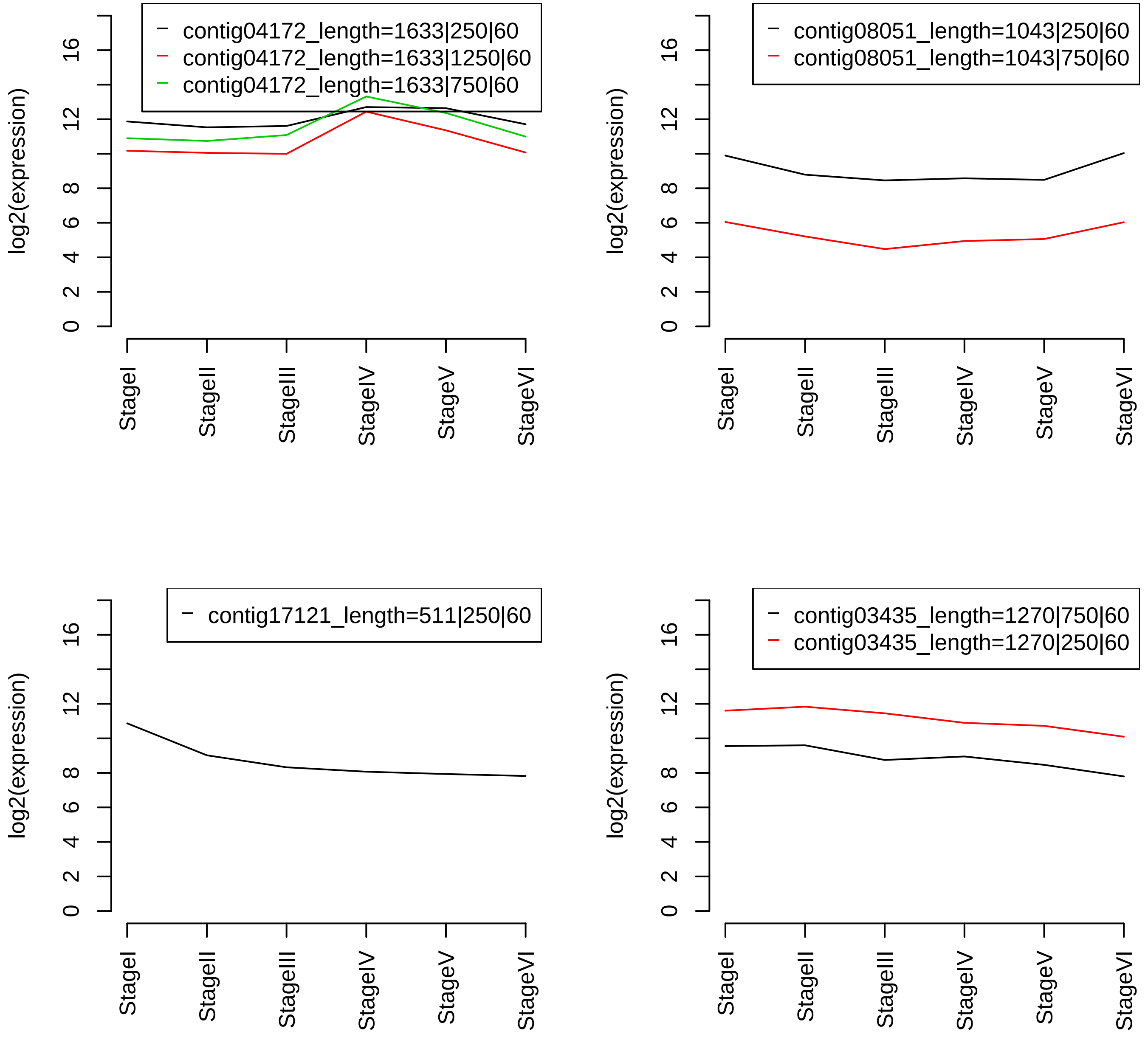
contig04172_length=1633|250|60
contig04172_length=1633|1250|60
contig04172_length=1633|750|60
log2(expression)
0
2
4
6
8
12
16
StageI
StageII
StageIII
StageIV
StageV
StageVI
contig08051_length=1043|250|60
contig08051_length=1043|750|60
contig17121_length=511|250|60
contig03435_length=1270|750|60
contig03435_length=1270|250|60

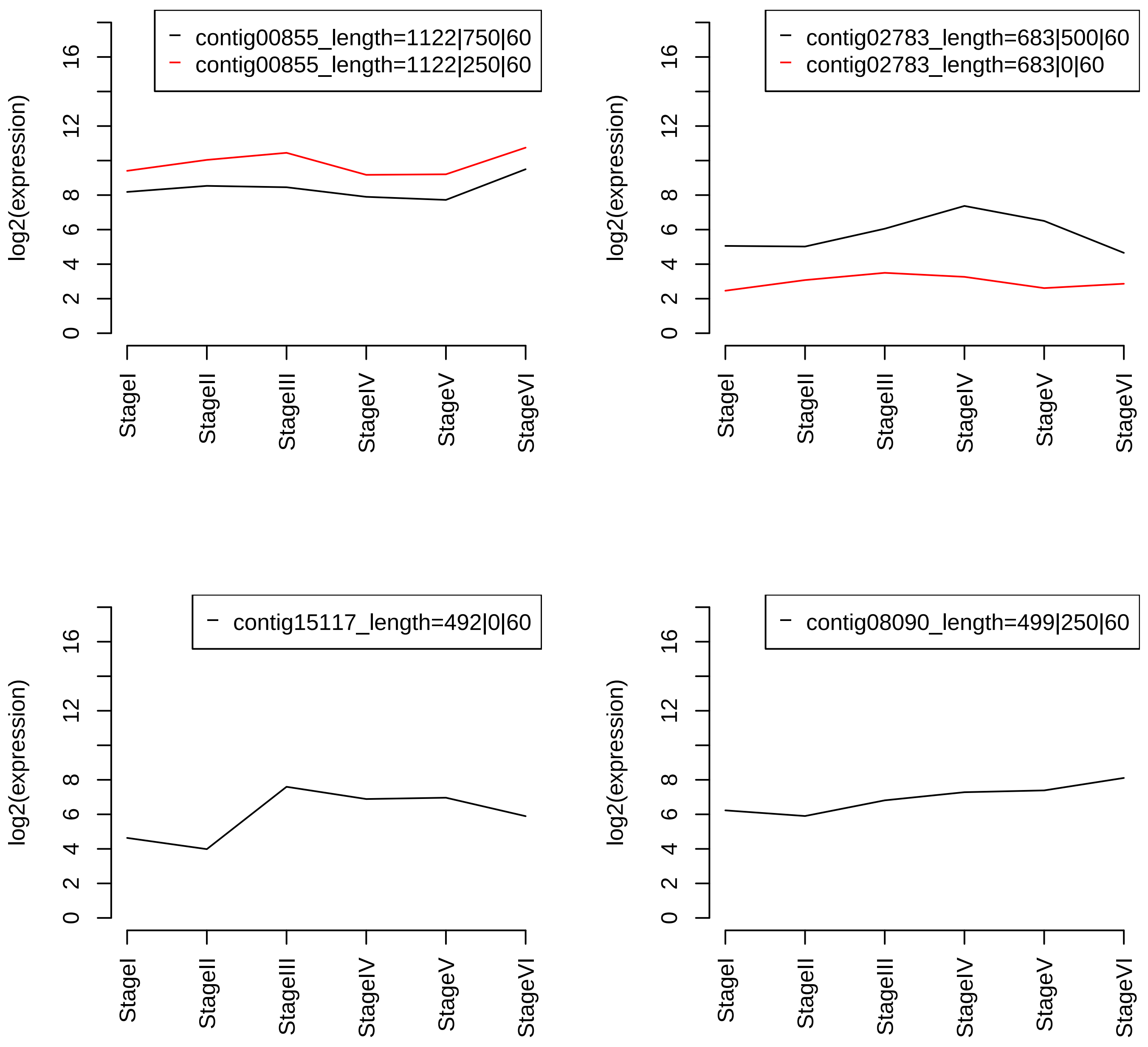
contig00855_length=1122|750|60
contig00855_length=1122|250|60
log2(expression)
0
2
4
6
8
12
16
StageI
StageII
StageIII
StageIV
StageV
StageVI
contig02783_length=683|500|60
contig02783_length=683|0|60
contig15117_length=492|0|60
contig08090_length=499|250|60

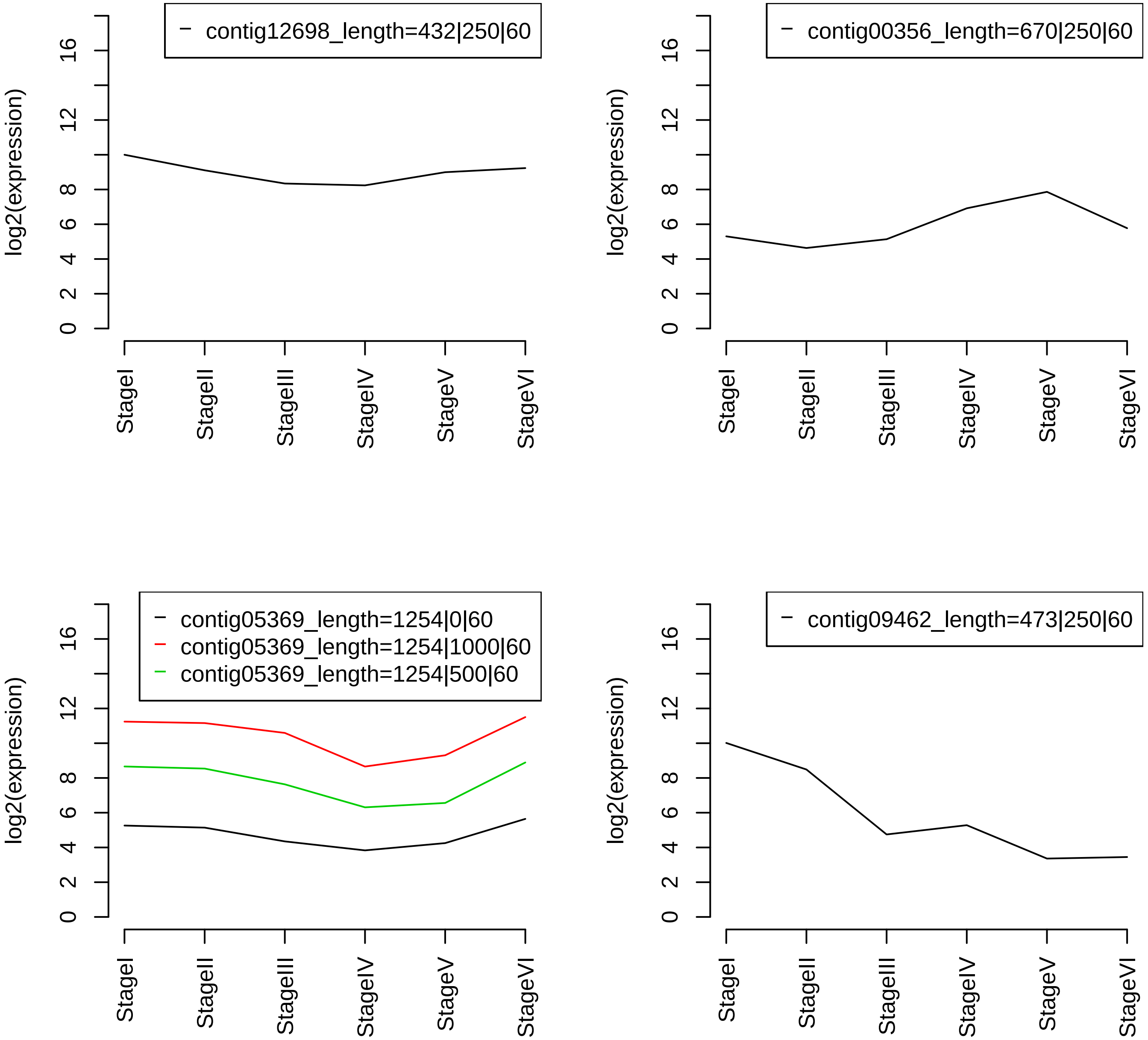
contig12698_length=432|250|60
contig00356_length=670|250|60
contig05369_length=1254|0|60
contig05369_length=1254|1000|60
contig05369_length=1254|500|60
contig09462_length=473|250|60
log2(expression)
0 2 4 6 8 12 16
StageI StageII StageIII StageIV StageV StageVI

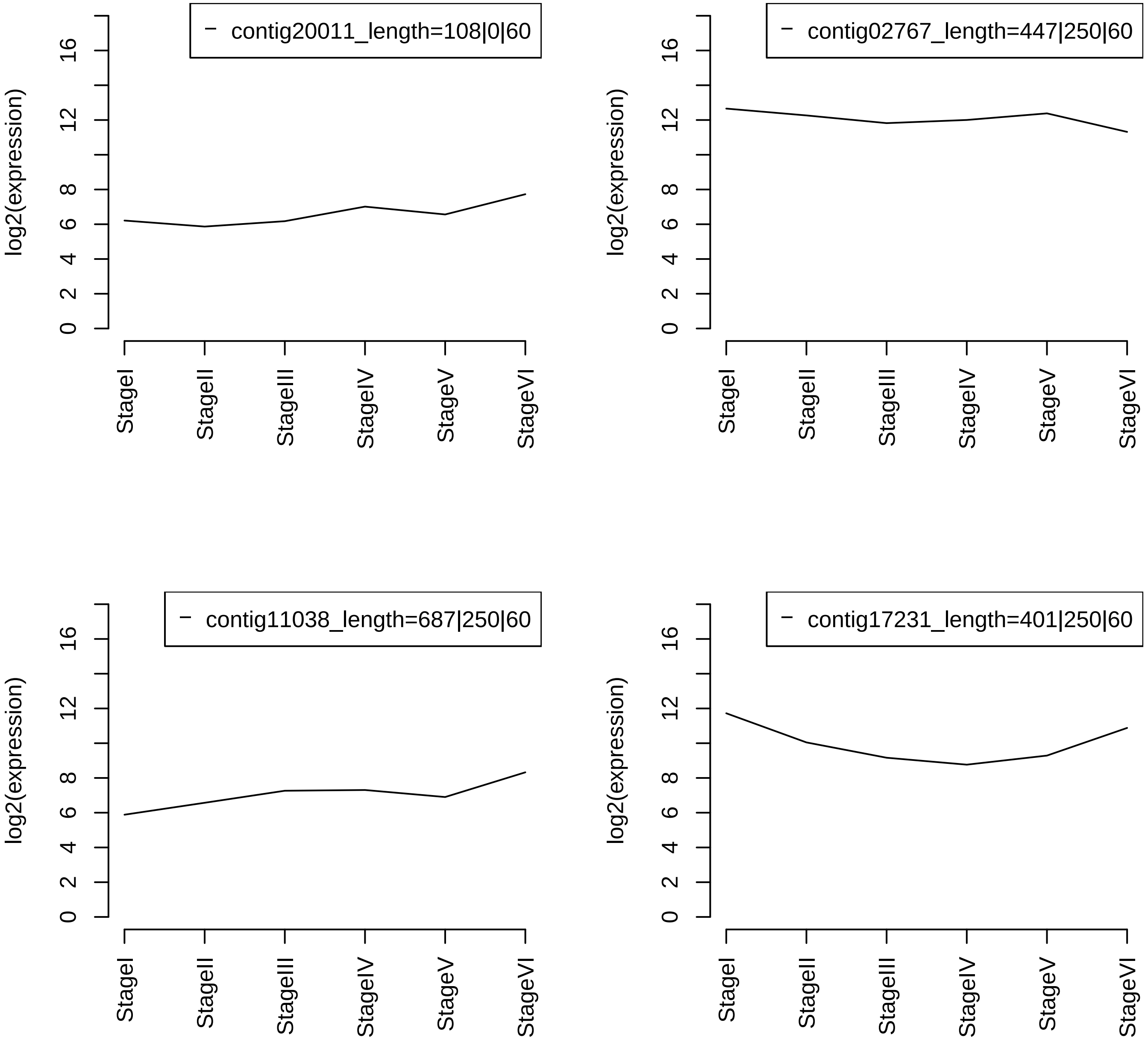

contig20011_length=108|0|60
contig02767_length=447|250|60
contig11038_length=687|250|60
contig17231_length=401|250|60
log2(expression)
0
2
4
6
8
12
16
StageI
StageII
StageIII
StageIV
StageV
StageVI

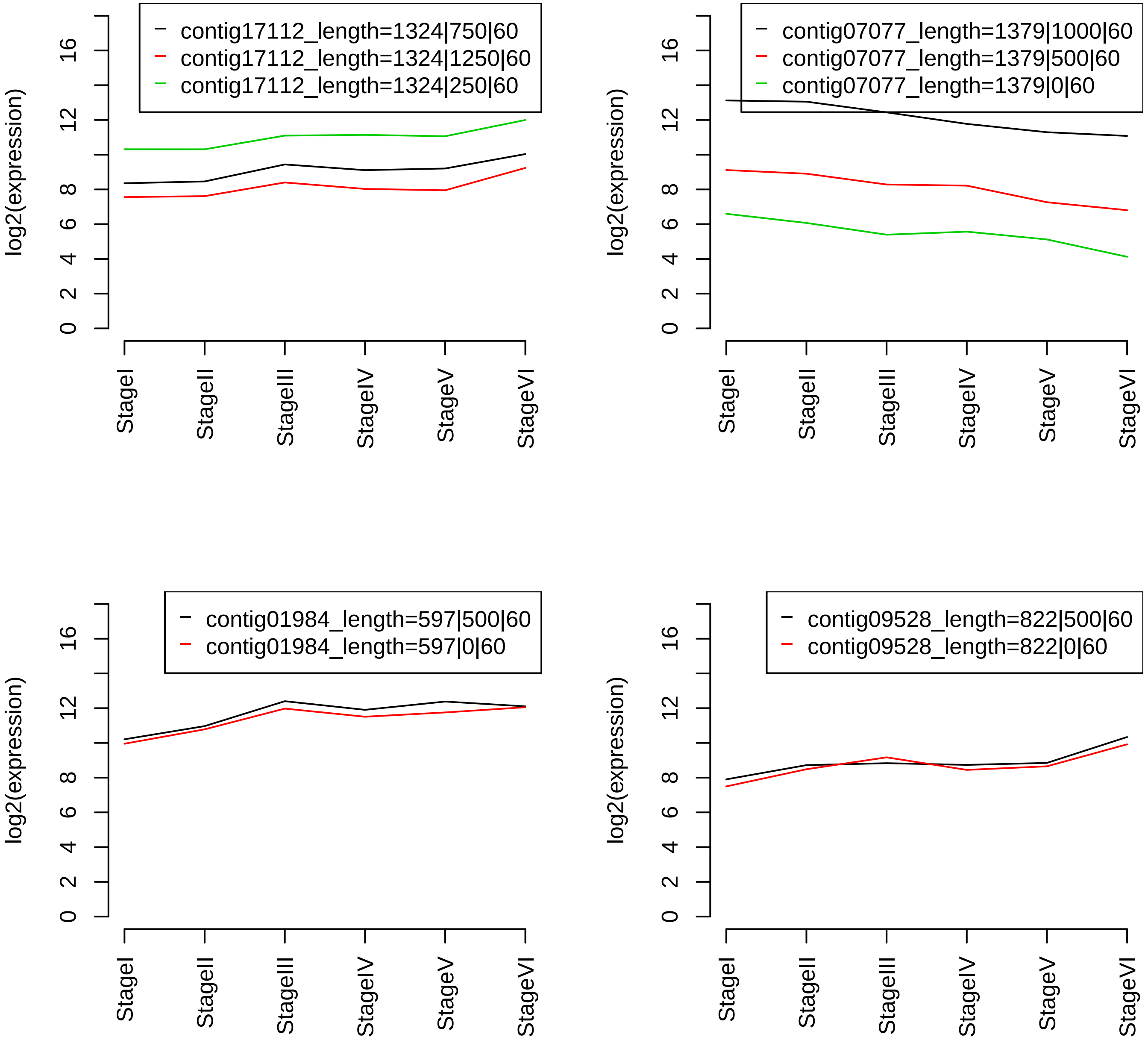

contig17112_length=1324|750|60
contig17112_length=1324|1250|60
contig17112_length=1324|250|60
log2(expression)
0
2
4
6
8
12
16
StageI
StageII
StageIII
StageIV
StageV
StageVI
contig07077_length=1379|1000|60
contig07077_length=1379|500|60
contig07077_length=1379|0|60
contig01984_length=597|500|60
contig01984_length=597|0|60
contig09528_length=822|500|60
contig09528_length=822|0|60

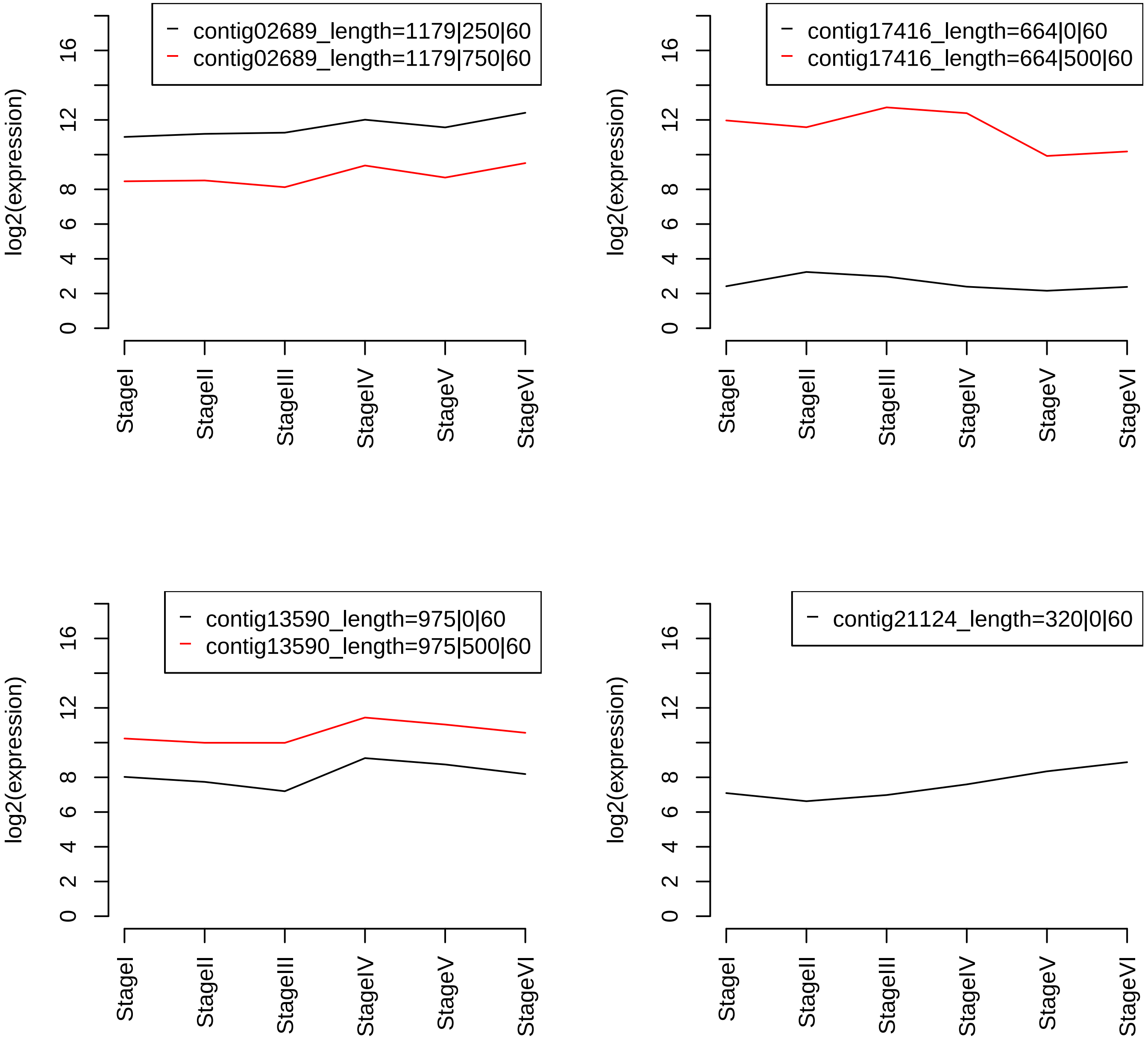

contig02689_length=1179|250|60
contig02689_length=1179|750|60
contig17416_length=664|0|60
contig17416_length=664|500|60
contig13590_length=975|0|60
contig13590_length=975|500|60
contig21124_length=320|0|60
log2(expression)
0
2
4
6
8
12
16
StageI
StageII
StageIII
StageIV
StageV
StageVI

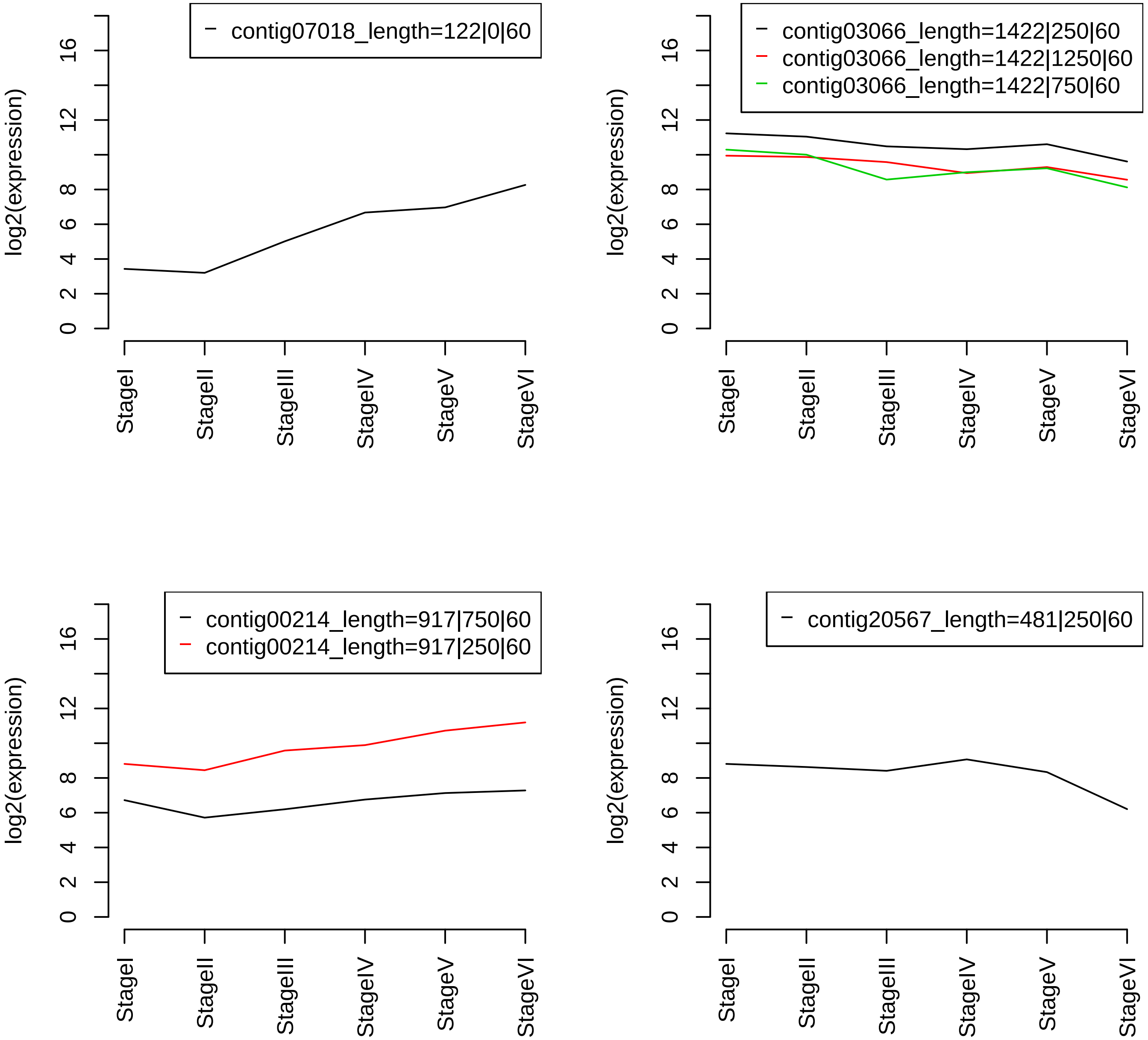

contig07018_length=122|0|60
contig03066_length=1422|250|60
contig03066_length=1422|1250|60
contig03066_length=1422|750|60
contig00214_length=917|750|60
contig00214_length=917|250|60
contig20567_length=481|250|60
log2(expression)
0
2
4
6
8
12
16
StageI
StageII
StageIII
StageIV
StageV
StageVI

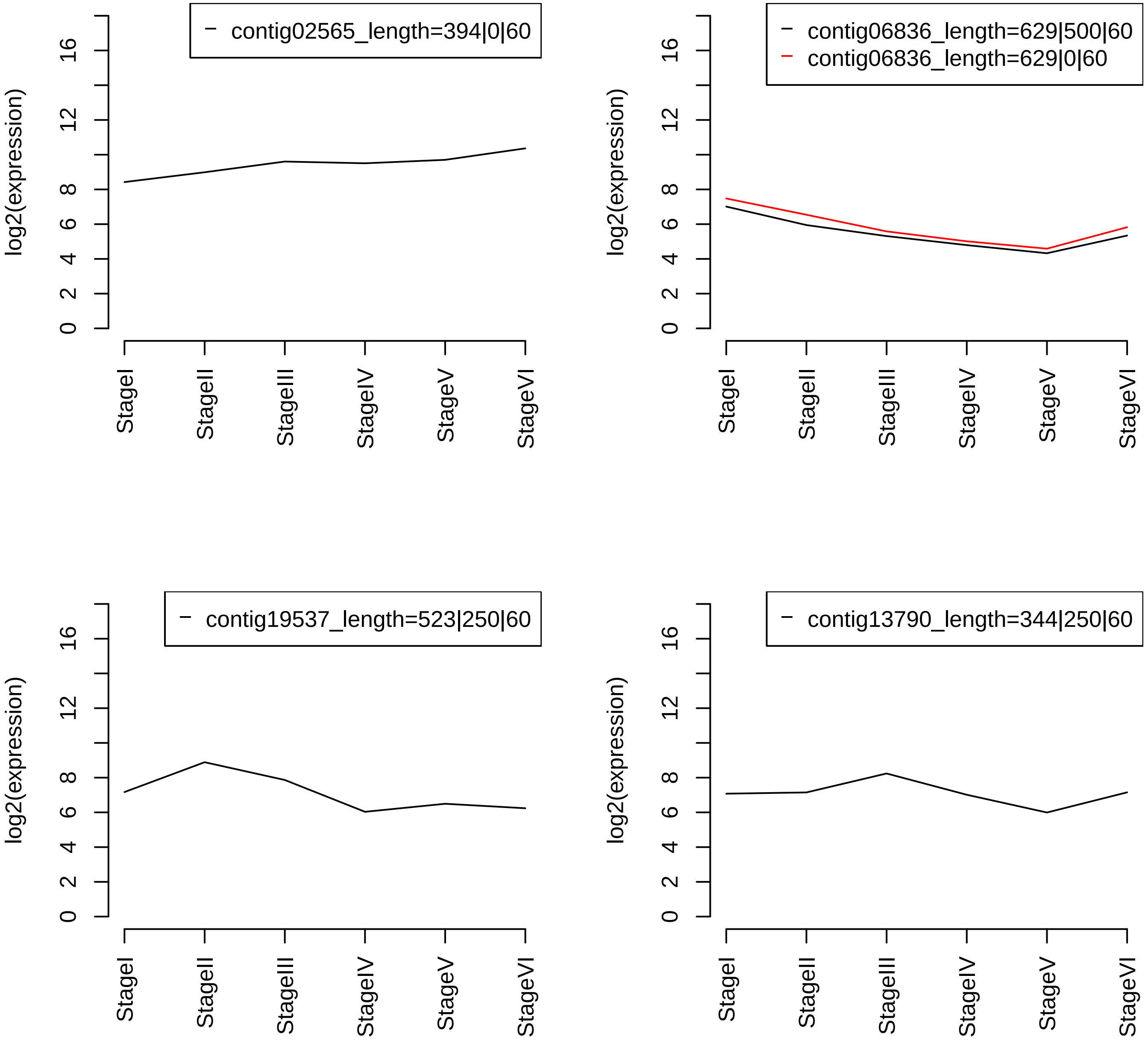
contig02565_length=394|0|60
contig06836_length=629|500|60
contig06836_length=629|0|60
contig19537_length=523|250|60
contig13790_length=344|250|60
log2(expression)
0
2
4
6
8
12
16
StageI
StageII
StageIII
StageIV
StageV
StageVI

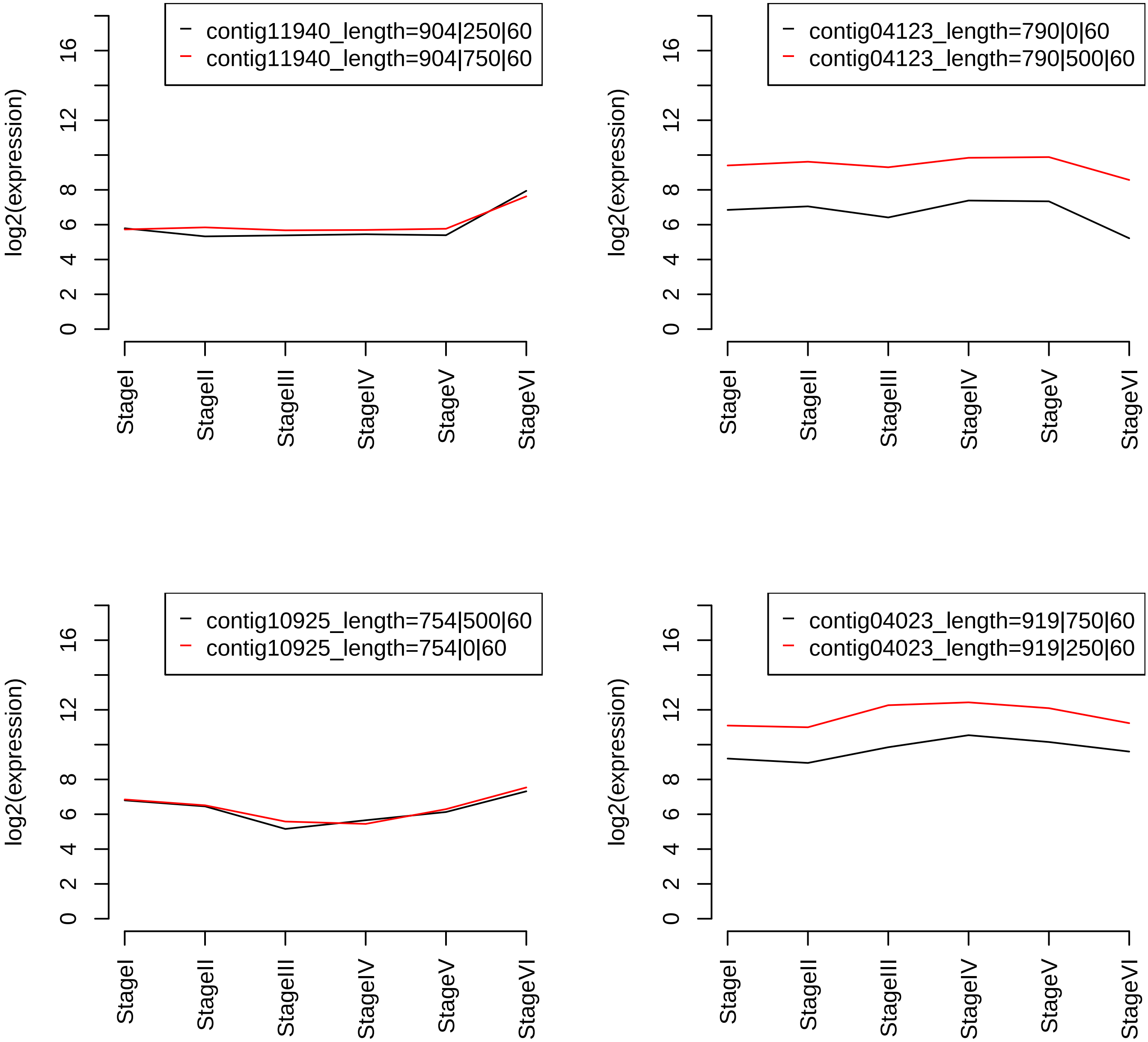

contig11940_length=904|250|60
contig11940_length=904|750|60
log2(expression)
0 2 4 6 8 12 16
StageI StageII StageIII StageIV StageV StageVI
contig04123_length=790|0|60
contig04123_length=790|500|60
log2(expression)
0 2 4 6 8 12 16
StageI StageII StageIII StageIV StageV StageVI
contig10925_length=754|500|60
contig10925_length=754|0|60
log2(expression)
0 2 4 6 8 12 16
StageI StageII StageIII StageIV StageV StageVI
contig04023_length=919|750|60
contig04023_length=919|250|60
log2(expression)
0 2 4 6 8 12 16
StageI StageII StageIII StageIV StageV StageVI

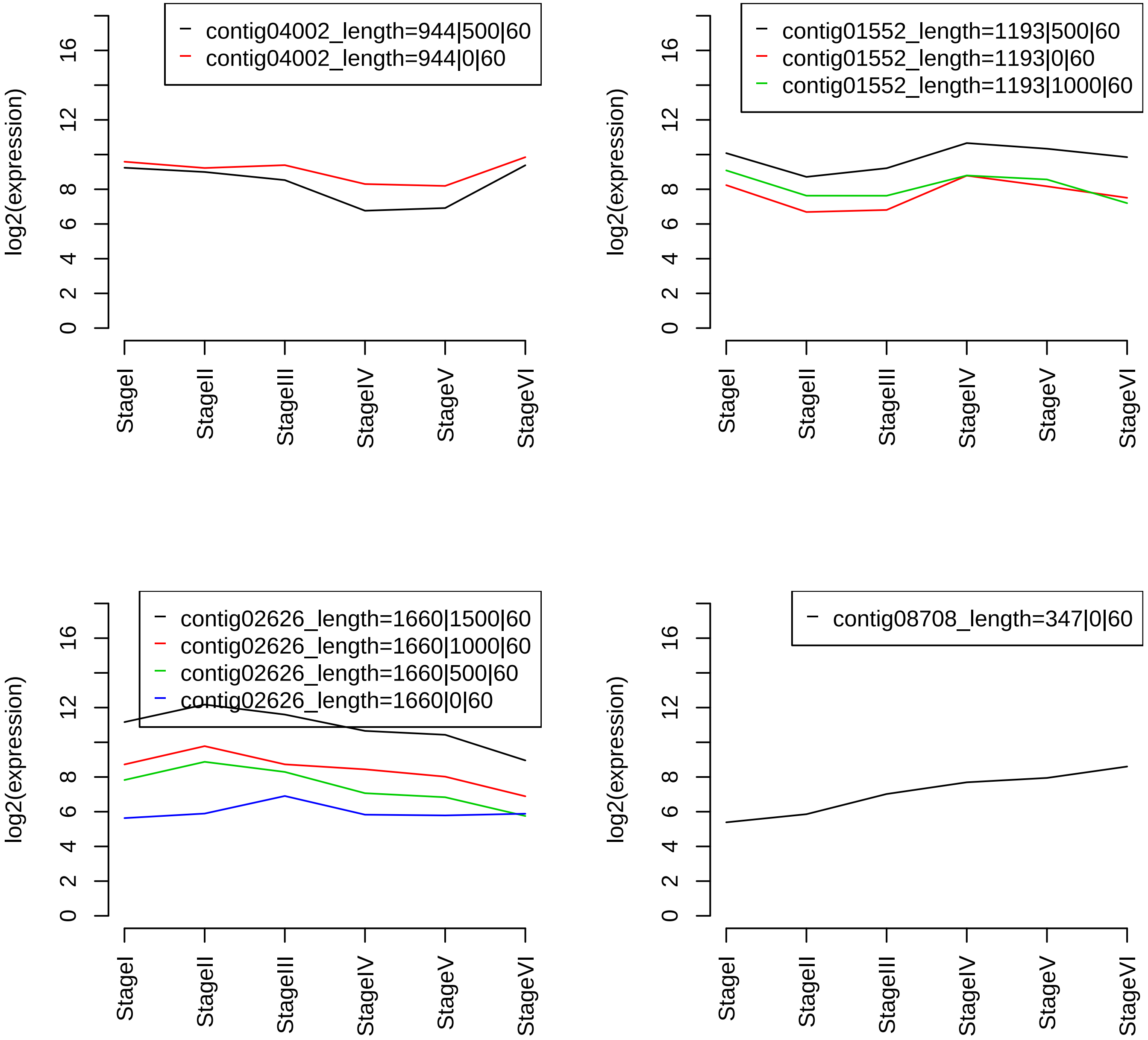
contig04002_length=944|500|60
contig04002_length=944|0|60
contig01552_length=1193|500|60
contig01552_length=1193|0|60
contig01552_length=1193|1000|60
contig02626_length=1660|1500|60
contig02626_length=1660|1000|60
contig02626_length=1660|500|60
contig02626_length=1660|0|60
contig08708_length=347|0|60
log2(expression)
0
2
4
6
8
12
16
StageI
StageII
StageIII
StageIV
StageV
StageVI

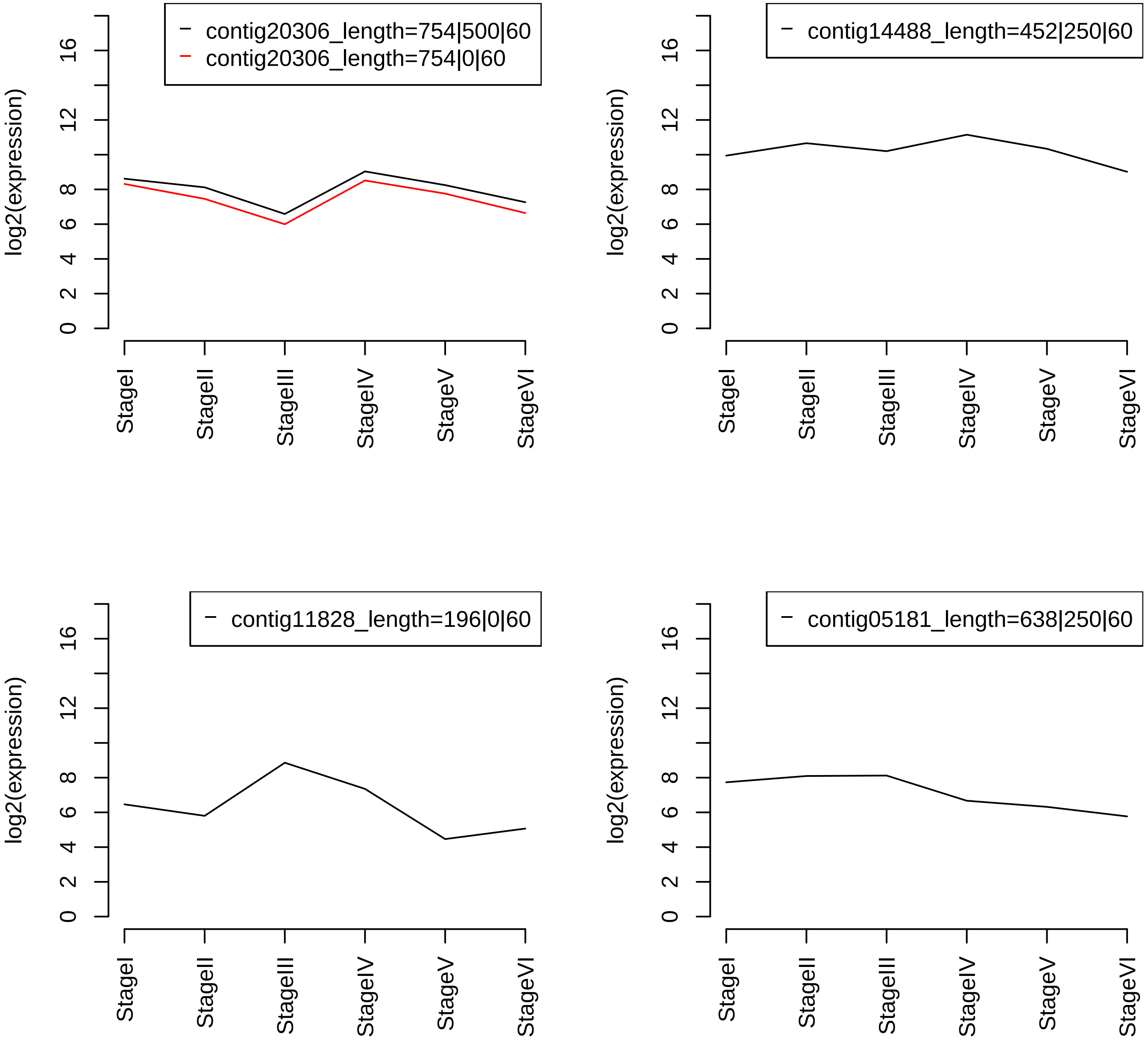
contig20306_length=754|500|60
contig20306_length=754|0|60
contig14488_length=452|250|60
contig11828_length=196|0|60
contig05181_length=638|250|60
log2(expression)
0
2
4
6
8
12
16
StageI
StageII
StageIII
StageIV
StageV
StageVI

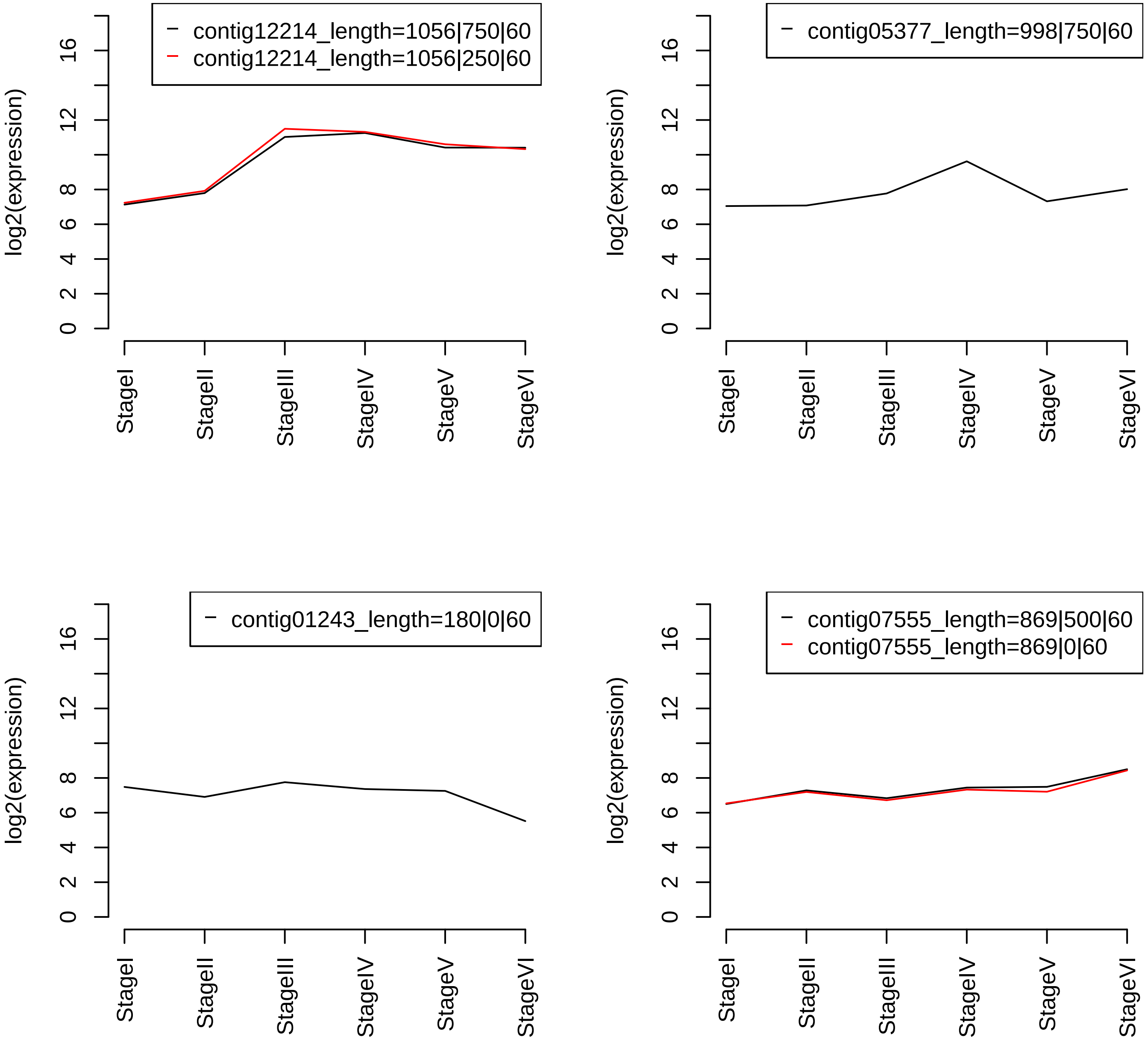
contig12214_length=1056|750|60
contig12214_length=1056|250|60
contig05377_length=998|750|60
contig01243_length=180|0|60
contig07555_length=869|500|60
contig07555_length=869|0|60
log2(expression)
0
2
4
6
8
12
16
StageI
StageII
StageIII
StageIV
StageV
StageVI

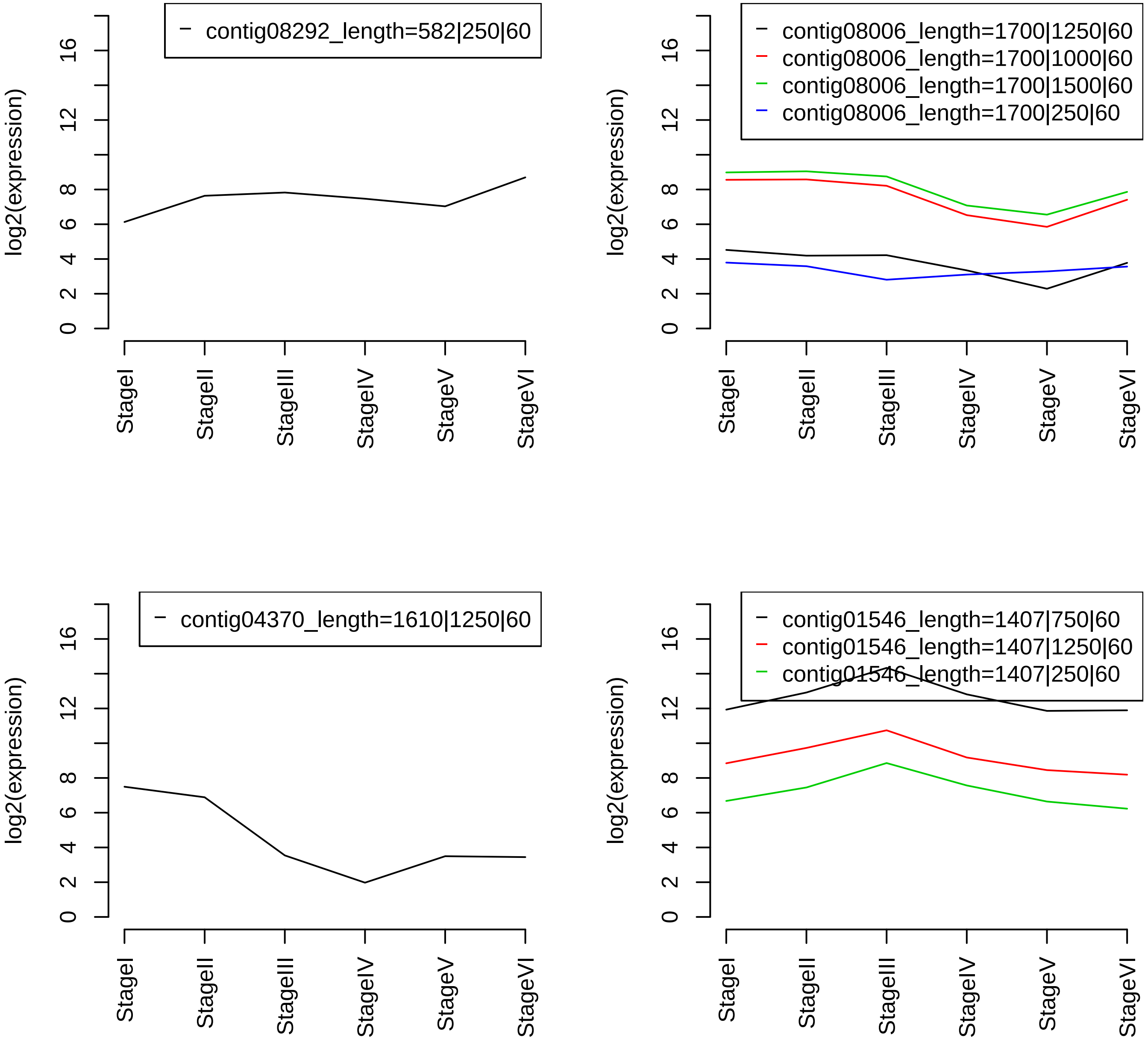

contig08292_length=582|250|60
contig08006_length=1700|1250|60
contig08006_length=1700|1000|60
contig08006_length=1700|1500|60
contig08006_length=1700|250|60
contig04370_length=1610|1250|60
contig01546_length=1407|750|60
contig01546_length=1407|1250|60
contig01546_length=1407|250|60
log2(expression)
0 2 4 6 8 12 16
StageI StageII StageIII StageIV StageV StageVI

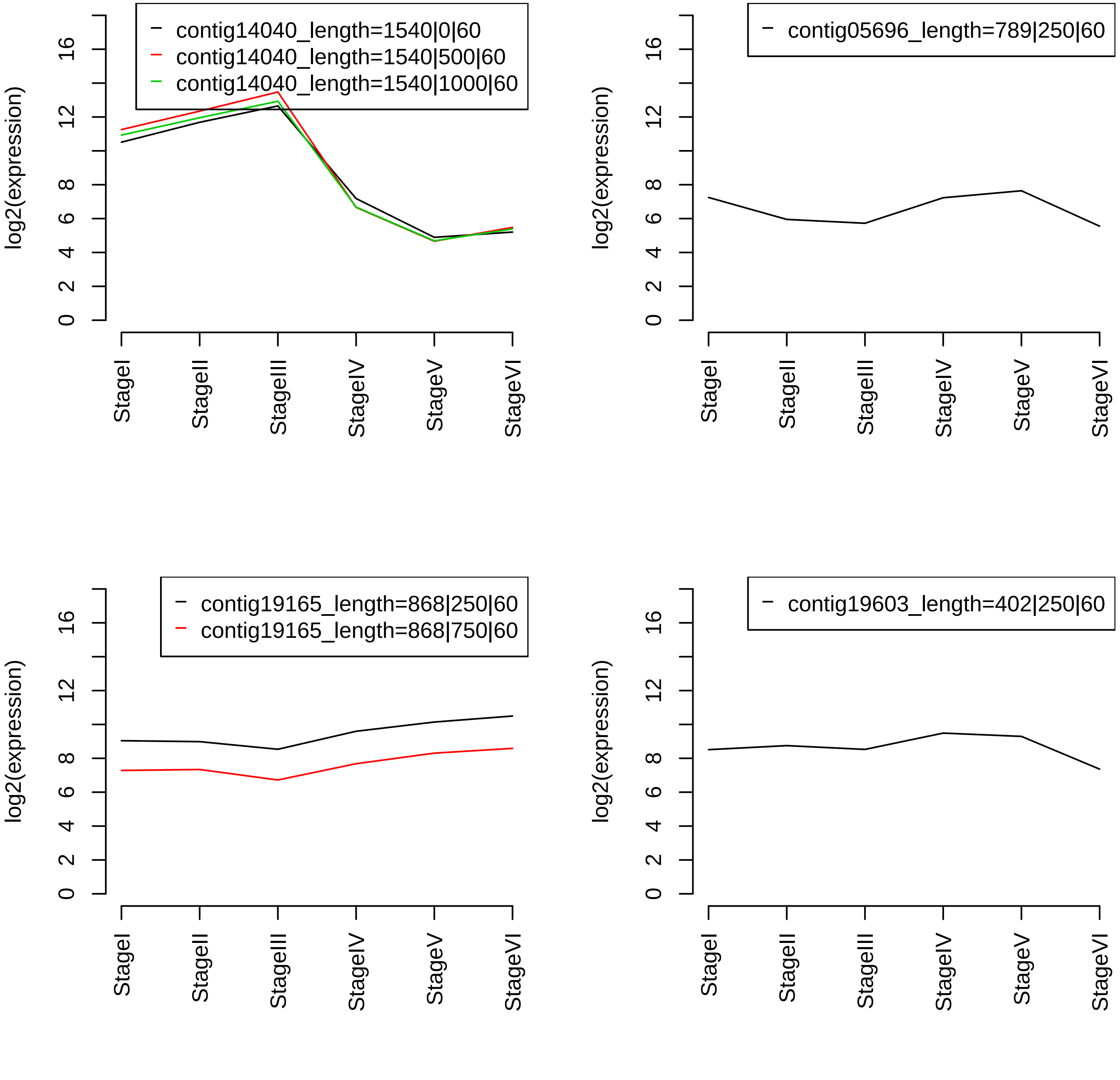
contig14040_length=1540|0|60
contig14040_length=1540|500|60
contig14040_length=1540|1000|60
contig05696_length=789|250|60
contig19165_length=868|250|60
contig19165_length=868|750|60
contig19603_length=402|250|60
log2(expression)
0
2
4
6
8
12
16
StageI
StageII
StageIII
StageIV
StageV
StageVI

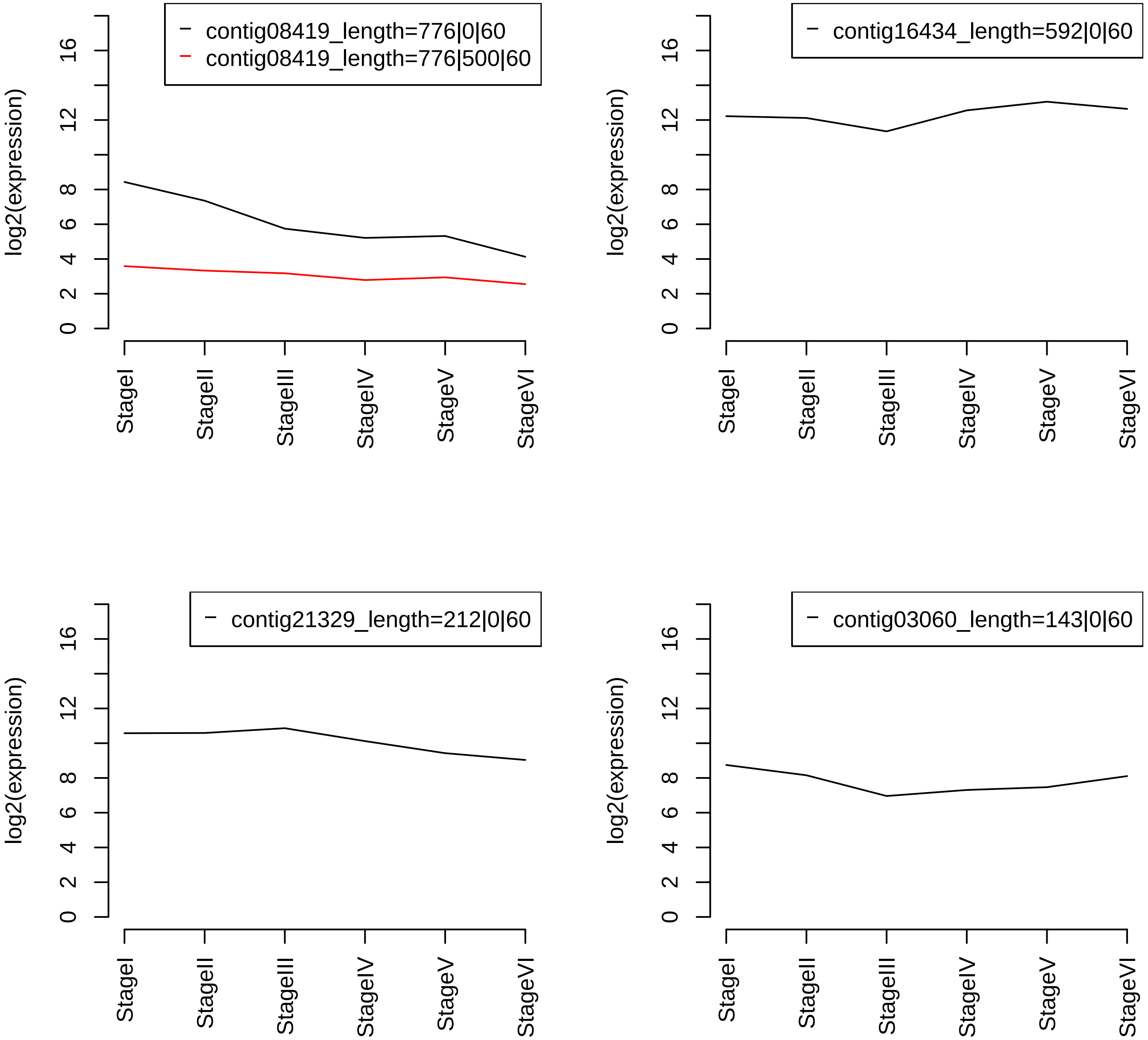

contig08419_length=776|0|60
contig08419_length=776|500|60
contig16434_length=592|0|60
contig21329_length=212|0|60
contig03060_length=143|0|60
log2(expression)
0
2
4
6
8
12
16
StageI
StageII
StageIII
StageIV
StageV
StageVI

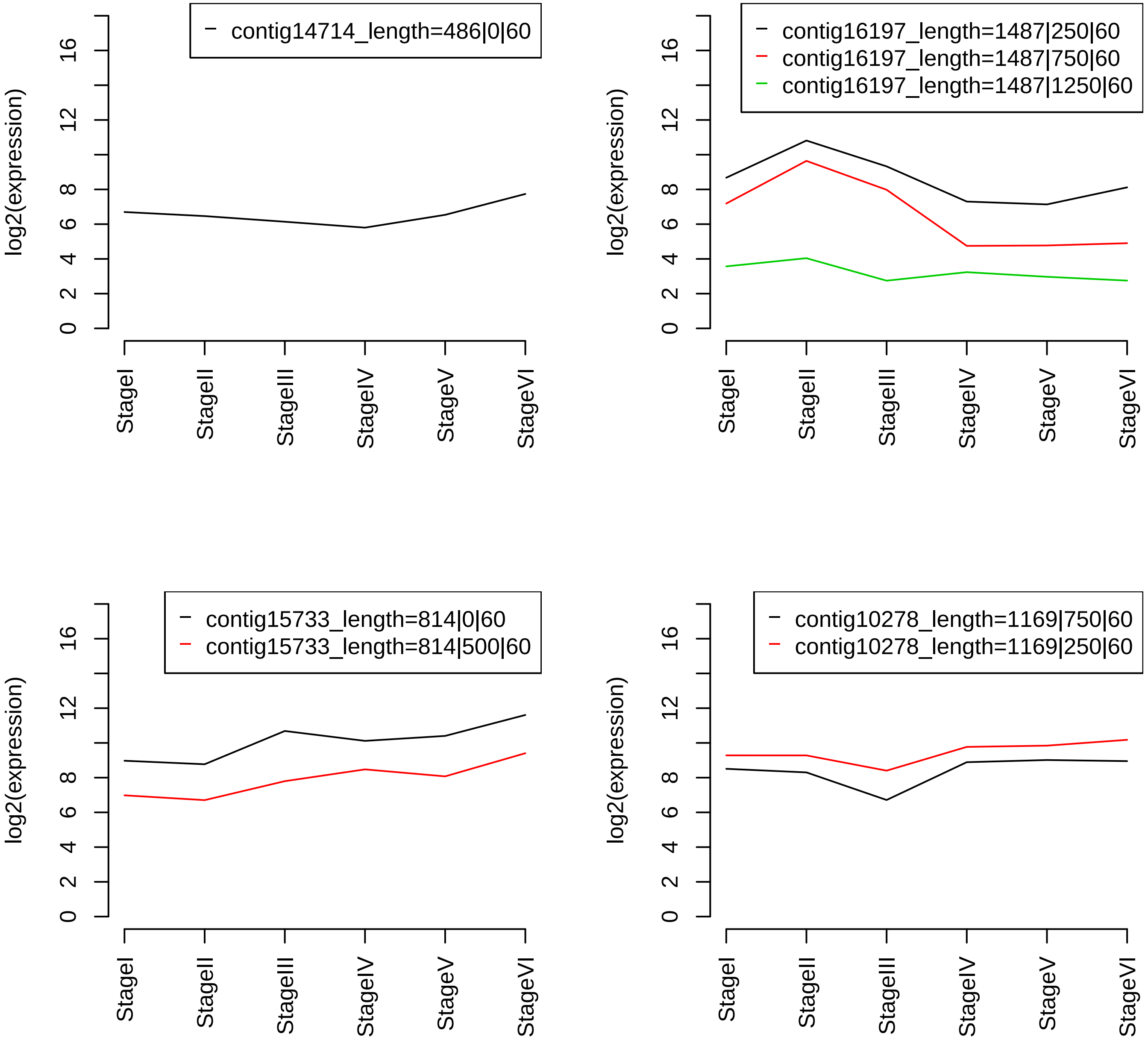
contig14714_length=486|0|60
contig16197_length=1487|250|60
contig16197_length=1487|750|60
contig16197_length=1487|1250|60
contig15733_length=814|0|60
contig15733_length=814|500|60
contig10278_length=1169|750|60
contig10278_length=1169|250|60
log2(expression)
0 2 4 6 8 12 16
StageI
StageII
StageIII
StageIV
StageV
StageVI

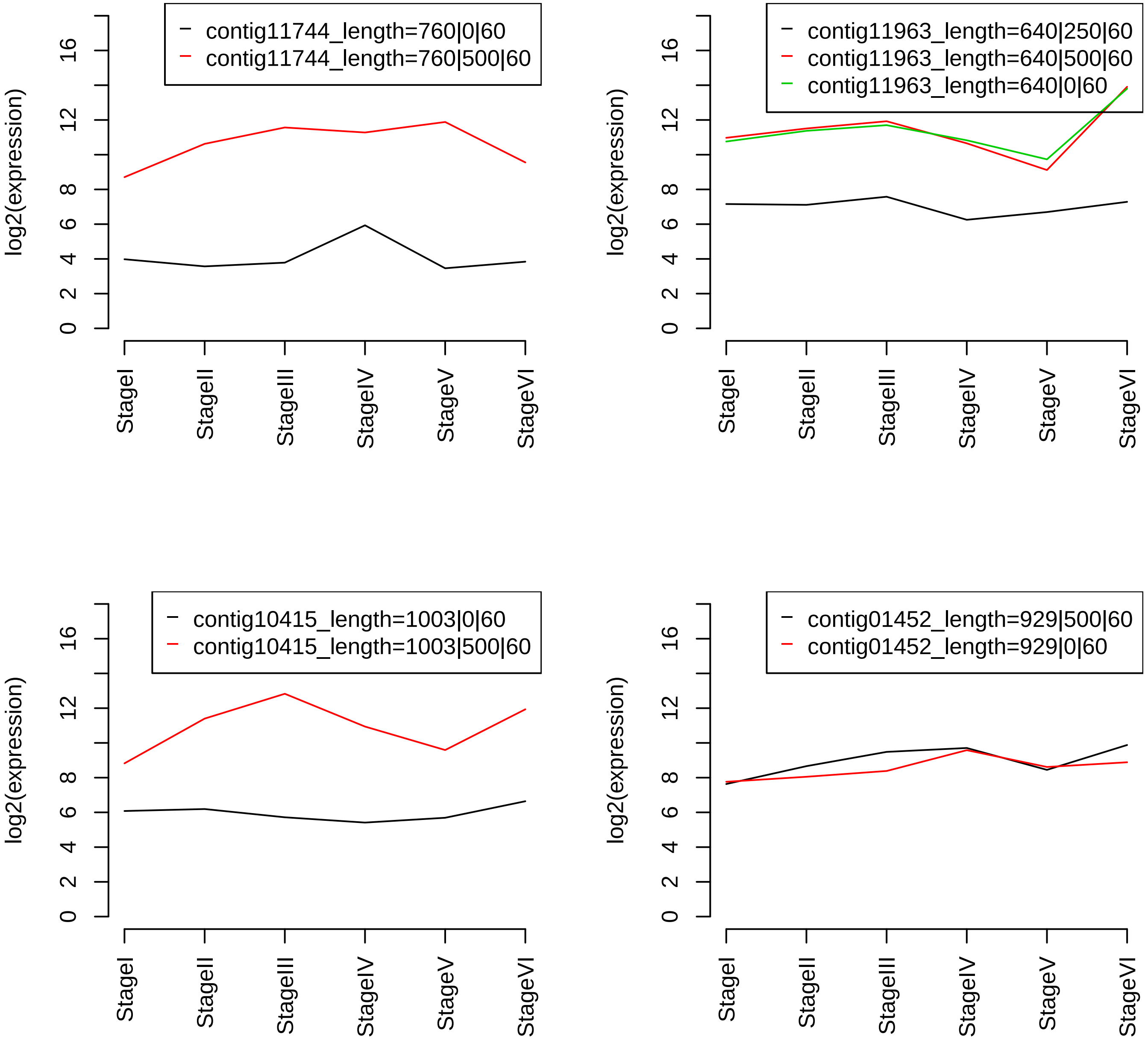
contig11744_length=760|0|60
contig11744_length=760|500|60
contig11963_length=640|250|60
contig11963_length=640|500|60
contig11963_length=640|0|60
contig10415_length=1003|0|60
contig10415_length=1003|500|60
contig01452_length=929|500|60
contig01452_length=929|0|60
log2(expression)
0
2
4
6
8
12
16
StageI
StageII
StageIII
StageIV
StageV
StageVI

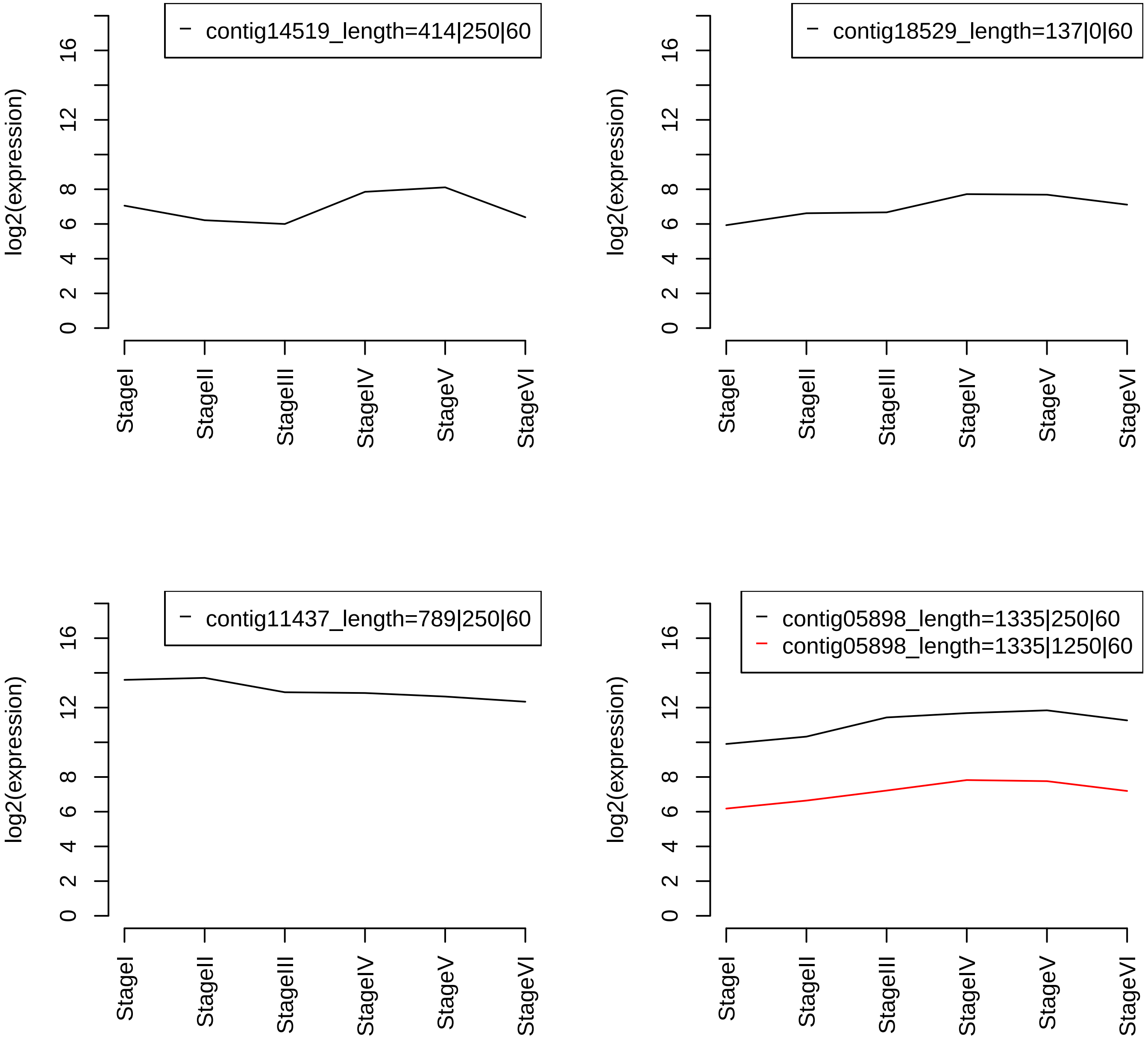
contig14519_length=414|250|60
contig18529_length=137|0|60
contig11437_length=789|250|60
contig05898_length=1335|250|60
contig05898_length=1335|1250|60
log2(expression)
0
2
4
6
8
12
16
StageI
StageII
StageIII
StageIV
StageV
StageVI

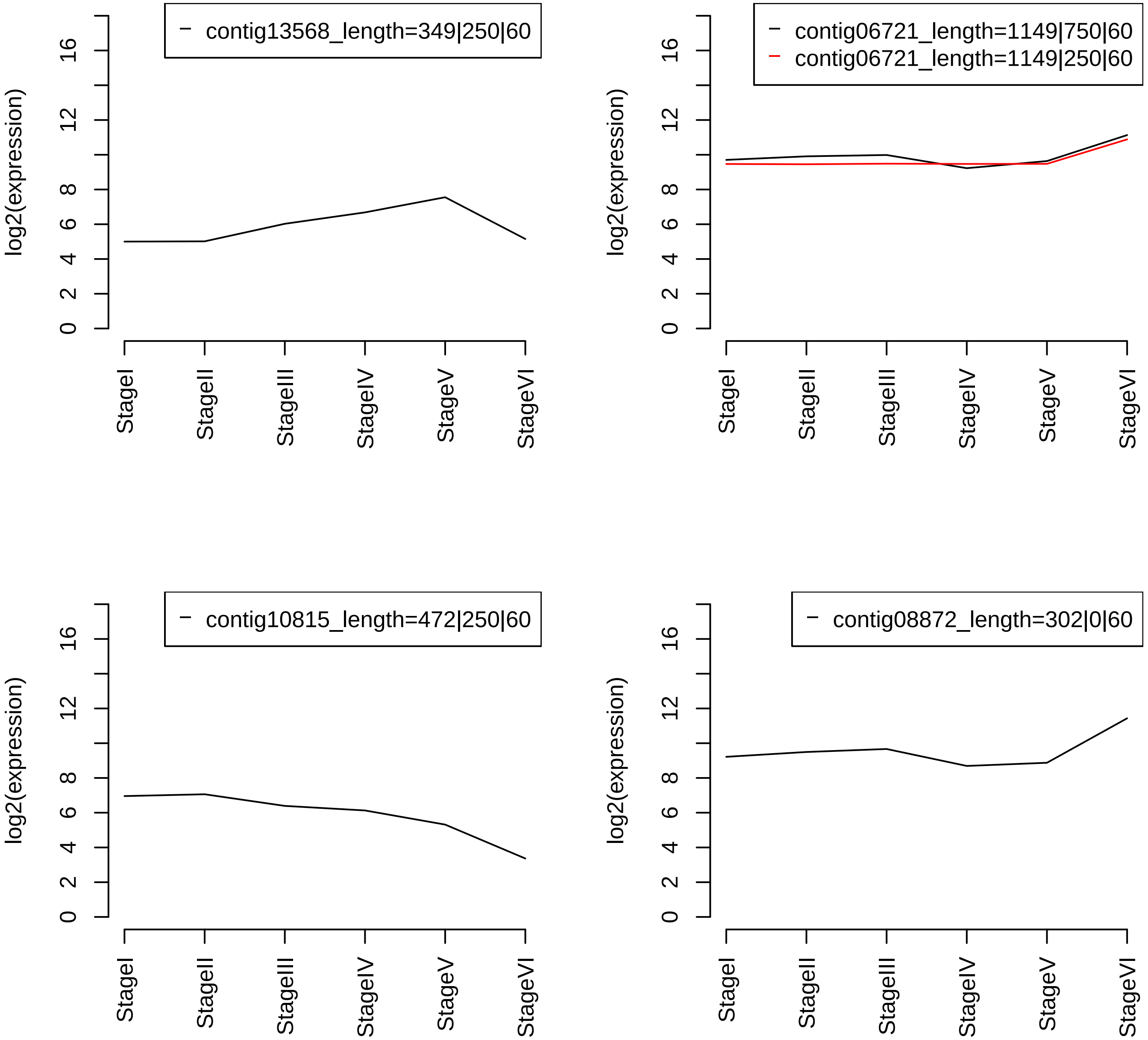

contig13568_length=349|250|60
contig06721_length=1149|750|60
contig06721_length=1149|250|60
contig10815_length=472|250|60
contig08872_length=302|0|60
log2(expression)
0
2
4
6
8
12
16
StageI
StageII
StageIII
StageIV
StageV
StageVI

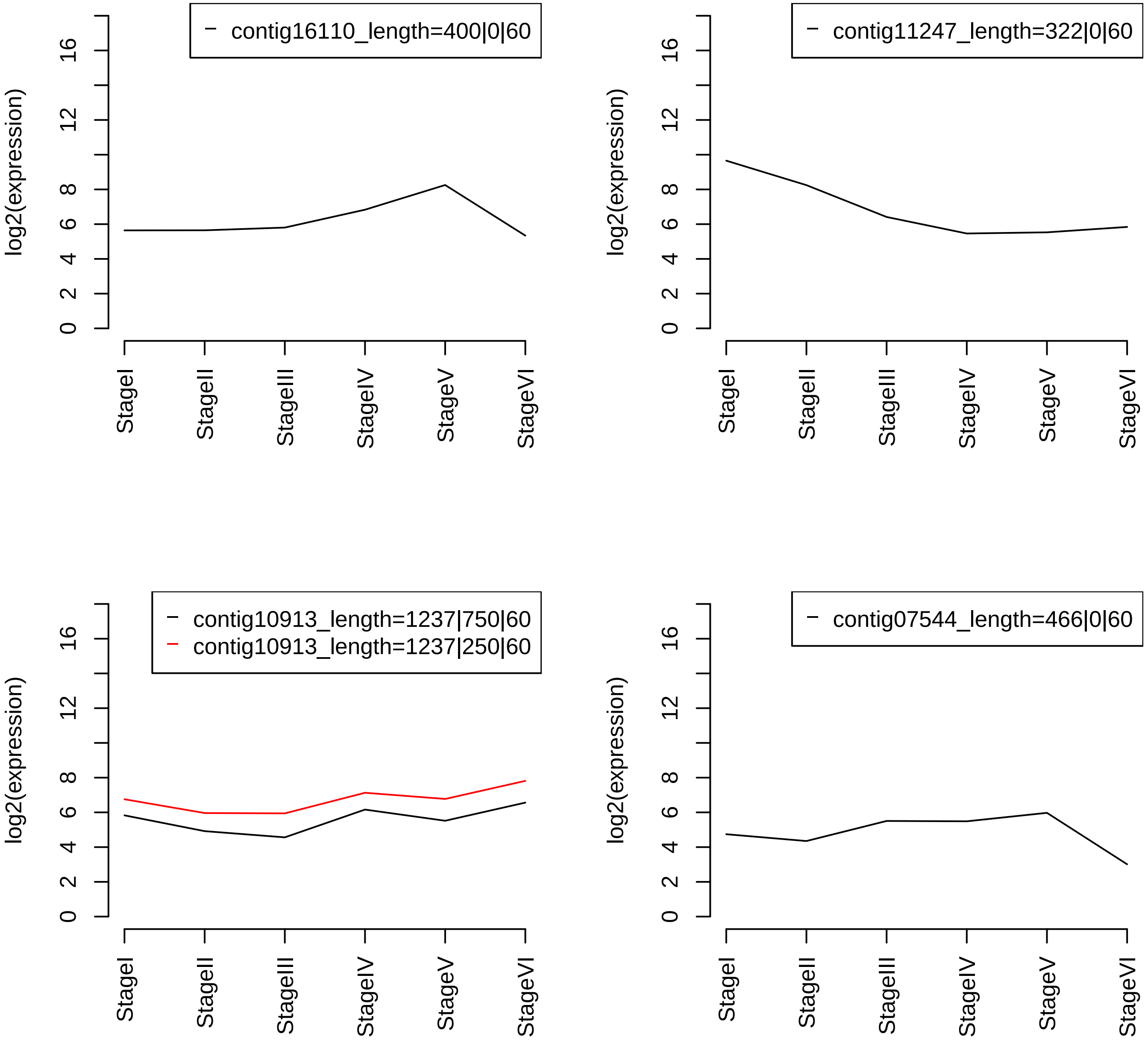
contig16110_length=400|0|60
contig11247_length=322|0|60
contig10913_length=1237|750|60
contig10913_length=1237|250|60
contig07544_length=466|0|60
log2(expression)
0
2
4
6
8
12
16
StageI
StageII
StageIII
StageIV
StageV
StageVI

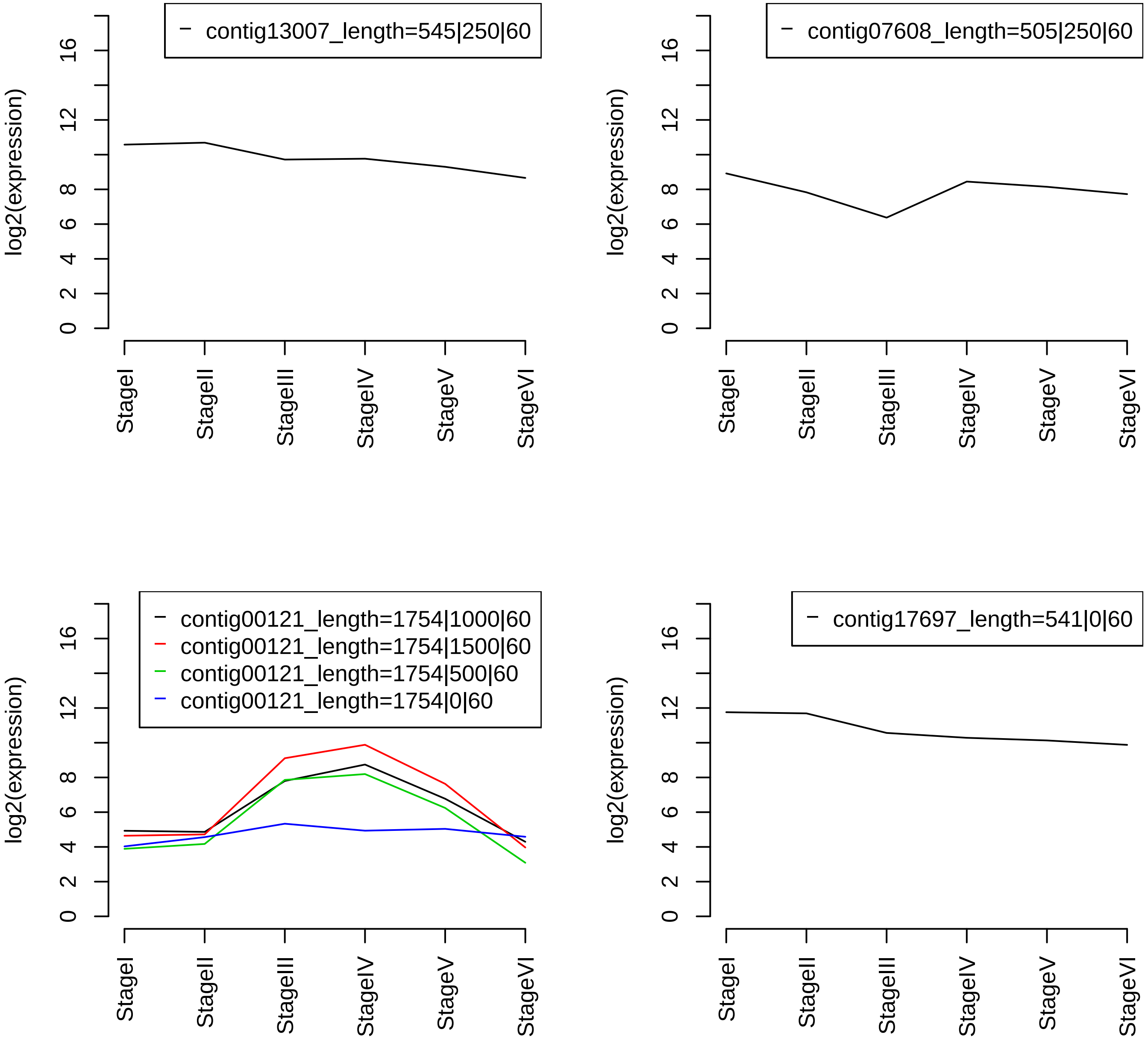
contig13007_length=545|250|60
contig07608_length=505|250|60
contig00121_length=1754|1000|60
contig00121_length=1754|1500|60
contig00121_length=1754|500|60
contig00121_length=1754|0|60
contig17697_length=541|0|60
log2(expression)
0
2
4
6
8
12
16
StageI
StageII
StageIII
StageIV
StageV
StageVI

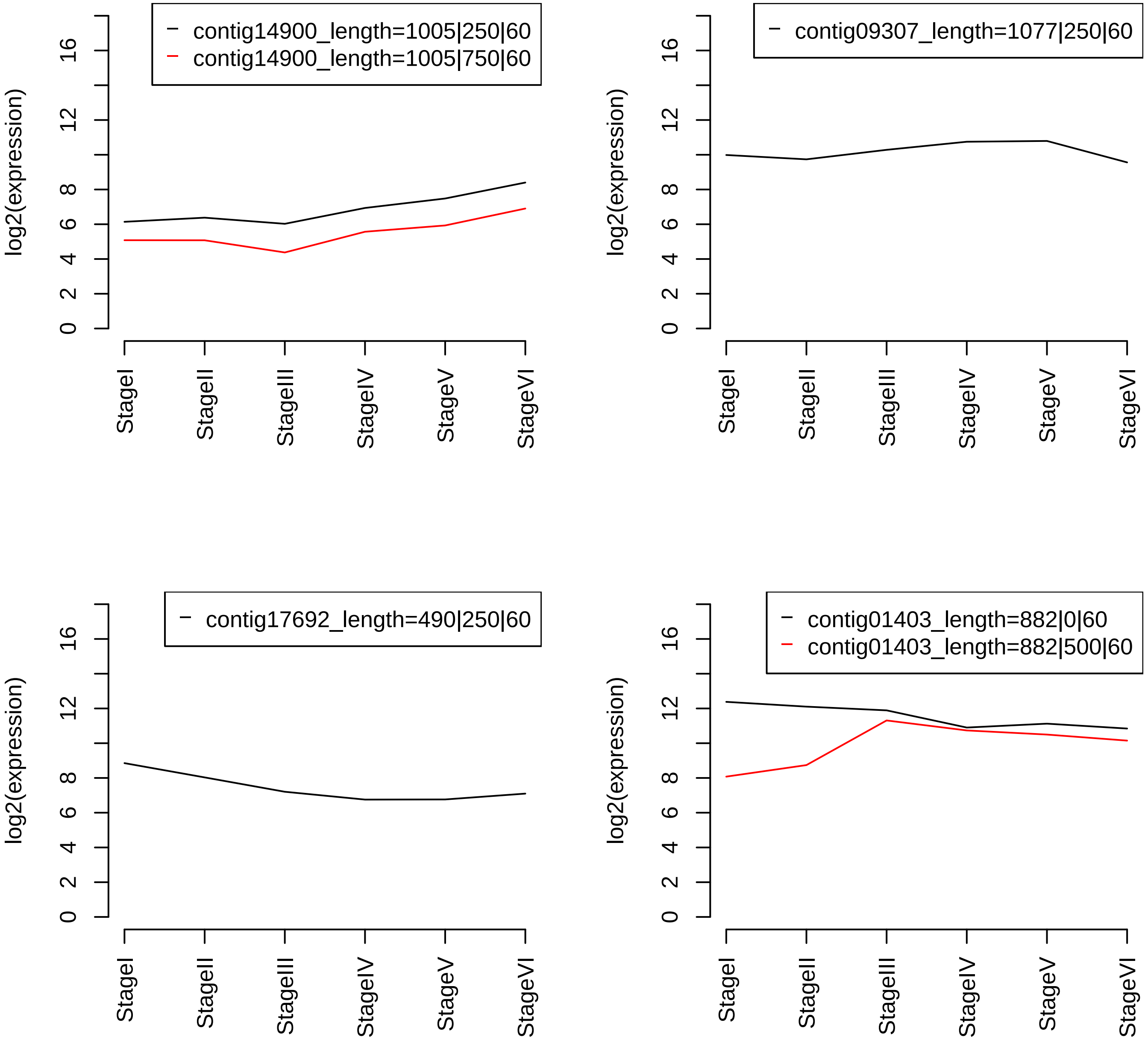
contig14900_length=1005|250|60
contig14900_length=1005|750|60
contig09307_length=1077|250|60
contig17692_length=490|250|60
contig01403_length=882|0|60
contig01403_length=882|500|60
log2(expression)
0
2
4
6
8
12
16
StageI
StageII
StageIII
StageIV
StageV
StageVI

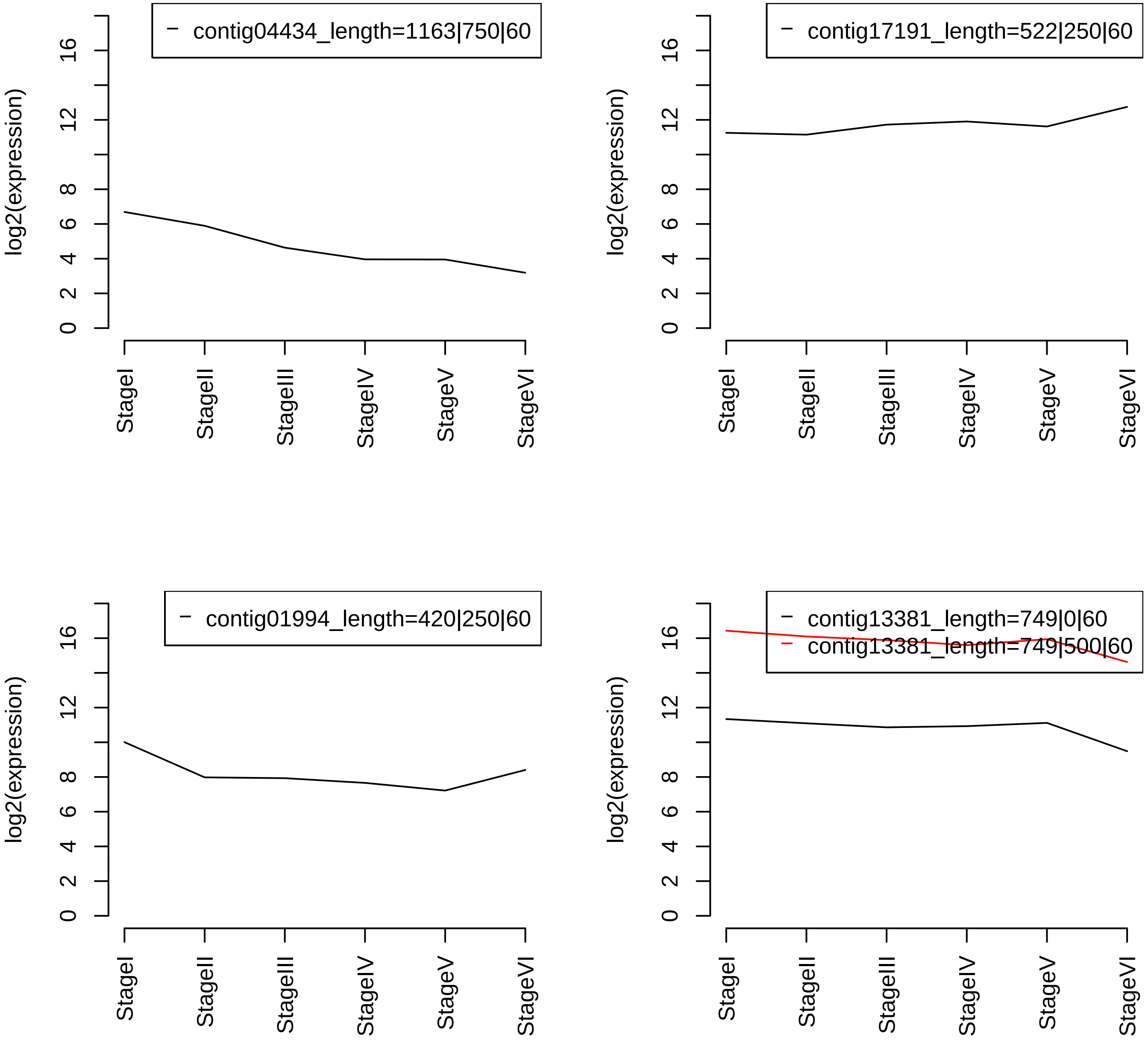
contig04434_length=1163|750|60
contig17191_length=522|250|60
contig01994_length=420|250|60
contig13381_length=749|0|60
contig13381_length=749|500|60
log2(expression)
0
2
4
6
8
12
16
StageI
StageII
StageIII
StageIV
StageV
StageVI

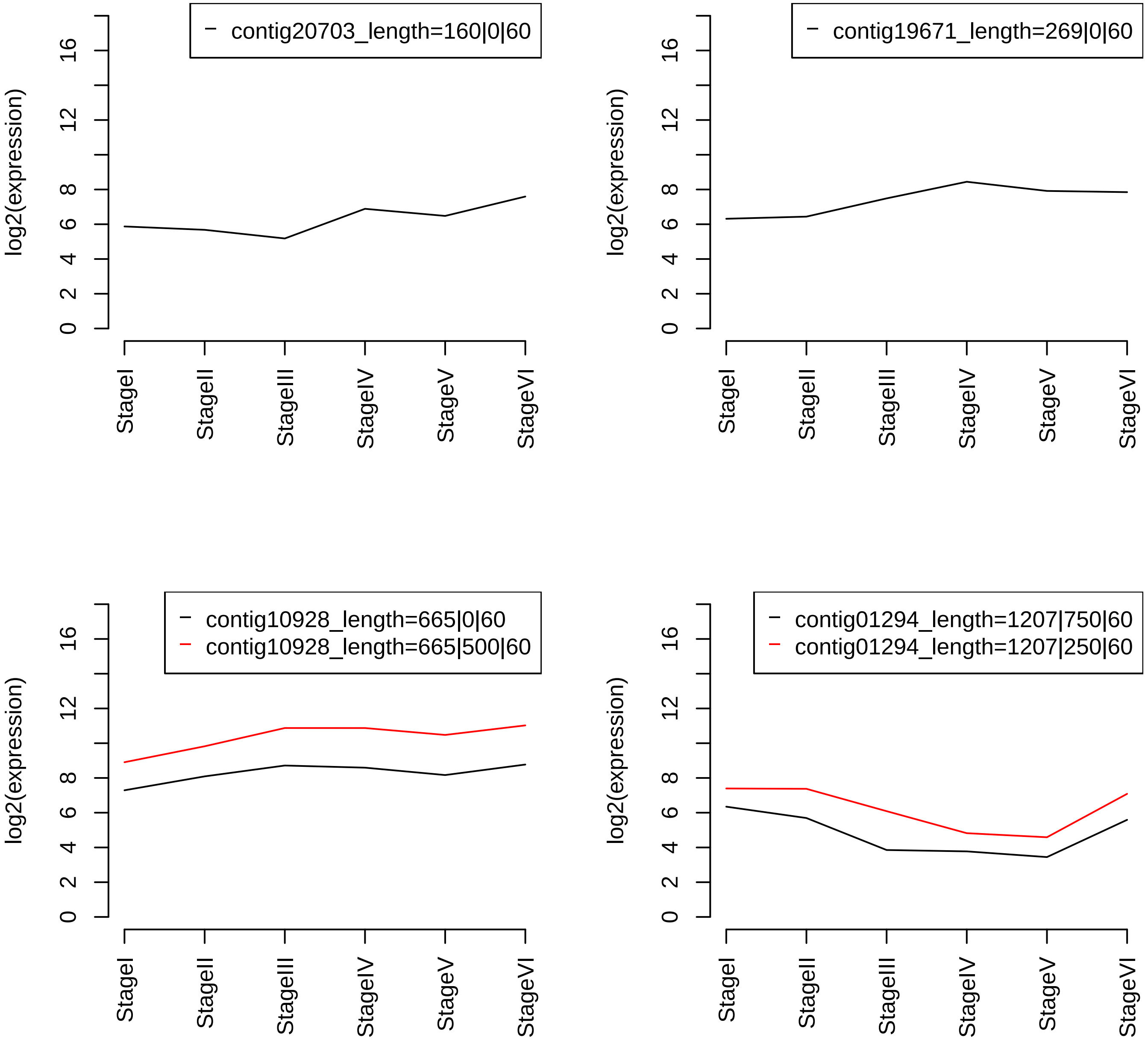

contig20703_length=160|0|60
contig19671_length=269|0|60
contig10928_length=665|0|60
contig10928_length=665|500|60
contig01294_length=1207|750|60
contig01294_length=1207|250|60
log2(expression)
0
2
4
6
8
12
16
StageI
StageII
StageIII
StageIV
StageV
StageVI

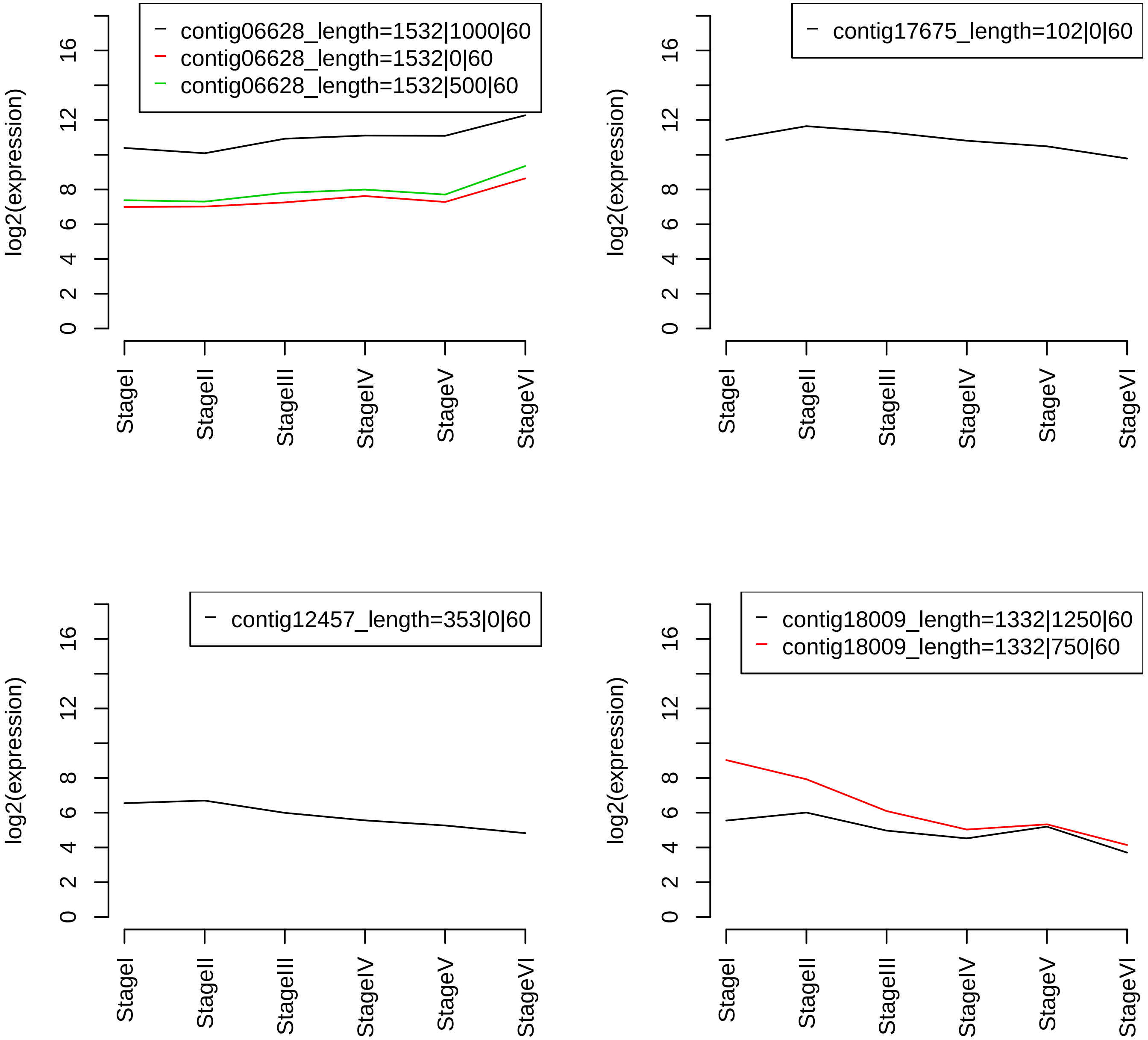

contig06628_length=1532|1000|60
contig06628_length=1532|0|60
contig06628_length=1532|500|60
contig17675_length=102|0|60
contig12457_length=353|0|60
contig18009_length=1332|1250|60
contig18009_length=1332|750|60
log2(expression)
0
2
4
6
8
12
16
StageI
StageII
StageIII
StageIV
StageV
StageVI

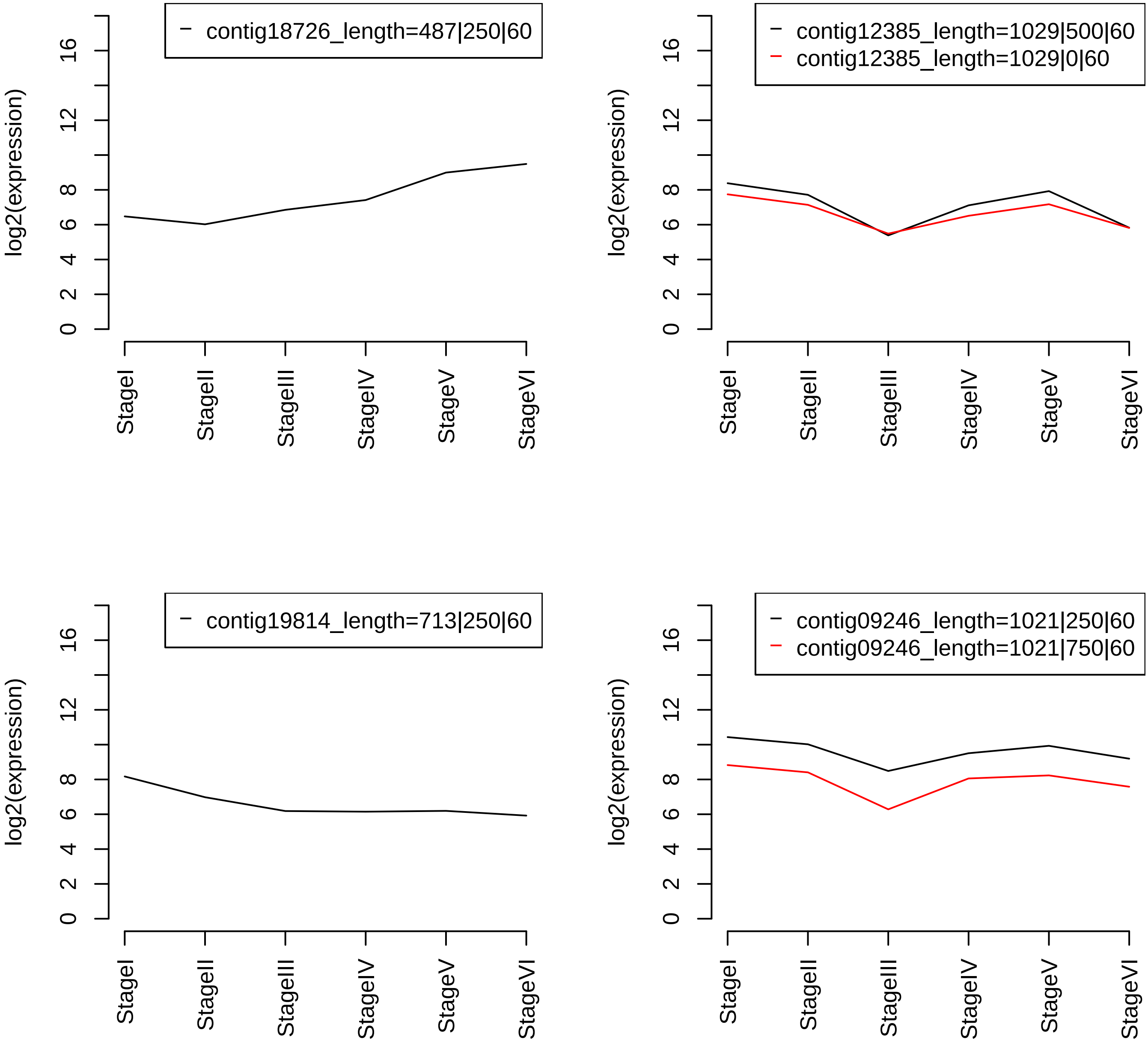
contig18726_length=487|250|60
contig12385_length=1029|500|60
contig12385_length=1029|0|60
contig19814_length=713|250|60
contig09246_length=1021|250|60
contig09246_length=1021|750|60
log2(expression)
0
2
4
6
8
12
16
StageI
StageII
StageIII
StageIV
StageV
StageVI

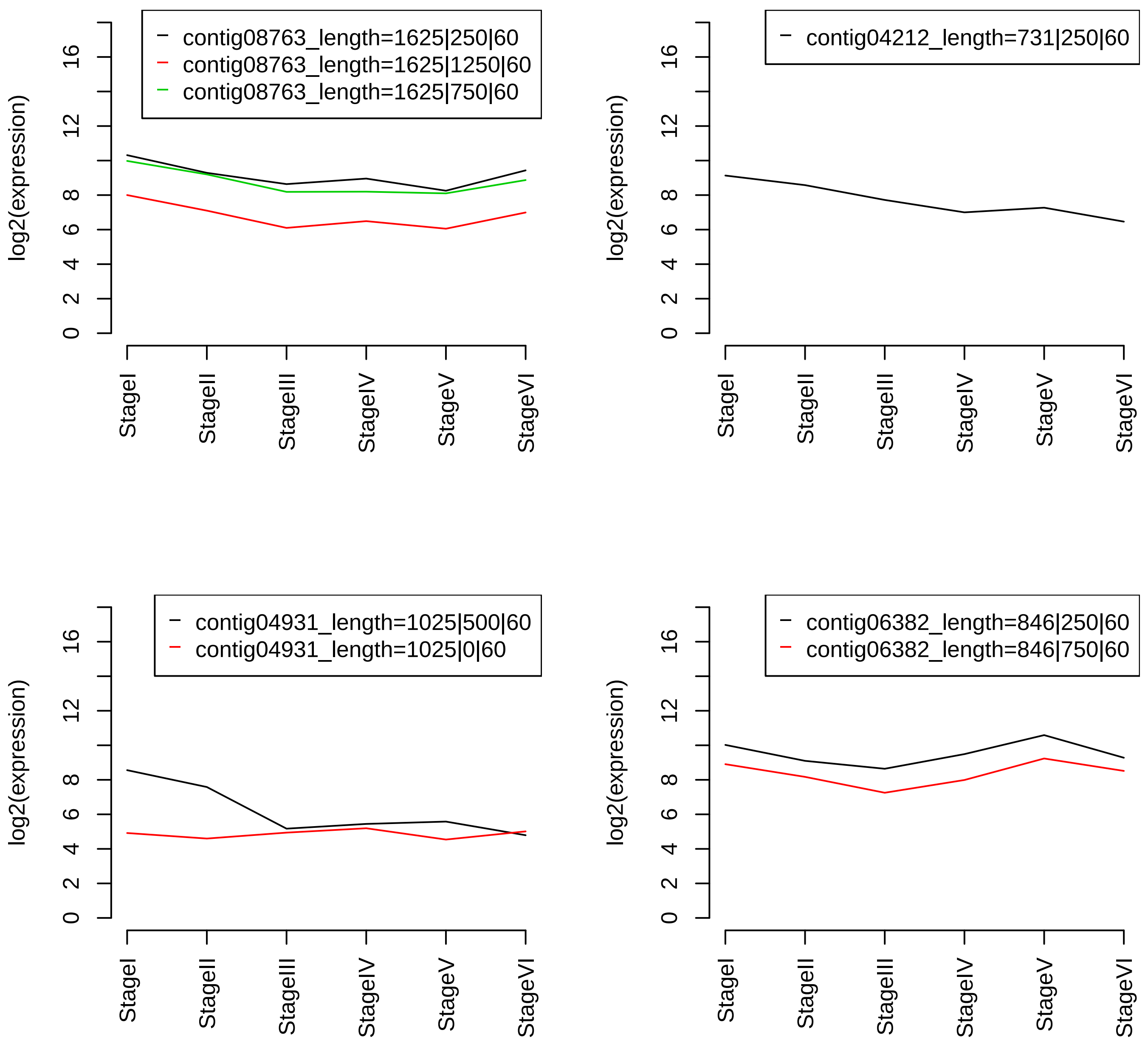
contig08763_length=1625|250|60
contig08763_length=1625|1250|60
contig08763_length=1625|750|60
contig04212_length=731|250|60
contig04931_length=1025|500|60
contig04931_length=1025|0|60
contig06382_length=846|250|60
contig06382_length=846|750|60
log2(expression)
StageI
StageII
StageIII
StageIV
StageV
StageVI

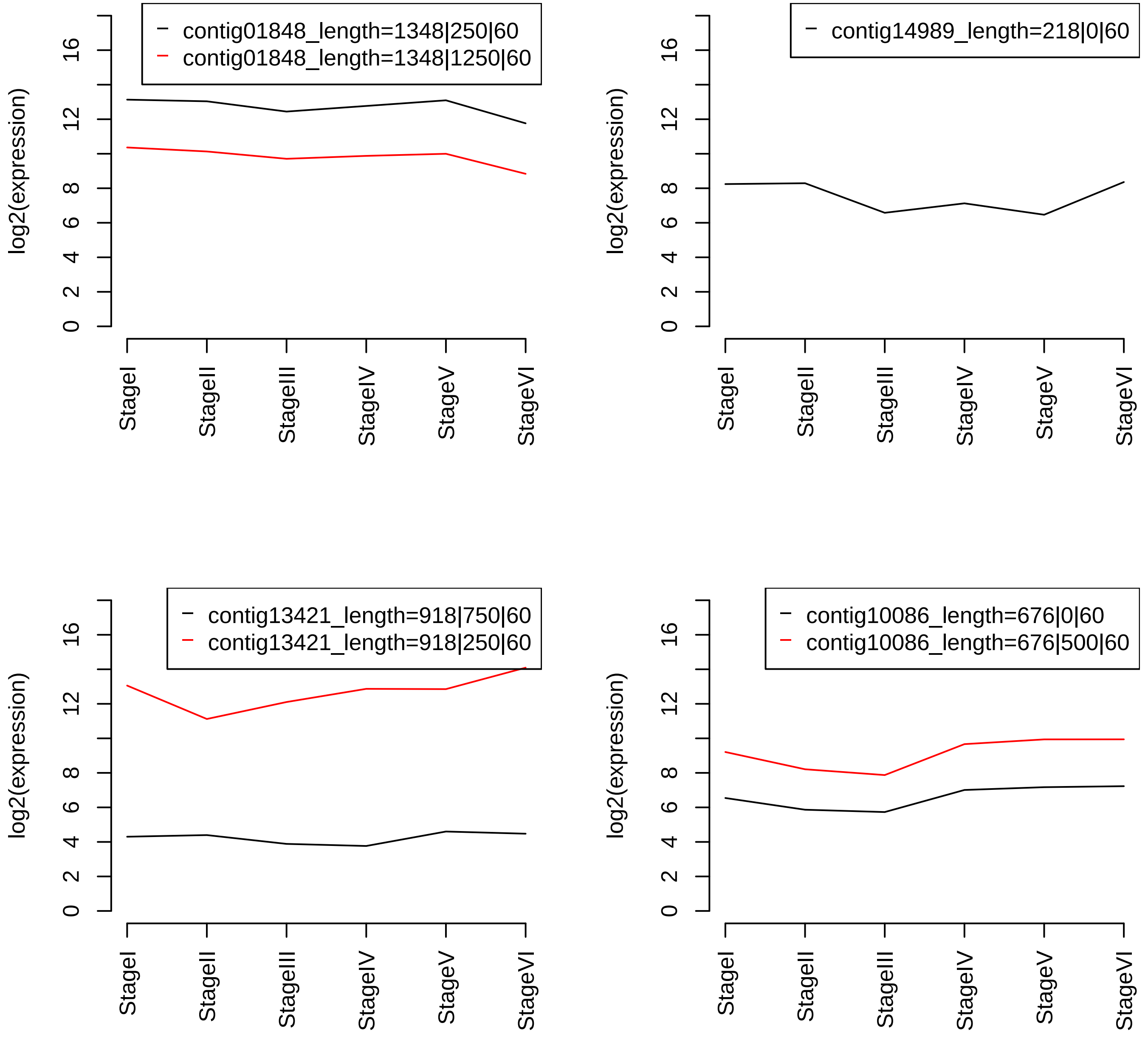

contig01848_length=1348|250|60
contig01848_length=1348|1250|60
contig14989_length=218|0|60
contig13421_length=918|750|60
contig13421_length=918|250|60
contig10086_length=676|0|60
contig10086_length=676|500|60
log2(expression)
0
2
4
6
8
12
16
StageI
StageII
StageIII
StageIV
StageV
StageVI

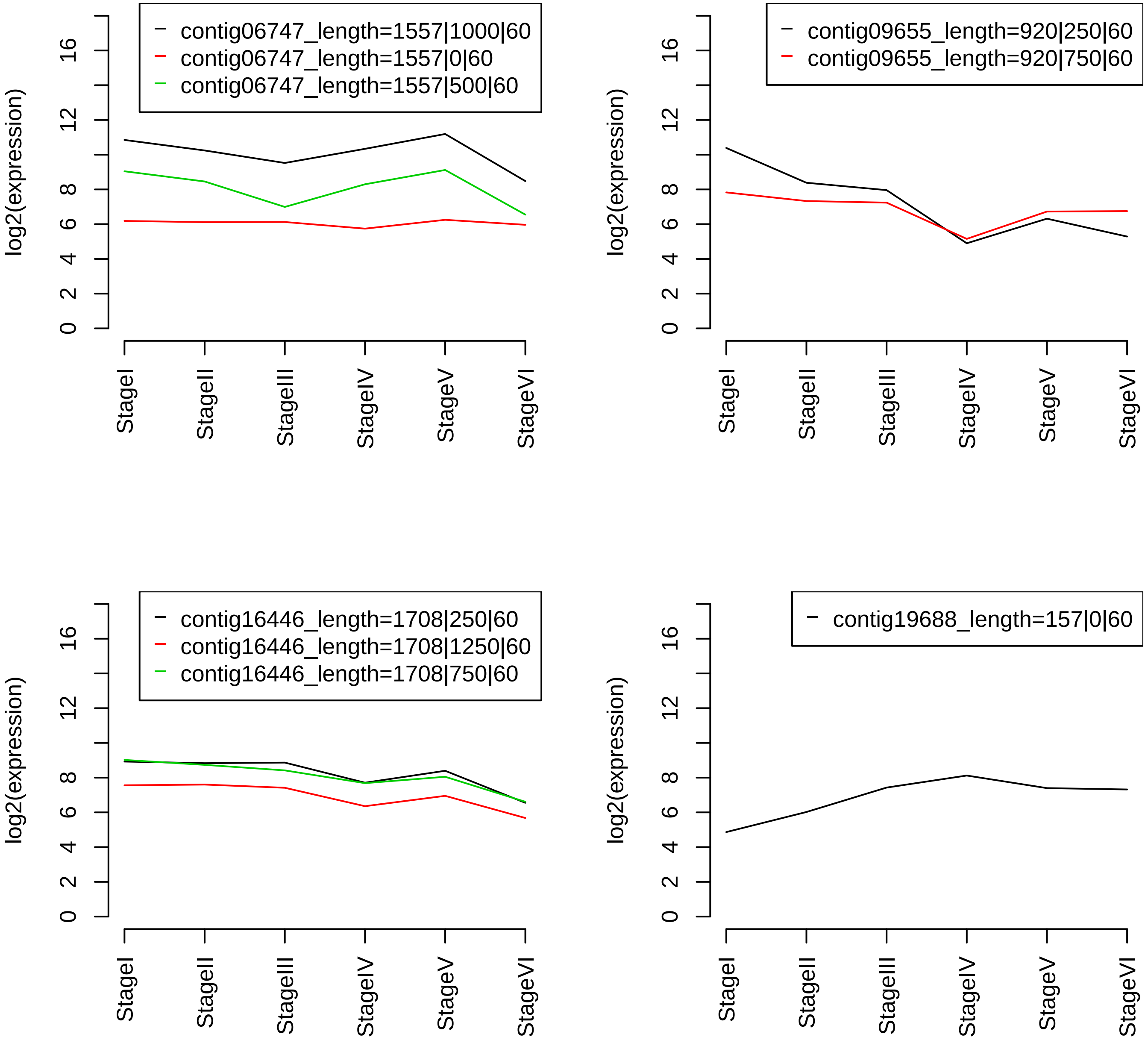
contig06747_length=1557|1000|60
contig06747_length=1557|0|60
contig06747_length=1557|500|60
contig09655_length=920|250|60
contig09655_length=920|750|60
contig16446_length=1708|250|60
contig16446_length=1708|1250|60
contig16446_length=1708|750|60
contig19688_length=157|0|60
log2(expression)
StageI
StageII
StageIII
StageIV
StageV
StageVI

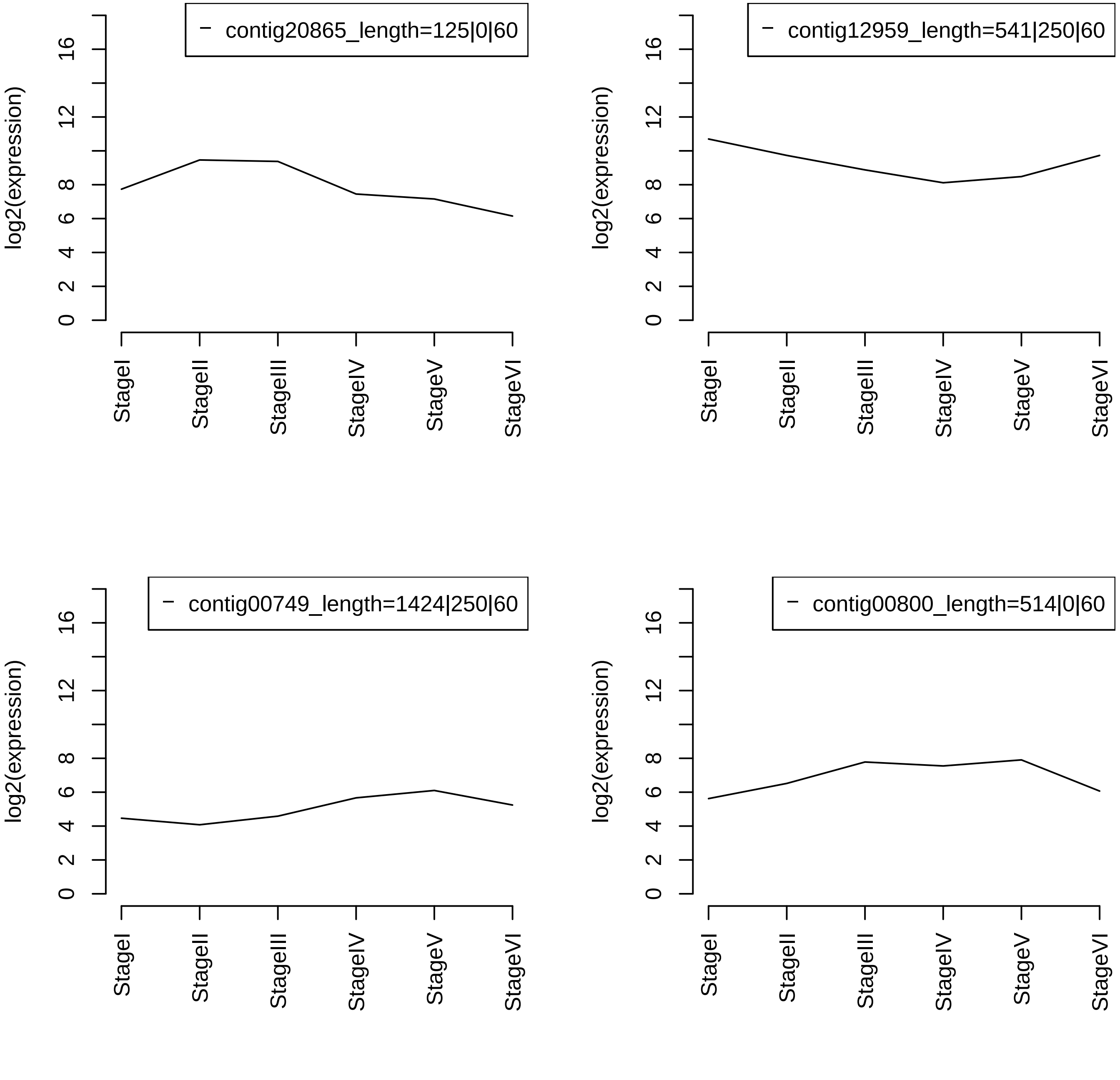
contig20865_length=125|0|60
contig12959_length=541|250|60
contig00749_length=1424|250|60
contig00800_length=514|0|60
log2(expression)
0
2
4
6
8
12
16
StageI
StageII
StageIII
StageIV
StageV
StageVI

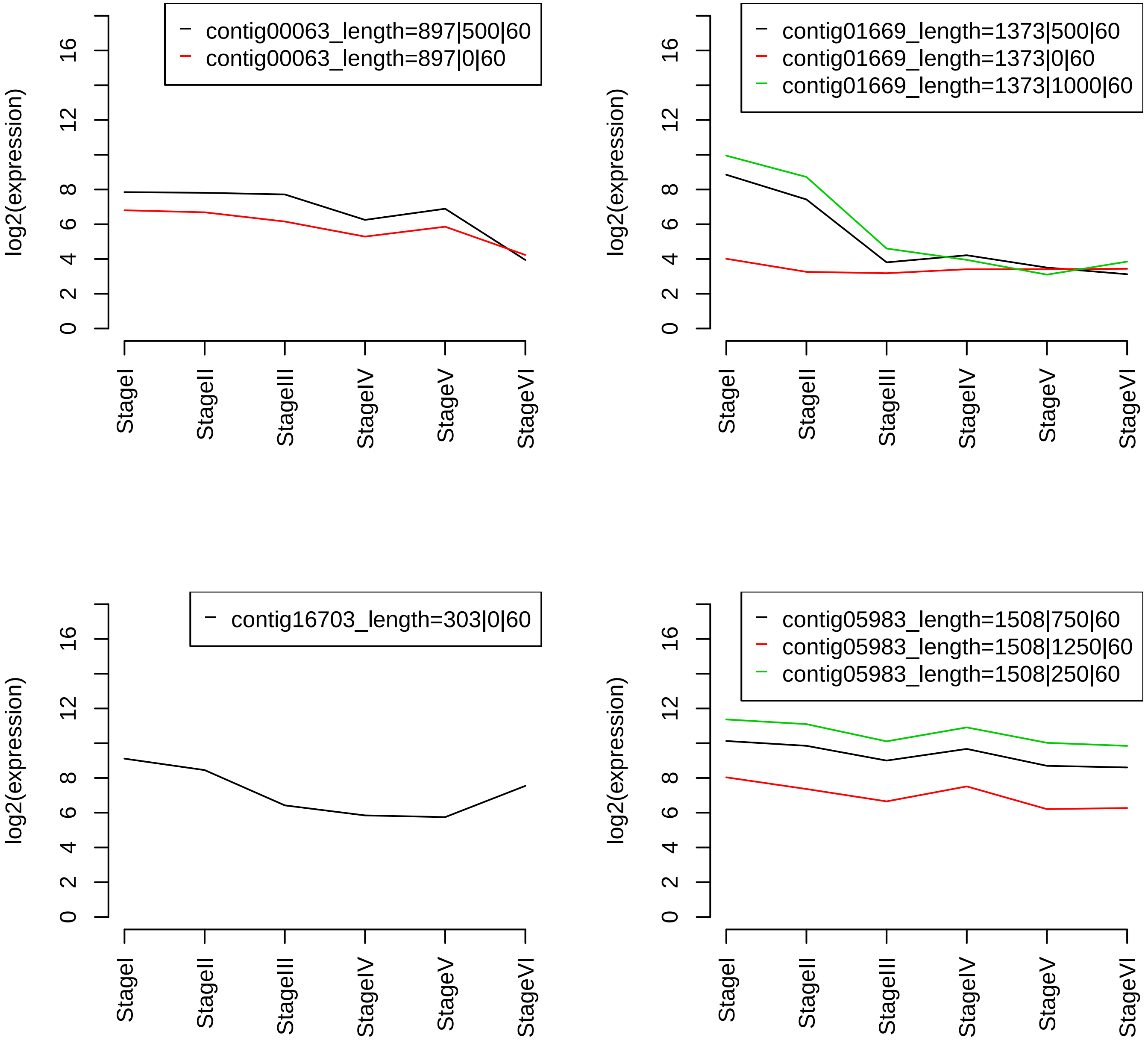

contig00063_length=897|500|60
contig00063_length=897|0|60
contig01669_length=1373|500|60
contig01669_length=1373|0|60
contig01669_length=1373|1000|60
contig16703_length=303|0|60
contig05983_length=1508|750|60
contig05983_length=1508|1250|60
contig05983_length=1508|250|60
log2(expression)
0
2
4
6
8
12
16
StageI
StageII
StageIII
StageIV
StageV
StageVI

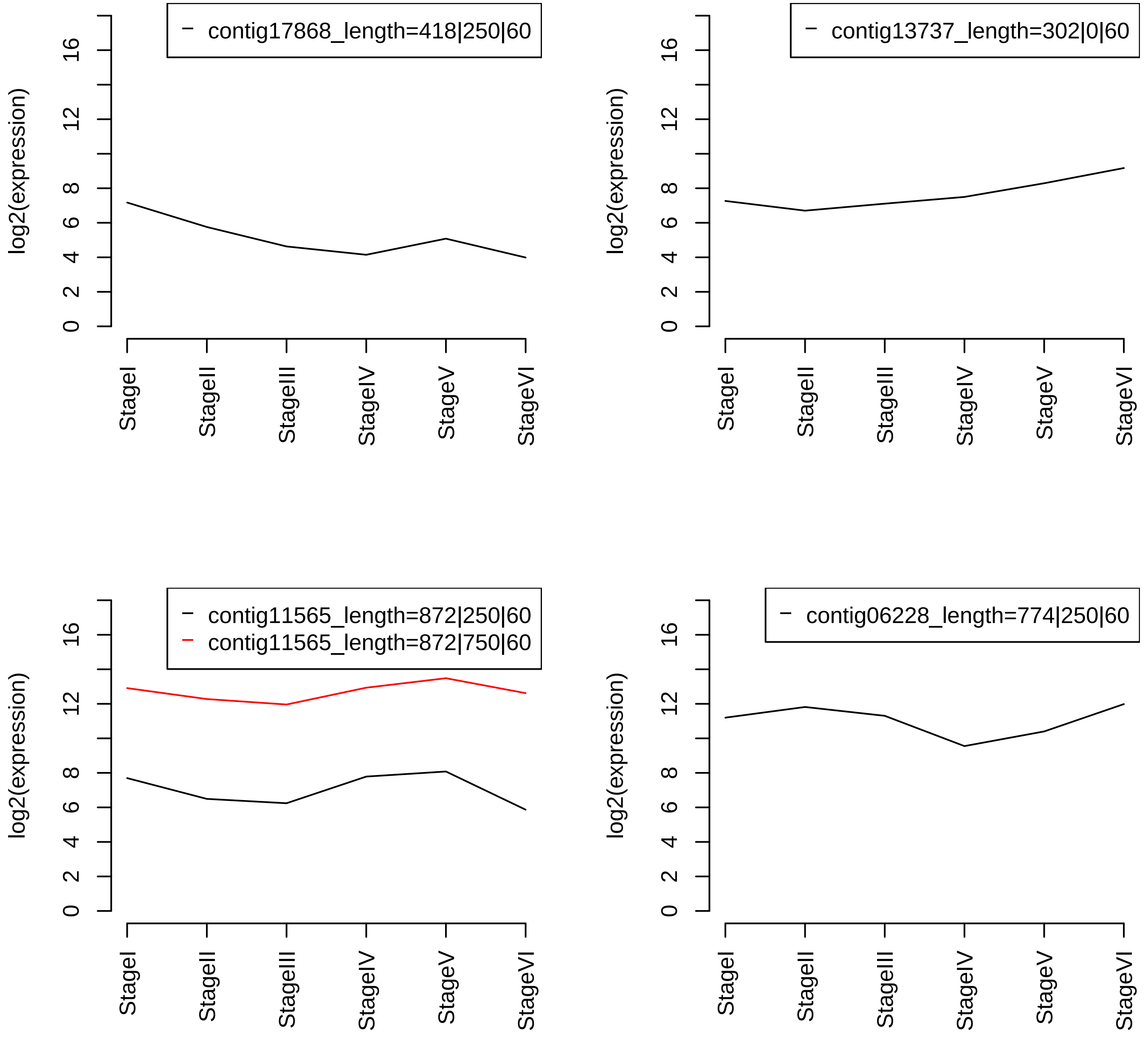

contig17868_length=418|250|60
contig13737_length=302|0|60
contig11565_length=872|250|60
contig11565_length=872|750|60
contig06228_length=774|250|60
log2(expression)
0 2 4 6 8 12 16
StageI
StageII
StageIII
StageIV
StageV
StageVI

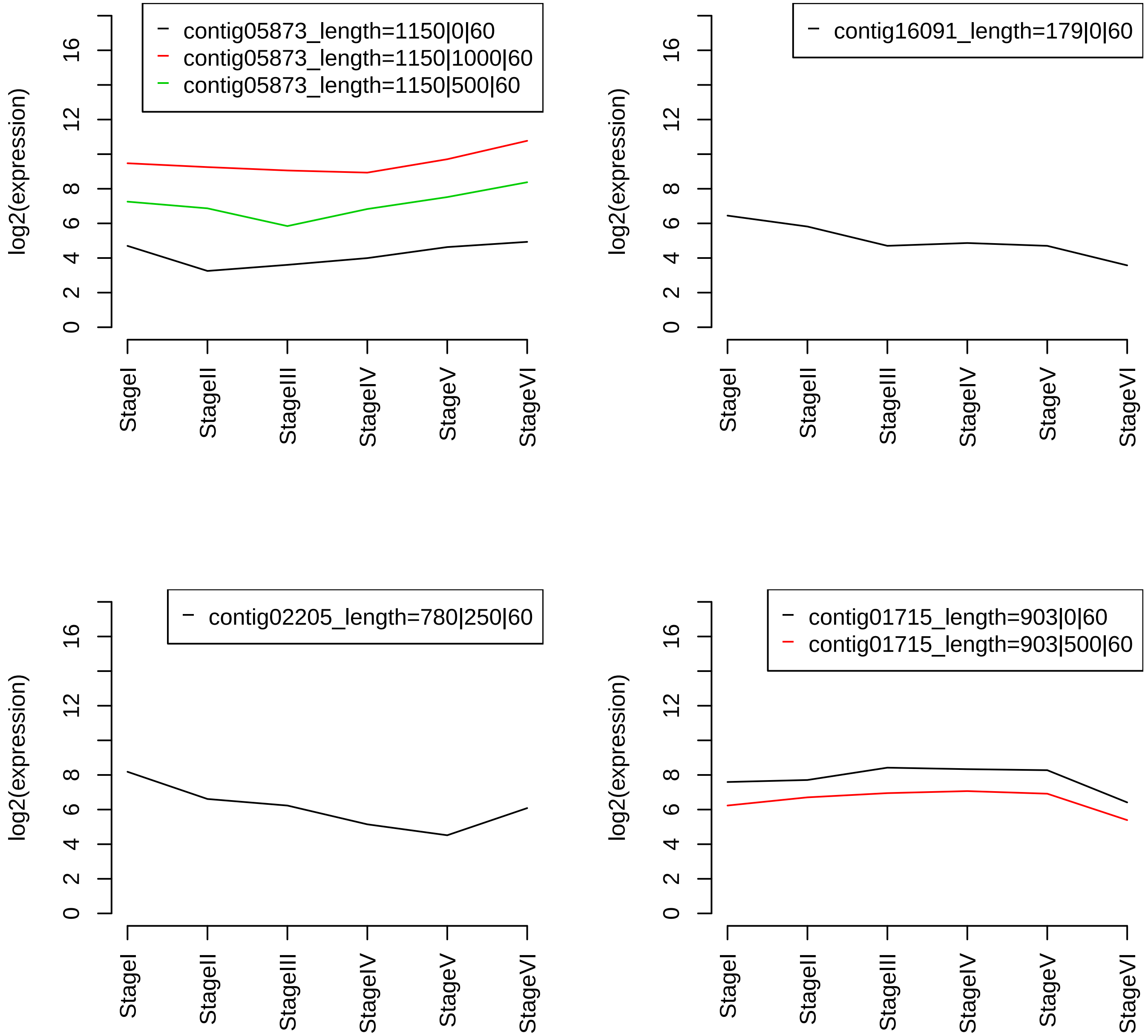

contig05873_length=1150|0|60
contig05873_length=1150|1000|60
contig05873_length=1150|500|60
contig16091_length=179|0|60
contig02205_length=780|250|60
contig01715_length=903|0|60
contig01715_length=903|500|60
log2(expression)
0
2
4
6
8
12
16
StageI
StageII
StageIII
StageIV
StageV
StageVI

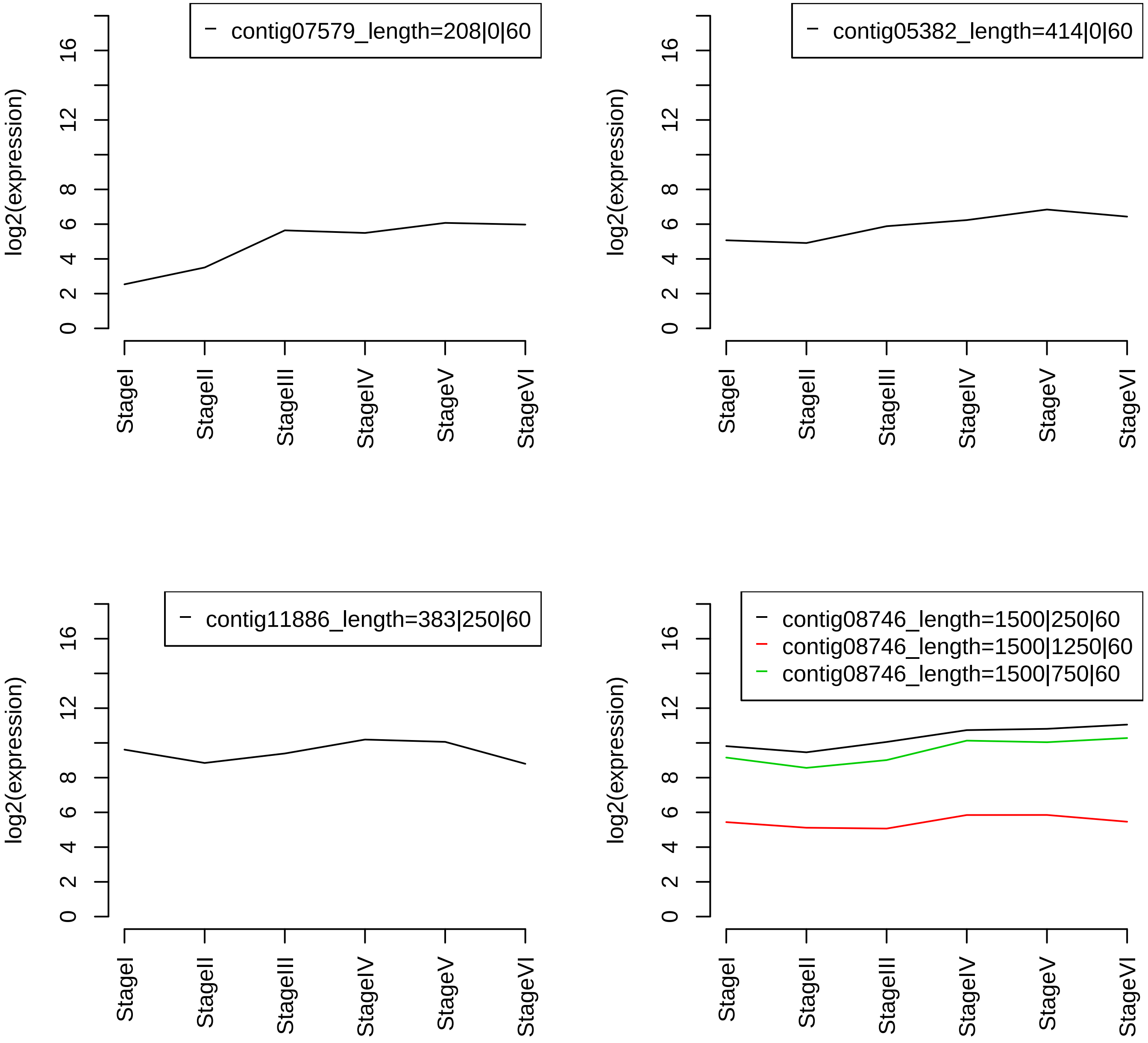
contig07579_length=208|0|60
contig05382_length=414|0|60
contig11886_length=383|250|60
contig08746_length=1500|250|60
contig08746_length=1500|1250|60
contig08746_length=1500|750|60
log2(expression)
0
2
4
6
8
12
16
StageI
StageII
StageIII
StageIV
StageV
StageVI

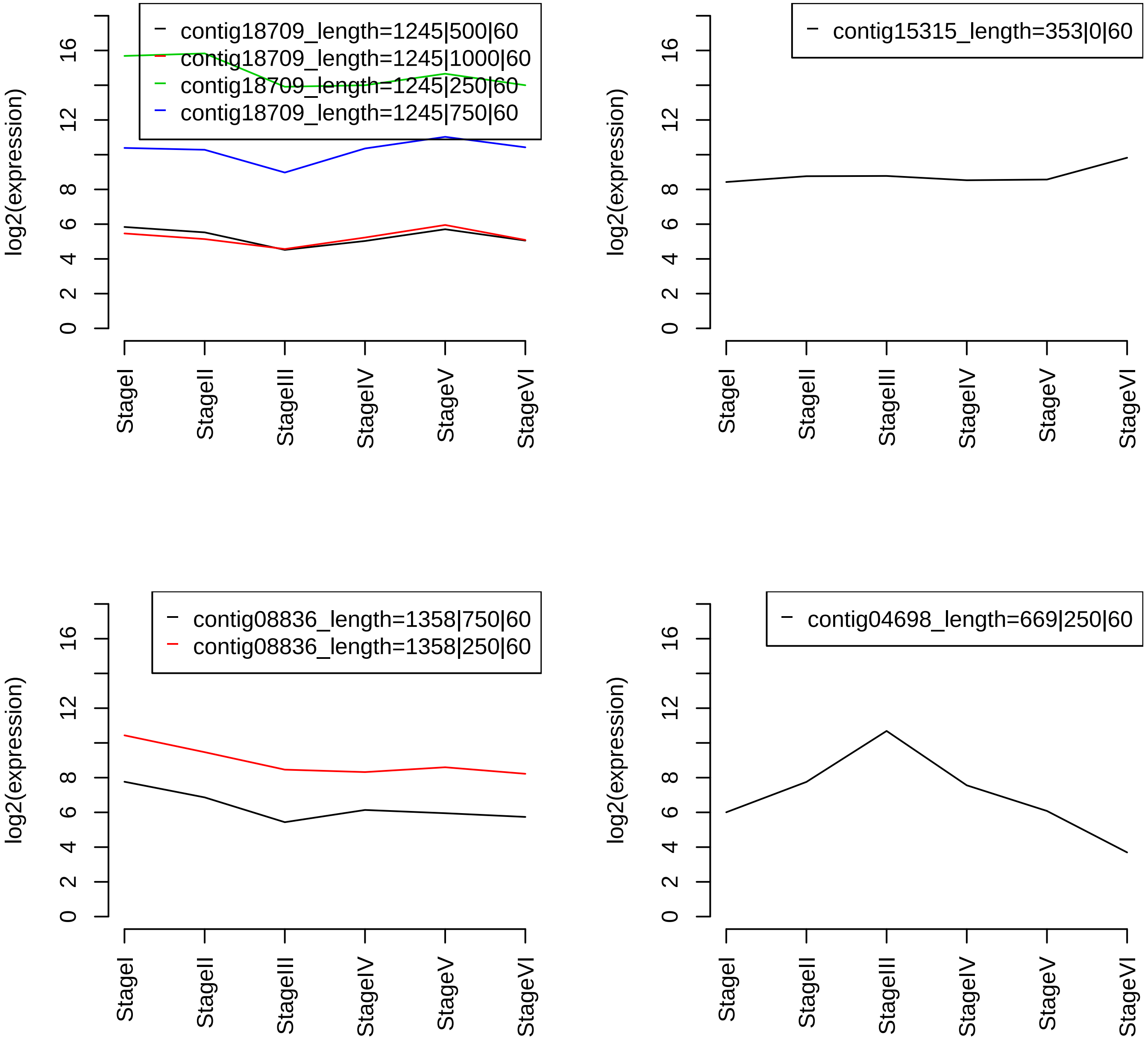

contig18709_length=1245|500|60
contig18709_length=1245|1000|60
contig18709_length=1245|250|60
contig18709_length=1245|750|60
contig15315_length=353|0|60
contig08836_length=1358|750|60
contig08836_length=1358|250|60
contig04698_length=669|250|60
log2(expression)
0
2
4
6
8
12
16
StageI
StageII
StageIII
StageIV
StageV
StageVI

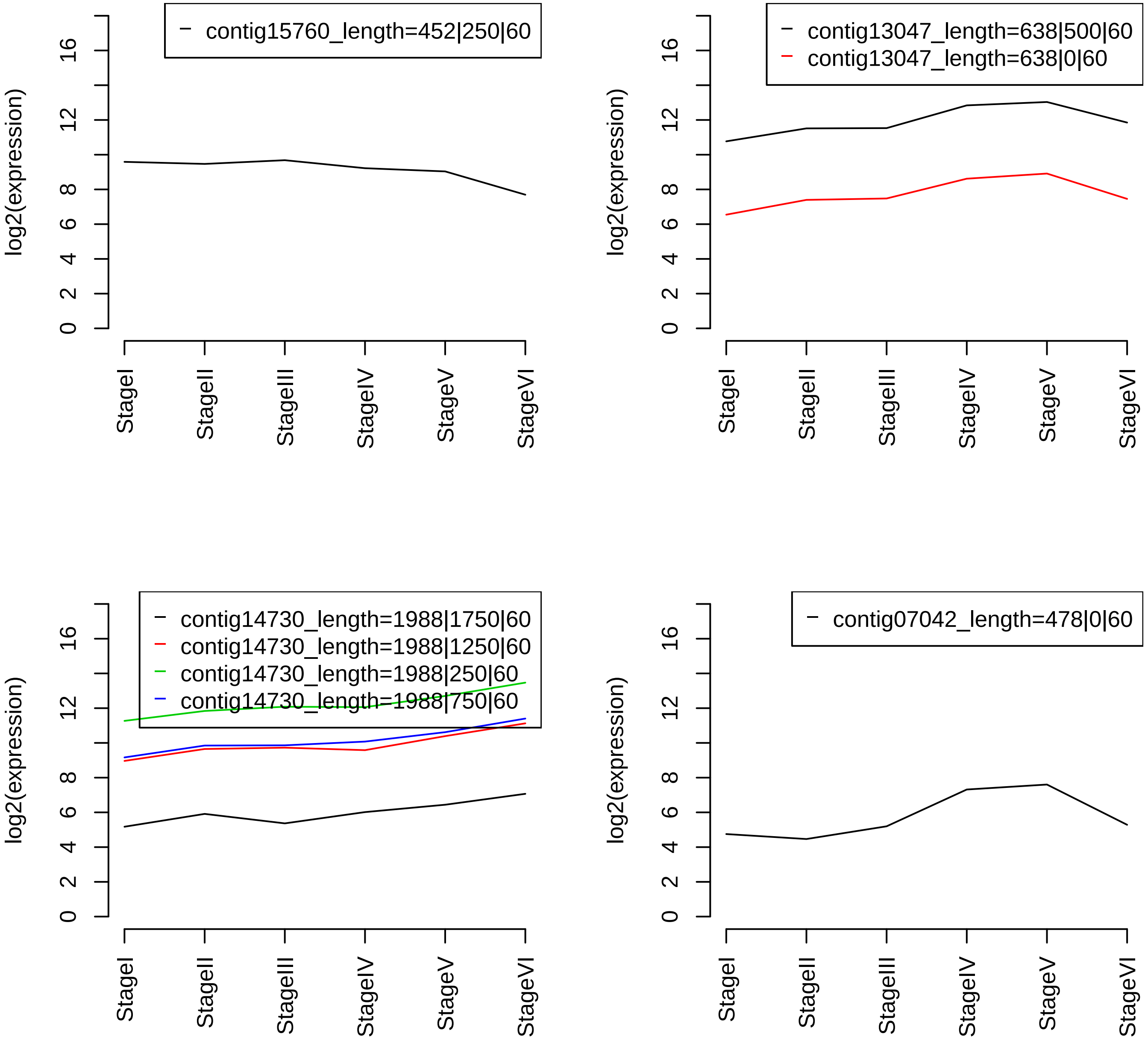

contig15760_length=452|250|60
contig13047_length=638|500|60
contig13047_length=638|0|60
contig14730_length=1988|1750|60
contig14730_length=1988|1250|60
contig14730_length=1988|250|60
contig14730_length=1988|750|60
contig07042_length=478|0|60
log2(expression)
0 2 4 6 8 12 16
StageI
StageII
StageIII
StageIV
StageV
StageVI

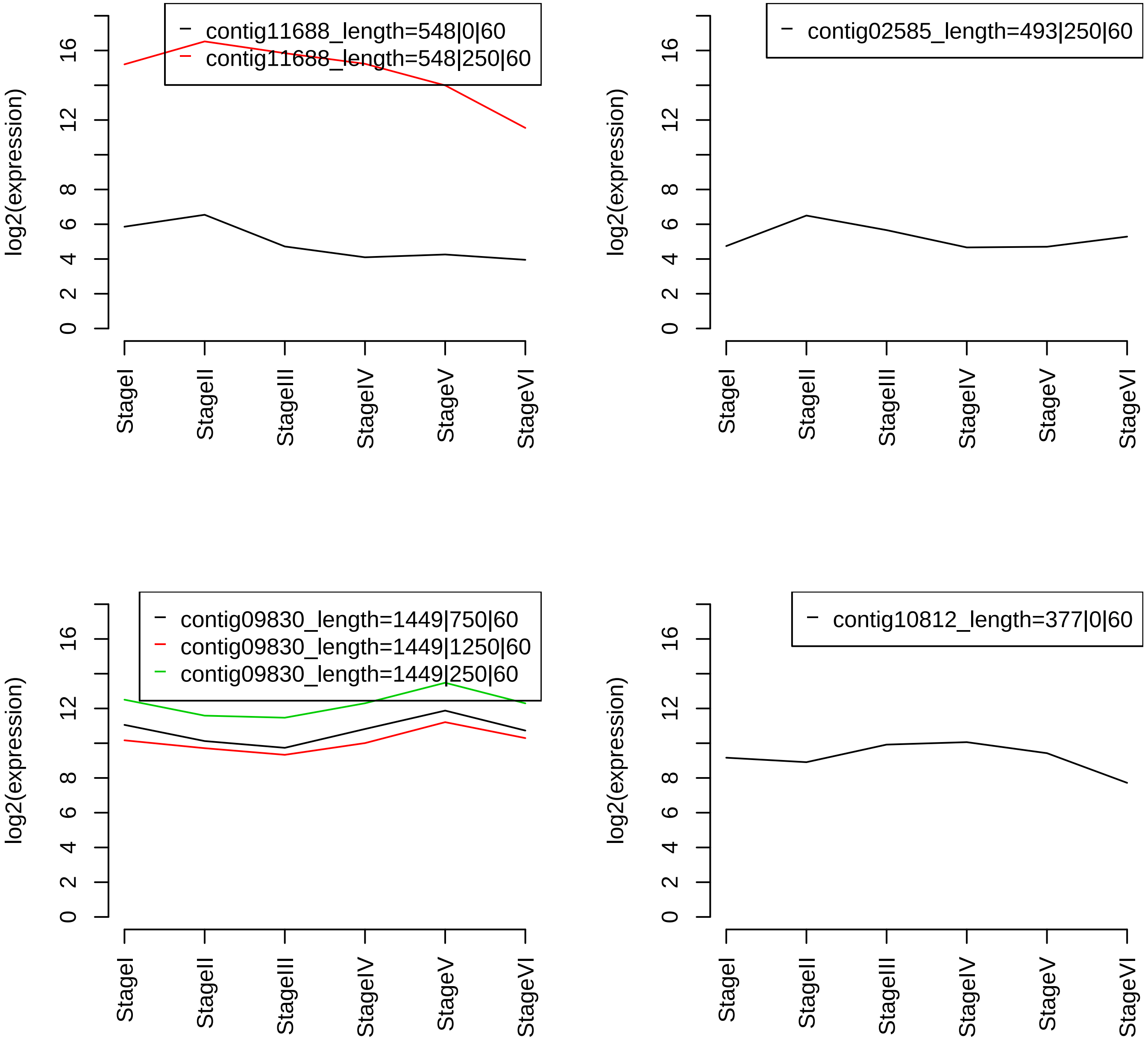

contig11688_length=548|0|60
contig11688_length=548|250|60
contig02585_length=493|250|60
contig09830_length=1449|750|60
contig09830_length=1449|1250|60
contig09830_length=1449|250|60
contig10812_length=377|0|60
log2(expression)
0
2
4
6
8
12
16
StageI
StageII
StageIII
StageIV
StageV
StageVI

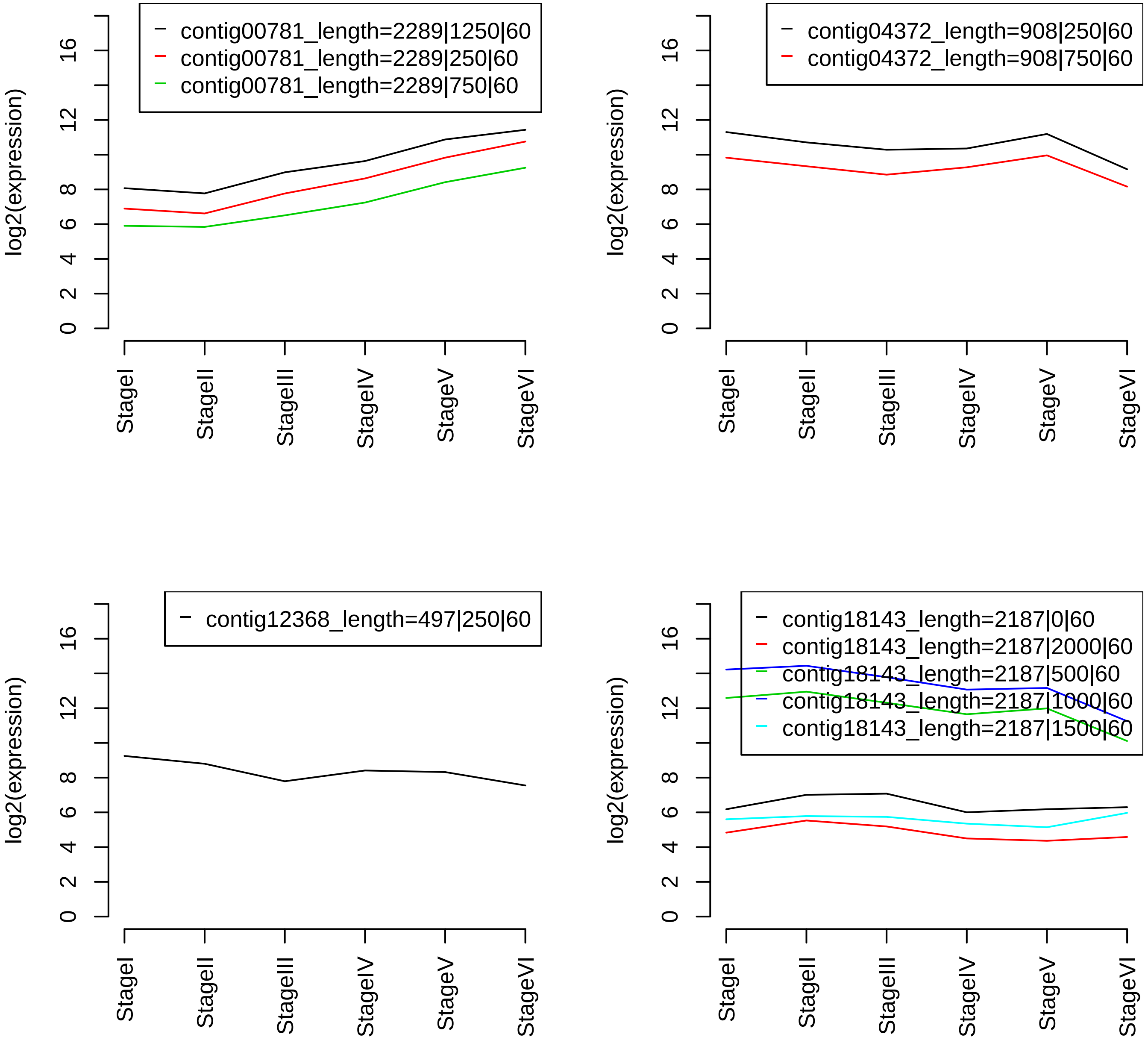
contig00781_length=2289|1250|60
contig00781_length=2289|250|60
contig00781_length=2289|750|60
log2(expression)
0
2
4
6
8
12
16
StageI
StageII
StageIII
StageIV
StageV
StageVI
contig04372_length=908|250|60
contig04372_length=908|750|60
contig12368_length=497|250|60
contig18143_length=2187|0|60
contig18143_length=2187|2000|60
contig18143_length=2187|500|60
contig18143_length=2187|1000|60
contig18143_length=2187|1500|60

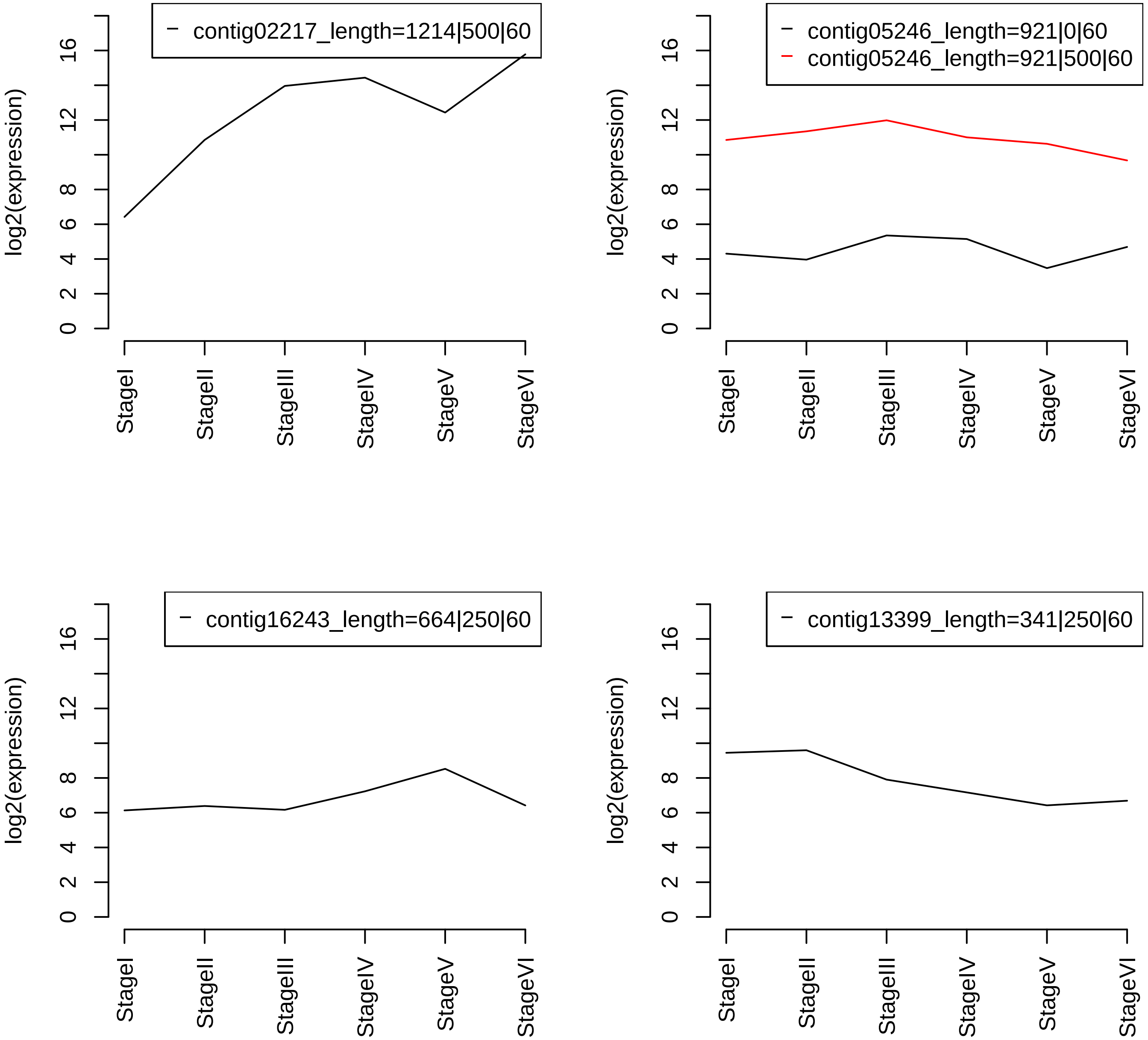

contig02217_length=1214|500|60
contig05246_length=921|0|60
contig05246_length=921|500|60
contig16243_length=664|250|60
contig13399_length=341|250|60
log2(expression)
0
2
4
6
8
12
16
StageI
StageII
StageIII
StageIV
StageV
StageVI

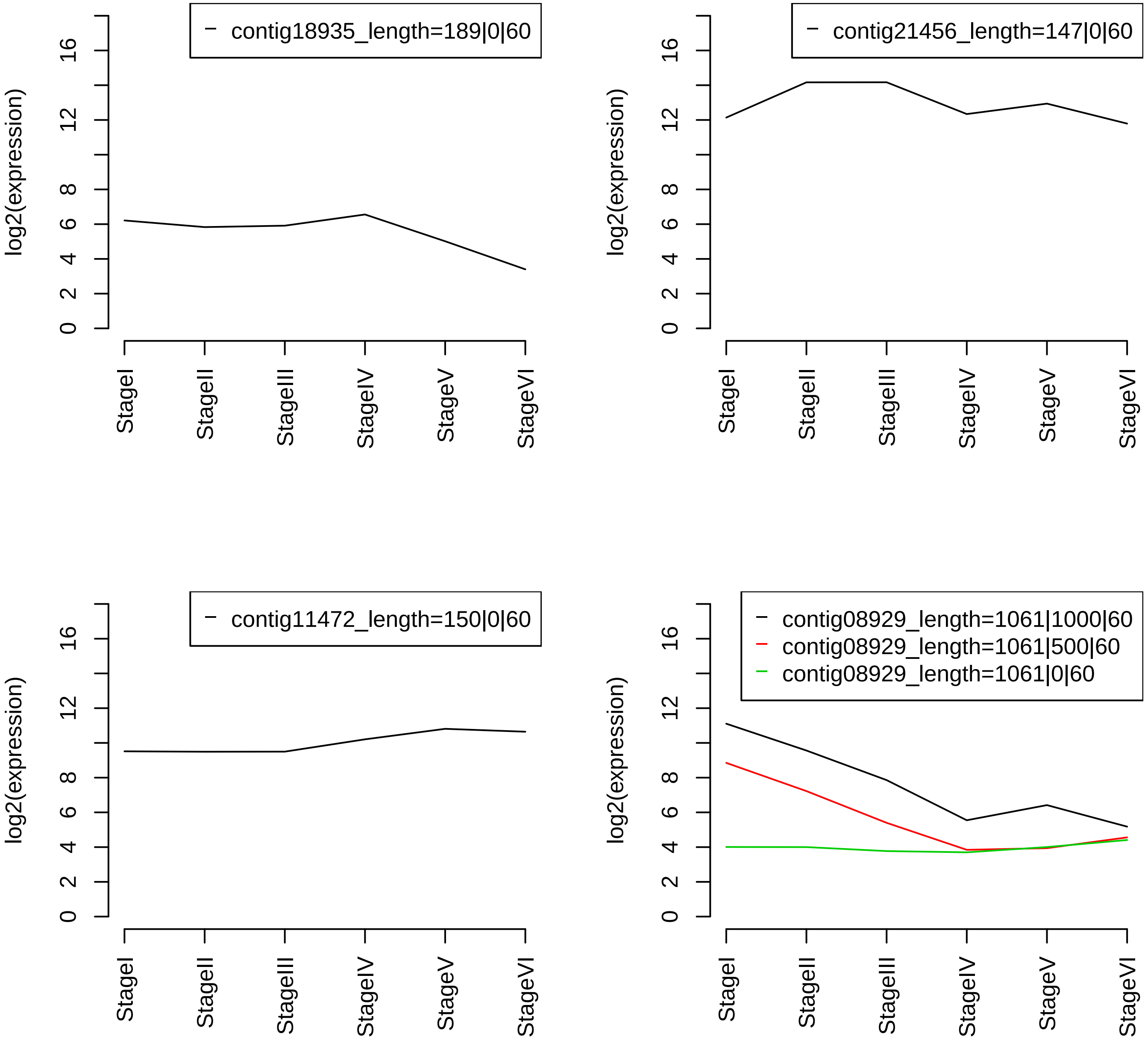
contig18935_length=189|0|60
contig21456_length=147|0|60
contig11472_length=150|0|60
contig08929_length=1061|1000|60
contig08929_length=1061|500|60
contig08929_length=1061|0|60
log2(expression)
0
2
4
6
8
12
16
StageI
StageII
StageIII
StageIV
StageV
StageVI

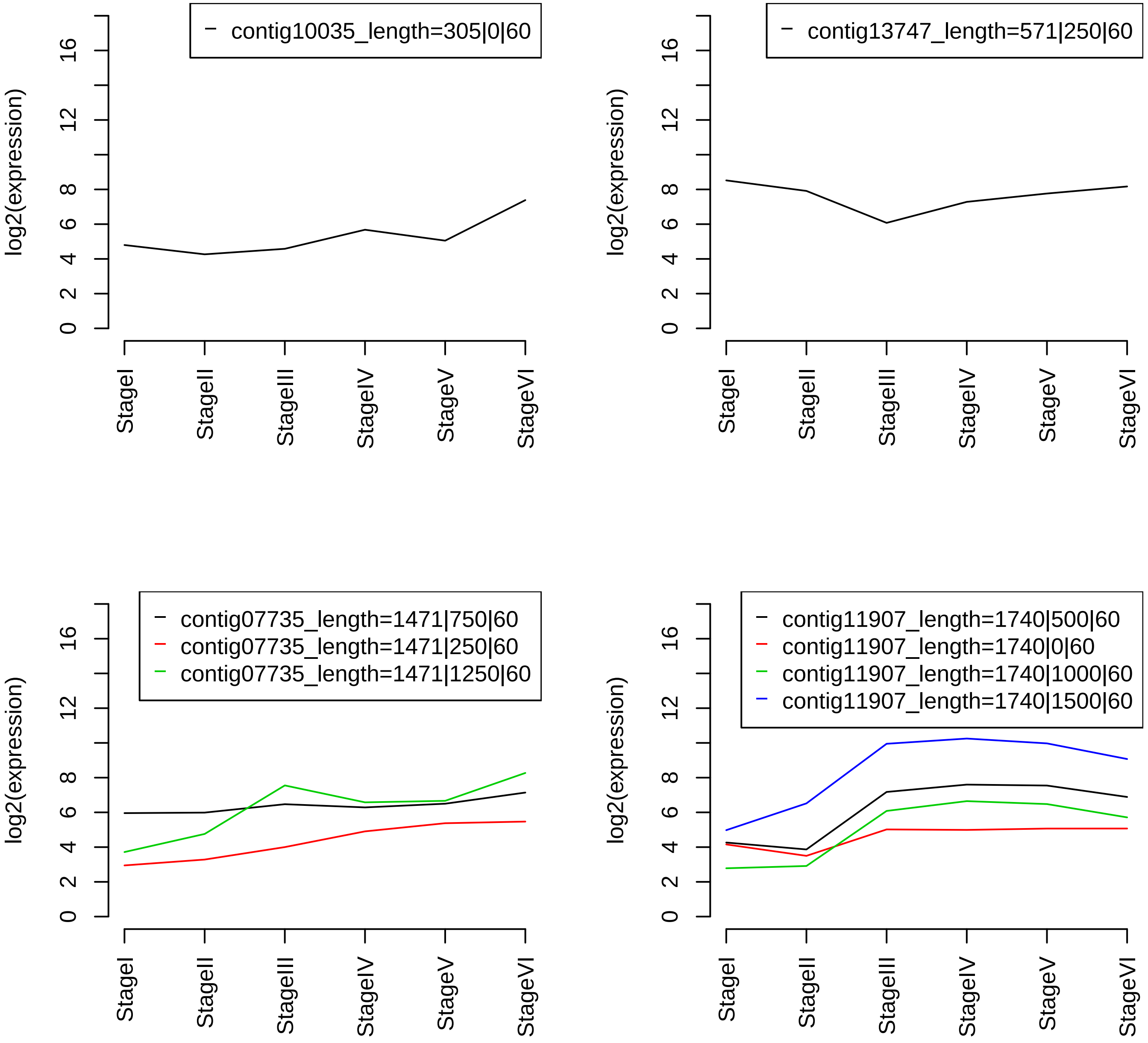

contig10035_length=305|0|60
contig13747_length=571|250|60
contig07735_length=1471|750|60
contig07735_length=1471|250|60
contig07735_length=1471|1250|60
contig11907_length=1740|500|60
contig11907_length=1740|0|60
contig11907_length=1740|1000|60
contig11907_length=1740|1500|60
log2(expression)
0 2 4 6 8 12 16
StageI StageII StageIII StageIV StageV StageVI

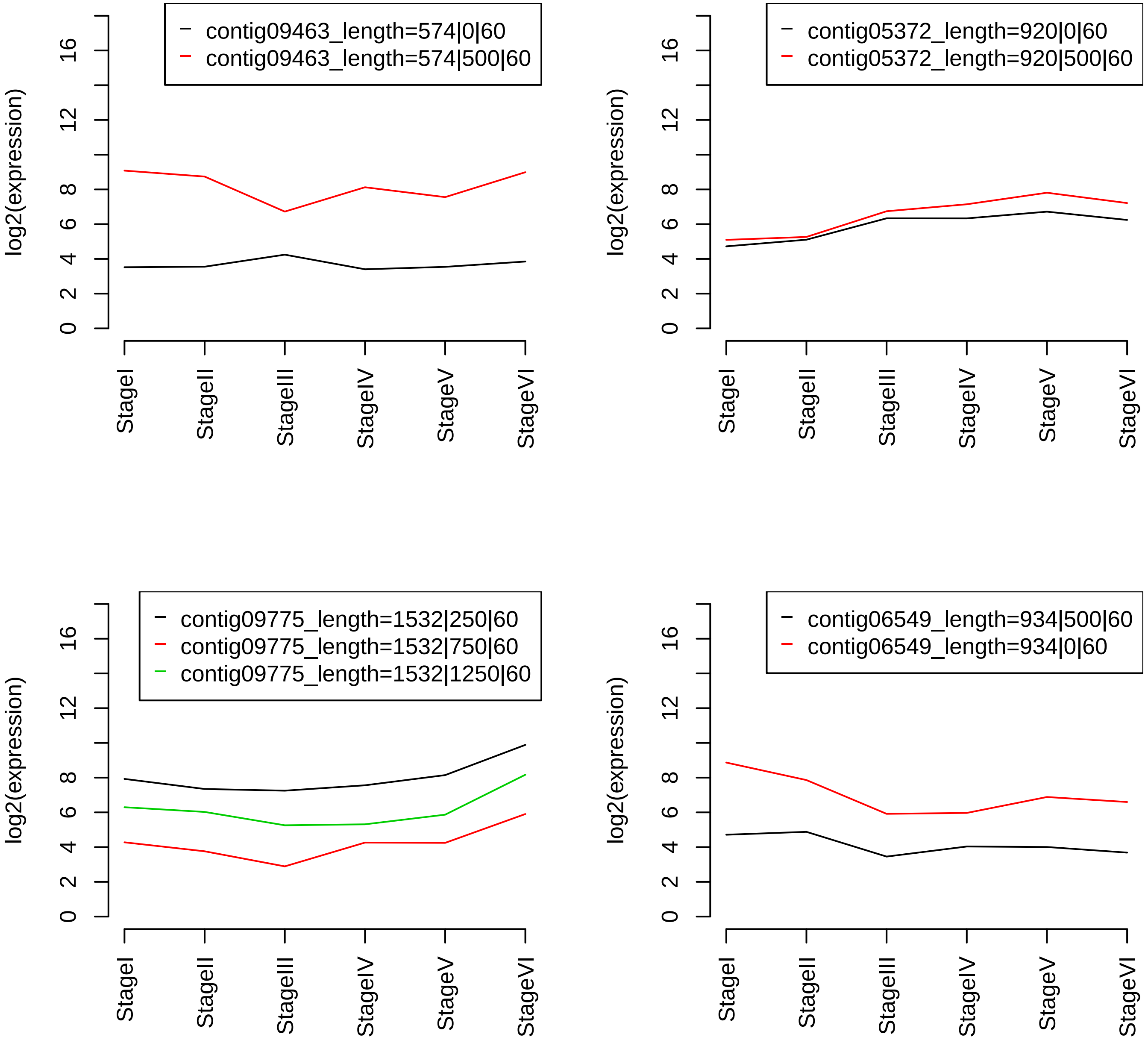
contig09463_length=574|0|60
contig09463_length=574|500|60
log2(expression)
0
2
4
6
8
12
16
StageI
StageII
StageIII
StageIV
StageV
StageVI
contig05372_length=920|0|60
contig05372_length=920|500|60
contig09775_length=1532|250|60
contig09775_length=1532|750|60
contig09775_length=1532|1250|60
contig06549_length=934|500|60
contig06549_length=934|0|60

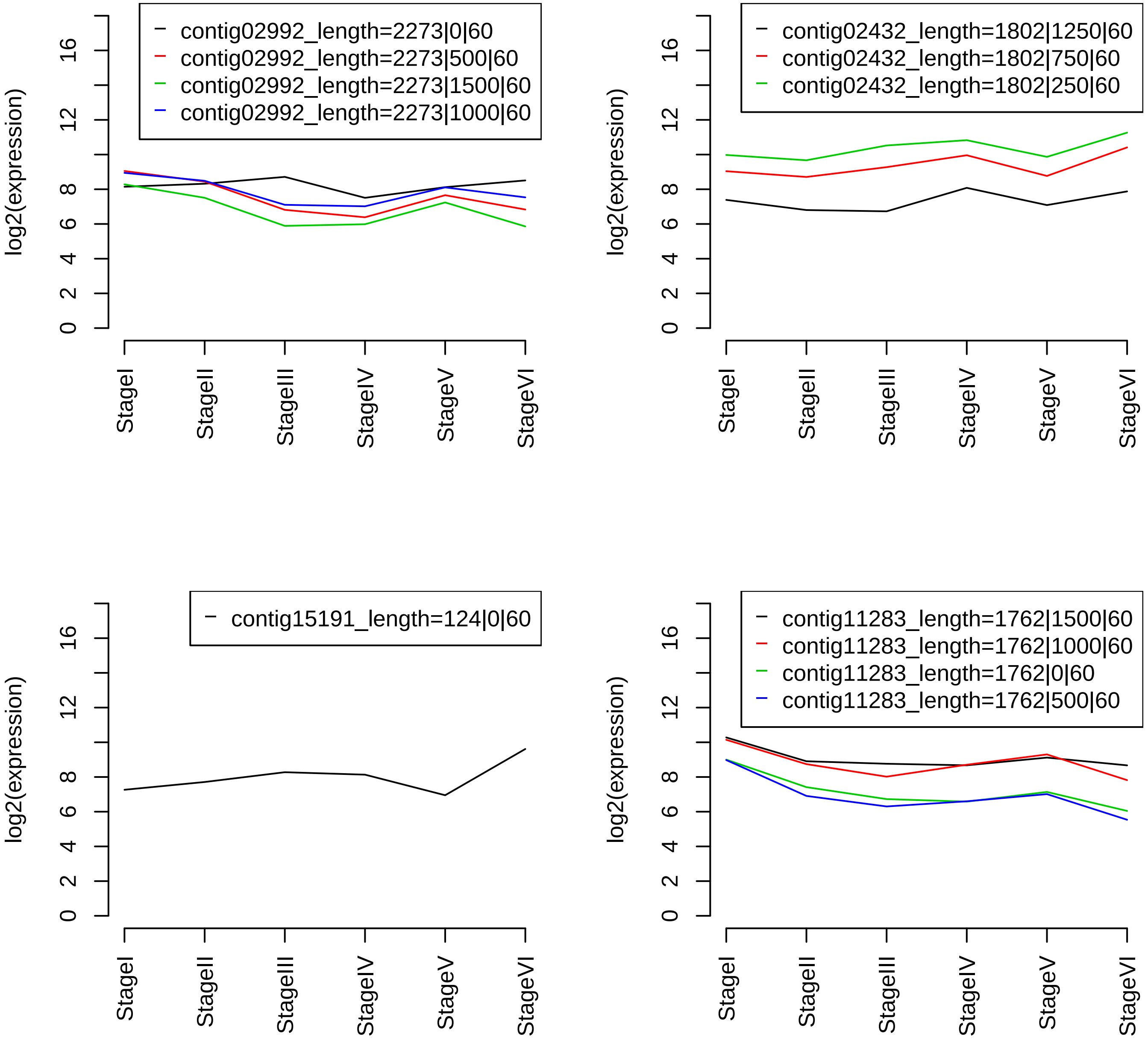

contig02992_length=2273|0|60
contig02992_length=2273|500|60
contig02992_length=2273|1500|60
contig02992_length=2273|1000|60
contig02432_length=1802|1250|60
contig02432_length=1802|750|60
contig02432_length=1802|250|60
contig15191_length=124|0|60
contig11283_length=1762|1500|60
contig11283_length=1762|1000|60
contig11283_length=1762|0|60
contig11283_length=1762|500|60
log2(expression)
0
2
4
6
8
12
16
StageI
StageII
StageIII
StageIV
StageV
StageVI

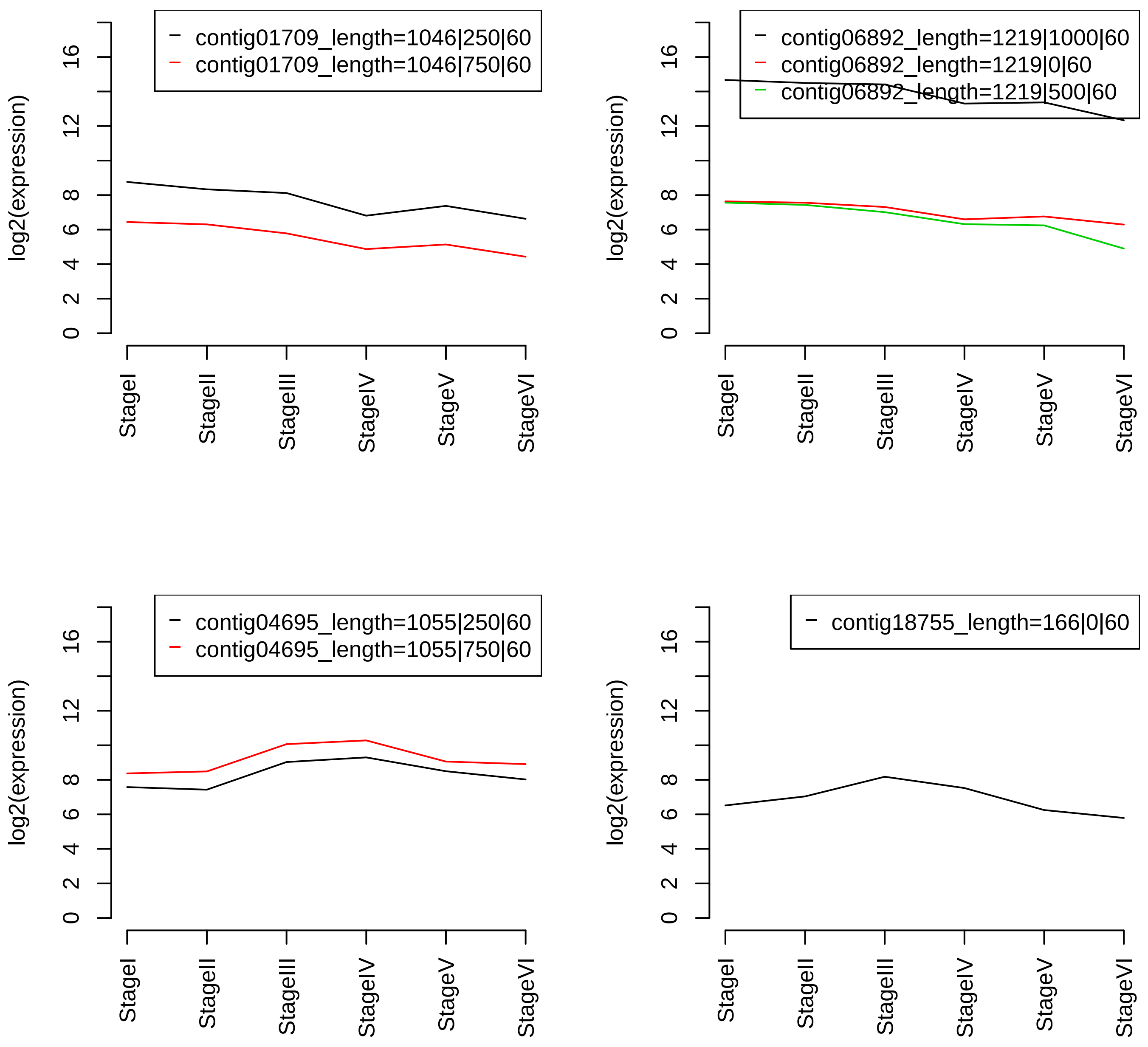

contig01709_length=1046|250|60
contig01709_length=1046|750|60
contig06892_length=1219|1000|60
contig06892_length=1219|0|60
contig06892_length=1219|500|60
contig04695_length=1055|250|60
contig04695_length=1055|750|60
contig18755_length=166|0|60
log2(expression)
StageI
StageII
StageIII
StageIV
StageV
StageVI

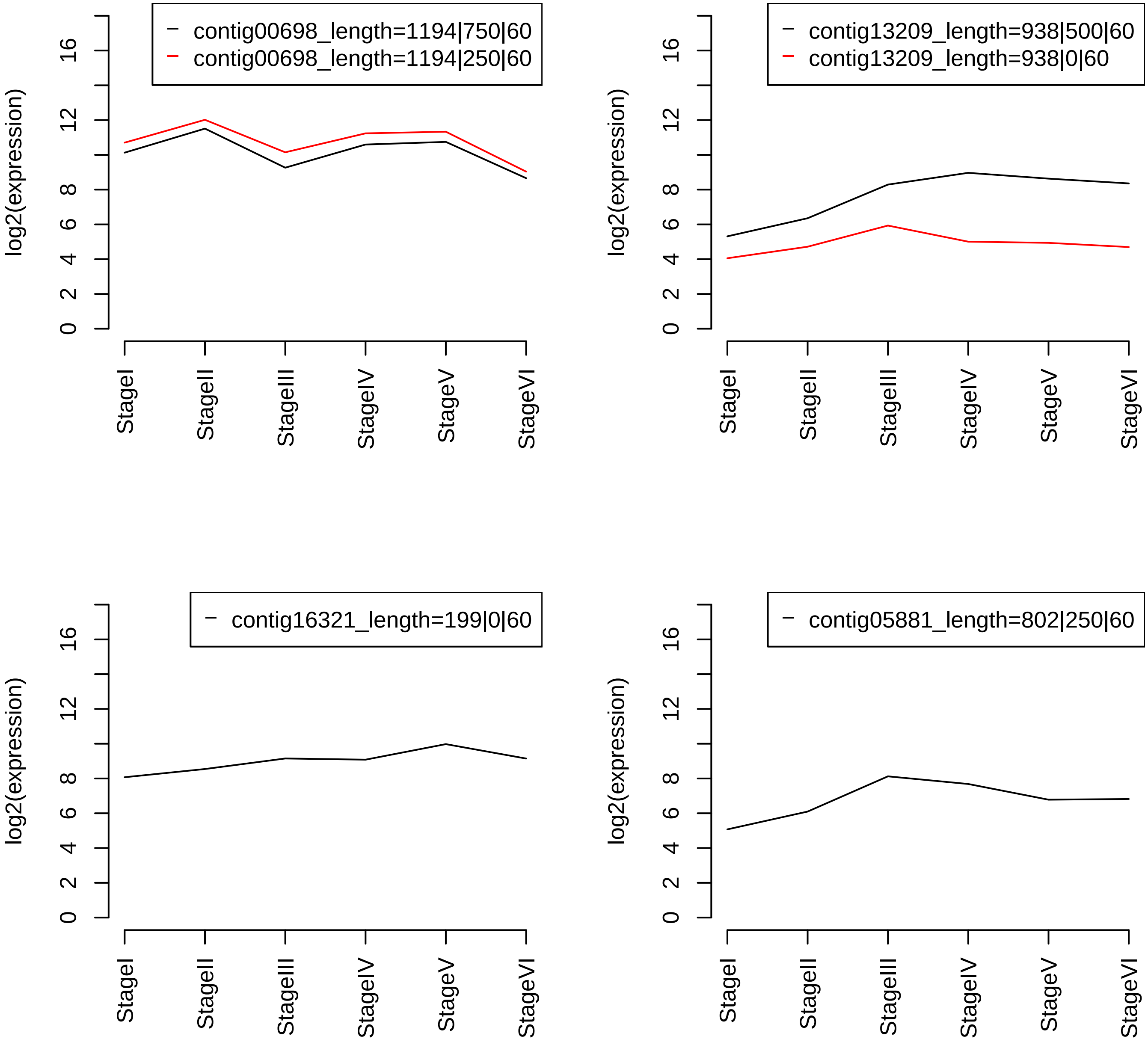
contig00698_length=1194|750|60
contig00698_length=1194|250|60
contig13209_length=938|500|60
contig13209_length=938|0|60
contig16321_length=199|0|60
contig05881_length=802|250|60
log2(expression)
0
2
4
6
8
12
16
StageI
StageII
StageIII
StageIV
StageV
StageVI

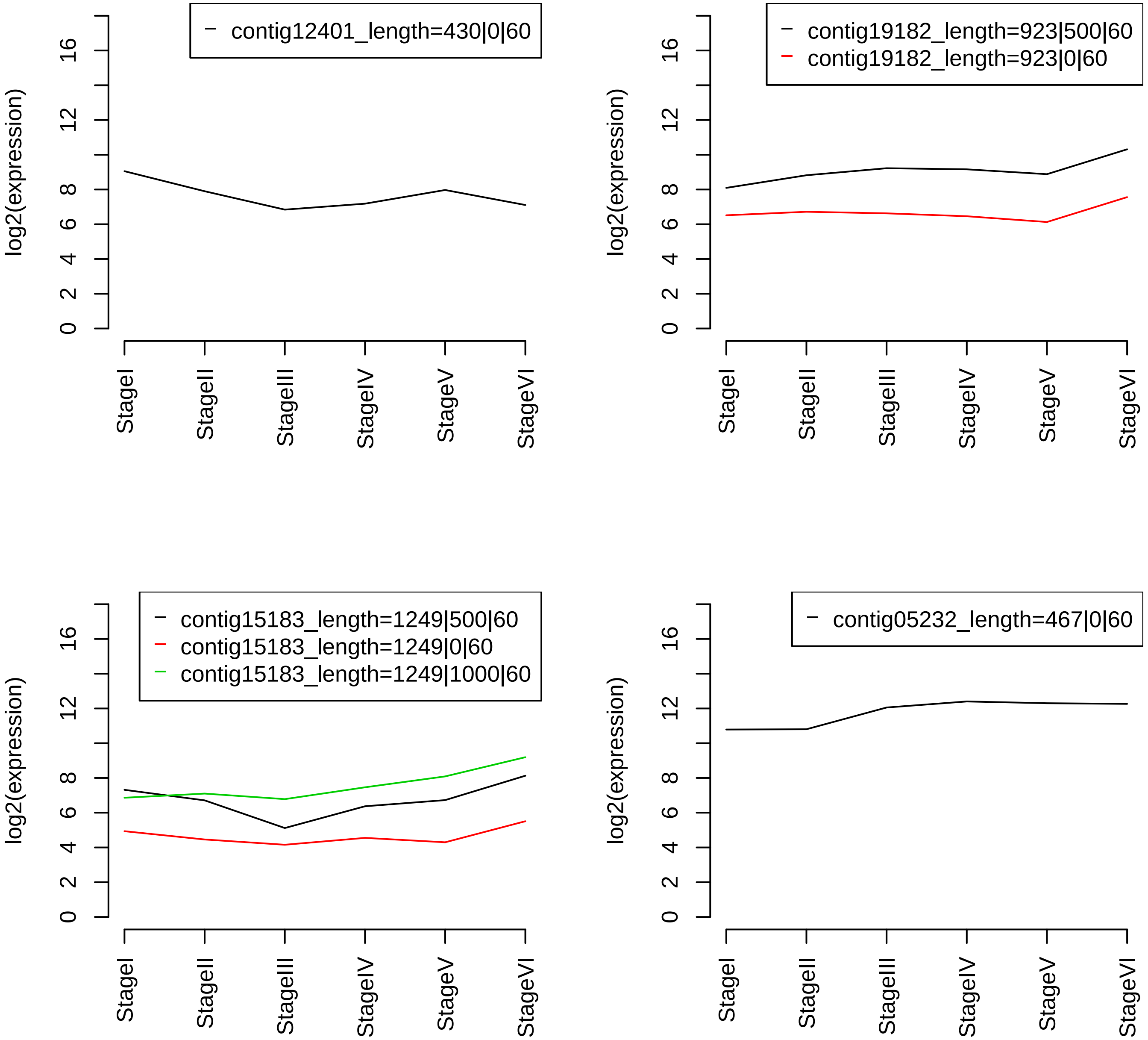

contig12401_length=430|0|60
contig19182_length=923|500|60
contig19182_length=923|0|60
contig15183_length=1249|500|60
contig15183_length=1249|0|60
contig15183_length=1249|1000|60
contig05232_length=467|0|60
log2(expression)
StageI
StageII
StageIII
StageIV
StageV
StageVI

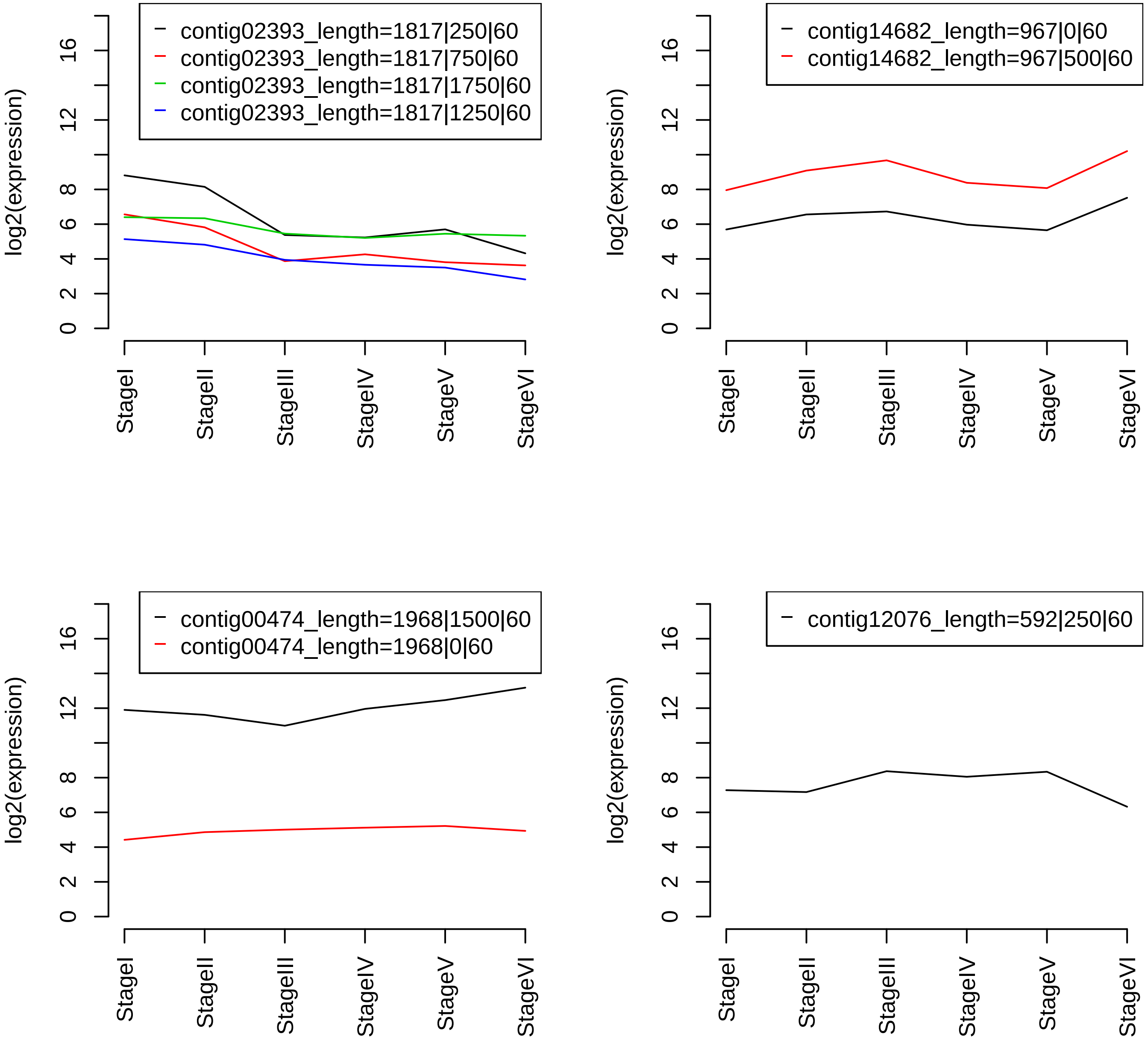

contig02393_length=1817|250|60
contig02393_length=1817|750|60
contig02393_length=1817|1750|60
contig02393_length=1817|1250|60
contig14682_length=967|0|60
contig14682_length=967|500|60
contig00474_length=1968|1500|60
contig00474_length=1968|0|60
contig12076_length=592|250|60
log2(expression)
0
2
4
6
8
12
16
StageI
StageII
StageIII
StageIV
StageV
StageVI

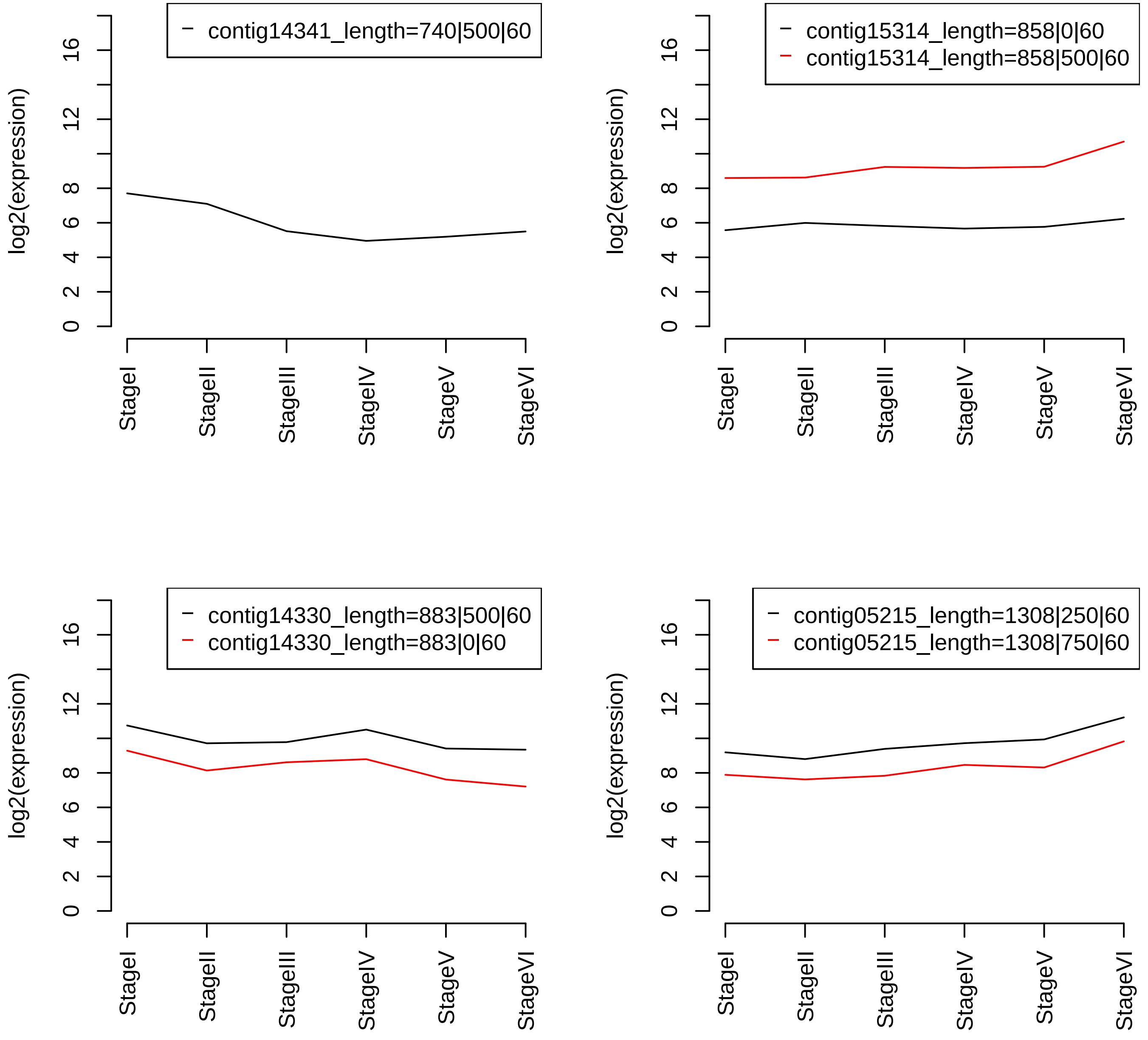

contig14341_length=740|500|60
contig15314_length=858|0|60
contig15314_length=858|500|60
contig14330_length=883|500|60
contig14330_length=883|0|60
contig05215_length=1308|250|60
contig05215_length=1308|750|60
log2(expression)
0
2
4
6
8
12
16
StageI
StageII
StageIII
StageIV
StageV
StageVI

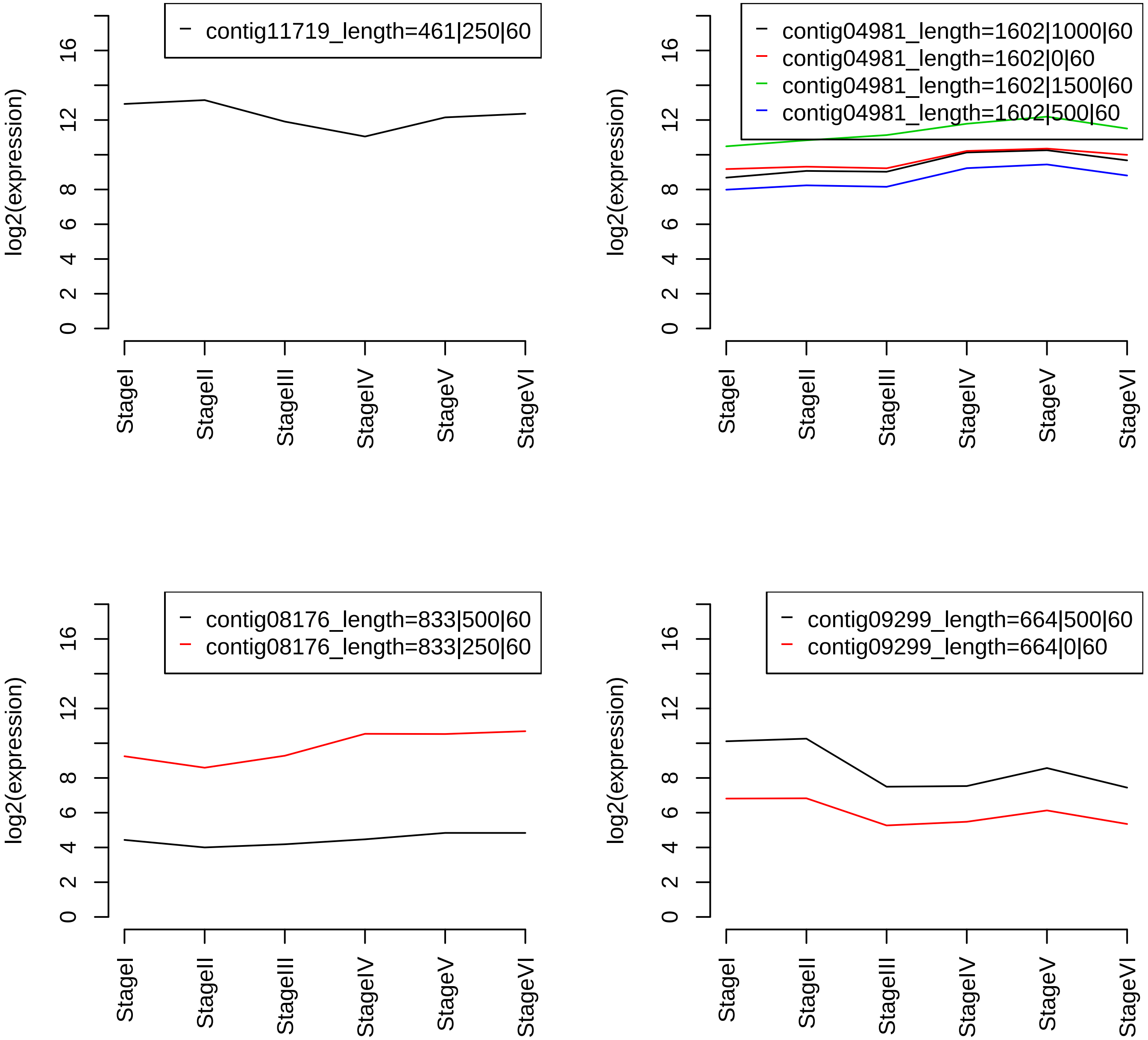

contig11719_length=461|250|60
contig04981_length=1602|1000|60
contig04981_length=1602|0|60
contig04981_length=1602|1500|60
contig04981_length=1602|500|60
contig08176_length=833|500|60
contig08176_length=833|250|60
contig09299_length=664|500|60
contig09299_length=664|0|60
log2(expression)
0 2 4 6 8 12 16
StageI StageII StageIII StageIV StageV StageVI

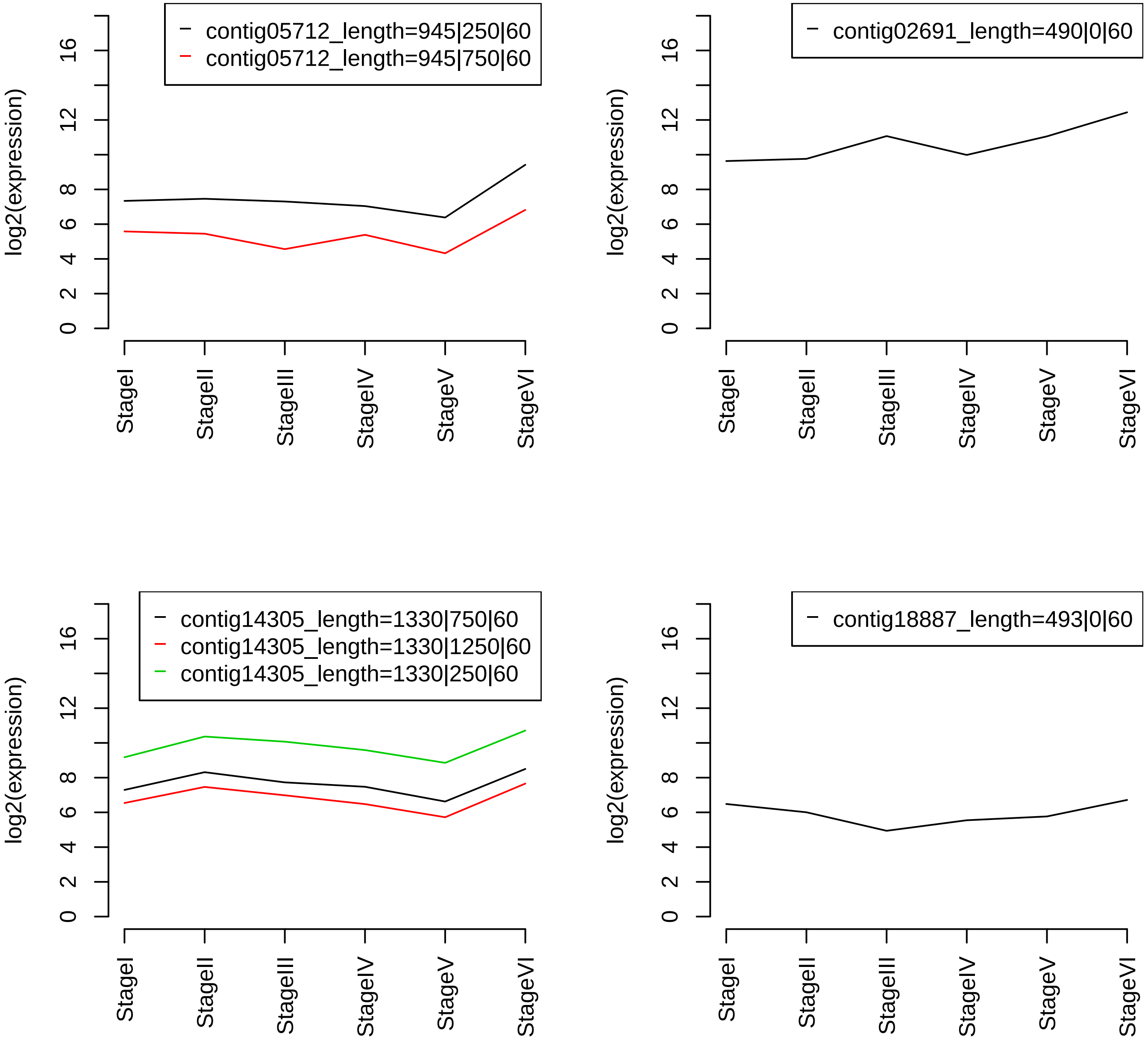
contig05712_length=945|250|60
contig05712_length=945|750|60
contig02691_length=490|0|60
contig14305_length=1330|750|60
contig14305_length=1330|1250|60
contig14305_length=1330|250|60
contig18887_length=493|0|60
log2(expression)
0
2
4
6
8
12
16
StageI
StageII
StageIII
StageIV
StageV
StageVI

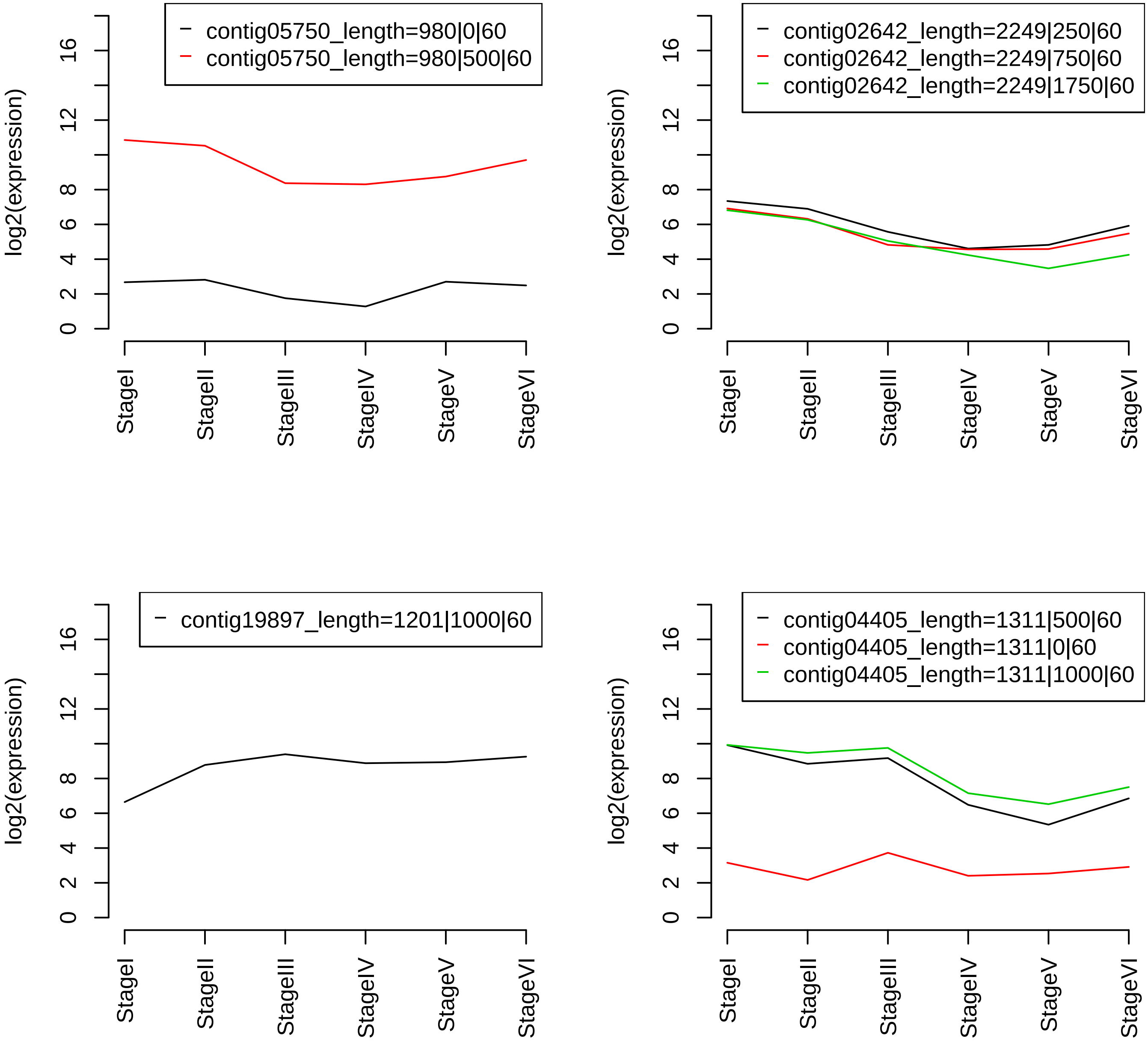

contig05750_length=980|0|60
contig05750_length=980|500|60
contig02642_length=2249|250|60
contig02642_length=2249|750|60
contig02642_length=2249|1750|60
contig19897_length=1201|1000|60
contig04405_length=1311|500|60
contig04405_length=1311|0|60
contig04405_length=1311|1000|60
log2(expression)
0
2
4
6
8
12
16
StageI
StageII
StageIII
StageIV
StageV
StageVI

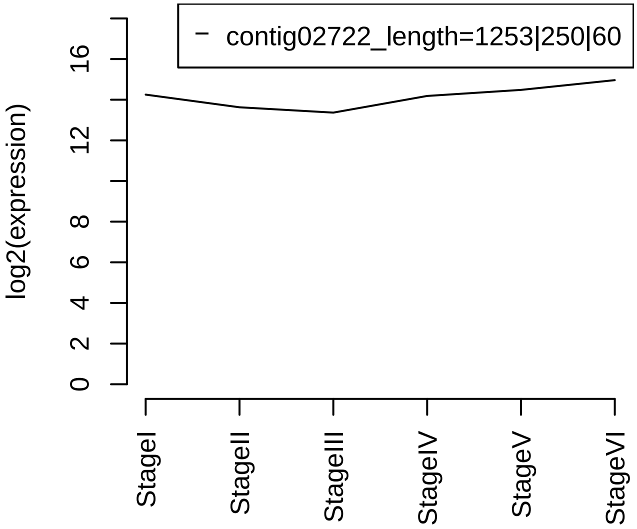
contig02722_length=1253|250|60
log2(expression)
0
2
4
6
8
12
16
StageI
StageII
StageIII
StageIV
StageV
StageVI

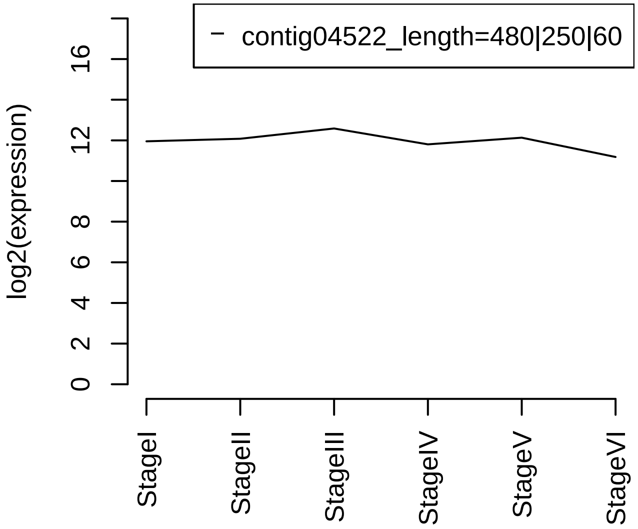
contig04522_length=480|250|60
log2(expression)
0
2
4
6
8
12
16
StageI
StageII
StageIII
StageIV
StageV
StageVI

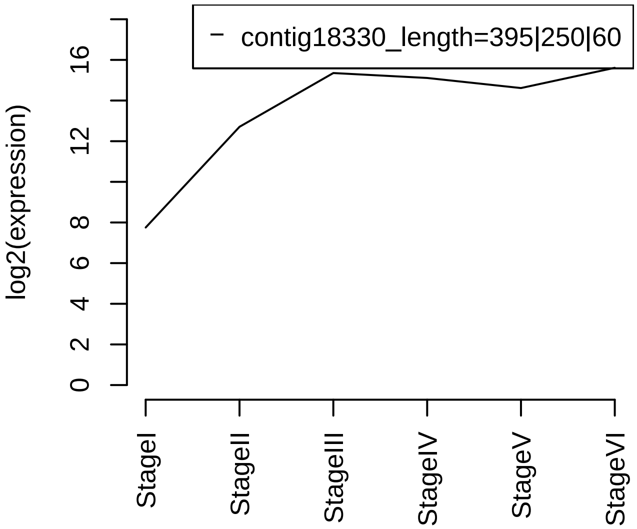
contig18330_length=395|250|60
log2(expression)
0
2
4
6
8
12
16
StageI
StageII
StageIII
StageIV
StageV
StageVI

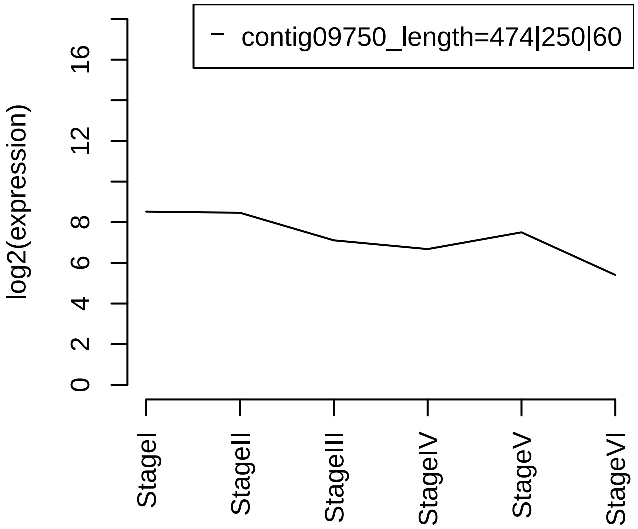
contig09750_length=474|250|60
log2(expression)
0
2
4
6
8
12
16
StageI
StageII
StageIII
StageIV
StageV
StageVI

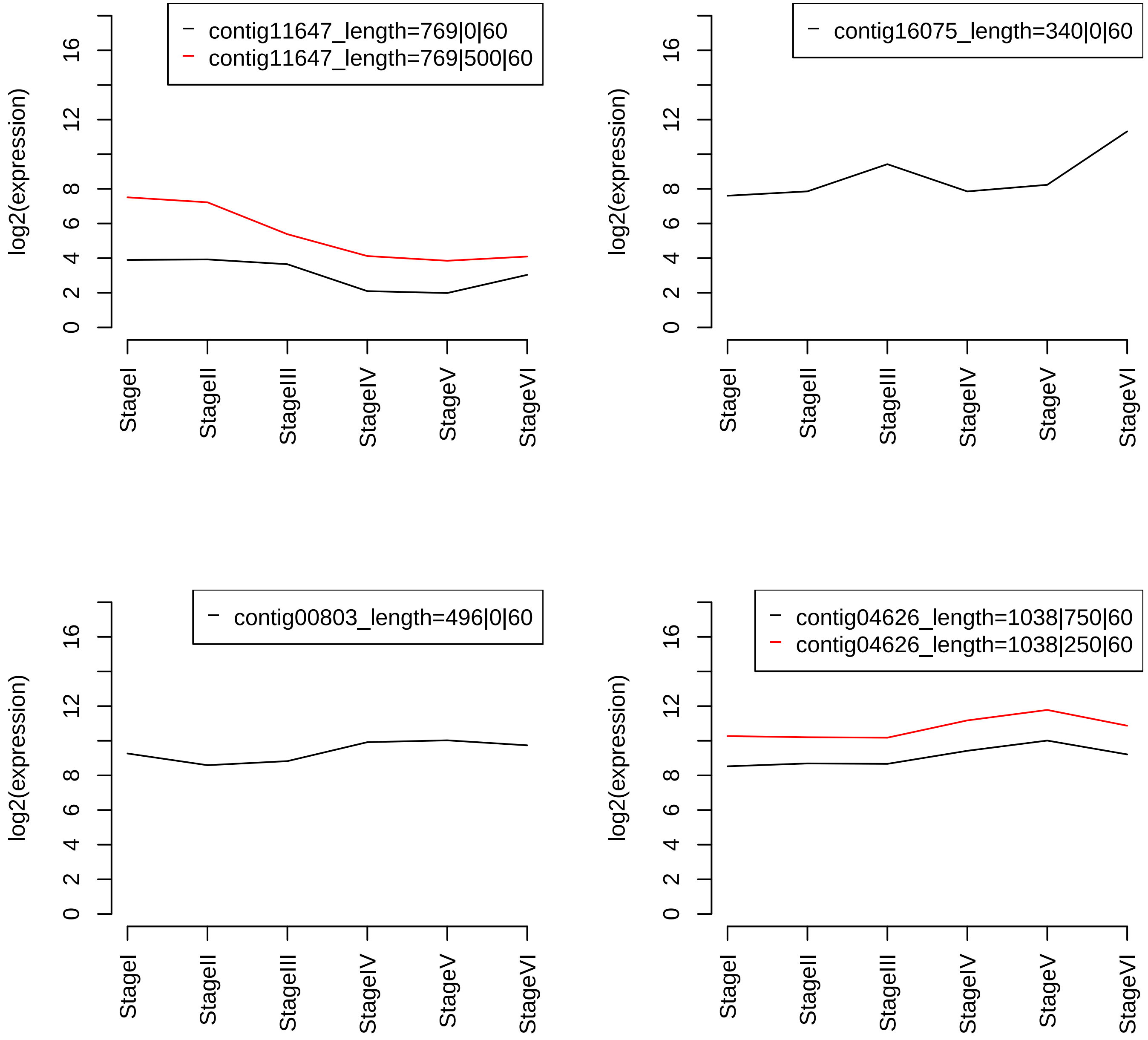
contig11647_length=769|0|60
contig11647_length=769|500|60
log2(expression)
0
2
4
6
8
12
16
StageI
StageII
StageIII
StageIV
StageV
StageVI
contig16075_length=340|0|60
log2(expression)
StageI
StageII
StageIII
StageIV
StageV
StageVI
contig00803_length=496|0|60
log2(expression)
StageI
StageII
StageIII
StageIV
StageV
StageVI
contig04626_length=1038|750|60
contig04626_length=1038|250|60
log2(expression)
StageI
StageII
StageIII
StageIV
StageV
StageVI

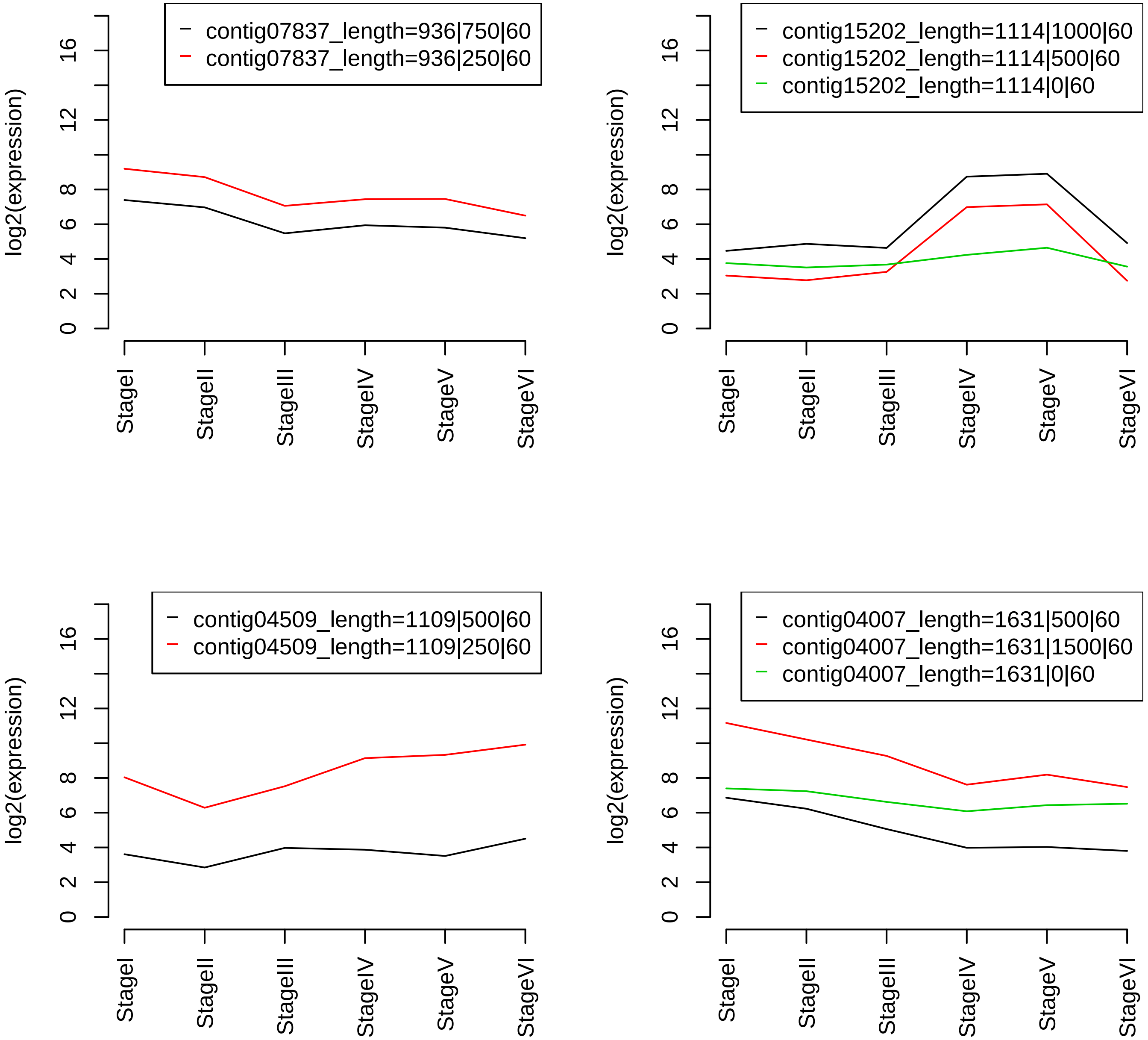
contig07837_length=936|750|60
contig07837_length=936|250|60
contig15202_length=1114|1000|60
contig15202_length=1114|500|60
contig15202_length=1114|0|60
contig04509_length=1109|500|60
contig04509_length=1109|250|60
contig04007_length=1631|500|60
contig04007_length=1631|1500|60
contig04007_length=1631|0|60
log2(expression)
0
2
4
6
8
12
16
StageI
StageII
StageIII
StageIV
StageV
StageVI

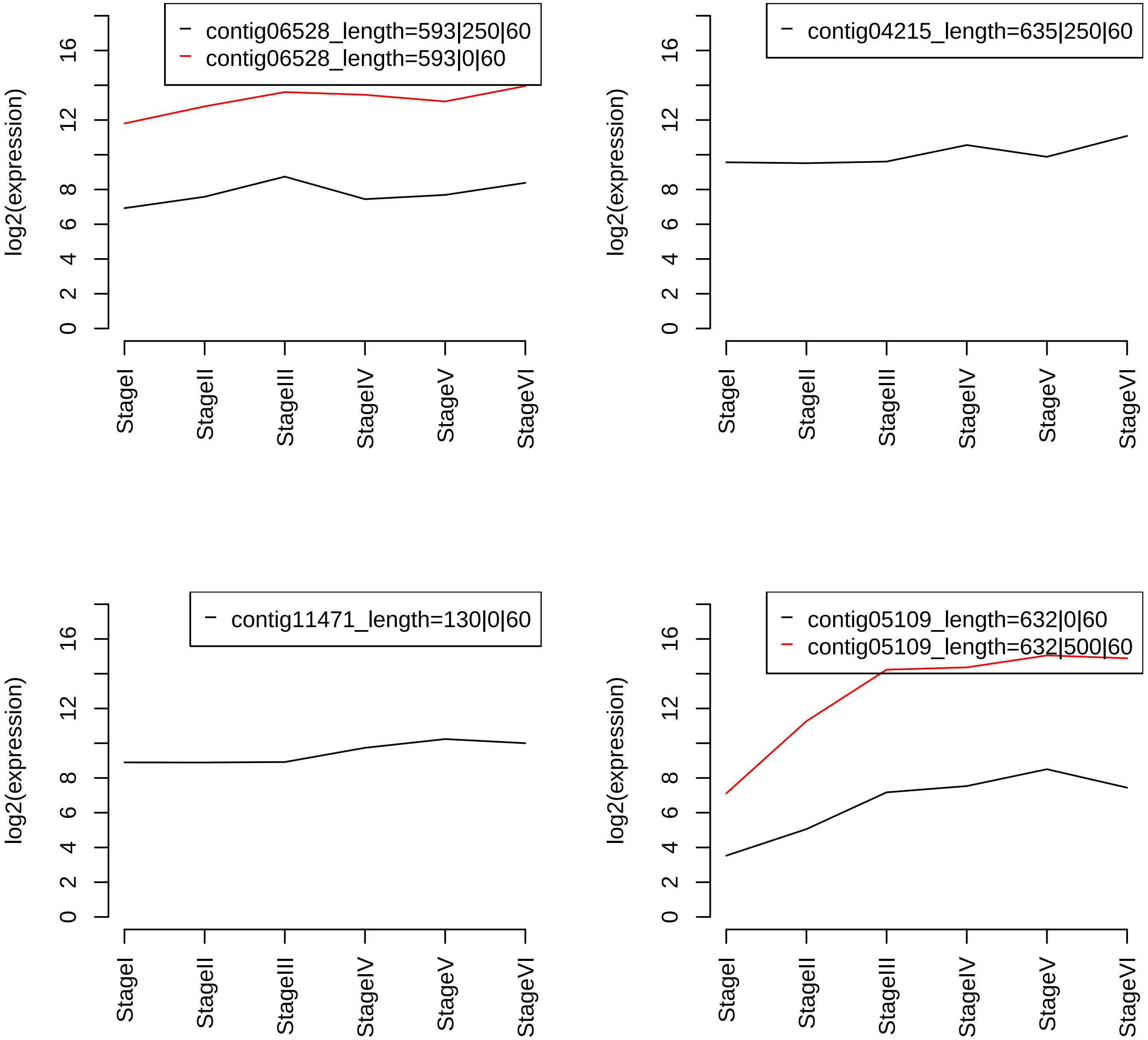

contig06528_length=593|250|60
contig06528_length=593|0|60
log2(expression)
0
2
4
6
8
12
16
StageI
StageII
StageIII
StageIV
StageV
StageVI
contig04215_length=635|250|60
log2(expression)
contig11471_length=130|0|60
log2(expression)
contig05109_length=632|0|60
contig05109_length=632|500|60
log2(expression)

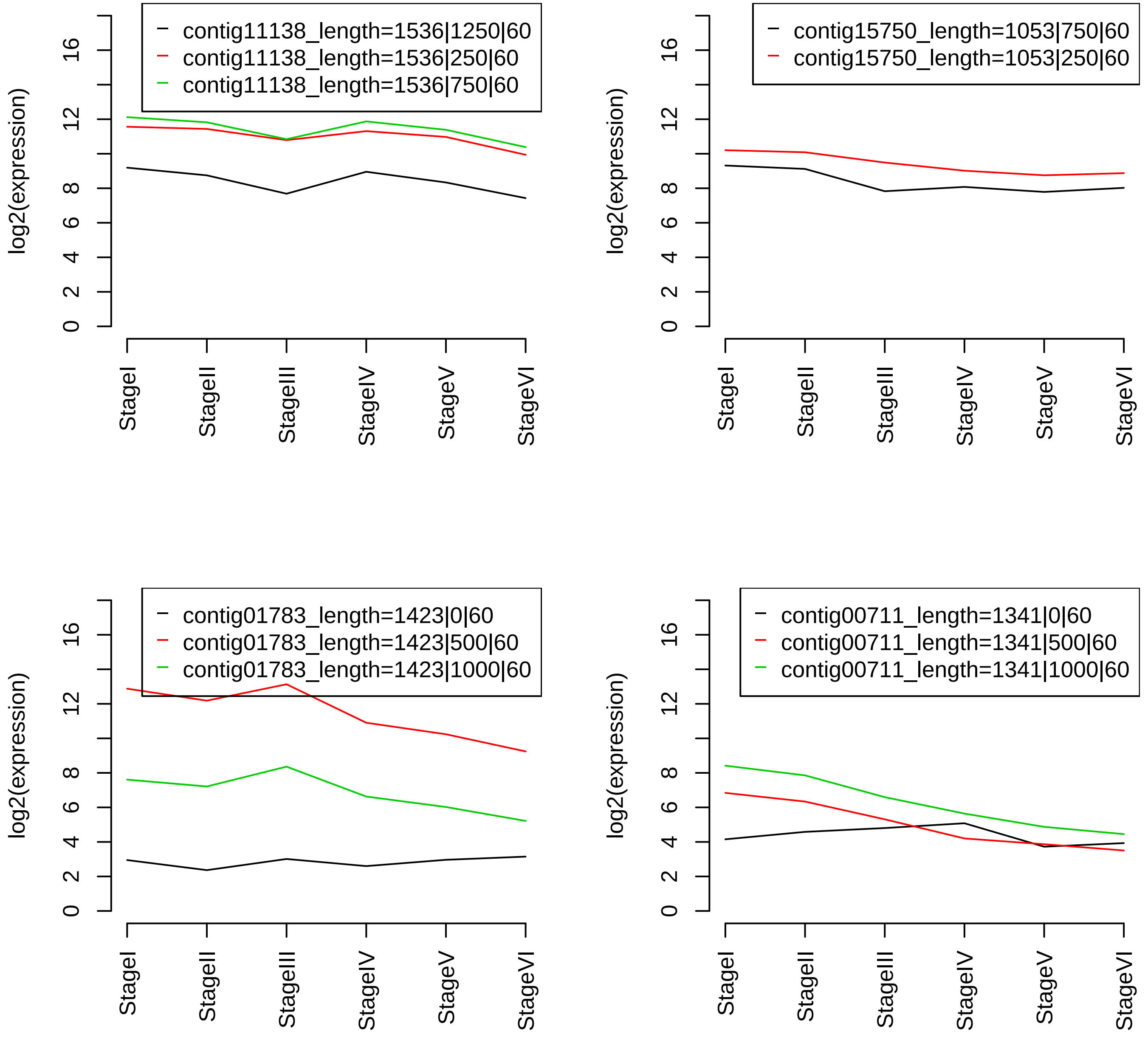

contig11138_length=1536|1250|60
contig11138_length=1536|250|60
contig11138_length=1536|750|60
contig15750_length=1053|750|60
contig15750_length=1053|250|60
contig01783_length=1423|0|60
contig01783_length=1423|500|60
contig01783_length=1423|1000|60
contig00711_length=1341|0|60
contig00711_length=1341|500|60
contig00711_length=1341|1000|60
log2(expression)
0
2
4
6
8
12
16
StageI
StageII
StageIII
StageIV
StageV
StageVI

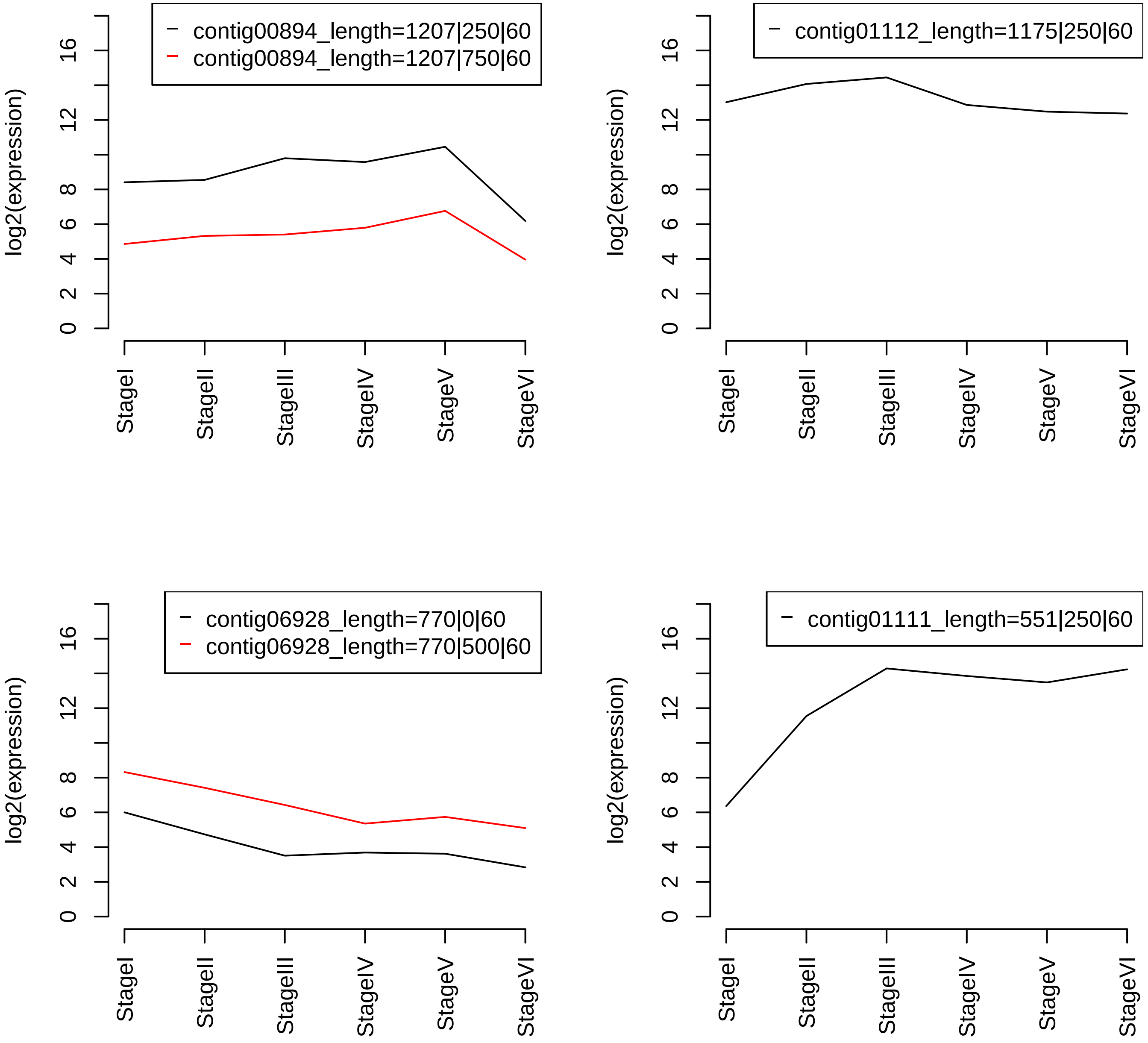

contig00894_length=1207|250|60
contig00894_length=1207|750|60
contig01112_length=1175|250|60
contig06928_length=770|0|60
contig06928_length=770|500|60
contig01111_length=551|250|60
log2(expression)
0
2
4
6
8
12
16
StageI
StageII
StageIII
StageIV
StageV
StageVI

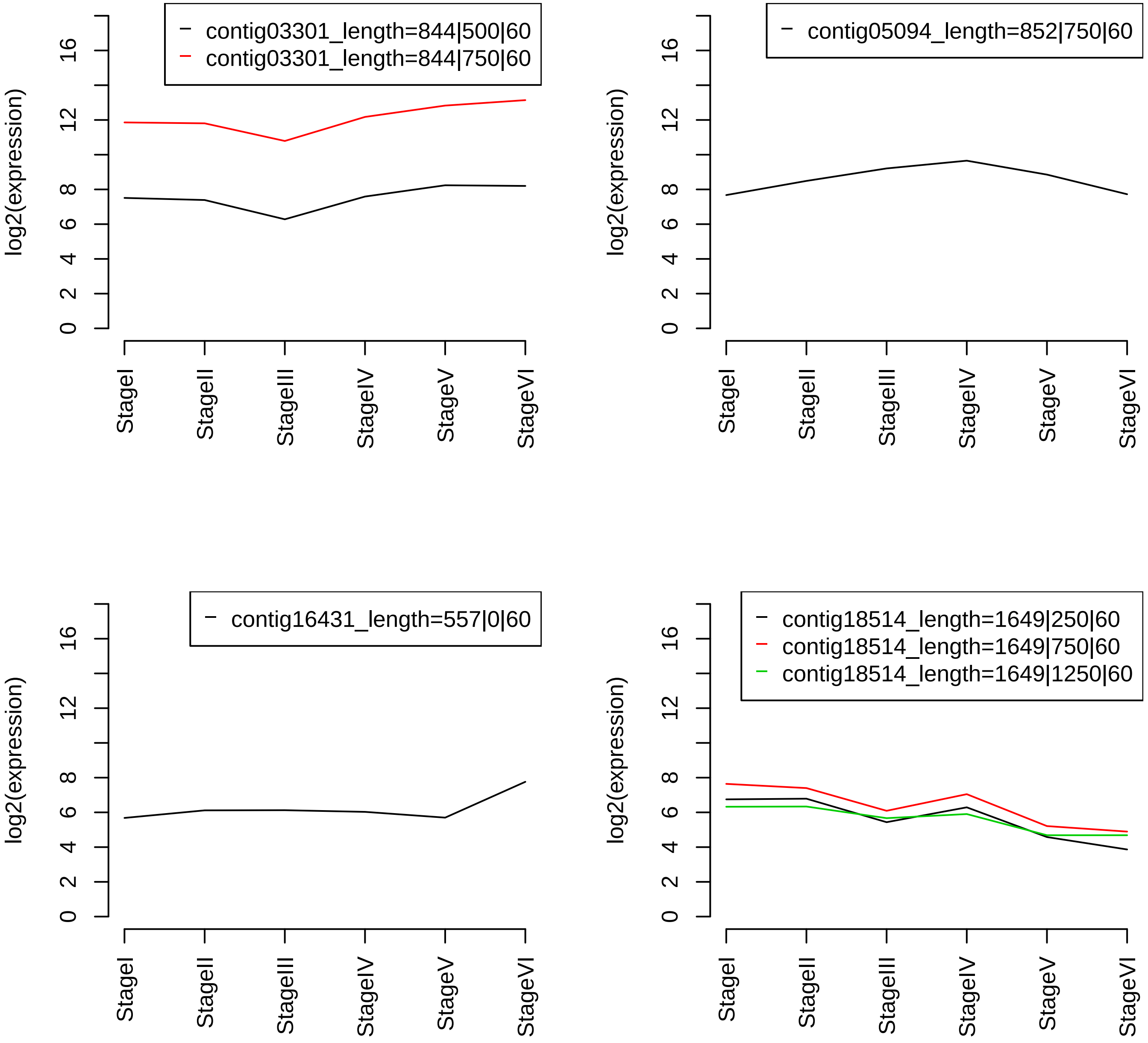
contig03301_length=844|500|60
contig03301_length=844|750|60
contig05094_length=852|750|60
contig16431_length=557|0|60
contig18514_length=1649|250|60
contig18514_length=1649|750|60
contig18514_length=1649|1250|60
log2(expression)
0
2
4
6
8
12
16
StageI
StageII
StageIII
StageIV
StageV
StageVI

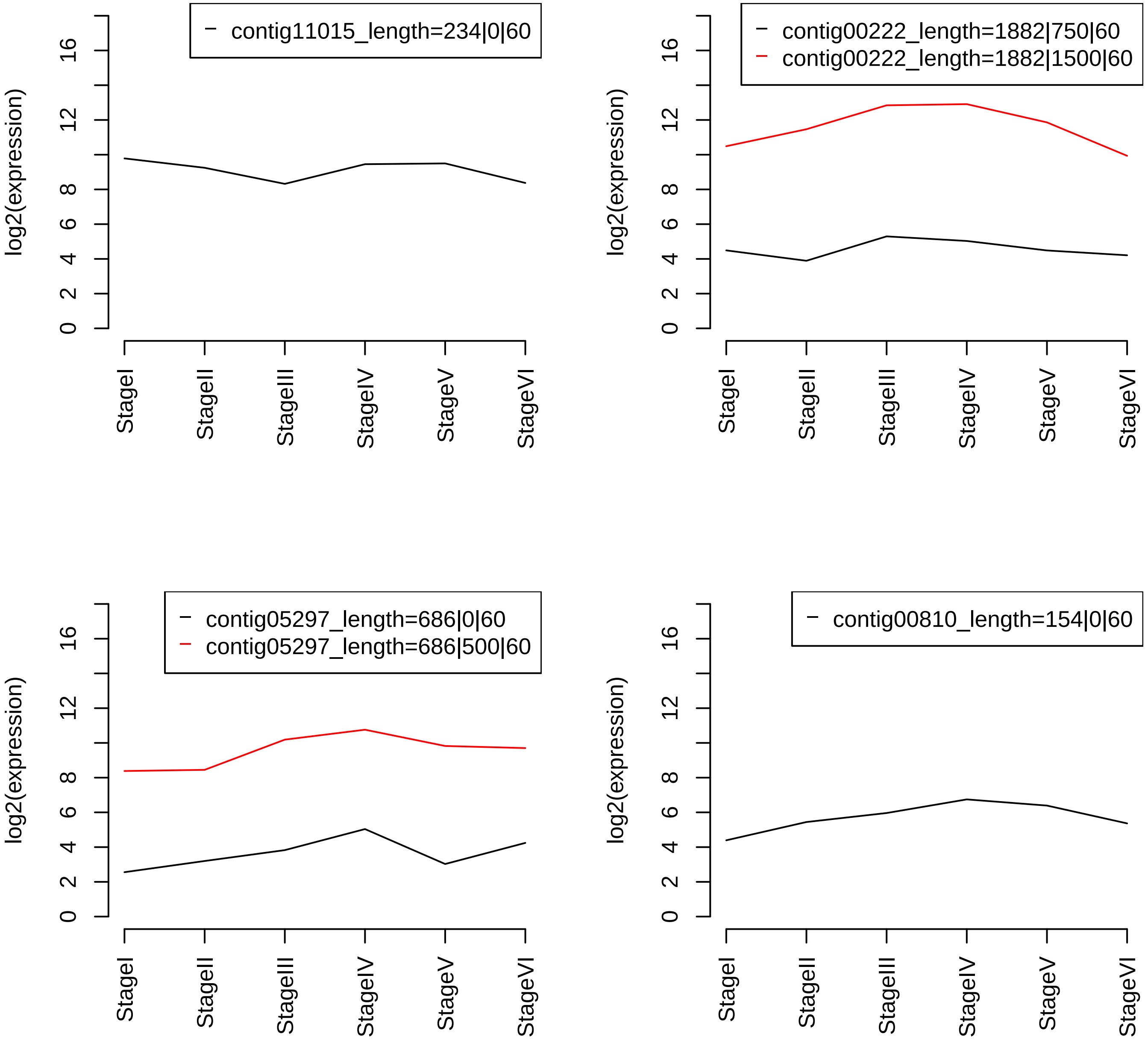
contig11015_length=234|0|60
contig00222_length=1882|750|60
contig00222_length=1882|1500|60
contig05297_length=686|0|60
contig05297_length=686|500|60
contig00810_length=154|0|60
log2(expression)
0
2
4
6
8
12
16
StageI
StageII
StageIII
StageIV
StageV
StageVI

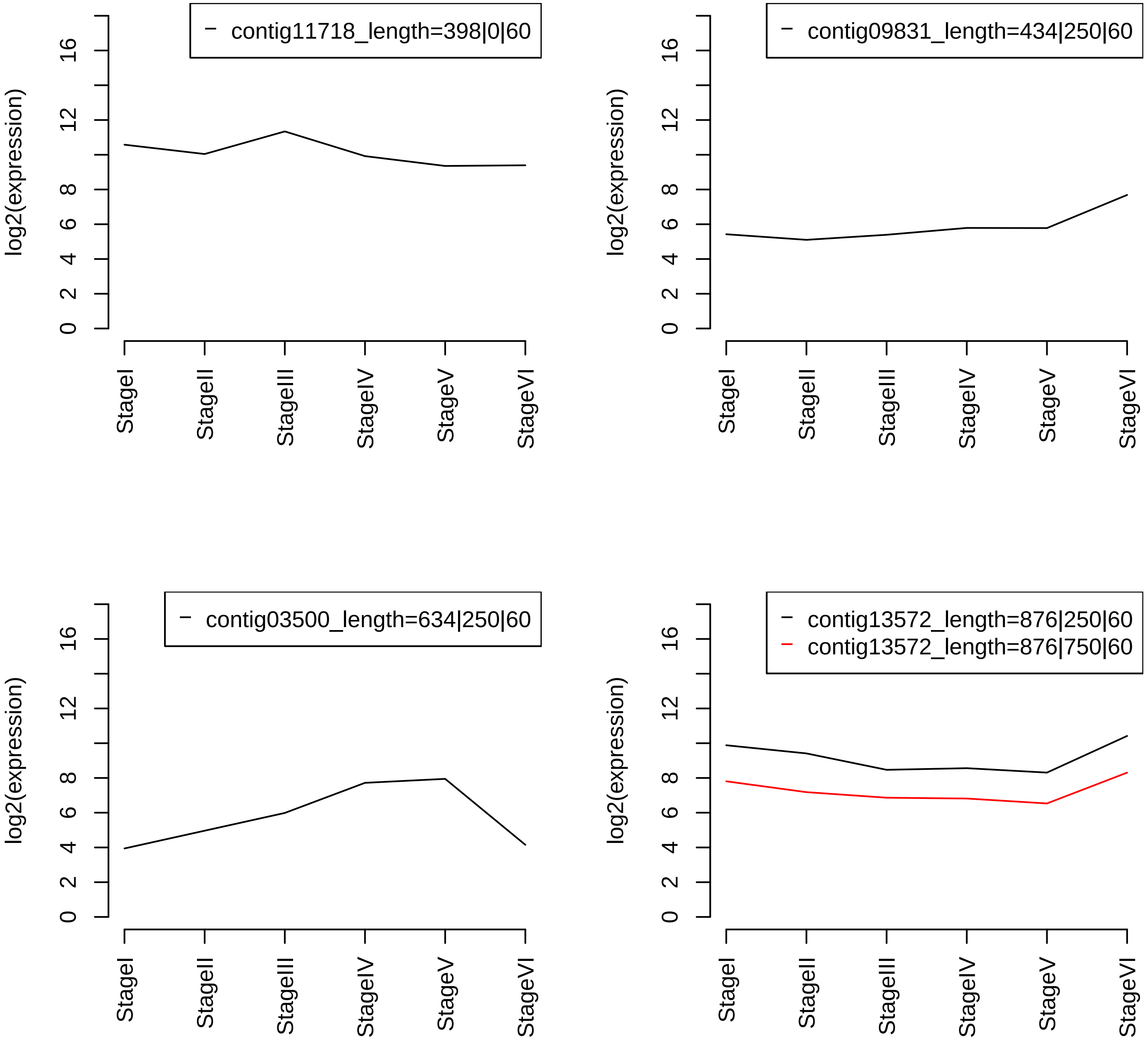

contig11718_length=398|0|60
contig09831_length=434|250|60
contig03500_length=634|250|60
contig13572_length=876|250|60
contig13572_length=876|750|60
log2(expression)
0
2
4
6
8
12
16
StageI
StageII
StageIII
StageIV
StageV
StageVI

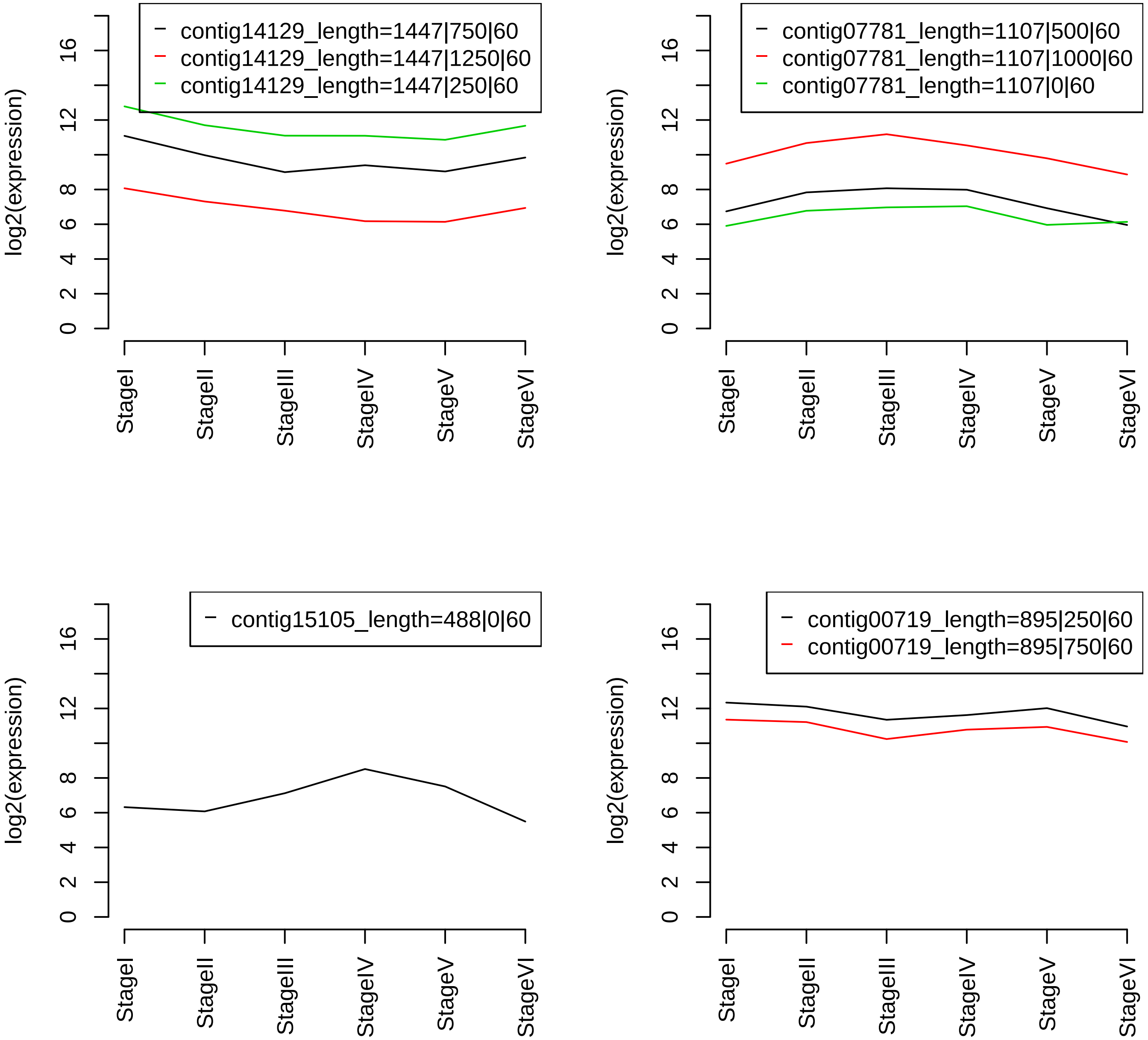
contig14129_length=1447|750|60
contig14129_length=1447|1250|60
contig14129_length=1447|250|60
contig07781_length=1107|500|60
contig07781_length=1107|1000|60
contig07781_length=1107|0|60
contig15105_length=488|0|60
contig00719_length=895|250|60
contig00719_length=895|750|60
log2(expression)
0
2
4
6
8
12
16
StageI
StageII
StageIII
StageIV
StageV
StageVI

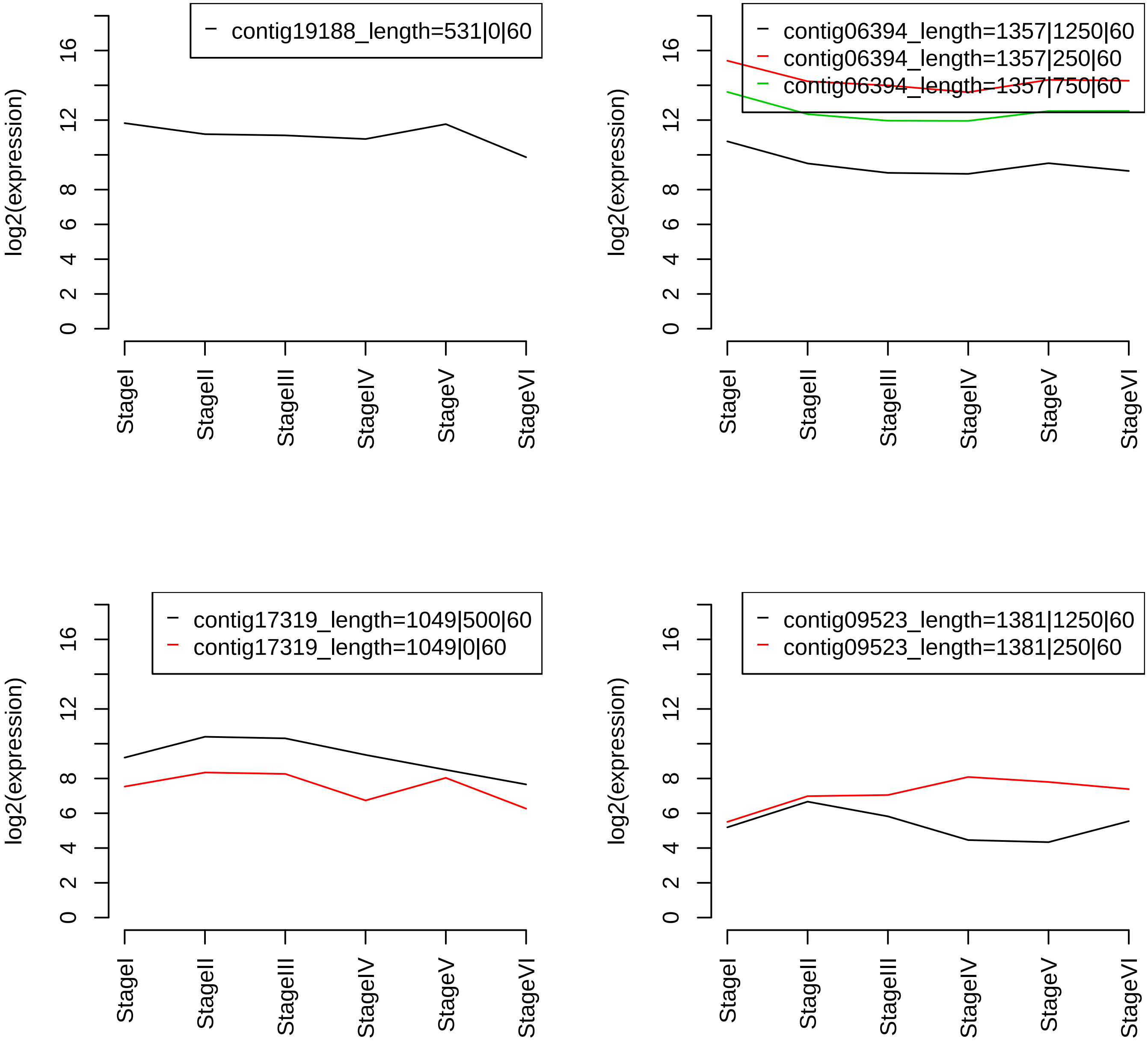
contig19188_length=531|0|60
contig06394_length=1357|1250|60
contig06394_length=1357|250|60
contig06394_length=1357|750|60
contig17319_length=1049|500|60
contig17319_length=1049|0|60
contig09523_length=1381|1250|60
contig09523_length=1381|250|60
log2(expression)
0
2
4
6
8
12
16
StageI
StageII
StageIII
StageIV
StageV
StageVI

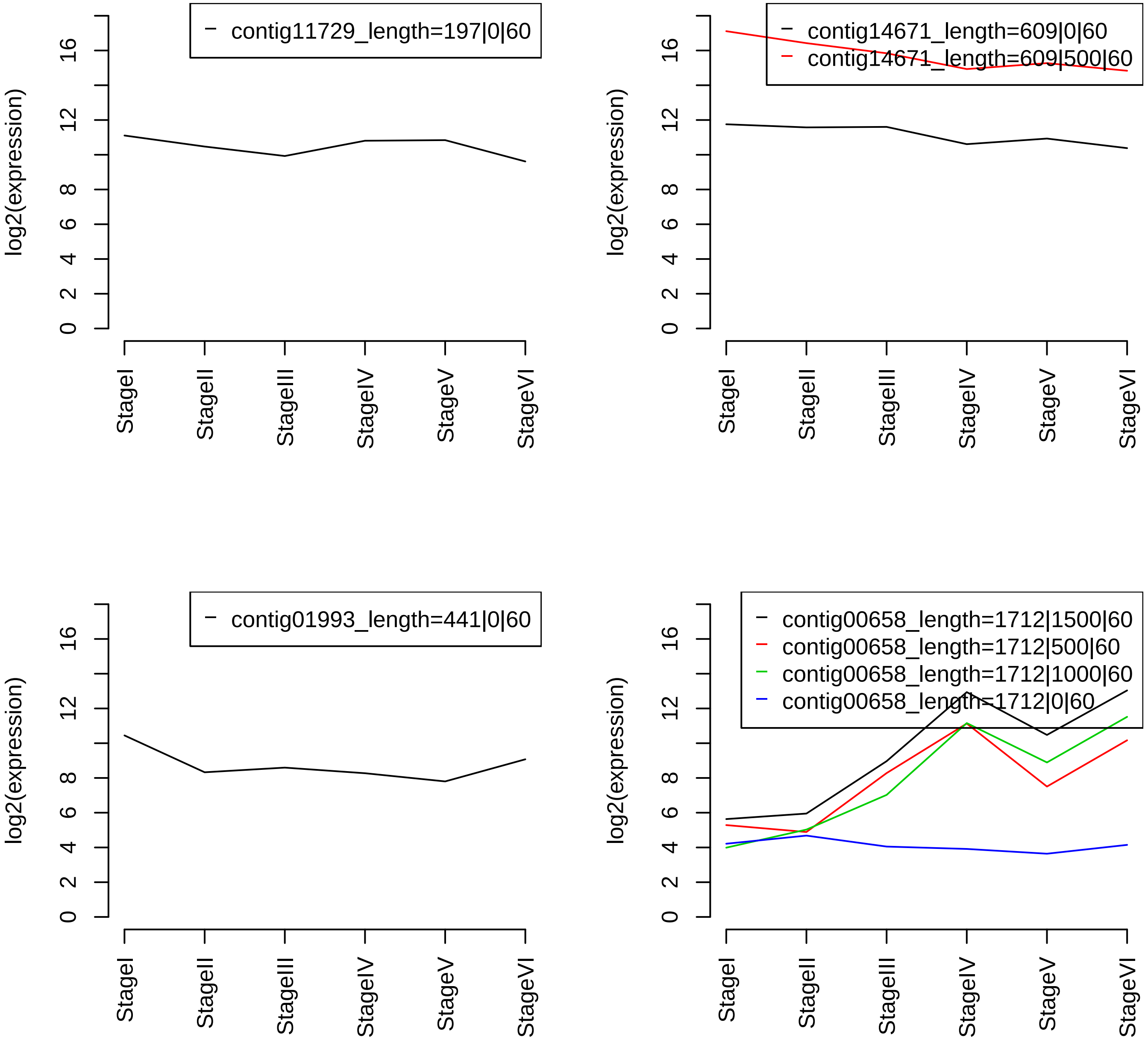

contig11729_length=197|0|60
contig14671_length=609|0|60
contig14671_length=609|500|60
contig01993_length=441|0|60
contig00658_length=1712|1500|60
contig00658_length=1712|500|60
contig00658_length=1712|1000|60
contig00658_length=1712|0|60
log2(expression)
0
2
4
6
8
12
16
StageI
StageII
StageIII
StageIV
StageV
StageVI

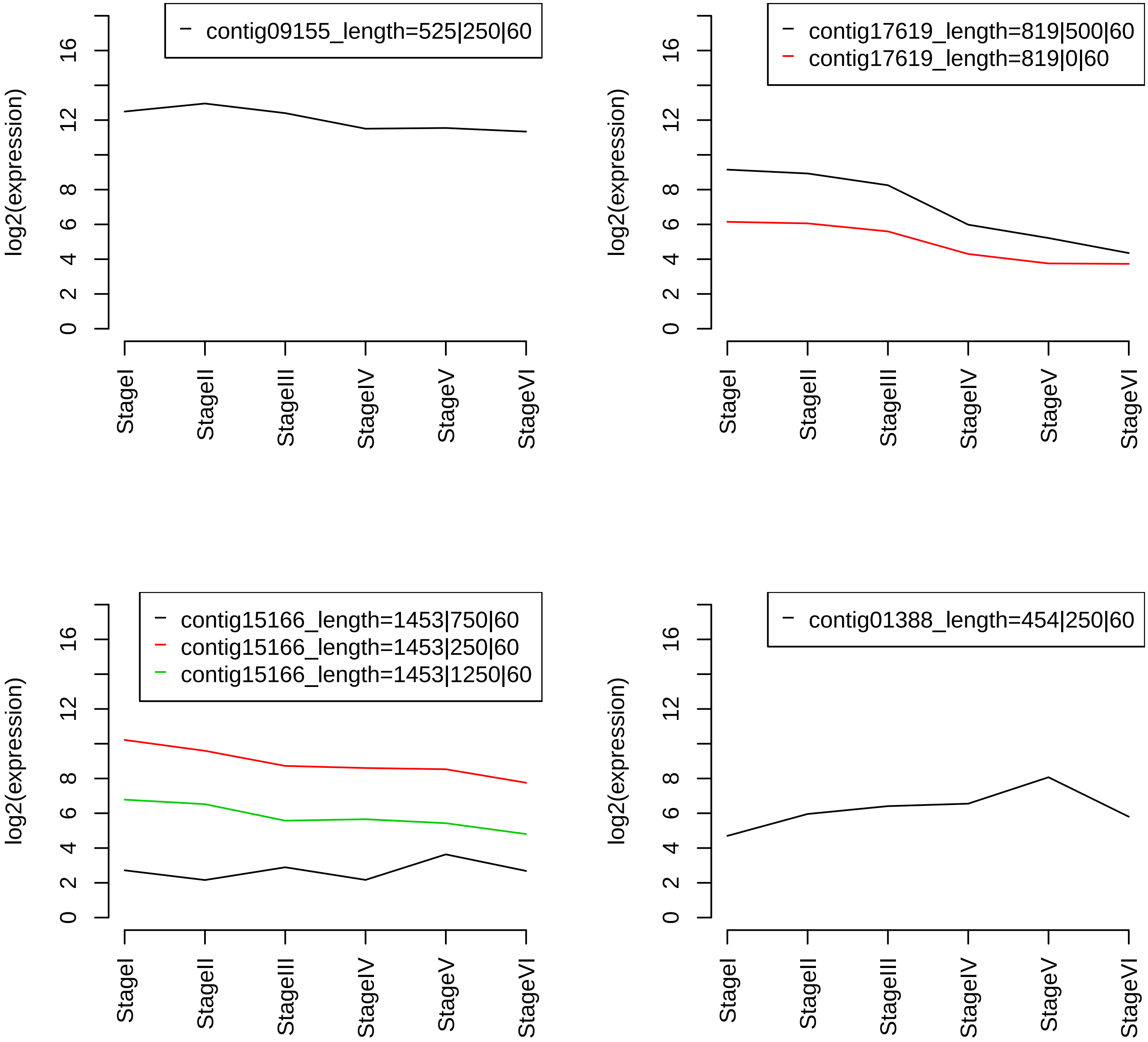
contig09155_length=525|250|60
contig17619_length=819|500|60
contig17619_length=819|0|60
contig15166_length=1453|750|60
contig15166_length=1453|250|60
contig15166_length=1453|1250|60
contig01388_length=454|250|60
log2(expression)
0 2 4 6 8 12 16
StageI StageII StageIII StageIV StageV StageVI

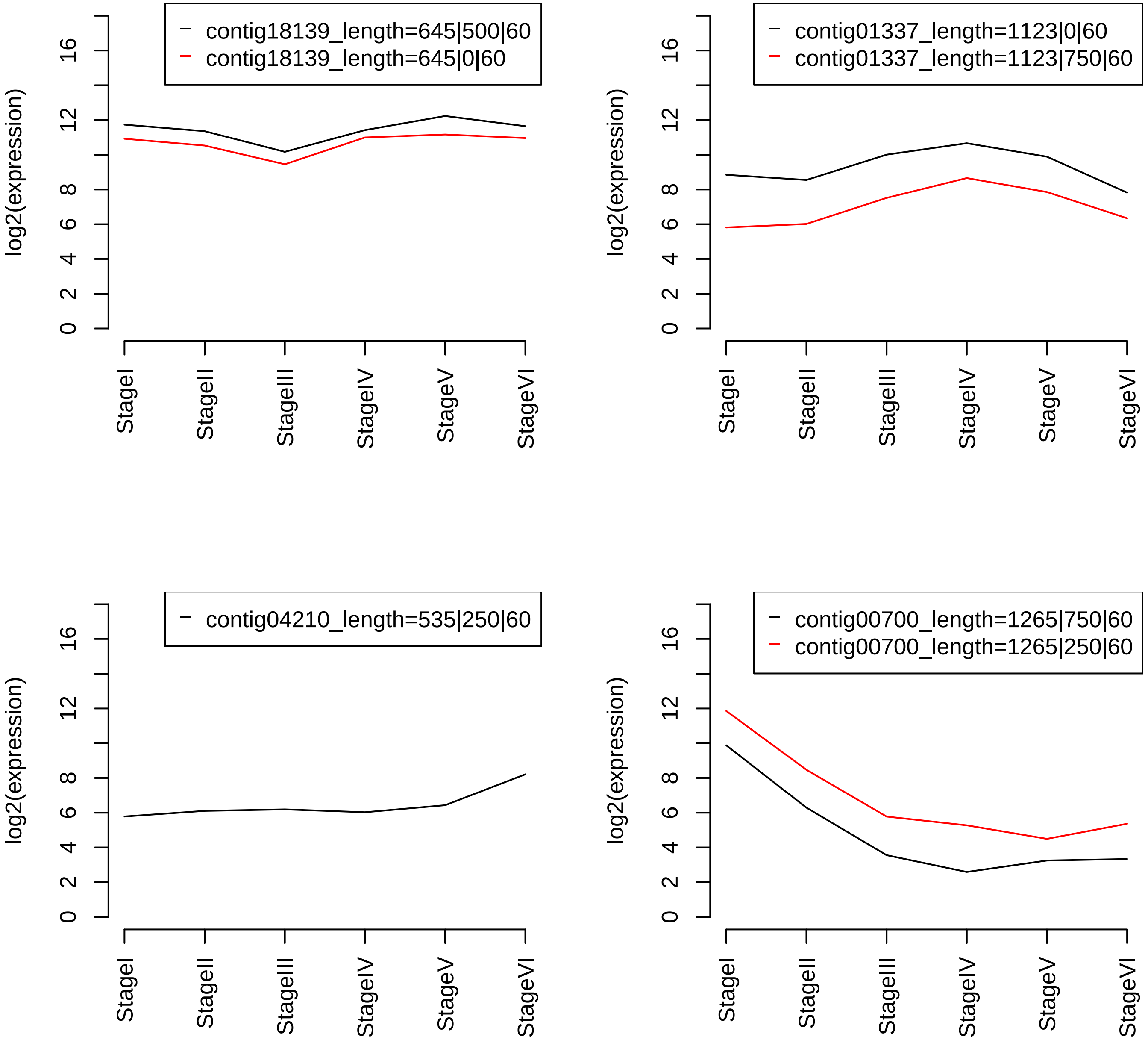

contig18139_length=645|500|60
contig18139_length=645|0|60
contig01337_length=1123|0|60
contig01337_length=1123|750|60
contig04210_length=535|250|60
contig00700_length=1265|750|60
contig00700_length=1265|250|60
log2(expression)
0
2
4
6
8
12
16
StageI
StageII
StageIII
StageIV
StageV
StageVI

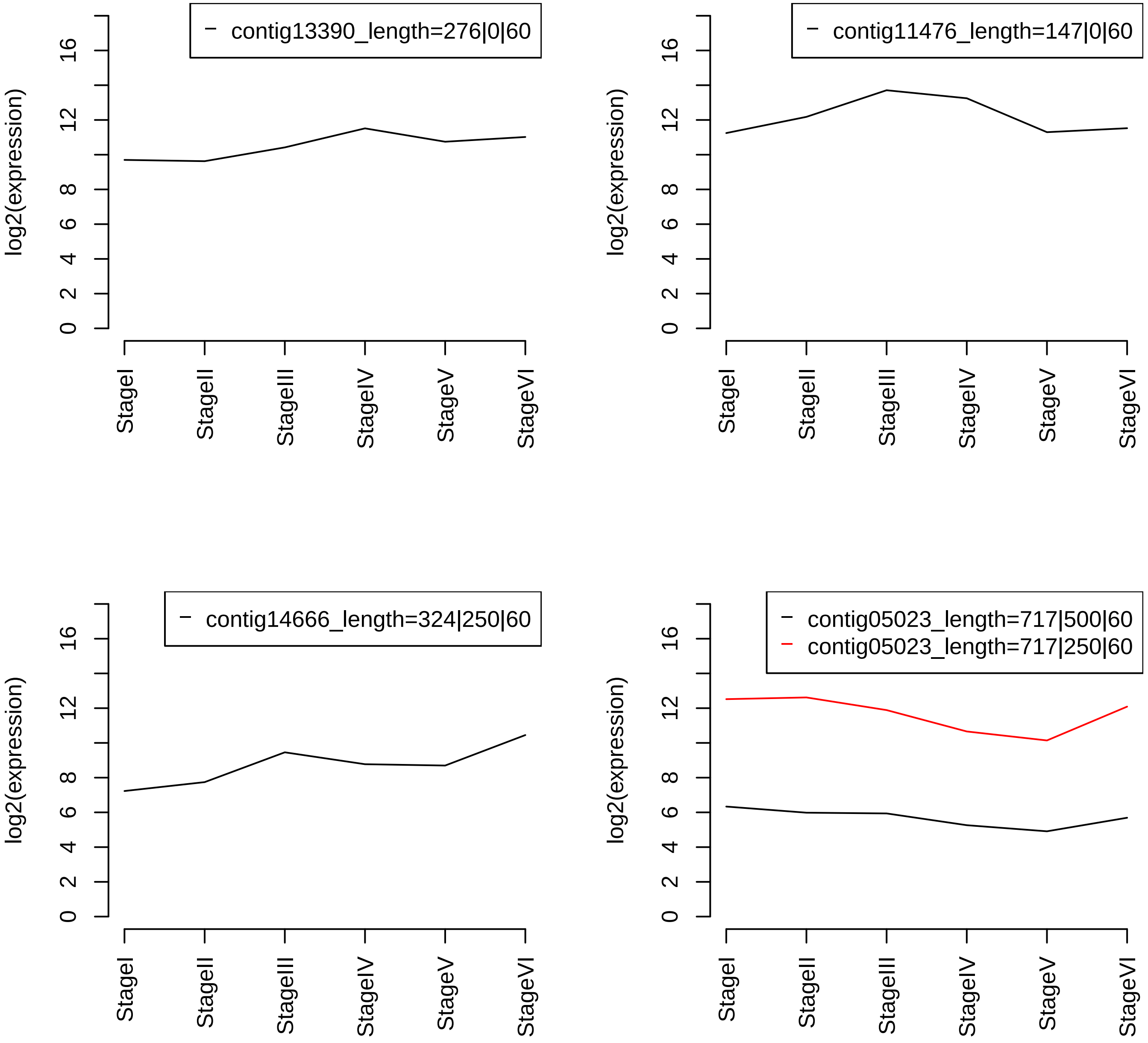

contig13390_length=276|0|60
contig11476_length=147|0|60
contig14666_length=324|250|60
contig05023_length=717|500|60
contig05023_length=717|250|60
log2(expression)
0
2
4
6
8
12
16
StageI
StageII
StageIII
StageIV
StageV
StageVI

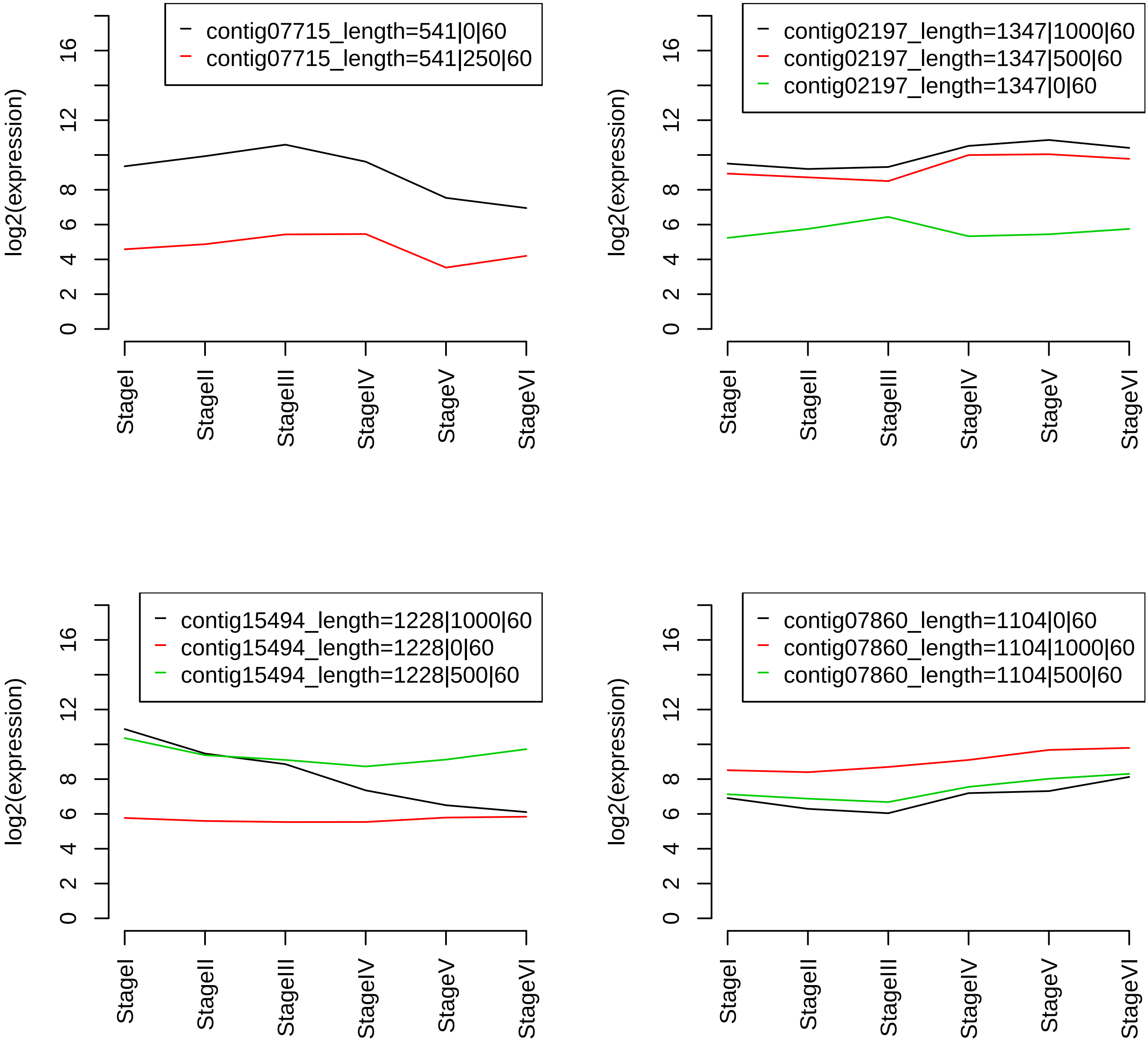
contig07715_length=541|0|60
contig07715_length=541|250|60
contig02197_length=1347|1000|60
contig02197_length=1347|500|60
contig02197_length=1347|0|60
contig15494_length=1228|1000|60
contig15494_length=1228|0|60
contig15494_length=1228|500|60
contig07860_length=1104|0|60
contig07860_length=1104|1000|60
contig07860_length=1104|500|60
log2(expression)
0 2 4 6 8 12 16
StageI
StageII
StageIII
StageIV
StageV
StageVI

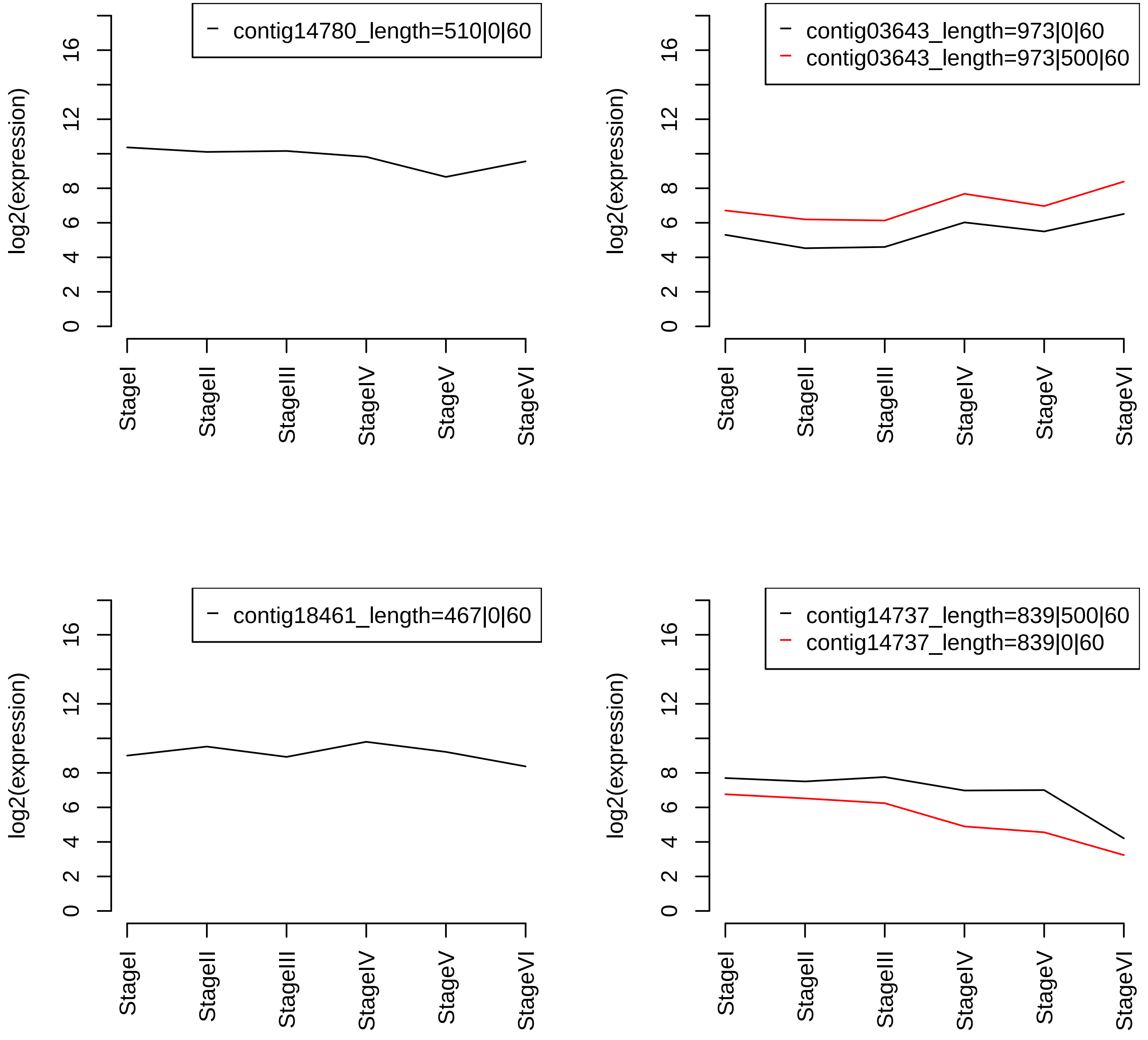

contig14780_length=510|0|60
contig03643_length=973|0|60
contig03643_length=973|500|60
contig18461_length=467|0|60
contig14737_length=839|500|60
contig14737_length=839|0|60
log2(expression)
0
2
4
6
8
12
16
StageI
StageII
StageIII
StageIV
StageV
StageVI

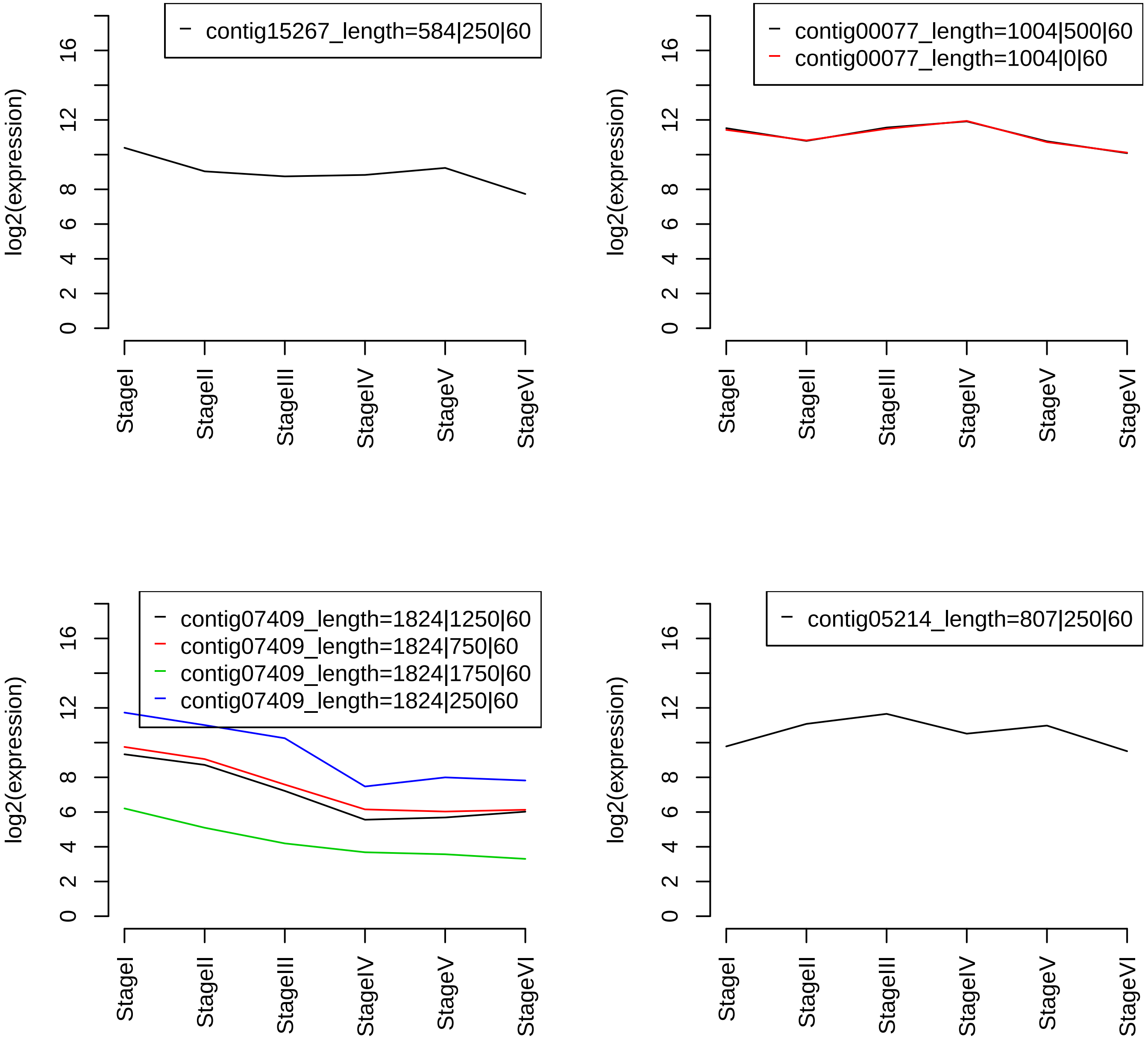
contig15267_length=584|250|60
contig00077_length=1004|500|60
contig00077_length=1004|0|60
contig07409_length=1824|1250|60
contig07409_length=1824|750|60
contig07409_length=1824|1750|60
contig07409_length=1824|250|60
contig05214_length=807|250|60
log2(expression)
0
2
4
6
8
12
16
StageI
StageII
StageIII
StageIV
StageV
StageVI

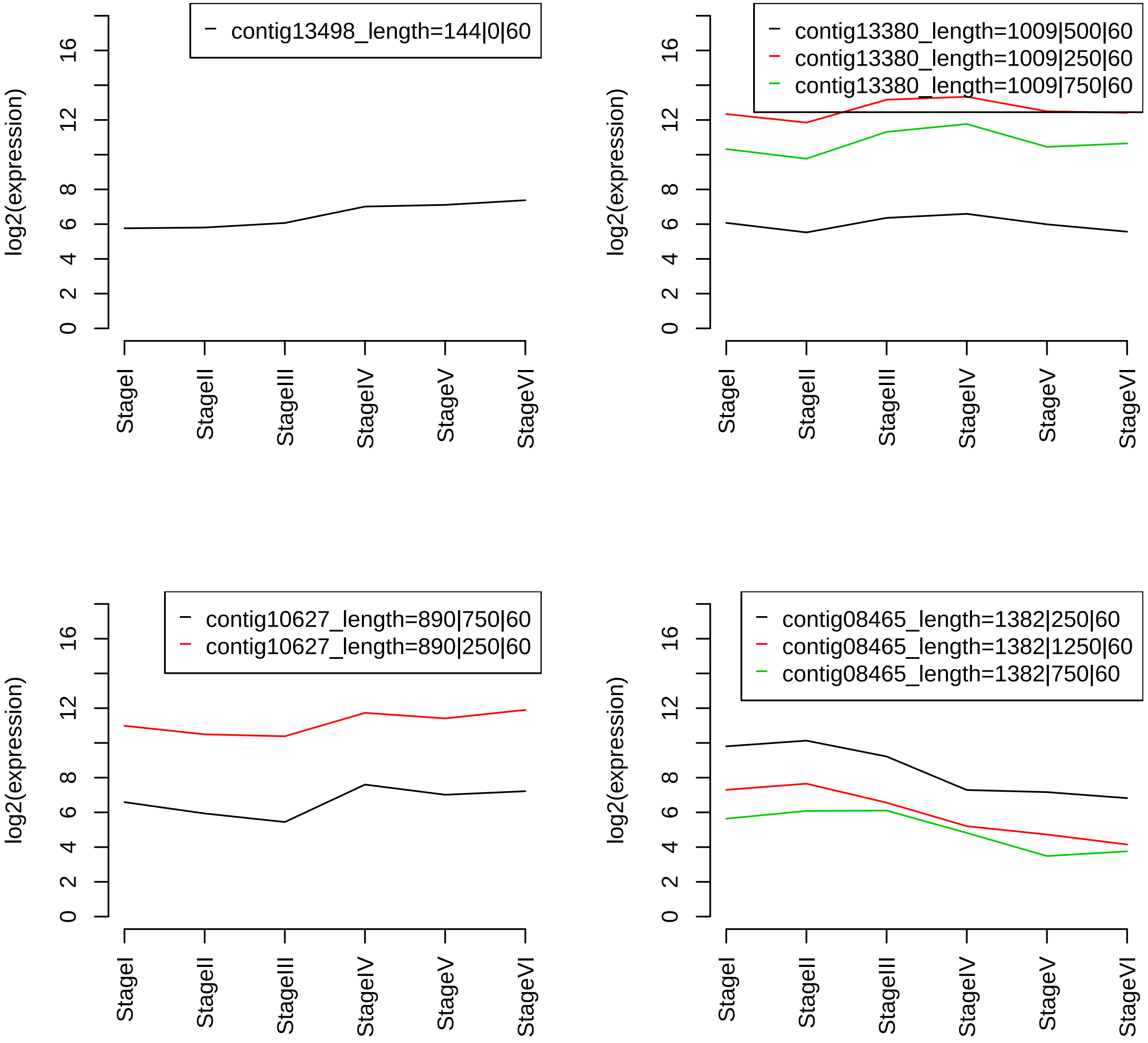
contig13498_length=144|0|60
contig13380_length=1009|500|60
contig13380_length=1009|250|60
contig13380_length=1009|750|60
contig10627_length=890|750|60
contig10627_length=890|250|60
contig08465_length=1382|250|60
contig08465_length=1382|1250|60
contig08465_length=1382|750|60
log2(expression)
0 2 4 6 8 12 16
StageI StageII StageIII StageIV StageV StageVI

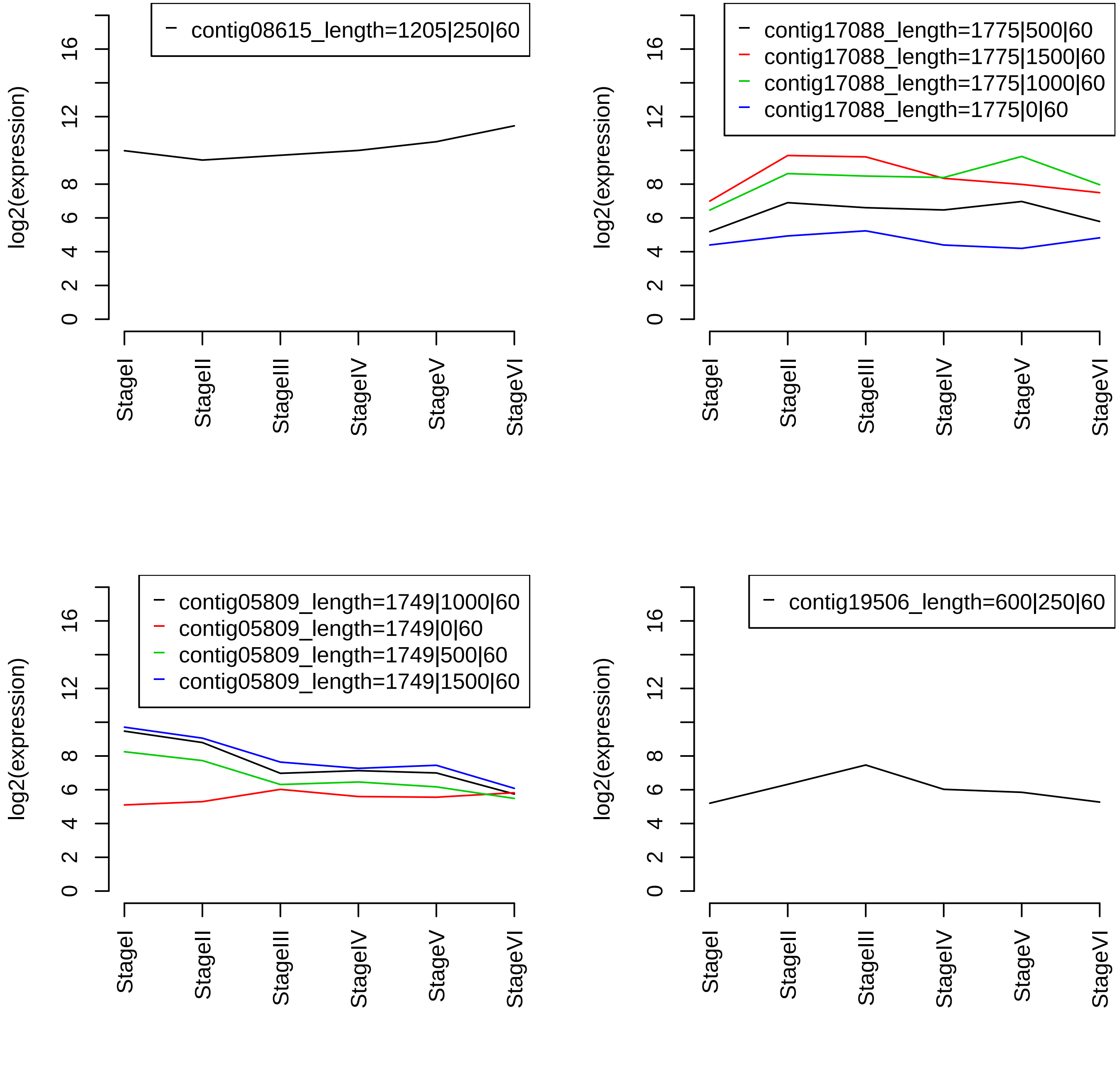

contig08615_length=1205|250|60
contig17088_length=1775|500|60
contig17088_length=1775|1500|60
contig17088_length=1775|1000|60
contig17088_length=1775|0|60
contig05809_length=1749|1000|60
contig05809_length=1749|0|60
contig05809_length=1749|500|60
contig05809_length=1749|1500|60
contig19506_length=600|250|60
log2(expression)
0 2 4 6 8 12 16
StageI StageII StageIII StageIV StageV StageVI

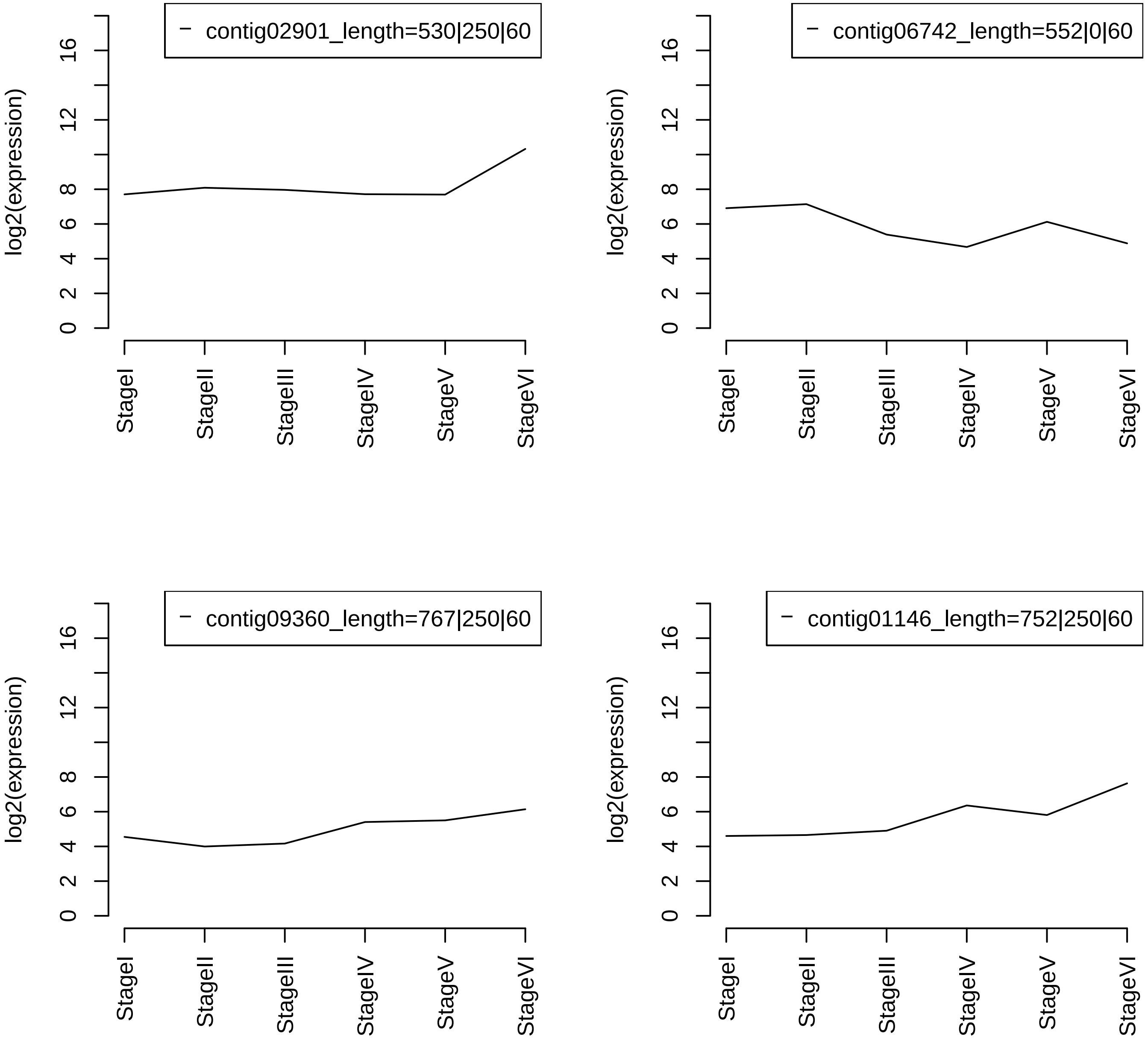
contig02901_length=530|250|60
contig06742_length=552|0|60
contig09360_length=767|250|60
contig01146_length=752|250|60
log2(expression)
0
2
4
6
8
12
16
StageI
StageII
StageIII
StageIV
StageV
StageVI

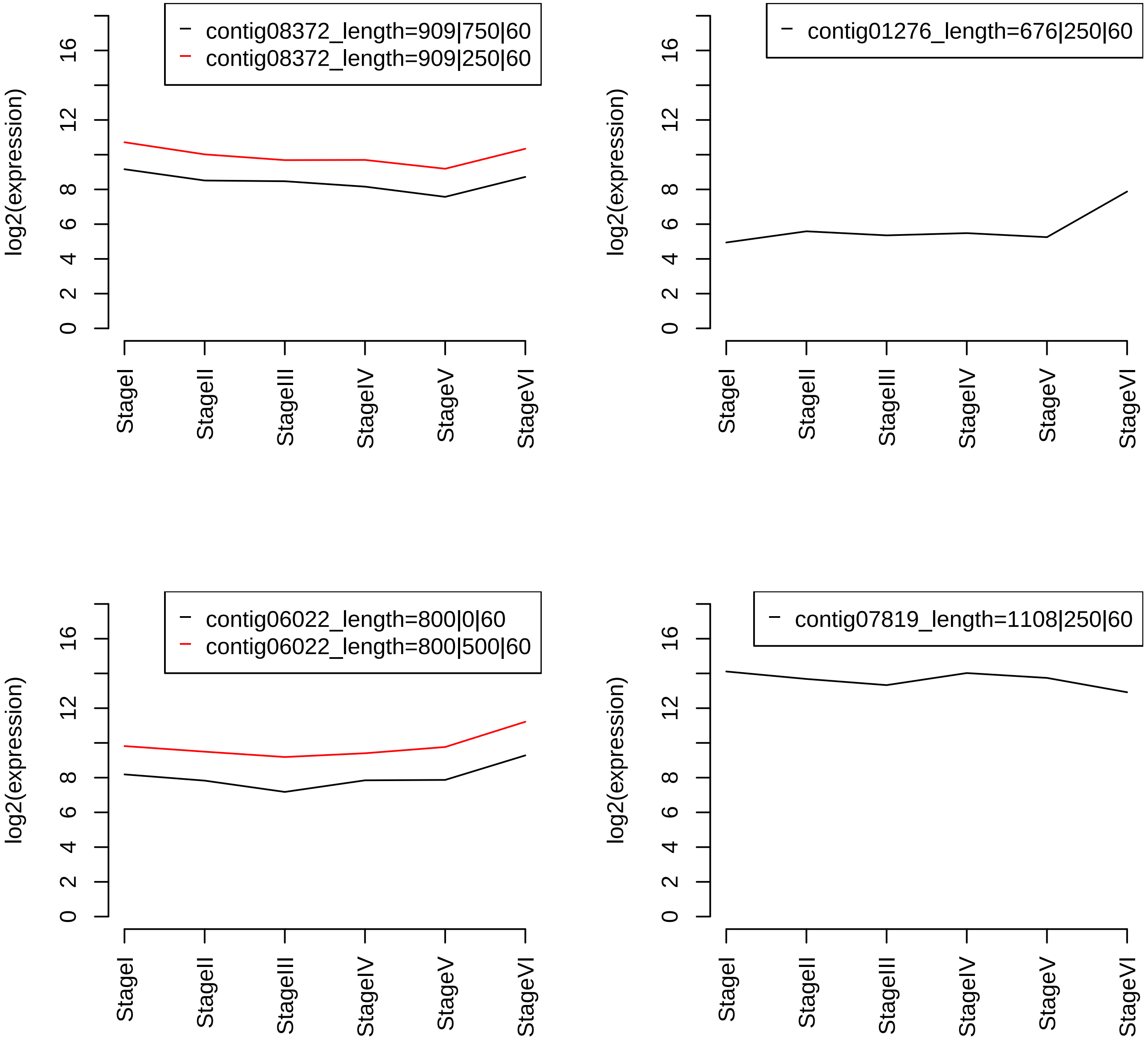
contig08372_length=909|750|60
contig08372_length=909|250|60
contig01276_length=676|250|60
contig06022_length=800|0|60
contig06022_length=800|500|60
contig07819_length=1108|250|60
log2(expression)
0
2
4
6
8
12
16
StageI
StageII
StageIII
StageIV
StageV
StageVI

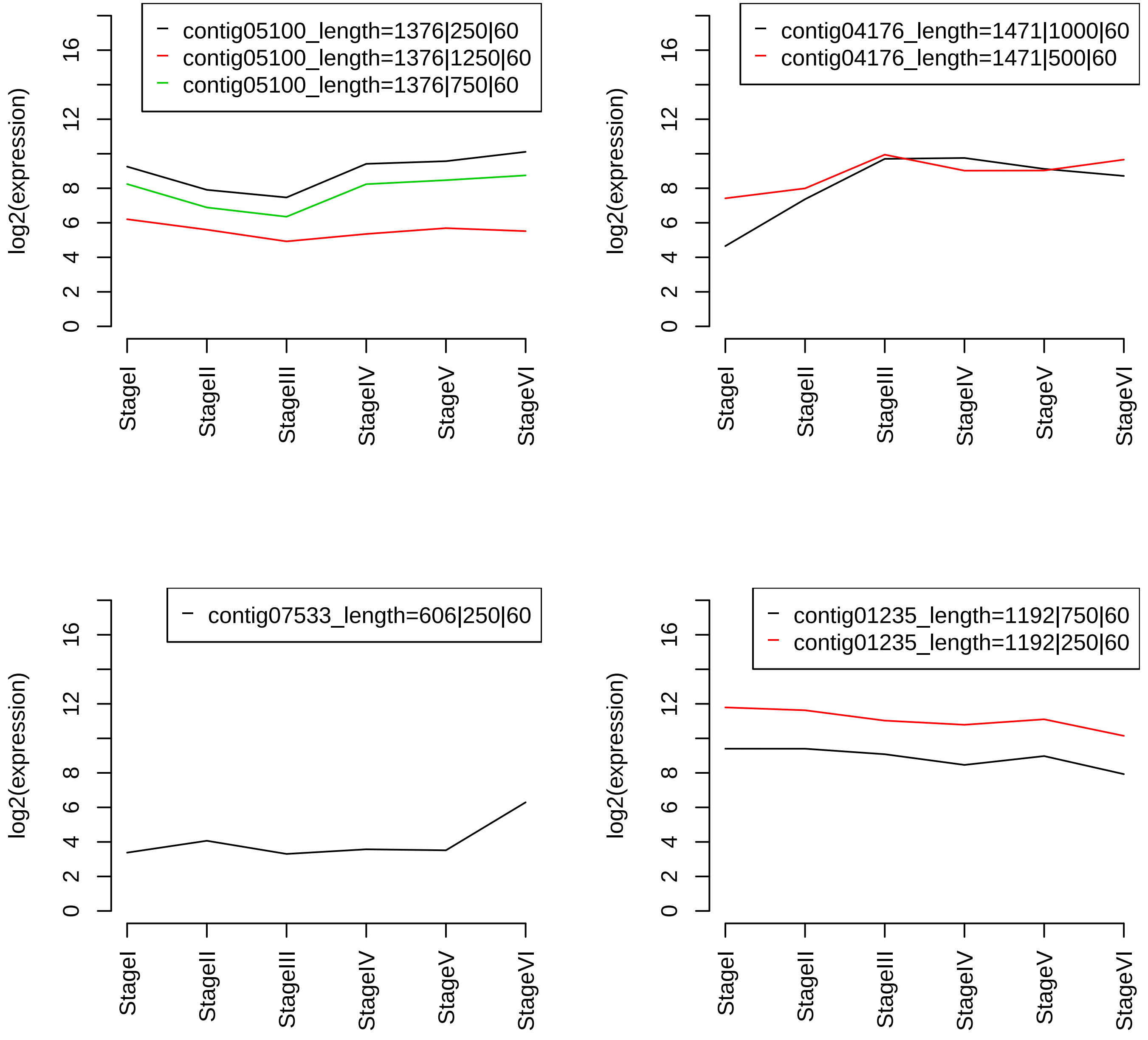

contig05100_length=1376|250|60
contig05100_length=1376|1250|60
contig05100_length=1376|750|60
contig04176_length=1471|1000|60
contig04176_length=1471|500|60
contig07533_length=606|250|60
contig01235_length=1192|750|60
contig01235_length=1192|250|60
log2(expression)
0
2
4
6
8
12
16
StageI
StageII
StageIII
StageIV
StageV
StageVI

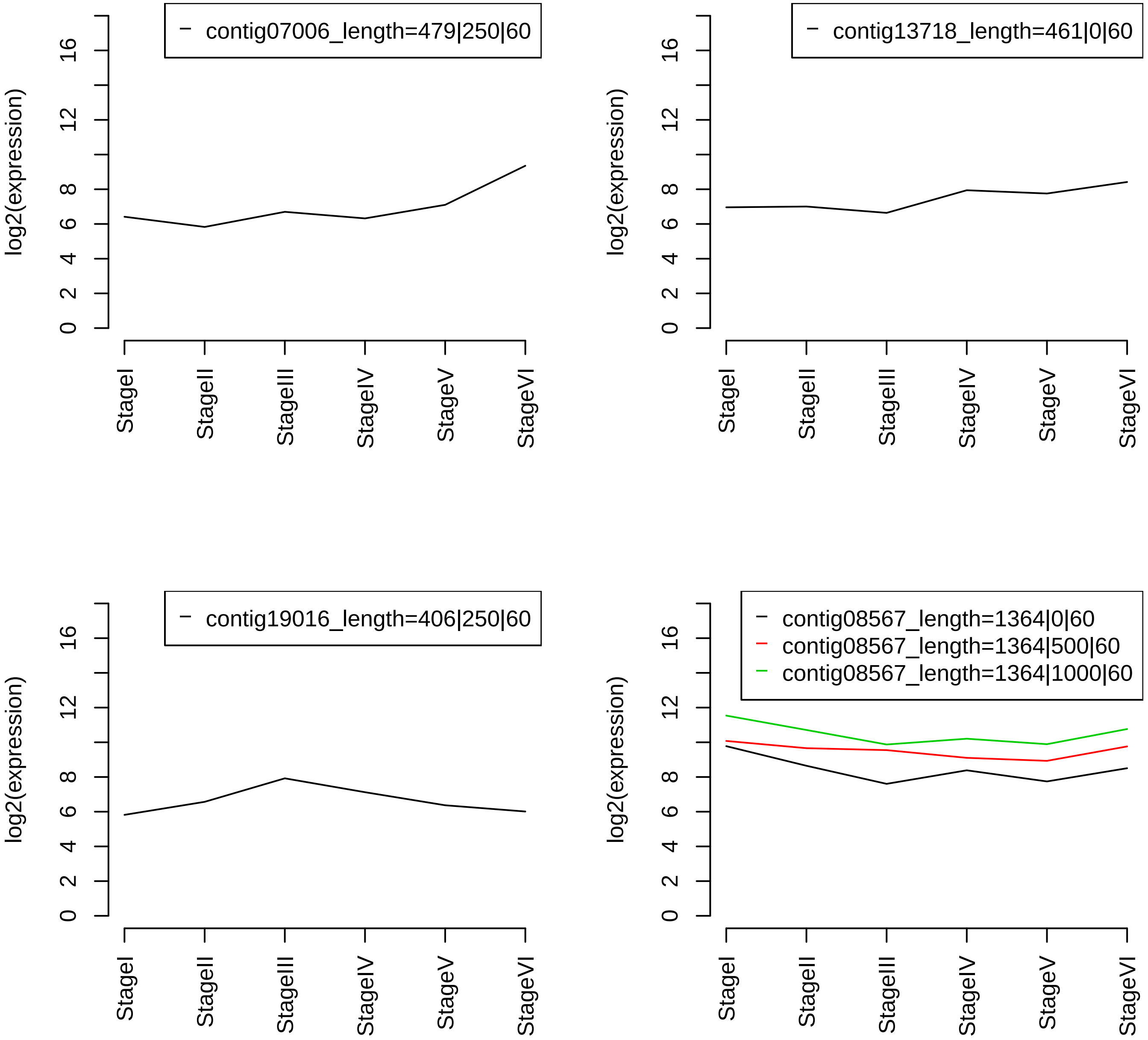
contig07006_length=479|250|60
contig13718_length=461|0|60
contig19016_length=406|250|60
contig08567_length=1364|0|60
contig08567_length=1364|500|60
contig08567_length=1364|1000|60
log2(expression)
0
2
4
6
8
12
16
StageI
StageII
StageIII
StageIV
StageV
StageVI

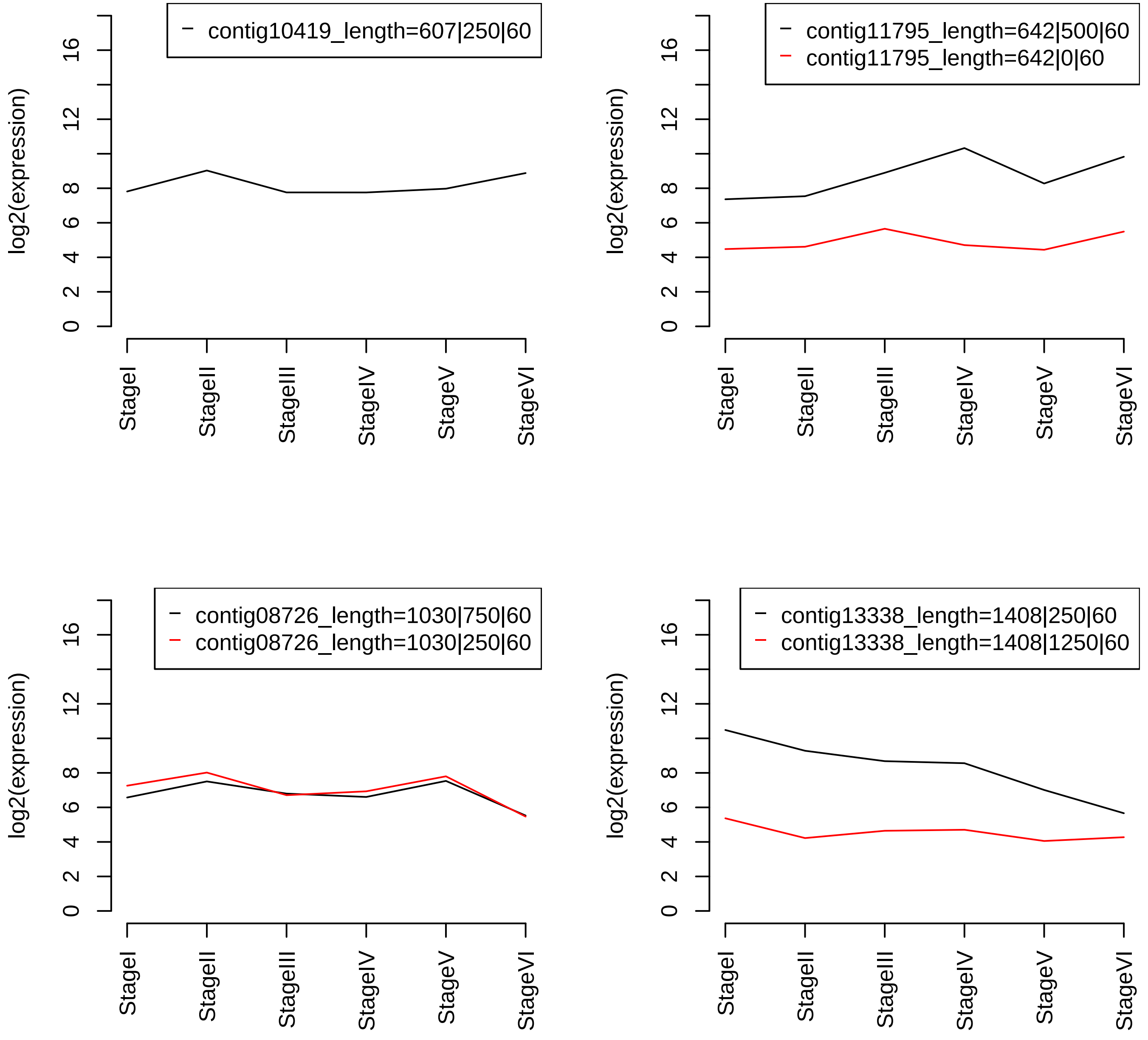

contig10419_length=607|250|60
contig11795_length=642|500|60
contig11795_length=642|0|60
contig08726_length=1030|750|60
contig08726_length=1030|250|60
contig13338_length=1408|250|60
contig13338_length=1408|1250|60
log2(expression)
0
2
4
6
8
12
16
StageI
StageII
StageIII
StageIV
StageV
StageVI

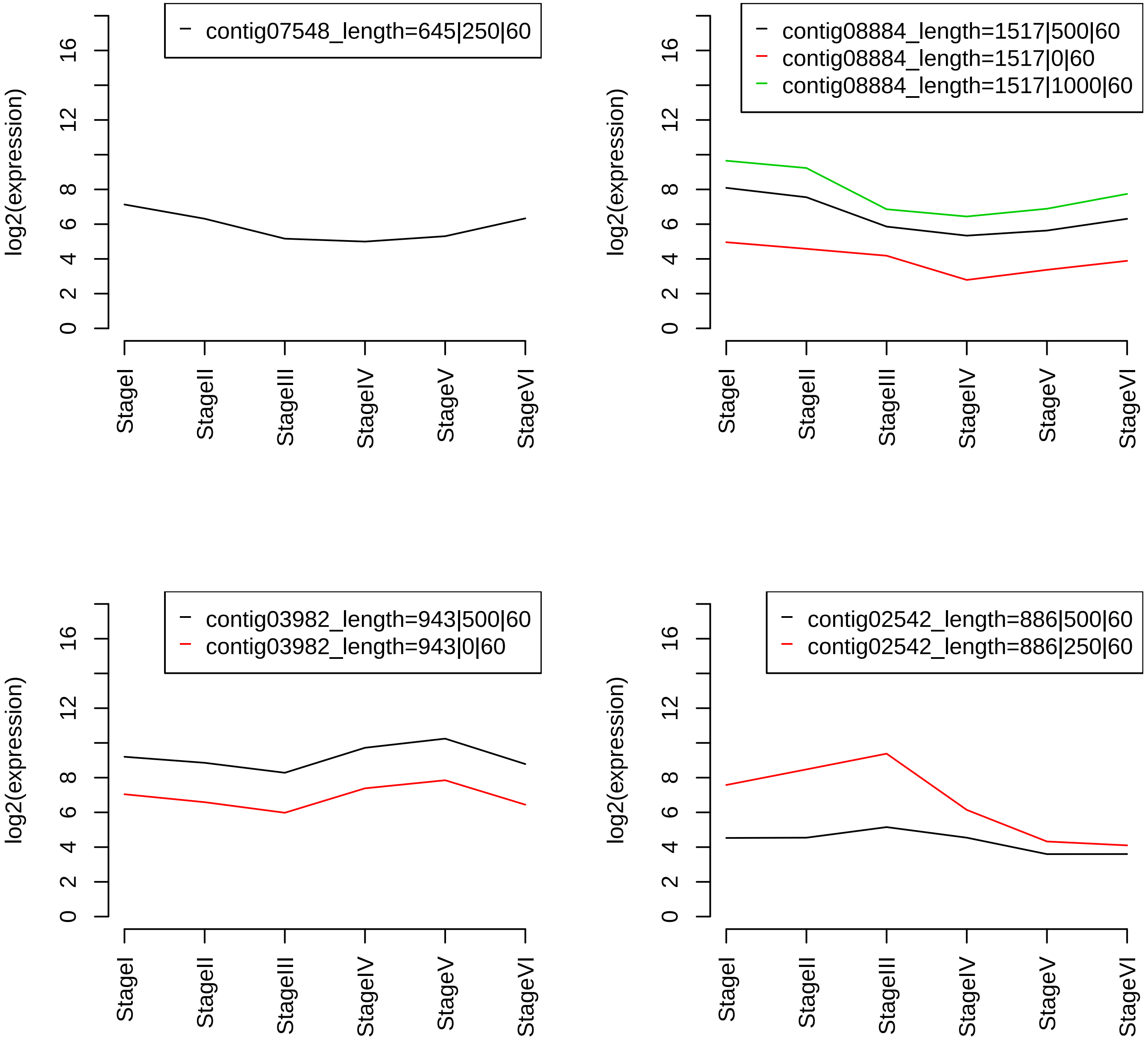

contig07548_length=645|250|60
contig08884_length=1517|500|60
contig08884_length=1517|0|60
contig08884_length=1517|1000|60
contig03982_length=943|500|60
contig03982_length=943|0|60
contig02542_length=886|500|60
contig02542_length=886|250|60
log2(expression)
0
2
4
6
8
12
16
StageI
StageII
StageIII
StageIV
StageV
StageVI

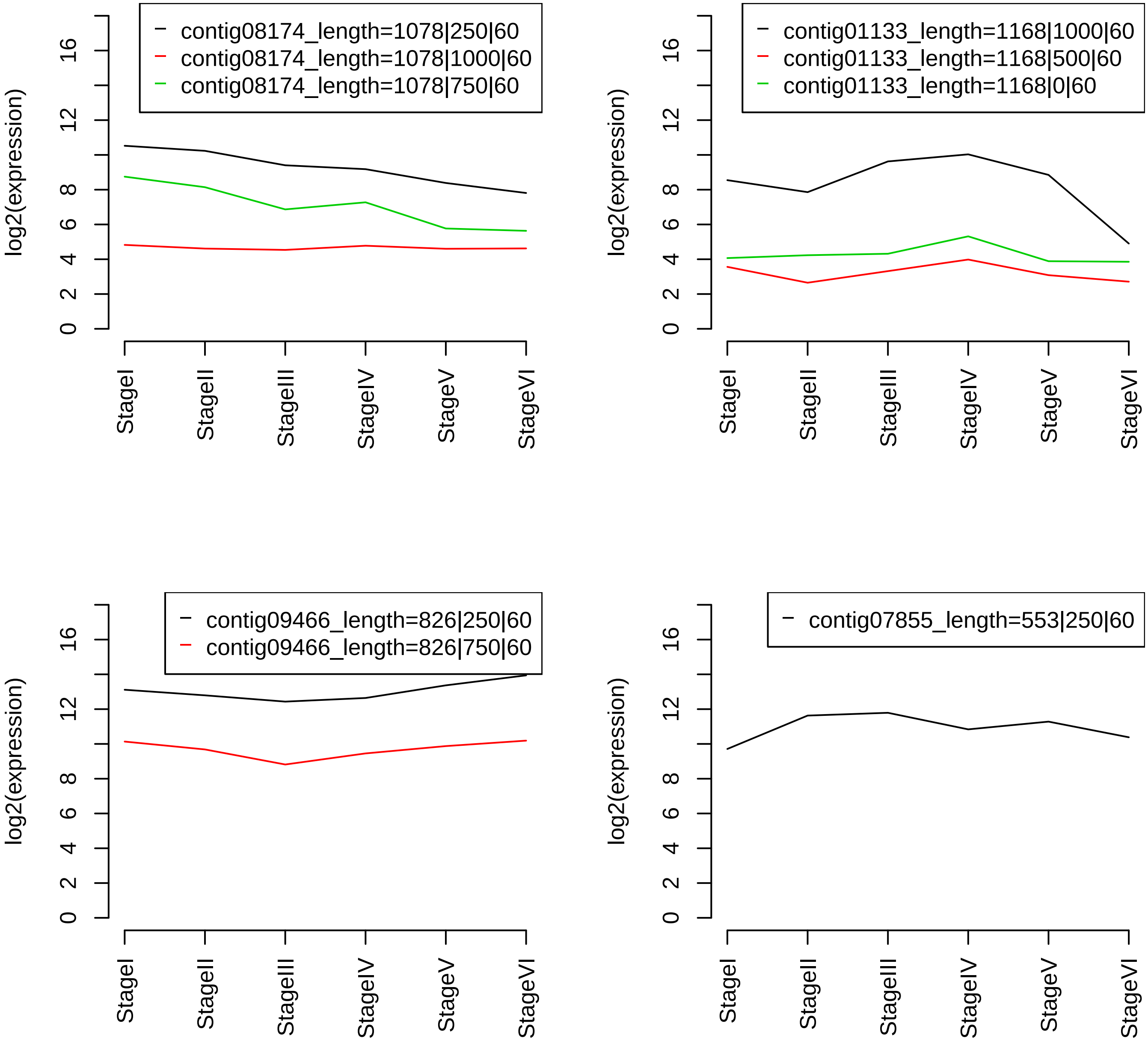

contig08174_length=1078|250|60
contig08174_length=1078|1000|60
contig08174_length=1078|750|60
contig01133_length=1168|1000|60
contig01133_length=1168|500|60
contig01133_length=1168|0|60
contig09466_length=826|250|60
contig09466_length=826|750|60
contig07855_length=553|250|60
log2(expression)
0
2
4
6
8
12
16
StageI
StageII
StageIII
StageIV
StageV
StageVI

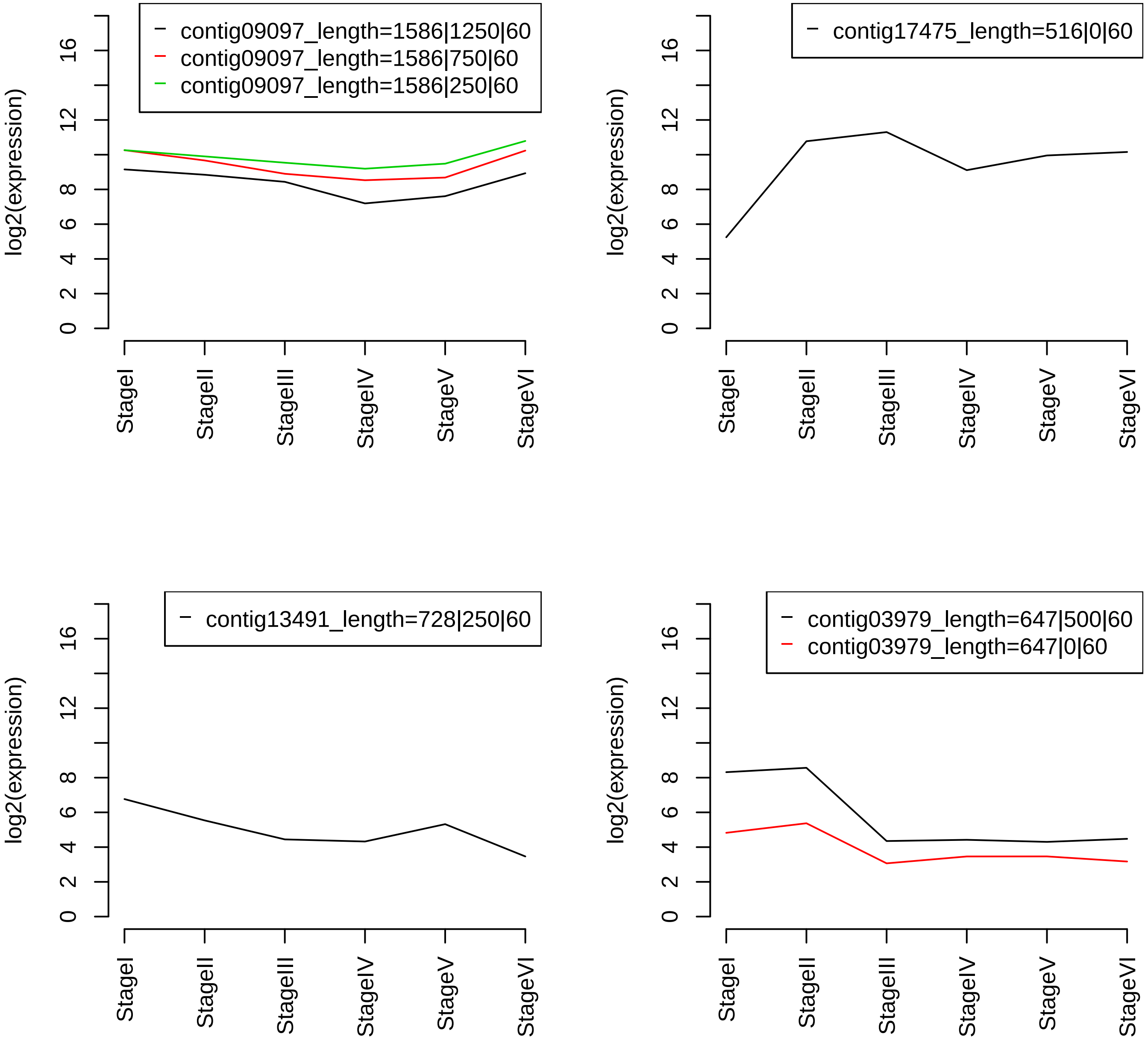

contig09097_length=1586|1250|60
contig09097_length=1586|750|60
contig09097_length=1586|250|60
contig17475_length=516|0|60
contig13491_length=728|250|60
contig03979_length=647|500|60
contig03979_length=647|0|60
log2(expression)
0
2
4
6
8
12
16
StageI
StageII
StageIII
StageIV
StageV
StageVI

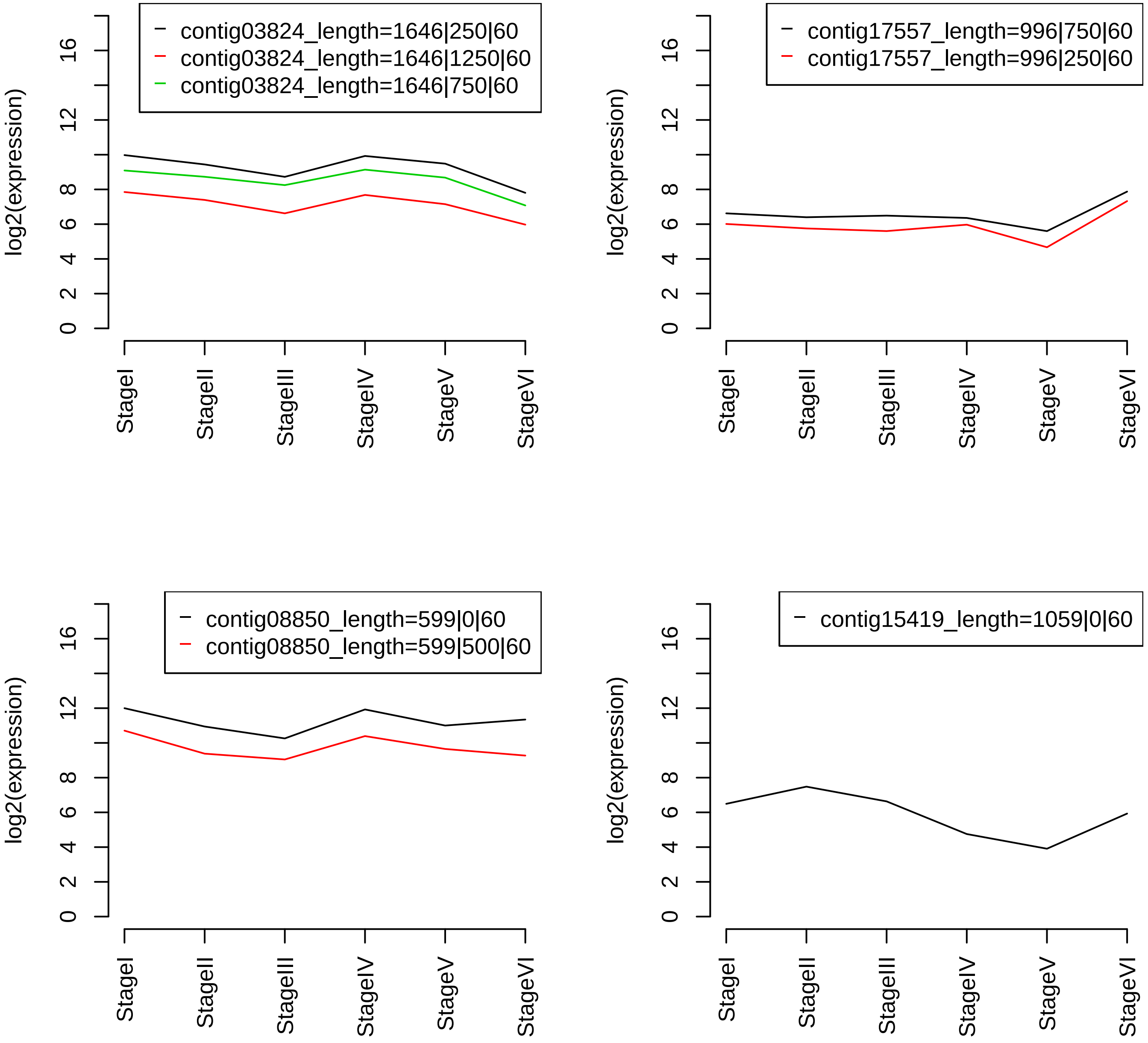
contig03824_length=1646|250|60
contig03824_length=1646|1250|60
contig03824_length=1646|750|60
contig17557_length=996|750|60
contig17557_length=996|250|60
contig08850_length=599|0|60
contig08850_length=599|500|60
contig15419_length=1059|0|60
log2(expression)
0
2
4
6
8
12
16
StageI
StageII
StageIII
StageIV
StageV
StageVI

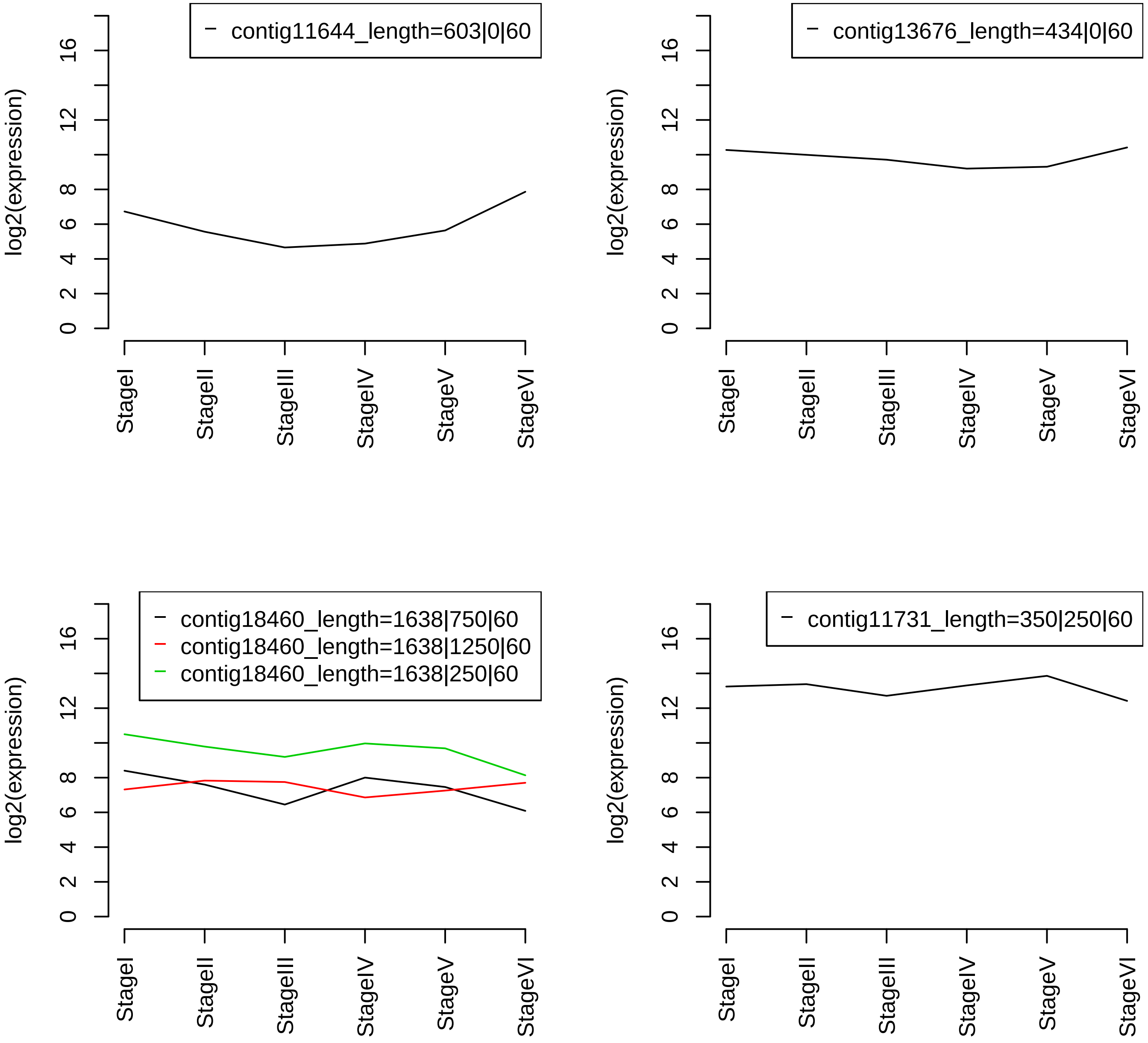

contig11644_length=603|0|60
log2(expression)
0
2
4
6
8
12
16
StageI
StageII
StageIII
StageIV
StageV
StageVI
contig13676_length=434|0|60
contig18460_length=1638|750|60
contig18460_length=1638|1250|60
contig18460_length=1638|250|60
contig11731_length=350|250|60

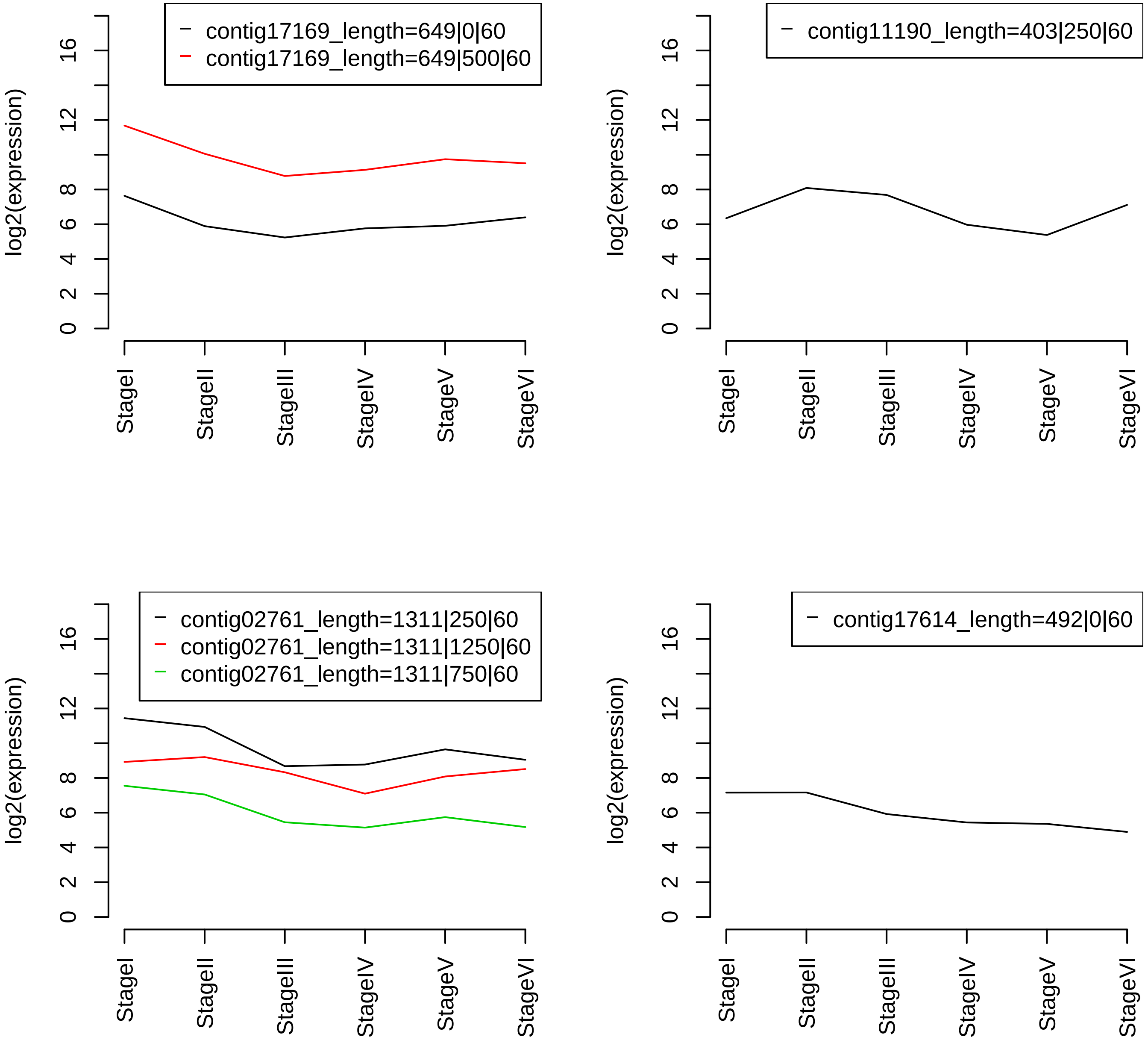
contig17169_length=649|0|60
contig17169_length=649|500|60
contig11190_length=403|250|60
contig02761_length=1311|250|60
contig02761_length=1311|1250|60
contig02761_length=1311|750|60
contig17614_length=492|0|60
log2(expression)
0
2
4
6
8
12
16
StageI
StageII
StageIII
StageIV
StageV
StageVI

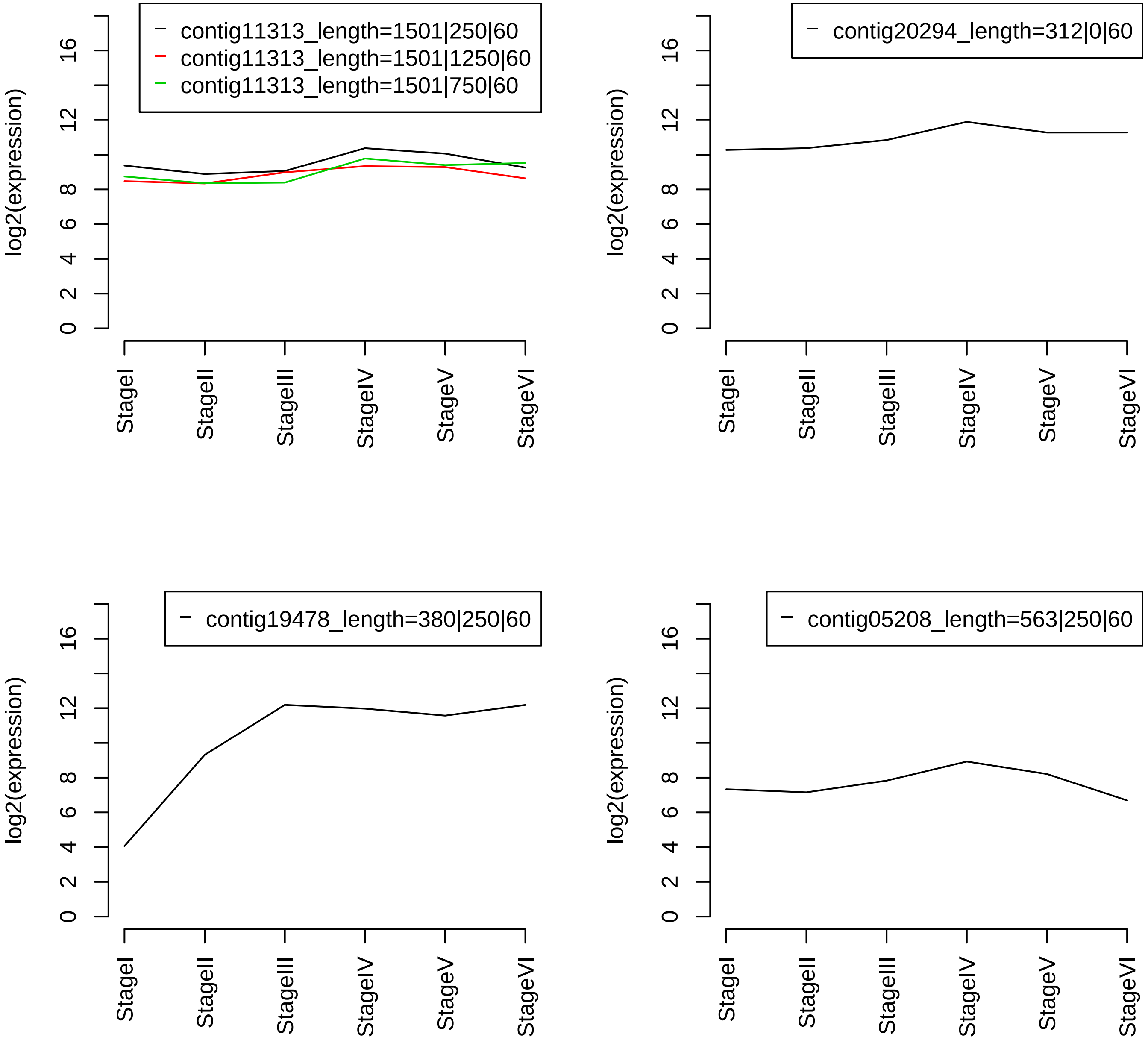
contig11313_length=1501|250|60
contig11313_length=1501|1250|60
contig11313_length=1501|750|60
contig20294_length=312|0|60
contig19478_length=380|250|60
contig05208_length=563|250|60
log2(expression)
0
2
4
6
8
12
16
StageI
StageII
StageIII
StageIV
StageV
StageVI

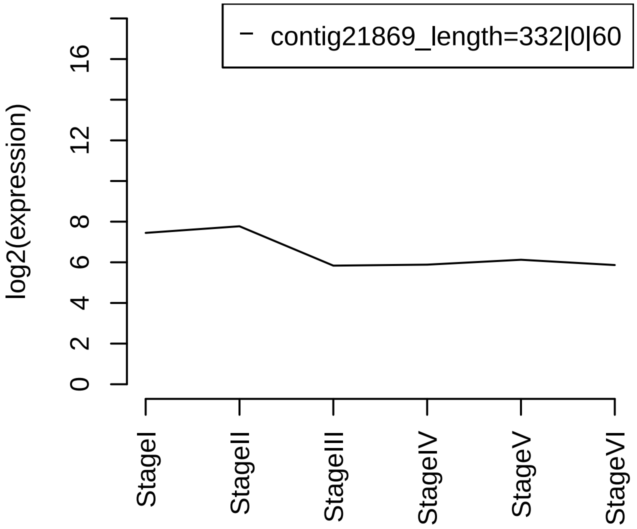
contig21869_length=332|0|60
log2(expression)
0
2
4
6
8
12
16
StageI
StageII
StageIII
StageIV
StageV
StageVI

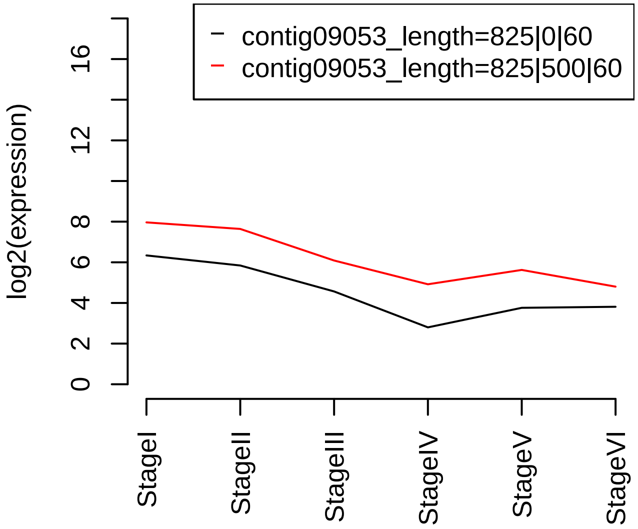
contig09053_length=825|0|60
contig09053_length=825|500|60
log2(expression)
0
2
4
6
8
12
16
StageI
StageII
StageIII
StageIV
StageV
StageVI

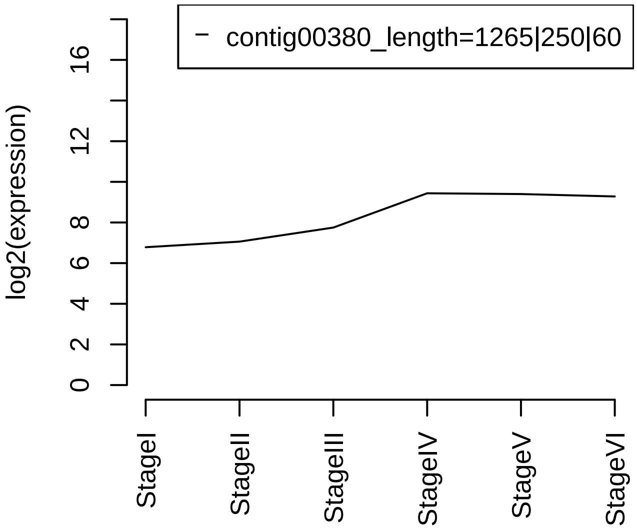
contig00380_length=1265|250|60
log2(expression)
0
2
4
6
8
12
16
StageI
StageII
StageIII
StageIV
StageV
StageVI

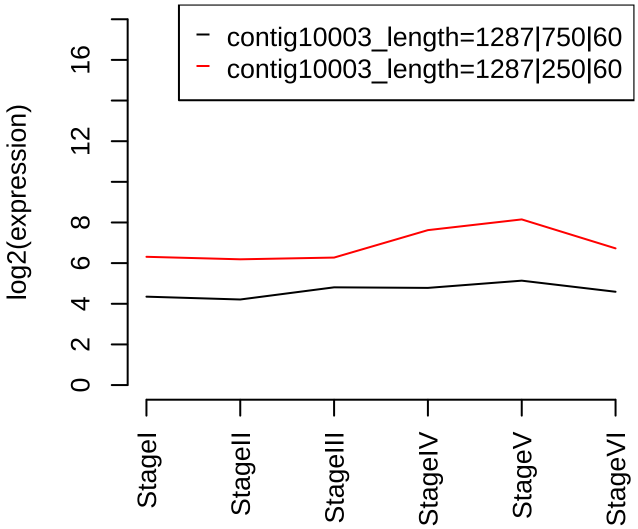
contig10003_length=1287|750|60
contig10003_length=1287|250|60
log2(expression)
0
2
4
6
8
12
16
StageI
StageII
StageIII
StageIV
StageV
StageVI

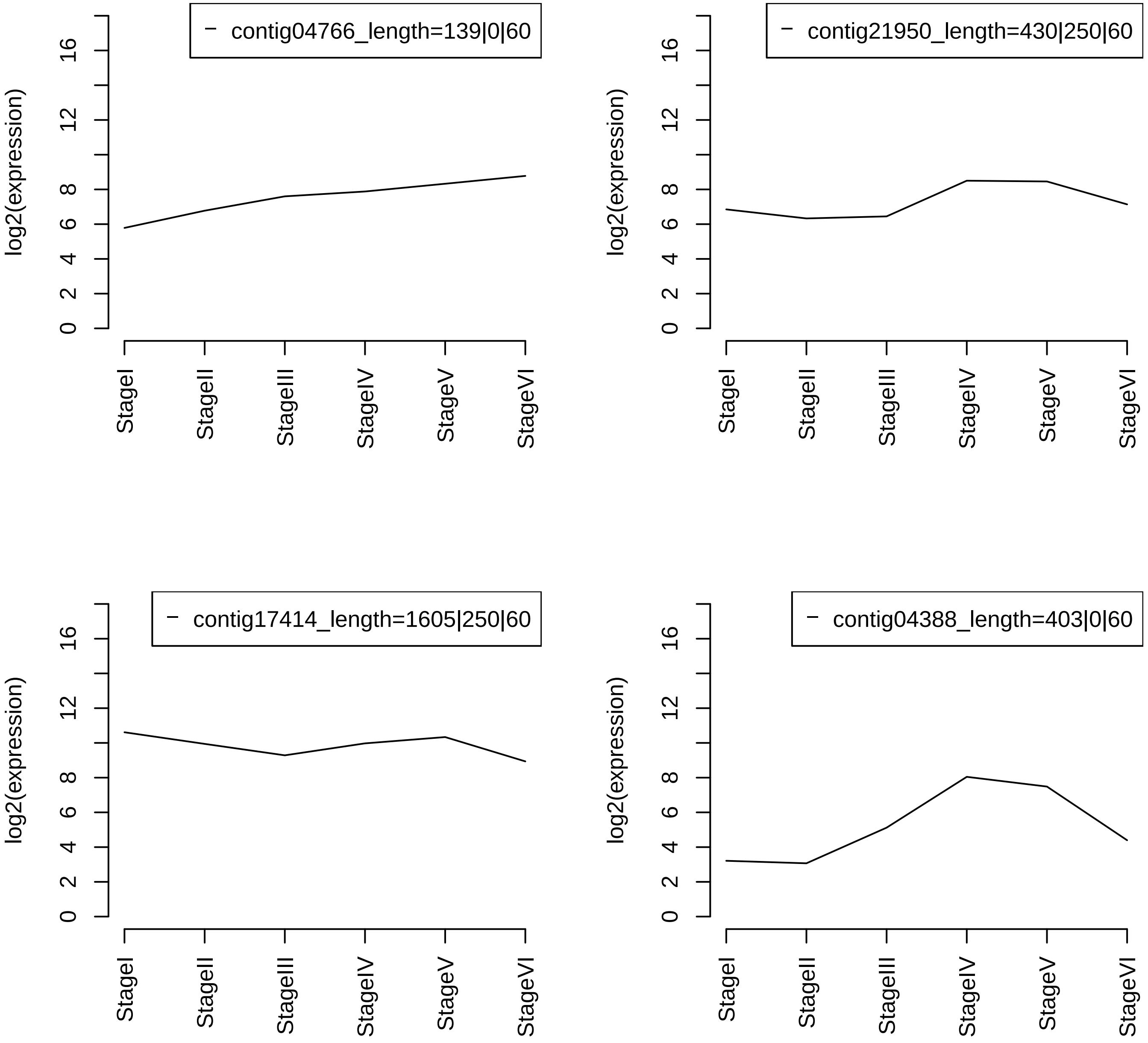

contig04766_length=139|0|60
contig21950_length=430|250|60
contig17414_length=1605|250|60
contig04388_length=403|0|60
log2(expression)
0
2
4
6
8
12
16
StageI
StageII
StageIII
StageIV
StageV
StageVI

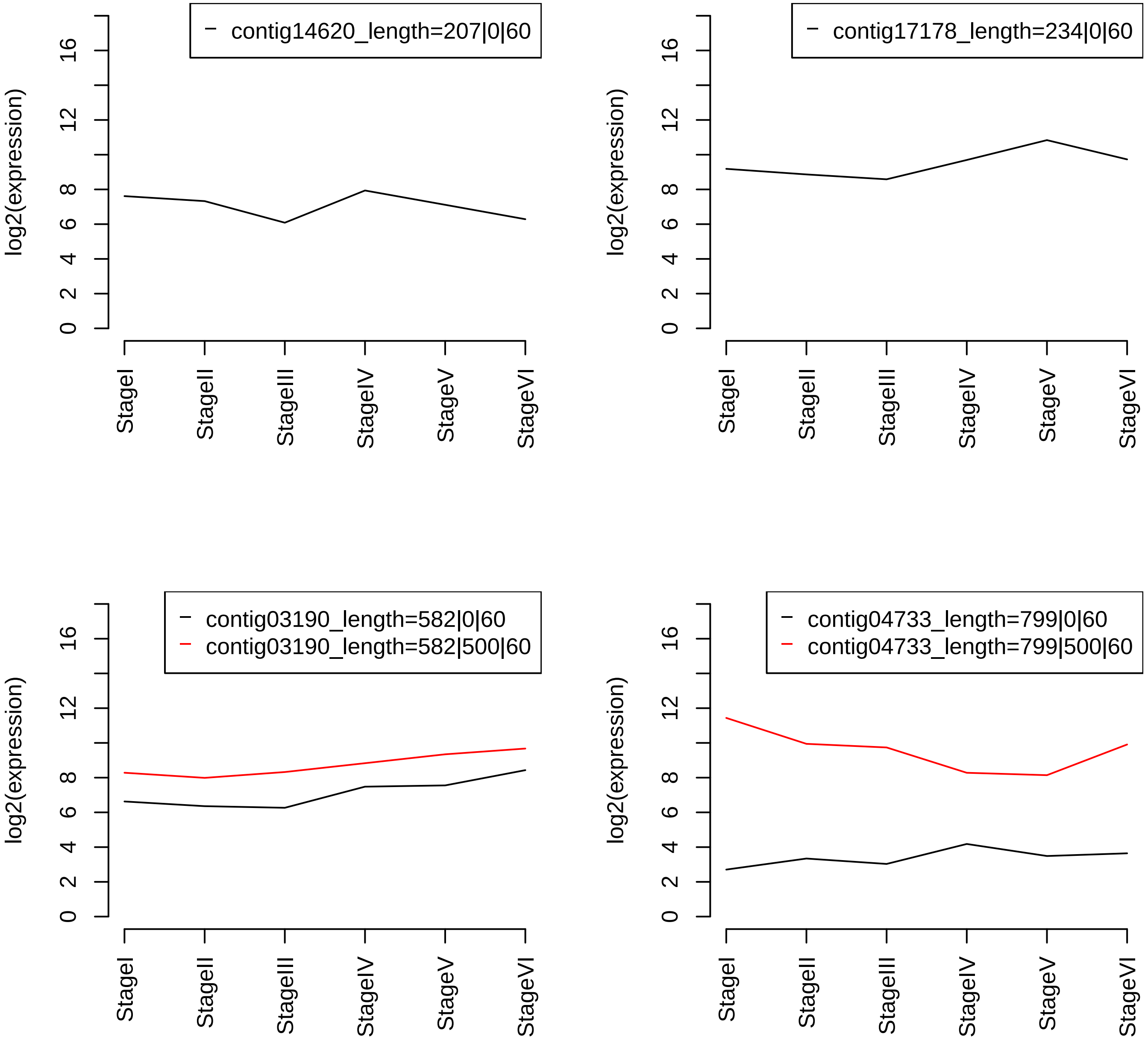
contig14620_length=207|0|60
contig17178_length=234|0|60
contig03190_length=582|0|60
contig03190_length=582|500|60
contig04733_length=799|0|60
contig04733_length=799|500|60
log2(expression)
0
2
4
6
8
12
16
StageI
StageII
StageIII
StageIV
StageV
StageVI

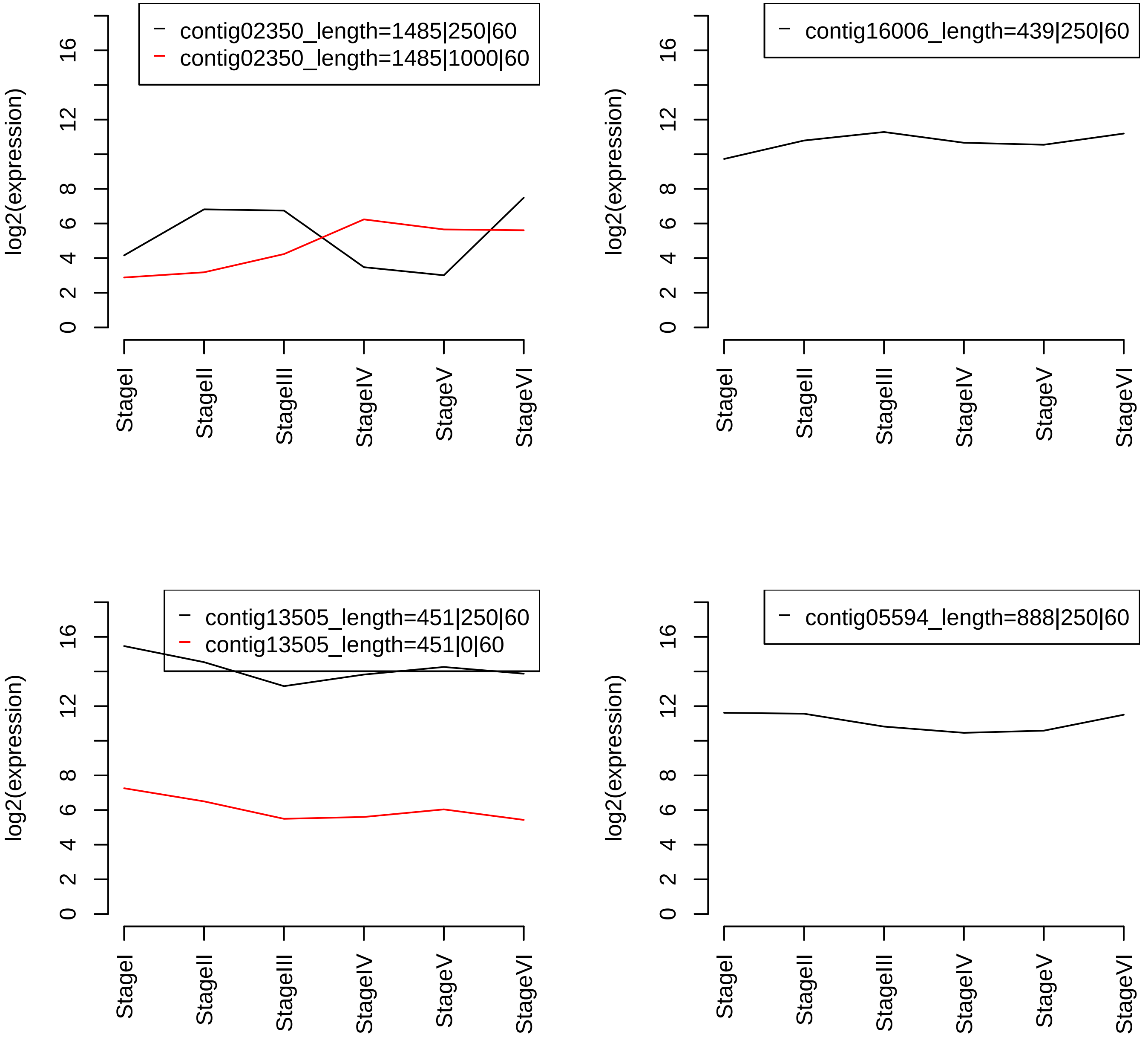
contig02350_length=1485|250|60
contig02350_length=1485|1000|60
contig16006_length=439|250|60
contig13505_length=451|250|60
contig13505_length=451|0|60
contig05594_length=888|250|60
log2(expression)
0
2
4
6
8
12
16
StageI
StageII
StageIII
StageIV
StageV
StageVI

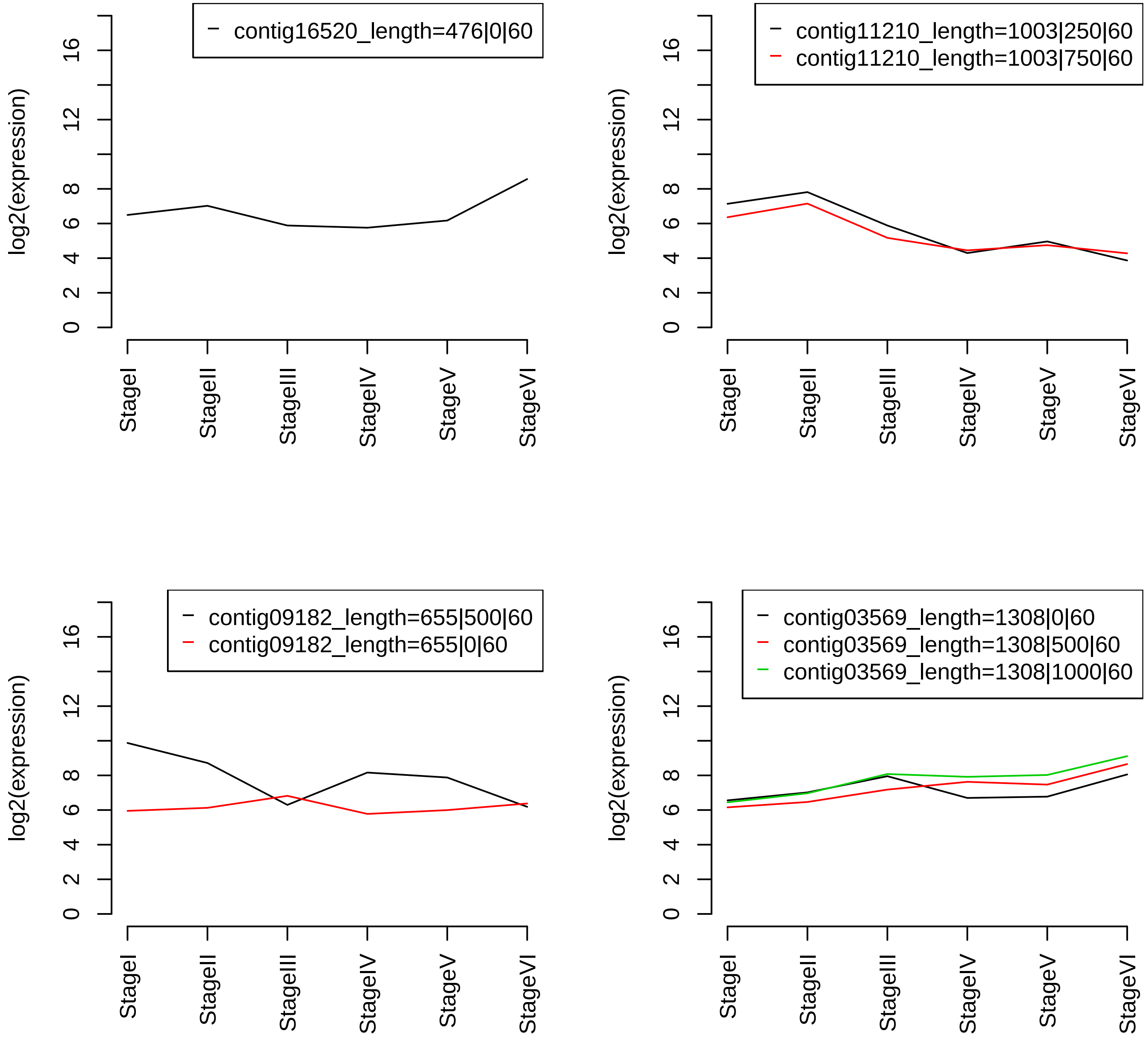
contig16520_length=476|0|60
contig11210_length=1003|250|60
contig11210_length=1003|750|60
contig09182_length=655|500|60
contig09182_length=655|0|60
contig03569_length=1308|0|60
contig03569_length=1308|500|60
contig03569_length=1308|1000|60
log2(expression)
0
2
4
6
8
12
16
StageI
StageII
StageIII
StageIV
StageV
StageVI

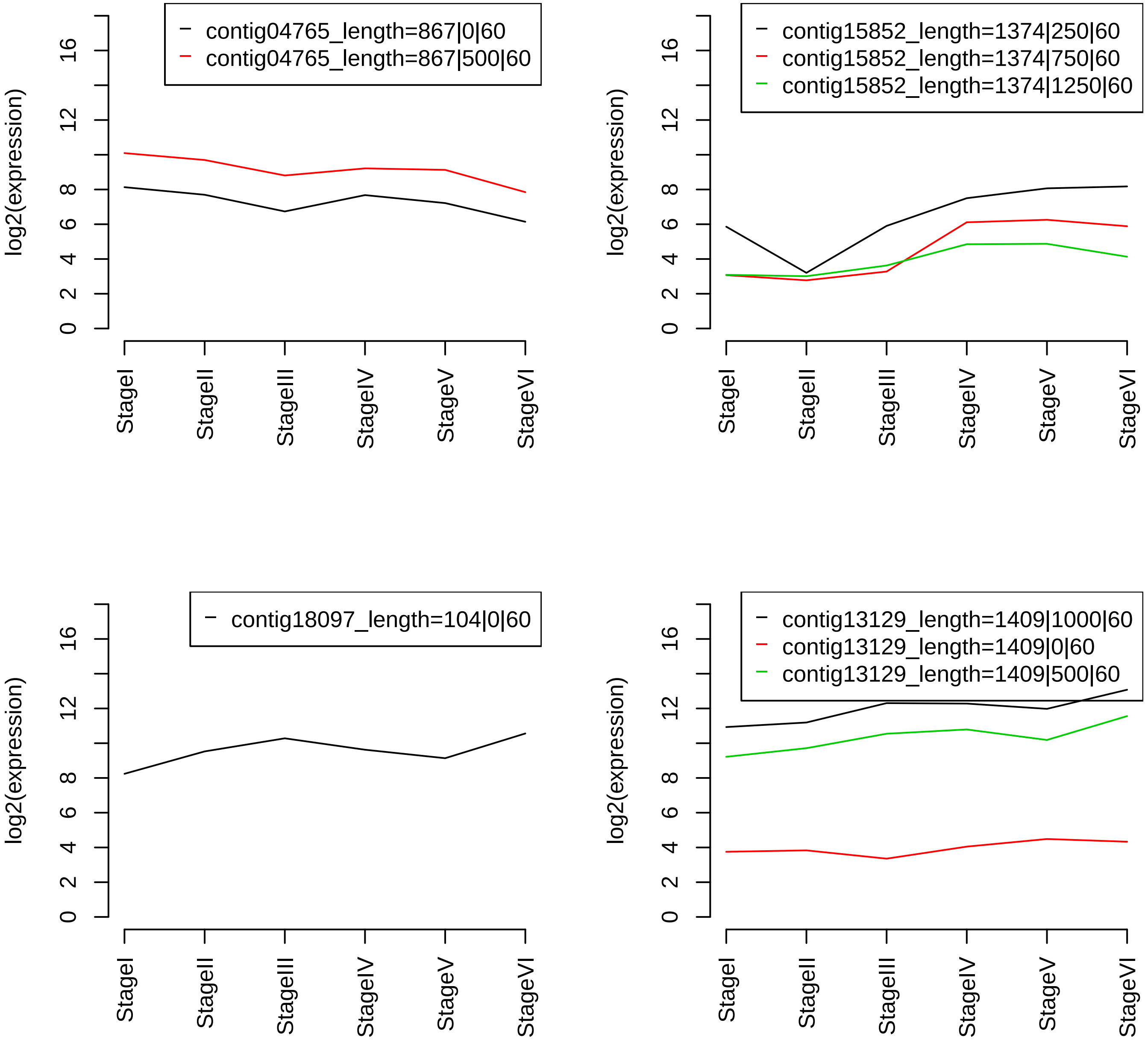

contig04765_length=867|0|60
contig04765_length=867|500|60
contig15852_length=1374|250|60
contig15852_length=1374|750|60
contig15852_length=1374|1250|60
contig18097_length=104|0|60
contig13129_length=1409|1000|60
contig13129_length=1409|0|60
contig13129_length=1409|500|60
log2(expression)
0
2
4
6
8
12
16
StageI
StageII
StageIII
StageIV
StageV
StageVI

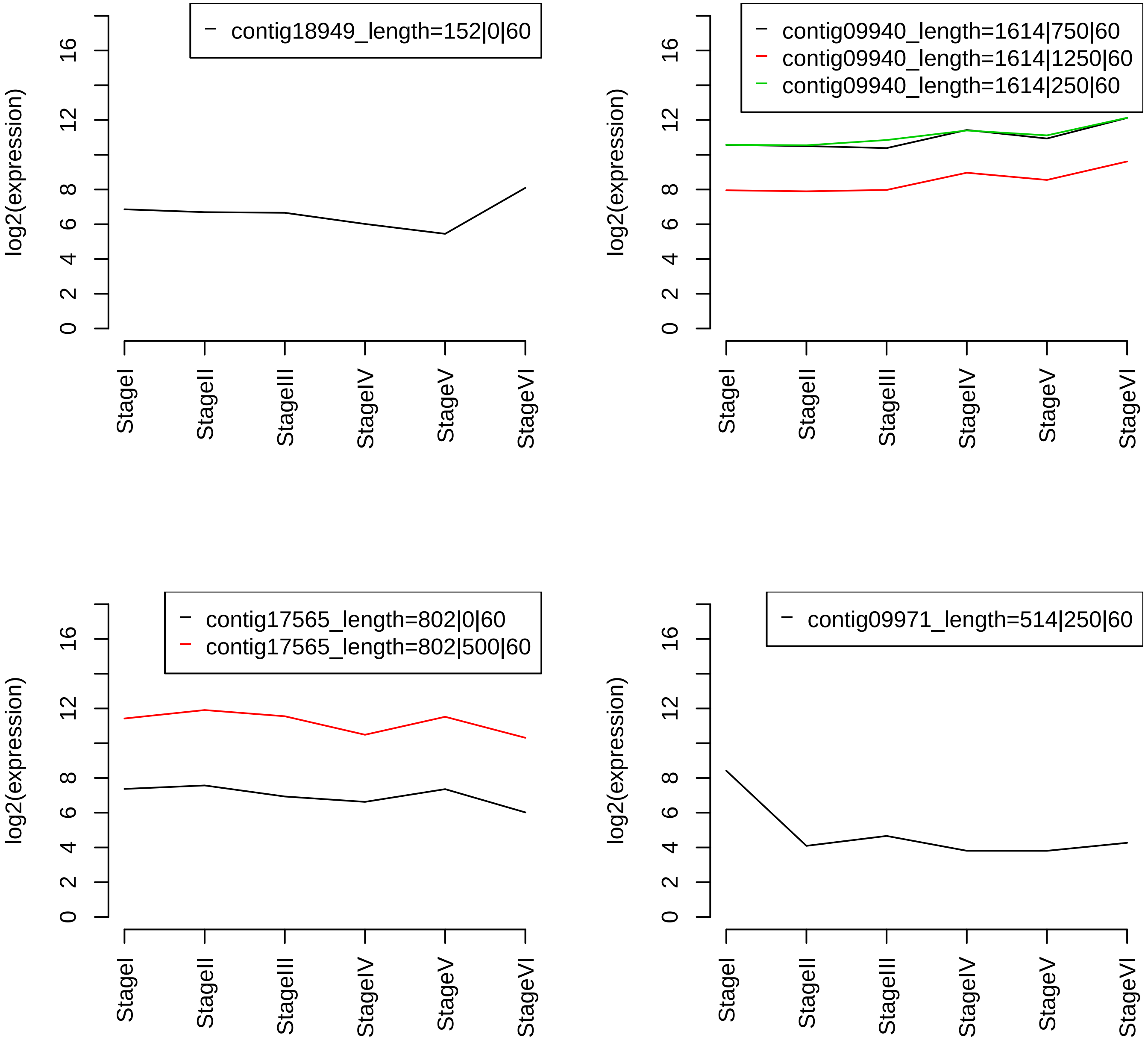

contig18949_length=152|0|60
contig09940_length=1614|750|60
contig09940_length=1614|1250|60
contig09940_length=1614|250|60
contig17565_length=802|0|60
contig17565_length=802|500|60
contig09971_length=514|250|60
log2(expression)
0 2 4 6 8 12 16
StageI StageII StageIII StageIV StageV StageVI

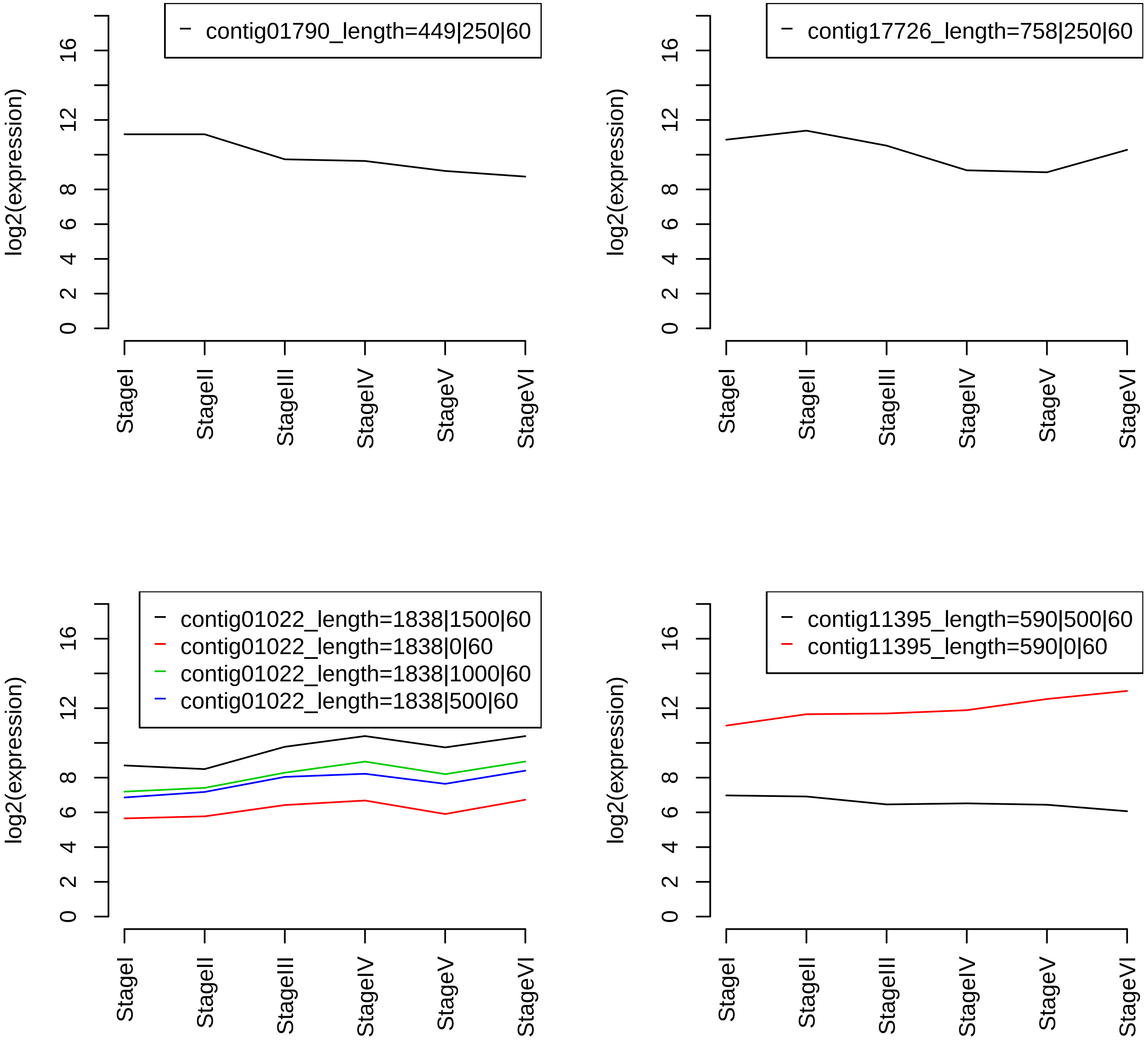

contig01790_length=449|250|60
contig17726_length=758|250|60
contig01022_length=1838|1500|60
contig01022_length=1838|0|60
contig01022_length=1838|1000|60
contig01022_length=1838|500|60
contig11395_length=590|500|60
contig11395_length=590|0|60
log2(expression)
0 2 4 6 8 12 16
StageI
StageII
StageIII
StageIV
StageV
StageVI

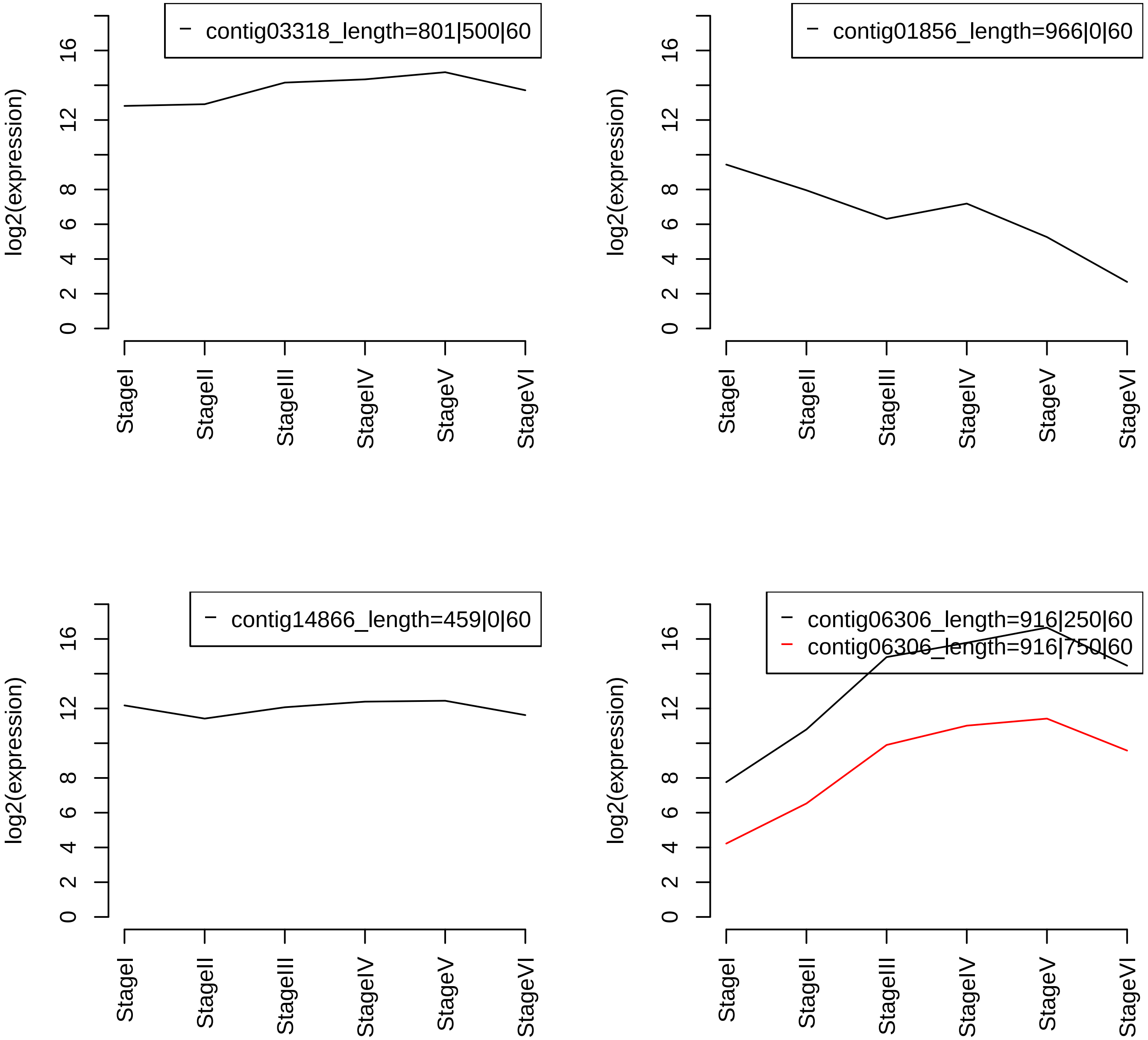

contig03318_length=801|500|60
contig01856_length=966|0|60
contig14866_length=459|0|60
contig06306_length=916|250|60
contig06306_length=916|750|60
log2(expression)
0
2
4
6
8
12
16
StageI
StageII
StageIII
StageIV
StageV
StageVI

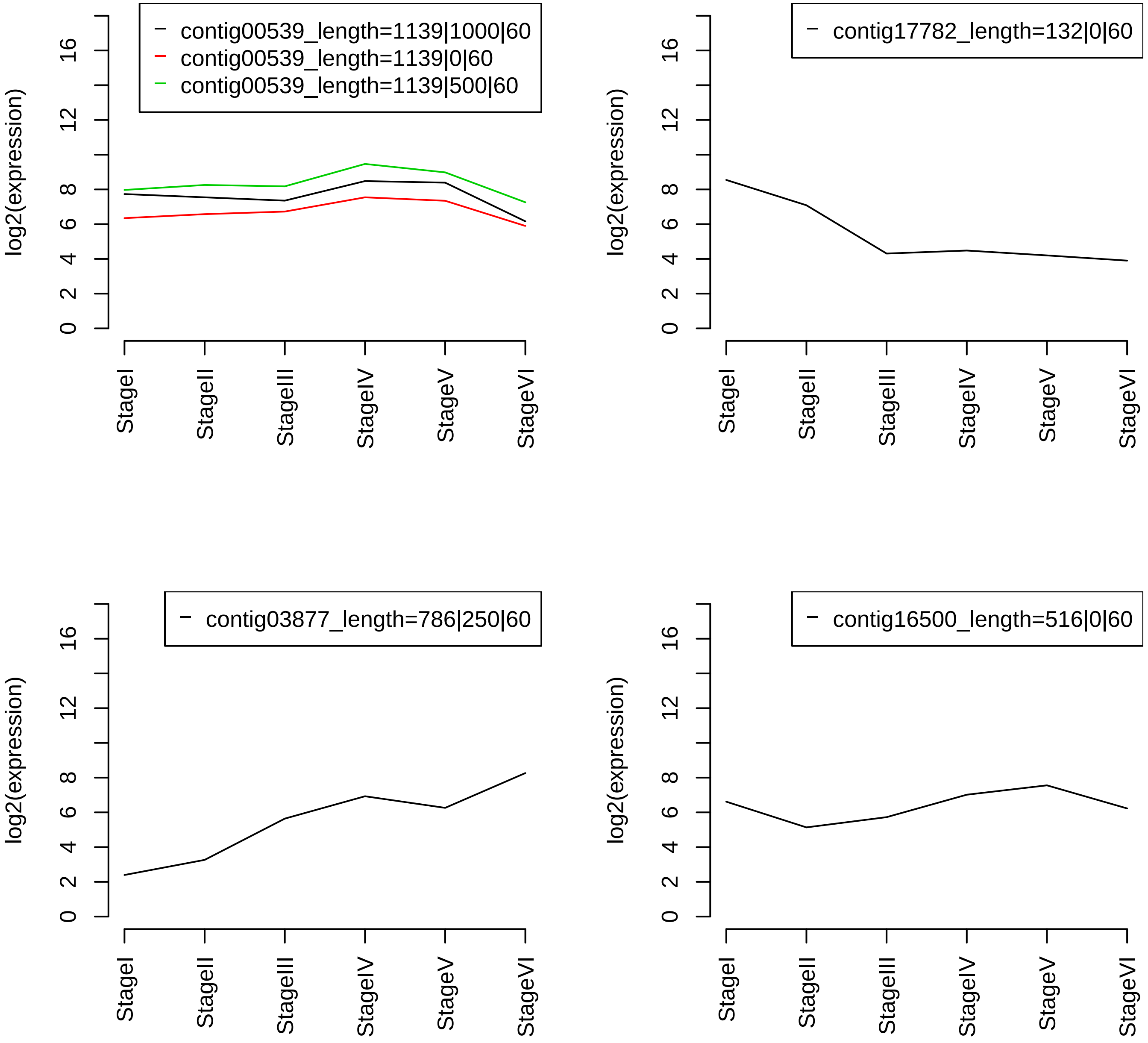
contig00539_length=1139|1000|60
contig00539_length=1139|0|60
contig00539_length=1139|500|60
contig17782_length=132|0|60
contig03877_length=786|250|60
contig16500_length=516|0|60
log2(expression)
StageI
StageII
StageIII
StageIV
StageV
StageVI

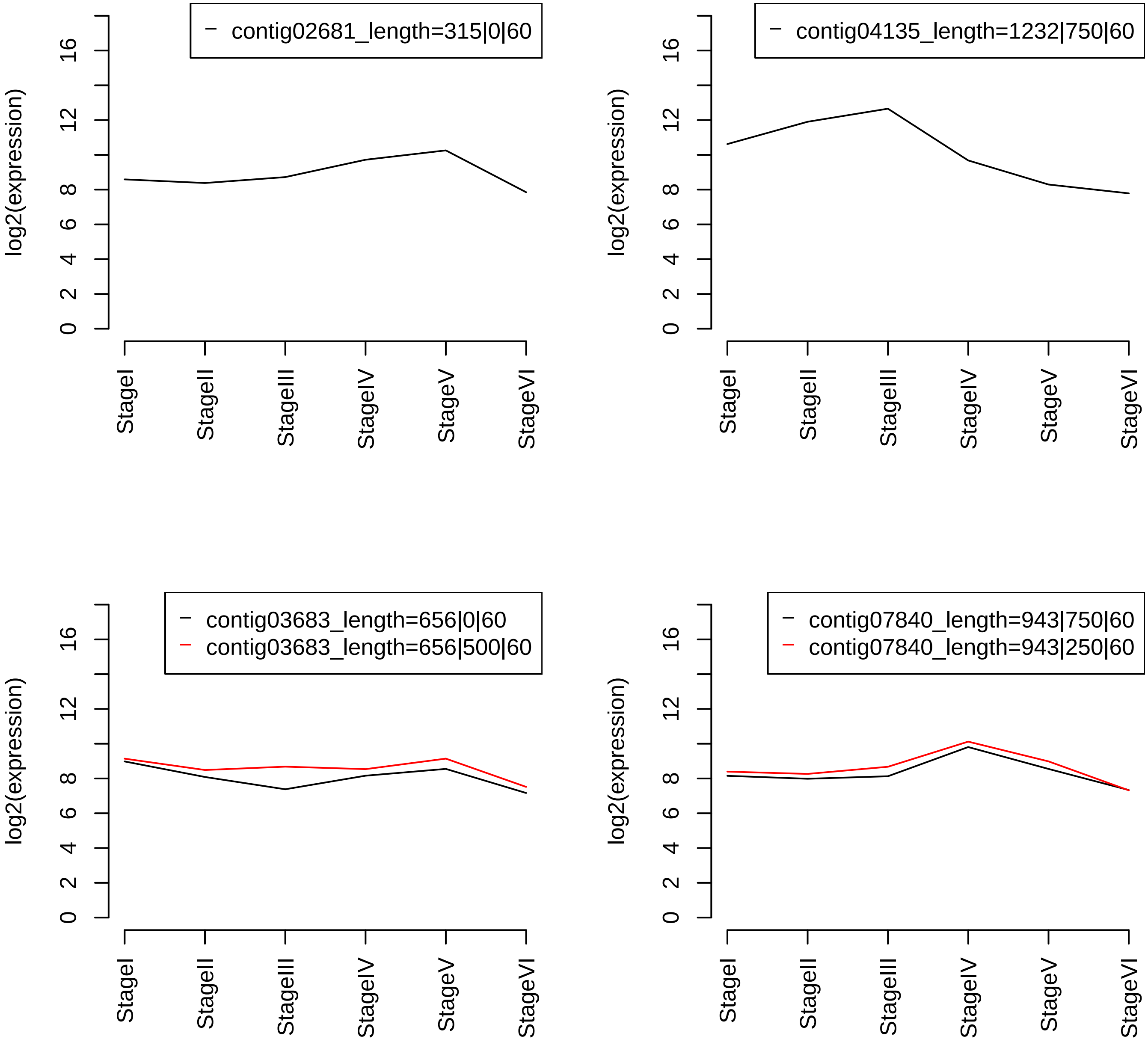
contig02681_length=315|0|60
contig04135_length=1232|750|60
contig03683_length=656|0|60
contig03683_length=656|500|60
contig07840_length=943|750|60
contig07840_length=943|250|60
log2(expression)
0
2
4
6
8
12
16
StageI
StageII
StageIII
StageIV
StageV
StageVI

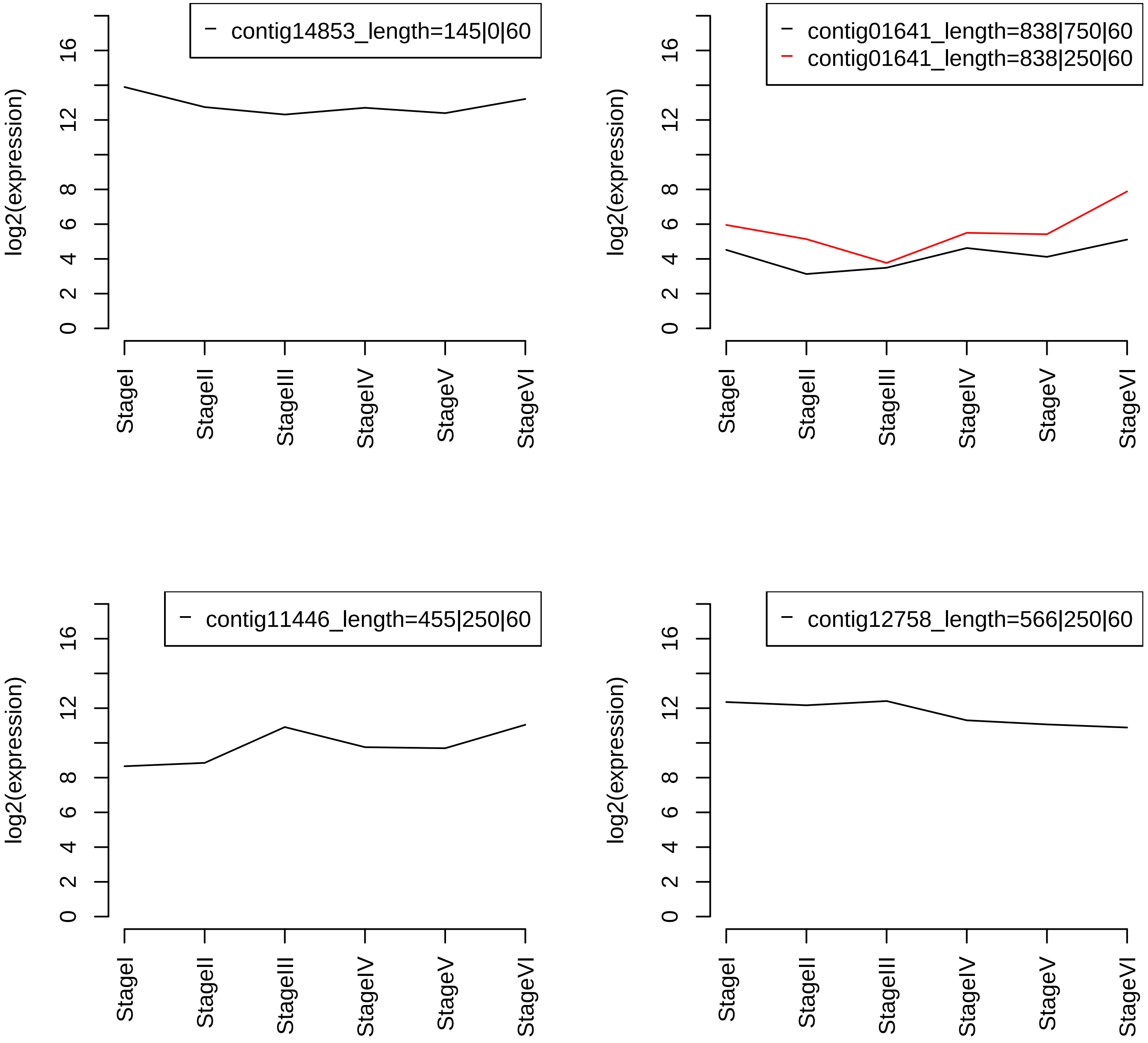

contig14853_length=145|0|60
contig01641_length=838|750|60
contig01641_length=838|250|60
contig11446_length=455|250|60
contig12758_length=566|250|60
log2(expression)
0
2
4
6
8
12
16
StageI
StageII
StageIII
StageIV
StageV
StageVI

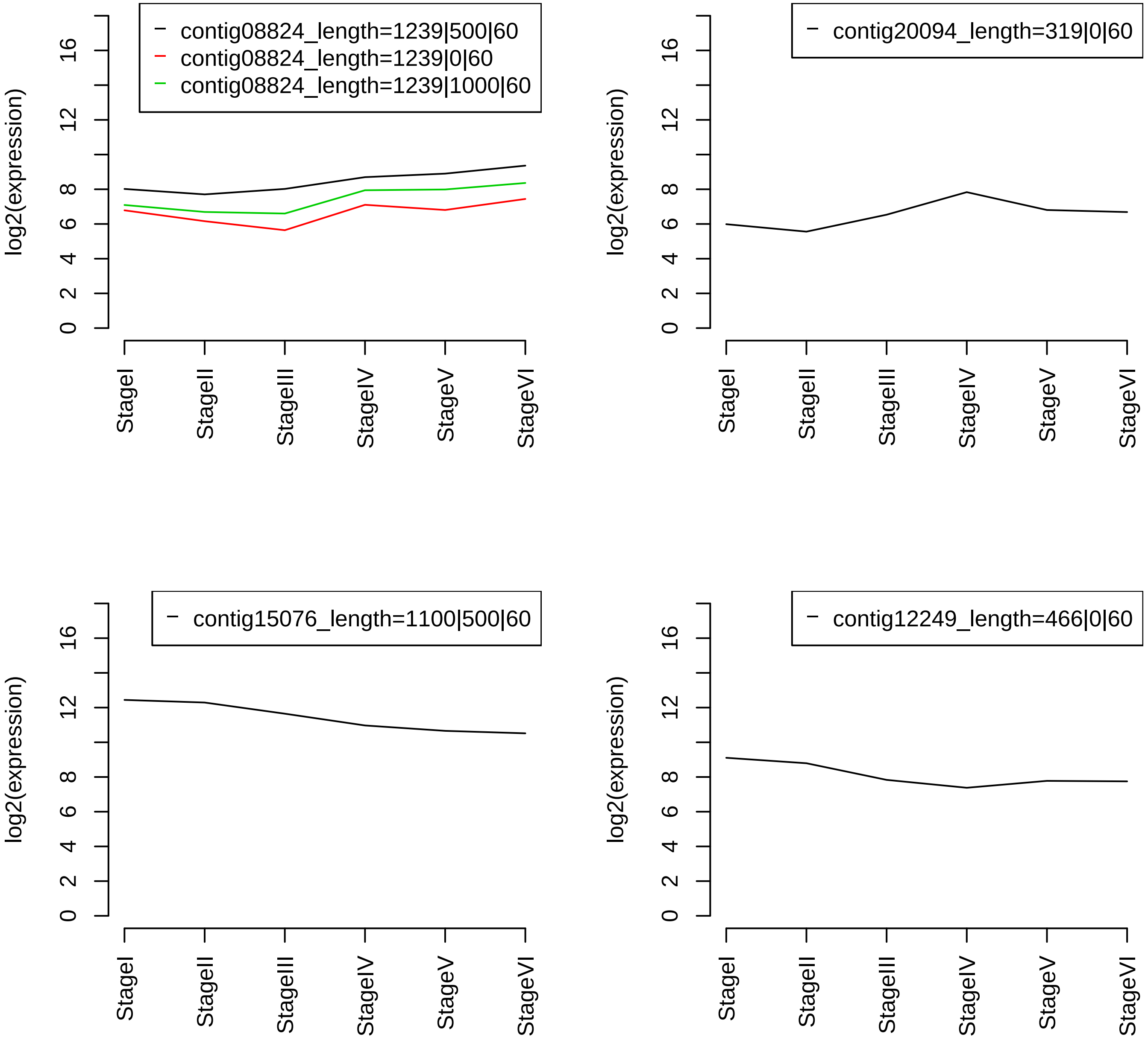
contig08824_length=1239|500|60
contig08824_length=1239|0|60
contig08824_length=1239|1000|60
contig20094_length=319|0|60
contig15076_length=1100|500|60
contig12249_length=466|0|60
log2(expression)
0
2
4
6
8
12
16
StageI
StageII
StageIII
StageIV
StageV
StageVI

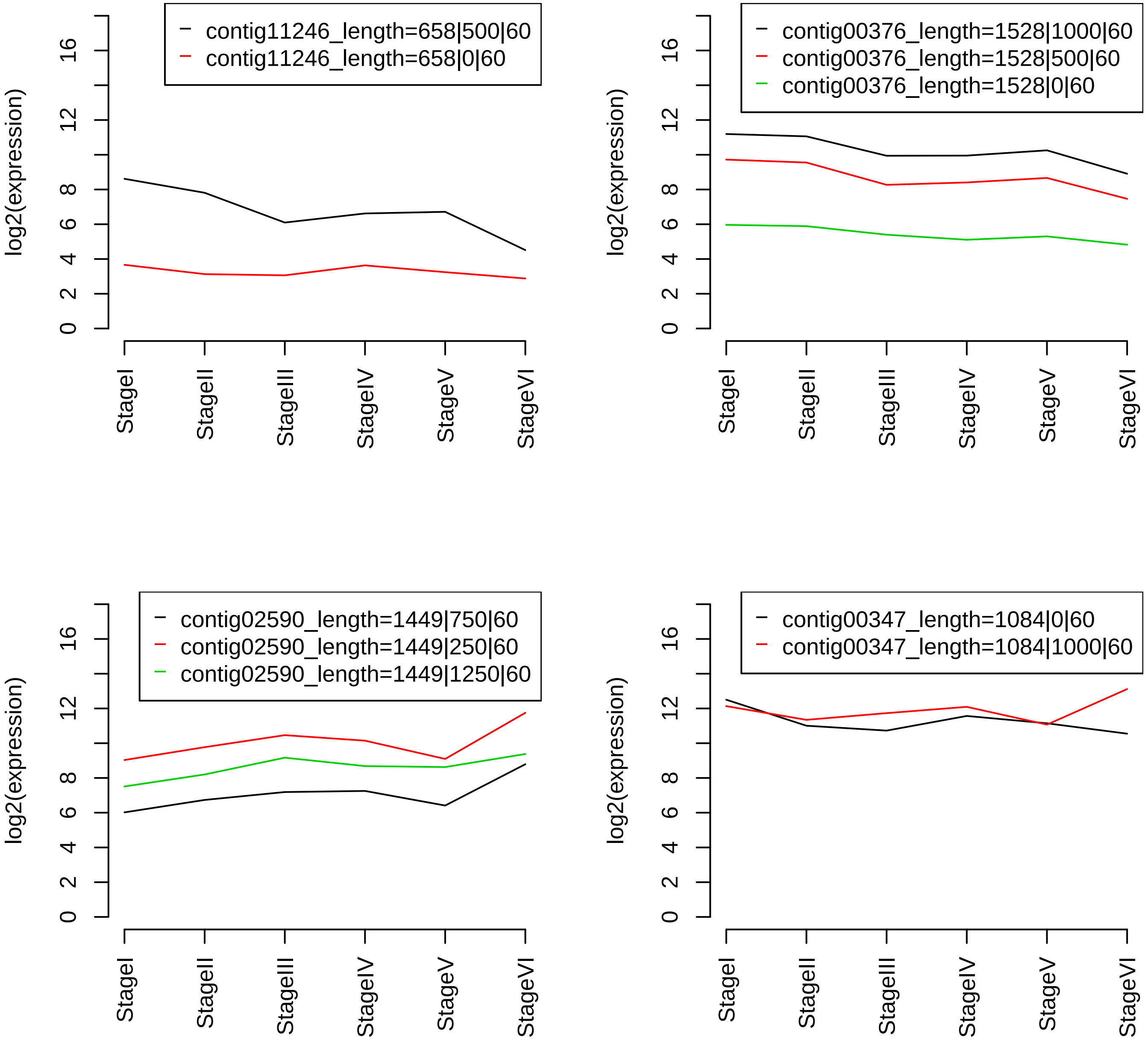

contig11246_length=658|500|60
contig11246_length=658|0|60
contig00376_length=1528|1000|60
contig00376_length=1528|500|60
contig00376_length=1528|0|60
contig02590_length=1449|750|60
contig02590_length=1449|250|60
contig02590_length=1449|1250|60
contig00347_length=1084|0|60
contig00347_length=1084|1000|60
log2(expression)
0
2
4
6
8
12
16
StageI
StageII
StageIII
StageIV
StageV
StageVI

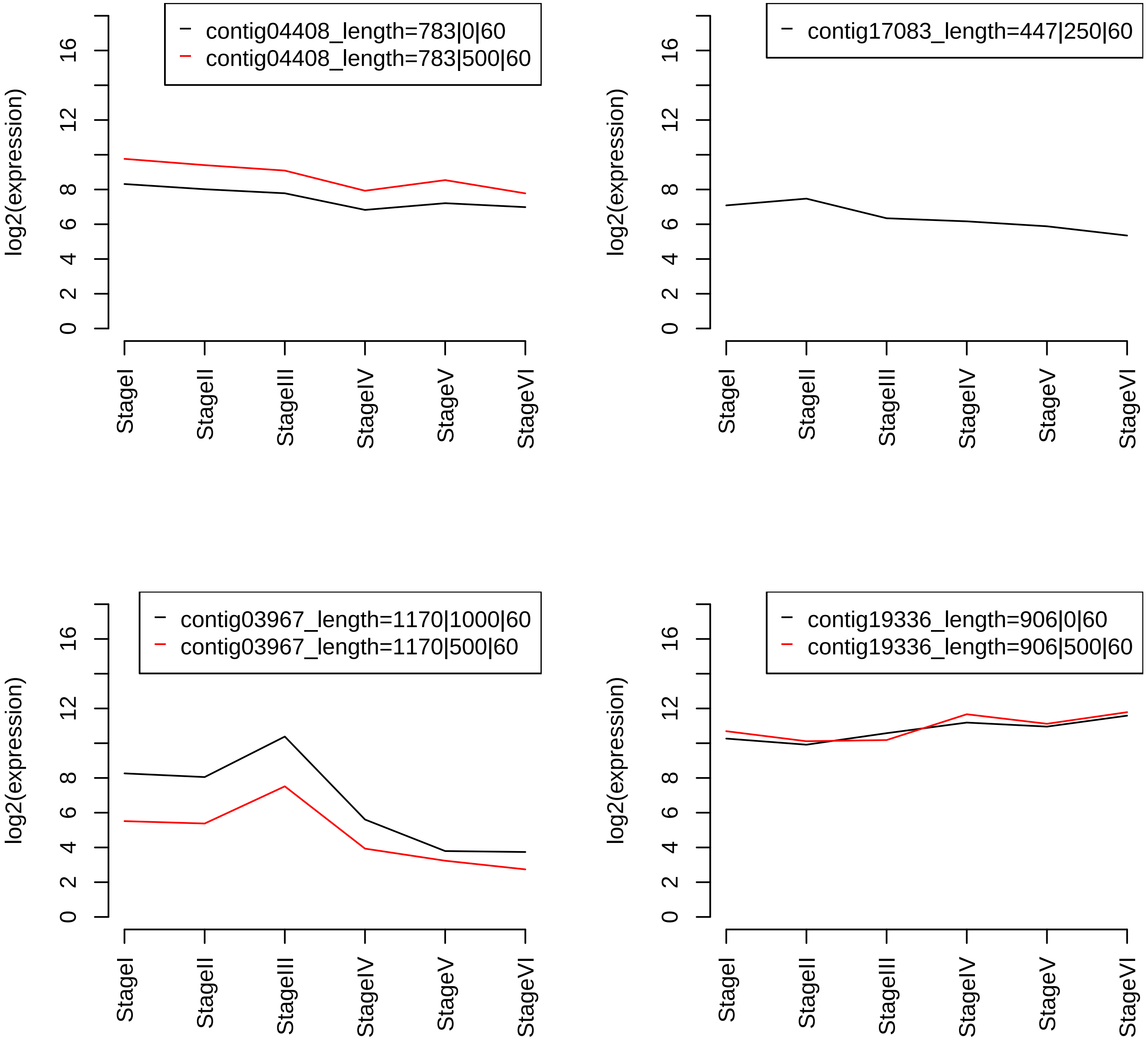

contig04408_length=783|0|60
contig04408_length=783|500|60
contig17083_length=447|250|60
contig03967_length=1170|1000|60
contig03967_length=1170|500|60
contig19336_length=906|0|60
contig19336_length=906|500|60
log2(expression)
0
2
4
6
8
12
16
StageI
StageII
StageIII
StageIV
StageV
StageVI

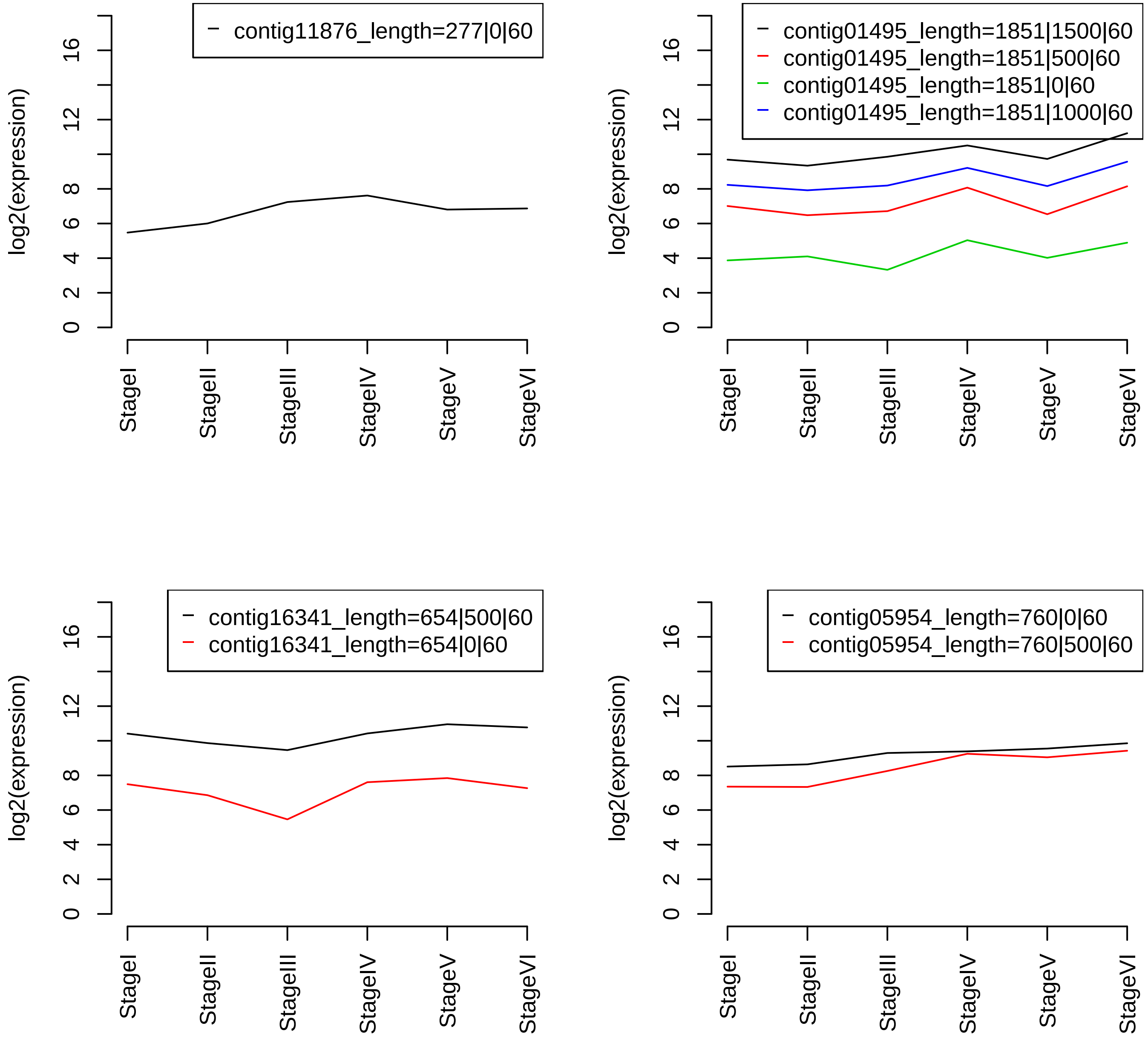
contig11876_length=277|0|60
contig01495_length=1851|1500|60
contig01495_length=1851|500|60
contig01495_length=1851|0|60
contig01495_length=1851|1000|60
contig16341_length=654|500|60
contig16341_length=654|0|60
contig05954_length=760|0|60
contig05954_length=760|500|60
log2(expression)
0
2
4
6
8
12
16
StageI
StageII
StageIII
StageIV
StageV
StageVI

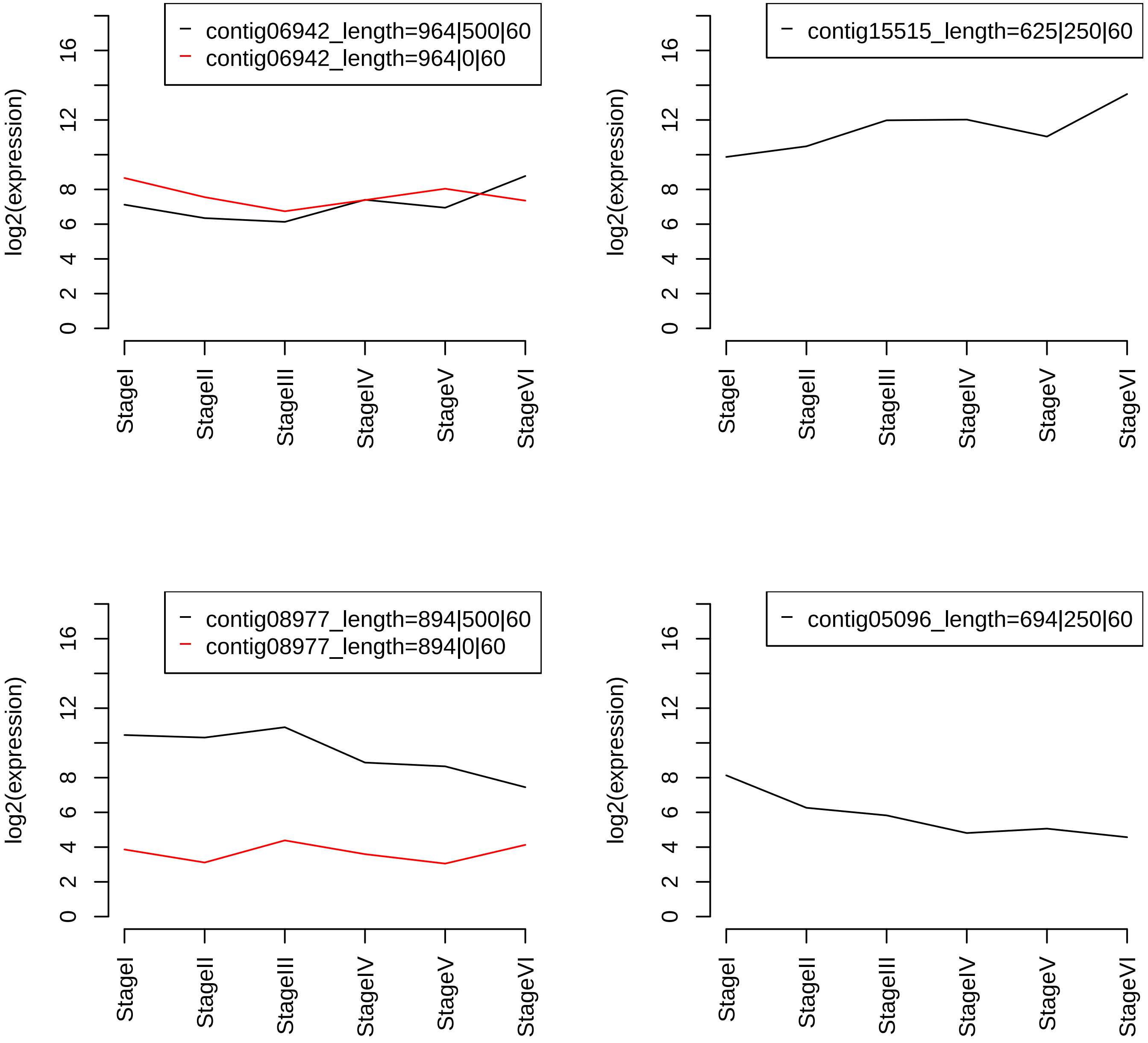

contig06942_length=964|500|60
contig06942_length=964|0|60
contig15515_length=625|250|60
contig08977_length=894|500|60
contig08977_length=894|0|60
contig05096_length=694|250|60
log2(expression)
0
2
4
6
8
12
16
StageI
StageII
StageIII
StageIV
StageV
StageVI

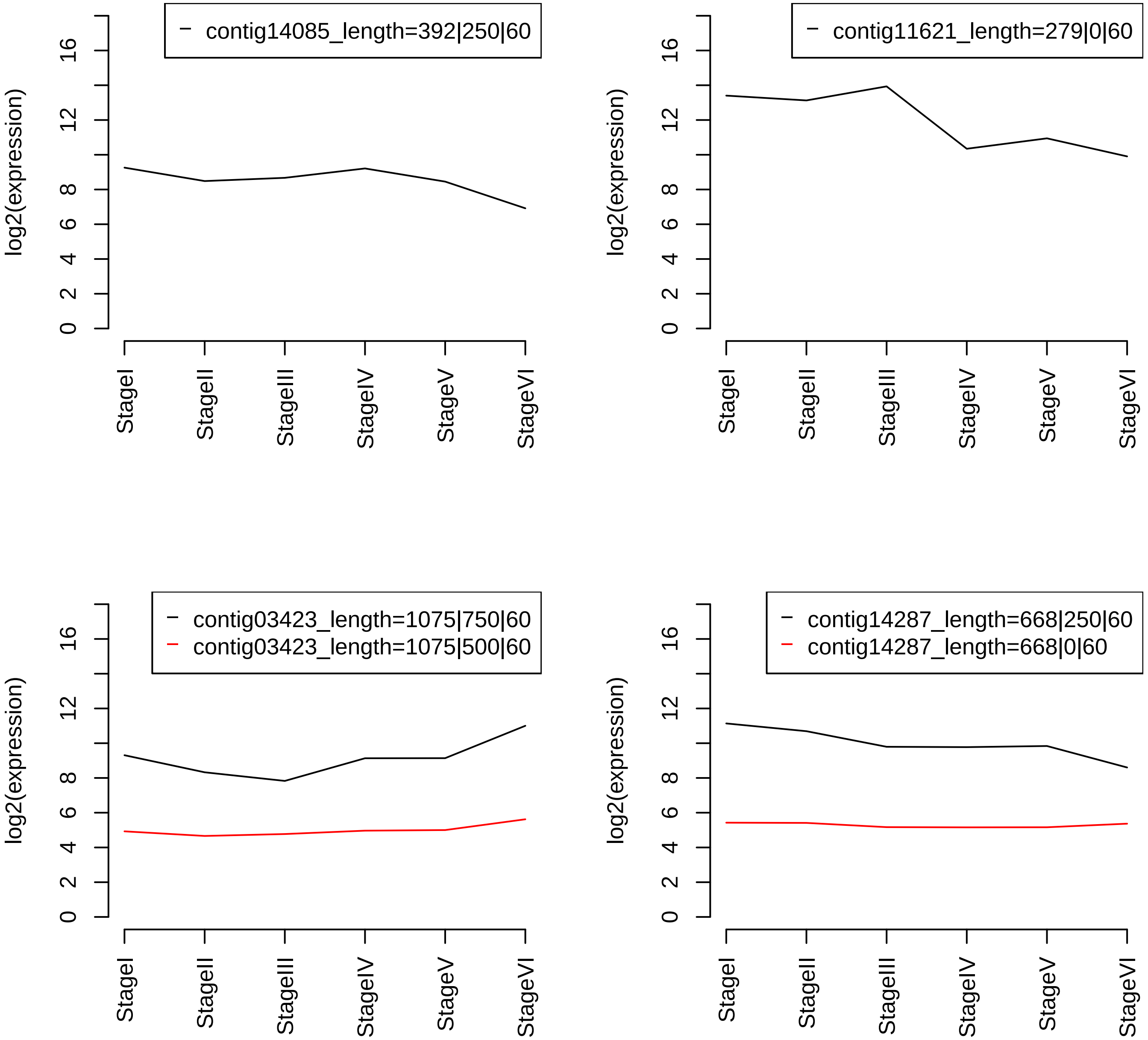

contig14085_length=392|250|60
contig11621_length=279|0|60
contig03423_length=1075|750|60
contig03423_length=1075|500|60
contig14287_length=668|250|60
contig14287_length=668|0|60
log2(expression)
0 2 4 6 8 12 16
StageI StageII StageIII StageIV StageV StageVI

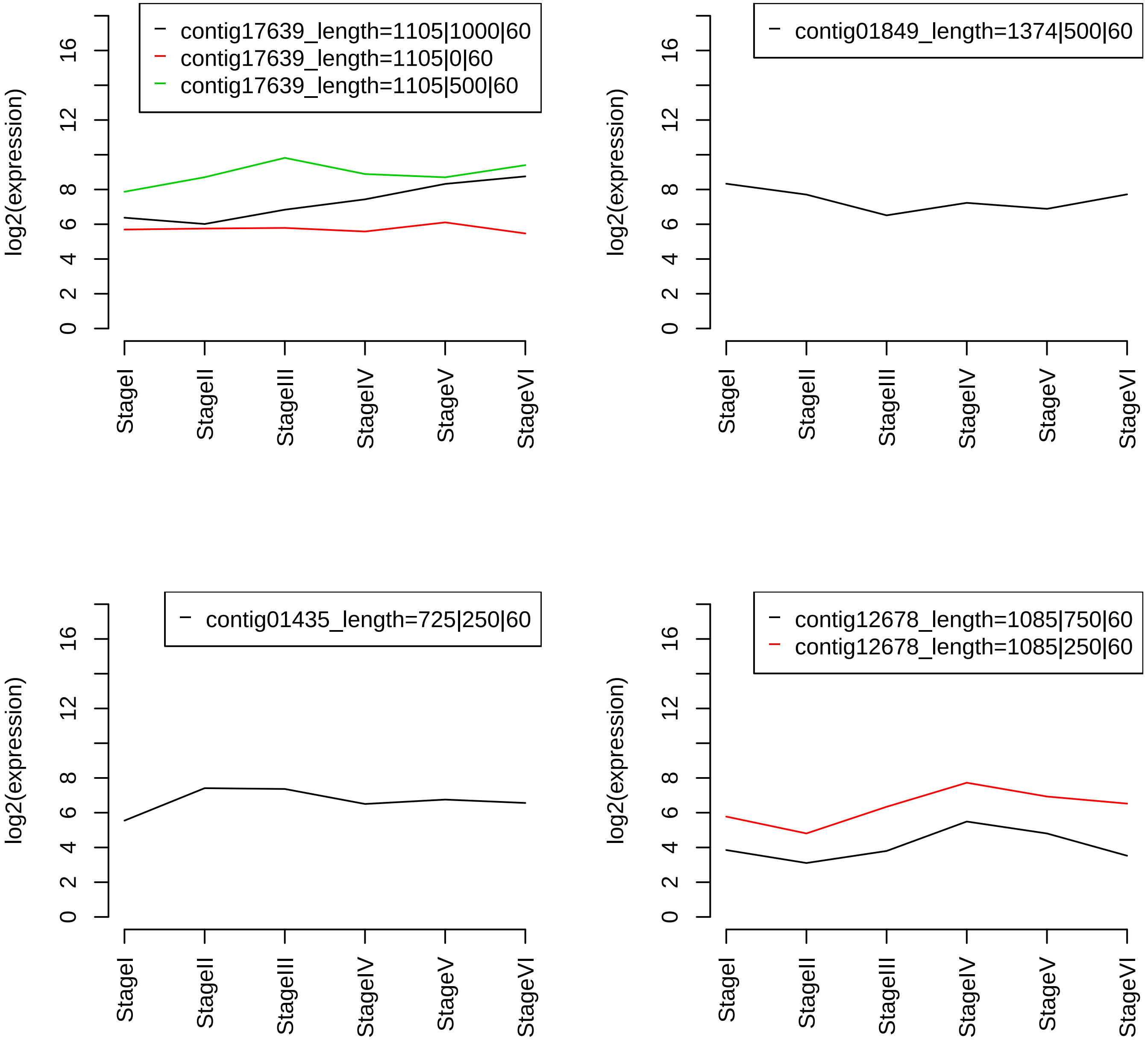
log2(expression)
0
2
4
6
8
12
16
contig17639_length=1105|1000|60
contig17639_length=1105|0|60
contig17639_length=1105|500|60
StageI
StageII
StageIII
StageIV
StageV
StageVI
contig01849_length=1374|500|60
contig01435_length=725|250|60
contig12678_length=1085|750|60
contig12678_length=1085|250|60

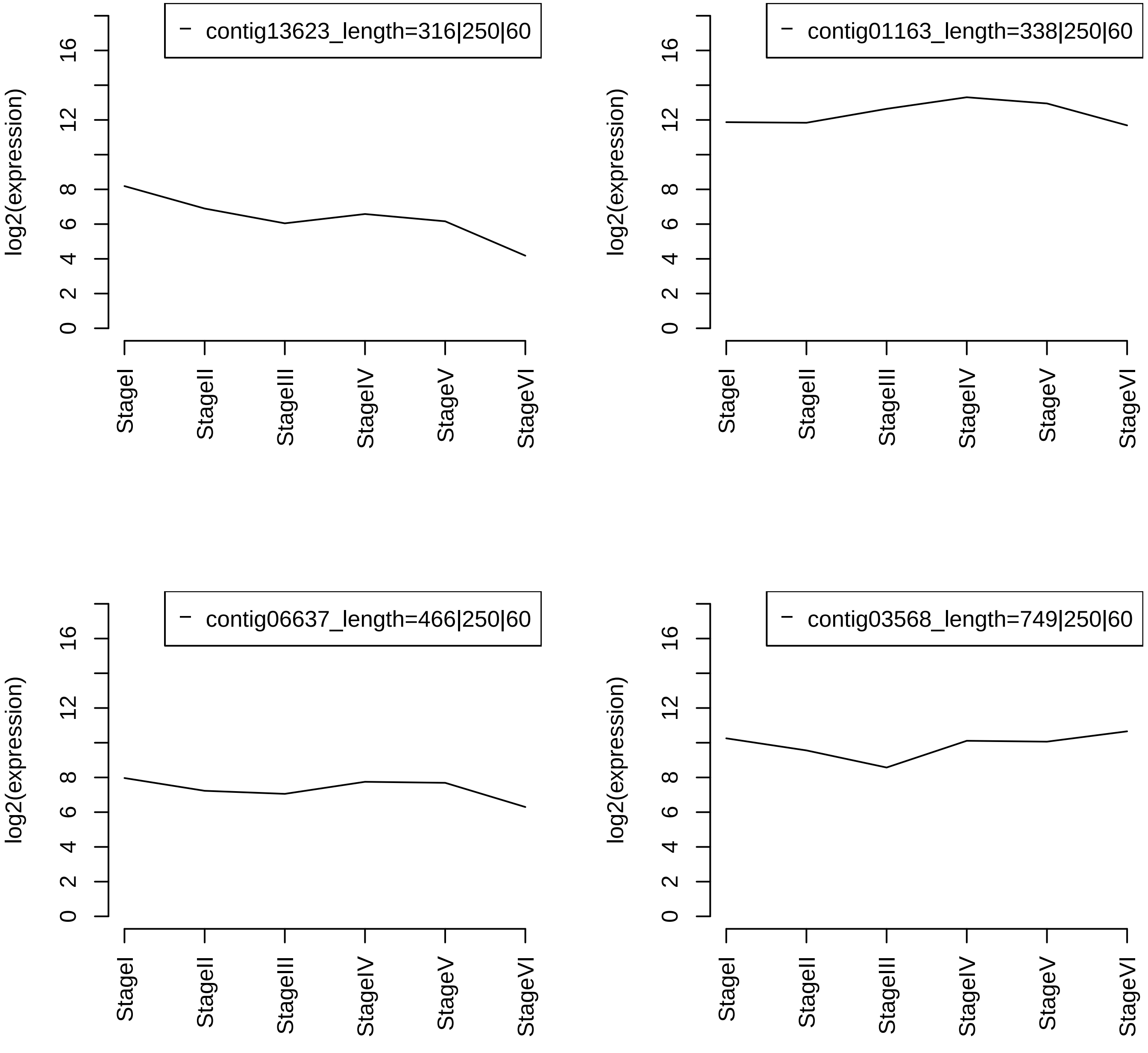
contig13623_length=316|250|60
contig01163_length=338|250|60
contig06637_length=466|250|60
contig03568_length=749|250|60
log2(expression)
0
2
4
6
8
12
16
StageI
StageII
StageIII
StageIV
StageV
StageVI

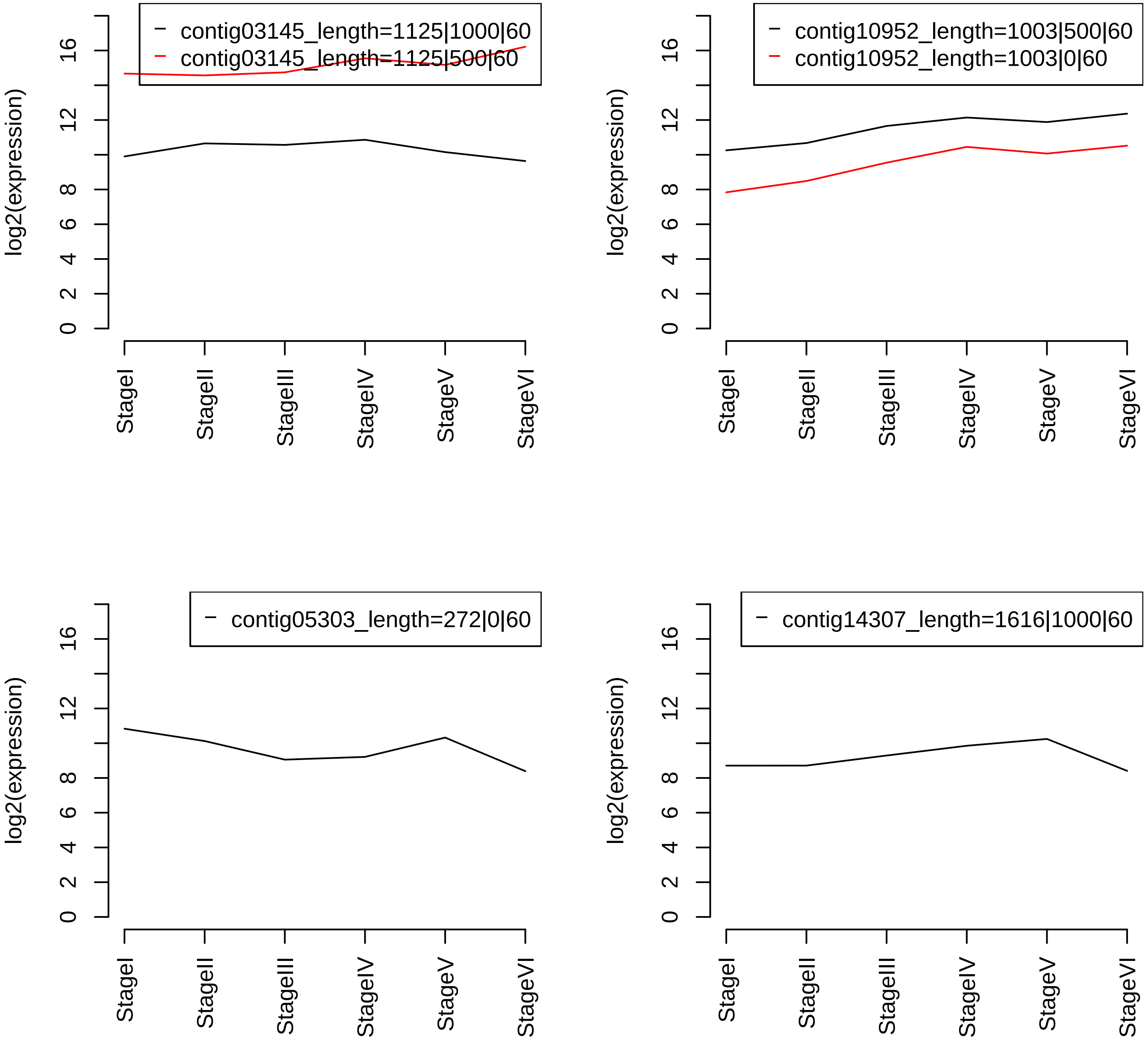

contig03145_length=1125|1000|60
contig03145_length=1125|500|60
contig10952_length=1003|500|60
contig10952_length=1003|0|60
contig05303_length=272|0|60
contig14307_length=1616|1000|60
log2(expression)
0
2
4
6
8
12
16
StageI
StageII
StageIII
StageIV
StageV
StageVI

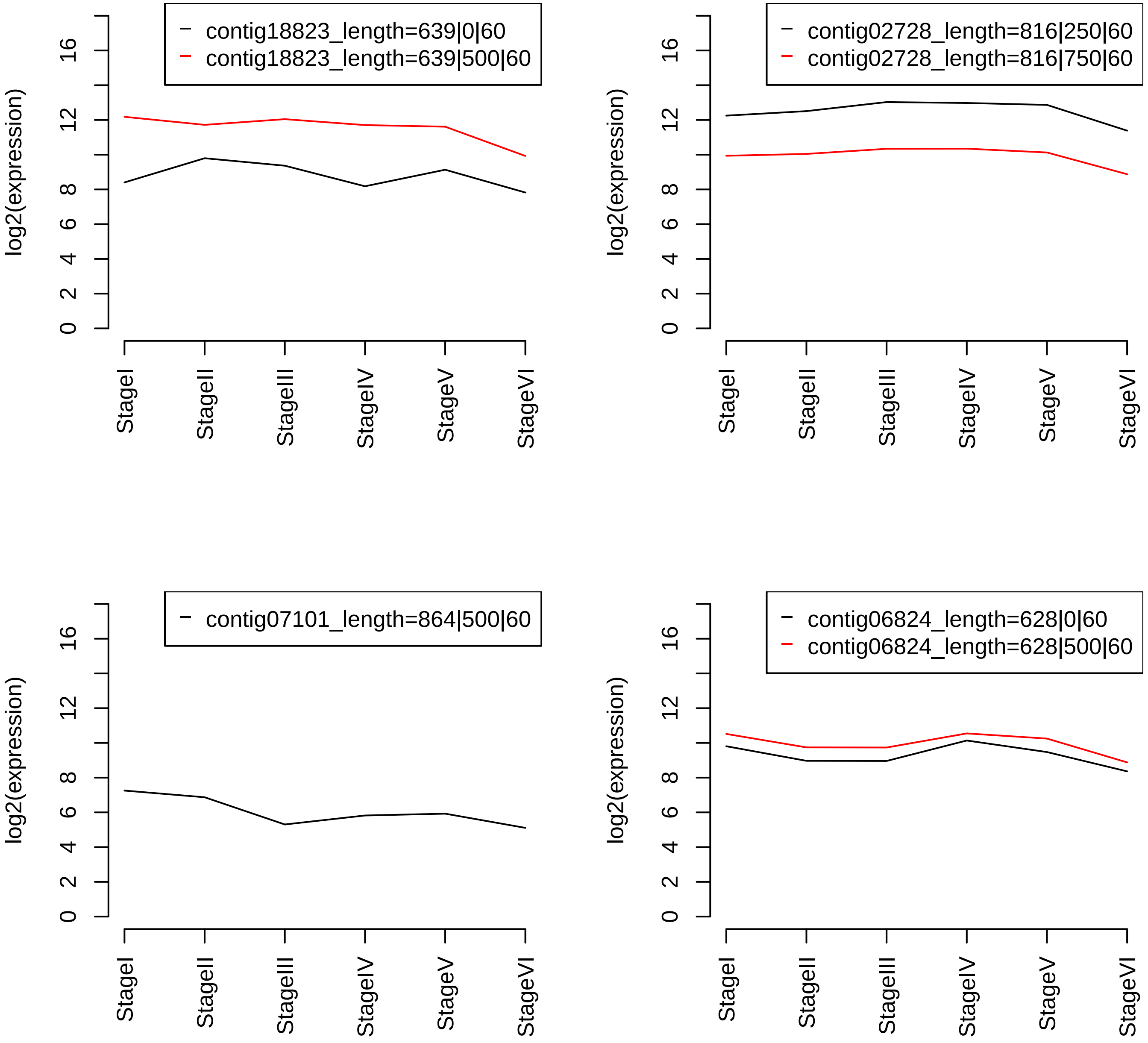

contig18823_length=639|0|60
contig18823_length=639|500|60
log2(expression)
0
2
4
6
8
12
16
StageI
StageII
StageIII
StageIV
StageV
StageVI
contig02728_length=816|250|60
contig02728_length=816|750|60
contig07101_length=864|500|60
contig06824_length=628|0|60
contig06824_length=628|500|60

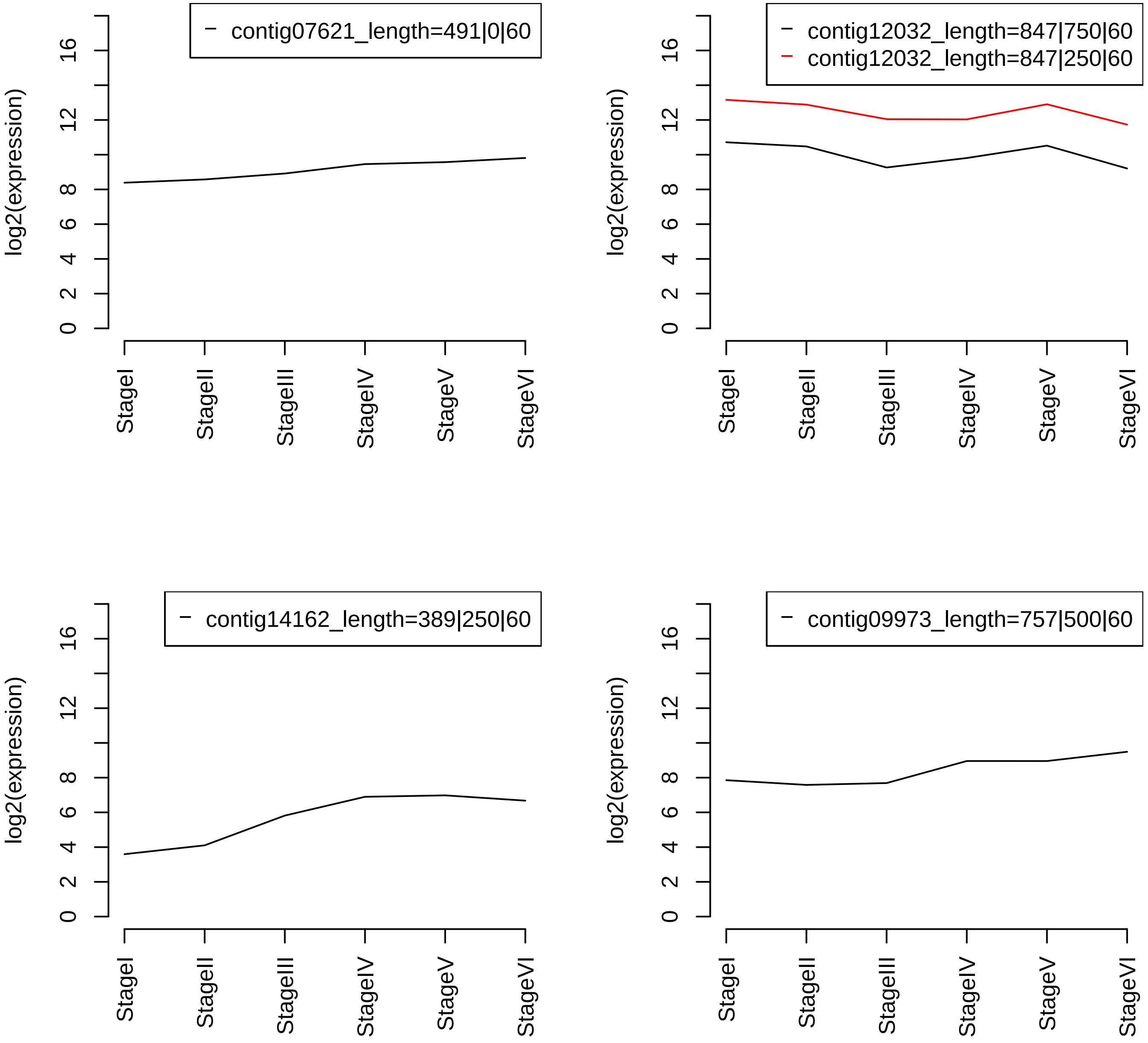
contig07621_length=491|0|60
contig12032_length=847|750|60
contig12032_length=847|250|60
contig14162_length=389|250|60
contig09973_length=757|500|60
log2(expression)
0
2
4
6
8
12
16
StageI
StageII
StageIII
StageIV
StageV
StageVI

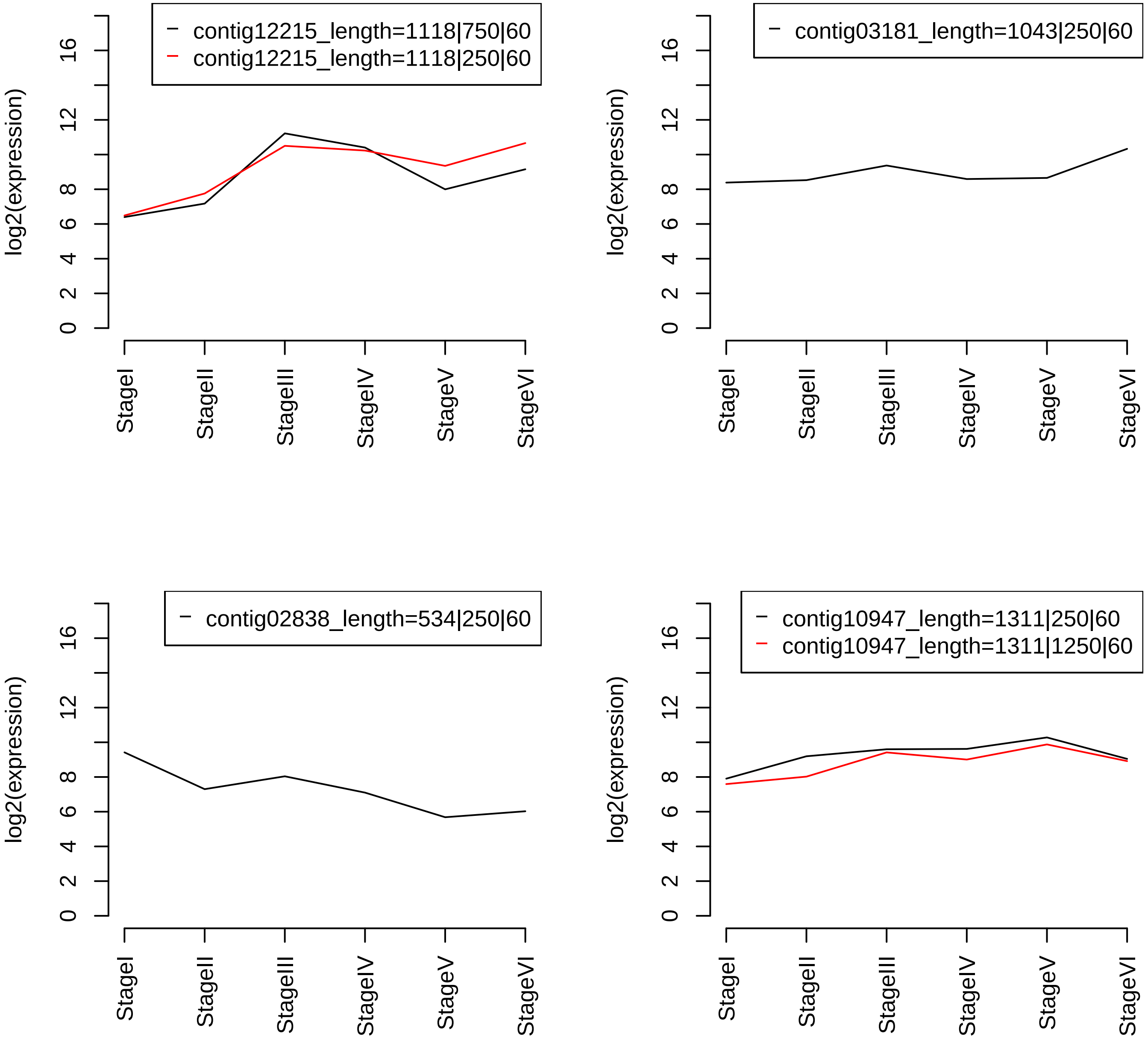

contig12215_length=1118|750|60
contig12215_length=1118|250|60
contig03181_length=1043|250|60
contig02838_length=534|250|60
contig10947_length=1311|250|60
contig10947_length=1311|1250|60
log2(expression)
0
2
4
6
8
12
16
StageI
StageII
StageIII
StageIV
StageV
StageVI

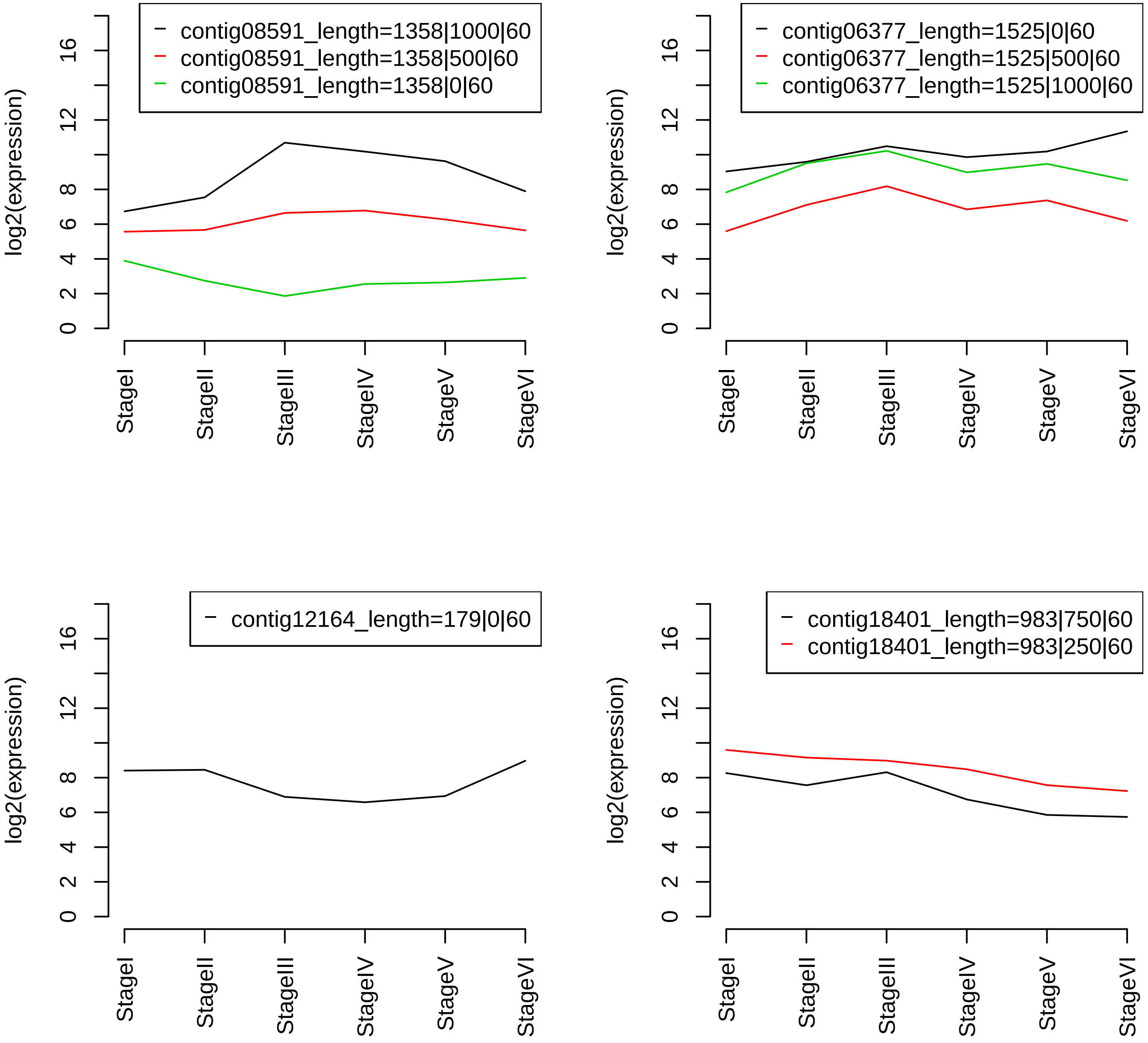
contig08591_length=1358|1000|60
contig08591_length=1358|500|60
contig08591_length=1358|0|60
contig06377_length=1525|0|60
contig06377_length=1525|500|60
contig06377_length=1525|1000|60
contig12164_length=179|0|60
contig18401_length=983|750|60
contig18401_length=983|250|60
log2(expression)
0
2
4
6
8
12
16
StageI
StageII
StageIII
StageIV
StageV
StageVI

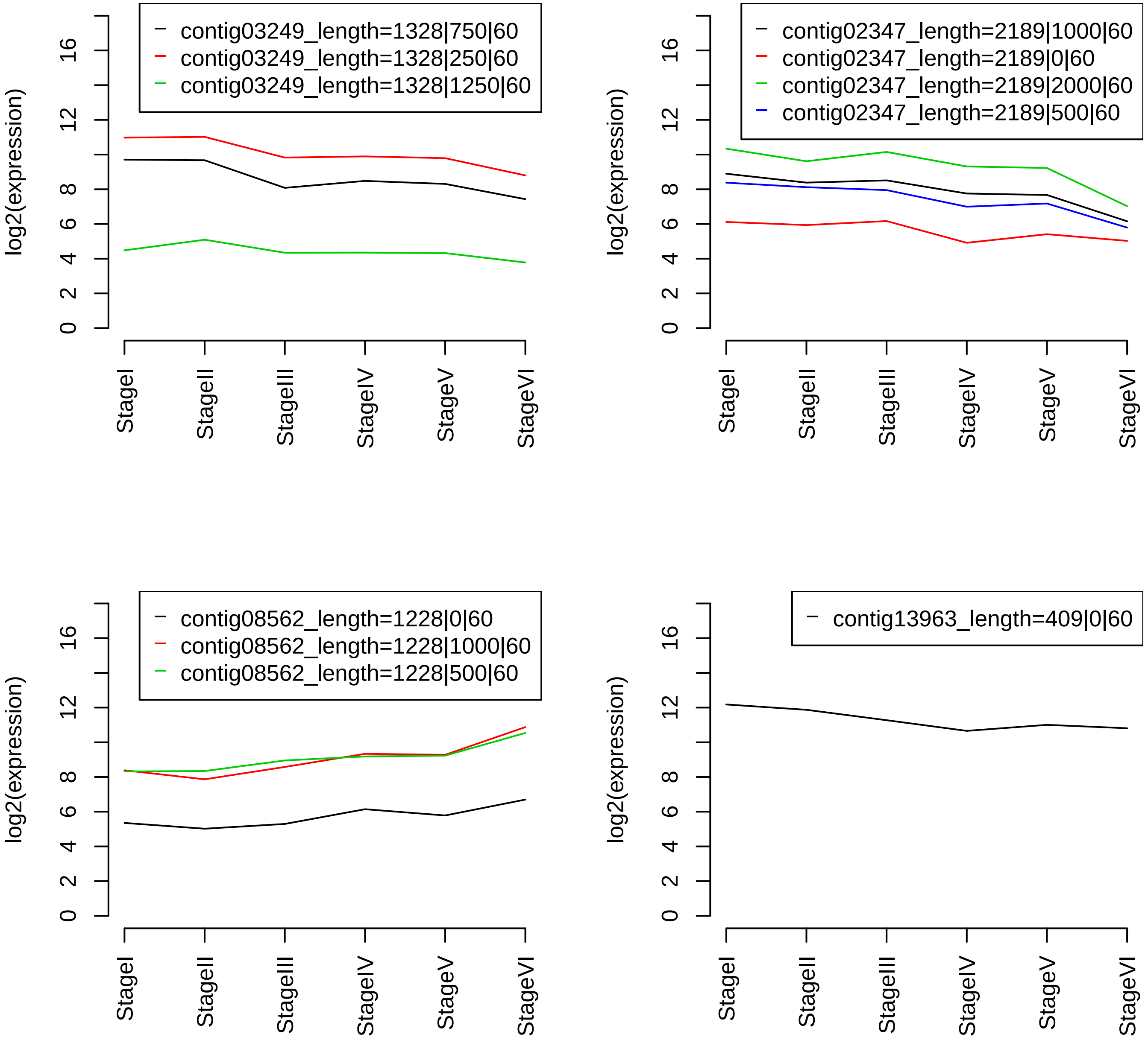

contig03249_length=1328|750|60
contig03249_length=1328|250|60
contig03249_length=1328|1250|60
contig02347_length=2189|1000|60
contig02347_length=2189|0|60
contig02347_length=2189|2000|60
contig02347_length=2189|500|60
contig08562_length=1228|0|60
contig08562_length=1228|1000|60
contig08562_length=1228|500|60
contig13963_length=409|0|60
log2(expression)
0 2 4 6 8 12 16
StageI StageII StageIII StageIV StageV StageVI

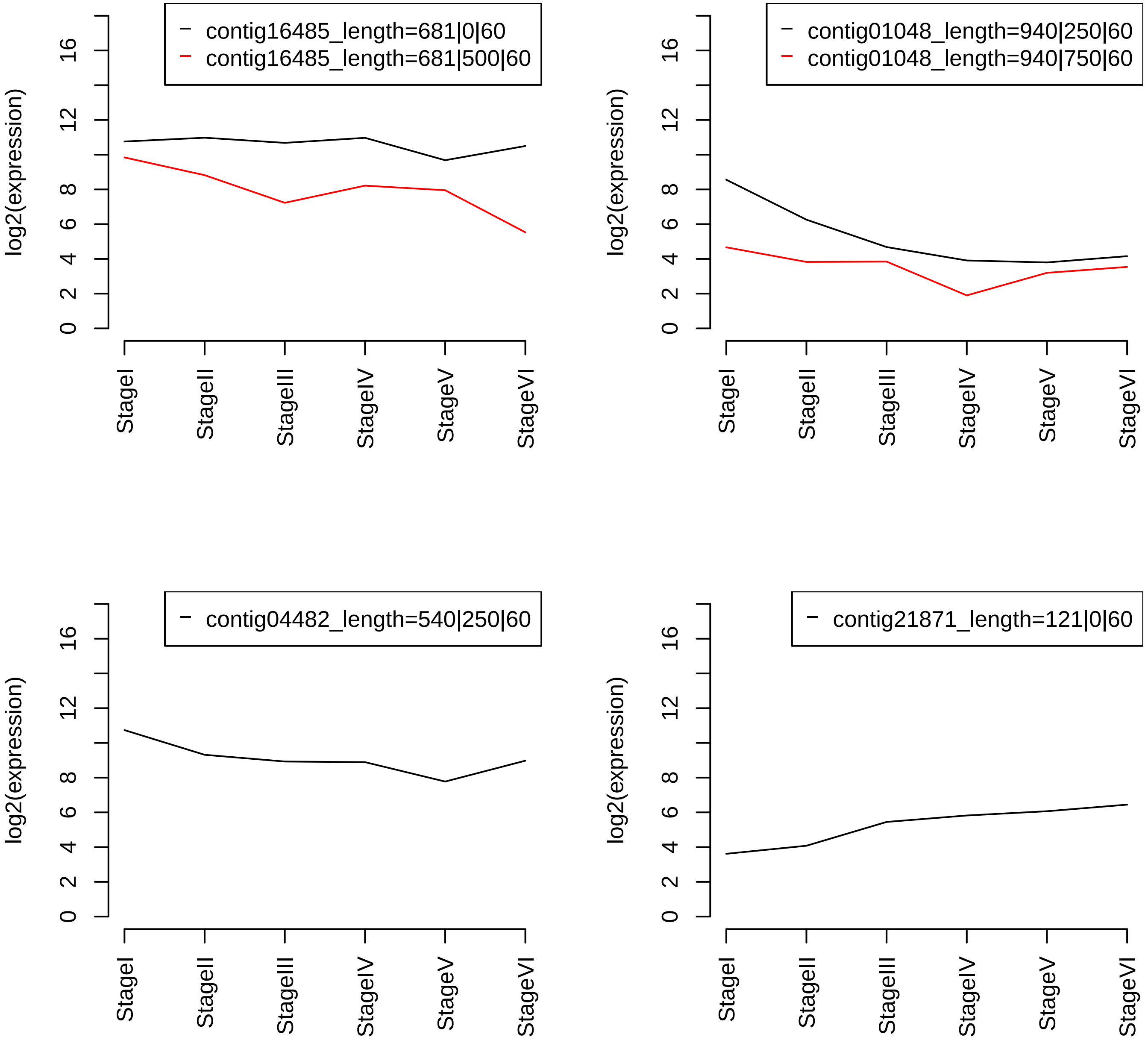
contig16485_length=681|0|60
contig16485_length=681|500|60
contig01048_length=940|250|60
contig01048_length=940|750|60
contig04482_length=540|250|60
contig21871_length=121|0|60
log2(expression)
0
2
4
6
8
12
16
StageI
StageII
StageIII
StageIV
StageV
StageVI

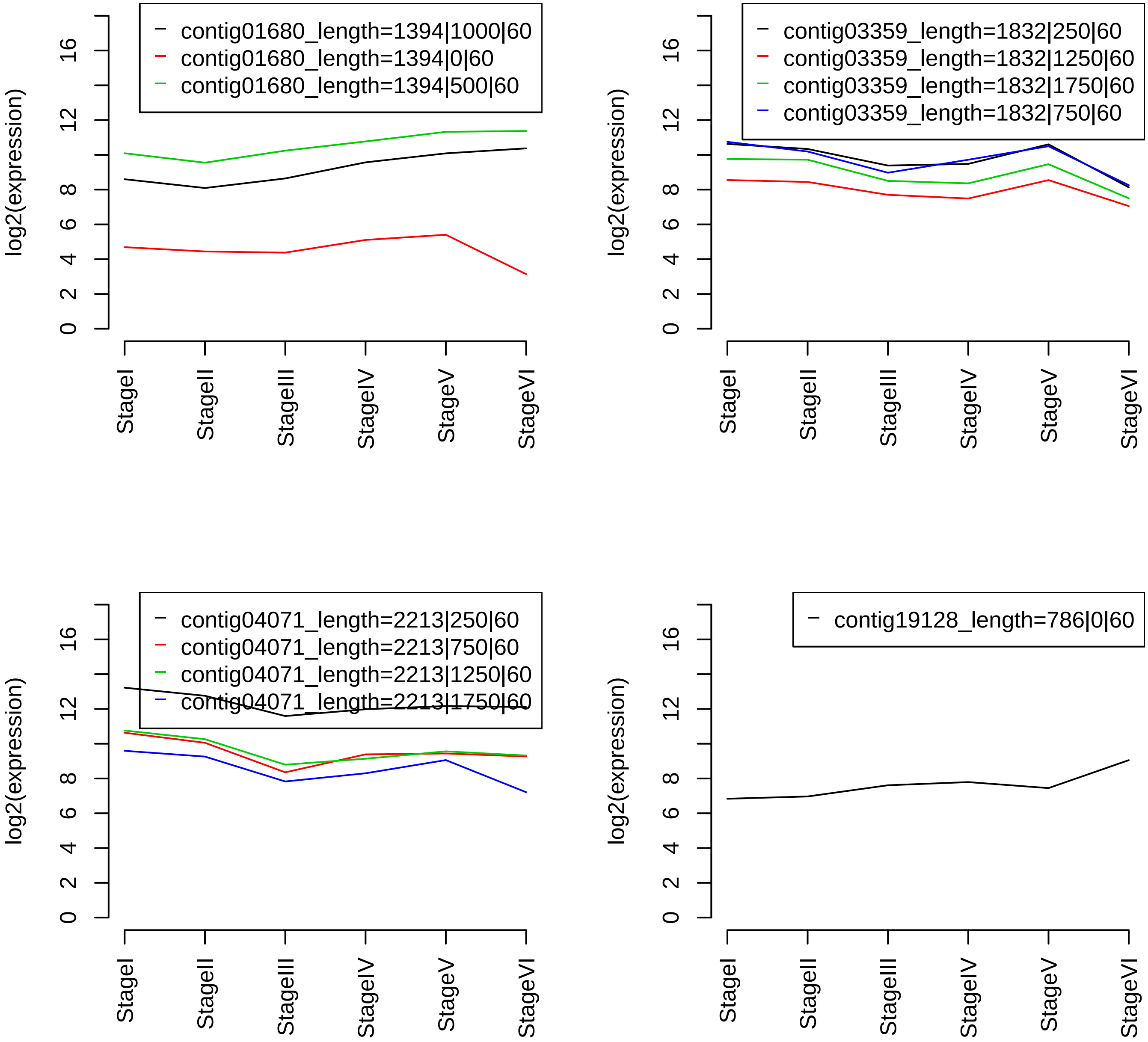
contig01680_length=1394|1000|60
contig01680_length=1394|0|60
contig01680_length=1394|500|60
log2(expression)
0
2
4
6
8
12
16
StageI
StageII
StageIII
StageIV
StageV
StageVI
contig03359_length=1832|250|60
contig03359_length=1832|1250|60
contig03359_length=1832|1750|60
contig03359_length=1832|750|60
log2(expression)
0
2
4
6
8
12
16
StageI
StageII
StageIII
StageIV
StageV
StageVI
contig04071_length=2213|250|60
contig04071_length=2213|750|60
contig04071_length=2213|1250|60
contig04071_length=2213|1750|60
log2(expression)
0
2
4
6
8
12
16
StageI
StageII
StageIII
StageIV
StageV
StageVI
contig19128_length=786|0|60
log2(expression)
0
2
4
6
8
12
16
StageI
StageII
StageIII
StageIV
StageV
StageVI

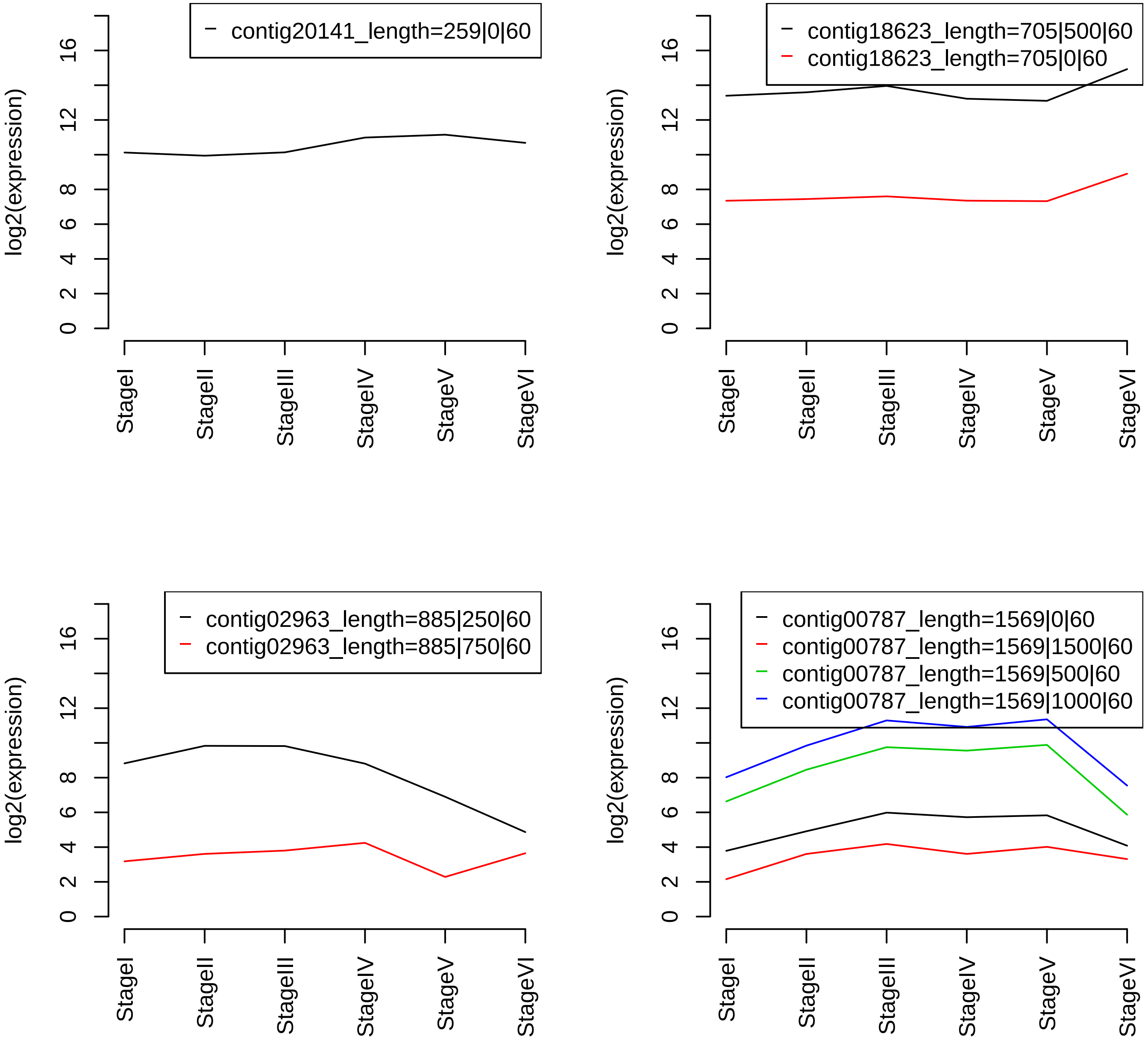

contig20141_length=259|0|60
contig18623_length=705|500|60
contig18623_length=705|0|60
contig02963_length=885|250|60
contig02963_length=885|750|60
contig00787_length=1569|0|60
contig00787_length=1569|1500|60
contig00787_length=1569|500|60
contig00787_length=1569|1000|60
log2(expression)
0
2
4
6
8
12
16
StageI
StageII
StageIII
StageIV
StageV
StageVI

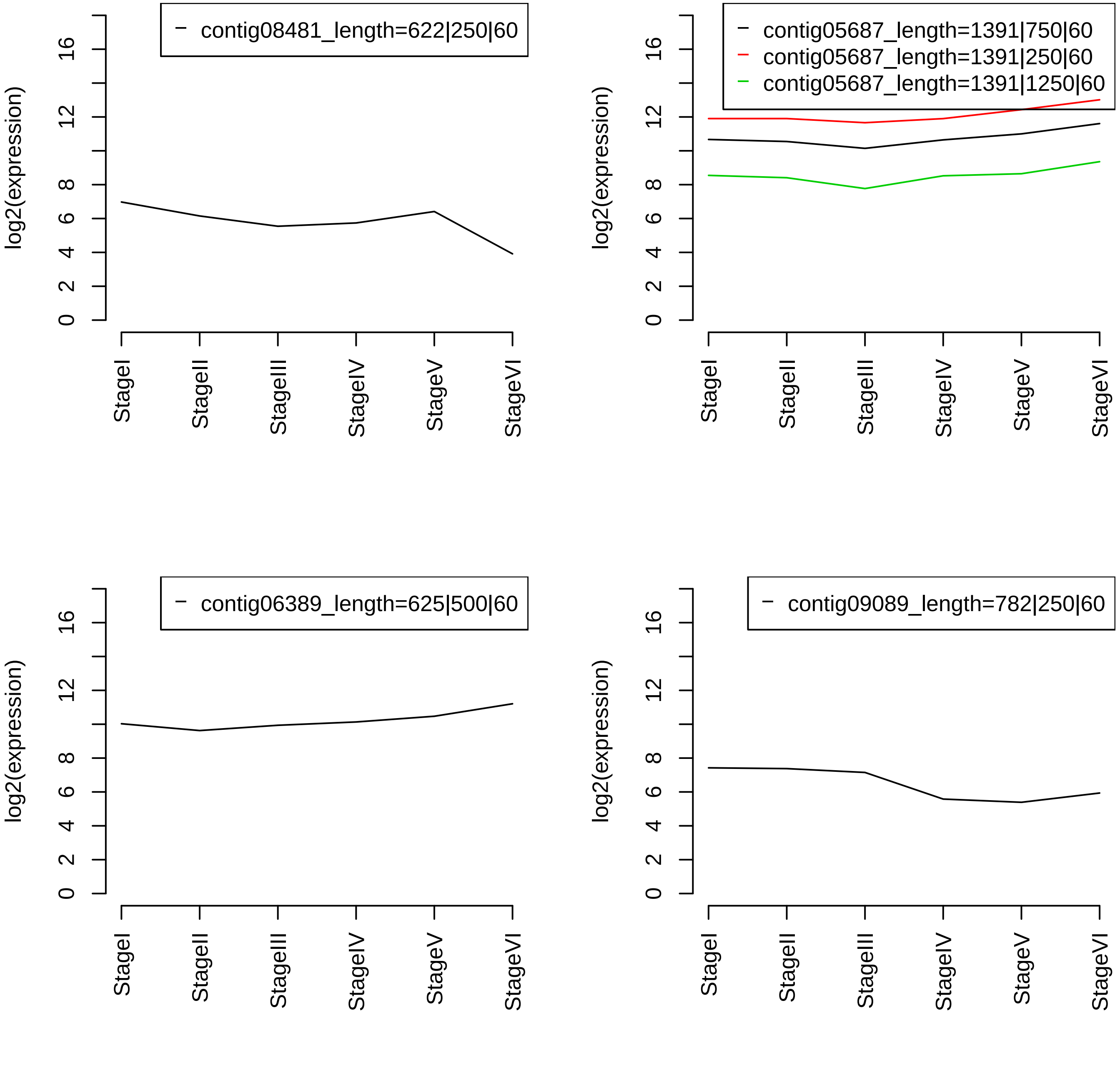

contig08481_length=622|250|60
contig05687_length=1391|750|60
contig05687_length=1391|250|60
contig05687_length=1391|1250|60
contig06389_length=625|500|60
contig09089_length=782|250|60
log2(expression)
0
2
4
6
8
12
16
StageI
StageII
StageIII
StageIV
StageV
StageVI

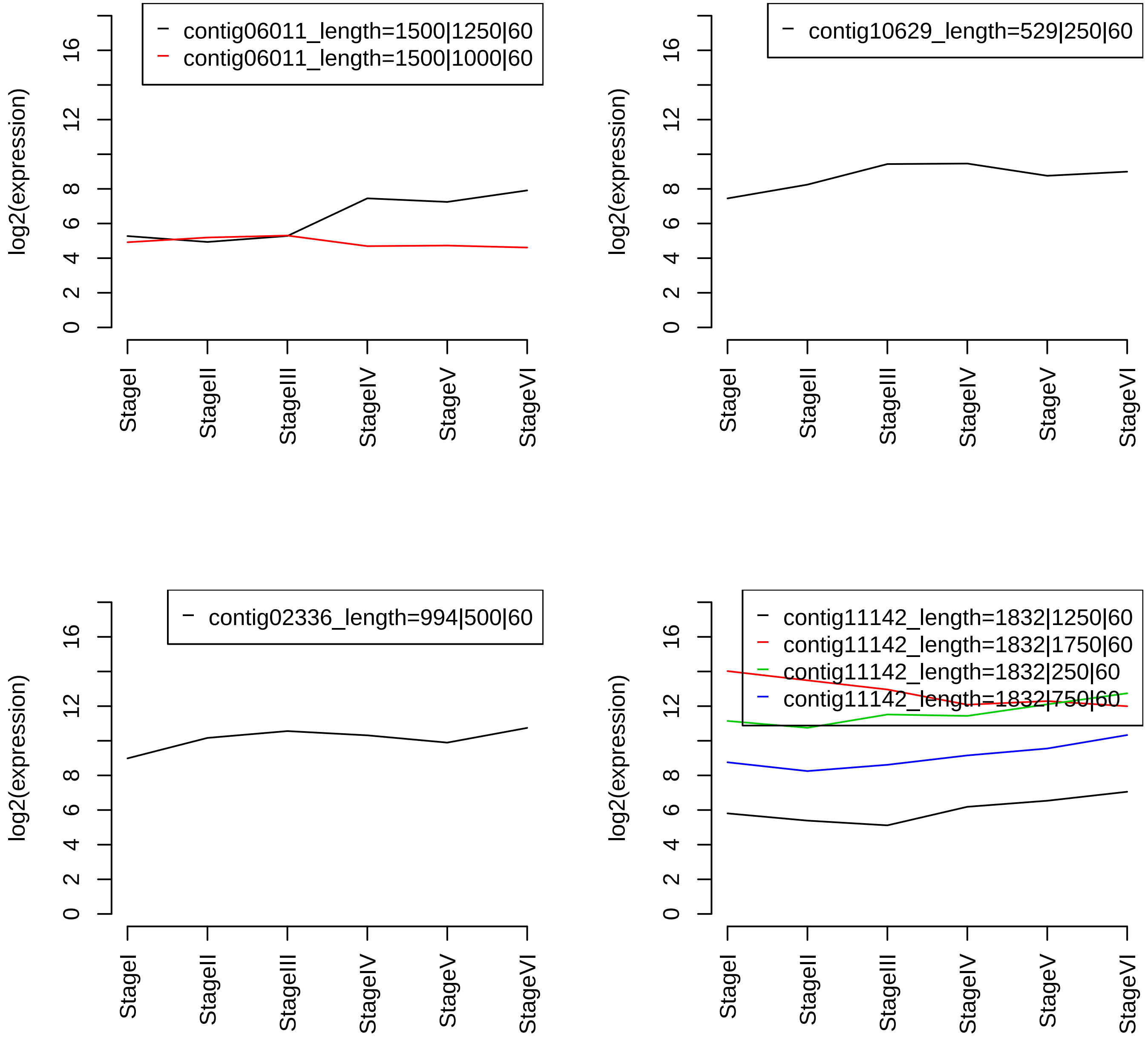

contig06011_length=1500|1250|60
contig06011_length=1500|1000|60
contig10629_length=529|250|60
contig02336_length=994|500|60
contig11142_length=1832|1250|60
contig11142_length=1832|1750|60
contig11142_length=1832|250|60
contig11142_length=1832|750|60
log2(expression)
0
2
4
6
8
12
16
StageI
StageII
StageIII
StageIV
StageV
StageVI

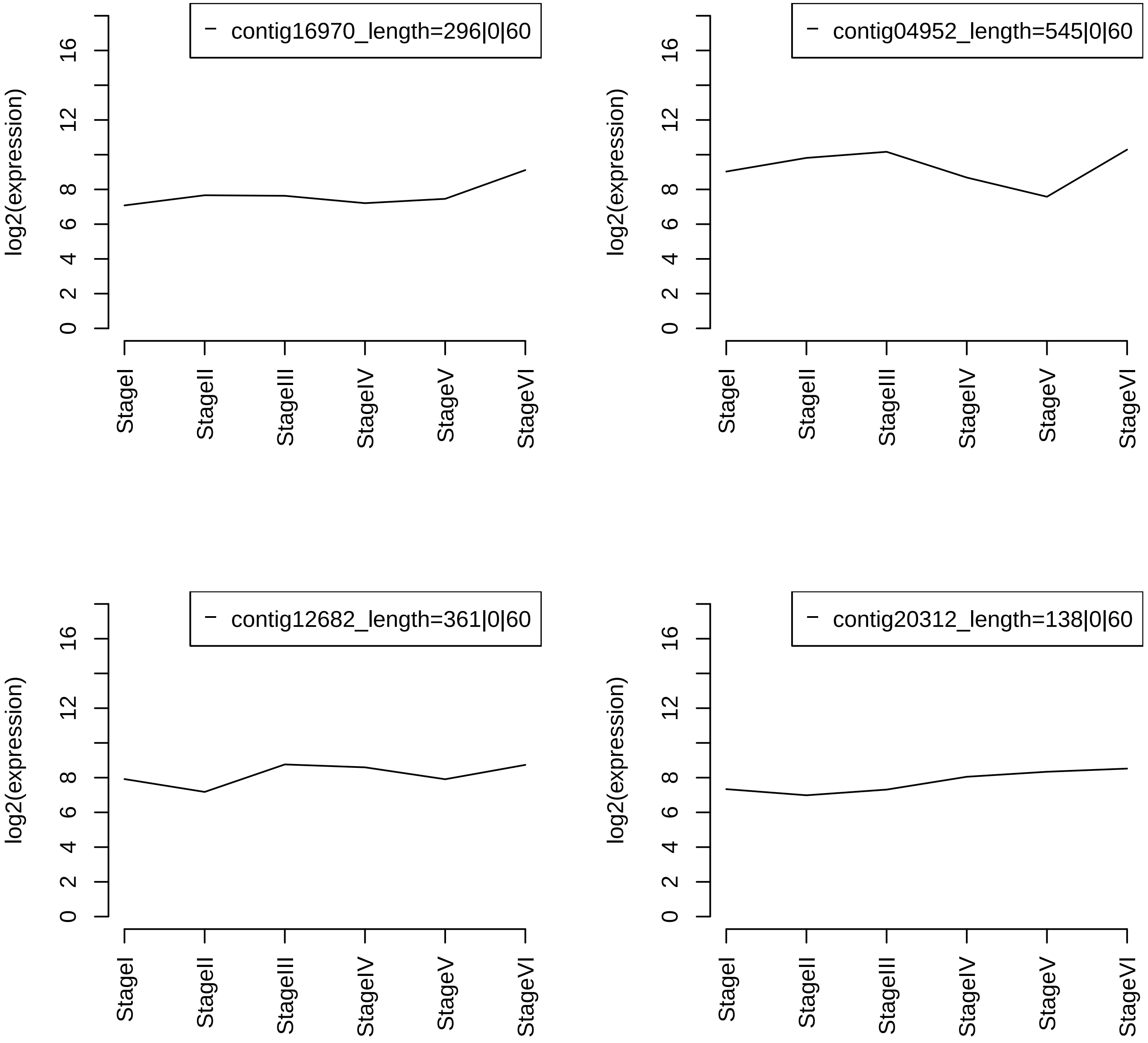
contig16970_length=296|0|60
contig04952_length=545|0|60
contig12682_length=361|0|60
contig20312_length=138|0|60
log2(expression)
0
2
4
6
8
12
16
StageI
StageII
StageIII
StageIV
StageV
StageVI

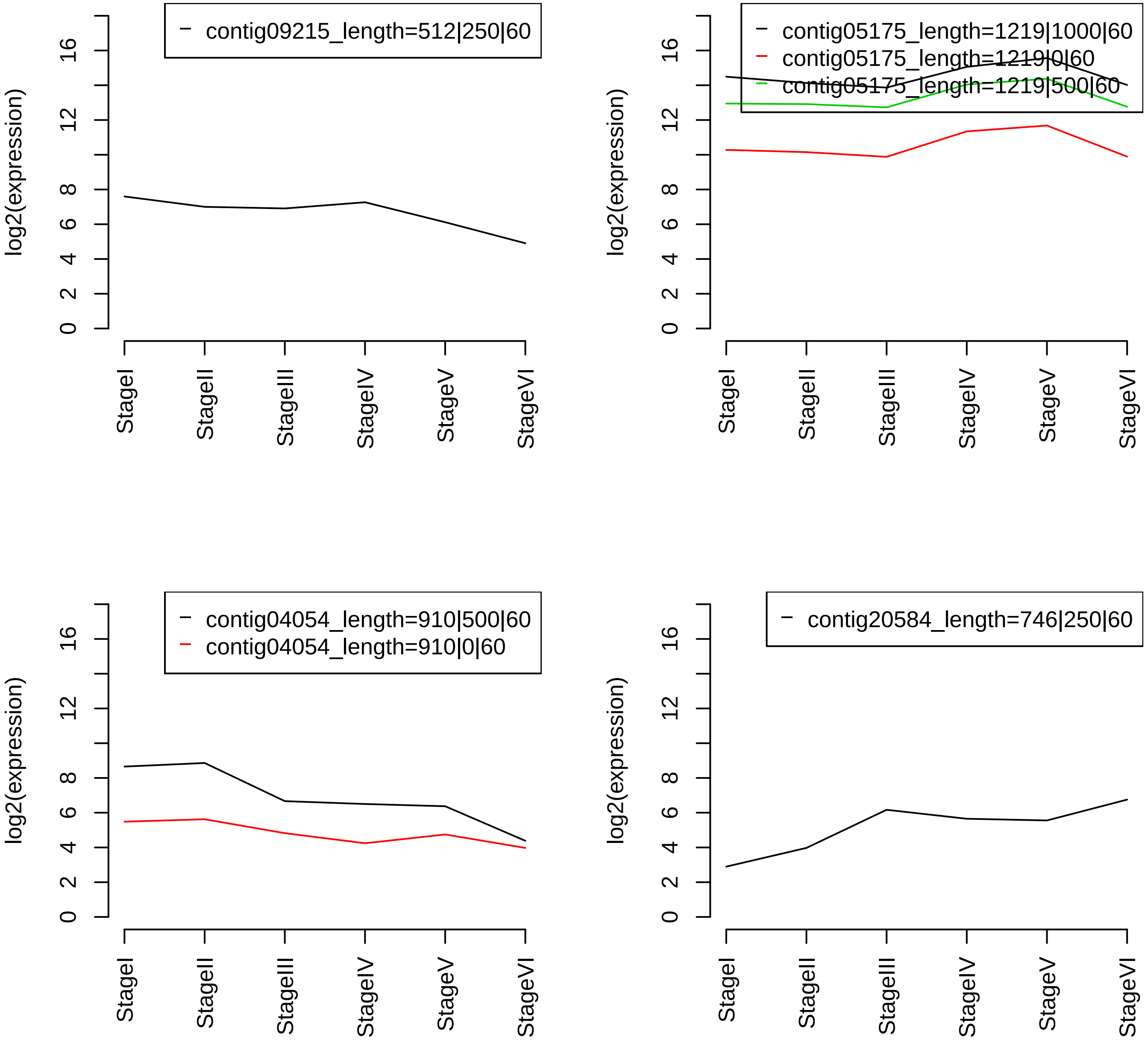
contig09215_length=512|250|60
contig05175_length=1219|1000|60
contig05175_length=1219|0|60
contig05175_length=1219|500|60
contig04054_length=910|500|60
contig04054_length=910|0|60
contig20584_length=746|250|60
log2(expression)
0
2
4
6
8
12
16
StageI
StageII
StageIII
StageIV
StageV
StageVI

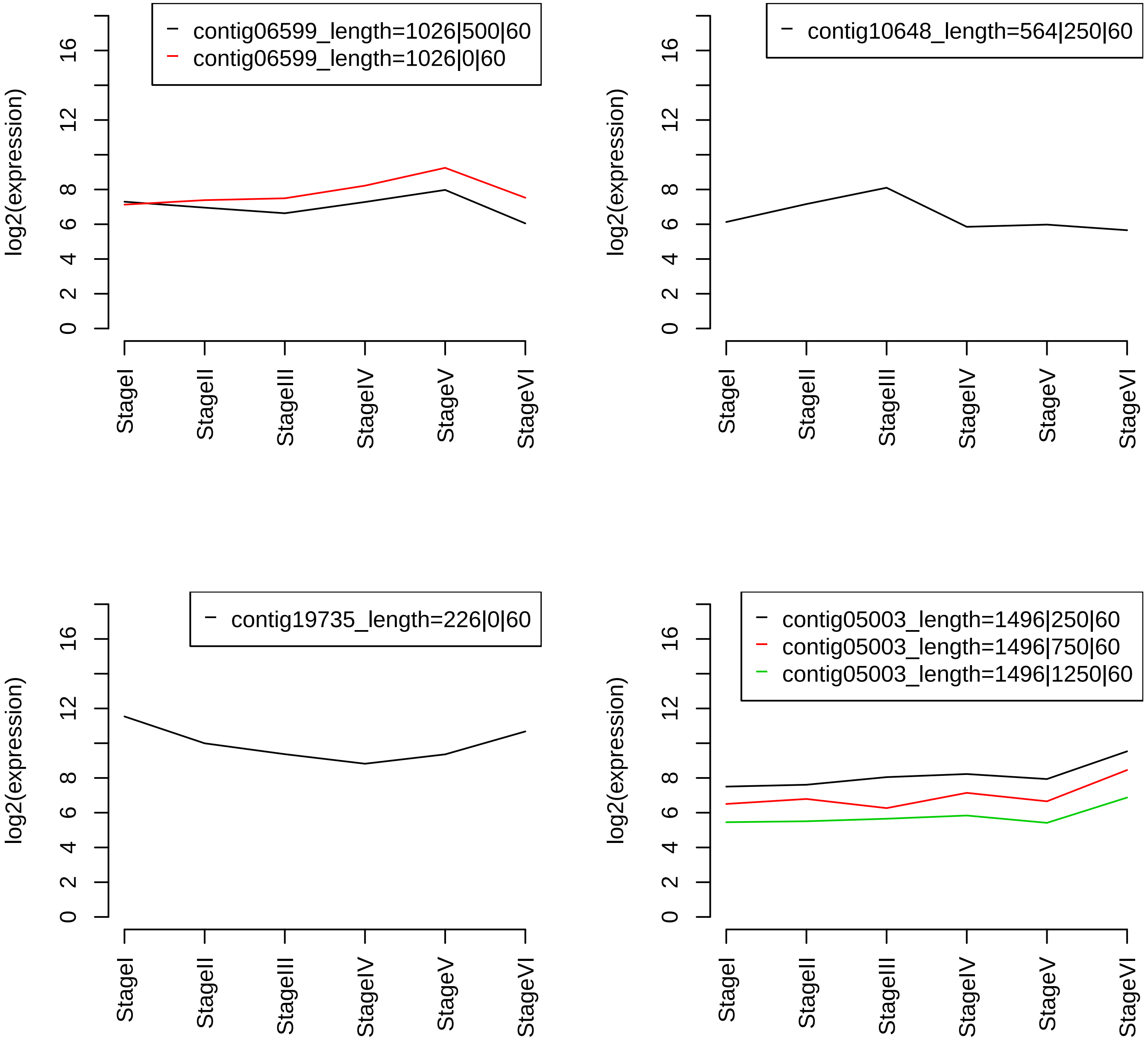

contig06599_length=1026|500|60
contig06599_length=1026|0|60
contig10648_length=564|250|60
contig19735_length=226|0|60
contig05003_length=1496|250|60
contig05003_length=1496|750|60
contig05003_length=1496|1250|60
log2(expression)
0
2
4
6
8
12
16
StageI
StageII
StageIII
StageIV
StageV
StageVI

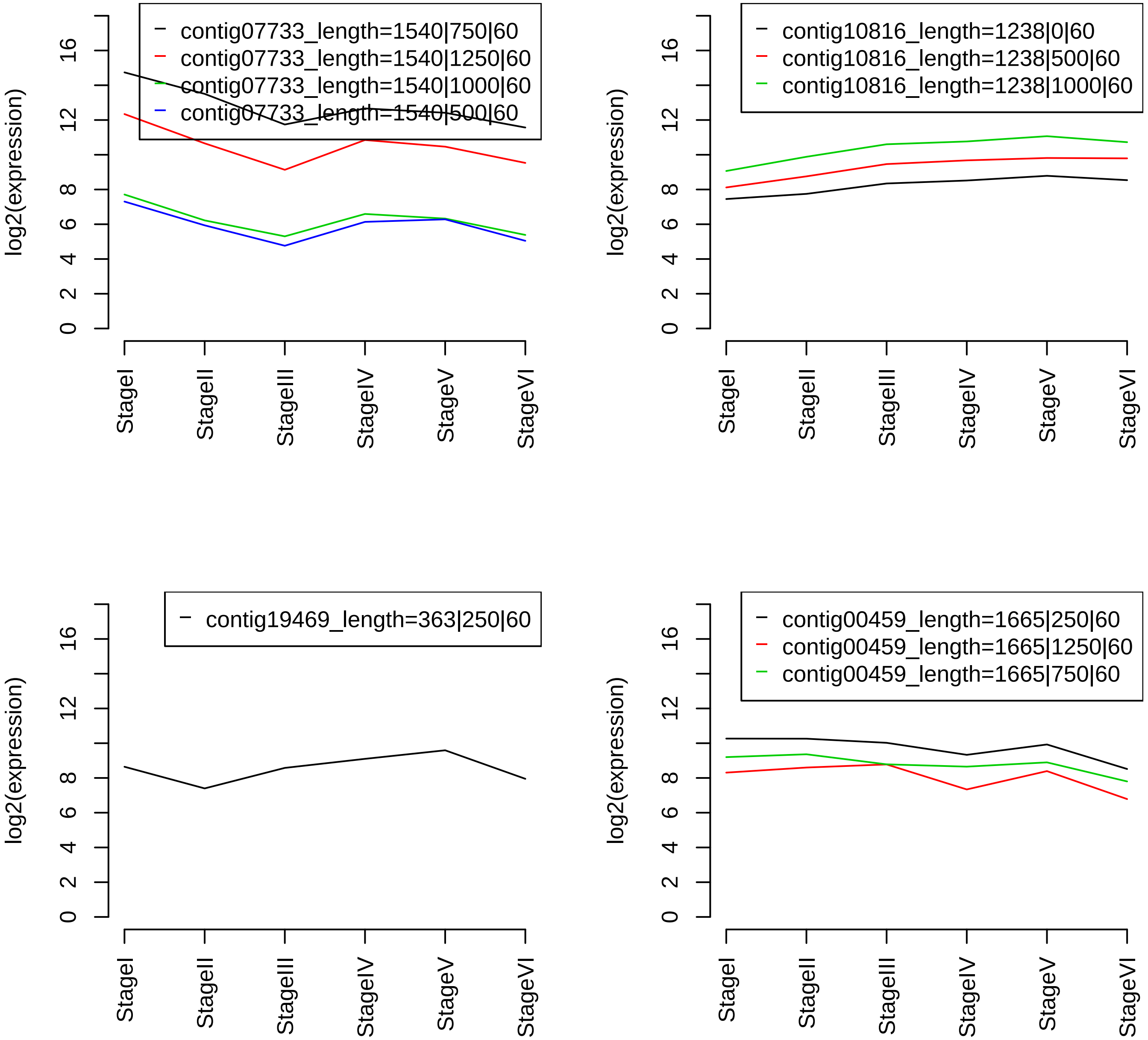

contig07733_length=1540|750|60
contig07733_length=1540|1250|60
contig07733_length=1540|1000|60
contig07733_length=1540|500|60
contig10816_length=1238|0|60
contig10816_length=1238|500|60
contig10816_length=1238|1000|60
contig19469_length=363|250|60
contig00459_length=1665|250|60
contig00459_length=1665|1250|60
contig00459_length=1665|750|60
log2(expression)
0 2 4 6 8 12 16
StageI StageII StageIII StageIV StageV StageVI

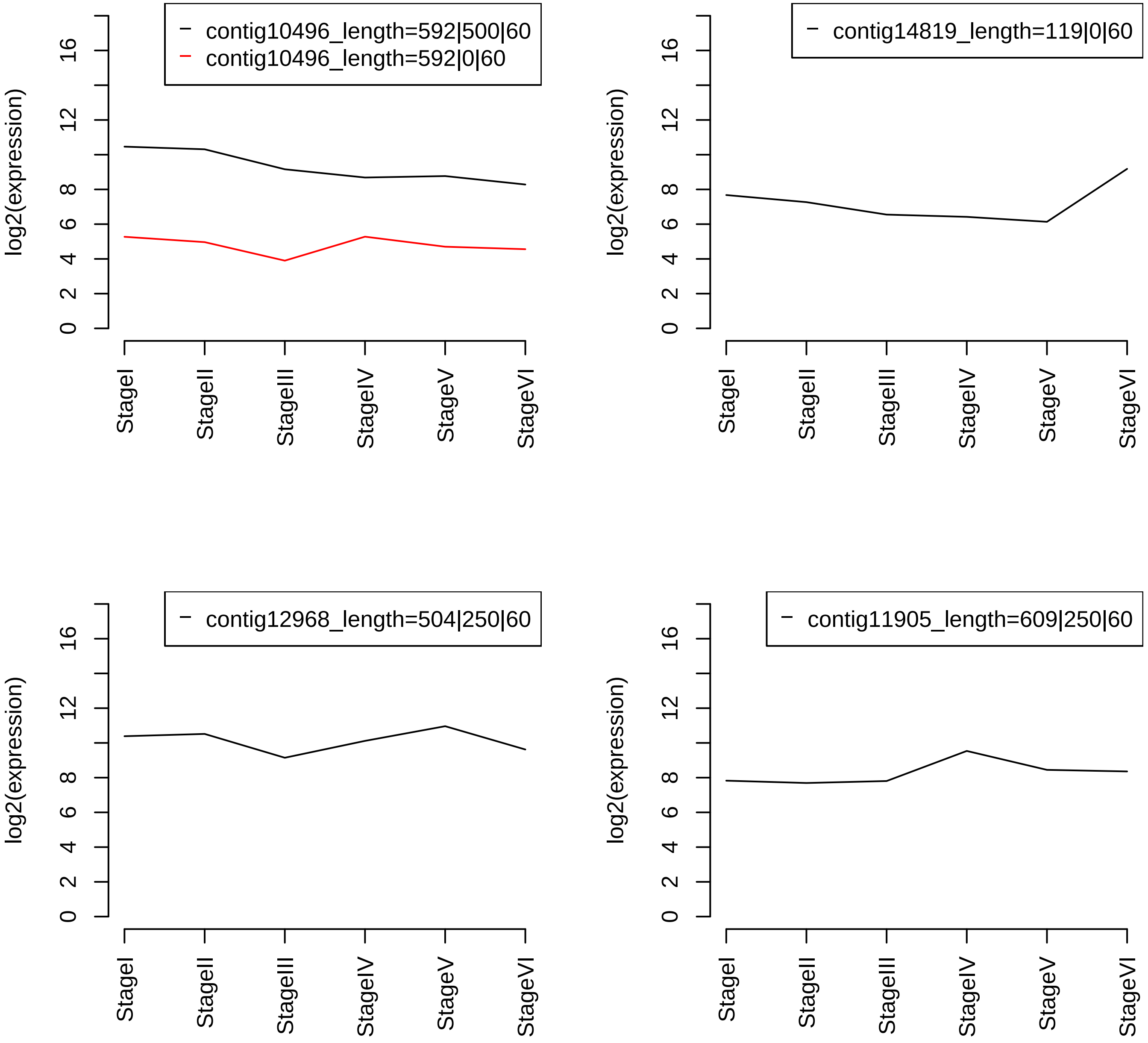

contig10496_length=592|500|60
contig10496_length=592|0|60
contig14819_length=119|0|60
contig12968_length=504|250|60
contig11905_length=609|250|60
log2(expression)
0
2
4
6
8
12
16
StageI
StageII
StageIII
StageIV
StageV
StageVI

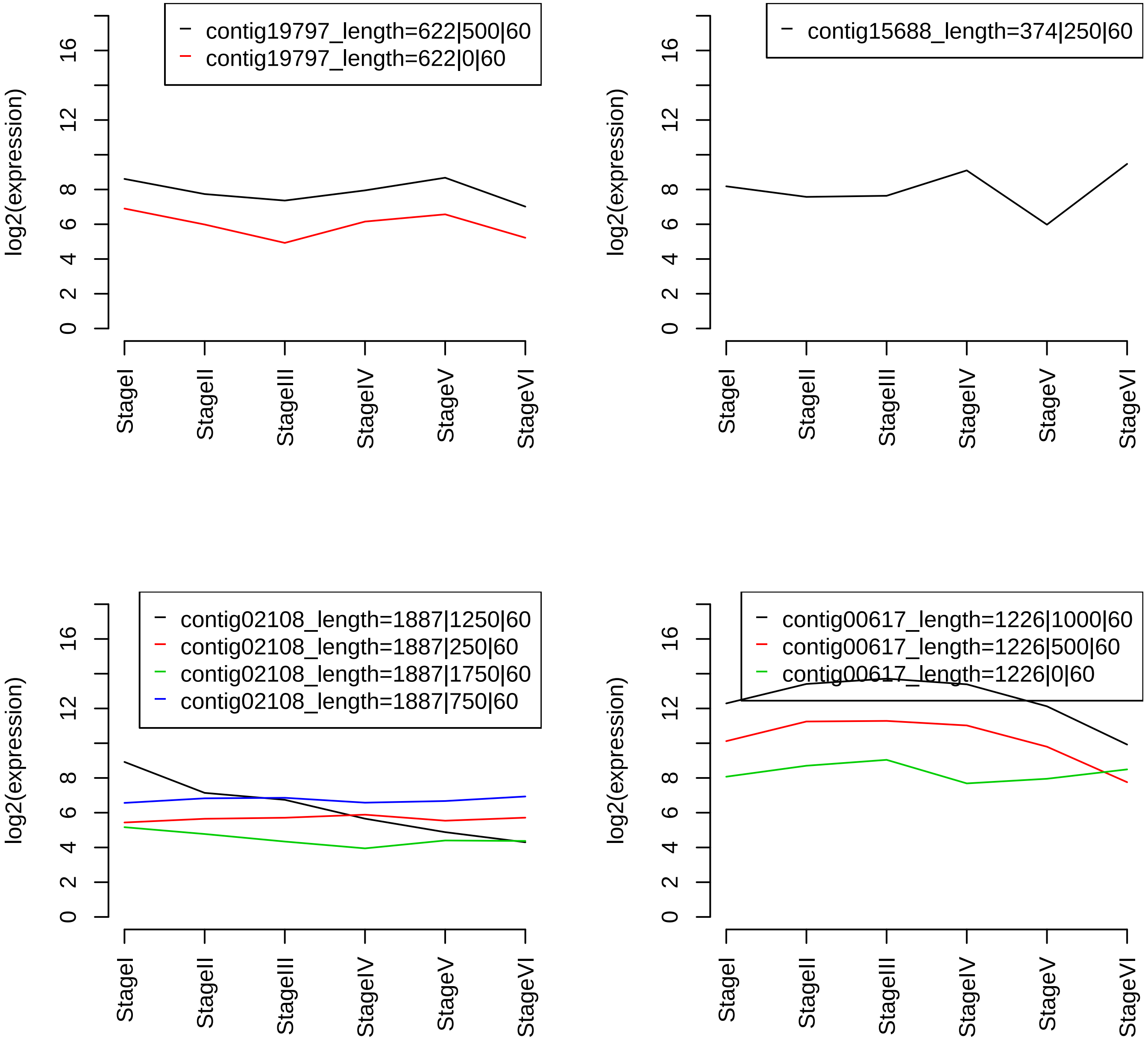

contig19797_length=622|500|60
contig19797_length=622|0|60
contig15688_length=374|250|60
contig02108_length=1887|1250|60
contig02108_length=1887|250|60
contig02108_length=1887|1750|60
contig02108_length=1887|750|60
contig00617_length=1226|1000|60
contig00617_length=1226|500|60
contig00617_length=1226|0|60
log2(expression)
0
2
4
6
8
12
16
StageI
StageII
StageIII
StageIV
StageV
StageVI

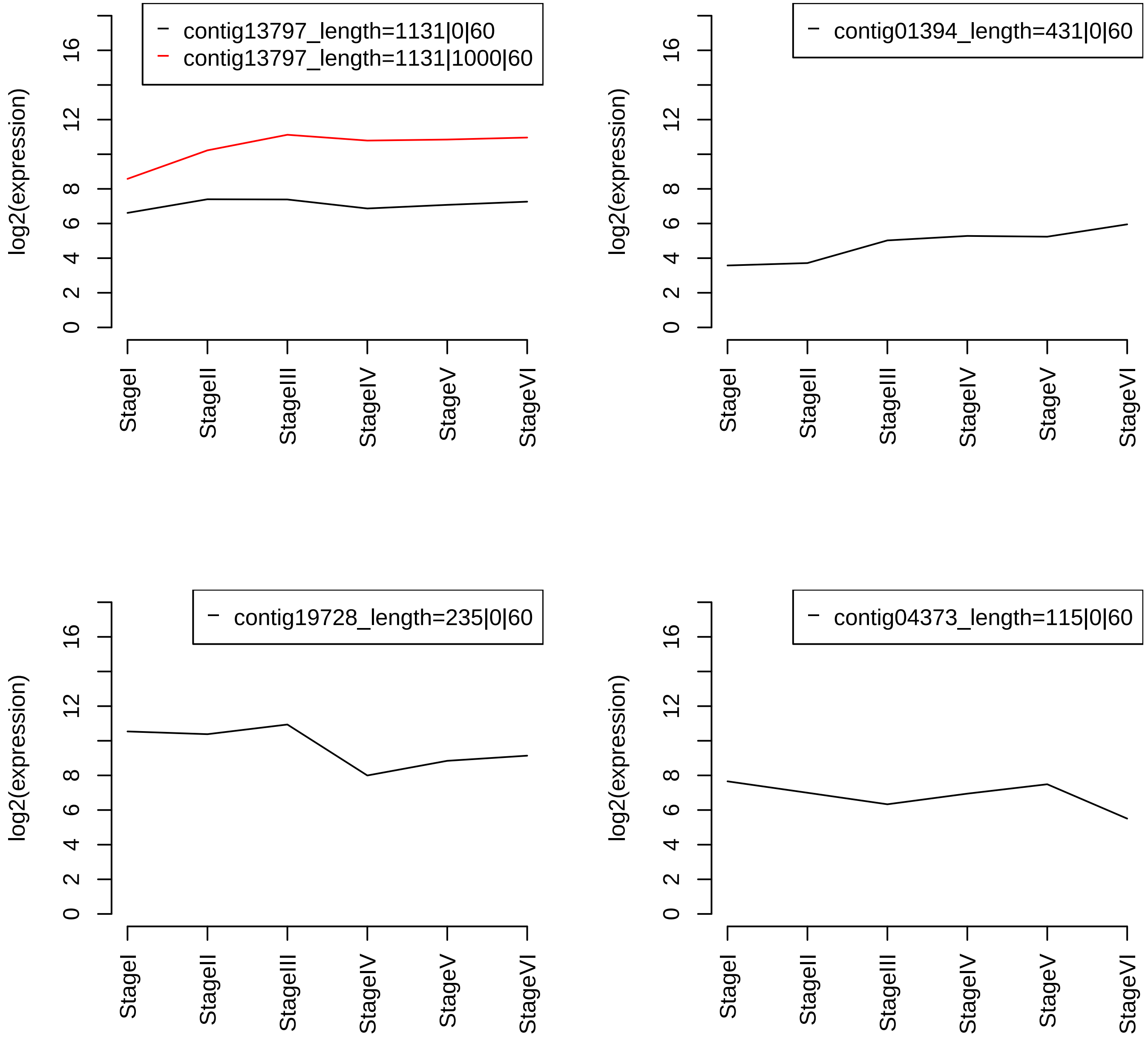
contig13797_length=1131|0|60
contig13797_length=1131|1000|60
contig01394_length=431|0|60
contig19728_length=235|0|60
contig04373_length=115|0|60
log2(expression)
0
2
4
6
8
12
16
StageI
StageII
StageIII
StageIV
StageV
StageVI

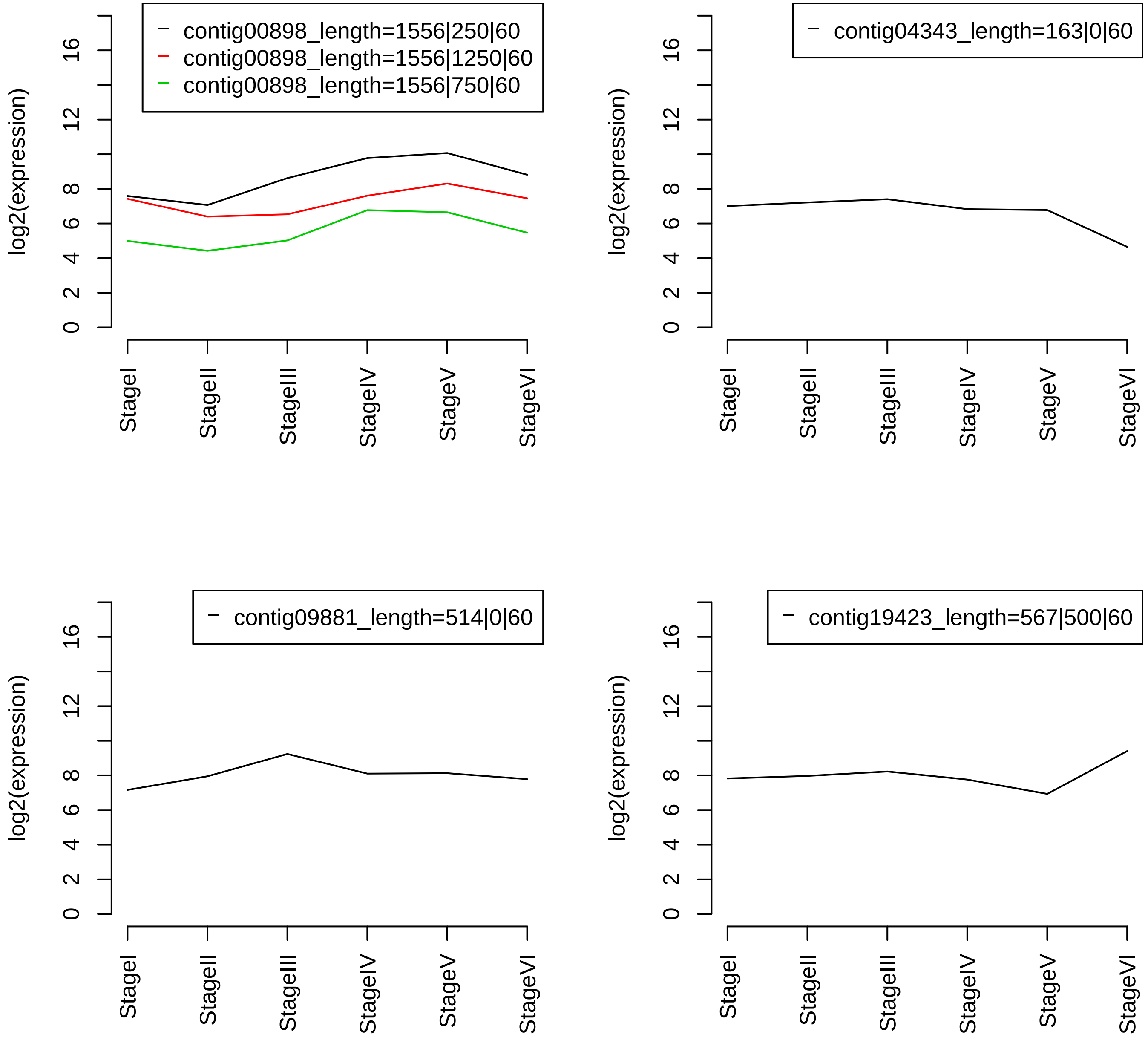

contig00898_length=1556|250|60
contig00898_length=1556|1250|60
contig00898_length=1556|750|60
contig04343_length=163|0|60
contig09881_length=514|0|60
contig19423_length=567|500|60
log2(expression)
0
2
4
6
8
12
16
StageI
StageII
StageIII
StageIV
StageV
StageVI

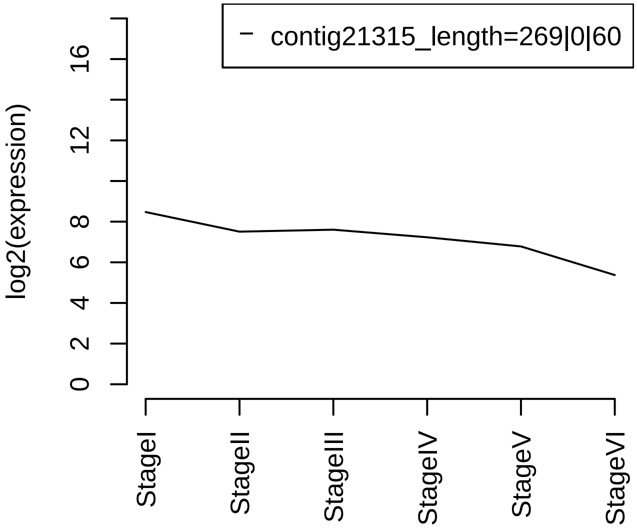
contig21315_length=269|0|60
log2(expression)
0
2
4
6
8
12
16
StageI
StageII
StageIII
StageIV
StageV
StageVI

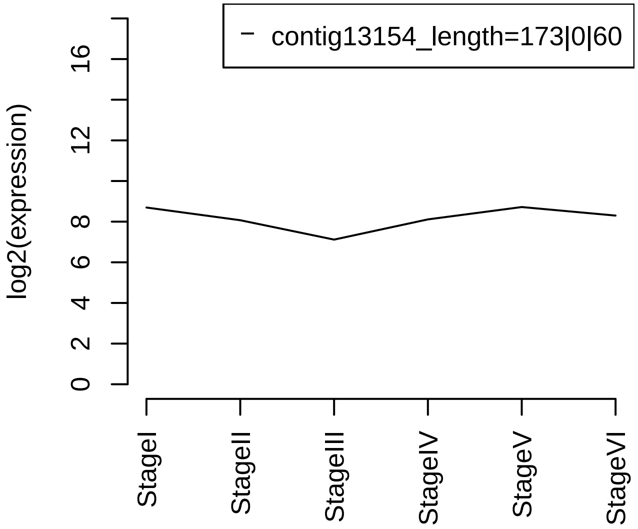
contig13154_length=173|0|60
log2(expression)
0
2
4
6
8
12
16
StageI
StageII
StageIII
StageIV
StageV
StageVI

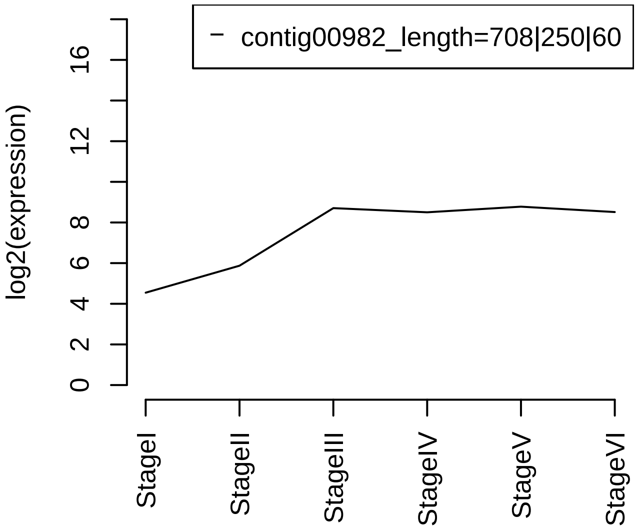
contig00982_length=708|250|60
log2(expression)
0
2
4
6
8
12
16
StageI
StageII
StageIII
StageIV
StageV
StageVI

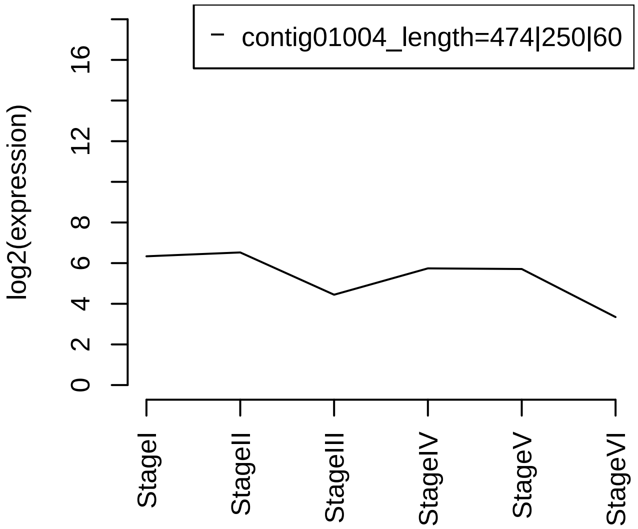
contig01004_length=474|250|60
log2(expression)
0
2
4
6
8
12
16
StageI
StageII
StageIII
StageIV
StageV
StageVI

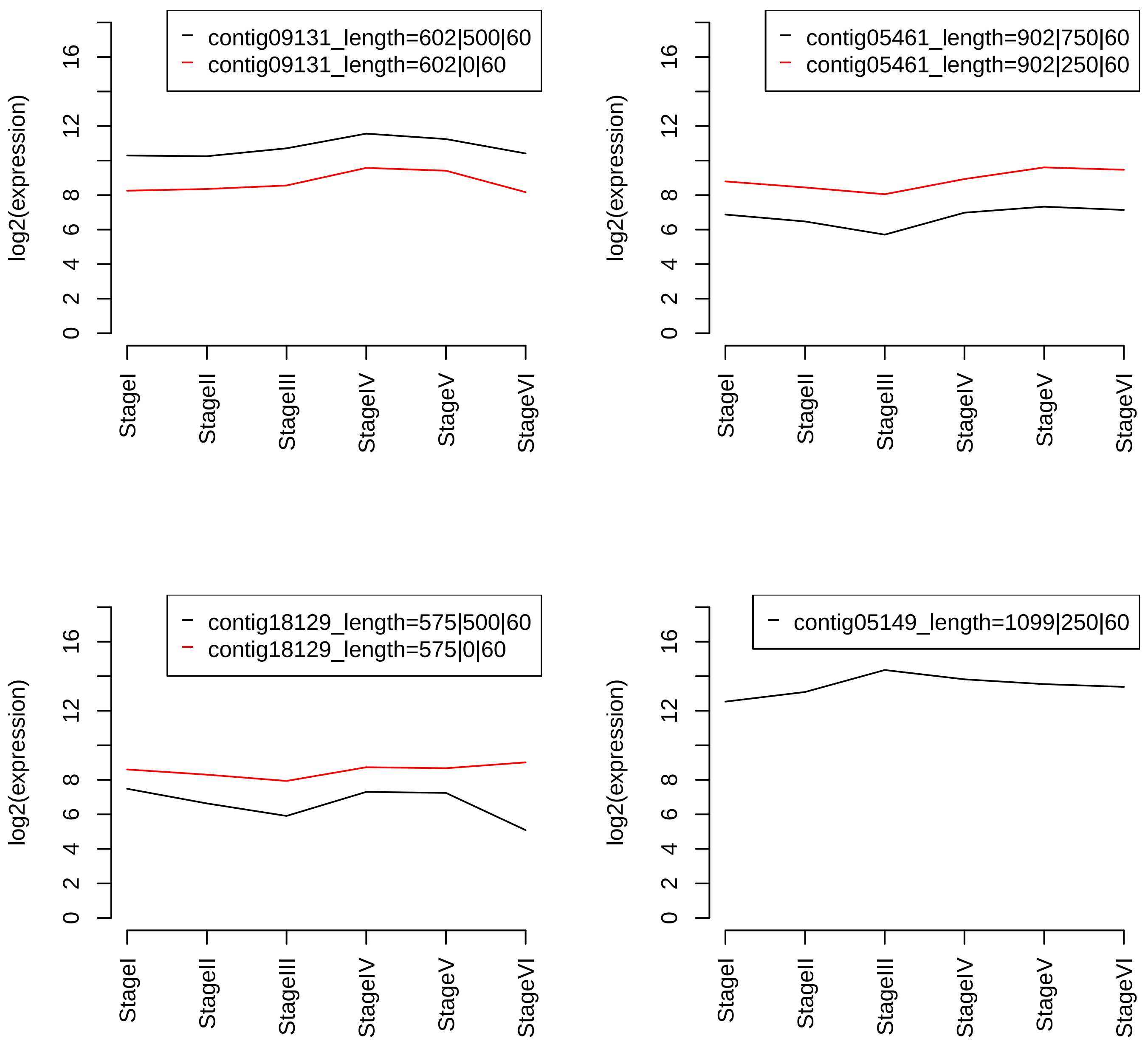

contig09131_length=602|500|60
contig09131_length=602|0|60
contig05461_length=902|750|60
contig05461_length=902|250|60
contig18129_length=575|500|60
contig18129_length=575|0|60
contig05149_length=1099|250|60
log2(expression)
0
2
4
6
8
12
16
StageI
StageII
StageIII
StageIV
StageV
StageVI

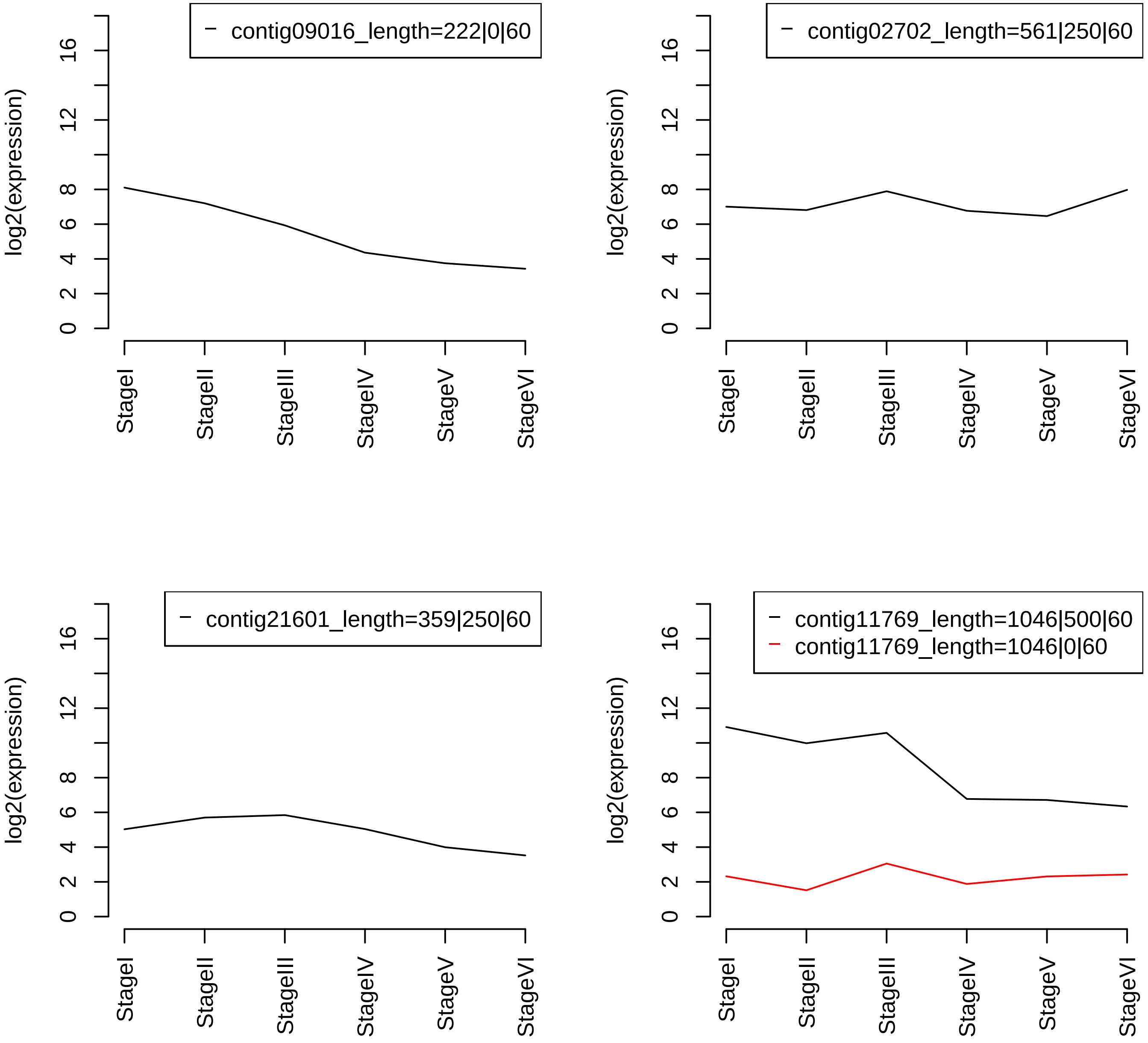
contig09016_length=222|0|60
contig02702_length=561|250|60
contig21601_length=359|250|60
contig11769_length=1046|500|60
contig11769_length=1046|0|60
log2(expression)
0
2
4
6
8
12
16
StageI
StageII
StageIII
StageIV
StageV
StageVI

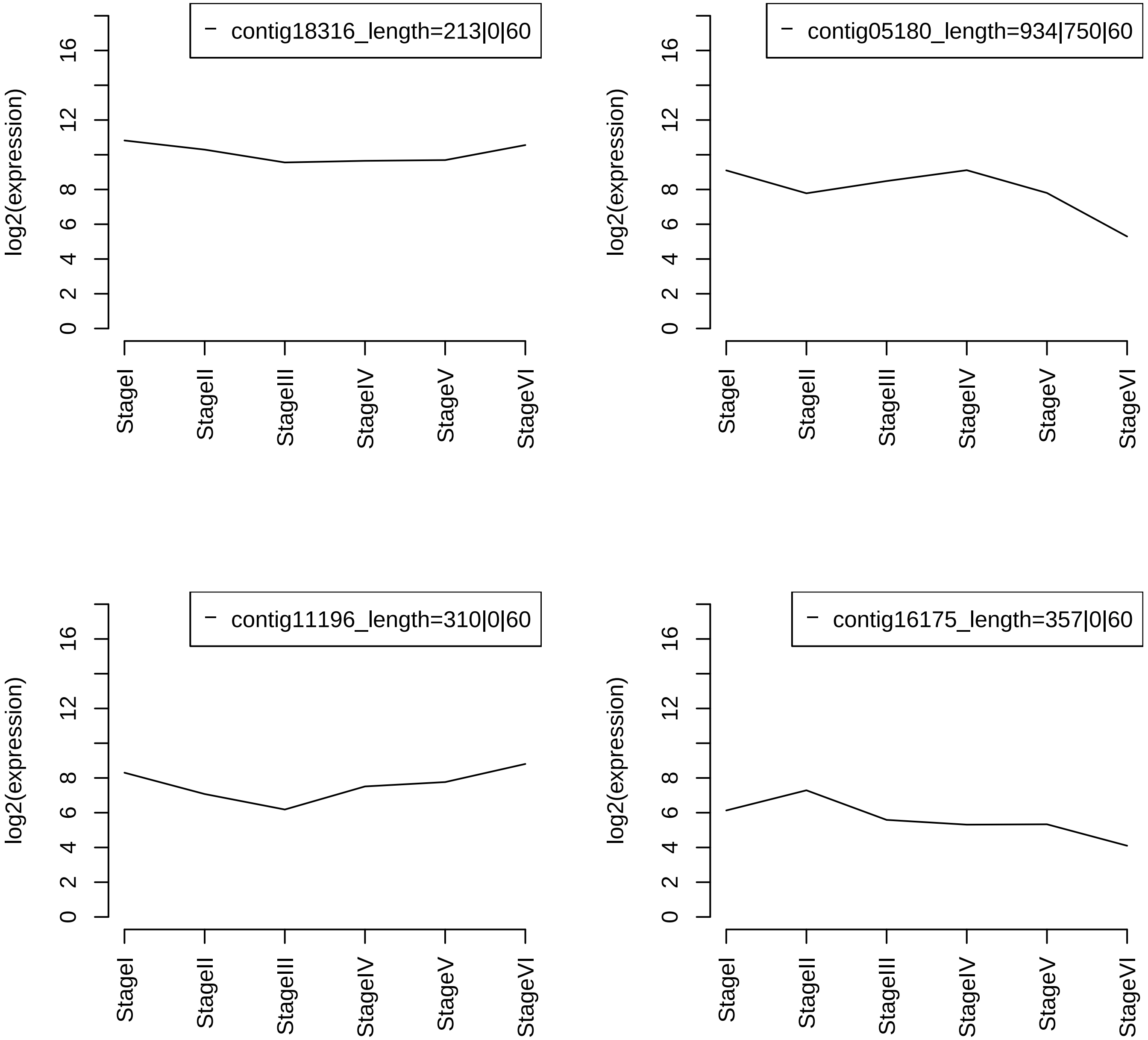
contig18316_length=213|0|60
contig05180_length=934|750|60
contig11196_length=310|0|60
contig16175_length=357|0|60
log2(expression)
0
2
4
6
8
12
16
StageI
StageII
StageIII
StageIV
StageV
StageVI

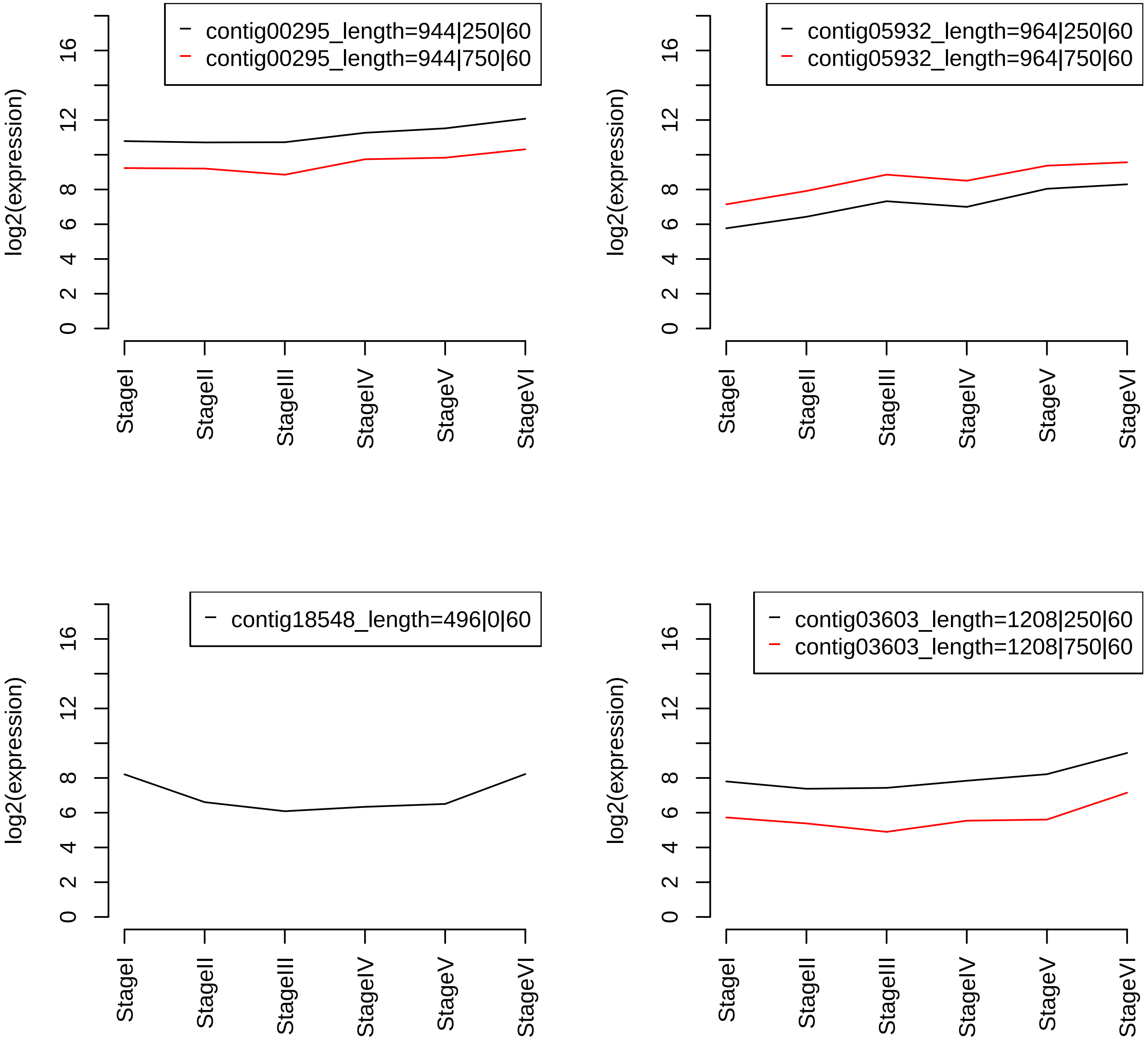

contig00295_length=944|250|60
contig00295_length=944|750|60
contig05932_length=964|250|60
contig05932_length=964|750|60
contig18548_length=496|0|60
contig03603_length=1208|250|60
contig03603_length=1208|750|60
log2(expression)
0
2
4
6
8
12
16
StageI
StageII
StageIII
StageIV
StageV
StageVI

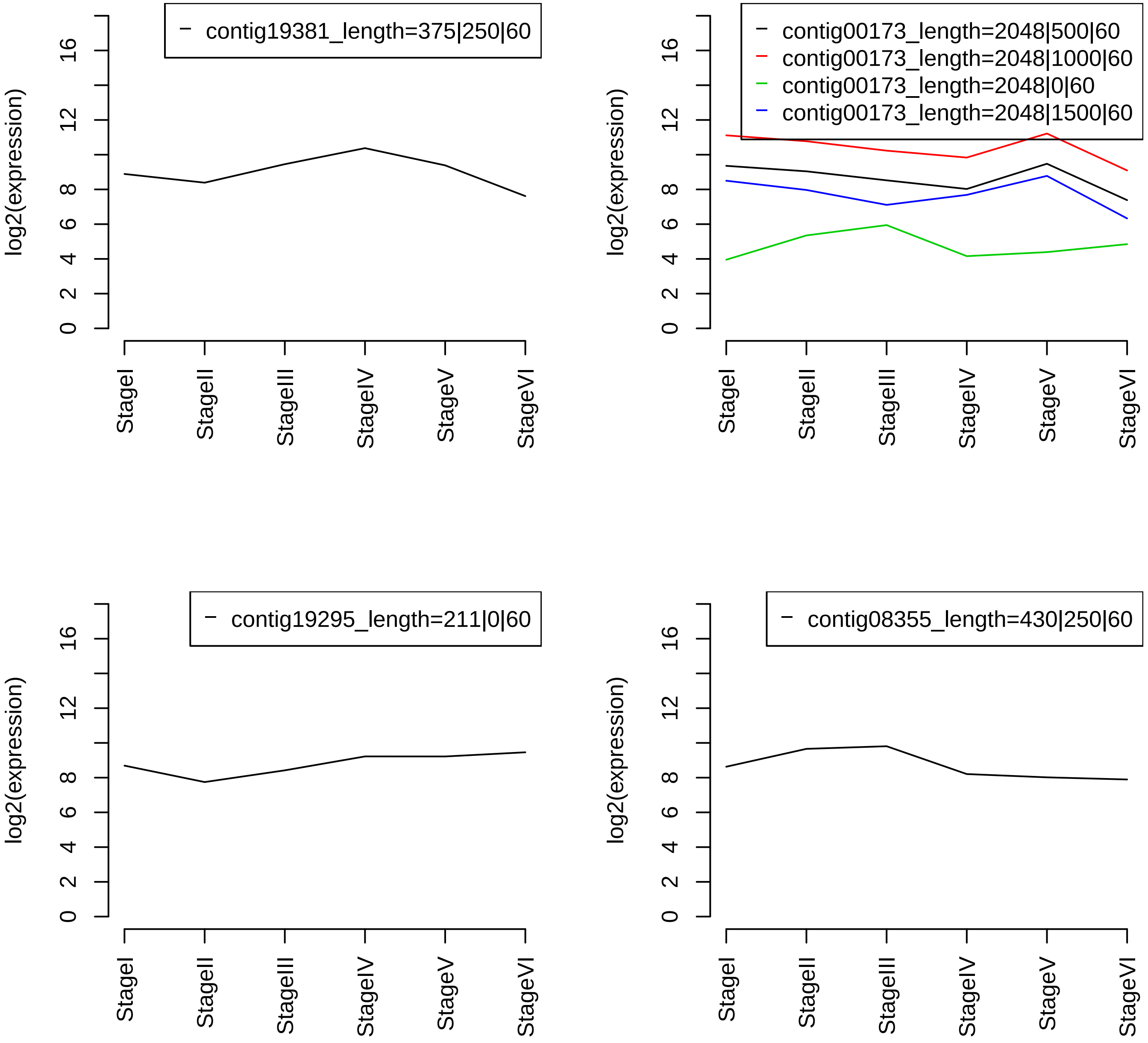
contig19381_length=375|250|60
contig00173_length=2048|500|60
contig00173_length=2048|1000|60
contig00173_length=2048|0|60
contig00173_length=2048|1500|60
contig19295_length=211|0|60
contig08355_length=430|250|60
log2(expression)
0
2
4
6
8
12
16
StageI
StageII
StageIII
StageIV
StageV
StageVI

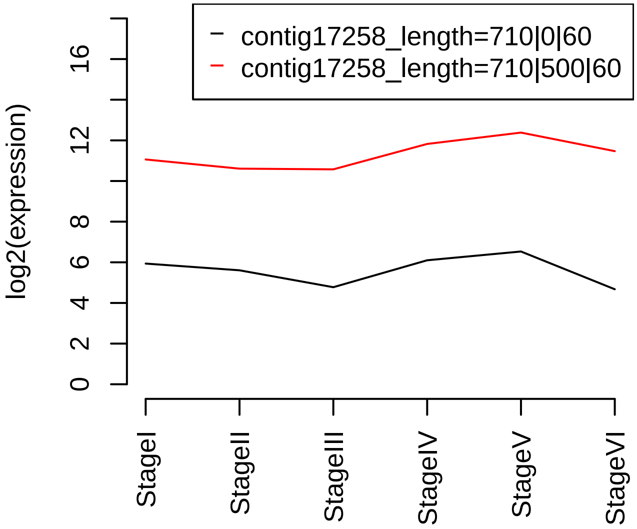

contig17258_length=710|0|60
contig17258_length=710|500|60
log2(expression)
0
2
4
6
8
12
16
StageI
StageII
StageIII
StageIV
StageV
StageVI

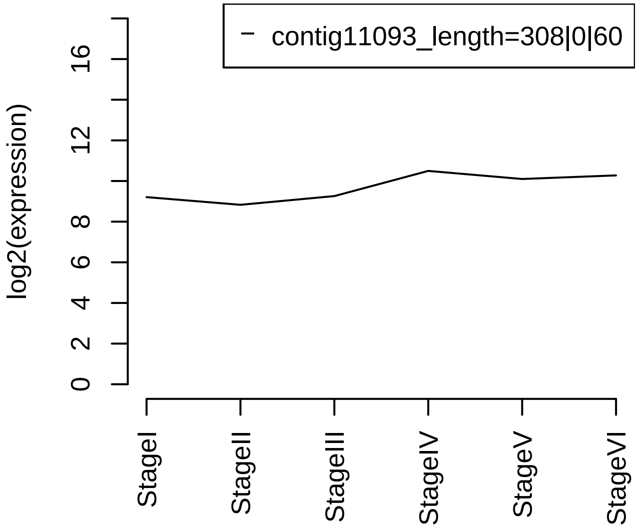

contig11093_length=308|0|60
log2(expression)
0
2
4
6
8
12
16
StageI
StageII
StageIII
StageIV
StageV
StageVI

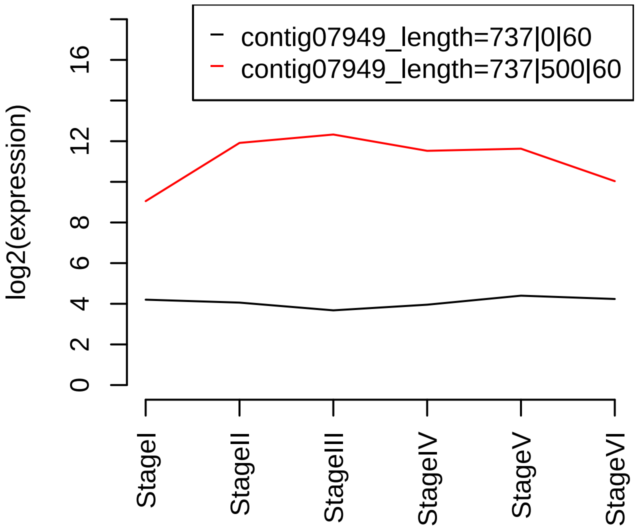

contig07949_length=737|0|60
contig07949_length=737|500|60
log2(expression)
0
2
4
6
8
12
16
StageI
StageII
StageIII
StageIV
StageV
StageVI

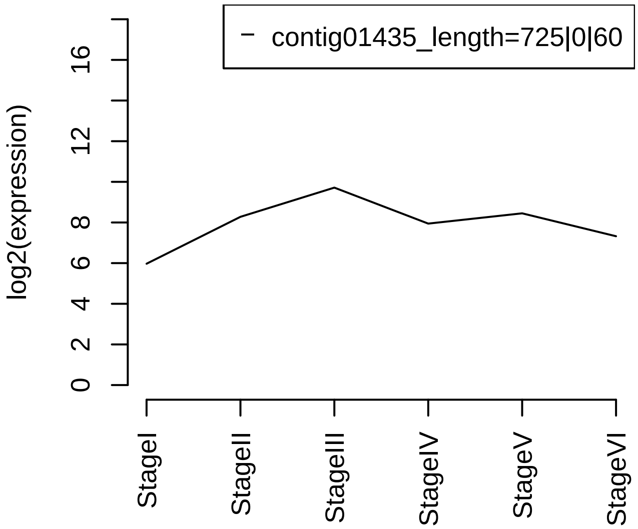

contig01435_length=725|0|60
log2(expression)
0
2
4
6
8
12
16
StageI
StageII
StageIII
StageIV
StageV
StageVI

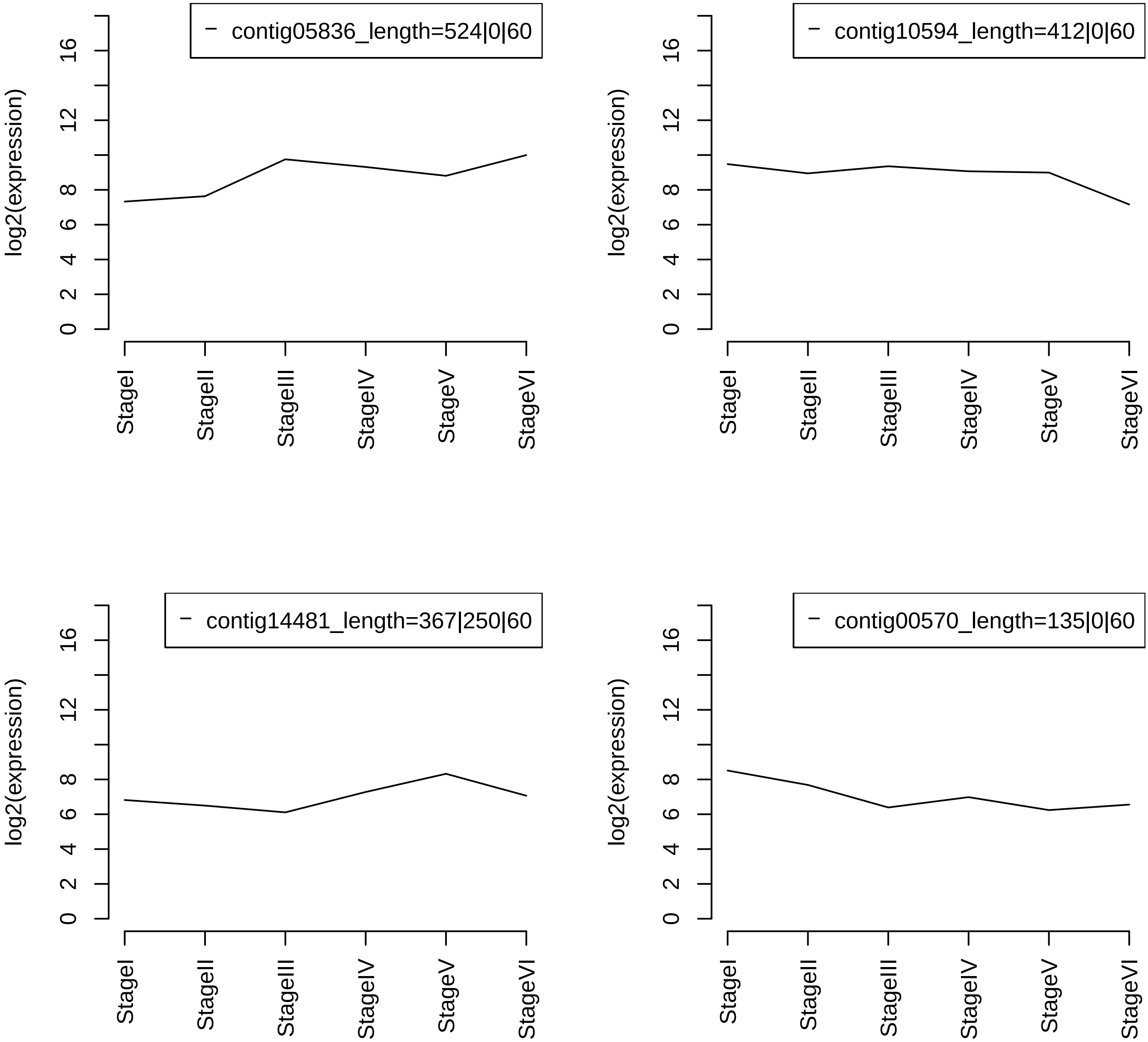

contig05836_length=524|0|60
contig10594_length=412|0|60
contig14481_length=367|250|60
contig00570_length=135|0|60
log2(expression)
0
2
4
6
8
12
16
StageI
StageII
StageIII
StageIV
StageV
StageVI

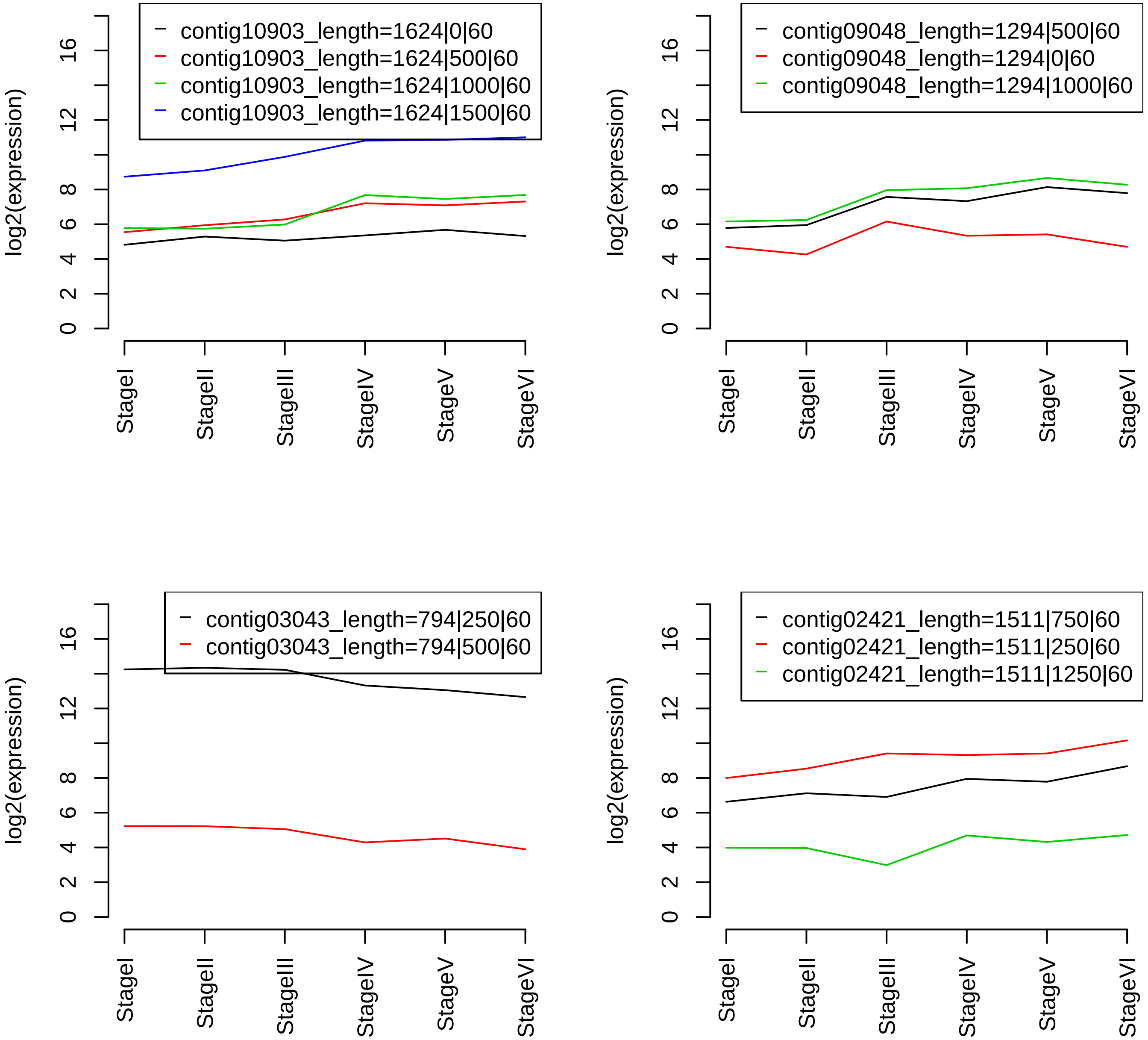
contig10903_length=1624|0|60
contig10903_length=1624|500|60
contig10903_length=1624|1000|60
contig10903_length=1624|1500|60
contig09048_length=1294|500|60
contig09048_length=1294|0|60
contig09048_length=1294|1000|60
contig03043_length=794|250|60
contig03043_length=794|500|60
contig02421_length=1511|750|60
contig02421_length=1511|250|60
contig02421_length=1511|1250|60
log2(expression)
0 2 4 6 8 12 16
StageI StageII StageIII StageIV StageV StageVI

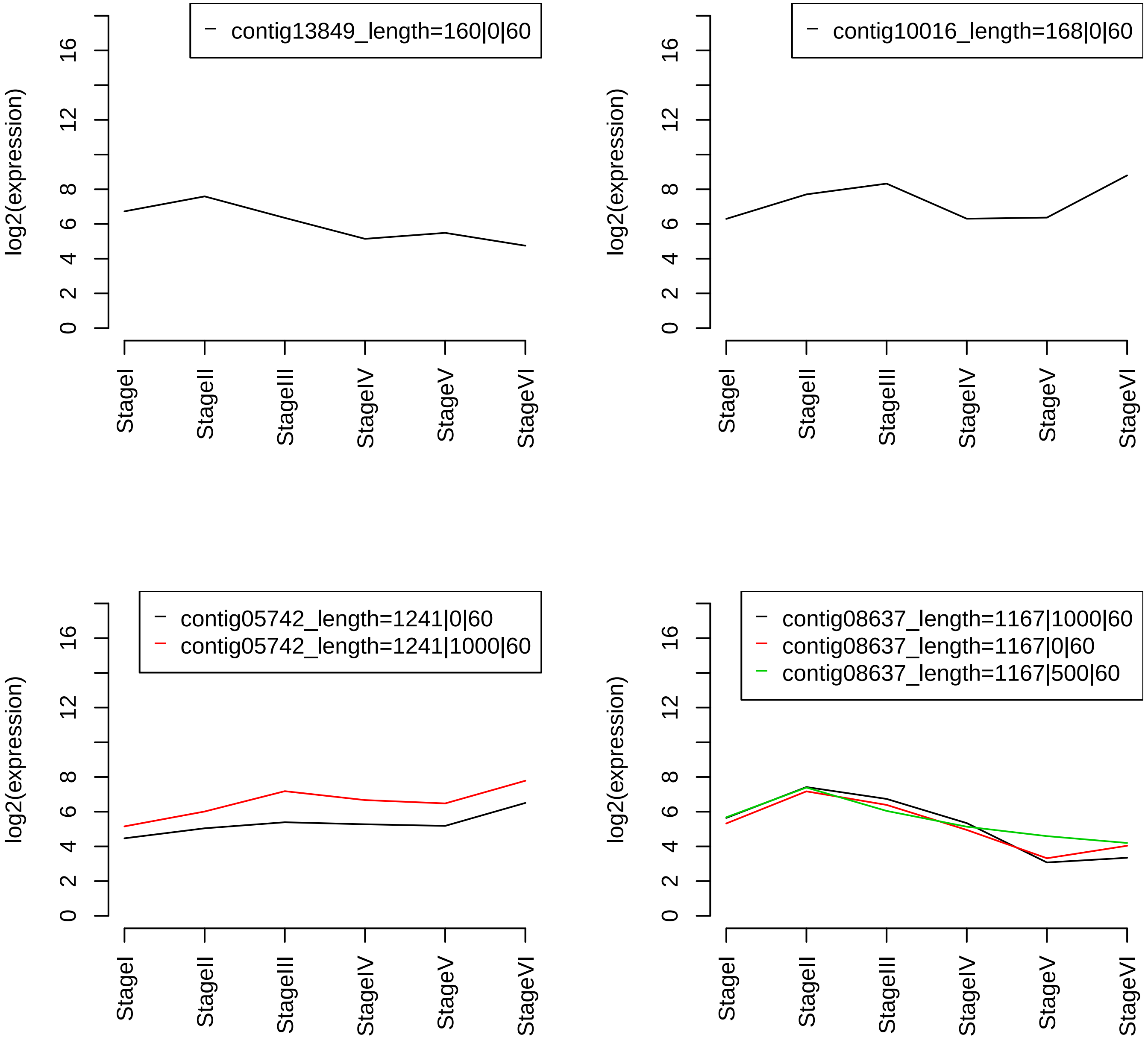
contig13849_length=160|0|60
contig10016_length=168|0|60
contig05742_length=1241|0|60
contig05742_length=1241|1000|60
contig08637_length=1167|1000|60
contig08637_length=1167|0|60
contig08637_length=1167|500|60
log2(expression)
0
2
4
6
8
12
16
StageI
StageII
StageIII
StageIV
StageV
StageVI

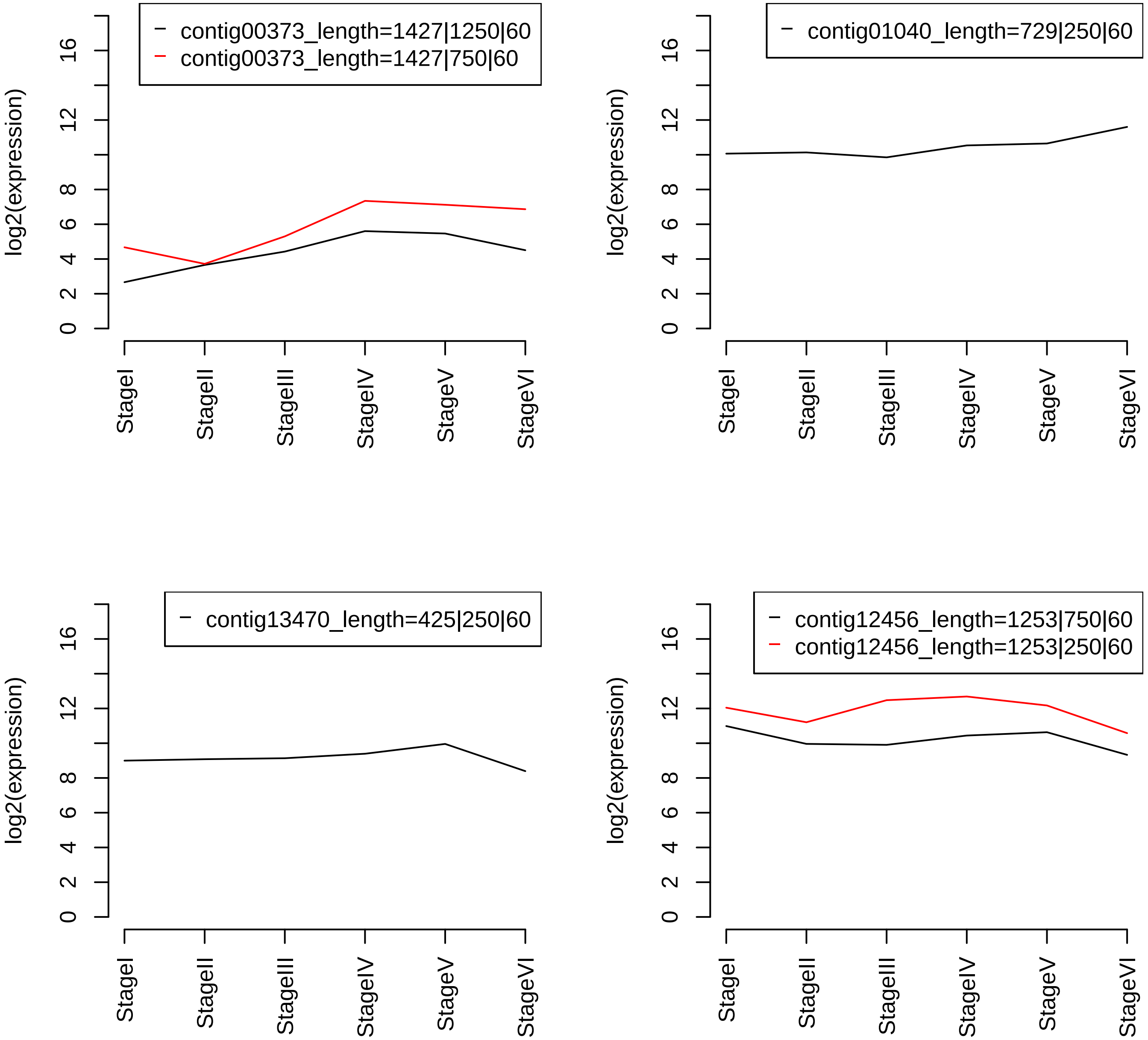
contig00373_length=1427|1250|60
contig00373_length=1427|750|60
contig01040_length=729|250|60
contig13470_length=425|250|60
contig12456_length=1253|750|60
contig12456_length=1253|250|60
log2(expression)
0
2
4
6
8
12
16
StageI
StageII
StageIII
StageIV
StageV
StageVI

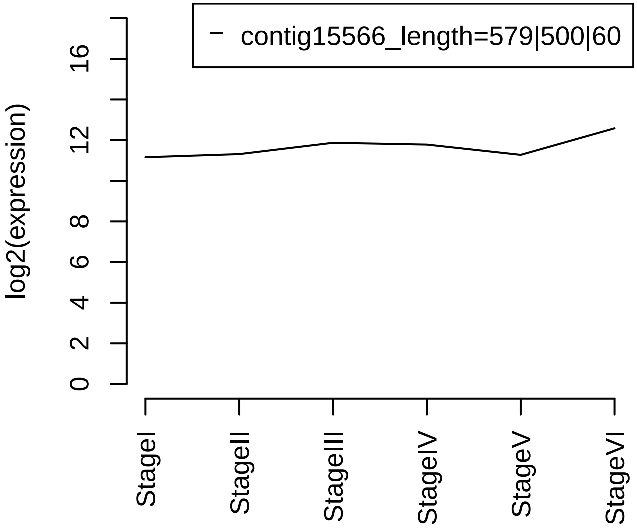
contig15566_length=579|500|60
log2(expression)
0
2
4
6
8
12
16
StageI
StageII
StageIII
StageIV
StageV
StageVI

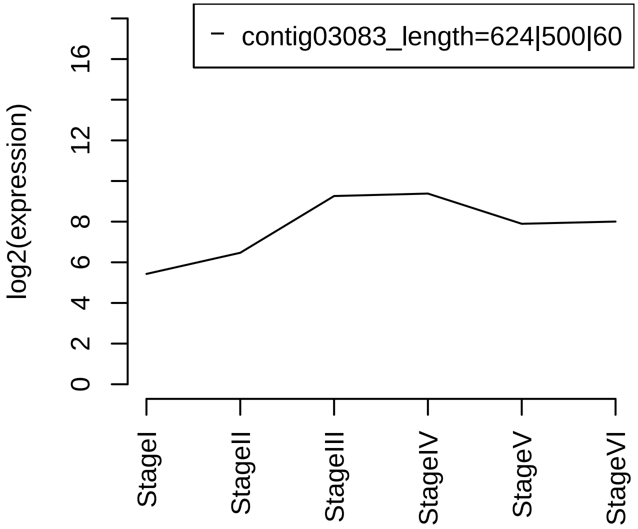
contig03083_length=624|500|60
log2(expression)
0
2
4
6
8
12
16
StageI
StageII
StageIII
StageIV
StageV
StageVI

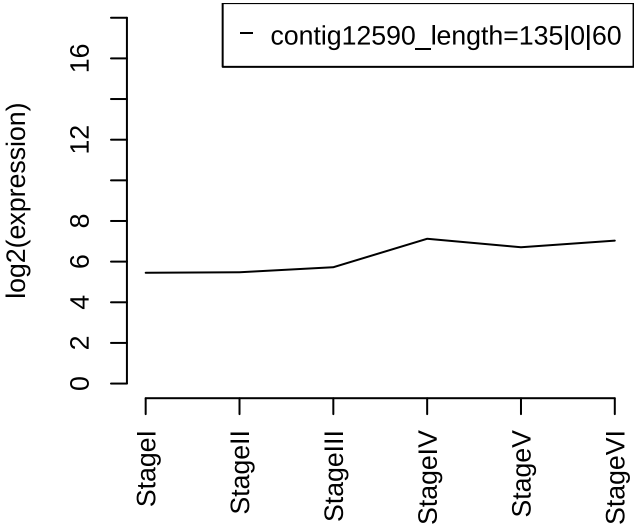
contig12590_length=135|0|60
log2(expression)
0
2
4
6
8
12
16
StageI
StageII
StageIII
StageIV
StageV
StageVI

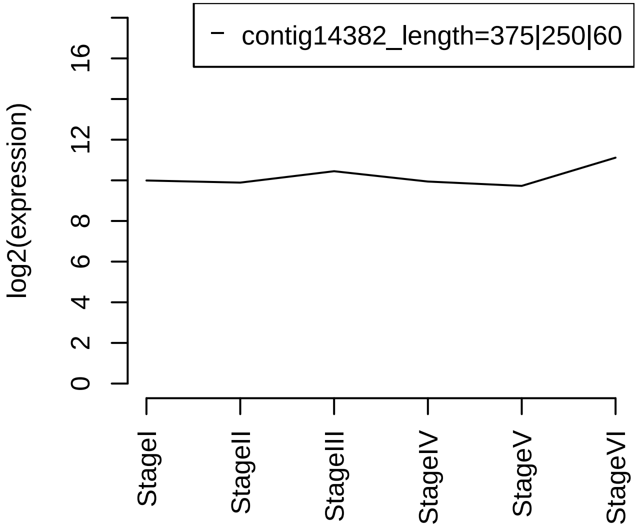
contig14382_length=375|250|60
log2(expression)
0
2
4
6
8
12
16
StageI
StageII
StageIII
StageIV
StageV
StageVI

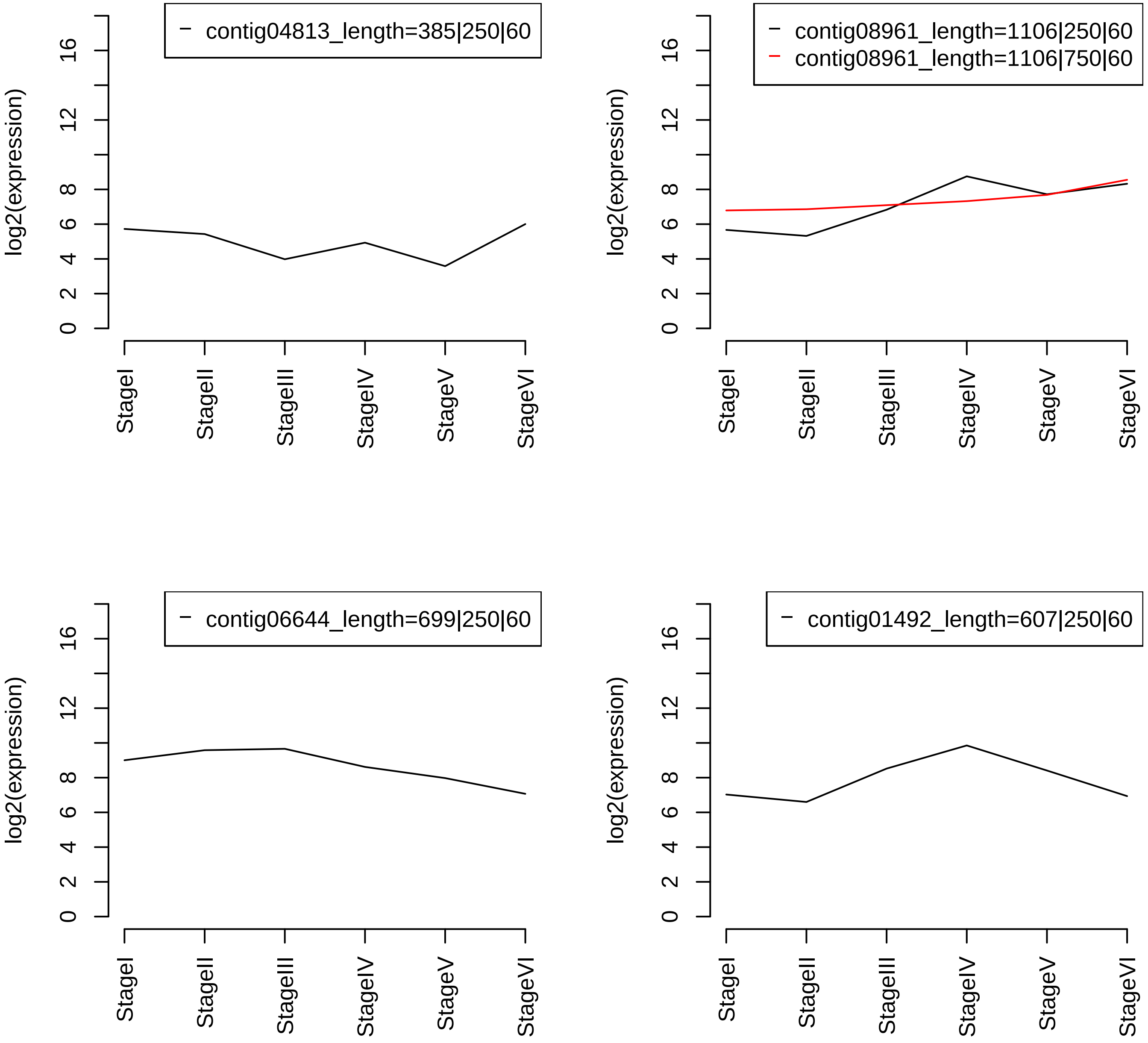
contig04813_length=385|250|60
contig08961_length=1106|250|60
contig08961_length=1106|750|60
contig06644_length=699|250|60
contig01492_length=607|250|60
log2(expression)
0
2
4
6
8
12
16
StageI
StageII
StageIII
StageIV
StageV
StageVI

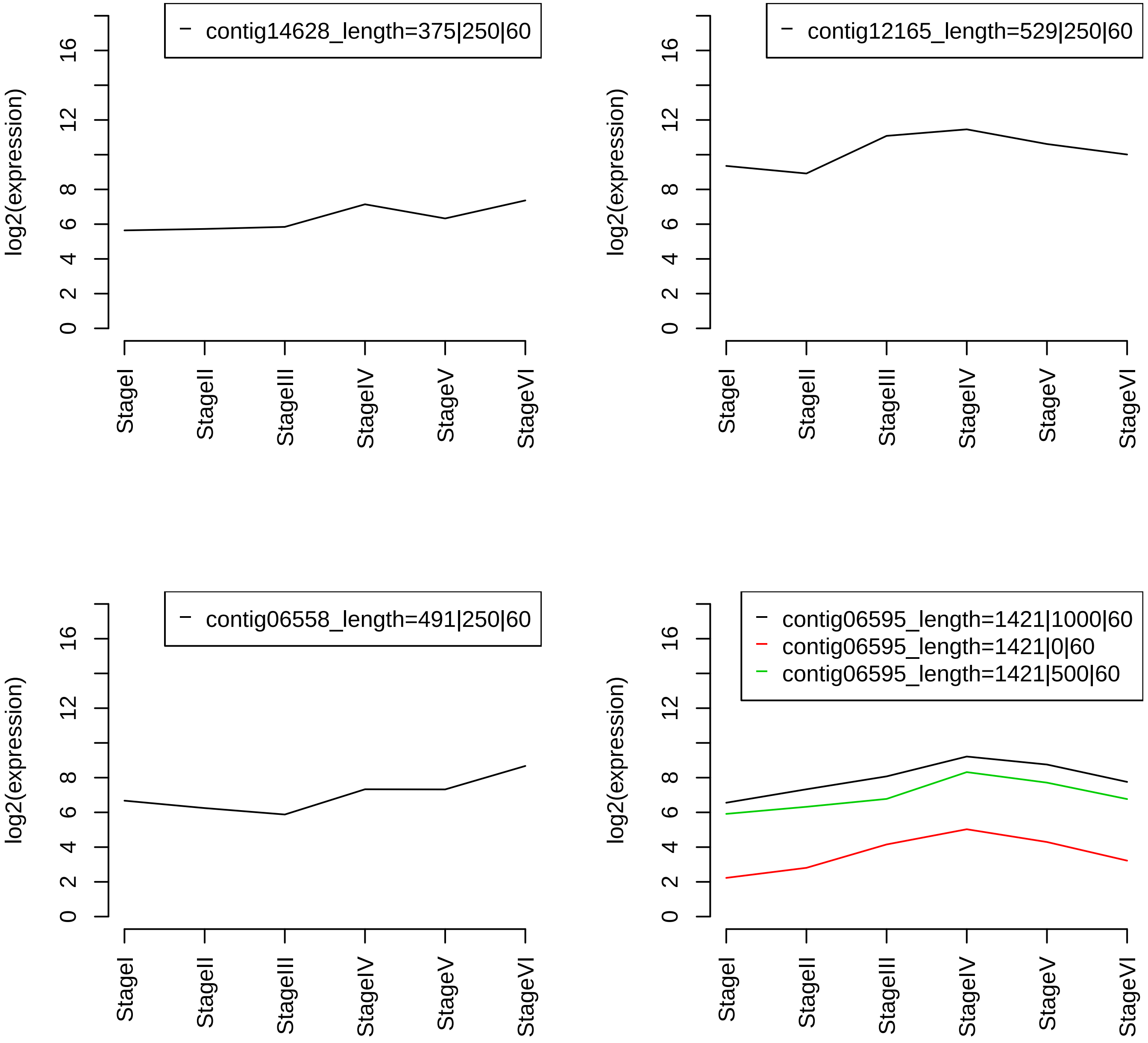
contig14628_length=375|250|60
contig12165_length=529|250|60
contig06558_length=491|250|60
contig06595_length=1421|1000|60
contig06595_length=1421|0|60
contig06595_length=1421|500|60
log2(expression)
0
2
4
6
8
12
16
StageI
StageII
StageIII
StageIV
StageV
StageVI

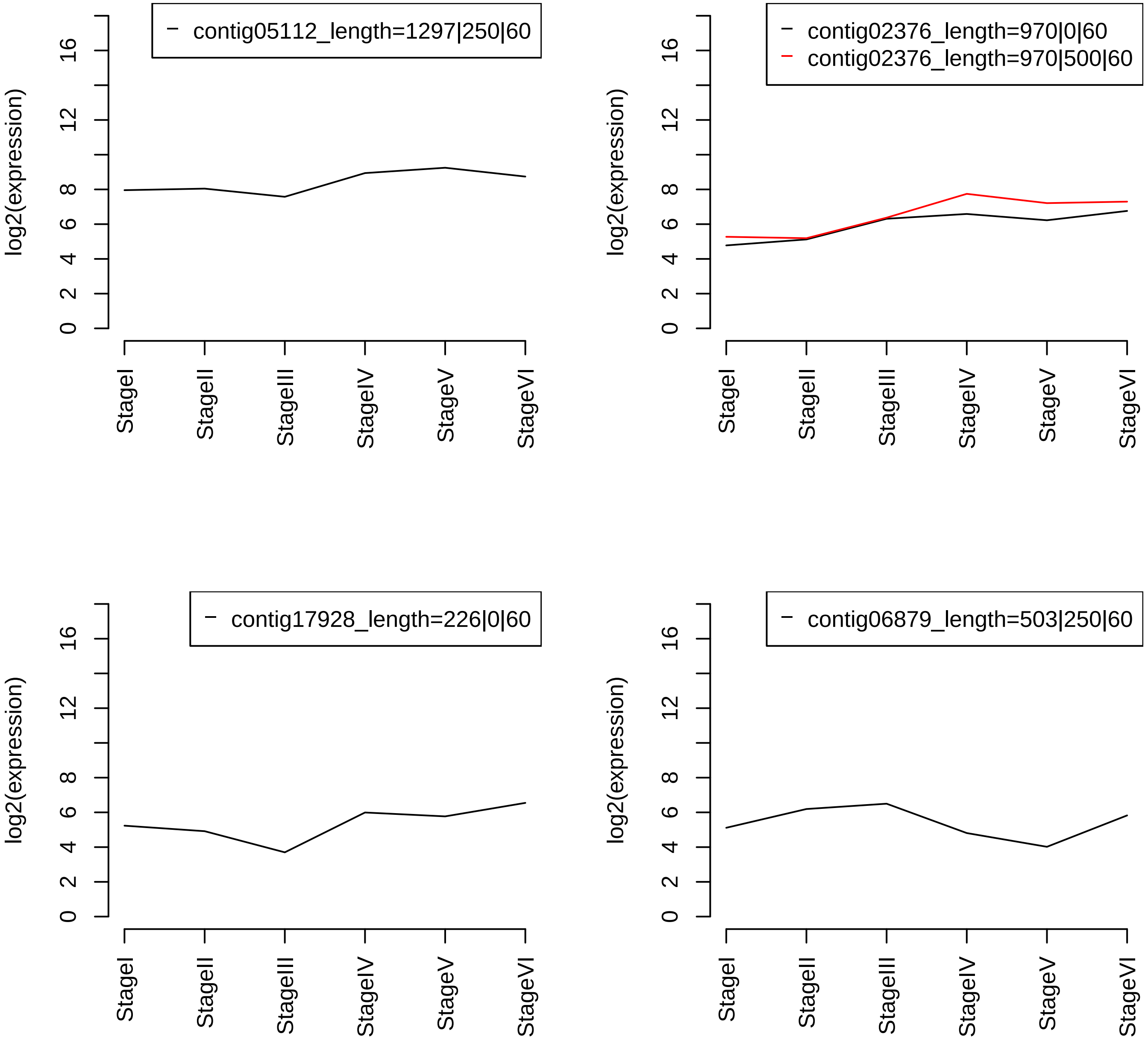

contig05112_length=1297|250|60
contig02376_length=970|0|60
contig02376_length=970|500|60
contig17928_length=226|0|60
contig06879_length=503|250|60
log2(expression)
0
2
4
6
8
12
16
StageI
StageII
StageIII
StageIV
StageV
StageVI

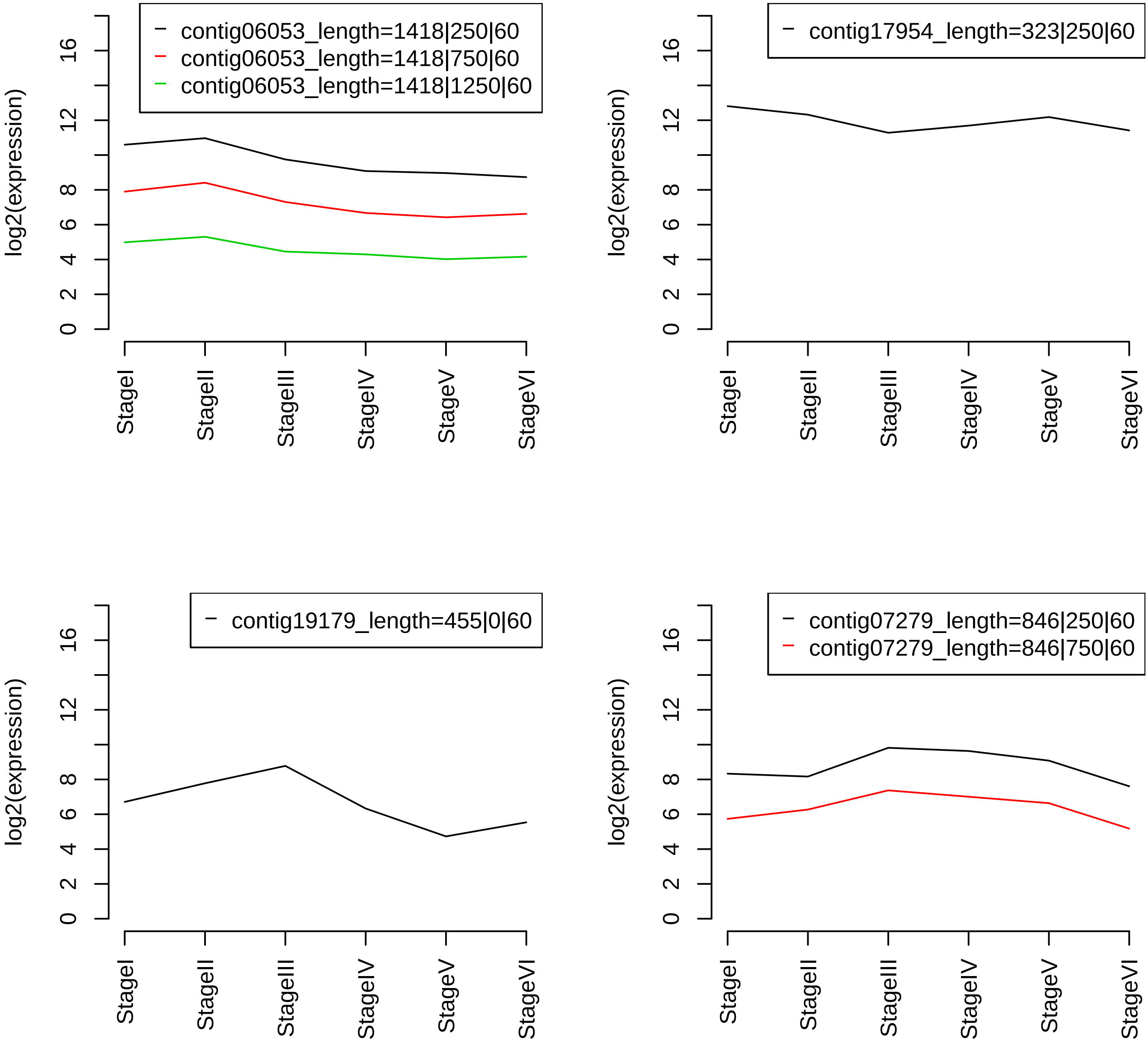

contig06053_length=1418|250|60
contig06053_length=1418|750|60
contig06053_length=1418|1250|60
contig17954_length=323|250|60
contig19179_length=455|0|60
contig07279_length=846|250|60
contig07279_length=846|750|60
log2(expression)
0
2
4
6
8
12
16
StageI
StageII
StageIII
StageIV
StageV
StageVI

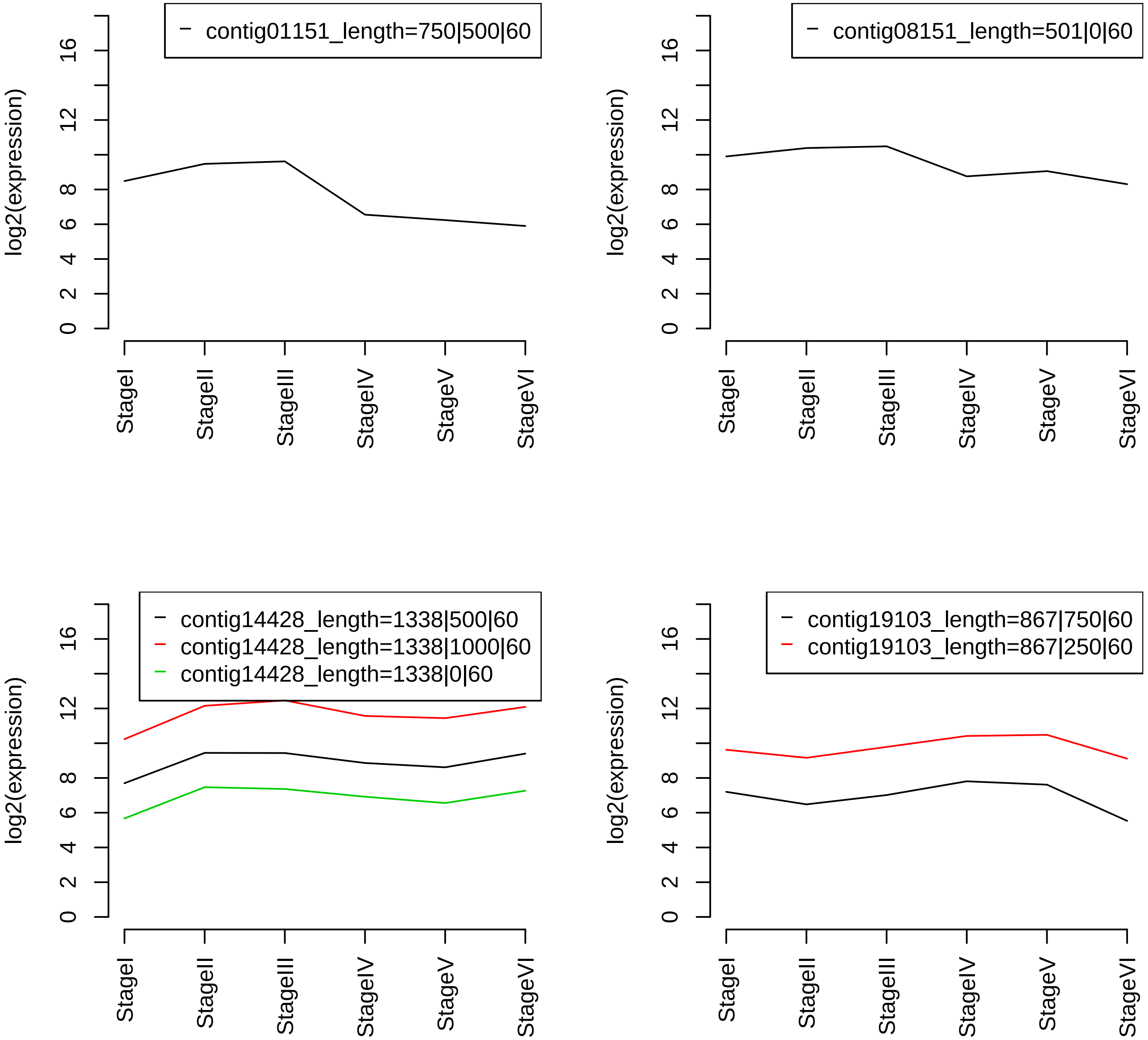
contig01151_length=750|500|60
contig08151_length=501|0|60
contig14428_length=1338|500|60
contig14428_length=1338|1000|60
contig14428_length=1338|0|60
contig19103_length=867|750|60
contig19103_length=867|250|60
log2(expression)
0
2
4
6
8
12
16
StageI
StageII
StageIII
StageIV
StageV
StageVI

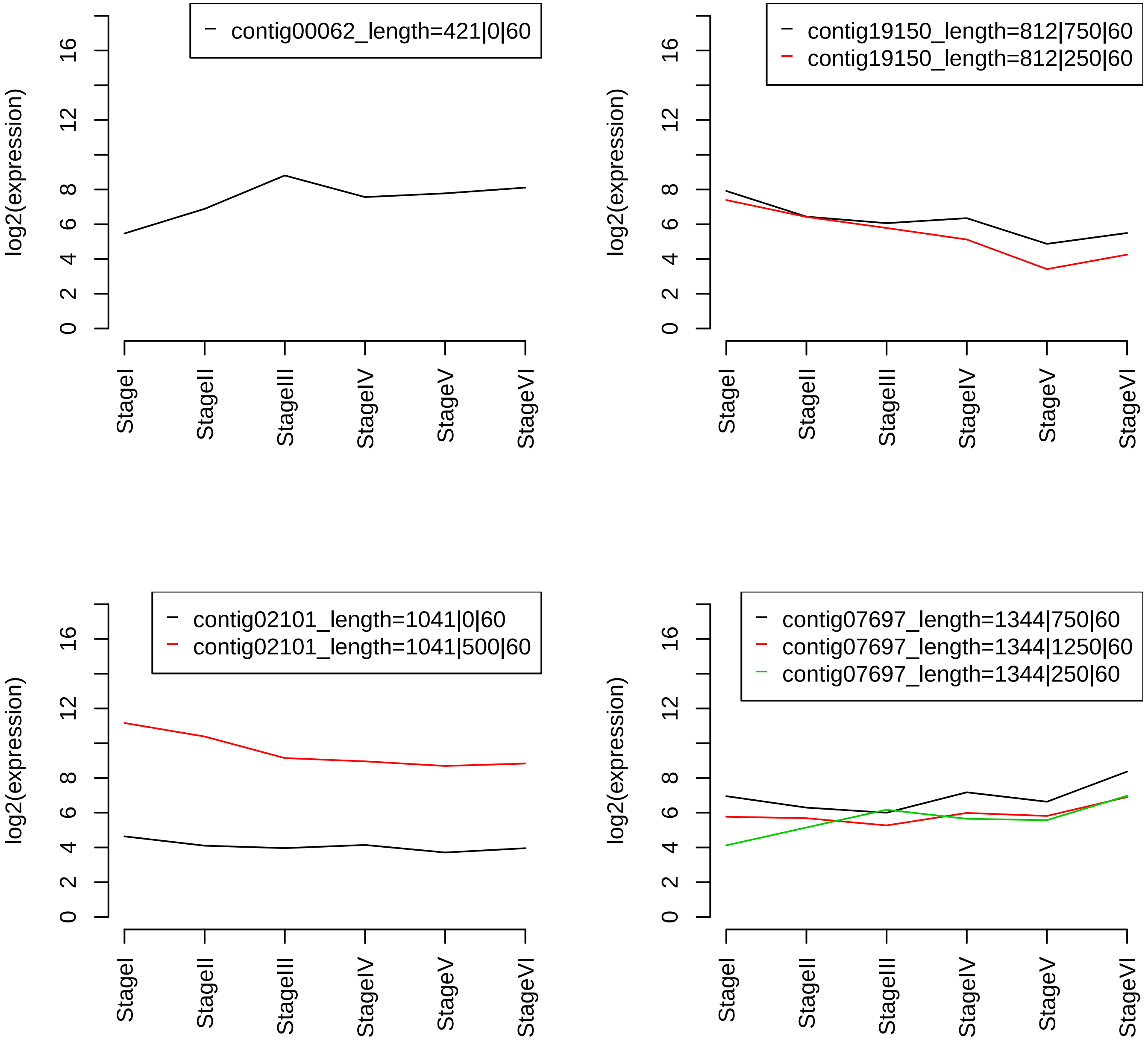

contig00062_length=421|0|60
contig19150_length=812|750|60
contig19150_length=812|250|60
contig02101_length=1041|0|60
contig02101_length=1041|500|60
contig07697_length=1344|750|60
contig07697_length=1344|1250|60
contig07697_length=1344|250|60
log2(expression)
0 2 4 6 8 12 16
StageI StageII StageIII StageIV StageV StageVI

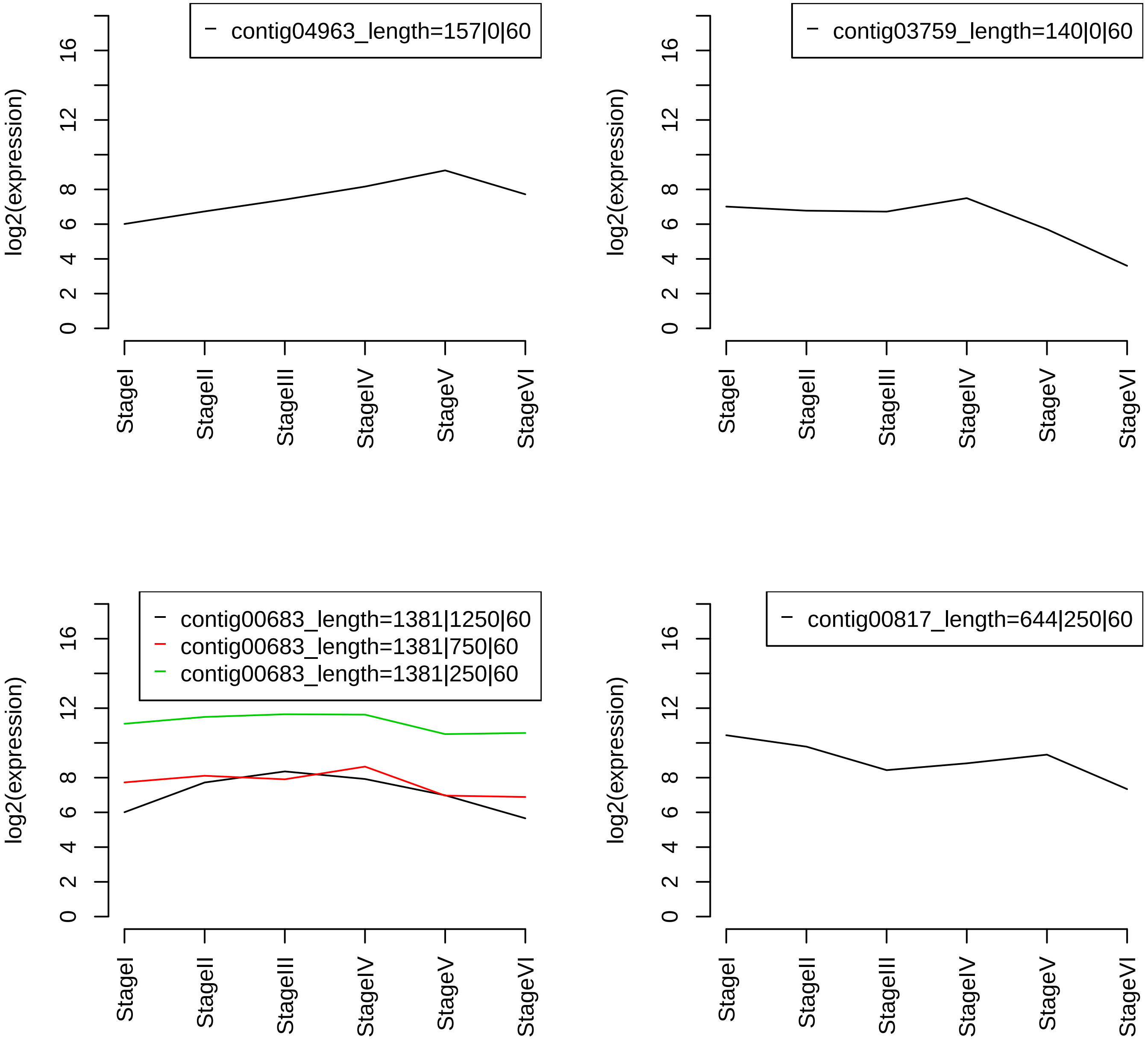

contig04963_length=157|0|60
contig03759_length=140|0|60
contig00683_length=1381|1250|60
contig00683_length=1381|750|60
contig00683_length=1381|250|60
contig00817_length=644|250|60
log2(expression)
0
2
4
6
8
12
16
StageI
StageII
StageIII
StageIV
StageV
StageVI

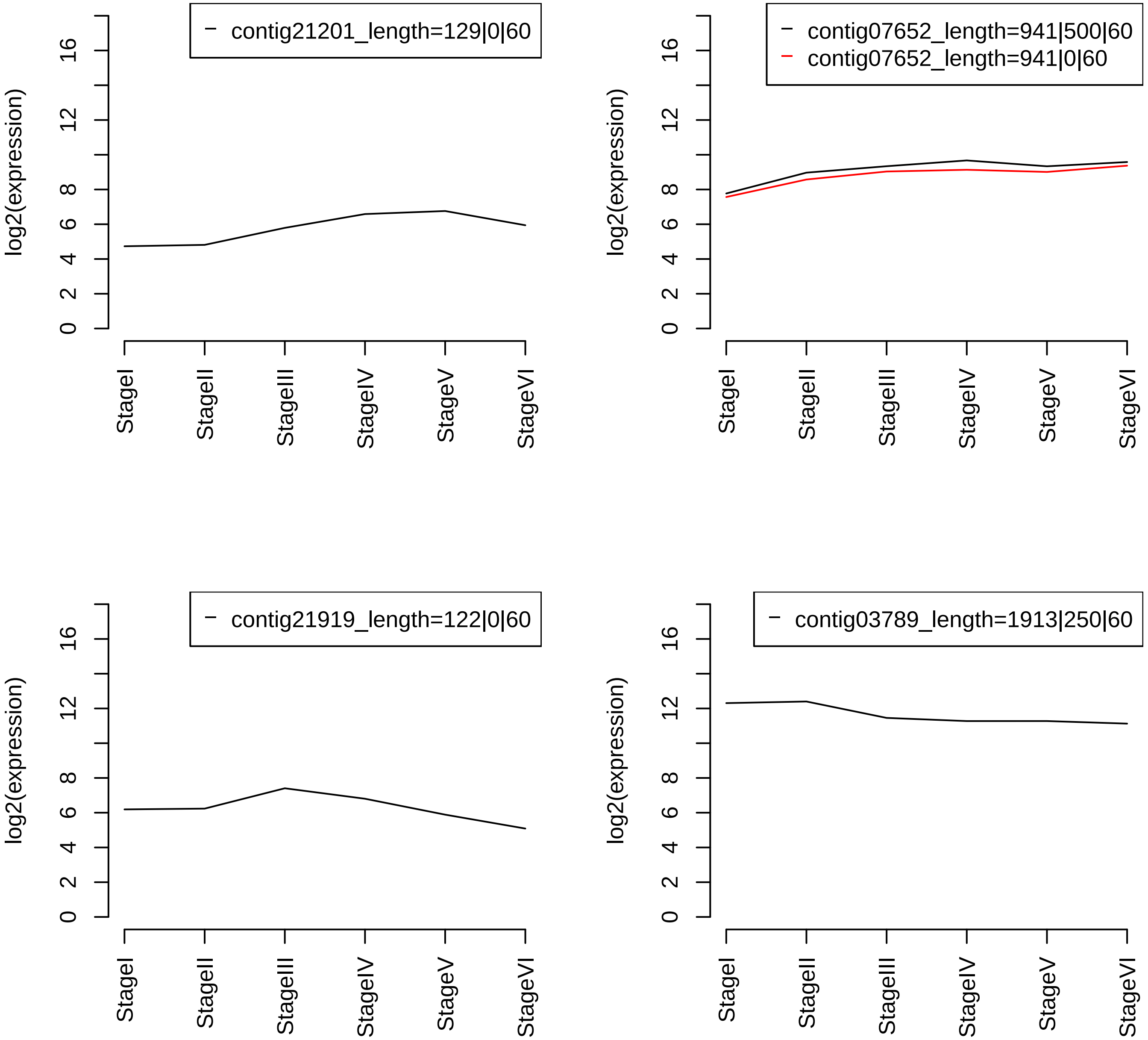
contig21201_length=129|0|60
contig07652_length=941|500|60
contig07652_length=941|0|60
contig21919_length=122|0|60
contig03789_length=1913|250|60
log2(expression)
0
2
4
6
8
12
16
StageI
StageII
StageIII
StageIV
StageV
StageVI

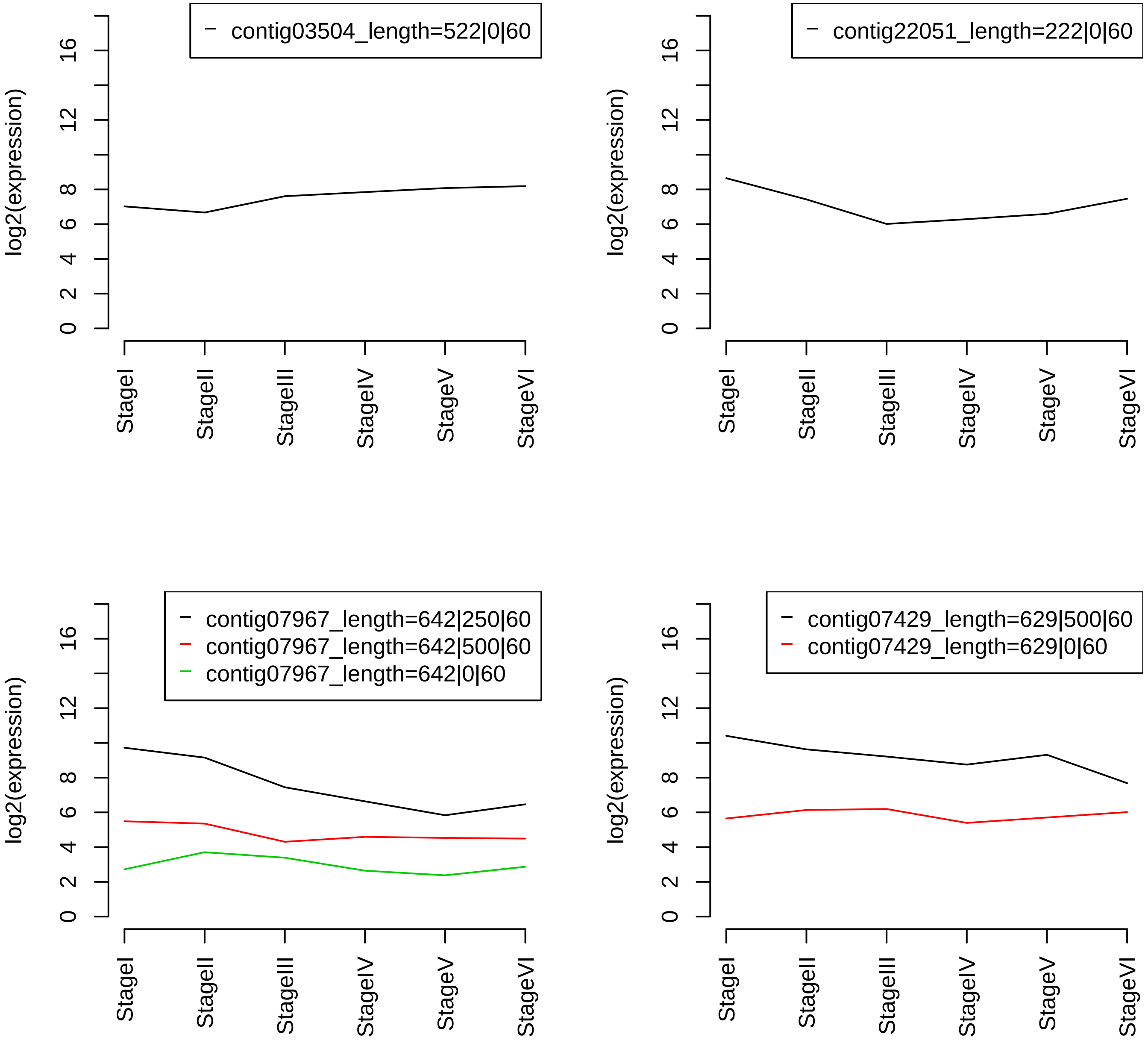
contig03504_length=522|0|60
contig22051_length=222|0|60
contig07967_length=642|250|60
contig07967_length=642|500|60
contig07967_length=642|0|60
contig07429_length=629|500|60
contig07429_length=629|0|60
log2(expression)
0
2
4
6
8
12
16
StageI
StageII
StageIII
StageIV
StageV
StageVI

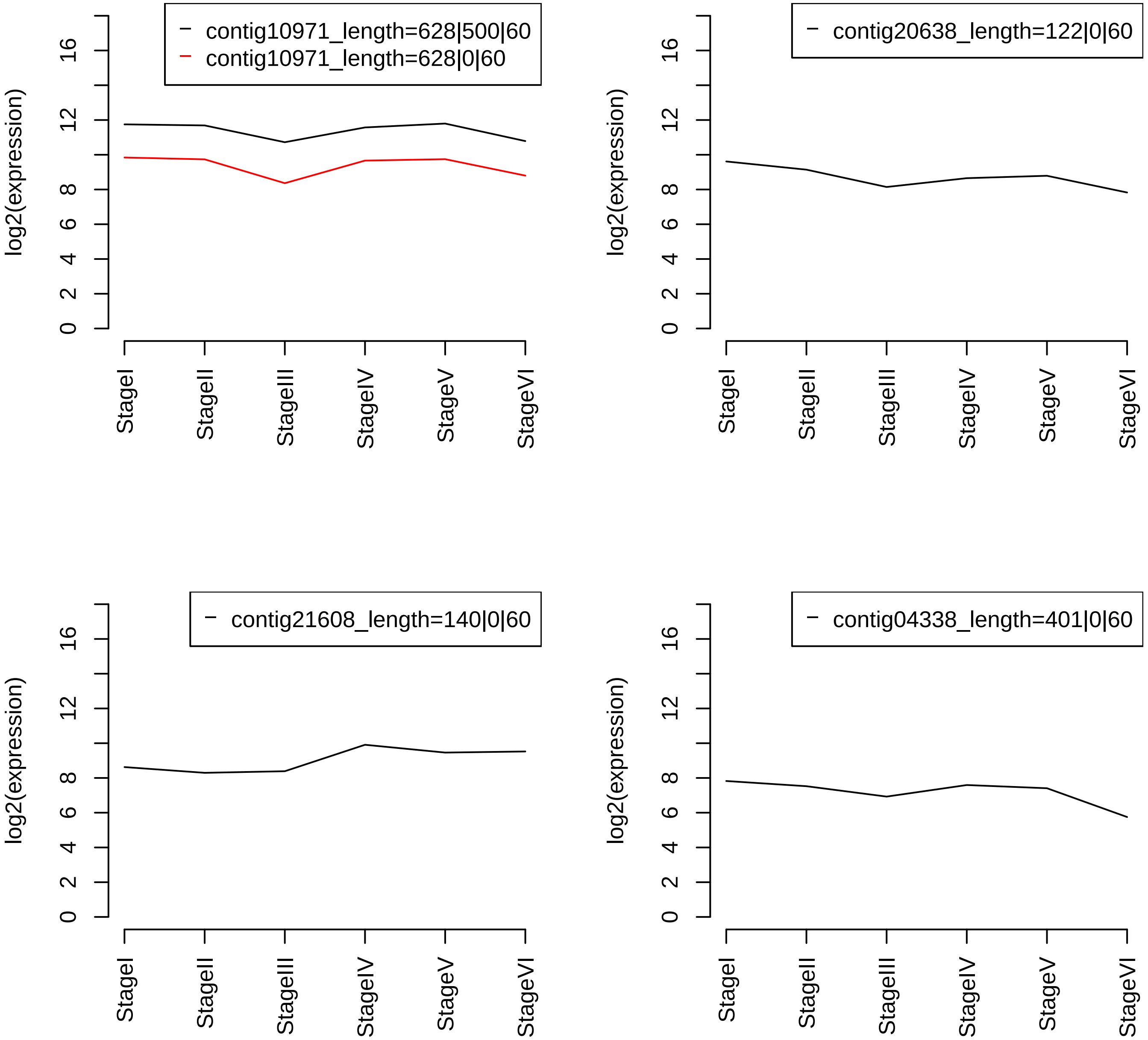
contig10971_length=628|500|60
contig10971_length=628|0|60
contig20638_length=122|0|60
contig21608_length=140|0|60
contig04338_length=401|0|60
log2(expression)
0
2
4
6
8
12
16
StageI
StageII
StageIII
StageIV
StageV
StageVI

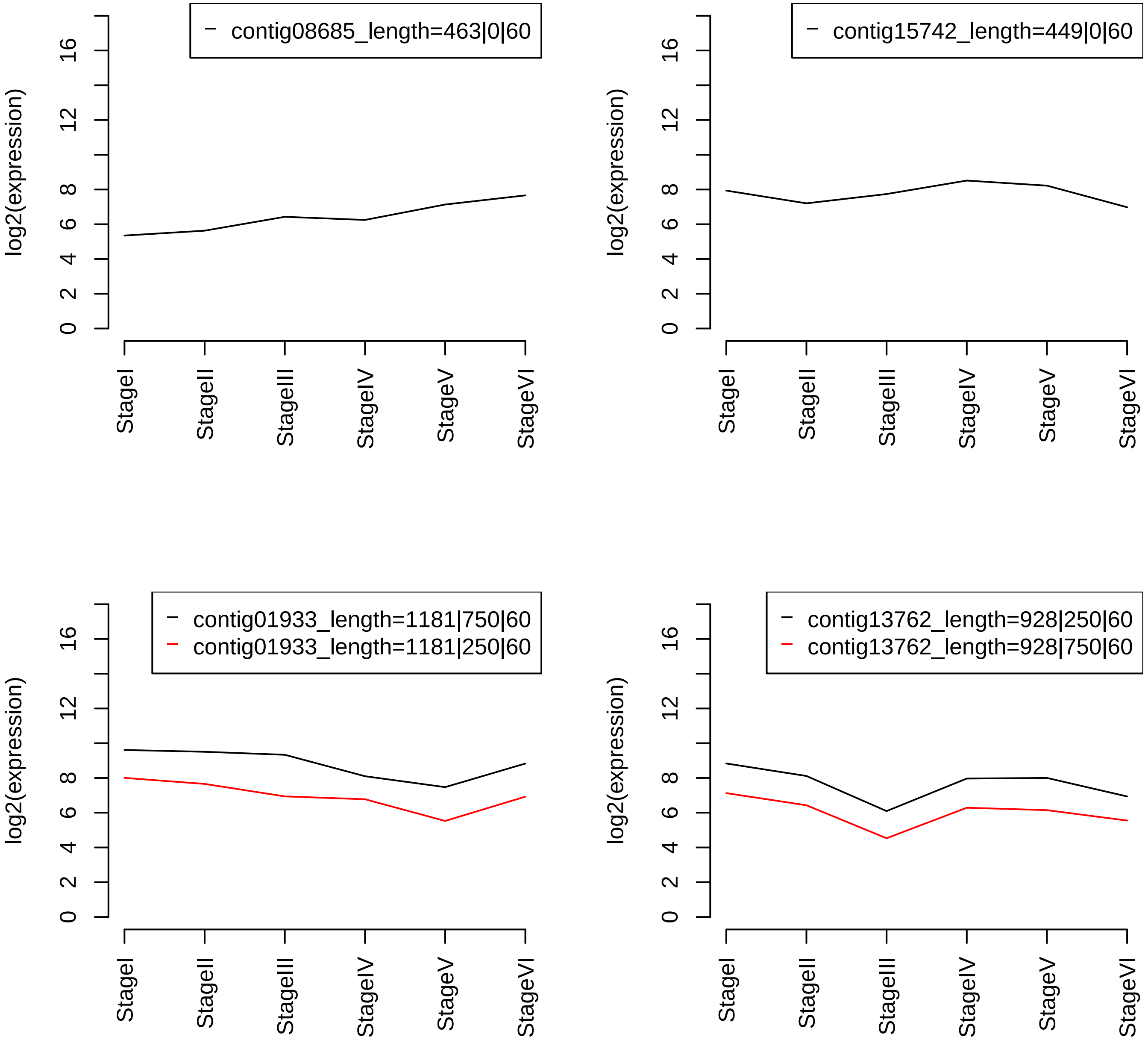
contig08685_length=463|0|60
contig15742_length=449|0|60
contig01933_length=1181|750|60
contig01933_length=1181|250|60
contig13762_length=928|250|60
contig13762_length=928|750|60
log2(expression)
0
2
4
6
8
12
16
StageI
StageII
StageIII
StageIV
StageV
StageVI

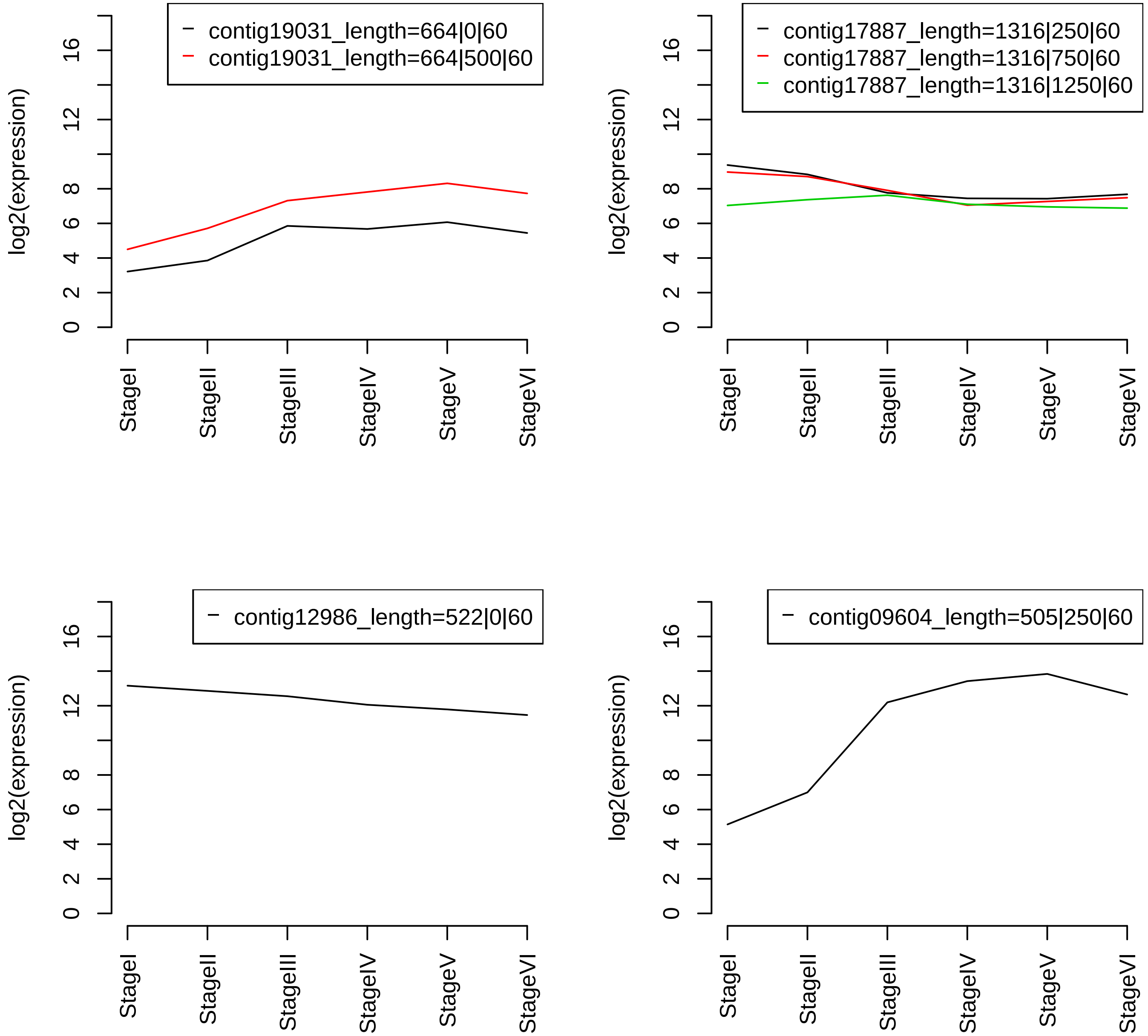
contig19031_length=664|0|60
contig19031_length=664|500|60
contig17887_length=1316|250|60
contig17887_length=1316|750|60
contig17887_length=1316|1250|60
contig12986_length=522|0|60
contig09604_length=505|250|60
log2(expression)
0 2 4 6 8 12 16
StageI StageII StageIII StageIV StageV StageVI

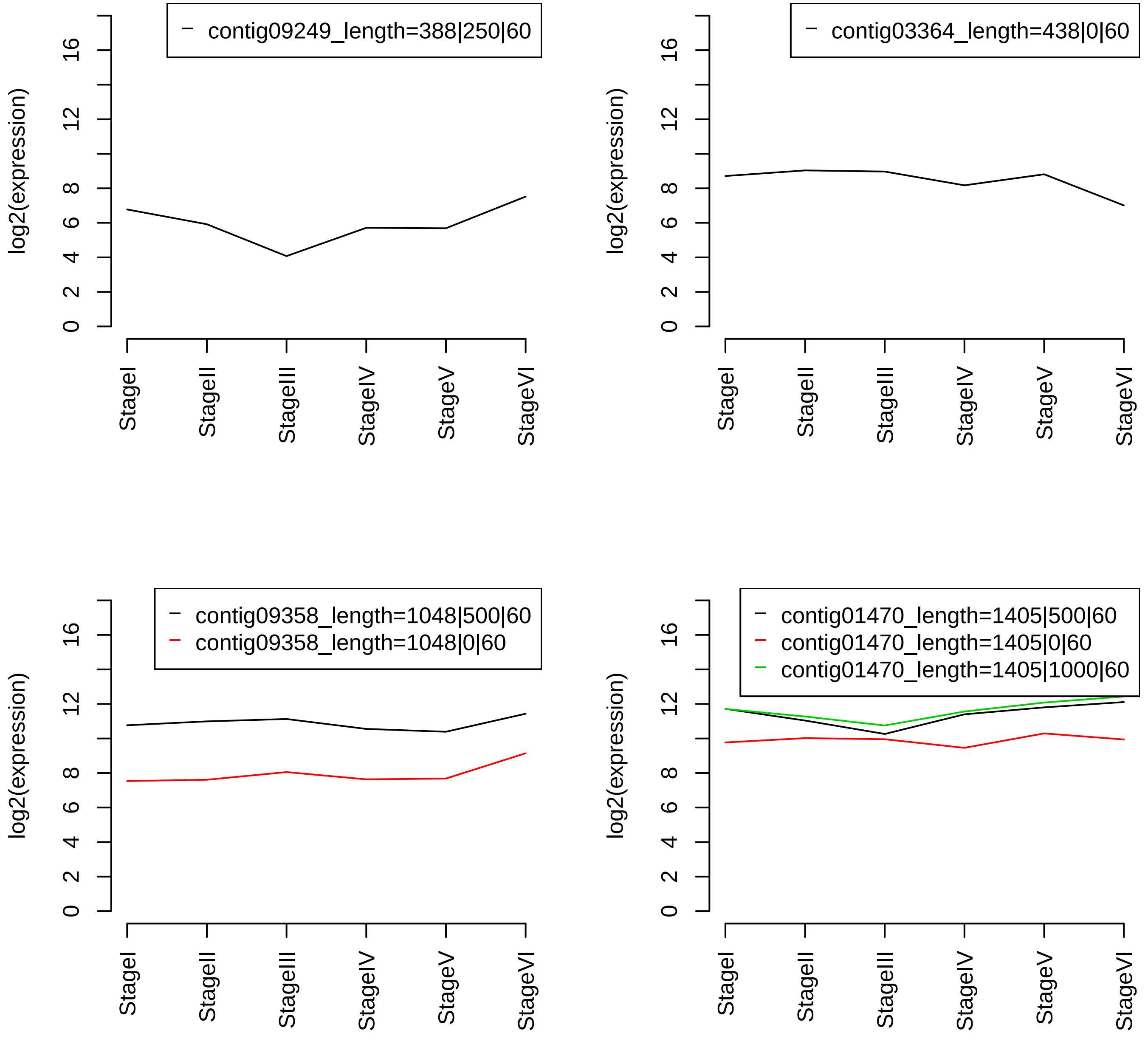

contig09249_length=388|250|60
contig03364_length=438|0|60
contig09358_length=1048|500|60
contig09358_length=1048|0|60
contig01470_length=1405|500|60
contig01470_length=1405|0|60
contig01470_length=1405|1000|60
log2(expression)
0
2
4
6
8
12
16
StageI
StageII
StageIII
StageIV
StageV
StageVI

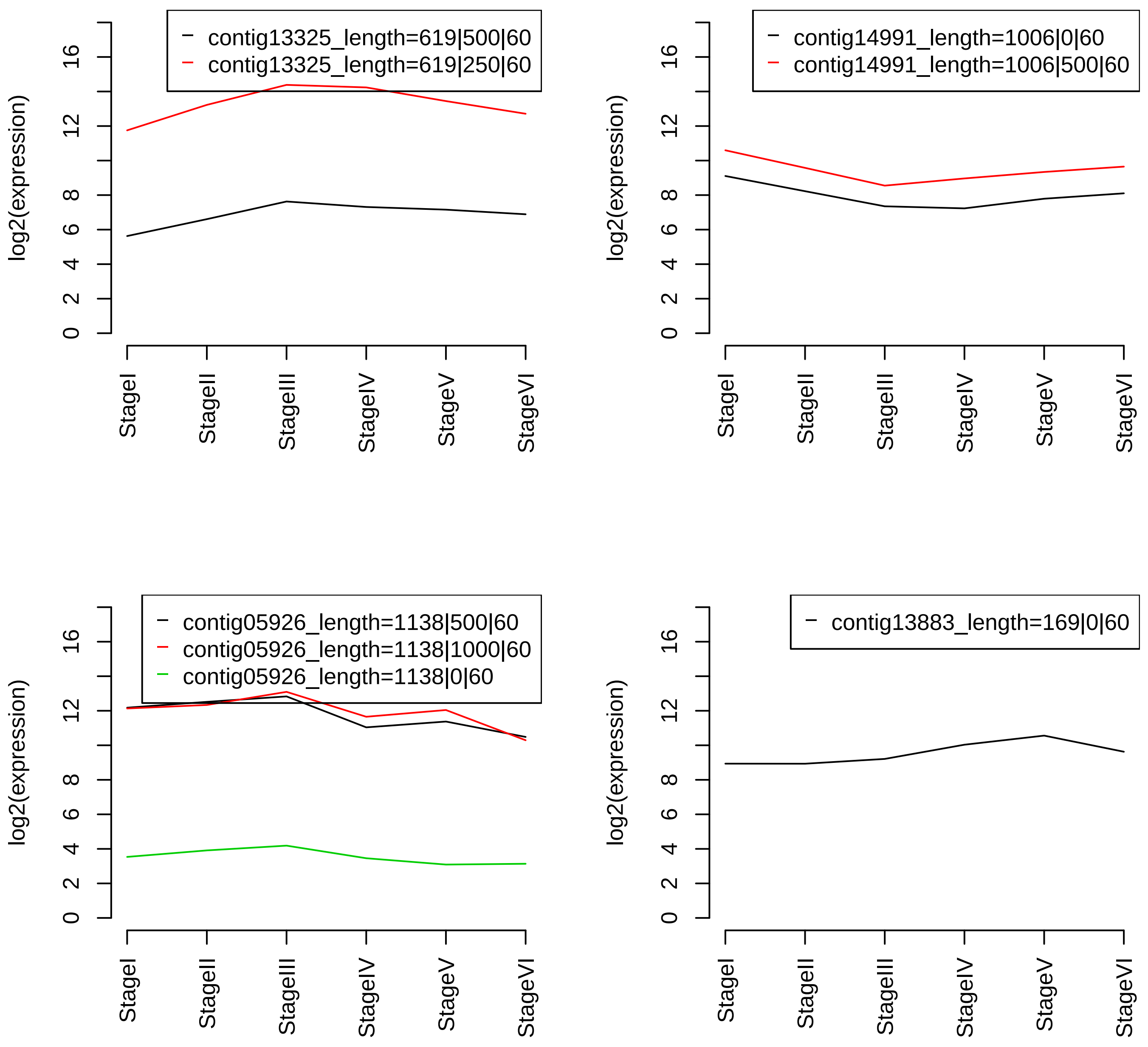
contig13325_length=619|500|60
contig13325_length=619|250|60
log2(expression)
0
2
4
6
8
12
16
StageI
StageII
StageIII
StageIV
StageV
StageVI
contig14991_length=1006|0|60
contig14991_length=1006|500|60
log2(expression)
contig05926_length=1138|500|60
contig05926_length=1138|1000|60
contig05926_length=1138|0|60
log2(expression)
contig13883_length=169|0|60
log2(expression)

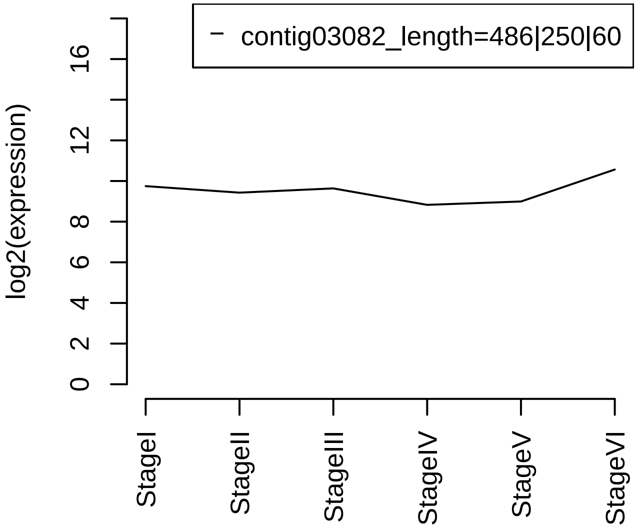
contig03082_length=486|250|60
log2(expression)
0
2
4
6
8
12
16
StageI
StageII
StageIII
StageIV
StageV
StageVI

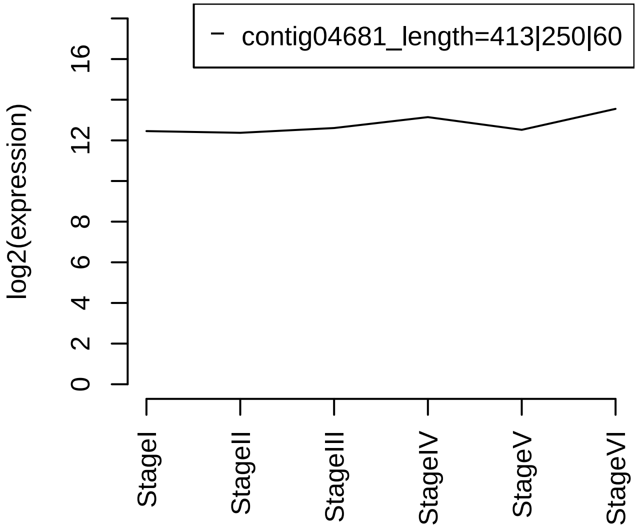
contig04681_length=413|250|60
log2(expression)
0
2
4
6
8
12
16
StageI
StageII
StageIII
StageIV
StageV
StageVI

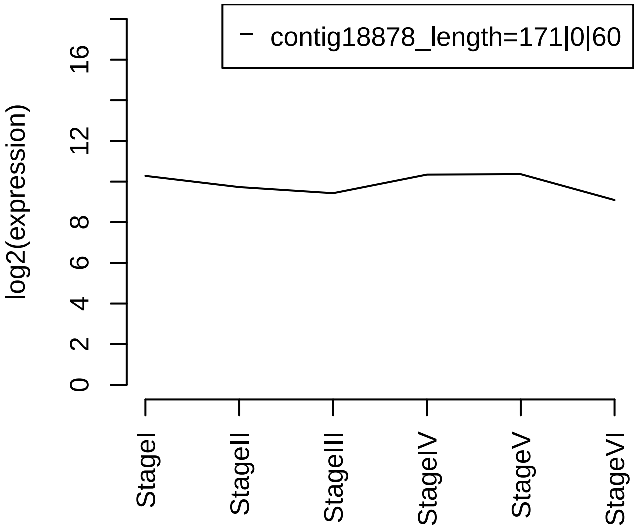
contig18878_length=171|0|60
log2(expression)
0
2
4
6
8
12
16
StageI
StageII
StageIII
StageIV
StageV
StageVI

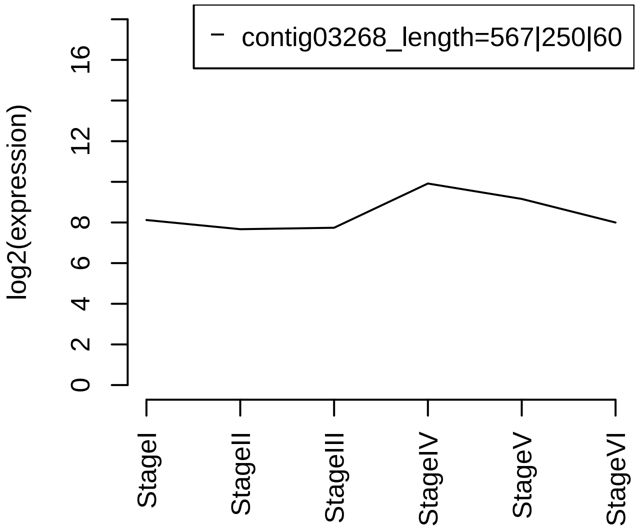
contig03268_length=567|250|60
log2(expression)
0
2
4
6
8
12
16
StageI
StageII
StageIII
StageIV
StageV
StageVI

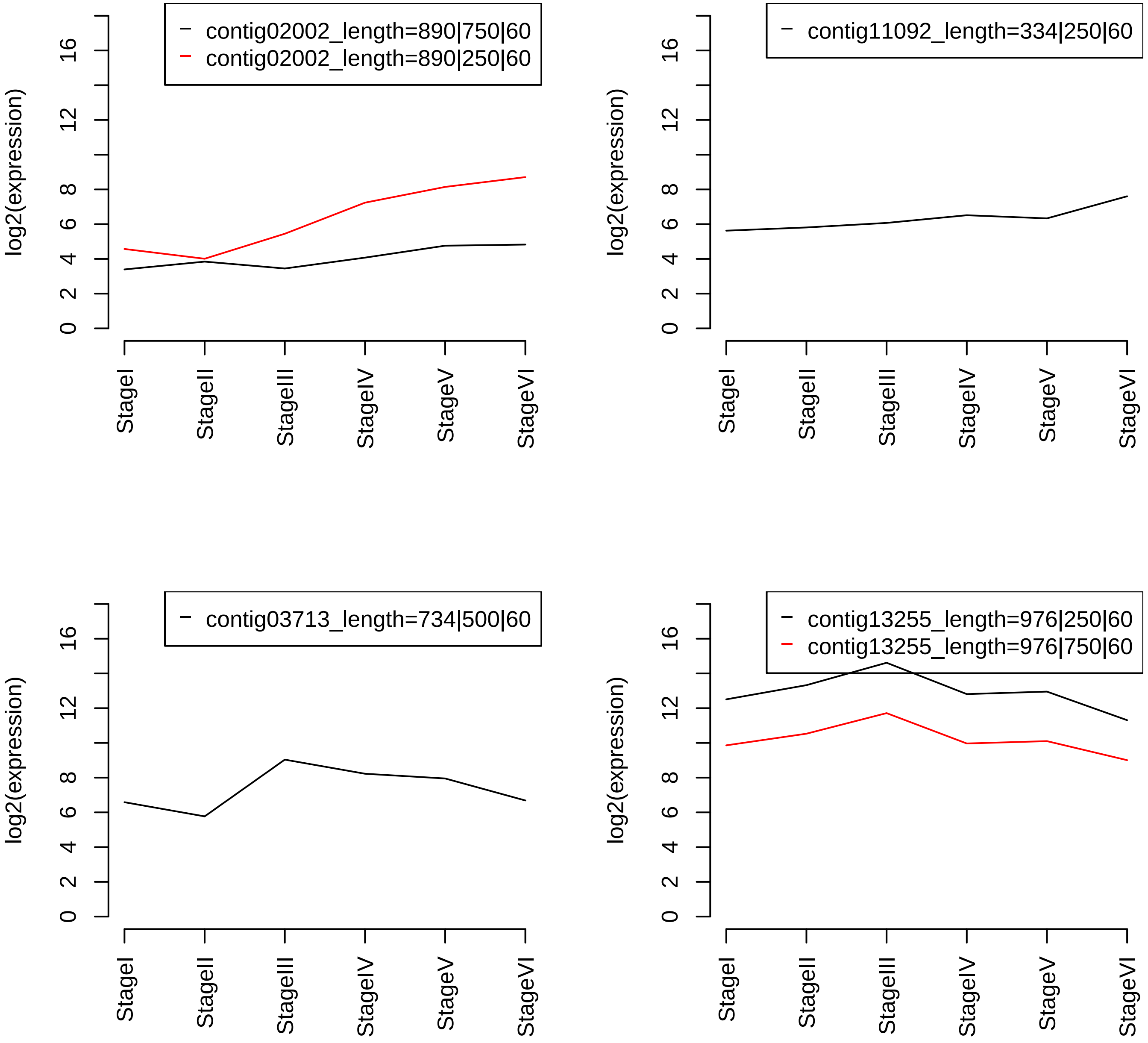
contig02002_length=890|750|60
contig02002_length=890|250|60
contig11092_length=334|250|60
contig03713_length=734|500|60
contig13255_length=976|250|60
contig13255_length=976|750|60
log2(expression)
0
2
4
6
8
12
16
StageI
StageII
StageIII
StageIV
StageV
StageVI

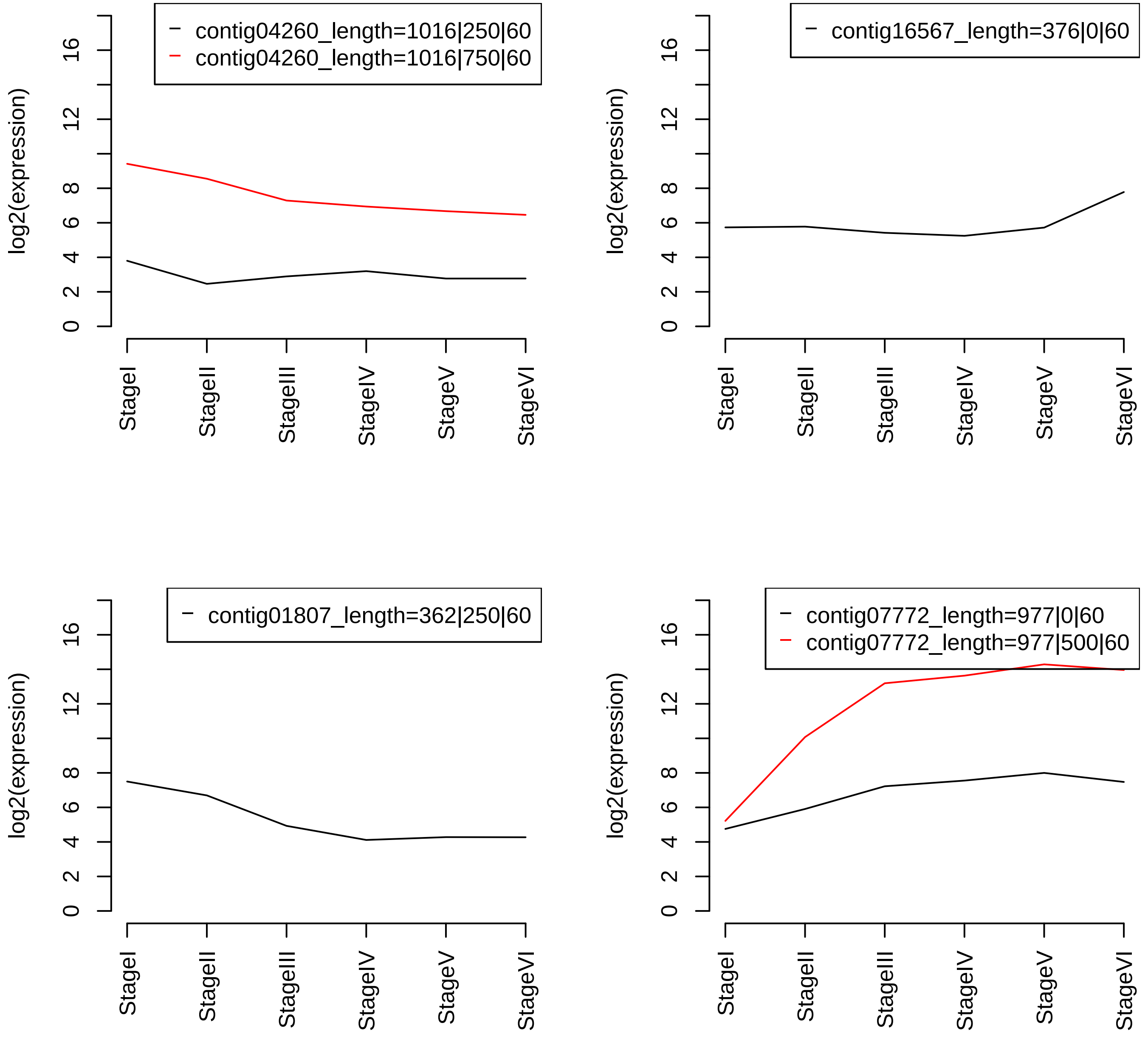

contig04260_length=1016|250|60
contig04260_length=1016|750|60
contig16567_length=376|0|60
contig01807_length=362|250|60
contig07772_length=977|0|60
contig07772_length=977|500|60
log2(expression)
0
2
4
6
8
12
16
StageI
StageII
StageIII
StageIV
StageV
StageVI

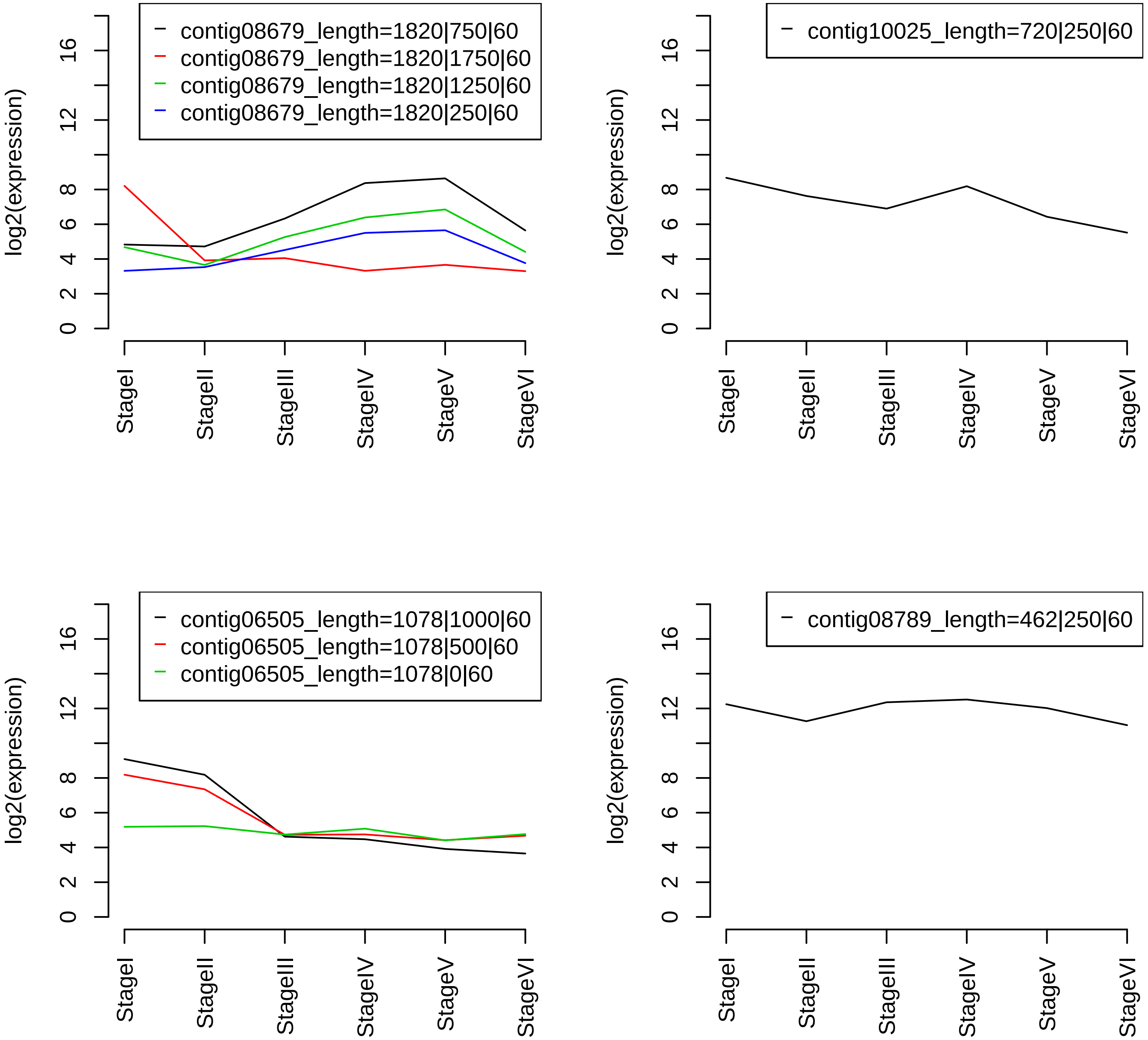
contig08679_length=1820|750|60
contig08679_length=1820|1750|60
contig08679_length=1820|1250|60
contig08679_length=1820|250|60
log2(expression)
StageI
StageII
StageIII
StageIV
StageV
StageVI
contig10025_length=720|250|60
contig06505_length=1078|1000|60
contig06505_length=1078|500|60
contig06505_length=1078|0|60
contig08789_length=462|250|60

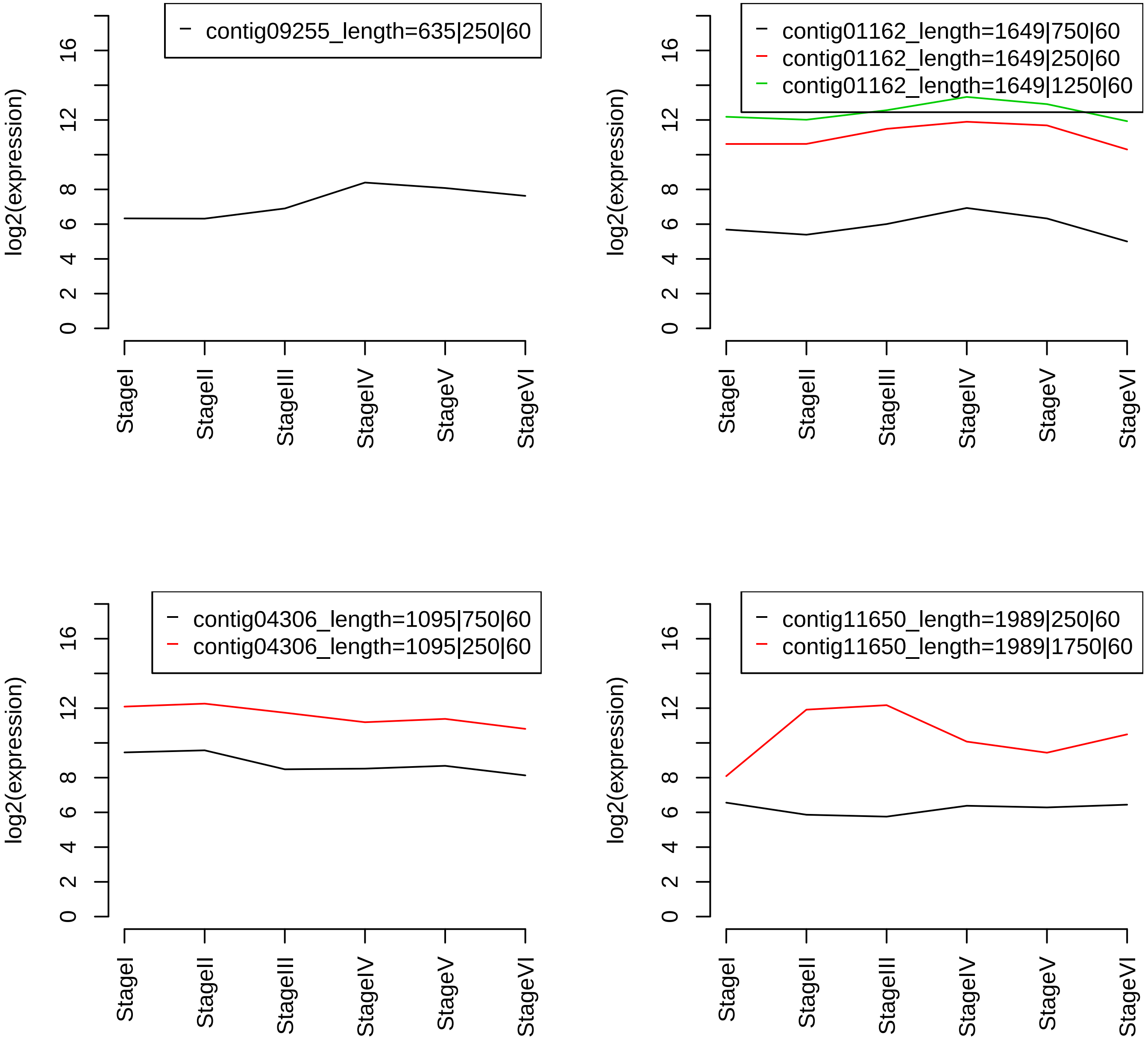
contig09255_length=635|250|60
contig01162_length=1649|750|60
contig01162_length=1649|250|60
contig01162_length=1649|1250|60
contig04306_length=1095|750|60
contig04306_length=1095|250|60
contig11650_length=1989|250|60
contig11650_length=1989|1750|60
log2(expression)
0
2
4
6
8
12
16
StageI
StageII
StageIII
StageIV
StageV
StageVI

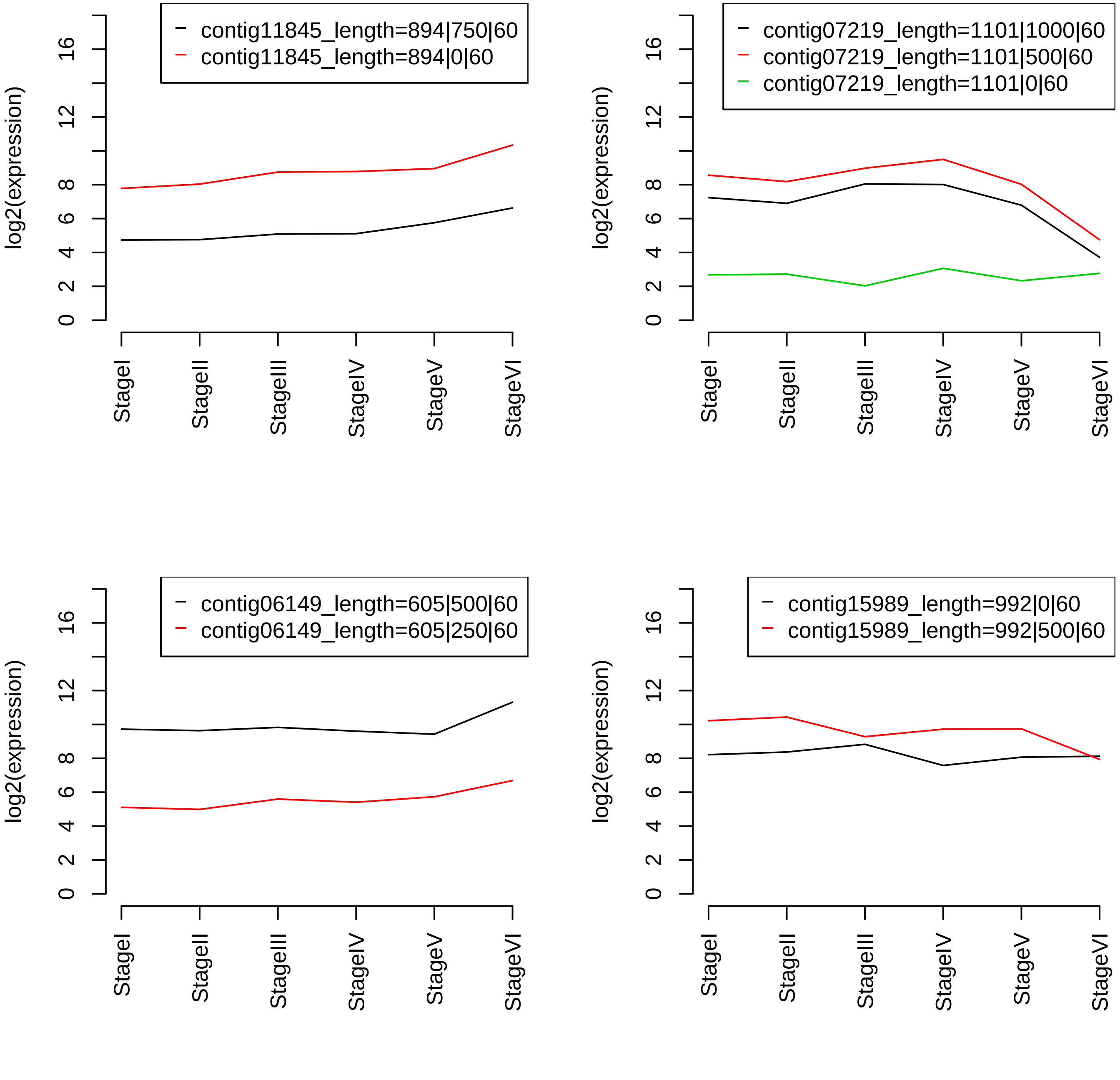
contig11845_length=894|750|60
contig11845_length=894|0|60
contig07219_length=1101|1000|60
contig07219_length=1101|500|60
contig07219_length=1101|0|60
contig06149_length=605|500|60
contig06149_length=605|250|60
contig15989_length=992|0|60
contig15989_length=992|500|60
log2(expression)
0 2 4 6 8 12 16
StageI StageII StageIII StageIV StageV StageVI

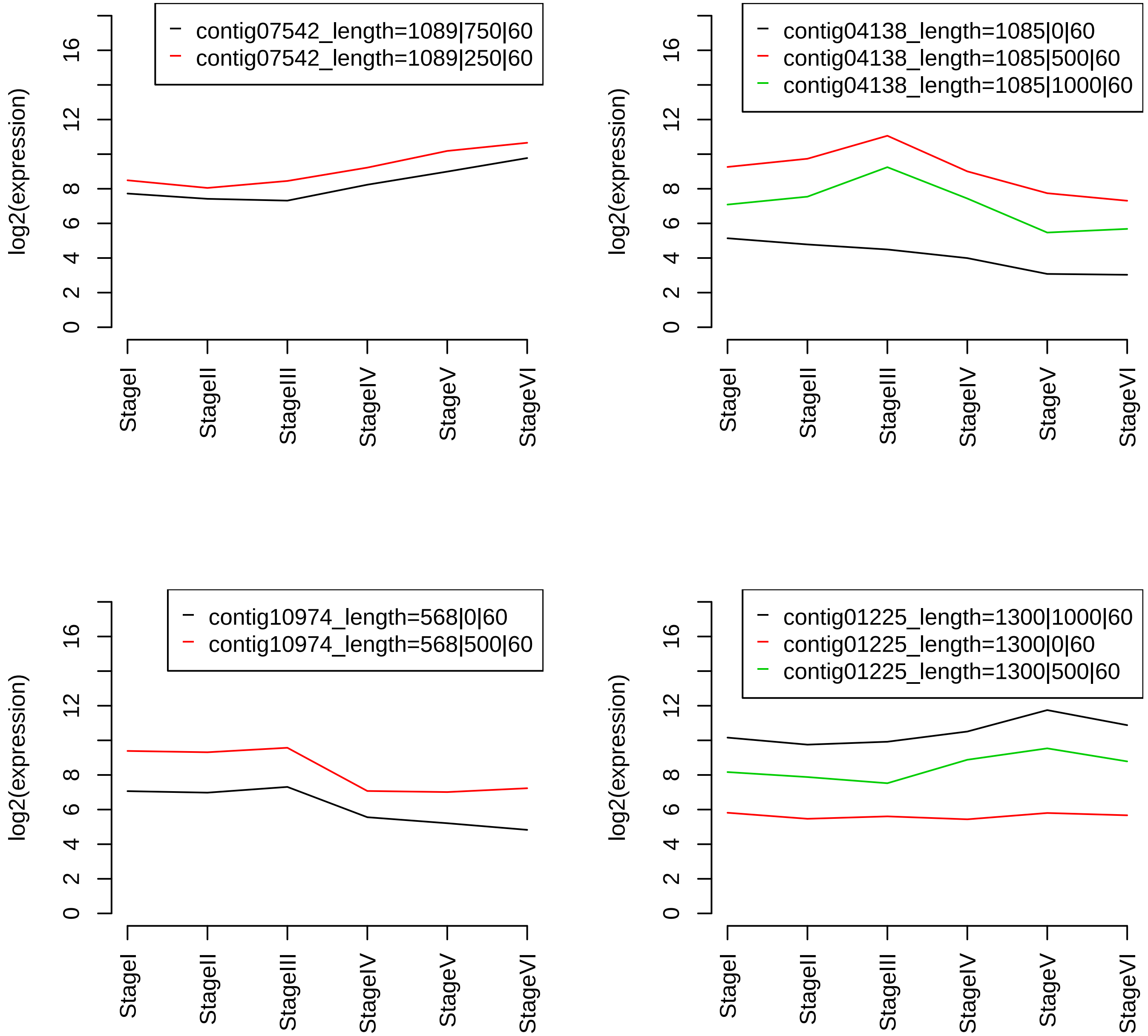
contig07542_length=1089|750|60
contig07542_length=1089|250|60
contig04138_length=1085|0|60
contig04138_length=1085|500|60
contig04138_length=1085|1000|60
contig10974_length=568|0|60
contig10974_length=568|500|60
contig01225_length=1300|1000|60
contig01225_length=1300|0|60
contig01225_length=1300|500|60
log2(expression)
0
2
4
6
8
12
16
StageI
StageII
StageIII
StageIV
StageV
StageVI

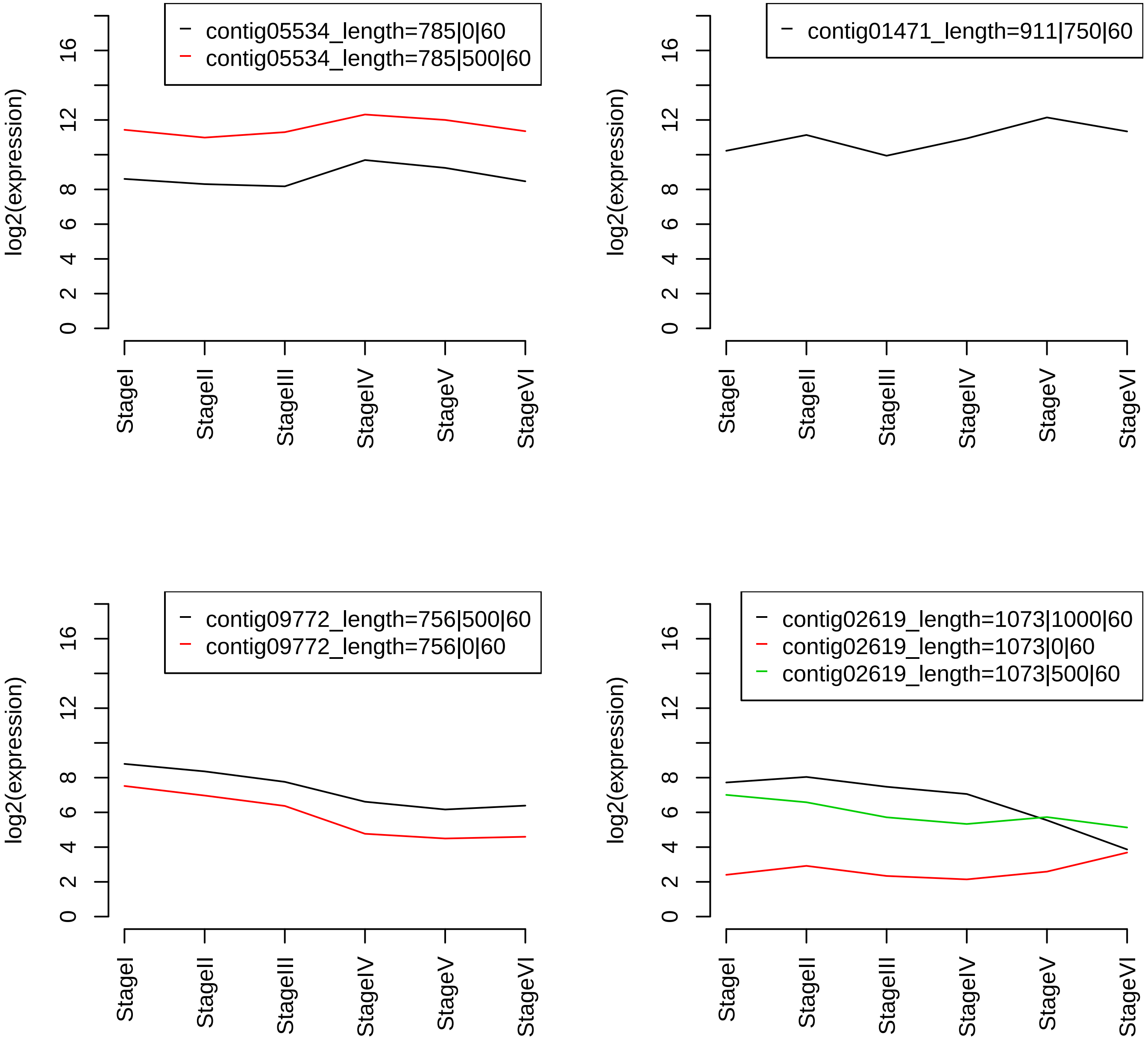
contig05534_length=785|0|60
contig05534_length=785|500|60
contig01471_length=911|750|60
contig09772_length=756|500|60
contig09772_length=756|0|60
contig02619_length=1073|1000|60
contig02619_length=1073|0|60
contig02619_length=1073|500|60
log2(expression)
0
2
4
6
8
12
16
StageI
StageII
StageIII
StageIV
StageV
StageVI

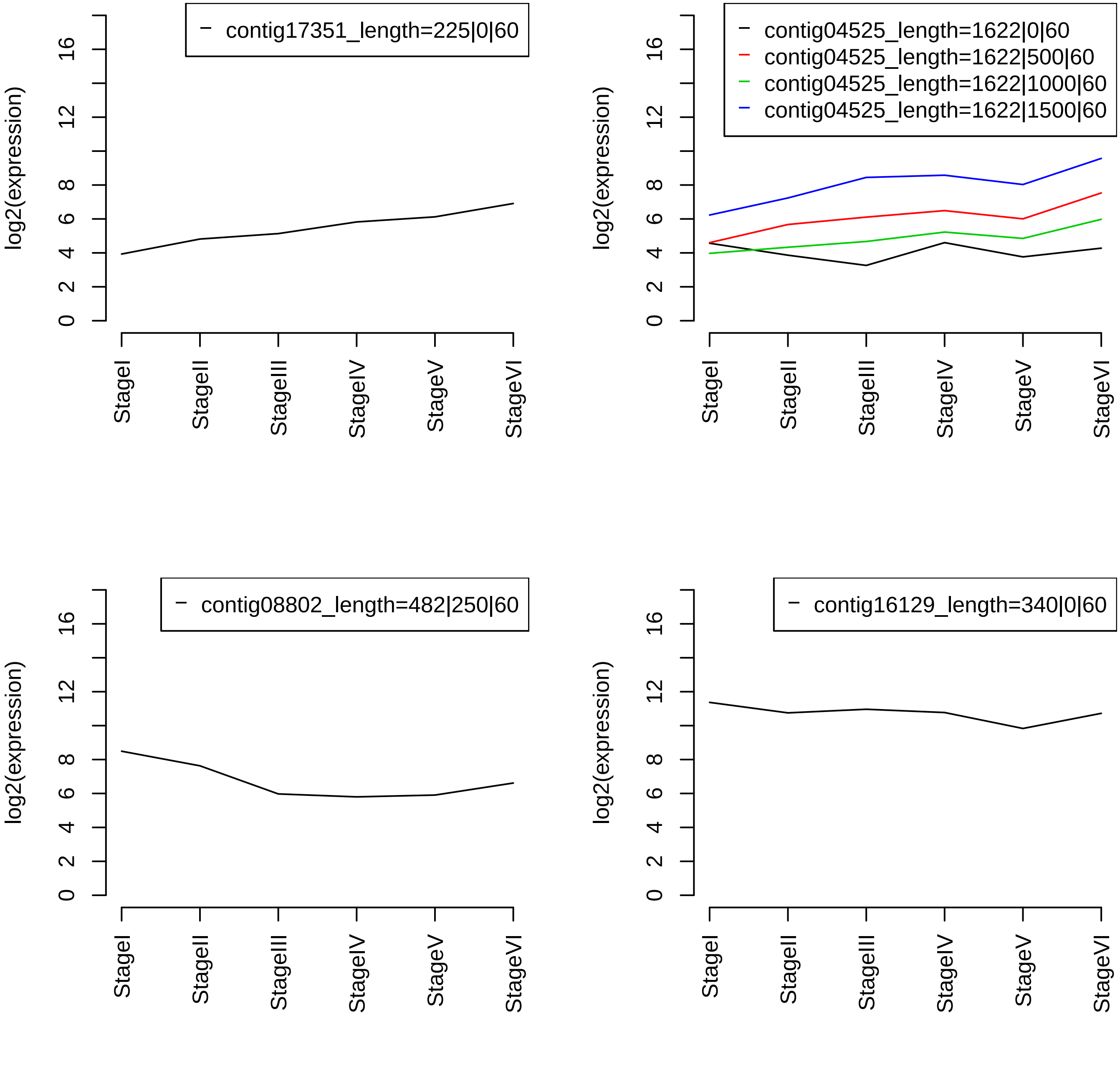
contig17351_length=225|0|60
contig04525_length=1622|0|60
contig04525_length=1622|500|60
contig04525_length=1622|1000|60
contig04525_length=1622|1500|60
contig08802_length=482|250|60
contig16129_length=340|0|60
log2(expression)
0
2
4
6
8
12
16
StageI
StageII
StageIII
StageIV
StageV
StageVI

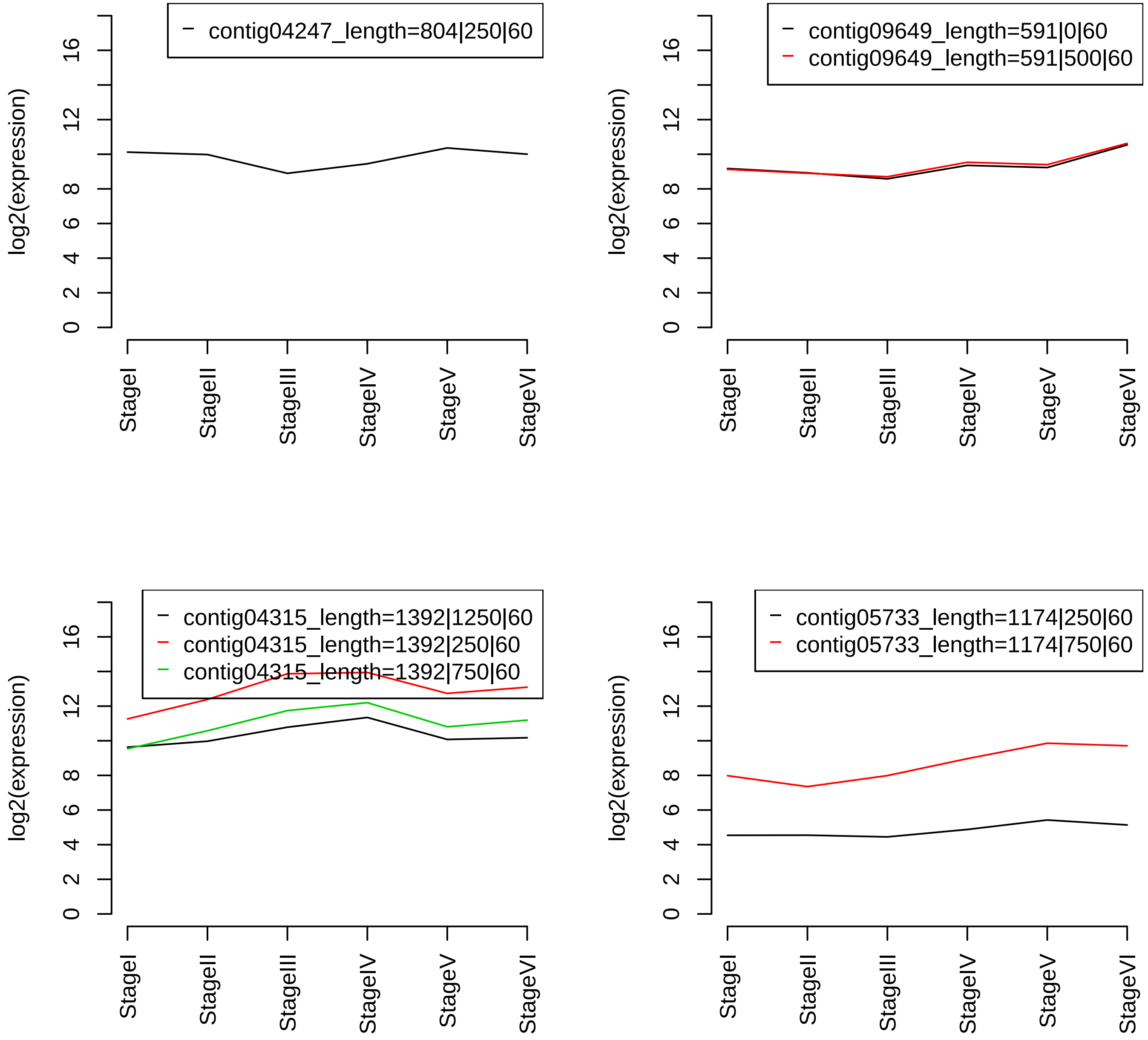
contig04247_length=804|250|60
contig09649_length=591|0|60
contig09649_length=591|500|60
contig04315_length=1392|1250|60
contig04315_length=1392|250|60
contig04315_length=1392|750|60
contig05733_length=1174|250|60
contig05733_length=1174|750|60
log2(expression)
0
2
4
6
8
12
16
StageI
StageII
StageIII
StageIV
StageV
StageVI

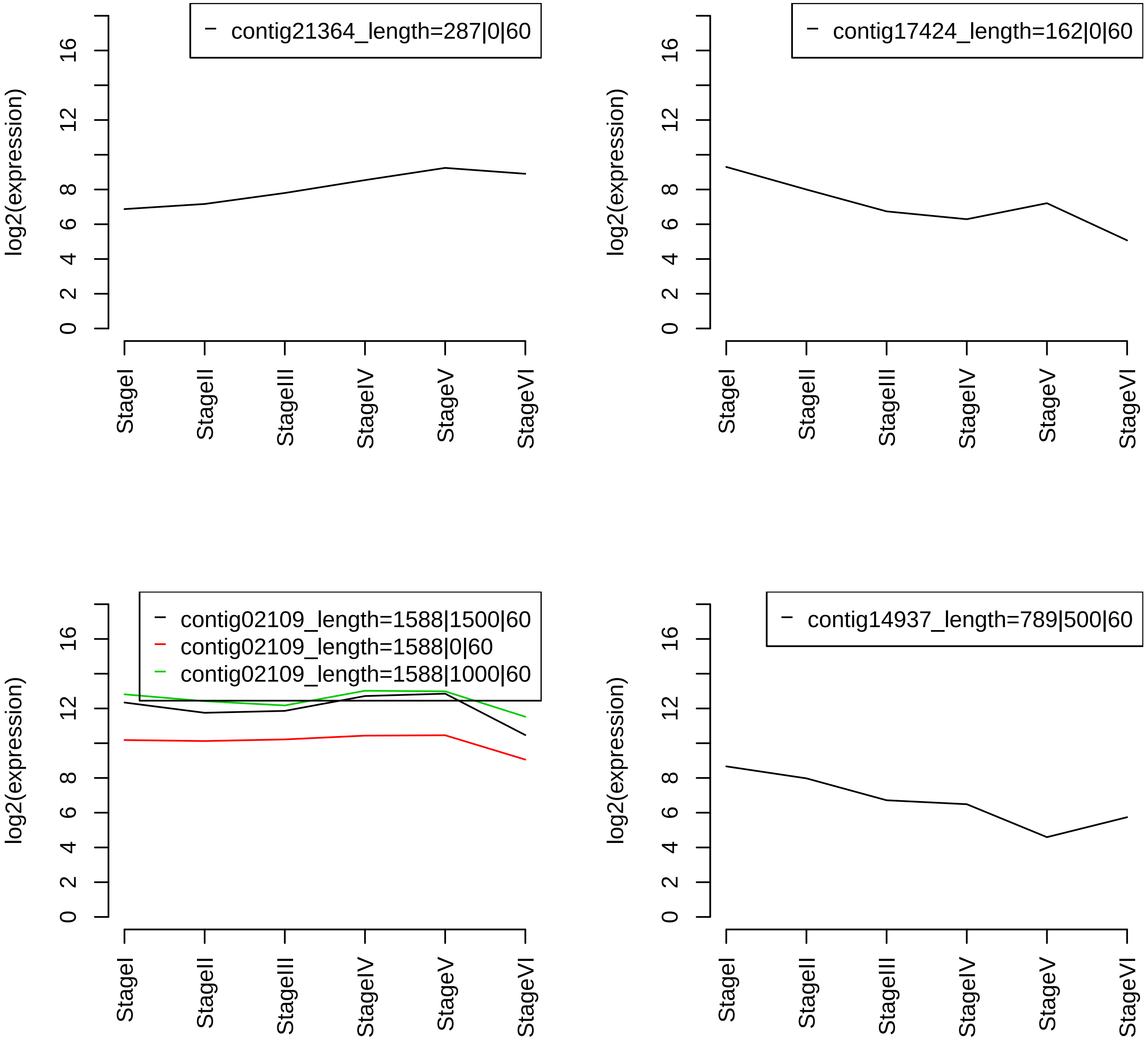
contig21364_length=287|0|60
contig17424_length=162|0|60
contig02109_length=1588|1500|60
contig02109_length=1588|0|60
contig02109_length=1588|1000|60
contig14937_length=789|500|60
log2(expression)
0
2
4
6
8
12
16
StageI
StageII
StageIII
StageIV
StageV
StageVI

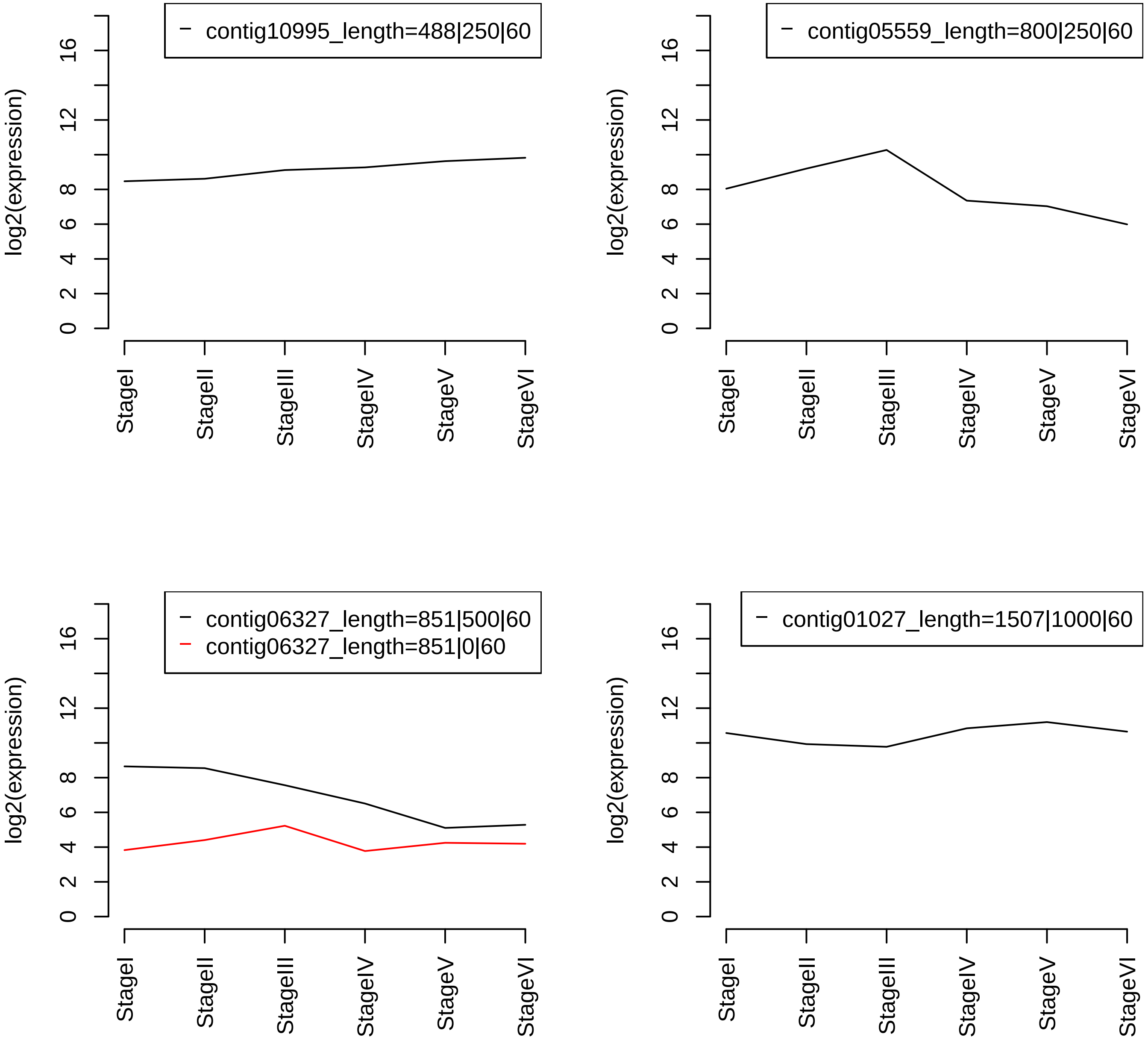
contig10995_length=488|250|60
contig05559_length=800|250|60
contig06327_length=851|500|60
contig06327_length=851|0|60
contig01027_length=1507|1000|60
log2(expression)
0
2
4
6
8
12
16
StageI
StageII
StageIII
StageIV
StageV
StageVI

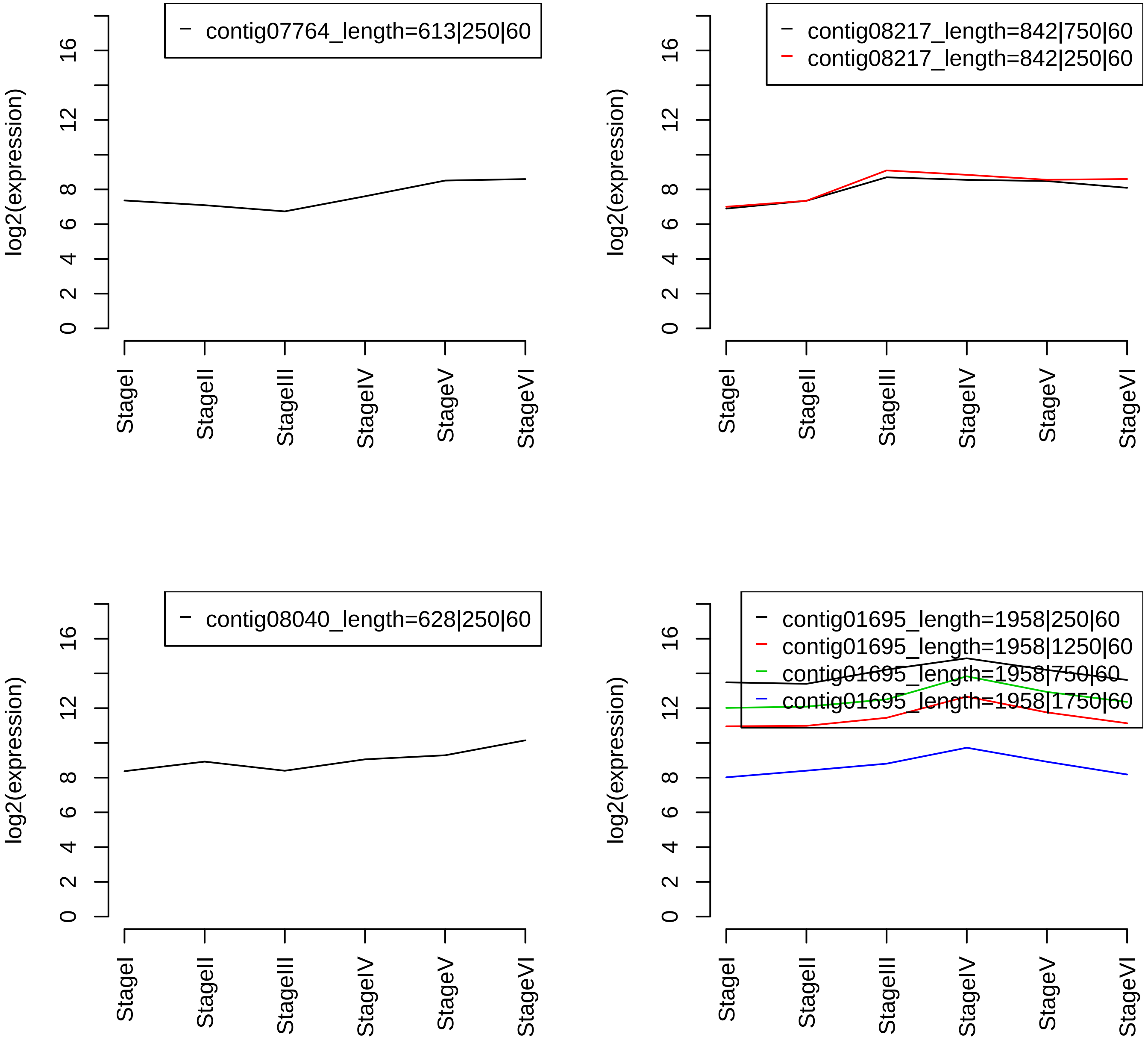

contig07764_length=613|250|60
contig08217_length=842|750|60
contig08217_length=842|250|60
contig08040_length=628|250|60
contig01695_length=1958|250|60
contig01695_length=1958|1250|60
contig01695_length=1958|750|60
contig01695_length=1958|1750|60
log2(expression)
0
2
4
6
8
12
16
StageI
StageII
StageIII
StageIV
StageV
StageVI

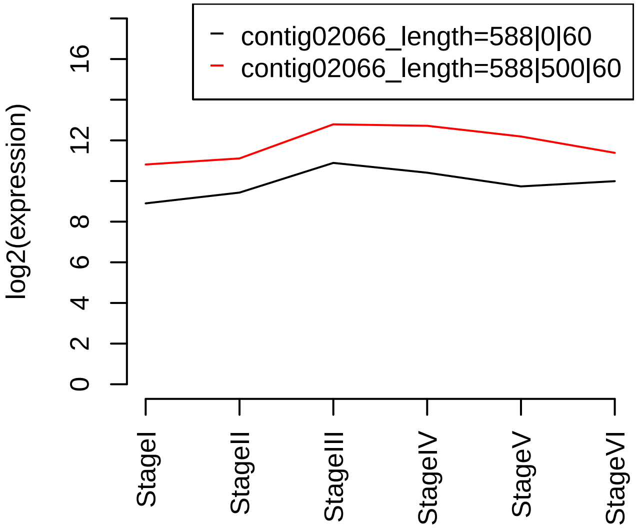

contig02066_length=588|0|60
contig02066_length=588|500|60
log2(expression)
0
2
4
6
8
12
16
StageI
StageII
StageIII
StageIV
StageV
StageVI

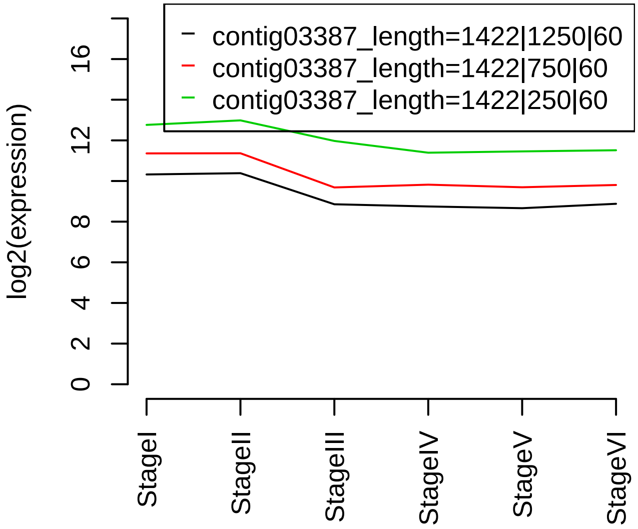

contig03387_length=1422|1250|60
contig03387_length=1422|750|60
contig03387_length=1422|250|60
log2(expression)
0
2
4
6
8
12
16
StageI
StageII
StageIII
StageIV
StageV
StageVI

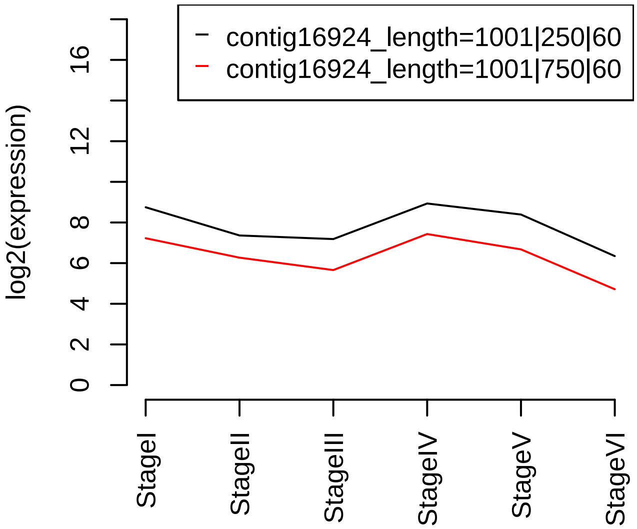

contig16924_length=1001|250|60
contig16924_length=1001|750|60
log2(expression)
0
2
4
6
8
12
16
StageI
StageII
StageIII
StageIV
StageV
StageVI

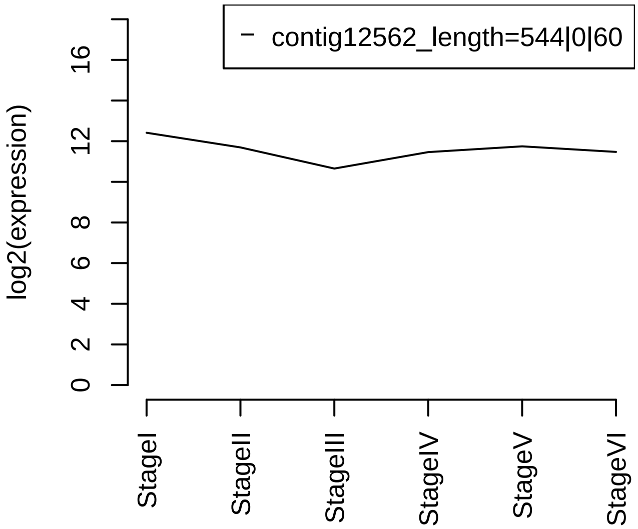

contig12562_length=544|0|60
log2(expression)
0
2
4
6
8
12
16
StageI
StageII
StageIII
StageIV
StageV
StageVI

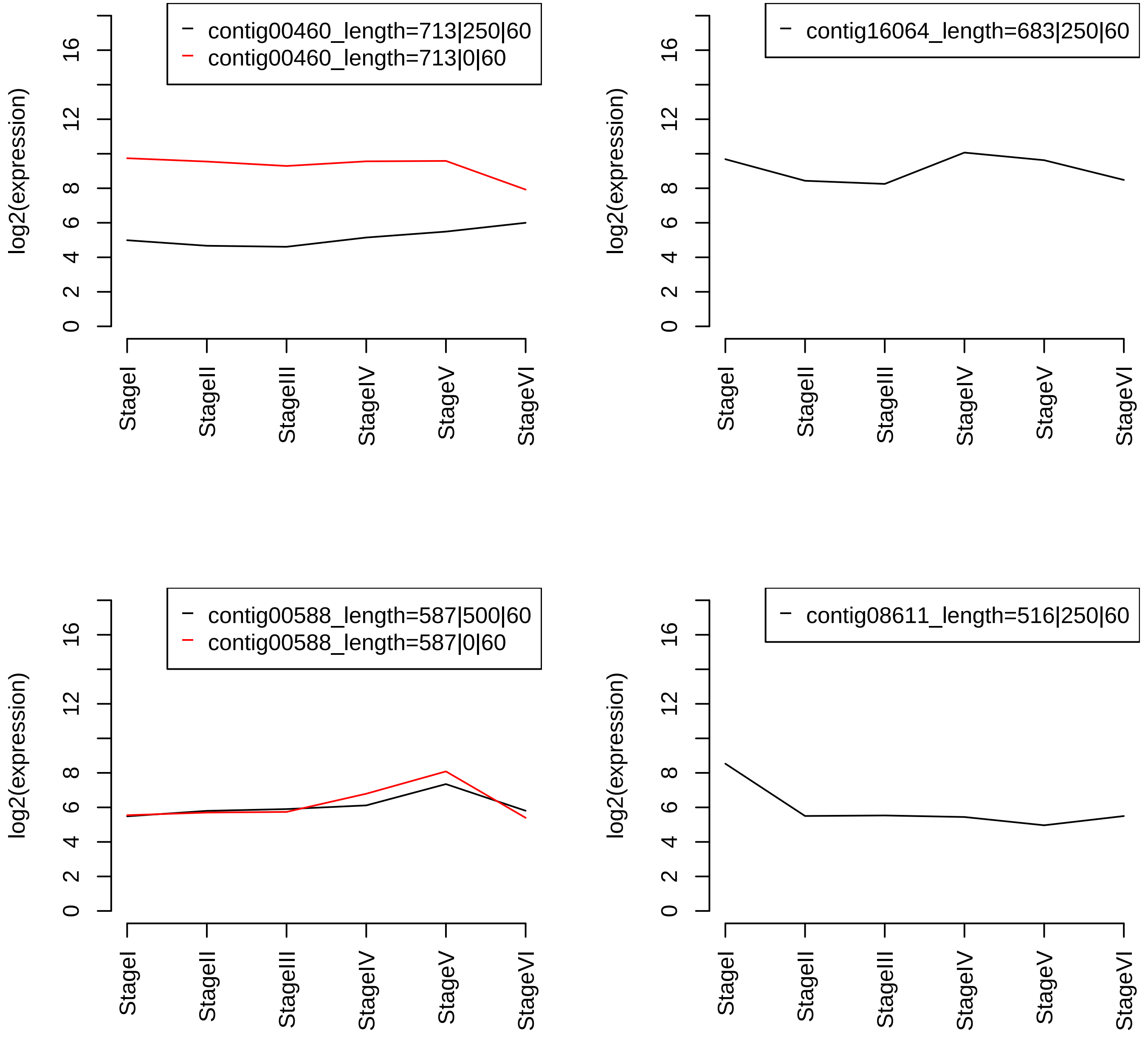

contig00460_length=713|250|60
contig00460_length=713|0|60
contig16064_length=683|250|60
contig00588_length=587|500|60
contig00588_length=587|0|60
contig08611_length=516|250|60
log2(expression)
0
2
4
6
8
12
16
StageI
StageII
StageIII
StageIV
StageV
StageVI

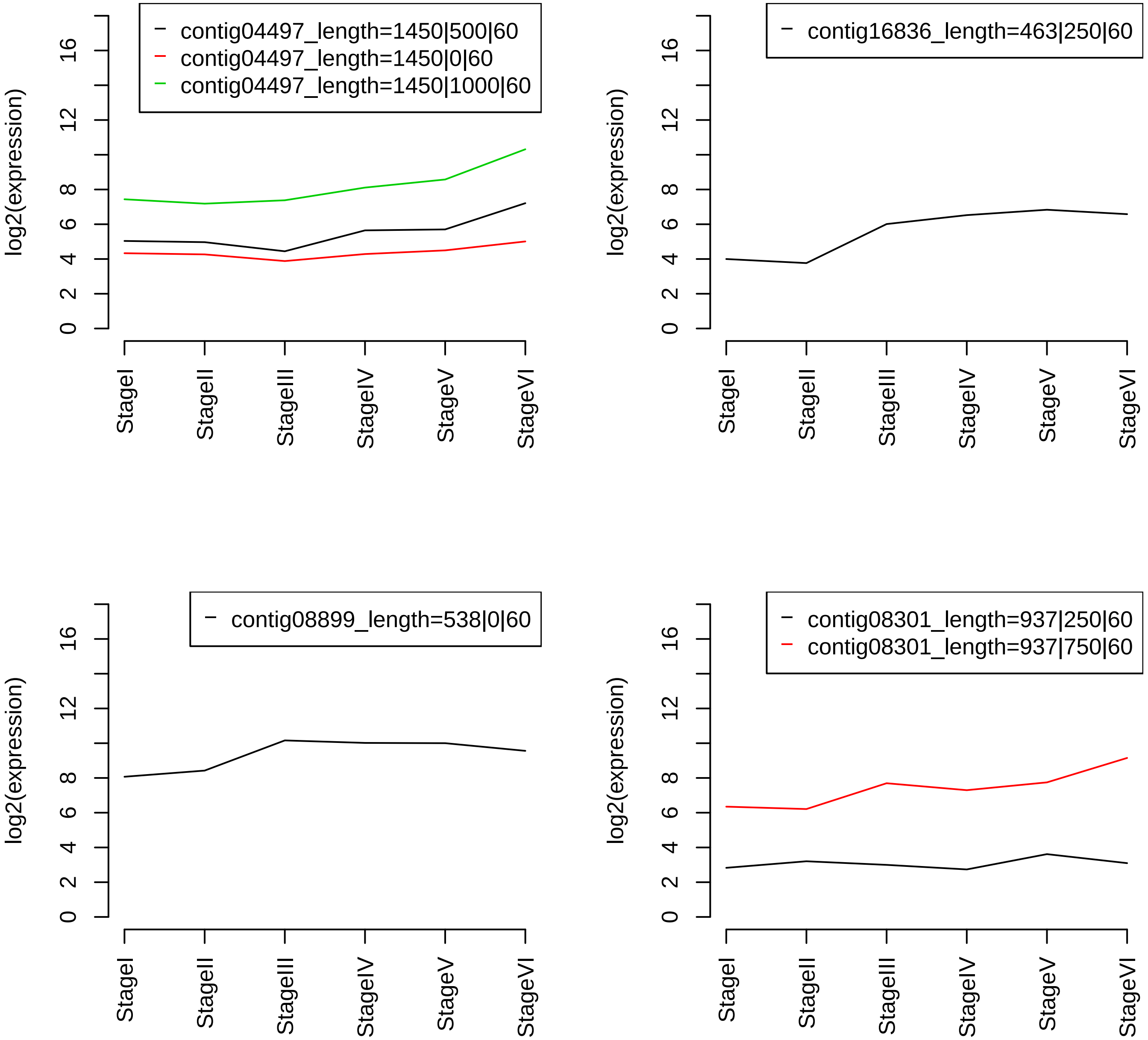

contig04497_length=1450|500|60
contig04497_length=1450|0|60
contig04497_length=1450|1000|60
contig16836_length=463|250|60
contig08899_length=538|0|60
contig08301_length=937|250|60
contig08301_length=937|750|60
log2(expression)
0
2
4
6
8
12
16
StageI
StageII
StageIII
StageIV
StageV
StageVI

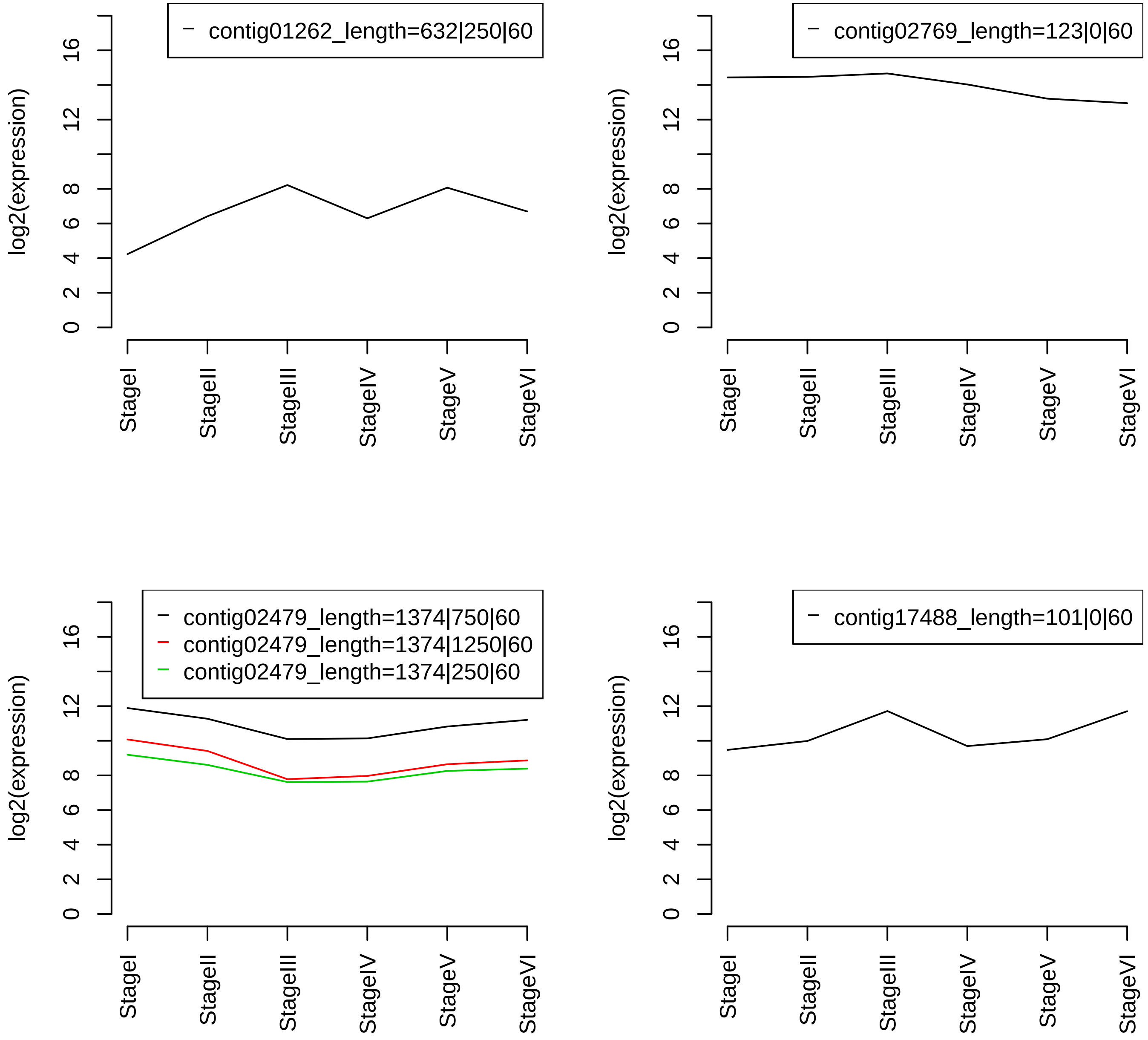
contig01262_length=632|250|60
contig02769_length=123|0|60
contig02479_length=1374|750|60
contig02479_length=1374|1250|60
contig02479_length=1374|250|60
contig17488_length=101|0|60
log2(expression)
0
2
4
6
8
12
16
StageI
StageII
StageIII
StageIV
StageV
StageVI

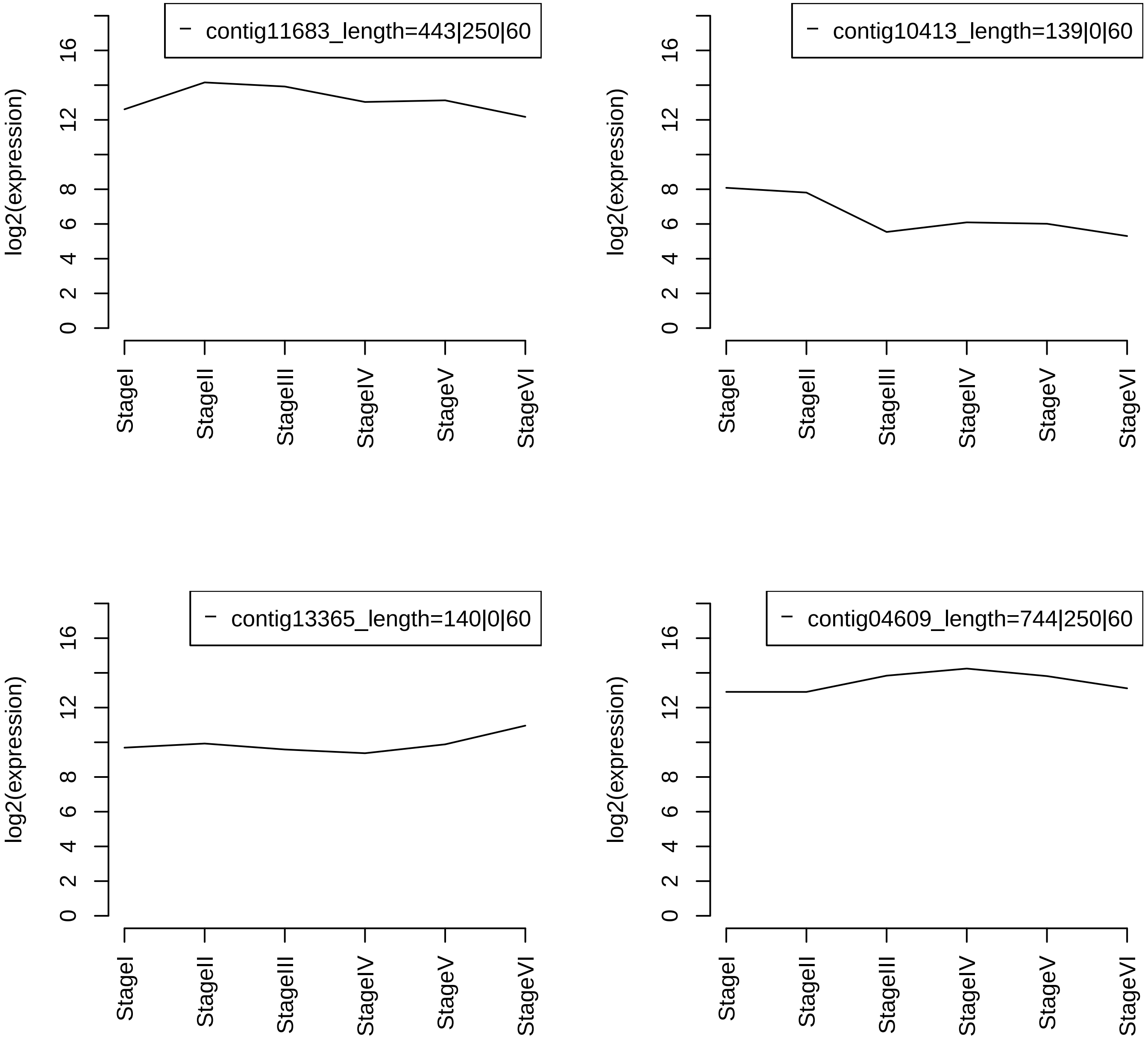
contig11683_length=443|250|60
contig10413_length=139|0|60
contig13365_length=140|0|60
contig04609_length=744|250|60
log2(expression)
0
2
4
6
8
12
16
StageI
StageII
StageIII
StageIV
StageV
StageVI

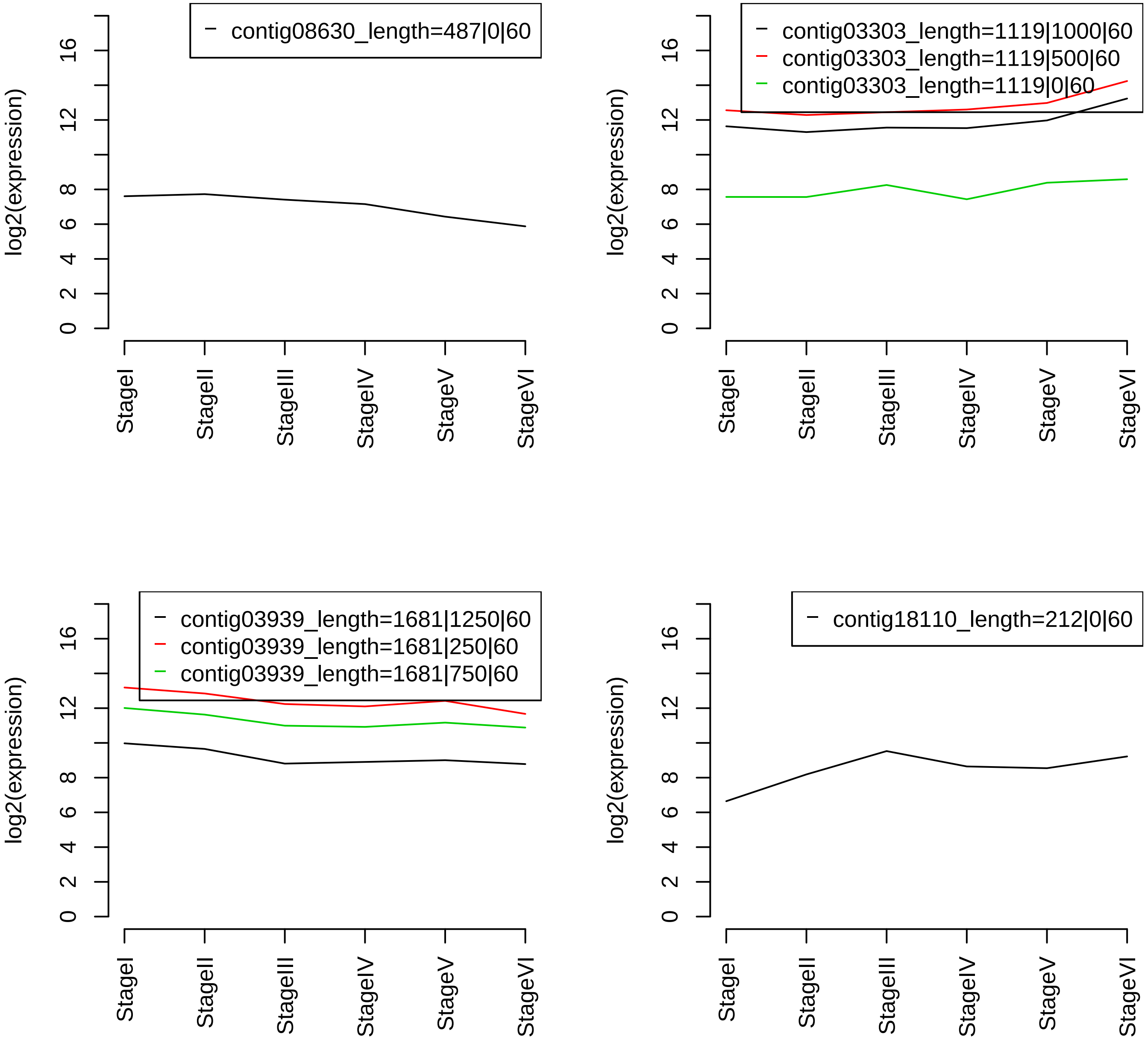

contig08630_length=487|0|60
contig03303_length=1119|1000|60
contig03303_length=1119|500|60
contig03303_length=1119|0|60
contig03939_length=1681|1250|60
contig03939_length=1681|250|60
contig03939_length=1681|750|60
contig18110_length=212|0|60
log2(expression)
0 2 4 6 8 12 16
StageI
StageII
StageIII
StageIV
StageV
StageVI

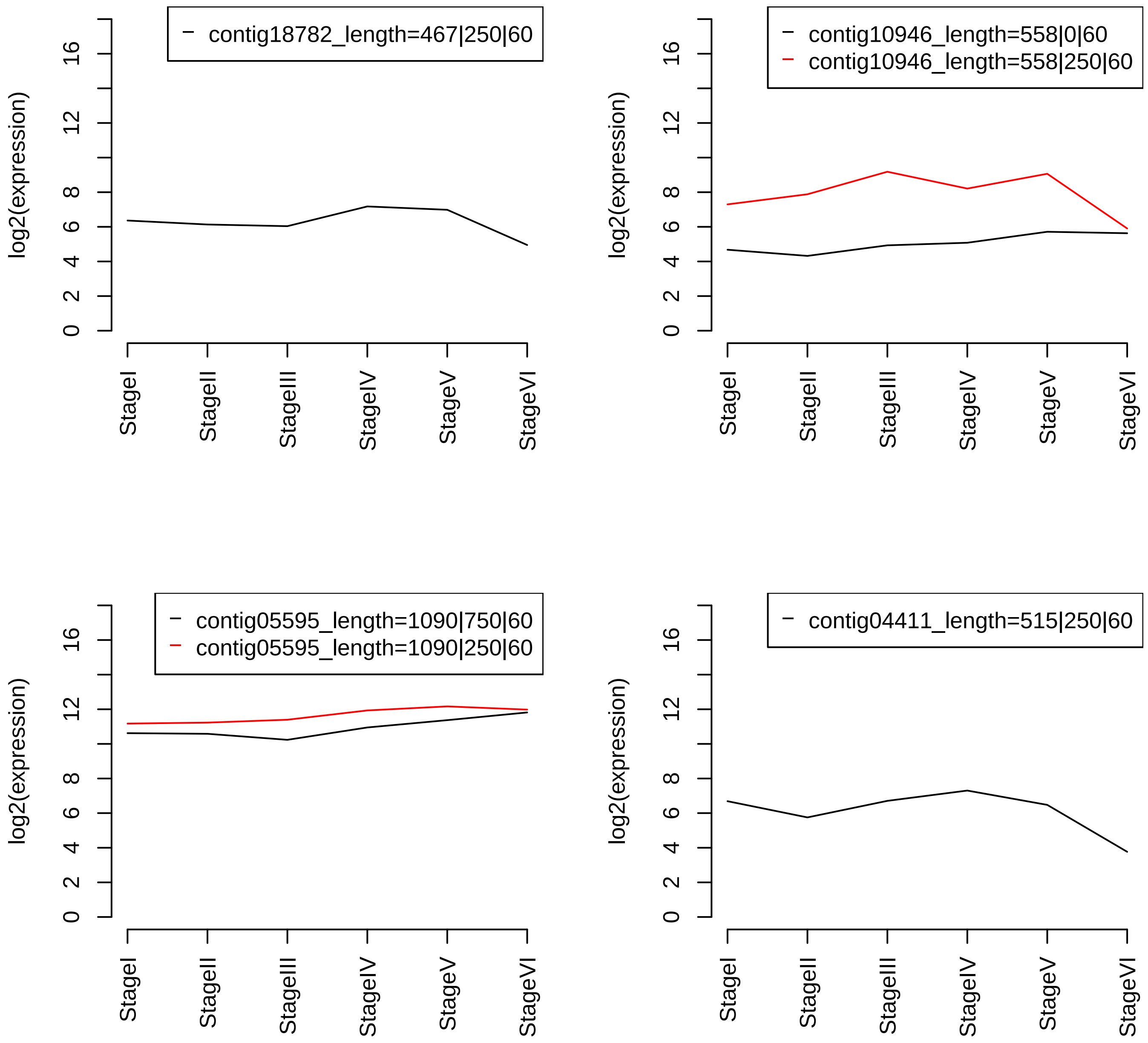
contig18782_length=467|250|60
contig10946_length=558|0|60
contig10946_length=558|250|60
contig05595_length=1090|750|60
contig05595_length=1090|250|60
contig04411_length=515|250|60
log2(expression)
StageI
StageII
StageIII
StageIV
StageV
StageVI

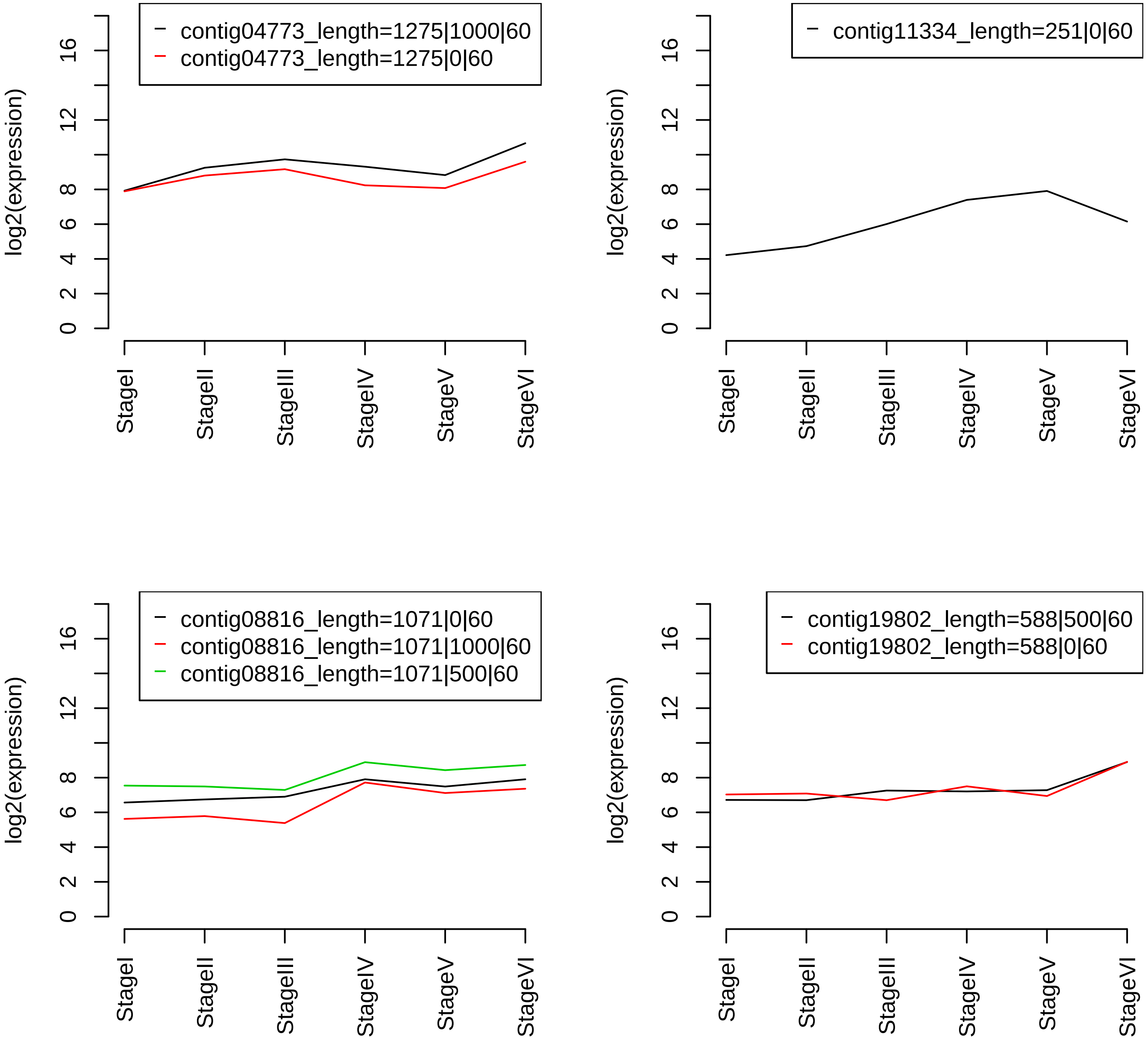
contig04773_length=1275|1000|60
contig04773_length=1275|0|60
contig11334_length=251|0|60
contig08816_length=1071|0|60
contig08816_length=1071|1000|60
contig08816_length=1071|500|60
contig19802_length=588|500|60
contig19802_length=588|0|60
log2(expression)
0 2 4 6 8 12 16
StageI StageII StageIII StageIV StageV StageVI

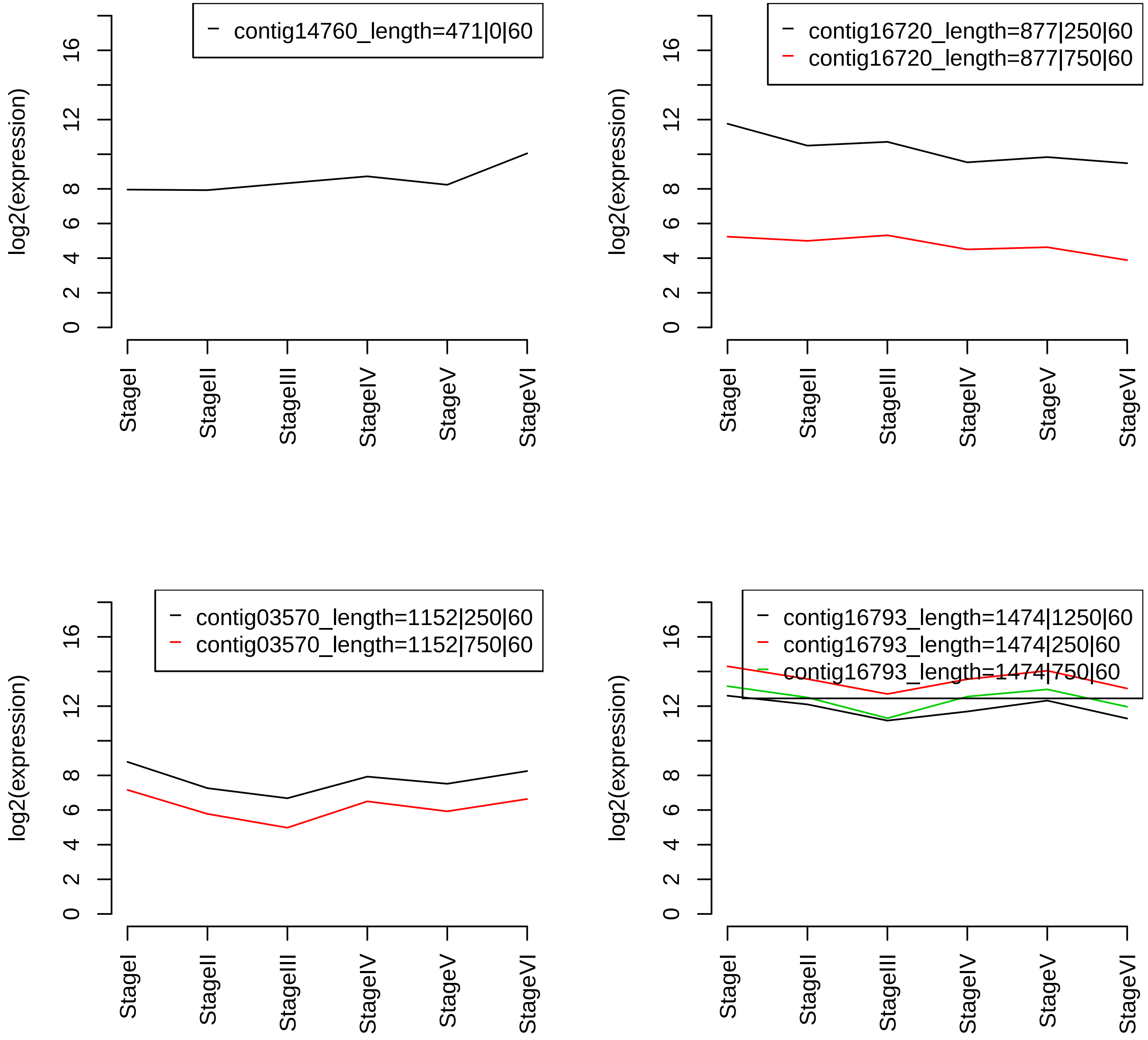
contig14760_length=471|0|60
contig16720_length=877|250|60
contig16720_length=877|750|60
contig03570_length=1152|250|60
contig03570_length=1152|750|60
contig16793_length=1474|1250|60
contig16793_length=1474|250|60
contig16793_length=1474|750|60
log2(expression)
0 2 4 6 8 12 16
StageI StageII StageIII StageIV StageV StageVI

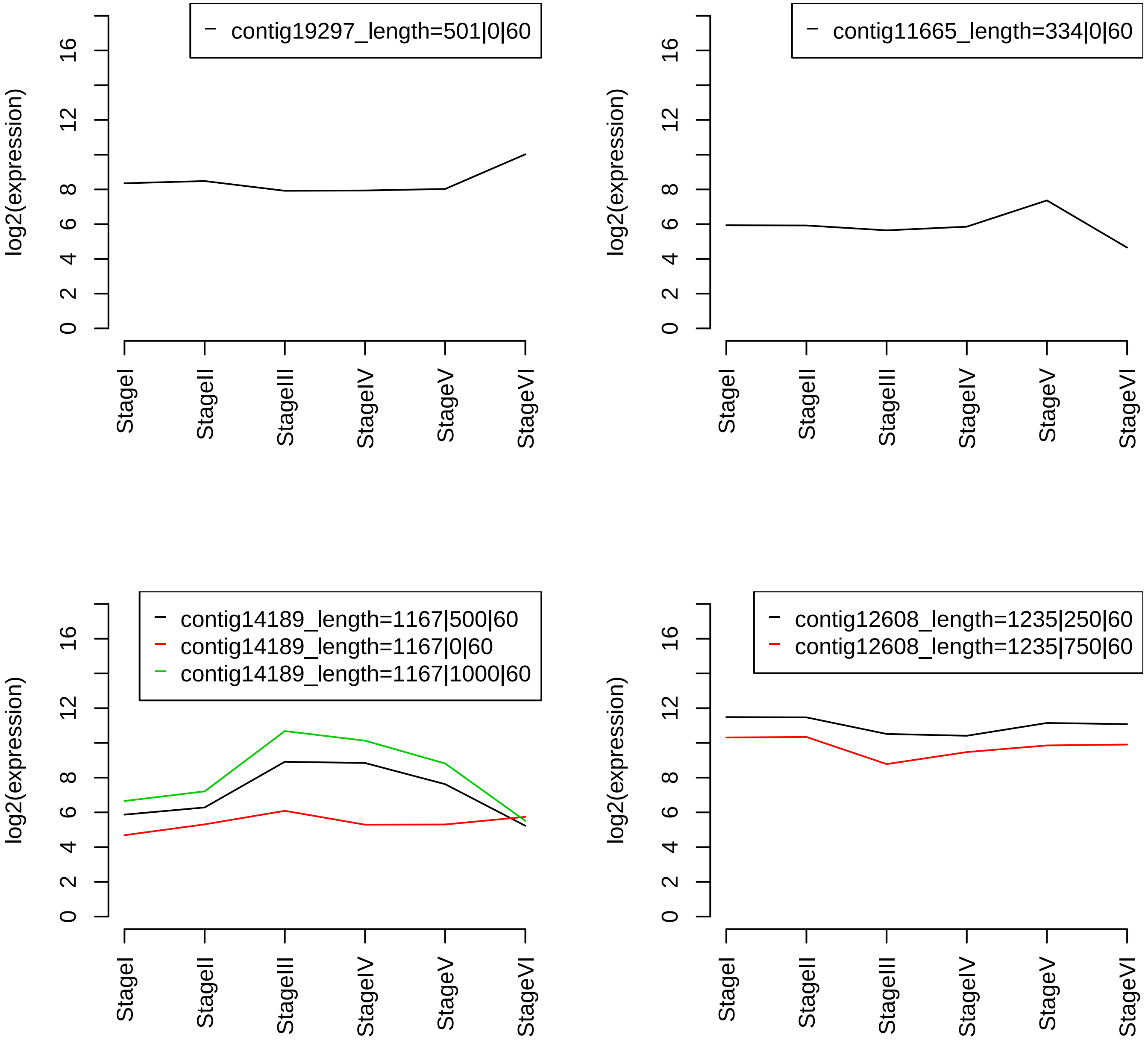

contig19297_length=501|0|60
contig11665_length=334|0|60
contig14189_length=1167|500|60
contig14189_length=1167|0|60
contig14189_length=1167|1000|60
contig12608_length=1235|250|60
contig12608_length=1235|750|60
log2(expression)
0
2
4
6
8
12
16
StageI
StageII
StageIII
StageIV
StageV
StageVI

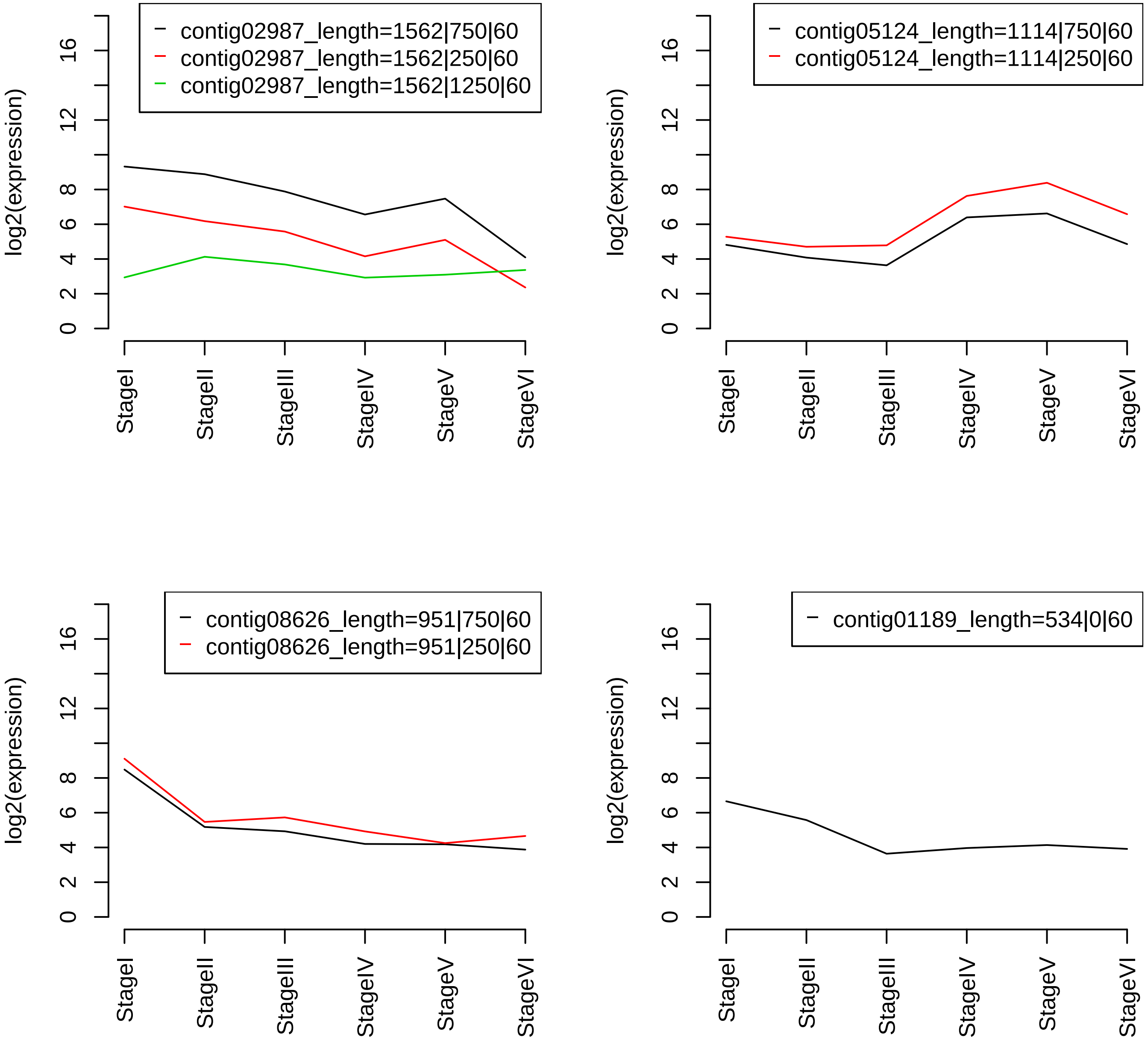

contig02987_length=1562|750|60
contig02987_length=1562|250|60
contig02987_length=1562|1250|60
contig05124_length=1114|750|60
contig05124_length=1114|250|60
contig08626_length=951|750|60
contig08626_length=951|250|60
contig01189_length=534|0|60
log2(expression)
0
2
4
6
8
12
16
StageI
StageII
StageIII
StageIV
StageV
StageVI

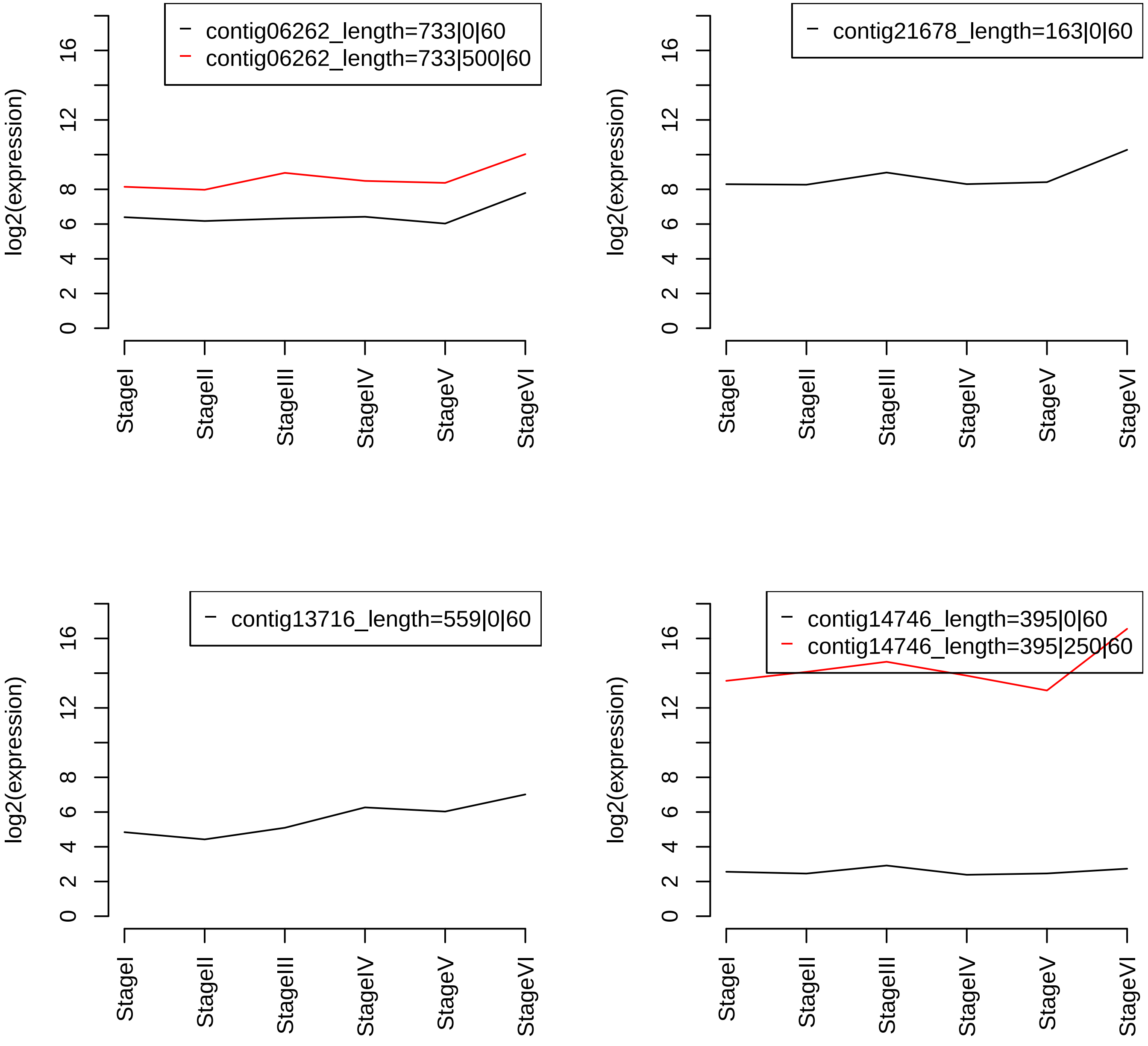

contig06262_length=733|0|60
contig06262_length=733|500|60
contig21678_length=163|0|60
contig13716_length=559|0|60
contig14746_length=395|0|60
contig14746_length=395|250|60
log2(expression)
0
2
4
6
8
12
16
StageI
StageII
StageIII
StageIV
StageV
StageVI

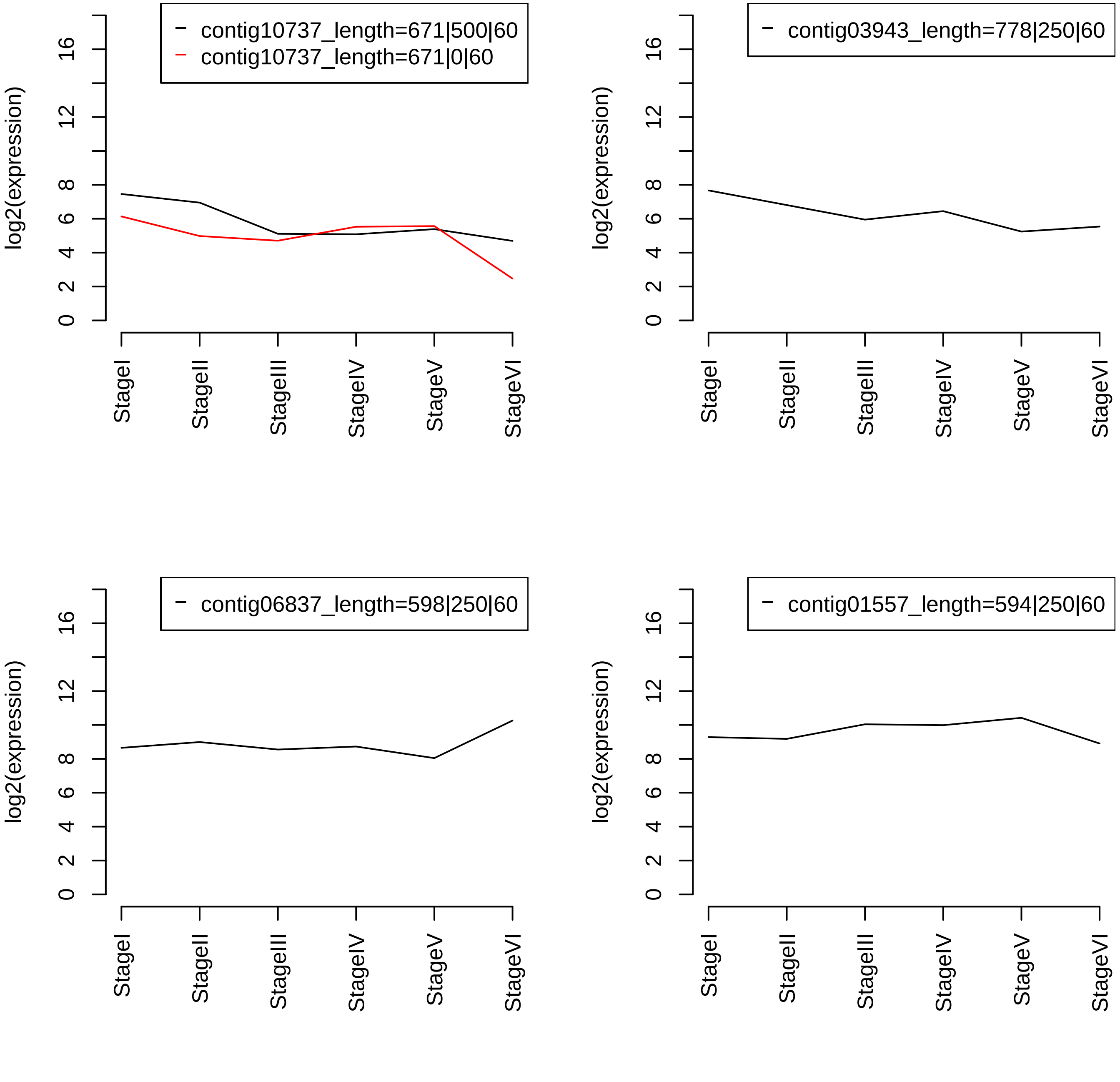
contig10737_length=671|500|60
contig10737_length=671|0|60
contig03943_length=778|250|60
contig06837_length=598|250|60
contig01557_length=594|250|60
log2(expression)
0
2
4
6
8
12
16
StageI
StageII
StageIII
StageIV
StageV
StageVI

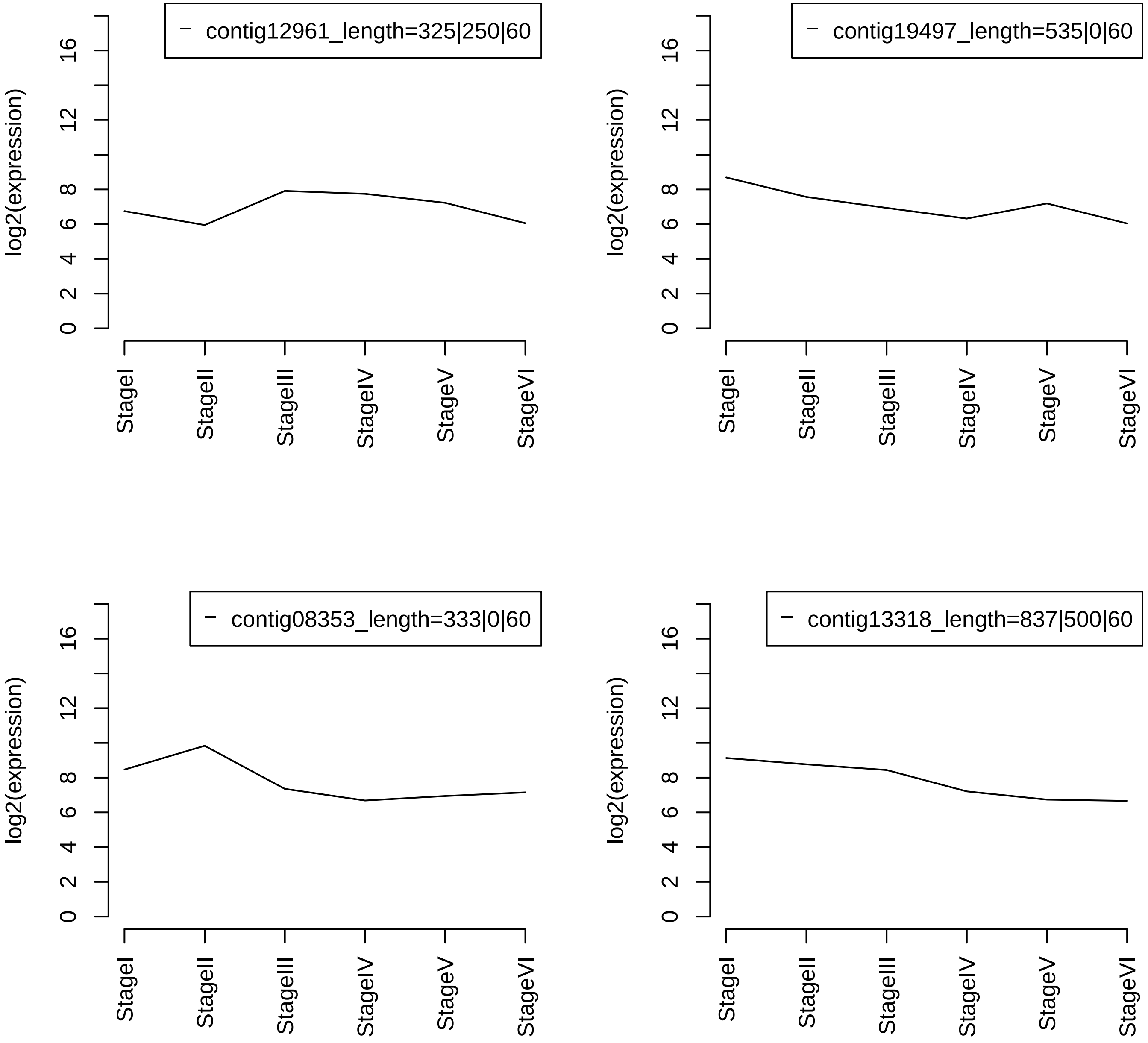

contig12961_length=325|250|60
contig19497_length=535|0|60
contig08353_length=333|0|60
contig13318_length=837|500|60
log2(expression)
0
2
4
6
8
12
16
StageI
StageII
StageIII
StageIV
StageV
StageVI

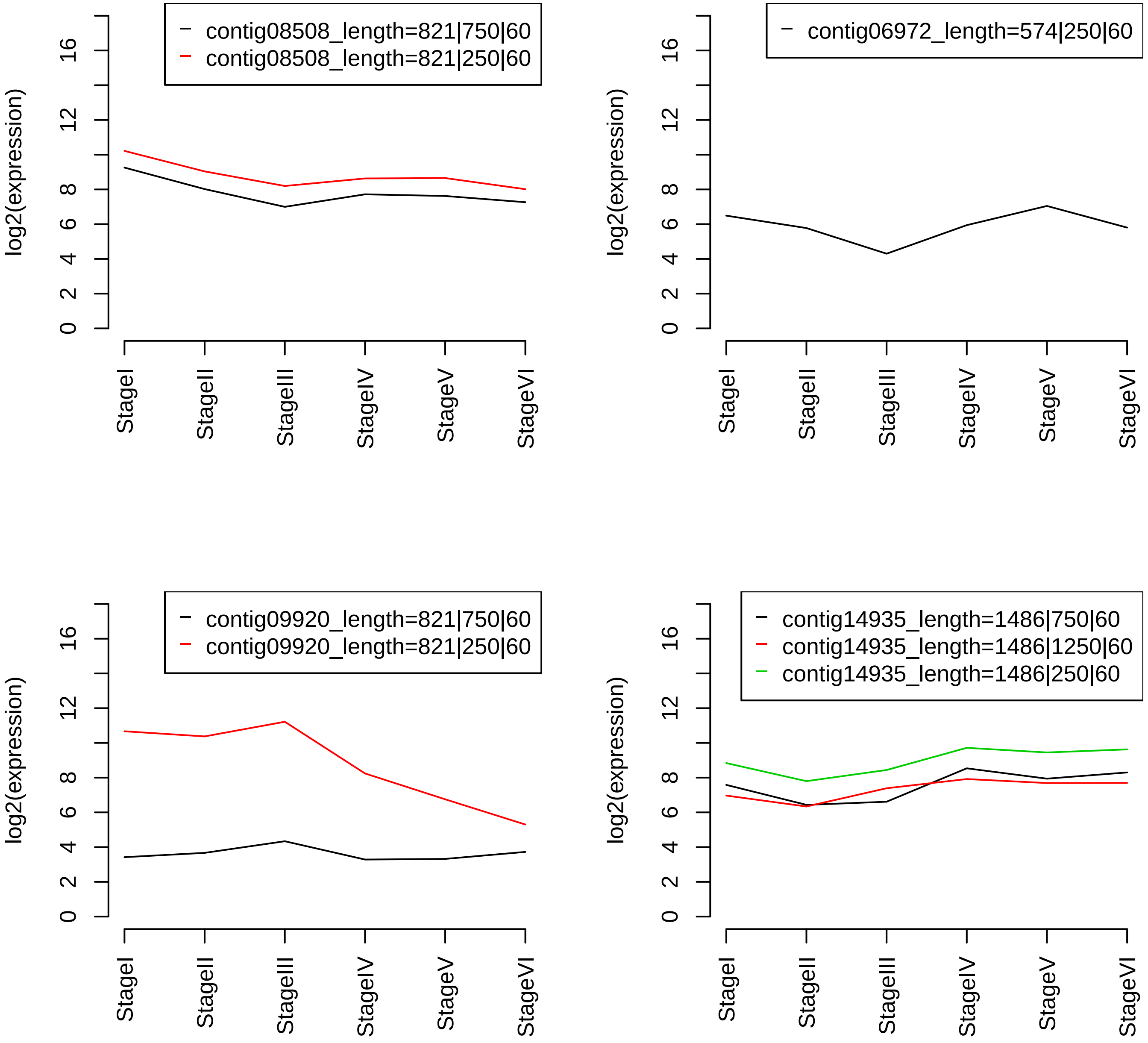

contig08508_length=821|750|60
contig08508_length=821|250|60
contig06972_length=574|250|60
contig09920_length=821|750|60
contig09920_length=821|250|60
contig14935_length=1486|750|60
contig14935_length=1486|1250|60
contig14935_length=1486|250|60
log2(expression)
0
2
4
6
8
12
16
StageI
StageII
StageIII
StageIV
StageV
StageVI

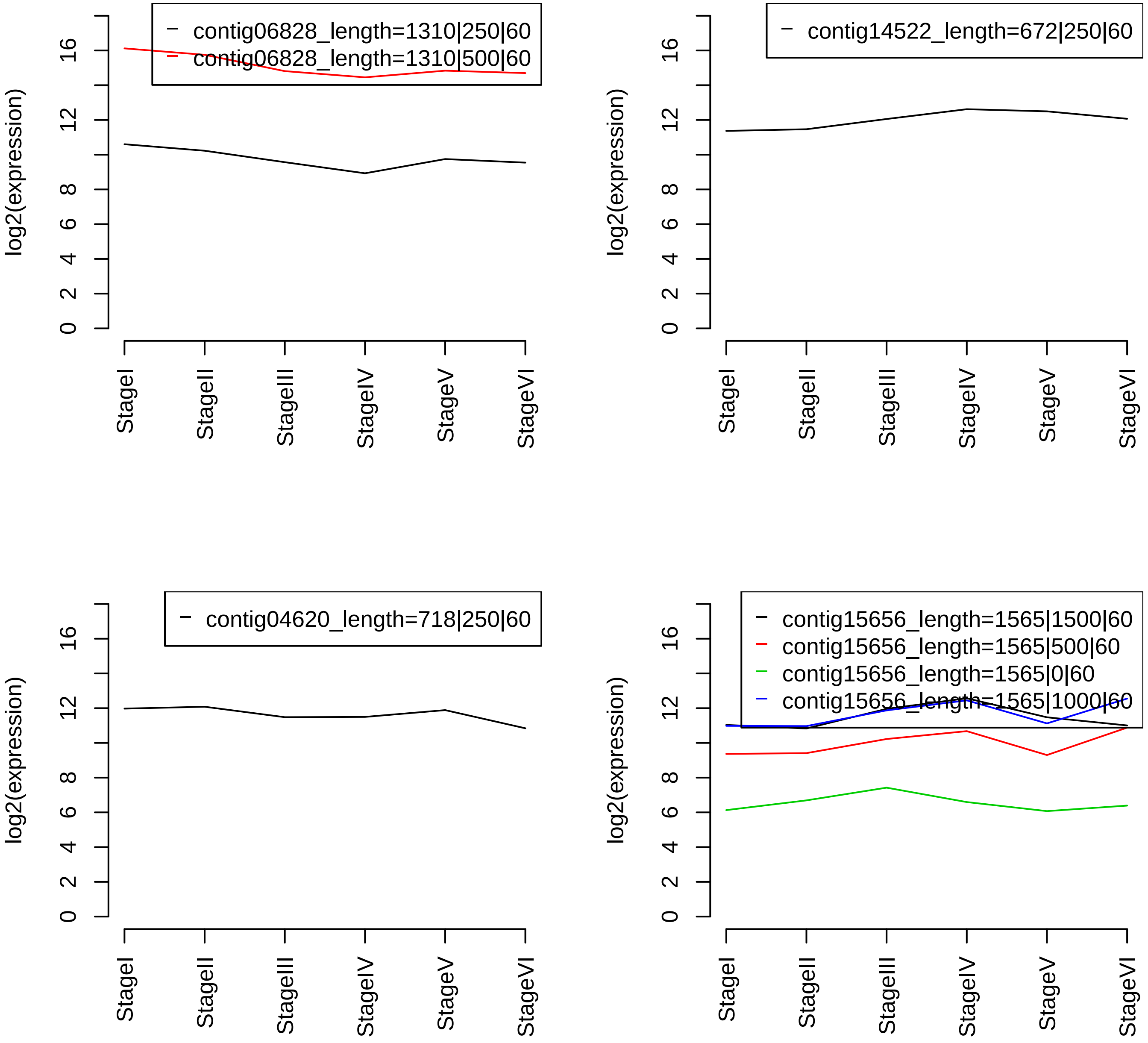
contig06828_length=1310|250|60
contig06828_length=1310|500|60
contig14522_length=672|250|60
contig04620_length=718|250|60
contig15656_length=1565|1500|60
contig15656_length=1565|500|60
contig15656_length=1565|0|60
contig15656_length=1565|1000|60
log2(expression)
0
2
4
6
8
12
16
StageI
StageII
StageIII
StageIV
StageV
StageVI

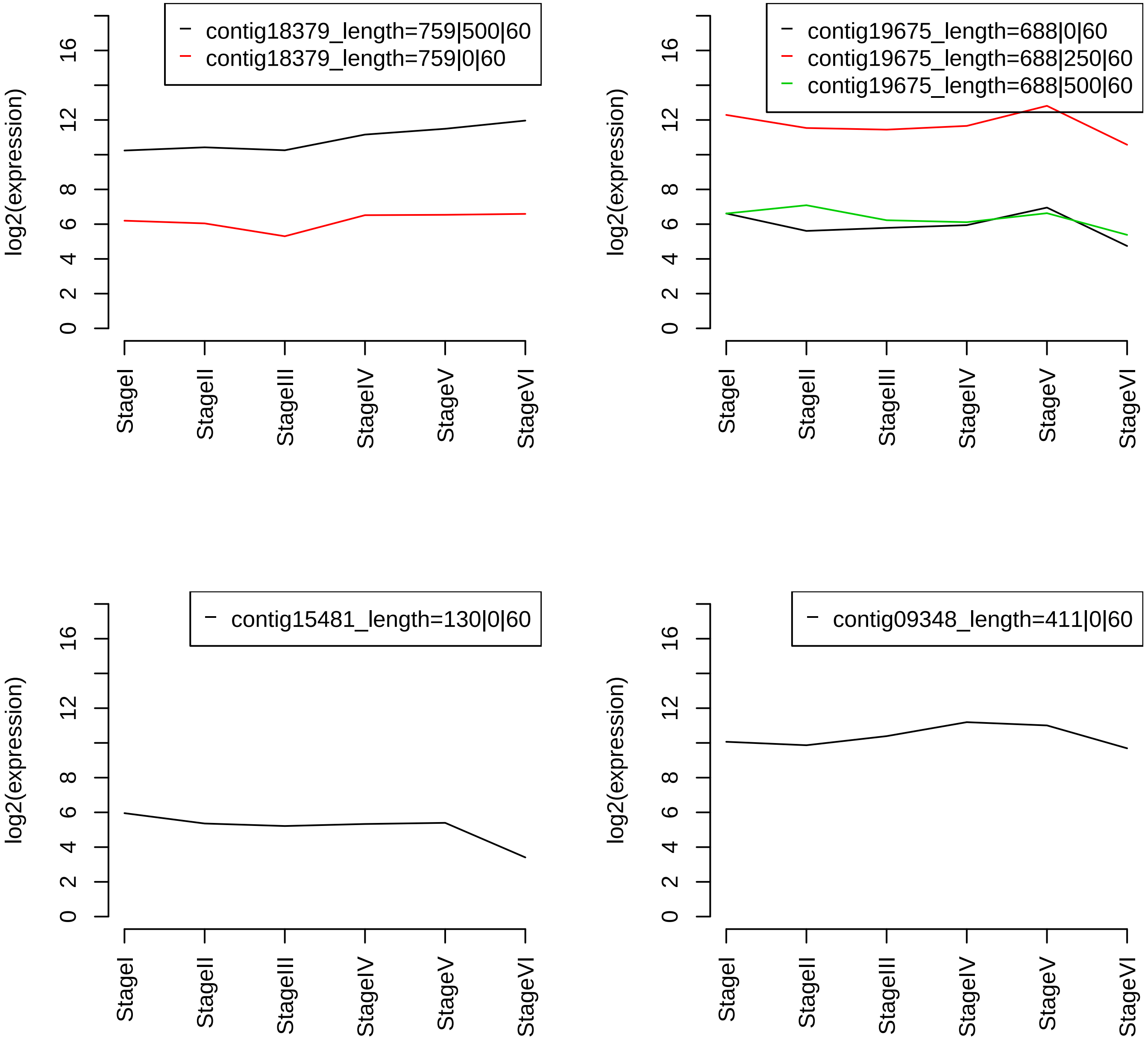

contig18379_length=759|500|60
contig18379_length=759|0|60
contig19675_length=688|0|60
contig19675_length=688|250|60
contig19675_length=688|500|60
contig15481_length=130|0|60
contig09348_length=411|0|60
log2(expression)
0
2
4
6
8
12
16
StageI
StageII
StageIII
StageIV
StageV
StageVI

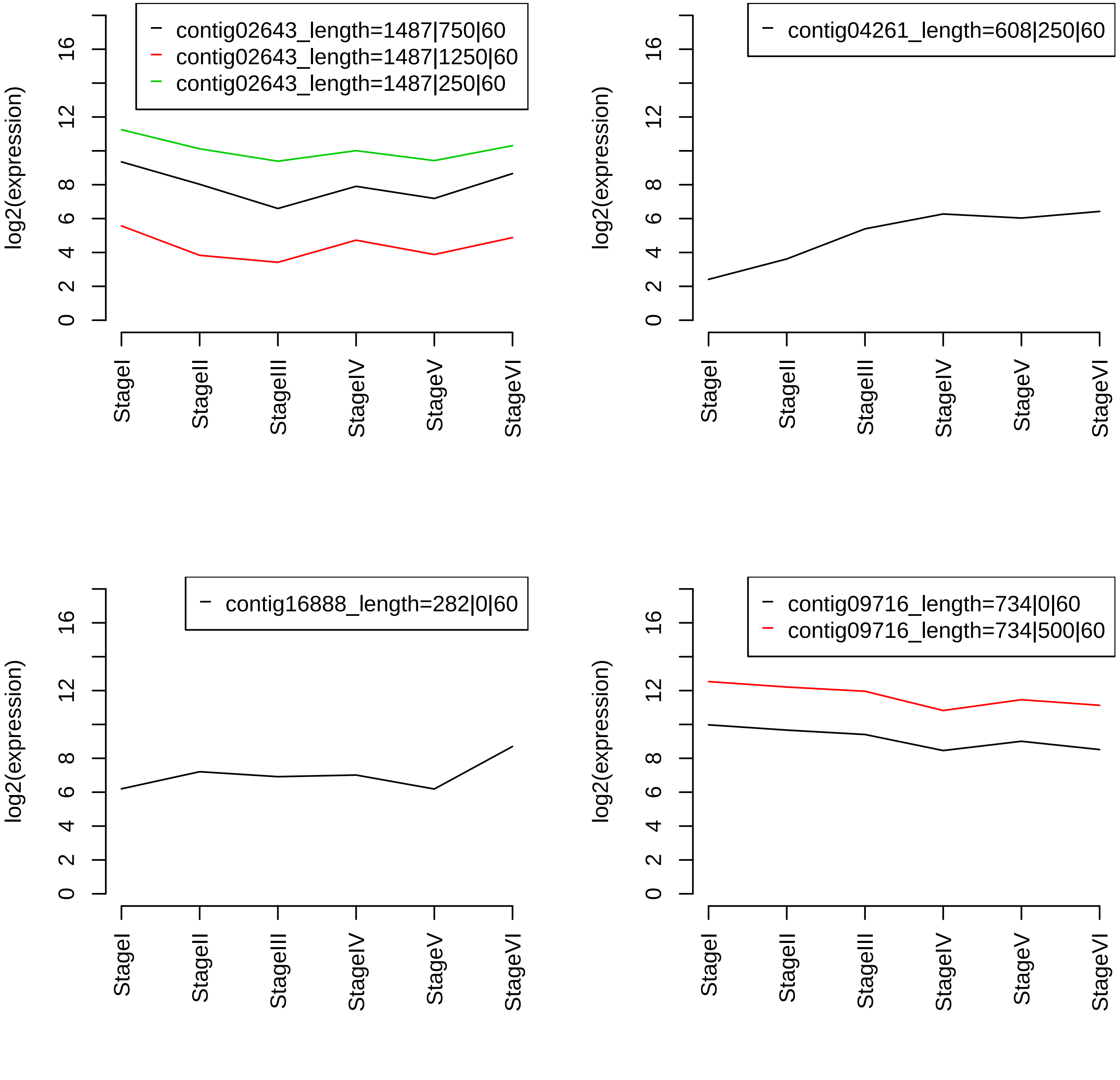
contig02643_length=1487|750|60
contig02643_length=1487|1250|60
contig02643_length=1487|250|60
contig04261_length=608|250|60
contig16888_length=282|0|60
contig09716_length=734|0|60
contig09716_length=734|500|60
log2(expression)
0
2
4
6
8
12
16
StageI
StageII
StageIII
StageIV
StageV
StageVI

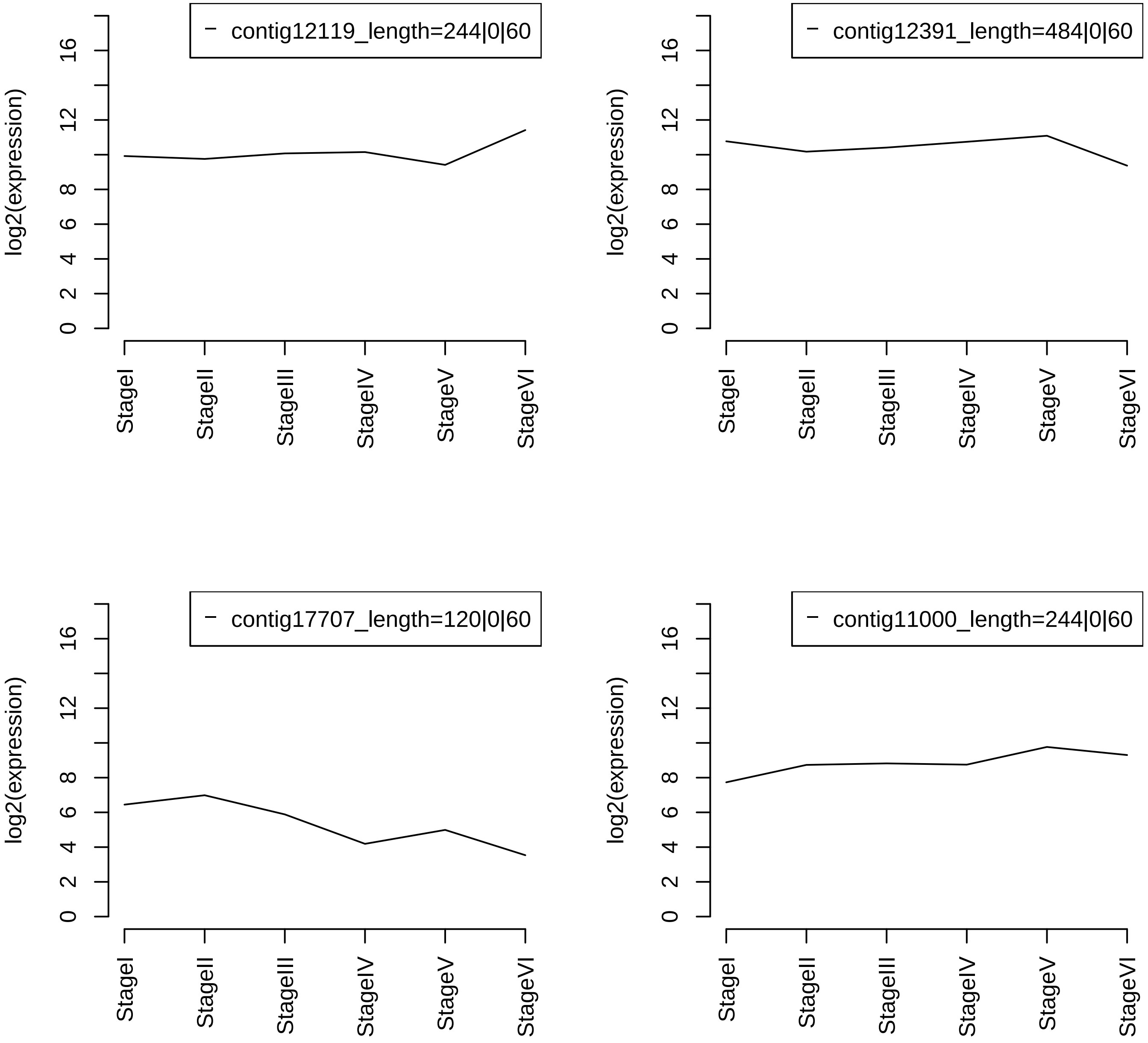
contig12119_length=244|0|60
contig12391_length=484|0|60
contig17707_length=120|0|60
contig11000_length=244|0|60
log2(expression)
0
2
4
6
8
12
16
StageI
StageII
StageIII
StageIV
StageV
StageVI

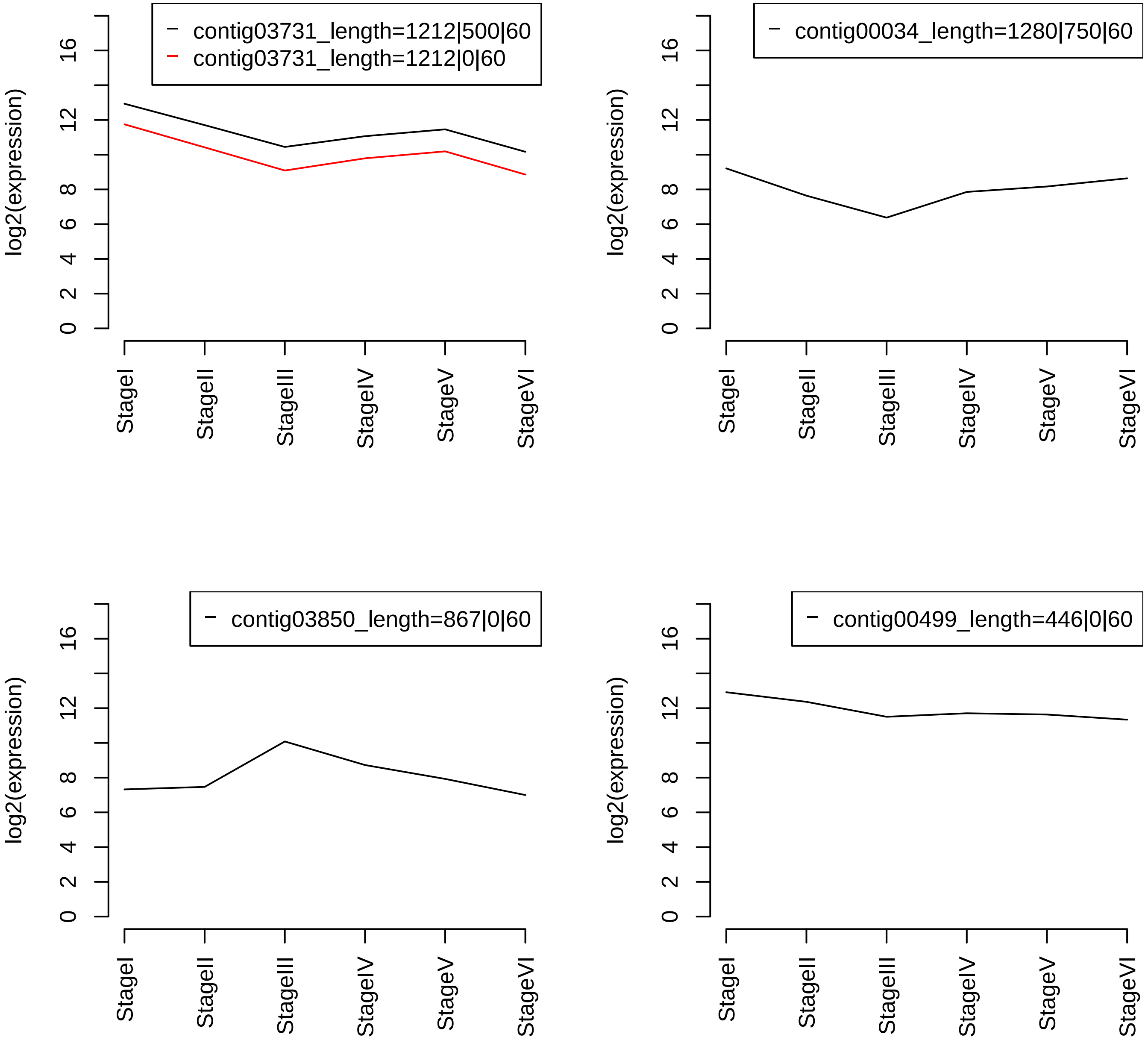
contig03731_length=1212|500|60
contig03731_length=1212|0|60
contig00034_length=1280|750|60
contig03850_length=867|0|60
contig00499_length=446|0|60
log2(expression)
0
2
4
6
8
12
16
StageI
StageII
StageIII
StageIV
StageV
StageVI

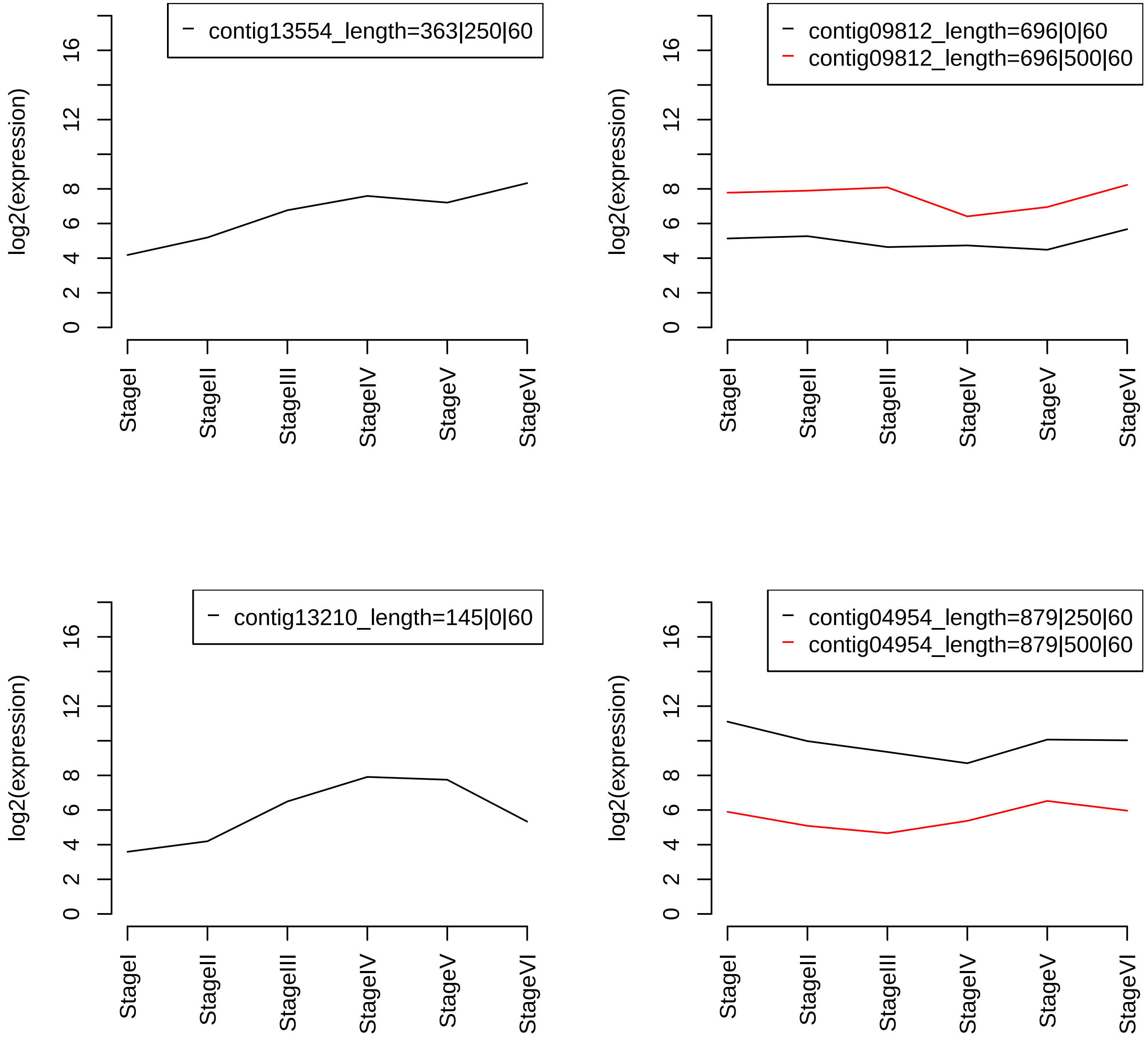
contig13554_length=363|250|60
contig09812_length=696|0|60
contig09812_length=696|500|60
contig13210_length=145|0|60
contig04954_length=879|250|60
contig04954_length=879|500|60
log2(expression)
0
2
4
6
8
12
16
StageI
StageII
StageIII
StageIV
StageV
StageVI

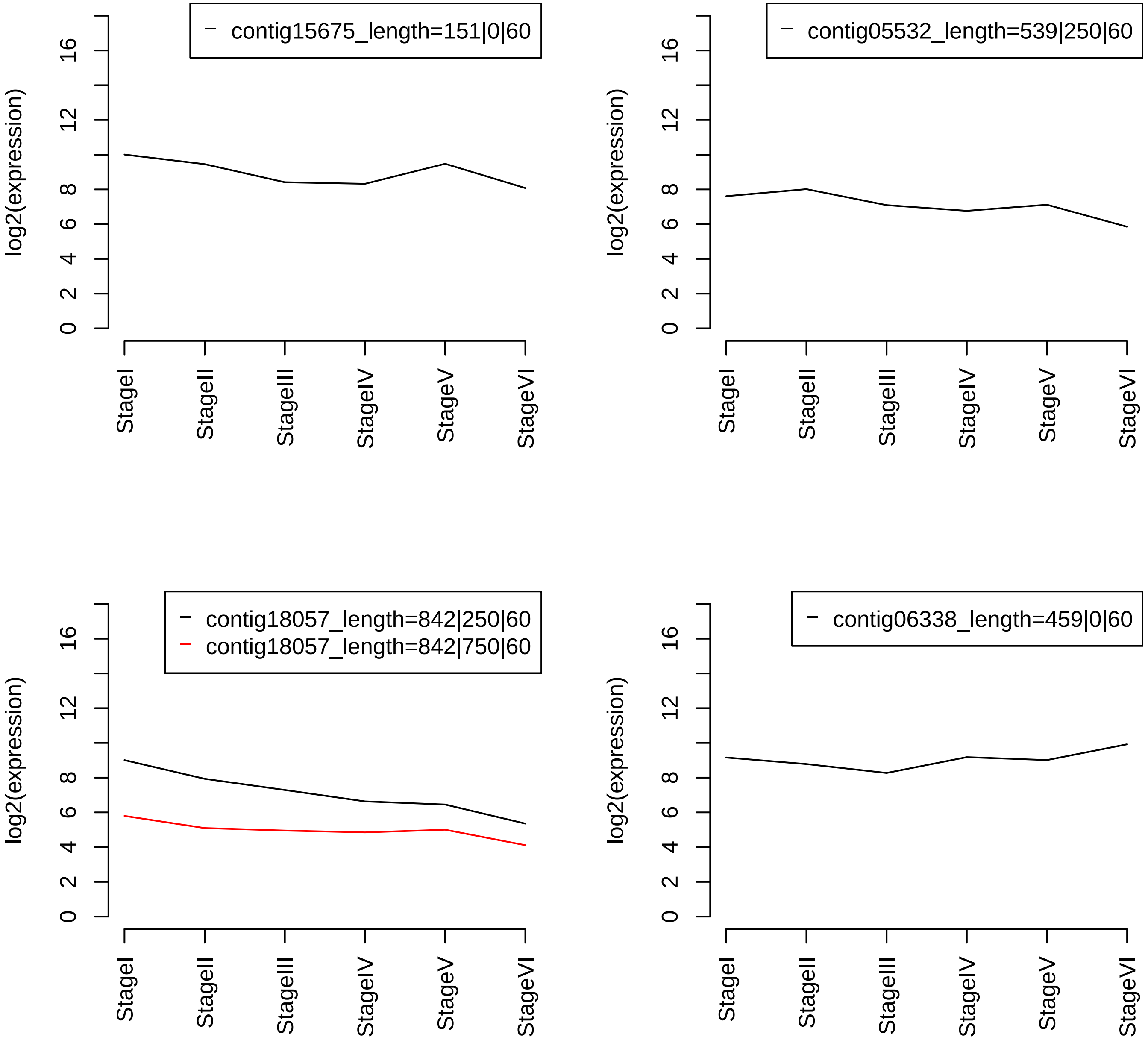
contig15675_length=151|0|60
contig05532_length=539|250|60
contig18057_length=842|250|60
contig18057_length=842|750|60
contig06338_length=459|0|60
log2(expression)
0 2 4 6 8 12 16
StageI
StageII
StageIII
StageIV
StageV
StageVI

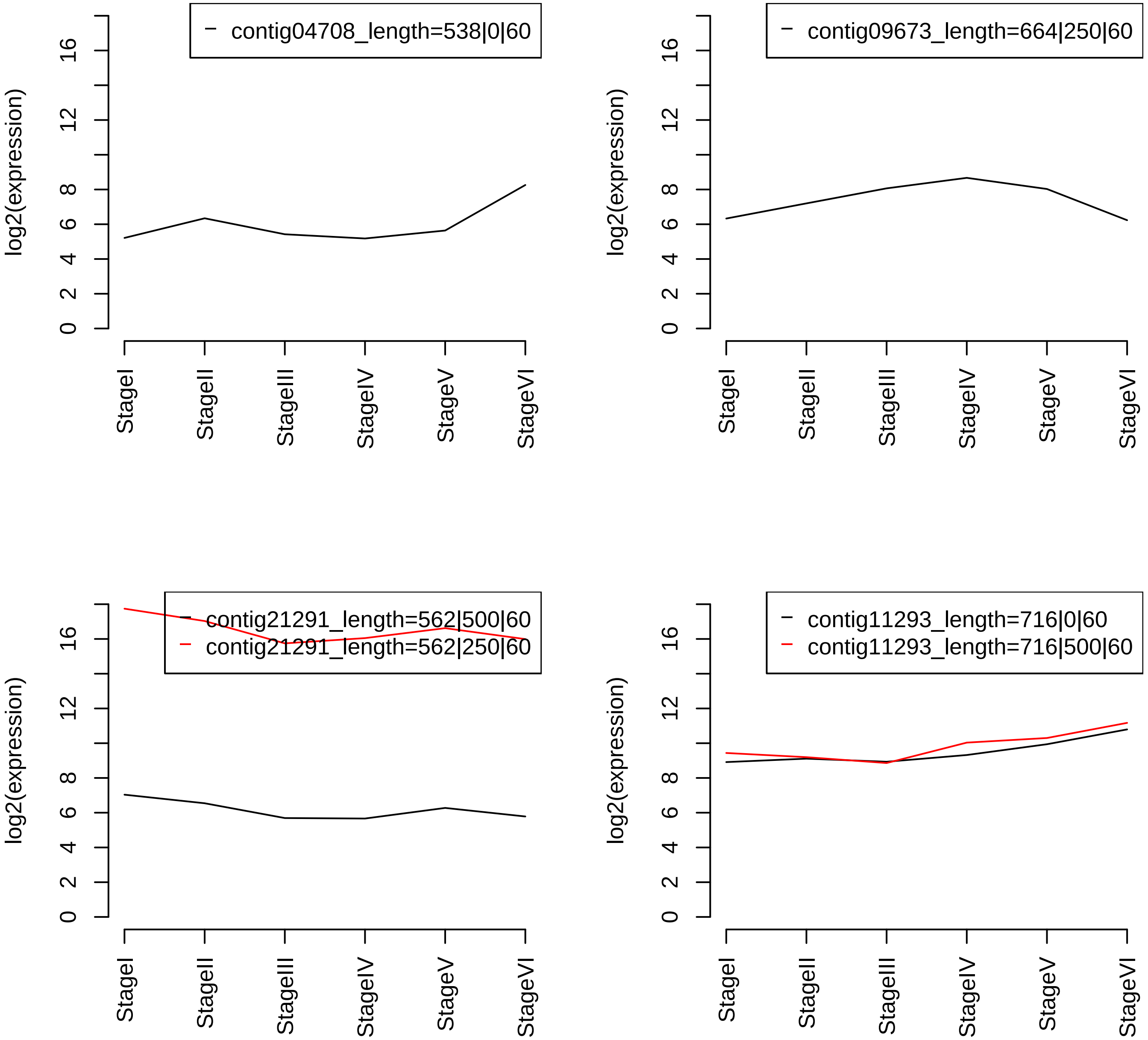
contig04708_length=538|0|60
contig09673_length=664|250|60
contig21291_length=562|500|60
contig21291_length=562|250|60
contig11293_length=716|0|60
contig11293_length=716|500|60
log2(expression)
0
2
4
6
8
12
16
StageI
StageII
StageIII
StageIV
StageV
StageVI

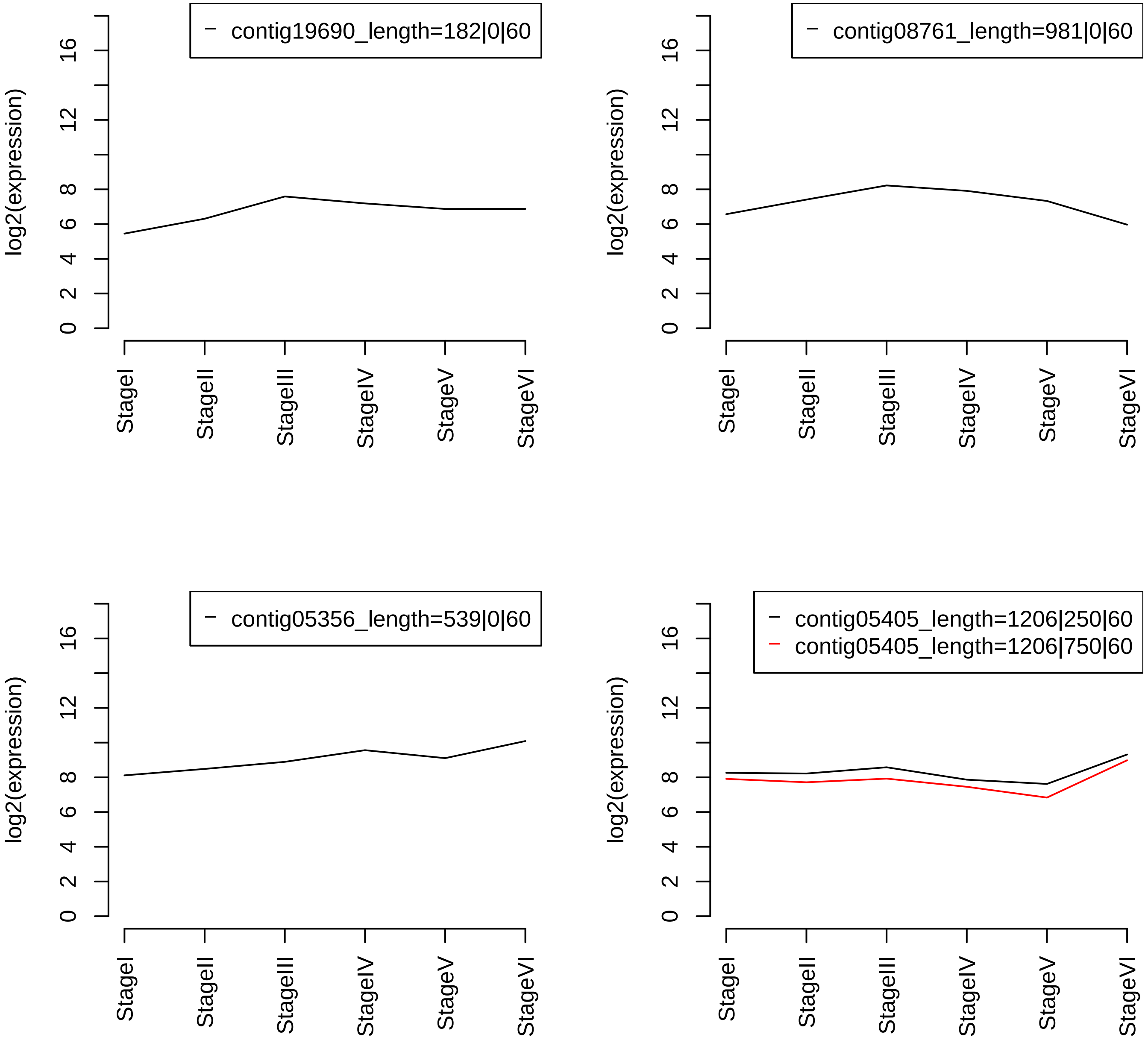

contig19690_length=182|0|60
contig08761_length=981|0|60
contig05356_length=539|0|60
contig05405_length=1206|250|60
contig05405_length=1206|750|60
log2(expression)
0
2
4
6
8
12
16
StageI
StageII
StageIII
StageIV
StageV
StageVI

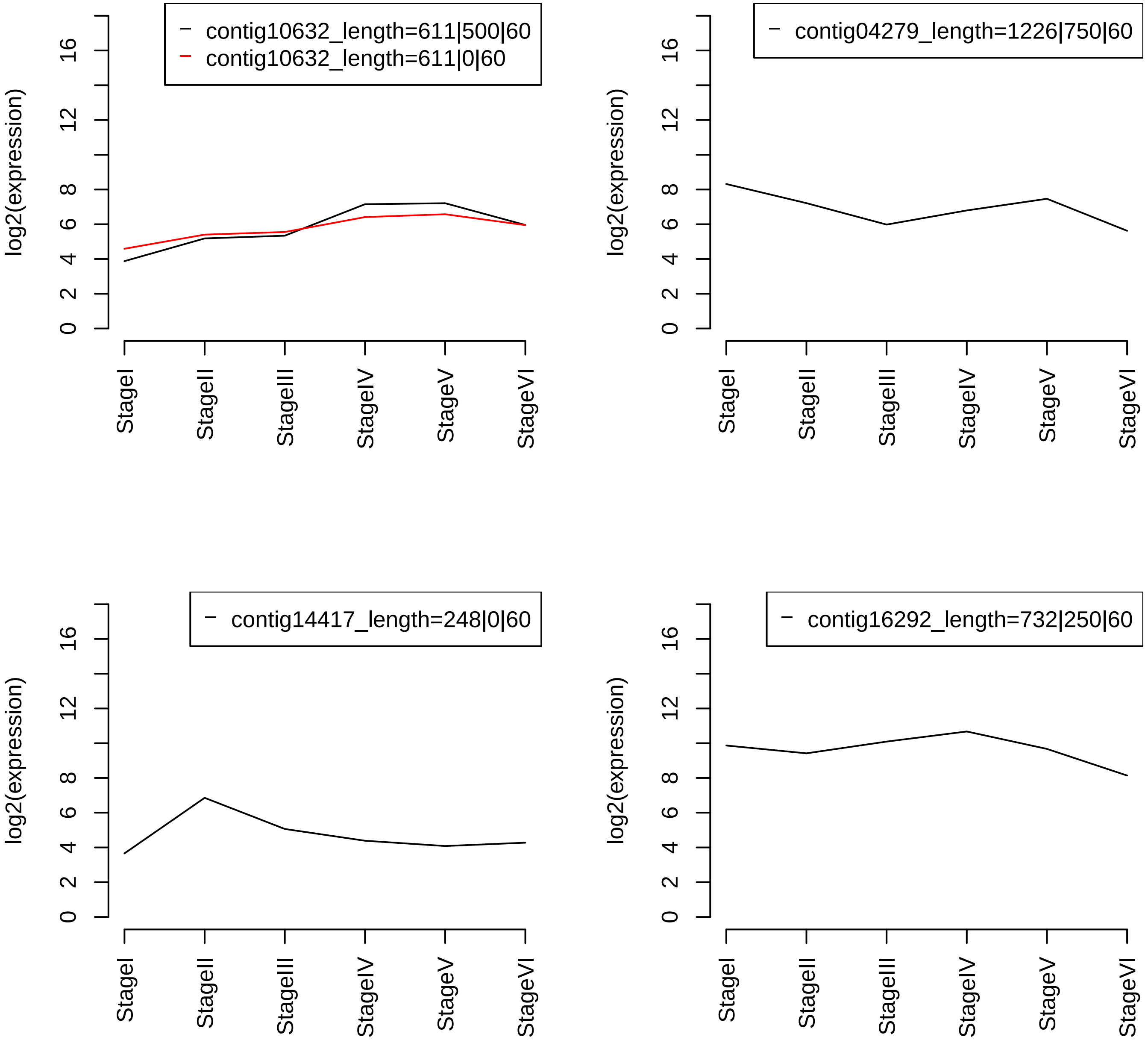

contig10632_length=611|500|60
contig10632_length=611|0|60
contig04279_length=1226|750|60
contig14417_length=248|0|60
contig16292_length=732|250|60
log2(expression)
0
2
4
6
8
12
16
StageI
StageII
StageIII
StageIV
StageV
StageVI

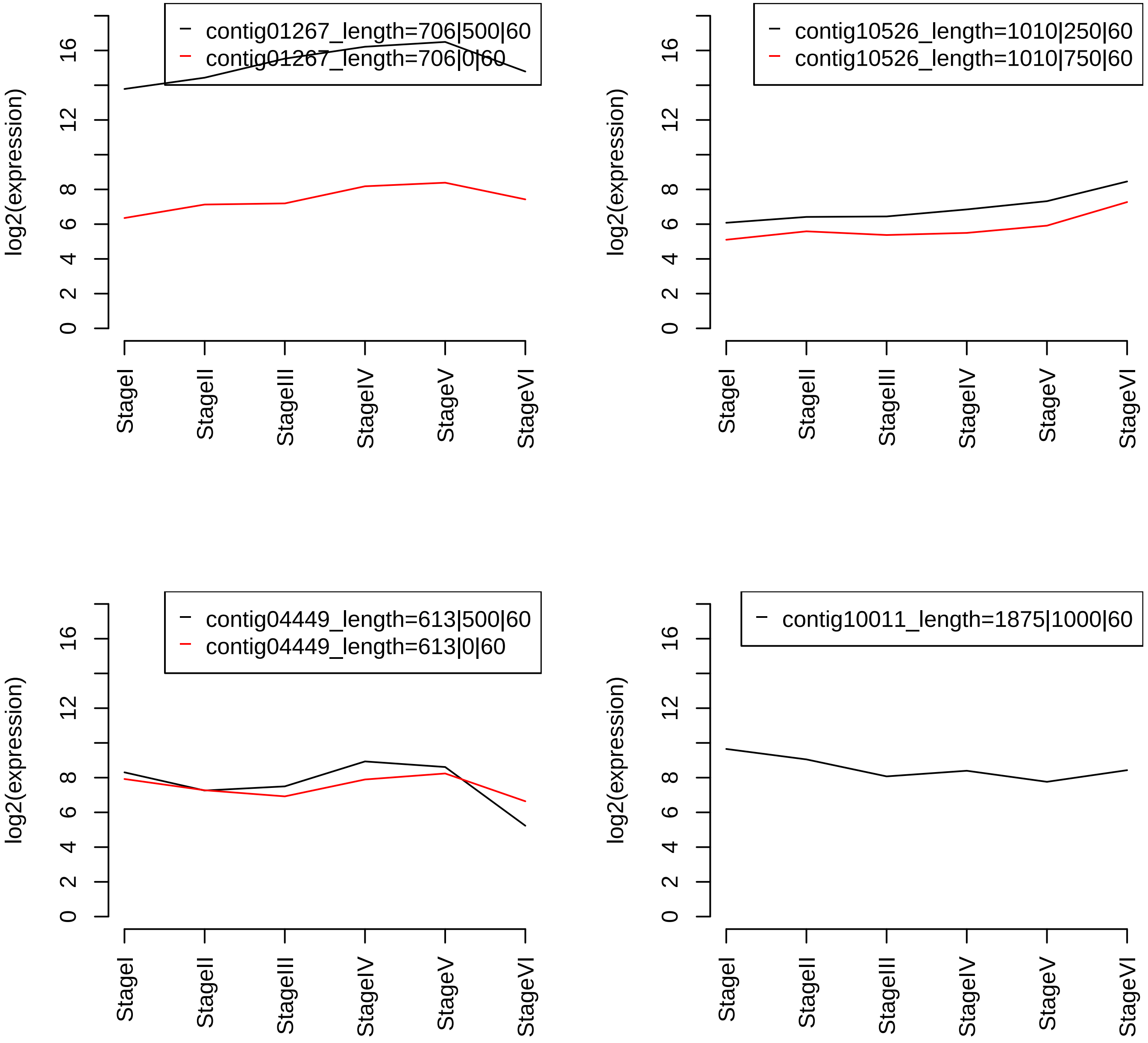
contig01267_length=706|500|60
contig01267_length=706|0|60
contig10526_length=1010|250|60
contig10526_length=1010|750|60
contig04449_length=613|500|60
contig04449_length=613|0|60
contig10011_length=1875|1000|60
log2(expression)
StageI
StageII
StageIII
StageIV
StageV
StageVI

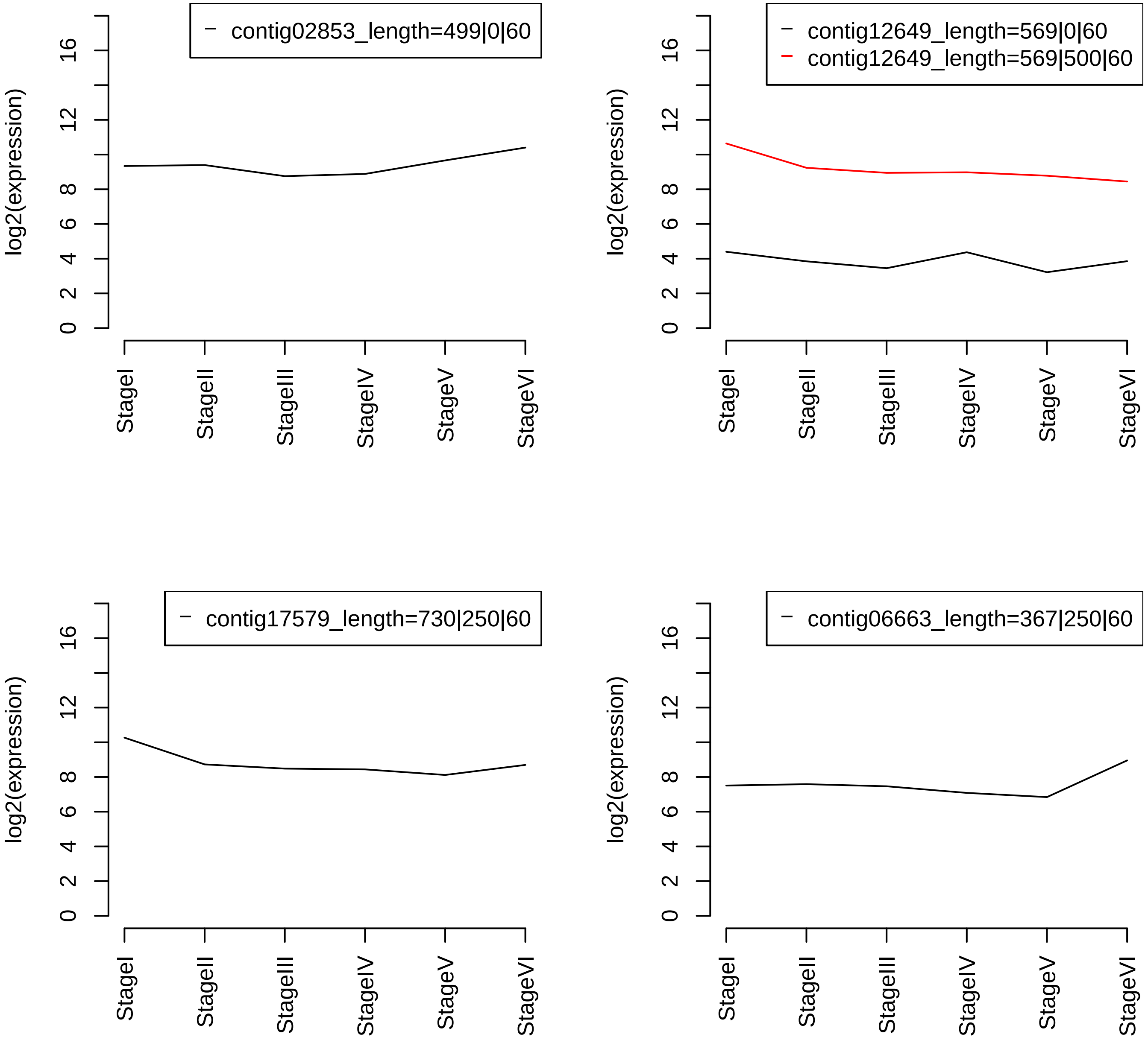
contig02853_length=499|0|60
contig12649_length=569|0|60
contig12649_length=569|500|60
contig17579_length=730|250|60
contig06663_length=367|250|60
log2(expression)
0 2 4 6 8 12 16
StageI StageII StageIII StageIV StageV StageVI

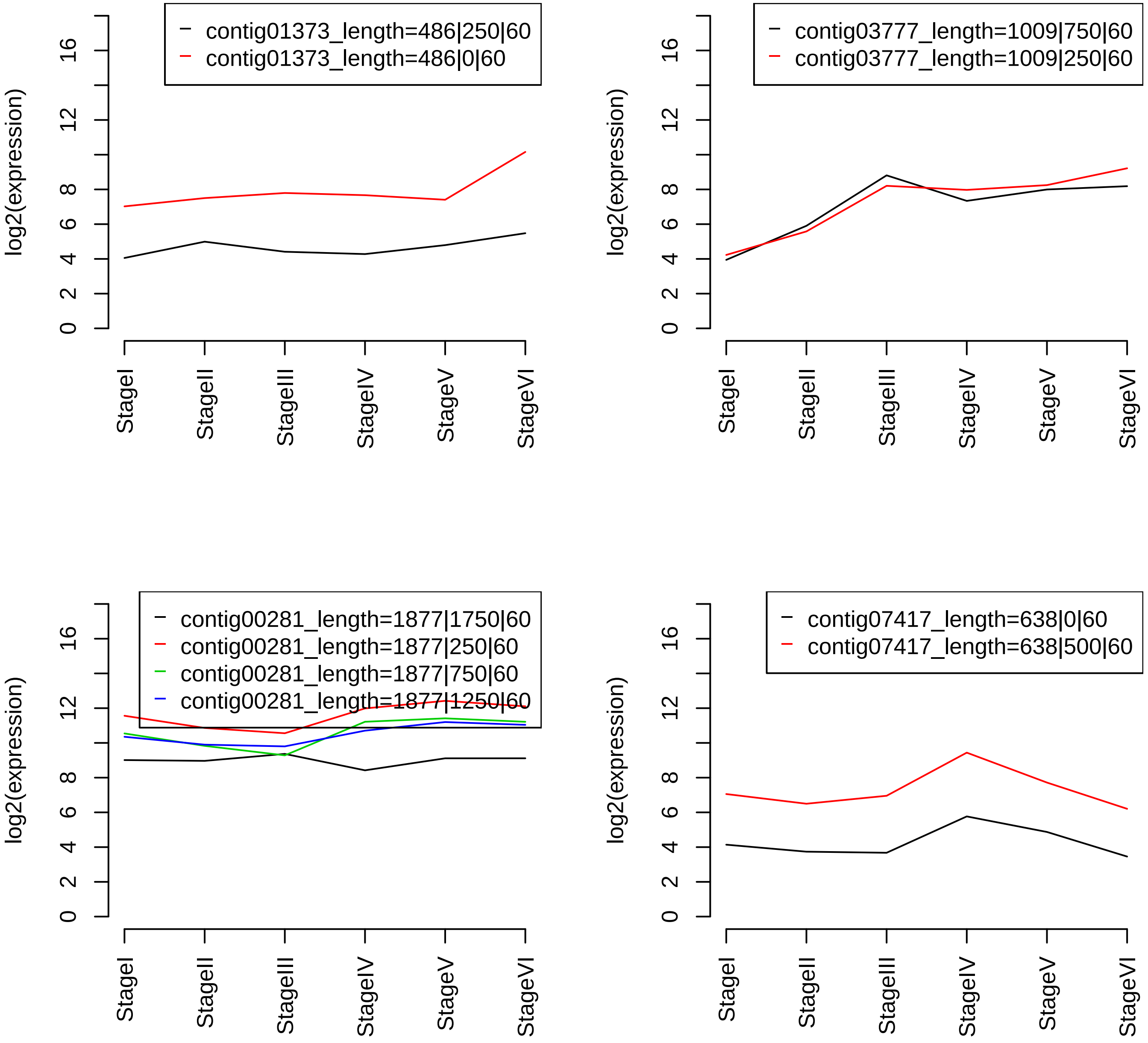
contig01373_length=486|250|60
contig01373_length=486|0|60
contig03777_length=1009|750|60
contig03777_length=1009|250|60
contig00281_length=1877|1750|60
contig00281_length=1877|250|60
contig00281_length=1877|750|60
contig00281_length=1877|1250|60
contig07417_length=638|0|60
contig07417_length=638|500|60
log2(expression)
0 2 4 6 8 12 16
StageI StageII StageIII StageIV StageV StageVI

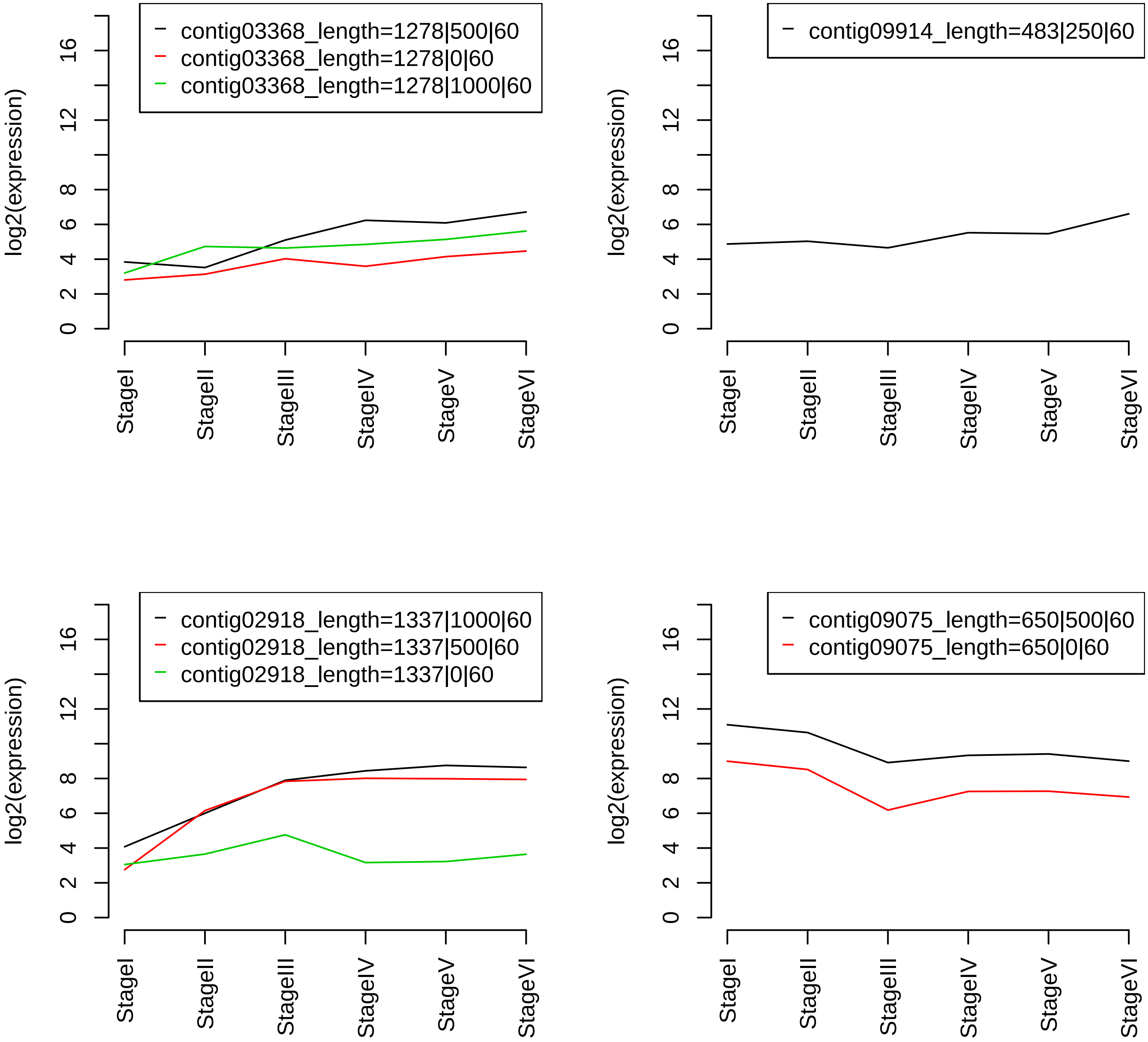

contig03368_length=1278|500|60
contig03368_length=1278|0|60
contig03368_length=1278|1000|60
contig09914_length=483|250|60
contig02918_length=1337|1000|60
contig02918_length=1337|500|60
contig02918_length=1337|0|60
contig09075_length=650|500|60
contig09075_length=650|0|60
log2(expression)
0
2
4
6
8
12
16
StageI
StageII
StageIII
StageIV
StageV
StageVI

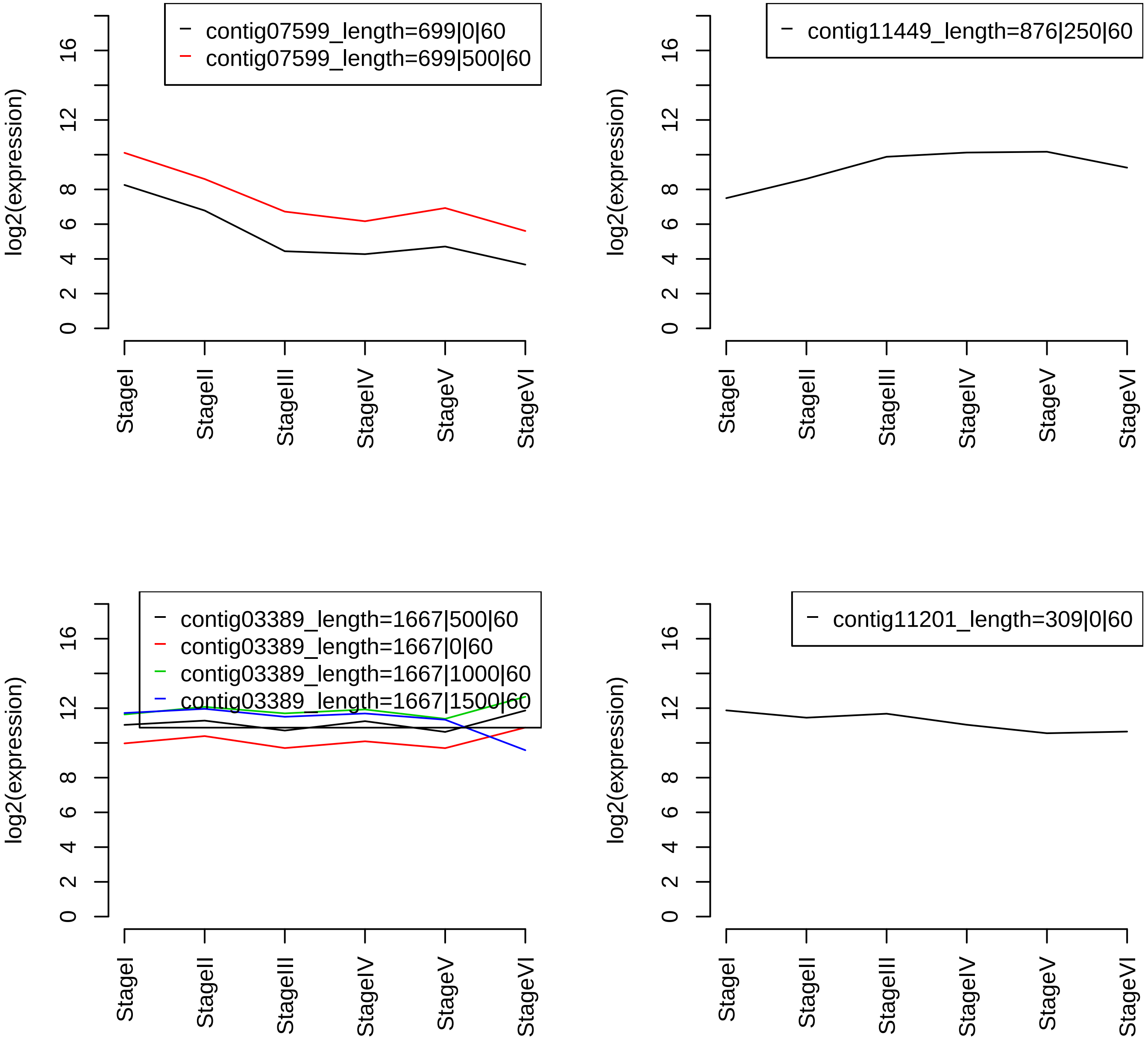
contig07599_length=699|0|60
contig07599_length=699|500|60
contig11449_length=876|250|60
contig03389_length=1667|500|60
contig03389_length=1667|0|60
contig03389_length=1667|1000|60
contig03389_length=1667|1500|60
contig11201_length=309|0|60
log2(expression)
0
2
4
6
8
12
16
StageI
StageII
StageIII
StageIV
StageV
StageVI

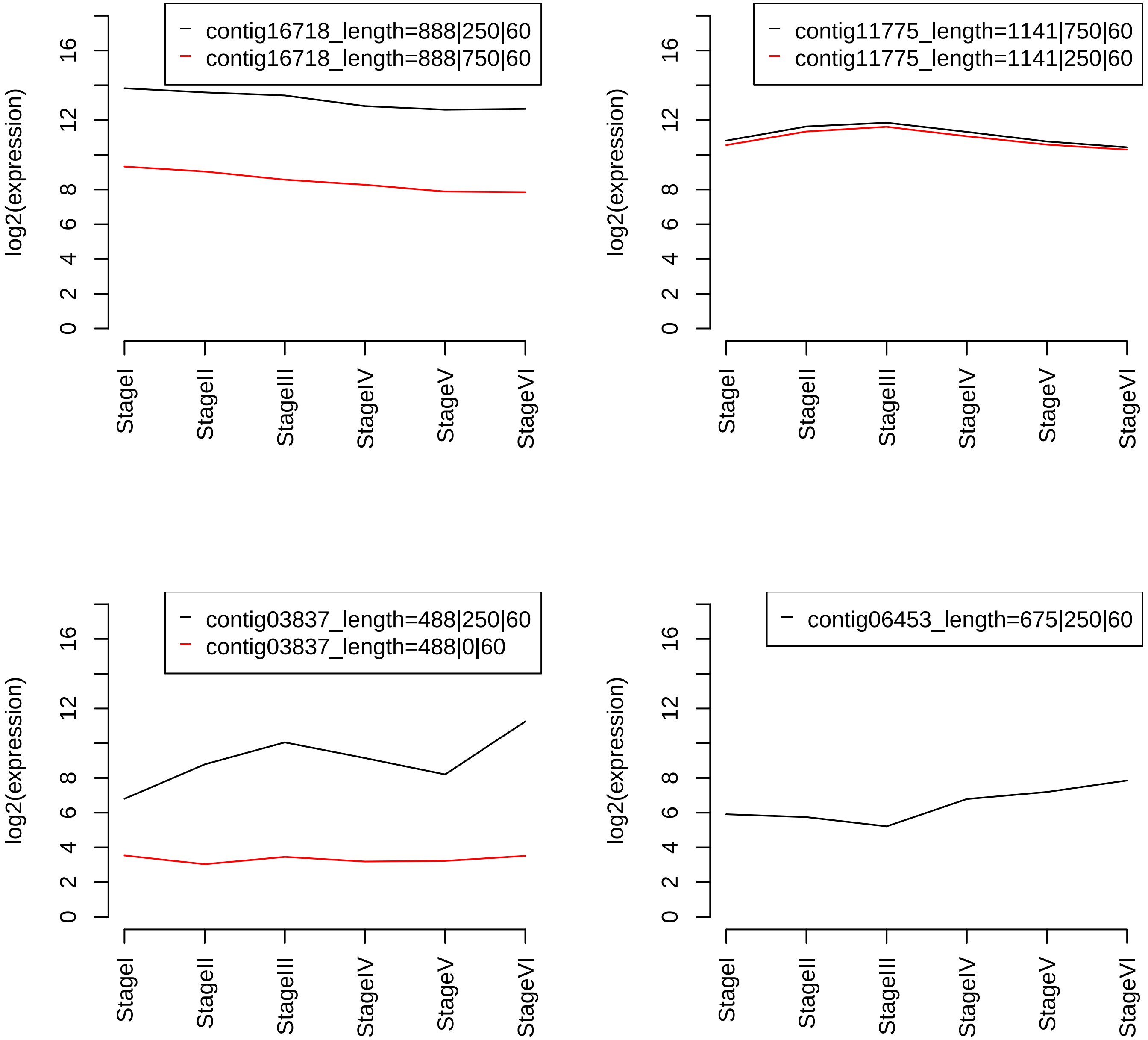
contig16718_length=888|250|60
contig16718_length=888|750|60
contig11775_length=1141|750|60
contig11775_length=1141|250|60
contig03837_length=488|250|60
contig03837_length=488|0|60
contig06453_length=675|250|60
log2(expression)
0
2
4
6
8
12
16
StageI
StageII
StageIII
StageIV
StageV
StageVI

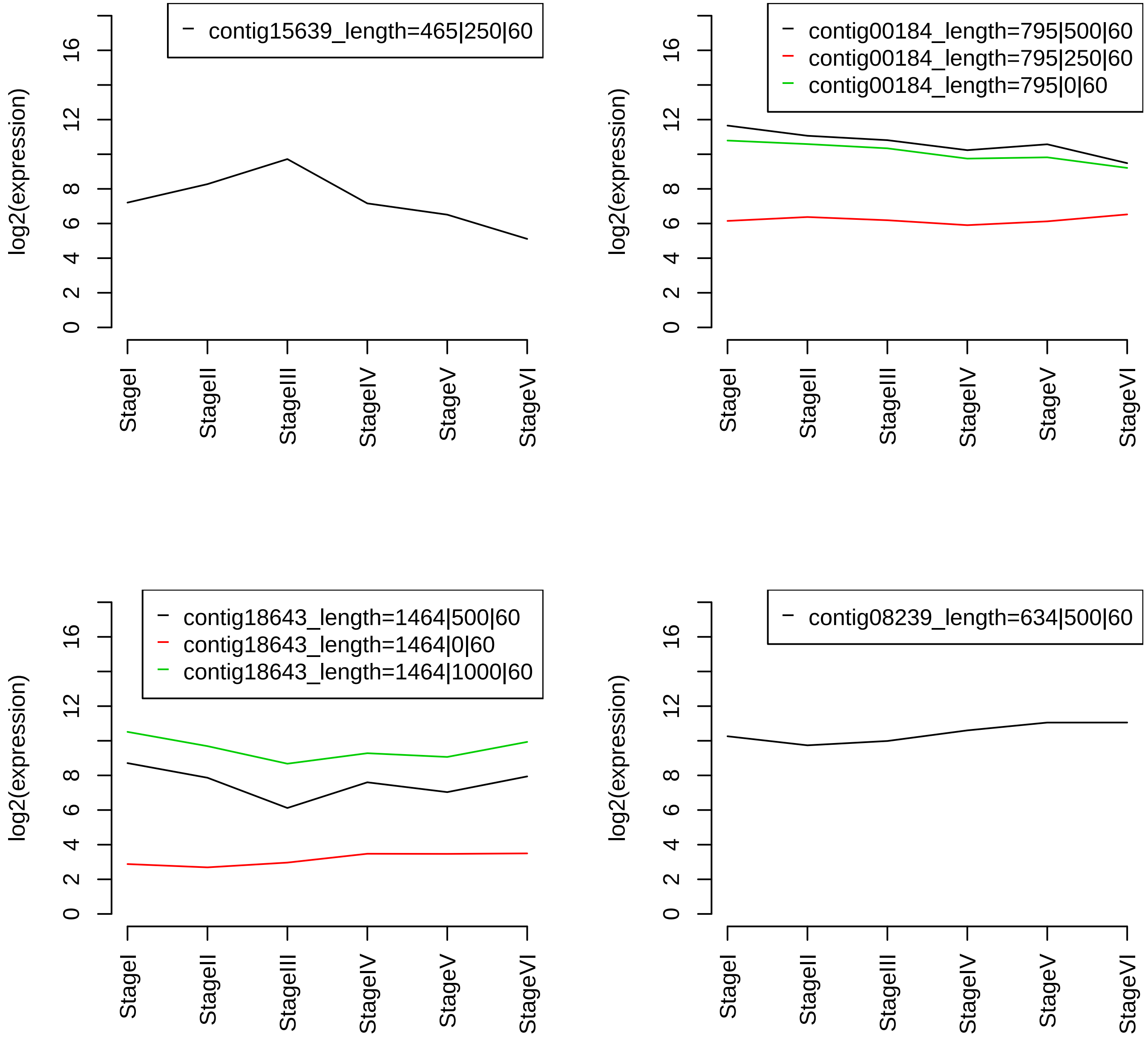

contig15639_length=465|250|60
contig00184_length=795|500|60
contig00184_length=795|250|60
contig00184_length=795|0|60
contig18643_length=1464|500|60
contig18643_length=1464|0|60
contig18643_length=1464|1000|60
contig08239_length=634|500|60
log2(expression)
0 2 4 6 8 12 16
StageI StageII StageIII StageIV StageV StageVI

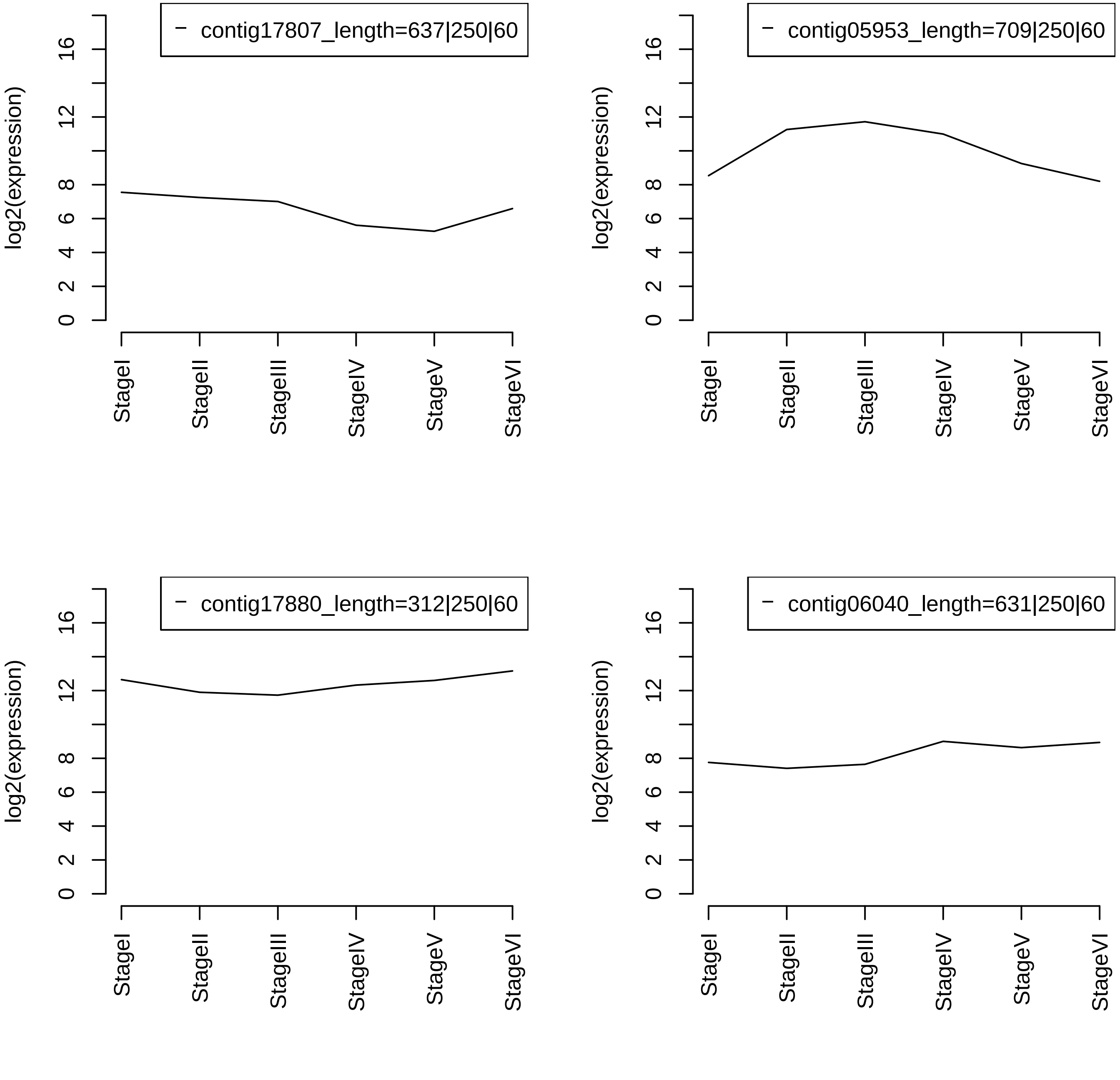
contig17807_length=637|250|60
contig05953_length=709|250|60
contig17880_length=312|250|60
contig06040_length=631|250|60
log2(expression)
0
2
4
6
8
12
16
StageI
StageII
StageIII
StageIV
StageV
StageVI

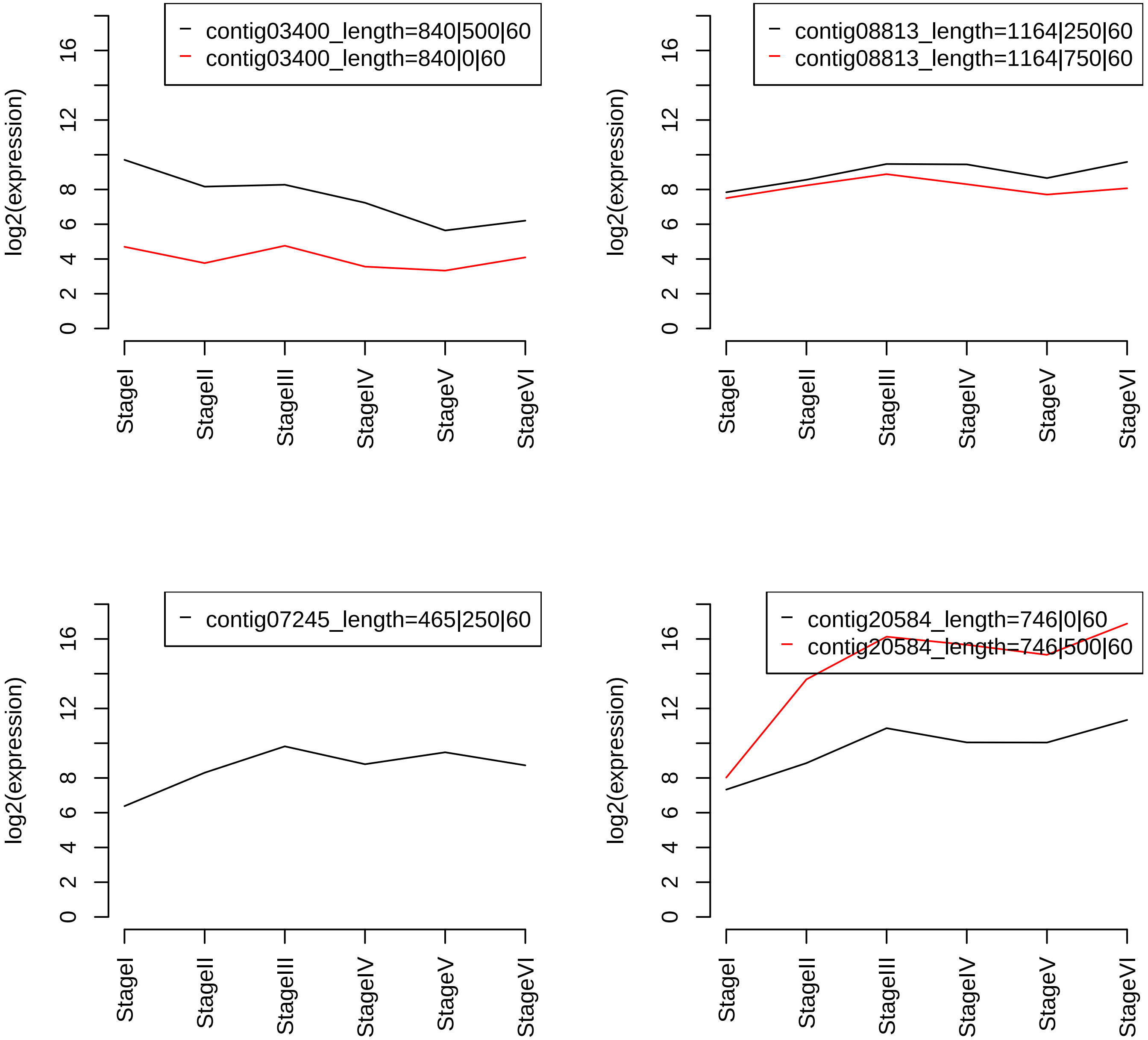

contig03400_length=840|500|60
contig03400_length=840|0|60
contig08813_length=1164|250|60
contig08813_length=1164|750|60
contig07245_length=465|250|60
contig20584_length=746|0|60
contig20584_length=746|500|60
log2(expression)
0
2
4
6
8
12
16
StageI
StageII
StageIII
StageIV
StageV
StageVI

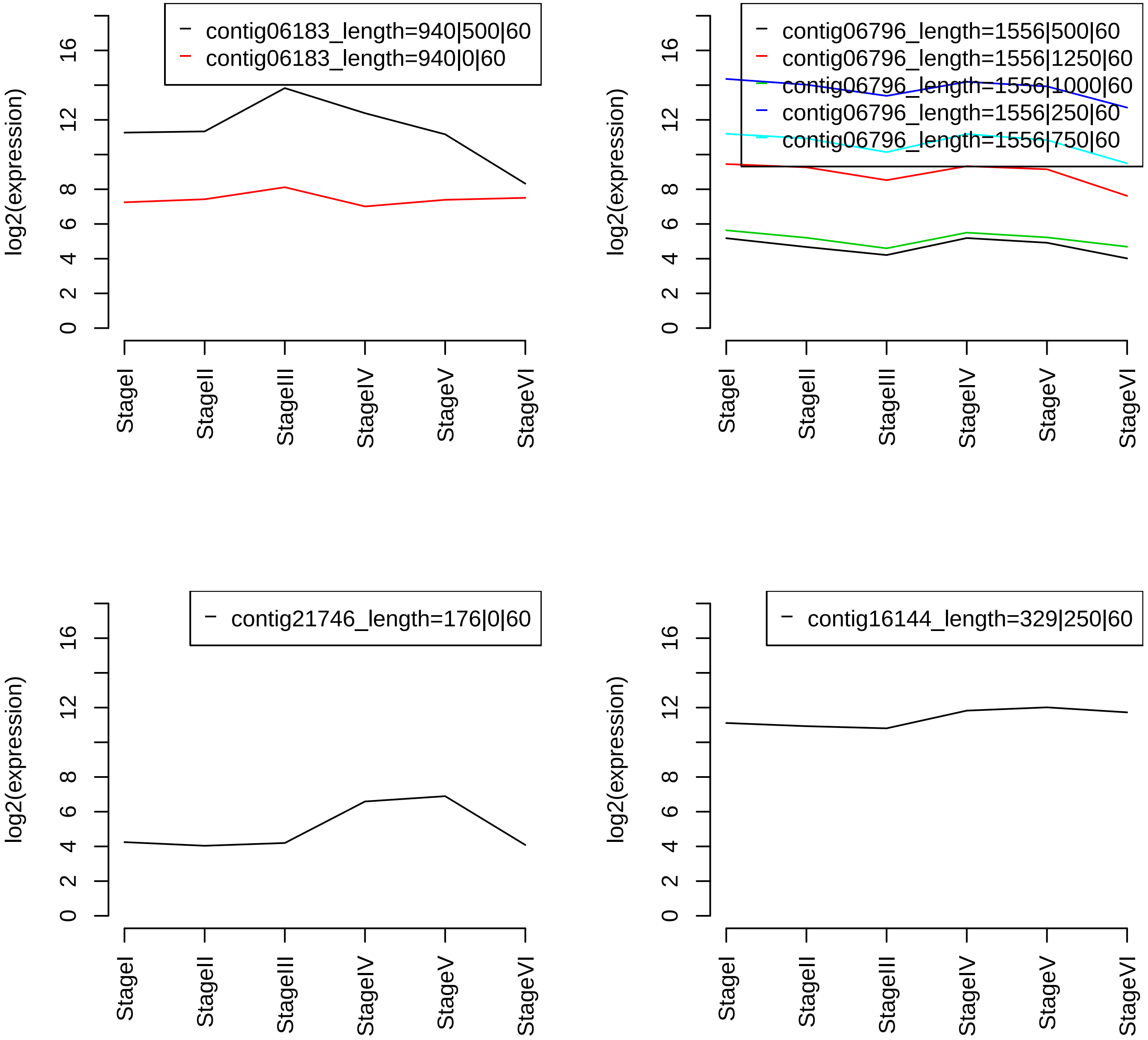

contig06183_length=940|500|60
contig06183_length=940|0|60
contig06796_length=1556|500|60
contig06796_length=1556|1250|60
contig06796_length=1556|1000|60
contig06796_length=1556|250|60
contig06796_length=1556|750|60
contig21746_length=176|0|60
contig16144_length=329|250|60
log2(expression)
0
2
4
6
8
12
16
StageI
StageII
StageIII
StageIV
StageV
StageVI

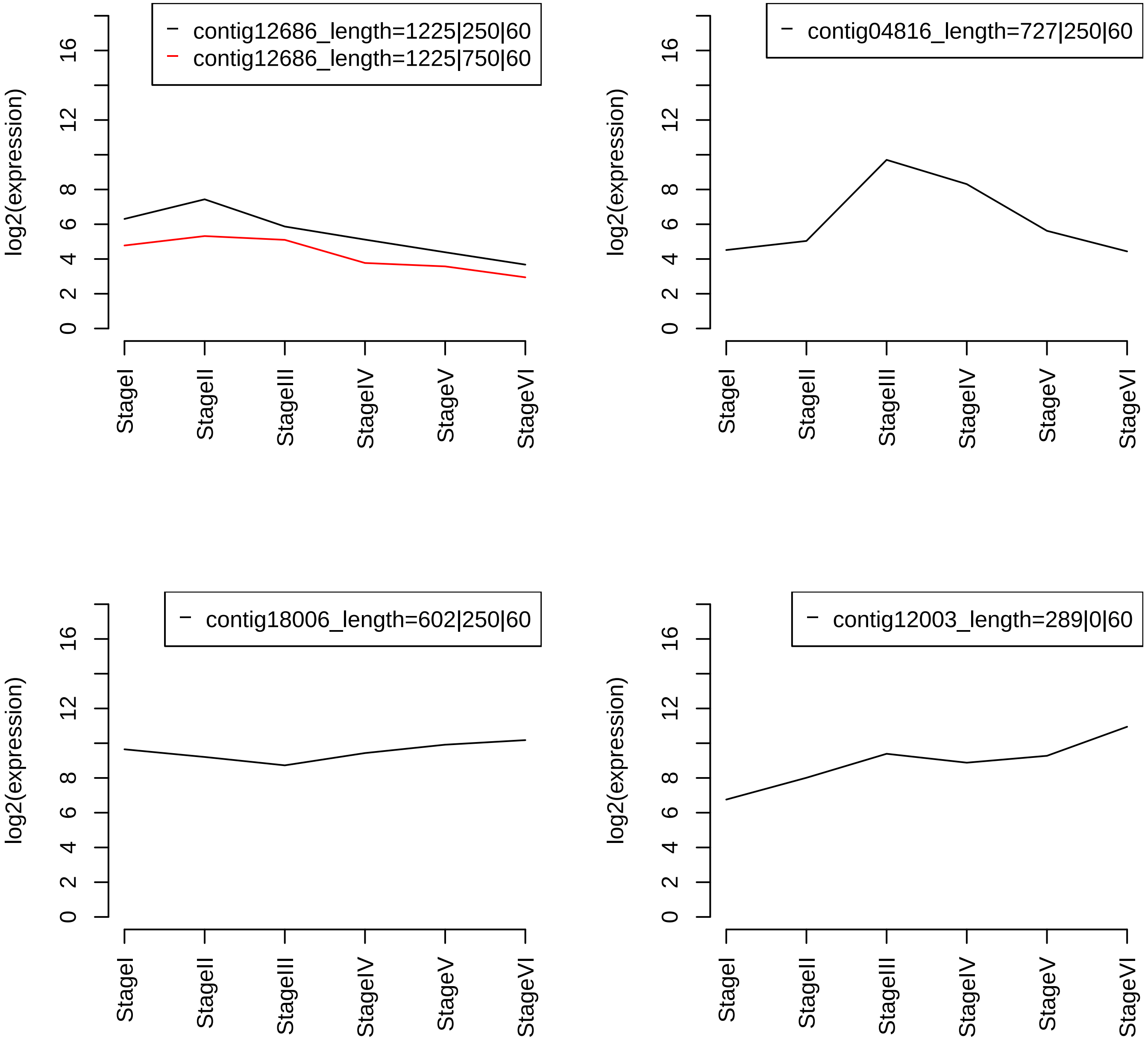
contig12686_length=1225|250|60
contig12686_length=1225|750|60
contig04816_length=727|250|60
contig18006_length=602|250|60
contig12003_length=289|0|60
log2(expression)
0
2
4
6
8
12
16
StageI
StageII
StageIII
StageIV
StageV
StageVI

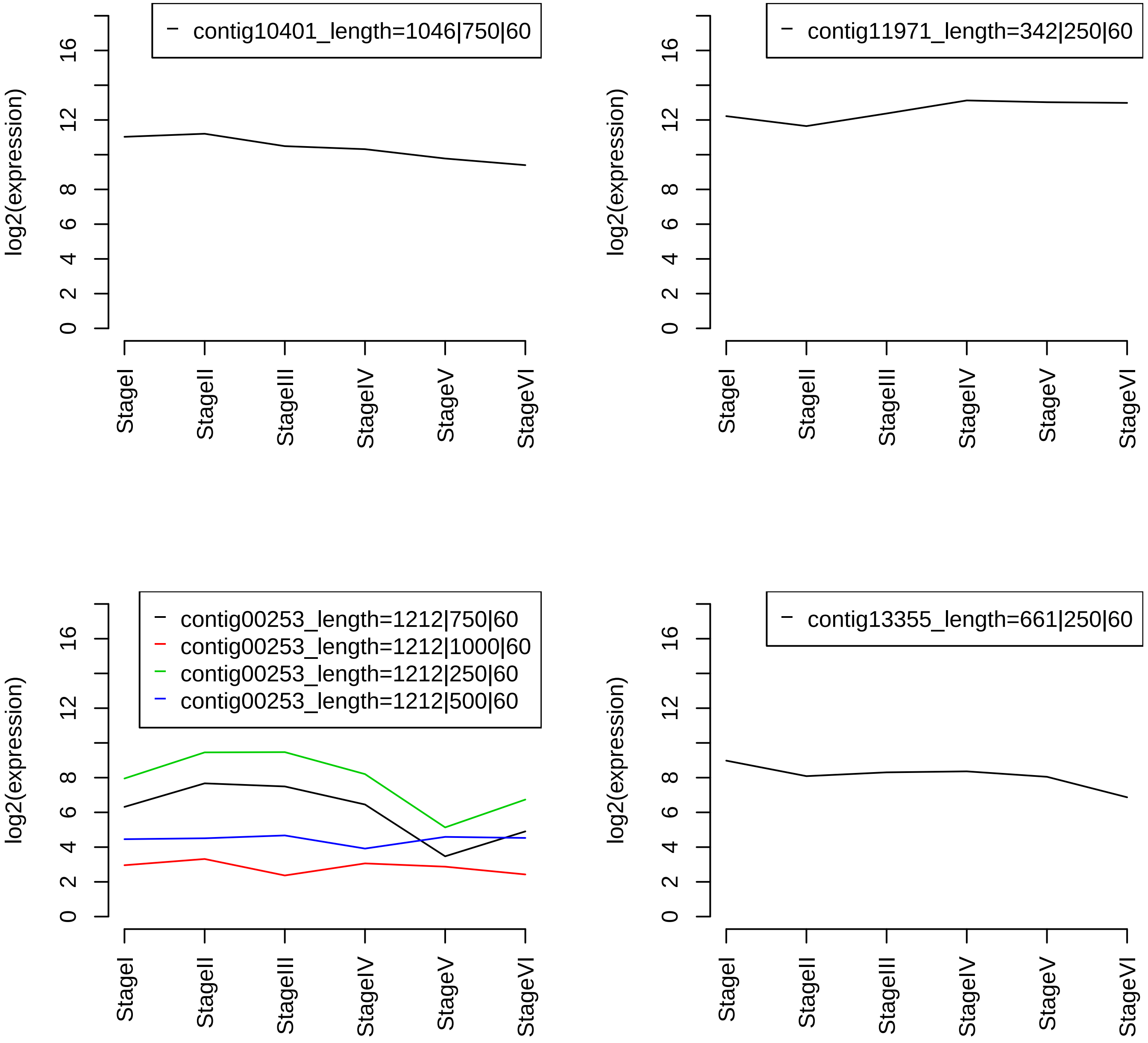

contig10401_length=1046|750|60
contig11971_length=342|250|60
contig00253_length=1212|750|60
contig00253_length=1212|1000|60
contig00253_length=1212|250|60
contig00253_length=1212|500|60
contig13355_length=661|250|60
log2(expression)
0
2
4
6
8
12
16
StageI
StageII
StageIII
StageIV
StageV
StageVI

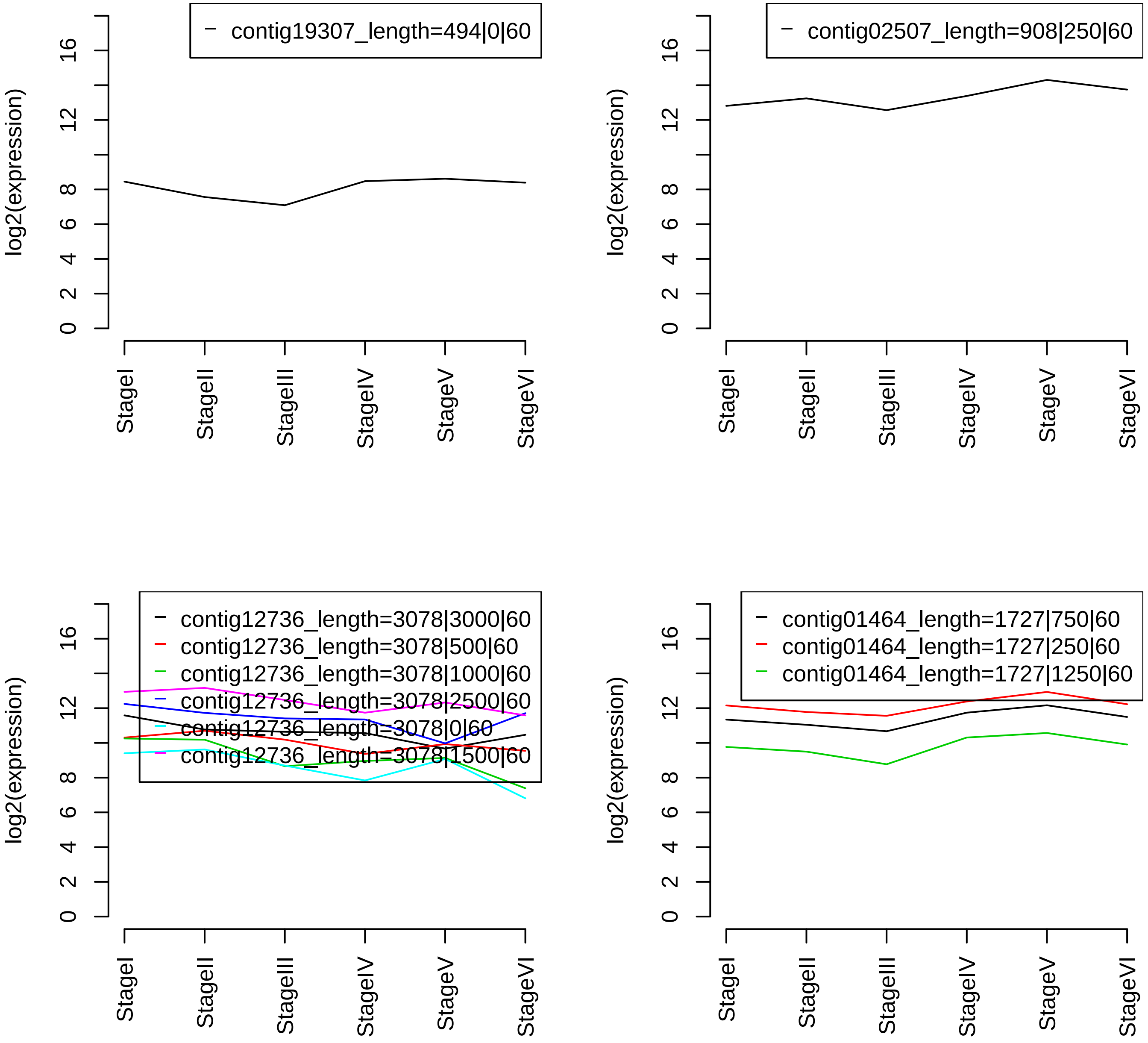
contig19307_length=494|0|60
contig02507_length=908|250|60
contig12736_length=3078|3000|60
contig12736_length=3078|500|60
contig12736_length=3078|1000|60
contig12736_length=3078|2500|60
contig12736_length=3078|0|60
contig12736_length=3078|1500|60
contig01464_length=1727|750|60
contig01464_length=1727|250|60
contig01464_length=1727|1250|60
log2(expression)
0
2
4
6
8
12
16
StageI
StageII
StageIII
StageIV
StageV
StageVI

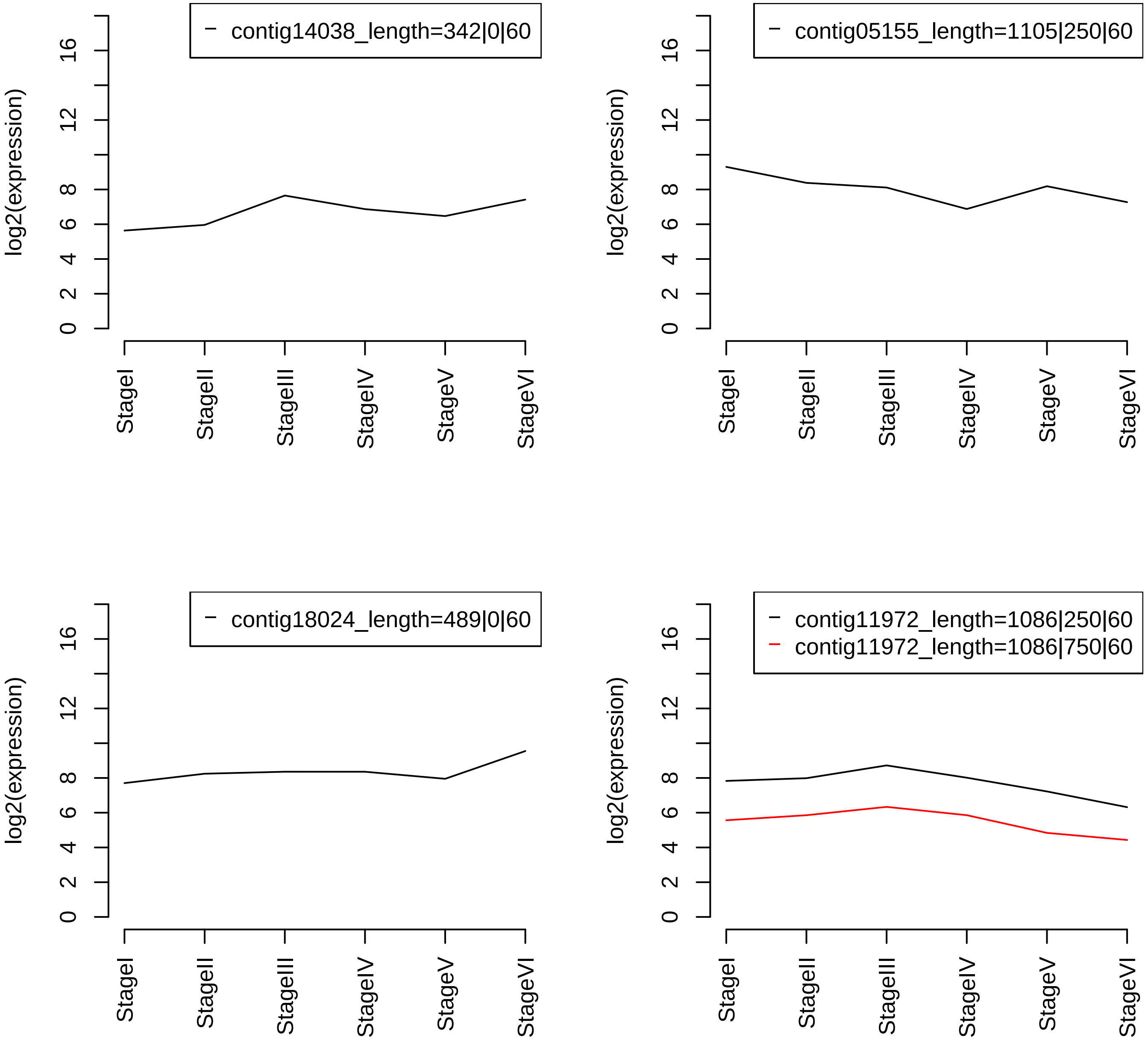

contig14038_length=342|0|60
contig05155_length=1105|250|60
contig18024_length=489|0|60
contig11972_length=1086|250|60
contig11972_length=1086|750|60
log2(expression)
0
2
4
6
8
12
16
StageI
StageII
StageIII
StageIV
StageV
StageVI

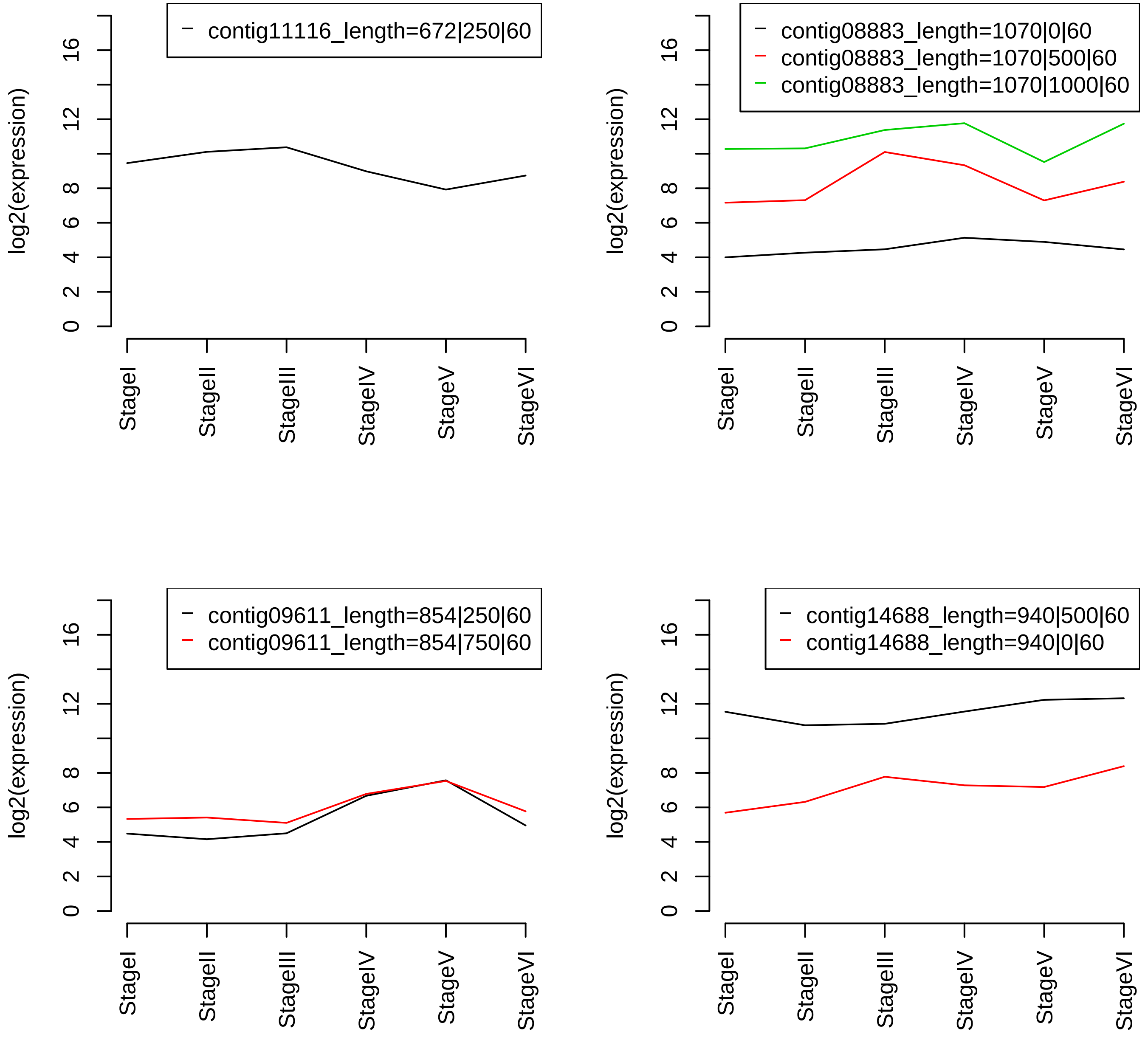

contig11116_length=672|250|60
contig08883_length=1070|0|60
contig08883_length=1070|500|60
contig08883_length=1070|1000|60
contig09611_length=854|250|60
contig09611_length=854|750|60
contig14688_length=940|500|60
contig14688_length=940|0|60
log2(expression)
0 2 4 6 8 12 16
StageI StageII StageIII StageIV StageV StageVI

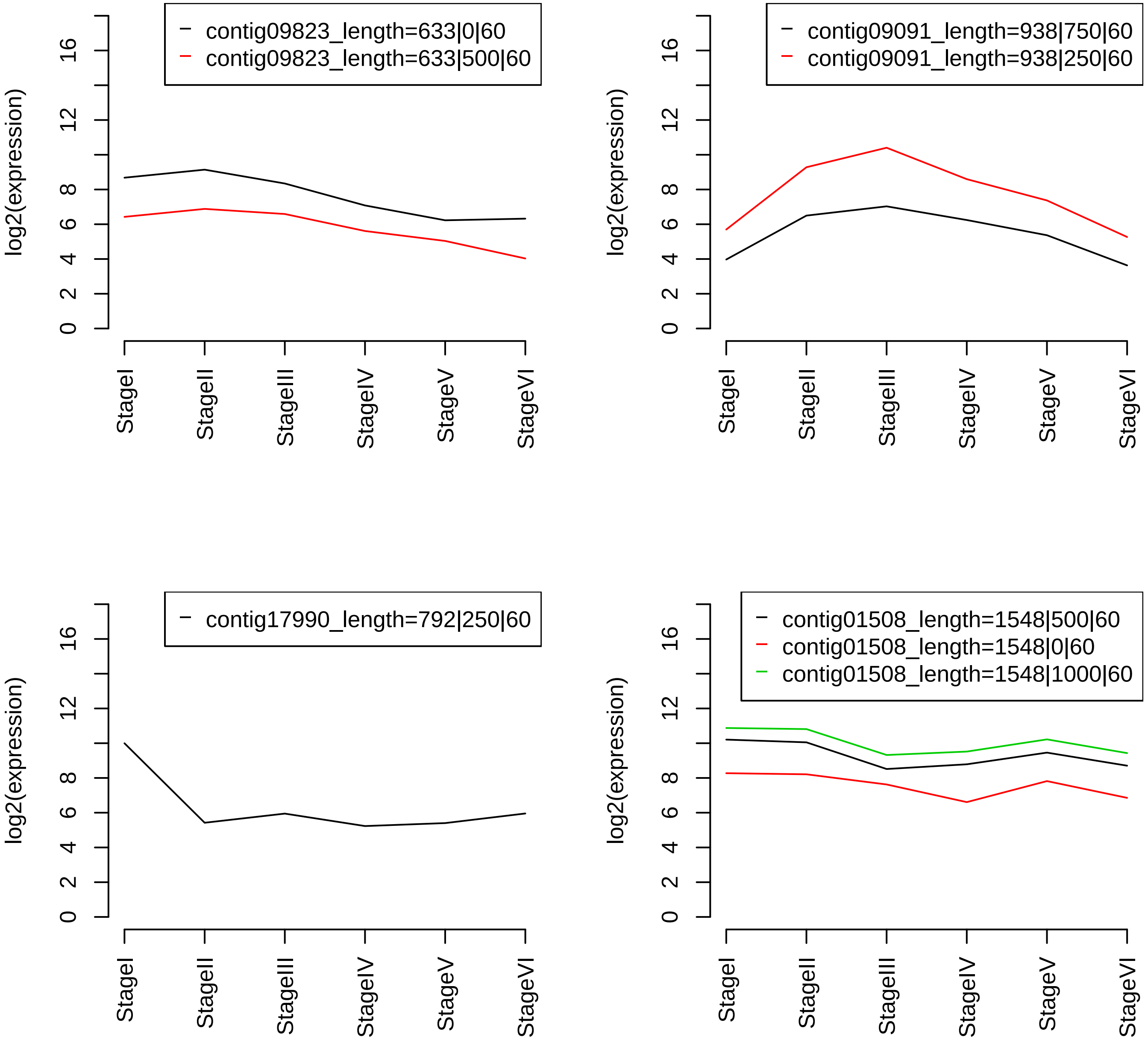

contig09823_length=633|0|60
contig09823_length=633|500|60
contig09091_length=938|750|60
contig09091_length=938|250|60
contig17990_length=792|250|60
contig01508_length=1548|500|60
contig01508_length=1548|0|60
contig01508_length=1548|1000|60
log2(expression)
0
2
4
6
8
12
16
StageI
StageII
StageIII
StageIV
StageV
StageVI

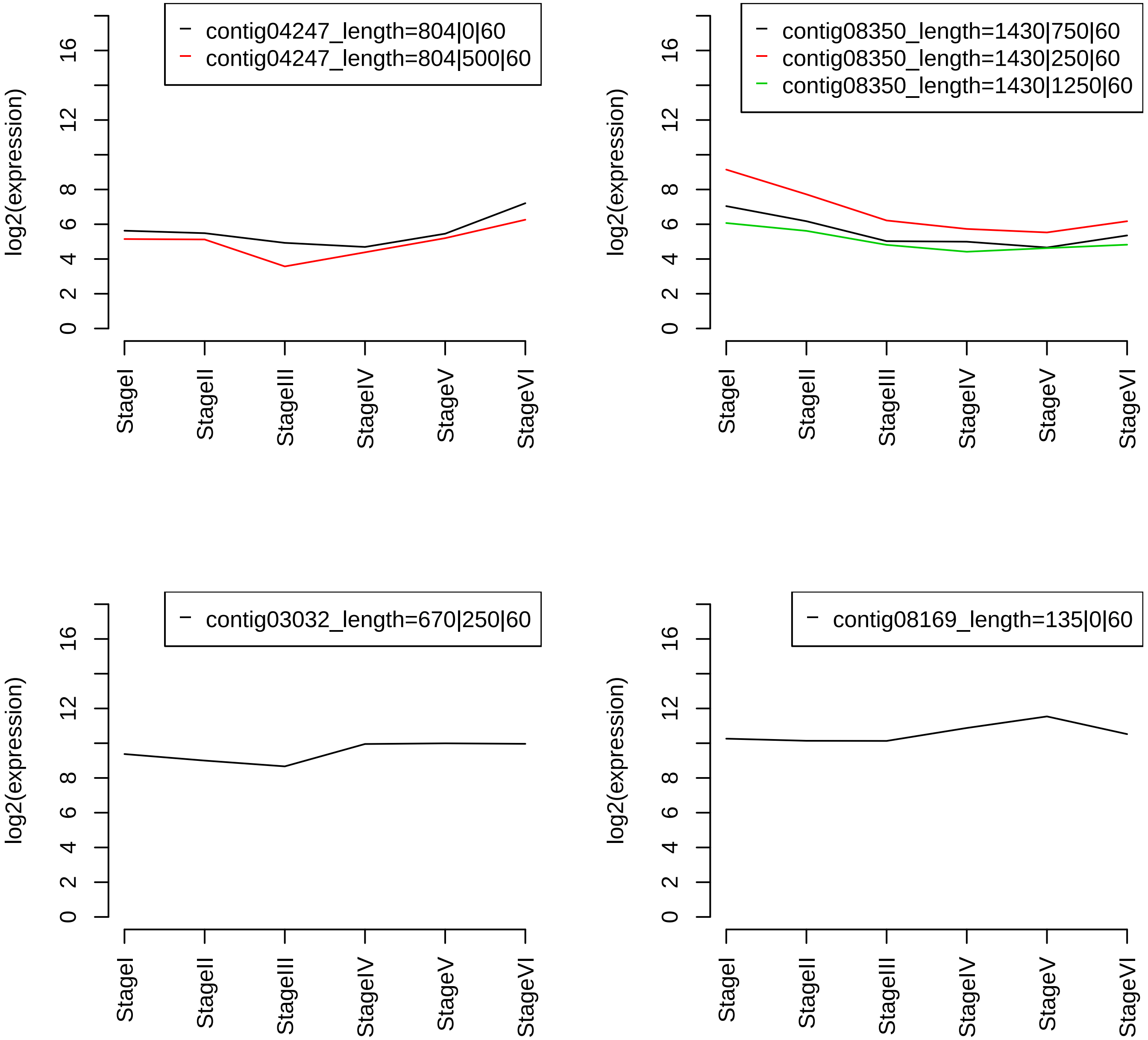
contig04247_length=804|0|60
contig04247_length=804|500|60
contig08350_length=1430|750|60
contig08350_length=1430|250|60
contig08350_length=1430|1250|60
contig03032_length=670|250|60
contig08169_length=135|0|60
log2(expression)
0 2 4 6 8 12 16
StageI
StageII
StageIII
StageIV
StageV
StageVI

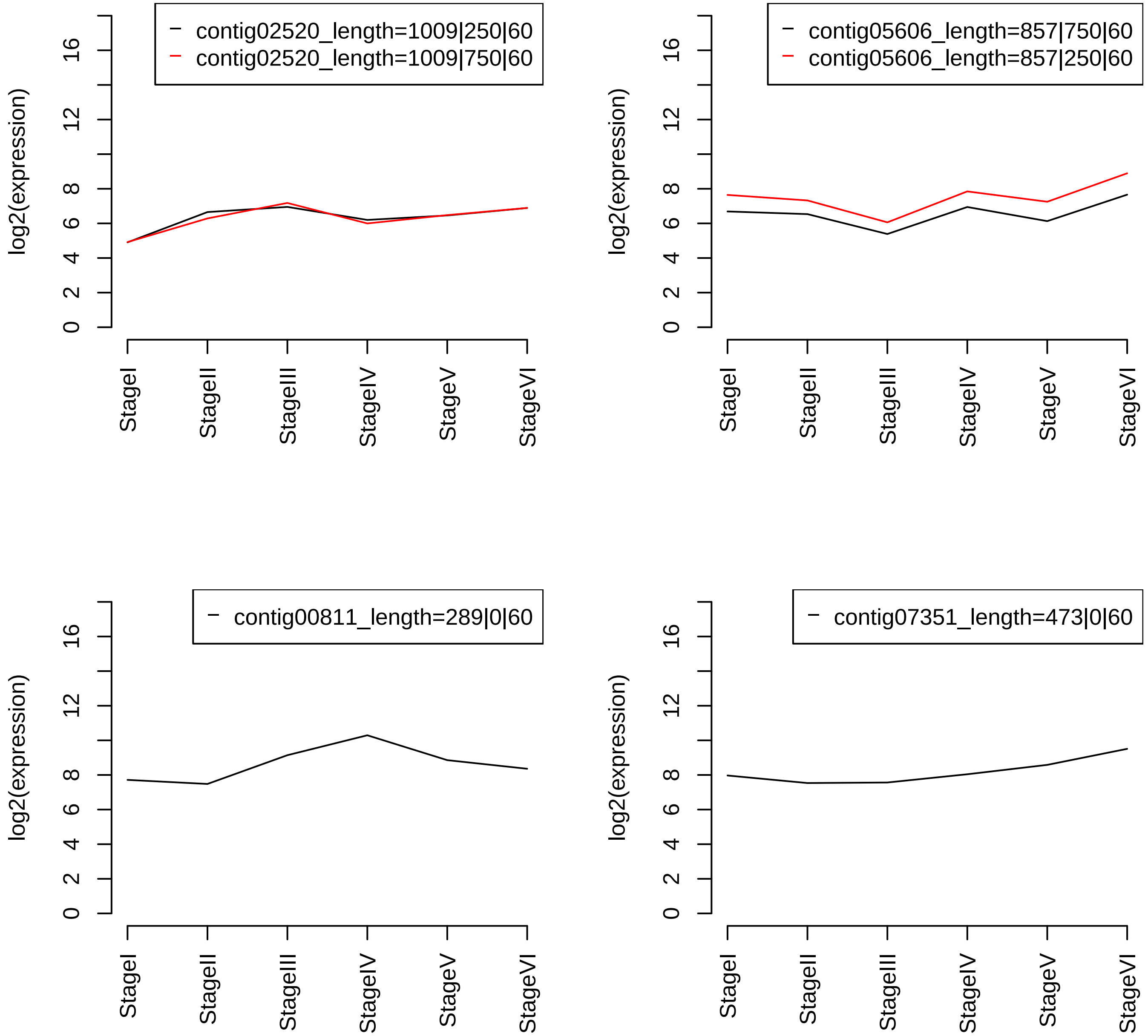

contig02520_length=1009|250|60
contig02520_length=1009|750|60
contig05606_length=857|750|60
contig05606_length=857|250|60
contig00811_length=289|0|60
contig07351_length=473|0|60
log2(expression)
0
2
4
6
8
12
16
StageI
StageII
StageIII
StageIV
StageV
StageVI

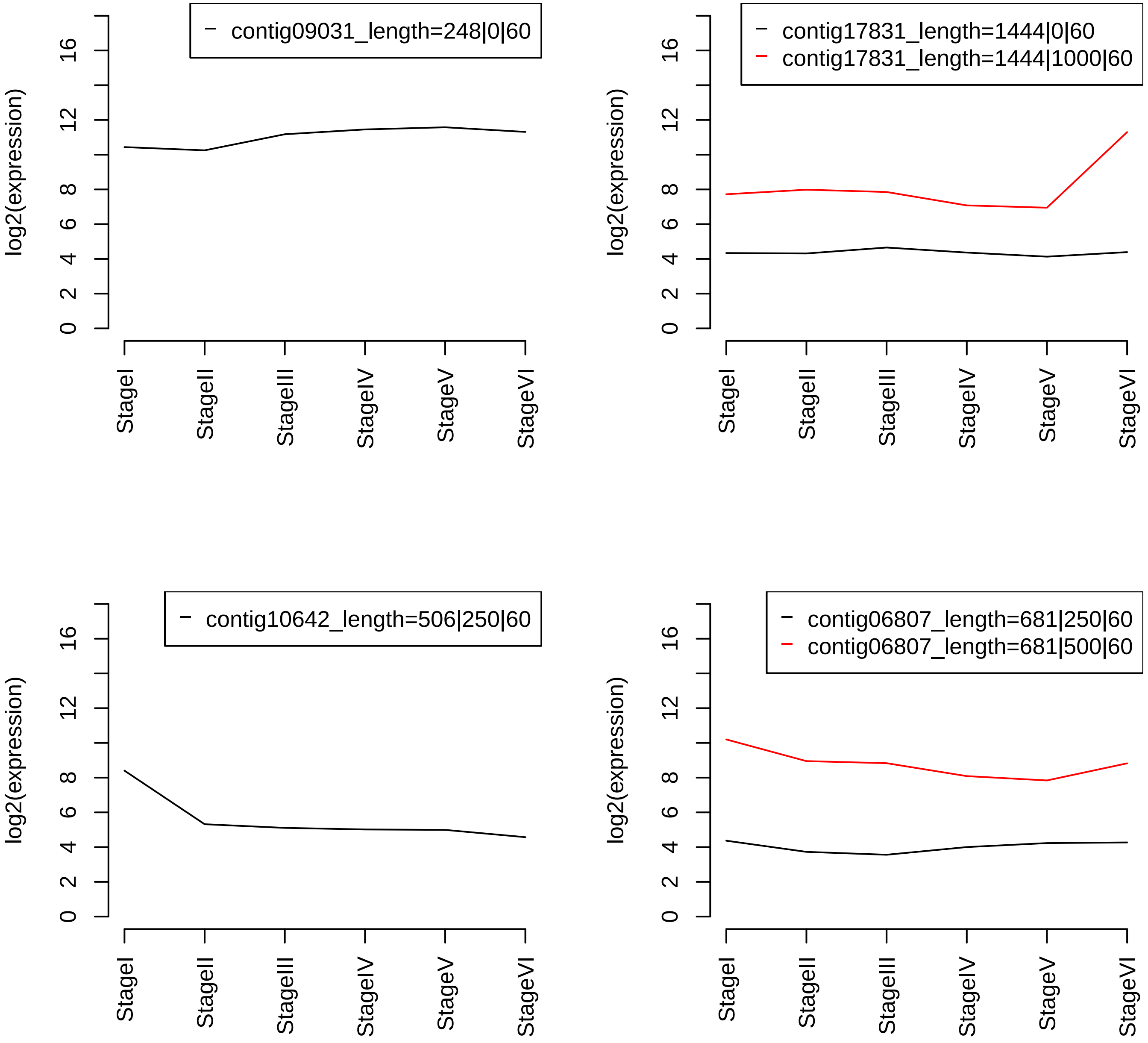
contig09031_length=248|0|60
contig17831_length=1444|0|60
contig17831_length=1444|1000|60
contig10642_length=506|250|60
contig06807_length=681|250|60
contig06807_length=681|500|60
log2(expression)
0
2
4
6
8
12
16
StageI
StageII
StageIII
StageIV
StageV
StageVI

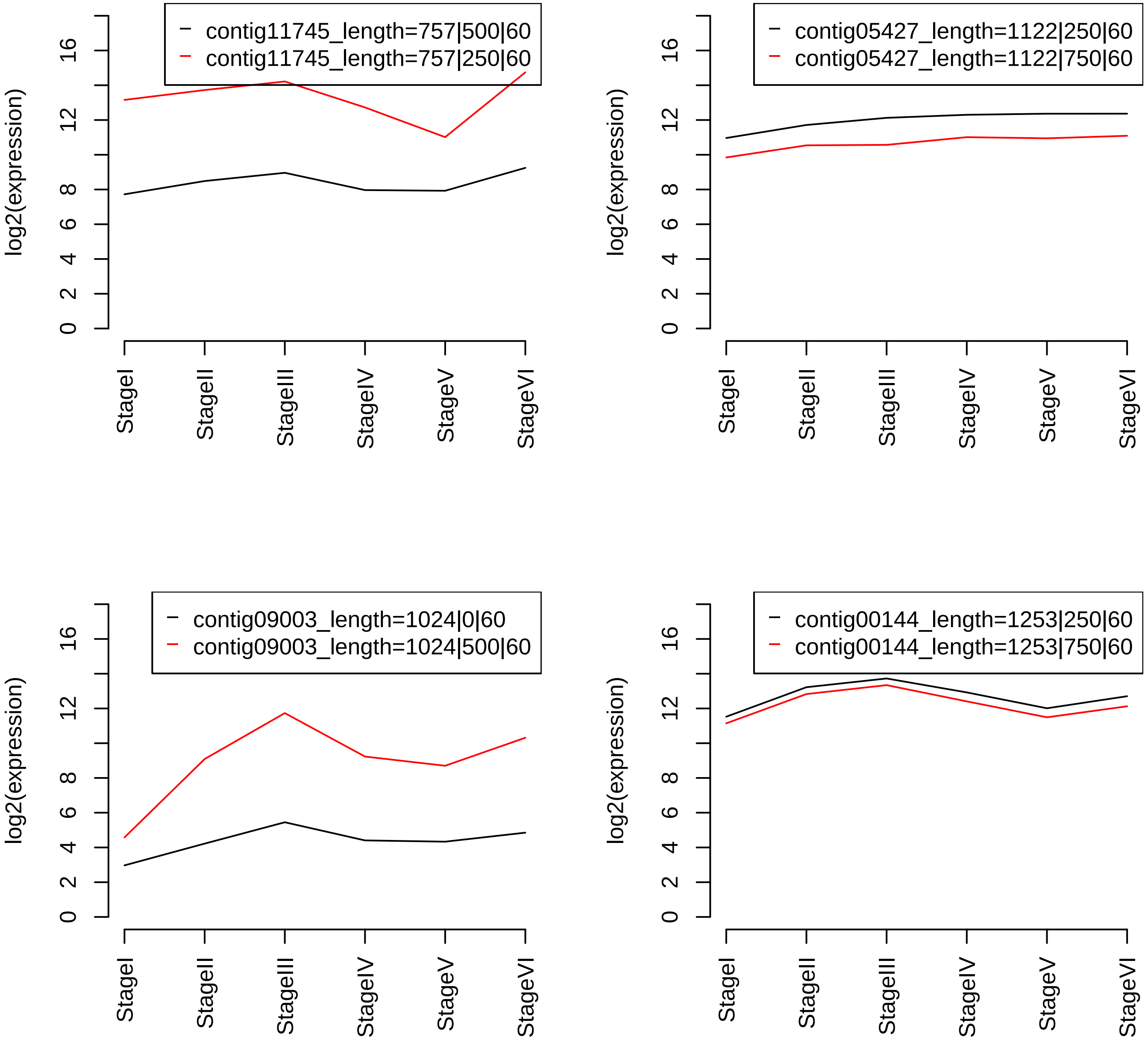

contig11745_length=757|500|60
contig11745_length=757|250|60
contig05427_length=1122|250|60
contig05427_length=1122|750|60
contig09003_length=1024|0|60
contig09003_length=1024|500|60
contig00144_length=1253|250|60
contig00144_length=1253|750|60
log2(expression)
0
2
4
6
8
12
16
StageI
StageII
StageIII
StageIV
StageV
StageVI

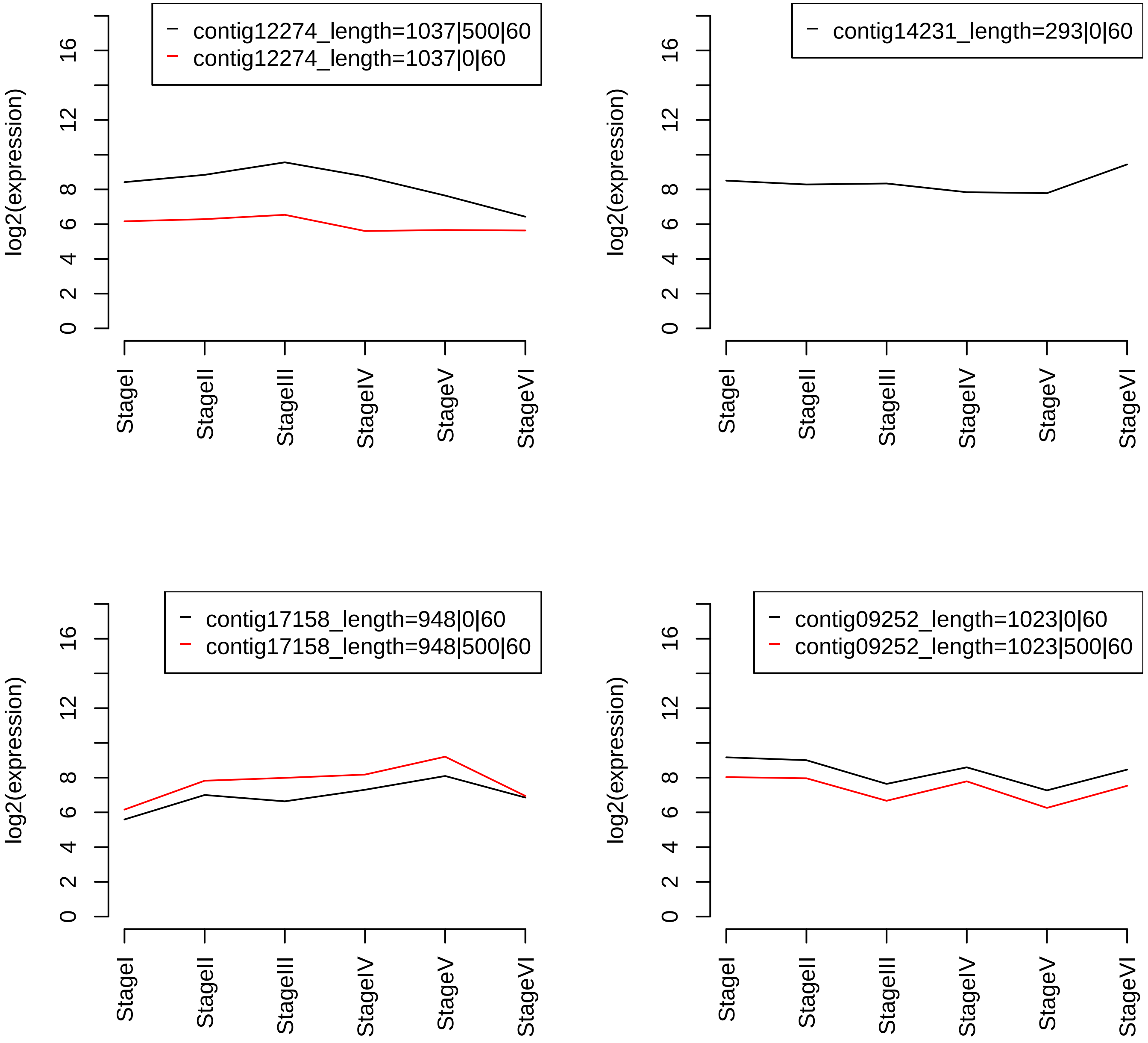

log2(expression)
0
2
4
6
8
12
16
StageI
StageII
StageIII
StageIV
StageV
StageVI
contig12274_length=1037|500|60
contig12274_length=1037|0|60
contig14231_length=293|0|60
contig17158_length=948|0|60
contig17158_length=948|500|60
contig09252_length=1023|0|60
contig09252_length=1023|500|60

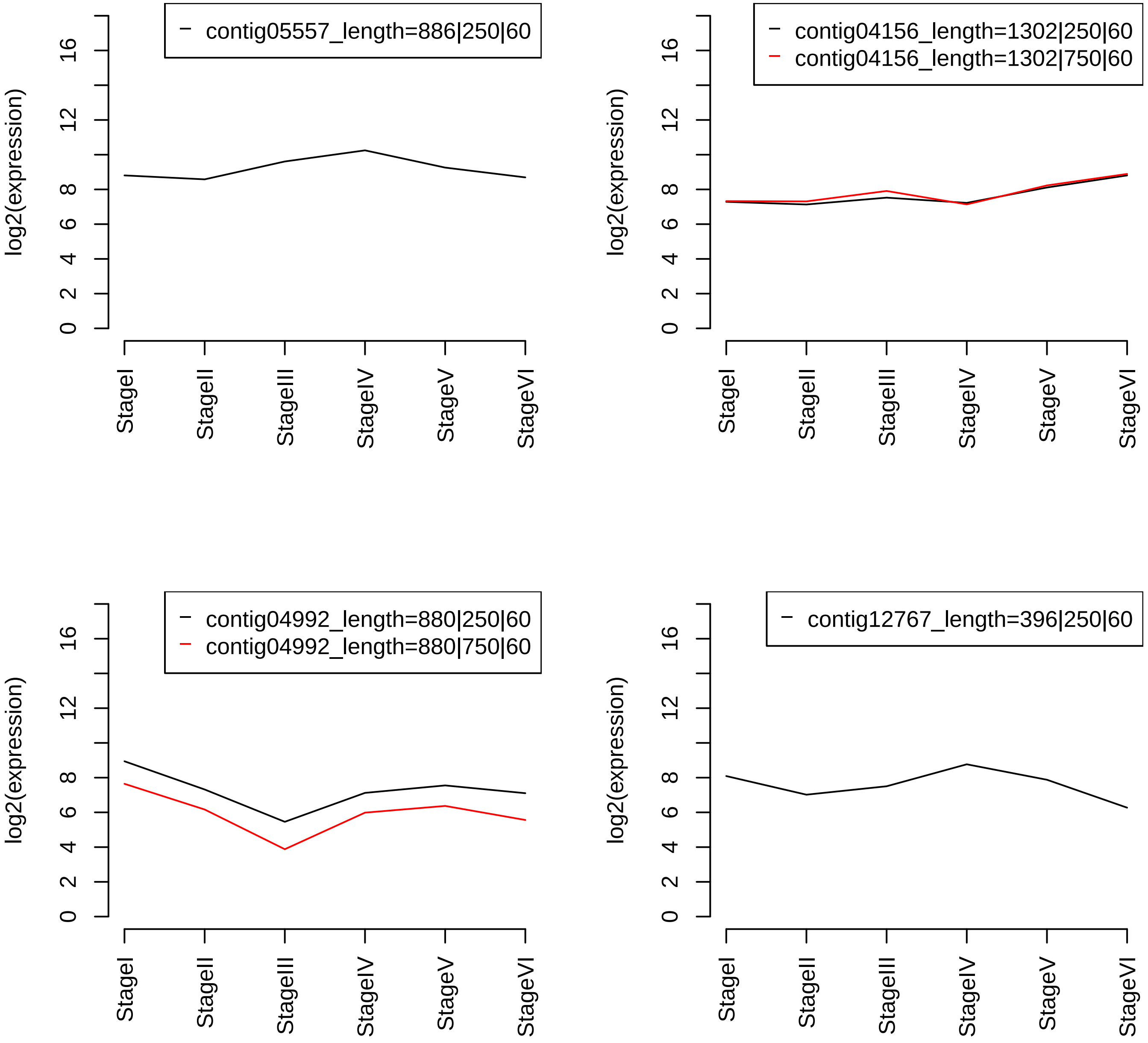

contig05557_length=886|250|60
contig04156_length=1302|250|60
contig04156_length=1302|750|60
contig04992_length=880|250|60
contig04992_length=880|750|60
contig12767_length=396|250|60
log2(expression)
0
2
4
6
8
12
16
StageI
StageII
StageIII
StageIV
StageV
StageVI

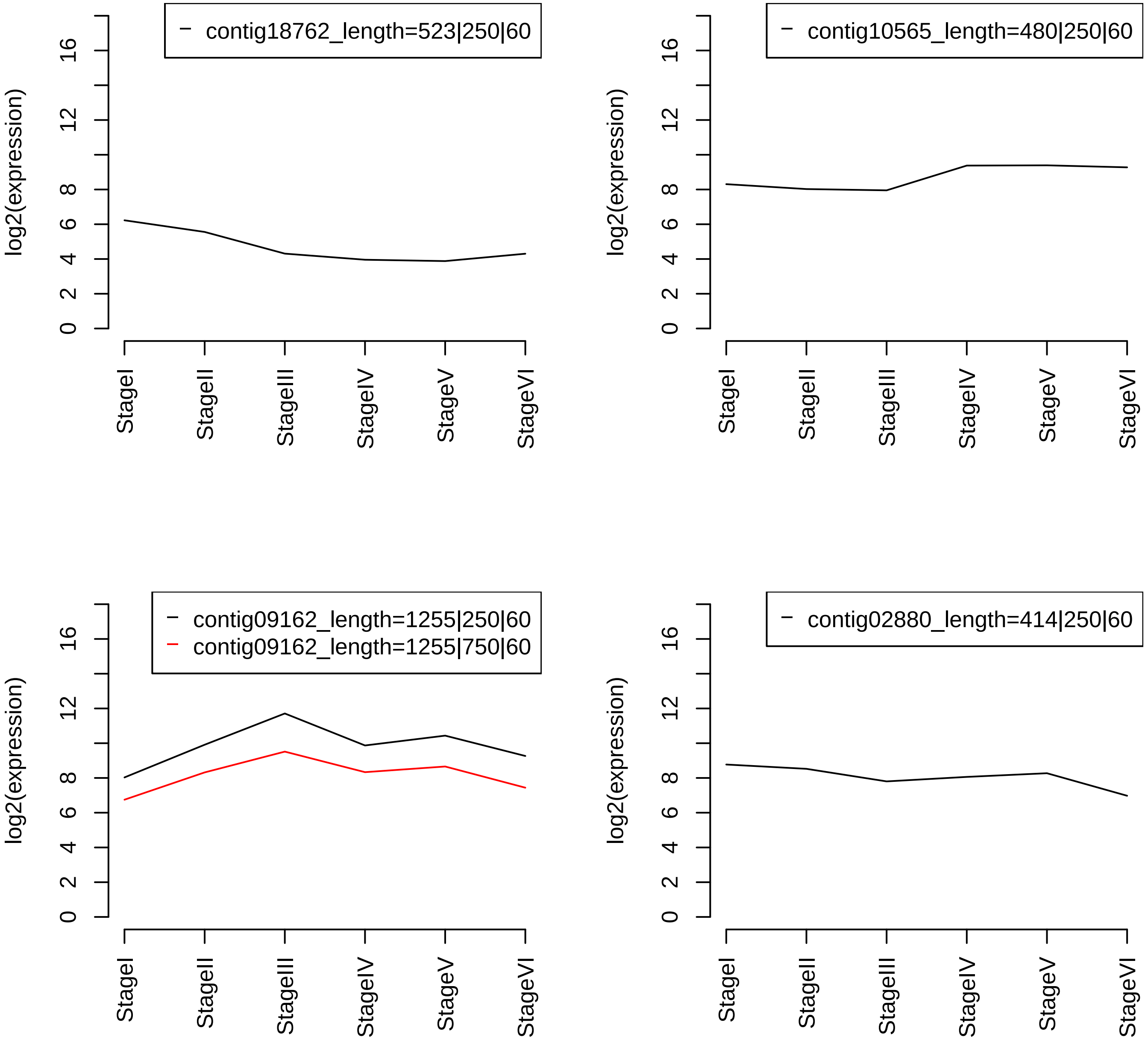

contig18762_length=523|250|60
contig10565_length=480|250|60
contig09162_length=1255|250|60
contig09162_length=1255|750|60
contig02880_length=414|250|60
log2(expression)
0
2
4
6
8
12
16
StageI
StageII
StageIII
StageIV
StageV
StageVI

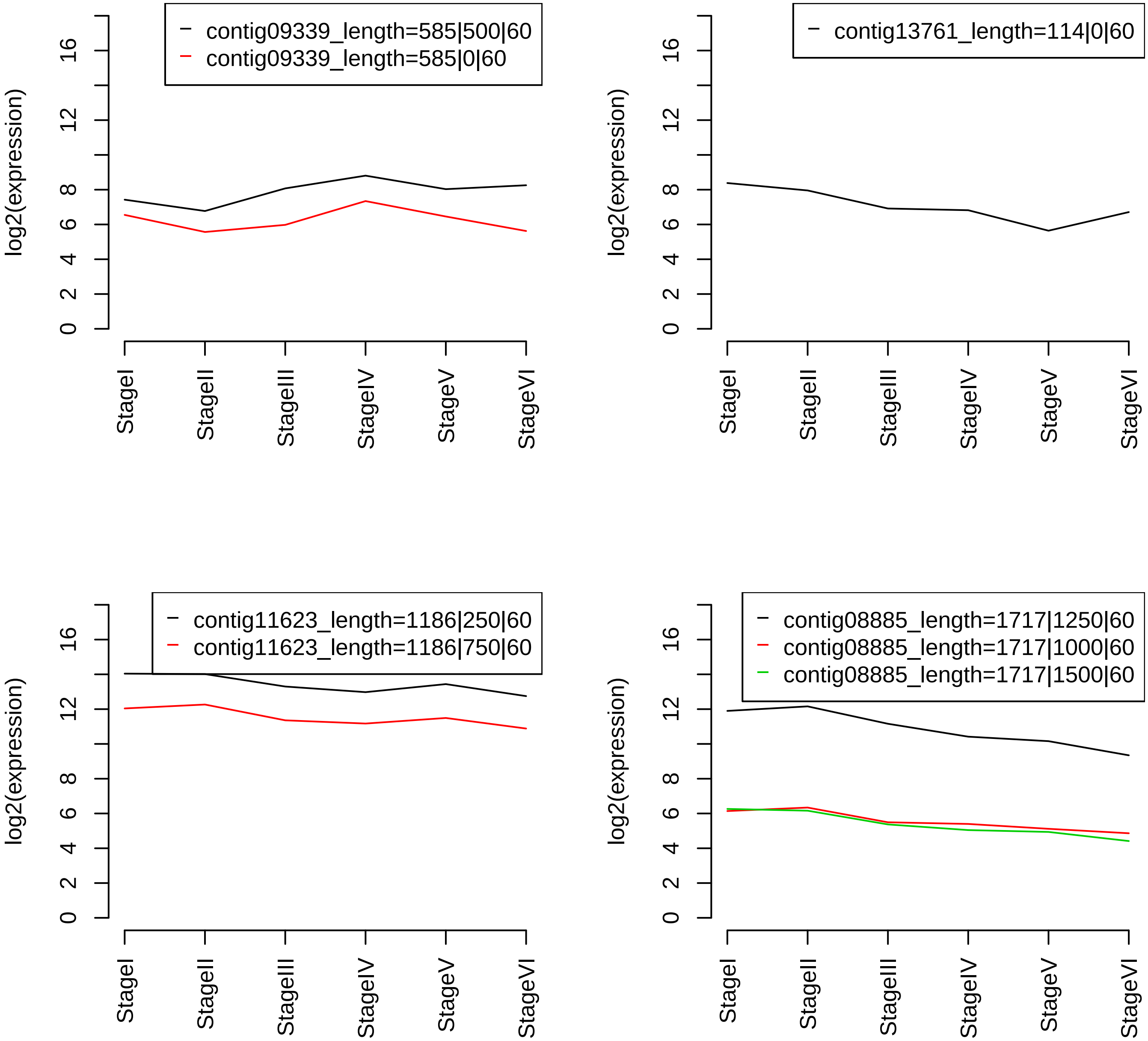
contig09339_length=585|500|60
contig09339_length=585|0|60
contig13761_length=114|0|60
contig11623_length=1186|250|60
contig11623_length=1186|750|60
contig08885_length=1717|1250|60
contig08885_length=1717|1000|60
contig08885_length=1717|1500|60
log2(expression)
0
2
4
6
8
12
16
StageI
StageII
StageIII
StageIV
StageV
StageVI

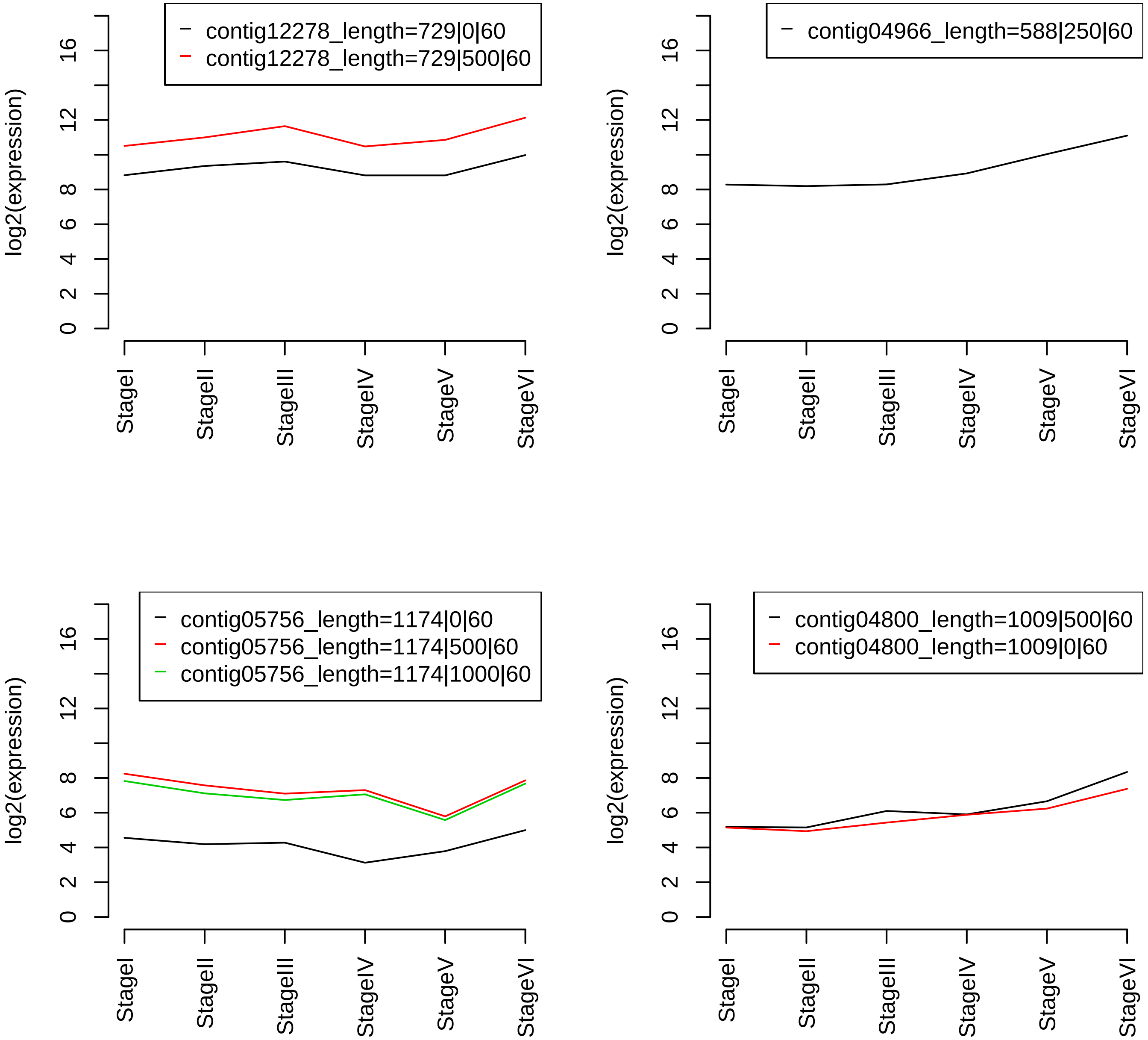

contig12278_length=729|0|60
contig12278_length=729|500|60
log2(expression)
0
2
4
6
8
12
16
StageI
StageII
StageIII
StageIV
StageV
StageVI
contig04966_length=588|250|60
log2(expression)
contig05756_length=1174|0|60
contig05756_length=1174|500|60
contig05756_length=1174|1000|60
log2(expression)
contig04800_length=1009|500|60
contig04800_length=1009|0|60
log2(expression)

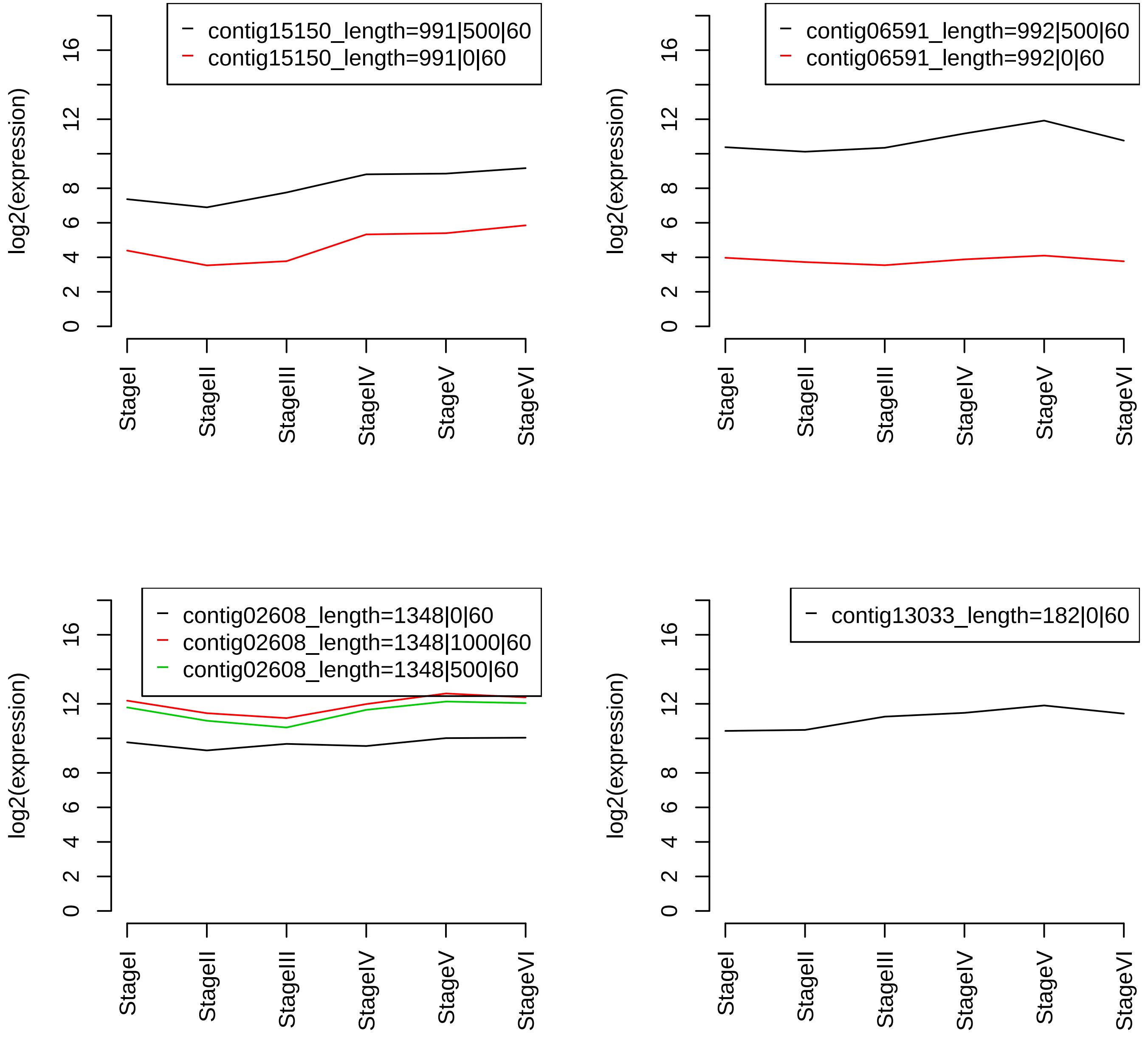
contig15150_length=991|500|60
contig15150_length=991|0|60
contig06591_length=992|500|60
contig06591_length=992|0|60
contig02608_length=1348|0|60
contig02608_length=1348|1000|60
contig02608_length=1348|500|60
contig13033_length=182|0|60
log2(expression)
0
2
4
6
8
12
16
StageI
StageII
StageIII
StageIV
StageV
StageVI

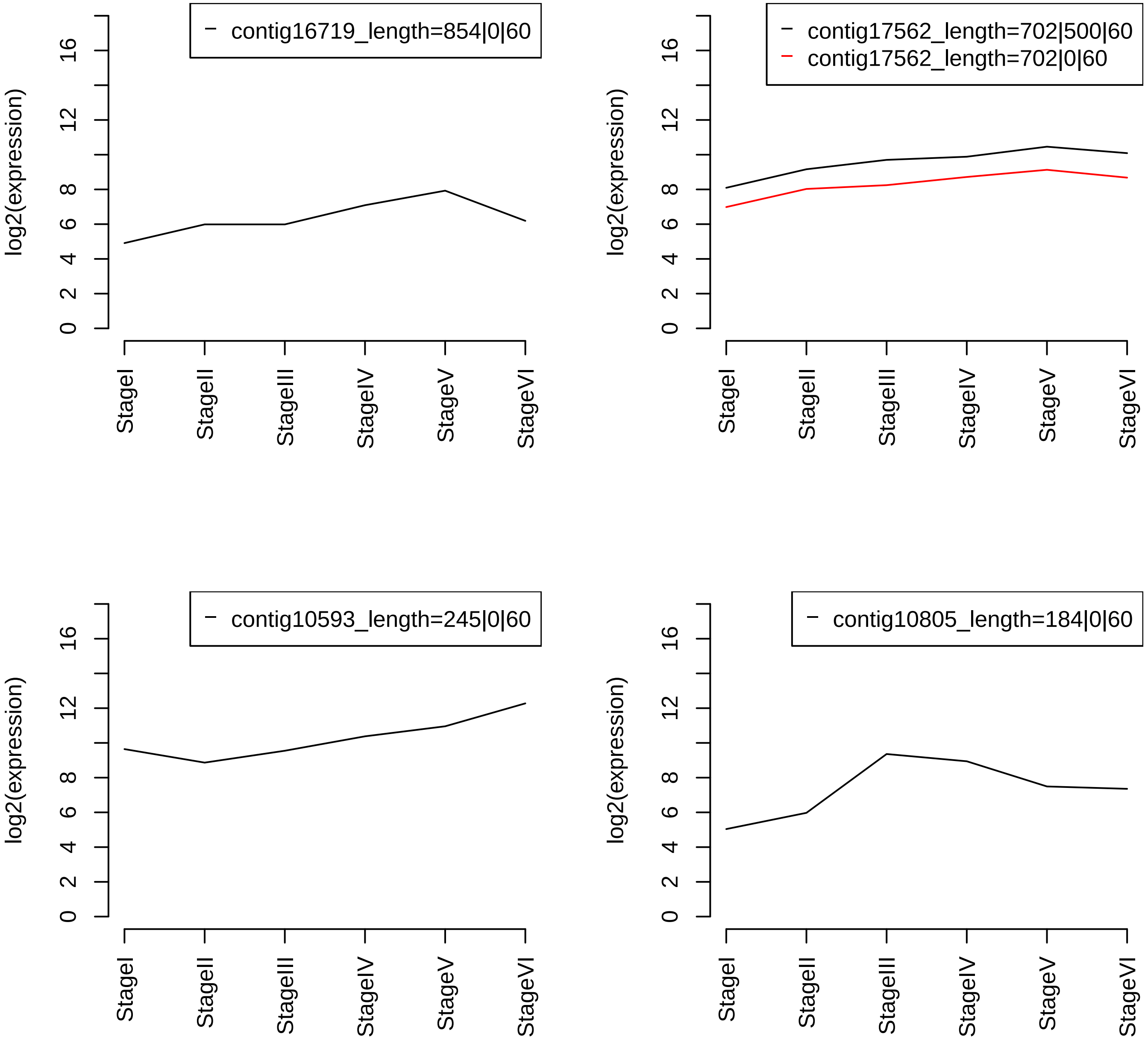
contig16719_length=854|0|60
contig17562_length=702|500|60
contig17562_length=702|0|60
contig10593_length=245|0|60
contig10805_length=184|0|60
log2(expression)
0
2
4
6
8
12
16
StageI
StageII
StageIII
StageIV
StageV
StageVI

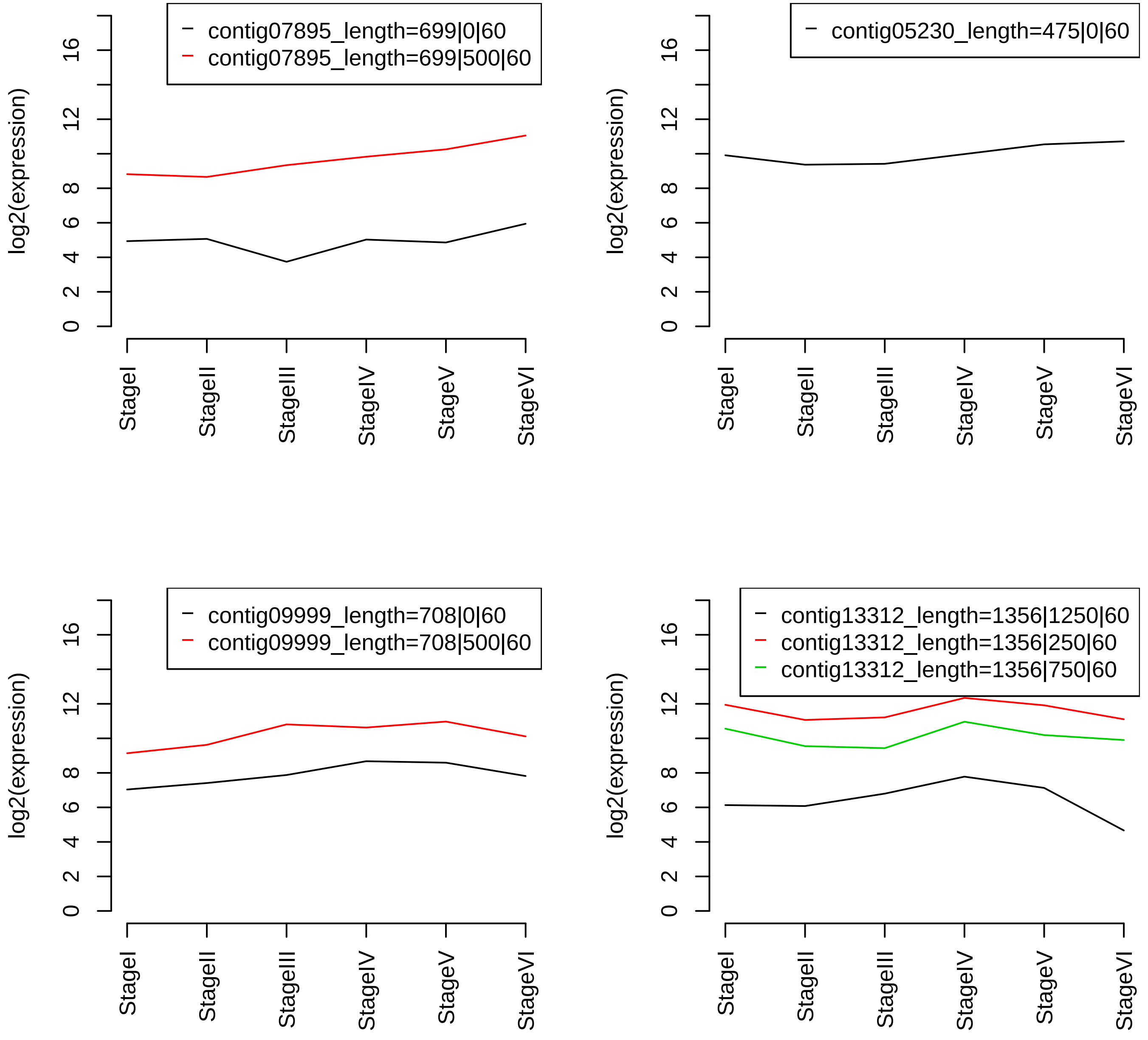
contig07895_length=699|0|60
contig07895_length=699|500|60
contig05230_length=475|0|60
contig09999_length=708|0|60
contig09999_length=708|500|60
contig13312_length=1356|1250|60
contig13312_length=1356|250|60
contig13312_length=1356|750|60
log2(expression)
0
2
4
6
8
12
16
StageI
StageII
StageIII
StageIV
StageV
StageVI

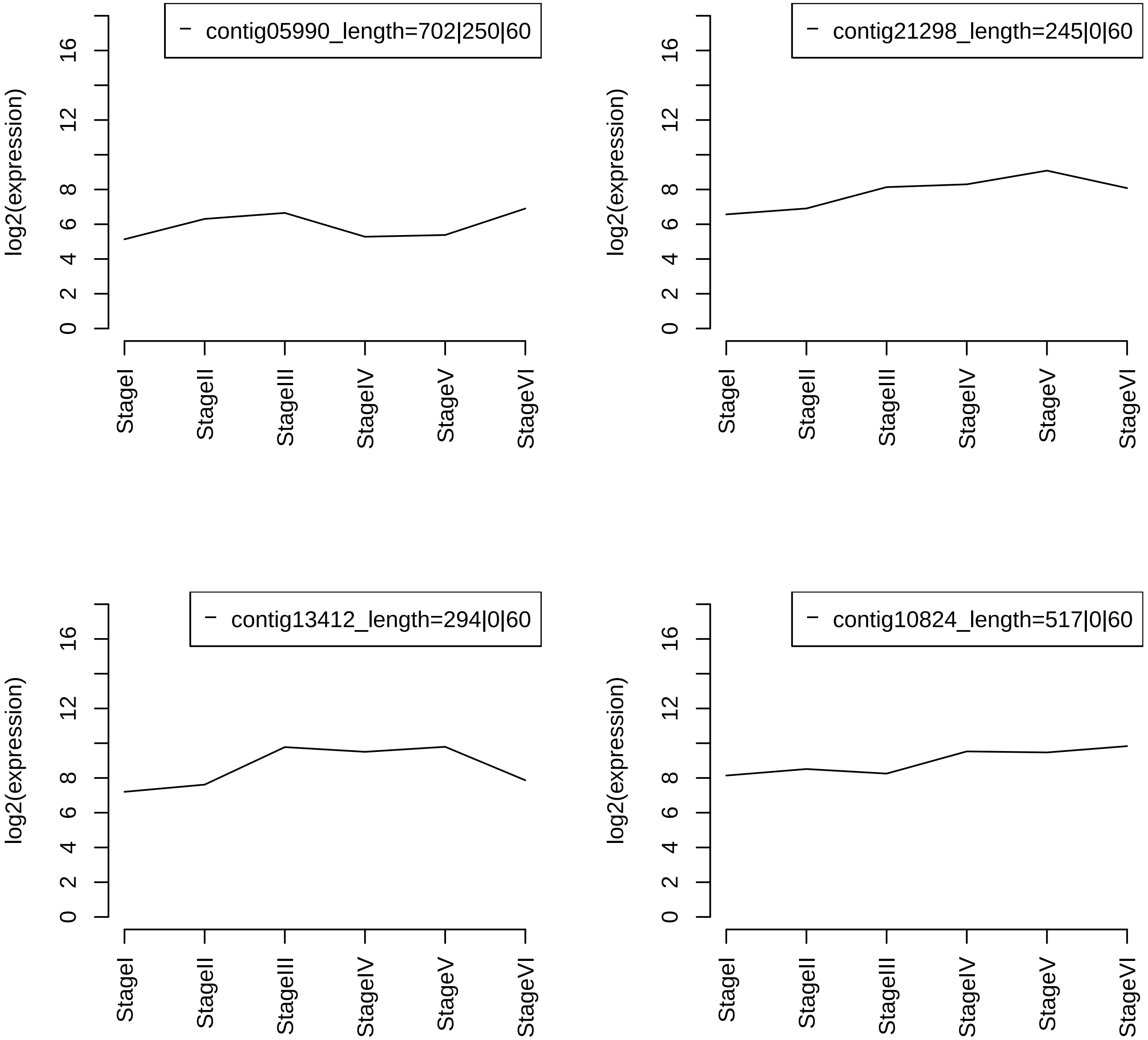
contig05990_length=702|250|60
contig21298_length=245|0|60
contig13412_length=294|0|60
contig10824_length=517|0|60
log2(expression)
0
2
4
6
8
12
16
StageI
StageII
StageIII
StageIV
StageV
StageVI

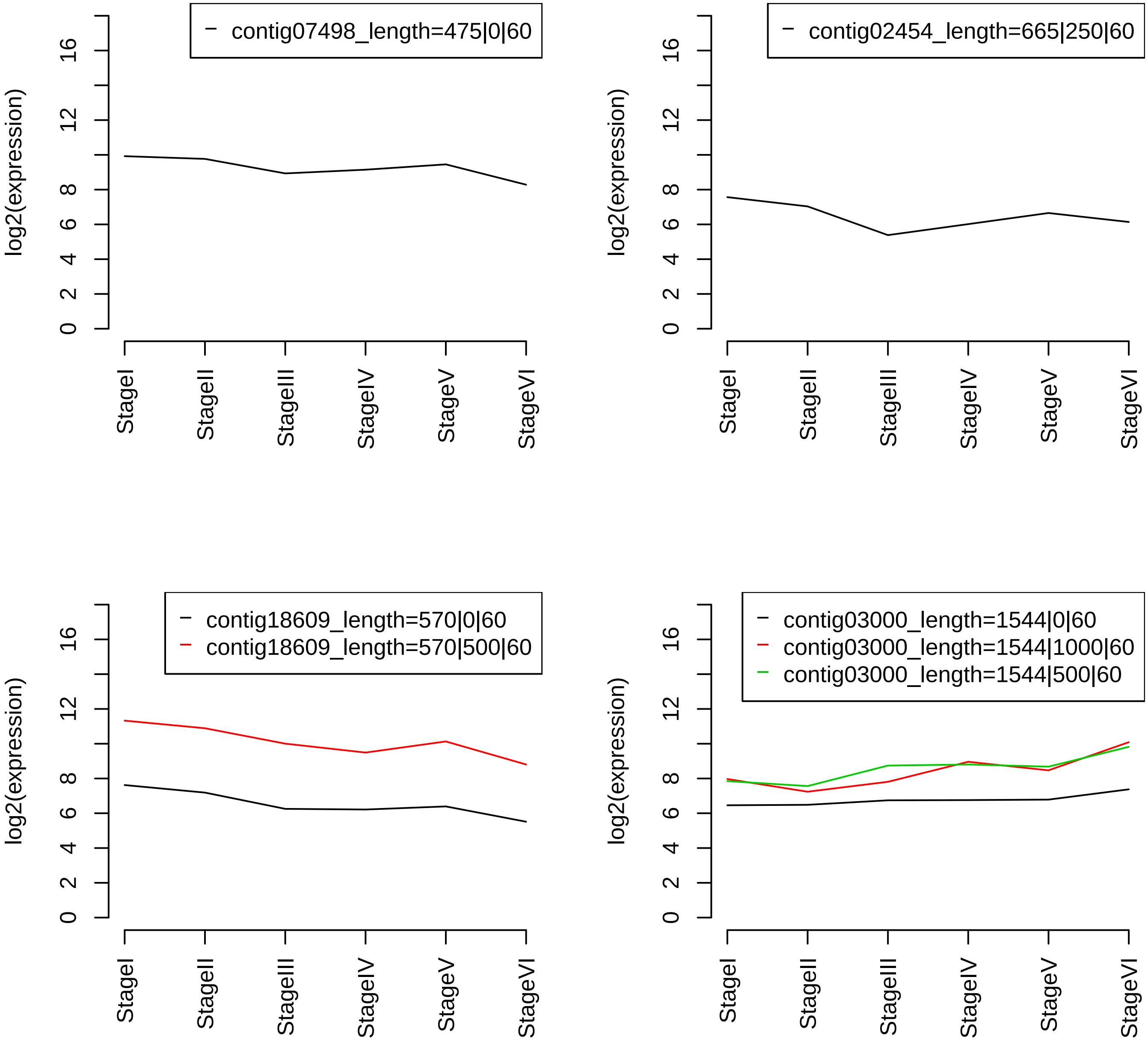
contig07498_length=475|0|60
contig02454_length=665|250|60
contig18609_length=570|0|60
contig18609_length=570|500|60
contig03000_length=1544|0|60
contig03000_length=1544|1000|60
contig03000_length=1544|500|60
log2(expression)
0
2
4
6
8
12
16
StageI
StageII
StageIII
StageIV
StageV
StageVI

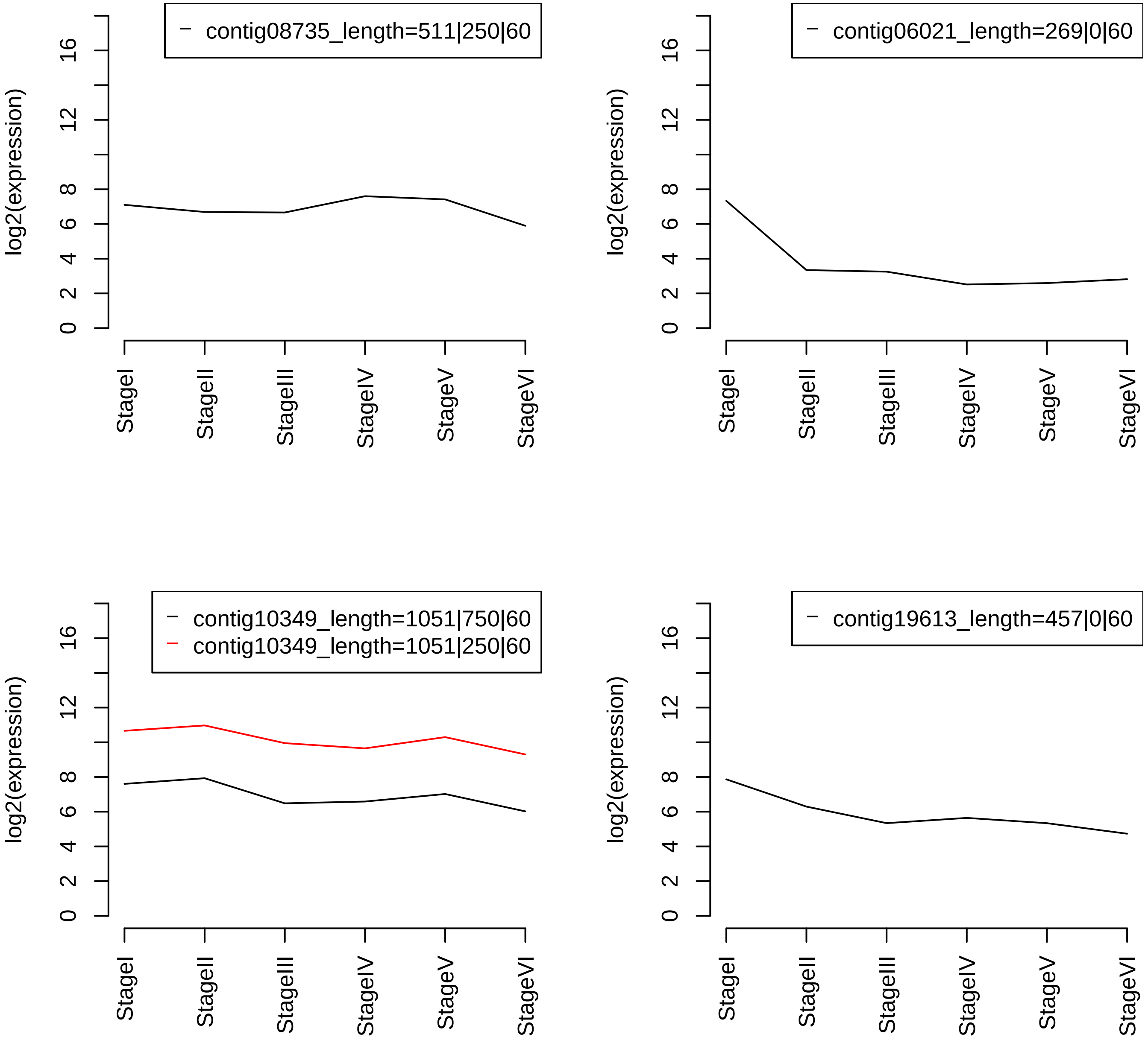
contig08735_length=511|250|60
contig06021_length=269|0|60
contig10349_length=1051|750|60
contig10349_length=1051|250|60
contig19613_length=457|0|60
log2(expression)
0 2 4 6 8 12 16
StageI StageII StageIII StageIV StageV StageVI

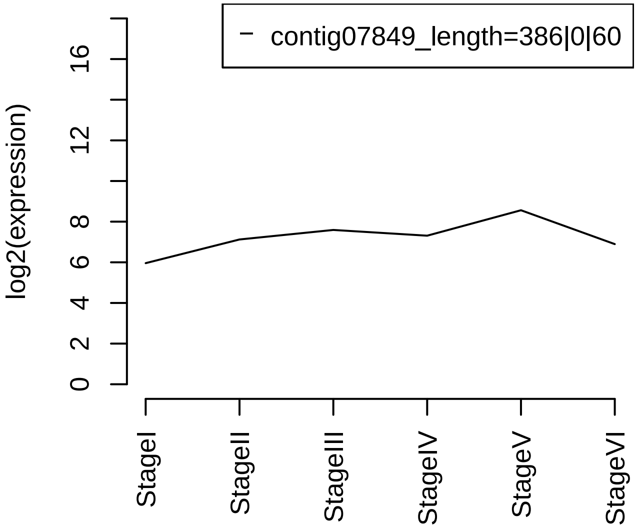

contig07849_length=386|0|60
log2(expression)
0
2
4
6
8
12
16
StageI
StageII
StageIII
StageIV
StageV
StageVI

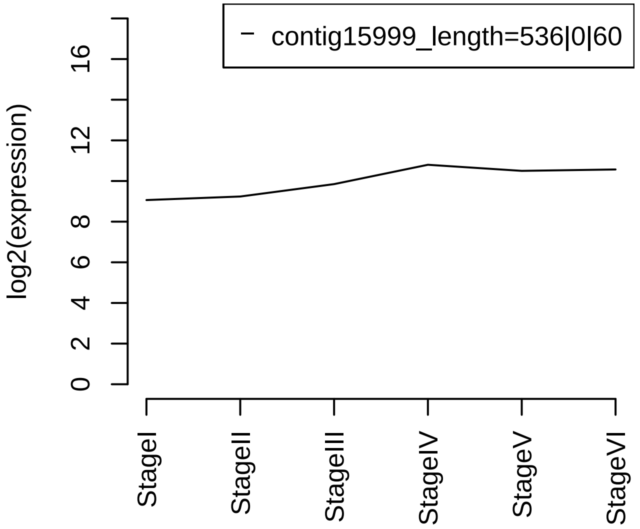

contig15999_length=536|0|60
log2(expression)
0
2
4
6
8
12
16
StageI
StageII
StageIII
StageIV
StageV
StageVI

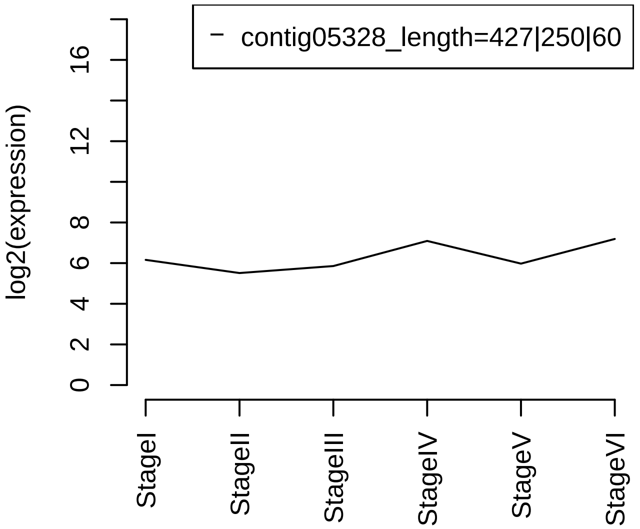

contig05328_length=427|250|60
log2(expression)
0
2
4
6
8
12
16
StageI
StageII
StageIII
StageIV
StageV
StageVI

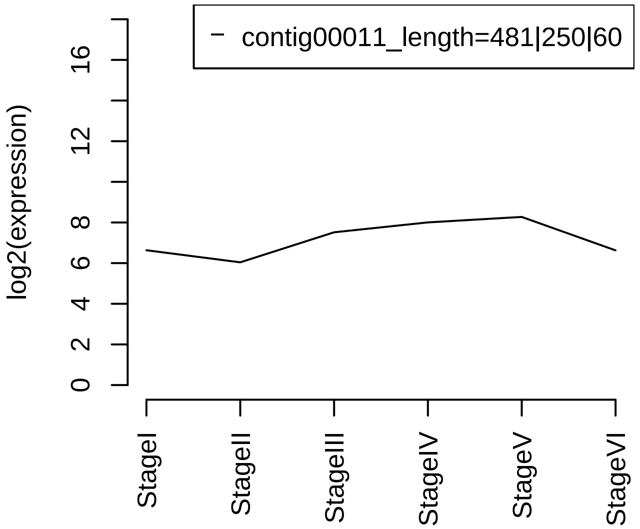

contig00011_length=481|250|60
log2(expression)
0
2
4
6
8
12
16
StageI
StageII
StageIII
StageIV
StageV
StageVI

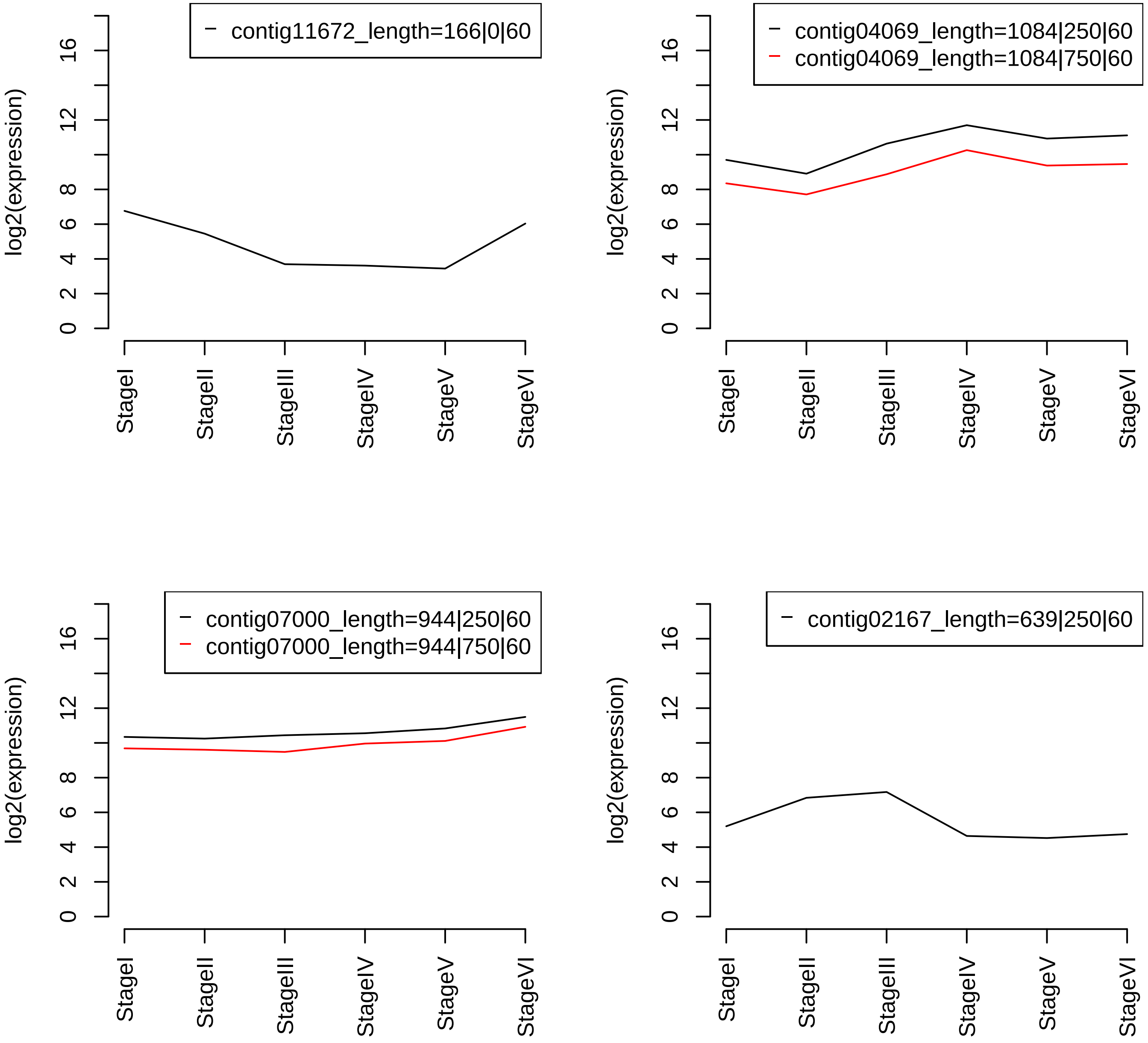

contig11672_length=166|0|60
contig04069_length=1084|250|60
contig04069_length=1084|750|60
contig07000_length=944|250|60
contig07000_length=944|750|60
contig02167_length=639|250|60
log2(expression)
0
2
4
6
8
12
16
StageI
StageII
StageIII
StageIV
StageV
StageVI

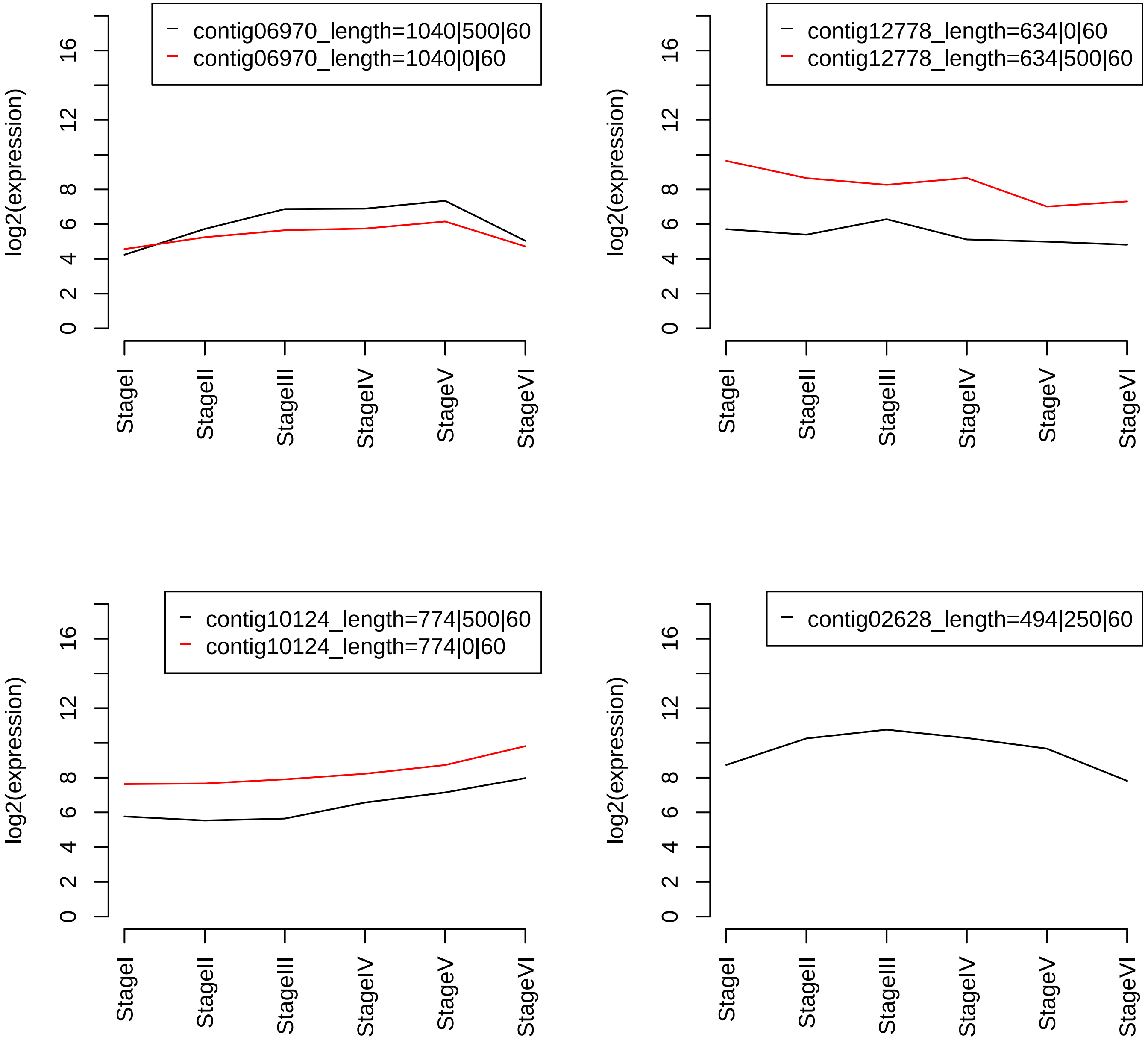
contig06970_length=1040|500|60
contig06970_length=1040|0|60
contig12778_length=634|0|60
contig12778_length=634|500|60
contig10124_length=774|500|60
contig10124_length=774|0|60
contig02628_length=494|250|60
log2(expression)
0
2
4
6
8
12
16
StageI
StageII
StageIII
StageIV
StageV
StageVI

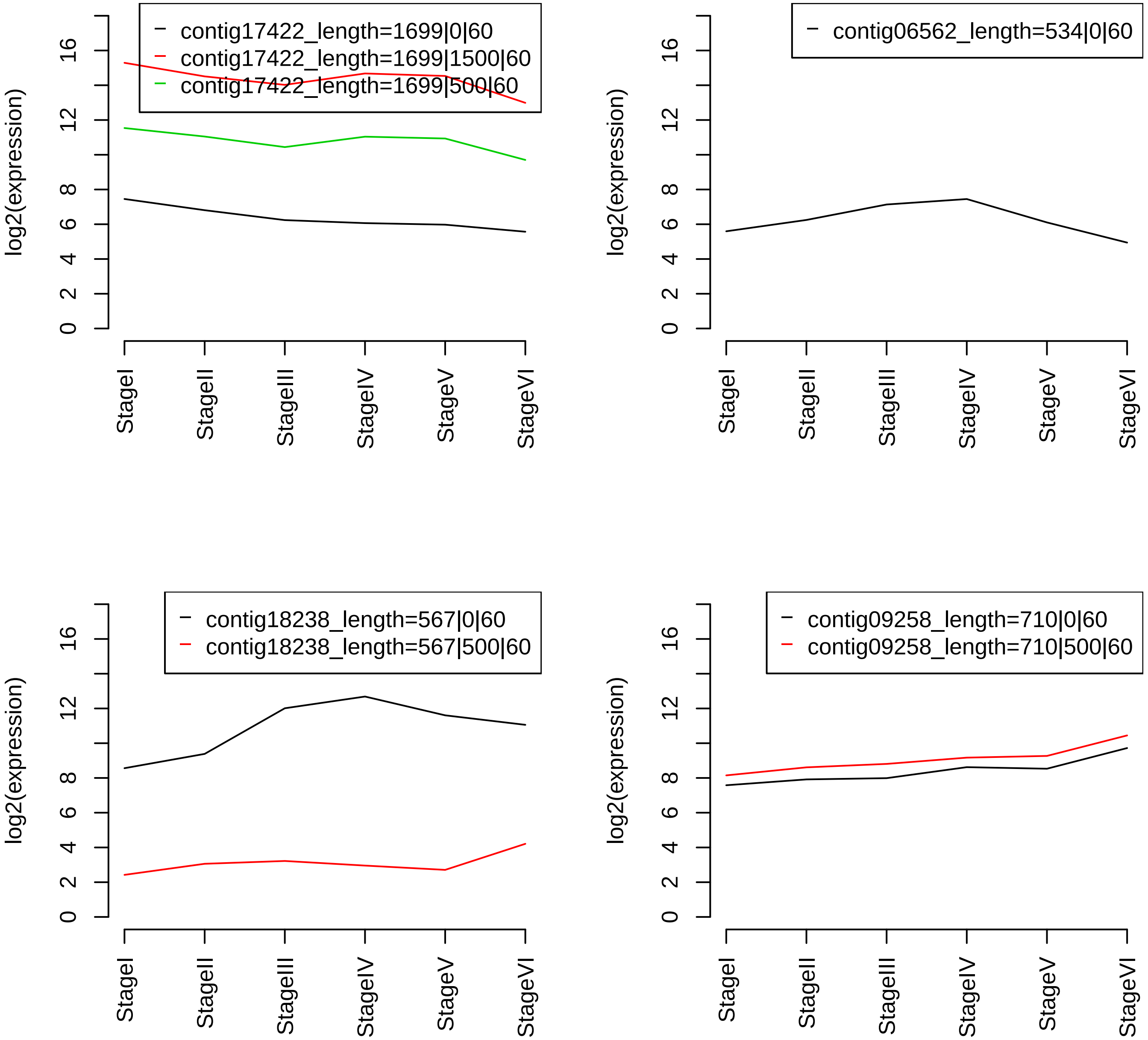

contig17422_length=1699|0|60
contig17422_length=1699|1500|60
contig17422_length=1699|500|60
contig06562_length=534|0|60
contig18238_length=567|0|60
contig18238_length=567|500|60
contig09258_length=710|0|60
contig09258_length=710|500|60
log2(expression)
0
2
4
6
8
12
16
StageI
StageII
StageIII
StageIV
StageV
StageVI

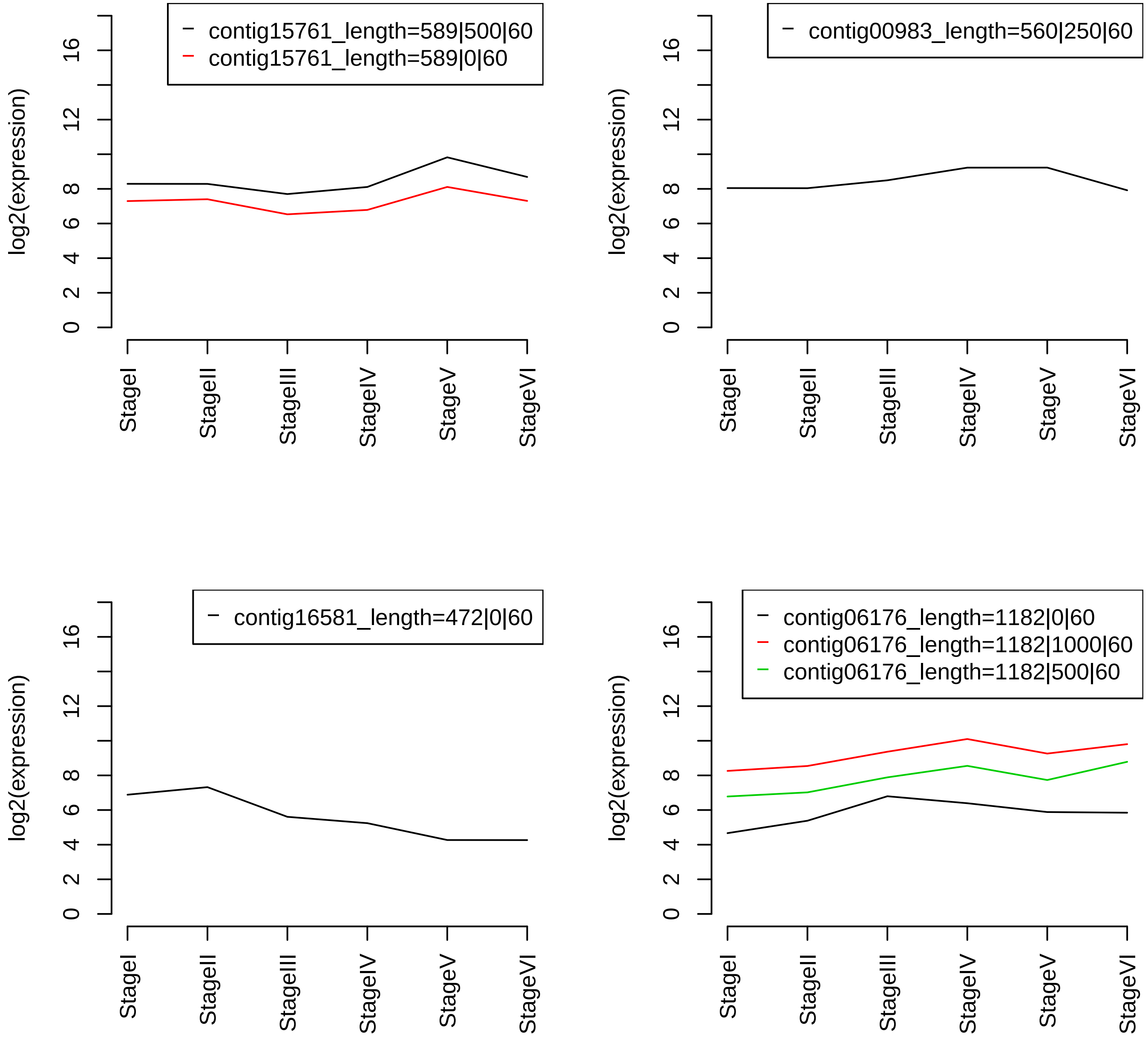
contig15761_length=589|500|60
contig15761_length=589|0|60
contig00983_length=560|250|60
contig16581_length=472|0|60
contig06176_length=1182|0|60
contig06176_length=1182|1000|60
contig06176_length=1182|500|60
log2(expression)
0
2
4
6
8
12
16
StageI
StageII
StageIII
StageIV
StageV
StageVI

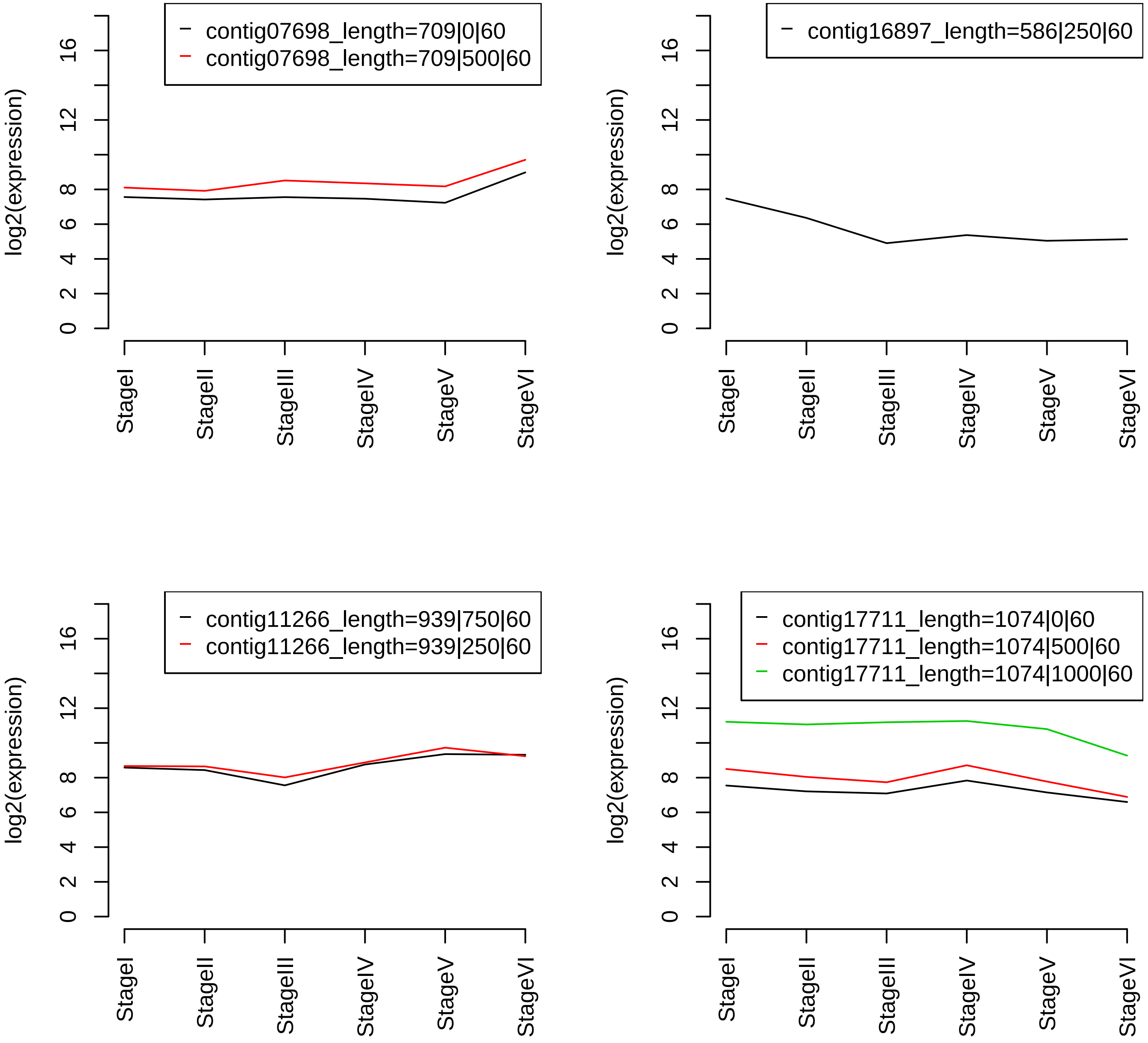

contig07698_length=709|0|60
contig07698_length=709|500|60
log2(expression)
0
2
4
6
8
12
16
StageI
StageII
StageIII
StageIV
StageV
StageVI
contig16897_length=586|250|60
log2(expression)
0
2
4
6
8
12
16
StageI
StageII
StageIII
StageIV
StageV
StageVI
contig11266_length=939|750|60
contig11266_length=939|250|60
log2(expression)
0
2
4
6
8
12
16
StageI
StageII
StageIII
StageIV
StageV
StageVI
contig17711_length=1074|0|60
contig17711_length=1074|500|60
contig17711_length=1074|1000|60
log2(expression)
0
2
4
6
8
12
16
StageI
StageII
StageIII
StageIV
StageV
StageVI

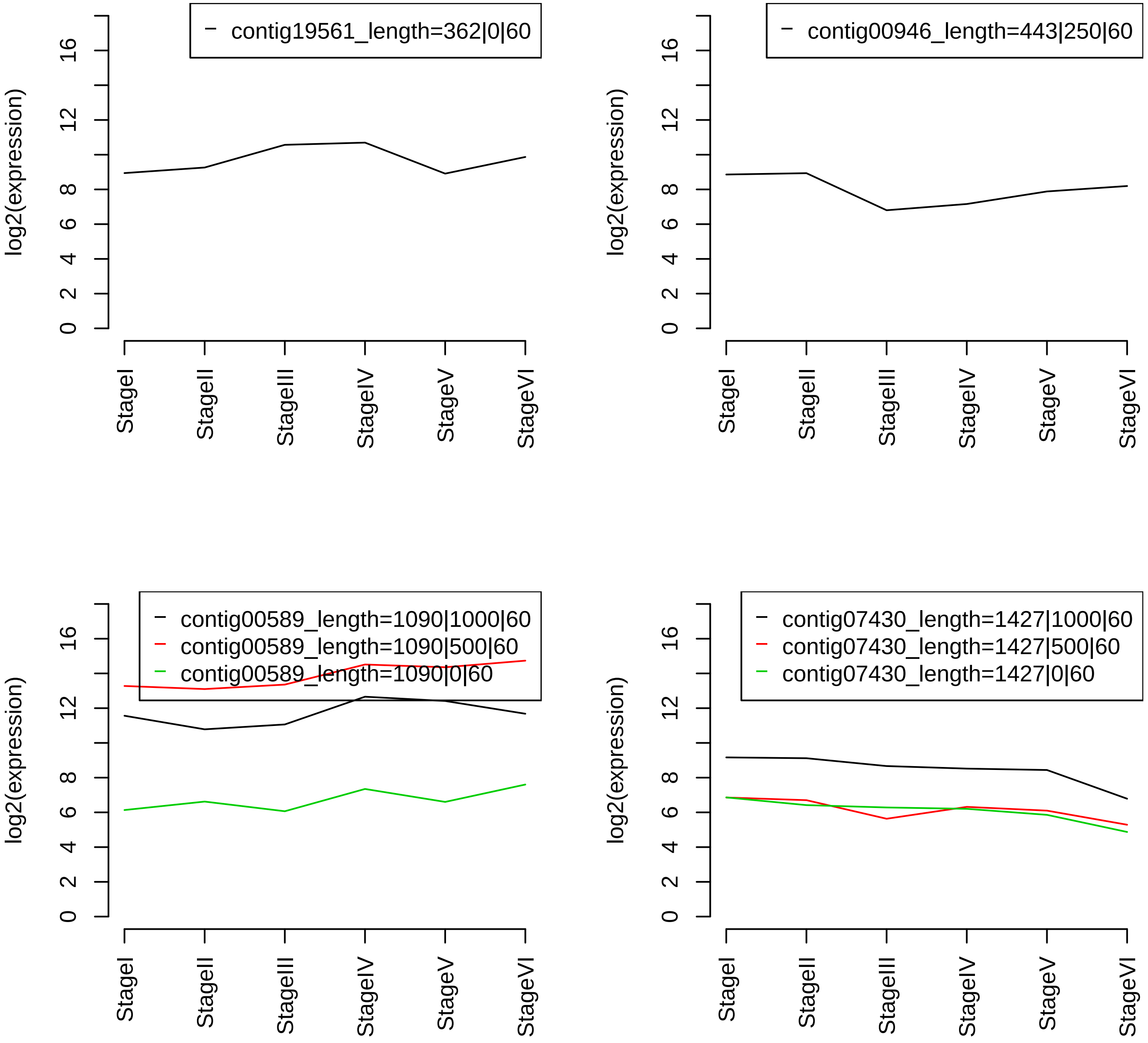

contig19561_length=362|0|60
contig00946_length=443|250|60
contig00589_length=1090|1000|60
contig00589_length=1090|500|60
contig00589_length=1090|0|60
contig07430_length=1427|1000|60
contig07430_length=1427|500|60
contig07430_length=1427|0|60
log2(expression)
0
2
4
6
8
12
16
StageI
StageII
StageIII
StageIV
StageV
StageVI

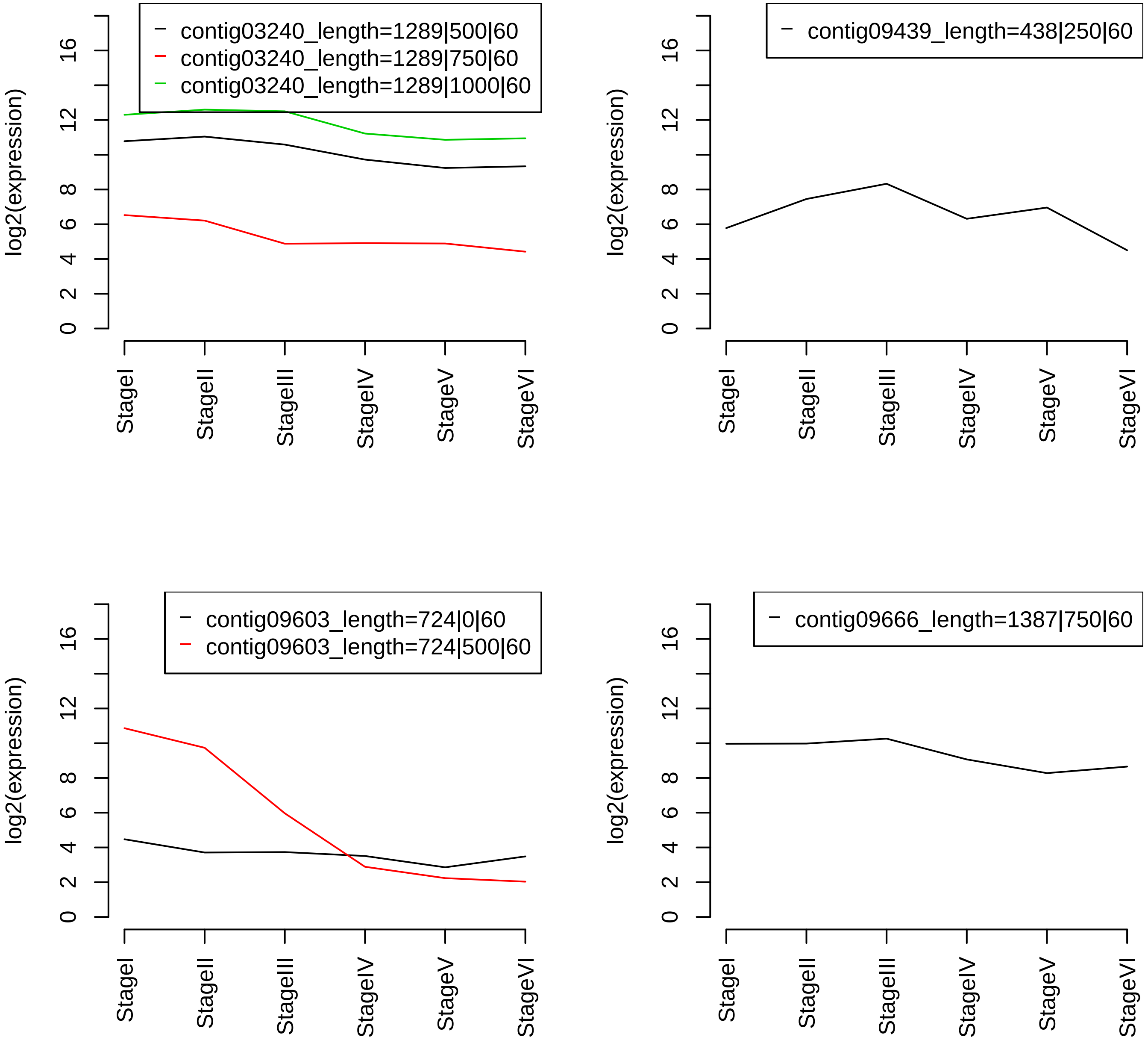
contig03240_length=1289|500|60
contig03240_length=1289|750|60
contig03240_length=1289|1000|60
log2(expression)
0
2
4
6
8
12
16
StageI
StageII
StageIII
StageIV
StageV
StageVI
contig09439_length=438|250|60
contig09603_length=724|0|60
contig09603_length=724|500|60
contig09666_length=1387|750|60

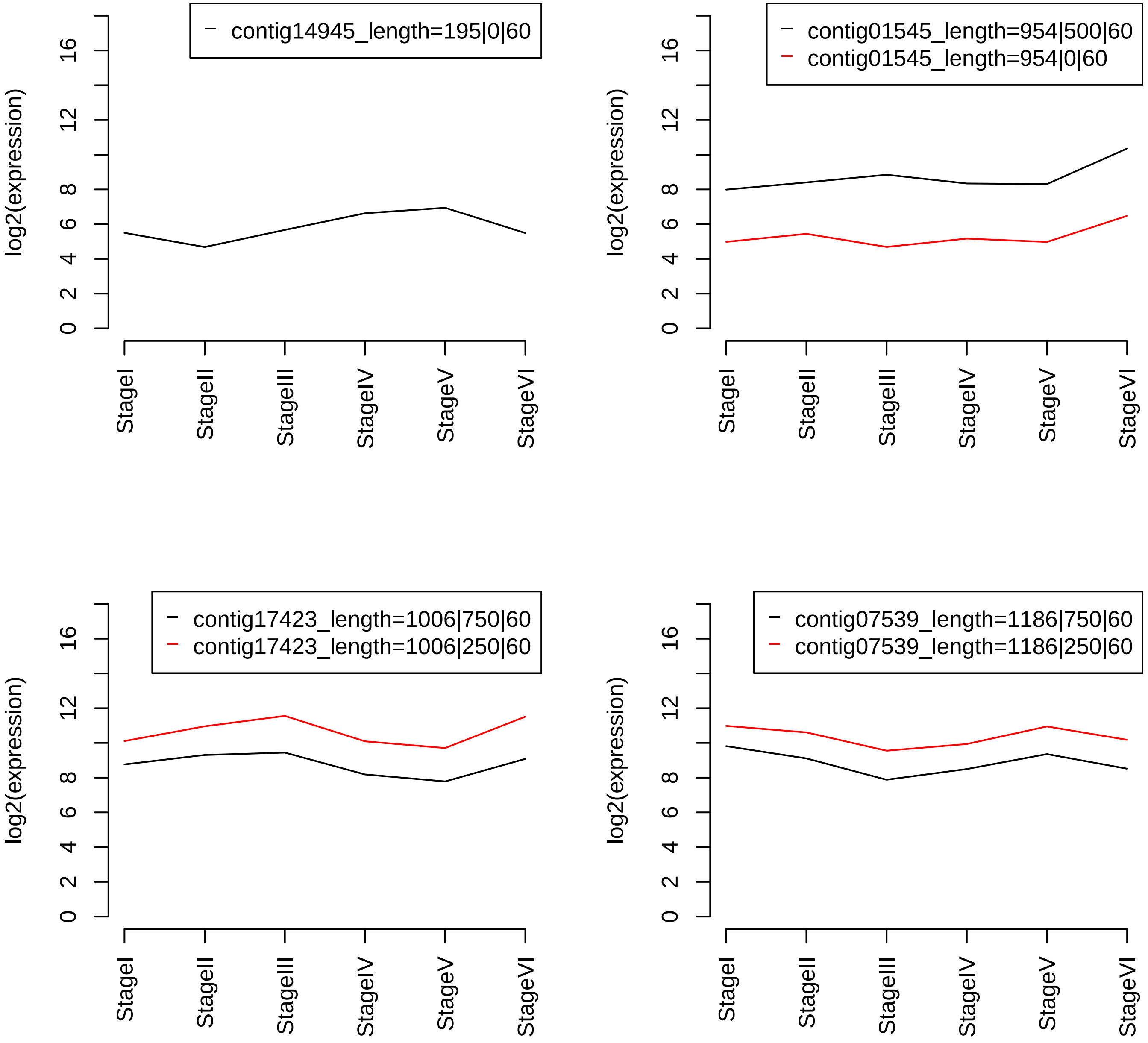
contig14945_length=195|0|60
contig01545_length=954|500|60
contig01545_length=954|0|60
contig17423_length=1006|750|60
contig17423_length=1006|250|60
contig07539_length=1186|750|60
contig07539_length=1186|250|60
log2(expression)
0
2
4
6
8
12
16
StageI
StageII
StageIII
StageIV
StageV
StageVI

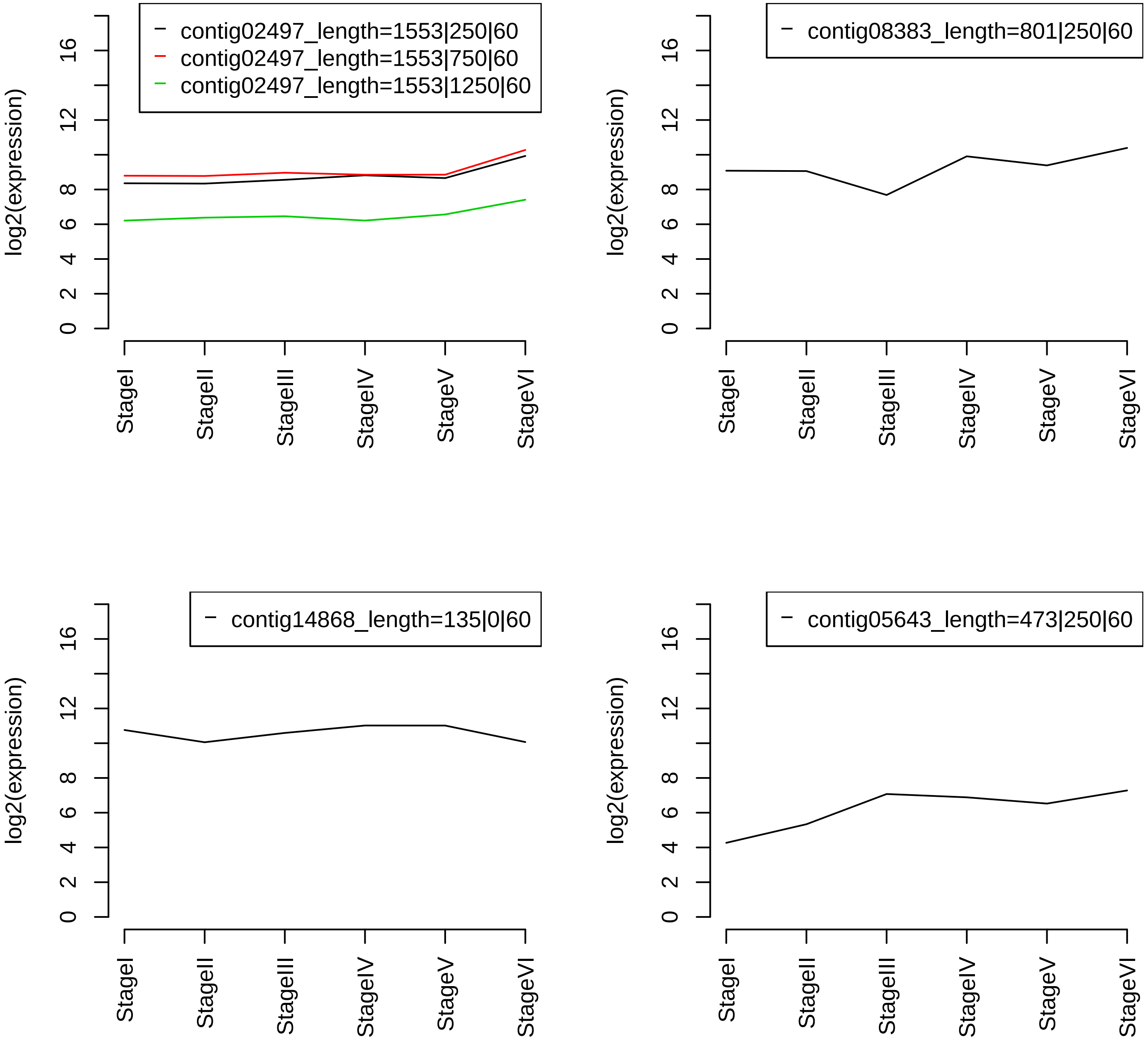
contig02497_length=1553|250|60
contig02497_length=1553|750|60
contig02497_length=1553|1250|60
contig08383_length=801|250|60
contig14868_length=135|0|60
contig05643_length=473|250|60
log2(expression)
StageI
StageII
StageIII
StageIV
StageV
StageVI

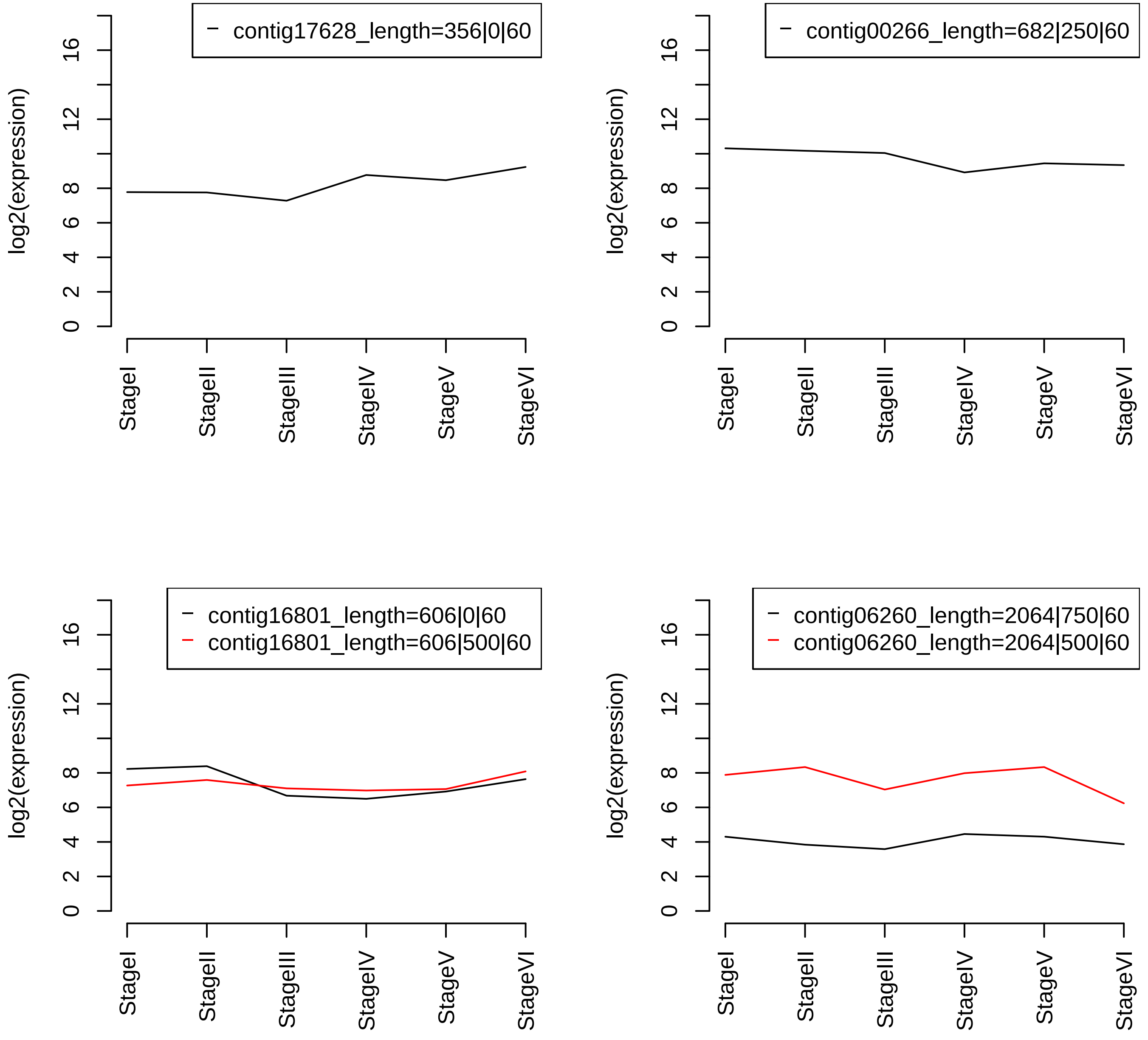
contig17628_length=356|0|60
contig00266_length=682|250|60
contig16801_length=606|0|60
contig16801_length=606|500|60
contig06260_length=2064|750|60
contig06260_length=2064|500|60
log2(expression)
0
2
4
6
8
12
16
StageI
StageII
StageIII
StageIV
StageV
StageVI

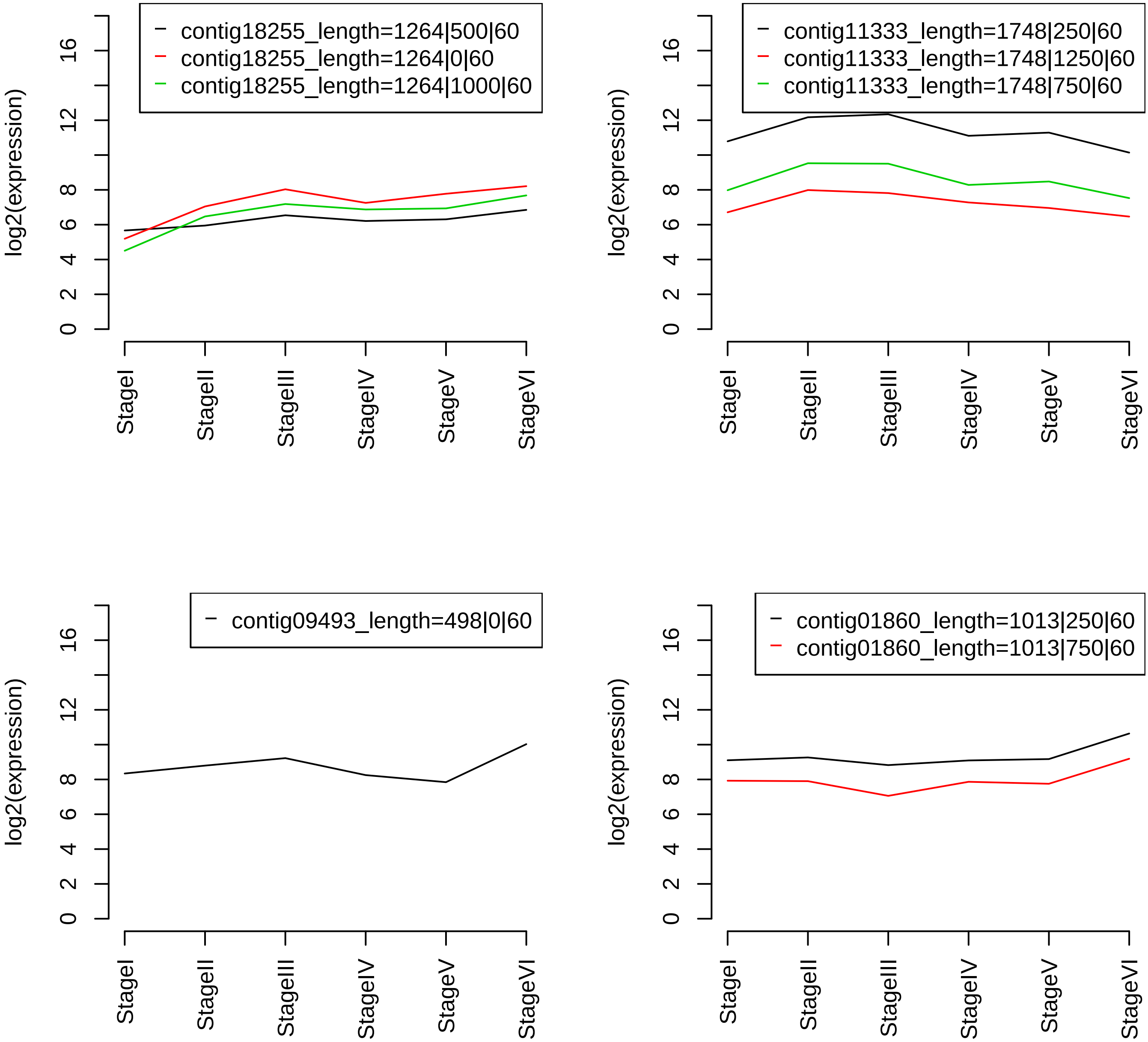

contig18255_length=1264|500|60
contig18255_length=1264|0|60
contig18255_length=1264|1000|60
contig11333_length=1748|250|60
contig11333_length=1748|1250|60
contig11333_length=1748|750|60
contig09493_length=498|0|60
contig01860_length=1013|250|60
contig01860_length=1013|750|60
log2(expression)
0
2
4
6
8
12
16
StageI
StageII
StageIII
StageIV
StageV
StageVI

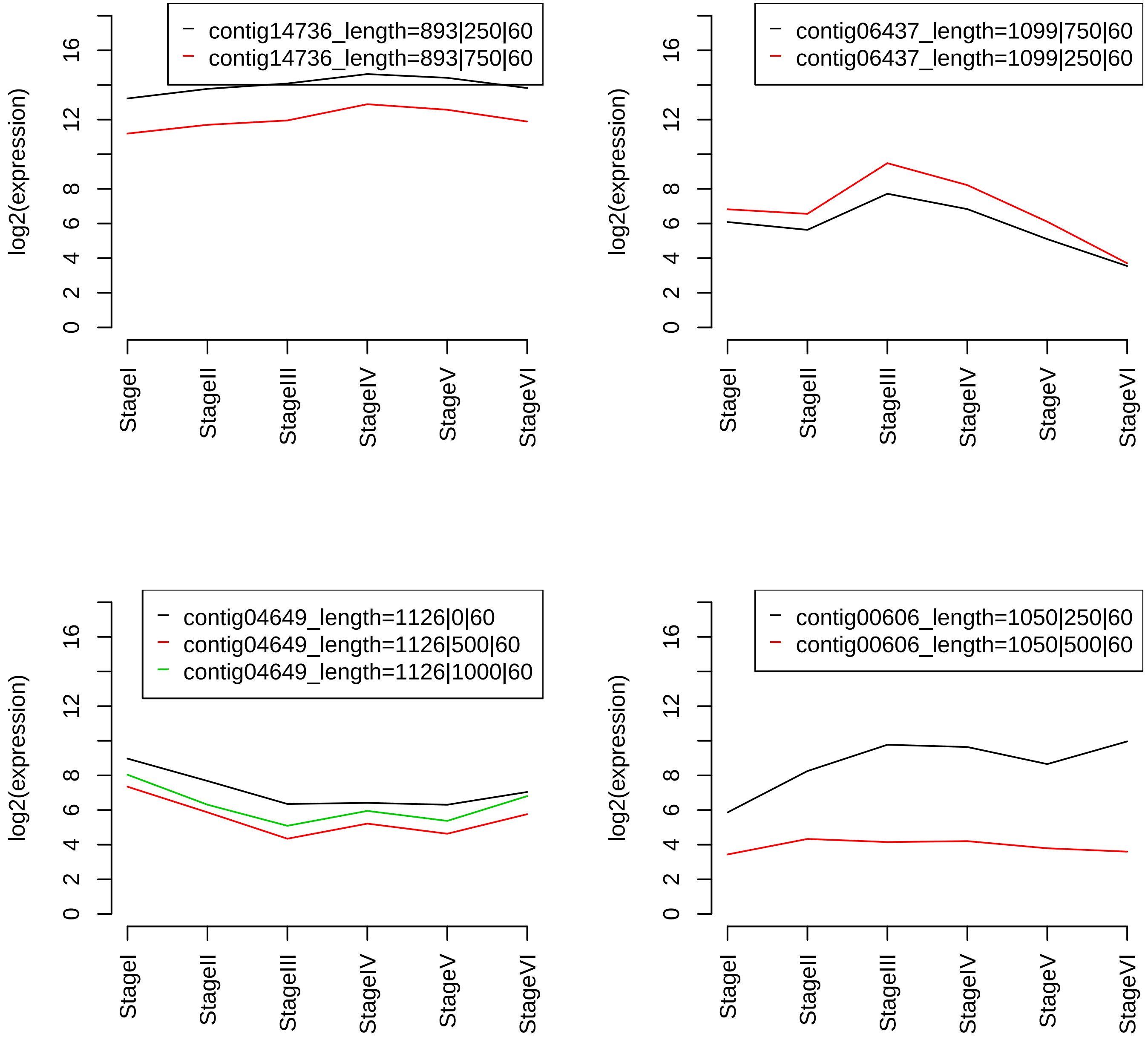
contig14736_length=893|250|60
contig14736_length=893|750|60
contig06437_length=1099|750|60
contig06437_length=1099|250|60
contig04649_length=1126|0|60
contig04649_length=1126|500|60
contig04649_length=1126|1000|60
contig00606_length=1050|250|60
contig00606_length=1050|500|60
log2(expression)
StageI
StageII
StageIII
StageIV
StageV
StageVI

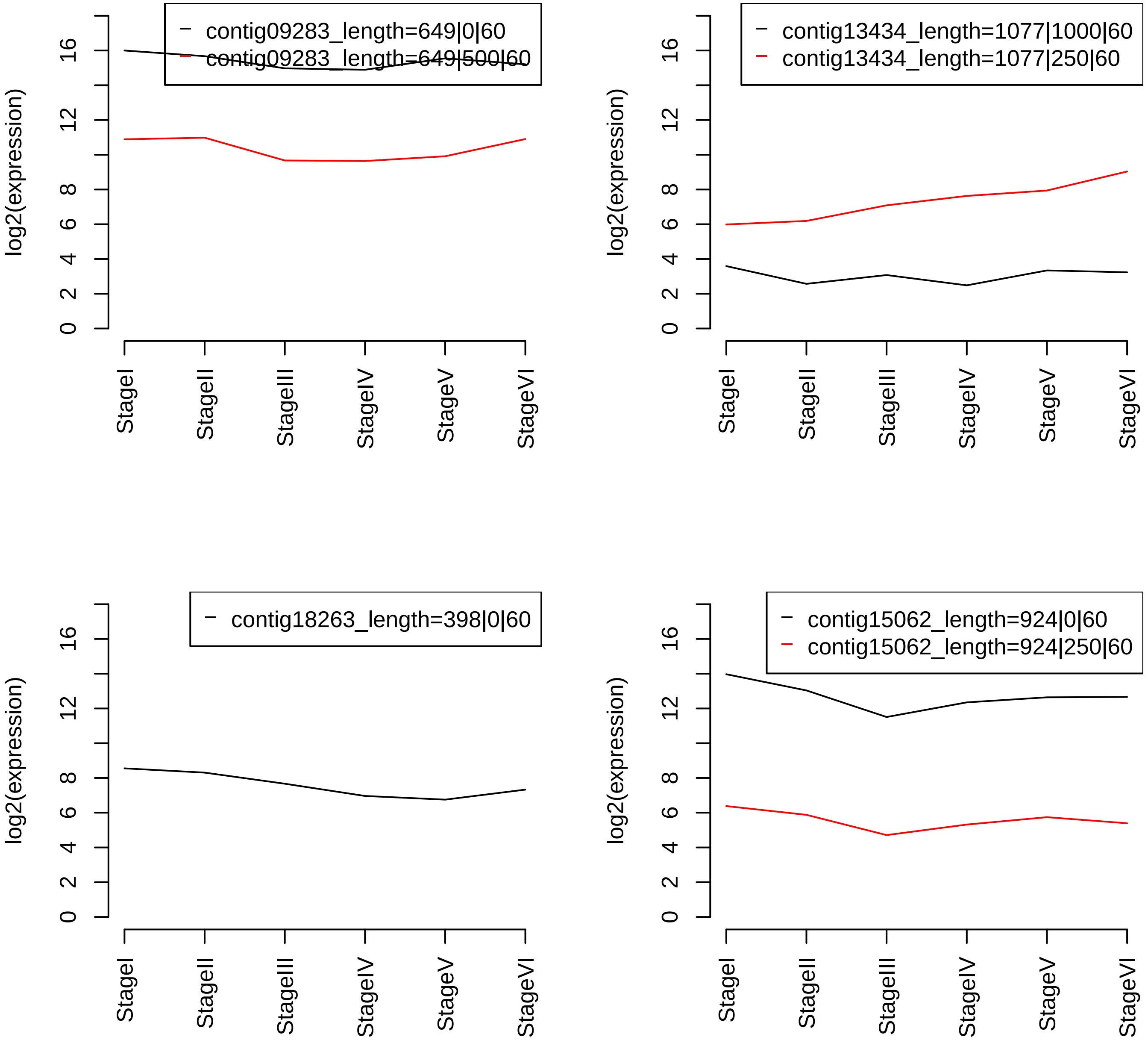

contig09283_length=649|0|60
contig09283_length=649|500|60
contig13434_length=1077|1000|60
contig13434_length=1077|250|60
contig18263_length=398|0|60
contig15062_length=924|0|60
contig15062_length=924|250|60
log2(expression)
0
2
4
6
8
12
16
StageI
StageII
StageIII
StageIV
StageV
StageVI

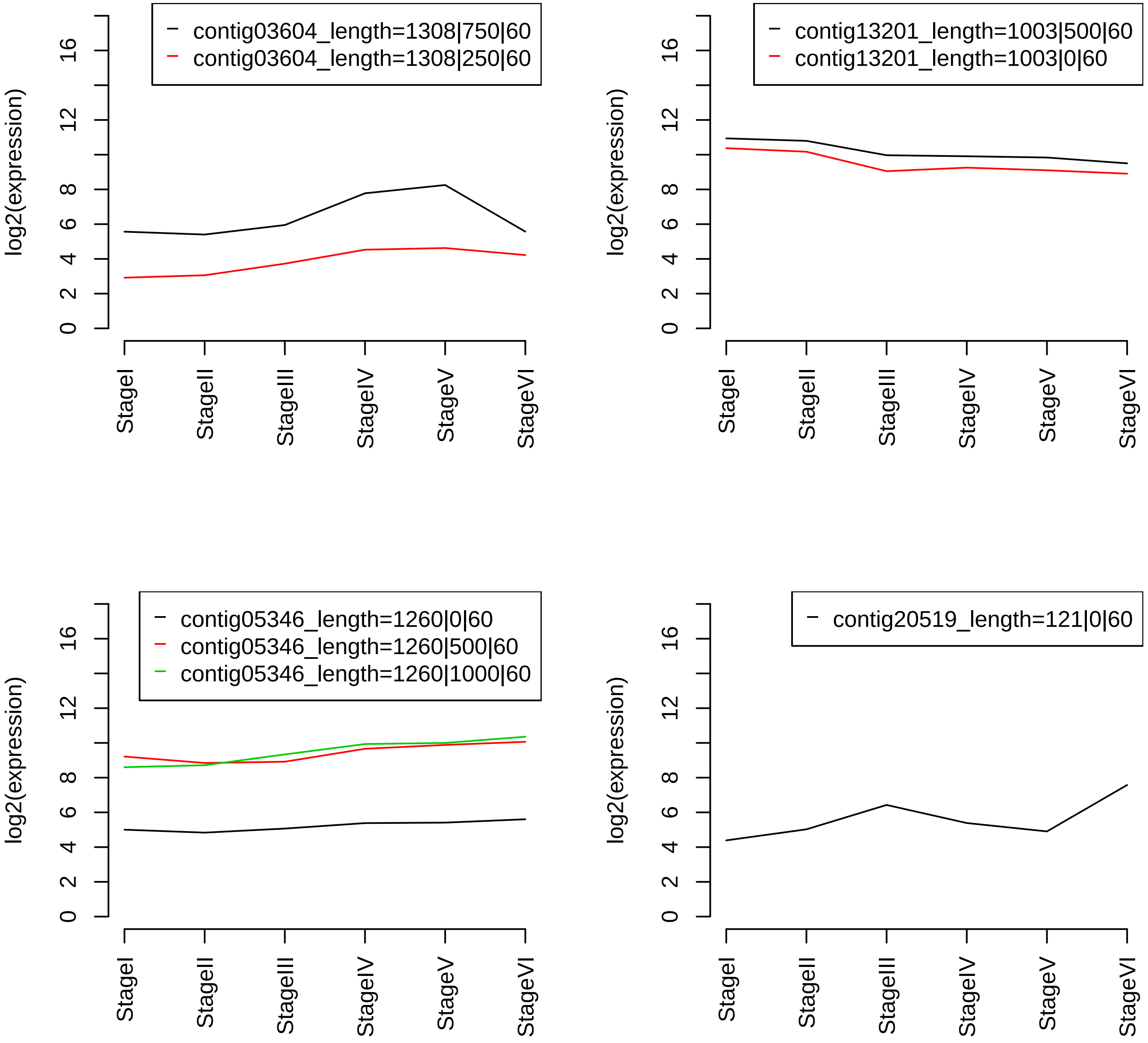

contig03604_length=1308|750|60
contig03604_length=1308|250|60
log2(expression)
0
2
4
6
8
12
16
StageI
StageII
StageIII
StageIV
StageV
StageVI
contig13201_length=1003|500|60
contig13201_length=1003|0|60
log2(expression)
contig05346_length=1260|0|60
contig05346_length=1260|500|60
contig05346_length=1260|1000|60
log2(expression)
contig20519_length=121|0|60
log2(expression)

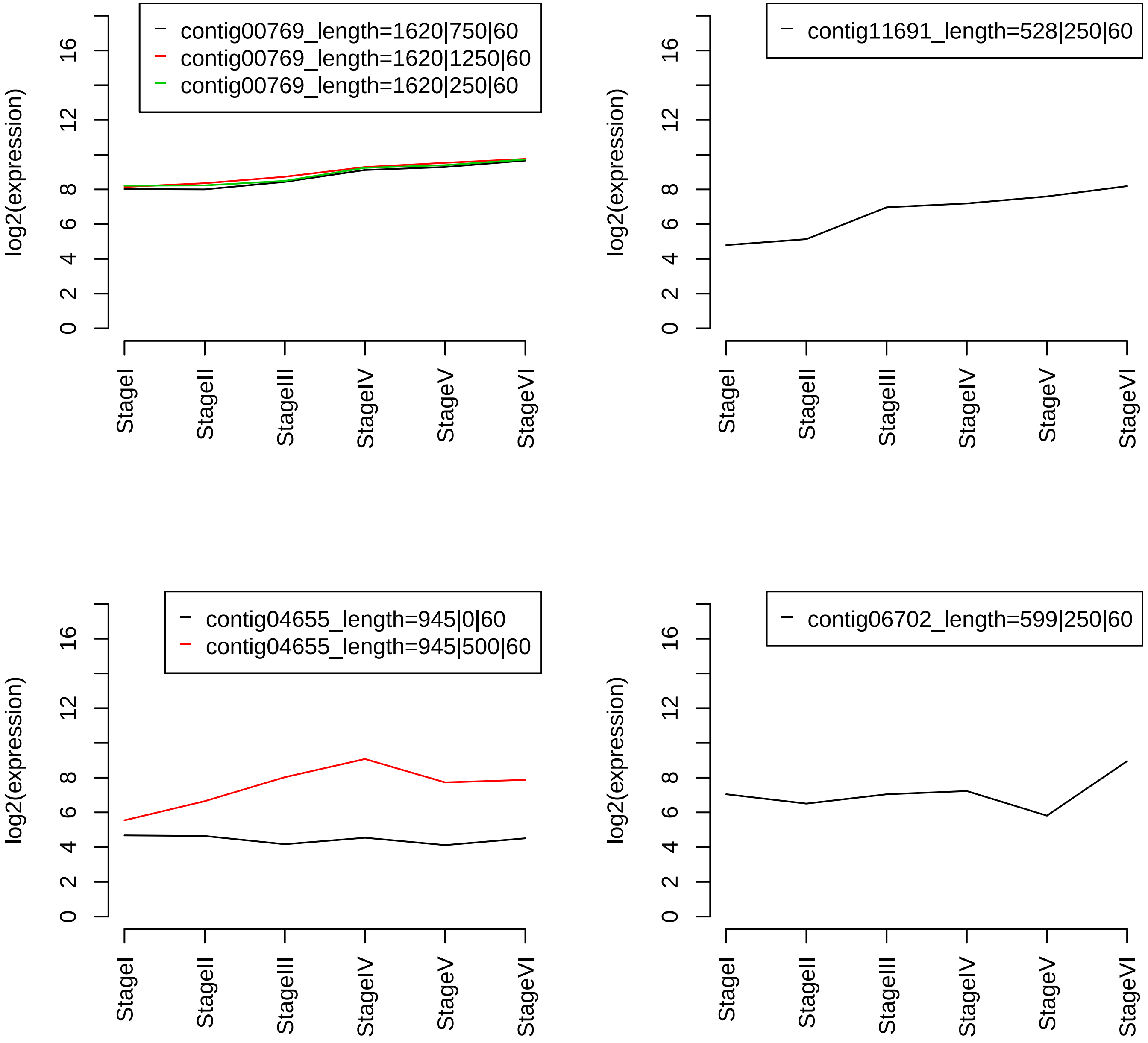

contig00769_length=1620|750|60
contig00769_length=1620|1250|60
contig00769_length=1620|250|60
contig11691_length=528|250|60
contig04655_length=945|0|60
contig04655_length=945|500|60
contig06702_length=599|250|60
log2(expression)
0
2
4
6
8
12
16
StageI
StageII
StageIII
StageIV
StageV
StageVI

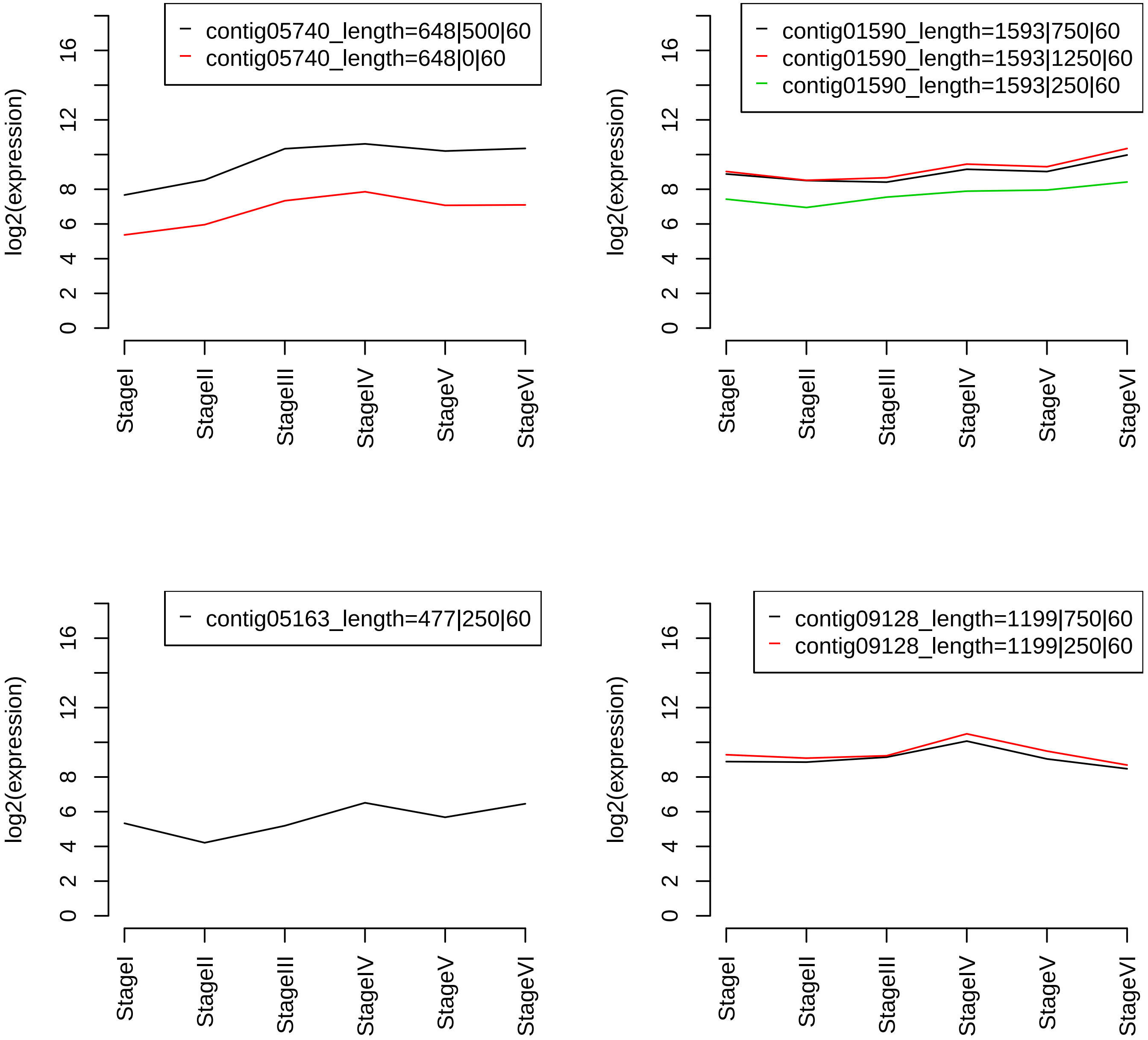
contig05740_length=648|500|60
contig05740_length=648|0|60
contig01590_length=1593|750|60
contig01590_length=1593|1250|60
contig01590_length=1593|250|60
contig05163_length=477|250|60
contig09128_length=1199|750|60
contig09128_length=1199|250|60
log2(expression)
0
2
4
6
8
12
16
StageI
StageII
StageIII
StageIV
StageV
StageVI

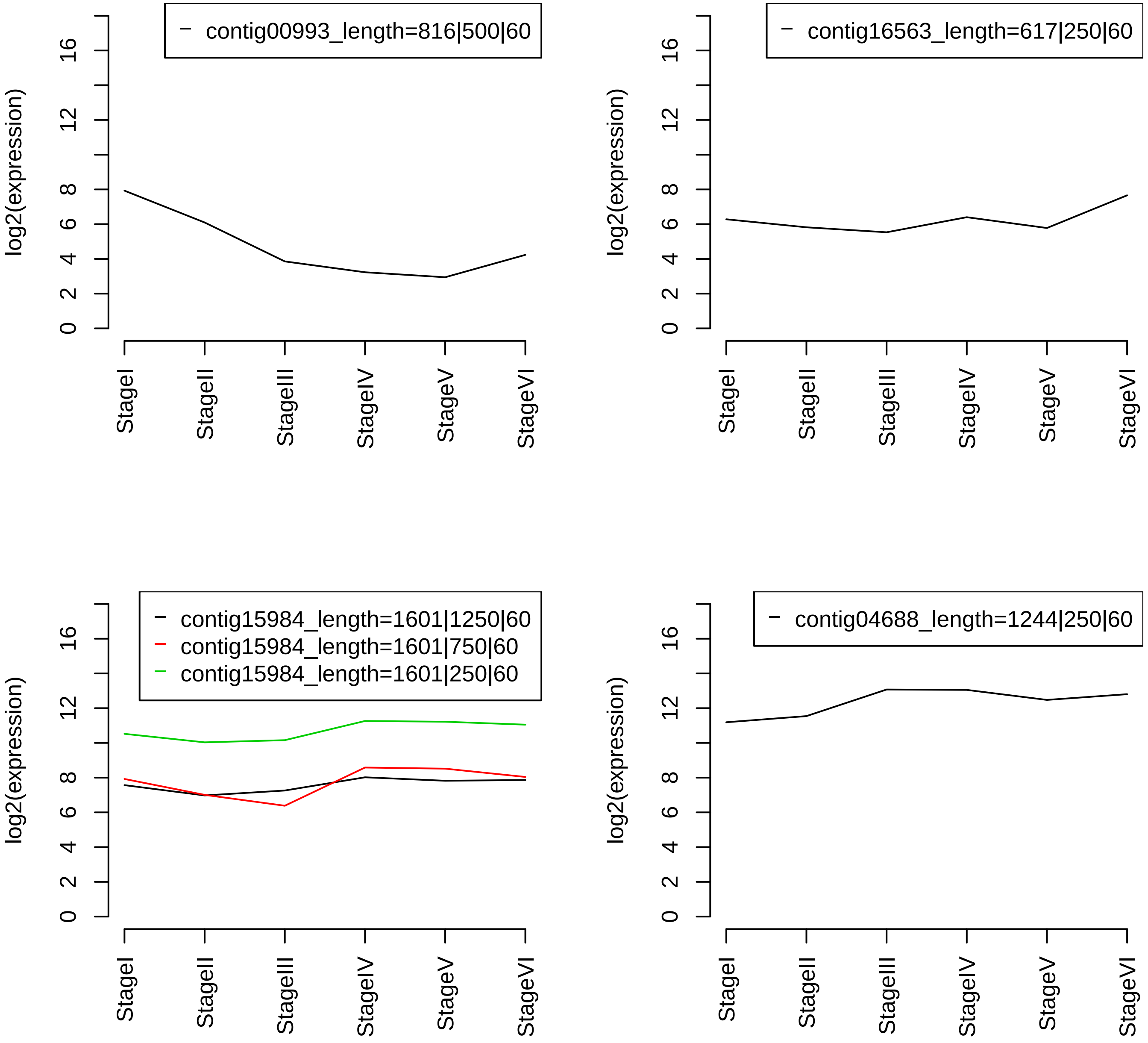

contig00993_length=816|500|60
contig16563_length=617|250|60
contig15984_length=1601|1250|60
contig15984_length=1601|750|60
contig15984_length=1601|250|60
contig04688_length=1244|250|60
log2(expression)
0
2
4
6
8
12
16
StageI
StageII
StageIII
StageIV
StageV
StageVI

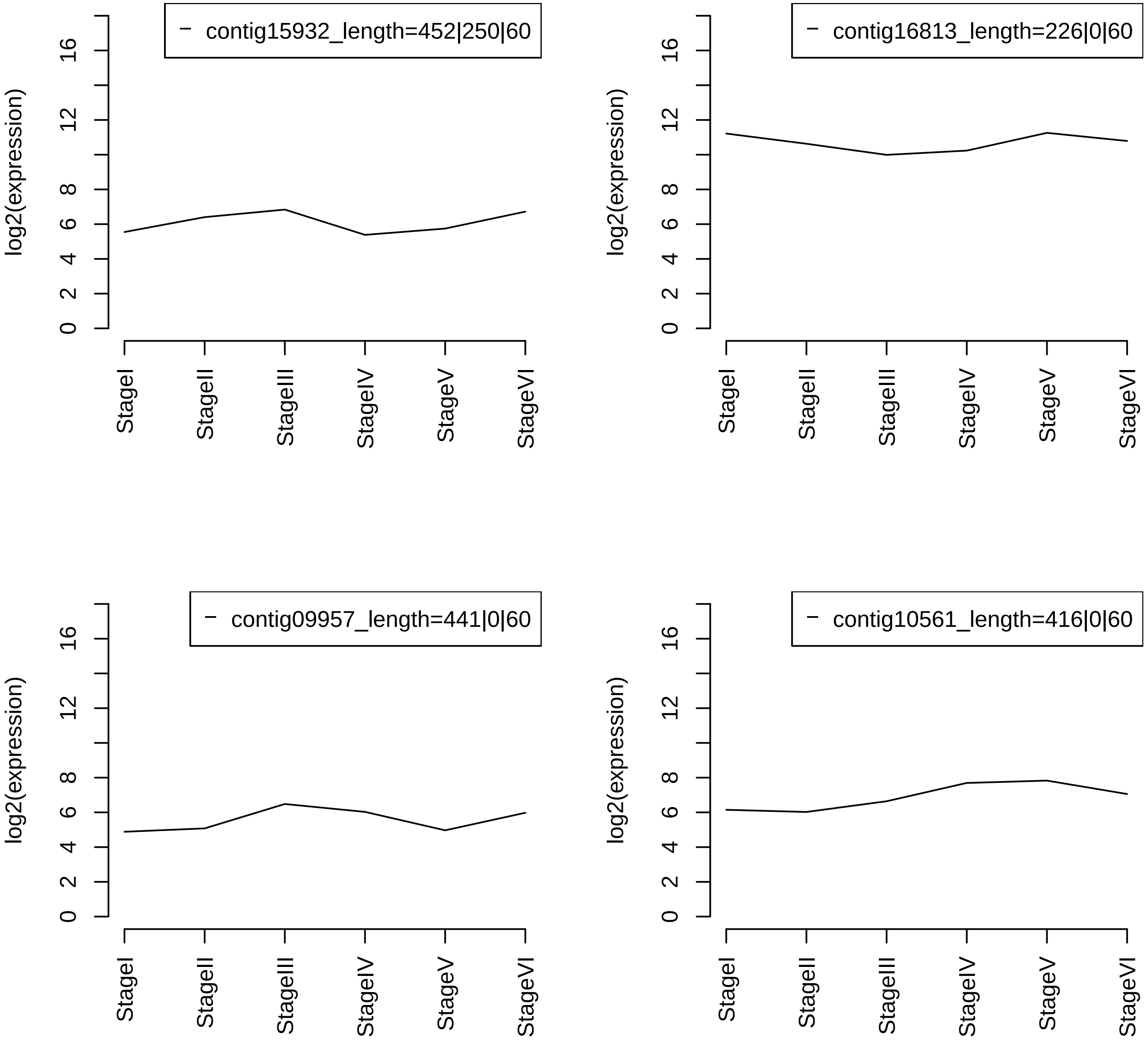

contig15932_length=452|250|60
contig16813_length=226|0|60
contig09957_length=441|0|60
contig10561_length=416|0|60
log2(expression)
0
2
4
6
8
12
16
StageI
StageII
StageIII
StageIV
StageV
StageVI

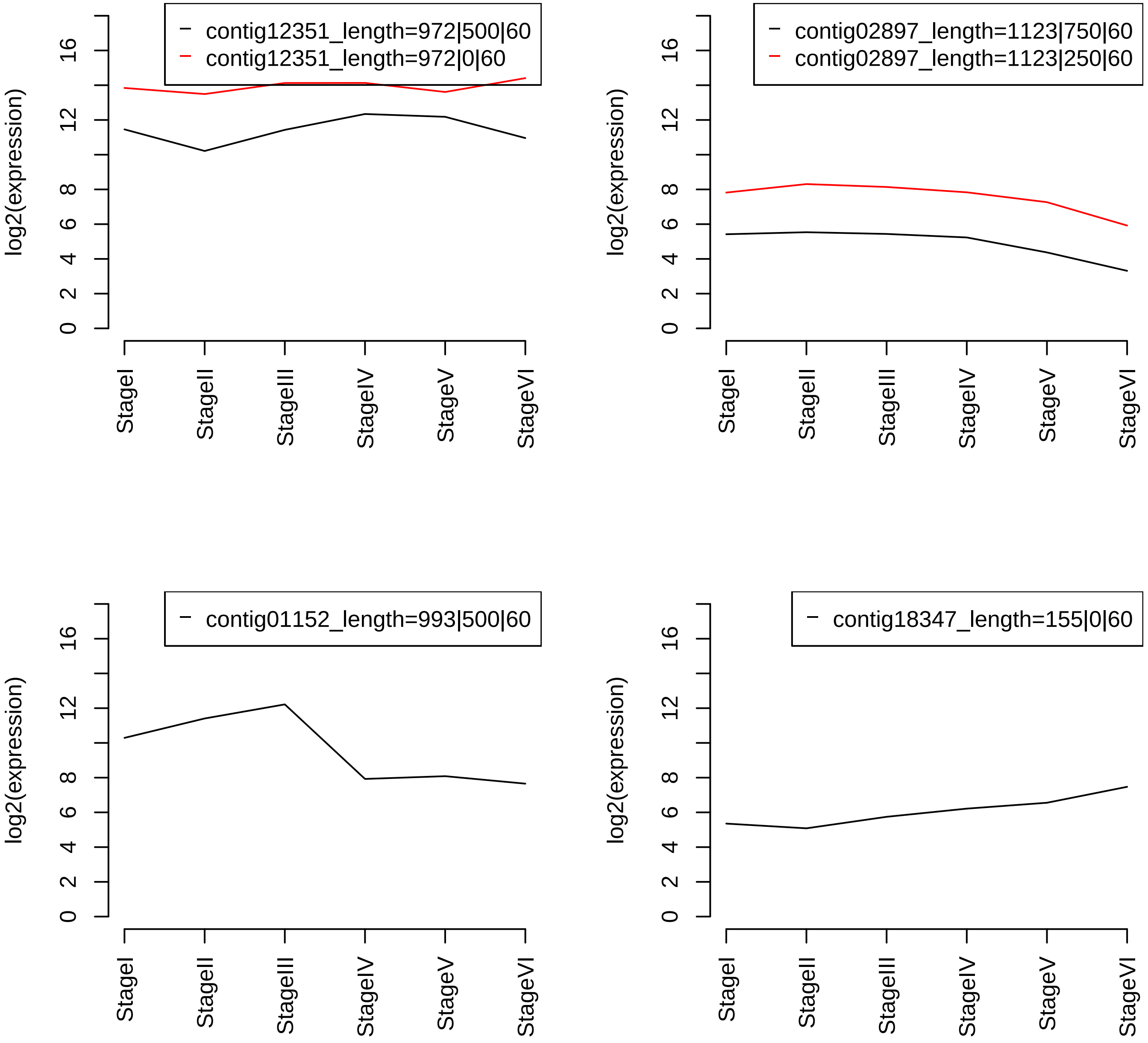

contig12351_length=972|500|60
contig12351_length=972|0|60
contig02897_length=1123|750|60
contig02897_length=1123|250|60
contig01152_length=993|500|60
contig18347_length=155|0|60
log2(expression)
0
2
4
6
8
12
16
StageI
StageII
StageIII
StageIV
StageV
StageVI

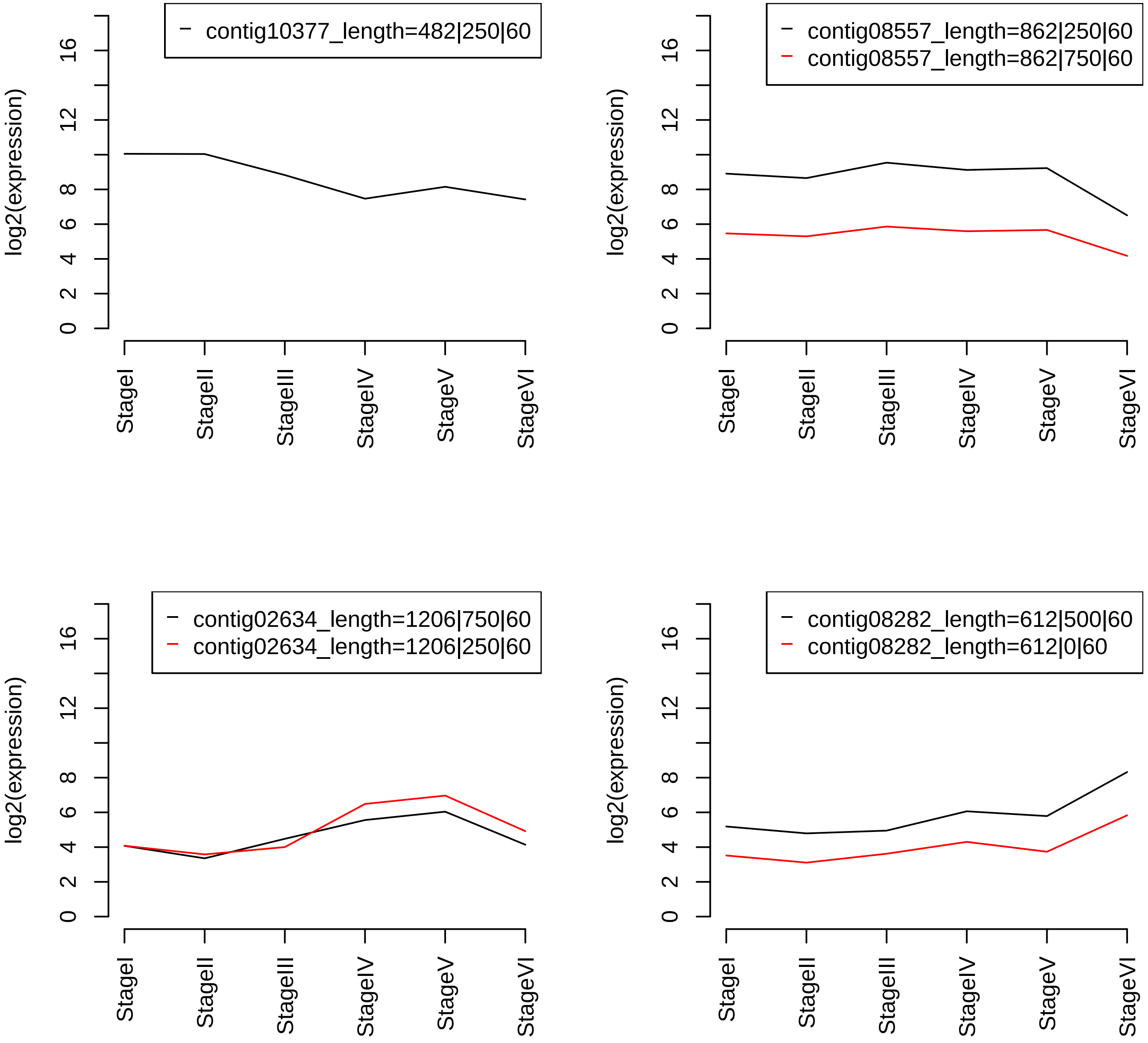
contig10377_length=482|250|60
contig08557_length=862|250|60
contig08557_length=862|750|60
contig02634_length=1206|750|60
contig02634_length=1206|250|60
contig08282_length=612|500|60
contig08282_length=612|0|60
log2(expression)
0
2
4
6
8
12
16
StageI
StageII
StageIII
StageIV
StageV
StageVI

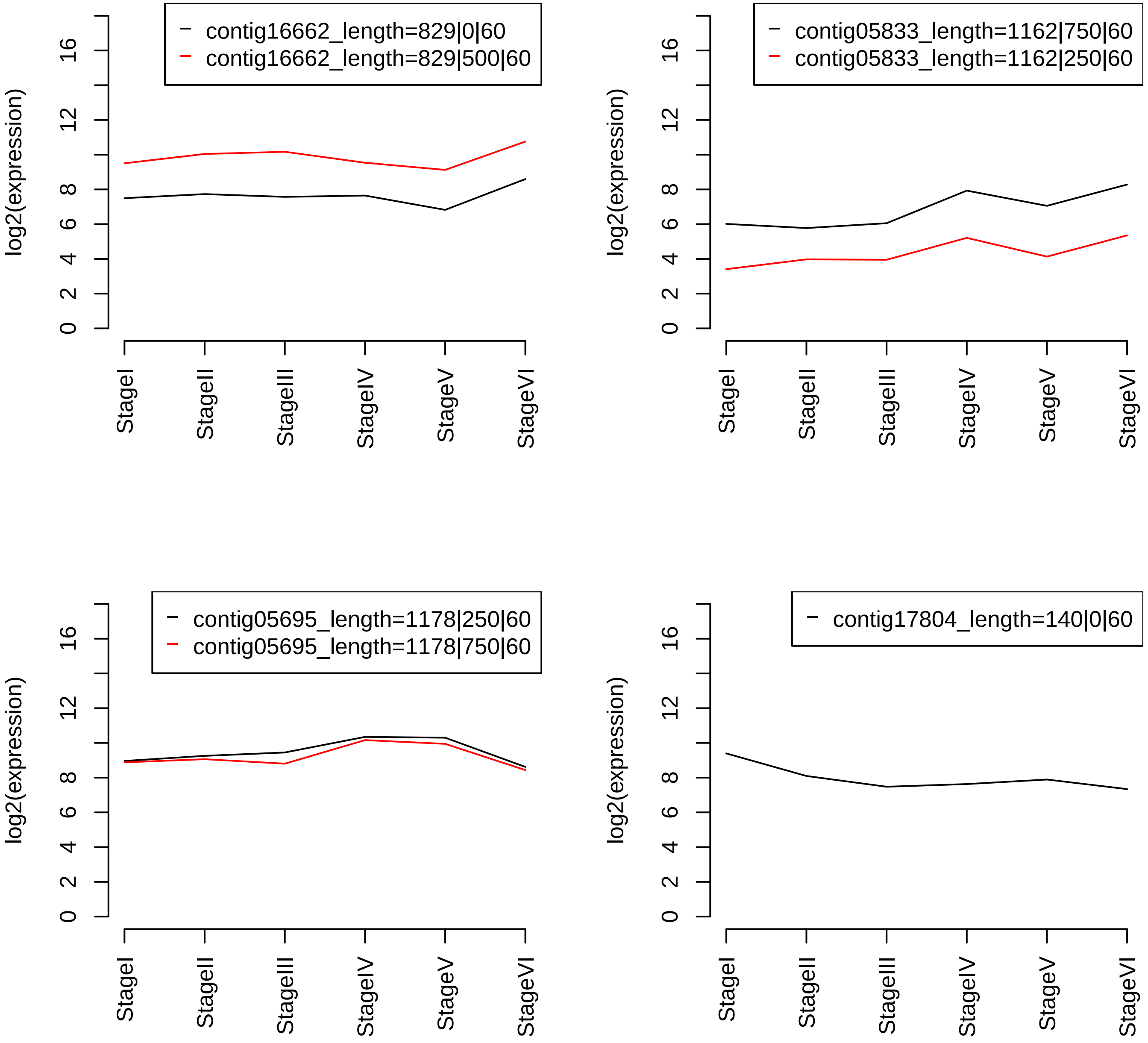
contig16662_length=829|0|60
contig16662_length=829|500|60
contig05833_length=1162|750|60
contig05833_length=1162|250|60
contig05695_length=1178|250|60
contig05695_length=1178|750|60
contig17804_length=140|0|60
log2(expression)
0
2
4
6
8
12
16
StageI
StageII
StageIII
StageIV
StageV
StageVI

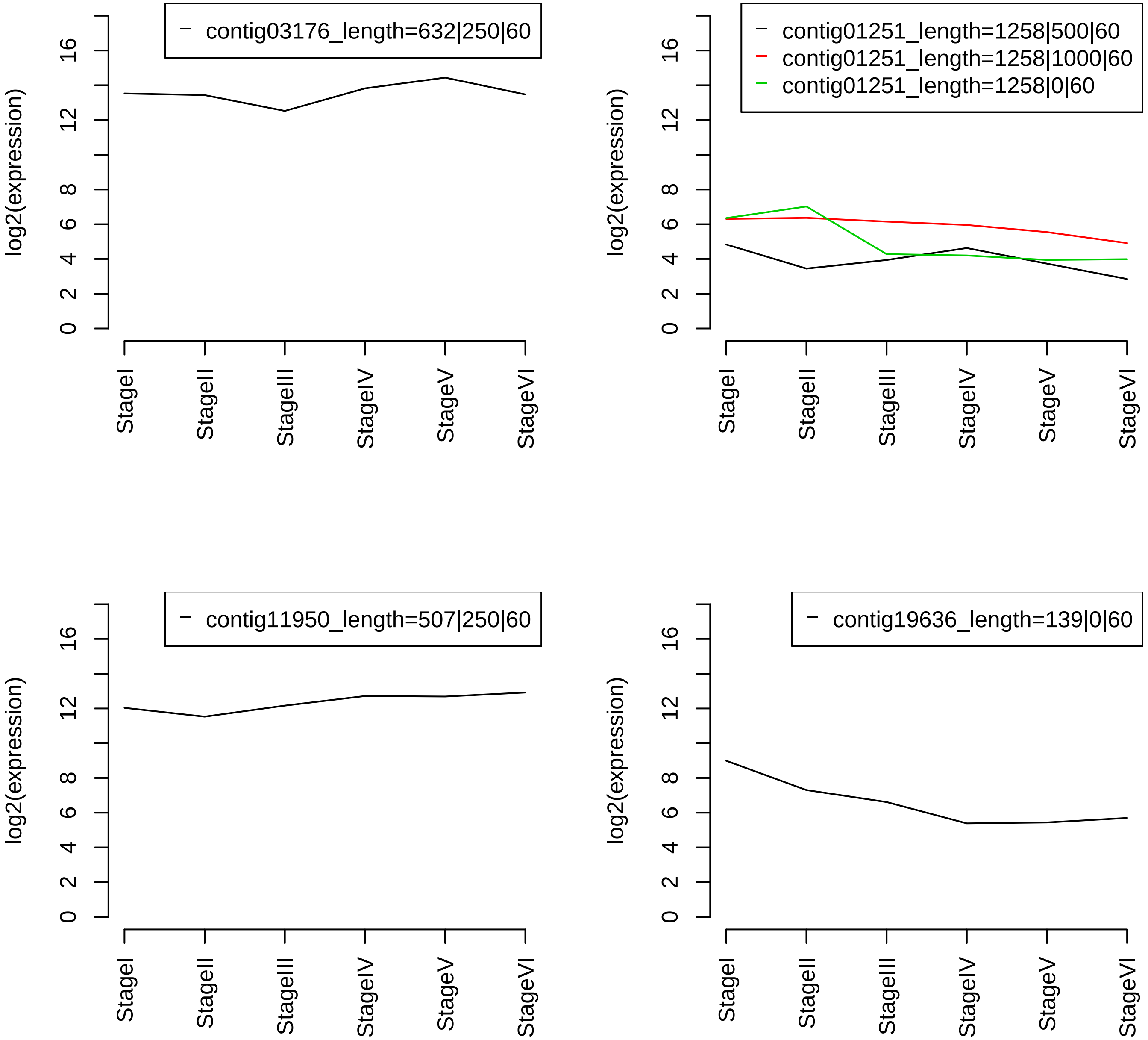
contig03176_length=632|250|60
contig01251_length=1258|500|60
contig01251_length=1258|1000|60
contig01251_length=1258|0|60
contig11950_length=507|250|60
contig19636_length=139|0|60
log2(expression)
0 2 4 6 8 12 16
StageI StageII StageIII StageIV StageV StageVI

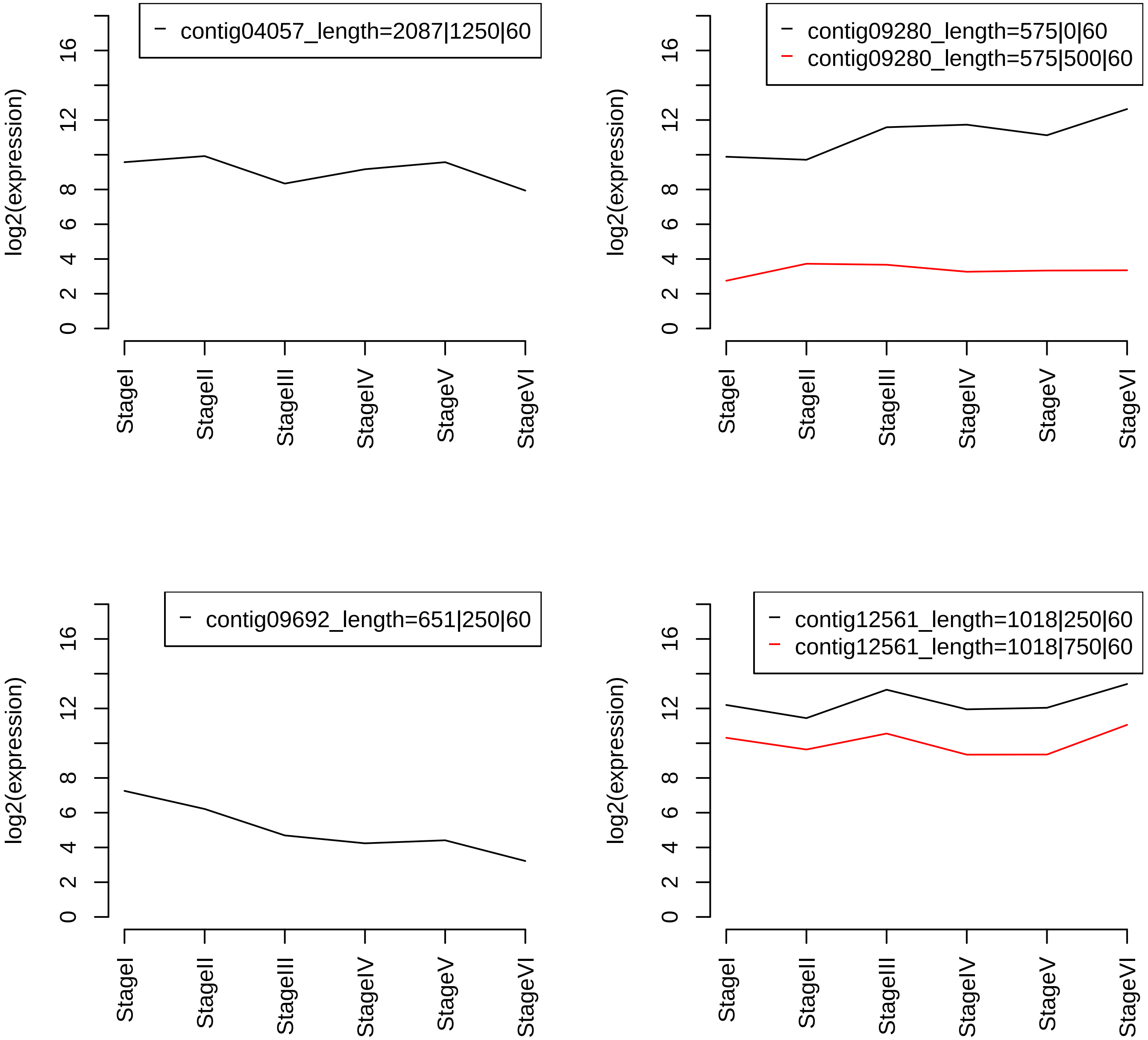
contig04057_length=2087|1250|60
contig09280_length=575|0|60
contig09280_length=575|500|60
contig09692_length=651|250|60
contig12561_length=1018|250|60
contig12561_length=1018|750|60
log2(expression)
0
2
4
6
8
12
16
StageI
StageII
StageIII
StageIV
StageV
StageVI

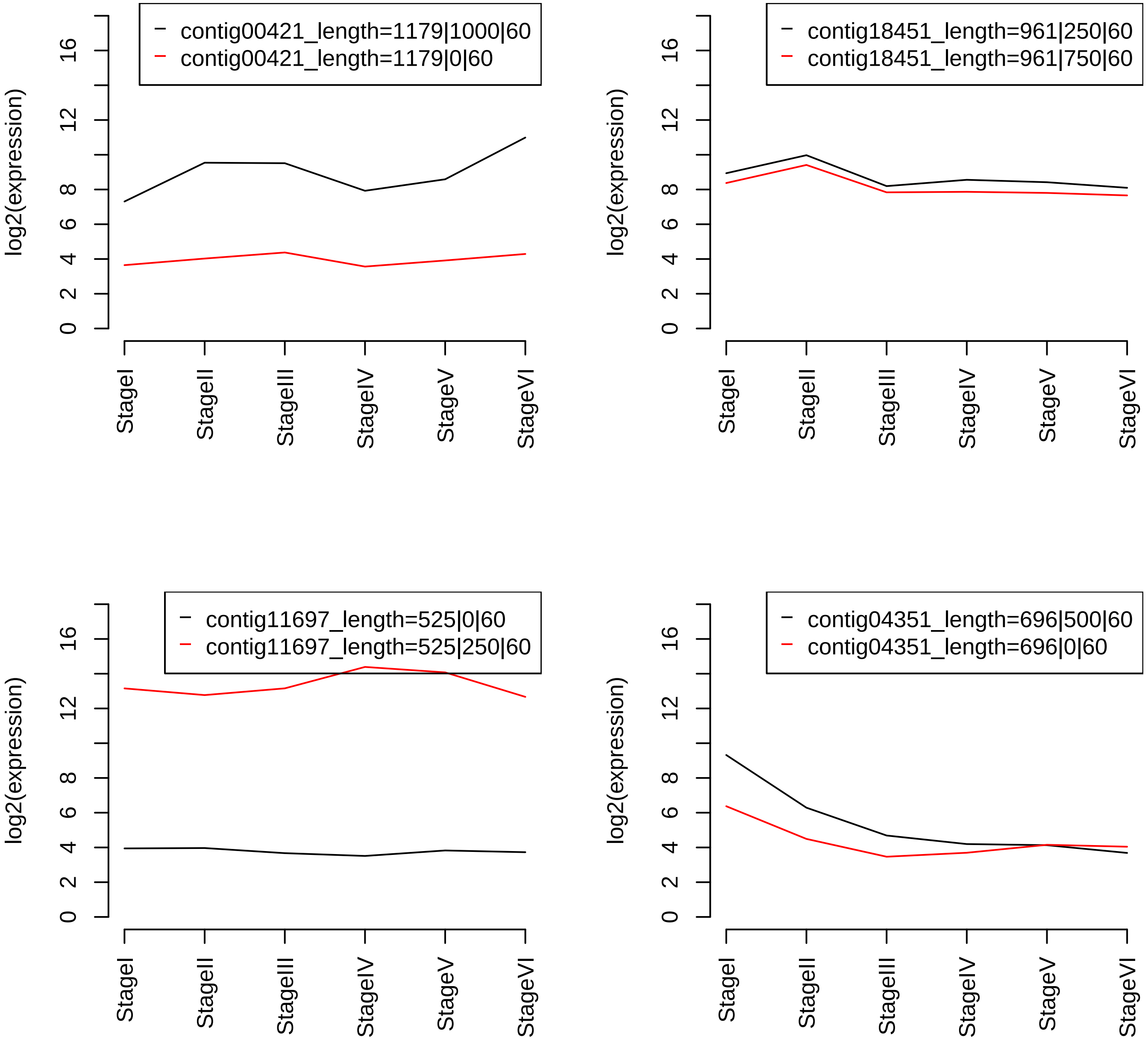

contig00421_length=1179|1000|60
contig00421_length=1179|0|60
contig18451_length=961|250|60
contig18451_length=961|750|60
contig11697_length=525|0|60
contig11697_length=525|250|60
contig04351_length=696|500|60
contig04351_length=696|0|60
log2(expression)
0
2
4
6
8
12
16
StageI
StageII
StageIII
StageIV
StageV
StageVI

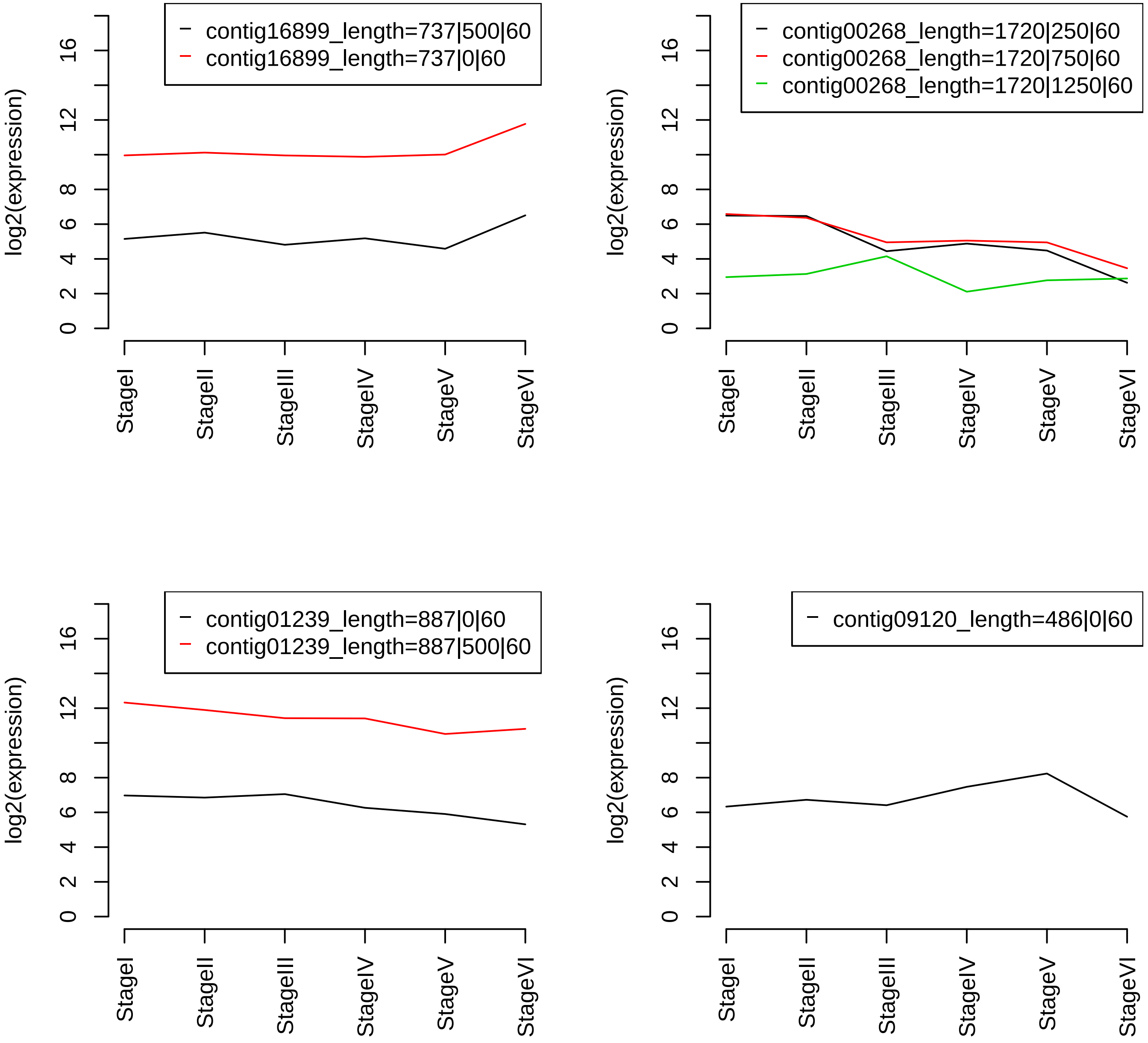
contig16899_length=737|500|60
contig16899_length=737|0|60
contig00268_length=1720|250|60
contig00268_length=1720|750|60
contig00268_length=1720|1250|60
contig01239_length=887|0|60
contig01239_length=887|500|60
contig09120_length=486|0|60
log2(expression)
0
2
4
6
8
12
16
StageI
StageII
StageIII
StageIV
StageV
StageVI

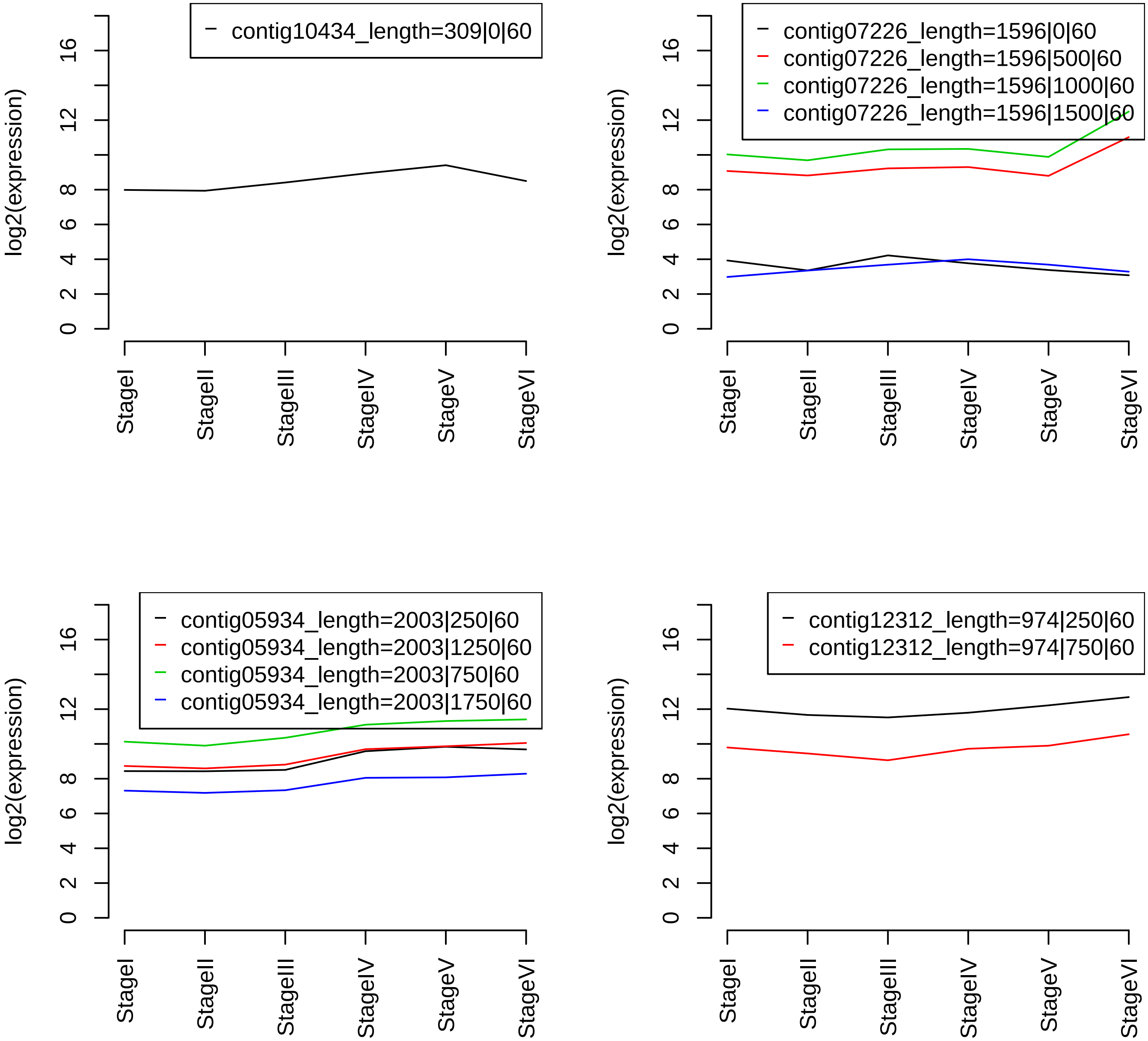
contig10434_length=309|0|60
contig07226_length=1596|0|60
contig07226_length=1596|500|60
contig07226_length=1596|1000|60
contig07226_length=1596|1500|60
contig05934_length=2003|250|60
contig05934_length=2003|1250|60
contig05934_length=2003|750|60
contig05934_length=2003|1750|60
contig12312_length=974|250|60
contig12312_length=974|750|60
log2(expression)
StageI
StageII
StageIII
StageIV
StageV
StageVI

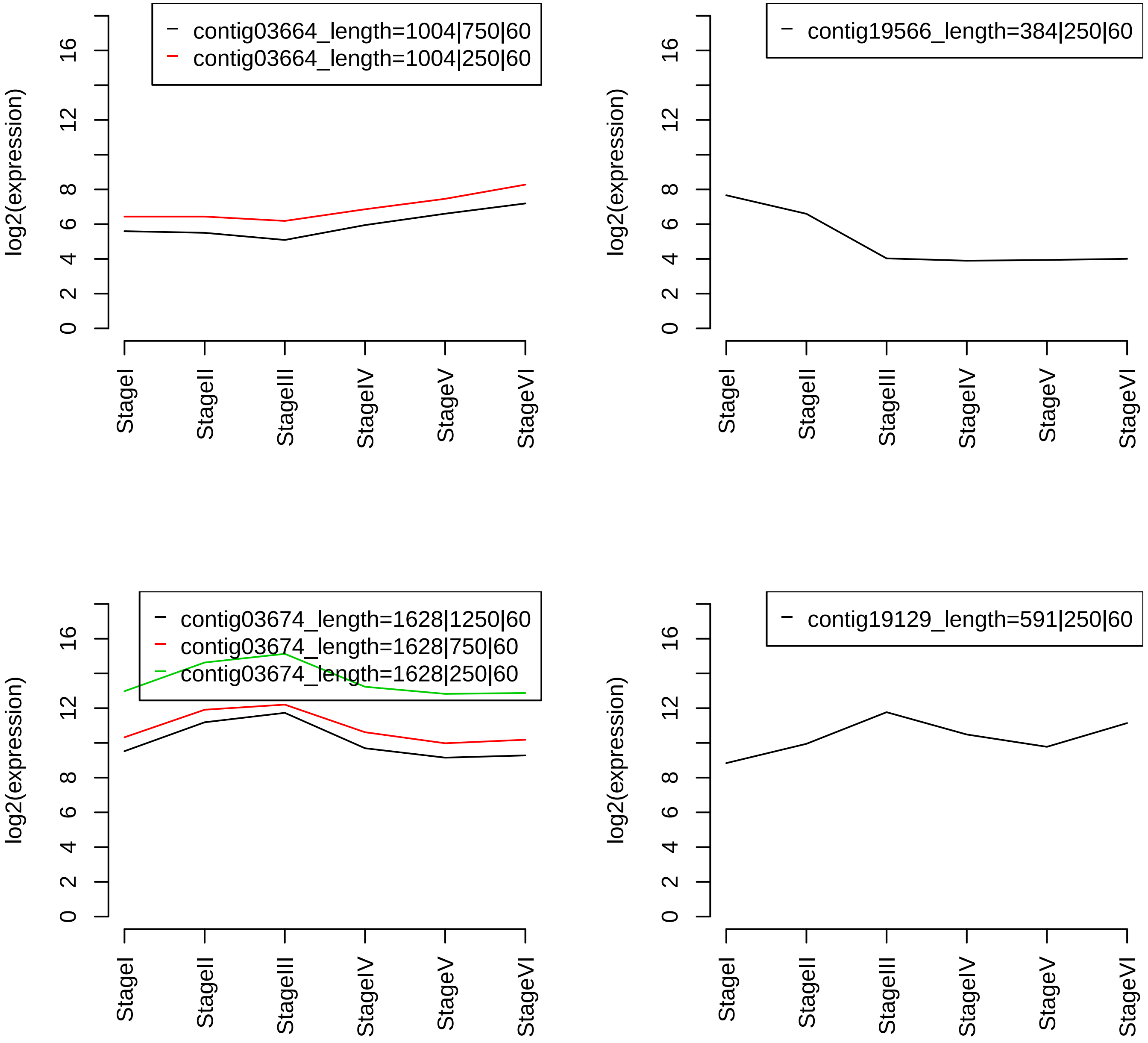

contig03664_length=1004|750|60
contig03664_length=1004|250|60
contig19566_length=384|250|60
contig03674_length=1628|1250|60
contig03674_length=1628|750|60
contig03674_length=1628|250|60
contig19129_length=591|250|60
log2(expression)
0
2
4
6
8
12
16
StageI
StageII
StageIII
StageIV
StageV
StageVI

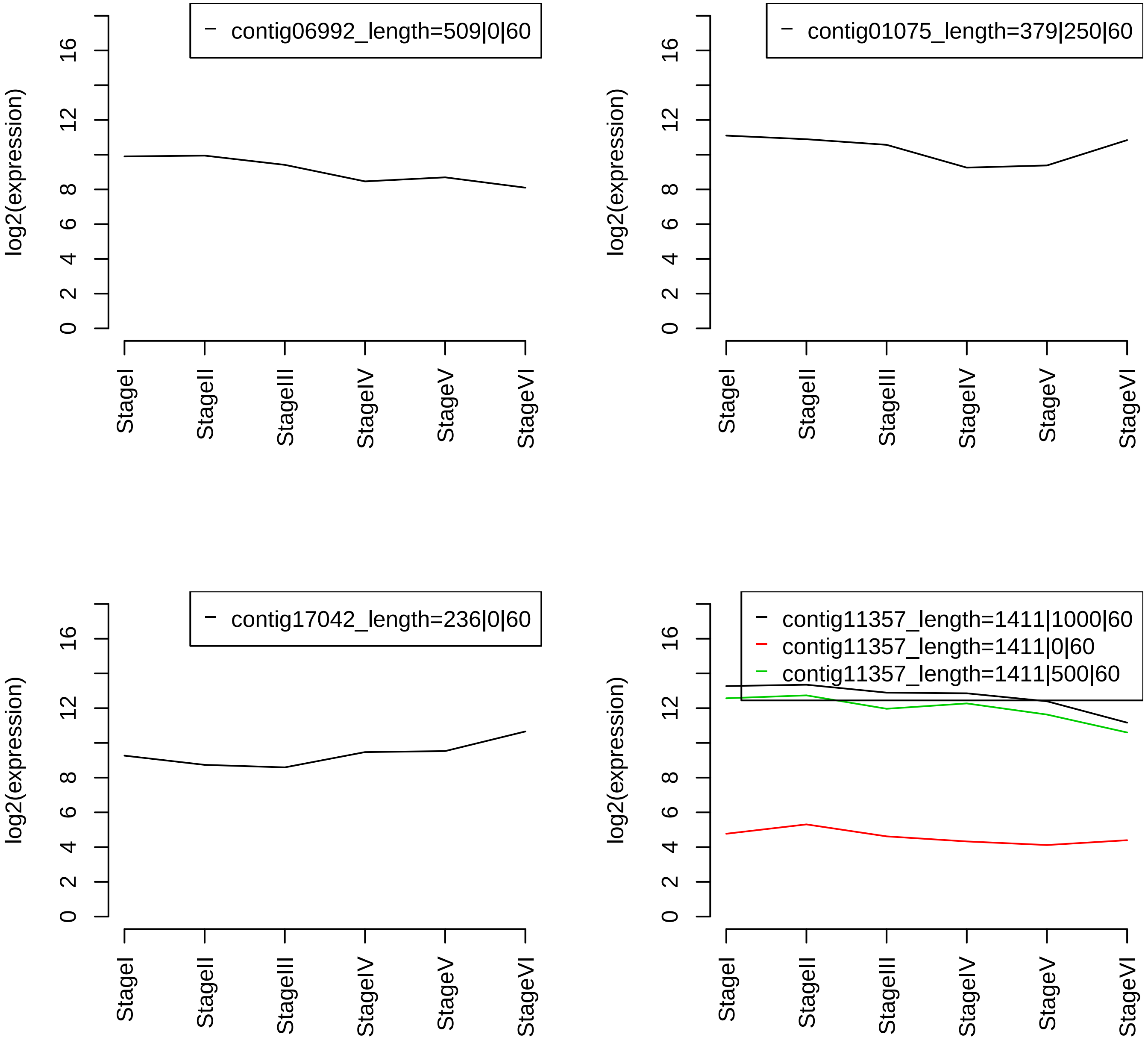
contig06992_length=509|0|60
contig01075_length=379|250|60
contig17042_length=236|0|60
contig11357_length=1411|1000|60
contig11357_length=1411|0|60
contig11357_length=1411|500|60
log2(expression)
0
2
4
6
8
12
16
StageI
StageII
StageIII
StageIV
StageV
StageVI

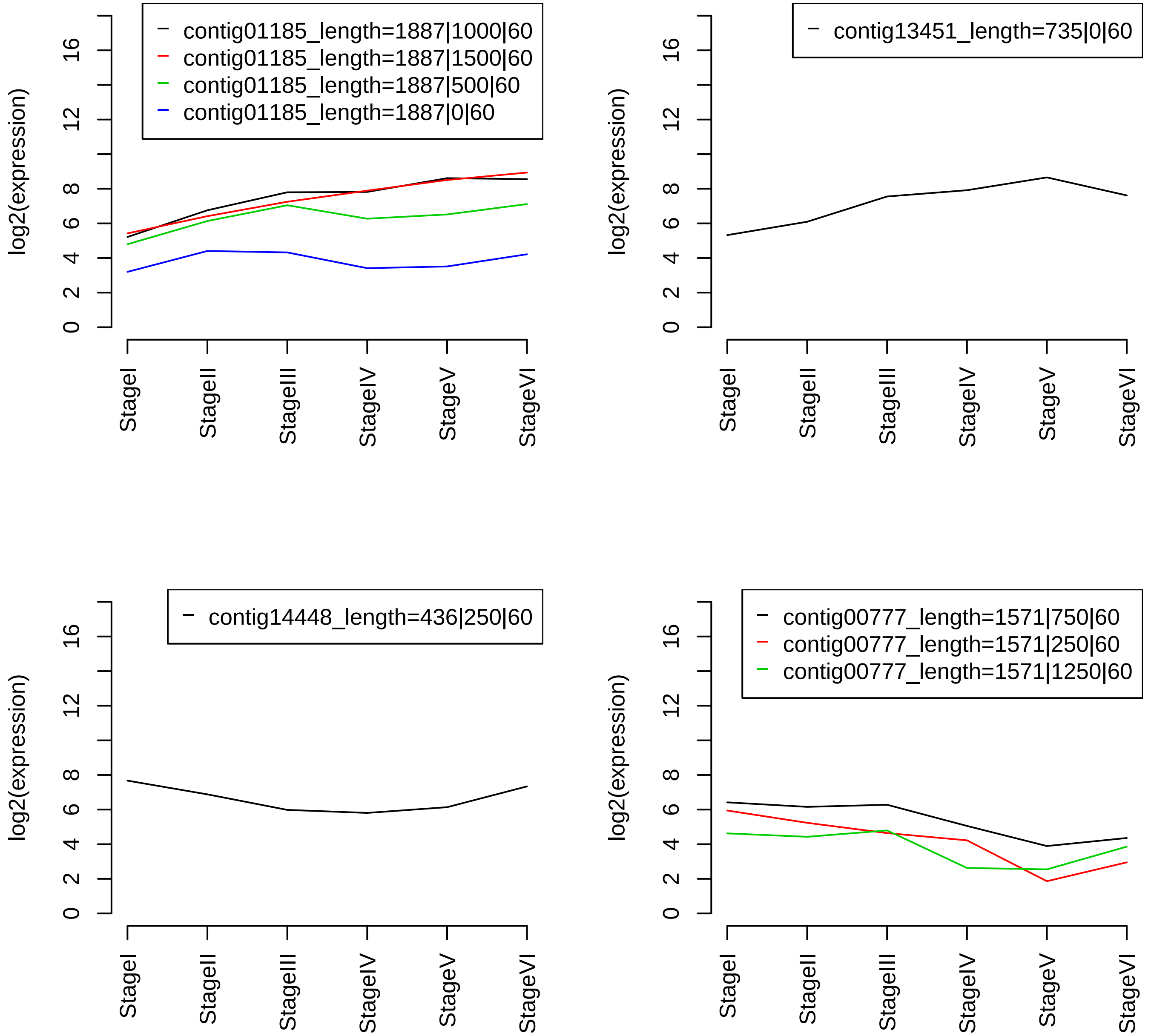

contig01185_length=1887|1000|60
contig01185_length=1887|1500|60
contig01185_length=1887|500|60
contig01185_length=1887|0|60
contig13451_length=735|0|60
contig14448_length=436|250|60
contig00777_length=1571|750|60
contig00777_length=1571|250|60
contig00777_length=1571|1250|60
log2(expression)
0 2 4 6 8 12 16
StageI StageII StageIII StageIV StageV StageVI

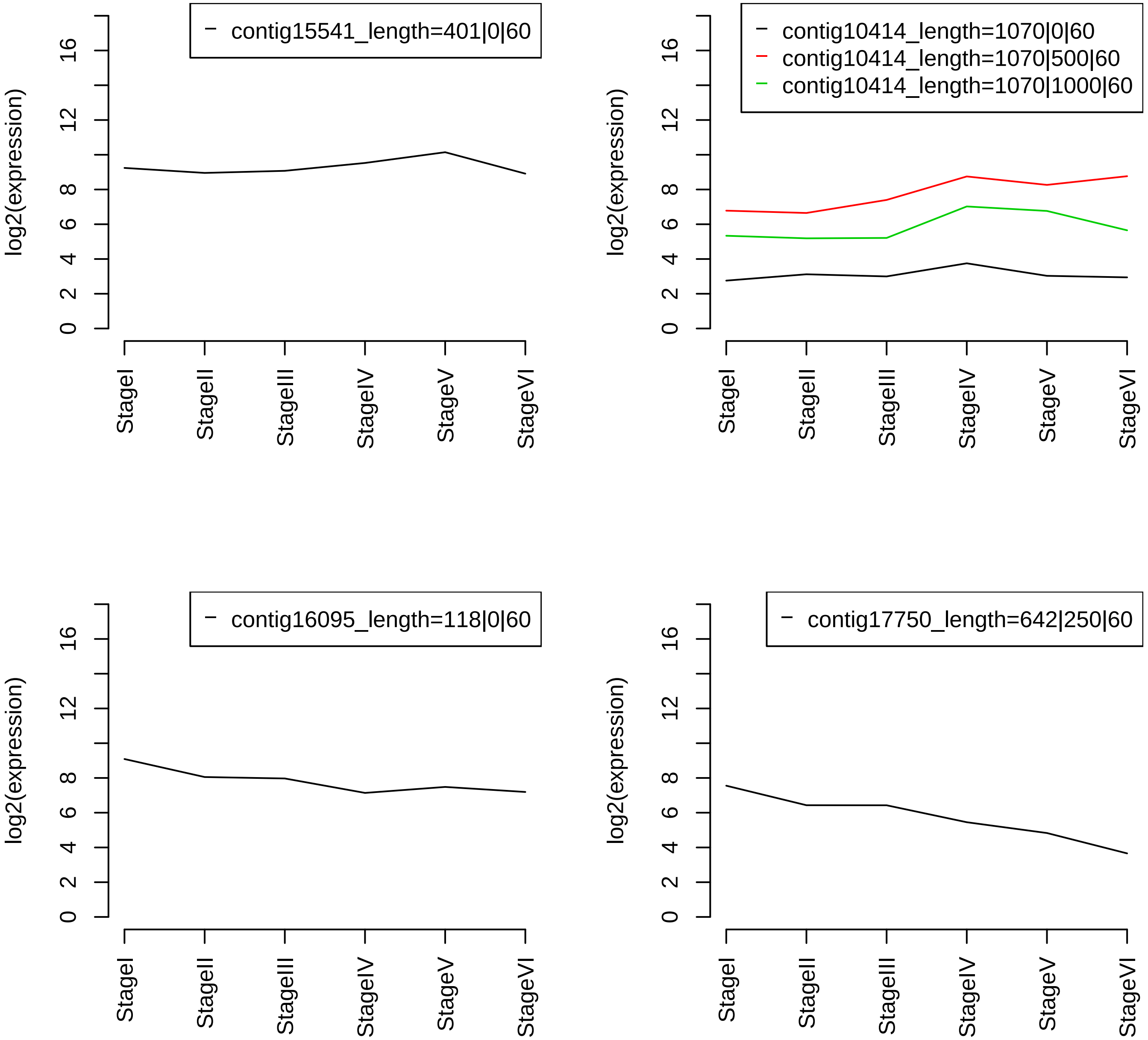

contig15541_length=401|0|60
contig10414_length=1070|0|60
contig10414_length=1070|500|60
contig10414_length=1070|1000|60
contig16095_length=118|0|60
contig17750_length=642|250|60
log2(expression)
0
2
4
6
8
12
16
StageI
StageII
StageIII
StageIV
StageV
StageVI

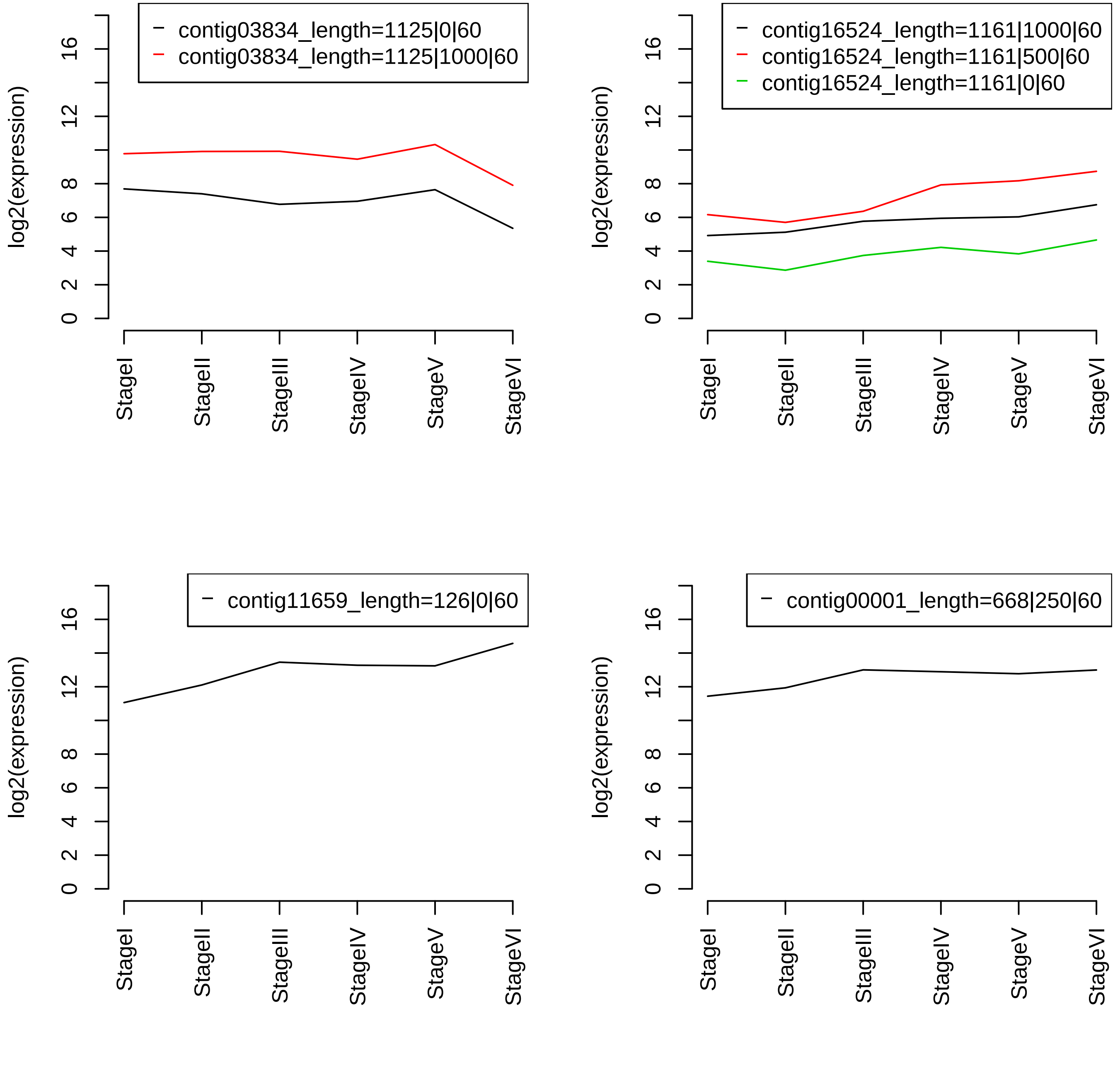

contig03834_length=1125|0|60
contig03834_length=1125|1000|60
contig16524_length=1161|1000|60
contig16524_length=1161|500|60
contig16524_length=1161|0|60
contig11659_length=126|0|60
contig00001_length=668|250|60
log2(expression)
0 2 4 6 8 12 16
StageI StageII StageIII StageIV StageV StageVI

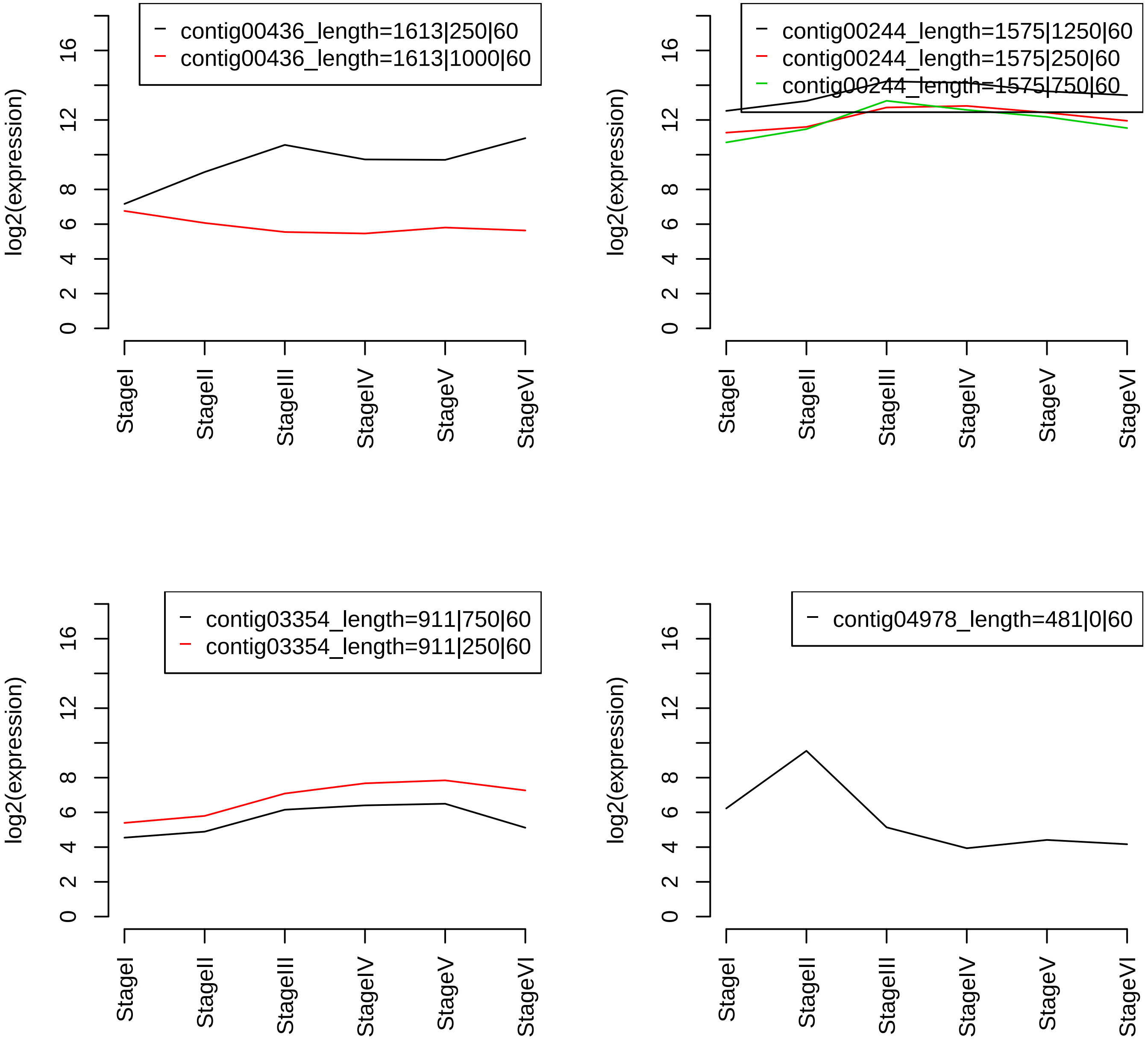
contig00436_length=1613|250|60
contig00436_length=1613|1000|60
contig00244_length=1575|1250|60
contig00244_length=1575|250|60
contig00244_length=1575|750|60
contig03354_length=911|750|60
contig03354_length=911|250|60
contig04978_length=481|0|60
log2(expression)
0
2
4
6
8
12
16
StageI
StageII
StageIII
StageIV
StageV
StageVI

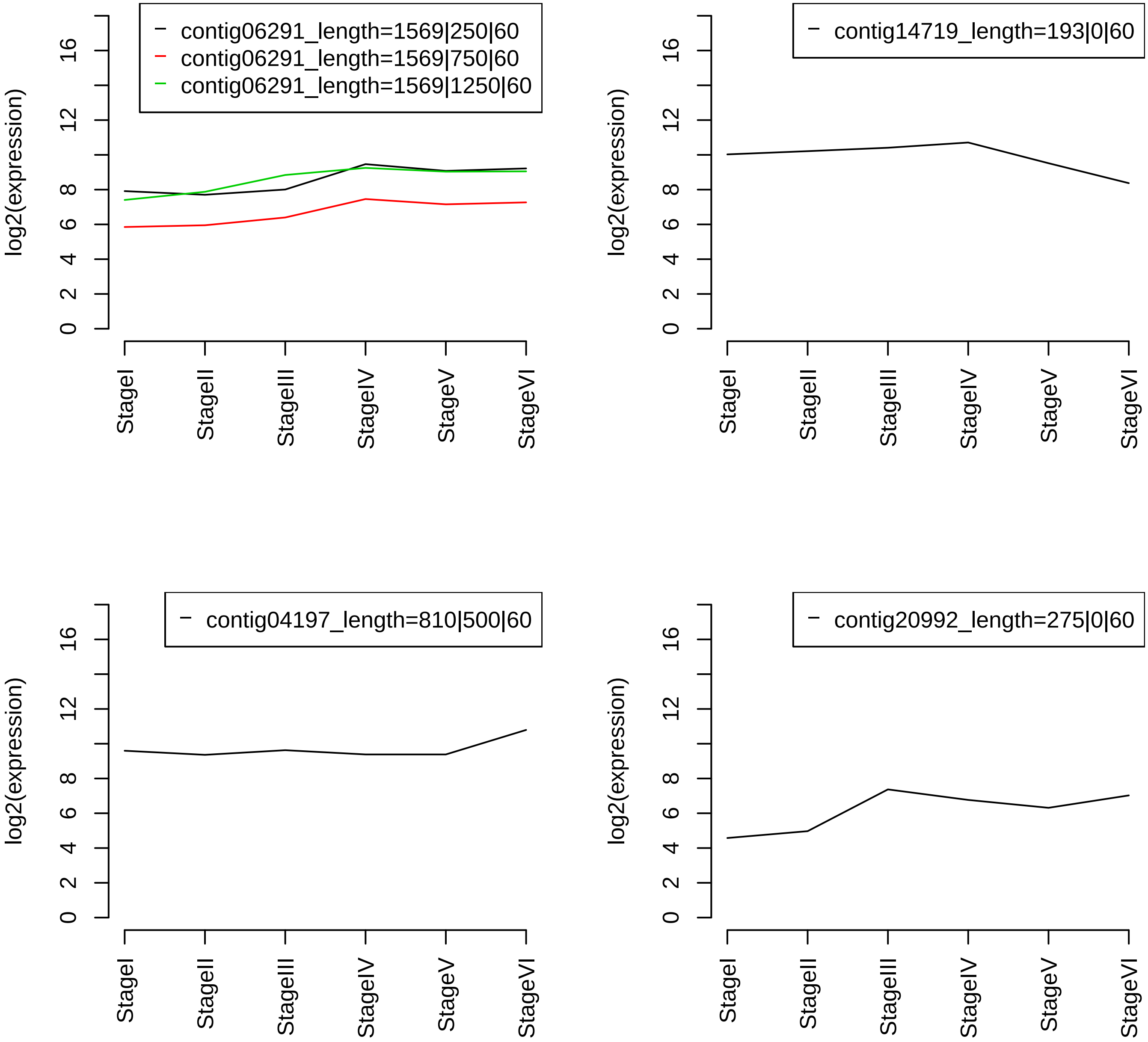

contig06291_length=1569|250|60
contig06291_length=1569|750|60
contig06291_length=1569|1250|60
contig14719_length=193|0|60
contig04197_length=810|500|60
contig20992_length=275|0|60
log2(expression)
0
2
4
6
8
12
16
StageI
StageII
StageIII
StageIV
StageV
StageVI

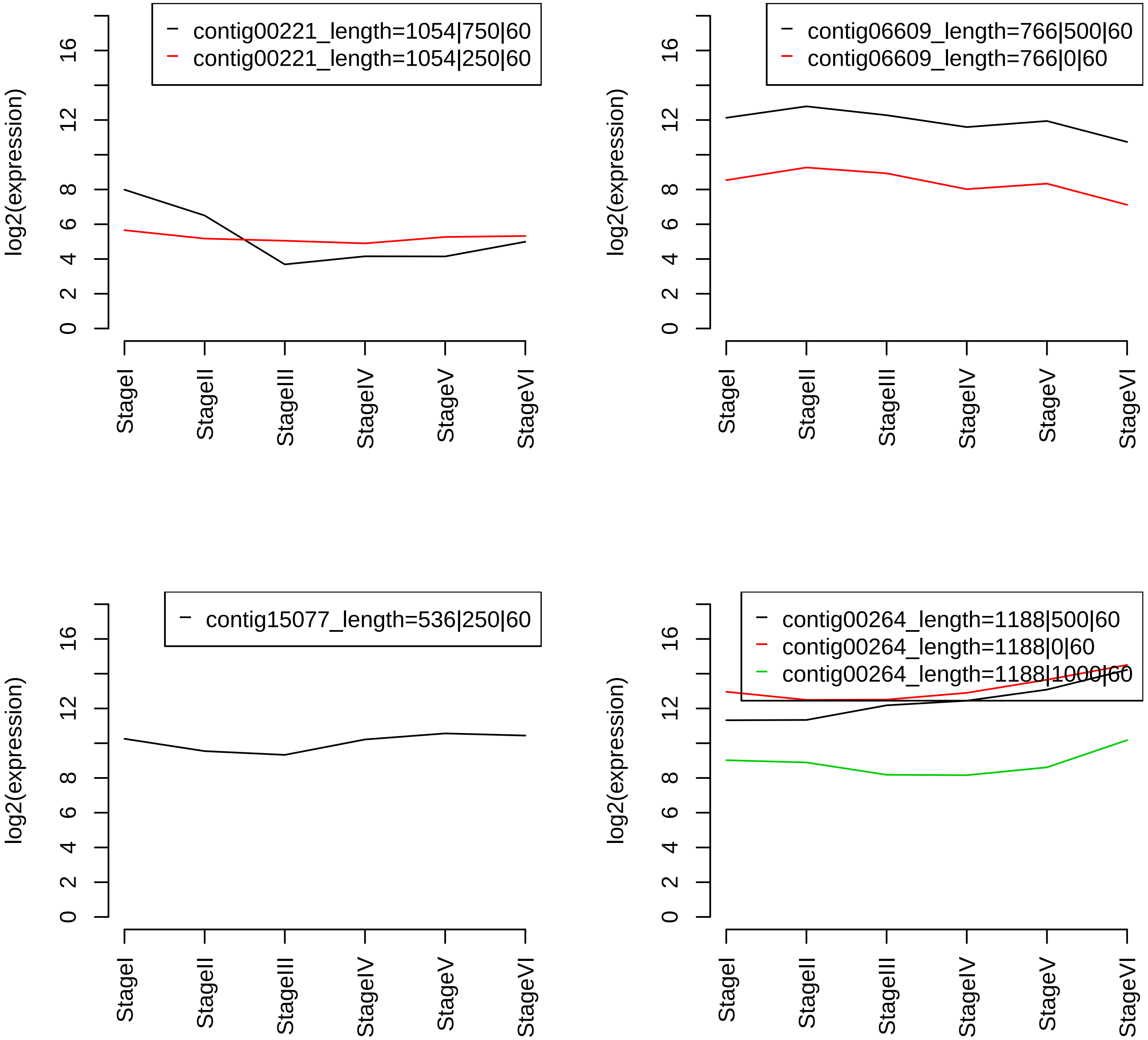
contig00221_length=1054|750|60
contig00221_length=1054|250|60
contig06609_length=766|500|60
contig06609_length=766|0|60
contig15077_length=536|250|60
contig00264_length=1188|500|60
contig00264_length=1188|0|60
contig00264_length=1188|1000|60
log2(expression)
StageI
StageII
StageIII
StageIV
StageV
StageVI

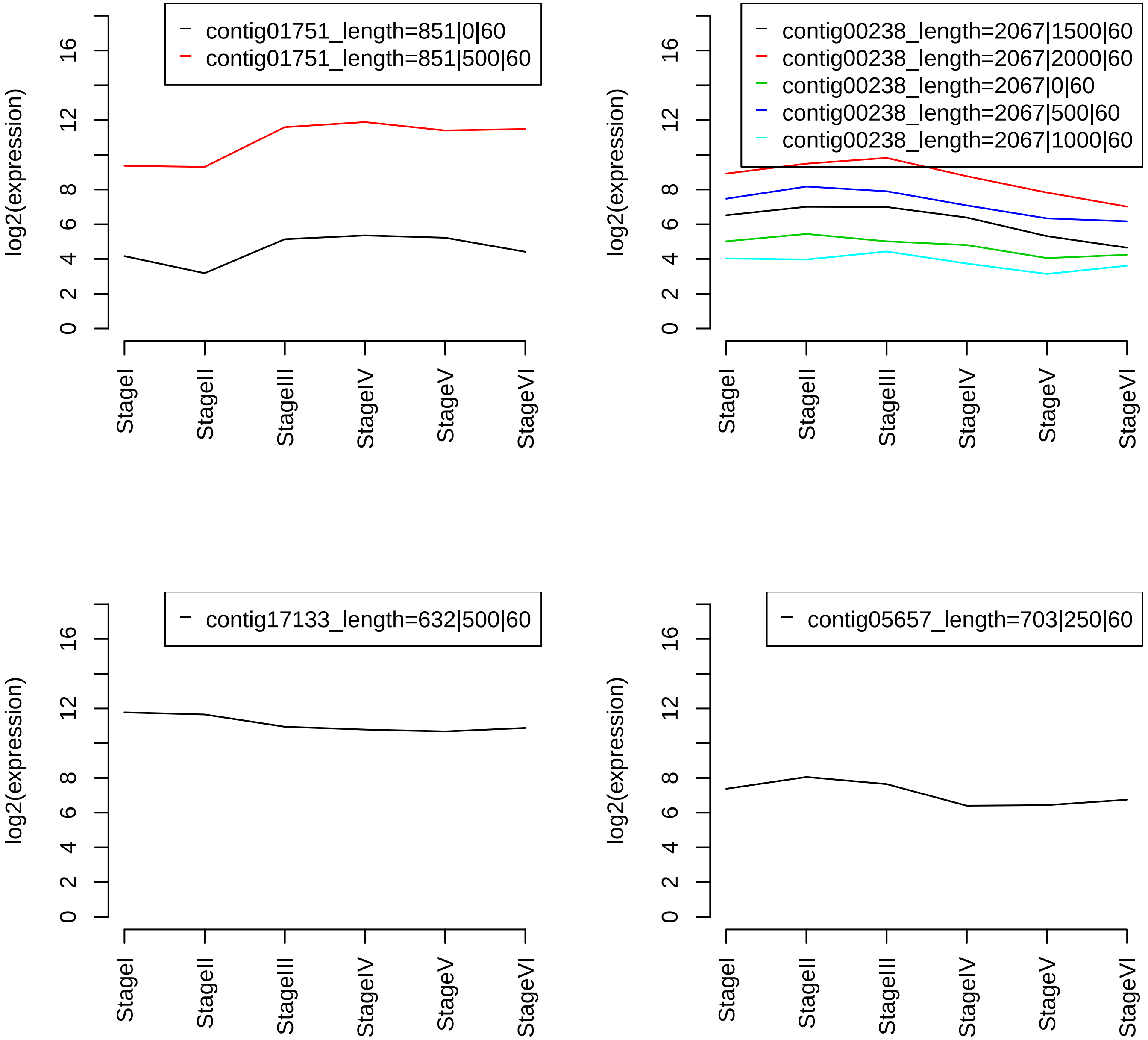

contig01751_length=851|0|60
contig01751_length=851|500|60
contig00238_length=2067|1500|60
contig00238_length=2067|2000|60
contig00238_length=2067|0|60
contig00238_length=2067|500|60
contig00238_length=2067|1000|60
contig17133_length=632|500|60
contig05657_length=703|250|60
log2(expression)
0
2
4
6
8
12
16
StageI
StageII
StageIII
StageIV
StageV
StageVI

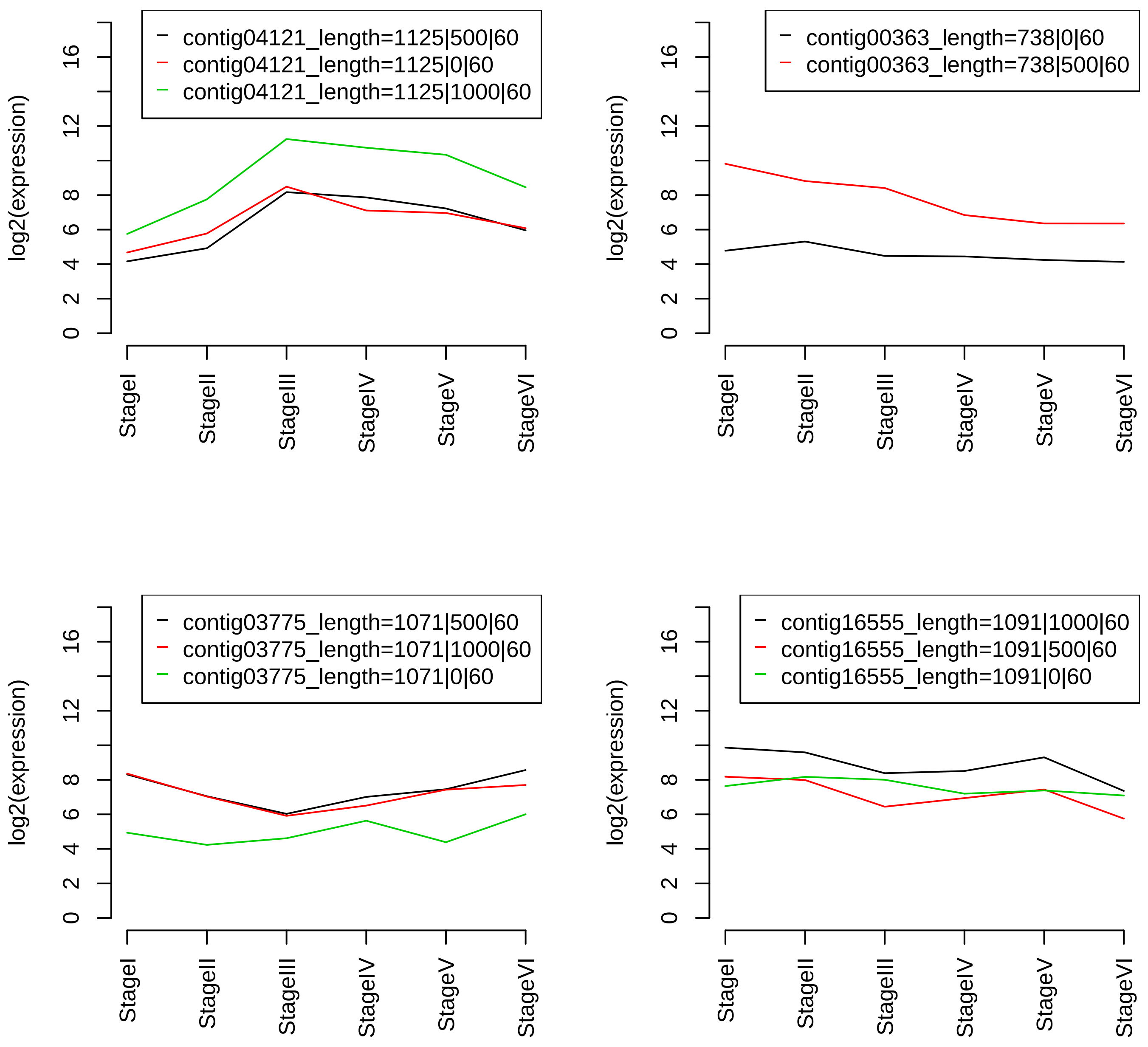

contig04121_length=1125|500|60
contig04121_length=1125|0|60
contig04121_length=1125|1000|60
contig00363_length=738|0|60
contig00363_length=738|500|60
contig03775_length=1071|500|60
contig03775_length=1071|1000|60
contig03775_length=1071|0|60
contig16555_length=1091|1000|60
contig16555_length=1091|500|60
contig16555_length=1091|0|60
log2(expression)
0
2
4
6
8
12
16
StageI
StageII
StageIII
StageIV
StageV
StageVI

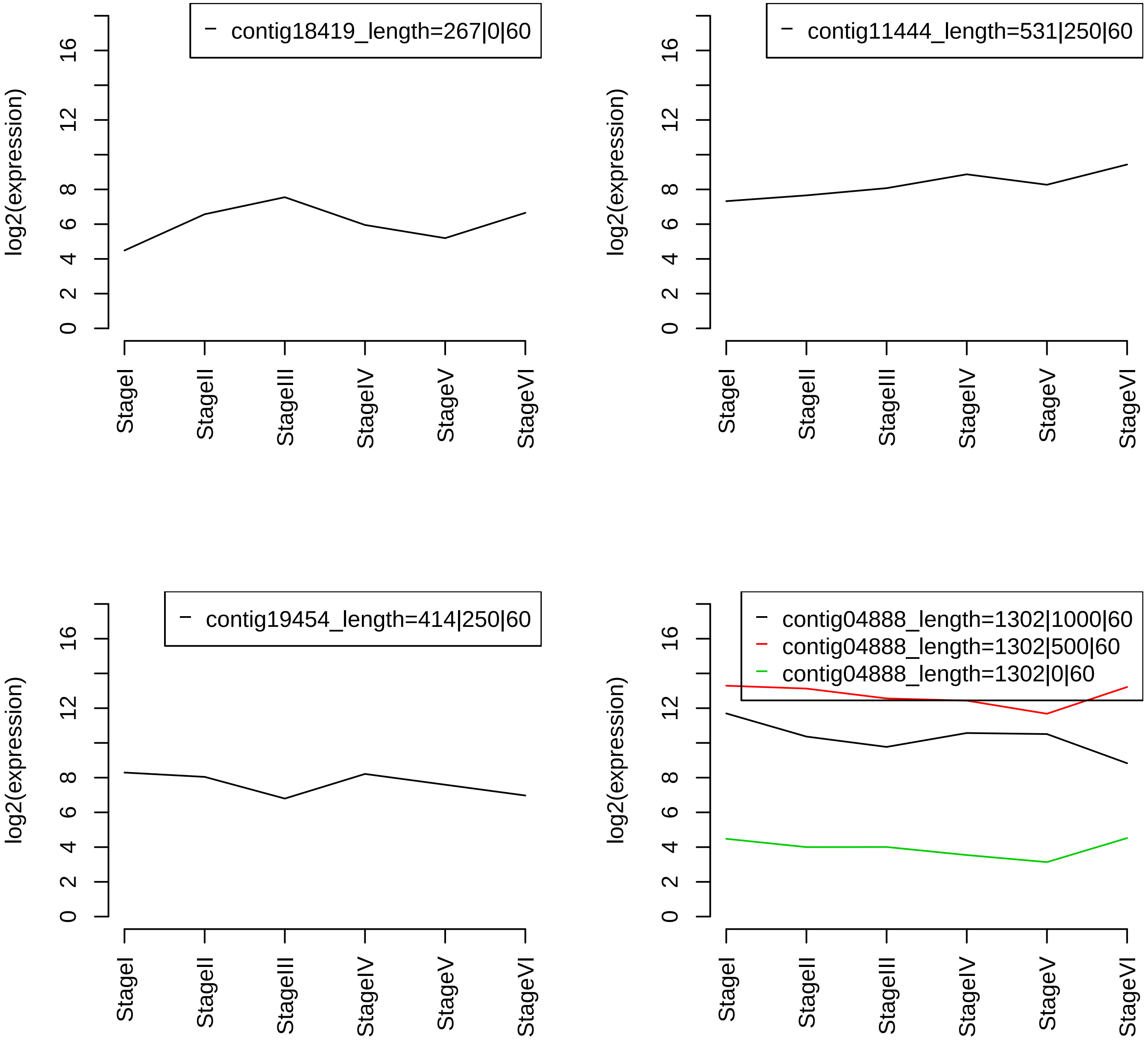

contig18419_length=267|0|60
contig11444_length=531|250|60
contig19454_length=414|250|60
contig04888_length=1302|1000|60
contig04888_length=1302|500|60
contig04888_length=1302|0|60
log2(expression)
0
2
4
6
8
12
16
StageI
StageII
StageIII
StageIV
StageV
StageVI

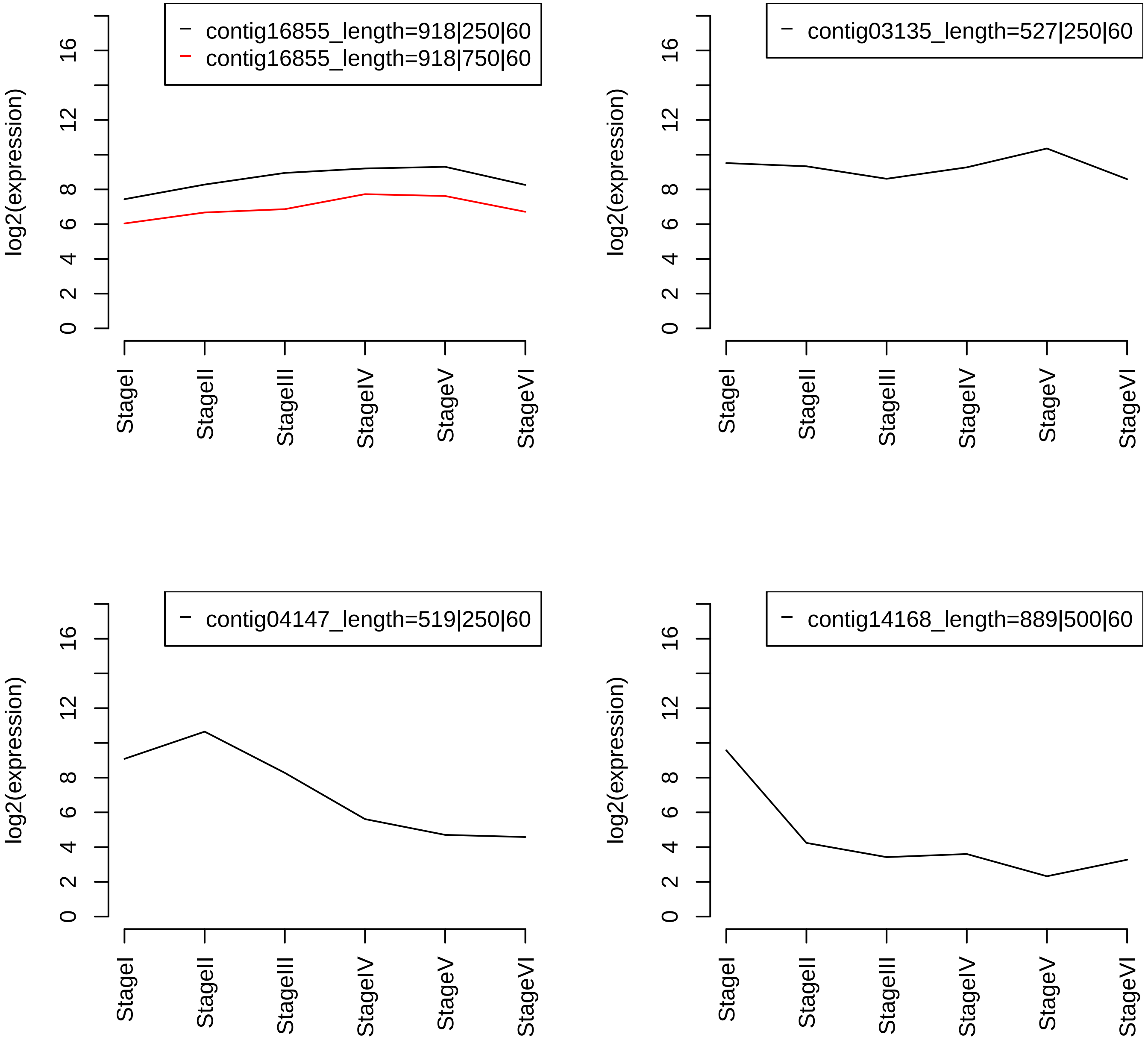
contig16855_length=918|250|60
contig16855_length=918|750|60
contig03135_length=527|250|60
contig04147_length=519|250|60
contig14168_length=889|500|60
log2(expression)
0
2
4
6
8
12
16
StageI
StageII
StageIII
StageIV
StageV
StageVI

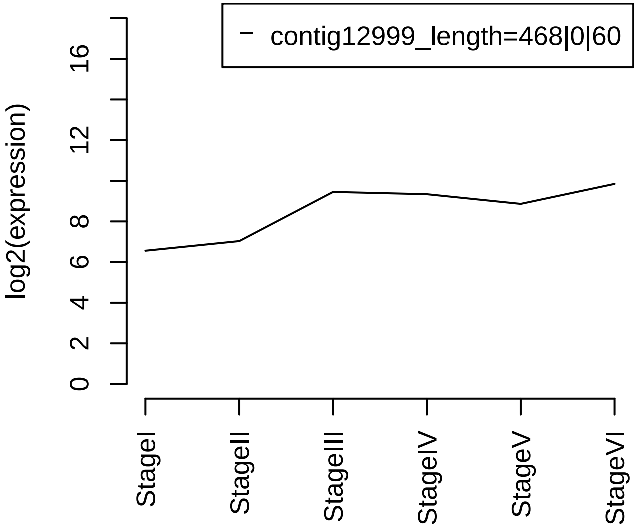
contig12999_length=468|0|60
log2(expression)
0
2
4
6
8
12
16
StageI
StageII
StageIII
StageIV
StageV
StageVI

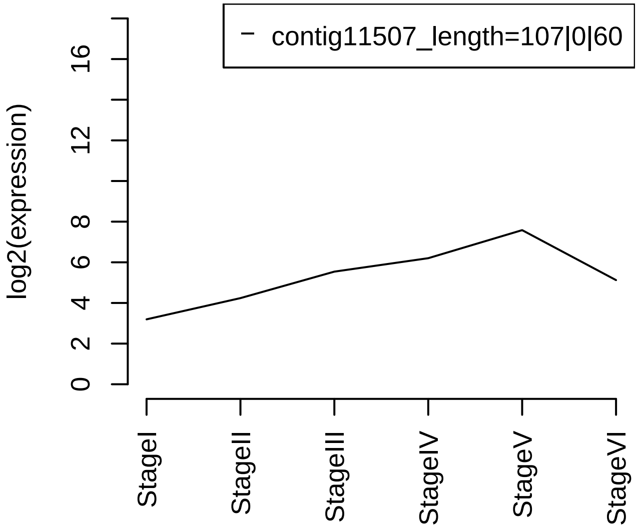
contig11507_length=107|0|60
log2(expression)
0
2
4
6
8
12
16
StageI
StageII
StageIII
StageIV
StageV
StageVI

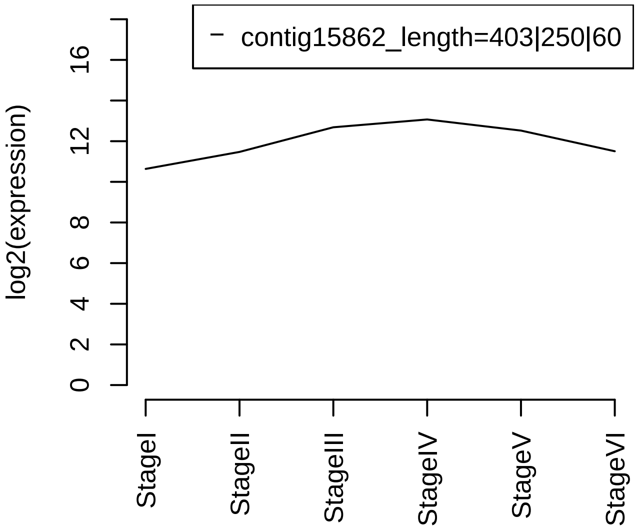
contig15862_length=403|250|60
log2(expression)
0
2
4
6
8
12
16
StageI
StageII
StageIII
StageIV
StageV
StageVI

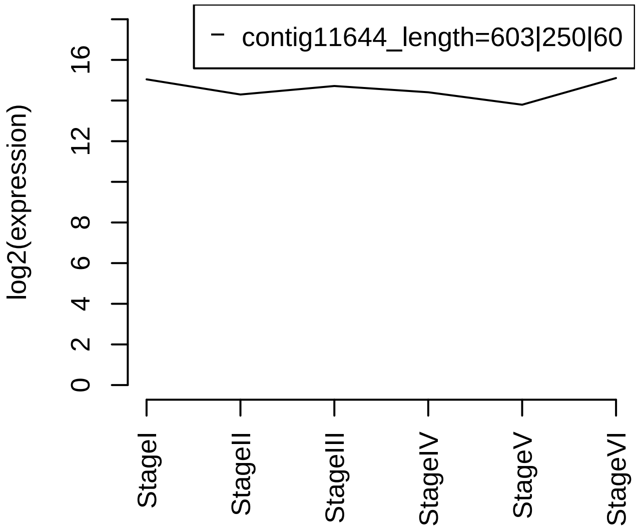
contig11644_length=603|250|60
log2(expression)
0
2
4
6
8
12
16
StageI
StageII
StageIII
StageIV
StageV
StageVI

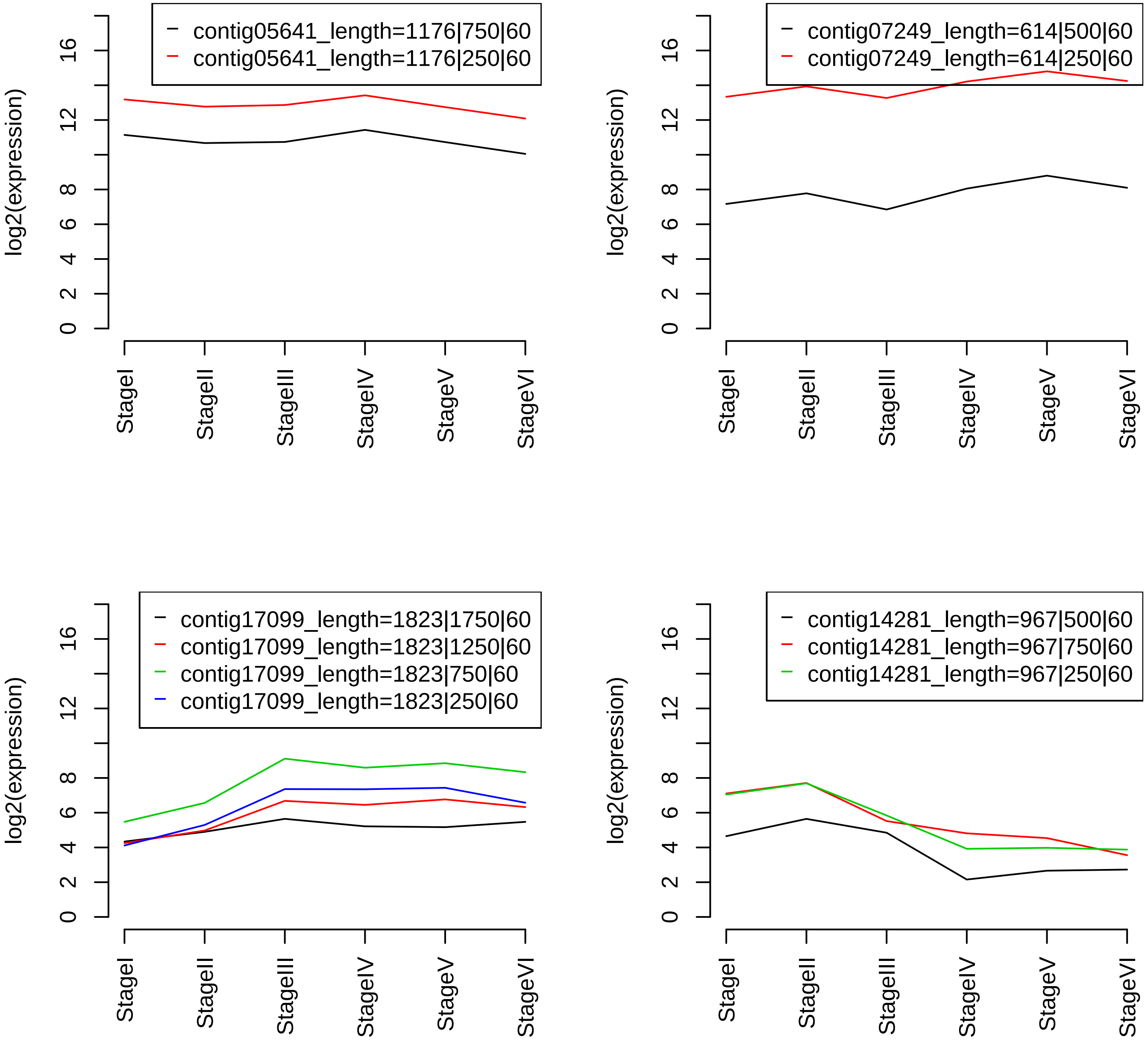

contig05641_length=1176|750|60
contig05641_length=1176|250|60
contig07249_length=614|500|60
contig07249_length=614|250|60
contig17099_length=1823|1750|60
contig17099_length=1823|1250|60
contig17099_length=1823|750|60
contig17099_length=1823|250|60
contig14281_length=967|500|60
contig14281_length=967|750|60
contig14281_length=967|250|60
log2(expression)
0
2
4
6
8
12
16
StageI
StageII
StageIII
StageIV
StageV
StageVI

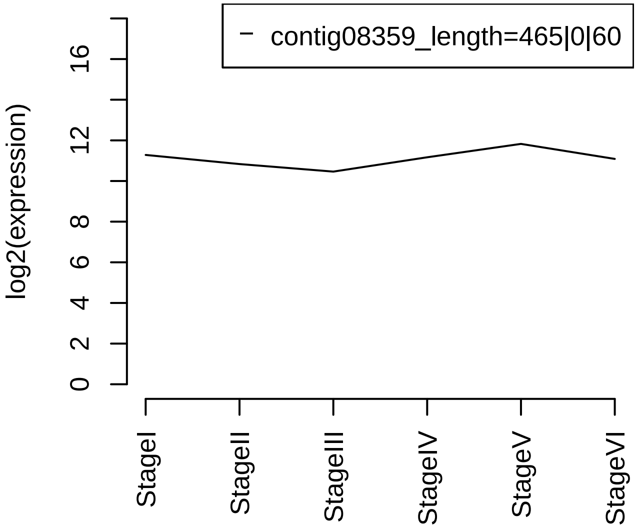

contig08359_length=465|0|60
log2(expression)
0
2
4
6
8
12
16
StageI
StageII
StageIII
StageIV
StageV
StageVI

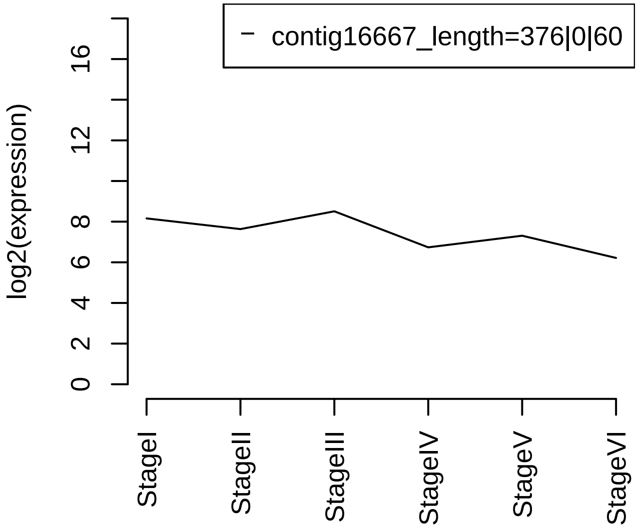

contig16667_length=376|0|60
log2(expression)
0
2
4
6
8
12
16
StageI
StageII
StageIII
StageIV
StageV
StageVI

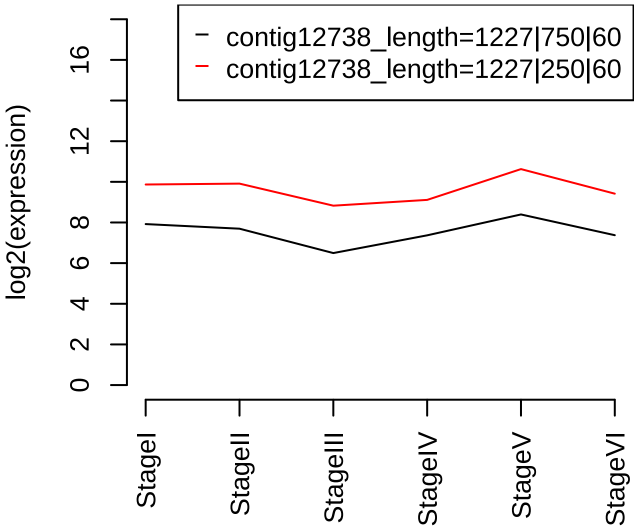

contig12738_length=1227|750|60
contig12738_length=1227|250|60
log2(expression)
0
2
4
6
8
12
16
StageI
StageII
StageIII
StageIV
StageV
StageVI

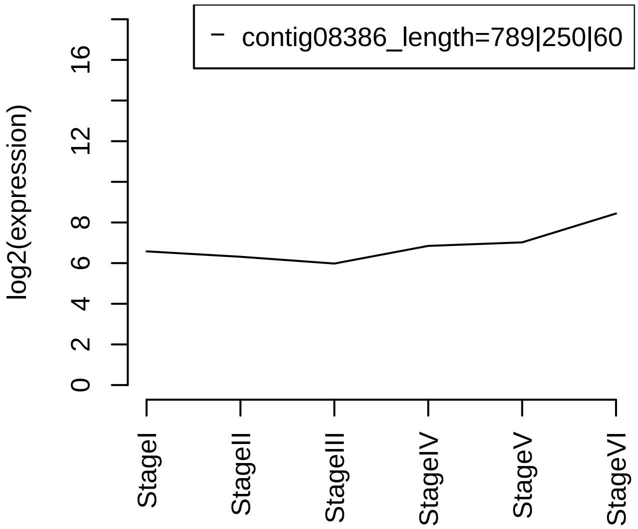

contig08386_length=789|250|60
log2(expression)
0
2
4
6
8
12
16
StageI
StageII
StageIII
StageIV
StageV
StageVI

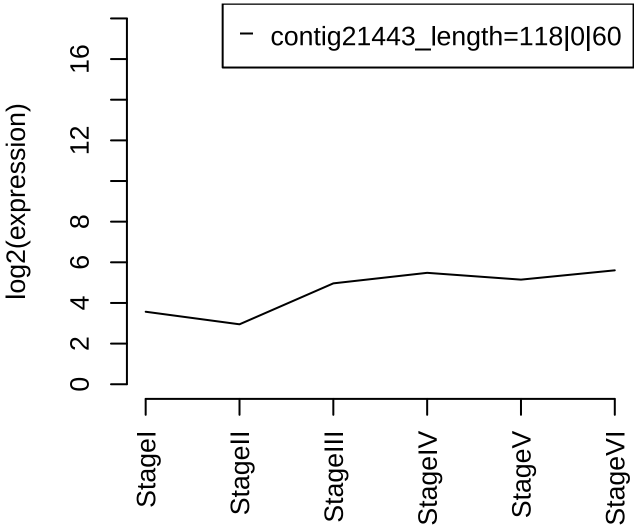

contig21443_length=118|0|60
log2(expression)
0
2
4
6
8
12
16
StageI
StageII
StageIII
StageIV
StageV
StageVI

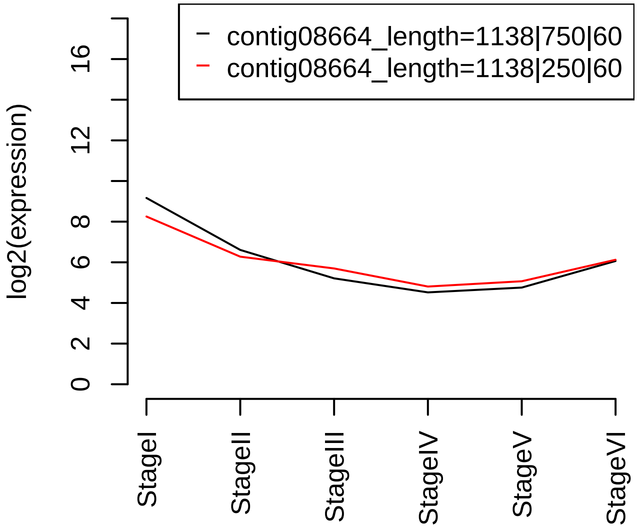

contig08664_length=1138|750|60
contig08664_length=1138|250|60
log2(expression)
0
2
4
6
8
12
16
StageI
StageII
StageIII
StageIV
StageV
StageVI

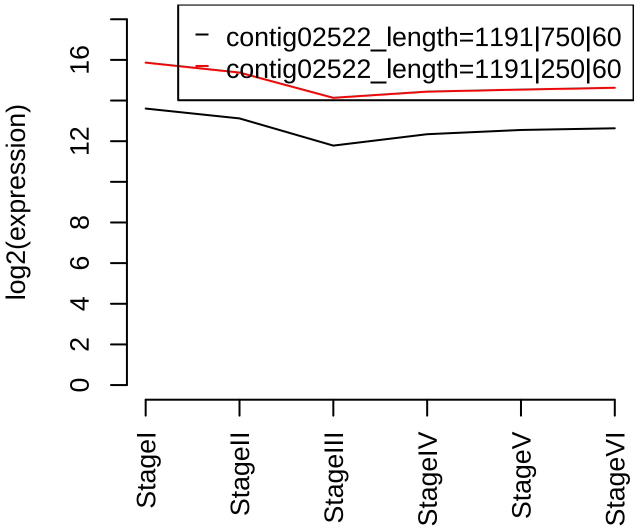

contig02522_length=1191|750|60
contig02522_length=1191|250|60
log2(expression)
0
2
4
6
8
12
16
StageI
StageII
StageIII
StageIV
StageV
StageVI

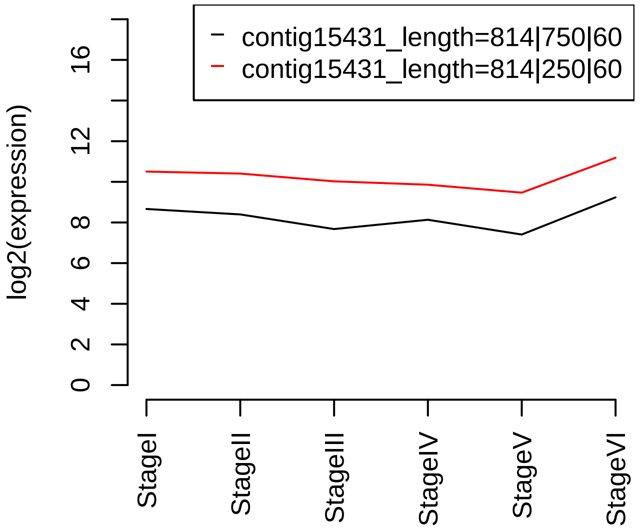

contig15431_length=814|750|60
contig15431_length=814|250|60
log2(expression)
0
2
4
6
8
12
16
StageI
StageII
StageIII
StageIV
StageV
StageVI

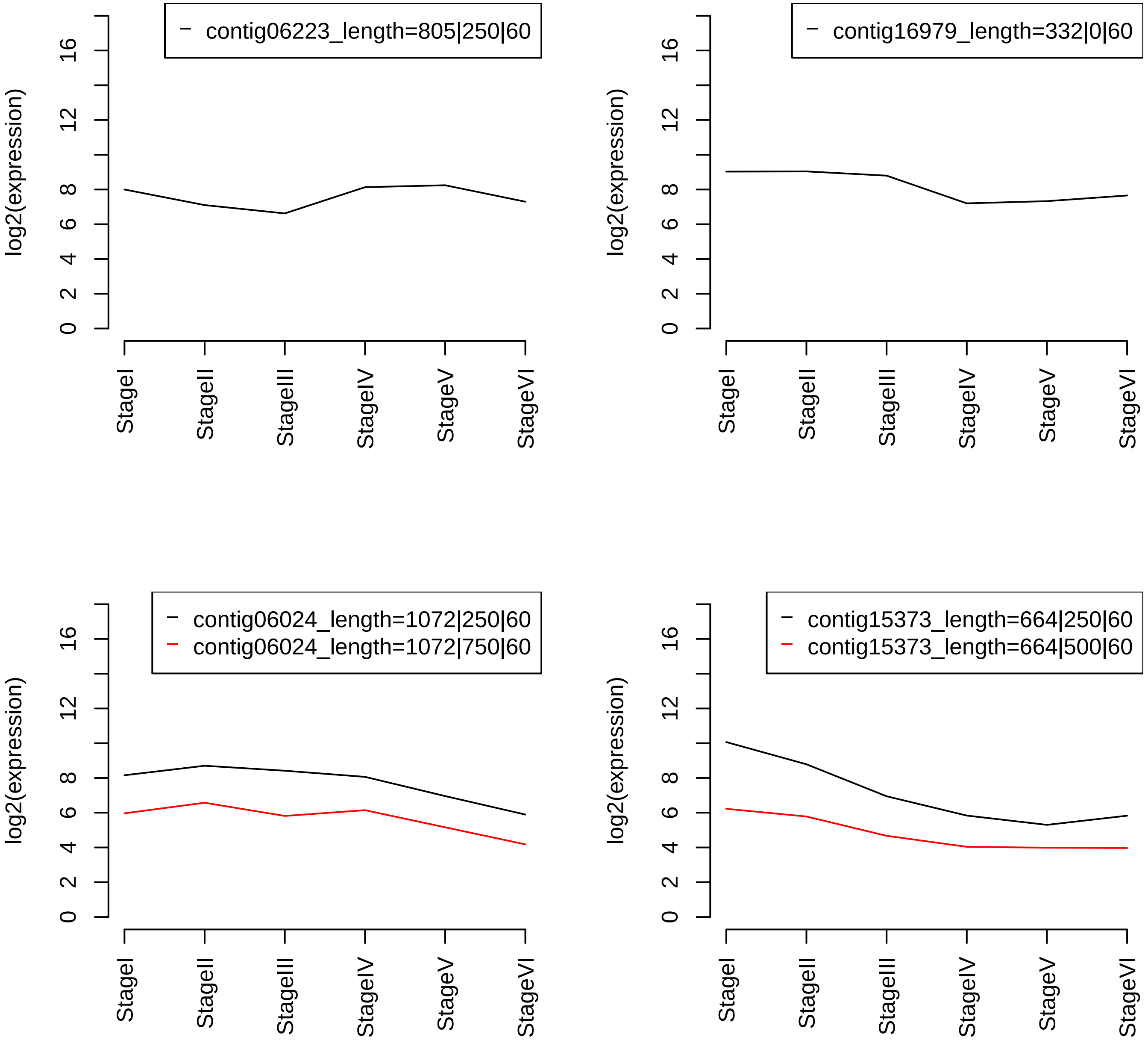

contig06223_length=805|250|60
contig16979_length=332|0|60
contig06024_length=1072|250|60
contig06024_length=1072|750|60
contig15373_length=664|250|60
contig15373_length=664|500|60
log2(expression)
0 2 4 6 8 12 16
StageI
StageII
StageIII
StageIV
StageV
StageVI

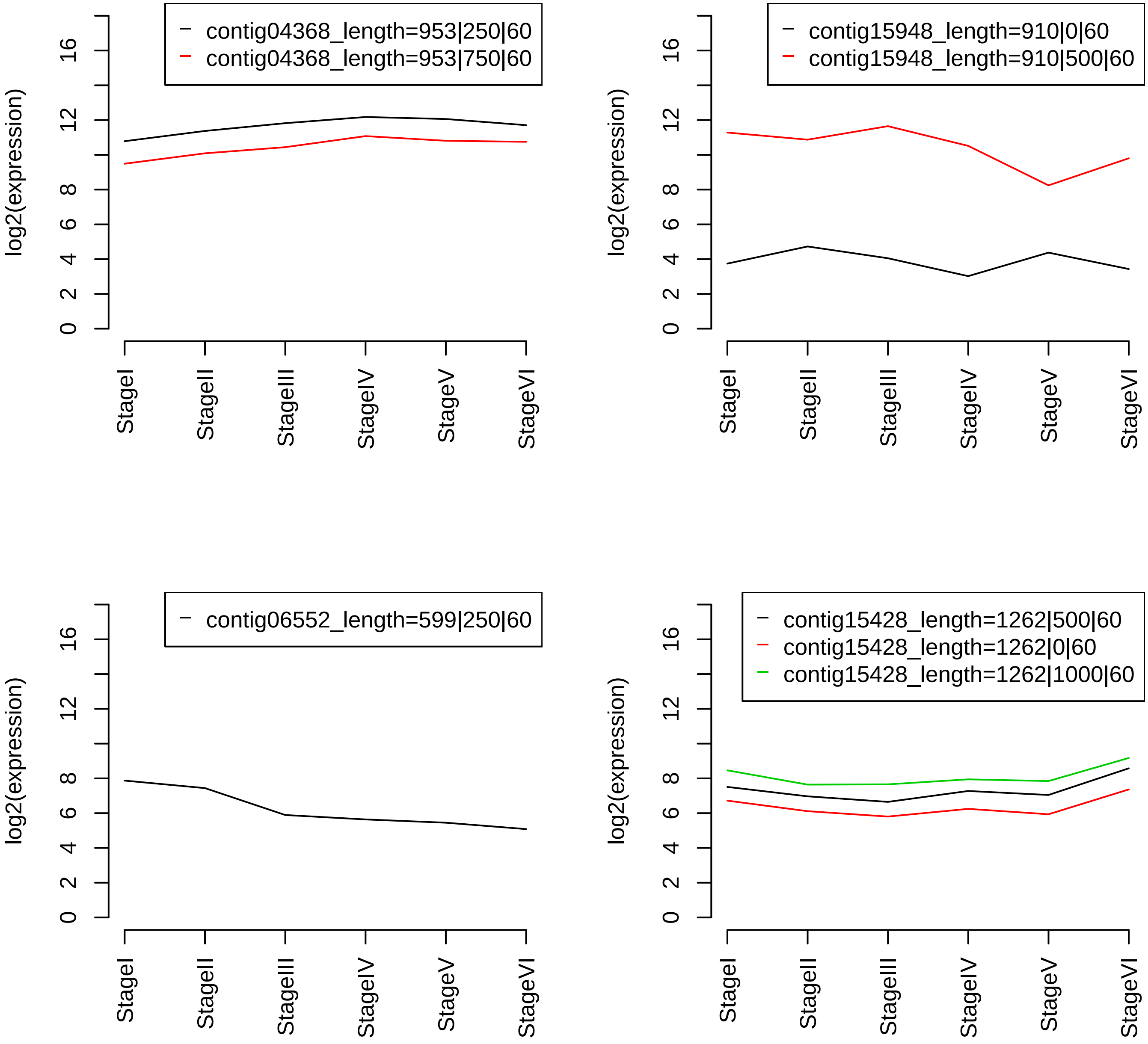

contig04368_length=953|250|60
contig04368_length=953|750|60
contig15948_length=910|0|60
contig15948_length=910|500|60
contig06552_length=599|250|60
contig15428_length=1262|500|60
contig15428_length=1262|0|60
contig15428_length=1262|1000|60
log2(expression)
0
2
4
6
8
12
16
StageI
StageII
StageIII
StageIV
StageV
StageVI

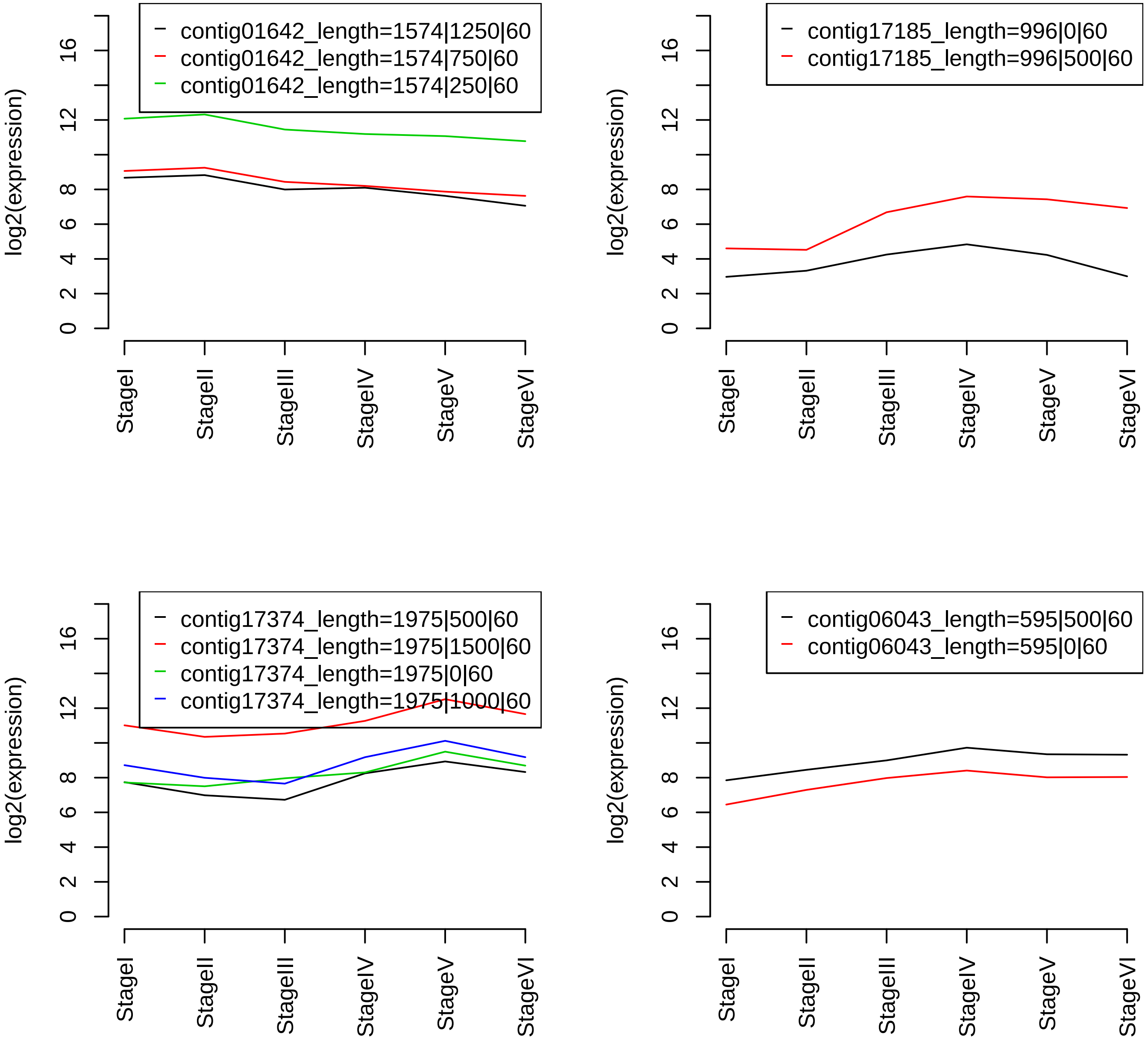
contig01642_length=1574|1250|60
contig01642_length=1574|750|60
contig01642_length=1574|250|60
log2(expression)
0
2
4
6
8
12
16
StageI
StageII
StageIII
StageIV
StageV
StageVI
contig17185_length=996|0|60
contig17185_length=996|500|60
contig17374_length=1975|500|60
contig17374_length=1975|1500|60
contig17374_length=1975|0|60
contig17374_length=1975|1000|60
contig06043_length=595|500|60
contig06043_length=595|0|60

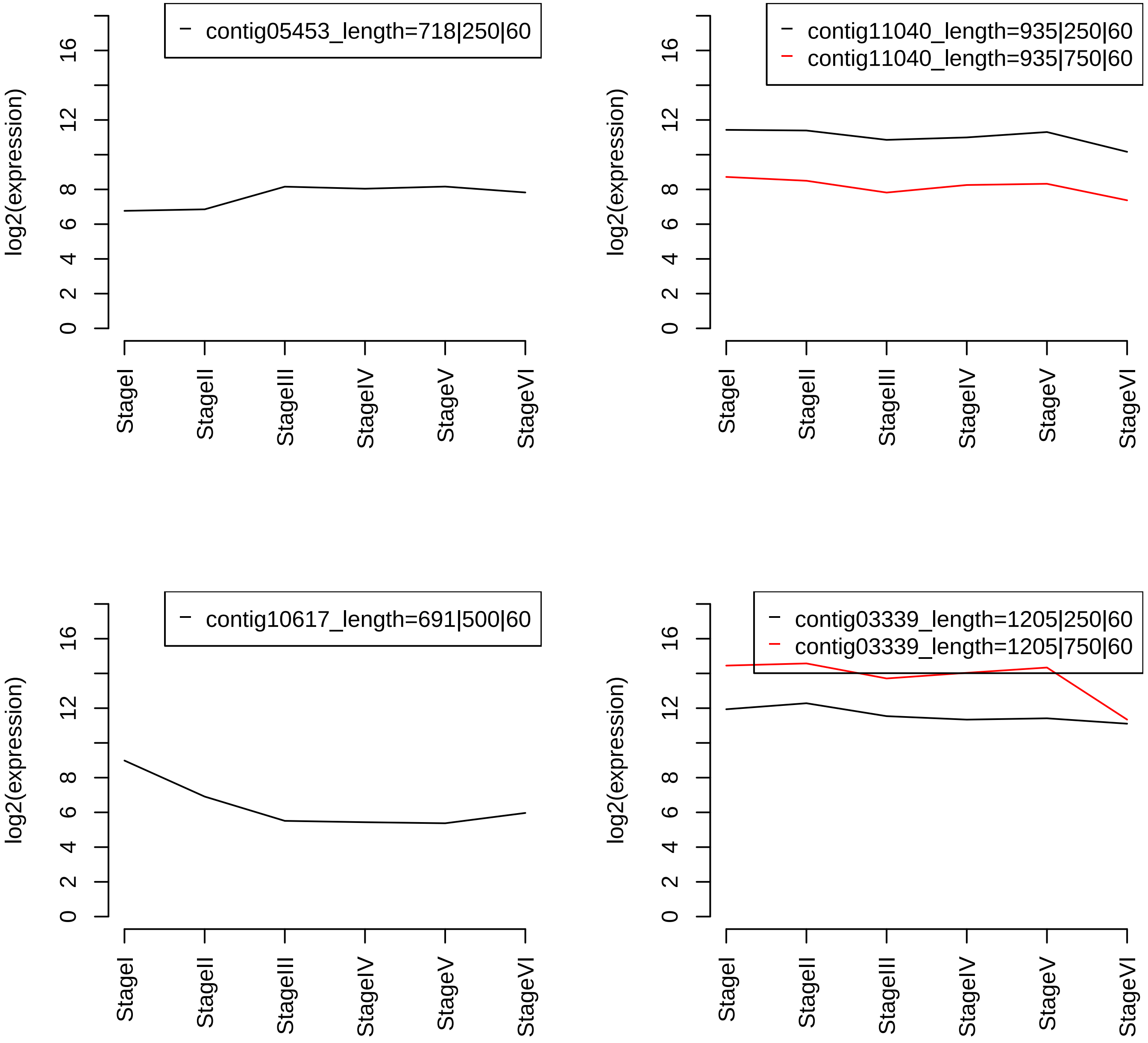
contig05453_length=718|250|60
contig11040_length=935|250|60
contig11040_length=935|750|60
contig10617_length=691|500|60
contig03339_length=1205|250|60
contig03339_length=1205|750|60
log2(expression)
0
2
4
6
8
12
16
StageI
StageII
StageIII
StageIV
StageV
StageVI

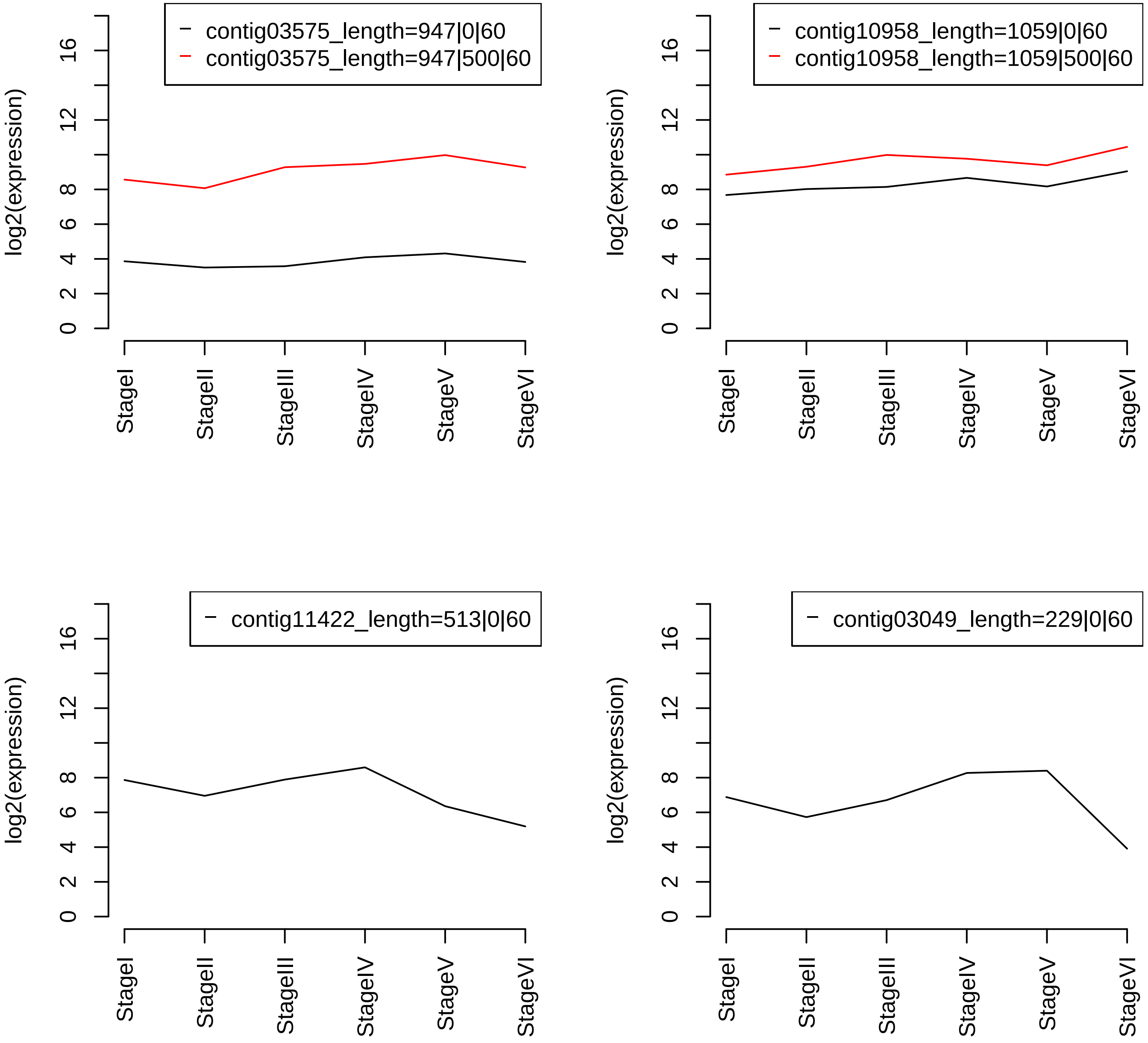

contig03575_length=947|0|60
contig03575_length=947|500|60
contig10958_length=1059|0|60
contig10958_length=1059|500|60
contig11422_length=513|0|60
contig03049_length=229|0|60
log2(expression)
0
2
4
6
8
12
16
StageI
StageII
StageIII
StageIV
StageV
StageVI

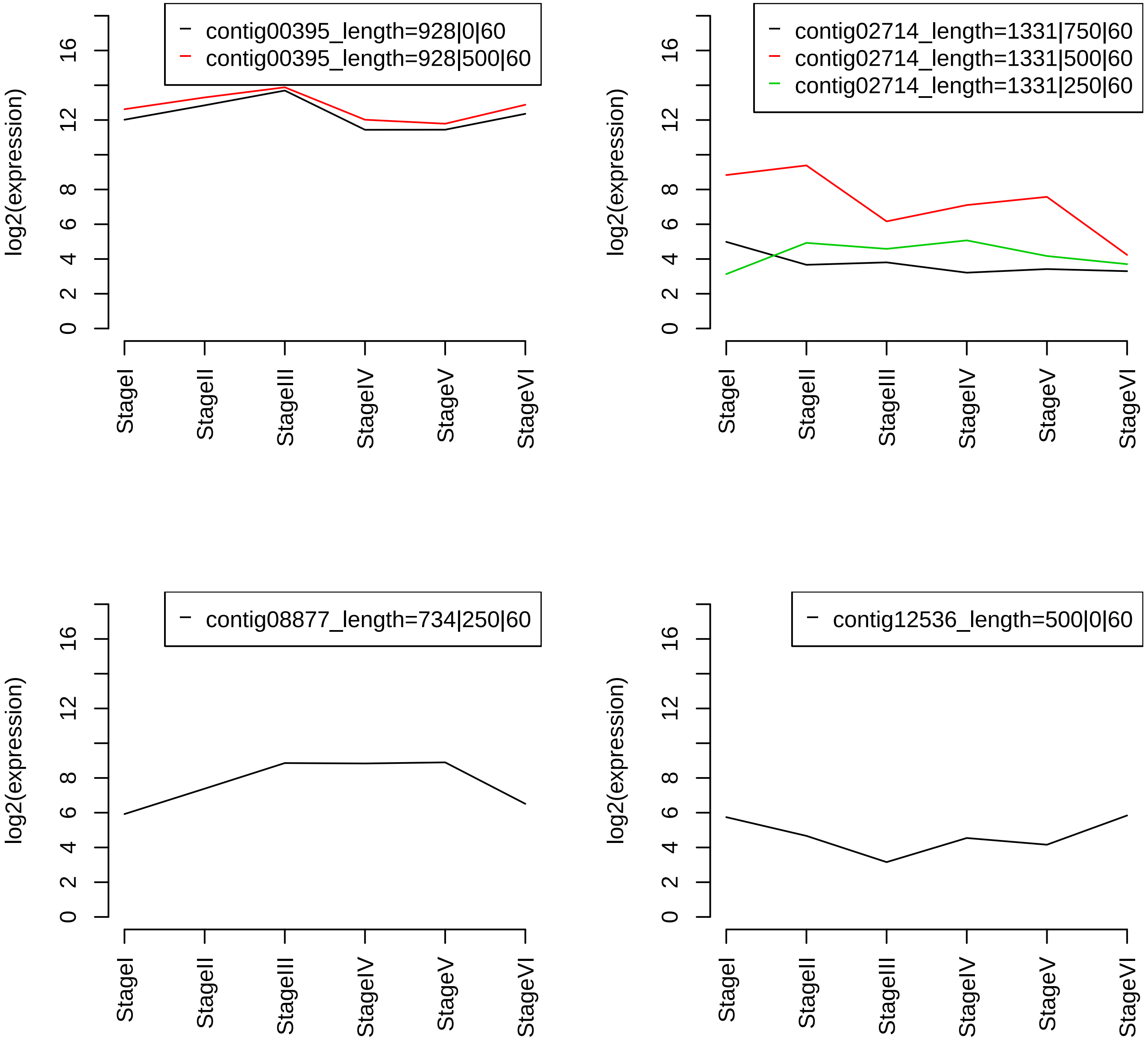
contig00395_length=928|0|60
contig00395_length=928|500|60
contig02714_length=1331|750|60
contig02714_length=1331|500|60
contig02714_length=1331|250|60
contig08877_length=734|250|60
contig12536_length=500|0|60
log2(expression)
0
2
4
6
8
12
16
StageI
StageII
StageIII
StageIV
StageV
StageVI

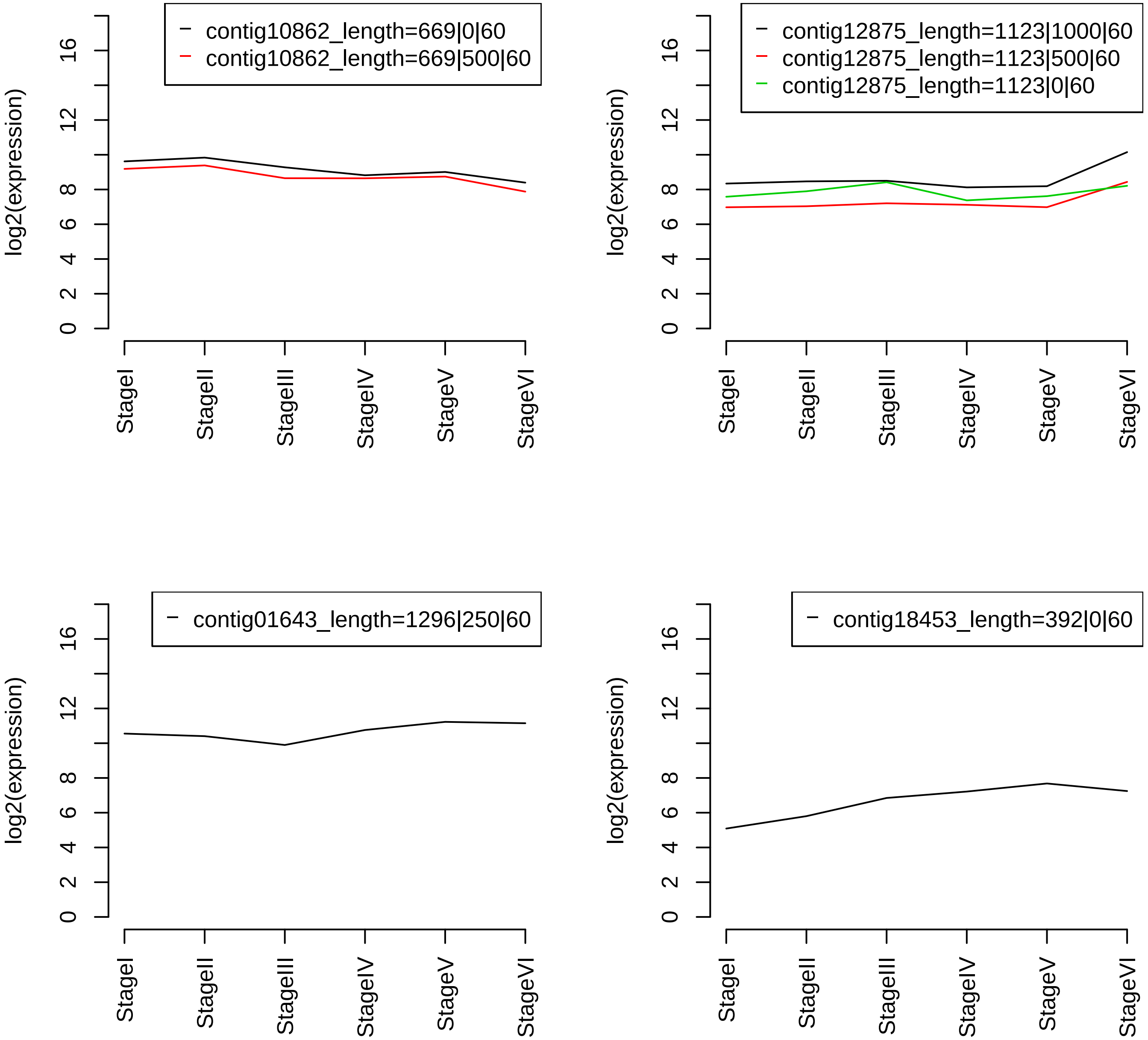

contig10862_length=669|0|60
contig10862_length=669|500|60
log2(expression)
0
2
4
6
8
12
16
StageI
StageII
StageIII
StageIV
StageV
StageVI
contig12875_length=1123|1000|60
contig12875_length=1123|500|60
contig12875_length=1123|0|60
log2(expression)
0
2
4
6
8
12
16
StageI
StageII
StageIII
StageIV
StageV
StageVI
contig01643_length=1296|250|60
log2(expression)
0
2
4
6
8
12
16
StageI
StageII
StageIII
StageIV
StageV
StageVI
contig18453_length=392|0|60
log2(expression)
0
2
4
6
8
12
16
StageI
StageII
StageIII
StageIV
StageV
StageVI

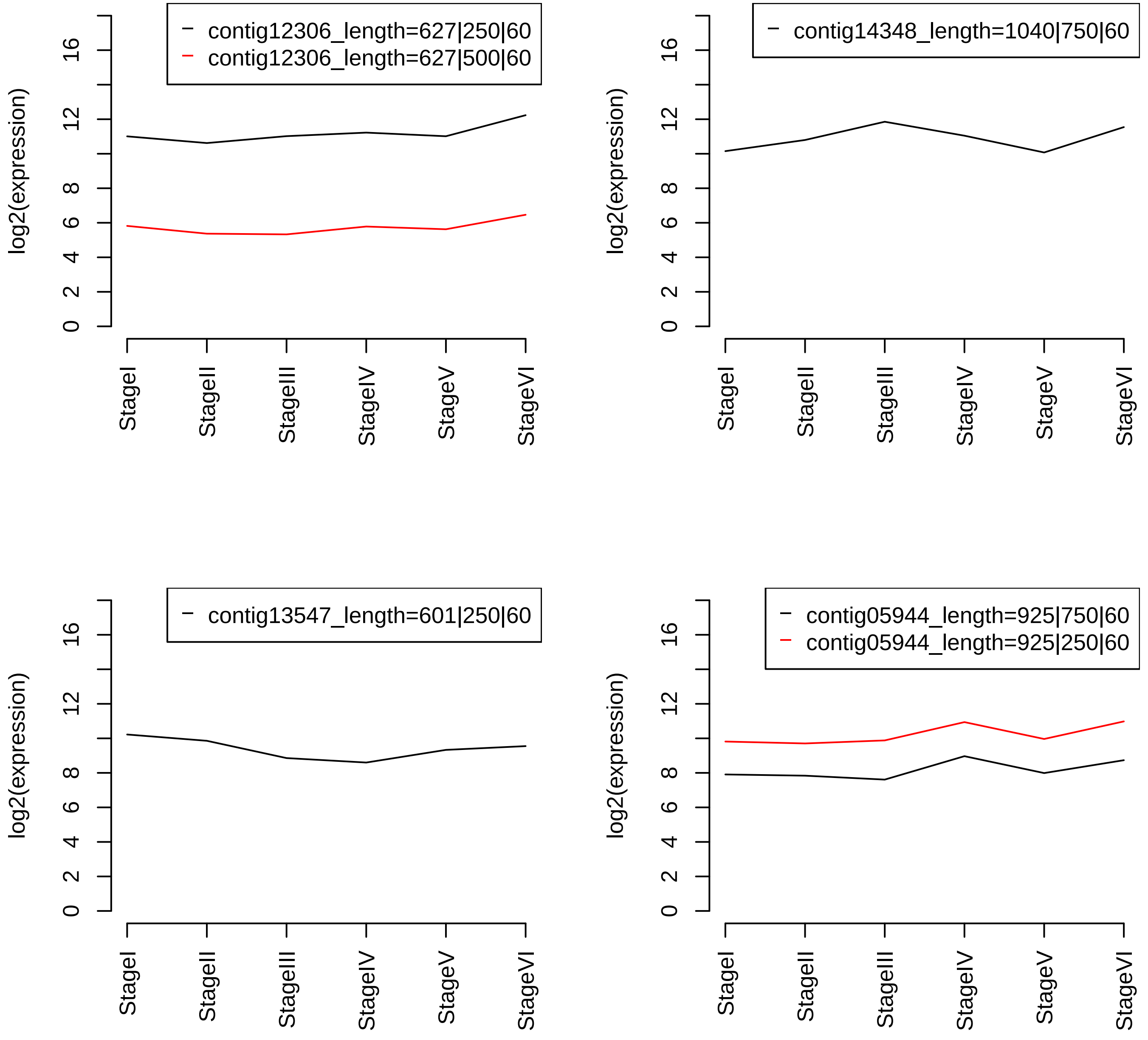

contig12306_length=627|250|60
contig12306_length=627|500|60
contig14348_length=1040|750|60
contig13547_length=601|250|60
contig05944_length=925|750|60
contig05944_length=925|250|60
log2(expression)
0
2
4
6
8
12
16
StageI
StageII
StageIII
StageIV
StageV
StageVI

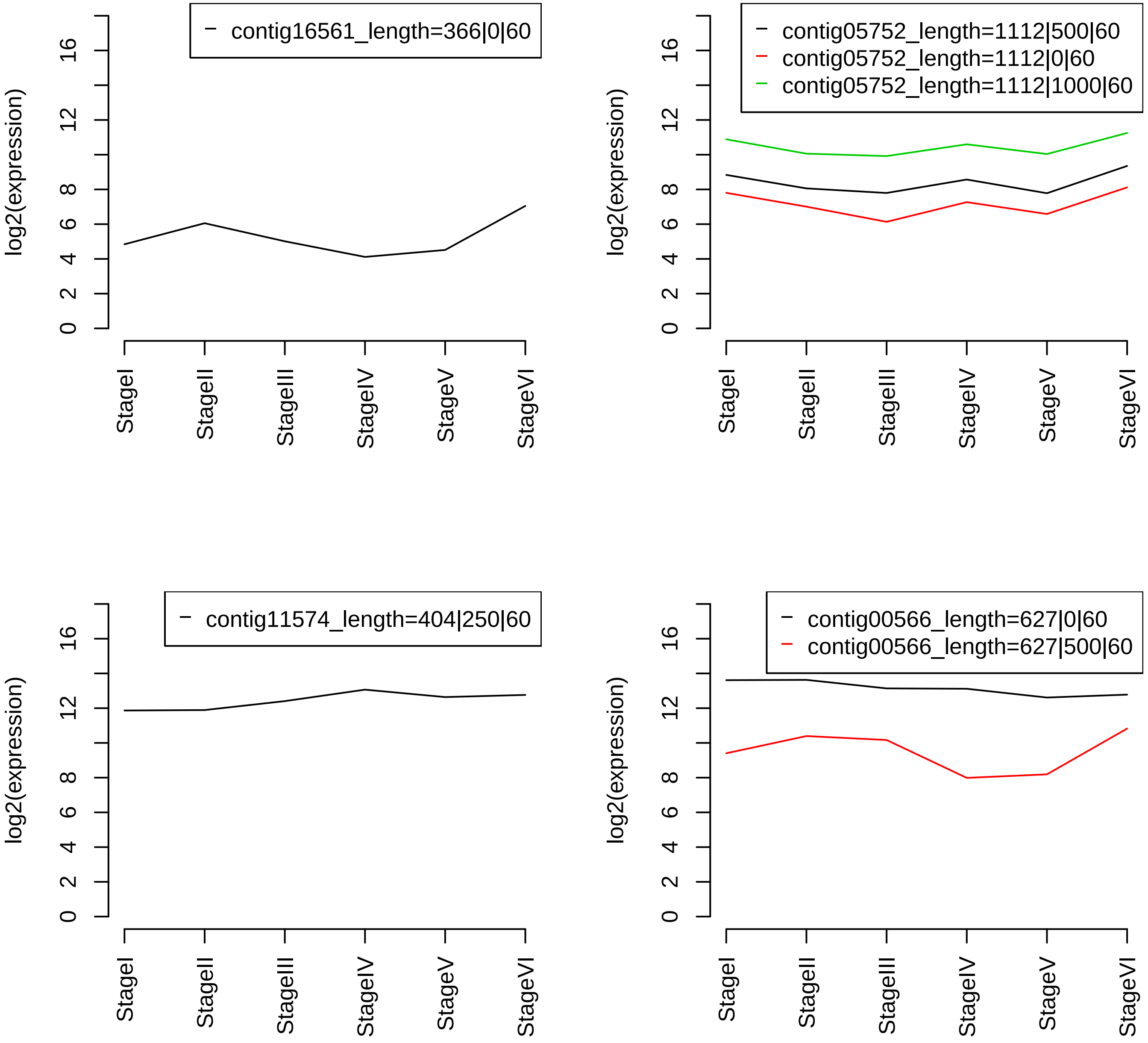
contig16561_length=366|0|60
contig05752_length=1112|500|60
contig05752_length=1112|0|60
contig05752_length=1112|1000|60
contig11574_length=404|250|60
contig00566_length=627|0|60
contig00566_length=627|500|60
log2(expression)
0
2
4
6
8
12
16
StageI
StageII
StageIII
StageIV
StageV
StageVI

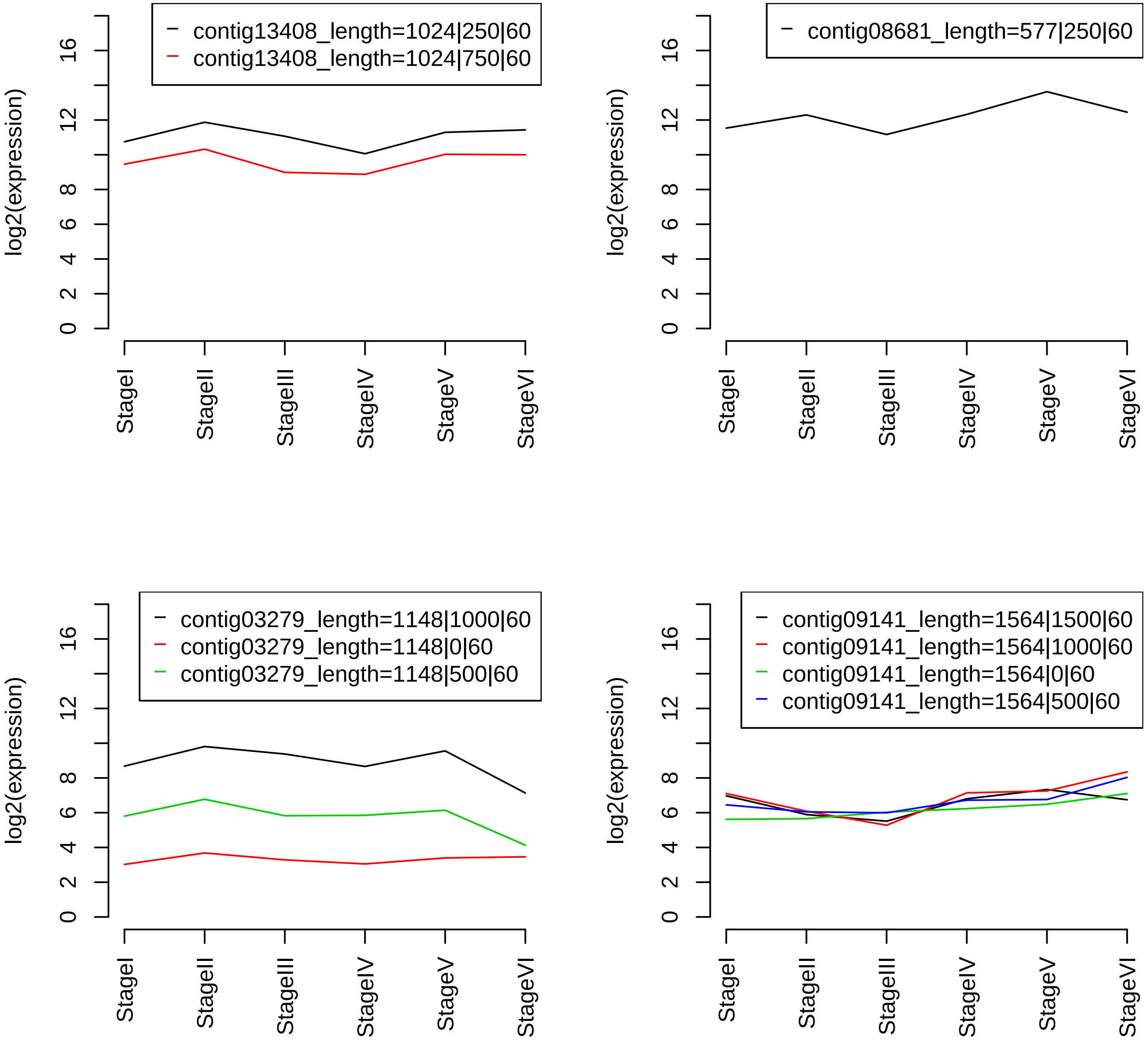

contig13408_length=1024|250|60
contig13408_length=1024|750|60
contig08681_length=577|250|60
contig03279_length=1148|1000|60
contig03279_length=1148|0|60
contig03279_length=1148|500|60
contig09141_length=1564|1500|60
contig09141_length=1564|1000|60
contig09141_length=1564|0|60
contig09141_length=1564|500|60
log2(expression)
0
2
4
6
8
12
16
StageI
StageII
StageIII
StageIV
StageV
StageVI

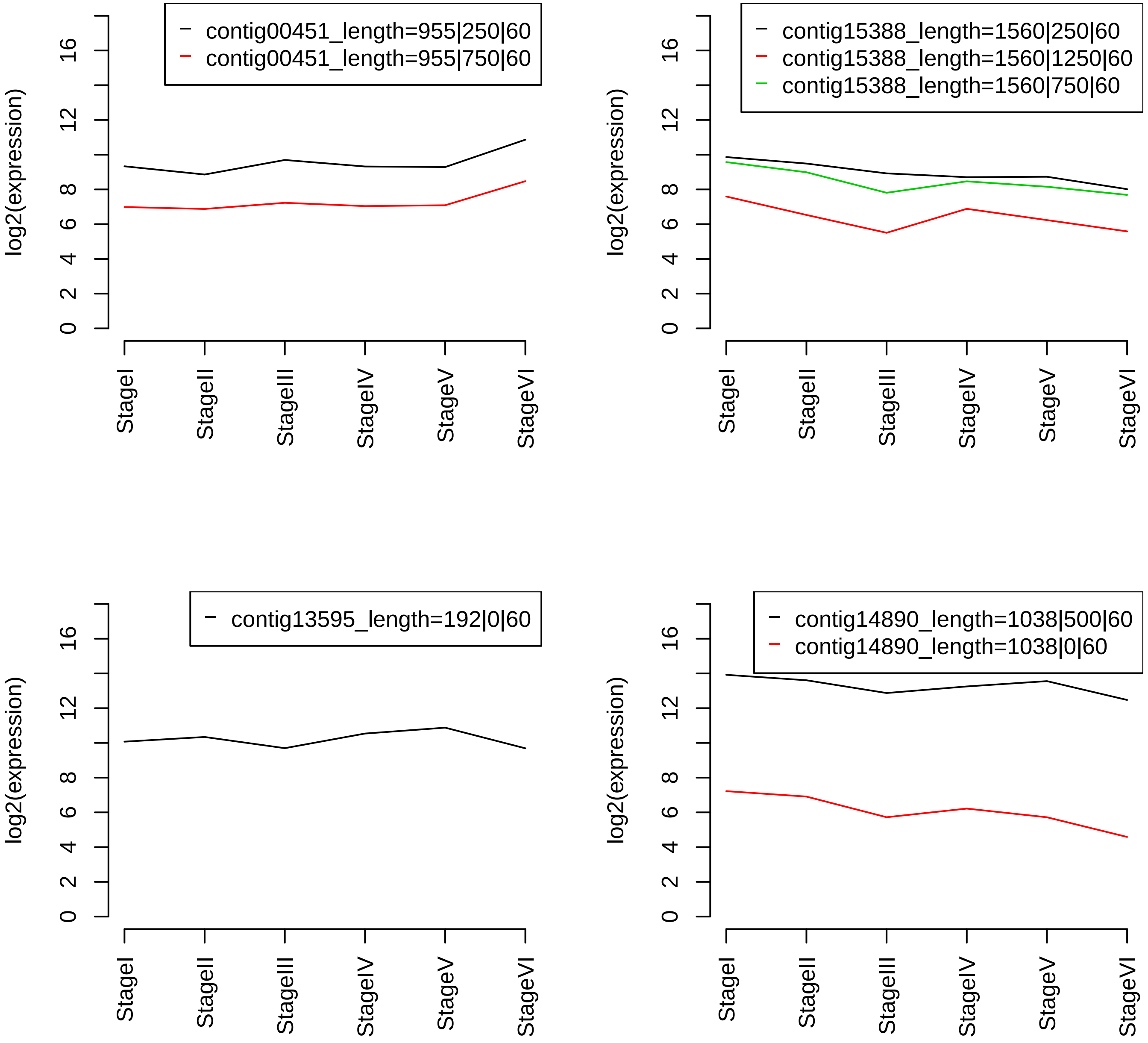
contig00451_length=955|250|60
contig00451_length=955|750|60
contig15388_length=1560|250|60
contig15388_length=1560|1250|60
contig15388_length=1560|750|60
contig13595_length=192|0|60
contig14890_length=1038|500|60
contig14890_length=1038|0|60
log2(expression)
0
2
4
6
8
12
16
StageI
StageII
StageIII
StageIV
StageV
StageVI

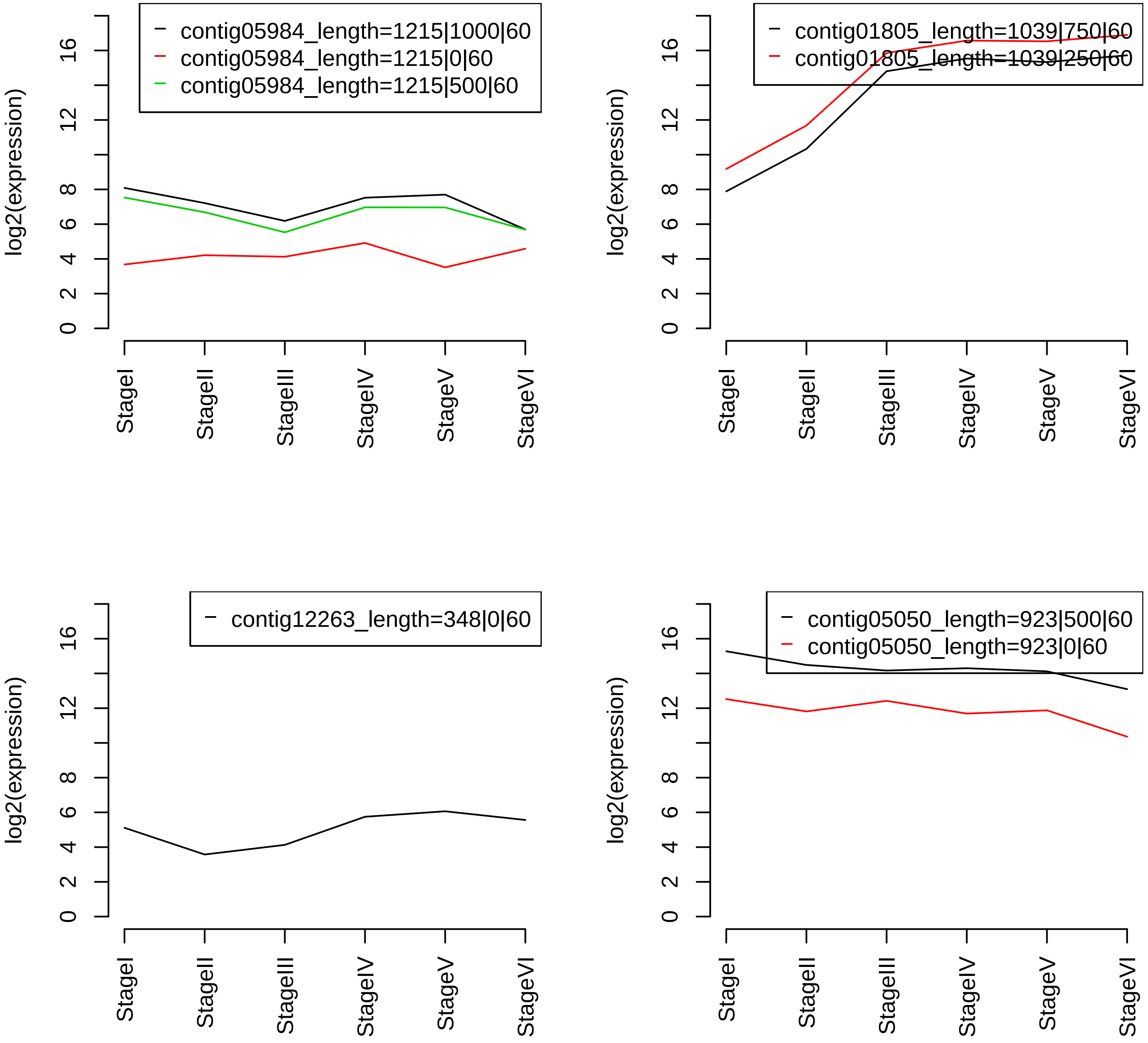
contig05984_length=1215|1000|60
contig05984_length=1215|0|60
contig05984_length=1215|500|60
contig01805_length=1039|750|60
contig01805_length=1039|250|60
contig12263_length=348|0|60
contig05050_length=923|500|60
contig05050_length=923|0|60
log2(expression)
0 2 4 6 8 12 16
StageI StageII StageIII StageIV StageV StageVI

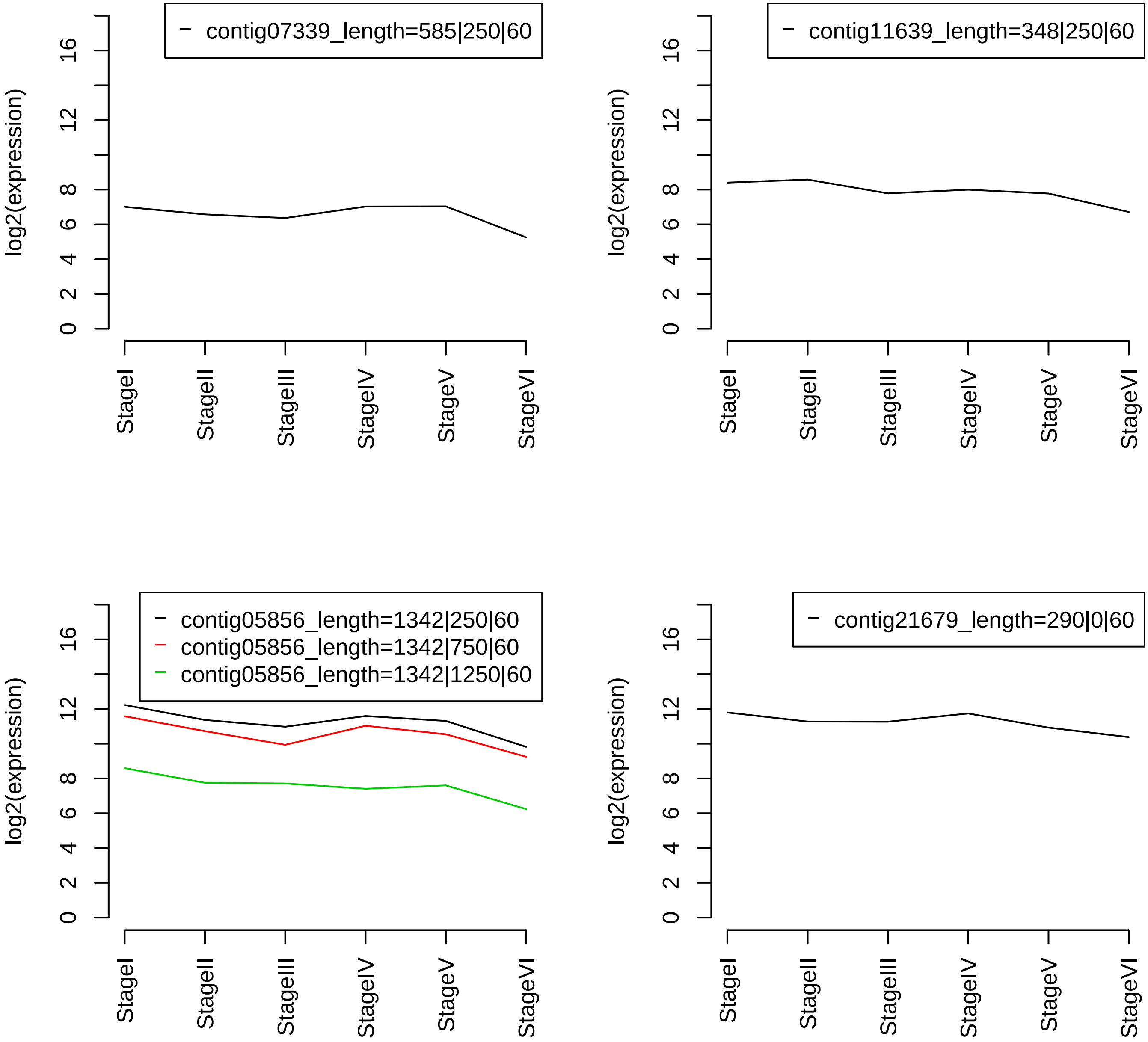
contig07339_length=585|250|60
contig11639_length=348|250|60
contig05856_length=1342|250|60
contig05856_length=1342|750|60
contig05856_length=1342|1250|60
contig21679_length=290|0|60
log2(expression)
0 2 4 6 8 12 16
StageI StageII StageIII StageIV StageV StageVI

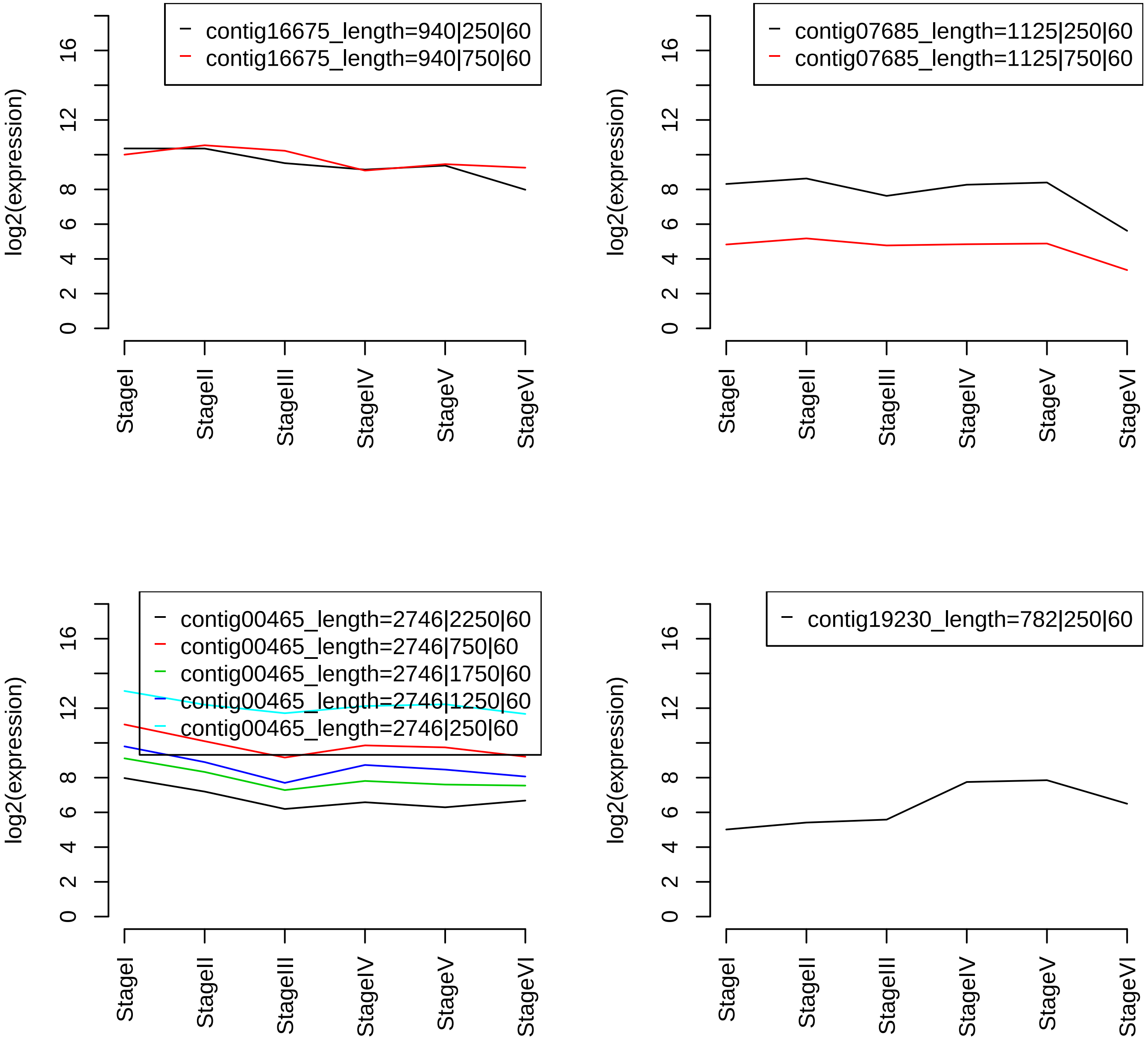
contig16675_length=940|250|60
contig16675_length=940|750|60
contig07685_length=1125|250|60
contig07685_length=1125|750|60
contig00465_length=2746|2250|60
contig00465_length=2746|750|60
contig00465_length=2746|1750|60
contig00465_length=2746|1250|60
contig00465_length=2746|250|60
contig19230_length=782|250|60
log2(expression)
0
2
4
6
8
12
16
StageI
StageII
StageIII
StageIV
StageV
StageVI

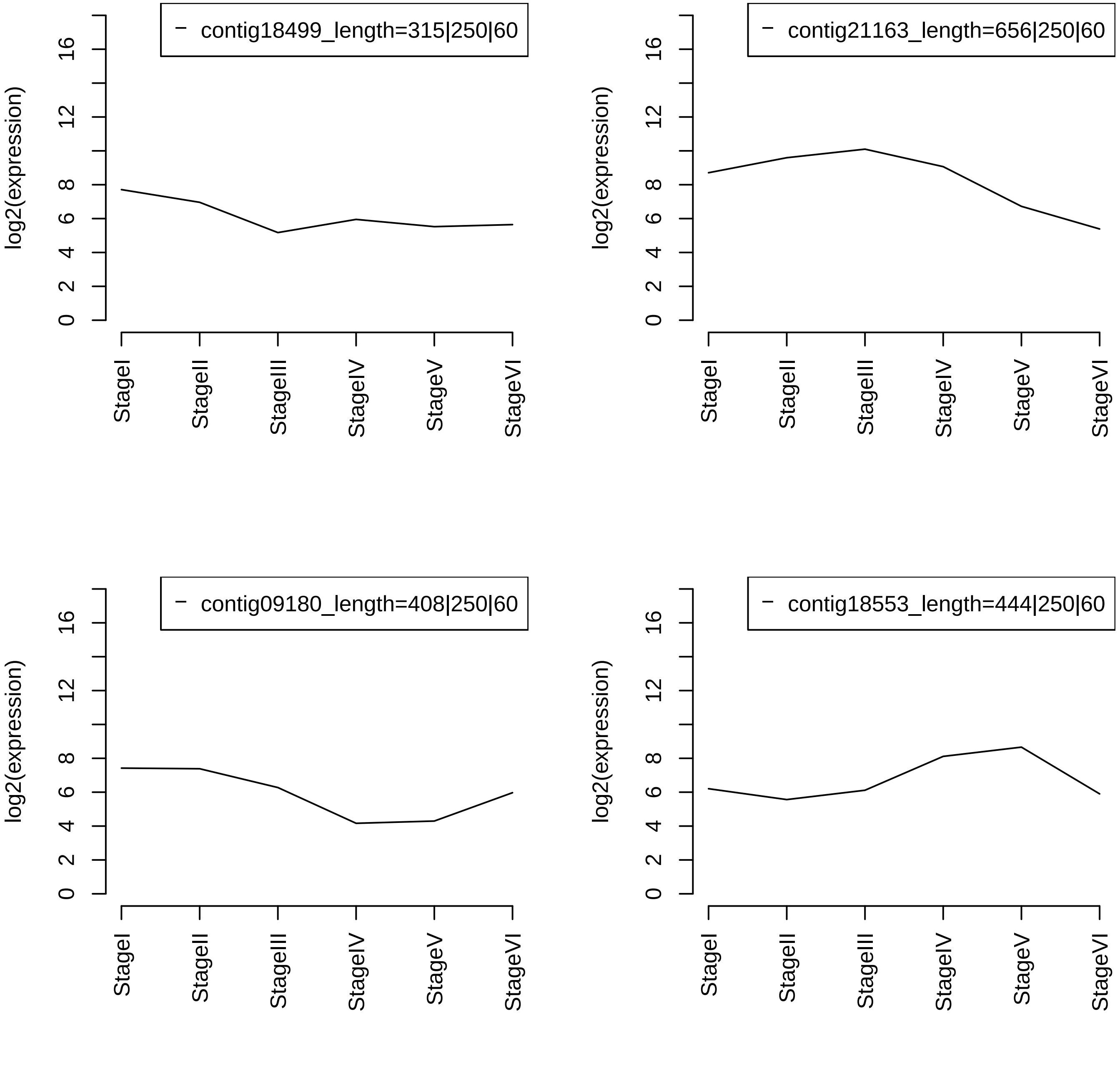
contig18499_length=315|250|60
contig21163_length=656|250|60
contig09180_length=408|250|60
contig18553_length=444|250|60
log2(expression)
0
2
4
6
8
12
16
StageI
StageII
StageIII
StageIV
StageV
StageVI

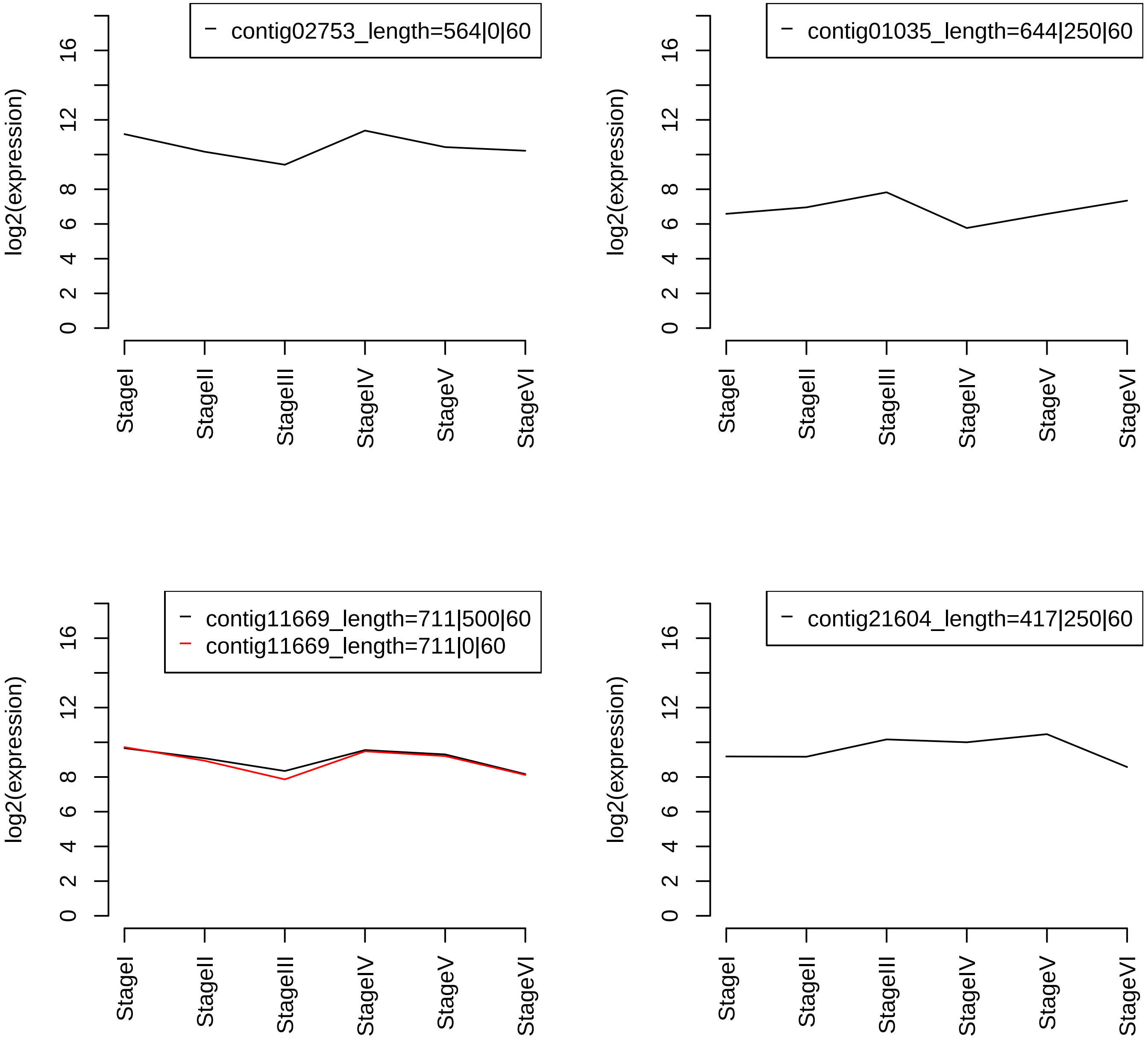

contig02753_length=564|0|60
contig01035_length=644|250|60
contig11669_length=711|500|60
contig11669_length=711|0|60
contig21604_length=417|250|60
log2(expression)
0
2
4
6
8
12
16
StageI
StageII
StageIII
StageIV
StageV
StageVI

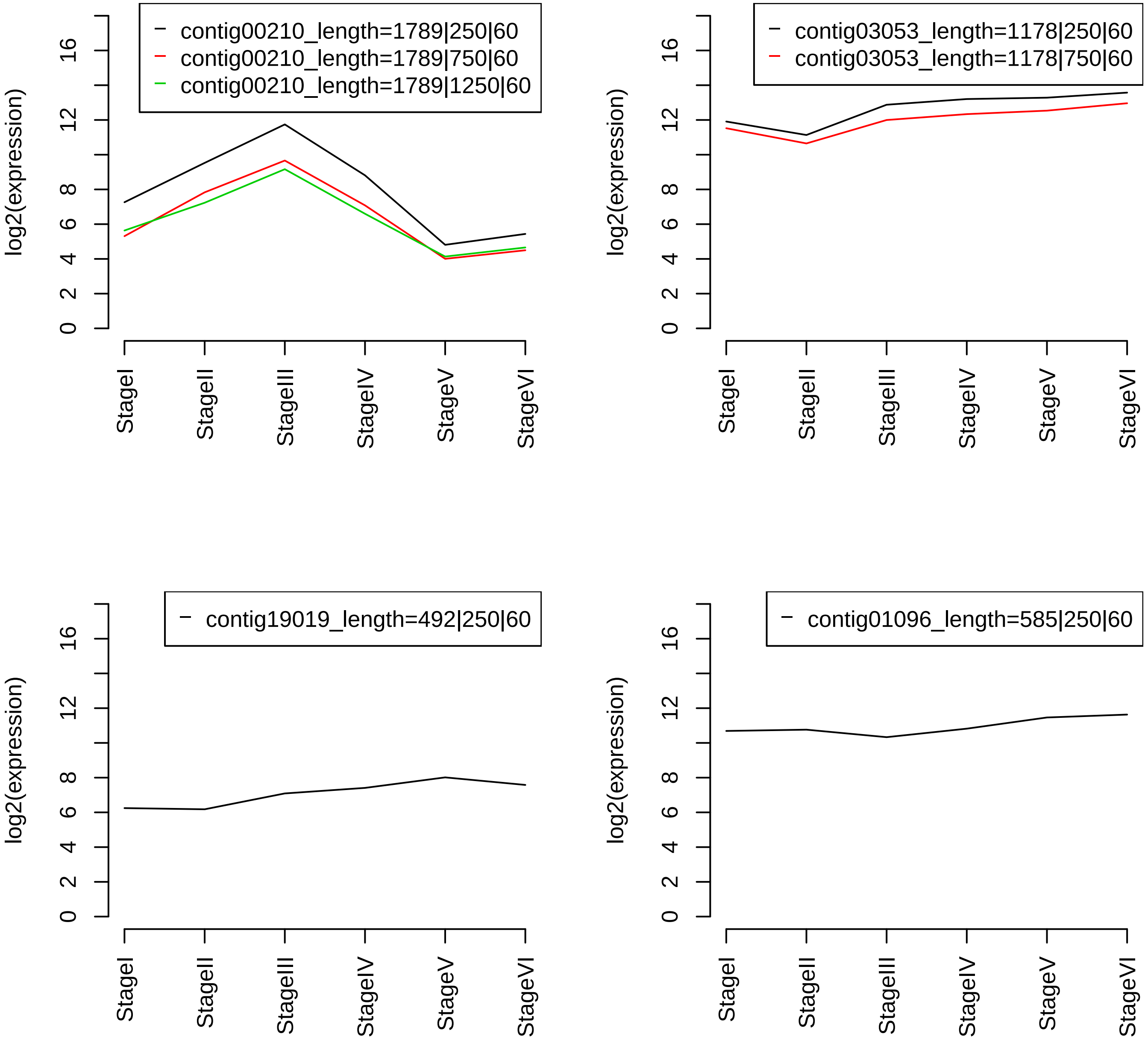
contig00210_length=1789|250|60
contig00210_length=1789|750|60
contig00210_length=1789|1250|60
log2(expression)
0
2
4
6
8
12
16
StageI
StageII
StageIII
StageIV
StageV
StageVI
contig03053_length=1178|250|60
contig03053_length=1178|750|60
contig19019_length=492|250|60
contig01096_length=585|250|60

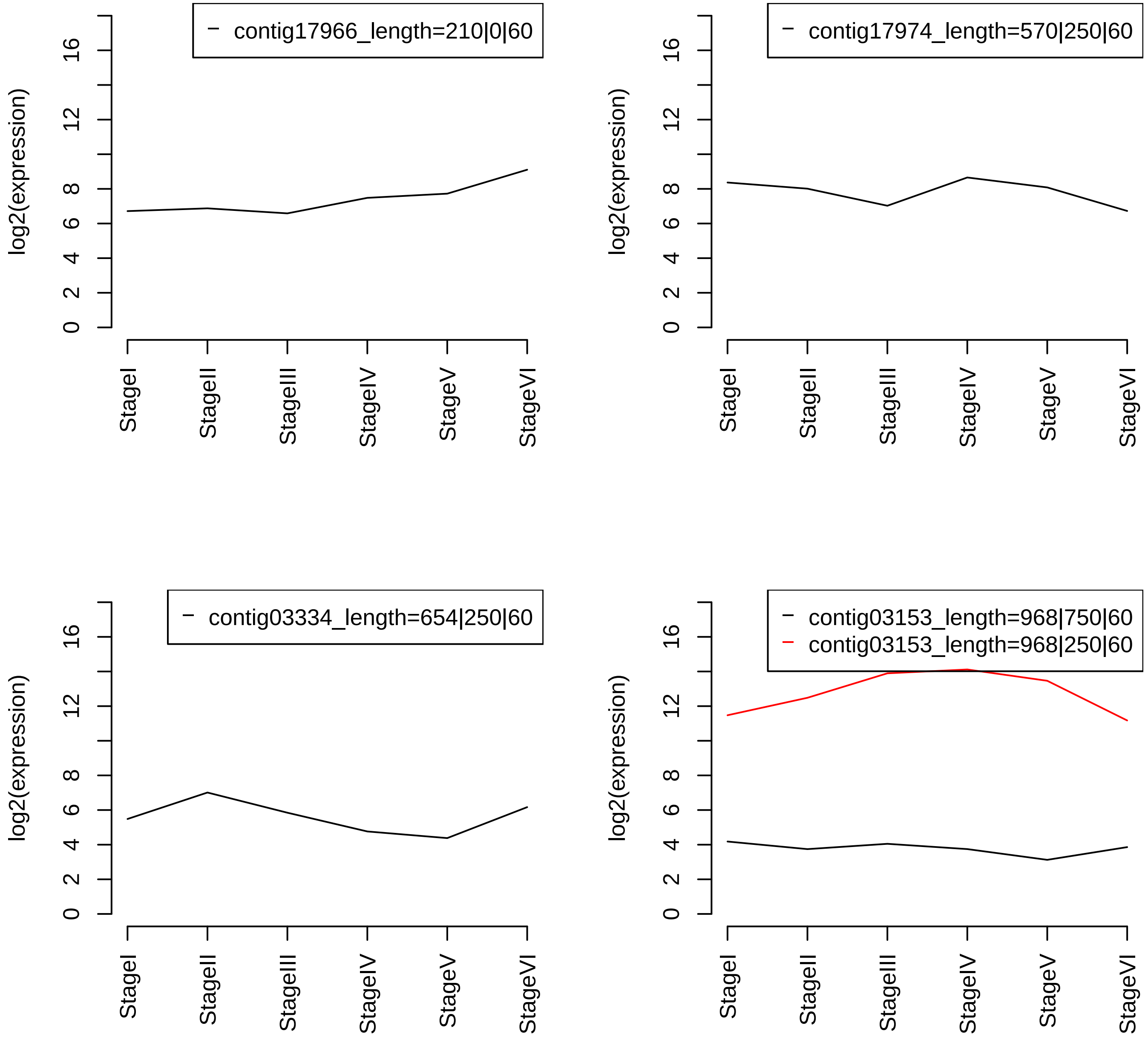

contig17966_length=210|0|60
log2(expression)
0
2
4
6
8
12
16
StageI
StageII
StageIII
StageIV
StageV
StageVI
contig17974_length=570|250|60
log2(expression)
0
2
4
6
8
12
16
StageI
StageII
StageIII
StageIV
StageV
StageVI
contig03334_length=654|250|60
log2(expression)
0
2
4
6
8
12
16
StageI
StageII
StageIII
StageIV
StageV
StageVI
contig03153_length=968|750|60
contig03153_length=968|250|60
log2(expression)
0
2
4
6
8
12
16
StageI
StageII
StageIII
StageIV
StageV
StageVI

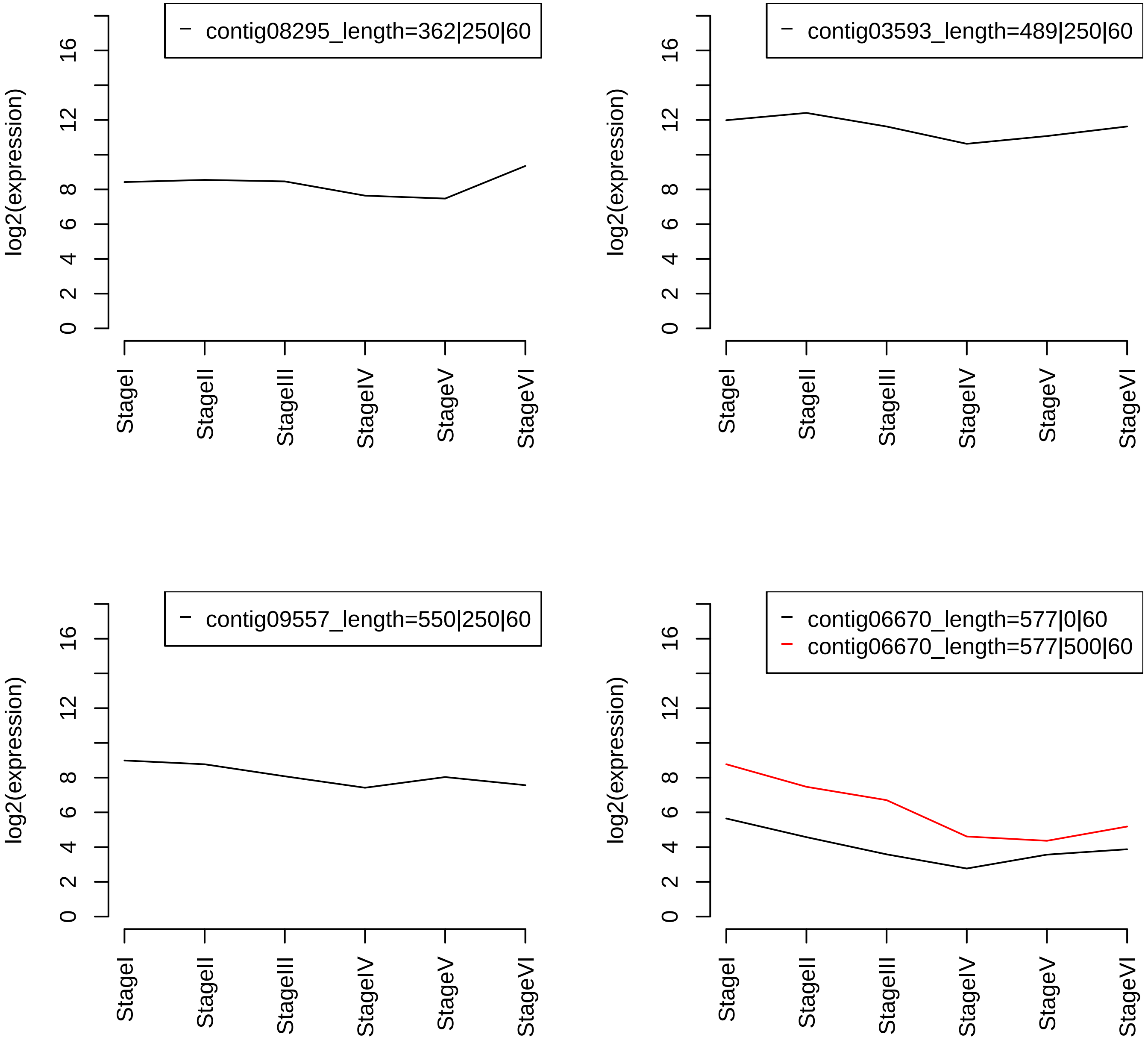
contig08295_length=362|250|60
contig03593_length=489|250|60
contig09557_length=550|250|60
contig06670_length=577|0|60
contig06670_length=577|500|60
log2(expression)
0
2
4
6
8
12
16
StageI
StageII
StageIII
StageIV
StageV
StageVI

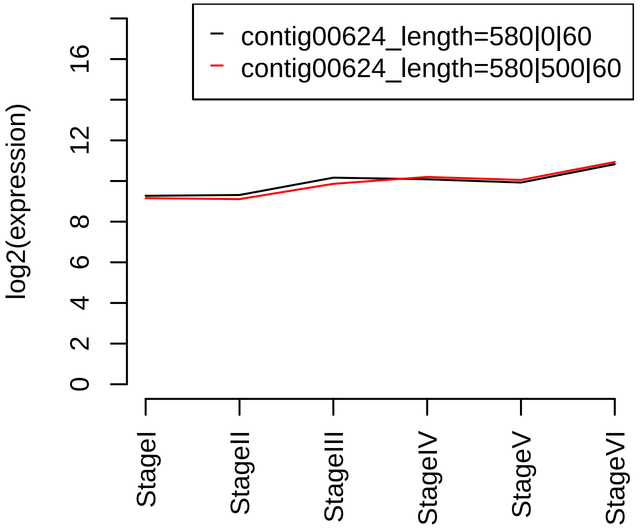
contig00624_length=580|0|60
contig00624_length=580|500|60
log2(expression)
0
2
4
6
8
12
16
StageI
StageII
StageIII
StageIV
StageV
StageVI

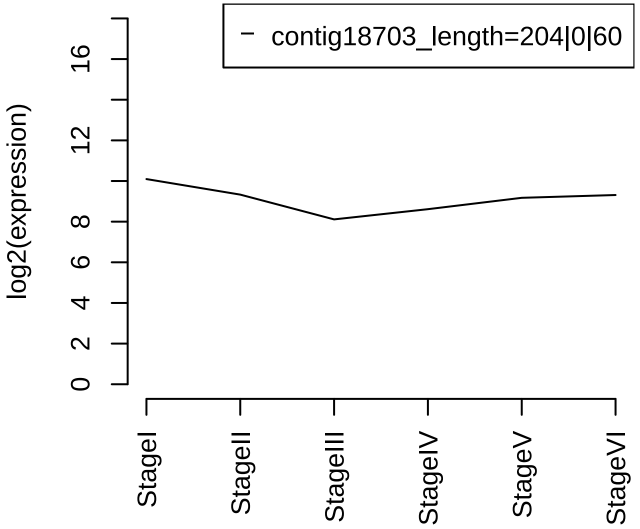
contig18703_length=204|0|60
log2(expression)
0
2
4
6
8
12
16
StageI
StageII
StageIII
StageIV
StageV
StageVI

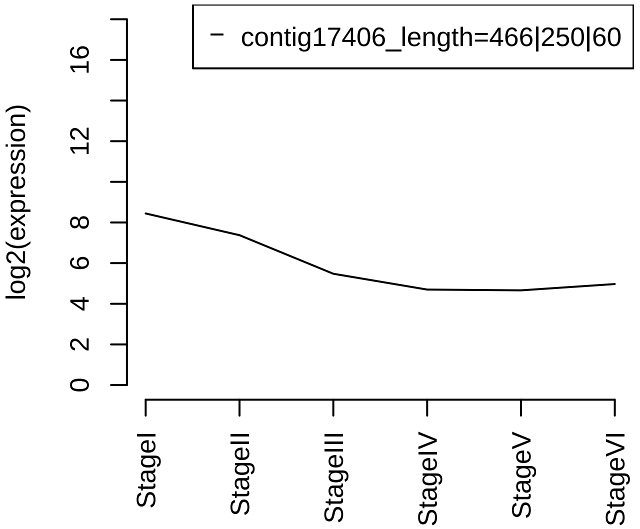
contig17406_length=466|250|60
log2(expression)
0
2
4
6
8
12
16
StageI
StageII
StageIII
StageIV
StageV
StageVI

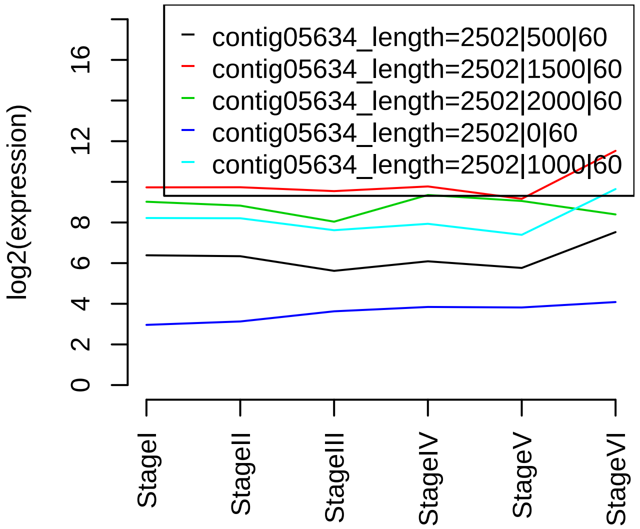
contig05634_length=2502|500|60
contig05634_length=2502|1500|60
contig05634_length=2502|2000|60
contig05634_length=2502|0|60
contig05634_length=2502|1000|60
log2(expression)
0
2
4
6
8
12
16
StageI
StageII
StageIII
StageIV
StageV
StageVI

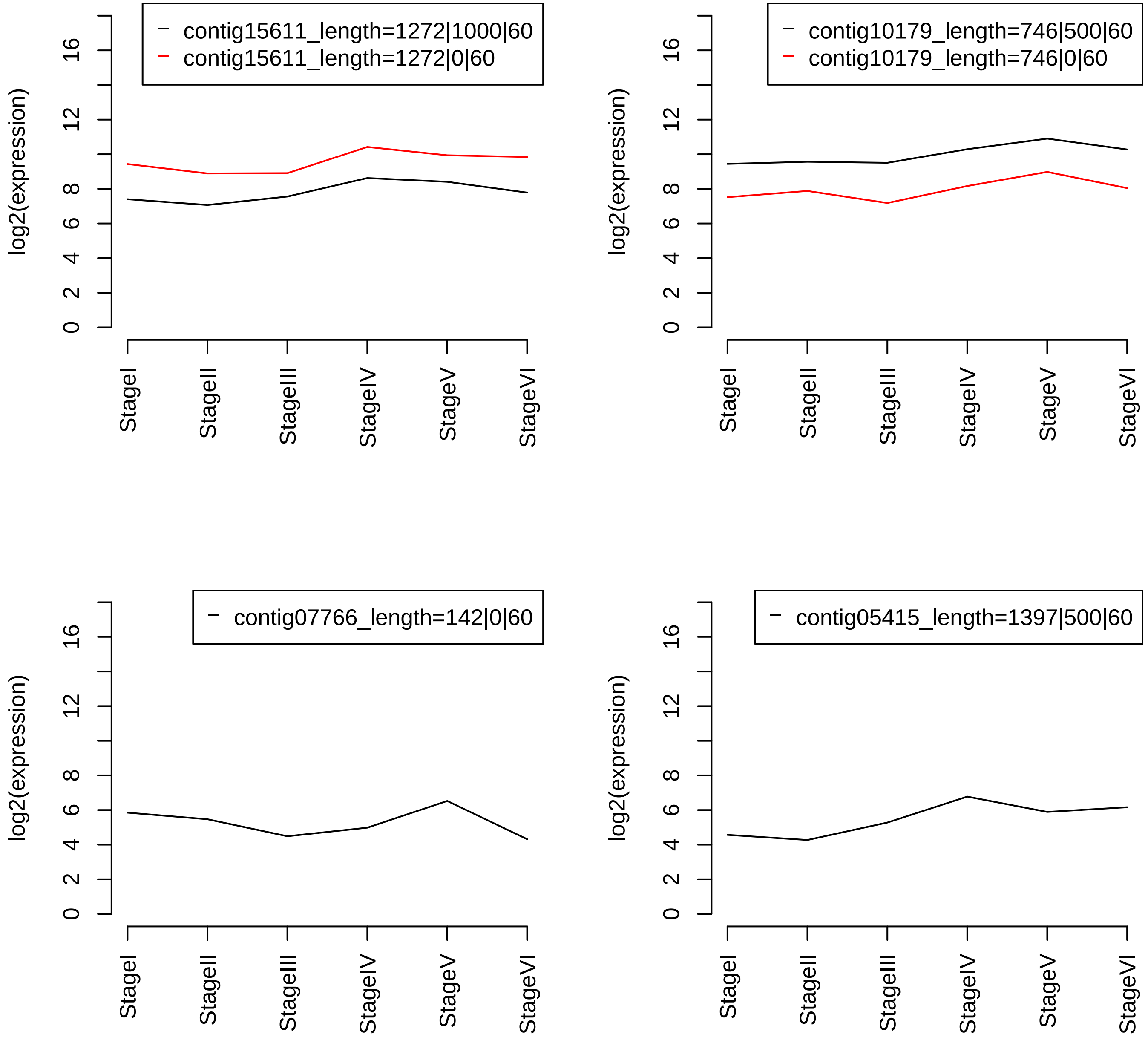

contig15611_length=1272|1000|60
contig15611_length=1272|0|60
contig10179_length=746|500|60
contig10179_length=746|0|60
contig07766_length=142|0|60
contig05415_length=1397|500|60
log2(expression)
0
2
4
6
8
12
16
StageI
StageII
StageIII
StageIV
StageV
StageVI

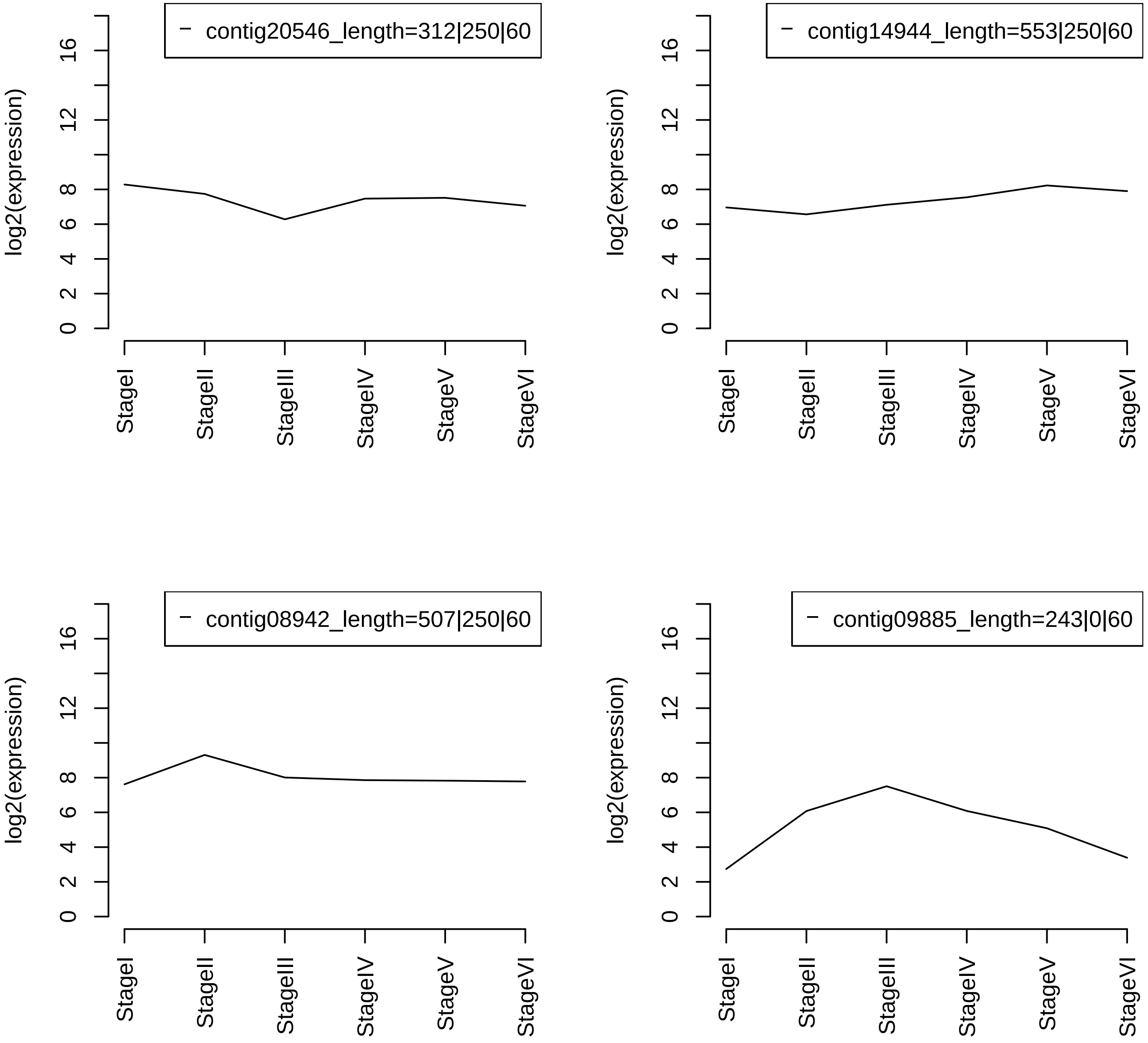
contig20546_length=312|250|60
contig14944_length=553|250|60
contig08942_length=507|250|60
contig09885_length=243|0|60
log2(expression)
0
2
4
6
8
12
16
StageI
StageII
StageIII
StageIV
StageV
StageVI

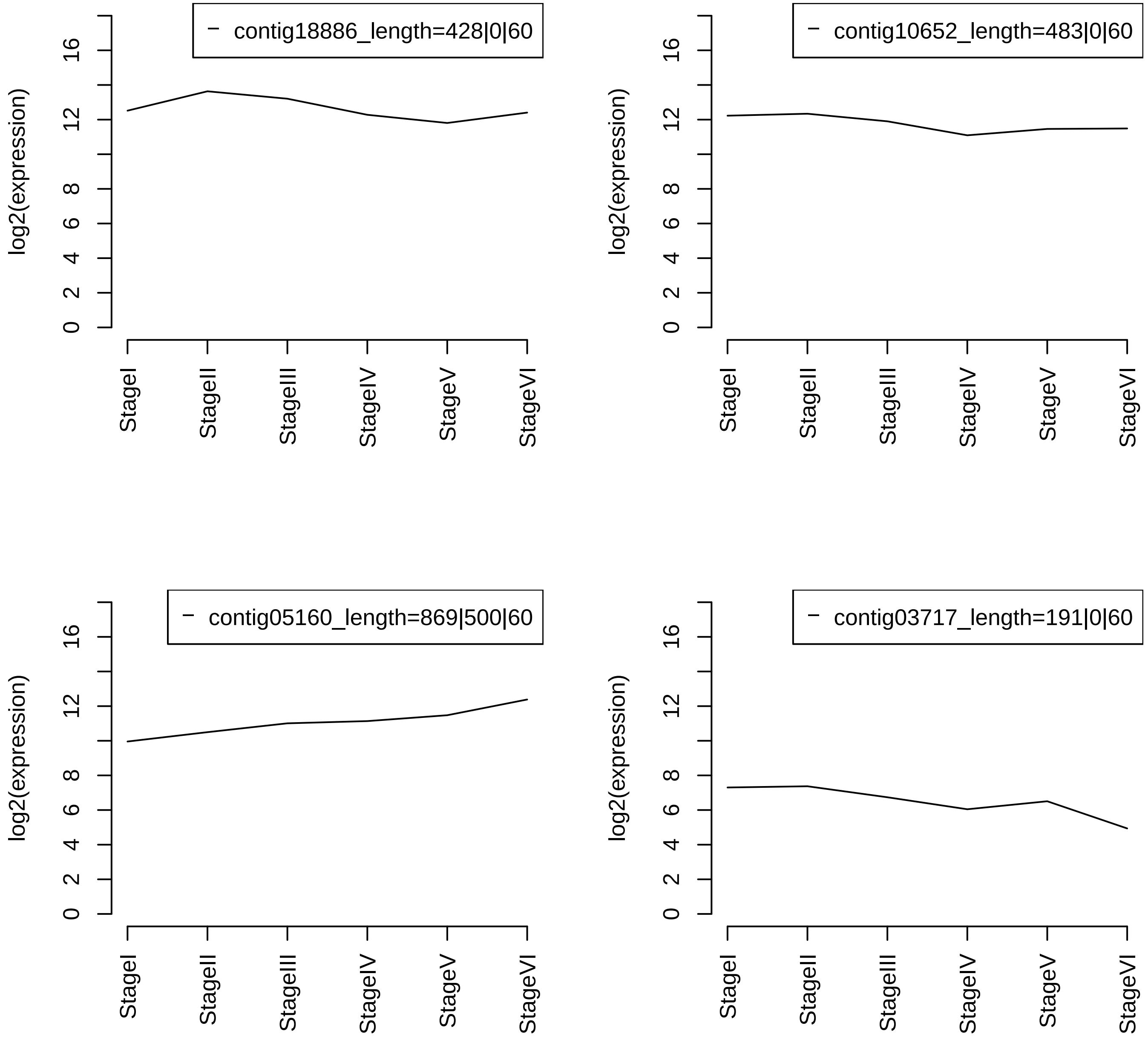
contig18886_length=428|0|60
contig10652_length=483|0|60
contig05160_length=869|500|60
contig03717_length=191|0|60
log2(expression)
0
2
4
6
8
12
16
StageI
StageII
StageIII
StageIV
StageV
StageVI

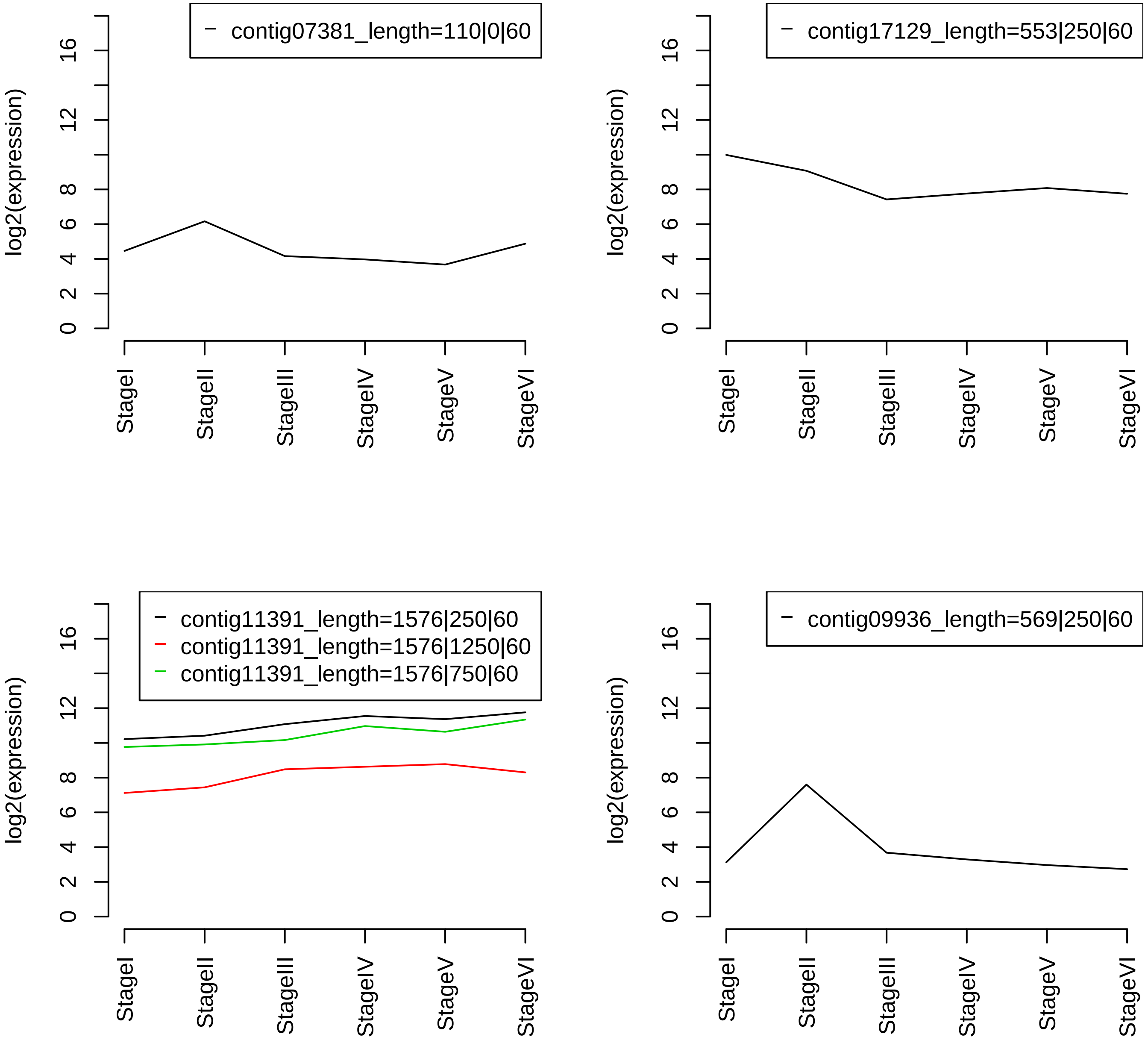
contig07381_length=110|0|60
contig17129_length=553|250|60
contig11391_length=1576|250|60
contig11391_length=1576|1250|60
contig11391_length=1576|750|60
contig09936_length=569|250|60
log2(expression)
0
2
4
6
8
12
16
StageI
StageII
StageIII
StageIV
StageV
StageVI

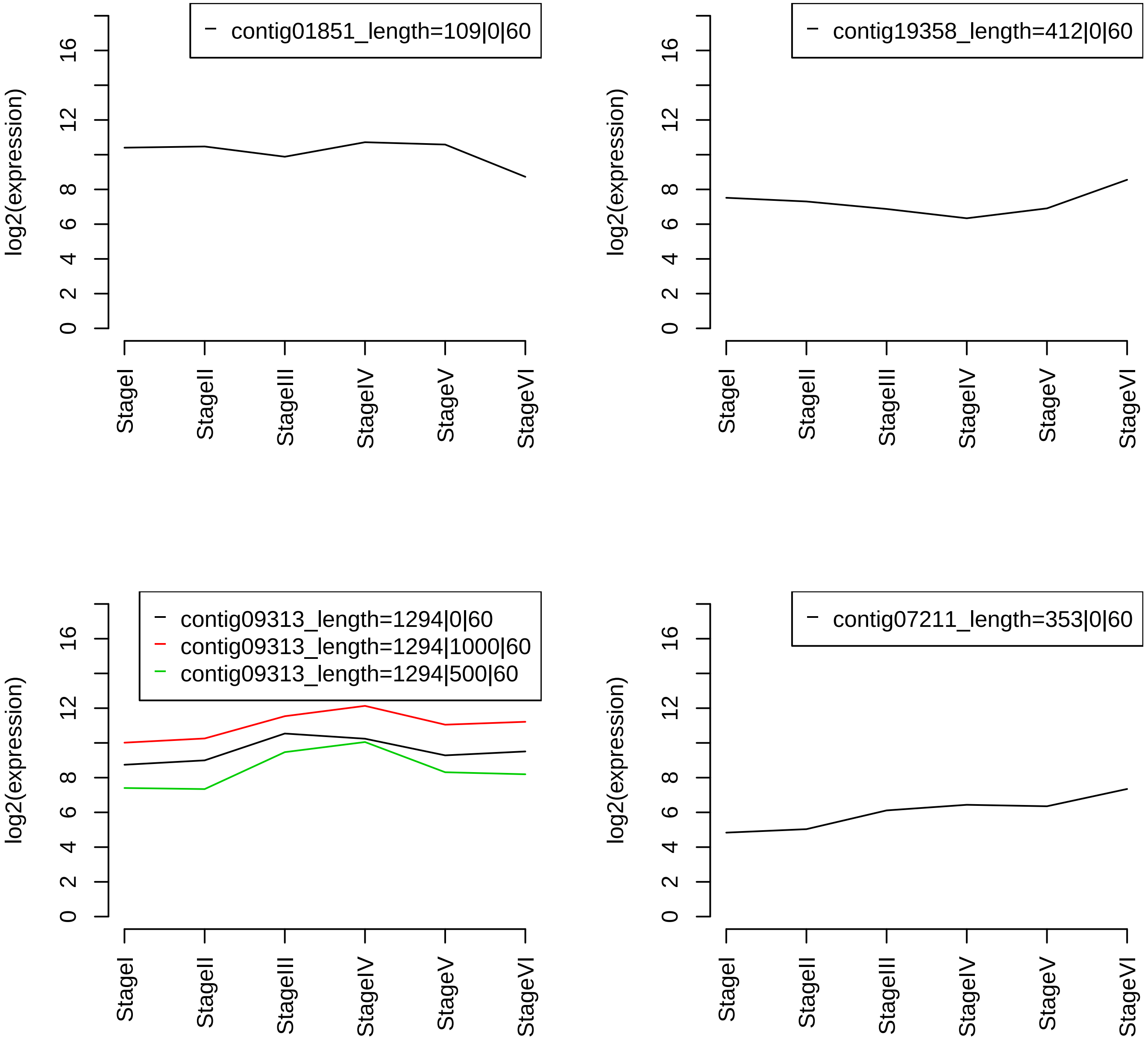

contig01851_length=109|0|60
contig19358_length=412|0|60
contig09313_length=1294|0|60
contig09313_length=1294|1000|60
contig09313_length=1294|500|60
contig07211_length=353|0|60
log2(expression)
0
2
4
6
8
12
16
StageI
StageII
StageIII
StageIV
StageV
StageVI

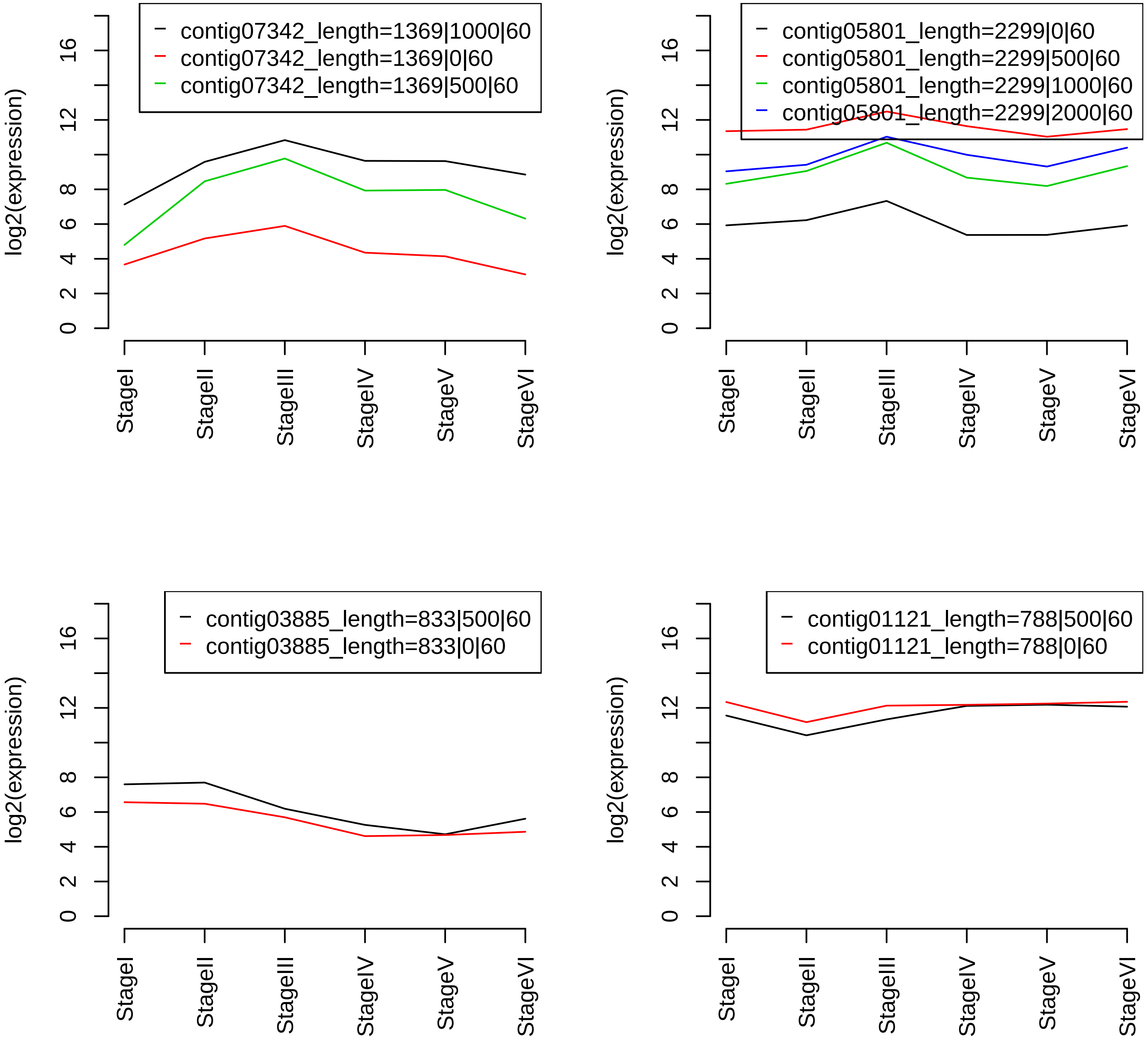
contig07342_length=1369|1000|60
contig07342_length=1369|0|60
contig07342_length=1369|500|60
contig05801_length=2299|0|60
contig05801_length=2299|500|60
contig05801_length=2299|1000|60
contig05801_length=2299|2000|60
contig03885_length=833|500|60
contig03885_length=833|0|60
contig01121_length=788|500|60
contig01121_length=788|0|60
log2(expression)
0
2
4
6
8
12
16
StageI
StageII
StageIII
StageIV
StageV
StageVI

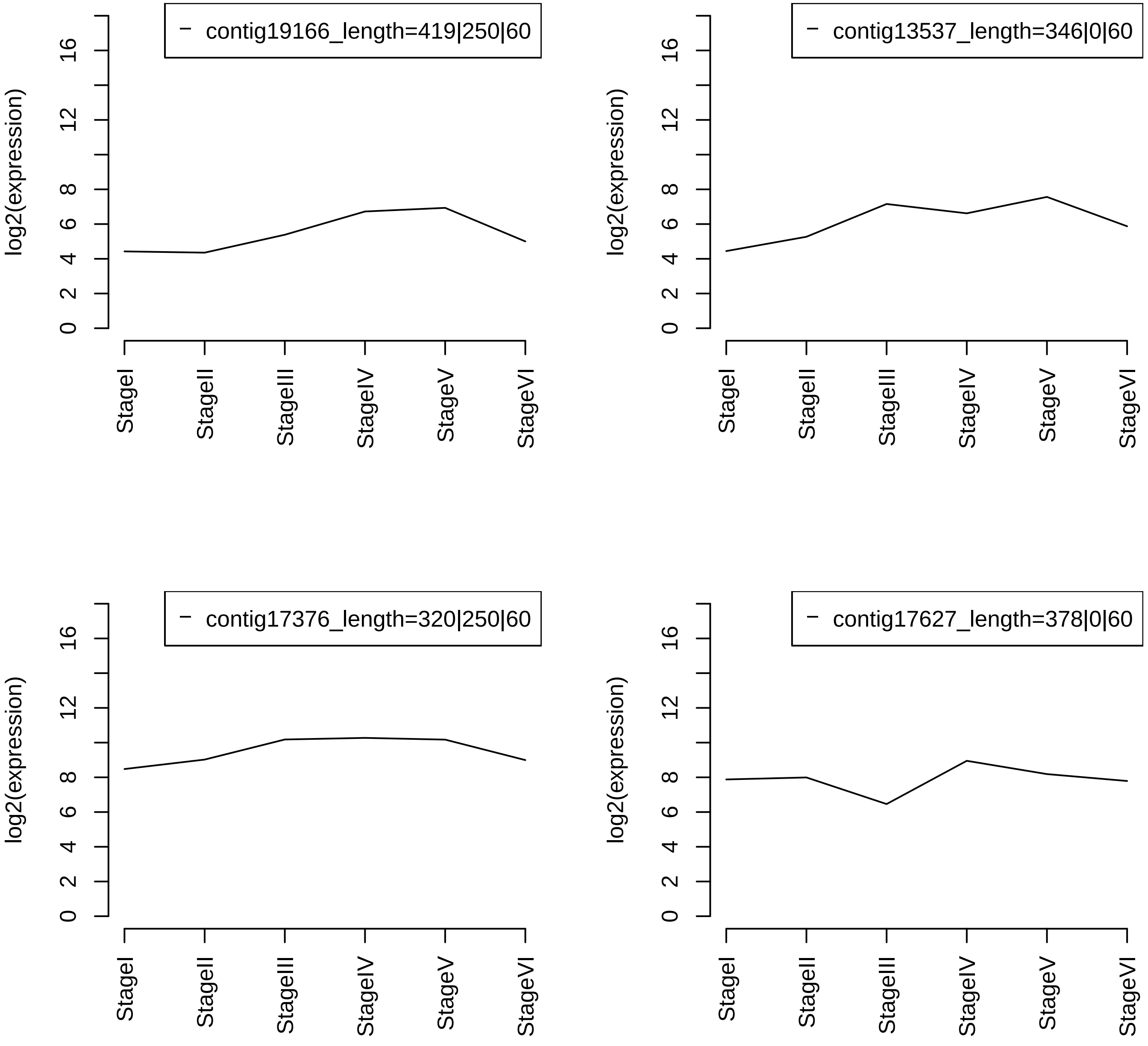
contig19166_length=419|250|60
contig13537_length=346|0|60
contig17376_length=320|250|60
contig17627_length=378|0|60
log2(expression)
0
2
4
6
8
12
16
StageI
StageII
StageIII
StageIV
StageV
StageVI

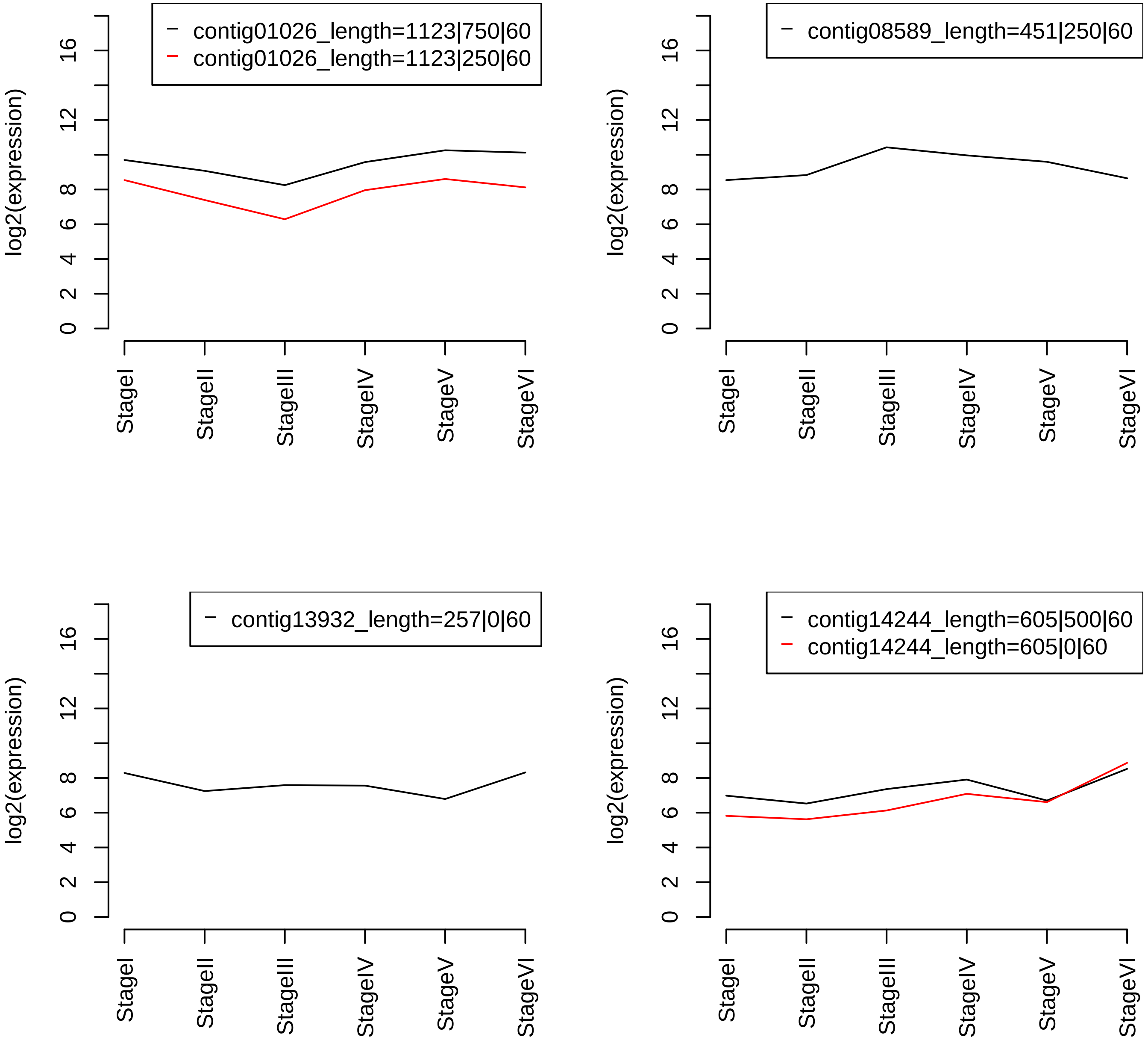
contig01026_length=1123|750|60
contig01026_length=1123|250|60
contig08589_length=451|250|60
contig13932_length=257|0|60
contig14244_length=605|500|60
contig14244_length=605|0|60
log2(expression)
StageI
StageII
StageIII
StageIV
StageV
StageVI

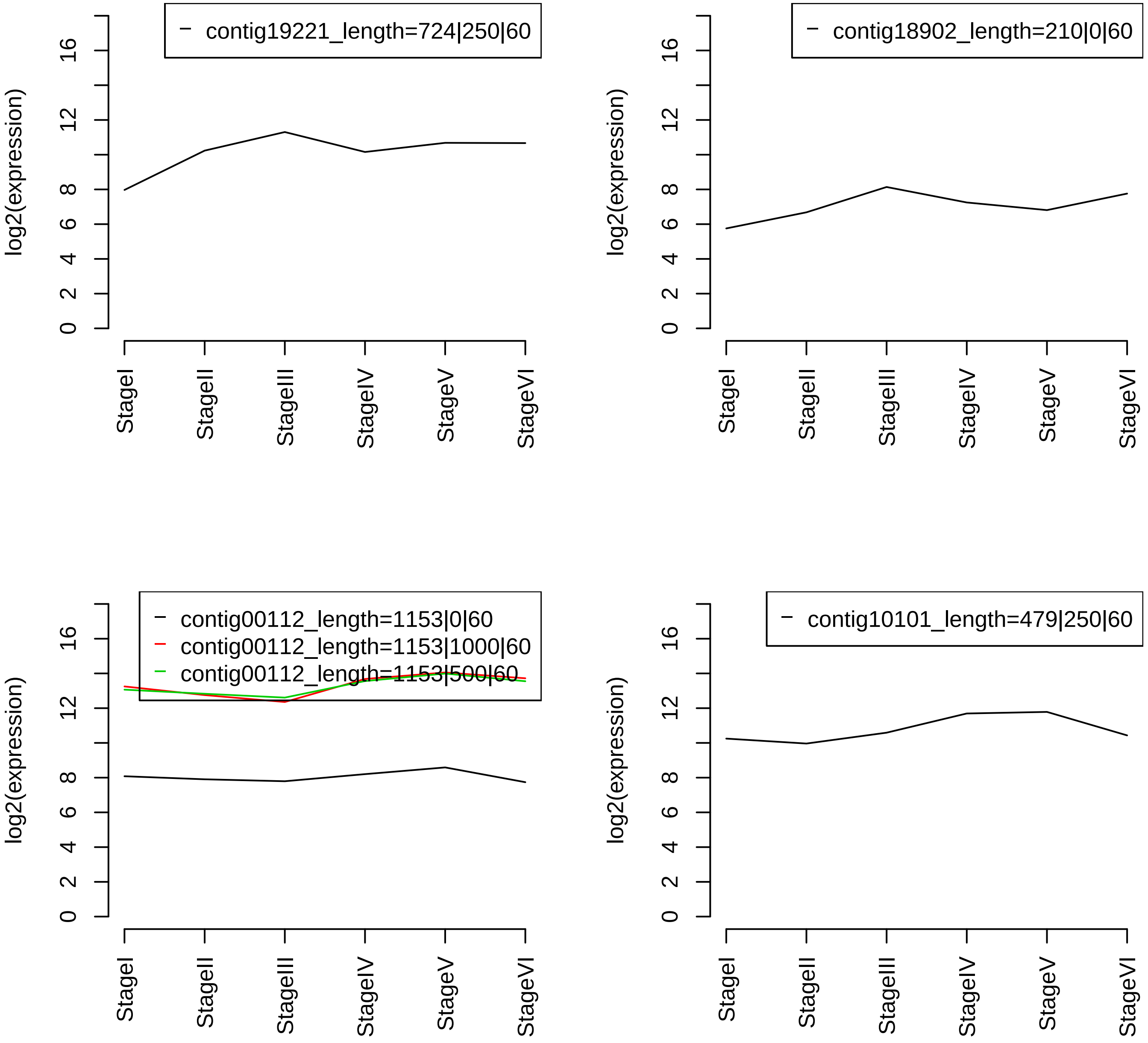
contig19221_length=724|250|60
contig18902_length=210|0|60
contig00112_length=1153|0|60
contig00112_length=1153|1000|60
contig00112_length=1153|500|60
contig10101_length=479|250|60
log2(expression)
0
2
4
6
8
12
16
StageI
StageII
StageIII
StageIV
StageV
StageVI

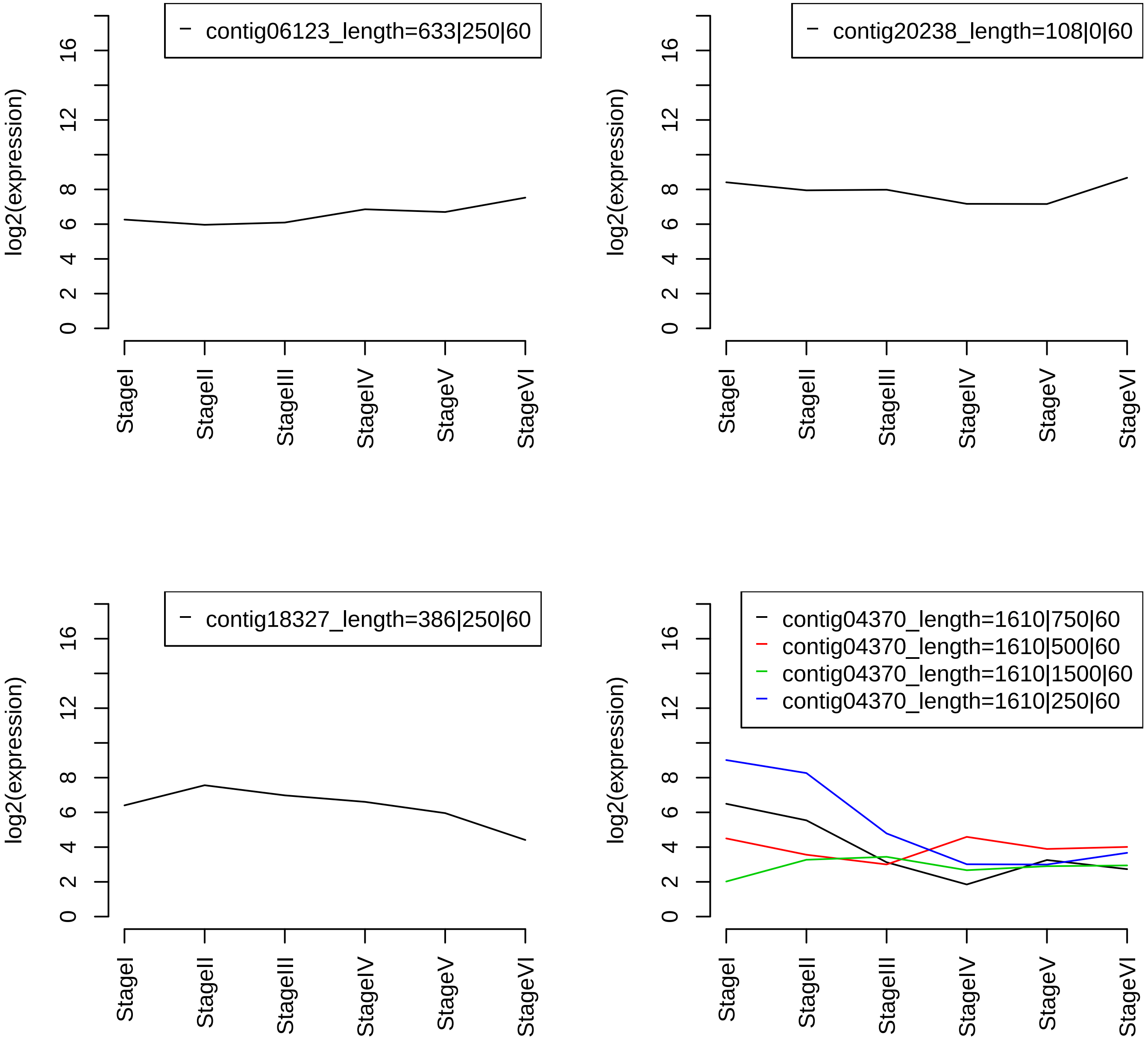

contig06123_length=633|250|60
contig20238_length=108|0|60
contig18327_length=386|250|60
contig04370_length=1610|750|60
contig04370_length=1610|500|60
contig04370_length=1610|1500|60
contig04370_length=1610|250|60
log2(expression)
0
2
4
6
8
12
16
StageI
StageII
StageIII
StageIV
StageV
StageVI

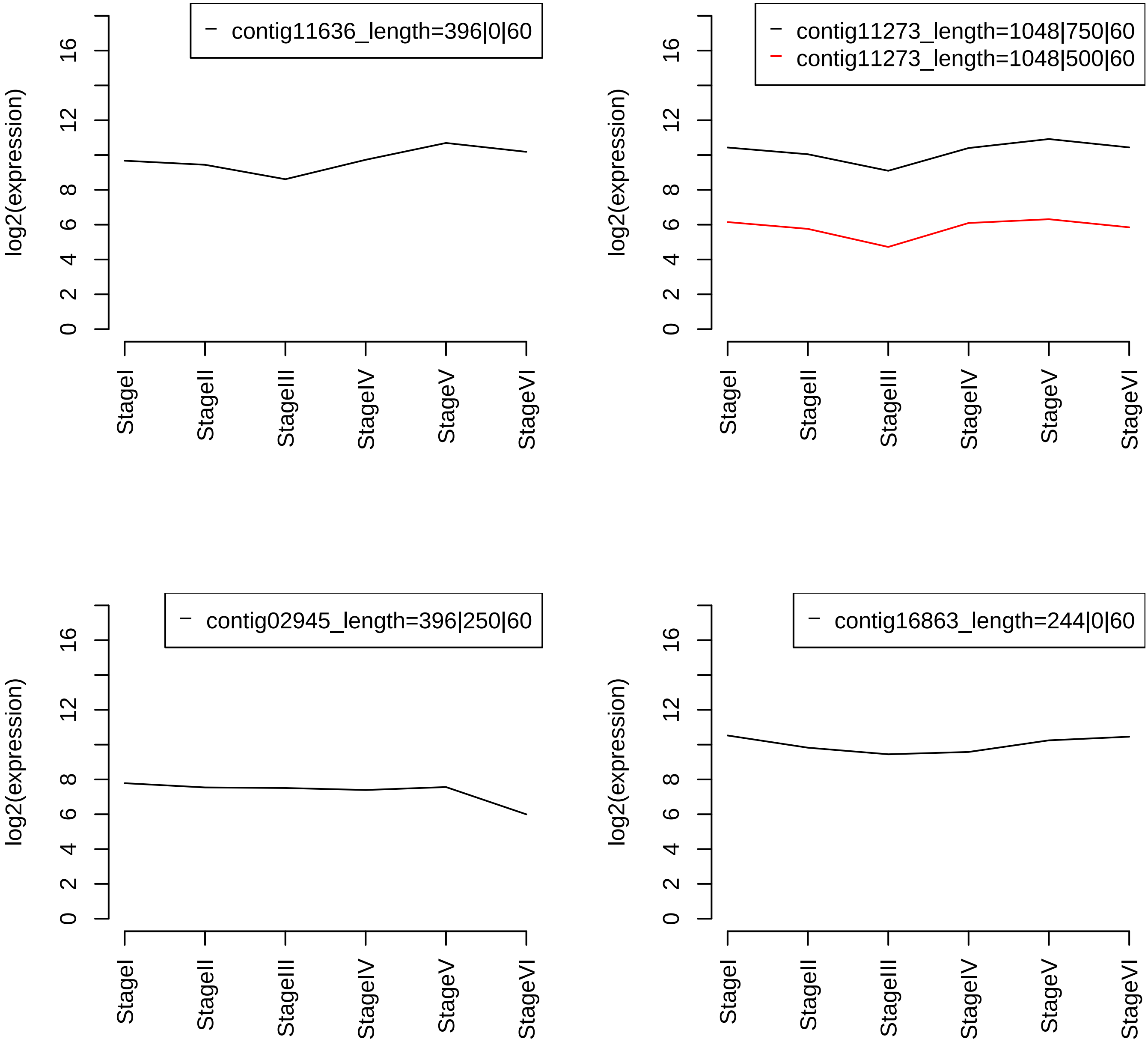
contig11636_length=396|0|60
contig11273_length=1048|750|60
contig11273_length=1048|500|60
contig02945_length=396|250|60
contig16863_length=244|0|60
log2(expression)
StageI
StageII
StageIII
StageIV
StageV
StageVI

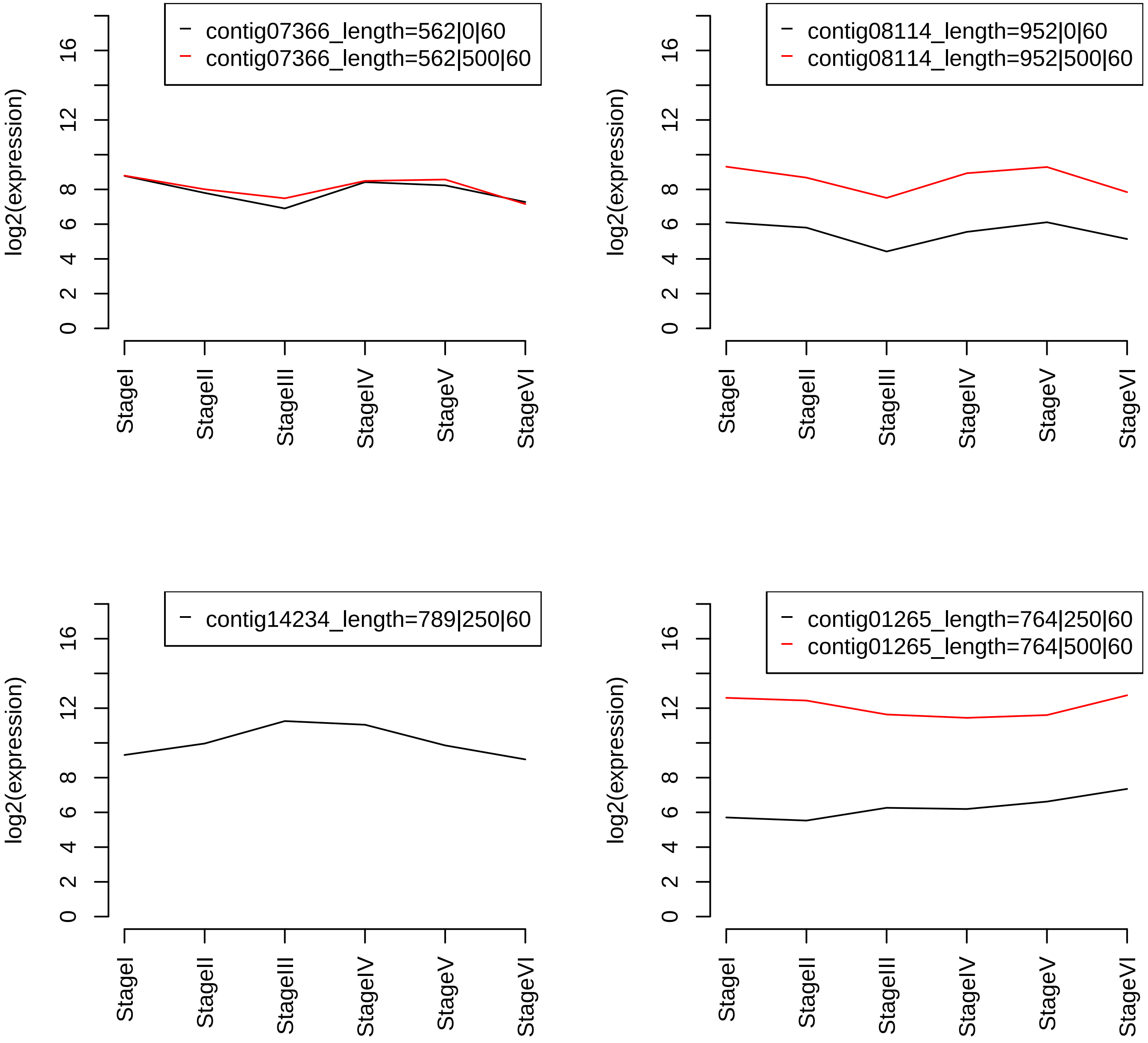
contig07366_length=562|0|60
contig07366_length=562|500|60
contig08114_length=952|0|60
contig08114_length=952|500|60
contig14234_length=789|250|60
contig01265_length=764|250|60
contig01265_length=764|500|60
log2(expression)
0 2 4 6 8 12 16
StageI StageII StageIII StageIV StageV StageVI

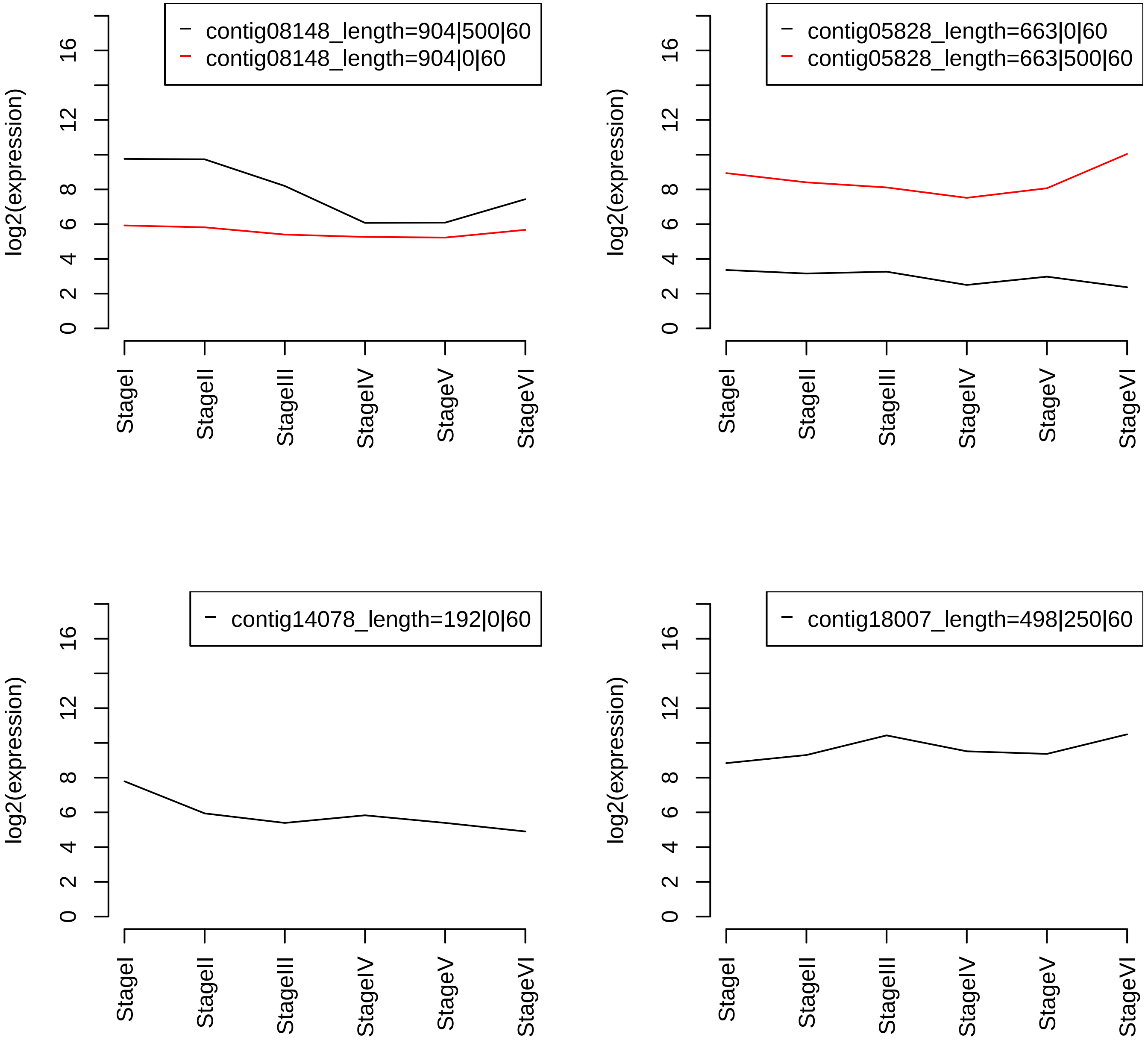
contig08148_length=904|500|60
contig08148_length=904|0|60
contig05828_length=663|0|60
contig05828_length=663|500|60
contig14078_length=192|0|60
contig18007_length=498|250|60
log2(expression)
0
2
4
6
8
12
16
StageI
StageII
StageIII
StageIV
StageV
StageVI

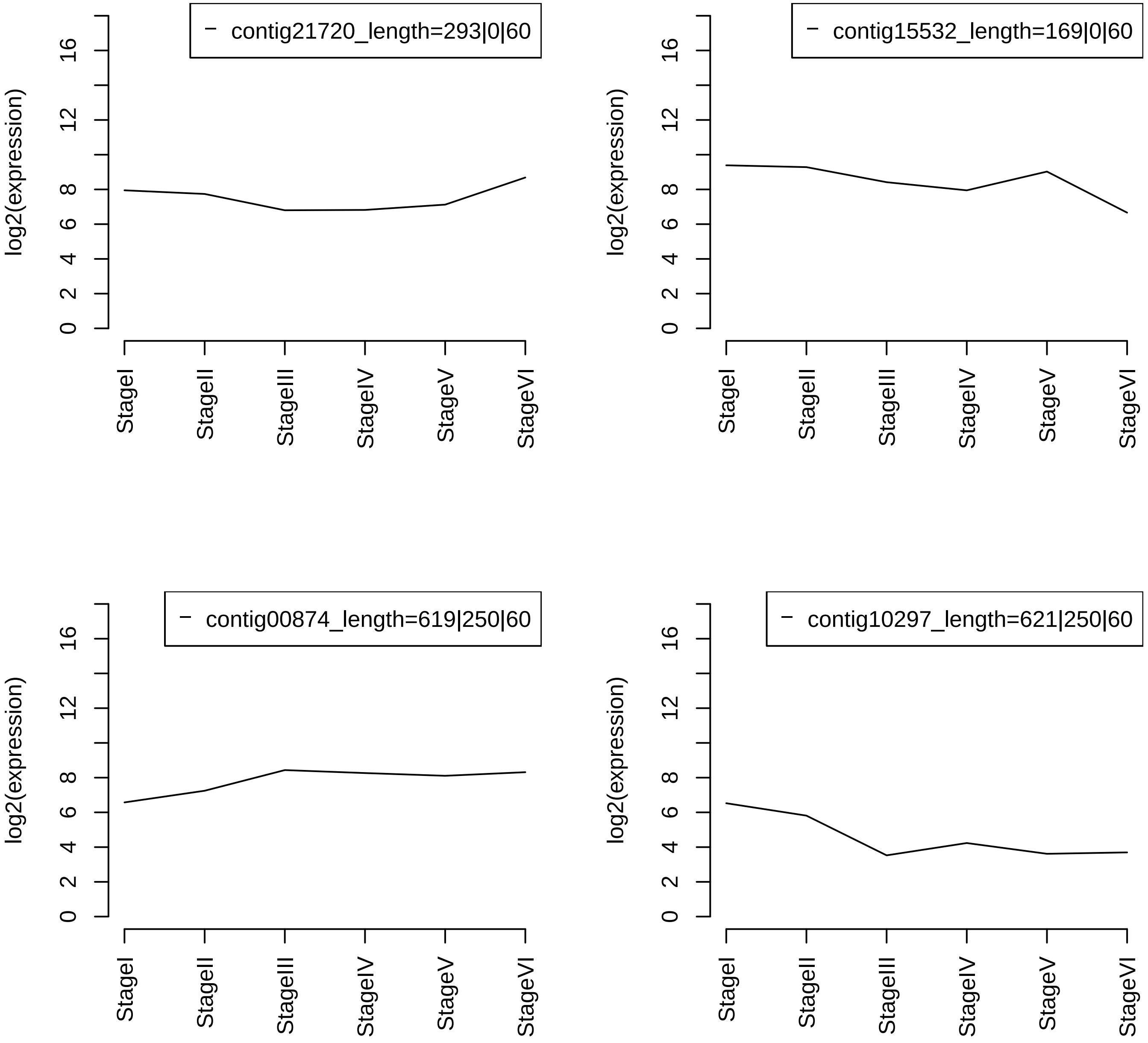
contig21720_length=293|0|60
contig15532_length=169|0|60
contig00874_length=619|250|60
contig10297_length=621|250|60
log2(expression)
0
2
4
6
8
12
16
StageI
StageII
StageIII
StageIV
StageV
StageVI

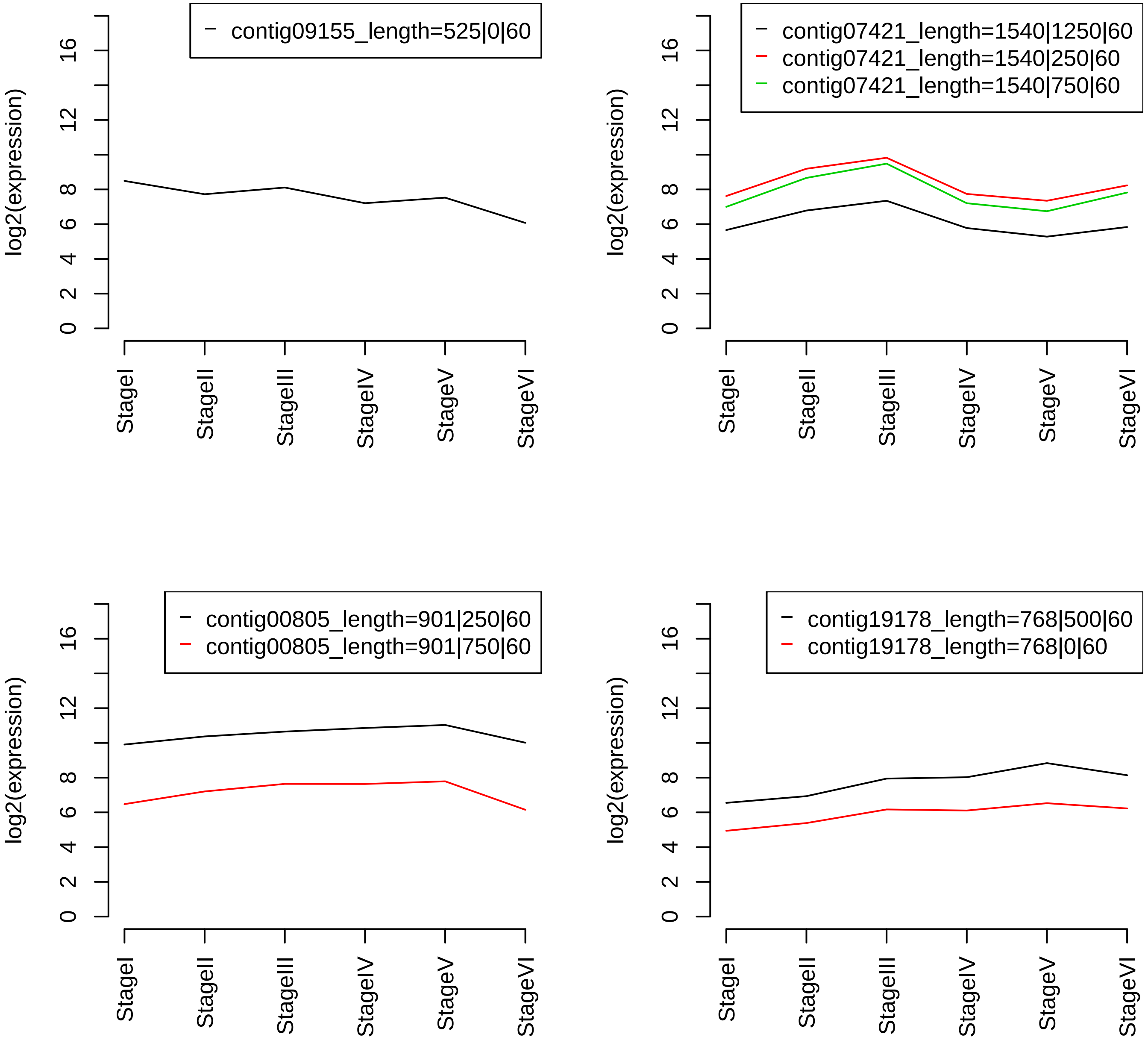

contig09155_length=525|0|60
log2(expression)
0
2
4
6
8
12
16
StageI
StageII
StageIII
StageIV
StageV
StageVI
contig07421_length=1540|1250|60
contig07421_length=1540|250|60
contig07421_length=1540|750|60
log2(expression)
StageI
StageII
StageIII
StageIV
StageV
StageVI
contig00805_length=901|250|60
contig00805_length=901|750|60
log2(expression)
StageI
StageII
StageIII
StageIV
StageV
StageVI
contig19178_length=768|500|60
contig19178_length=768|0|60
log2(expression)
StageI
StageII
StageIII
StageIV
StageV
StageVI

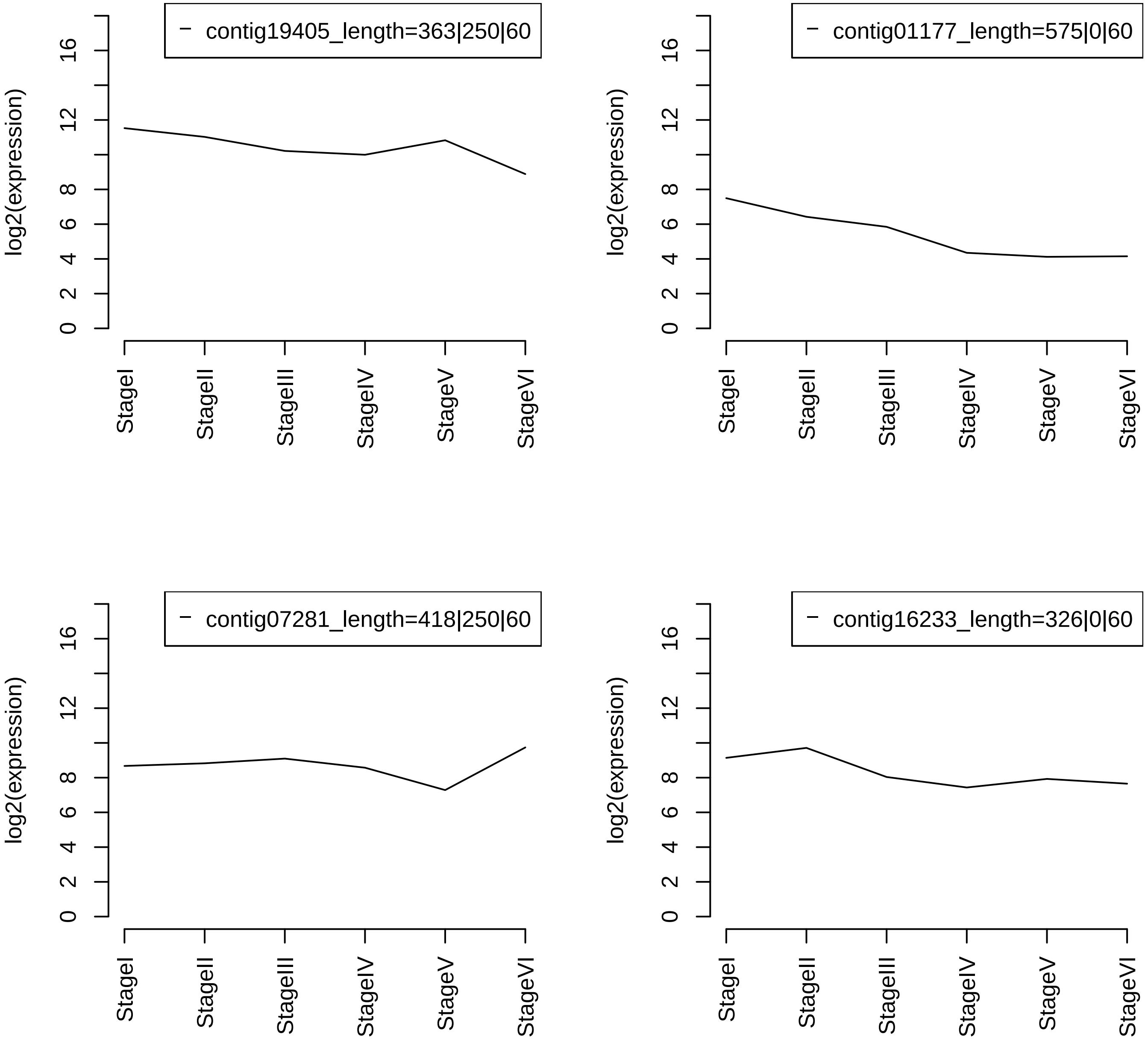
contig19405_length=363|250|60
contig01177_length=575|0|60
contig07281_length=418|250|60
contig16233_length=326|0|60
log2(expression)
0
2
4
6
8
12
16
StageI
StageII
StageIII
StageIV
StageV
StageVI

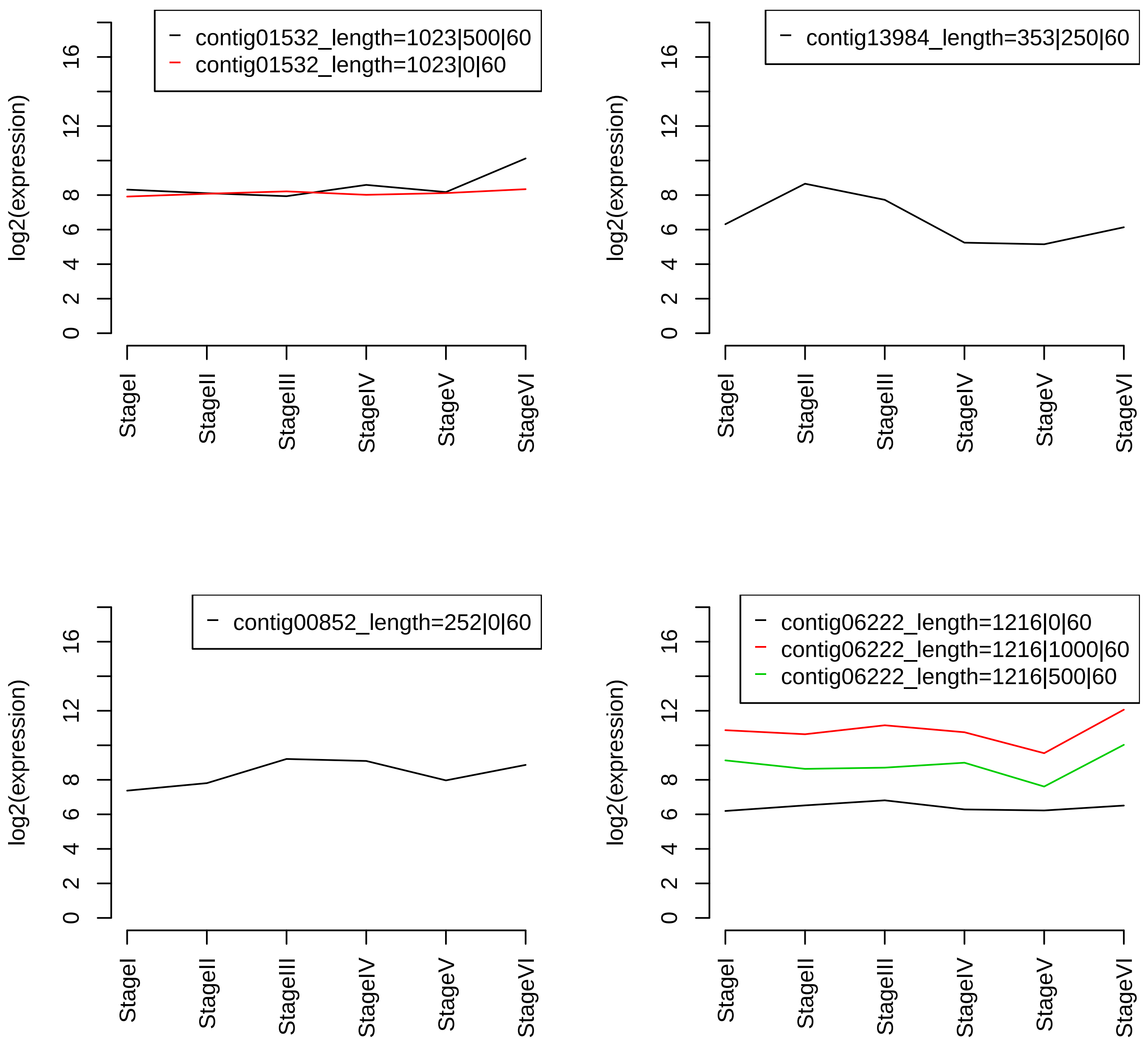
contig01532_length=1023|500|60
contig01532_length=1023|0|60
contig13984_length=353|250|60
contig00852_length=252|0|60
contig06222_length=1216|0|60
contig06222_length=1216|1000|60
contig06222_length=1216|500|60
log2(expression)
0
2
4
6
8
12
16
StageI
StageII
StageIII
StageIV
StageV
StageVI

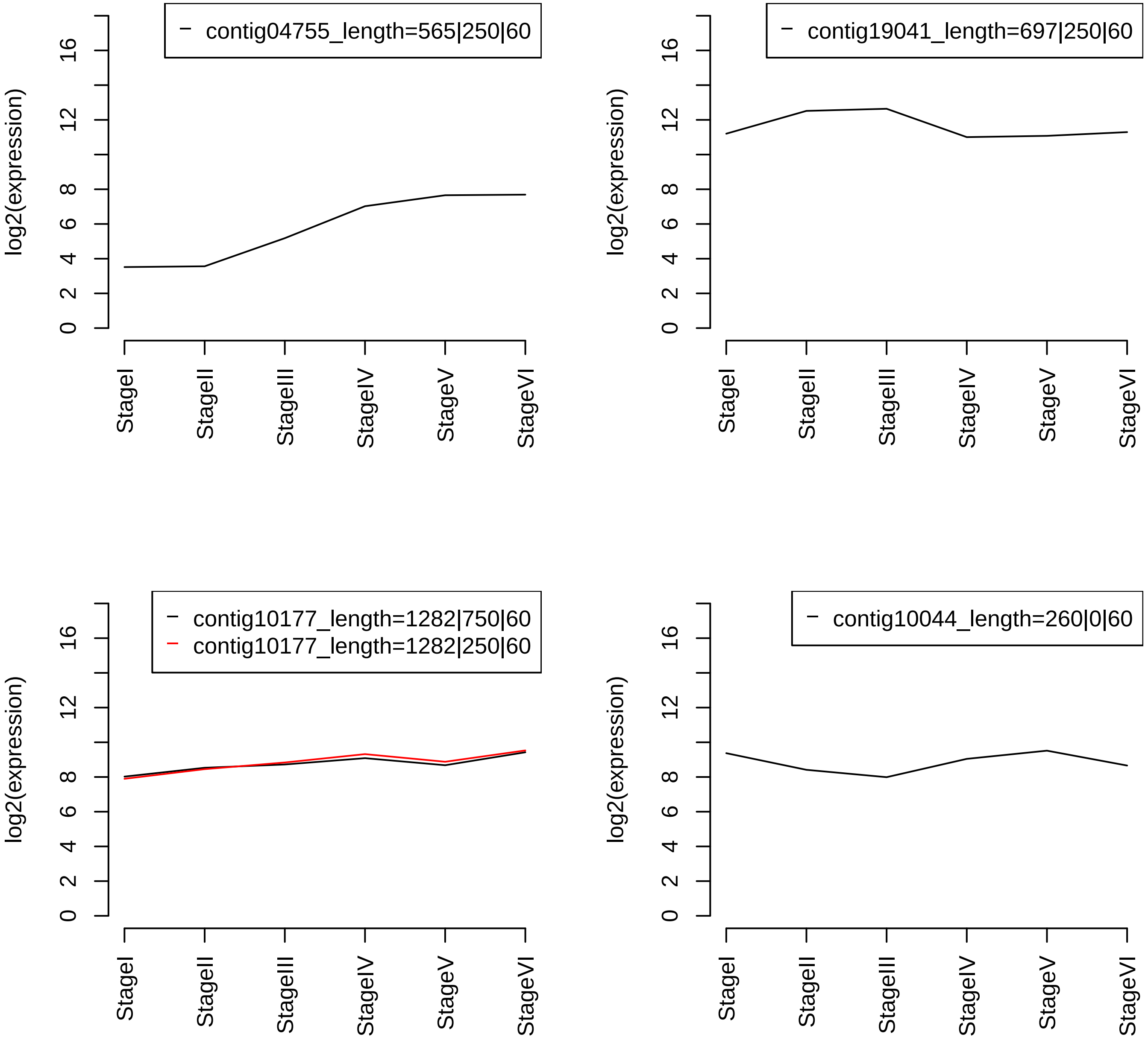
contig04755_length=565|250|60
contig19041_length=697|250|60
contig10177_length=1282|750|60
contig10177_length=1282|250|60
contig10044_length=260|0|60
log2(expression)
0
2
4
6
8
12
16
StageI
StageII
StageIII
StageIV
StageV
StageVI

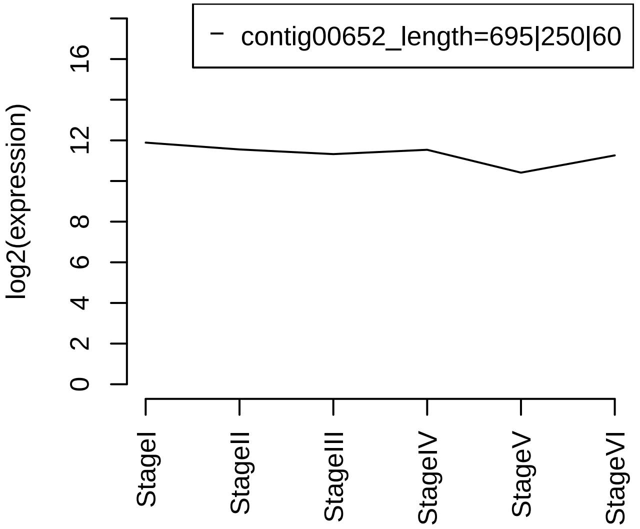

contig00652_length=695|250|60
log2(expression)
StageI
StageII
StageIII
StageIV
StageV
StageVI

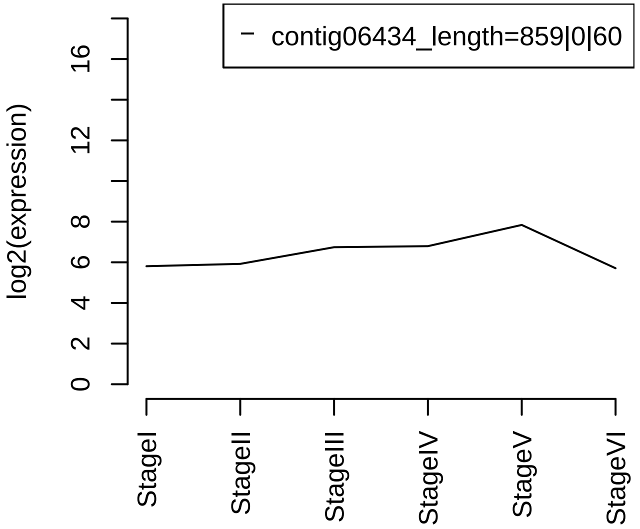

contig06434_length=859|0|60
log2(expression)
StageI
StageII
StageIII
StageIV
StageV
StageVI

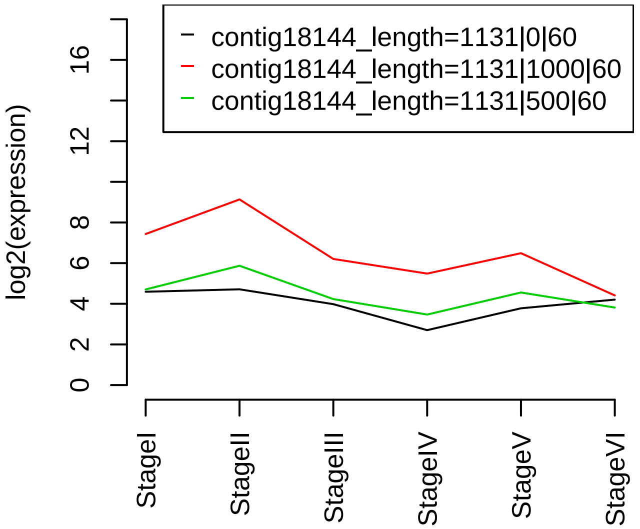

contig18144_length=1131|0|60
contig18144_length=1131|1000|60
contig18144_length=1131|500|60
log2(expression)
StageI
StageII
StageIII
StageIV
StageV
StageVI

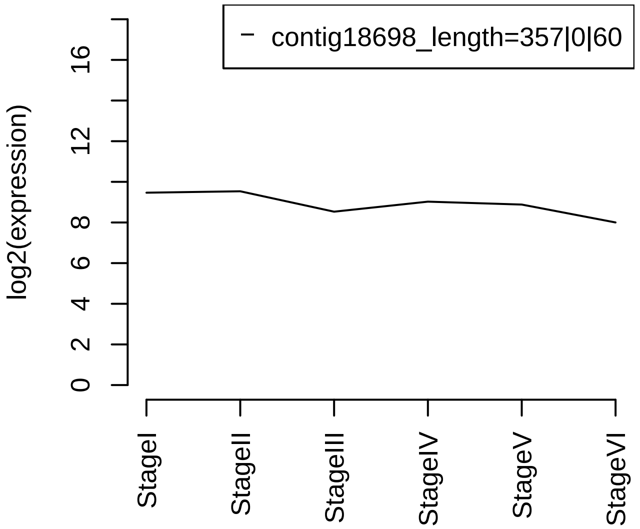

contig18698_length=357|0|60
log2(expression)
StageI
StageII
StageIII
StageIV
StageV
StageVI

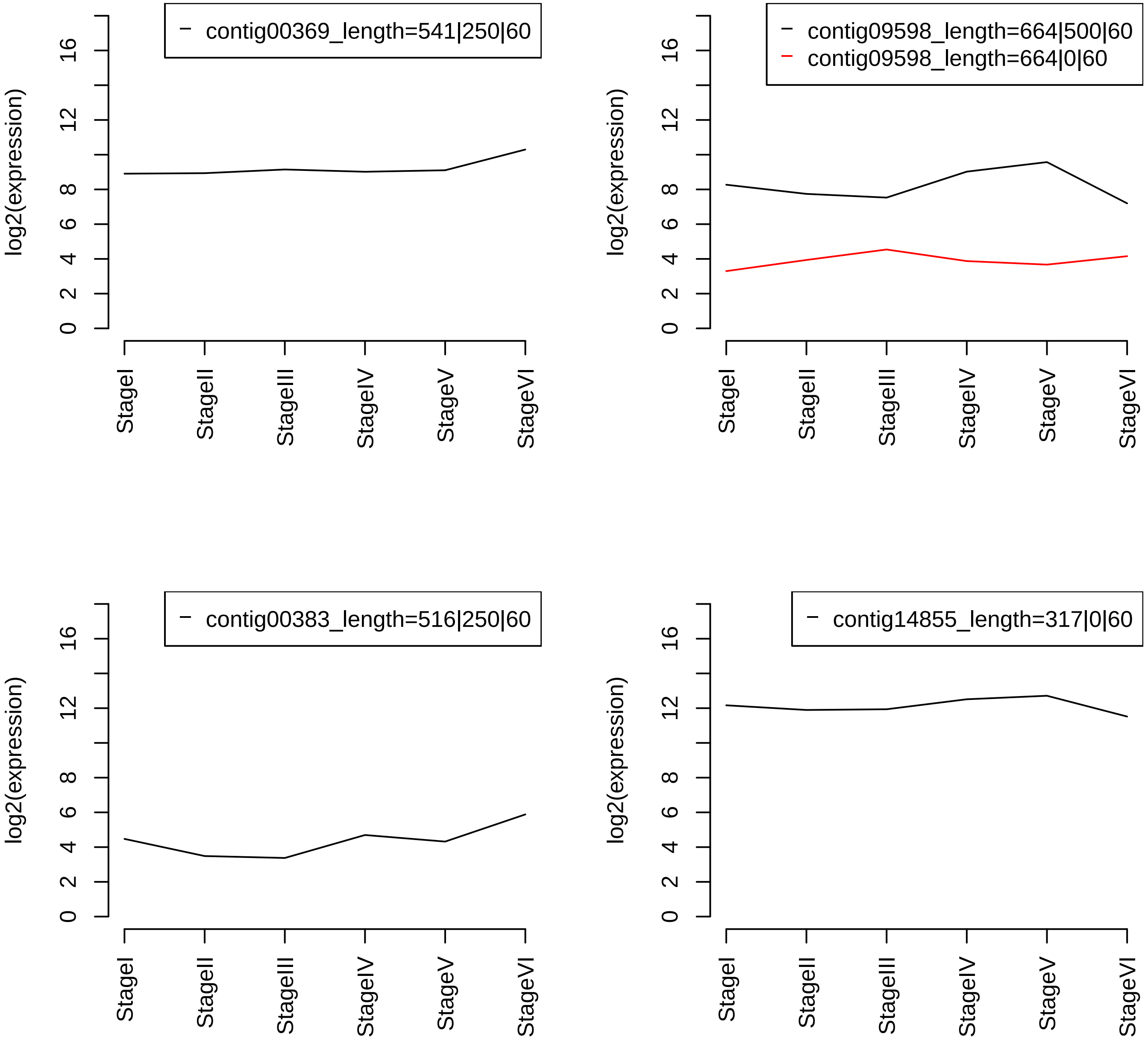

contig00369_length=541|250|60
contig09598_length=664|500|60
contig09598_length=664|0|60
contig00383_length=516|250|60
contig14855_length=317|0|60
log2(expression)
0
2
4
6
8
12
16
StageI
StageII
StageIII
StageIV
StageV
StageVI

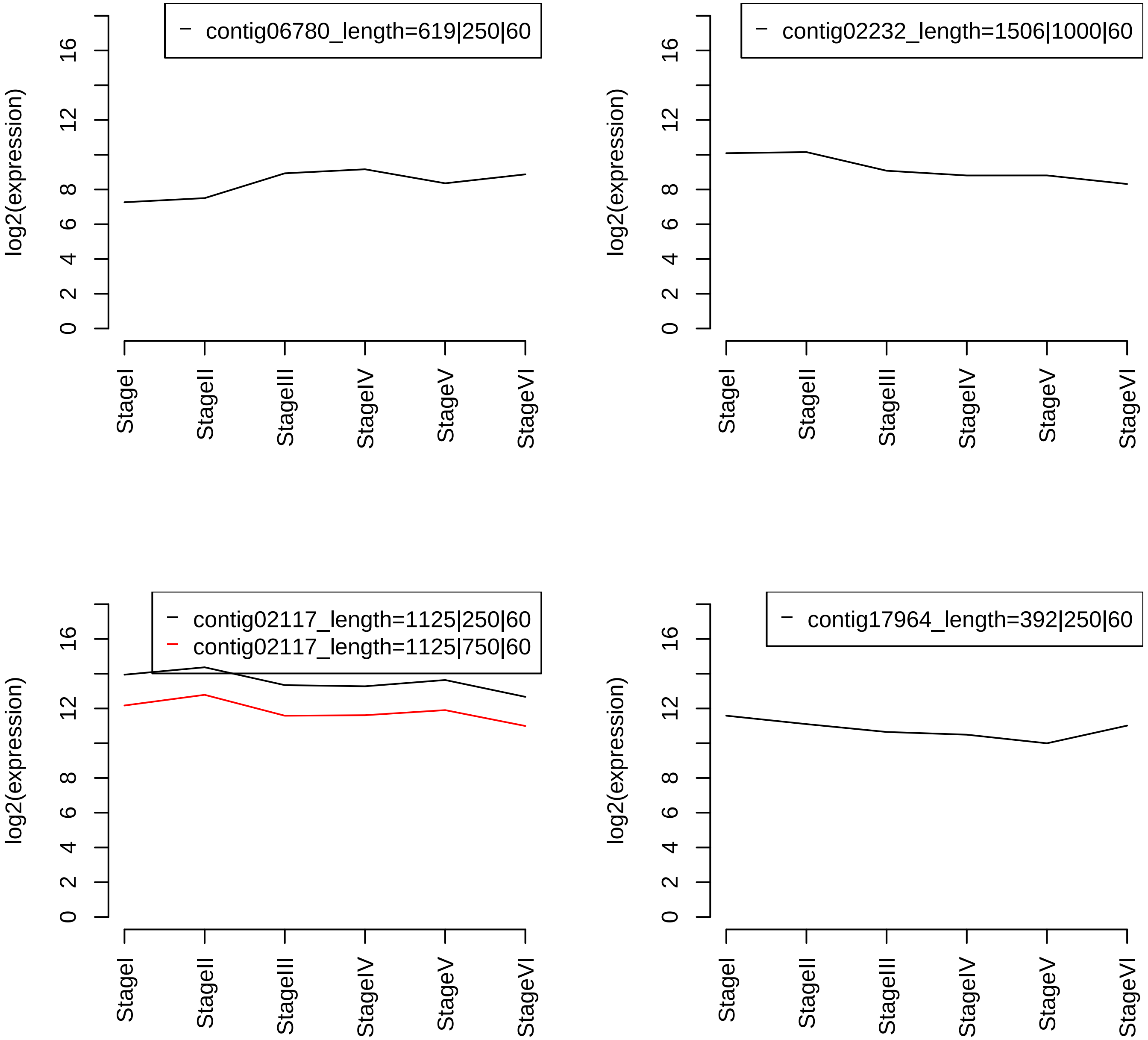

contig06780_length=619|250|60
contig02232_length=1506|1000|60
contig02117_length=1125|250|60
contig02117_length=1125|750|60
contig17964_length=392|250|60
log2(expression)
0
2
4
6
8
12
16
StageI
StageII
StageIII
StageIV
StageV
StageVI

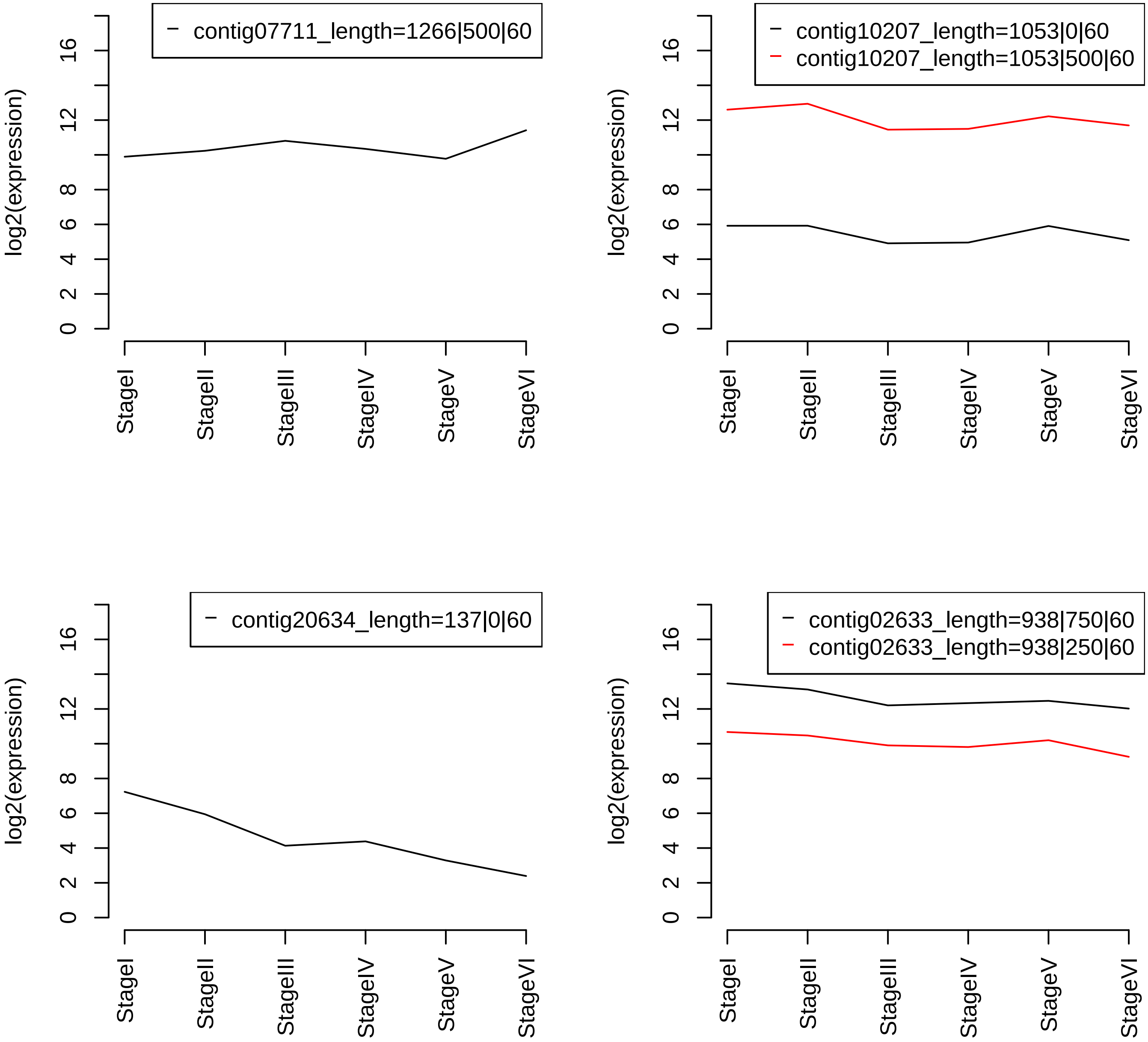
contig07711_length=1266|500|60
contig10207_length=1053|0|60
contig10207_length=1053|500|60
contig20634_length=137|0|60
contig02633_length=938|750|60
contig02633_length=938|250|60
log2(expression)
0
2
4
6
8
12
16
StageI
StageII
StageIII
StageIV
StageV
StageVI

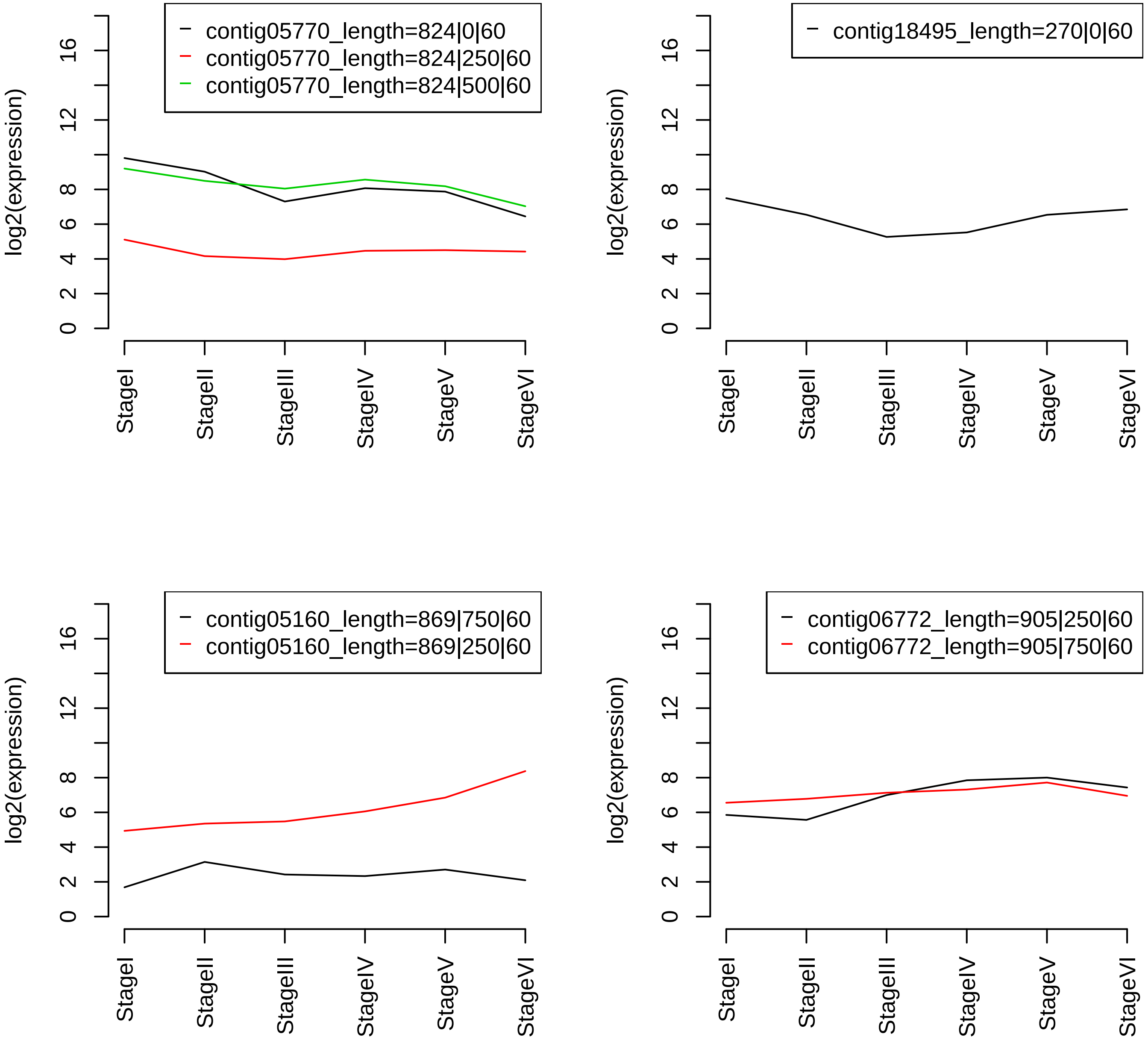
contig05770_length=824|0|60
contig05770_length=824|250|60
contig05770_length=824|500|60
contig18495_length=270|0|60
contig05160_length=869|750|60
contig05160_length=869|250|60
contig06772_length=905|250|60
contig06772_length=905|750|60
log2(expression)
0
2
4
6
8
12
16
StageI
StageII
StageIII
StageIV
StageV
StageVI

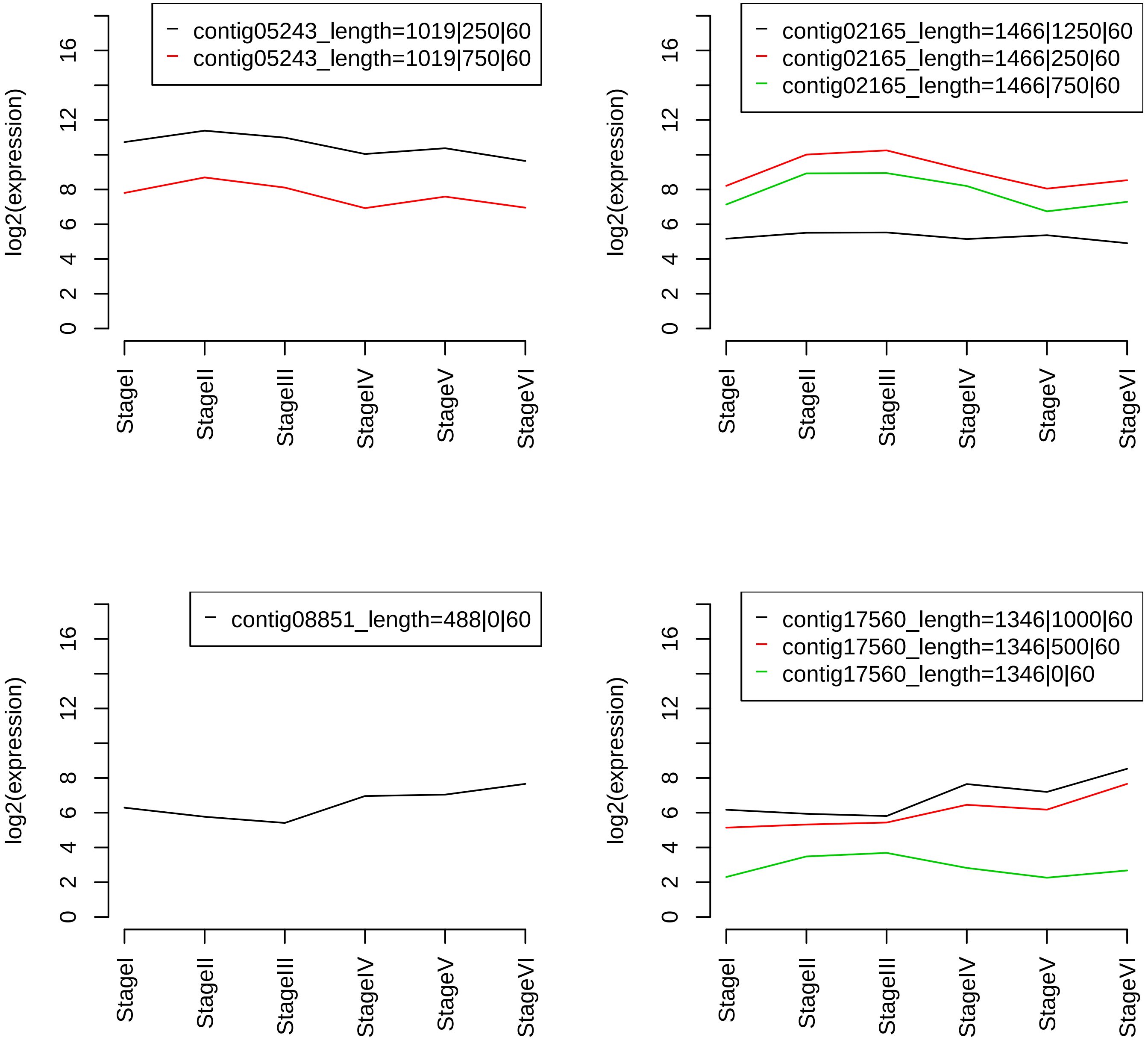
contig05243_length=1019|250|60
contig05243_length=1019|750|60
contig02165_length=1466|1250|60
contig02165_length=1466|250|60
contig02165_length=1466|750|60
contig08851_length=488|0|60
contig17560_length=1346|1000|60
contig17560_length=1346|500|60
contig17560_length=1346|0|60
log2(expression)
0 2 4 6 8 12 16
StageI StageII StageIII StageIV StageV StageVI

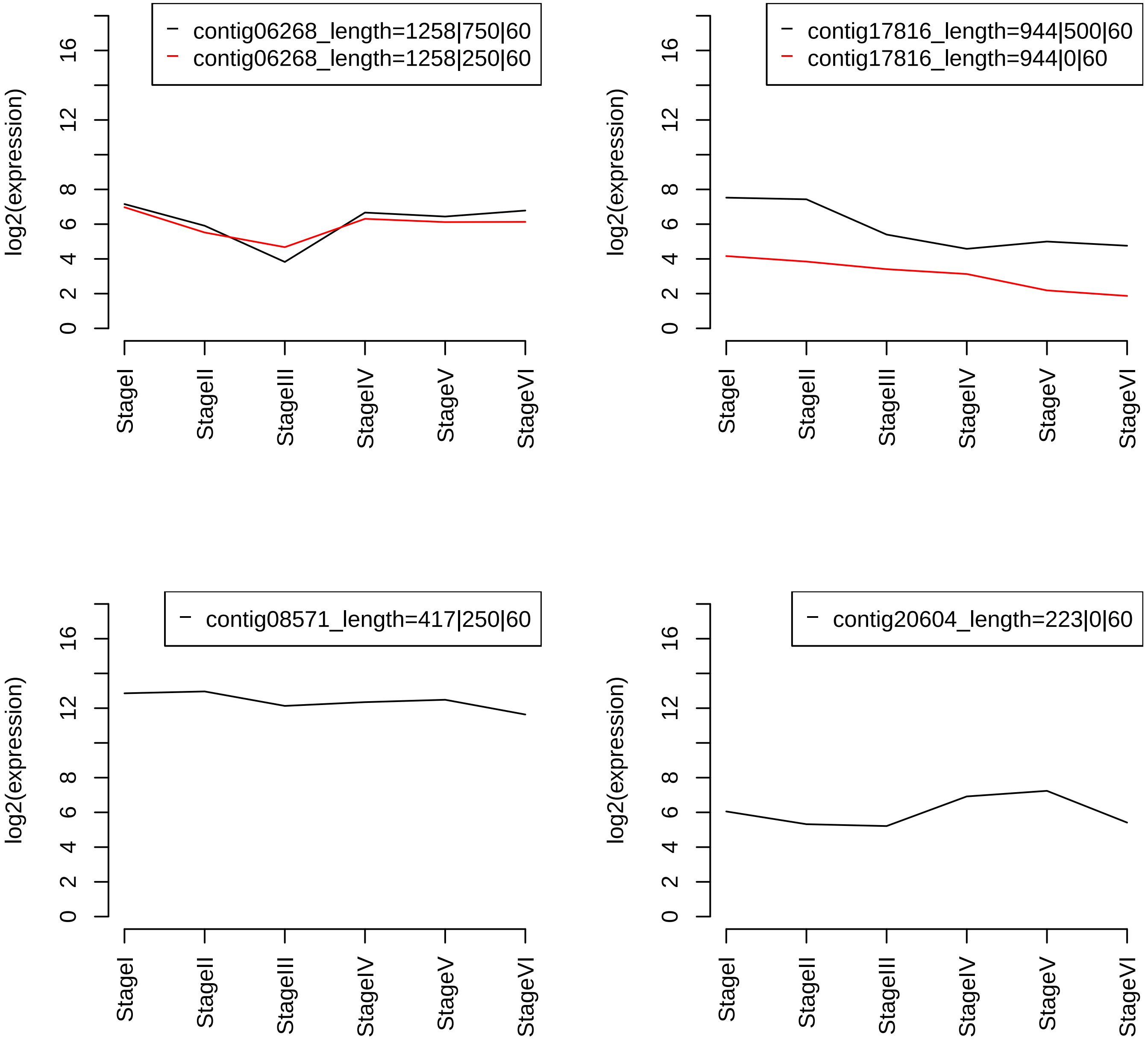

contig06268_length=1258|750|60
contig06268_length=1258|250|60
contig17816_length=944|500|60
contig17816_length=944|0|60
contig08571_length=417|250|60
contig20604_length=223|0|60
log2(expression)
0
2
4
6
8
12
16
StageI
StageII
StageIII
StageIV
StageV
StageVI

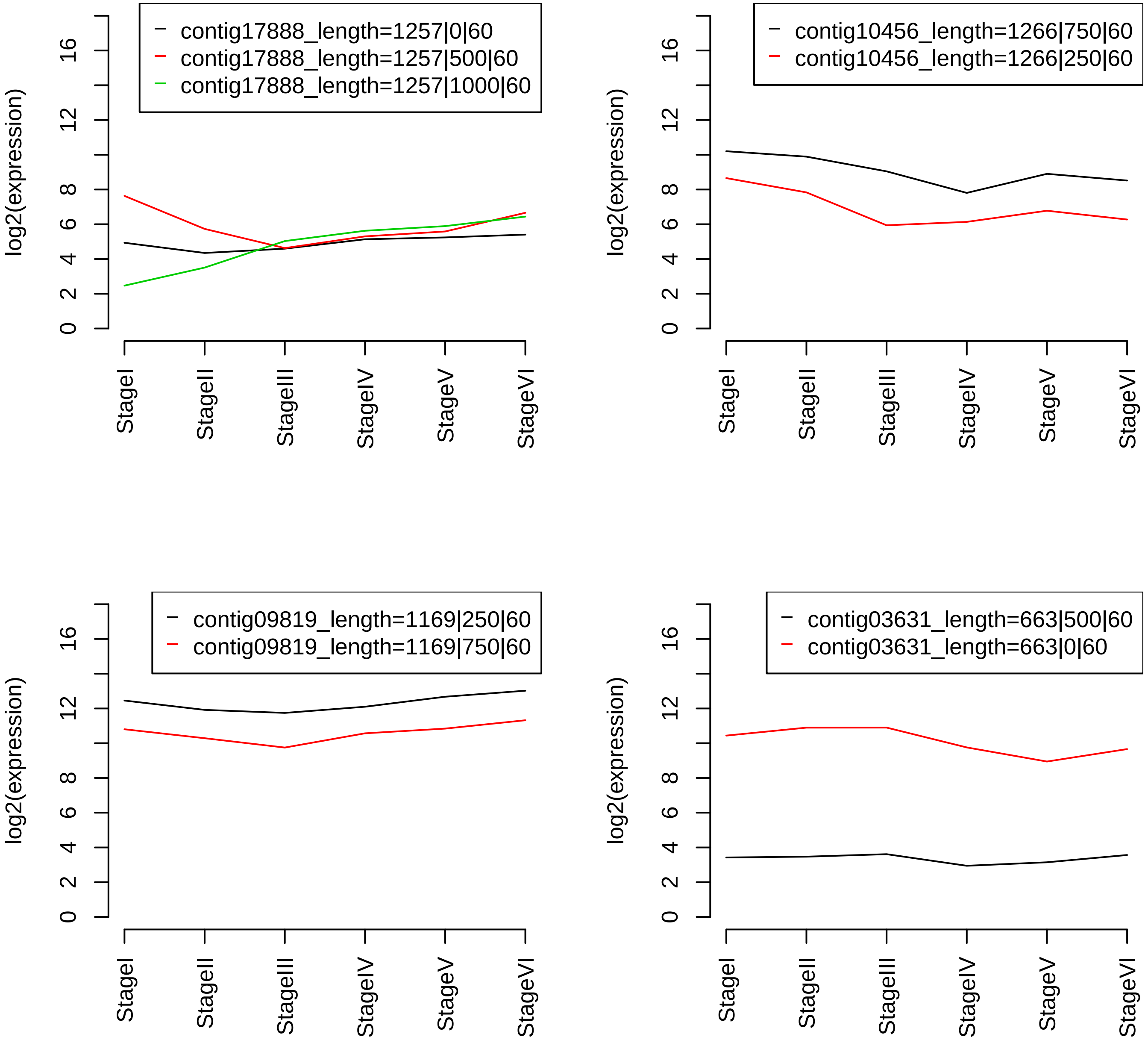

contig17888_length=1257|0|60
contig17888_length=1257|500|60
contig17888_length=1257|1000|60
contig10456_length=1266|750|60
contig10456_length=1266|250|60
contig09819_length=1169|250|60
contig09819_length=1169|750|60
contig03631_length=663|500|60
contig03631_length=663|0|60
log2(expression)
0
2
4
6
8
12
16
StageI
StageII
StageIII
StageIV
StageV
StageVI

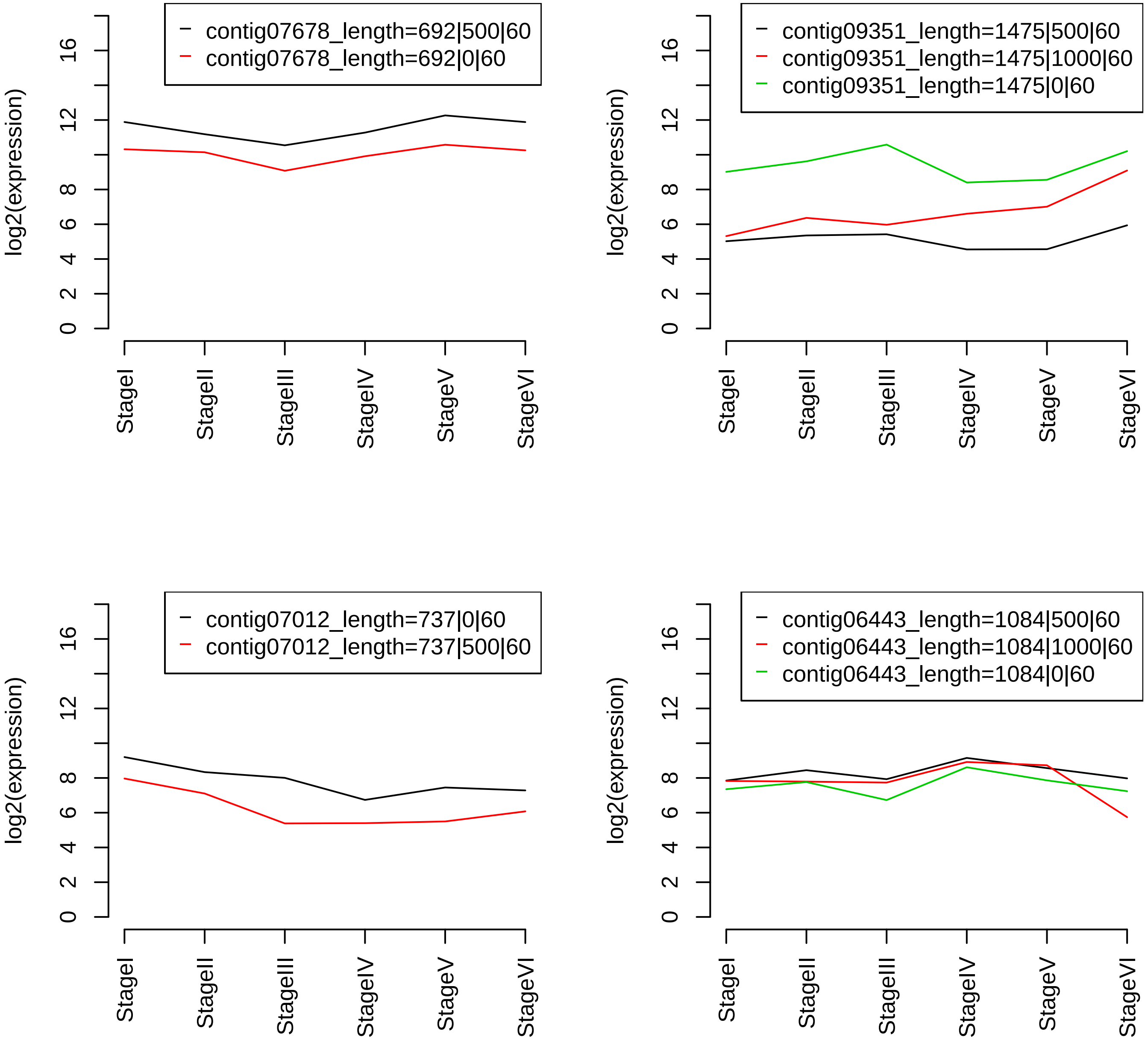

contig07678_length=692|500|60
contig07678_length=692|0|60
contig09351_length=1475|500|60
contig09351_length=1475|1000|60
contig09351_length=1475|0|60
contig07012_length=737|0|60
contig07012_length=737|500|60
contig06443_length=1084|500|60
contig06443_length=1084|1000|60
contig06443_length=1084|0|60
log2(expression)
0
2
4
6
8
12
16
StageI
StageII
StageIII
StageIV
StageV
StageVI

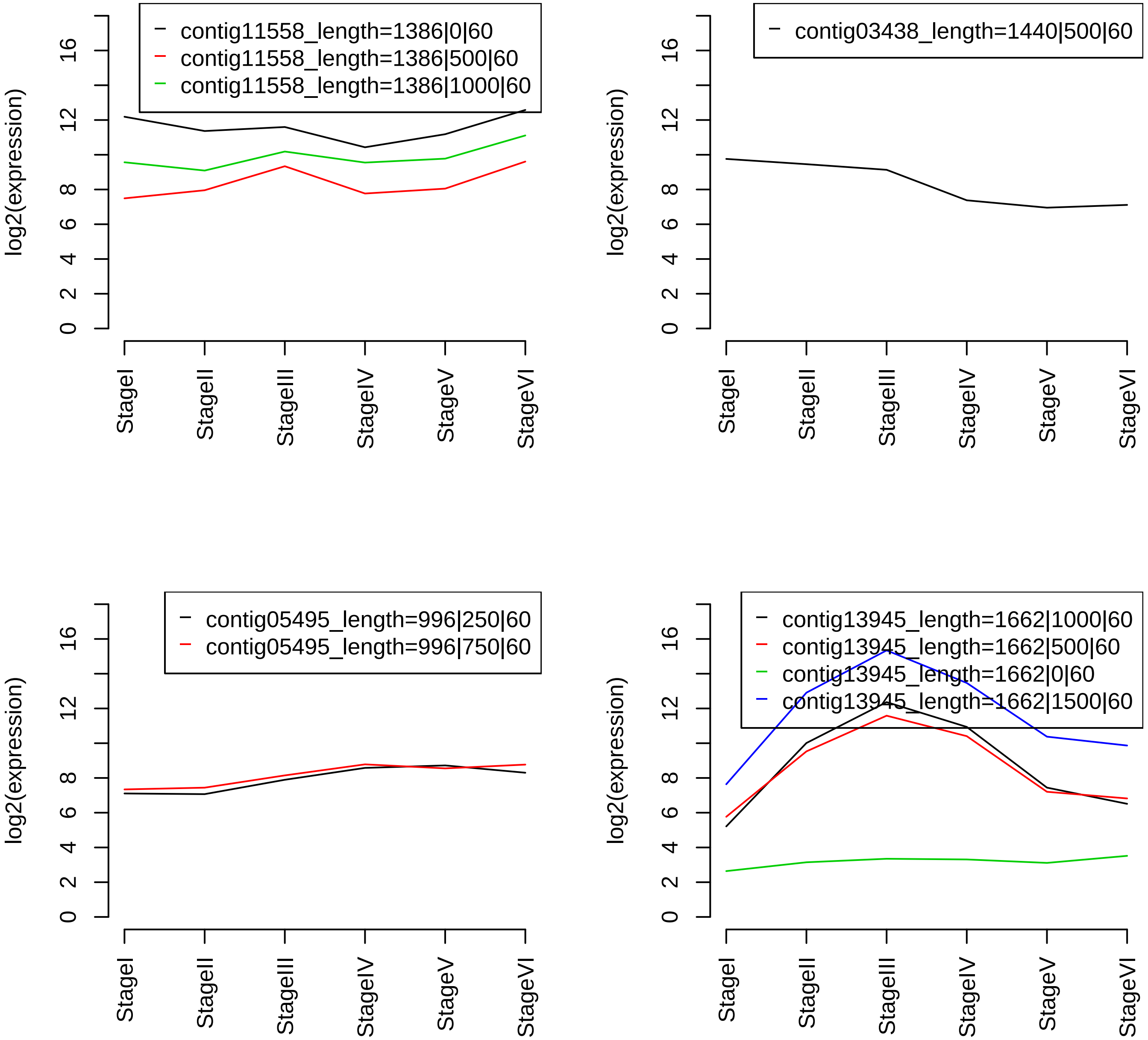

contig11558_length=1386|0|60
contig11558_length=1386|500|60
contig11558_length=1386|1000|60
contig03438_length=1440|500|60
contig05495_length=996|250|60
contig05495_length=996|750|60
contig13945_length=1662|1000|60
contig13945_length=1662|500|60
contig13945_length=1662|0|60
contig13945_length=1662|1500|60
log2(expression)
0
2
4
6
8
12
16
StageI
StageII
StageIII
StageIV
StageV
StageVI

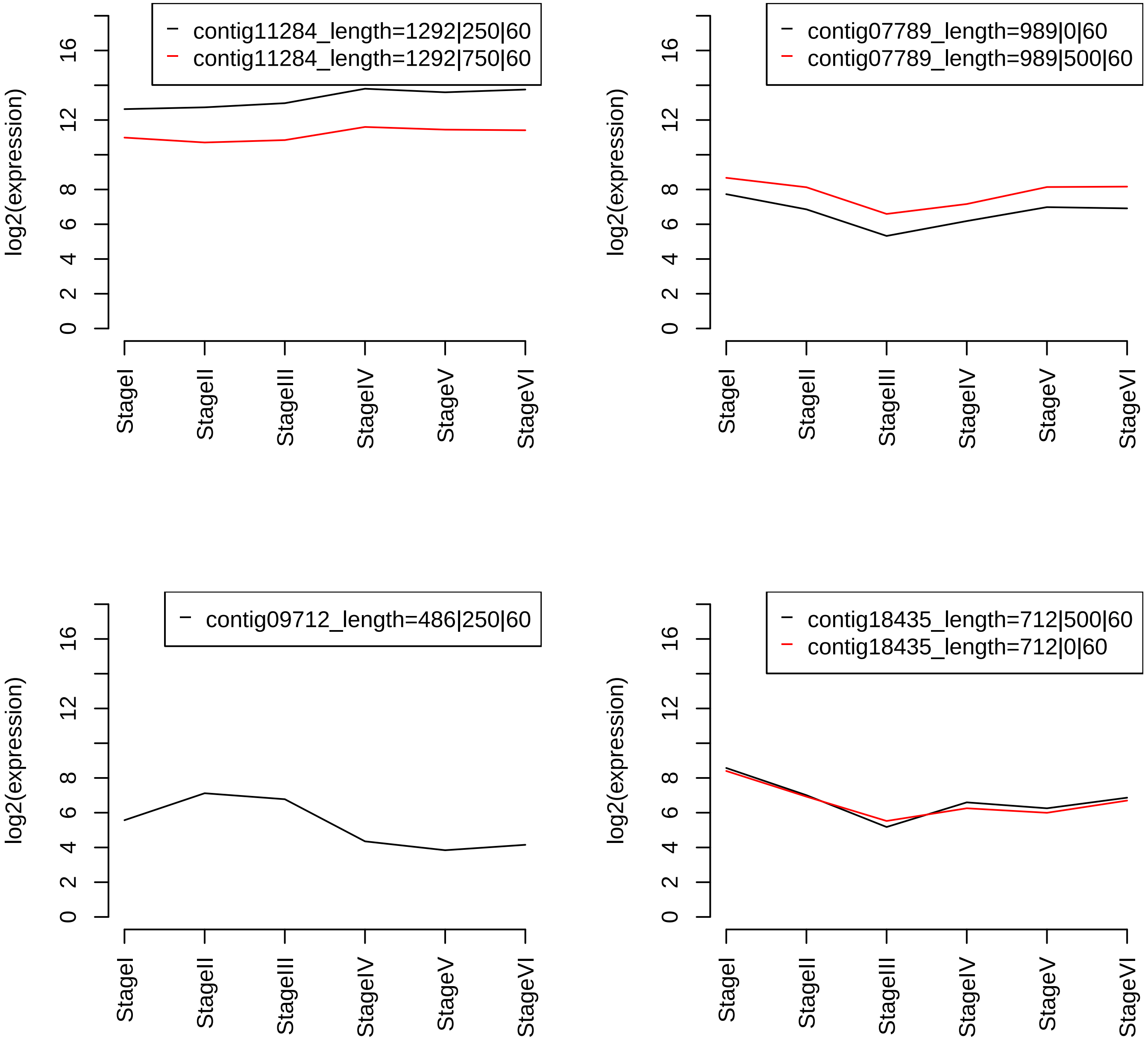
contig11284_length=1292|250|60
contig11284_length=1292|750|60
contig07789_length=989|0|60
contig07789_length=989|500|60
contig09712_length=486|250|60
contig18435_length=712|500|60
contig18435_length=712|0|60
log2(expression)
0
2
4
6
8
12
16
StageI
StageII
StageIII
StageIV
StageV
StageVI

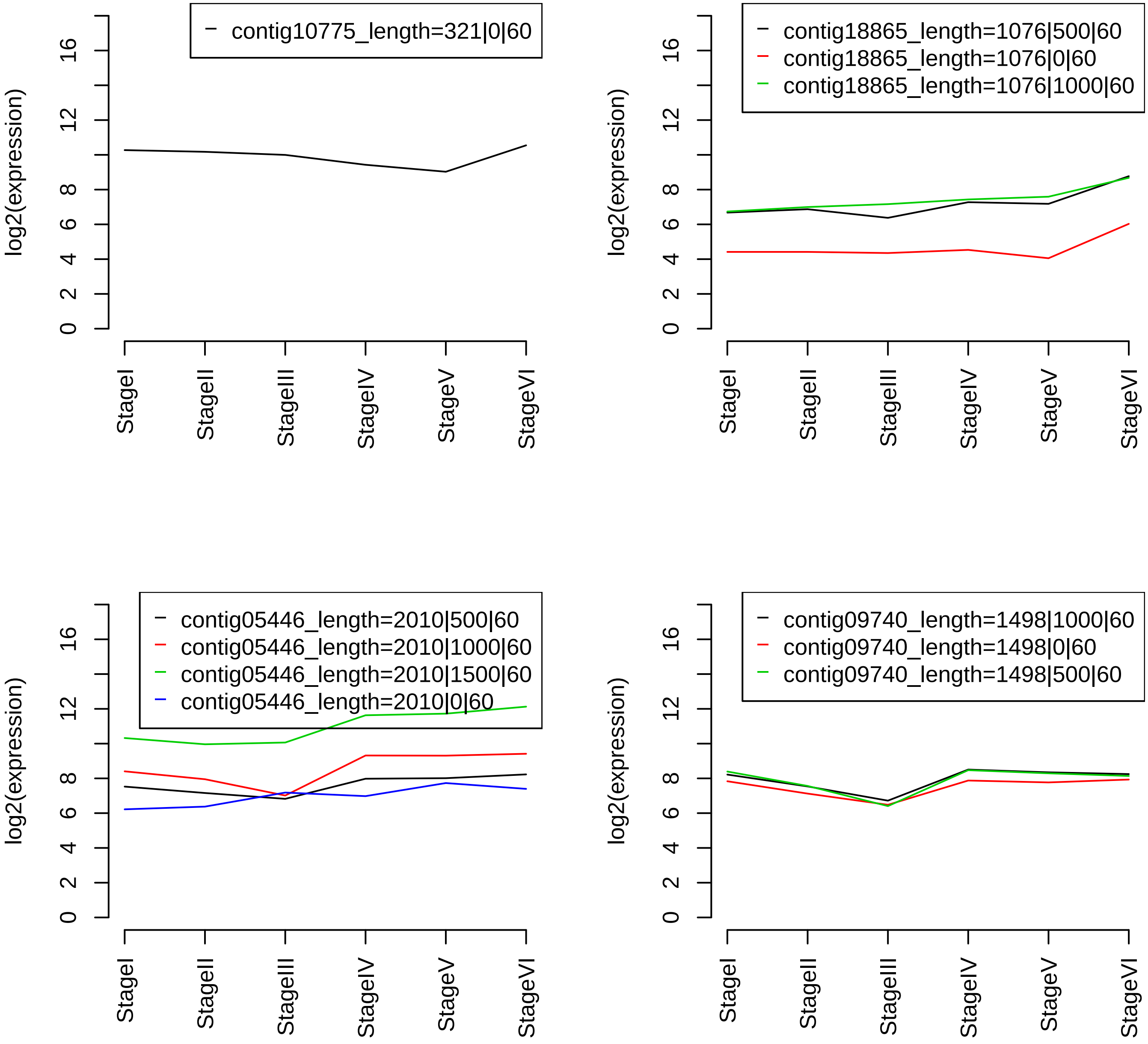

contig10775_length=321|0|60
contig18865_length=1076|500|60
contig18865_length=1076|0|60
contig18865_length=1076|1000|60
contig05446_length=2010|500|60
contig05446_length=2010|1000|60
contig05446_length=2010|1500|60
contig05446_length=2010|0|60
contig09740_length=1498|1000|60
contig09740_length=1498|0|60
contig09740_length=1498|500|60
log2(expression)
0
2
4
6
8
12
16
StageI
StageII
StageIII
StageIV
StageV
StageVI

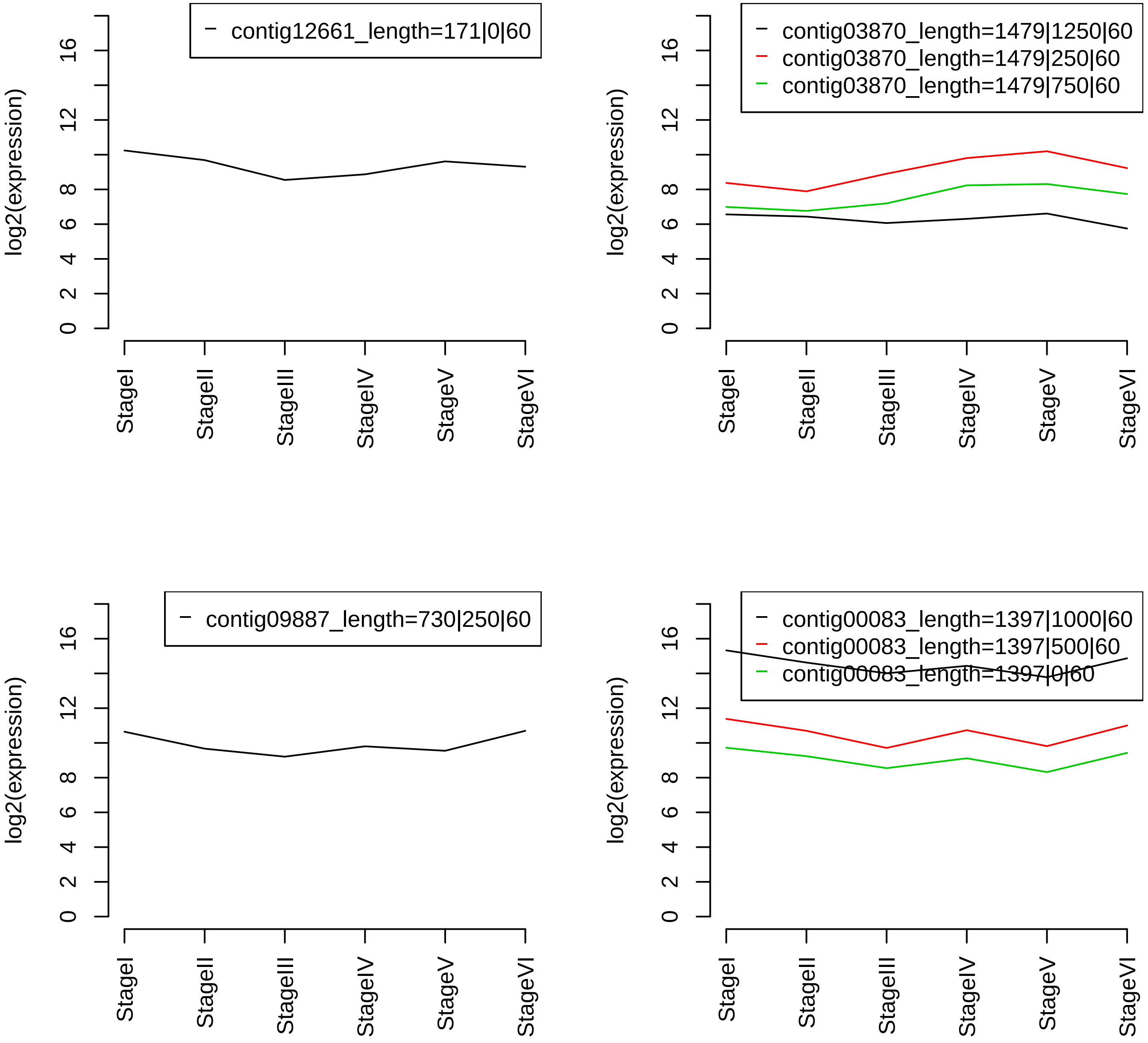

contig12661_length=171|0|60
contig03870_length=1479|1250|60
contig03870_length=1479|250|60
contig03870_length=1479|750|60
contig09887_length=730|250|60
contig00083_length=1397|1000|60
contig00083_length=1397|500|60
contig00083_length=1397|0|60
log2(expression)
0
2
4
6
8
12
16
StageI
StageII
StageIII
StageIV
StageV
StageVI

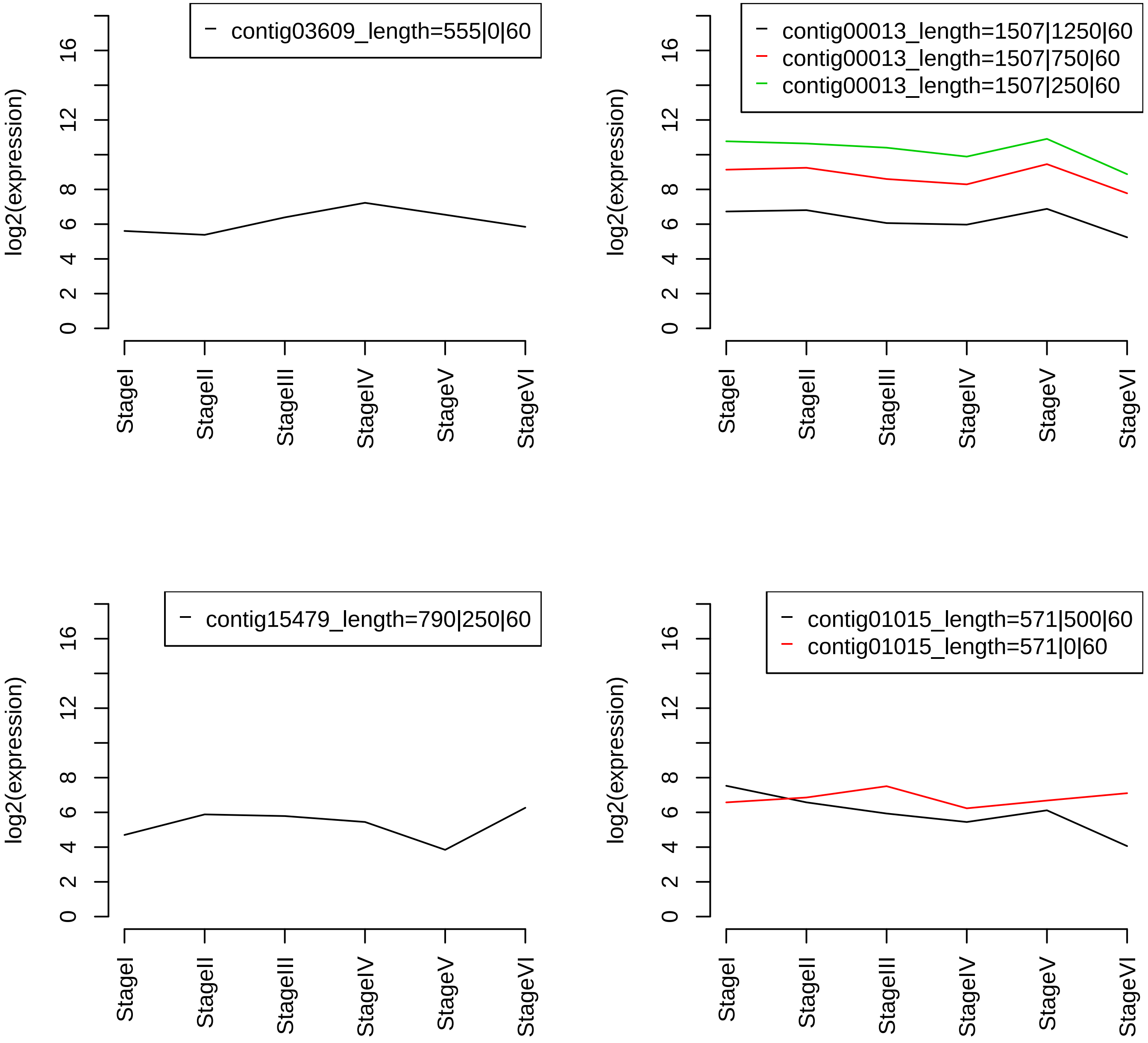

contig03609_length=555|0|60
contig00013_length=1507|1250|60
contig00013_length=1507|750|60
contig00013_length=1507|250|60
contig15479_length=790|250|60
contig01015_length=571|500|60
contig01015_length=571|0|60
log2(expression)
0 2 4 6 8 12 16
StageI
StageII
StageIII
StageIV
StageV
StageVI

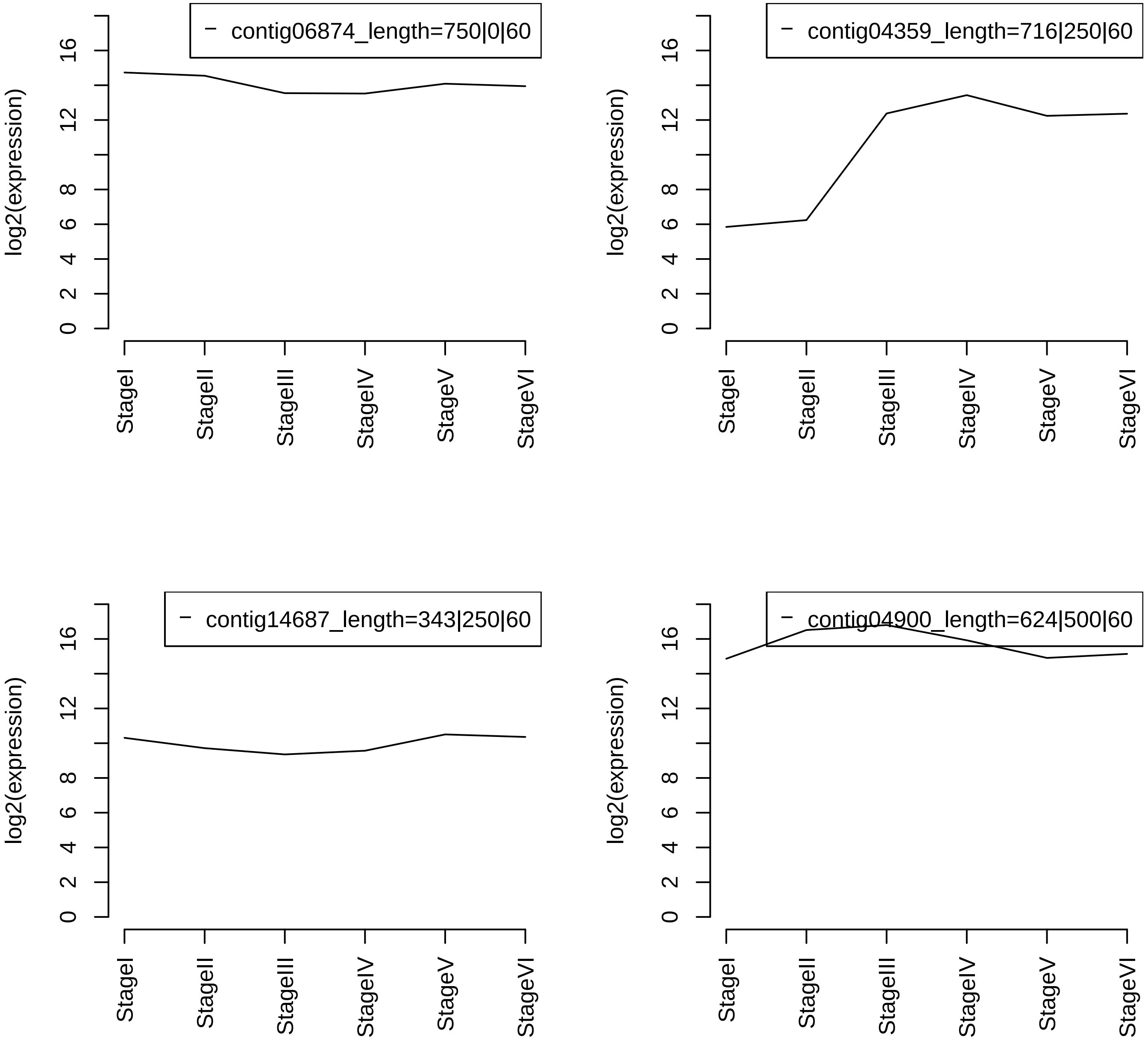
contig06874_length=750|0|60
contig04359_length=716|250|60
contig14687_length=343|250|60
contig04900_length=624|500|60
log2(expression)
0
2
4
6
8
12
16
StageI
StageII
StageIII
StageIV
StageV
StageVI

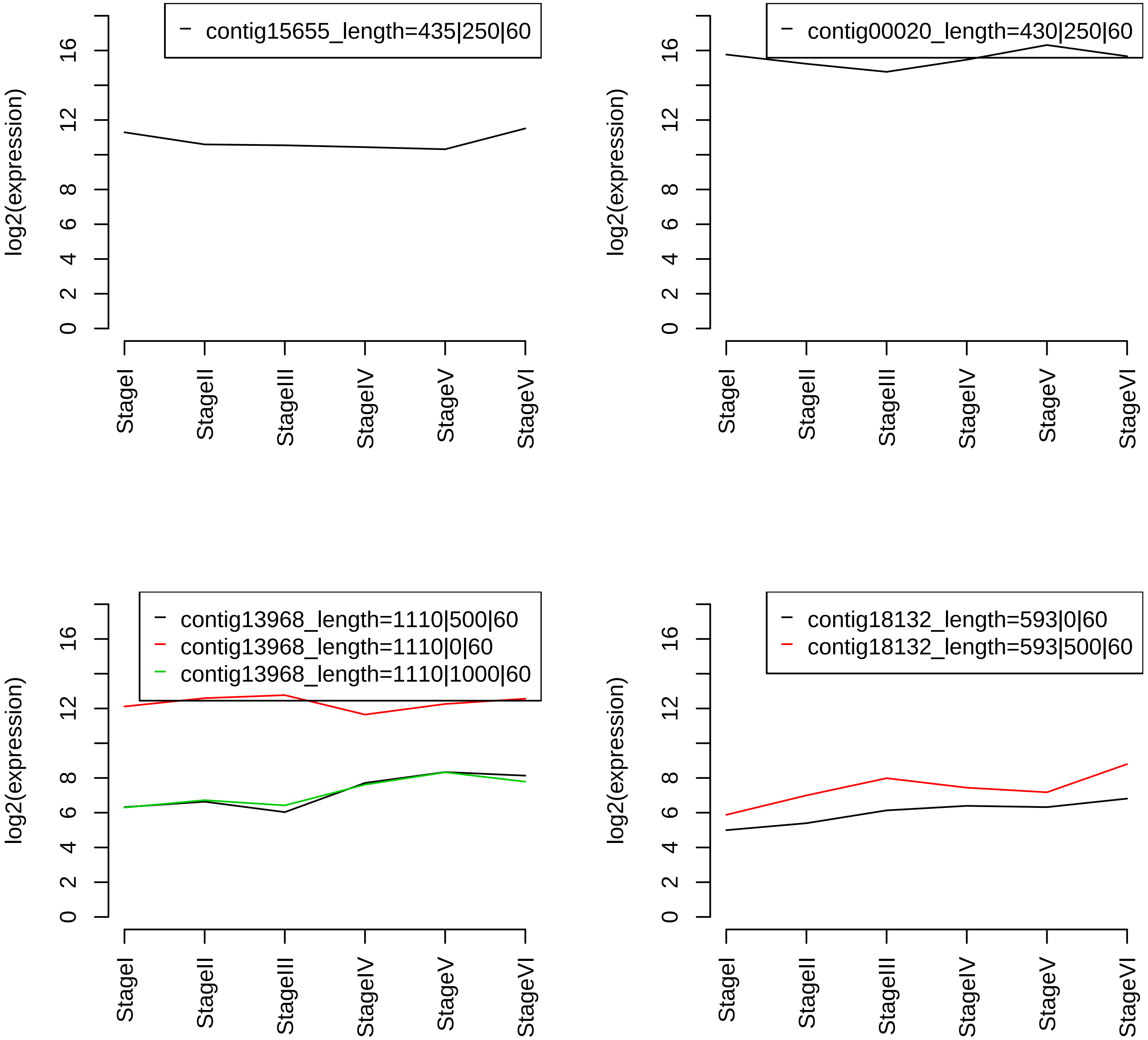
contig15655_length=435|250|60
contig00020_length=430|250|60
contig13968_length=1110|500|60
contig13968_length=1110|0|60
contig13968_length=1110|1000|60
contig18132_length=593|0|60
contig18132_length=593|500|60
log2(expression)
0
2
4
6
8
12
16
StageI
StageII
StageIII
StageIV
StageV
StageVI

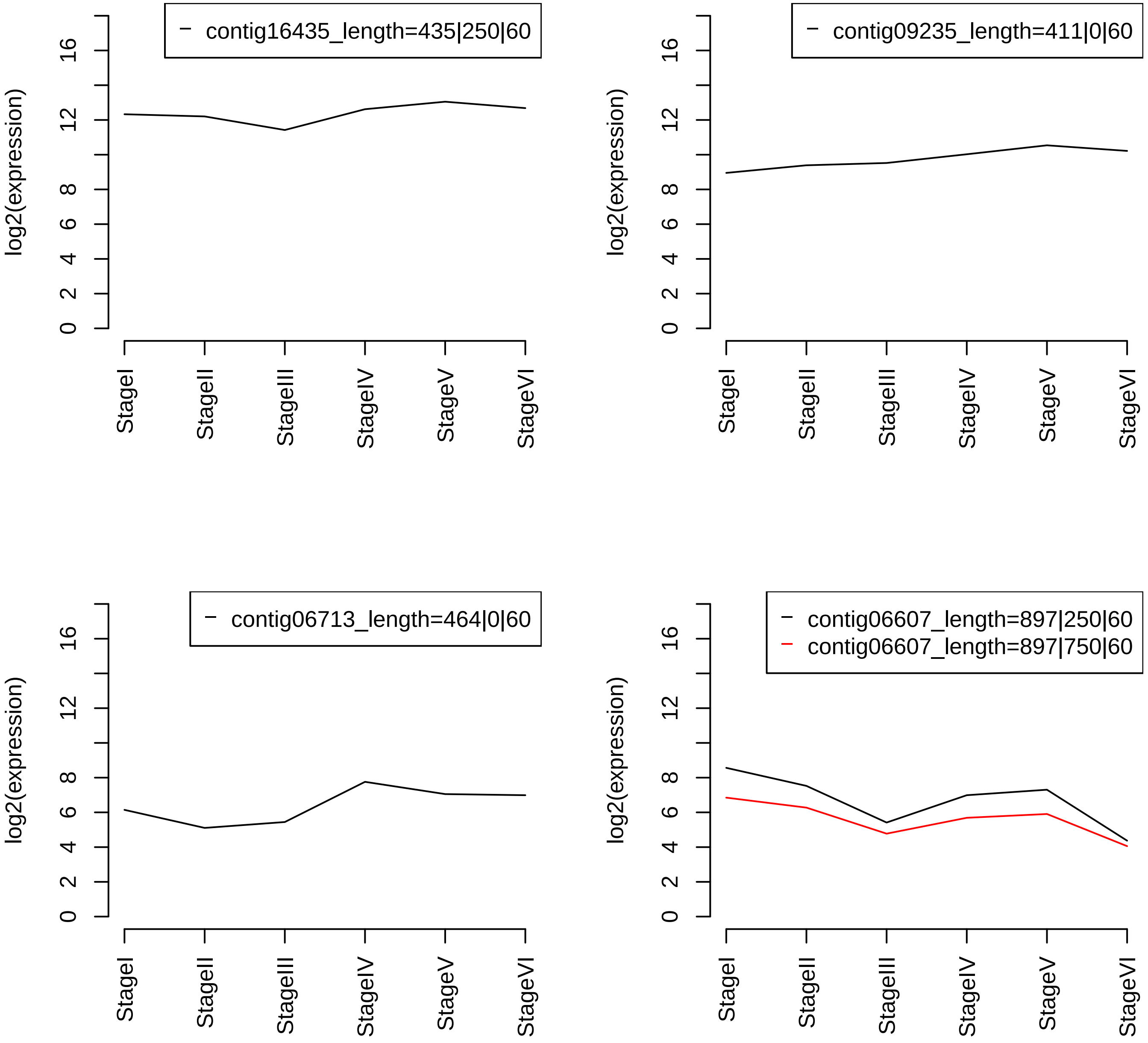
contig16435_length=435|250|60
contig09235_length=411|0|60
contig06713_length=464|0|60
contig06607_length=897|250|60
contig06607_length=897|750|60
log2(expression)
0
2
4
6
8
12
16
StageI
StageII
StageIII
StageIV
StageV
StageVI

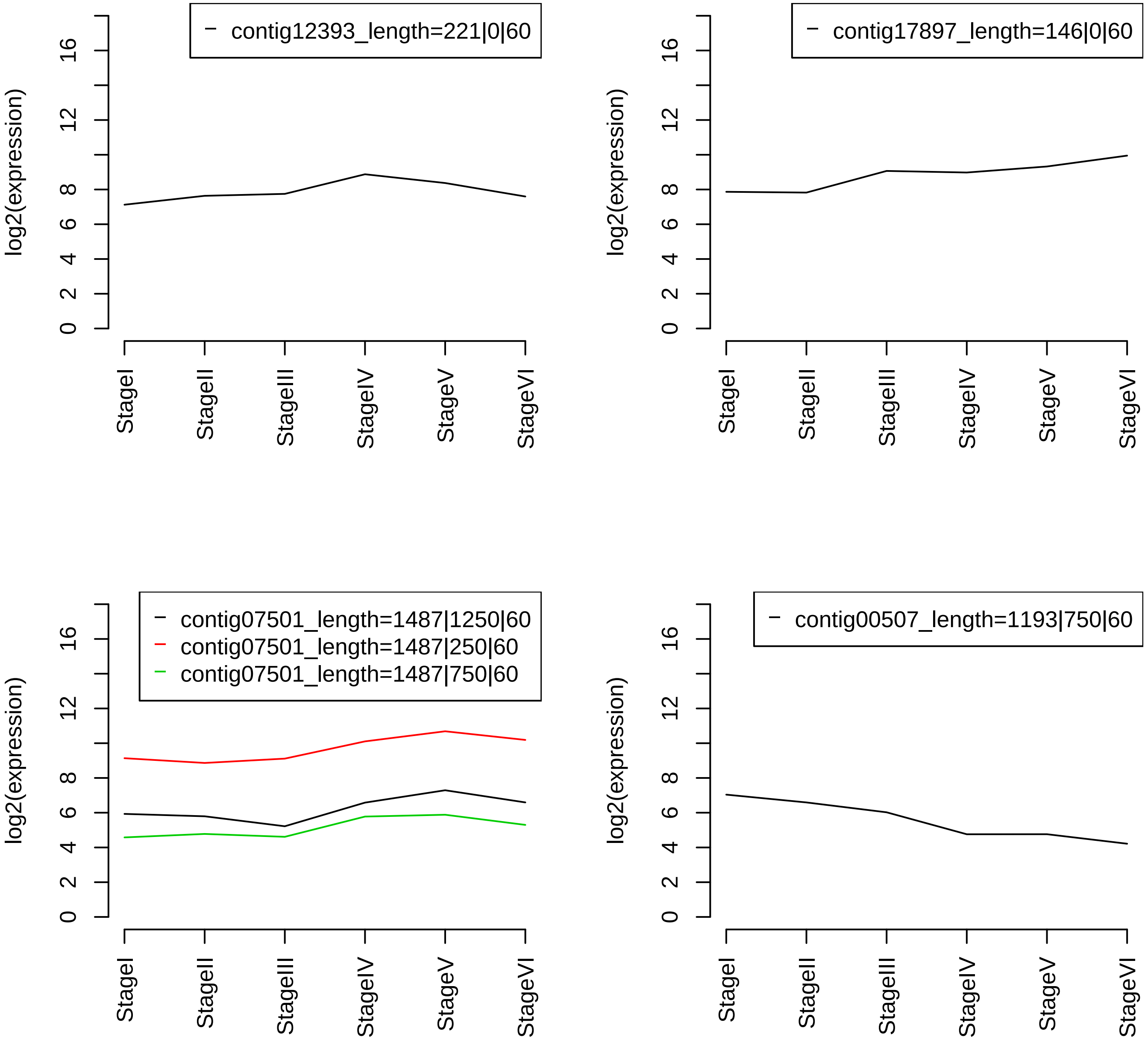

contig12393_length=221|0|60
contig17897_length=146|0|60
contig07501_length=1487|1250|60
contig07501_length=1487|250|60
contig07501_length=1487|750|60
contig00507_length=1193|750|60
log2(expression)
0
2
4
6
8
12
16
StageI
StageII
StageIII
StageIV
StageV
StageVI

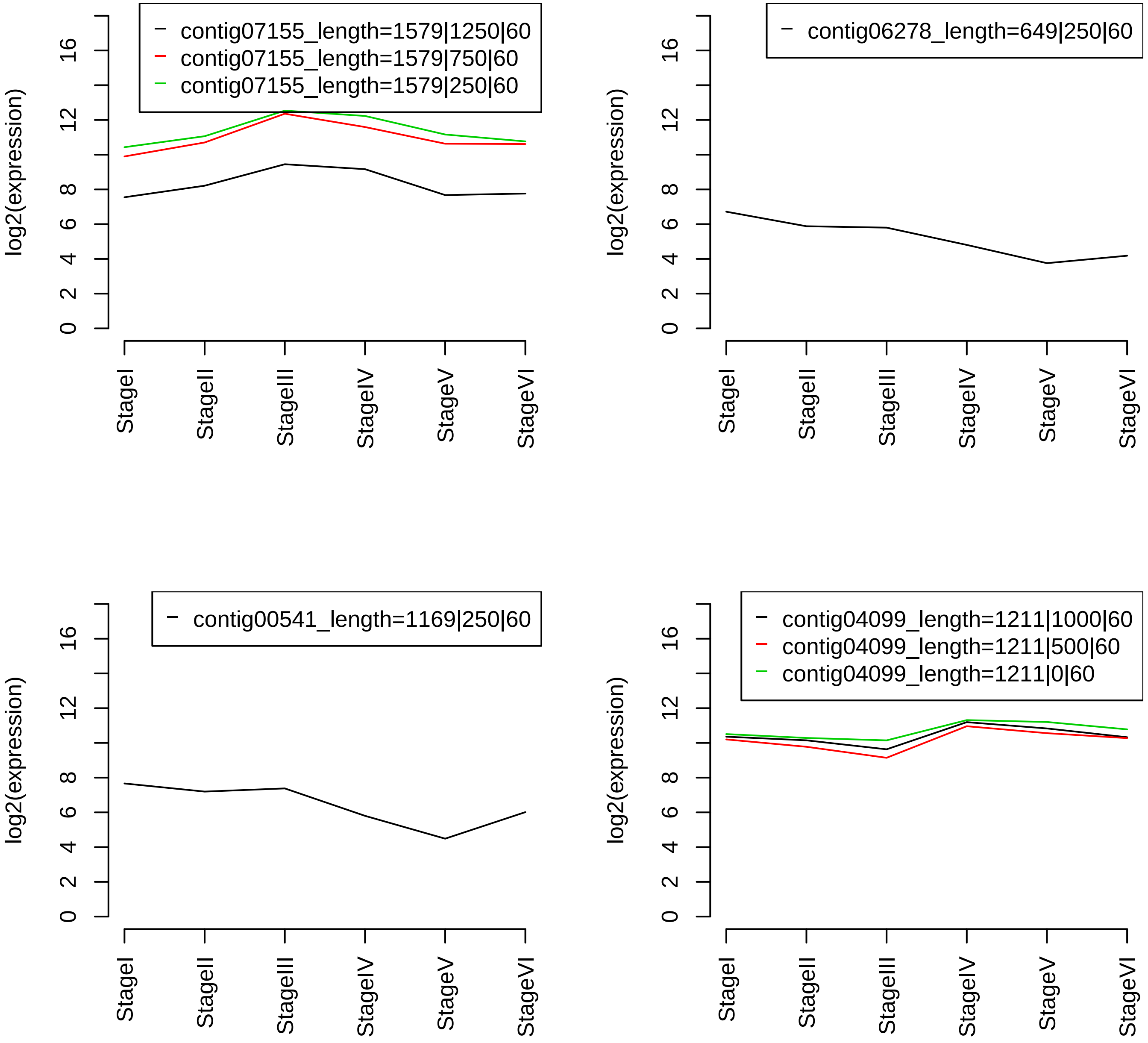
contig07155_length=1579|1250|60
contig07155_length=1579|750|60
contig07155_length=1579|250|60
contig06278_length=649|250|60
contig00541_length=1169|250|60
contig04099_length=1211|1000|60
contig04099_length=1211|500|60
contig04099_length=1211|0|60
log2(expression)
0
2
4
6
8
12
16
StageI
StageII
StageIII
StageIV
StageV
StageVI

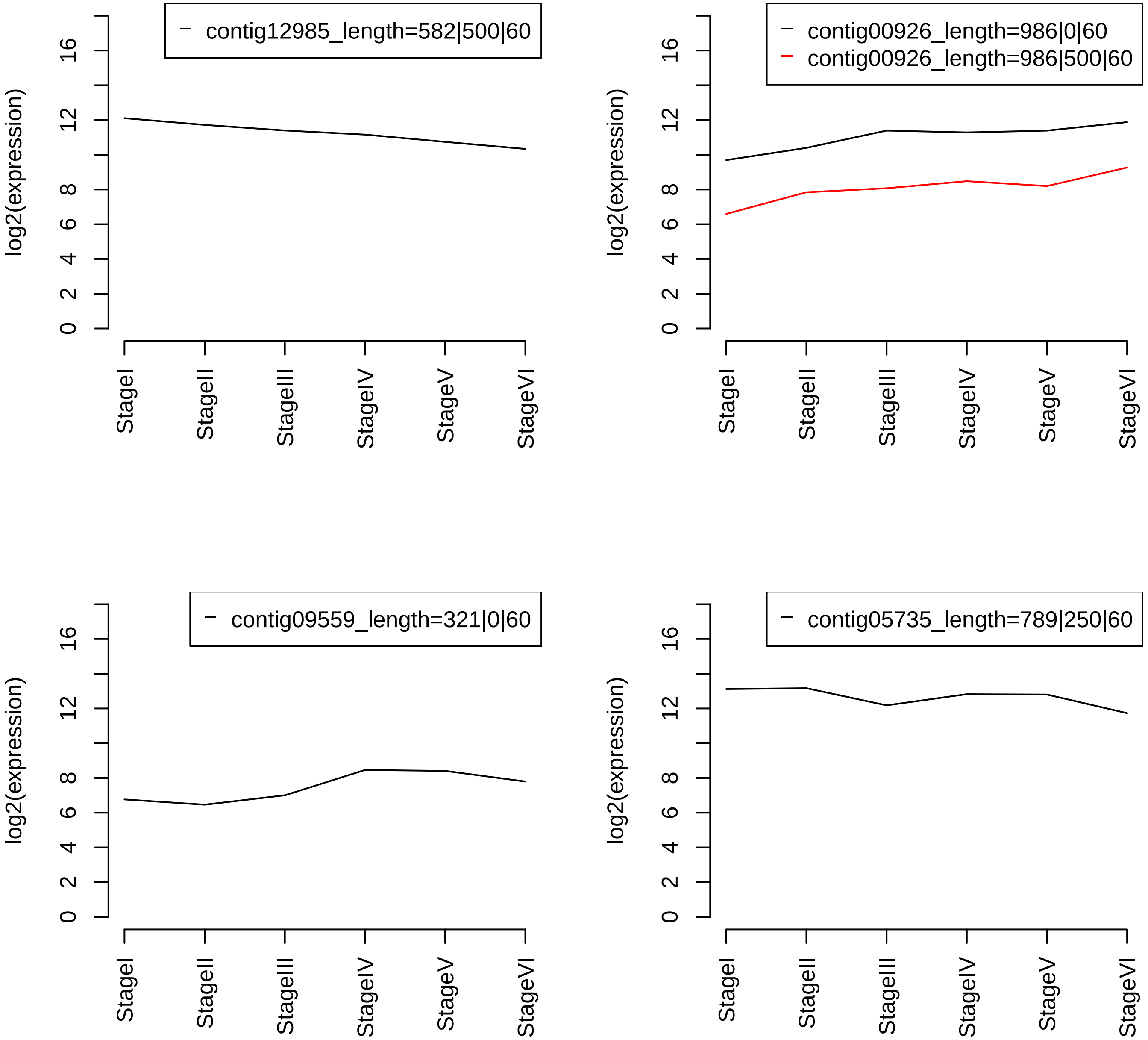

contig12985_length=582|500|60
contig00926_length=986|0|60
contig00926_length=986|500|60
contig09559_length=321|0|60
contig05735_length=789|250|60
log2(expression)
0
2
4
6
8
12
16
StageI
StageII
StageIII
StageIV
StageV
StageVI

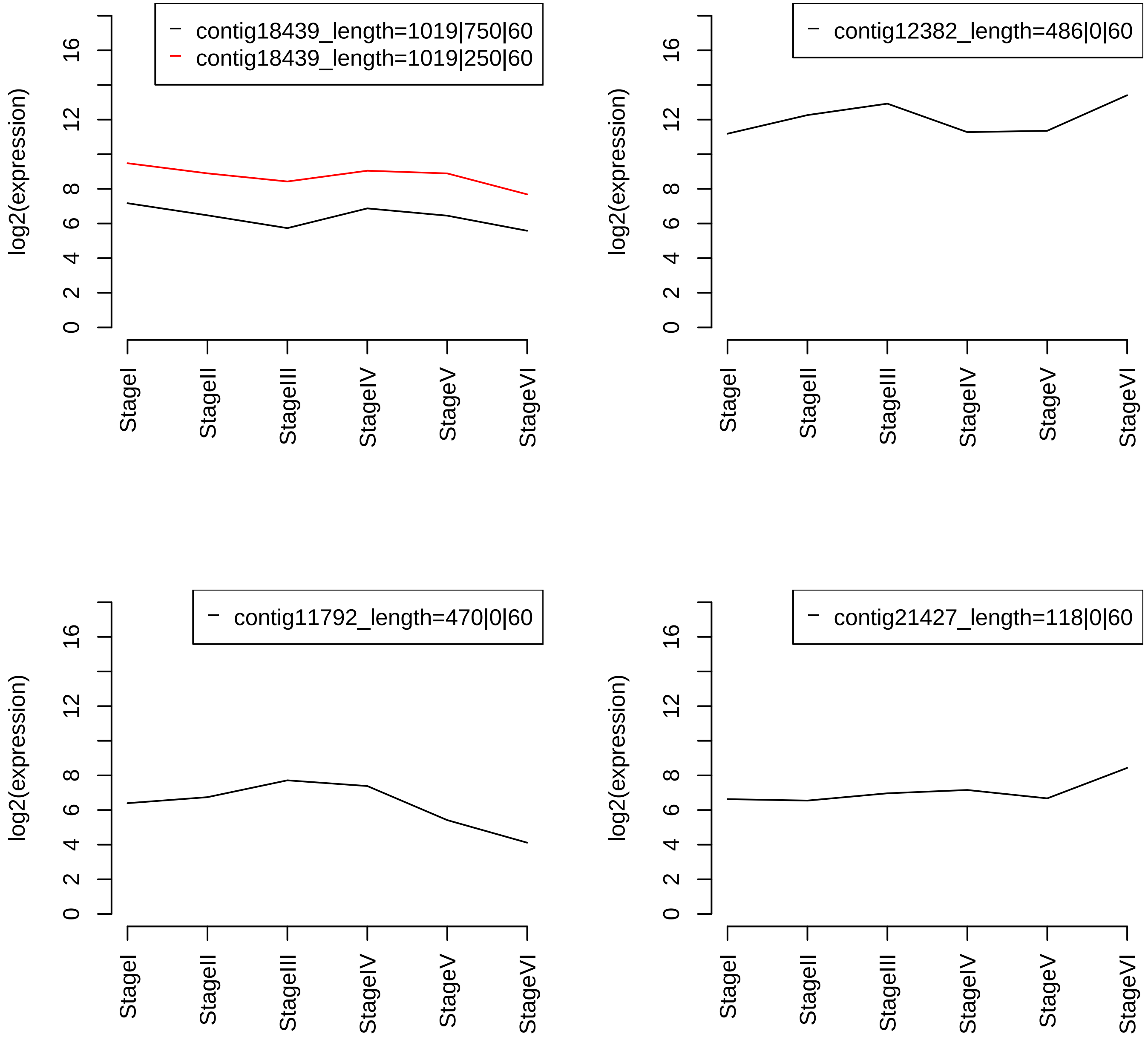

contig18439_length=1019|750|60
contig18439_length=1019|250|60
contig12382_length=486|0|60
contig11792_length=470|0|60
contig21427_length=118|0|60
log2(expression)
0
2
4
6
8
12
16
StageI
StageII
StageIII
StageIV
StageV
StageVI

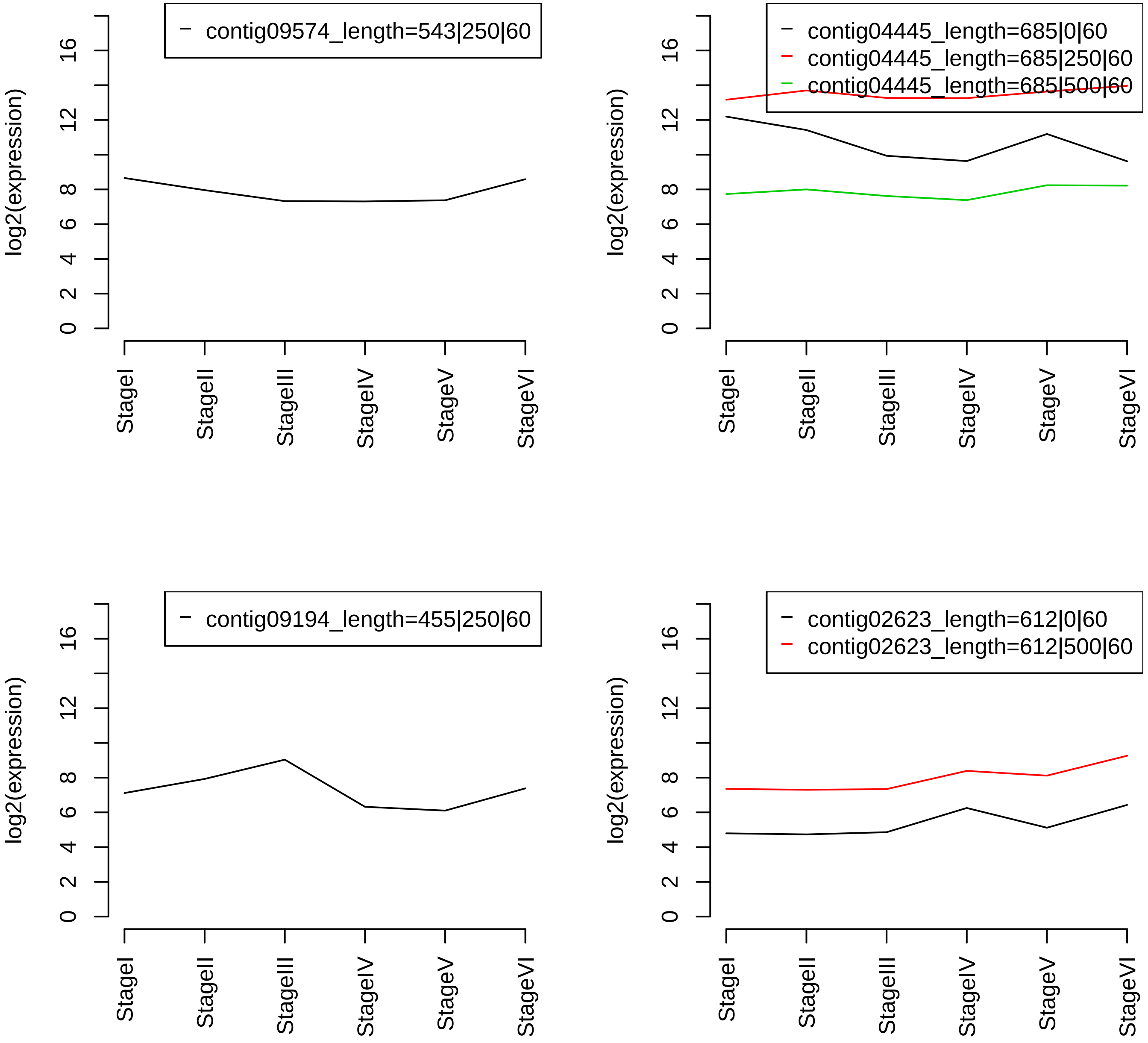

contig09574_length=543|250|60
contig04445_length=685|0|60
contig04445_length=685|250|60
contig04445_length=685|500|60
contig09194_length=455|250|60
contig02623_length=612|0|60
contig02623_length=612|500|60
log2(expression)
0
2
4
6
8
12
16
StageI
StageII
StageIII
StageIV
StageV
StageVI

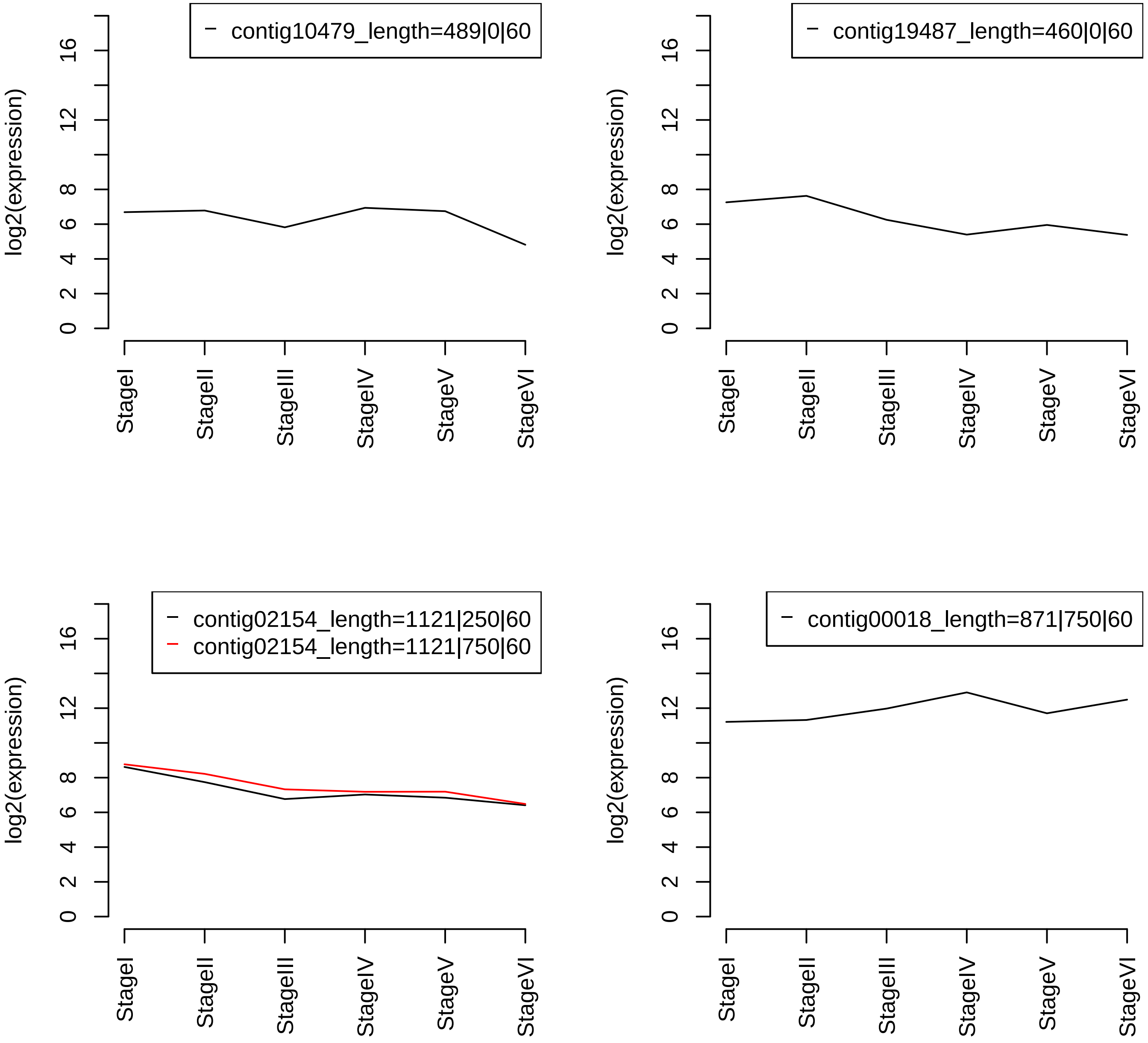
contig10479_length=489|0|60
contig19487_length=460|0|60
contig02154_length=1121|250|60
contig02154_length=1121|750|60
contig00018_length=871|750|60
log2(expression)
StageI
StageII
StageIII
StageIV
StageV
StageVI

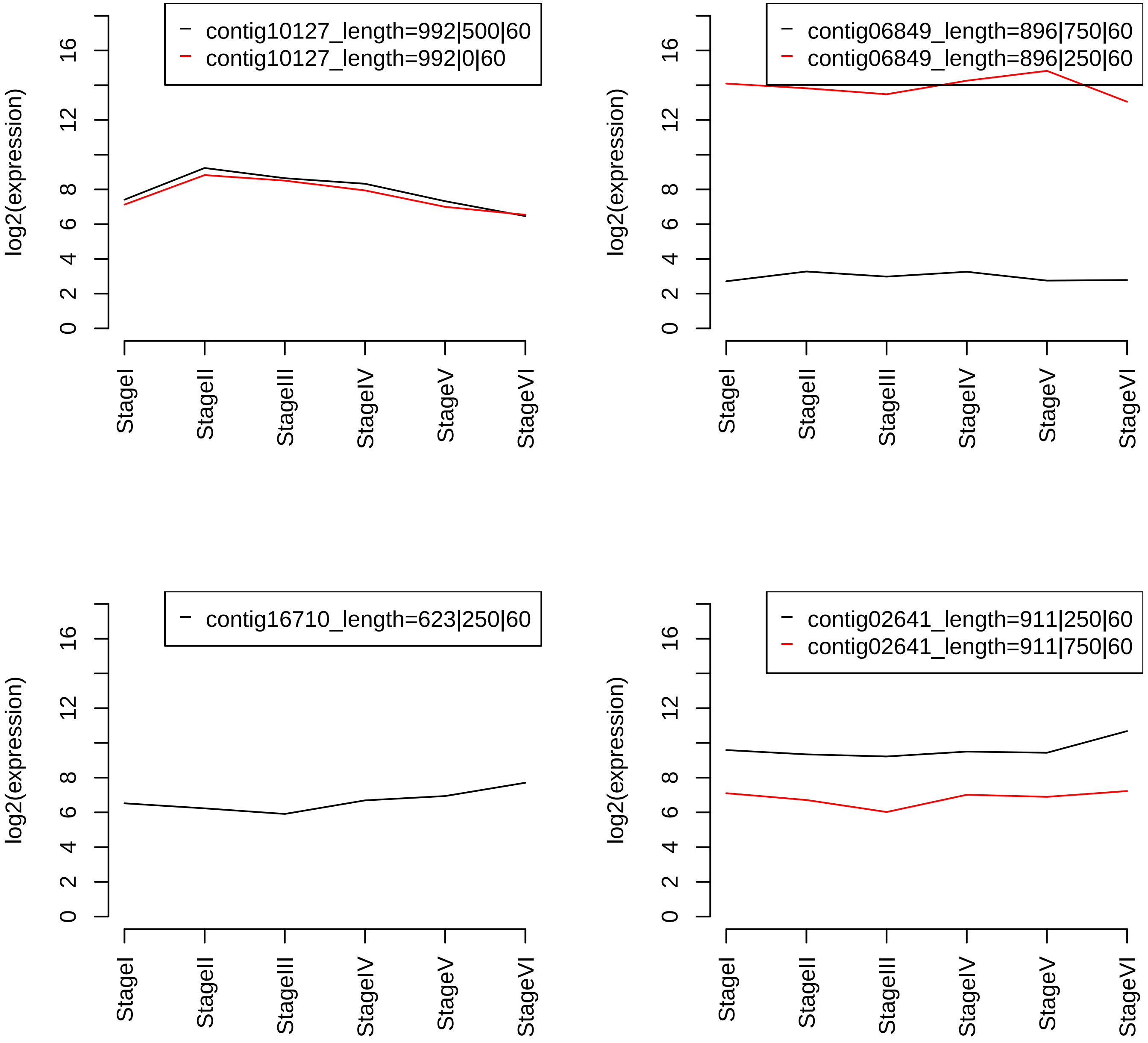

contig10127_length=992|500|60
contig10127_length=992|0|60
contig06849_length=896|750|60
contig06849_length=896|250|60
contig16710_length=623|250|60
contig02641_length=911|250|60
contig02641_length=911|750|60
log2(expression)
0 2 4 6 8 12 16
StageI StageII StageIII StageIV StageV StageVI

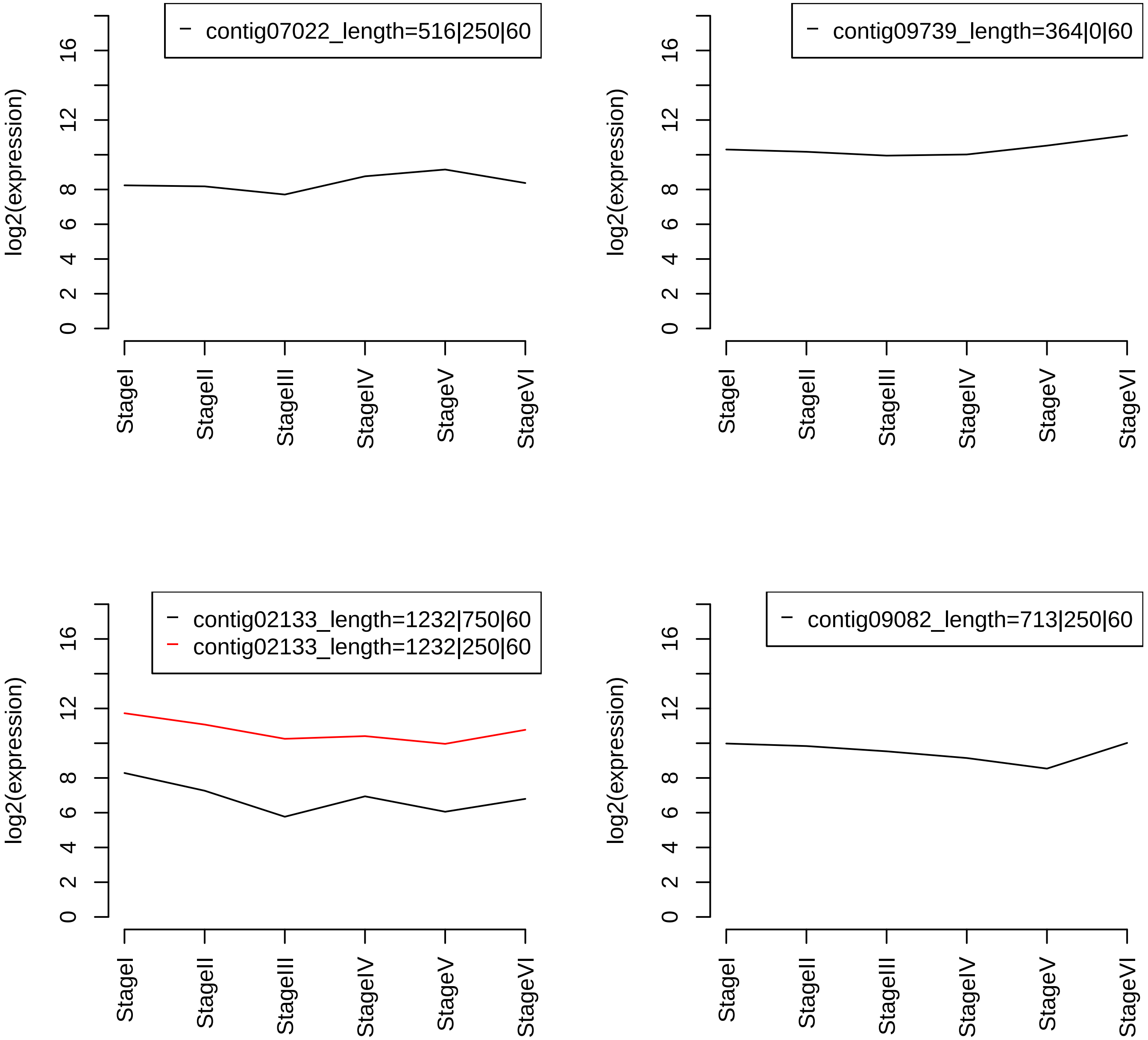

contig07022_length=516|250|60
contig09739_length=364|0|60
contig02133_length=1232|750|60
contig02133_length=1232|250|60
contig09082_length=713|250|60
log2(expression)
0
2
4
6
8
12
16
StageI
StageII
StageIII
StageIV
StageV
StageVI

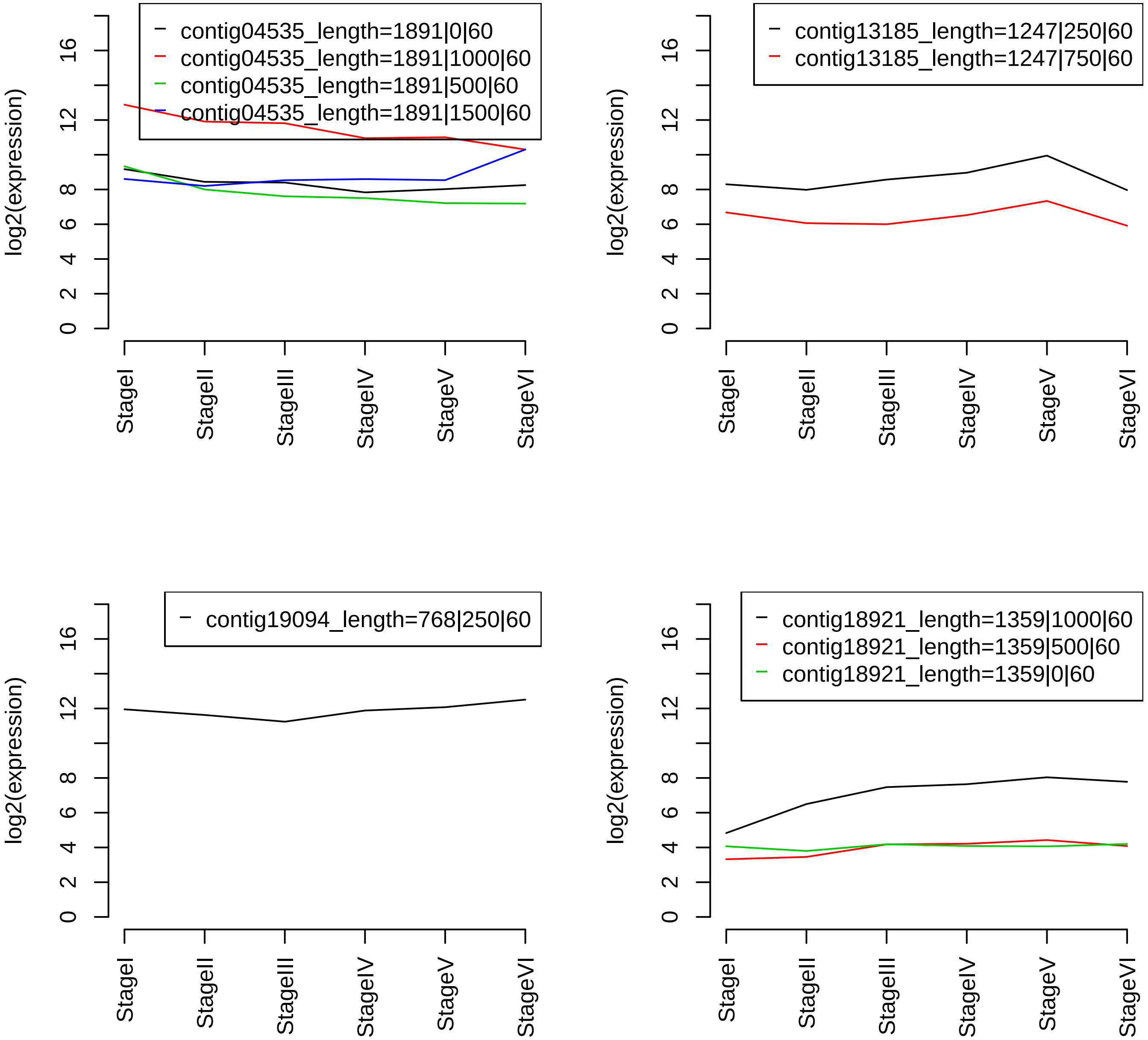

contig04535_length=1891|0|60
contig04535_length=1891|1000|60
contig04535_length=1891|500|60
contig04535_length=1891|1500|60
contig13185_length=1247|250|60
contig13185_length=1247|750|60
contig19094_length=768|250|60
contig18921_length=1359|1000|60
contig18921_length=1359|500|60
contig18921_length=1359|0|60
log2(expression)
0 2 4 6 8 12 16
StageI StageII StageIII StageIV StageV StageVI

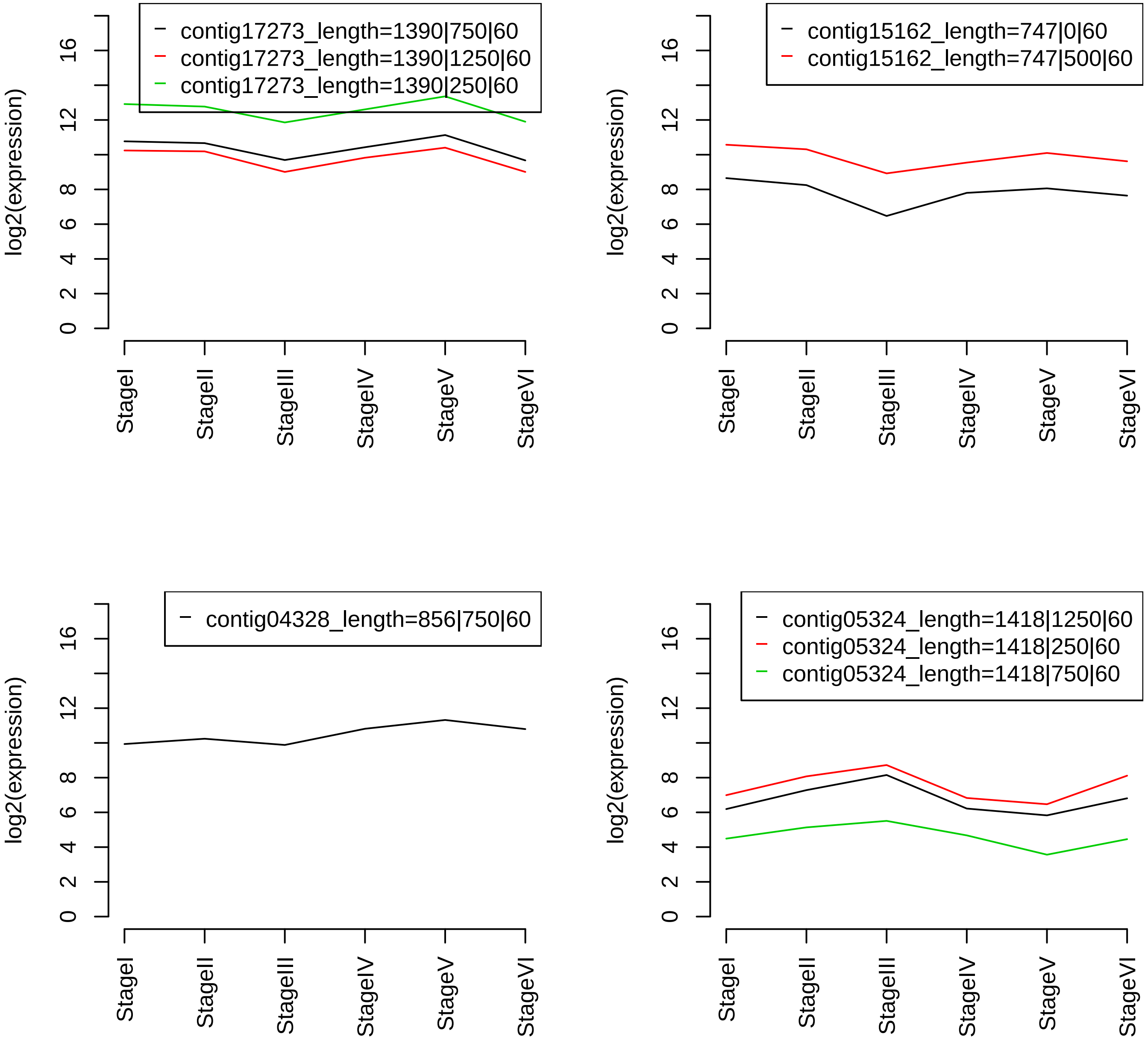
contig17273_length=1390|750|60
contig17273_length=1390|1250|60
contig17273_length=1390|250|60
contig15162_length=747|0|60
contig15162_length=747|500|60
contig04328_length=856|750|60
contig05324_length=1418|1250|60
contig05324_length=1418|250|60
contig05324_length=1418|750|60
log2(expression)
0 2 4 6 8 12 16
StageI
StageII
StageIII
StageIV
StageV
StageVI

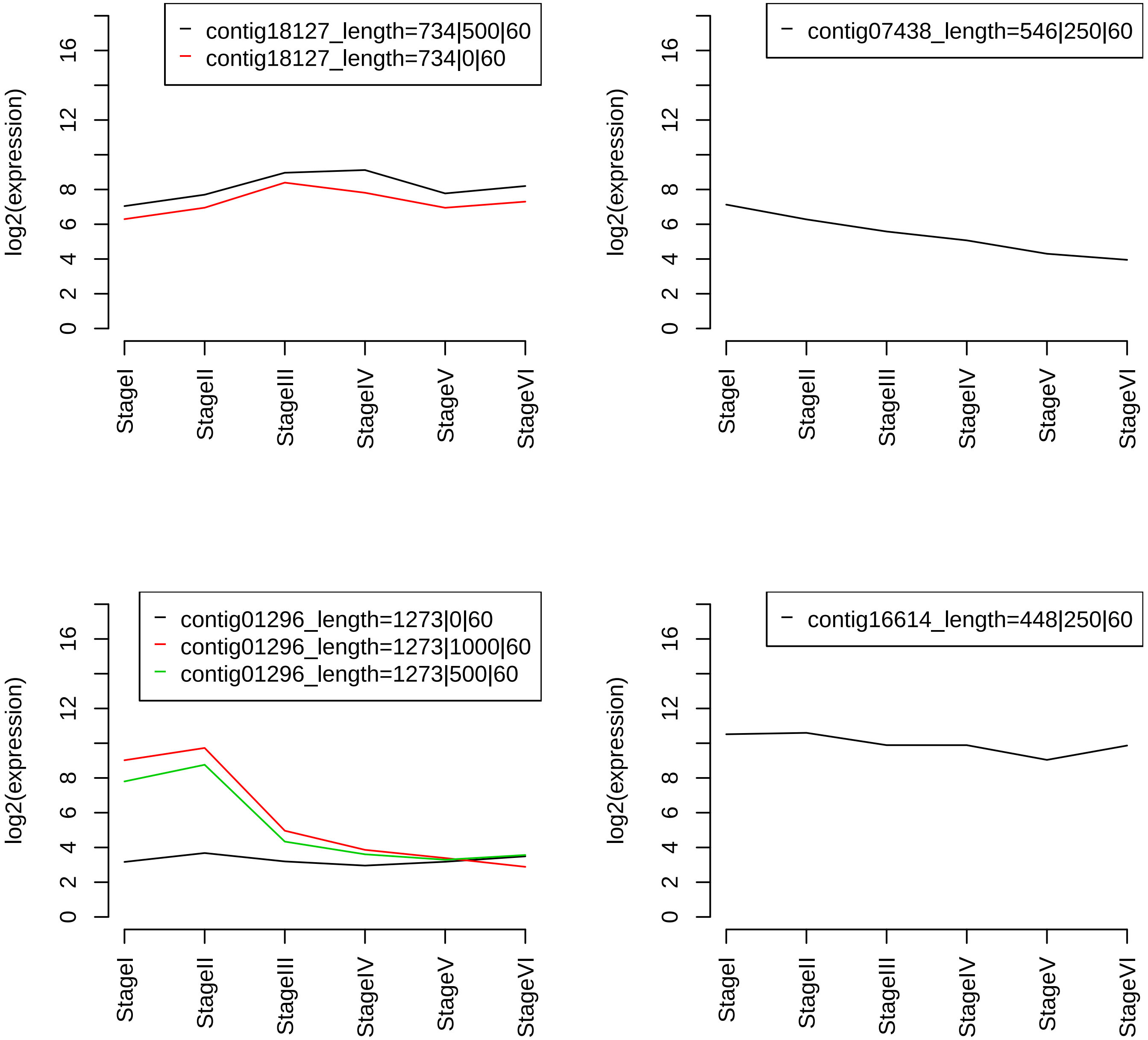
contig18127_length=734|500|60
contig18127_length=734|0|60
contig07438_length=546|250|60
contig01296_length=1273|0|60
contig01296_length=1273|1000|60
contig01296_length=1273|500|60
contig16614_length=448|250|60
log2(expression)
0
2
4
6
8
12
16
StageI
StageII
StageIII
StageIV
StageV
StageVI

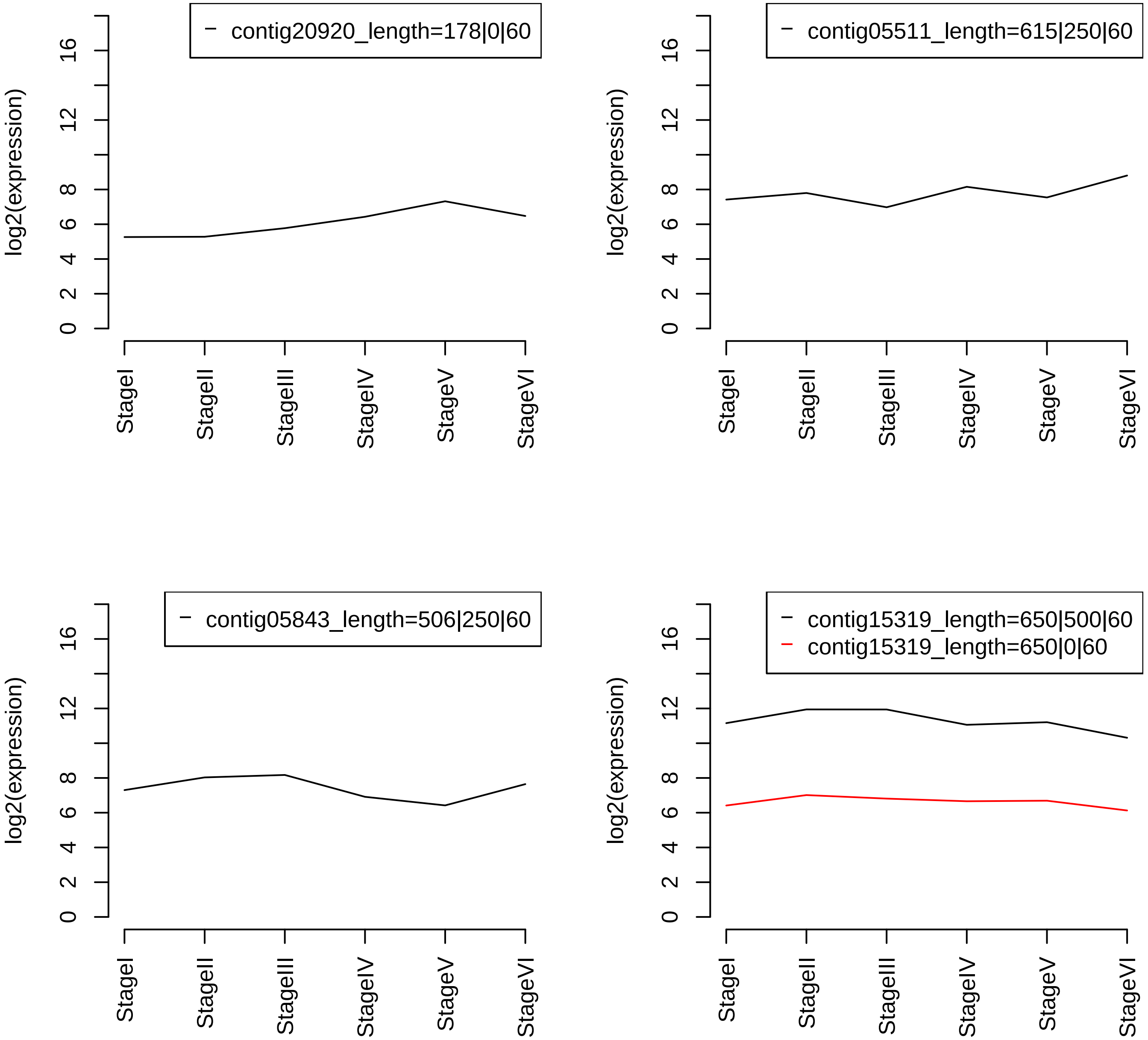
contig20920_length=178|0|60
contig05511_length=615|250|60
contig05843_length=506|250|60
contig15319_length=650|500|60
contig15319_length=650|0|60
log2(expression)
0
2
4
6
8
12
16
StageI
StageII
StageIII
StageIV
StageV
StageVI

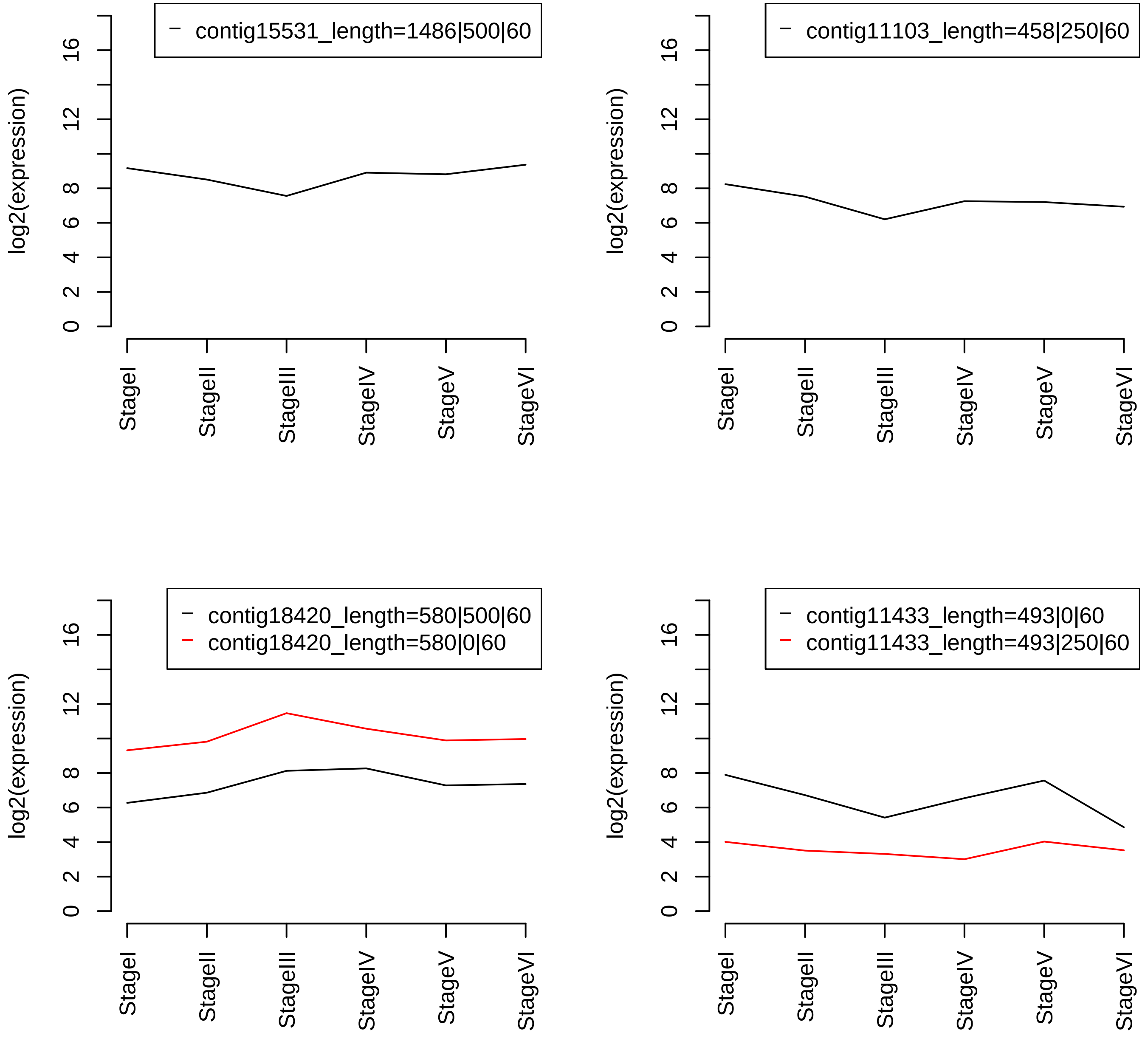
contig15531_length=1486|500|60
contig11103_length=458|250|60
contig18420_length=580|500|60
contig18420_length=580|0|60
contig11433_length=493|0|60
contig11433_length=493|250|60
log2(expression)
0
2
4
6
8
12
16
StageI
StageII
StageIII
StageIV
StageV
StageVI

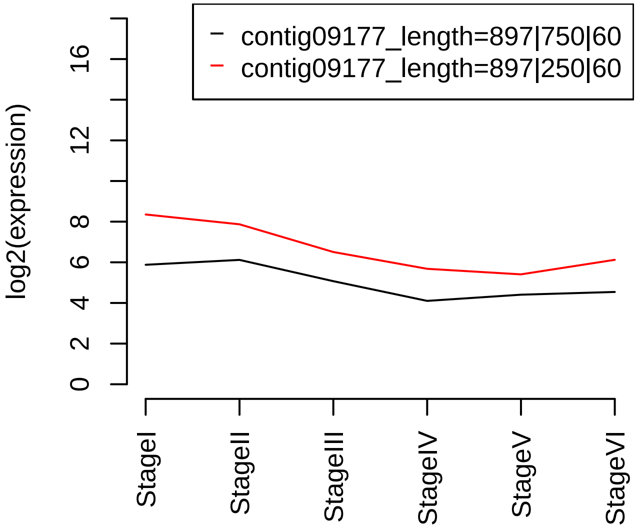
contig09177_length=897|750|60
contig09177_length=897|250|60
log2(expression)
0
2
4
6
8
12
16
StageI
StageII
StageIII
StageIV
StageV
StageVI

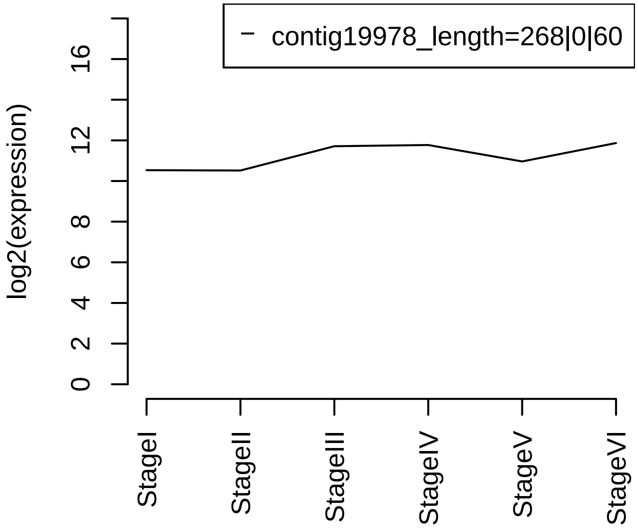
contig19978_length=268|0|60
log2(expression)
0
2
4
6
8
12
16
StageI
StageII
StageIII
StageIV
StageV
StageVI

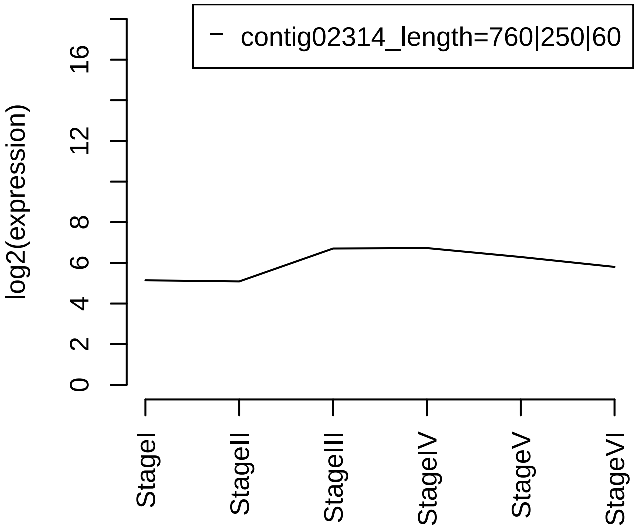
contig02314_length=760|250|60
log2(expression)
0
2
4
6
8
12
16
StageI
StageII
StageIII
StageIV
StageV
StageVI

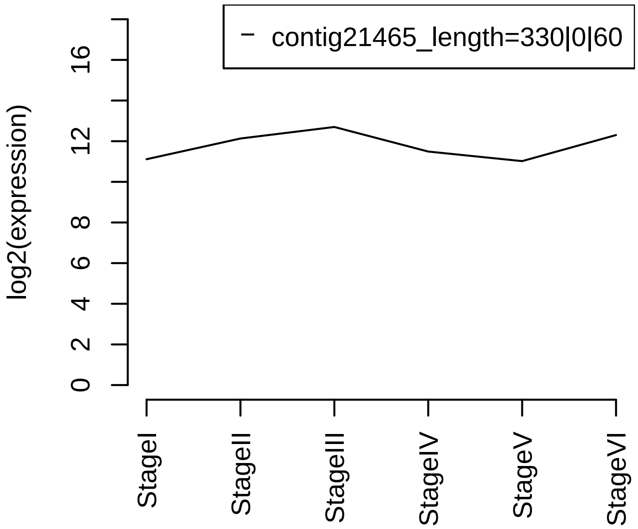
contig21465_length=330|0|60
log2(expression)
0
2
4
6
8
12
16
StageI
StageII
StageIII
StageIV
StageV
StageVI

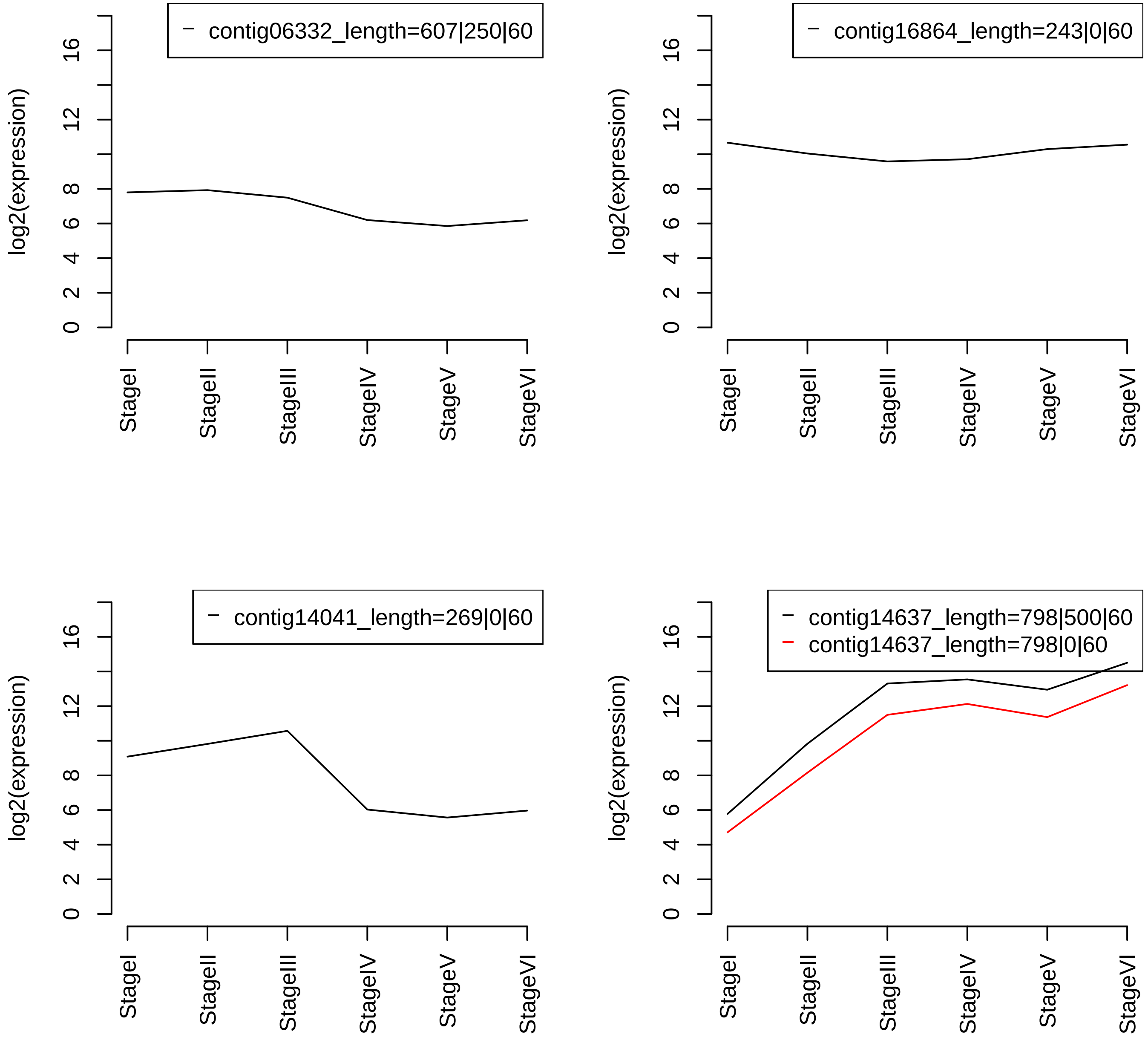
contig06332_length=607|250|60
contig16864_length=243|0|60
contig14041_length=269|0|60
contig14637_length=798|500|60
contig14637_length=798|0|60
log2(expression)
0
2
4
6
8
12
16
StageI
StageII
StageIII
StageIV
StageV
StageVI

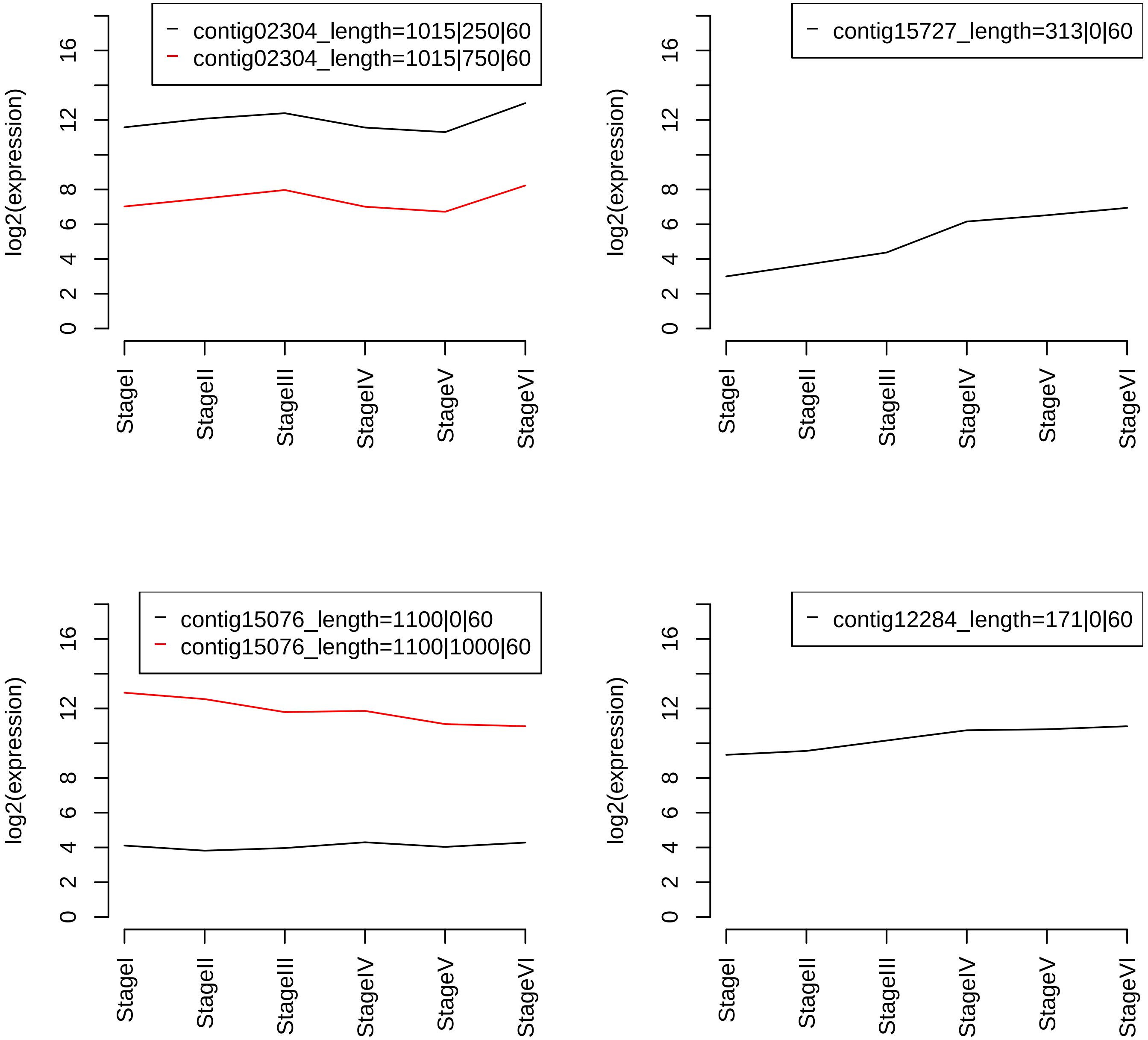
contig02304_length=1015|250|60
contig02304_length=1015|750|60
contig15727_length=313|0|60
contig15076_length=1100|0|60
contig15076_length=1100|1000|60
contig12284_length=171|0|60
log2(expression)
0
2
4
6
8
12
16
StageI
StageII
StageIII
StageIV
StageV
StageVI

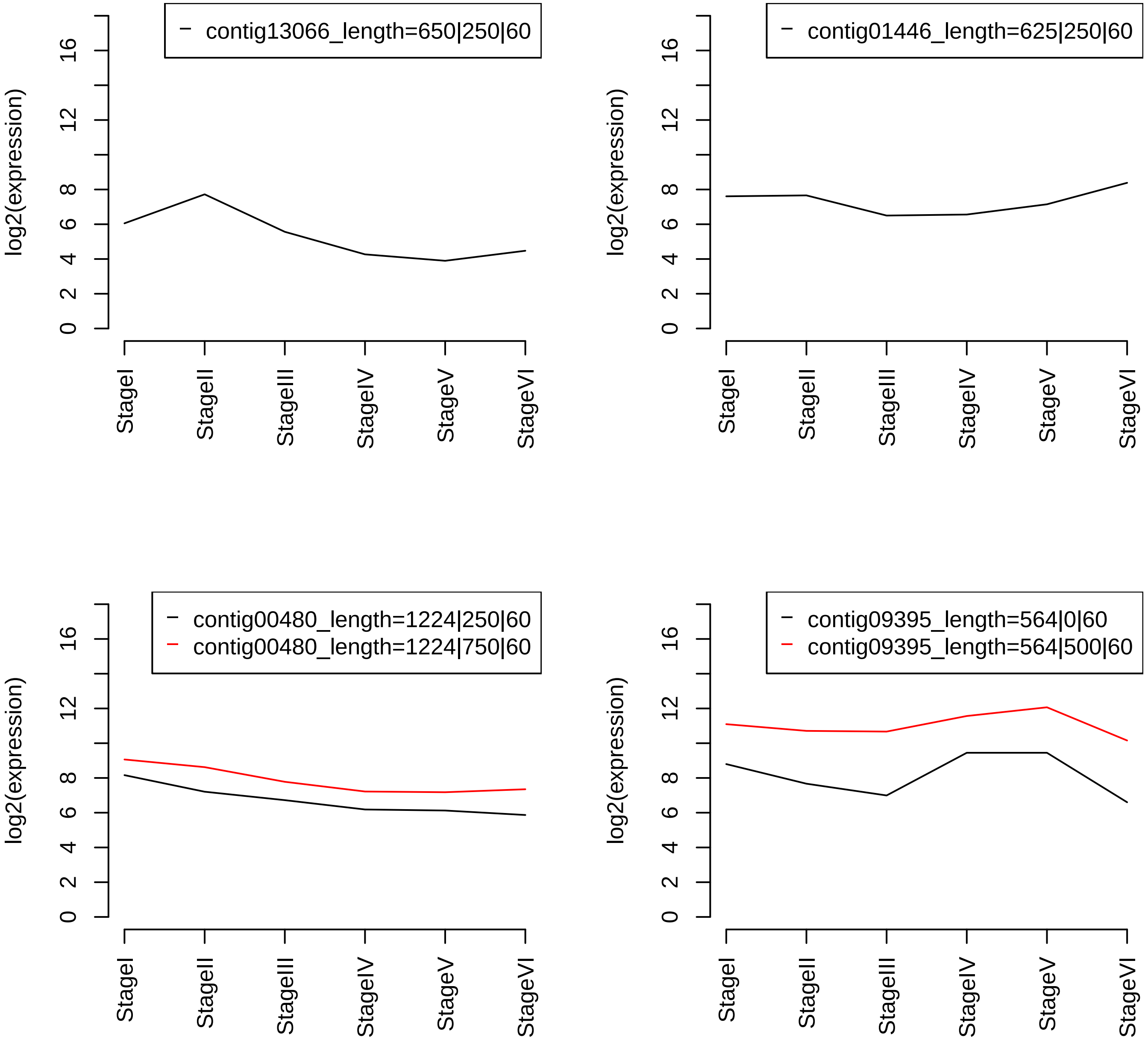
contig13066_length=650|250|60
contig01446_length=625|250|60
contig00480_length=1224|250|60
contig00480_length=1224|750|60
contig09395_length=564|0|60
contig09395_length=564|500|60
log2(expression)
StageI
StageII
StageIII
StageIV
StageV
StageVI

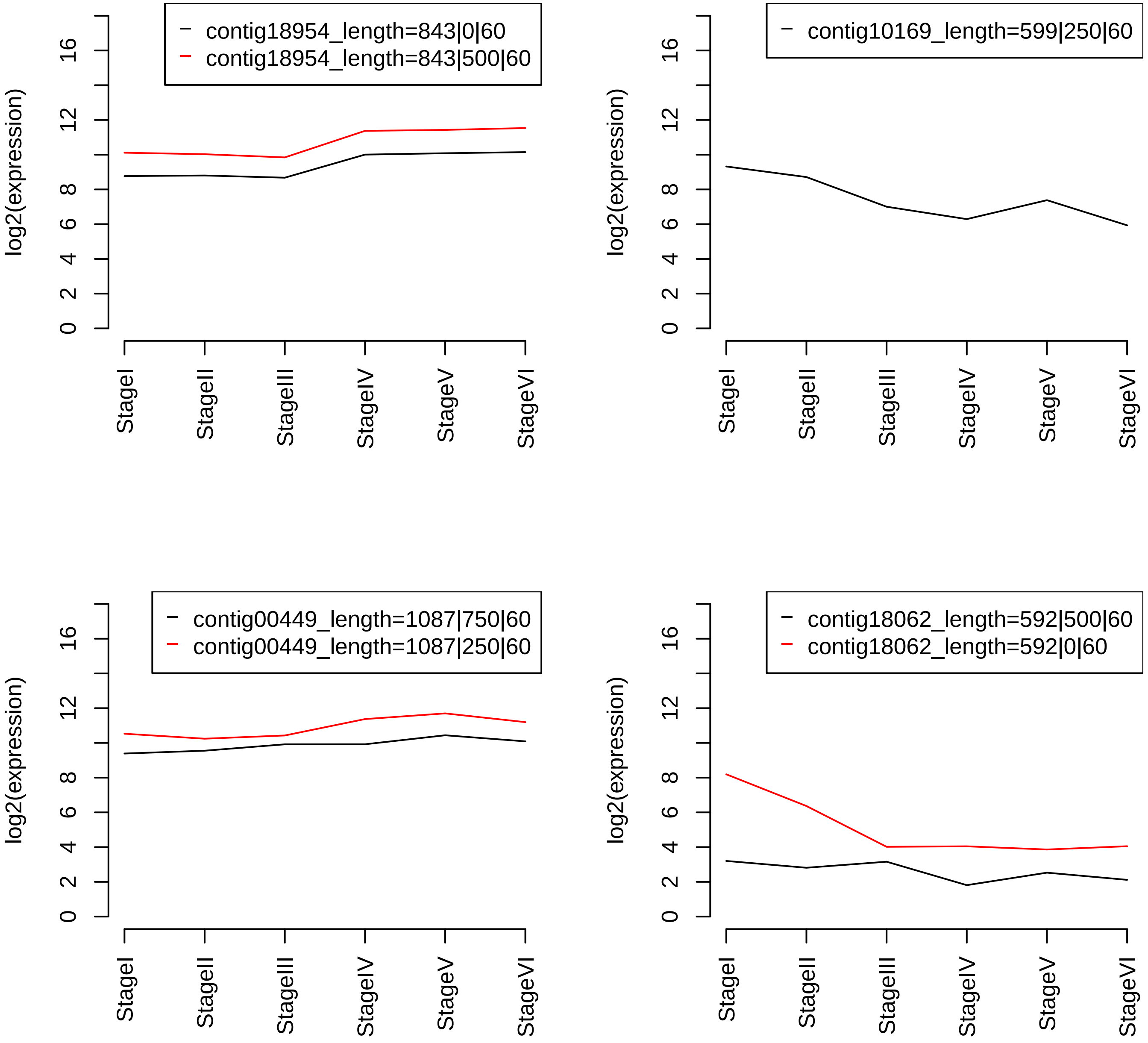

contig18954_length=843|0|60
contig18954_length=843|500|60
contig10169_length=599|250|60
contig00449_length=1087|750|60
contig00449_length=1087|250|60
contig18062_length=592|500|60
contig18062_length=592|0|60
log2(expression)
StageI
StageII
StageIII
StageIV
StageV
StageVI

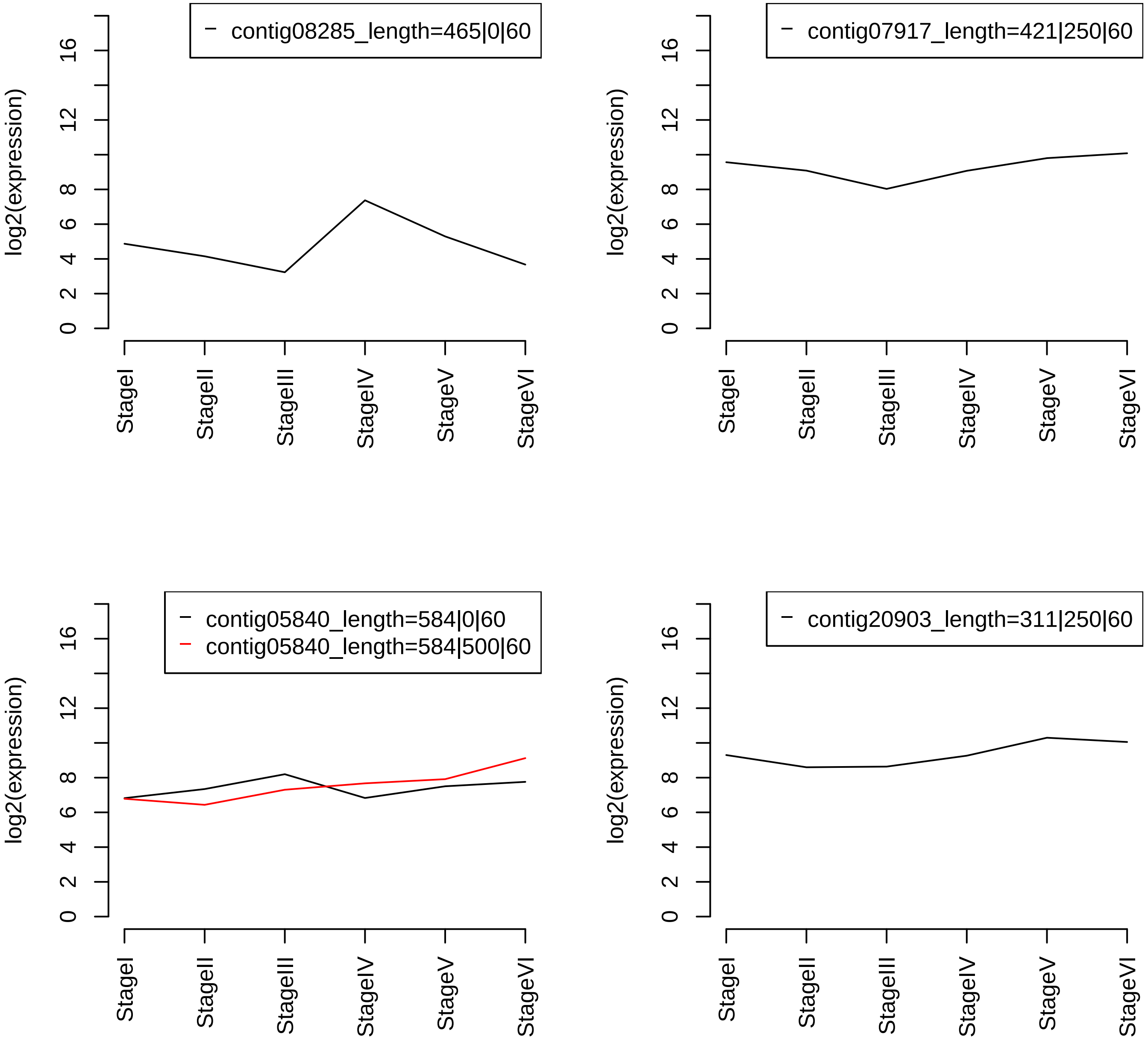
contig08285_length=465|0|60
contig07917_length=421|250|60
contig05840_length=584|0|60
contig05840_length=584|500|60
contig20903_length=311|250|60
log2(expression)
0
2
4
6
8
12
16
StageI
StageII
StageIII
StageIV
StageV
StageVI

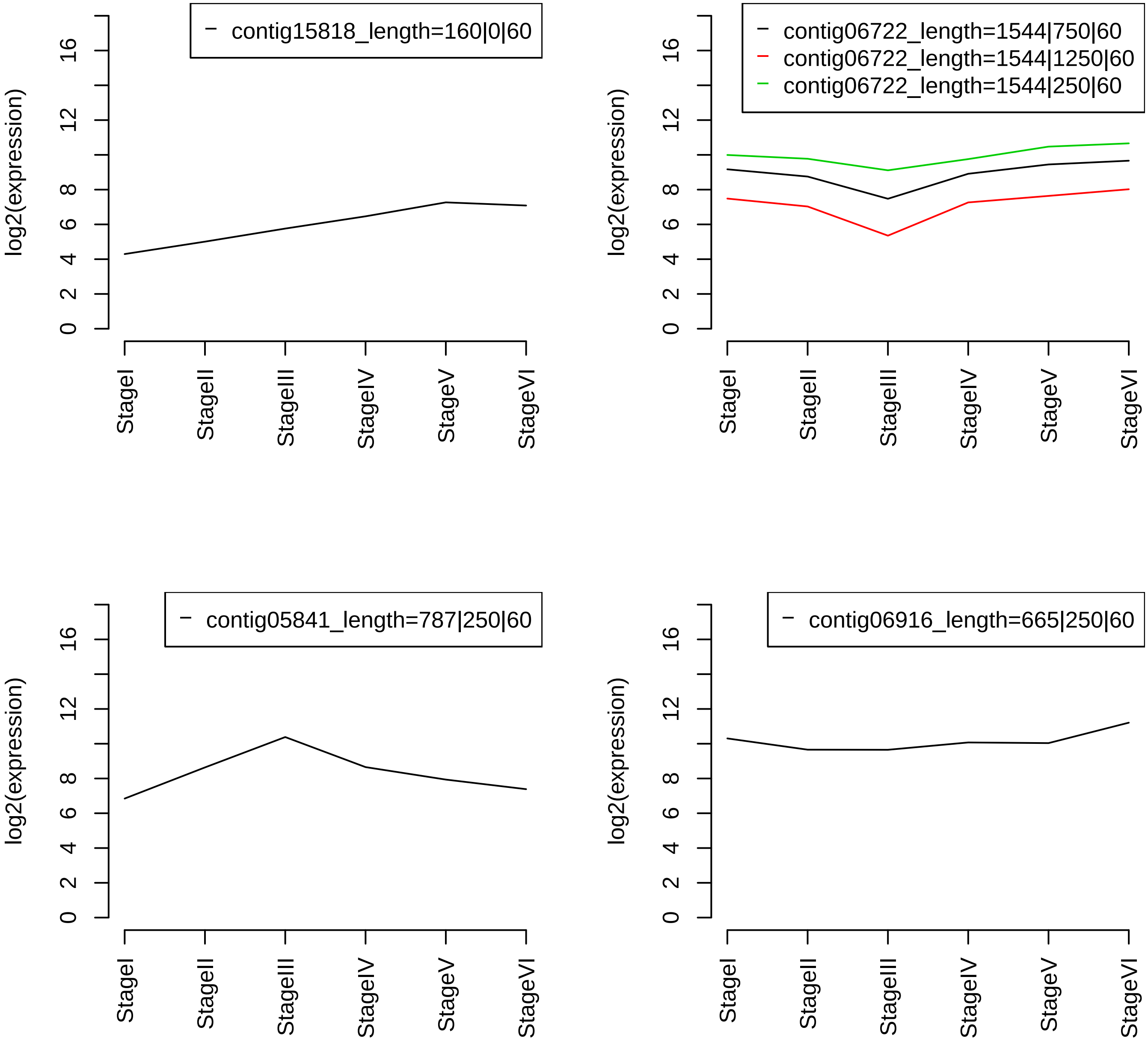
contig15818_length=160|0|60
contig06722_length=1544|750|60
contig06722_length=1544|1250|60
contig06722_length=1544|250|60
contig05841_length=787|250|60
contig06916_length=665|250|60
log2(expression)
0
2
4
6
8
12
16
StageI
StageII
StageIII
StageIV
StageV
StageVI

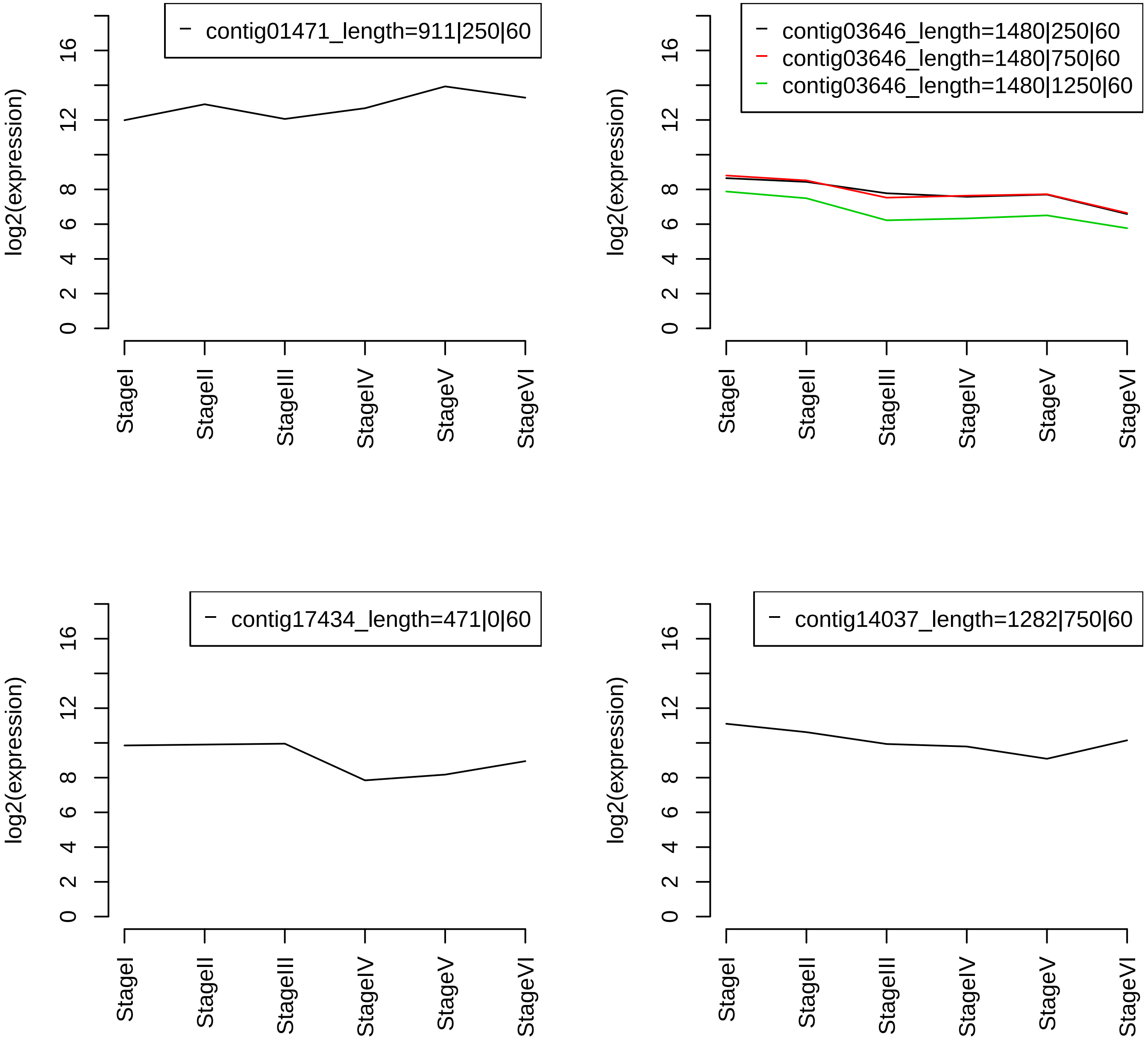
contig01471_length=911|250|60
contig03646_length=1480|250|60
contig03646_length=1480|750|60
contig03646_length=1480|1250|60
contig17434_length=471|0|60
contig14037_length=1282|750|60
log2(expression)
0
2
4
6
8
12
16
StageI
StageII
StageIII
StageIV
StageV
StageVI

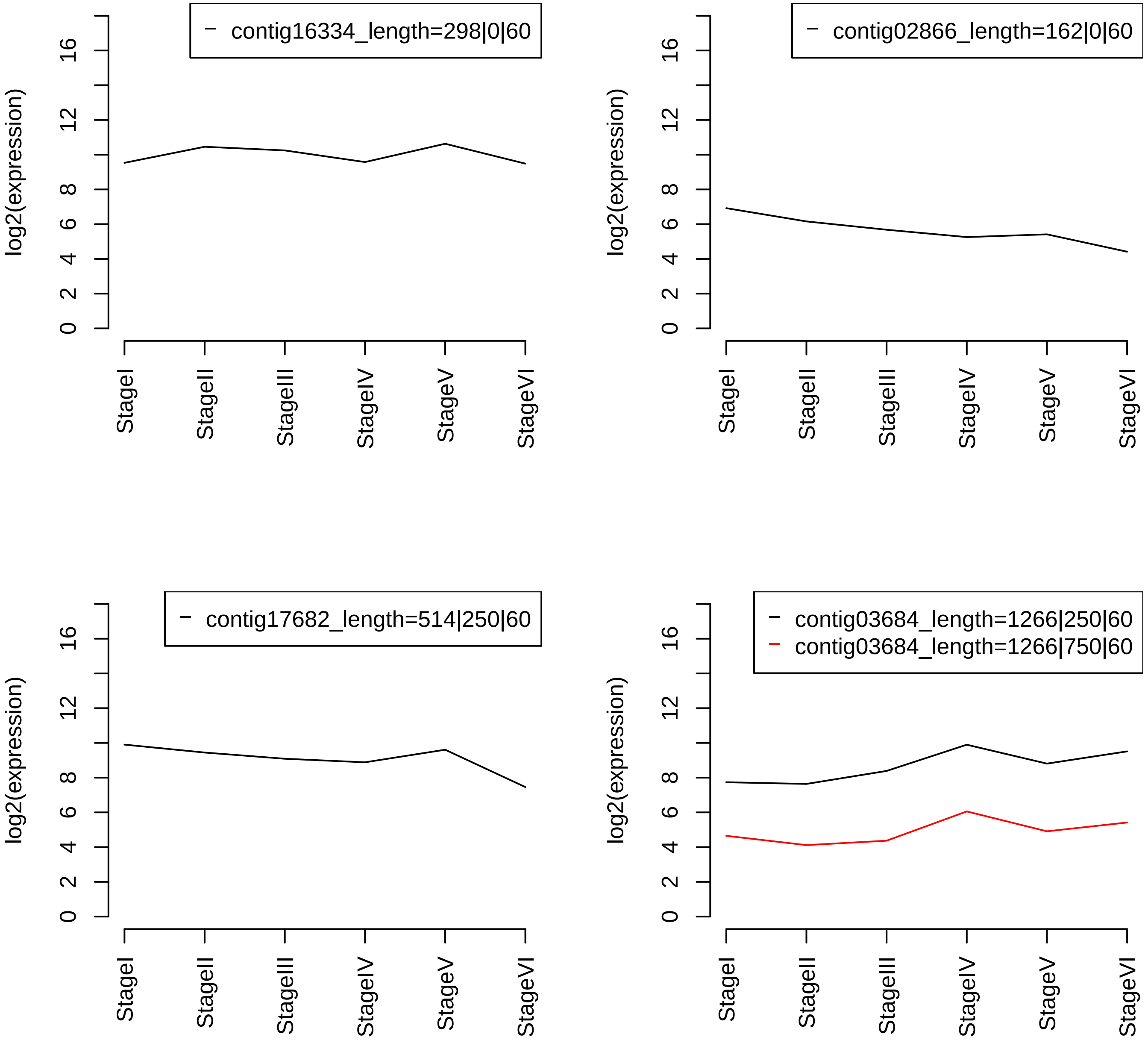

contig16334_length=298|0|60
contig02866_length=162|0|60
contig17682_length=514|250|60
contig03684_length=1266|250|60
contig03684_length=1266|750|60
log2(expression)
0
2
4
6
8
12
16
StageI
StageII
StageIII
StageIV
StageV
StageVI

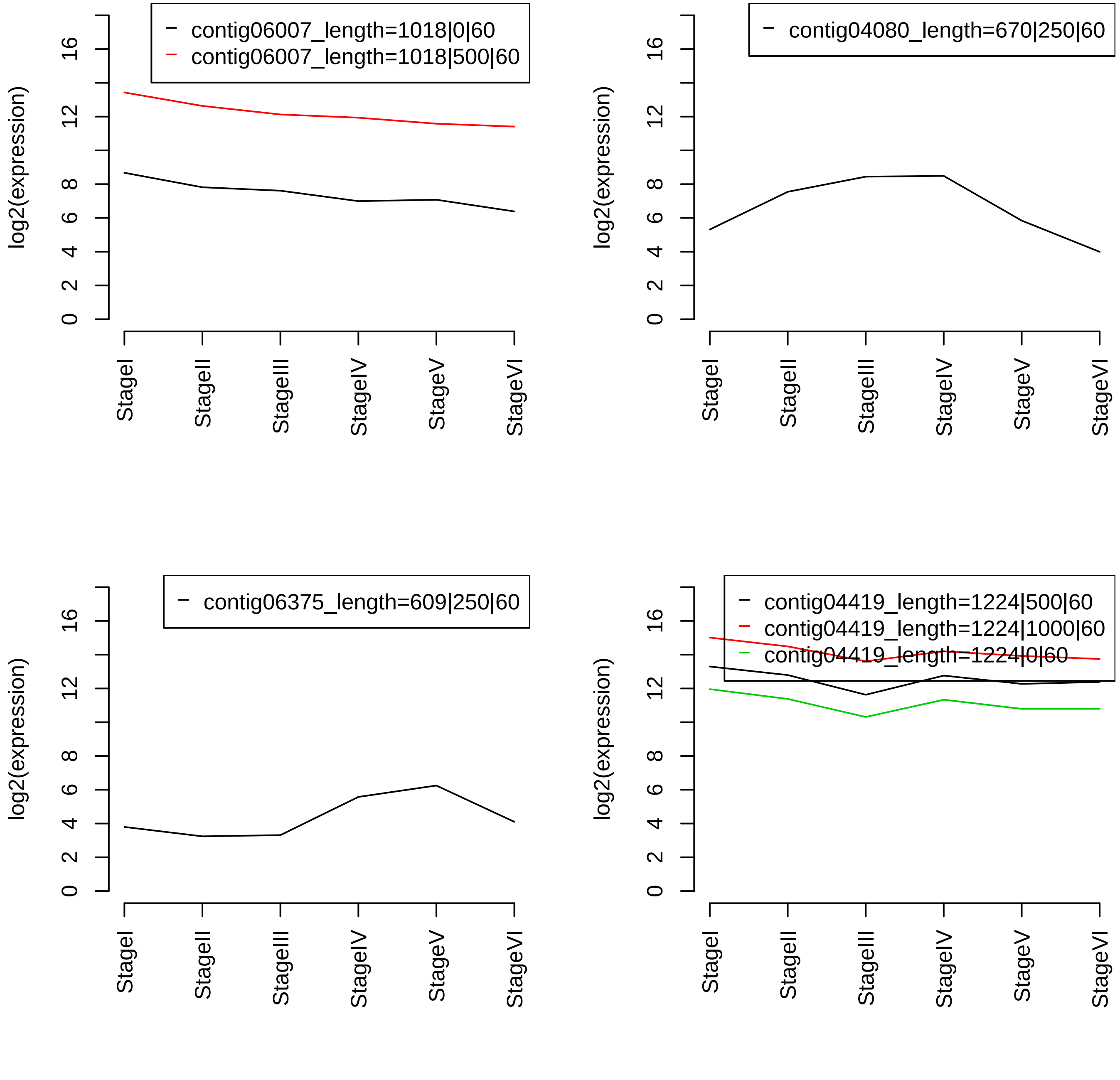

contig06007_length=1018|0|60
contig06007_length=1018|500|60
log2(expression)
0
2
4
6
8
12
16
StageI
StageII
StageIII
StageIV
StageV
StageVI
contig04080_length=670|250|60
log2(expression)
0
2
4
6
8
12
16
StageI
StageII
StageIII
StageIV
StageV
StageVI
contig06375_length=609|250|60
log2(expression)
0
2
4
6
8
12
16
StageI
StageII
StageIII
StageIV
StageV
StageVI
contig04419_length=1224|500|60
contig04419_length=1224|1000|60
contig04419_length=1224|0|60
log2(expression)
0
2
4
6
8
12
16
StageI
StageII
StageIII
StageIV
StageV
StageVI

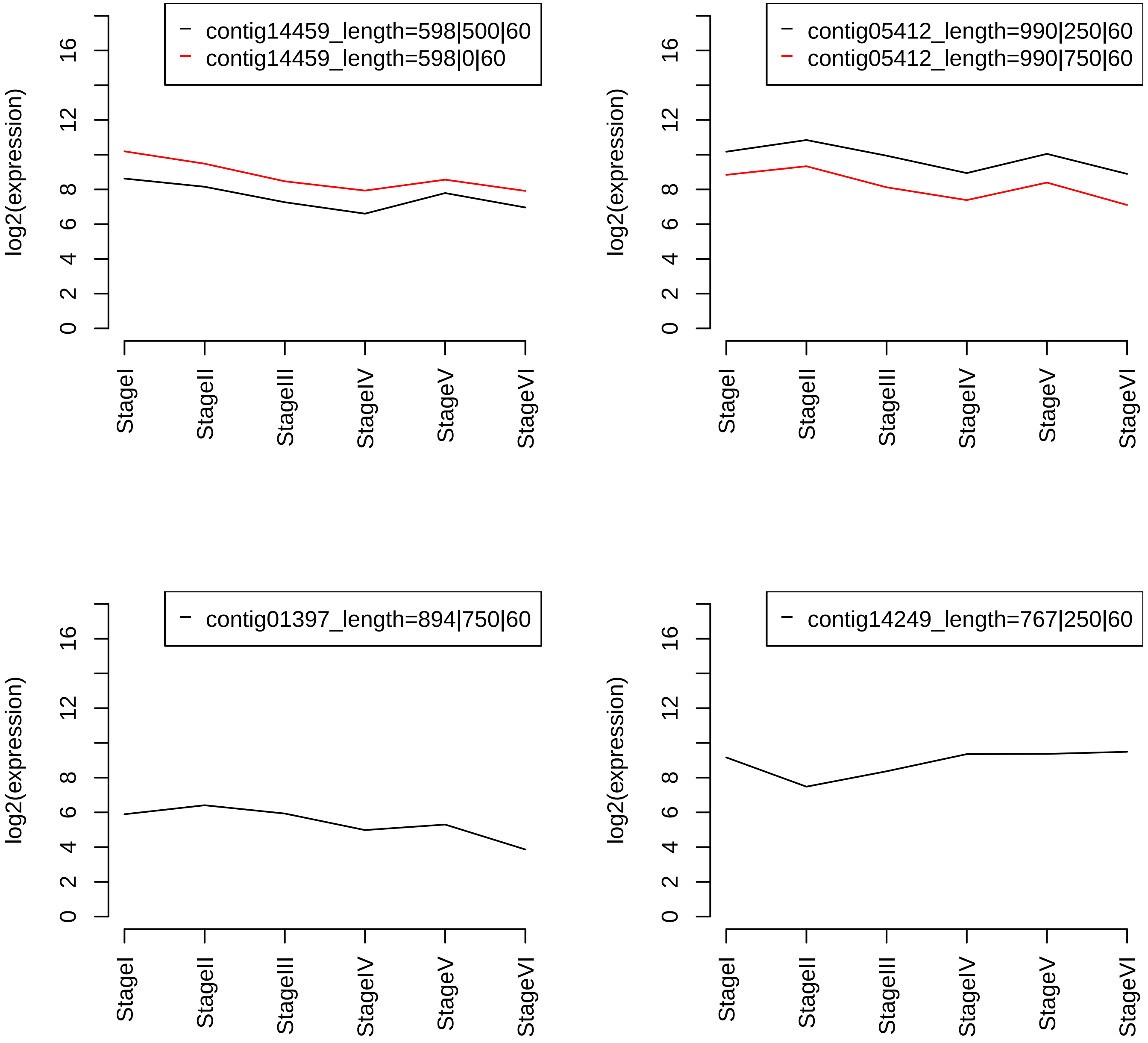
contig14459_length=598|500|60
contig14459_length=598|0|60
contig05412_length=990|250|60
contig05412_length=990|750|60
contig01397_length=894|750|60
contig14249_length=767|250|60
log2(expression)
0 2 4 6 8 12 16
StageI
StageII
StageIII
StageIV
StageV
StageVI

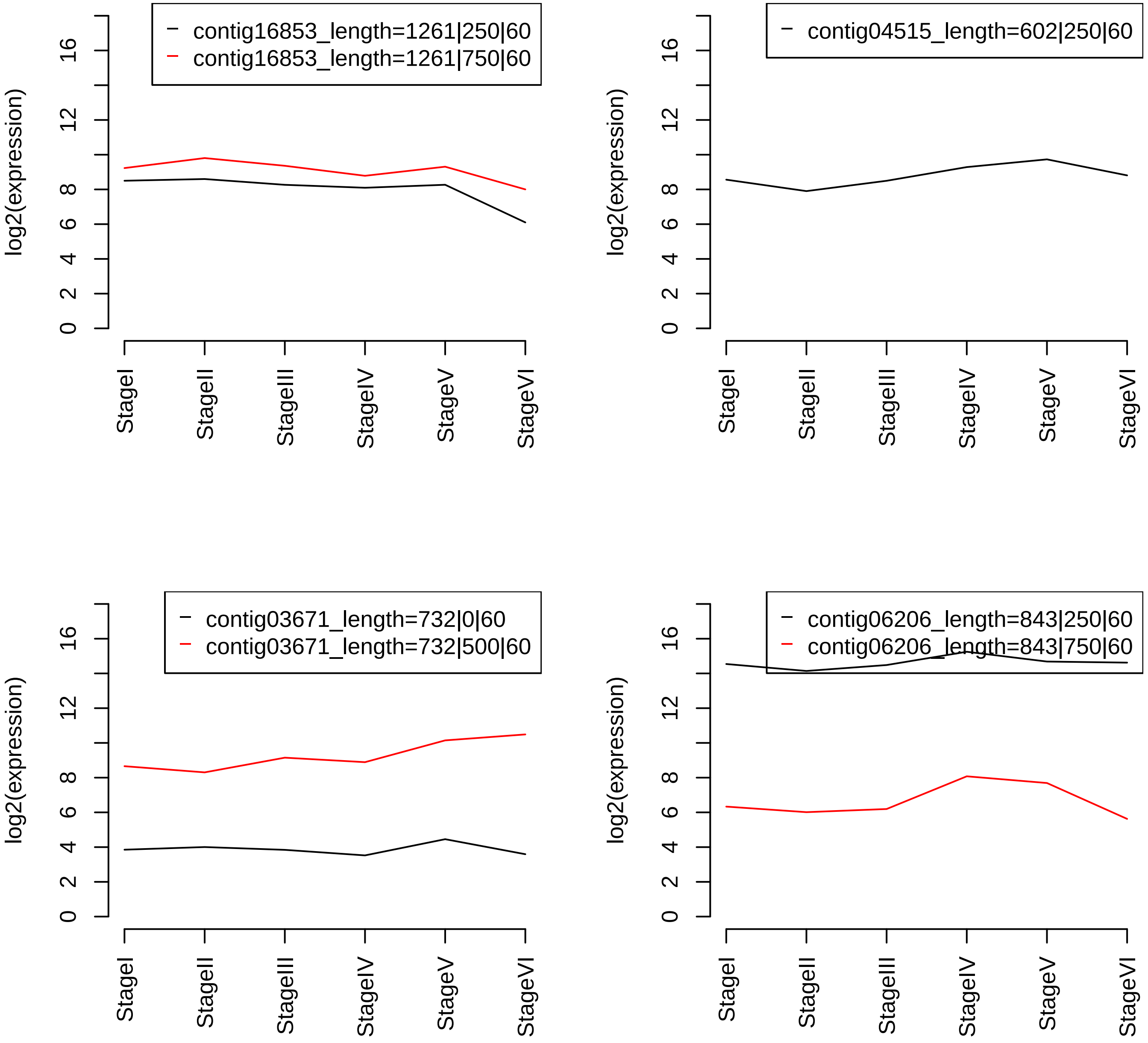

contig16853_length=1261|250|60
contig16853_length=1261|750|60
log2(expression)
0
2
4
6
8
12
16
StageI
StageII
StageIII
StageIV
StageV
StageVI
contig04515_length=602|250|60
log2(expression)
0
2
4
6
8
12
16
StageI
StageII
StageIII
StageIV
StageV
StageVI
contig03671_length=732|0|60
contig03671_length=732|500|60
log2(expression)
0
2
4
6
8
12
16
StageI
StageII
StageIII
StageIV
StageV
StageVI
contig06206_length=843|250|60
contig06206_length=843|750|60
log2(expression)
0
2
4
6
8
12
16
StageI
StageII
StageIII
StageIV
StageV
StageVI

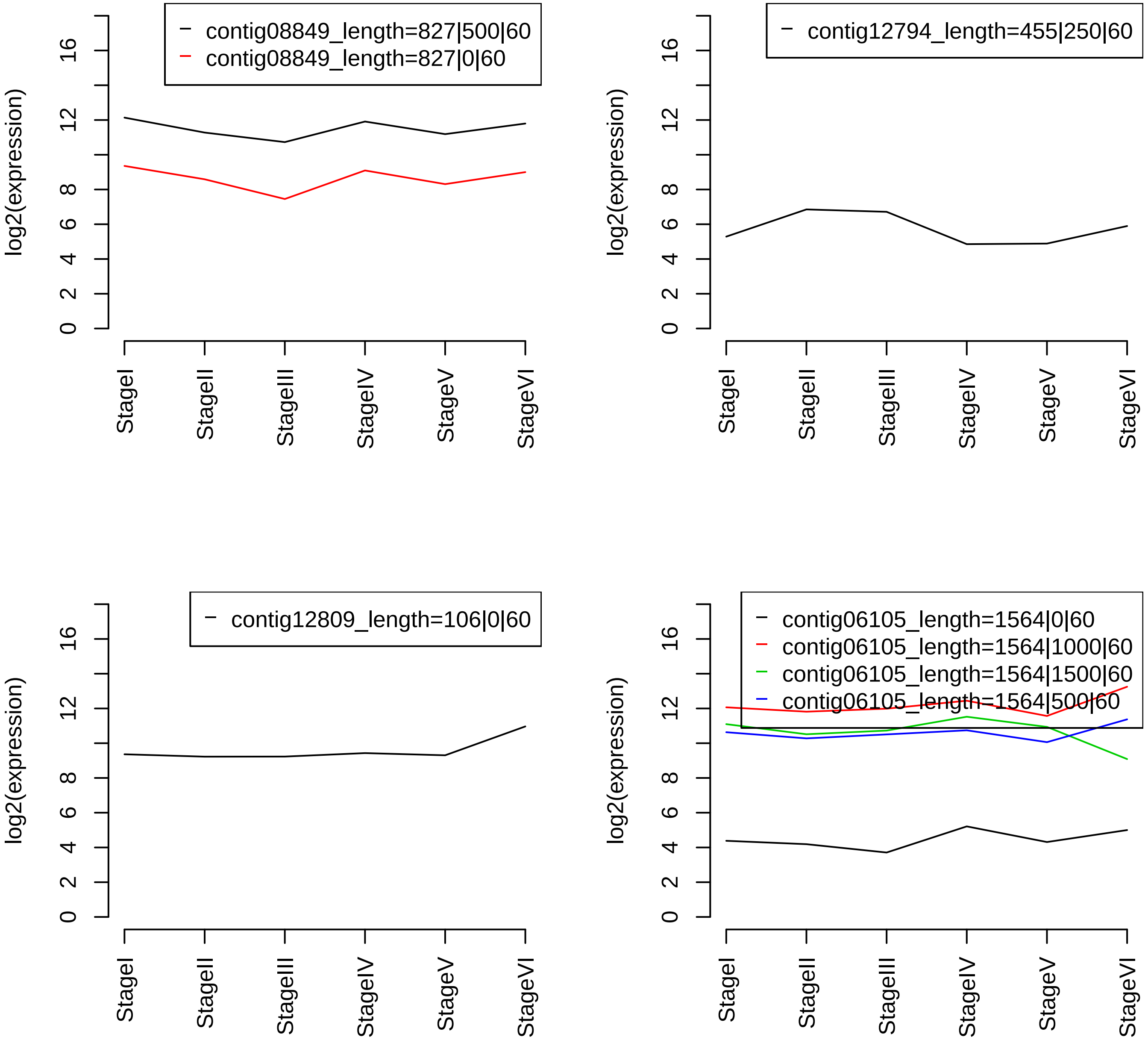

contig08849_length=827|500|60
contig08849_length=827|0|60
log2(expression)
0
2
4
6
8
12
16
StageI
StageII
StageIII
StageIV
StageV
StageVI
contig12794_length=455|250|60
contig12809_length=106|0|60
contig06105_length=1564|0|60
contig06105_length=1564|1000|60
contig06105_length=1564|1500|60
contig06105_length=1564|500|60

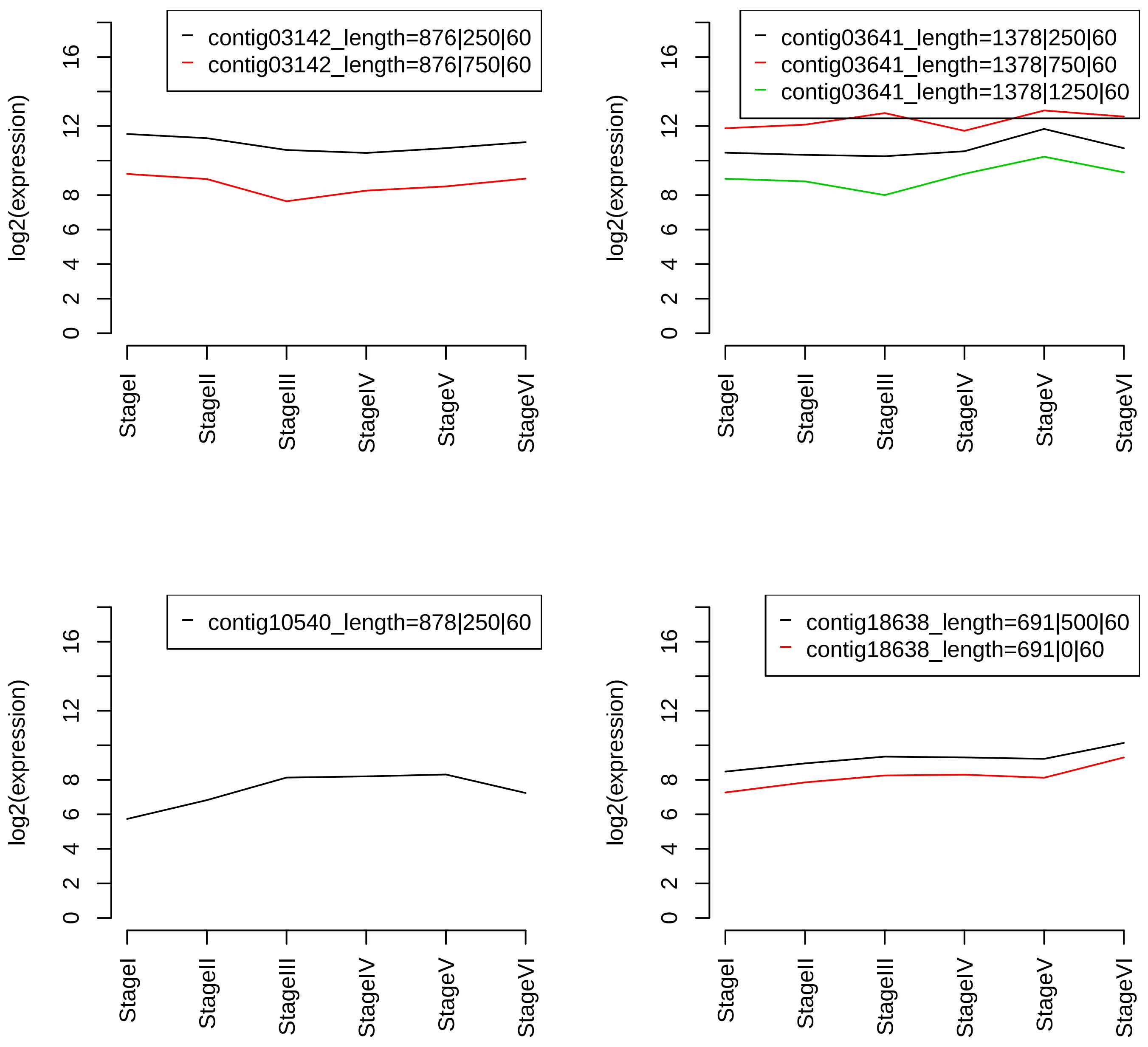
contig03142_length=876|250|60
contig03142_length=876|750|60
contig03641_length=1378|250|60
contig03641_length=1378|750|60
contig03641_length=1378|1250|60
contig10540_length=878|250|60
contig18638_length=691|500|60
contig18638_length=691|0|60
log2(expression)
0
2
4
6
8
12
16
StageI
StageII
StageIII
StageIV
StageV
StageVI

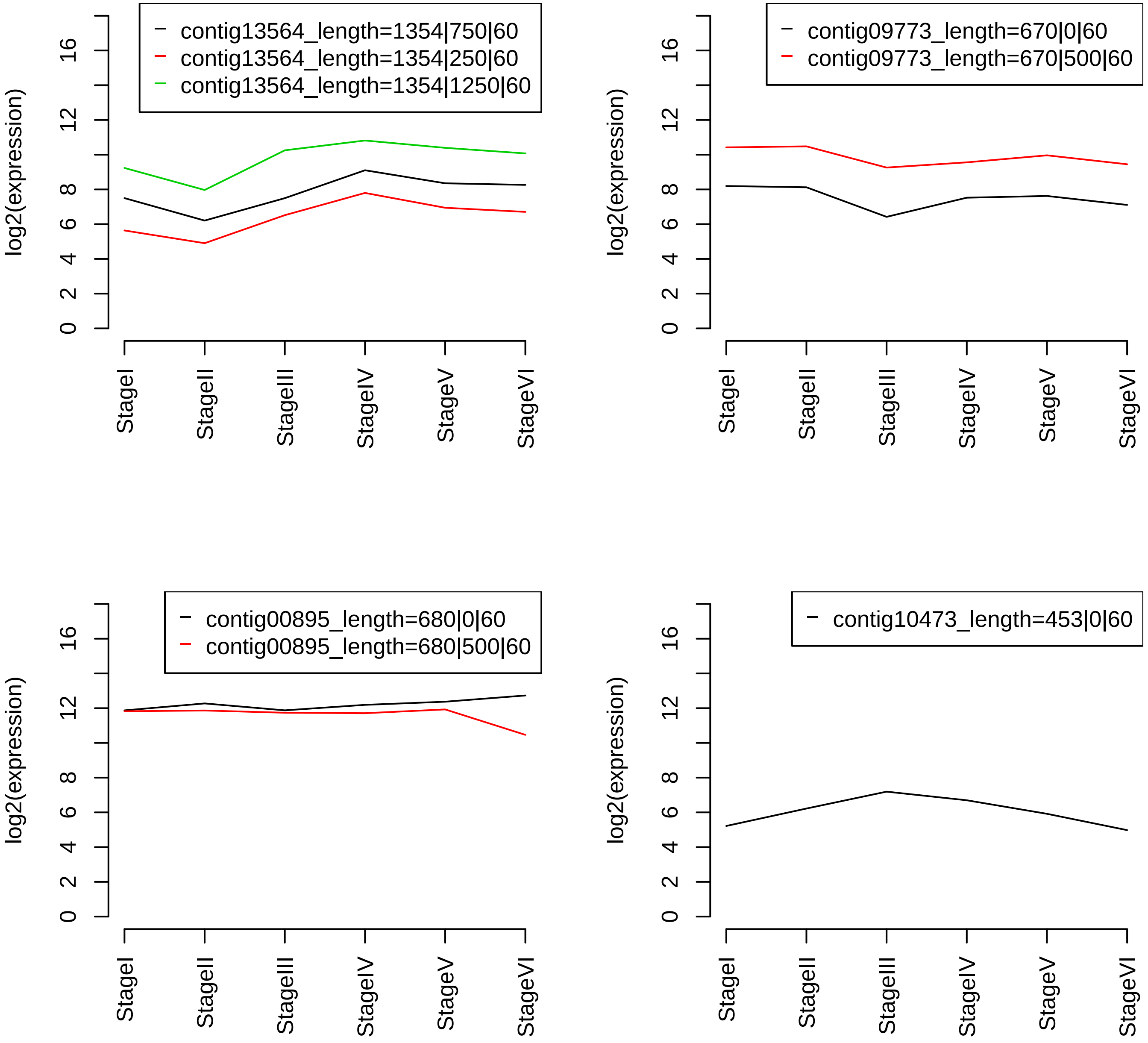

contig13564_length=1354|750|60
contig13564_length=1354|250|60
contig13564_length=1354|1250|60
contig09773_length=670|0|60
contig09773_length=670|500|60
contig00895_length=680|0|60
contig00895_length=680|500|60
contig10473_length=453|0|60
log2(expression)
0
2
4
6
8
12
16
StageI
StageII
StageIII
StageIV
StageV
StageVI

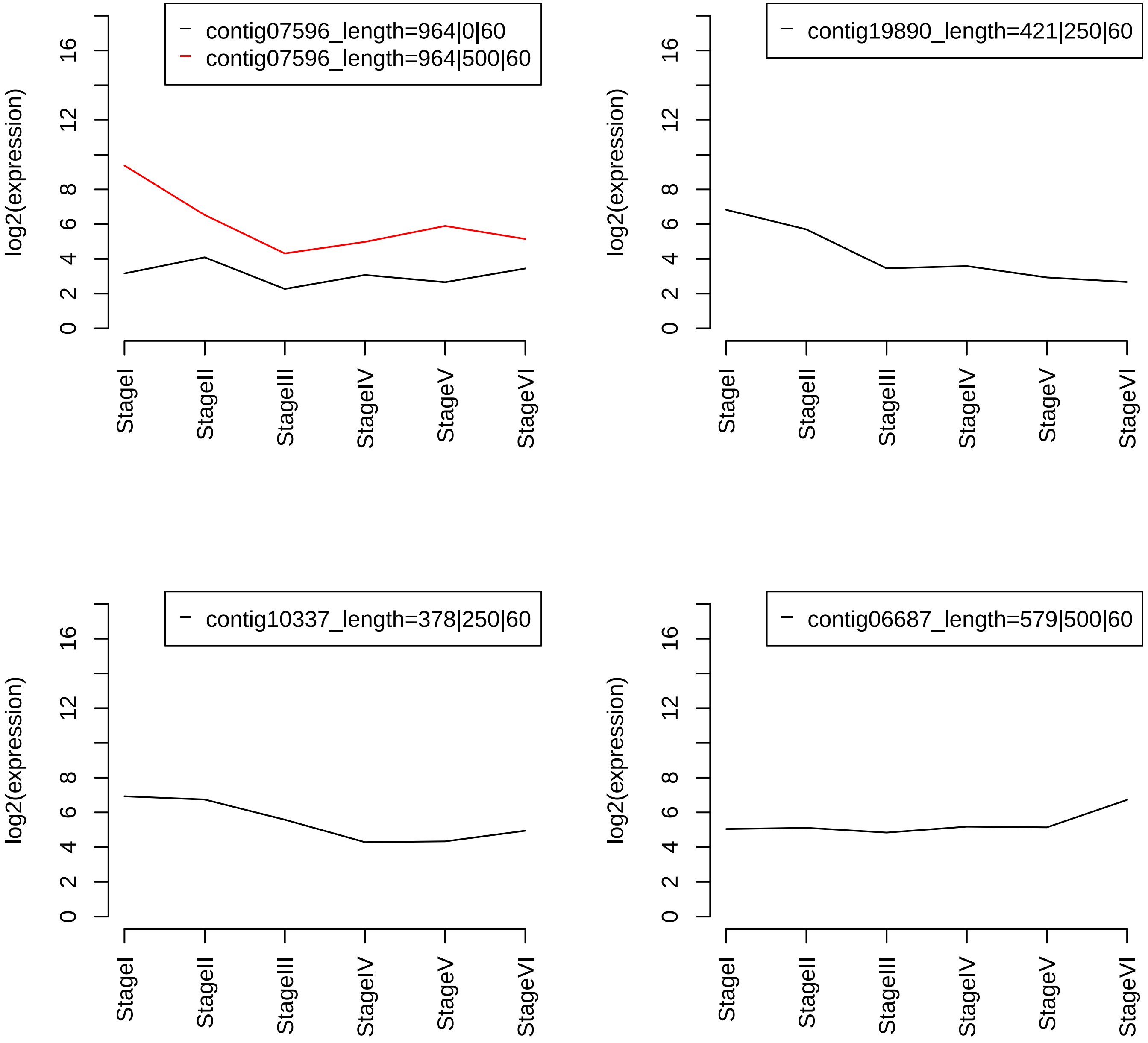

contig07596_length=964|0|60
contig07596_length=964|500|60
contig19890_length=421|250|60
contig10337_length=378|250|60
contig06687_length=579|500|60
log2(expression)
0
2
4
6
8
12
16
StageI
StageII
StageIII
StageIV
StageV
StageVI

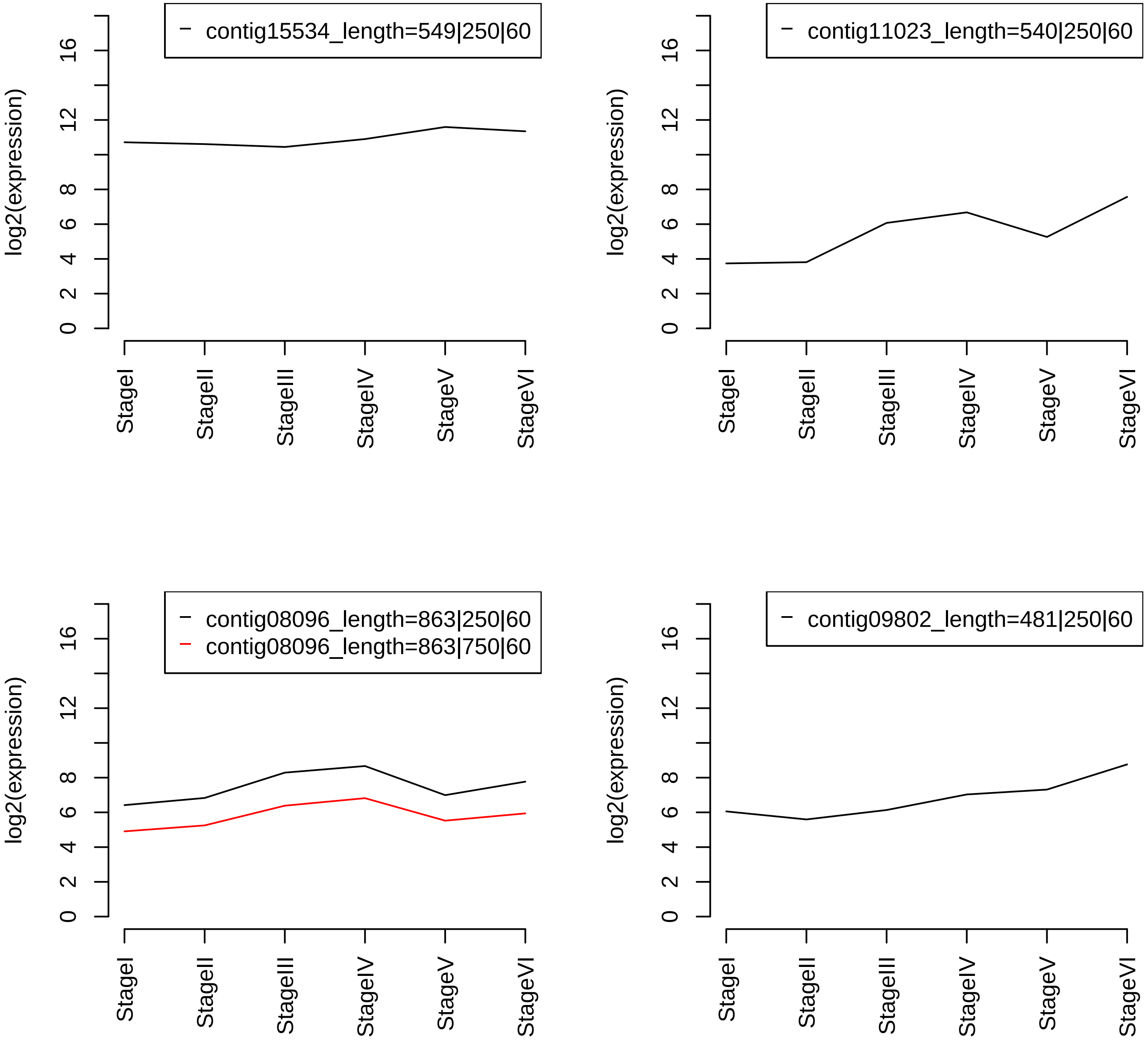
contig15534_length=549|250|60
contig11023_length=540|250|60
contig08096_length=863|250|60
contig08096_length=863|750|60
contig09802_length=481|250|60
log2(expression)
0
2
4
6
8
12
16
StageI
StageII
StageIII
StageIV
StageV
StageVI

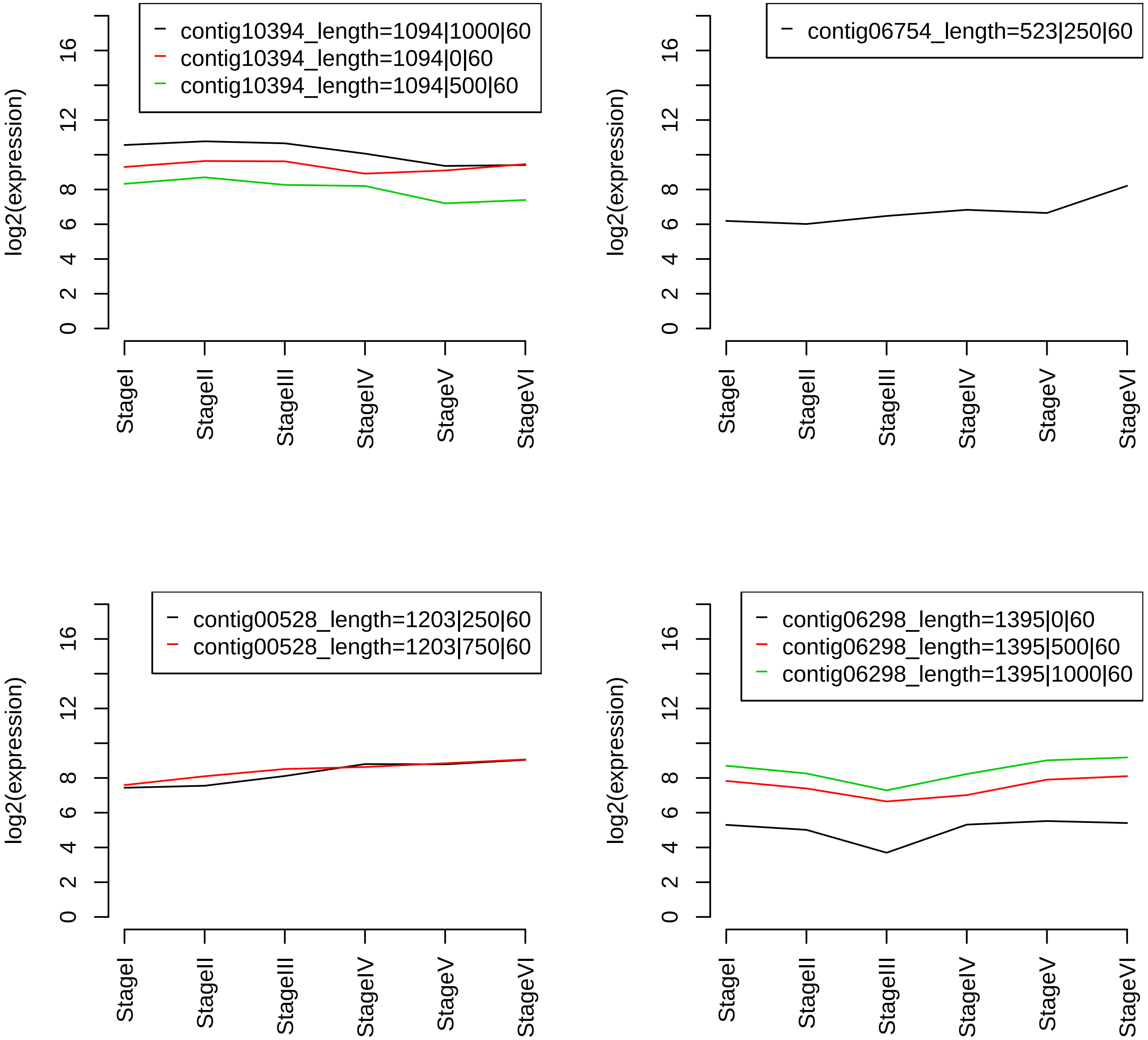

contig10394_length=1094|1000|60
contig10394_length=1094|0|60
contig10394_length=1094|500|60
contig06754_length=523|250|60
contig00528_length=1203|250|60
contig00528_length=1203|750|60
contig06298_length=1395|0|60
contig06298_length=1395|500|60
contig06298_length=1395|1000|60
log2(expression)
0 2 4 6 8 12 16
StageI
StageII
StageIII
StageIV
StageV
StageVI

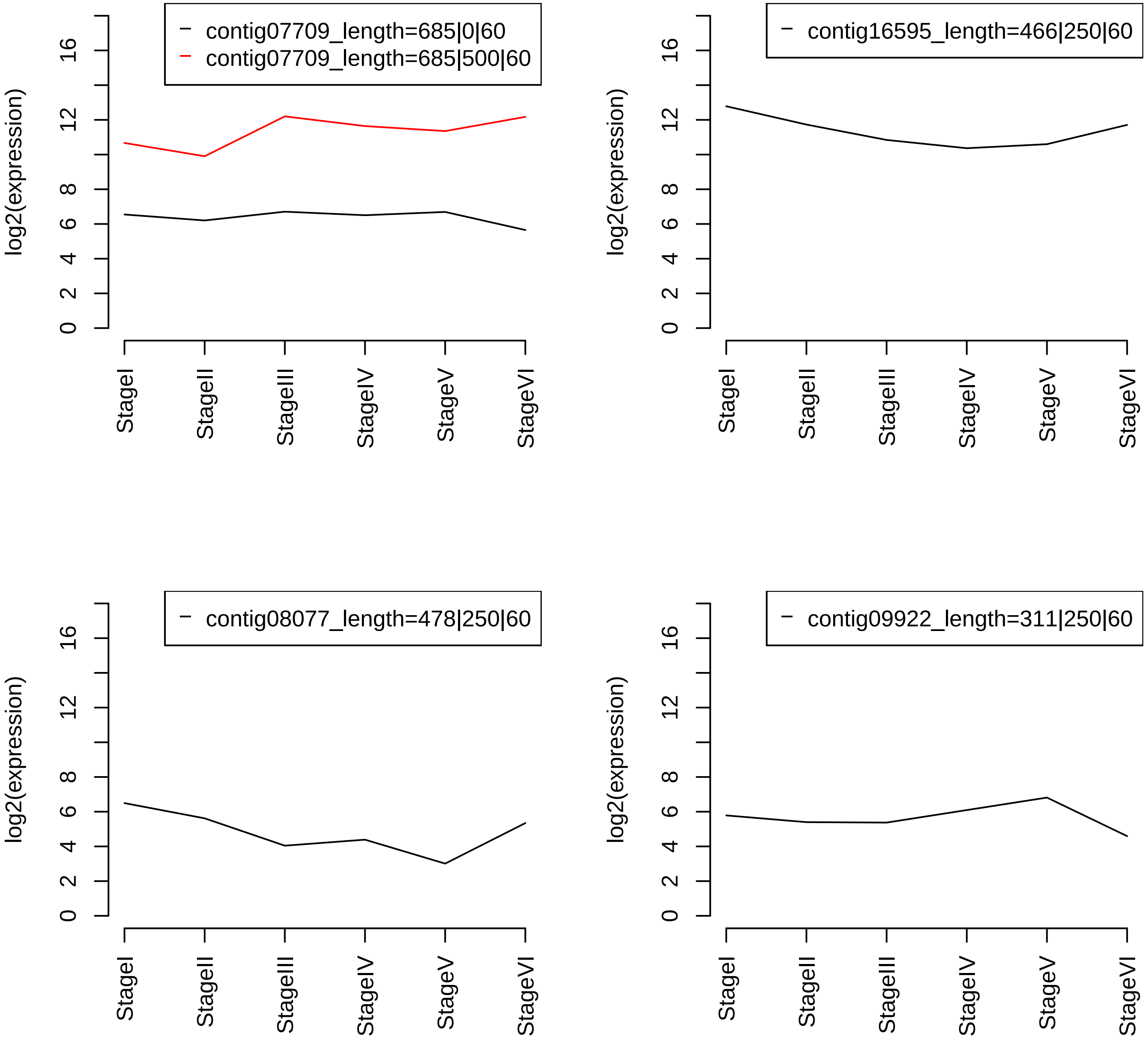
contig07709_length=685|0|60
contig07709_length=685|500|60
contig16595_length=466|250|60
contig08077_length=478|250|60
contig09922_length=311|250|60
log2(expression)
0
2
4
6
8
12
16
StageI
StageII
StageIII
StageIV
StageV
StageVI

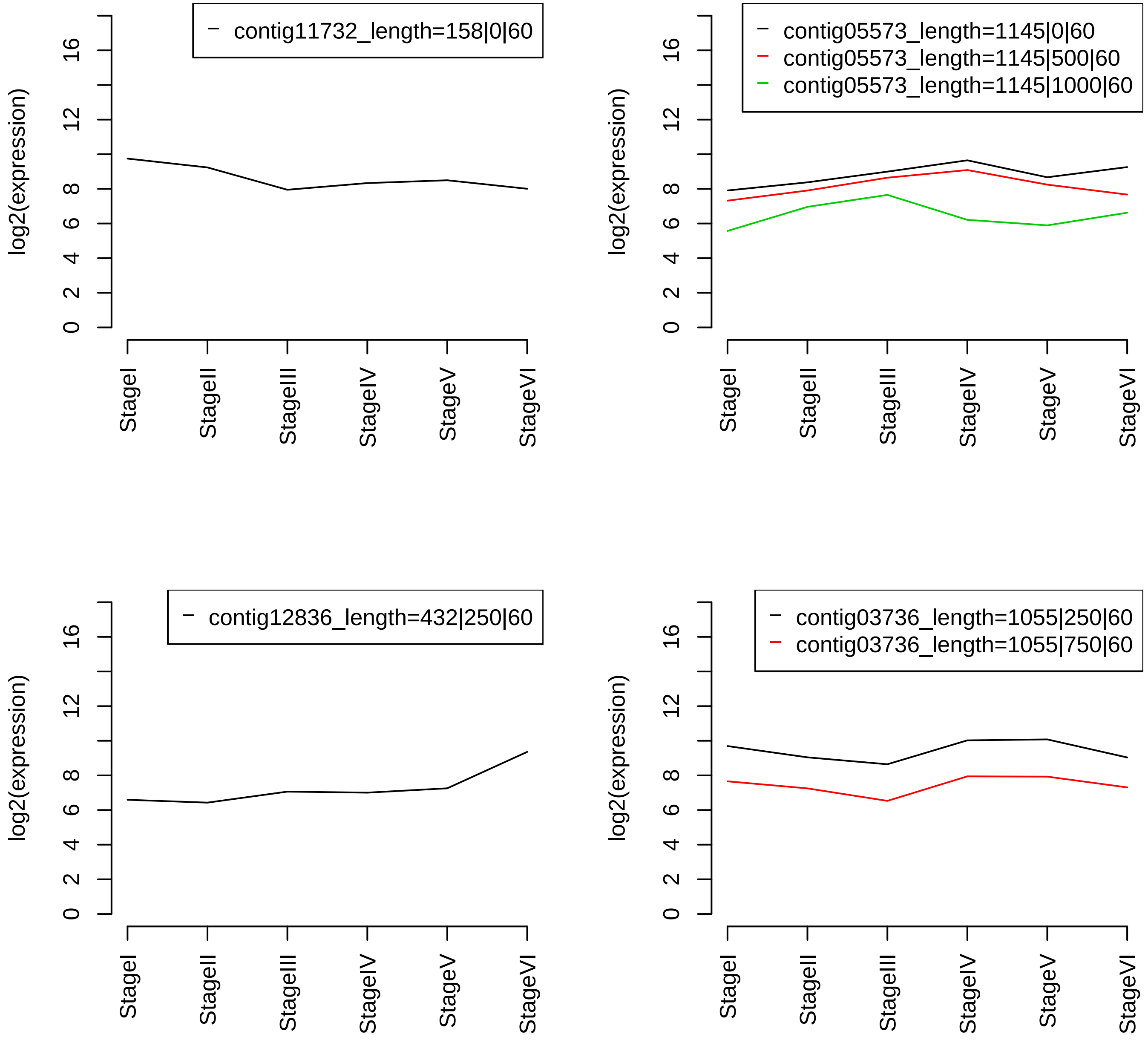
contig11732_length=158|0|60
contig05573_length=1145|0|60
contig05573_length=1145|500|60
contig05573_length=1145|1000|60
contig12836_length=432|250|60
contig03736_length=1055|250|60
contig03736_length=1055|750|60
log2(expression)
0 2 4 6 8 12 16
StageI StageII StageIII StageIV StageV StageVI

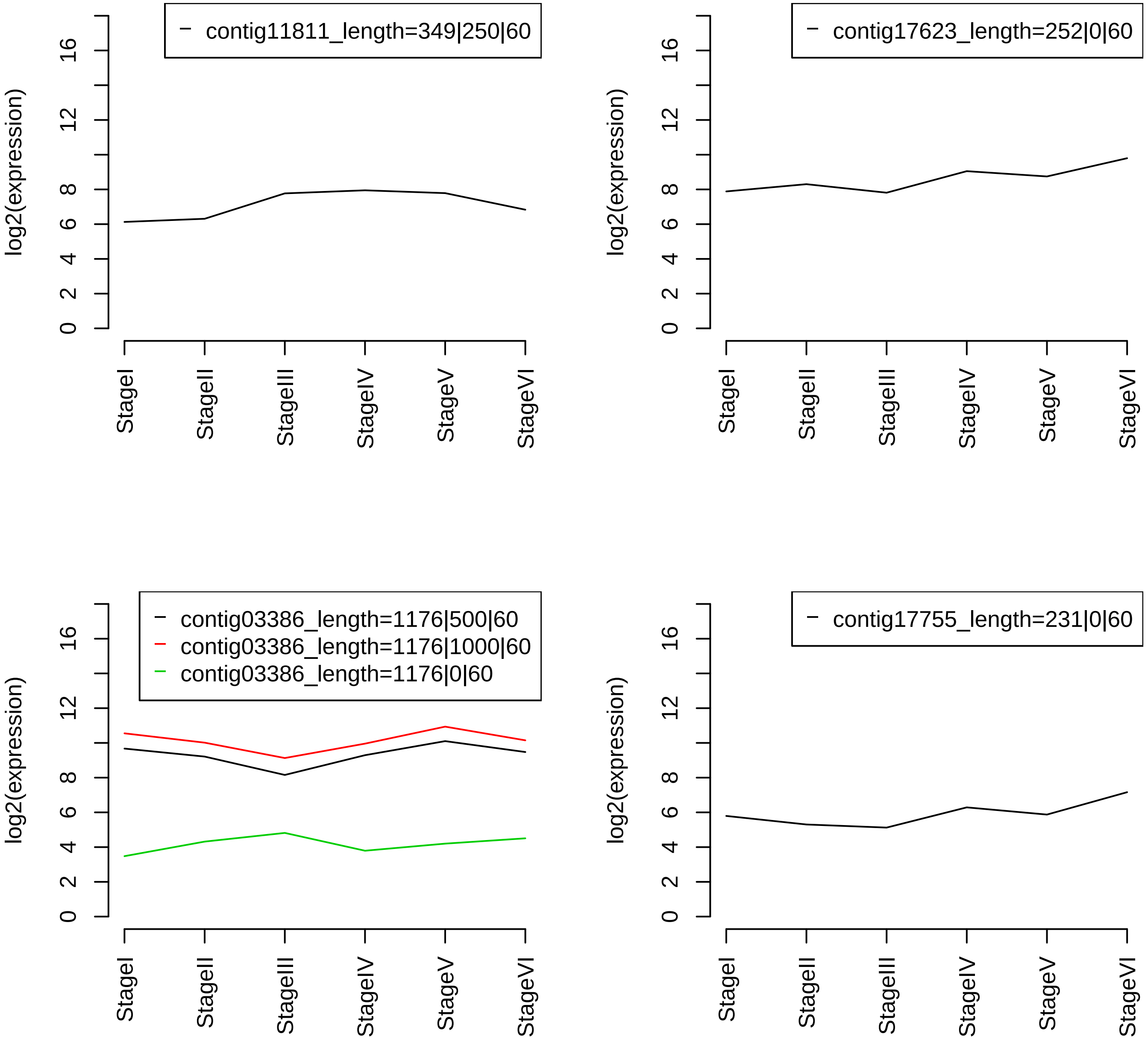
contig11811_length=349|250|60
log2(expression)
0
2
4
6
8
12
16
StageI
StageII
StageIII
StageIV
StageV
StageVI
contig17623_length=252|0|60
log2(expression)
0
2
4
6
8
12
16
StageI
StageII
StageIII
StageIV
StageV
StageVI
contig03386_length=1176|500|60
contig03386_length=1176|1000|60
contig03386_length=1176|0|60
log2(expression)
0
2
4
6
8
12
16
StageI
StageII
StageIII
StageIV
StageV
StageVI
contig17755_length=231|0|60
log2(expression)
0
2
4
6
8
12
16
StageI
StageII
StageIII
StageIV
StageV
StageVI

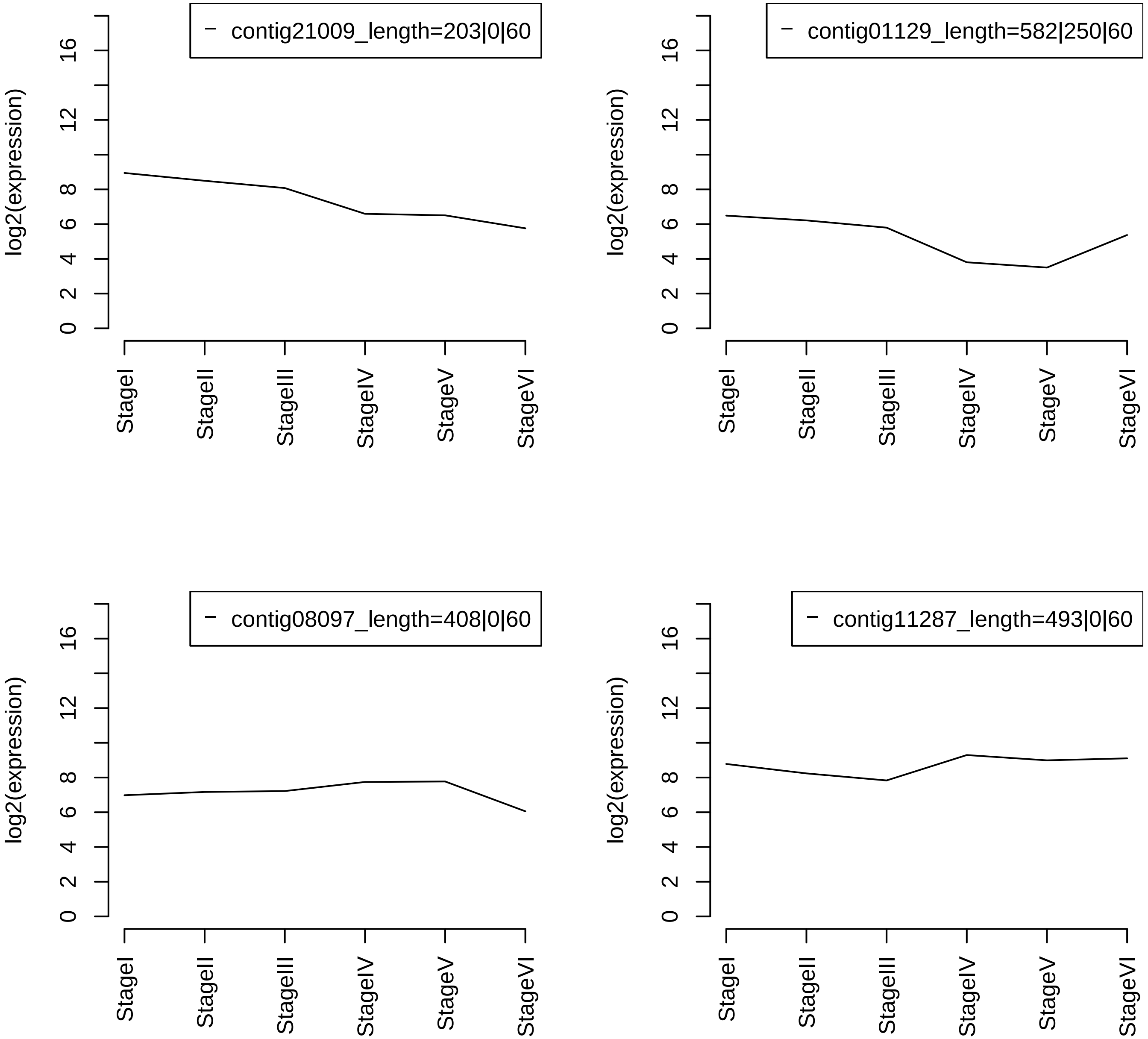
contig21009_length=203|0|60
contig01129_length=582|250|60
contig08097_length=408|0|60
contig11287_length=493|0|60
log2(expression)
0
2
4
6
8
12
16
StageI
StageII
StageIII
StageIV
StageV
StageVI

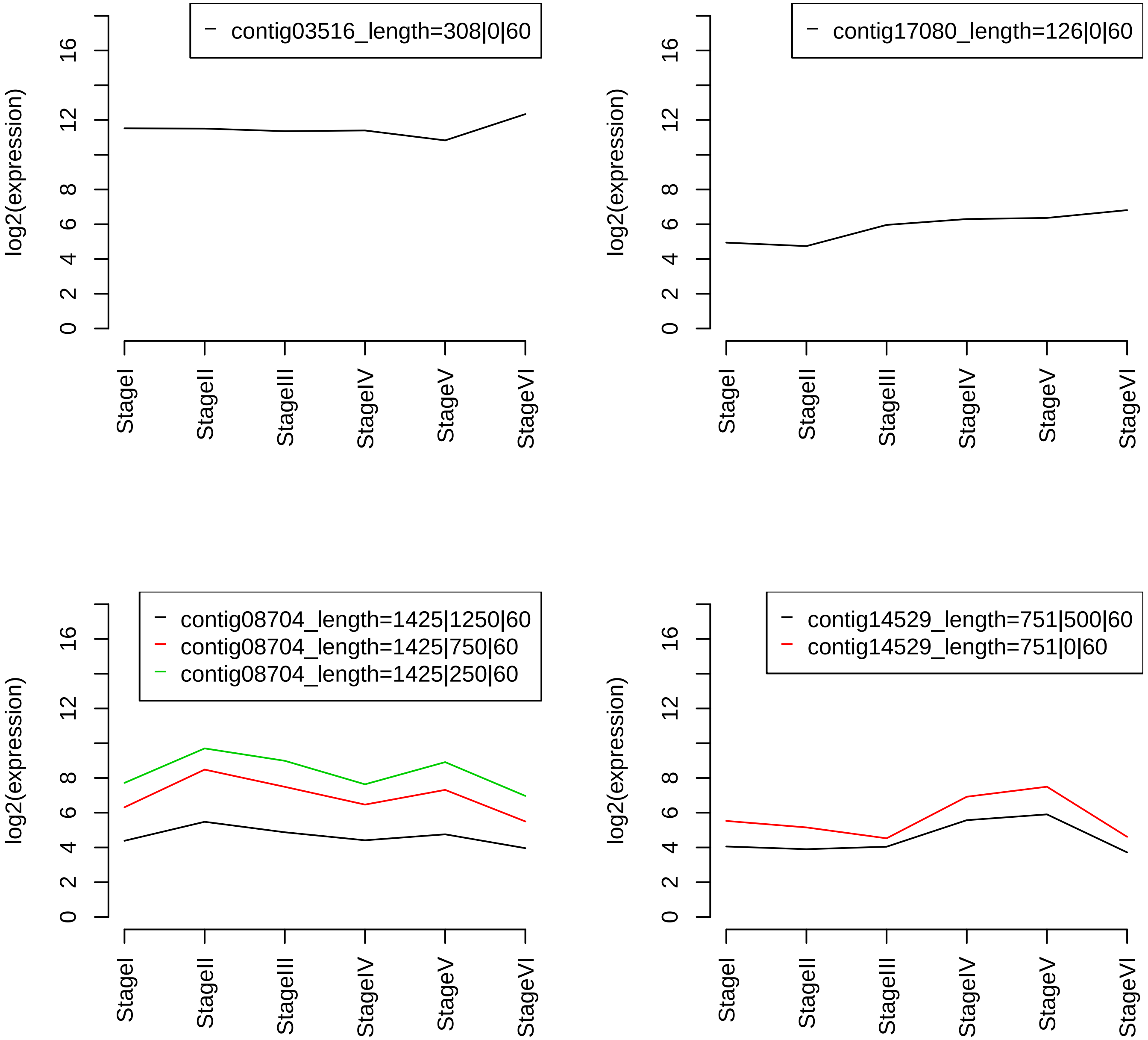
contig03516_length=308|0|60
contig17080_length=126|0|60
contig08704_length=1425|1250|60
contig08704_length=1425|750|60
contig08704_length=1425|250|60
contig14529_length=751|500|60
contig14529_length=751|0|60
log2(expression)
0
2
4
6
8
12
16
StageI
StageII
StageIII
StageIV
StageV
StageVI

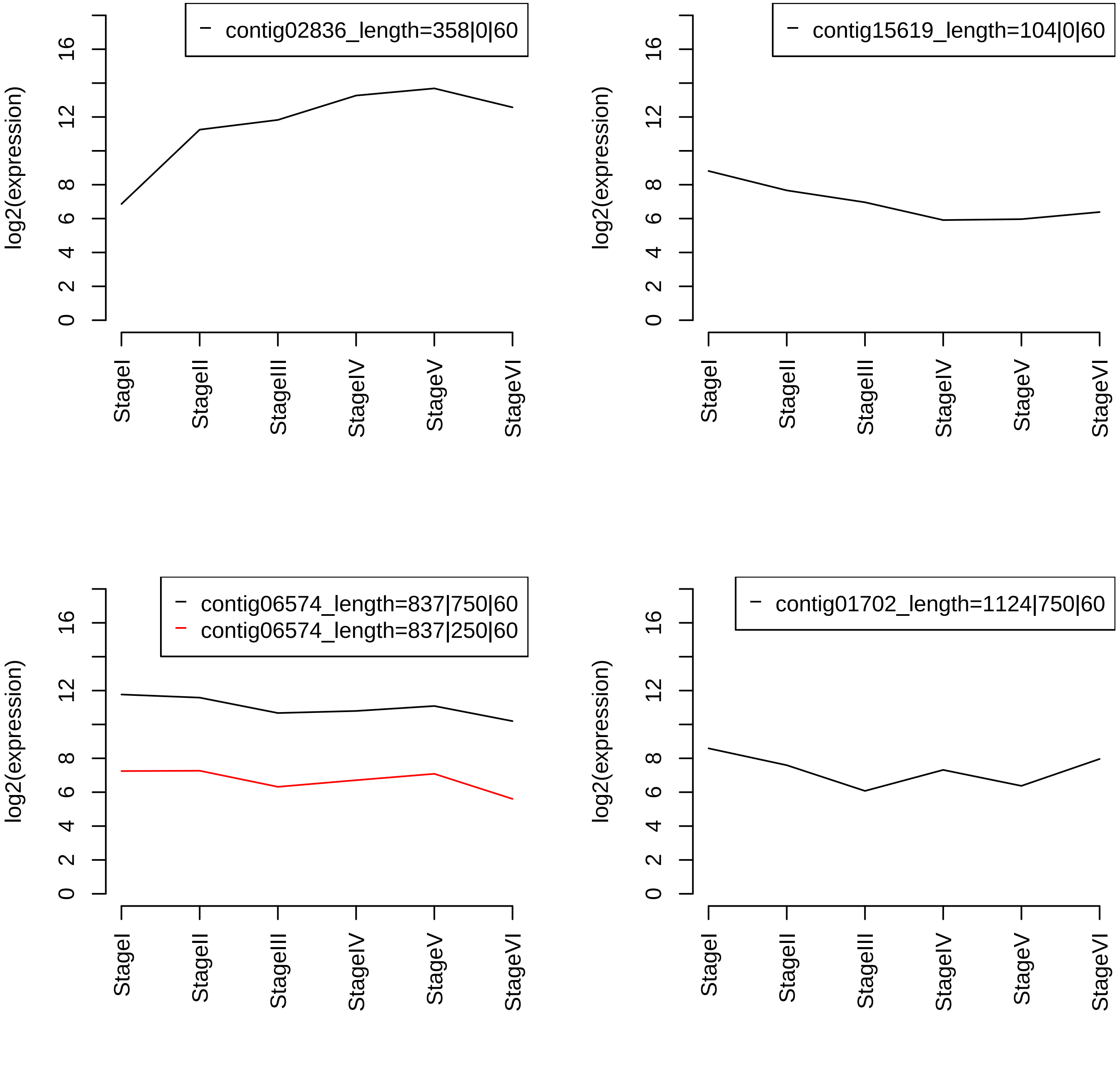
contig02836_length=358|0|60
contig15619_length=104|0|60
contig06574_length=837|750|60
contig06574_length=837|250|60
contig01702_length=1124|750|60
log2(expression)
0
2
4
6
8
12
16
StageI
StageII
StageIII
StageIV
StageV
StageVI

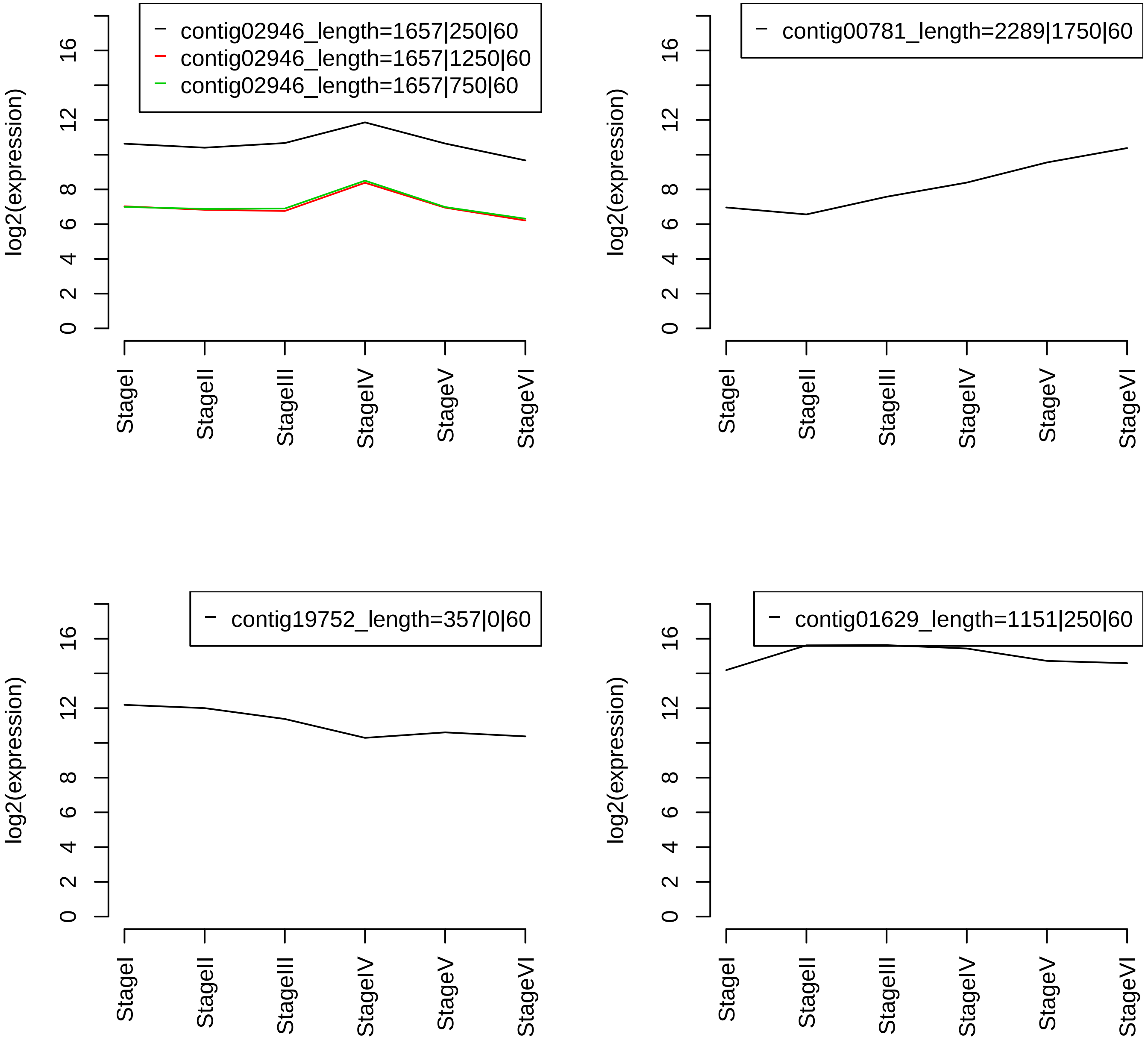

contig02946_length=1657|250|60
contig02946_length=1657|1250|60
contig02946_length=1657|750|60
contig00781_length=2289|1750|60
contig19752_length=357|0|60
contig01629_length=1151|250|60
log2(expression)
0
2
4
6
8
12
16
StageI
StageII
StageIII
StageIV
StageV
StageVI

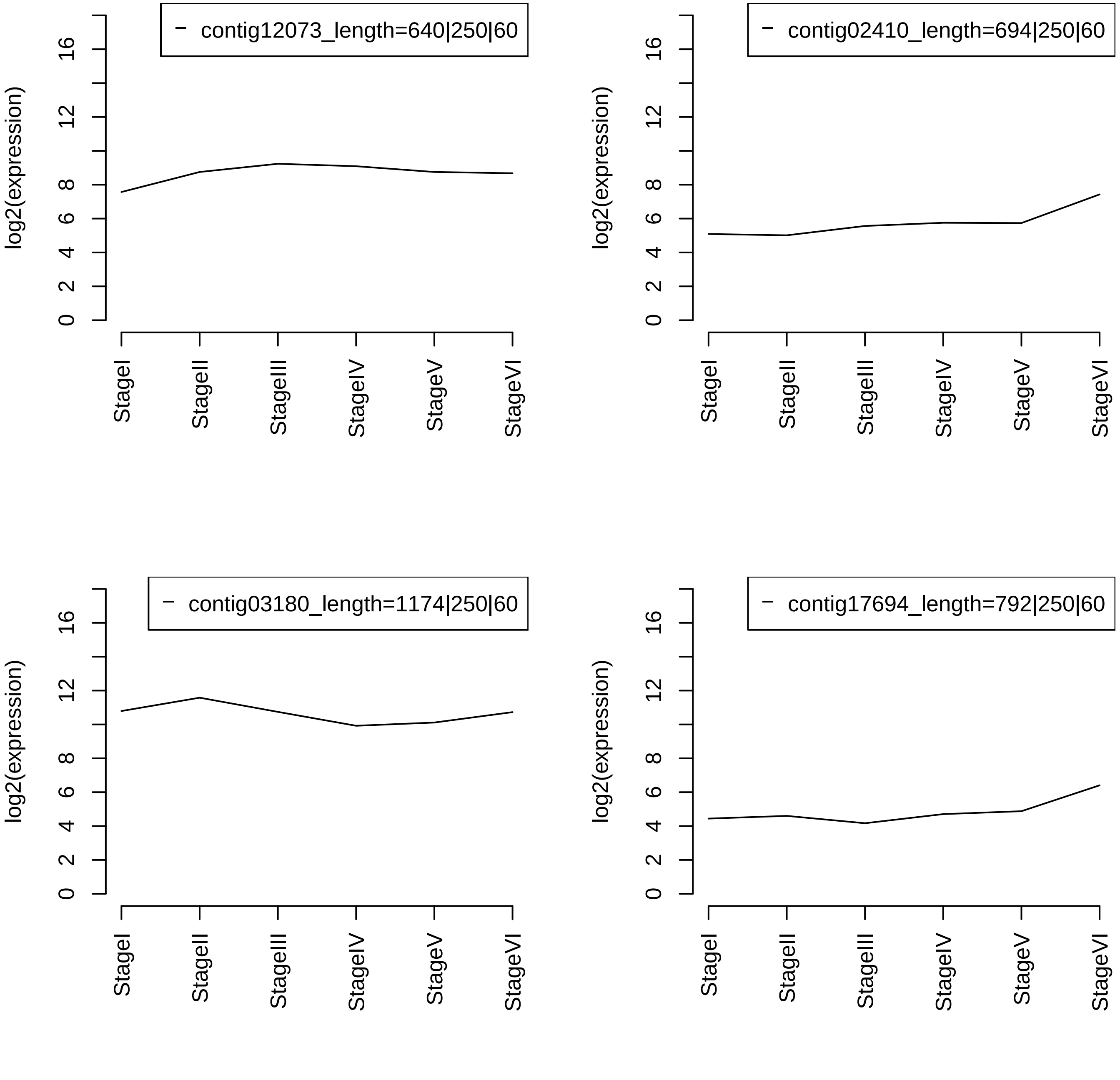
contig12073_length=640|250|60
contig02410_length=694|250|60
contig03180_length=1174|250|60
contig17694_length=792|250|60
log2(expression)
0
2
4
6
8
12
16
StageI
StageII
StageIII
StageIV
StageV
StageVI

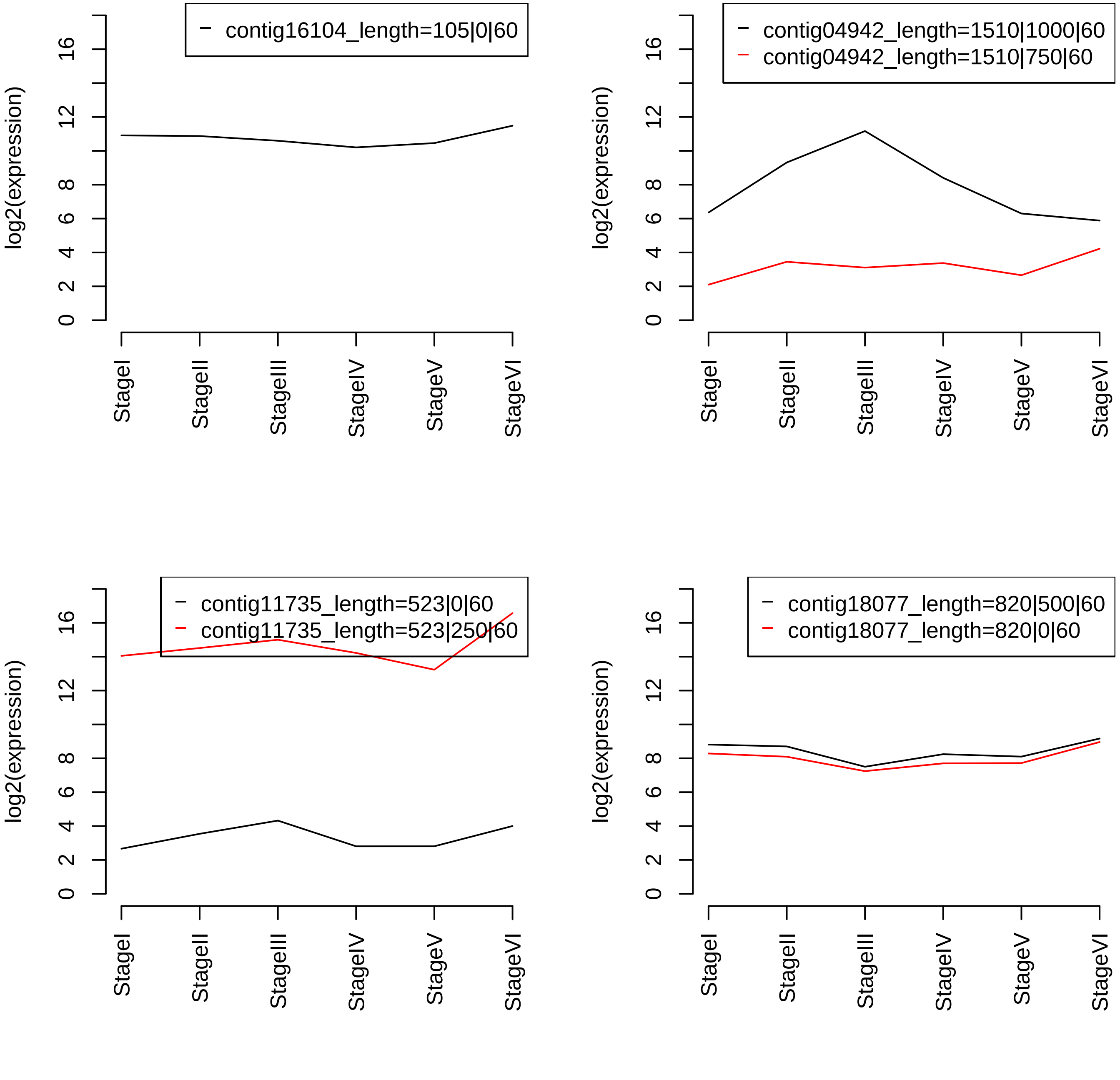
contig16104_length=105|0|60
log2(expression)
0
2
4
6
8
12
16
StageI
StageII
StageIII
StageIV
StageV
StageVI
contig04942_length=1510|1000|60
contig04942_length=1510|750|60
contig11735_length=523|0|60
contig11735_length=523|250|60
contig18077_length=820|500|60
contig18077_length=820|0|60

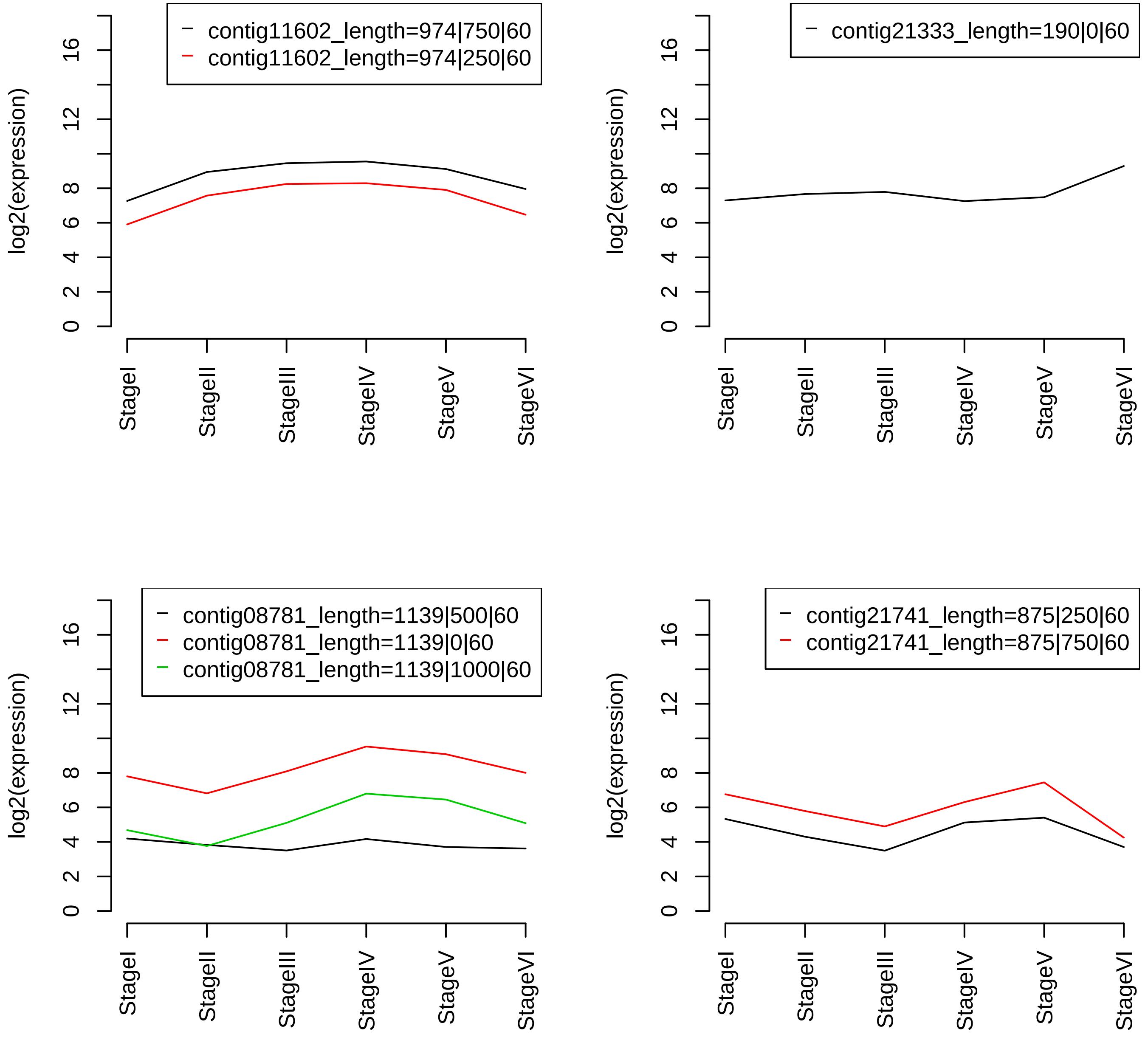
contig11602_length=974|750|60
contig11602_length=974|250|60
contig21333_length=190|0|60
contig08781_length=1139|500|60
contig08781_length=1139|0|60
contig08781_length=1139|1000|60
contig21741_length=875|250|60
contig21741_length=875|750|60
log2(expression)
0
2
4
6
8
12
16
StageI
StageII
StageIII
StageIV
StageV
StageVI

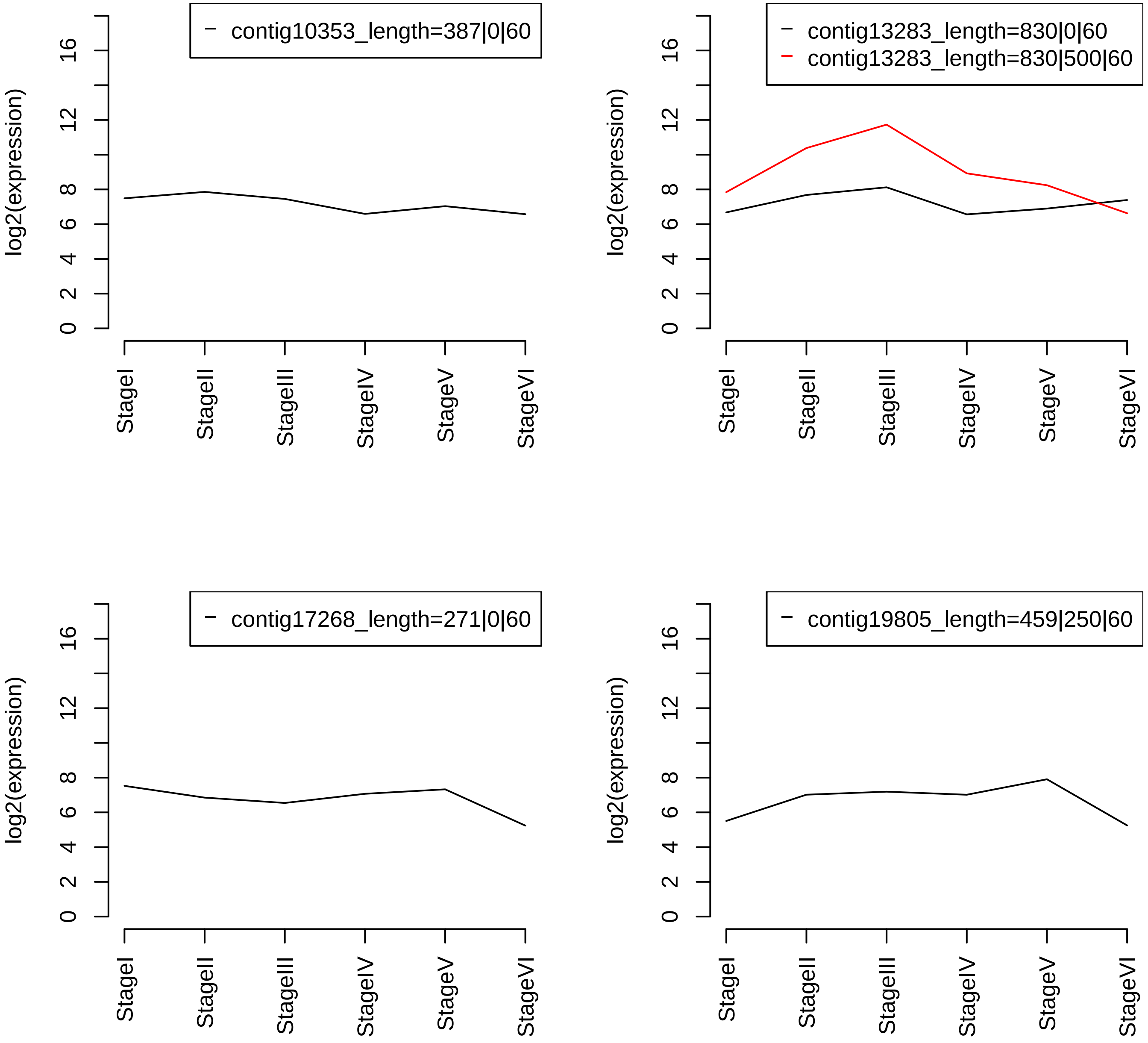
contig10353_length=387|0|60
contig13283_length=830|0|60
contig13283_length=830|500|60
contig17268_length=271|0|60
contig19805_length=459|250|60
log2(expression)
0
2
4
6
8
12
16
StageI
StageII
StageIII
StageIV
StageV
StageVI

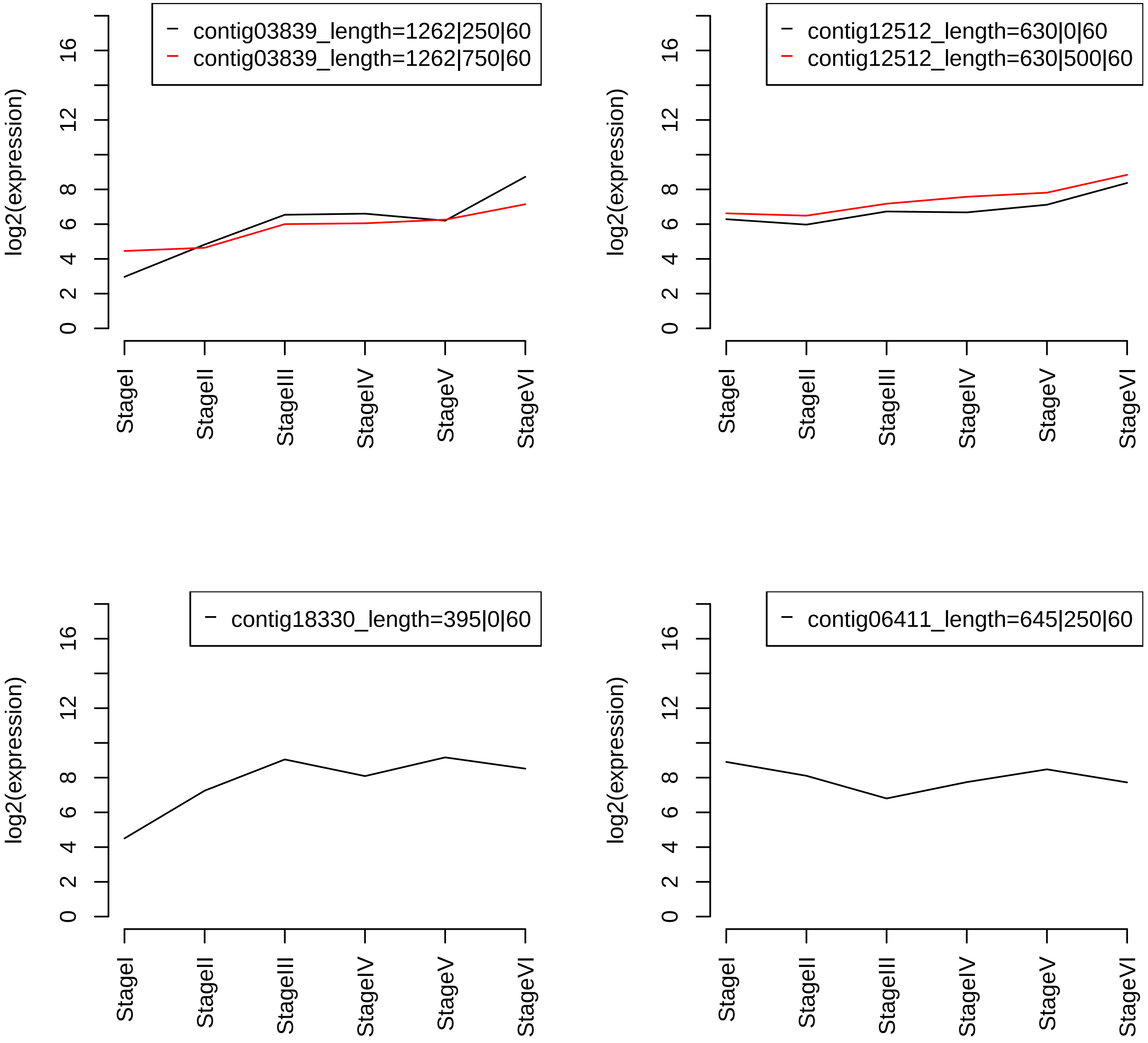
contig03839_length=1262|250|60
contig03839_length=1262|750|60
contig12512_length=630|0|60
contig12512_length=630|500|60
contig18330_length=395|0|60
contig06411_length=645|250|60
log2(expression)
0
2
4
6
8
12
16
StageI
StageII
StageIII
StageIV
StageV
StageVI

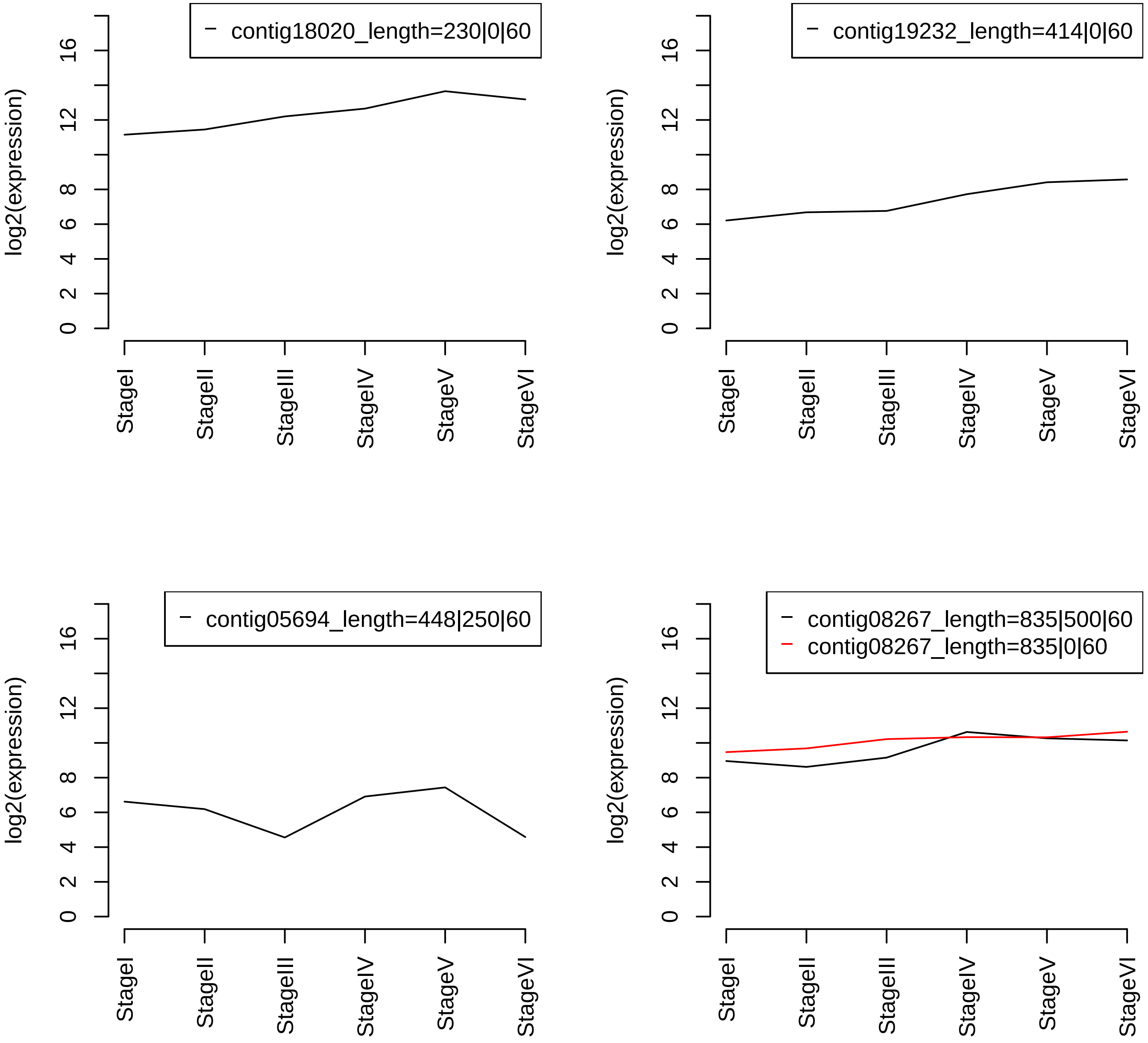
contig18020_length=230|0|60
contig19232_length=414|0|60
contig05694_length=448|250|60
contig08267_length=835|500|60
contig08267_length=835|0|60
log2(expression)
0
2
4
6
8
12
16
StageI
StageII
StageIII
StageIV
StageV
StageVI

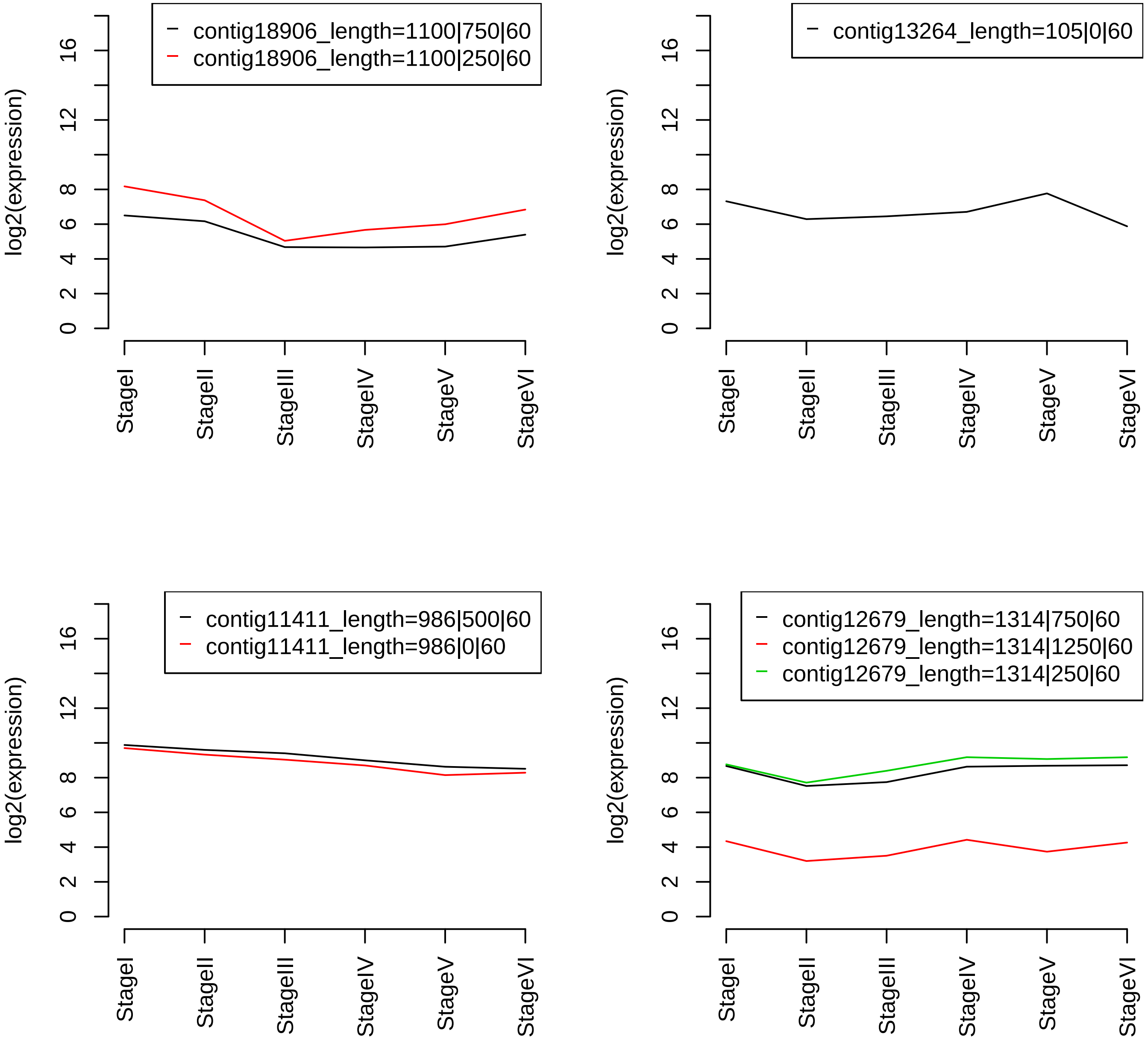

contig18906_length=1100|750|60
contig18906_length=1100|250|60
contig13264_length=105|0|60
contig11411_length=986|500|60
contig11411_length=986|0|60
contig12679_length=1314|750|60
contig12679_length=1314|1250|60
contig12679_length=1314|250|60
log2(expression)
0
2
4
6
8
12
16
StageI
StageII
StageIII
StageIV
StageV
StageVI

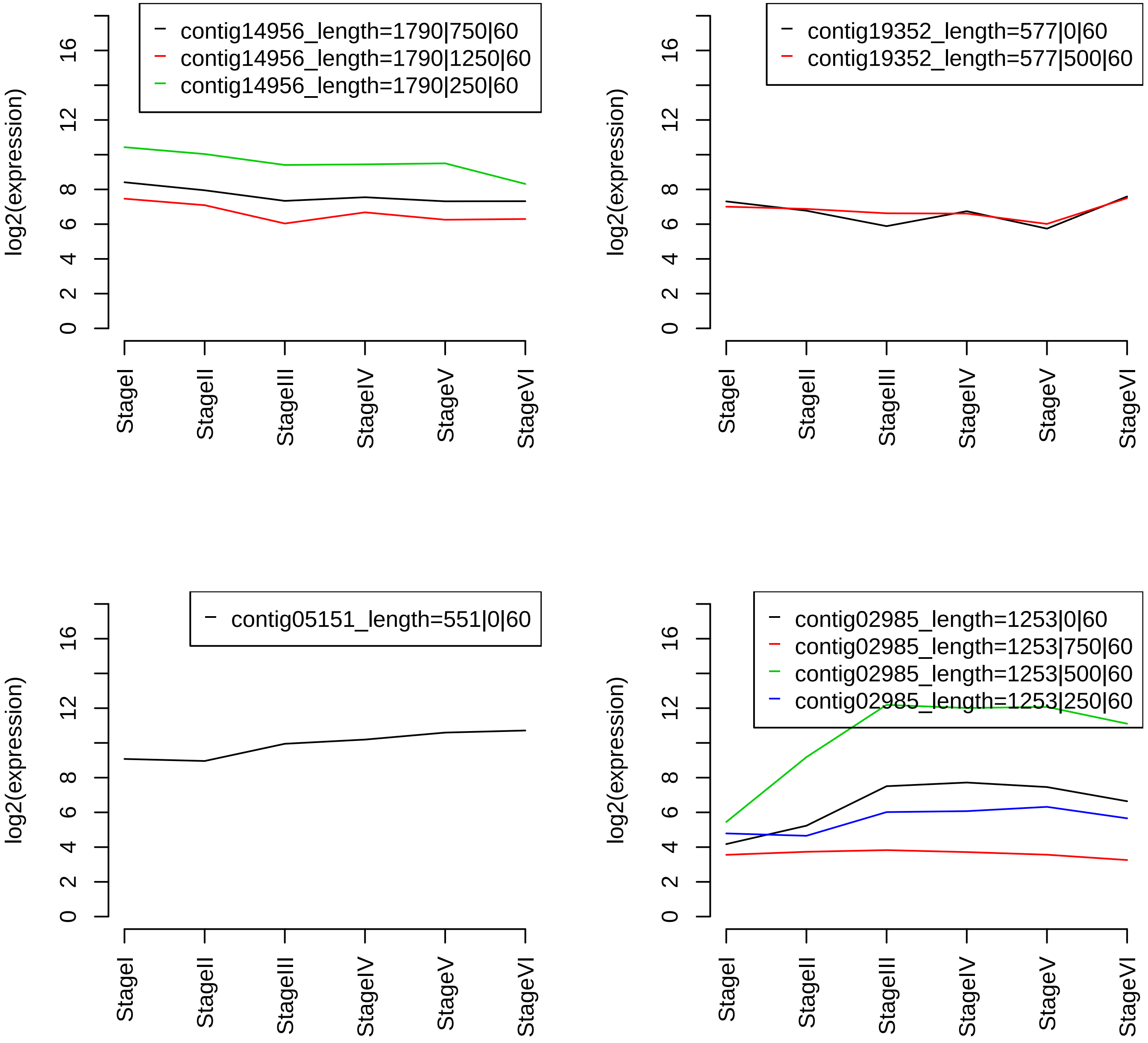
contig14956_length=1790|750|60
contig14956_length=1790|1250|60
contig14956_length=1790|250|60
contig19352_length=577|0|60
contig19352_length=577|500|60
contig05151_length=551|0|60
contig02985_length=1253|0|60
contig02985_length=1253|750|60
contig02985_length=1253|500|60
contig02985_length=1253|250|60
log2(expression)
0
2
4
6
8
12
16
StageI
StageII
StageIII
StageIV
StageV
StageVI

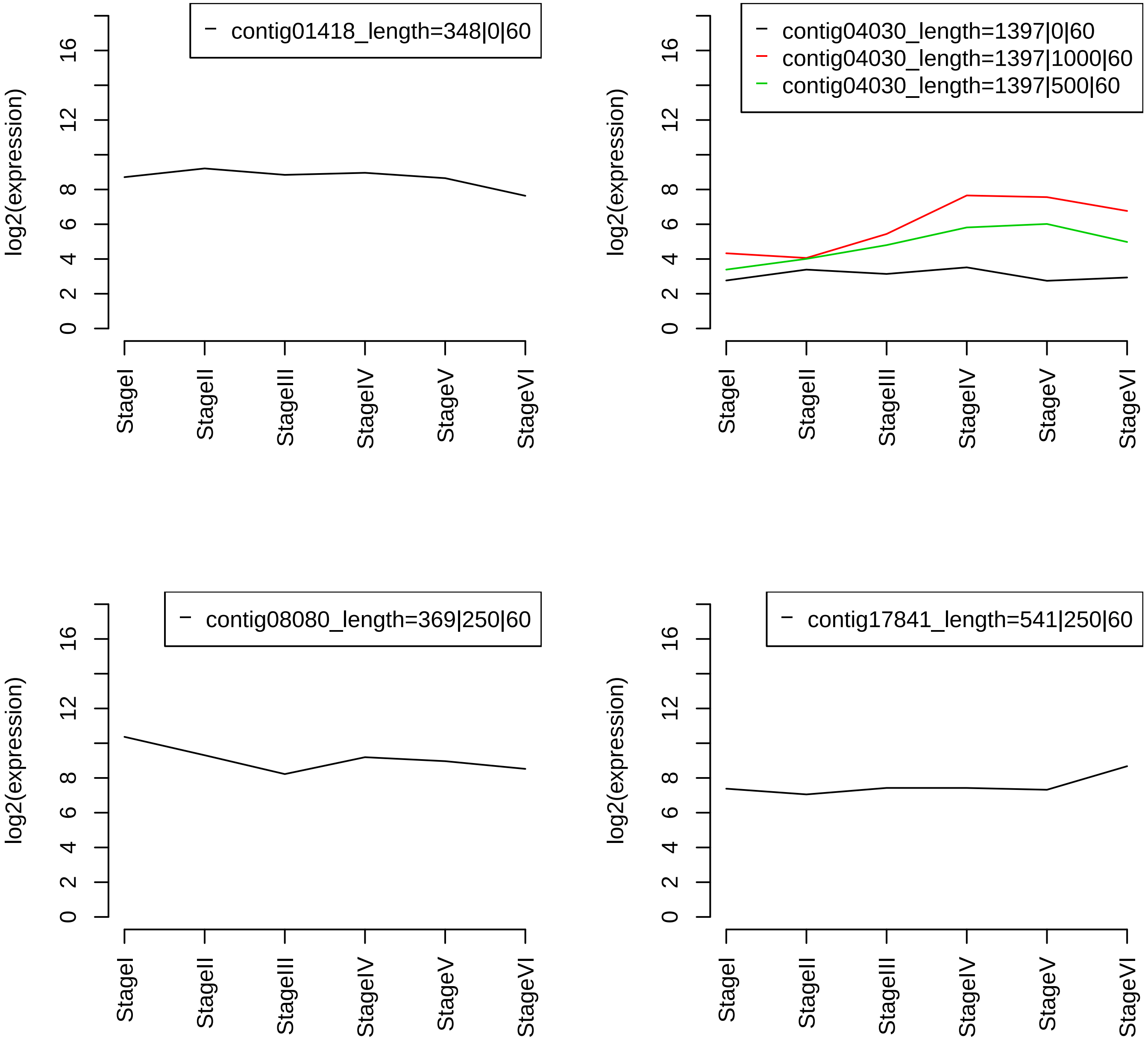
contig01418_length=348|0|60
contig04030_length=1397|0|60
contig04030_length=1397|1000|60
contig04030_length=1397|500|60
contig08080_length=369|250|60
contig17841_length=541|250|60
log2(expression)
0
2
4
6
8
12
16
StageI
StageII
StageIII
StageIV
StageV
StageVI

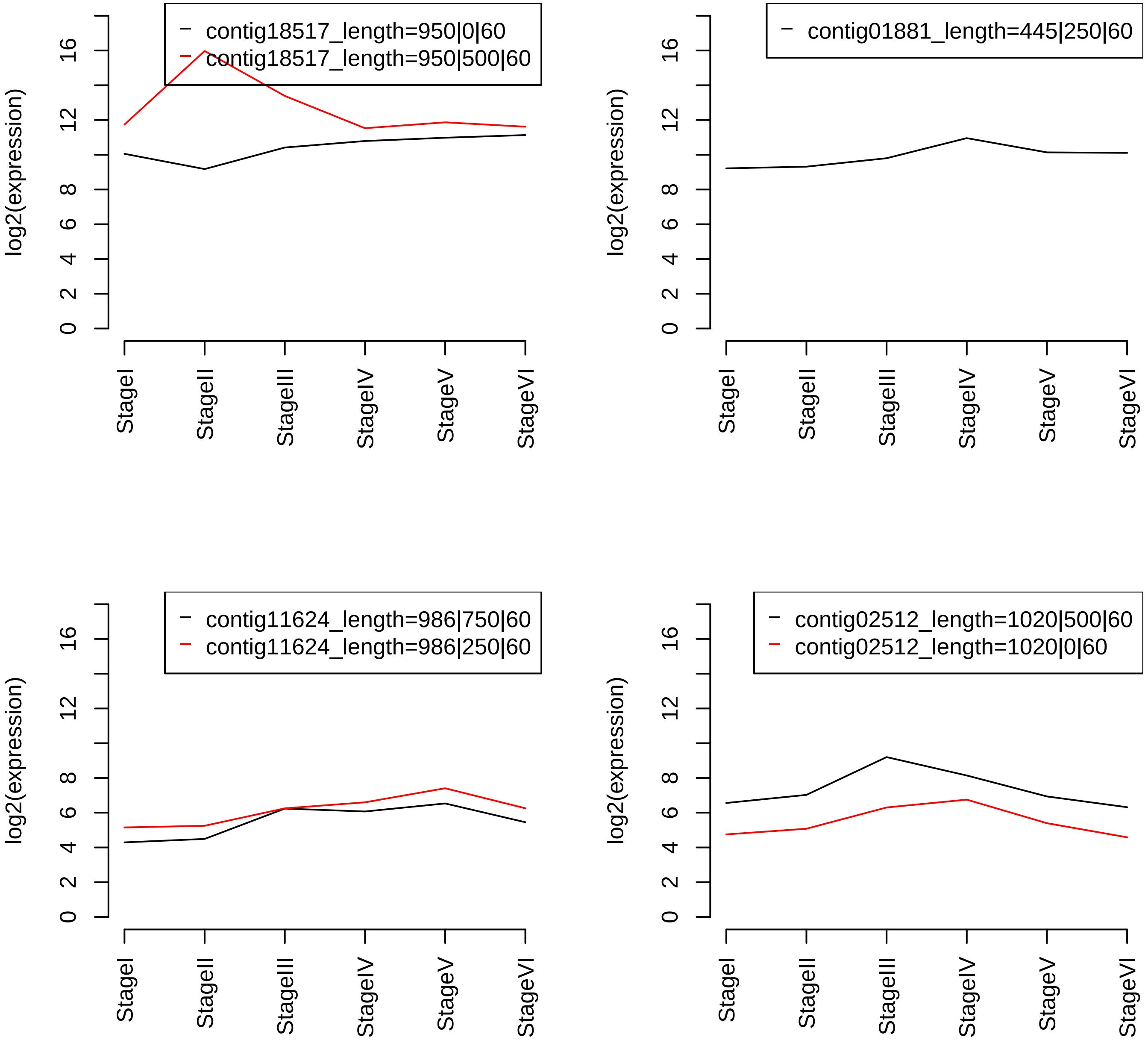
contig18517_length=950|0|60
contig18517_length=950|500|60
contig01881_length=445|250|60
contig11624_length=986|750|60
contig11624_length=986|250|60
contig02512_length=1020|500|60
contig02512_length=1020|0|60
log2(expression)
0
2
4
6
8
12
16
StageI
StageII
StageIII
StageIV
StageV
StageVI

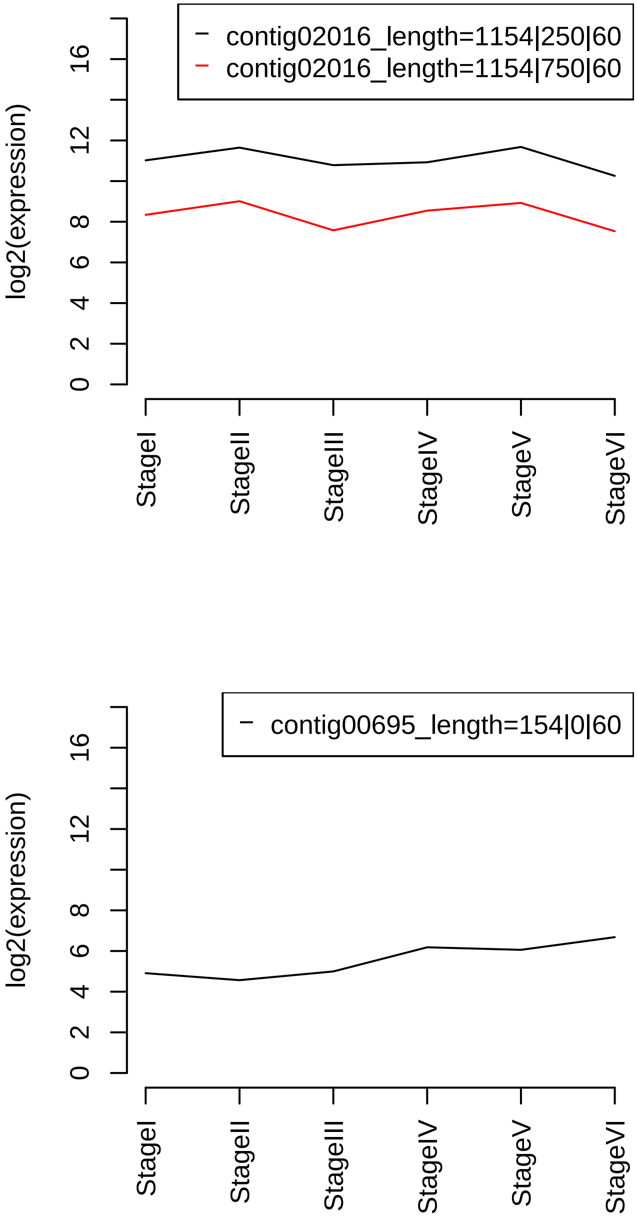
contig02016_length=1154|250|60
contig02016_length=1154|750|60
contig14920_length=565|250|60
contig00695_length=154|0|60
contig06769_length=1154|750|60
contig06769_length=1154|250|60
log2(expression)
0
2
4
6
8
12
16
StageI
StageII
StageIII
StageIV
StageV
StageVI

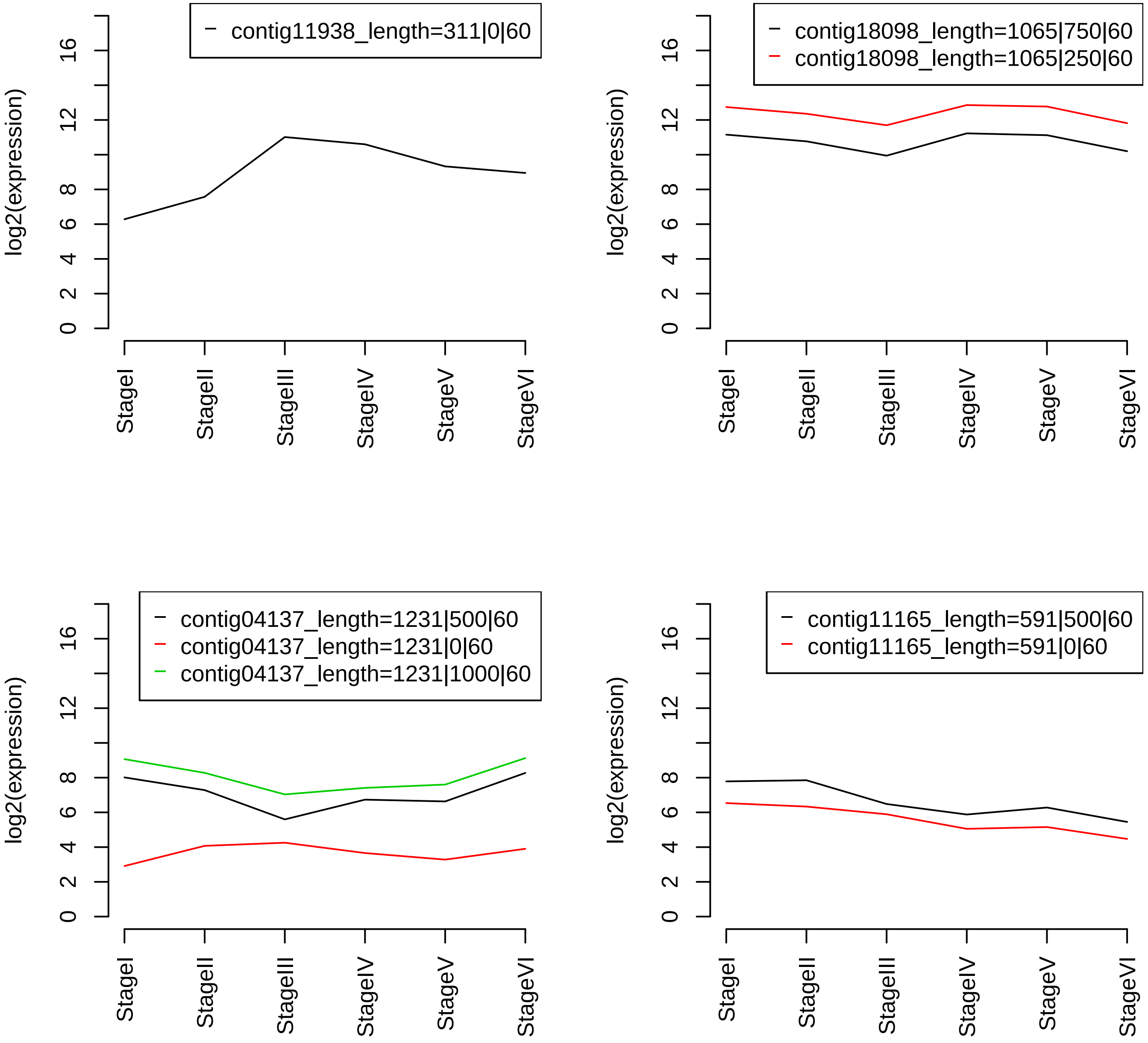

contig11938_length=311|0|60
contig18098_length=1065|750|60
contig18098_length=1065|250|60
contig04137_length=1231|500|60
contig04137_length=1231|0|60
contig04137_length=1231|1000|60
contig11165_length=591|500|60
contig11165_length=591|0|60
log2(expression)
0
2
4
6
8
12
16
StageI
StageII
StageIII
StageIV
StageV
StageVI

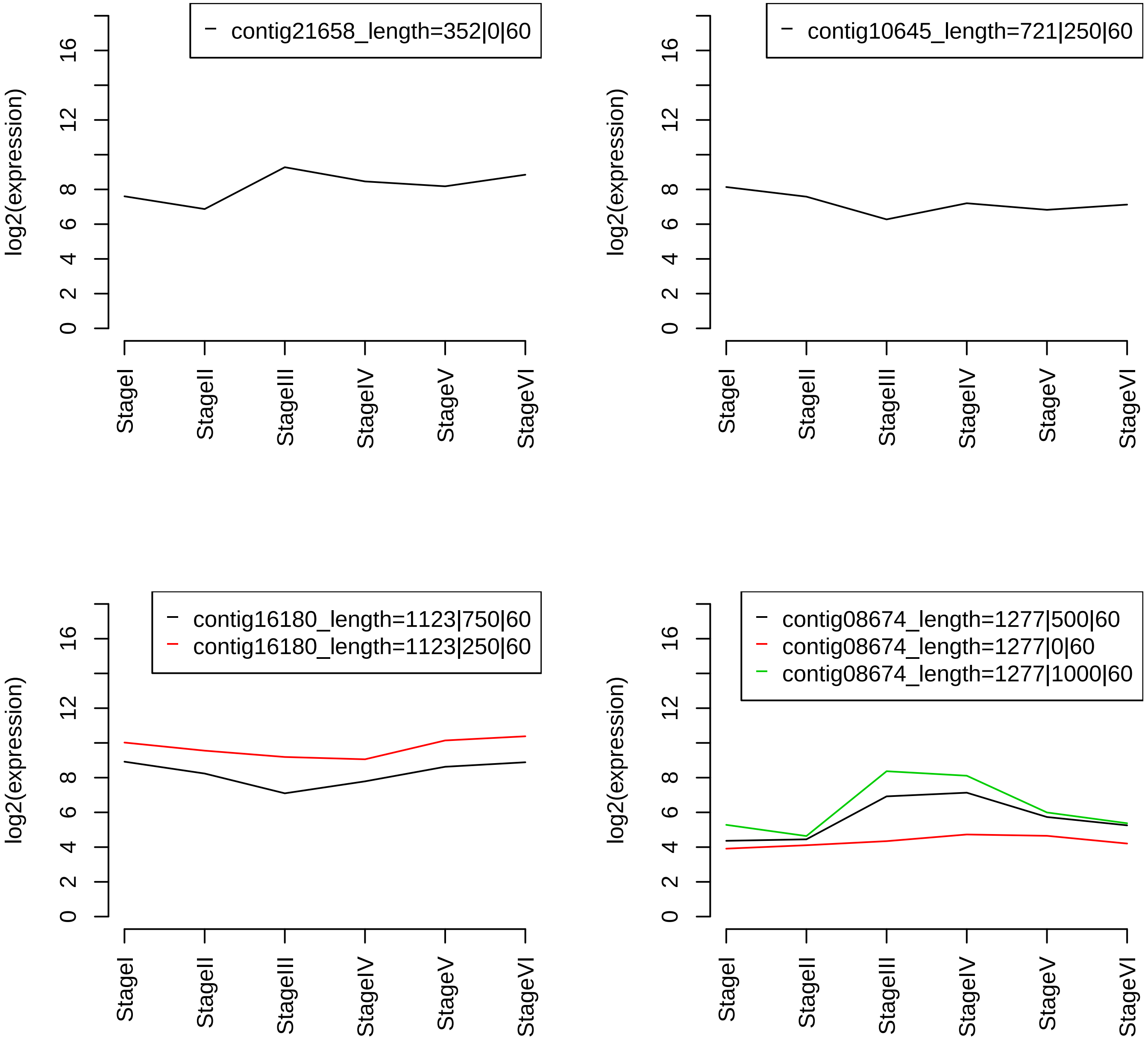

contig21658_length=352|0|60
contig10645_length=721|250|60
contig16180_length=1123|750|60
contig16180_length=1123|250|60
contig08674_length=1277|500|60
contig08674_length=1277|0|60
contig08674_length=1277|1000|60
log2(expression)
0
2
4
6
8
12
16
StageI
StageII
StageIII
StageIV
StageV
StageVI

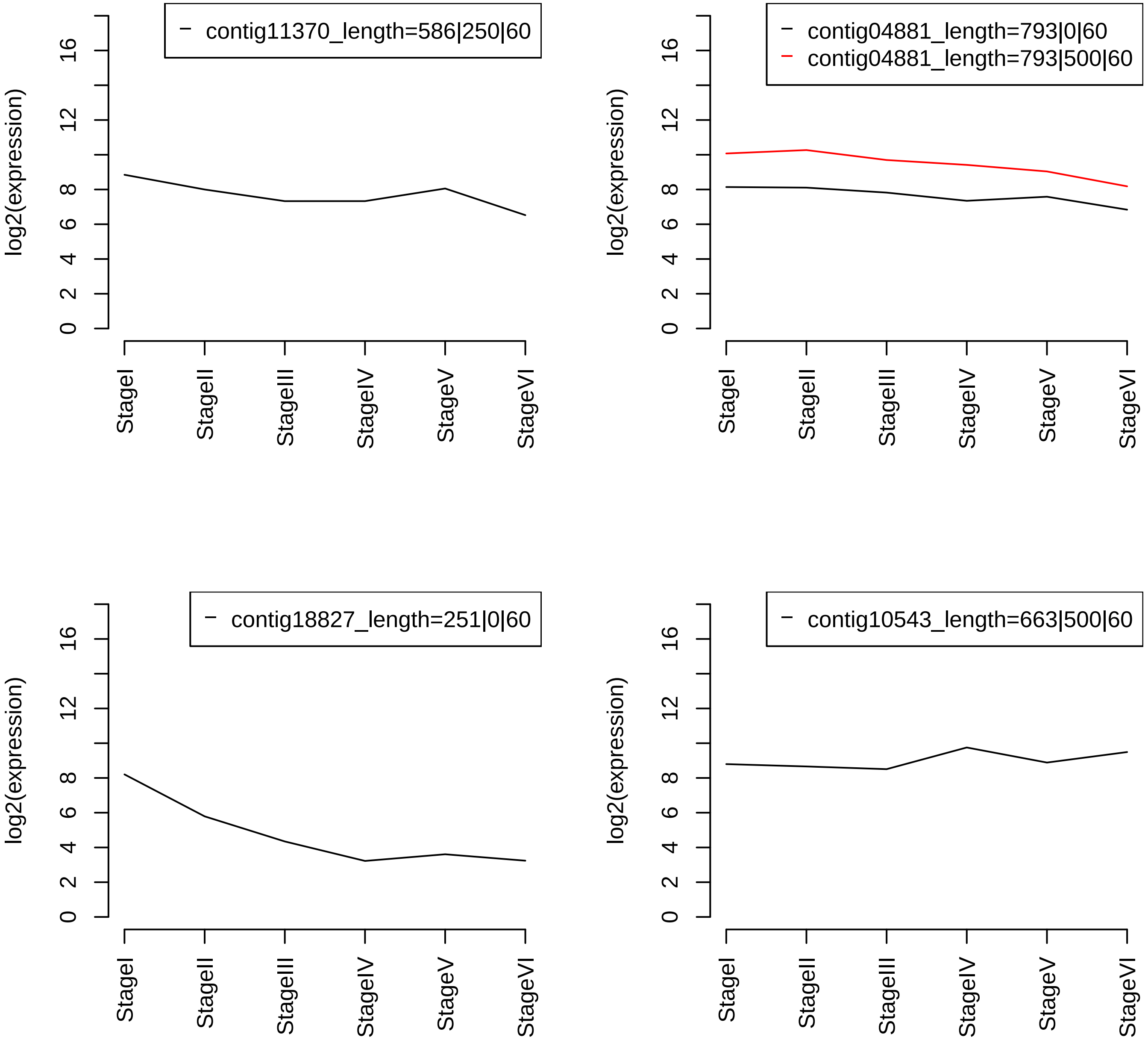
contig11370_length=586|250|60
contig04881_length=793|0|60
contig04881_length=793|500|60
contig18827_length=251|0|60
contig10543_length=663|500|60
log2(expression)
0
2
4
6
8
12
16
StageI
StageII
StageIII
StageIV
StageV
StageVI

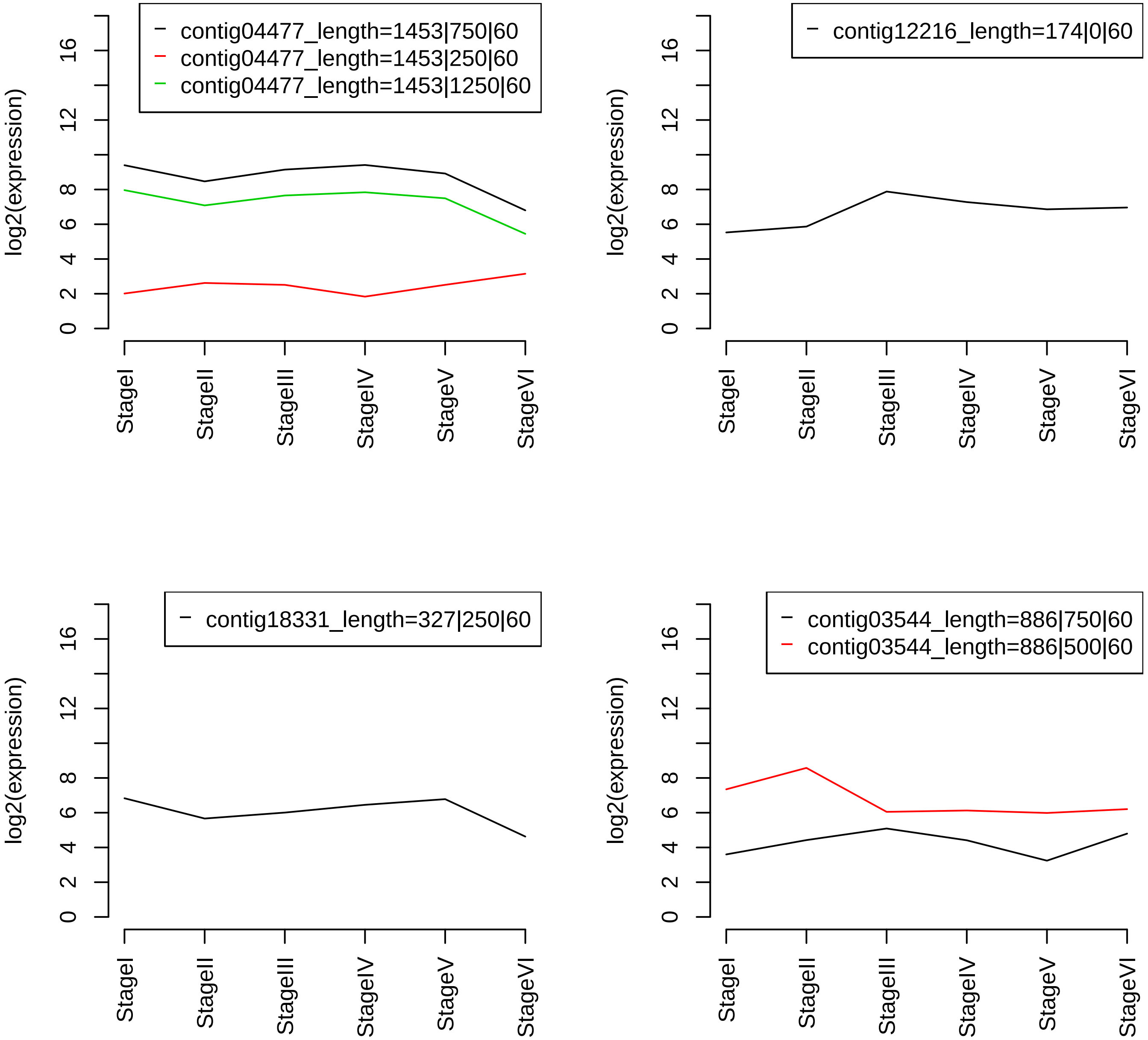

contig04477_length=1453|750|60
contig04477_length=1453|250|60
contig04477_length=1453|1250|60
contig12216_length=174|0|60
contig18331_length=327|250|60
contig03544_length=886|750|60
contig03544_length=886|500|60
log2(expression)
0
2
4
6
8
12
16
StageI
StageII
StageIII
StageIV
StageV
StageVI

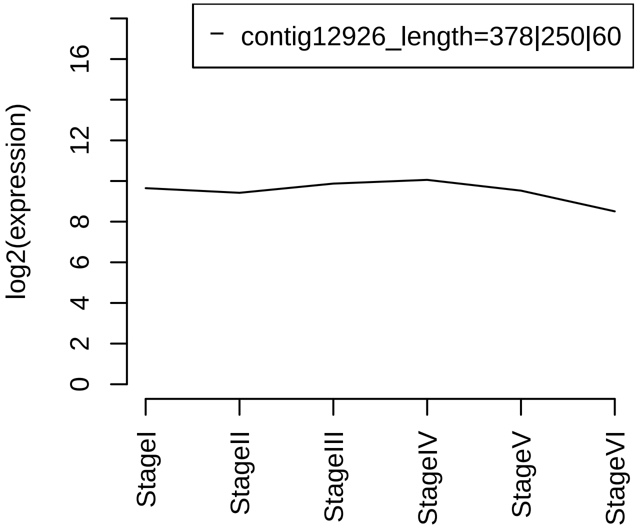
contig12926_length=378|250|60
log2(expression)
0
2
4
6
8
12
16
StageI
StageII
StageIII
StageIV
StageV
StageVI

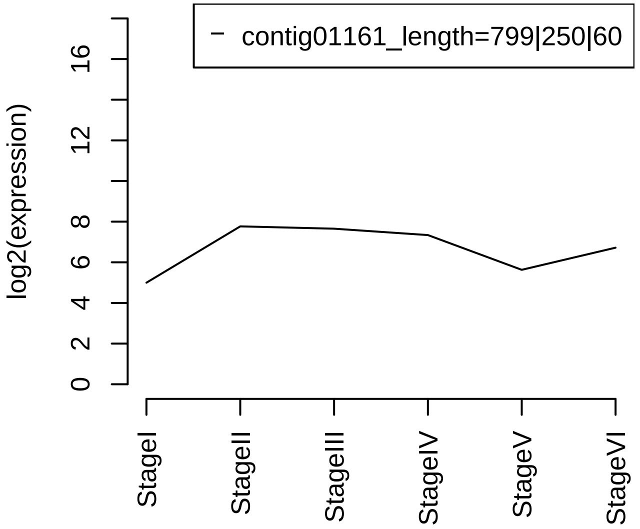
contig01161_length=799|250|60
log2(expression)
0
2
4
6
8
12
16
StageI
StageII
StageIII
StageIV
StageV
StageVI

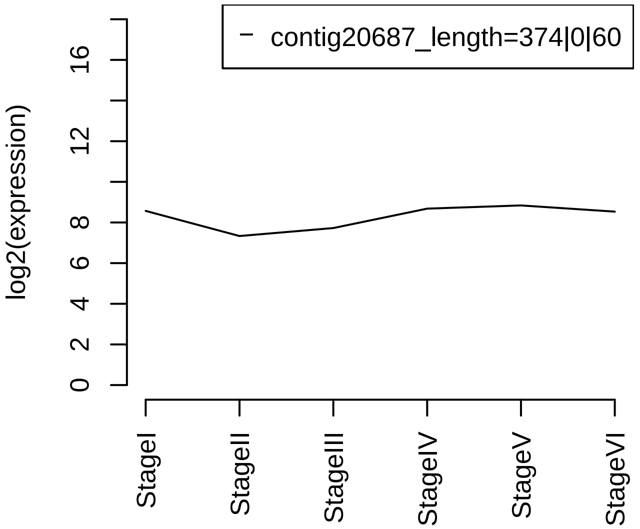
contig20687_length=374|0|60
log2(expression)
0
2
4
6
8
12
16
StageI
StageII
StageIII
StageIV
StageV
StageVI

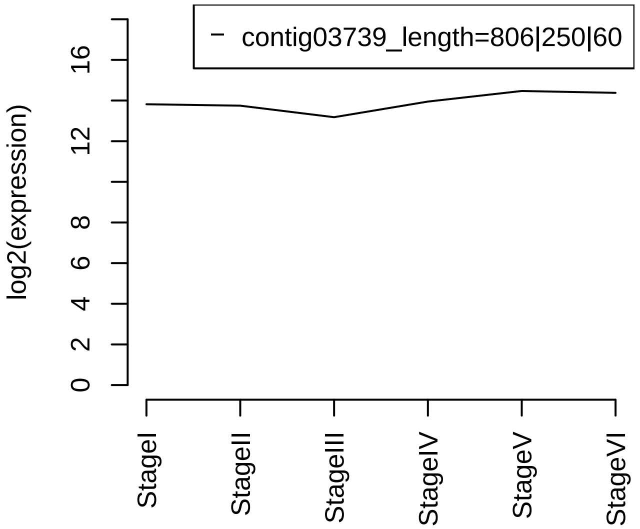
contig03739_length=806|250|60
log2(expression)
0
2
4
6
8
12
16
StageI
StageII
StageIII
StageIV
StageV
StageVI

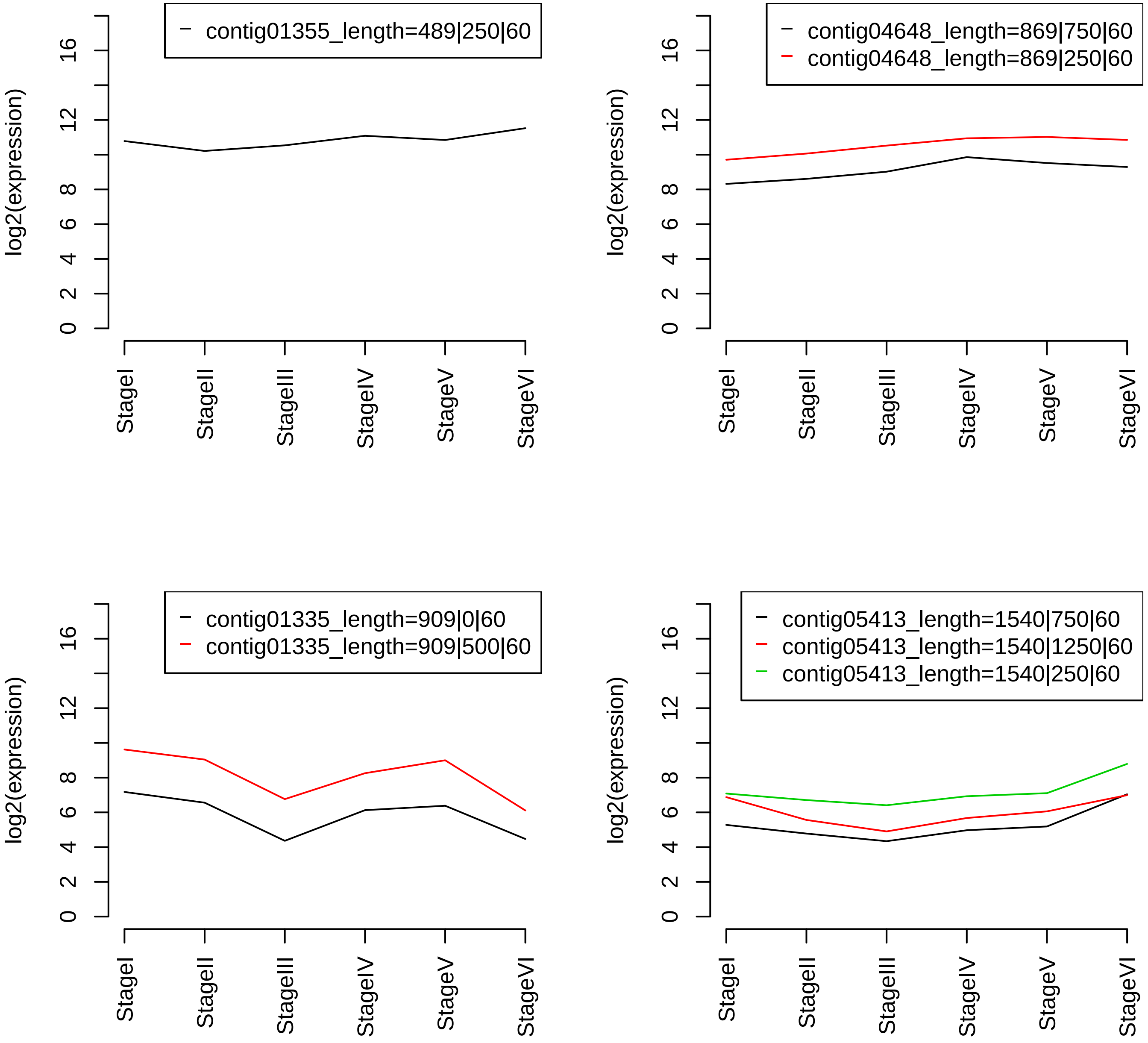

contig01355_length=489|250|60
contig04648_length=869|750|60
contig04648_length=869|250|60
contig01335_length=909|0|60
contig01335_length=909|500|60
contig05413_length=1540|750|60
contig05413_length=1540|1250|60
contig05413_length=1540|250|60
log2(expression)
0
2
4
6
8
12
16
StageI
StageII
StageIII
StageIV
StageV
StageVI

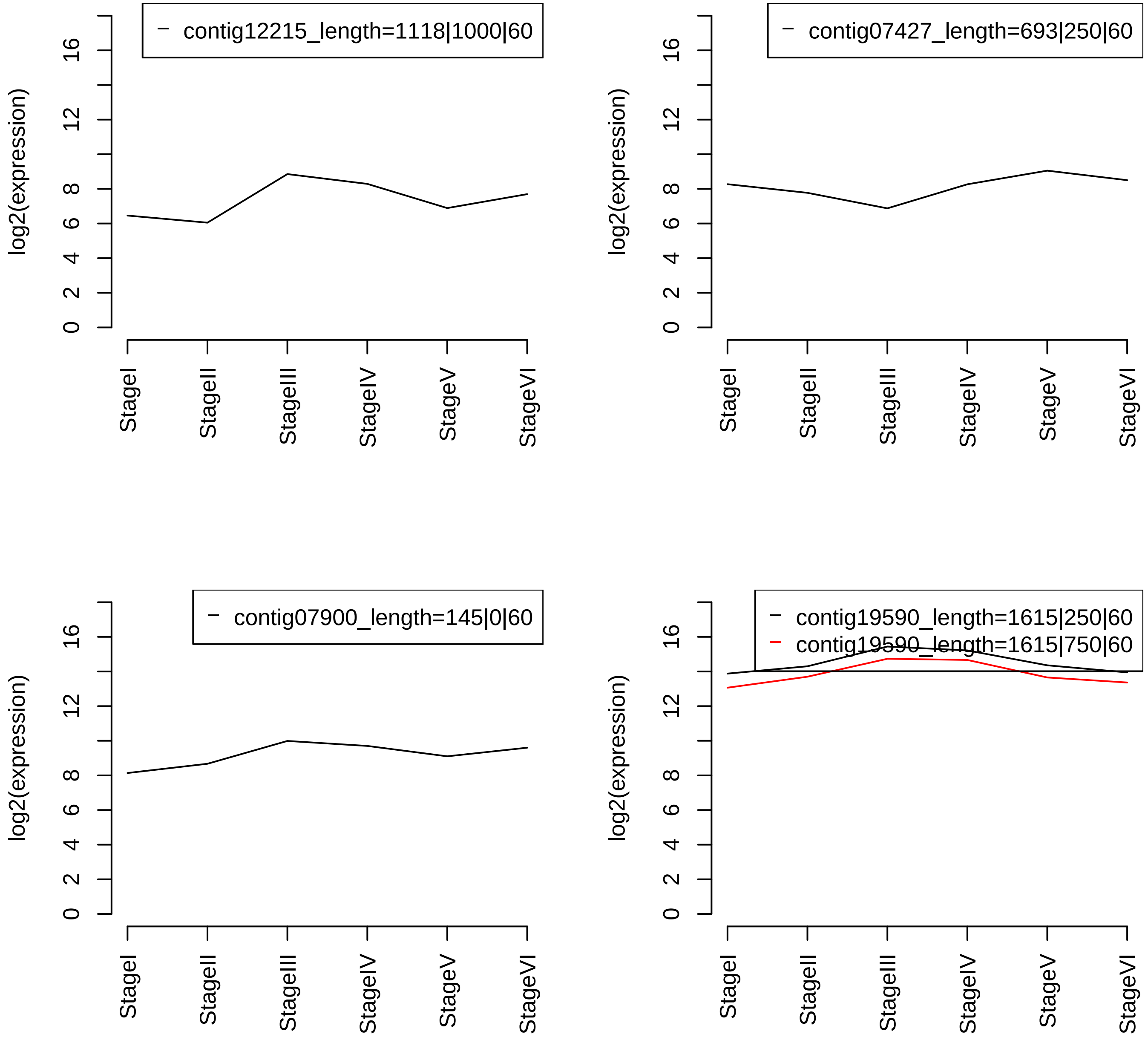
contig12215_length=1118|1000|60
contig07427_length=693|250|60
contig07900_length=145|0|60
contig19590_length=1615|250|60
contig19590_length=1615|750|60
log2(expression)
0
2
4
6
8
12
16
StageI
StageII
StageIII
StageIV
StageV
StageVI

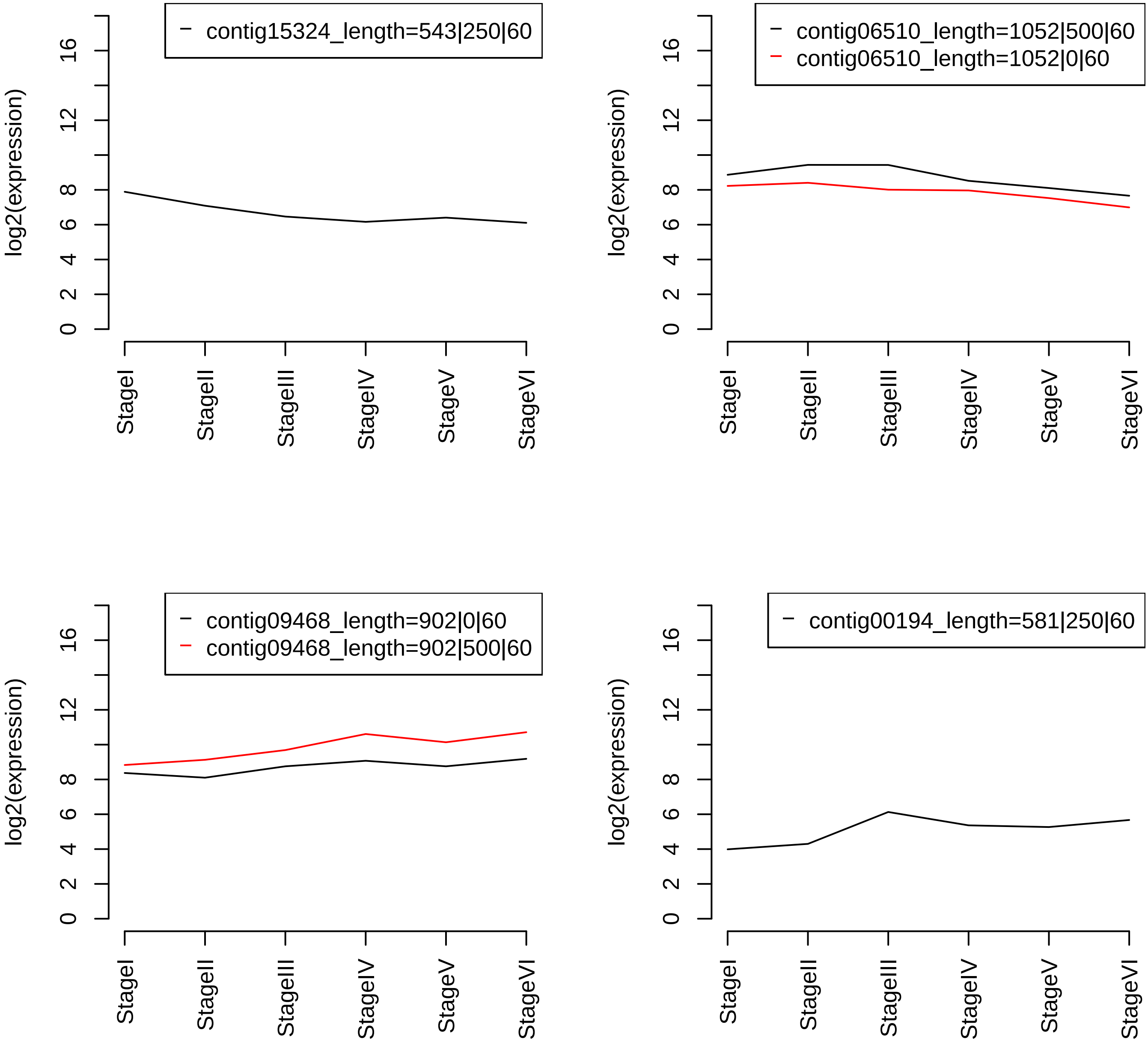
contig15324_length=543|250|60
contig06510_length=1052|500|60
contig06510_length=1052|0|60
contig09468_length=902|0|60
contig09468_length=902|500|60
contig00194_length=581|250|60
log2(expression)
0
2
4
6
8
12
16
StageI
StageII
StageIII
StageIV
StageV
StageVI

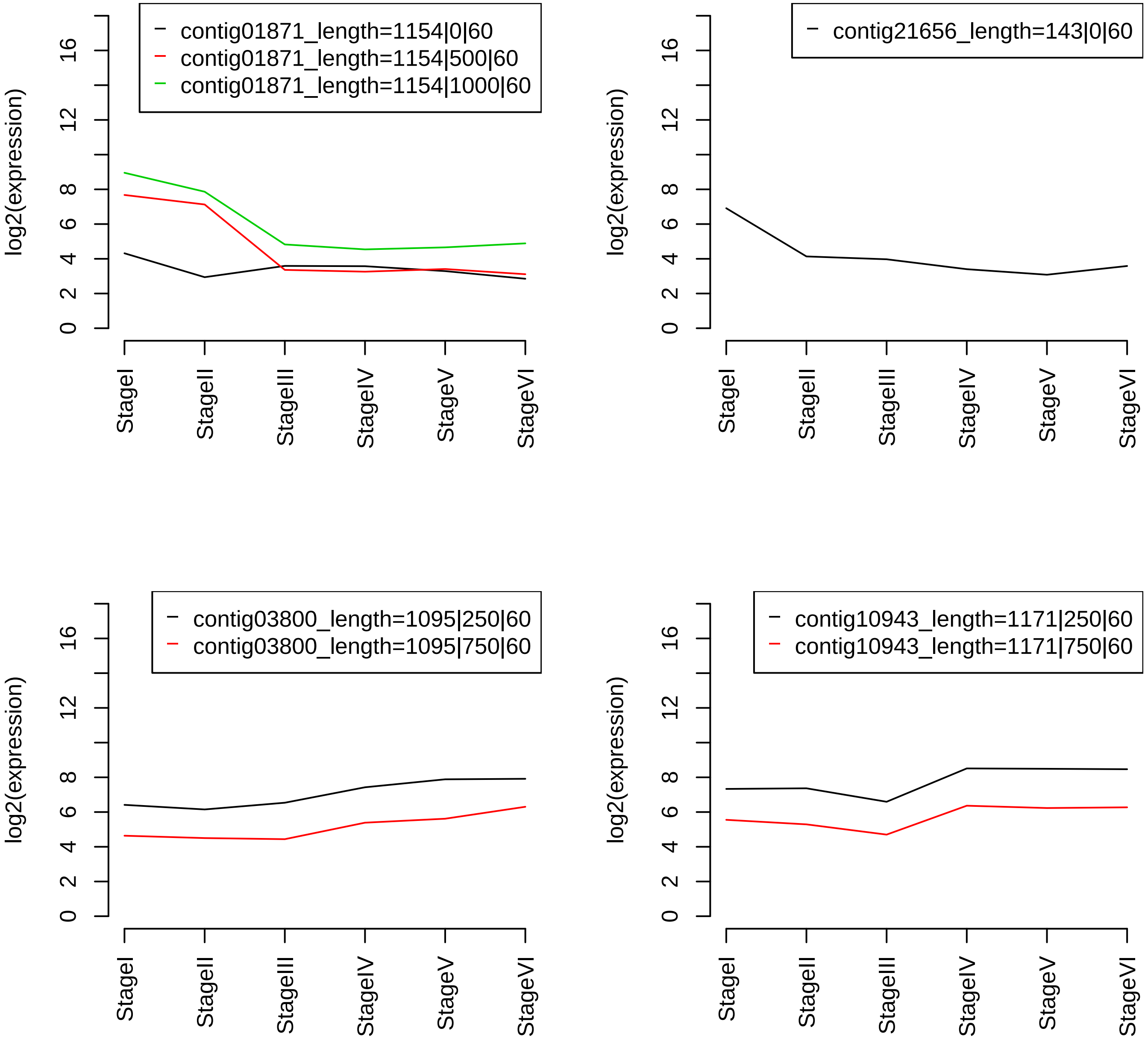

contig01871_length=1154|0|60
contig01871_length=1154|500|60
contig01871_length=1154|1000|60
contig21656_length=143|0|60
contig03800_length=1095|250|60
contig03800_length=1095|750|60
contig10943_length=1171|250|60
contig10943_length=1171|750|60
log2(expression)
0
2
4
6
8
12
16
StageI
StageII
StageIII
StageIV
StageV
StageVI

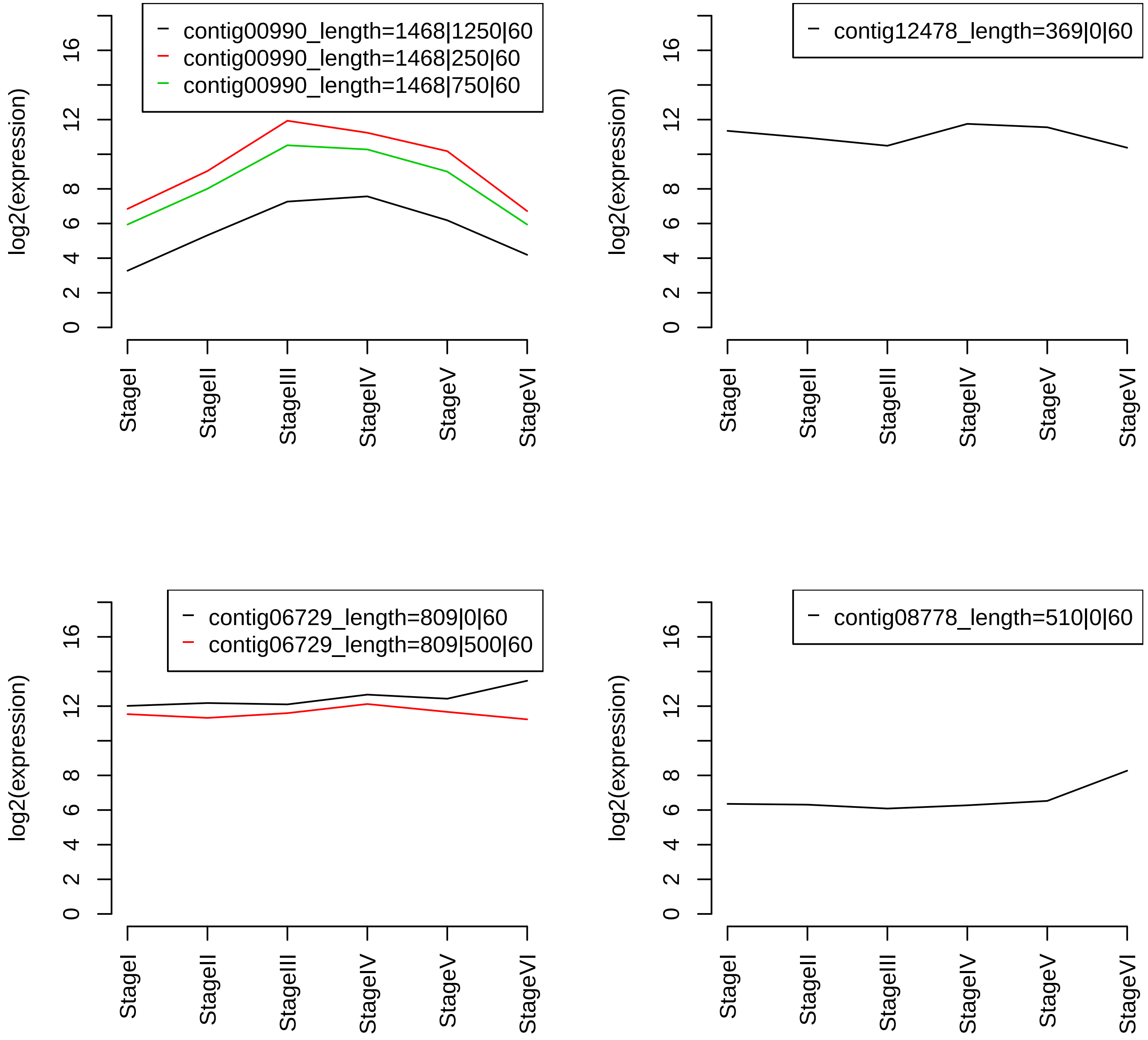

contig00990_length=1468|1250|60
contig00990_length=1468|250|60
contig00990_length=1468|750|60
contig12478_length=369|0|60
contig06729_length=809|0|60
contig06729_length=809|500|60
contig08778_length=510|0|60
log2(expression)
0
2
4
6
8
12
16
StageI
StageII
StageIII
StageIV
StageV
StageVI

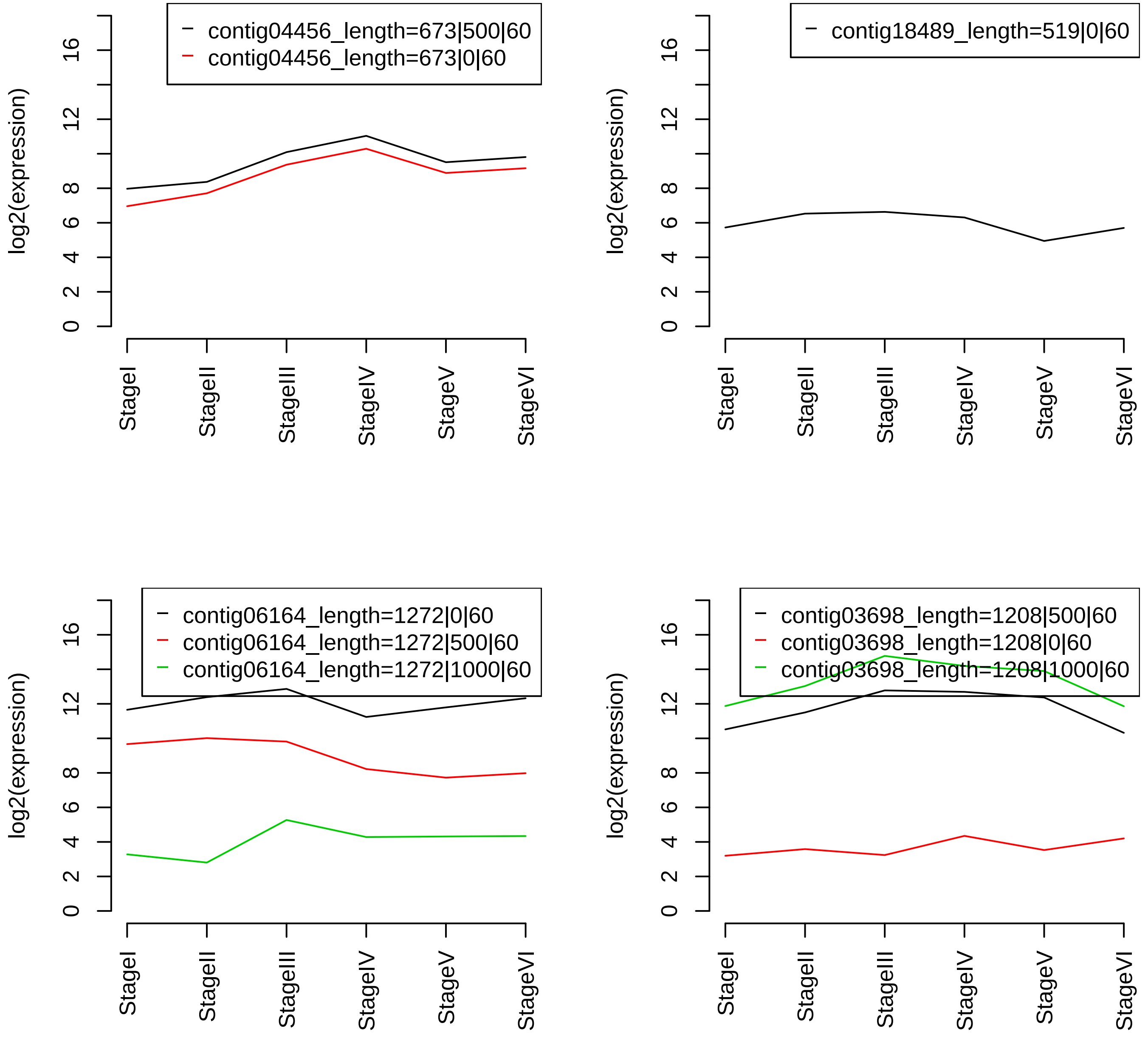
contig04456_length=673|500|60
contig04456_length=673|0|60
log2(expression)
0
2
4
6
8
12
16
StageI
StageII
StageIII
StageIV
StageV
StageVI
contig18489_length=519|0|60
contig06164_length=1272|0|60
contig06164_length=1272|500|60
contig06164_length=1272|1000|60
contig03698_length=1208|500|60
contig03698_length=1208|0|60
contig03698_length=1208|1000|60

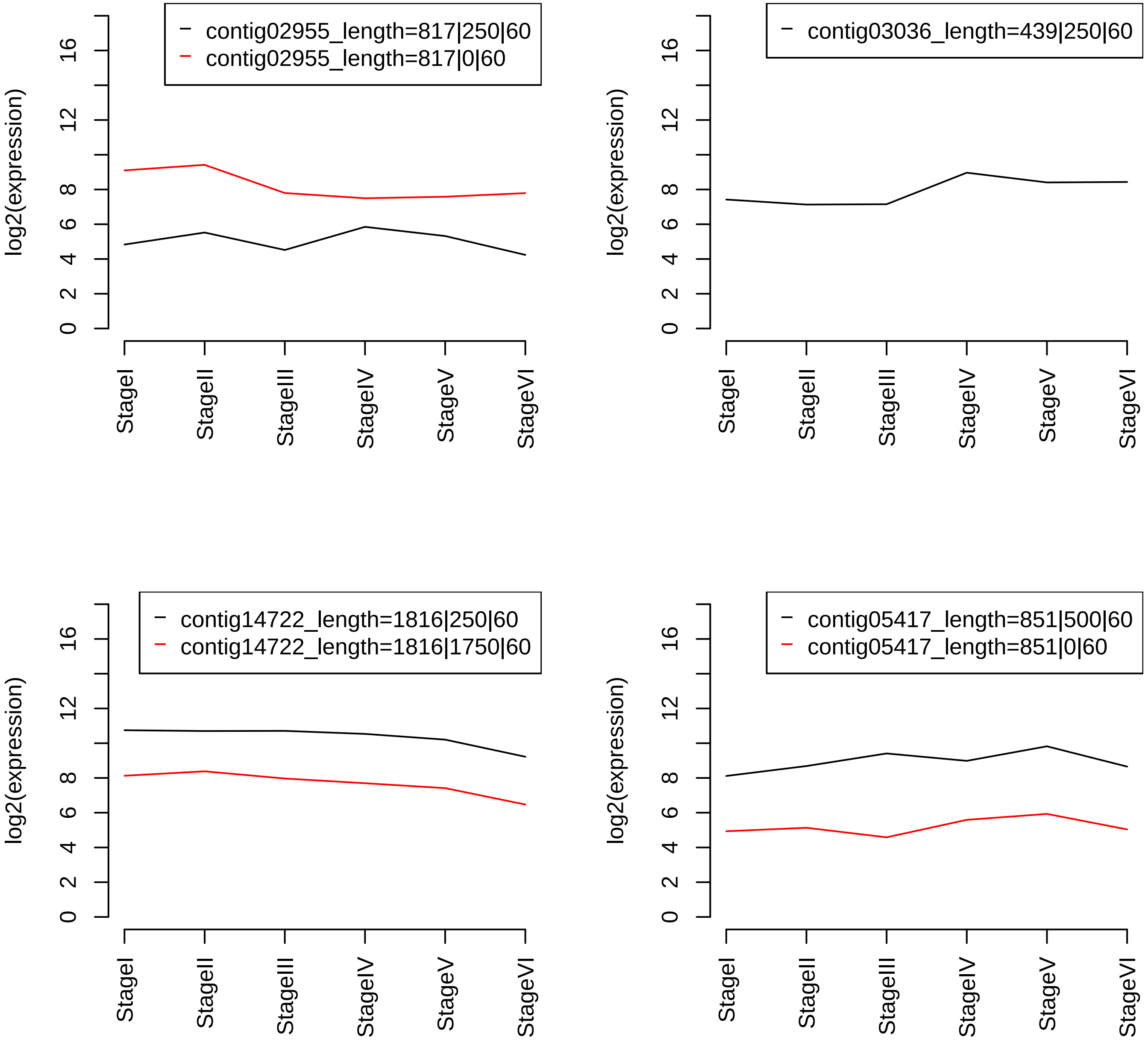

contig02955_length=817|250|60
contig02955_length=817|0|60
contig03036_length=439|250|60
contig14722_length=1816|250|60
contig14722_length=1816|1750|60
contig05417_length=851|500|60
contig05417_length=851|0|60
log2(expression)
0
2
4
6
8
12
16
StageI
StageII
StageIII
StageIV
StageV
StageVI

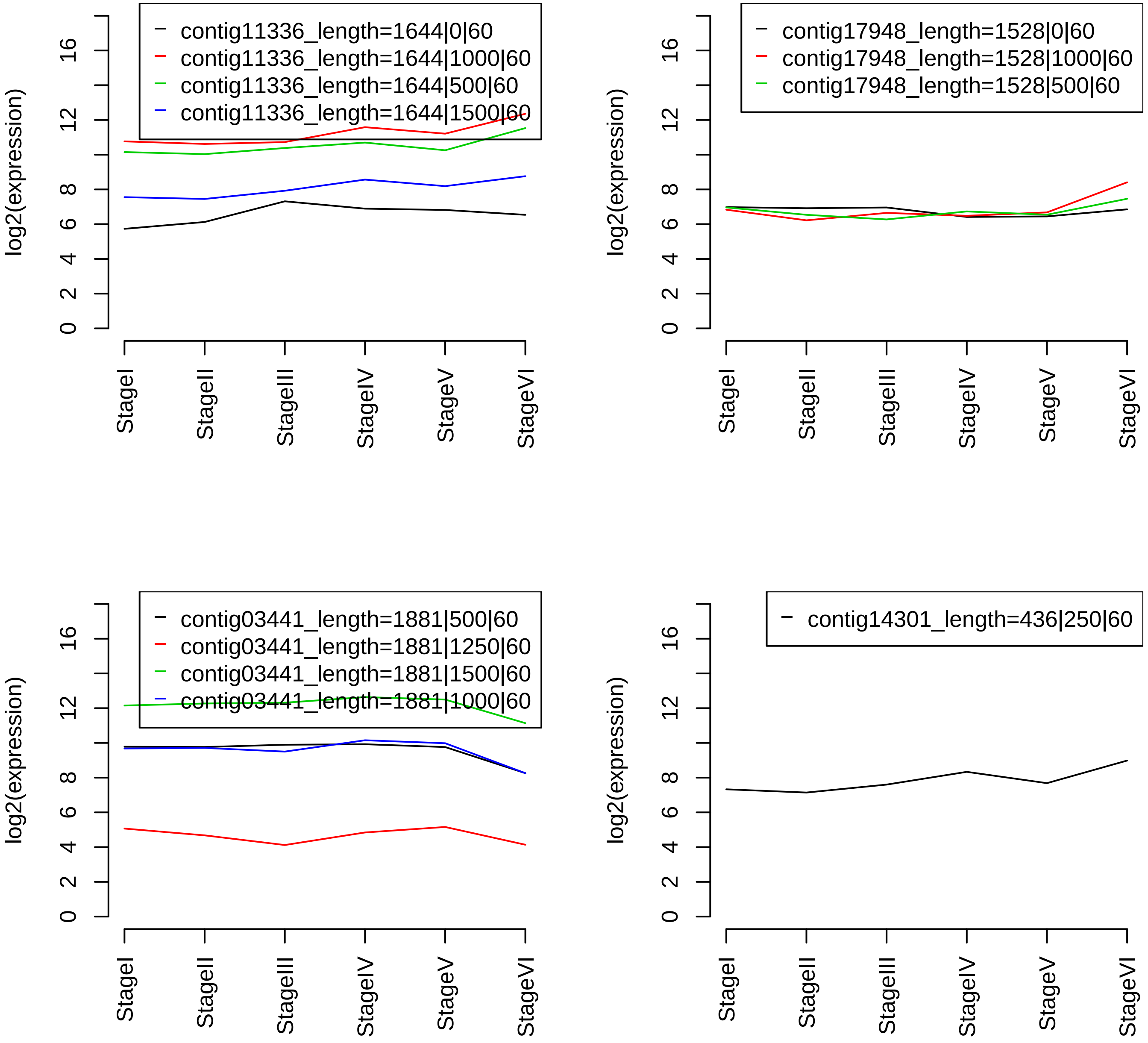
contig11336_length=1644|0|60
contig11336_length=1644|1000|60
contig11336_length=1644|500|60
contig11336_length=1644|1500|60
contig17948_length=1528|0|60
contig17948_length=1528|1000|60
contig17948_length=1528|500|60
contig03441_length=1881|500|60
contig03441_length=1881|1250|60
contig03441_length=1881|1500|60
contig03441_length=1881|1000|60
contig14301_length=436|250|60
log2(expression)
0
2
4
6
8
12
16
StageI
StageII
StageIII
StageIV
StageV
StageVI

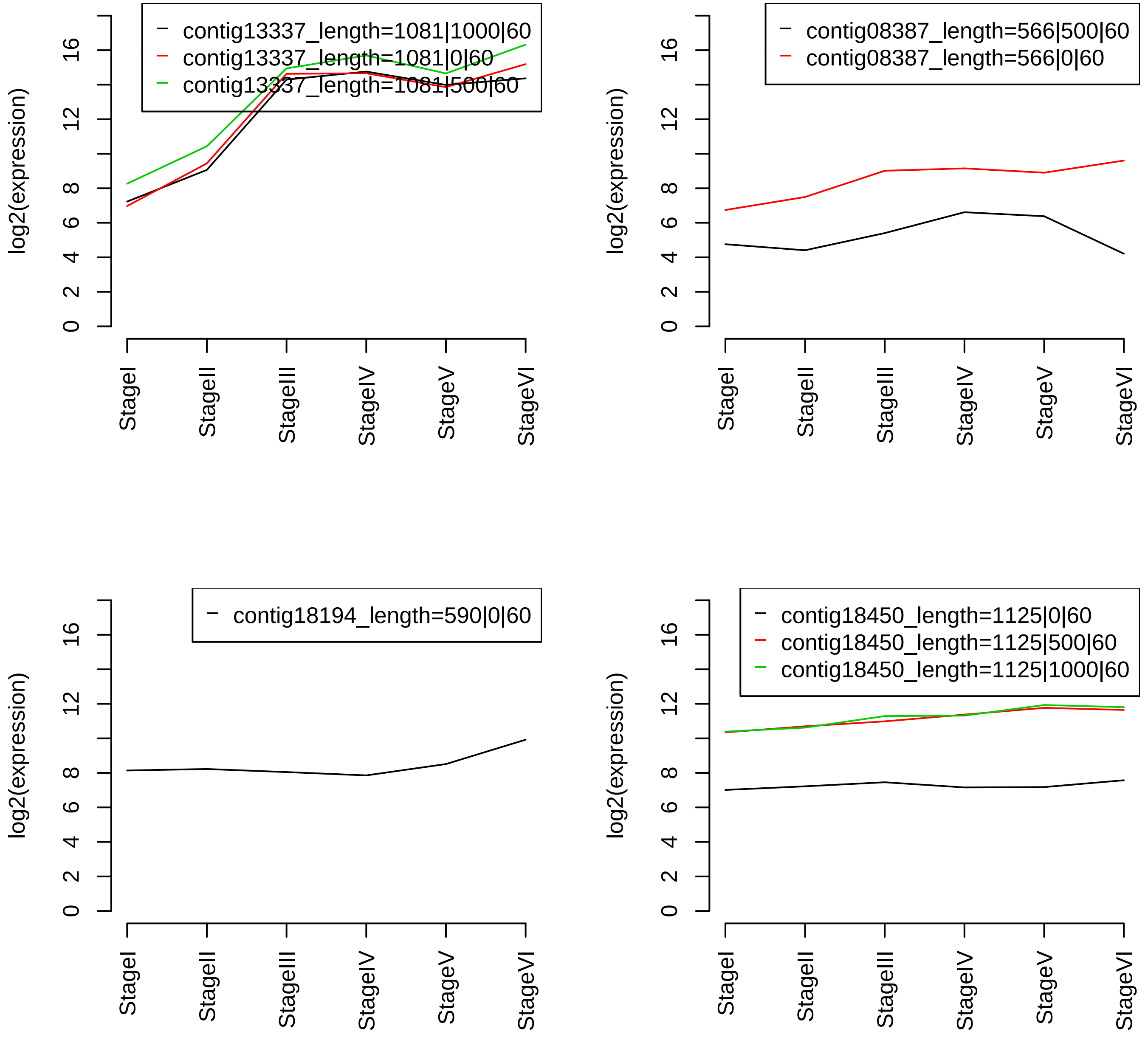

contig13337_length=1081|1000|60
contig13337_length=1081|0|60
contig13337_length=1081|500|60
contig08387_length=566|500|60
contig08387_length=566|0|60
contig18194_length=590|0|60
contig18450_length=1125|0|60
contig18450_length=1125|500|60
contig18450_length=1125|1000|60
log2(expression)
0
2
4
6
8
12
16
StageI
StageII
StageIII
StageIV
StageV
StageVI

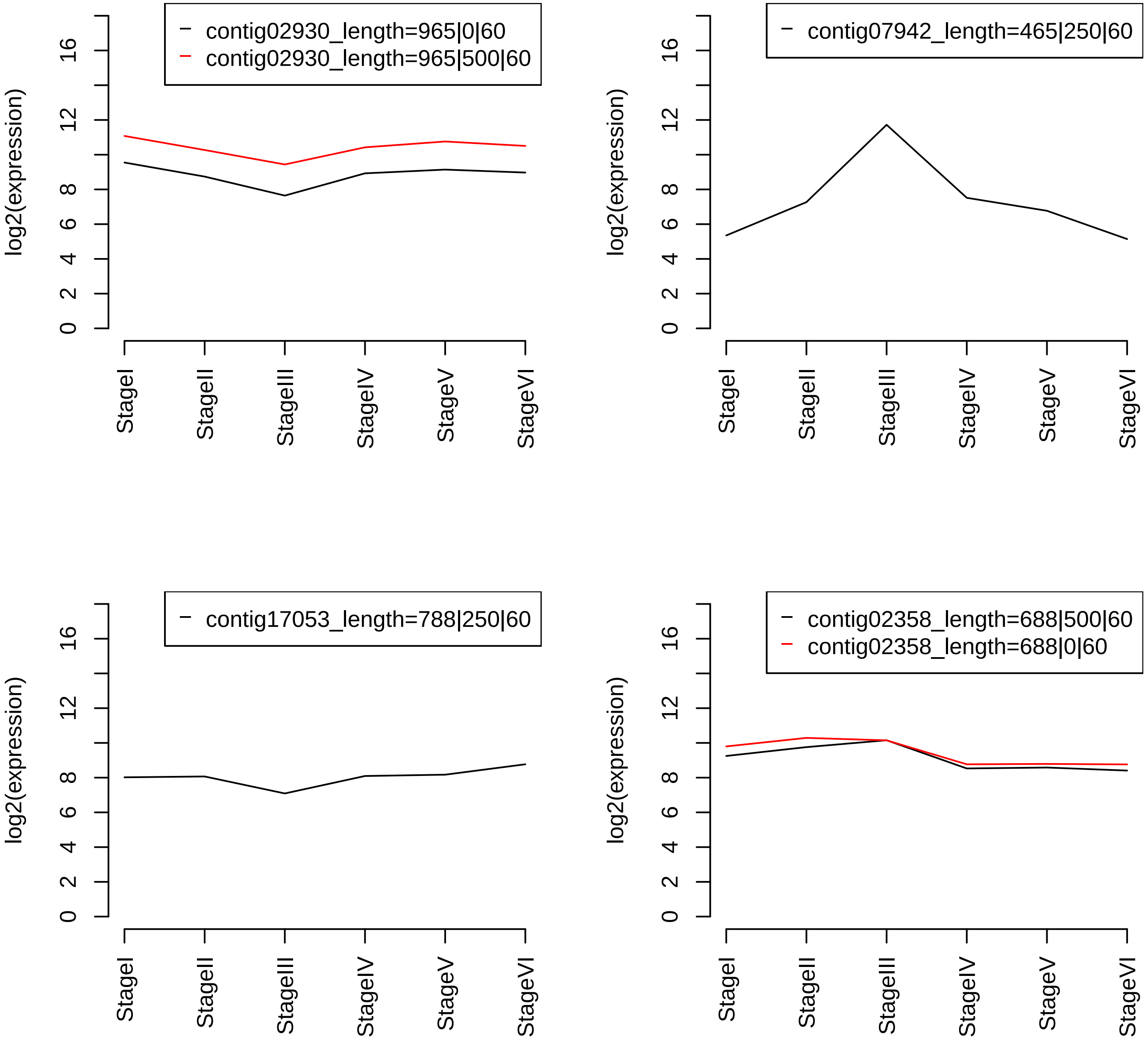
contig02930_length=965|0|60
contig02930_length=965|500|60
contig07942_length=465|250|60
contig17053_length=788|250|60
contig02358_length=688|500|60
contig02358_length=688|0|60
log2(expression)
0
2
4
6
8
12
16
StageI
StageII
StageIII
StageIV
StageV
StageVI

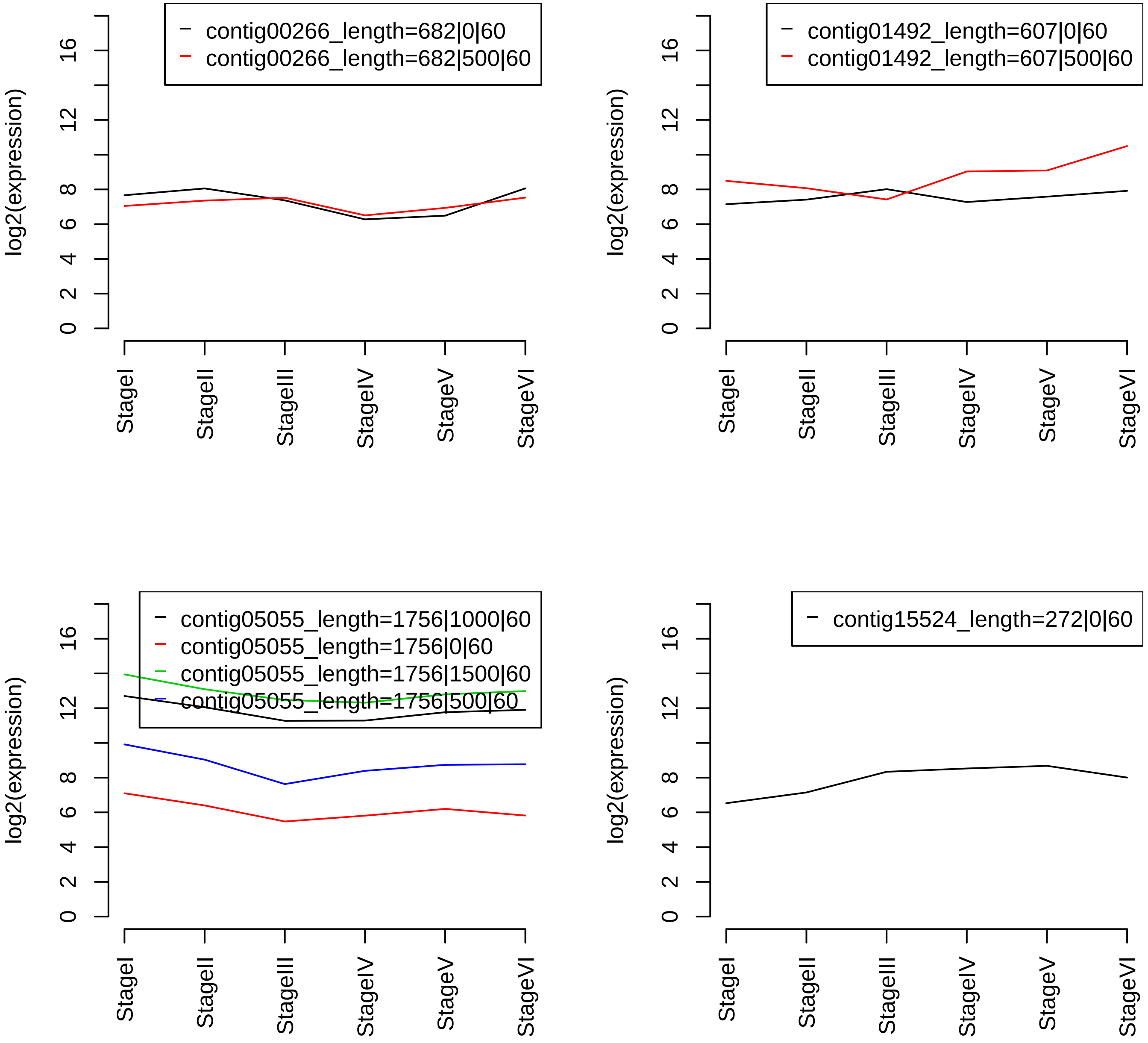
contig00266_length=682|0|60
contig00266_length=682|500|60
log2(expression)
0
2
4
6
8
12
16
StageI
StageII
StageIII
StageIV
StageV
StageVI
contig01492_length=607|0|60
contig01492_length=607|500|60
log2(expression)
contig05055_length=1756|1000|60
contig05055_length=1756|0|60
contig05055_length=1756|1500|60
contig05055_length=1756|500|60
log2(expression)
contig15524_length=272|0|60
log2(expression)

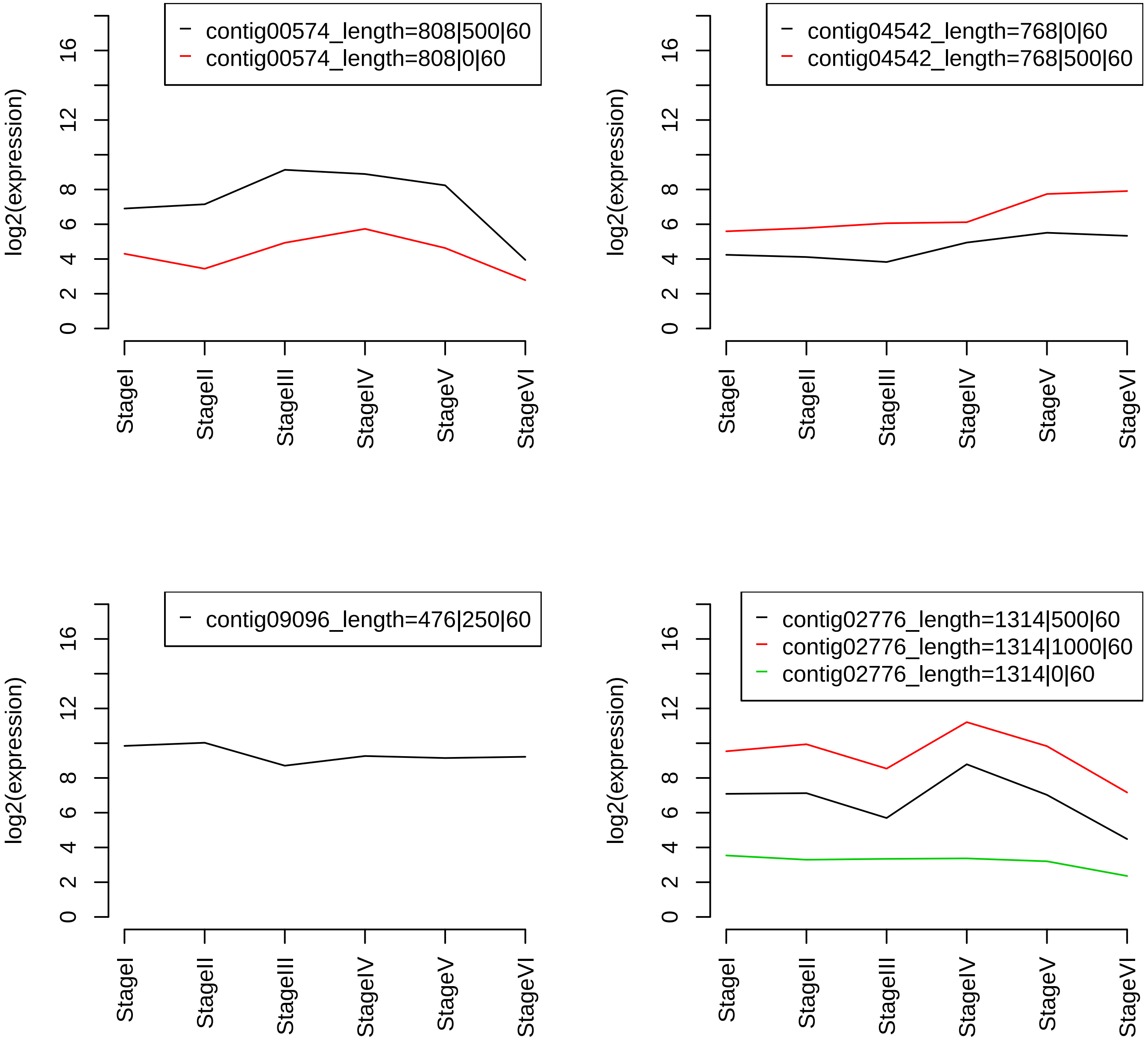

contig00574_length=808|500|60
contig00574_length=808|0|60
contig04542_length=768|0|60
contig04542_length=768|500|60
contig09096_length=476|250|60
contig02776_length=1314|500|60
contig02776_length=1314|1000|60
contig02776_length=1314|0|60
log2(expression)
0
2
4
6
8
12
16
StageI
StageII
StageIII
StageIV
StageV
StageVI

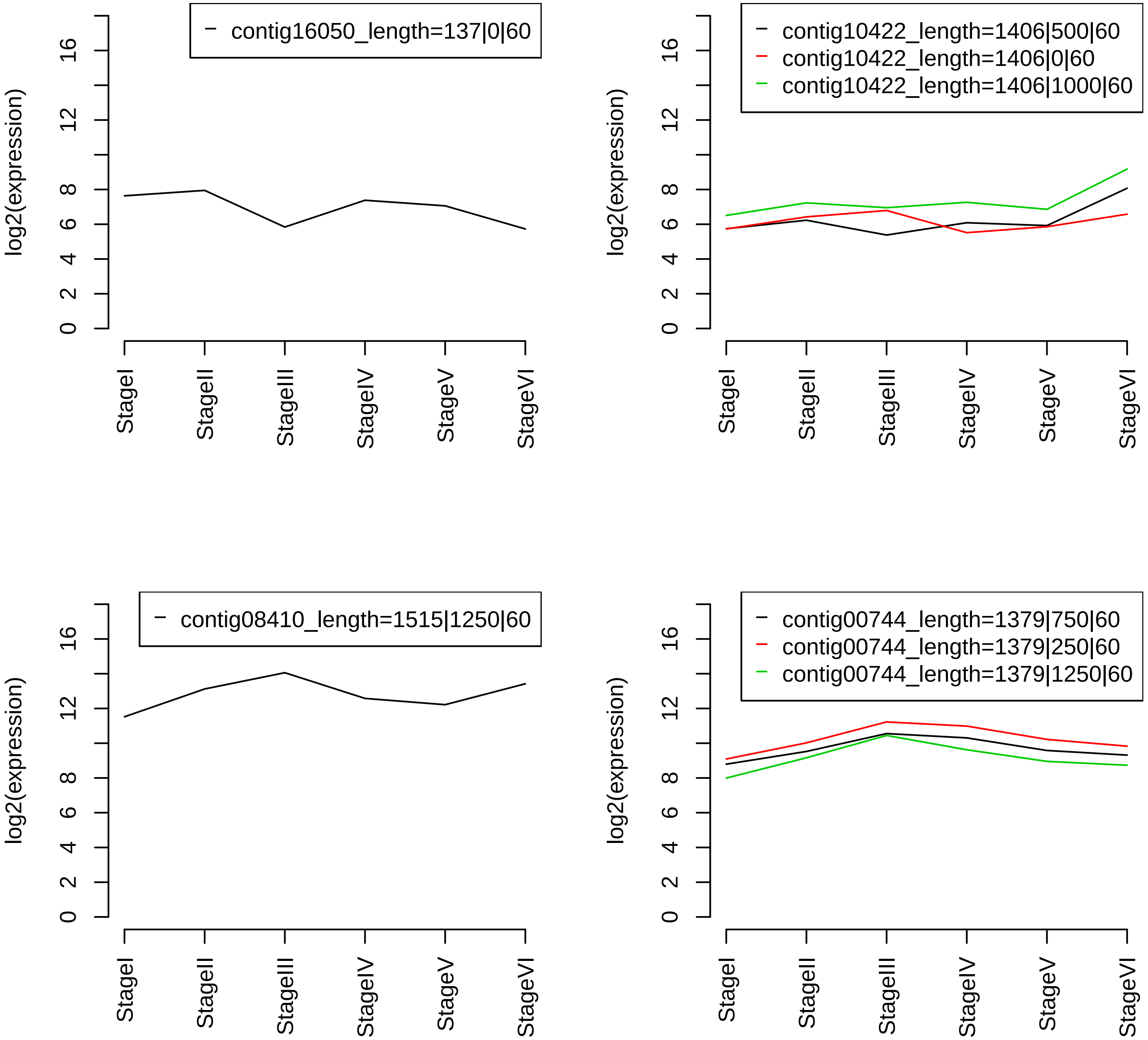
contig16050_length=137|0|60
contig10422_length=1406|500|60
contig10422_length=1406|0|60
contig10422_length=1406|1000|60
contig08410_length=1515|1250|60
contig00744_length=1379|750|60
contig00744_length=1379|250|60
contig00744_length=1379|1250|60
log2(expression)
0 2 4 6 8 12 16
StageI StageII StageIII StageIV StageV StageVI

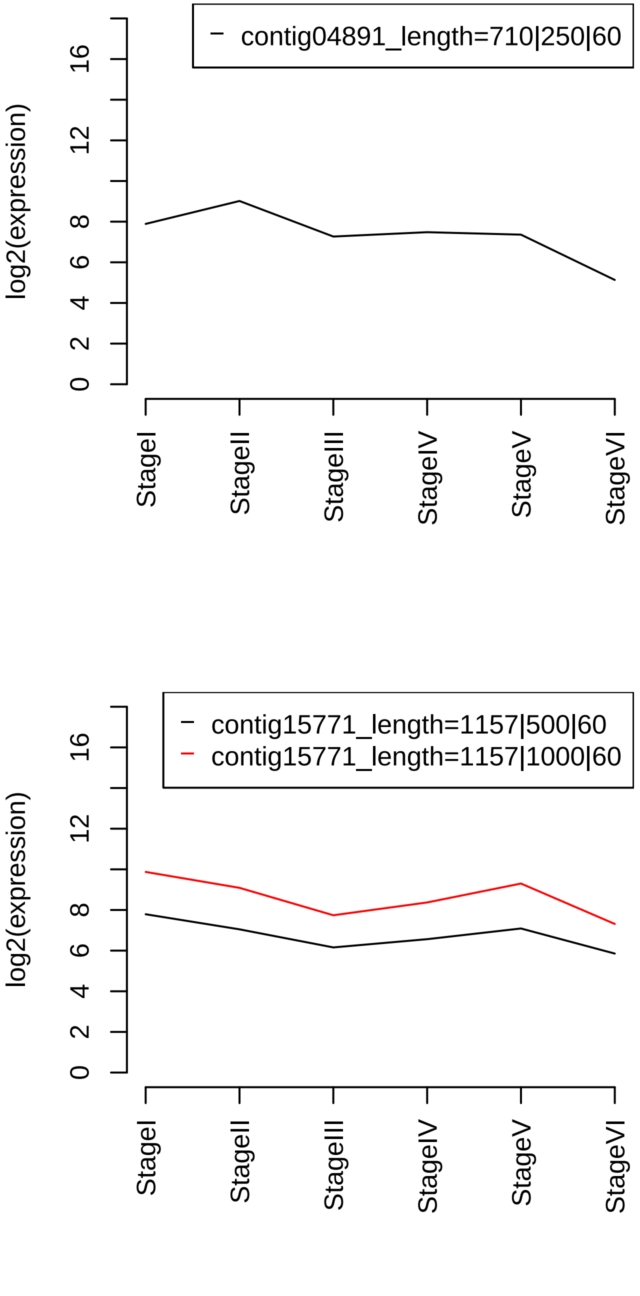
contig04891_length=710|250|60
contig08321_length=779|250|60
contig15771_length=1157|500|60
contig15771_length=1157|1000|60
contig08882_length=784|500|60
contig08882_length=784|0|60
log2(expression)
0
2
4
6
8
12
16
StageI
StageII
StageIII
StageIV
StageV
StageVI

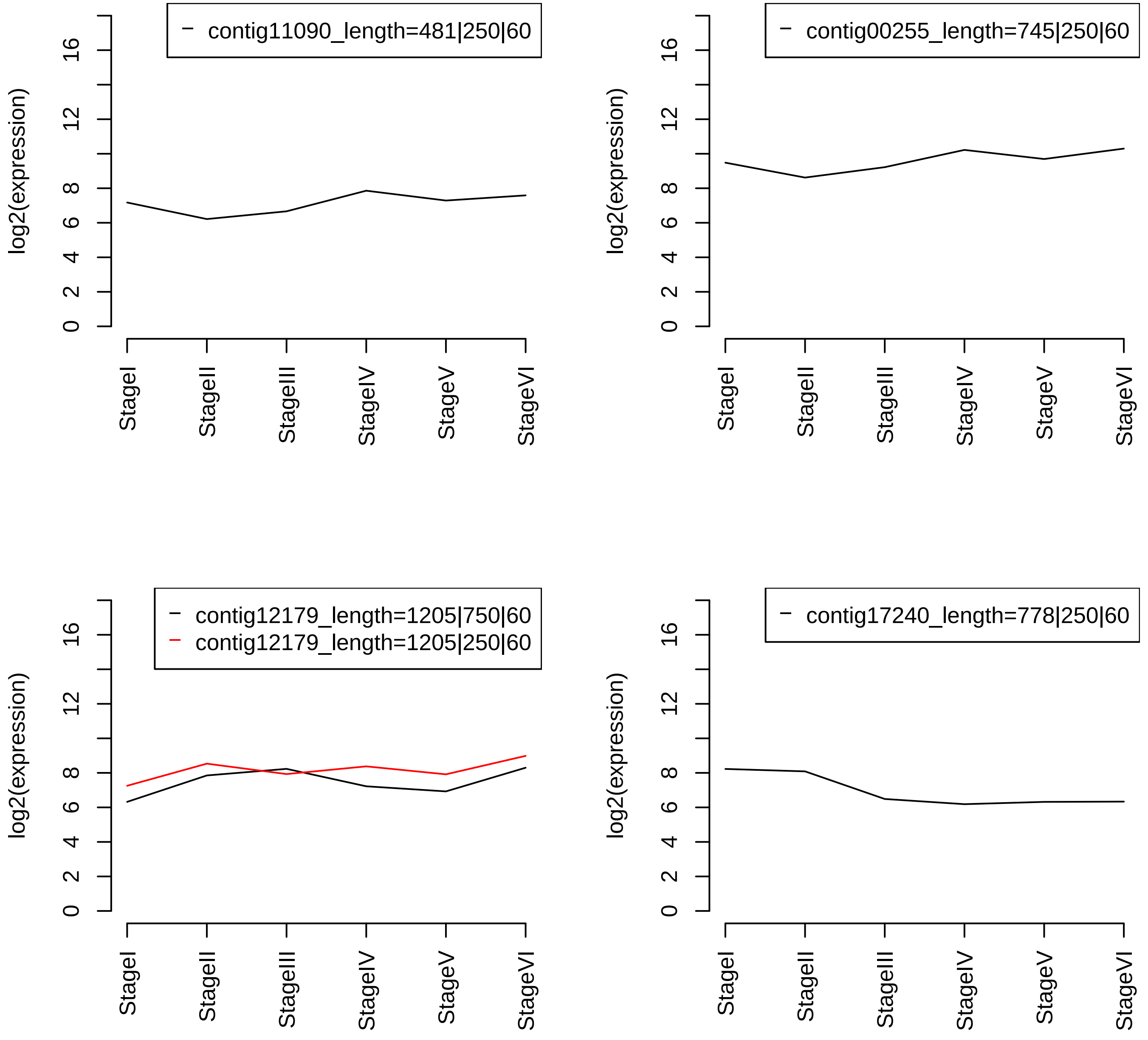

contig11090_length=481|250|60
contig00255_length=745|250|60
contig12179_length=1205|750|60
contig12179_length=1205|250|60
contig17240_length=778|250|60
log2(expression)
0
2
4
6
8
12
16
StageI
StageII
StageIII
StageIV
StageV
StageVI

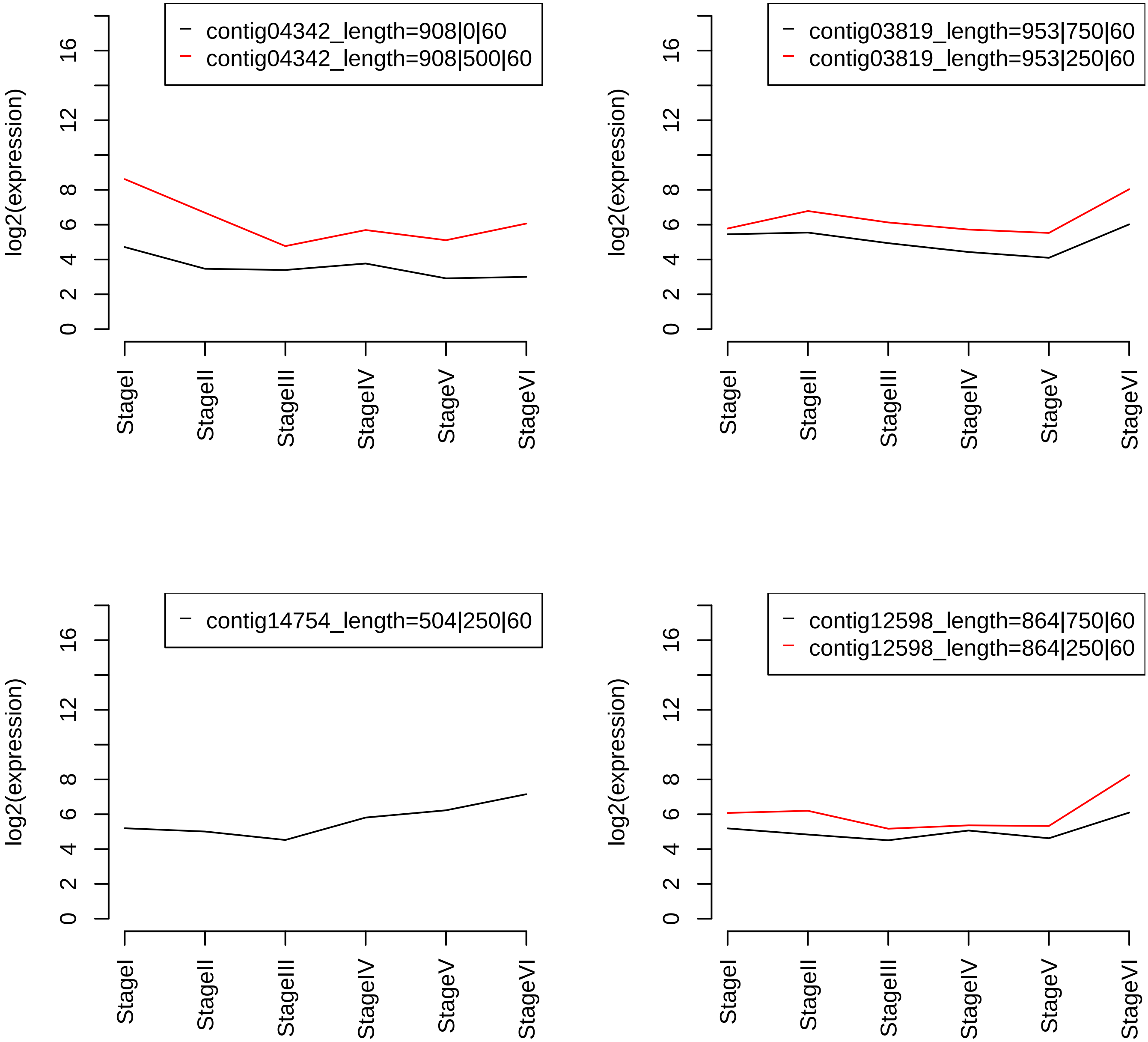

contig04342_length=908|0|60
contig04342_length=908|500|60
log2(expression)
0
2
4
6
8
12
16
StageI
StageII
StageIII
StageIV
StageV
StageVI
contig03819_length=953|750|60
contig03819_length=953|250|60
contig14754_length=504|250|60
contig12598_length=864|750|60
contig12598_length=864|250|60

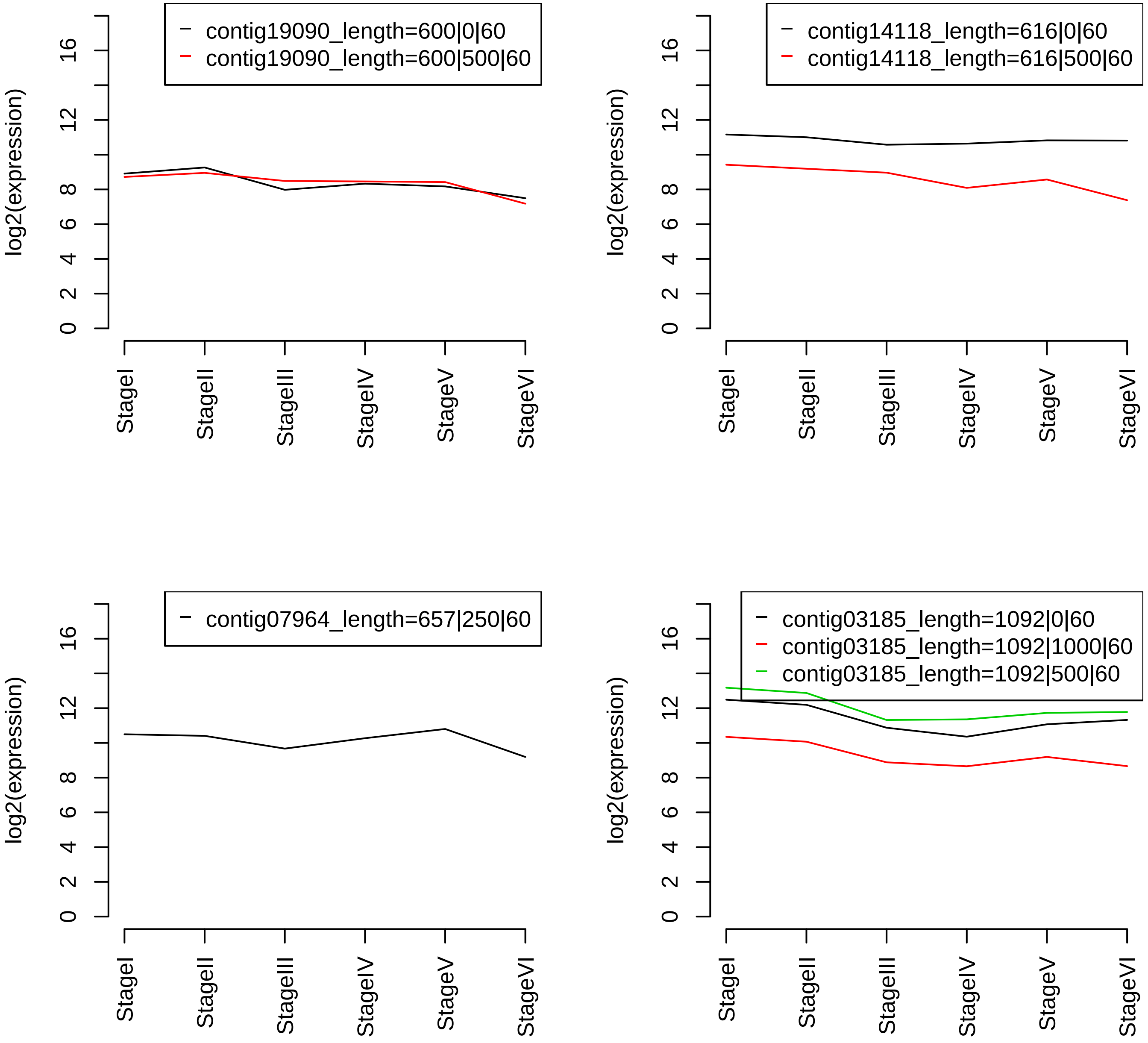
contig19090_length=600|0|60
contig19090_length=600|500|60
contig14118_length=616|0|60
contig14118_length=616|500|60
contig07964_length=657|250|60
contig03185_length=1092|0|60
contig03185_length=1092|1000|60
contig03185_length=1092|500|60
log2(expression)
0 2 4 6 8 12 16
StageI StageII StageIII StageIV StageV StageVI

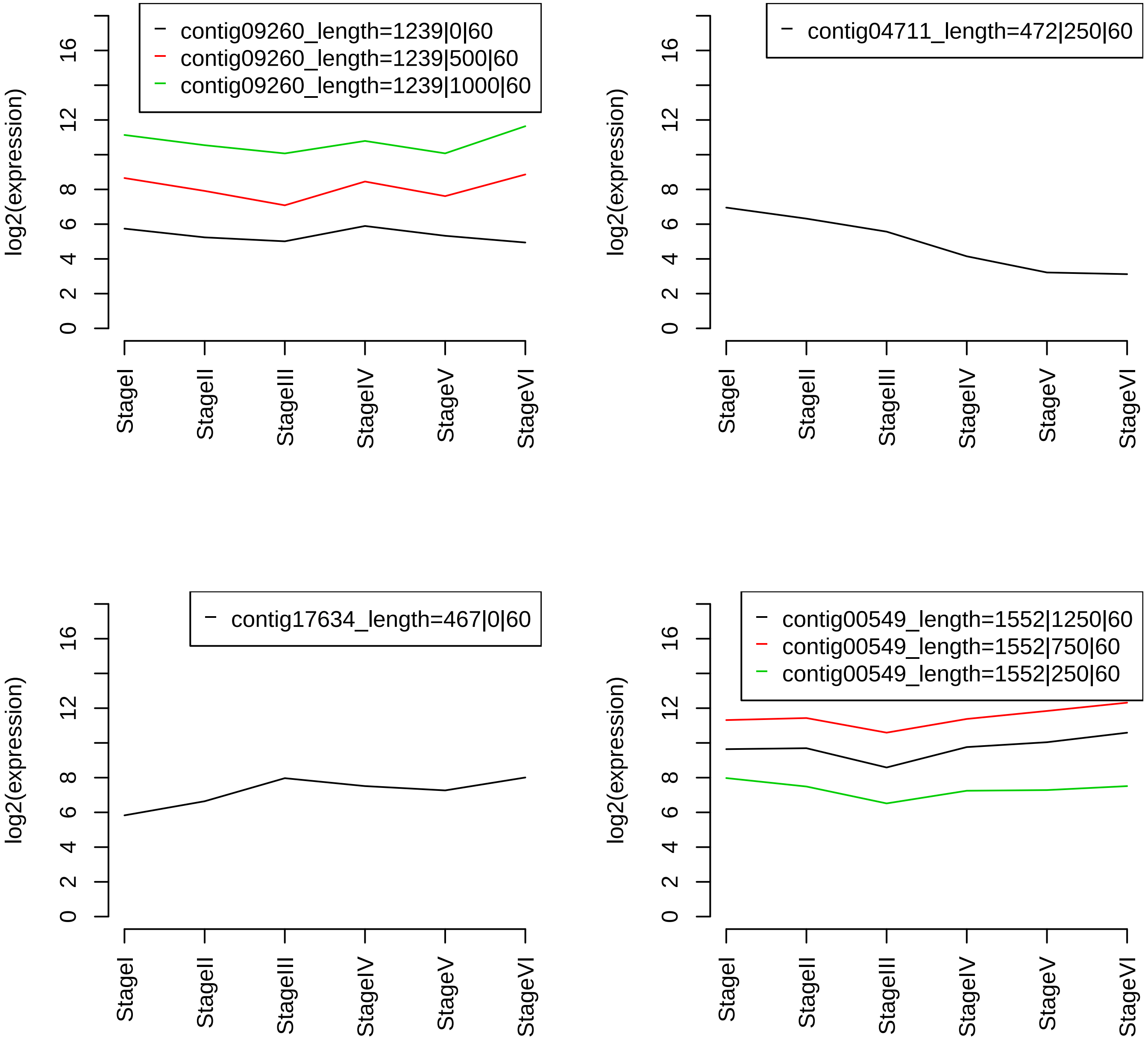

contig09260_length=1239|0|60
contig09260_length=1239|500|60
contig09260_length=1239|1000|60
log2(expression)
0
2
4
6
8
12
16
StageI
StageII
StageIII
StageIV
StageV
StageVI
contig04711_length=472|250|60
log2(expression)
0
2
4
6
8
12
16
StageI
StageII
StageIII
StageIV
StageV
StageVI
contig17634_length=467|0|60
log2(expression)
0
2
4
6
8
12
16
StageI
StageII
StageIII
StageIV
StageV
StageVI
contig00549_length=1552|1250|60
contig00549_length=1552|750|60
contig00549_length=1552|250|60
log2(expression)
0
2
4
6
8
12
16
StageI
StageII
StageIII
StageIV
StageV
StageVI

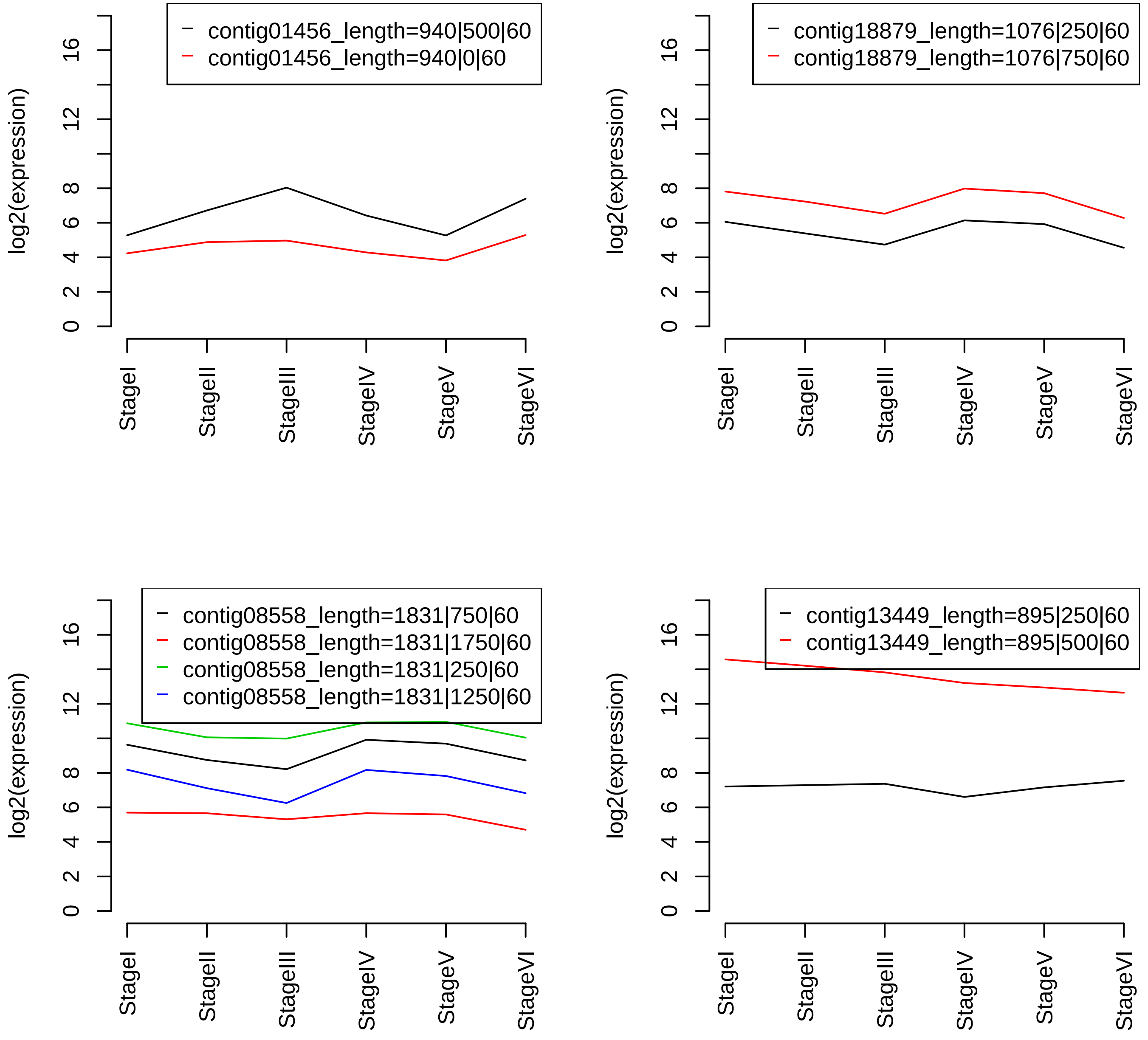
contig01456_length=940|500|60
contig01456_length=940|0|60
contig18879_length=1076|250|60
contig18879_length=1076|750|60
contig08558_length=1831|750|60
contig08558_length=1831|1750|60
contig08558_length=1831|250|60
contig08558_length=1831|1250|60
contig13449_length=895|250|60
contig13449_length=895|500|60
log2(expression)
0
2
4
6
8
12
16
StageI
StageII
StageIII
StageIV
StageV
StageVI

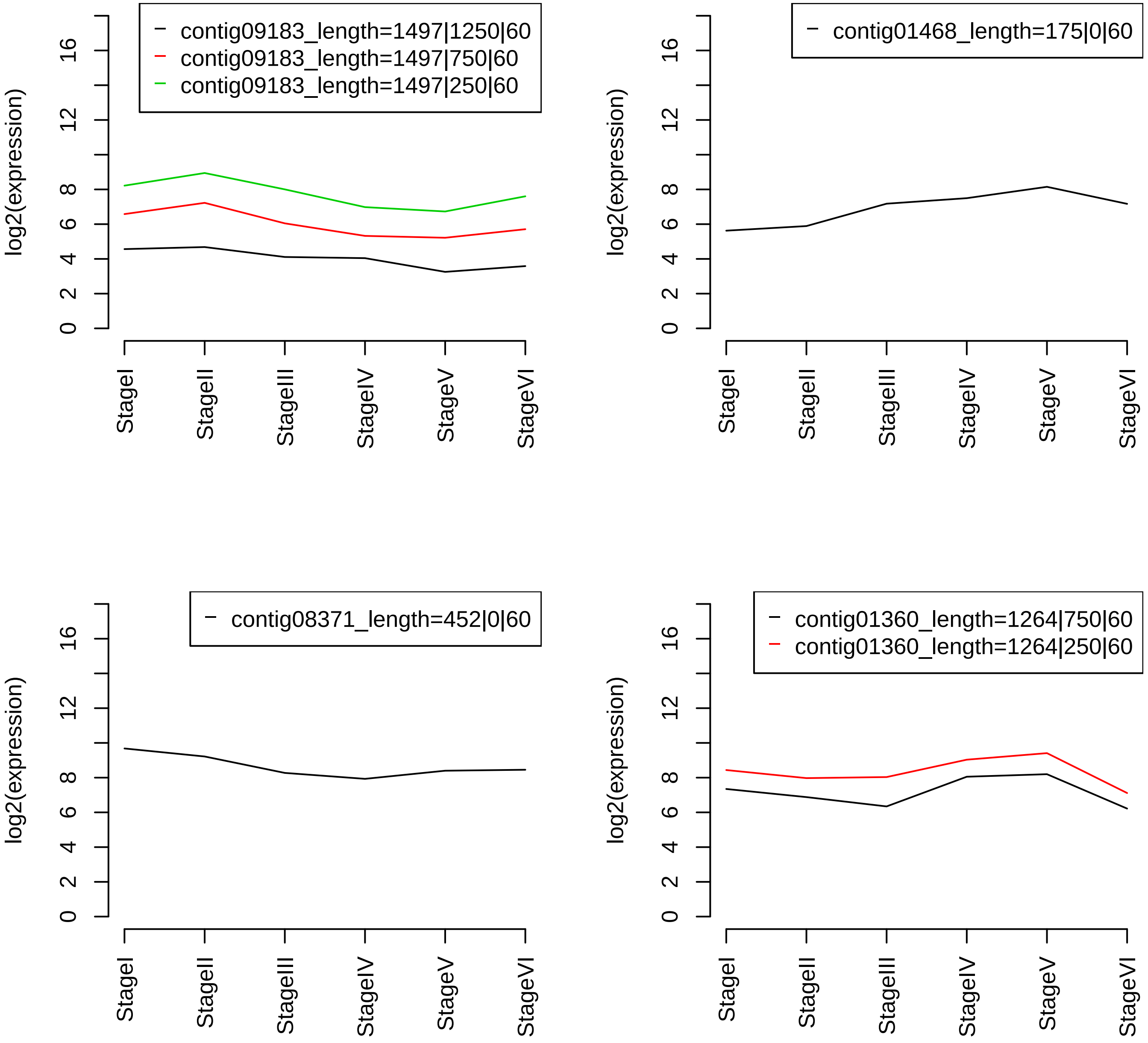

contig09183_length=1497|1250|60
contig09183_length=1497|750|60
contig09183_length=1497|250|60
contig01468_length=175|0|60
contig08371_length=452|0|60
contig01360_length=1264|750|60
contig01360_length=1264|250|60
log2(expression)
0 2 4 6 8 12 16
StageI
StageII
StageIII
StageIV
StageV
StageVI

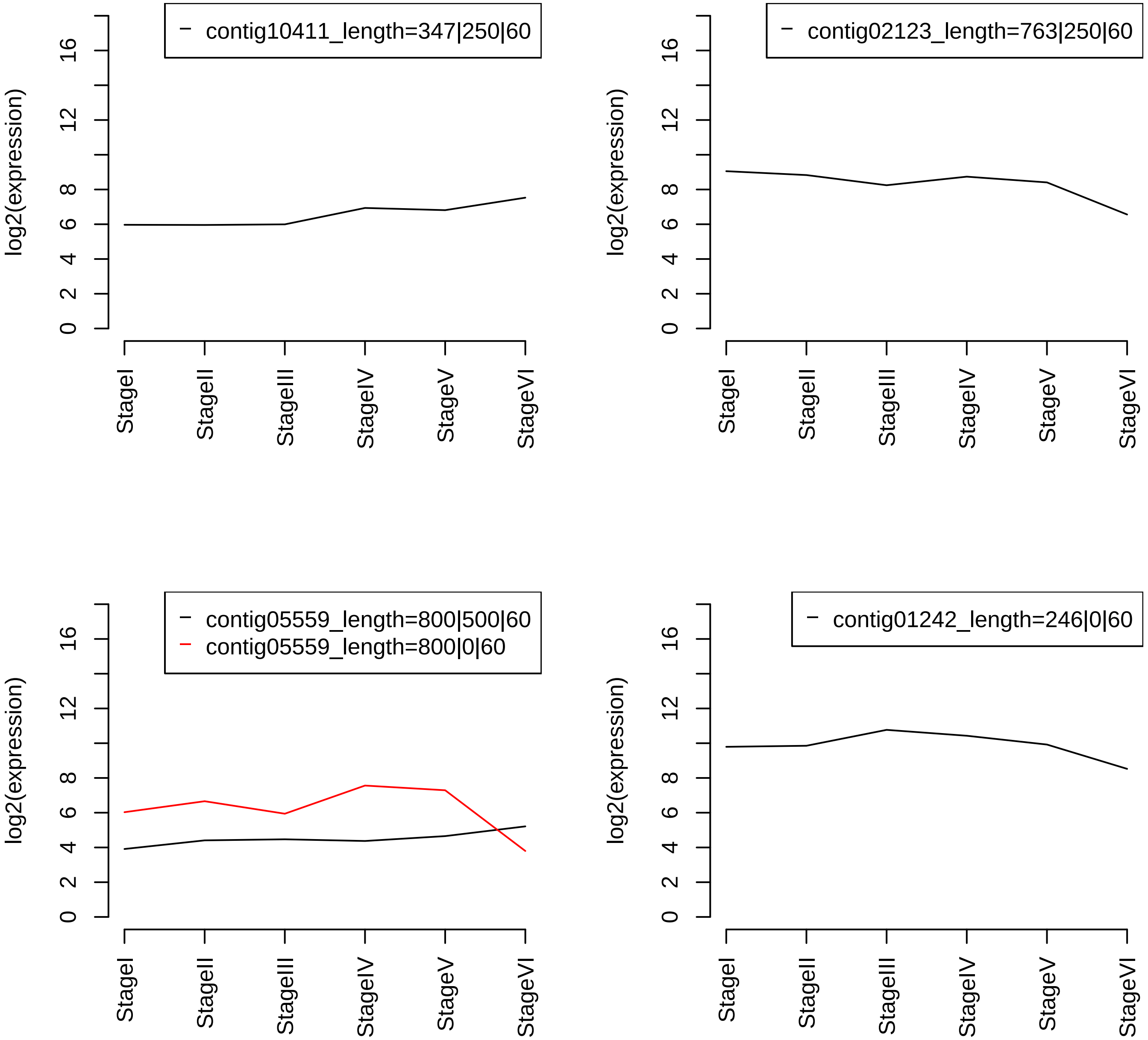

contig10411_length=347|250|60
contig02123_length=763|250|60
contig05559_length=800|500|60
contig05559_length=800|0|60
contig01242_length=246|0|60
log2(expression)
0
2
4
6
8
12
16
StageI
StageII
StageIII
StageIV
StageV
StageVI

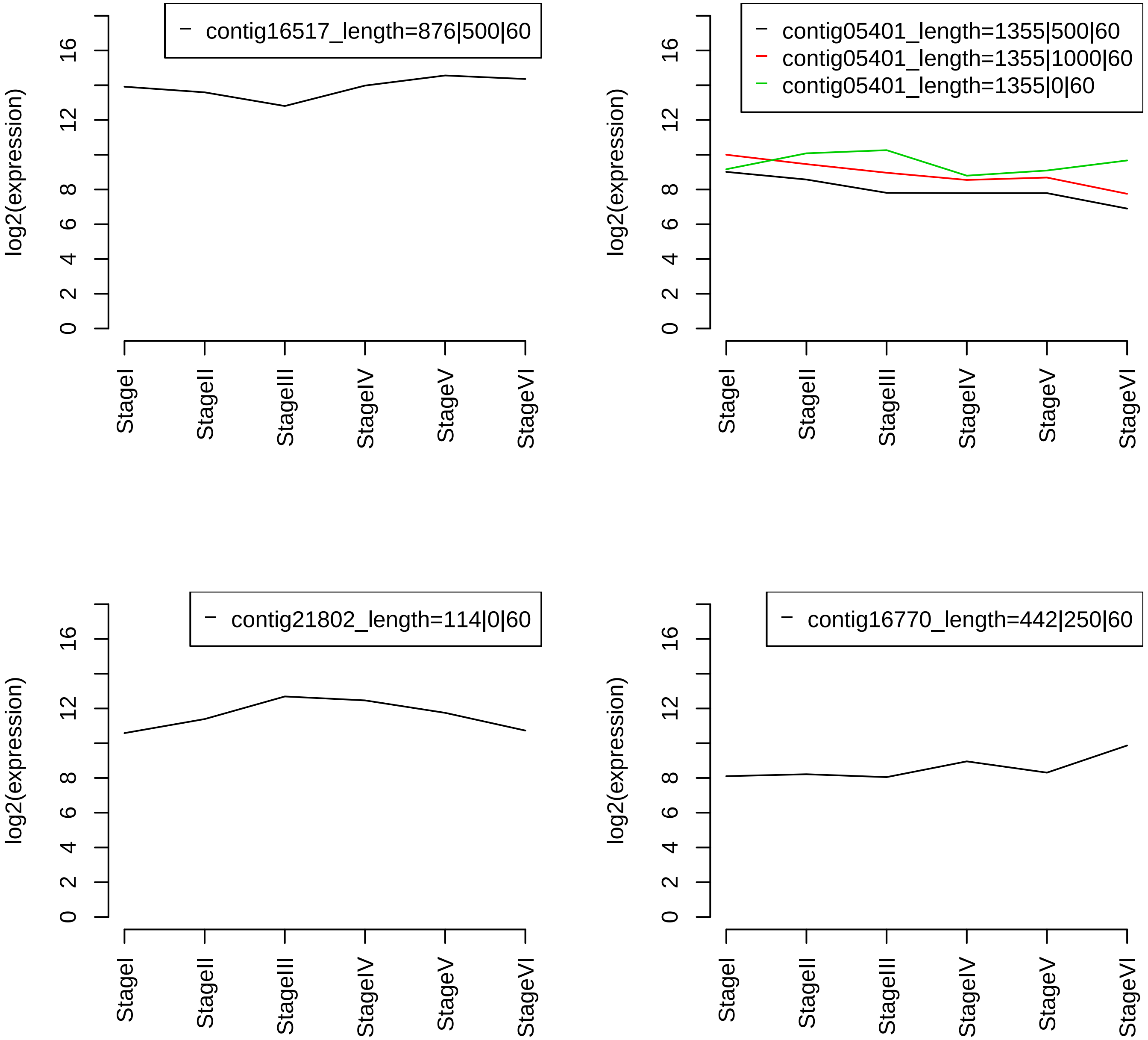
contig16517_length=876|500|60
contig05401_length=1355|500|60
contig05401_length=1355|1000|60
contig05401_length=1355|0|60
contig21802_length=114|0|60
contig16770_length=442|250|60
log2(expression)
0
2
4
6
8
12
16
StageI
StageII
StageIII
StageIV
StageV
StageVI

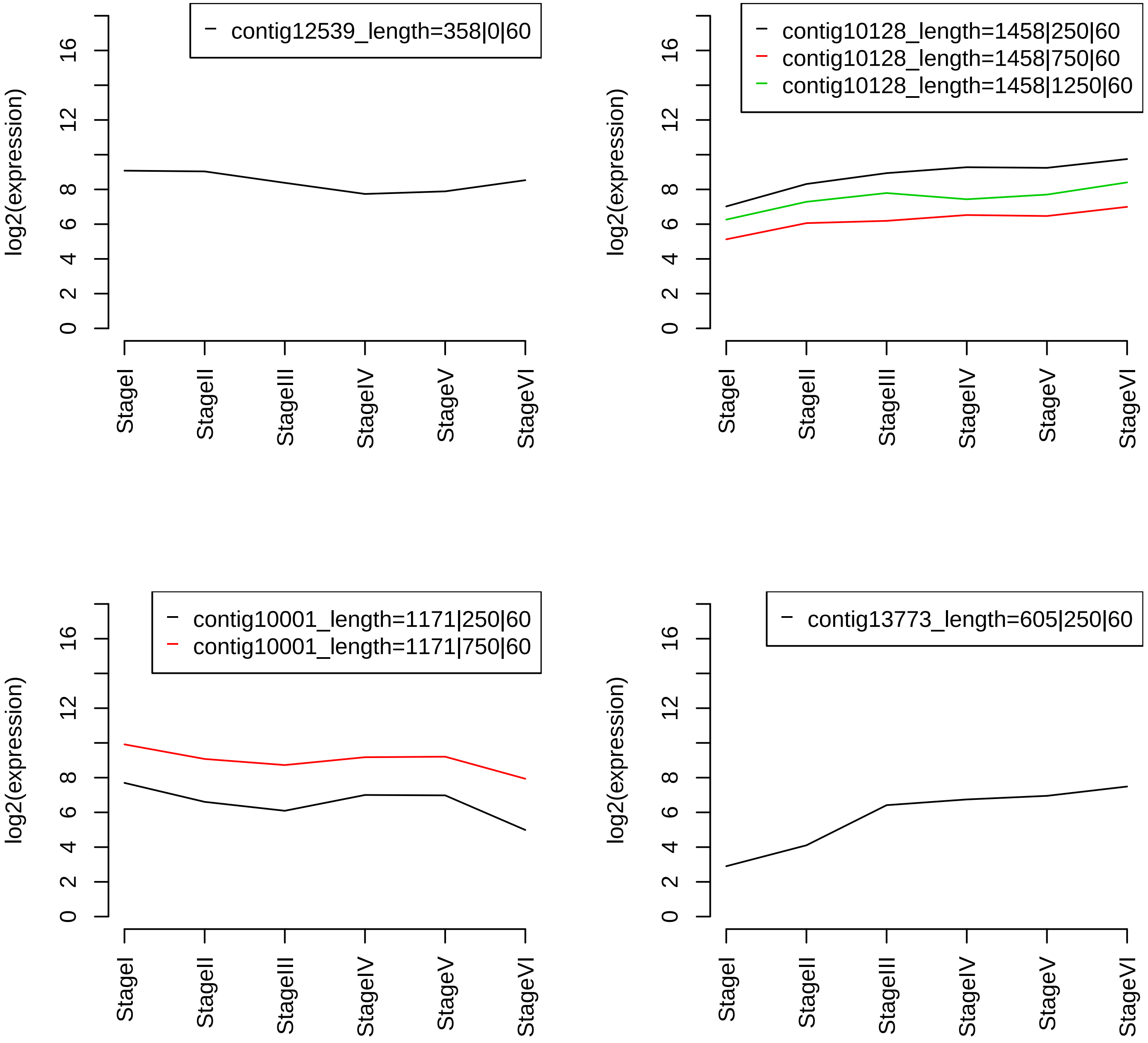

contig12539_length=358|0|60
contig10128_length=1458|250|60
contig10128_length=1458|750|60
contig10128_length=1458|1250|60
contig10001_length=1171|250|60
contig10001_length=1171|750|60
contig13773_length=605|250|60
log2(expression)
StageI
StageII
StageIII
StageIV
StageV
StageVI

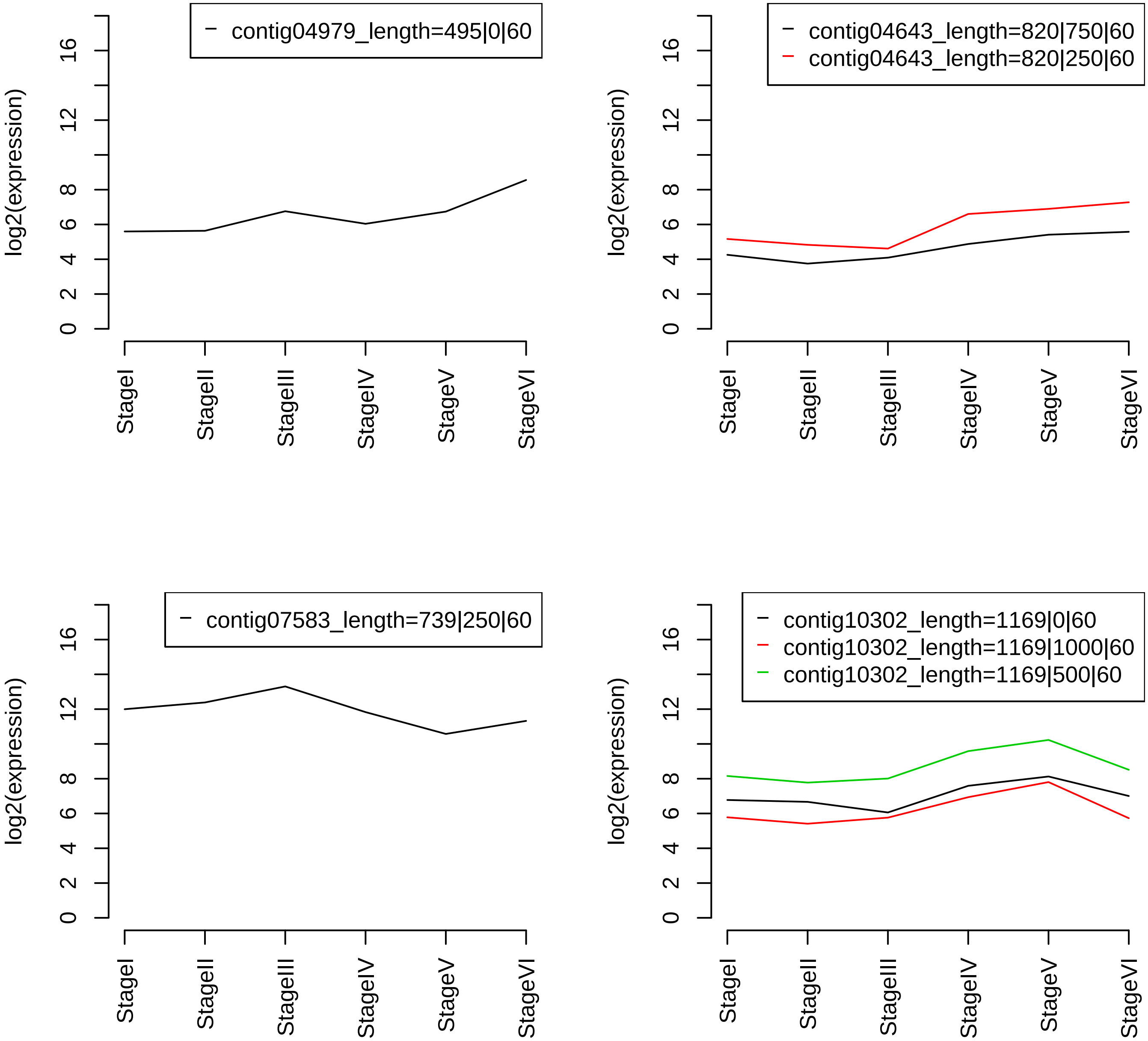
contig04979_length=495|0|60
contig04643_length=820|750|60
contig04643_length=820|250|60
contig07583_length=739|250|60
contig10302_length=1169|0|60
contig10302_length=1169|1000|60
contig10302_length=1169|500|60
log2(expression)
0
2
4
6
8
12
16
StageI
StageII
StageIII
StageIV
StageV
StageVI

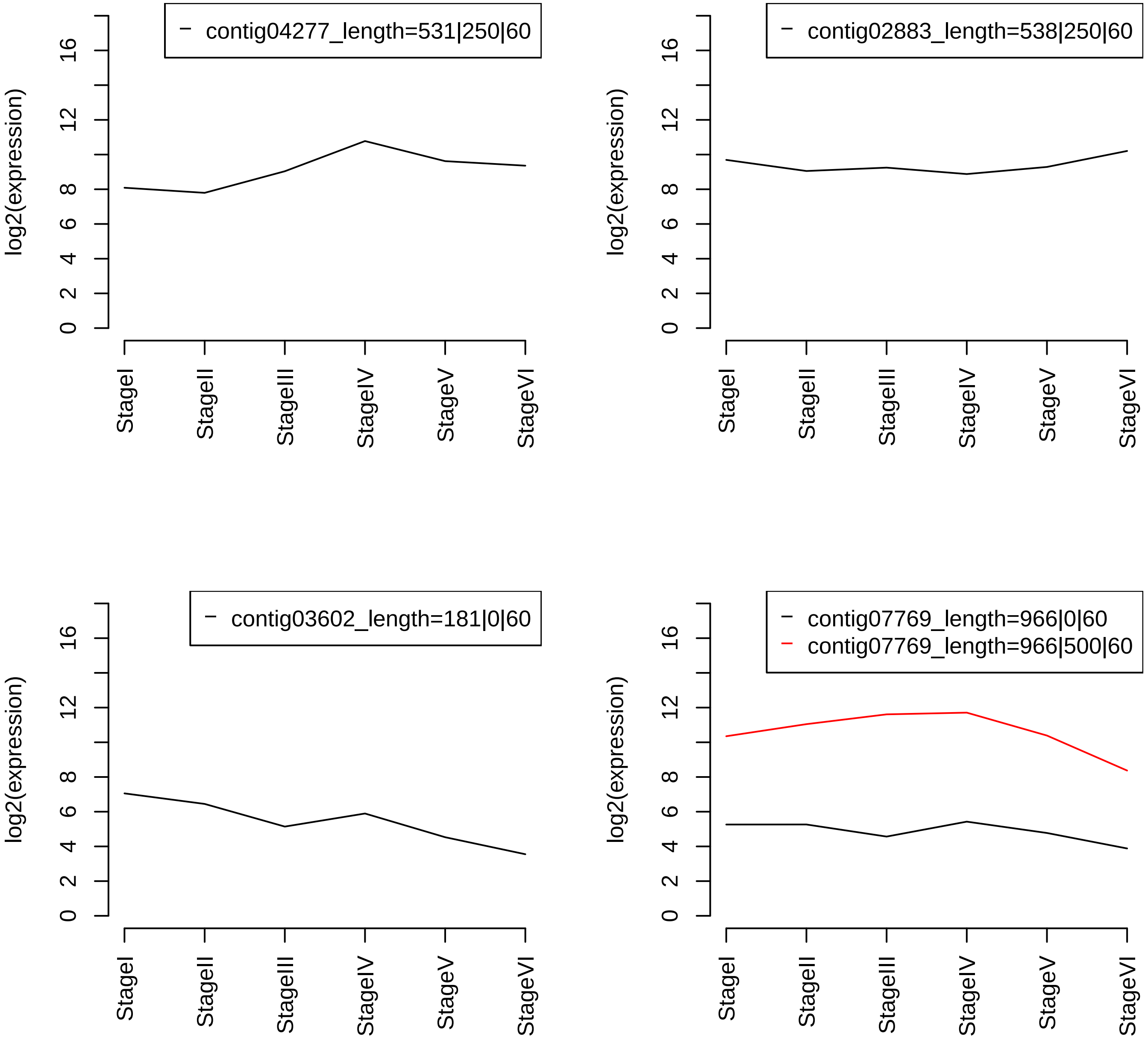
contig04277_length=531|250|60
contig02883_length=538|250|60
contig03602_length=181|0|60
contig07769_length=966|0|60
contig07769_length=966|500|60
log2(expression)
0
2
4
6
8
12
16
StageI
StageII
StageIII
StageIV
StageV
StageVI

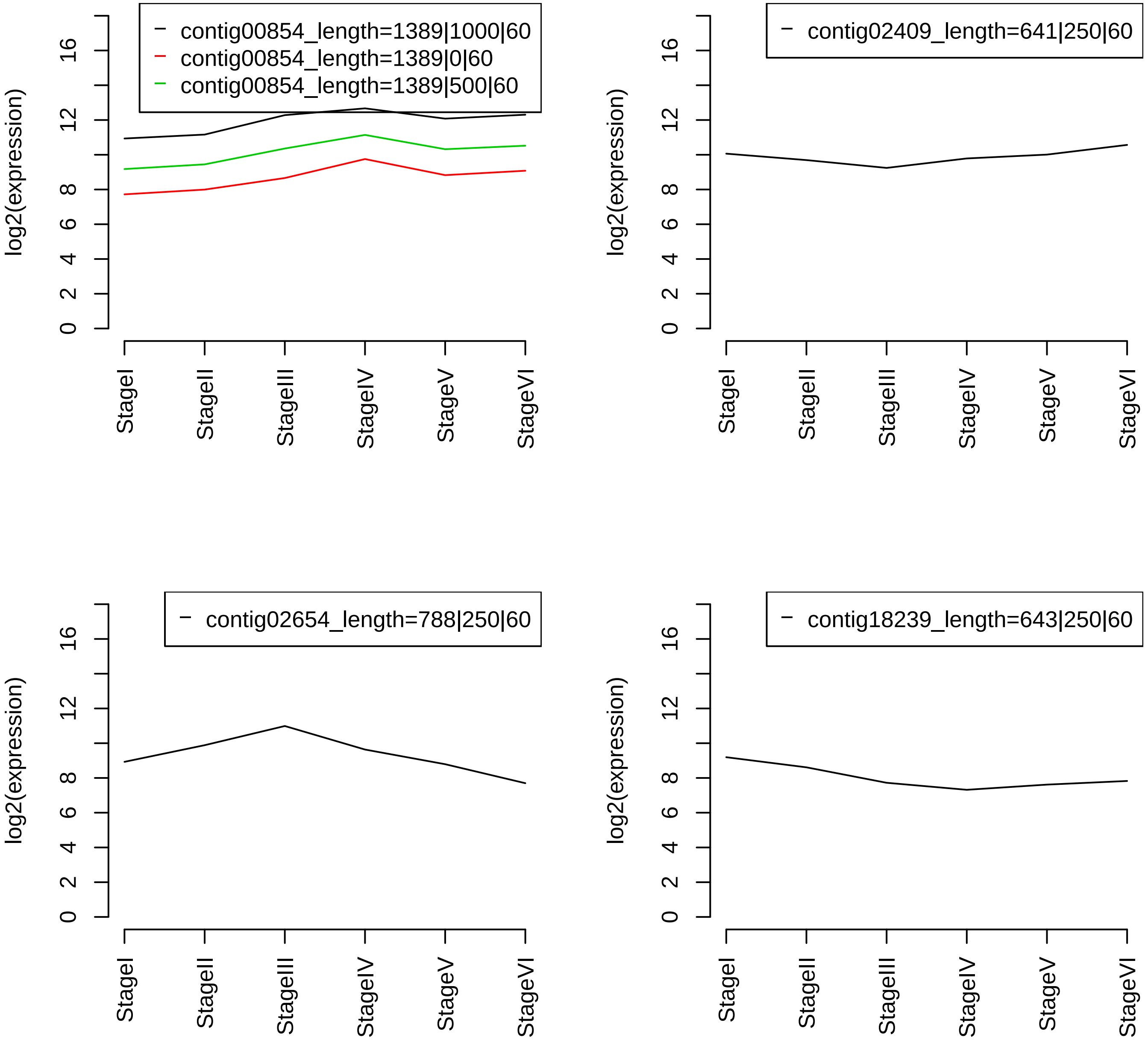
contig00854_length=1389|1000|60
contig00854_length=1389|0|60
contig00854_length=1389|500|60
contig02409_length=641|250|60
contig02654_length=788|250|60
contig18239_length=643|250|60
log2(expression)
0
2
4
6
8
12
16
StageI
StageII
StageIII
StageIV
StageV
StageVI

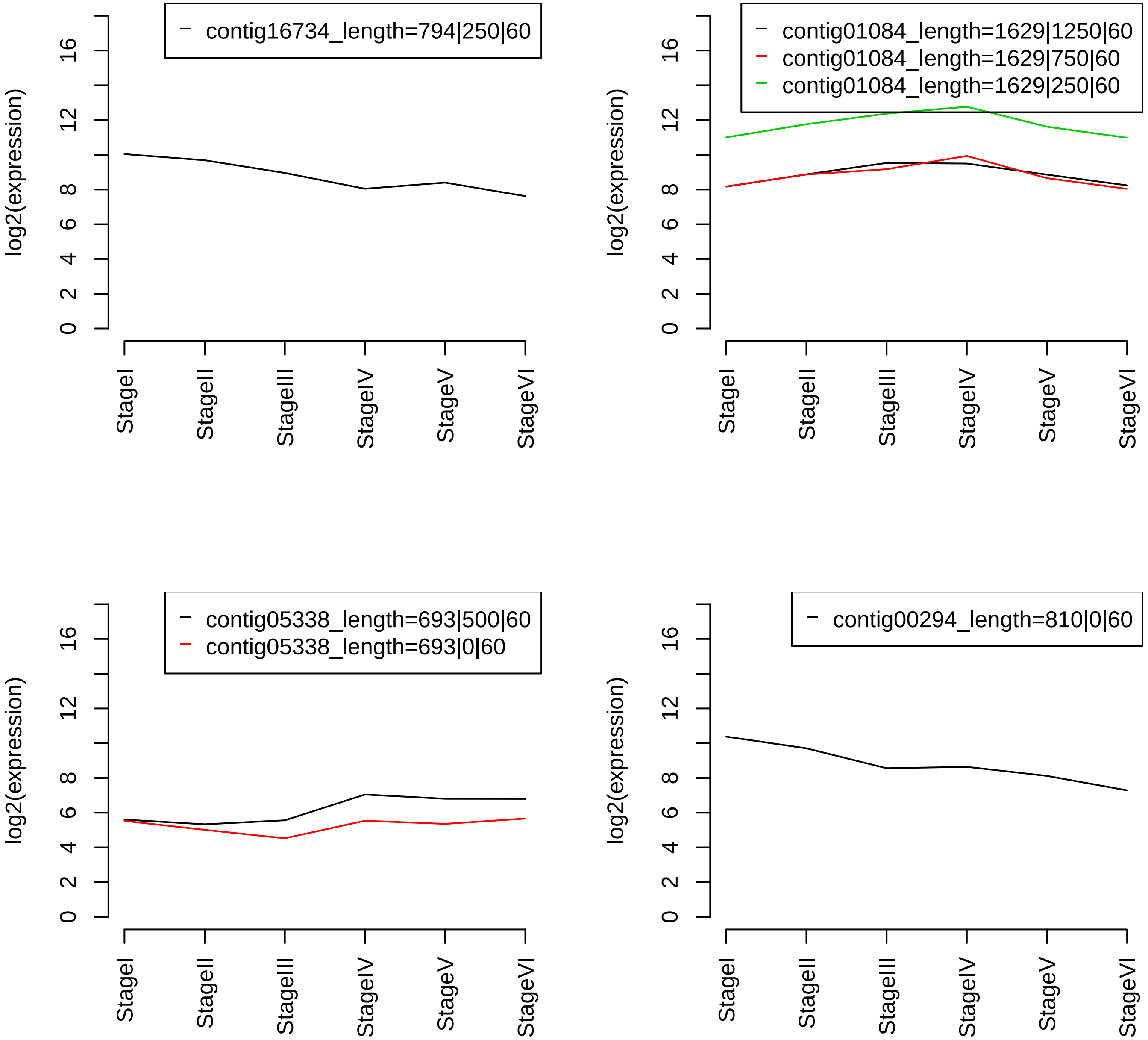

contig16734_length=794|250|60
contig01084_length=1629|1250|60
contig01084_length=1629|750|60
contig01084_length=1629|250|60
contig05338_length=693|500|60
contig05338_length=693|0|60
contig00294_length=810|0|60
log2(expression)
0
2
4
6
8
12
16
StageI
StageII
StageIII
StageIV
StageV
StageVI

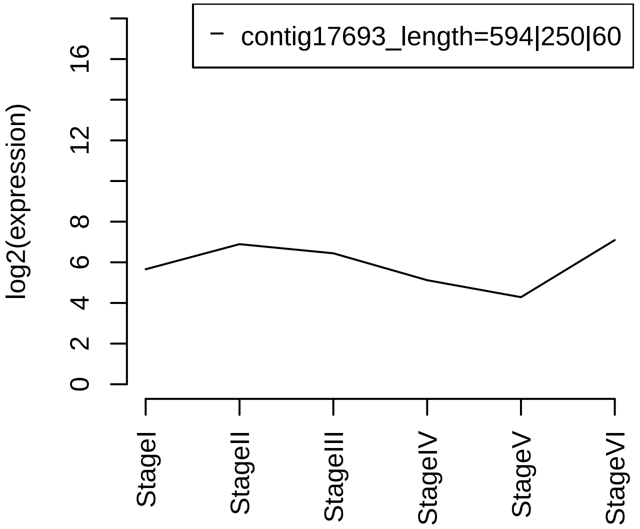

contig17693_length=594|250|60
log2(expression)
0
2
4
6
8
12
16
StageI
StageII
StageIII
StageIV
StageV
StageVI

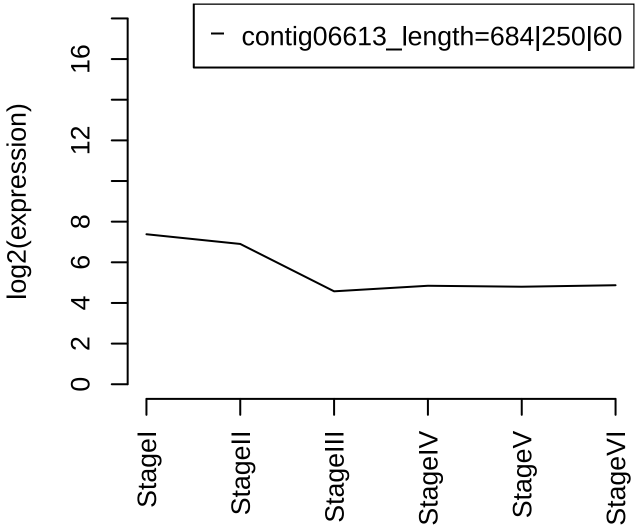

contig06613_length=684|250|60
log2(expression)
0
2
4
6
8
12
16
StageI
StageII
StageIII
StageIV
StageV
StageVI

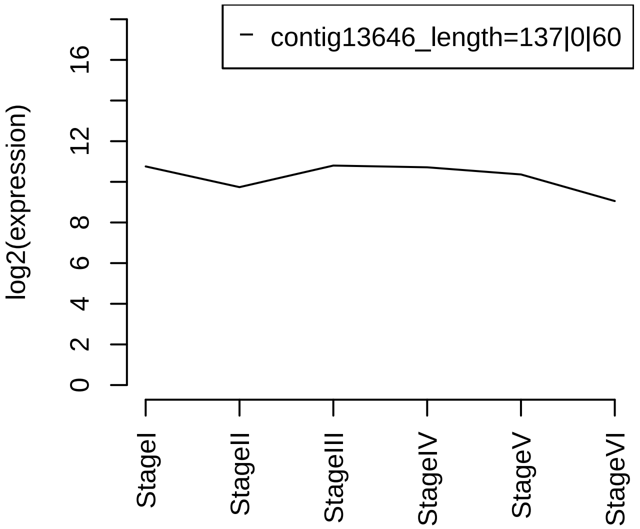

contig13646_length=137|0|60
log2(expression)
0
2
4
6
8
12
16
StageI
StageII
StageIII
StageIV
StageV
StageVI

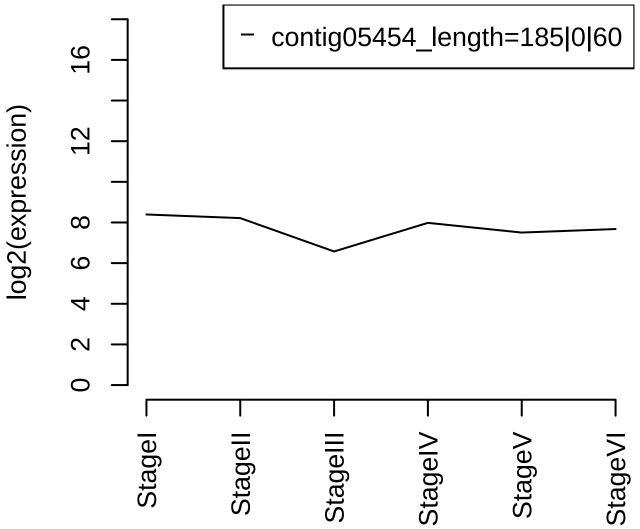

contig05454_length=185|0|60
log2(expression)
0
2
4
6
8
12
16
StageI
StageII
StageIII
StageIV
StageV
StageVI

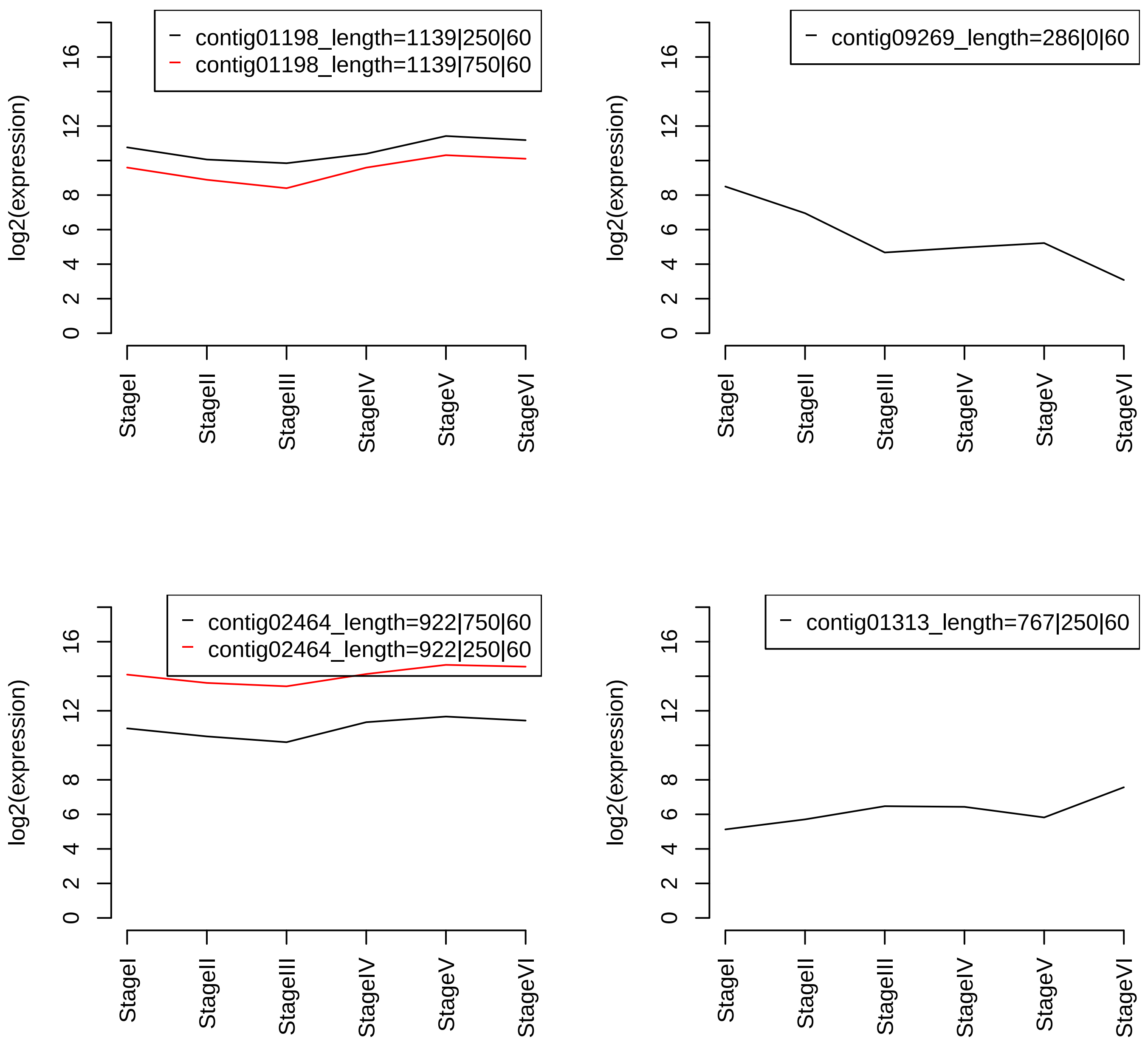
contig01198_length=1139|250|60
contig01198_length=1139|750|60
contig09269_length=286|0|60
contig02464_length=922|750|60
contig02464_length=922|250|60
contig01313_length=767|250|60
log2(expression)
0
2
4
6
8
12
16
StageI
StageII
StageIII
StageIV
StageV
StageVI

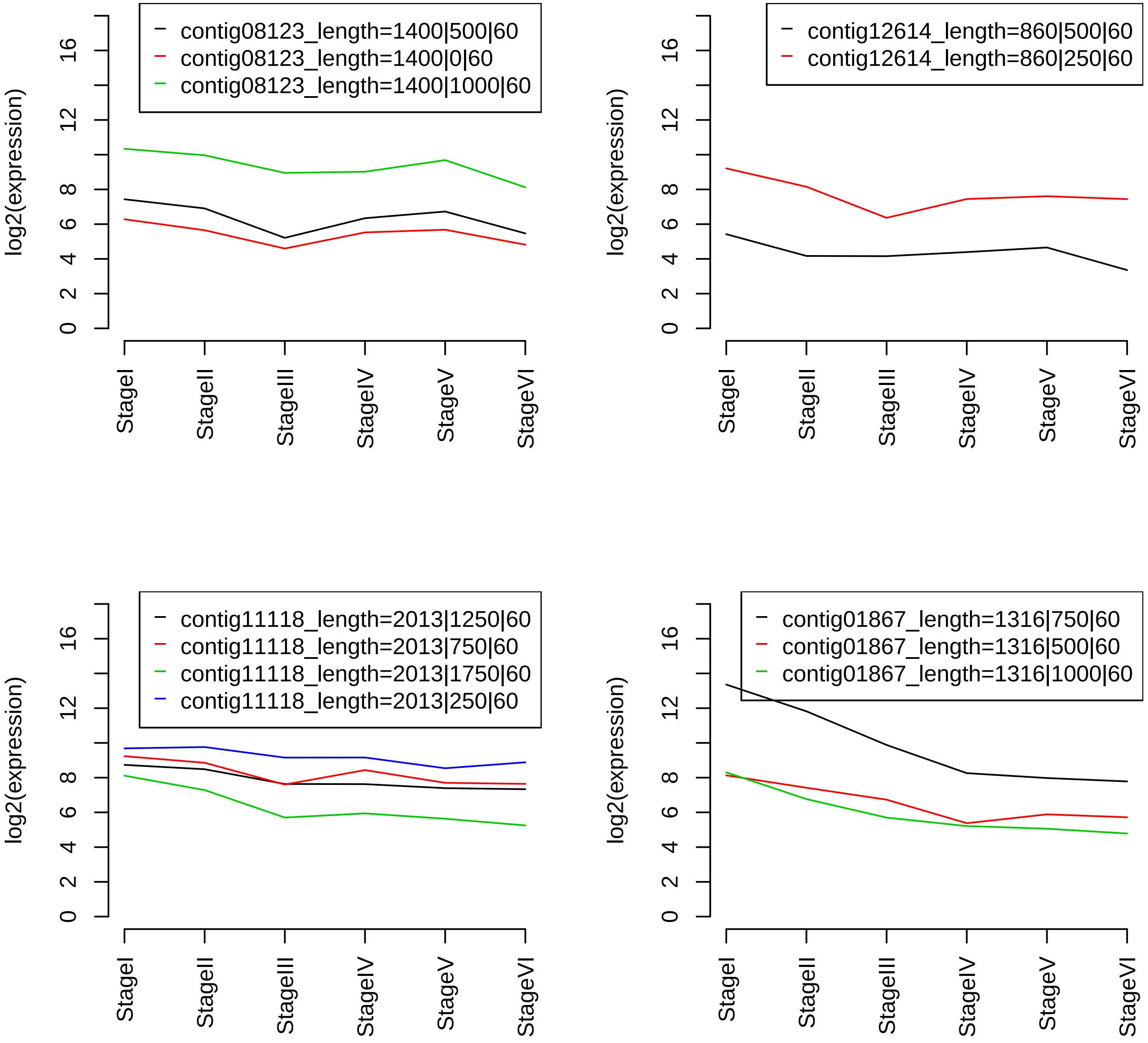

contig08123_length=1400|500|60
contig08123_length=1400|0|60
contig08123_length=1400|1000|60
contig12614_length=860|500|60
contig12614_length=860|250|60
contig11118_length=2013|1250|60
contig11118_length=2013|750|60
contig11118_length=2013|1750|60
contig11118_length=2013|250|60
contig01867_length=1316|750|60
contig01867_length=1316|500|60
contig01867_length=1316|1000|60
log2(expression)
0 2 4 6 8 12 16
StageI
StageII
StageIII
StageIV
StageV
StageVI

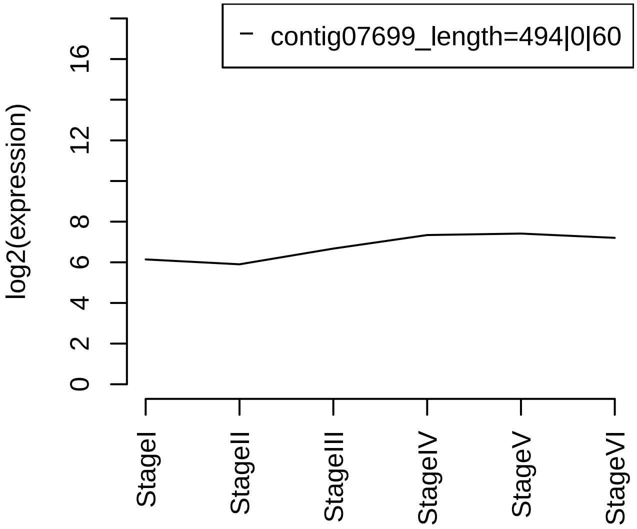
contig07699_length=494|0|60
log2(expression)
0
2
4
6
8
12
16
StageI
StageII
StageIII
StageIV
StageV
StageVI

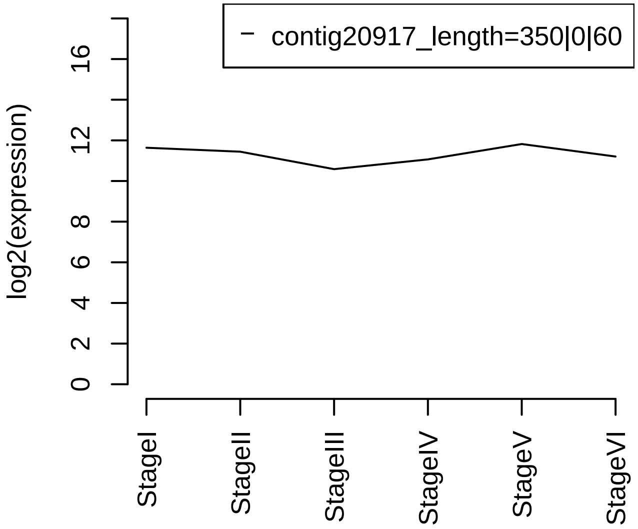
contig20917_length=350|0|60
log2(expression)
0
2
4
6
8
12
16
StageI
StageII
StageIII
StageIV
StageV
StageVI

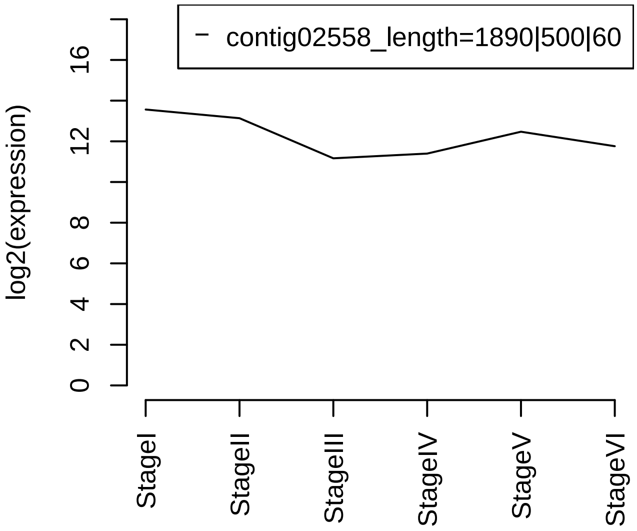
contig02558_length=1890|500|60
log2(expression)
0
2
4
6
8
12
16
StageI
StageII
StageIII
StageIV
StageV
StageVI

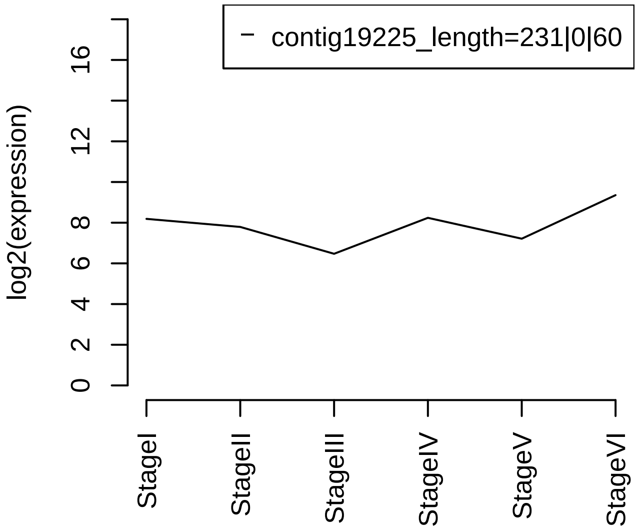
contig19225_length=231|0|60
log2(expression)
0
2
4
6
8
12
16
StageI
StageII
StageIII
StageIV
StageV
StageVI

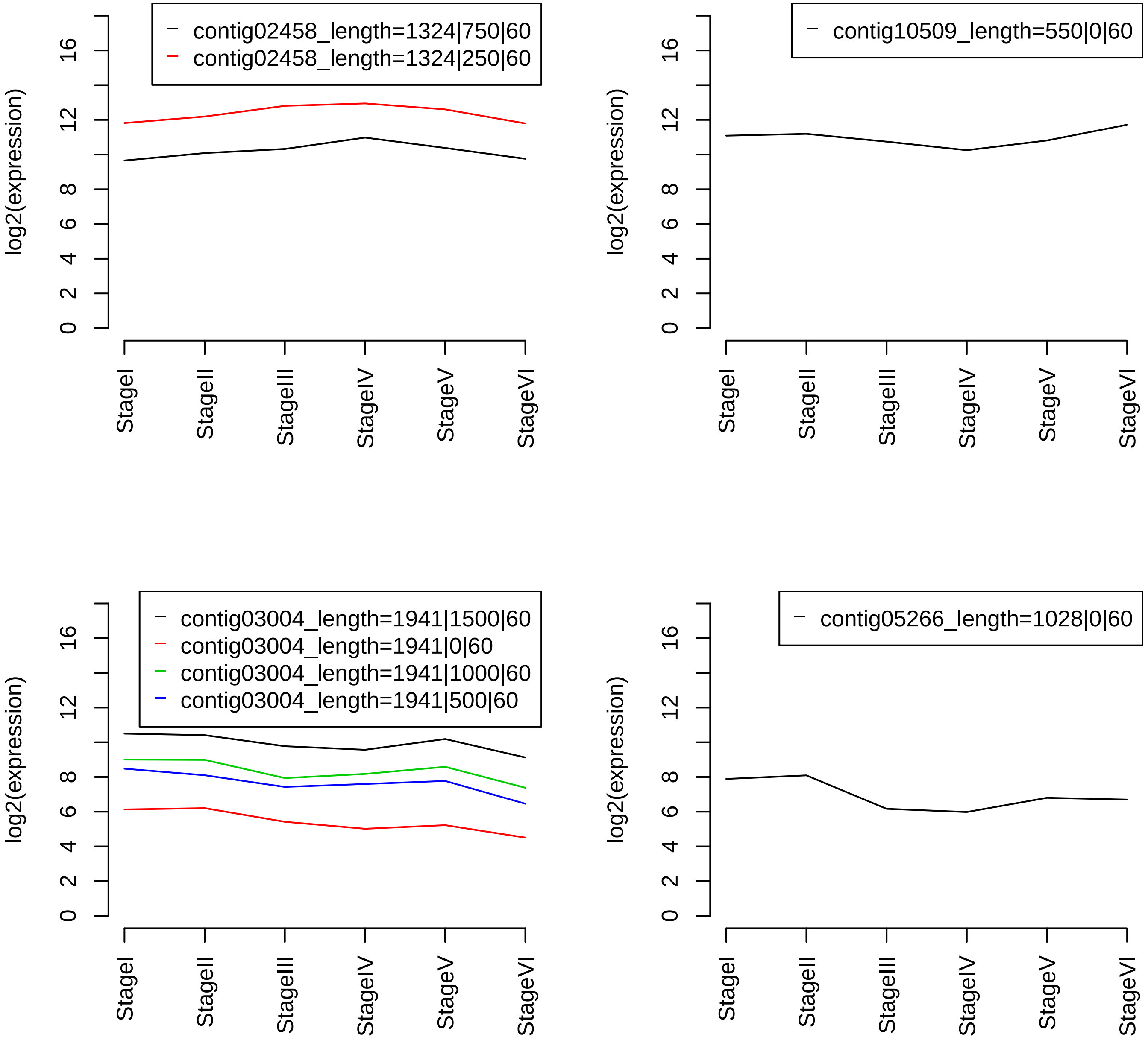
contig02458_length=1324|750|60
contig02458_length=1324|250|60
log2(expression)
0
2
4
6
8
12
16
StageI
StageII
StageIII
StageIV
StageV
StageVI
contig10509_length=550|0|60
log2(expression)
0
2
4
6
8
12
16
StageI
StageII
StageIII
StageIV
StageV
StageVI
contig03004_length=1941|1500|60
contig03004_length=1941|0|60
contig03004_length=1941|1000|60
contig03004_length=1941|500|60
log2(expression)
0
2
4
6
8
12
16
StageI
StageII
StageIII
StageIV
StageV
StageVI
contig05266_length=1028|0|60
log2(expression)
0
2
4
6
8
12
16
StageI
StageII
StageIII
StageIV
StageV
StageVI

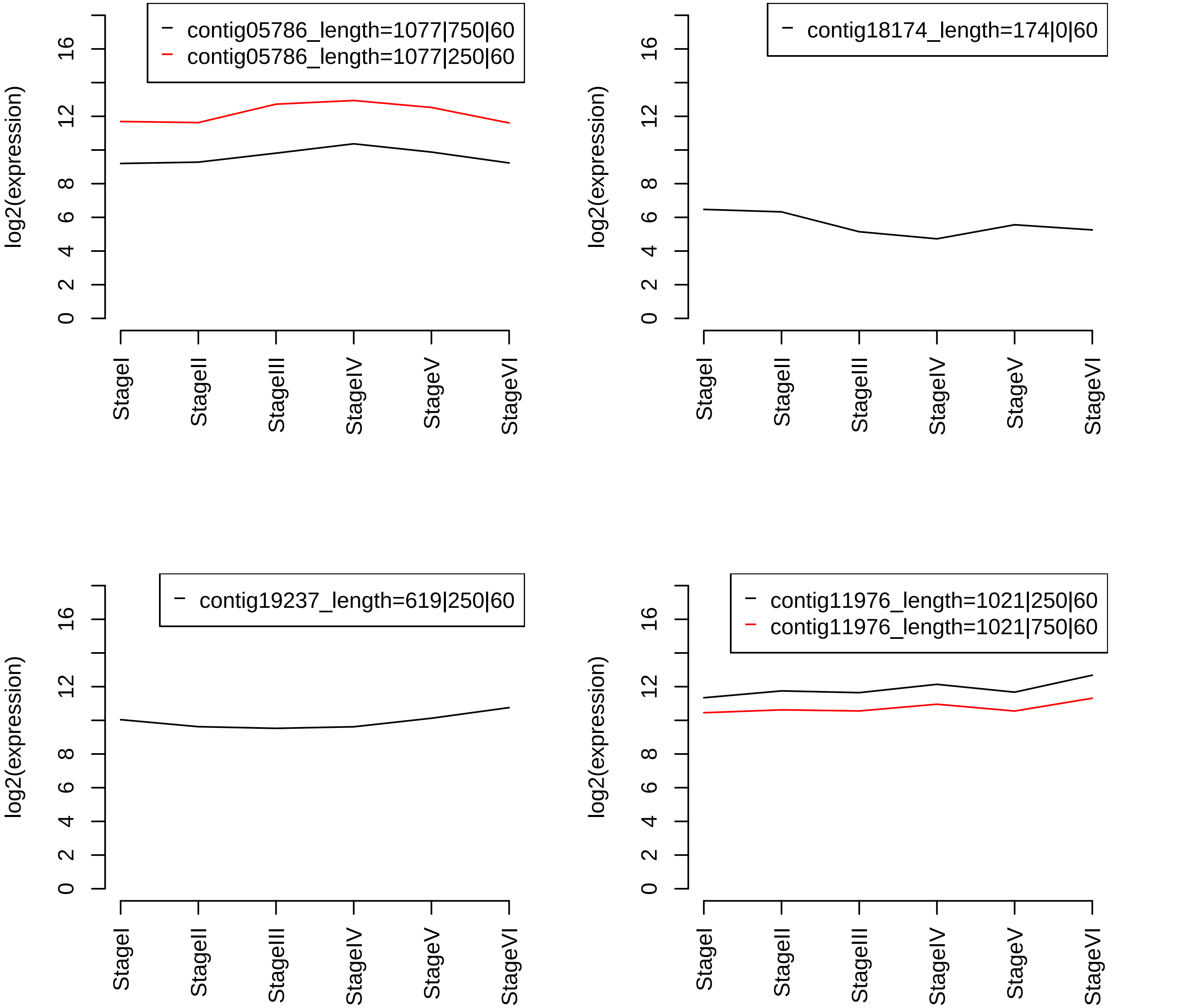
contig05786_length=1077|750|60
contig05786_length=1077|250|60
contig18174_length=174|0|60
contig19237_length=619|250|60
contig11976_length=1021|250|60
contig11976_length=1021|750|60
log2(expression)
0
2
4
6
8
12
16
StageI
StageII
StageIII
StageIV
StageV
StageVI

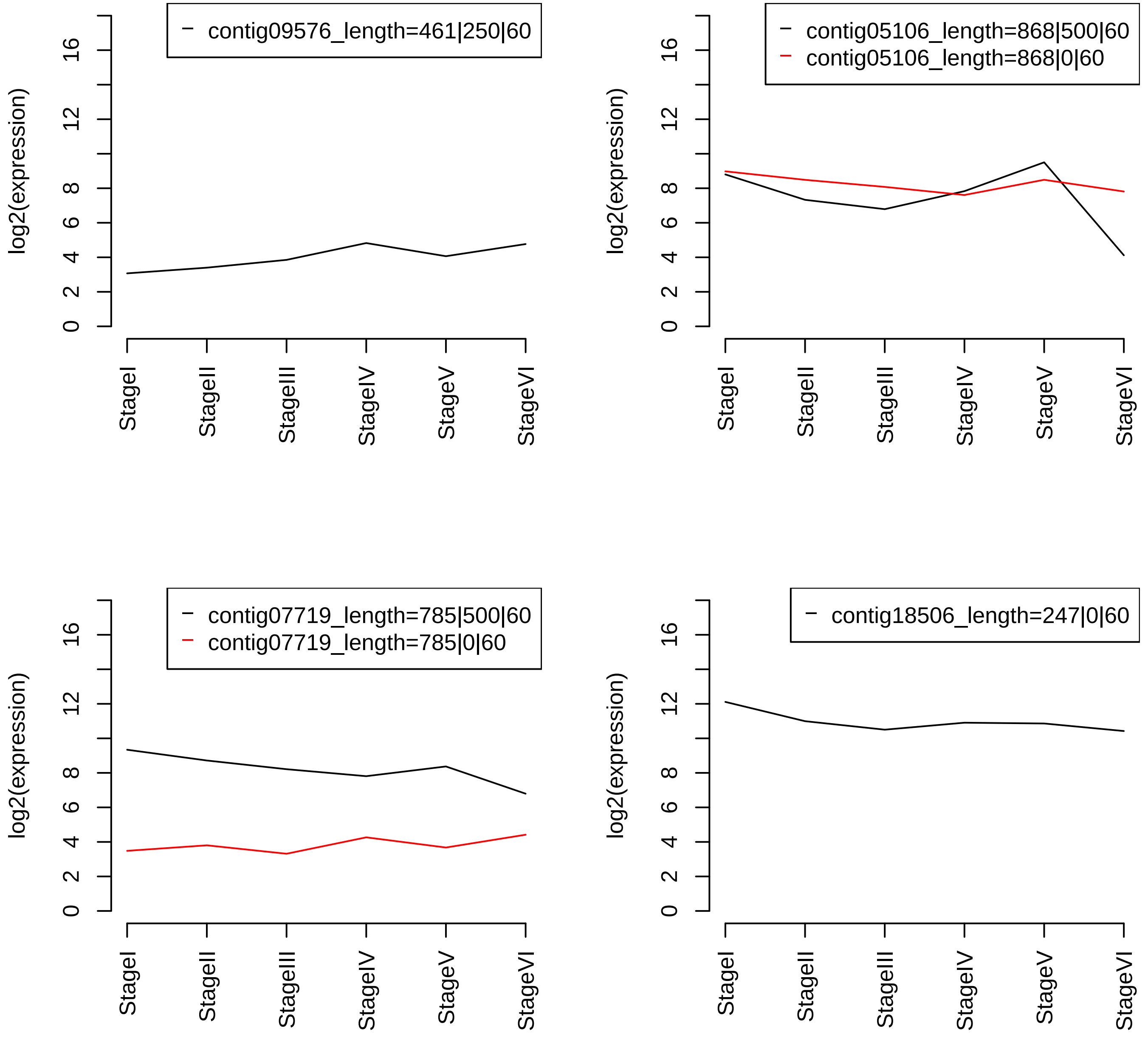
contig09576_length=461|250|60
contig05106_length=868|500|60
contig05106_length=868|0|60
contig07719_length=785|500|60
contig07719_length=785|0|60
contig18506_length=247|0|60
log2(expression)
0
2
4
6
8
12
16
StageI
StageII
StageIII
StageIV
StageV
StageVI

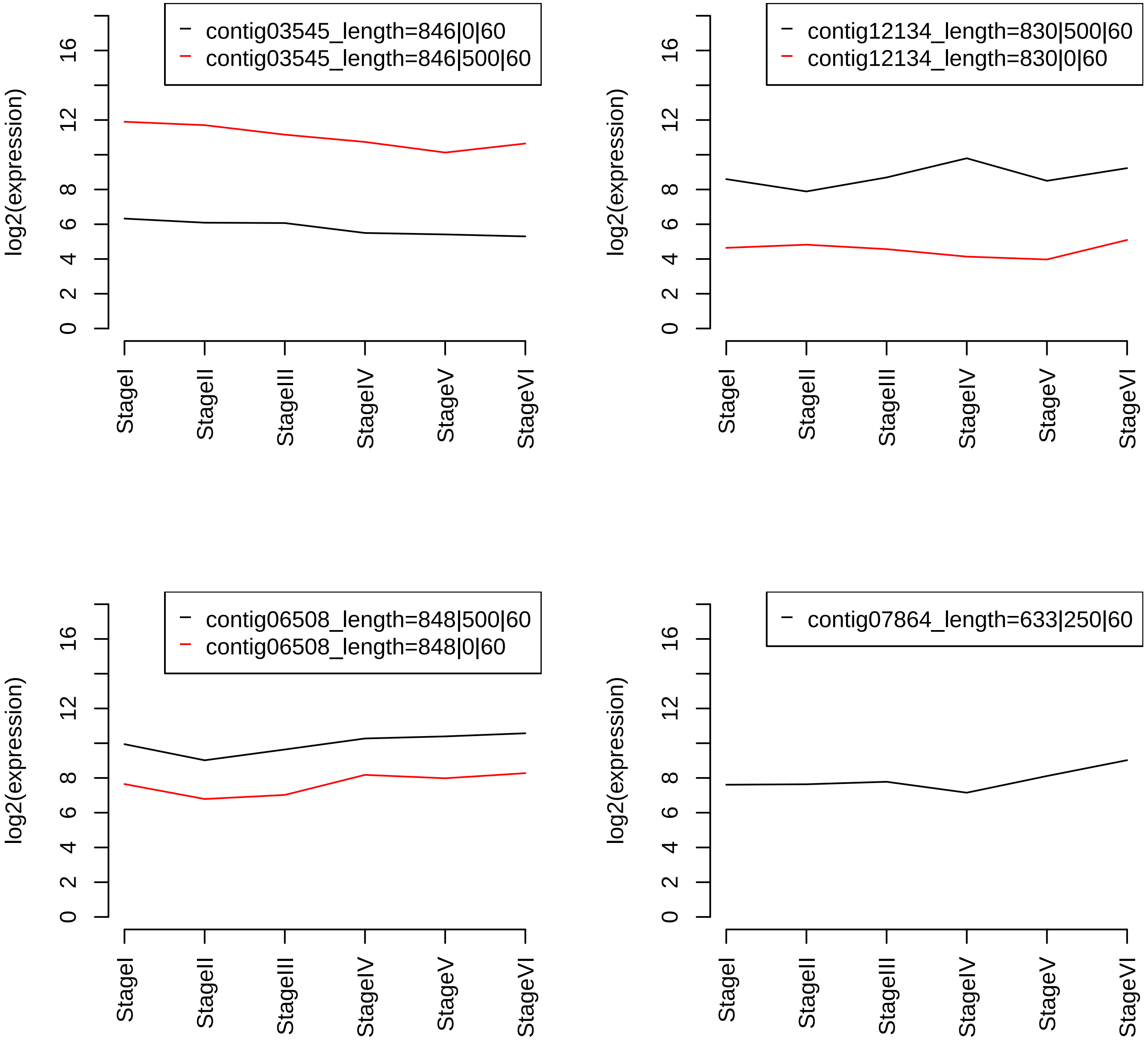
contig03545_length=846|0|60
contig03545_length=846|500|60
contig12134_length=830|500|60
contig12134_length=830|0|60
contig06508_length=848|500|60
contig06508_length=848|0|60
contig07864_length=633|250|60
log2(expression)
0
2
4
6
8
12
16
StageI
StageII
StageIII
StageIV
StageV
StageVI

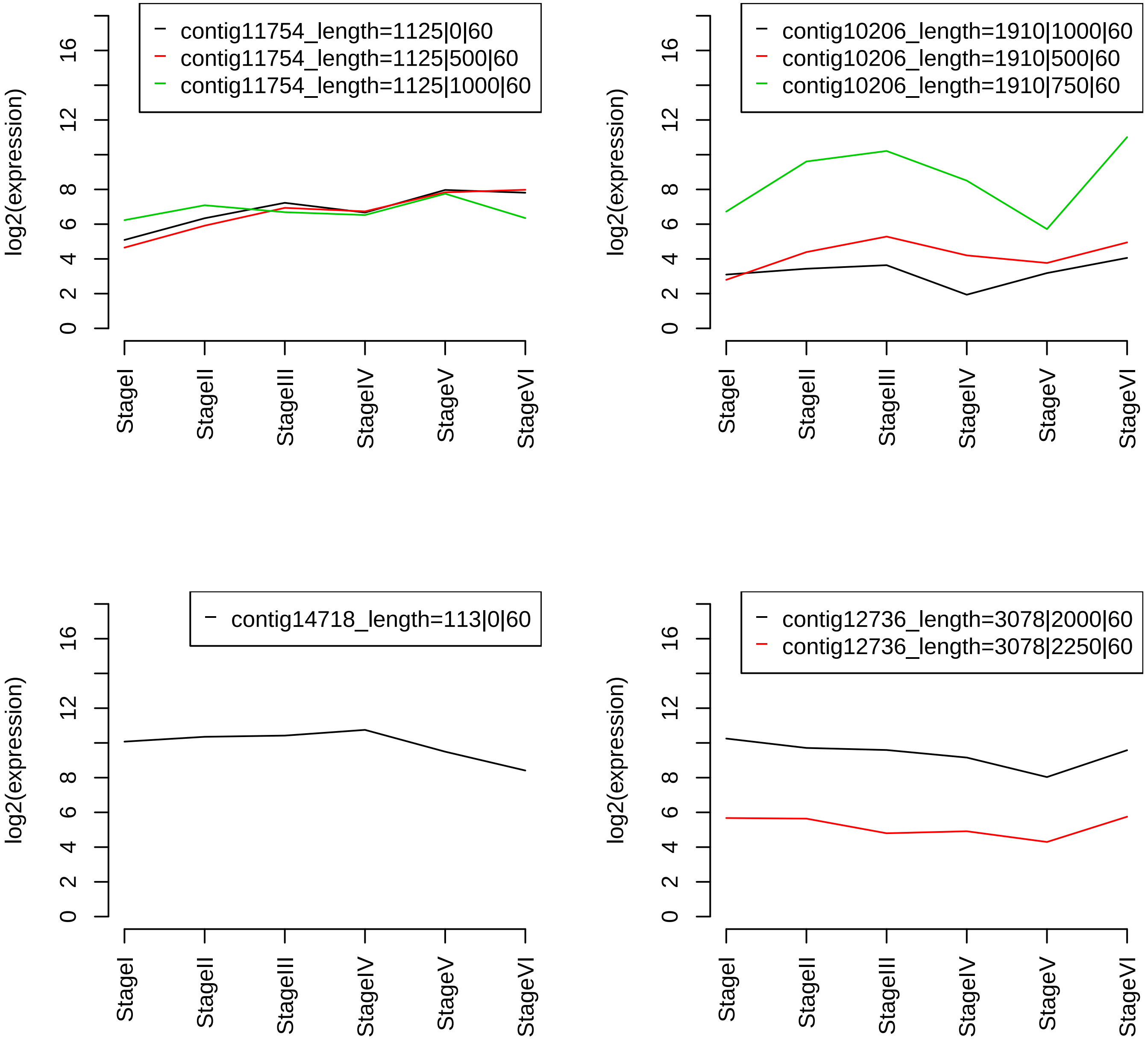
contig11754_length=1125|0|60
contig11754_length=1125|500|60
contig11754_length=1125|1000|60
contig10206_length=1910|1000|60
contig10206_length=1910|500|60
contig10206_length=1910|750|60
contig14718_length=113|0|60
contig12736_length=3078|2000|60
contig12736_length=3078|2250|60
log2(expression)
StageI
StageII
StageIII
StageIV
StageV
StageVI

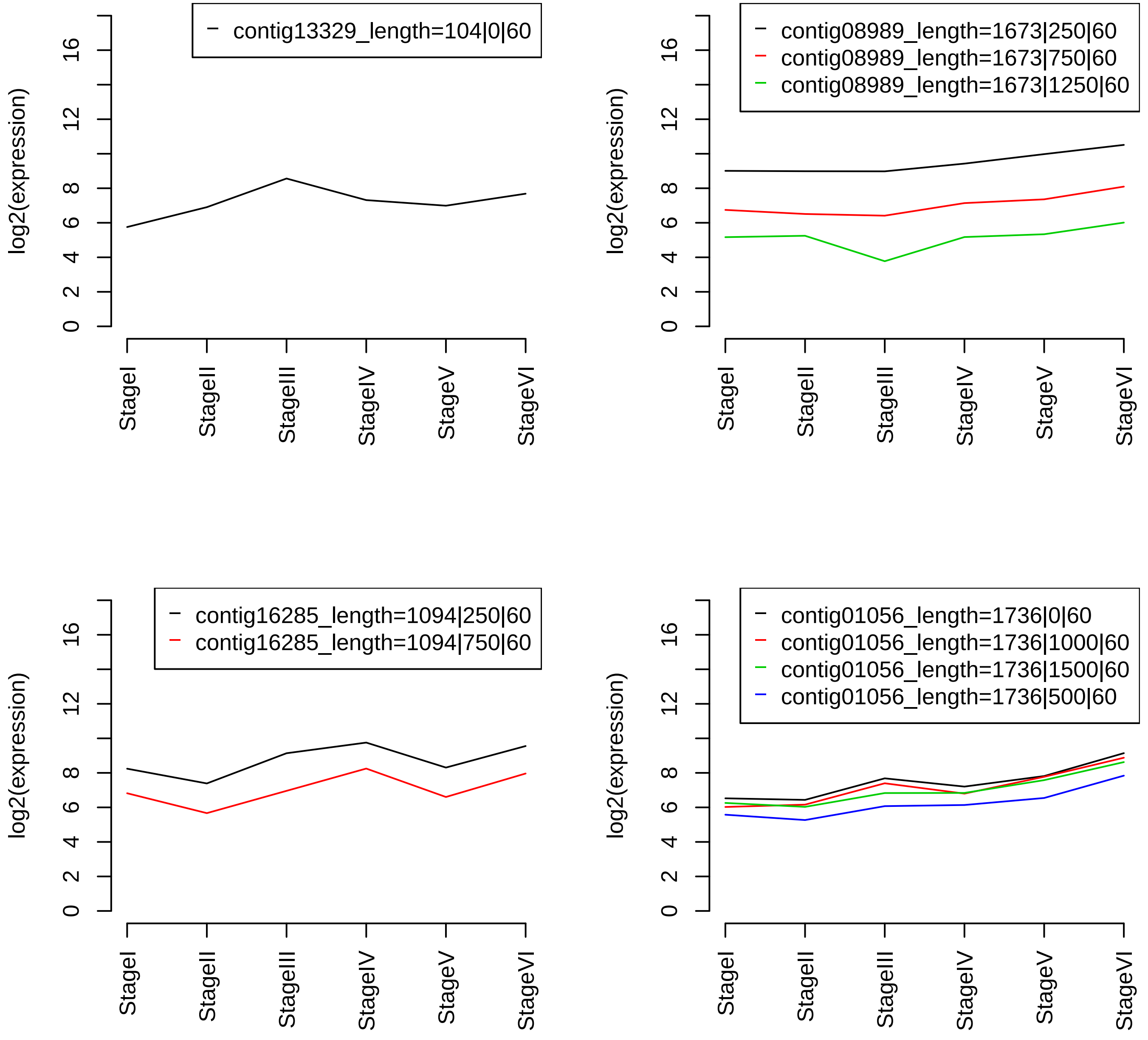

contig13329_length=104|0|60
contig08989_length=1673|250|60
contig08989_length=1673|750|60
contig08989_length=1673|1250|60
contig16285_length=1094|250|60
contig16285_length=1094|750|60
contig01056_length=1736|0|60
contig01056_length=1736|1000|60
contig01056_length=1736|1500|60
contig01056_length=1736|500|60
log2(expression)
0
2
4
6
8
12
16
StageI
StageII
StageIII
StageIV
StageV
StageVI

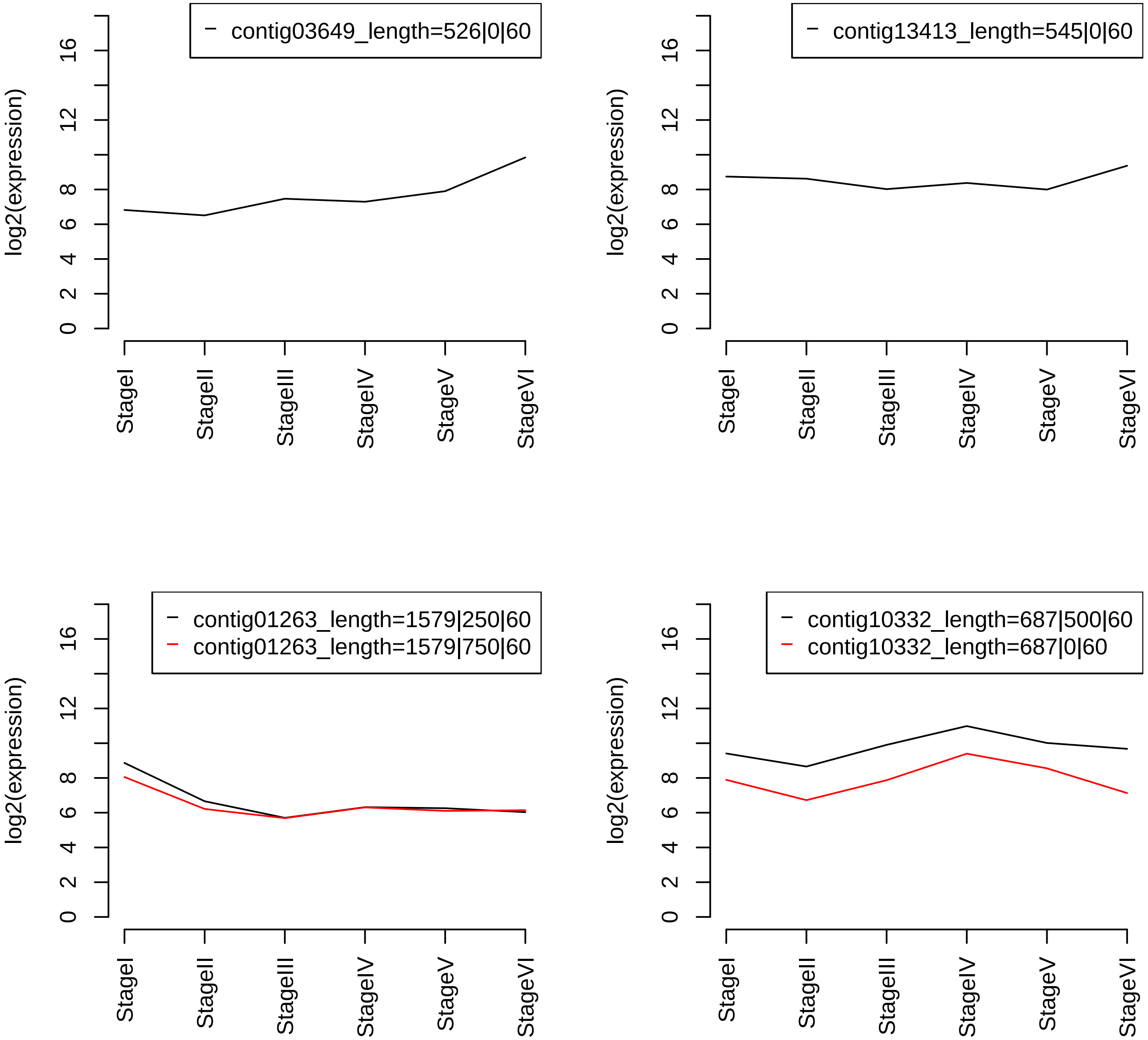

contig03649_length=526|0|60
contig13413_length=545|0|60
contig01263_length=1579|250|60
contig01263_length=1579|750|60
contig10332_length=687|500|60
contig10332_length=687|0|60
log2(expression)
0 2 4 6 8 12 16
StageI
StageII
StageIII
StageIV
StageV
StageVI

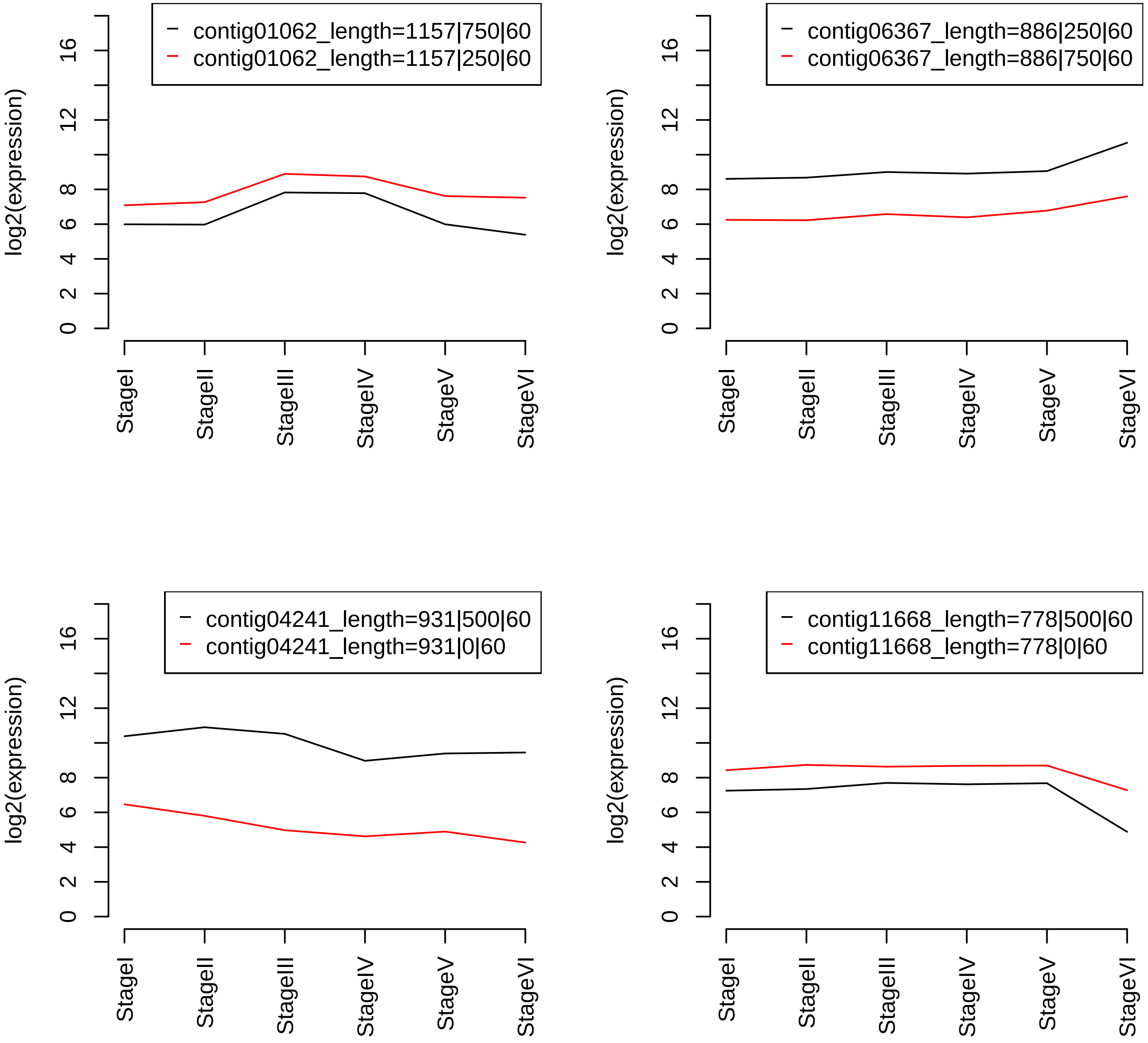
contig01062_length=1157|750|60
contig01062_length=1157|250|60
contig06367_length=886|250|60
contig06367_length=886|750|60
contig04241_length=931|500|60
contig04241_length=931|0|60
contig11668_length=778|500|60
contig11668_length=778|0|60
log2(expression)
0
2
4
6
8
12
16
StageI
StageII
StageIII
StageIV
StageV
StageVI

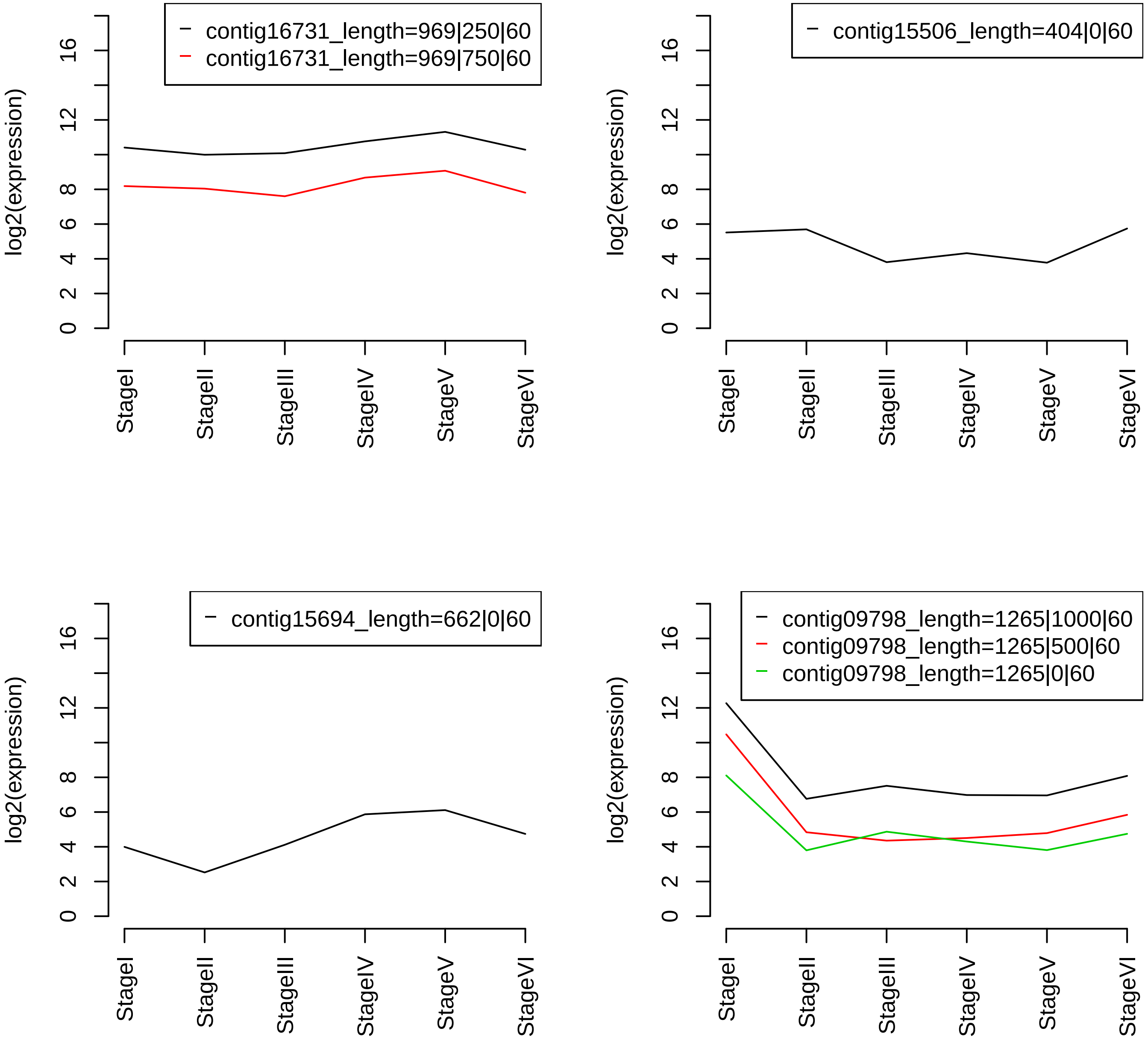
contig16731_length=969|250|60
contig16731_length=969|750|60
contig15506_length=404|0|60
contig15694_length=662|0|60
contig09798_length=1265|1000|60
contig09798_length=1265|500|60
contig09798_length=1265|0|60
log2(expression)
StageI
StageII
StageIII
StageIV
StageV
StageVI

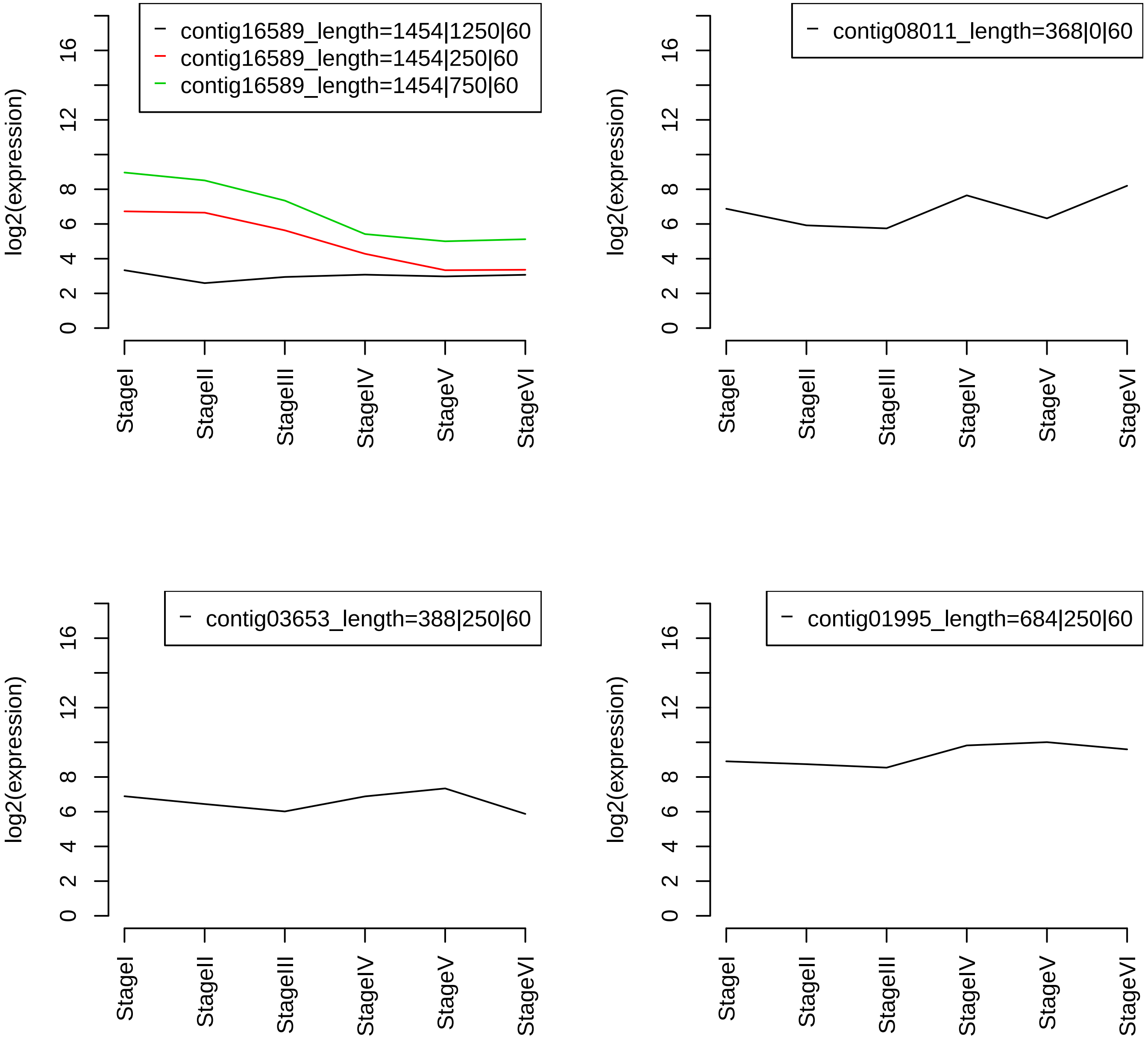
contig16589_length=1454|1250|60
contig16589_length=1454|250|60
contig16589_length=1454|750|60
contig08011_length=368|0|60
contig03653_length=388|250|60
contig01995_length=684|250|60
log2(expression)
0
2
4
6
8
12
16
StageI
StageII
StageIII
StageIV
StageV
StageVI

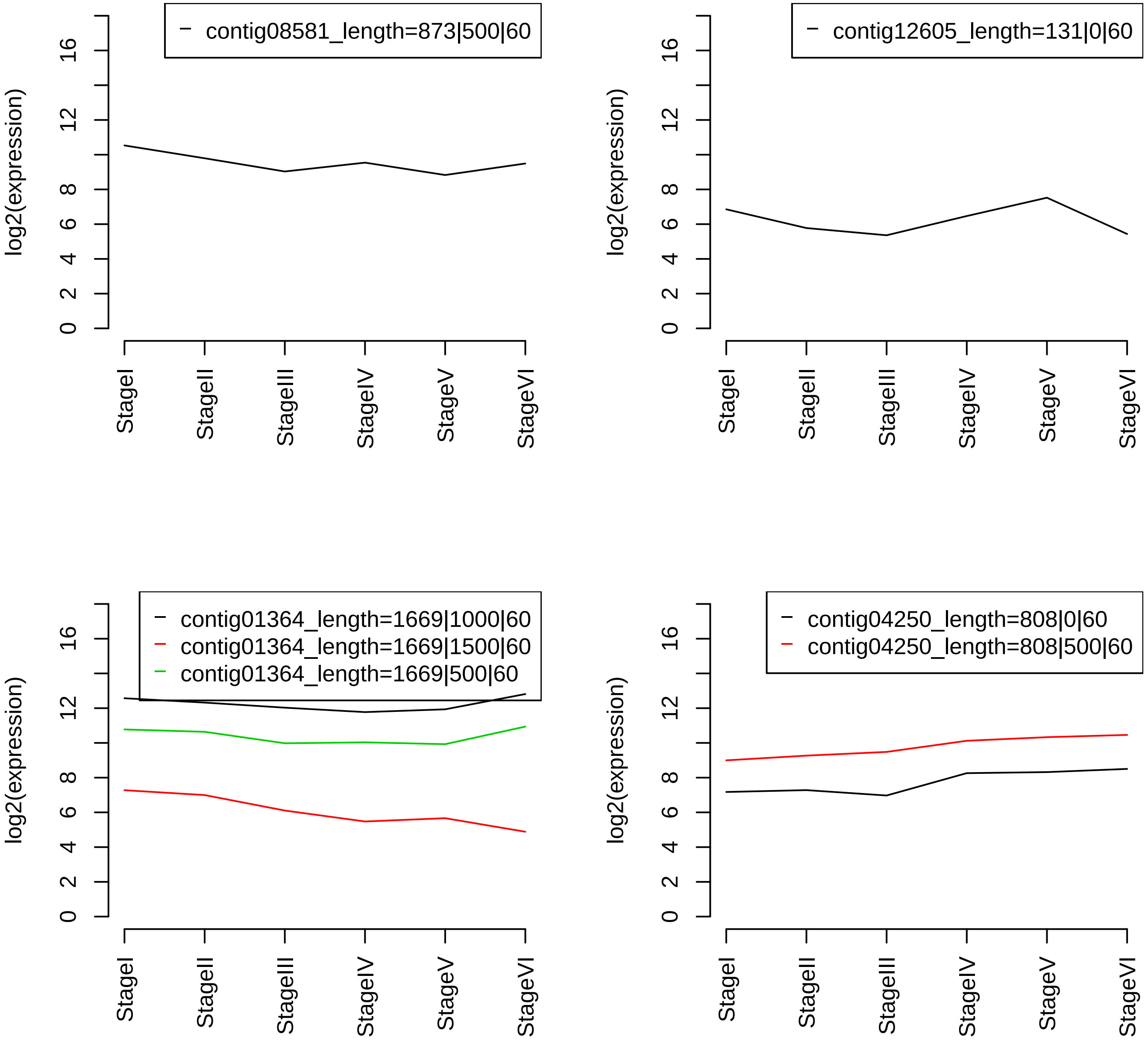

contig08581_length=873|500|60
contig12605_length=131|0|60
contig01364_length=1669|1000|60
contig01364_length=1669|1500|60
contig01364_length=1669|500|60
contig04250_length=808|0|60
contig04250_length=808|500|60
log2(expression)
0
2
4
6
8
12
16
StageI
StageII
StageIII
StageIV
StageV
StageVI

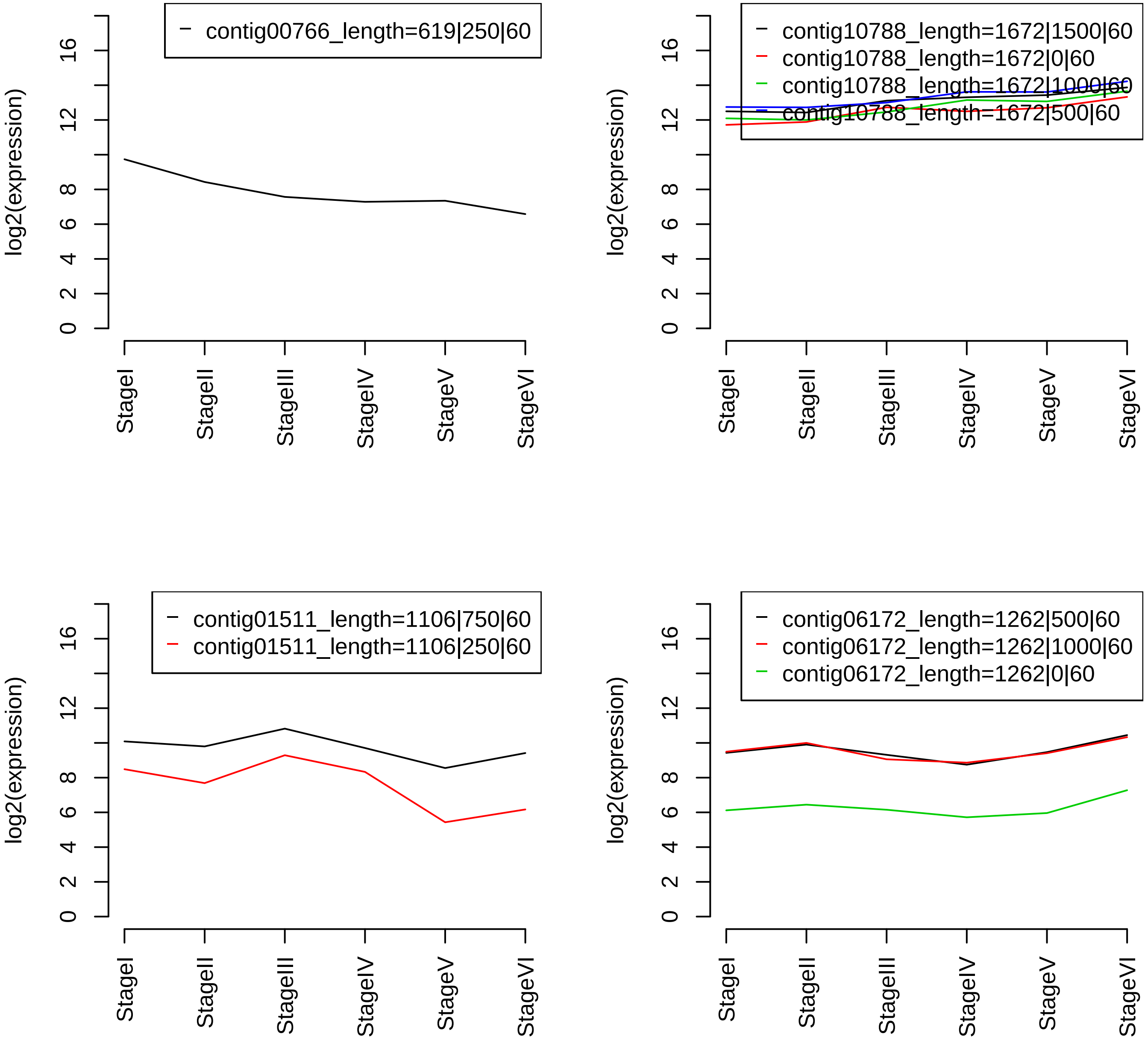

contig00766_length=619|250|60
log2(expression)
0
2
4
6
8
12
16
StageI
StageII
StageIII
StageIV
StageV
StageVI
contig10788_length=1672|1500|60
contig10788_length=1672|0|60
contig10788_length=1672|1000|60
contig10788_length=1672|500|60
contig01511_length=1106|750|60
contig01511_length=1106|250|60
contig06172_length=1262|500|60
contig06172_length=1262|1000|60
contig06172_length=1262|0|60

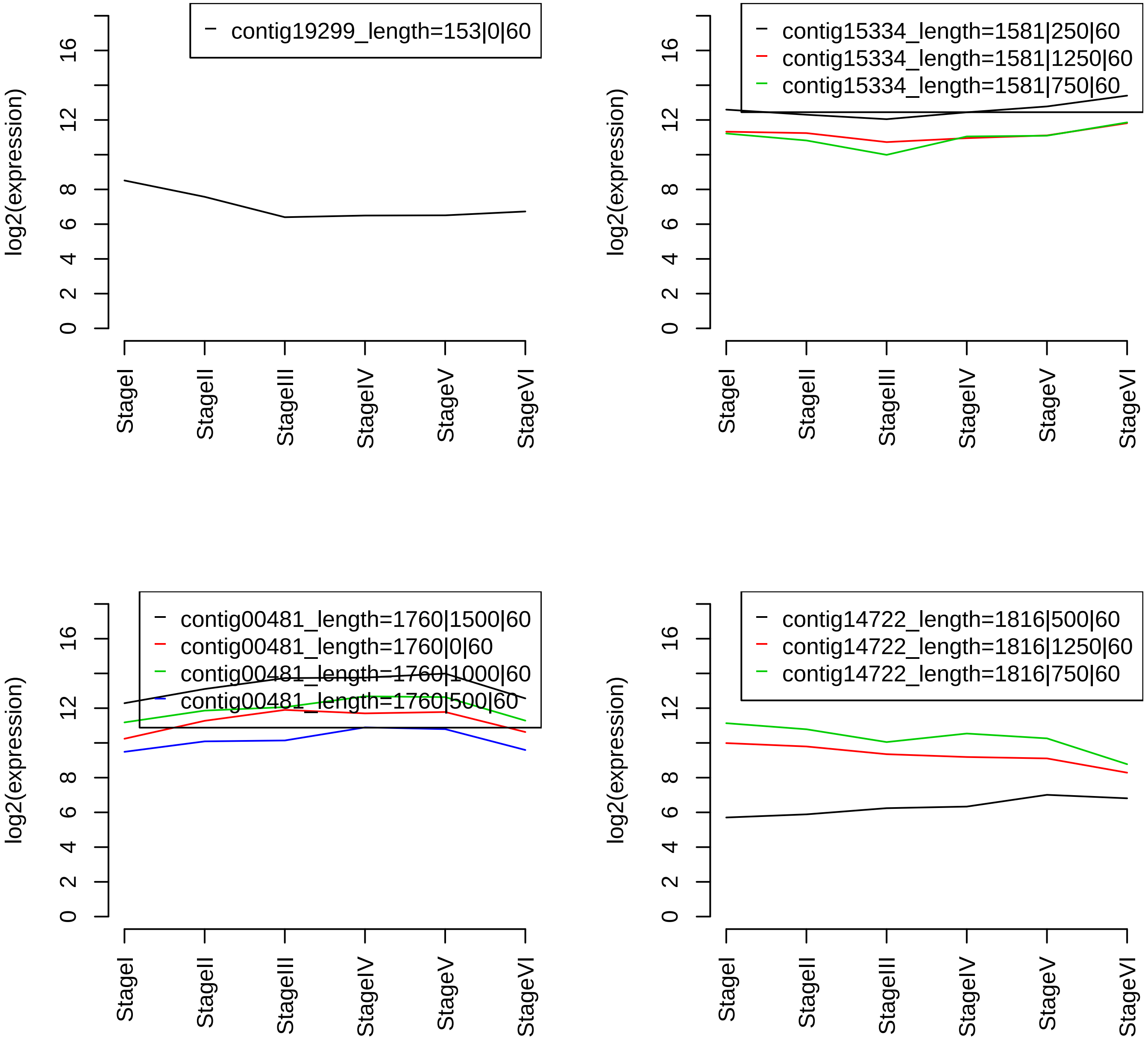

contig19299_length=153|0|60
contig15334_length=1581|250|60
contig15334_length=1581|1250|60
contig15334_length=1581|750|60
contig00481_length=1760|1500|60
contig00481_length=1760|0|60
contig00481_length=1760|1000|60
contig00481_length=1760|500|60
contig14722_length=1816|500|60
contig14722_length=1816|1250|60
contig14722_length=1816|750|60
log2(expression)
0
2
4
6
8
12
16
StageI
StageII
StageIII
StageIV
StageV
StageVI

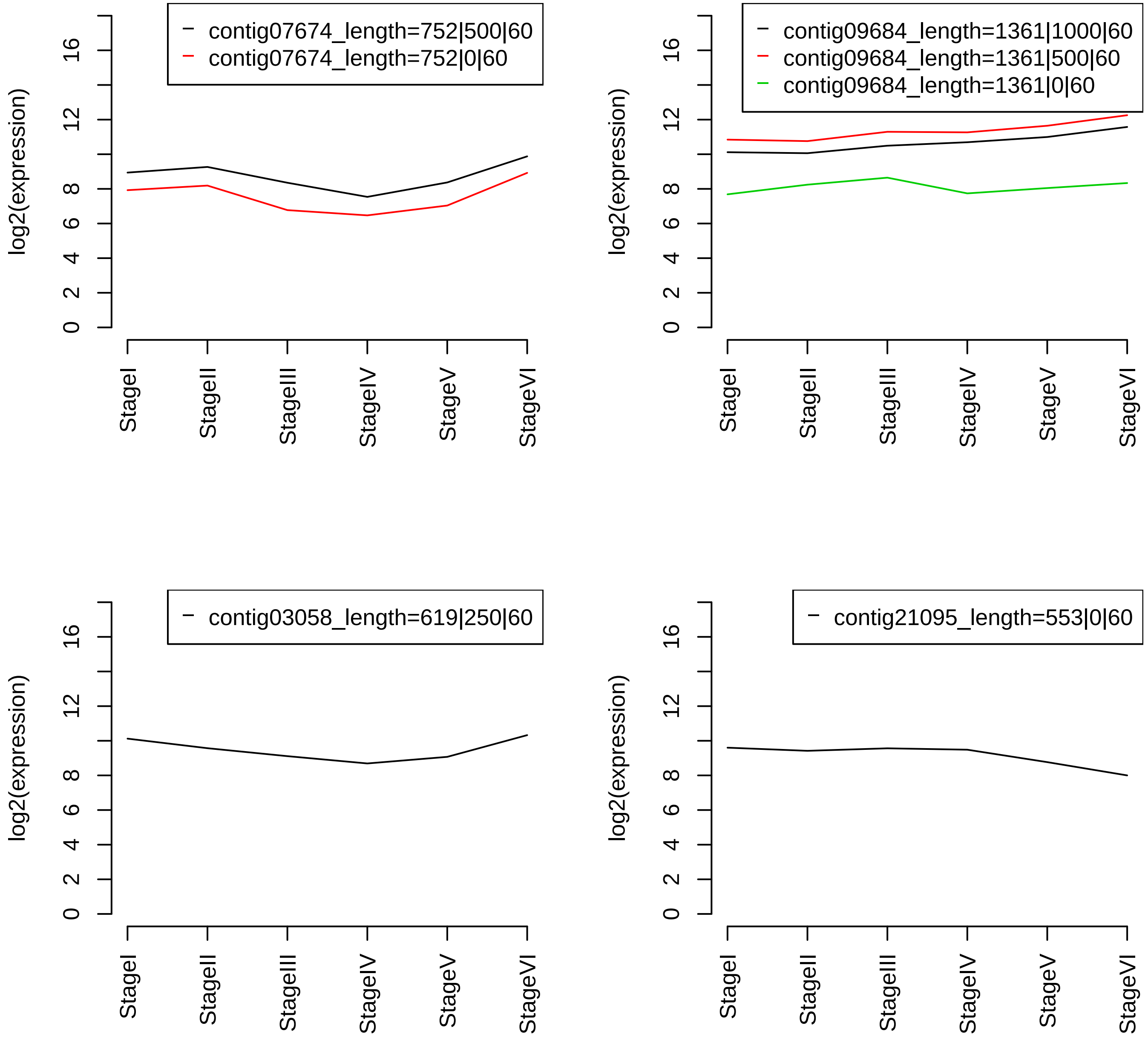

contig07674_length=752|500|60
contig07674_length=752|0|60
contig09684_length=1361|1000|60
contig09684_length=1361|500|60
contig09684_length=1361|0|60
contig03058_length=619|250|60
contig21095_length=553|0|60
log2(expression)
0
2
4
6
8
12
16
StageI
StageII
StageIII
StageIV
StageV
StageVI

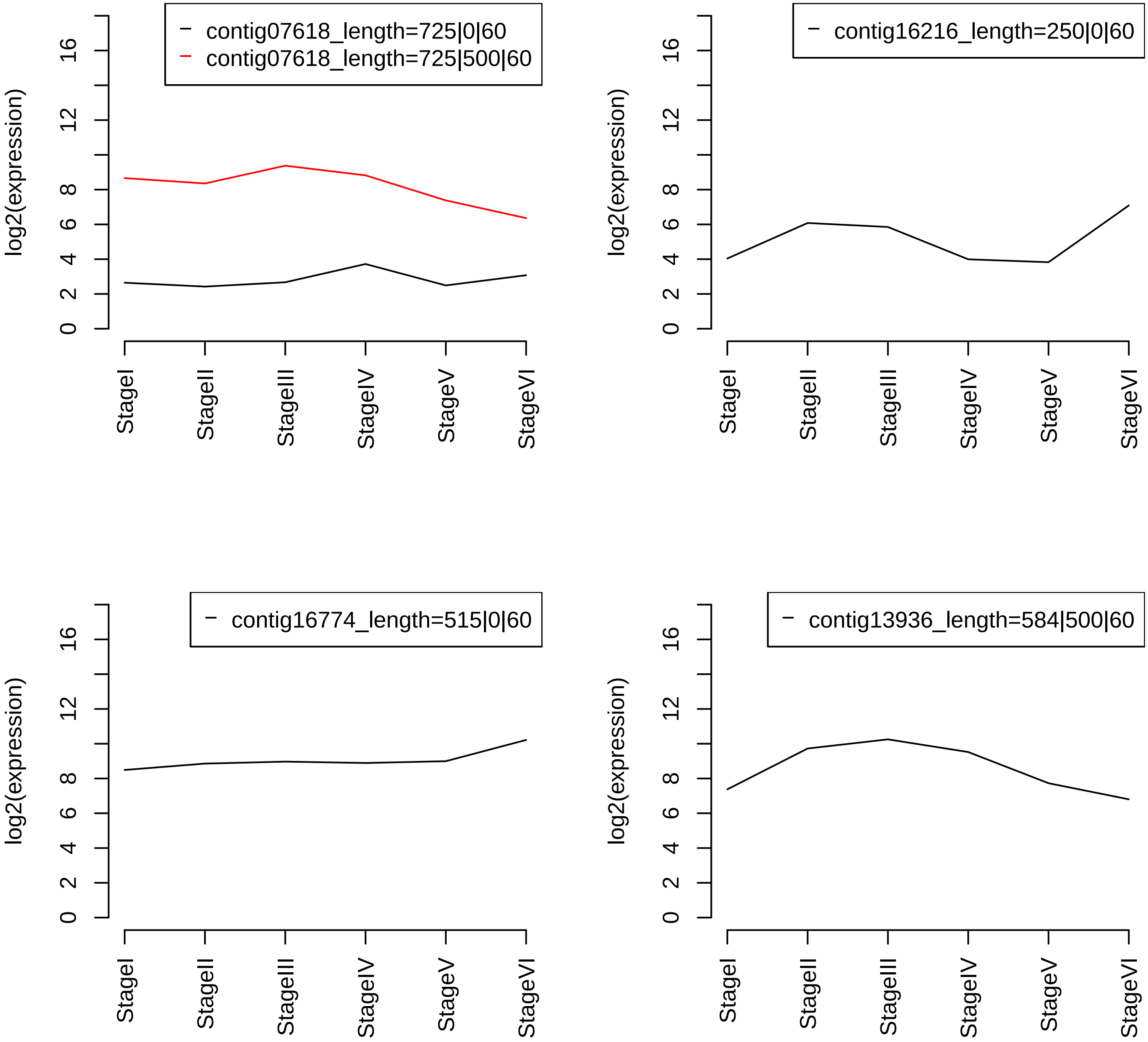
contig07618_length=725|0|60
contig07618_length=725|500|60
contig16216_length=250|0|60
contig16774_length=515|0|60
contig13936_length=584|500|60
log2(expression)
0
2
4
6
8
12
16
StageI
StageII
StageIII
StageIV
StageV
StageVI

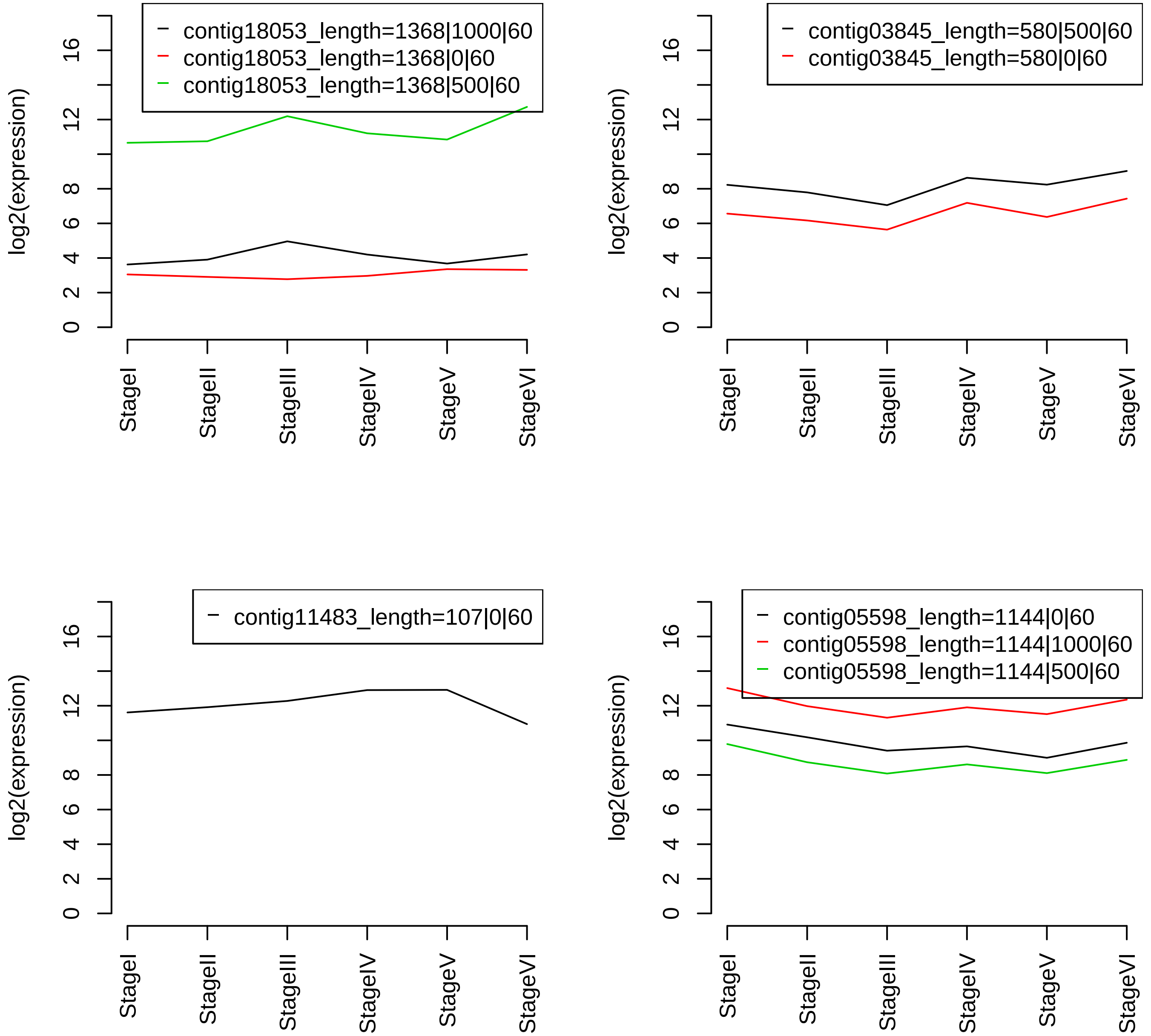

contig18053_length=1368|1000|60
contig18053_length=1368|0|60
contig18053_length=1368|500|60
log2(expression)
0
2
4
6
8
12
16
StageI
StageII
StageIII
StageIV
StageV
StageVI
contig03845_length=580|500|60
contig03845_length=580|0|60
contig11483_length=107|0|60
contig05598_length=1144|0|60
contig05598_length=1144|1000|60
contig05598_length=1144|500|60

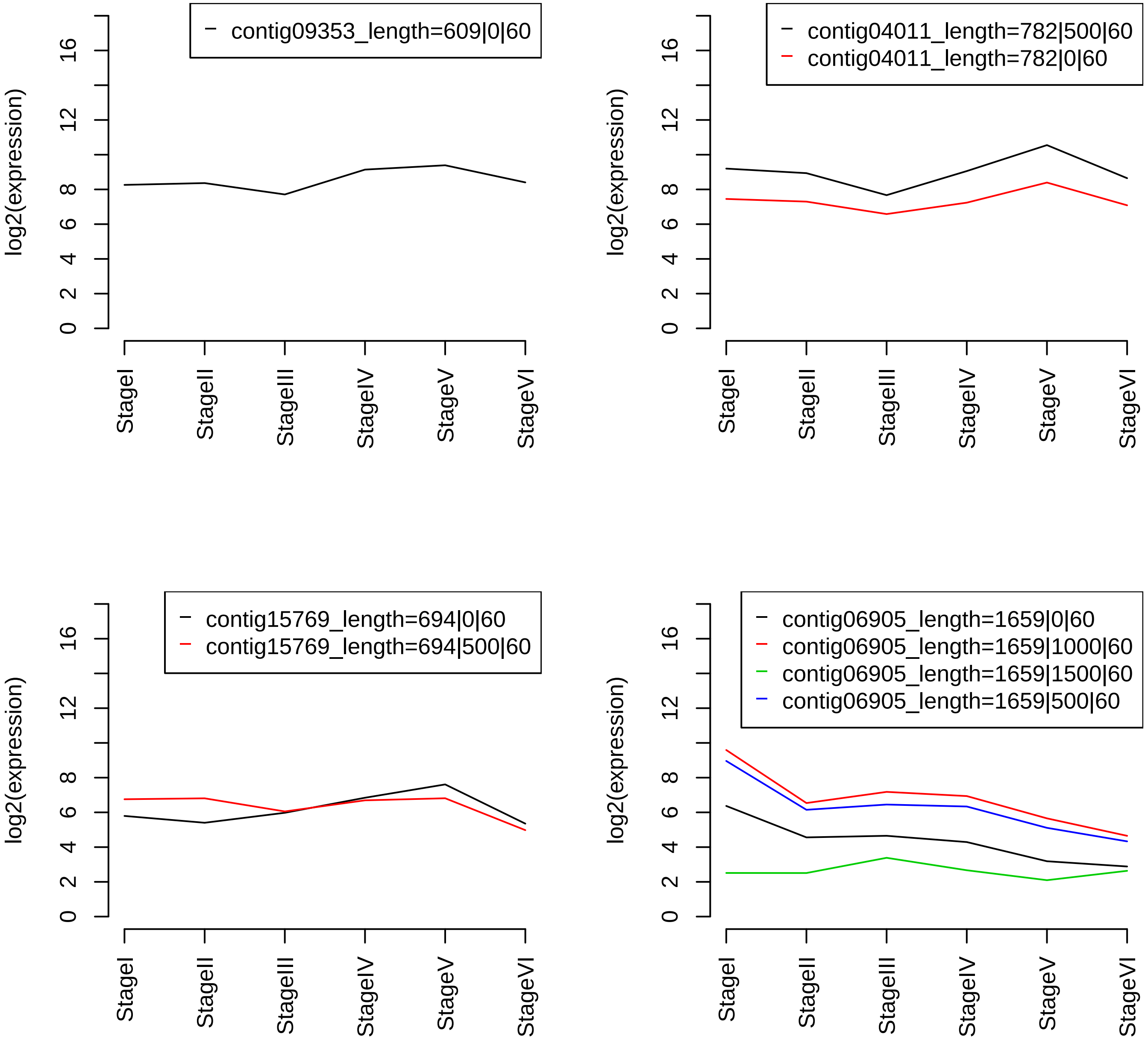

contig09353_length=609|0|60
contig04011_length=782|500|60
contig04011_length=782|0|60
contig15769_length=694|0|60
contig15769_length=694|500|60
contig06905_length=1659|0|60
contig06905_length=1659|1000|60
contig06905_length=1659|1500|60
contig06905_length=1659|500|60
log2(expression)
0
2
4
6
8
12
16
StageI
StageII
StageIII
StageIV
StageV
StageVI

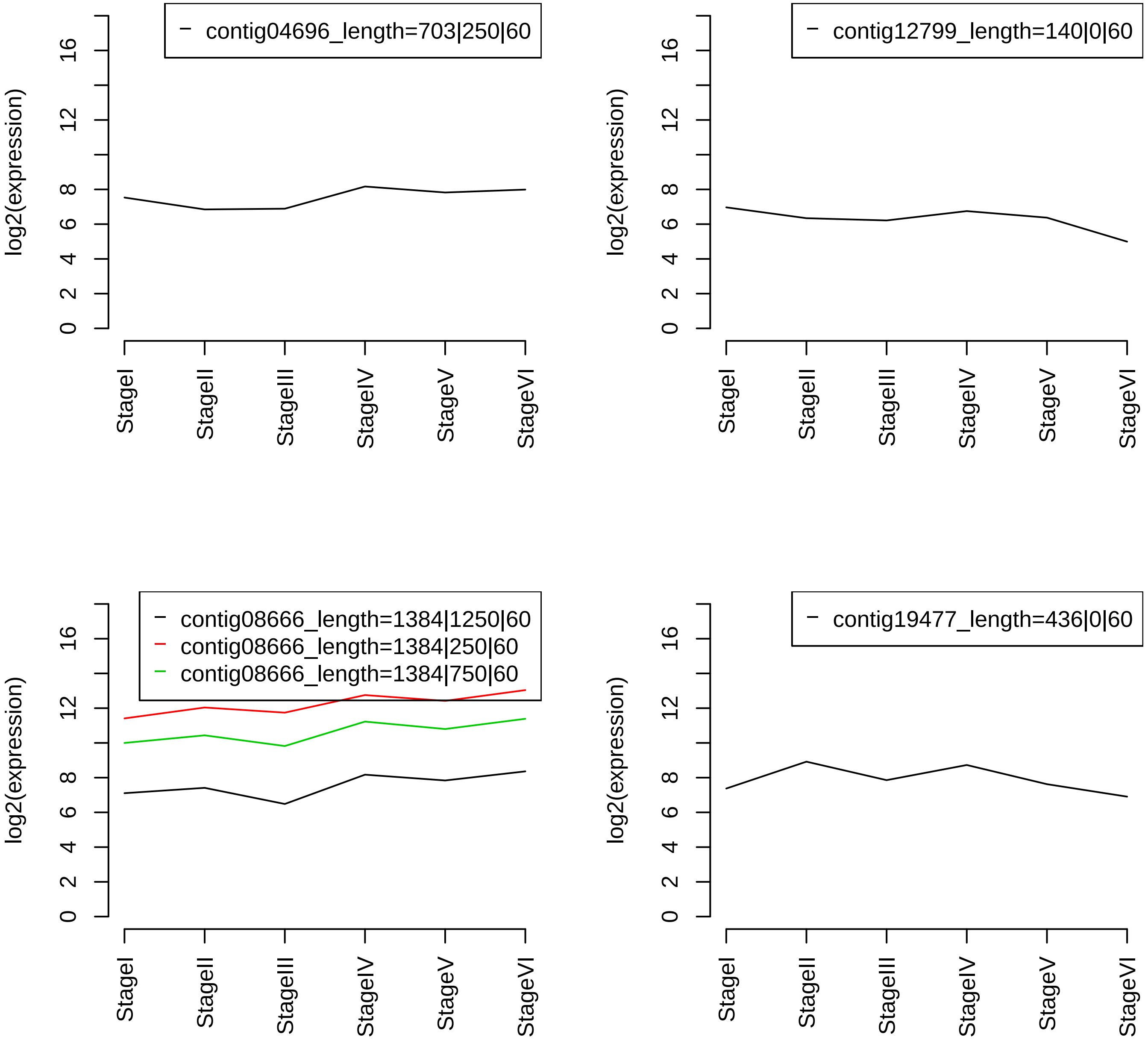

contig04696_length=703|250|60
contig12799_length=140|0|60
contig08666_length=1384|1250|60
contig08666_length=1384|250|60
contig08666_length=1384|750|60
contig19477_length=436|0|60
log2(expression)
0
2
4
6
8
12
16
StageI
StageII
StageIII
StageIV
StageV
StageVI

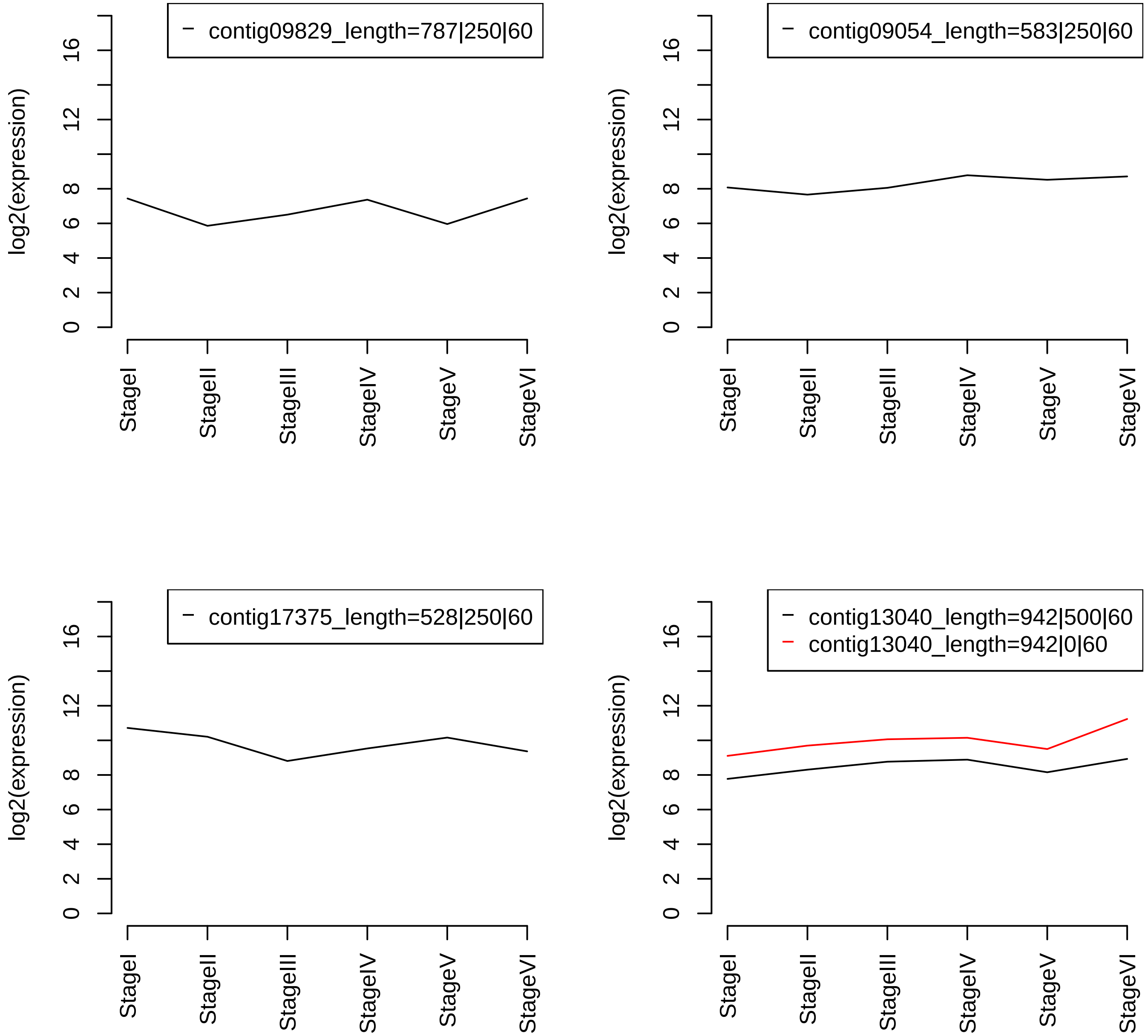
contig09829_length=787|250|60
contig09054_length=583|250|60
contig17375_length=528|250|60
contig13040_length=942|500|60
contig13040_length=942|0|60
log2(expression)
0
2
4
6
8
12
16
StageI
StageII
StageIII
StageIV
StageV
StageVI

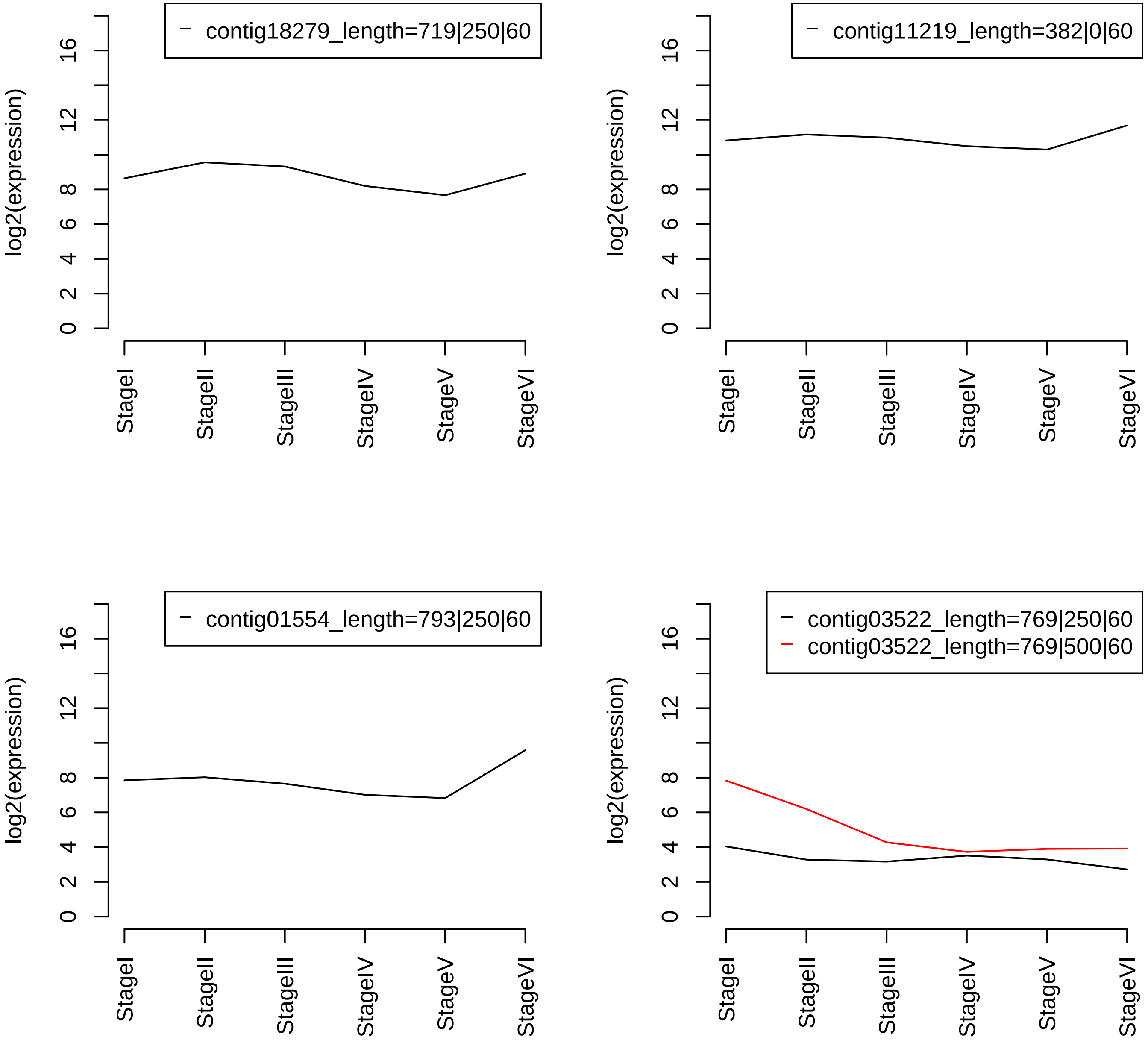
contig18279_length=719|250|60
contig11219_length=382|0|60
contig01554_length=793|250|60
contig03522_length=769|250|60
contig03522_length=769|500|60
log2(expression)
0
2
4
6
8
12
16
StageI
StageII
StageIII
StageIV
StageV
StageVI

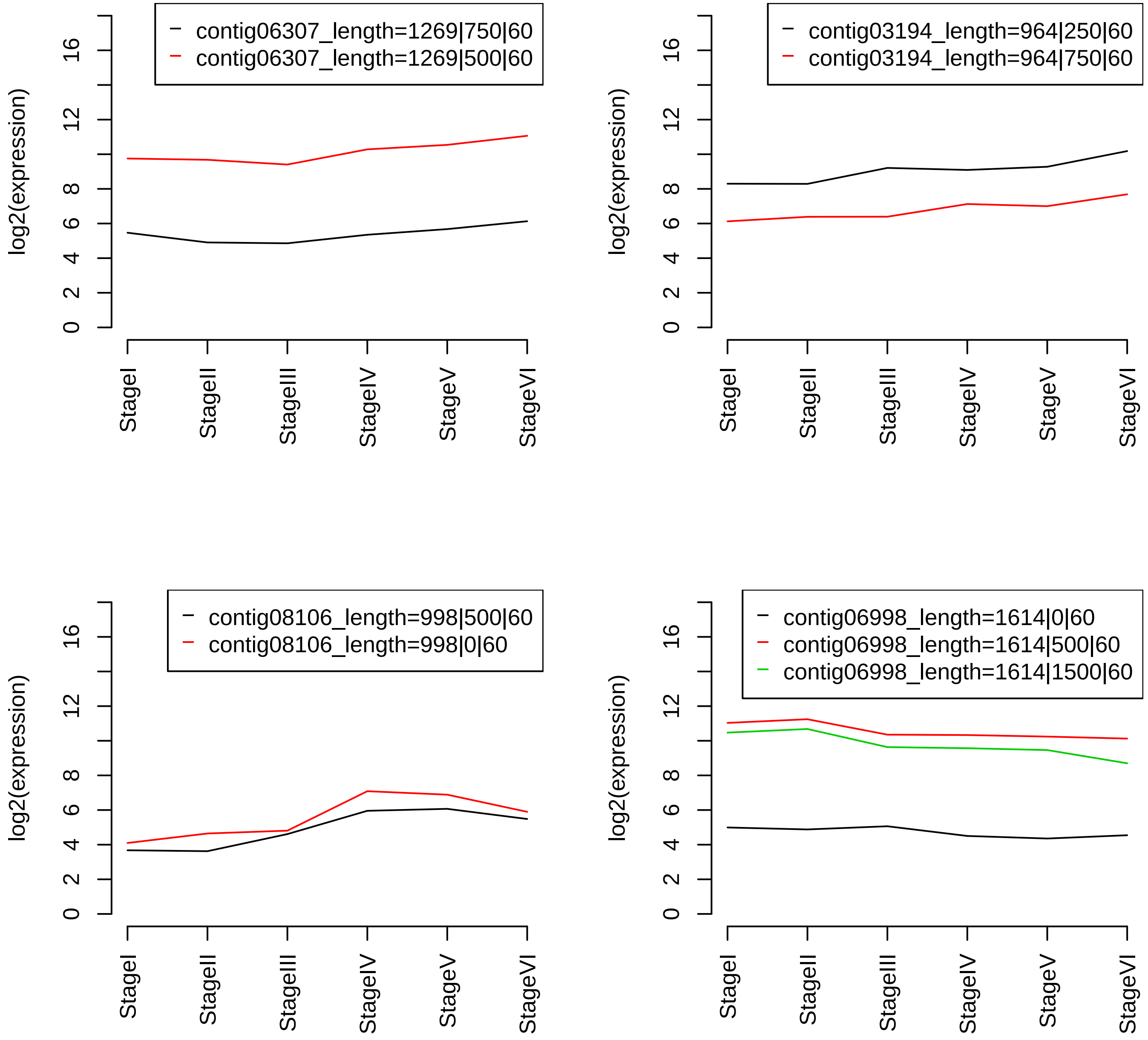
contig06307_length=1269|750|60
contig06307_length=1269|500|60
contig03194_length=964|250|60
contig03194_length=964|750|60
contig08106_length=998|500|60
contig08106_length=998|0|60
contig06998_length=1614|0|60
contig06998_length=1614|500|60
contig06998_length=1614|1500|60
log2(expression)
0
2
4
6
8
12
16
StageI
StageII
StageIII
StageIV
StageV
StageVI

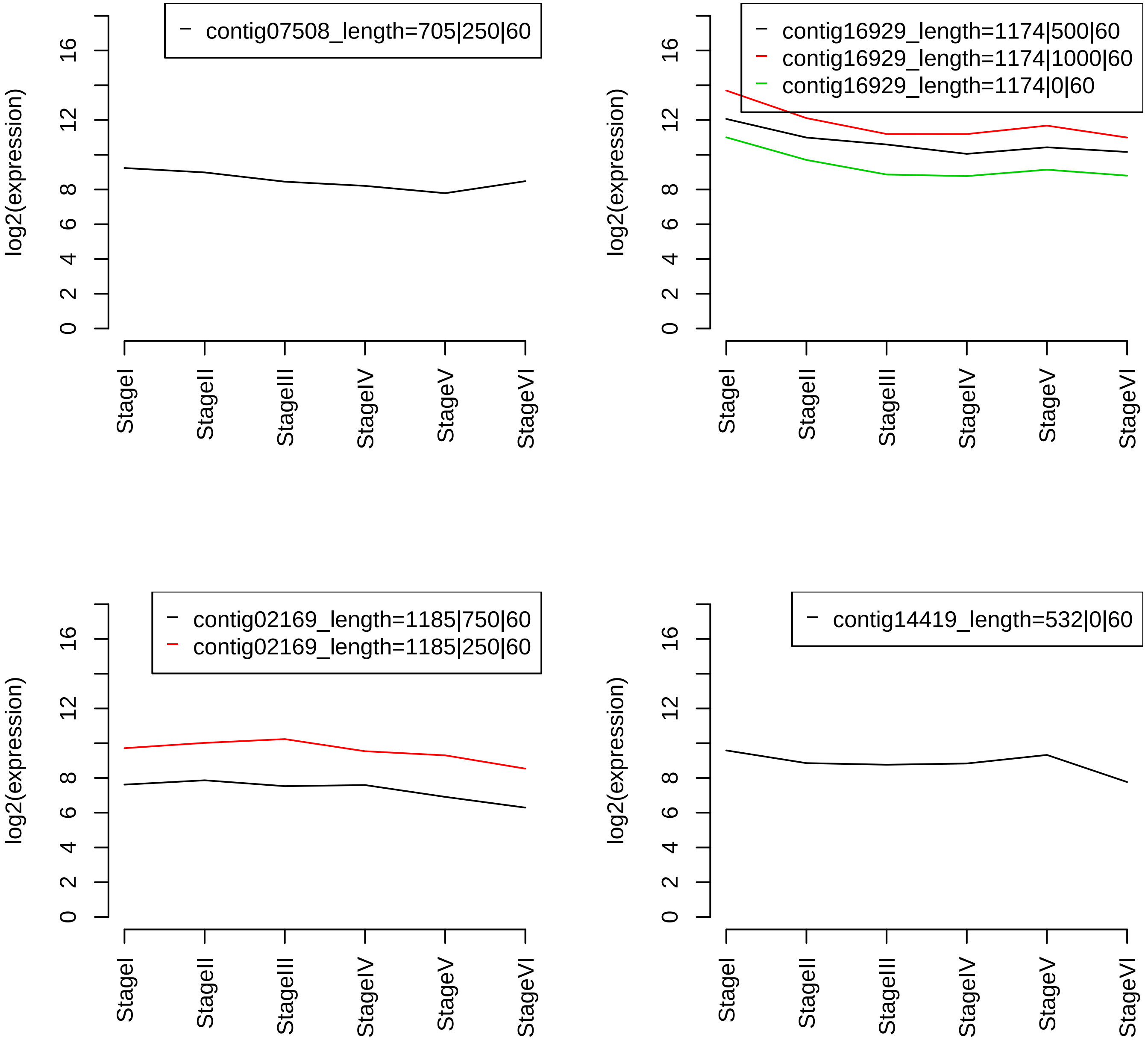

contig07508_length=705|250|60
contig16929_length=1174|500|60
contig16929_length=1174|1000|60
contig16929_length=1174|0|60
contig02169_length=1185|750|60
contig02169_length=1185|250|60
contig14419_length=532|0|60
log2(expression)
0 2 4 6 8 12 16
StageI StageII StageIII StageIV StageV StageVI

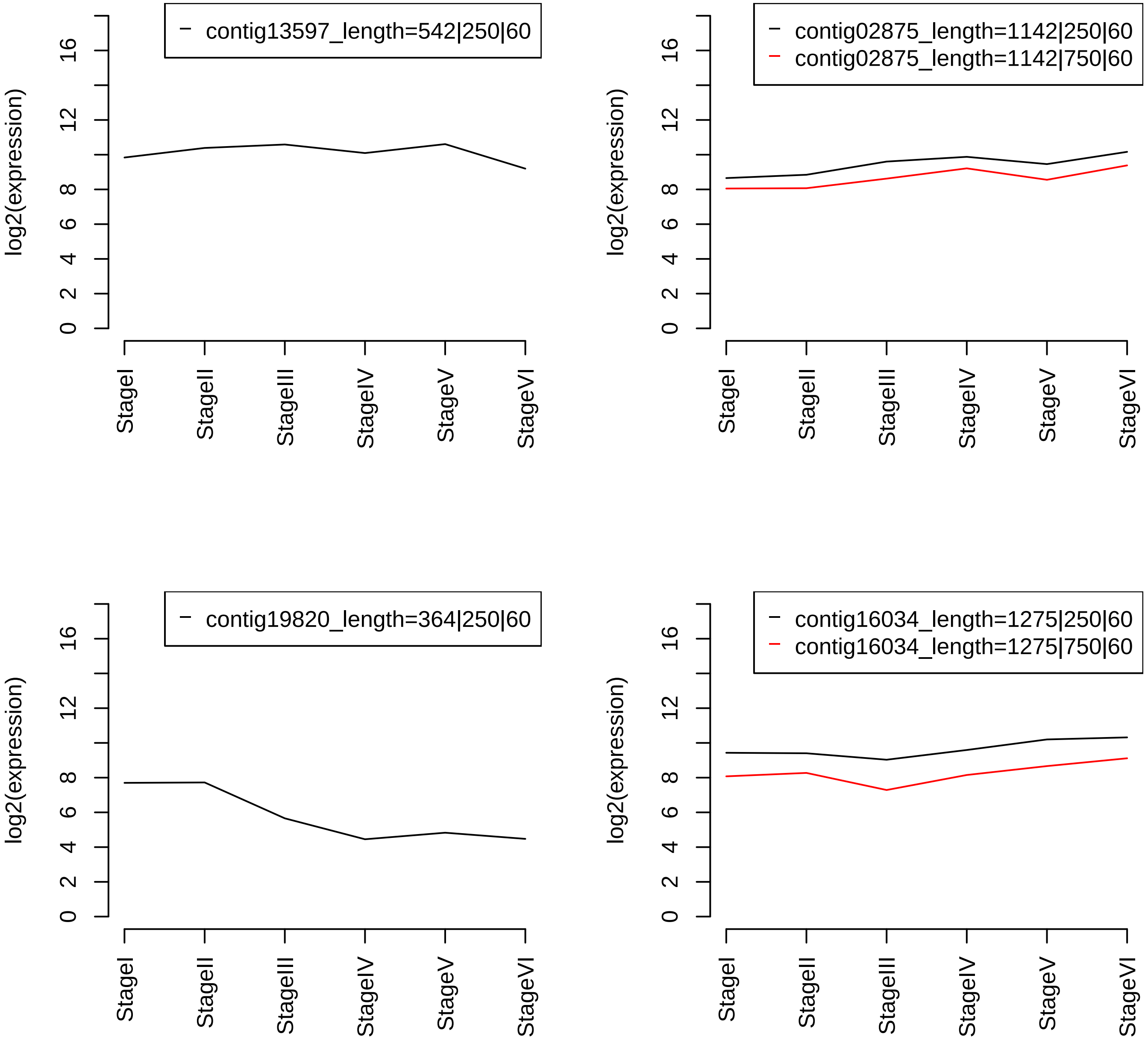
contig13597_length=542|250|60
contig02875_length=1142|250|60
contig02875_length=1142|750|60
contig19820_length=364|250|60
contig16034_length=1275|250|60
contig16034_length=1275|750|60
log2(expression)
0
2
4
6
8
12
16
StageI
StageII
StageIII
StageIV
StageV
StageVI

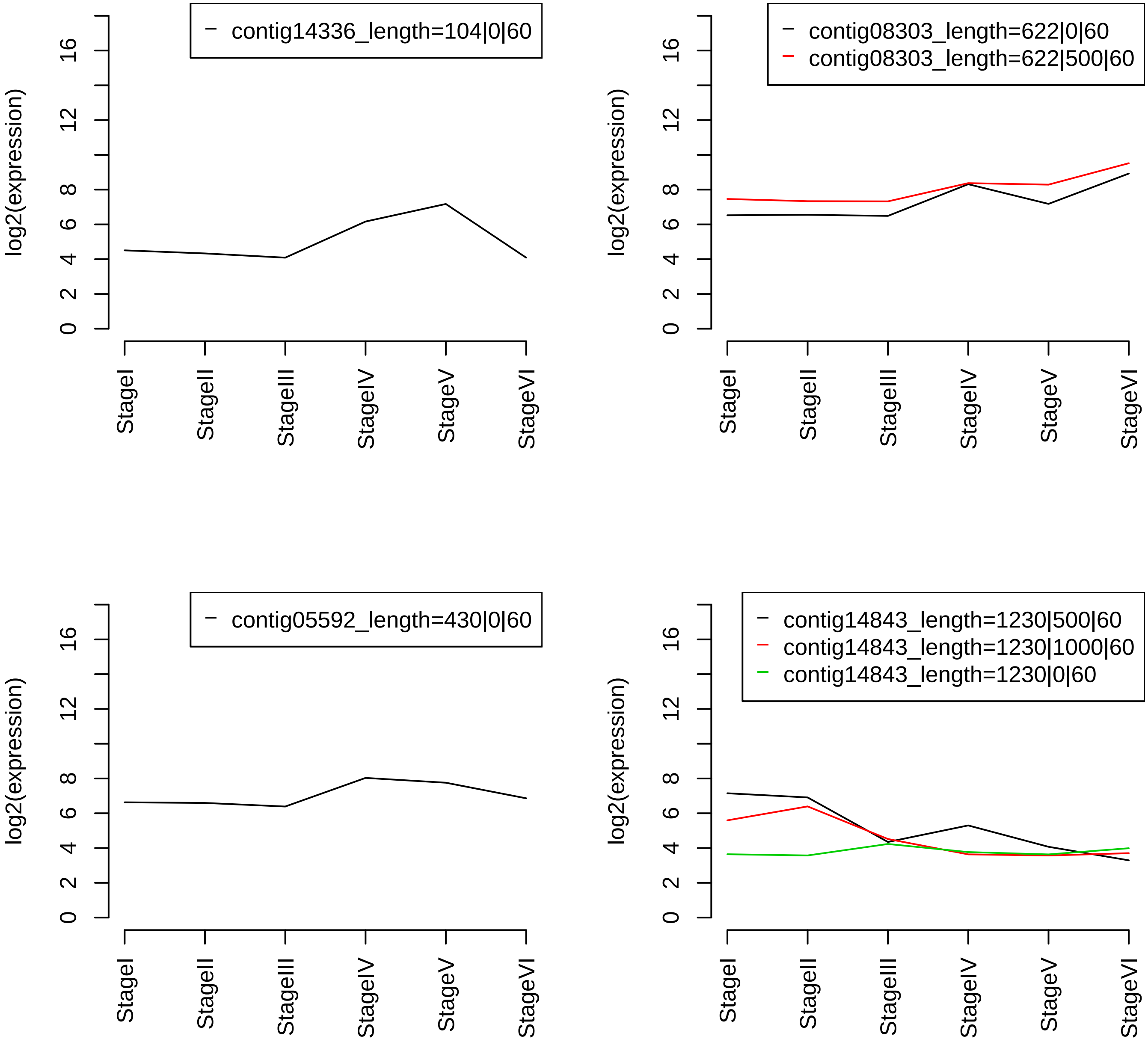
contig14336_length=104|0|60
contig08303_length=622|0|60
contig08303_length=622|500|60
contig05592_length=430|0|60
contig14843_length=1230|500|60
contig14843_length=1230|1000|60
contig14843_length=1230|0|60
log2(expression)
0
2
4
6
8
12
16
StageI
StageII
StageIII
StageIV
StageV
StageVI

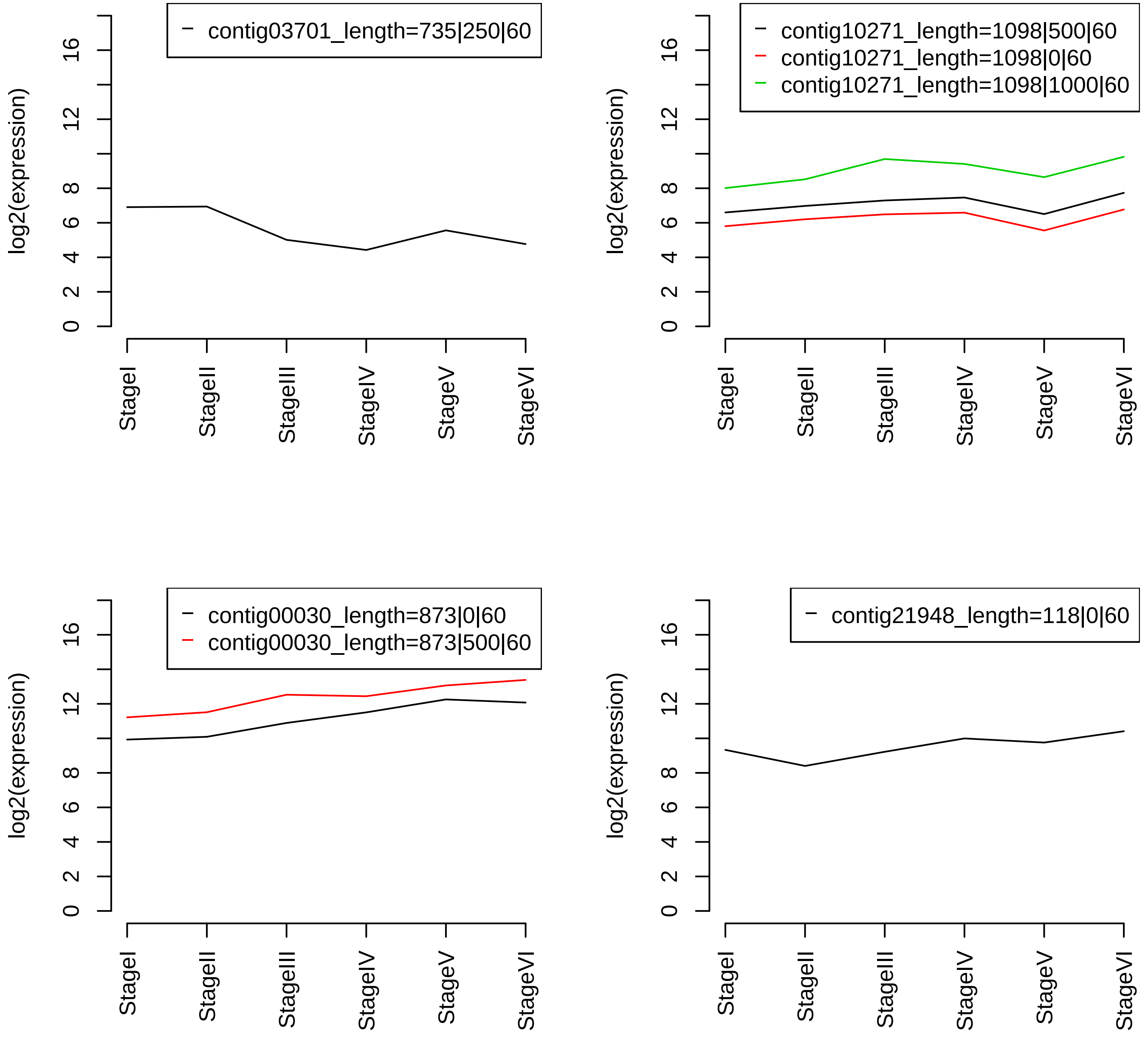
contig03701_length=735|250|60
contig10271_length=1098|500|60
contig10271_length=1098|0|60
contig10271_length=1098|1000|60
contig00030_length=873|0|60
contig00030_length=873|500|60
contig21948_length=118|0|60
log2(expression)
0
2
4
6
8
12
16
StageI
StageII
StageIII
StageIV
StageV
StageVI

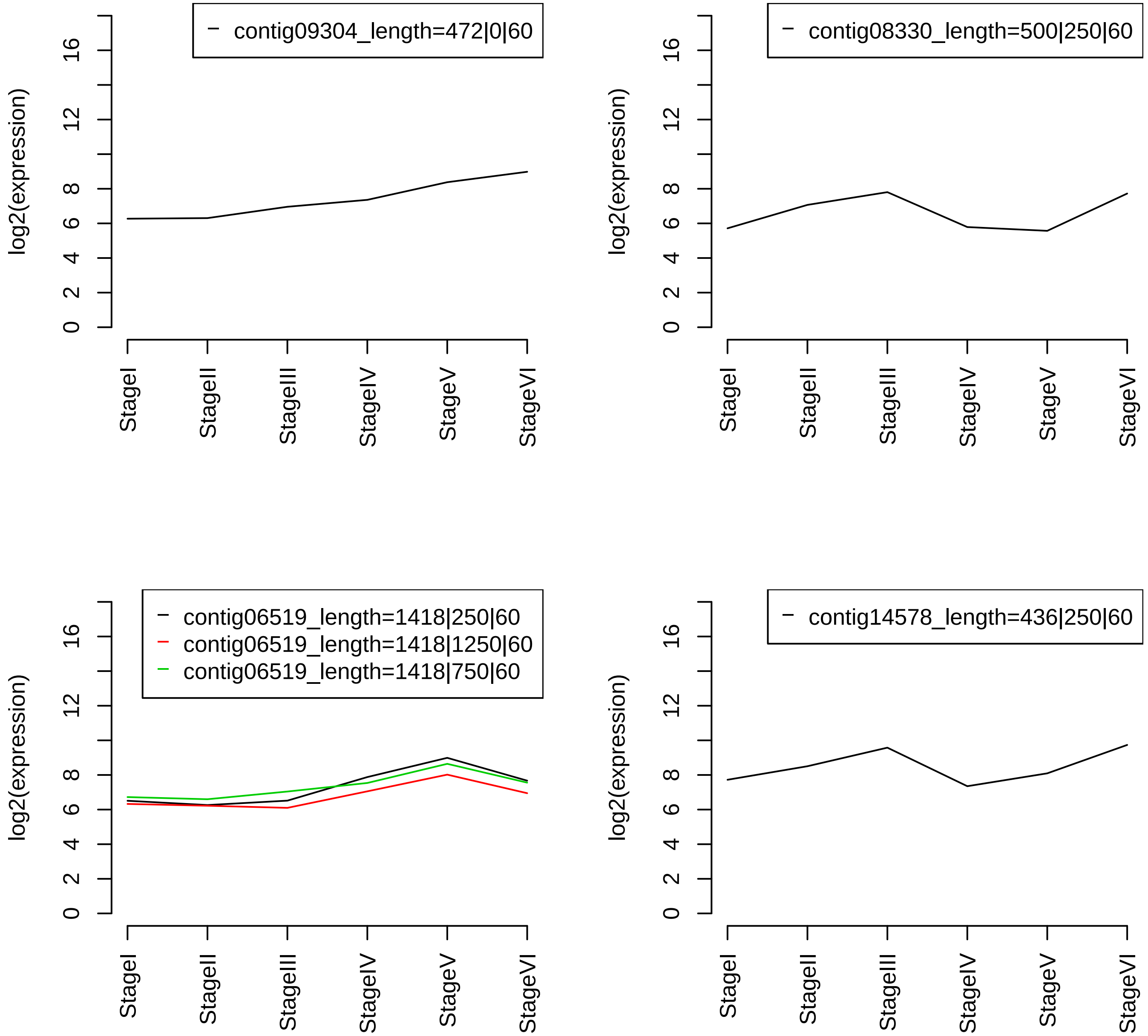
contig09304_length=472|0|60
contig08330_length=500|250|60
contig06519_length=1418|250|60
contig06519_length=1418|1250|60
contig06519_length=1418|750|60
contig14578_length=436|250|60
log2(expression)
0
2
4
6
8
12
16
StageI
StageII
StageIII
StageIV
StageV
StageVI

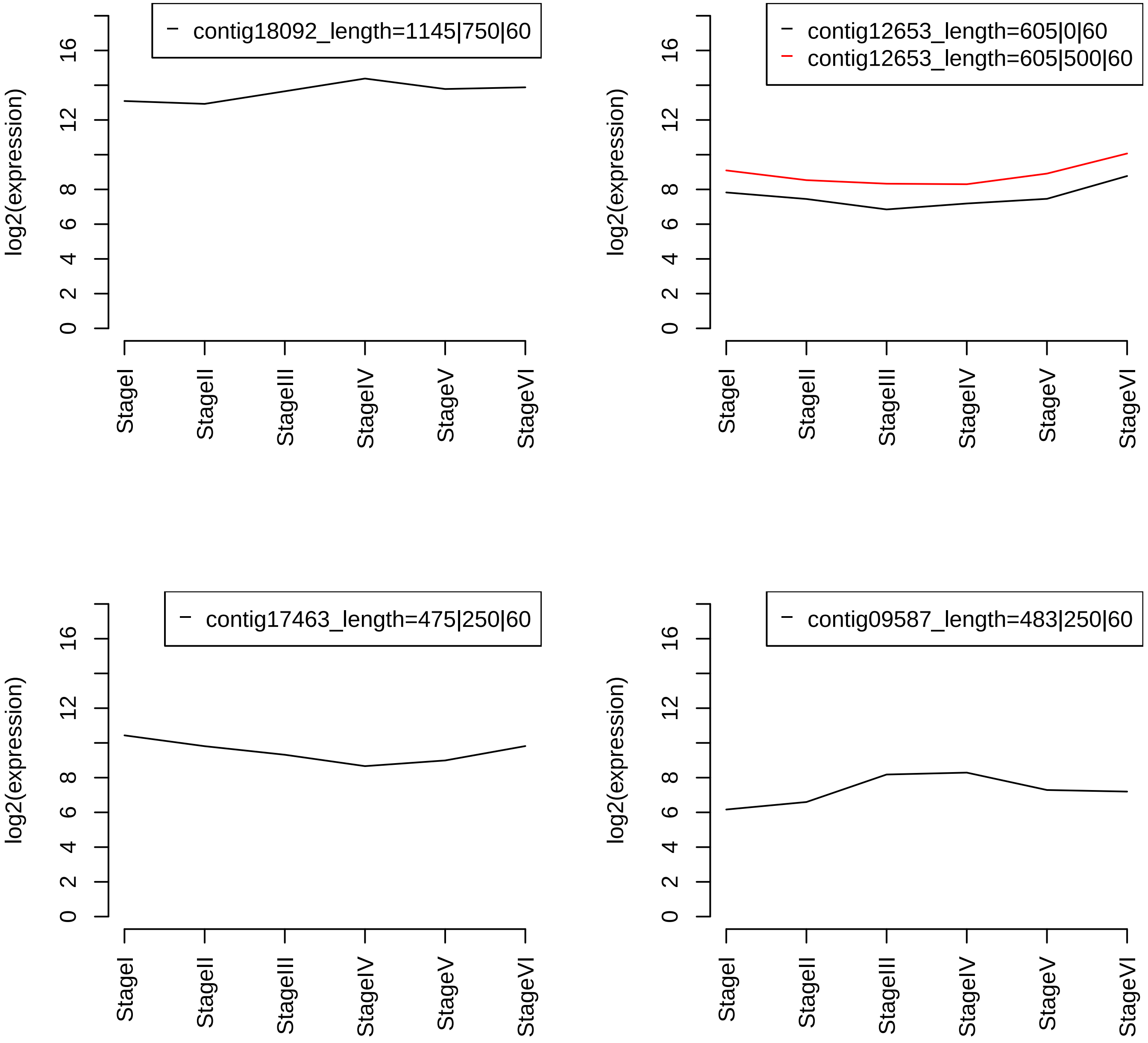
contig18092_length=1145|750|60
contig12653_length=605|0|60
contig12653_length=605|500|60
contig17463_length=475|250|60
contig09587_length=483|250|60
log2(expression)
0
2
4
6
8
12
16
StageI
StageII
StageIII
StageIV
StageV
StageVI

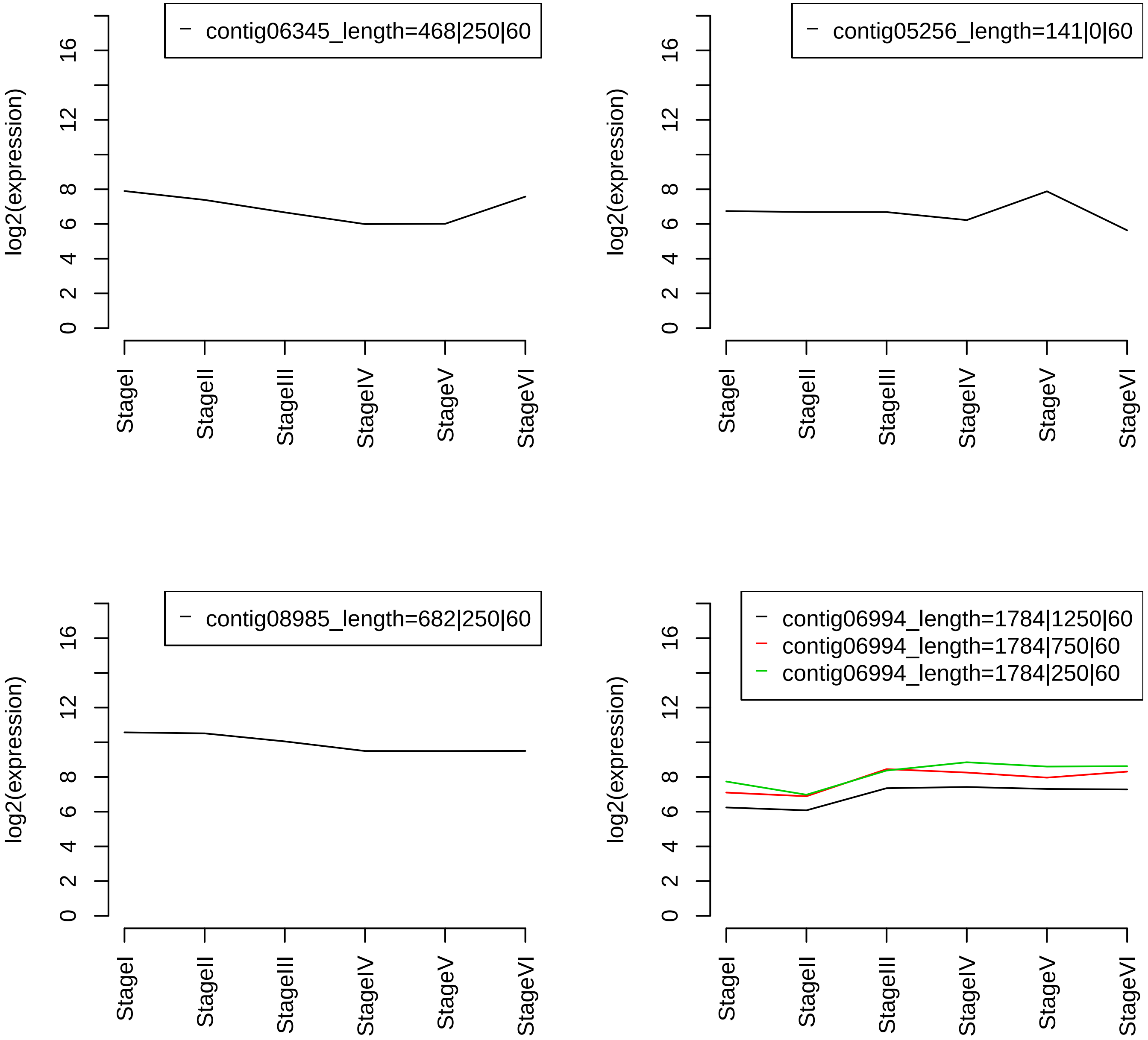
contig06345_length=468|250|60
contig05256_length=141|0|60
contig08985_length=682|250|60
contig06994_length=1784|1250|60
contig06994_length=1784|750|60
contig06994_length=1784|250|60
log2(expression)
0
2
4
6
8
12
16
StageI
StageII
StageIII
StageIV
StageV
StageVI

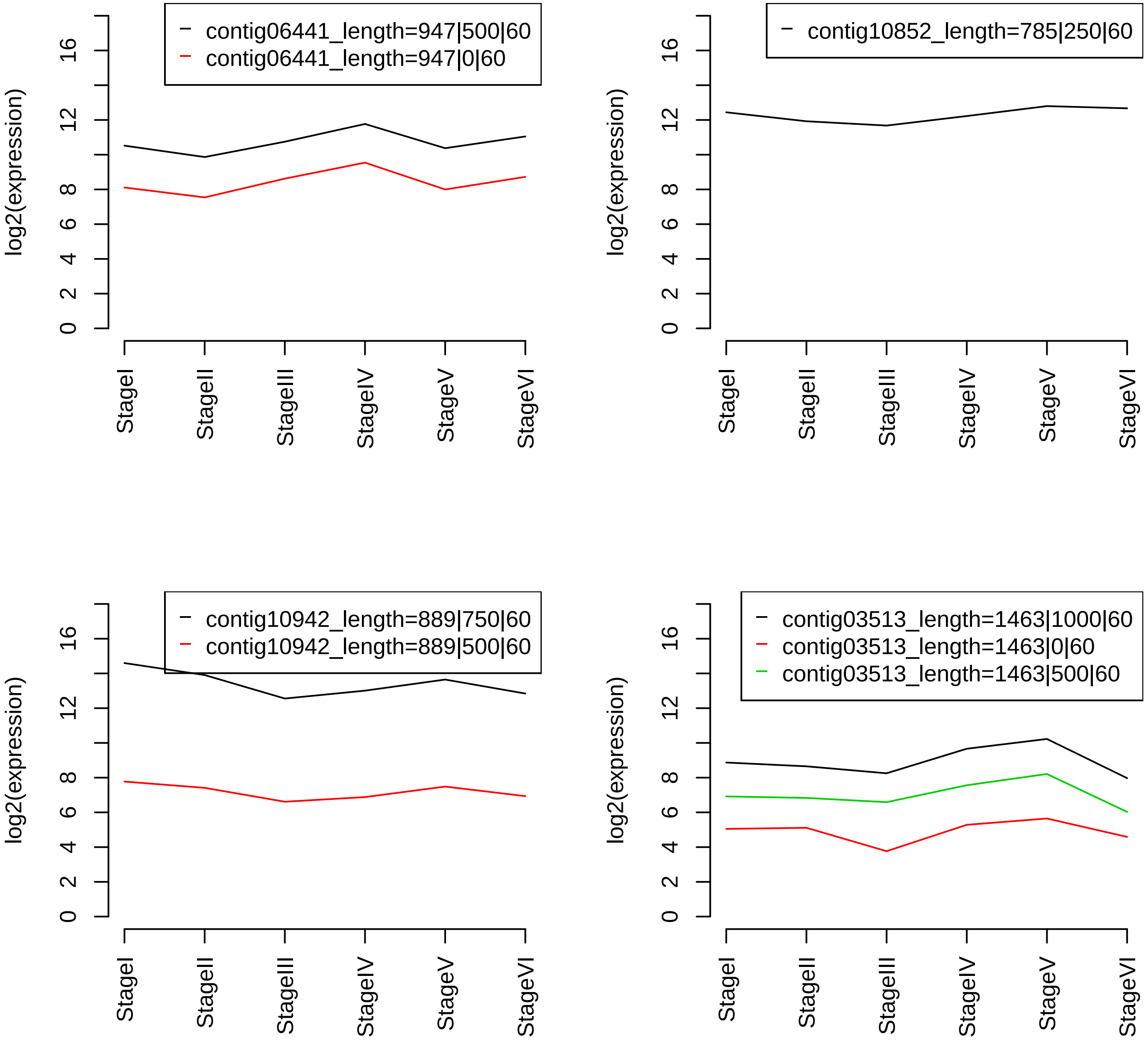

contig06441_length=947|500|60
contig06441_length=947|0|60
contig10852_length=785|250|60
contig10942_length=889|750|60
contig10942_length=889|500|60
contig03513_length=1463|1000|60
contig03513_length=1463|0|60
contig03513_length=1463|500|60
log2(expression)
0
2
4
6
8
12
16
StageI
StageII
StageIII
StageIV
StageV
StageVI

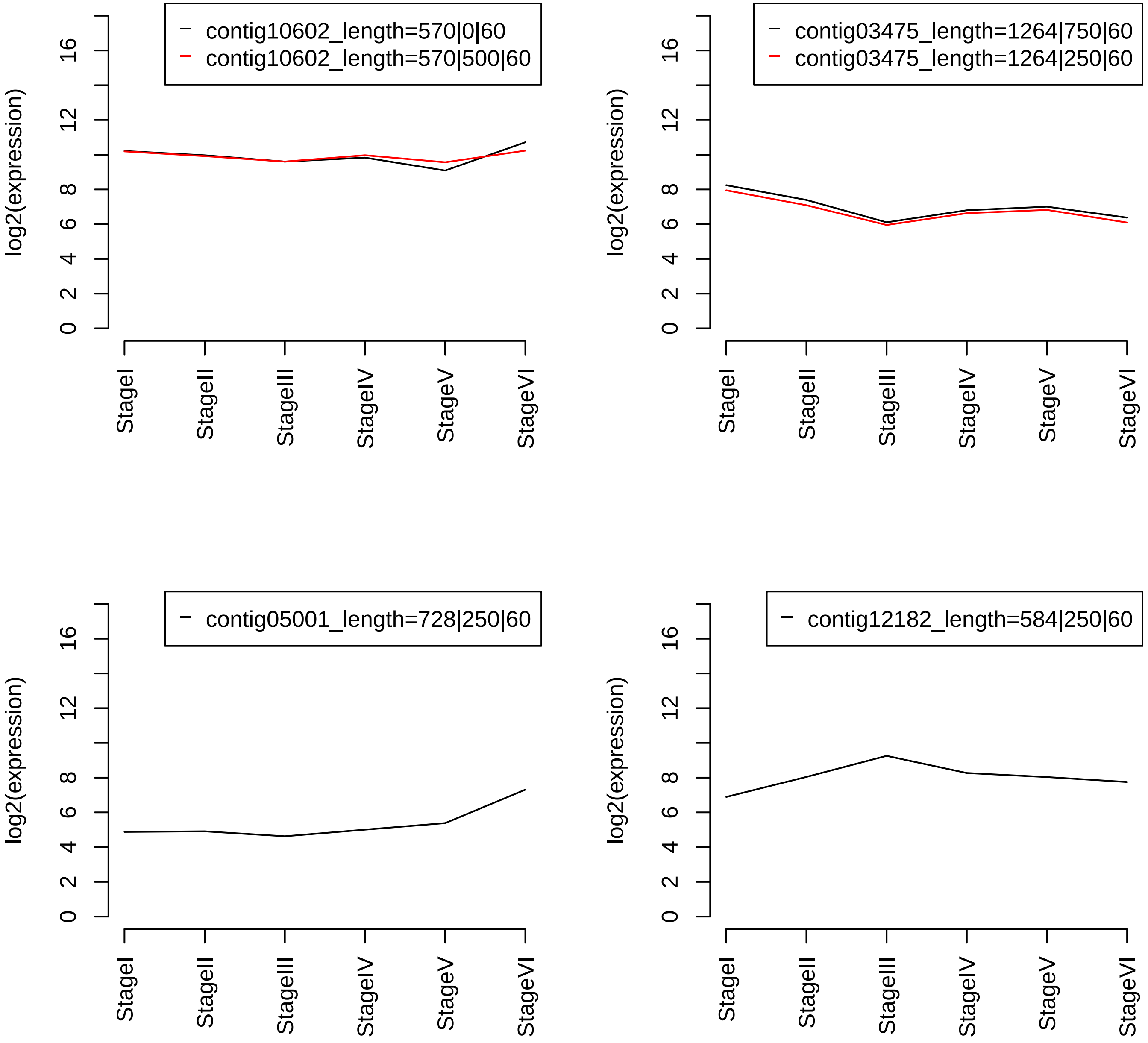

contig10602_length=570|0|60
contig10602_length=570|500|60
contig03475_length=1264|750|60
contig03475_length=1264|250|60
contig05001_length=728|250|60
contig12182_length=584|250|60
log2(expression)
0
2
4
6
8
12
16
StageI
StageII
StageIII
StageIV
StageV
StageVI

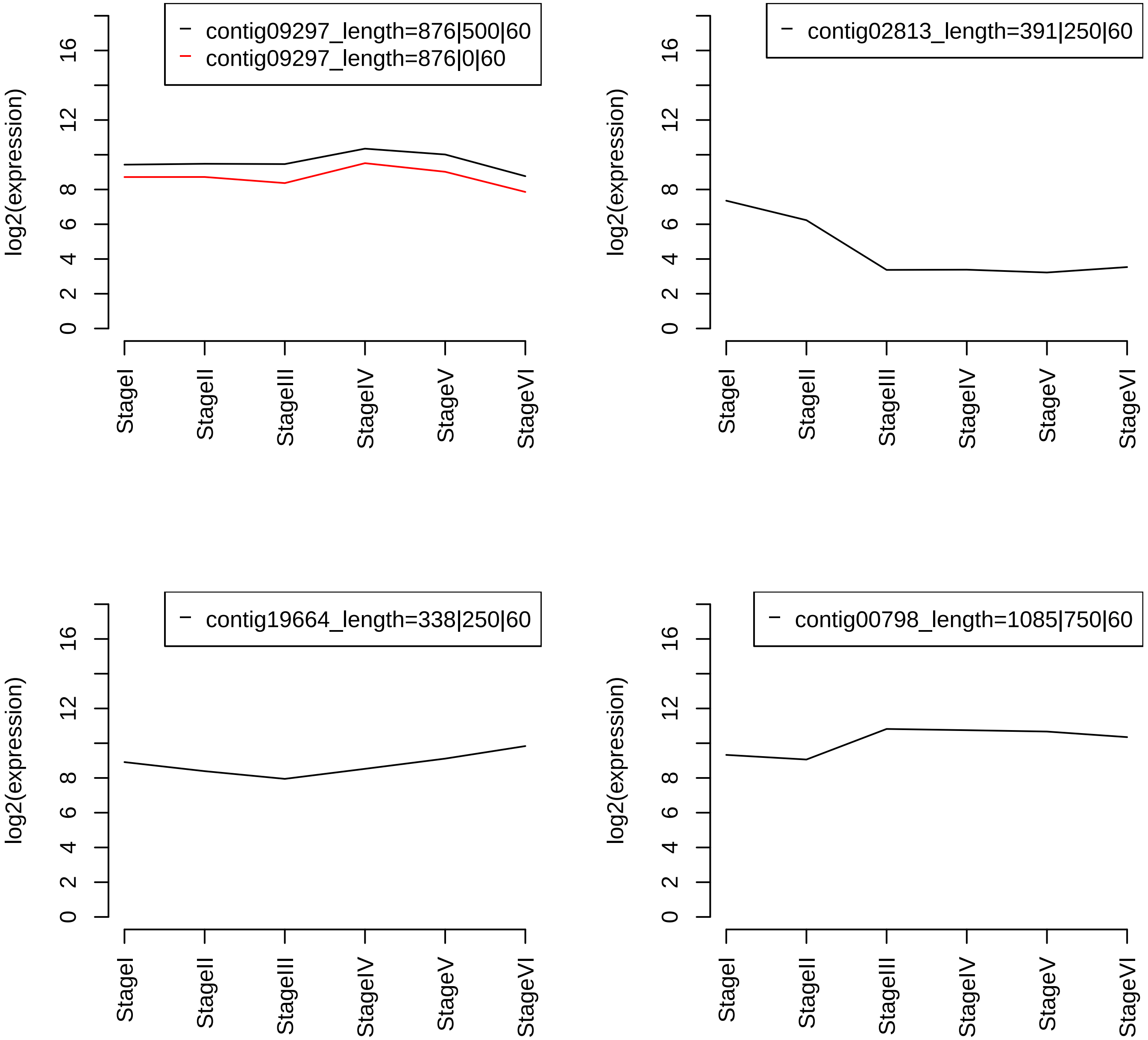

contig09297_length=876|500|60
contig09297_length=876|0|60
contig02813_length=391|250|60
contig19664_length=338|250|60
contig00798_length=1085|750|60
log2(expression)
0
2
4
6
8
12
16
StageI
StageII
StageIII
StageIV
StageV
StageVI

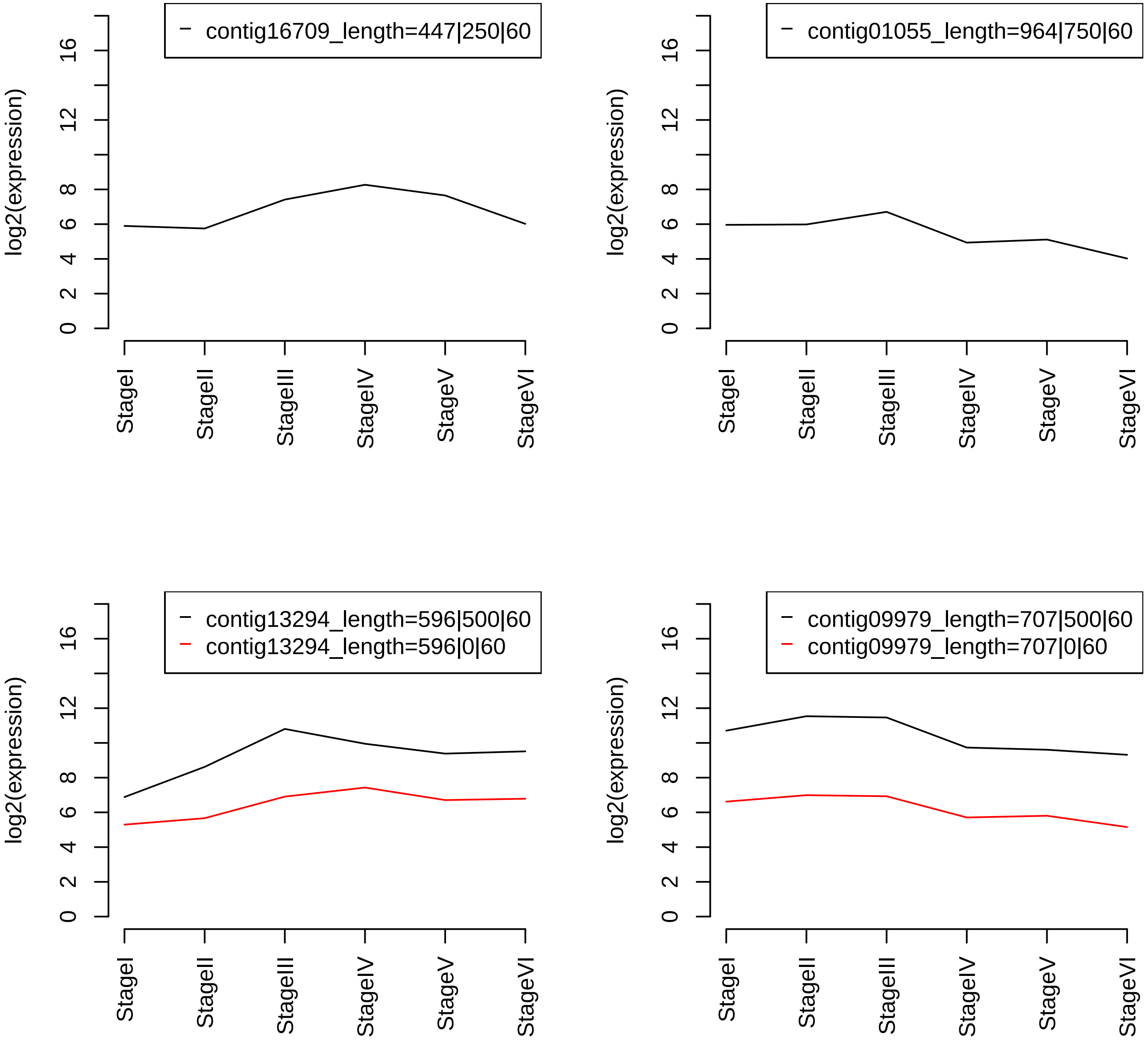
contig16709_length=447|250|60
contig01055_length=964|750|60
contig13294_length=596|500|60
contig13294_length=596|0|60
contig09979_length=707|500|60
contig09979_length=707|0|60
log2(expression)
0
2
4
6
8
12
16
StageI
StageII
StageIII
StageIV
StageV
StageVI

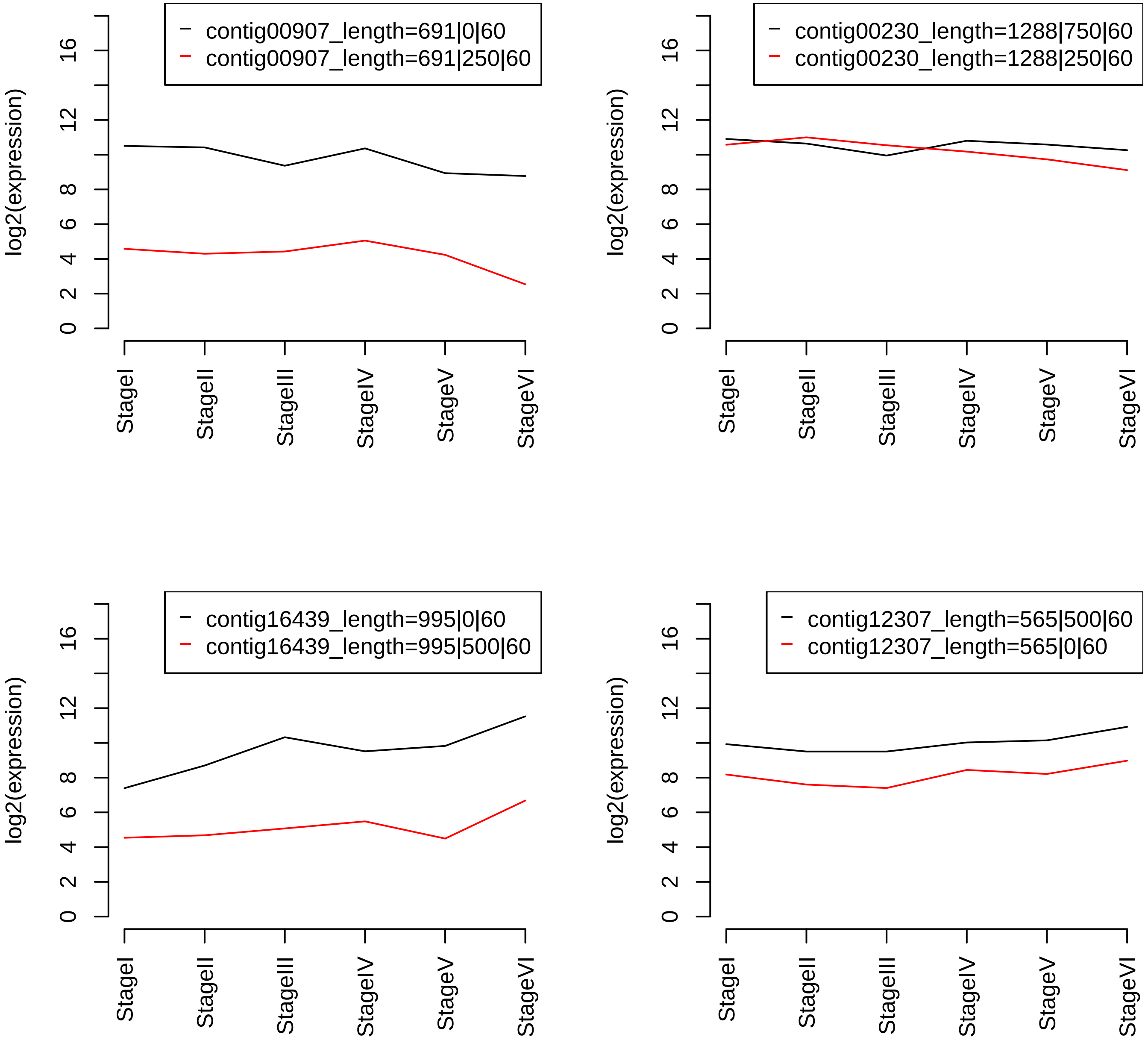

contig00907_length=691|0|60
contig00907_length=691|250|60
contig00230_length=1288|750|60
contig00230_length=1288|250|60
contig16439_length=995|0|60
contig16439_length=995|500|60
contig12307_length=565|500|60
contig12307_length=565|0|60
log2(expression)
StageI
StageII
StageIII
StageIV
StageV
StageVI

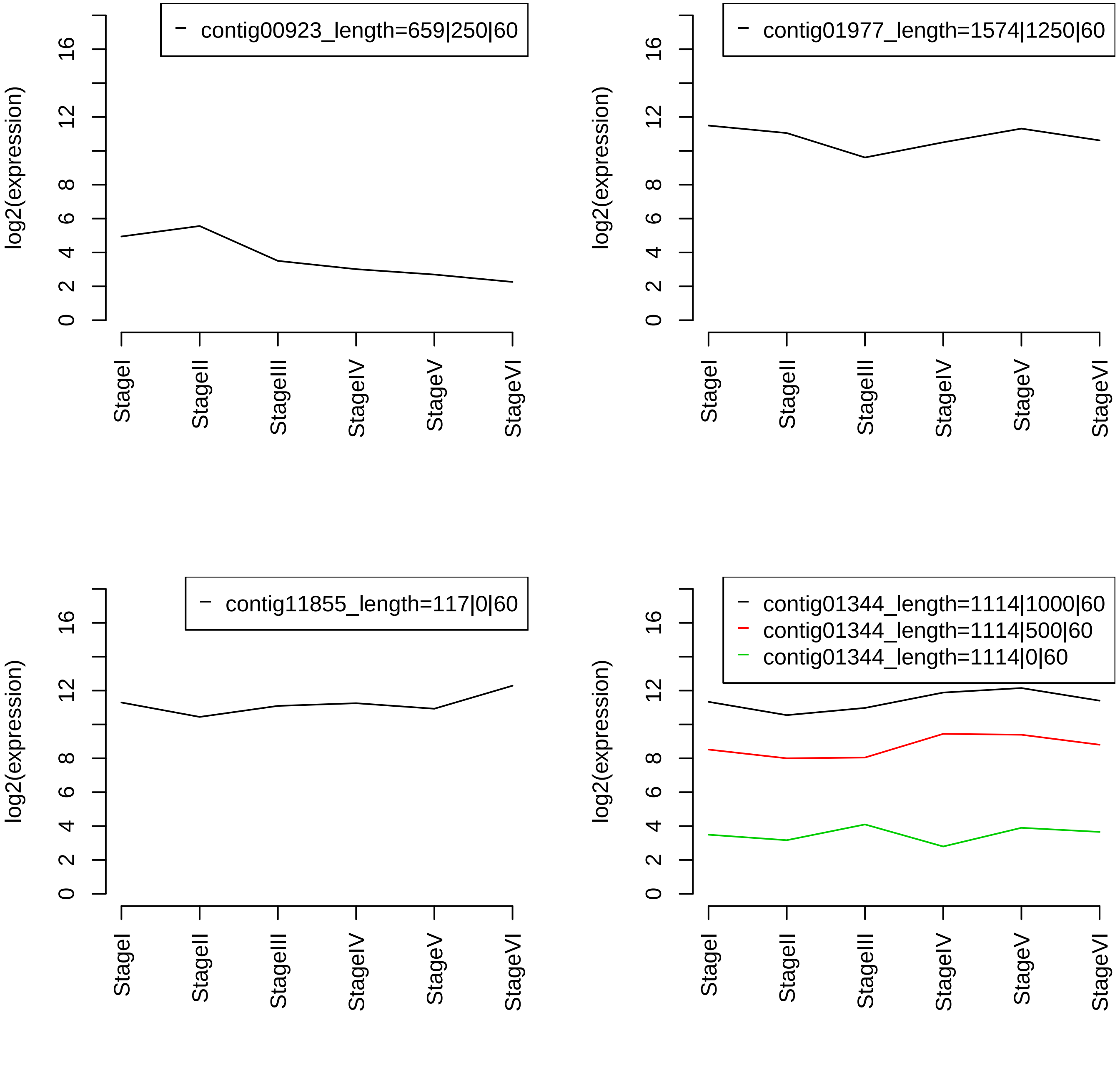
contig00923_length=659|250|60
contig01977_length=1574|1250|60
contig11855_length=117|0|60
contig01344_length=1114|1000|60
contig01344_length=1114|500|60
contig01344_length=1114|0|60
log2(expression)
0
2
4
6
8
12
16
StageI
StageII
StageIII
StageIV
StageV
StageVI

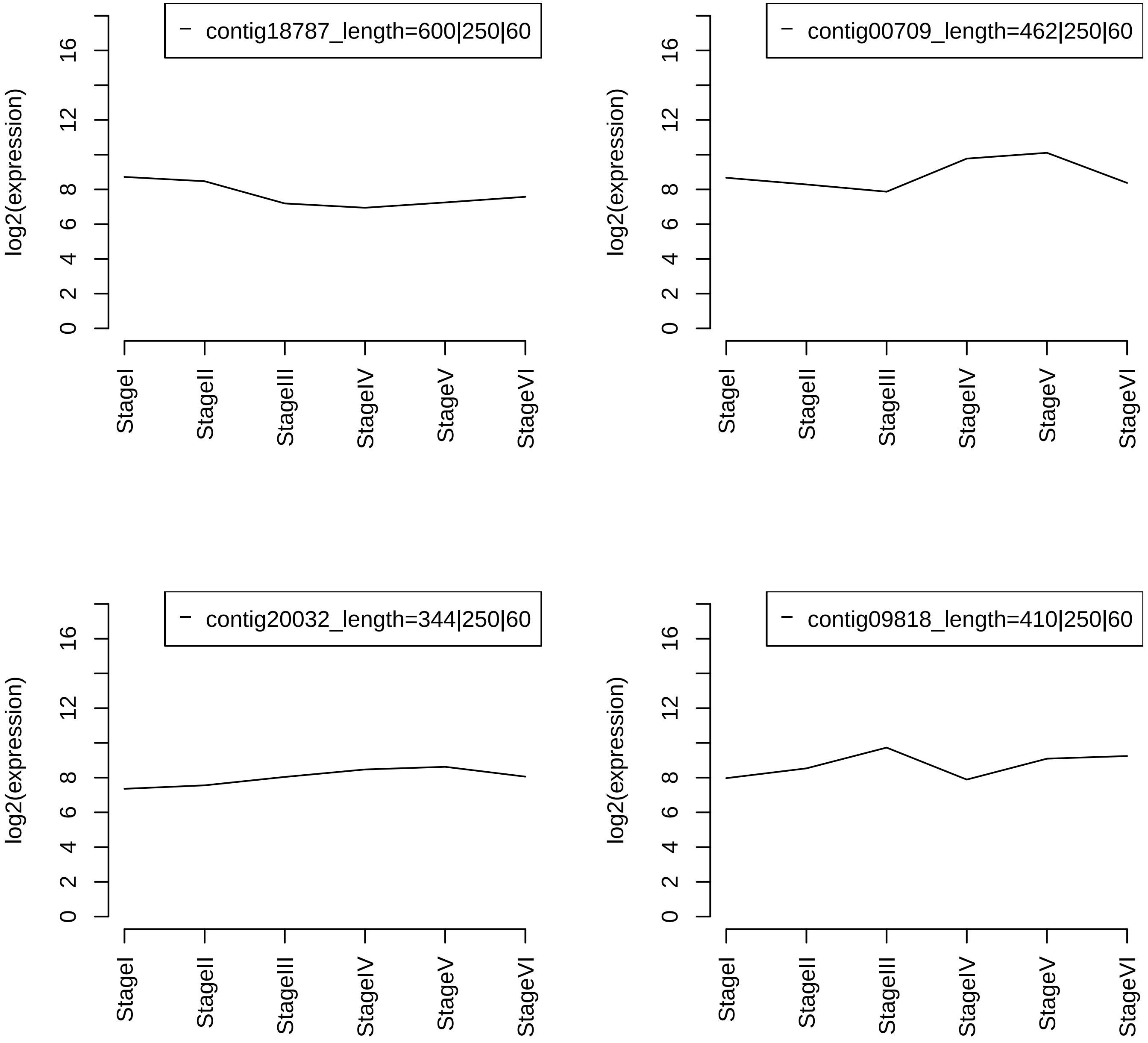

contig18787_length=600|250|60
contig00709_length=462|250|60
contig20032_length=344|250|60
contig09818_length=410|250|60
log2(expression)
0
2
4
6
8
12
16
StageI
StageII
StageIII
StageIV
StageV
StageVI

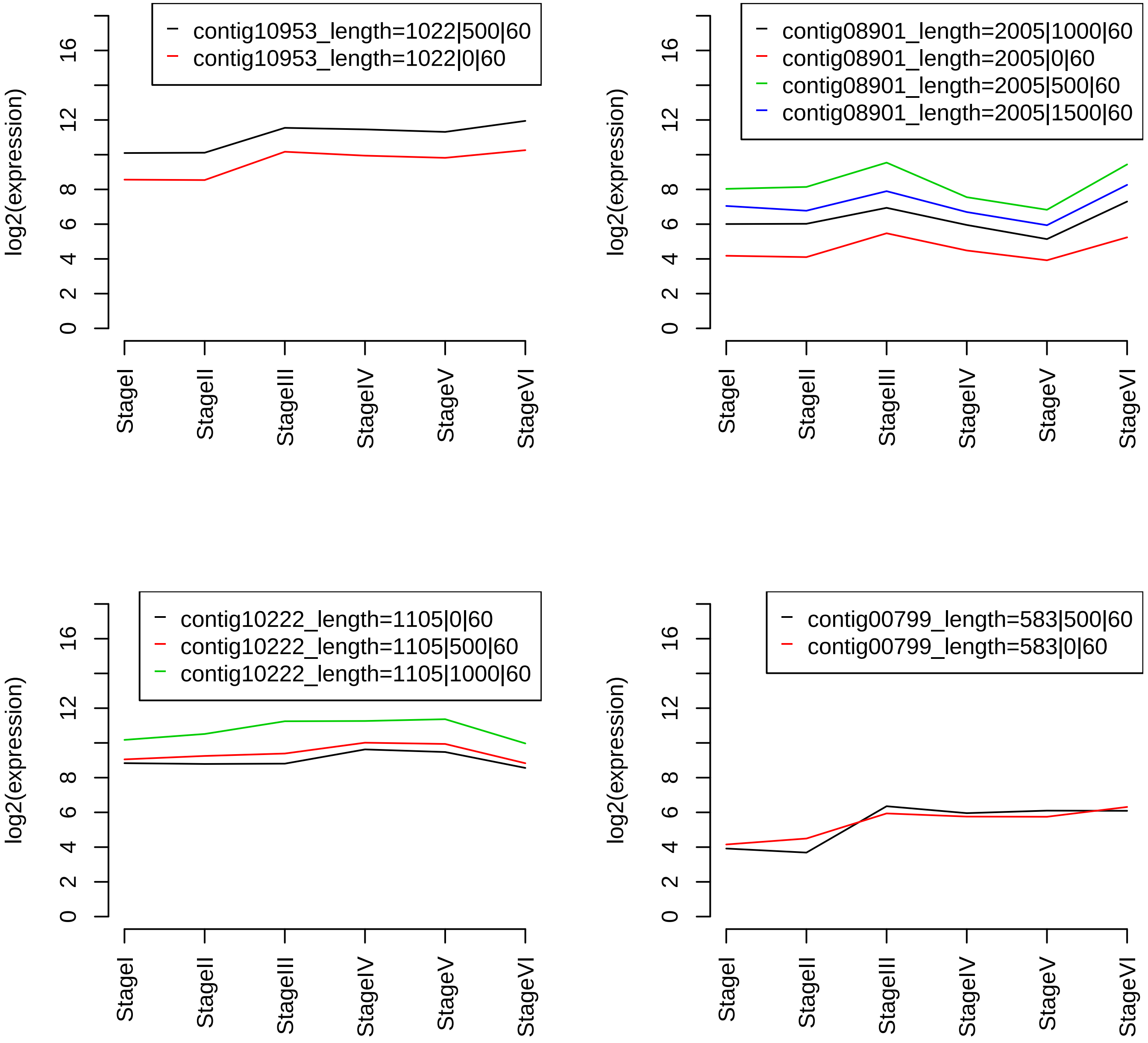
contig10953_length=1022|500|60
contig10953_length=1022|0|60
log2(expression)
0
2
4
6
8
12
16
StageI
StageII
StageIII
StageIV
StageV
StageVI
contig08901_length=2005|1000|60
contig08901_length=2005|0|60
contig08901_length=2005|500|60
contig08901_length=2005|1500|60
contig10222_length=1105|0|60
contig10222_length=1105|500|60
contig10222_length=1105|1000|60
contig00799_length=583|500|60
contig00799_length=583|0|60

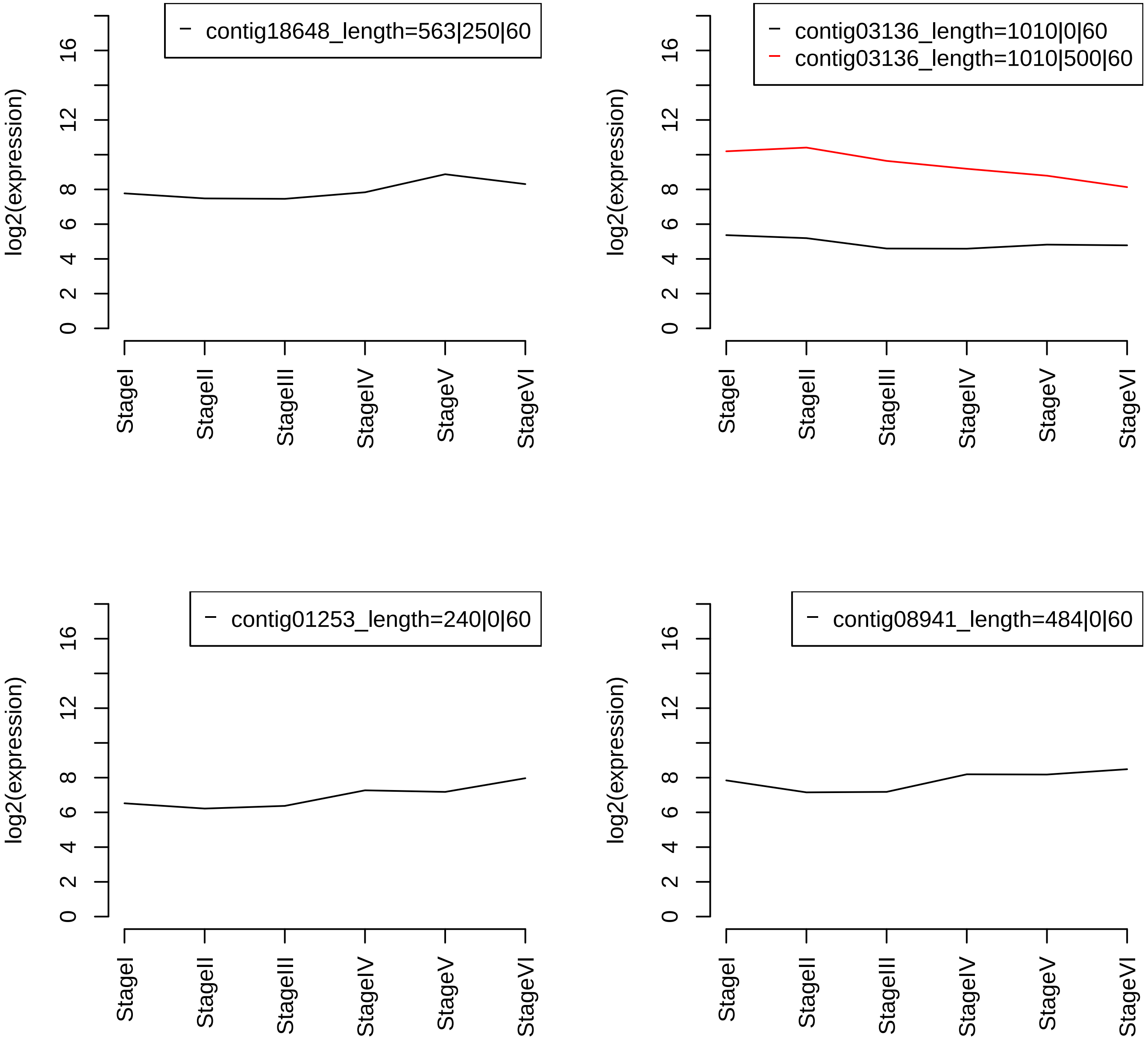
contig18648_length=563|250|60
contig03136_length=1010|0|60
contig03136_length=1010|500|60
contig01253_length=240|0|60
contig08941_length=484|0|60
log2(expression)
0
2
4
6
8
12
16
StageI
StageII
StageIII
StageIV
StageV
StageVI

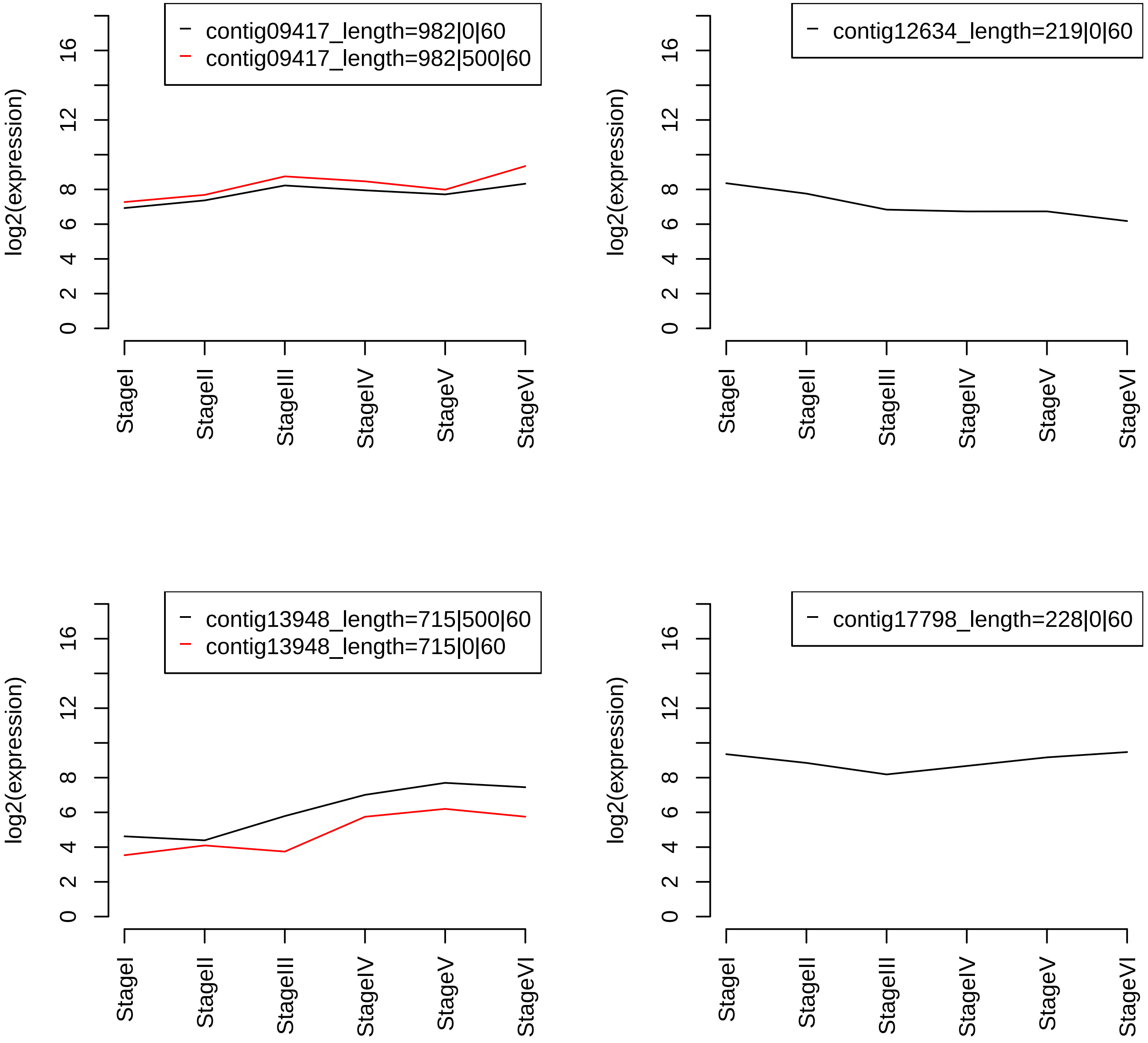
contig09417_length=982|0|60
contig09417_length=982|500|60
contig12634_length=219|0|60
contig13948_length=715|500|60
contig13948_length=715|0|60
contig17798_length=228|0|60
log2(expression)
0
2
4
6
8
12
16
StageI
StageII
StageIII
StageIV
StageV
StageVI

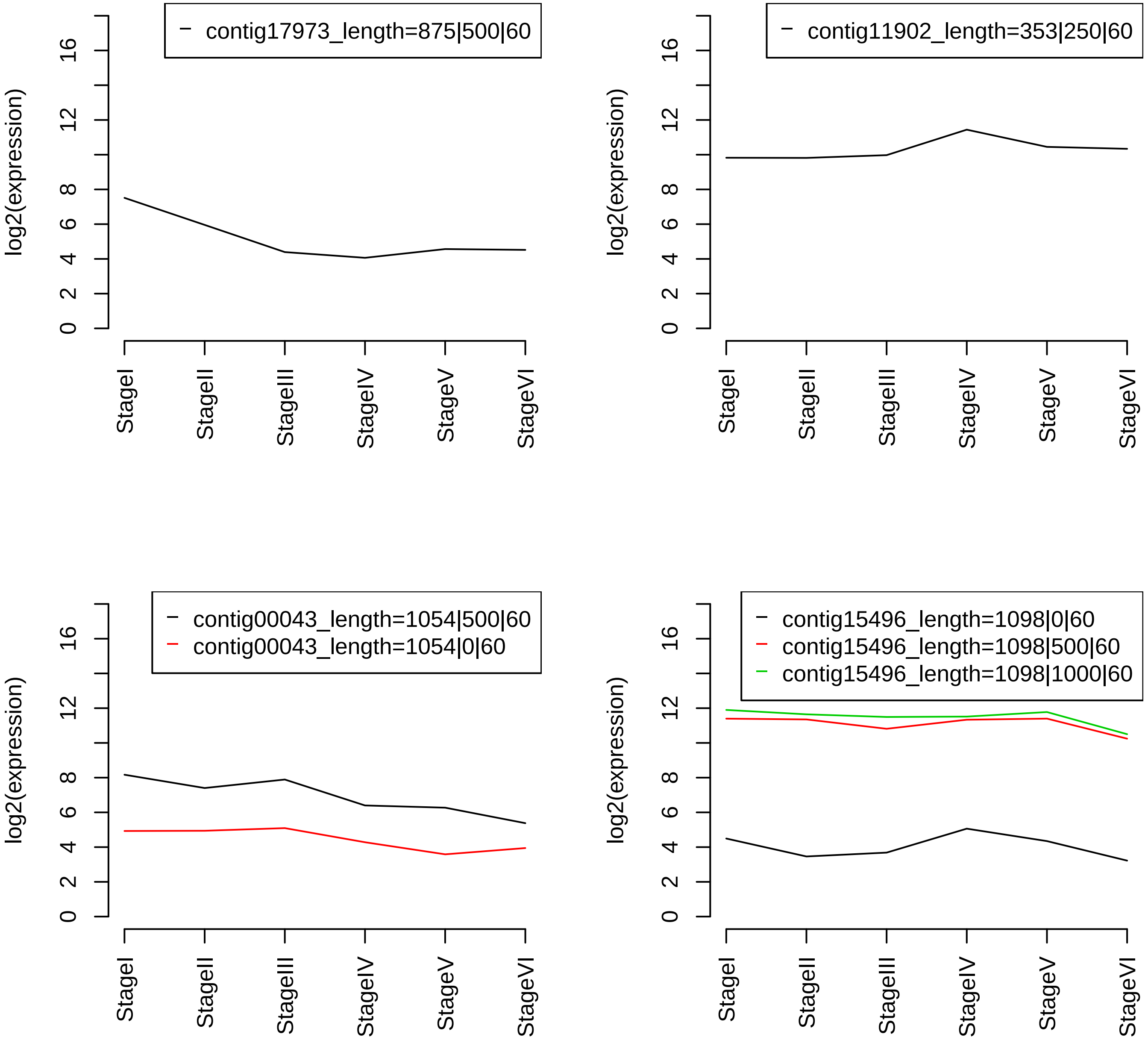
contig17973_length=875|500|60
contig11902_length=353|250|60
contig00043_length=1054|500|60
contig00043_length=1054|0|60
contig15496_length=1098|0|60
contig15496_length=1098|500|60
contig15496_length=1098|1000|60
log2(expression)
0
2
4
6
8
12
16
StageI
StageII
StageIII
StageIV
StageV
StageVI

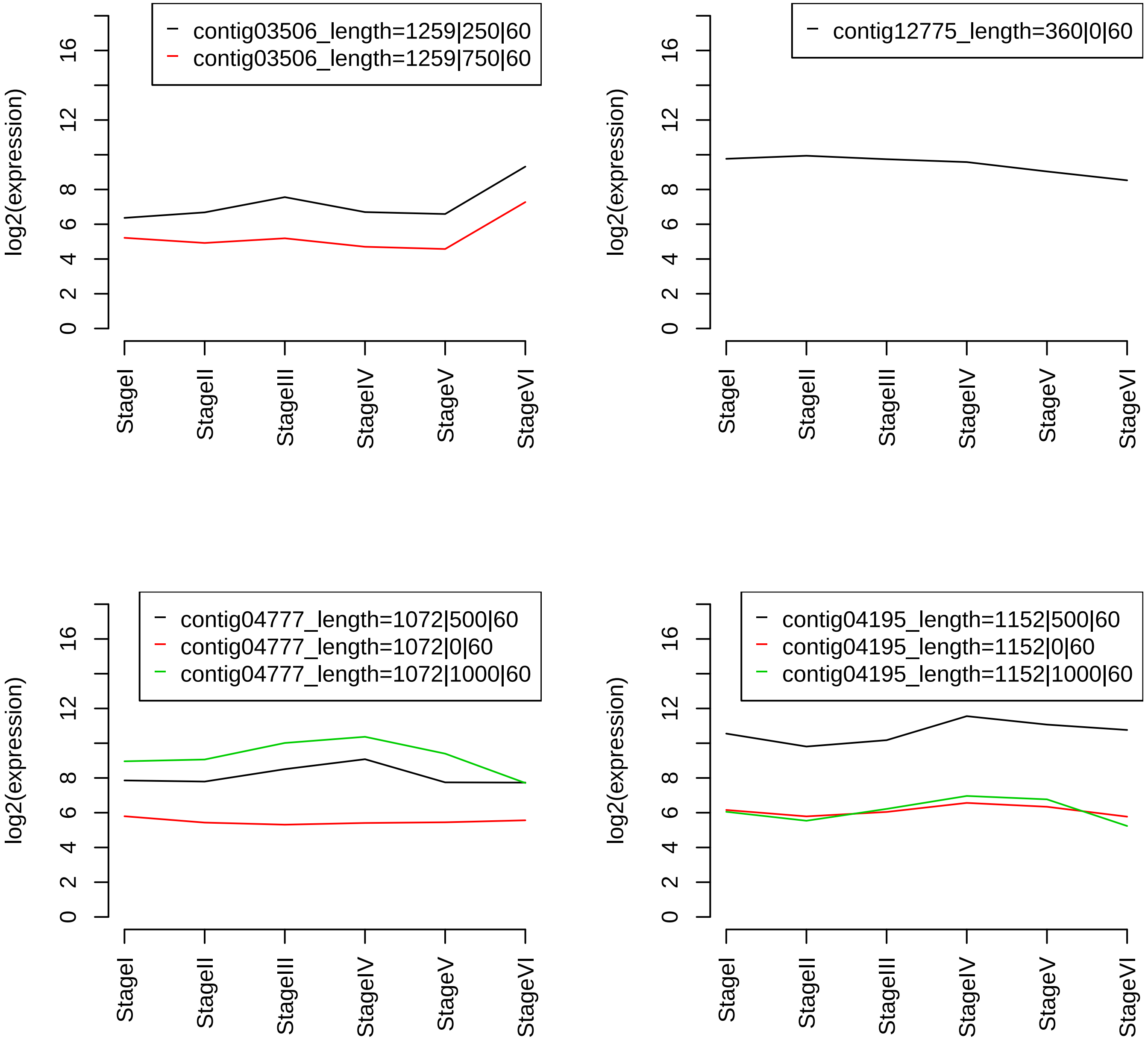
contig03506_length=1259|250|60
contig03506_length=1259|750|60
contig12775_length=360|0|60
contig04777_length=1072|500|60
contig04777_length=1072|0|60
contig04777_length=1072|1000|60
contig04195_length=1152|500|60
contig04195_length=1152|0|60
contig04195_length=1152|1000|60
log2(expression)
0
2
4
6
8
12
16
StageI
StageII
StageIII
StageIV
StageV
StageVI

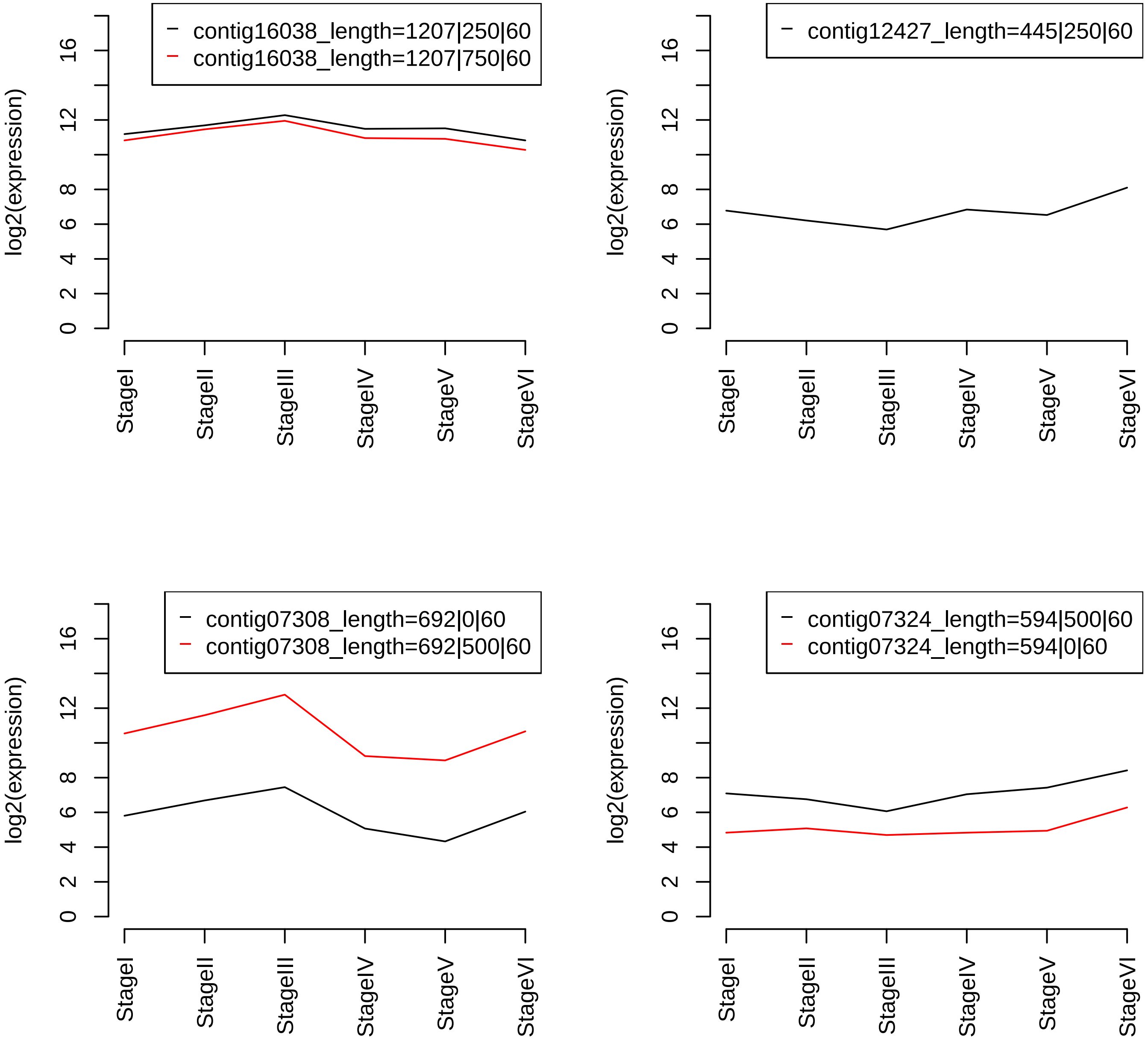

contig16038_length=1207|250|60
contig16038_length=1207|750|60
log2(expression)
0
2
4
6
8
12
16
StageI
StageII
StageIII
StageIV
StageV
StageVI
contig12427_length=445|250|60
contig07308_length=692|0|60
contig07308_length=692|500|60
contig07324_length=594|500|60
contig07324_length=594|0|60

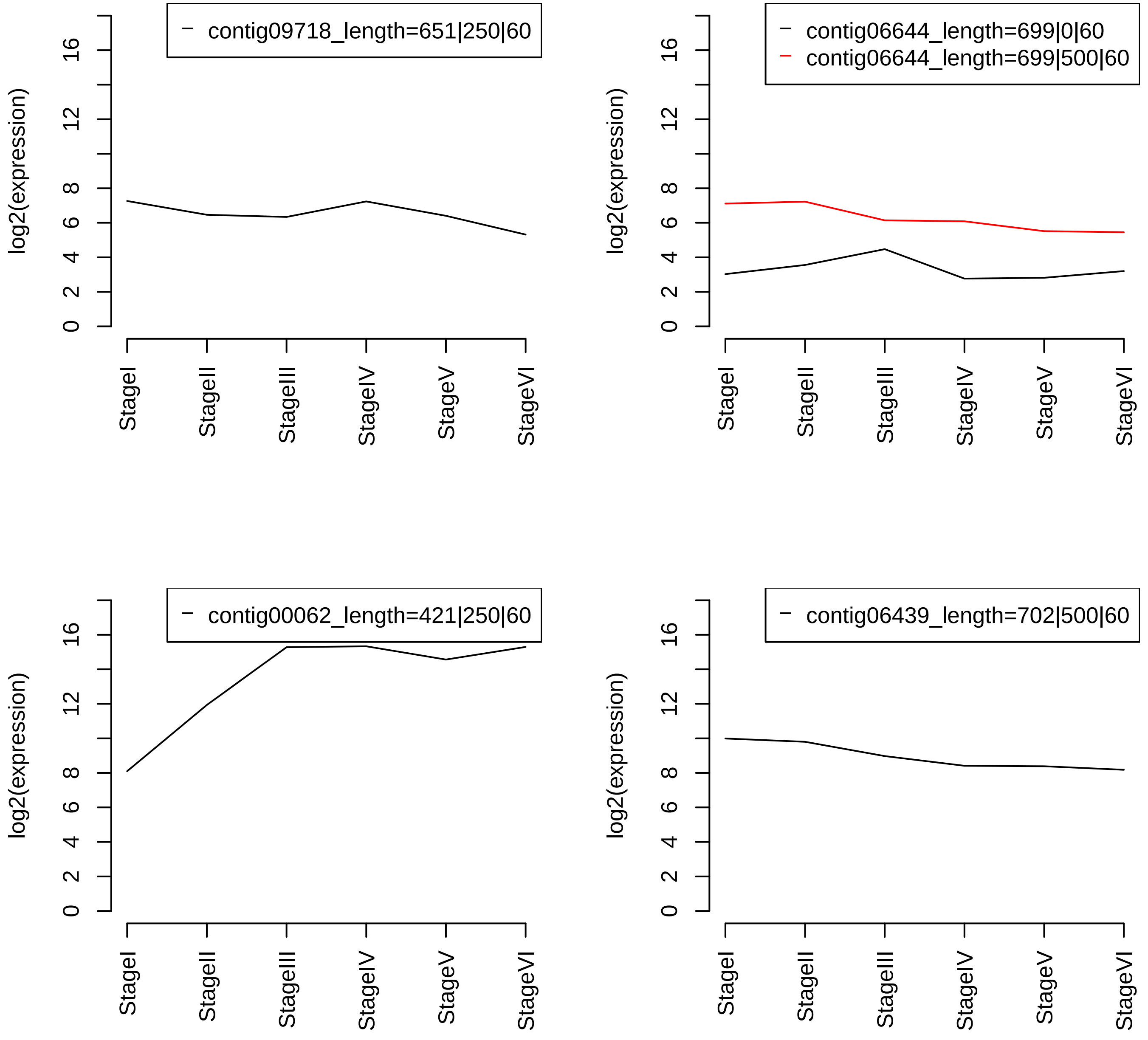
contig09718_length=651|250|60
contig06644_length=699|0|60
contig06644_length=699|500|60
contig00062_length=421|250|60
contig06439_length=702|500|60
log2(expression)
0
2
4
6
8
12
16
StageI
StageII
StageIII
StageIV
StageV
StageVI

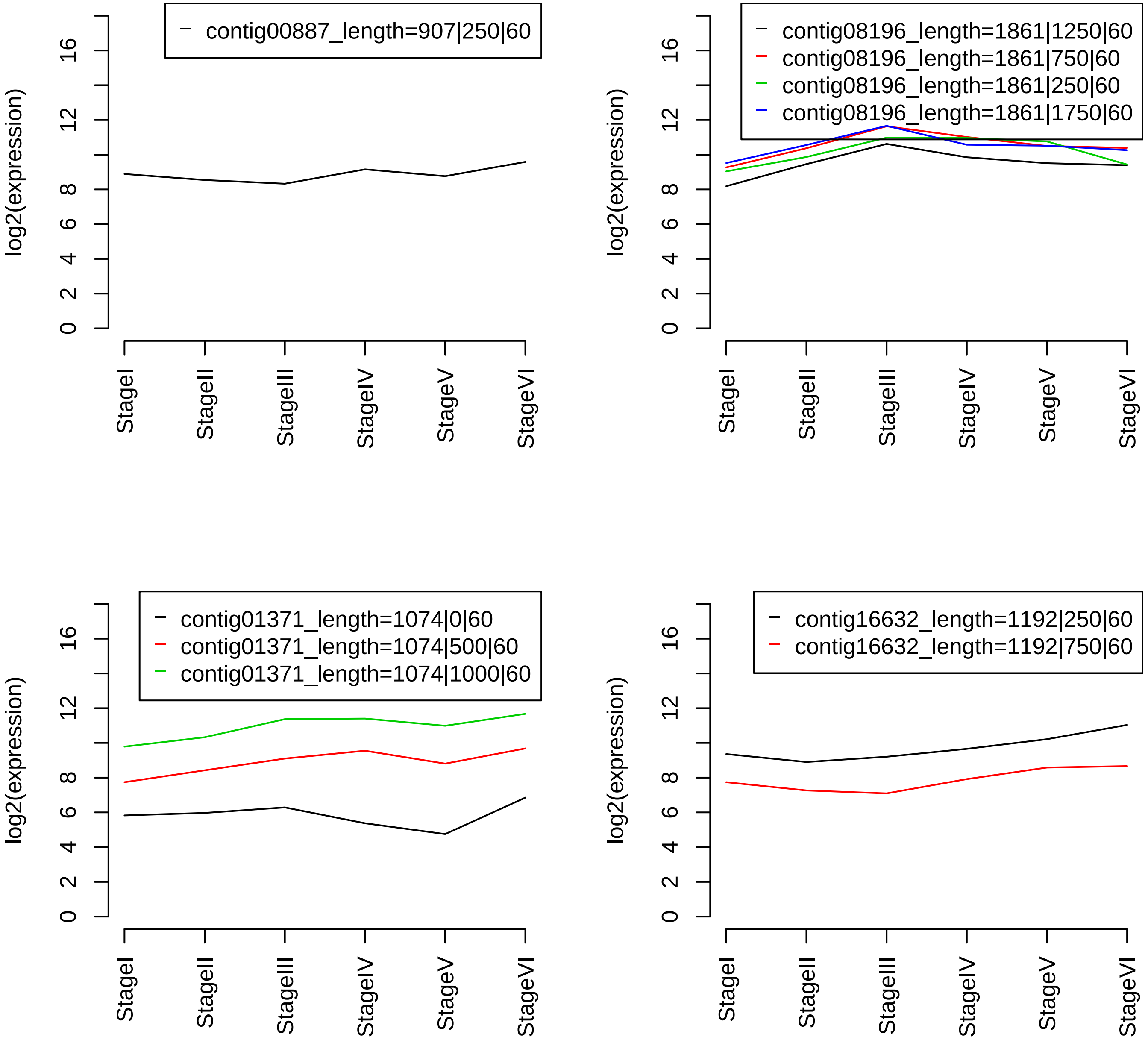

contig00887_length=907|250|60
contig08196_length=1861|1250|60
contig08196_length=1861|750|60
contig08196_length=1861|250|60
contig08196_length=1861|1750|60
contig01371_length=1074|0|60
contig01371_length=1074|500|60
contig01371_length=1074|1000|60
contig16632_length=1192|250|60
contig16632_length=1192|750|60
log2(expression)
0 2 4 6 8 12 16
StageI StageII StageIII StageIV StageV StageVI

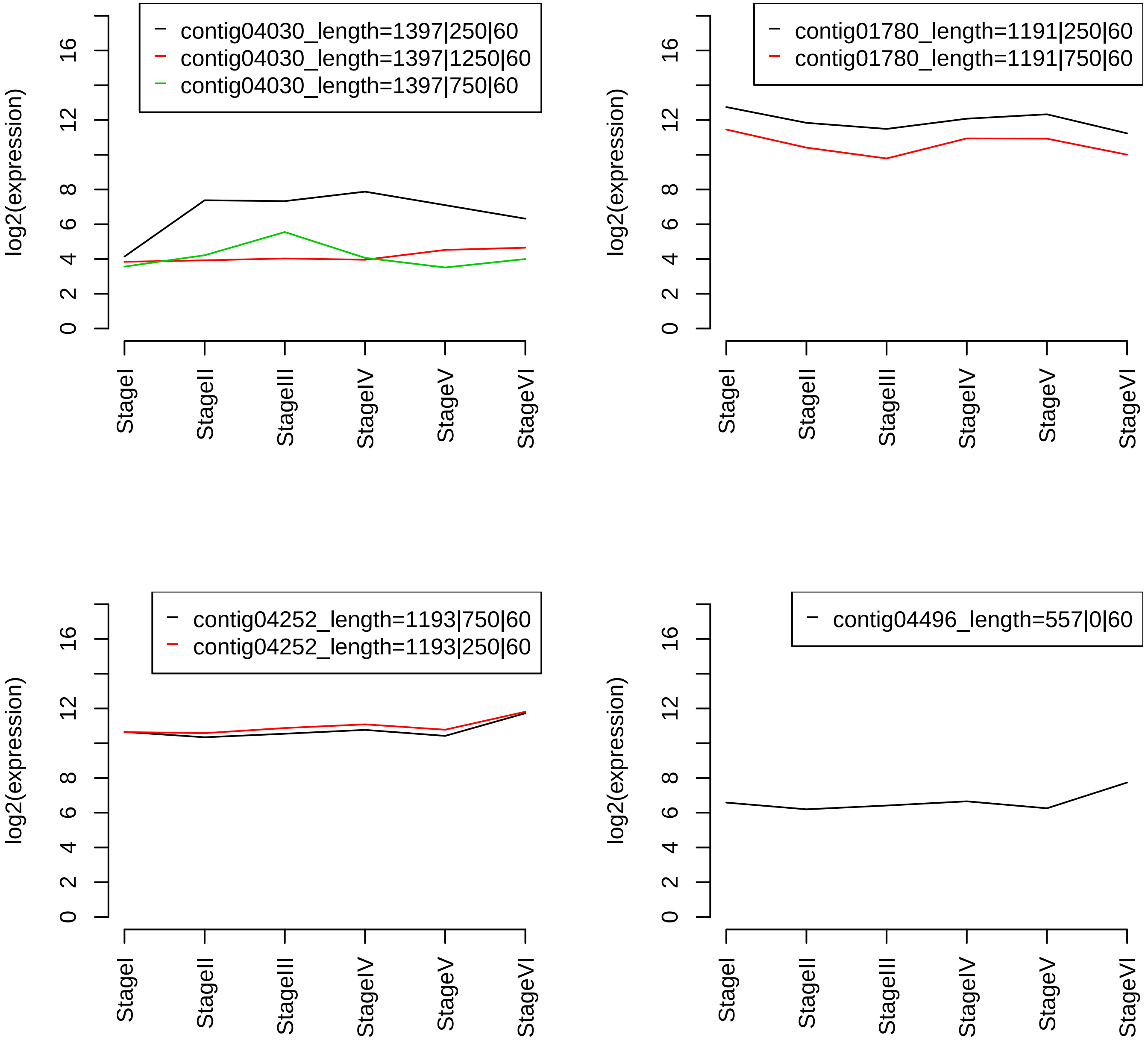

contig04030_length=1397|250|60
contig04030_length=1397|1250|60
contig04030_length=1397|750|60
log2(expression)
0
2
4
6
8
12
16
StageI
StageII
StageIII
StageIV
StageV
StageVI
contig01780_length=1191|250|60
contig01780_length=1191|750|60
contig04252_length=1193|750|60
contig04252_length=1193|250|60
contig04496_length=557|0|60

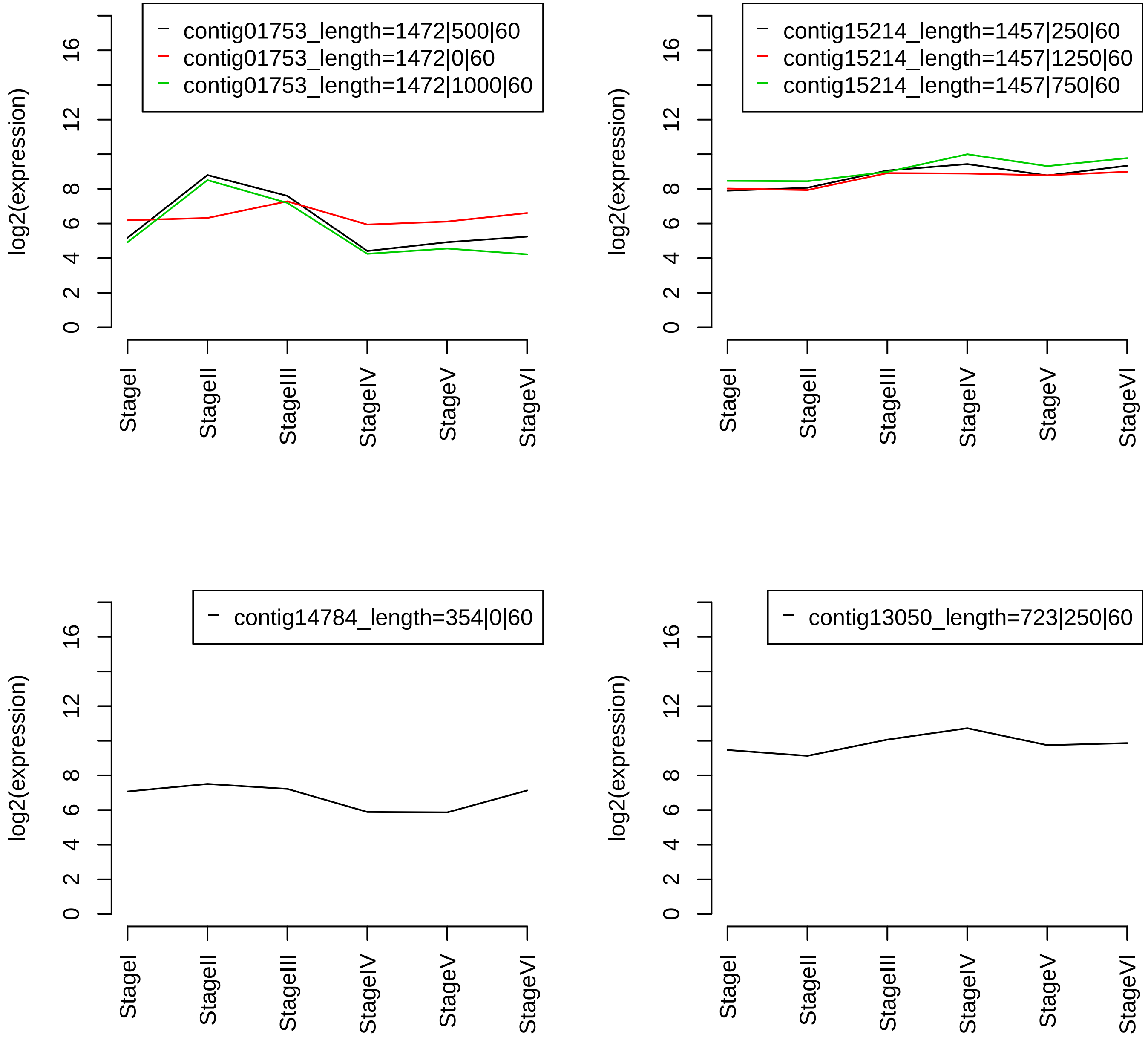

contig01753_length=1472|500|60
contig01753_length=1472|0|60
contig01753_length=1472|1000|60
contig15214_length=1457|250|60
contig15214_length=1457|1250|60
contig15214_length=1457|750|60
contig14784_length=354|0|60
contig13050_length=723|250|60
log2(expression)
0
2
4
6
8
12
16
StageI
StageII
StageIII
StageIV
StageV
StageVI

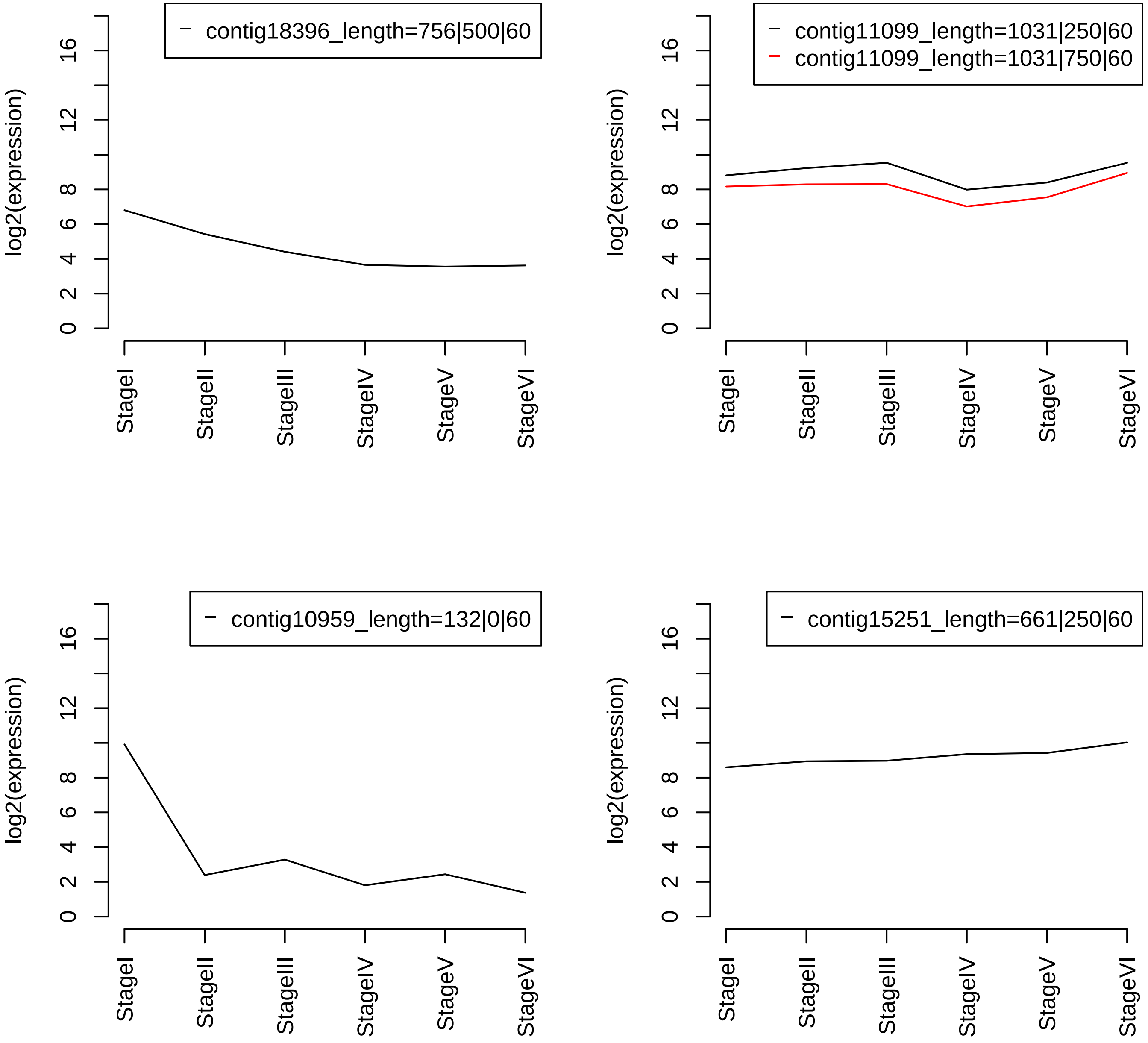

contig18396_length=756|500|60
contig11099_length=1031|250|60
contig11099_length=1031|750|60
contig10959_length=132|0|60
contig15251_length=661|250|60
log2(expression)
0
2
4
6
8
12
16
StageI
StageII
StageIII
StageIV
StageV
StageVI

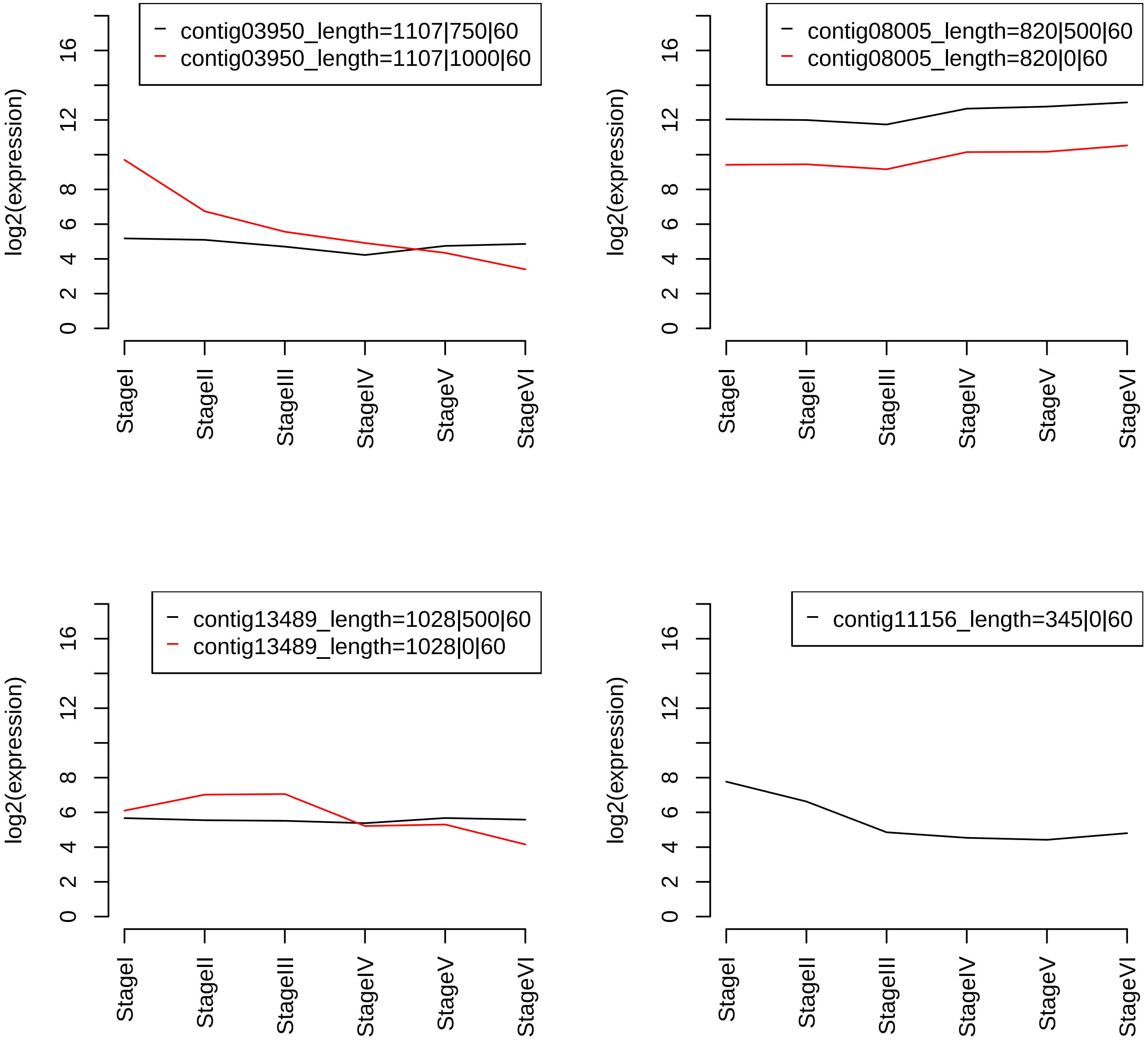

contig03950_length=1107|750|60
contig03950_length=1107|1000|60
contig08005_length=820|500|60
contig08005_length=820|0|60
contig13489_length=1028|500|60
contig13489_length=1028|0|60
contig11156_length=345|0|60
log2(expression)
0
2
4
6
8
12
16
StageI
StageII
StageIII
StageIV
StageV
StageVI

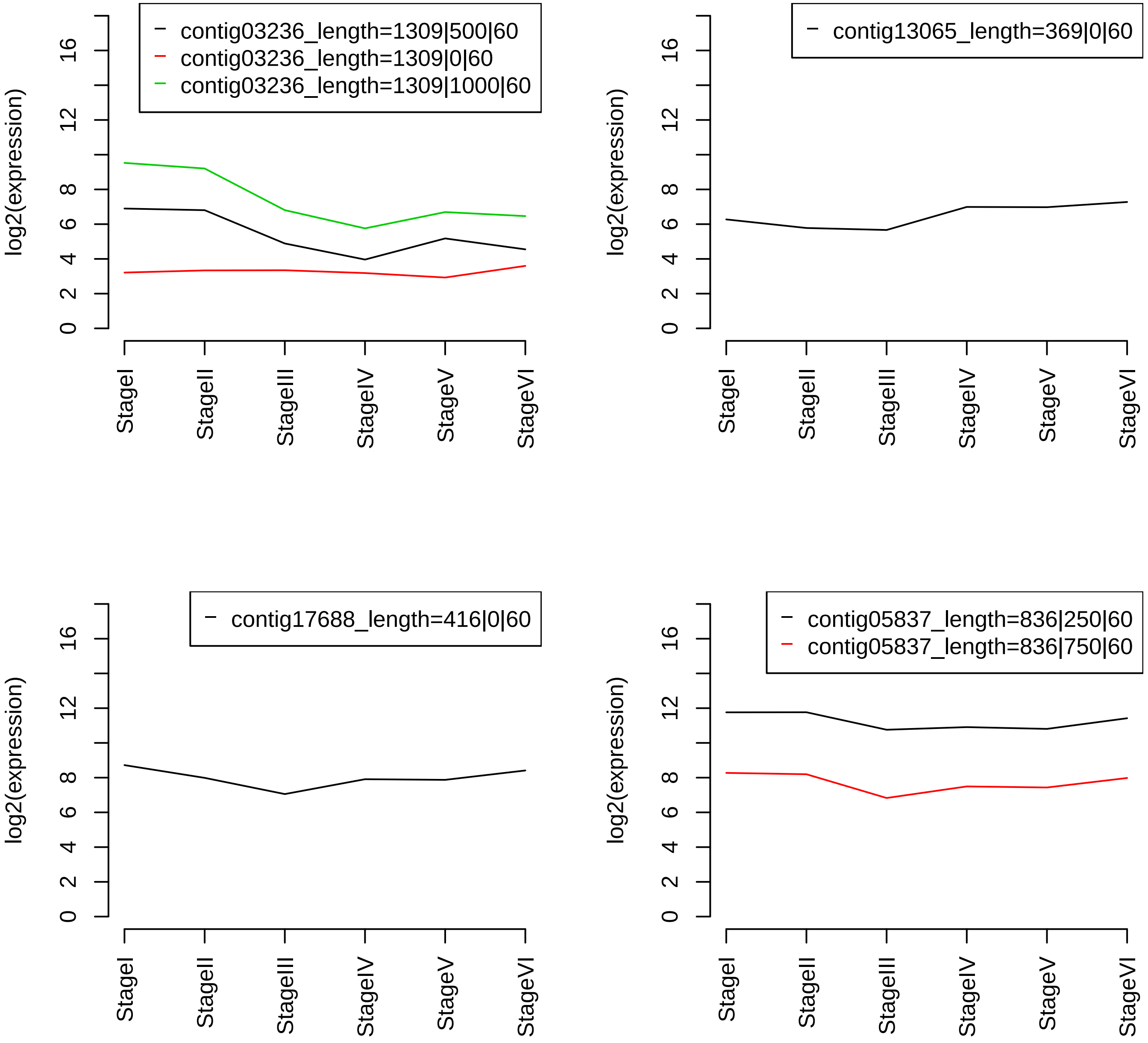

contig03236_length=1309|500|60
contig03236_length=1309|0|60
contig03236_length=1309|1000|60
contig13065_length=369|0|60
contig17688_length=416|0|60
contig05837_length=836|250|60
contig05837_length=836|750|60
log2(expression)
0
2
4
6
8
12
16
StageI
StageII
StageIII
StageIV
StageV
StageVI

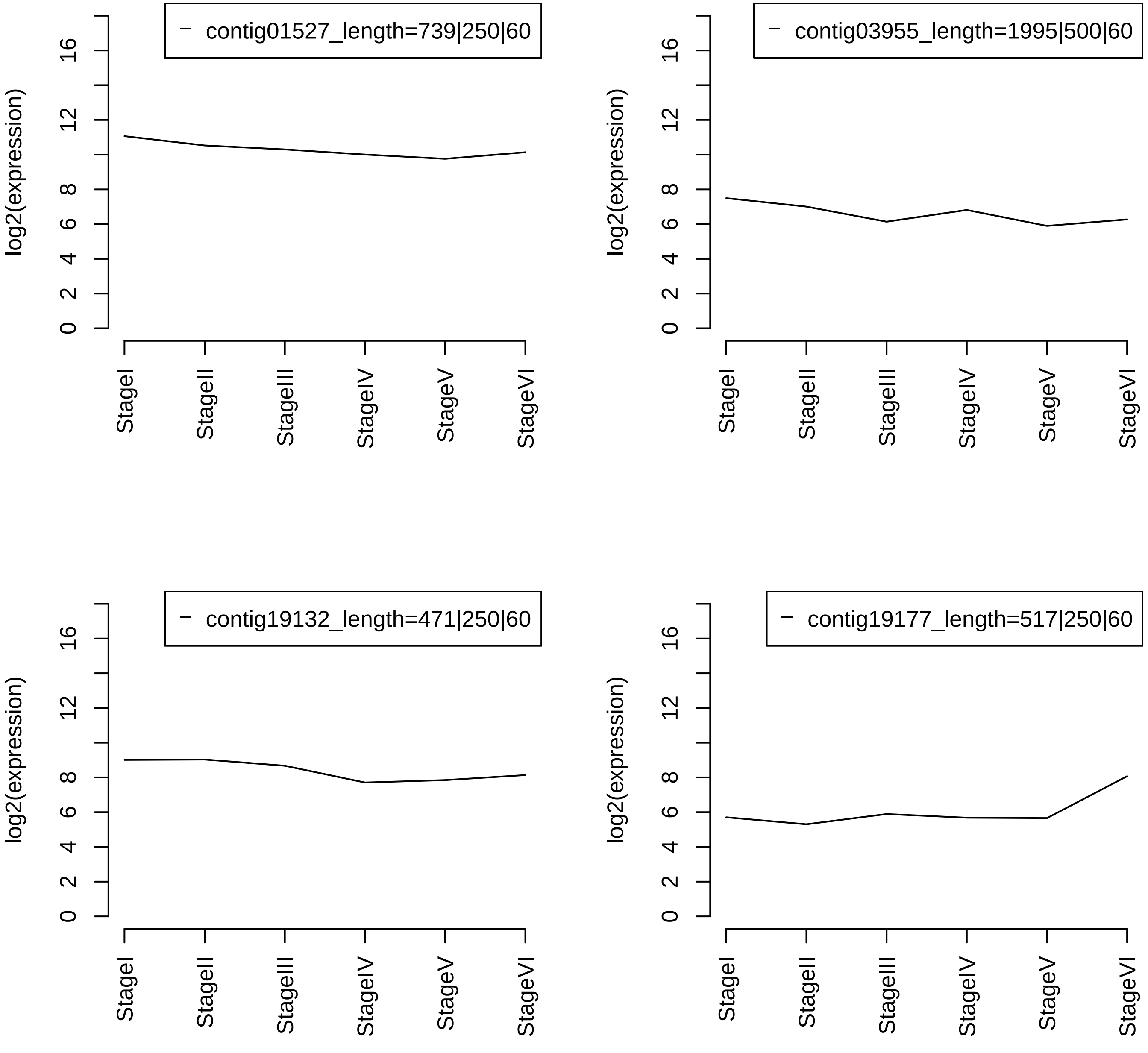
contig01527_length=739|250|60
contig03955_length=1995|500|60
contig19132_length=471|250|60
contig19177_length=517|250|60
log2(expression)
0
2
4
6
8
12
16
StageI
StageII
StageIII
StageIV
StageV
StageVI

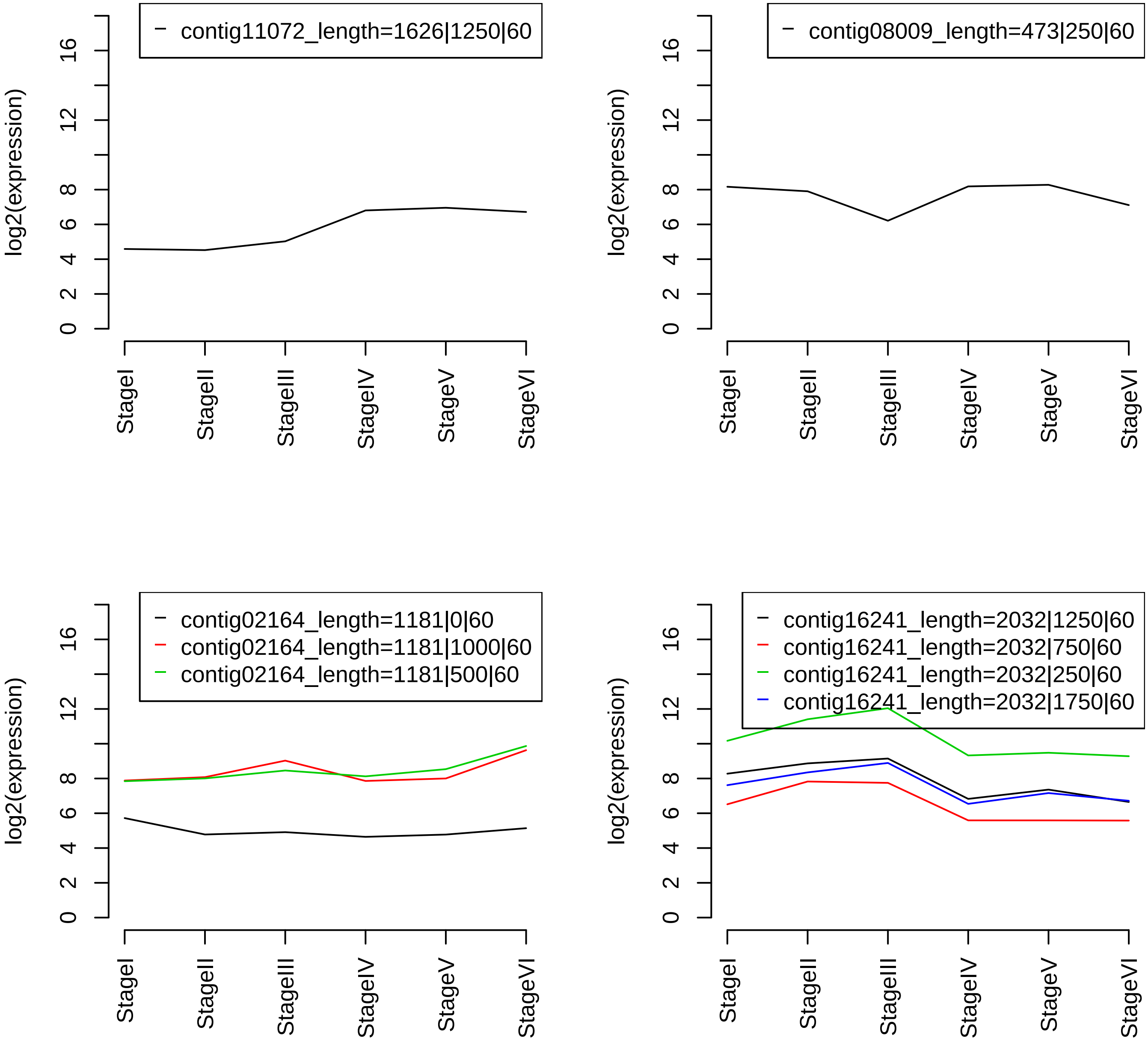

contig11072_length=1626|1250|60
contig08009_length=473|250|60
contig02164_length=1181|0|60
contig02164_length=1181|1000|60
contig02164_length=1181|500|60
contig16241_length=2032|1250|60
contig16241_length=2032|750|60
contig16241_length=2032|250|60
contig16241_length=2032|1750|60
log2(expression)
0
2
4
6
8
12
16
StageI
StageII
StageIII
StageIV
StageV
StageVI

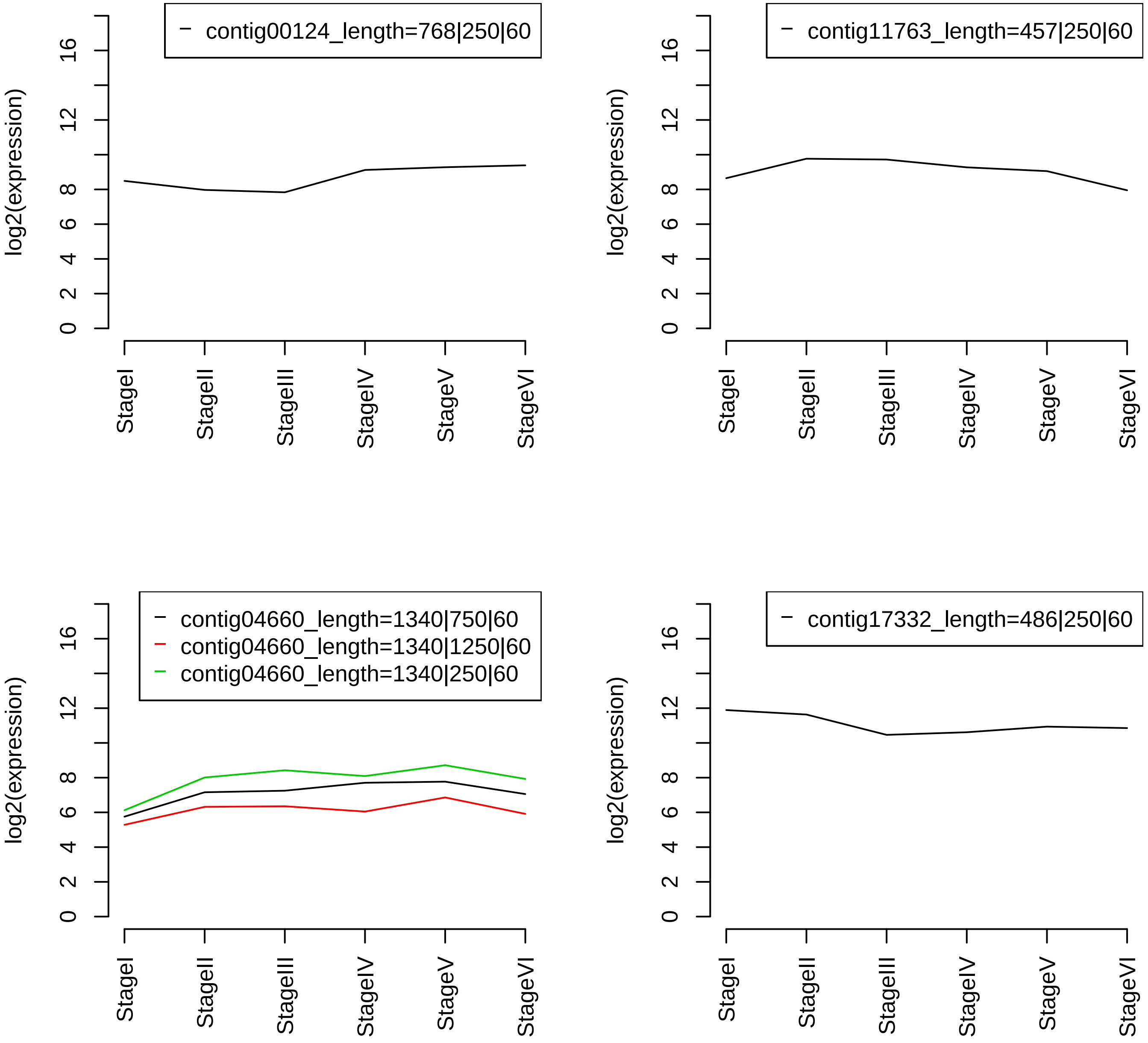
contig00124_length=768|250|60
contig11763_length=457|250|60
contig04660_length=1340|750|60
contig04660_length=1340|1250|60
contig04660_length=1340|250|60
contig17332_length=486|250|60
log2(expression)
0
2
4
6
8
12
16
StageI
StageII
StageIII
StageIV
StageV
StageVI

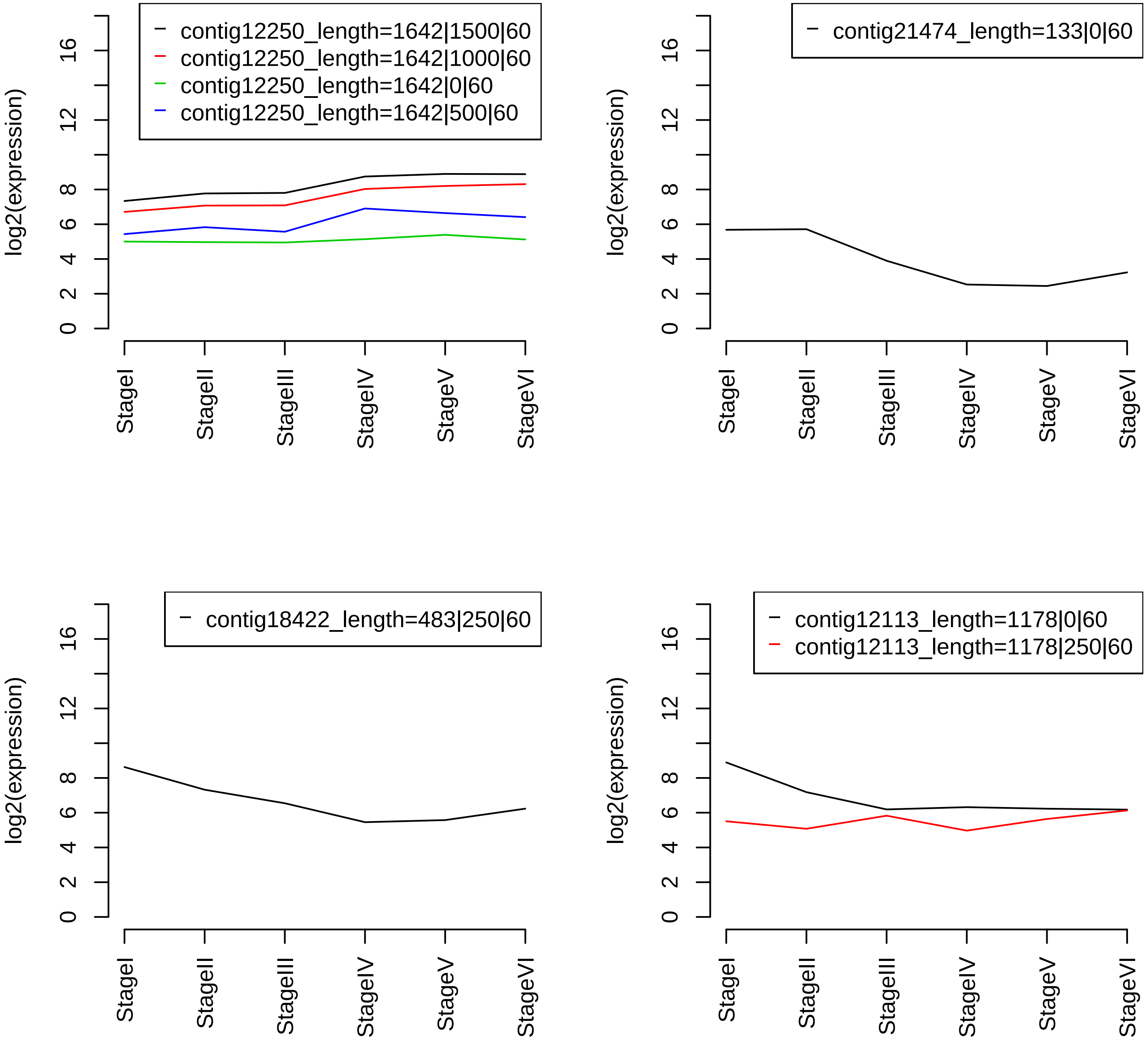

contig12250_length=1642|1500|60
contig12250_length=1642|1000|60
contig12250_length=1642|0|60
contig12250_length=1642|500|60
contig21474_length=133|0|60
contig18422_length=483|250|60
contig12113_length=1178|0|60
contig12113_length=1178|250|60
log2(expression)
0
2
4
6
8
12
16
StageI
StageII
StageIII
StageIV
StageV
StageVI

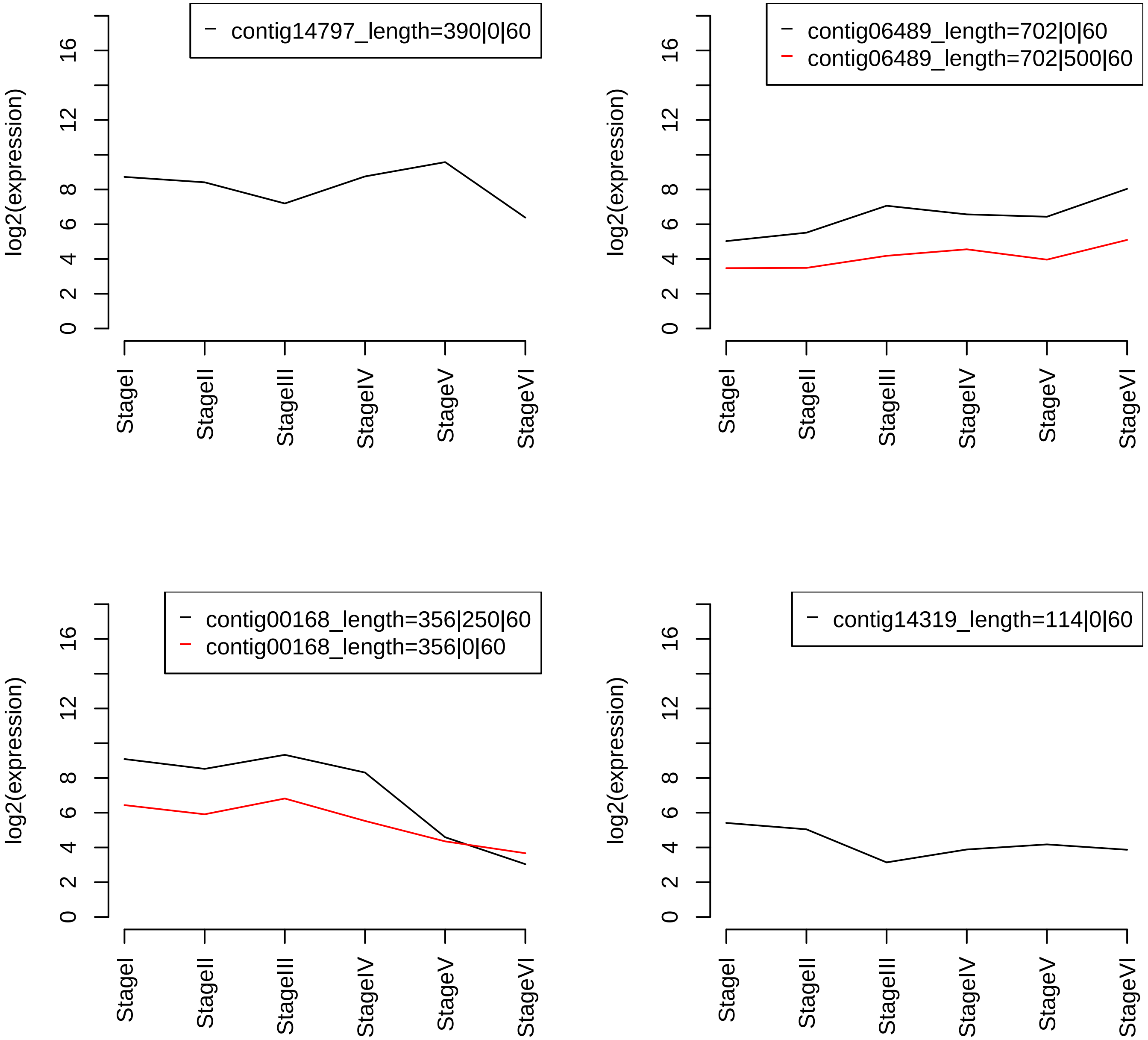

contig14797_length=390|0|60
contig06489_length=702|0|60
contig06489_length=702|500|60
contig00168_length=356|250|60
contig00168_length=356|0|60
contig14319_length=114|0|60
log2(expression)
0
2
4
6
8
12
16
StageI
StageII
StageIII
StageIV
StageV
StageVI

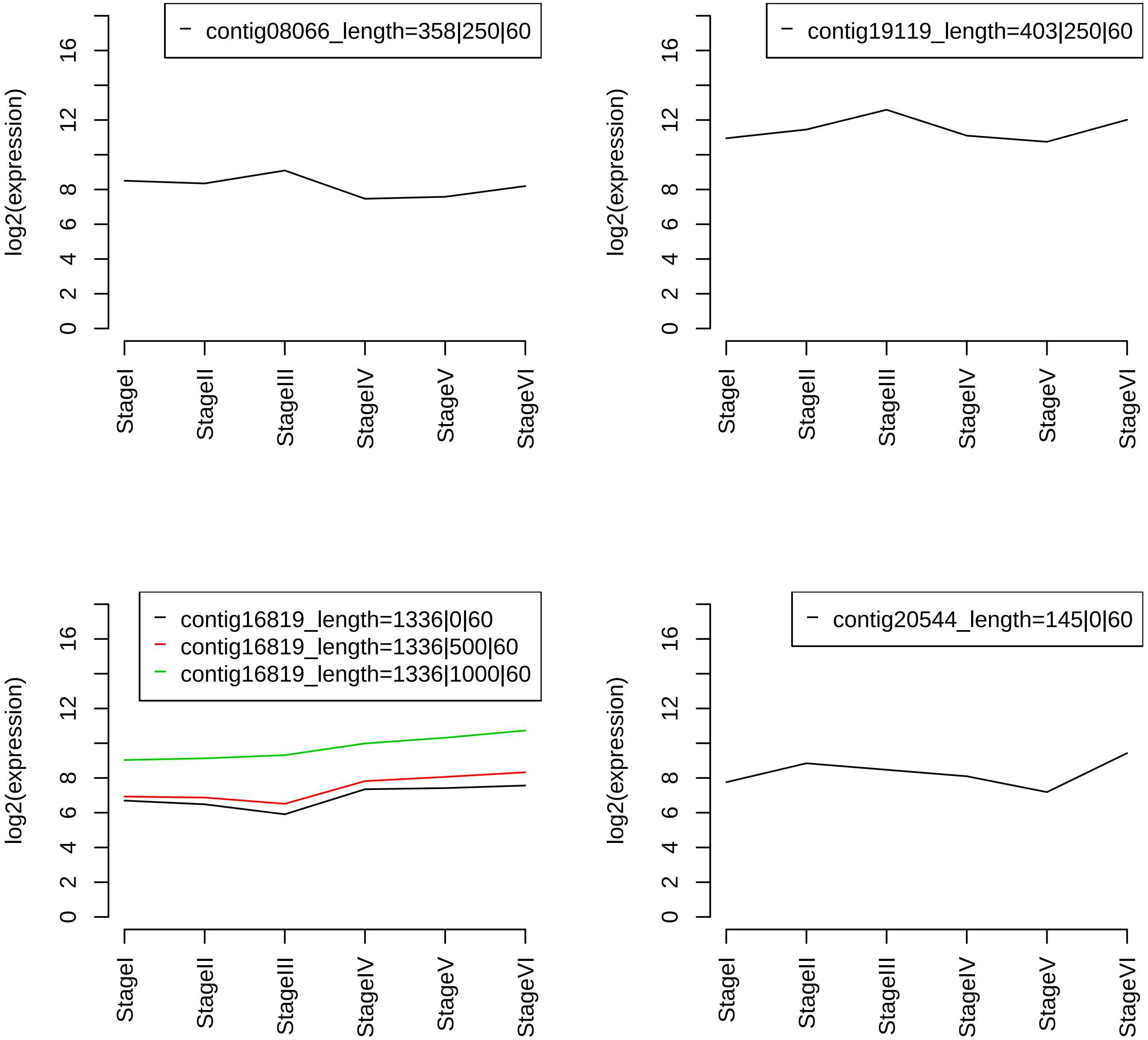
contig08066_length=358|250|60
contig19119_length=403|250|60
contig16819_length=1336|0|60
contig16819_length=1336|500|60
contig16819_length=1336|1000|60
contig20544_length=145|0|60
log2(expression)
0
2
4
6
8
12
16
StageI
StageII
StageIII
StageIV
StageV
StageVI

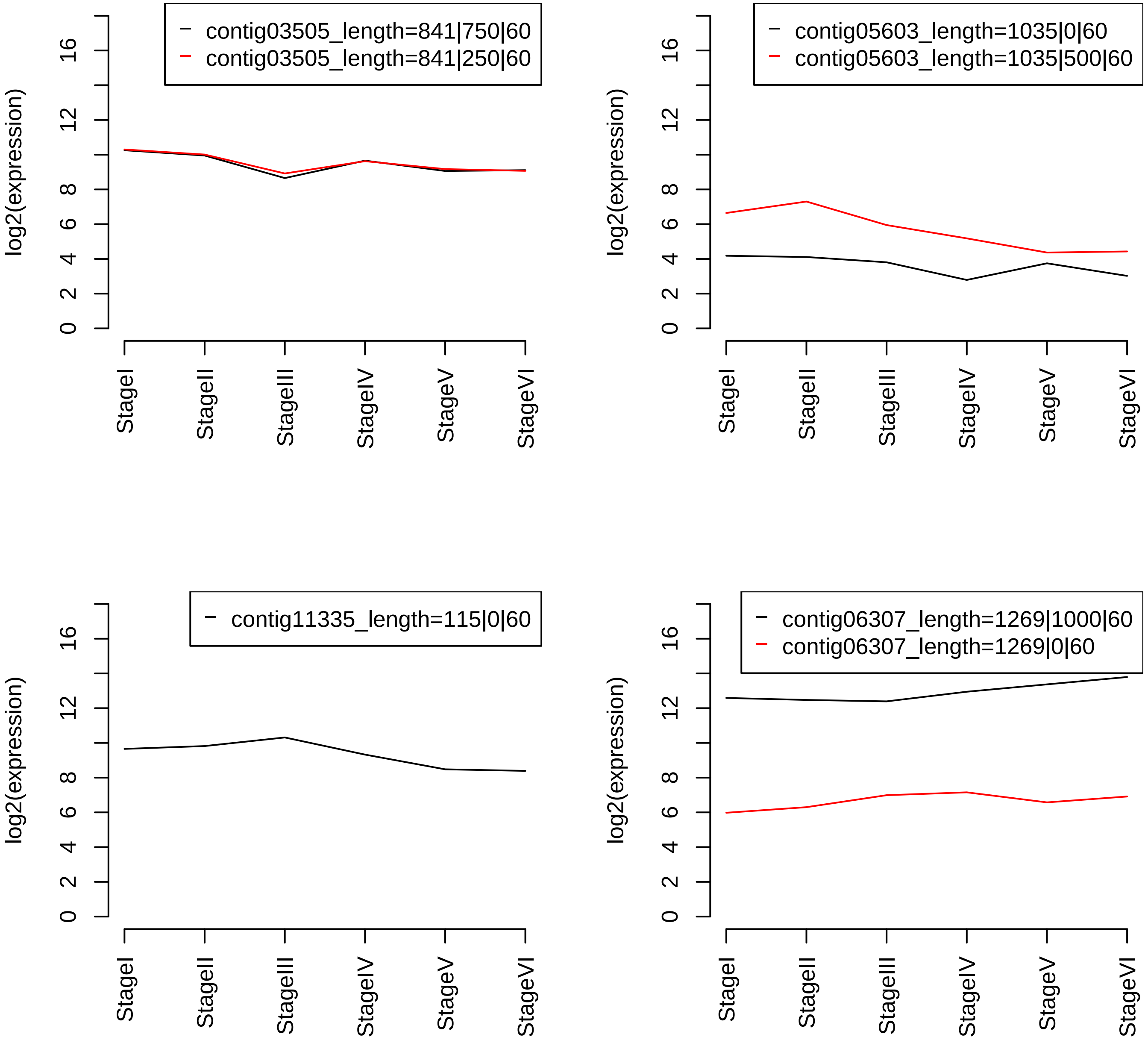

contig03505_length=841|750|60
contig03505_length=841|250|60
contig05603_length=1035|0|60
contig05603_length=1035|500|60
contig11335_length=115|0|60
contig06307_length=1269|1000|60
contig06307_length=1269|0|60
log2(expression)
0
2
4
6
8
12
16
StageI
StageII
StageIII
StageIV
StageV
StageVI

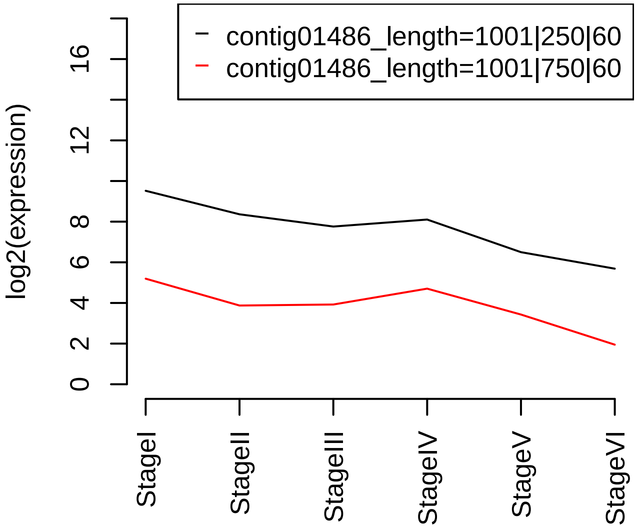

contig01486_length=1001|250|60
contig01486_length=1001|750|60
log2(expression)
0
2
4
6
8
12
16
StageI
StageII
StageIII
StageIV
StageV
StageVI

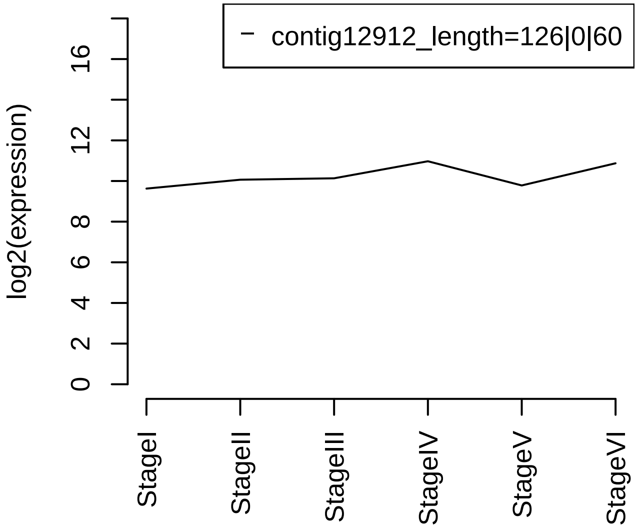

contig12912_length=126|0|60
log2(expression)
0
2
4
6
8
12
16
StageI
StageII
StageIII
StageIV
StageV
StageVI

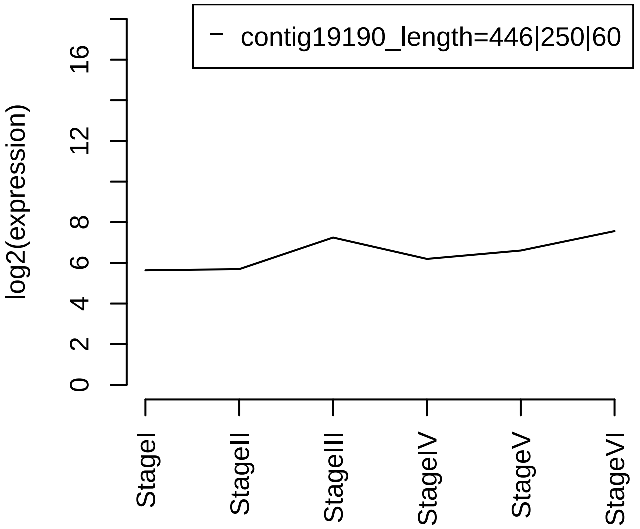

contig19190_length=446|250|60
log2(expression)
0
2
4
6
8
12
16
StageI
StageII
StageIII
StageIV
StageV
StageVI

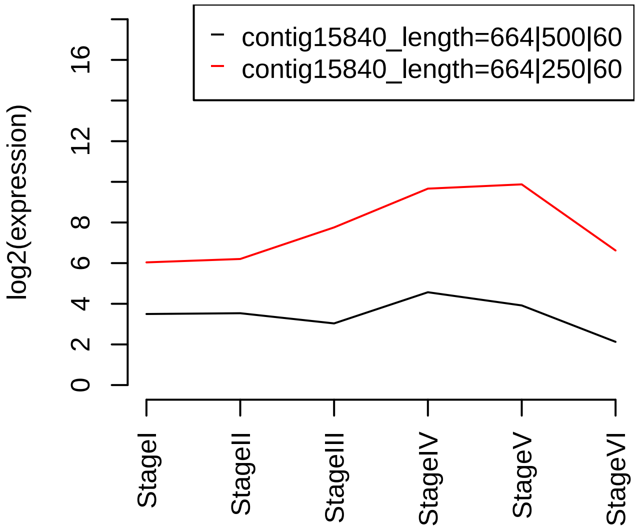

contig15840_length=664|500|60
contig15840_length=664|250|60
log2(expression)
0
2
4
6
8
12
16
StageI
StageII
StageIII
StageIV
StageV
StageVI

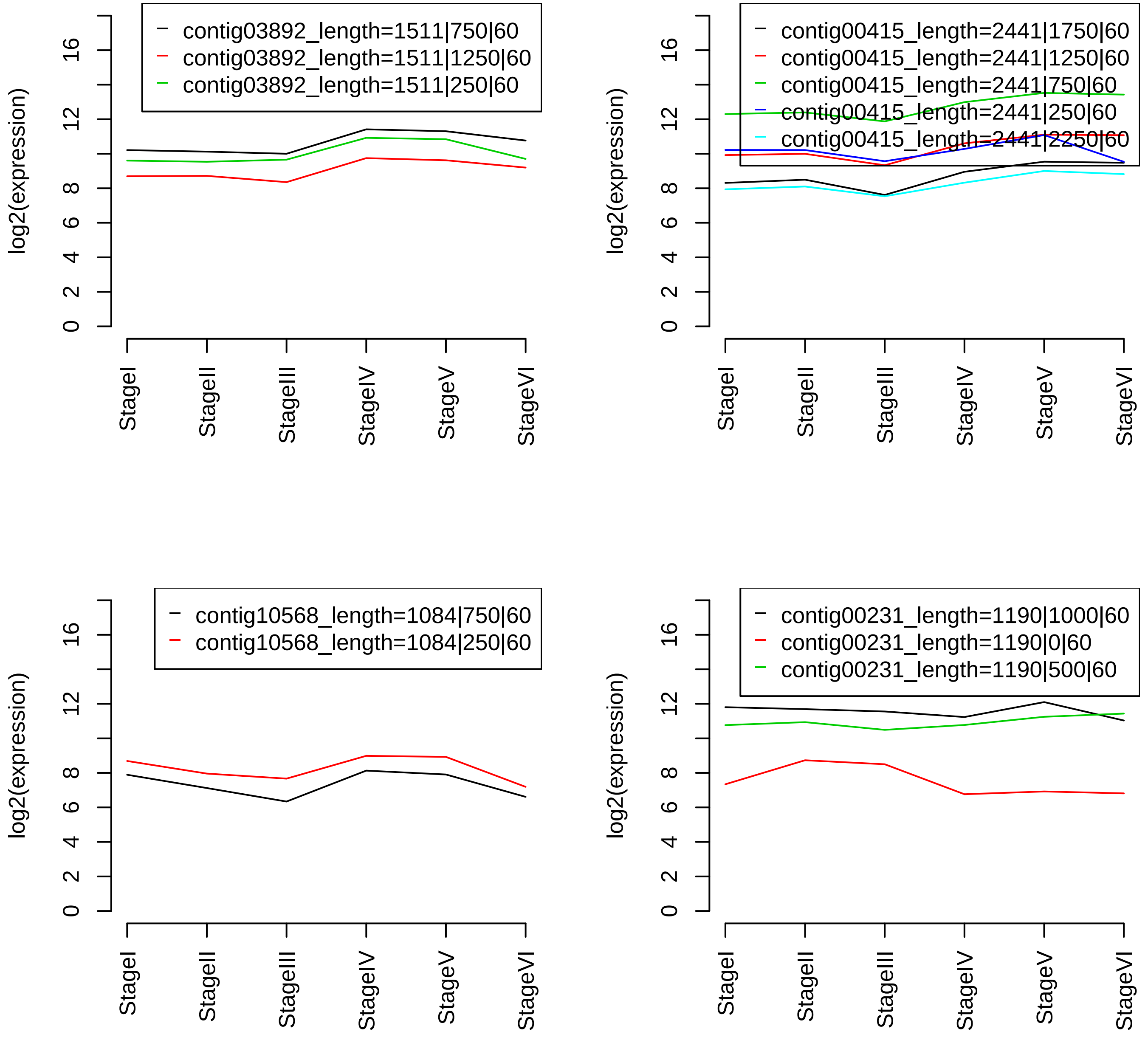

contig03892_length=1511|750|60
contig03892_length=1511|1250|60
contig03892_length=1511|250|60
contig00415_length=2441|1750|60
contig00415_length=2441|1250|60
contig00415_length=2441|750|60
contig00415_length=2441|250|60
contig00415_length=2441|2250|60
contig10568_length=1084|750|60
contig10568_length=1084|250|60
contig00231_length=1190|1000|60
contig00231_length=1190|0|60
contig00231_length=1190|500|60
log2(expression)
0
2
4
6
8
12
16
StageI
StageII
StageIII
StageIV
StageV
StageVI

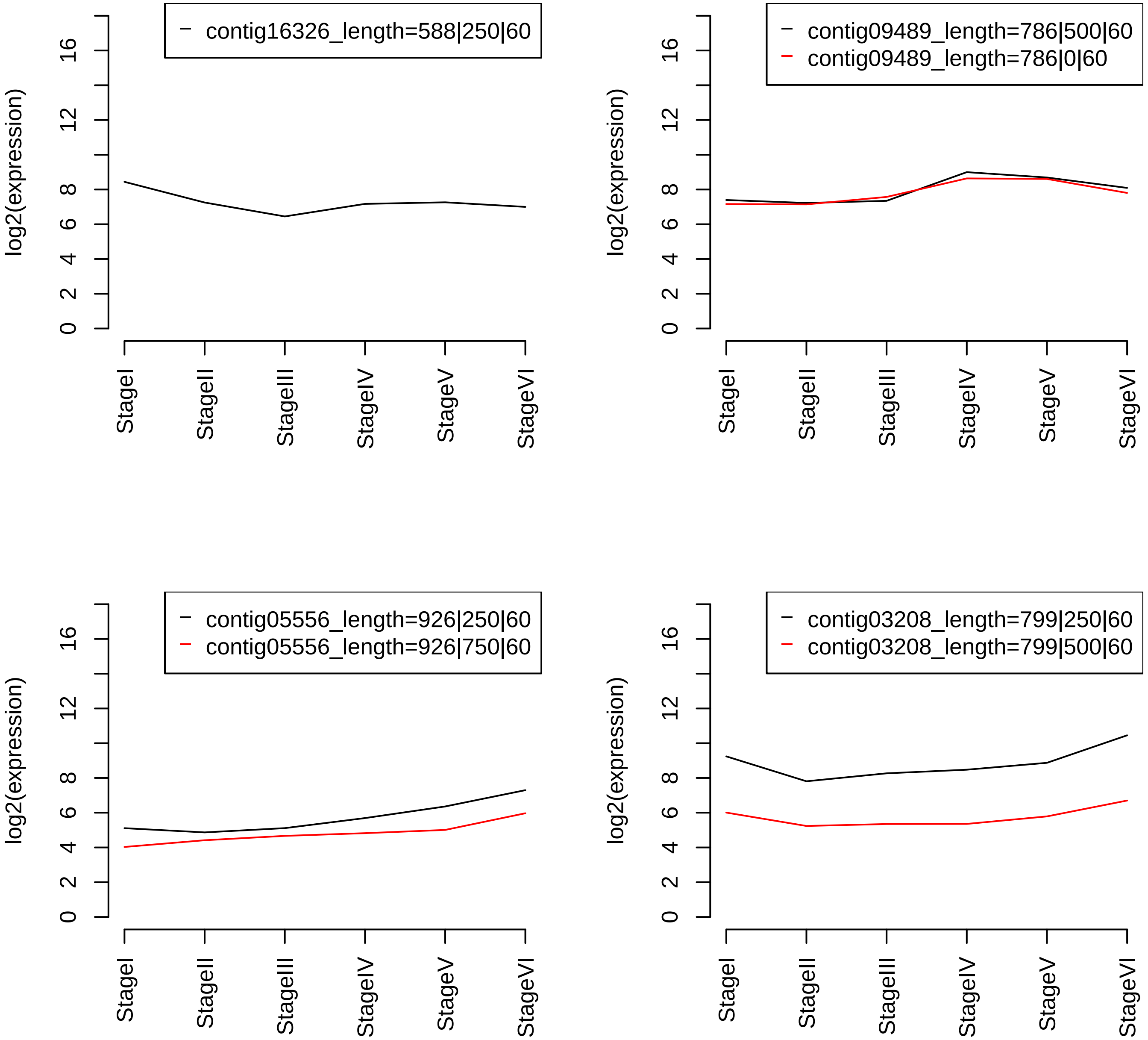

contig16326_length=588|250|60
contig09489_length=786|500|60
contig09489_length=786|0|60
contig05556_length=926|250|60
contig05556_length=926|750|60
contig03208_length=799|250|60
contig03208_length=799|500|60
log2(expression)
0
2
4
6
8
12
16
StageI
StageII
StageIII
StageIV
StageV
StageVI

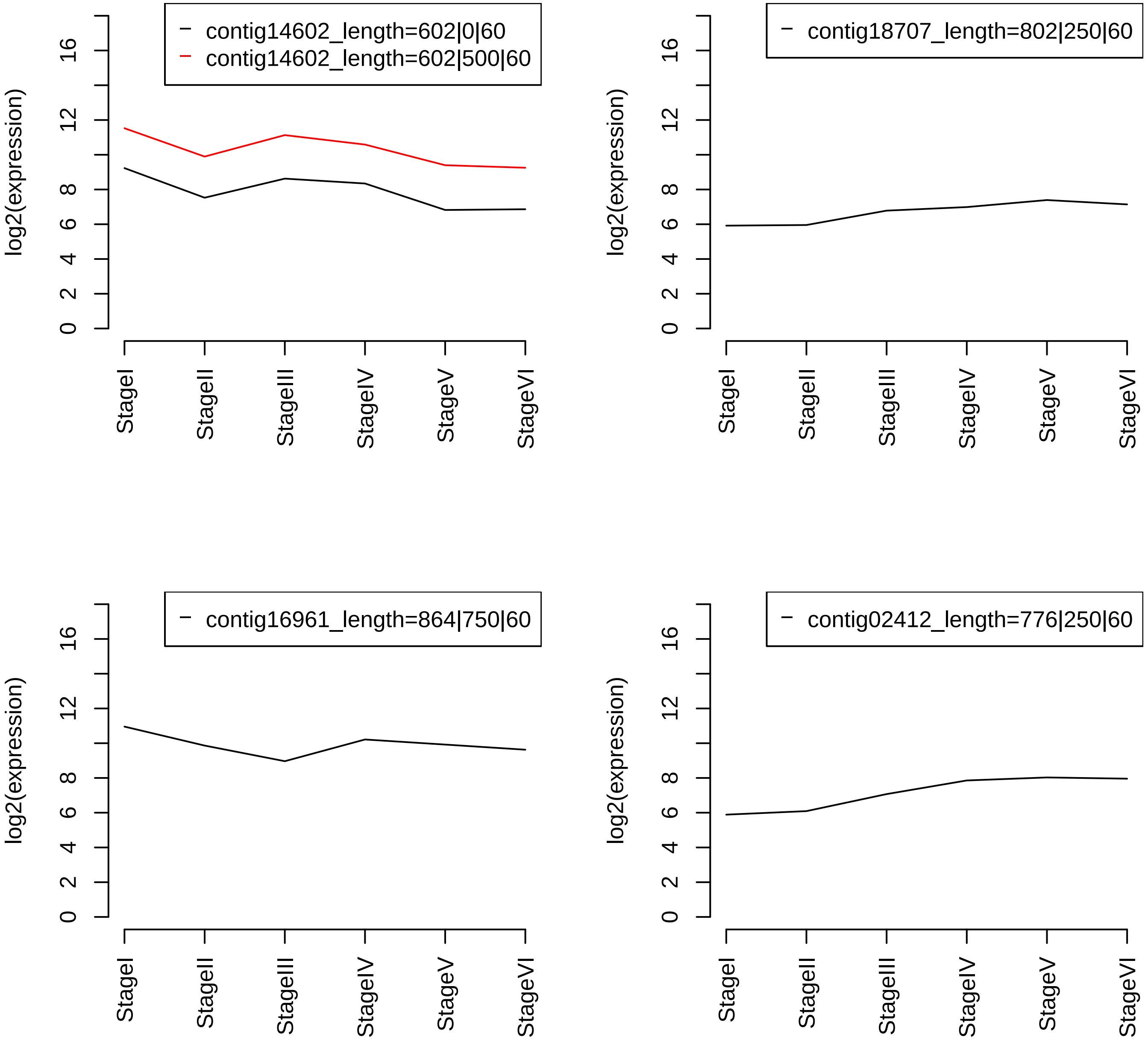

contig14602_length=602|0|60
contig14602_length=602|500|60
contig18707_length=802|250|60
contig16961_length=864|750|60
contig02412_length=776|250|60
log2(expression)
0
2
4
6
8
12
16
StageI
StageII
StageIII
StageIV
StageV
StageVI

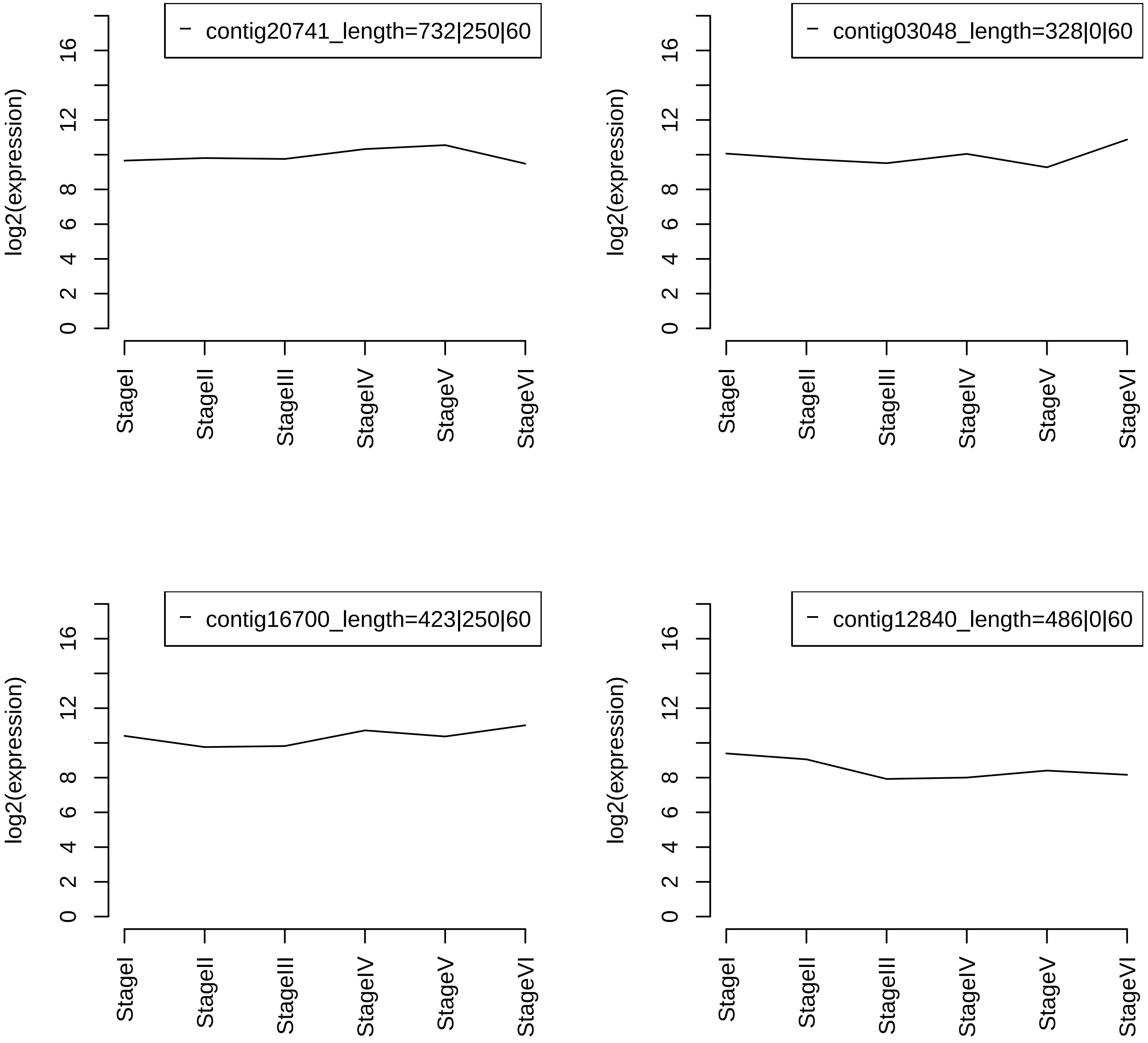
contig20741_length=732|250|60
contig03048_length=328|0|60
contig16700_length=423|250|60
contig12840_length=486|0|60
log2(expression)
0
2
4
6
8
12
16
StageI
StageII
StageIII
StageIV
StageV
StageVI

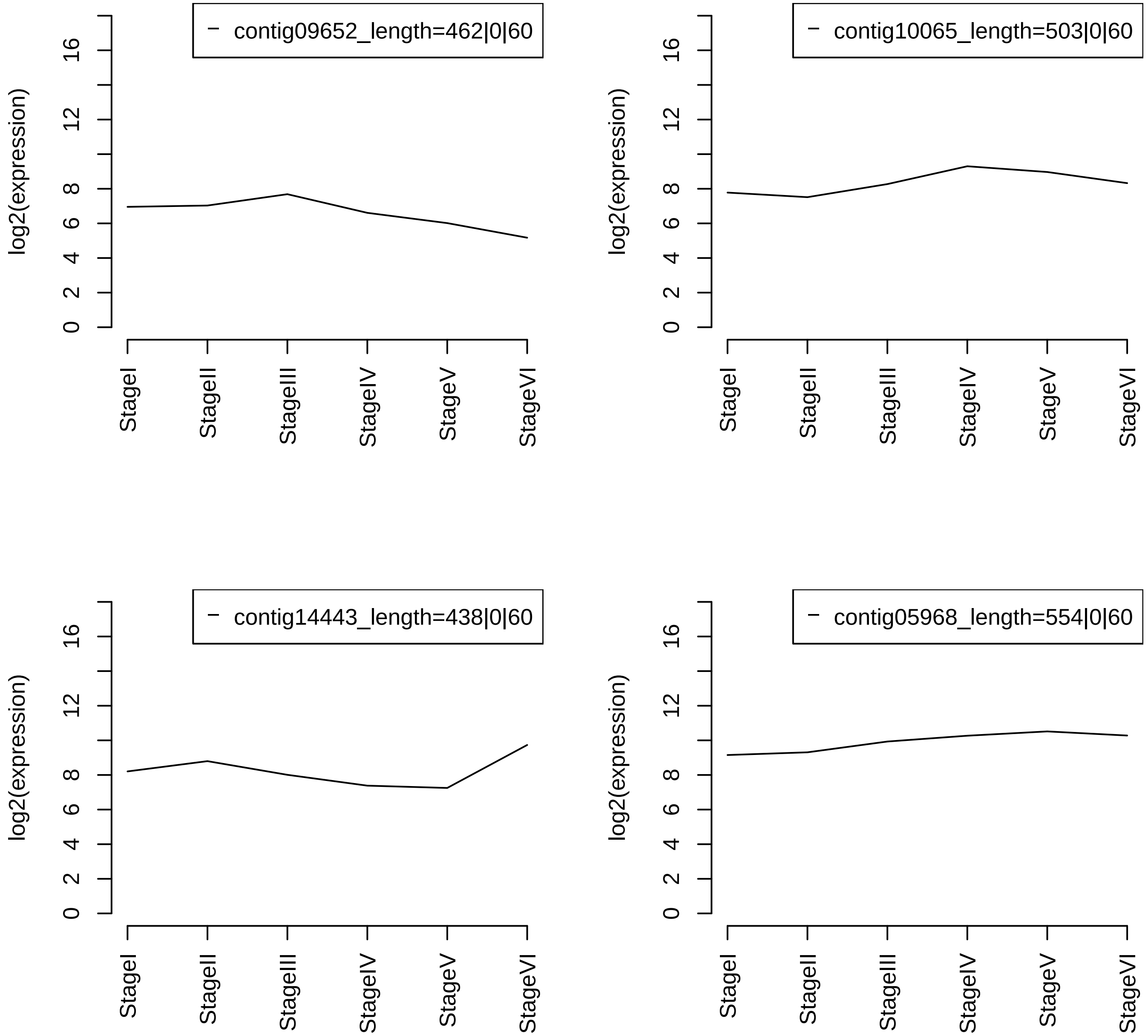
contig09652_length=462|0|60
contig10065_length=503|0|60
contig14443_length=438|0|60
contig05968_length=554|0|60
log2(expression)
0
2
4
6
8
12
16
StageI
StageII
StageIII
StageIV
StageV
StageVI

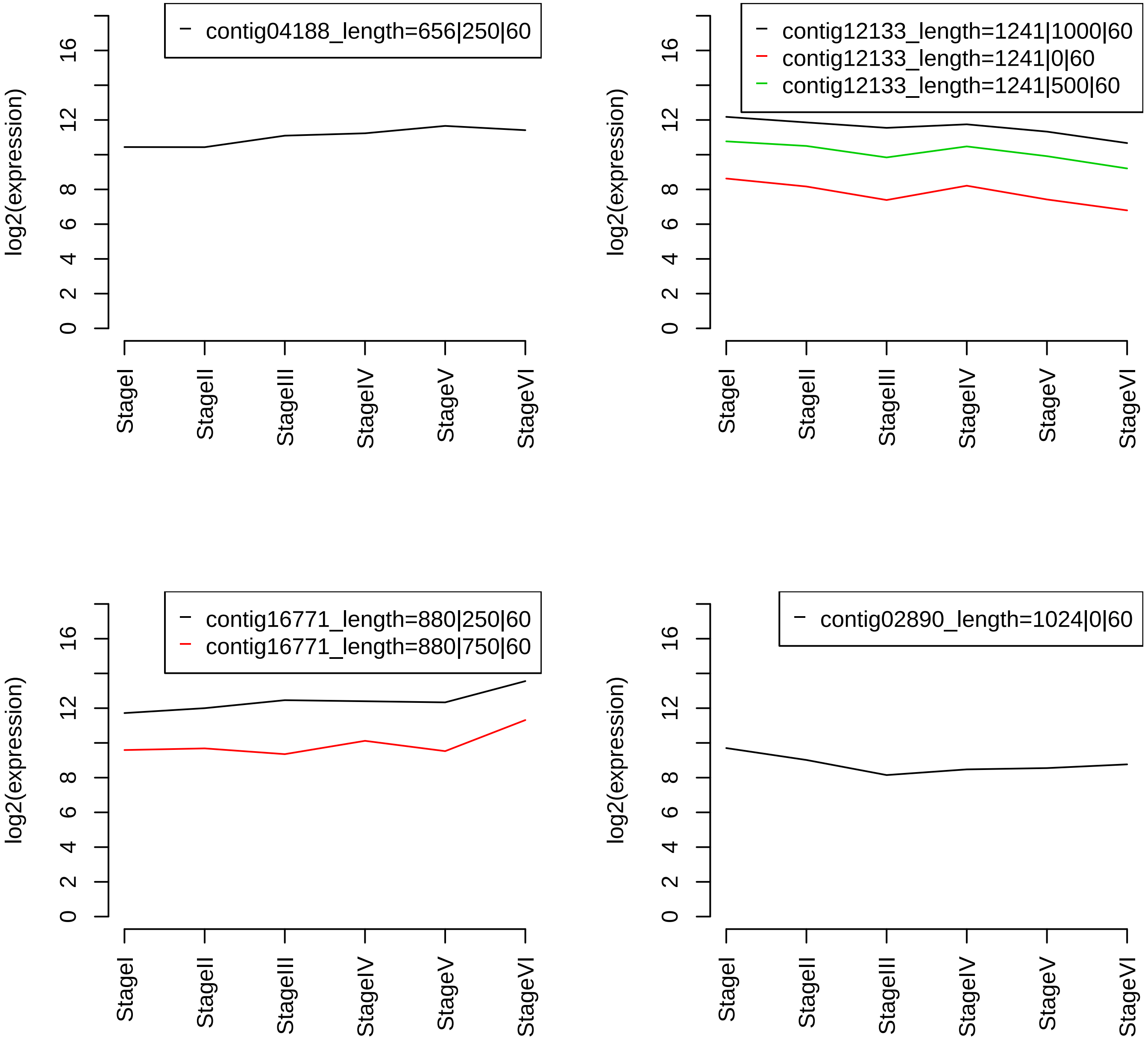

contig04188_length=656|250|60
contig12133_length=1241|1000|60
contig12133_length=1241|0|60
contig12133_length=1241|500|60
contig16771_length=880|250|60
contig16771_length=880|750|60
contig02890_length=1024|0|60
log2(expression)
0
2
4
6
8
12
16
StageI
StageII
StageIII
StageIV
StageV
StageVI

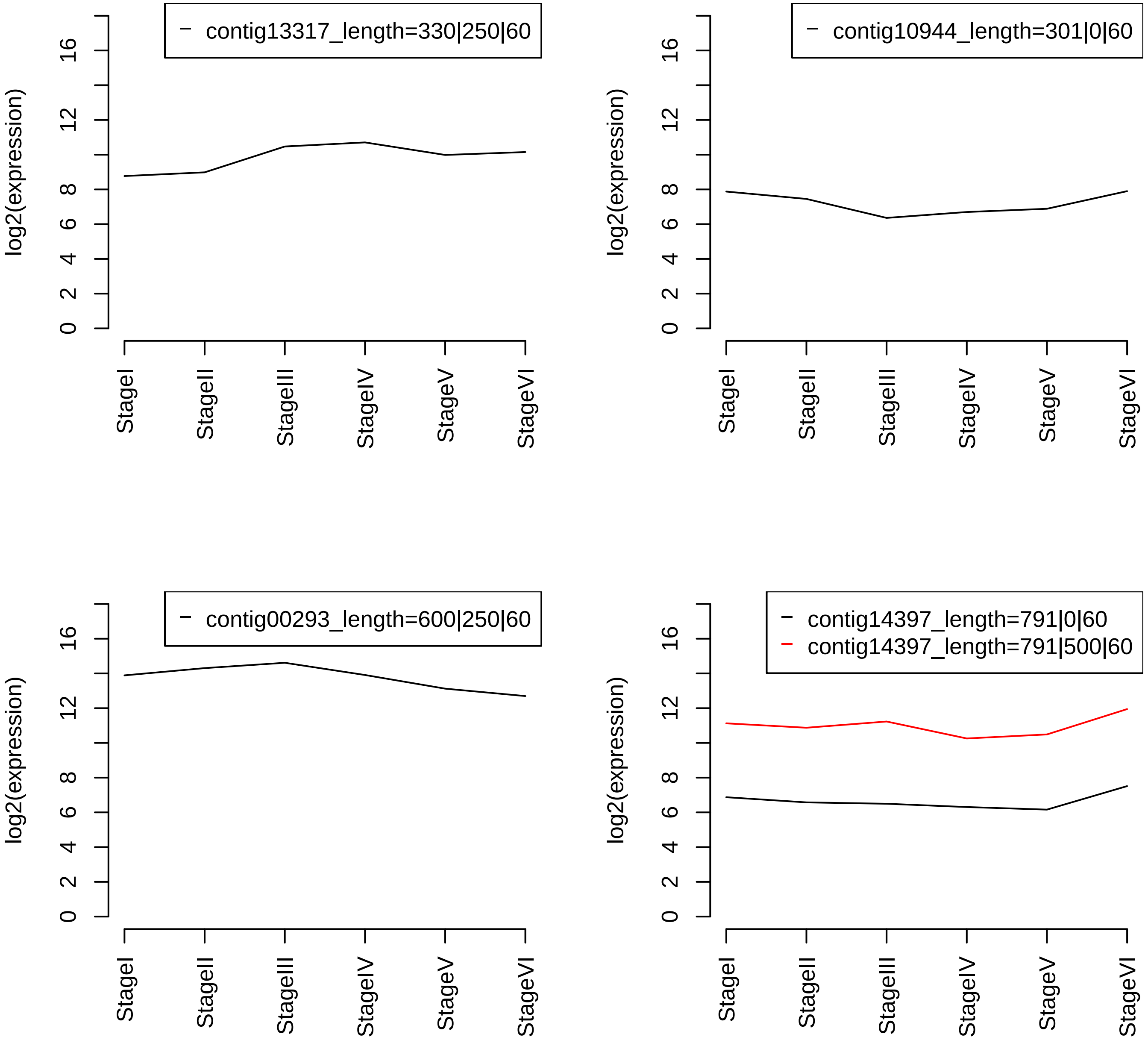
contig13317_length=330|250|60
contig10944_length=301|0|60
contig00293_length=600|250|60
contig14397_length=791|0|60
contig14397_length=791|500|60
log2(expression)
0
2
4
6
8
12
16
StageI
StageII
StageIII
StageIV
StageV
StageVI

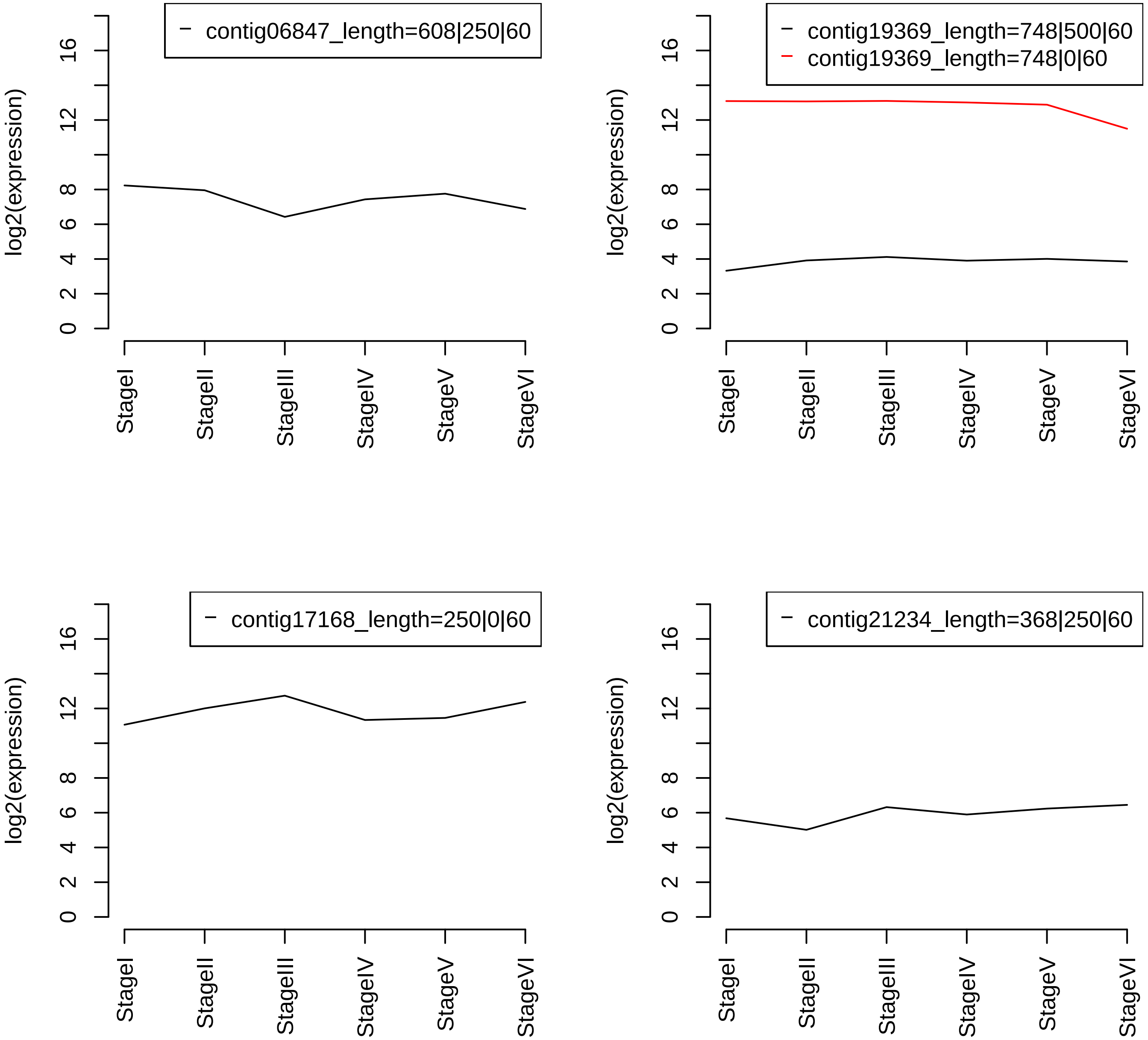
contig06847_length=608|250|60
contig19369_length=748|500|60
contig19369_length=748|0|60
contig17168_length=250|0|60
contig21234_length=368|250|60
log2(expression)
StageI
StageII
StageIII
StageIV
StageV
StageVI

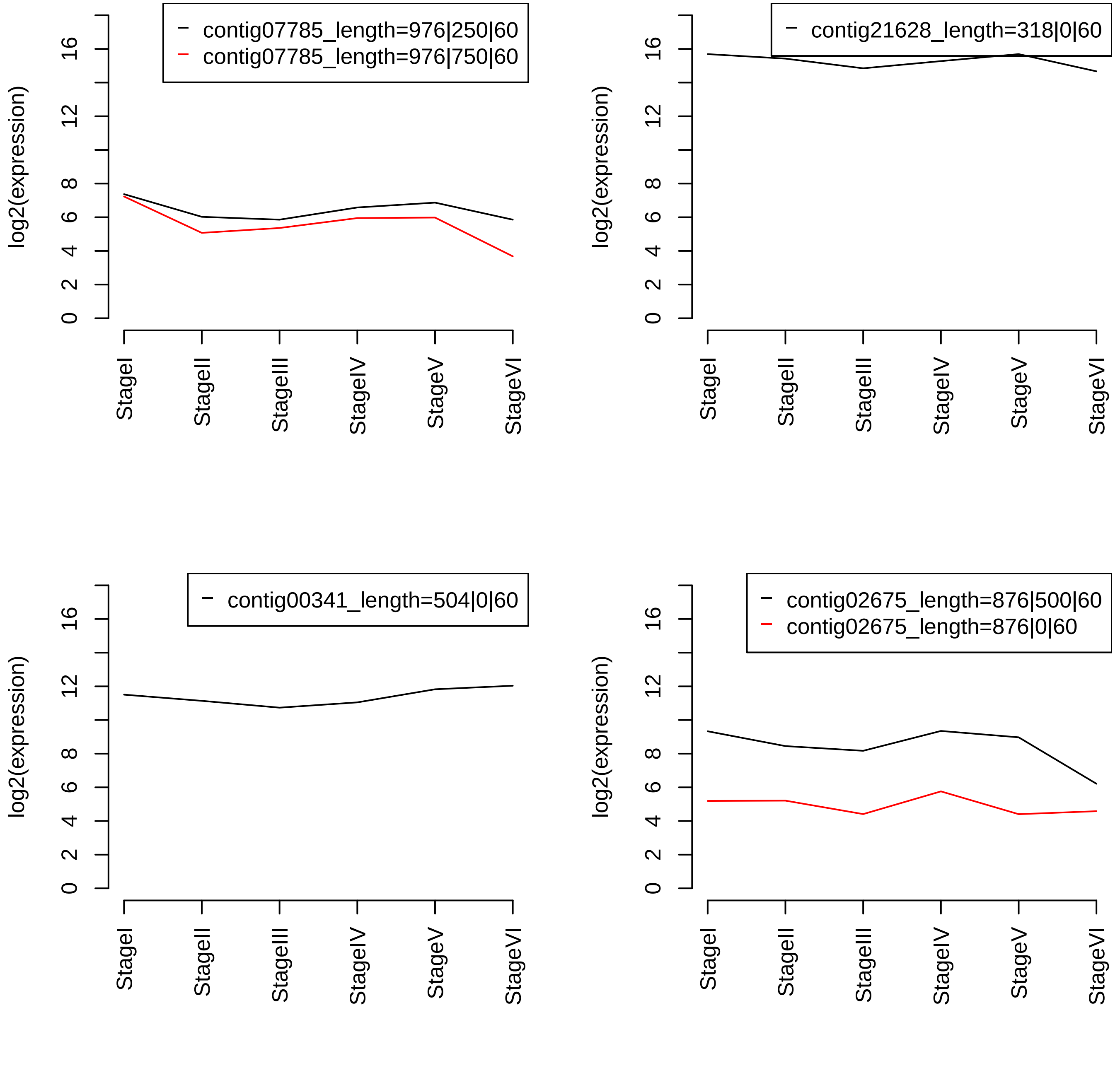

contig07785_length=976|250|60
contig07785_length=976|750|60
contig21628_length=318|0|60
contig00341_length=504|0|60
contig02675_length=876|500|60
contig02675_length=876|0|60
log2(expression)
0
2
4
6
8
12
16
StageI
StageII
StageIII
StageIV
StageV
StageVI

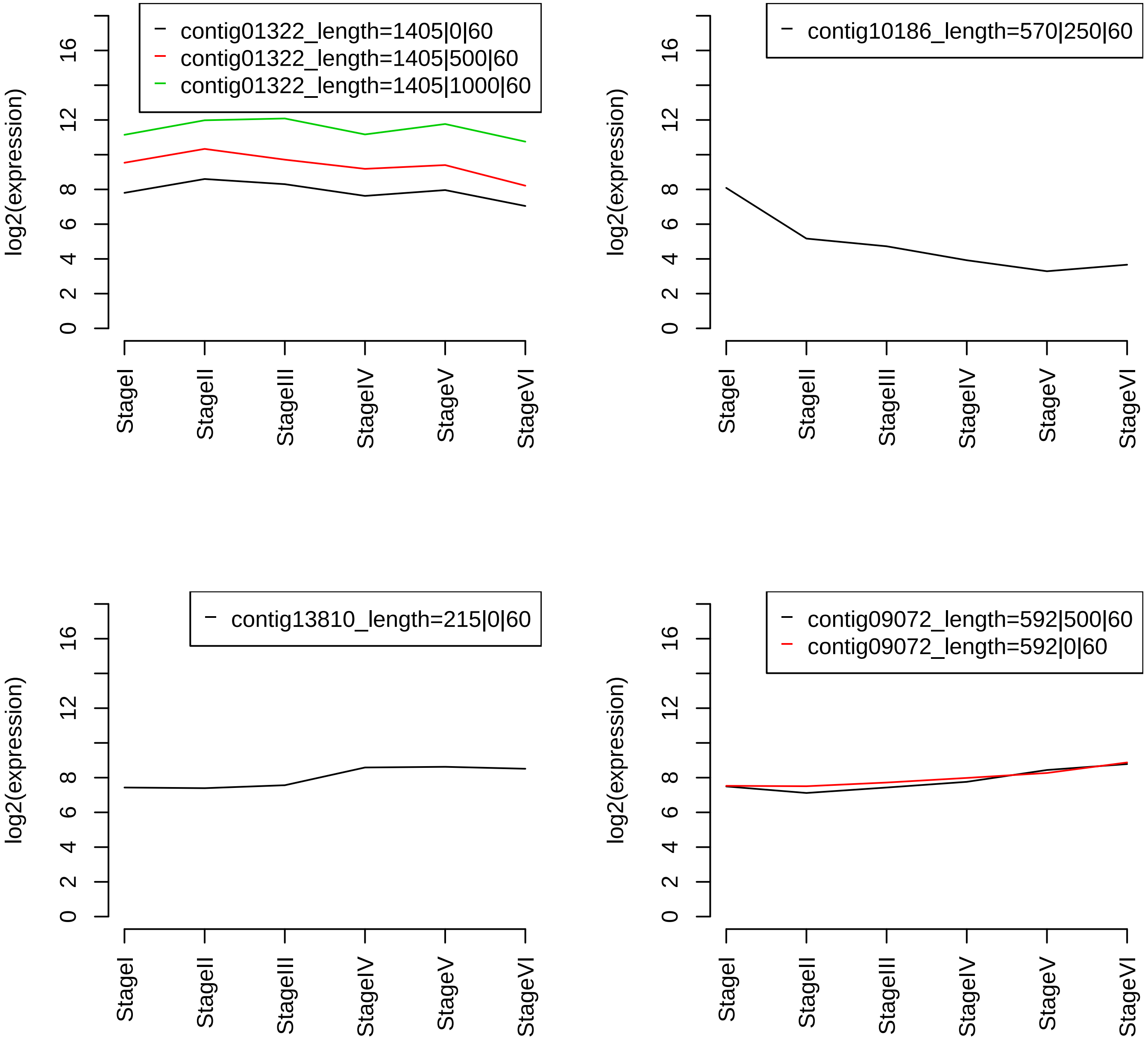
contig01322_length=1405|0|60
contig01322_length=1405|500|60
contig01322_length=1405|1000|60
contig10186_length=570|250|60
contig13810_length=215|0|60
contig09072_length=592|500|60
contig09072_length=592|0|60
log2(expression)
StageI
StageII
StageIII
StageIV
StageV
StageVI
0
2
4
6
8
12
16

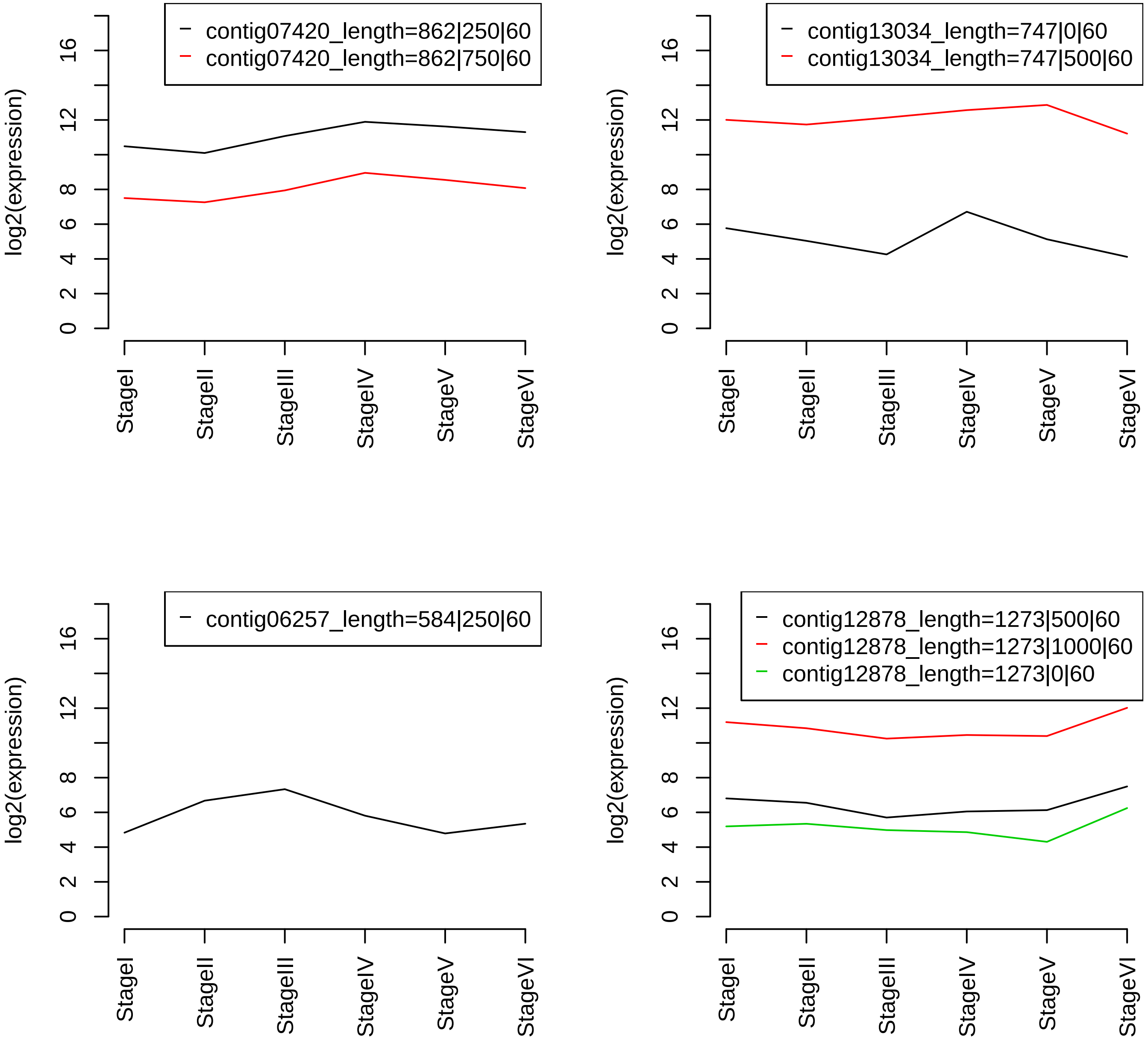
contig07420_length=862|250|60
contig07420_length=862|750|60
contig13034_length=747|0|60
contig13034_length=747|500|60
contig06257_length=584|250|60
contig12878_length=1273|500|60
contig12878_length=1273|1000|60
contig12878_length=1273|0|60
log2(expression)
0
2
4
6
8
12
16
StageI
StageII
StageIII
StageIV
StageV
StageVI

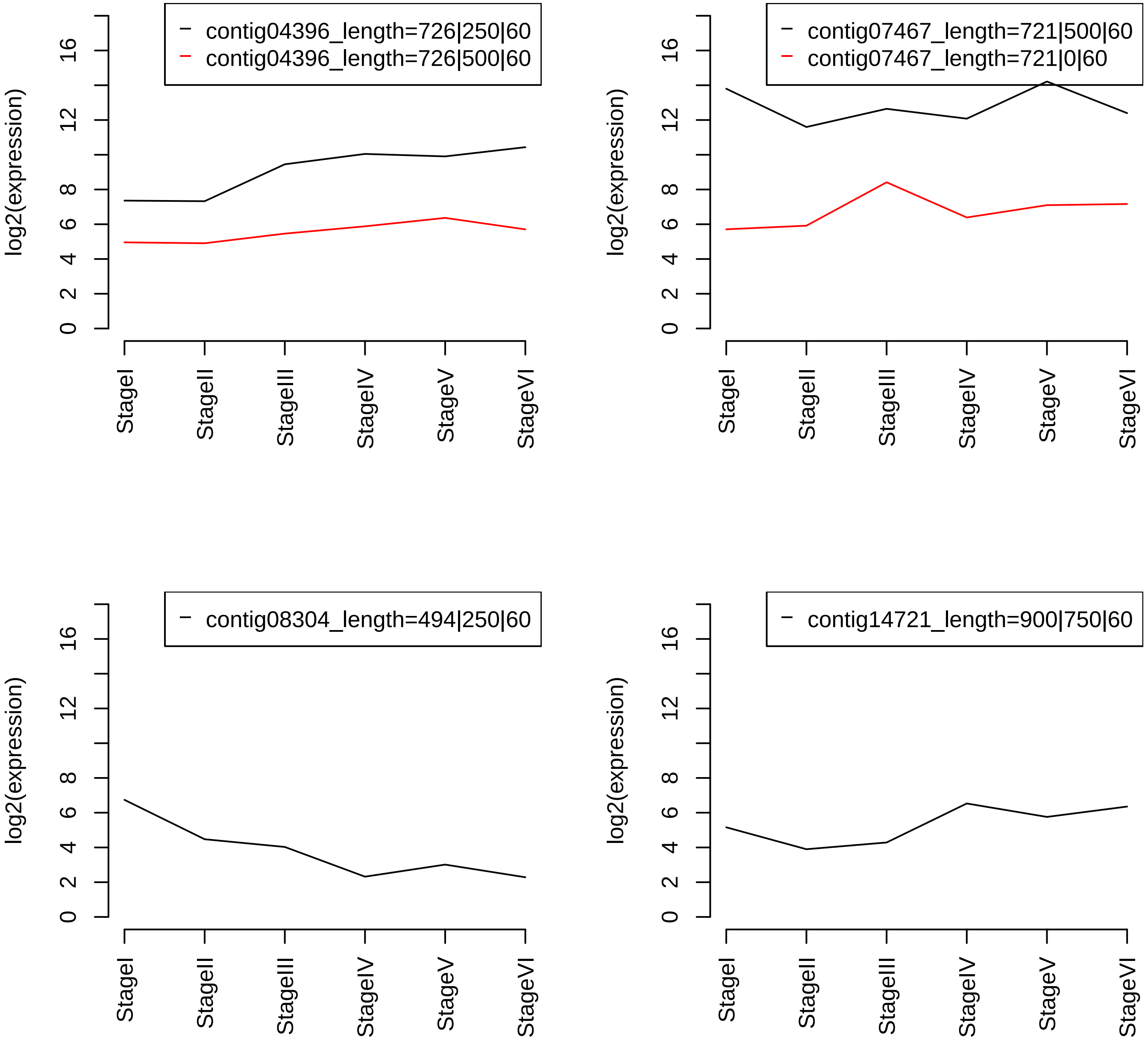

contig04396_length=726|250|60
contig04396_length=726|500|60
contig07467_length=721|500|60
contig07467_length=721|0|60
contig08304_length=494|250|60
contig14721_length=900|750|60
log2(expression)
0
2
4
6
8
12
16
StageI
StageII
StageIII
StageIV
StageV
StageVI

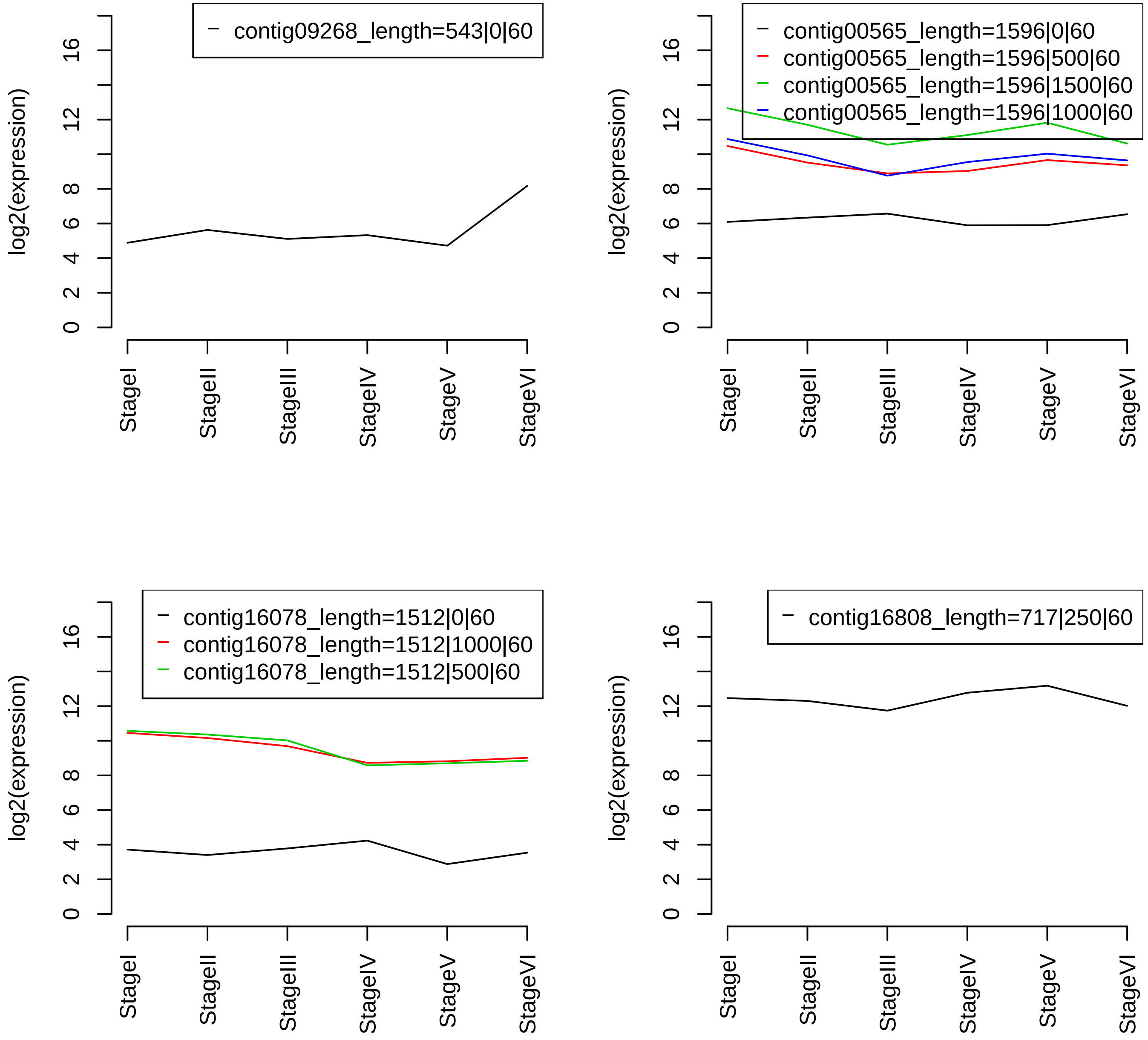

contig09268_length=543|0|60
contig00565_length=1596|0|60
contig00565_length=1596|500|60
contig00565_length=1596|1500|60
contig00565_length=1596|1000|60
contig16078_length=1512|0|60
contig16078_length=1512|1000|60
contig16078_length=1512|500|60
contig16808_length=717|250|60
log2(expression)
0
2
4
6
8
12
16
StageI
StageII
StageIII
StageIV
StageV
StageVI

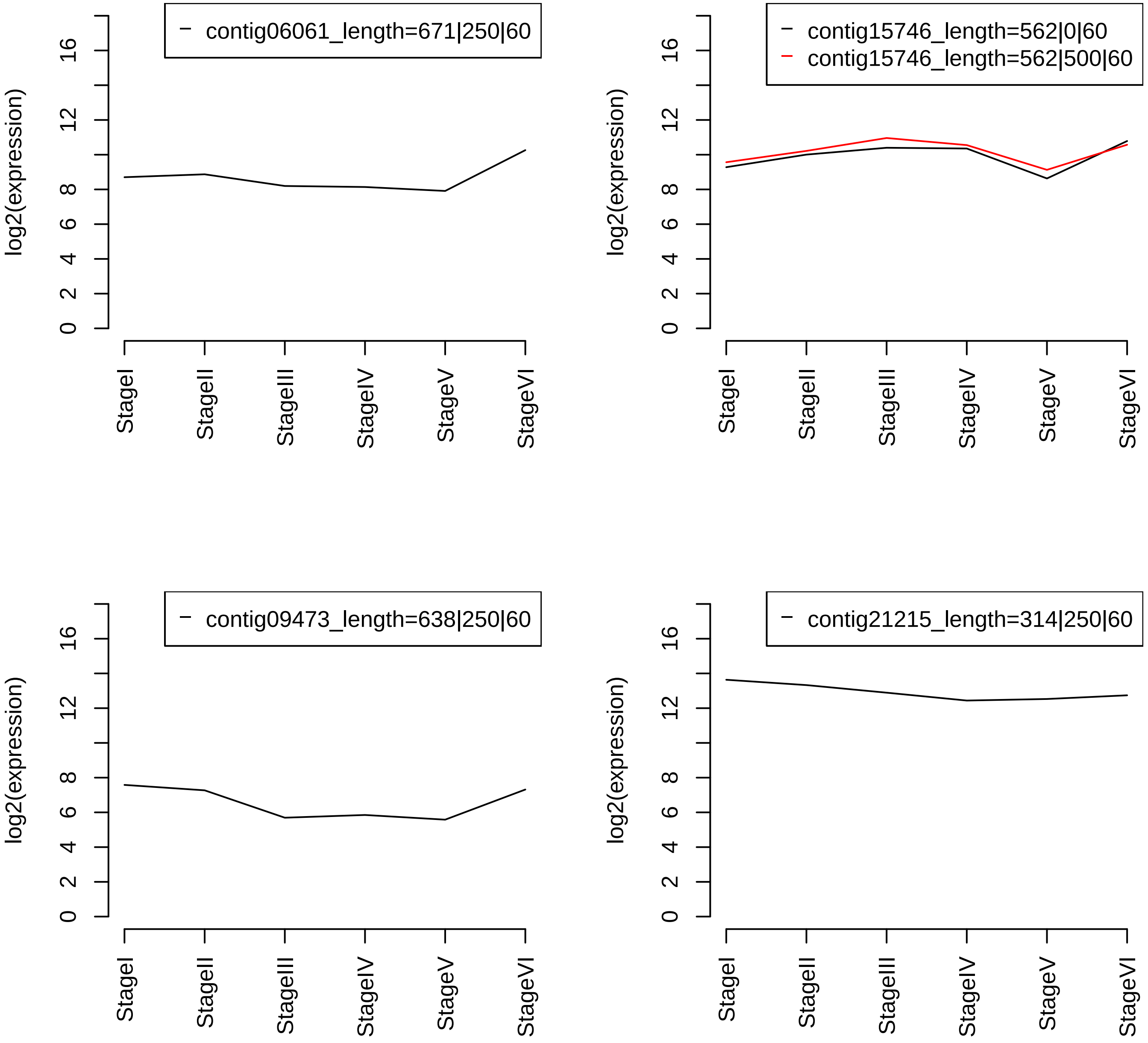
contig06061_length=671|250|60
contig15746_length=562|0|60
contig15746_length=562|500|60
contig09473_length=638|250|60
contig21215_length=314|250|60
log2(expression)
0
2
4
6
8
12
16
StageI
StageII
StageIII
StageIV
StageV
StageVI

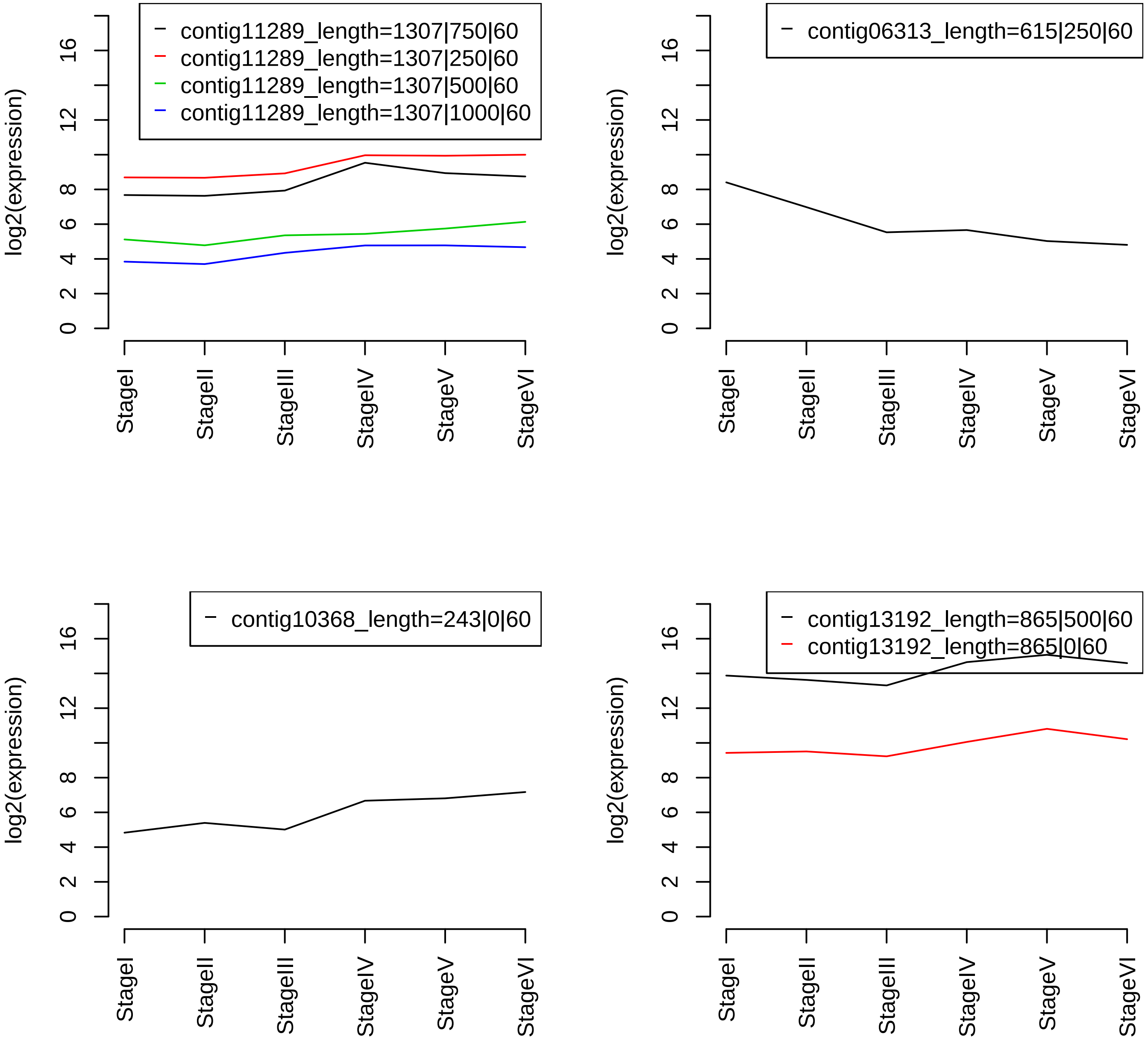

contig11289_length=1307|750|60
contig11289_length=1307|250|60
contig11289_length=1307|500|60
contig11289_length=1307|1000|60
contig06313_length=615|250|60
contig10368_length=243|0|60
contig13192_length=865|500|60
contig13192_length=865|0|60
log2(expression)
0
2
4
6
8
12
16
StageI
StageII
StageIII
StageIV
StageV
StageVI

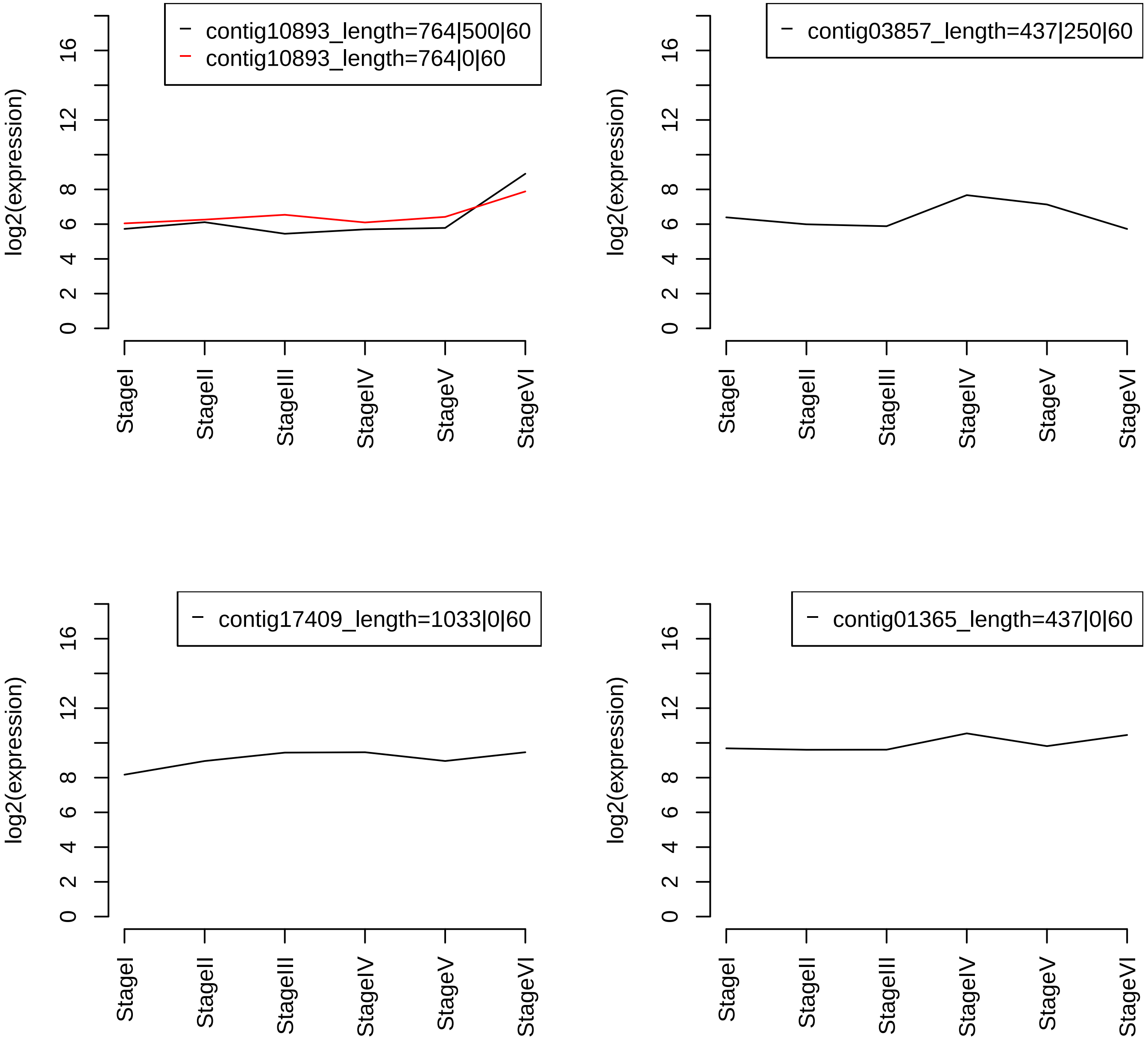

contig10893_length=764|500|60
contig10893_length=764|0|60
contig03857_length=437|250|60
contig17409_length=1033|0|60
contig01365_length=437|0|60
log2(expression)
0
2
4
6
8
12
16
StageI
StageII
StageIII
StageIV
StageV
StageVI

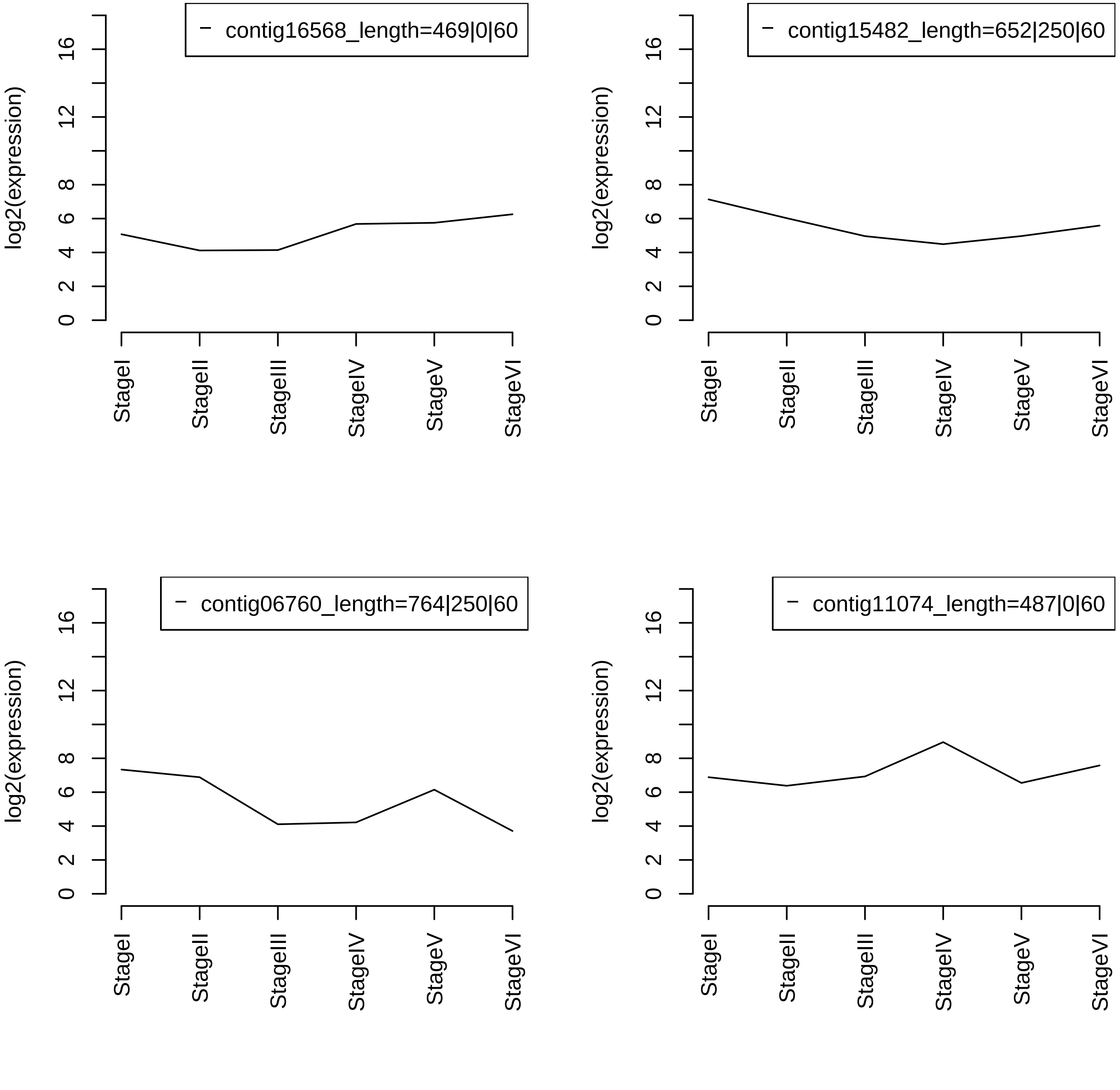
contig16568_length=469|0|60
contig15482_length=652|250|60
contig06760_length=764|250|60
contig11074_length=487|0|60
log2(expression)
0
2
4
6
8
12
16
StageI
StageII
StageIII
StageIV
StageV
StageVI

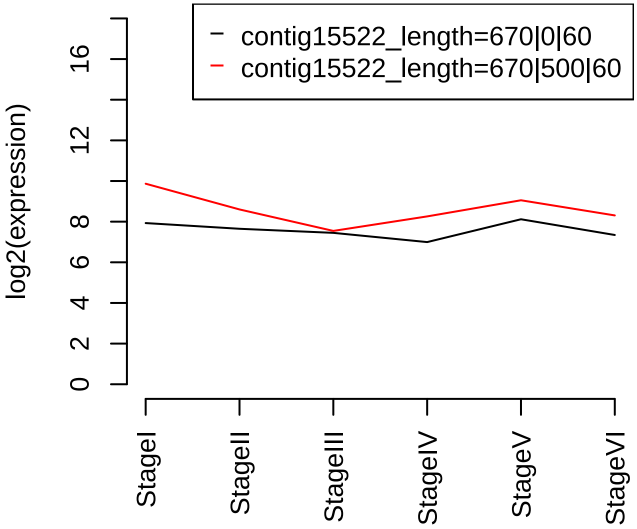

contig15522_length=670|0|60
contig15522_length=670|500|60
log2(expression)
0
2
4
6
8
12
16
StageI
StageII
StageIII
StageIV
StageV
StageVI

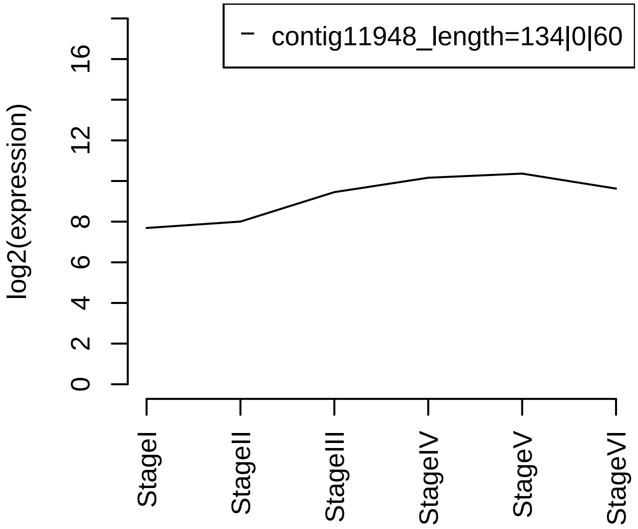

contig11948_length=134|0|60
log2(expression)
0
2
4
6
8
12
16
StageI
StageII
StageIII
StageIV
StageV
StageVI

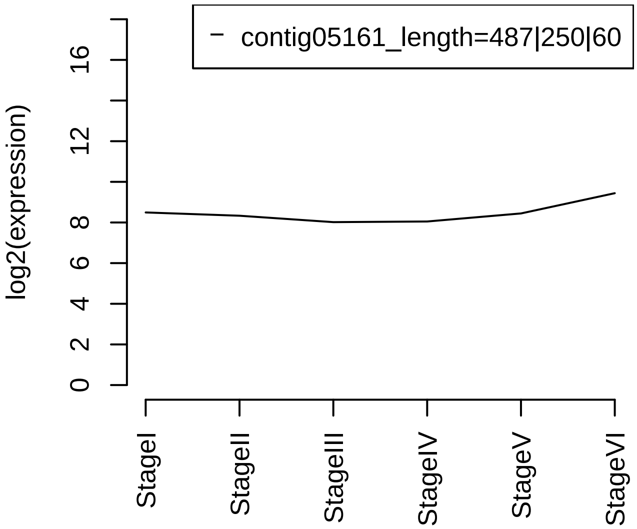

contig05161_length=487|250|60
log2(expression)
0
2
4
6
8
12
16
StageI
StageII
StageIII
StageIV
StageV
StageVI

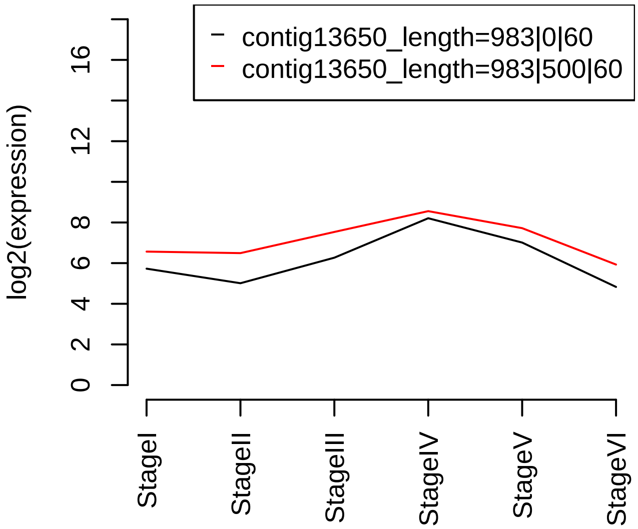

contig13650_length=983|0|60
contig13650_length=983|500|60
log2(expression)
0
2
4
6
8
12
16
StageI
StageII
StageIII
StageIV
StageV
StageVI

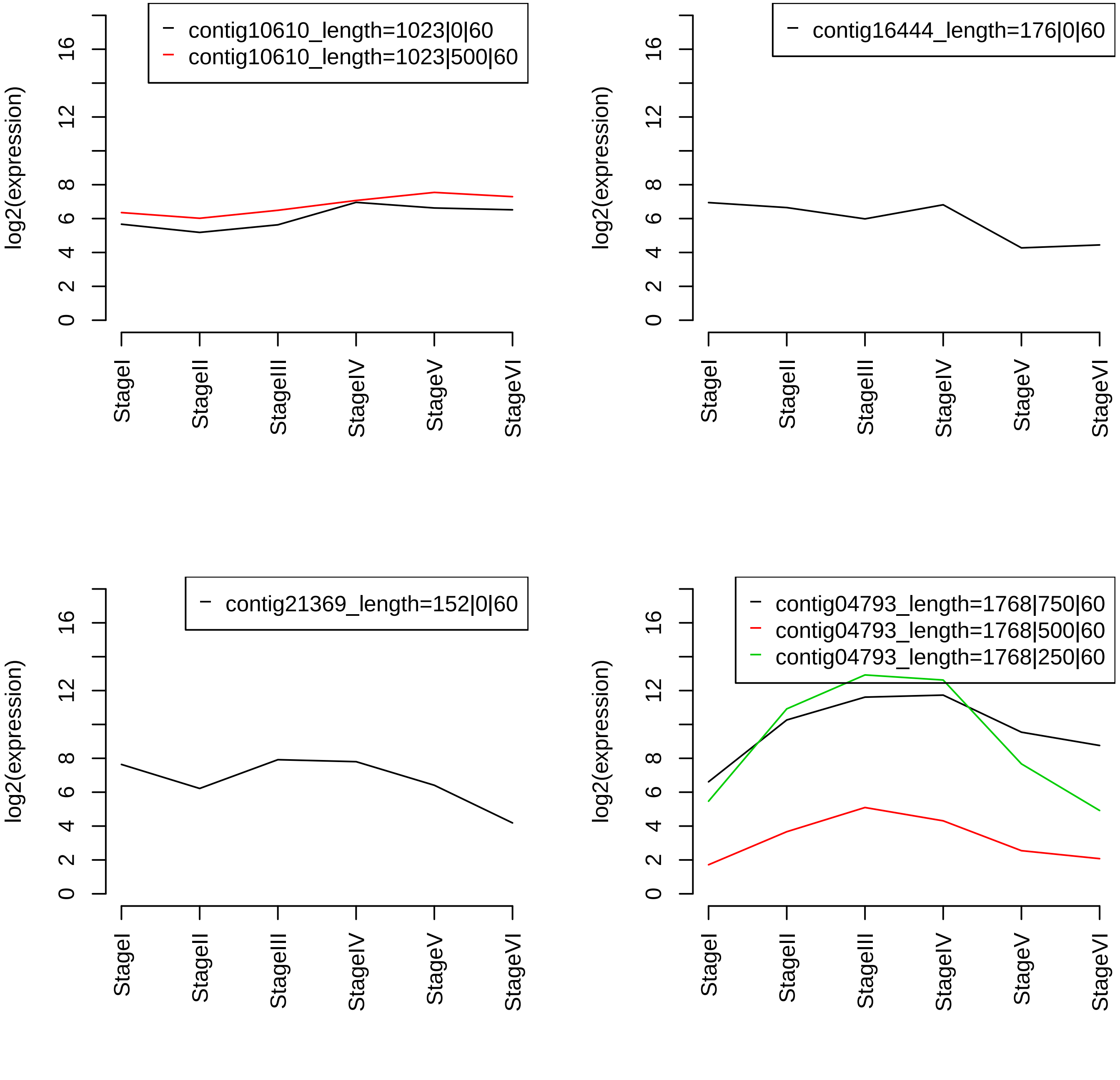

contig10610_length=1023|0|60
contig10610_length=1023|500|60
log2(expression)
0
2
4
6
8
12
16
StageI
StageII
StageIII
StageIV
StageV
StageVI
contig16444_length=176|0|60
contig21369_length=152|0|60
contig04793_length=1768|750|60
contig04793_length=1768|500|60
contig04793_length=1768|250|60

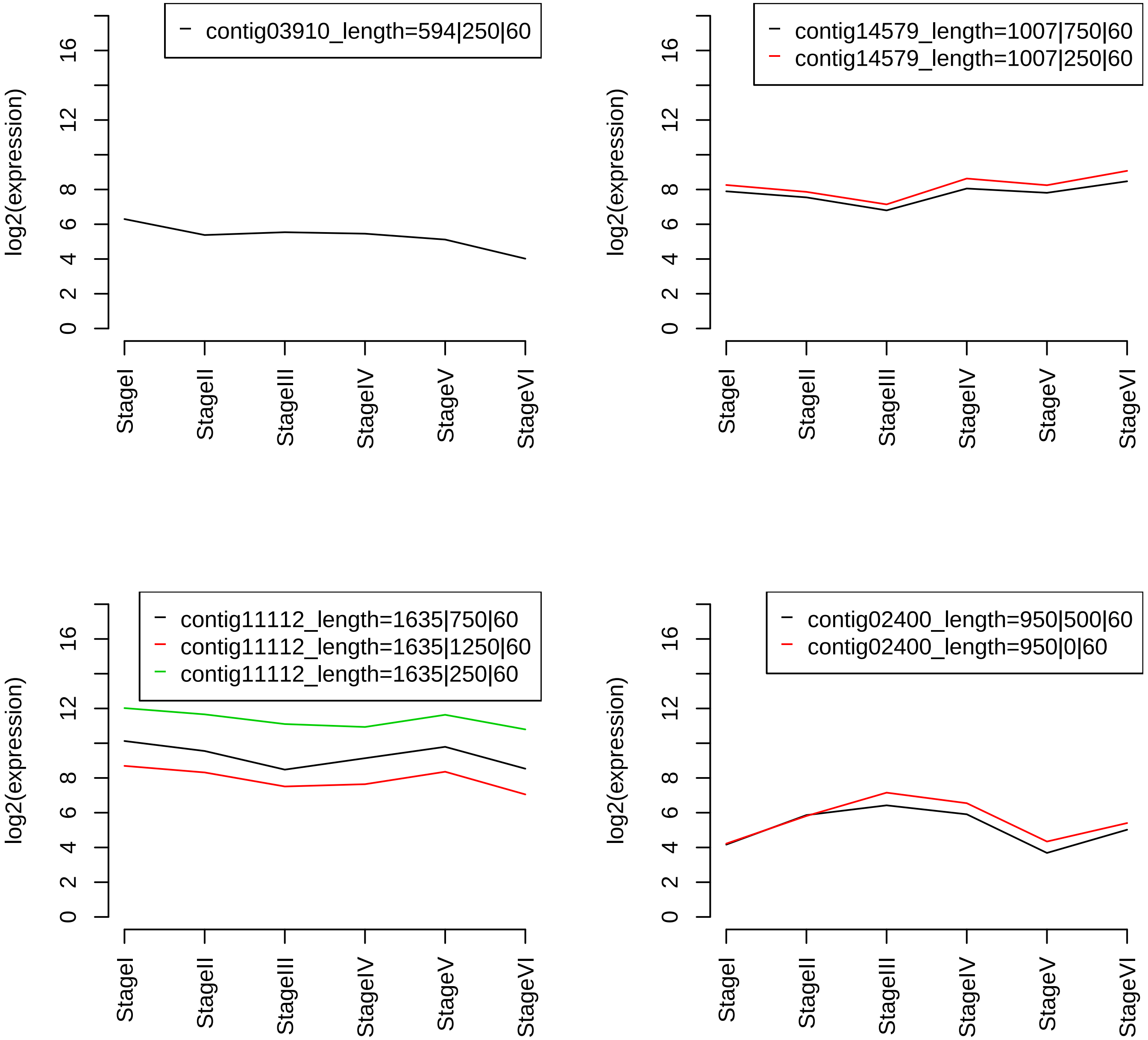

contig03910_length=594|250|60
contig14579_length=1007|750|60
contig14579_length=1007|250|60
contig11112_length=1635|750|60
contig11112_length=1635|1250|60
contig11112_length=1635|250|60
contig02400_length=950|500|60
contig02400_length=950|0|60
log2(expression)
0
2
4
6
8
12
16
StageI
StageII
StageIII
StageIV
StageV
StageVI

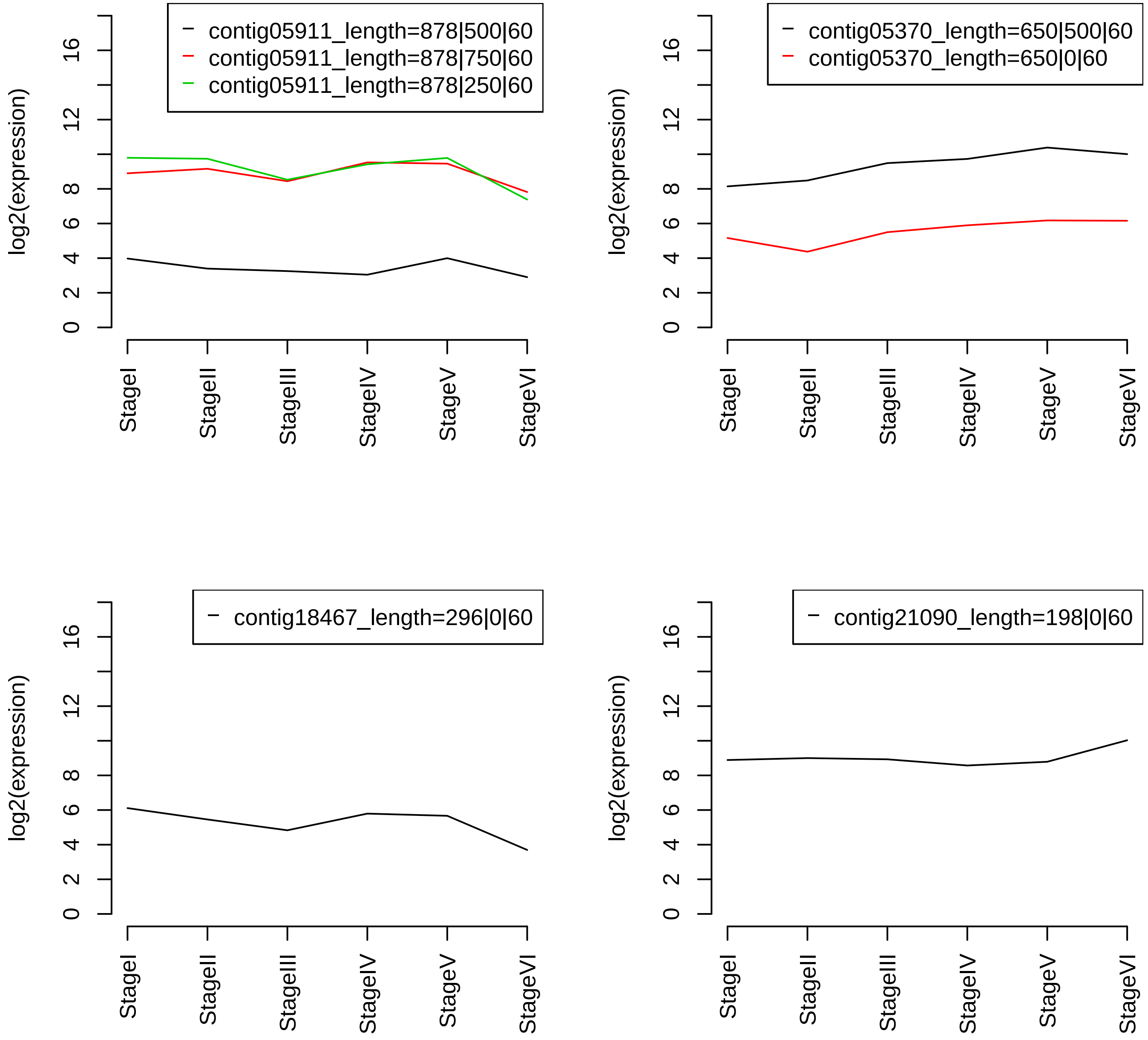

contig05911_length=878|500|60
contig05911_length=878|750|60
contig05911_length=878|250|60
contig05370_length=650|500|60
contig05370_length=650|0|60
contig18467_length=296|0|60
contig21090_length=198|0|60
log2(expression)
0
2
4
6
8
12
16
StageI
StageII
StageIII
StageIV
StageV
StageVI

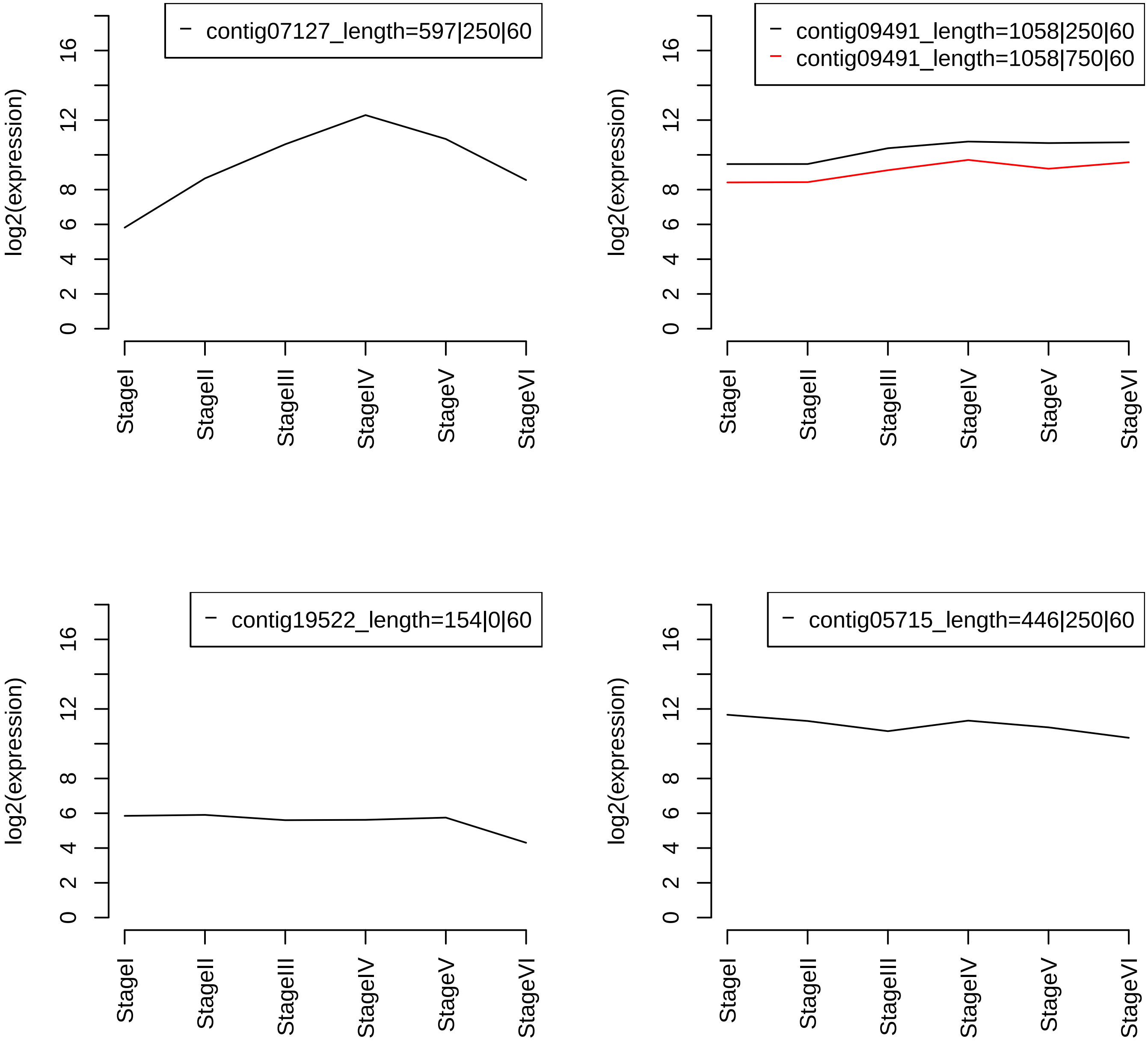
contig07127_length=597|250|60
contig09491_length=1058|250|60
contig09491_length=1058|750|60
contig19522_length=154|0|60
contig05715_length=446|250|60
log2(expression)
0
2
4
6
8
12
16
StageI
StageII
StageIII
StageIV
StageV
StageVI

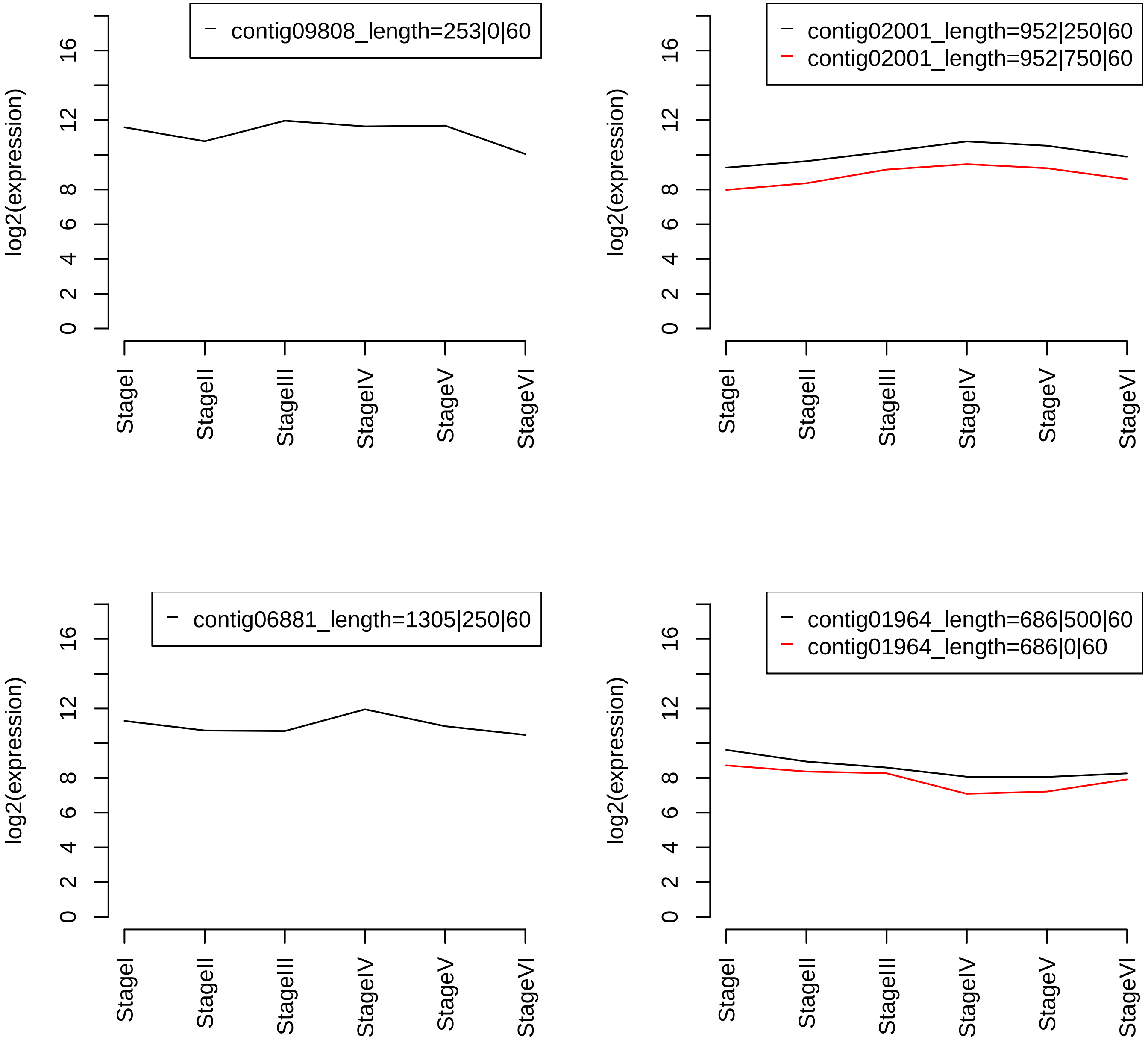
contig09808_length=253|0|60
contig02001_length=952|250|60
contig02001_length=952|750|60
contig06881_length=1305|250|60
contig01964_length=686|500|60
contig01964_length=686|0|60
log2(expression)
0
2
4
6
8
12
16
StageI
StageII
StageIII
StageIV
StageV
StageVI

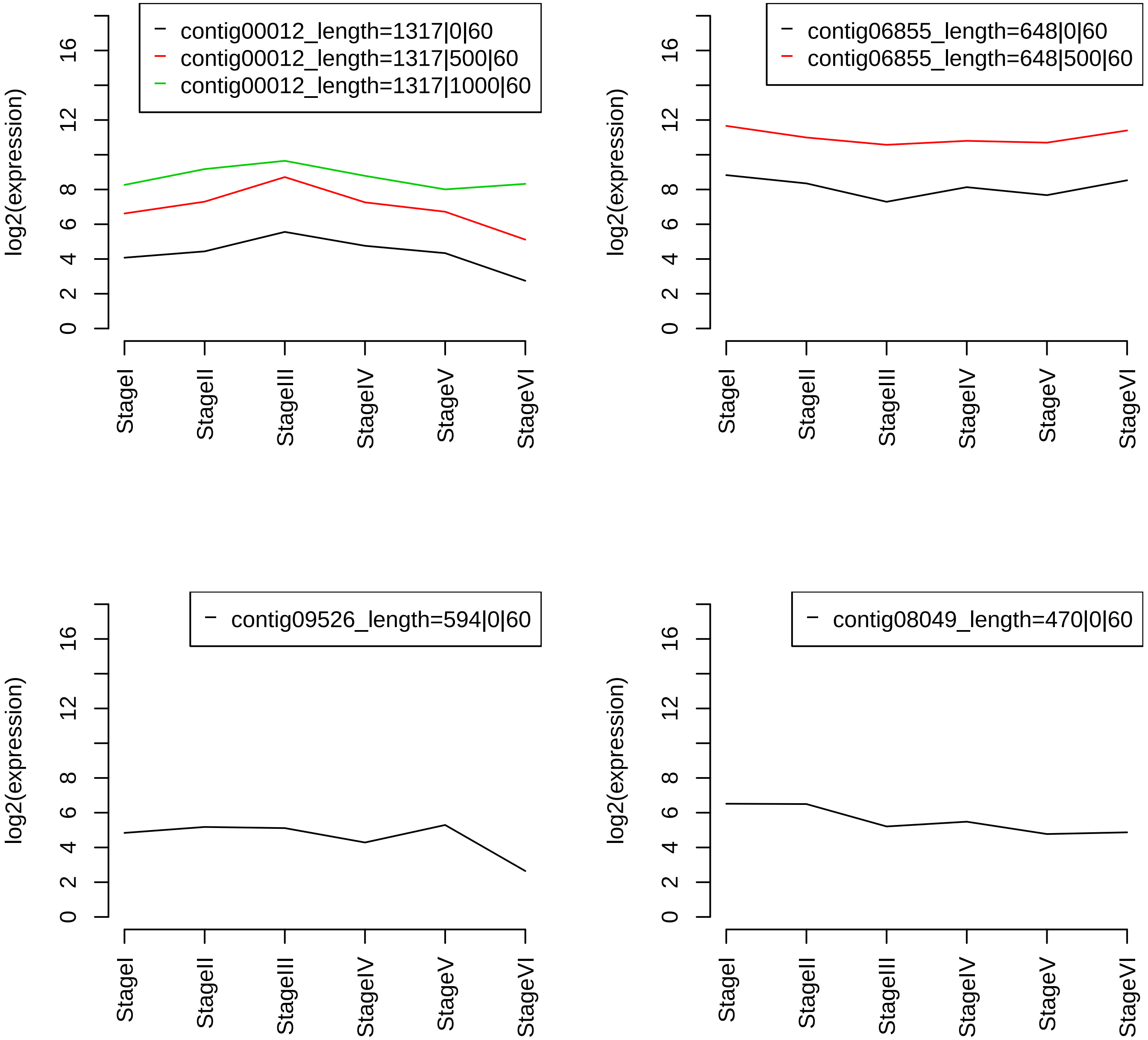
contig00012_length=1317|0|60
contig00012_length=1317|500|60
contig00012_length=1317|1000|60
contig06855_length=648|0|60
contig06855_length=648|500|60
contig09526_length=594|0|60
contig08049_length=470|0|60
log2(expression)
0
2
4
6
8
12
16
StageI
StageII
StageIII
StageIV
StageV
StageVI

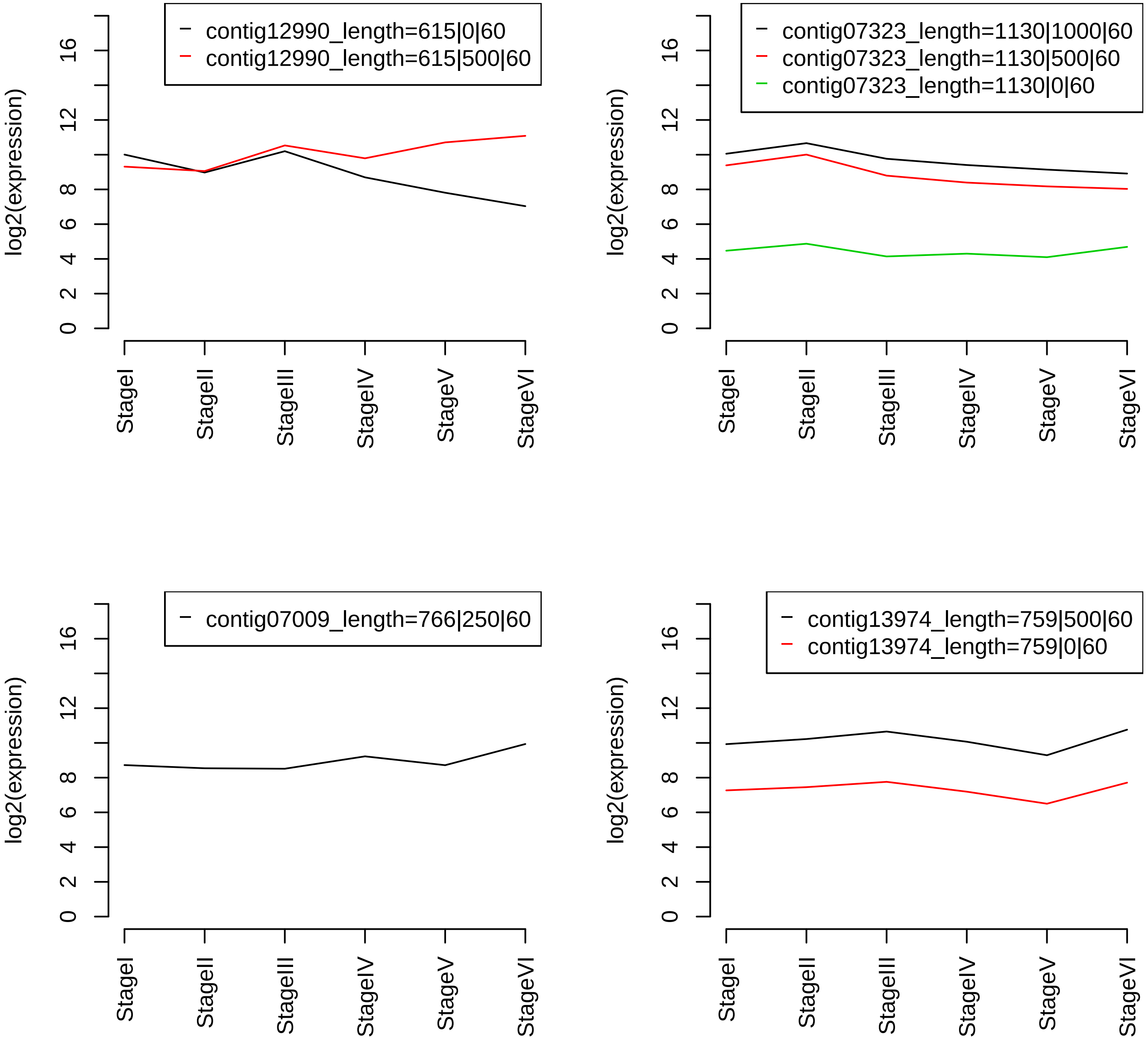
contig12990_length=615|0|60
contig12990_length=615|500|60
contig07323_length=1130|1000|60
contig07323_length=1130|500|60
contig07323_length=1130|0|60
contig07009_length=766|250|60
contig13974_length=759|500|60
contig13974_length=759|0|60
log2(expression)
0
2
4
6
8
12
16
StageI
StageII
StageIII
StageIV
StageV
StageVI

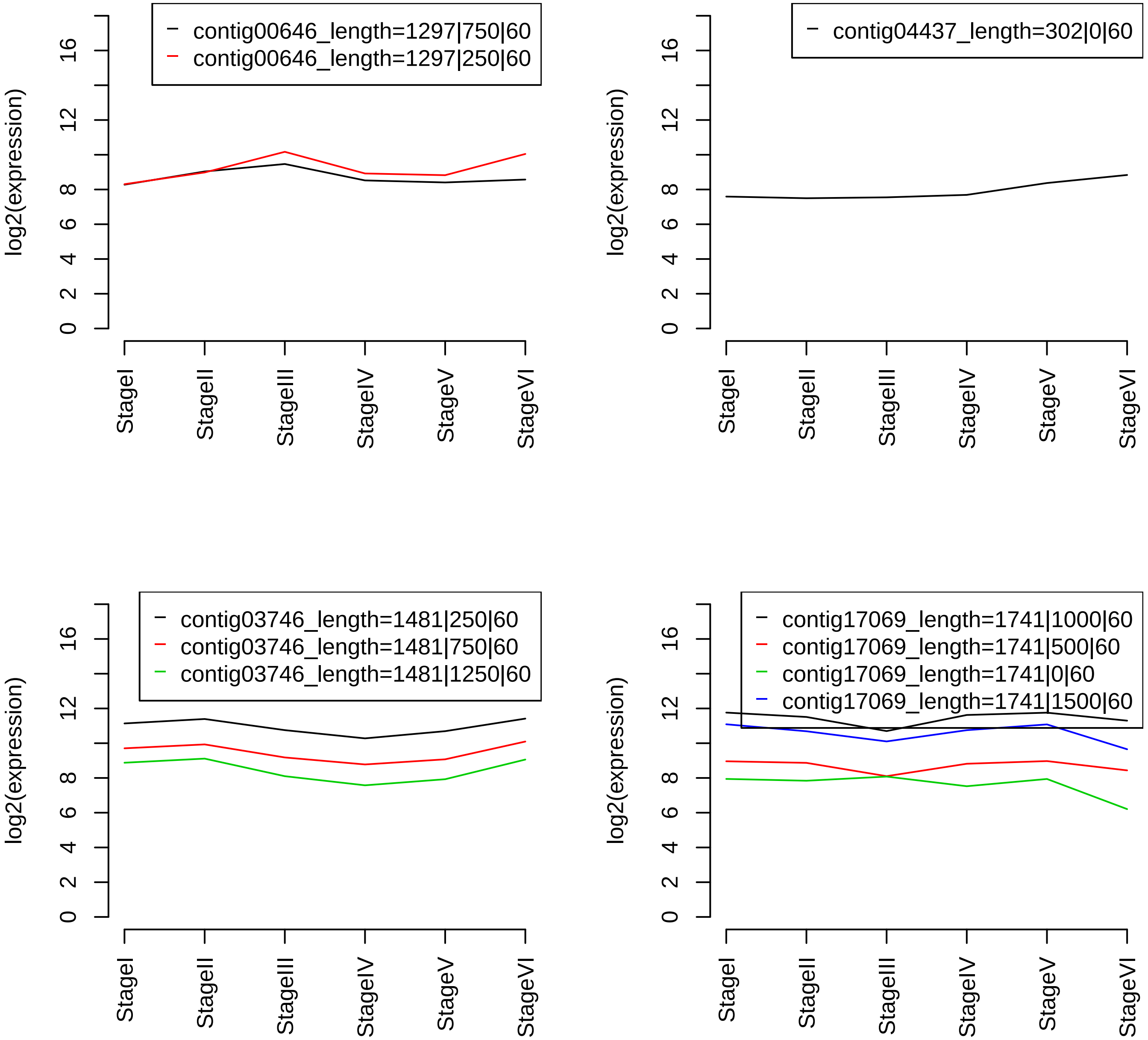
contig00646_length=1297|750|60
contig00646_length=1297|250|60
contig04437_length=302|0|60
contig03746_length=1481|250|60
contig03746_length=1481|750|60
contig03746_length=1481|1250|60
contig17069_length=1741|1000|60
contig17069_length=1741|500|60
contig17069_length=1741|0|60
contig17069_length=1741|1500|60
log2(expression)
0
2
4
6
8
12
16
StageI
StageII
StageIII
StageIV
StageV
StageVI

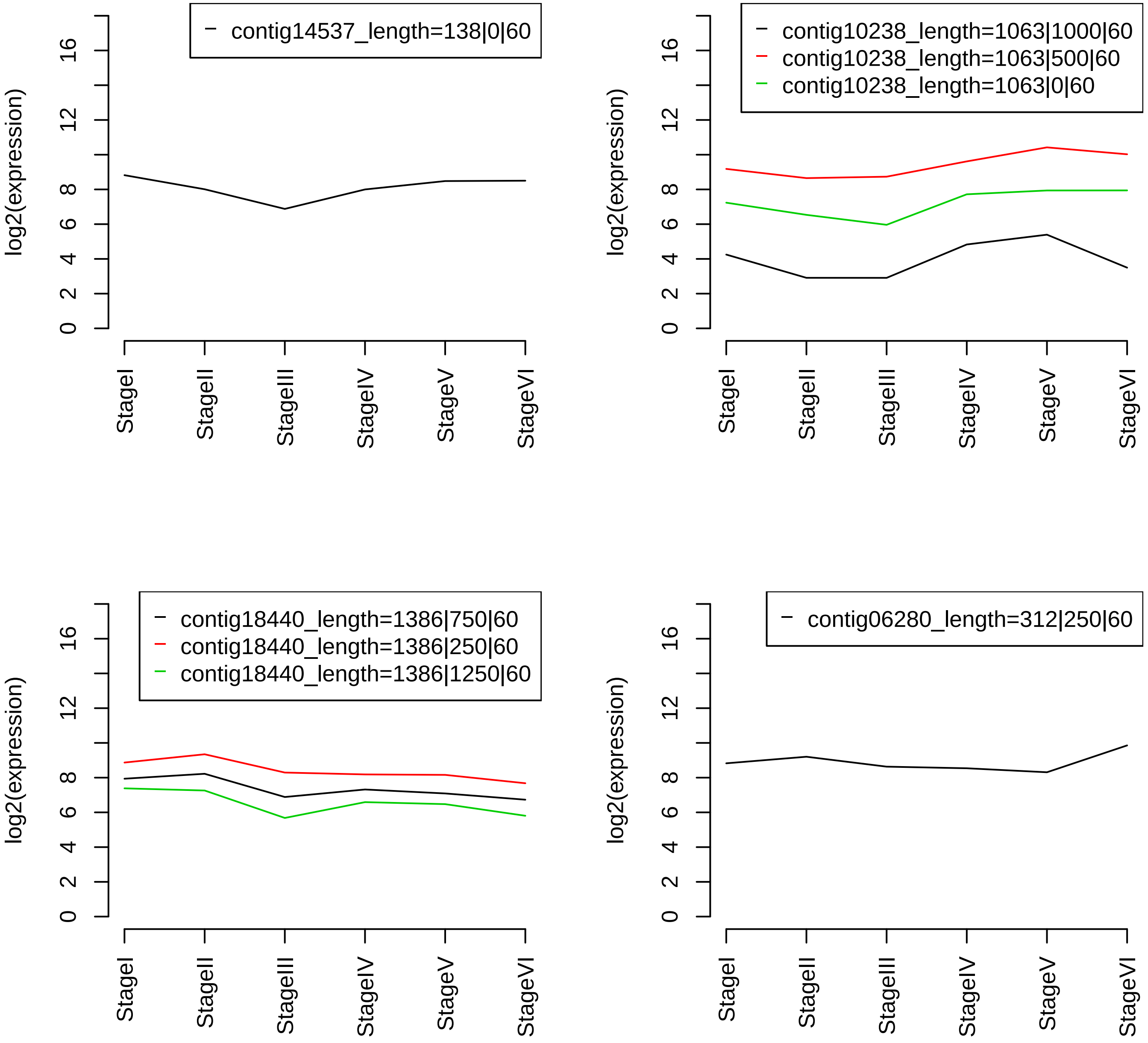
contig14537_length=138|0|60
contig10238_length=1063|1000|60
contig10238_length=1063|500|60
contig10238_length=1063|0|60
contig18440_length=1386|750|60
contig18440_length=1386|250|60
contig18440_length=1386|1250|60
contig06280_length=312|250|60
log2(expression)
0
2
4
6
8
12
16
StageI
StageII
StageIII
StageIV
StageV
StageVI

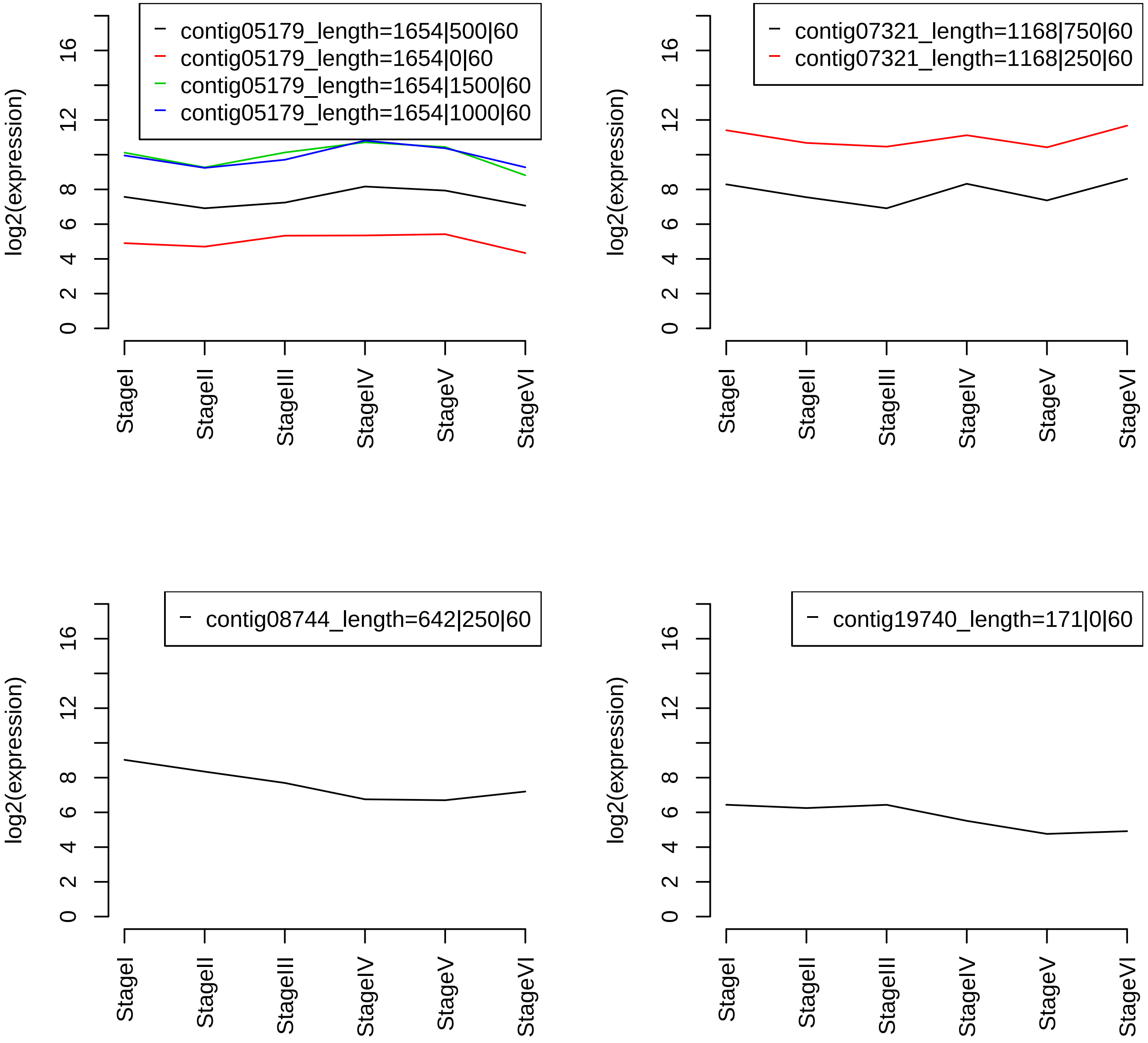

contig05179_length=1654|500|60
contig05179_length=1654|0|60
contig05179_length=1654|1500|60
contig05179_length=1654|1000|60
contig07321_length=1168|750|60
contig07321_length=1168|250|60
contig08744_length=642|250|60
contig19740_length=171|0|60
log2(expression)
0 2 4 6 8 12 16
StageI
StageII
StageIII
StageIV
StageV
StageVI

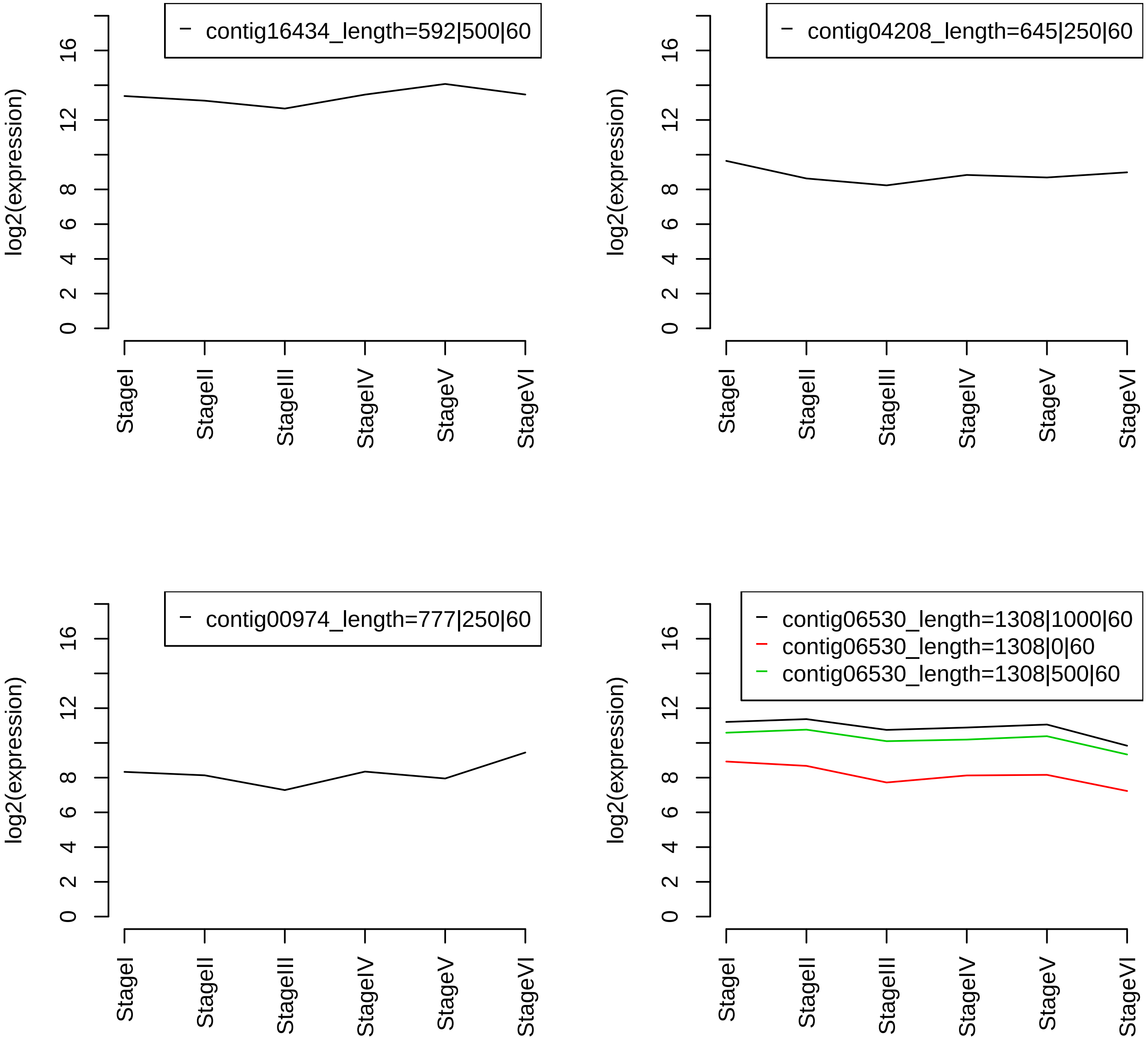

contig16434_length=592|500|60
log2(expression)
0
2
4
6
8
12
16
StageI
StageII
StageIII
StageIV
StageV
StageVI
contig04208_length=645|250|60
contig00974_length=777|250|60
contig06530_length=1308|1000|60
contig06530_length=1308|0|60
contig06530_length=1308|500|60

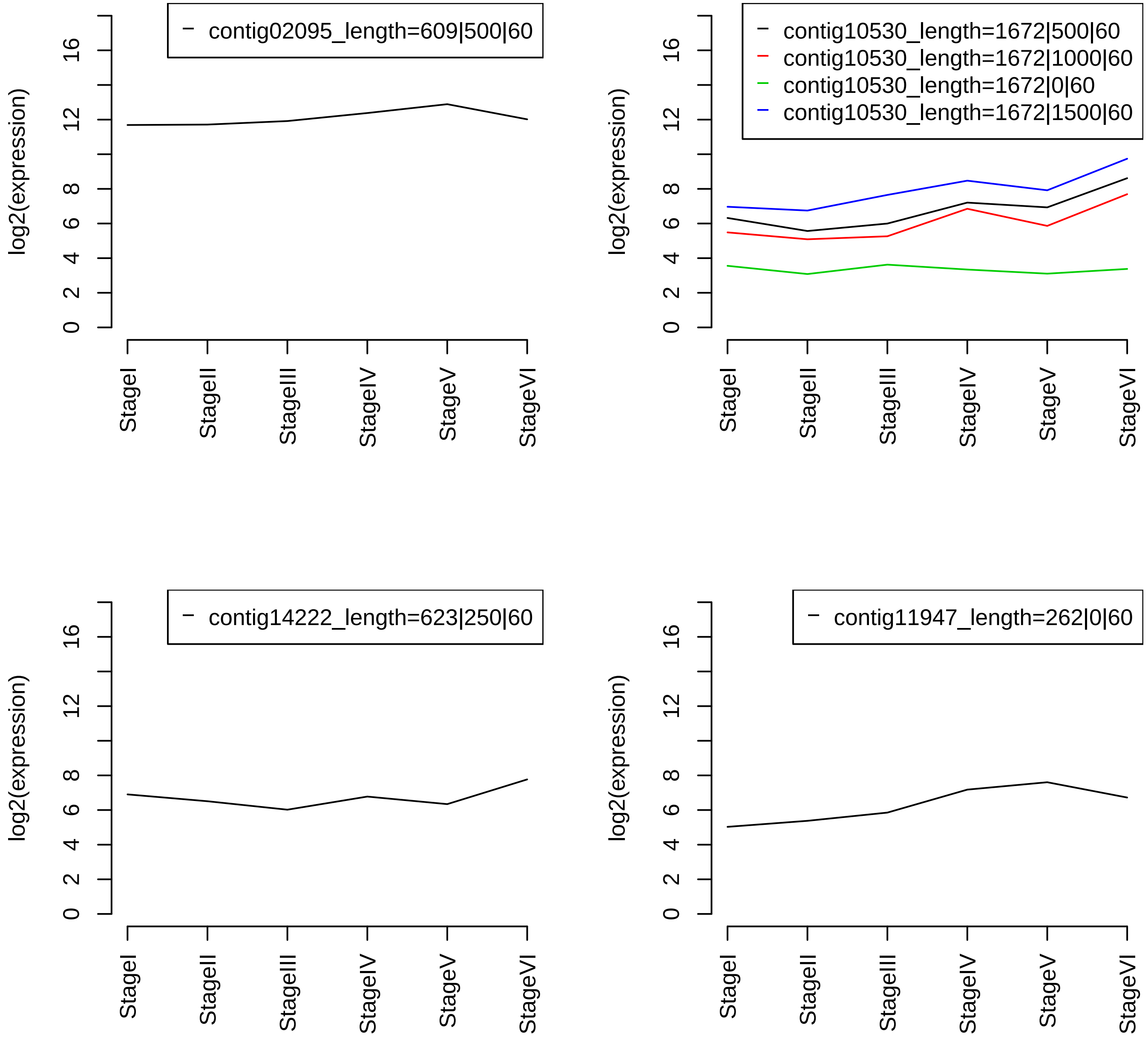

contig02095_length=609|500|60
contig10530_length=1672|500|60
contig10530_length=1672|1000|60
contig10530_length=1672|0|60
contig10530_length=1672|1500|60
contig14222_length=623|250|60
contig11947_length=262|0|60
log2(expression)
0
2
4
6
8
12
16
StageI
StageII
StageIII
StageIV
StageV
StageVI

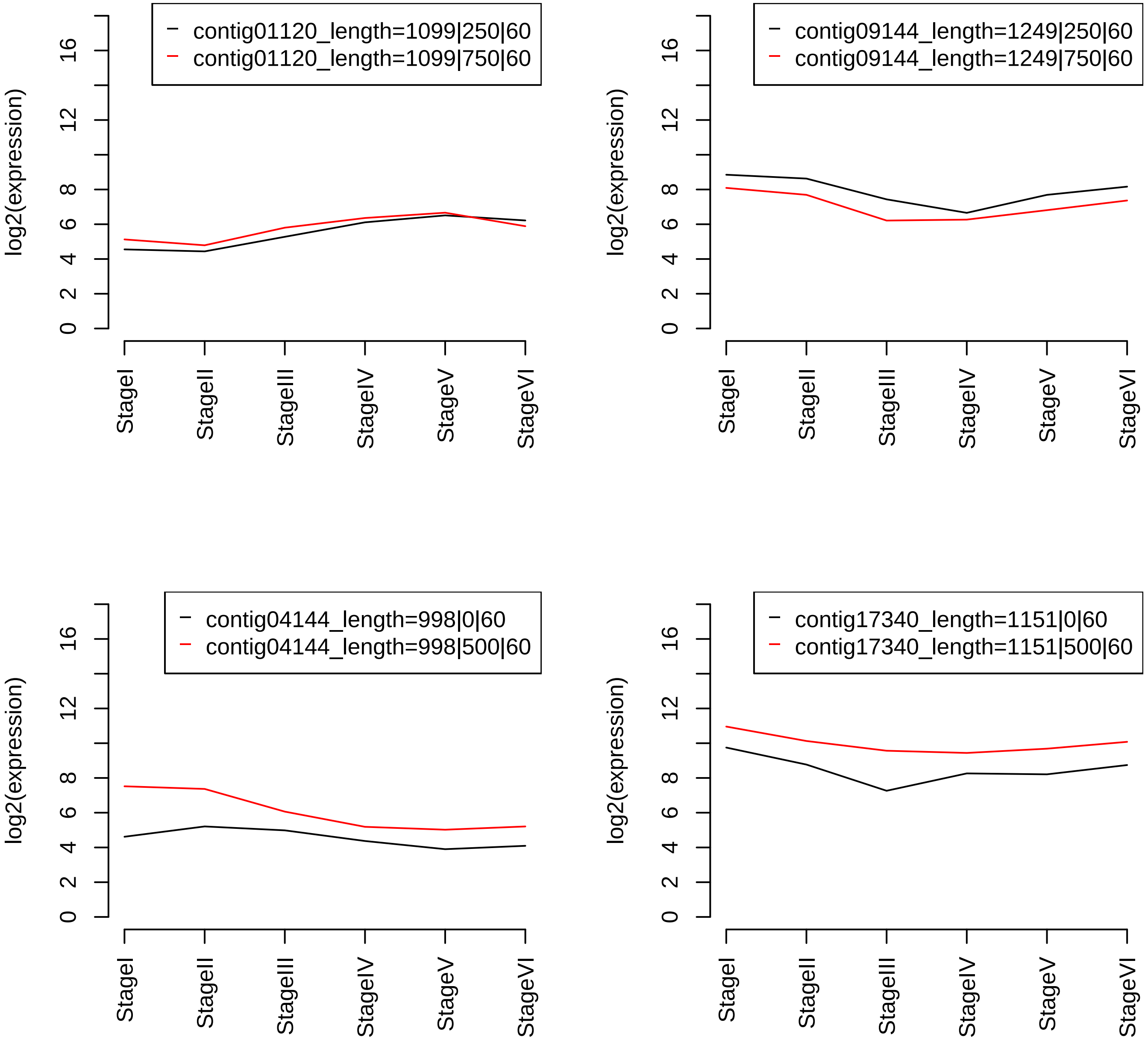

contig01120_length=1099|250|60
contig01120_length=1099|750|60
contig09144_length=1249|250|60
contig09144_length=1249|750|60
contig04144_length=998|0|60
contig04144_length=998|500|60
contig17340_length=1151|0|60
contig17340_length=1151|500|60
log2(expression)
0
2
4
6
8
12
16
StageI
StageII
StageIII
StageIV
StageV
StageVI

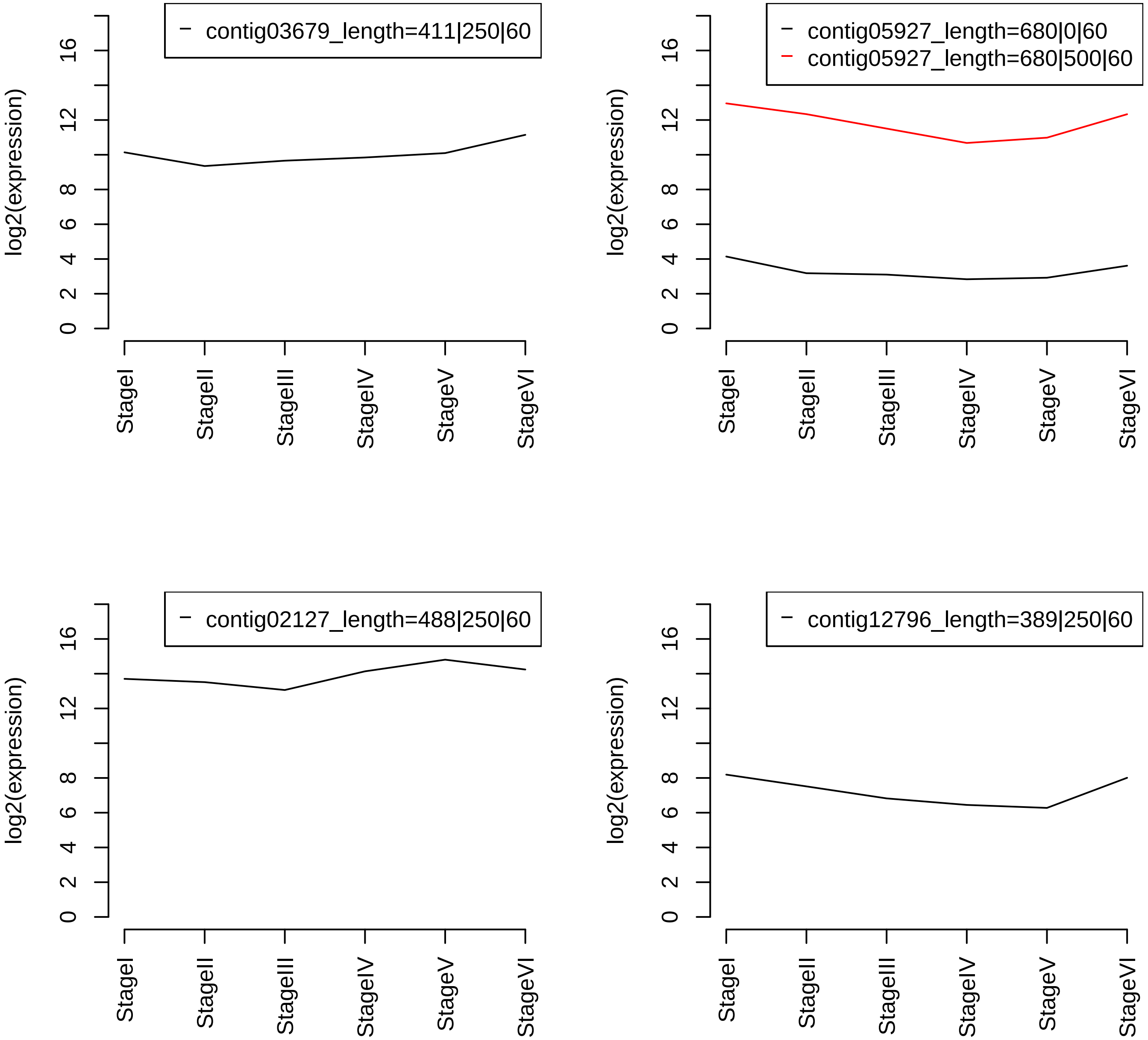
contig03679_length=411|250|60
contig05927_length=680|0|60
contig05927_length=680|500|60
contig02127_length=488|250|60
contig12796_length=389|250|60
log2(expression)
0
2
4
6
8
12
16
StageI
StageII
StageIII
StageIV
StageV
StageVI

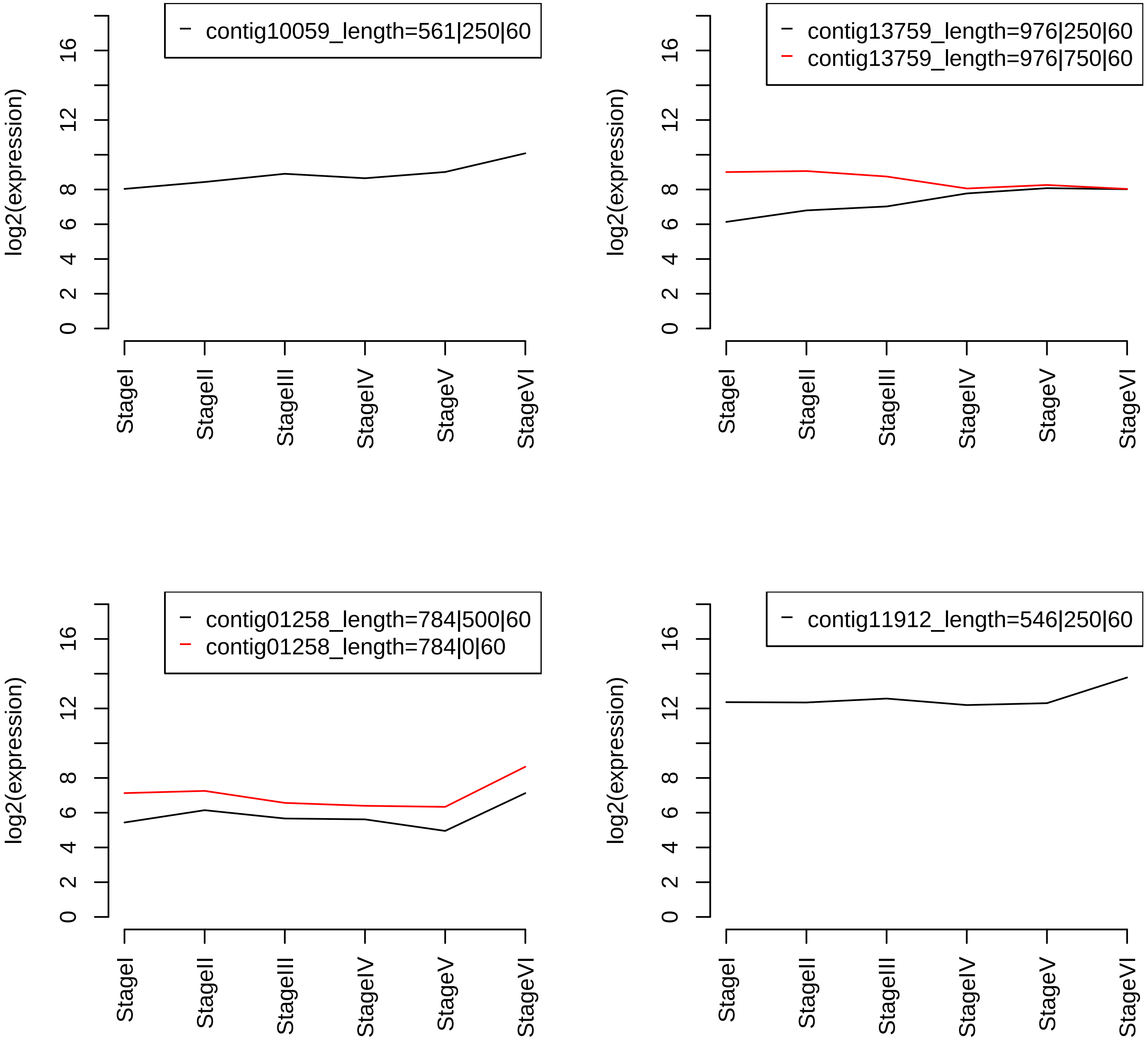

contig10059_length=561|250|60
contig13759_length=976|250|60
contig13759_length=976|750|60
contig01258_length=784|500|60
contig01258_length=784|0|60
contig11912_length=546|250|60
log2(expression)
0
2
4
6
8
12
16
StageI
StageII
StageIII
StageIV
StageV
StageVI

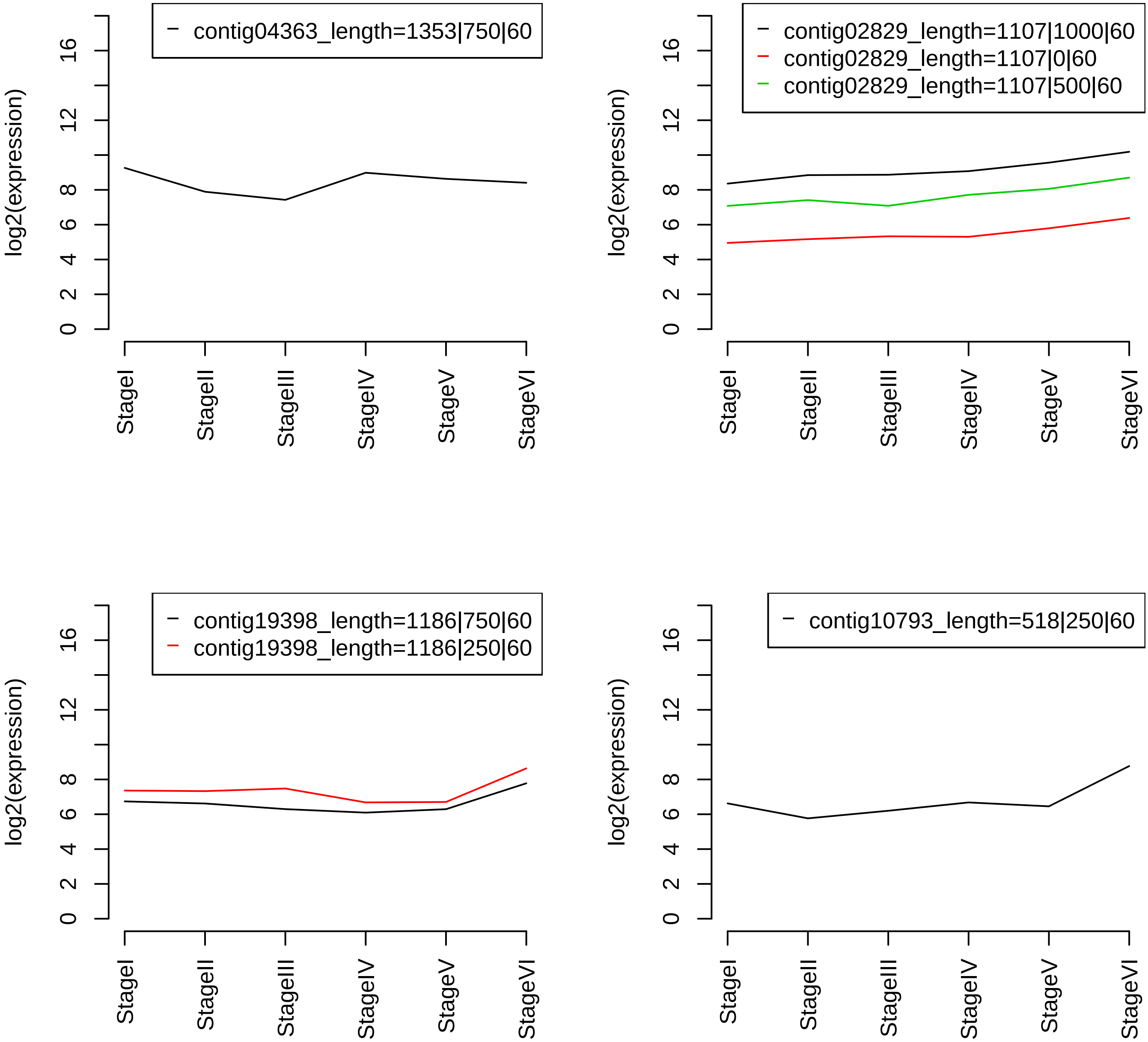

contig04363_length=1353|750|60
contig02829_length=1107|1000|60
contig02829_length=1107|0|60
contig02829_length=1107|500|60
contig19398_length=1186|750|60
contig19398_length=1186|250|60
contig10793_length=518|250|60
log2(expression)
0
2
4
6
8
12
16
StageI
StageII
StageIII
StageIV
StageV
StageVI

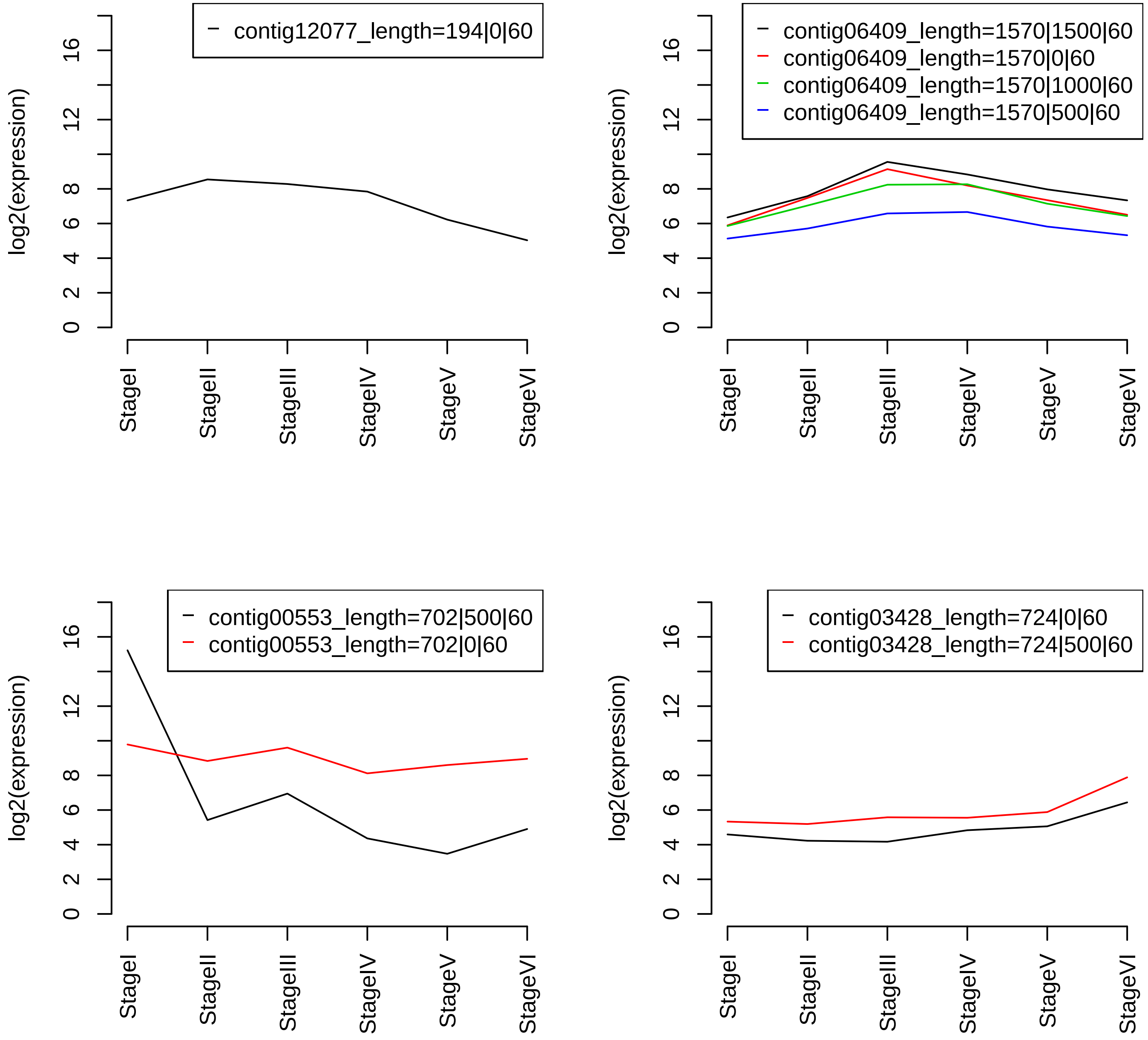

contig12077_length=194|0|60
contig06409_length=1570|1500|60
contig06409_length=1570|0|60
contig06409_length=1570|1000|60
contig06409_length=1570|500|60
contig00553_length=702|500|60
contig00553_length=702|0|60
contig03428_length=724|0|60
contig03428_length=724|500|60
log2(expression)
0
2
4
6
8
12
16
StageI
StageII
StageIII
StageIV
StageV
StageVI

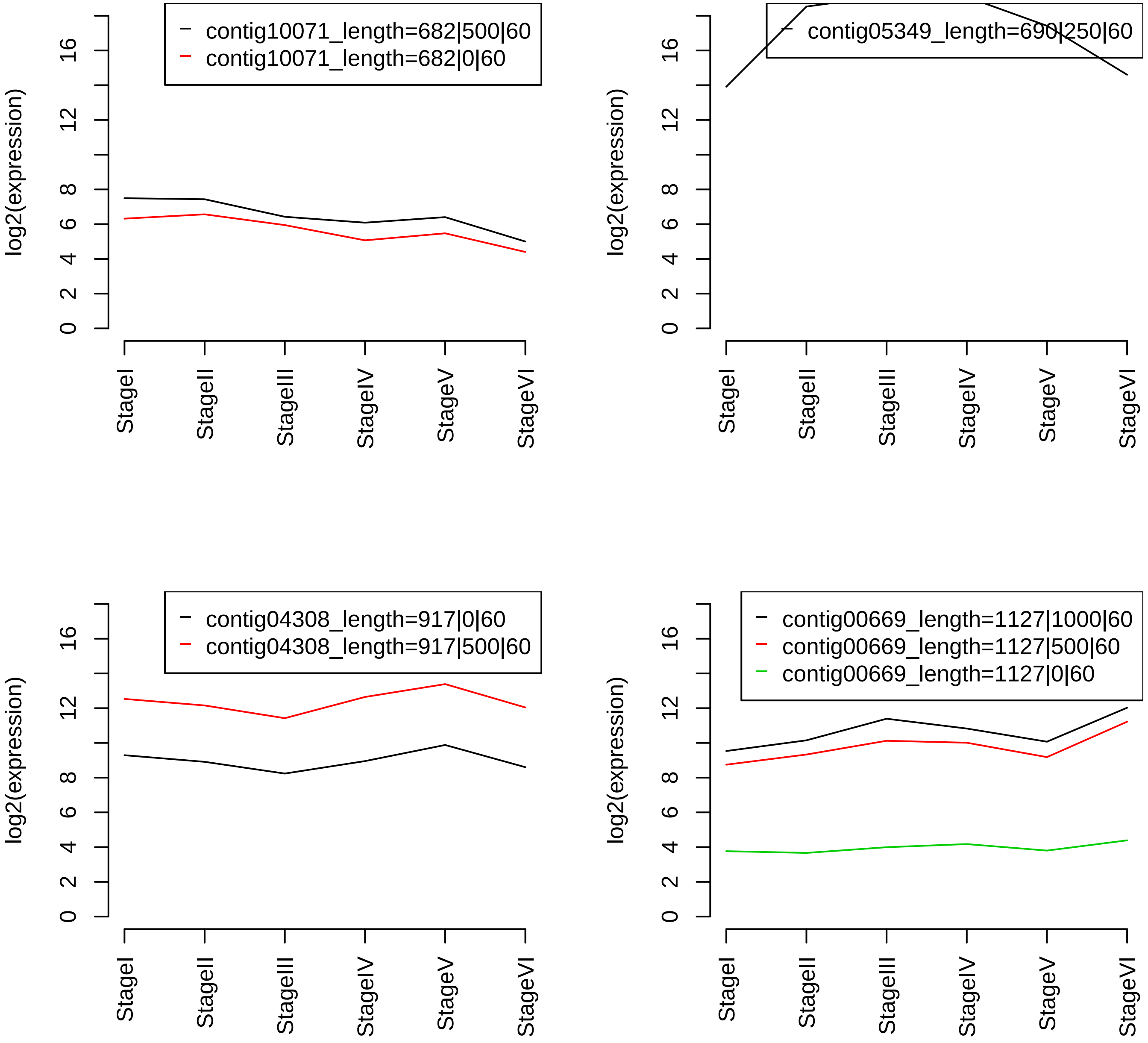

contig10071_length=682|500|60
contig10071_length=682|0|60
contig05349_length=690|250|60
contig04308_length=917|0|60
contig04308_length=917|500|60
contig00669_length=1127|1000|60
contig00669_length=1127|500|60
contig00669_length=1127|0|60
log2(expression)
0
2
4
6
8
12
16
StageI
StageII
StageIII
StageIV
StageV
StageVI

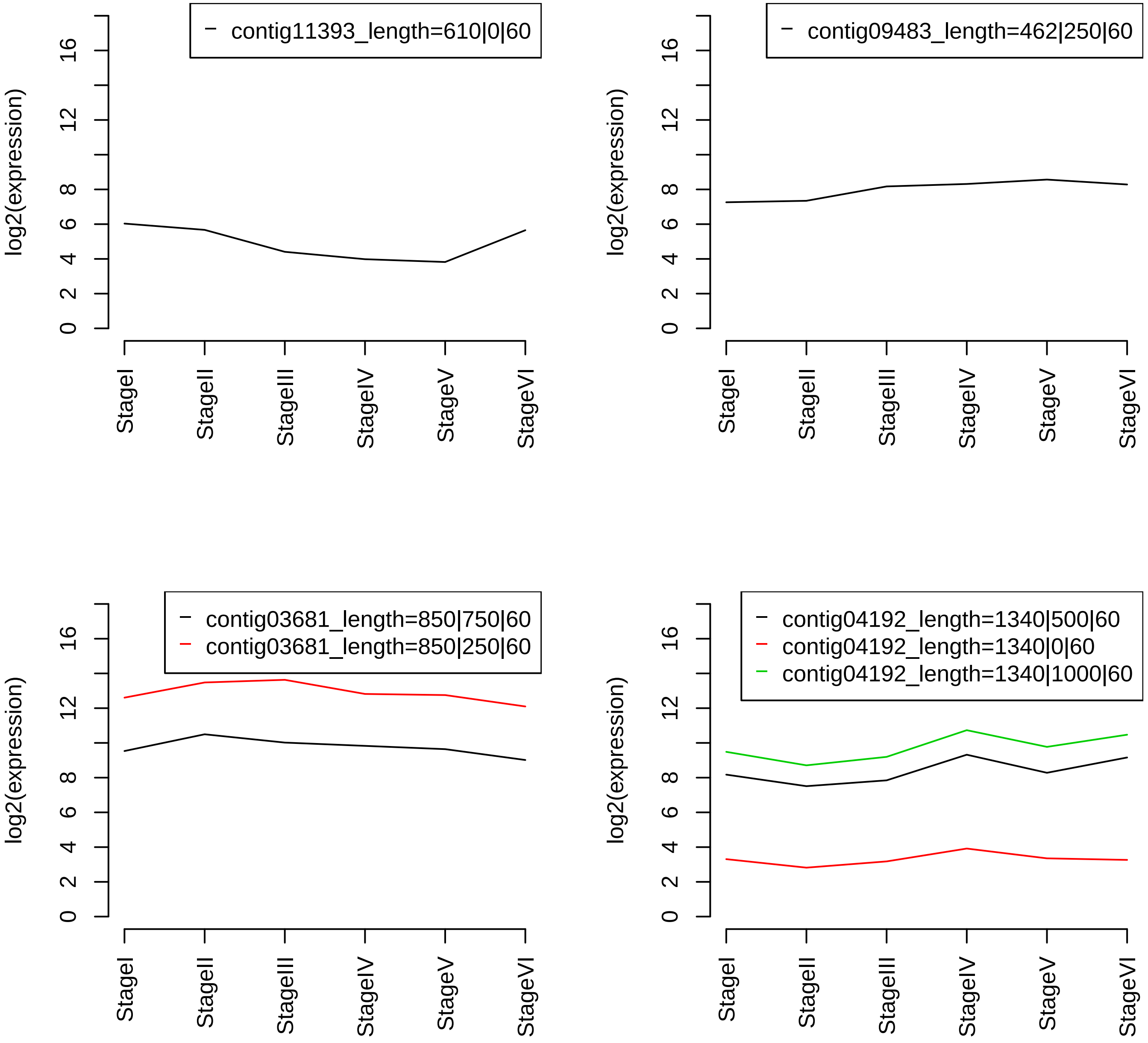
contig11393_length=610|0|60
contig09483_length=462|250|60
contig03681_length=850|750|60
contig03681_length=850|250|60
contig04192_length=1340|500|60
contig04192_length=1340|0|60
contig04192_length=1340|1000|60
log2(expression)
0 2 4 6 8 12 16
StageI
StageII
StageIII
StageIV
StageV
StageVI

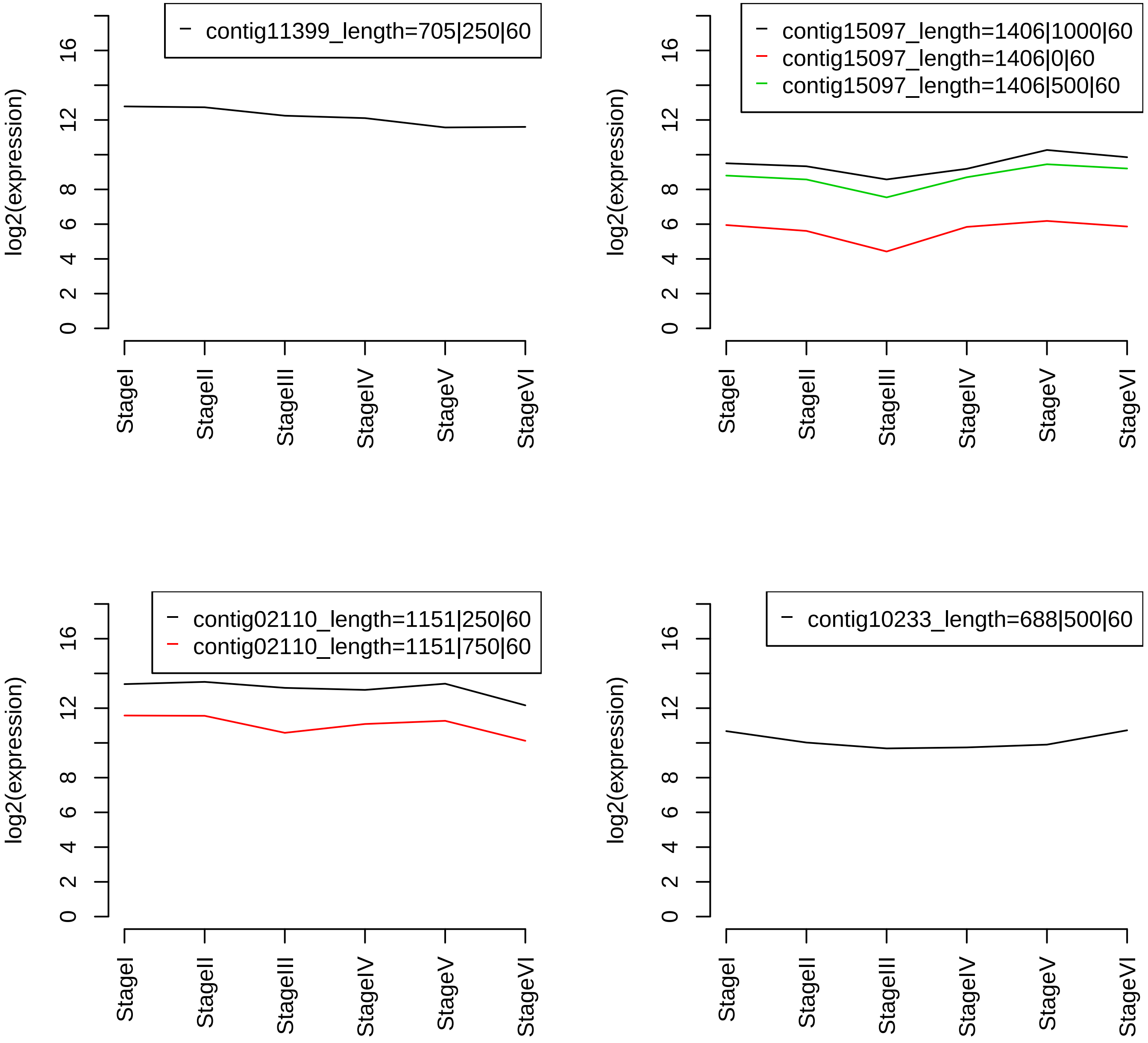
contig11399_length=705|250|60
contig15097_length=1406|1000|60
contig15097_length=1406|0|60
contig15097_length=1406|500|60
contig02110_length=1151|250|60
contig02110_length=1151|750|60
contig10233_length=688|500|60
log2(expression)
0
2
4
6
8
12
16
StageI
StageII
StageIII
StageIV
StageV
StageVI

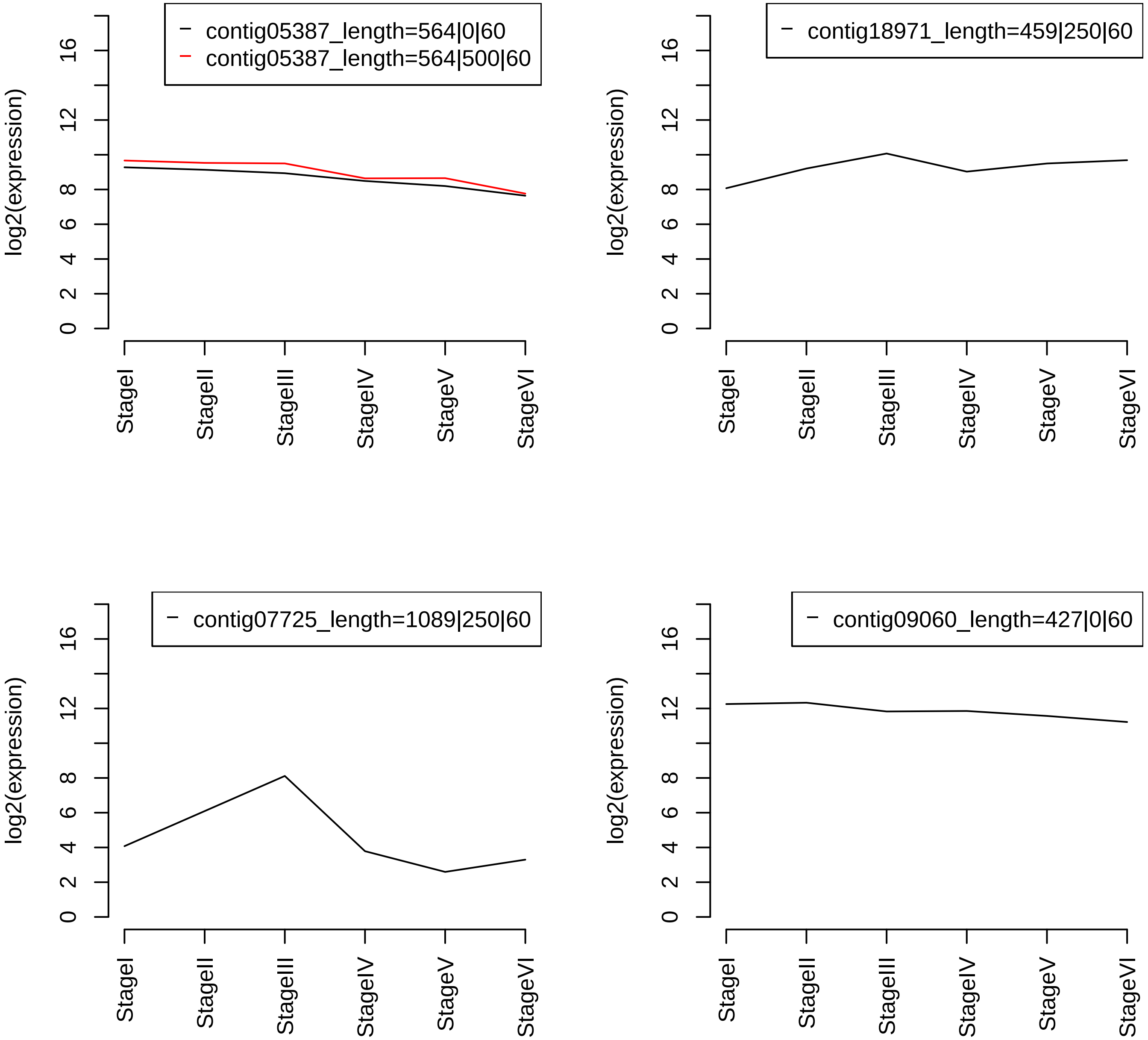
contig05387_length=564|0|60
contig05387_length=564|500|60
contig18971_length=459|250|60
contig07725_length=1089|250|60
contig09060_length=427|0|60
log2(expression)
0
2
4
6
8
12
16
StageI
StageII
StageIII
StageIV
StageV
StageVI

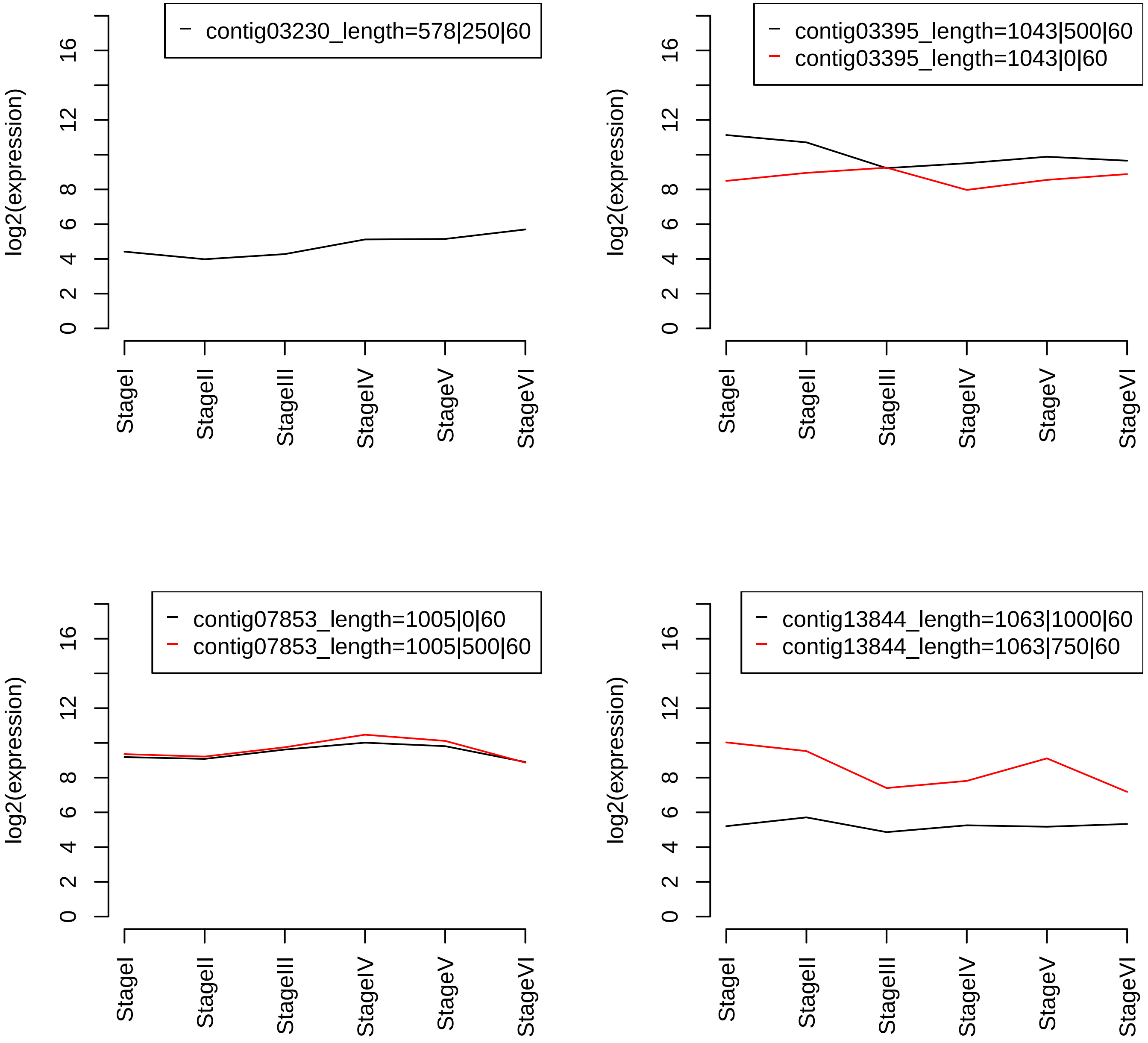
contig03230_length=578|250|60
contig03395_length=1043|500|60
contig03395_length=1043|0|60
contig07853_length=1005|0|60
contig07853_length=1005|500|60
contig13844_length=1063|1000|60
contig13844_length=1063|750|60
log2(expression)
0
2
4
6
8
12
16
StageI
StageII
StageIII
StageIV
StageV
StageVI

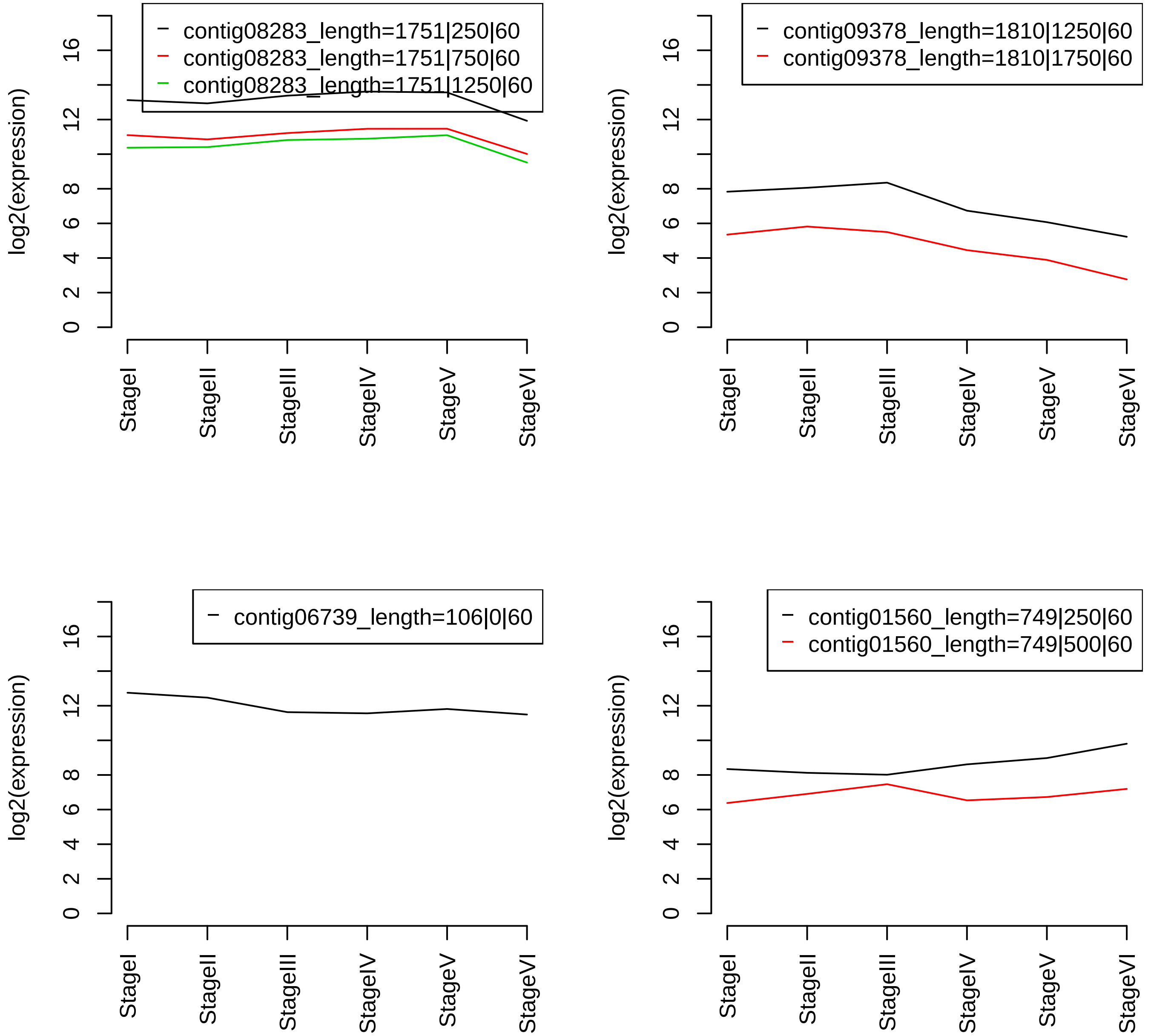

contig08283_length=1751|250|60
contig08283_length=1751|750|60
contig08283_length=1751|1250|60
log2(expression)
0
2
4
6
8
12
16
StageI
StageII
StageIII
StageIV
StageV
StageVI
contig09378_length=1810|1250|60
contig09378_length=1810|1750|60
log2(expression)
0
2
4
6
8
12
16
StageI
StageII
StageIII
StageIV
StageV
StageVI
contig06739_length=106|0|60
log2(expression)
0
2
4
6
8
12
16
StageI
StageII
StageIII
StageIV
StageV
StageVI
contig01560_length=749|250|60
contig01560_length=749|500|60
log2(expression)
0
2
4
6
8
12
16
StageI
StageII
StageIII
StageIV
StageV
StageVI

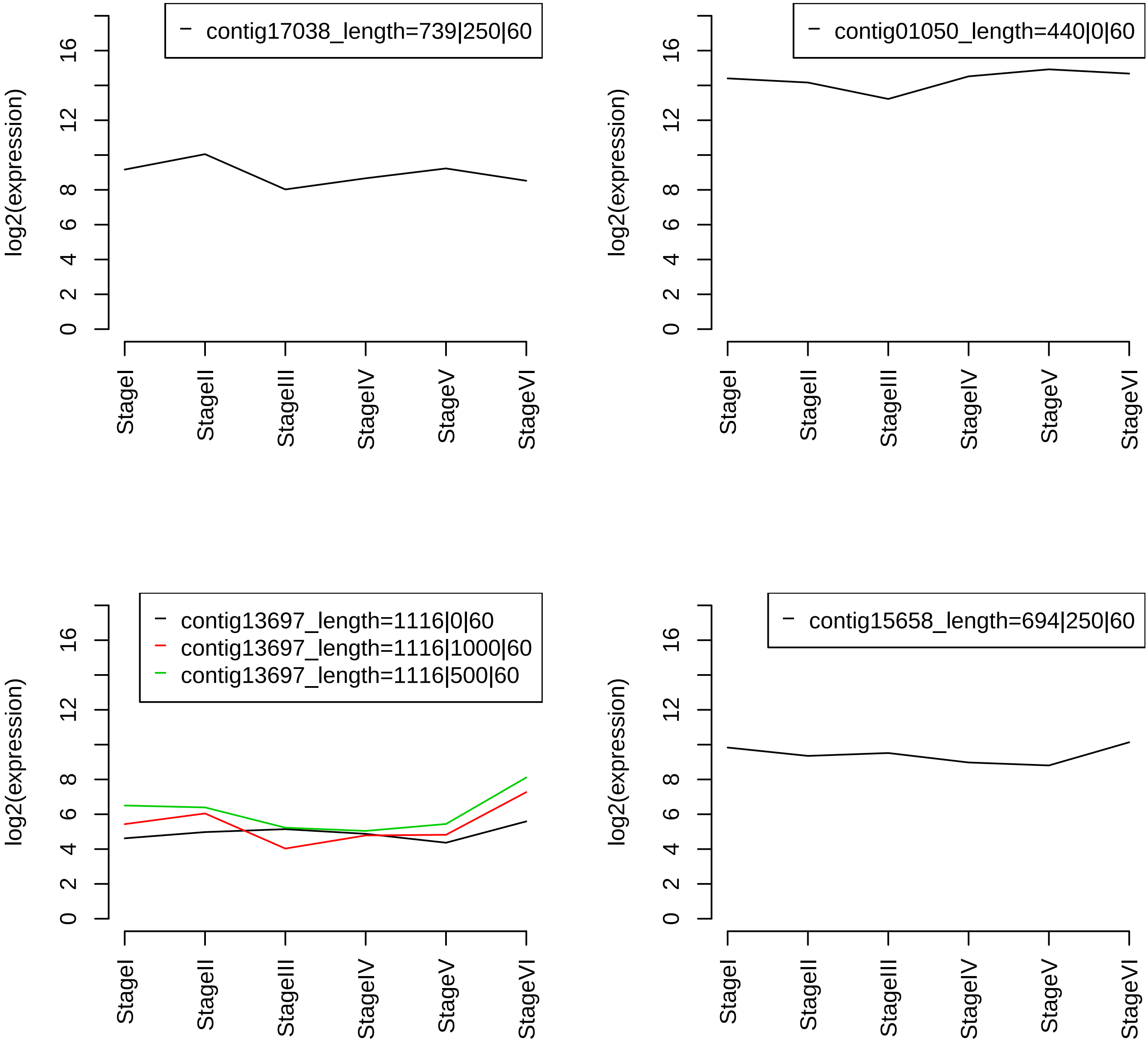
contig17038_length=739|250|60
contig01050_length=440|0|60
contig13697_length=1116|0|60
contig13697_length=1116|1000|60
contig13697_length=1116|500|60
contig15658_length=694|250|60
log2(expression)
0 2 4 6 8 12 16
StageI
StageII
StageIII
StageIV
StageV
StageVI

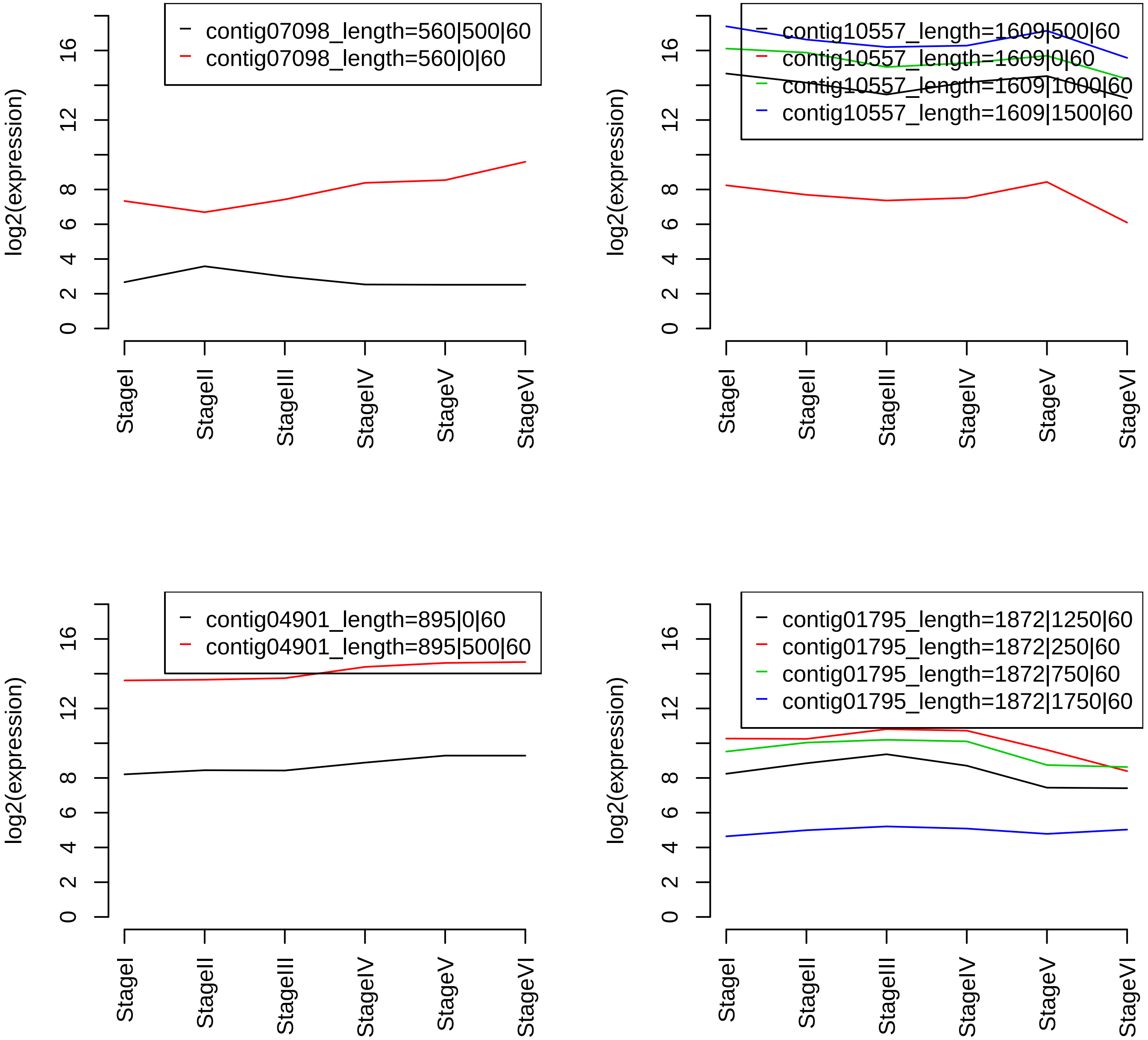

contig07098_length=560|500|60
contig07098_length=560|0|60
contig10557_length=1609|500|60
contig10557_length=1609|0|60
contig10557_length=1609|1000|60
contig10557_length=1609|1500|60
contig04901_length=895|0|60
contig04901_length=895|500|60
contig01795_length=1872|1250|60
contig01795_length=1872|250|60
contig01795_length=1872|750|60
contig01795_length=1872|1750|60
log2(expression)
0
2
4
6
8
12
16
StageI
StageII
StageIII
StageIV
StageV
StageVI

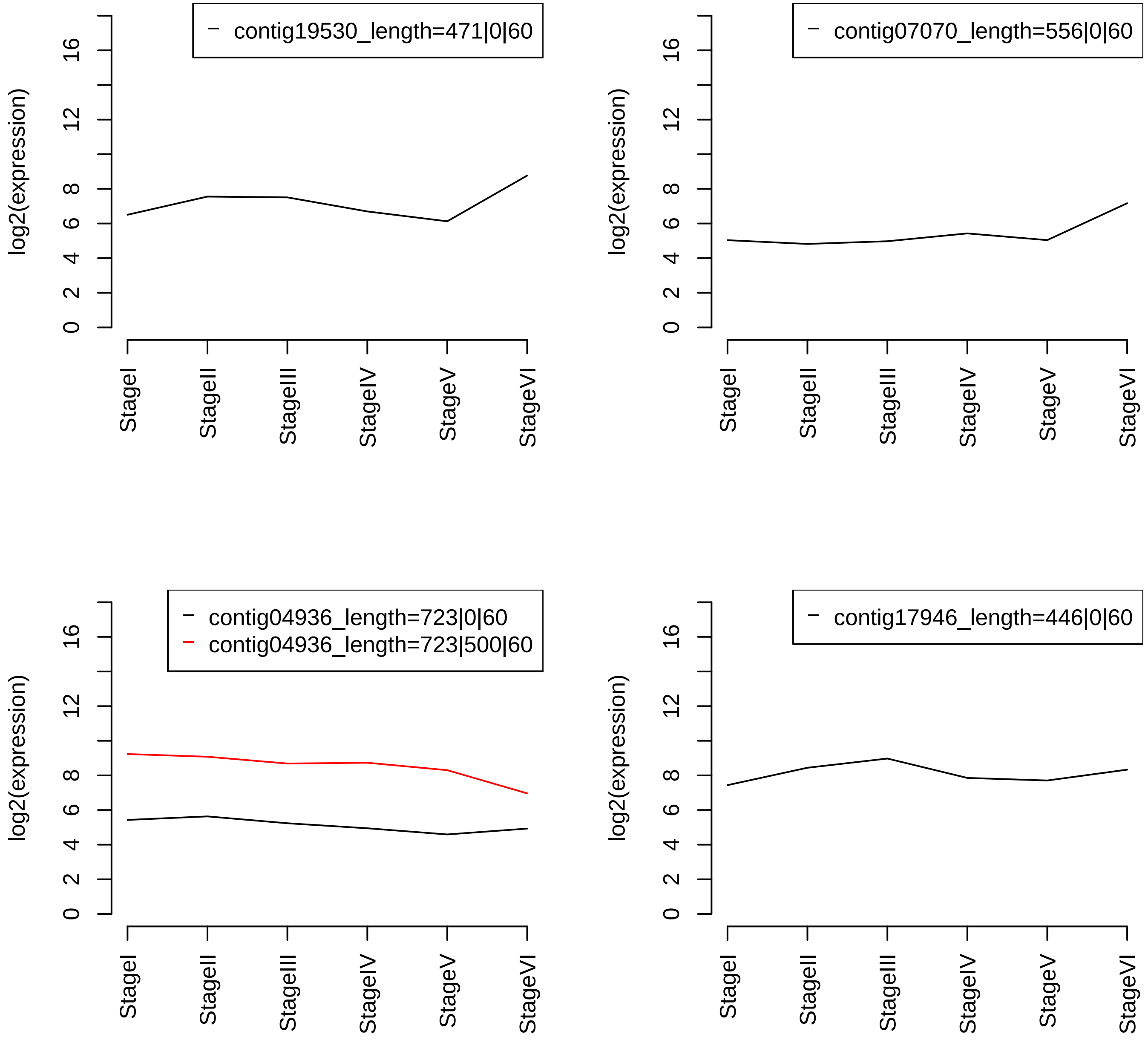

contig19530_length=471|0|60
contig07070_length=556|0|60
contig04936_length=723|0|60
contig04936_length=723|500|60
contig17946_length=446|0|60
log2(expression)
0
2
4
6
8
12
16
StageI
StageII
StageIII
StageIV
StageV
StageVI

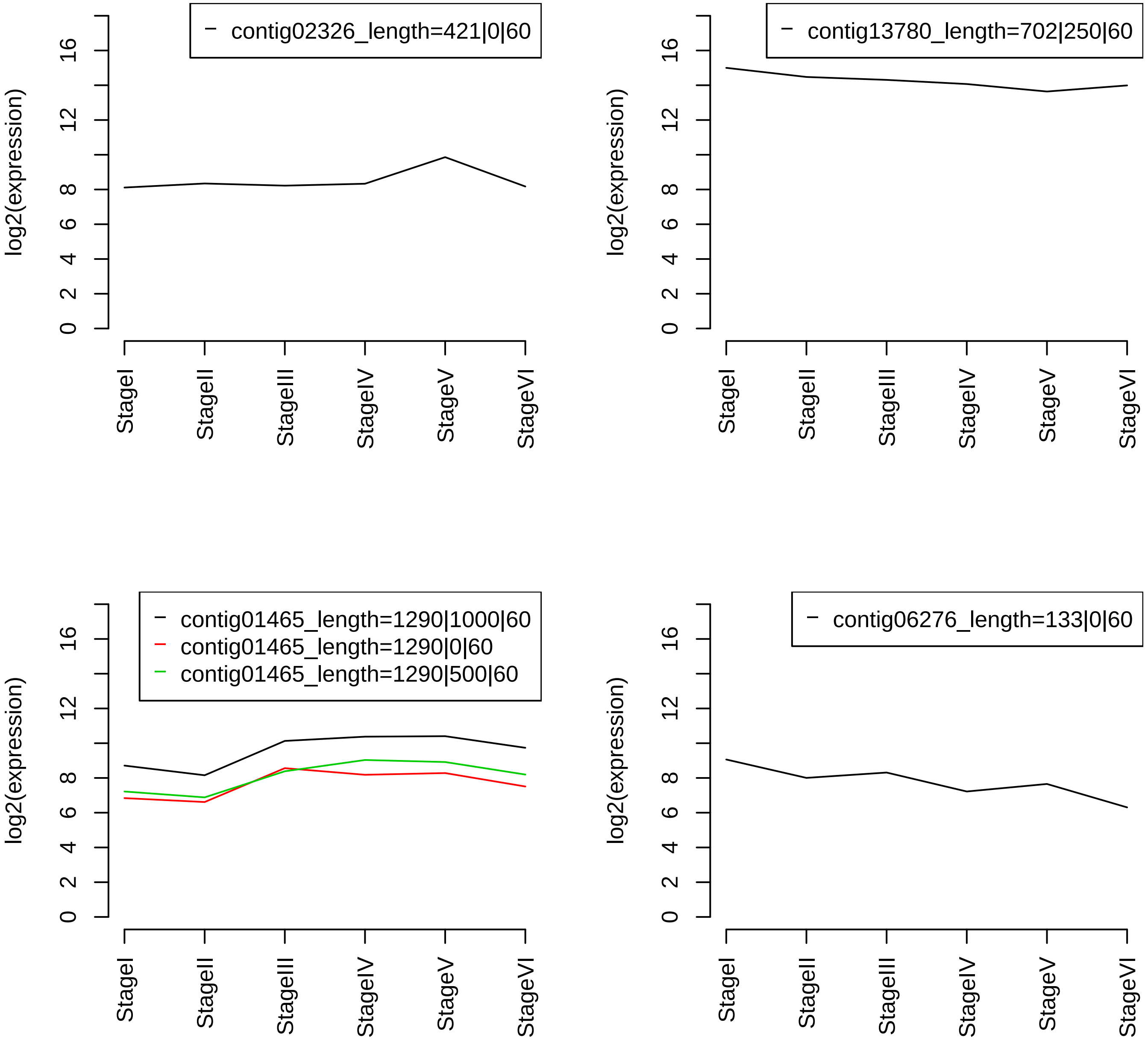

contig02326_length=421|0|60
contig13780_length=702|250|60
contig01465_length=1290|1000|60
contig01465_length=1290|0|60
contig01465_length=1290|500|60
contig06276_length=133|0|60
log2(expression)
0
2
4
6
8
12
16
StageI
StageII
StageIII
StageIV
StageV
StageVI

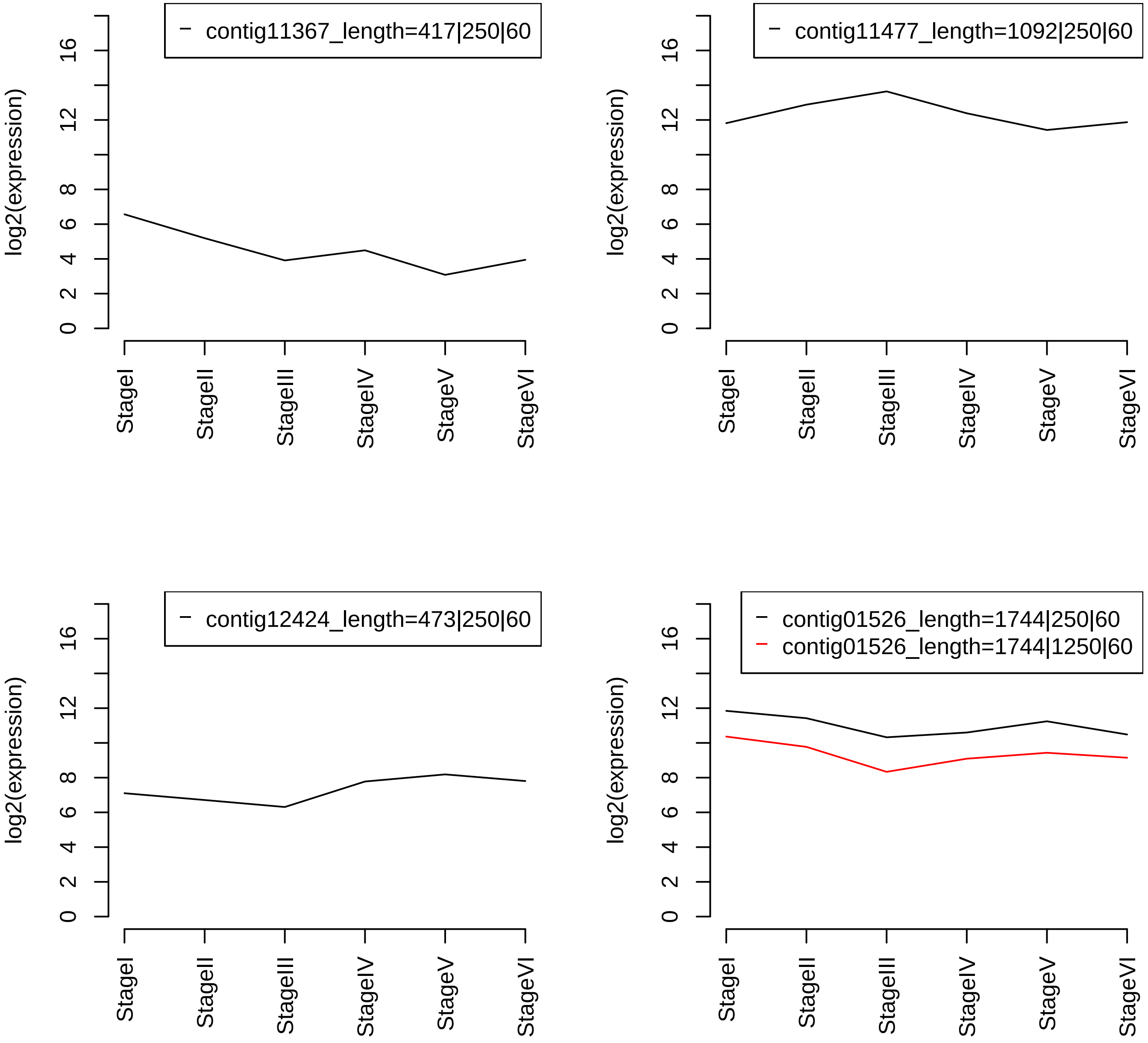
contig11367_length=417|250|60
contig11477_length=1092|250|60
contig12424_length=473|250|60
contig01526_length=1744|250|60
contig01526_length=1744|1250|60
log2(expression)
0
2
4
6
8
12
16
StageI
StageII
StageIII
StageIV
StageV
StageVI

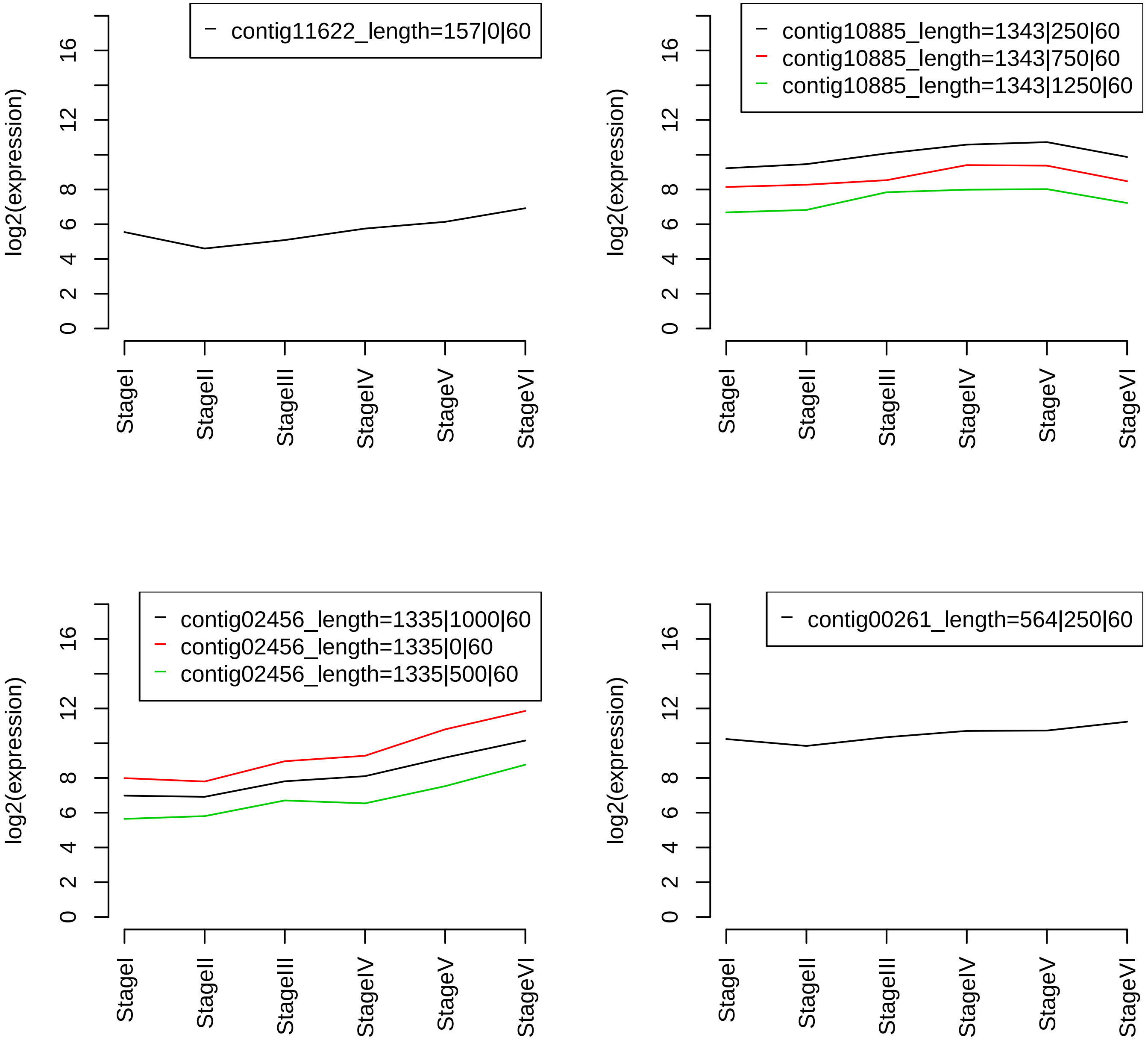

contig11622_length=157|0|60
contig10885_length=1343|250|60
contig10885_length=1343|750|60
contig10885_length=1343|1250|60
contig02456_length=1335|1000|60
contig02456_length=1335|0|60
contig02456_length=1335|500|60
contig00261_length=564|250|60
log2(expression)
0 2 4 6 8 12 16
StageI StageII StageIII StageIV StageV StageVI

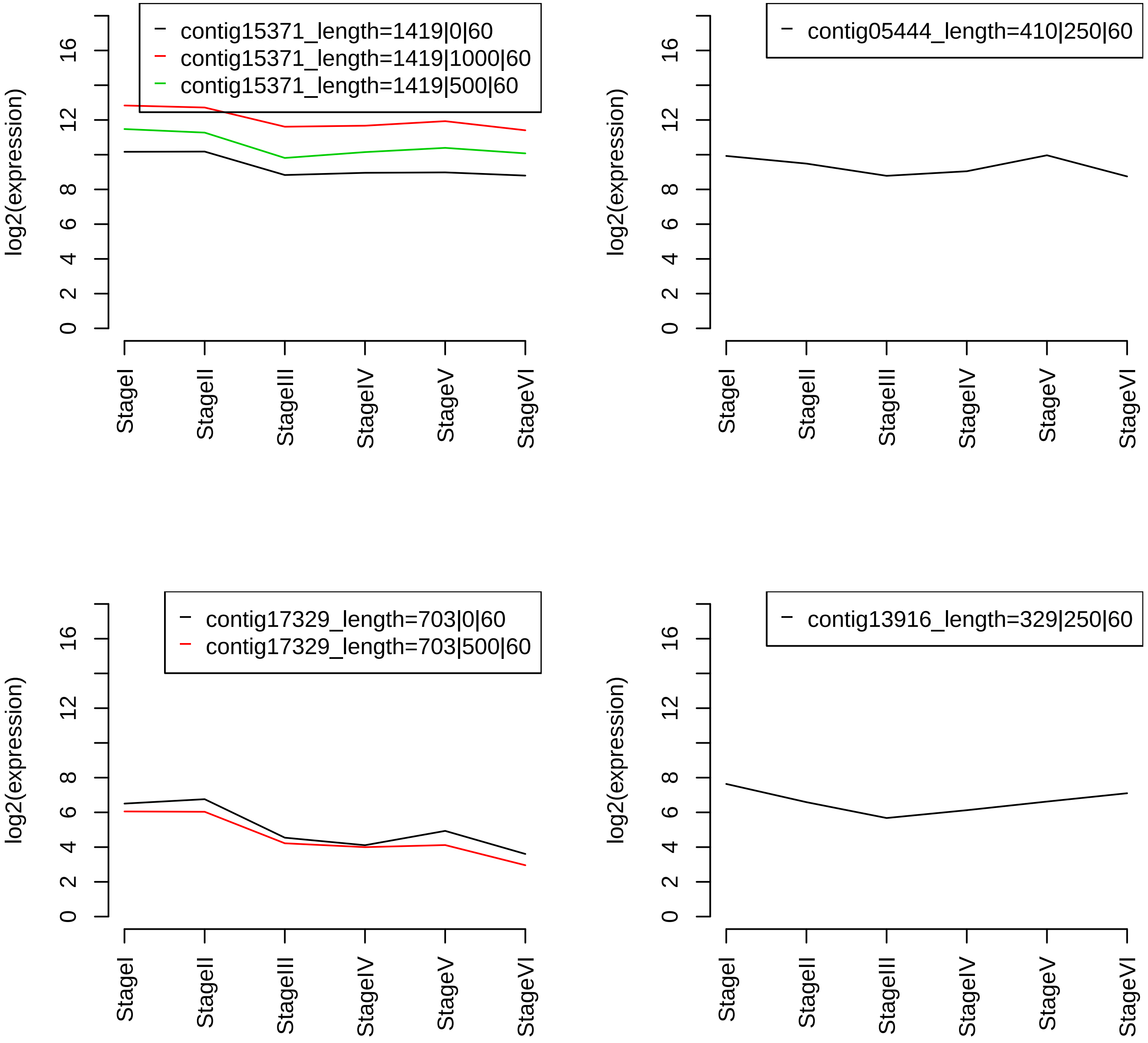

contig15371_length=1419|0|60
contig15371_length=1419|1000|60
contig15371_length=1419|500|60
contig05444_length=410|250|60
contig17329_length=703|0|60
contig17329_length=703|500|60
contig13916_length=329|250|60
log2(expression)
0
2
4
6
8
12
16
StageI
StageII
StageIII
StageIV
StageV
StageVI

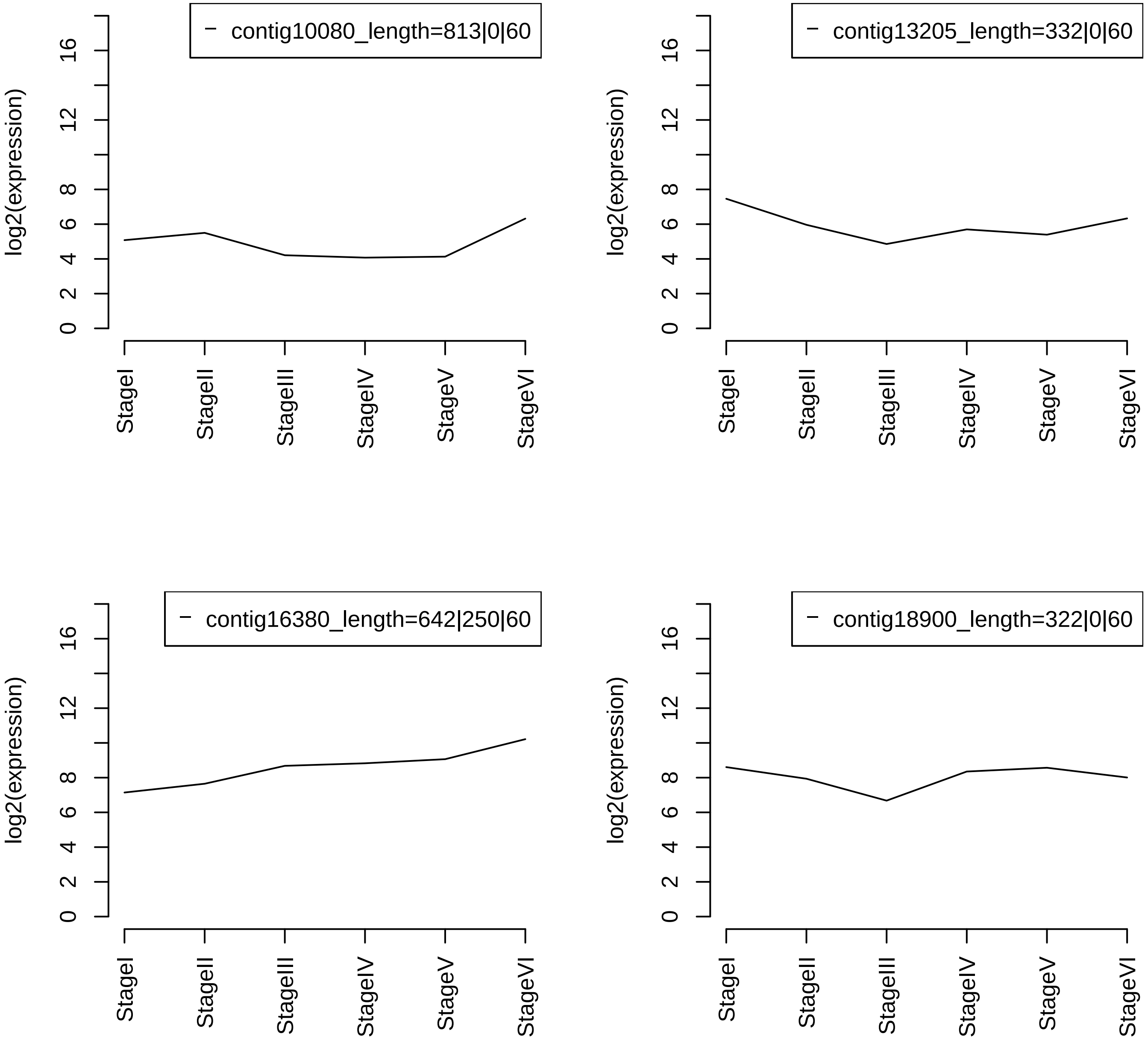

contig10080_length=813|0|60
contig13205_length=332|0|60
contig16380_length=642|250|60
contig18900_length=322|0|60
log2(expression)
0
2
4
6
8
12
16
StageI
StageII
StageIII
StageIV
StageV
StageVI

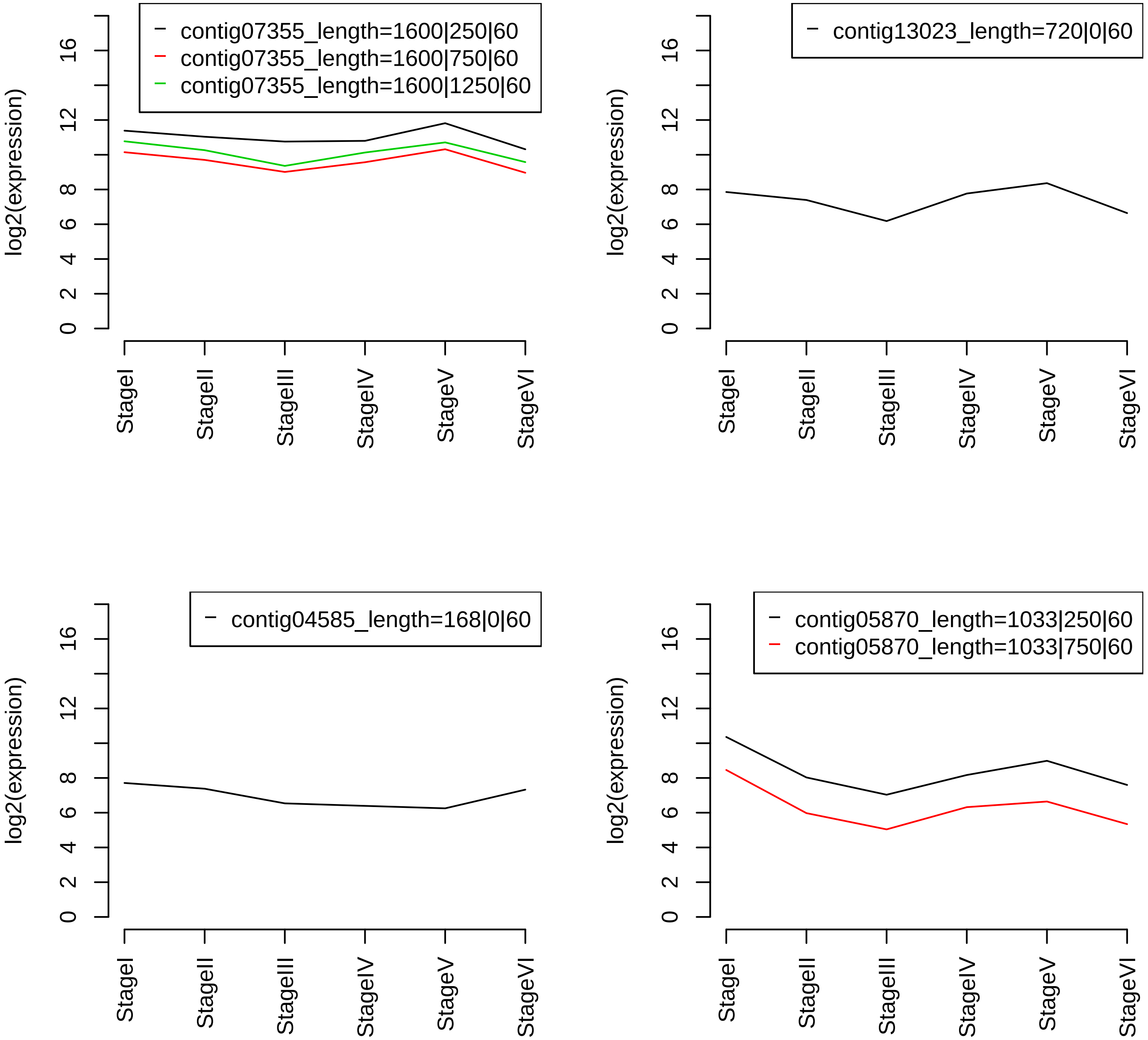
contig07355_length=1600|250|60
contig07355_length=1600|750|60
contig07355_length=1600|1250|60
contig13023_length=720|0|60
contig04585_length=168|0|60
contig05870_length=1033|250|60
contig05870_length=1033|750|60
log2(expression)
StageI
StageII
StageIII
StageIV
StageV
StageVI

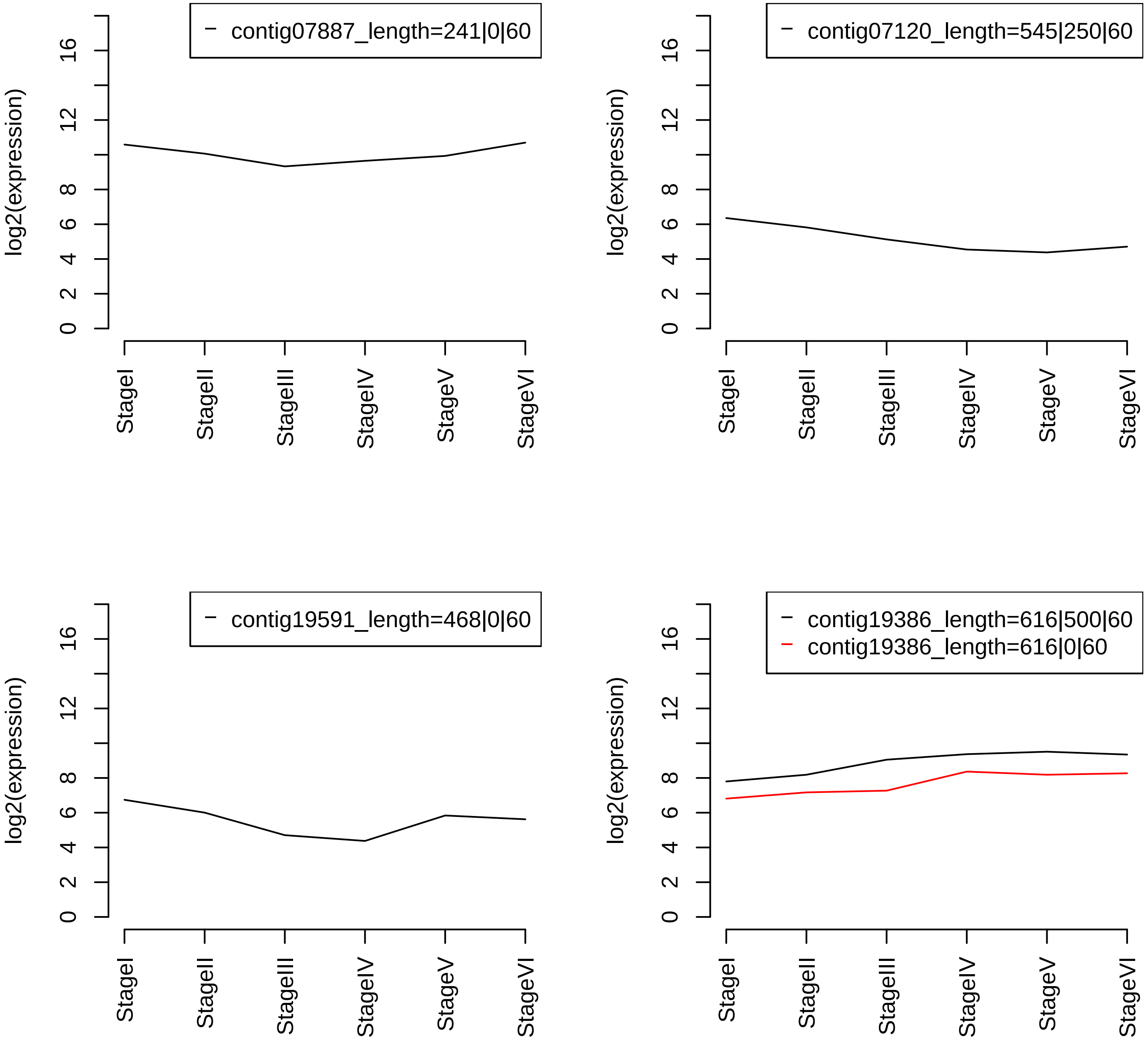
contig07887_length=241|0|60
contig07120_length=545|250|60
contig19591_length=468|0|60
contig19386_length=616|500|60
contig19386_length=616|0|60
log2(expression)
0
2
4
6
8
12
16
StageI
StageII
StageIII
StageIV
StageV
StageVI

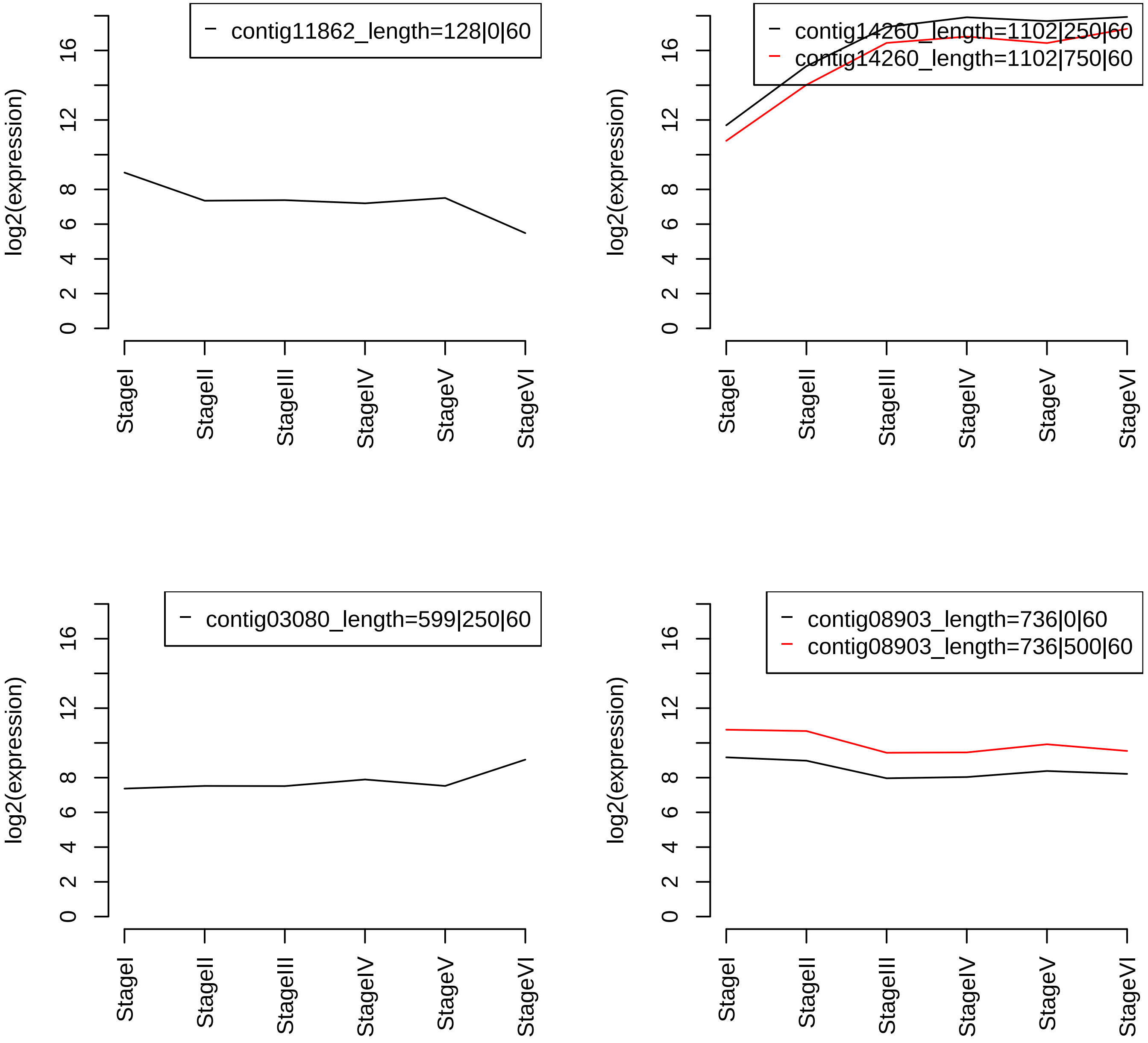

contig11862_length=128|0|60
contig14260_length=1102|250|60
contig14260_length=1102|750|60
contig03080_length=599|250|60
contig08903_length=736|0|60
contig08903_length=736|500|60
log2(expression)
0
2
4
6
8
12
16
StageI
StageII
StageIII
StageIV
StageV
StageVI

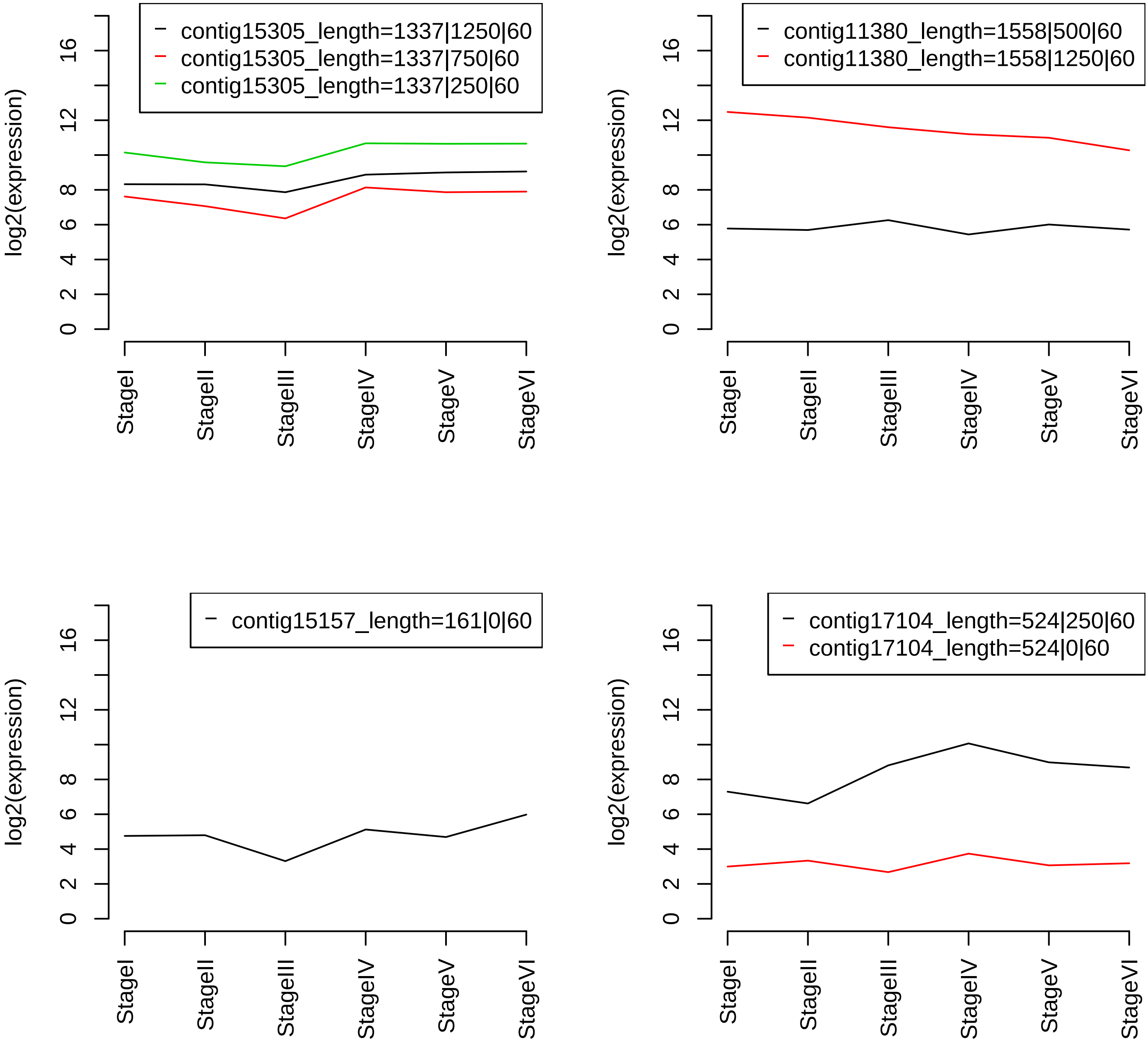
contig15305_length=1337|1250|60
contig15305_length=1337|750|60
contig15305_length=1337|250|60
log2(expression)
0
2
4
6
8
12
16
StageI
StageII
StageIII
StageIV
StageV
StageVI
contig11380_length=1558|500|60
contig11380_length=1558|1250|60
contig15157_length=161|0|60
contig17104_length=524|250|60
contig17104_length=524|0|60

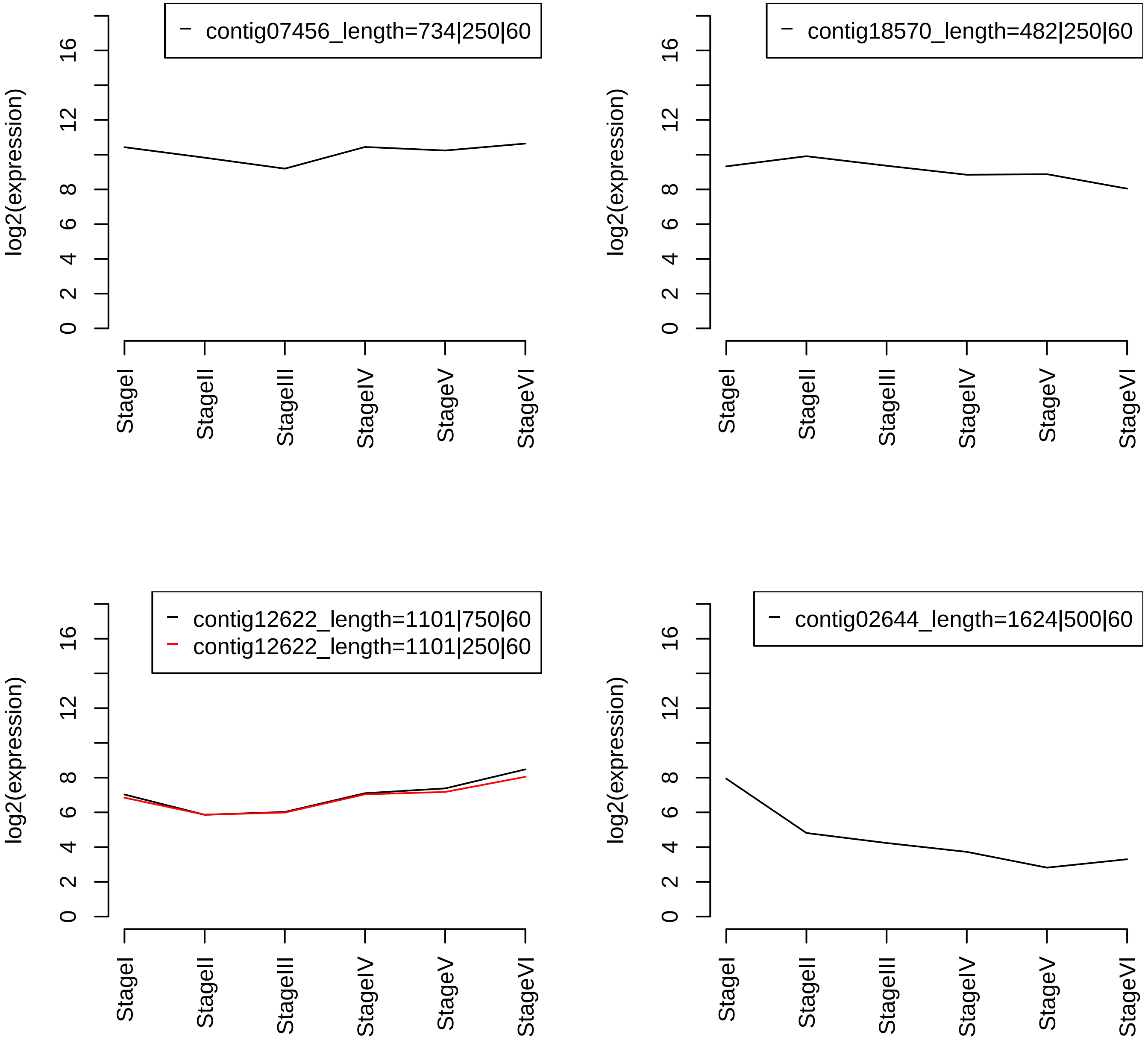

contig07456_length=734|250|60
contig18570_length=482|250|60
contig12622_length=1101|750|60
contig12622_length=1101|250|60
contig02644_length=1624|500|60
log2(expression)
0
2
4
6
8
12
16
StageI
StageII
StageIII
StageIV
StageV
StageVI

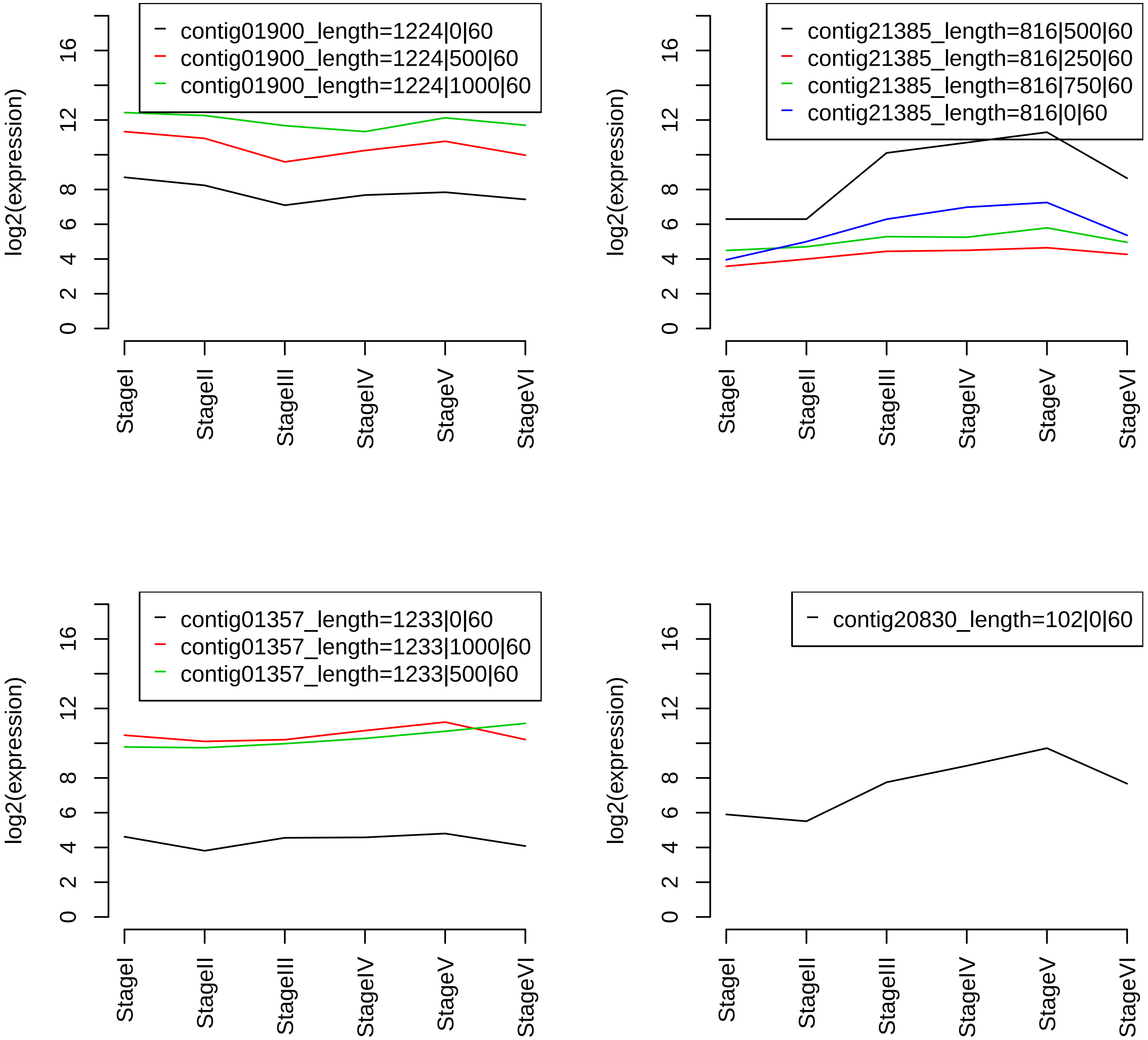

contig01900_length=1224|0|60
contig01900_length=1224|500|60
contig01900_length=1224|1000|60
contig21385_length=816|500|60
contig21385_length=816|250|60
contig21385_length=816|750|60
contig21385_length=816|0|60
contig01357_length=1233|0|60
contig01357_length=1233|1000|60
contig01357_length=1233|500|60
contig20830_length=102|0|60
log2(expression)
0
2
4
6
8
12
16
StageI
StageII
StageIII
StageIV
StageV
StageVI

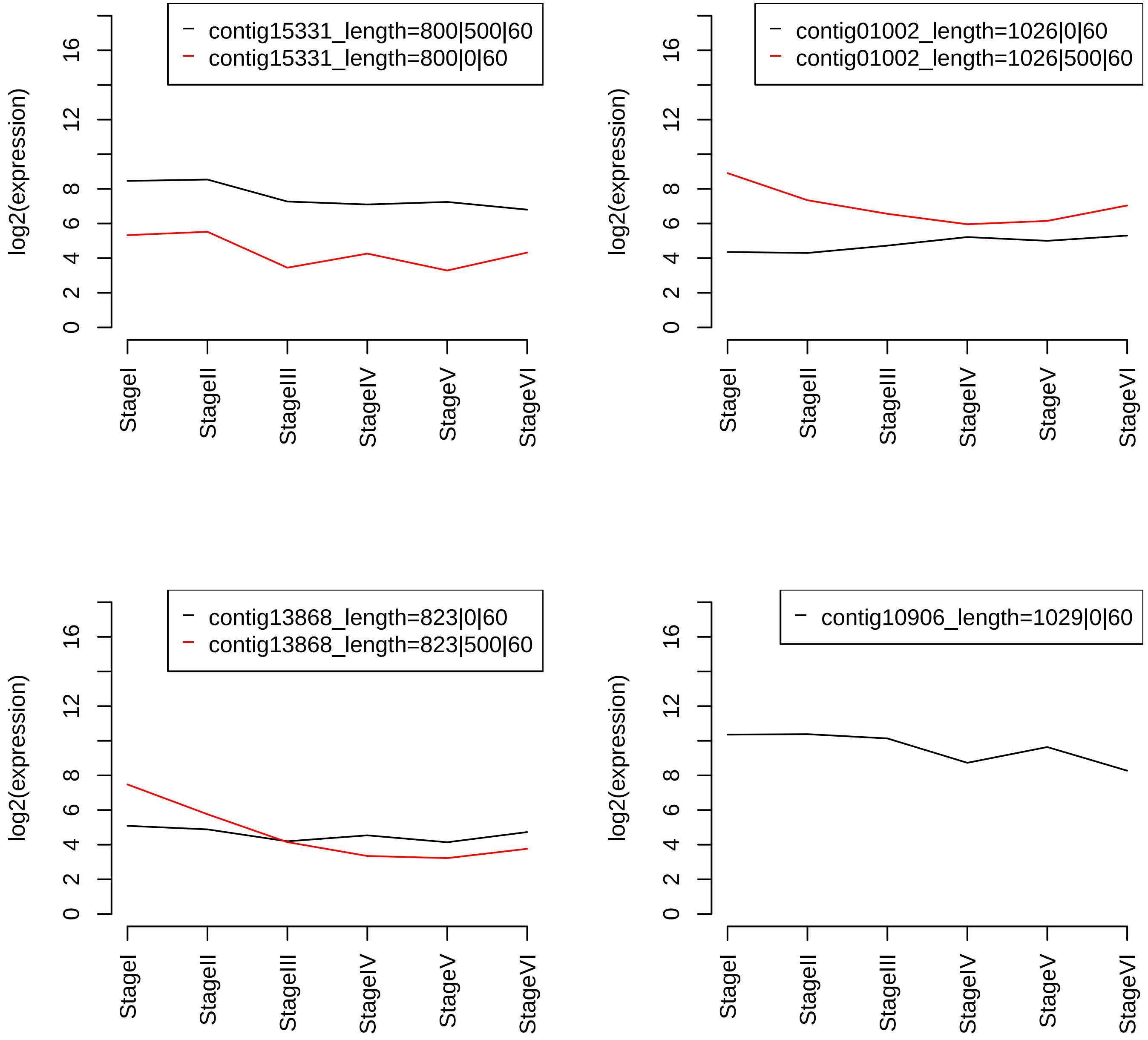

contig15331_length=800|500|60
contig15331_length=800|0|60
contig01002_length=1026|0|60
contig01002_length=1026|500|60
contig13868_length=823|0|60
contig13868_length=823|500|60
contig10906_length=1029|0|60
log2(expression)
0
2
4
6
8
12
16
StageI
StageII
StageIII
StageIV
StageV
StageVI

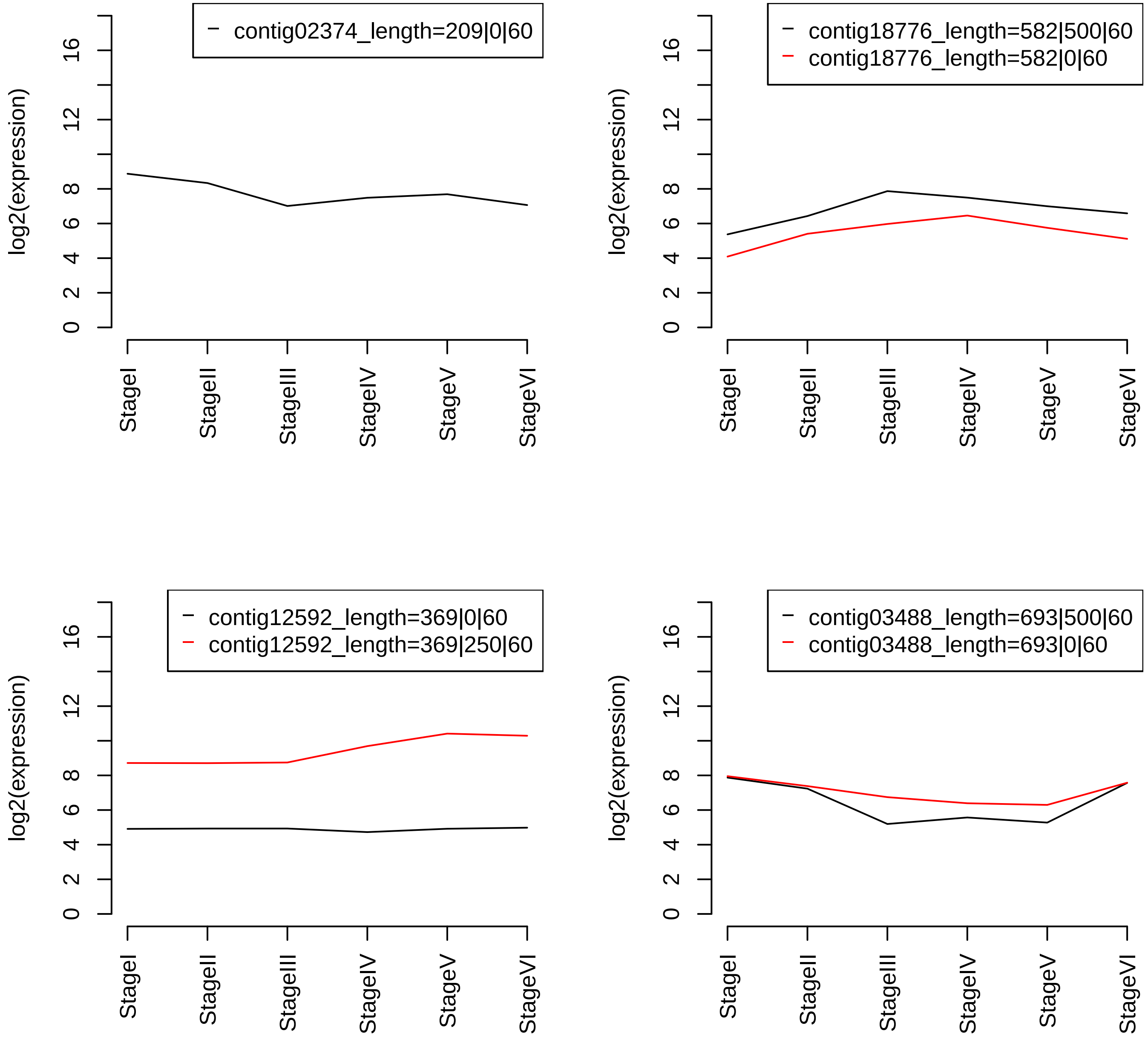
contig02374_length=209|0|60
contig18776_length=582|500|60
contig18776_length=582|0|60
contig12592_length=369|0|60
contig12592_length=369|250|60
contig03488_length=693|500|60
contig03488_length=693|0|60
log2(expression)
0
2
4
6
8
12
16
StageI
StageII
StageIII
StageIV
StageV
StageVI

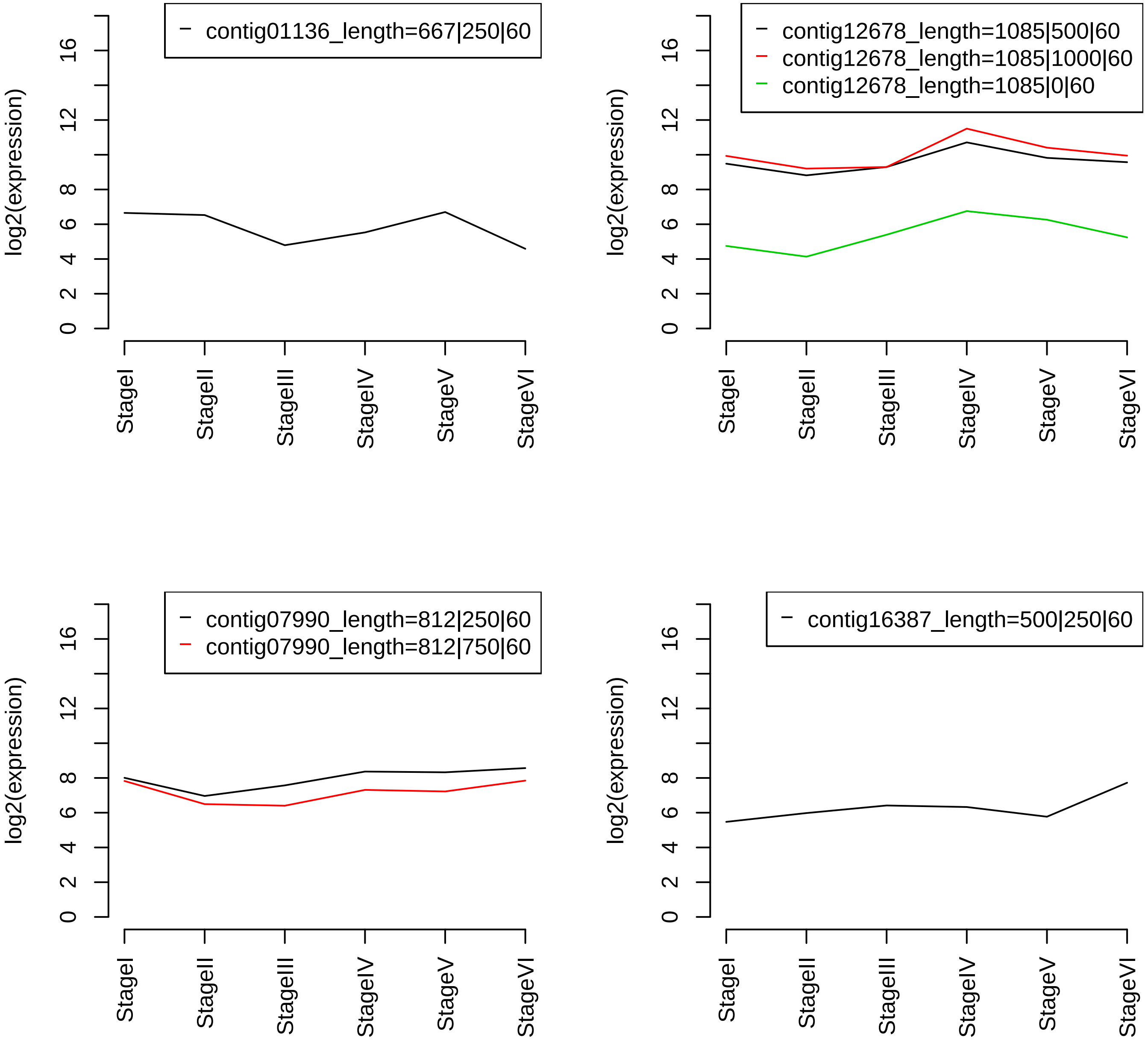

contig01136_length=667|250|60
contig12678_length=1085|500|60
contig12678_length=1085|1000|60
contig12678_length=1085|0|60
contig07990_length=812|250|60
contig07990_length=812|750|60
contig16387_length=500|250|60
log2(expression)
0
2
4
6
8
12
16
StageI
StageII
StageIII
StageIV
StageV
StageVI

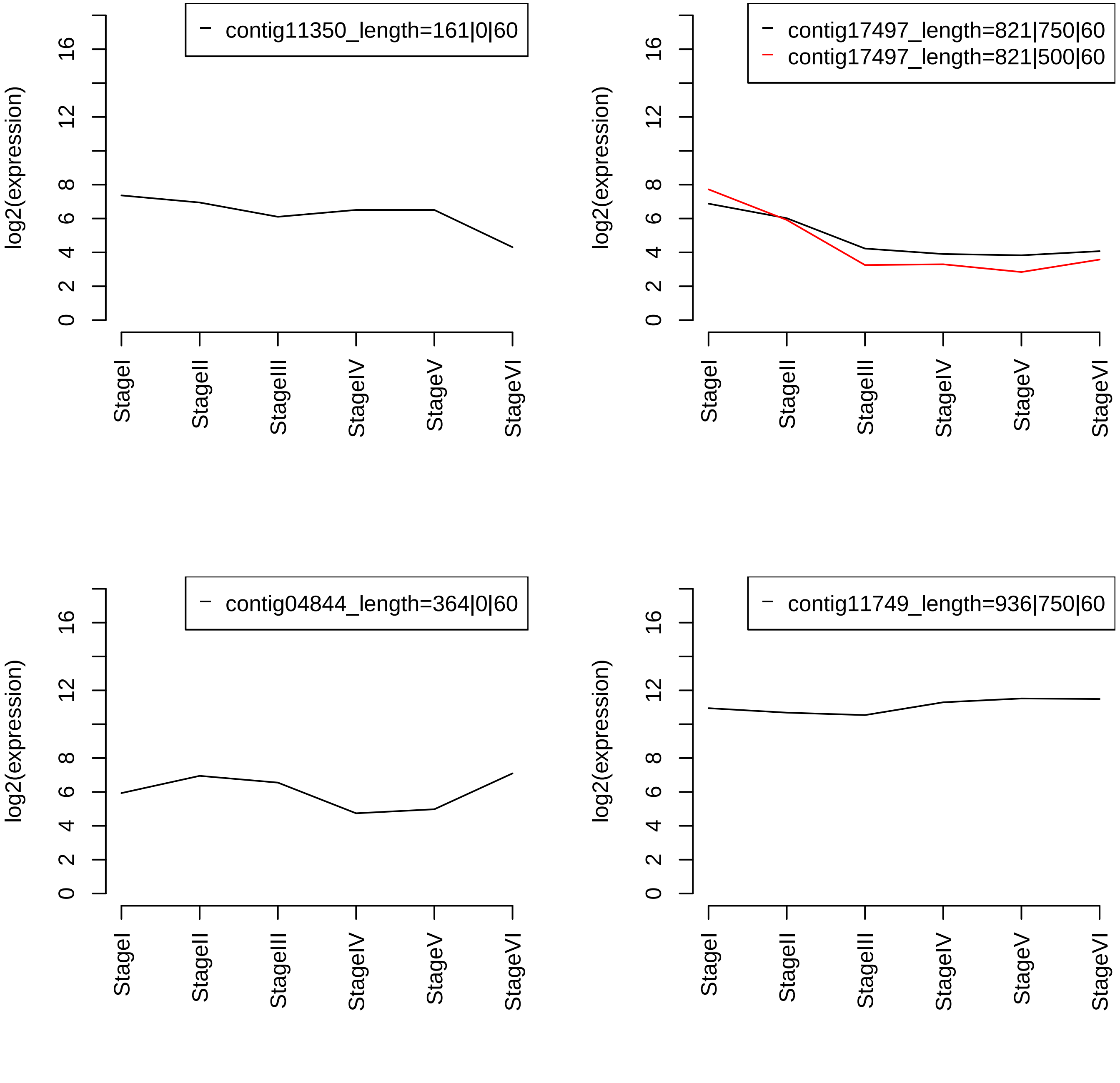

contig11350_length=161|0|60
contig17497_length=821|750|60
contig17497_length=821|500|60
contig04844_length=364|0|60
contig11749_length=936|750|60
log2(expression)
0
2
4
6
8
12
16
StageI
StageII
StageIII
StageIV
StageV
StageVI

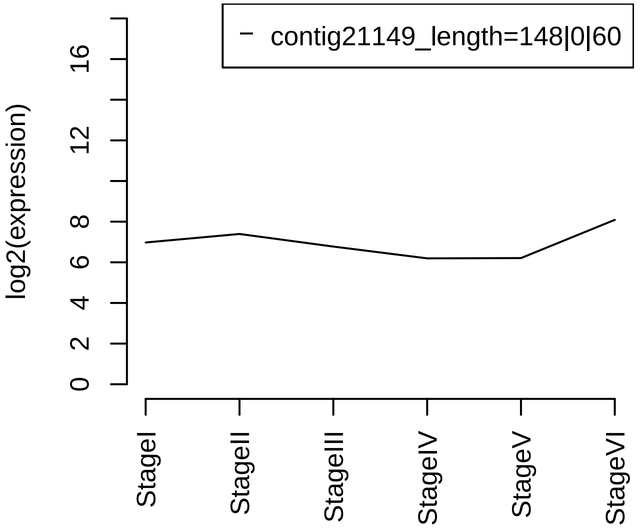

contig21149_length=148|0|60
log2(expression)
0
2
4
6
8
12
16
StageI
StageII
StageIII
StageIV
StageV
StageVI

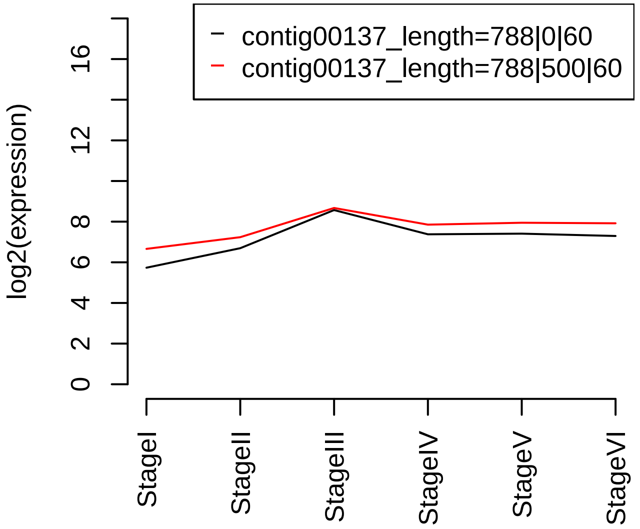

contig00137_length=788|0|60
contig00137_length=788|500|60
log2(expression)
0
2
4
6
8
12
16
StageI
StageII
StageIII
StageIV
StageV
StageVI

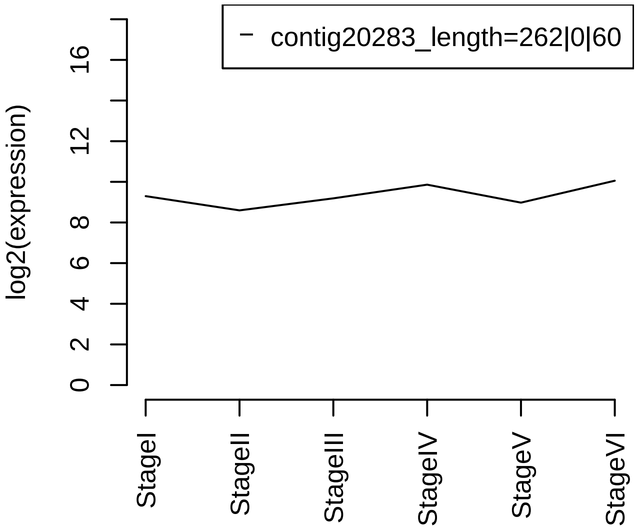

contig20283_length=262|0|60
log2(expression)
0
2
4
6
8
12
16
StageI
StageII
StageIII
StageIV
StageV
StageVI

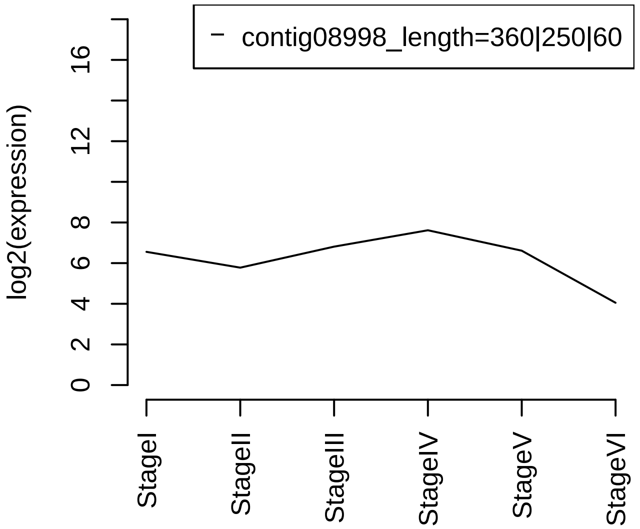

contig08998_length=360|250|60
log2(expression)
0
2
4
6
8
12
16
StageI
StageII
StageIII
StageIV
StageV
StageVI

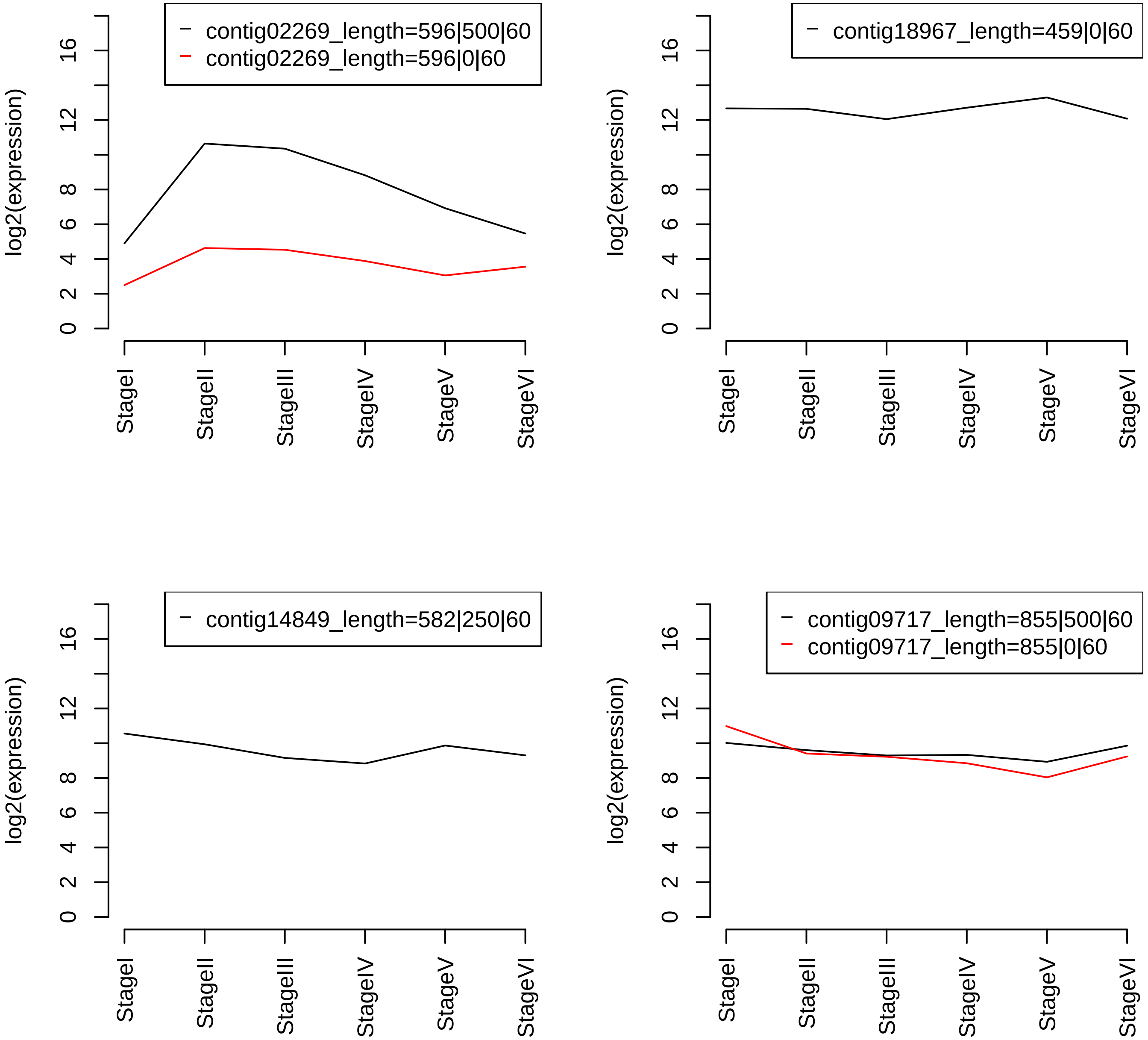

contig02269_length=596|500|60
contig02269_length=596|0|60
contig18967_length=459|0|60
contig14849_length=582|250|60
contig09717_length=855|500|60
contig09717_length=855|0|60
log2(expression)
0 2 4 6 8 12 16
StageI StageII StageIII StageIV StageV StageVI

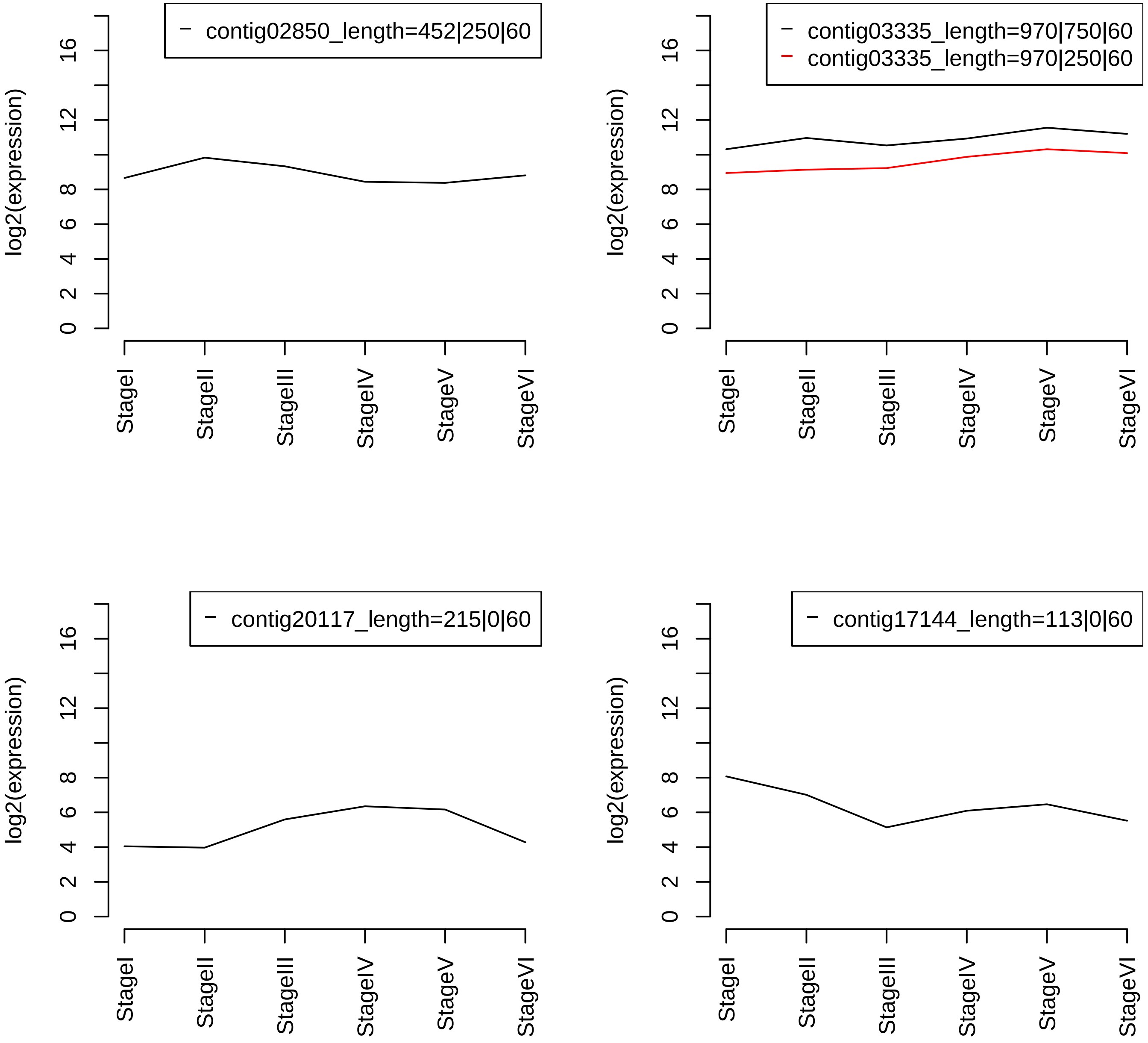
contig02850_length=452|250|60
contig03335_length=970|750|60
contig03335_length=970|250|60
contig20117_length=215|0|60
contig17144_length=113|0|60
log2(expression)
0
2
4
6
8
12
16
StageI
StageII
StageIII
StageIV
StageV
StageVI

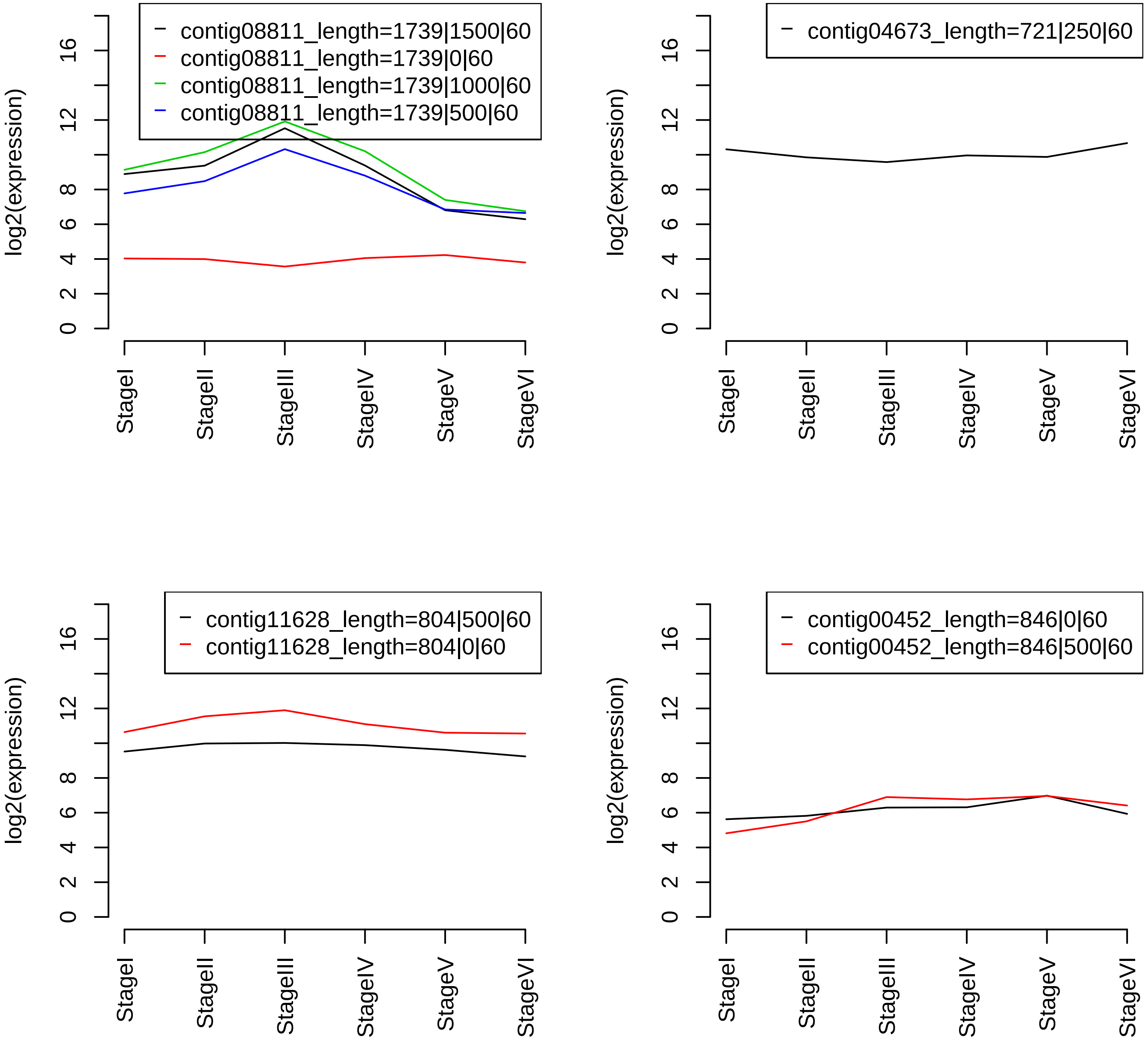

contig08811_length=1739|1500|60
contig08811_length=1739|0|60
contig08811_length=1739|1000|60
contig08811_length=1739|500|60
log2(expression)
0 2 4 6 8 12 16
StageI StageII StageIII StageIV StageV StageVI
contig04673_length=721|250|60
log2(expression)
0 2 4 6 8 12 16
StageI StageII StageIII StageIV StageV StageVI
contig11628_length=804|500|60
contig11628_length=804|0|60
log2(expression)
0 2 4 6 8 12 16
StageI StageII StageIII StageIV StageV StageVI
contig00452_length=846|0|60
contig00452_length=846|500|60
log2(expression)
0 2 4 6 8 12 16
StageI StageII StageIII StageIV StageV StageVI

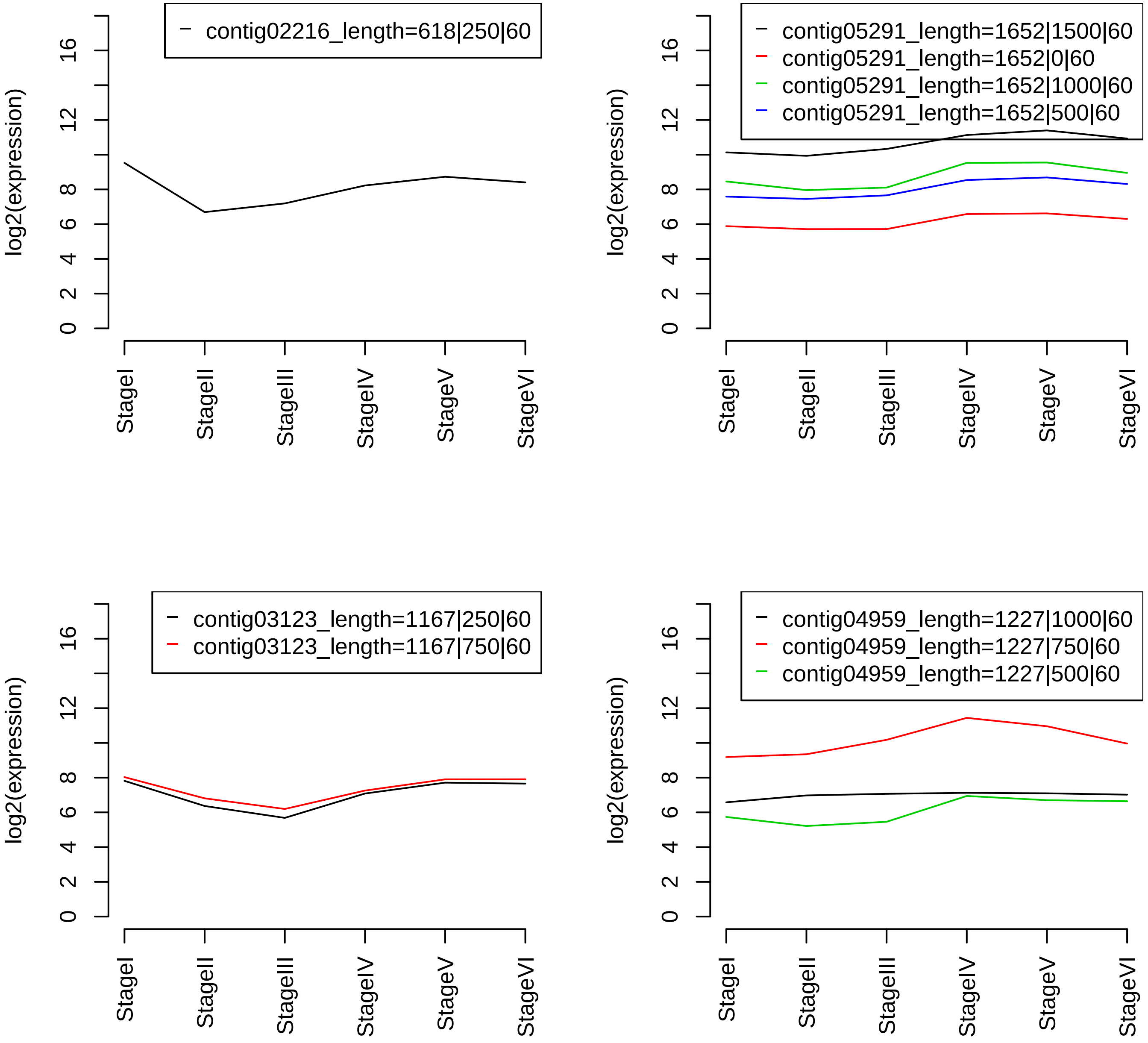

log2(expression)
0
2
4
6
8
12
16
contig02216_length=618|250|60
StageI
StageII
StageIII
StageIV
StageV
StageVI
contig05291_length=1652|1500|60
contig05291_length=1652|0|60
contig05291_length=1652|1000|60
contig05291_length=1652|500|60
contig03123_length=1167|250|60
contig03123_length=1167|750|60
contig04959_length=1227|1000|60
contig04959_length=1227|750|60
contig04959_length=1227|500|60

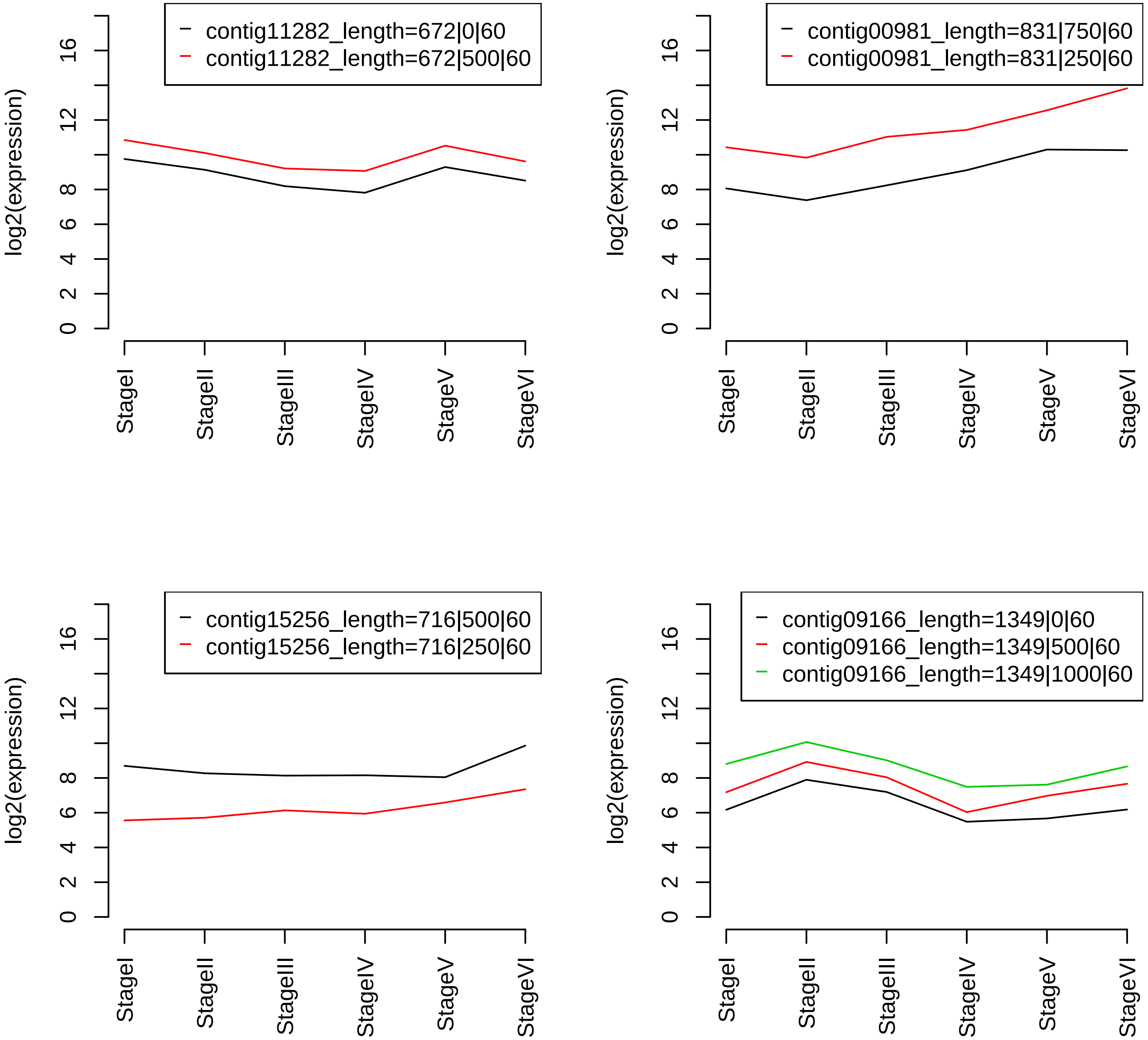
contig11282_length=672|0|60
contig11282_length=672|500|60
contig00981_length=831|750|60
contig00981_length=831|250|60
contig15256_length=716|500|60
contig15256_length=716|250|60
contig09166_length=1349|0|60
contig09166_length=1349|500|60
contig09166_length=1349|1000|60
log2(expression)
0 2 4 6 8 12 16
StageI StageII StageIII StageIV StageV StageVI

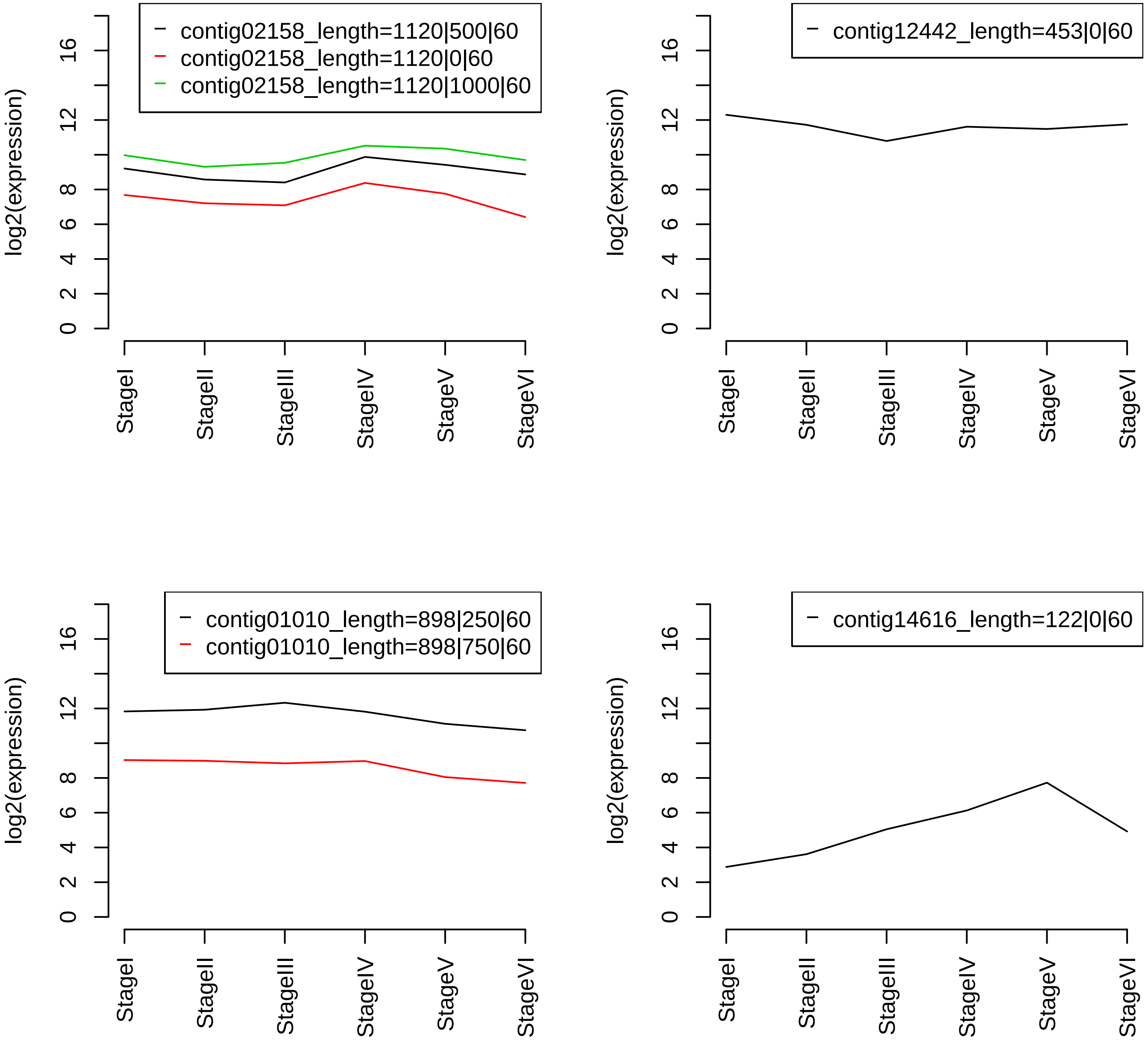

contig02158_length=1120|500|60
contig02158_length=1120|0|60
contig02158_length=1120|1000|60
log2(expression)
0
2
4
6
8
12
16
StageI
StageII
StageIII
StageIV
StageV
StageVI
contig12442_length=453|0|60
log2(expression)
contig01010_length=898|250|60
contig01010_length=898|750|60
log2(expression)
contig14616_length=122|0|60
log2(expression)

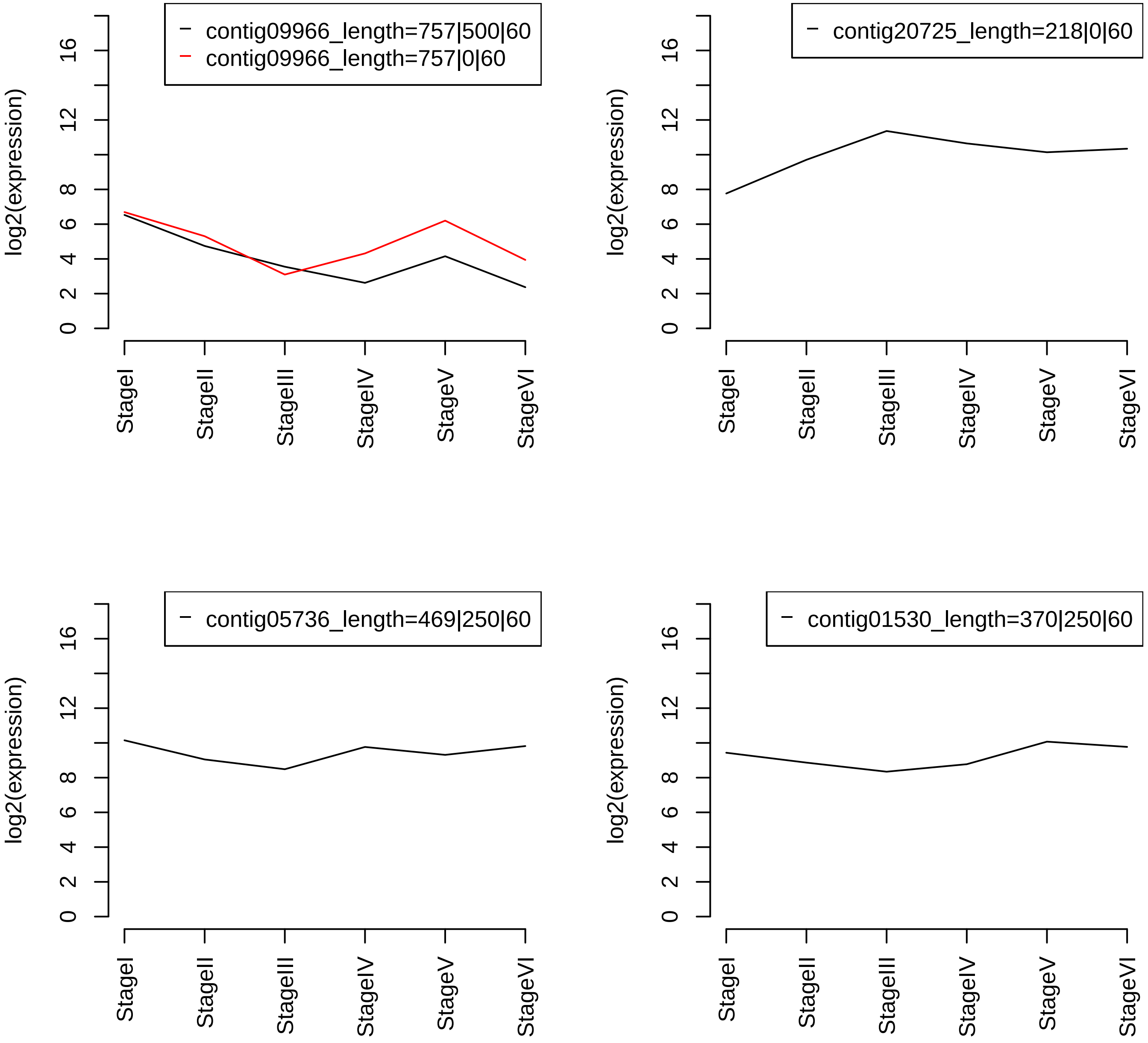

contig09966_length=757|500|60
contig09966_length=757|0|60
contig20725_length=218|0|60
contig05736_length=469|250|60
contig01530_length=370|250|60
log2(expression)
0
2
4
6
8
12
16
StageI
StageII
StageIII
StageIV
StageV
StageVI

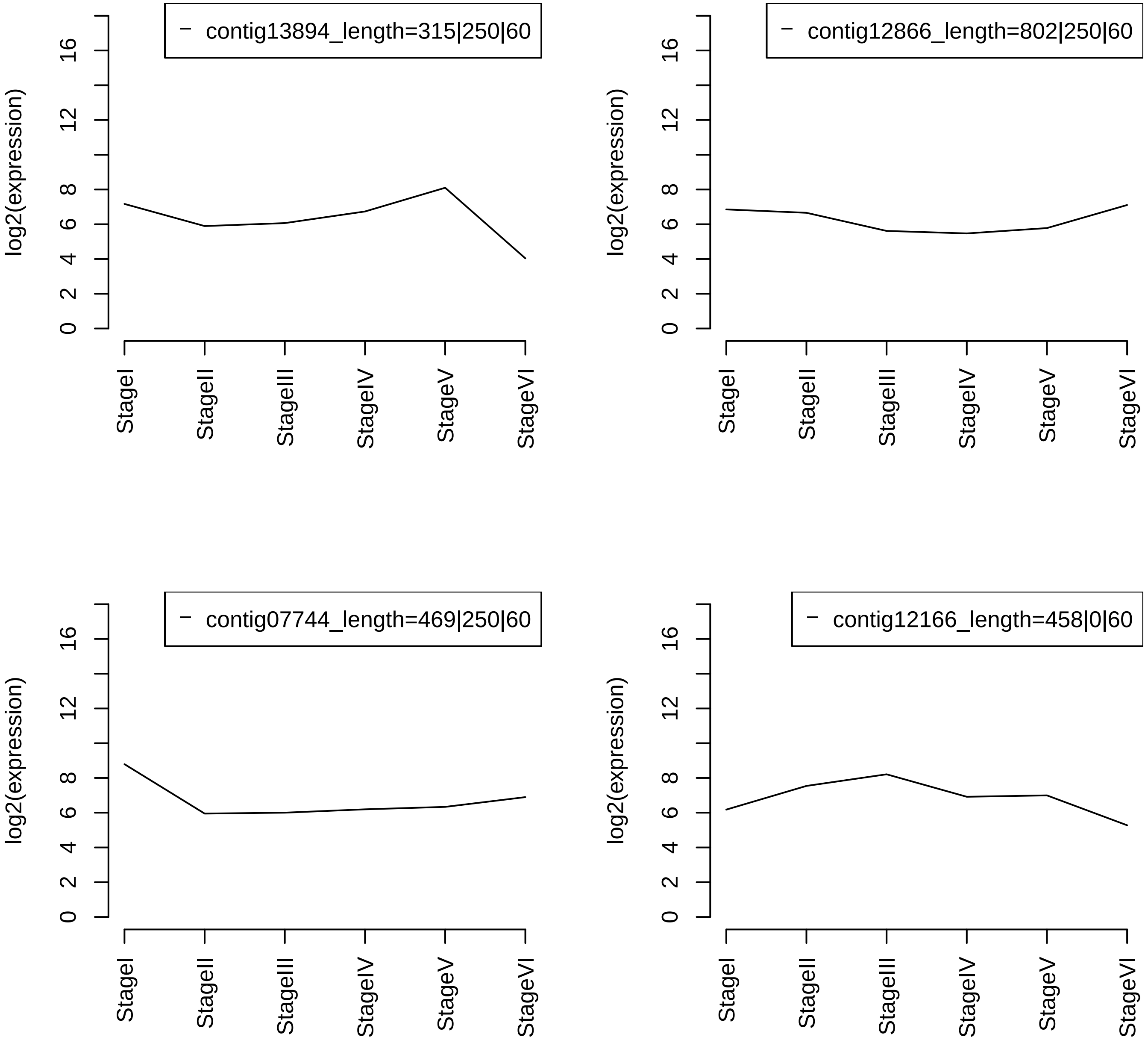
contig13894_length=315|250|60
contig12866_length=802|250|60
contig07744_length=469|250|60
contig12166_length=458|0|60
log2(expression)
0
2
4
6
8
12
16
StageI
StageII
StageIII
StageIV
StageV
StageVI

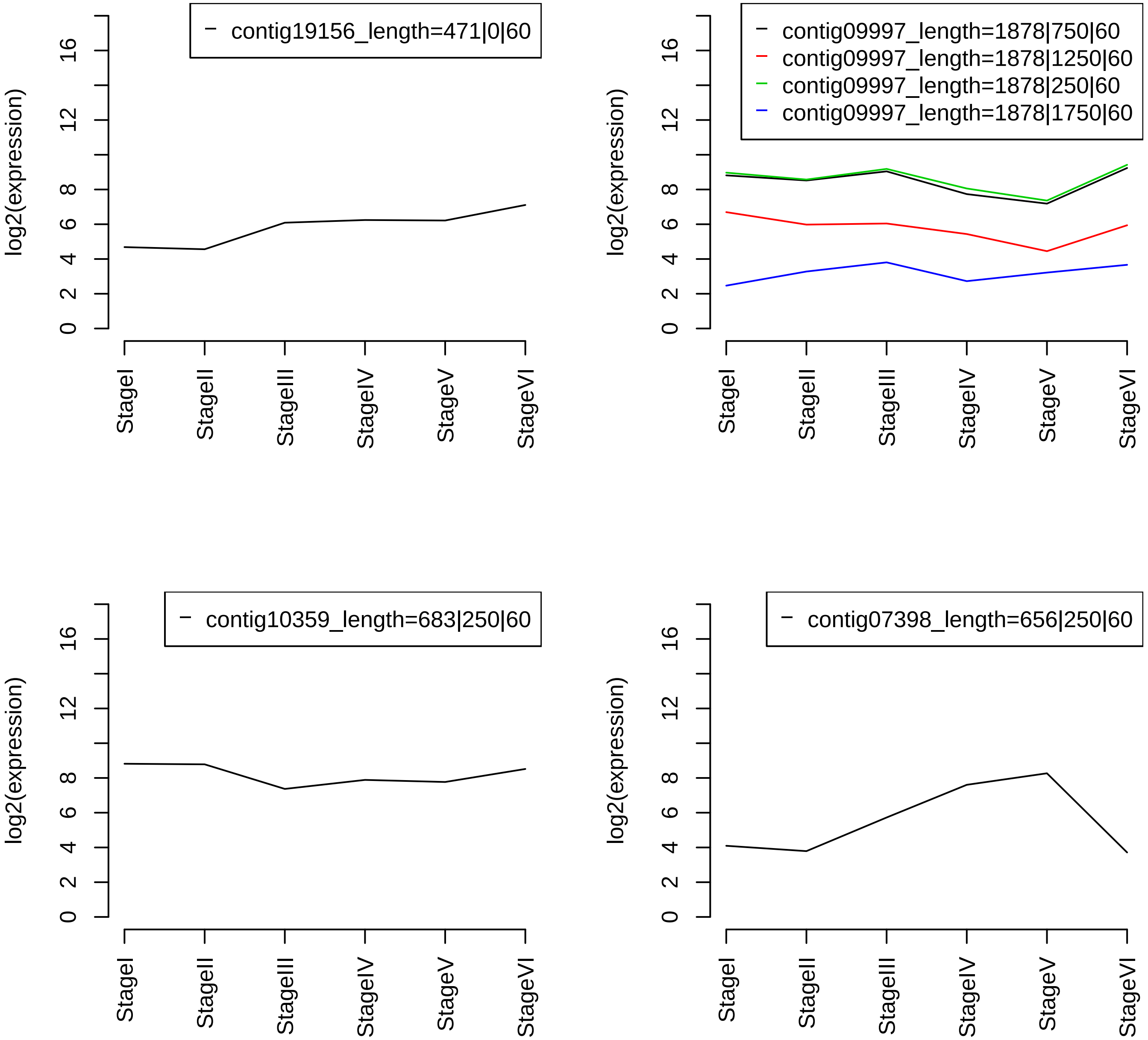

contig19156_length=471|0|60
contig09997_length=1878|750|60
contig09997_length=1878|1250|60
contig09997_length=1878|250|60
contig09997_length=1878|1750|60
contig10359_length=683|250|60
contig07398_length=656|250|60
log2(expression)
0
2
4
6
8
12
16
StageI
StageII
StageIII
StageIV
StageV
StageVI

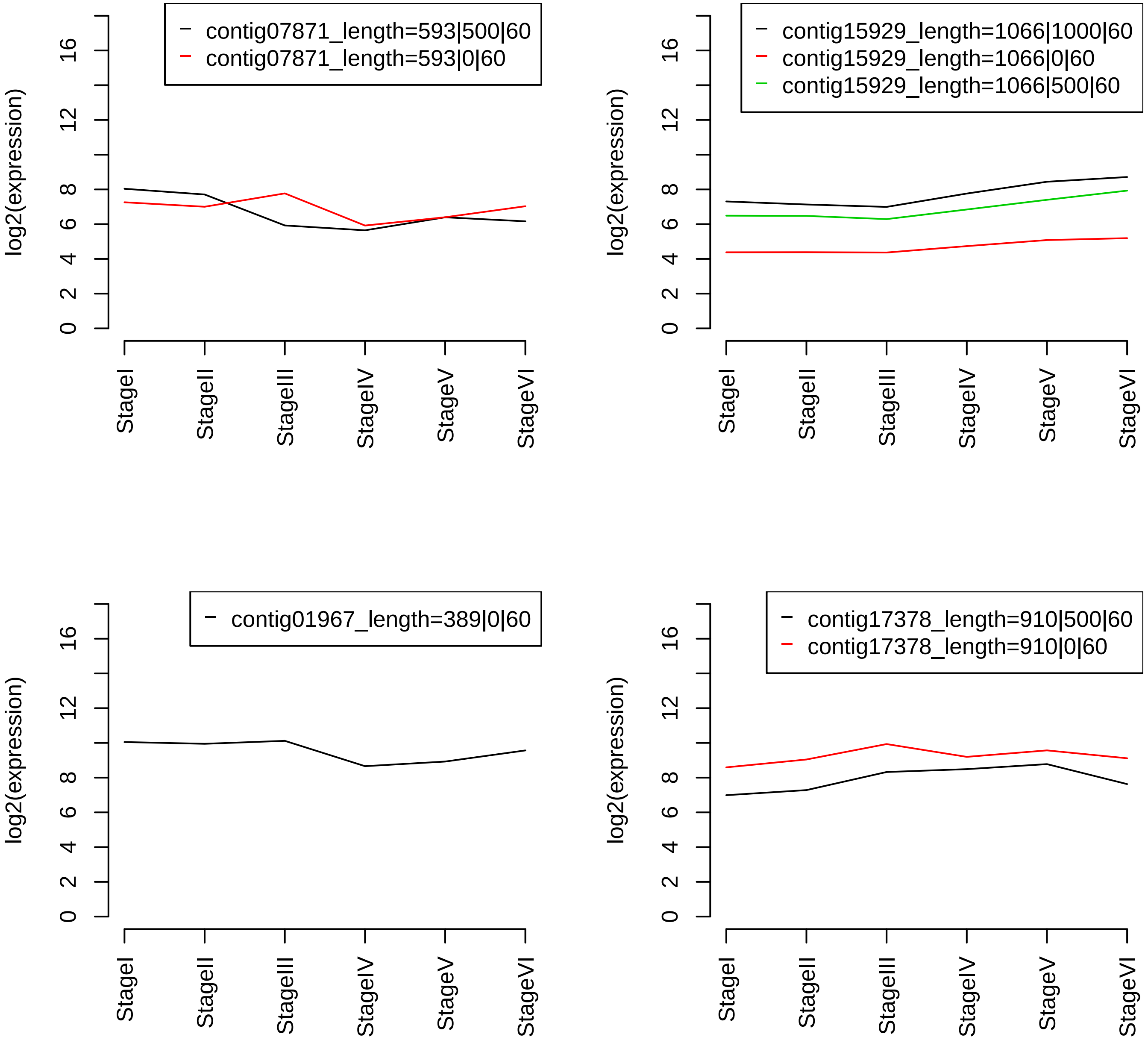
contig07871_length=593|500|60
contig07871_length=593|0|60
contig15929_length=1066|1000|60
contig15929_length=1066|0|60
contig15929_length=1066|500|60
contig01967_length=389|0|60
contig17378_length=910|500|60
contig17378_length=910|0|60
log2(expression)
0
2
4
6
8
12
16
StageI
StageII
StageIII
StageIV
StageV
StageVI

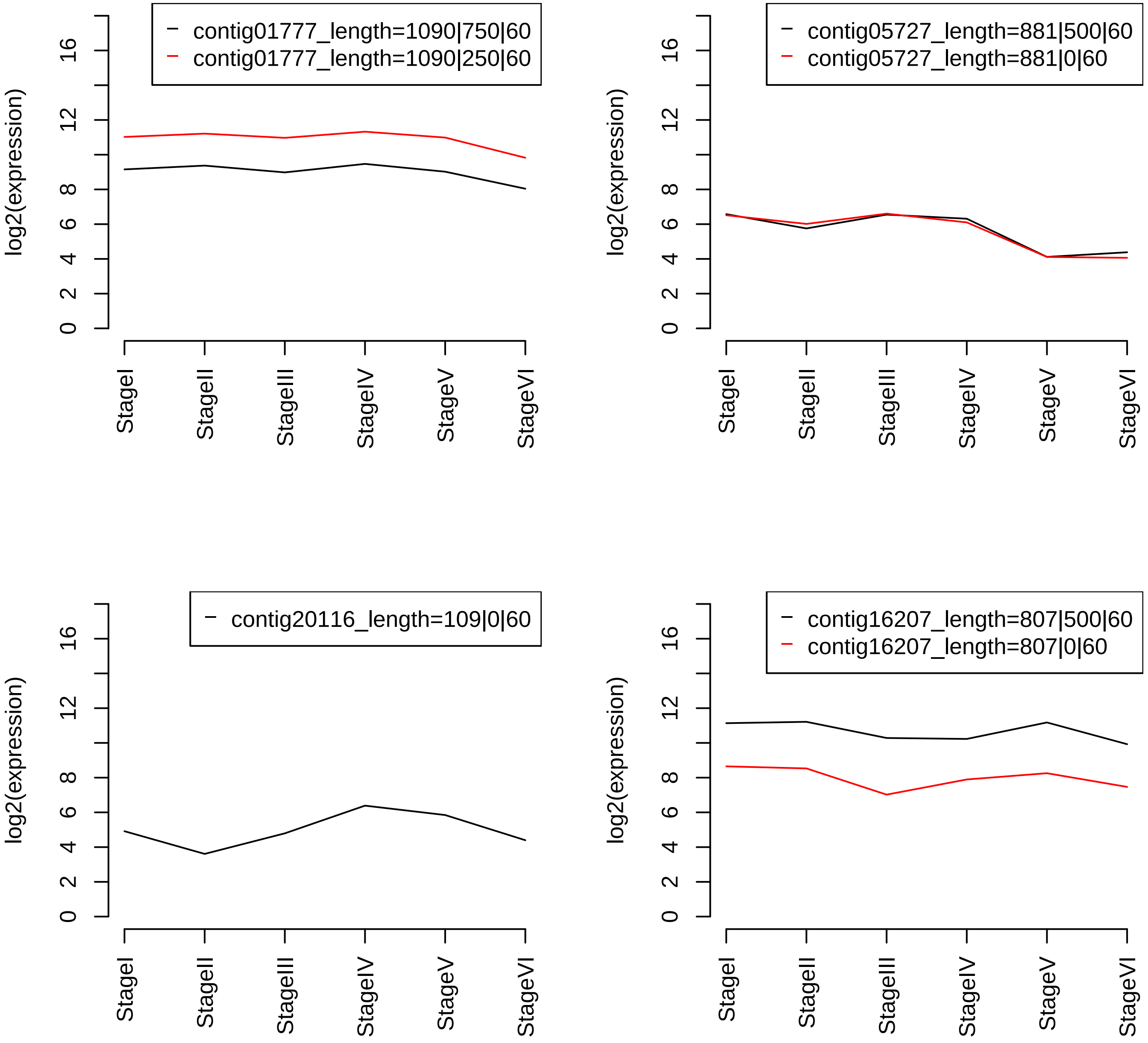
contig01777_length=1090|750|60
contig01777_length=1090|250|60
contig05727_length=881|500|60
contig05727_length=881|0|60
contig20116_length=109|0|60
contig16207_length=807|500|60
contig16207_length=807|0|60
log2(expression)
0 2 4 6 8 12 16
StageI StageII StageIII StageIV StageV StageVI

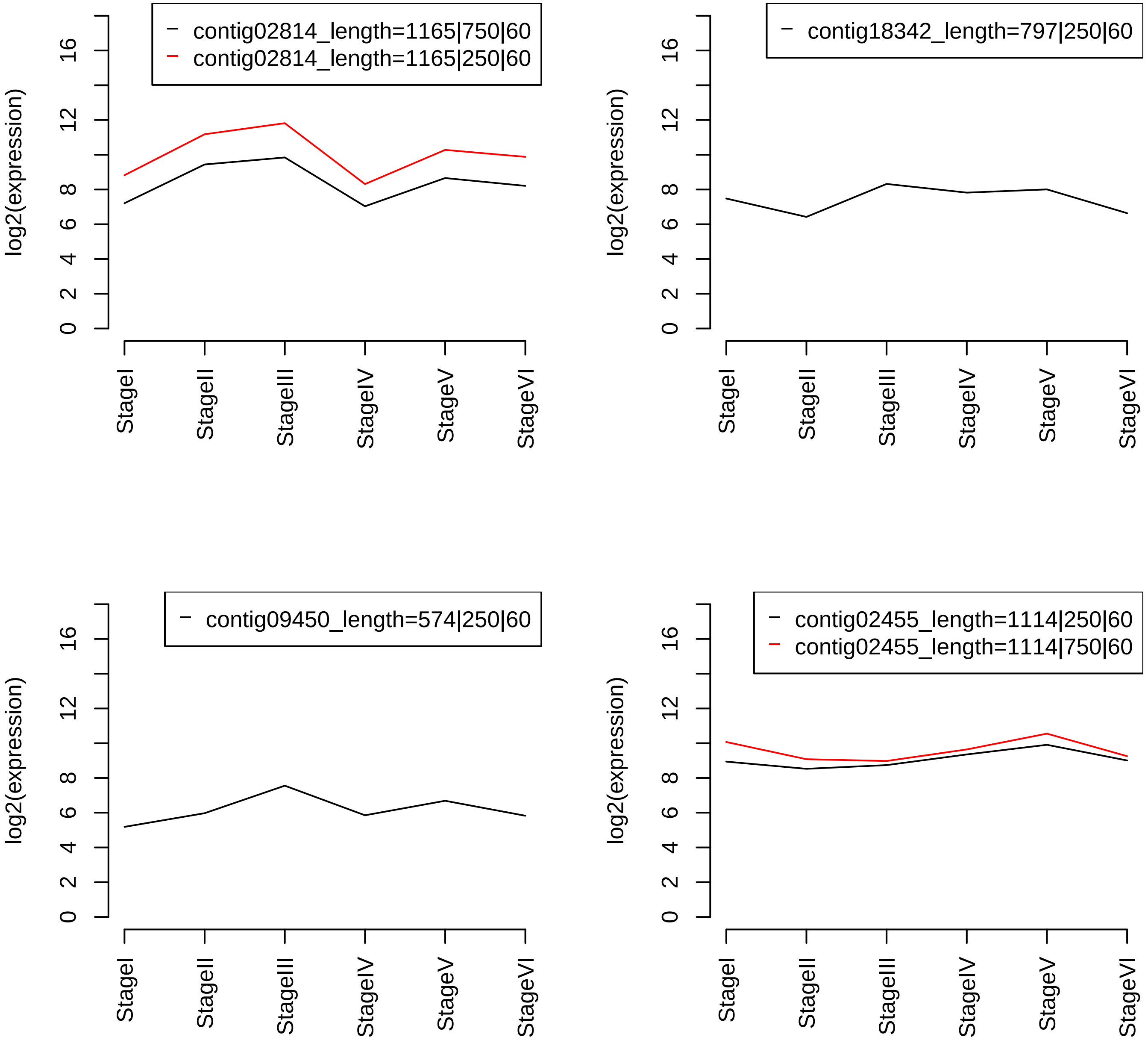

contig02814_length=1165|750|60
contig02814_length=1165|250|60
contig18342_length=797|250|60
contig09450_length=574|250|60
contig02455_length=1114|250|60
contig02455_length=1114|750|60
log2(expression)
0
2
4
6
8
12
16
StageI
StageII
StageIII
StageIV
StageV
StageVI

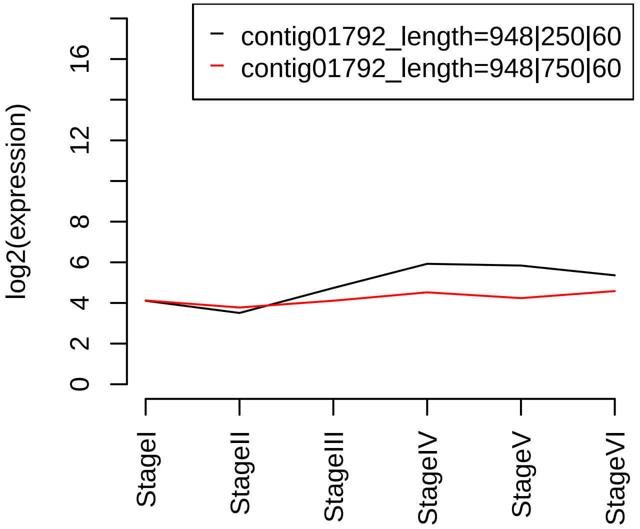

contig01792_length=948|250|60
contig01792_length=948|750|60
log2(expression)
0
2
4
6
8
12
16
StageI
StageII
StageIII
StageIV
StageV
StageVI

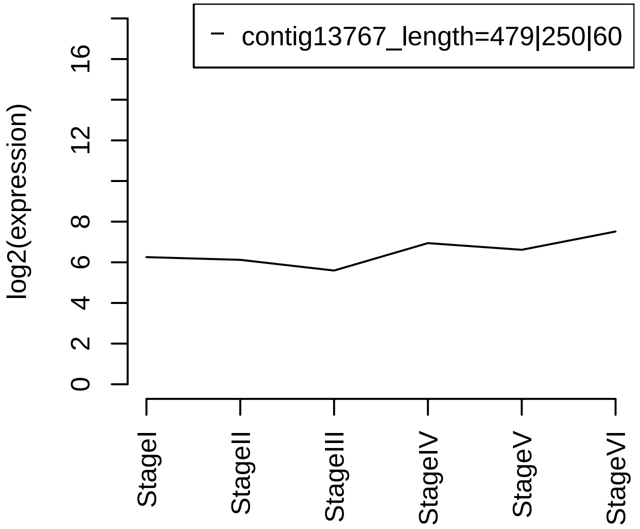

contig13767_length=479|250|60
log2(expression)
0
2
4
6
8
12
16
StageI
StageII
StageIII
StageIV
StageV
StageVI

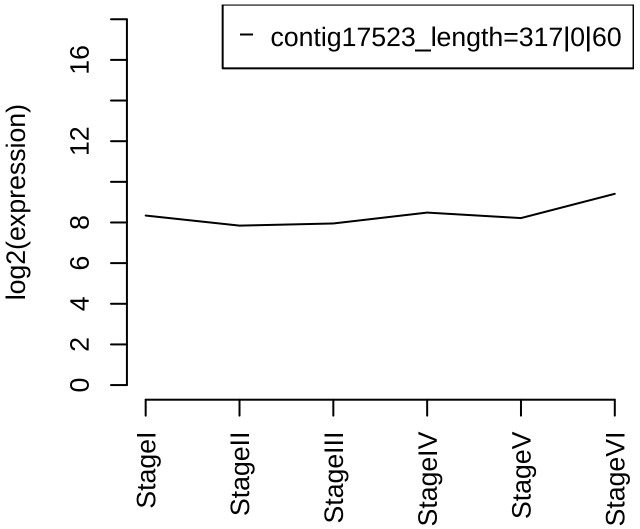

contig17523_length=317|0|60
log2(expression)
0
2
4
6
8
12
16
StageI
StageII
StageIII
StageIV
StageV
StageVI

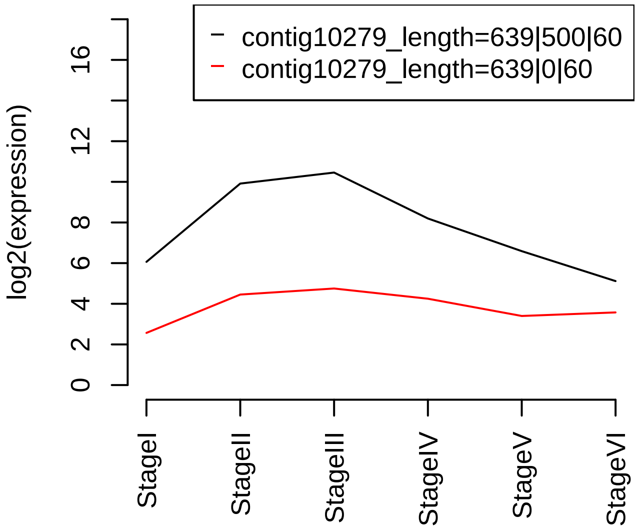

contig10279_length=639|500|60
contig10279_length=639|0|60
log2(expression)
0
2
4
6
8
12
16
StageI
StageII
StageIII
StageIV
StageV
StageVI

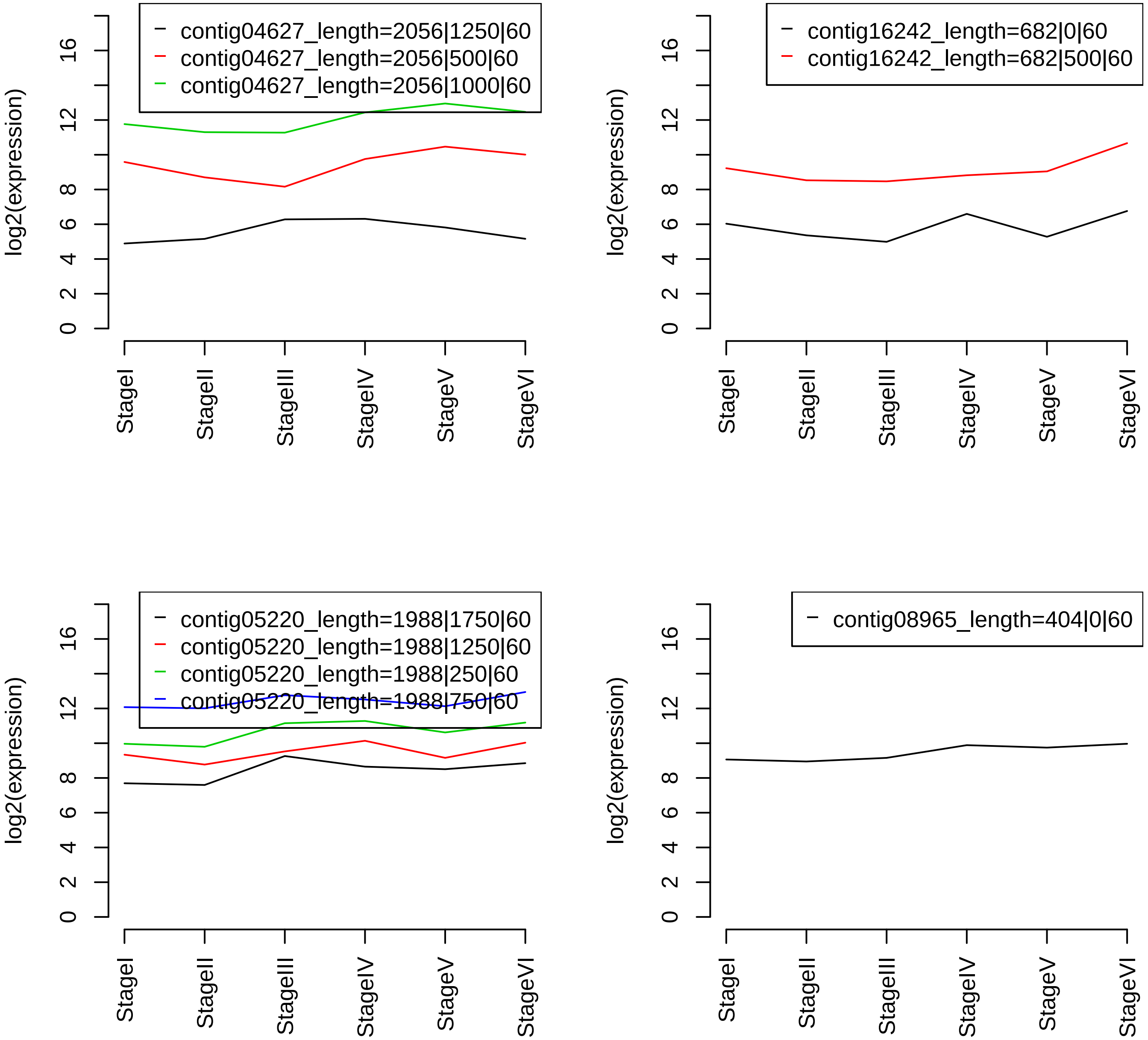
contig04627_length=2056|1250|60
contig04627_length=2056|500|60
contig04627_length=2056|1000|60
contig16242_length=682|0|60
contig16242_length=682|500|60
contig05220_length=1988|1750|60
contig05220_length=1988|1250|60
contig05220_length=1988|250|60
contig05220_length=1988|750|60
contig08965_length=404|0|60
log2(expression)
0 2 4 6 8 12 16
StageI StageII StageIII StageIV StageV StageVI

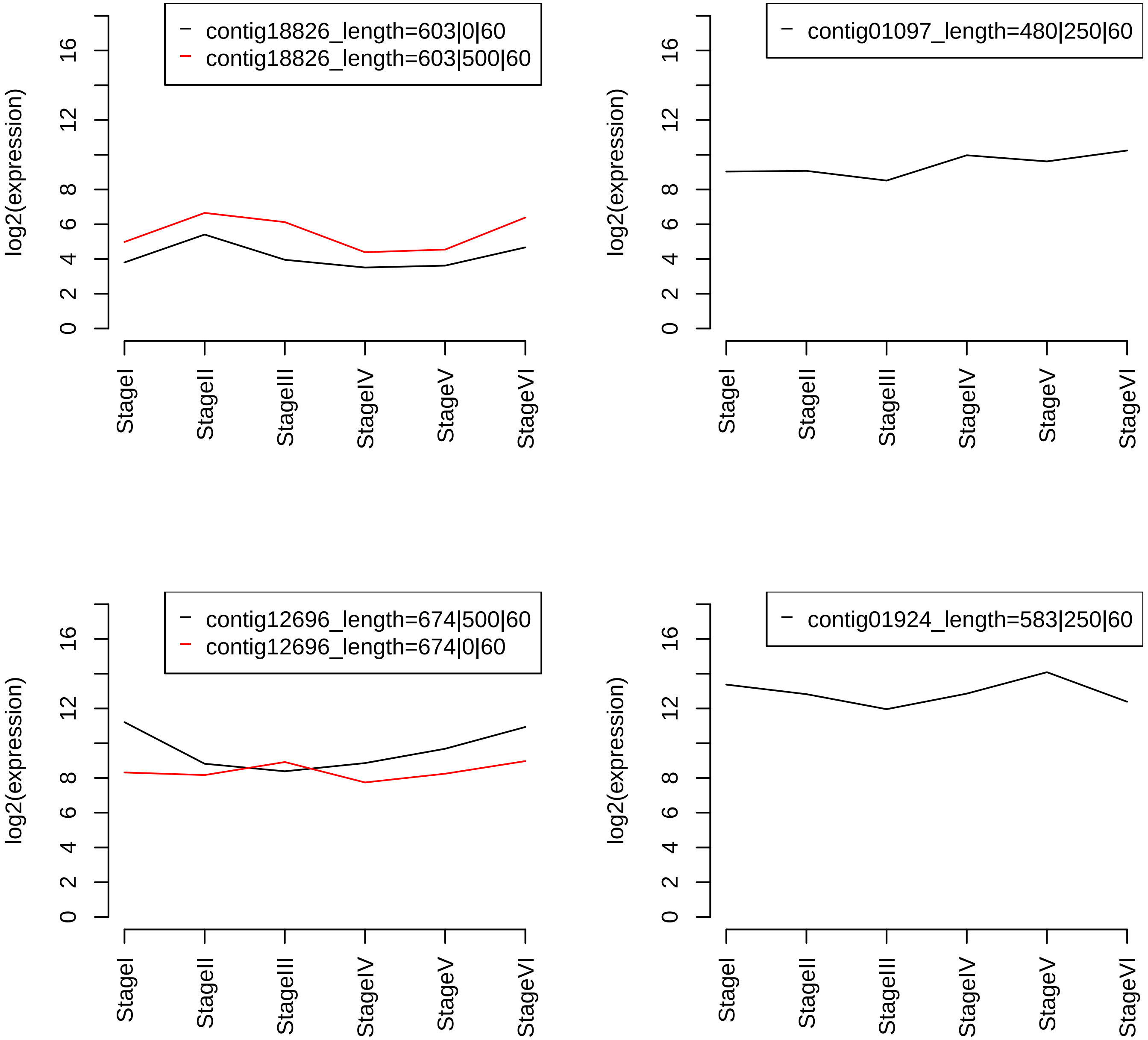

contig18826_length=603|0|60
contig18826_length=603|500|60
log2(expression)
0
2
4
6
8
12
16
StageI
StageII
StageIII
StageIV
StageV
StageVI
contig01097_length=480|250|60
contig12696_length=674|500|60
contig12696_length=674|0|60
contig01924_length=583|250|60

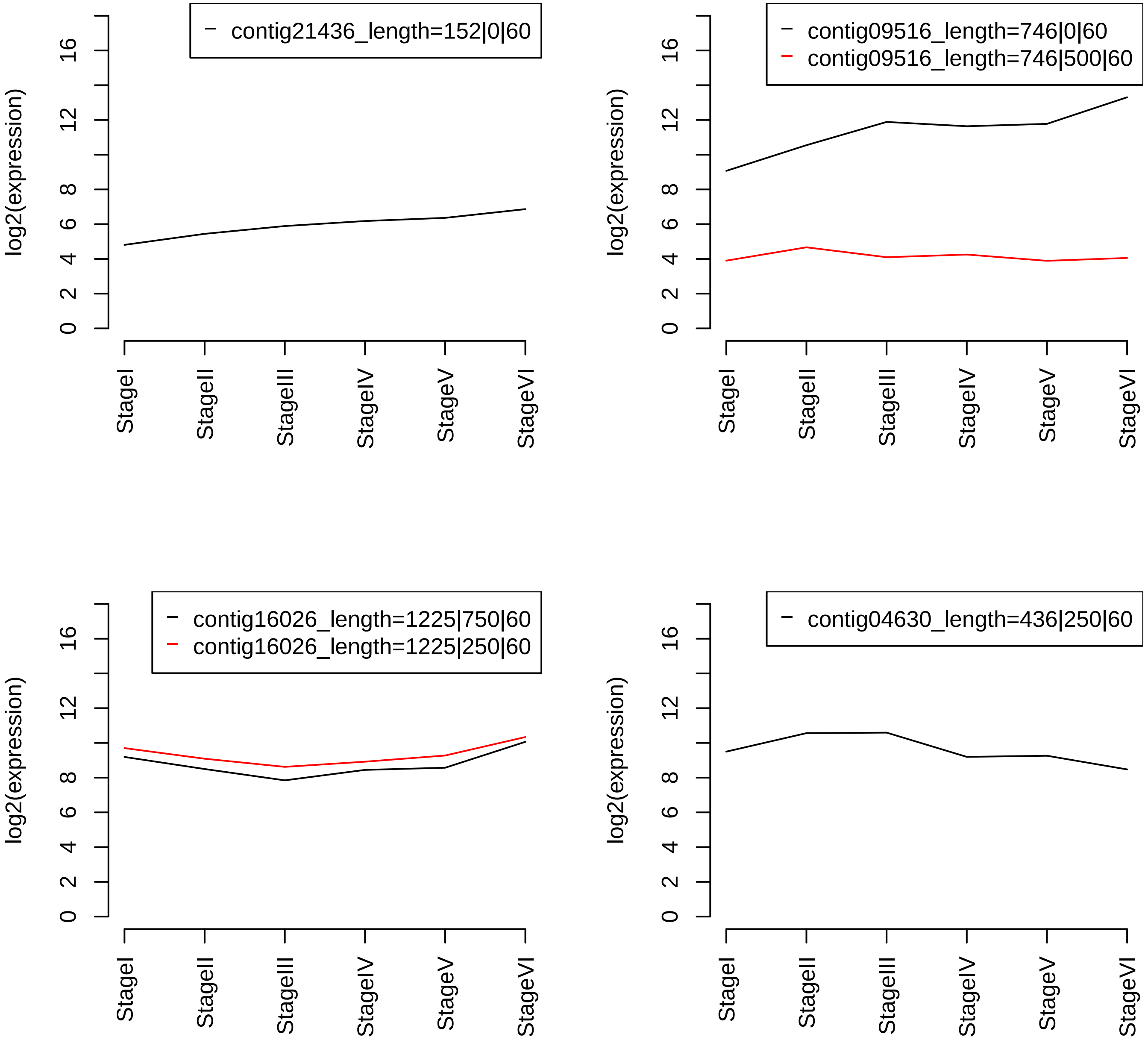
contig21436_length=152|0|60
contig09516_length=746|0|60
contig09516_length=746|500|60
contig16026_length=1225|750|60
contig16026_length=1225|250|60
contig04630_length=436|250|60
log2(expression)
0
2
4
6
8
12
16
StageI
StageII
StageIII
StageIV
StageV
StageVI

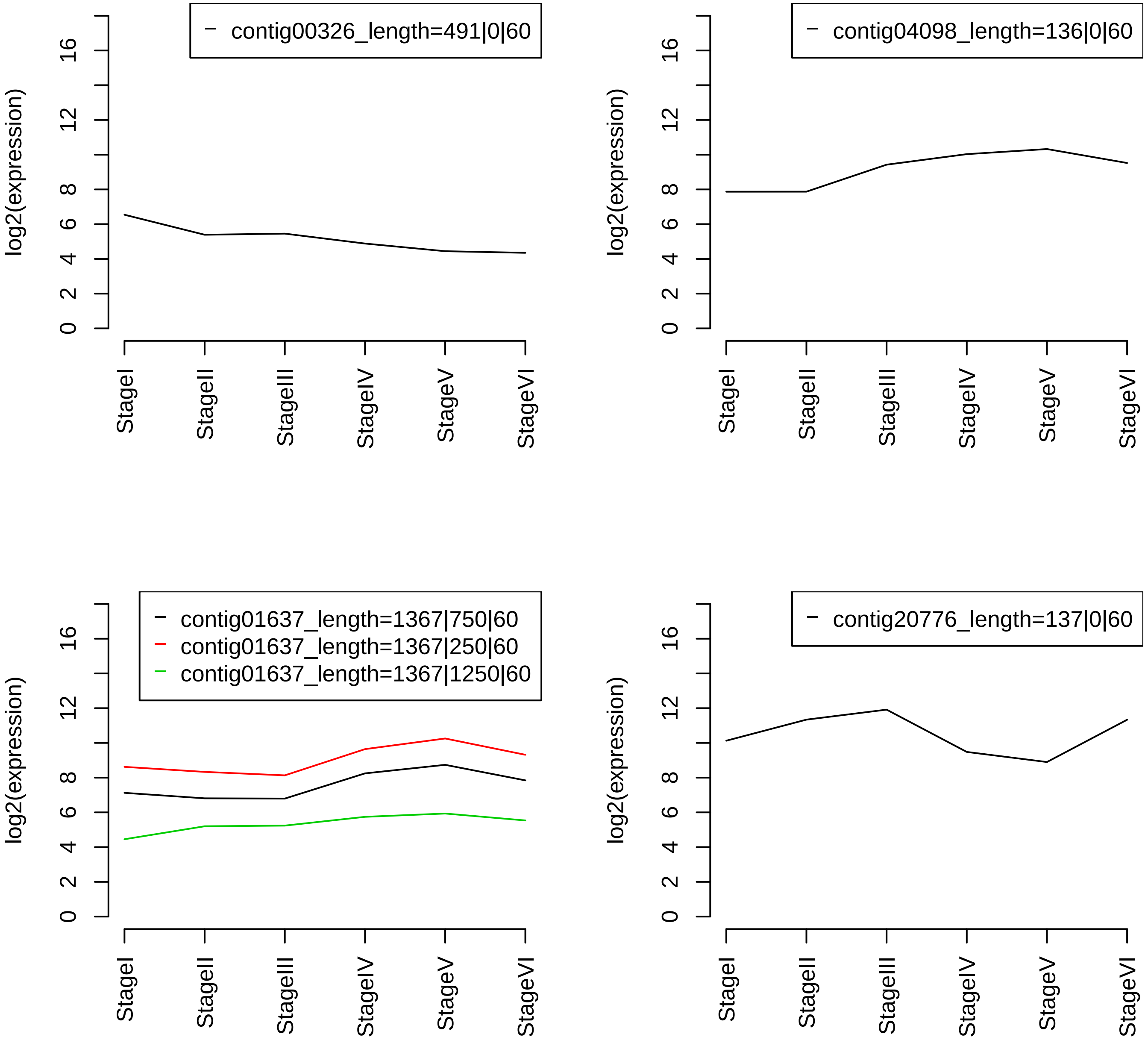

contig00326_length=491|0|60
contig04098_length=136|0|60
contig01637_length=1367|750|60
contig01637_length=1367|250|60
contig01637_length=1367|1250|60
contig20776_length=137|0|60
log2(expression)
0
2
4
6
8
12
16
StageI
StageII
StageIII
StageIV
StageV
StageVI

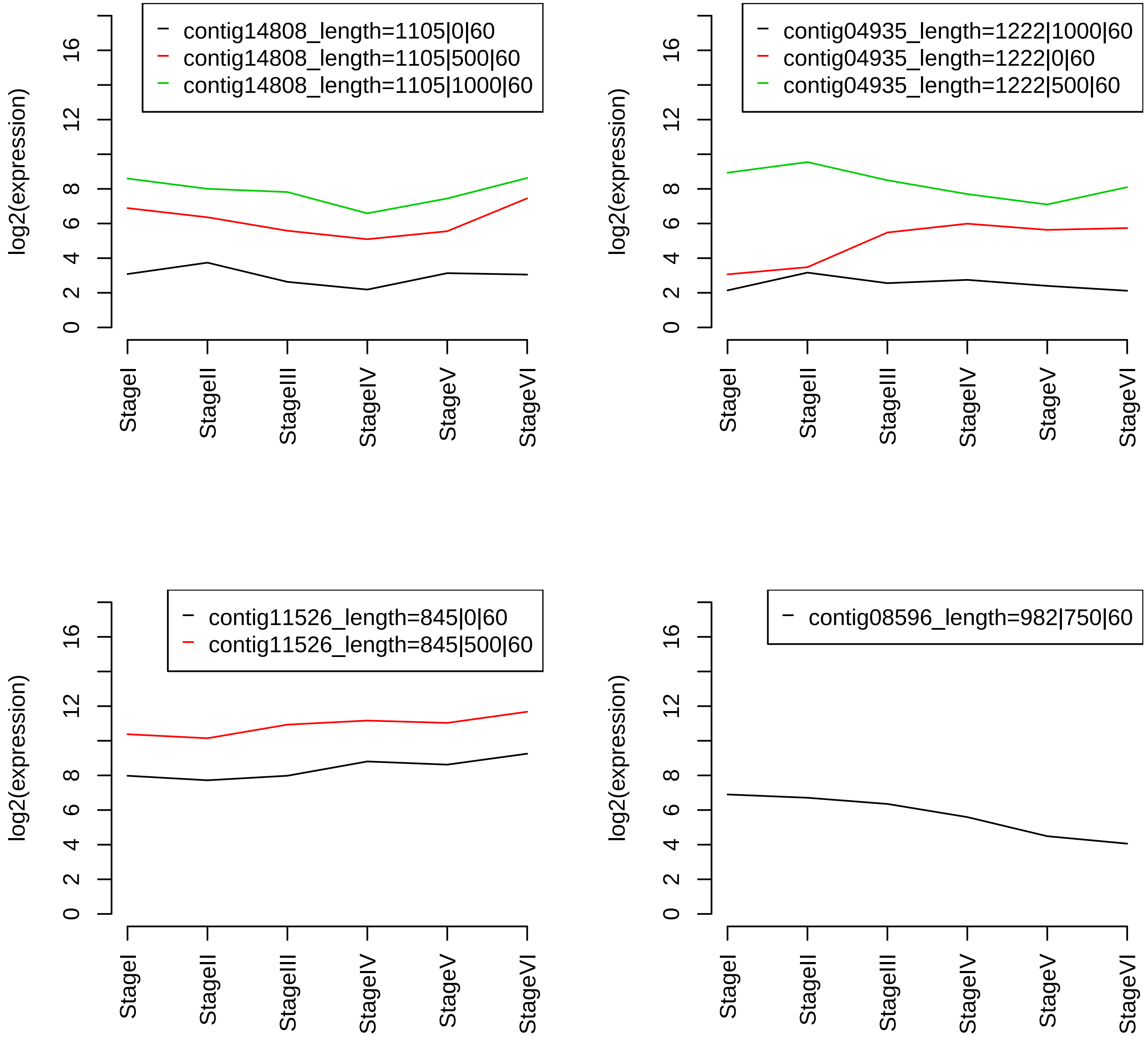

contig14808_length=1105|0|60
contig14808_length=1105|500|60
contig14808_length=1105|1000|60
contig04935_length=1222|1000|60
contig04935_length=1222|0|60
contig04935_length=1222|500|60
contig11526_length=845|0|60
contig11526_length=845|500|60
contig08596_length=982|750|60
log2(expression)
0
2
4
6
8
12
16
StageI
StageII
StageIII
StageIV
StageV
StageVI

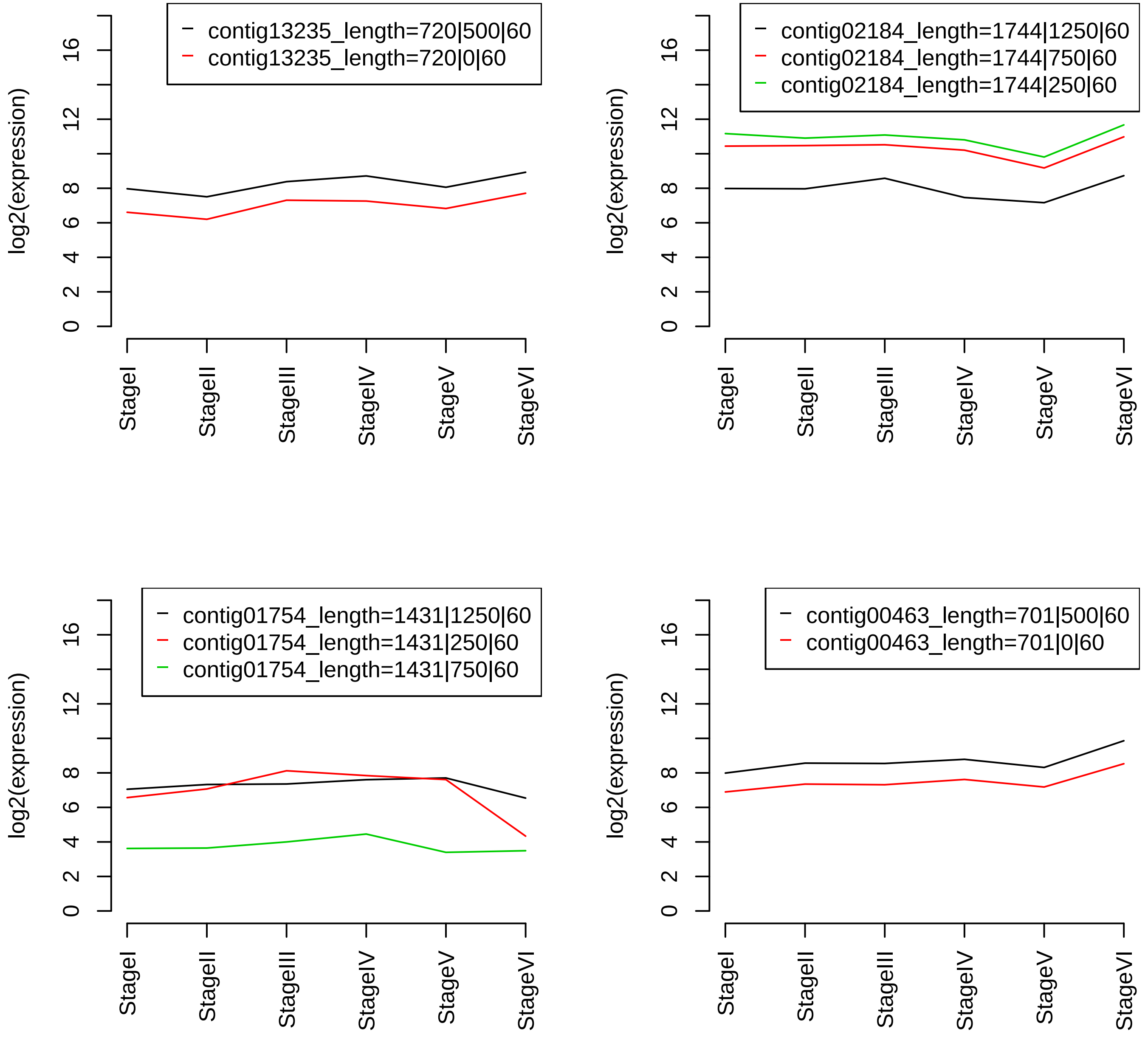

contig13235_length=720|500|60
contig13235_length=720|0|60
log2(expression)
0
2
4
6
8
12
16
StageI
StageII
StageIII
StageIV
StageV
StageVI
contig02184_length=1744|1250|60
contig02184_length=1744|750|60
contig02184_length=1744|250|60
log2(expression)
contig01754_length=1431|1250|60
contig01754_length=1431|250|60
contig01754_length=1431|750|60
log2(expression)
contig00463_length=701|500|60
contig00463_length=701|0|60
log2(expression)

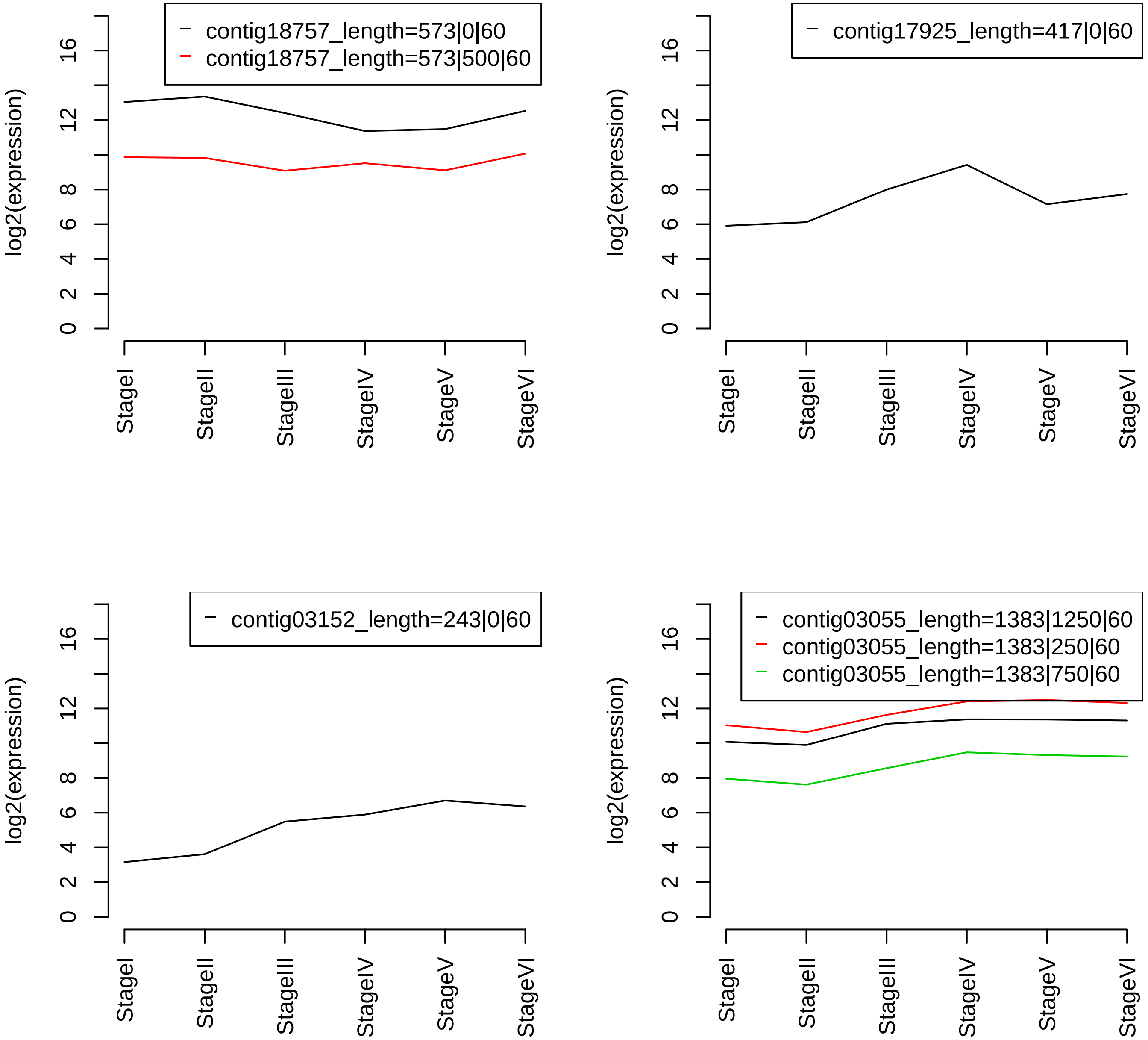

contig18757_length=573|0|60
contig18757_length=573|500|60
contig17925_length=417|0|60
contig03152_length=243|0|60
contig03055_length=1383|1250|60
contig03055_length=1383|250|60
contig03055_length=1383|750|60
log2(expression)
0
2
4
6
8
12
16
StageI
StageII
StageIII
StageIV
StageV
StageVI

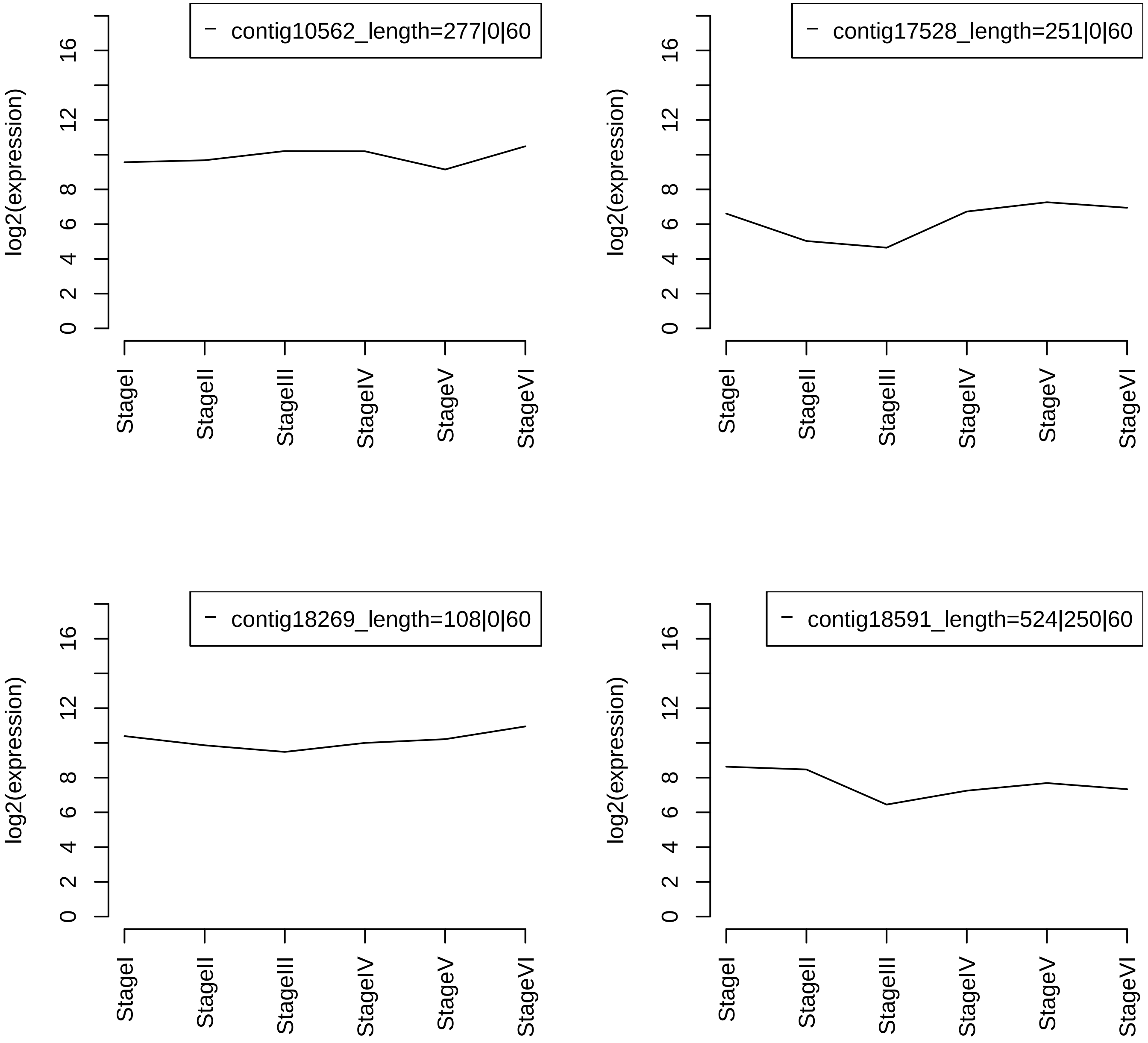

contig10562_length=277|0|60
contig17528_length=251|0|60
contig18269_length=108|0|60
contig18591_length=524|250|60
log2(expression)
0
2
4
6
8
12
16
StageI
StageII
StageIII
StageIV
StageV
StageVI

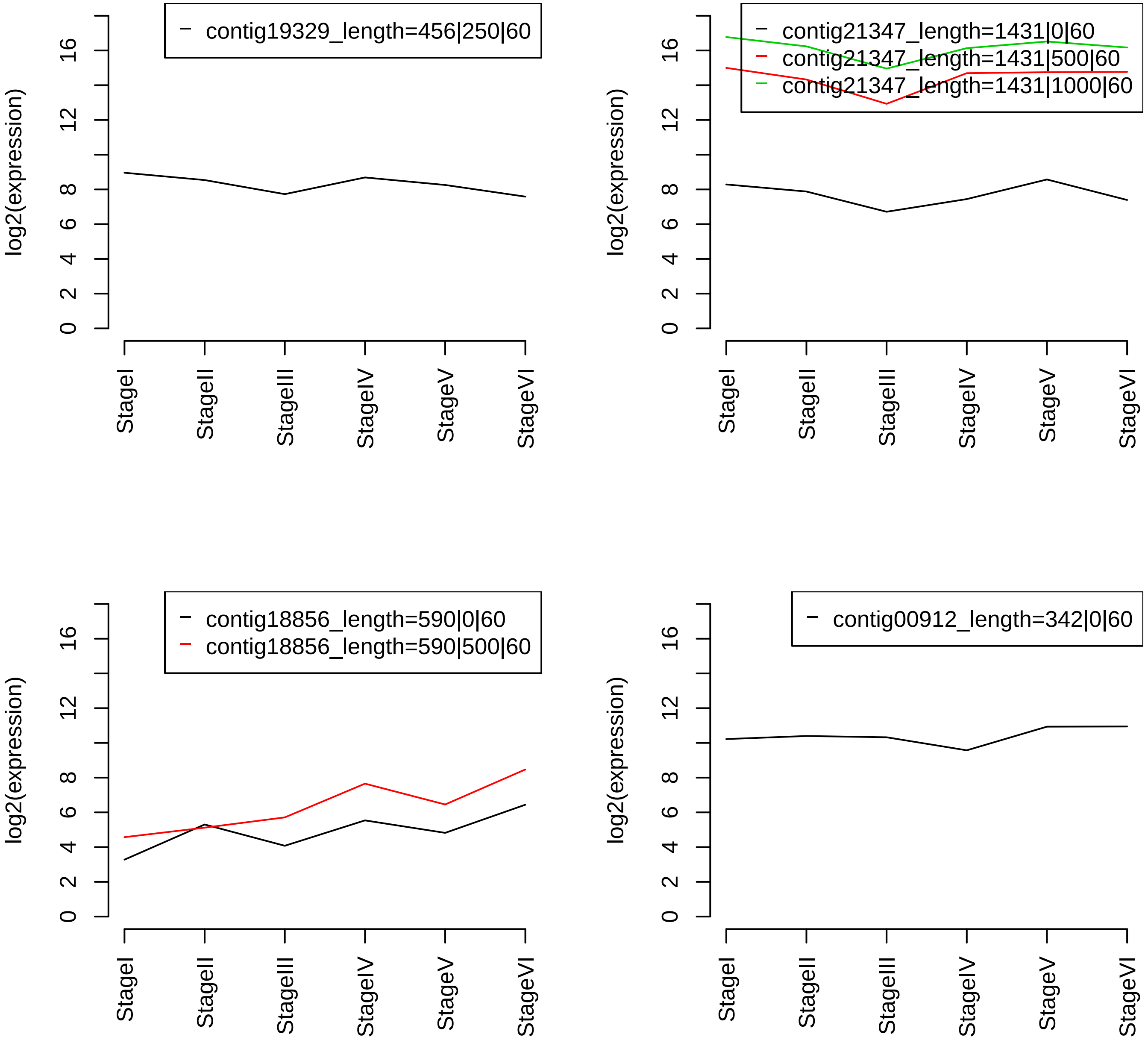
contig19329_length=456|250|60
contig21347_length=1431|0|60
contig21347_length=1431|500|60
contig21347_length=1431|1000|60
contig18856_length=590|0|60
contig18856_length=590|500|60
contig00912_length=342|0|60
log2(expression)
0
2
4
6
8
12
16
StageI
StageII
StageIII
StageIV
StageV
StageVI

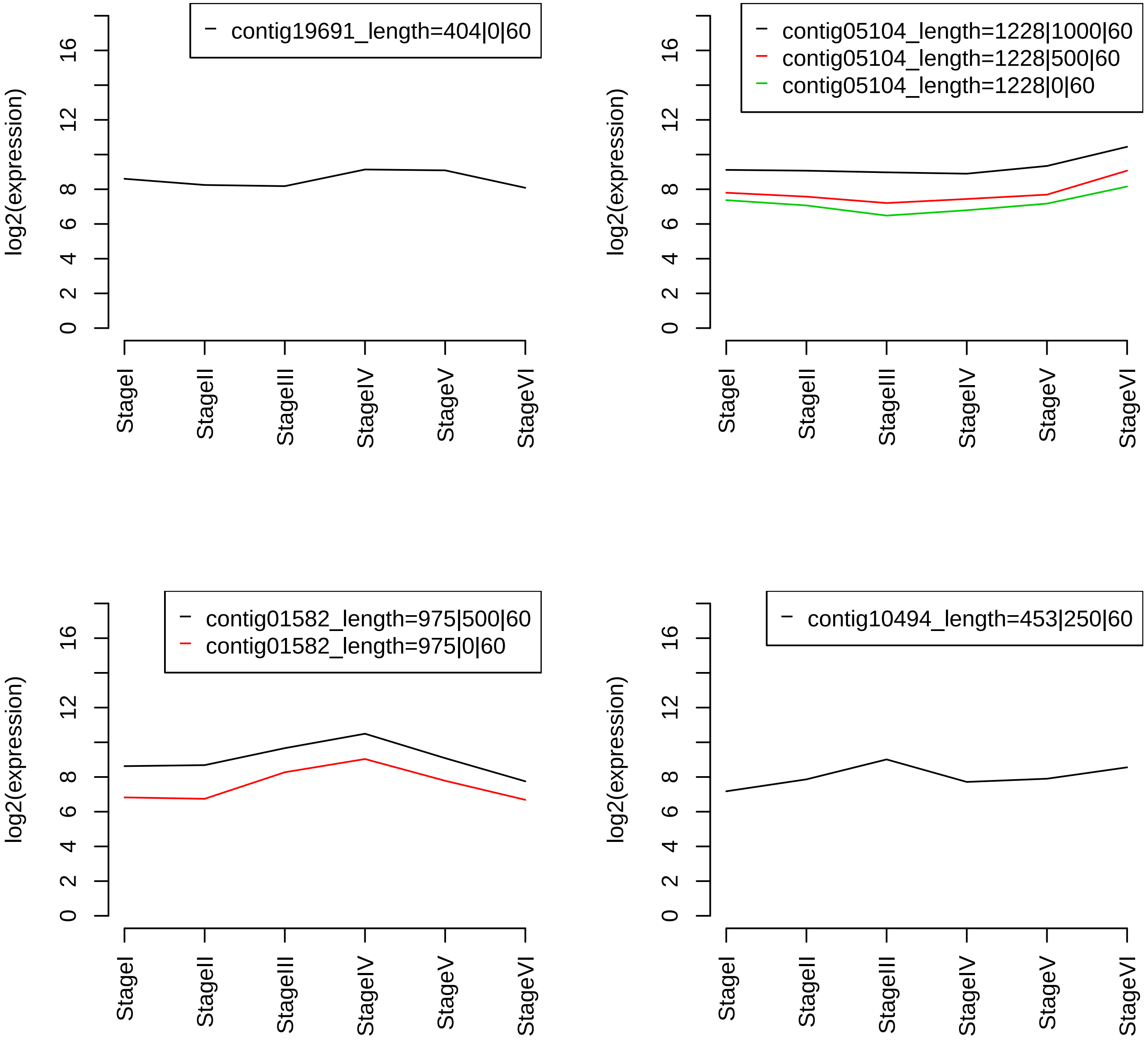
contig19691_length=404|0|60
contig05104_length=1228|1000|60
contig05104_length=1228|500|60
contig05104_length=1228|0|60
contig01582_length=975|500|60
contig01582_length=975|0|60
contig10494_length=453|250|60
log2(expression)
0
2
4
6
8
12
16
StageI
StageII
StageIII
StageIV
StageV
StageVI

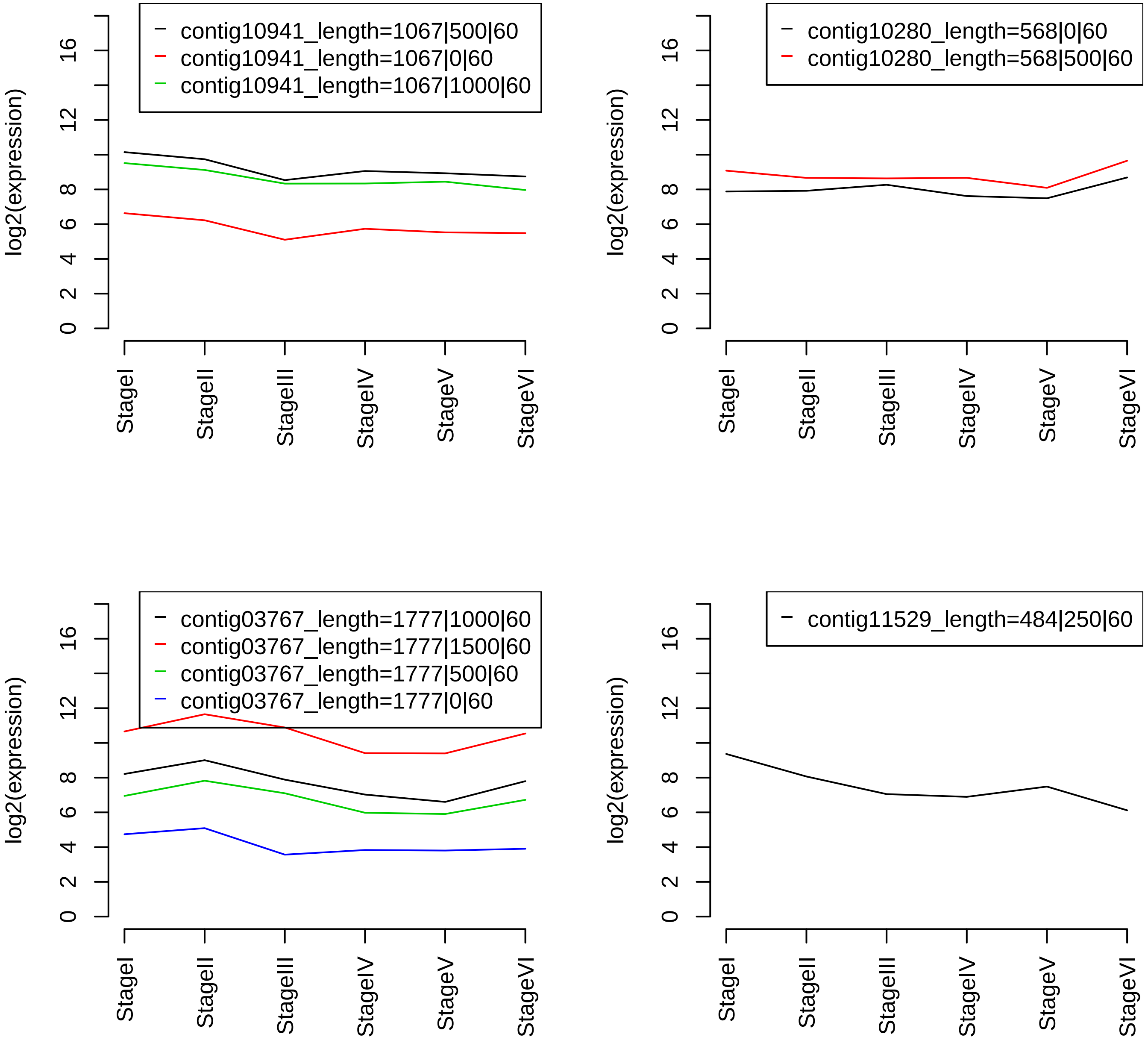
contig10941_length=1067|500|60
contig10941_length=1067|0|60
contig10941_length=1067|1000|60
contig10280_length=568|0|60
contig10280_length=568|500|60
contig03767_length=1777|1000|60
contig03767_length=1777|1500|60
contig03767_length=1777|500|60
contig03767_length=1777|0|60
contig11529_length=484|250|60
log2(expression)
StageI
StageII
StageIII
StageIV
StageV
StageVI
0
2
4
6
8
12
16

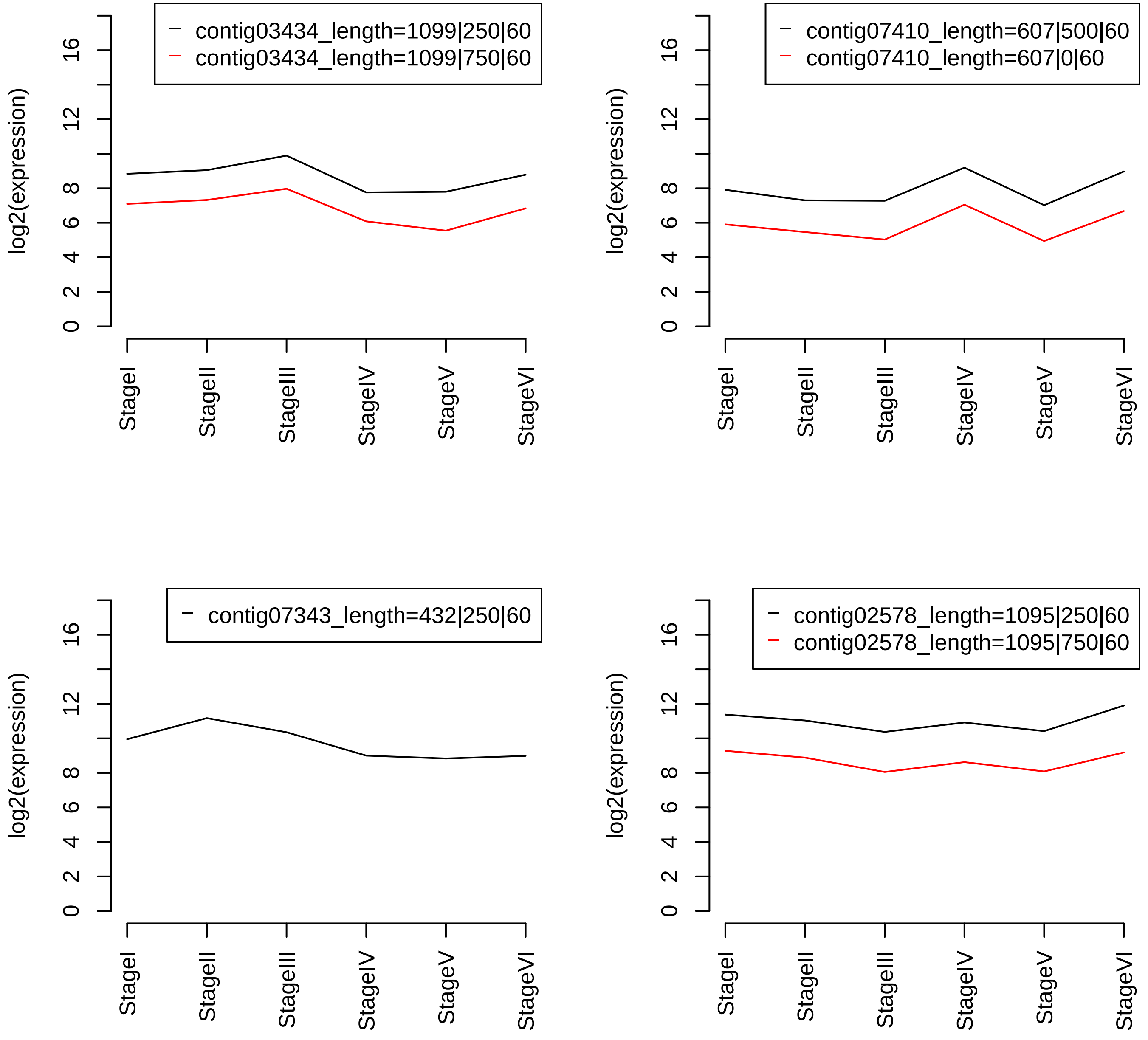
contig03434_length=1099|250|60
contig03434_length=1099|750|60
contig07410_length=607|500|60
contig07410_length=607|0|60
contig07343_length=432|250|60
contig02578_length=1095|250|60
contig02578_length=1095|750|60
log2(expression)
0
2
4
6
8
12
16
StageI
StageII
StageIII
StageIV
StageV
StageVI

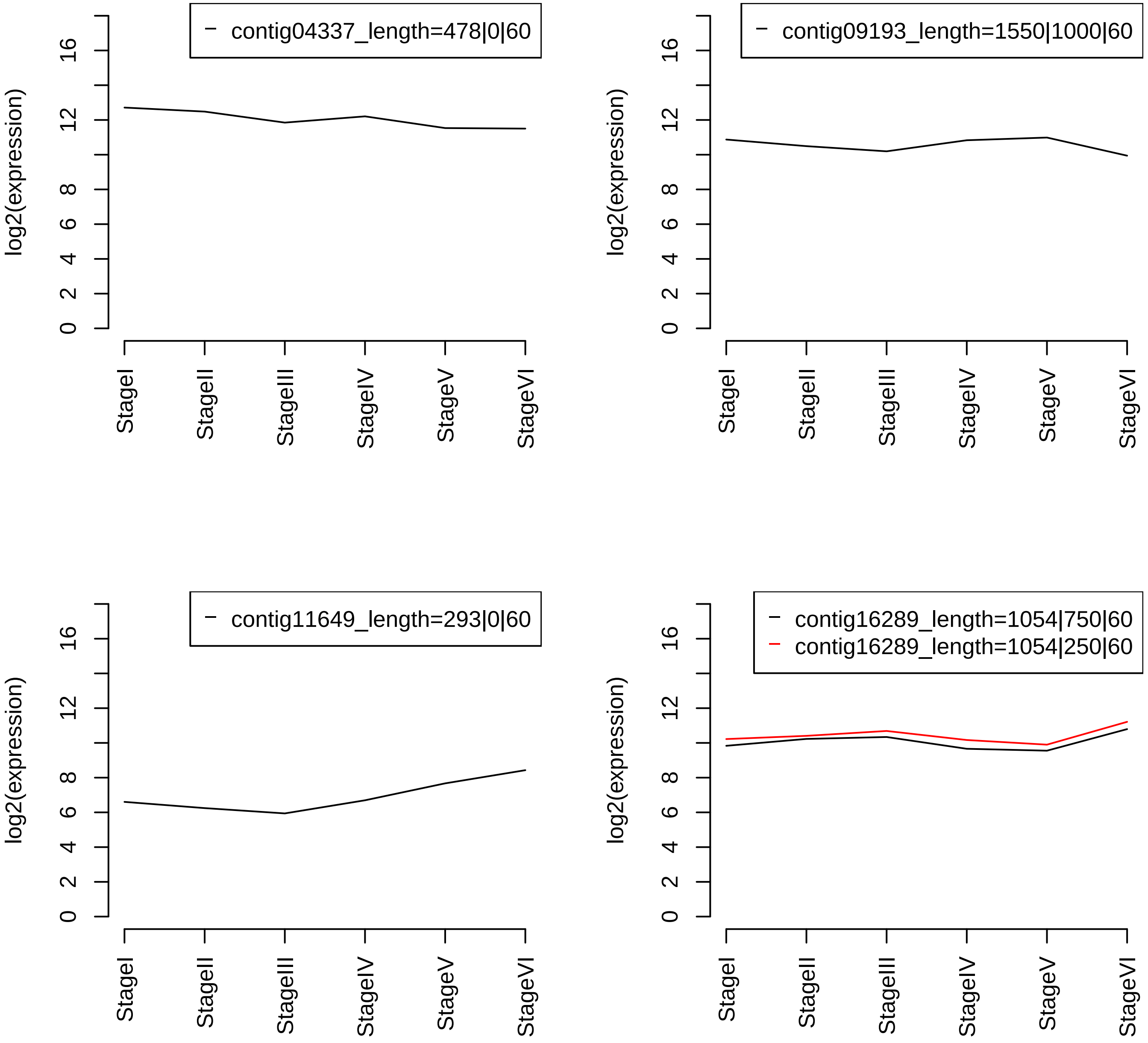
contig04337_length=478|0|60
contig09193_length=1550|1000|60
contig11649_length=293|0|60
contig16289_length=1054|750|60
contig16289_length=1054|250|60
log2(expression)
0
2
4
6
8
12
16
StageI
StageII
StageIII
StageIV
StageV
StageVI

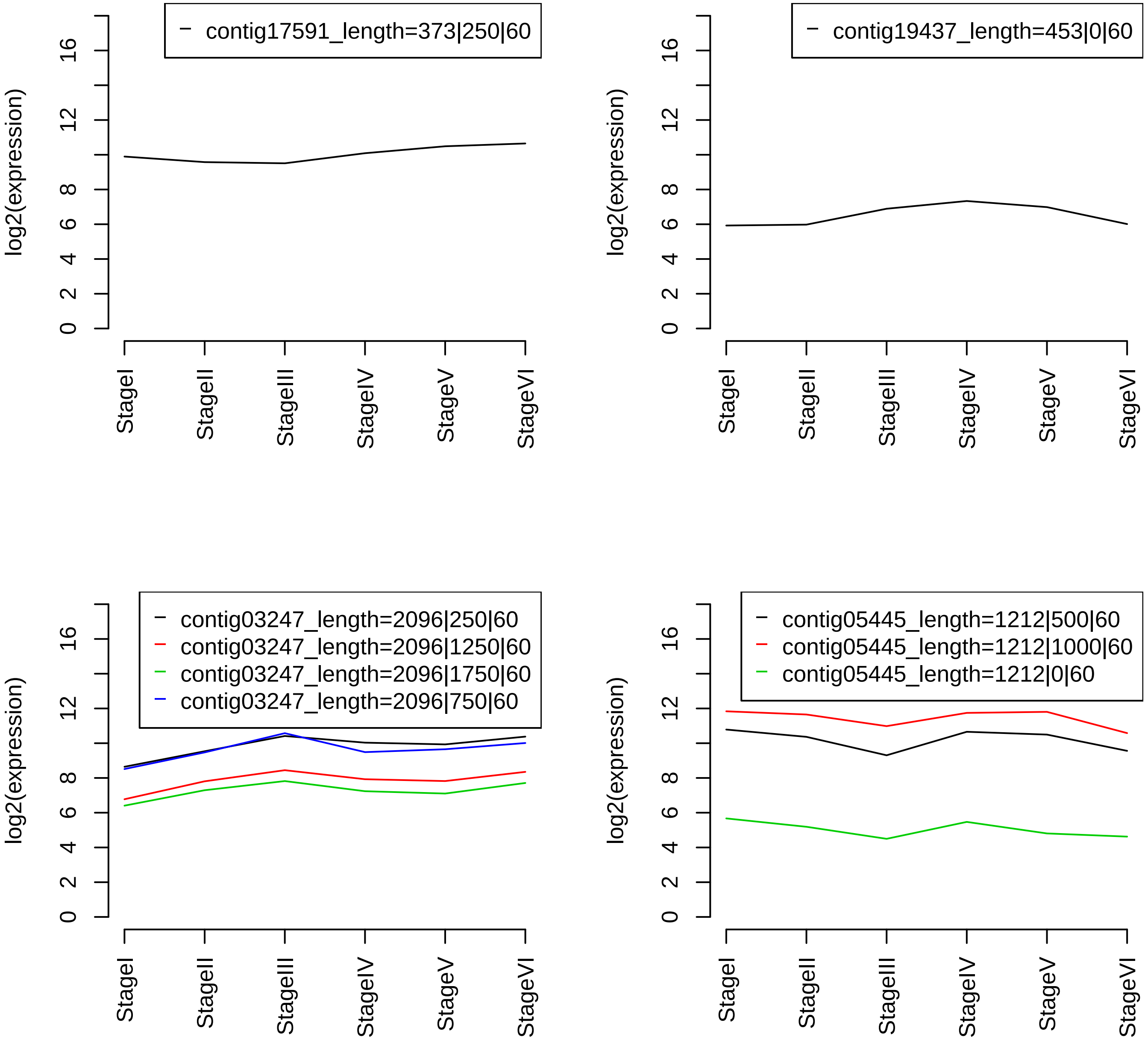
contig17591_length=373|250|60
contig19437_length=453|0|60
contig03247_length=2096|250|60
contig03247_length=2096|1250|60
contig03247_length=2096|1750|60
contig03247_length=2096|750|60
contig05445_length=1212|500|60
contig05445_length=1212|1000|60
contig05445_length=1212|0|60
log2(expression)
0
2
4
6
8
12
16
StageI
StageII
StageIII
StageIV
StageV
StageVI

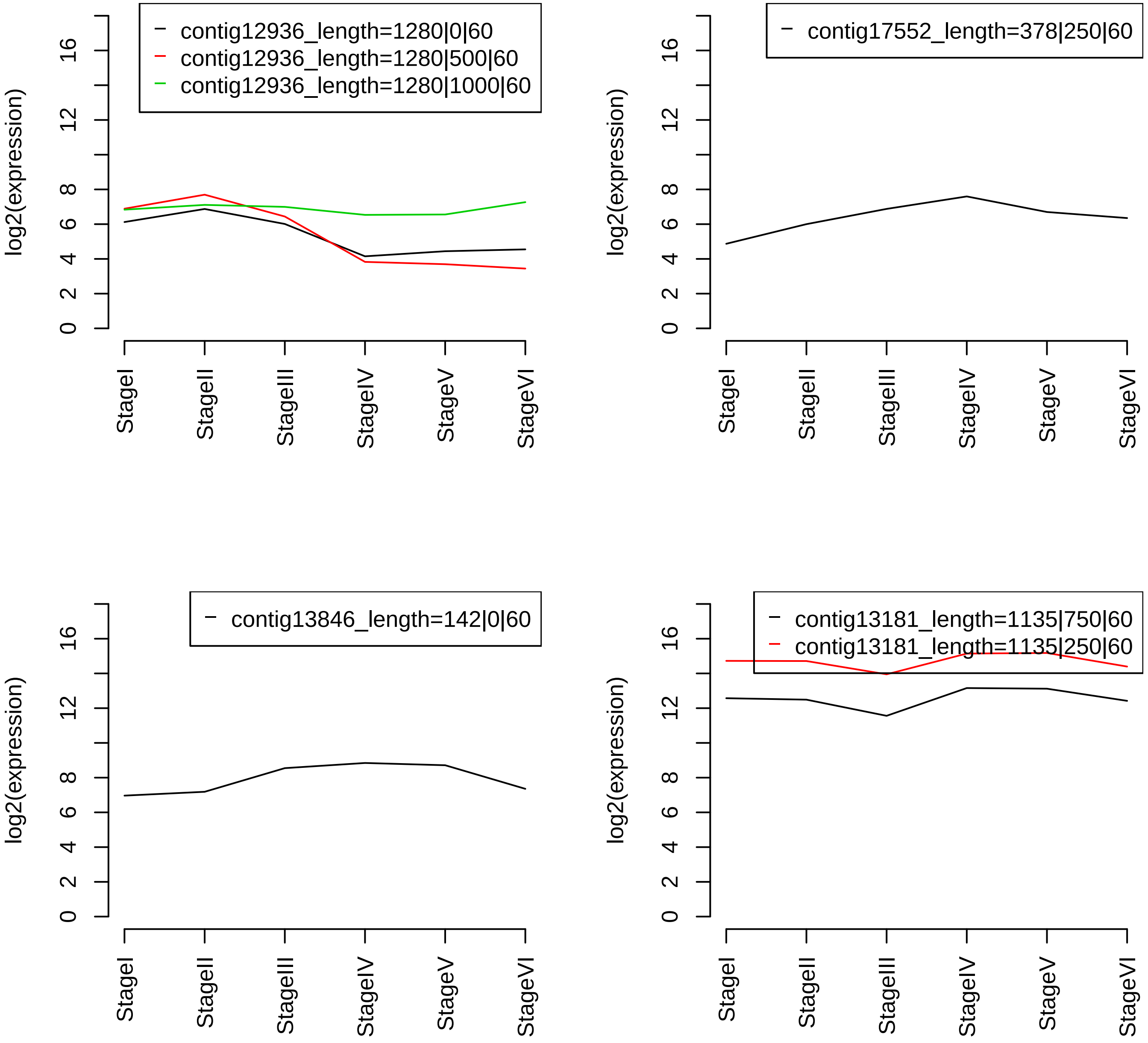
contig12936_length=1280|0|60
contig12936_length=1280|500|60
contig12936_length=1280|1000|60
contig17552_length=378|250|60
contig13846_length=142|0|60
contig13181_length=1135|750|60
contig13181_length=1135|250|60
log2(expression)
0 2 4 6 8 12 16
StageI StageII StageIII StageIV StageV StageVI

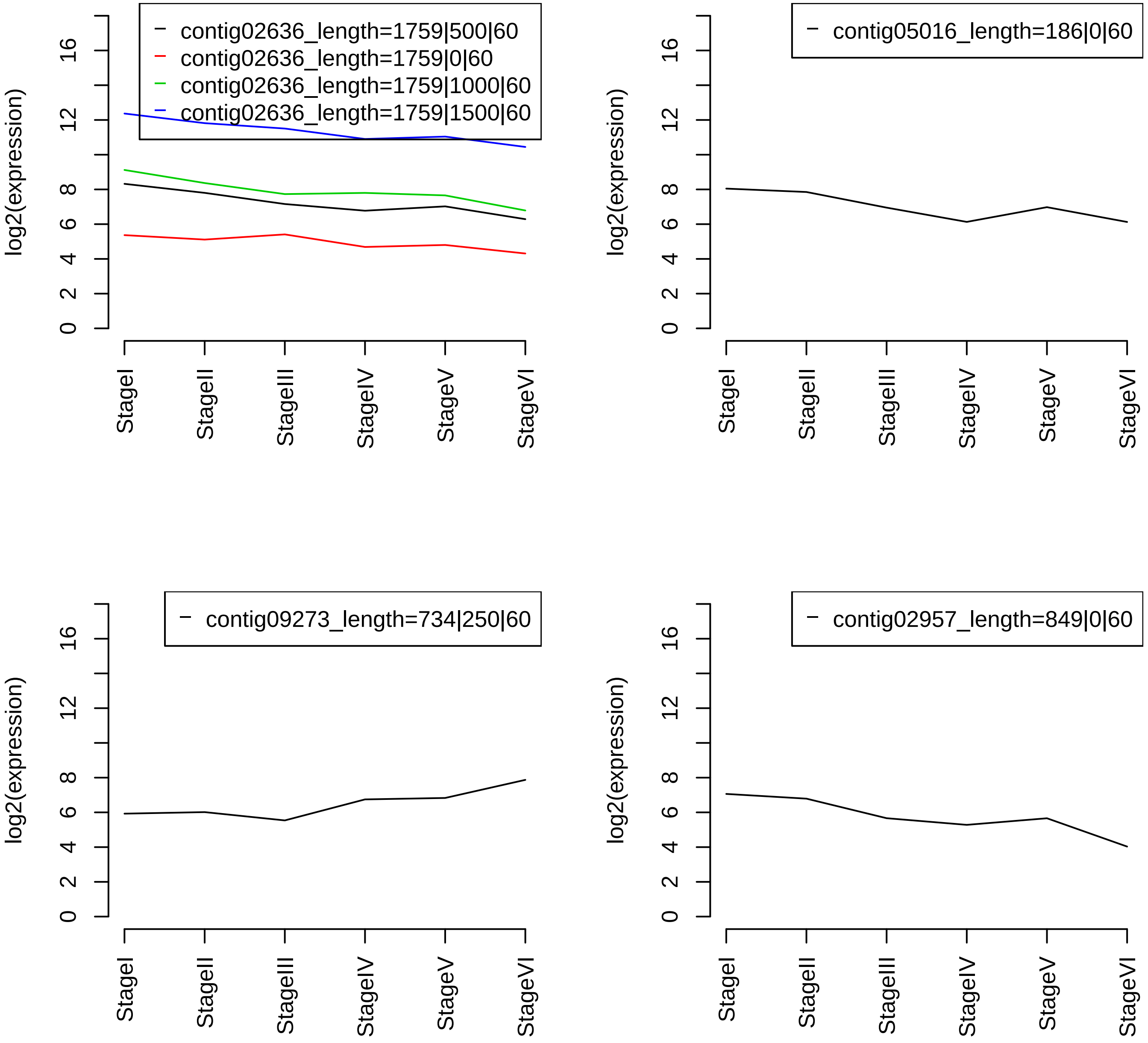
contig02636_length=1759|500|60
contig02636_length=1759|0|60
contig02636_length=1759|1000|60
contig02636_length=1759|1500|60
contig05016_length=186|0|60
contig09273_length=734|250|60
contig02957_length=849|0|60
log2(expression)
0
2
4
6
8
12
16
StageI
StageII
StageIII
StageIV
StageV
StageVI

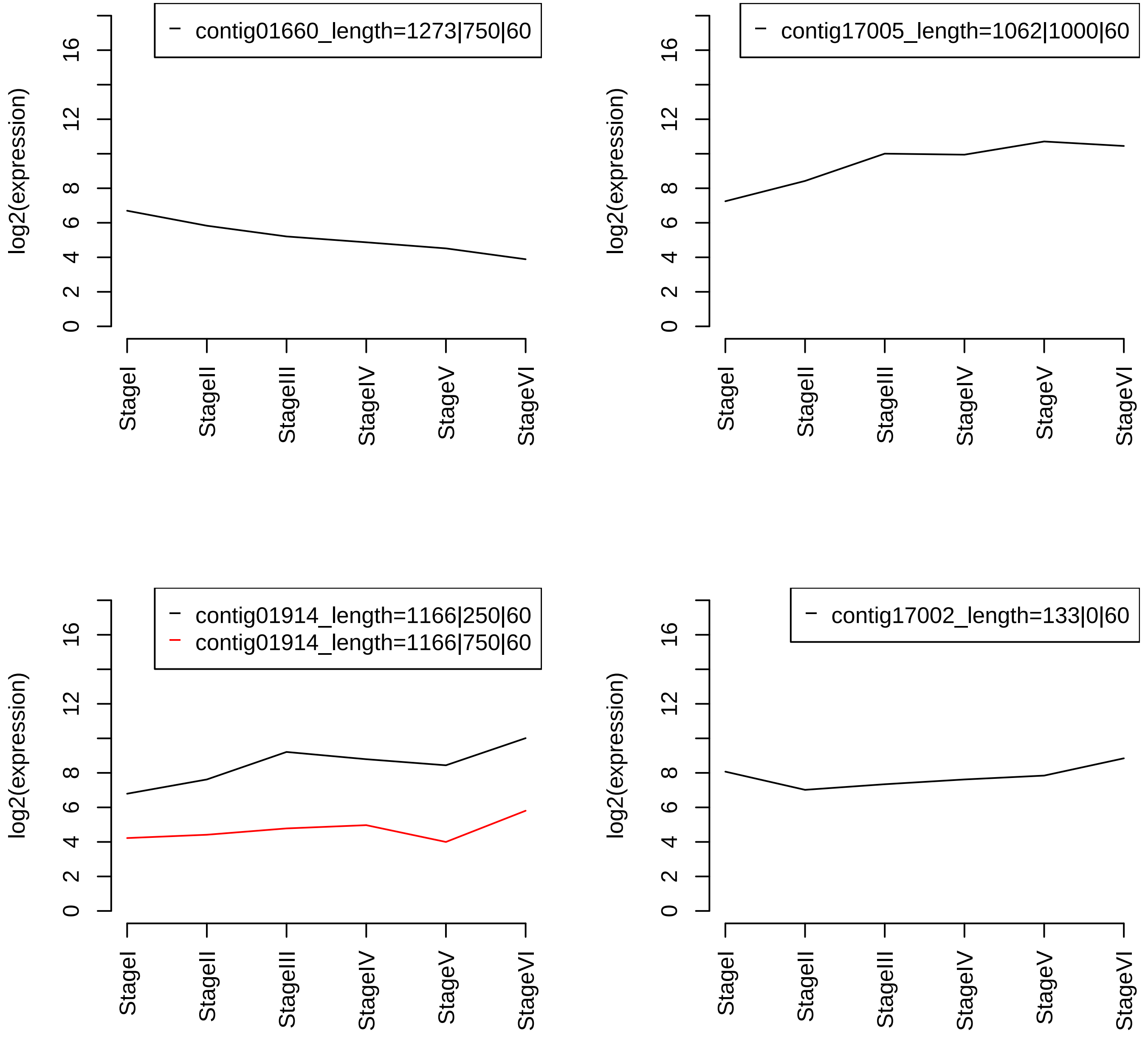

contig01660_length=1273|750|60
contig17005_length=1062|1000|60
contig01914_length=1166|250|60
contig01914_length=1166|750|60
contig17002_length=133|0|60
log2(expression)
0
2
4
6
8
12
16
StageI
StageII
StageIII
StageIV
StageV
StageVI

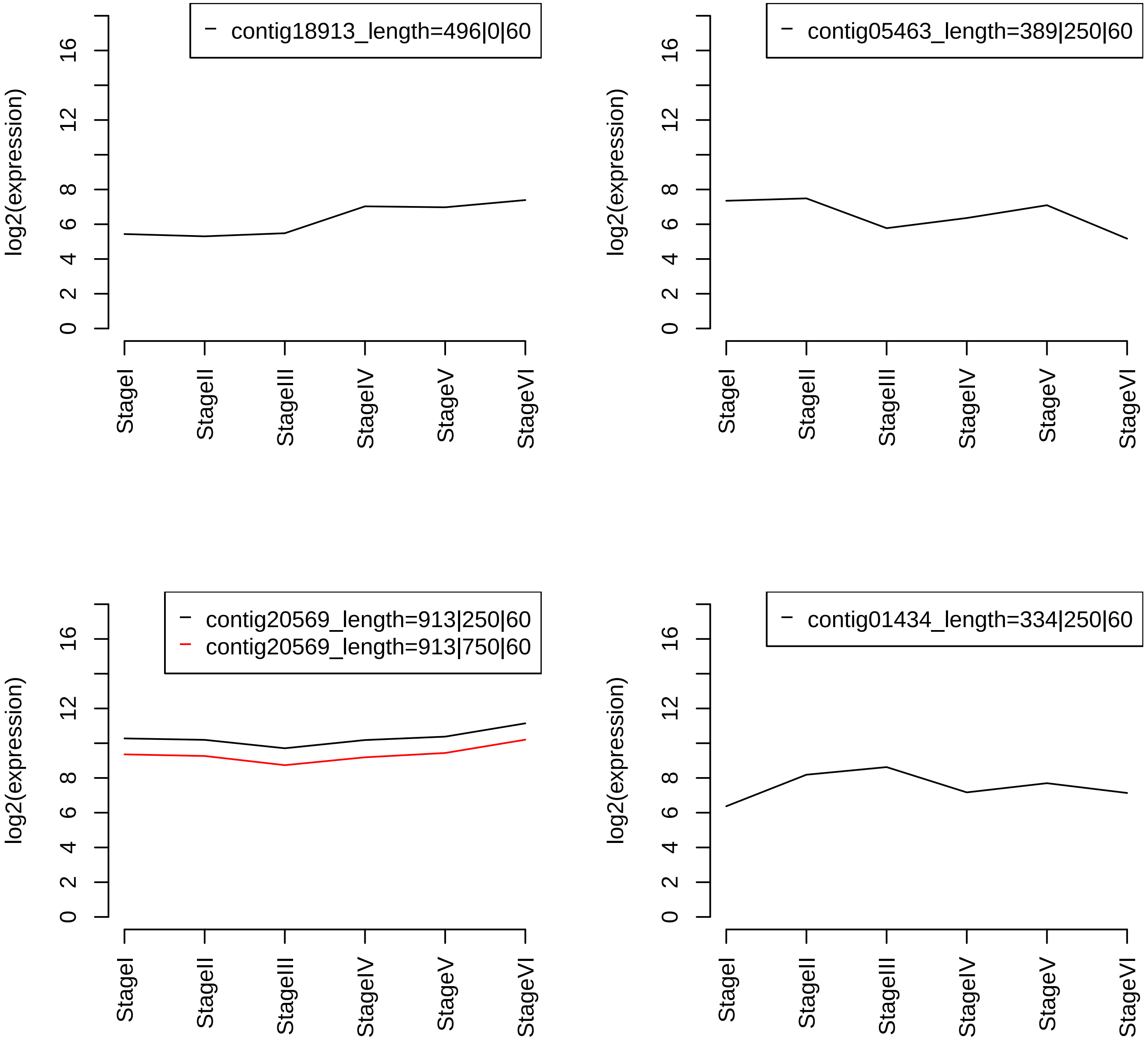
contig18913_length=496|0|60
contig05463_length=389|250|60
contig20569_length=913|250|60
contig20569_length=913|750|60
contig01434_length=334|250|60
log2(expression)
0
2
4
6
8
12
16
StageI
StageII
StageIII
StageIV
StageV
StageVI

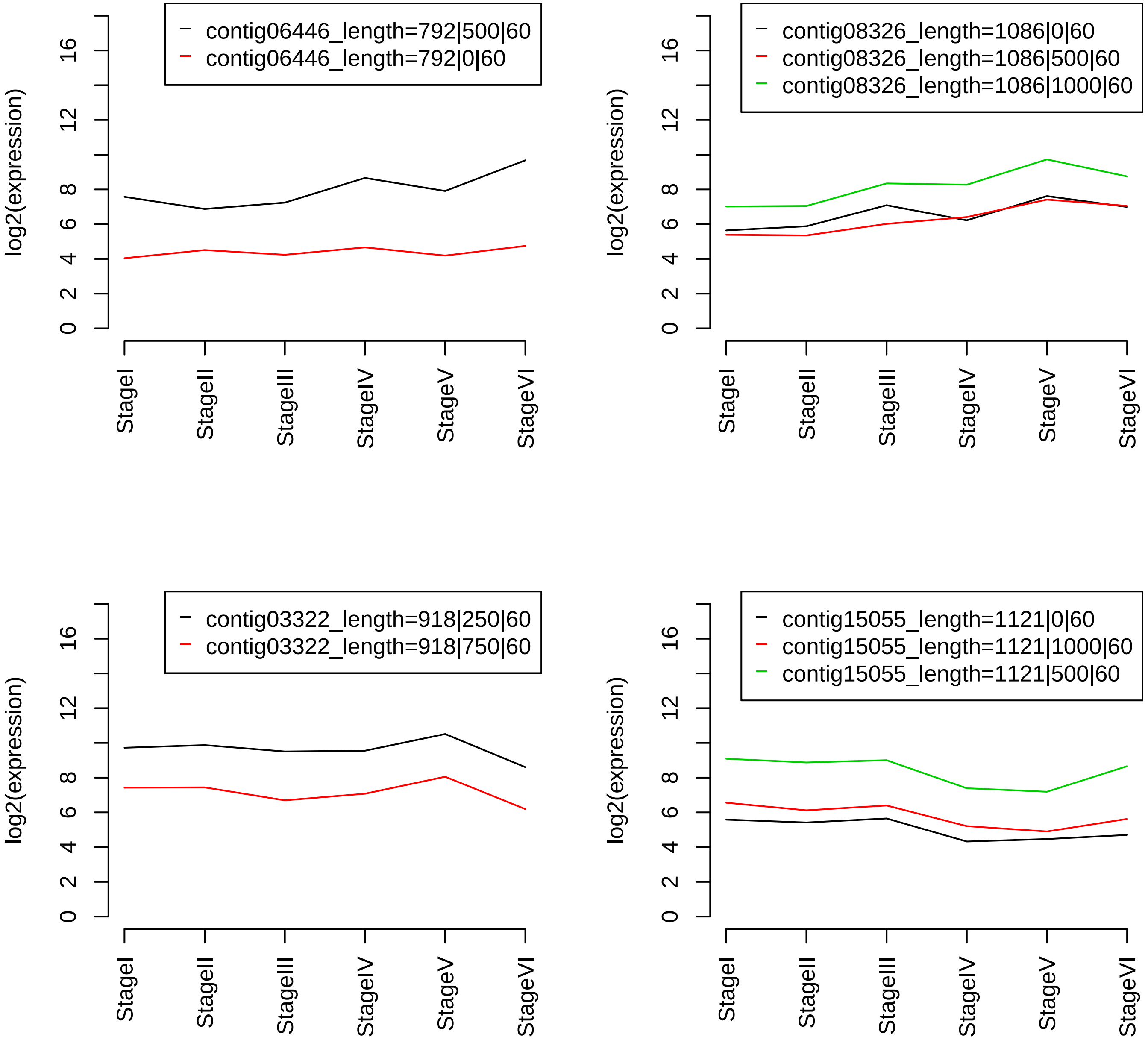
contig06446_length=792|500|60
contig06446_length=792|0|60
contig08326_length=1086|0|60
contig08326_length=1086|500|60
contig08326_length=1086|1000|60
contig03322_length=918|250|60
contig03322_length=918|750|60
contig15055_length=1121|0|60
contig15055_length=1121|1000|60
contig15055_length=1121|500|60
log2(expression)
0 2 4 6 8 12 16
StageI StageII StageIII StageIV StageV StageVI

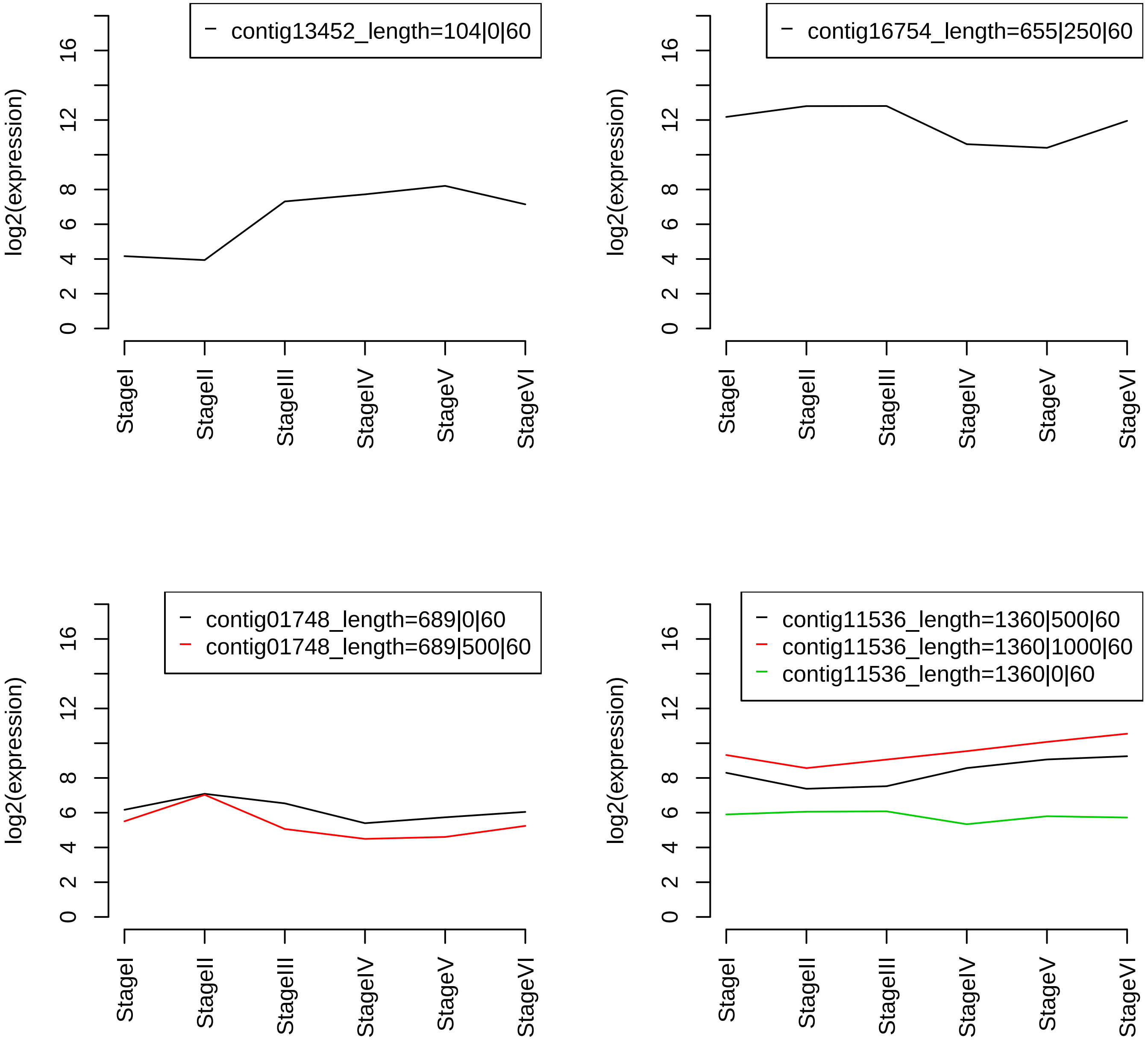
contig13452_length=104|0|60
contig16754_length=655|250|60
contig01748_length=689|0|60
contig01748_length=689|500|60
contig11536_length=1360|500|60
contig11536_length=1360|1000|60
contig11536_length=1360|0|60
log2(expression)
0
2
4
6
8
12
16
StageI
StageII
StageIII
StageIV
StageV
StageVI

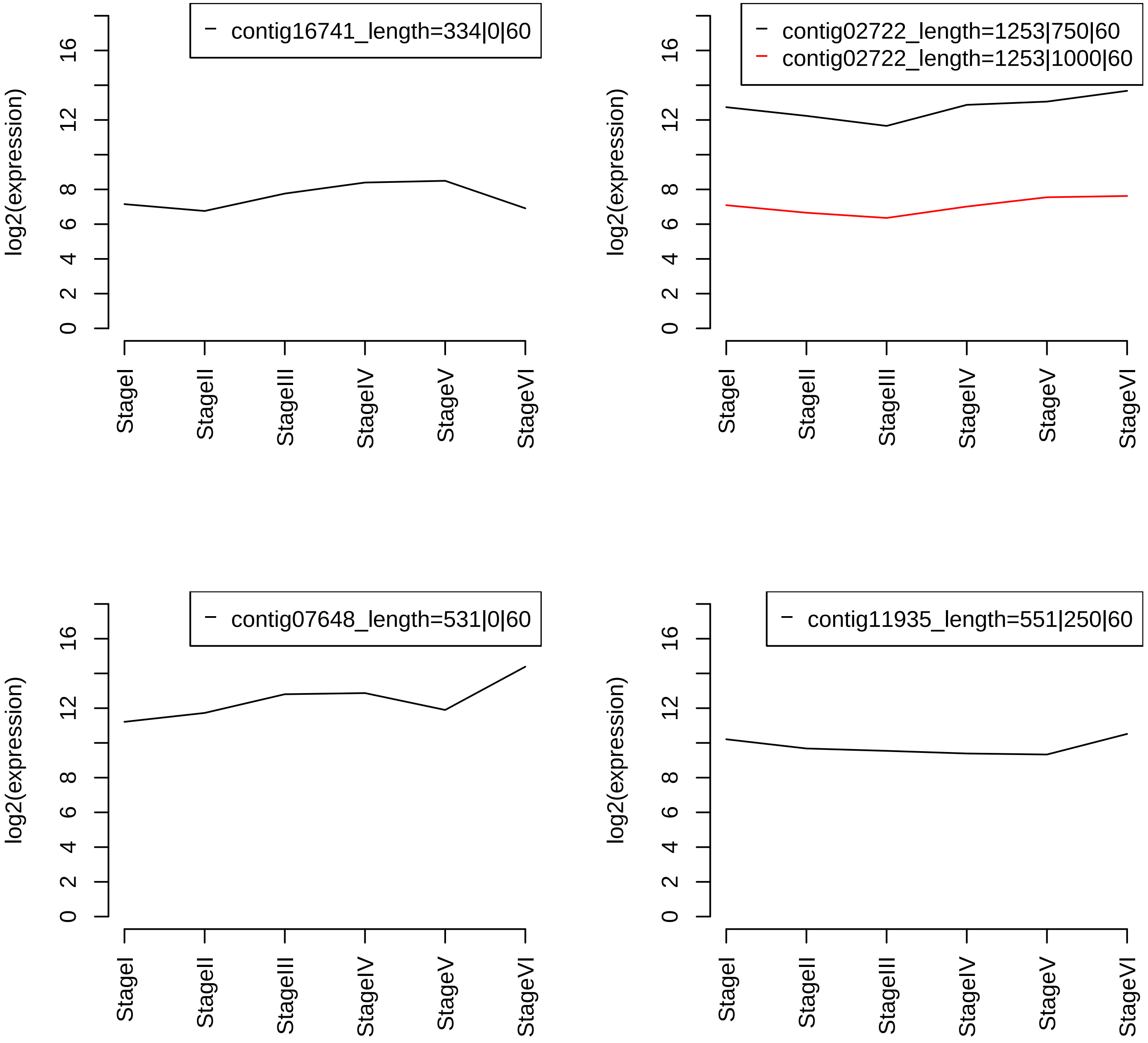
contig16741_length=334|0|60
contig02722_length=1253|750|60
contig02722_length=1253|1000|60
contig07648_length=531|0|60
contig11935_length=551|250|60
log2(expression)
0
2
4
6
8
12
16
StageI
StageII
StageIII
StageIV
StageV
StageVI

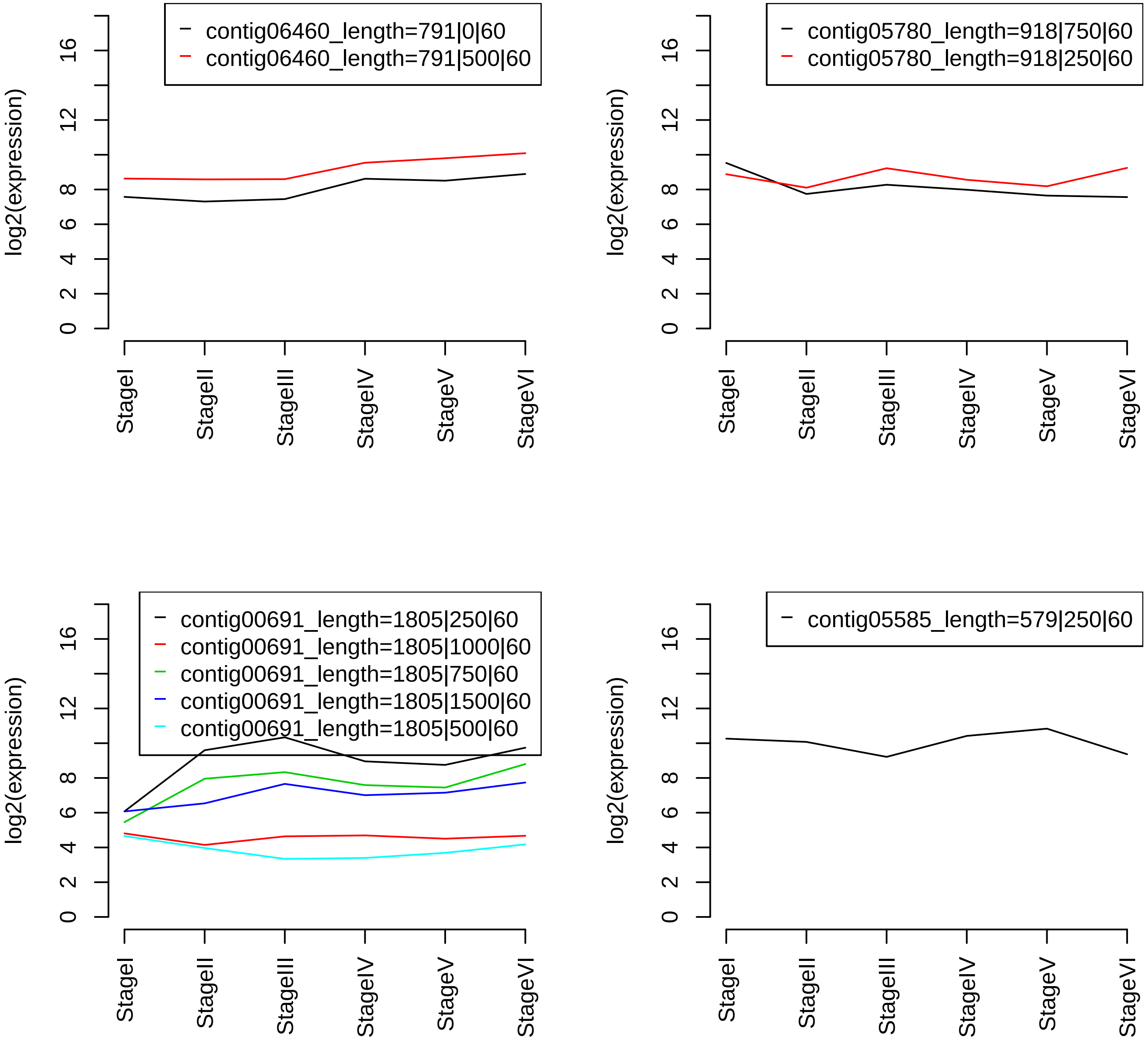
contig06460_length=791|0|60
contig06460_length=791|500|60
log2(expression)
0
2
4
6
8
12
16
StageI
StageII
StageIII
StageIV
StageV
StageVI
contig05780_length=918|750|60
contig05780_length=918|250|60
log2(expression)
0
2
4
6
8
12
16
StageI
StageII
StageIII
StageIV
StageV
StageVI
contig00691_length=1805|250|60
contig00691_length=1805|1000|60
contig00691_length=1805|750|60
contig00691_length=1805|1500|60
contig00691_length=1805|500|60
log2(expression)
0
2
4
6
8
12
16
StageI
StageII
StageIII
StageIV
StageV
StageVI
contig05585_length=579|250|60
log2(expression)
0
2
4
6
8
12
16
StageI
StageII
StageIII
StageIV
StageV
StageVI

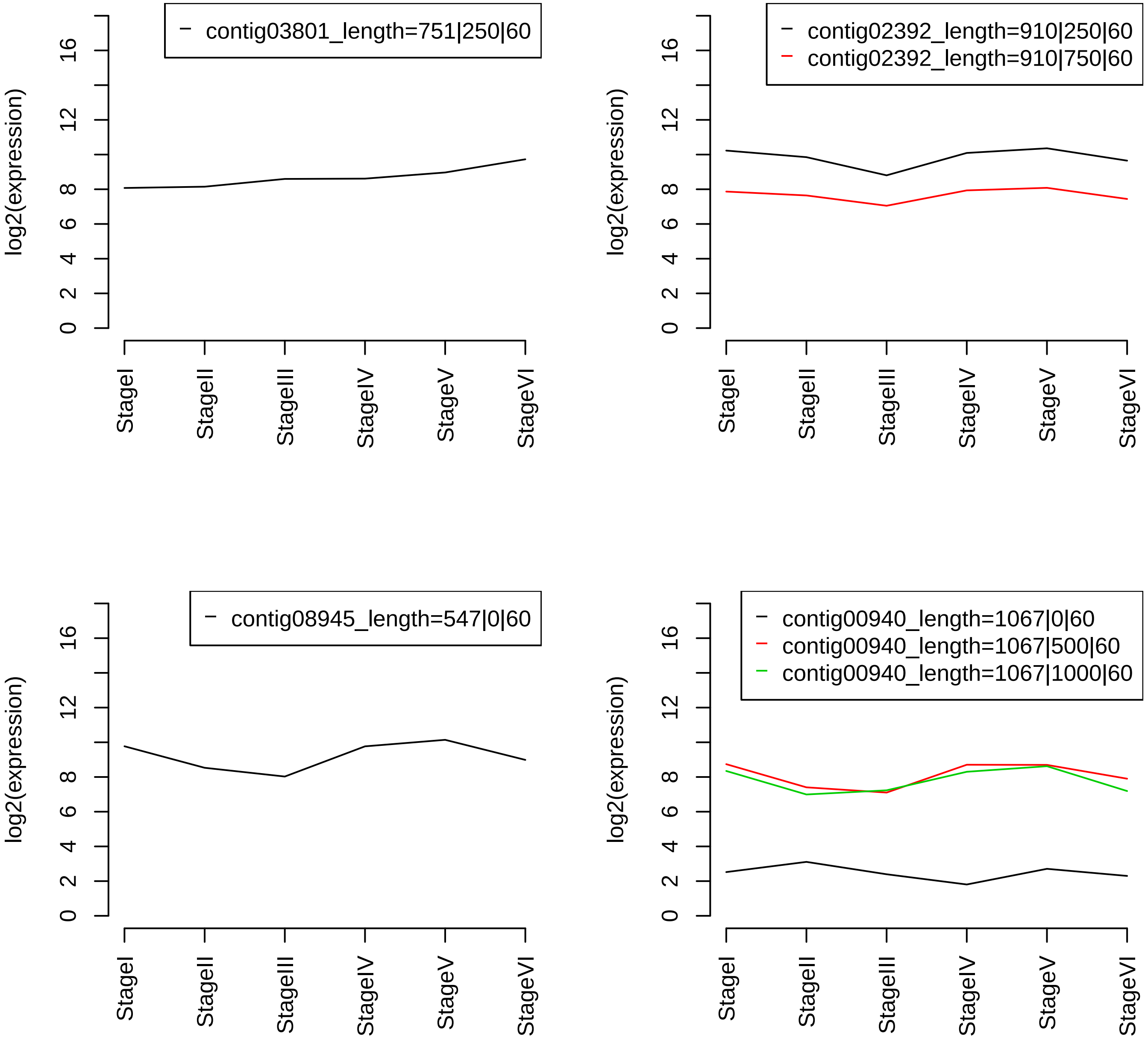
contig03801_length=751|250|60
contig02392_length=910|250|60
contig02392_length=910|750|60
contig08945_length=547|0|60
contig00940_length=1067|0|60
contig00940_length=1067|500|60
contig00940_length=1067|1000|60
log2(expression)
0
2
4
6
8
12
16
StageI
StageII
StageIII
StageIV
StageV
StageVI

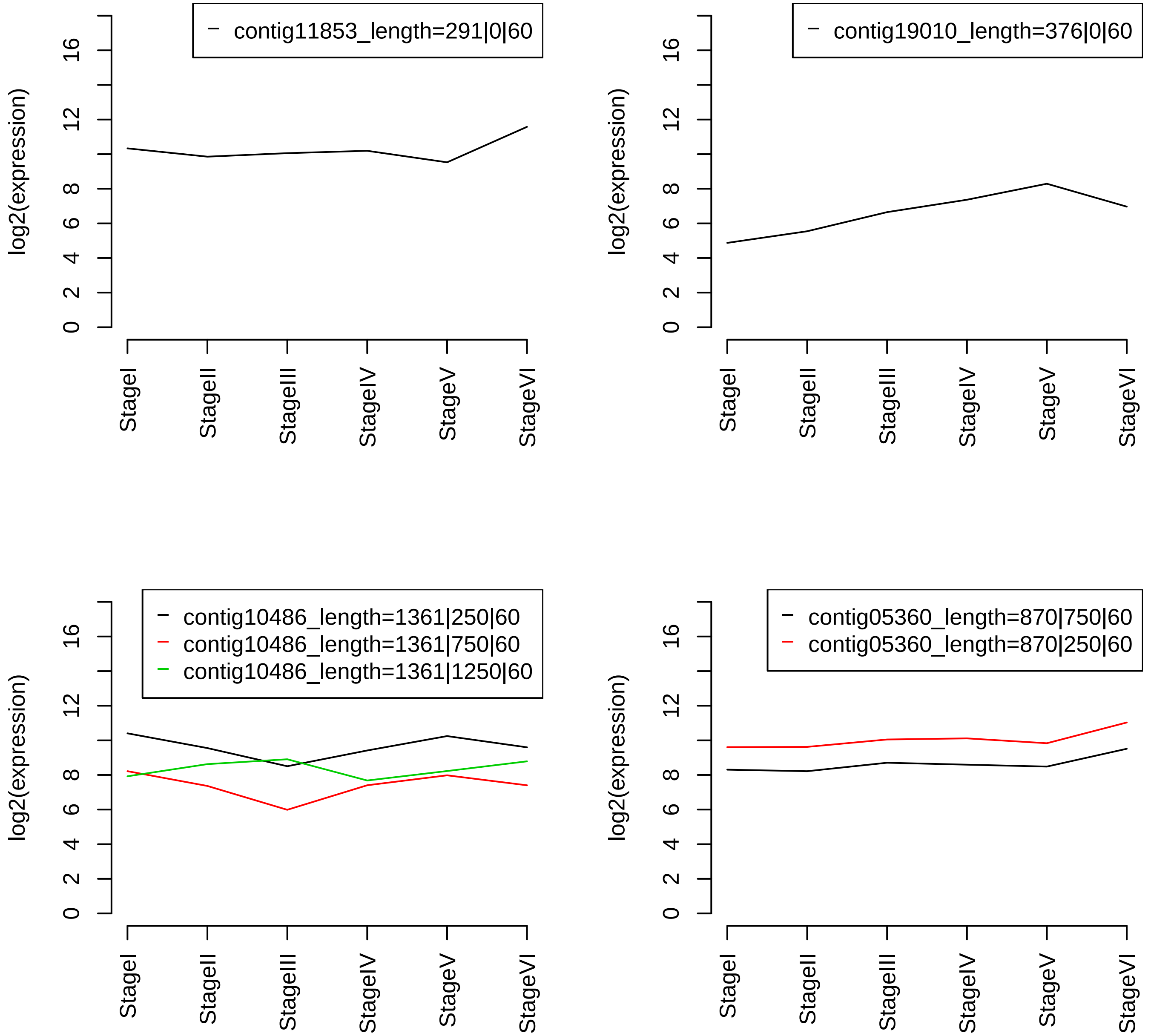
contig11853_length=291|0|60
contig19010_length=376|0|60
contig10486_length=1361|250|60
contig10486_length=1361|750|60
contig10486_length=1361|1250|60
contig05360_length=870|750|60
contig05360_length=870|250|60
log2(expression)
0
2
4
6
8
12
16
StageI
StageII
StageIII
StageIV
StageV
StageVI

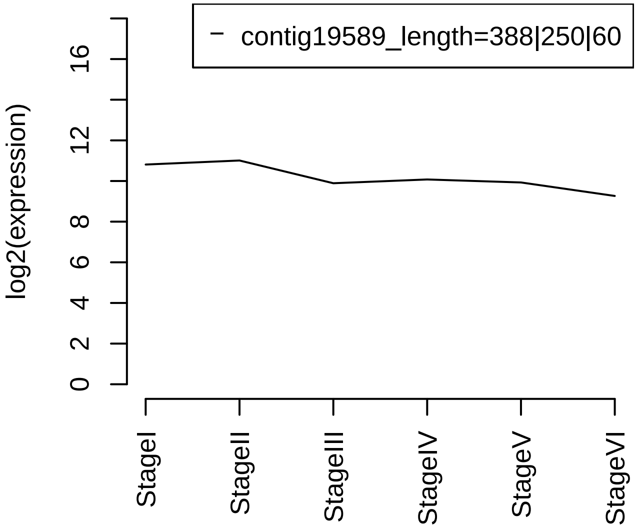
contig19589_length=388|250|60
log2(expression)
0
2
4
6
8
12
16
StageI
StageII
StageIII
StageIV
StageV
StageVI

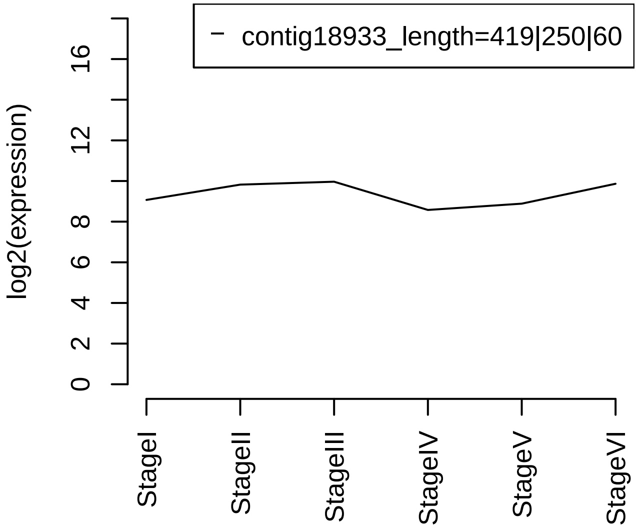
contig18933_length=419|250|60
log2(expression)
0
2
4
6
8
12
16
StageI
StageII
StageIII
StageIV
StageV
StageVI

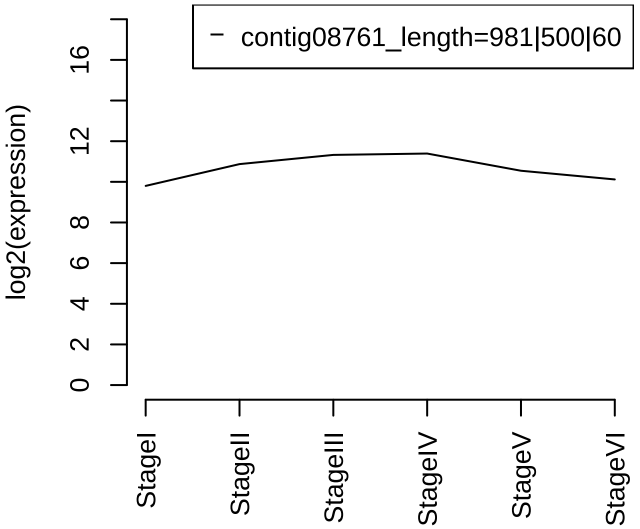
contig08761_length=981|500|60
log2(expression)
0
2
4
6
8
12
16
StageI
StageII
StageIII
StageIV
StageV
StageVI

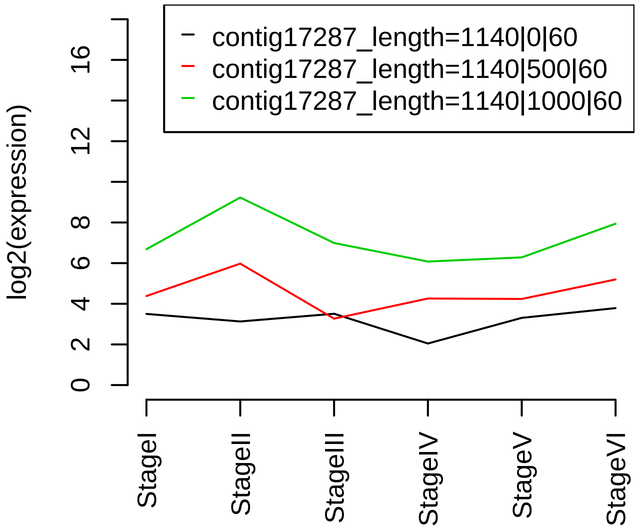
contig17287_length=1140|0|60
contig17287_length=1140|500|60
contig17287_length=1140|1000|60
log2(expression)
0
2
4
6
8
12
16
StageI
StageII
StageIII
StageIV
StageV
StageVI

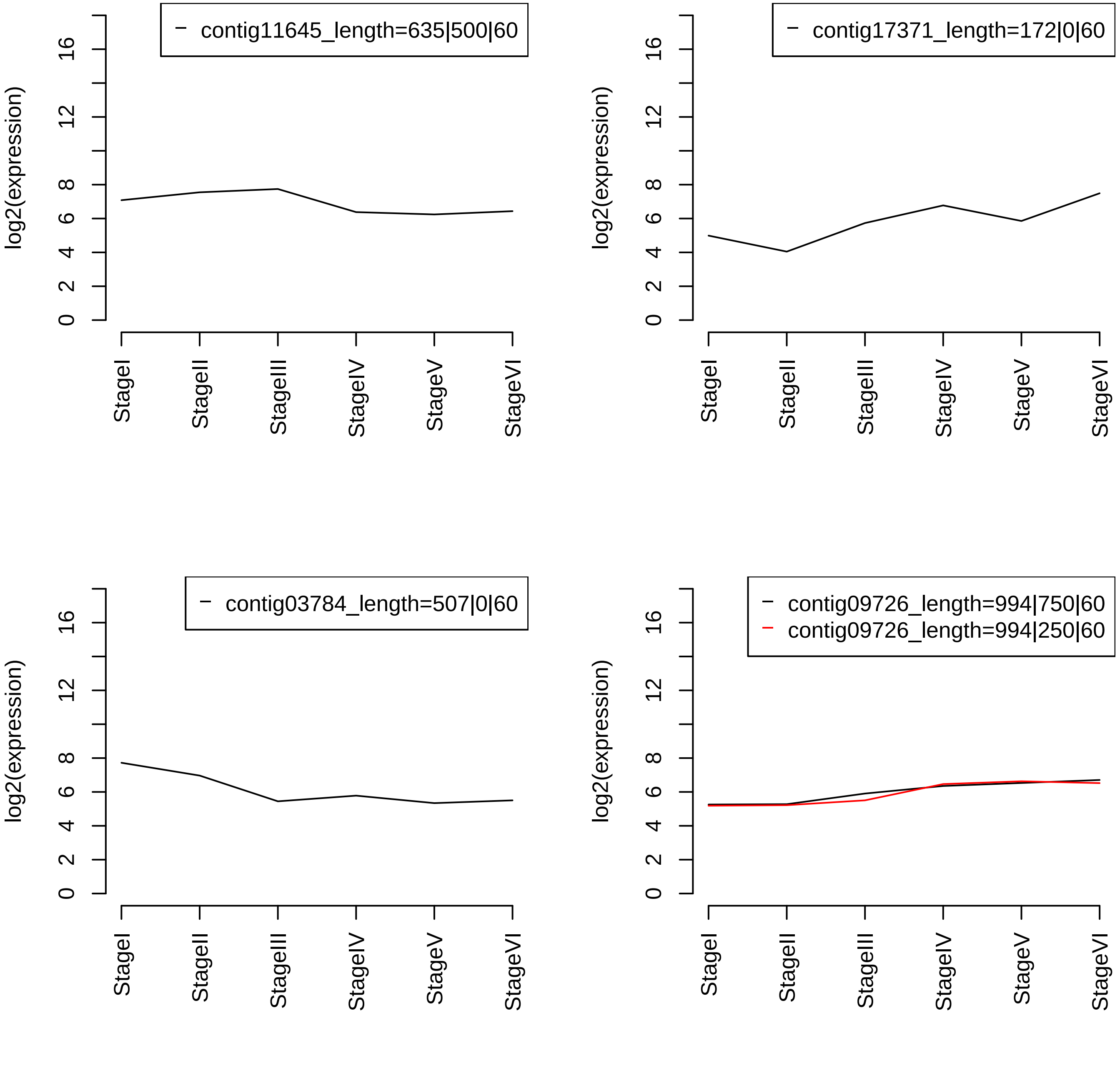
contig11645_length=635|500|60
contig17371_length=172|0|60
contig03784_length=507|0|60
contig09726_length=994|750|60
contig09726_length=994|250|60
log2(expression)
0
2
4
6
8
12
16
StageI
StageII
StageIII
StageIV
StageV
StageVI

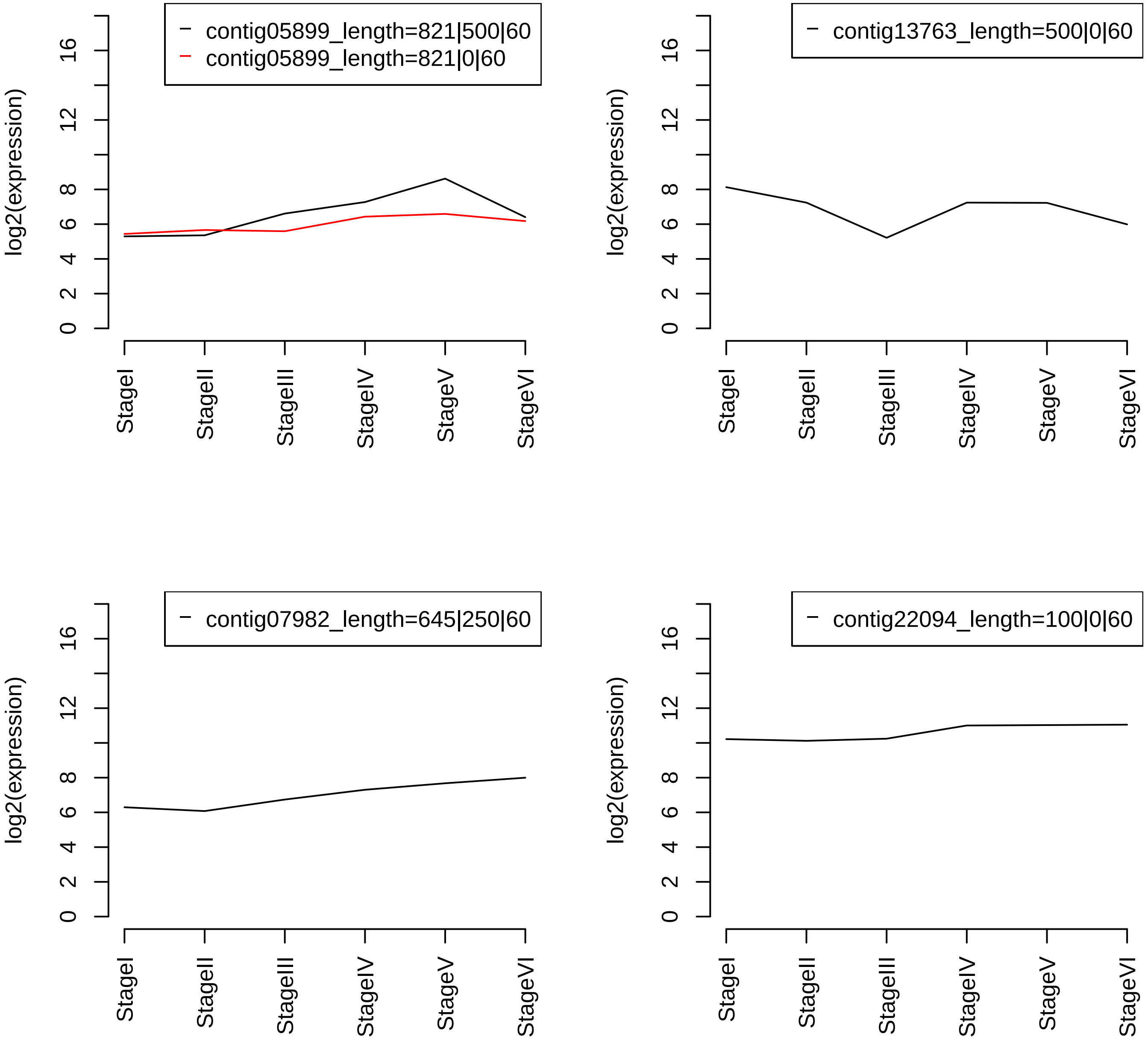
contig05899_length=821|500|60
contig05899_length=821|0|60
contig13763_length=500|0|60
contig07982_length=645|250|60
contig22094_length=100|0|60
log2(expression)
StageI
StageII
StageIII
StageIV
StageV
StageVI
0
2
4
6
8
12
16

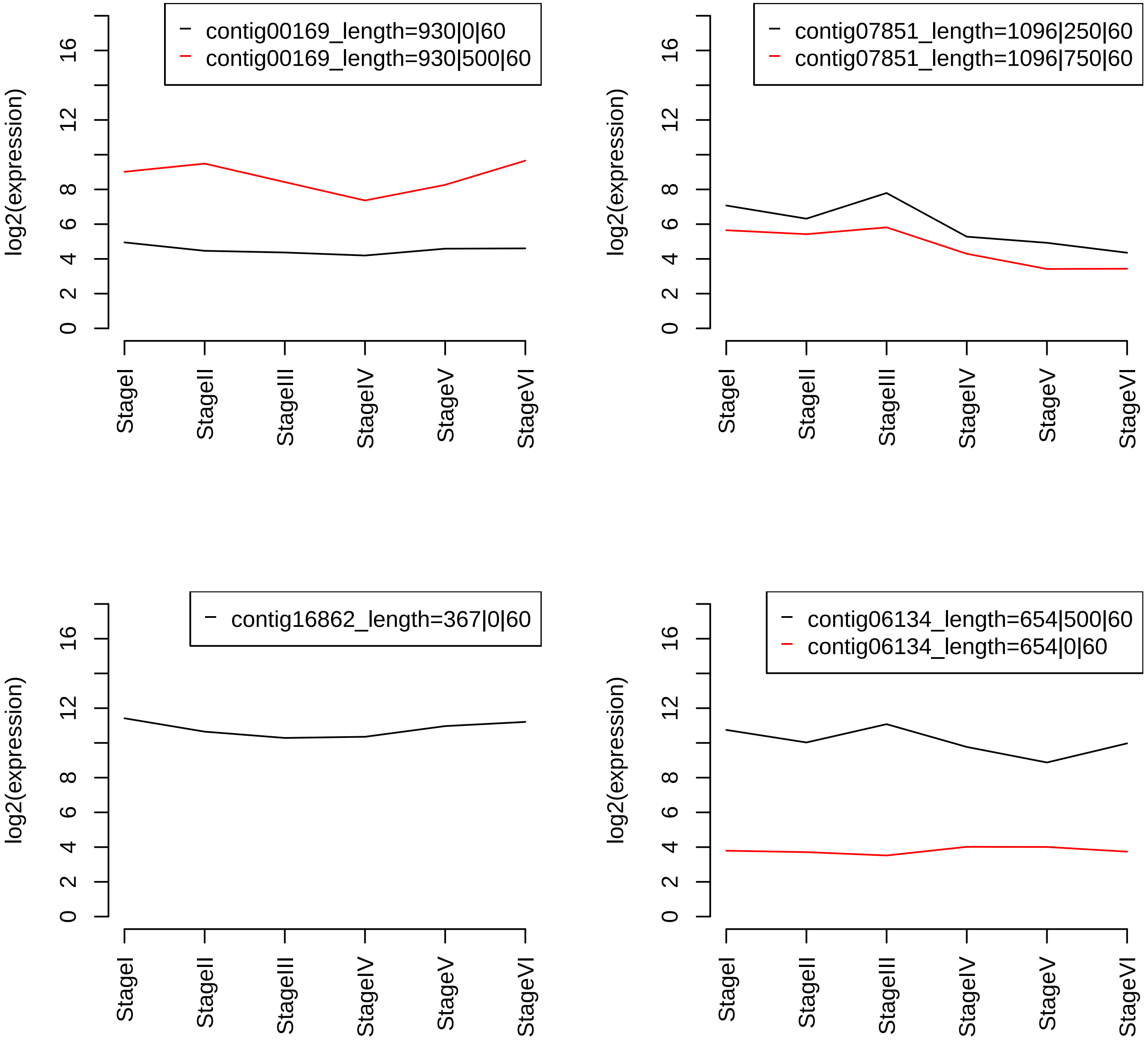
contig00169_length=930|0|60
contig00169_length=930|500|60
contig07851_length=1096|250|60
contig07851_length=1096|750|60
contig16862_length=367|0|60
contig06134_length=654|500|60
contig06134_length=654|0|60
log2(expression)
0
2
4
6
8
12
16
StageI
StageII
StageIII
StageIV
StageV
StageVI

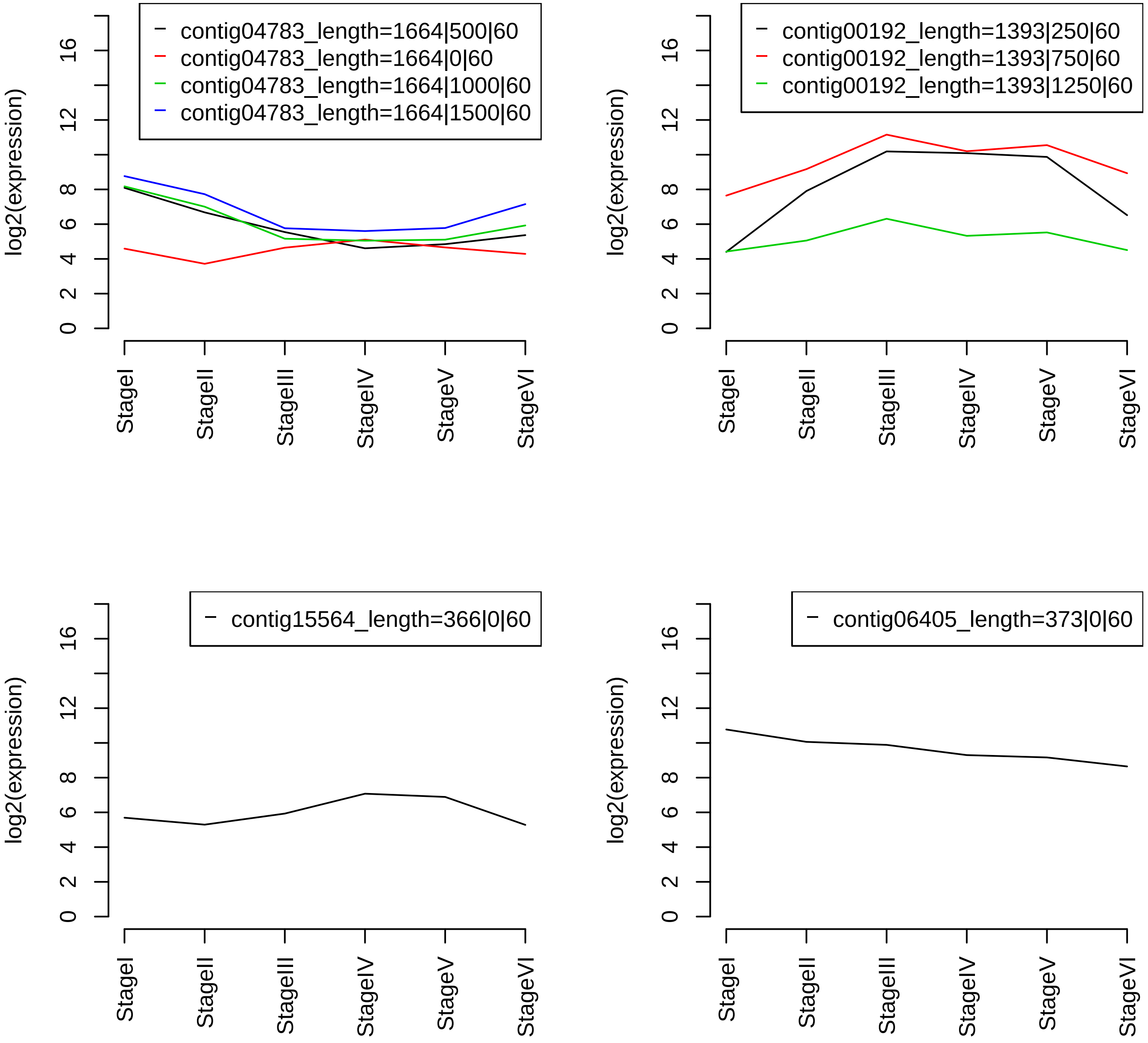
contig04783_length=1664|500|60
contig04783_length=1664|0|60
contig04783_length=1664|1000|60
contig04783_length=1664|1500|60
contig00192_length=1393|250|60
contig00192_length=1393|750|60
contig00192_length=1393|1250|60
contig15564_length=366|0|60
contig06405_length=373|0|60
log2(expression)
0
2
4
6
8
12
16
StageI
StageII
StageIII
StageIV
StageV
StageVI

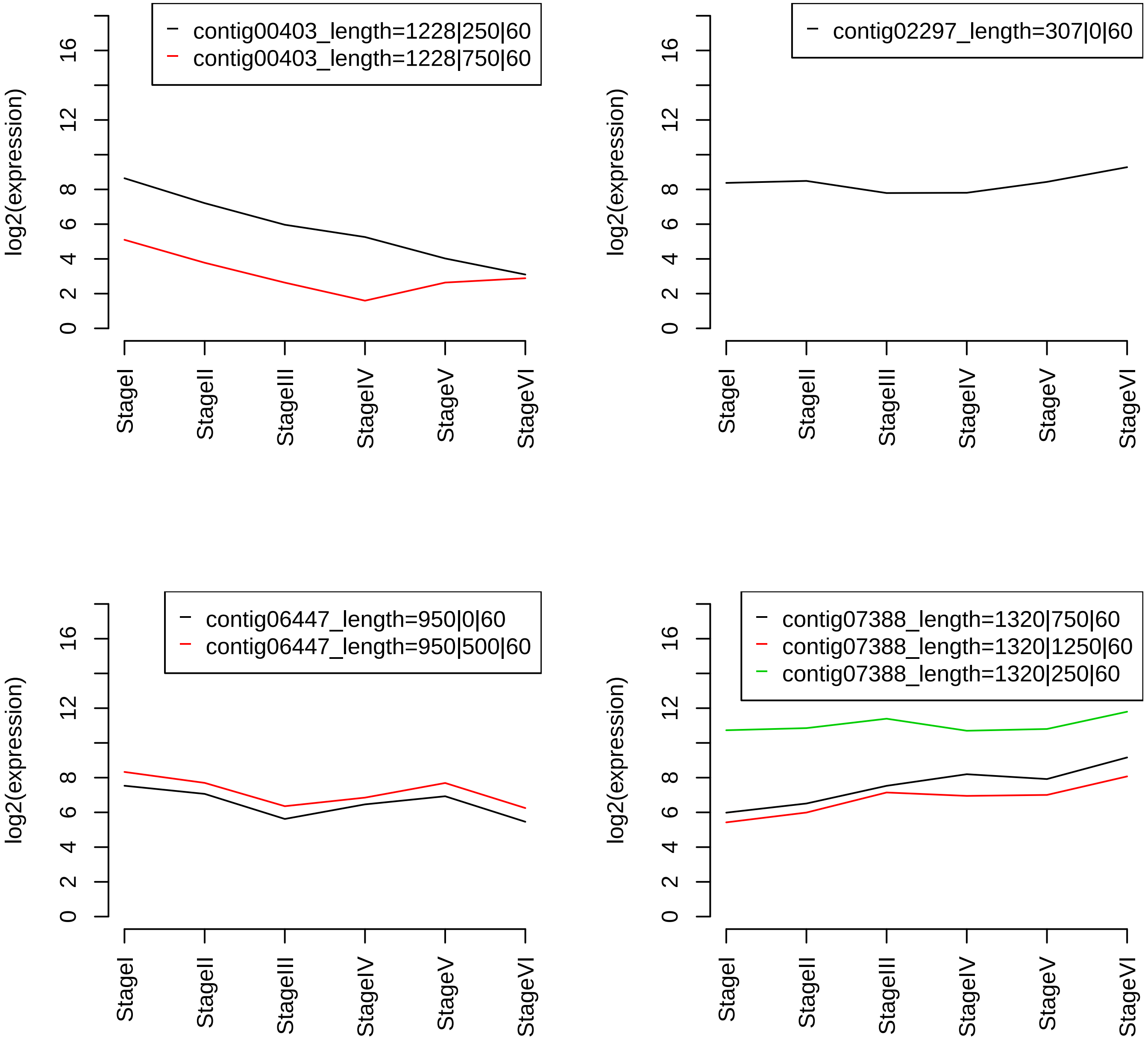
contig00403_length=1228|250|60
contig00403_length=1228|750|60
contig02297_length=307|0|60
contig06447_length=950|0|60
contig06447_length=950|500|60
contig07388_length=1320|750|60
contig07388_length=1320|1250|60
contig07388_length=1320|250|60
log2(expression)
0
2
4
6
8
12
16
StageI
StageII
StageIII
StageIV
StageV
StageVI

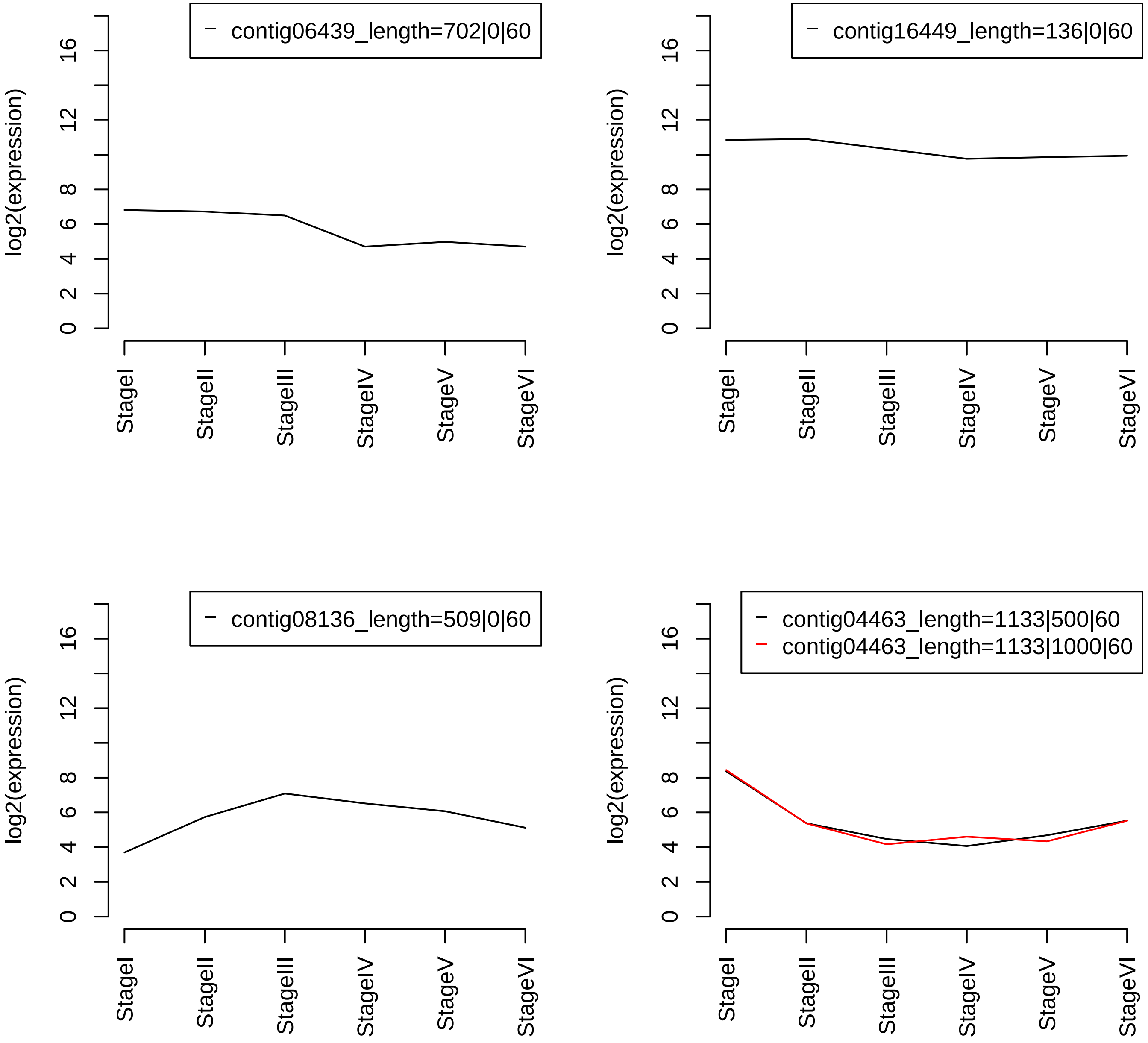

contig06439_length=702|0|60
contig16449_length=136|0|60
contig08136_length=509|0|60
contig04463_length=1133|500|60
contig04463_length=1133|1000|60
log2(expression)
0
2
4
6
8
12
16
StageI
StageII
StageIII
StageIV
StageV
StageVI

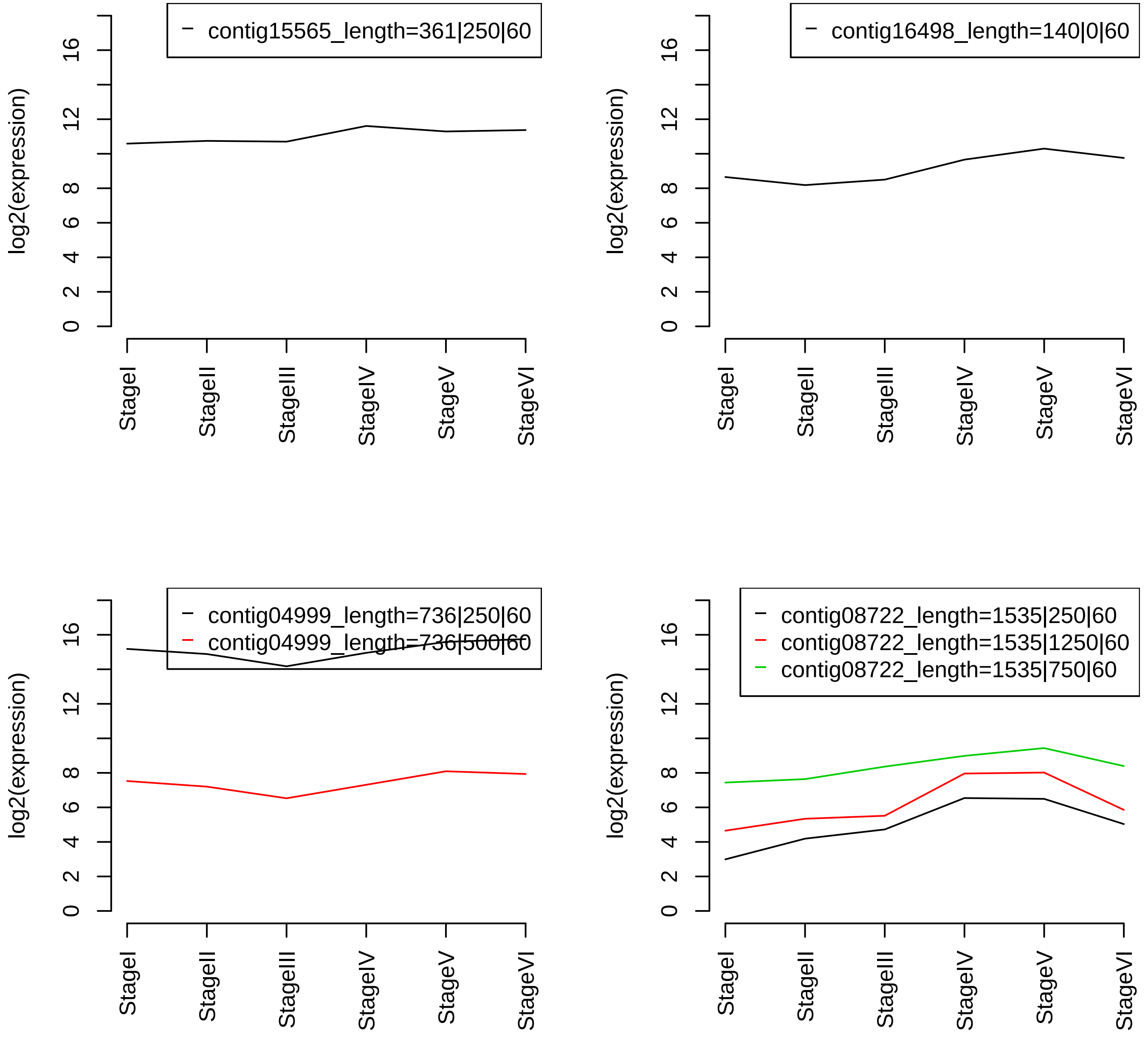
contig15565_length=361|250|60
contig16498_length=140|0|60
contig04999_length=736|250|60
contig04999_length=736|500|60
contig08722_length=1535|250|60
contig08722_length=1535|1250|60
contig08722_length=1535|750|60
log2(expression)
0
2
4
6
8
12
16
StageI
StageII
StageIII
StageIV
StageV
StageVI

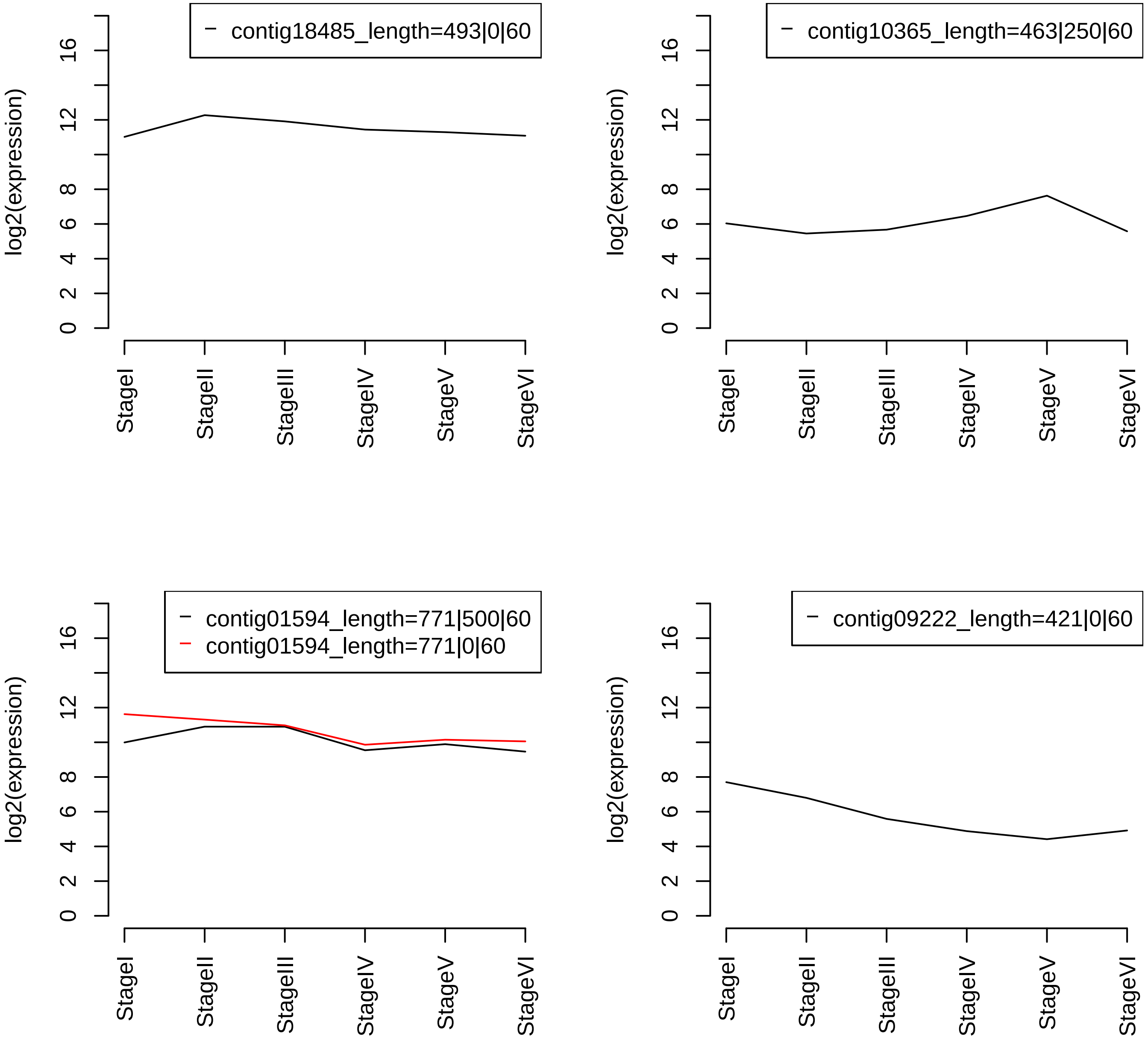
contig18485_length=493|0|60
contig10365_length=463|250|60
contig01594_length=771|500|60
contig01594_length=771|0|60
contig09222_length=421|0|60
log2(expression)
0
2
4
6
8
12
16
StageI
StageII
StageIII
StageIV
StageV
StageVI

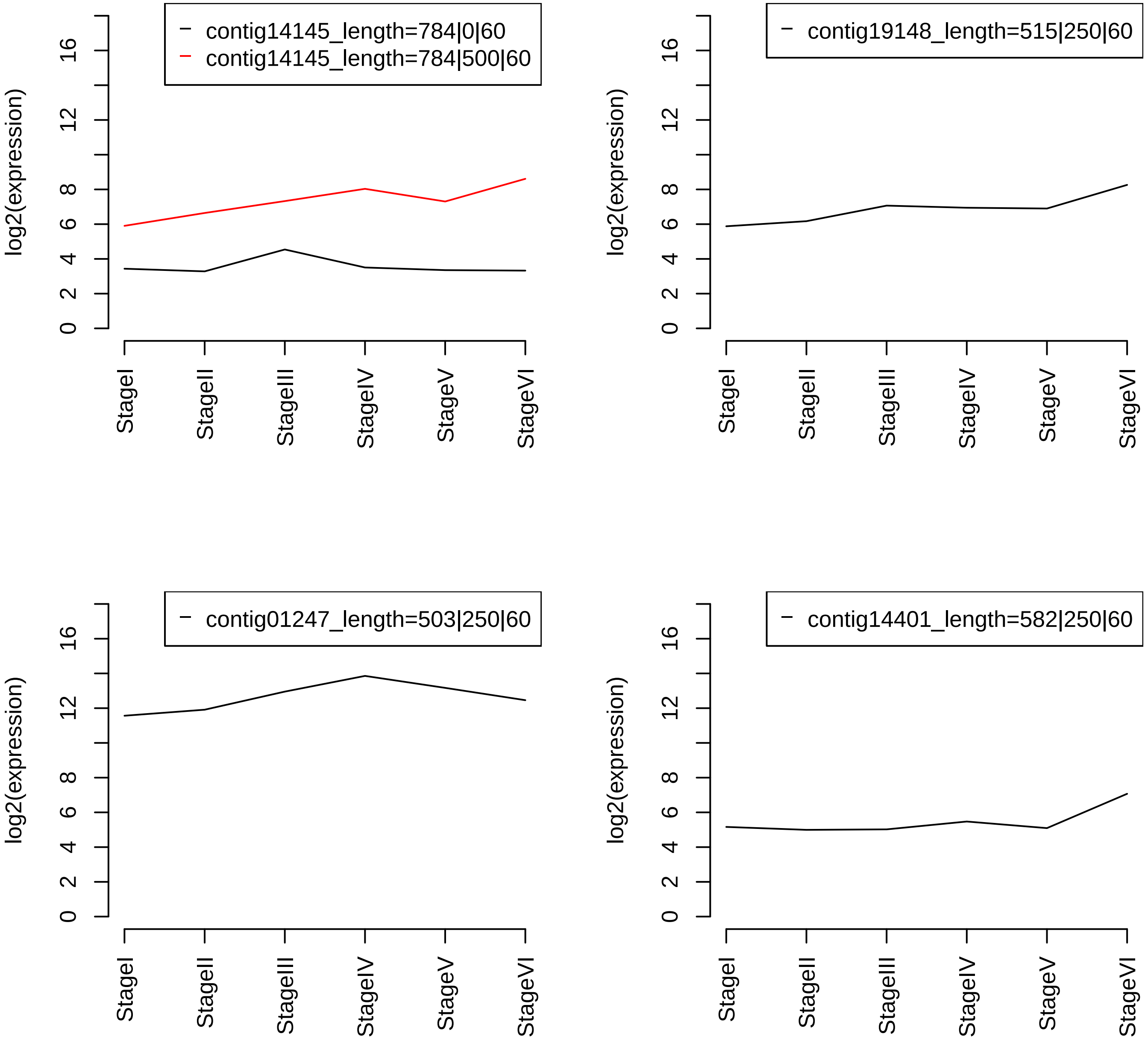

contig14145_length=784|0|60
contig14145_length=784|500|60
contig19148_length=515|250|60
contig01247_length=503|250|60
contig14401_length=582|250|60
log2(expression)
0
2
4
6
8
12
16
StageI
StageII
StageIII
StageIV
StageV
StageVI

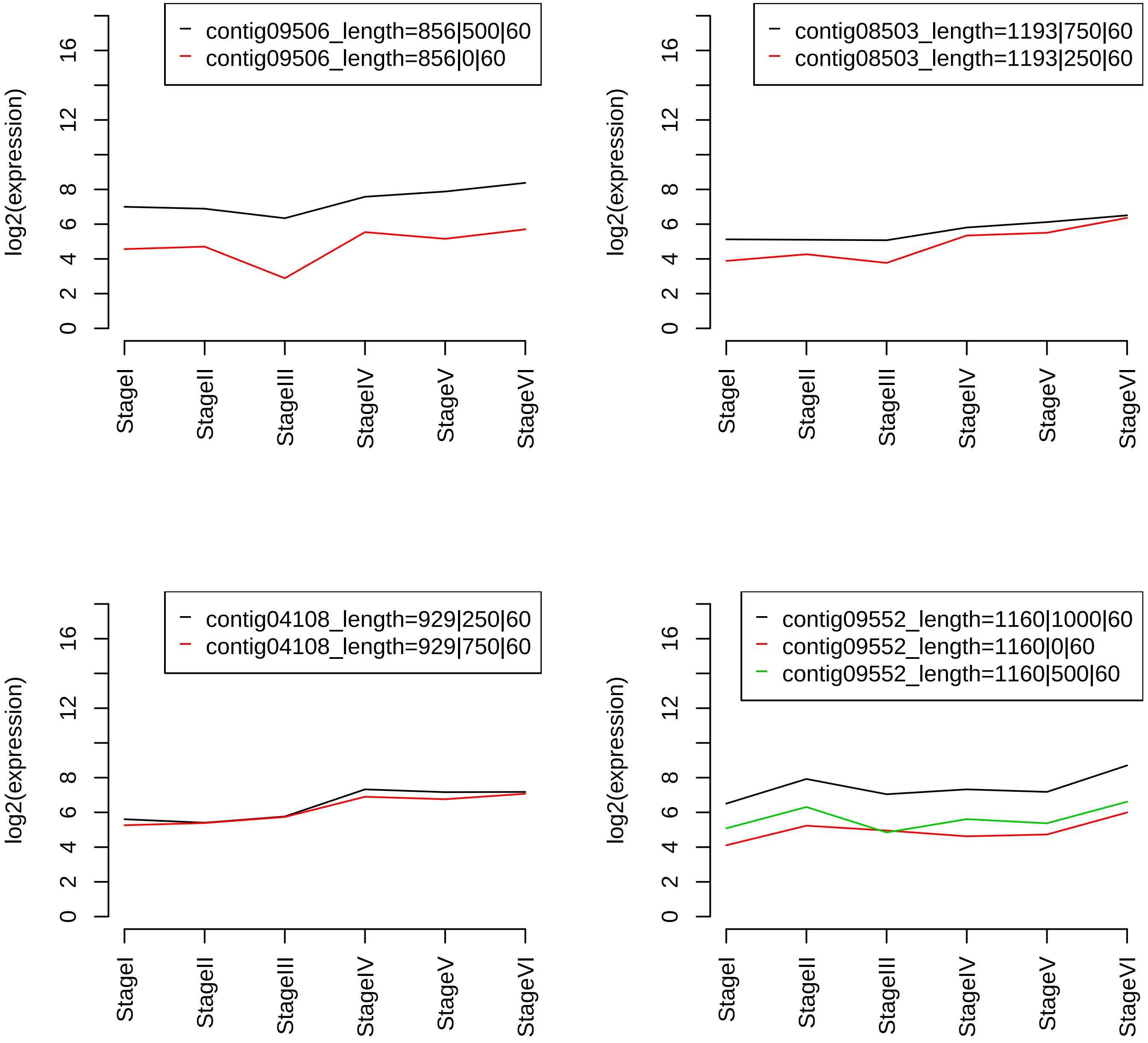
contig09506_length=856|500|60
contig09506_length=856|0|60
contig08503_length=1193|750|60
contig08503_length=1193|250|60
contig04108_length=929|250|60
contig04108_length=929|750|60
contig09552_length=1160|1000|60
contig09552_length=1160|0|60
contig09552_length=1160|500|60
log2(expression)
0
2
4
6
8
12
16
StageI
StageII
StageIII
StageIV
StageV
StageVI

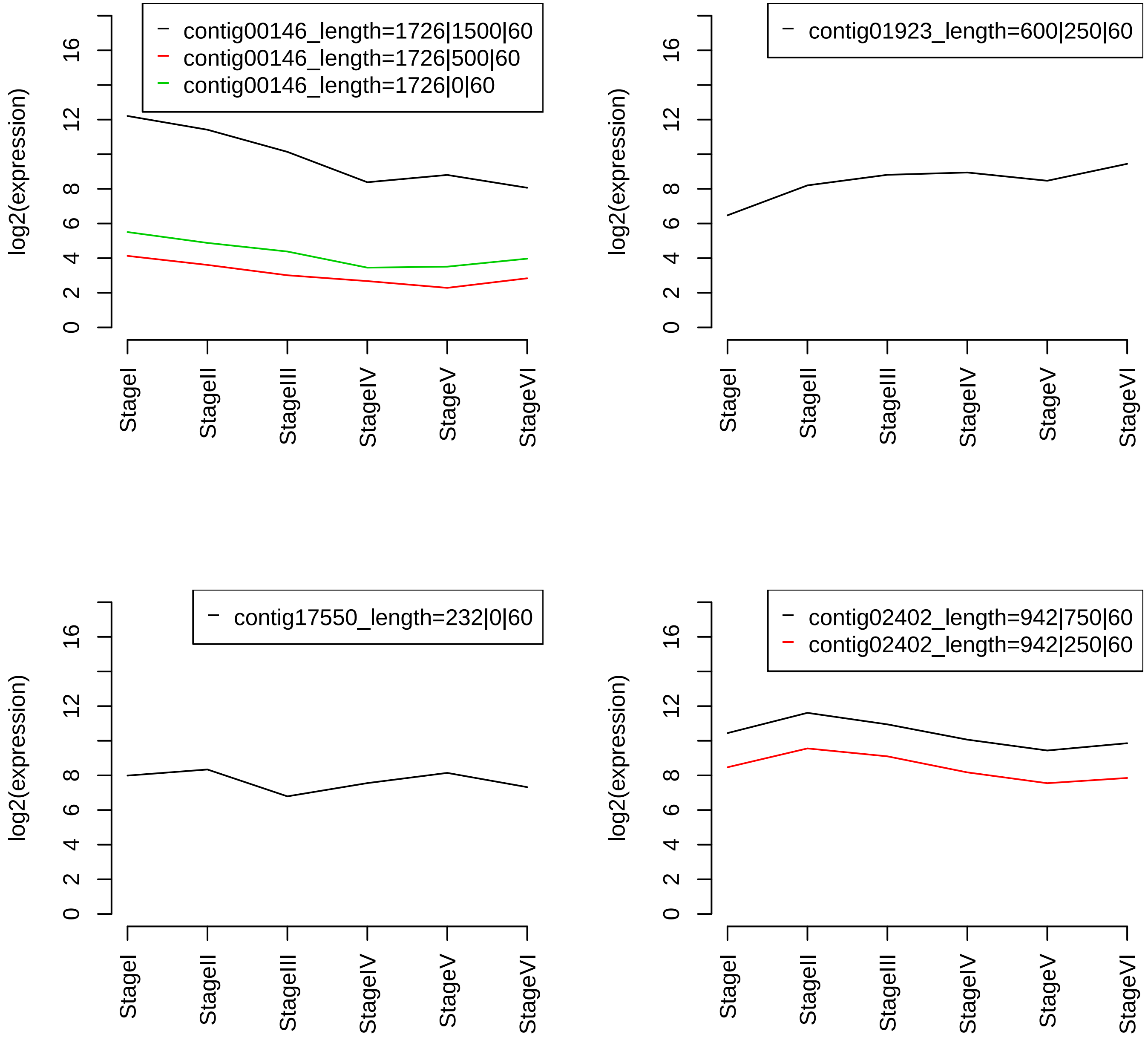

contig00146_length=1726|1500|60
contig00146_length=1726|500|60
contig00146_length=1726|0|60
contig01923_length=600|250|60
contig17550_length=232|0|60
contig02402_length=942|750|60
contig02402_length=942|250|60
log2(expression)
0
2
4
6
8
12
16
StageI
StageII
StageIII
StageIV
StageV
StageVI

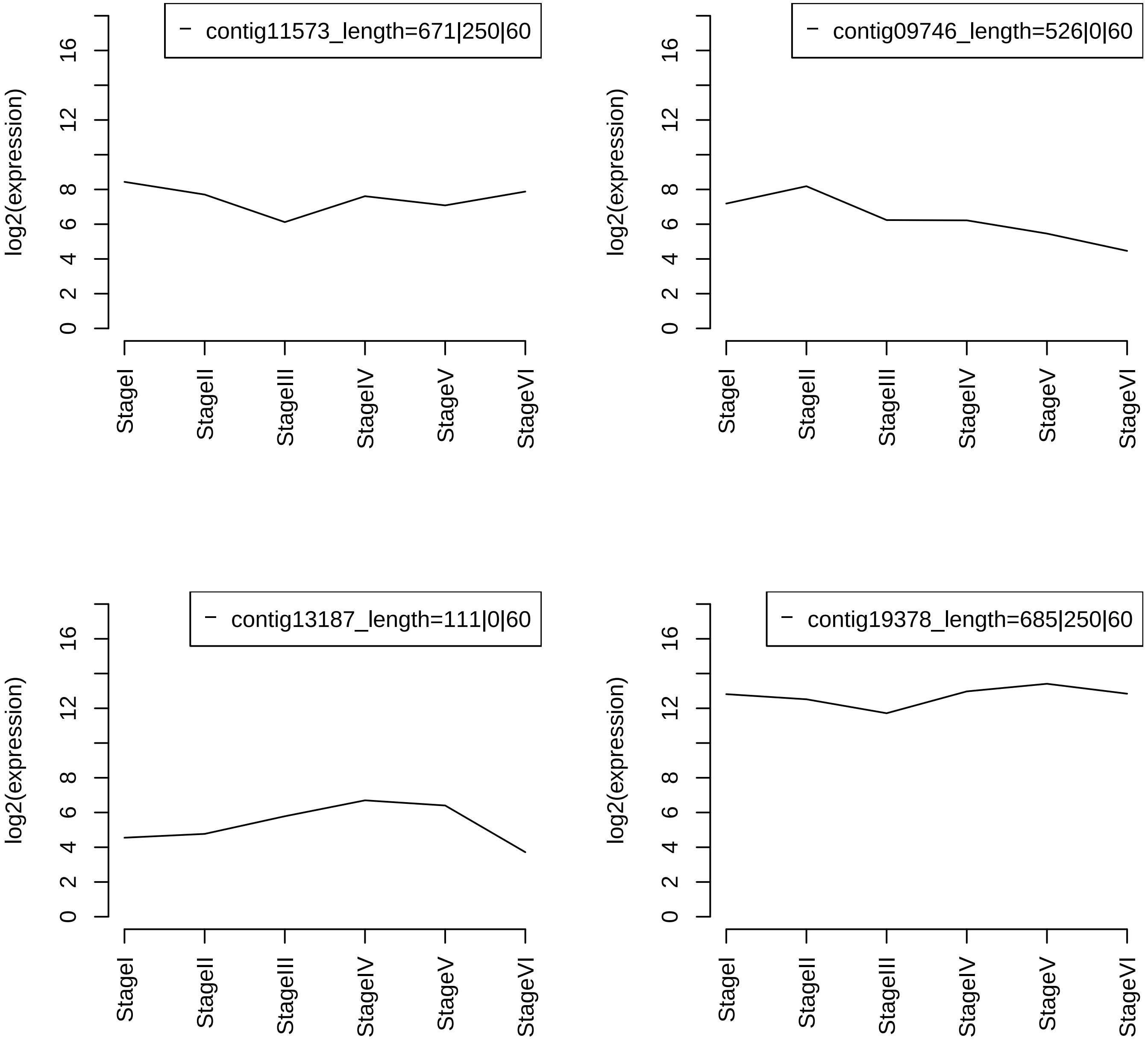

contig11573_length=671|250|60
contig09746_length=526|0|60
contig13187_length=111|0|60
contig19378_length=685|250|60
log2(expression)
0
2
4
6
8
12
16
StageI
StageII
StageIII
StageIV
StageV
StageVI

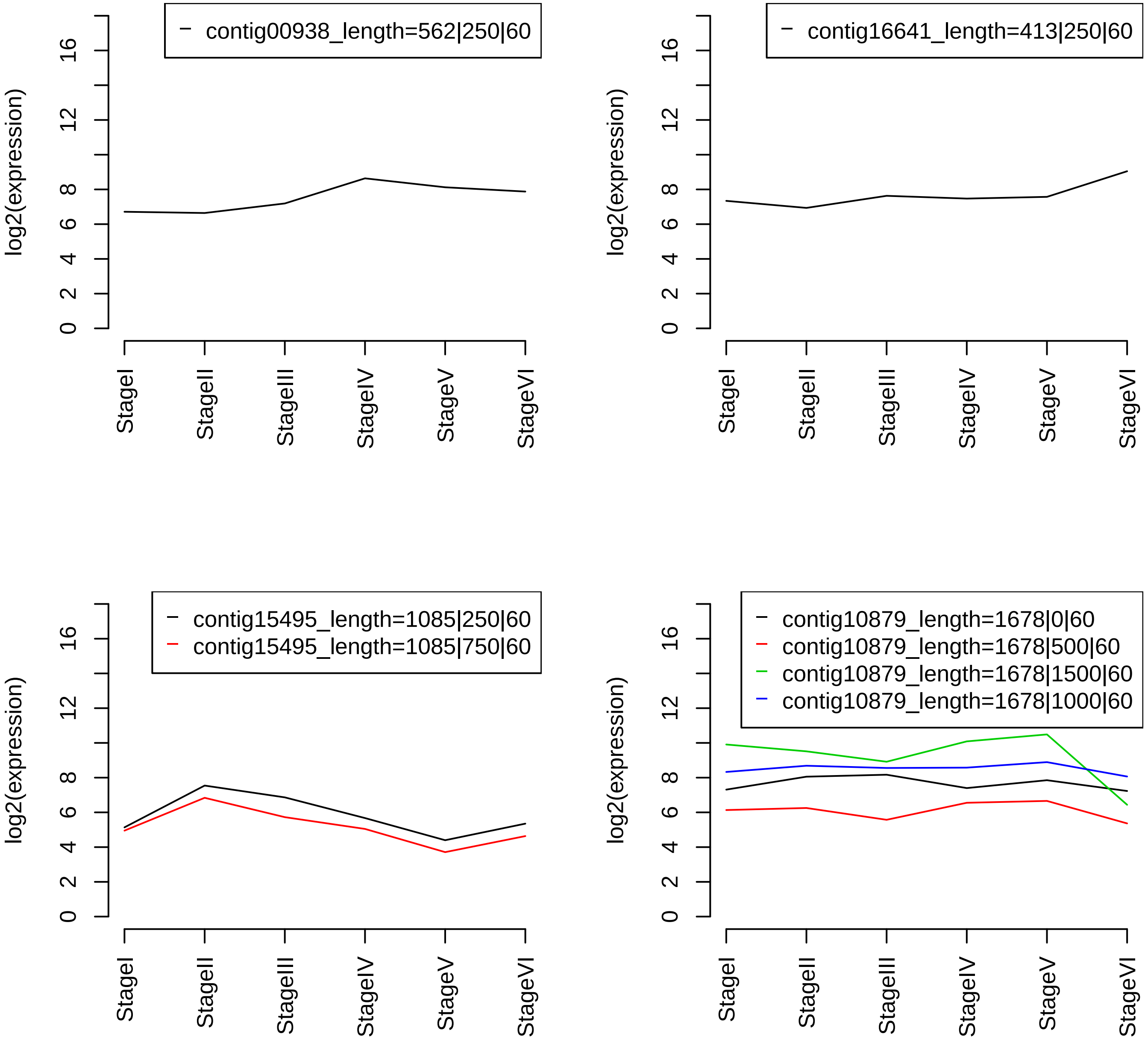

contig00938_length=562|250|60
contig16641_length=413|250|60
contig15495_length=1085|250|60
contig15495_length=1085|750|60
contig10879_length=1678|0|60
contig10879_length=1678|500|60
contig10879_length=1678|1500|60
contig10879_length=1678|1000|60
log2(expression)
0
2
4
6
8
12
16
StageI
StageII
StageIII
StageIV
StageV
StageVI

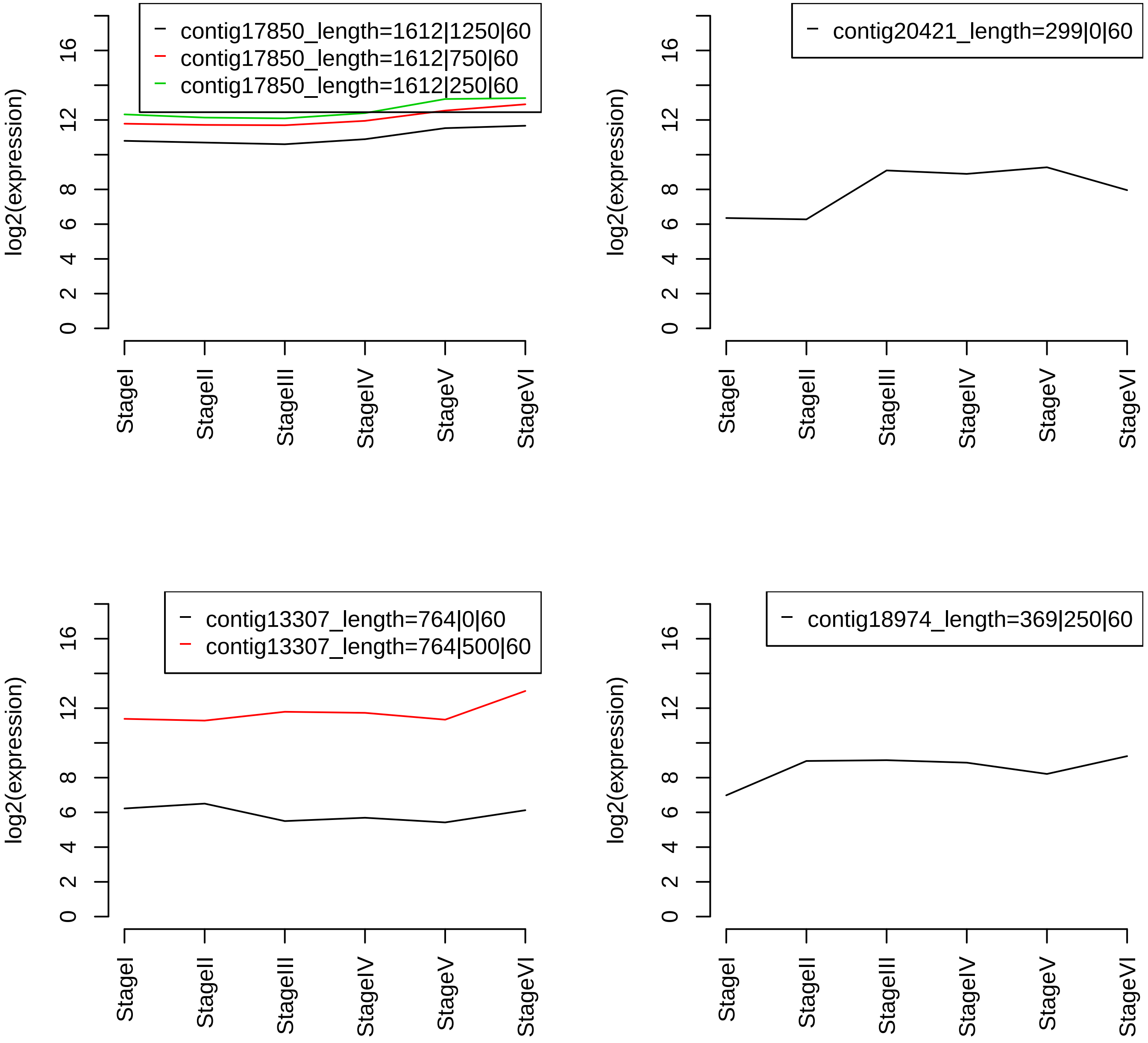
contig17850_length=1612|1250|60
contig17850_length=1612|750|60
contig17850_length=1612|250|60
contig20421_length=299|0|60
contig13307_length=764|0|60
contig13307_length=764|500|60
contig18974_length=369|250|60
log2(expression)
0
2
4
6
8
12
16
StageI
StageII
StageIII
StageIV
StageV
StageVI

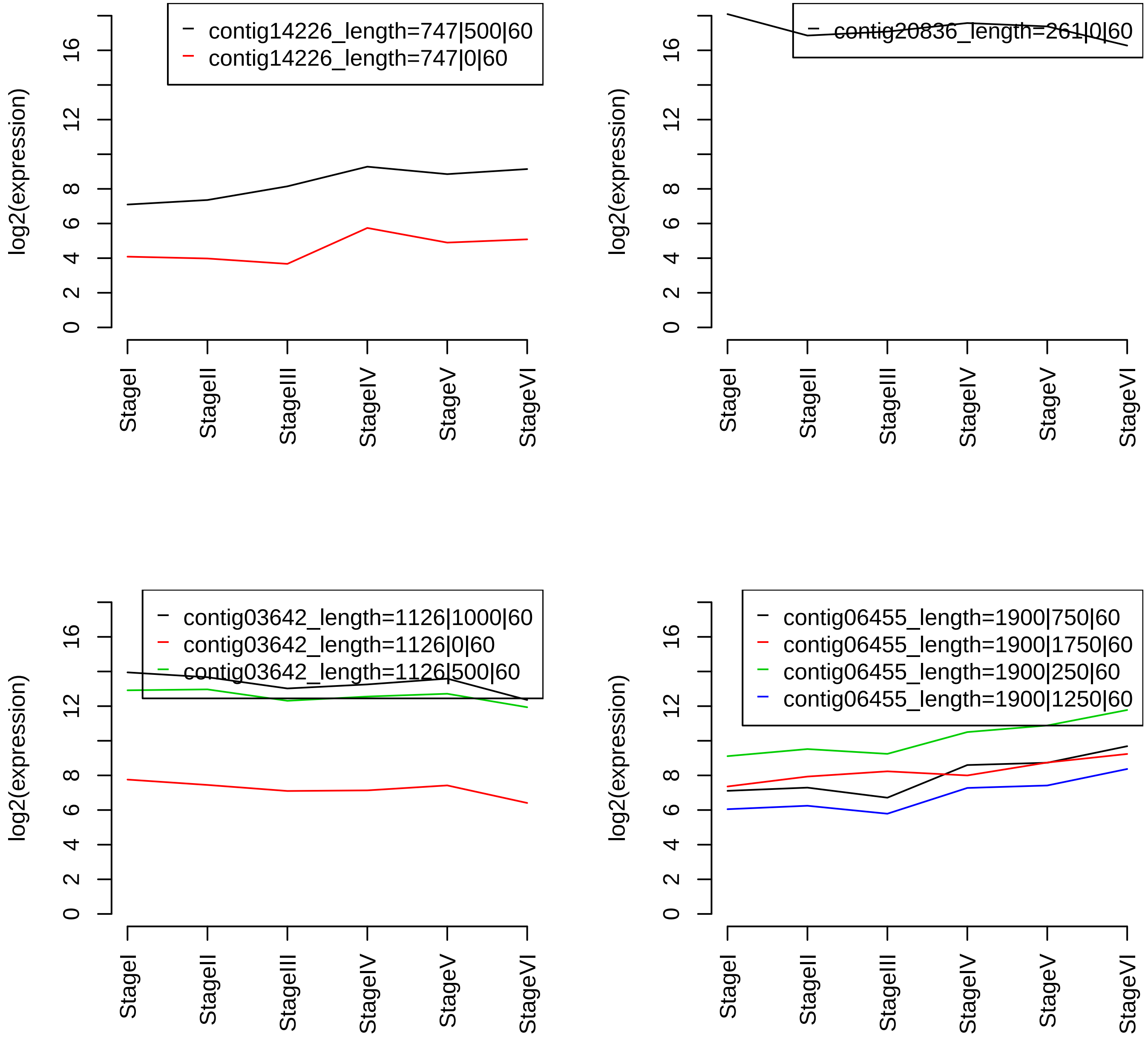

contig14226_length=747|500|60
contig14226_length=747|0|60
log2(expression)
0
2
4
6
8
12
16
StageI
StageII
StageIII
StageIV
StageV
StageVI
contig20836_length=261|0|60
contig03642_length=1126|1000|60
contig03642_length=1126|0|60
contig03642_length=1126|500|60
contig06455_length=1900|750|60
contig06455_length=1900|1750|60
contig06455_length=1900|250|60
contig06455_length=1900|1250|60

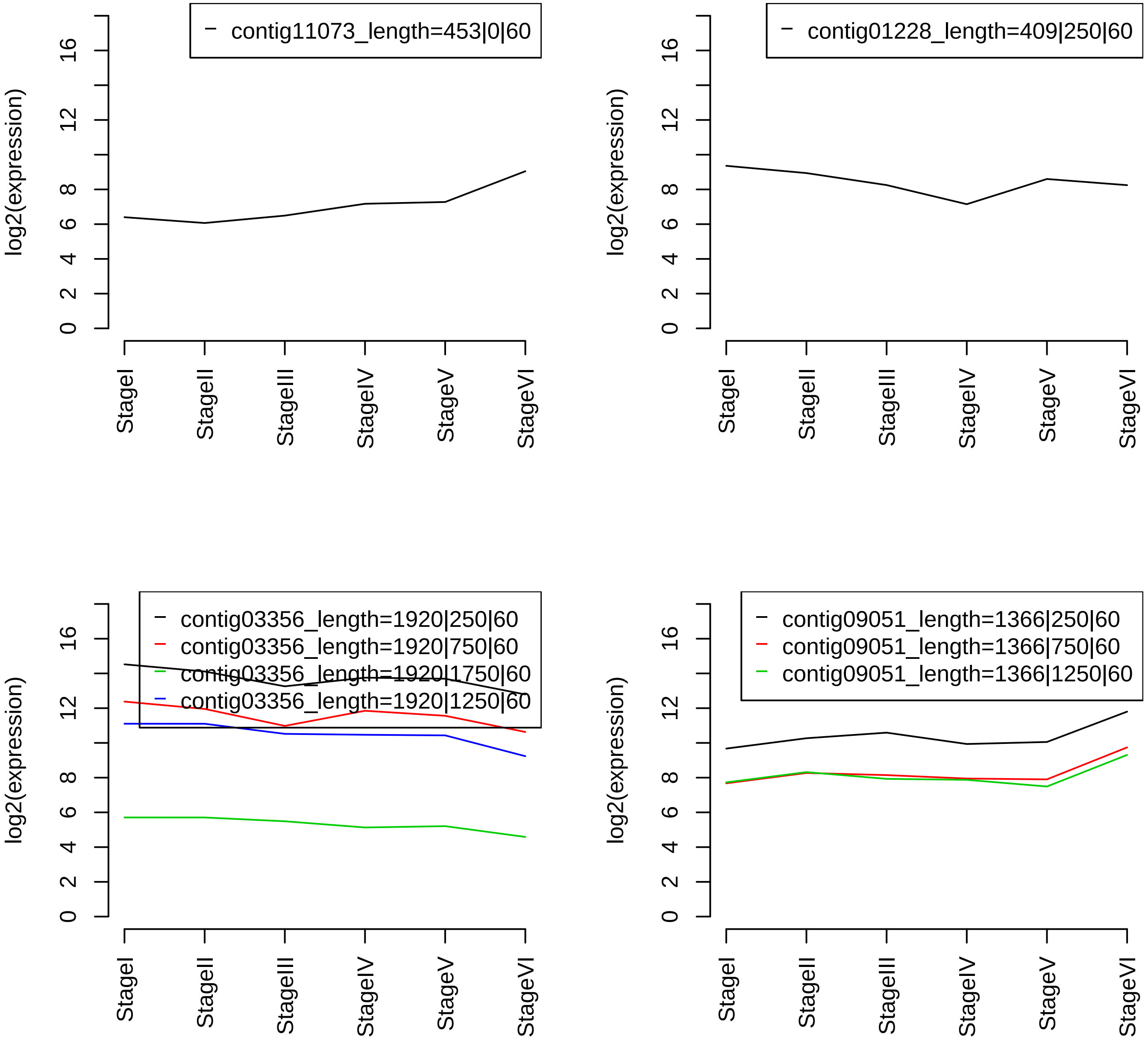
contig11073_length=453|0|60
contig01228_length=409|250|60
contig03356_length=1920|250|60
contig03356_length=1920|750|60
contig03356_length=1920|1750|60
contig03356_length=1920|1250|60
contig09051_length=1366|250|60
contig09051_length=1366|750|60
contig09051_length=1366|1250|60
log2(expression)
0
2
4
6
8
12
16
StageI
StageII
StageIII
StageIV
StageV
StageVI

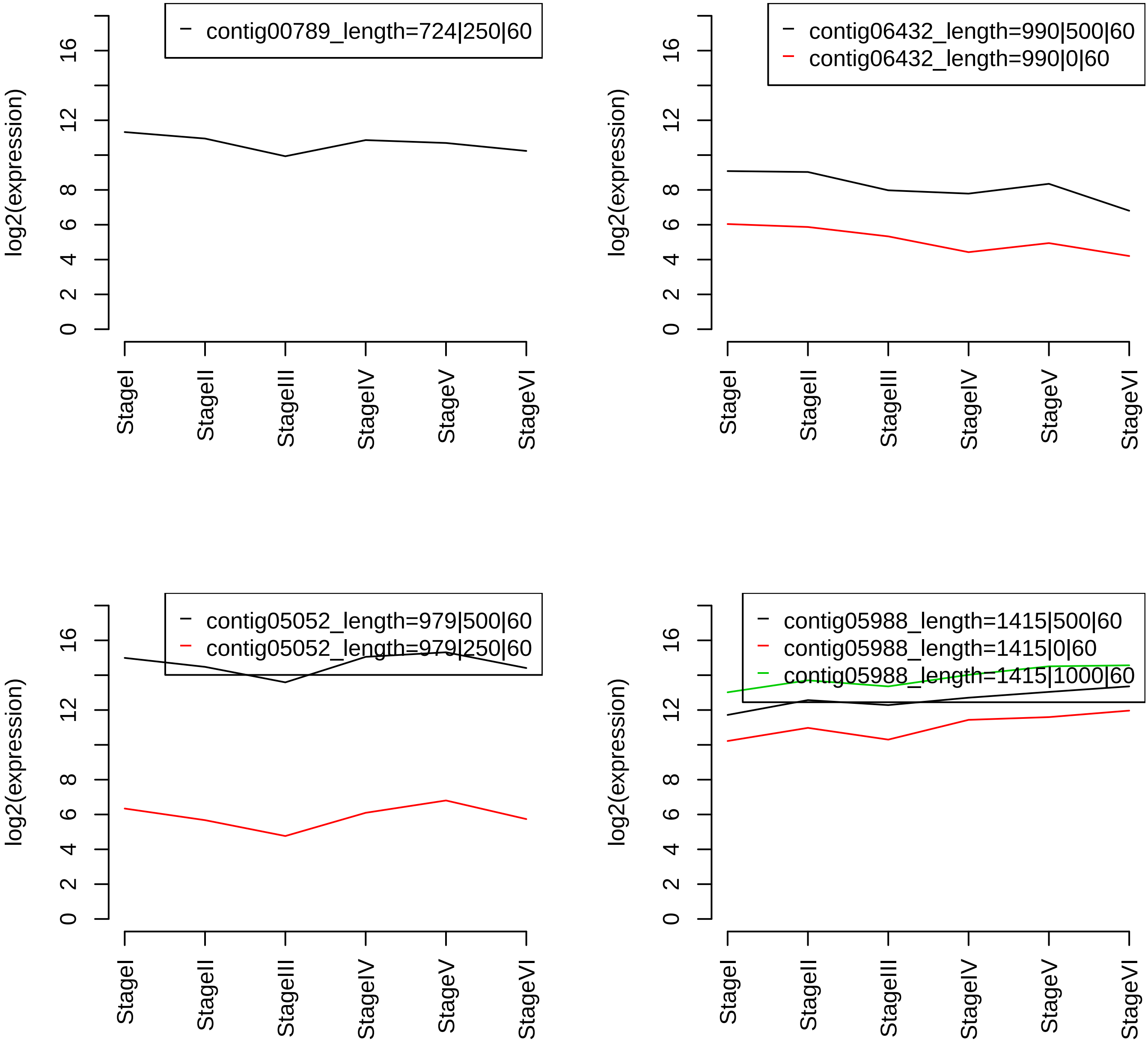
contig00789_length=724|250|60
contig06432_length=990|500|60
contig06432_length=990|0|60
contig05052_length=979|500|60
contig05052_length=979|250|60
contig05988_length=1415|500|60
contig05988_length=1415|0|60
contig05988_length=1415|1000|60
log2(expression)
0 2 4 6 8 12 16
StageI
StageII
StageIII
StageIV
StageV
StageVI

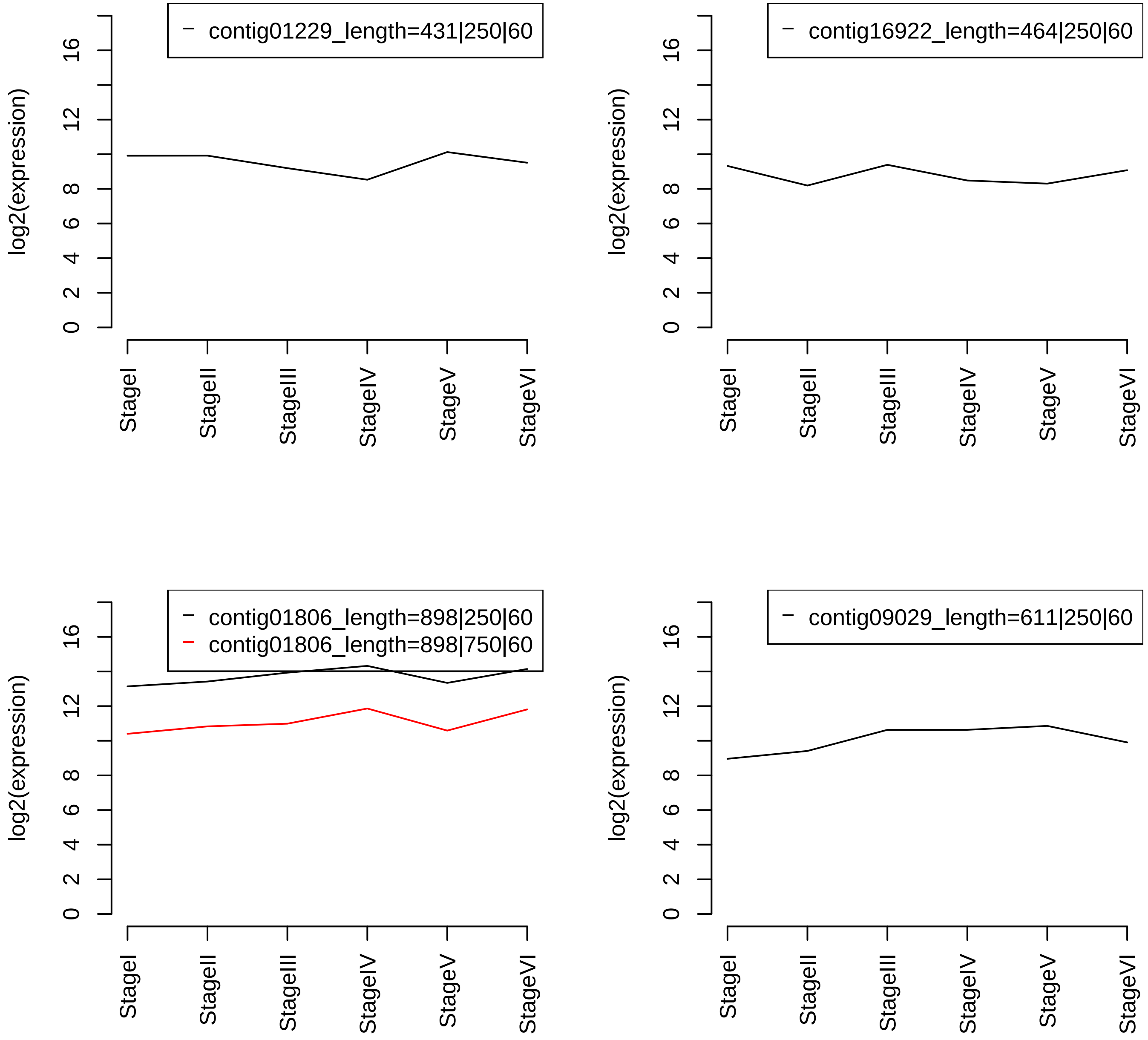
contig01229_length=431|250|60
contig16922_length=464|250|60
contig01806_length=898|250|60
contig01806_length=898|750|60
contig09029_length=611|250|60
log2(expression)
0
2
4
6
8
12
16
StageI
StageII
StageIII
StageIV
StageV
StageVI

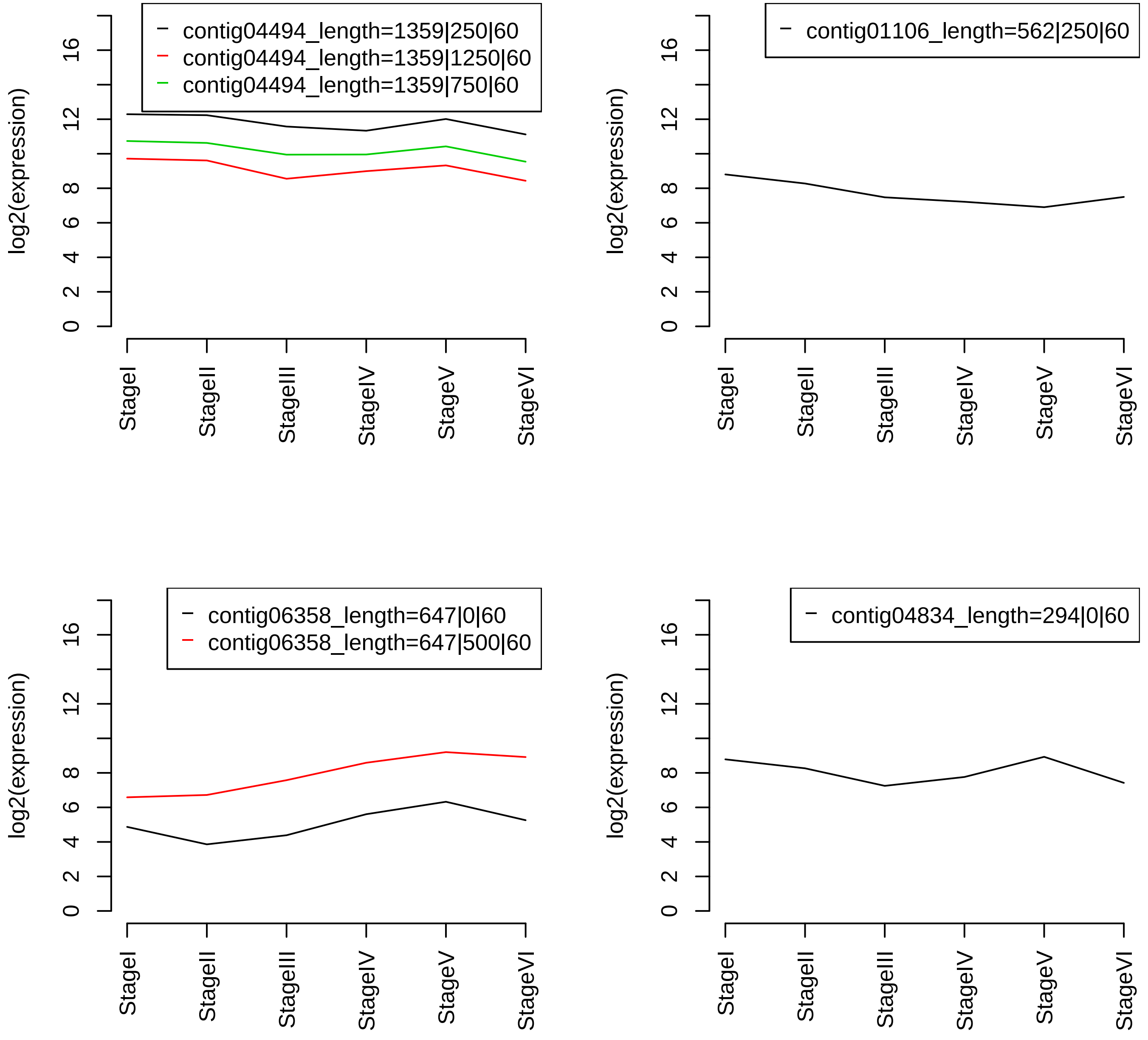

contig04494_length=1359|250|60
contig04494_length=1359|1250|60
contig04494_length=1359|750|60
contig01106_length=562|250|60
contig06358_length=647|0|60
contig06358_length=647|500|60
contig04834_length=294|0|60
log2(expression)
0
2
4
6
8
12
16
StageI
StageII
StageIII
StageIV
StageV
StageVI

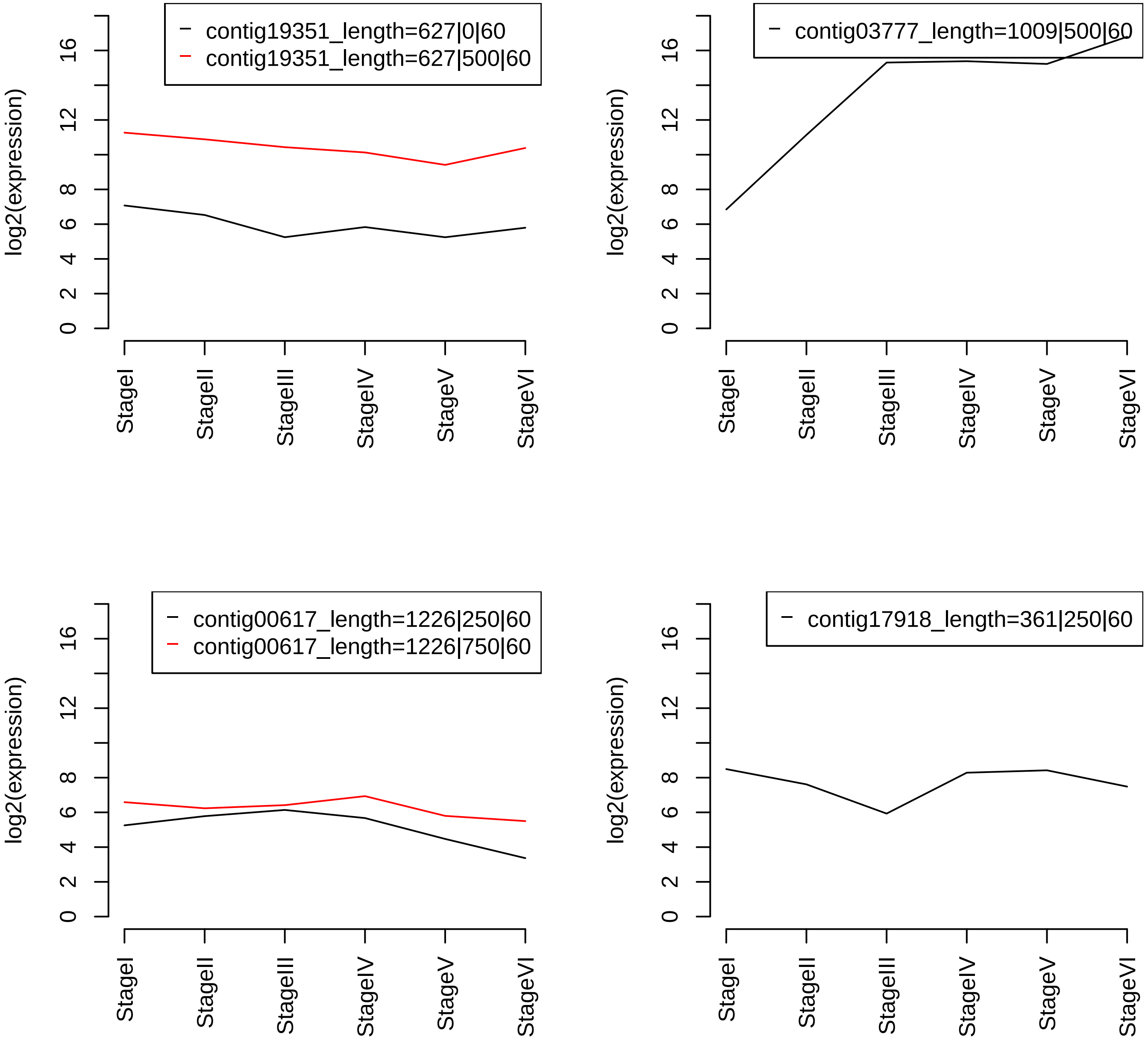

contig19351_length=627|0|60
contig19351_length=627|500|60
contig03777_length=1009|500|60
contig00617_length=1226|250|60
contig00617_length=1226|750|60
contig17918_length=361|250|60
log2(expression)
0
2
4
6
8
12
16
StageI
StageII
StageIII
StageIV
StageV
StageVI

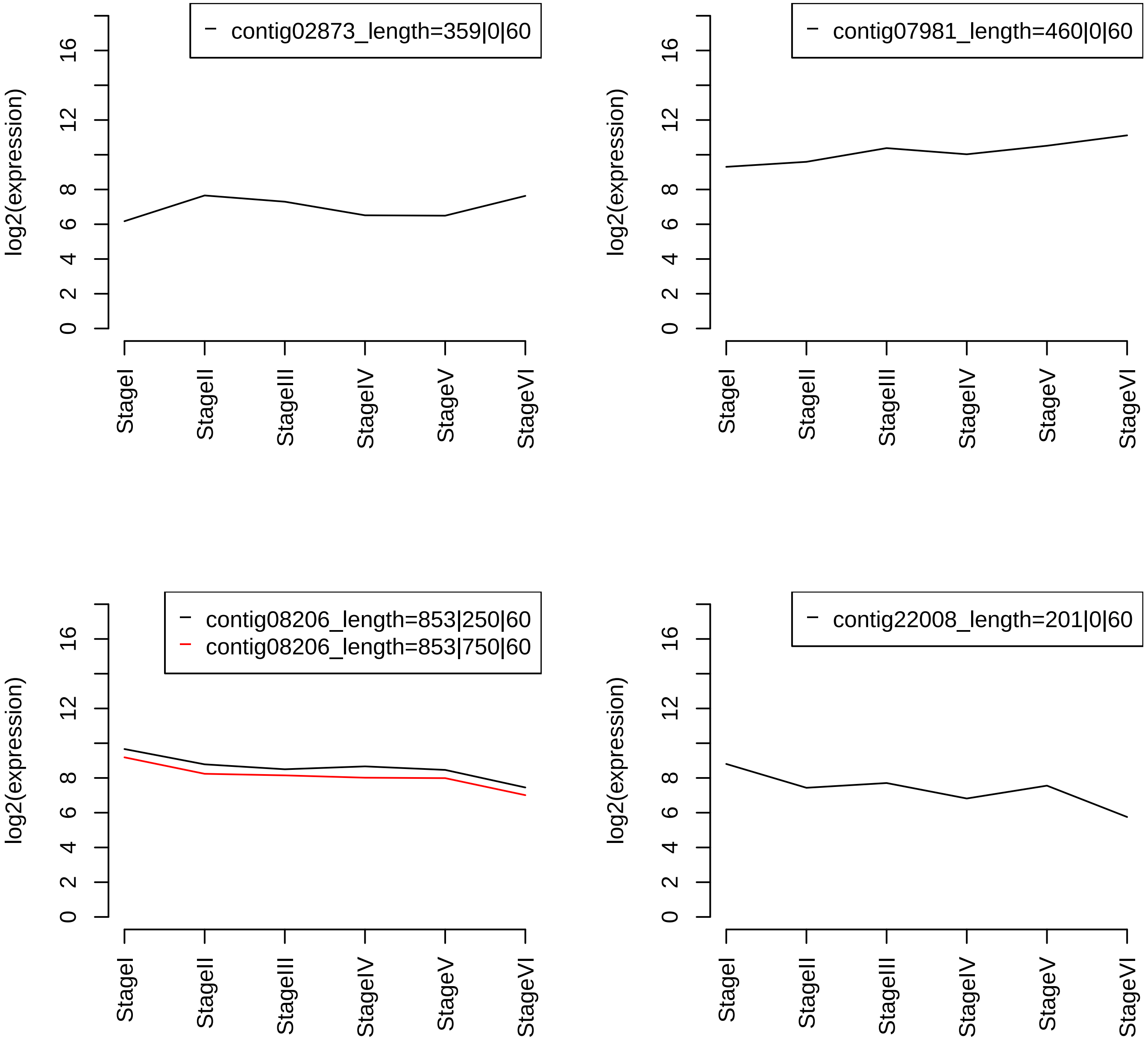
contig02873_length=359|0|60
contig07981_length=460|0|60
contig08206_length=853|250|60
contig08206_length=853|750|60
contig22008_length=201|0|60
log2(expression)
0
2
4
6
8
12
16
StageI
StageII
StageIII
StageIV
StageV
StageVI

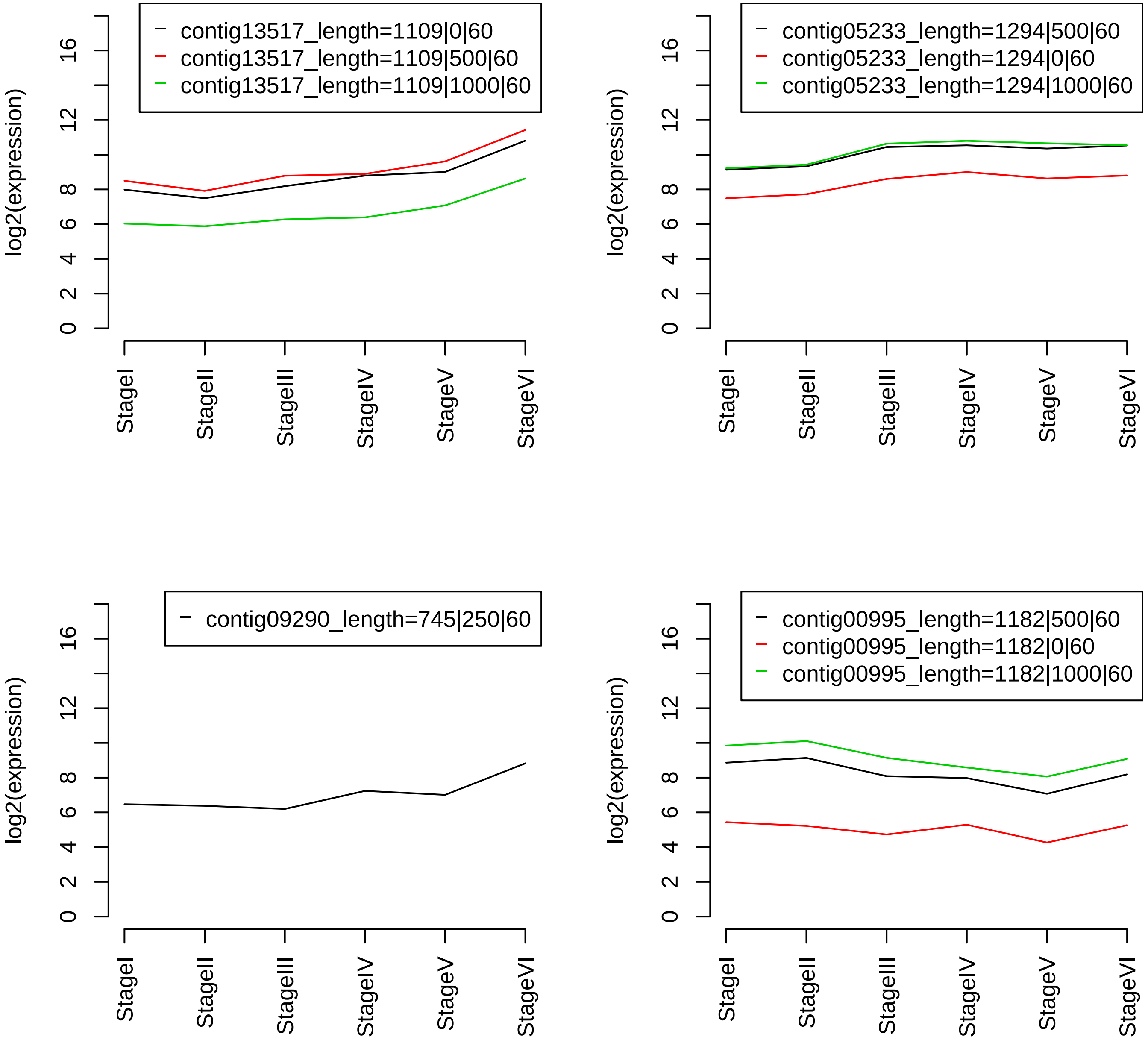

contig13517_length=1109|0|60
contig13517_length=1109|500|60
contig13517_length=1109|1000|60
log2(expression)
0
2
4
6
8
12
16
StageI
StageII
StageIII
StageIV
StageV
StageVI
contig05233_length=1294|500|60
contig05233_length=1294|0|60
contig05233_length=1294|1000|60
contig09290_length=745|250|60
contig00995_length=1182|500|60
contig00995_length=1182|0|60
contig00995_length=1182|1000|60

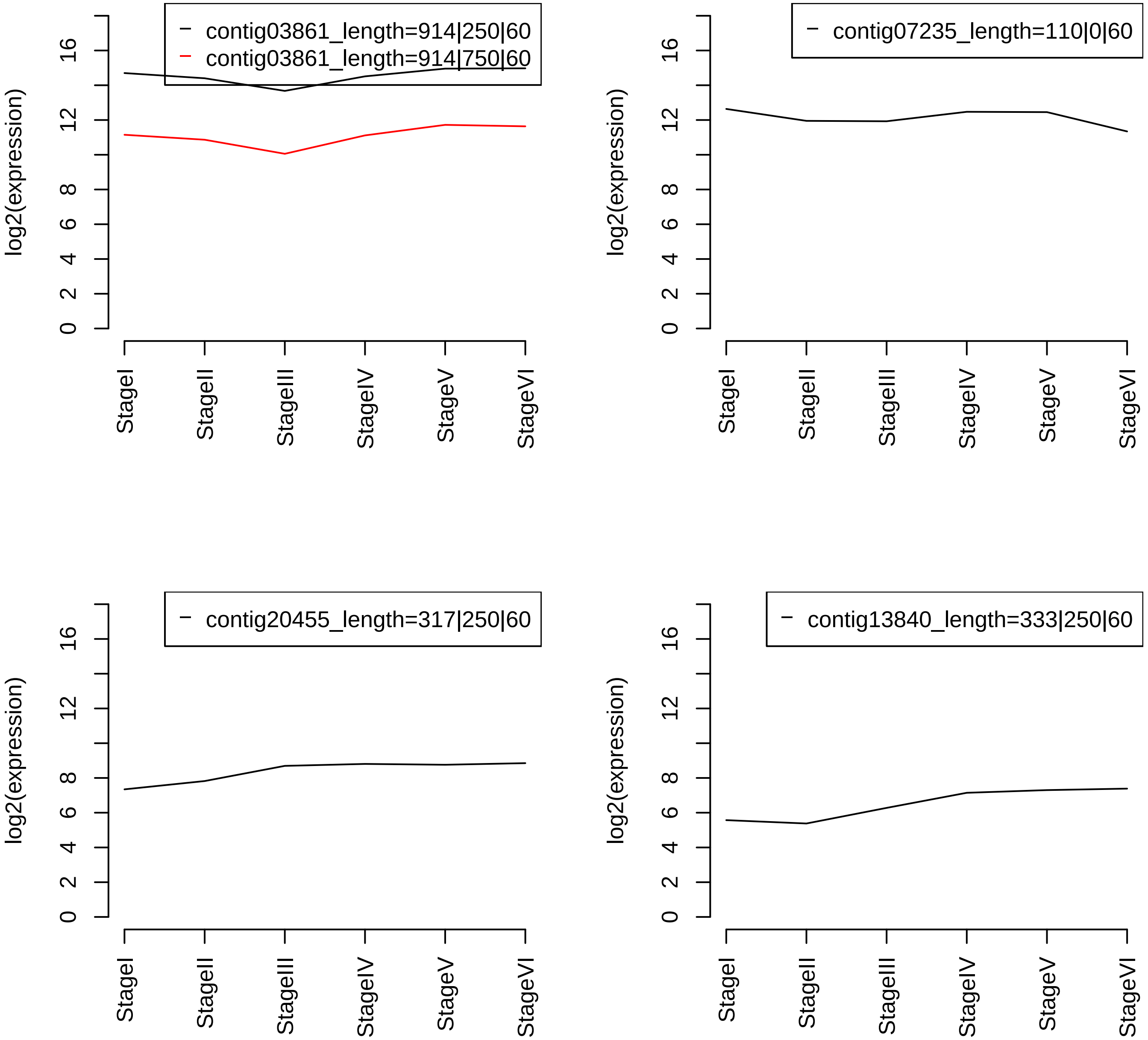

contig03861_length=914|250|60
contig03861_length=914|750|60
contig07235_length=110|0|60
contig20455_length=317|250|60
contig13840_length=333|250|60
log2(expression)
0
2
4
6
8
12
16
StageI
StageII
StageIII
StageIV
StageV
StageVI

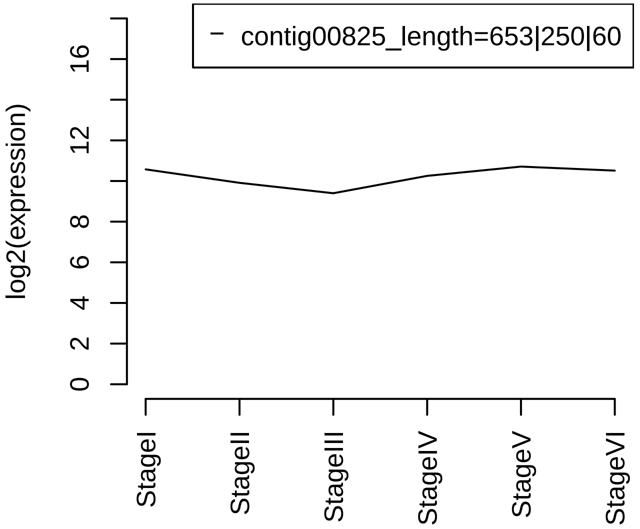
contig00825_length=653|250|60
log2(expression)
0
2
4
6
8
12
16
StageI
StageII
StageIII
StageIV
StageV
StageVI

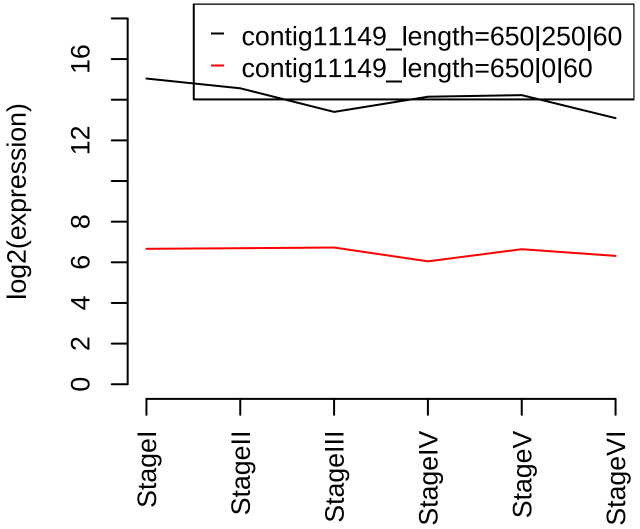
contig11149_length=650|250|60
contig11149_length=650|0|60
log2(expression)
0
2
4
6
8
12
16
StageI
StageII
StageIII
StageIV
StageV
StageVI

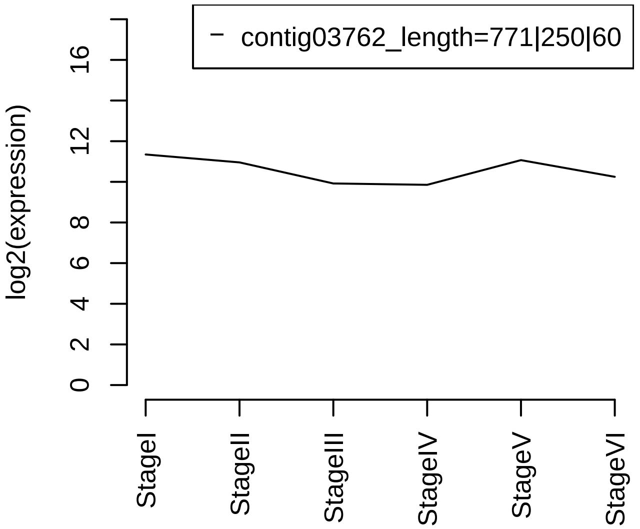
contig03762_length=771|250|60
log2(expression)
0
2
4
6
8
12
16
StageI
StageII
StageIII
StageIV
StageV
StageVI

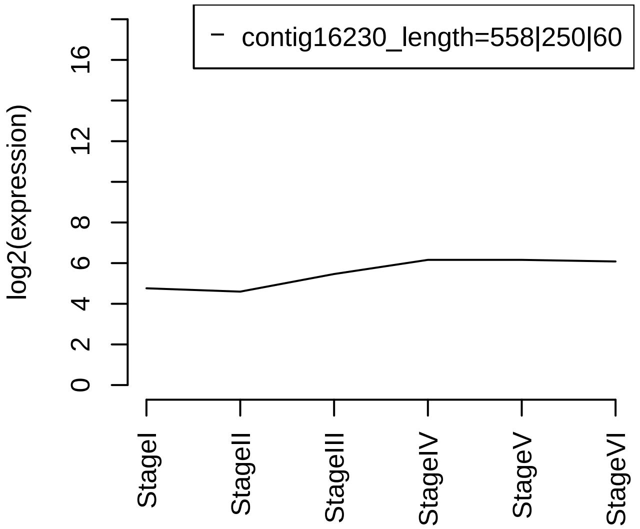
contig16230_length=558|250|60
log2(expression)
0
2
4
6
8
12
16
StageI
StageII
StageIII
StageIV
StageV
StageVI

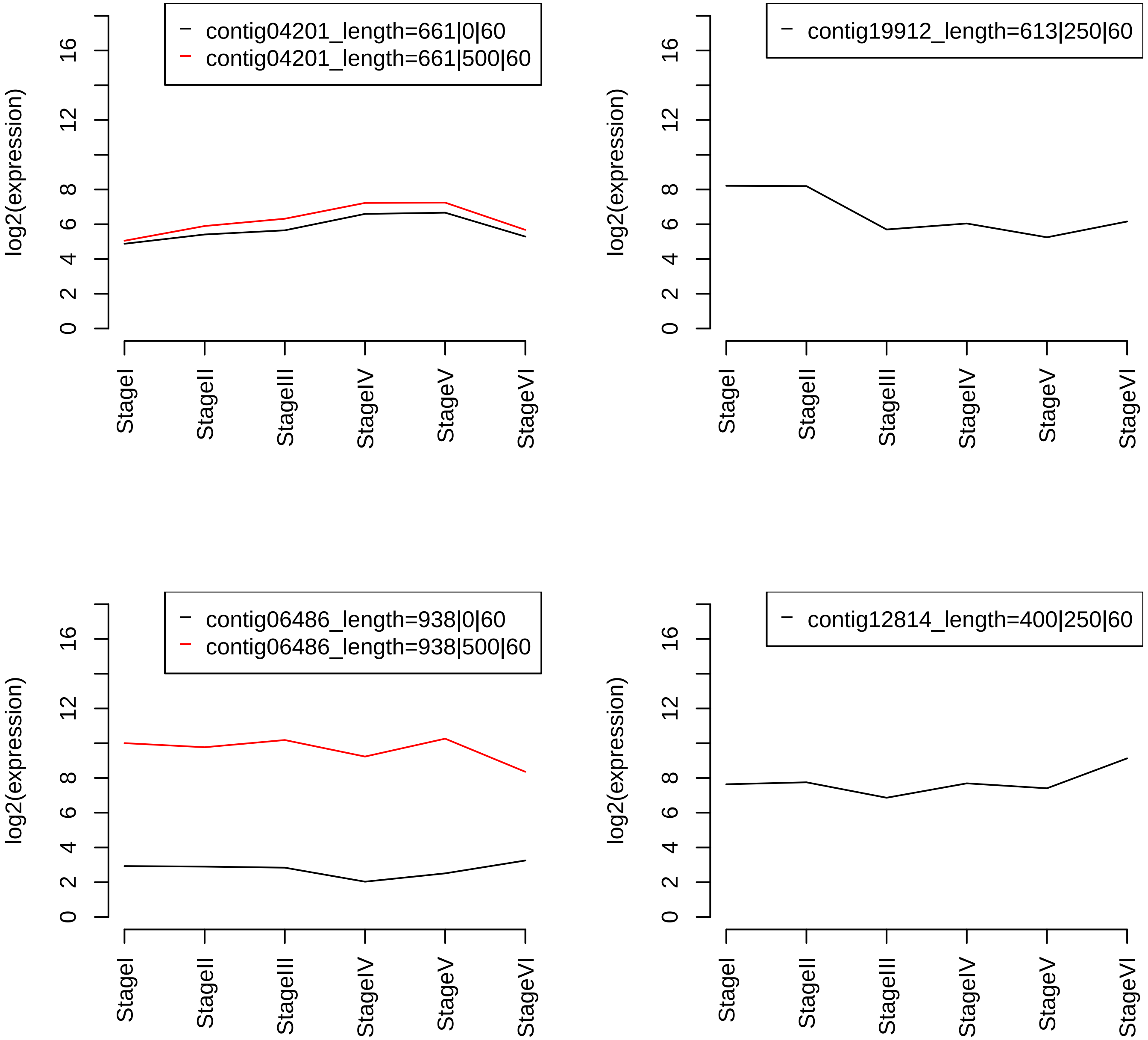

contig04201_length=661|0|60
contig04201_length=661|500|60
contig19912_length=613|250|60
contig06486_length=938|0|60
contig06486_length=938|500|60
contig12814_length=400|250|60
log2(expression)
0
2
4
6
8
12
16
StageI
StageII
StageIII
StageIV
StageV
StageVI

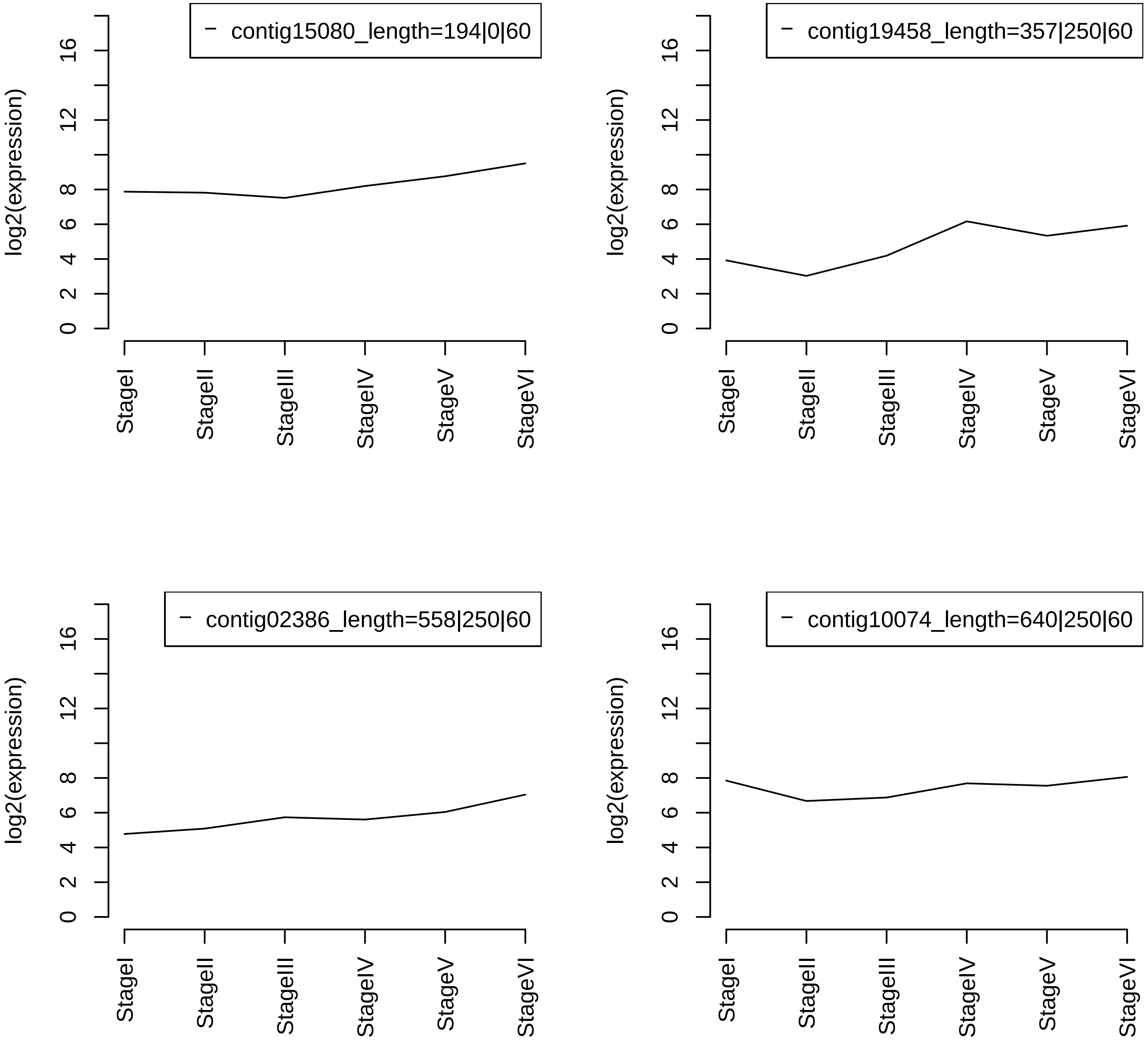

contig15080_length=194|0|60
contig19458_length=357|250|60
contig02386_length=558|250|60
contig10074_length=640|250|60
log2(expression)
0
2
4
6
8
12
16
StageI
StageII
StageIII
StageIV
StageV
StageVI

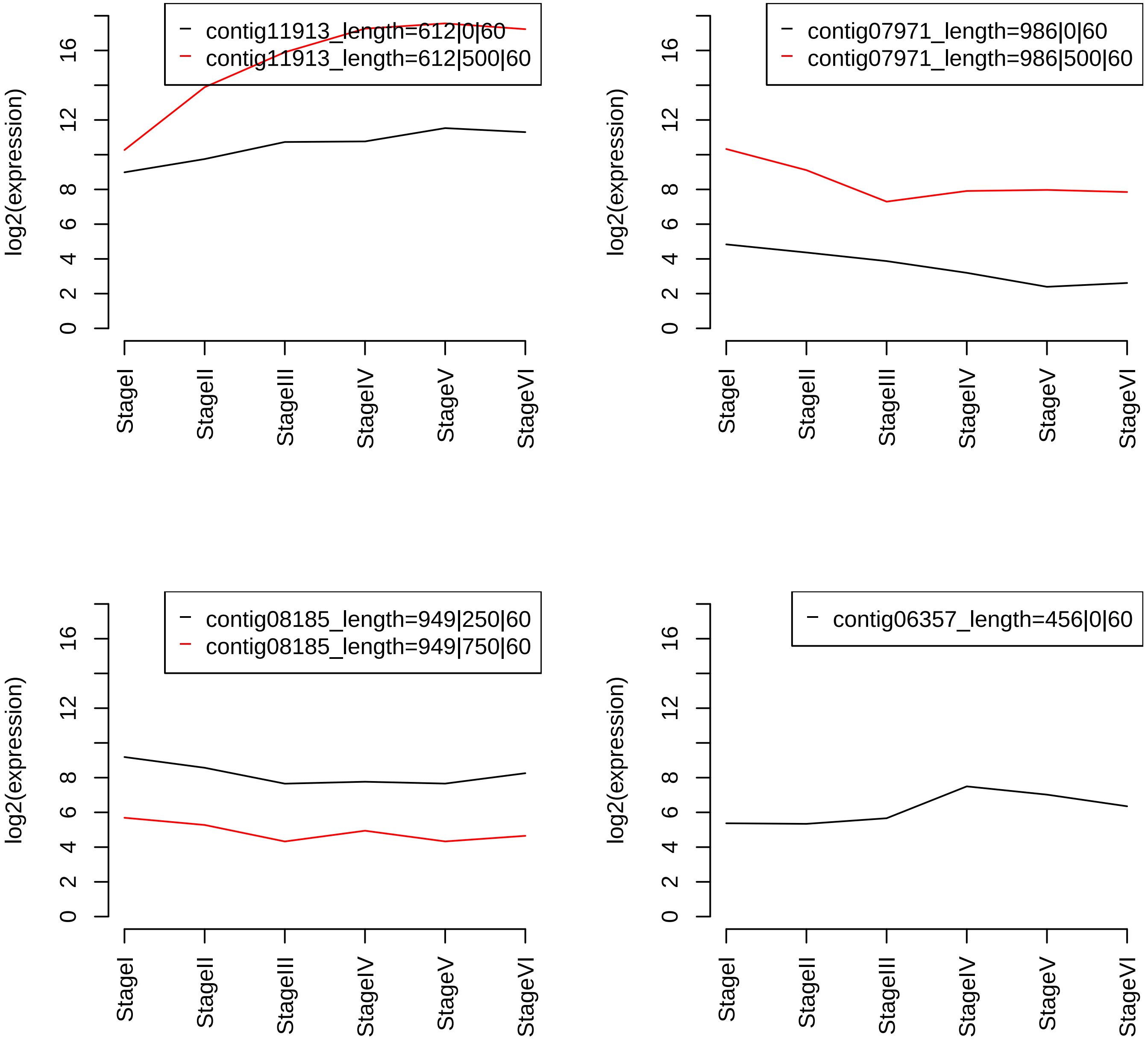

contig11913_length=612|0|60
contig11913_length=612|500|60
contig07971_length=986|0|60
contig07971_length=986|500|60
contig08185_length=949|250|60
contig08185_length=949|750|60
contig06357_length=456|0|60
log2(expression)
0
2
4
6
8
12
16
StageI
StageII
StageIII
StageIV
StageV
StageVI

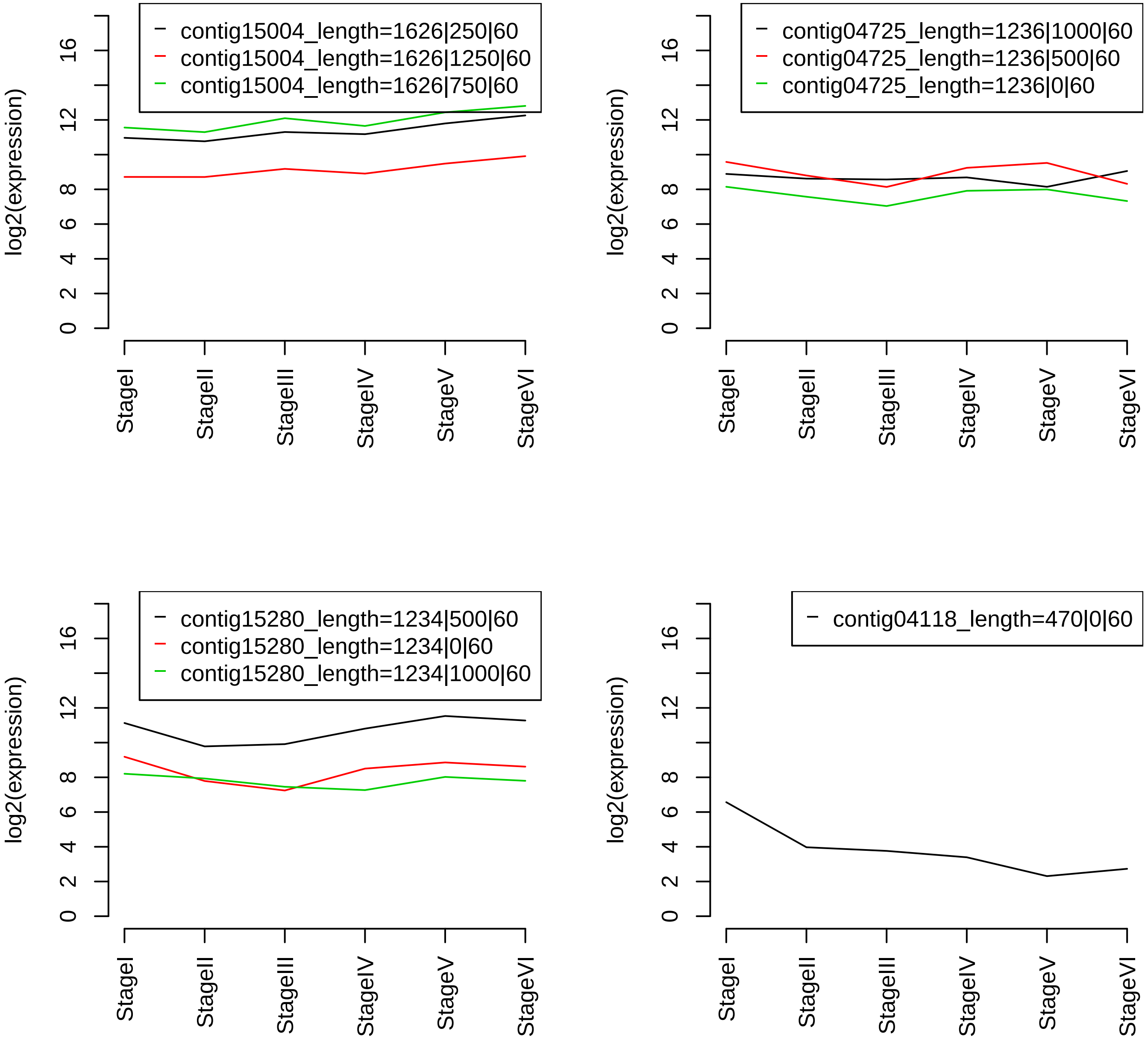

contig15004_length=1626|250|60
contig15004_length=1626|1250|60
contig15004_length=1626|750|60
contig04725_length=1236|1000|60
contig04725_length=1236|500|60
contig04725_length=1236|0|60
contig15280_length=1234|500|60
contig15280_length=1234|0|60
contig15280_length=1234|1000|60
contig04118_length=470|0|60
log2(expression)
StageI
StageII
StageIII
StageIV
StageV
StageVI

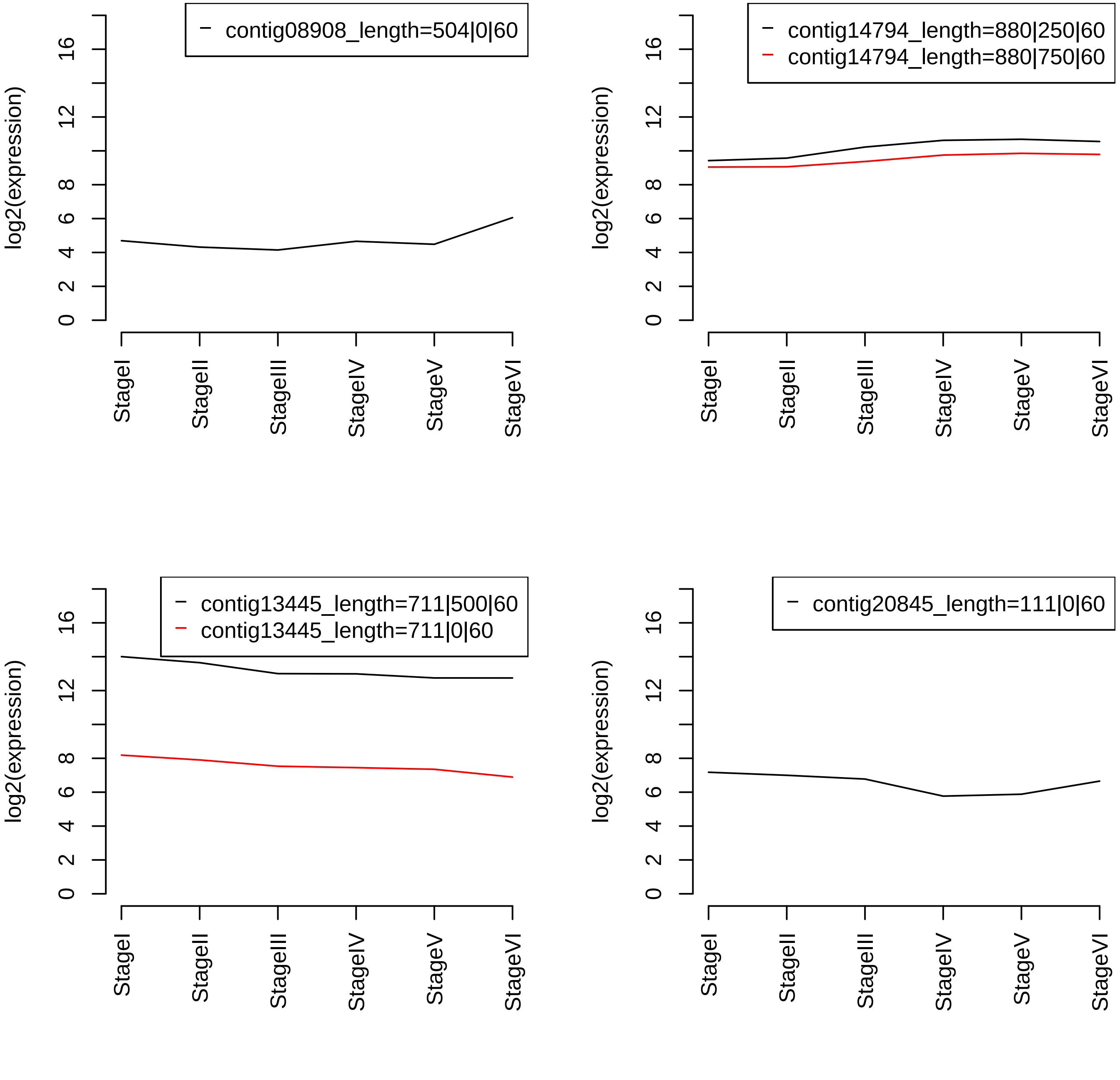

contig08908_length=504|0|60
contig14794_length=880|250|60
contig14794_length=880|750|60
contig13445_length=711|500|60
contig13445_length=711|0|60
contig20845_length=111|0|60
log2(expression)
0
2
4
6
8
12
16
StageI
StageII
StageIII
StageIV
StageV
StageVI

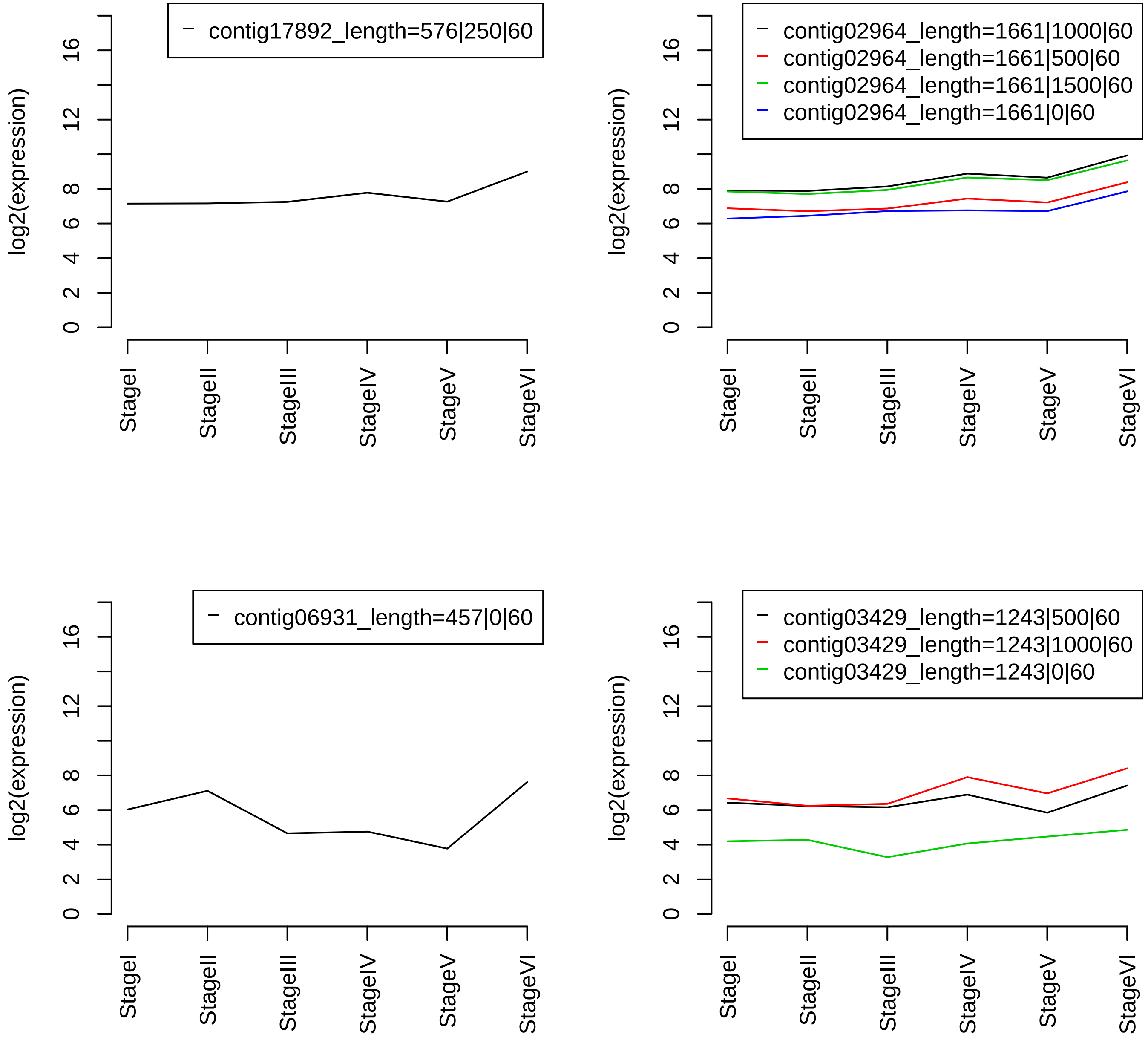

contig17892_length=576|250|60
contig02964_length=1661|1000|60
contig02964_length=1661|500|60
contig02964_length=1661|1500|60
contig02964_length=1661|0|60
contig06931_length=457|0|60
contig03429_length=1243|500|60
contig03429_length=1243|1000|60
contig03429_length=1243|0|60
log2(expression)
0
2
4
6
8
12
16
StageI
StageII
StageIII
StageIV
StageV
StageVI

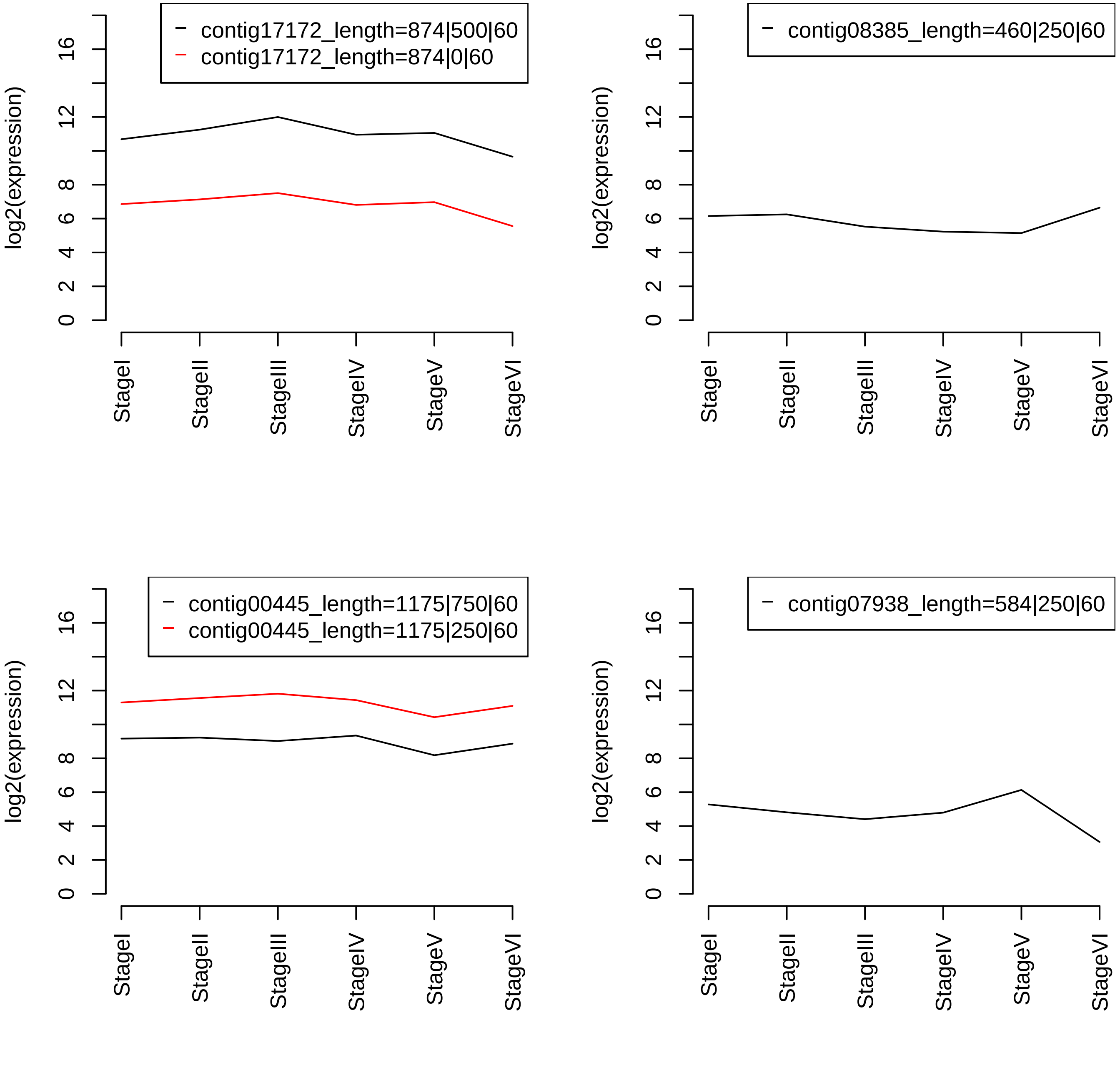

contig17172_length=874|500|60
contig17172_length=874|0|60
contig08385_length=460|250|60
contig00445_length=1175|750|60
contig00445_length=1175|250|60
contig07938_length=584|250|60
log2(expression)
0
2
4
6
8
12
16
StageI
StageII
StageIII
StageIV
StageV
StageVI

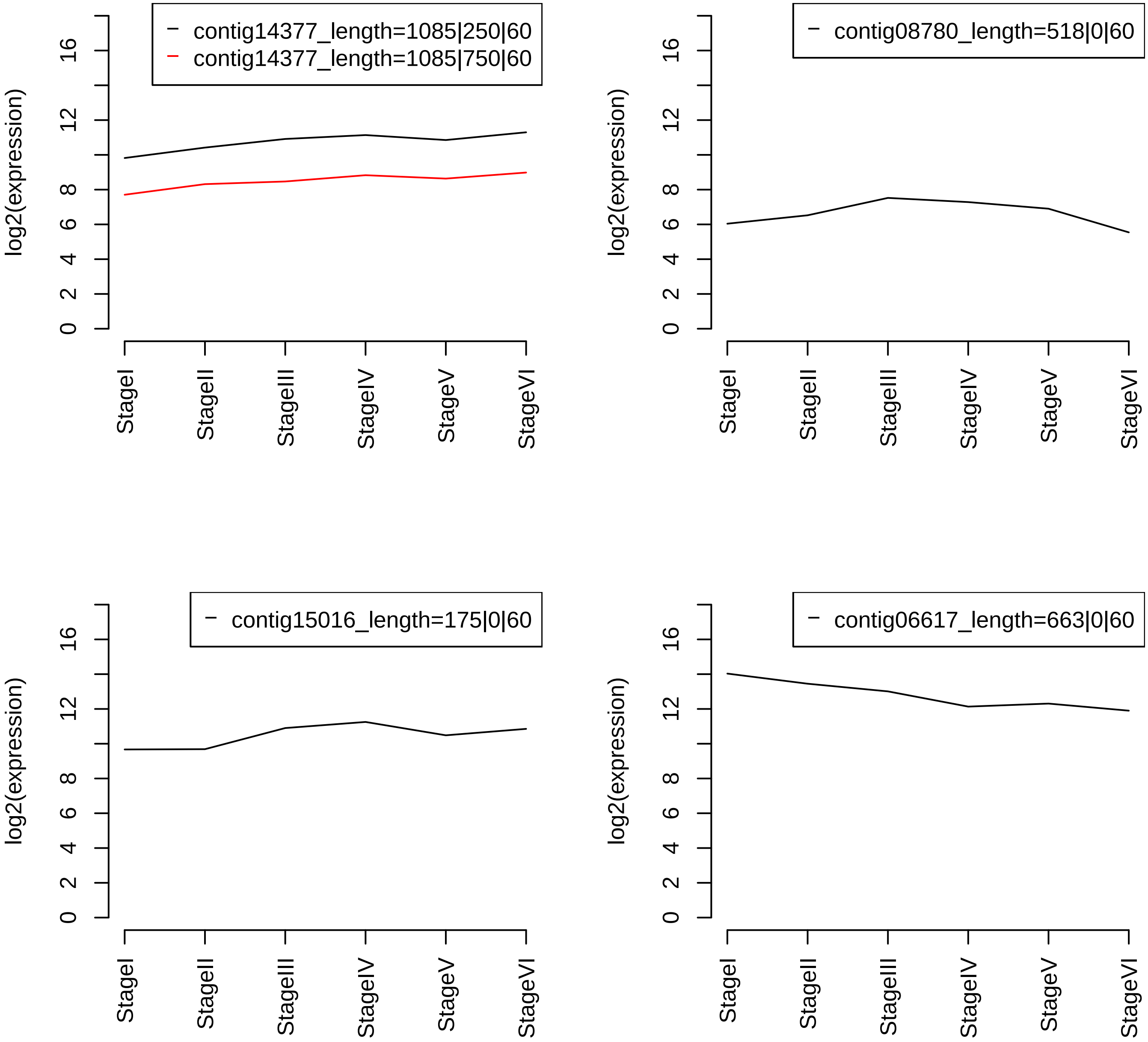

contig14377_length=1085|250|60
contig14377_length=1085|750|60
contig08780_length=518|0|60
contig15016_length=175|0|60
contig06617_length=663|0|60
log2(expression)
0
2
4
6
8
12
16
StageI
StageII
StageIII
StageIV
StageV
StageVI

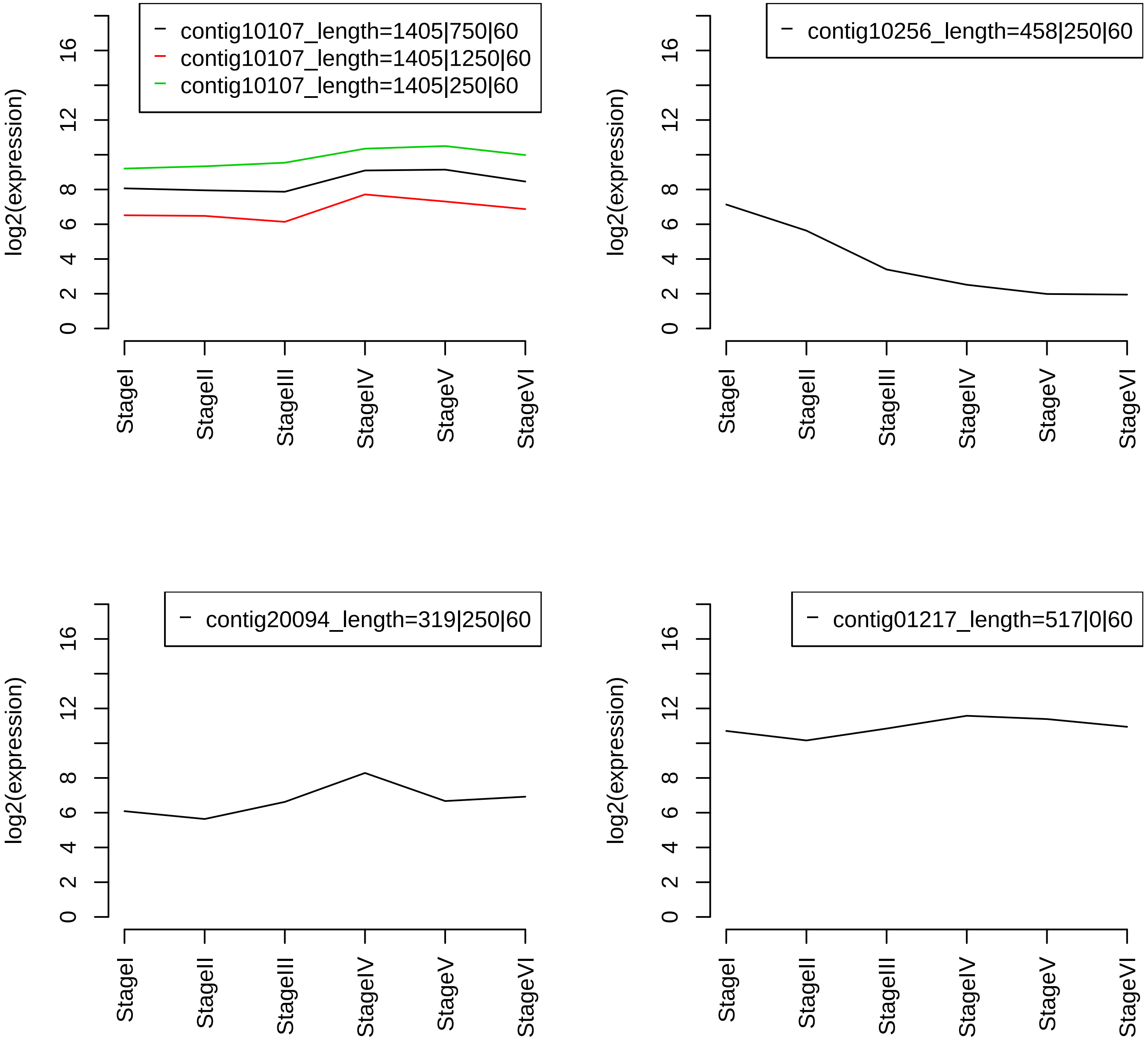
contig10107_length=1405|750|60
contig10107_length=1405|1250|60
contig10107_length=1405|250|60
contig10256_length=458|250|60
contig20094_length=319|250|60
contig01217_length=517|0|60
log2(expression)
0
2
4
6
8
12
16
StageI
StageII
StageIII
StageIV
StageV
StageVI

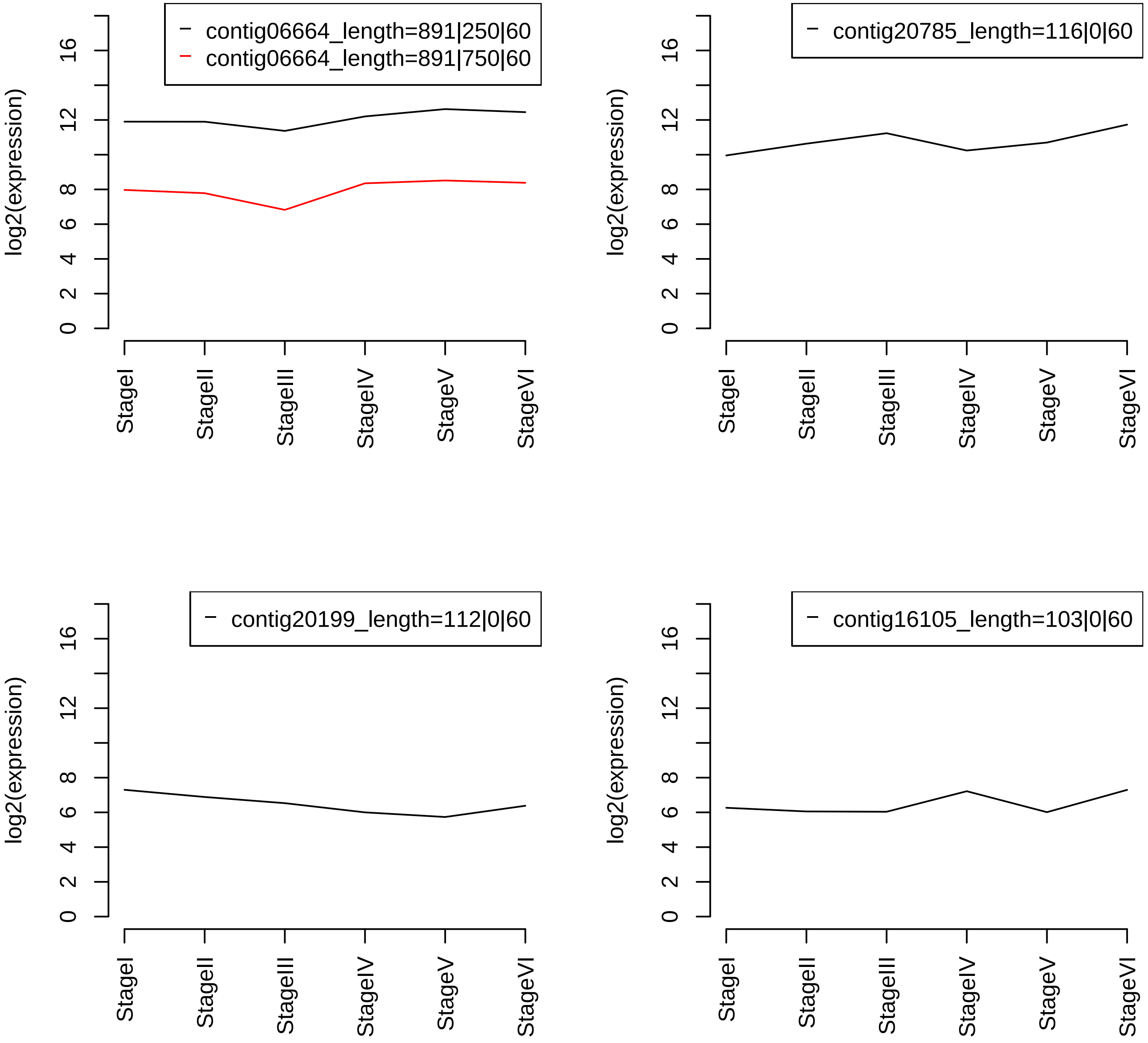

contig06664_length=891|250|60
contig06664_length=891|750|60
contig20785_length=116|0|60
contig20199_length=112|0|60
contig16105_length=103|0|60
log2(expression)
0
2
4
6
8
12
16
StageI
StageII
StageIII
StageIV
StageV
StageVI

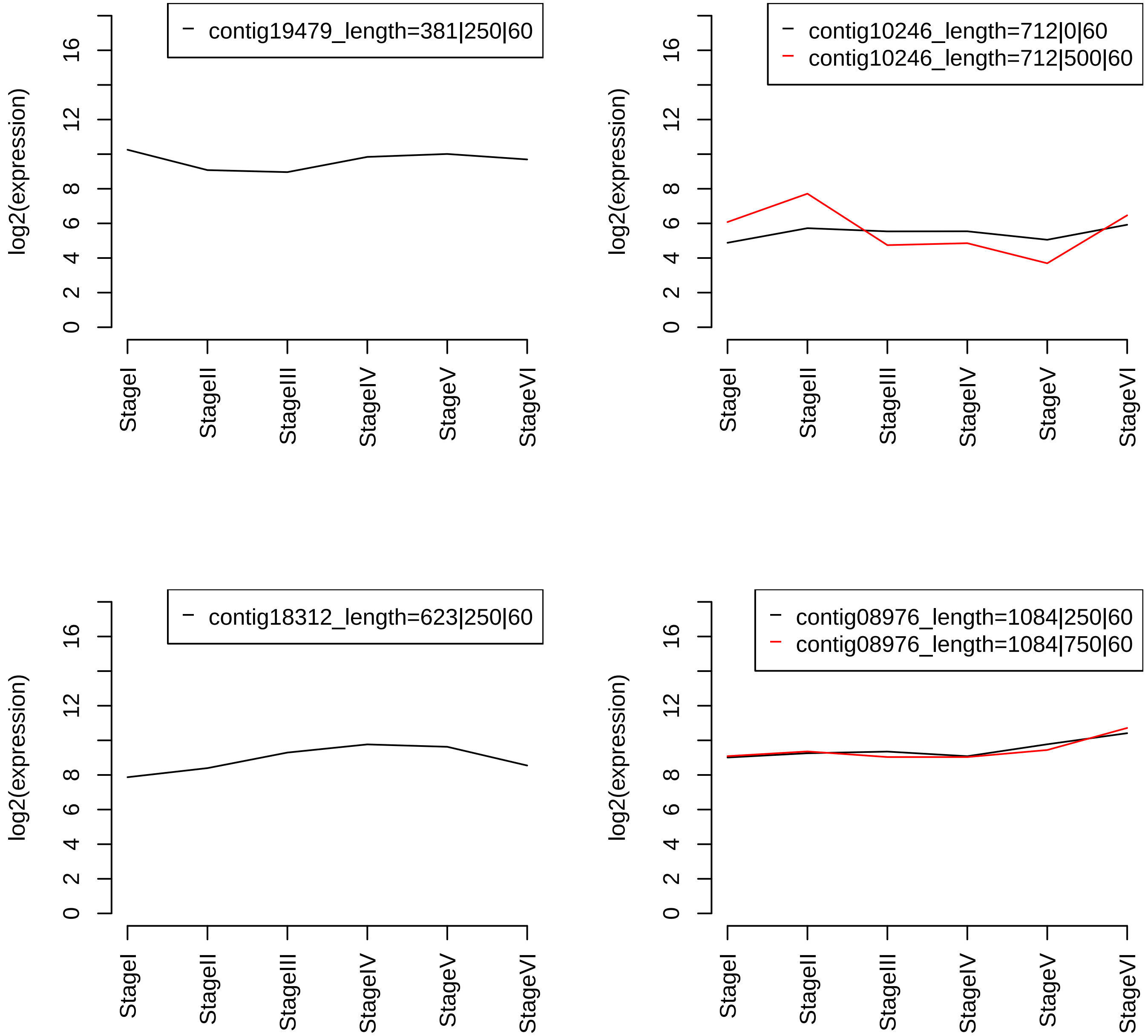
contig19479_length=381|250|60
contig10246_length=712|0|60
contig10246_length=712|500|60
contig18312_length=623|250|60
contig08976_length=1084|250|60
contig08976_length=1084|750|60
log2(expression)
0
2
4
6
8
12
16
StageI
StageII
StageIII
StageIV
StageV
StageVI

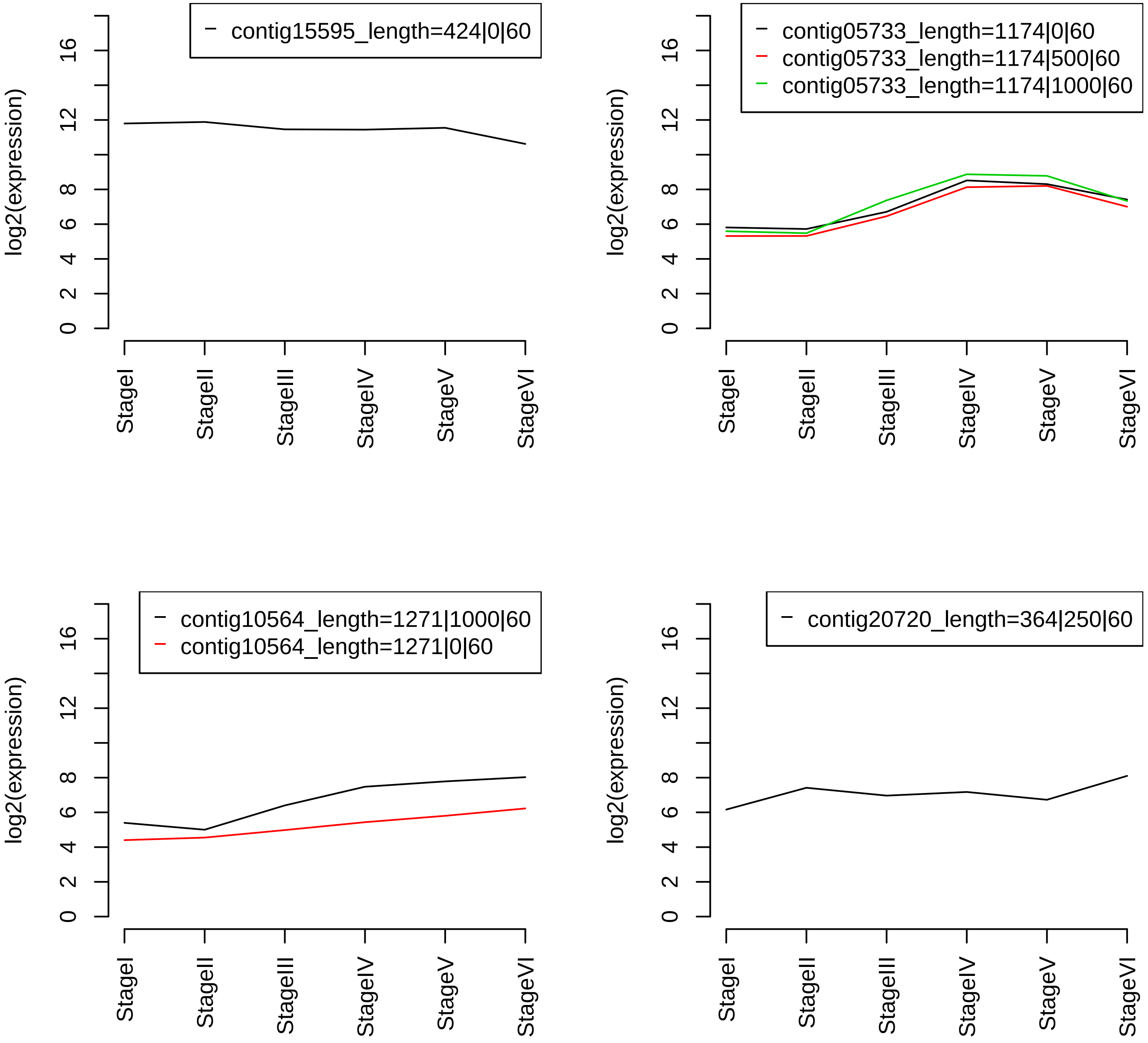

contig15595_length=424|0|60
log2(expression)
0
2
4
6
8
12
16
StageI
StageII
StageIII
StageIV
StageV
StageVI
contig05733_length=1174|0|60
contig05733_length=1174|500|60
contig05733_length=1174|1000|60
contig10564_length=1271|1000|60
contig10564_length=1271|0|60
contig20720_length=364|250|60

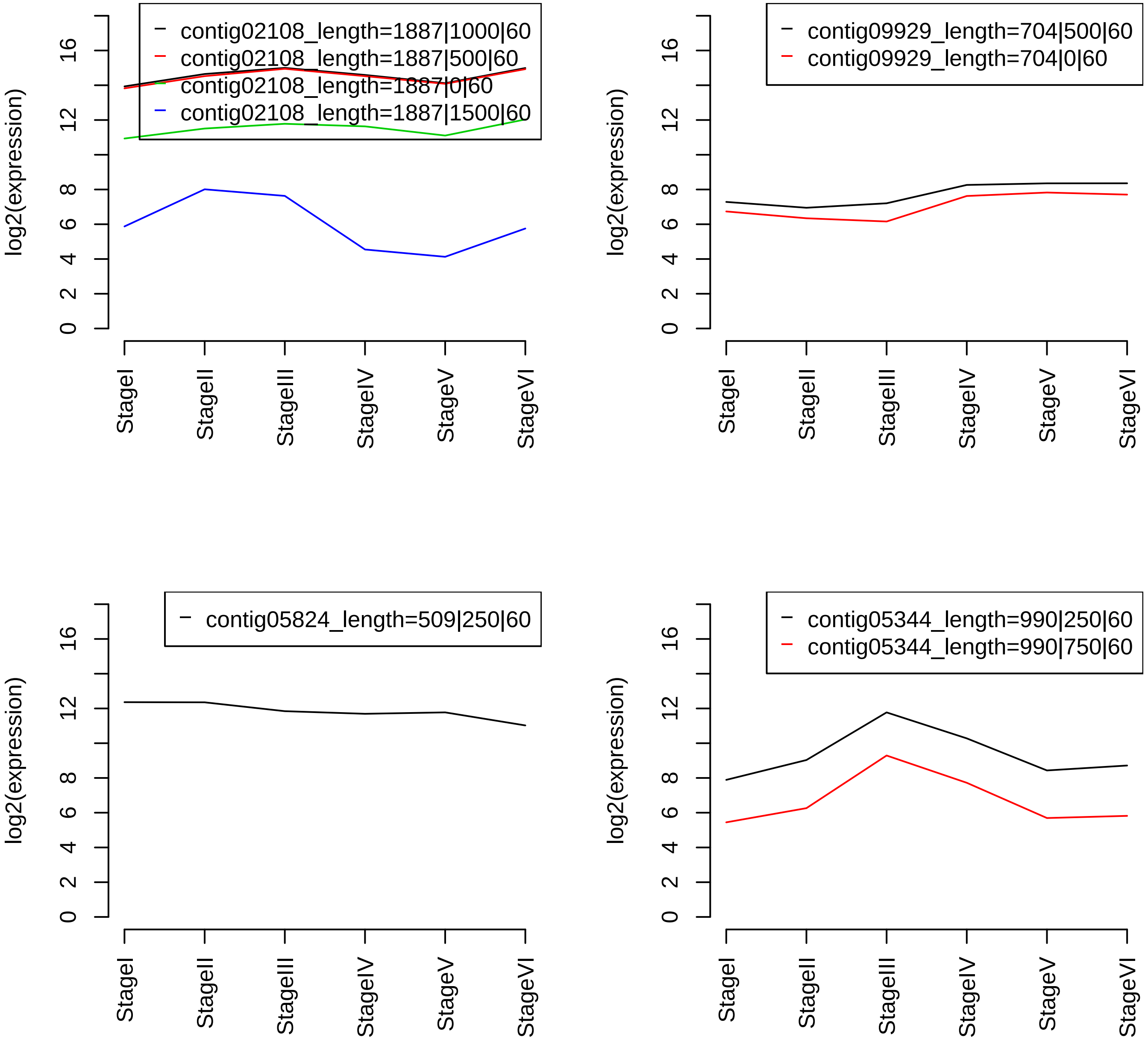

contig02108_length=1887|1000|60
contig02108_length=1887|500|60
contig02108_length=1887|0|60
contig02108_length=1887|1500|60
contig09929_length=704|500|60
contig09929_length=704|0|60
contig05824_length=509|250|60
contig05344_length=990|250|60
contig05344_length=990|750|60
log2(expression)
0
2
4
6
8
12
16
StageI
StageII
StageIII
StageIV
StageV
StageVI

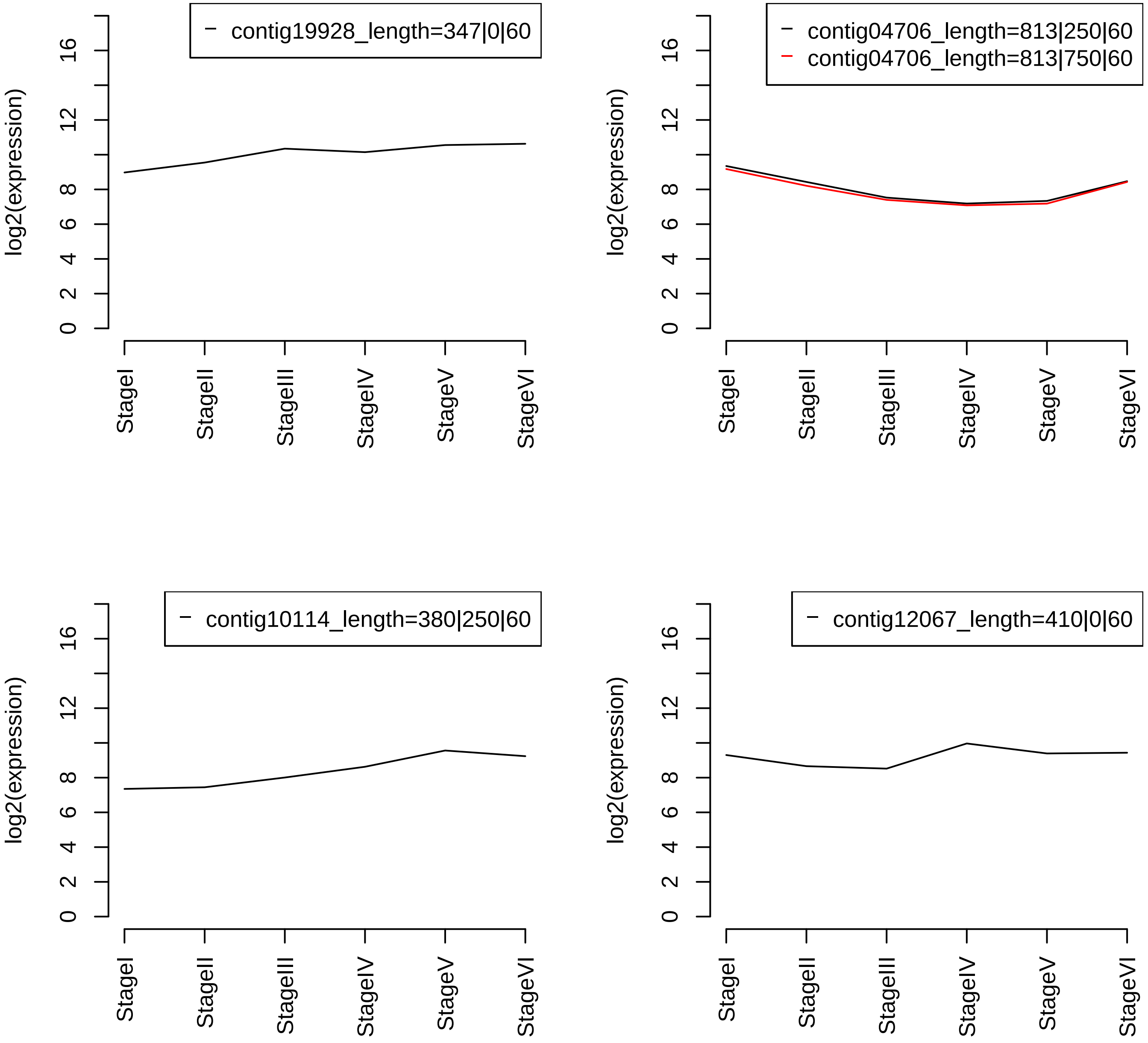

contig19928_length=347|0|60
contig04706_length=813|250|60
contig04706_length=813|750|60
contig10114_length=380|250|60
contig12067_length=410|0|60
log2(expression)
0
2
4
6
8
12
16
StageI
StageII
StageIII
StageIV
StageV
StageVI

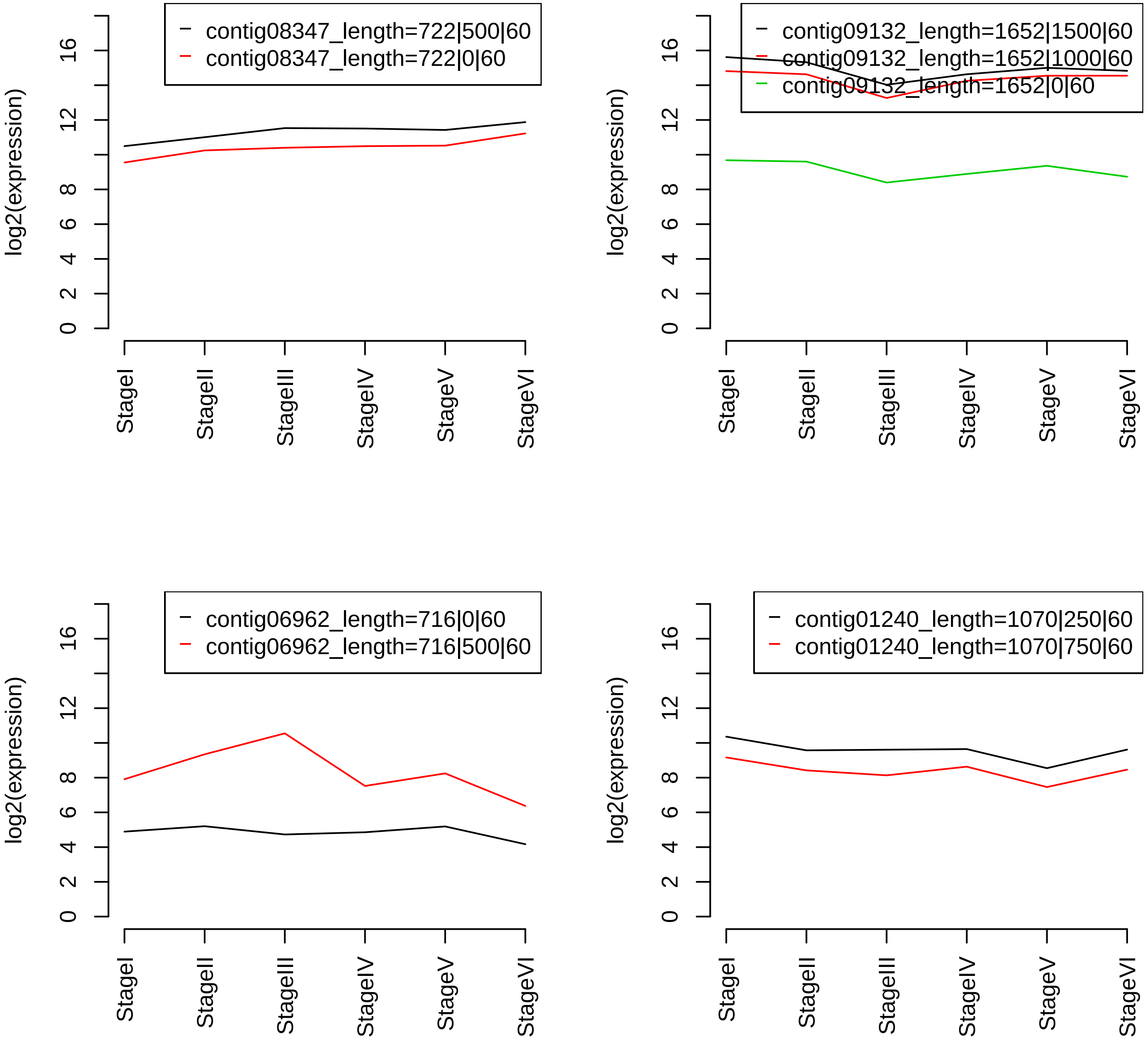
contig08347_length=722|500|60
contig08347_length=722|0|60
contig09132_length=1652|1500|60
contig09132_length=1652|1000|60
contig09132_length=1652|0|60
contig06962_length=716|0|60
contig06962_length=716|500|60
contig01240_length=1070|250|60
contig01240_length=1070|750|60
log2(expression)
0
2
4
6
8
12
16
StageI
StageII
StageIII
StageIV
StageV
StageVI

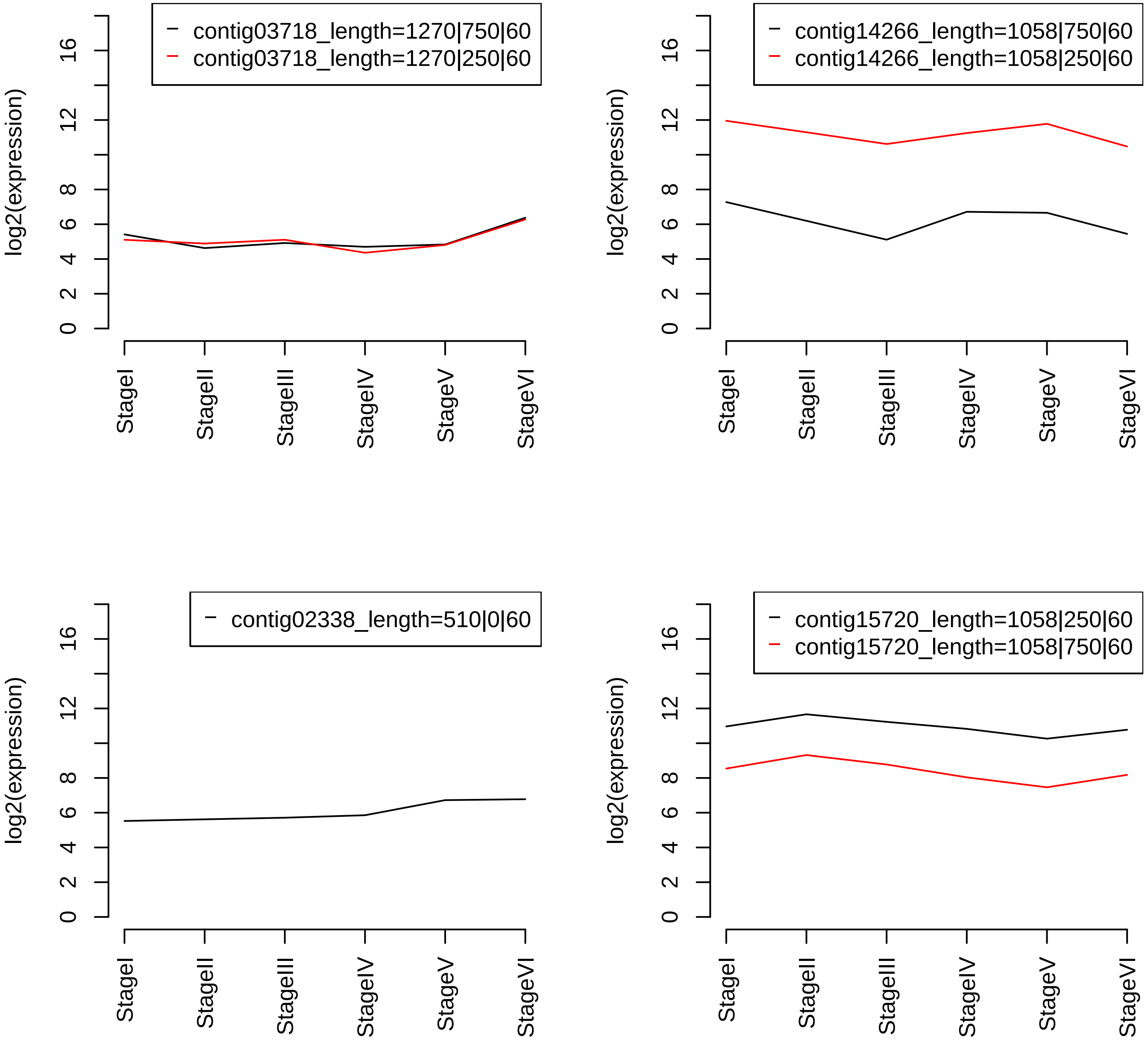

contig03718_length=1270|750|60
contig03718_length=1270|250|60
contig14266_length=1058|750|60
contig14266_length=1058|250|60
contig02338_length=510|0|60
contig15720_length=1058|250|60
contig15720_length=1058|750|60
log2(expression)
0
2
4
6
8
12
16
StageI
StageII
StageIII
StageIV
StageV
StageVI

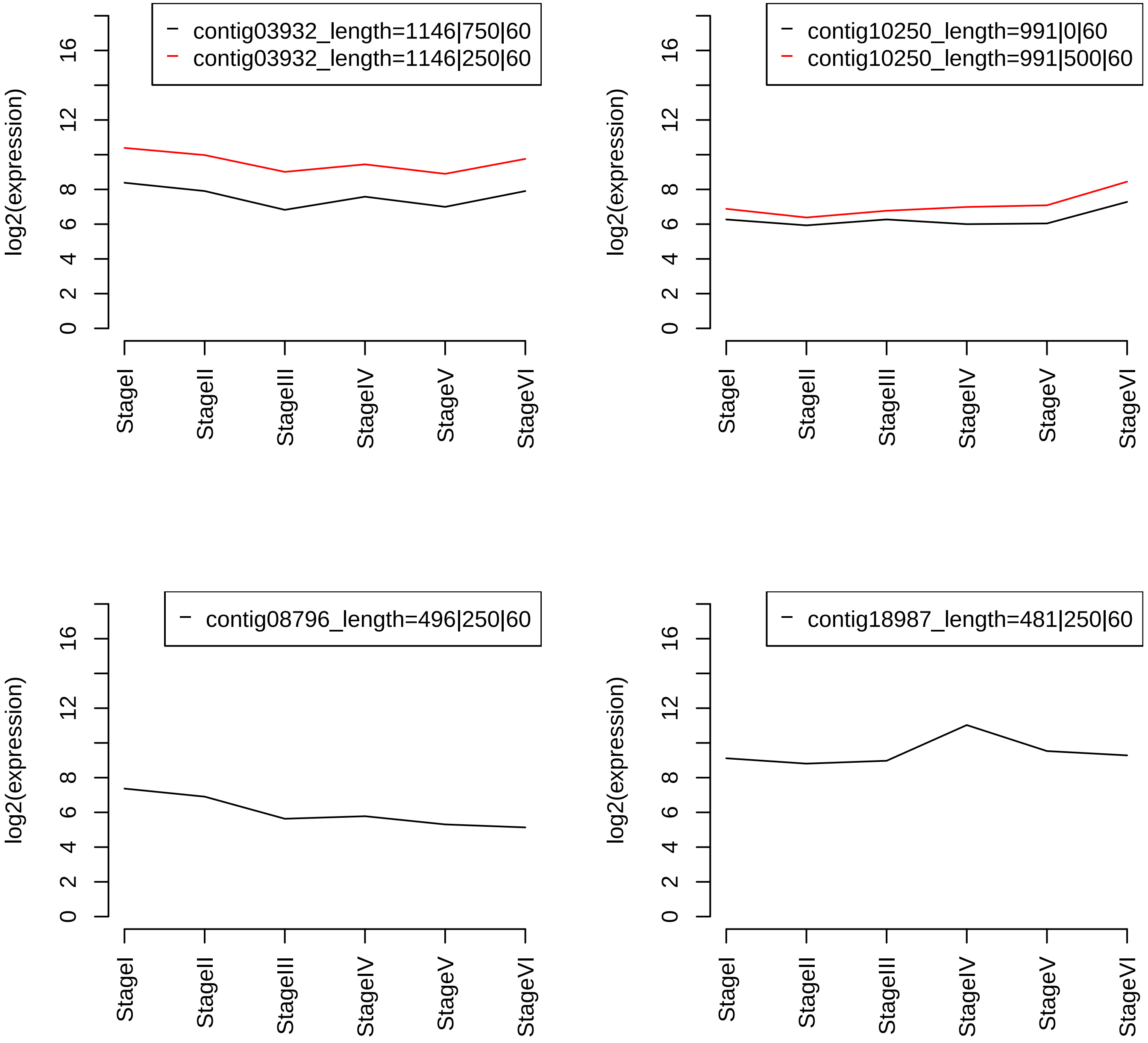

contig03932_length=1146|750|60
contig03932_length=1146|250|60
contig10250_length=991|0|60
contig10250_length=991|500|60
contig08796_length=496|250|60
contig18987_length=481|250|60
log2(expression)
0
2
4
6
8
12
16
StageI
StageII
StageIII
StageIV
StageV
StageVI

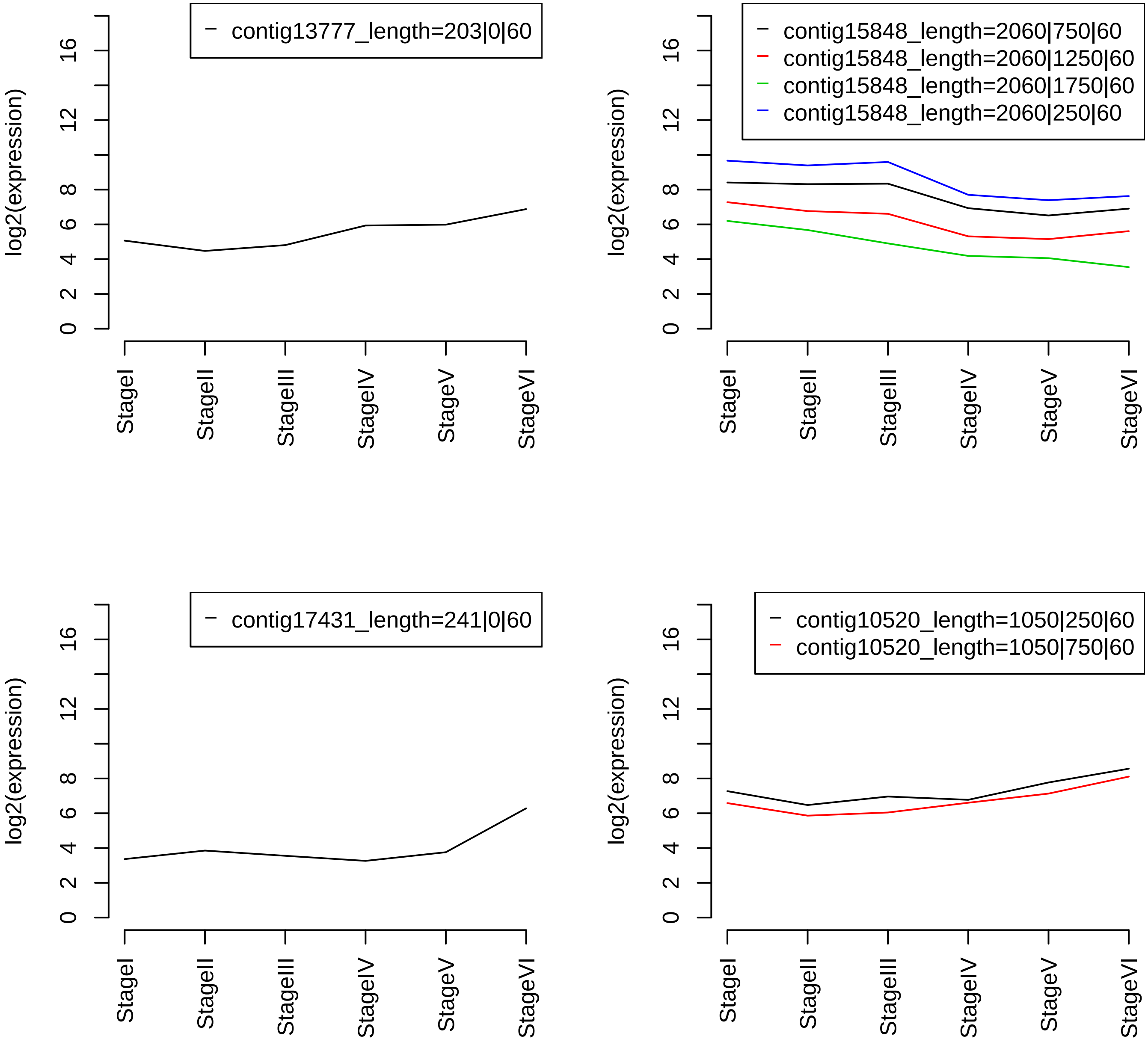
contig13777_length=203|0|60
contig15848_length=2060|750|60
contig15848_length=2060|1250|60
contig15848_length=2060|1750|60
contig15848_length=2060|250|60
contig17431_length=241|0|60
contig10520_length=1050|250|60
contig10520_length=1050|750|60
log2(expression)
0
2
4
6
8
12
16
StageI
StageII
StageIII
StageIV
StageV
StageVI

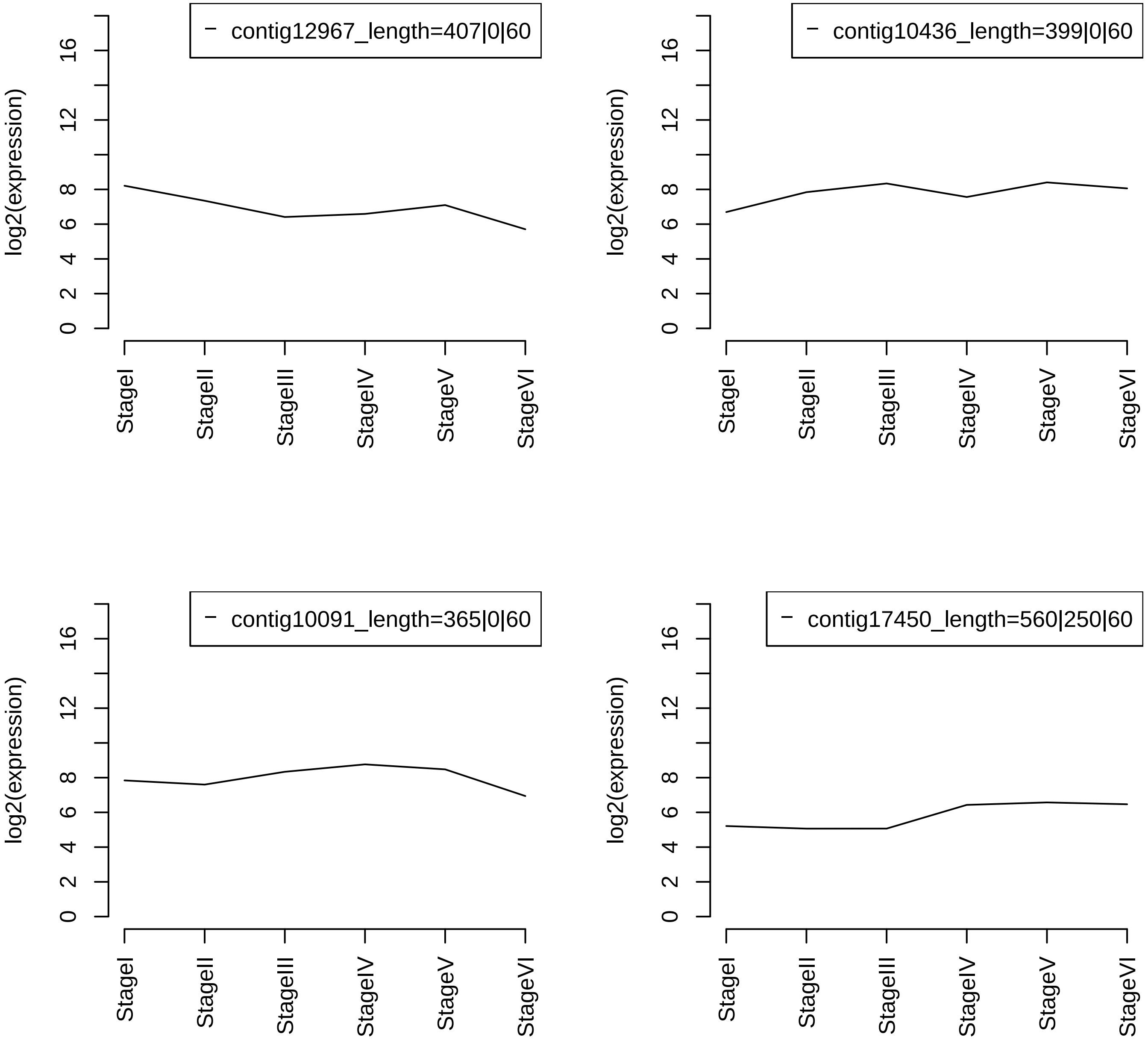
contig12967_length=407|0|60
contig10436_length=399|0|60
contig10091_length=365|0|60
contig17450_length=560|250|60
log2(expression)
0 2 4 6 8 12 16
StageI StageII StageIII StageIV StageV StageVI

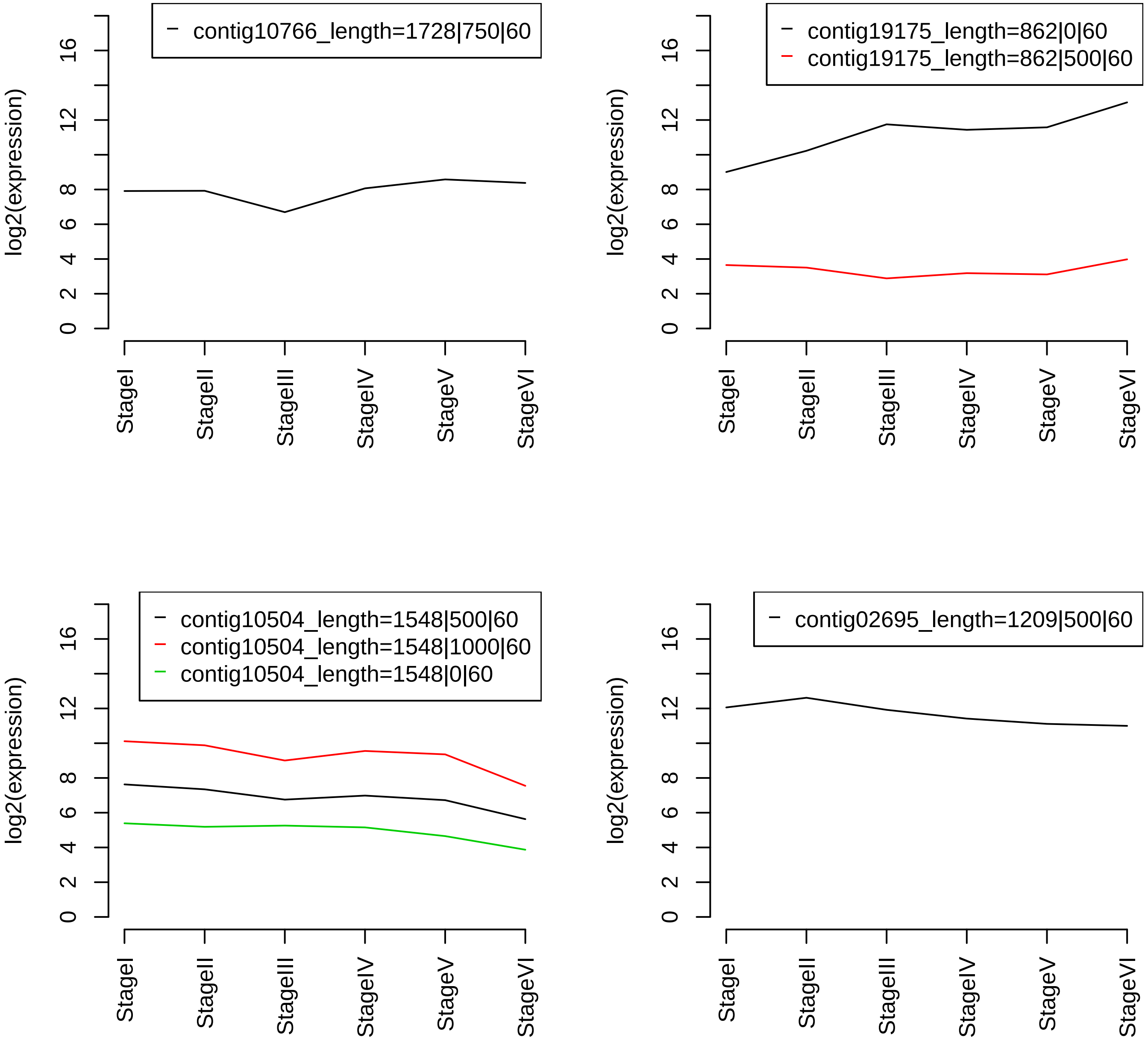

contig10766_length=1728|750|60
contig19175_length=862|0|60
contig19175_length=862|500|60
contig10504_length=1548|500|60
contig10504_length=1548|1000|60
contig10504_length=1548|0|60
contig02695_length=1209|500|60
log2(expression)
0
2
4
6
8
12
16
StageI
StageII
StageIII
StageIV
StageV
StageVI

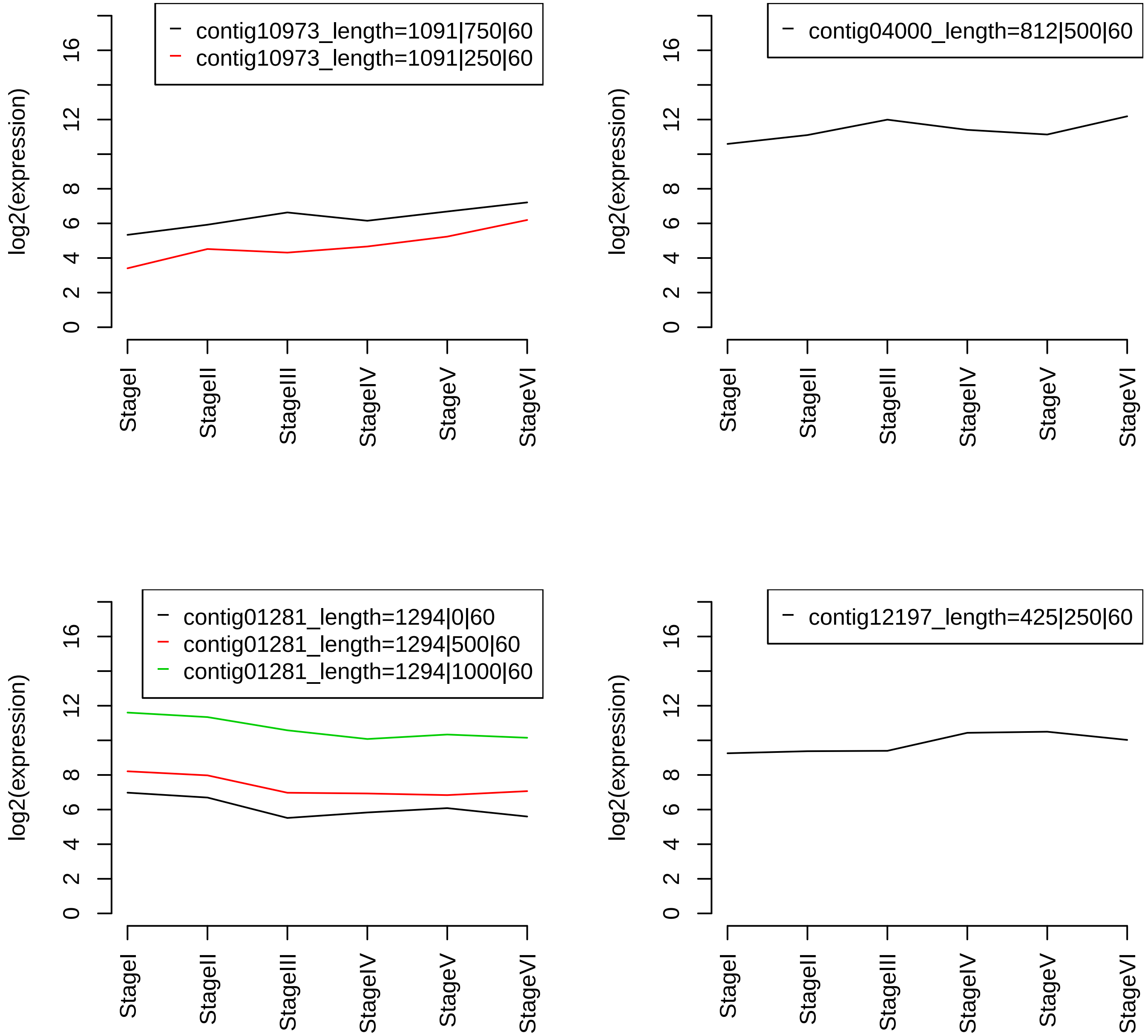
contig10973_length=1091|750|60
contig10973_length=1091|250|60
contig04000_length=812|500|60
contig01281_length=1294|0|60
contig01281_length=1294|500|60
contig01281_length=1294|1000|60
contig12197_length=425|250|60
log2(expression)
0
2
4
6
8
12
16
StageI
StageII
StageIII
StageIV
StageV
StageVI

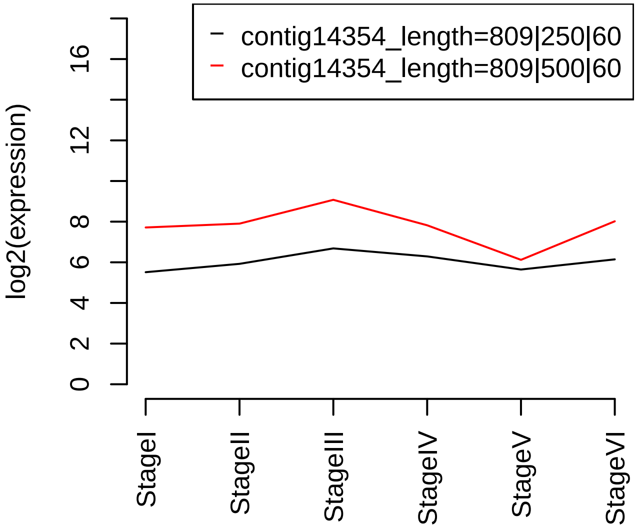
contig14354_length=809|250|60
contig14354_length=809|500|60
log2(expression)
StageI
StageII
StageIII
StageIV
StageV
StageVI

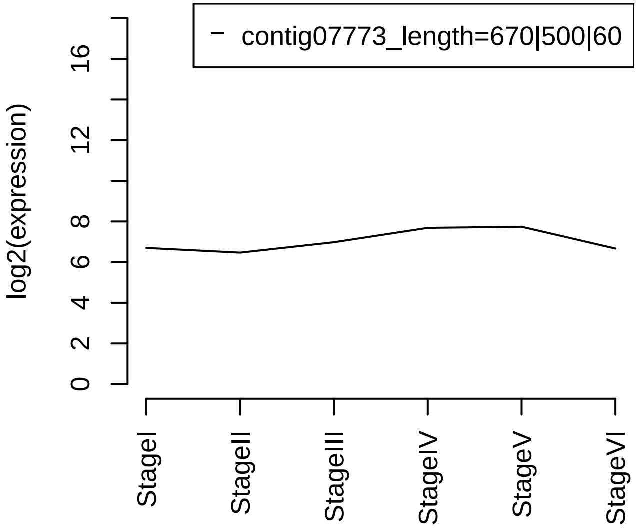
contig07773_length=670|500|60
log2(expression)
StageI
StageII
StageIII
StageIV
StageV
StageVI

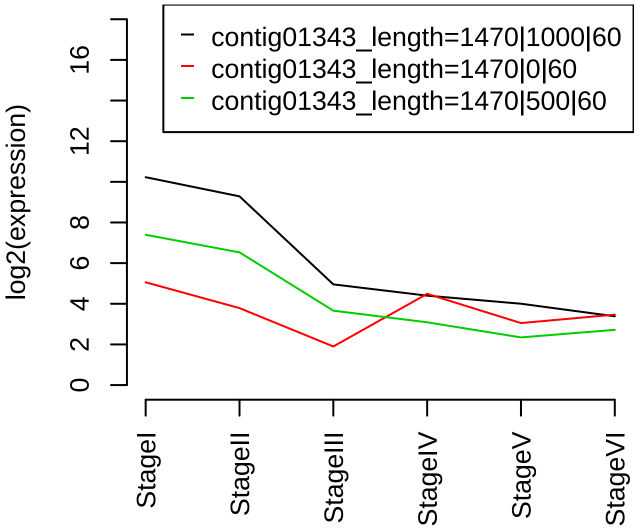
contig01343_length=1470|1000|60
contig01343_length=1470|0|60
contig01343_length=1470|500|60
log2(expression)
StageI
StageII
StageIII
StageIV
StageV
StageVI

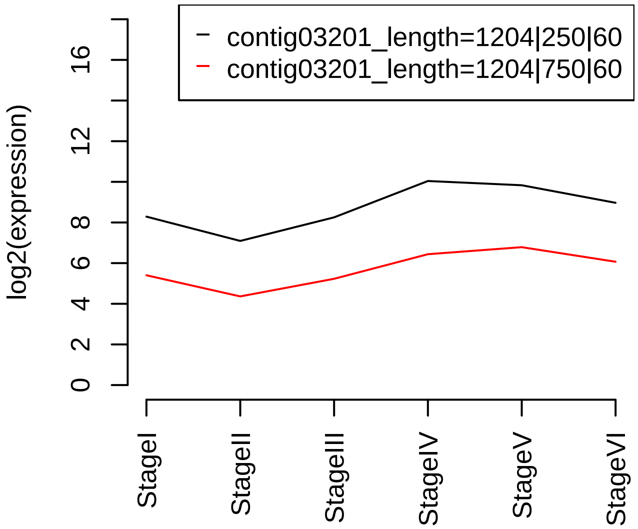
contig03201_length=1204|250|60
contig03201_length=1204|750|60
log2(expression)
StageI
StageII
StageIII
StageIV
StageV
StageVI

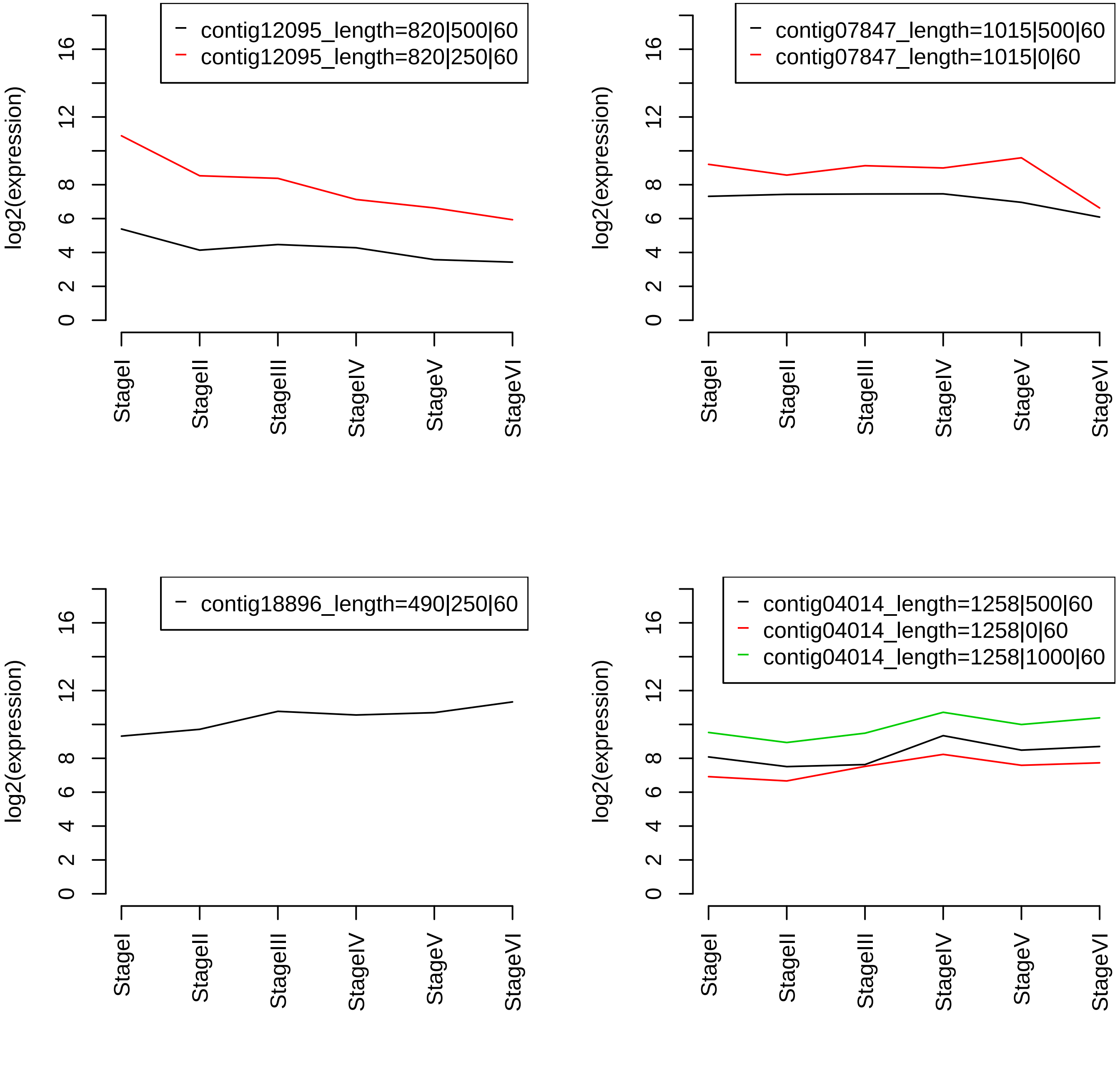

contig12095_length=820|500|60
contig12095_length=820|250|60
contig07847_length=1015|500|60
contig07847_length=1015|0|60
contig18896_length=490|250|60
contig04014_length=1258|500|60
contig04014_length=1258|0|60
contig04014_length=1258|1000|60
log2(expression)
0 2 4 6 8 12 16
StageI StageII StageIII StageIV StageV StageVI

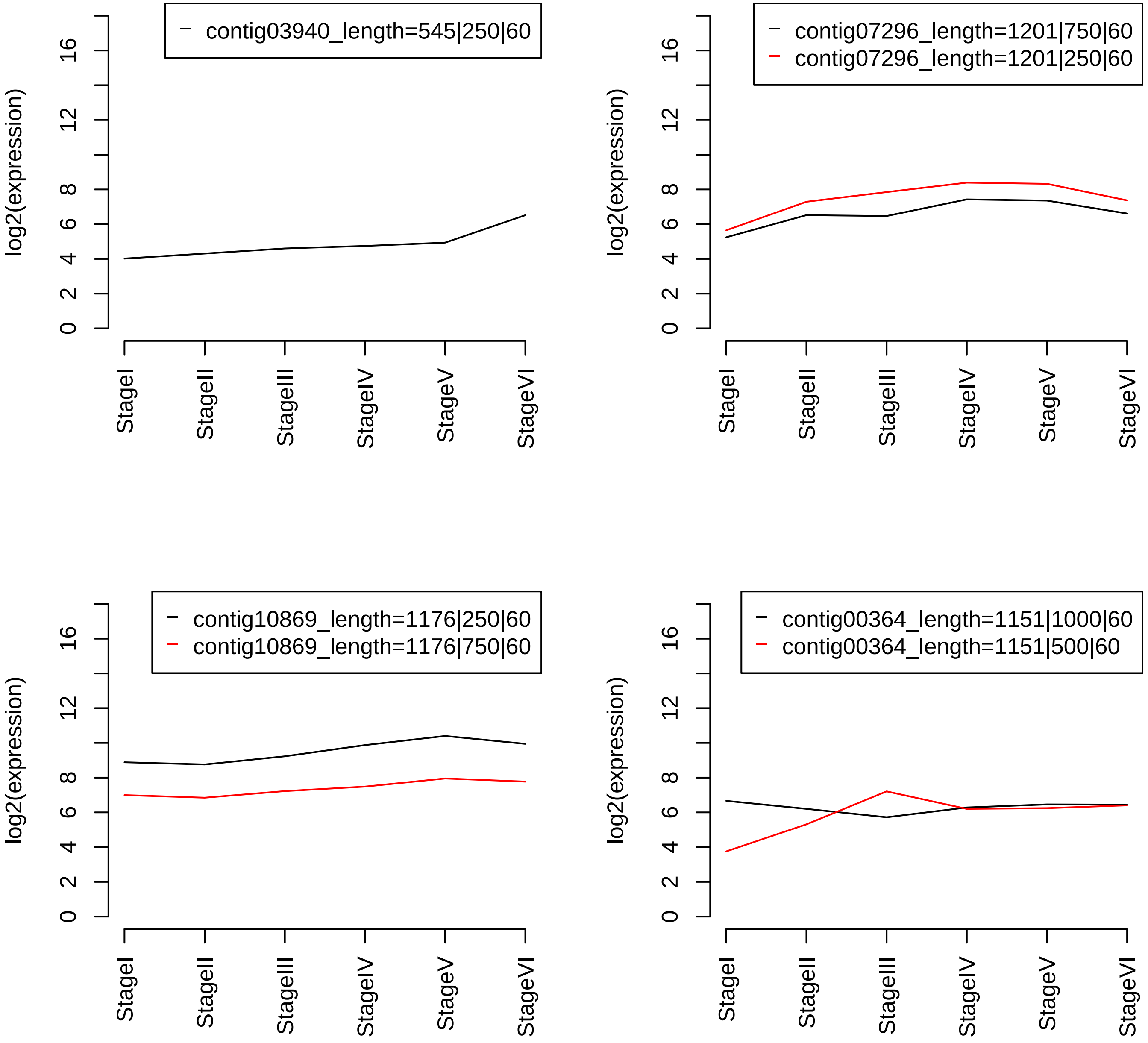

contig03940_length=545|250|60
contig07296_length=1201|750|60
contig07296_length=1201|250|60
contig10869_length=1176|250|60
contig10869_length=1176|750|60
contig00364_length=1151|1000|60
contig00364_length=1151|500|60
log2(expression)
0 2 4 6 8 12 16
StageI StageII StageIII StageIV StageV StageVI

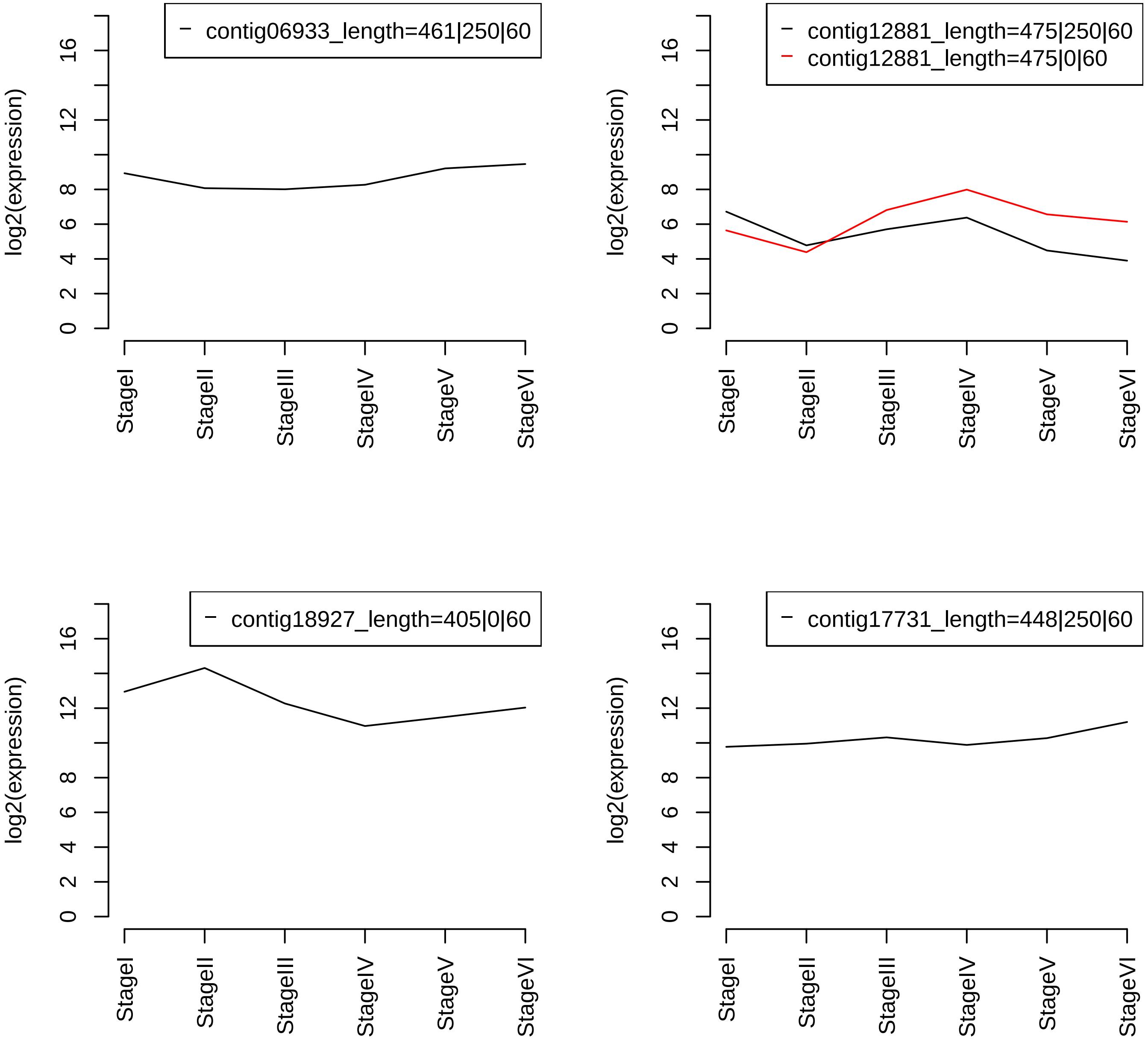

contig06933_length=461|250|60
contig12881_length=475|250|60
contig12881_length=475|0|60
contig18927_length=405|0|60
contig17731_length=448|250|60
log2(expression)
0
2
4
6
8
12
16
StageI
StageII
StageIII
StageIV
StageV
StageVI

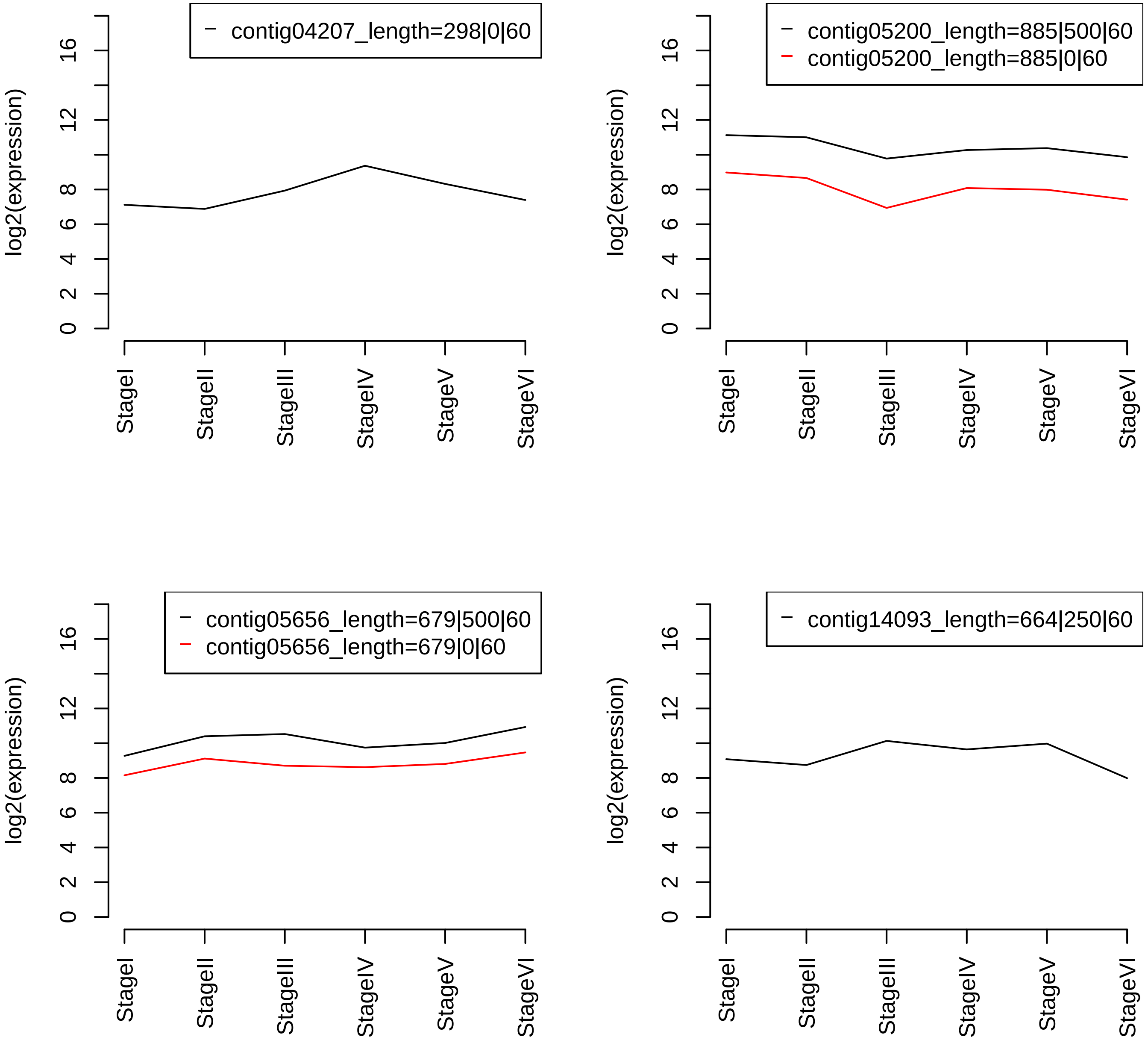
contig04207_length=298|0|60
contig05200_length=885|500|60
contig05200_length=885|0|60
contig05656_length=679|500|60
contig05656_length=679|0|60
contig14093_length=664|250|60
log2(expression)
0
2
4
6
8
12
16
StageI
StageII
StageIII
StageIV
StageV
StageVI

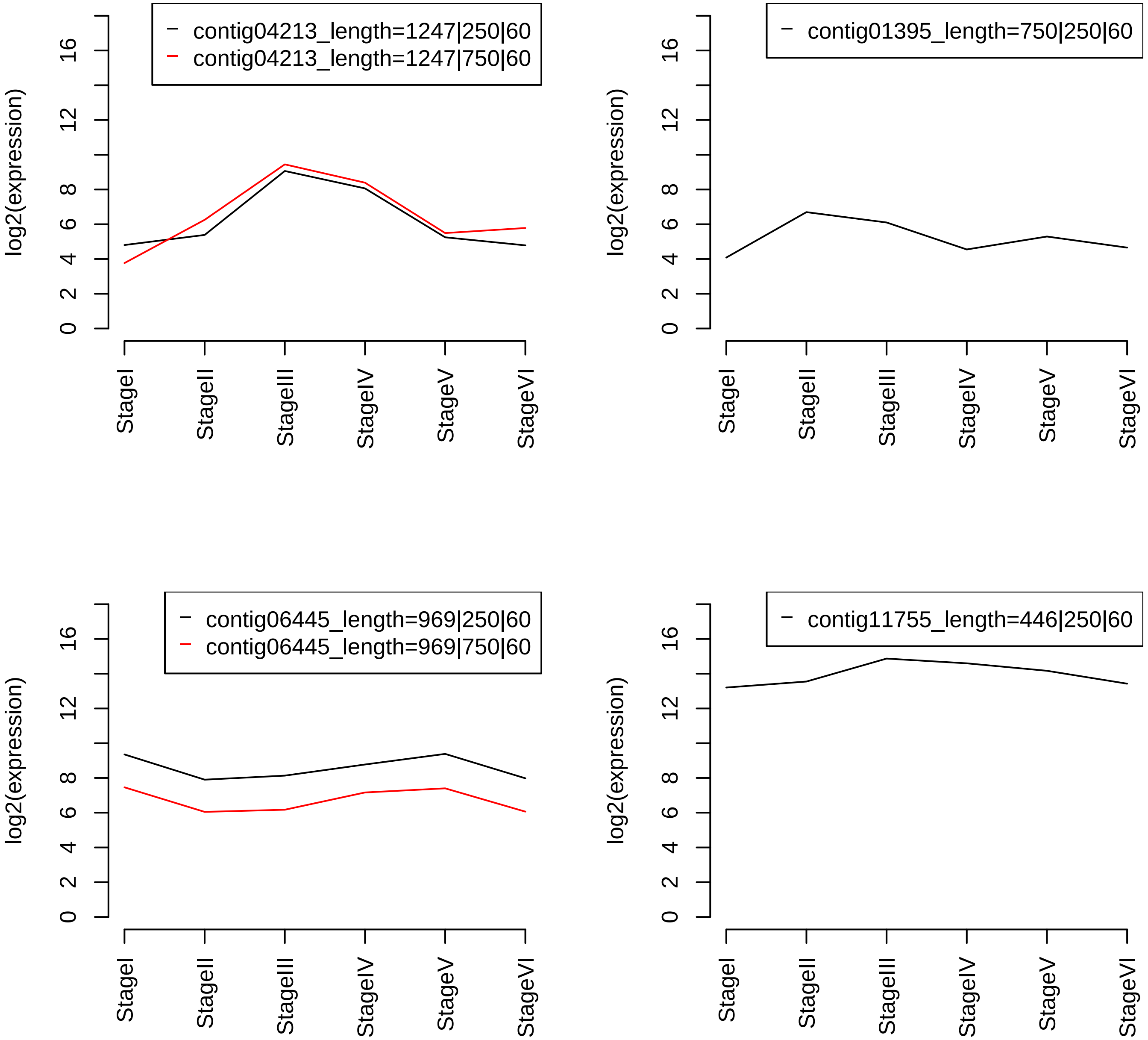
contig04213_length=1247|250|60
contig04213_length=1247|750|60
contig01395_length=750|250|60
contig06445_length=969|250|60
contig06445_length=969|750|60
contig11755_length=446|250|60
log2(expression)
0
2
4
6
8
12
16
StageI
StageII
StageIII
StageIV
StageV
StageVI

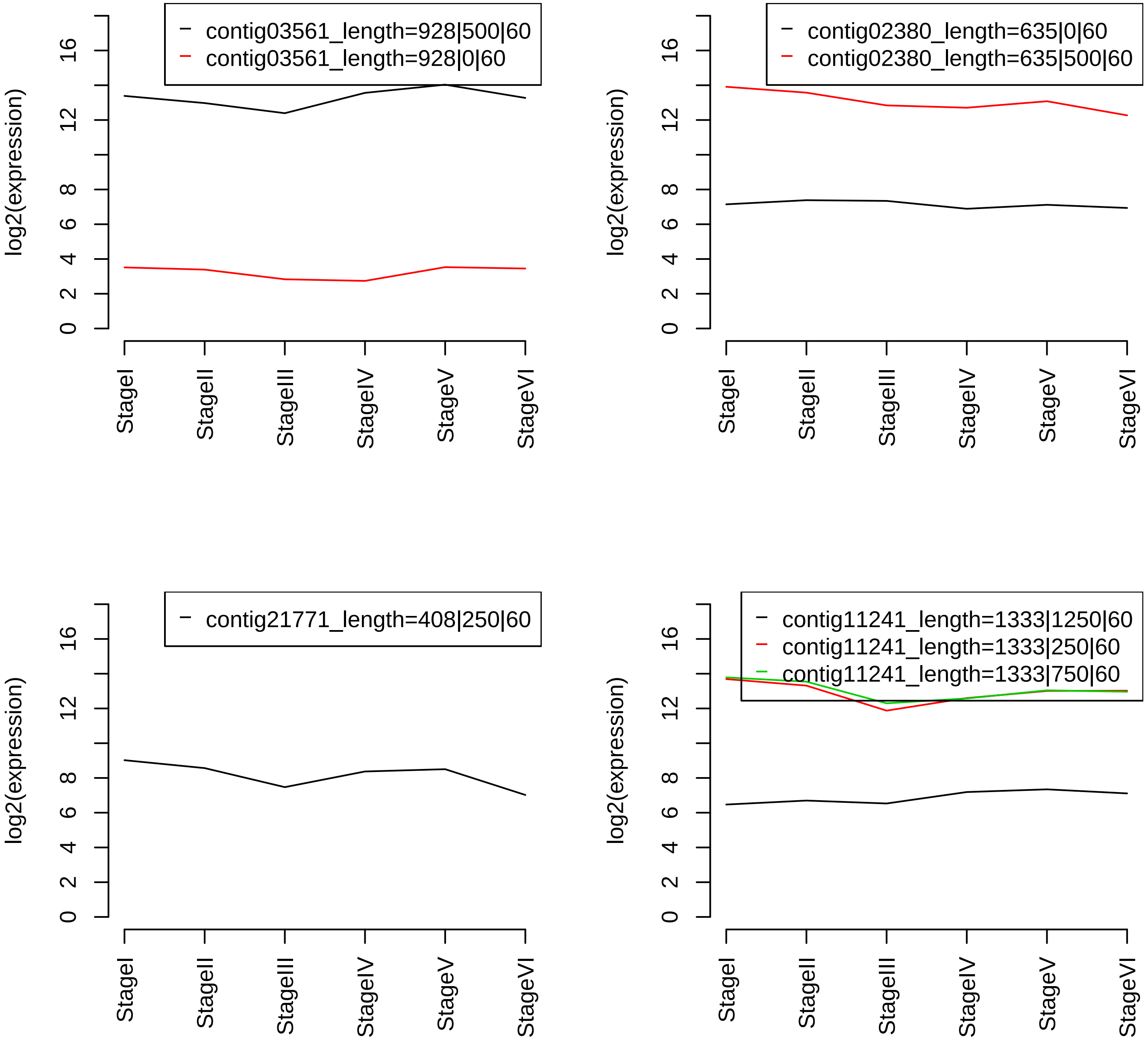

contig03561_length=928|500|60
contig03561_length=928|0|60
contig02380_length=635|0|60
contig02380_length=635|500|60
contig21771_length=408|250|60
contig11241_length=1333|1250|60
contig11241_length=1333|250|60
contig11241_length=1333|750|60
log2(expression)
0
2
4
6
8
12
16
StageI
StageII
StageIII
StageIV
StageV
StageVI

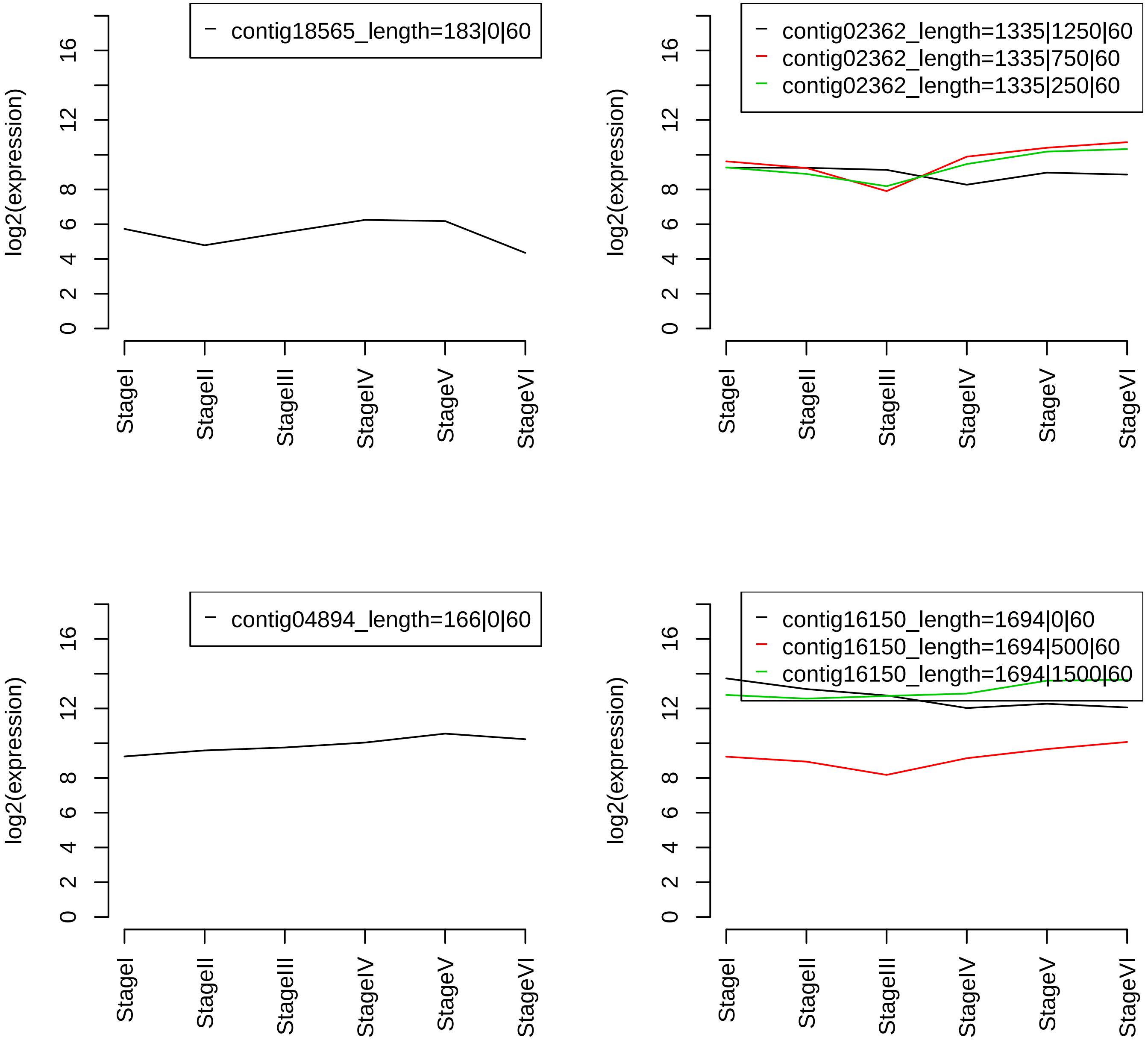
contig18565_length=183|0|60
contig02362_length=1335|1250|60
contig02362_length=1335|750|60
contig02362_length=1335|250|60
contig04894_length=166|0|60
contig16150_length=1694|0|60
contig16150_length=1694|500|60
contig16150_length=1694|1500|60
log2(expression)
0
2
4
6
8
12
16
StageI
StageII
StageIII
StageIV
StageV
StageVI

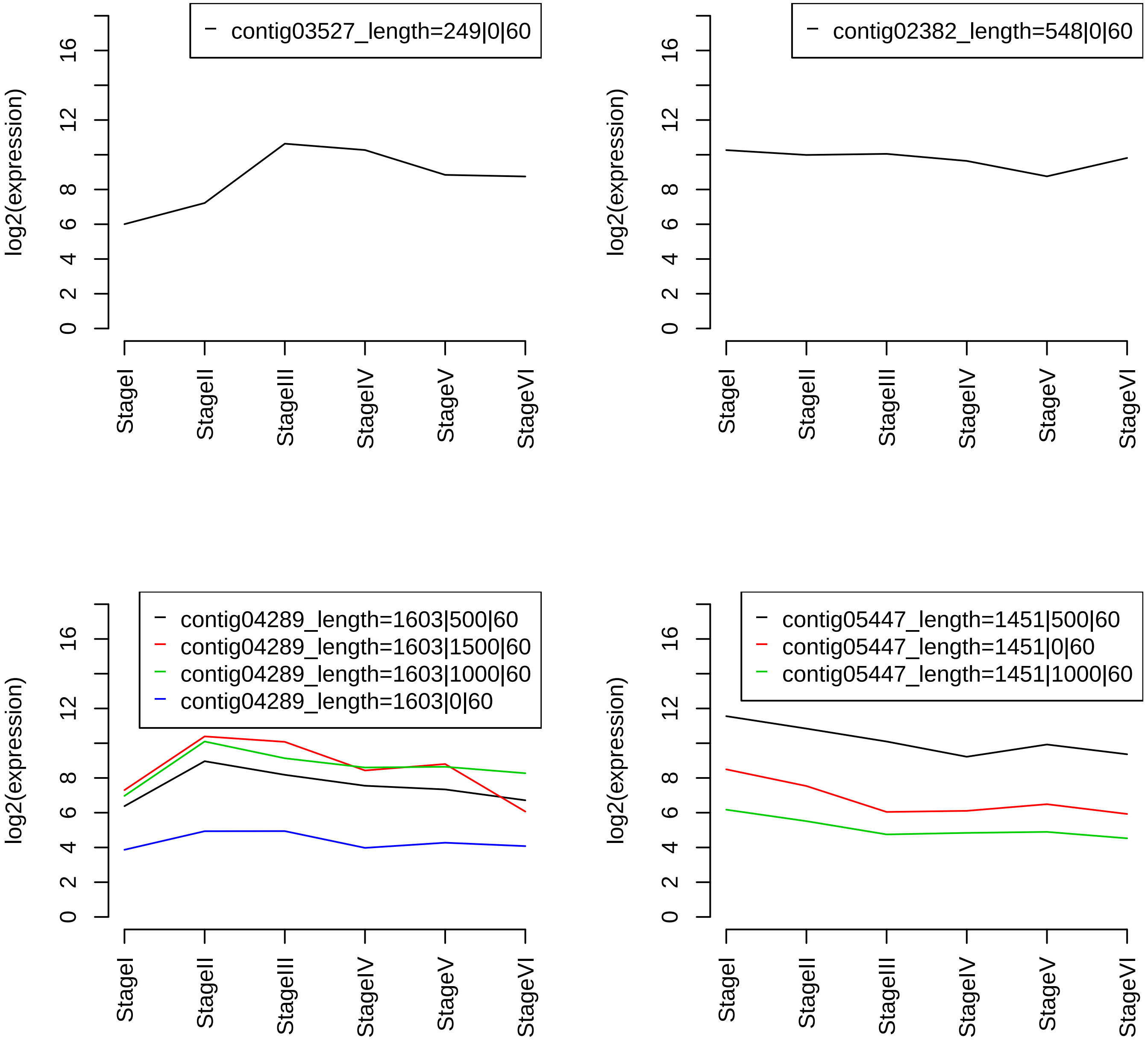

contig03527_length=249|0|60
contig02382_length=548|0|60
contig04289_length=1603|500|60
contig04289_length=1603|1500|60
contig04289_length=1603|1000|60
contig04289_length=1603|0|60
contig05447_length=1451|500|60
contig05447_length=1451|0|60
contig05447_length=1451|1000|60
log2(expression)
0
2
4
6
8
12
16
StageI
StageII
StageIII
StageIV
StageV
StageVI

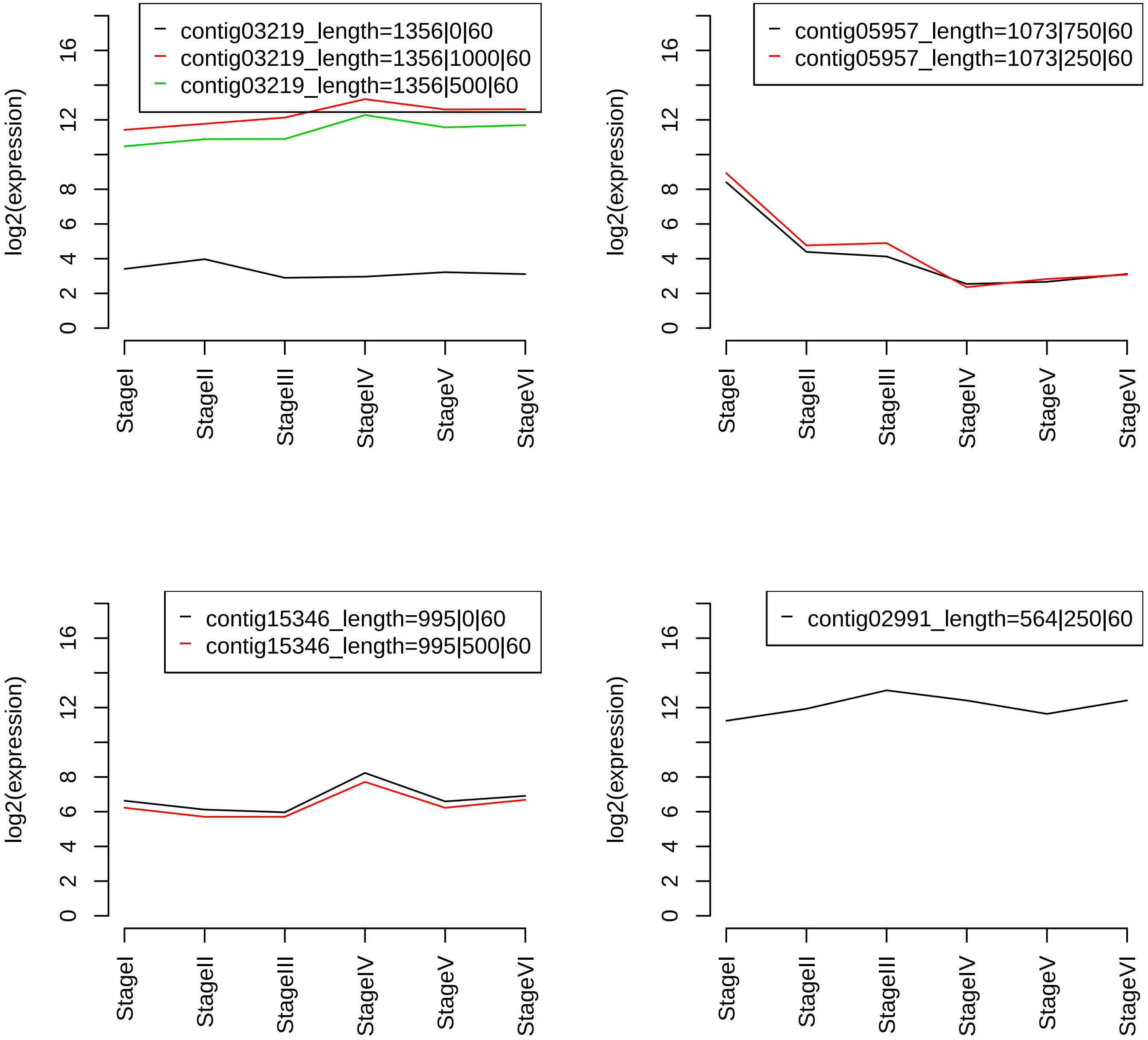
contig03219_length=1356|0|60
contig03219_length=1356|1000|60
contig03219_length=1356|500|60
contig05957_length=1073|750|60
contig05957_length=1073|250|60
contig15346_length=995|0|60
contig15346_length=995|500|60
contig02991_length=564|250|60
log2(expression)
0
2
4
6
8
12
16
StageI
StageII
StageIII
StageIV
StageV
StageVI

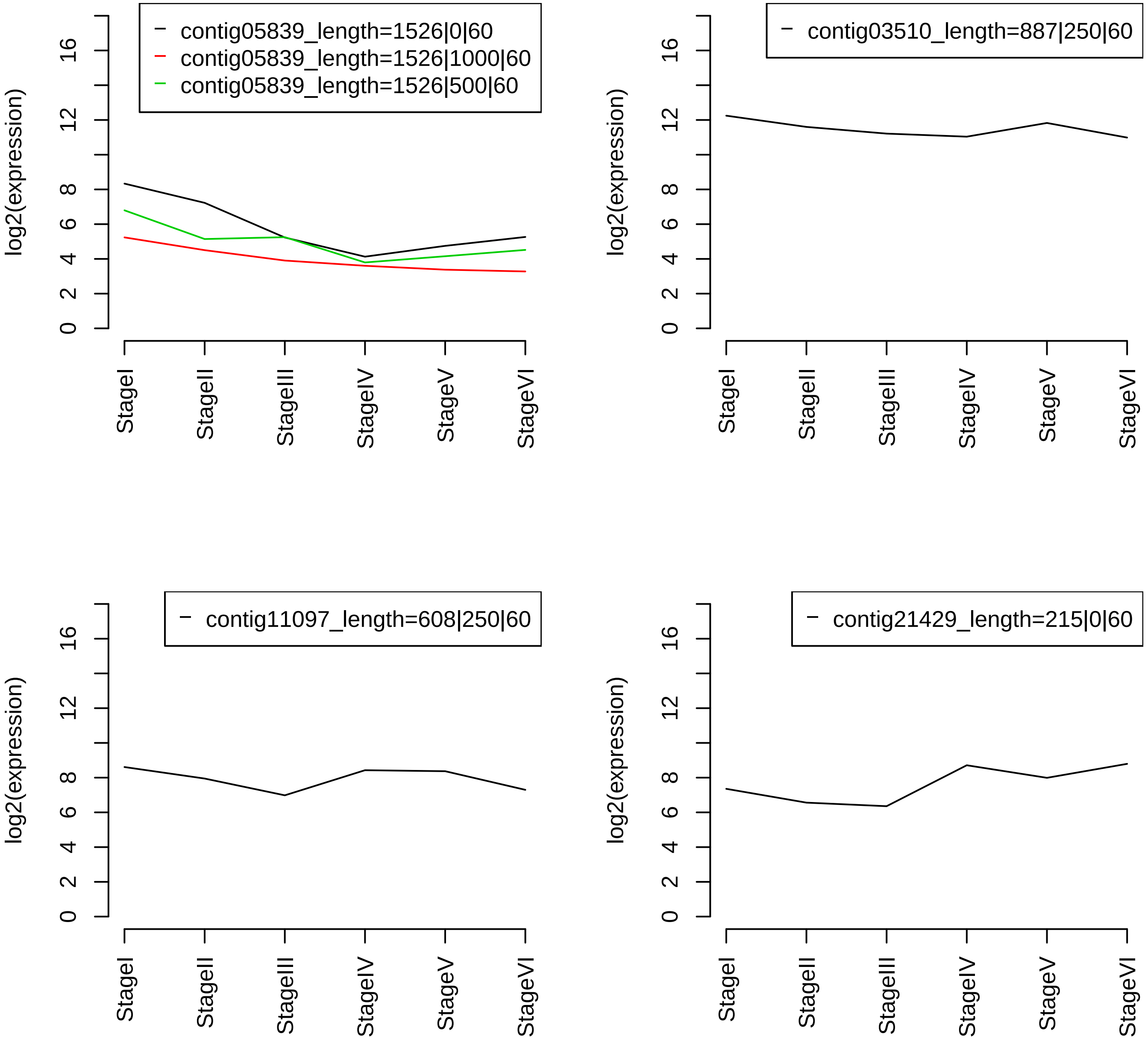
contig05839_length=1526|0|60
contig05839_length=1526|1000|60
contig05839_length=1526|500|60
contig03510_length=887|250|60
contig11097_length=608|250|60
contig21429_length=215|0|60
log2(expression)
0
2
4
6
8
12
16
StageI
StageII
StageIII
StageIV
StageV
StageVI

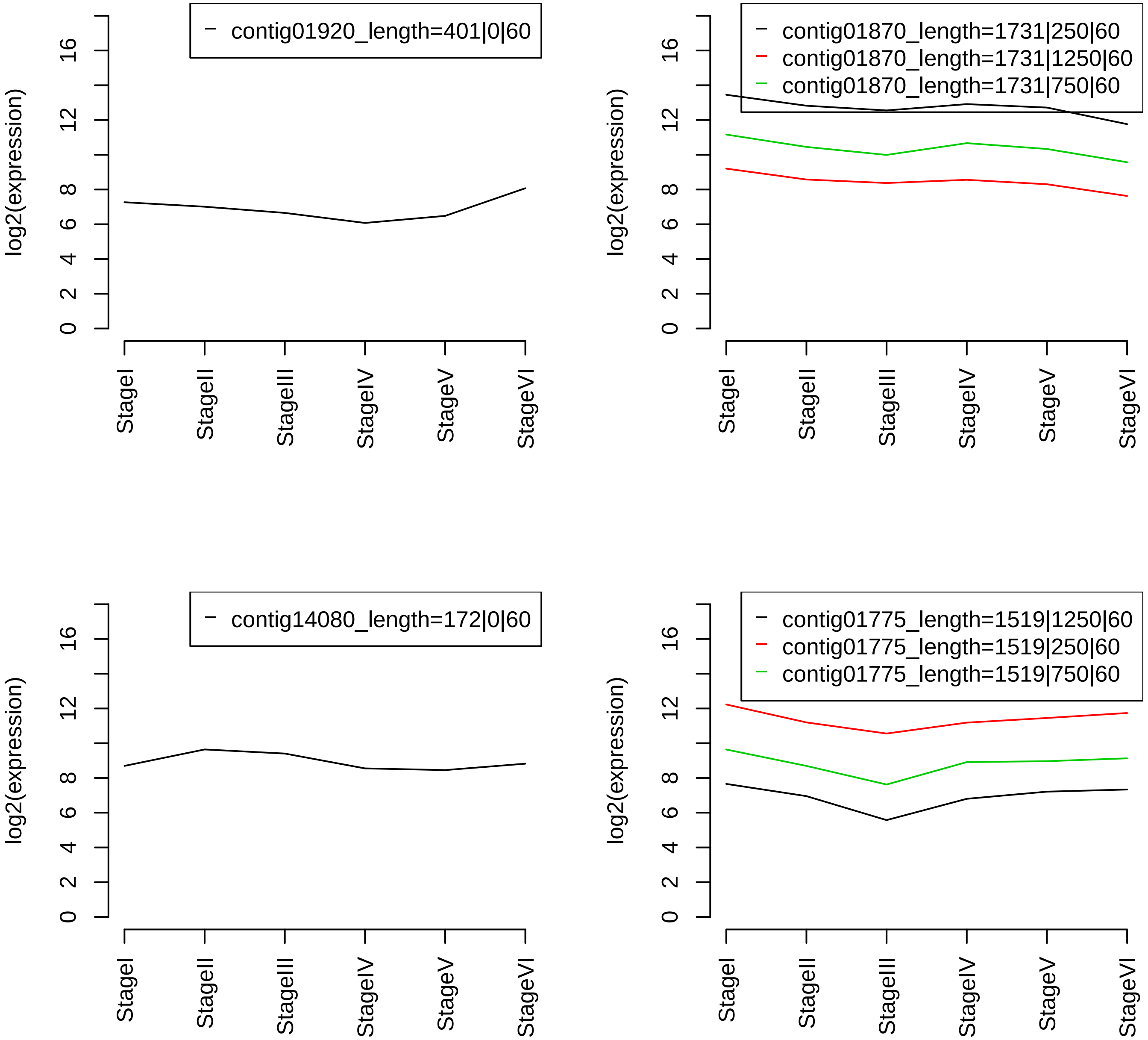

contig01920_length=401|0|60
contig01870_length=1731|250|60
contig01870_length=1731|1250|60
contig01870_length=1731|750|60
contig14080_length=172|0|60
contig01775_length=1519|1250|60
contig01775_length=1519|250|60
contig01775_length=1519|750|60
log2(expression)
0 2 4 6 8 12 16
StageI StageII StageIII StageIV StageV StageVI

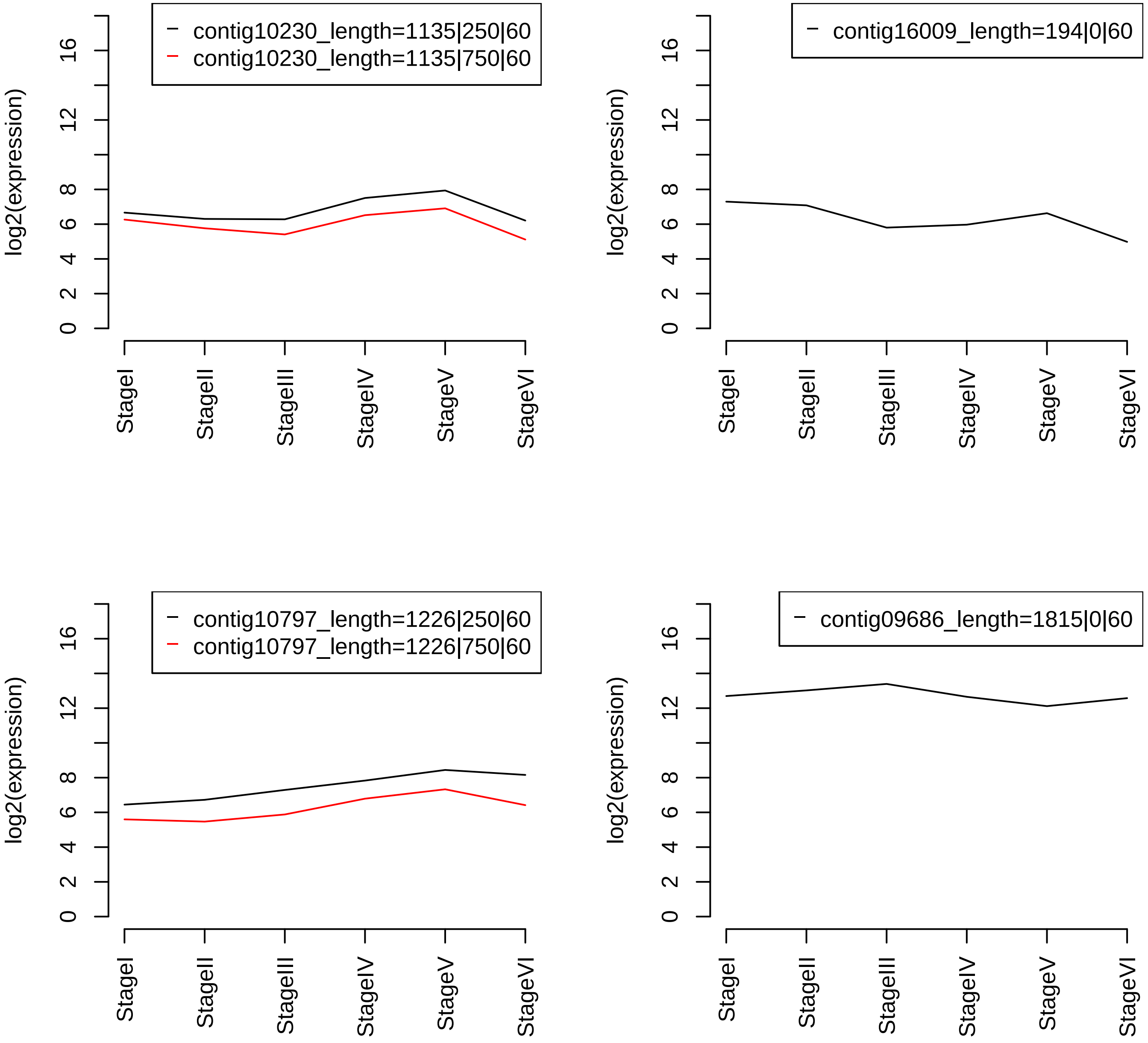
contig10230_length=1135|250|60
contig10230_length=1135|750|60
log2(expression)
0
2
4
6
8
12
16
StageI
StageII
StageIII
StageIV
StageV
StageVI
contig16009_length=194|0|60
contig10797_length=1226|250|60
contig10797_length=1226|750|60
contig09686_length=1815|0|60

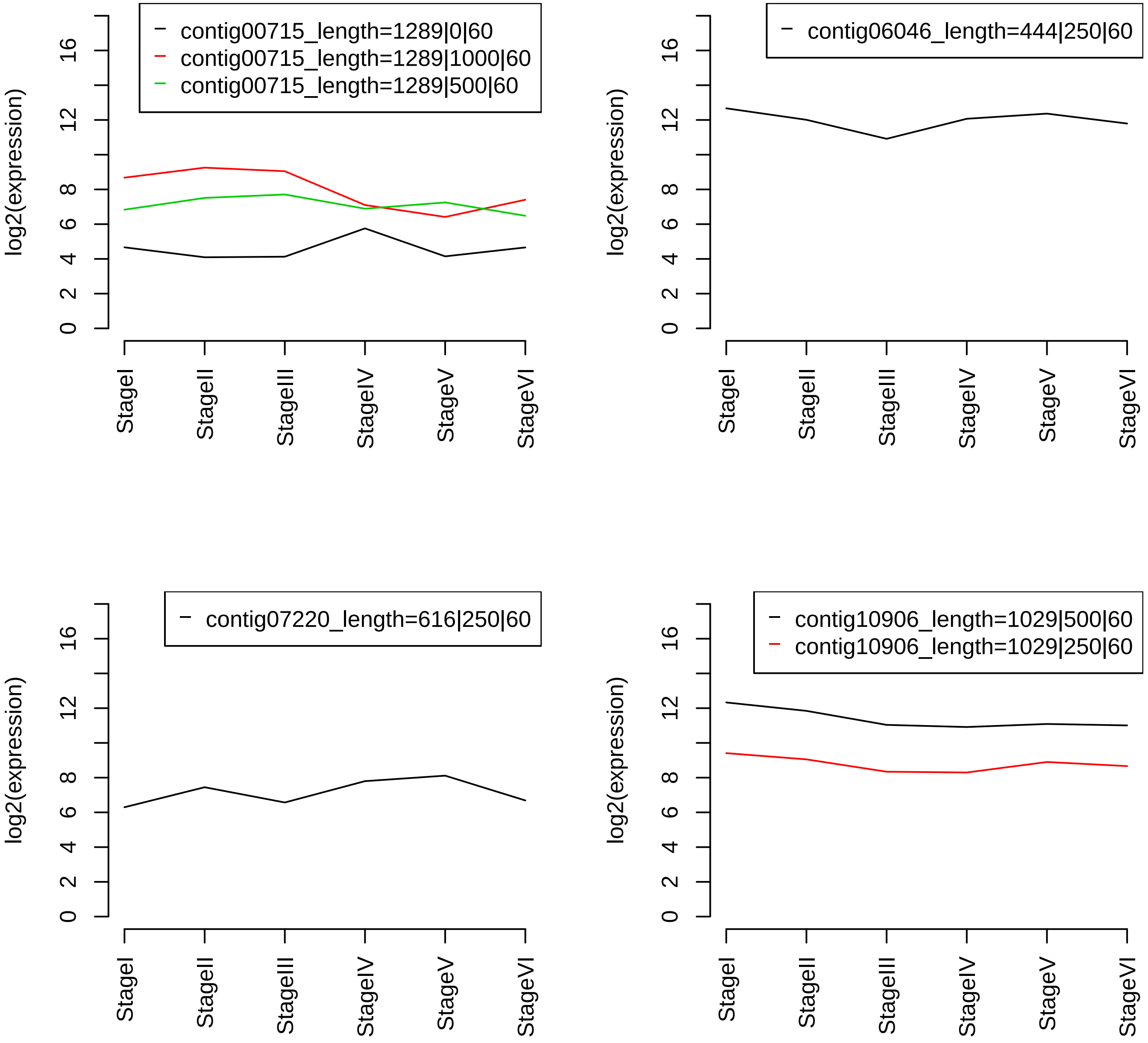
contig00715_length=1289|0|60
contig00715_length=1289|1000|60
contig00715_length=1289|500|60
log2(expression)
0
2
4
6
8
12
16
StageI
StageII
StageIII
StageIV
StageV
StageVI
contig06046_length=444|250|60
contig07220_length=616|250|60
contig10906_length=1029|500|60
contig10906_length=1029|250|60

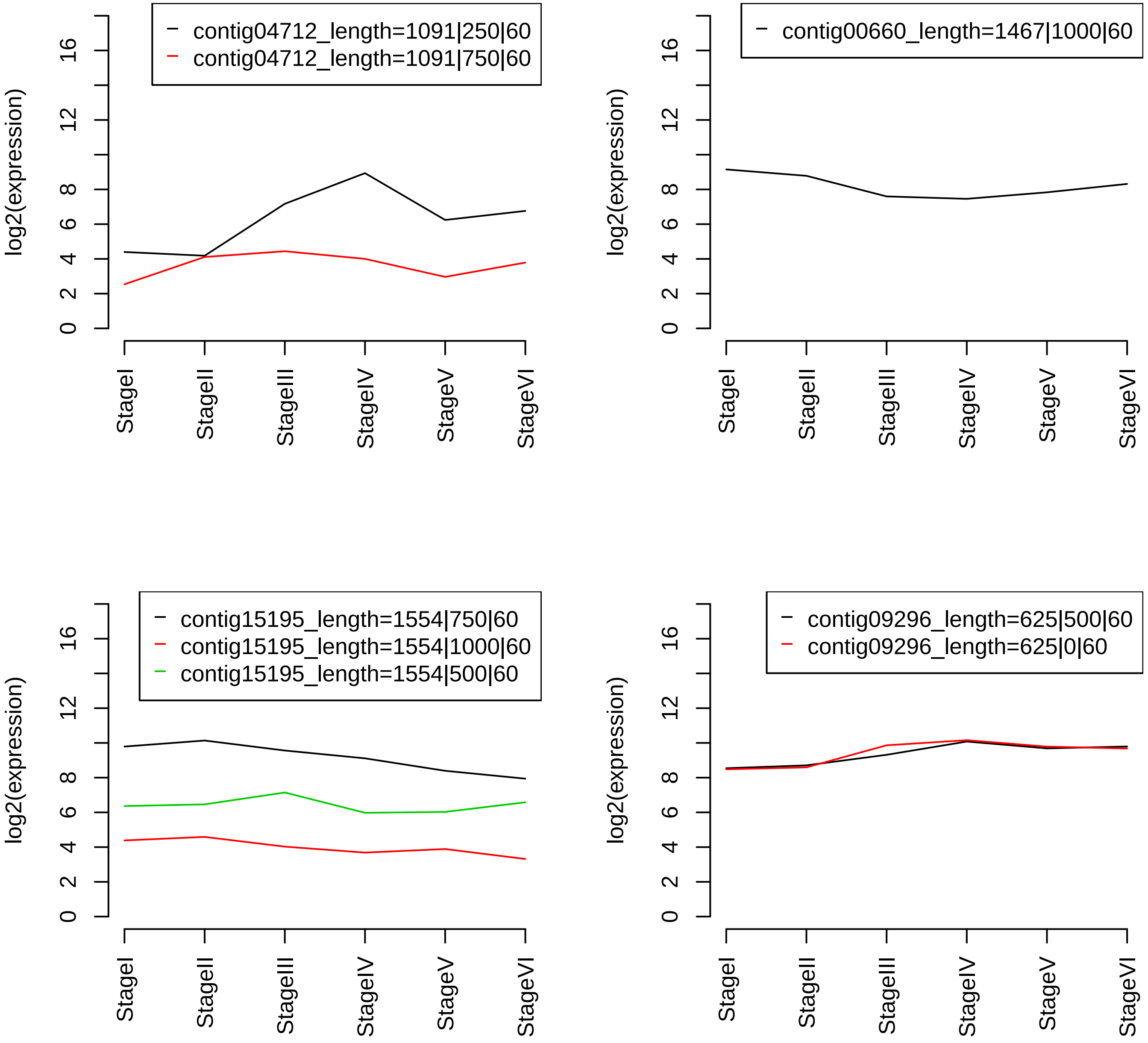
contig04712_length=1091|250|60
contig04712_length=1091|750|60
contig00660_length=1467|1000|60
contig15195_length=1554|750|60
contig15195_length=1554|1000|60
contig15195_length=1554|500|60
contig09296_length=625|500|60
contig09296_length=625|0|60
log2(expression)
0
2
4
6
8
12
16
StageI
StageII
StageIII
StageIV
StageV
StageVI

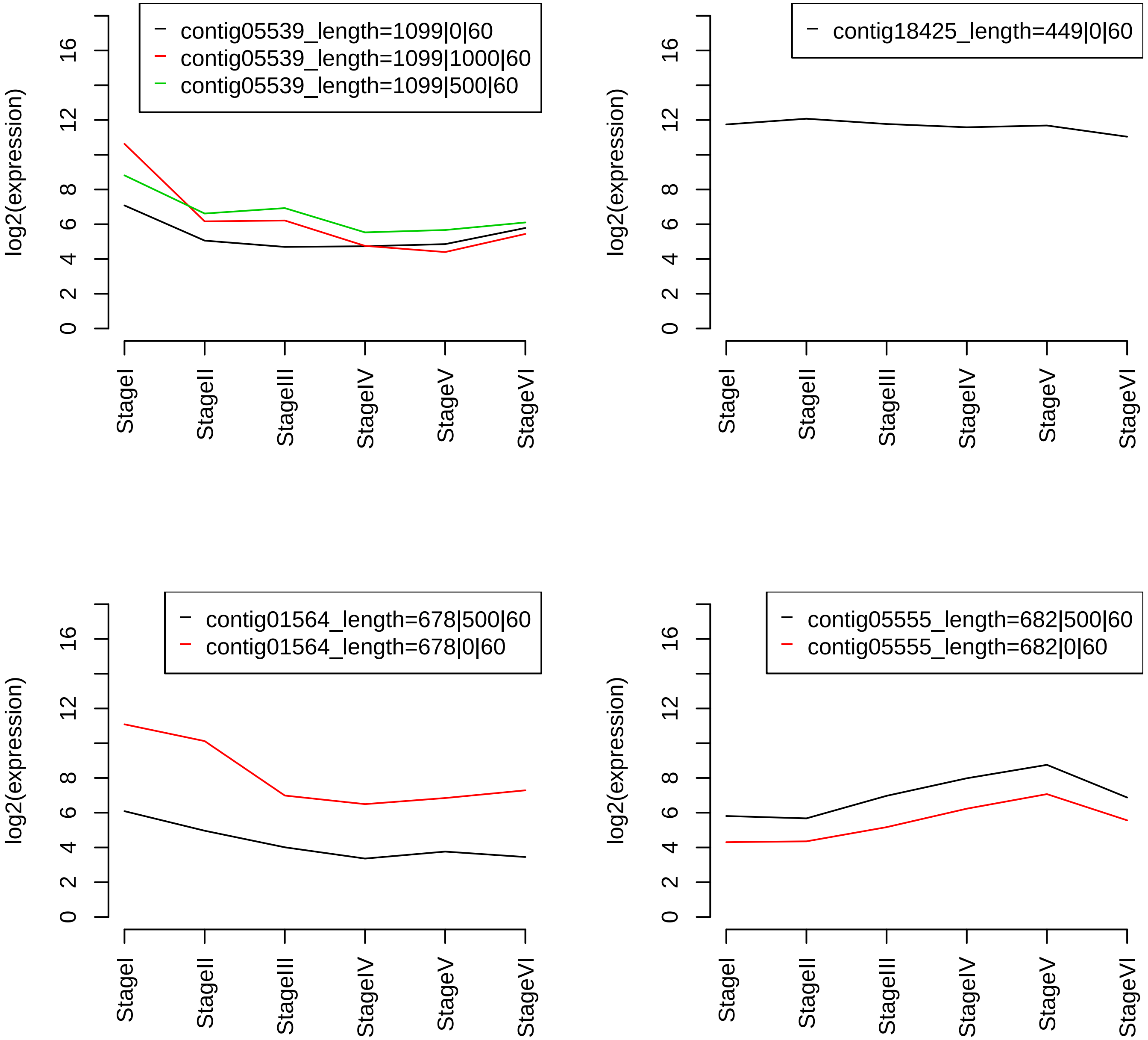

contig05539_length=1099|0|60
contig05539_length=1099|1000|60
contig05539_length=1099|500|60
contig18425_length=449|0|60
contig01564_length=678|500|60
contig01564_length=678|0|60
contig05555_length=682|500|60
contig05555_length=682|0|60
log2(expression)
0
2
4
6
8
12
16
StageI
StageII
StageIII
StageIV
StageV
StageVI

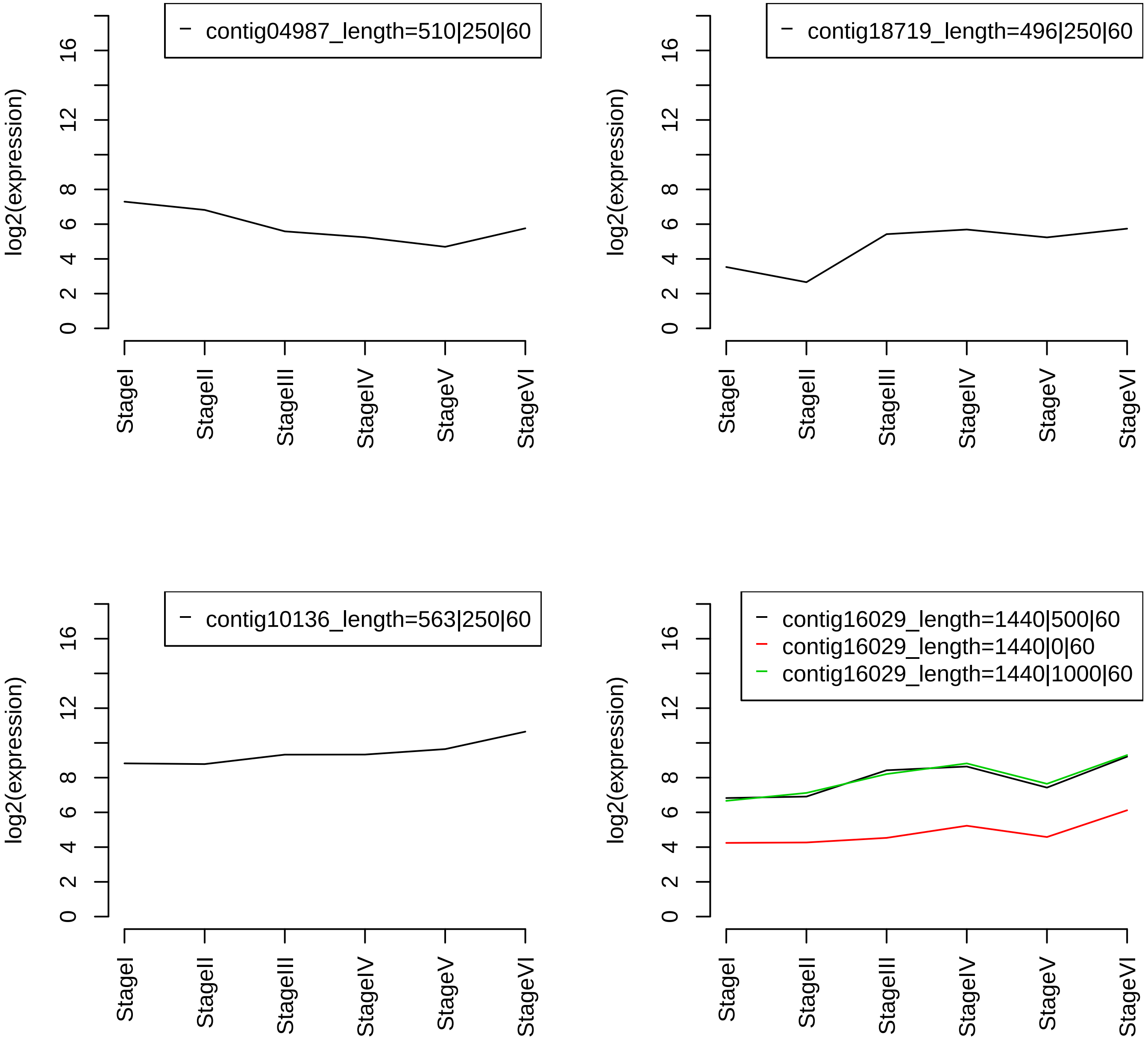
contig04987_length=510|250|60
contig18719_length=496|250|60
contig10136_length=563|250|60
contig16029_length=1440|500|60
contig16029_length=1440|0|60
contig16029_length=1440|1000|60
log2(expression)
0
2
4
6
8
12
16
StageI
StageII
StageIII
StageIV
StageV
StageVI

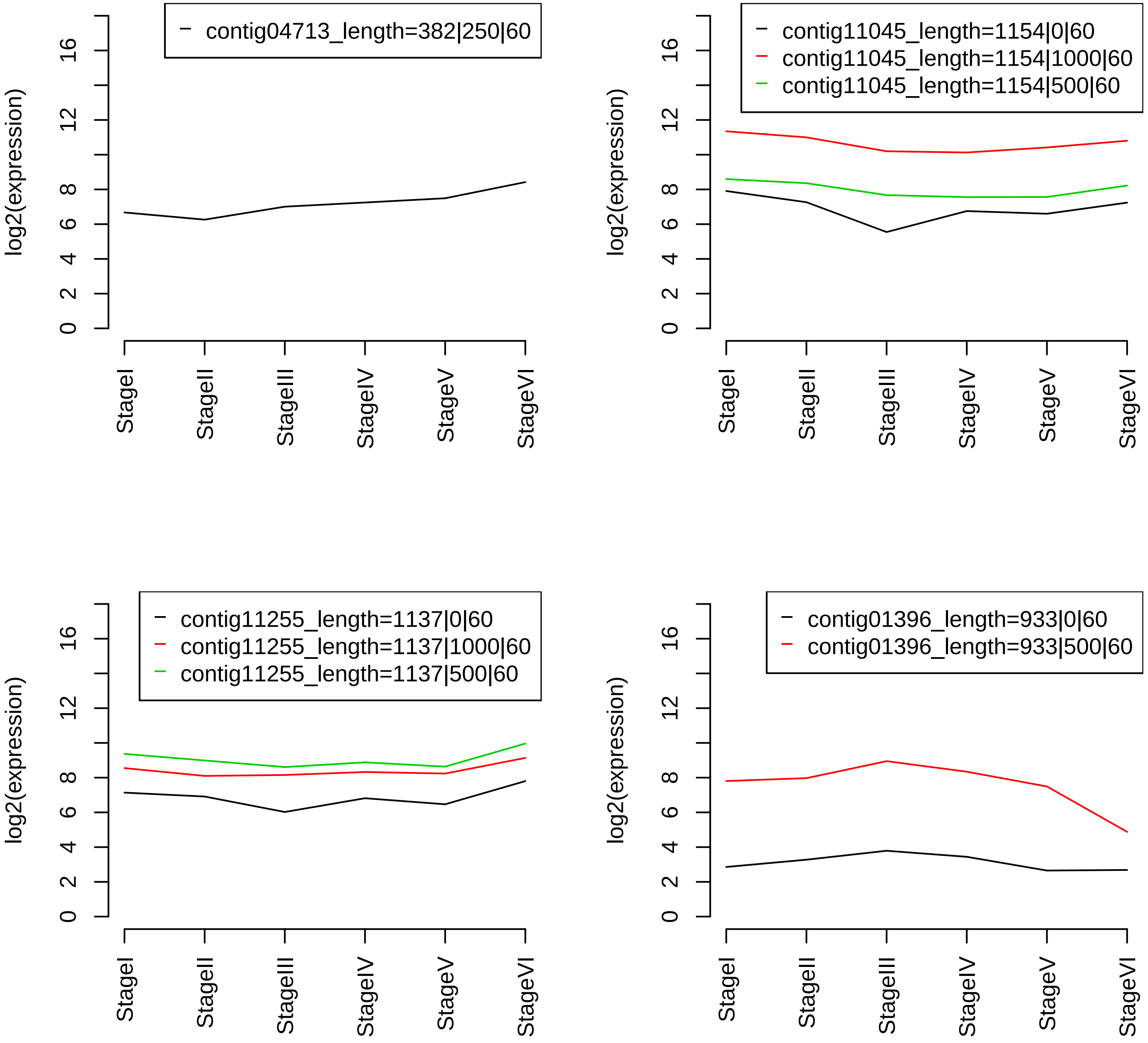
contig04713_length=382|250|60
contig11045_length=1154|0|60
contig11045_length=1154|1000|60
contig11045_length=1154|500|60
contig11255_length=1137|0|60
contig11255_length=1137|1000|60
contig11255_length=1137|500|60
contig01396_length=933|0|60
contig01396_length=933|500|60
log2(expression)
0 2 4 6 8 12 16
StageI
StageII
StageIII
StageIV
StageV
StageVI

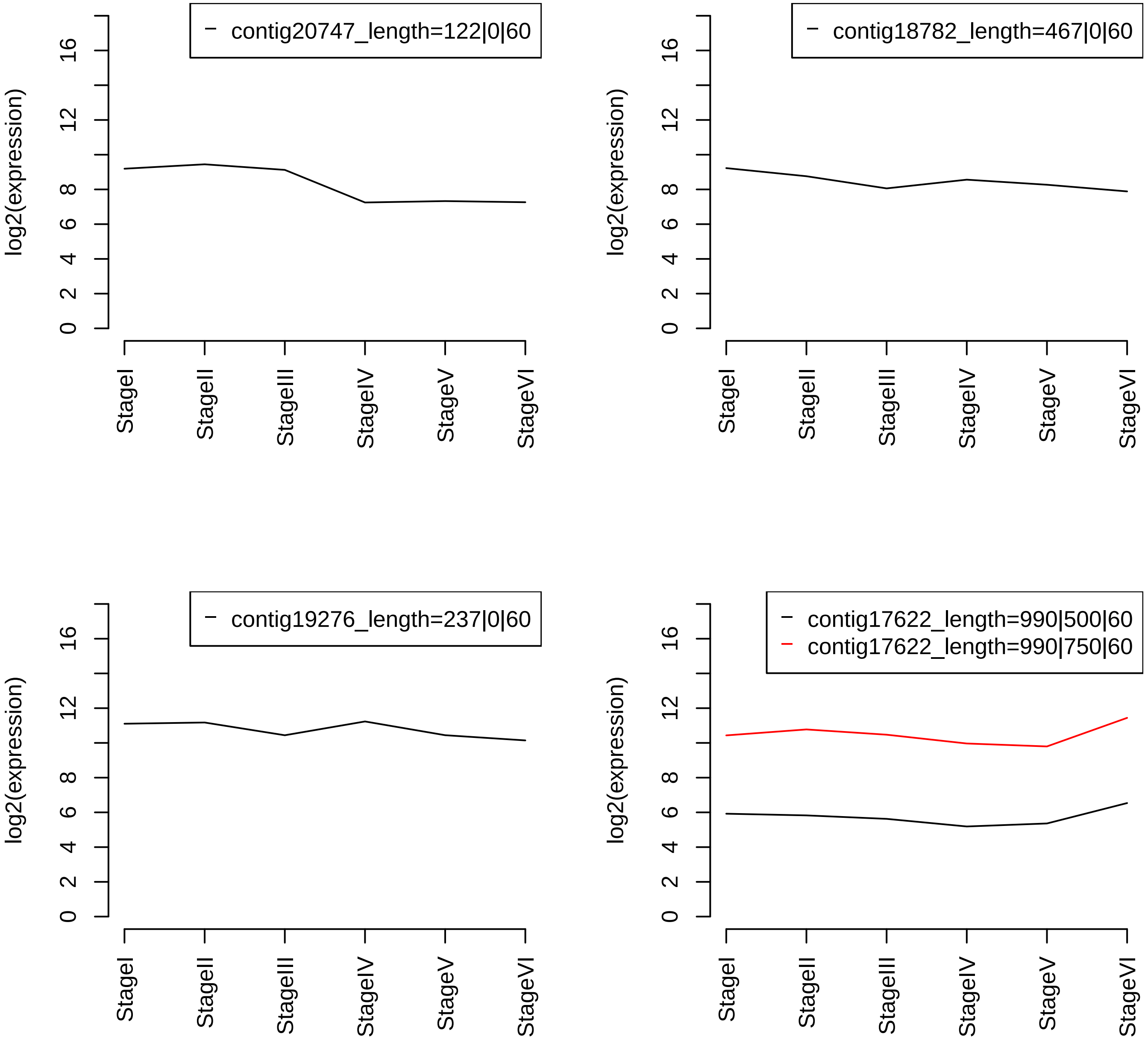
contig20747_length=122|0|60
contig18782_length=467|0|60
contig19276_length=237|0|60
contig17622_length=990|500|60
contig17622_length=990|750|60
log2(expression)
0
2
4
6
8
12
16
StageI
StageII
StageIII
StageIV
StageV
StageVI

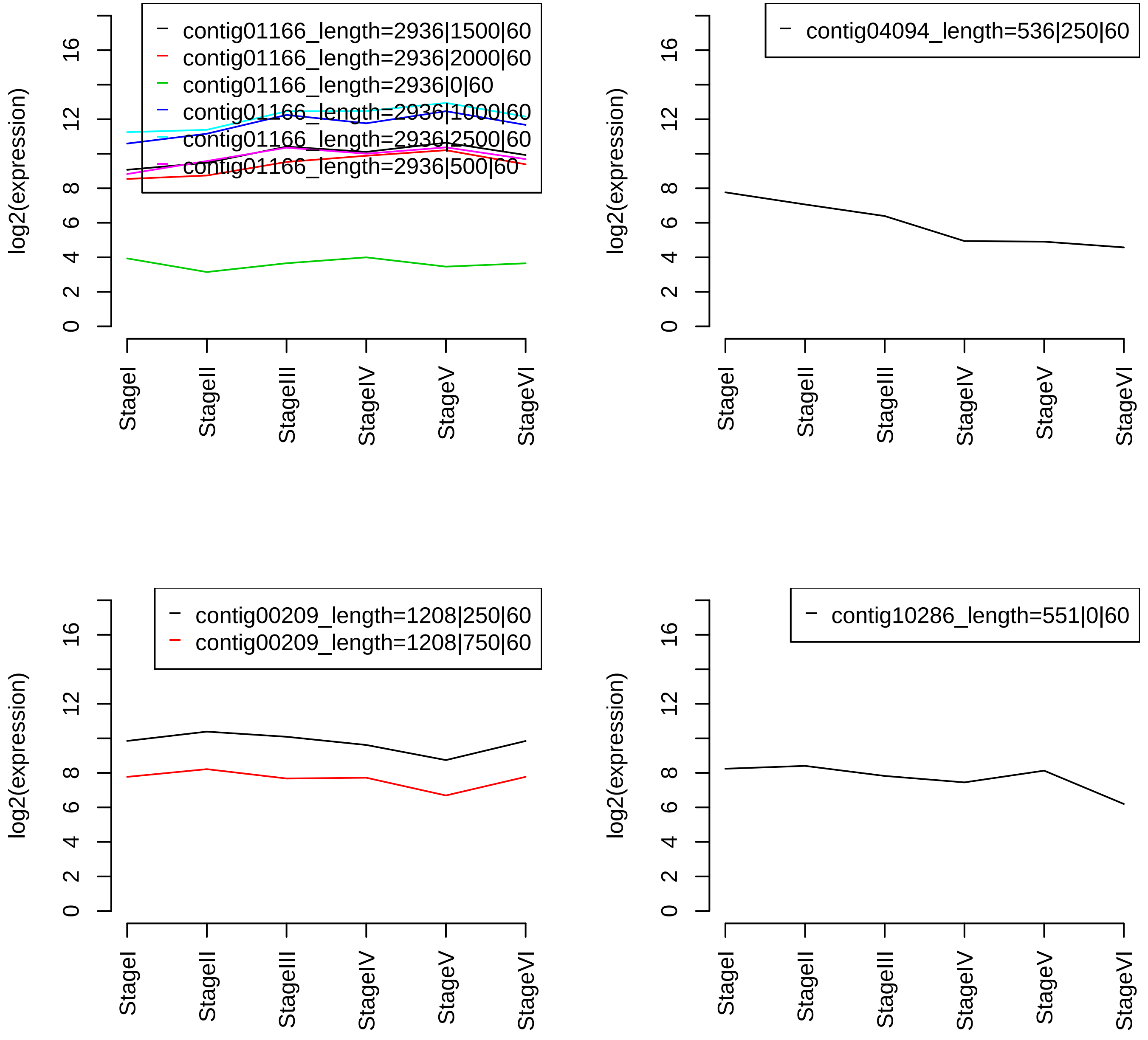
contig01166_length=2936|1500|60
contig01166_length=2936|2000|60
contig01166_length=2936|0|60
contig01166_length=2936|1000|60
contig01166_length=2936|2500|60
contig01166_length=2936|500|60
contig04094_length=536|250|60
contig00209_length=1208|250|60
contig00209_length=1208|750|60
contig10286_length=551|0|60
log2(expression)
0
2
4
6
8
12
16
StageI
StageII
StageIII
StageIV
StageV
StageVI

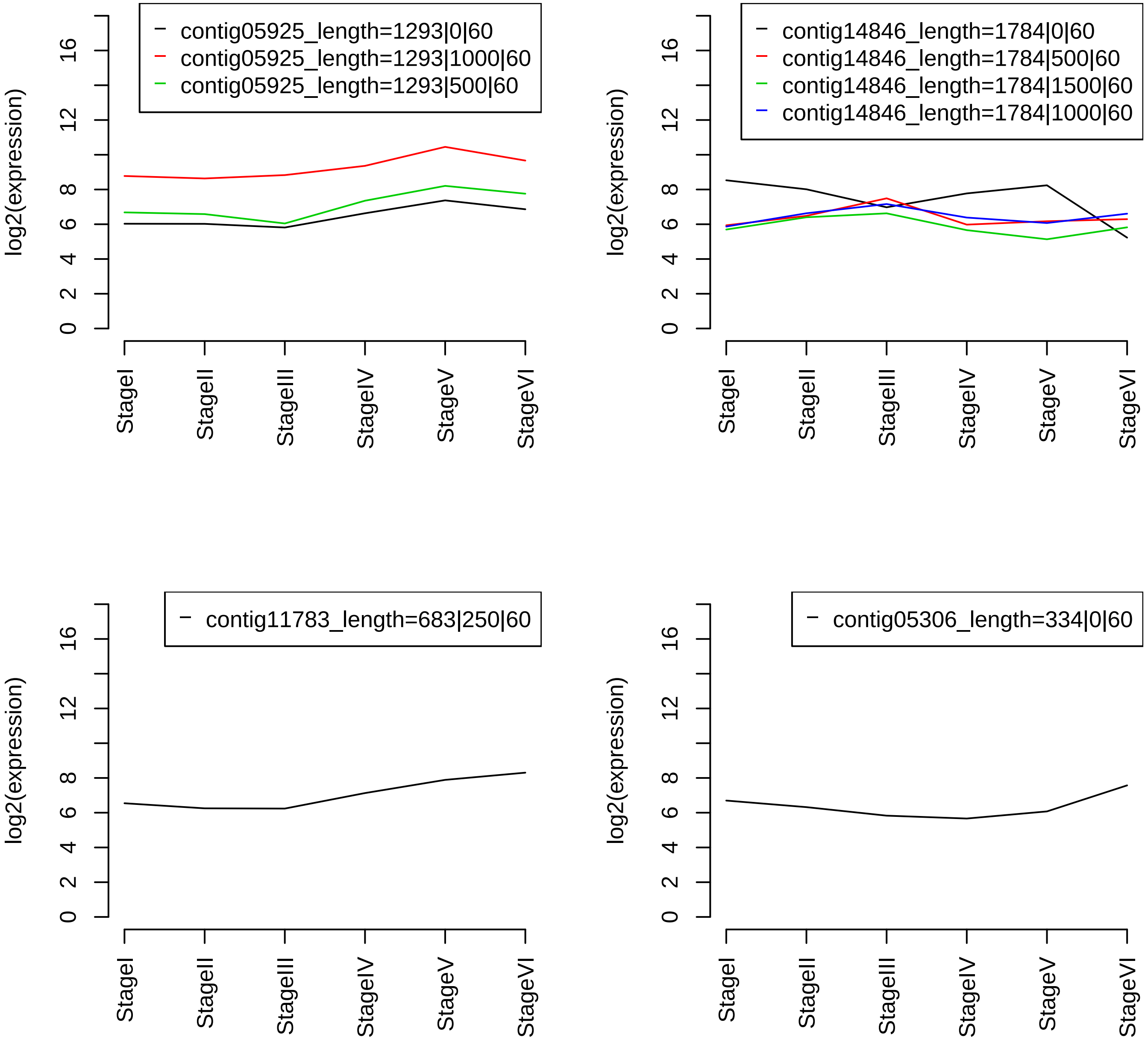
contig05925_length=1293|0|60
contig05925_length=1293|1000|60
contig05925_length=1293|500|60
contig14846_length=1784|0|60
contig14846_length=1784|500|60
contig14846_length=1784|1500|60
contig14846_length=1784|1000|60
contig11783_length=683|250|60
contig05306_length=334|0|60
log2(expression)
0 2 4 6 8 12 16
StageI
StageII
StageIII
StageIV
StageV
StageVI

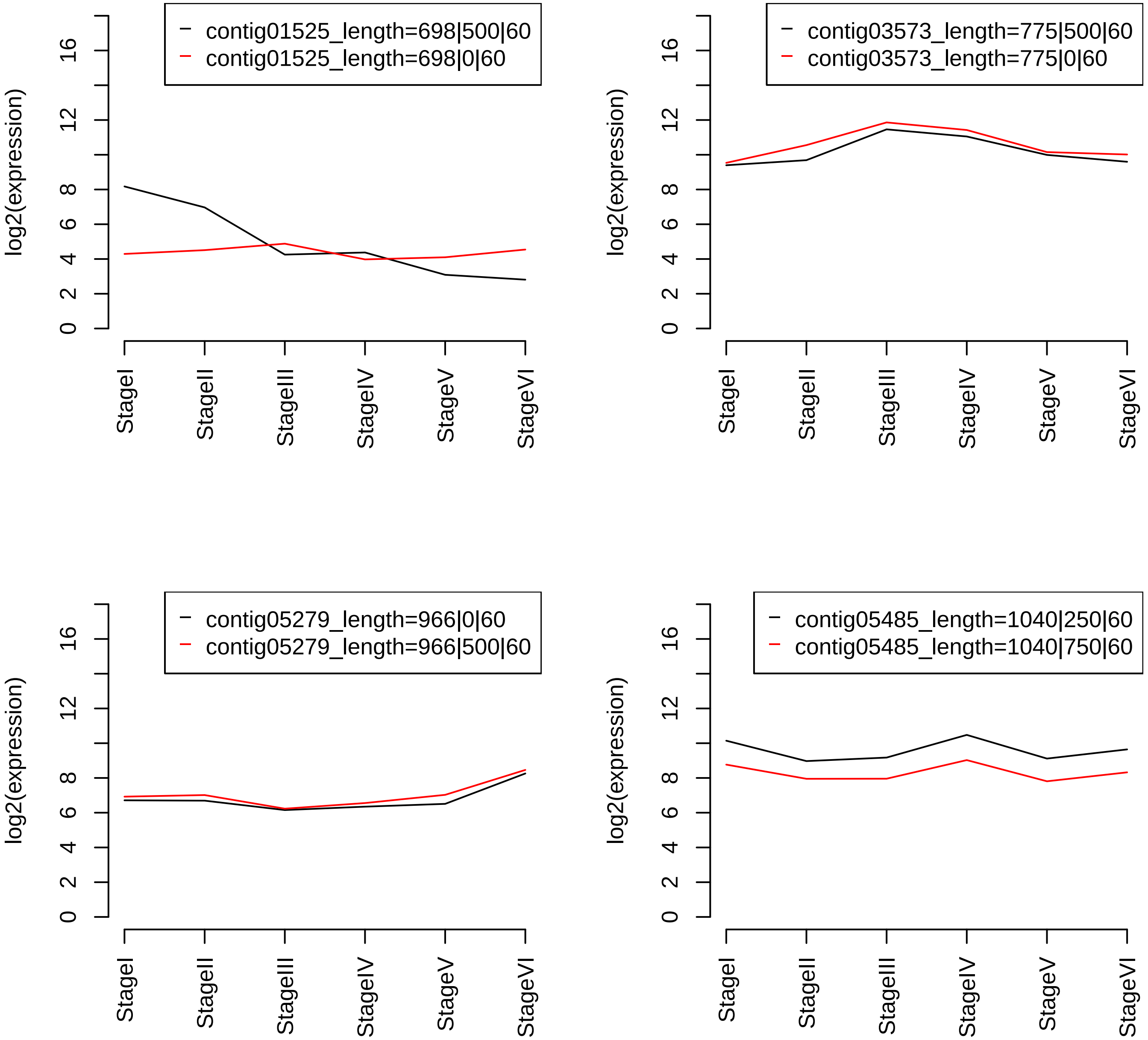

contig01525_length=698|500|60
contig01525_length=698|0|60
contig03573_length=775|500|60
contig03573_length=775|0|60
contig05279_length=966|0|60
contig05279_length=966|500|60
contig05485_length=1040|250|60
contig05485_length=1040|750|60
log2(expression)
StageI
StageII
StageIII
StageIV
StageV
StageVI

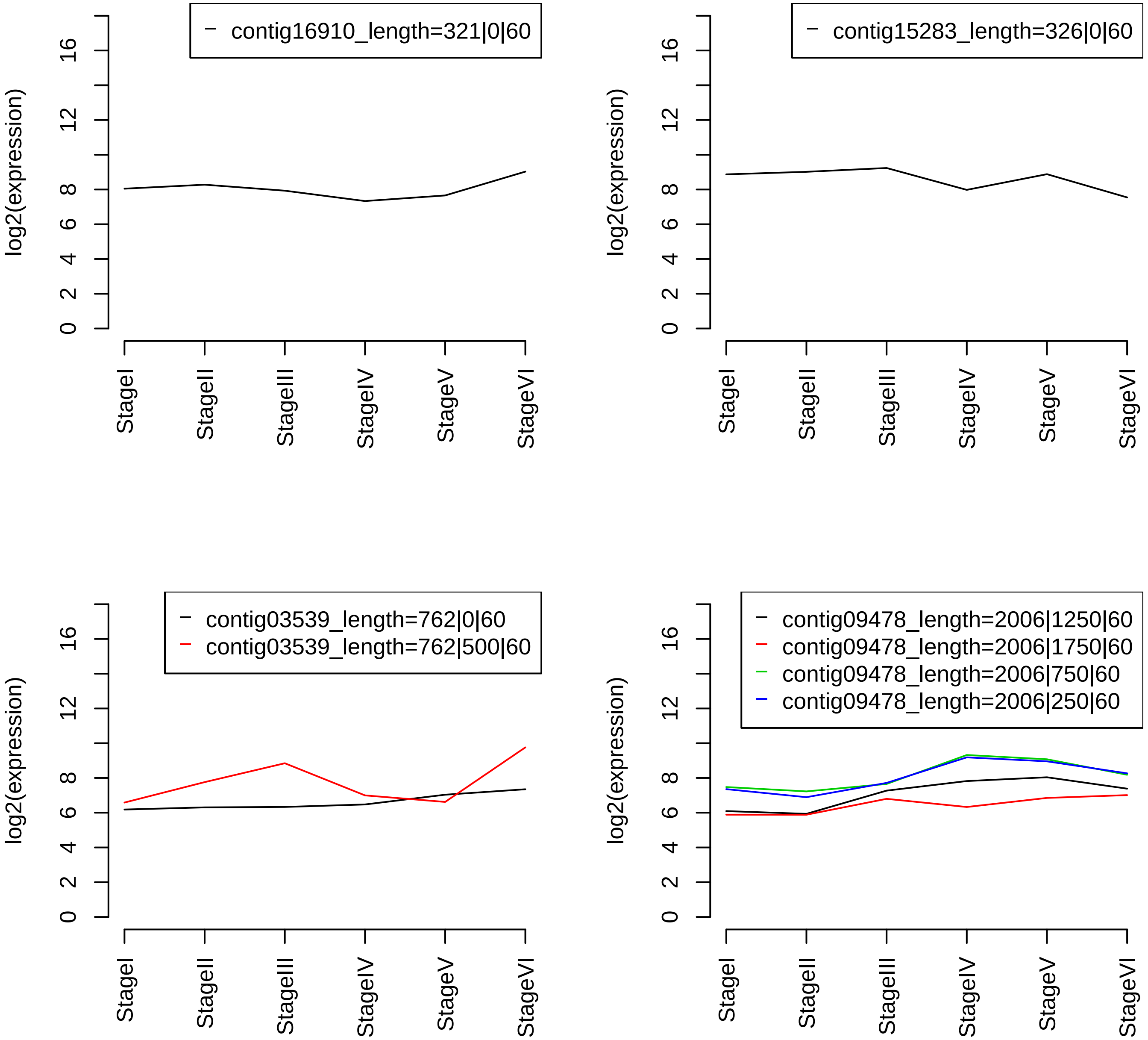
contig16910_length=321|0|60
contig15283_length=326|0|60
contig03539_length=762|0|60
contig03539_length=762|500|60
contig09478_length=2006|1250|60
contig09478_length=2006|1750|60
contig09478_length=2006|750|60
contig09478_length=2006|250|60
log2(expression)
0
2
4
6
8
12
16
StageI
StageII
StageIII
StageIV
StageV
StageVI

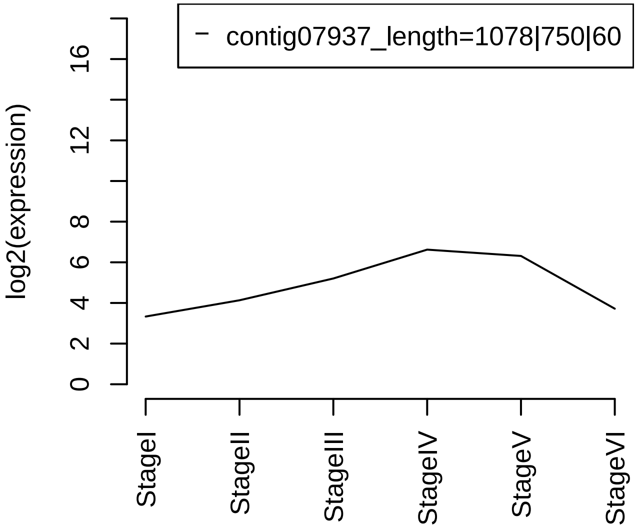
contig07937_length=1078|750|60
log2(expression)
0
2
4
6
8
12
16
StageI
StageII
StageIII
StageIV
StageV
StageVI

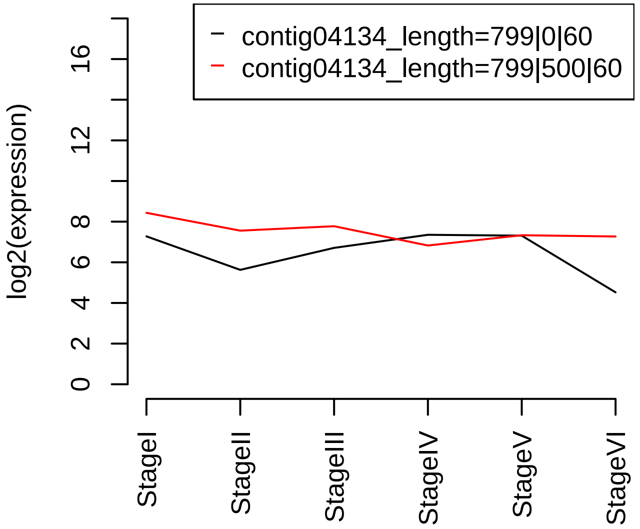
contig04134_length=799|0|60
contig04134_length=799|500|60
log2(expression)
0
2
4
6
8
12
16
StageI
StageII
StageIII
StageIV
StageV
StageVI

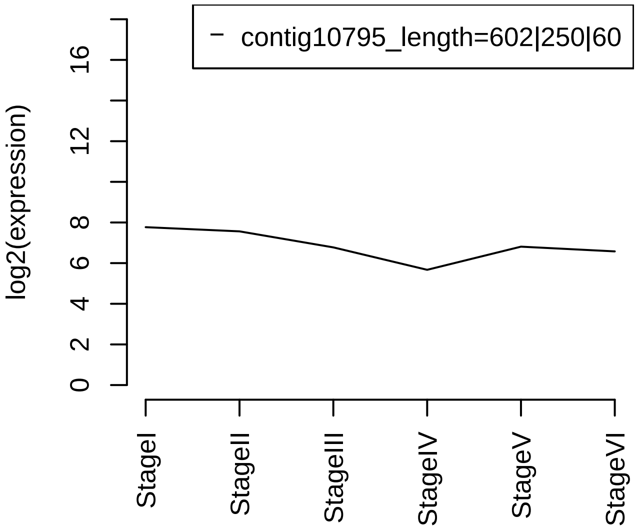
contig10795_length=602|250|60
log2(expression)
0
2
4
6
8
12
16
StageI
StageII
StageIII
StageIV
StageV
StageVI

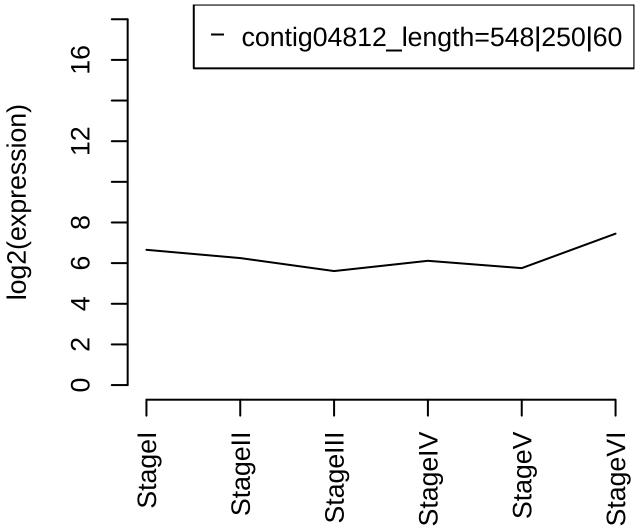
contig04812_length=548|250|60
log2(expression)
0
2
4
6
8
12
16
StageI
StageII
StageIII
StageIV
StageV
StageVI

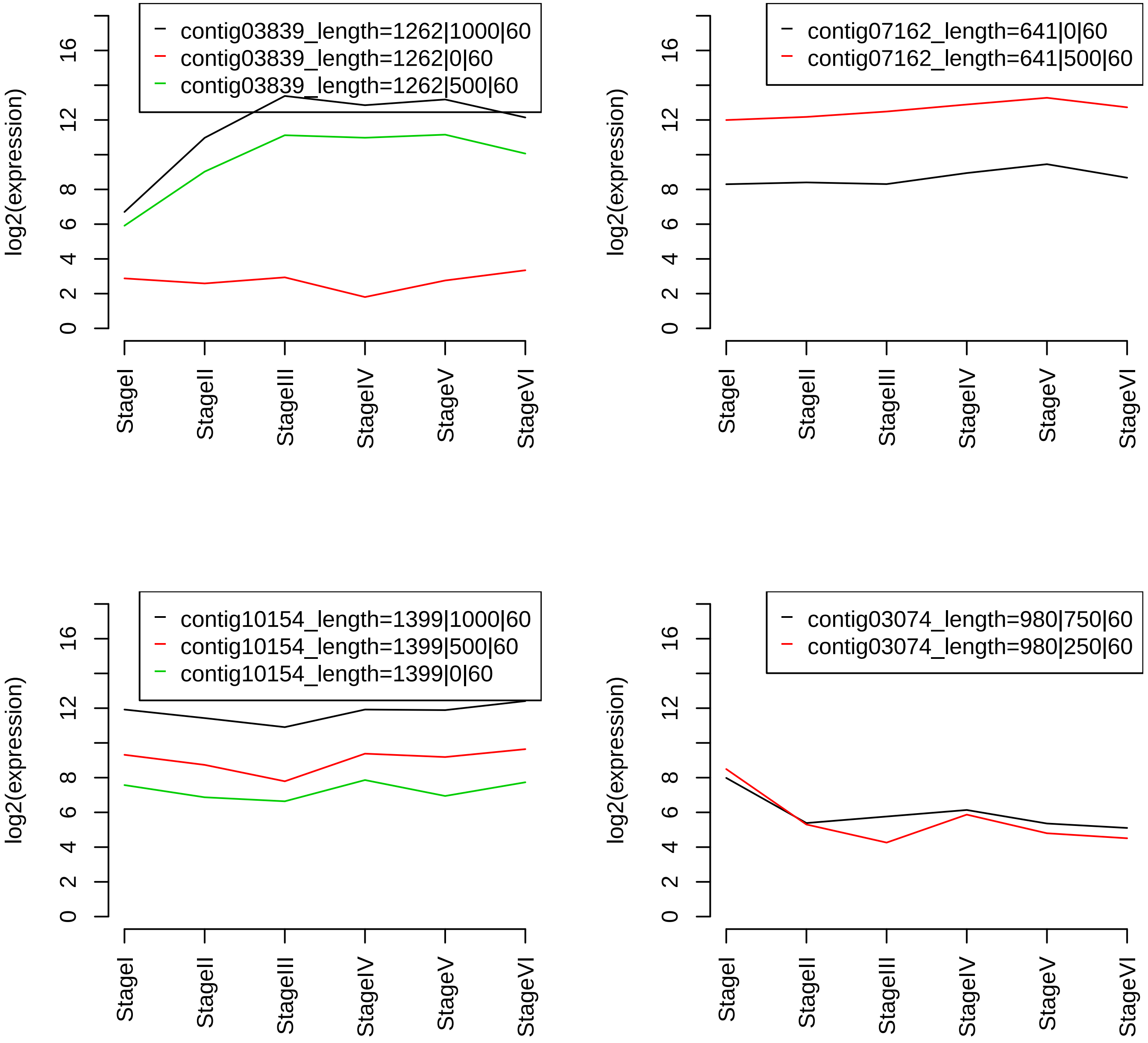
contig03839_length=1262|1000|60
contig03839_length=1262|0|60
contig03839_length=1262|500|60
contig07162_length=641|0|60
contig07162_length=641|500|60
contig10154_length=1399|1000|60
contig10154_length=1399|500|60
contig10154_length=1399|0|60
contig03074_length=980|750|60
contig03074_length=980|250|60
log2(expression)
0
2
4
6
8
12
16
StageI
StageII
StageIII
StageIV
StageV
StageVI

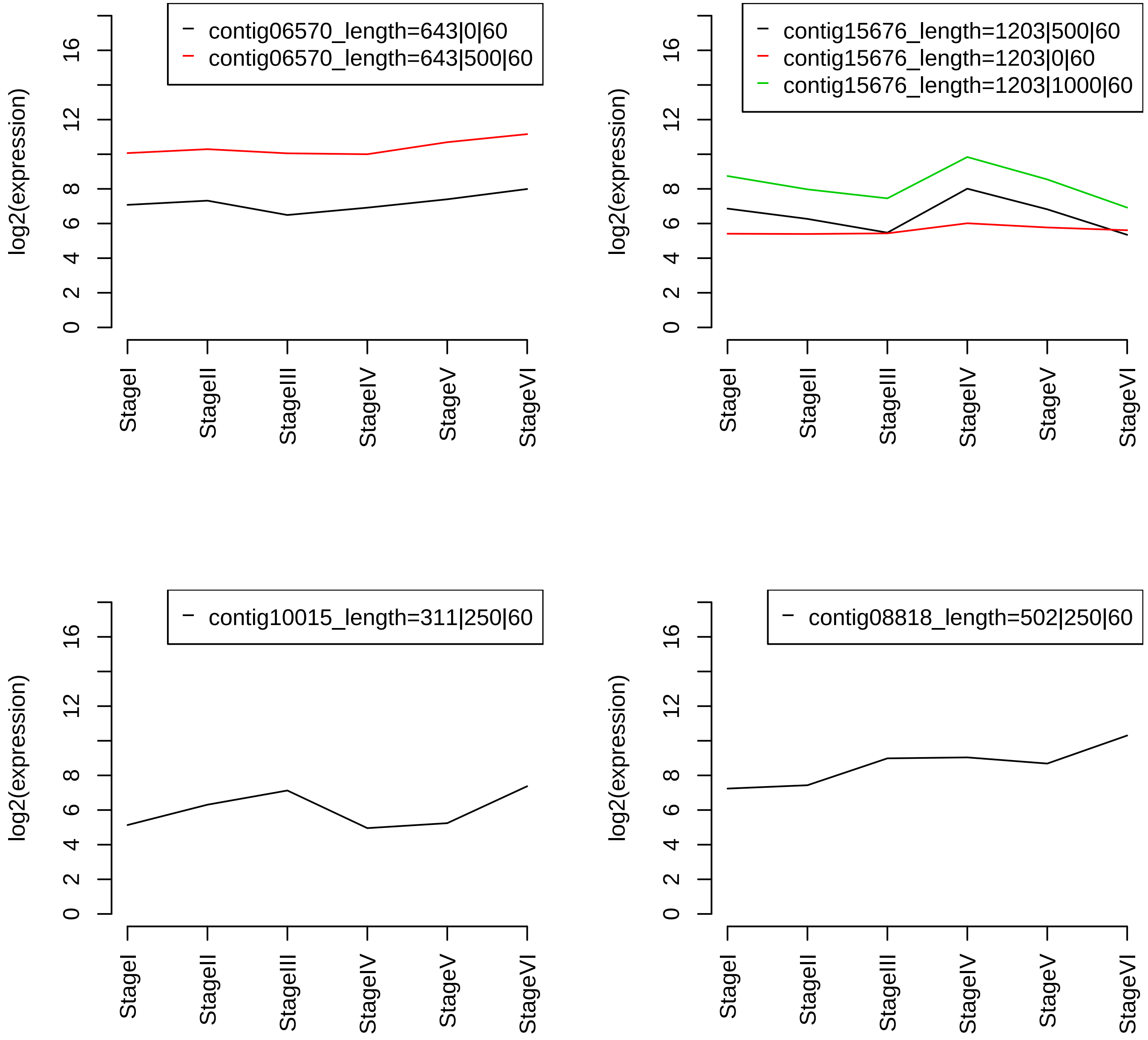
contig06570_length=643|0|60
contig06570_length=643|500|60
contig15676_length=1203|500|60
contig15676_length=1203|0|60
contig15676_length=1203|1000|60
contig10015_length=311|250|60
contig08818_length=502|250|60
log2(expression)
0 2 4 6 8 12 16
StageI StageII StageIII StageIV StageV StageVI

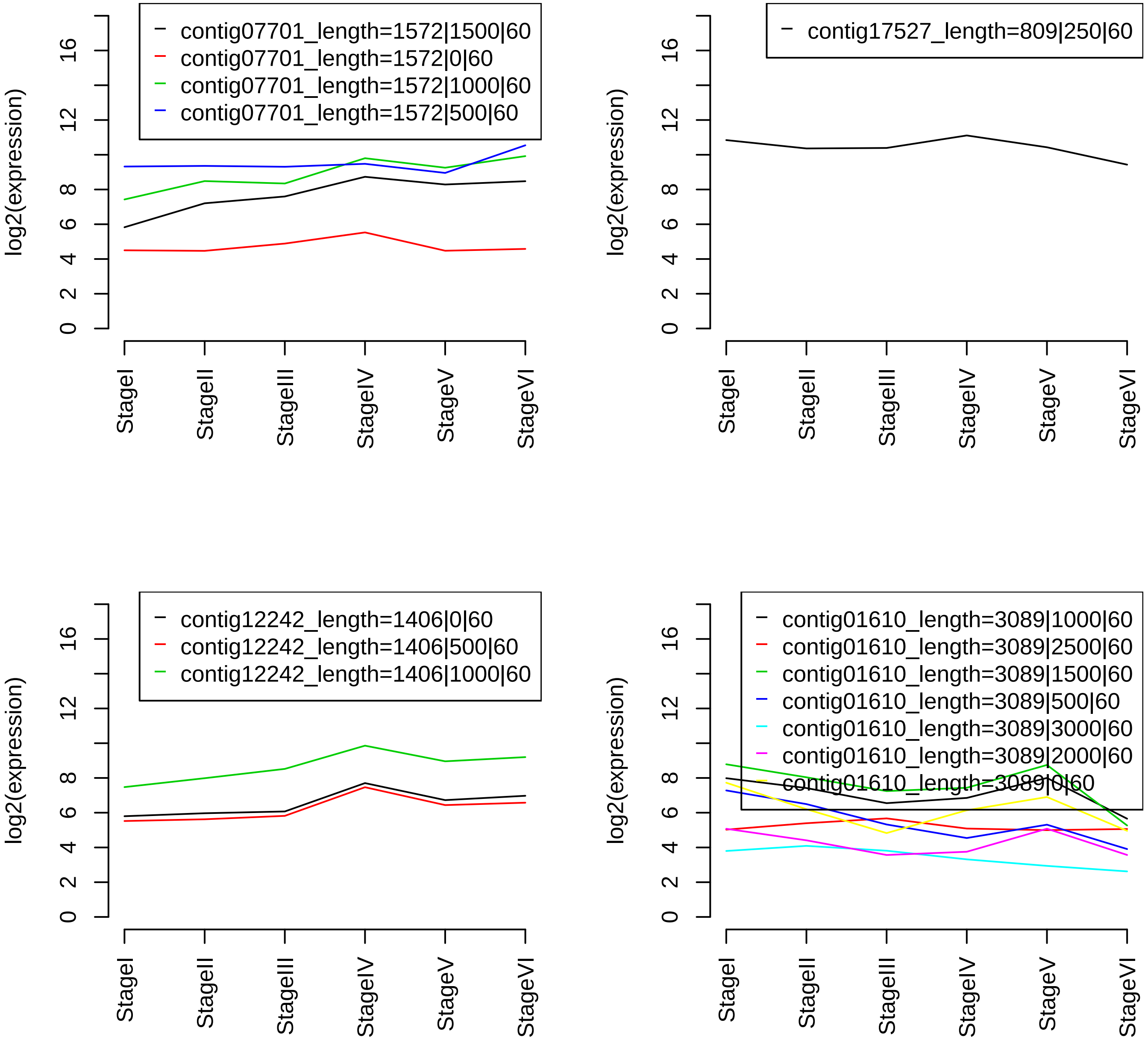

contig07701_length=1572|1500|60
contig07701_length=1572|0|60
contig07701_length=1572|1000|60
contig07701_length=1572|500|60
contig17527_length=809|250|60
contig12242_length=1406|0|60
contig12242_length=1406|500|60
contig12242_length=1406|1000|60
contig01610_length=3089|1000|60
contig01610_length=3089|2500|60
contig01610_length=3089|1500|60
contig01610_length=3089|500|60
contig01610_length=3089|3000|60
contig01610_length=3089|2000|60
contig01610_length=3089|0|60
log2(expression)
0
2
4
6
8
12
16
StageI
StageII
StageIII
StageIV
StageV
StageVI

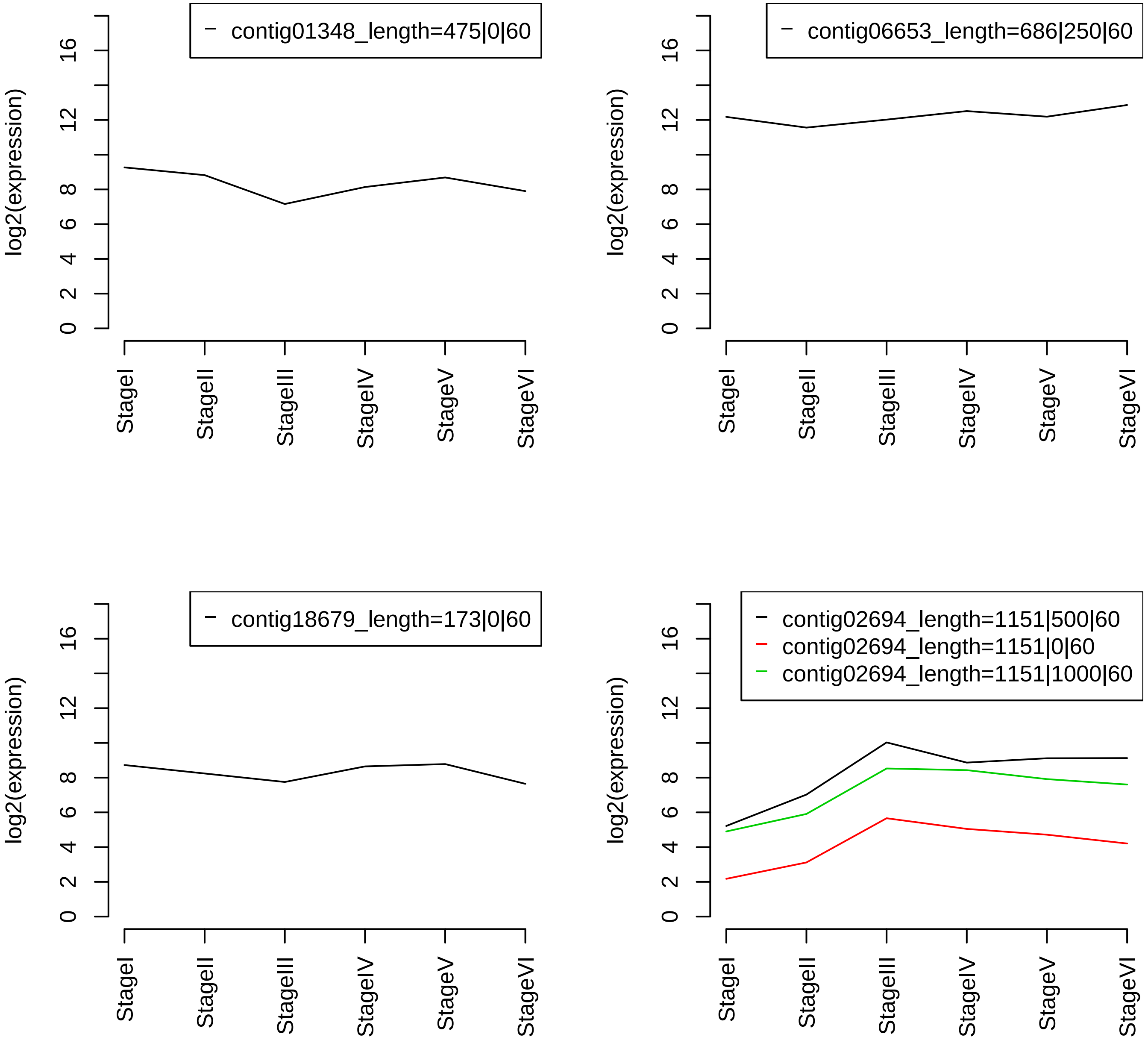
contig01348_length=475|0|60
contig06653_length=686|250|60
contig18679_length=173|0|60
contig02694_length=1151|500|60
contig02694_length=1151|0|60
contig02694_length=1151|1000|60
log2(expression)
0
2
4
6
8
12
16
StageI
StageII
StageIII
StageIV
StageV
StageVI

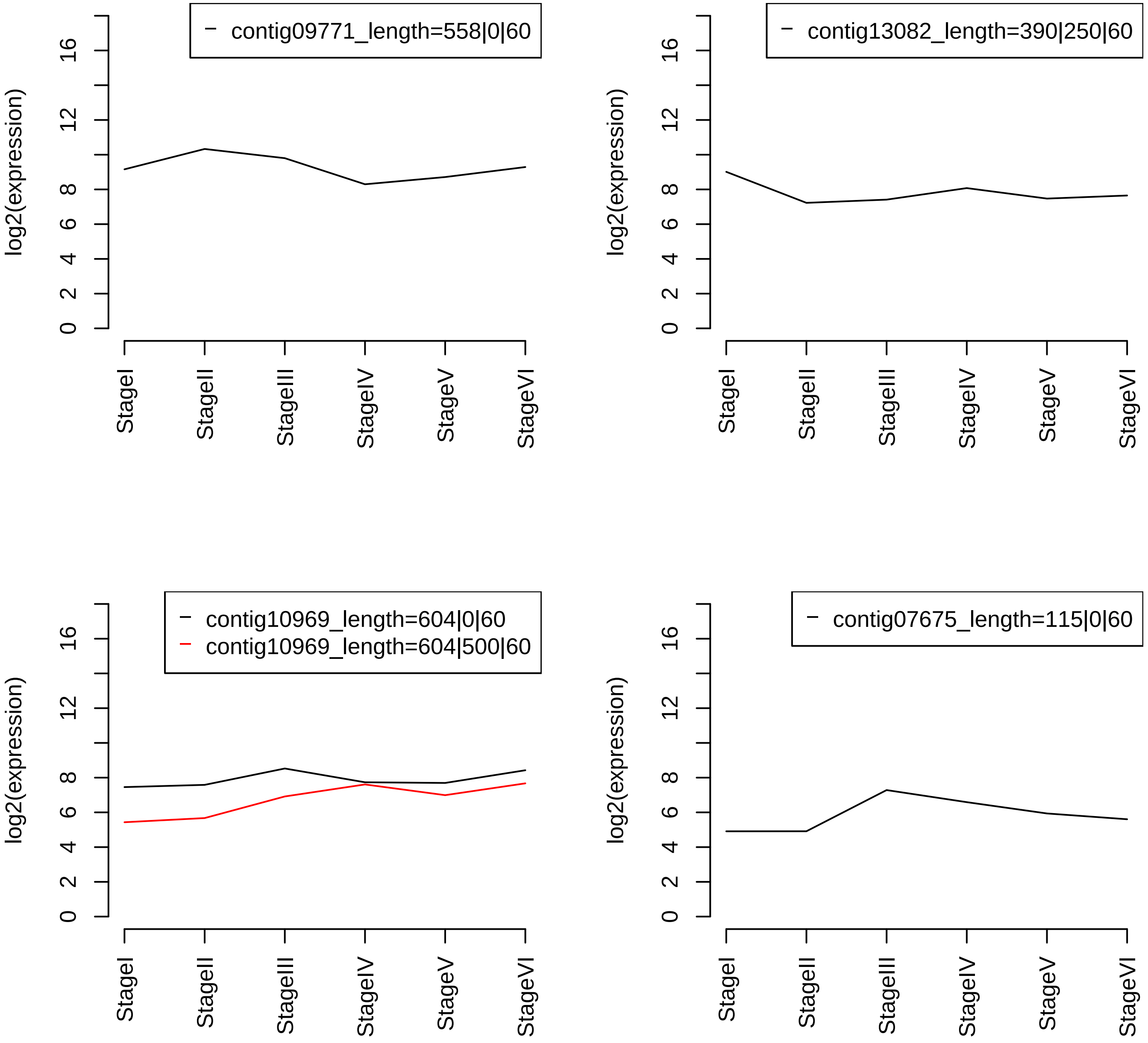

contig09771_length=558|0|60
contig13082_length=390|250|60
contig10969_length=604|0|60
contig10969_length=604|500|60
contig07675_length=115|0|60
log2(expression)
0
2
4
6
8
12
16
StageI
StageII
StageIII
StageIV
StageV
StageVI

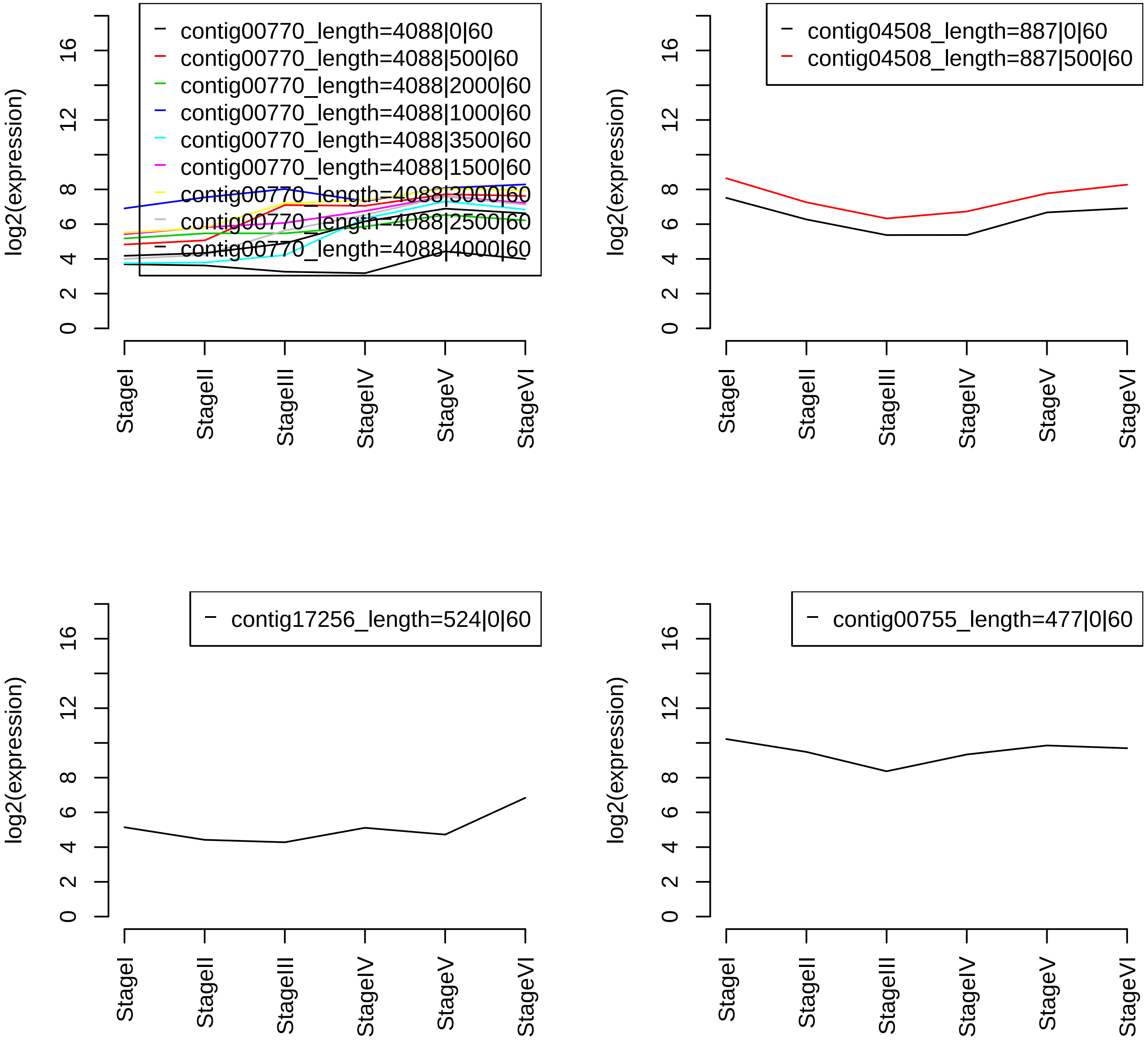

log2(expression)
contig00770_length=4088|0|60
contig00770_length=4088|500|60
contig00770_length=4088|2000|60
contig00770_length=4088|1000|60
contig00770_length=4088|3500|60
contig00770_length=4088|1500|60
contig00770_length=4088|3000|60
contig00770_length=4088|2500|60
contig00770_length=4088|4000|60
contig04508_length=887|0|60
contig04508_length=887|500|60
contig17256_length=524|0|60
contig00755_length=477|0|60
StageI
StageII
StageIII
StageIV
StageV
StageVI
0
2
4
6
8
12
16

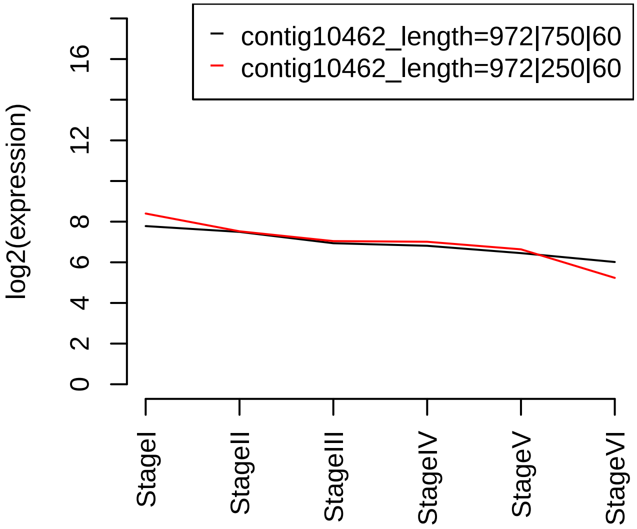

contig10462_length=972|750|60
contig10462_length=972|250|60
log2(expression)
0
2
4
6
8
12
16
StageI
StageII
StageIII
StageIV
StageV
StageVI

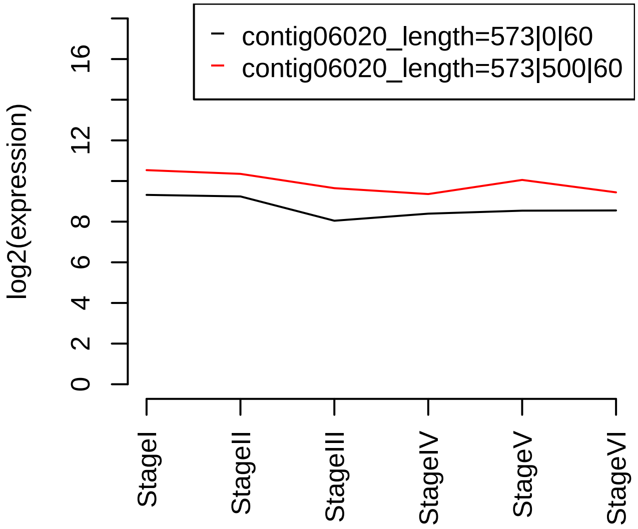

contig06020_length=573|0|60
contig06020_length=573|500|60
log2(expression)
0
2
4
6
8
12
16
StageI
StageII
StageIII
StageIV
StageV
StageVI

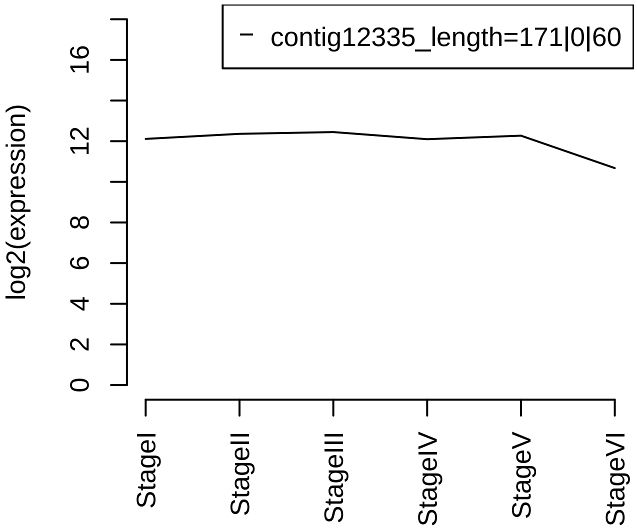

contig12335_length=171|0|60
log2(expression)
0
2
4
6
8
12
16
StageI
StageII
StageIII
StageIV
StageV
StageVI

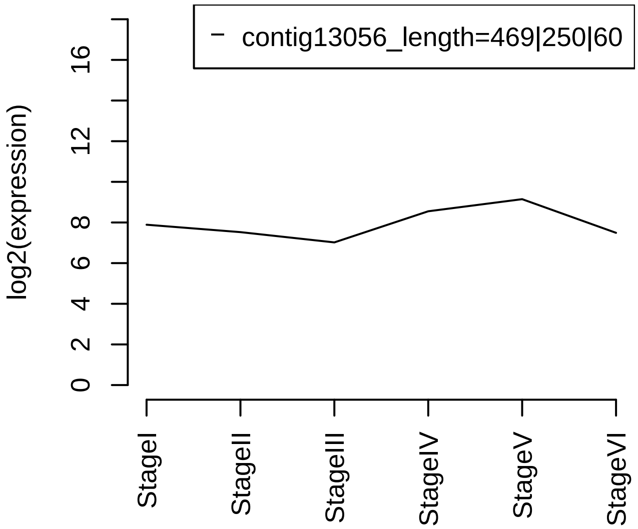

contig13056_length=469|250|60
log2(expression)
0
2
4
6
8
12
16
StageI
StageII
StageIII
StageIV
StageV
StageVI

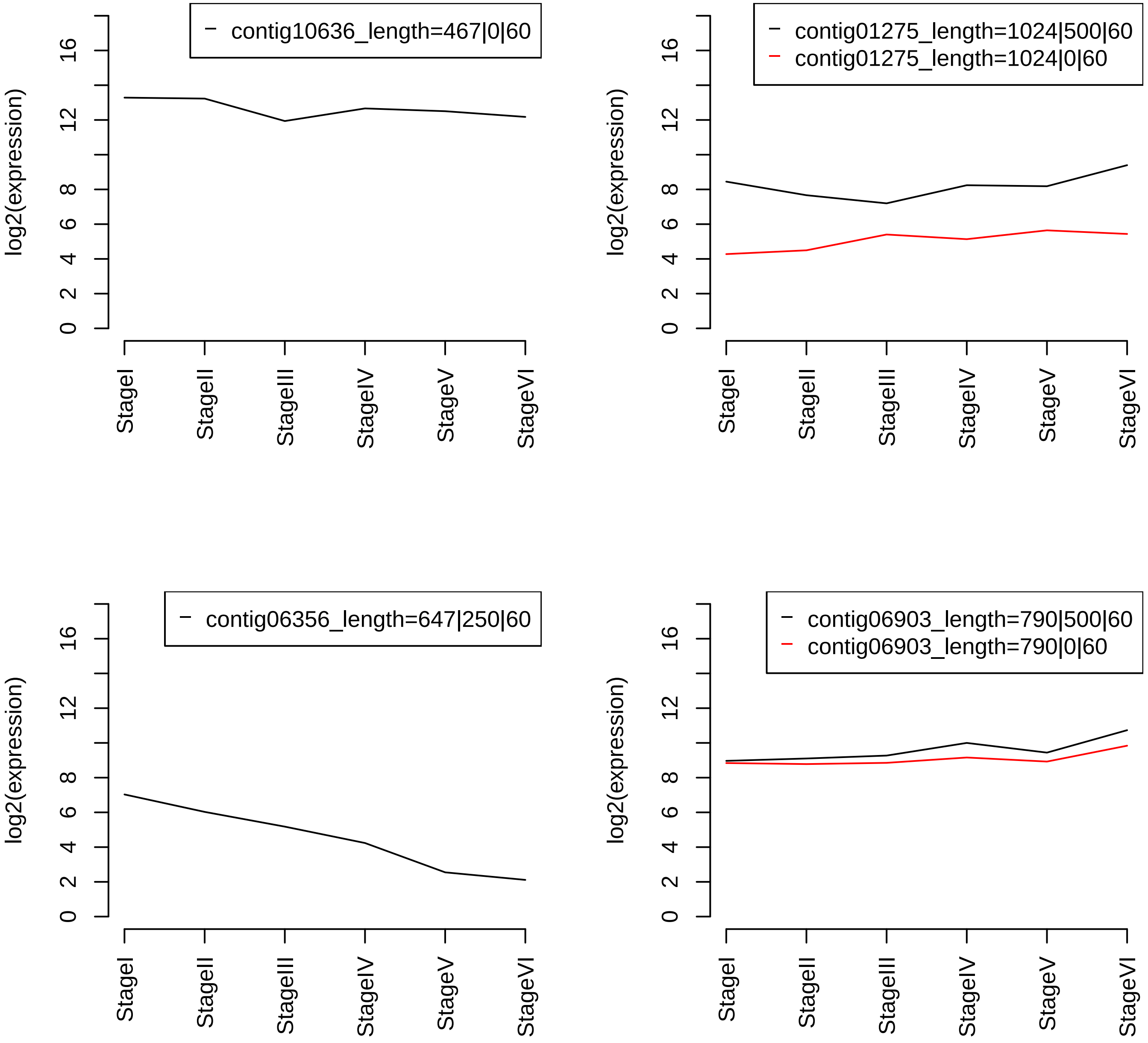

contig10636_length=467|0|60
contig01275_length=1024|500|60
contig01275_length=1024|0|60
contig06356_length=647|250|60
contig06903_length=790|500|60
contig06903_length=790|0|60
log2(expression)
0
2
4
6
8
12
16
StageI
StageII
StageIII
StageIV
StageV
StageVI

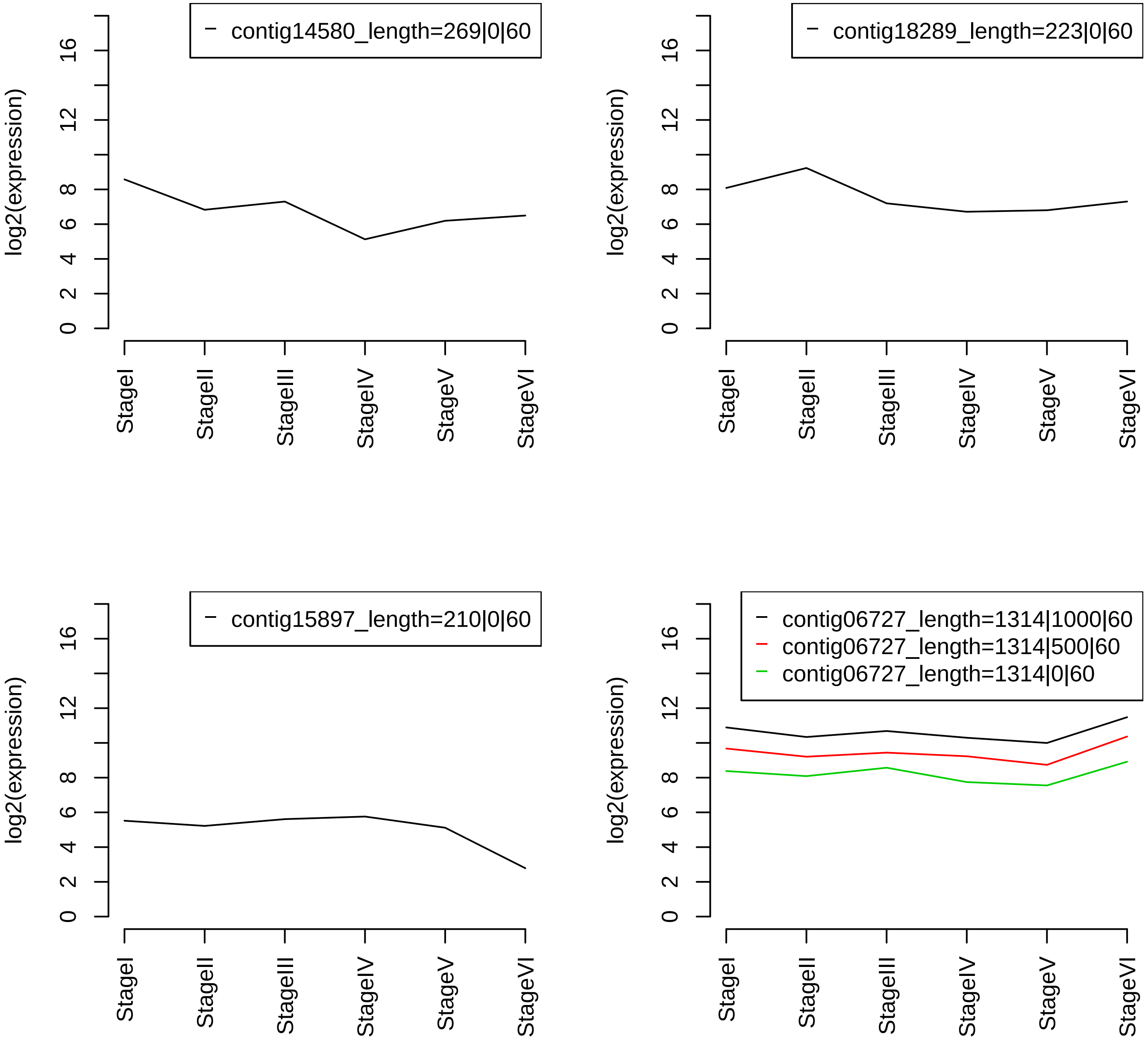
contig14580_length=269|0|60
contig18289_length=223|0|60
contig15897_length=210|0|60
contig06727_length=1314|1000|60
contig06727_length=1314|500|60
contig06727_length=1314|0|60
log2(expression)
0
2
4
6
8
12
16
StageI
StageII
StageIII
StageIV
StageV
StageVI

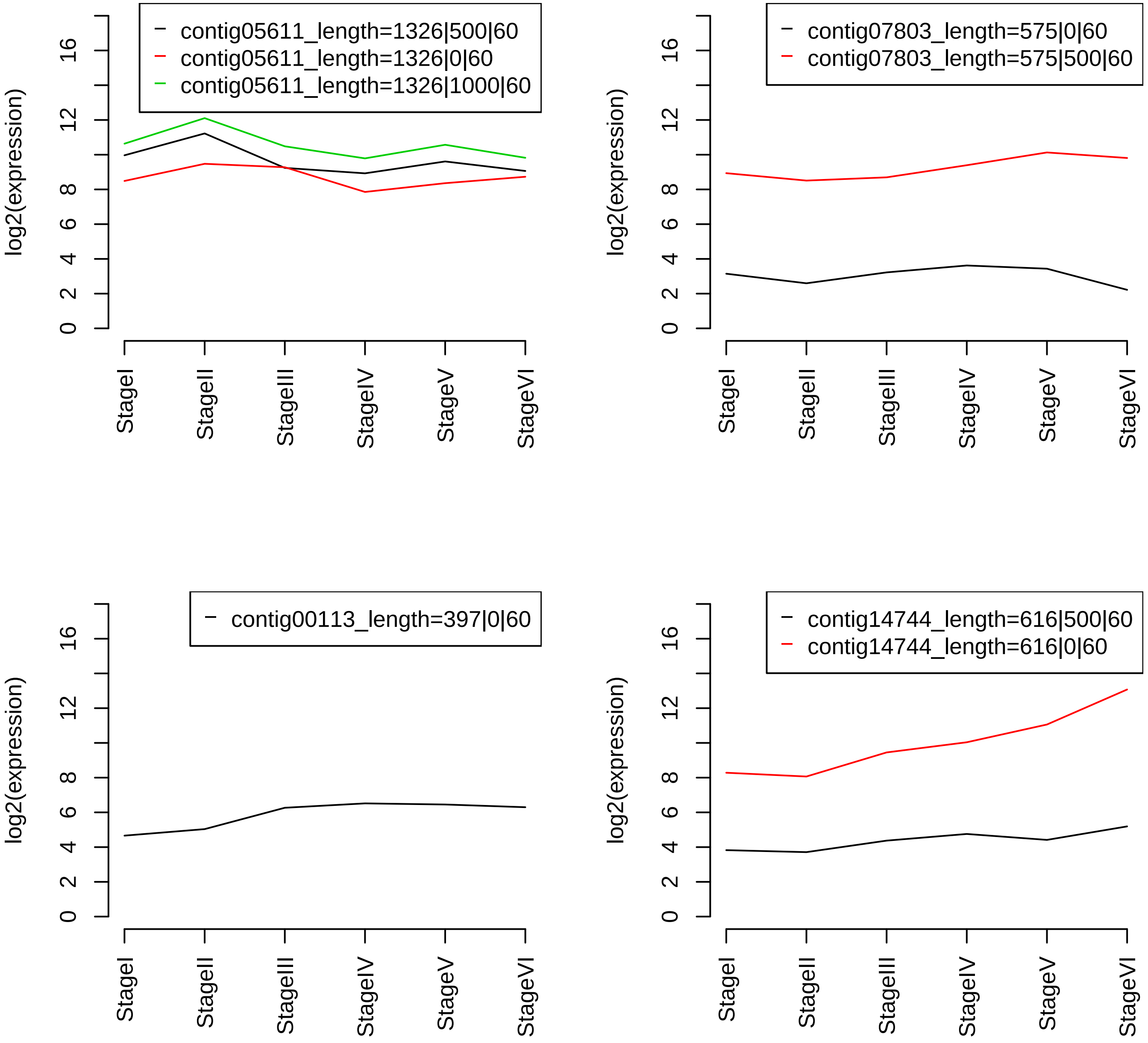
contig05611_length=1326|500|60
contig05611_length=1326|0|60
contig05611_length=1326|1000|60
contig07803_length=575|0|60
contig07803_length=575|500|60
contig00113_length=397|0|60
contig14744_length=616|500|60
contig14744_length=616|0|60
log2(expression)
StageI
StageII
StageIII
StageIV
StageV
StageVI

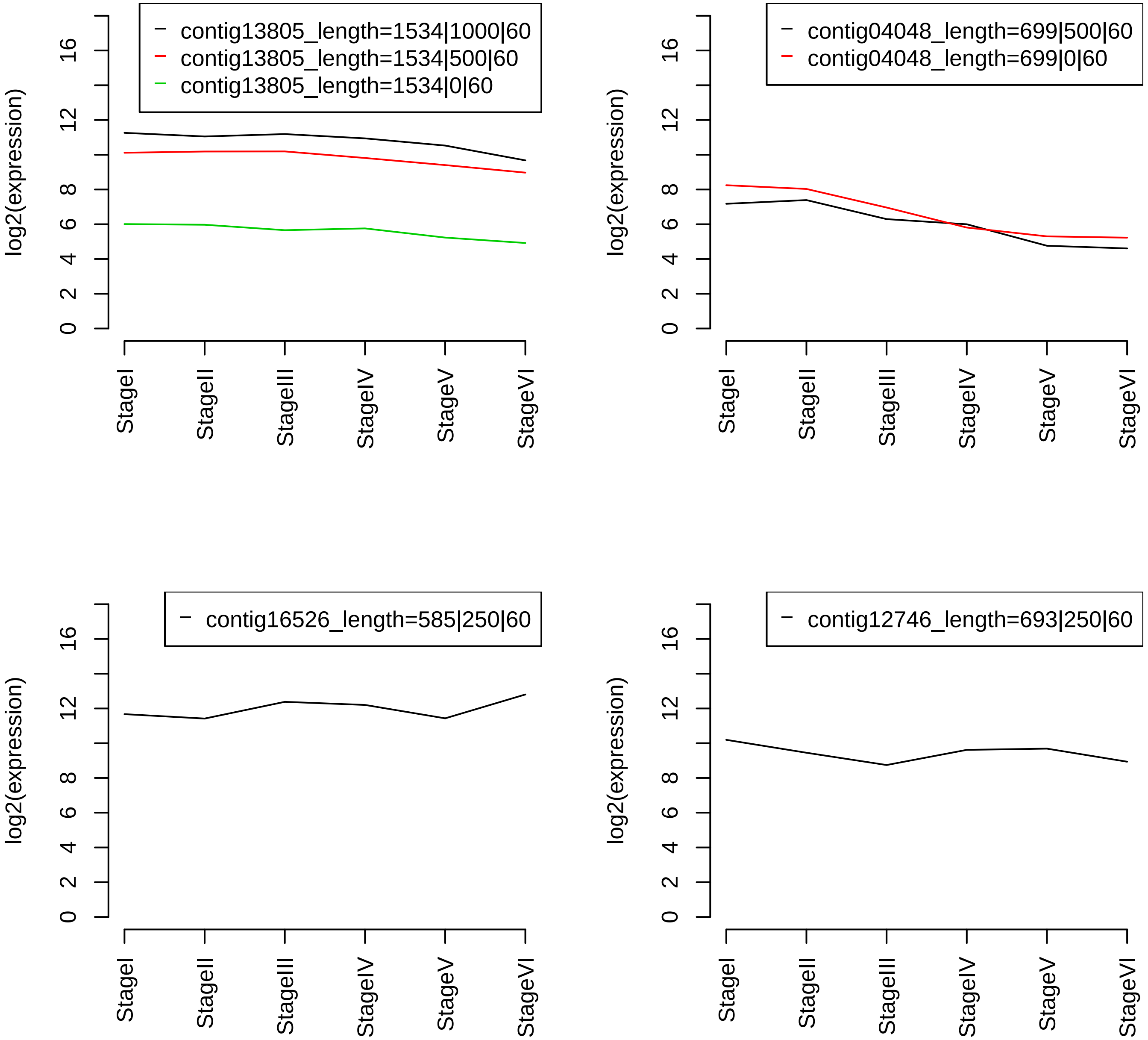

contig13805_length=1534|1000|60
contig13805_length=1534|500|60
contig13805_length=1534|0|60
contig04048_length=699|500|60
contig04048_length=699|0|60
contig16526_length=585|250|60
contig12746_length=693|250|60
log2(expression)
0
2
4
6
8
12
16
StageI
StageII
StageIII
StageIV
StageV
StageVI

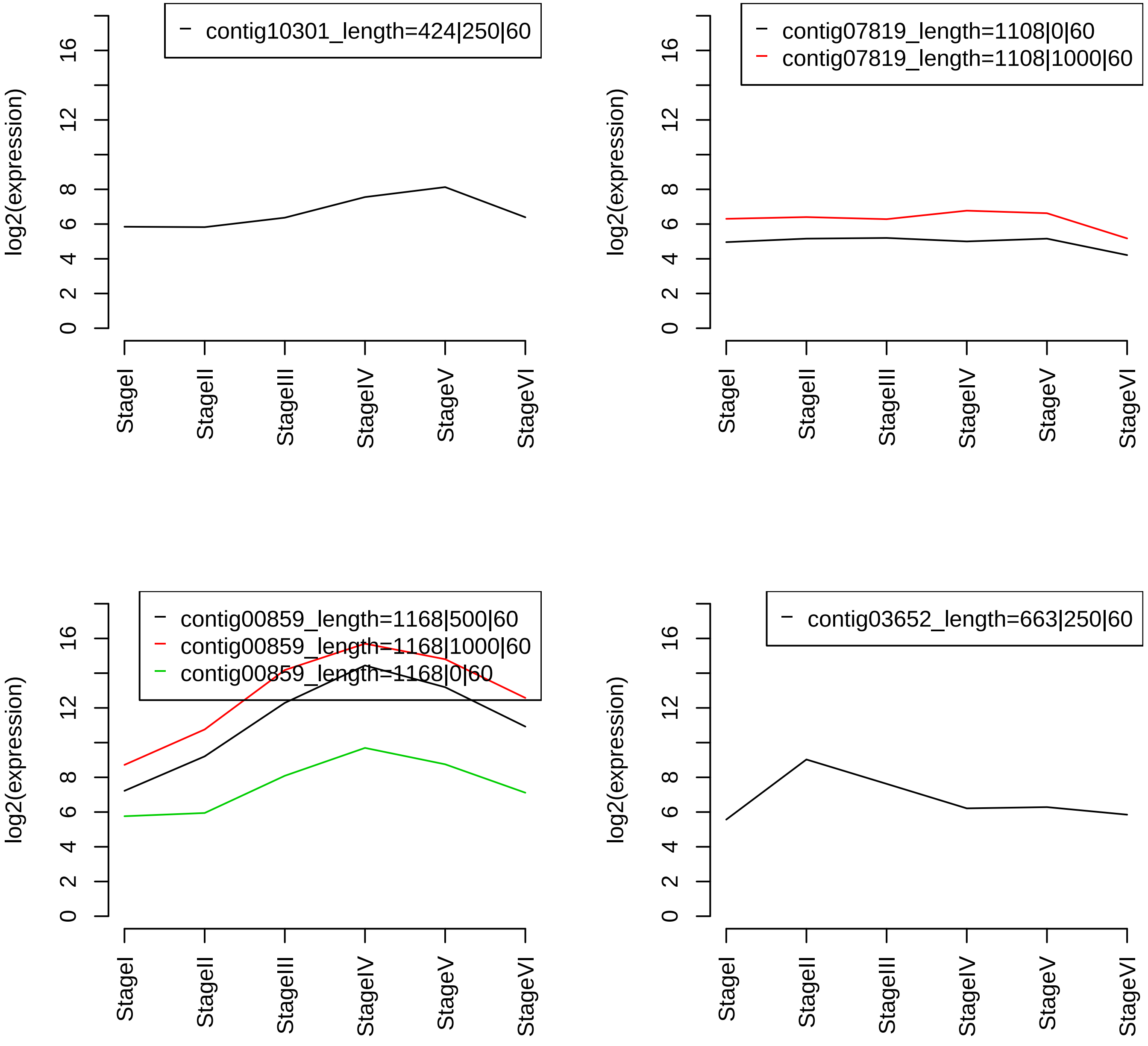

contig10301_length=424|250|60
contig07819_length=1108|0|60
contig07819_length=1108|1000|60
contig00859_length=1168|500|60
contig00859_length=1168|1000|60
contig00859_length=1168|0|60
contig03652_length=663|250|60
log2(expression)
0
2
4
6
8
12
16
StageI
StageII
StageIII
StageIV
StageV
StageVI

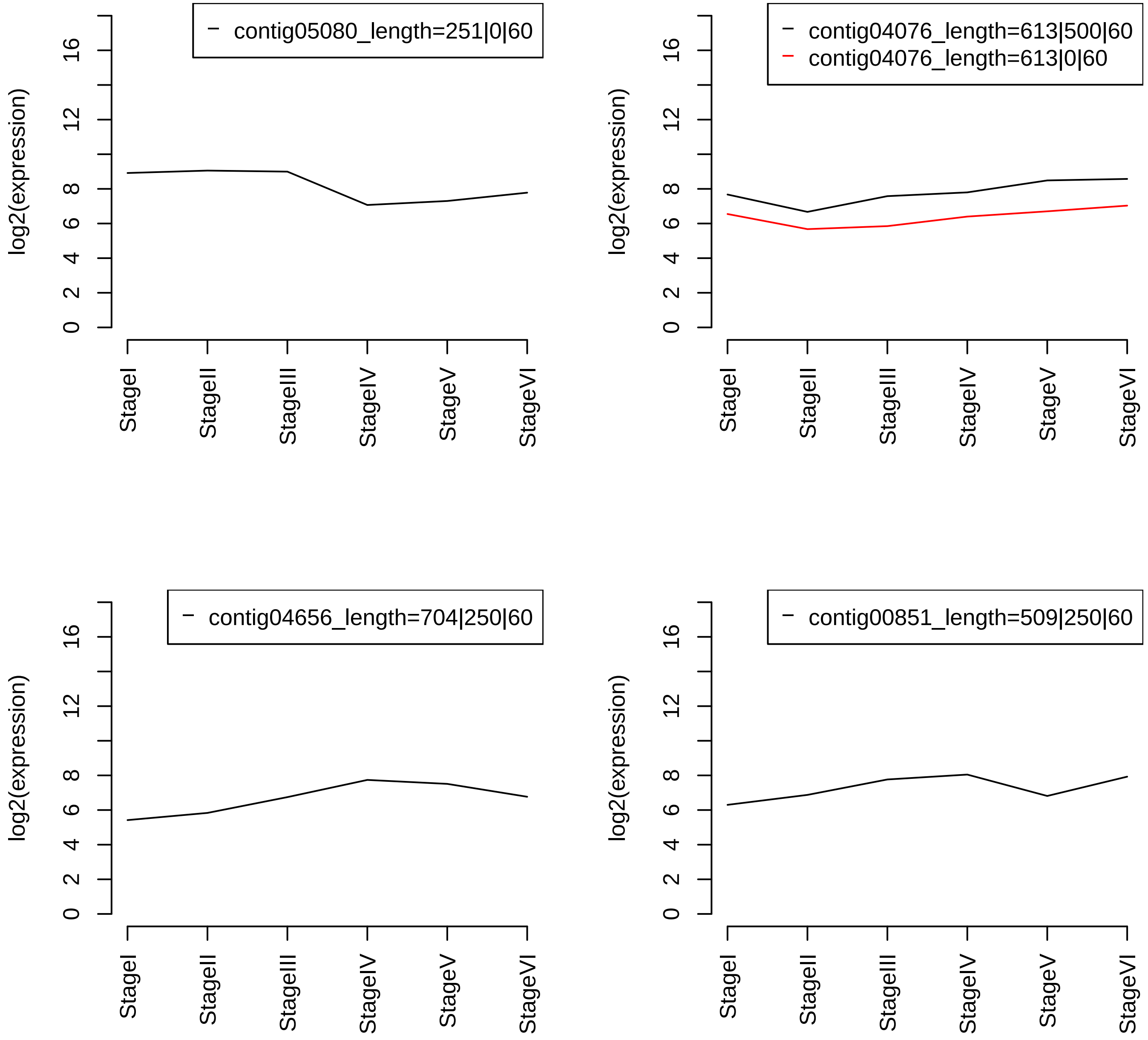

contig05080_length=251|0|60
contig04076_length=613|500|60
contig04076_length=613|0|60
contig04656_length=704|250|60
contig00851_length=509|250|60
log2(expression)
0
2
4
6
8
12
16
StageI
StageII
StageIII
StageIV
StageV
StageVI

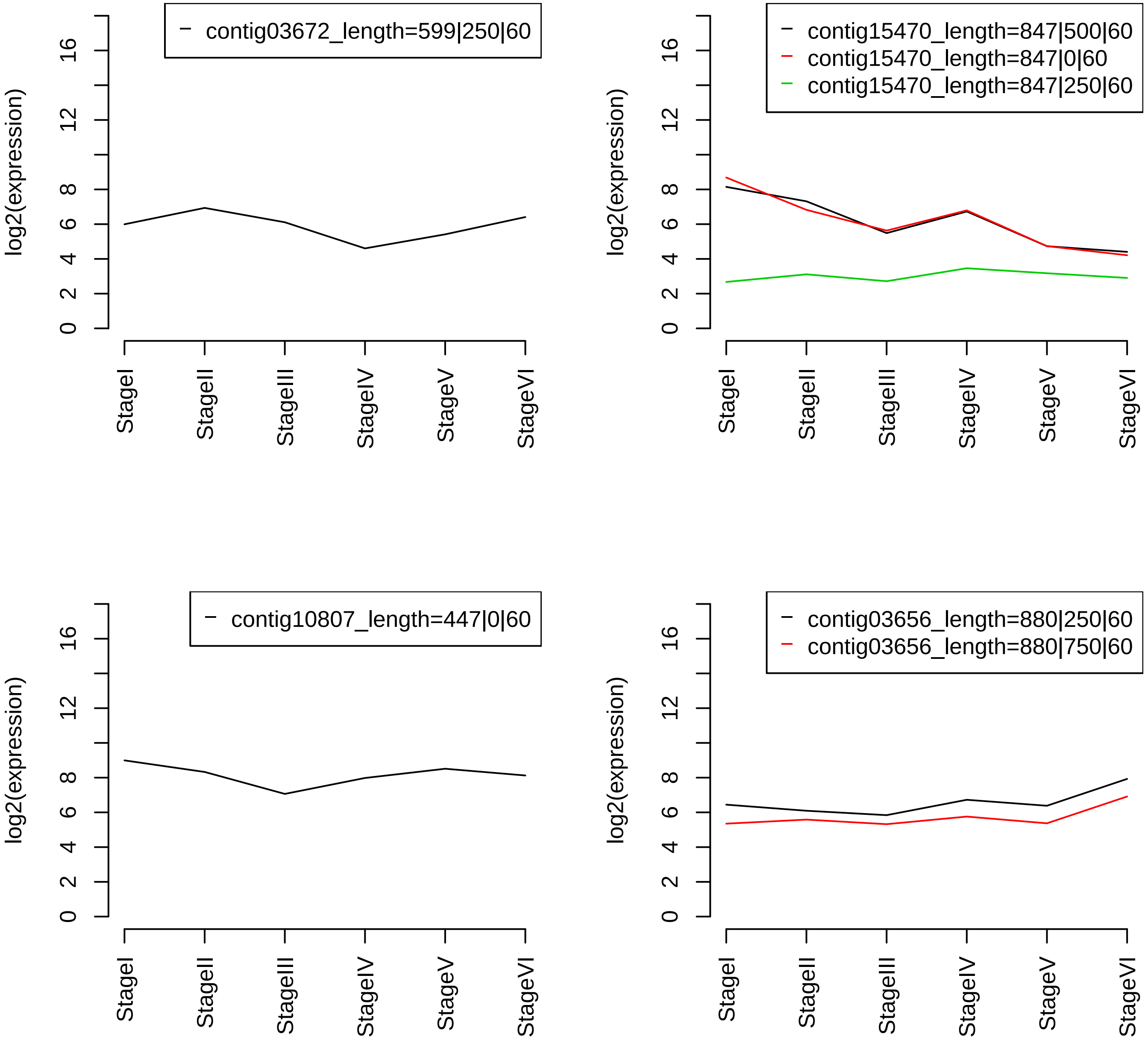
contig03672_length=599|250|60
contig15470_length=847|500|60
contig15470_length=847|0|60
contig15470_length=847|250|60
contig10807_length=447|0|60
contig03656_length=880|250|60
contig03656_length=880|750|60
log2(expression)
StageI
StageII
StageIII
StageIV
StageV
StageVI

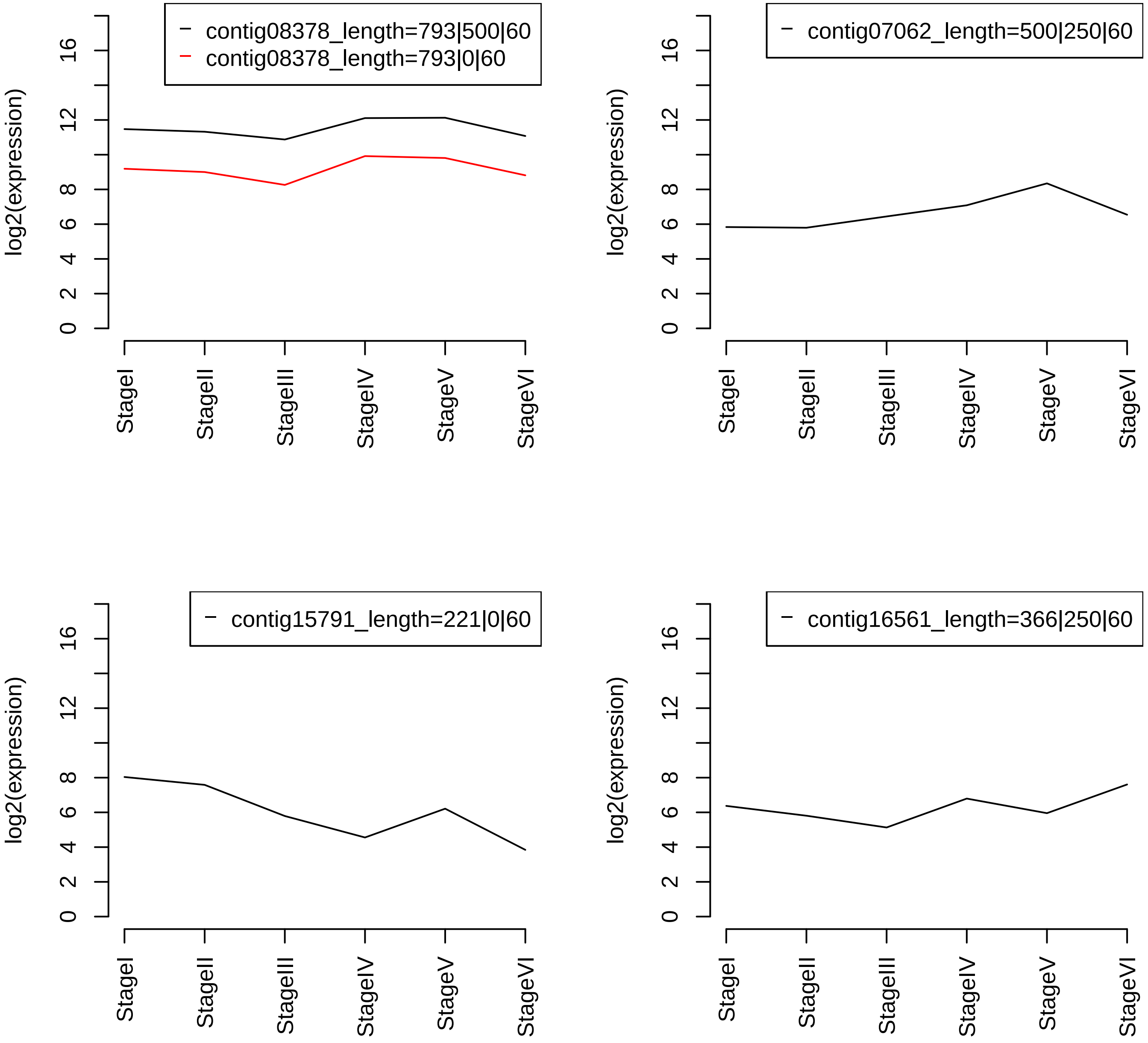
contig08378_length=793|500|60
contig08378_length=793|0|60
contig07062_length=500|250|60
contig15791_length=221|0|60
contig16561_length=366|250|60
log2(expression)
0
2
4
6
8
12
16
StageI
StageII
StageIII
StageIV
StageV
StageVI

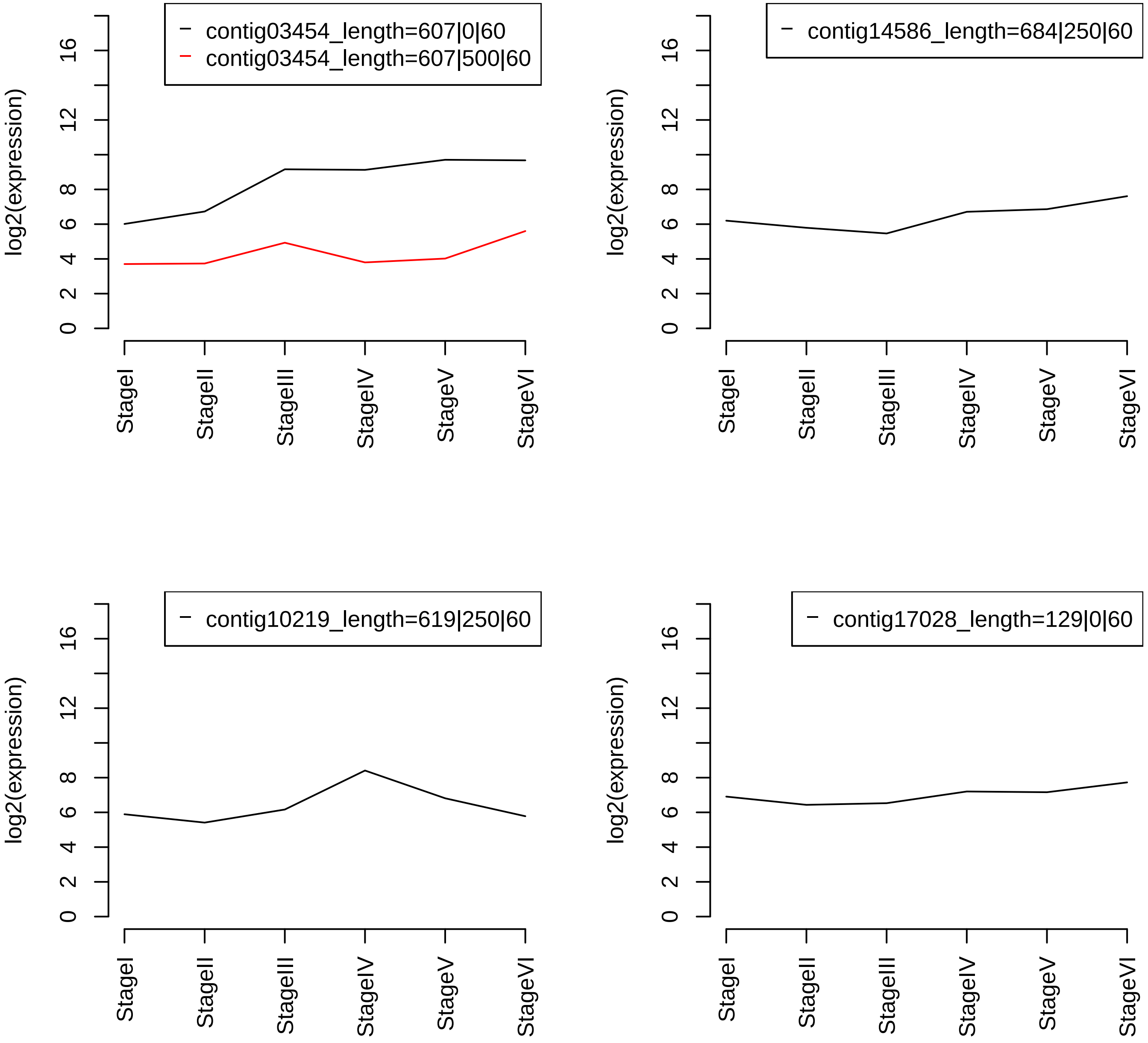

contig03454_length=607|0|60
contig03454_length=607|500|60
contig14586_length=684|250|60
contig10219_length=619|250|60
contig17028_length=129|0|60
log2(expression)
0
2
4
6
8
12
16
StageI
StageII
StageIII
StageIV
StageV
StageVI

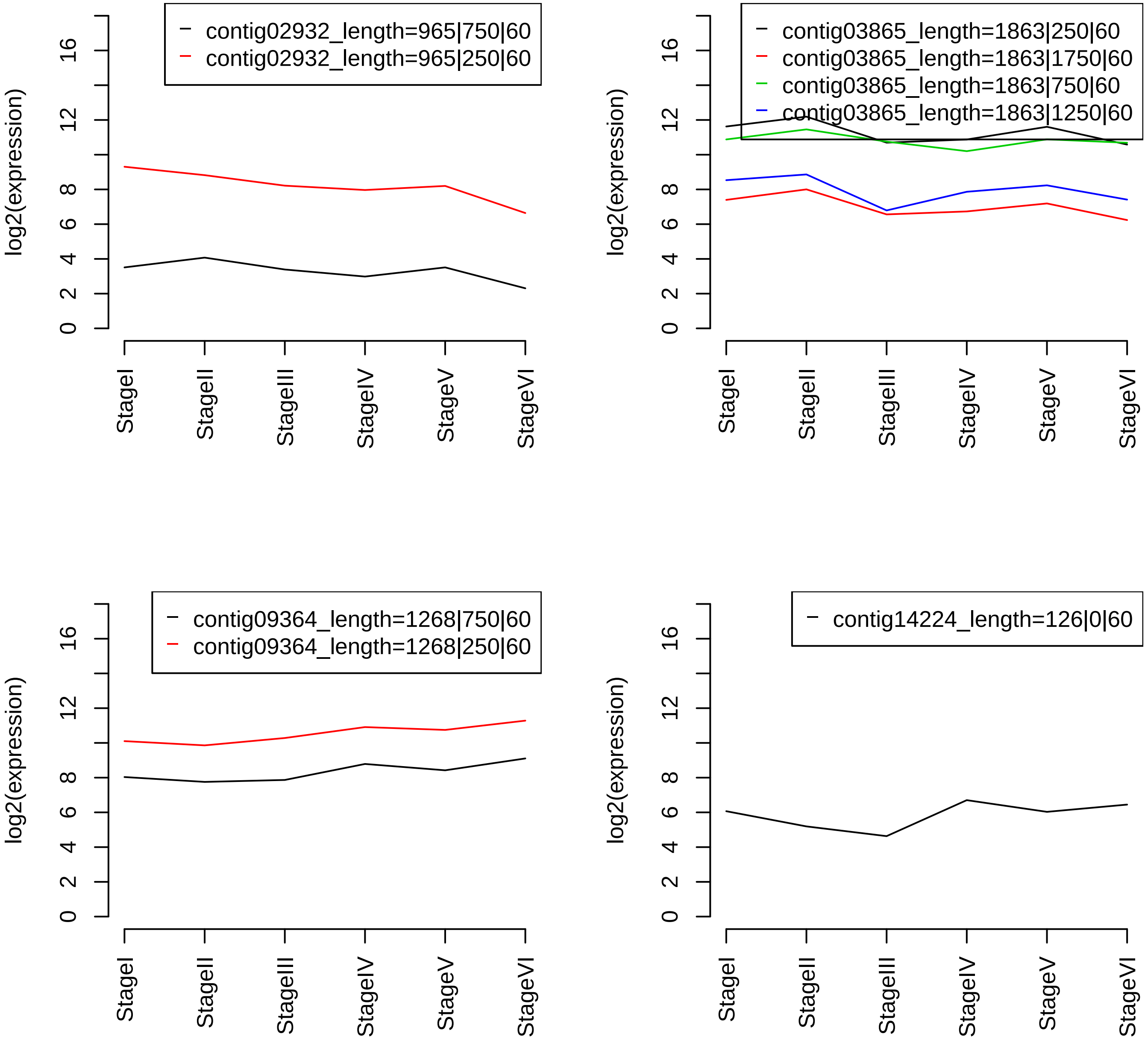

contig02932_length=965|750|60
contig02932_length=965|250|60
log2(expression)
0
2
4
6
8
12
16
StageI
StageII
StageIII
StageIV
StageV
StageVI
contig03865_length=1863|250|60
contig03865_length=1863|1750|60
contig03865_length=1863|750|60
contig03865_length=1863|1250|60
contig09364_length=1268|750|60
contig09364_length=1268|250|60
contig14224_length=126|0|60

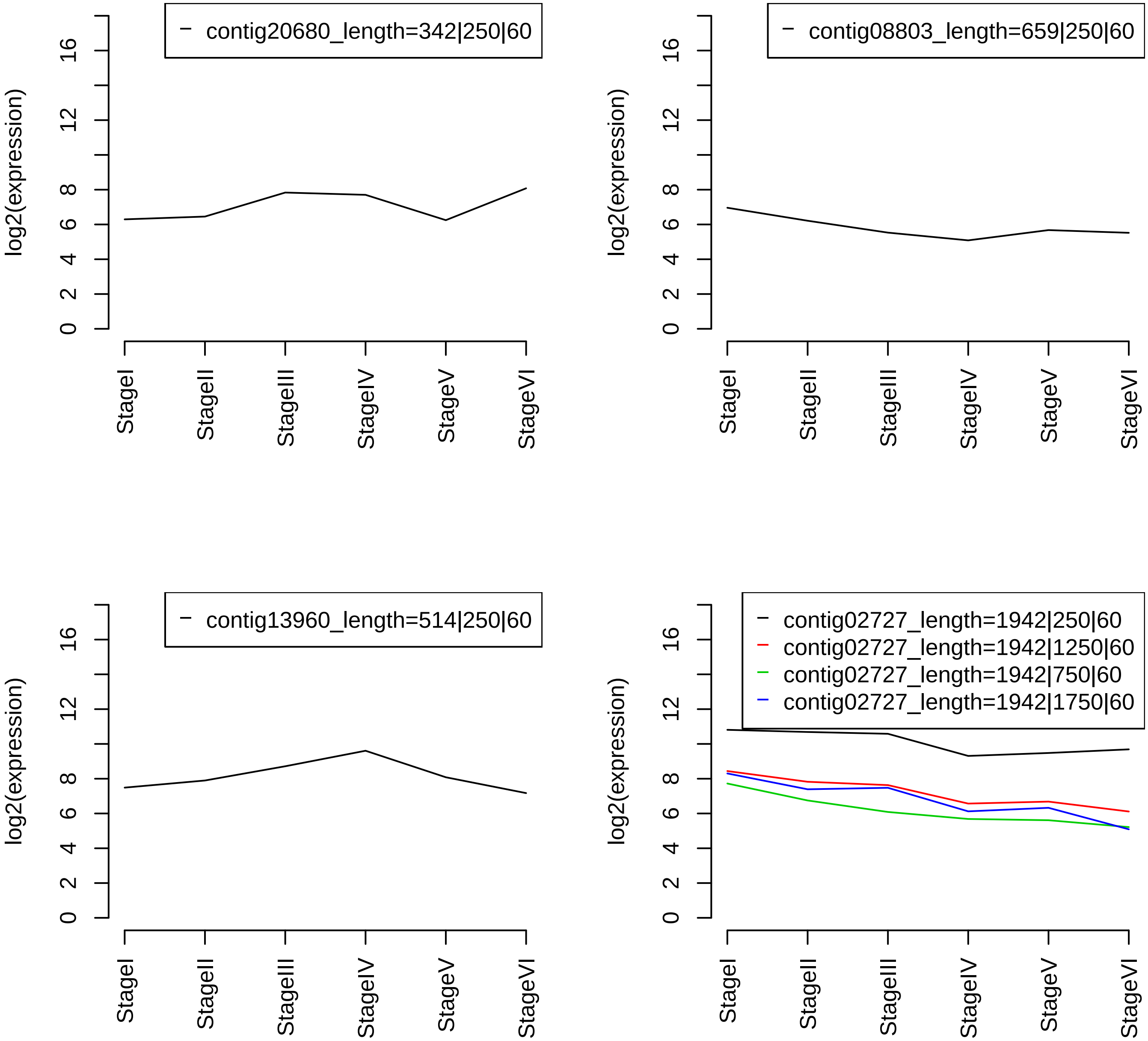

contig20680_length=342|250|60
contig08803_length=659|250|60
contig13960_length=514|250|60
contig02727_length=1942|250|60
contig02727_length=1942|1250|60
contig02727_length=1942|750|60
contig02727_length=1942|1750|60
log2(expression)
0
2
4
6
8
12
16
StageI
StageII
StageIII
StageIV
StageV
StageVI

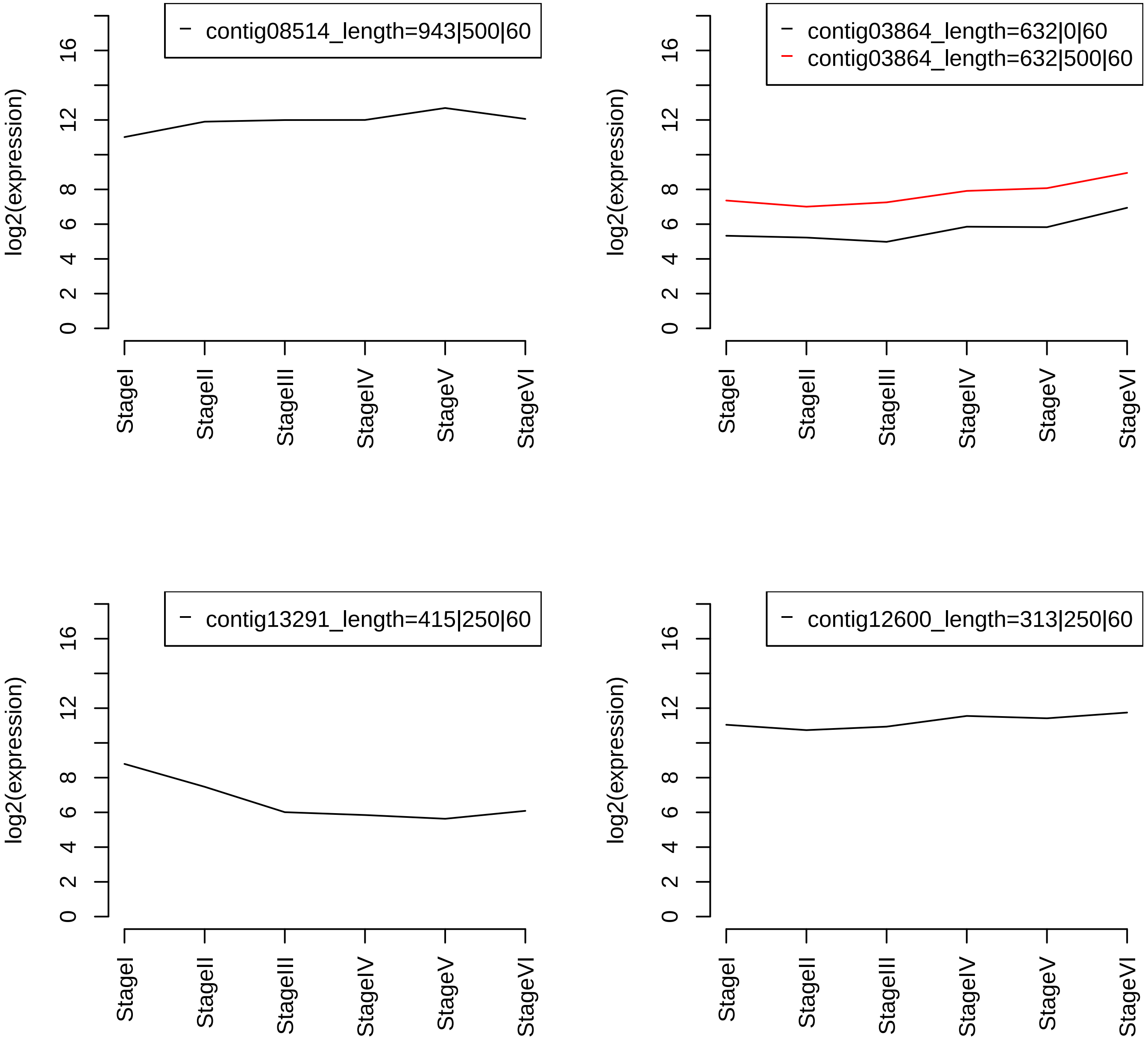
contig08514_length=943|500|60
contig03864_length=632|0|60
contig03864_length=632|500|60
contig13291_length=415|250|60
contig12600_length=313|250|60
log2(expression)
0
2
4
6
8
12
16
StageI
StageII
StageIII
StageIV
StageV
StageVI

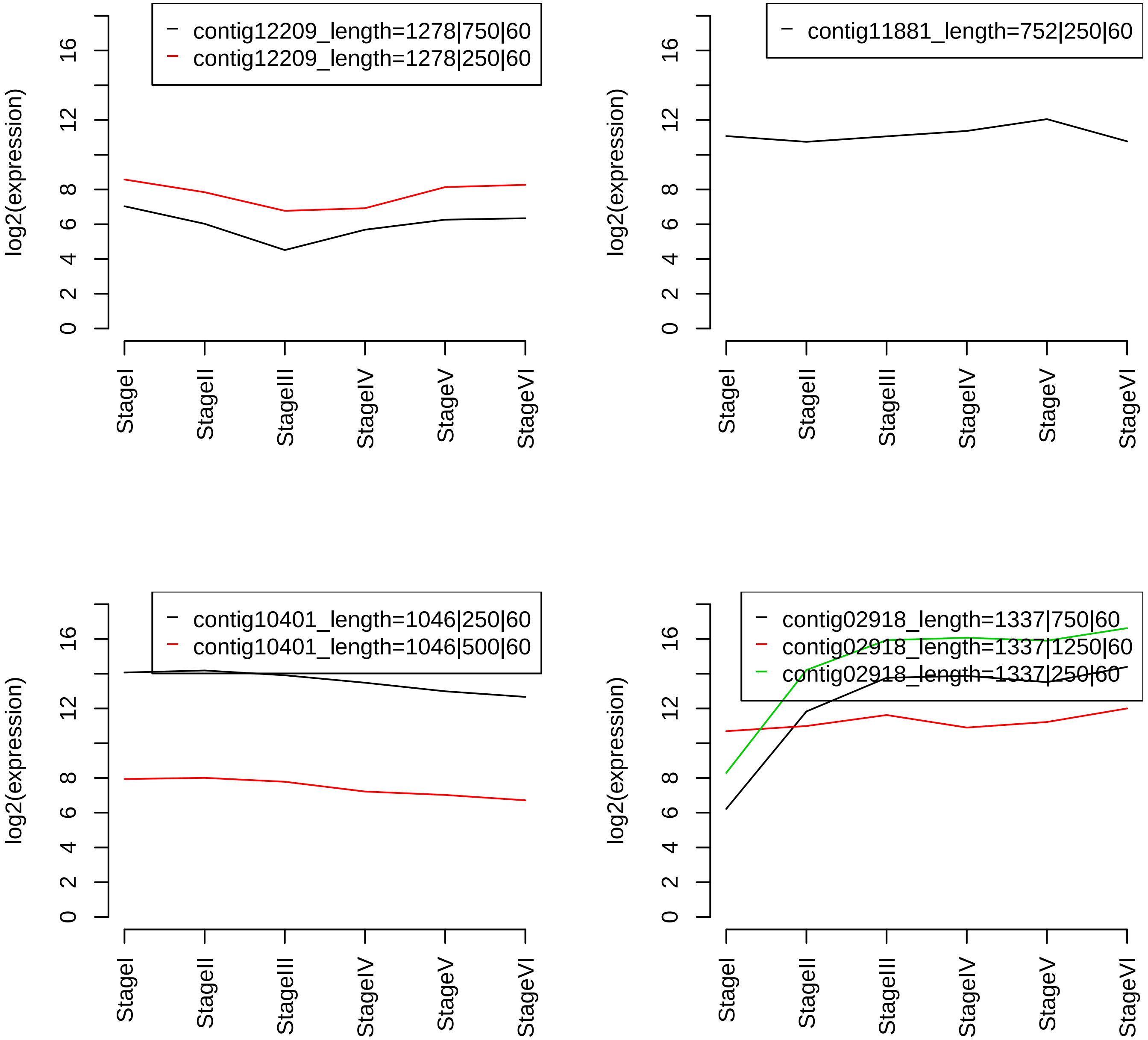

contig12209_length=1278|750|60
contig12209_length=1278|250|60
contig11881_length=752|250|60
contig10401_length=1046|250|60
contig10401_length=1046|500|60
contig02918_length=1337|750|60
contig02918_length=1337|1250|60
contig02918_length=1337|250|60
log2(expression)
0
2
4
6
8
12
16
StageI
StageII
StageIII
StageIV
StageV
StageVI

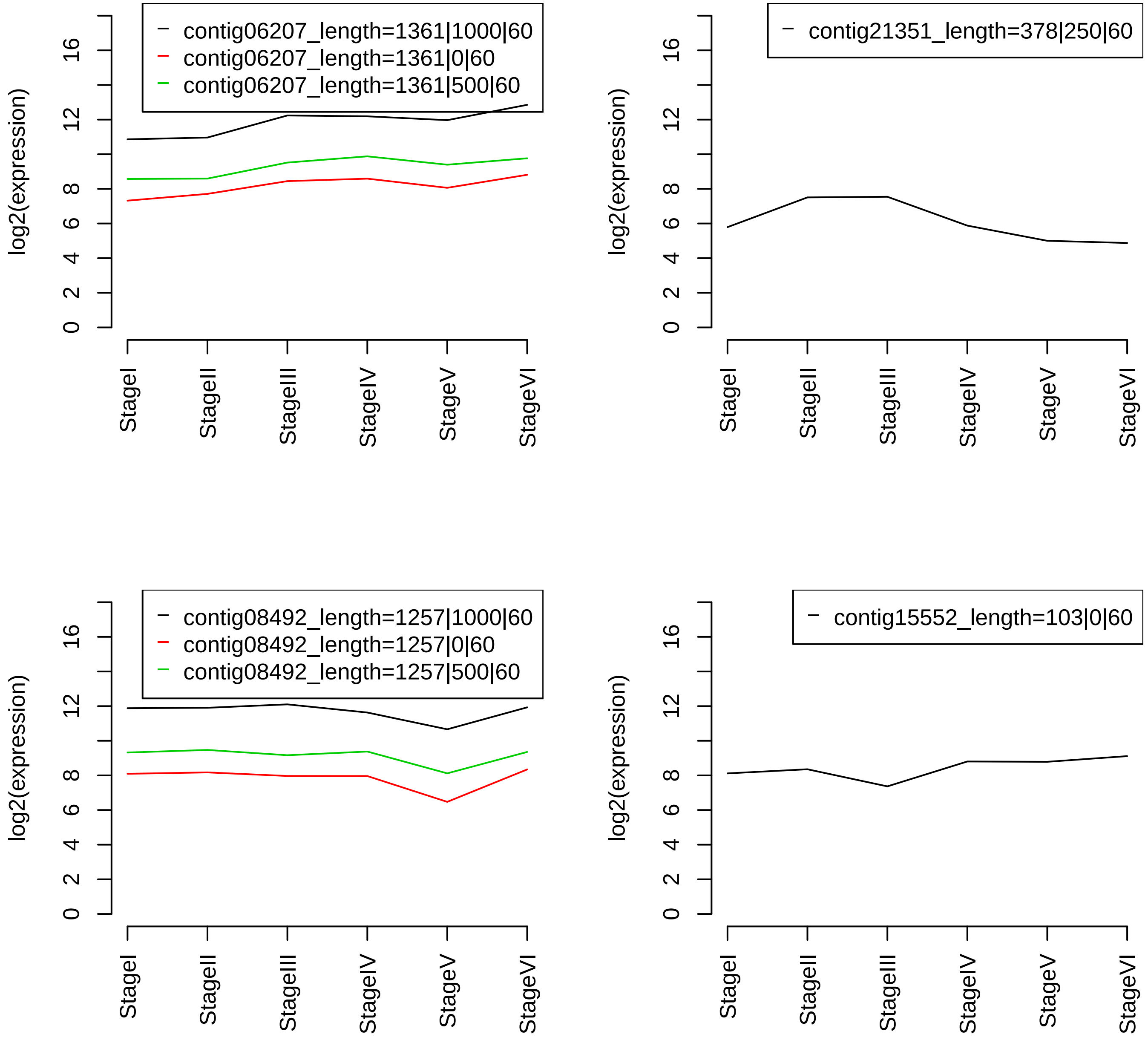

contig06207_length=1361|1000|60
contig06207_length=1361|0|60
contig06207_length=1361|500|60
log2(expression)
0
2
4
6
8
12
16
StageI
StageII
StageIII
StageIV
StageV
StageVI
contig21351_length=378|250|60
contig08492_length=1257|1000|60
contig08492_length=1257|0|60
contig08492_length=1257|500|60
contig15552_length=103|0|60

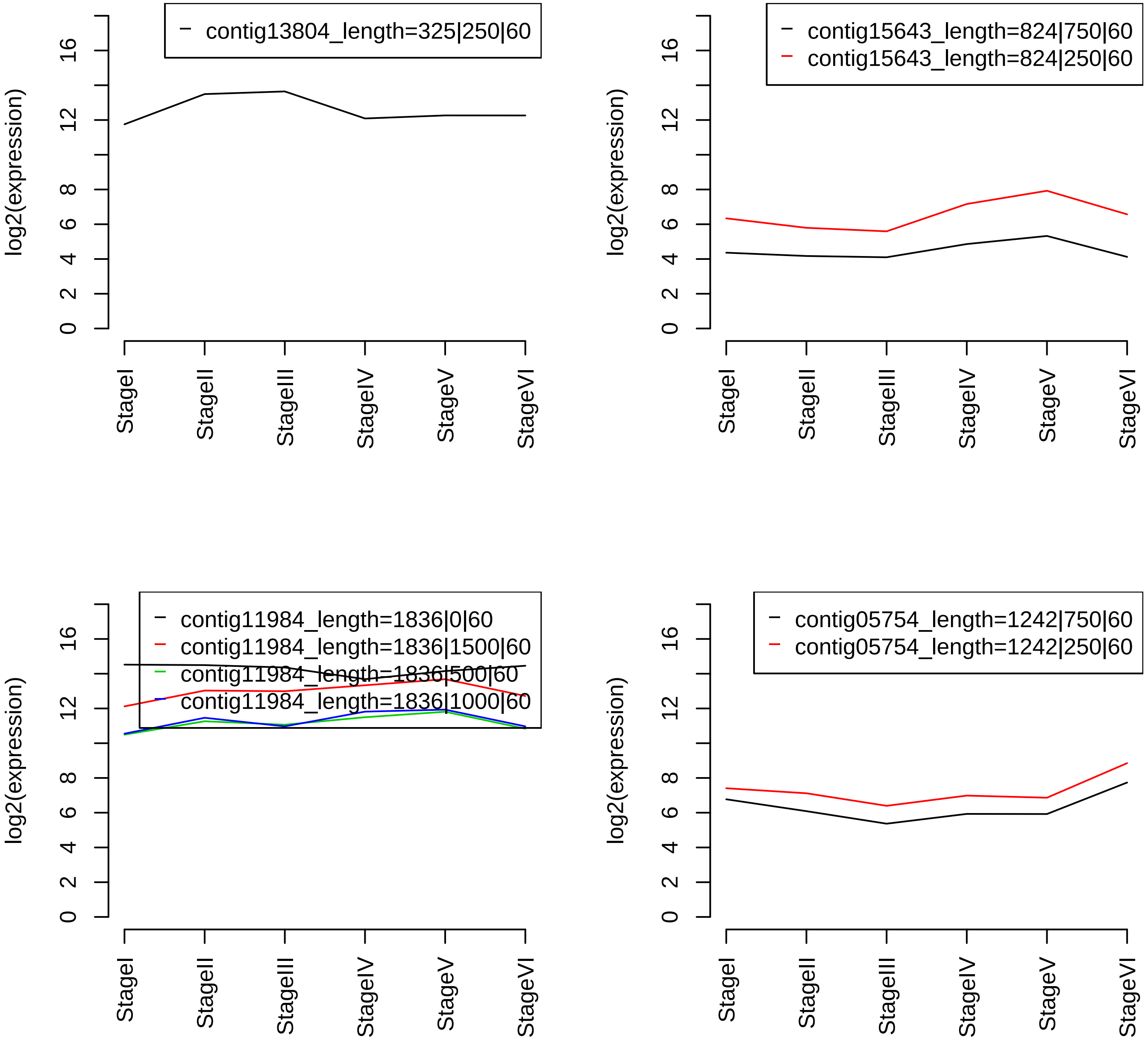

contig13804_length=325|250|60
contig15643_length=824|750|60
contig15643_length=824|250|60
contig11984_length=1836|0|60
contig11984_length=1836|1500|60
contig11984_length=1836|500|60
contig11984_length=1836|1000|60
contig05754_length=1242|750|60
contig05754_length=1242|250|60
log2(expression)
0
2
4
6
8
12
16
StageI
StageII
StageIII
StageIV
StageV
StageVI

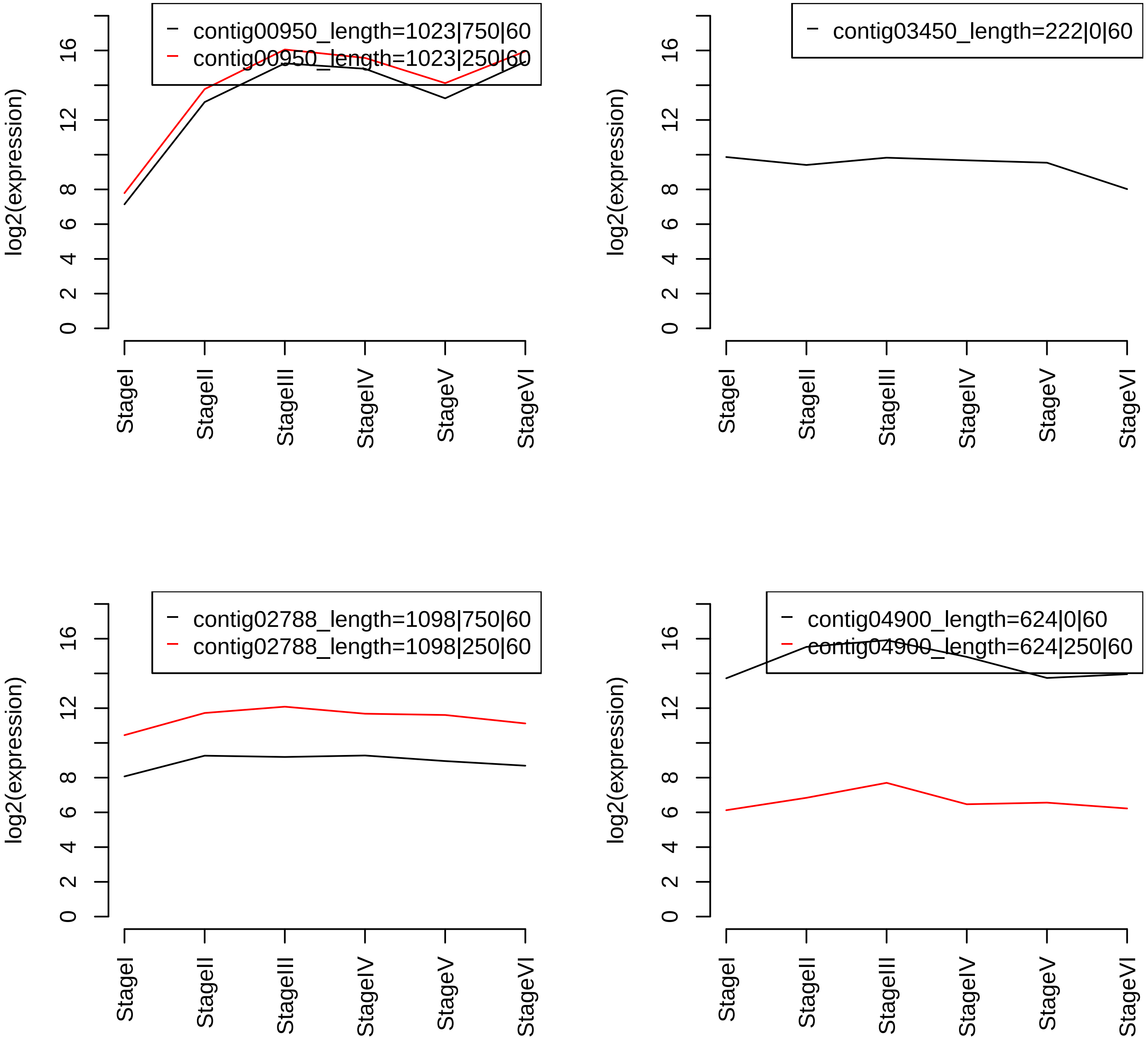
contig00950_length=1023|750|60
contig00950_length=1023|250|60
contig03450_length=222|0|60
contig02788_length=1098|750|60
contig02788_length=1098|250|60
contig04900_length=624|0|60
contig04900_length=624|250|60
log2(expression)
0
2
4
6
8
12
16
StageI
StageII
StageIII
StageIV
StageV
StageVI

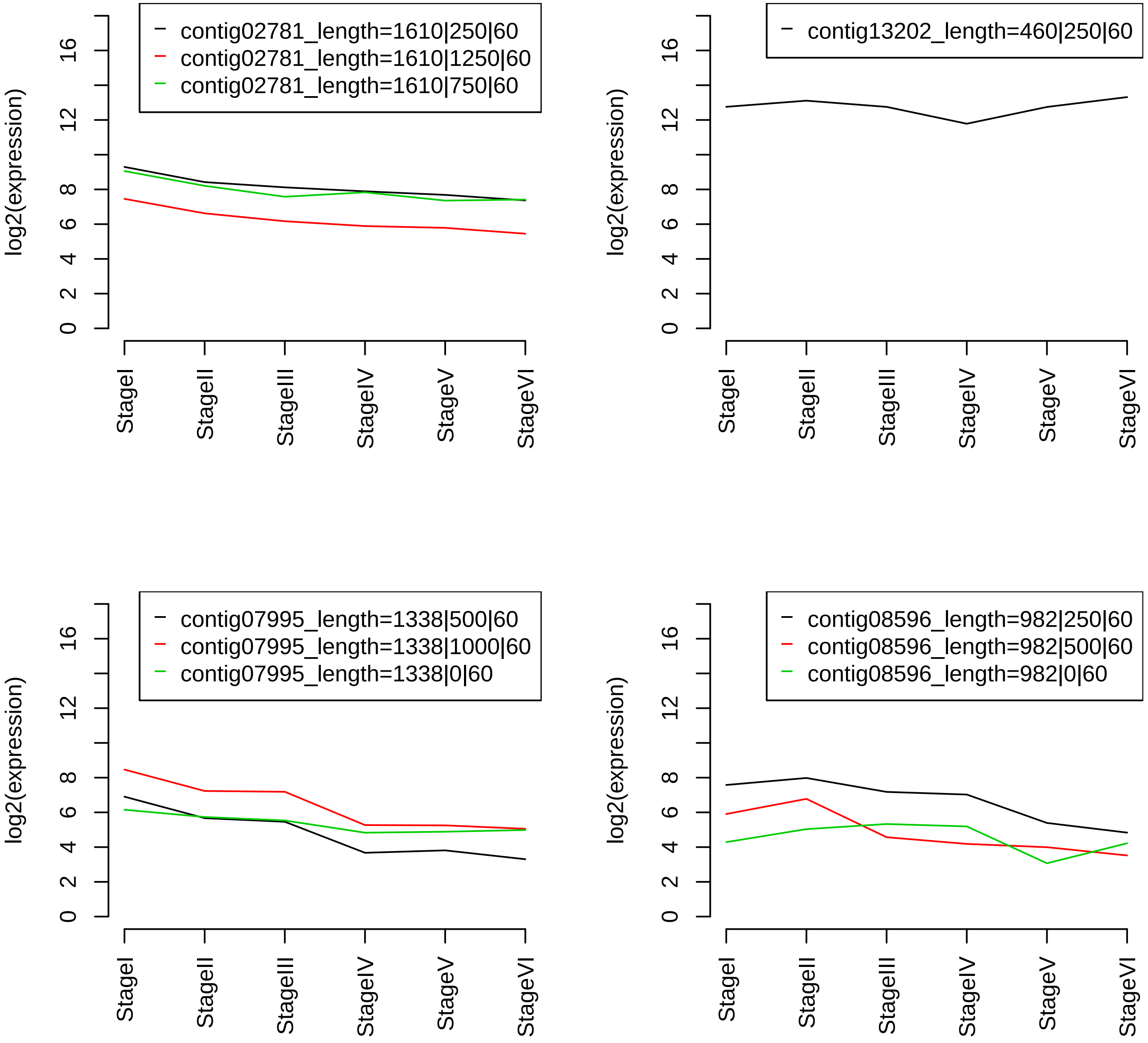
contig02781_length=1610|250|60
contig02781_length=1610|1250|60
contig02781_length=1610|750|60
contig13202_length=460|250|60
contig07995_length=1338|500|60
contig07995_length=1338|1000|60
contig07995_length=1338|0|60
contig08596_length=982|250|60
contig08596_length=982|500|60
contig08596_length=982|0|60
log2(expression)
StageI
StageII
StageIII
StageIV
StageV
StageVI

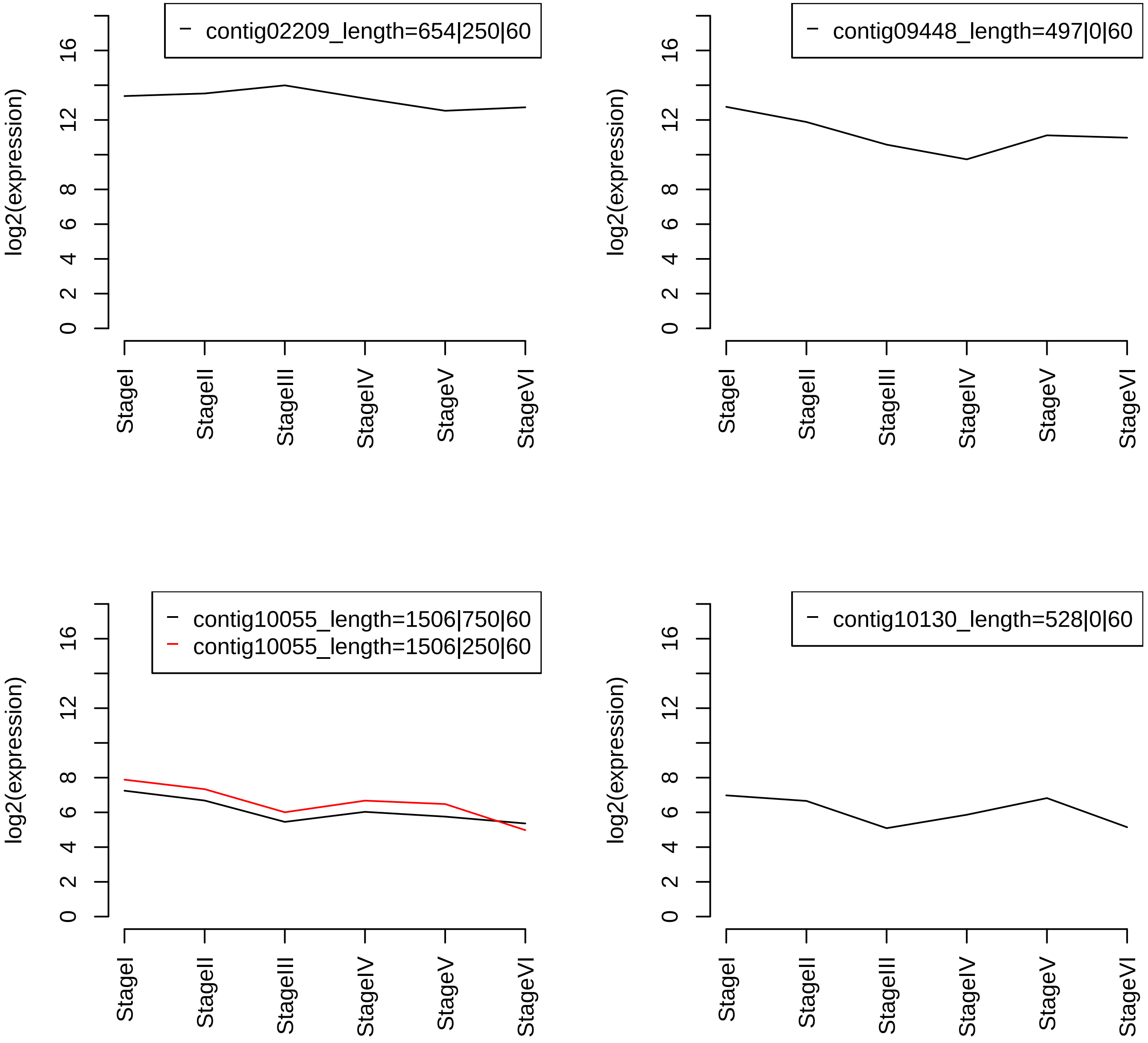
contig02209_length=654|250|60
contig09448_length=497|0|60
contig10055_length=1506|750|60
contig10055_length=1506|250|60
contig10130_length=528|0|60
log2(expression)
0
2
4
6
8
12
16
StageI
StageII
StageIII
StageIV
StageV
StageVI

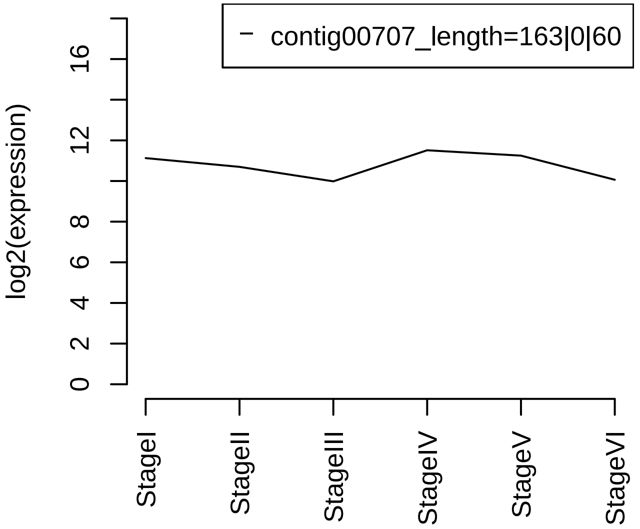

contig00707_length=163|0|60
log2(expression)
0
2
4
6
8
12
16
StageI
StageII
StageIII
StageIV
StageV
StageVI

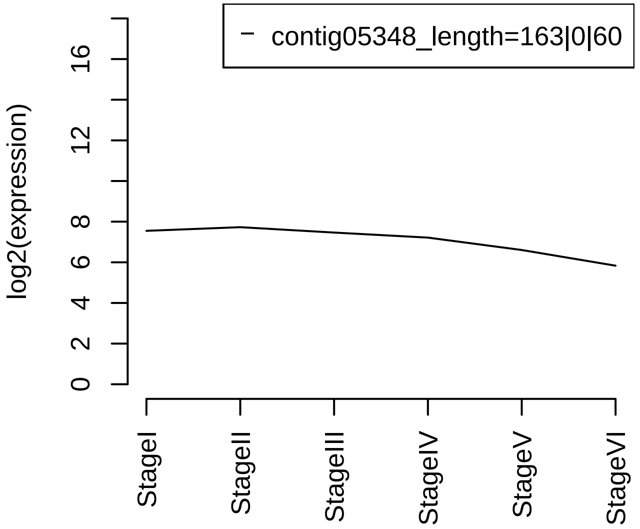

contig05348_length=163|0|60
log2(expression)
0
2
4
6
8
12
16
StageI
StageII
StageIII
StageIV
StageV
StageVI

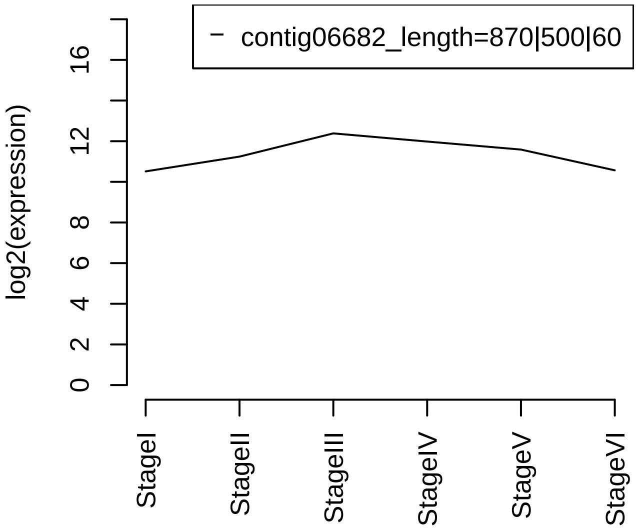

contig06682_length=870|500|60
log2(expression)
0
2
4
6
8
12
16
StageI
StageII
StageIII
StageIV
StageV
StageVI

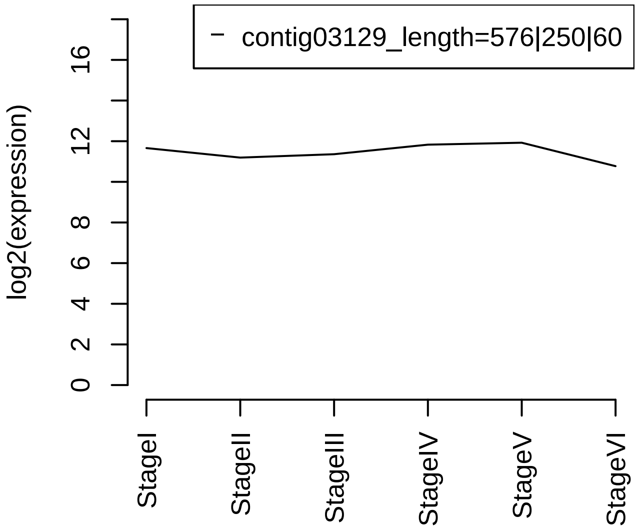

contig03129_length=576|250|60
log2(expression)
0
2
4
6
8
12
16
StageI
StageII
StageIII
StageIV
StageV
StageVI

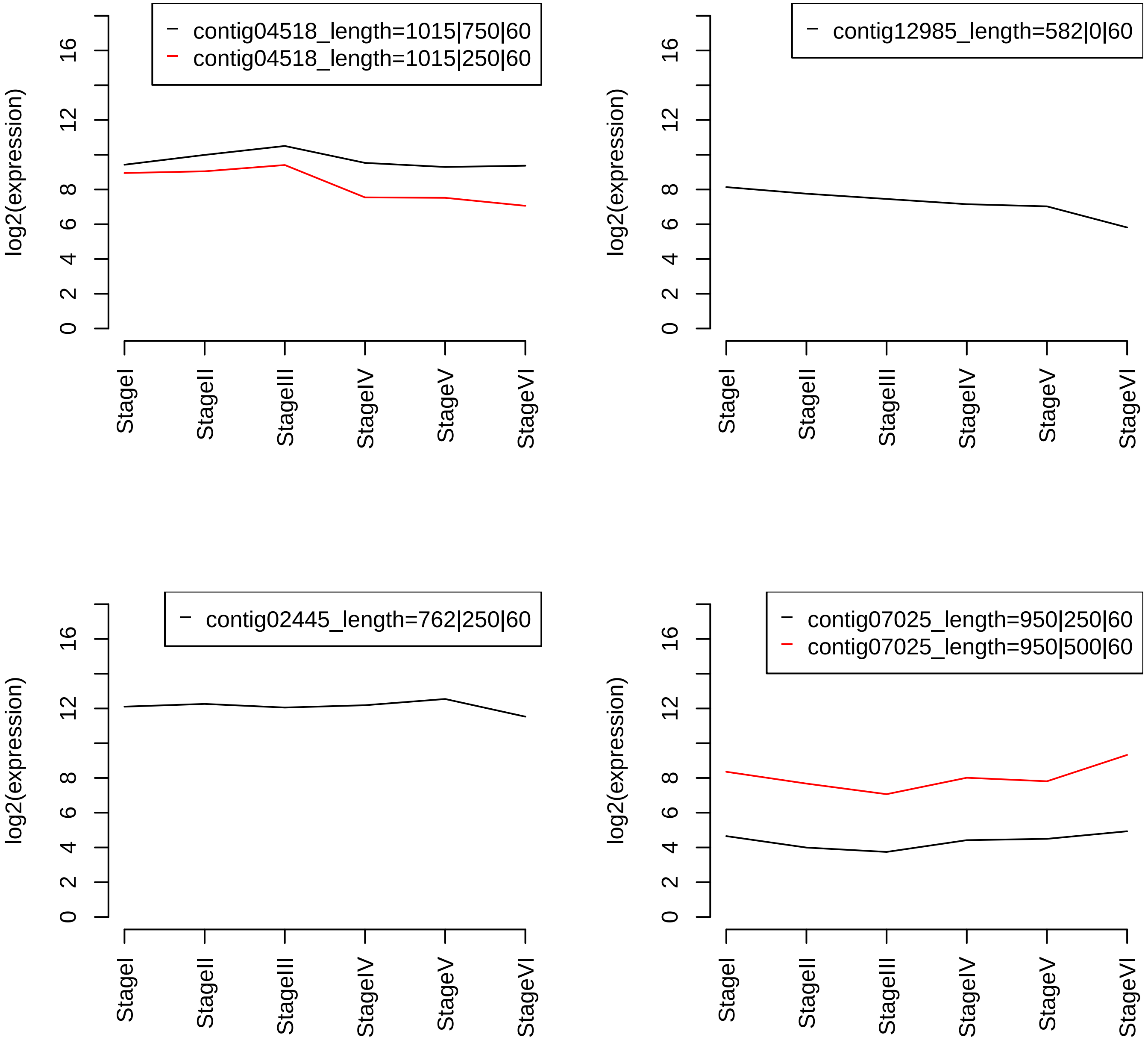

contig04518_length=1015|750|60
contig04518_length=1015|250|60
contig12985_length=582|0|60
contig02445_length=762|250|60
contig07025_length=950|250|60
contig07025_length=950|500|60
log2(expression)
0
2
4
6
8
12
16
StageI
StageII
StageIII
StageIV
StageV
StageVI

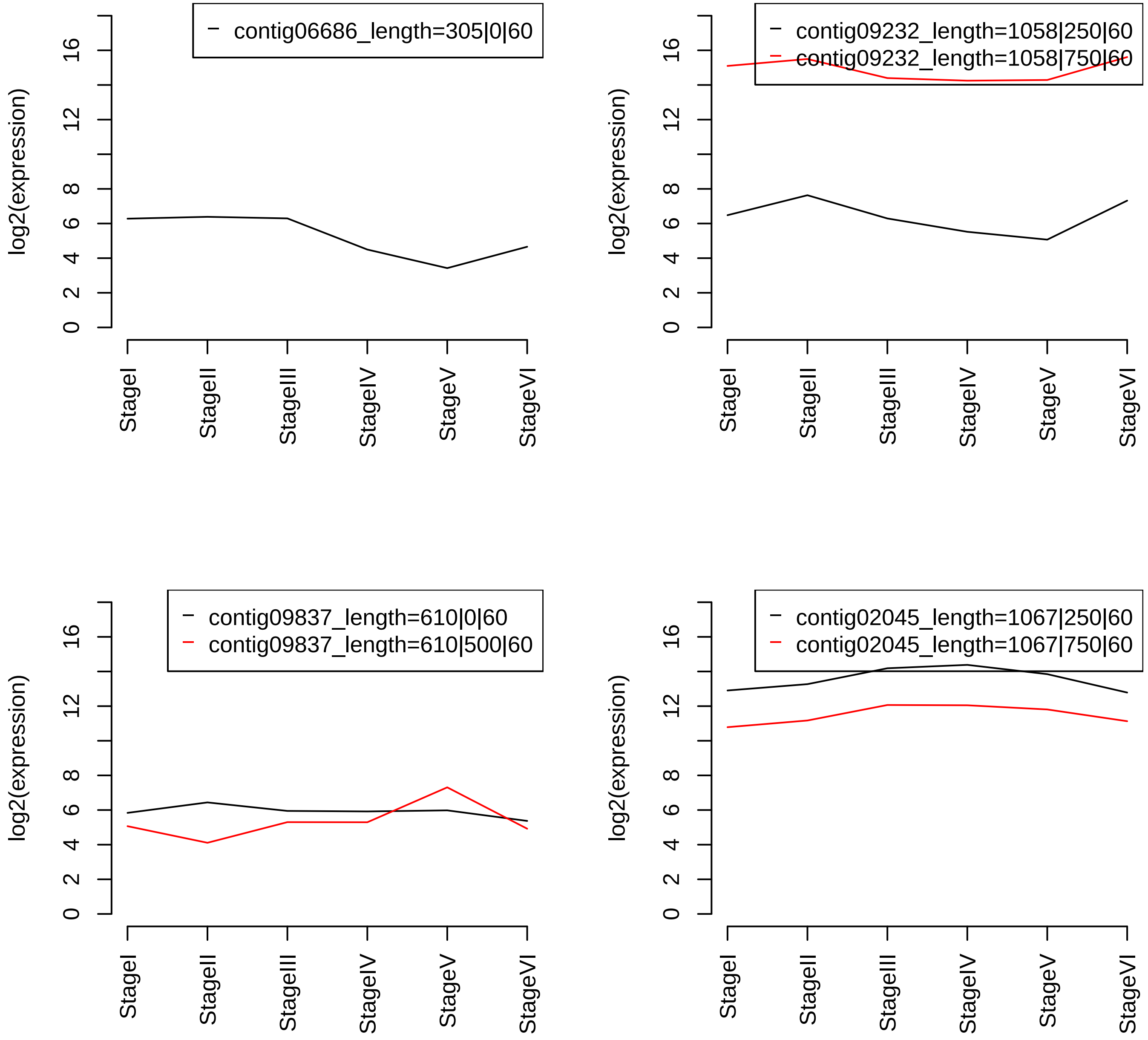
contig06686_length=305|0|60
contig09232_length=1058|250|60
contig09232_length=1058|750|60
contig09837_length=610|0|60
contig09837_length=610|500|60
contig02045_length=1067|250|60
contig02045_length=1067|750|60
log2(expression)
0
2
4
6
8
12
16
StageI
StageII
StageIII
StageIV
StageV
StageVI

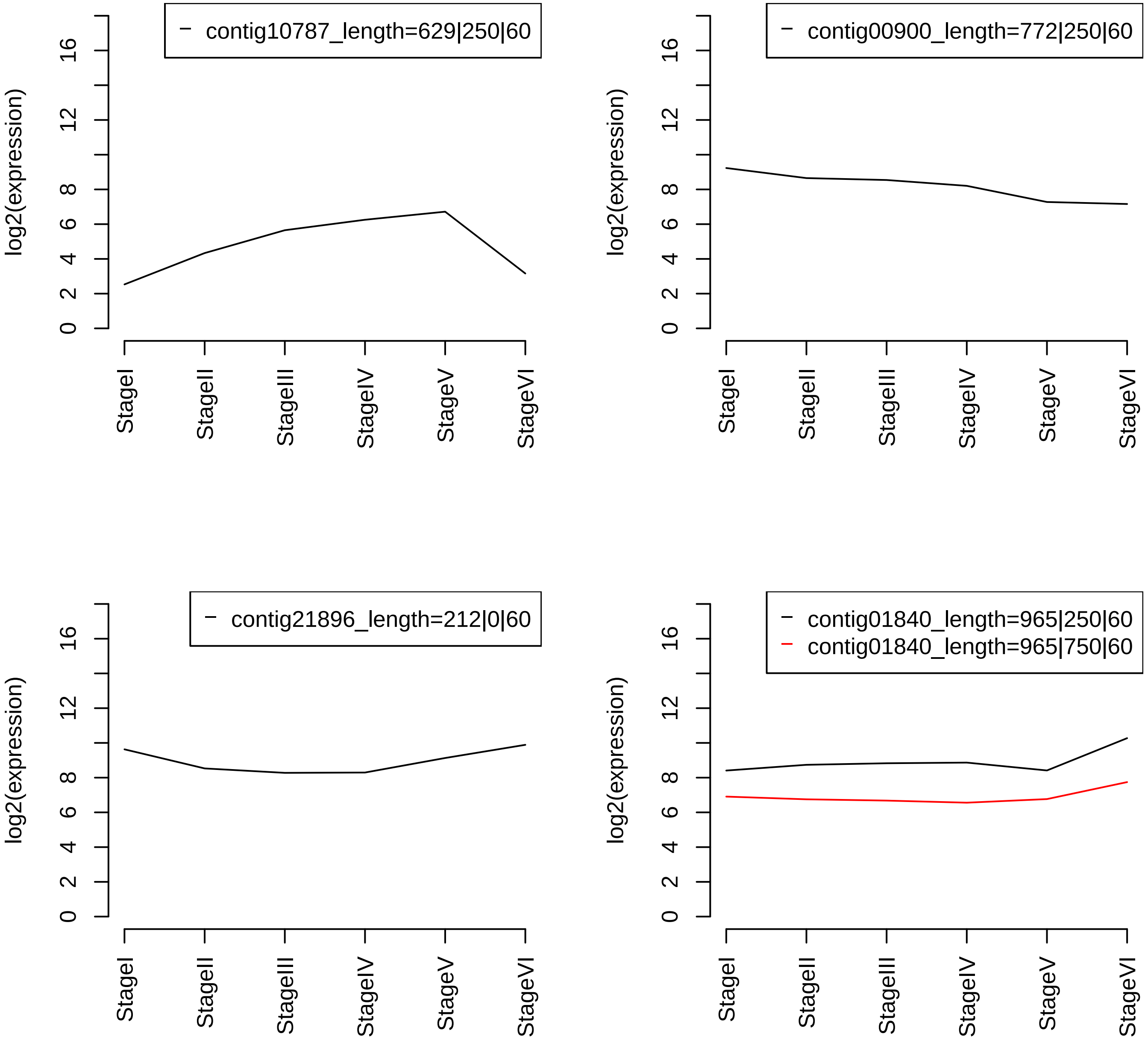

contig10787_length=629|250|60
log2(expression)
0
2
4
6
8
12
16
StageI
StageII
StageIII
StageIV
StageV
StageVI
contig00900_length=772|250|60
log2(expression)
0
2
4
6
8
12
16
StageI
StageII
StageIII
StageIV
StageV
StageVI
contig21896_length=212|0|60
log2(expression)
0
2
4
6
8
12
16
StageI
StageII
StageIII
StageIV
StageV
StageVI
contig01840_length=965|250|60
contig01840_length=965|750|60
log2(expression)
0
2
4
6
8
12
16
StageI
StageII
StageIII
StageIV
StageV
StageVI

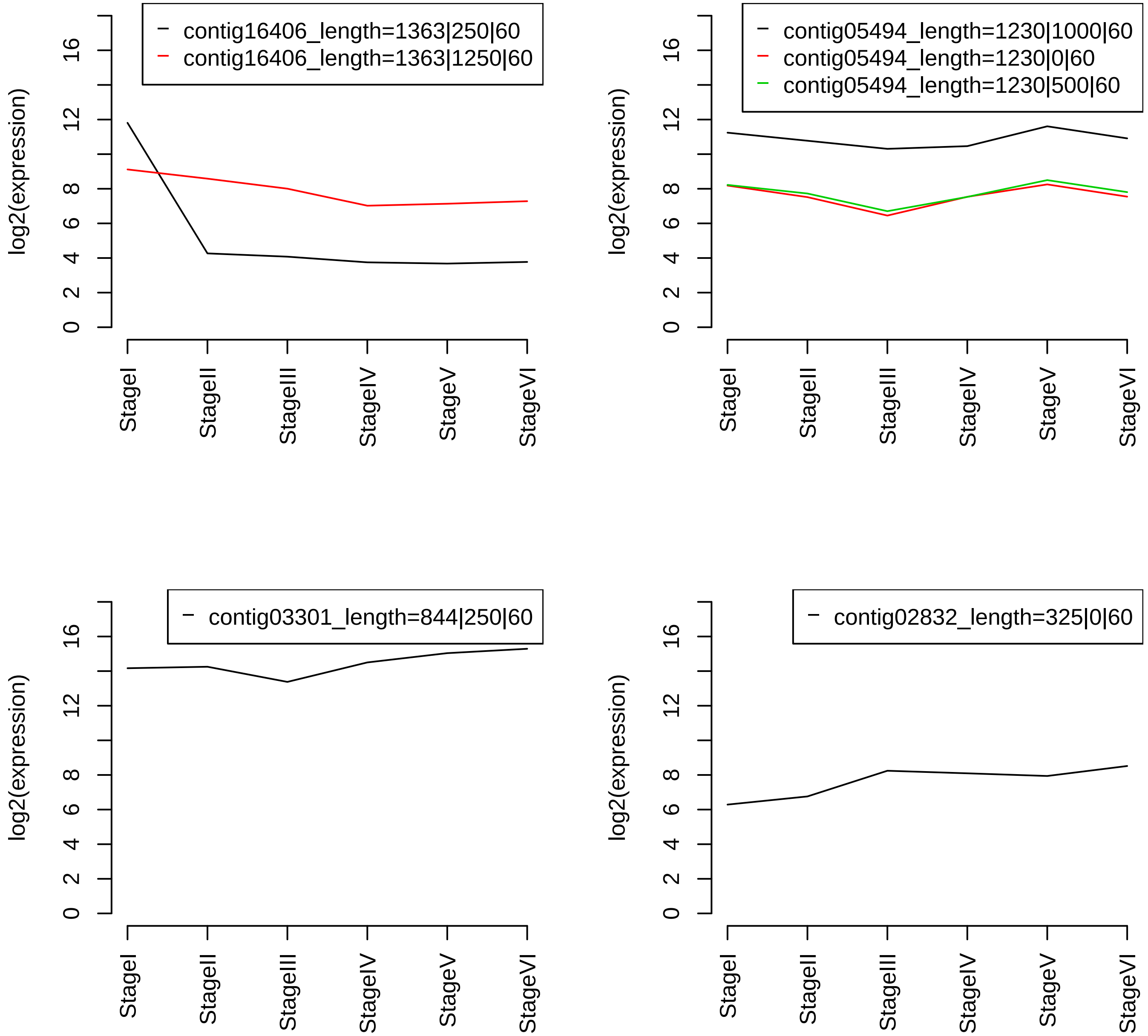
contig16406_length=1363|250|60
contig16406_length=1363|1250|60
contig05494_length=1230|1000|60
contig05494_length=1230|0|60
contig05494_length=1230|500|60
contig03301_length=844|250|60
contig02832_length=325|0|60
log2(expression)
0
2
4
6
8
12
16
StageI
StageII
StageIII
StageIV
StageV
StageVI

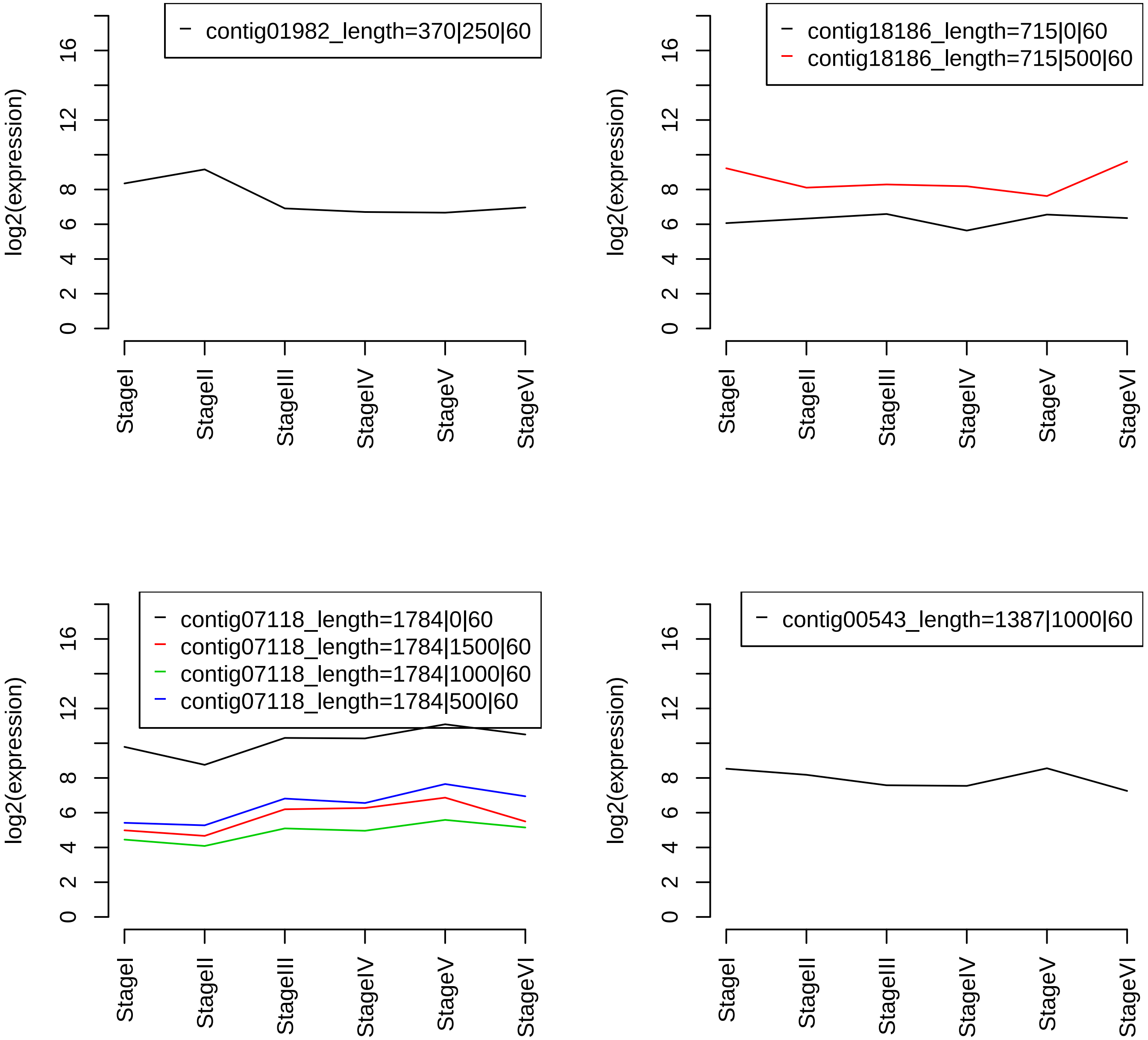
contig01982_length=370|250|60
contig18186_length=715|0|60
contig18186_length=715|500|60
contig07118_length=1784|0|60
contig07118_length=1784|1500|60
contig07118_length=1784|1000|60
contig07118_length=1784|500|60
contig00543_length=1387|1000|60
log2(expression)
0
2
4
6
8
12
16
StageI
StageII
StageIII
StageIV
StageV
StageVI

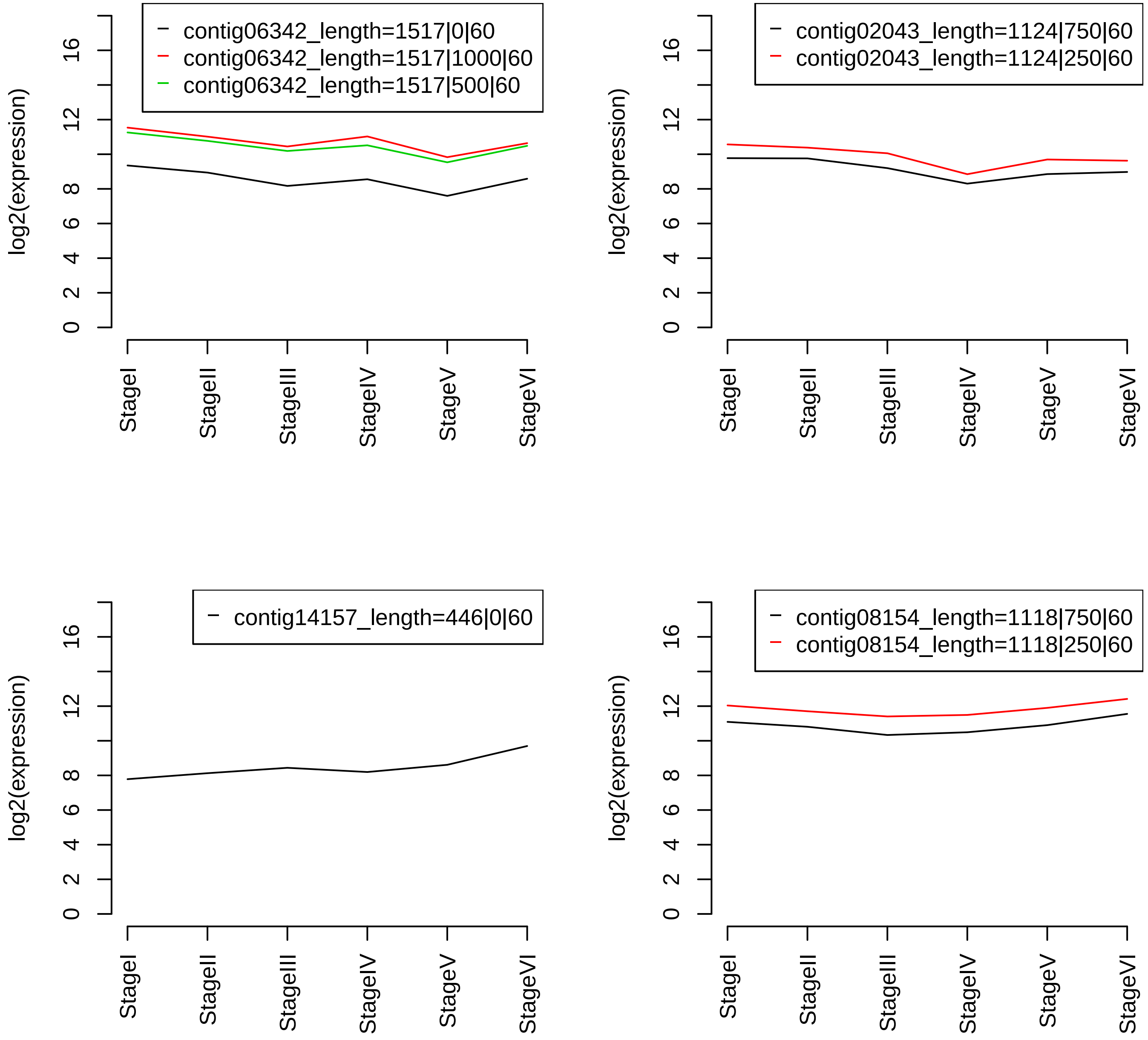

contig06342_length=1517|0|60
contig06342_length=1517|1000|60
contig06342_length=1517|500|60
contig02043_length=1124|750|60
contig02043_length=1124|250|60
contig14157_length=446|0|60
contig08154_length=1118|750|60
contig08154_length=1118|250|60
log2(expression)
0 2 4 6 8 12 16
StageI
StageII
StageIII
StageIV
StageV
StageVI

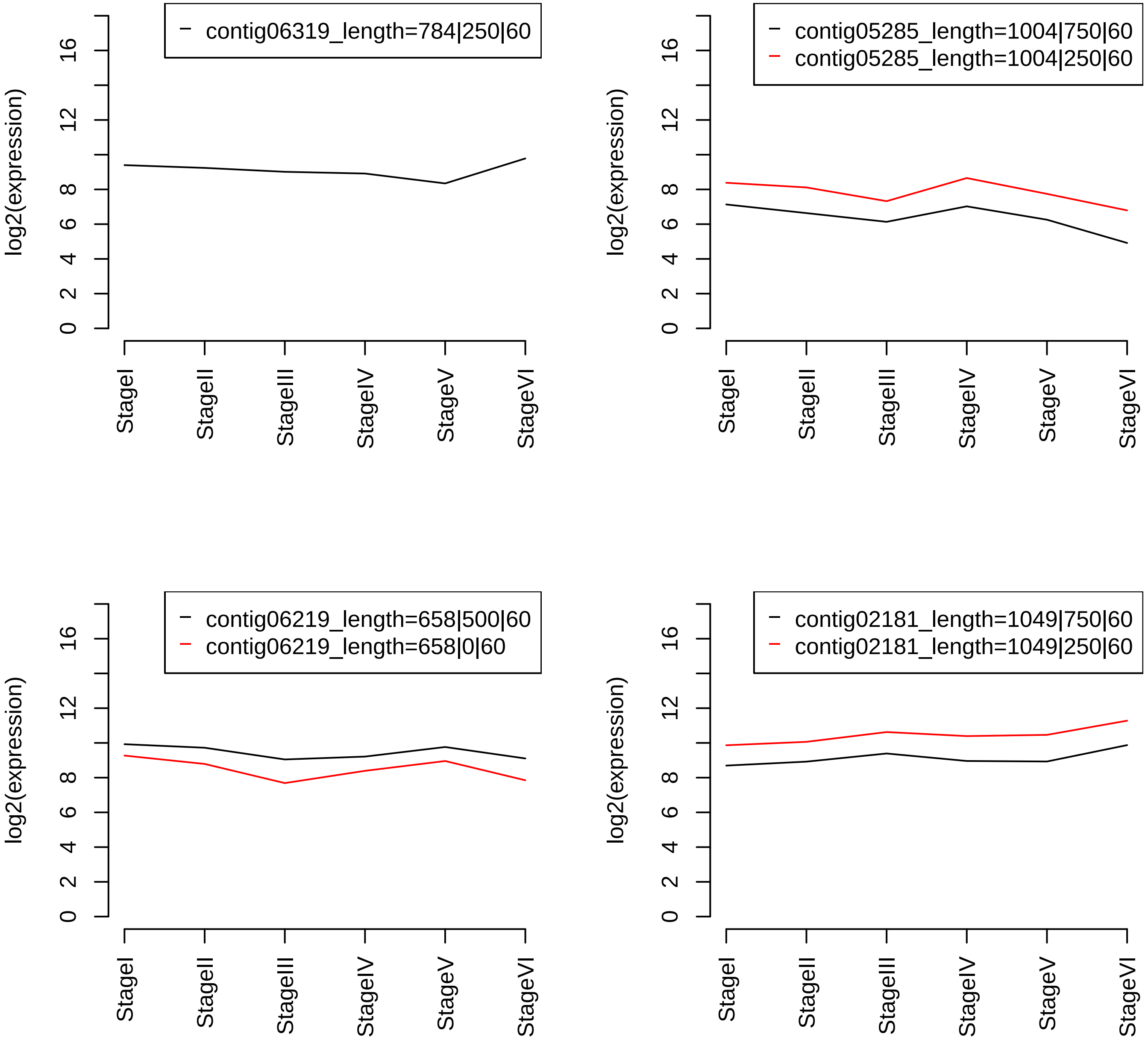

contig06319_length=784|250|60
contig05285_length=1004|750|60
contig05285_length=1004|250|60
contig06219_length=658|500|60
contig06219_length=658|0|60
contig02181_length=1049|750|60
contig02181_length=1049|250|60
log2(expression)
0
2
4
6
8
12
16
StageI
StageII
StageIII
StageIV
StageV
StageVI

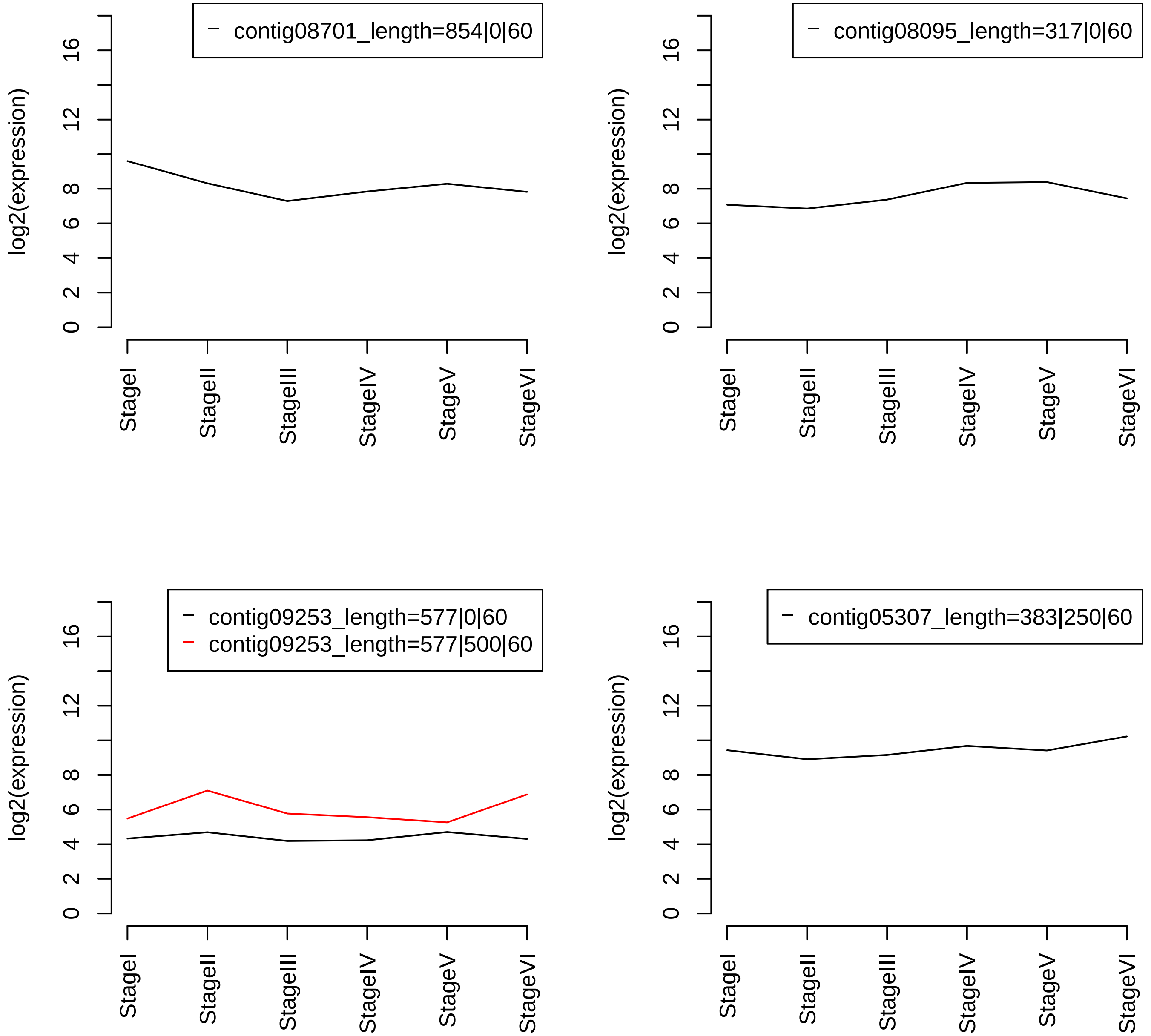

contig08701_length=854|0|60
contig08095_length=317|0|60
contig09253_length=577|0|60
contig09253_length=577|500|60
contig05307_length=383|250|60
log2(expression)
0
2
4
6
8
12
16
StageI
StageII
StageIII
StageIV
StageV
StageVI

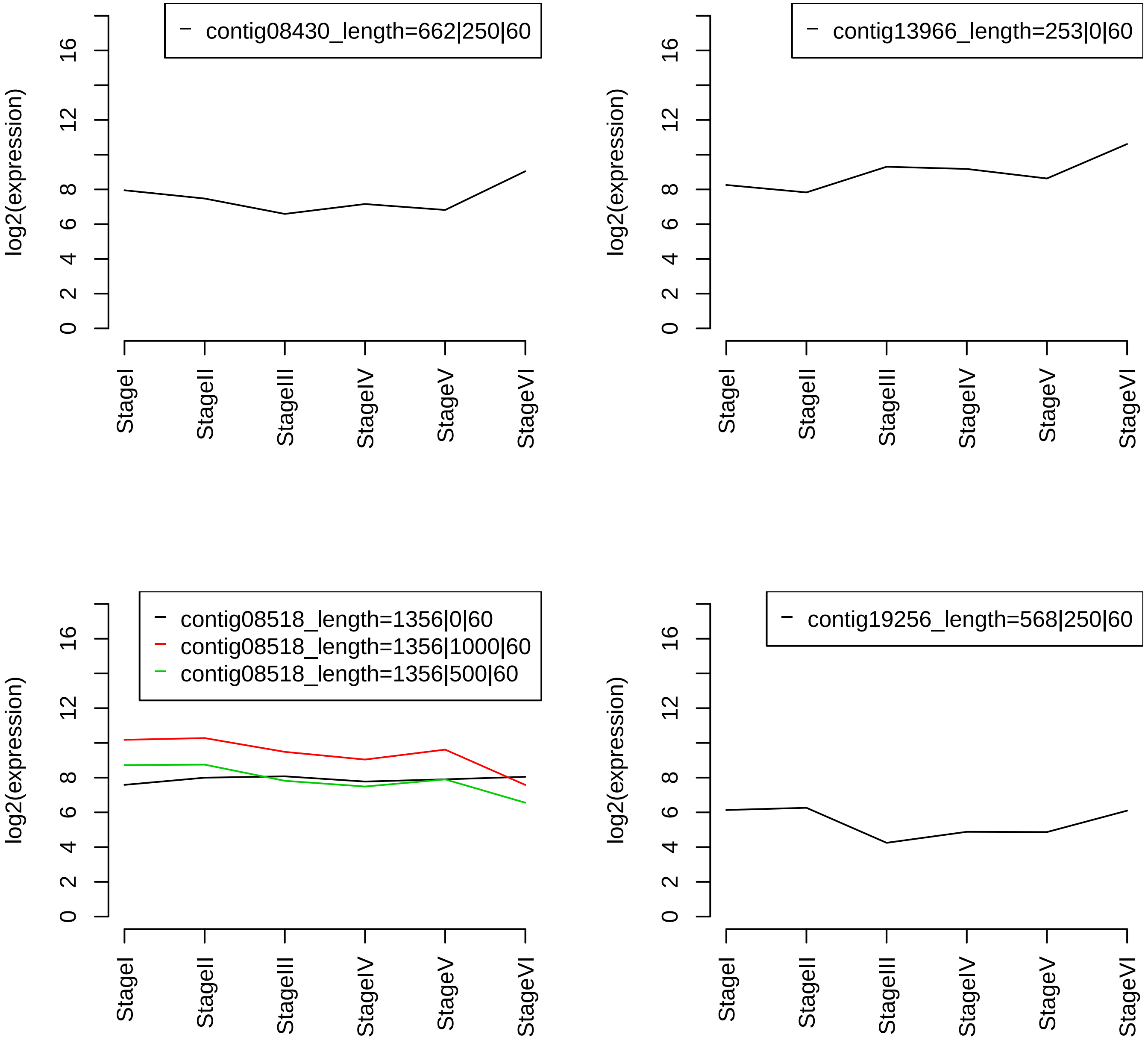

contig08430_length=662|250|60
contig13966_length=253|0|60
contig08518_length=1356|0|60
contig08518_length=1356|1000|60
contig08518_length=1356|500|60
contig19256_length=568|250|60
log2(expression)
0
2
4
6
8
12
16
StageI
StageII
StageIII
StageIV
StageV
StageVI

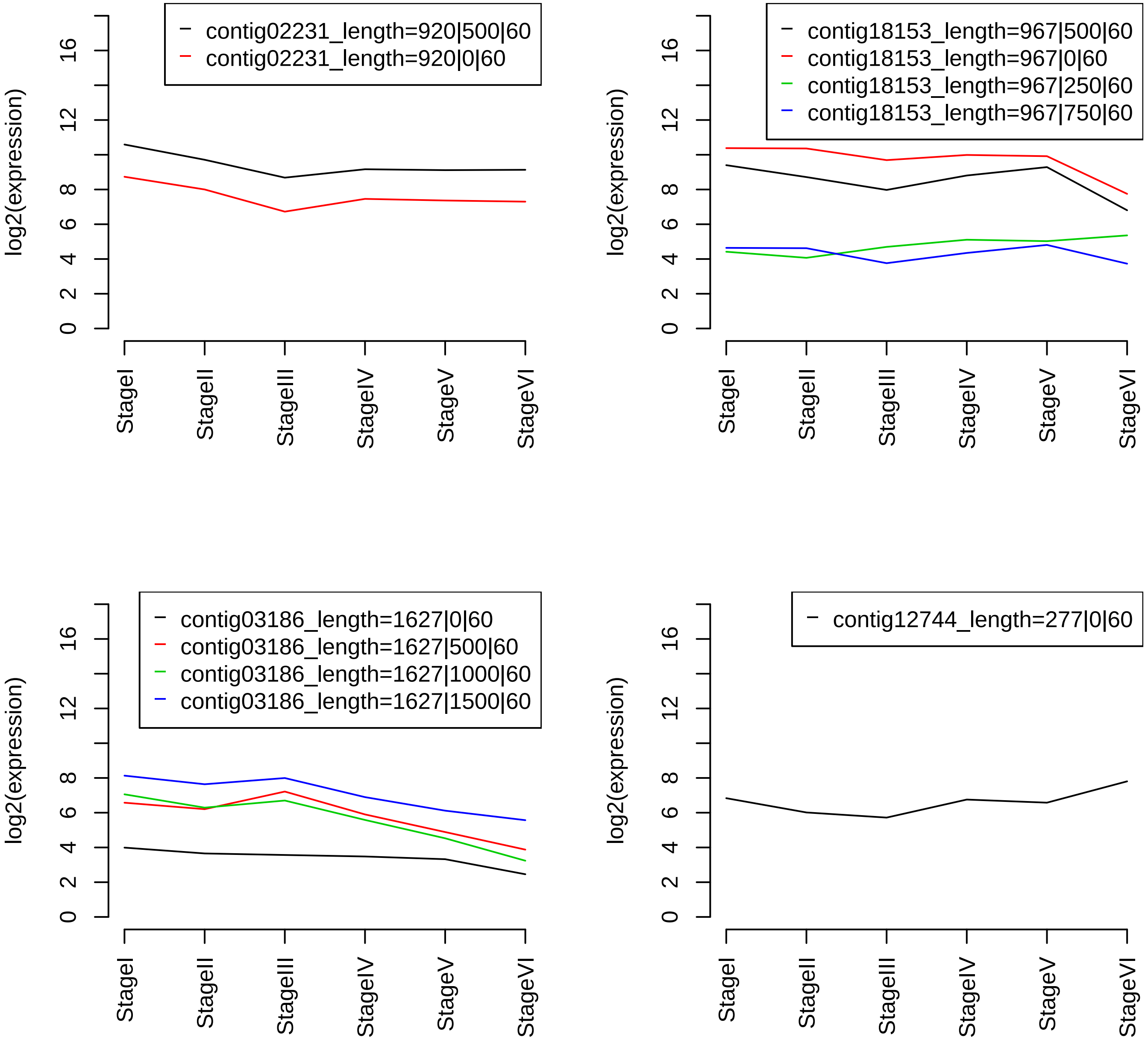
contig02231_length=920|500|60
contig02231_length=920|0|60
contig18153_length=967|500|60
contig18153_length=967|0|60
contig18153_length=967|250|60
contig18153_length=967|750|60
contig03186_length=1627|0|60
contig03186_length=1627|500|60
contig03186_length=1627|1000|60
contig03186_length=1627|1500|60
contig12744_length=277|0|60
log2(expression)
StageI
StageII
StageIII
StageIV
StageV
StageVI

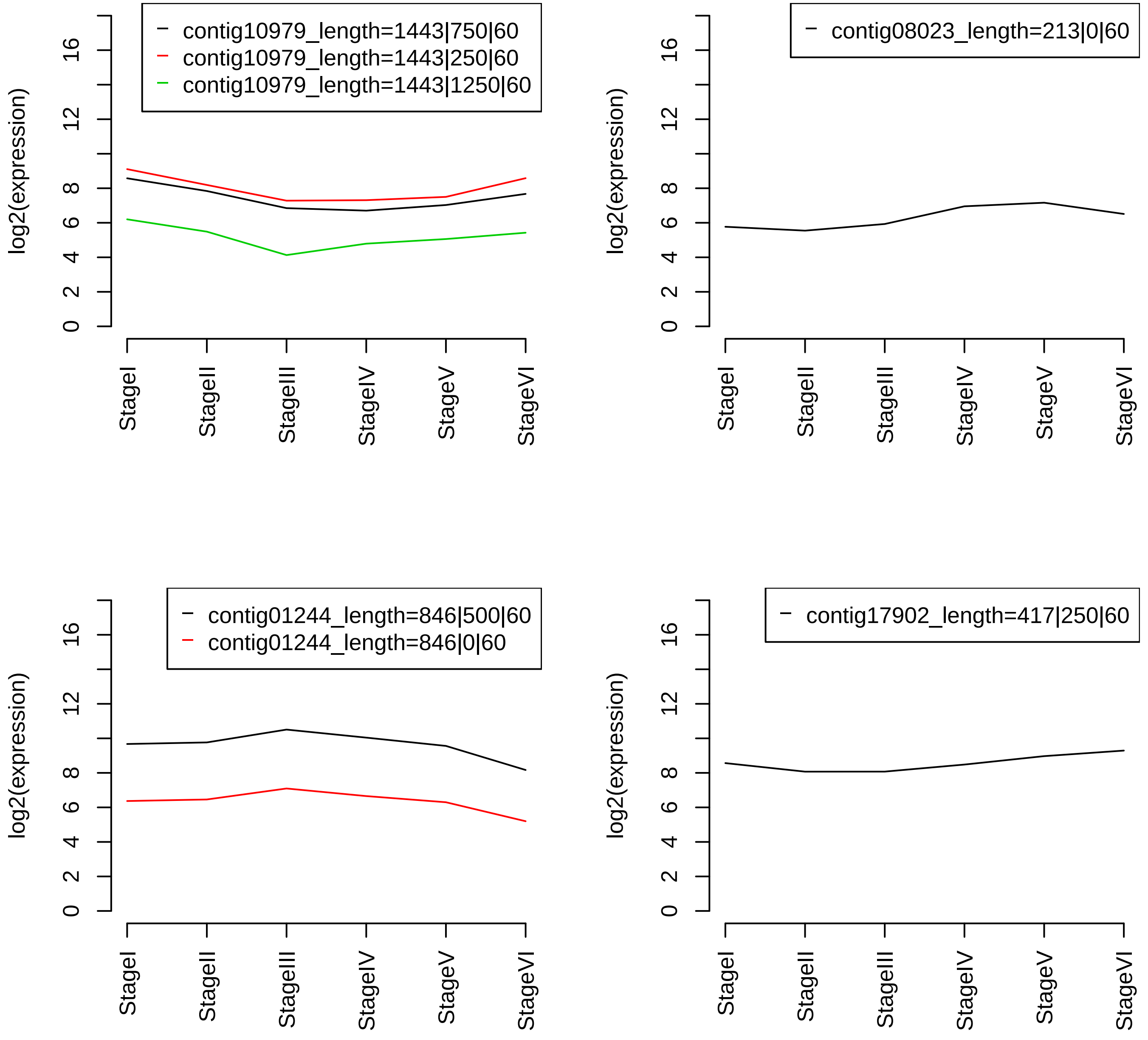

contig10979_length=1443|750|60
contig10979_length=1443|250|60
contig10979_length=1443|1250|60
log2(expression)
0
2
4
6
8
12
16
StageI
StageII
StageIII
StageIV
StageV
StageVI
contig08023_length=213|0|60
contig01244_length=846|500|60
contig01244_length=846|0|60
contig17902_length=417|250|60

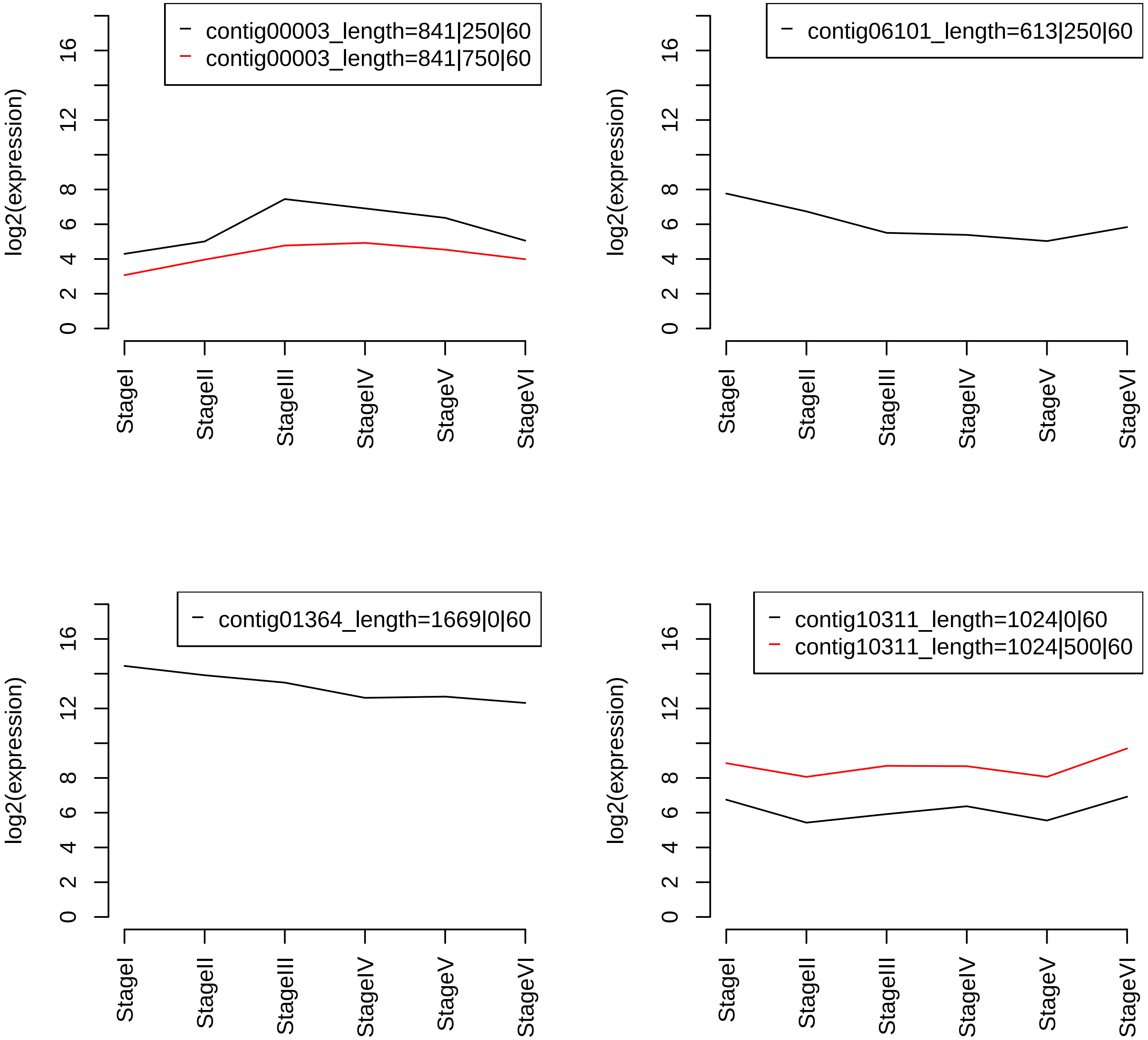

contig00003_length=841|250|60
contig00003_length=841|750|60
contig06101_length=613|250|60
contig01364_length=1669|0|60
contig10311_length=1024|0|60
contig10311_length=1024|500|60
log2(expression)
0
2
4
6
8
12
16
StageI
StageII
StageIII
StageIV
StageV
StageVI

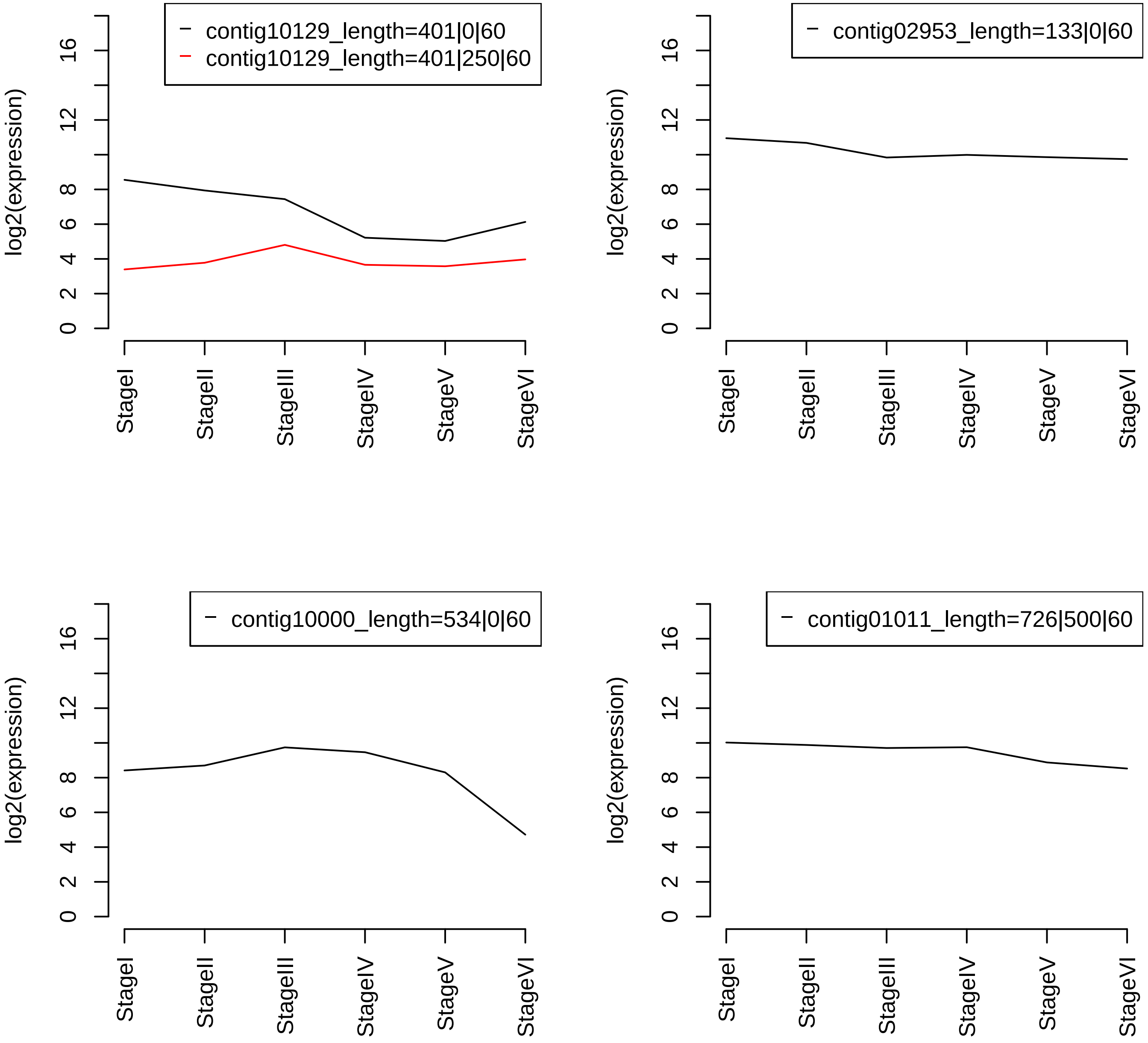

contig10129_length=401|0|60
contig10129_length=401|250|60
contig02953_length=133|0|60
contig10000_length=534|0|60
contig01011_length=726|500|60
log2(expression)
0
2
4
6
8
12
16
StageI
StageII
StageIII
StageIV
StageV
StageVI

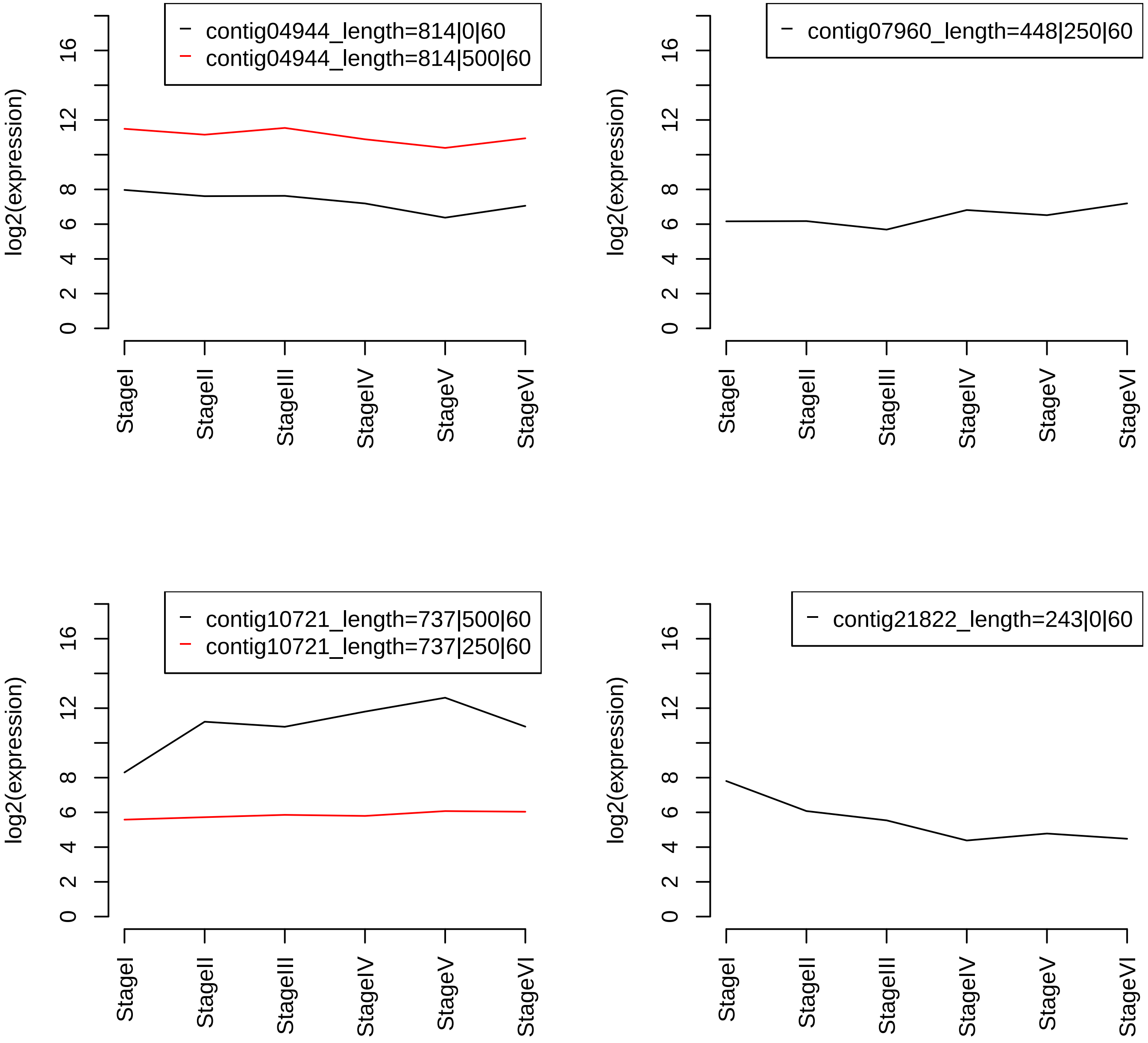

contig04944_length=814|0|60
contig04944_length=814|500|60
log2(expression)
0
2
4
6
8
12
16
StageI
StageII
StageIII
StageIV
StageV
StageVI
contig07960_length=448|250|60
contig10721_length=737|500|60
contig10721_length=737|250|60
contig21822_length=243|0|60

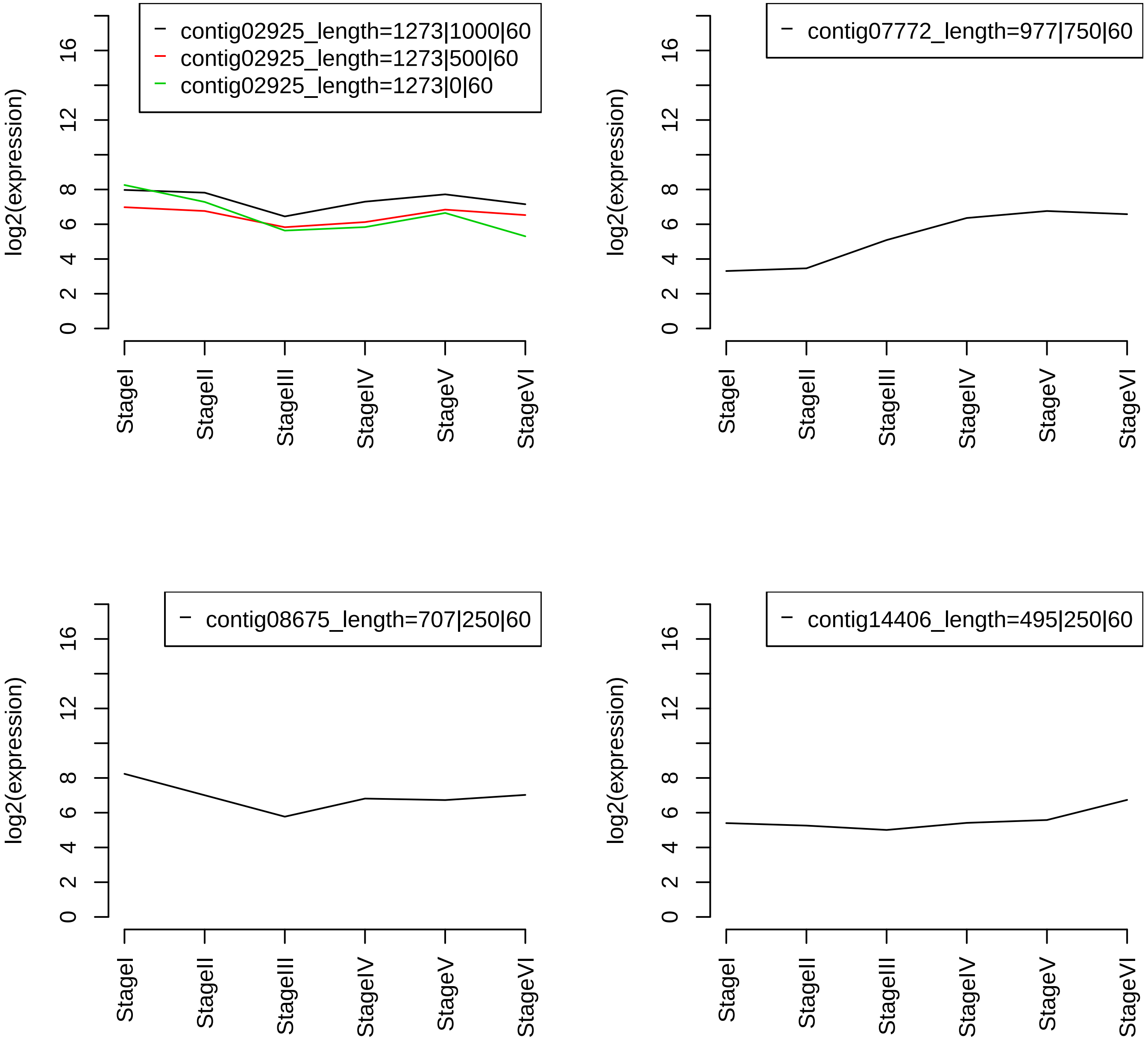
contig02925_length=1273|1000|60
contig02925_length=1273|500|60
contig02925_length=1273|0|60
contig07772_length=977|750|60
contig08675_length=707|250|60
contig14406_length=495|250|60
log2(expression)
0
2
4
6
8
12
16
StageI
StageII
StageIII
StageIV
StageV
StageVI

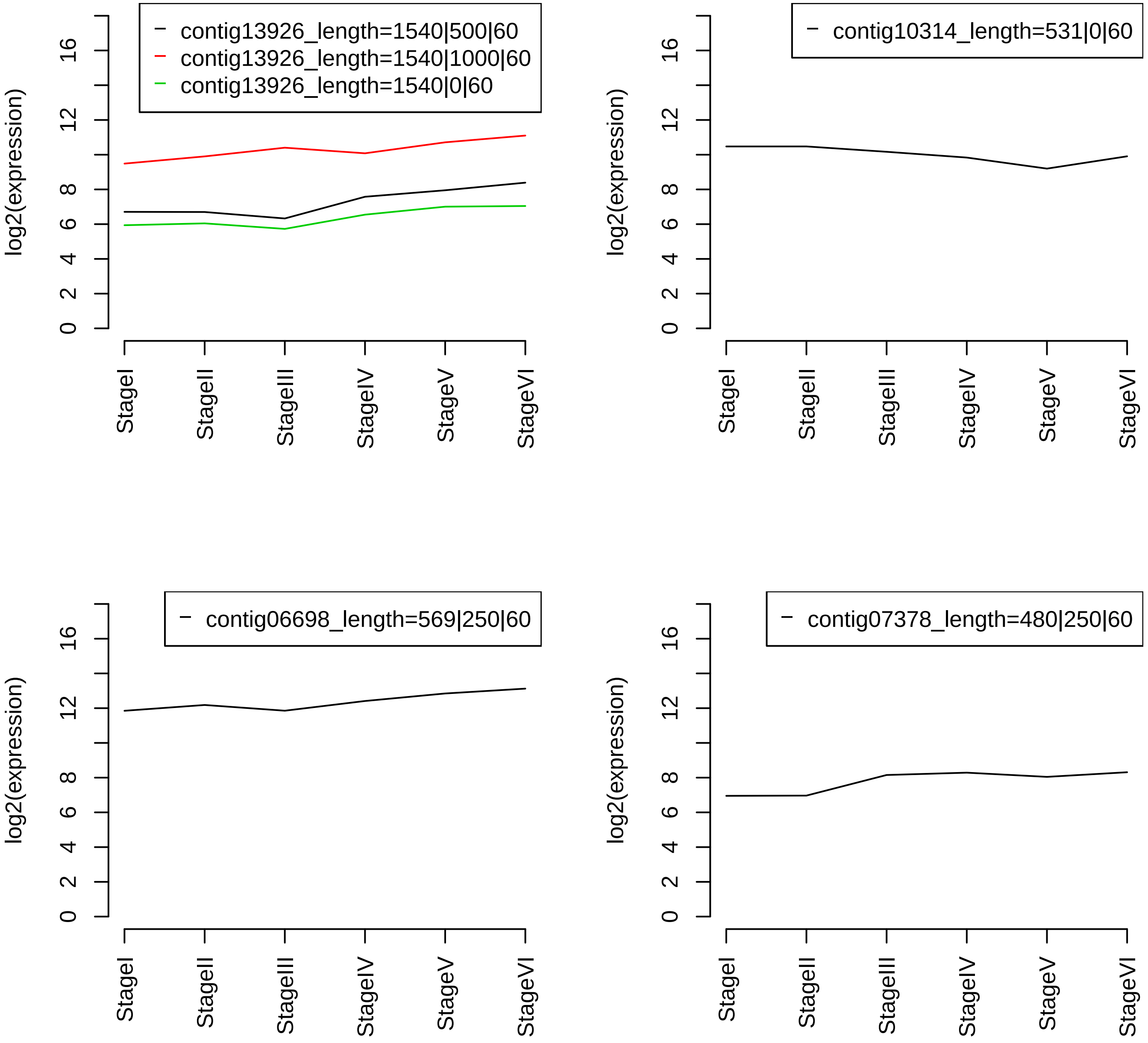
contig13926_length=1540|500|60
contig13926_length=1540|1000|60
contig13926_length=1540|0|60
contig10314_length=531|0|60
contig06698_length=569|250|60
contig07378_length=480|250|60
log2(expression)
0
2
4
6
8
12
16
StageI
StageII
StageIII
StageIV
StageV
StageVI

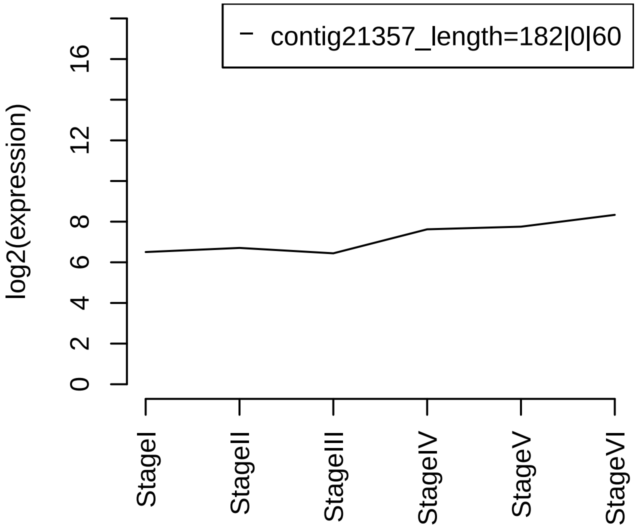
contig21357_length=182|0|60
log2(expression)
0
2
4
6
8
12
16
StageI
StageII
StageIII
StageIV
StageV
StageVI

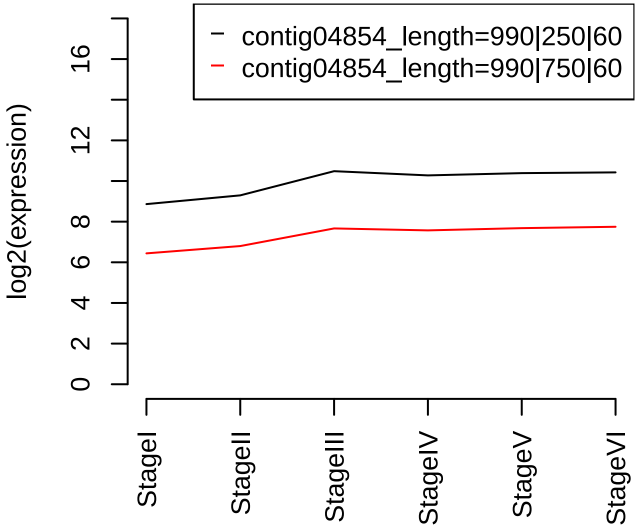
contig04854_length=990|250|60
contig04854_length=990|750|60
log2(expression)
0
2
4
6
8
12
16
StageI
StageII
StageIII
StageIV
StageV
StageVI

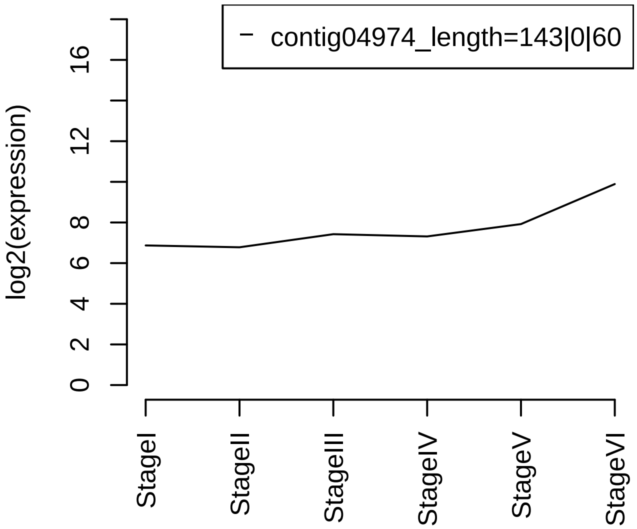
contig04974_length=143|0|60
log2(expression)
0
2
4
6
8
12
16
StageI
StageII
StageIII
StageIV
StageV
StageVI

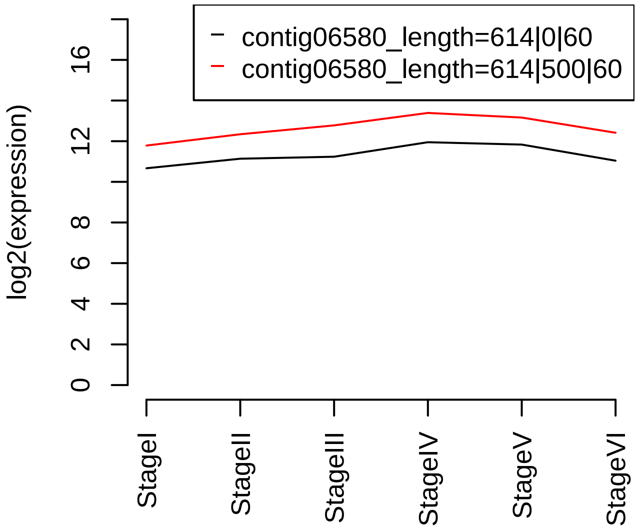
contig06580_length=614|0|60
contig06580_length=614|500|60
log2(expression)
0
2
4
6
8
12
16
StageI
StageII
StageIII
StageIV
StageV
StageVI

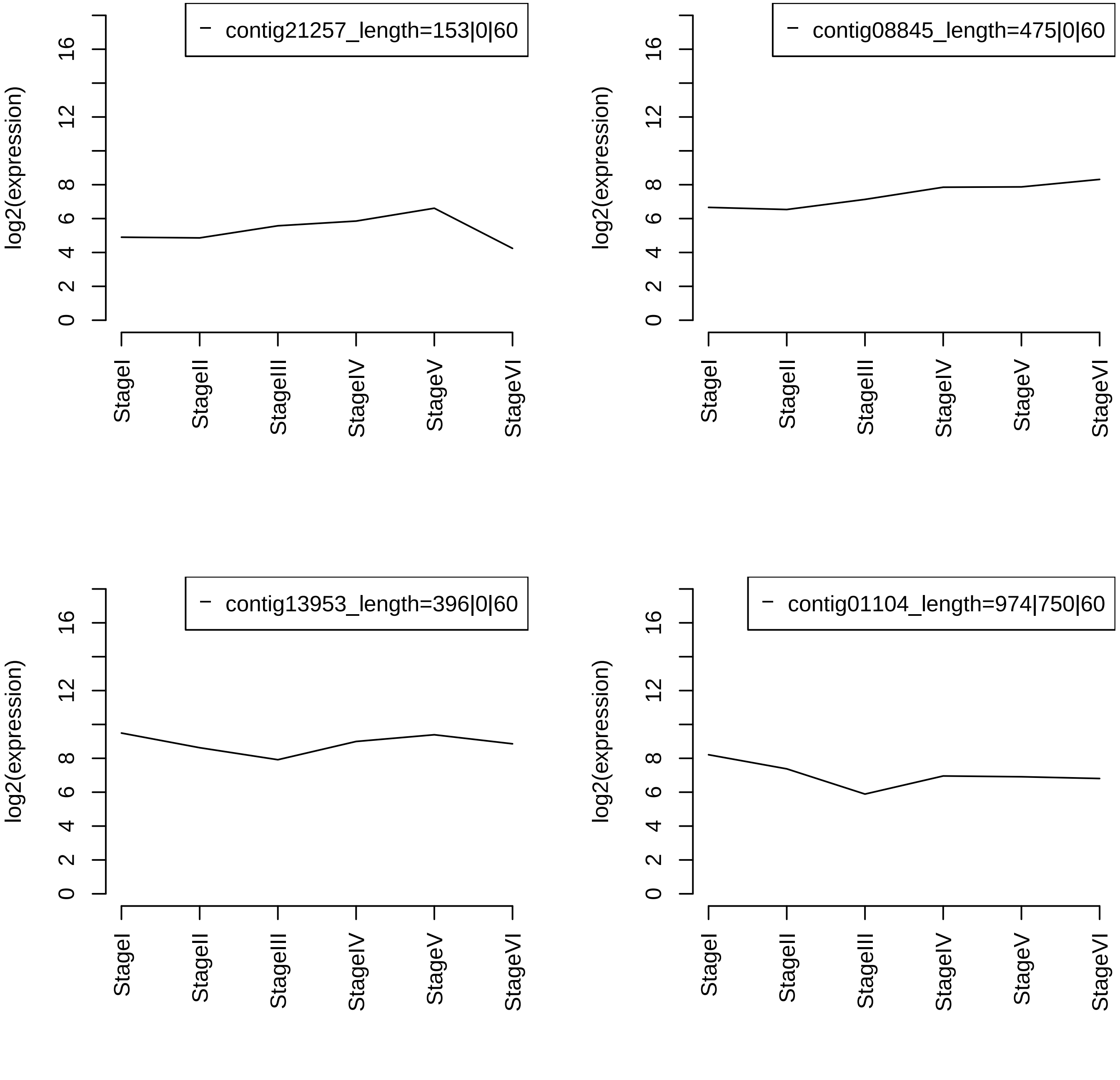
contig21257_length=153|0|60
contig08845_length=475|0|60
contig13953_length=396|0|60
contig01104_length=974|750|60
log2(expression)
0
2
4
6
8
12
16
StageI
StageII
StageIII
StageIV
StageV
StageVI

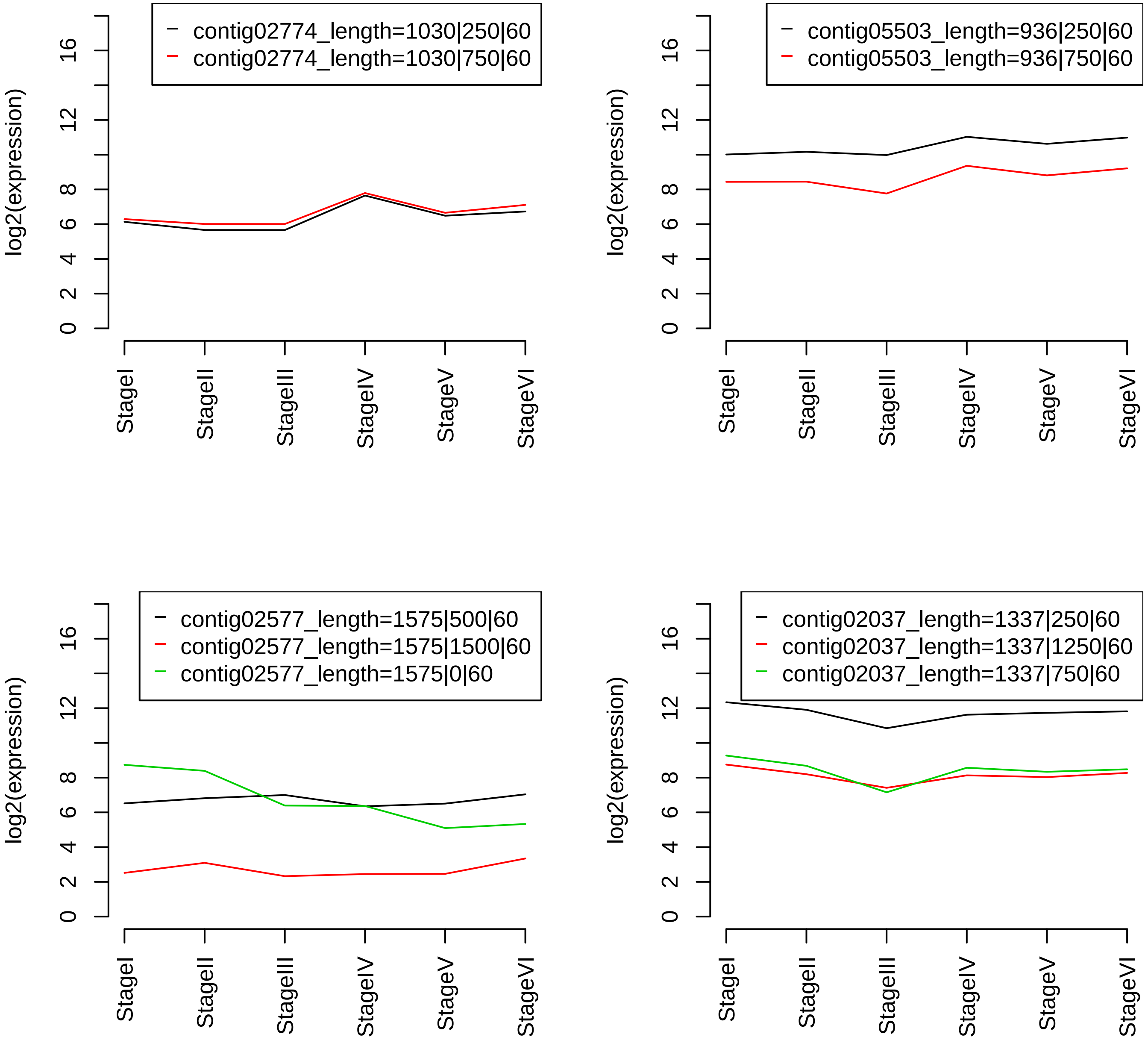

contig02774_length=1030|250|60
contig02774_length=1030|750|60
contig05503_length=936|250|60
contig05503_length=936|750|60
contig02577_length=1575|500|60
contig02577_length=1575|1500|60
contig02577_length=1575|0|60
contig02037_length=1337|250|60
contig02037_length=1337|1250|60
contig02037_length=1337|750|60
log2(expression)
0
2
4
6
8
12
16
StageI
StageII
StageIII
StageIV
StageV
StageVI

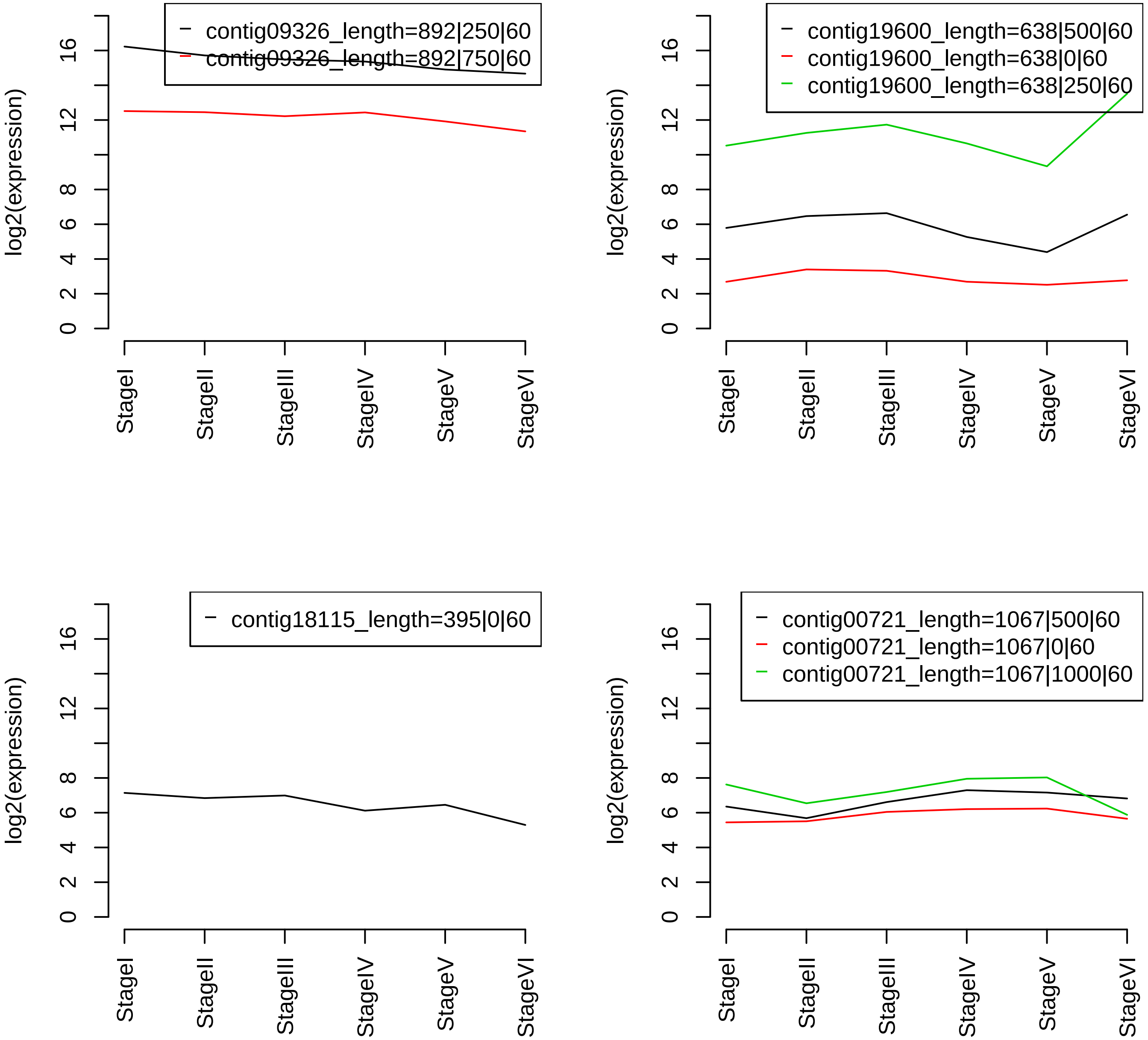

contig09326_length=892|250|60
contig09326_length=892|750|60
contig19600_length=638|500|60
contig19600_length=638|0|60
contig19600_length=638|250|60
contig18115_length=395|0|60
contig00721_length=1067|500|60
contig00721_length=1067|0|60
contig00721_length=1067|1000|60
log2(expression)
0
2
4
6
8
12
16
StageI
StageII
StageIII
StageIV
StageV
StageVI

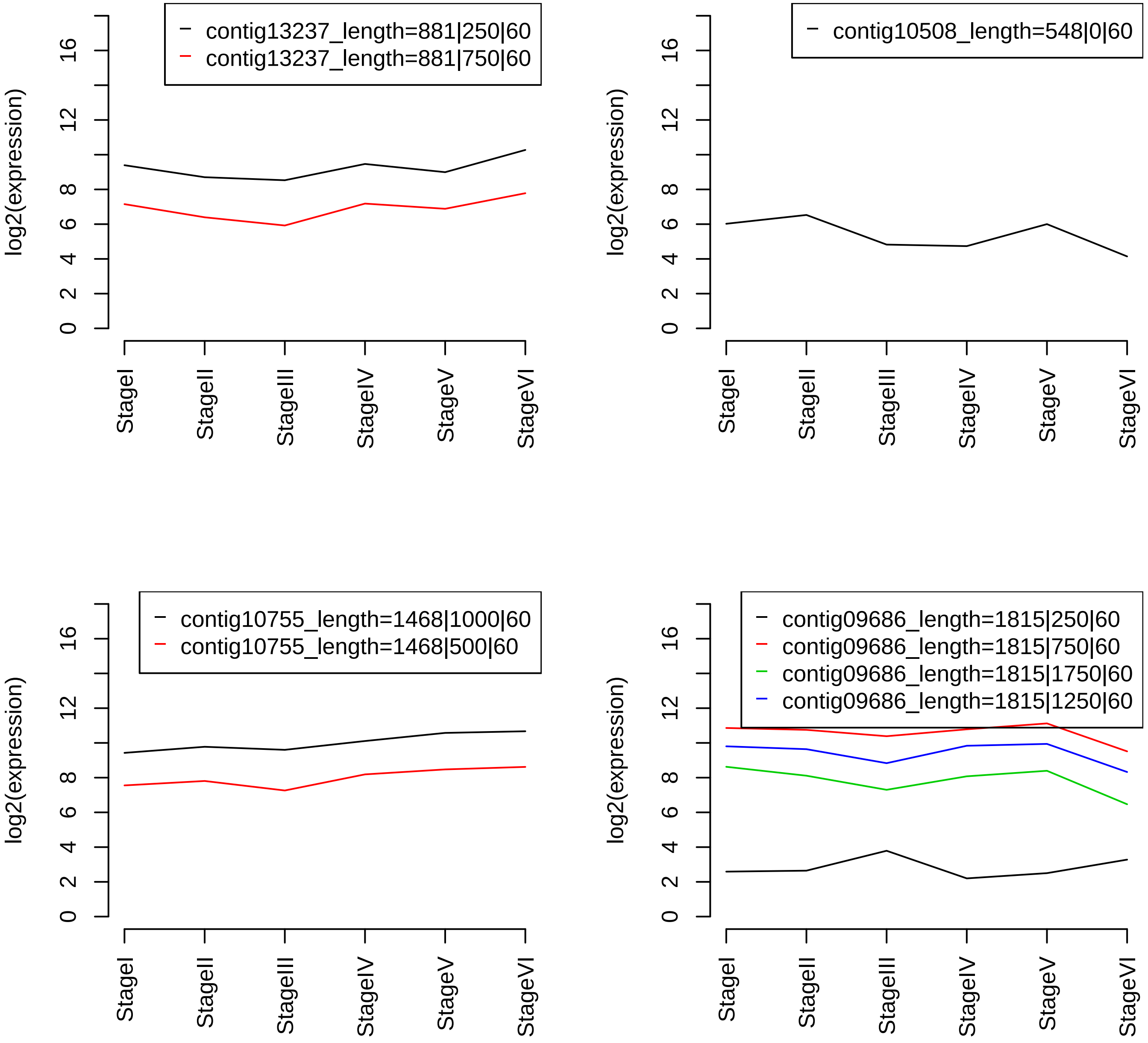
contig13237_length=881|250|60
contig13237_length=881|750|60
contig10508_length=548|0|60
contig10755_length=1468|1000|60
contig10755_length=1468|500|60
contig09686_length=1815|250|60
contig09686_length=1815|750|60
contig09686_length=1815|1750|60
contig09686_length=1815|1250|60
log2(expression)
StageI
StageII
StageIII
StageIV
StageV
StageVI

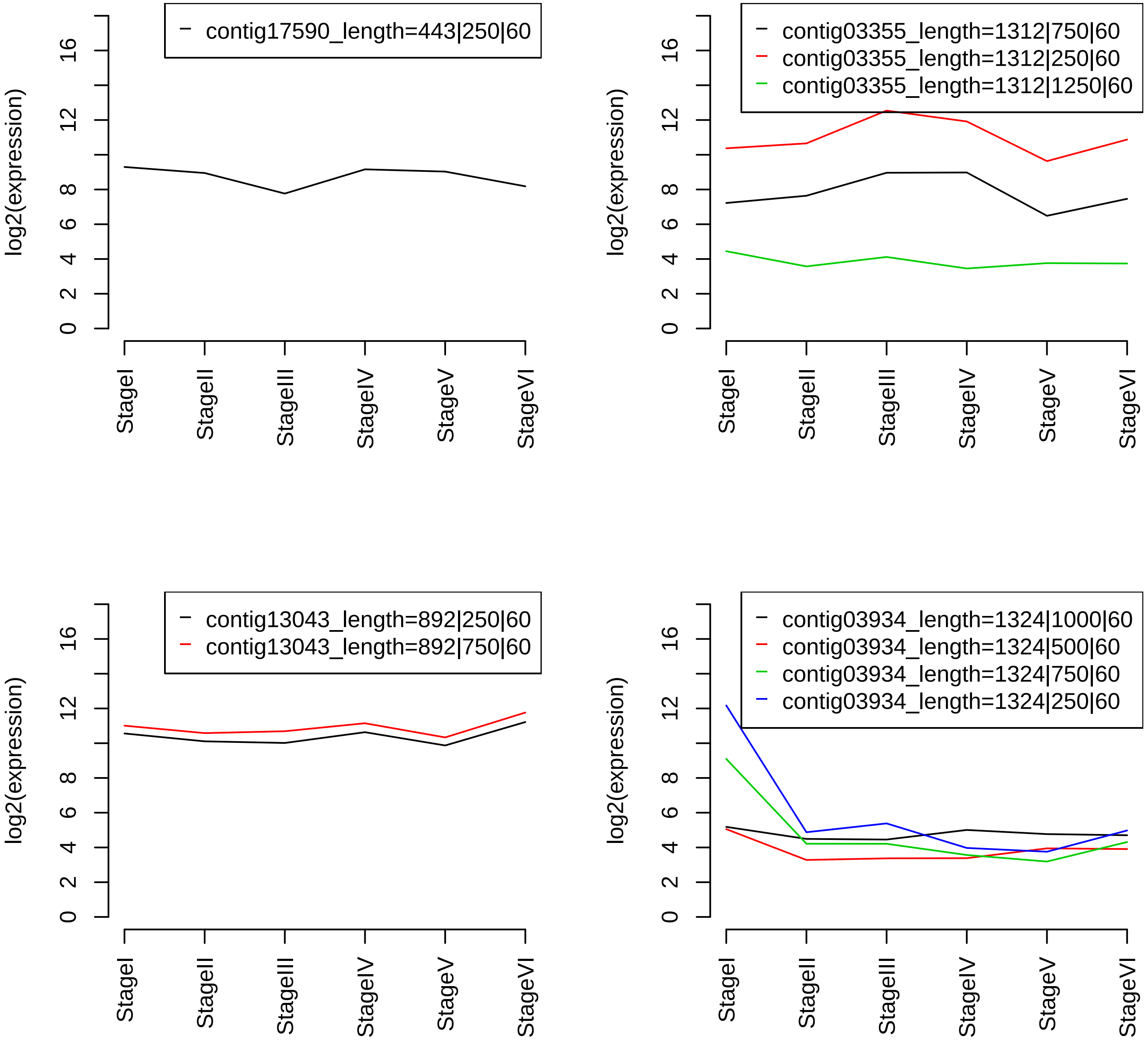
contig17590_length=443|250|60
contig03355_length=1312|750|60
contig03355_length=1312|250|60
contig03355_length=1312|1250|60
contig13043_length=892|250|60
contig13043_length=892|750|60
contig03934_length=1324|1000|60
contig03934_length=1324|500|60
contig03934_length=1324|750|60
contig03934_length=1324|250|60
log2(expression)
0
2
4
6
8
12
16
StageI
StageII
StageIII
StageIV
StageV
StageVI

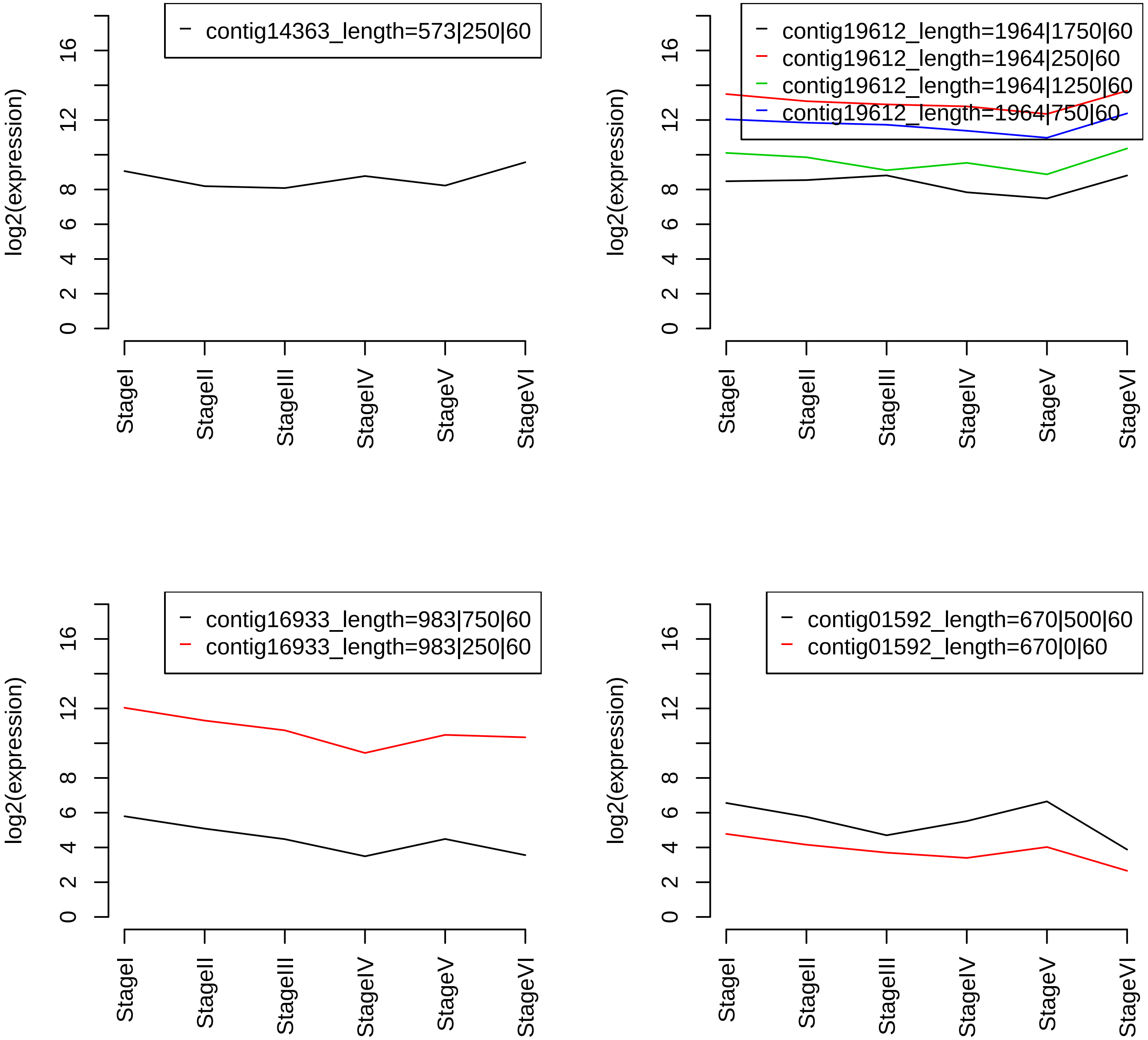

contig14363_length=573|250|60
contig19612_length=1964|1750|60
contig19612_length=1964|250|60
contig19612_length=1964|1250|60
contig19612_length=1964|750|60
contig16933_length=983|750|60
contig16933_length=983|250|60
contig01592_length=670|500|60
contig01592_length=670|0|60
log2(expression)
0
2
4
6
8
12
16
StageI
StageII
StageIII
StageIV
StageV
StageVI

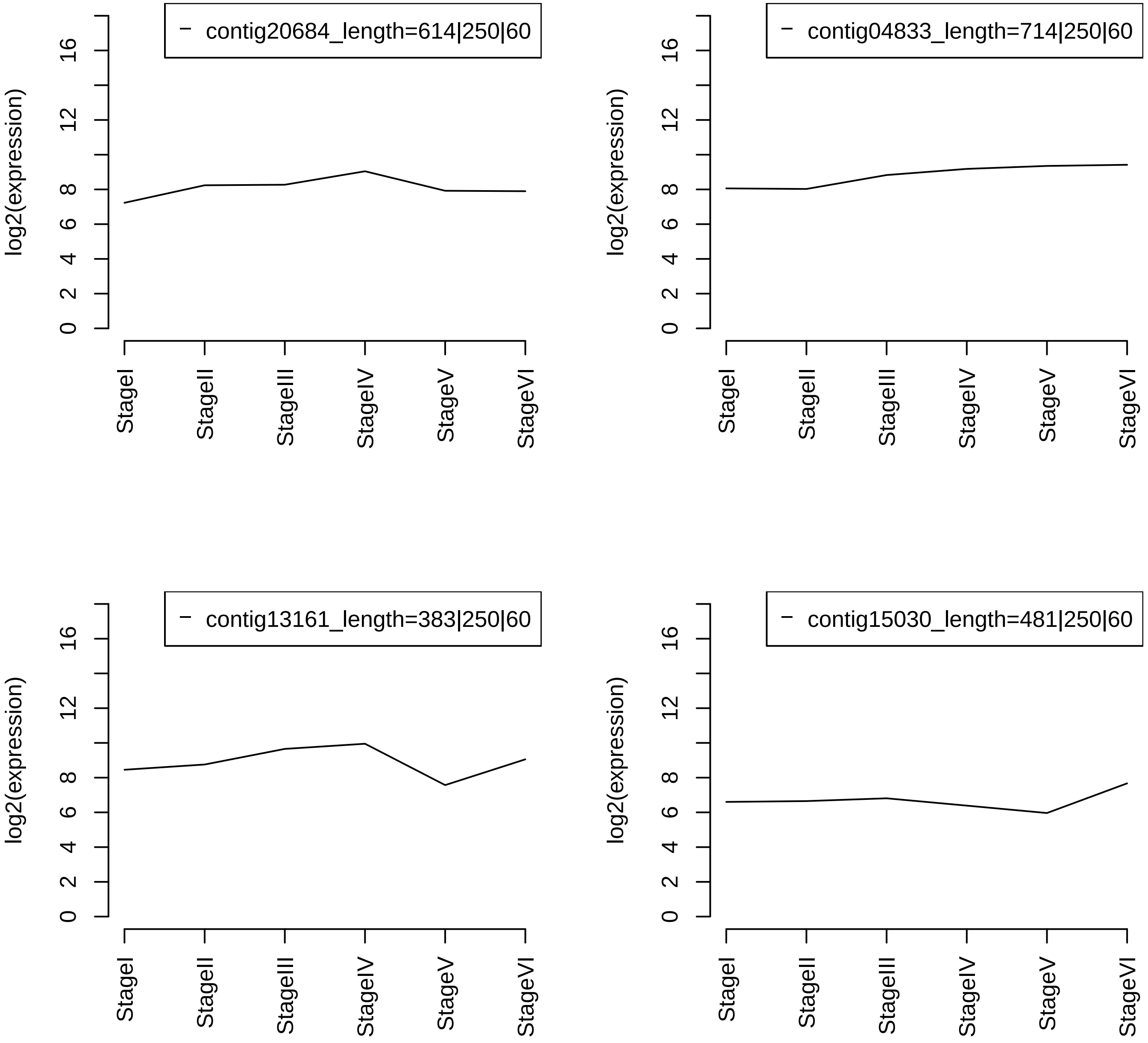
contig20684_length=614|250|60
contig04833_length=714|250|60
contig13161_length=383|250|60
contig15030_length=481|250|60
log2(expression)
0
2
4
6
8
12
16
StageI
StageII
StageIII
StageIV
StageV
StageVI

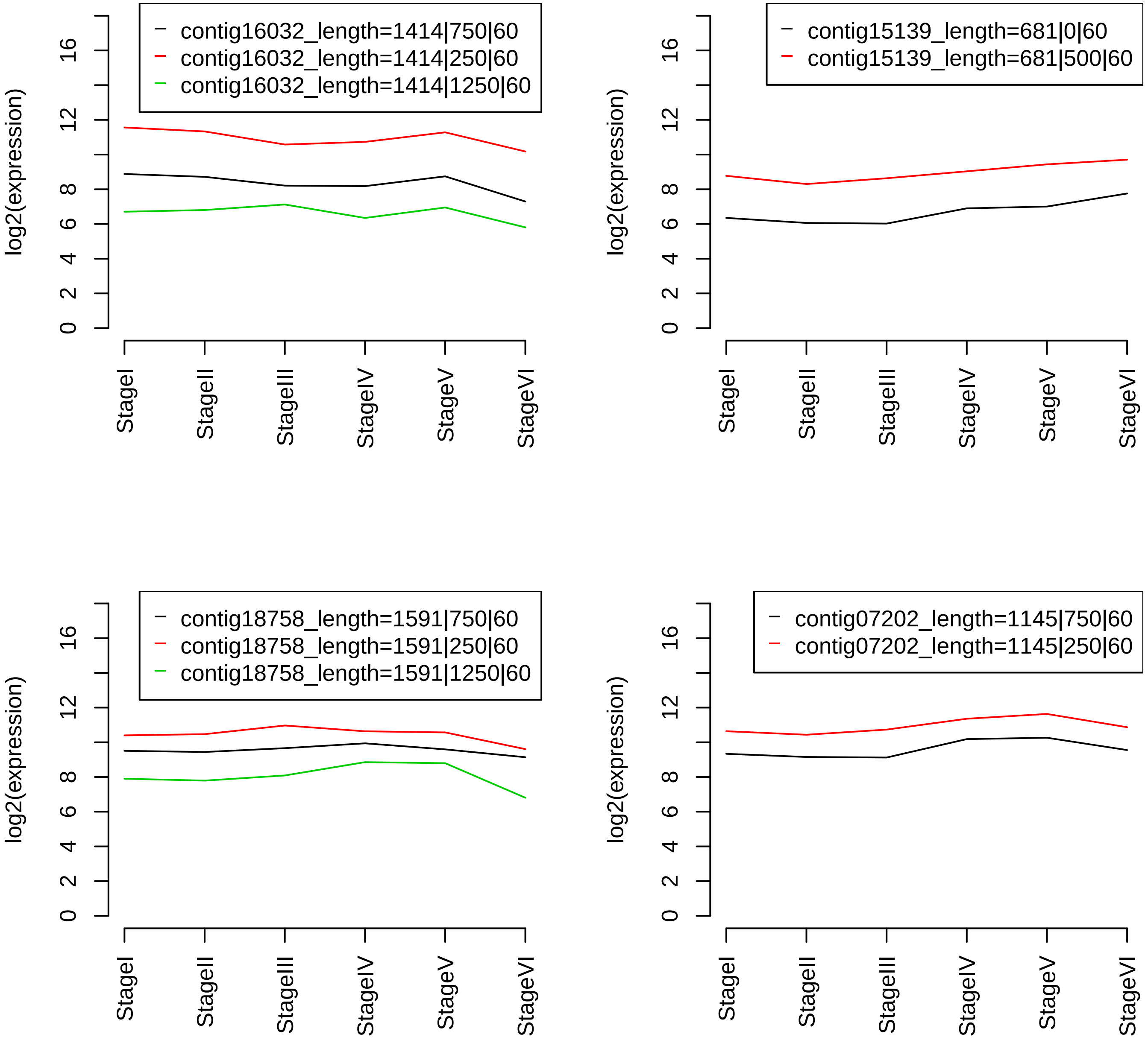

log2(expression)
0
2
4
6
8
12
16
StageI
StageII
StageIII
StageIV
StageV
StageVI
contig16032_length=1414|750|60
contig16032_length=1414|250|60
contig16032_length=1414|1250|60
contig15139_length=681|0|60
contig15139_length=681|500|60
contig18758_length=1591|750|60
contig18758_length=1591|250|60
contig18758_length=1591|1250|60
contig07202_length=1145|750|60
contig07202_length=1145|250|60

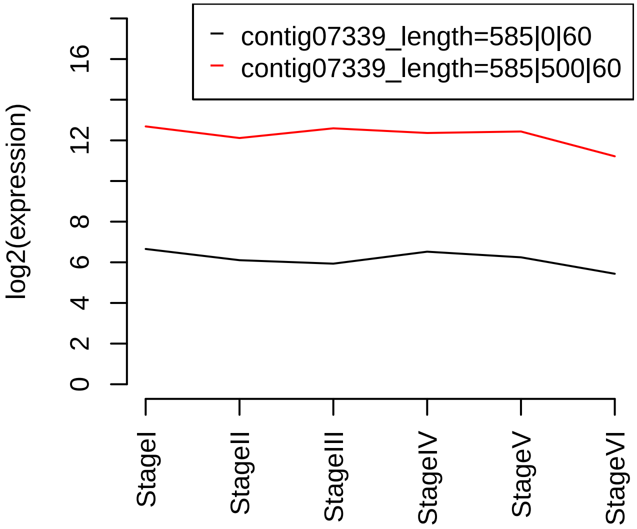
contig07339_length=585|0|60
contig07339_length=585|500|60
log2(expression)
0
2
4
6
8
12
16
StageI
StageII
StageIII
StageIV
StageV
StageVI

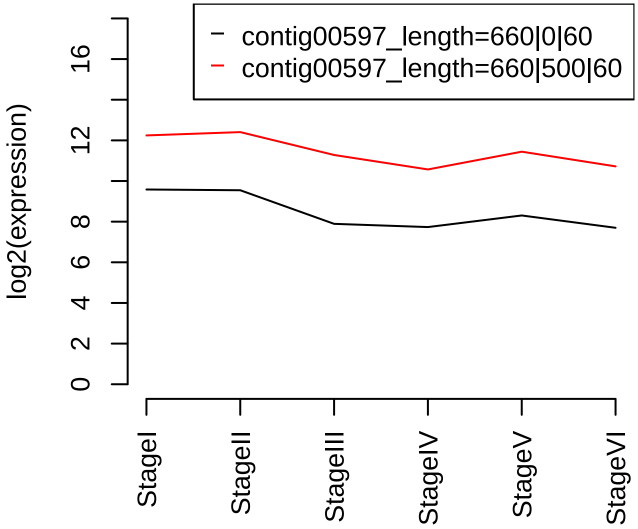
contig00597_length=660|0|60
contig00597_length=660|500|60
log2(expression)
0
2
4
6
8
12
16
StageI
StageII
StageIII
StageIV
StageV
StageVI

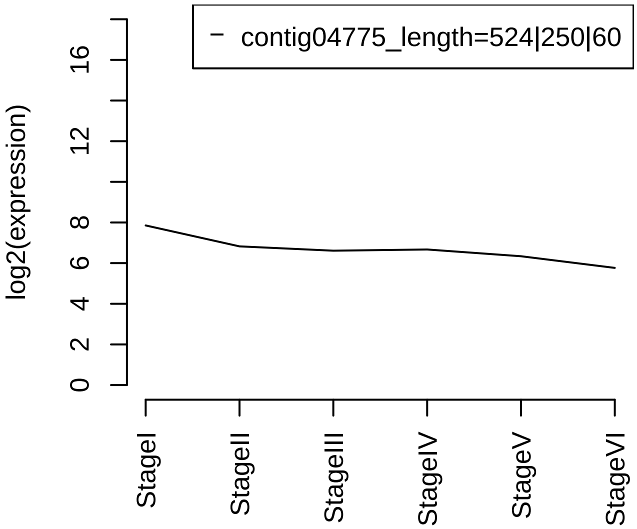
contig04775_length=524|250|60
log2(expression)
0
2
4
6
8
12
16
StageI
StageII
StageIII
StageIV
StageV
StageVI

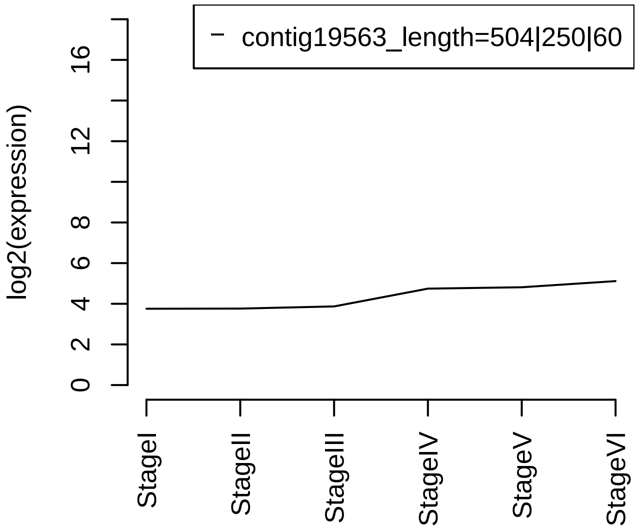
contig19563_length=504|250|60
log2(expression)
0
2
4
6
8
12
16
StageI
StageII
StageIII
StageIV
StageV
StageVI

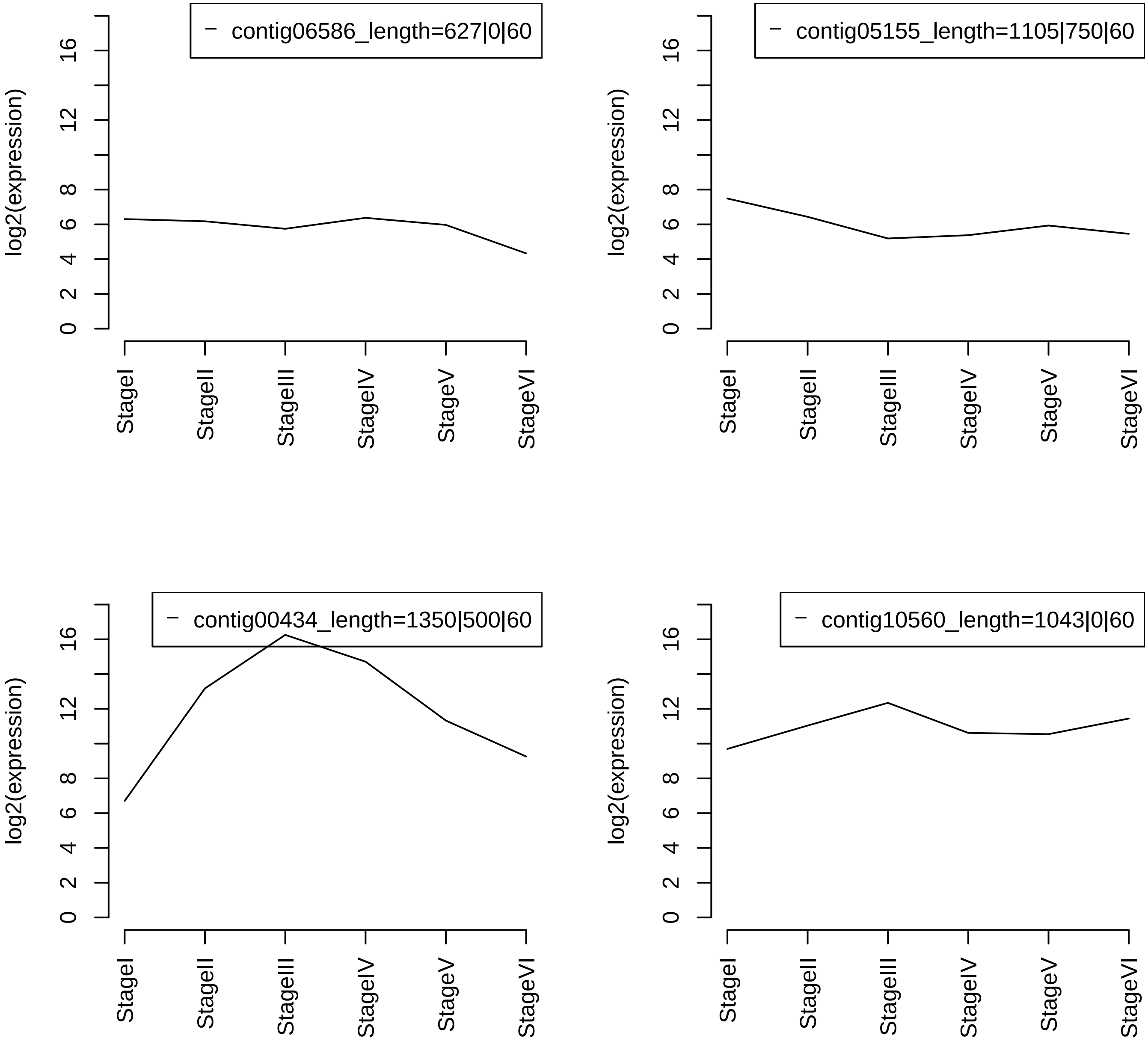
contig06586_length=627|0|60
contig05155_length=1105|750|60
contig00434_length=1350|500|60
contig10560_length=1043|0|60
log2(expression)
StageI
StageII
StageIII
StageIV
StageV
StageVI

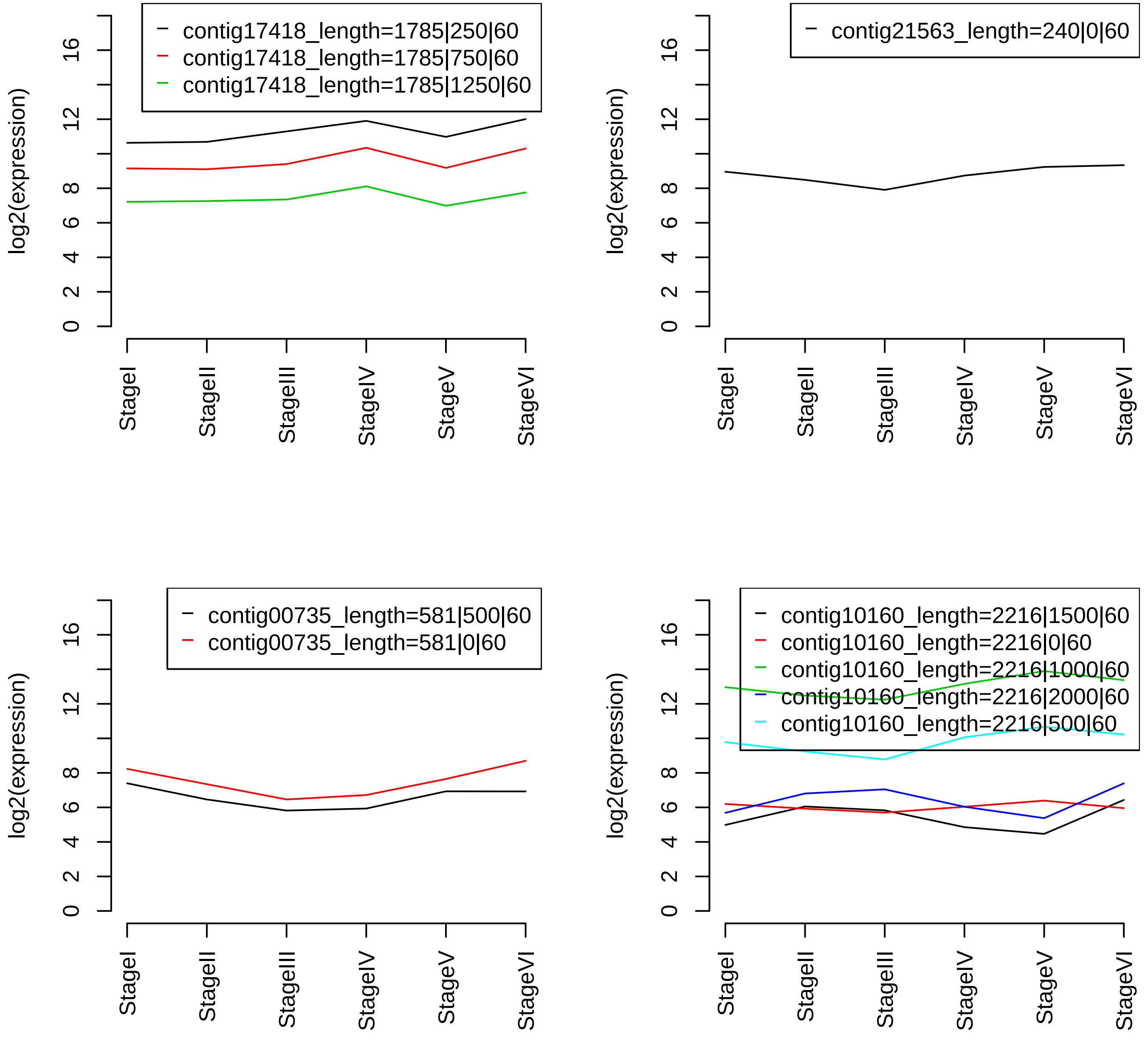
contig17418_length=1785|250|60
contig17418_length=1785|750|60
contig17418_length=1785|1250|60
contig21563_length=240|0|60
contig00735_length=581|500|60
contig00735_length=581|0|60
contig10160_length=2216|1500|60
contig10160_length=2216|0|60
contig10160_length=2216|1000|60
contig10160_length=2216|2000|60
contig10160_length=2216|500|60
log2(expression)
0
2
4
6
8
12
16
StageI
StageII
StageIII
StageIV
StageV
StageVI

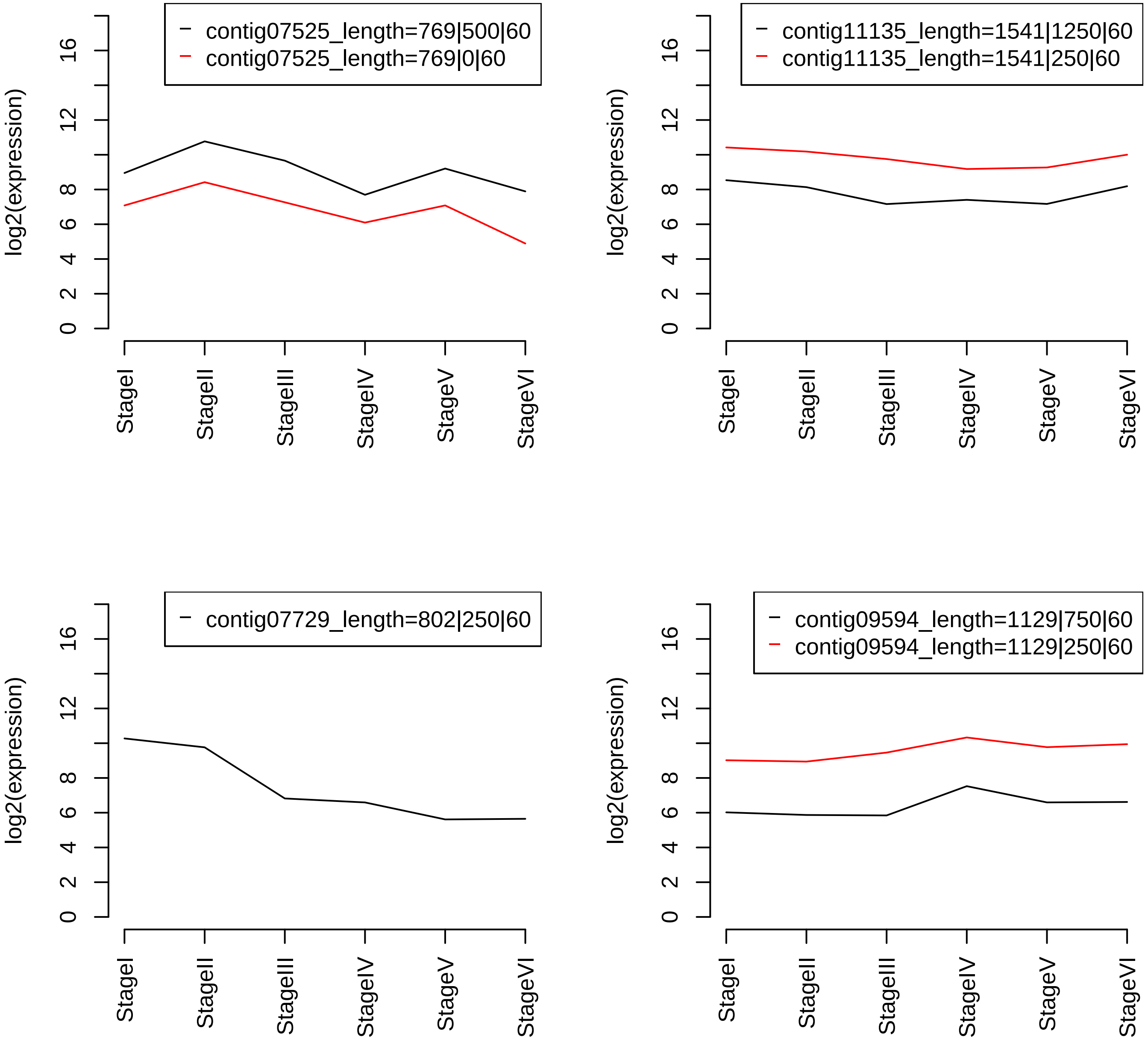

contig07525_length=769|500|60
contig07525_length=769|0|60
contig11135_length=1541|1250|60
contig11135_length=1541|250|60
contig07729_length=802|250|60
contig09594_length=1129|750|60
contig09594_length=1129|250|60
log2(expression)
0
2
4
6
8
12
16
StageI
StageII
StageIII
StageIV
StageV
StageVI

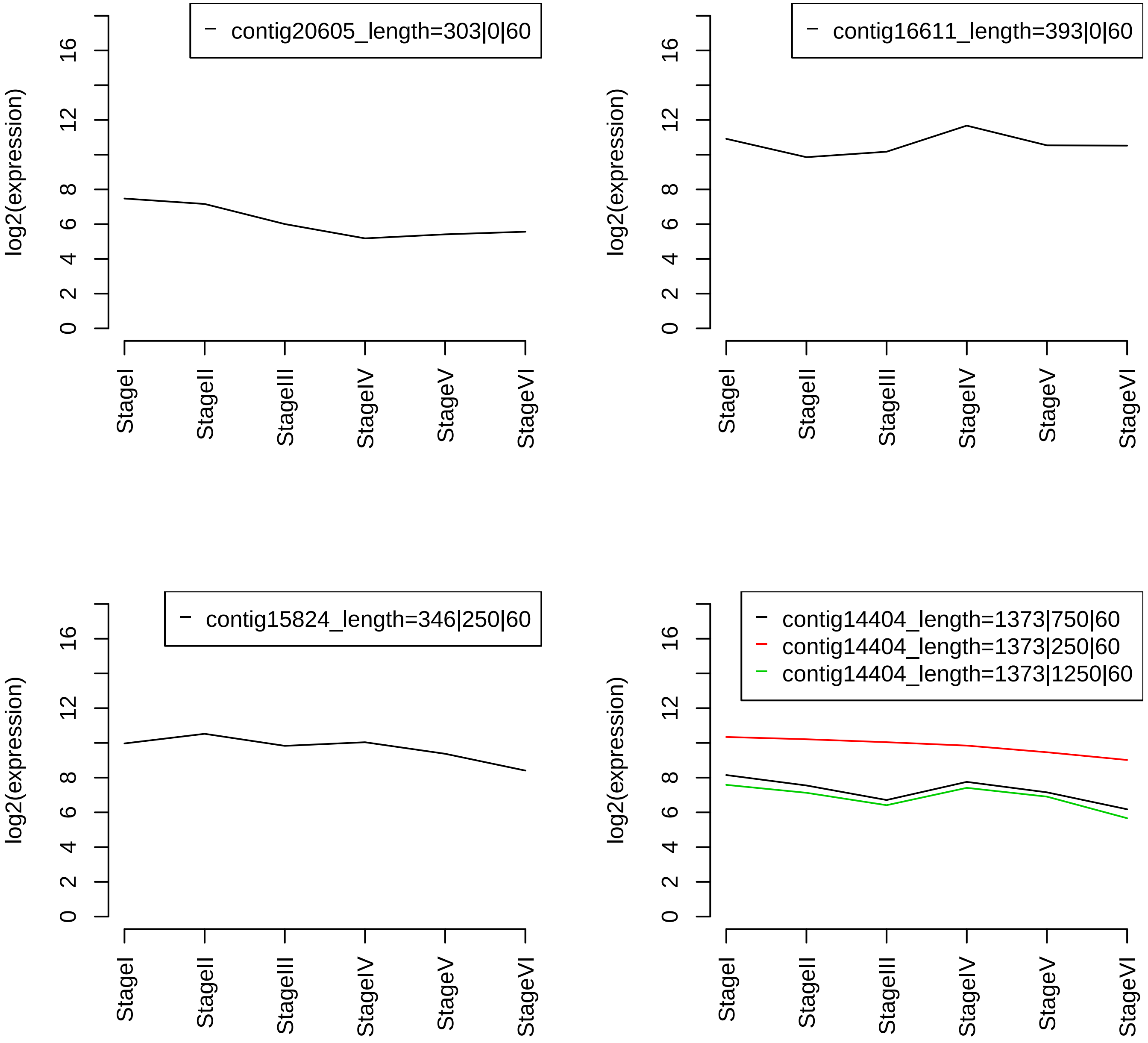

contig20605_length=303|0|60
contig16611_length=393|0|60
contig15824_length=346|250|60
contig14404_length=1373|750|60
contig14404_length=1373|250|60
contig14404_length=1373|1250|60
log2(expression)
0
2
4
6
8
12
16
StageI
StageII
StageIII
StageIV
StageV
StageVI

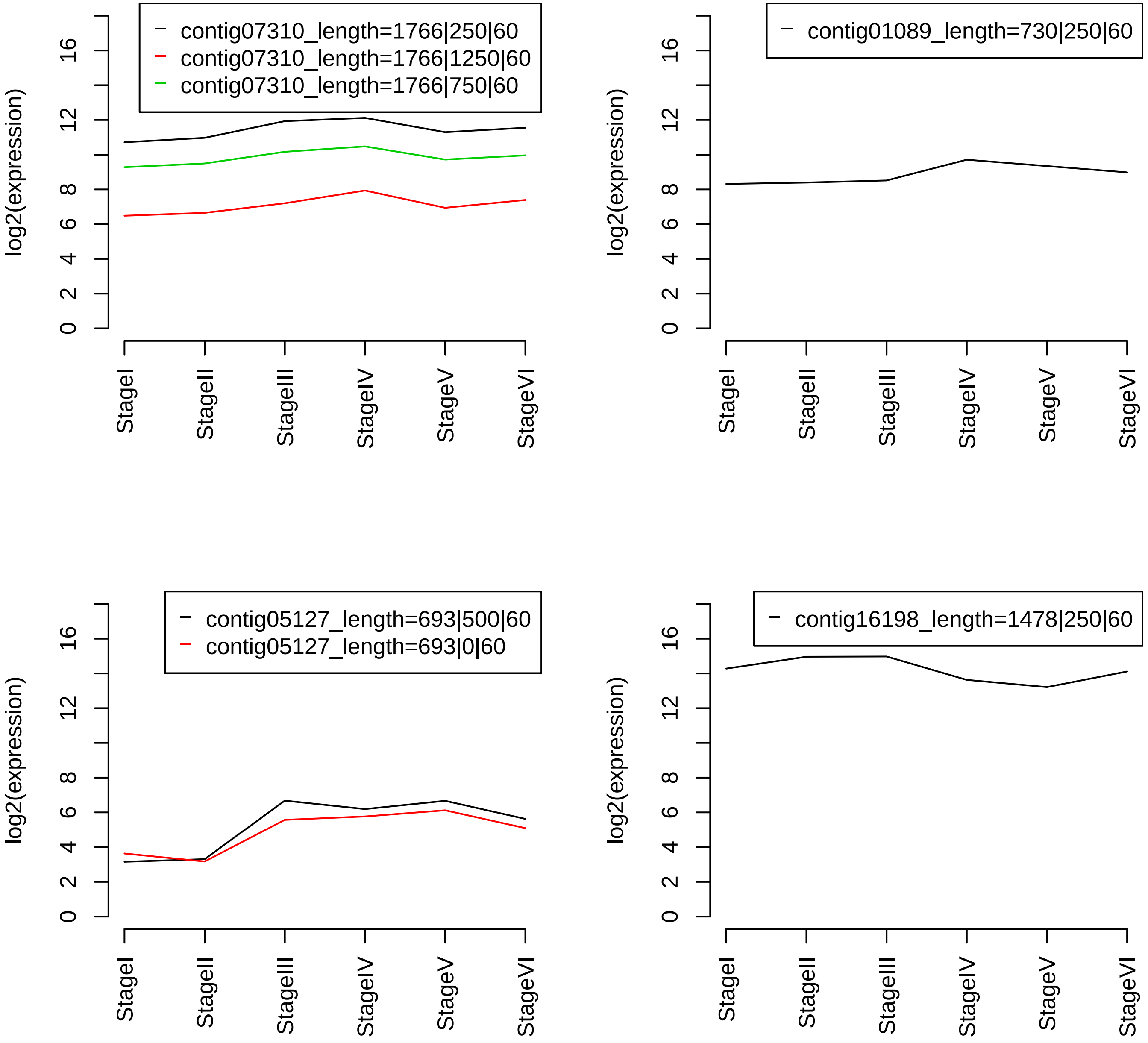
contig07310_length=1766|250|60
contig07310_length=1766|1250|60
contig07310_length=1766|750|60
contig01089_length=730|250|60
contig05127_length=693|500|60
contig05127_length=693|0|60
contig16198_length=1478|250|60
log2(expression)
0
2
4
6
8
12
16
StageI
StageII
StageIII
StageIV
StageV
StageVI

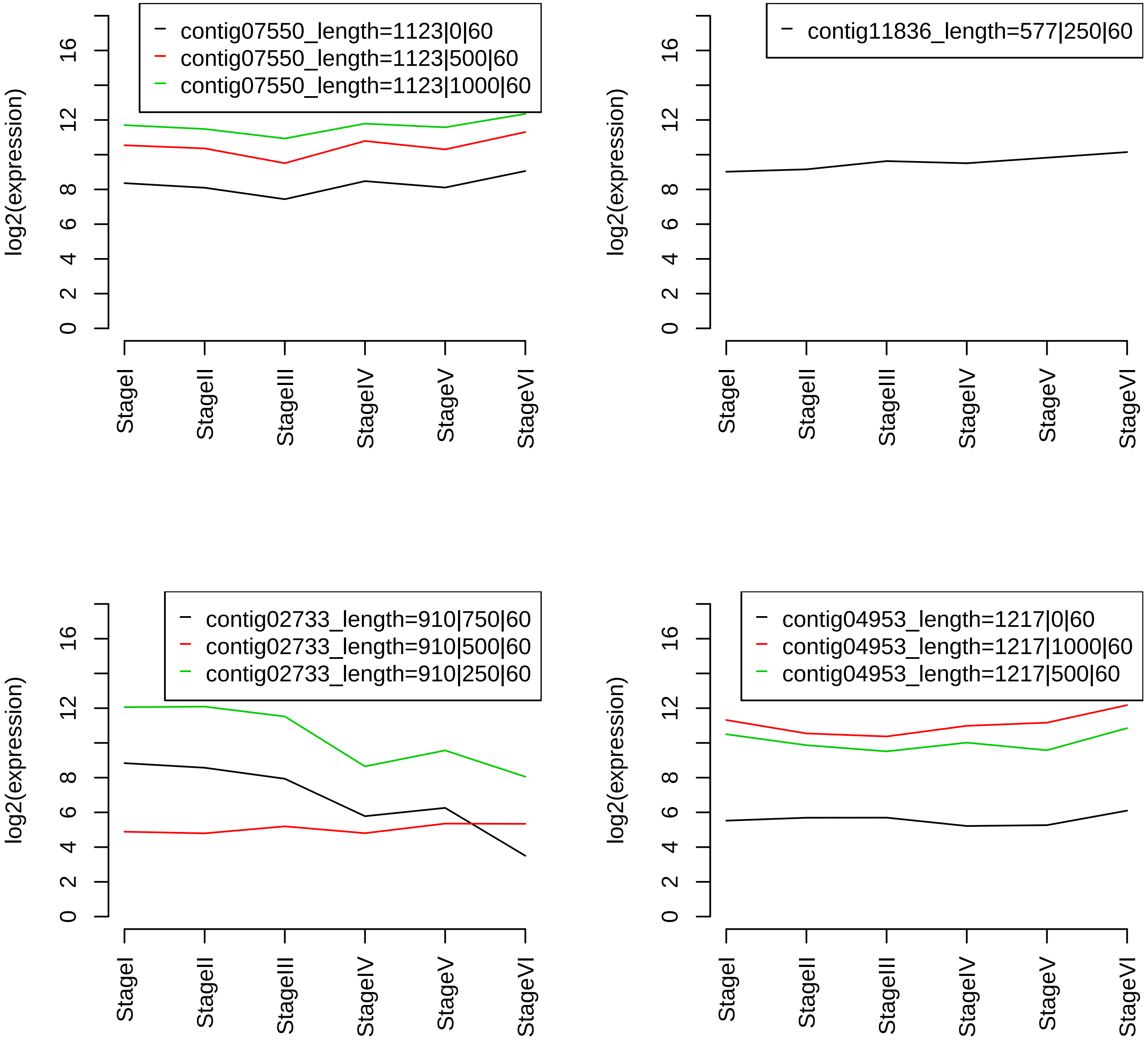

contig07550_length=1123|0|60
contig07550_length=1123|500|60
contig07550_length=1123|1000|60
log2(expression)
0
2
4
6
8
12
16
StageI
StageII
StageIII
StageIV
StageV
StageVI
contig11836_length=577|250|60
contig02733_length=910|750|60
contig02733_length=910|500|60
contig02733_length=910|250|60
contig04953_length=1217|0|60
contig04953_length=1217|1000|60
contig04953_length=1217|500|60

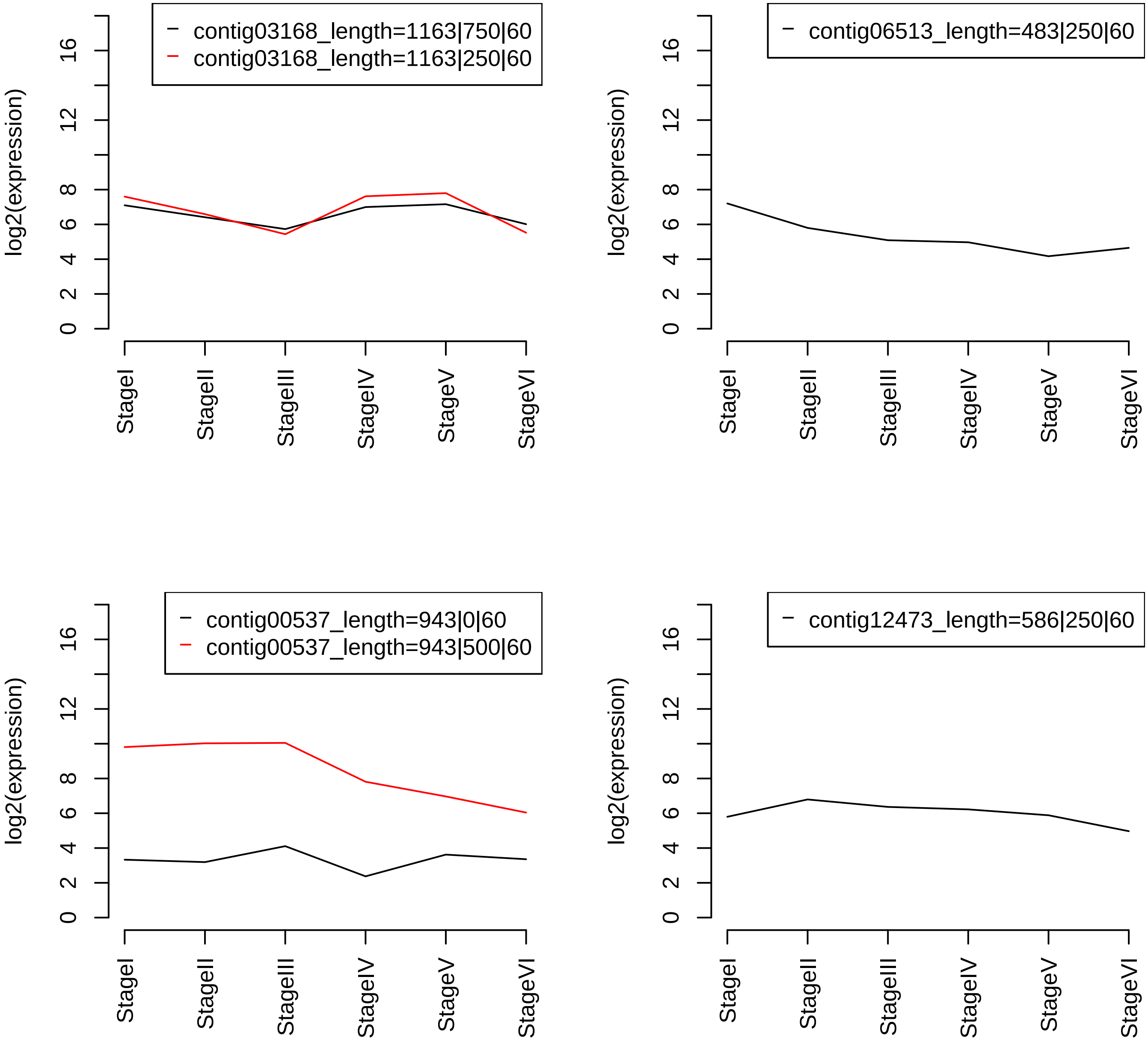

contig03168_length=1163|750|60
contig03168_length=1163|250|60
contig06513_length=483|250|60
contig00537_length=943|0|60
contig00537_length=943|500|60
contig12473_length=586|250|60
log2(expression)
0
2
4
6
8
12
16
StageI
StageII
StageIII
StageIV
StageV
StageVI

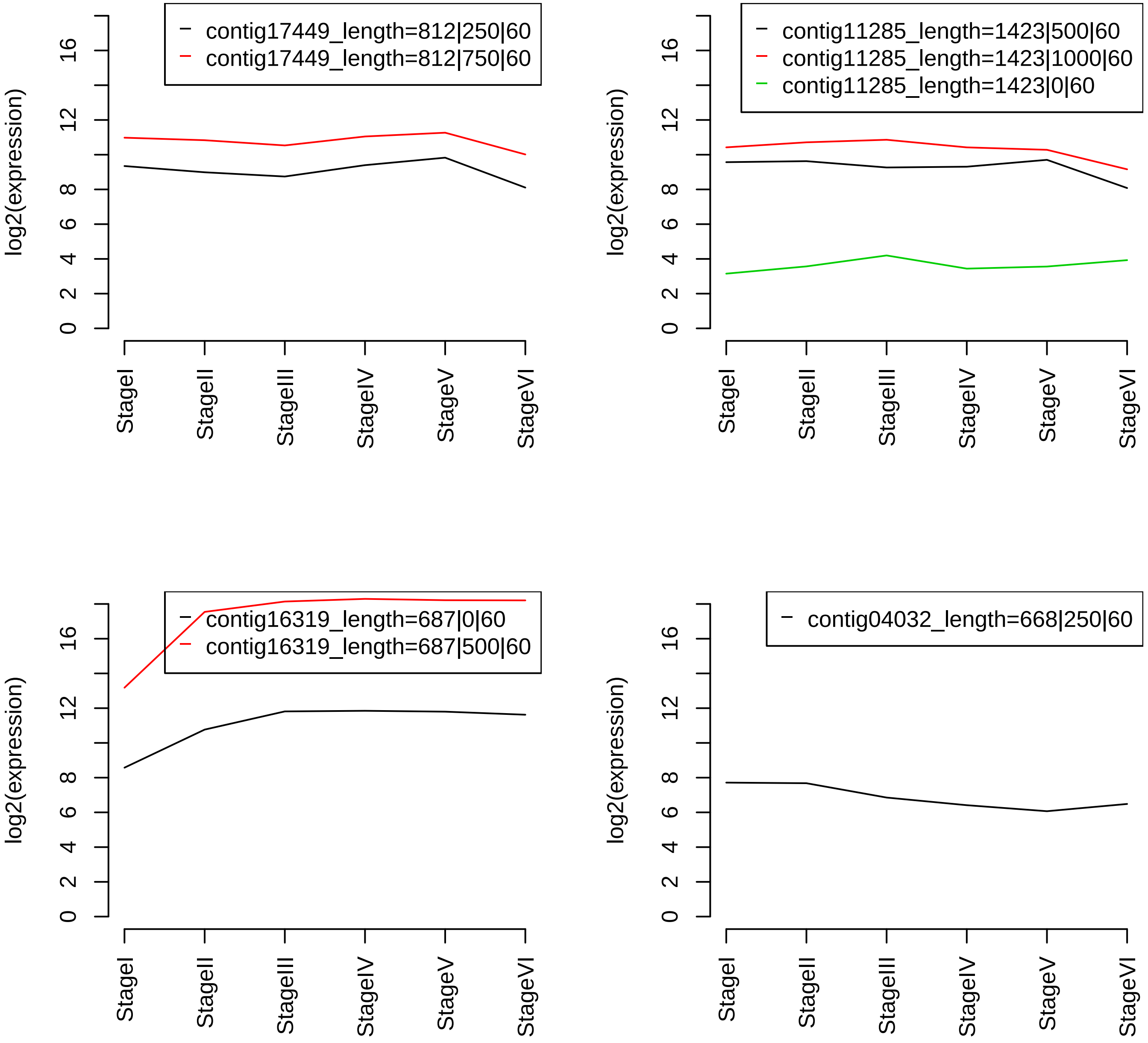

contig17449_length=812|250|60
contig17449_length=812|750|60
contig11285_length=1423|500|60
contig11285_length=1423|1000|60
contig11285_length=1423|0|60
contig16319_length=687|0|60
contig16319_length=687|500|60
contig04032_length=668|250|60
log2(expression)
0 2 4 6 8 12 16
StageI StageII StageIII StageIV StageV StageVI

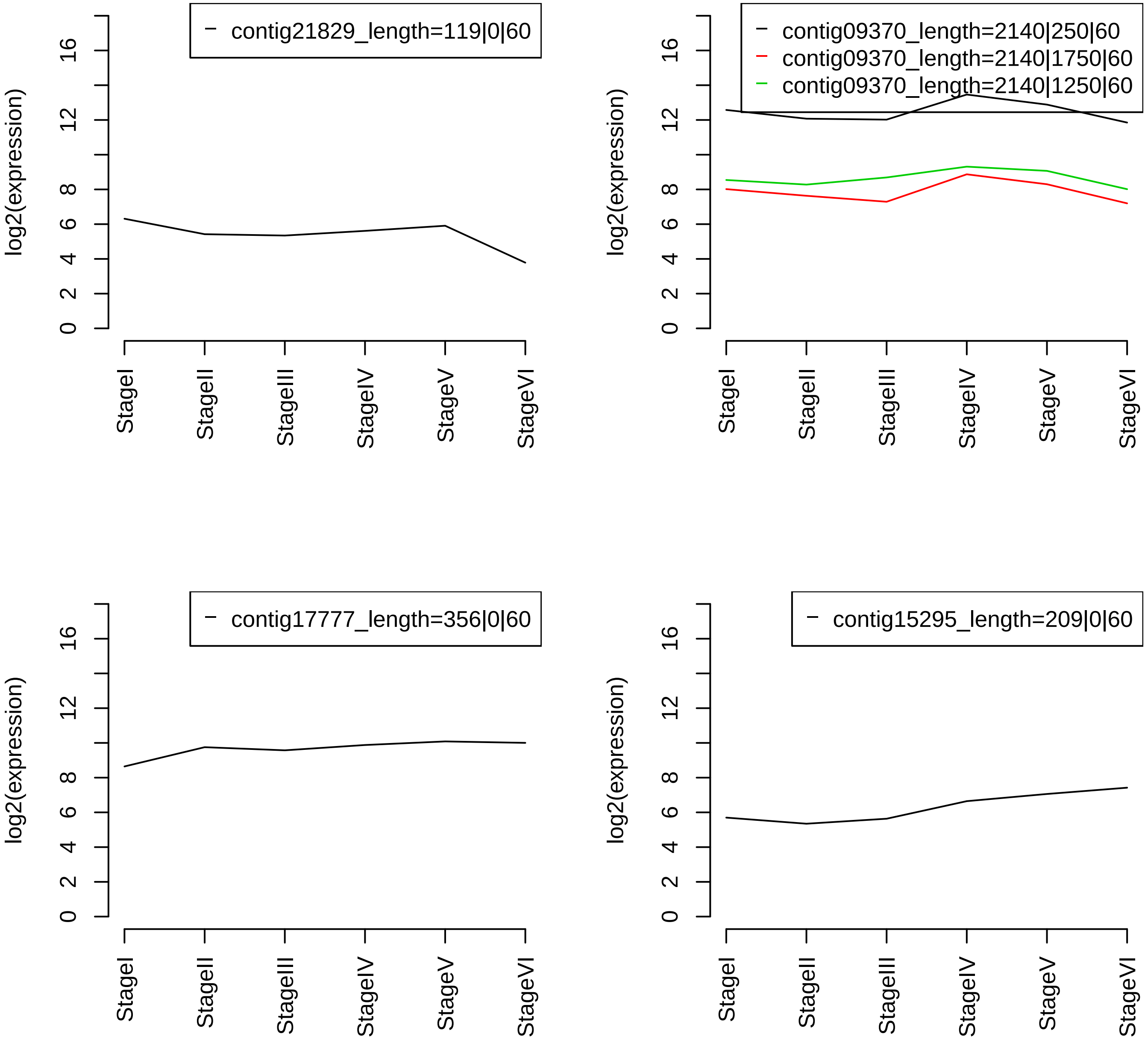
contig21829_length=119|0|60
contig09370_length=2140|250|60
contig09370_length=2140|1750|60
contig09370_length=2140|1250|60
contig17777_length=356|0|60
contig15295_length=209|0|60
log2(expression)
StageI
StageII
StageIII
StageIV
StageV
StageVI

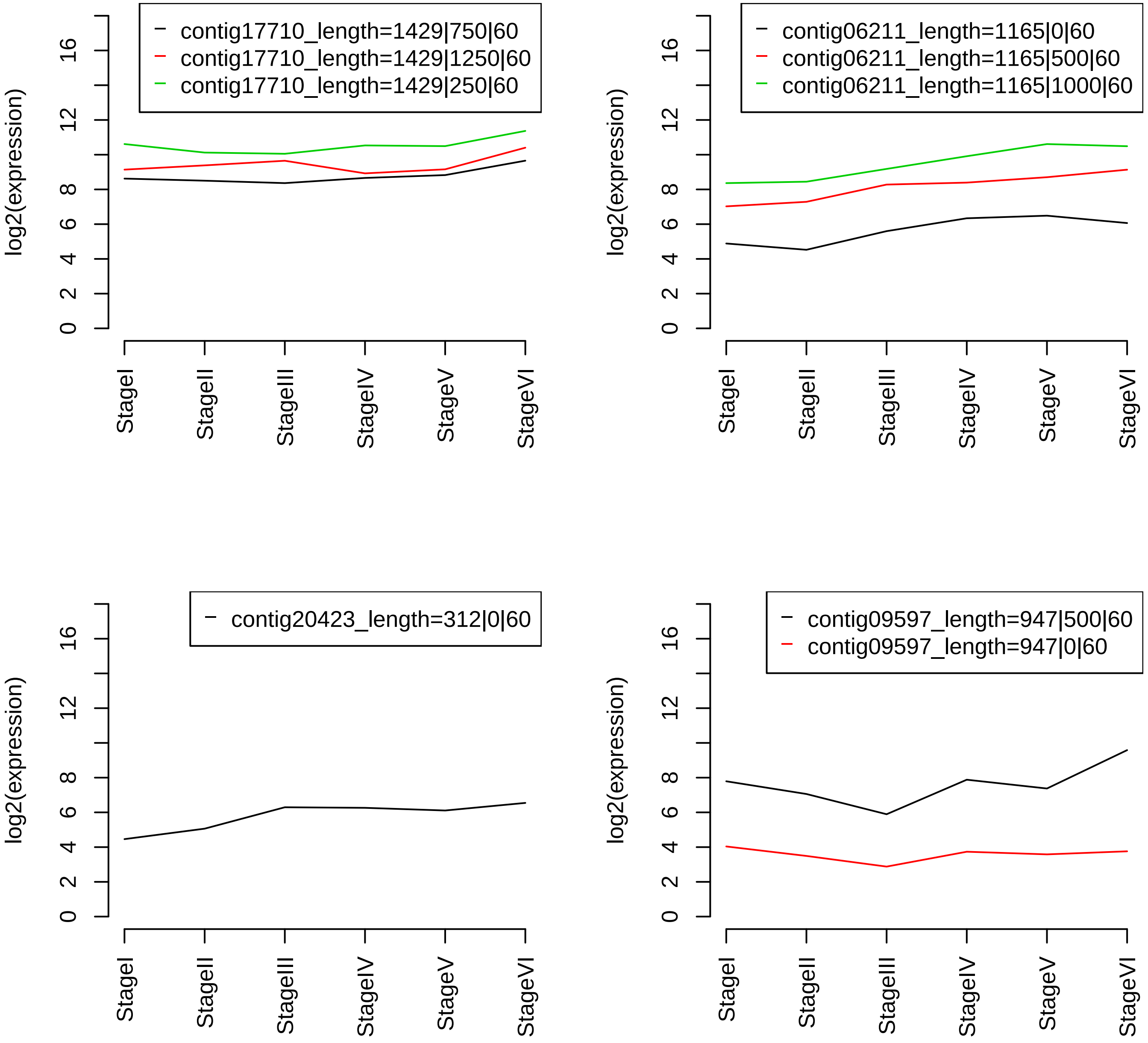

contig17710_length=1429|750|60
contig17710_length=1429|1250|60
contig17710_length=1429|250|60
contig06211_length=1165|0|60
contig06211_length=1165|500|60
contig06211_length=1165|1000|60
contig20423_length=312|0|60
contig09597_length=947|500|60
contig09597_length=947|0|60
log2(expression)
0
2
4
6
8
12
16
StageI
StageII
StageIII
StageIV
StageV
StageVI

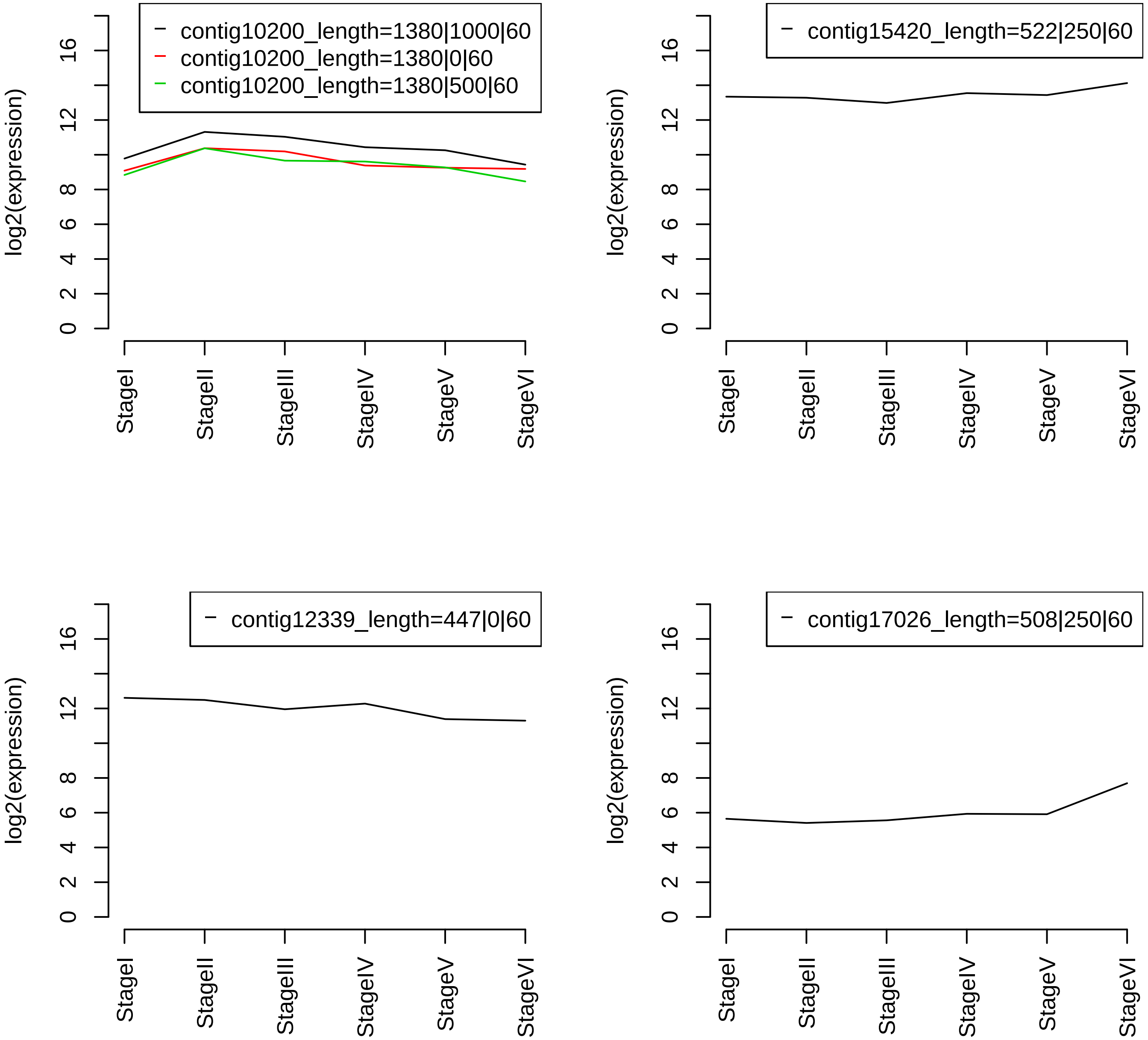

contig10200_length=1380|1000|60
contig10200_length=1380|0|60
contig10200_length=1380|500|60
contig15420_length=522|250|60
contig12339_length=447|0|60
contig17026_length=508|250|60
log2(expression)
0
2
4
6
8
12
16
StageI
StageII
StageIII
StageIV
StageV
StageVI

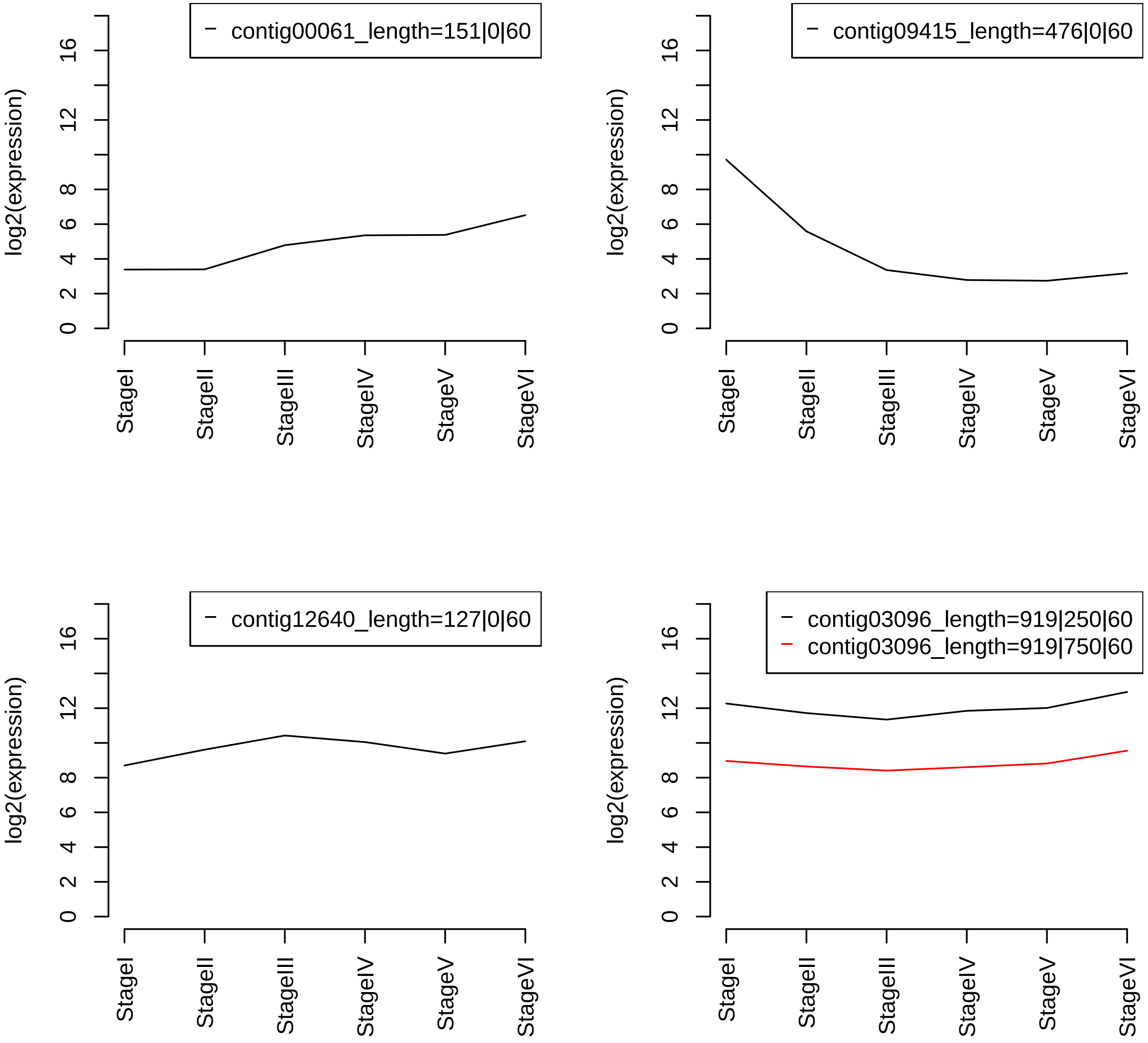
contig00061_length=151|0|60
contig09415_length=476|0|60
contig12640_length=127|0|60
contig03096_length=919|250|60
contig03096_length=919|750|60
log2(expression)
0
2
4
6
8
12
16
StageI
StageII
StageIII
StageIV
StageV
StageVI

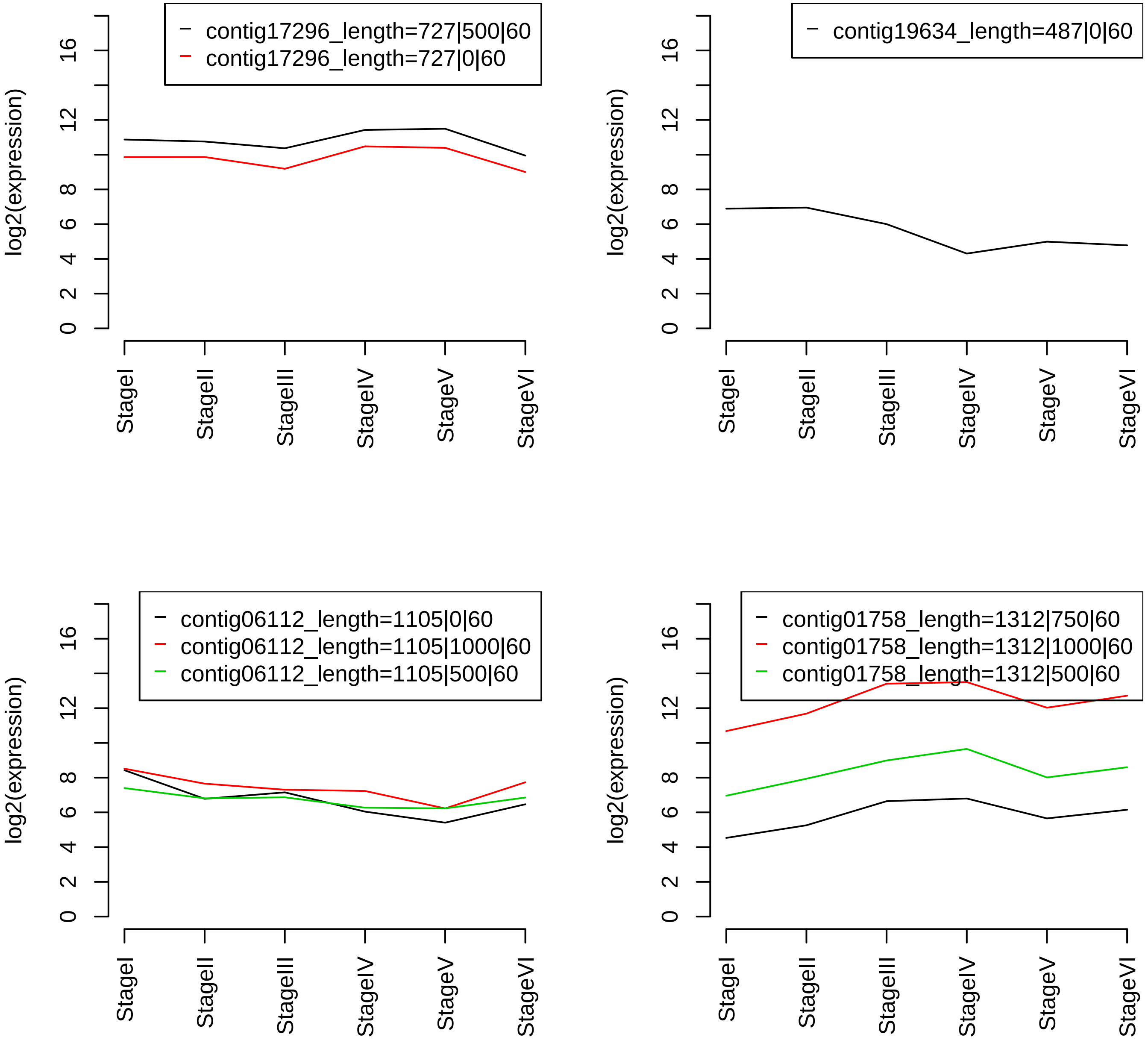

contig17296_length=727|500|60
contig17296_length=727|0|60
contig19634_length=487|0|60
contig06112_length=1105|0|60
contig06112_length=1105|1000|60
contig06112_length=1105|500|60
contig01758_length=1312|750|60
contig01758_length=1312|1000|60
contig01758_length=1312|500|60
log2(expression)
0
2
4
6
8
12
16
StageI
StageII
StageIII
StageIV
StageV
StageVI

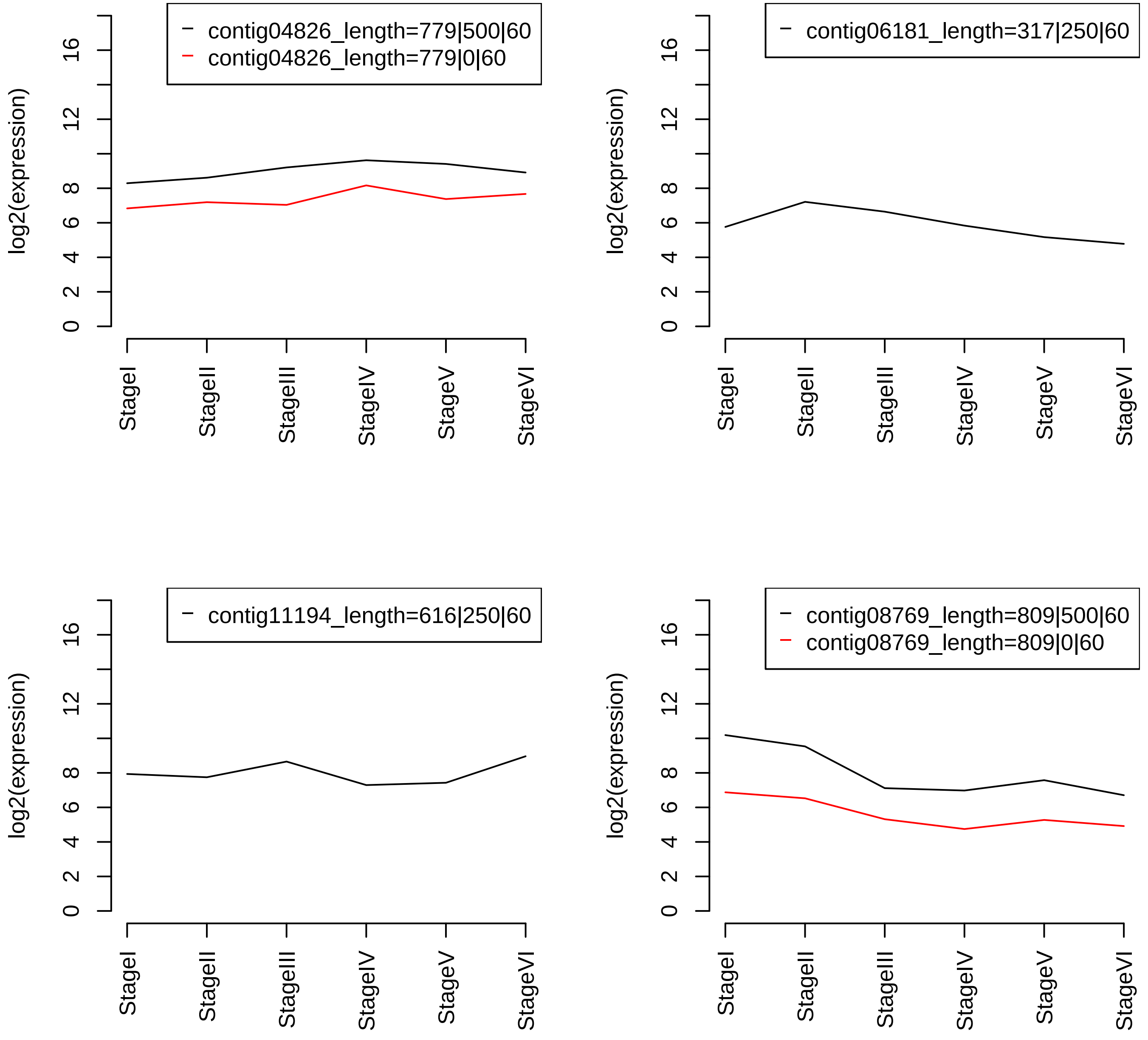

contig04826_length=779|500|60
contig04826_length=779|0|60
log2(expression)
0
2
4
6
8
12
16
StageI
StageII
StageIII
StageIV
StageV
StageVI
contig06181_length=317|250|60
log2(expression)
0
2
4
6
8
12
16
StageI
StageII
StageIII
StageIV
StageV
StageVI
contig11194_length=616|250|60
log2(expression)
0
2
4
6
8
12
16
StageI
StageII
StageIII
StageIV
StageV
StageVI
contig08769_length=809|500|60
contig08769_length=809|0|60
log2(expression)
0
2
4
6
8
12
16
StageI
StageII
StageIII
StageIV
StageV
StageVI

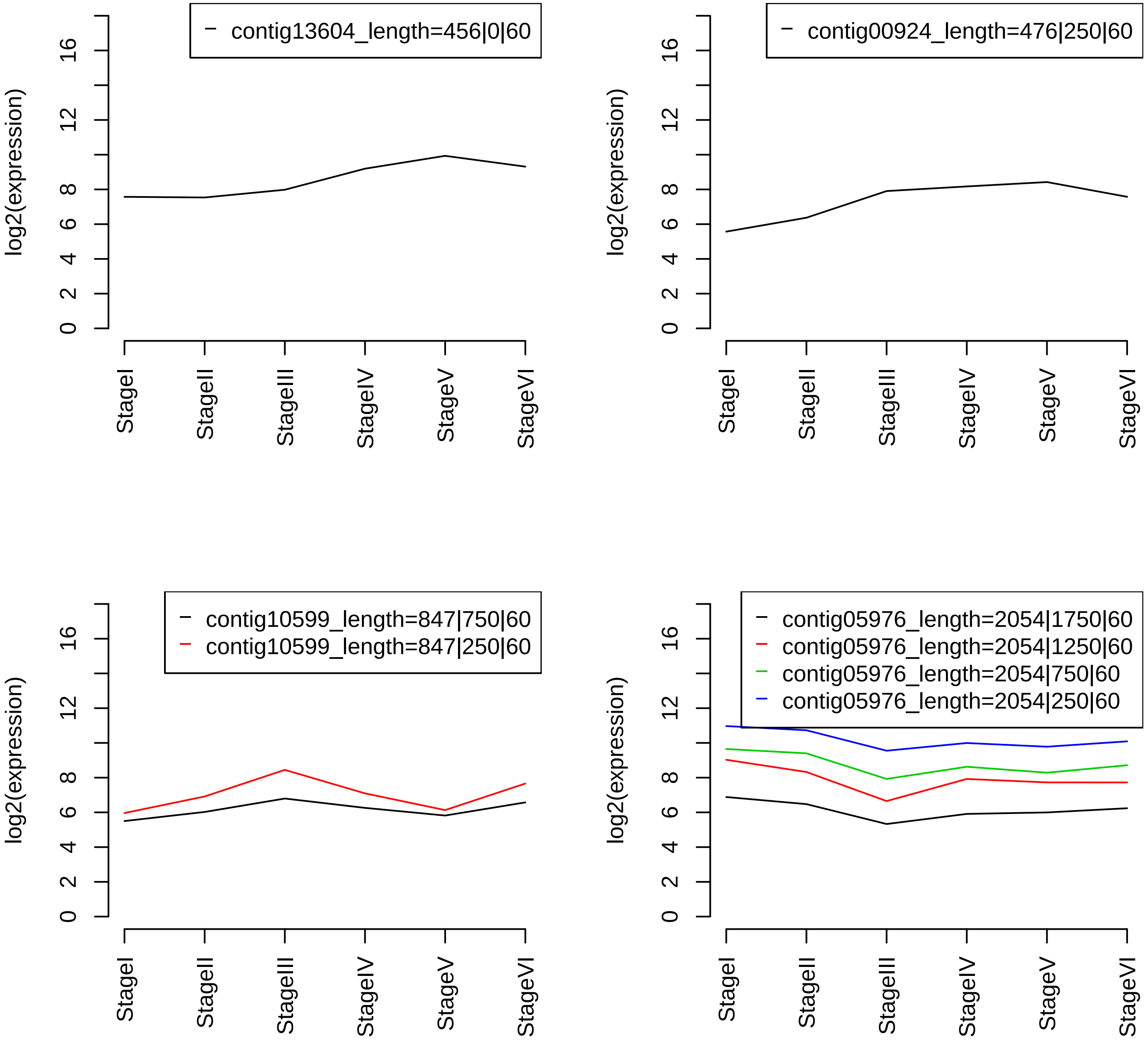
contig13604_length=456|0|60
contig00924_length=476|250|60
contig10599_length=847|750|60
contig10599_length=847|250|60
contig05976_length=2054|1750|60
contig05976_length=2054|1250|60
contig05976_length=2054|750|60
contig05976_length=2054|250|60
log2(expression)
0
2
4
6
8
12
16
StageI
StageII
StageIII
StageIV
StageV
StageVI

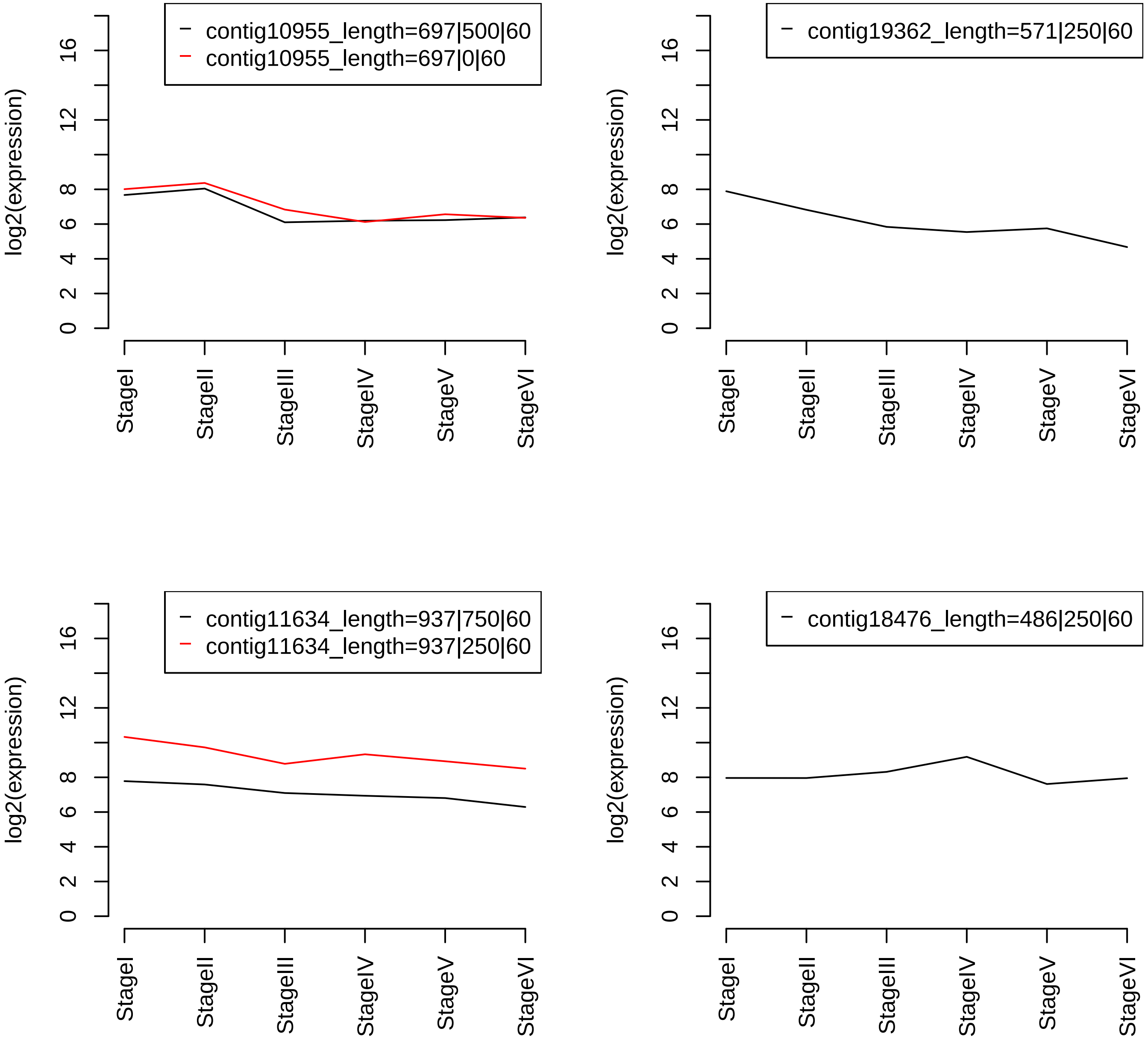
contig10955_length=697|500|60
contig10955_length=697|0|60
contig19362_length=571|250|60
contig11634_length=937|750|60
contig11634_length=937|250|60
contig18476_length=486|250|60
log2(expression)
0
2
4
6
8
12
16
StageI
StageII
StageIII
StageIV
StageV
StageVI

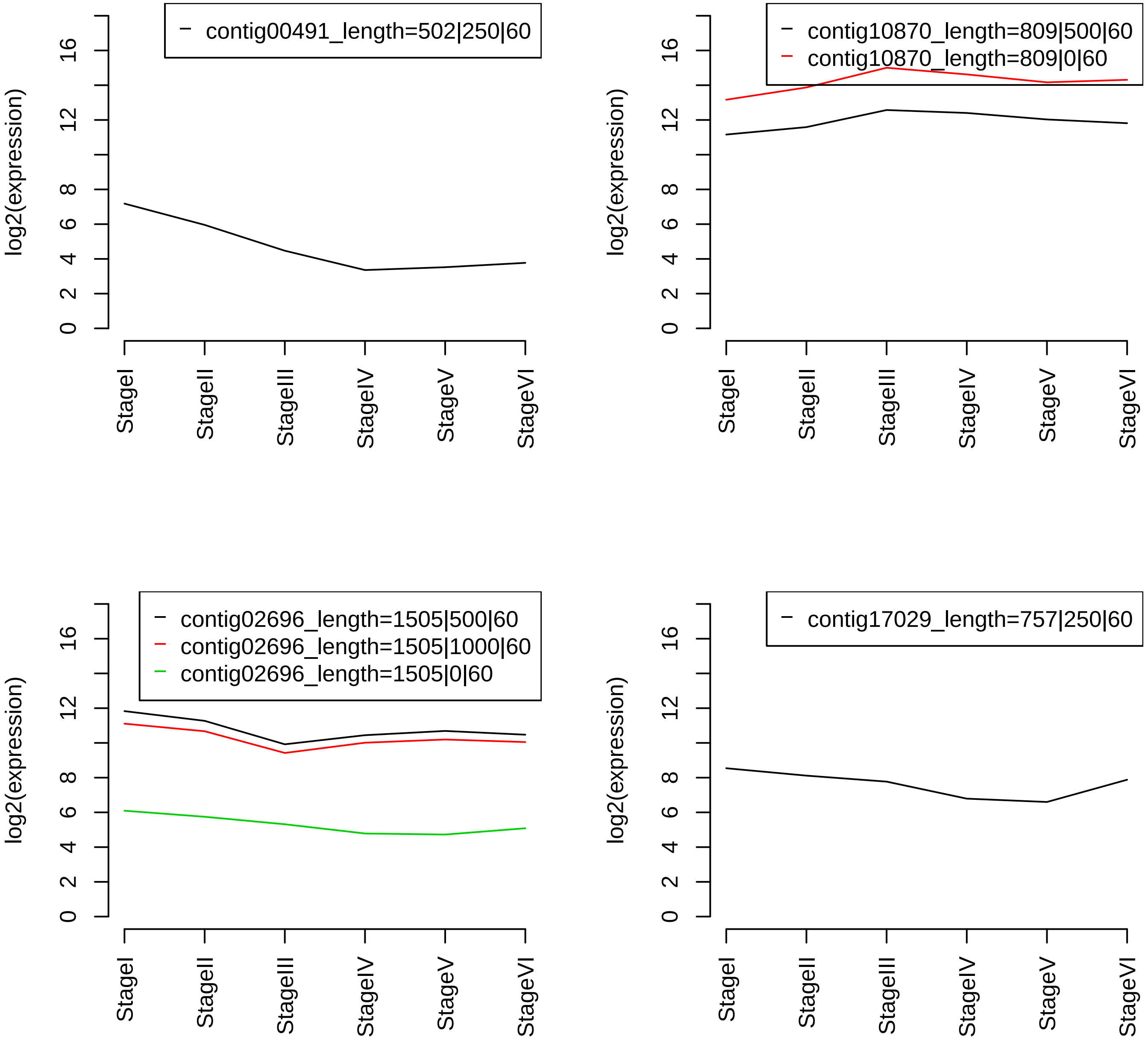
contig00491_length=502|250|60
contig10870_length=809|500|60
contig10870_length=809|0|60
contig02696_length=1505|500|60
contig02696_length=1505|1000|60
contig02696_length=1505|0|60
contig17029_length=757|250|60
log2(expression)
0
2
4
6
8
12
16
StageI
StageII
StageIII
StageIV
StageV
StageVI

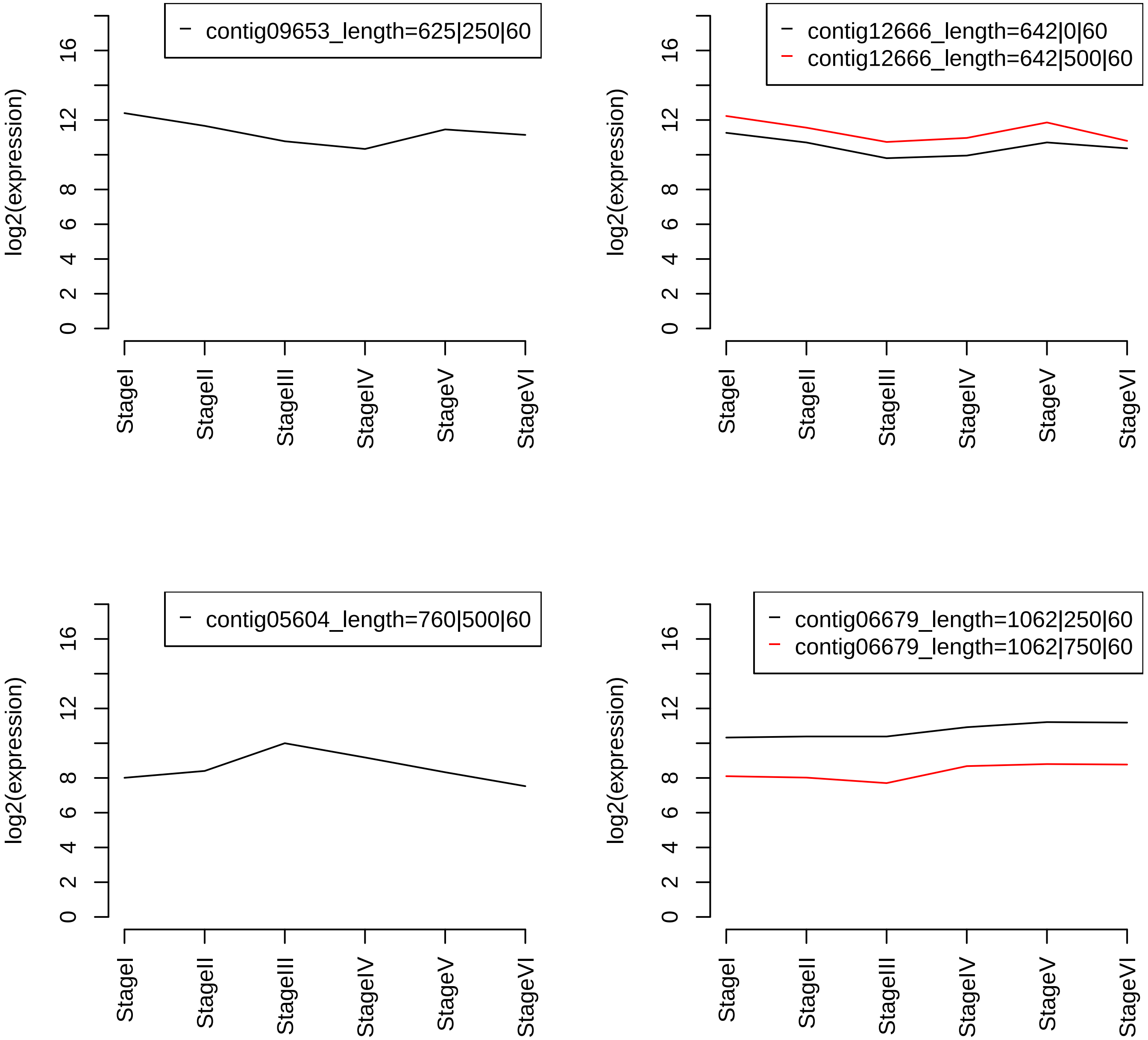
contig09653_length=625|250|60
contig12666_length=642|0|60
contig12666_length=642|500|60
contig05604_length=760|500|60
contig06679_length=1062|250|60
contig06679_length=1062|750|60
log2(expression)
0
2
4
6
8
12
16
StageI
StageII
StageIII
StageIV
StageV
StageVI

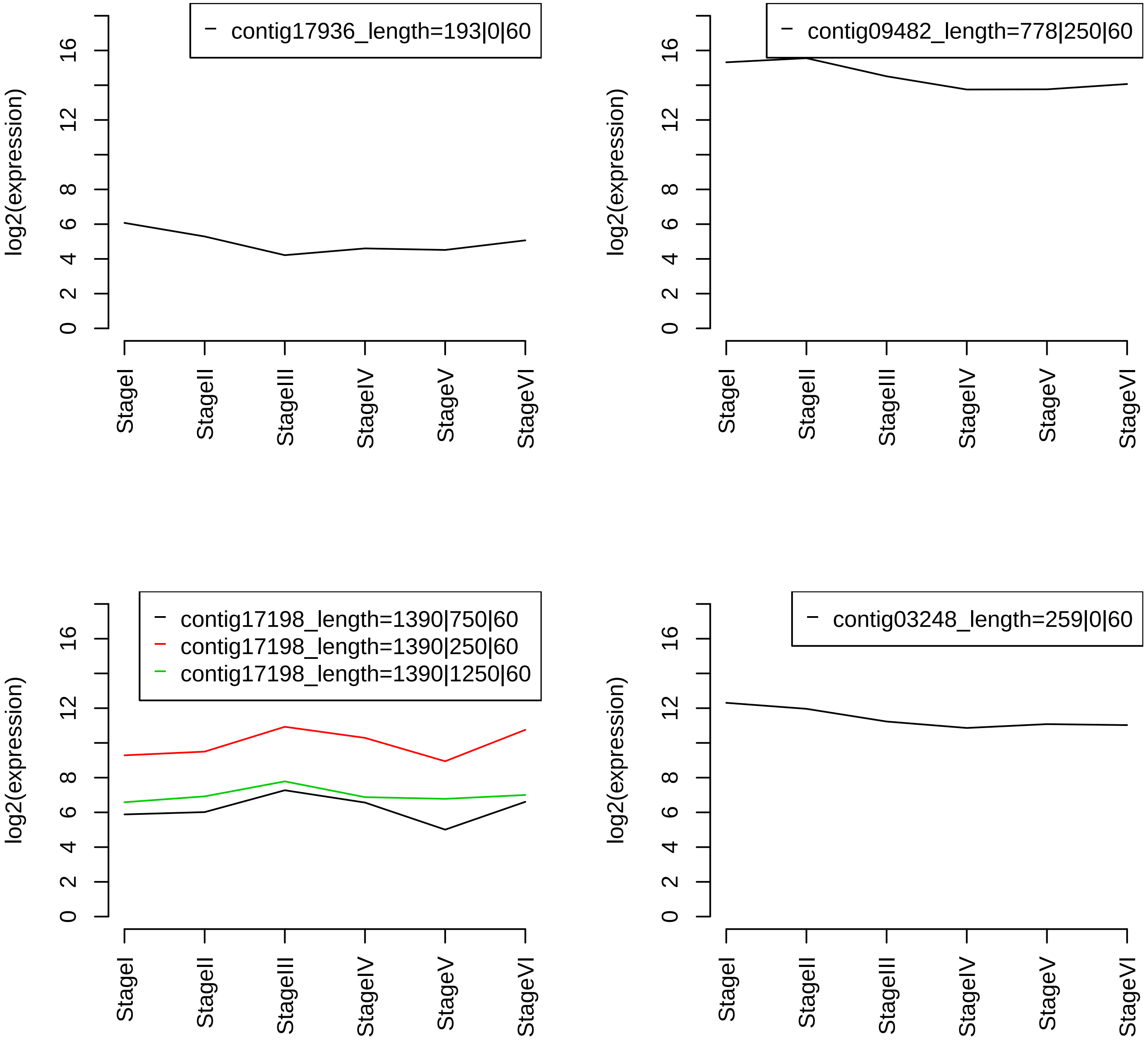

contig17936_length=193|0|60
contig09482_length=778|250|60
contig17198_length=1390|750|60
contig17198_length=1390|250|60
contig17198_length=1390|1250|60
contig03248_length=259|0|60
log2(expression)
0
2
4
6
8
12
16
StageI
StageII
StageIII
StageIV
StageV
StageVI

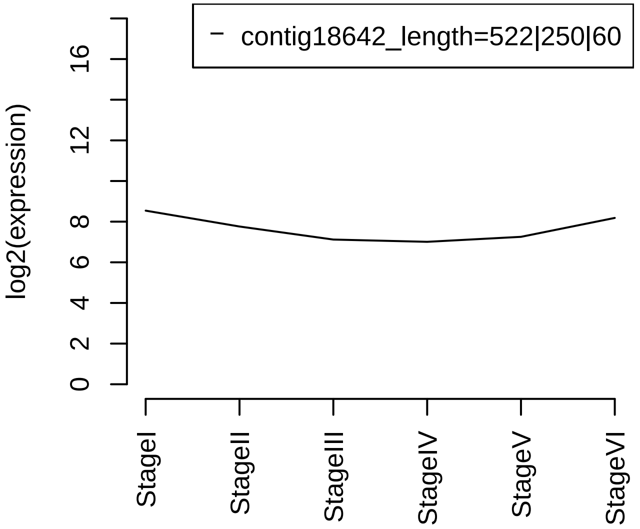
contig18642_length=522|250|60
log2(expression)
0
2
4
6
8
12
16
StageI
StageII
StageIII
StageIV
StageV
StageVI

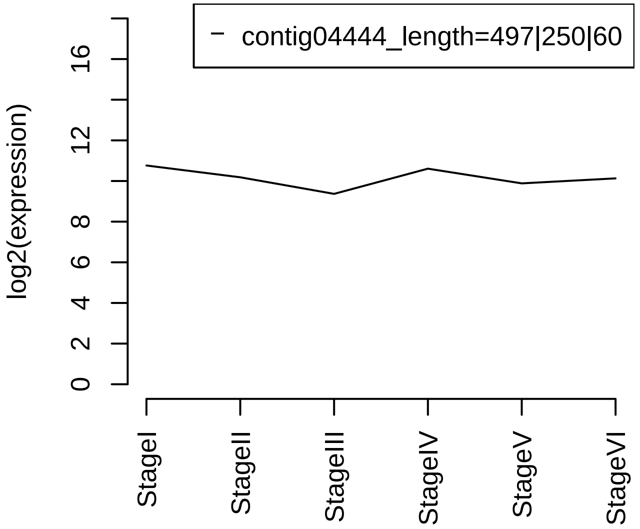
contig04444_length=497|250|60
log2(expression)
0
2
4
6
8
12
16
StageI
StageII
StageIII
StageIV
StageV
StageVI

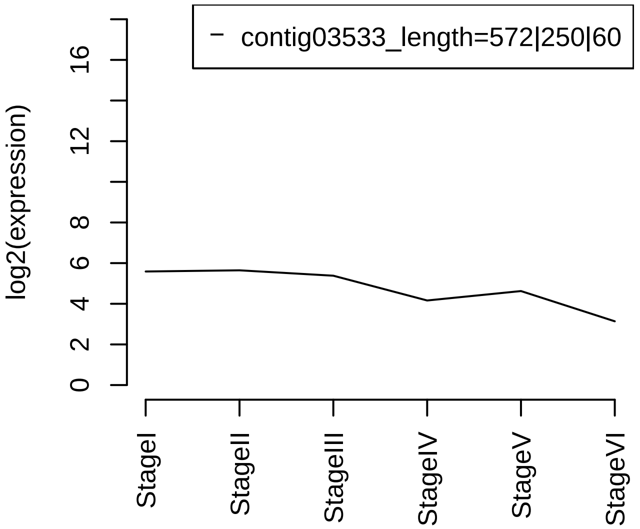
contig03533_length=572|250|60
log2(expression)
0
2
4
6
8
12
16
StageI
StageII
StageIII
StageIV
StageV
StageVI

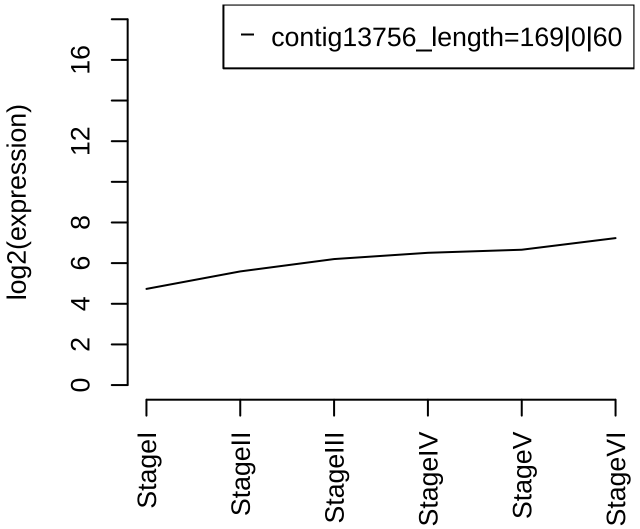
contig13756_length=169|0|60
log2(expression)
0
2
4
6
8
12
16
StageI
StageII
StageIII
StageIV
StageV
StageVI

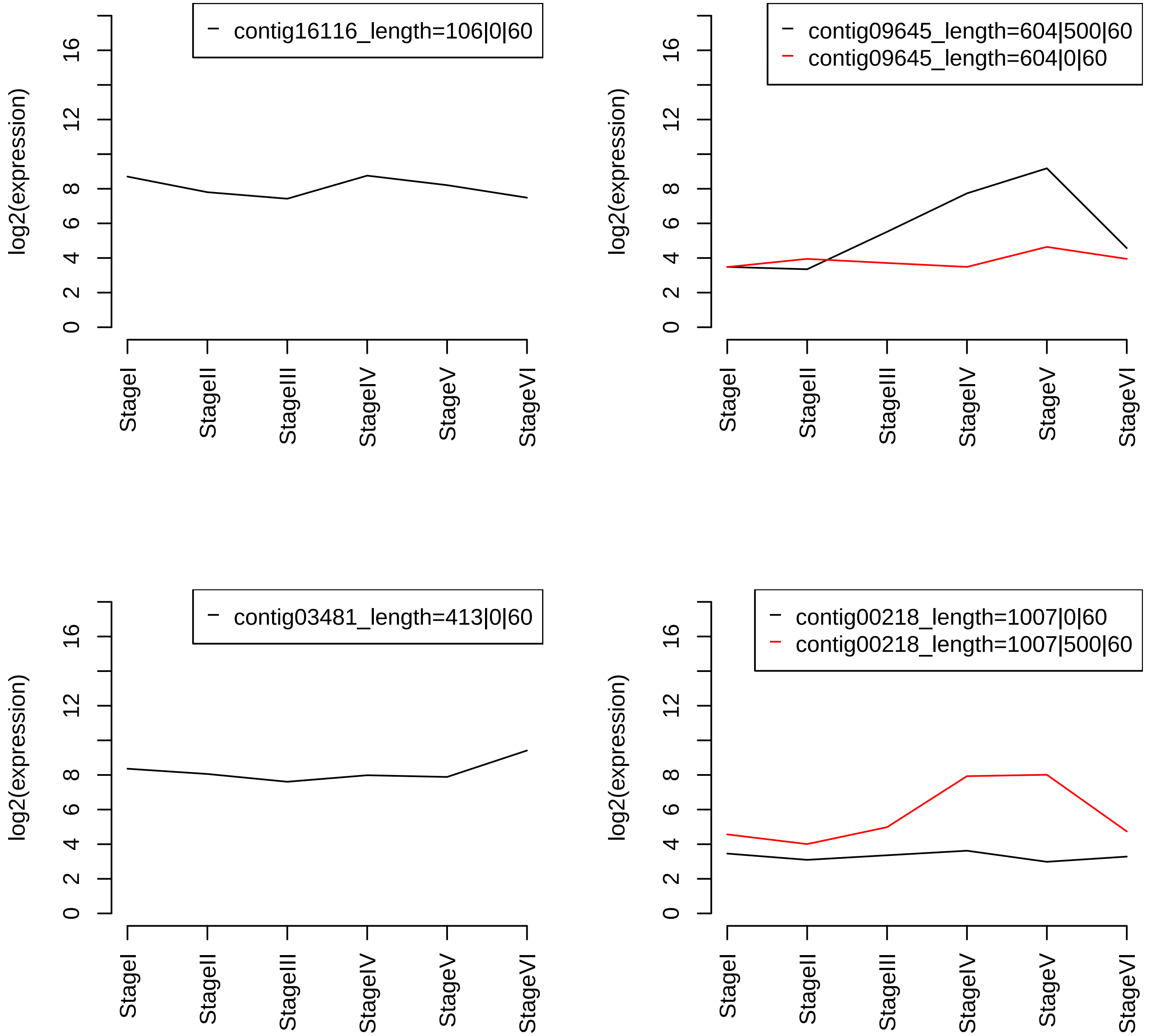

contig16116_length=106|0|60
contig09645_length=604|500|60
contig09645_length=604|0|60
contig03481_length=413|0|60
contig00218_length=1007|0|60
contig00218_length=1007|500|60
log2(expression)
0
2
4
6
8
12
16
StageI
StageII
StageIII
StageIV
StageV
StageVI

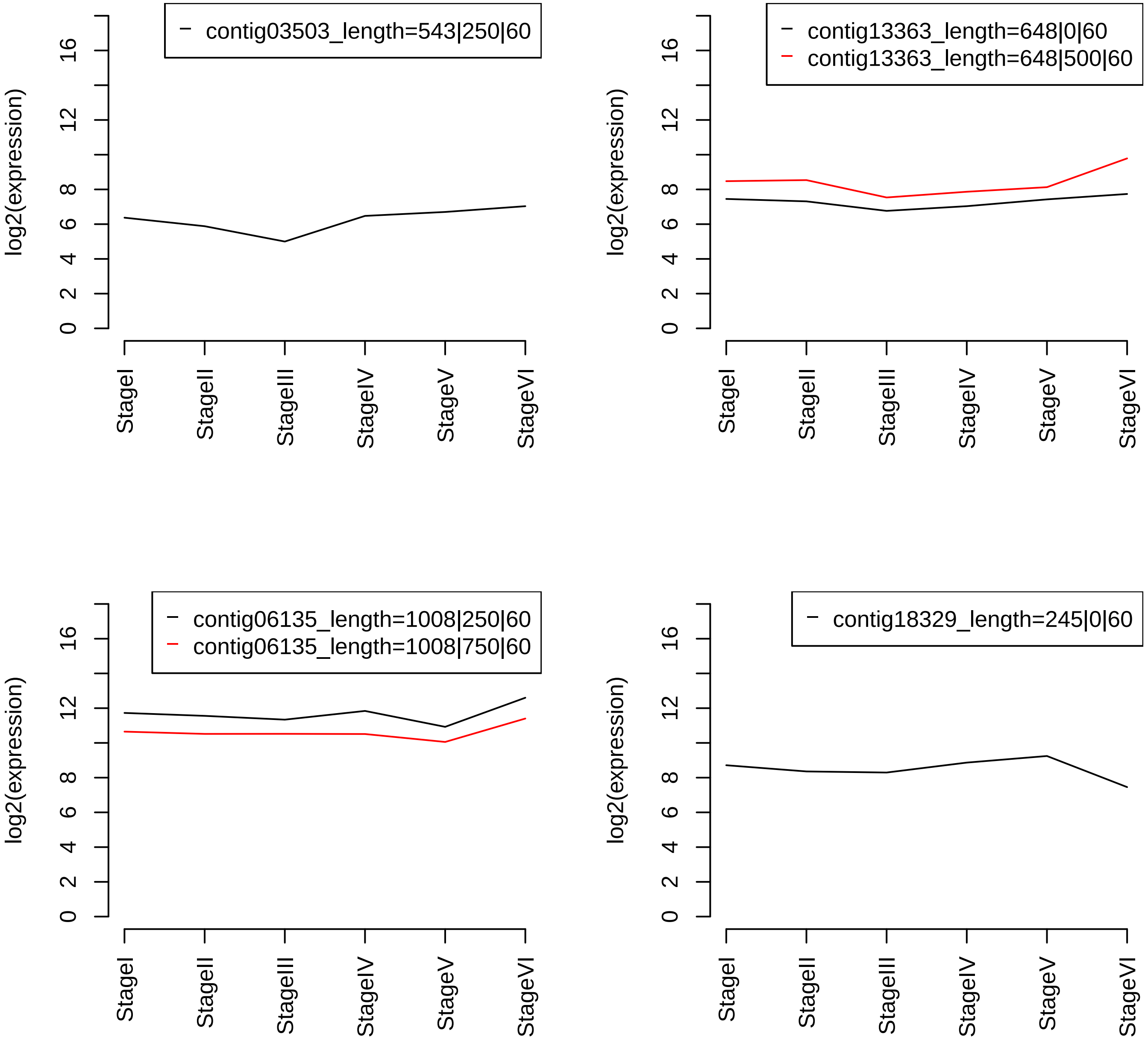

contig03503_length=543|250|60
contig13363_length=648|0|60
contig13363_length=648|500|60
contig06135_length=1008|250|60
contig06135_length=1008|750|60
contig18329_length=245|0|60
log2(expression)
0
2
4
6
8
12
16
StageI
StageII
StageIII
StageIV
StageV
StageVI

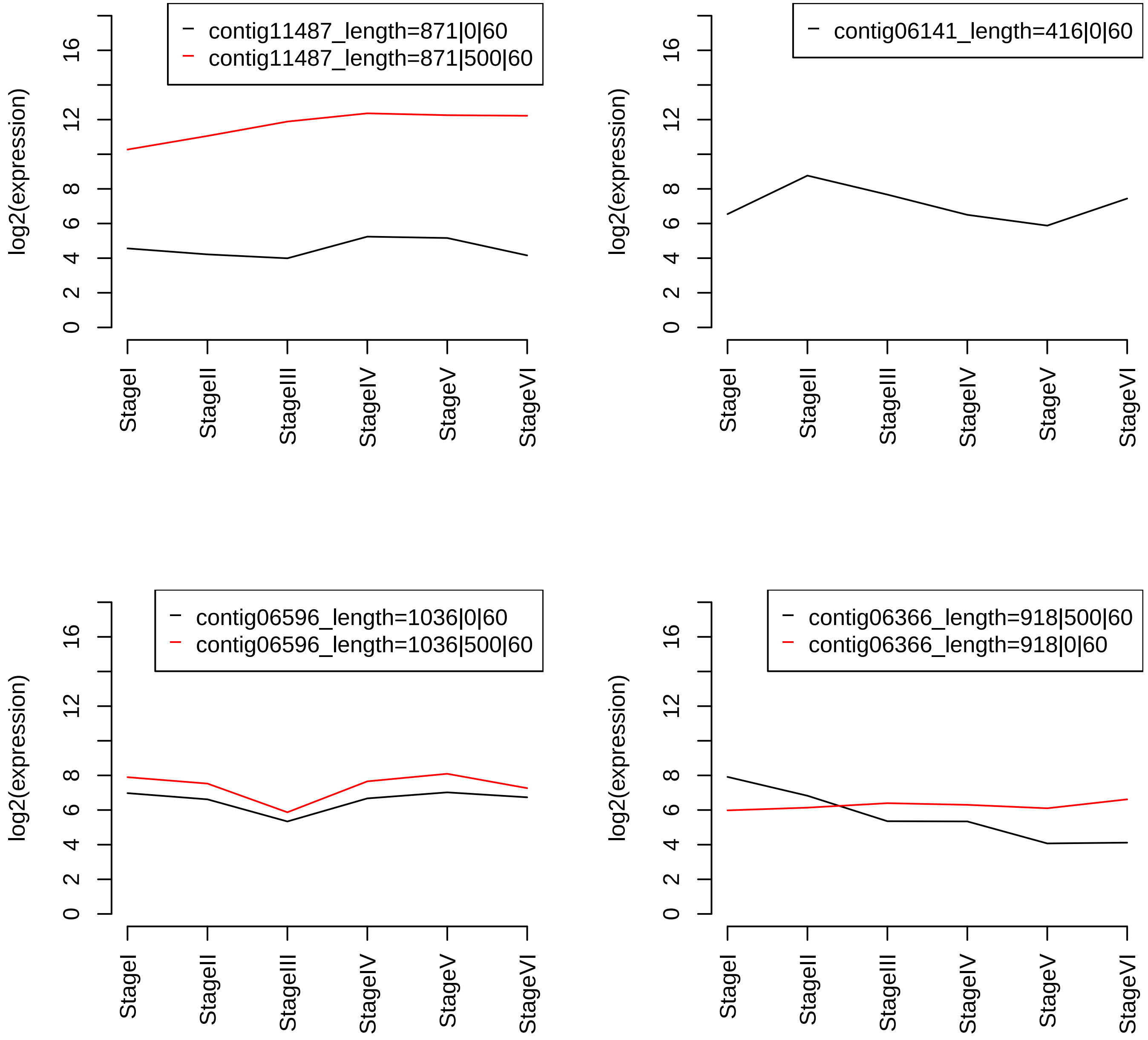
contig11487_length=871|0|60
contig11487_length=871|500|60
contig06141_length=416|0|60
contig06596_length=1036|0|60
contig06596_length=1036|500|60
contig06366_length=918|500|60
contig06366_length=918|0|60
log2(expression)
0
2
4
6
8
12
16
StageI
StageII
StageIII
StageIV
StageV
StageVI

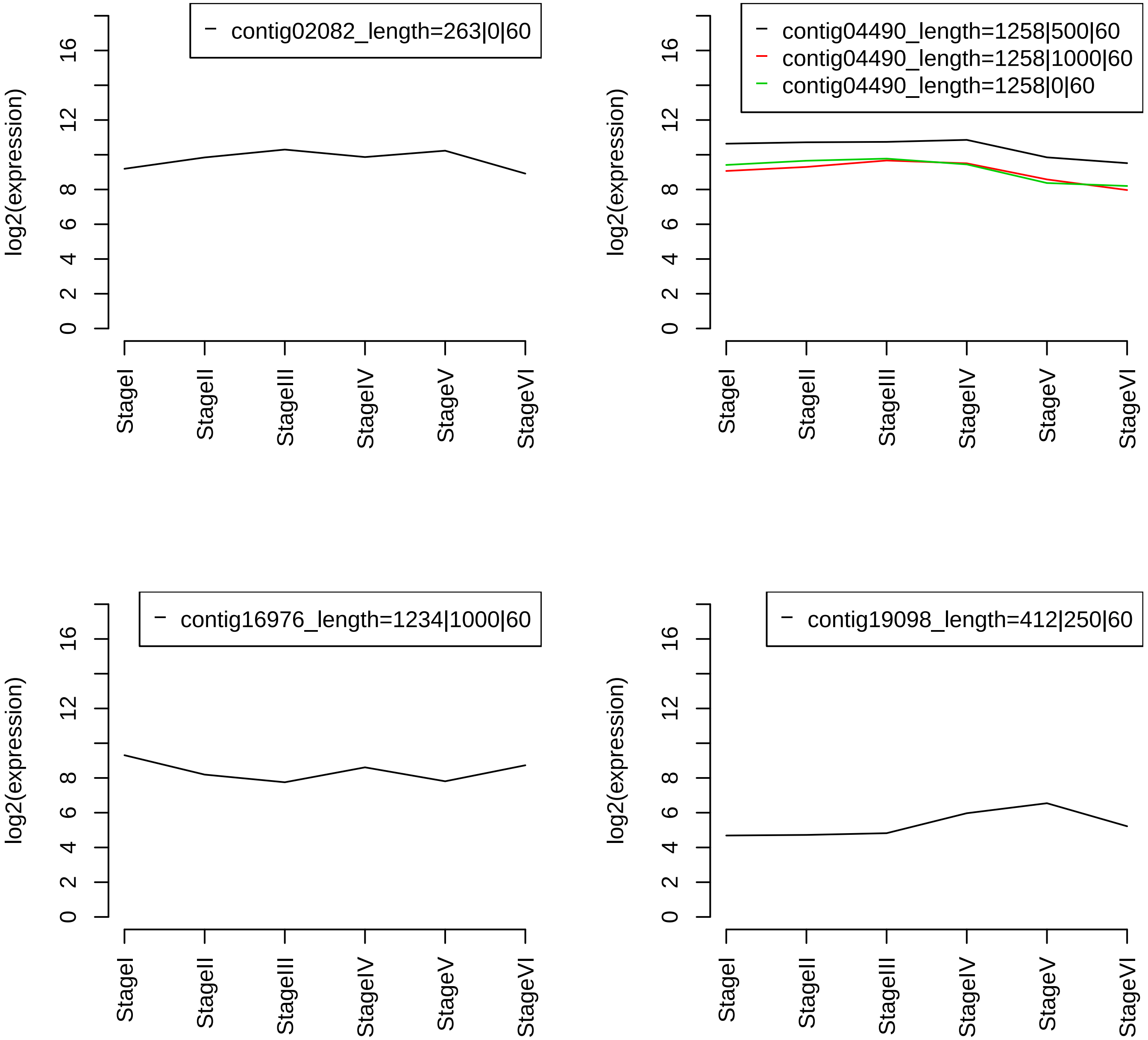

contig02082_length=263|0|60
contig04490_length=1258|500|60
contig04490_length=1258|1000|60
contig04490_length=1258|0|60
contig16976_length=1234|1000|60
contig19098_length=412|250|60
log2(expression)
0 2 4 6 8 12 16
StageI StageII StageIII StageIV StageV StageVI

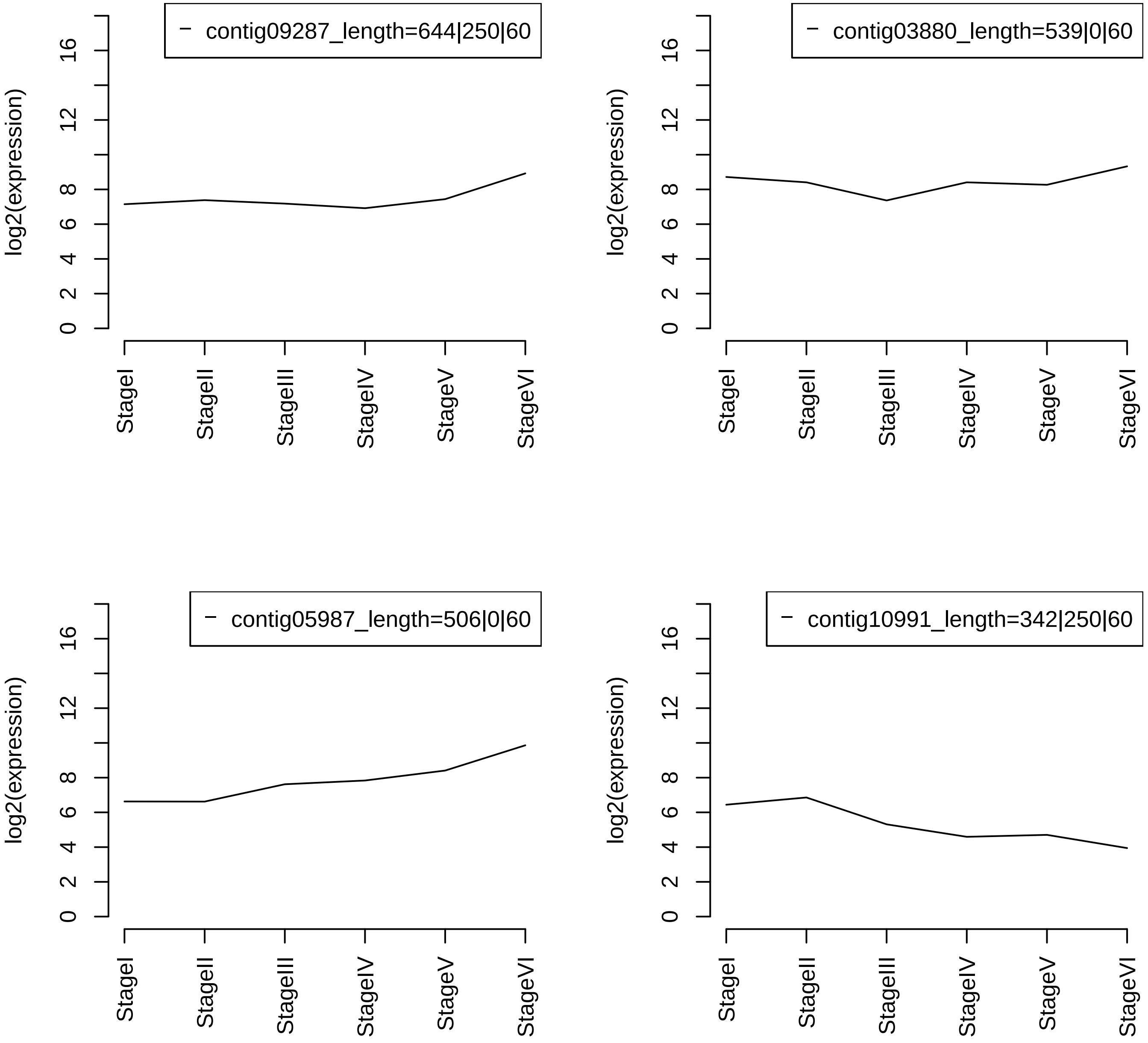

contig09287_length=644|250|60
contig03880_length=539|0|60
contig05987_length=506|0|60
contig10991_length=342|250|60
log2(expression)
0
2
4
6
8
12
16
StageI
StageII
StageIII
StageIV
StageV
StageVI

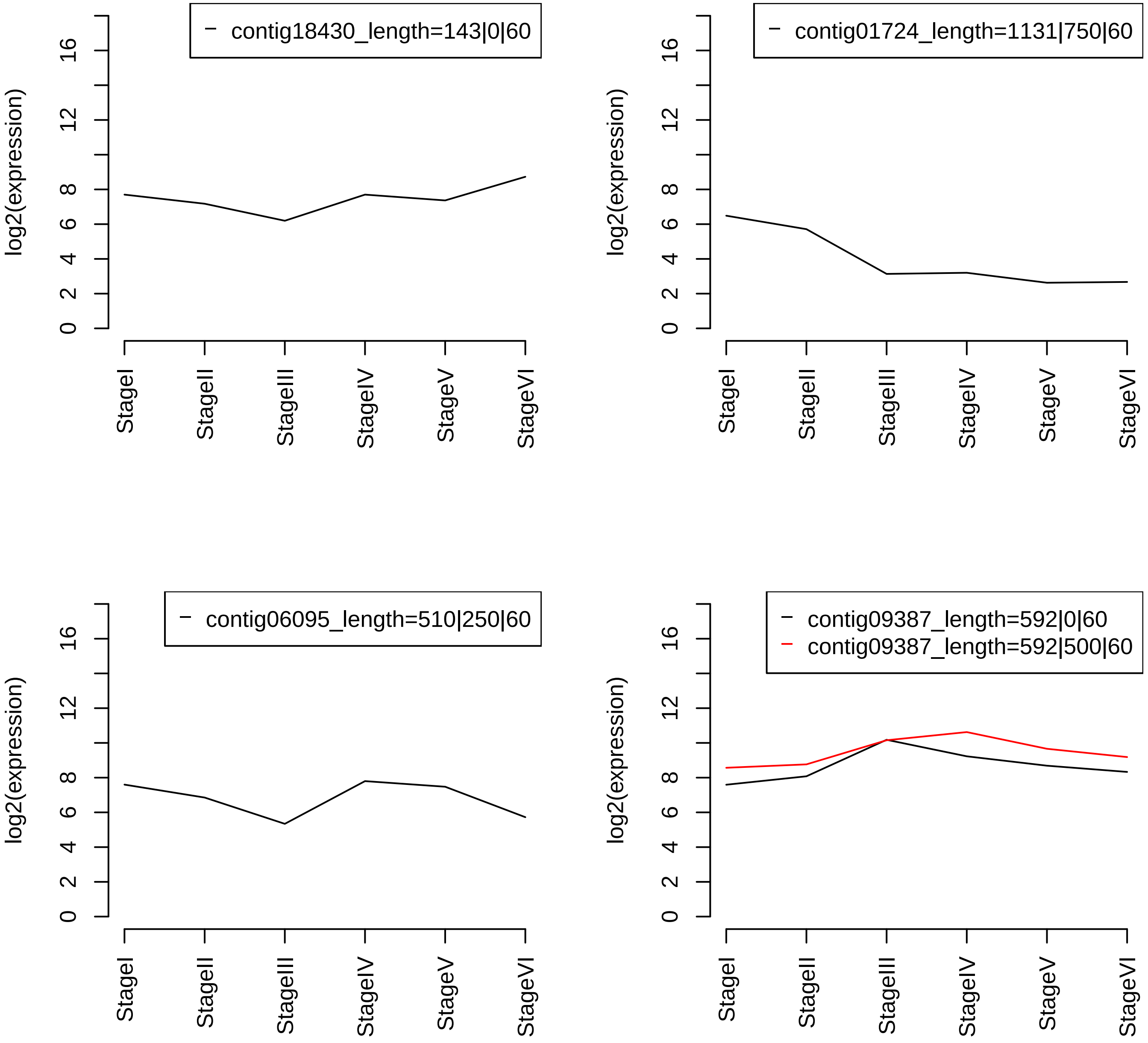
contig18430_length=143|0|60
contig01724_length=1131|750|60
contig06095_length=510|250|60
contig09387_length=592|0|60
contig09387_length=592|500|60
log2(expression)
0 2 4 6 8 12 16
StageI StageII StageIII StageIV StageV StageVI

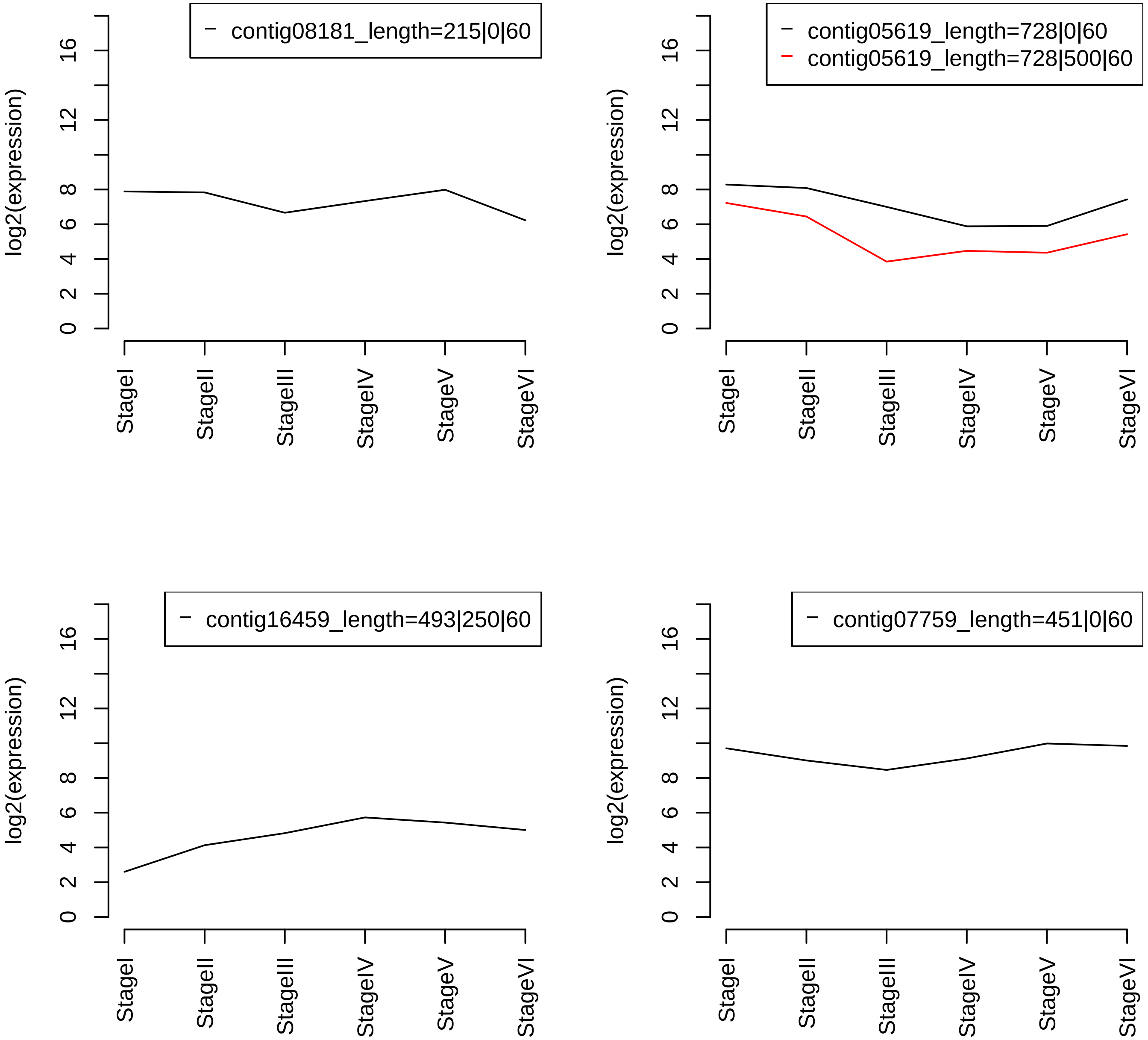

contig08181_length=215|0|60
contig05619_length=728|0|60
contig05619_length=728|500|60
contig16459_length=493|250|60
contig07759_length=451|0|60
log2(expression)
0
2
4
6
8
12
16
StageI
StageII
StageIII
StageIV
StageV
StageVI

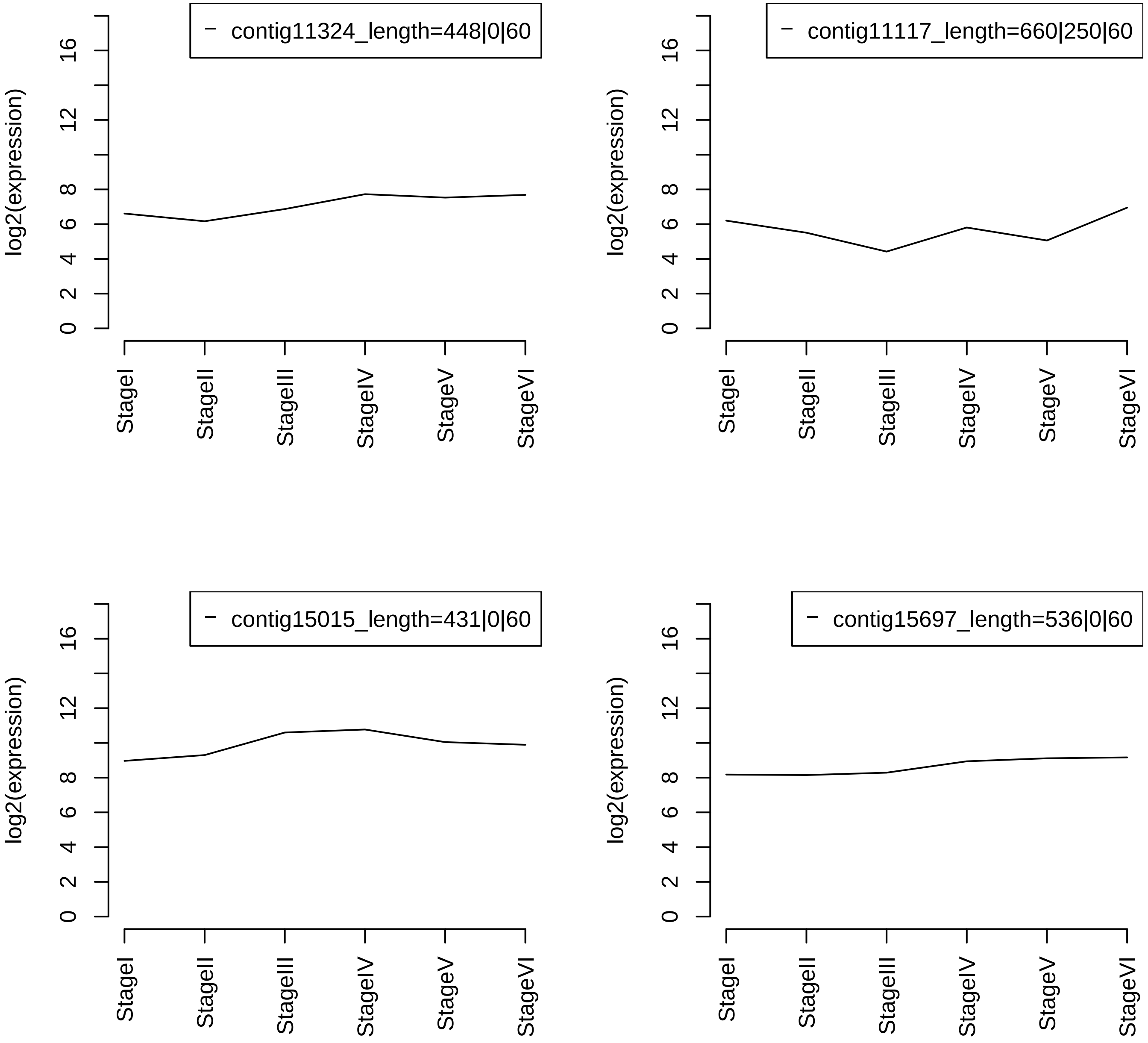
contig11324_length=448|0|60
contig11117_length=660|250|60
contig15015_length=431|0|60
contig15697_length=536|0|60
log2(expression)
0
2
4
6
8
12
16
StageI
StageII
StageIII
StageIV
StageV
StageVI

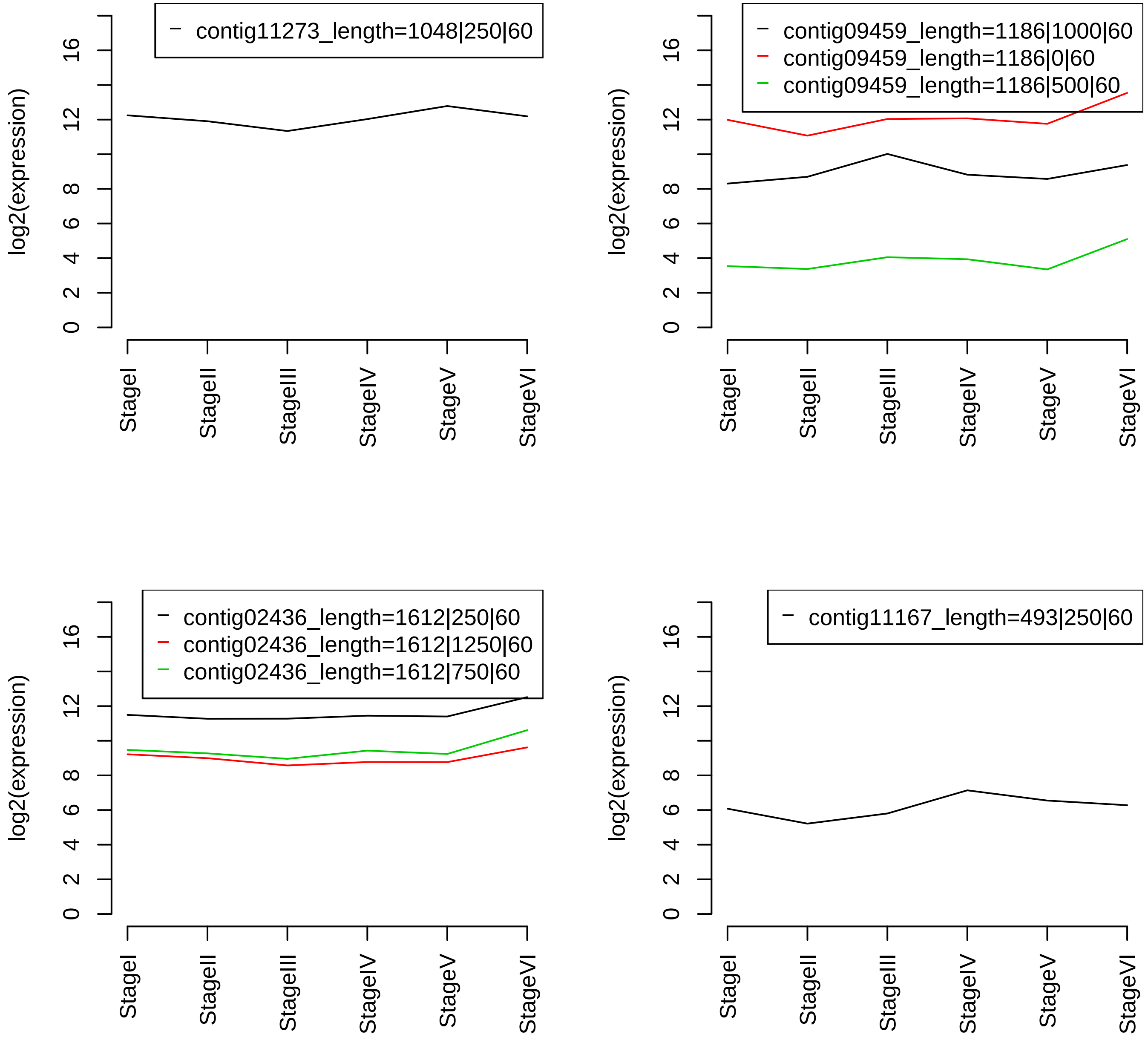

contig11273_length=1048|250|60
contig09459_length=1186|1000|60
contig09459_length=1186|0|60
contig09459_length=1186|500|60
contig02436_length=1612|250|60
contig02436_length=1612|1250|60
contig02436_length=1612|750|60
contig11167_length=493|250|60
log2(expression)
0
2
4
6
8
12
16
StageI
StageII
StageIII
StageIV
StageV
StageVI

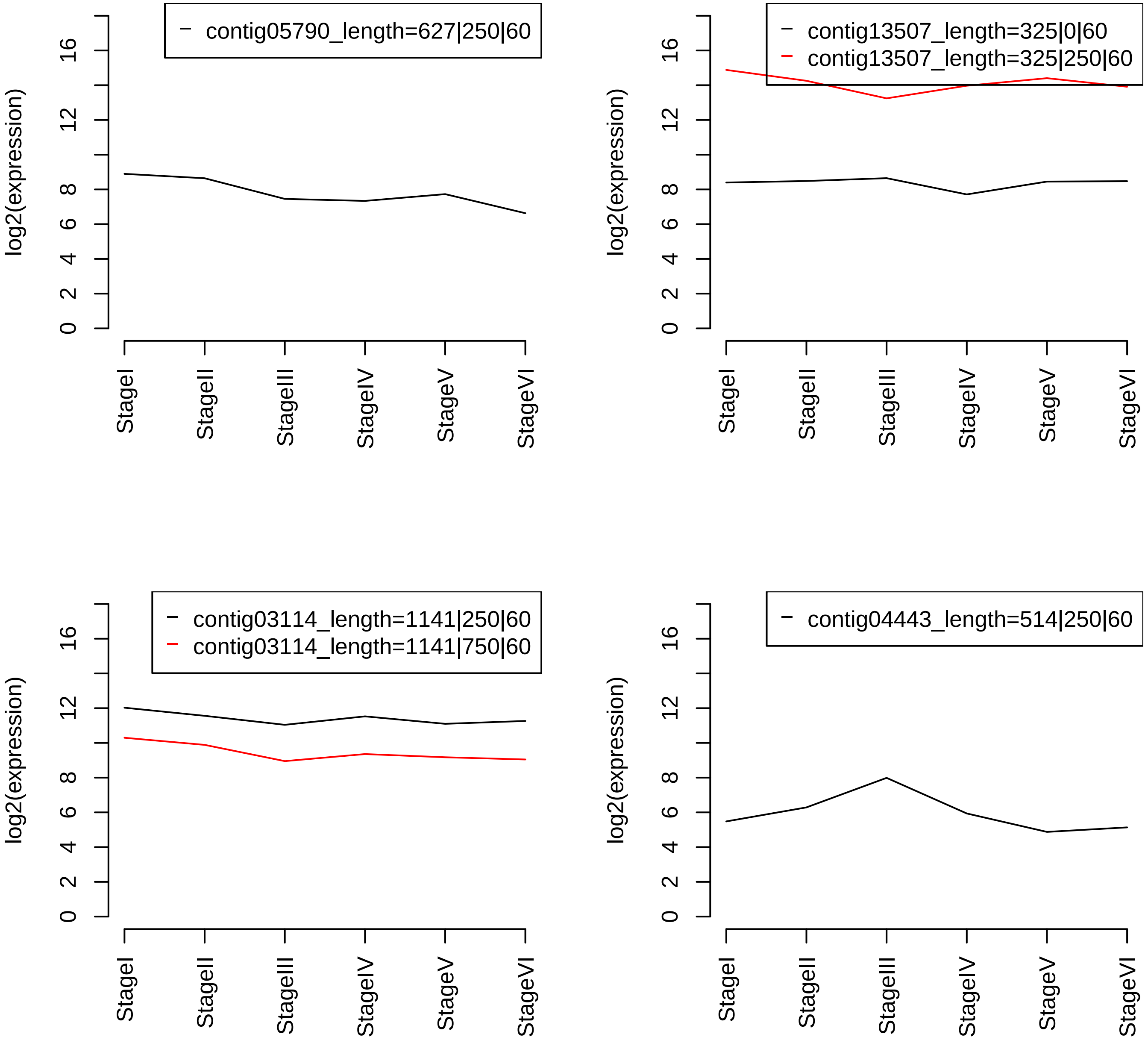

contig05790_length=627|250|60
contig13507_length=325|0|60
contig13507_length=325|250|60
contig03114_length=1141|250|60
contig03114_length=1141|750|60
contig04443_length=514|250|60
log2(expression)
0
2
4
6
8
12
16
StageI
StageII
StageIII
StageIV
StageV
StageVI

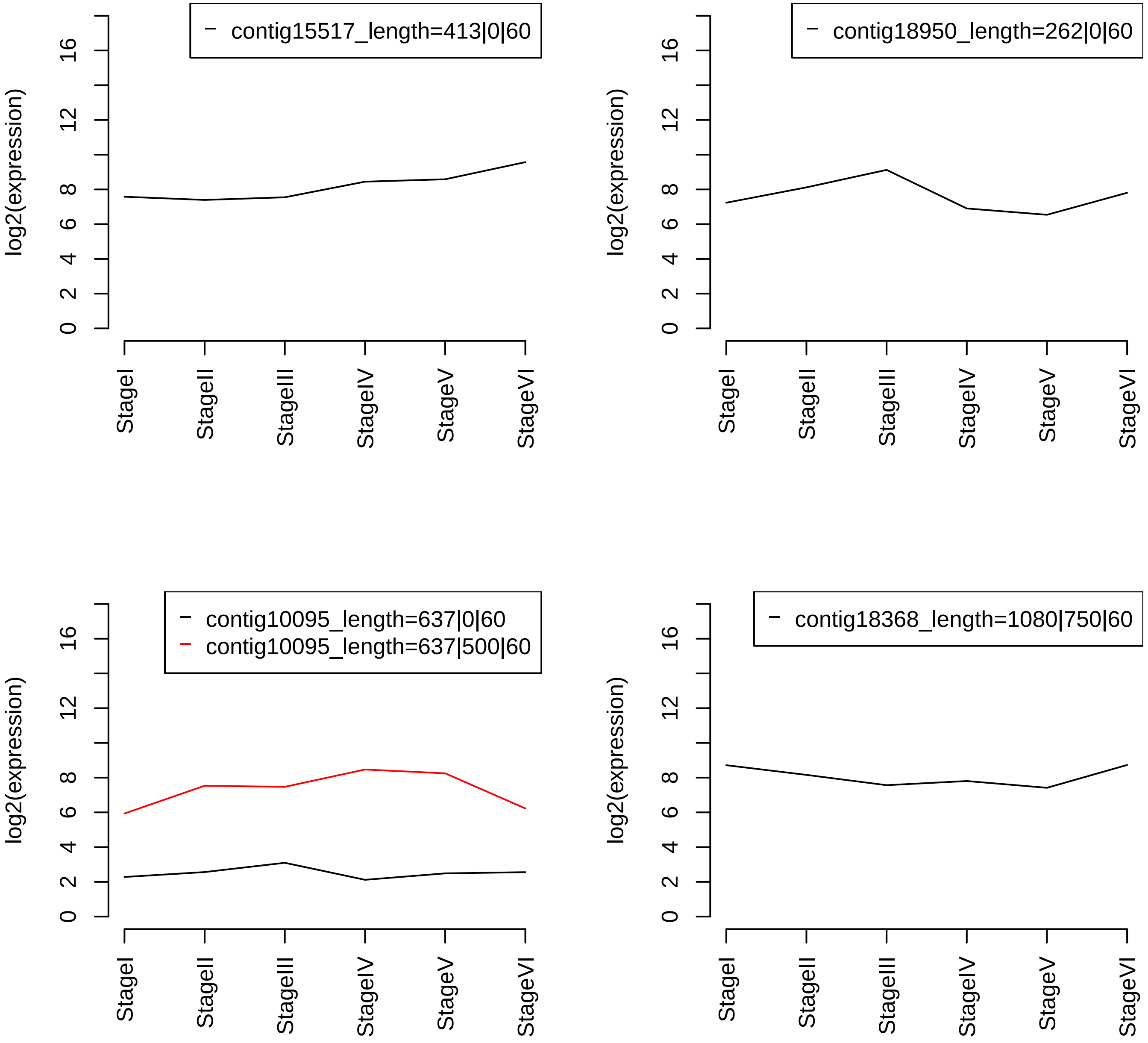
contig15517_length=413|0|60
contig18950_length=262|0|60
contig10095_length=637|0|60
contig10095_length=637|500|60
contig18368_length=1080|750|60
log2(expression)
0
2
4
6
8
12
16
StageI
StageII
StageIII
StageIV
StageV
StageVI

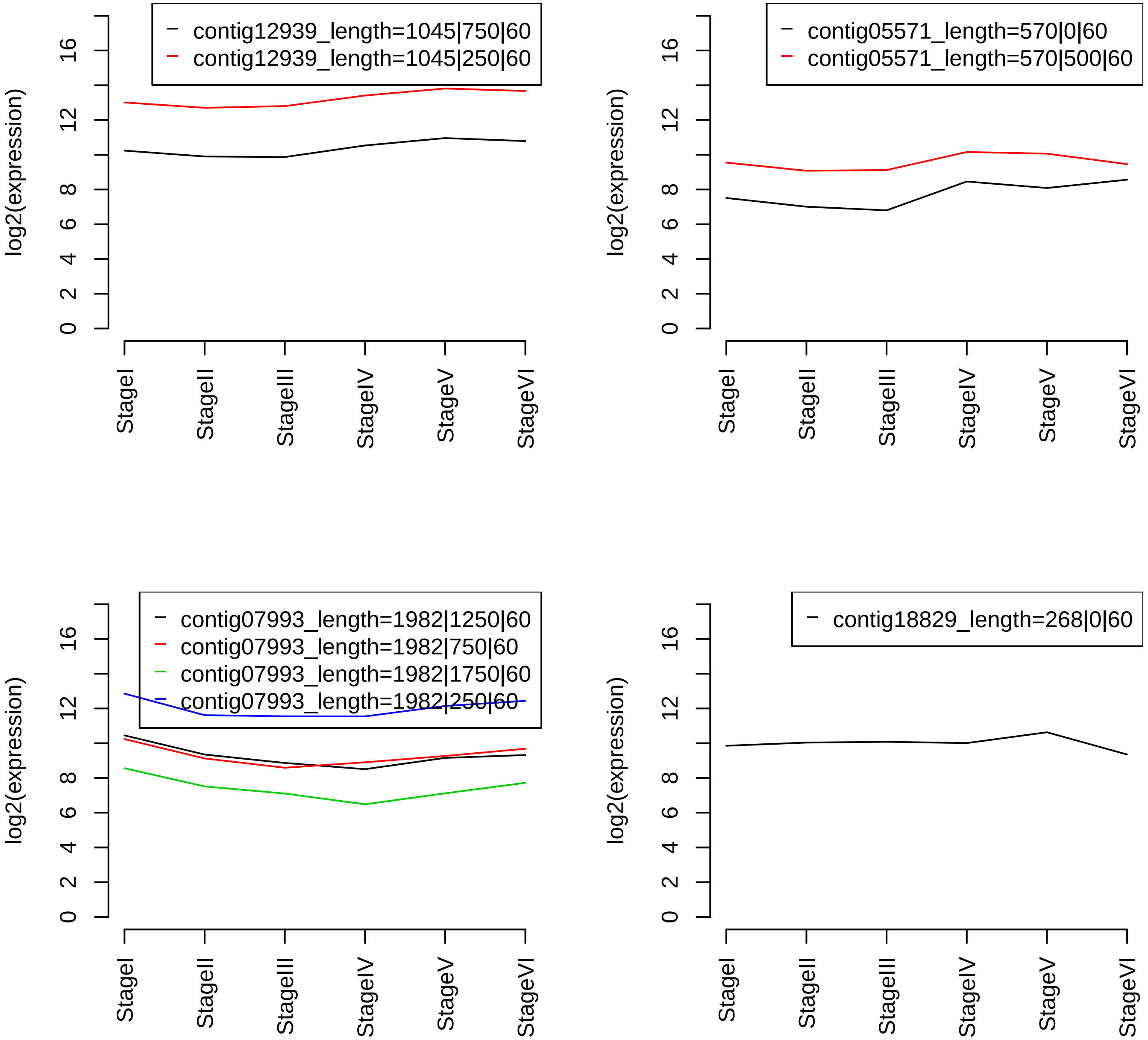

log2(expression)
0
2
4
6
8
12
16
contig12939_length=1045|750|60
contig12939_length=1045|250|60
StageI
StageII
StageIII
StageIV
StageV
StageVI
contig05571_length=570|0|60
contig05571_length=570|500|60
contig07993_length=1982|1250|60
contig07993_length=1982|750|60
contig07993_length=1982|1750|60
contig07993_length=1982|250|60
contig18829_length=268|0|60

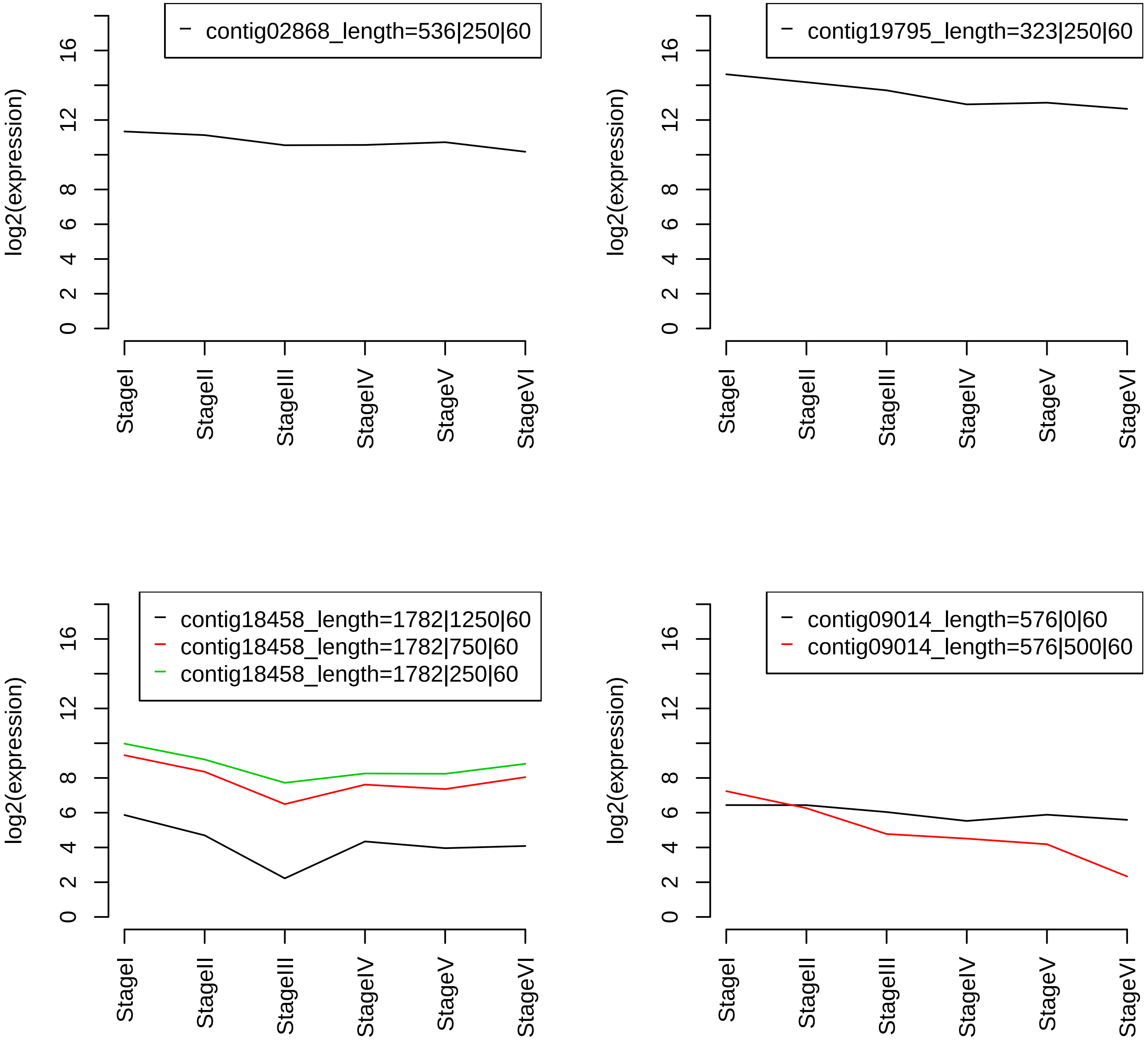
contig02868_length=536|250|60
contig19795_length=323|250|60
contig18458_length=1782|1250|60
contig18458_length=1782|750|60
contig18458_length=1782|250|60
contig09014_length=576|0|60
contig09014_length=576|500|60
log2(expression)
0
2
4
6
8
12
16
StageI
StageII
StageIII
StageIV
StageV
StageVI

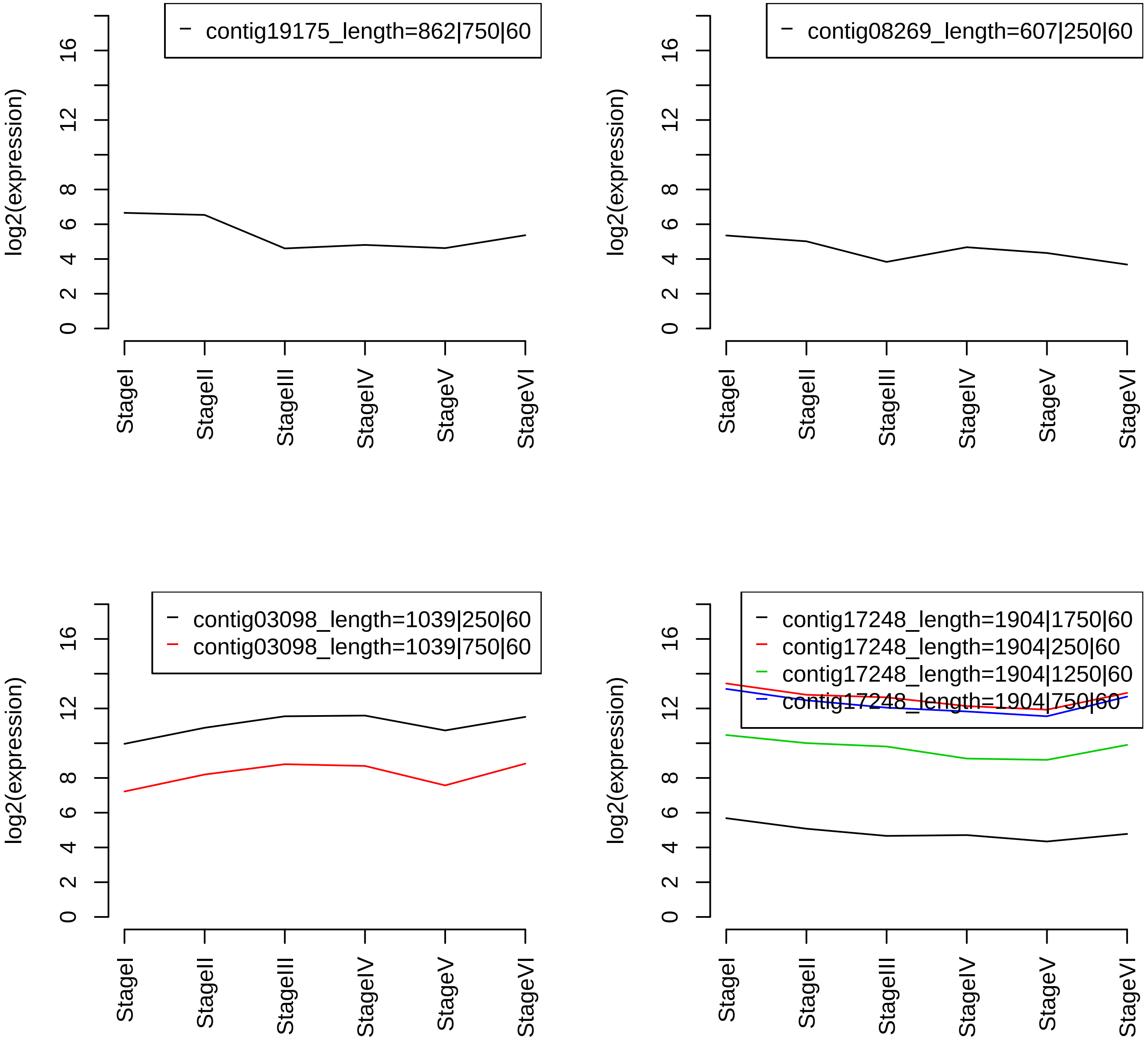

contig19175_length=862|750|60
contig08269_length=607|250|60
contig03098_length=1039|250|60
contig03098_length=1039|750|60
contig17248_length=1904|1750|60
contig17248_length=1904|250|60
contig17248_length=1904|1250|60
contig17248_length=1904|750|60
log2(expression)
0
2
4
6
8
12
16
StageI
StageII
StageIII
StageIV
StageV
StageVI

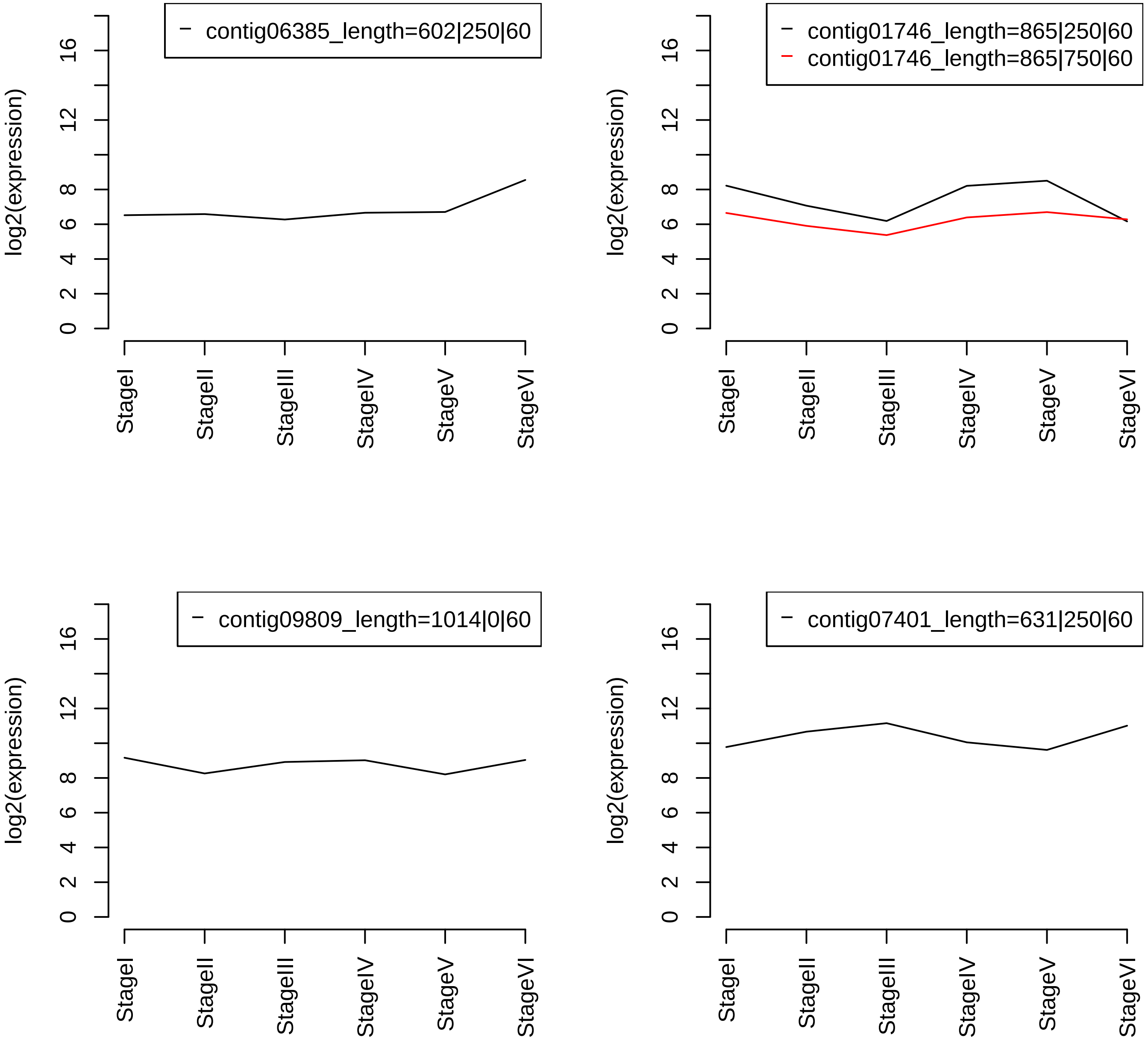
contig06385_length=602|250|60
contig01746_length=865|250|60
contig01746_length=865|750|60
contig09809_length=1014|0|60
contig07401_length=631|250|60
log2(expression)
0
2
4
6
8
12
16
StageI
StageII
StageIII
StageIV
StageV
StageVI

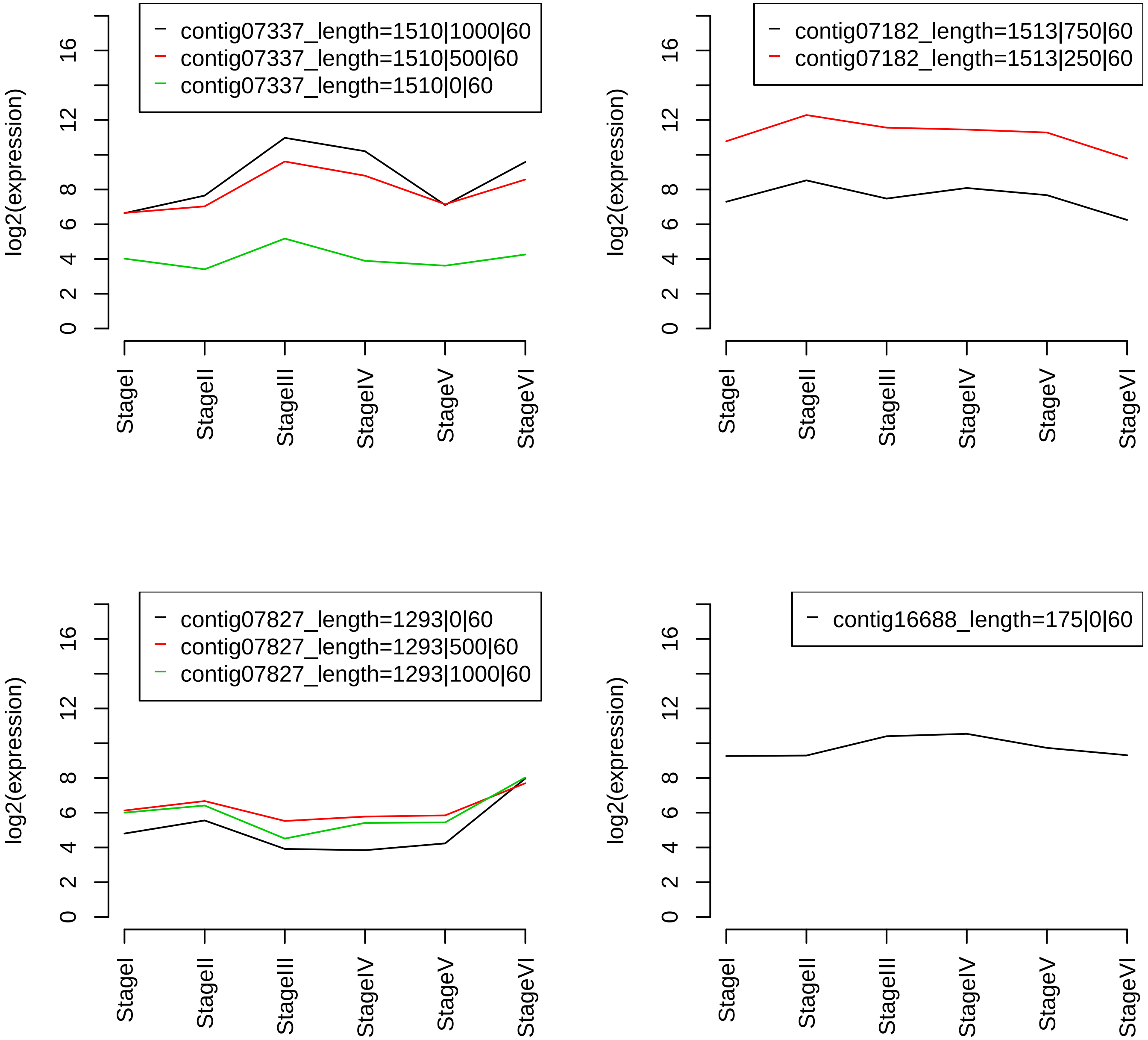
contig07337_length=1510|1000|60
contig07337_length=1510|500|60
contig07337_length=1510|0|60
contig07182_length=1513|750|60
contig07182_length=1513|250|60
contig07827_length=1293|0|60
contig07827_length=1293|500|60
contig07827_length=1293|1000|60
contig16688_length=175|0|60
log2(expression)
0 2 4 6 8 12 16
StageI StageII StageIII StageIV StageV StageVI

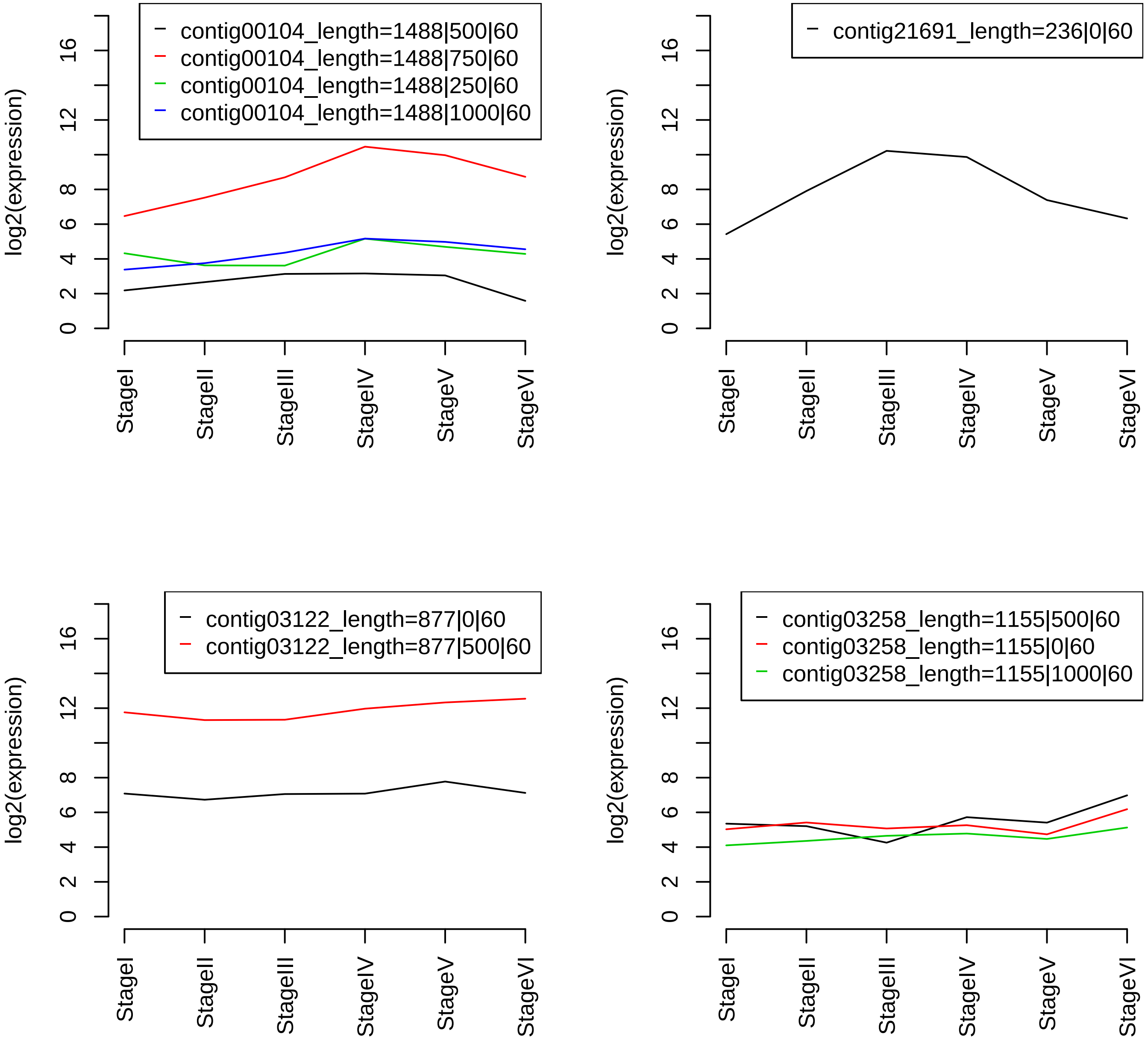

contig00104_length=1488|500|60
contig00104_length=1488|750|60
contig00104_length=1488|250|60
contig00104_length=1488|1000|60
log2(expression)
0
2
4
6
8
12
16
StageI
StageII
StageIII
StageIV
StageV
StageVI
contig21691_length=236|0|60
contig03122_length=877|0|60
contig03122_length=877|500|60
contig03258_length=1155|500|60
contig03258_length=1155|0|60
contig03258_length=1155|1000|60

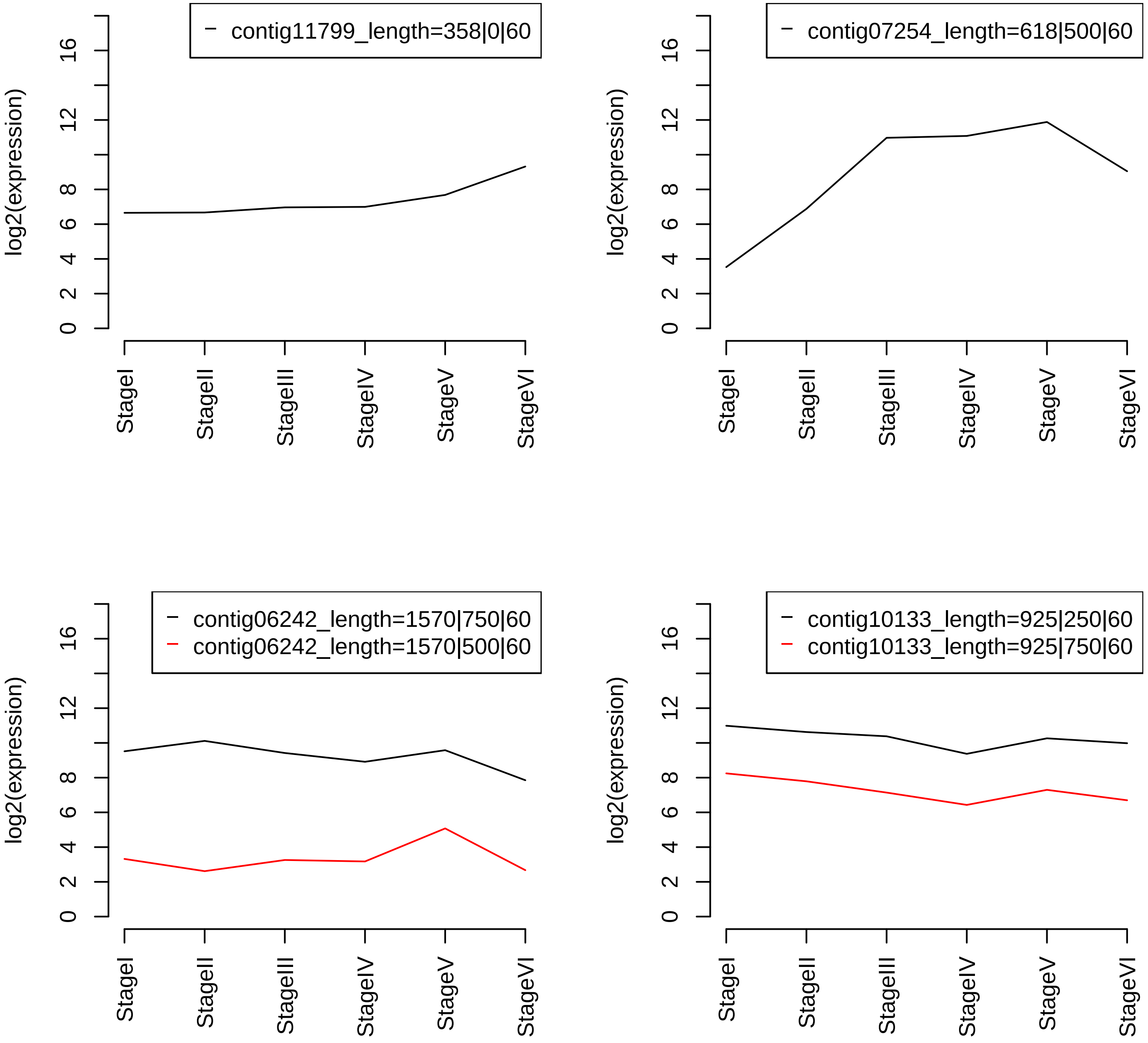
contig11799_length=358|0|60
contig07254_length=618|500|60
contig06242_length=1570|750|60
contig06242_length=1570|500|60
contig10133_length=925|250|60
contig10133_length=925|750|60
log2(expression)
0
2
4
6
8
12
16
StageI
StageII
StageIII
StageIV
StageV
StageVI

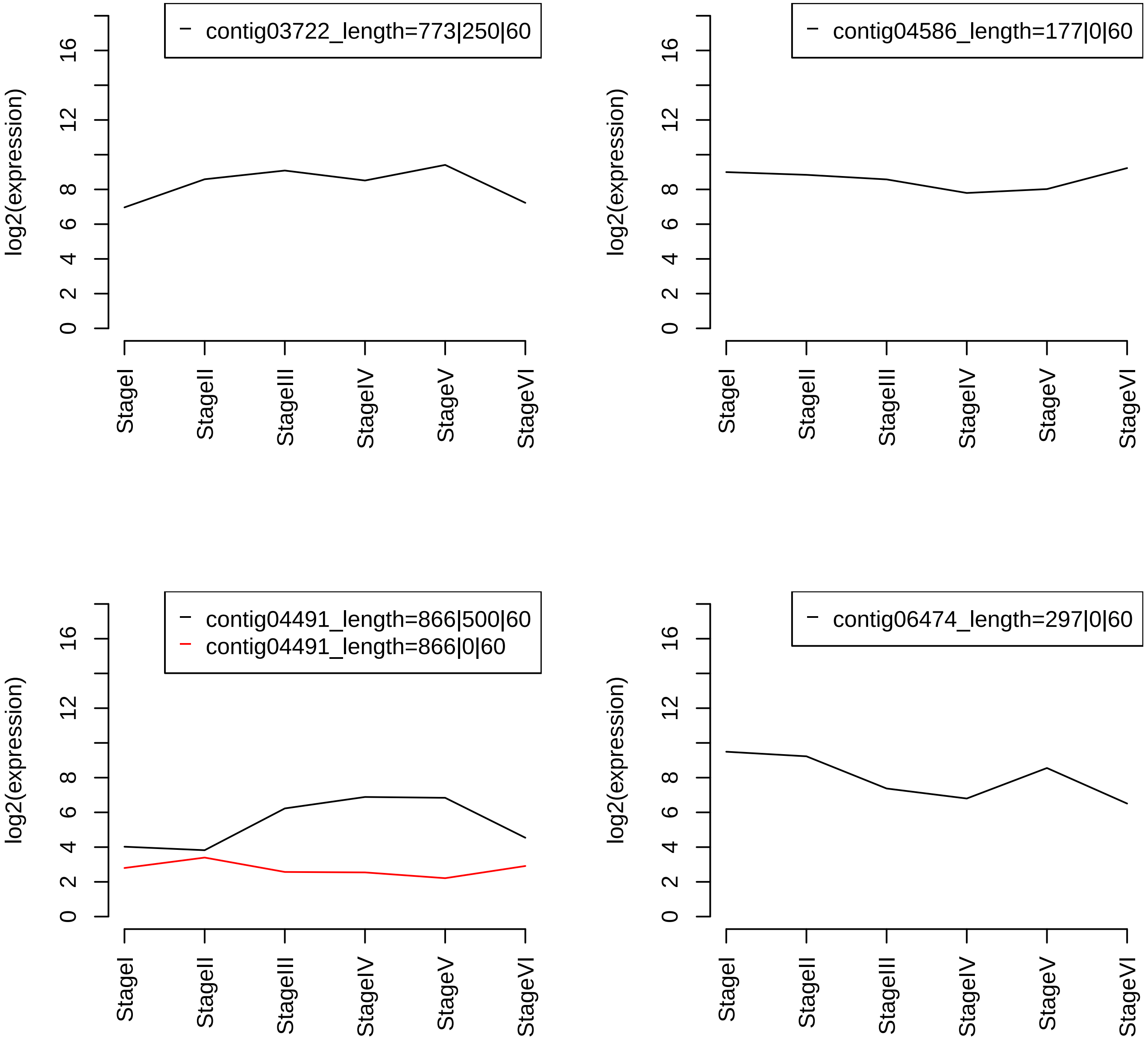
contig03722_length=773|250|60
contig04586_length=177|0|60
contig04491_length=866|500|60
contig04491_length=866|0|60
contig06474_length=297|0|60
log2(expression)
0
2
4
6
8
12
16
StageI
StageII
StageIII
StageIV
StageV
StageVI

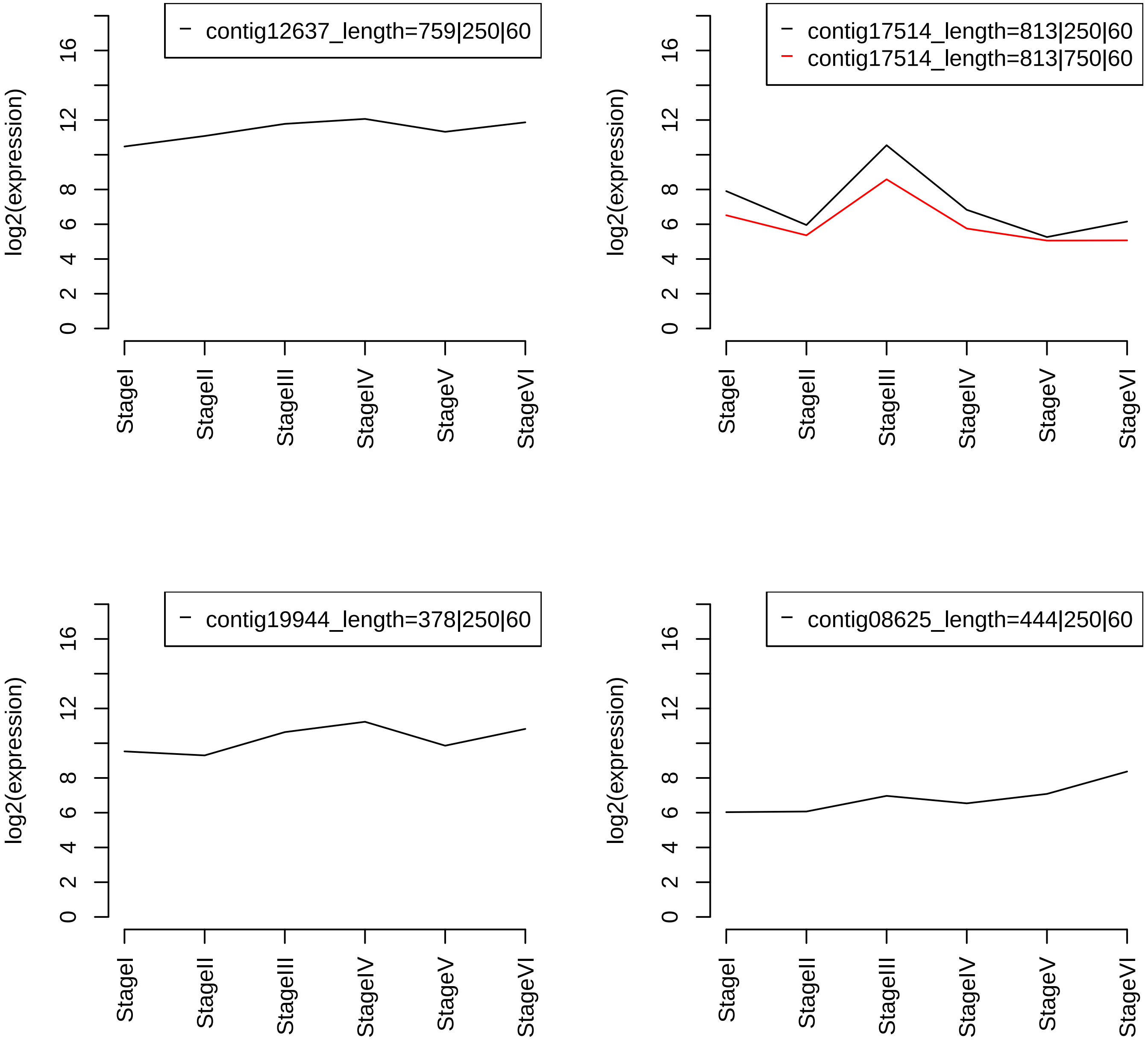

contig12637_length=759|250|60
log2(expression)
0
2
4
6
8
12
16
StageI
StageII
StageIII
StageIV
StageV
StageVI
contig17514_length=813|250|60
contig17514_length=813|750|60
contig19944_length=378|250|60
contig08625_length=444|250|60

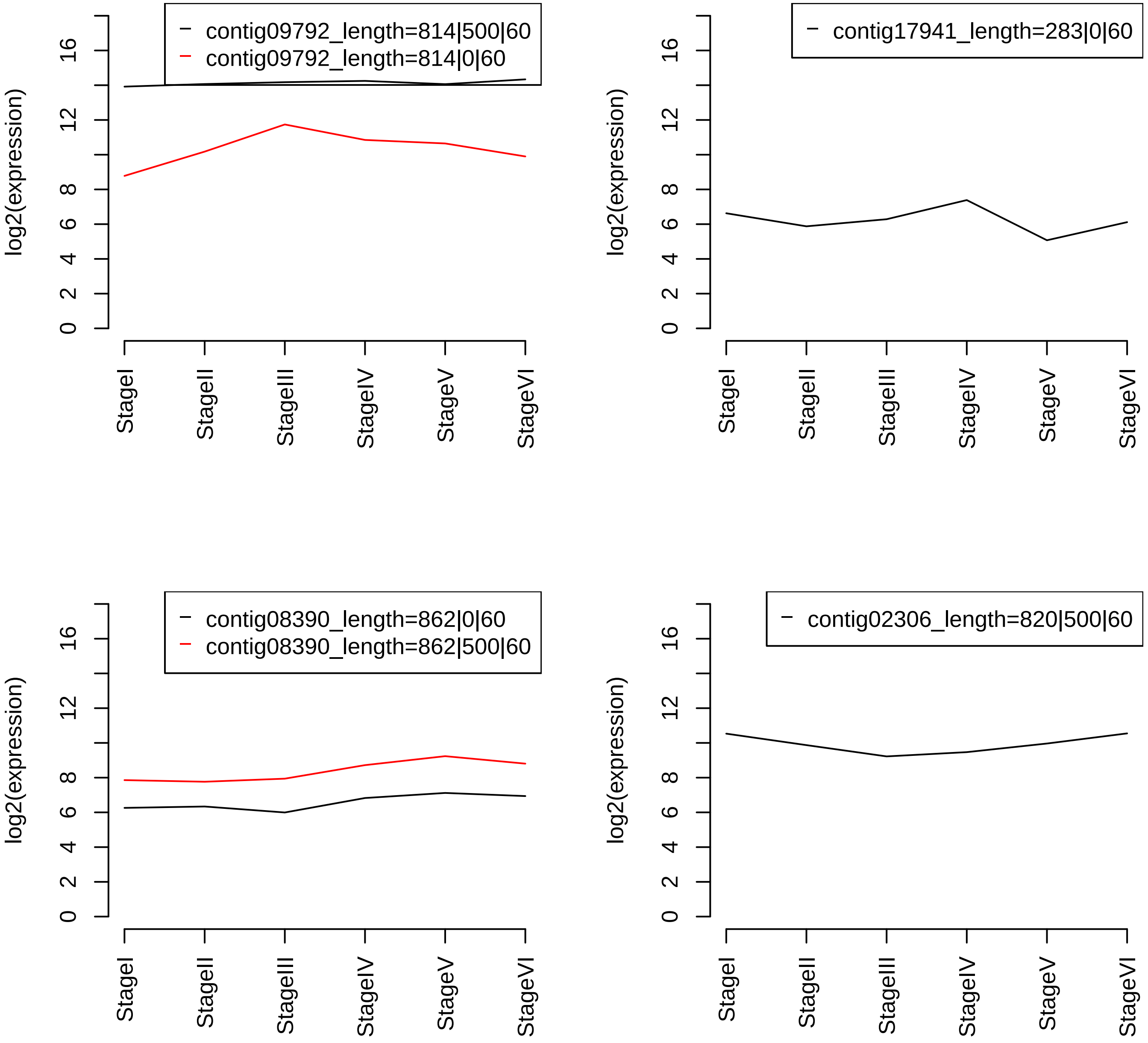
contig09792_length=814|500|60
contig09792_length=814|0|60
contig17941_length=283|0|60
contig08390_length=862|0|60
contig08390_length=862|500|60
contig02306_length=820|500|60
log2(expression)
0
2
4
6
8
12
16
StageI
StageII
StageIII
StageIV
StageV
StageVI

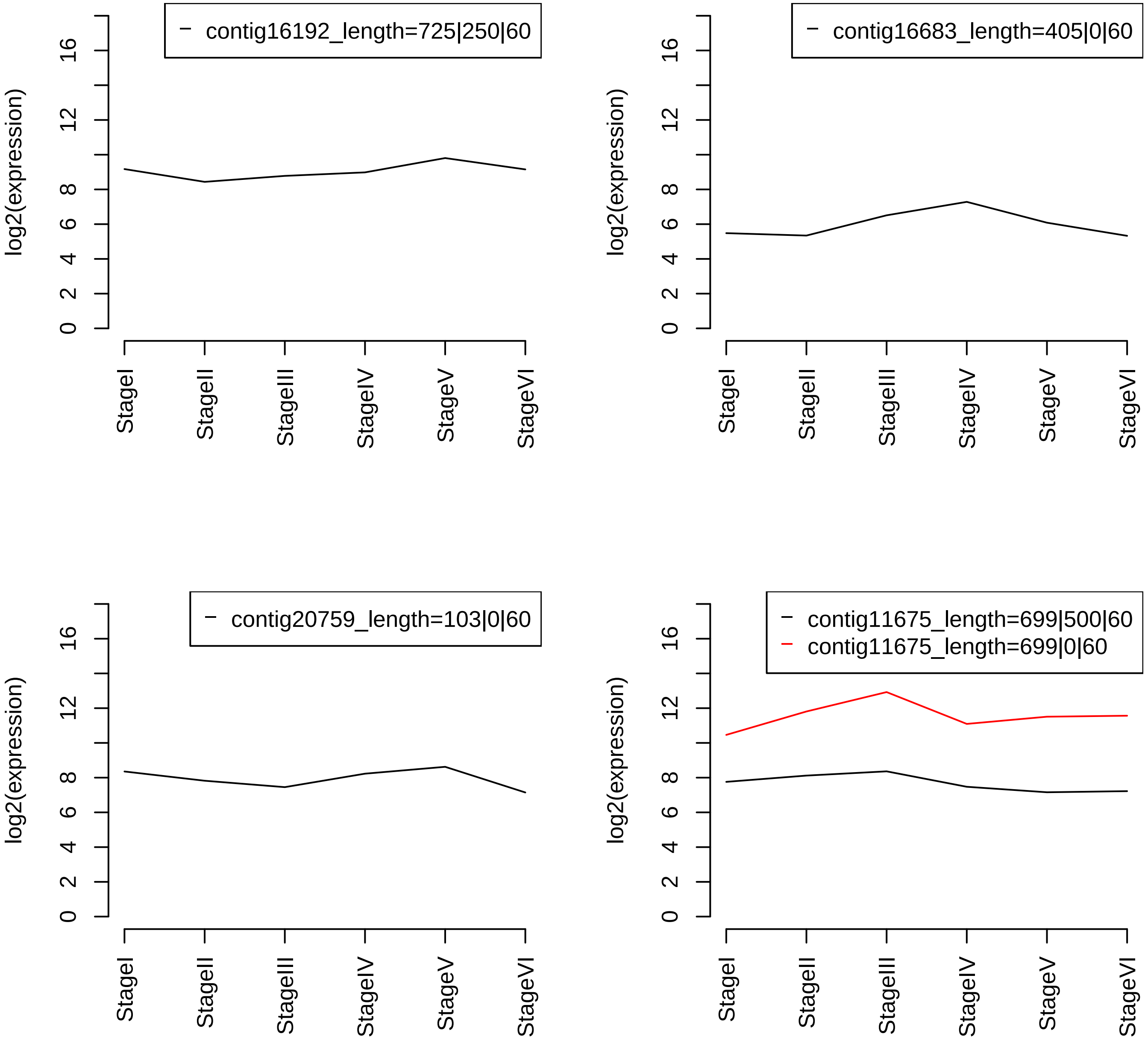
contig16192_length=725|250|60
contig16683_length=405|0|60
contig20759_length=103|0|60
contig11675_length=699|500|60
contig11675_length=699|0|60
log2(expression)
0
2
4
6
8
12
16
StageI
StageII
StageIII
StageIV
StageV
StageVI

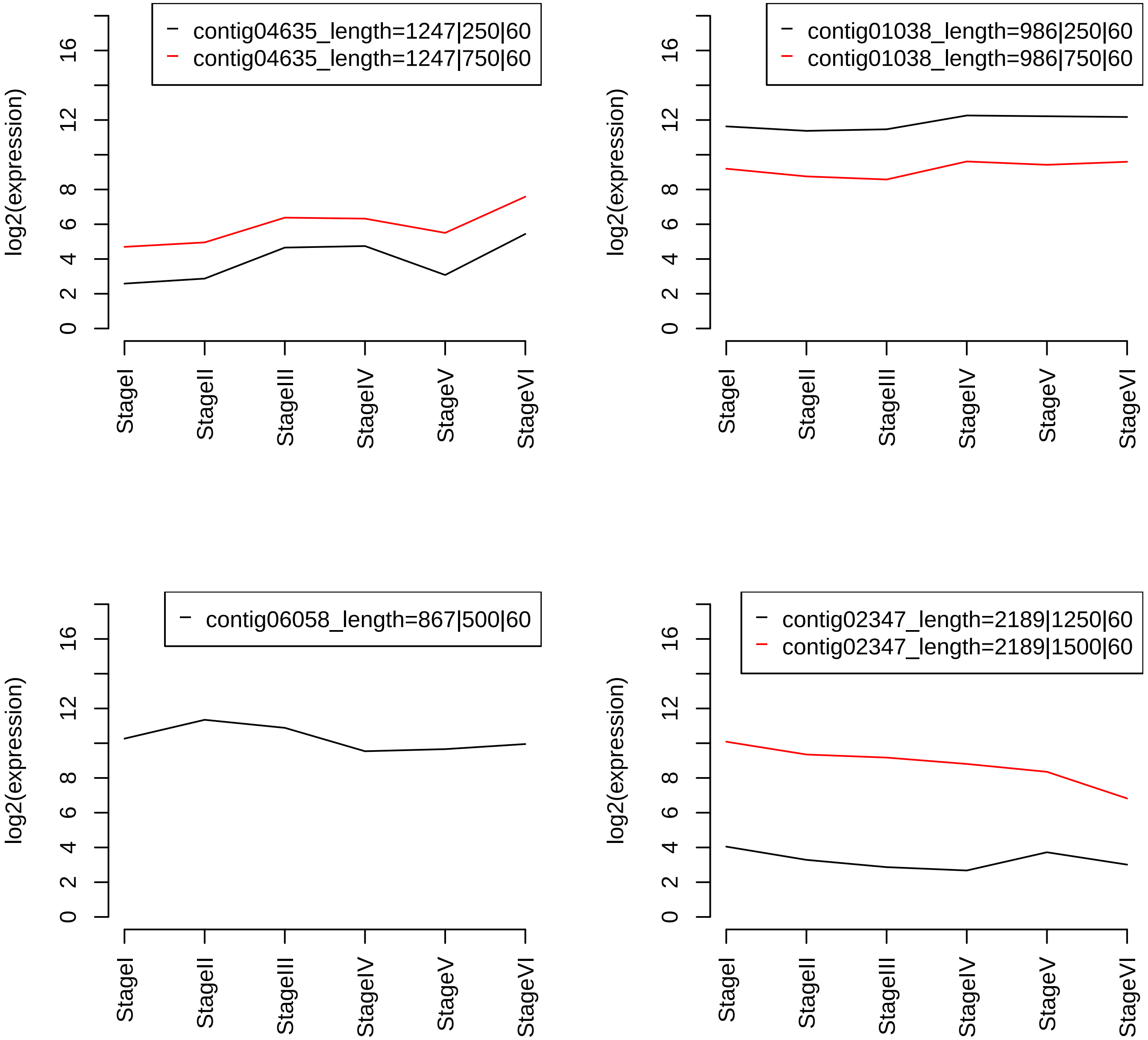
contig04635_length=1247|250|60
contig04635_length=1247|750|60
log2(expression)
0
2
4
6
8
12
16
StageI
StageII
StageIII
StageIV
StageV
StageVI
contig01038_length=986|250|60
contig01038_length=986|750|60
log2(expression)
StageI
StageII
StageIII
StageIV
StageV
StageVI
contig06058_length=867|500|60
log2(expression)
StageI
StageII
StageIII
StageIV
StageV
StageVI
contig02347_length=2189|1250|60
contig02347_length=2189|1500|60
log2(expression)
StageI
StageII
StageIII
StageIV
StageV
StageVI

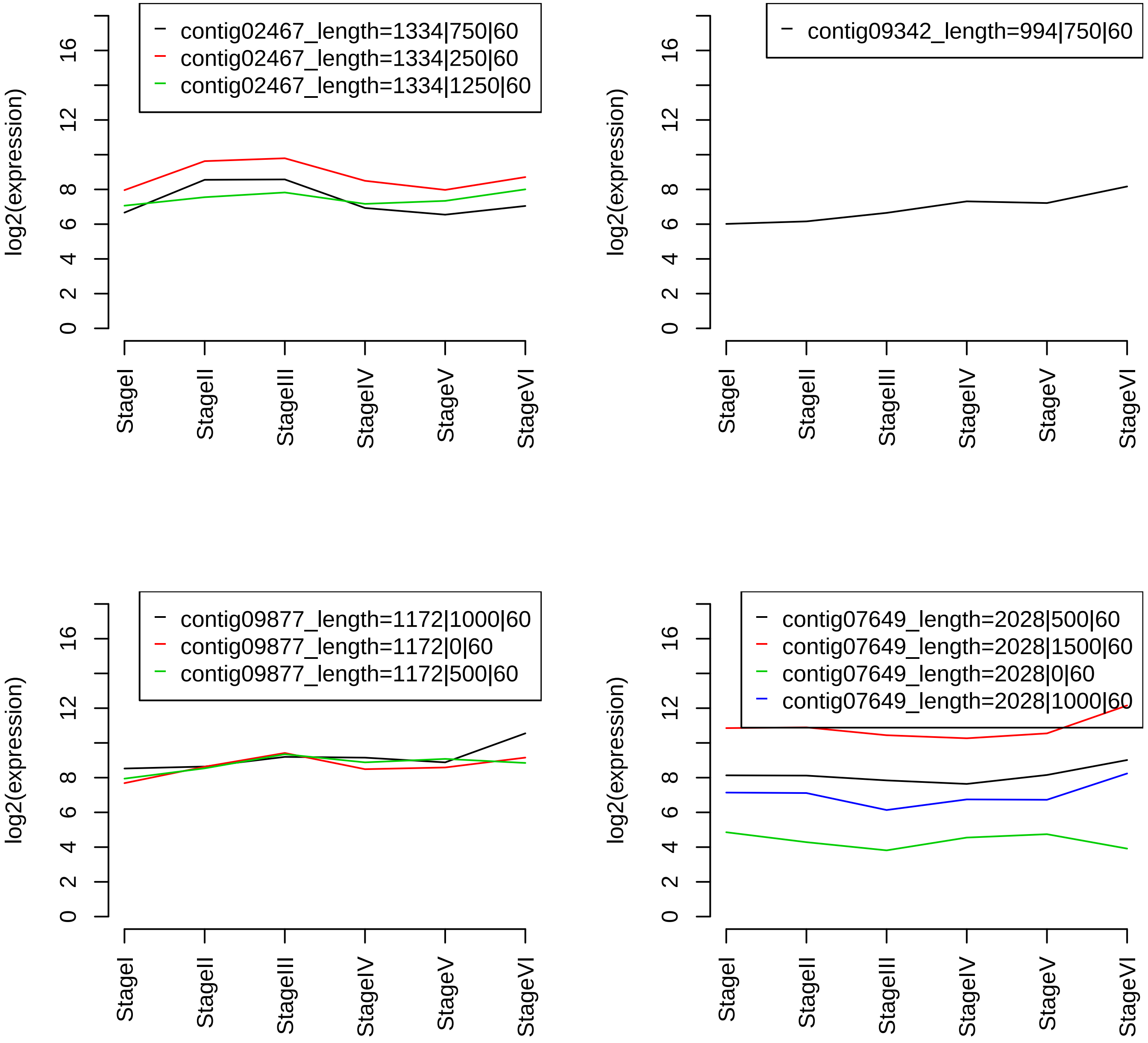

contig02467_length=1334|750|60
contig02467_length=1334|250|60
contig02467_length=1334|1250|60
contig09342_length=994|750|60
contig09877_length=1172|1000|60
contig09877_length=1172|0|60
contig09877_length=1172|500|60
contig07649_length=2028|500|60
contig07649_length=2028|1500|60
contig07649_length=2028|0|60
contig07649_length=2028|1000|60
log2(expression)
0
2
4
6
8
12
16
StageI
StageII
StageIII
StageIV
StageV
StageVI

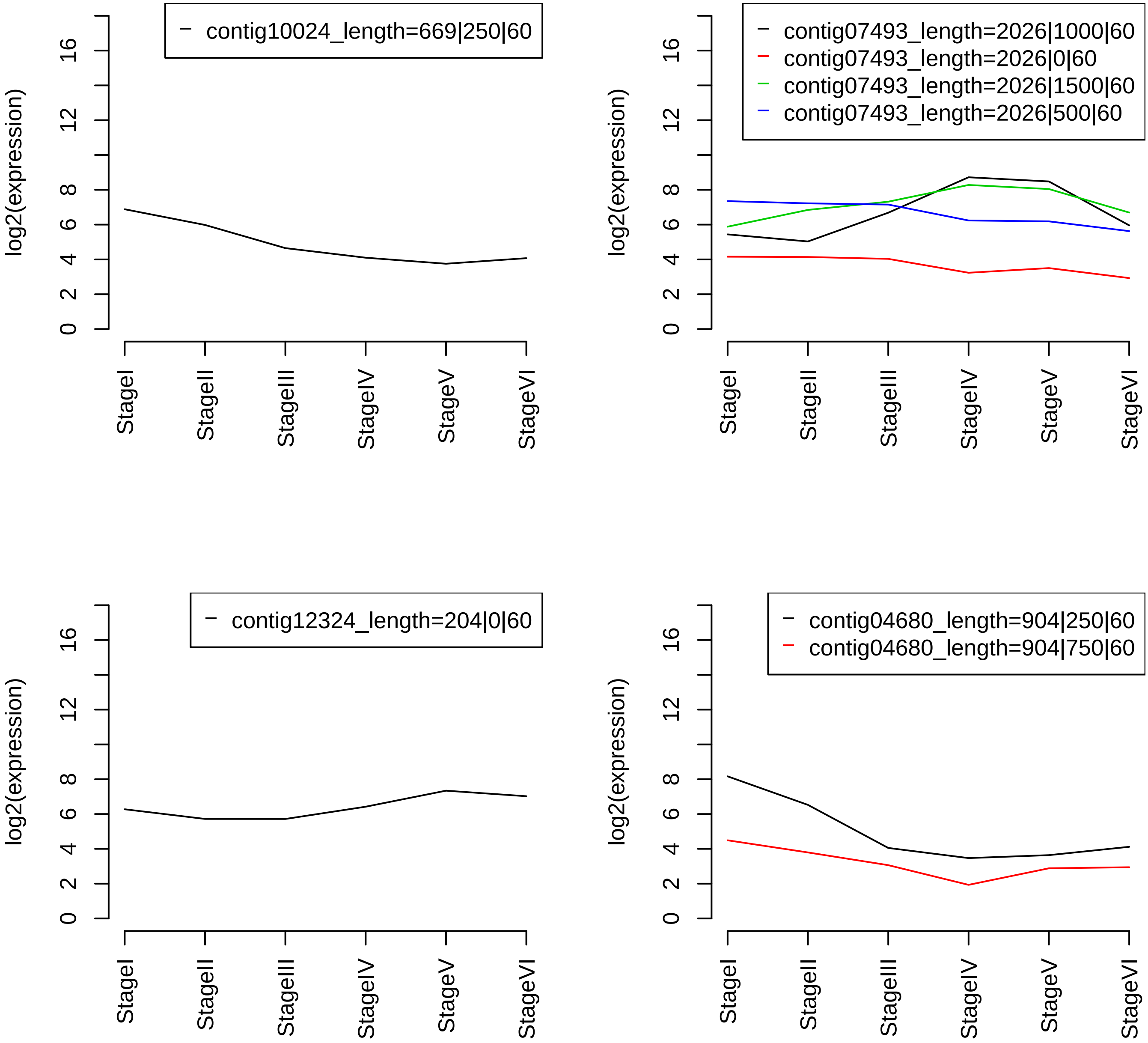

contig10024_length=669|250|60
contig07493_length=2026|1000|60
contig07493_length=2026|0|60
contig07493_length=2026|1500|60
contig07493_length=2026|500|60
contig12324_length=204|0|60
contig04680_length=904|250|60
contig04680_length=904|750|60
log2(expression)
0
2
4
6
8
12
16
StageI
StageII
StageIII
StageIV
StageV
StageVI

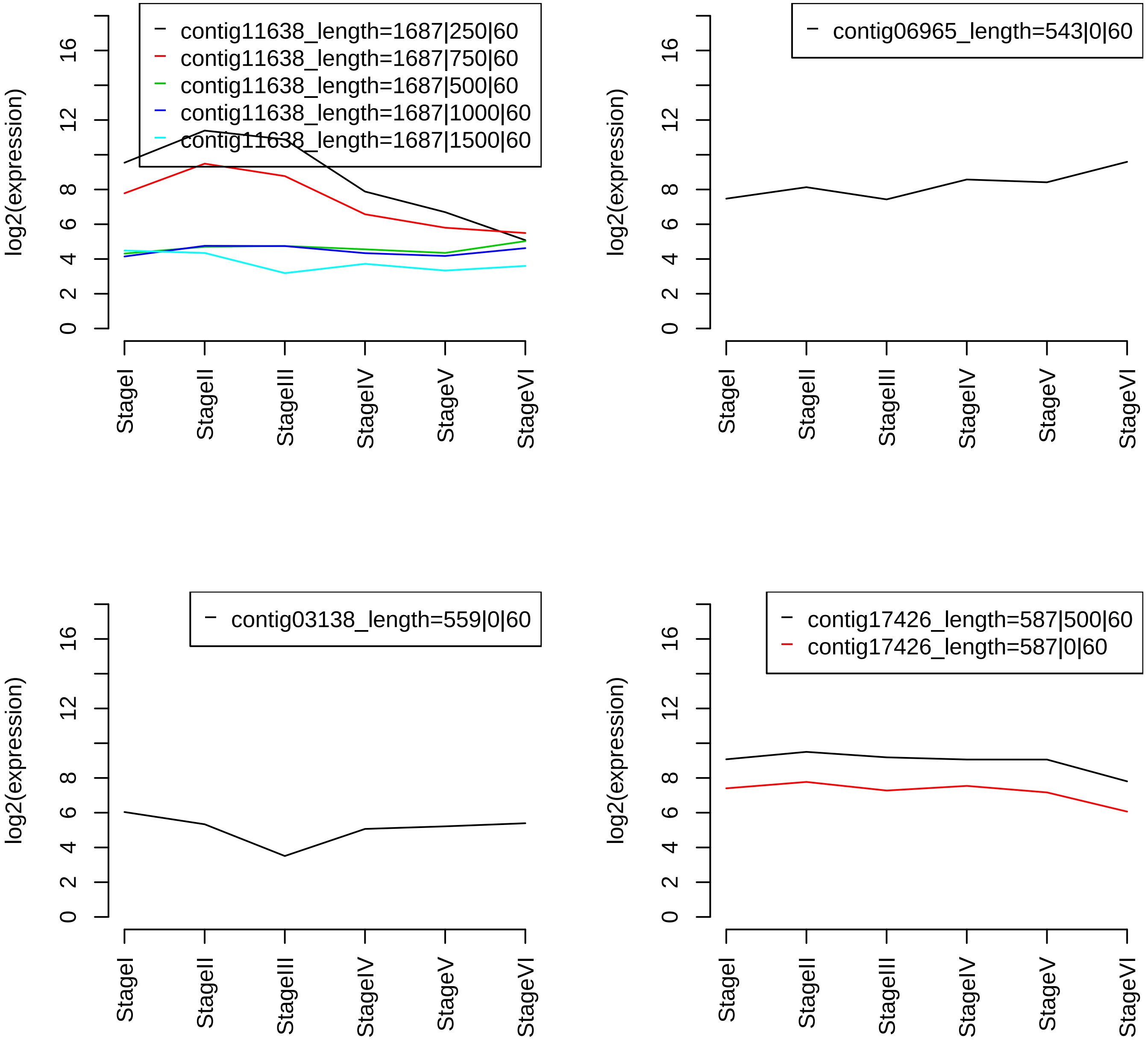
contig11638_length=1687|250|60
contig11638_length=1687|750|60
contig11638_length=1687|500|60
contig11638_length=1687|1000|60
contig11638_length=1687|1500|60
contig06965_length=543|0|60
contig03138_length=559|0|60
contig17426_length=587|500|60
contig17426_length=587|0|60
log2(expression)
0
2
4
6
8
12
16
StageI
StageII
StageIII
StageIV
StageV
StageVI

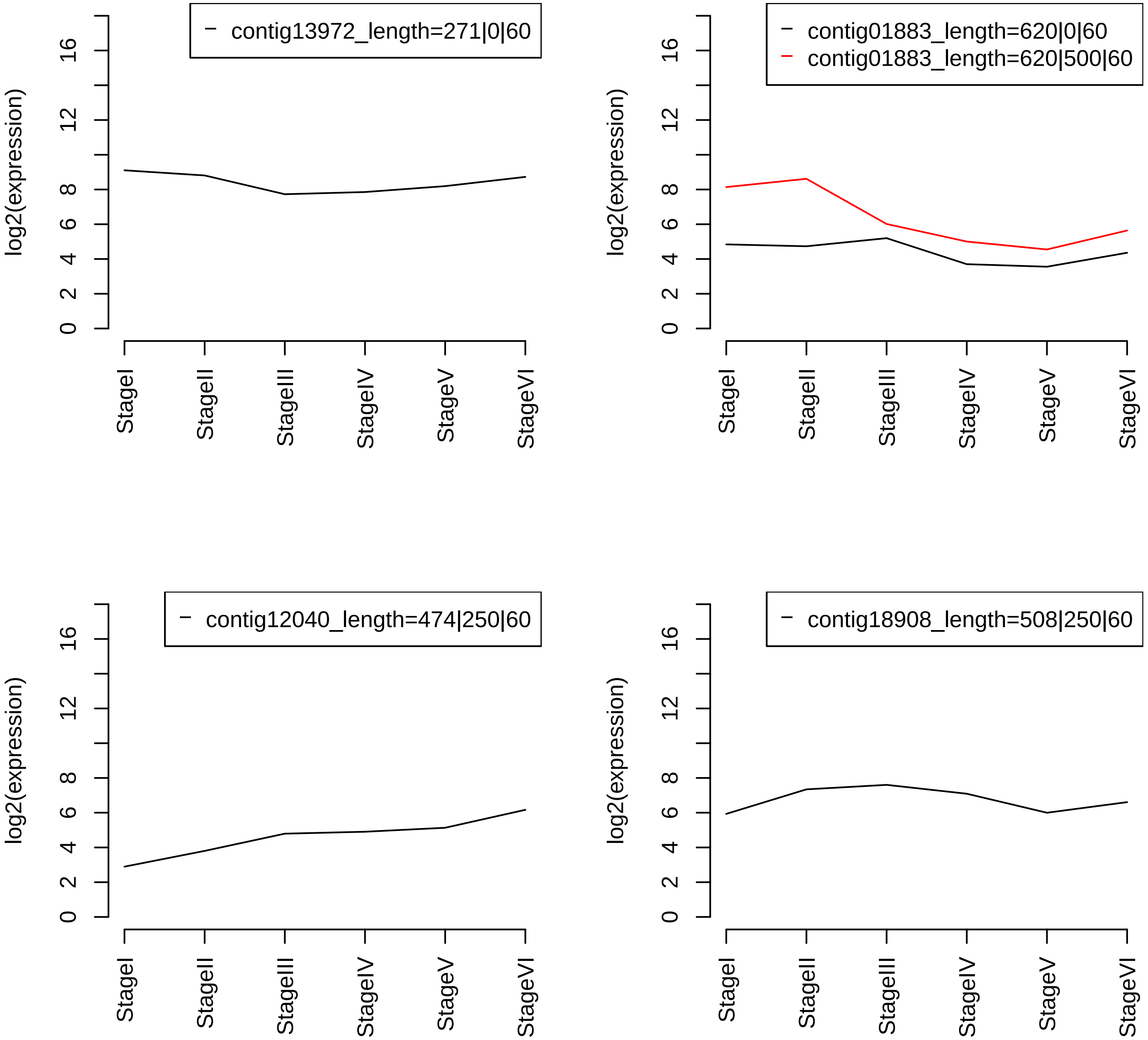

contig13972_length=271|0|60
contig01883_length=620|0|60
contig01883_length=620|500|60
contig12040_length=474|250|60
contig18908_length=508|250|60
log2(expression)
0
2
4
6
8
12
16
StageI
StageII
StageIII
StageIV
StageV
StageVI

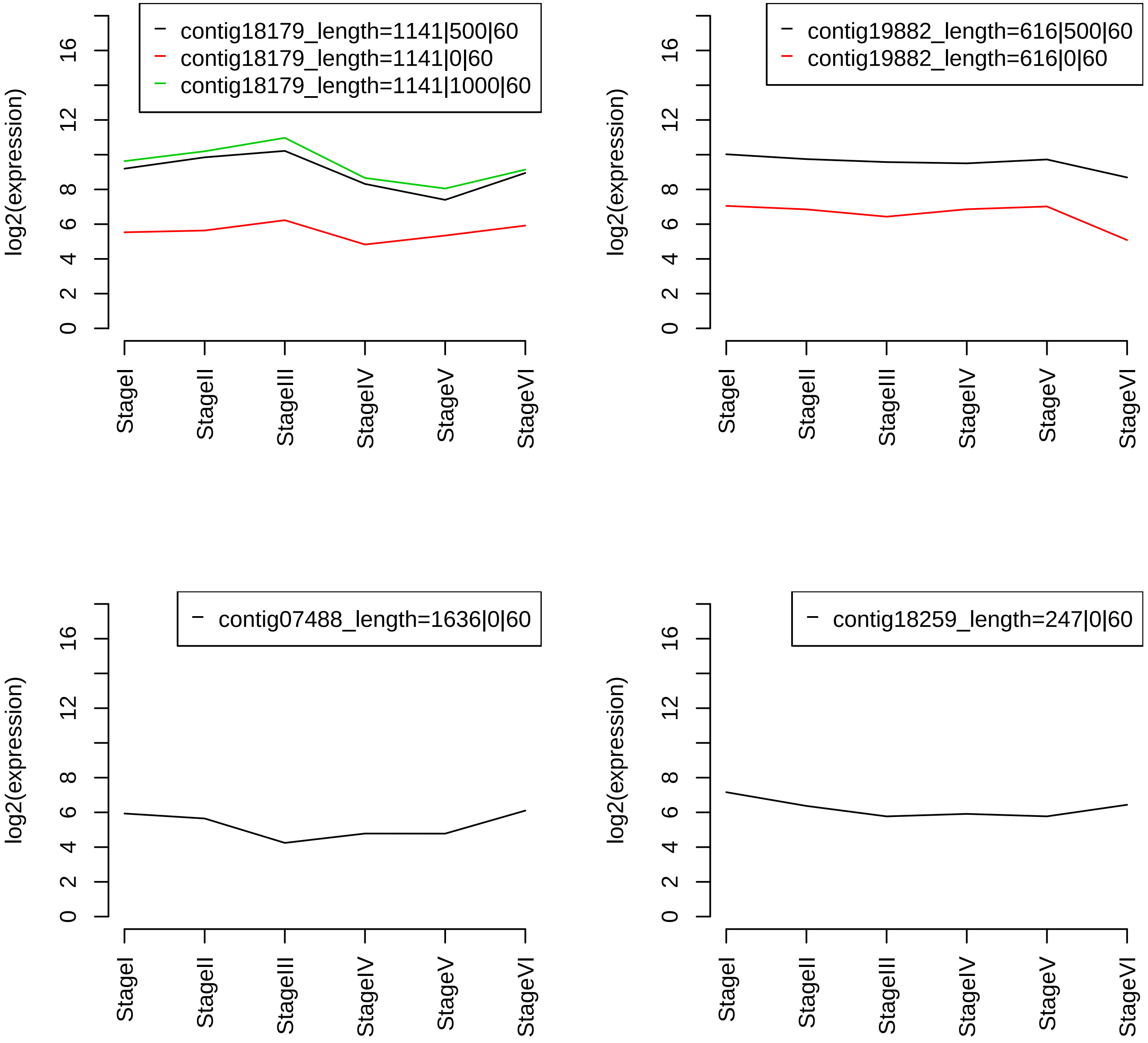
contig18179_length=1141|500|60
contig18179_length=1141|0|60
contig18179_length=1141|1000|60
contig19882_length=616|500|60
contig19882_length=616|0|60
contig07488_length=1636|0|60
contig18259_length=247|0|60
log2(expression)
StageI
StageII
StageIII
StageIV
StageV
StageVI

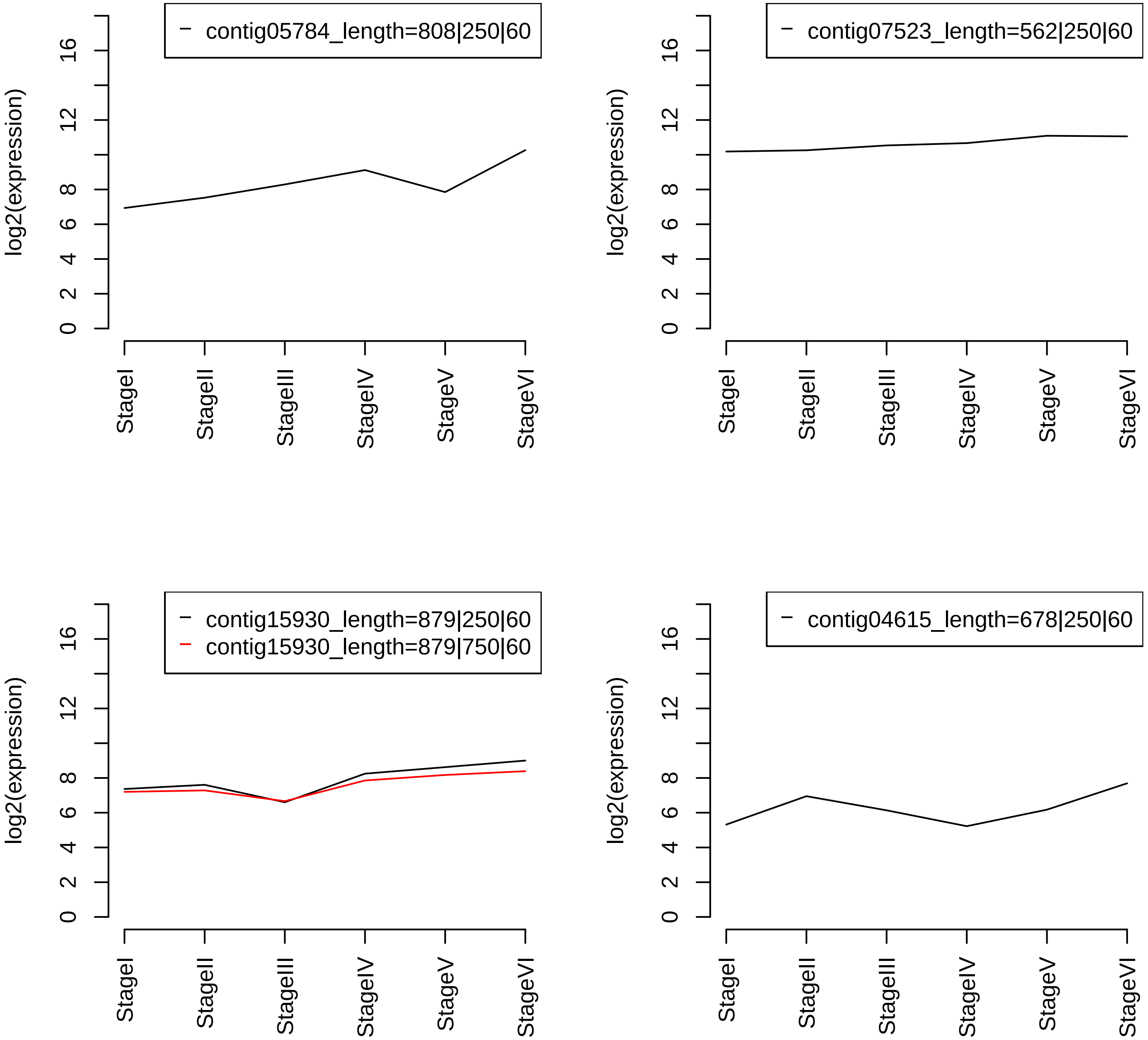

contig05784_length=808|250|60
contig07523_length=562|250|60
contig15930_length=879|250|60
contig15930_length=879|750|60
contig04615_length=678|250|60
log2(expression)
0
2
4
6
8
12
16
StageI
StageII
StageIII
StageIV
StageV
StageVI

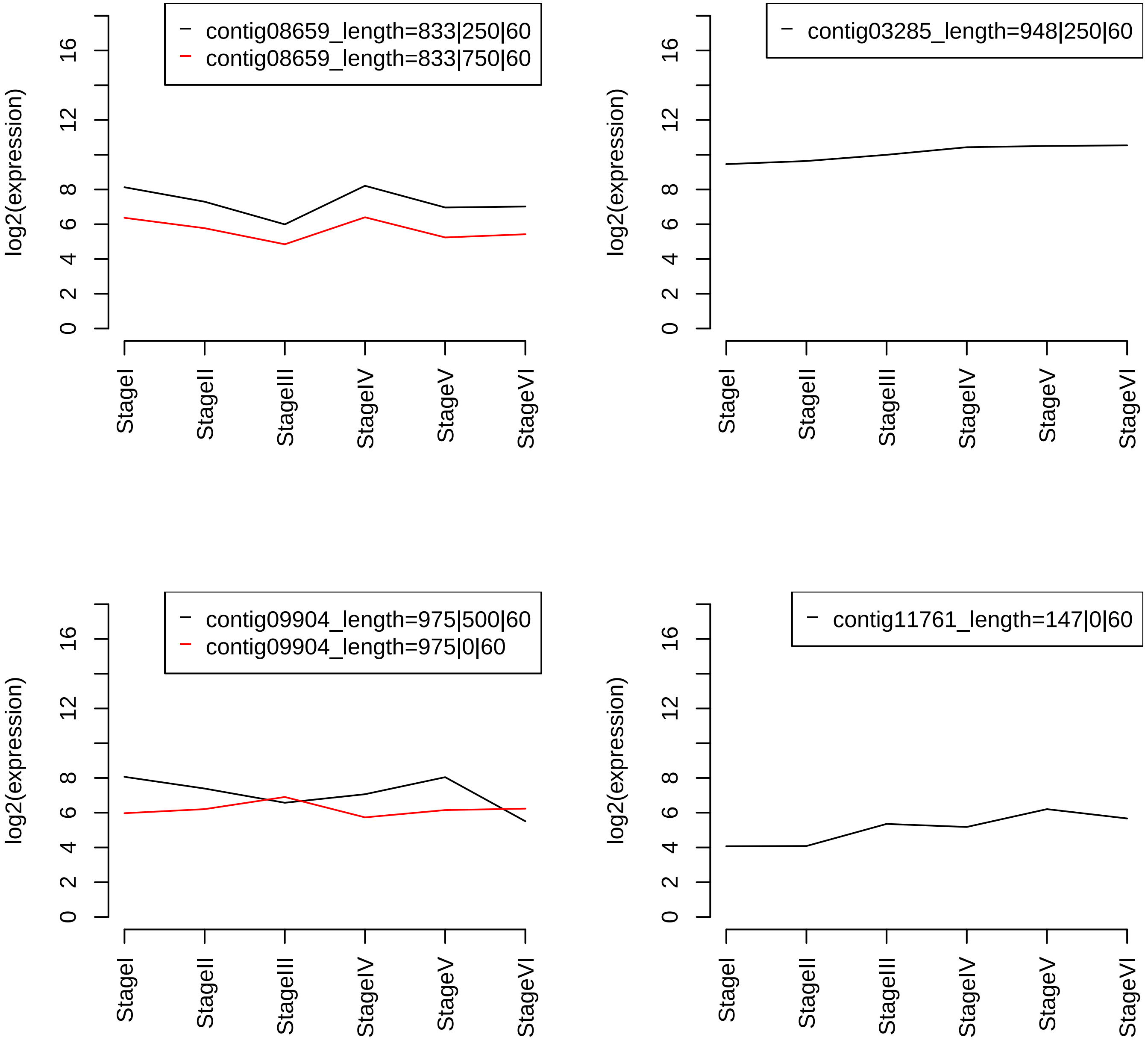
contig08659_length=833|250|60
contig08659_length=833|750|60
contig03285_length=948|250|60
contig09904_length=975|500|60
contig09904_length=975|0|60
contig11761_length=147|0|60
log2(expression)
0
2
4
6
8
12
16
StageI
StageII
StageIII
StageIV
StageV
StageVI

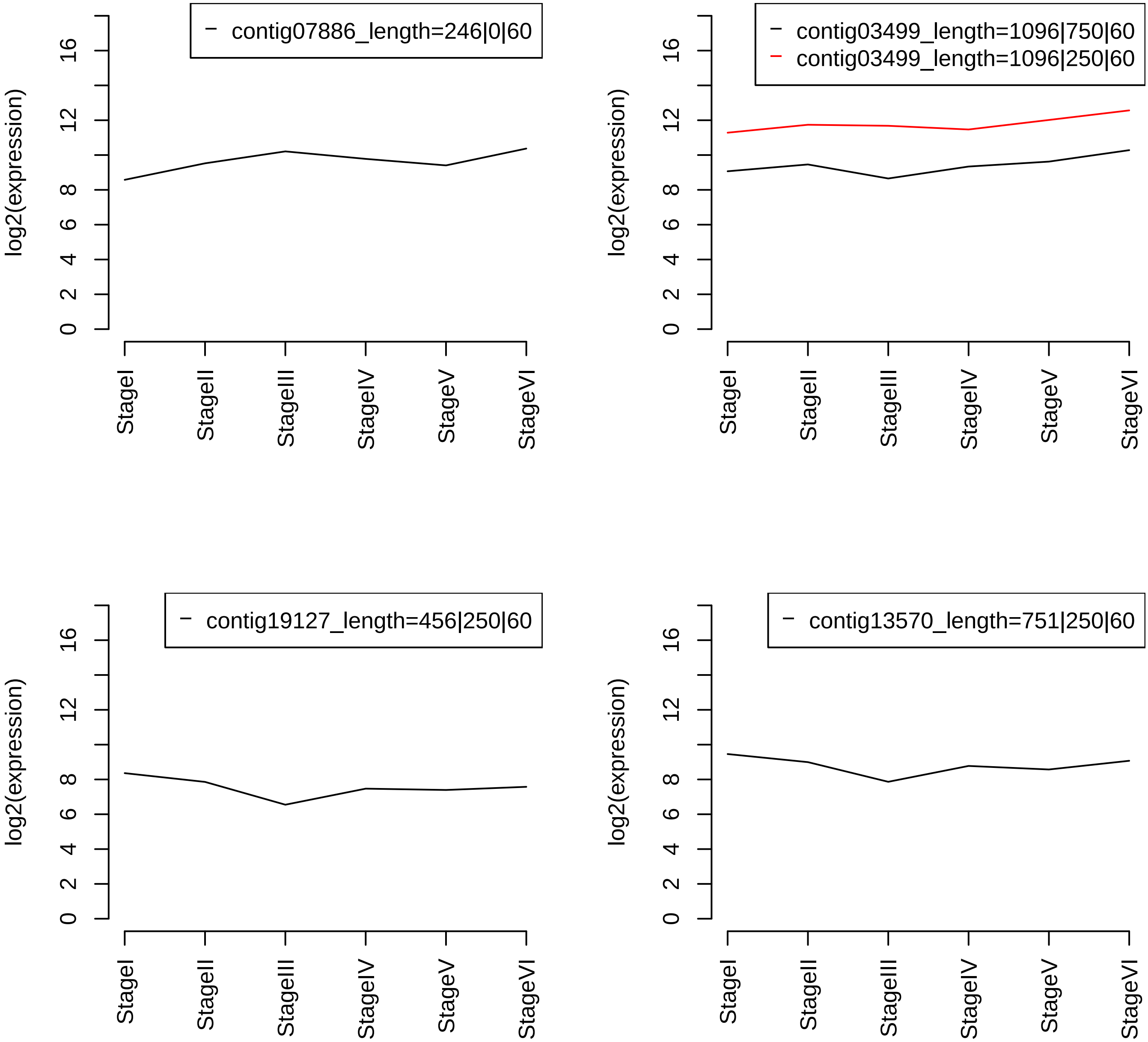
contig07886_length=246|0|60
contig03499_length=1096|750|60
contig03499_length=1096|250|60
contig19127_length=456|250|60
contig13570_length=751|250|60
log2(expression)
0
2
4
6
8
12
16
StageI
StageII
StageIII
StageIV
StageV
StageVI

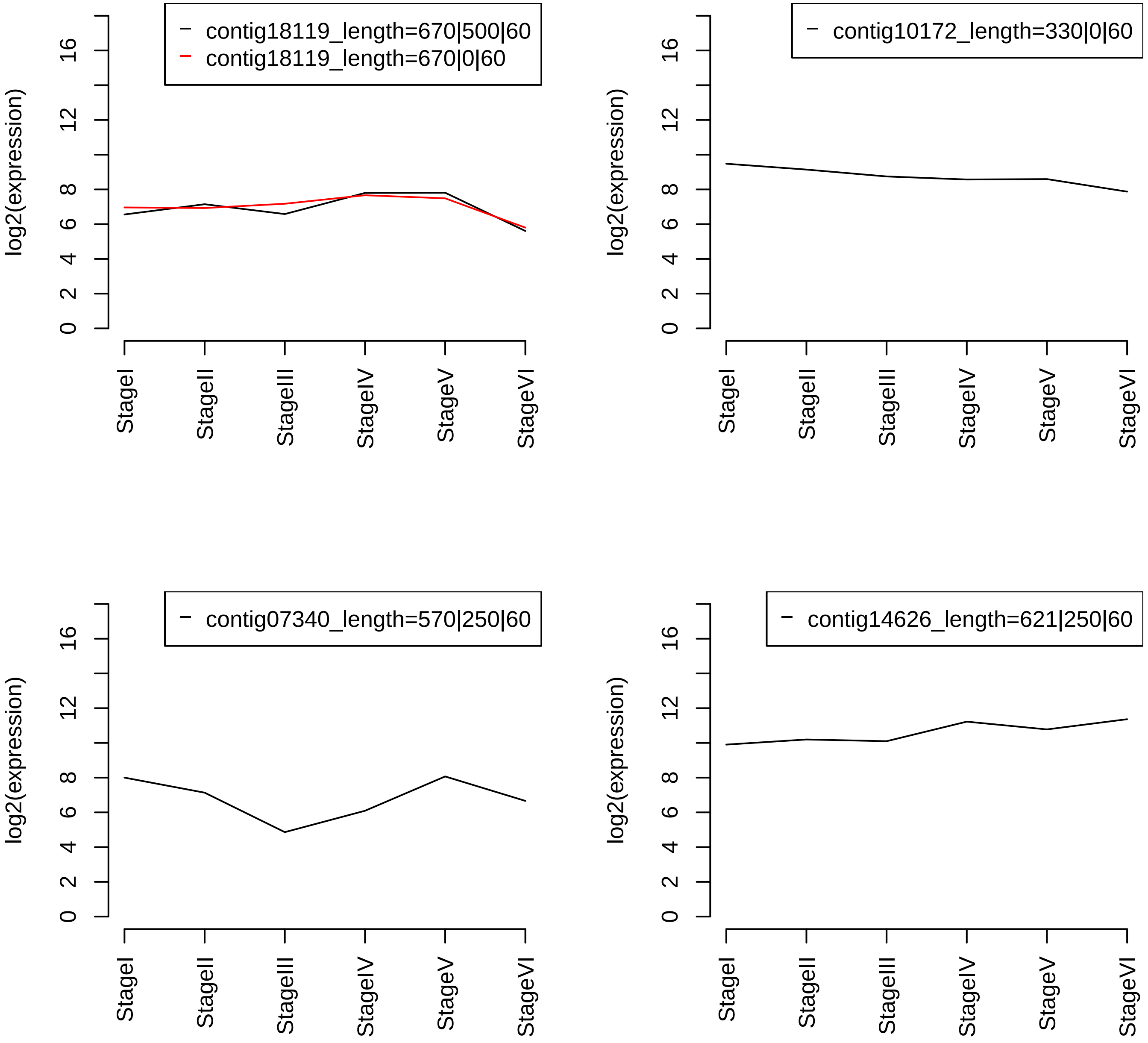
contig18119_length=670|500|60
contig18119_length=670|0|60
contig10172_length=330|0|60
contig07340_length=570|250|60
contig14626_length=621|250|60
log2(expression)
0
2
4
6
8
12
16
StageI
StageII
StageIII
StageIV
StageV
StageVI

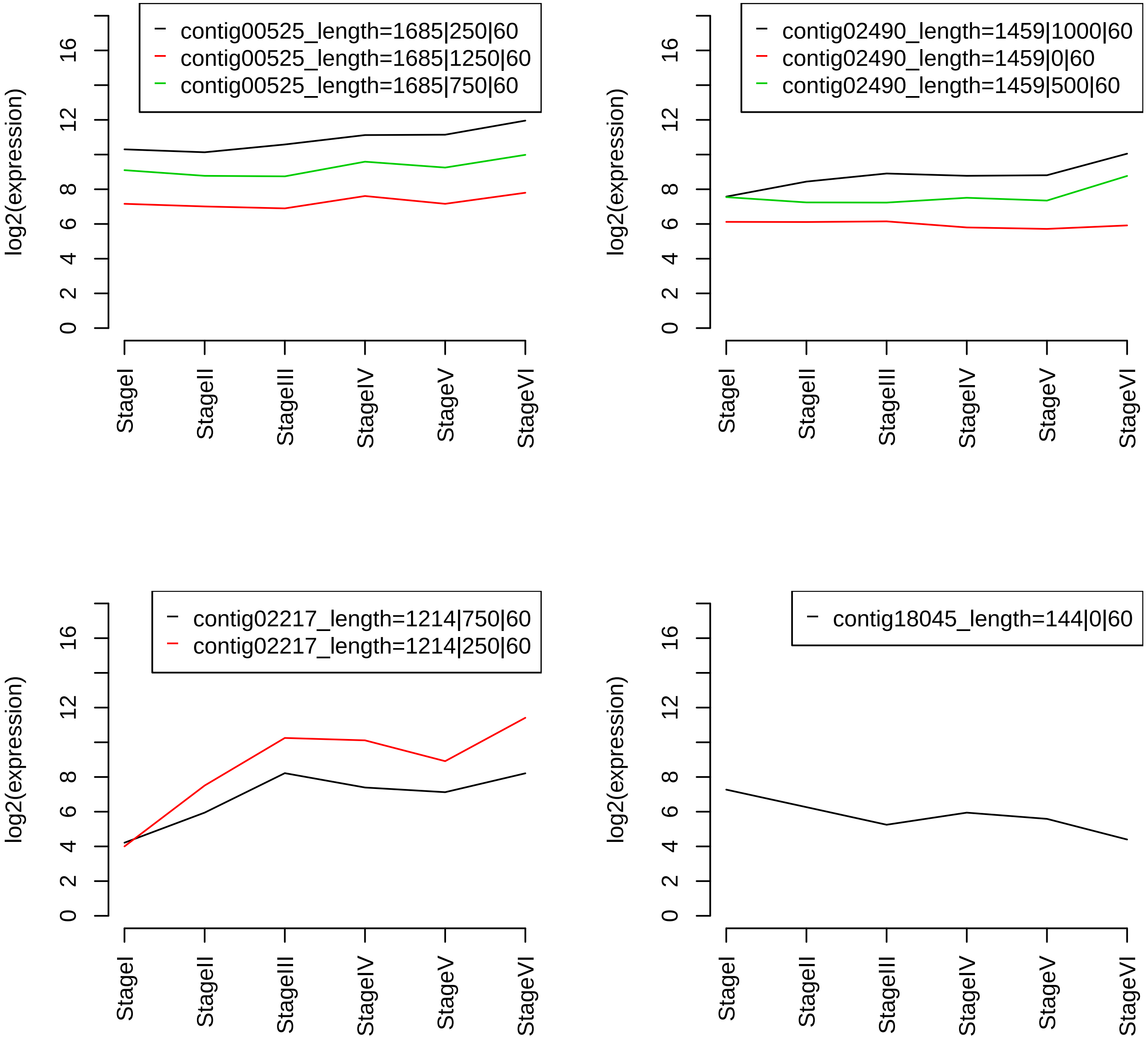
contig00525_length=1685|250|60
contig00525_length=1685|1250|60
contig00525_length=1685|750|60
contig02490_length=1459|1000|60
contig02490_length=1459|0|60
contig02490_length=1459|500|60
contig02217_length=1214|750|60
contig02217_length=1214|250|60
contig18045_length=144|0|60
log2(expression)
0
2
4
6
8
12
16
StageI
StageII
StageIII
StageIV
StageV
StageVI

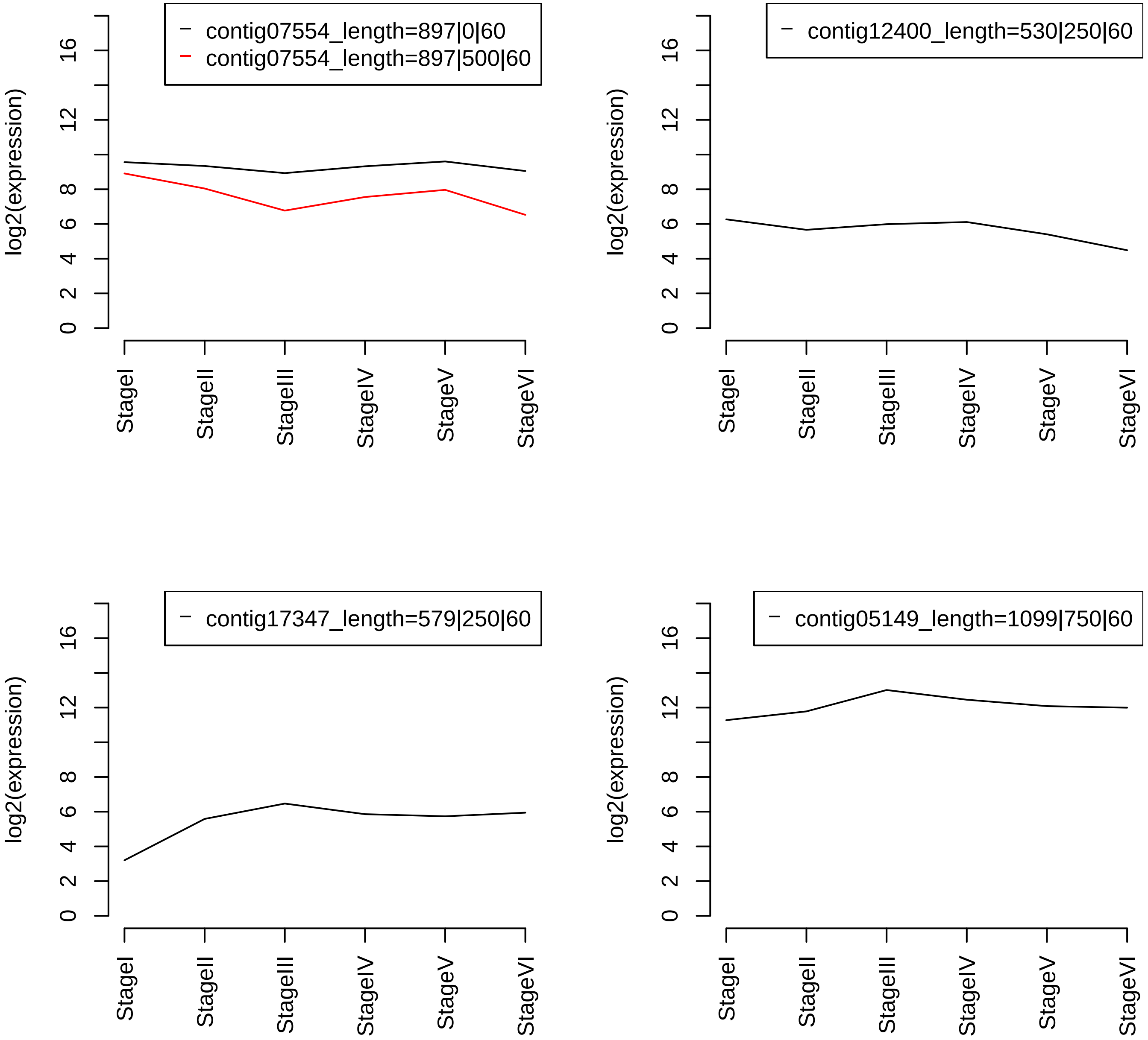
contig07554_length=897|0|60
contig07554_length=897|500|60
contig12400_length=530|250|60
contig17347_length=579|250|60
contig05149_length=1099|750|60
log2(expression)
0
2
4
6
8
12
16
StageI
StageII
StageIII
StageIV
StageV
StageVI

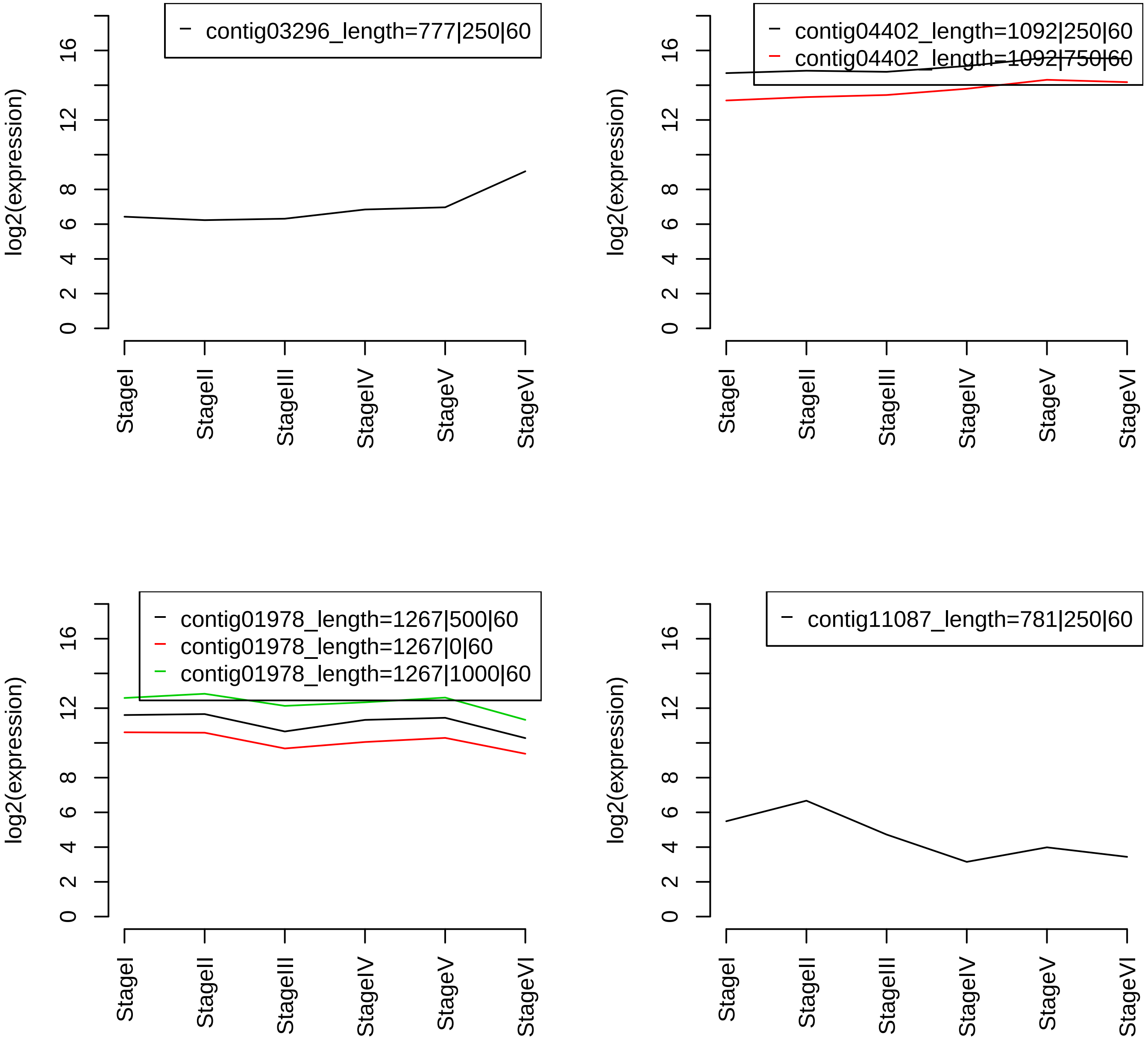
contig03296_length=777|250|60
contig04402_length=1092|250|60
contig04402_length=1092|750|60
contig01978_length=1267|500|60
contig01978_length=1267|0|60
contig01978_length=1267|1000|60
contig11087_length=781|250|60
log2(expression)
0
2
4
6
8
12
16
StageI
StageII
StageIII
StageIV
StageV
StageVI

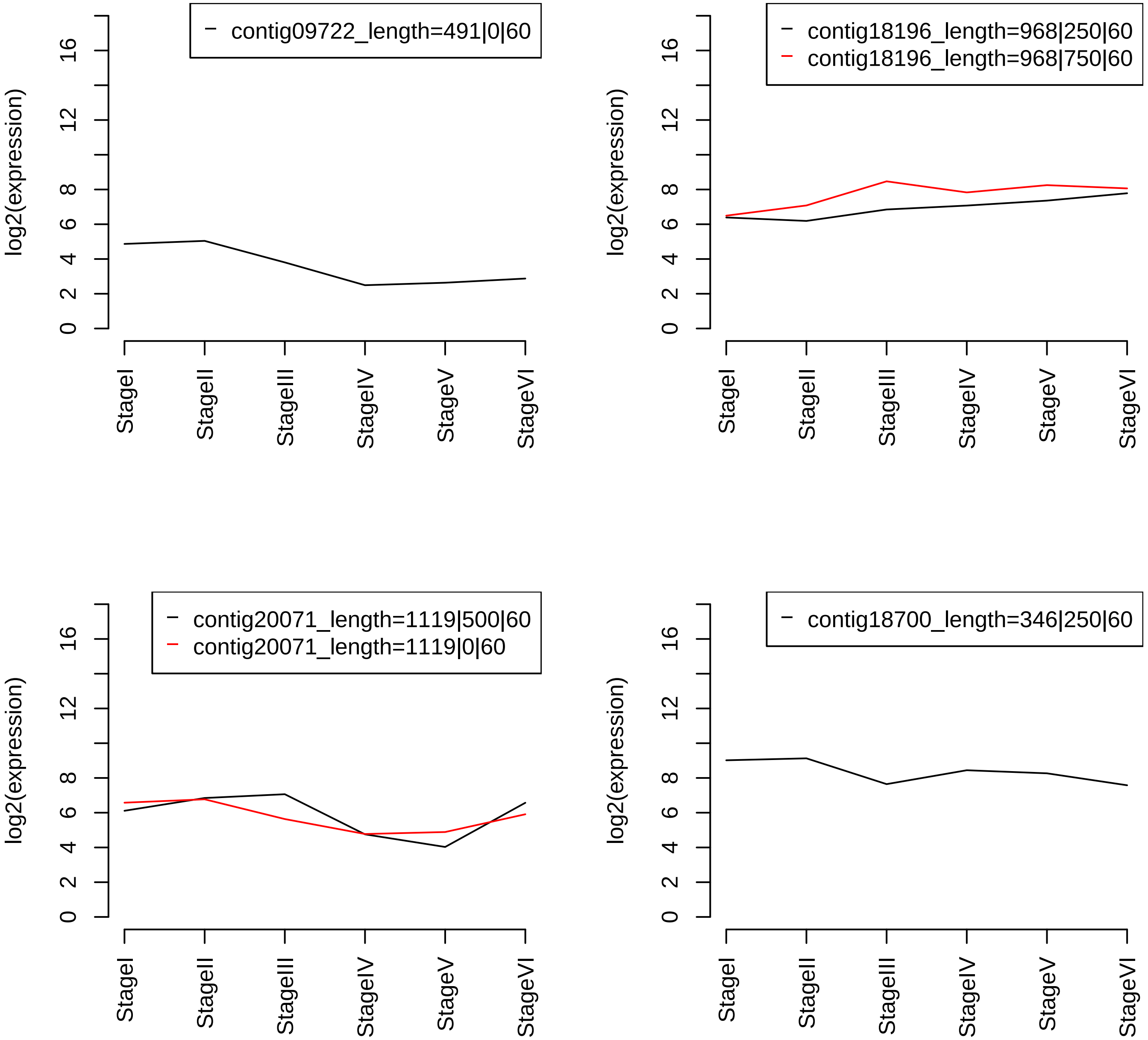
contig09722_length=491|0|60
contig18196_length=968|250|60
contig18196_length=968|750|60
contig20071_length=1119|500|60
contig20071_length=1119|0|60
contig18700_length=346|250|60
log2(expression)
0
2
4
6
8
12
16
StageI
StageII
StageIII
StageIV
StageV
StageVI

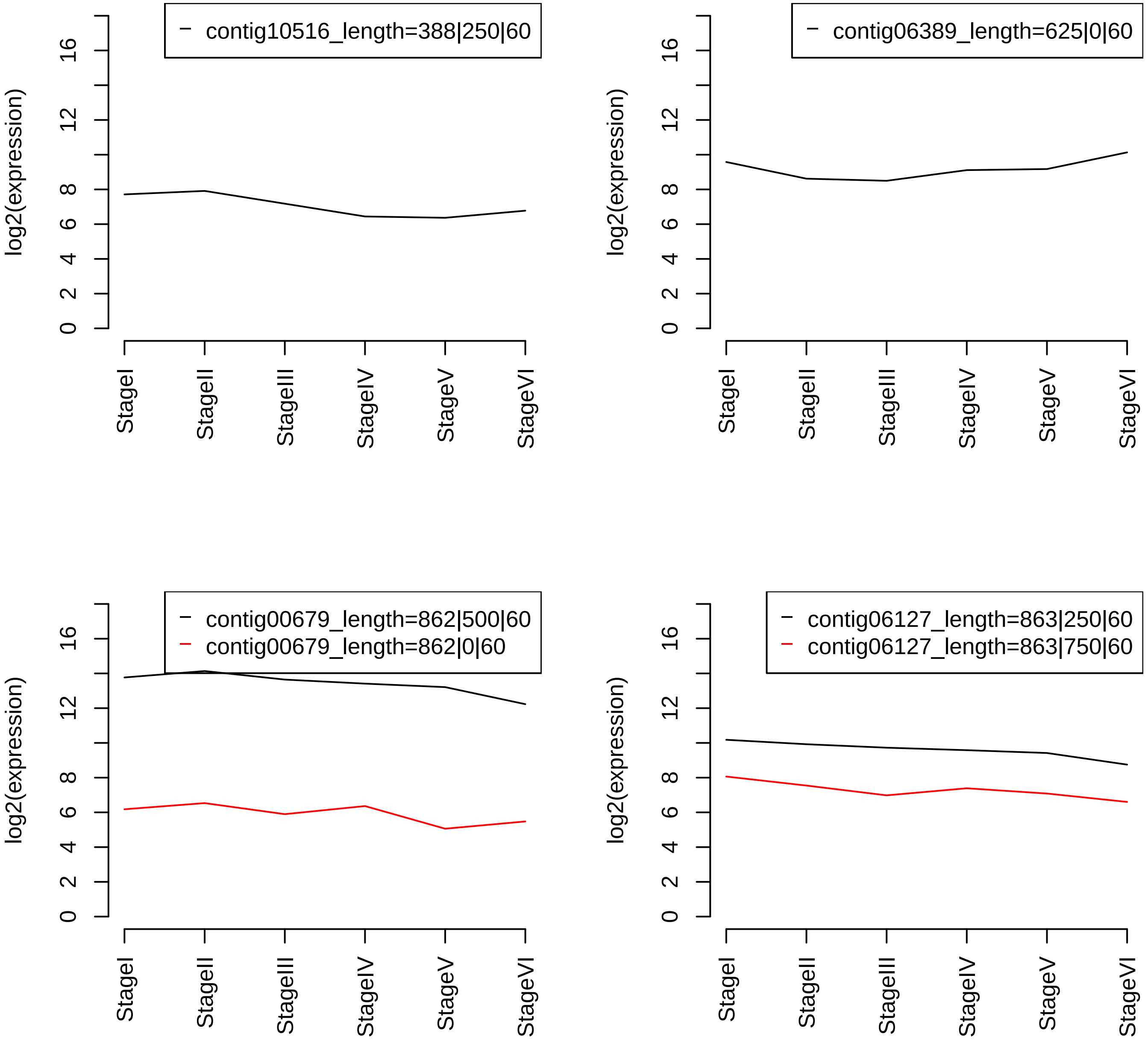

contig10516_length=388|250|60
contig06389_length=625|0|60
contig00679_length=862|500|60
contig00679_length=862|0|60
contig06127_length=863|250|60
contig06127_length=863|750|60
log2(expression)
0
2
4
6
8
12
16
StageI
StageII
StageIII
StageIV
StageV
StageVI

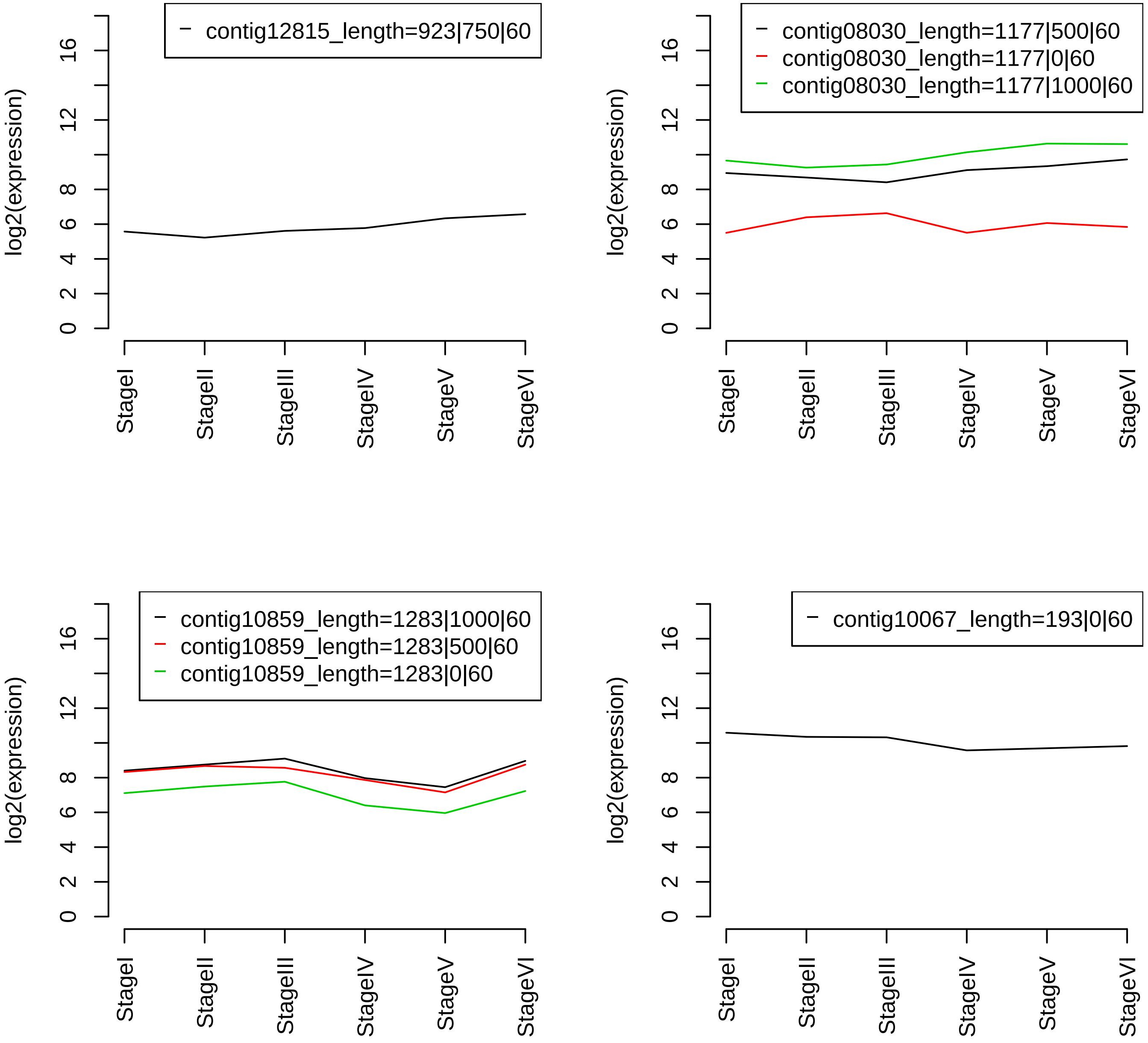
contig12815_length=923|750|60
contig08030_length=1177|500|60
contig08030_length=1177|0|60
contig08030_length=1177|1000|60
contig10859_length=1283|1000|60
contig10859_length=1283|500|60
contig10859_length=1283|0|60
contig10067_length=193|0|60
log2(expression)
0
2
4
6
8
12
16
StageI
StageII
StageIII
StageIV
StageV
StageVI

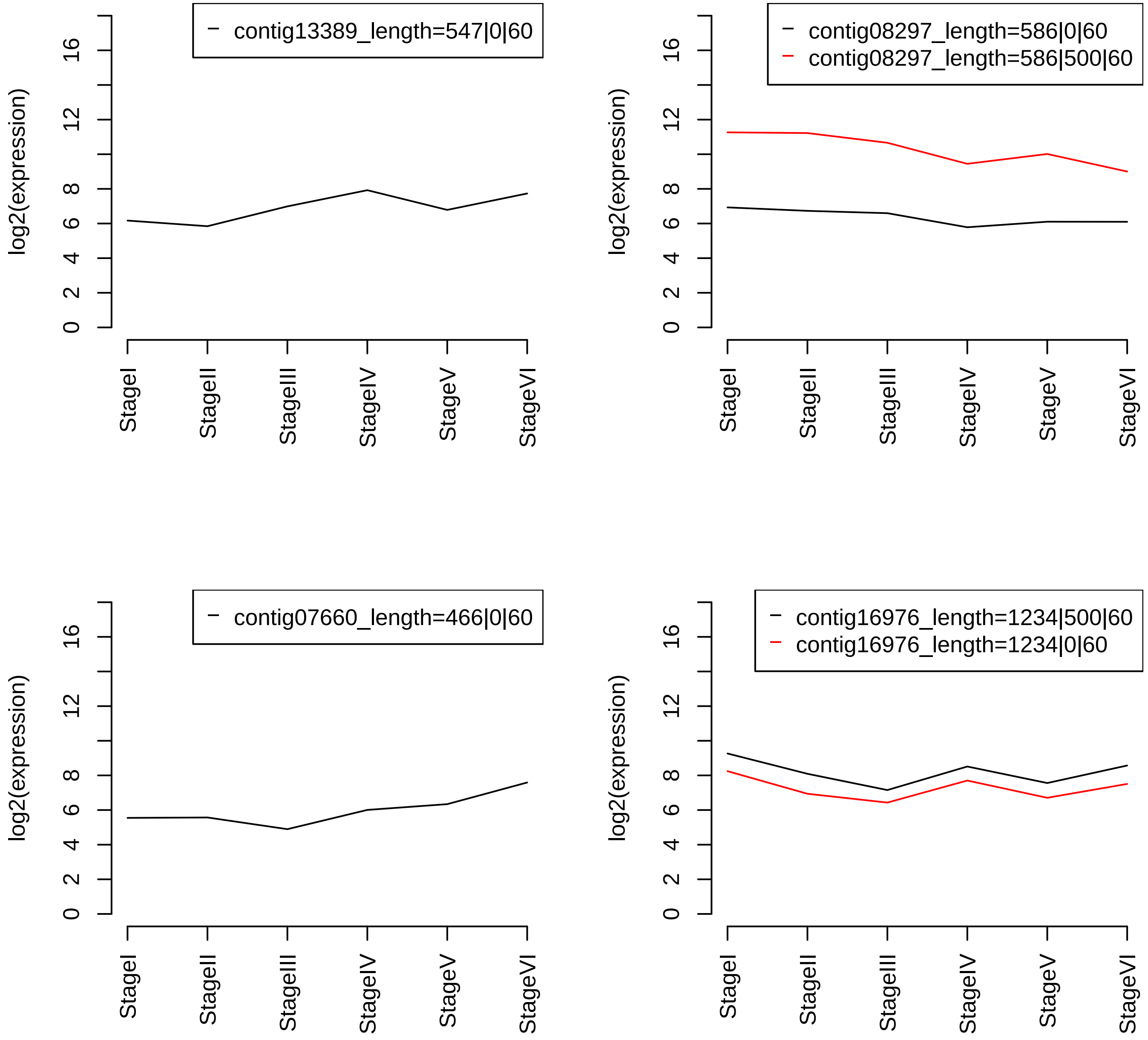
contig13389_length=547|0|60
contig08297_length=586|0|60
contig08297_length=586|500|60
contig07660_length=466|0|60
contig16976_length=1234|500|60
contig16976_length=1234|0|60
log2(expression)
0
2
4
6
8
12
16
StageI
StageII
StageIII
StageIV
StageV
StageVI

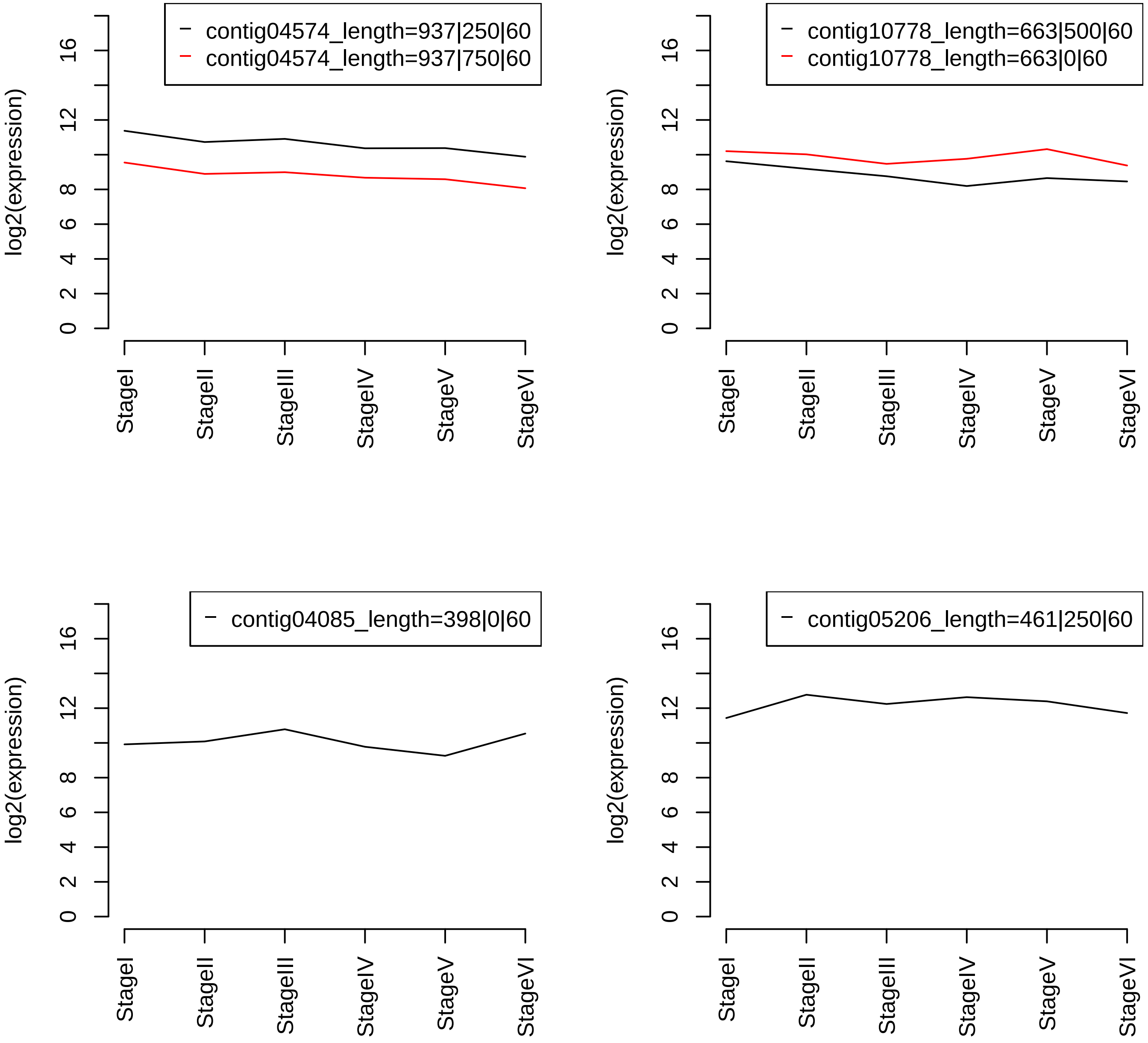
contig04574_length=937|250|60
contig04574_length=937|750|60
contig10778_length=663|500|60
contig10778_length=663|0|60
contig04085_length=398|0|60
contig05206_length=461|250|60
log2(expression)
0
2
4
6
8
12
16
StageI
StageII
StageIII
StageIV
StageV
StageVI

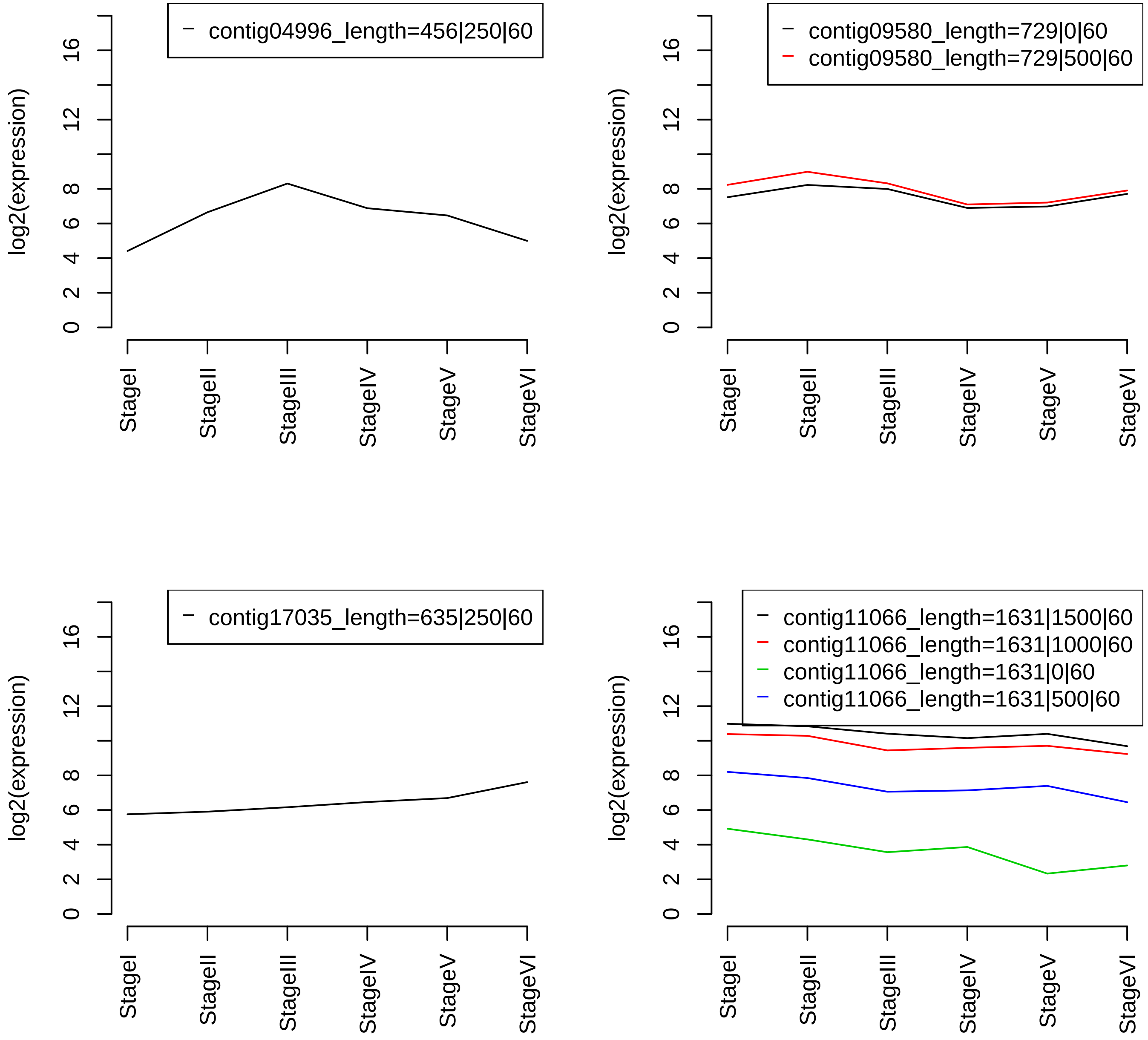
contig04996_length=456|250|60
contig09580_length=729|0|60
contig09580_length=729|500|60
contig17035_length=635|250|60
contig11066_length=1631|1500|60
contig11066_length=1631|1000|60
contig11066_length=1631|0|60
contig11066_length=1631|500|60
log2(expression)
0
2
4
6
8
12
16
StageI
StageII
StageIII
StageIV
StageV
StageVI

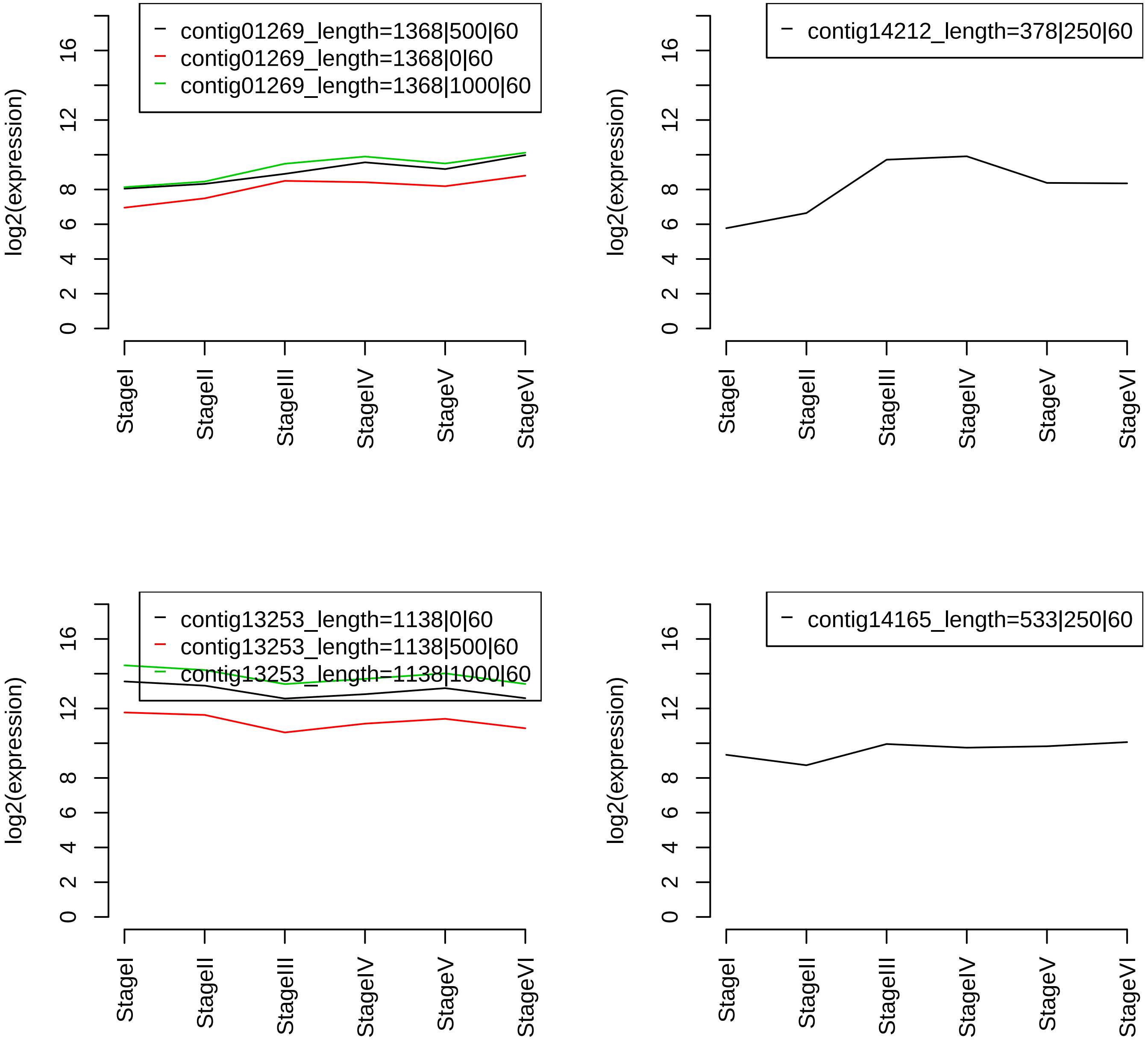

contig01269_length=1368|500|60
contig01269_length=1368|0|60
contig01269_length=1368|1000|60
log2(expression)
0
2
4
6
8
12
16
StageI
StageII
StageIII
StageIV
StageV
StageVI
contig14212_length=378|250|60
log2(expression)
0
2
4
6
8
12
16
StageI
StageII
StageIII
StageIV
StageV
StageVI
contig13253_length=1138|0|60
contig13253_length=1138|500|60
contig13253_length=1138|1000|60
log2(expression)
0
2
4
6
8
12
16
StageI
StageII
StageIII
StageIV
StageV
StageVI
contig14165_length=533|250|60
log2(expression)
0
2
4
6
8
12
16
StageI
StageII
StageIII
StageIV
StageV
StageVI

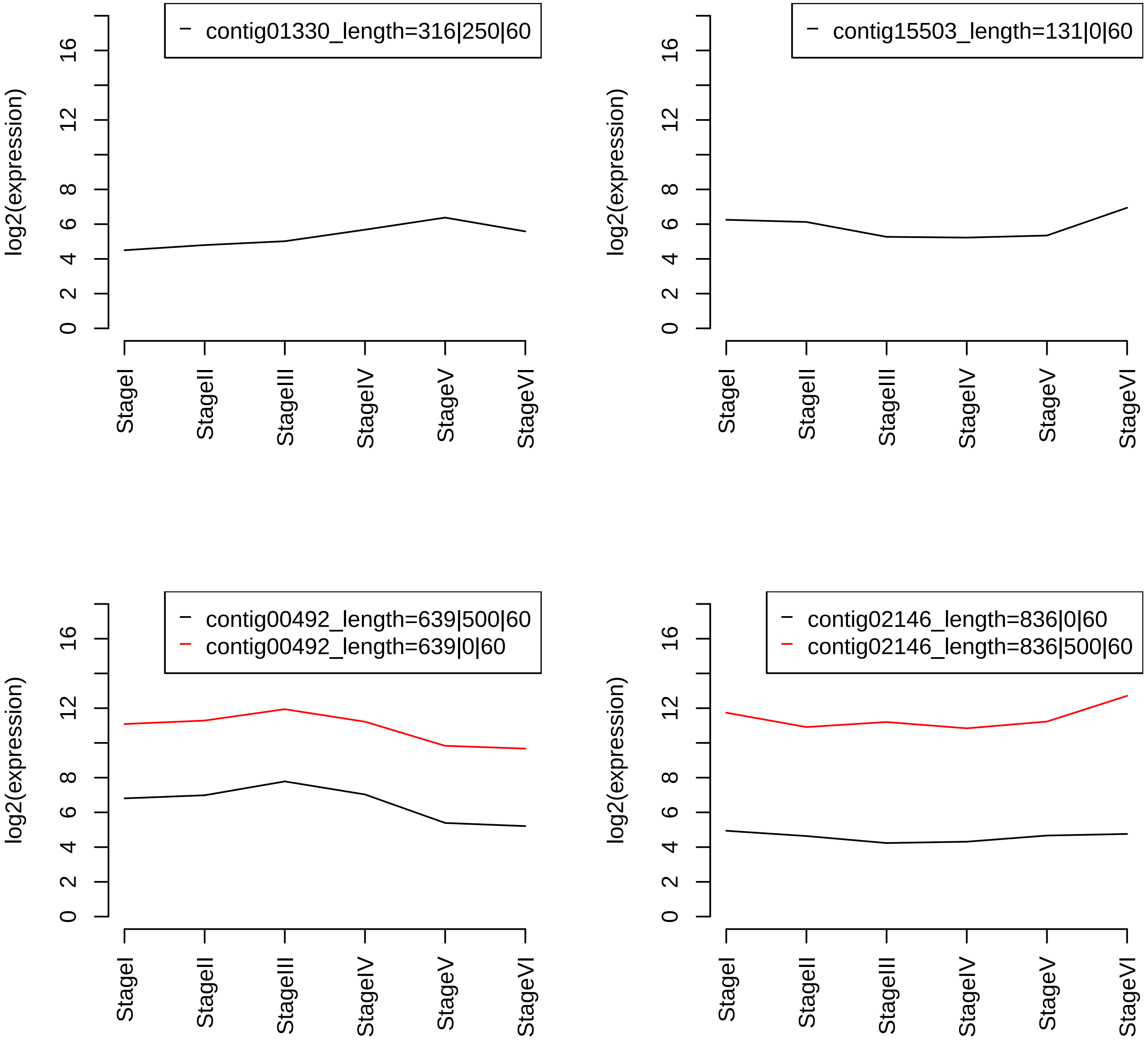
contig01330_length=316|250|60
contig15503_length=131|0|60
contig00492_length=639|500|60
contig00492_length=639|0|60
contig02146_length=836|0|60
contig02146_length=836|500|60
log2(expression)
0
2
4
6
8
12
16
StageI
StageII
StageIII
StageIV
StageV
StageVI

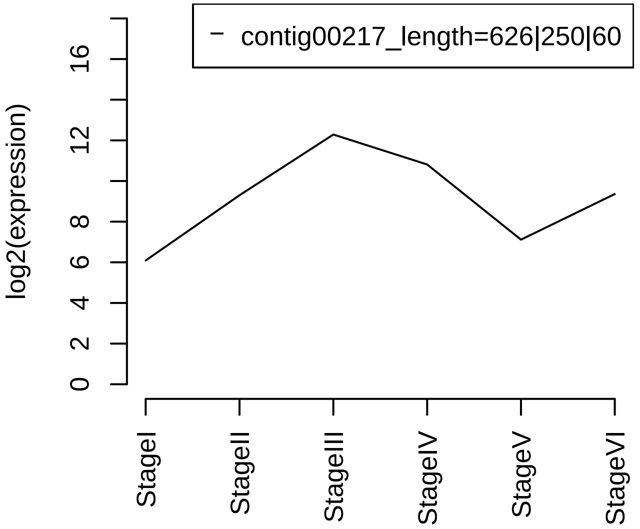

contig00217_length=626|250|60
log2(expression)
0 2 4 6 8 12 16
StageI StageII StageIII StageIV StageV StageVI

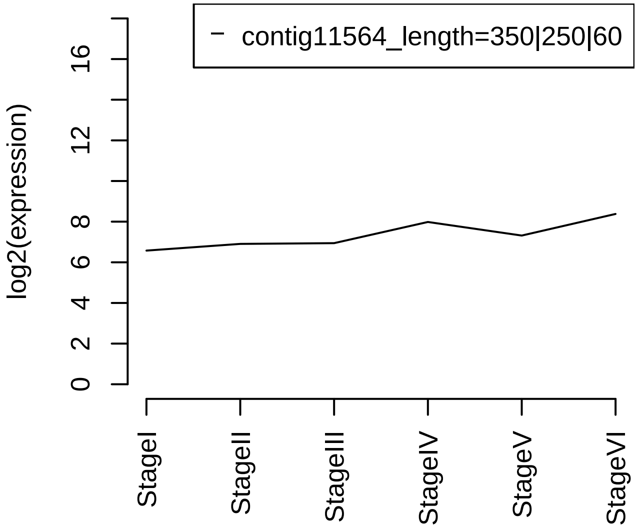

contig11564_length=350|250|60
log2(expression)
0 2 4 6 8 12 16
StageI StageII StageIII StageIV StageV StageVI

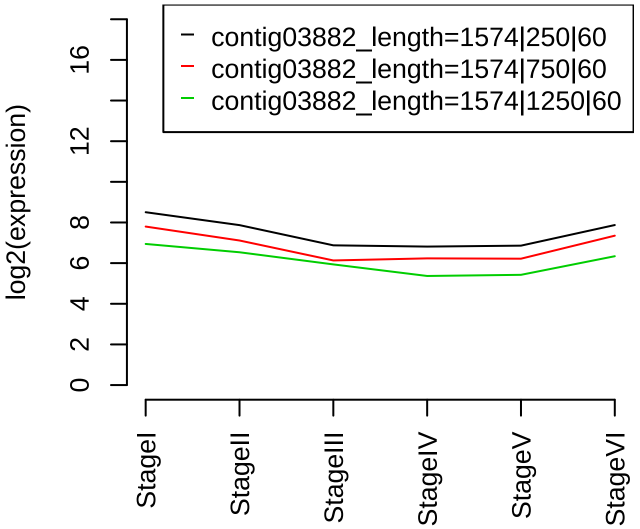

contig03882_length=1574|250|60
contig03882_length=1574|750|60
contig03882_length=1574|1250|60
log2(expression)
0 2 4 6 8 12 16
StageI StageII StageIII StageIV StageV StageVI

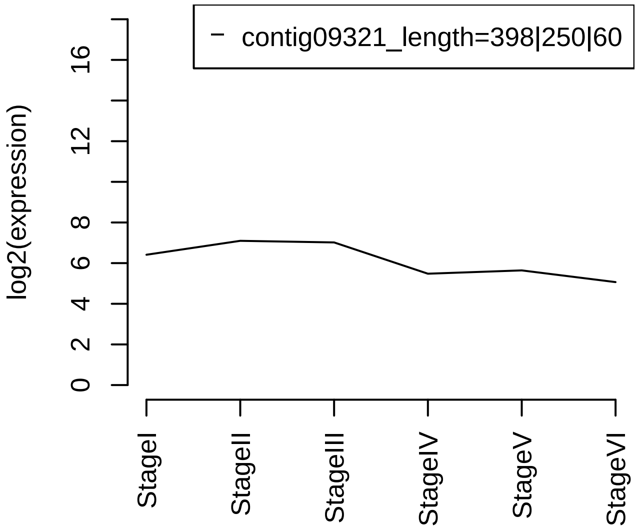

contig09321_length=398|250|60
log2(expression)
0 2 4 6 8 12 16
StageI StageII StageIII StageIV StageV StageVI

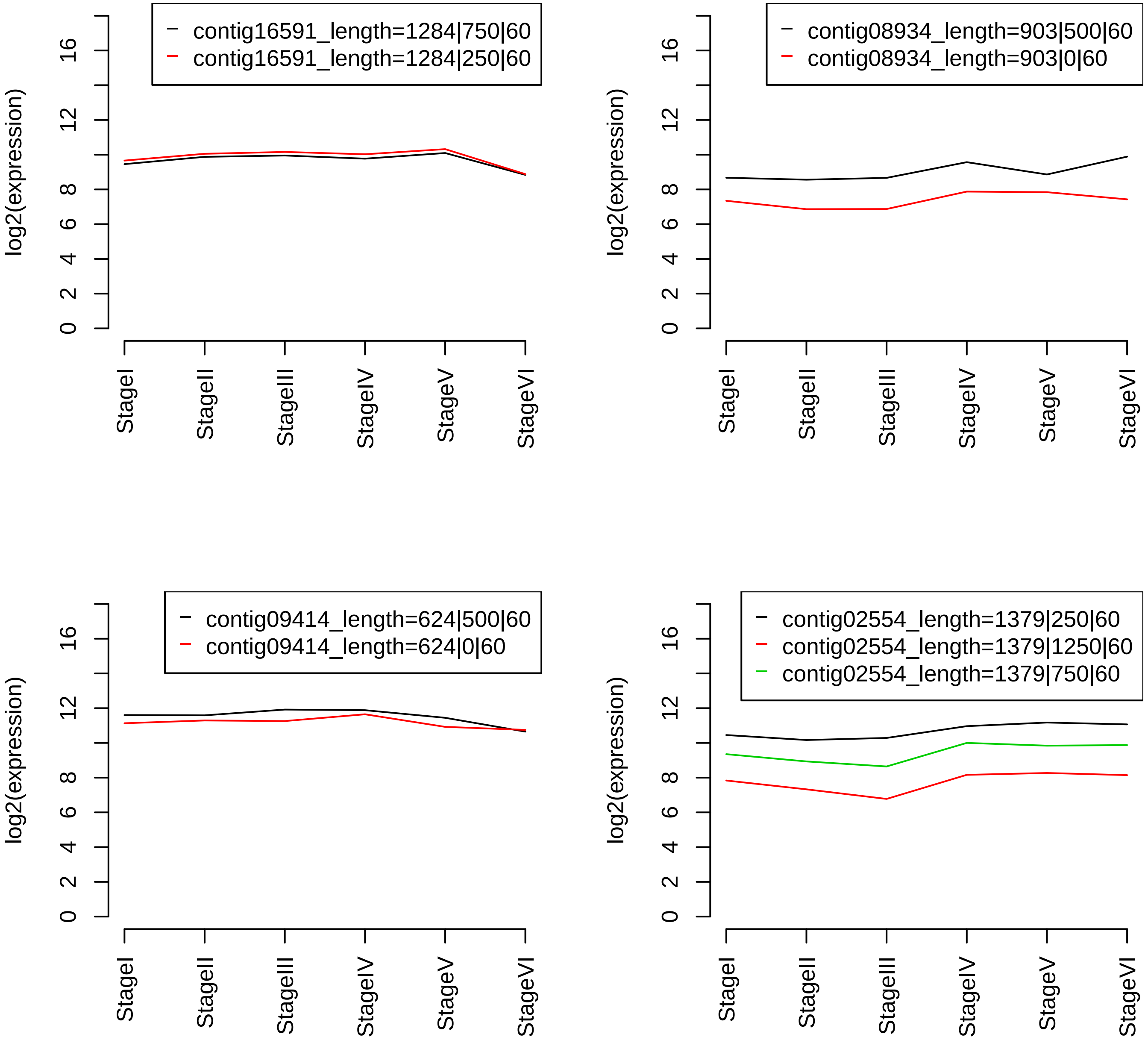

contig16591_length=1284|750|60
contig16591_length=1284|250|60
log2(expression)
0
2
4
6
8
12
16
StageI
StageII
StageIII
StageIV
StageV
StageVI
contig08934_length=903|500|60
contig08934_length=903|0|60
contig09414_length=624|500|60
contig09414_length=624|0|60
contig02554_length=1379|250|60
contig02554_length=1379|1250|60
contig02554_length=1379|750|60

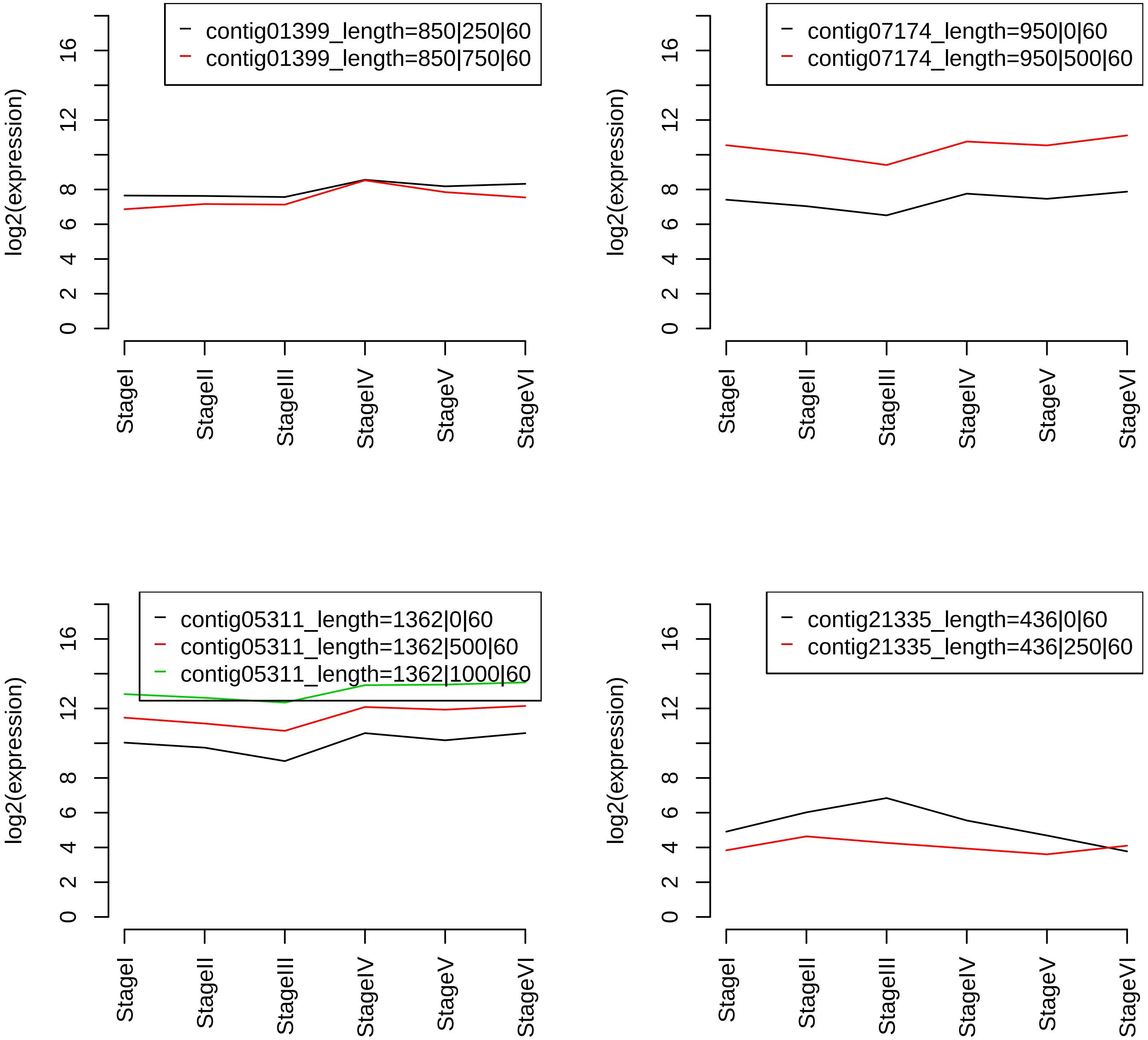
contig01399_length=850|250|60
contig01399_length=850|750|60
contig07174_length=950|0|60
contig07174_length=950|500|60
contig05311_length=1362|0|60
contig05311_length=1362|500|60
contig05311_length=1362|1000|60
contig21335_length=436|0|60
contig21335_length=436|250|60
log2(expression)
0
2
4
6
8
12
16
StageI
StageII
StageIII
StageIV
StageV
StageVI

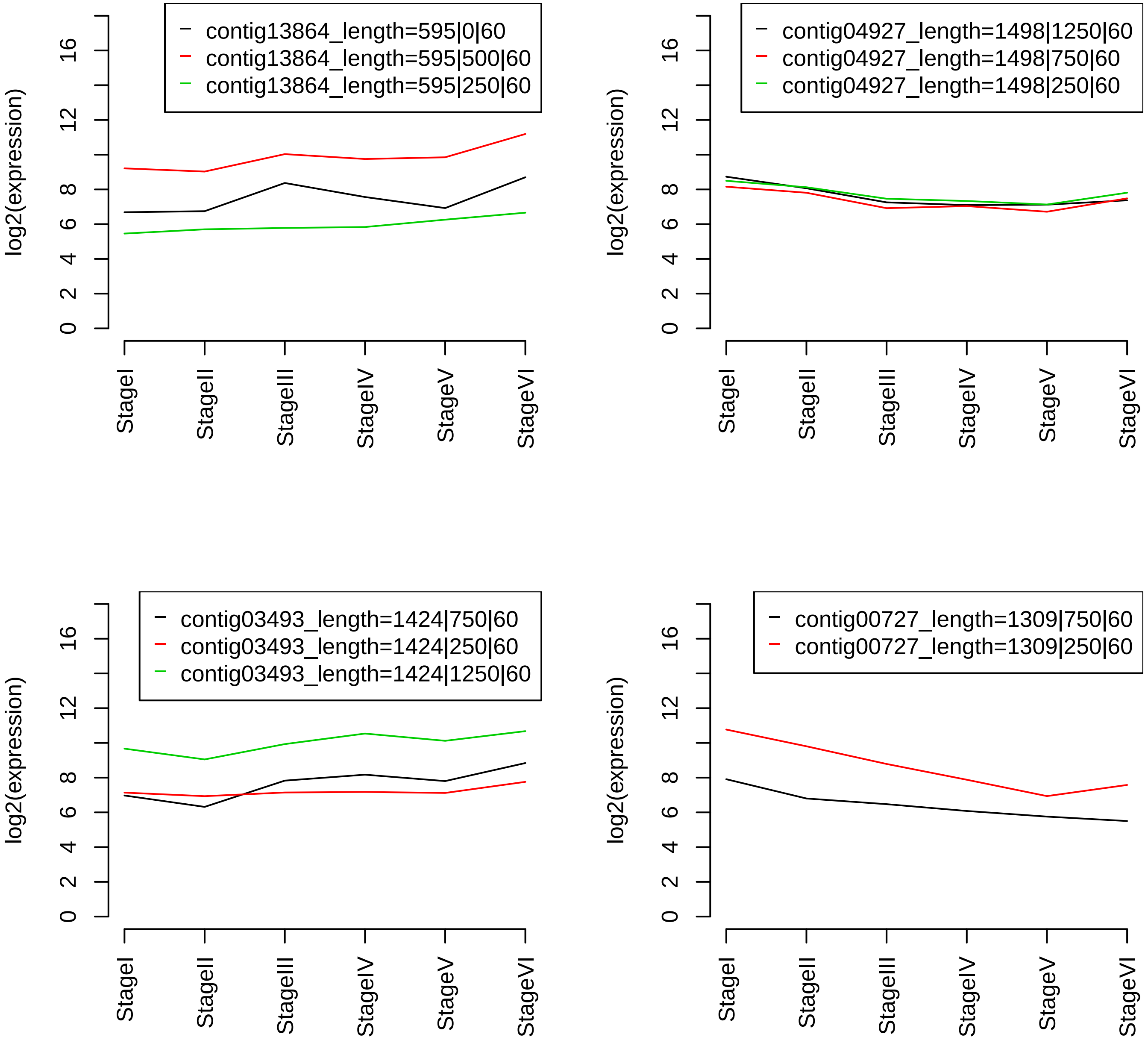

contig13864_length=595|0|60
contig13864_length=595|500|60
contig13864_length=595|250|60
contig04927_length=1498|1250|60
contig04927_length=1498|750|60
contig04927_length=1498|250|60
contig03493_length=1424|750|60
contig03493_length=1424|250|60
contig03493_length=1424|1250|60
contig00727_length=1309|750|60
contig00727_length=1309|250|60
log2(expression)
0
2
4
6
8
12
16
StageI
StageII
StageIII
StageIV
StageV
StageVI

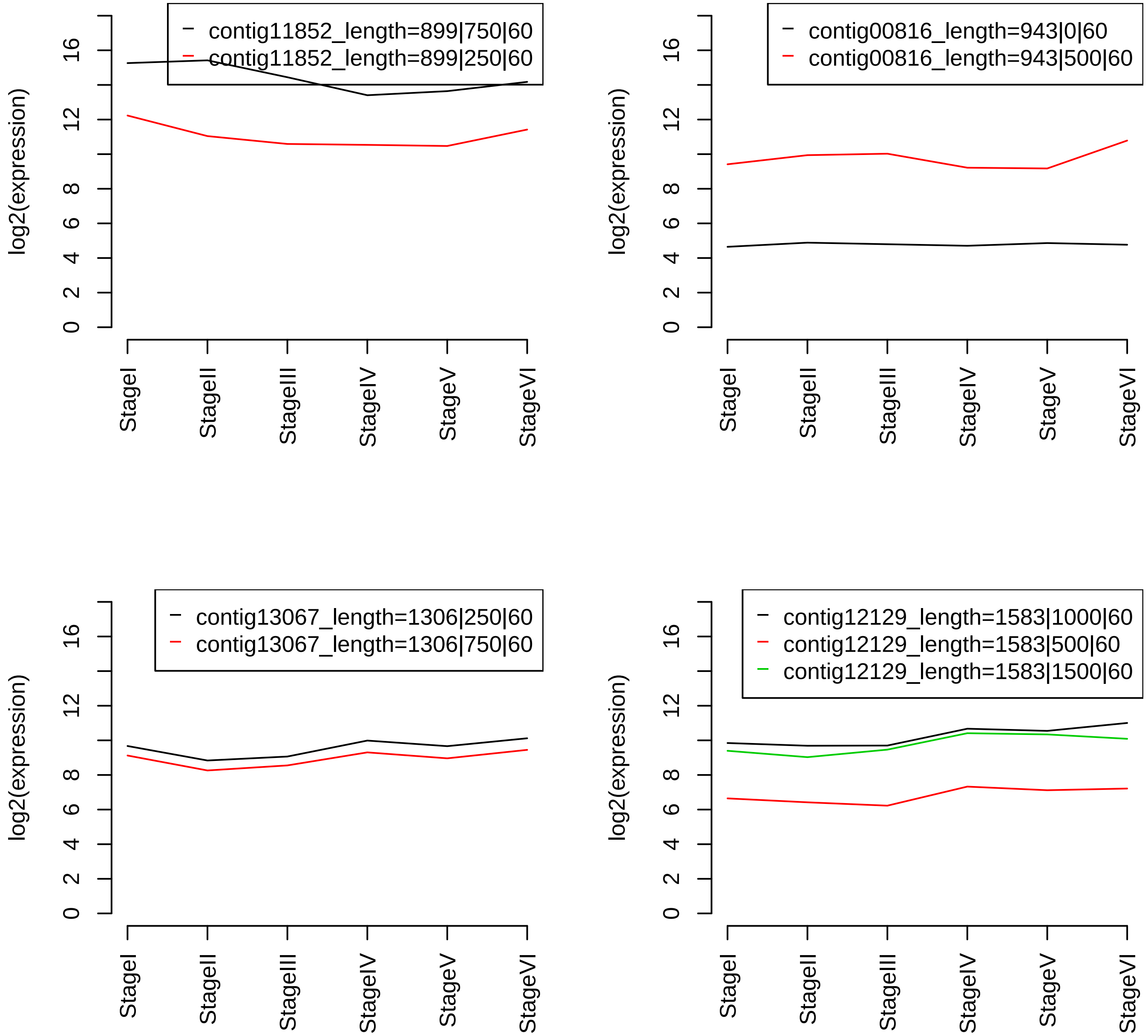
contig11852_length=899|750|60
contig11852_length=899|250|60
contig00816_length=943|0|60
contig00816_length=943|500|60
contig13067_length=1306|250|60
contig13067_length=1306|750|60
contig12129_length=1583|1000|60
contig12129_length=1583|500|60
contig12129_length=1583|1500|60
log2(expression)
0 2 4 6 8 12 16
StageI StageII StageIII StageIV StageV StageVI

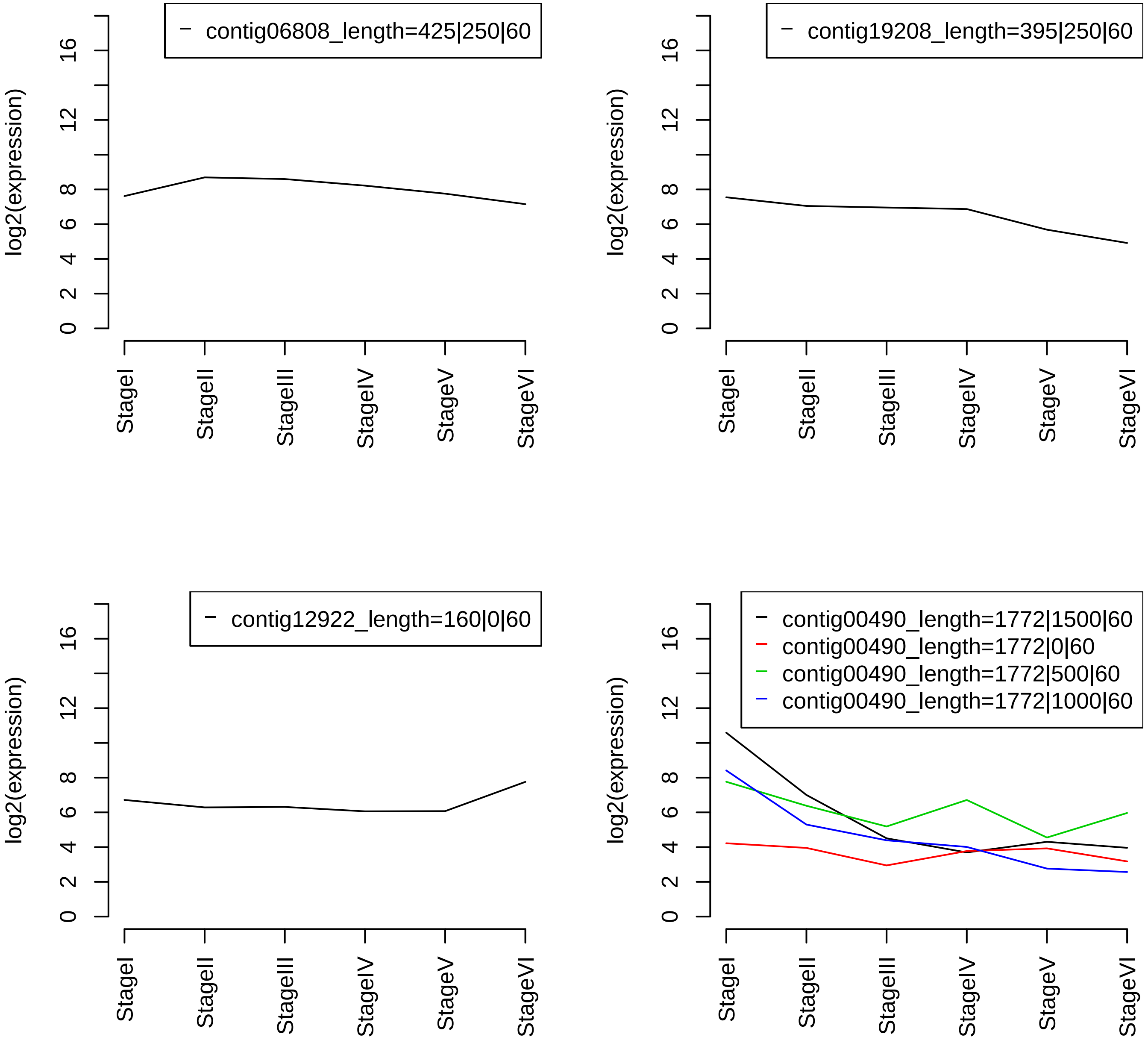

contig06808_length=425|250|60
contig19208_length=395|250|60
contig12922_length=160|0|60
contig00490_length=1772|1500|60
contig00490_length=1772|0|60
contig00490_length=1772|500|60
contig00490_length=1772|1000|60
log2(expression)
0
2
4
6
8
12
16
StageI
StageII
StageIII
StageIV
StageV
StageVI

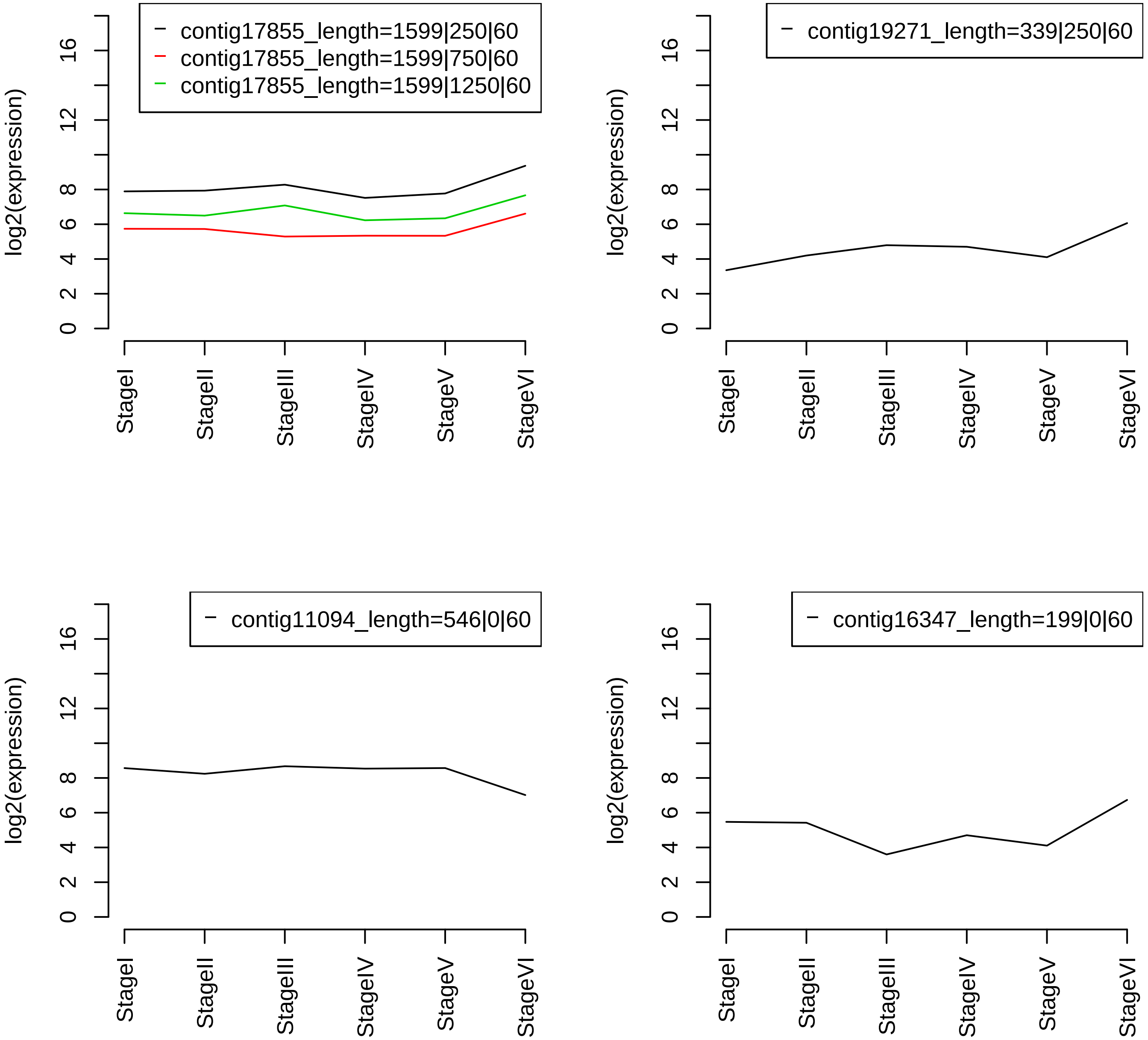

contig17855_length=1599|250|60
contig17855_length=1599|750|60
contig17855_length=1599|1250|60
contig19271_length=339|250|60
contig11094_length=546|0|60
contig16347_length=199|0|60
log2(expression)
0
2
4
6
8
12
16
StageI
StageII
StageIII
StageIV
StageV
StageVI

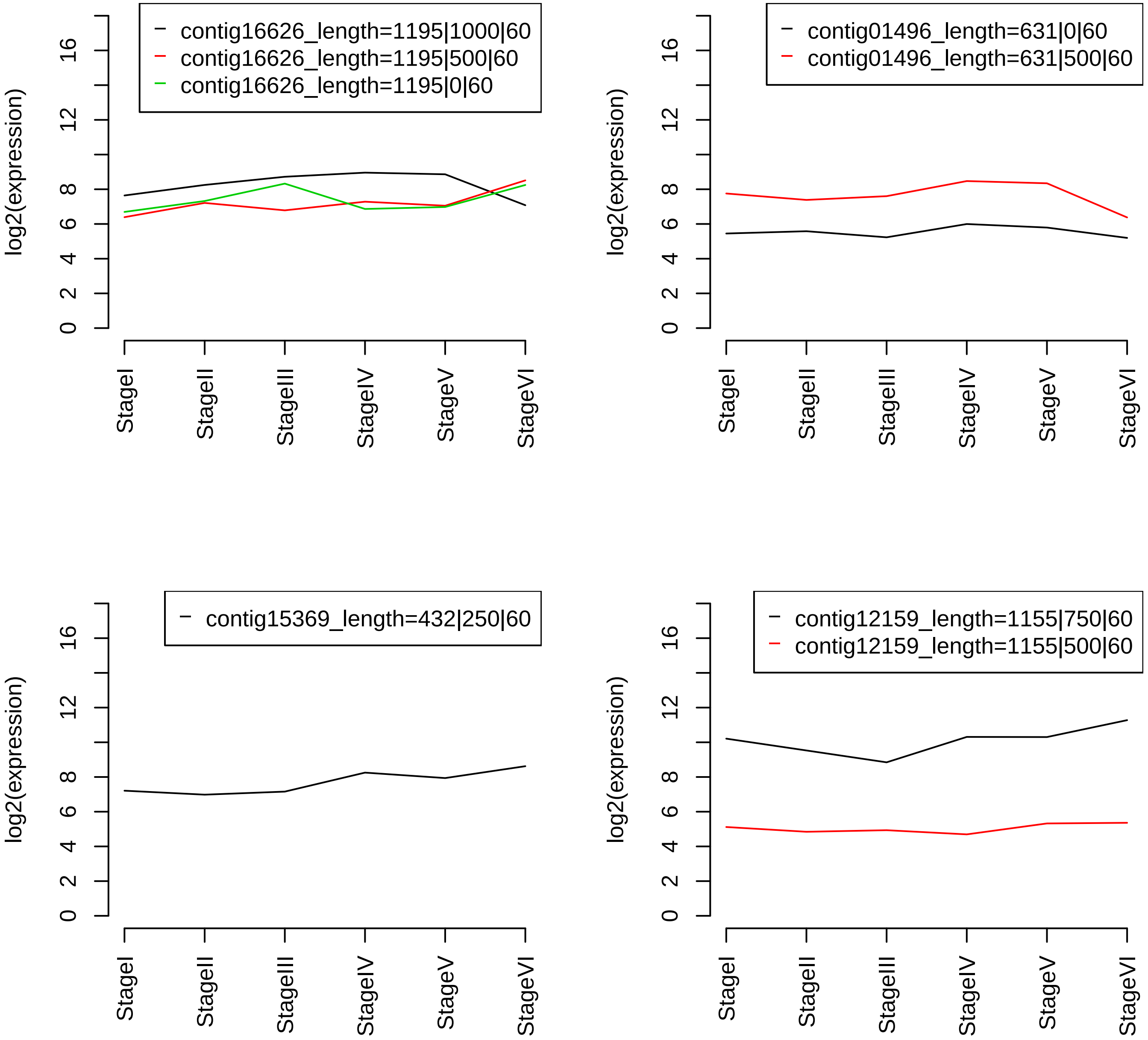
contig16626_length=1195|1000|60
contig16626_length=1195|500|60
contig16626_length=1195|0|60
contig01496_length=631|0|60
contig01496_length=631|500|60
contig15369_length=432|250|60
contig12159_length=1155|750|60
contig12159_length=1155|500|60
log2(expression)
0 2 4 6 8 12 16
StageI
StageII
StageIII
StageIV
StageV
StageVI

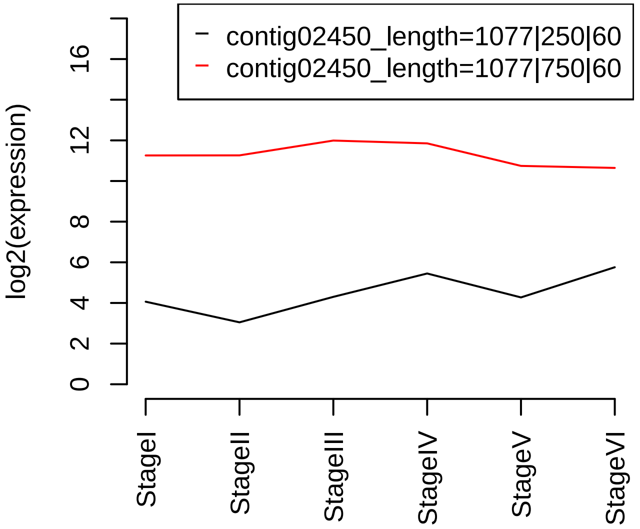

contig02450_length=1077|250|60
contig02450_length=1077|750|60
log2(expression)
0
2
4
6
8
12
16
StageI
StageII
StageIII
StageIV
StageV
StageVI

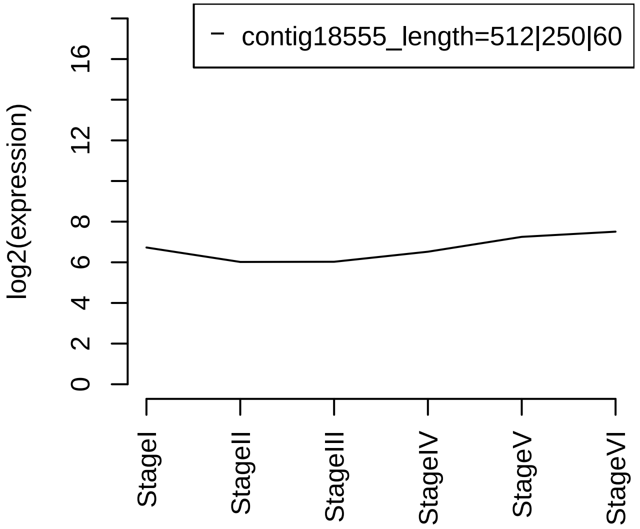

contig18555_length=512|250|60
log2(expression)
0
2
4
6
8
12
16
StageI
StageII
StageIII
StageIV
StageV
StageVI

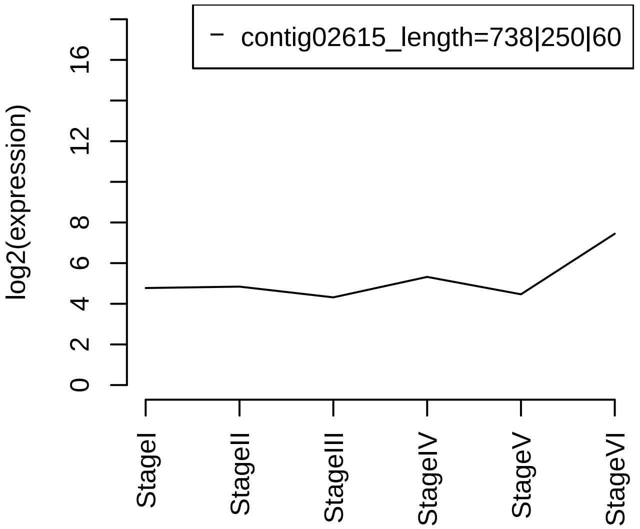

contig02615_length=738|250|60
log2(expression)
0
2
4
6
8
12
16
StageI
StageII
StageIII
StageIV
StageV
StageVI

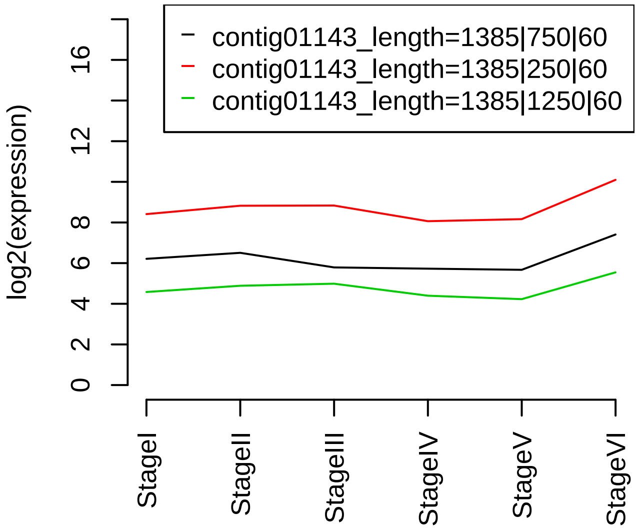

contig01143_length=1385|750|60
contig01143_length=1385|250|60
contig01143_length=1385|1250|60
log2(expression)
0
2
4
6
8
12
16
StageI
StageII
StageIII
StageIV
StageV
StageVI

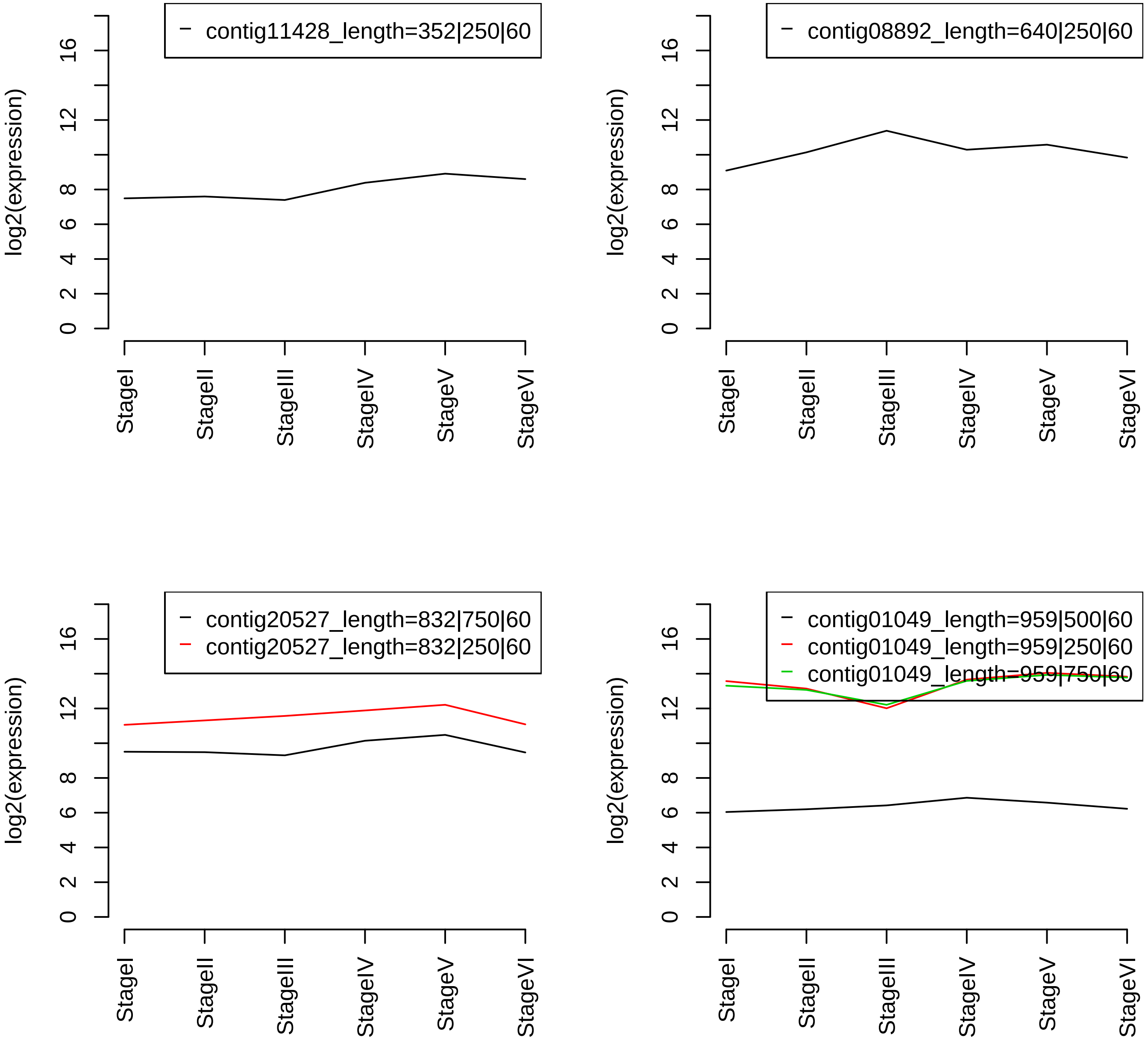

contig11428_length=352|250|60
contig08892_length=640|250|60
contig20527_length=832|750|60
contig20527_length=832|250|60
contig01049_length=959|500|60
contig01049_length=959|250|60
contig01049_length=959|750|60
log2(expression)
0
2
4
6
8
12
16
StageI
StageII
StageIII
StageIV
StageV
StageVI

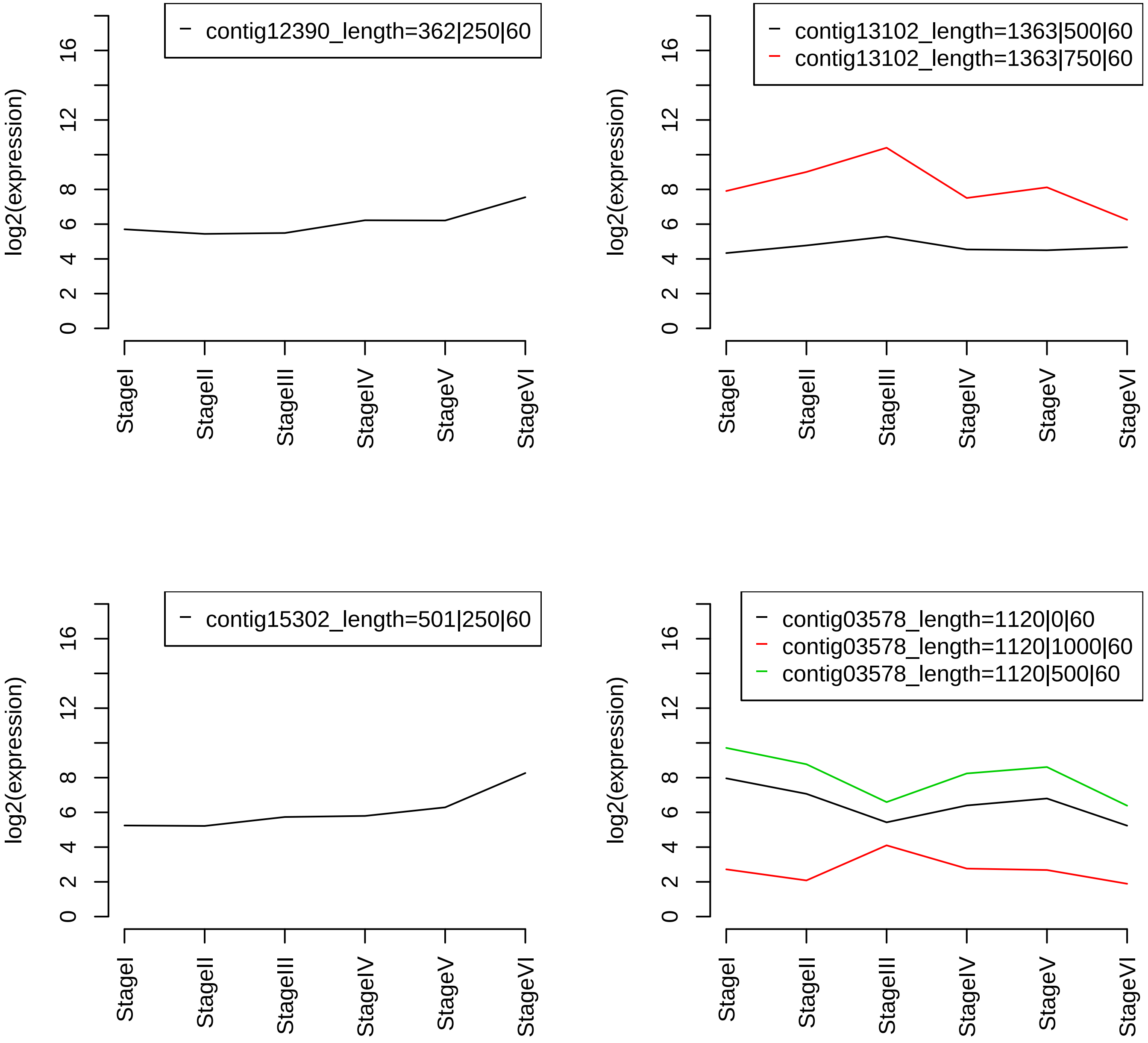

contig12390_length=362|250|60
contig13102_length=1363|500|60
contig13102_length=1363|750|60
contig15302_length=501|250|60
contig03578_length=1120|0|60
contig03578_length=1120|1000|60
contig03578_length=1120|500|60
log2(expression)
0
2
4
6
8
12
16
StageI
StageII
StageIII
StageIV
StageV
StageVI

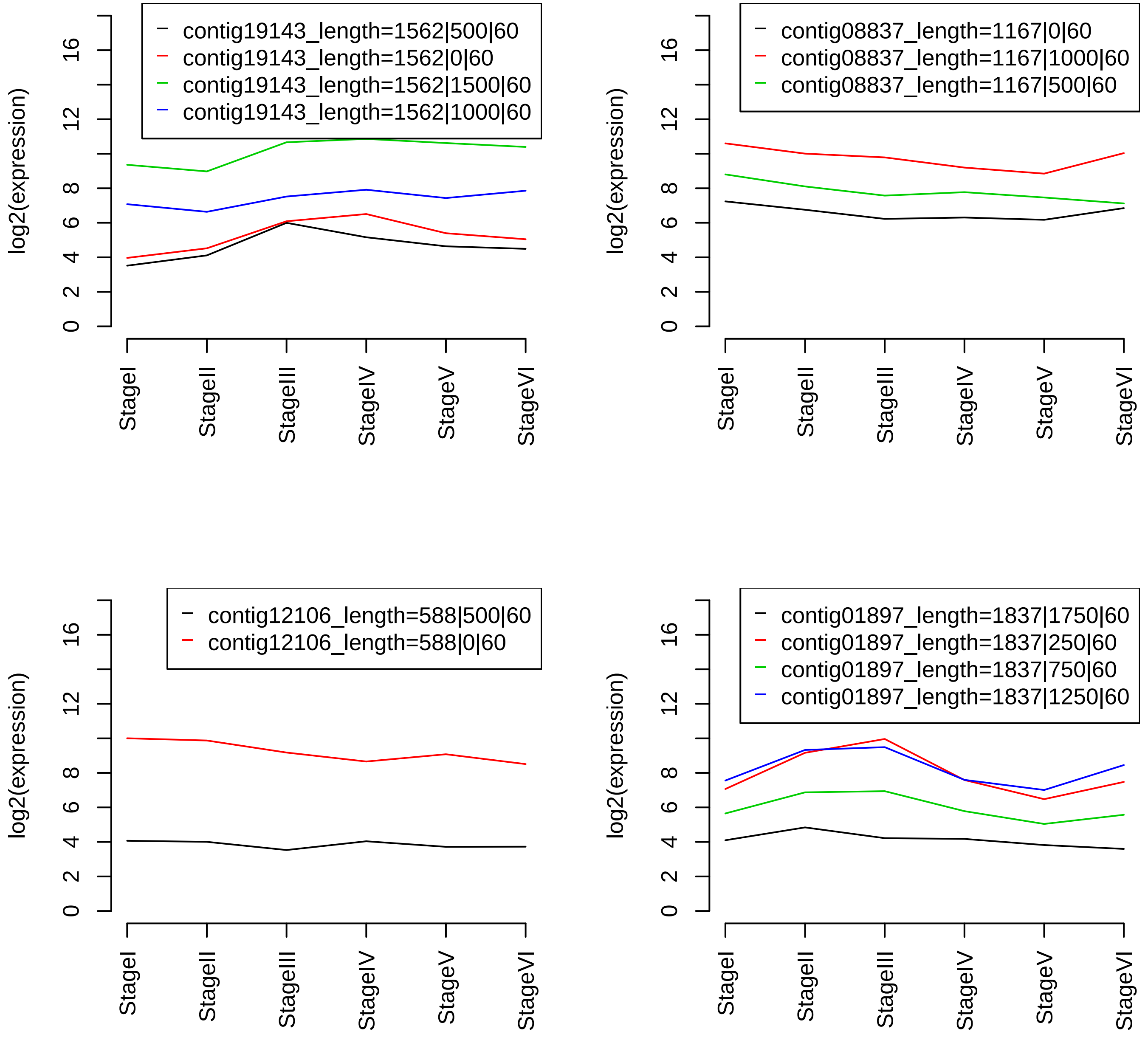
contig19143_length=1562|500|60
contig19143_length=1562|0|60
contig19143_length=1562|1500|60
contig19143_length=1562|1000|60
contig08837_length=1167|0|60
contig08837_length=1167|1000|60
contig08837_length=1167|500|60
contig12106_length=588|500|60
contig12106_length=588|0|60
contig01897_length=1837|1750|60
contig01897_length=1837|250|60
contig01897_length=1837|750|60
contig01897_length=1837|1250|60
log2(expression)
0 2 4 6 8 12 16
StageI StageII StageIII StageIV StageV StageVI

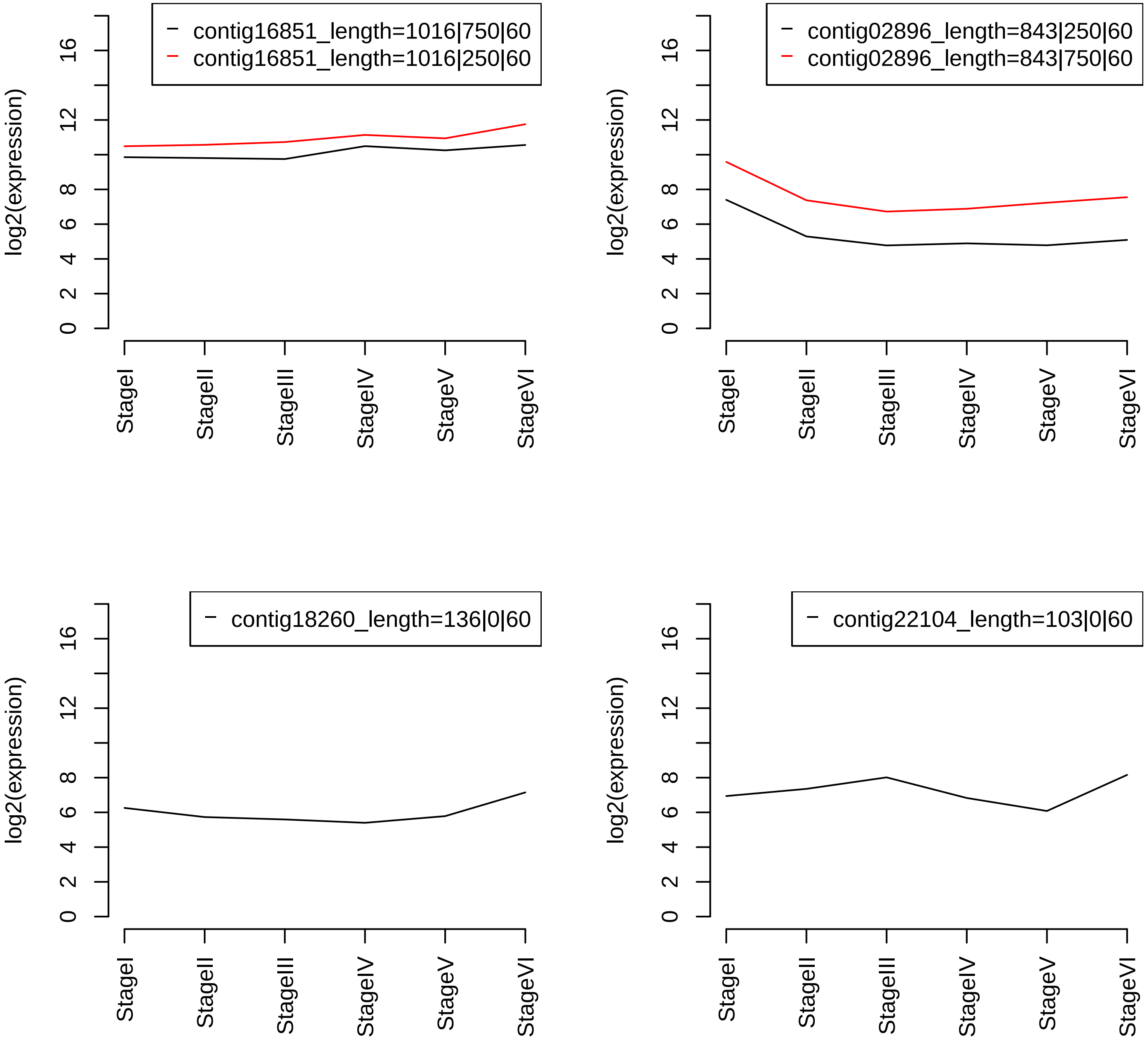
contig16851_length=1016|750|60
contig16851_length=1016|250|60
contig02896_length=843|250|60
contig02896_length=843|750|60
contig18260_length=136|0|60
contig22104_length=103|0|60
log2(expression)
0
2
4
6
8
12
16
StageI
StageII
StageIII
StageIV
StageV
StageVI

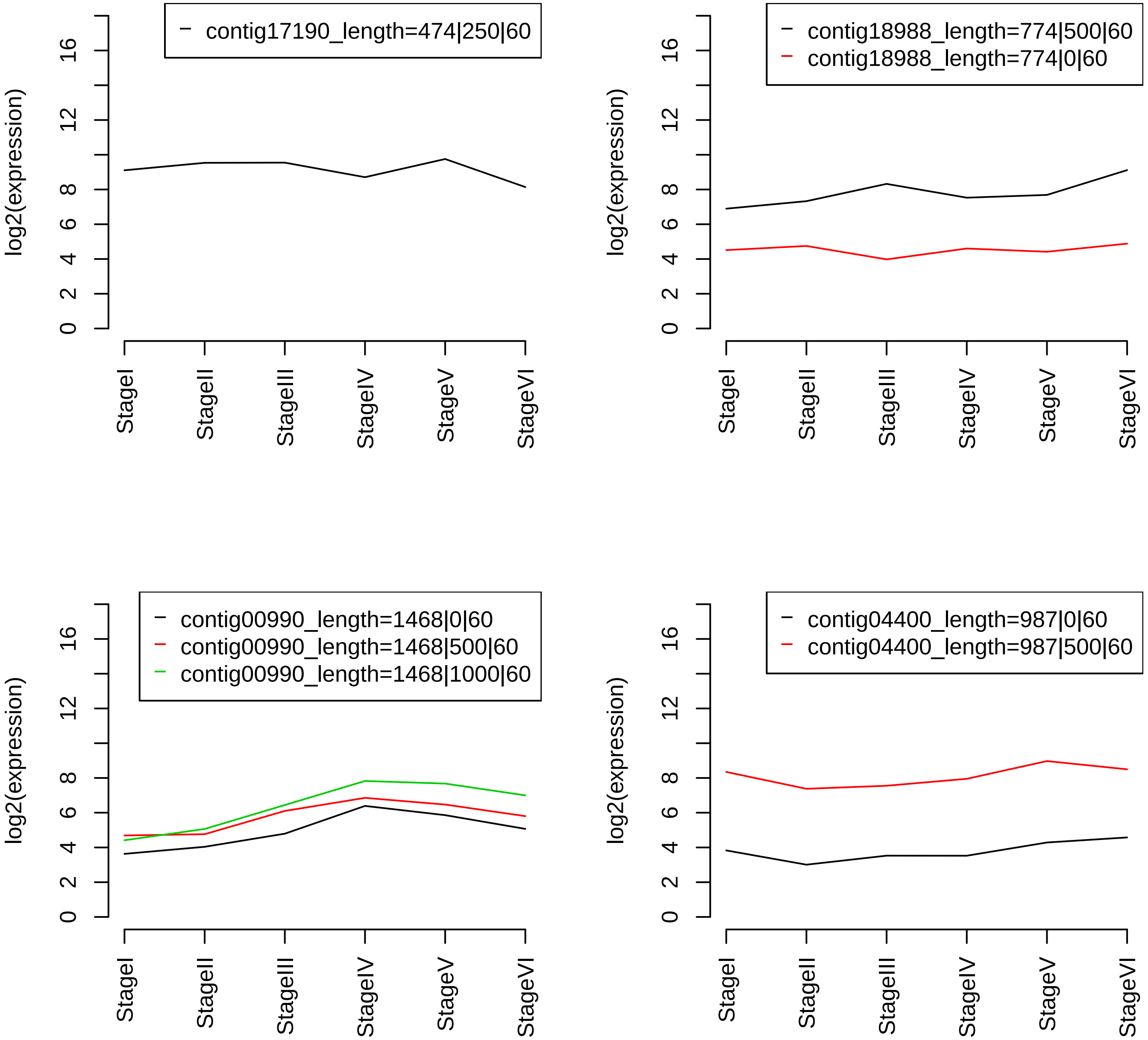
contig17190_length=474|250|60
contig18988_length=774|500|60
contig18988_length=774|0|60
contig00990_length=1468|0|60
contig00990_length=1468|500|60
contig00990_length=1468|1000|60
contig04400_length=987|0|60
contig04400_length=987|500|60
log2(expression)
0
2
4
6
8
12
16
StageI
StageII
StageIII
StageIV
StageV
StageVI

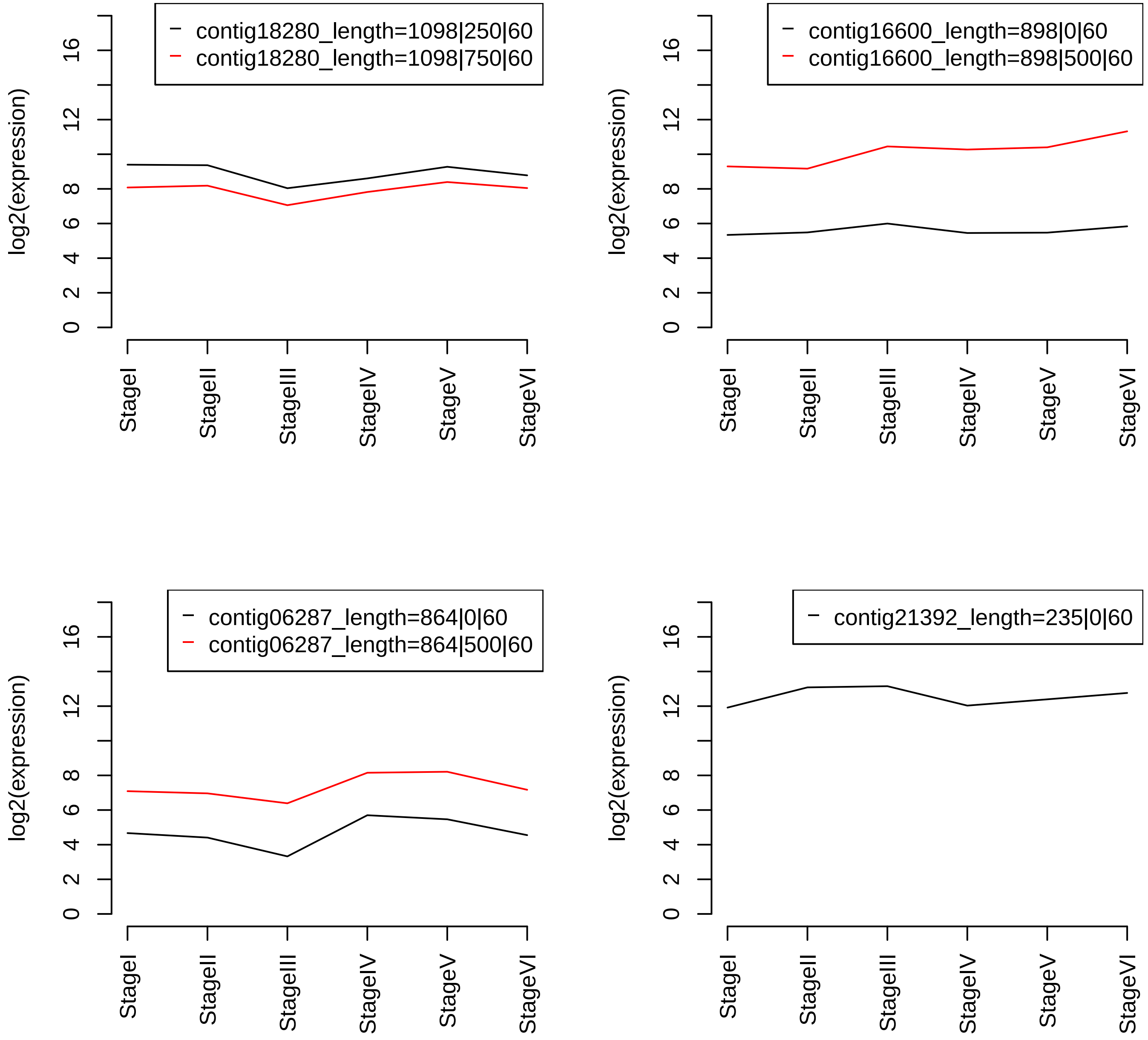

contig18280_length=1098|250|60
contig18280_length=1098|750|60
contig16600_length=898|0|60
contig16600_length=898|500|60
contig06287_length=864|0|60
contig06287_length=864|500|60
contig21392_length=235|0|60
log2(expression)
0 2 4 6 8 12 16
StageI StageII StageIII StageIV StageV StageVI

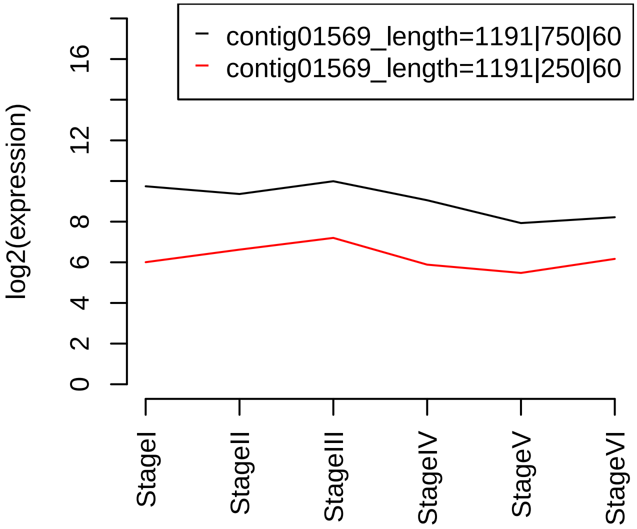

contig01569_length=1191|750|60
contig01569_length=1191|250|60
log2(expression)
0
2
4
6
8
12
16
StageI
StageII
StageIII
StageIV
StageV
StageVI

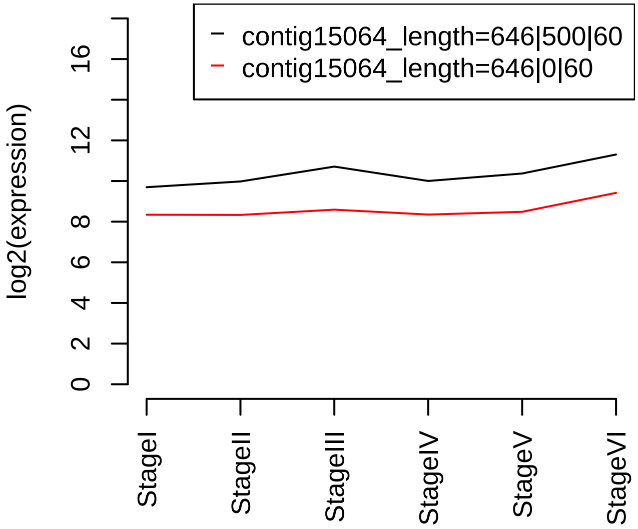

contig15064_length=646|500|60
contig15064_length=646|0|60
log2(expression)
0
2
4
6
8
12
16
StageI
StageII
StageIII
StageIV
StageV
StageVI

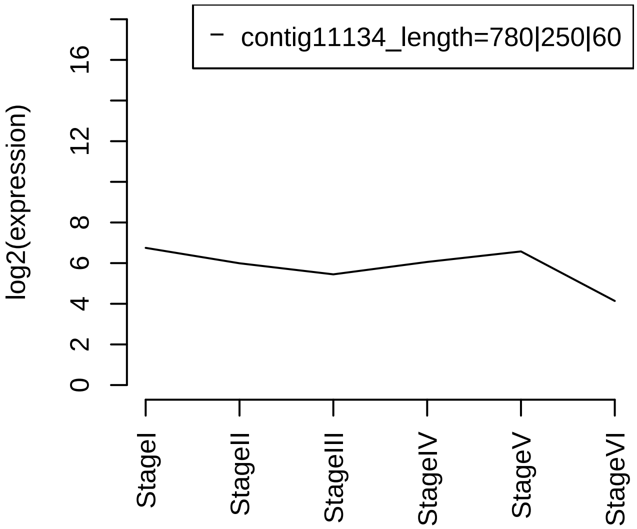

contig11134_length=780|250|60
log2(expression)
0
2
4
6
8
12
16
StageI
StageII
StageIII
StageIV
StageV
StageVI

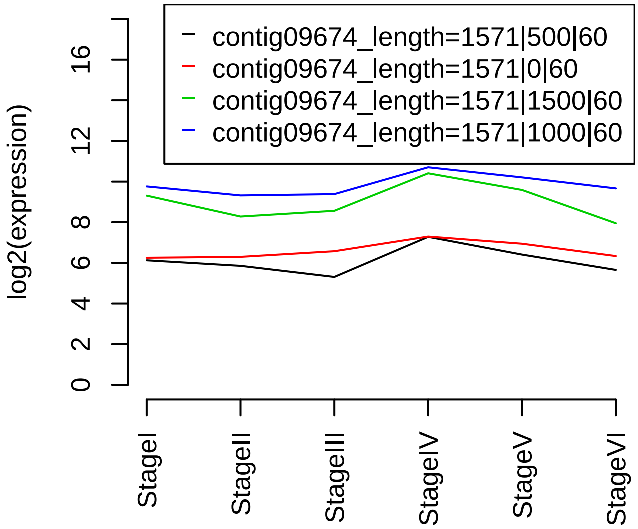

contig09674_length=1571|500|60
contig09674_length=1571|0|60
contig09674_length=1571|1500|60
contig09674_length=1571|1000|60
log2(expression)
0
2
4
6
8
12
16
StageI
StageII
StageIII
StageIV
StageV
StageVI

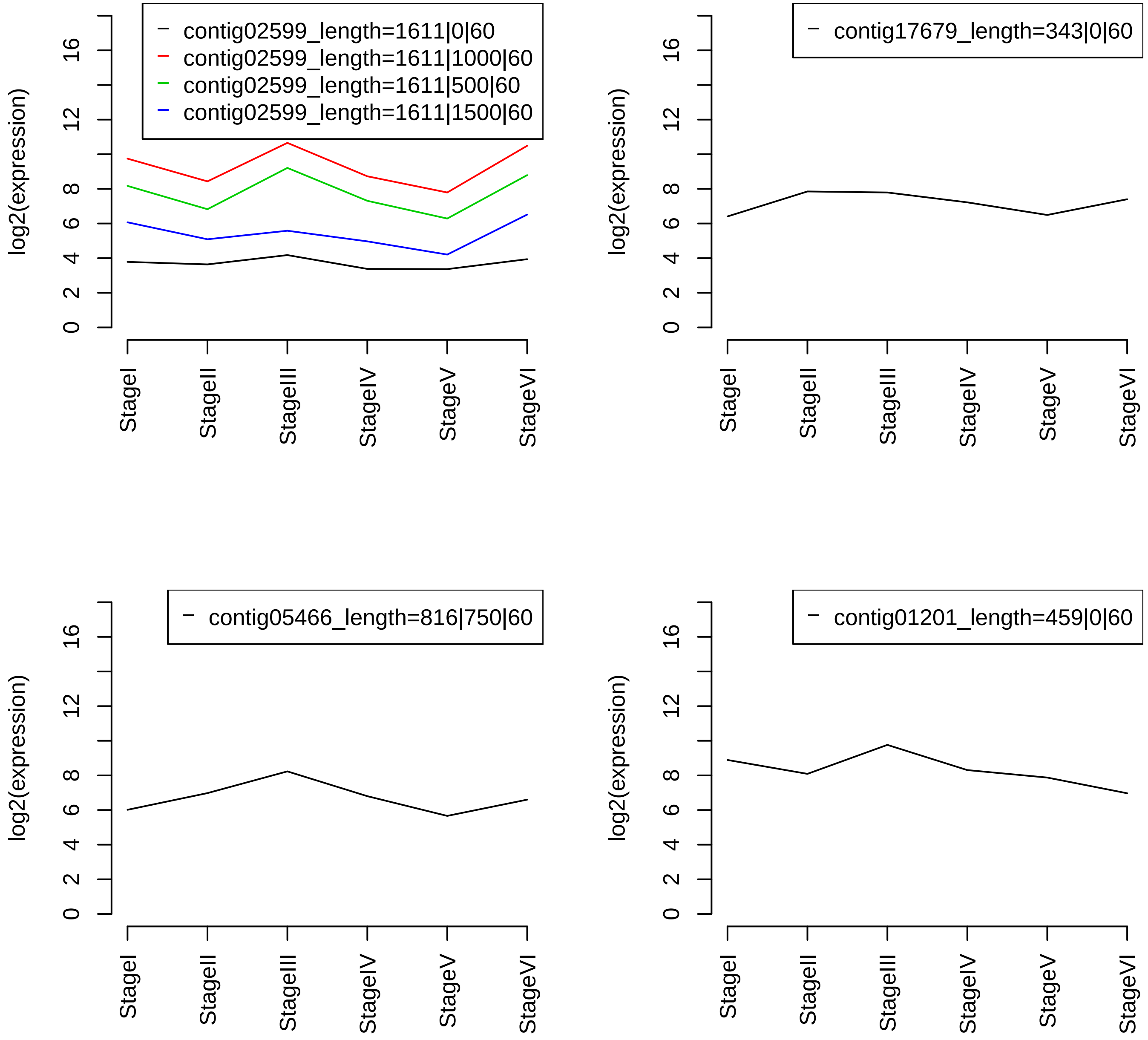

contig02599_length=1611|0|60
contig02599_length=1611|1000|60
contig02599_length=1611|500|60
contig02599_length=1611|1500|60
contig17679_length=343|0|60
contig05466_length=816|750|60
contig01201_length=459|0|60
log2(expression)
0
2
4
6
8
12
16
StageI
StageII
StageIII
StageIV
StageV
StageVI

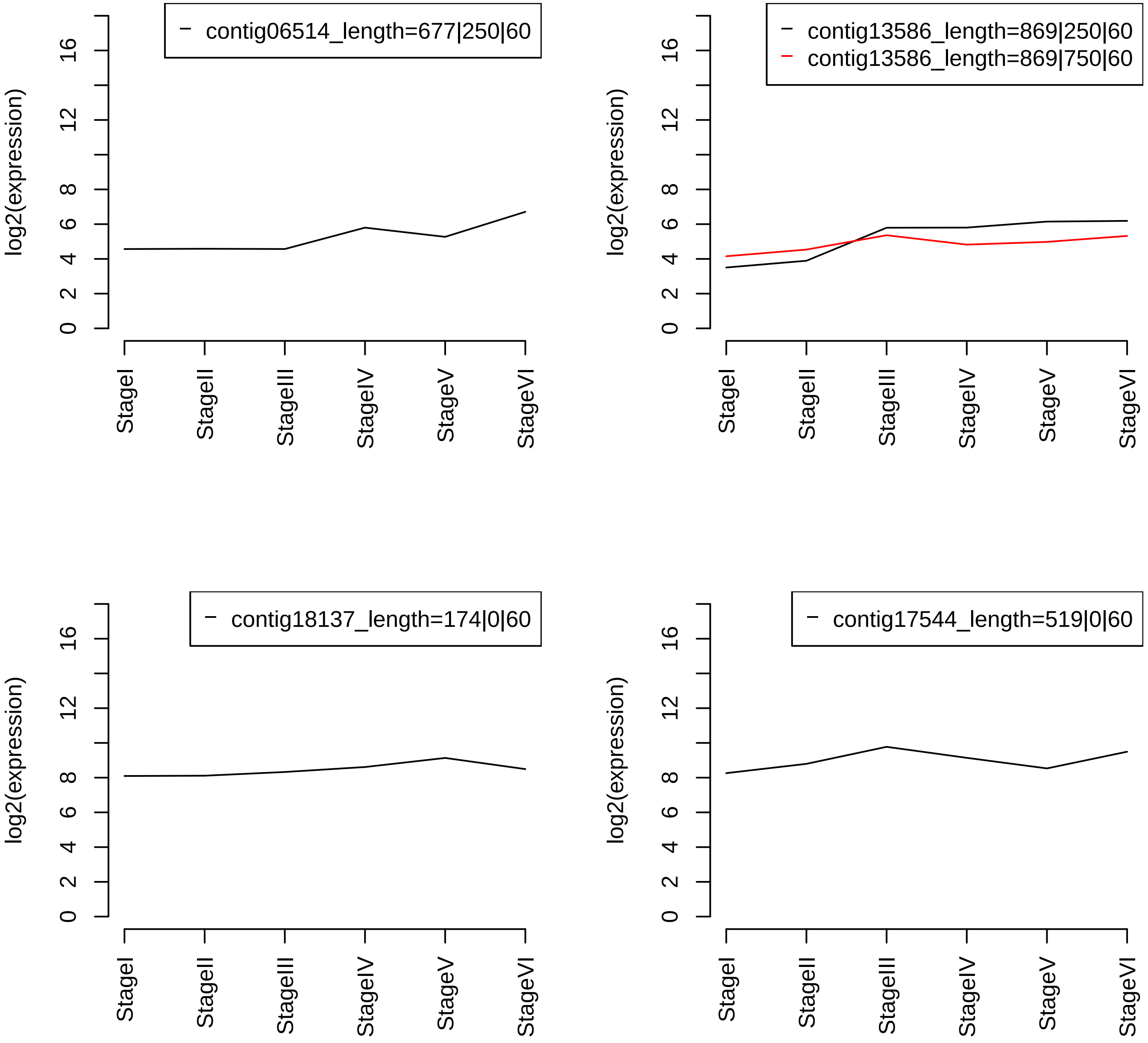

contig06514_length=677|250|60
contig13586_length=869|250|60
contig13586_length=869|750|60
contig18137_length=174|0|60
contig17544_length=519|0|60
log2(expression)
0 2 4 6 8 12 16
StageI
StageII
StageIII
StageIV
StageV
StageVI

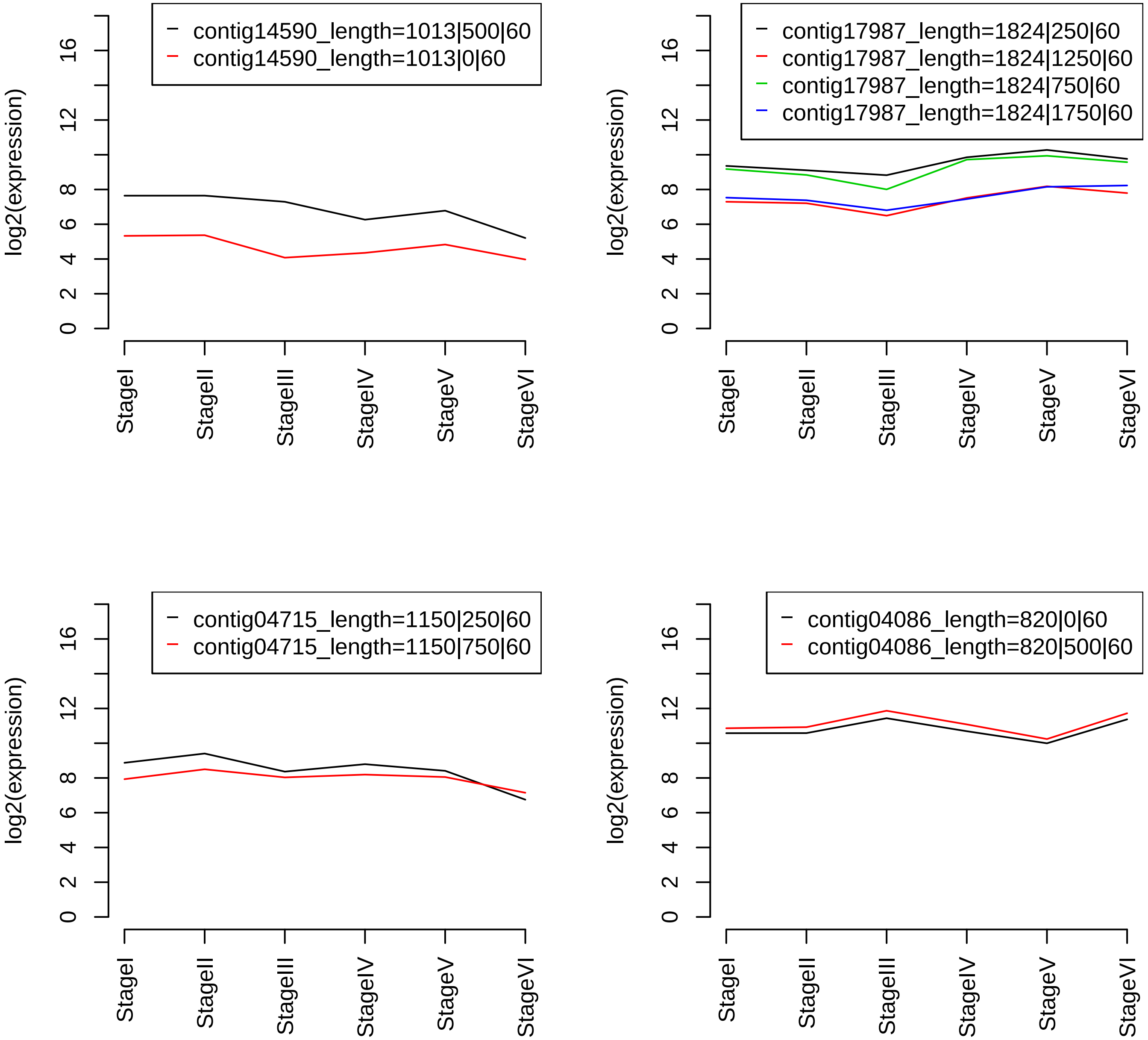

contig14590_length=1013|500|60
contig14590_length=1013|0|60
contig17987_length=1824|250|60
contig17987_length=1824|1250|60
contig17987_length=1824|750|60
contig17987_length=1824|1750|60
contig04715_length=1150|250|60
contig04715_length=1150|750|60
contig04086_length=820|0|60
contig04086_length=820|500|60
log2(expression)
0
2
4
6
8
12
16
StageI
StageII
StageIII
StageIV
StageV
StageVI

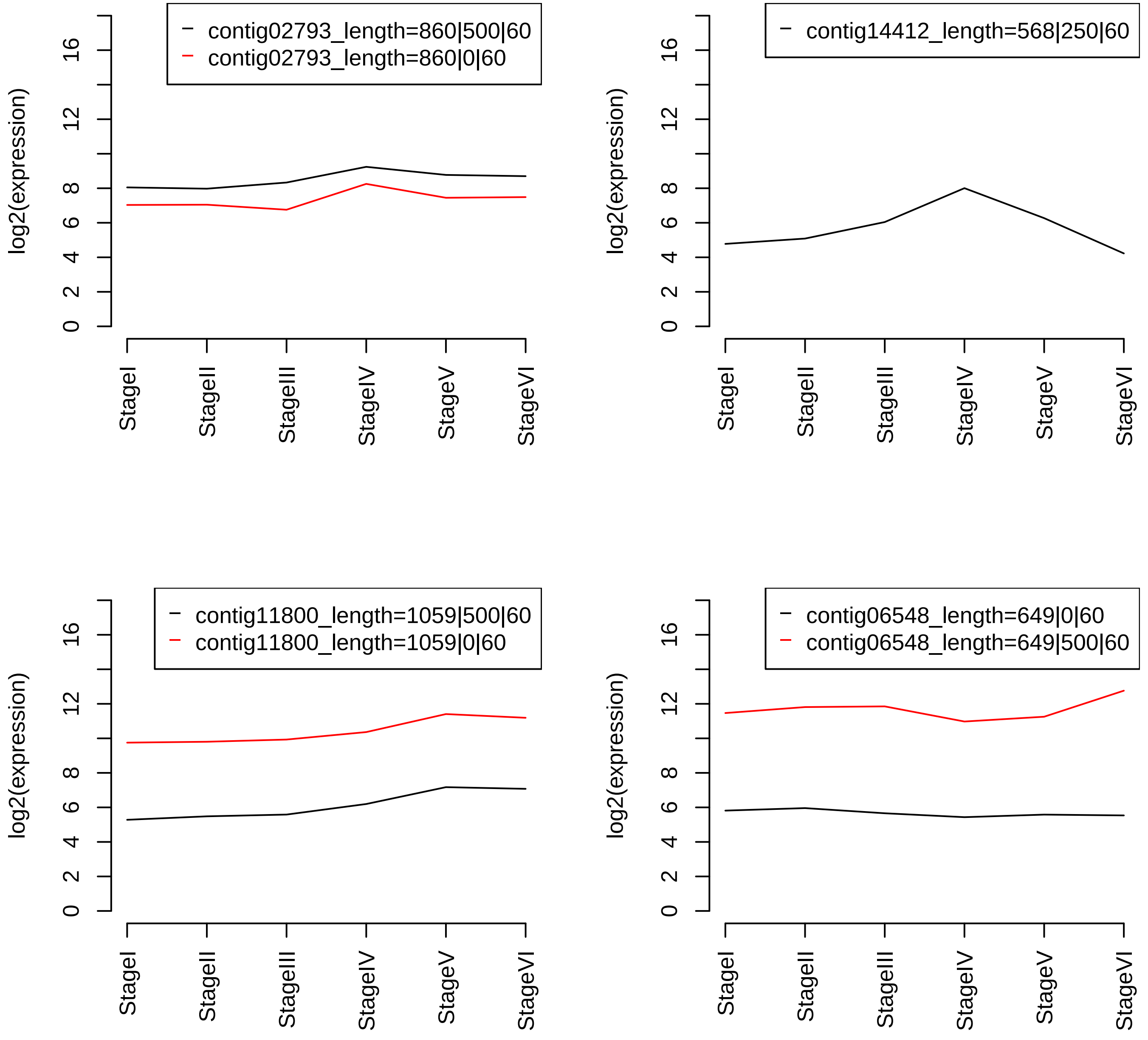
contig02793_length=860|500|60
contig02793_length=860|0|60
contig14412_length=568|250|60
contig11800_length=1059|500|60
contig11800_length=1059|0|60
contig06548_length=649|0|60
contig06548_length=649|500|60
log2(expression)
0
2
4
6
8
12
16
StageI
StageII
StageIII
StageIV
StageV
StageVI

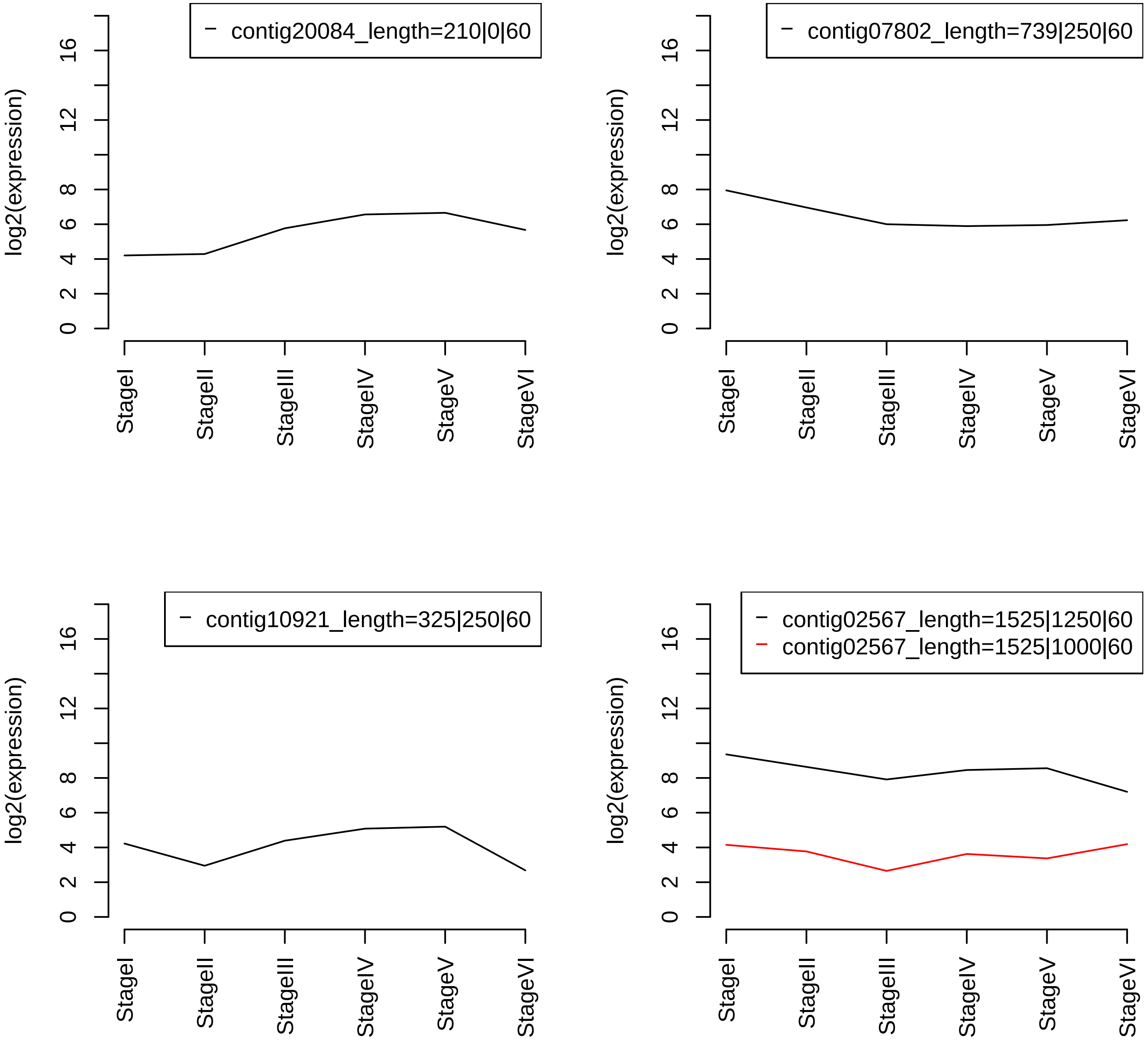

contig20084_length=210|0|60
contig07802_length=739|250|60
contig10921_length=325|250|60
contig02567_length=1525|1250|60
contig02567_length=1525|1000|60
log2(expression)
0 2 4 6 8 12 16
StageI StageII StageIII StageIV StageV StageVI

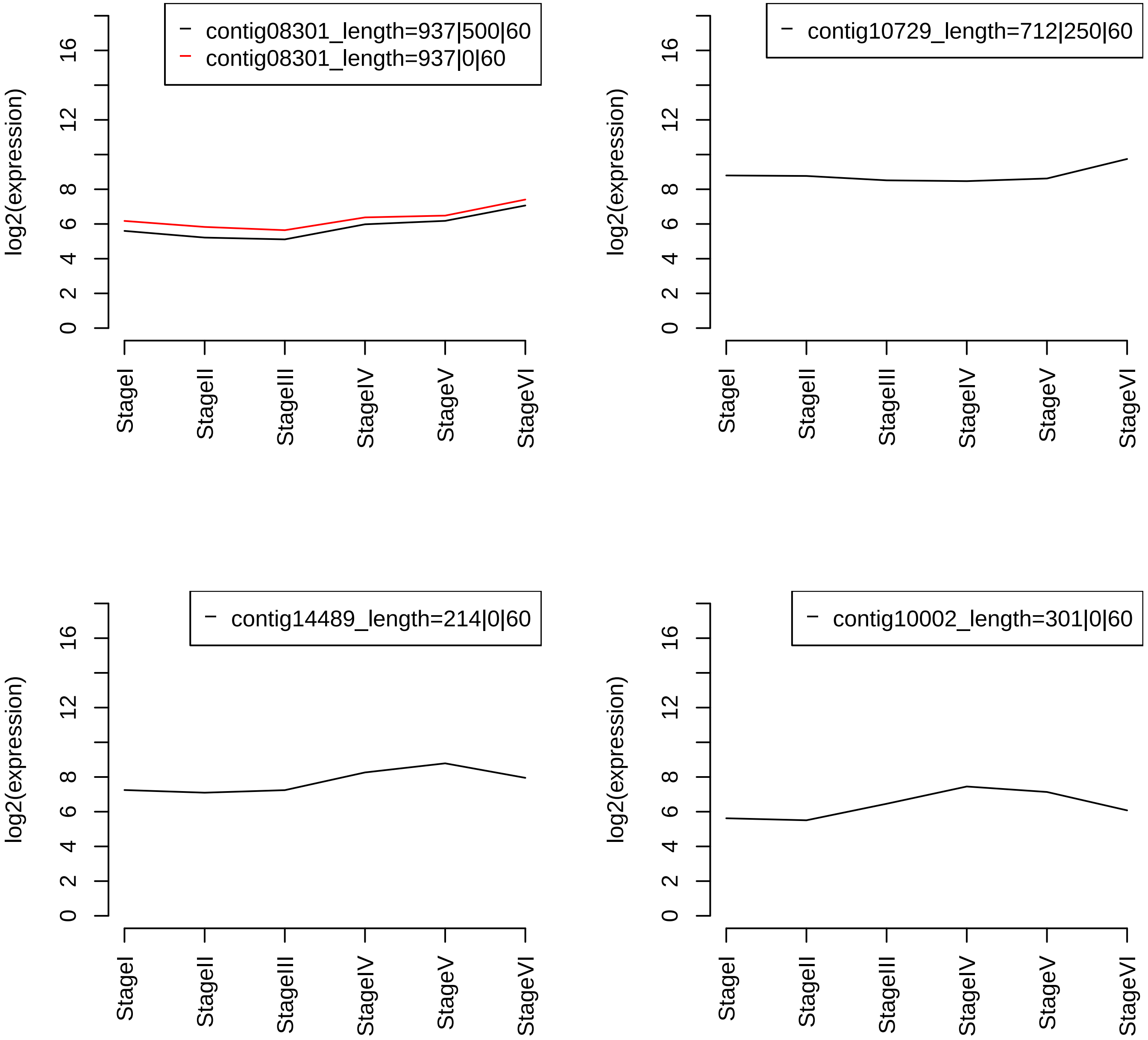
contig08301_length=937|500|60
contig08301_length=937|0|60
contig10729_length=712|250|60
contig14489_length=214|0|60
contig10002_length=301|0|60
log2(expression)
0
2
4
6
8
12
16
StageI
StageII
StageIII
StageIV
StageV
StageVI

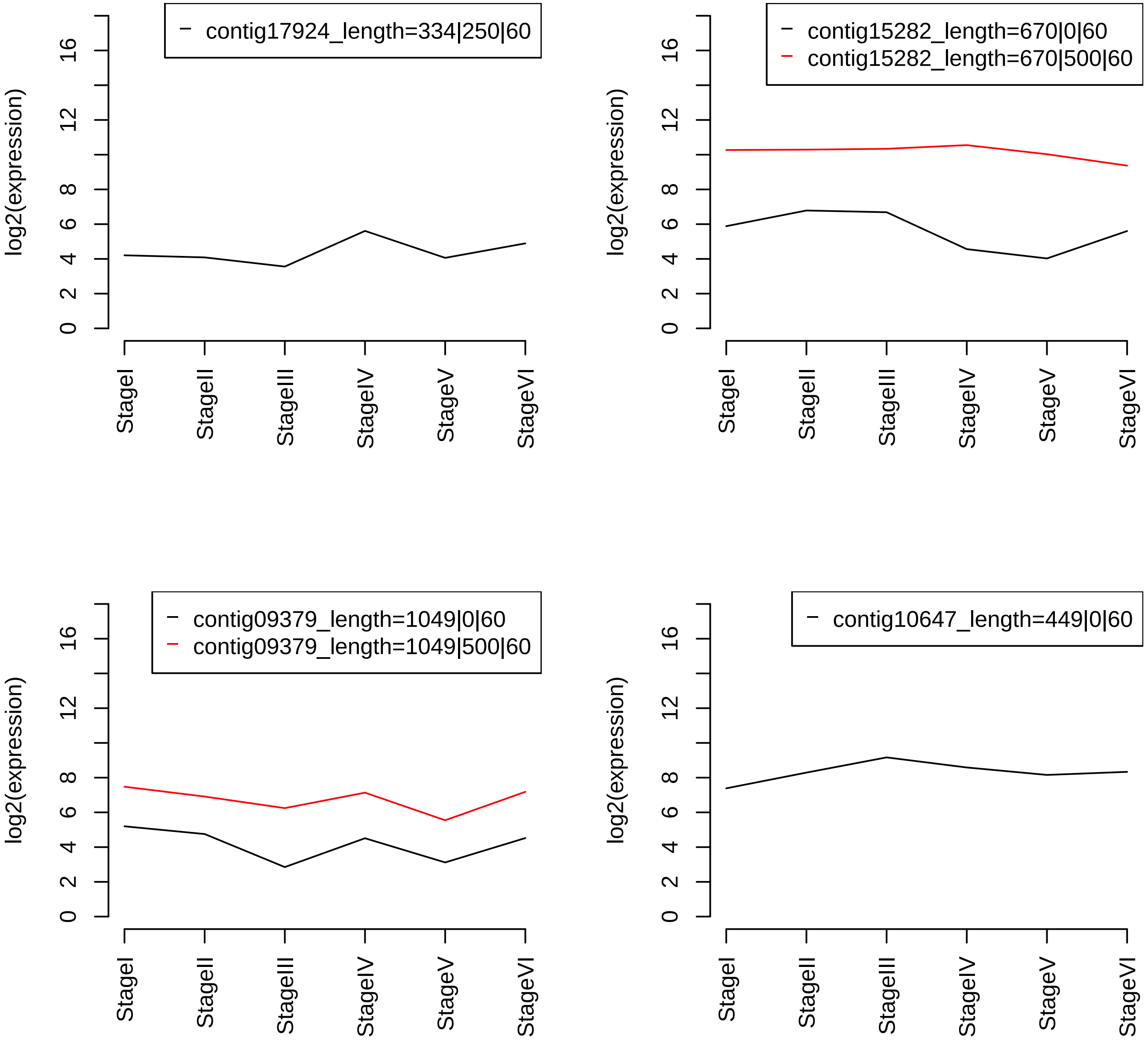
contig17924_length=334|250|60
contig15282_length=670|0|60
contig15282_length=670|500|60
contig09379_length=1049|0|60
contig09379_length=1049|500|60
contig10647_length=449|0|60
log2(expression)
StageI
StageII
StageIII
StageIV
StageV
StageVI

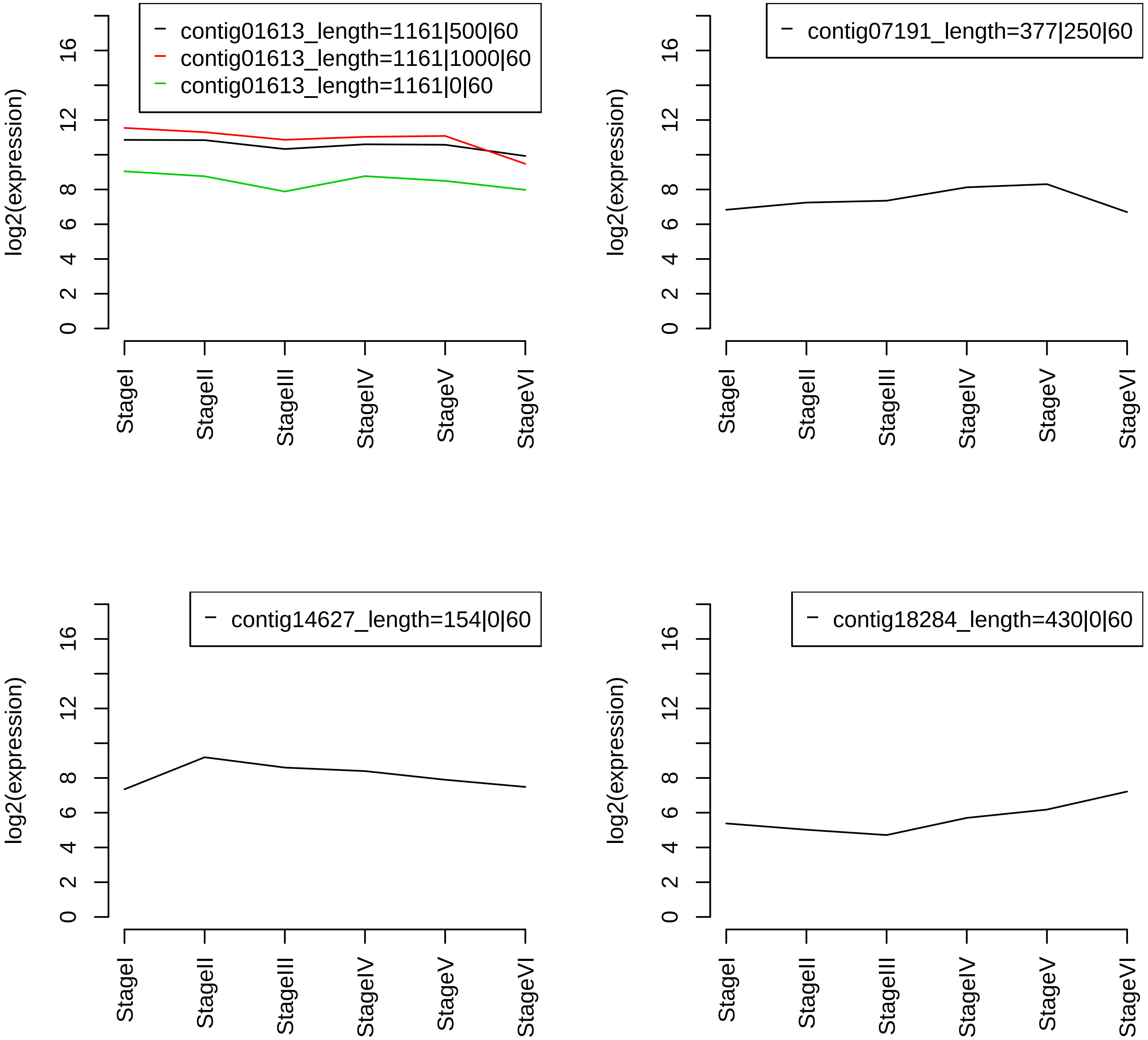

contig01613_length=1161|500|60
contig01613_length=1161|1000|60
contig01613_length=1161|0|60
contig07191_length=377|250|60
contig14627_length=154|0|60
contig18284_length=430|0|60
log2(expression)
StageI
StageII
StageIII
StageIV
StageV
StageVI

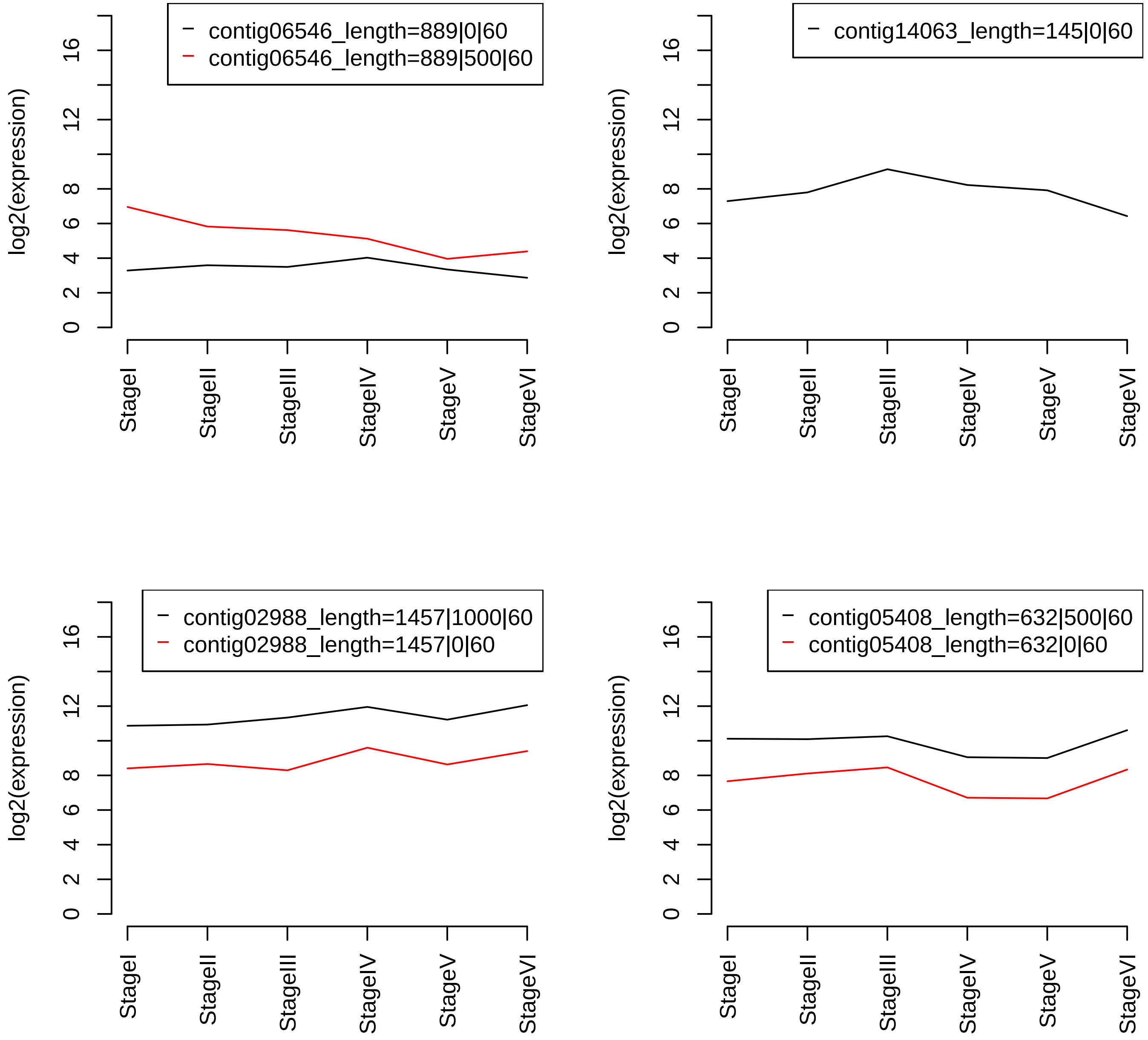
contig06546_length=889|0|60
contig06546_length=889|500|60
contig14063_length=145|0|60
contig02988_length=1457|1000|60
contig02988_length=1457|0|60
contig05408_length=632|500|60
contig05408_length=632|0|60
log2(expression)
0
2
4
6
8
12
16
StageI
StageII
StageIII
StageIV
StageV
StageVI

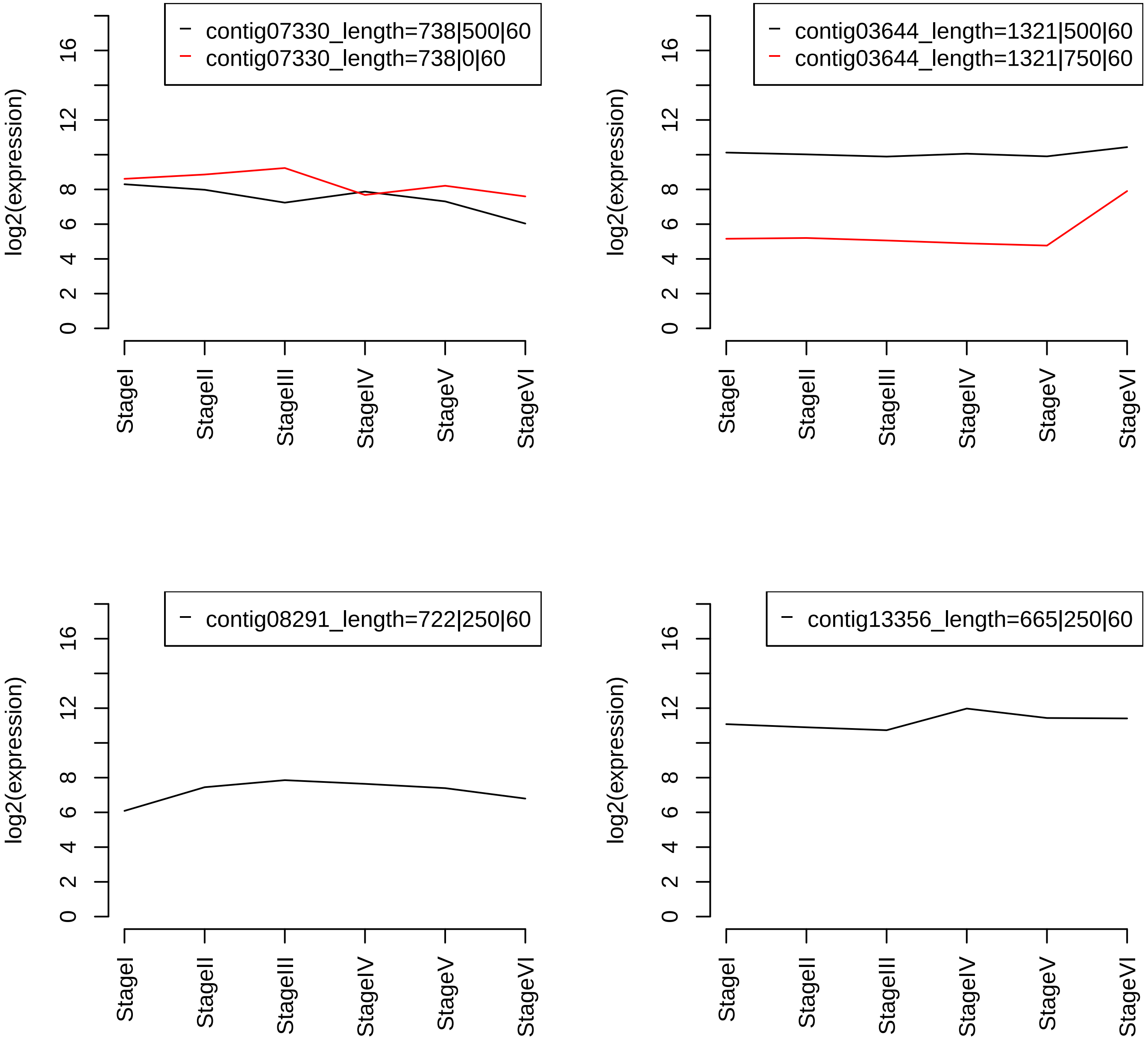

contig07330_length=738|500|60
contig07330_length=738|0|60
contig03644_length=1321|500|60
contig03644_length=1321|750|60
contig08291_length=722|250|60
contig13356_length=665|250|60
log2(expression)
StageI
StageII
StageIII
StageIV
StageV
StageVI

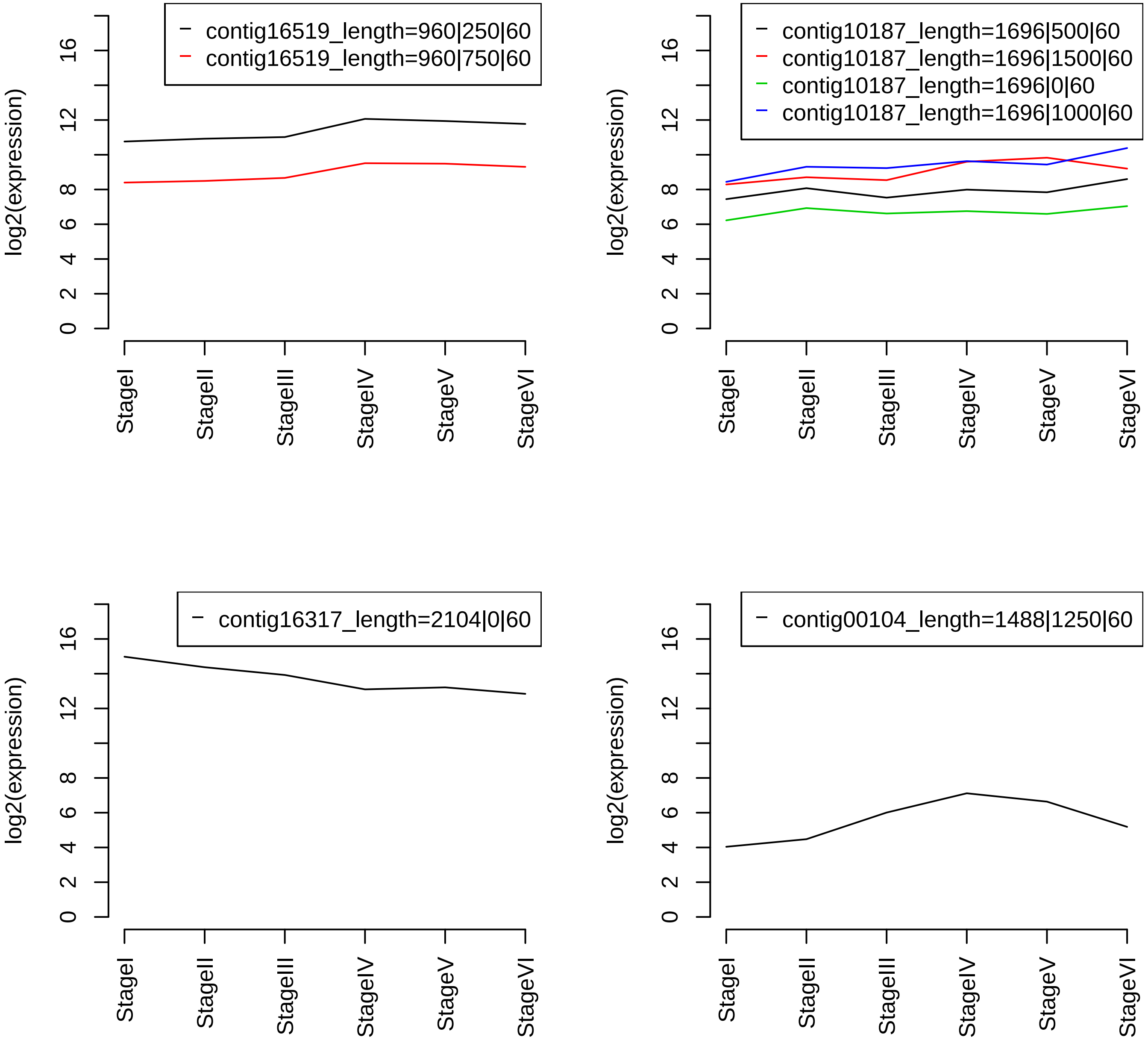
contig16519_length=960|250|60
contig16519_length=960|750|60
contig10187_length=1696|500|60
contig10187_length=1696|1500|60
contig10187_length=1696|0|60
contig10187_length=1696|1000|60
contig16317_length=2104|0|60
contig00104_length=1488|1250|60
log2(expression)
0
2
4
6
8
12
16
StageI
StageII
StageIII
StageIV
StageV
StageVI

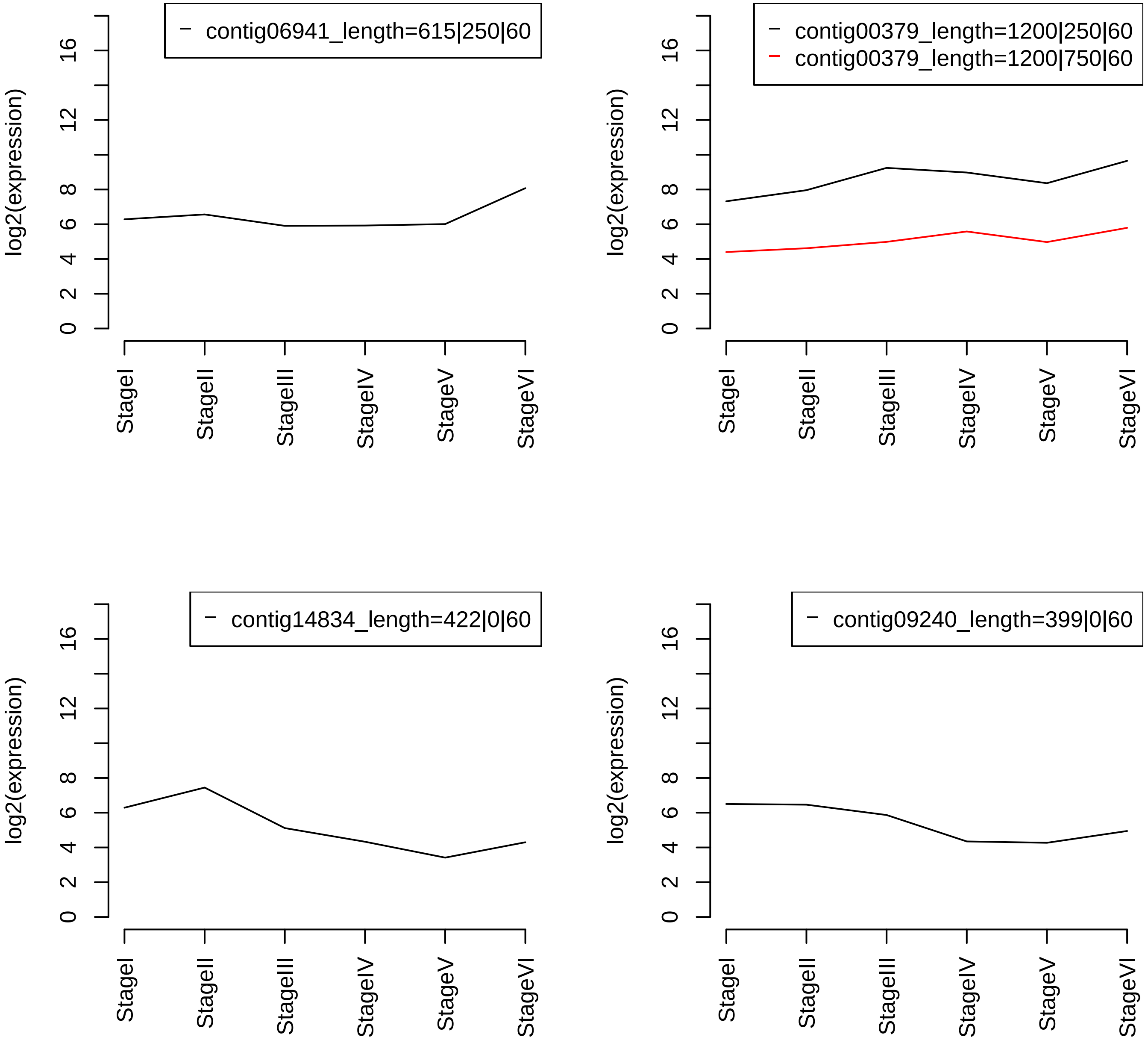

contig06941_length=615|250|60
contig00379_length=1200|250|60
contig00379_length=1200|750|60
contig14834_length=422|0|60
contig09240_length=399|0|60
log2(expression)
0
2
4
6
8
12
16
StageI
StageII
StageIII
StageIV
StageV
StageVI

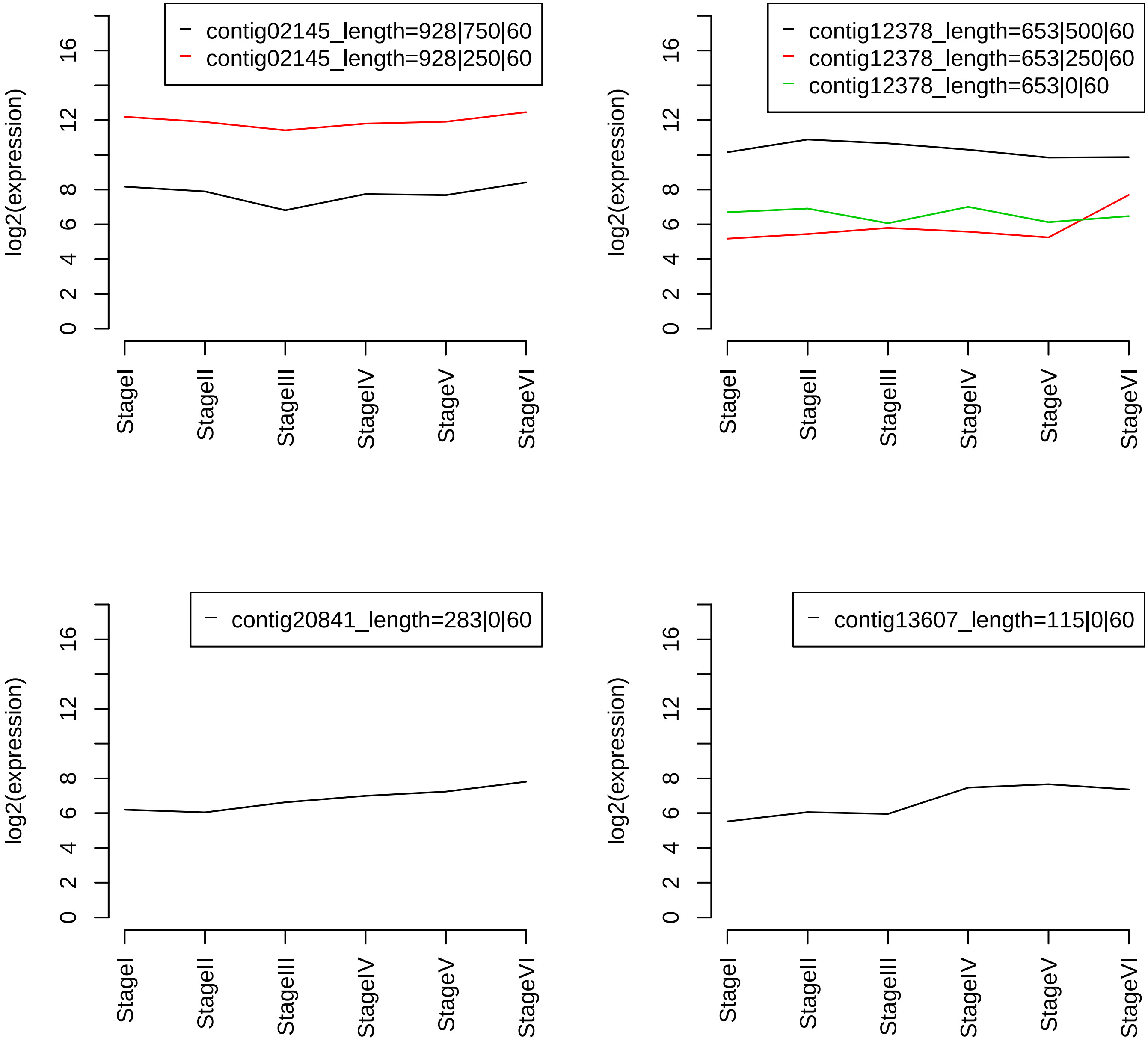

contig02145_length=928|750|60
contig02145_length=928|250|60
contig12378_length=653|500|60
contig12378_length=653|250|60
contig12378_length=653|0|60
contig20841_length=283|0|60
contig13607_length=115|0|60
log2(expression)
0
2
4
6
8
12
16
StageI
StageII
StageIII
StageIV
StageV
StageVI

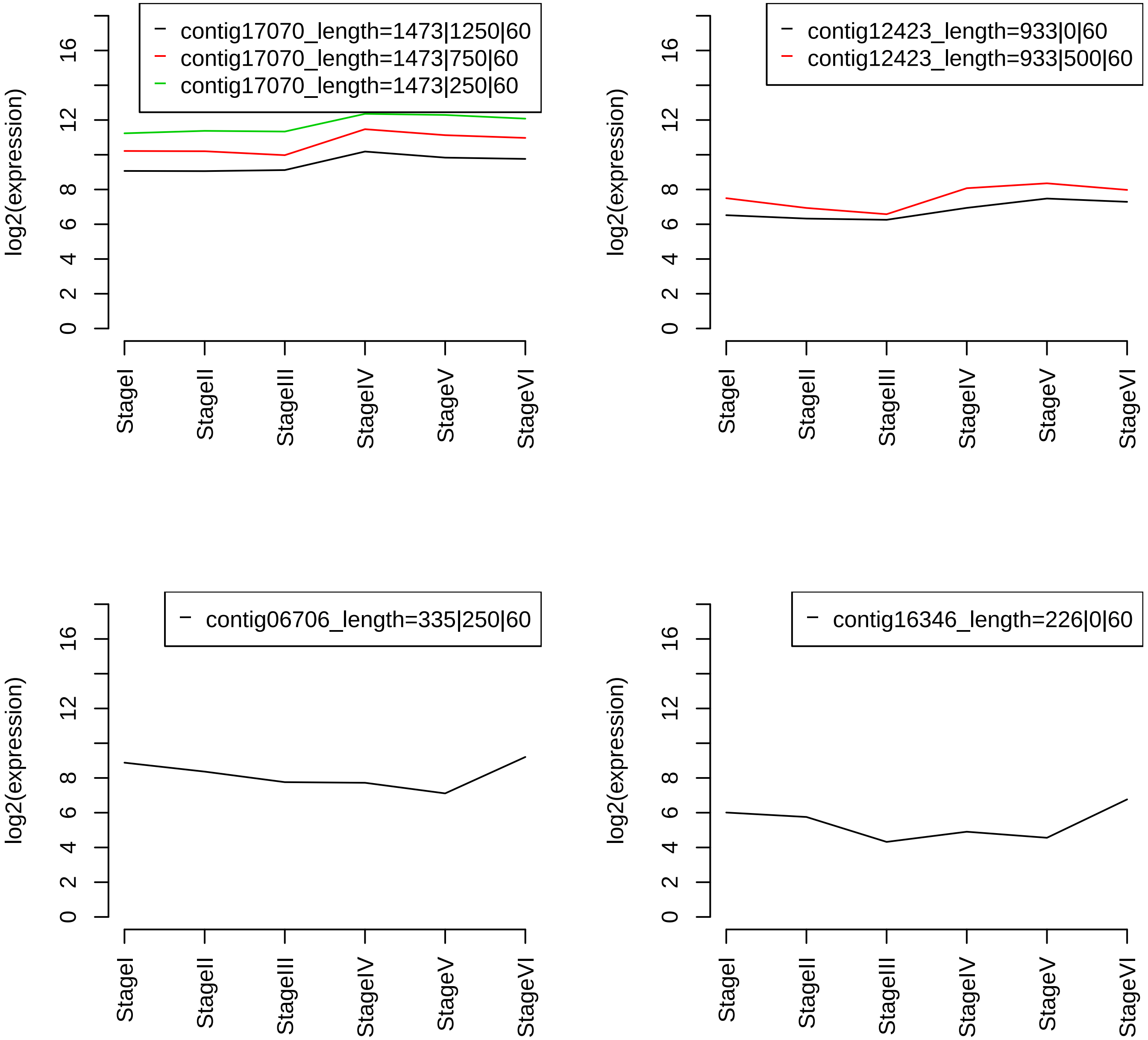
contig17070_length=1473|1250|60
contig17070_length=1473|750|60
contig17070_length=1473|250|60
contig12423_length=933|0|60
contig12423_length=933|500|60
contig06706_length=335|250|60
contig16346_length=226|0|60
log2(expression)
0
2
4
6
8
12
16
StageI
StageII
StageIII
StageIV
StageV
StageVI

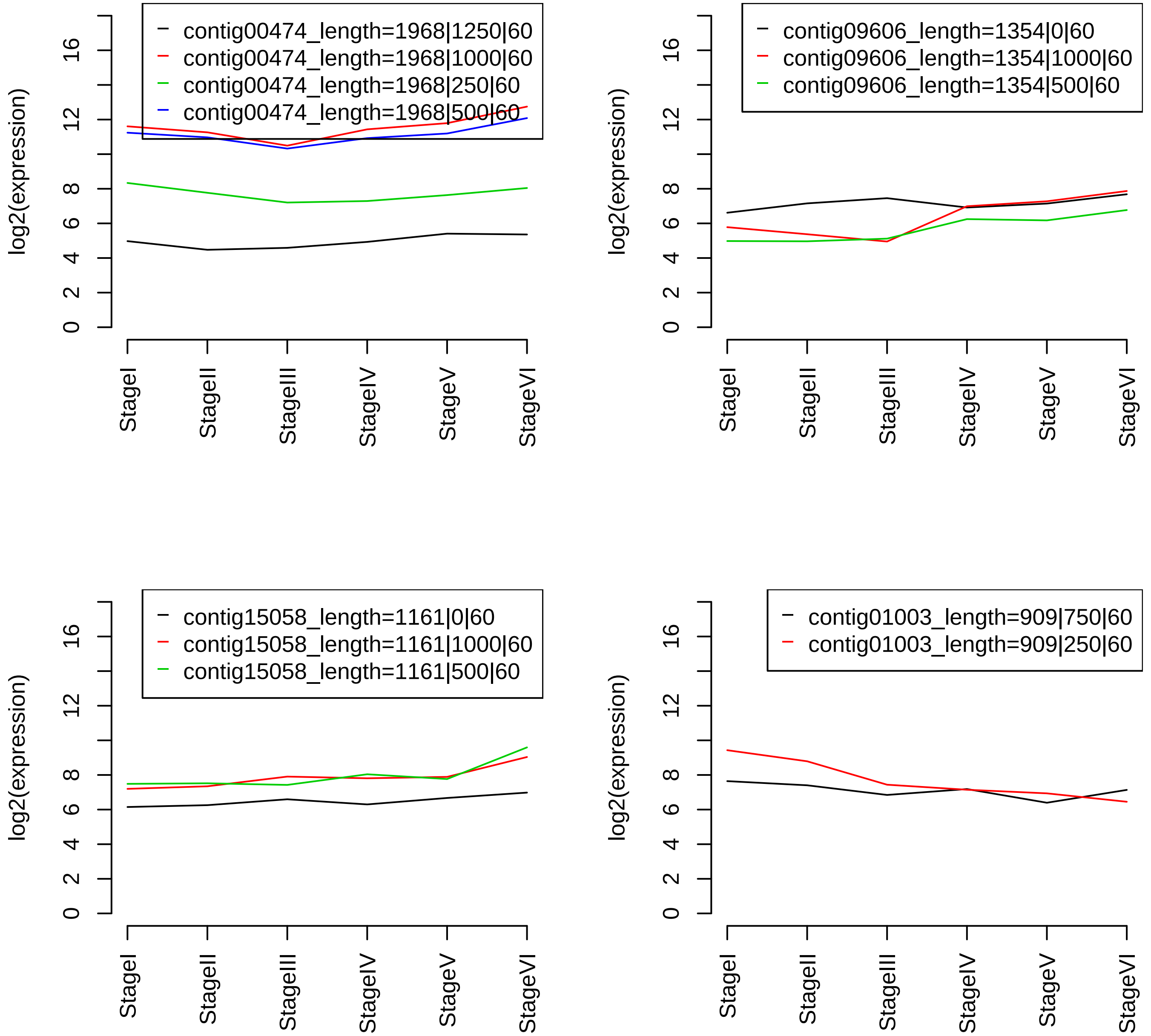

contig00474_length=1968|1250|60
contig00474_length=1968|1000|60
contig00474_length=1968|250|60
contig00474_length=1968|500|60
contig09606_length=1354|0|60
contig09606_length=1354|1000|60
contig09606_length=1354|500|60
contig15058_length=1161|0|60
contig15058_length=1161|1000|60
contig15058_length=1161|500|60
contig01003_length=909|750|60
contig01003_length=909|250|60
log2(expression)
0 2 4 6 8 12 16
StageI StageII StageIII StageIV StageV StageVI

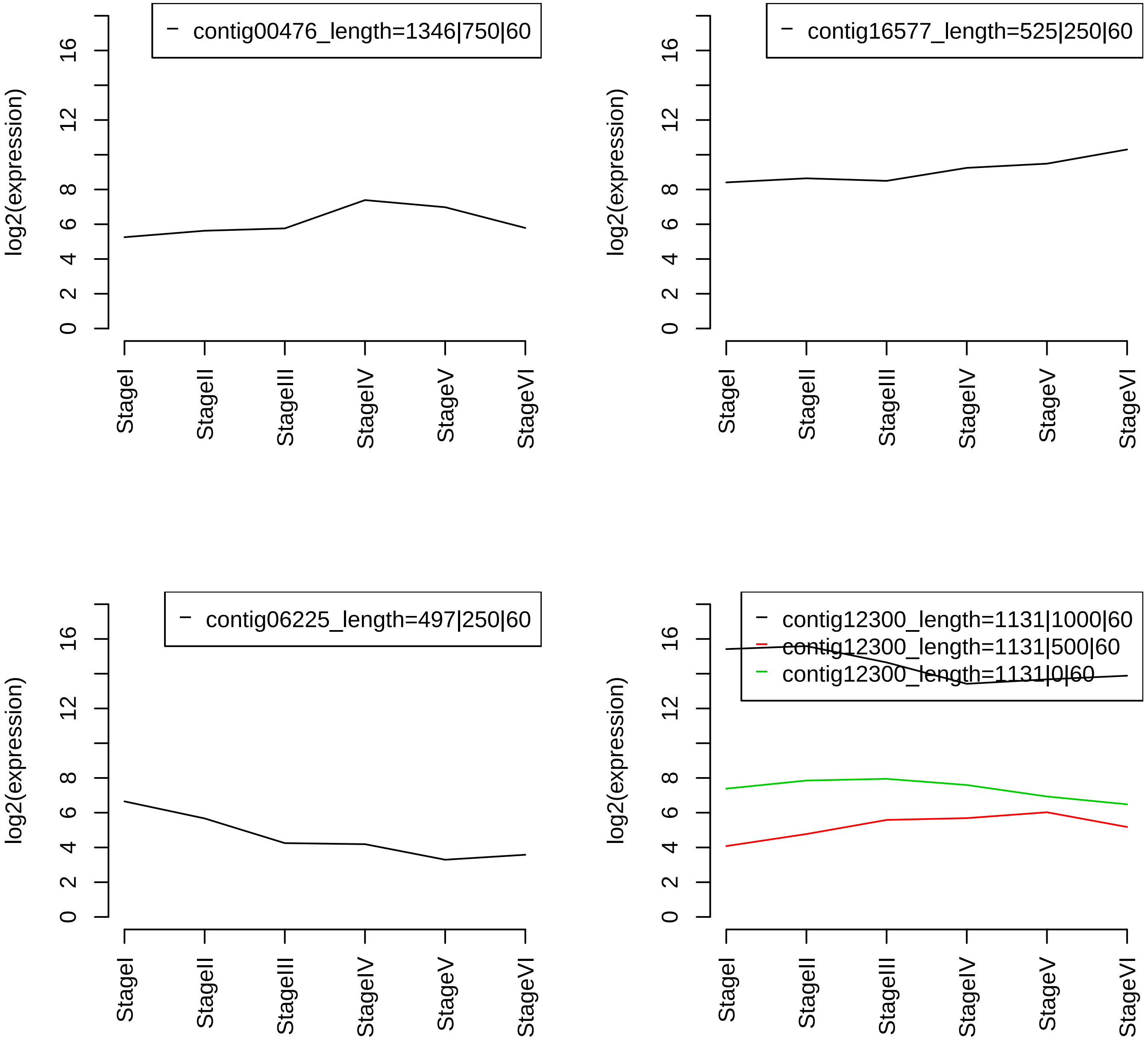

contig00476_length=1346|750|60
contig16577_length=525|250|60
contig06225_length=497|250|60
contig12300_length=1131|1000|60
contig12300_length=1131|500|60
contig12300_length=1131|0|60
log2(expression)
0
2
4
6
8
12
16
StageI
StageII
StageIII
StageIV
StageV
StageVI

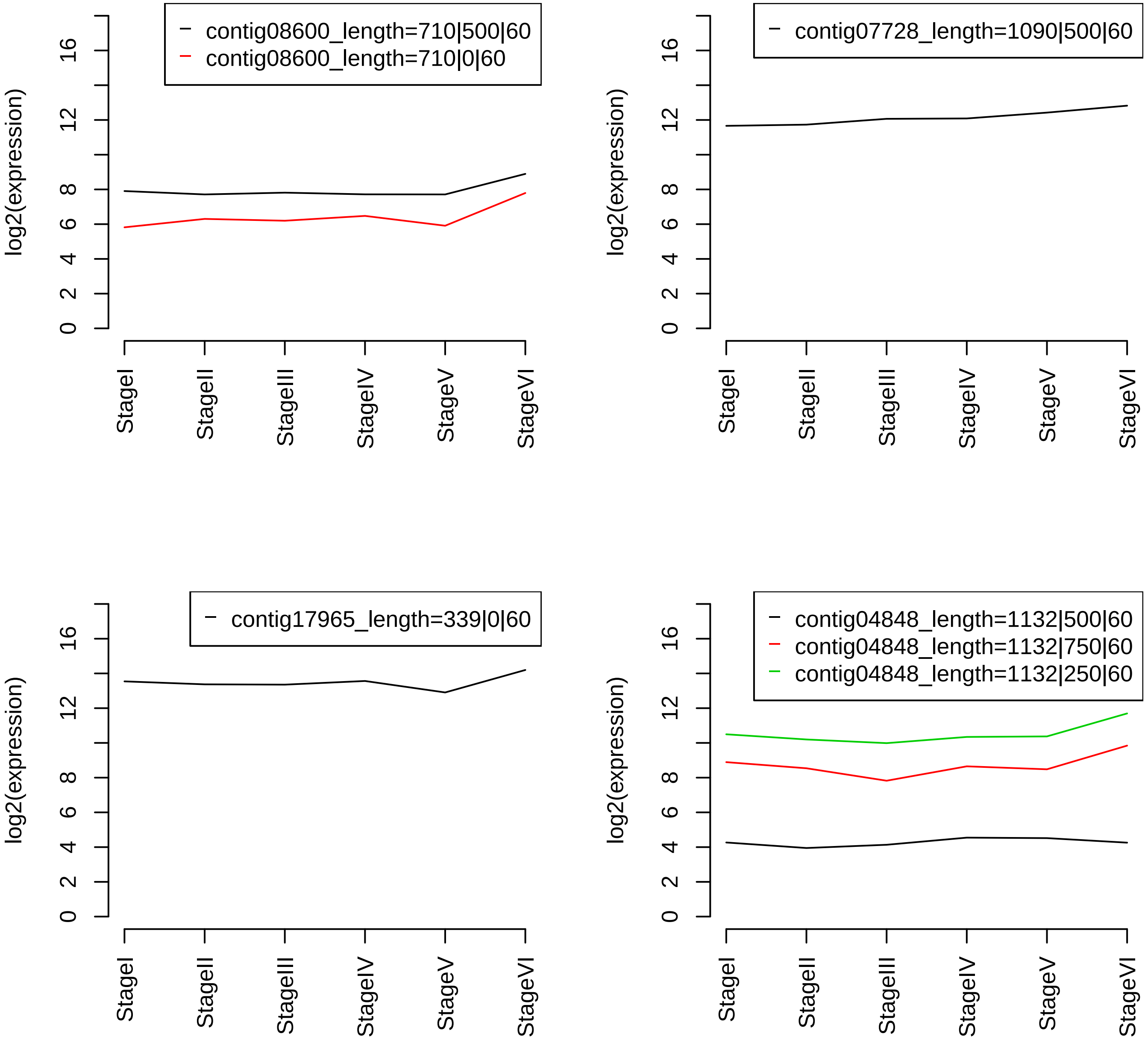
contig08600_length=710|500|60
contig08600_length=710|0|60
contig07728_length=1090|500|60
contig17965_length=339|0|60
contig04848_length=1132|500|60
contig04848_length=1132|750|60
contig04848_length=1132|250|60
log2(expression)
0
2
4
6
8
12
16
StageI
StageII
StageIII
StageIV
StageV
StageVI

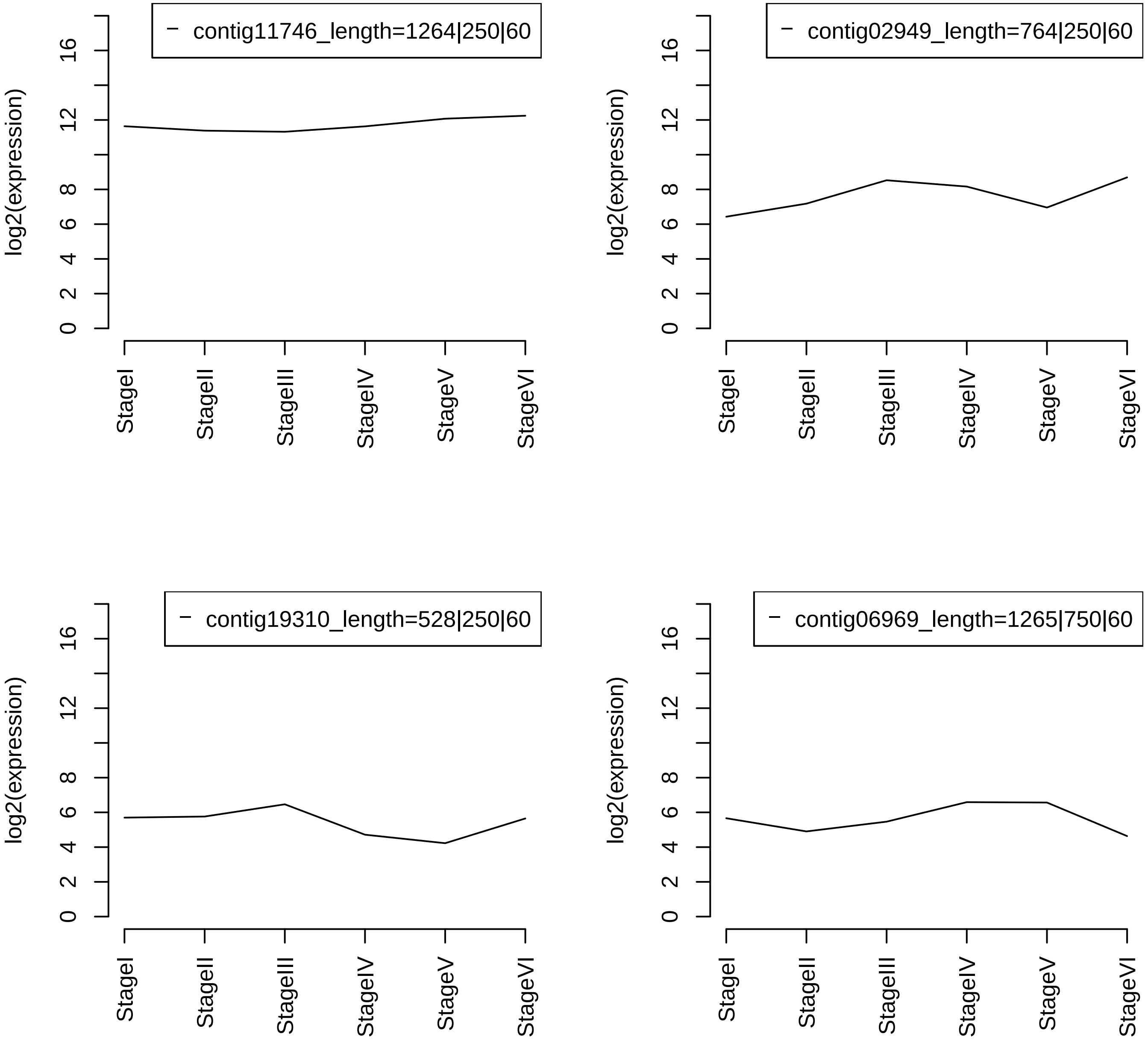
contig11746_length=1264|250|60
contig02949_length=764|250|60
contig19310_length=528|250|60
contig06969_length=1265|750|60
log2(expression)
0
2
4
6
8
12
16
StageI
StageII
StageIII
StageIV
StageV
StageVI

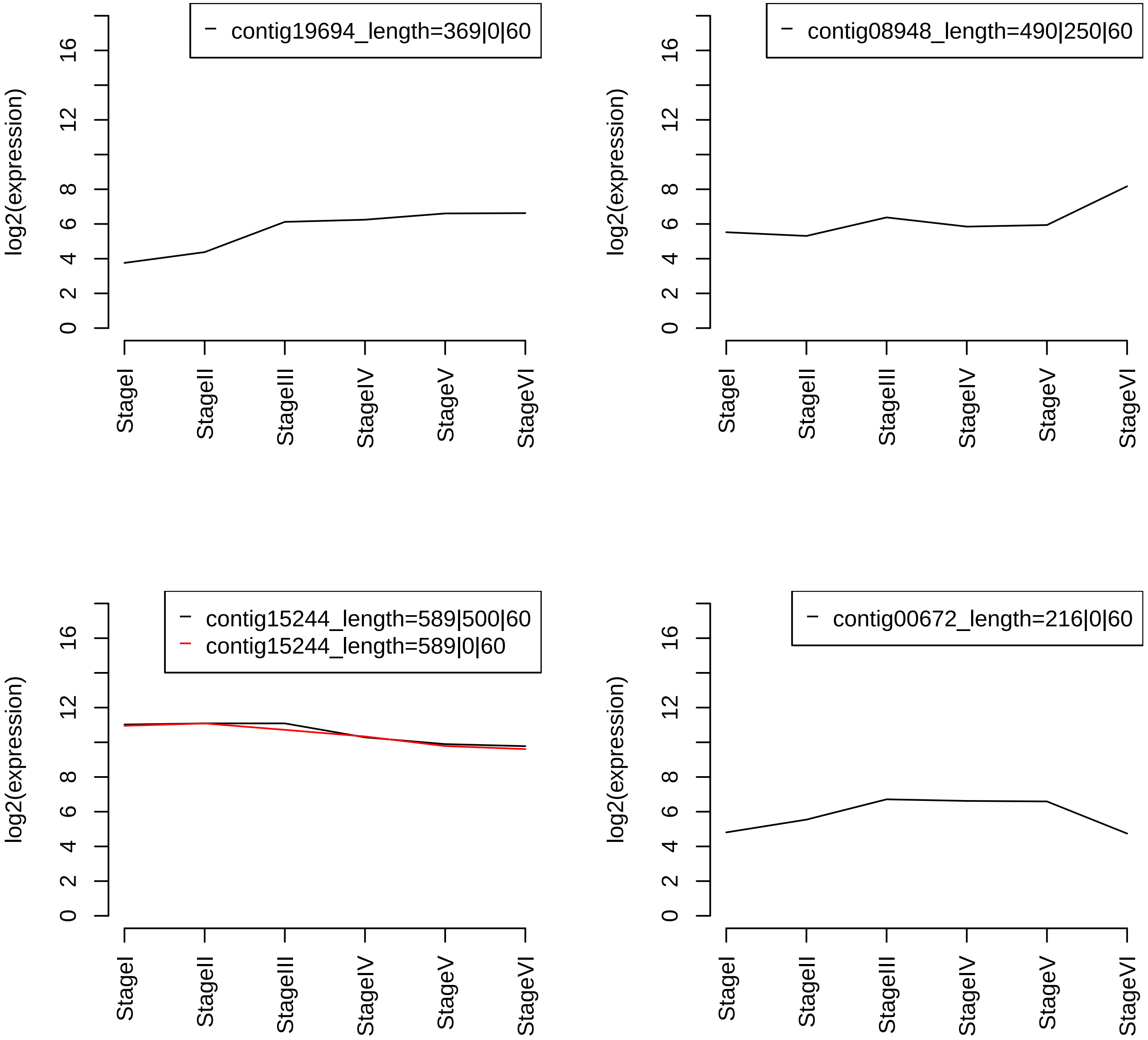
contig19694_length=369|0|60
contig08948_length=490|250|60
contig15244_length=589|500|60
contig15244_length=589|0|60
contig00672_length=216|0|60
log2(expression)
0
2
4
6
8
12
16
StageI
StageII
StageIII
StageIV
StageV
StageVI

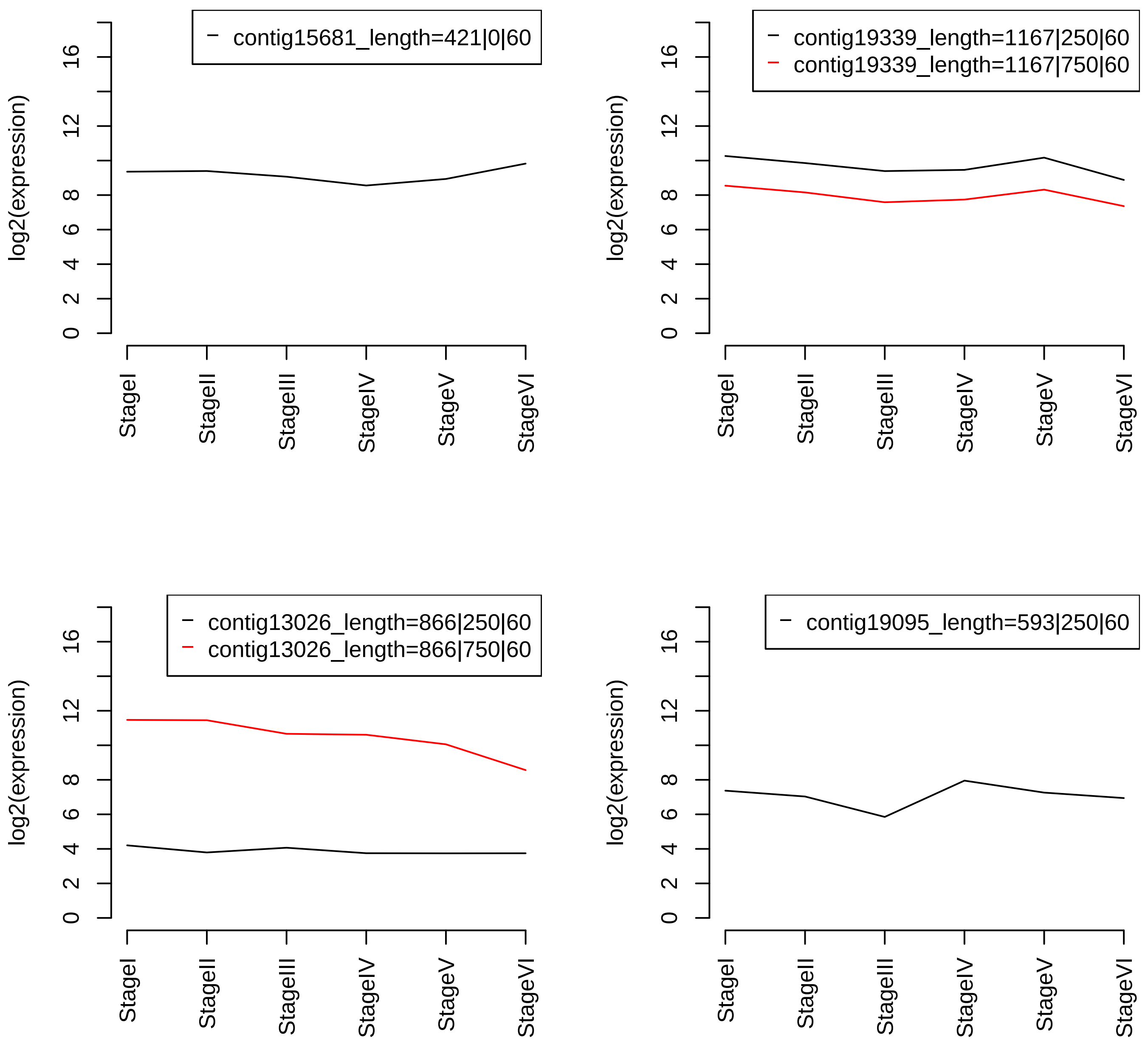
contig15681_length=421|0|60
contig19339_length=1167|250|60
contig19339_length=1167|750|60
contig13026_length=866|250|60
contig13026_length=866|750|60
contig19095_length=593|250|60
log2(expression)
StageI
StageII
StageIII
StageIV
StageV
StageVI

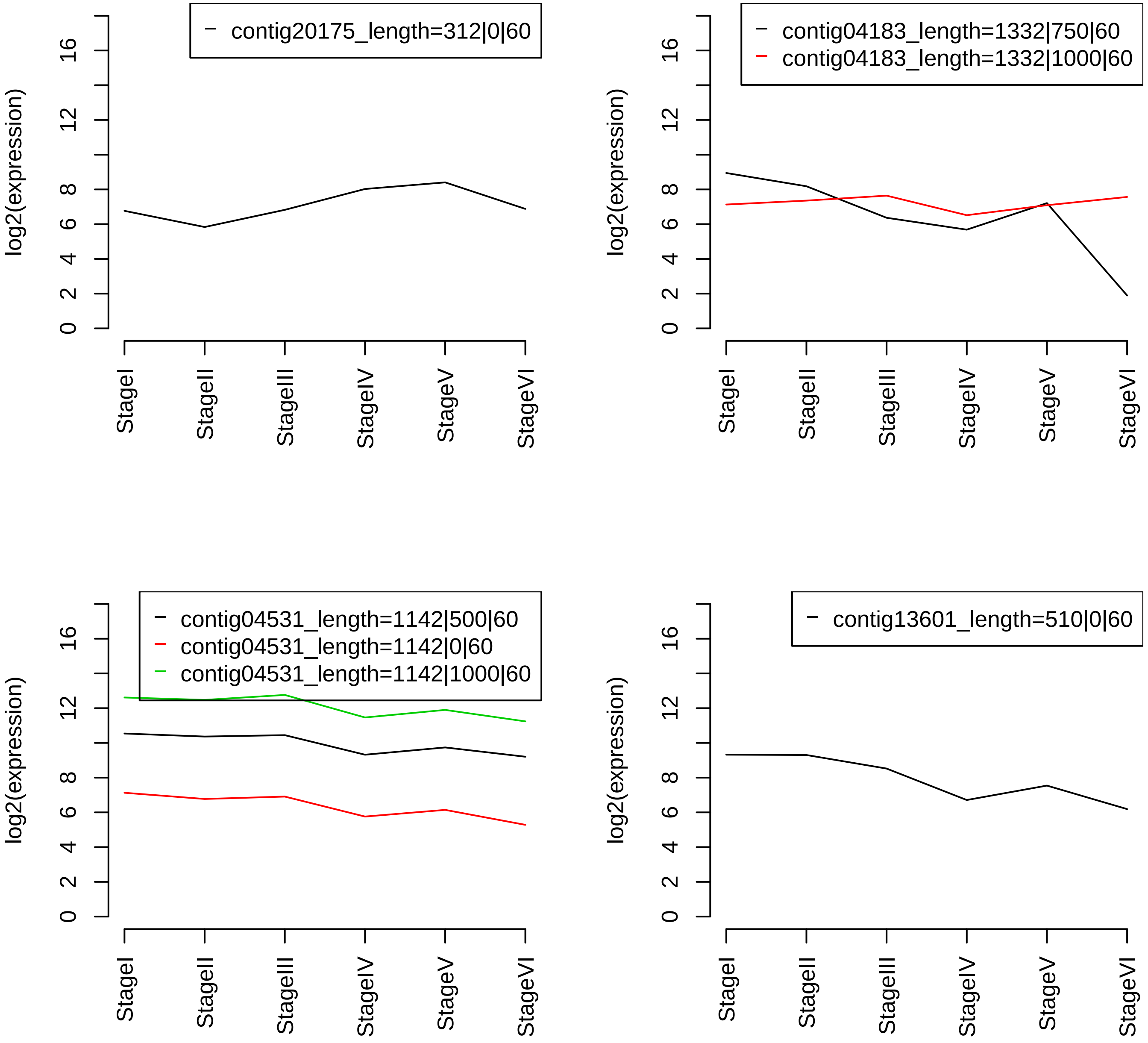

contig20175_length=312|0|60
contig04183_length=1332|750|60
contig04183_length=1332|1000|60
contig04531_length=1142|500|60
contig04531_length=1142|0|60
contig04531_length=1142|1000|60
contig13601_length=510|0|60
log2(expression)
0 2 4 6 8 12 16
StageI StageII StageIII StageIV StageV StageVI

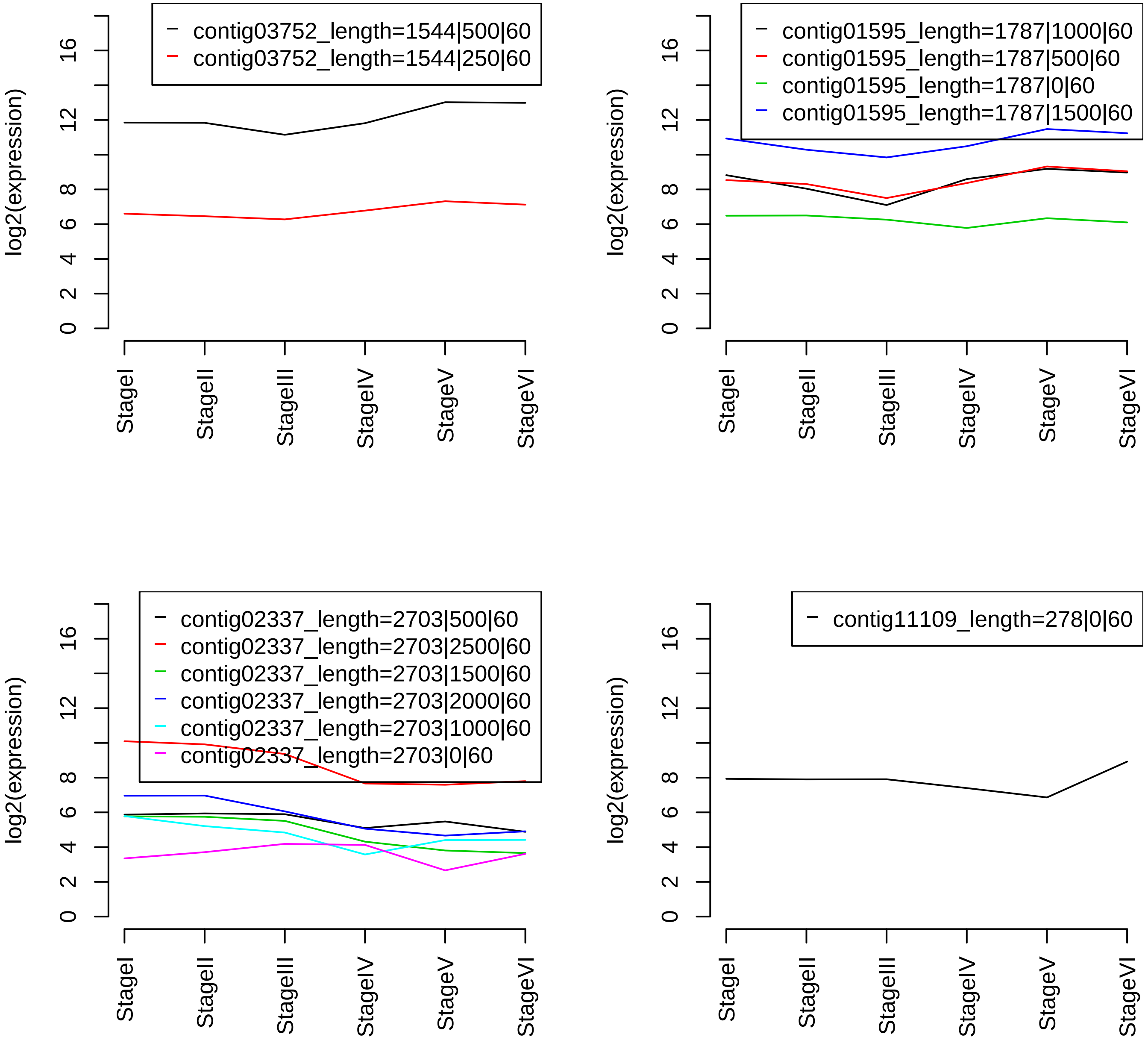
contig03752_length=1544|500|60
contig03752_length=1544|250|60
contig01595_length=1787|1000|60
contig01595_length=1787|500|60
contig01595_length=1787|0|60
contig01595_length=1787|1500|60
contig02337_length=2703|500|60
contig02337_length=2703|2500|60
contig02337_length=2703|1500|60
contig02337_length=2703|2000|60
contig02337_length=2703|1000|60
contig02337_length=2703|0|60
contig11109_length=278|0|60
log2(expression)
0 2 4 6 8 12 16
StageI
StageII
StageIII
StageIV
StageV
StageVI

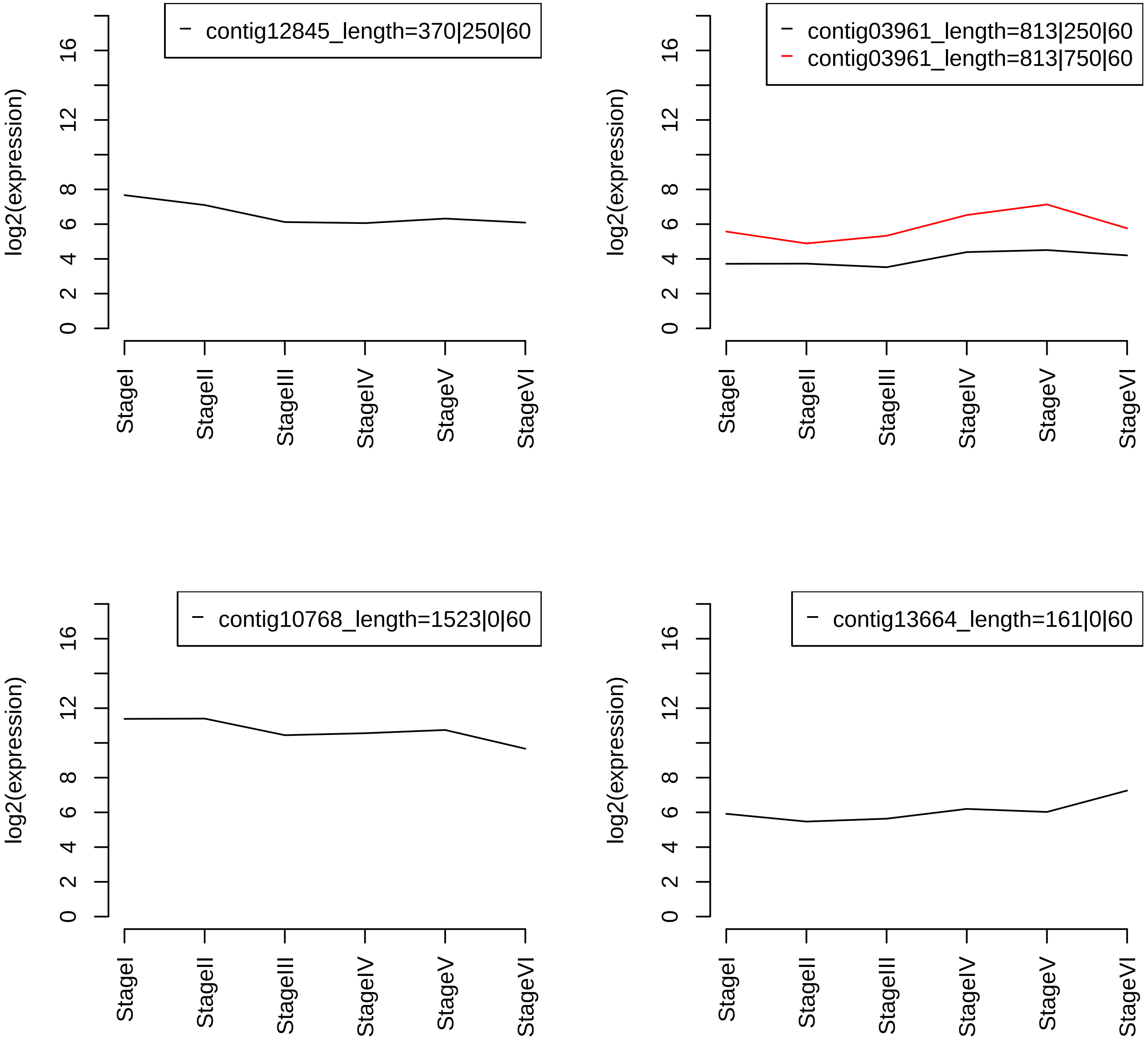
contig12845_length=370|250|60
contig03961_length=813|250|60
contig03961_length=813|750|60
contig10768_length=1523|0|60
contig13664_length=161|0|60
log2(expression)
0
2
4
6
8
12
16
StageI
StageII
StageIII
StageIV
StageV
StageVI

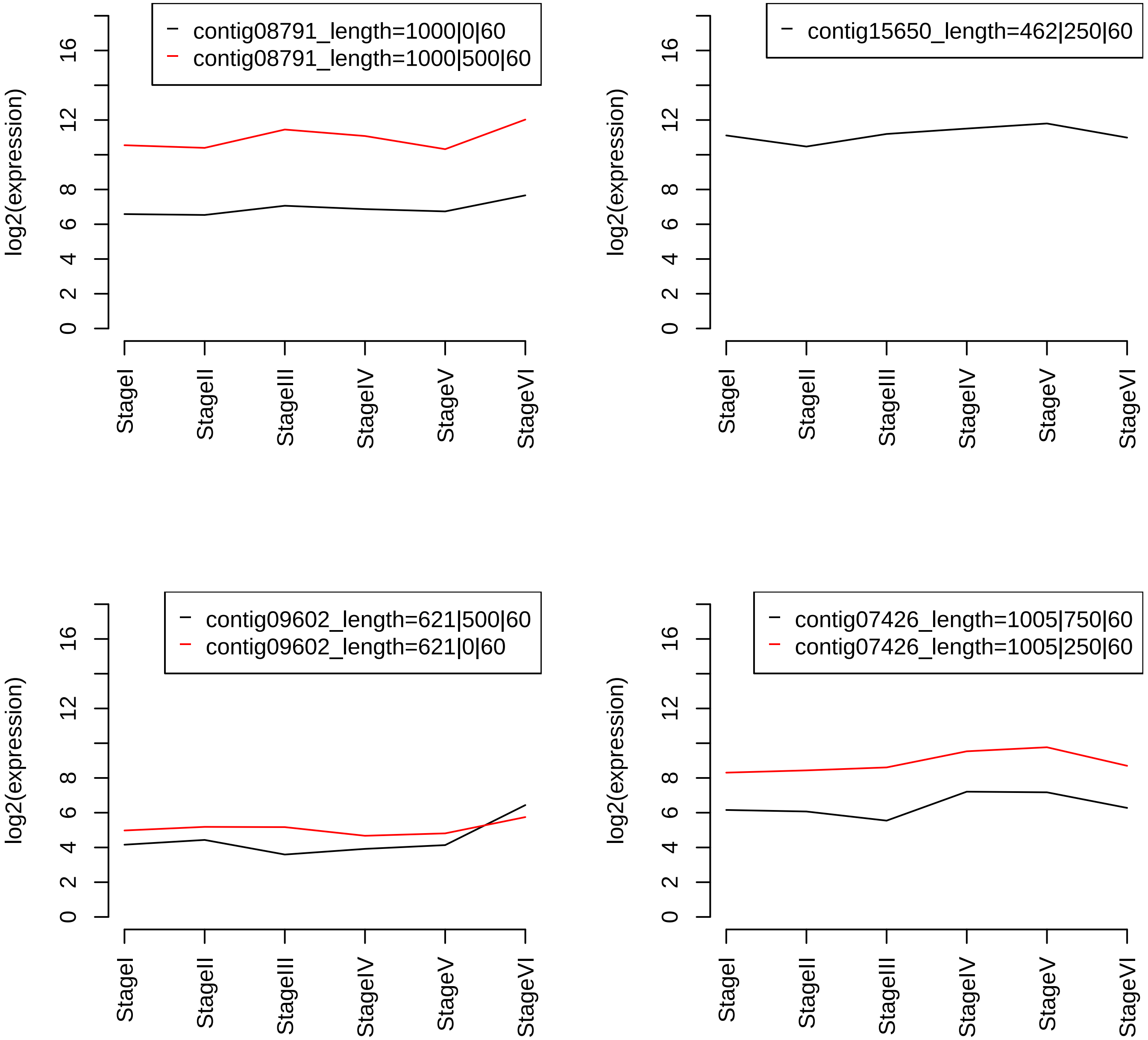
contig08791_length=1000|0|60
contig08791_length=1000|500|60
contig15650_length=462|250|60
contig09602_length=621|500|60
contig09602_length=621|0|60
contig07426_length=1005|750|60
contig07426_length=1005|250|60
log2(expression)
0 2 4 6 8 12 16
StageI StageII StageIII StageIV StageV StageVI

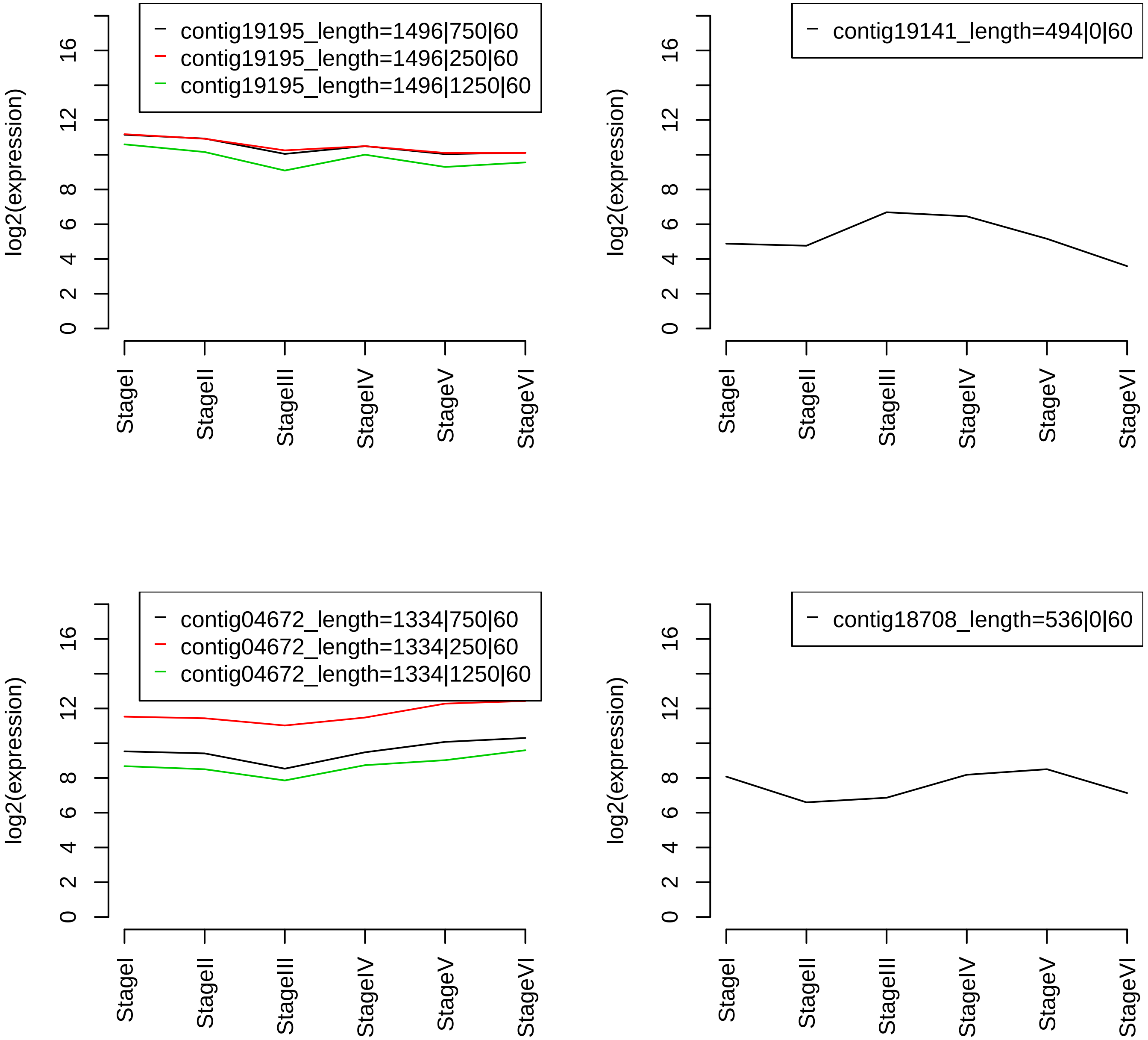
contig19195_length=1496|750|60
contig19195_length=1496|250|60
contig19195_length=1496|1250|60
contig19141_length=494|0|60
contig04672_length=1334|750|60
contig04672_length=1334|250|60
contig04672_length=1334|1250|60
contig18708_length=536|0|60
log2(expression)
0
2
4
6
8
12
16
StageI
StageII
StageIII
StageIV
StageV
StageVI

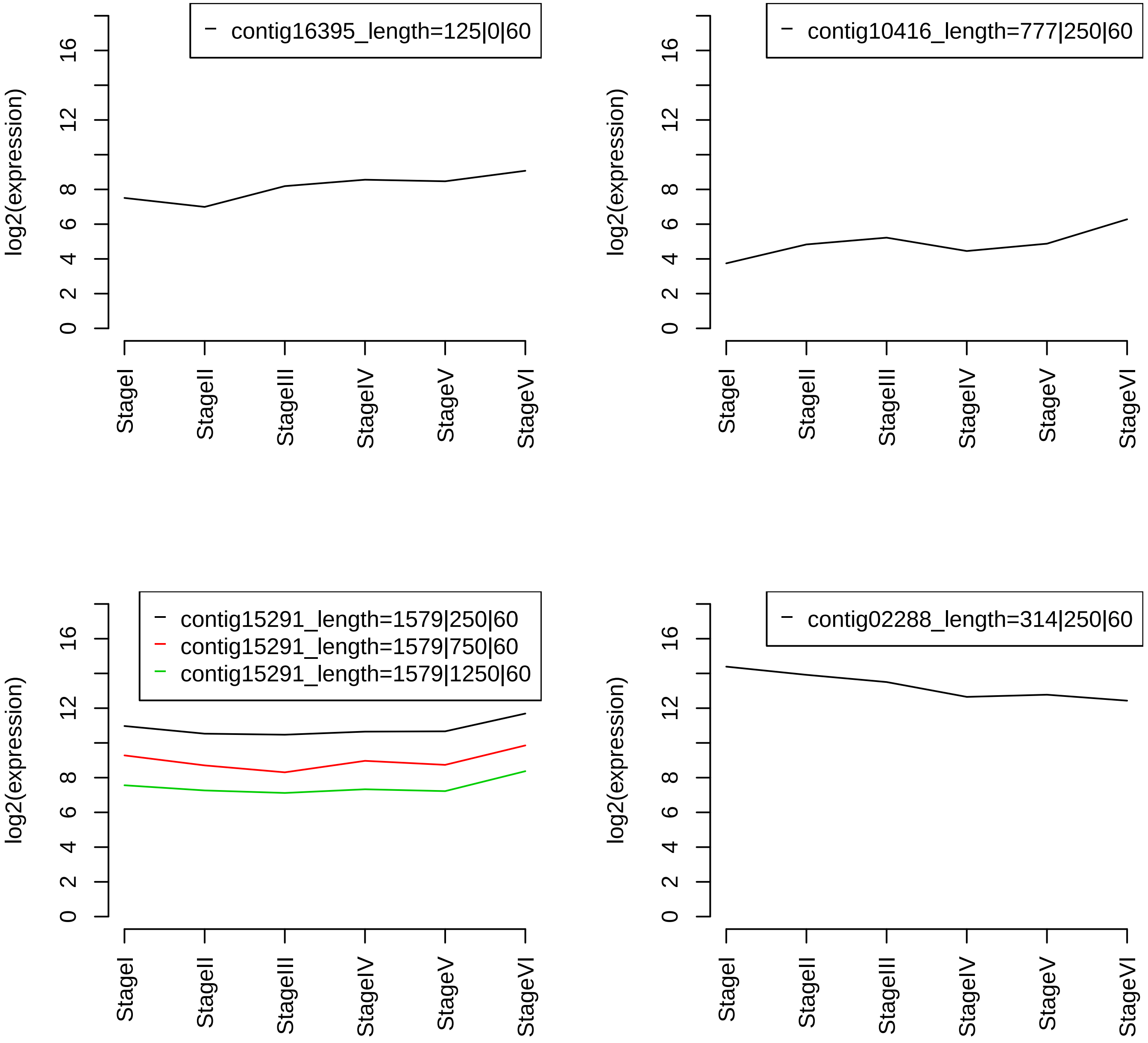
contig16395_length=125|0|60
contig10416_length=777|250|60
contig15291_length=1579|250|60
contig15291_length=1579|750|60
contig15291_length=1579|1250|60
contig02288_length=314|250|60
log2(expression)
0
2
4
6
8
12
16
StageI
StageII
StageIII
StageIV
StageV
StageVI

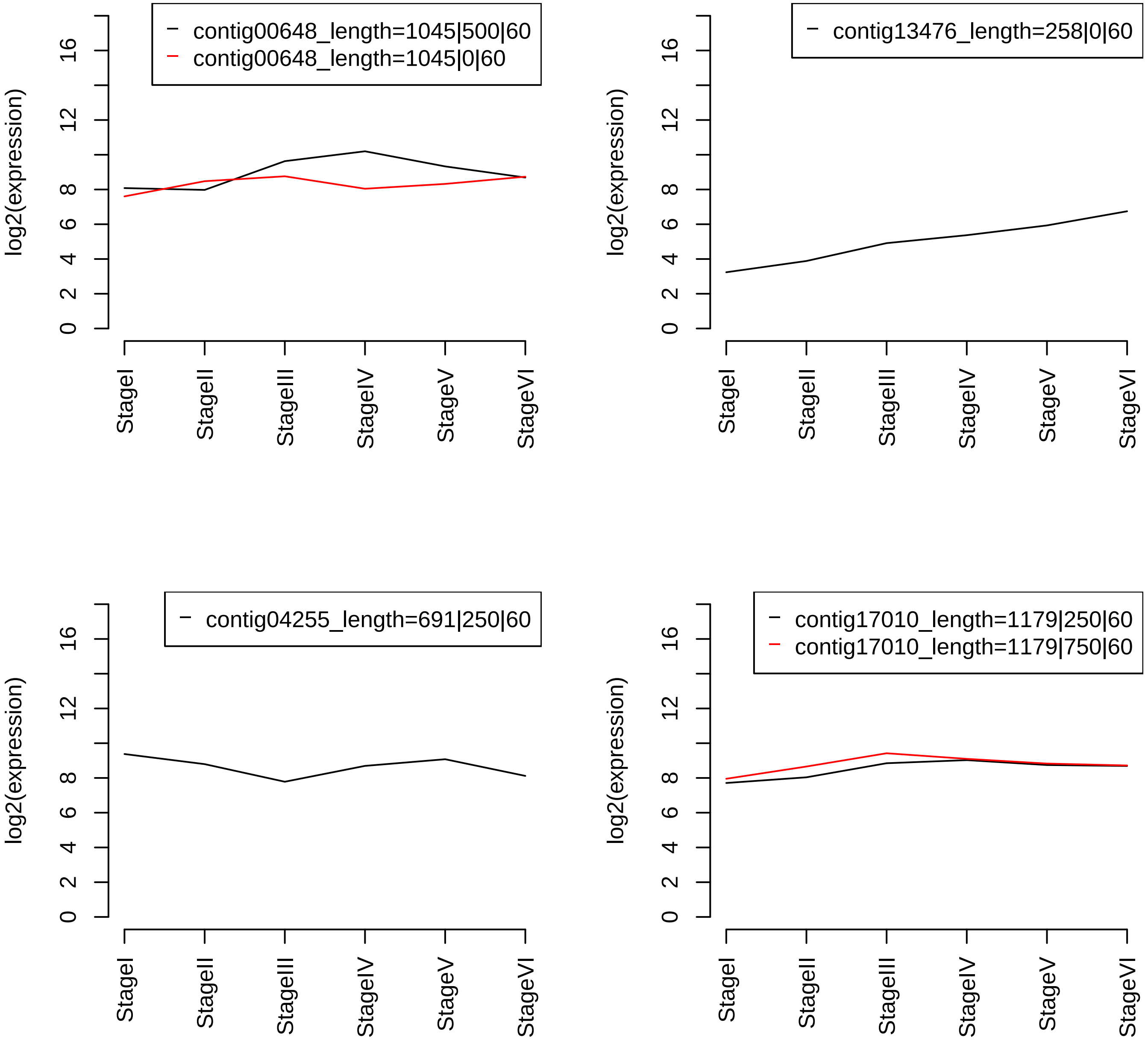
contig00648_length=1045|500|60
contig00648_length=1045|0|60
contig13476_length=258|0|60
contig04255_length=691|250|60
contig17010_length=1179|250|60
contig17010_length=1179|750|60
log2(expression)
0
2
4
6
8
12
16
StageI
StageII
StageIII
StageIV
StageV
StageVI

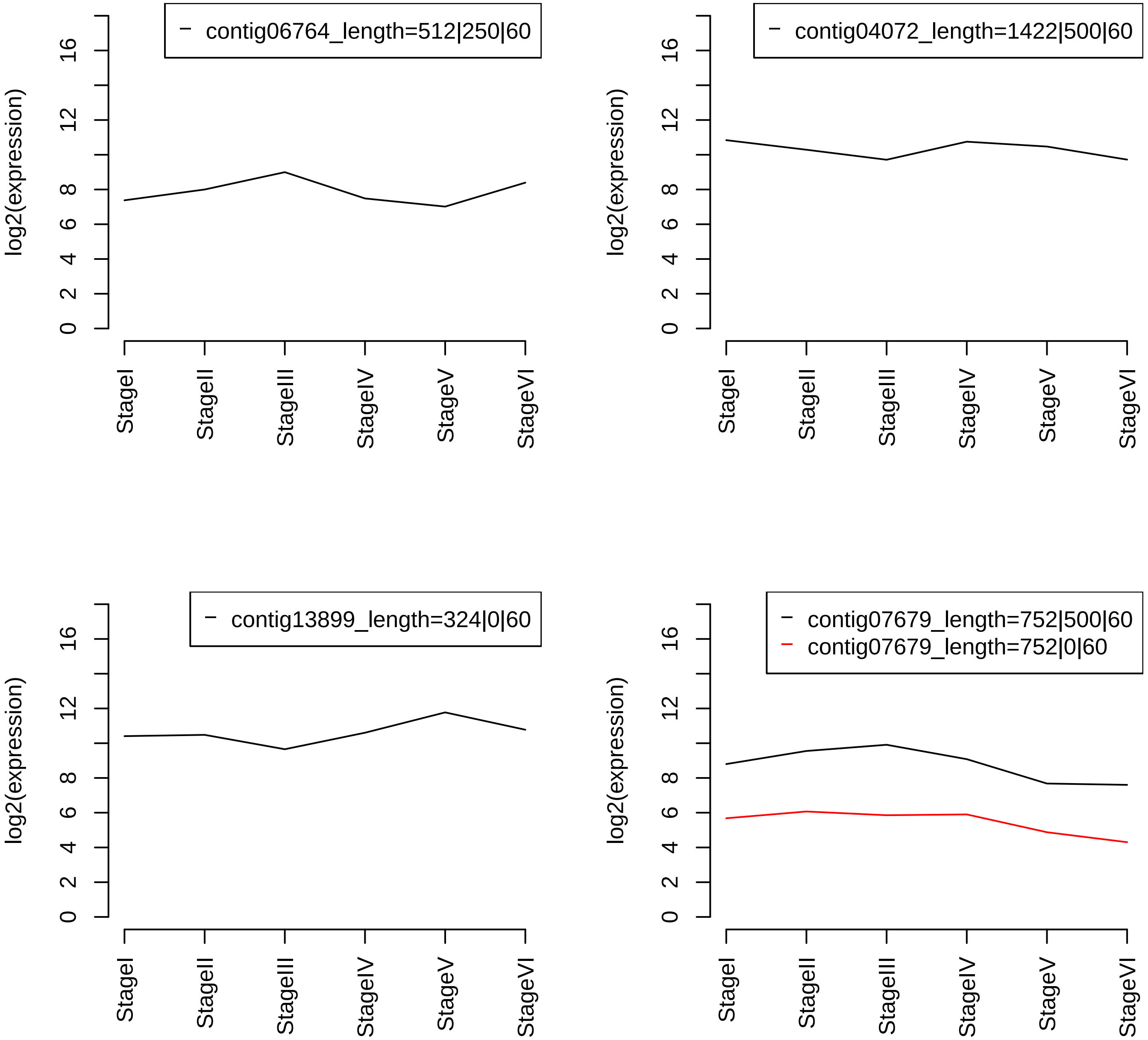

contig06764_length=512|250|60
contig04072_length=1422|500|60
contig13899_length=324|0|60
contig07679_length=752|500|60
contig07679_length=752|0|60
log2(expression)
0
2
4
6
8
12
16
StageI
StageII
StageIII
StageIV
StageV
StageVI

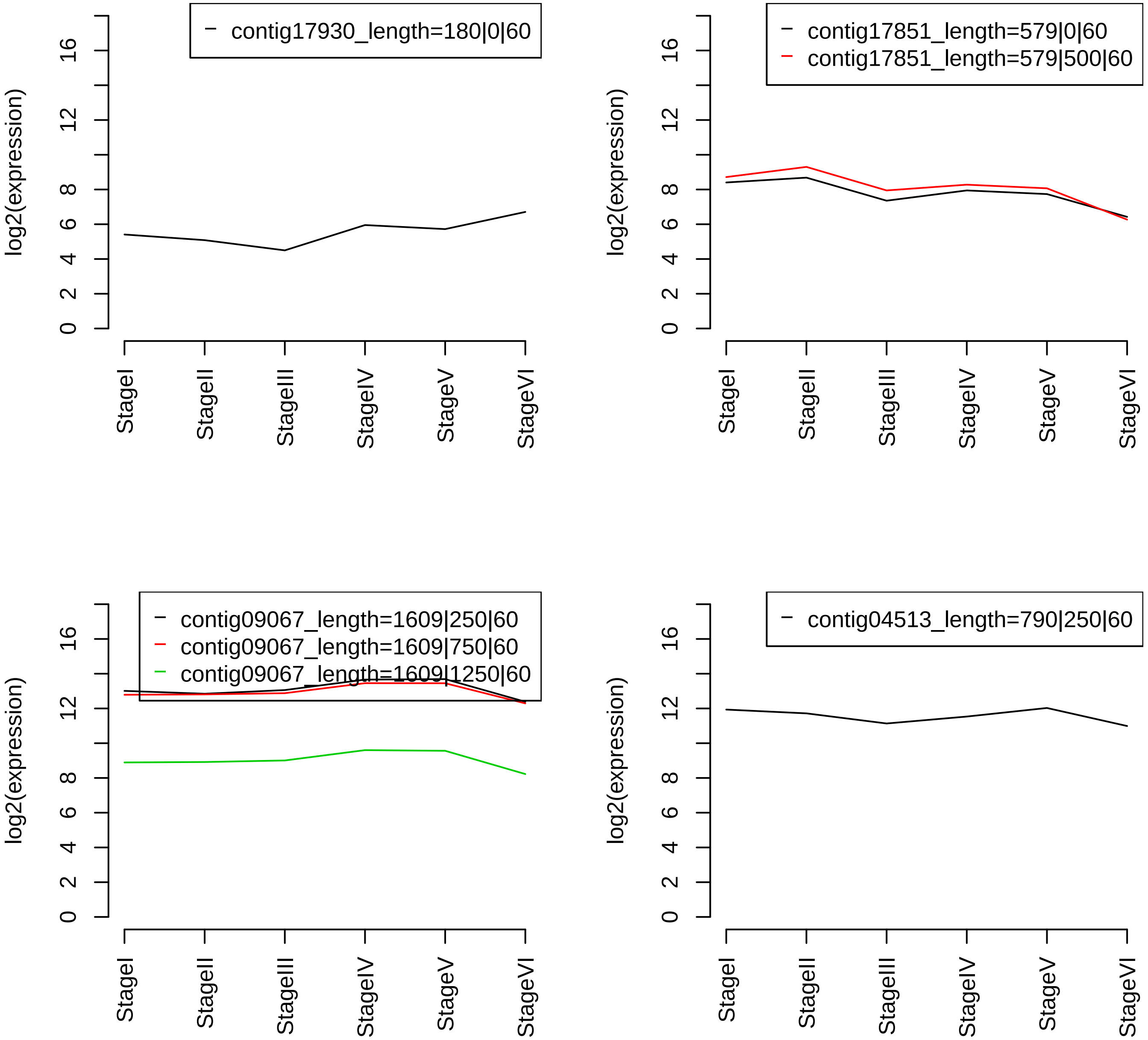

contig17930_length=180|0|60
contig17851_length=579|0|60
contig17851_length=579|500|60
contig09067_length=1609|250|60
contig09067_length=1609|750|60
contig09067_length=1609|1250|60
contig04513_length=790|250|60
log2(expression)
0
2
4
6
8
12
16
StageI
StageII
StageIII
StageIV
StageV
StageVI

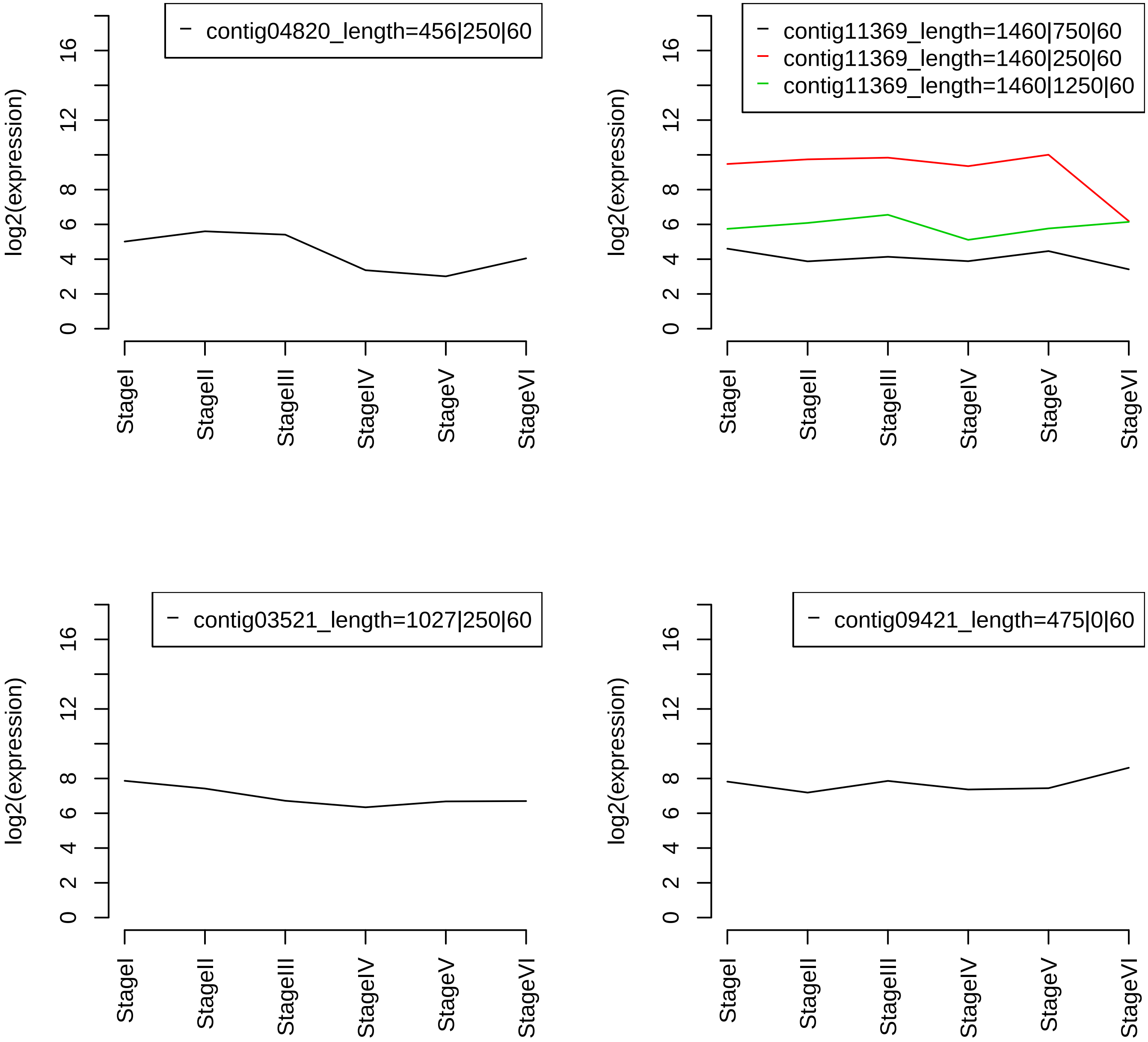
contig04820_length=456|250|60
contig11369_length=1460|750|60
contig11369_length=1460|250|60
contig11369_length=1460|1250|60
contig03521_length=1027|250|60
contig09421_length=475|0|60
log2(expression)
0
2
4
6
8
12
16
StageI
StageII
StageIII
StageIV
StageV
StageVI

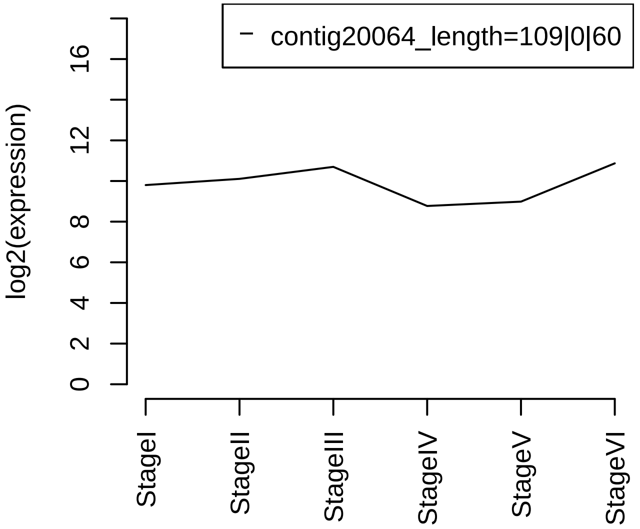

contig20064_length=109|0|60
log2(expression)
0
2
4
6
8
12
16
StageI
StageII
StageIII
StageIV
StageV
StageVI

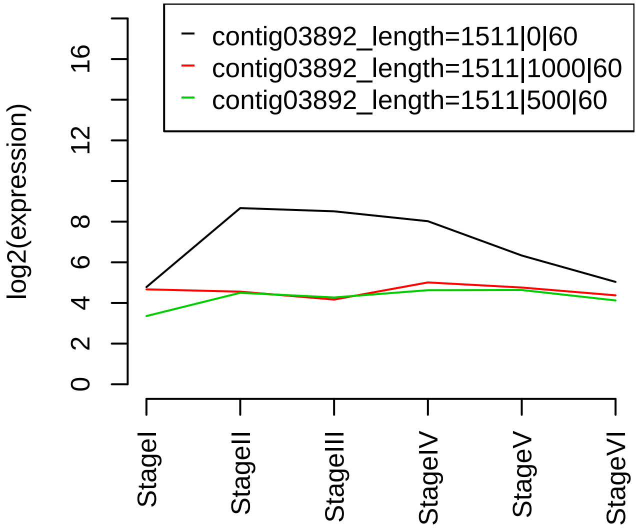

contig03892_length=1511|0|60
contig03892_length=1511|1000|60
contig03892_length=1511|500|60
log2(expression)
0
2
4
6
8
12
16
StageI
StageII
StageIII
StageIV
StageV
StageVI

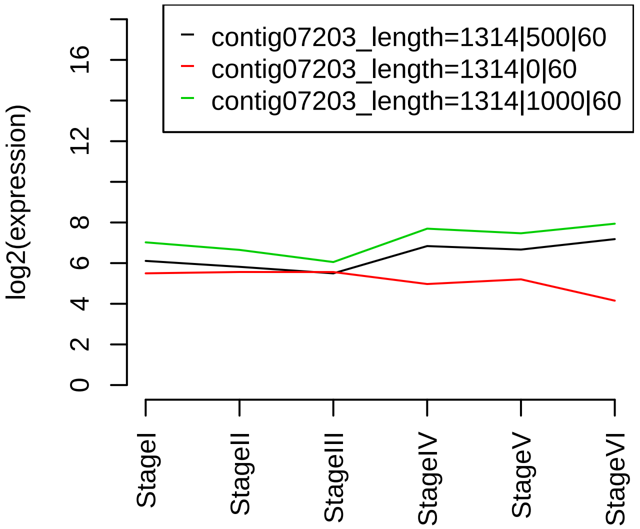

contig07203_length=1314|500|60
contig07203_length=1314|0|60
contig07203_length=1314|1000|60
log2(expression)
0
2
4
6
8
12
16
StageI
StageII
StageIII
StageIV
StageV
StageVI

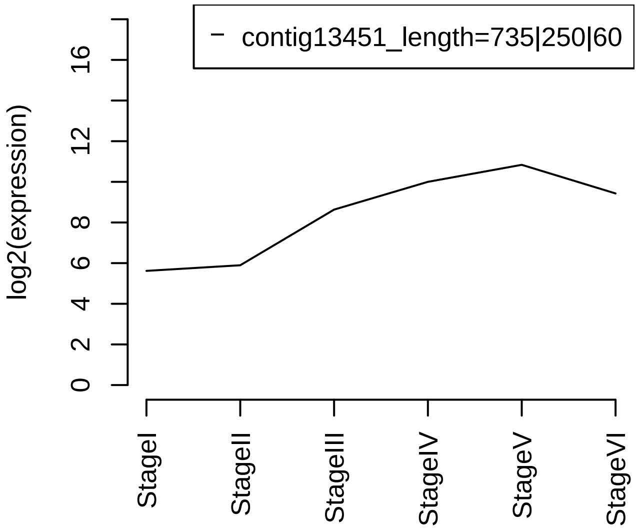

contig13451_length=735|250|60
log2(expression)
0
2
4
6
8
12
16
StageI
StageII
StageIII
StageIV
StageV
StageVI

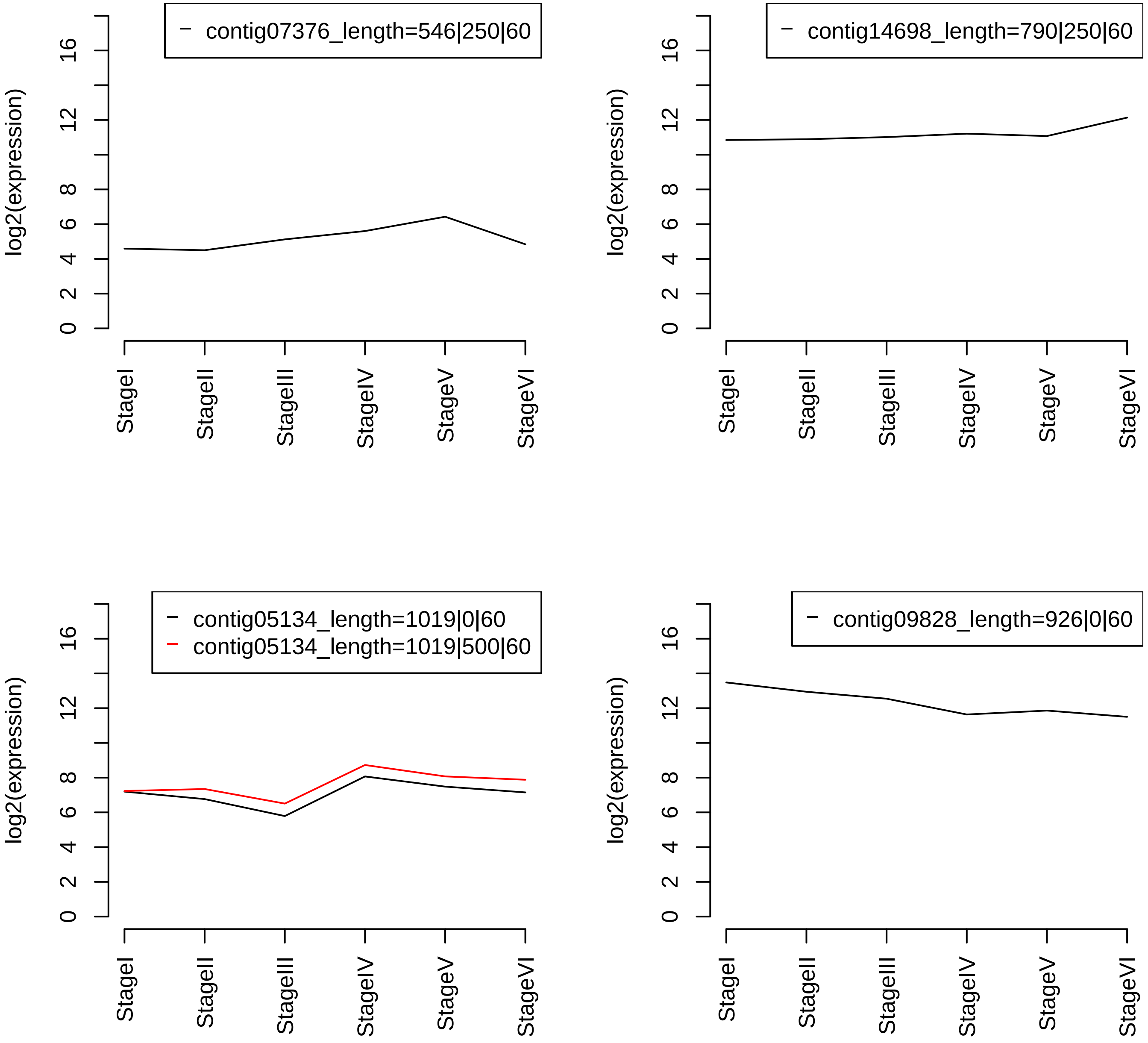
contig07376_length=546|250|60
contig14698_length=790|250|60
contig05134_length=1019|0|60
contig05134_length=1019|500|60
contig09828_length=926|0|60
log2(expression)
0
2
4
6
8
12
16
StageI
StageII
StageIII
StageIV
StageV
StageVI

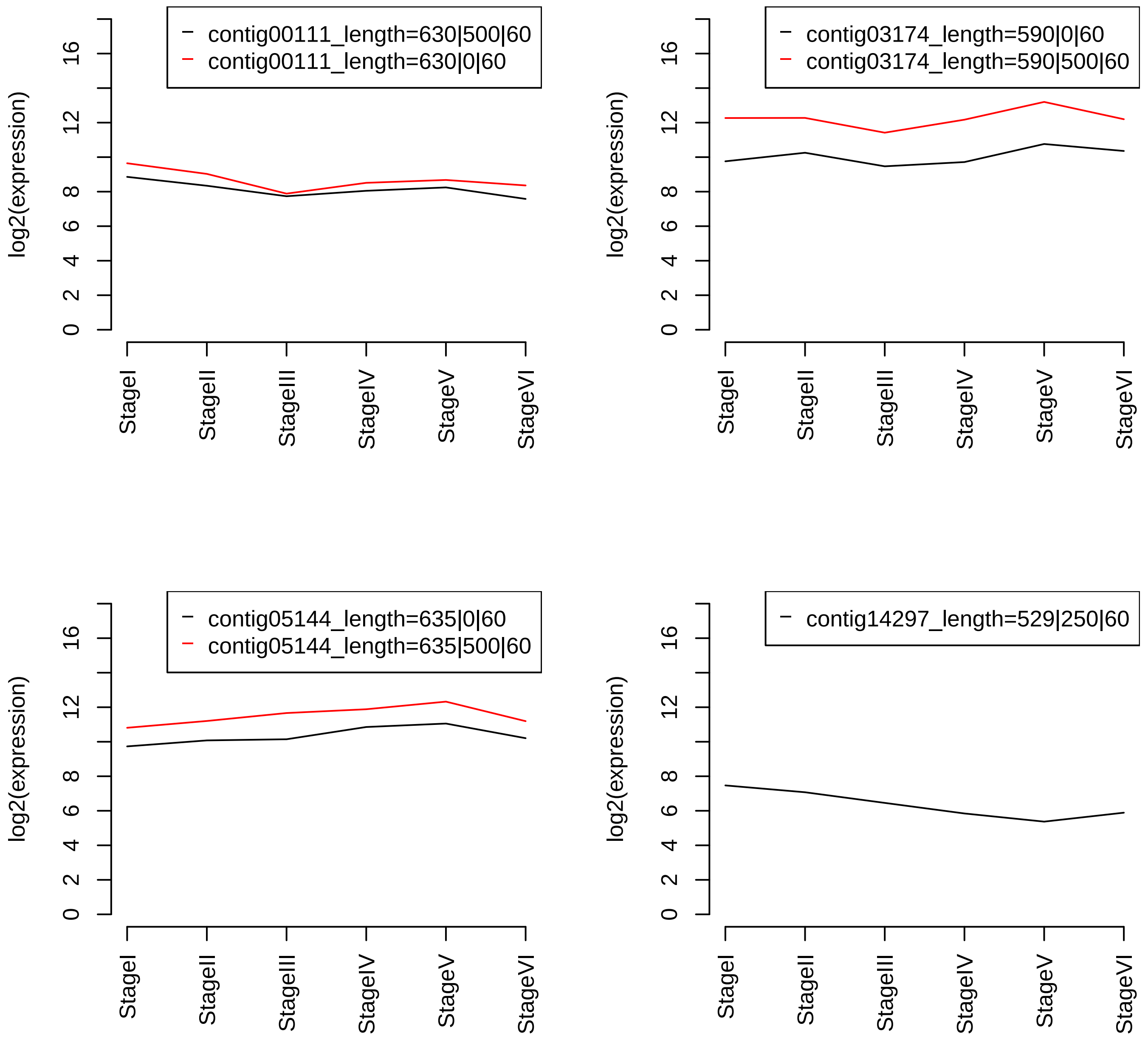
contig00111_length=630|500|60
contig00111_length=630|0|60
contig03174_length=590|0|60
contig03174_length=590|500|60
contig05144_length=635|0|60
contig05144_length=635|500|60
contig14297_length=529|250|60
log2(expression)
0
2
4
6
8
12
16
StageI
StageII
StageIII
StageIV
StageV
StageVI

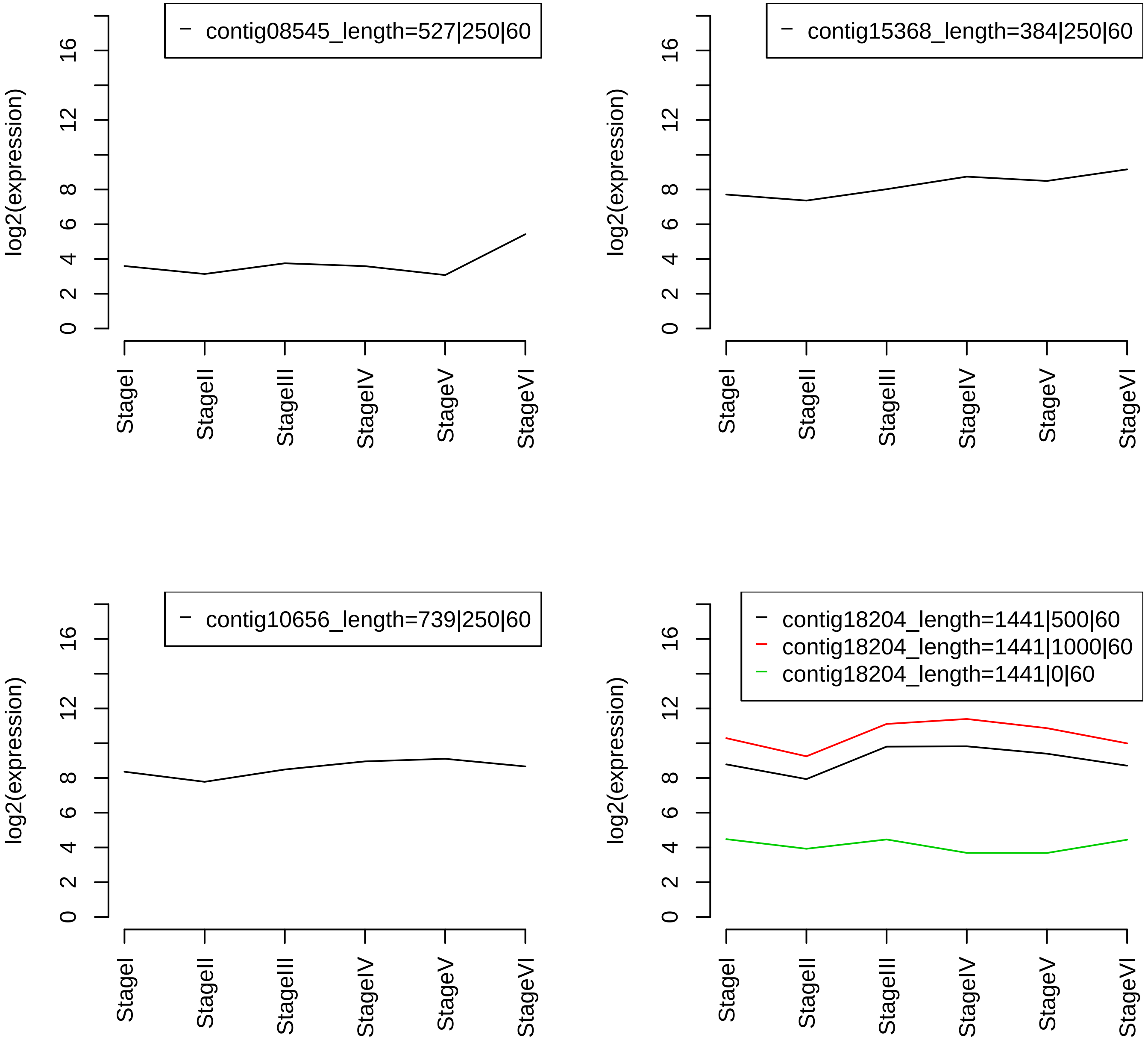

contig08545_length=527|250|60
contig15368_length=384|250|60
contig10656_length=739|250|60
contig18204_length=1441|500|60
contig18204_length=1441|1000|60
contig18204_length=1441|0|60
log2(expression)
0
2
4
6
8
12
16
StageI
StageII
StageIII
StageIV
StageV
StageVI

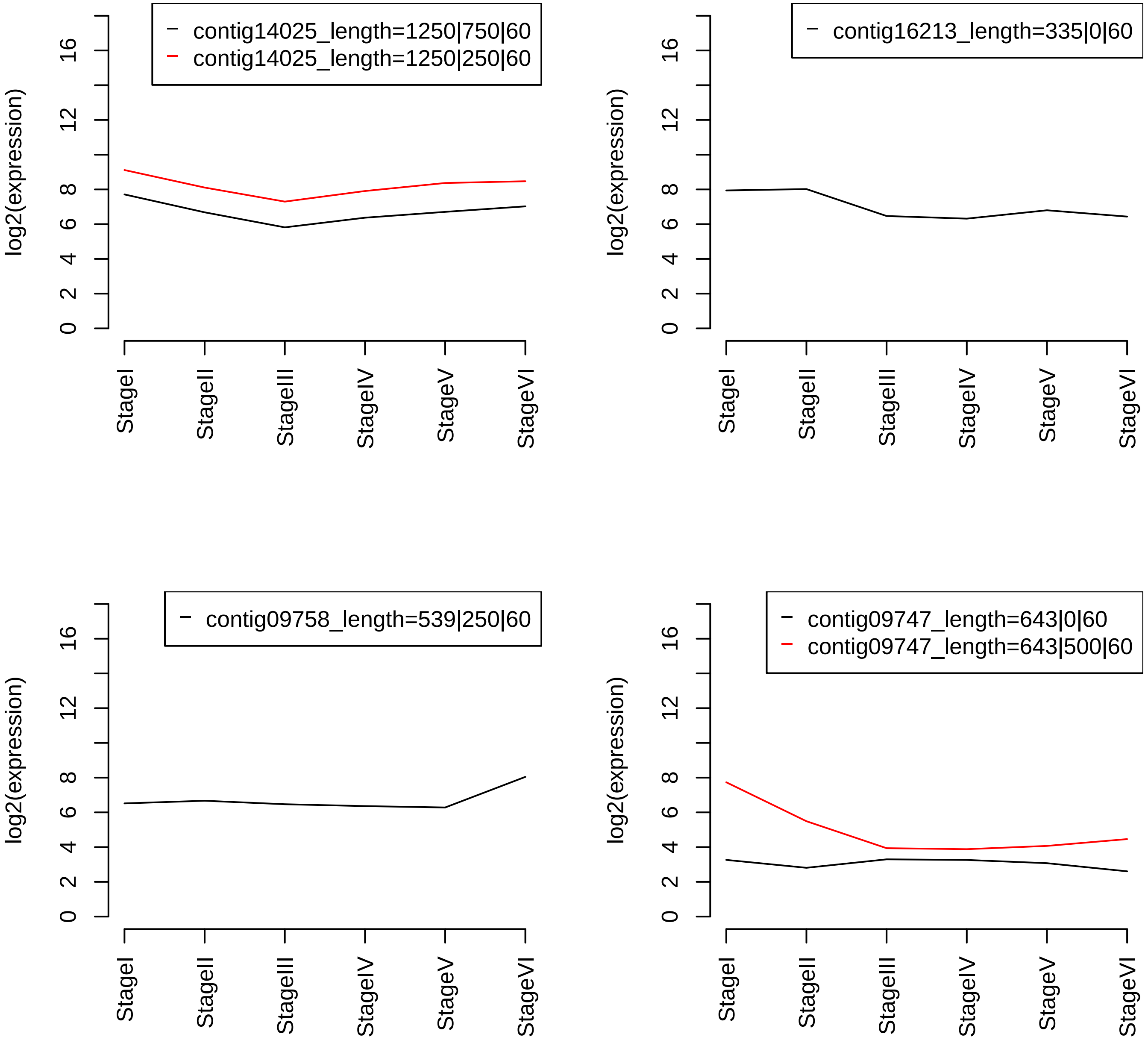

contig14025_length=1250|750|60
contig14025_length=1250|250|60
contig16213_length=335|0|60
contig09758_length=539|250|60
contig09747_length=643|0|60
contig09747_length=643|500|60
log2(expression)
0 2 4 6 8 12 16
StageI StageII StageIII StageIV StageV StageVI

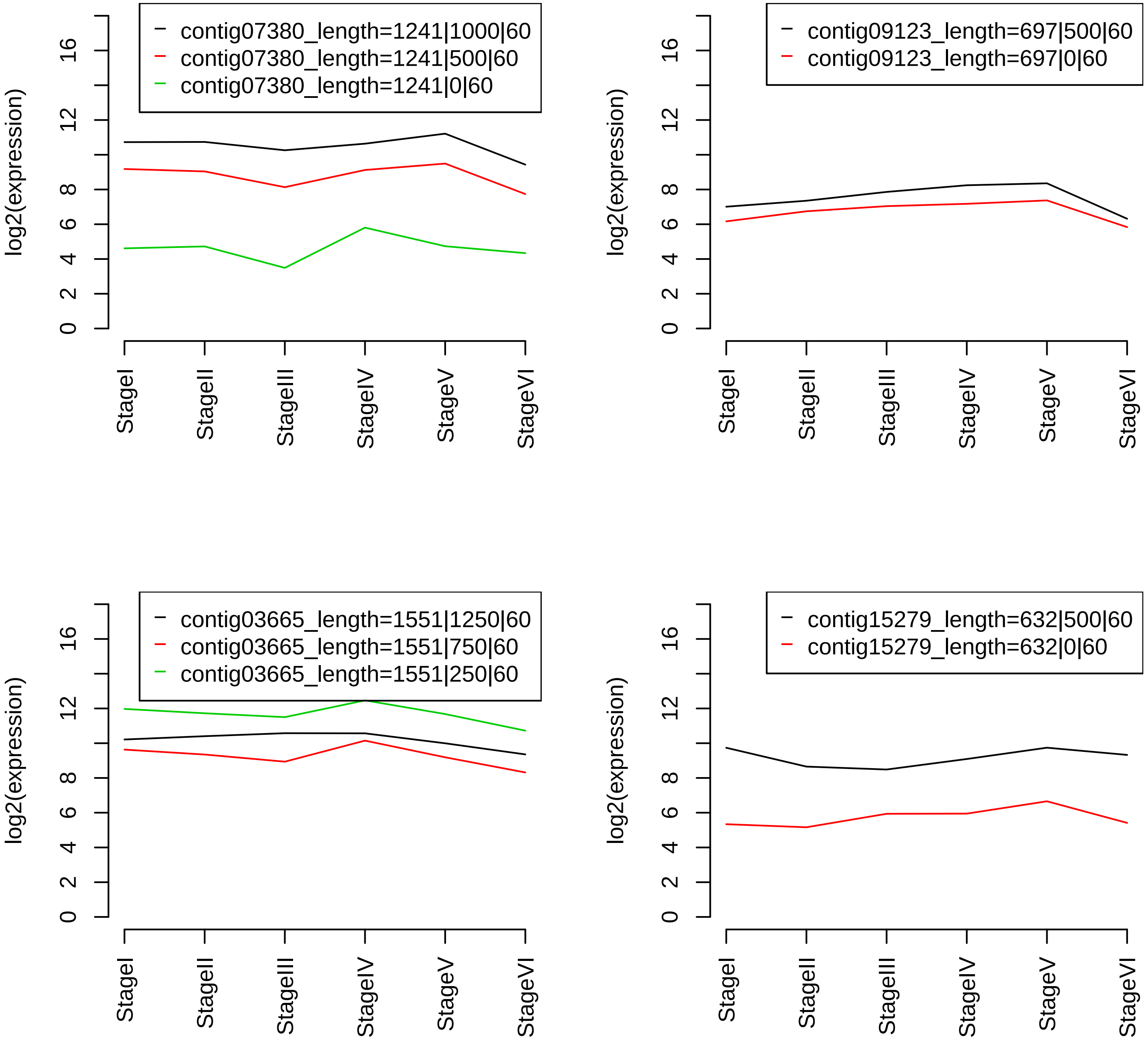
contig07380_length=1241|1000|60
contig07380_length=1241|500|60
contig07380_length=1241|0|60
contig09123_length=697|500|60
contig09123_length=697|0|60
contig03665_length=1551|1250|60
contig03665_length=1551|750|60
contig03665_length=1551|250|60
contig15279_length=632|500|60
contig15279_length=632|0|60
log2(expression)
0
2
4
6
8
12
16
StageI
StageII
StageIII
StageIV
StageV
StageVI

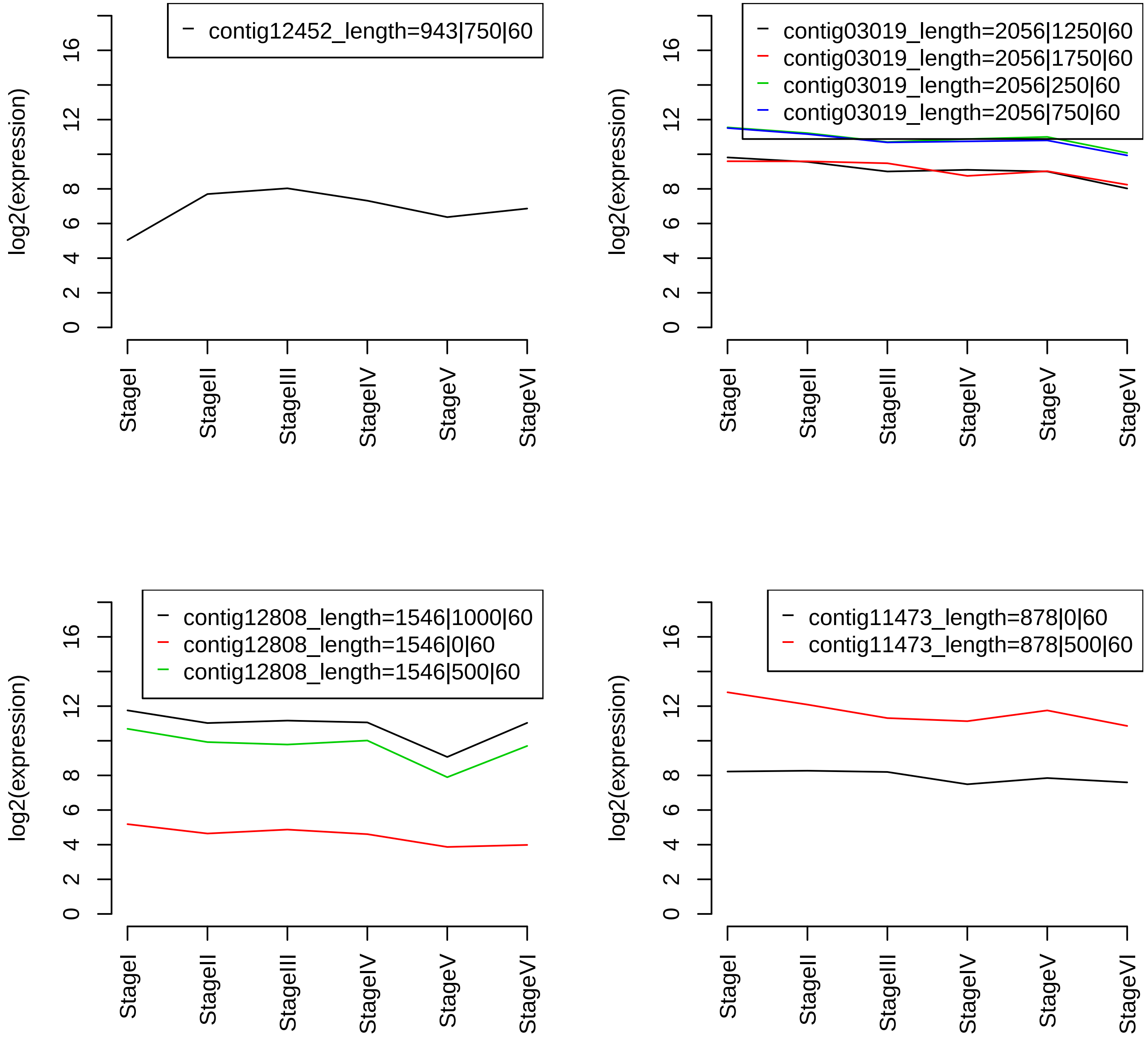

contig12452_length=943|750|60
contig03019_length=2056|1250|60
contig03019_length=2056|1750|60
contig03019_length=2056|250|60
contig03019_length=2056|750|60
contig12808_length=1546|1000|60
contig12808_length=1546|0|60
contig12808_length=1546|500|60
contig11473_length=878|0|60
contig11473_length=878|500|60
log2(expression)
0
2
4
6
8
12
16
StageI
StageII
StageIII
StageIV
StageV
StageVI

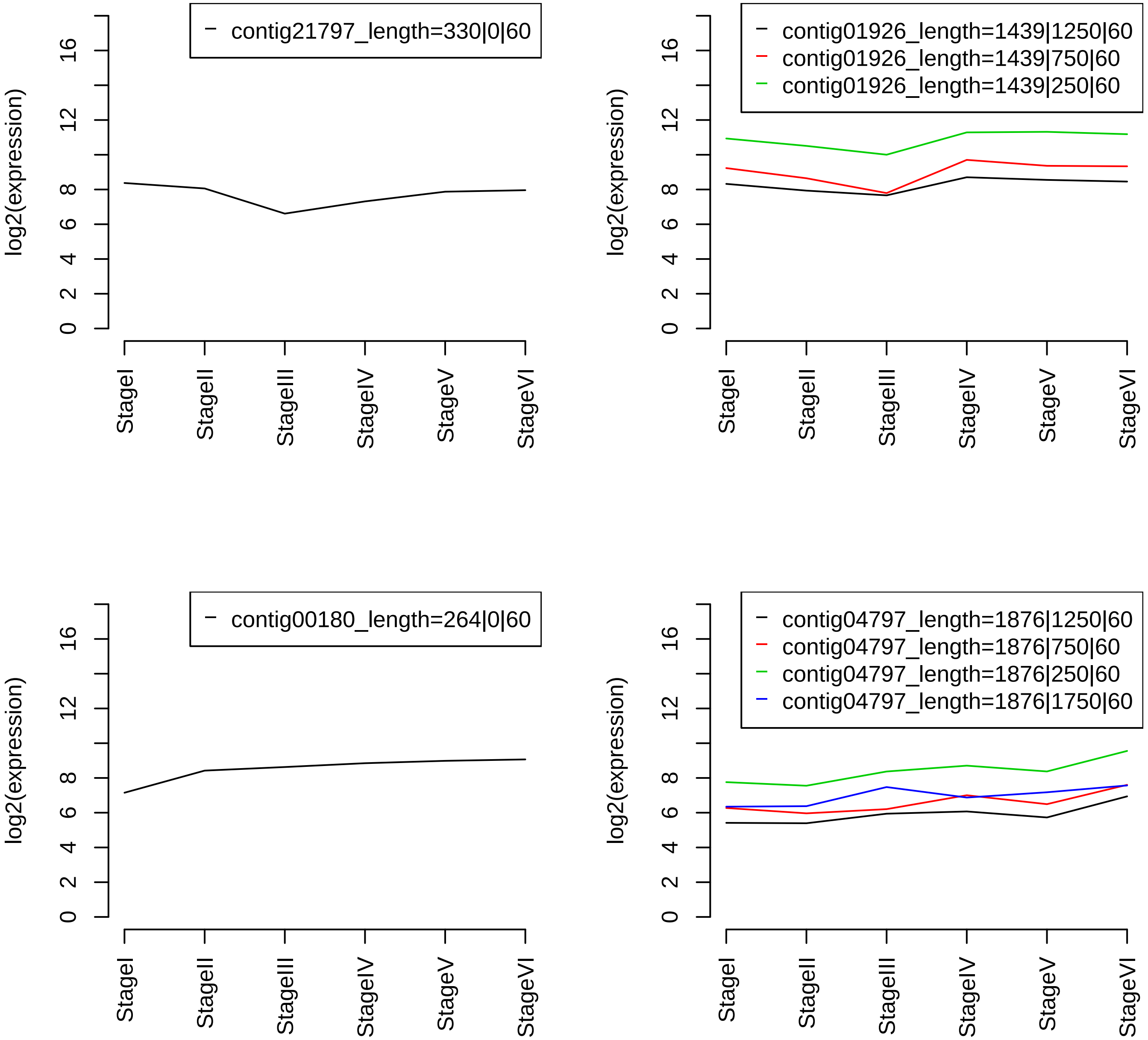
contig21797_length=330|0|60
contig01926_length=1439|1250|60
contig01926_length=1439|750|60
contig01926_length=1439|250|60
contig00180_length=264|0|60
contig04797_length=1876|1250|60
contig04797_length=1876|750|60
contig04797_length=1876|250|60
contig04797_length=1876|1750|60
log2(expression)
0
2
4
6
8
12
16
StageI
StageII
StageIII
StageIV
StageV
StageVI

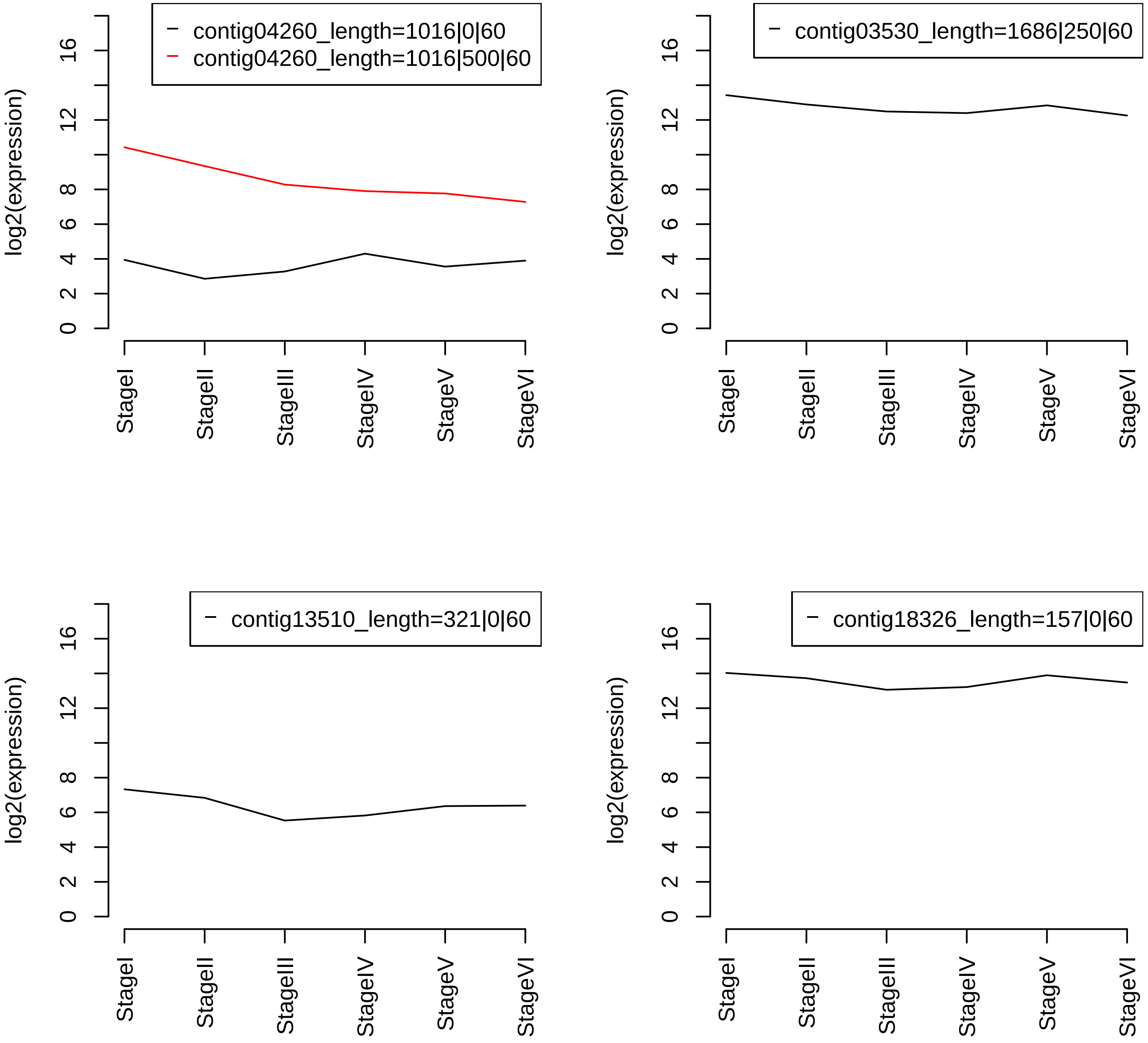
contig04260_length=1016|0|60
contig04260_length=1016|500|60
contig03530_length=1686|250|60
contig13510_length=321|0|60
contig18326_length=157|0|60
log2(expression)
0
2
4
6
8
12
16
StageI
StageII
StageIII
StageIV
StageV
StageVI

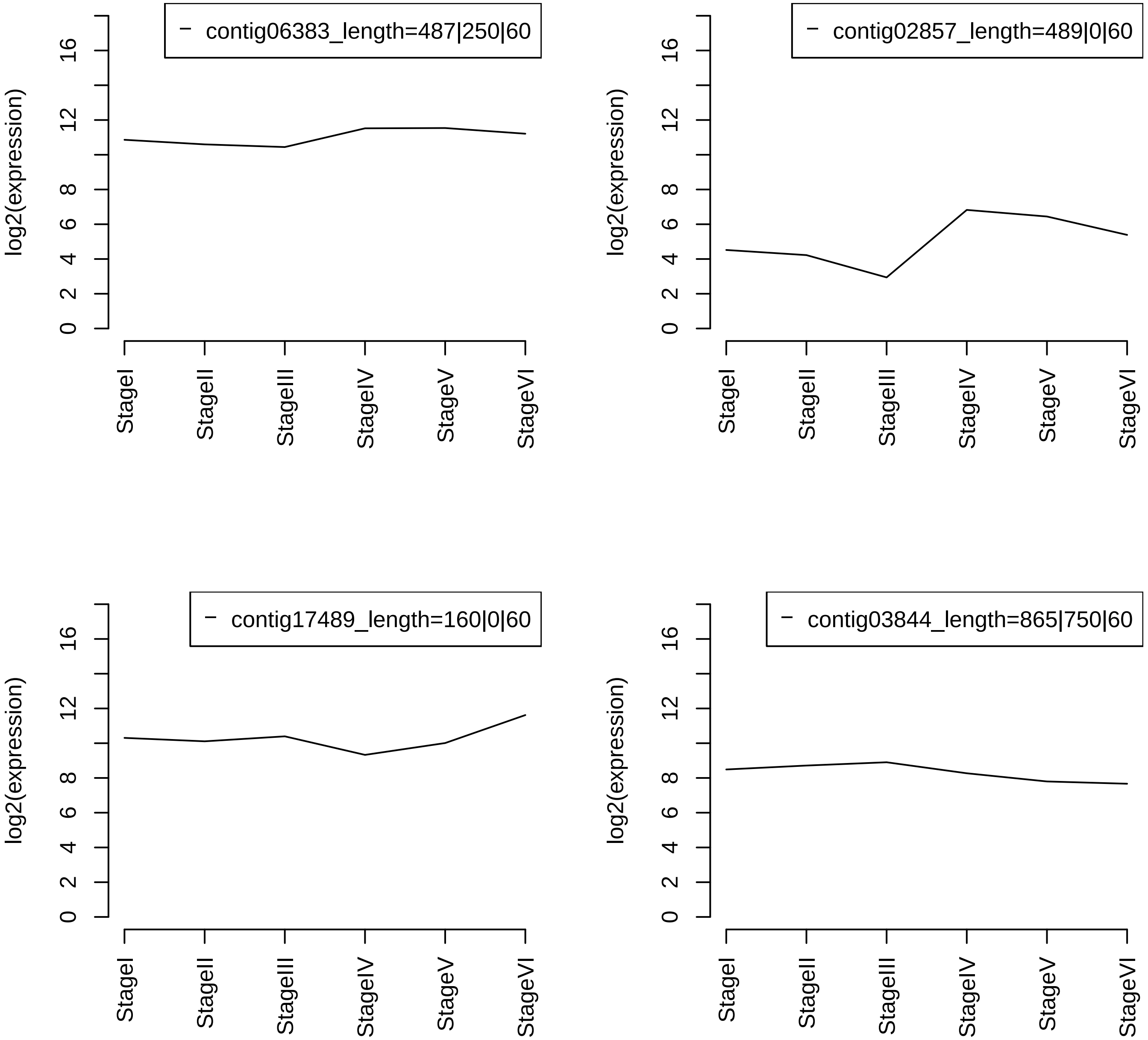

contig06383_length=487|250|60
contig02857_length=489|0|60
contig17489_length=160|0|60
contig03844_length=865|750|60
log2(expression)
0
2
4
6
8
12
16
StageI
StageII
StageIII
StageIV
StageV
StageVI

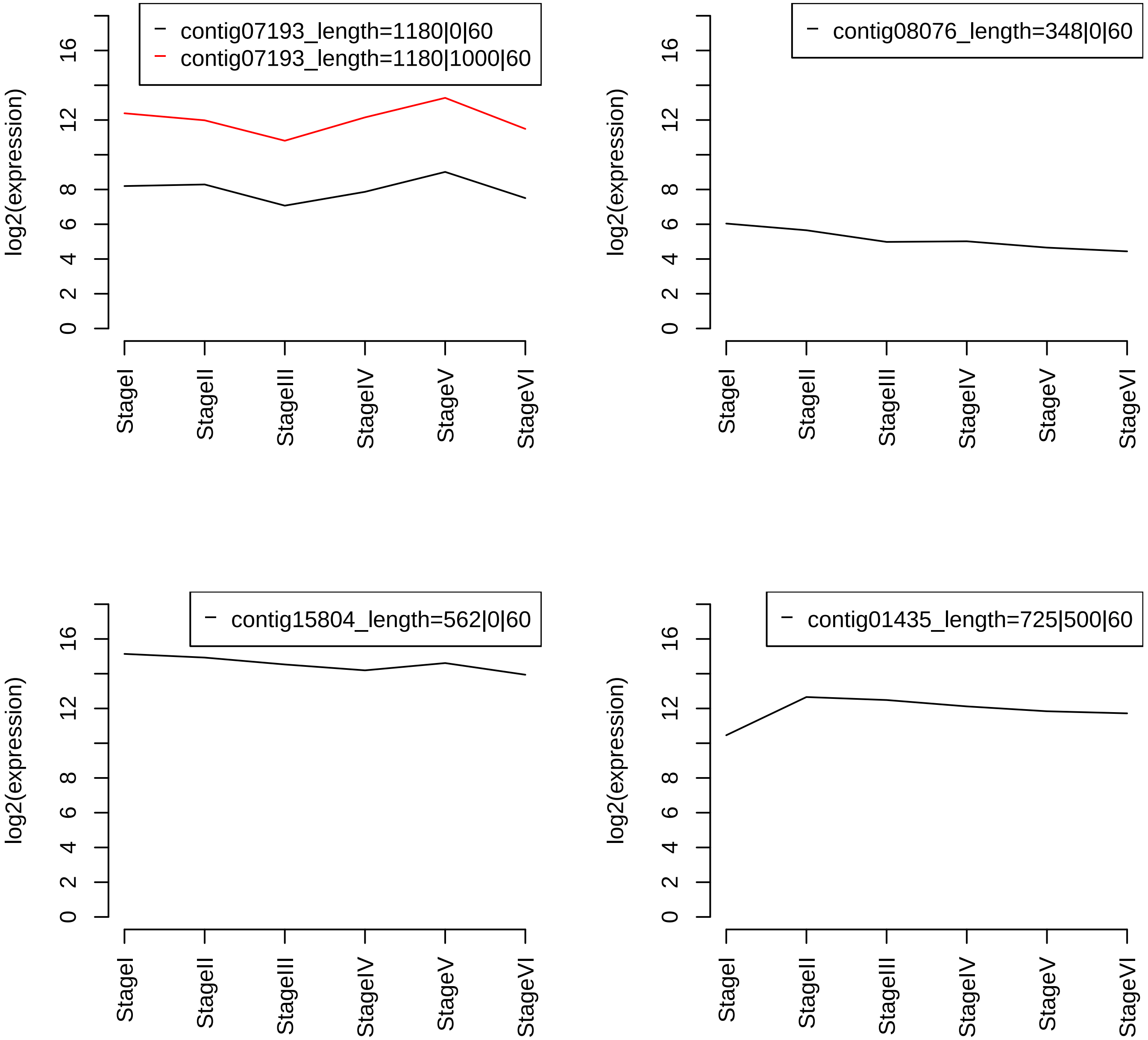
contig07193_length=1180|0|60
contig07193_length=1180|1000|60
contig08076_length=348|0|60
contig15804_length=562|0|60
contig01435_length=725|500|60
log2(expression)
0
2
4
6
8
12
16
StageI
StageII
StageIII
StageIV
StageV
StageVI

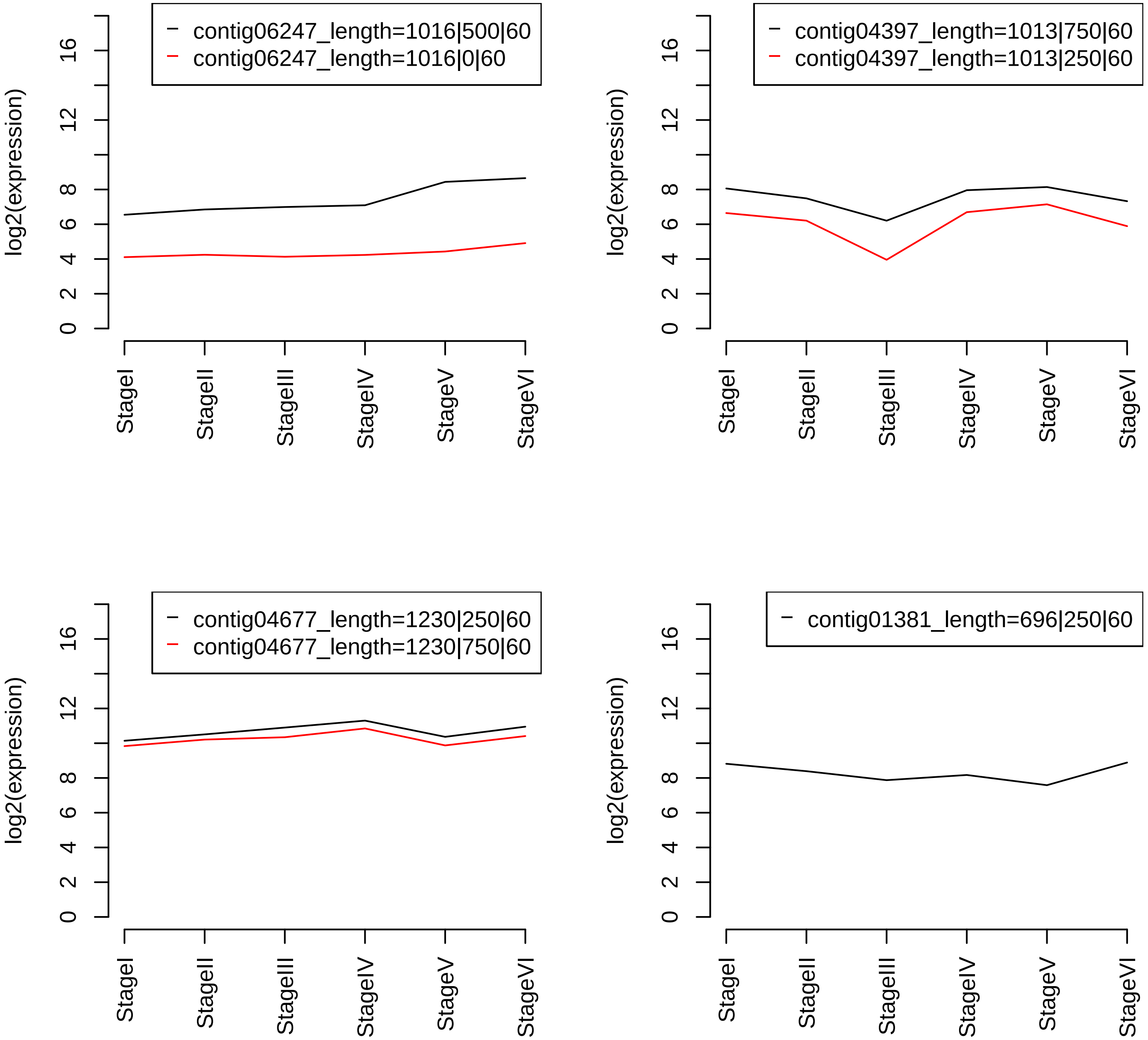
contig06247_length=1016|500|60
contig06247_length=1016|0|60
contig04397_length=1013|750|60
contig04397_length=1013|250|60
contig04677_length=1230|250|60
contig04677_length=1230|750|60
contig01381_length=696|250|60
log2(expression)
0
2
4
6
8
12
16
StageI
StageII
StageIII
StageIV
StageV
StageVI

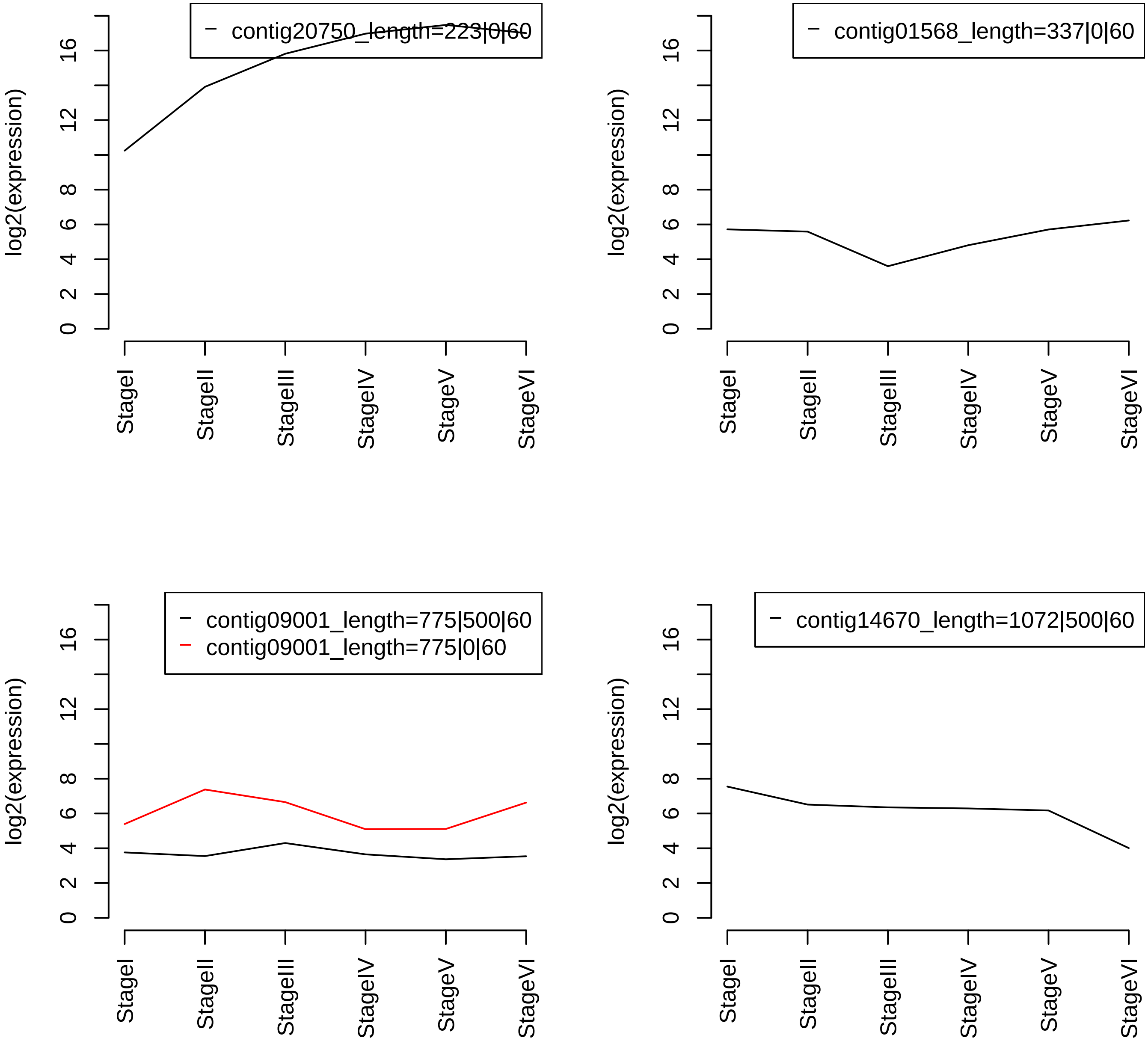

contig20750_length=223|0|60
contig01568_length=337|0|60
contig09001_length=775|500|60
contig09001_length=775|0|60
contig14670_length=1072|500|60
log2(expression)
0
2
4
6
8
12
16
StageI
StageII
StageIII
StageIV
StageV
StageVI

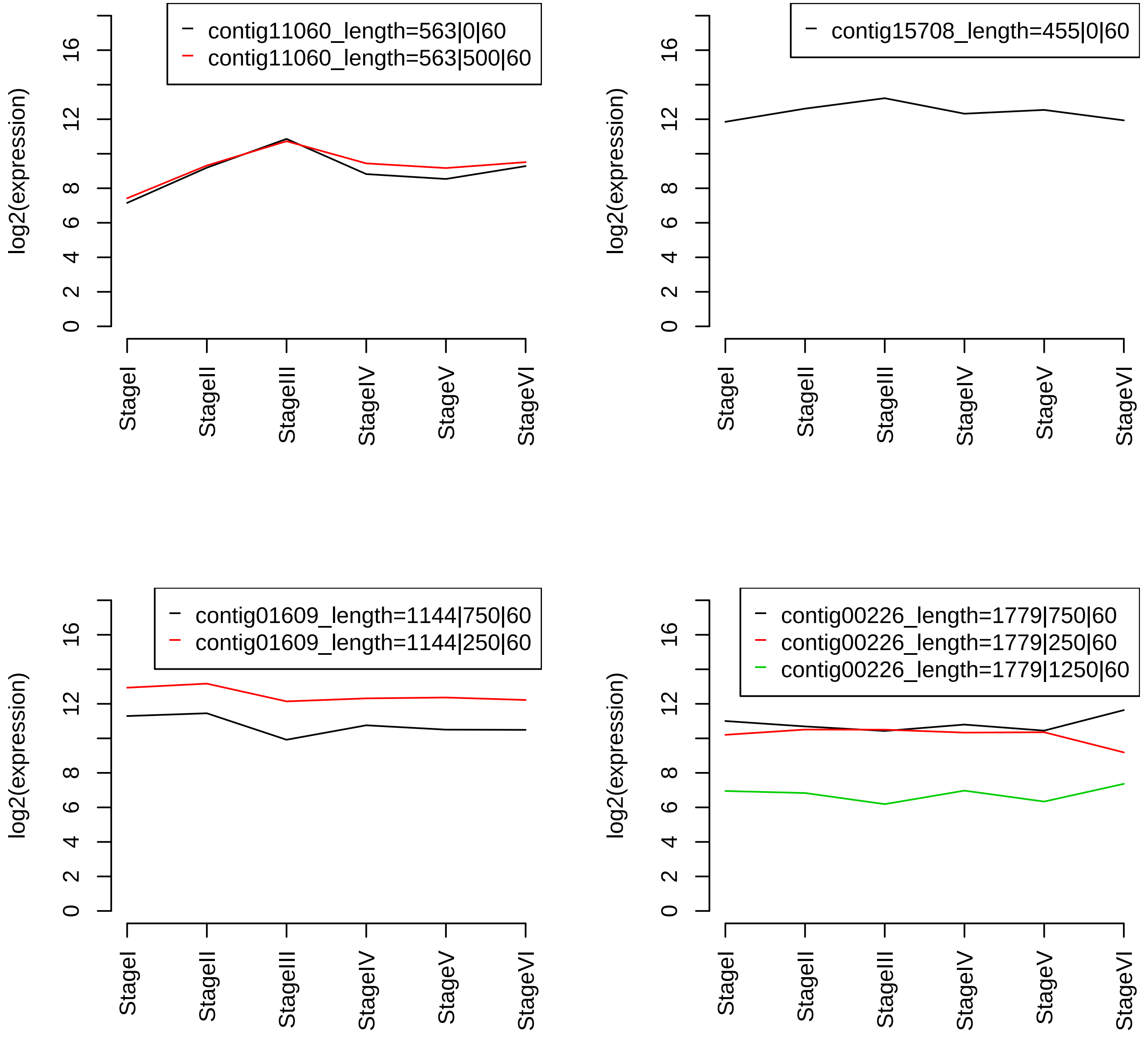
contig11060_length=563|0|60
contig11060_length=563|500|60
contig15708_length=455|0|60
contig01609_length=1144|750|60
contig01609_length=1144|250|60
contig00226_length=1779|750|60
contig00226_length=1779|250|60
contig00226_length=1779|1250|60
log2(expression)
StageI
StageII
StageIII
StageIV
StageV
StageVI

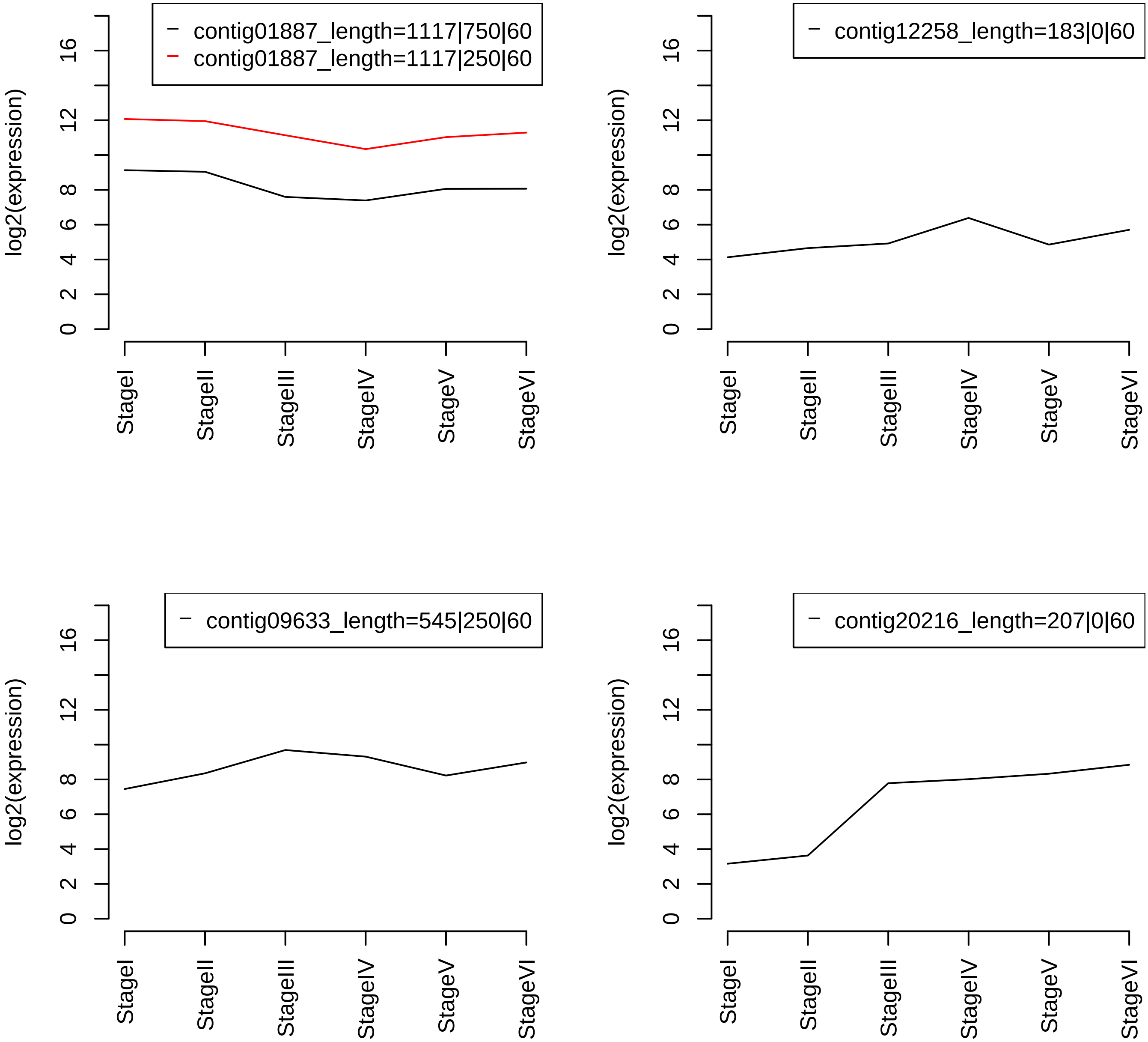

contig01887_length=1117|750|60
contig01887_length=1117|250|60
contig12258_length=183|0|60
contig09633_length=545|250|60
contig20216_length=207|0|60
log2(expression)
0
2
4
6
8
12
16
StageI
StageII
StageIII
StageIV
StageV
StageVI

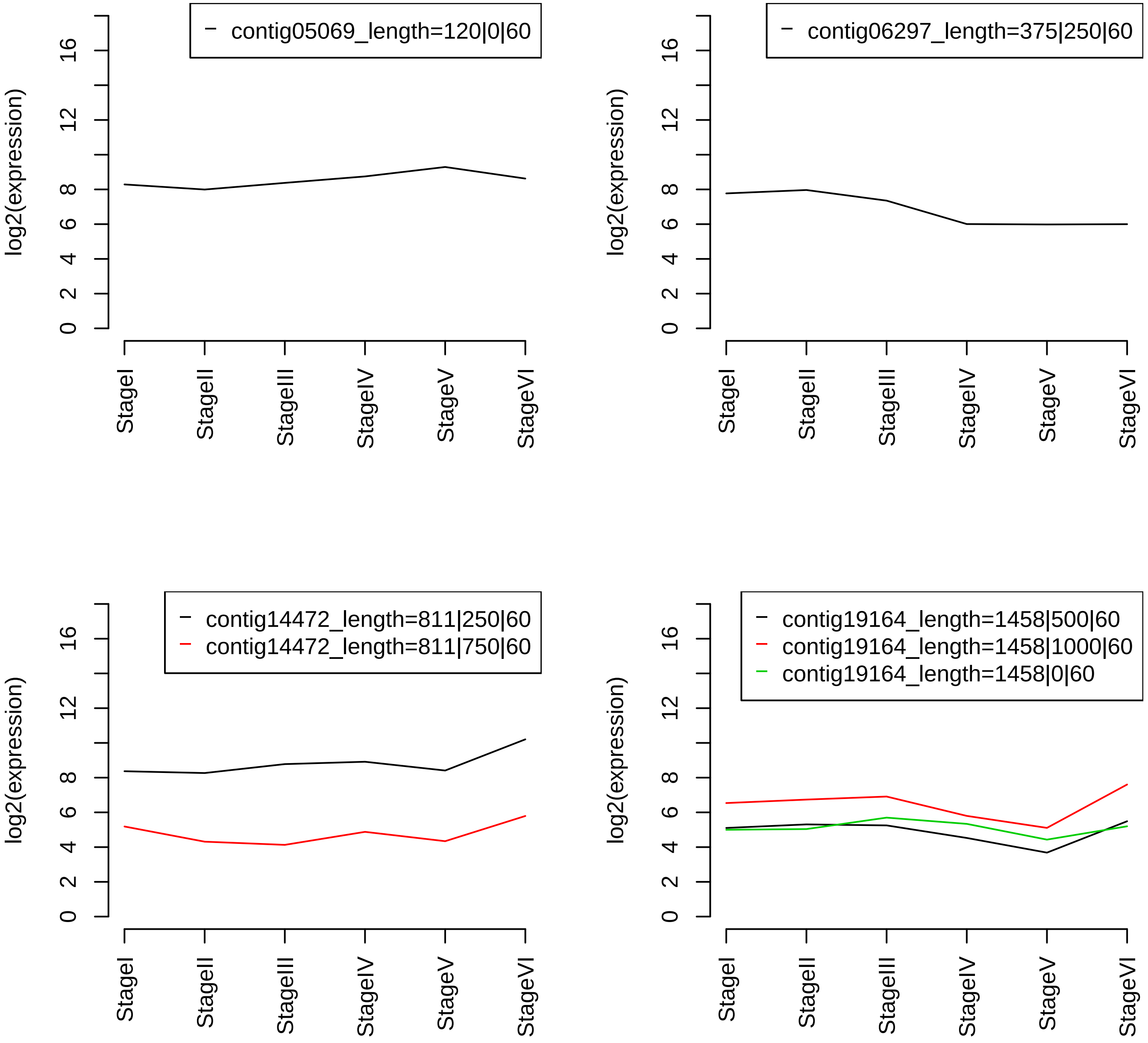
contig05069_length=120|0|60
contig06297_length=375|250|60
contig14472_length=811|250|60
contig14472_length=811|750|60
contig19164_length=1458|500|60
contig19164_length=1458|1000|60
contig19164_length=1458|0|60
log2(expression)
0
2
4
6
8
12
16
StageI
StageII
StageIII
StageIV
StageV
StageVI

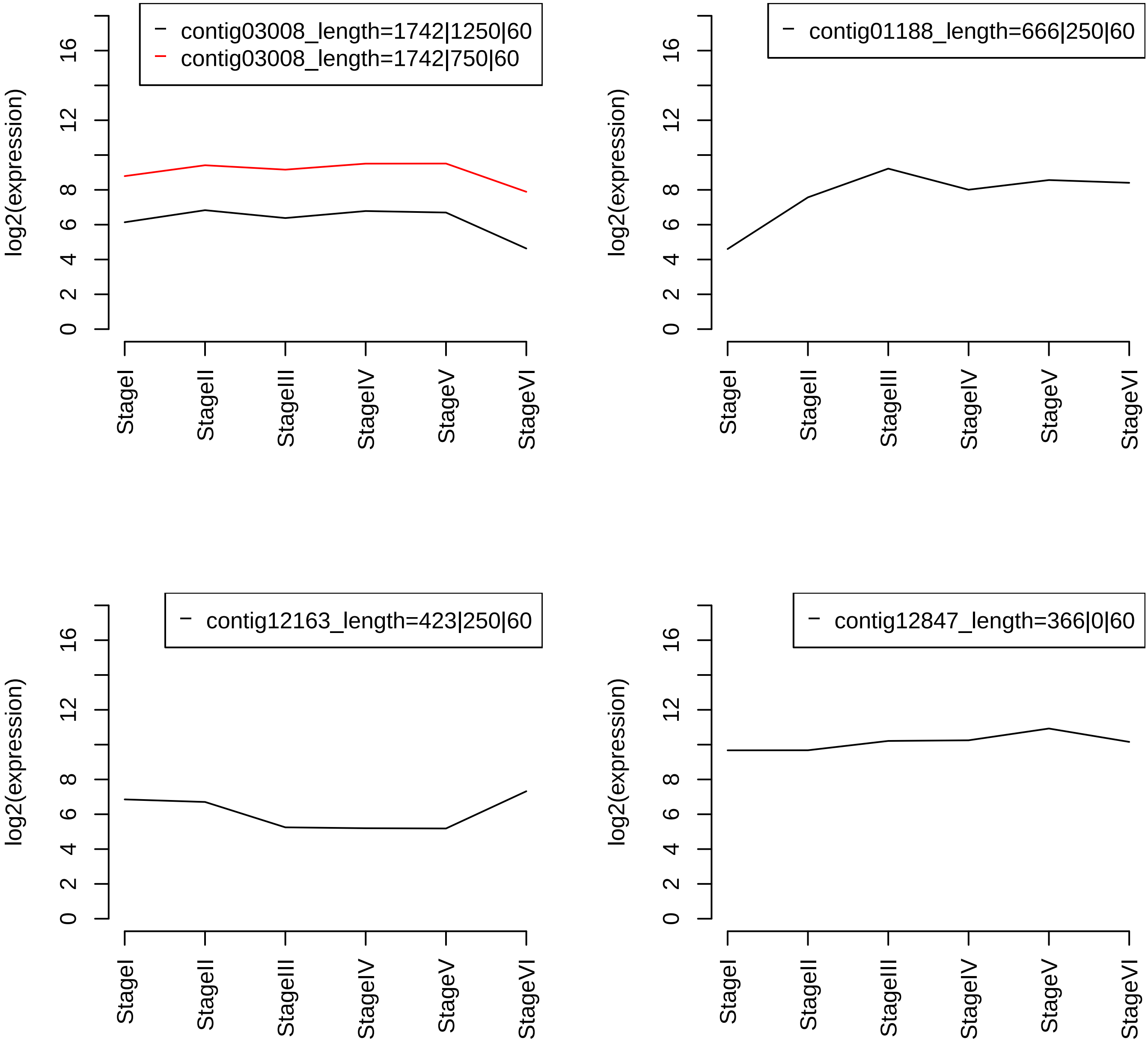
contig03008_length=1742|1250|60
contig03008_length=1742|750|60
contig01188_length=666|250|60
contig12163_length=423|250|60
contig12847_length=366|0|60
log2(expression)
0
2
4
6
8
12
16
StageI
StageII
StageIII
StageIV
StageV
StageVI

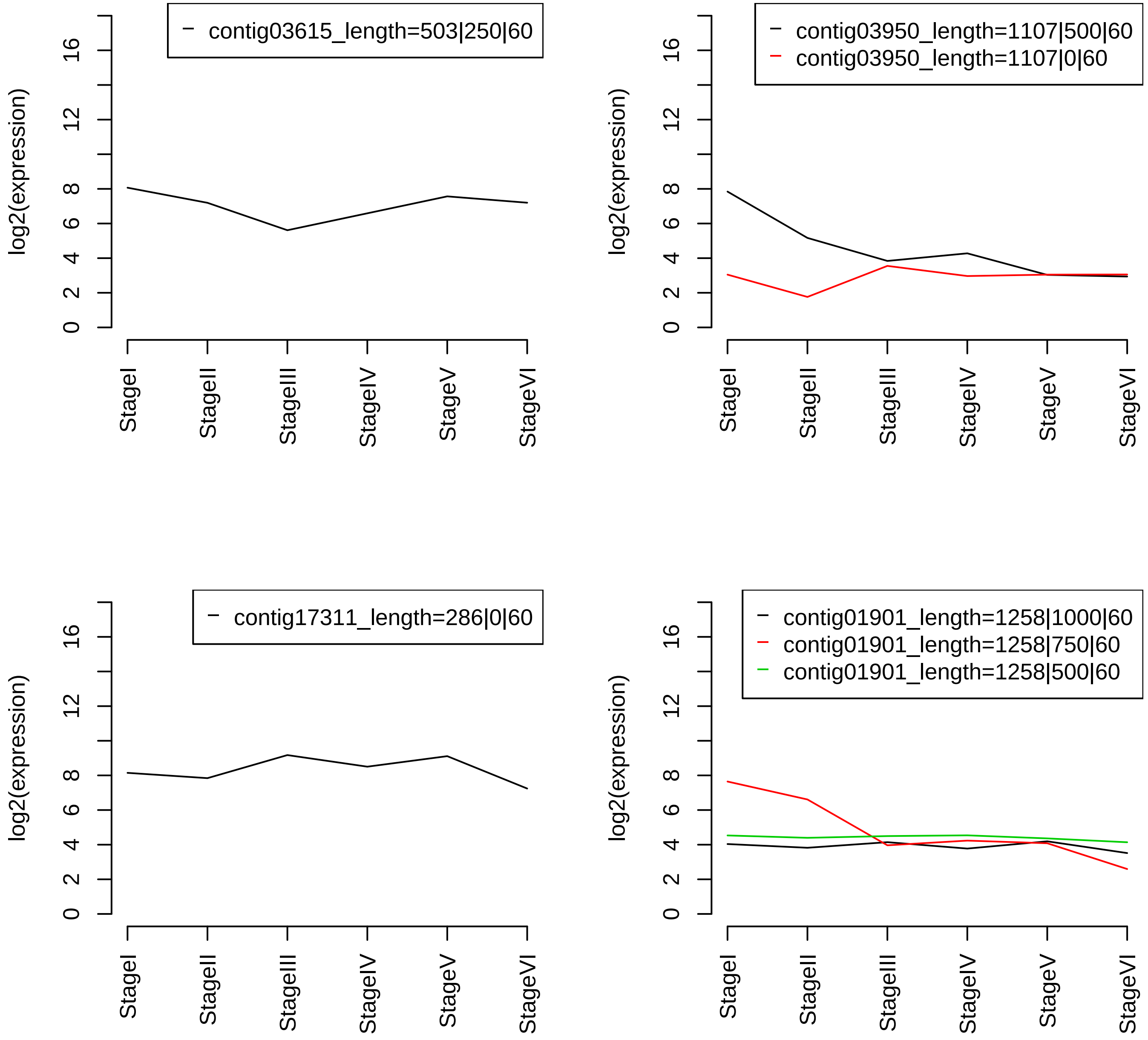

contig03615_length=503|250|60
contig03950_length=1107|500|60
contig03950_length=1107|0|60
contig17311_length=286|0|60
contig01901_length=1258|1000|60
contig01901_length=1258|750|60
contig01901_length=1258|500|60
log2(expression)
0
2
4
6
8
12
16
StageI
StageII
StageIII
StageIV
StageV
StageVI

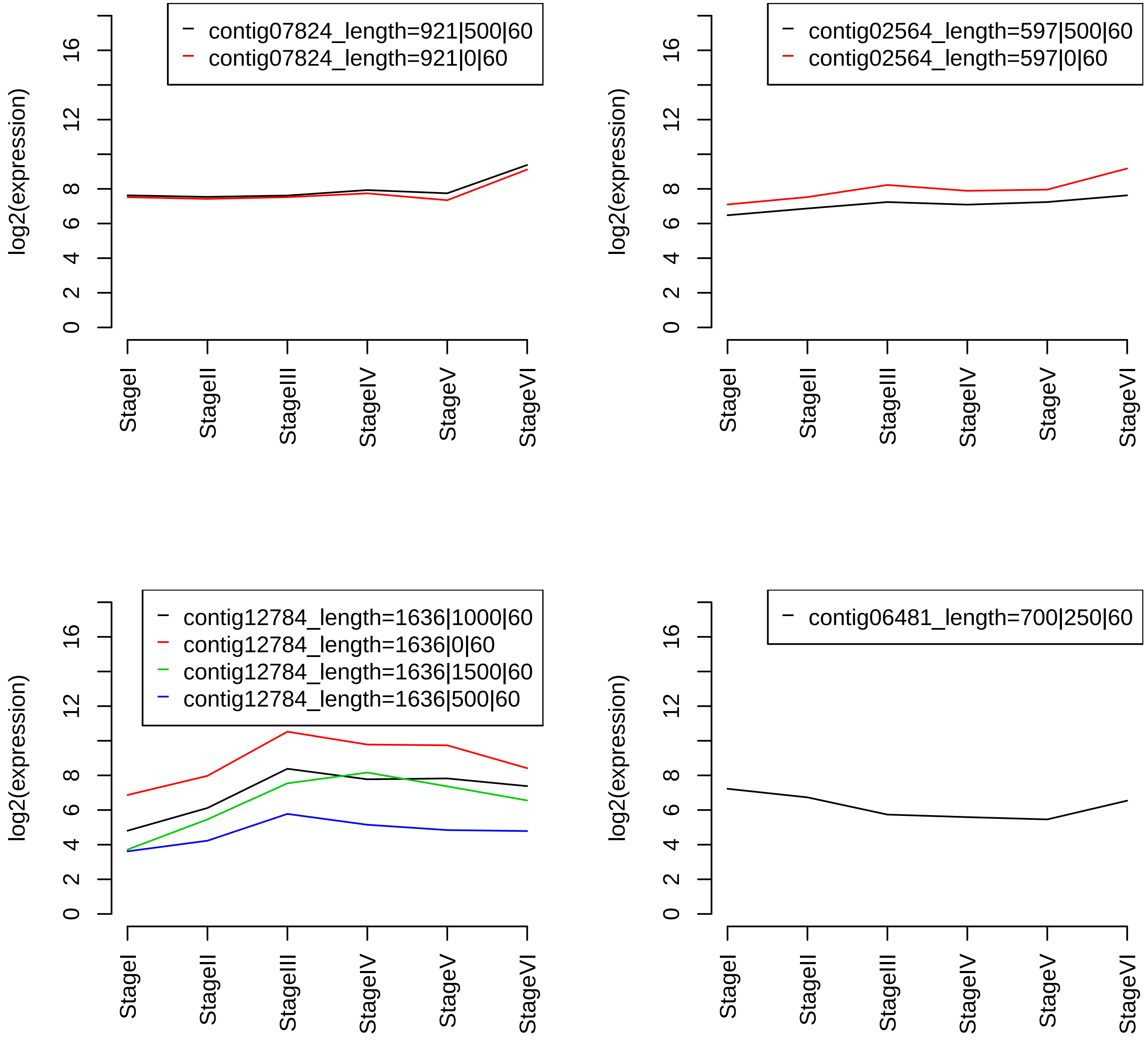
contig07824_length=921|500|60
contig07824_length=921|0|60
contig02564_length=597|500|60
contig02564_length=597|0|60
contig12784_length=1636|1000|60
contig12784_length=1636|0|60
contig12784_length=1636|1500|60
contig12784_length=1636|500|60
contig06481_length=700|250|60
log2(expression)
0
2
4
6
8
12
16
StageI
StageII
StageIII
StageIV
StageV
StageVI

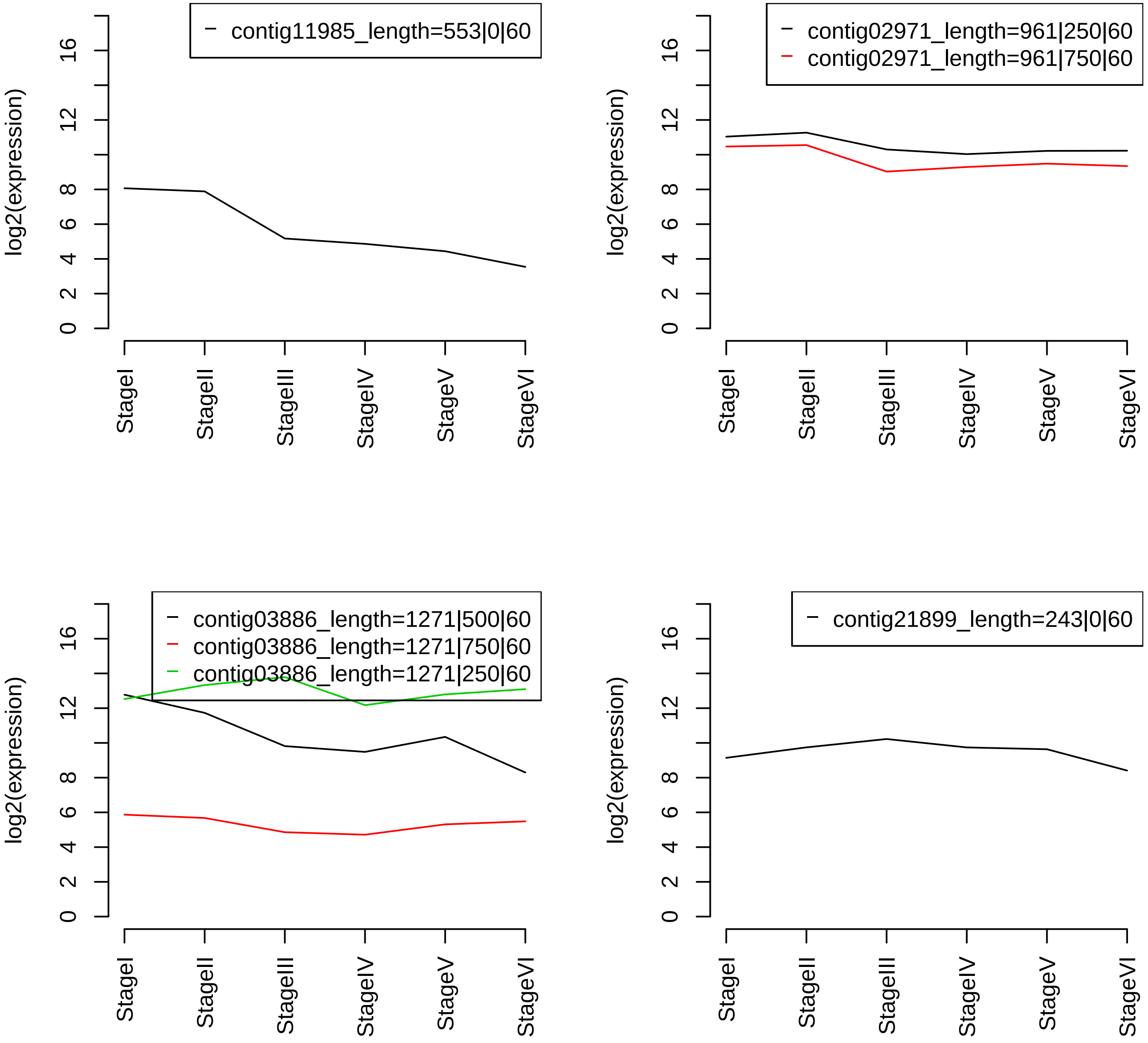
contig11985_length=553|0|60
contig02971_length=961|250|60
contig02971_length=961|750|60
contig03886_length=1271|500|60
contig03886_length=1271|750|60
contig03886_length=1271|250|60
contig21899_length=243|0|60
log2(expression)
0
2
4
6
8
12
16
StageI
StageII
StageIII
StageIV
StageV
StageVI

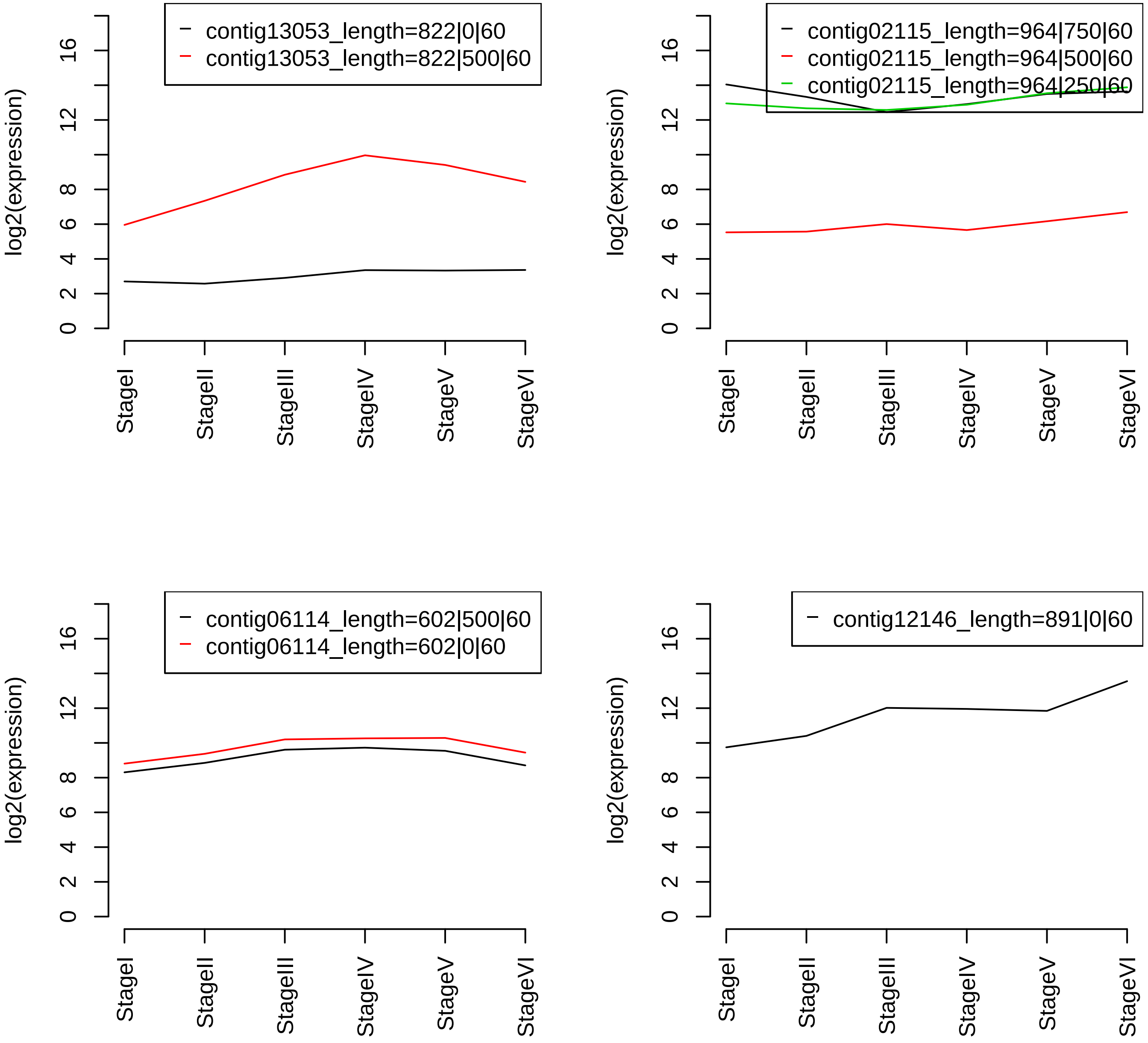

contig13053_length=822|0|60
contig13053_length=822|500|60
log2(expression)
0
2
4
6
8
12
16
StageI
StageII
StageIII
StageIV
StageV
StageVI
contig02115_length=964|750|60
contig02115_length=964|500|60
contig02115_length=964|250|60
contig06114_length=602|500|60
contig06114_length=602|0|60
contig12146_length=891|0|60

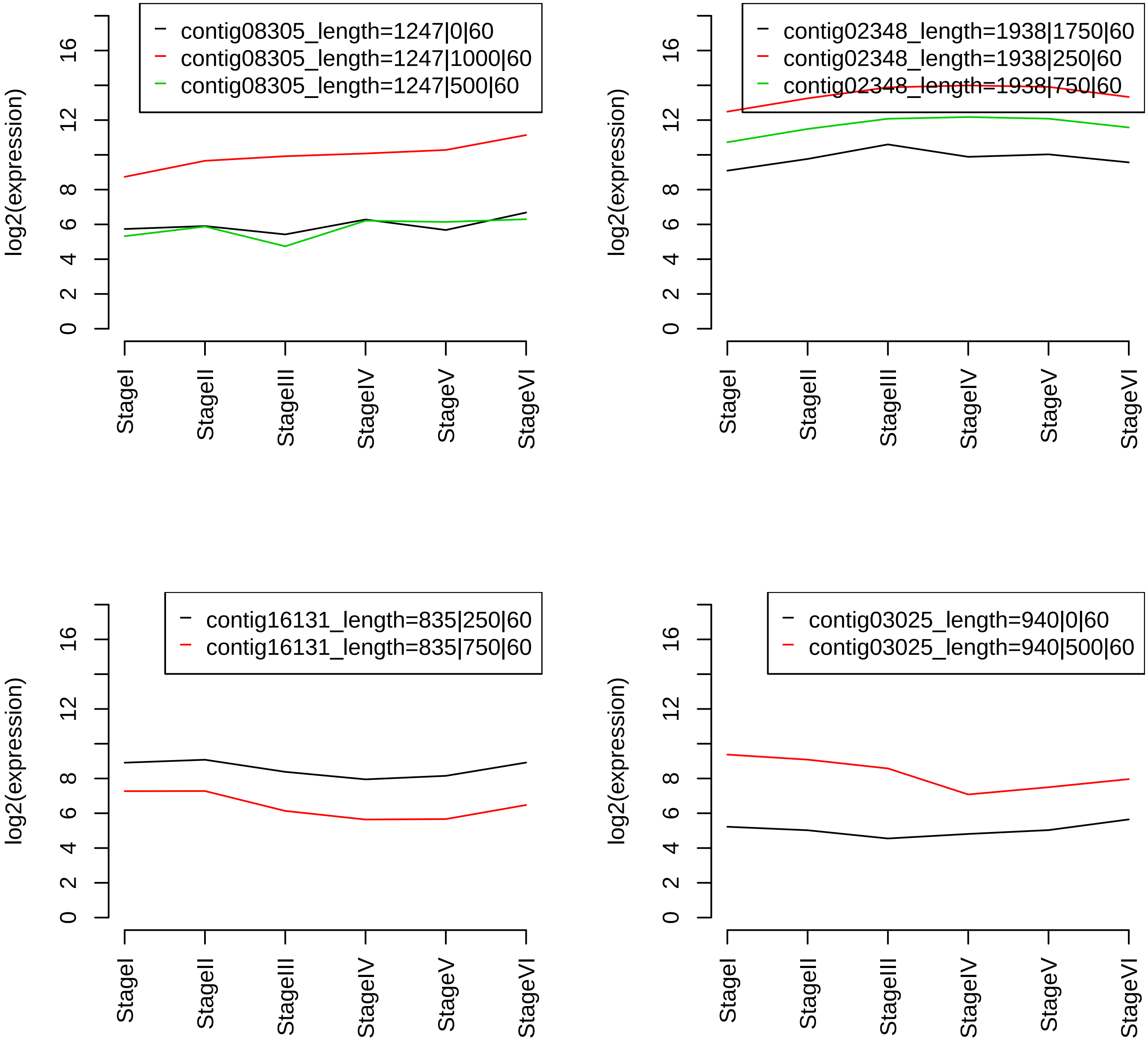

contig08305_length=1247|0|60
contig08305_length=1247|1000|60
contig08305_length=1247|500|60
contig02348_length=1938|1750|60
contig02348_length=1938|250|60
contig02348_length=1938|750|60
contig16131_length=835|250|60
contig16131_length=835|750|60
contig03025_length=940|0|60
contig03025_length=940|500|60
log2(expression)
0
2
4
6
8
12
16
StageI
StageII
StageIII
StageIV
StageV
StageVI

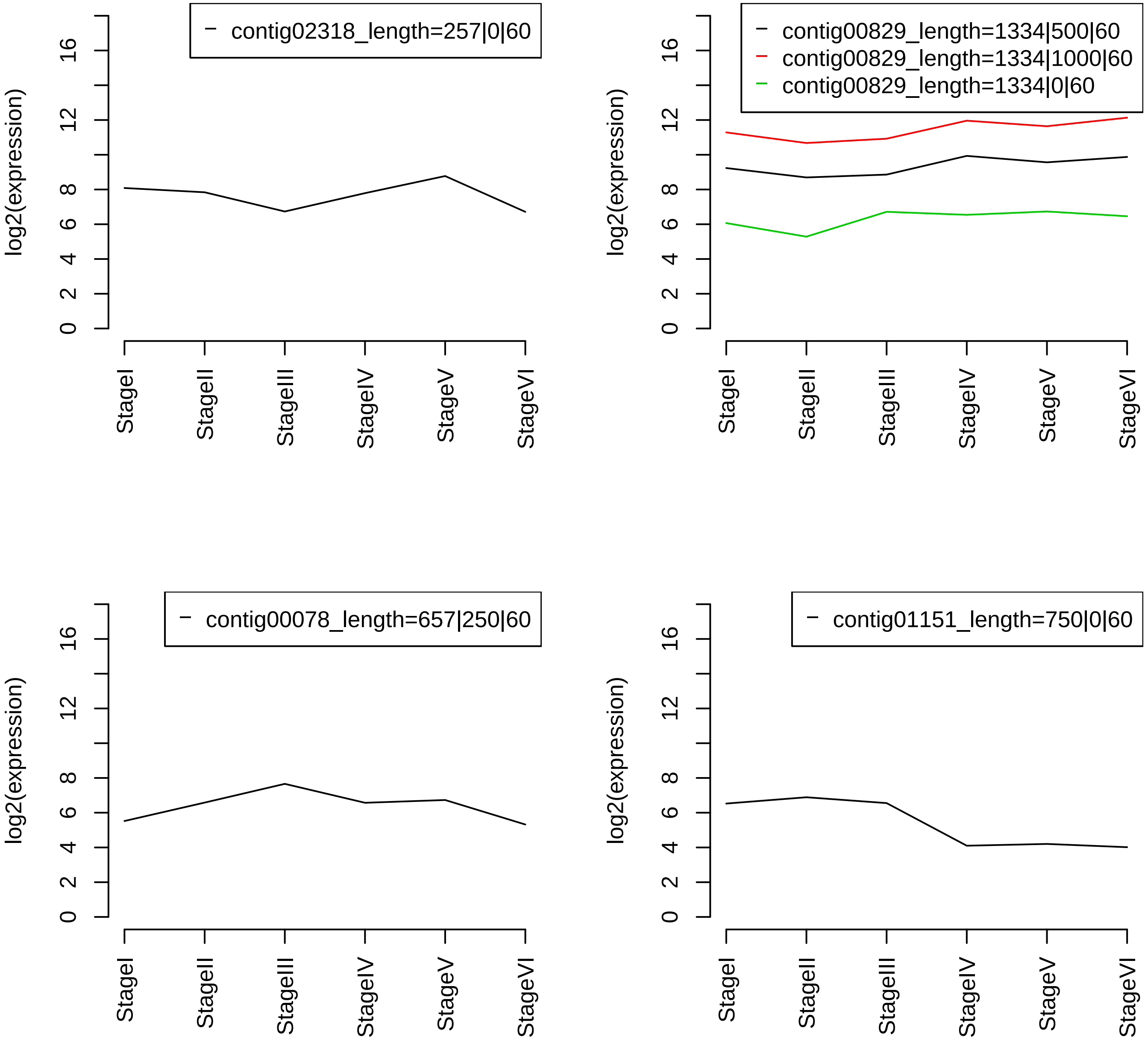
contig02318_length=257|0|60
contig00829_length=1334|500|60
contig00829_length=1334|1000|60
contig00829_length=1334|0|60
contig00078_length=657|250|60
contig01151_length=750|0|60
log2(expression)
0
2
4
6
8
12
16
StageI
StageII
StageIII
StageIV
StageV
StageVI

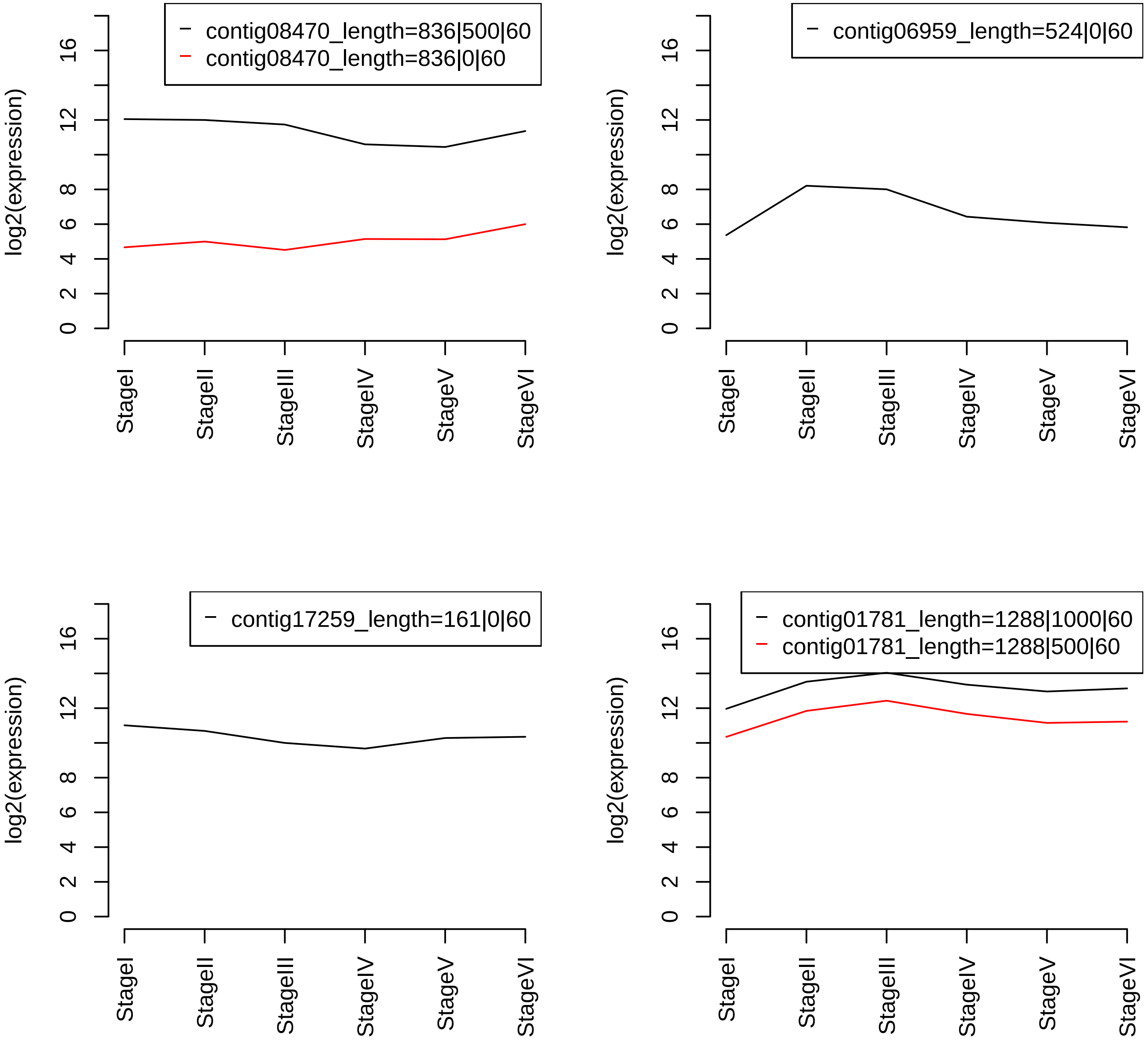

contig08470_length=836|500|60
contig08470_length=836|0|60
contig06959_length=524|0|60
contig17259_length=161|0|60
contig01781_length=1288|1000|60
contig01781_length=1288|500|60
log2(expression)
0
2
4
6
8
12
16
StageI
StageII
StageIII
StageIV
StageV
StageVI

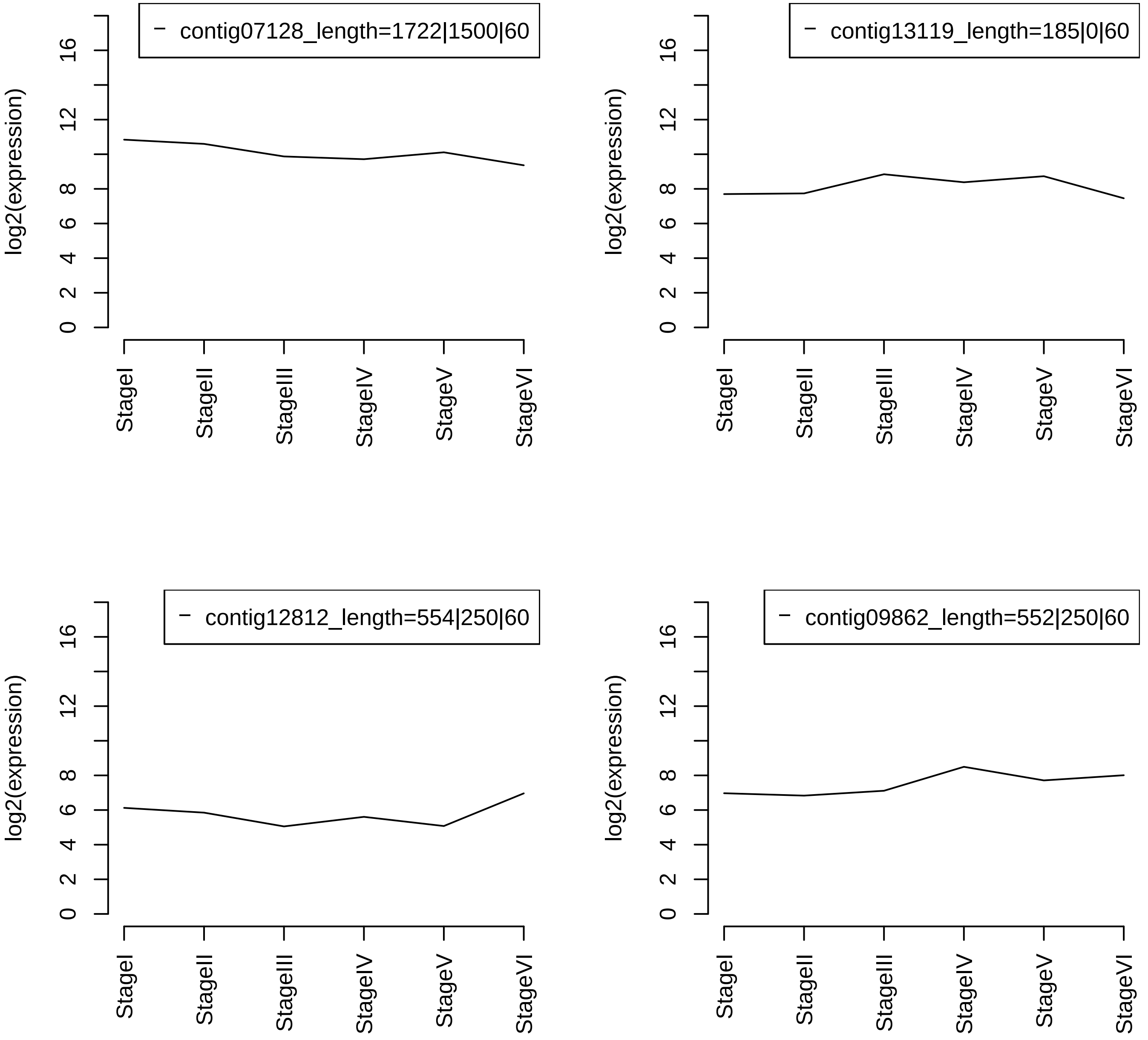

contig07128_length=1722|1500|60
contig13119_length=185|0|60
contig12812_length=554|250|60
contig09862_length=552|250|60
log2(expression)
0
2
4
6
8
12
16
StageI
StageII
StageIII
StageIV
StageV
StageVI

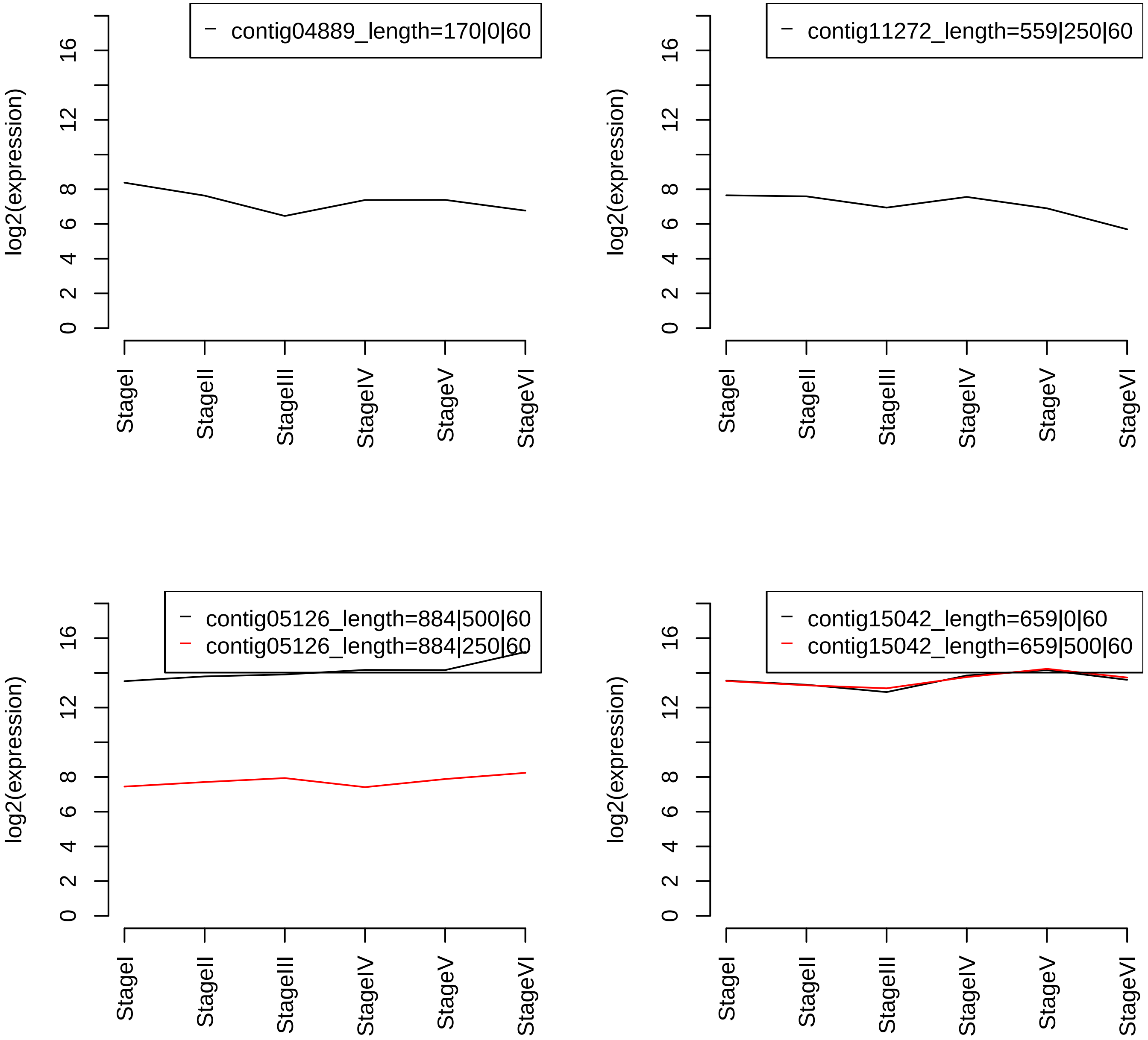

contig04889_length=170|0|60
log2(expression)
0
2
4
6
8
12
16
StageI
StageII
StageIII
StageIV
StageV
StageVI
contig11272_length=559|250|60
log2(expression)
0
2
4
6
8
12
16
StageI
StageII
StageIII
StageIV
StageV
StageVI
contig05126_length=884|500|60
contig05126_length=884|250|60
log2(expression)
0
2
4
6
8
12
16
StageI
StageII
StageIII
StageIV
StageV
StageVI
contig15042_length=659|0|60
contig15042_length=659|500|60
log2(expression)
0
2
4
6
8
12
16
StageI
StageII
StageIII
StageIV
StageV
StageVI

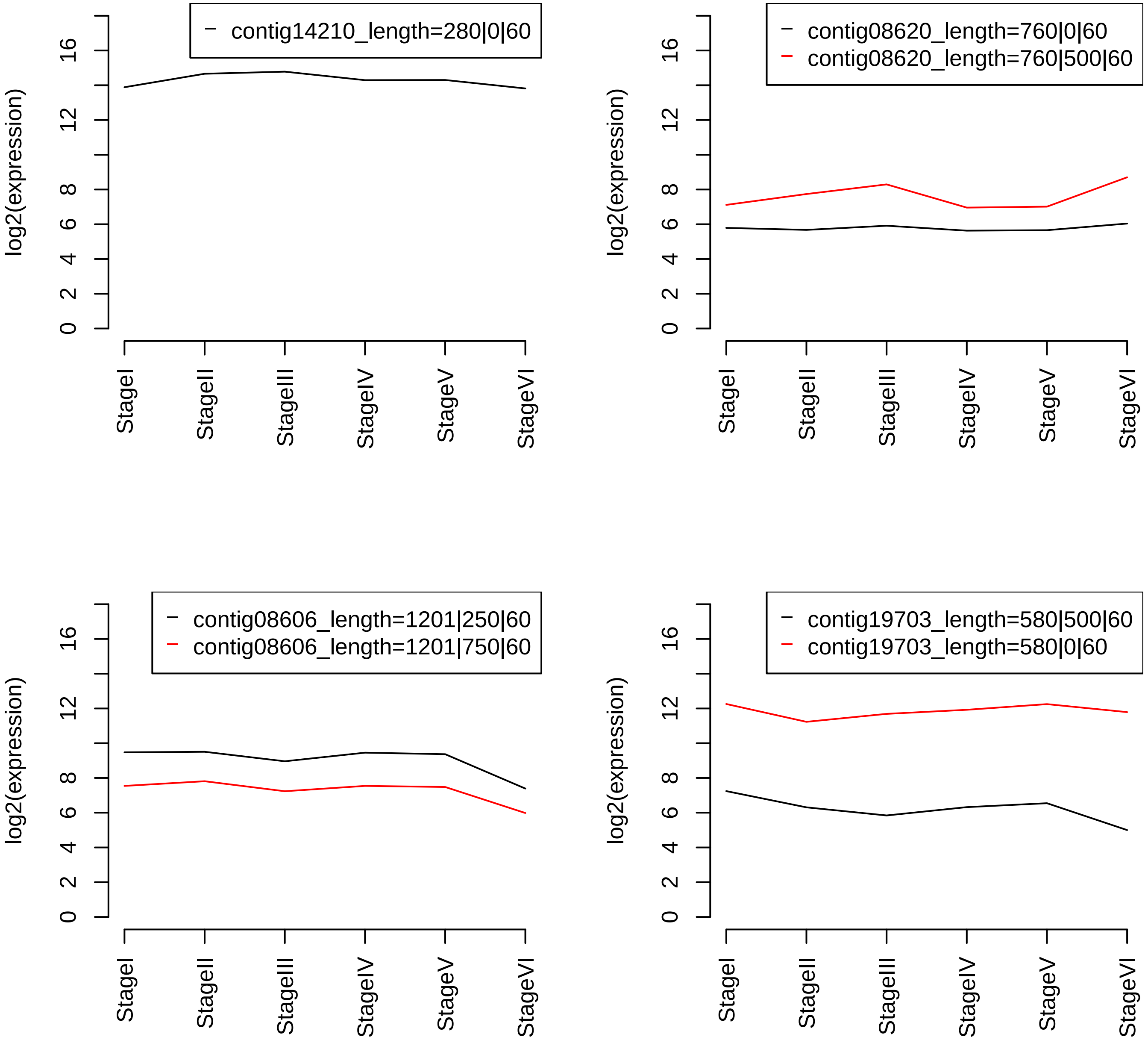

contig14210_length=280|0|60
contig08620_length=760|0|60
contig08620_length=760|500|60
contig08606_length=1201|250|60
contig08606_length=1201|750|60
contig19703_length=580|500|60
contig19703_length=580|0|60
log2(expression)
0
2
4
6
8
12
16
StageI
StageII
StageIII
StageIV
StageV
StageVI

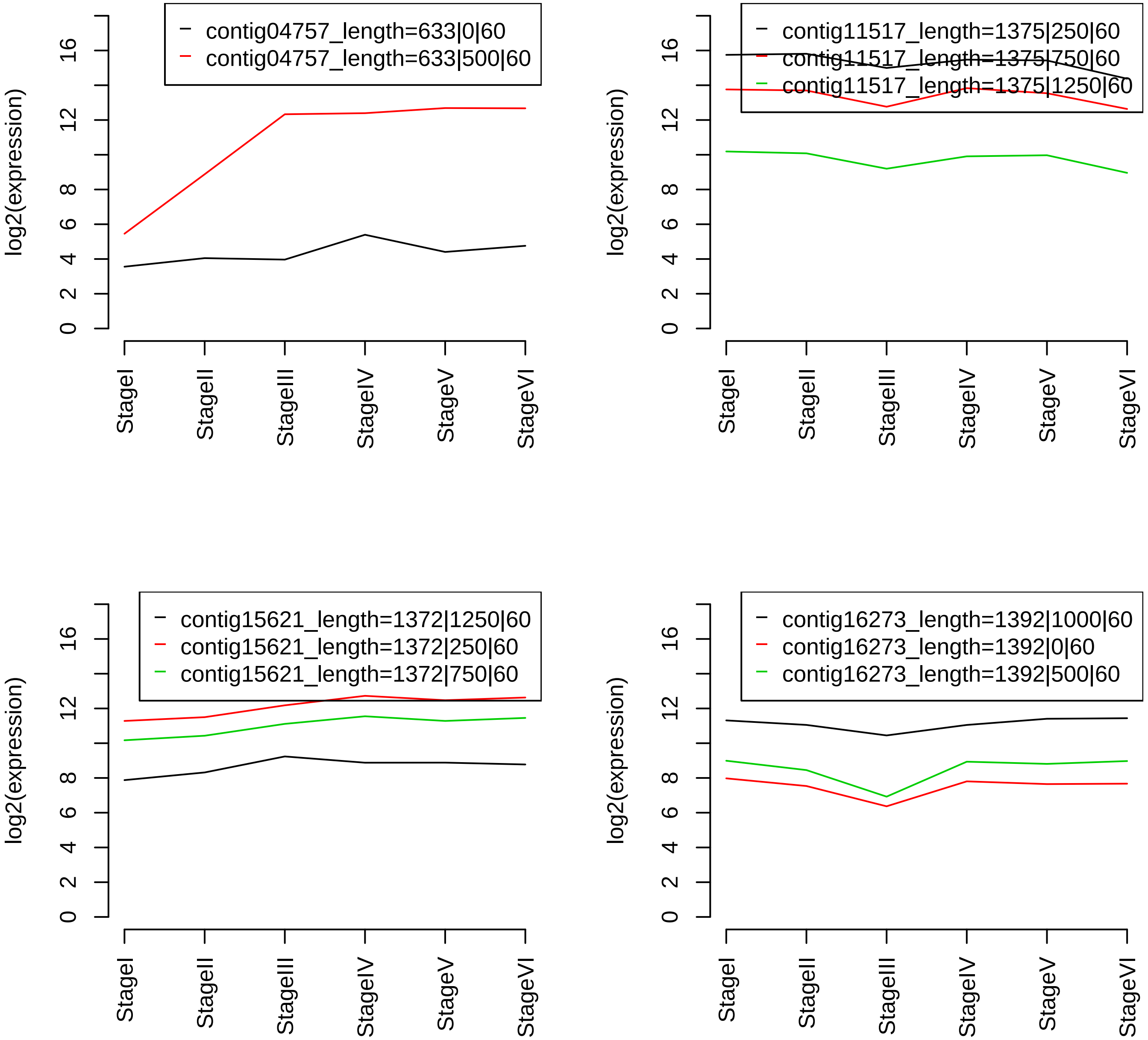

contig04757_length=633|0|60
contig04757_length=633|500|60
contig11517_length=1375|250|60
contig11517_length=1375|750|60
contig11517_length=1375|1250|60
contig15621_length=1372|1250|60
contig15621_length=1372|250|60
contig15621_length=1372|750|60
contig16273_length=1392|1000|60
contig16273_length=1392|0|60
contig16273_length=1392|500|60
log2(expression)
0
2
4
6
8
12
16
StageI
StageII
StageIII
StageIV
StageV
StageVI

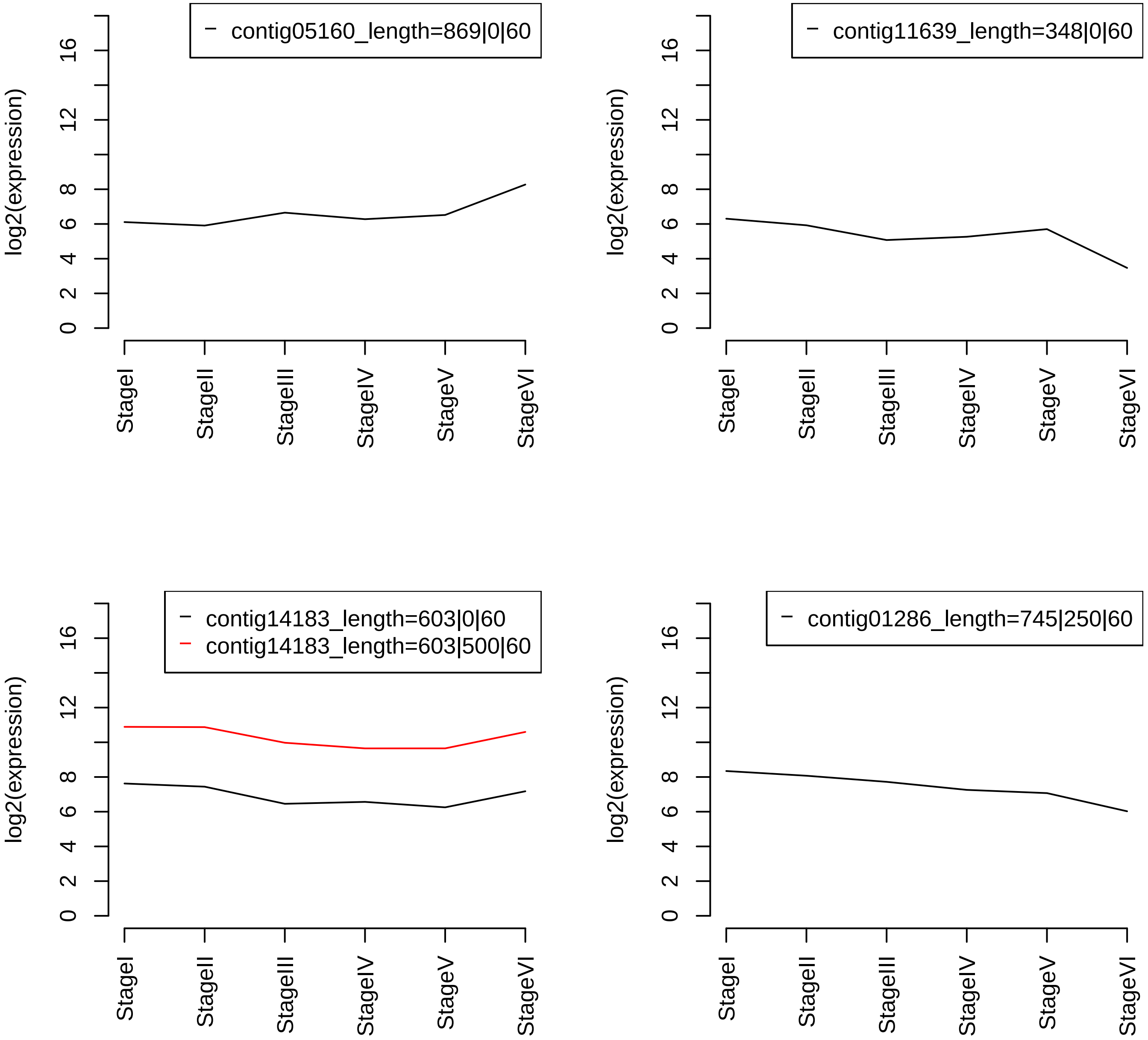
contig05160_length=869|0|60
contig11639_length=348|0|60
contig14183_length=603|0|60
contig14183_length=603|500|60
contig01286_length=745|250|60
log2(expression)
0
2
4
6
8
12
16
StageI
StageII
StageIII
StageIV
StageV
StageVI

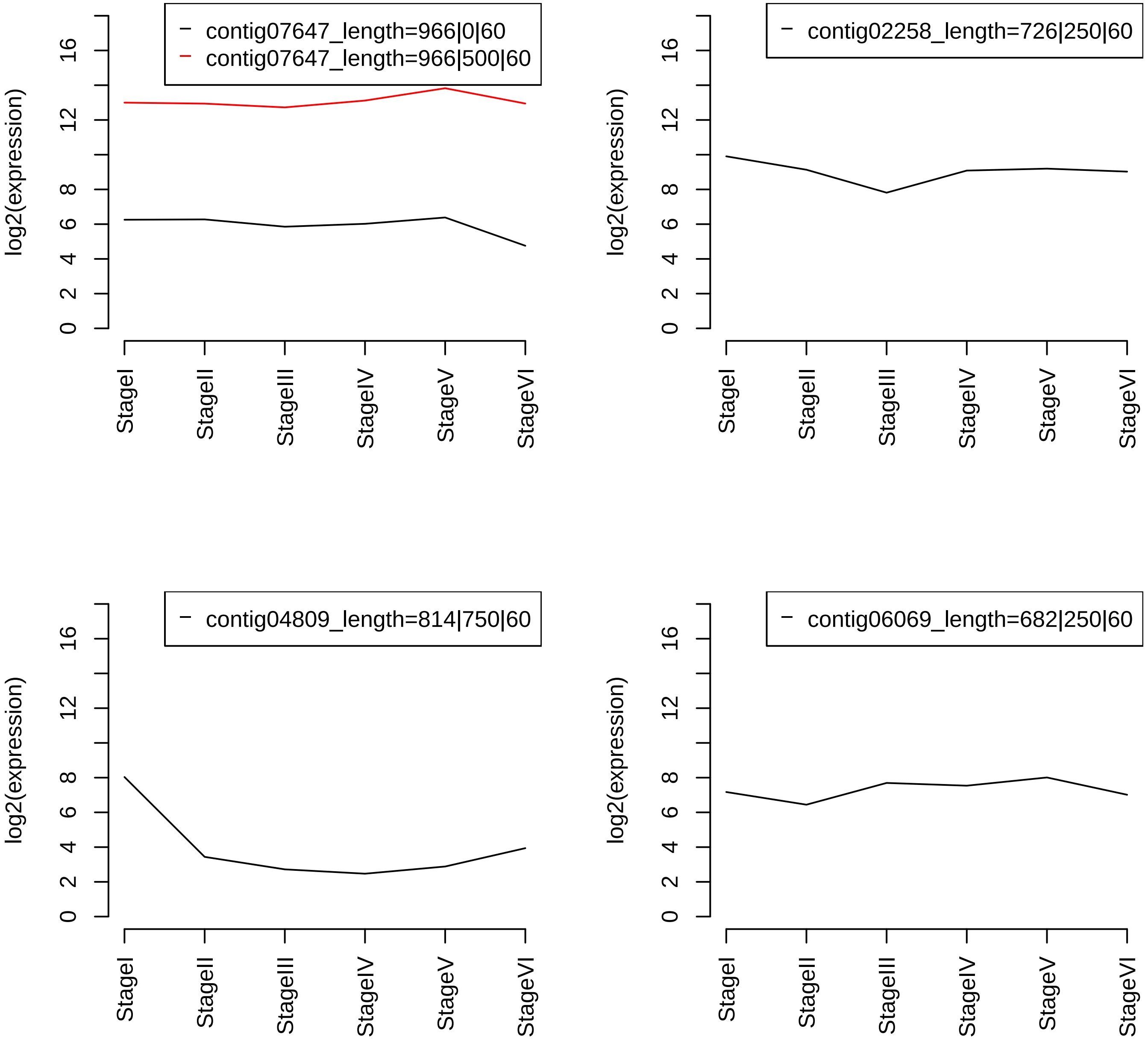
contig07647_length=966|0|60
contig07647_length=966|500|60
contig02258_length=726|250|60
contig04809_length=814|750|60
contig06069_length=682|250|60
log2(expression)
0
2
4
6
8
12
16
StageI
StageII
StageIII
StageIV
StageV
StageVI

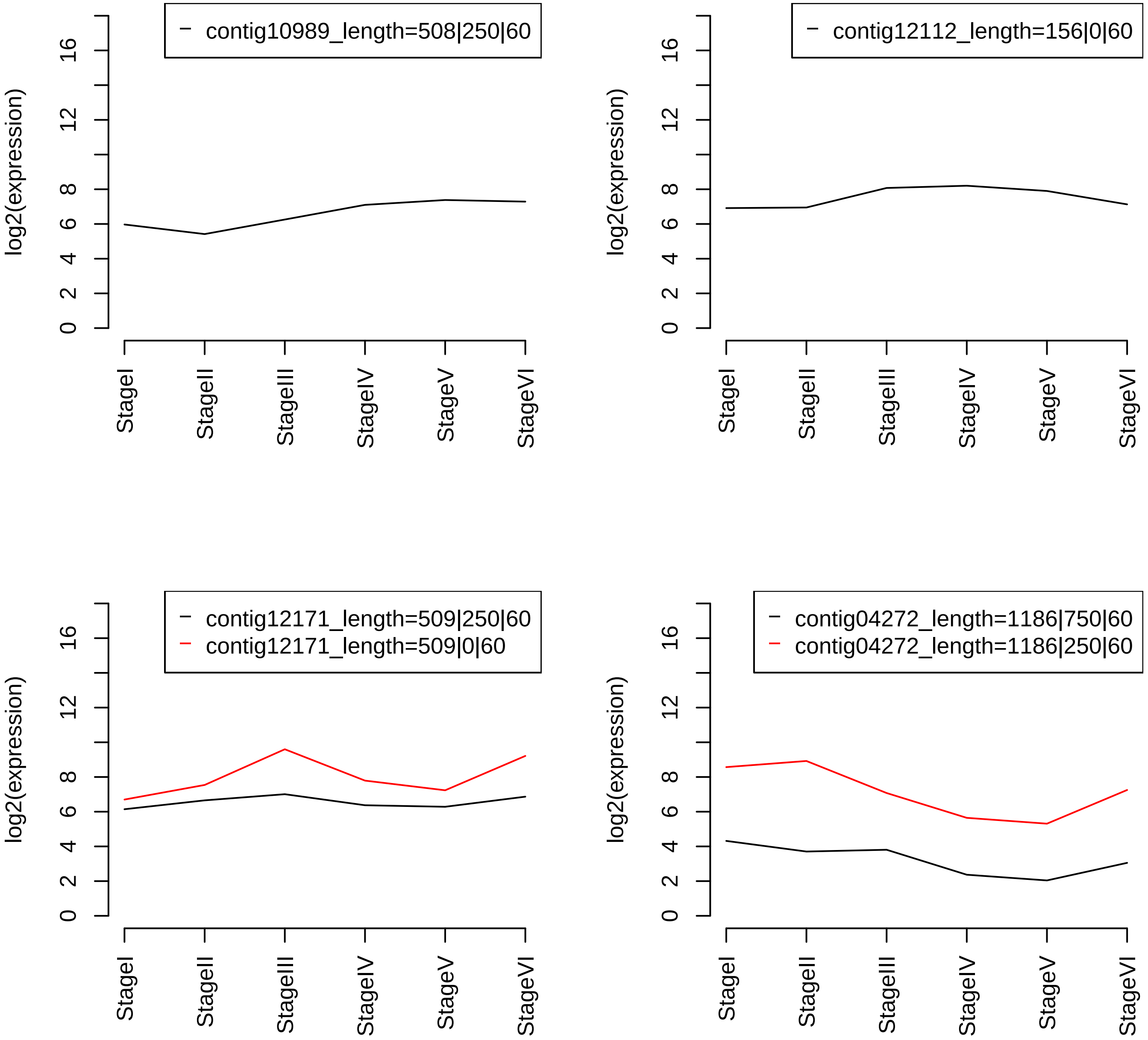
contig10989_length=508|250|60
contig12112_length=156|0|60
contig12171_length=509|250|60
contig12171_length=509|0|60
contig04272_length=1186|750|60
contig04272_length=1186|250|60
log2(expression)
0
2
4
6
8
12
16
StageI
StageII
StageIII
StageIV
StageV
StageVI

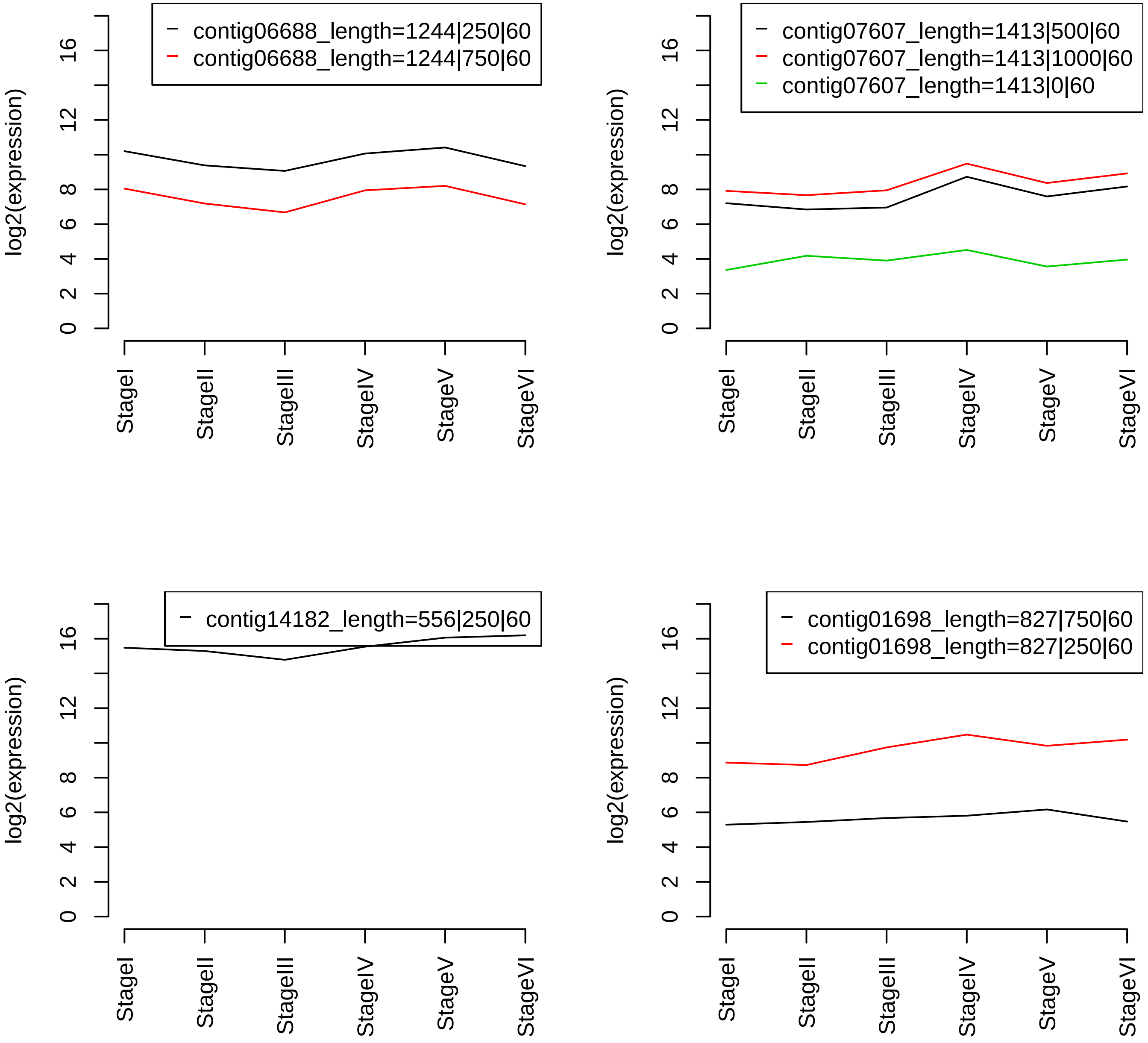
contig06688_length=1244|250|60
contig06688_length=1244|750|60
contig07607_length=1413|500|60
contig07607_length=1413|1000|60
contig07607_length=1413|0|60
contig14182_length=556|250|60
contig01698_length=827|750|60
contig01698_length=827|250|60
log2(expression)
0 2 4 6 8 12 16
StageI StageII StageIII StageIV StageV StageVI

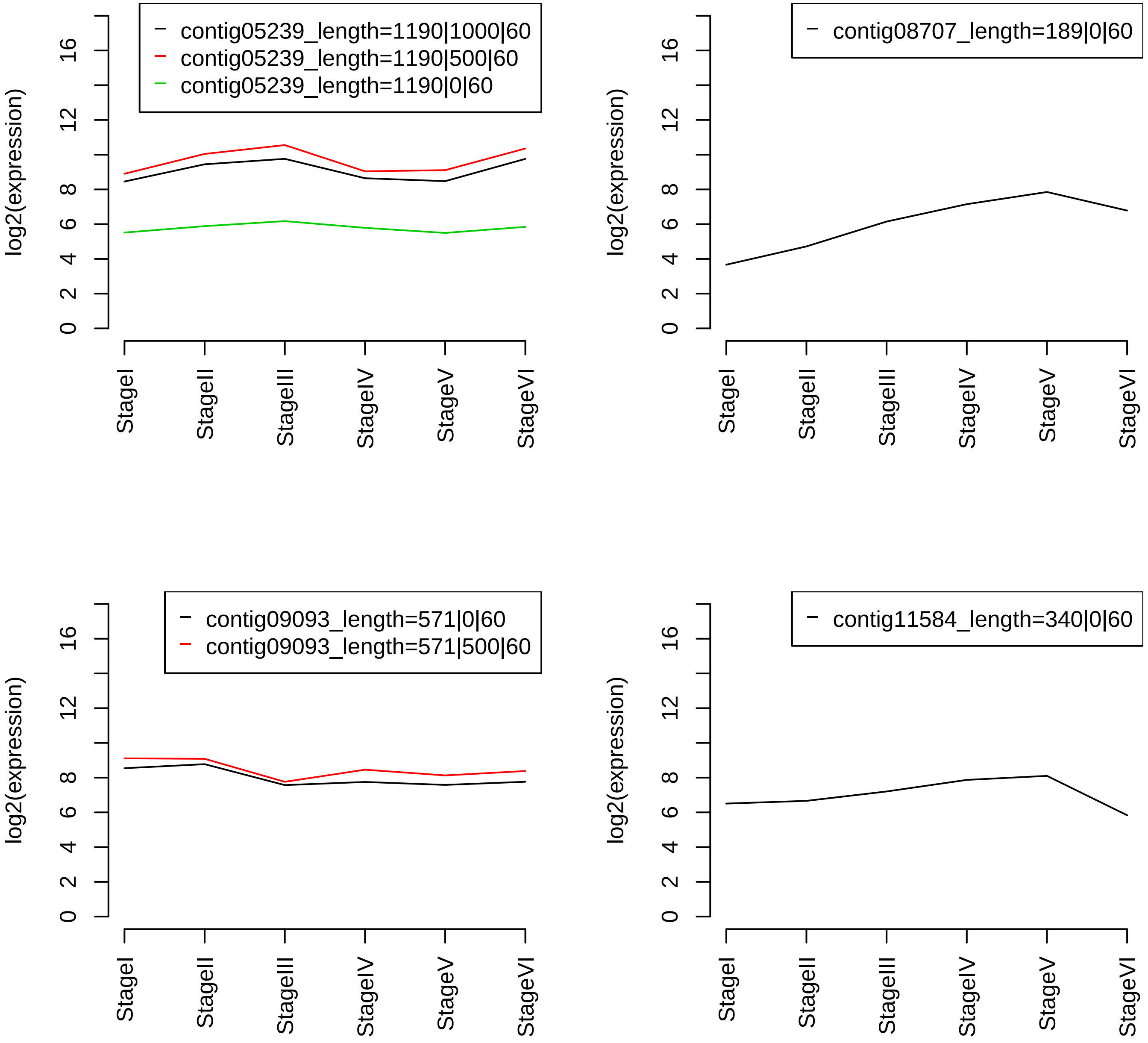
contig05239_length=1190|1000|60
contig05239_length=1190|500|60
contig05239_length=1190|0|60
contig08707_length=189|0|60
contig09093_length=571|0|60
contig09093_length=571|500|60
contig11584_length=340|0|60
log2(expression)
0
2
4
6
8
12
16
StageI
StageII
StageIII
StageIV
StageV
StageVI

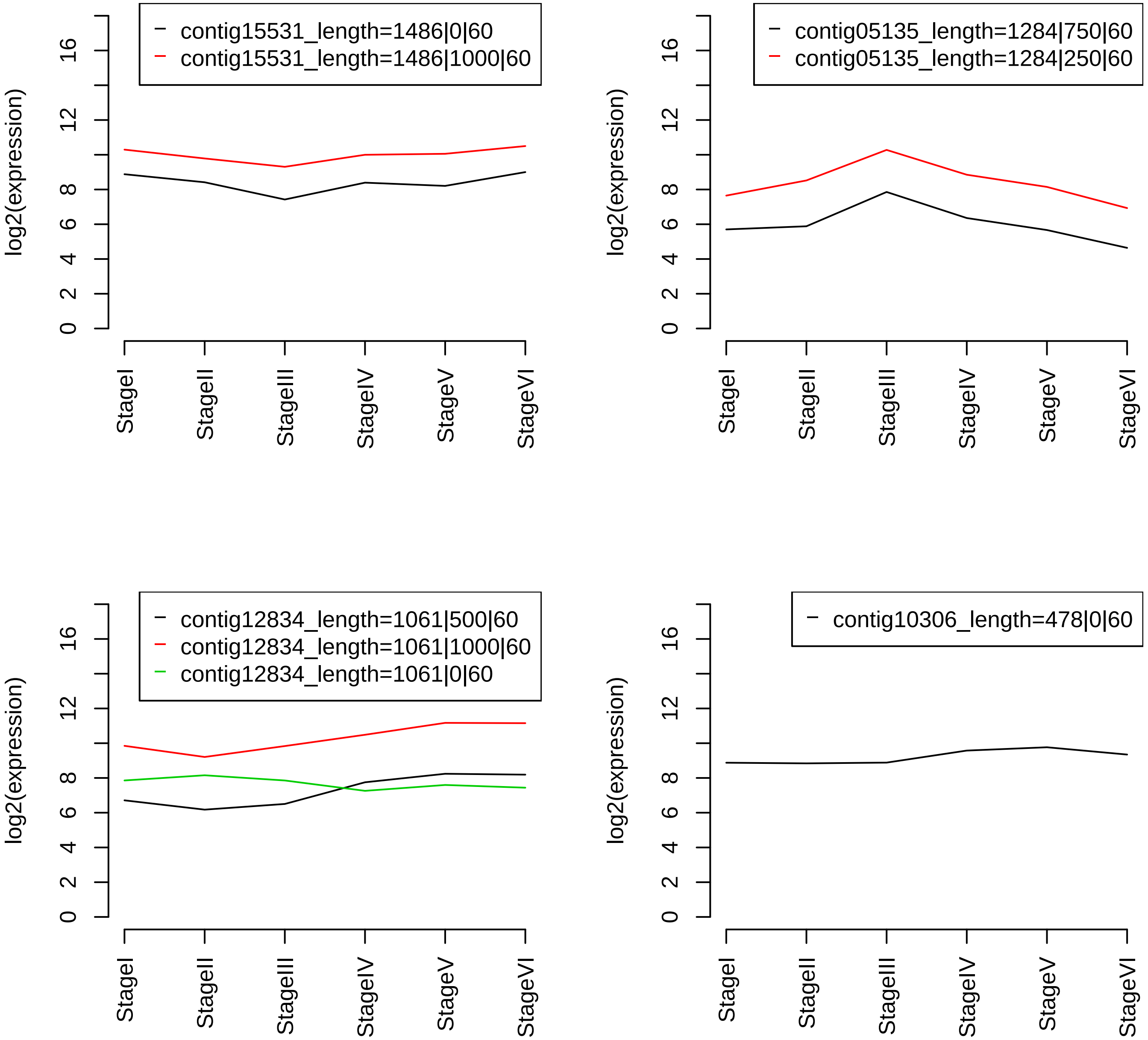

contig15531_length=1486|0|60
contig15531_length=1486|1000|60
log2(expression)
0
2
4
6
8
12
16
StageI
StageII
StageIII
StageIV
StageV
StageVI
contig05135_length=1284|750|60
contig05135_length=1284|250|60
contig12834_length=1061|500|60
contig12834_length=1061|1000|60
contig12834_length=1061|0|60
contig10306_length=478|0|60

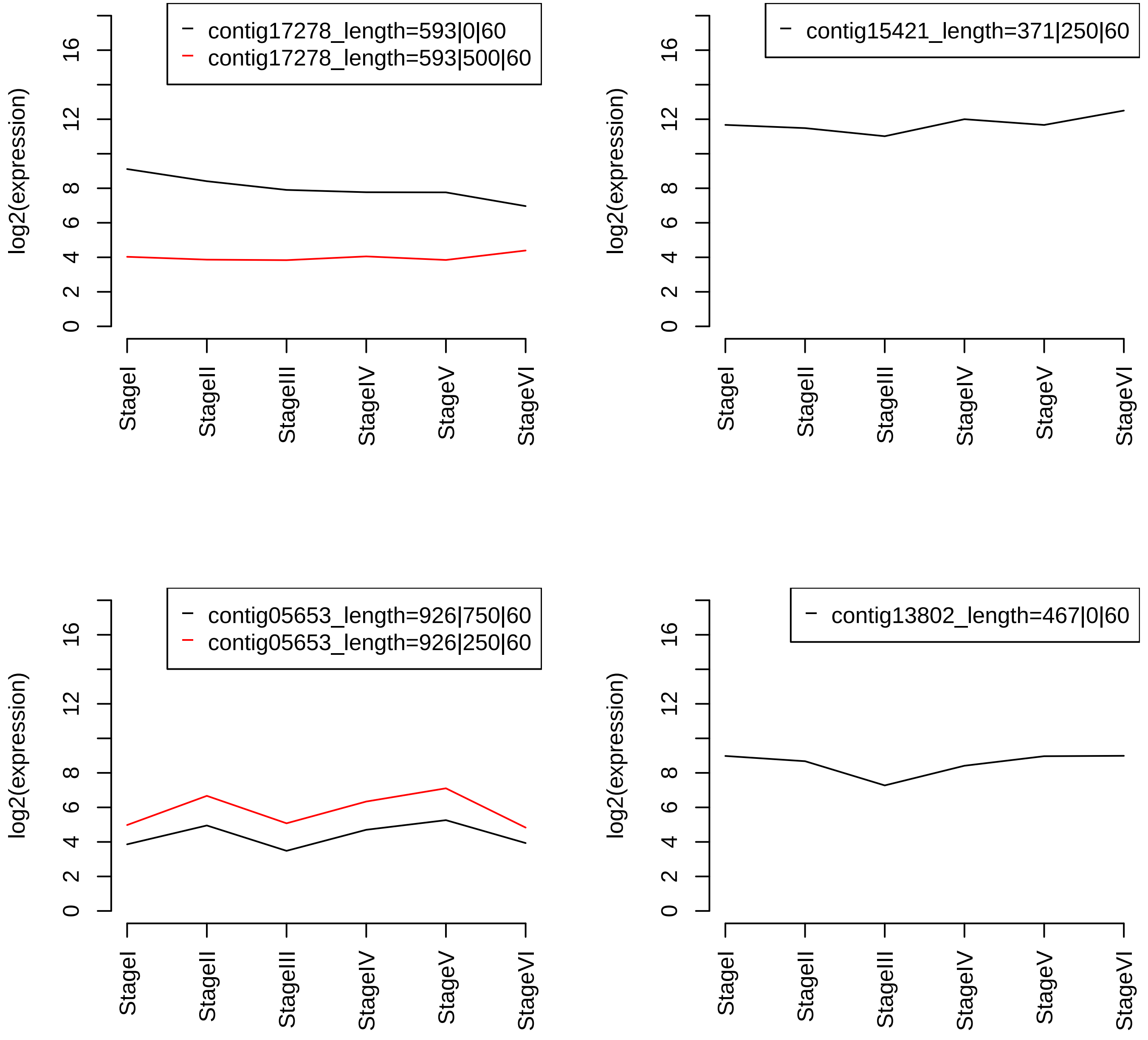

contig17278_length=593|0|60
contig17278_length=593|500|60
contig15421_length=371|250|60
contig05653_length=926|750|60
contig05653_length=926|250|60
contig13802_length=467|0|60
log2(expression)
0
2
4
6
8
12
16
StageI
StageII
StageIII
StageIV
StageV
StageVI

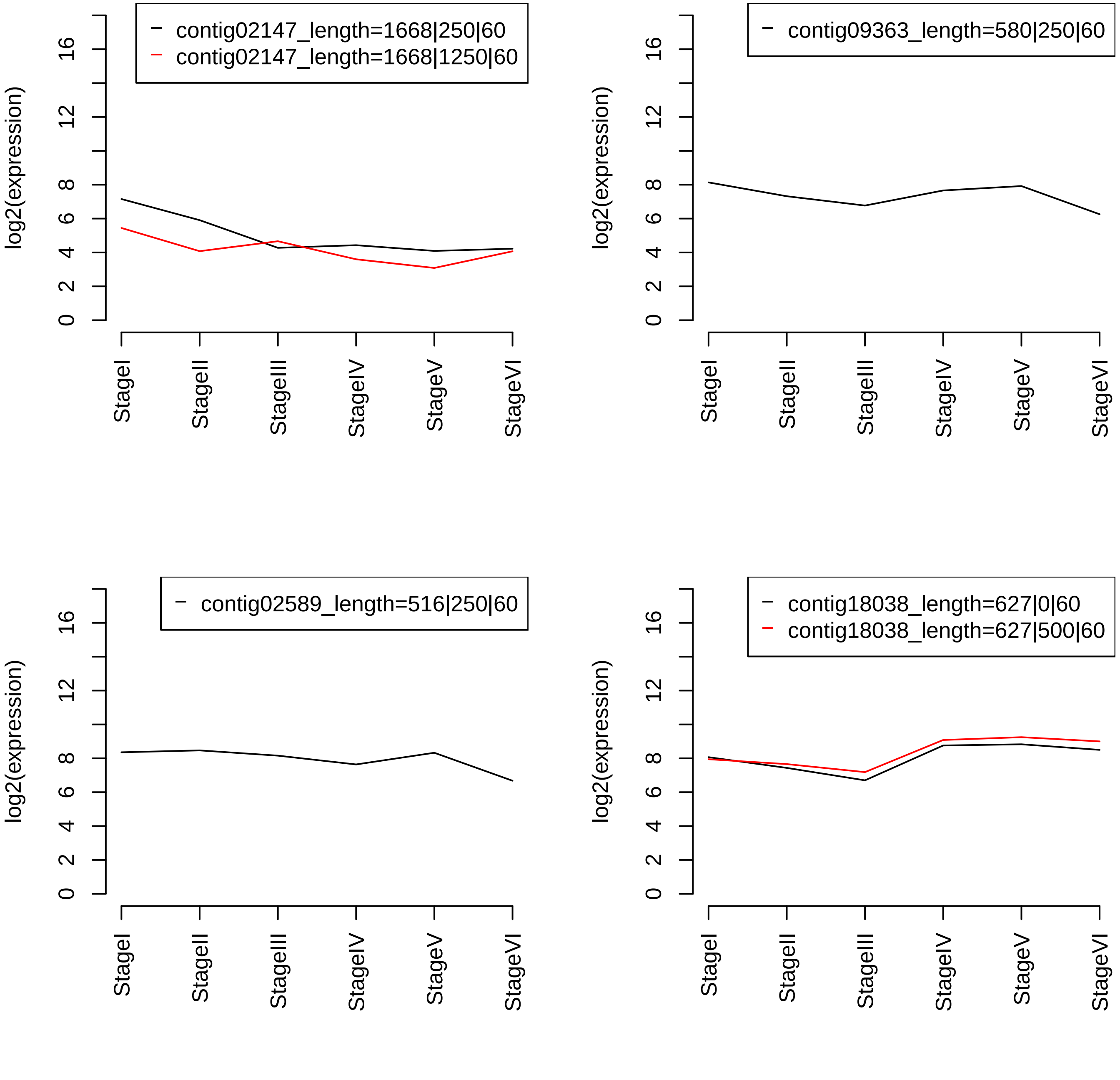
contig02147_length=1668|250|60
contig02147_length=1668|1250|60
contig09363_length=580|250|60
contig02589_length=516|250|60
contig18038_length=627|0|60
contig18038_length=627|500|60
log2(expression)
0
2
4
6
8
12
16
StageI
StageII
StageIII
StageIV
StageV
StageVI

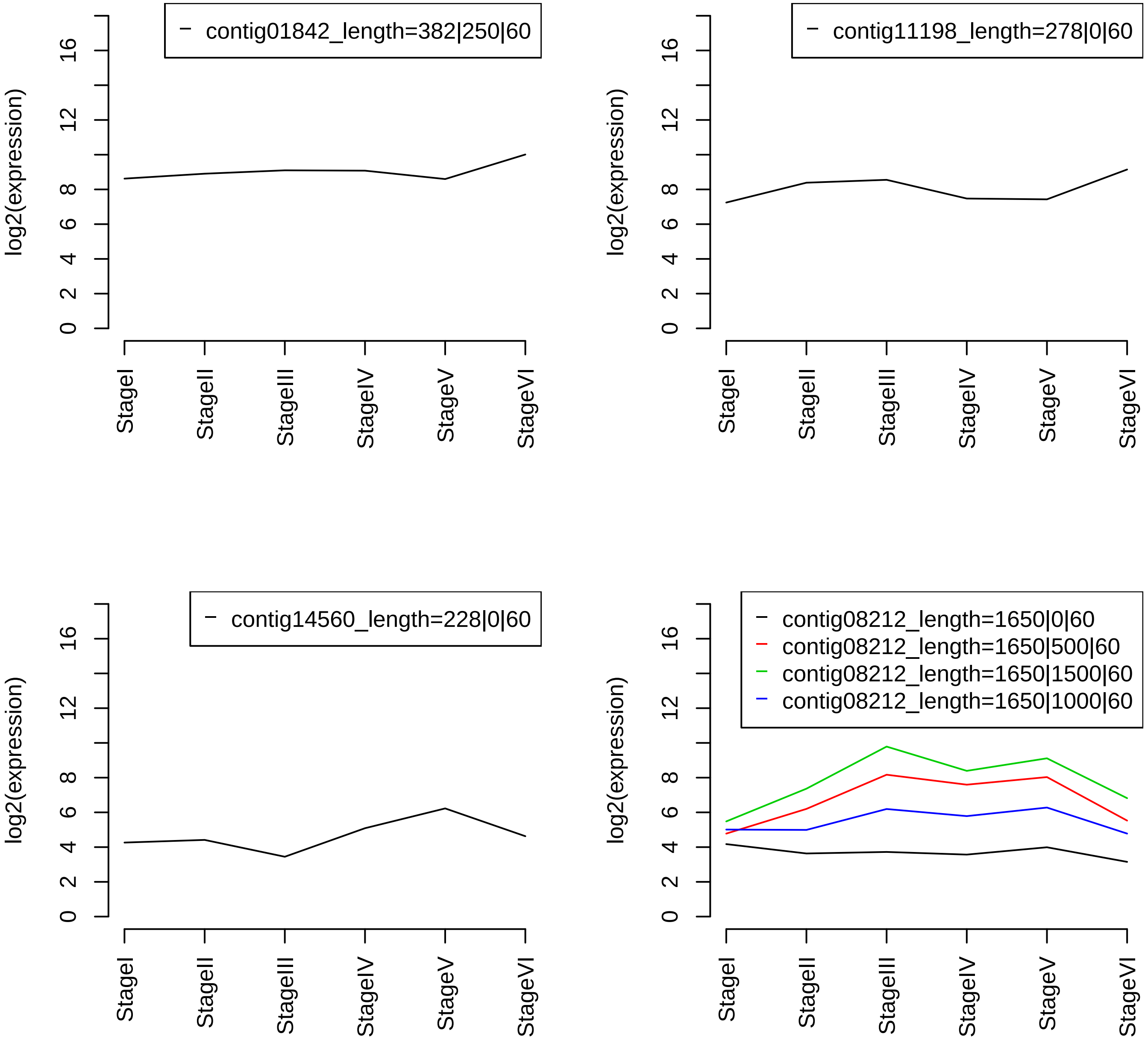
contig01842_length=382|250|60
contig11198_length=278|0|60
contig14560_length=228|0|60
contig08212_length=1650|0|60
contig08212_length=1650|500|60
contig08212_length=1650|1500|60
contig08212_length=1650|1000|60
log2(expression)
0
2
4
6
8
12
16
StageI
StageII
StageIII
StageIV
StageV
StageVI

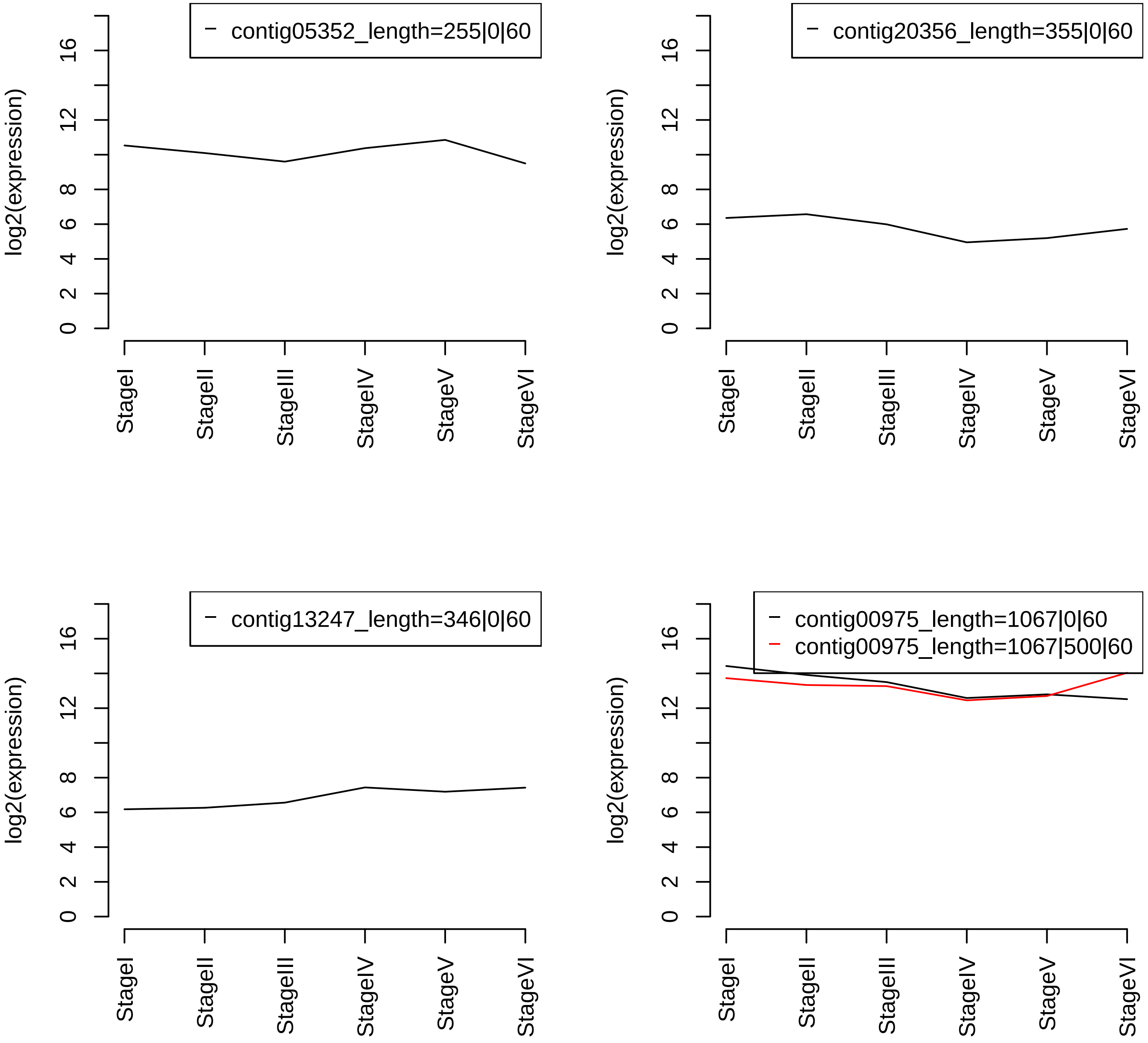
contig05352_length=255|0|60
contig20356_length=355|0|60
contig13247_length=346|0|60
contig00975_length=1067|0|60
contig00975_length=1067|500|60
log2(expression)
0
2
4
6
8
12
16
StageI
StageII
StageIII
StageIV
StageV
StageVI

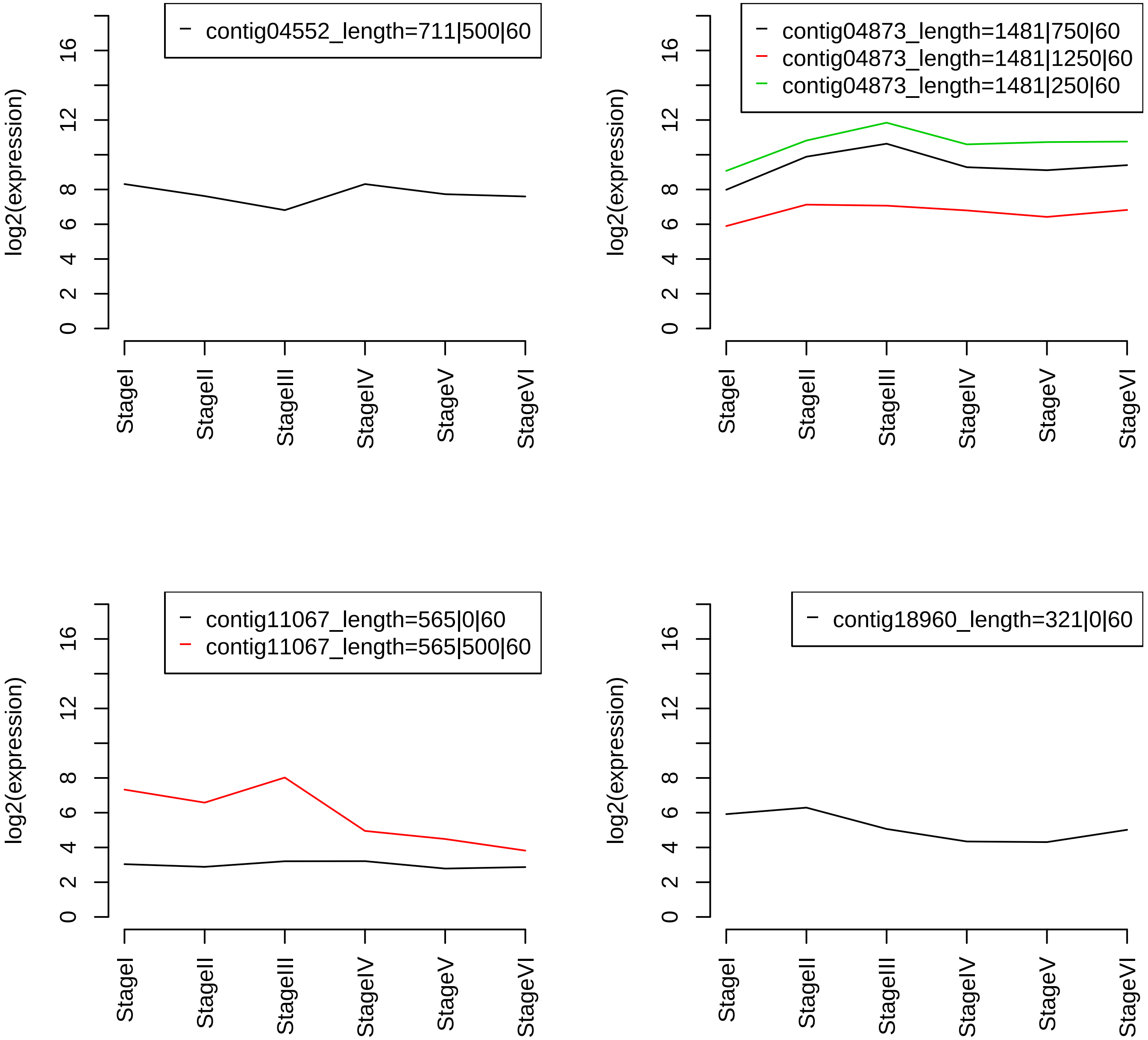
contig04552_length=711|500|60
contig04873_length=1481|750|60
contig04873_length=1481|1250|60
contig04873_length=1481|250|60
contig11067_length=565|0|60
contig11067_length=565|500|60
contig18960_length=321|0|60
log2(expression)
0
2
4
6
8
12
16
StageI
StageII
StageIII
StageIV
StageV
StageVI

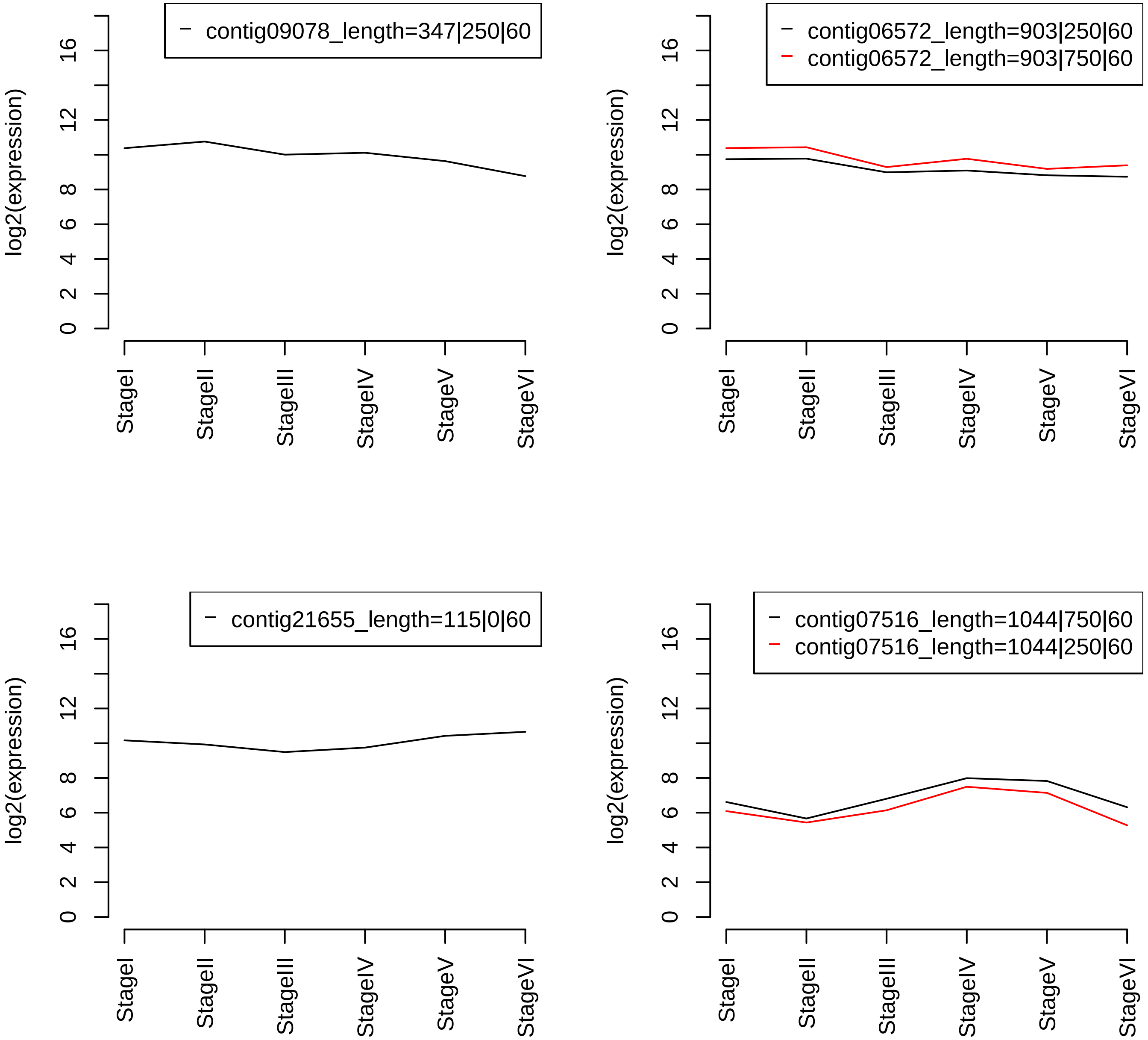

contig09078_length=347|250|60
contig06572_length=903|250|60
contig06572_length=903|750|60
contig21655_length=115|0|60
contig07516_length=1044|750|60
contig07516_length=1044|250|60
log2(expression)
0
2
4
6
8
12
16
StageI
StageII
StageIII
StageIV
StageV
StageVI

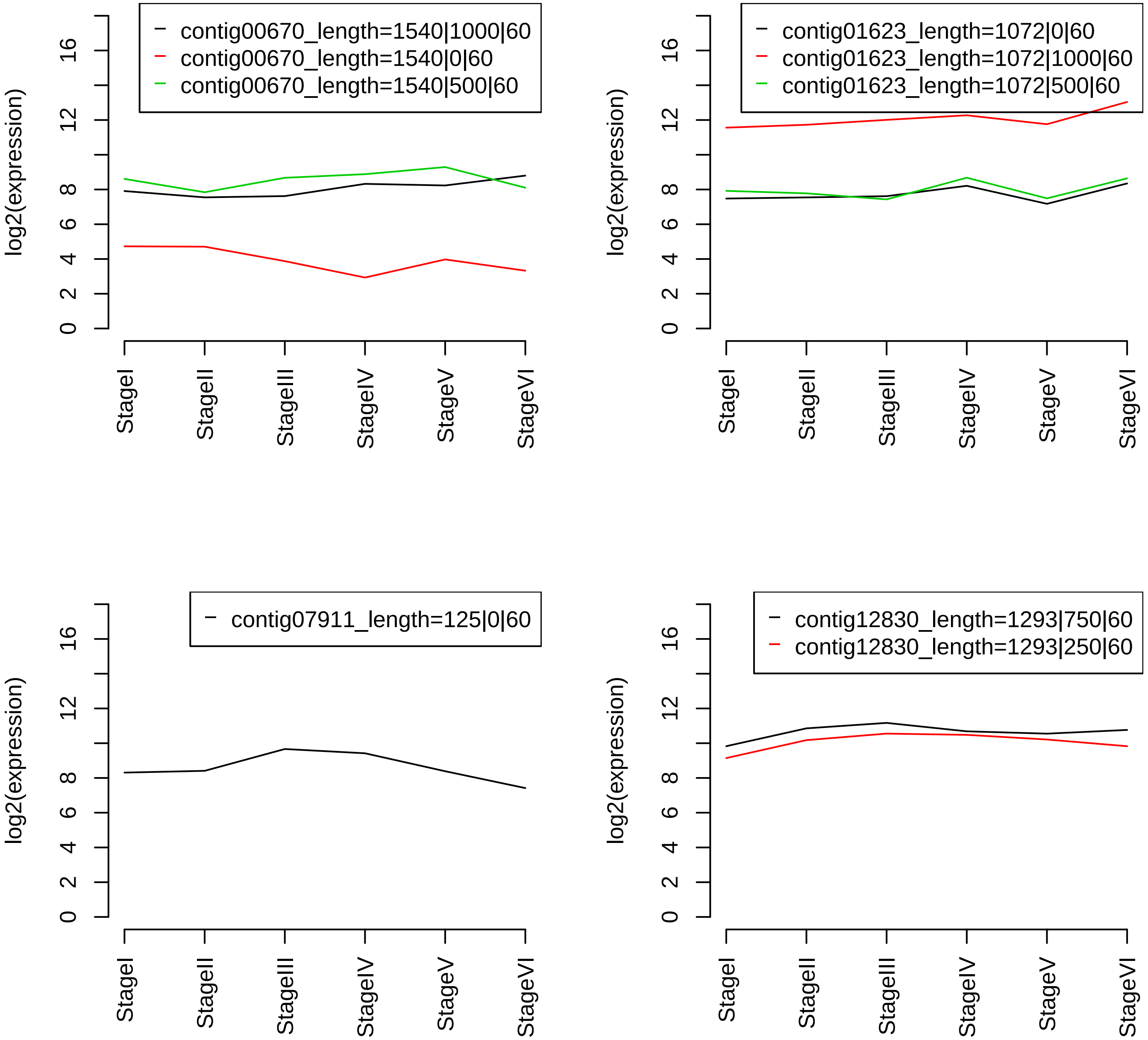

contig00670_length=1540|1000|60
contig00670_length=1540|0|60
contig00670_length=1540|500|60
log2(expression)
0
2
4
6
8
12
16
StageI
StageII
StageIII
StageIV
StageV
StageVI
contig01623_length=1072|0|60
contig01623_length=1072|1000|60
contig01623_length=1072|500|60
contig07911_length=125|0|60
contig12830_length=1293|750|60
contig12830_length=1293|250|60

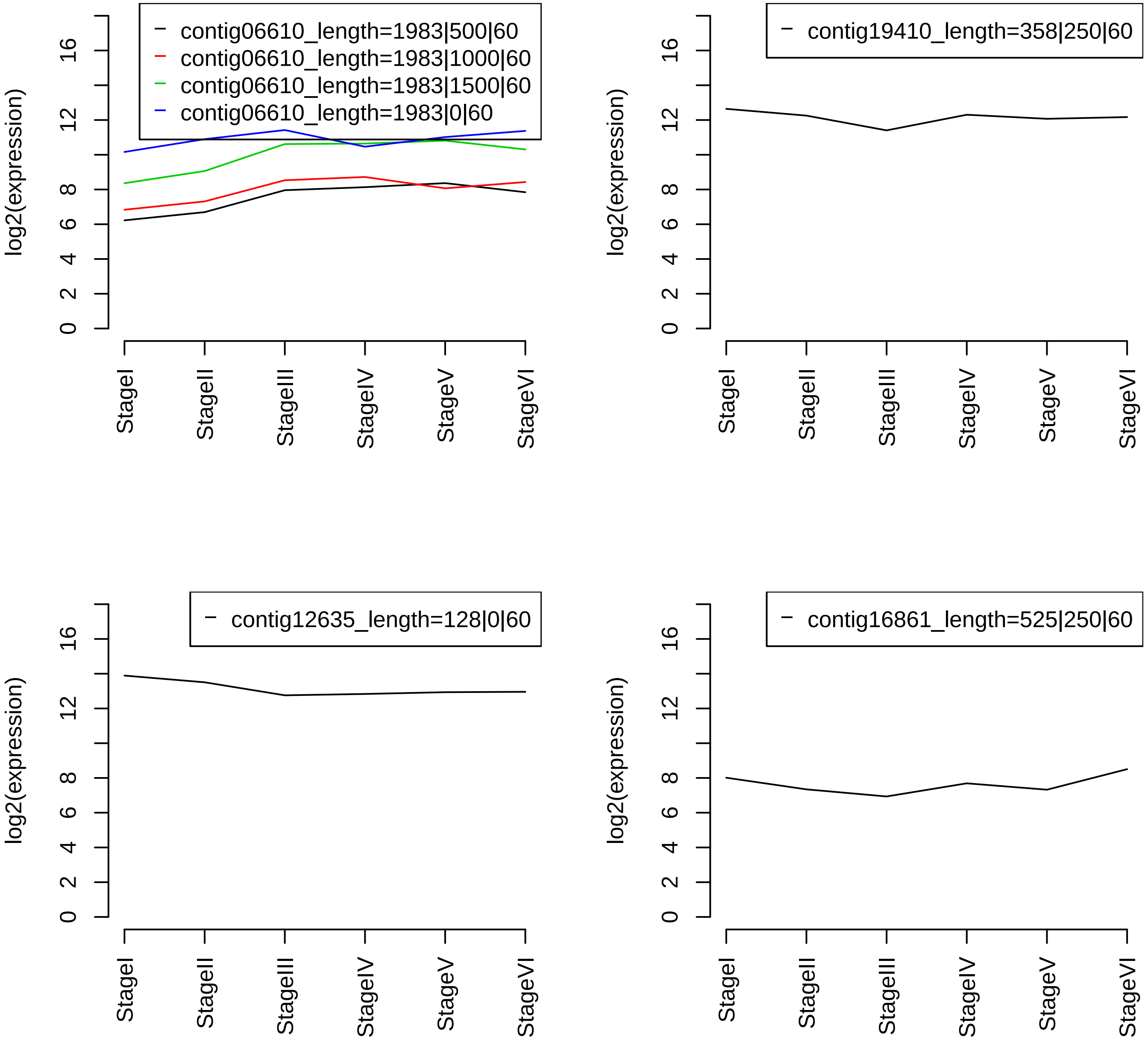
contig06610_length=1983|500|60
contig06610_length=1983|1000|60
contig06610_length=1983|1500|60
contig06610_length=1983|0|60
contig19410_length=358|250|60
contig12635_length=128|0|60
contig16861_length=525|250|60
log2(expression)
StageI
StageII
StageIII
StageIV
StageV
StageVI
0
2
4
6
8
12
16

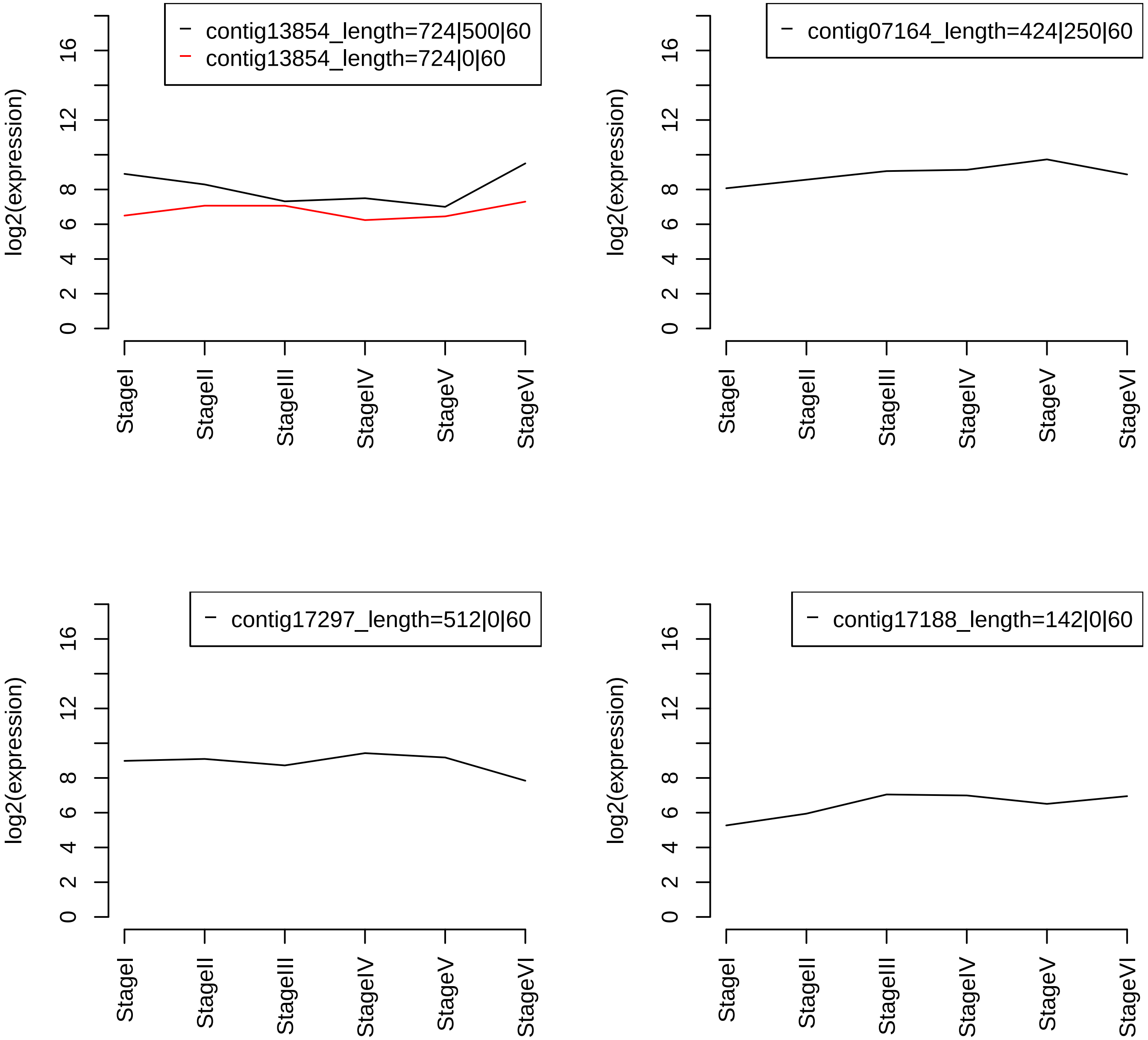

contig13854_length=724|500|60
contig13854_length=724|0|60
contig07164_length=424|250|60
contig17297_length=512|0|60
contig17188_length=142|0|60
log2(expression)
0
2
4
6
8
12
16
StageI
StageII
StageIII
StageIV
StageV
StageVI

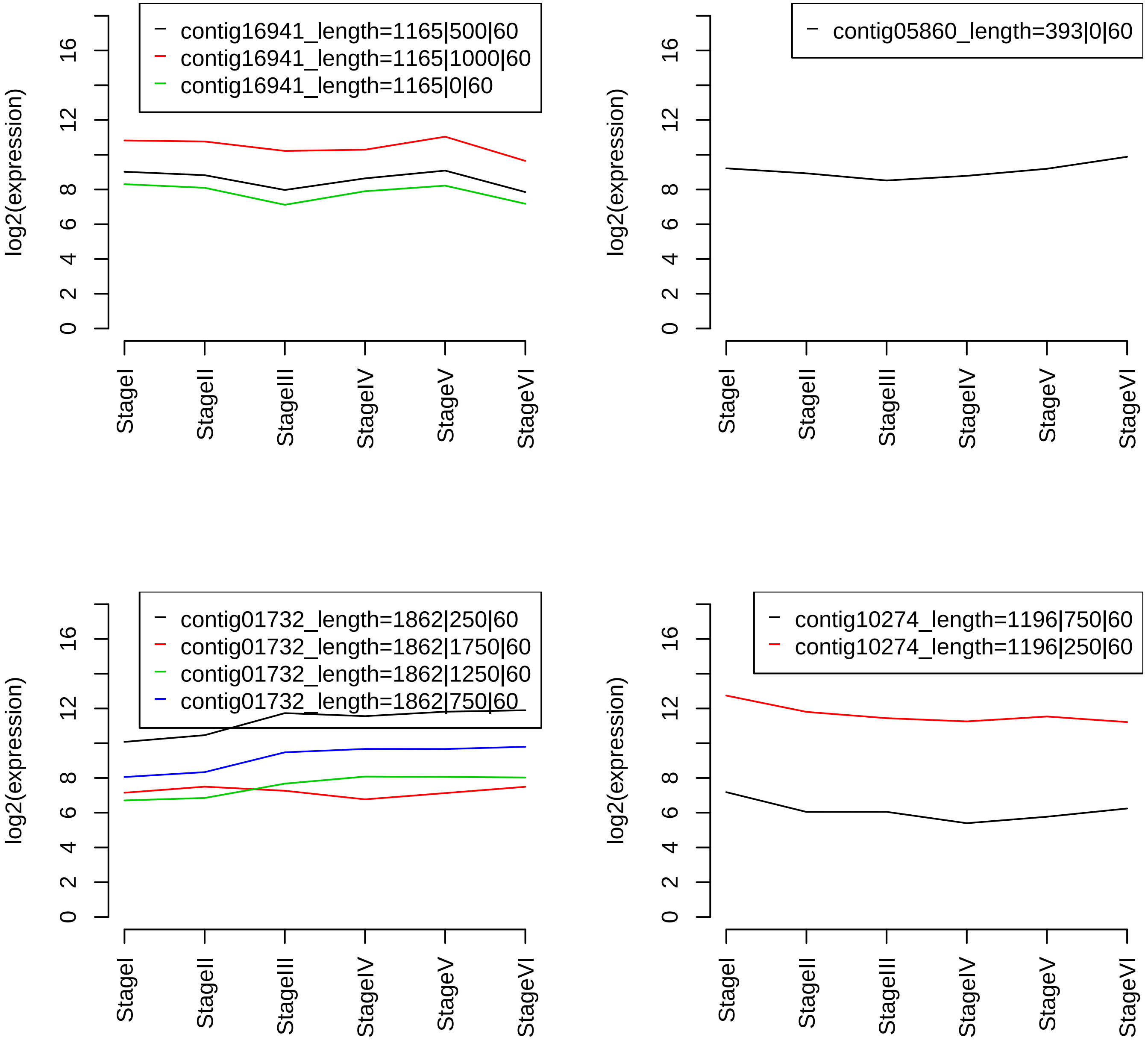
contig16941_length=1165|500|60
contig16941_length=1165|1000|60
contig16941_length=1165|0|60
log2(expression)
0
2
4
6
8
12
16
StageI
StageII
StageIII
StageIV
StageV
StageVI
contig05860_length=393|0|60
log2(expression)
0
2
4
6
8
12
16
StageI
StageII
StageIII
StageIV
StageV
StageVI
contig01732_length=1862|250|60
contig01732_length=1862|1750|60
contig01732_length=1862|1250|60
contig01732_length=1862|750|60
log2(expression)
0
2
4
6
8
12
16
StageI
StageII
StageIII
StageIV
StageV
StageVI
contig10274_length=1196|750|60
contig10274_length=1196|250|60
log2(expression)
0
2
4
6
8
12
16
StageI
StageII
StageIII
StageIV
StageV
StageVI

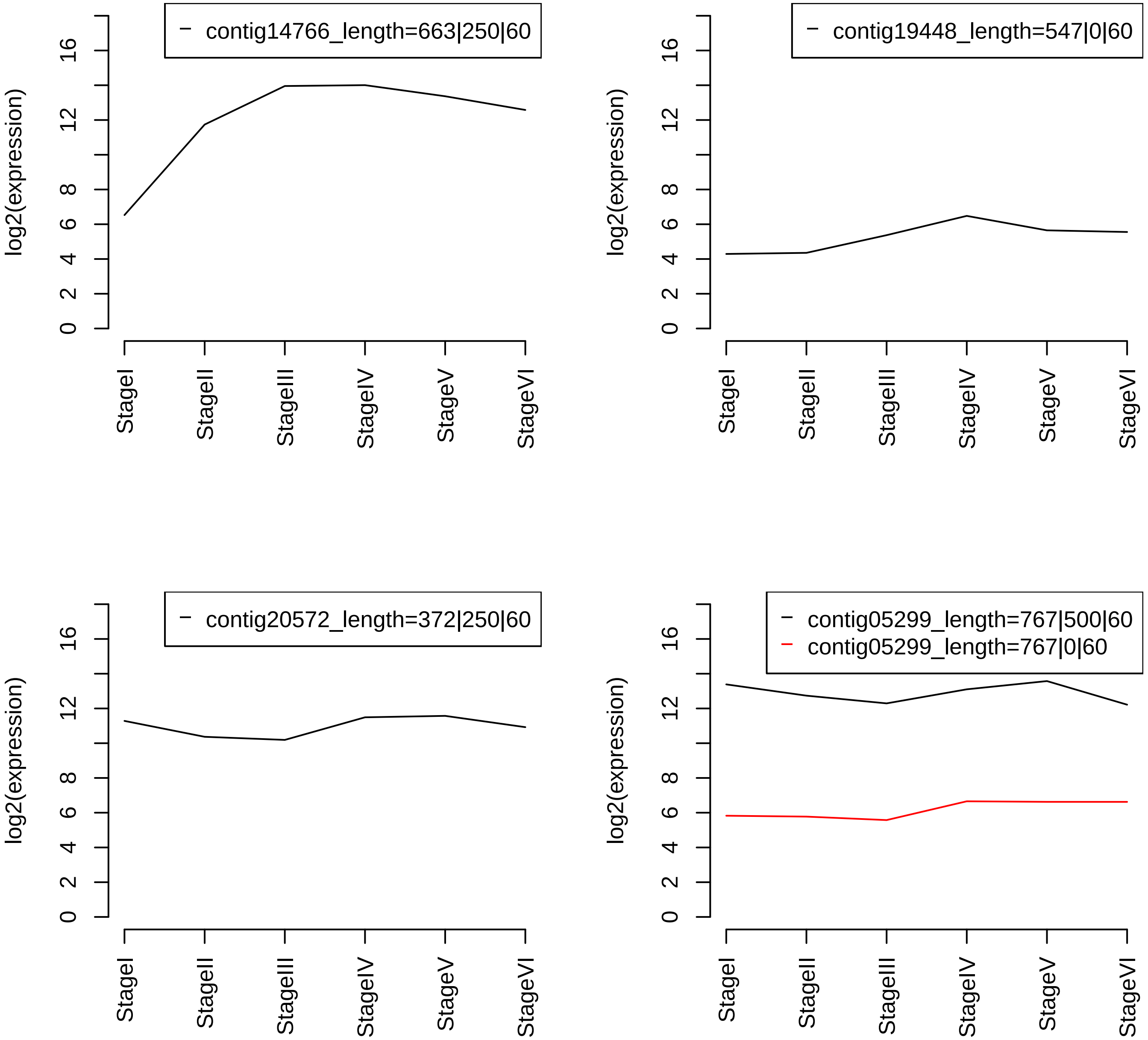
contig14766_length=663|250|60
contig19448_length=547|0|60
contig20572_length=372|250|60
contig05299_length=767|500|60
contig05299_length=767|0|60
log2(expression)
0
2
4
6
8
12
16
StageI
StageII
StageIII
StageIV
StageV
StageVI

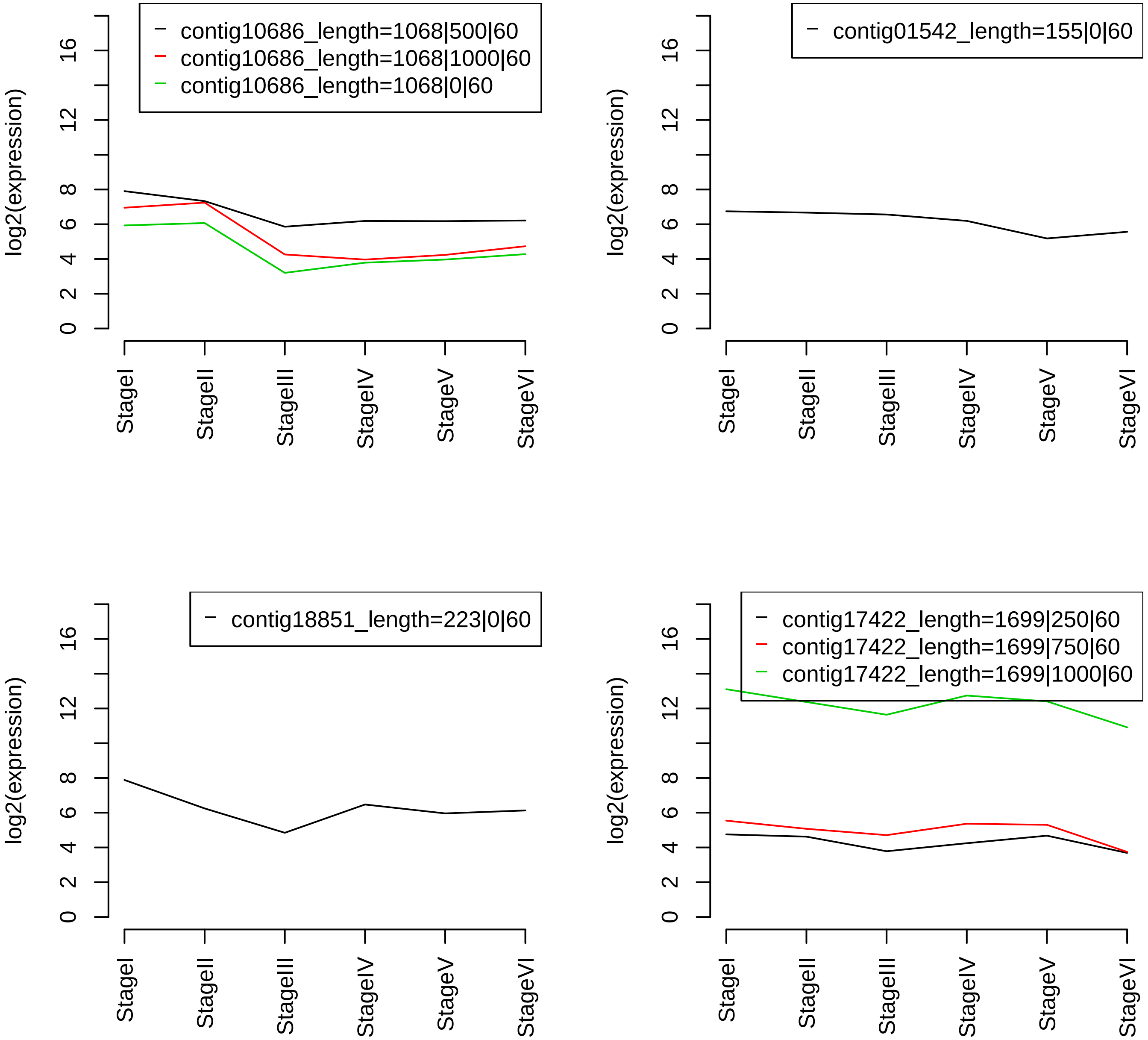

contig10686_length=1068|500|60
contig10686_length=1068|1000|60
contig10686_length=1068|0|60
contig01542_length=155|0|60
contig18851_length=223|0|60
contig17422_length=1699|250|60
contig17422_length=1699|750|60
contig17422_length=1699|1000|60
log2(expression)
0
2
4
6
8
12
16
StageI
StageII
StageIII
StageIV
StageV
StageVI

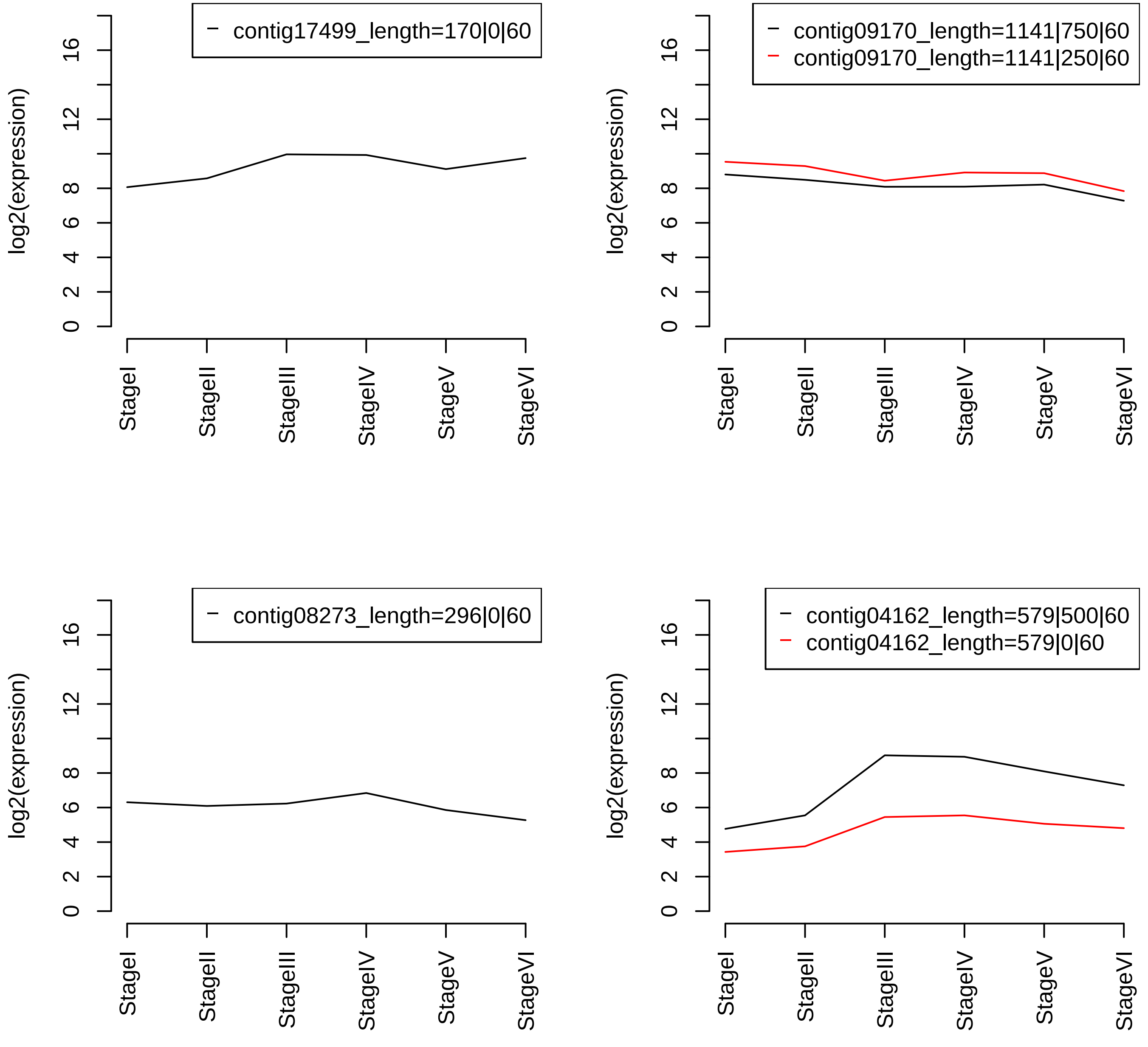
contig17499_length=170|0|60
contig09170_length=1141|750|60
contig09170_length=1141|250|60
contig08273_length=296|0|60
contig04162_length=579|500|60
contig04162_length=579|0|60
log2(expression)
0
2
4
6
8
12
16
StageI
StageII
StageIII
StageIV
StageV
StageVI

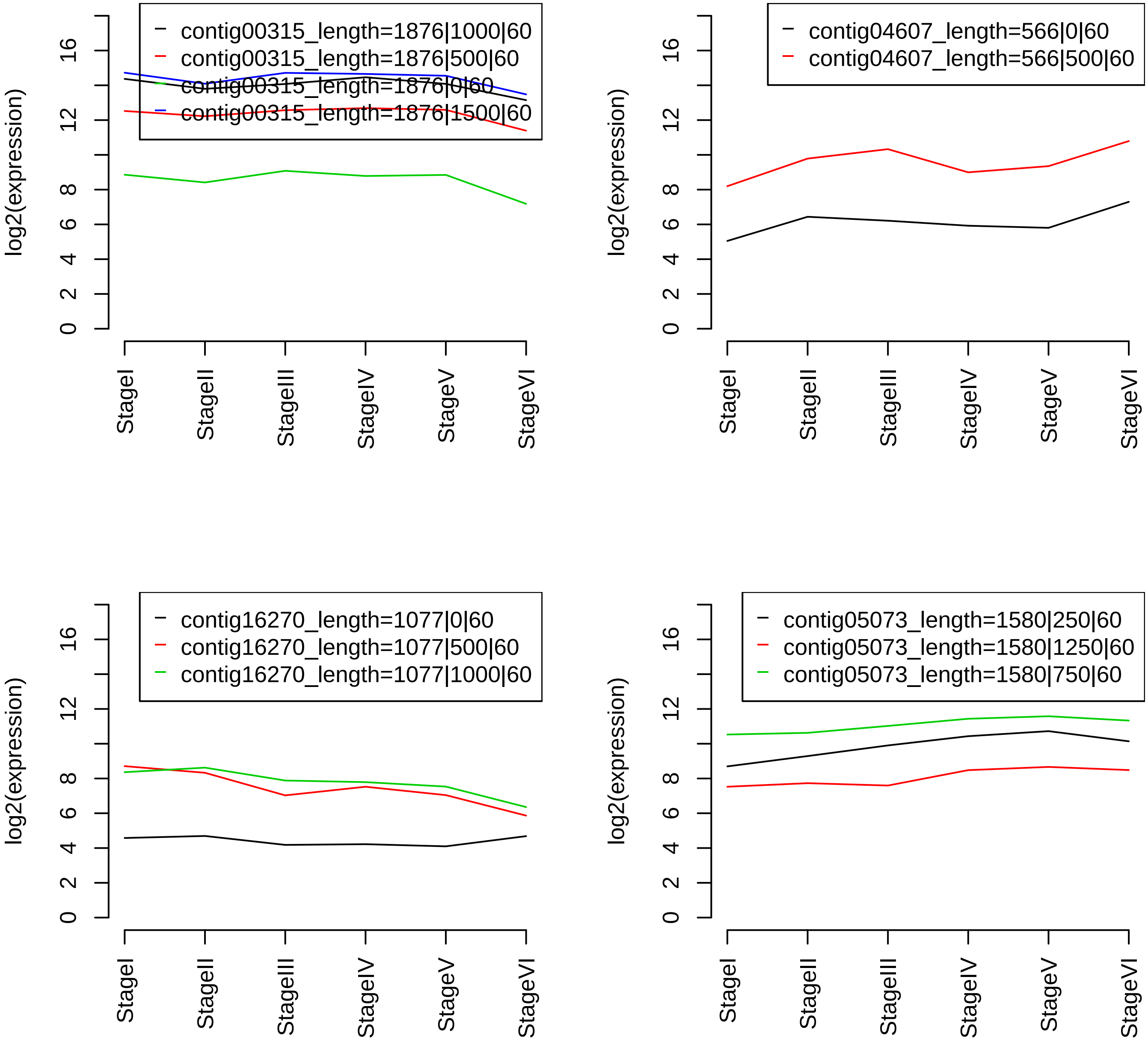
contig00315_length=1876|1000|60
contig00315_length=1876|500|60
contig00315_length=1876|0|60
contig00315_length=1876|1500|60
contig04607_length=566|0|60
contig04607_length=566|500|60
contig16270_length=1077|0|60
contig16270_length=1077|500|60
contig16270_length=1077|1000|60
contig05073_length=1580|250|60
contig05073_length=1580|1250|60
contig05073_length=1580|750|60
log2(expression)
0
2
4
6
8
12
16
StageI
StageII
StageIII
StageIV
StageV
StageVI

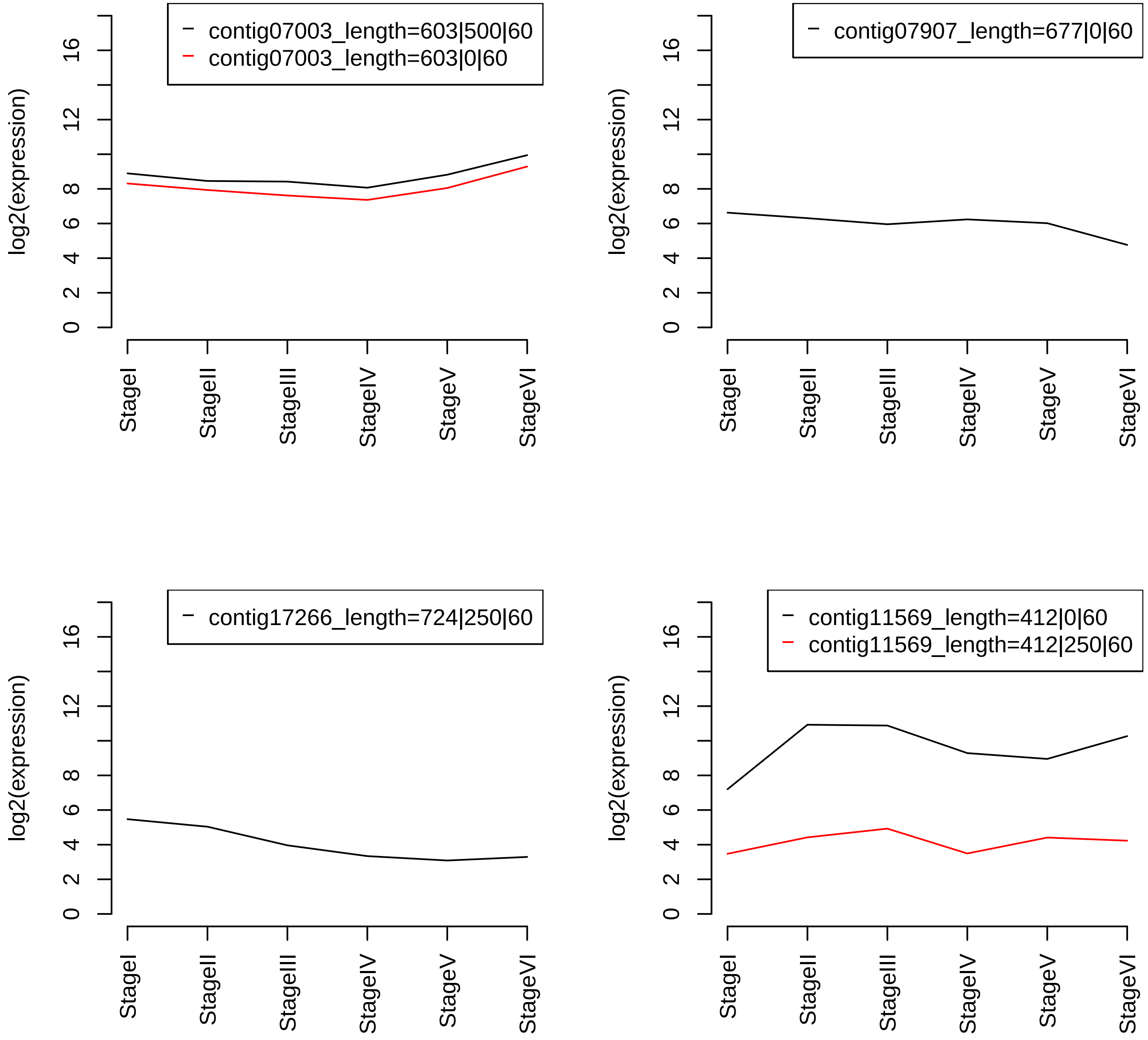

contig07003_length=603|500|60
contig07003_length=603|0|60
contig07907_length=677|0|60
contig17266_length=724|250|60
contig11569_length=412|0|60
contig11569_length=412|250|60
log2(expression)
0
2
4
6
8
12
16
StageI
StageII
StageIII
StageIV
StageV
StageVI

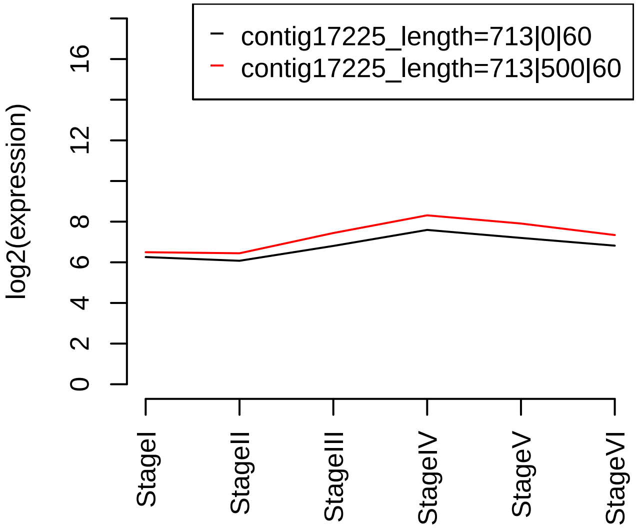

contig17225_length=713|0|60
contig17225_length=713|500|60
log2(expression)
0
2
4
6
8
12
16
StageI
StageII
StageIII
StageIV
StageV
StageVI

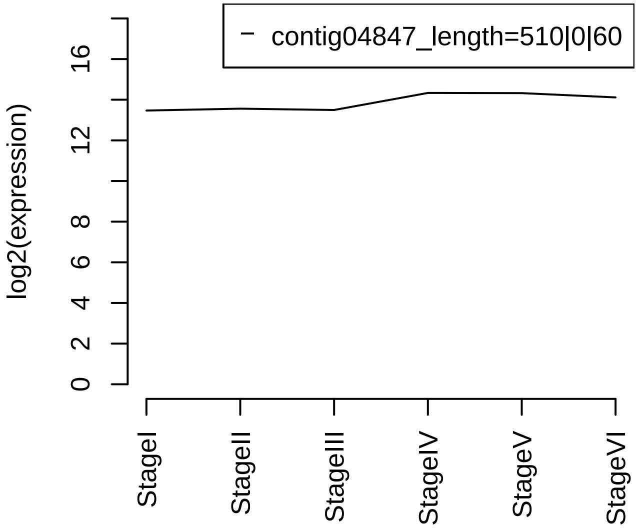

contig04847_length=510|0|60
log2(expression)
0
2
4
6
8
12
16
StageI
StageII
StageIII
StageIV
StageV
StageVI

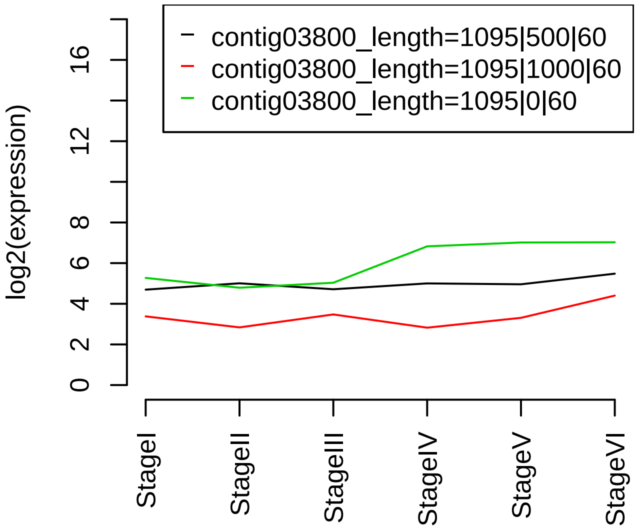

contig03800_length=1095|500|60
contig03800_length=1095|1000|60
contig03800_length=1095|0|60
log2(expression)
0
2
4
6
8
12
16
StageI
StageII
StageIII
StageIV
StageV
StageVI

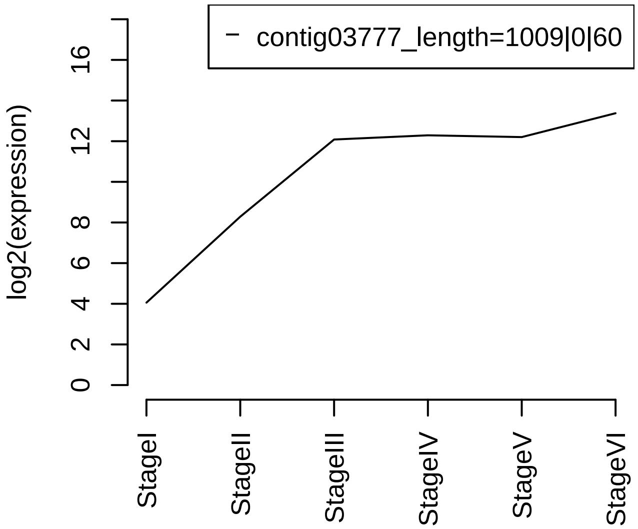

contig03777_length=1009|0|60
log2(expression)
0
2
4
6
8
12
16
StageI
StageII
StageIII
StageIV
StageV
StageVI

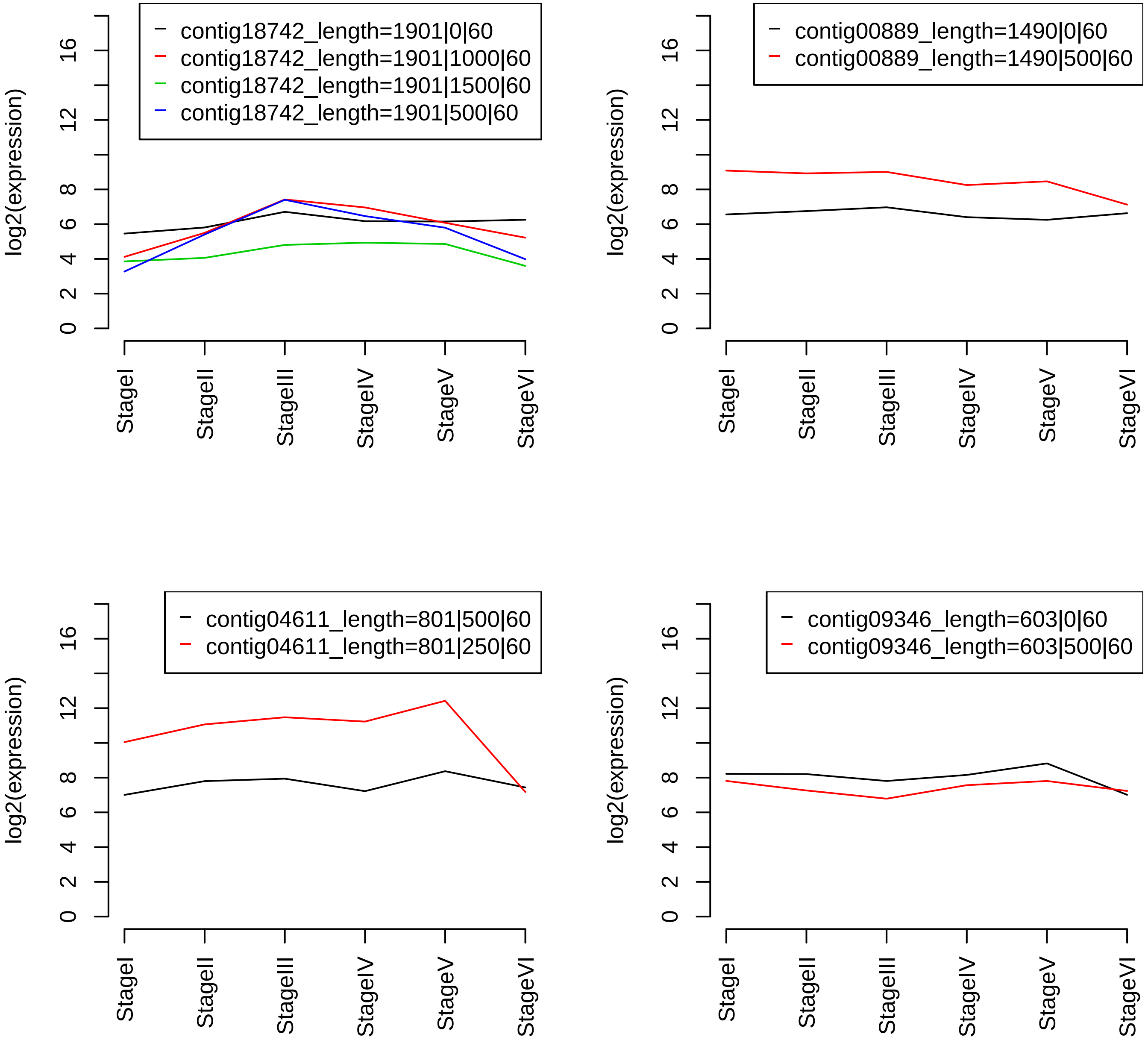

contig18742_length=1901|0|60
contig18742_length=1901|1000|60
contig18742_length=1901|1500|60
contig18742_length=1901|500|60
contig00889_length=1490|0|60
contig00889_length=1490|500|60
contig04611_length=801|500|60
contig04611_length=801|250|60
contig09346_length=603|0|60
contig09346_length=603|500|60
log2(expression)
0
2
4
6
8
12
16
StageI
StageII
StageIII
StageIV
StageV
StageVI

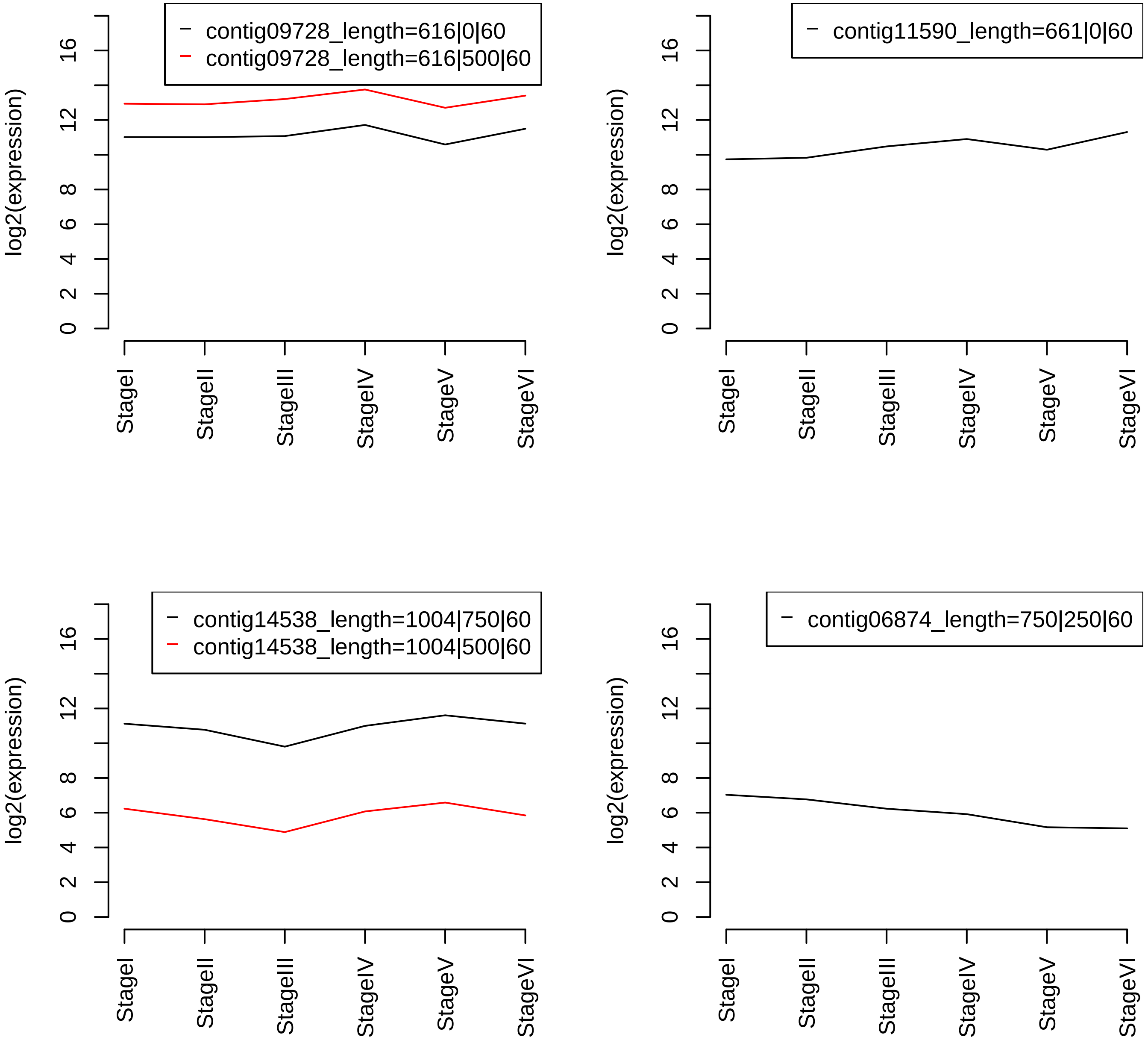

contig09728_length=616|0|60
contig09728_length=616|500|60
contig11590_length=661|0|60
contig14538_length=1004|750|60
contig14538_length=1004|500|60
contig06874_length=750|250|60
log2(expression)
0
2
4
6
8
12
16
StageI
StageII
StageIII
StageIV
StageV
StageVI

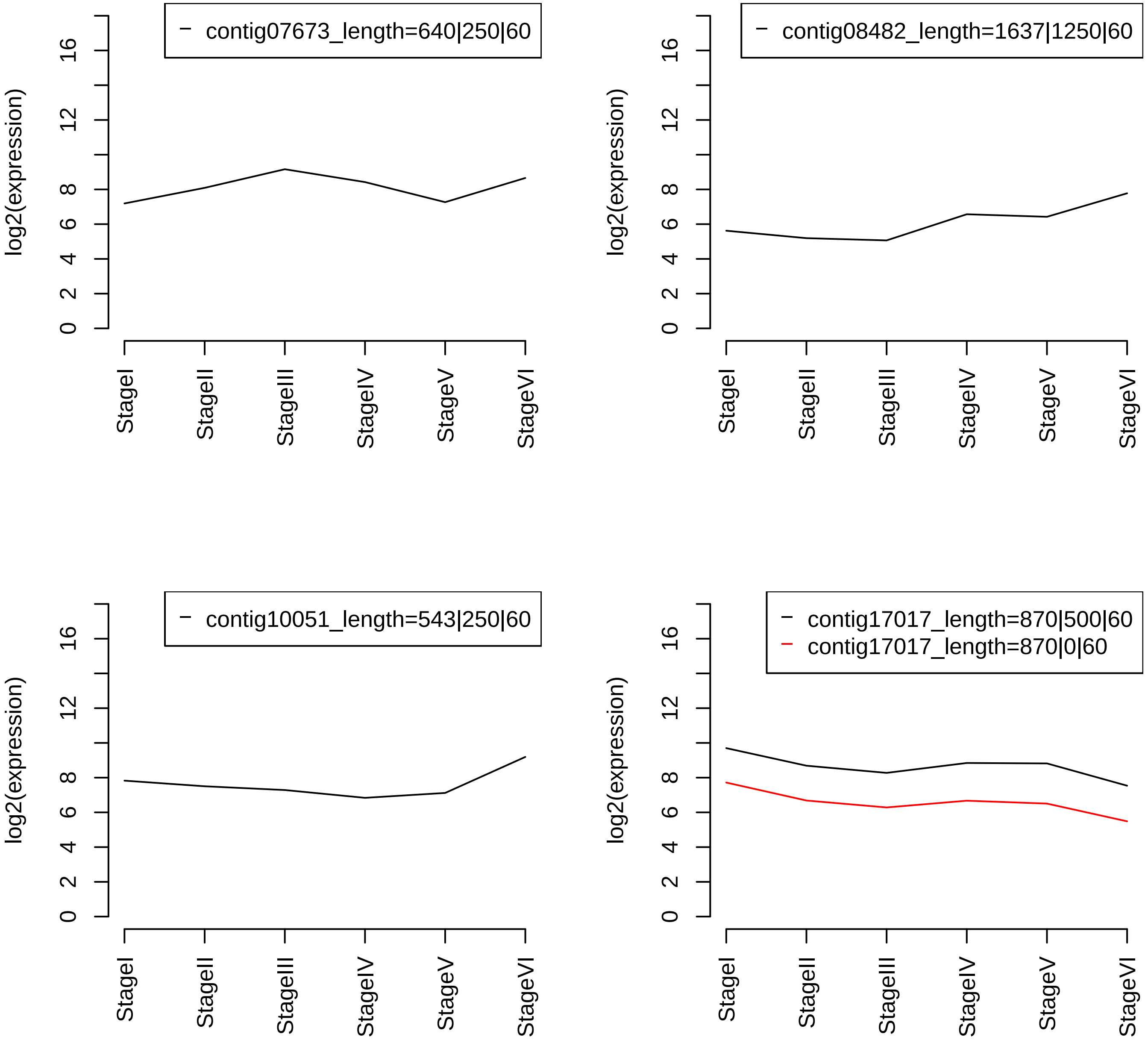

contig07673_length=640|250|60
contig08482_length=1637|1250|60
contig10051_length=543|250|60
contig17017_length=870|500|60
contig17017_length=870|0|60
log2(expression)
0
2
4
6
8
12
16
StageI
StageII
StageIII
StageIV
StageV
StageVI

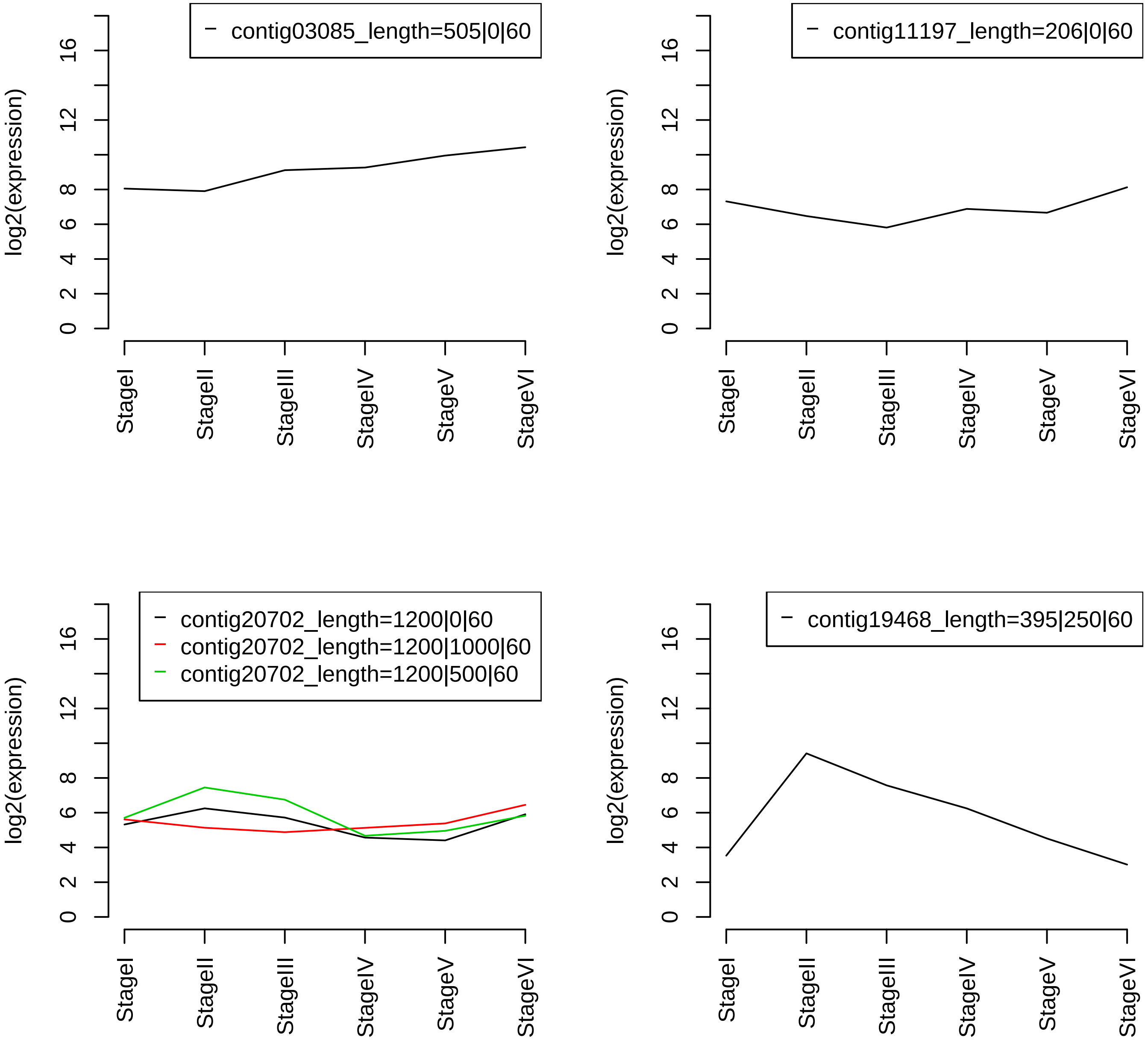
contig03085_length=505|0|60
contig11197_length=206|0|60
contig20702_length=1200|0|60
contig20702_length=1200|1000|60
contig20702_length=1200|500|60
contig19468_length=395|250|60
log2(expression)
StageI
StageII
StageIII
StageIV
StageV
StageVI

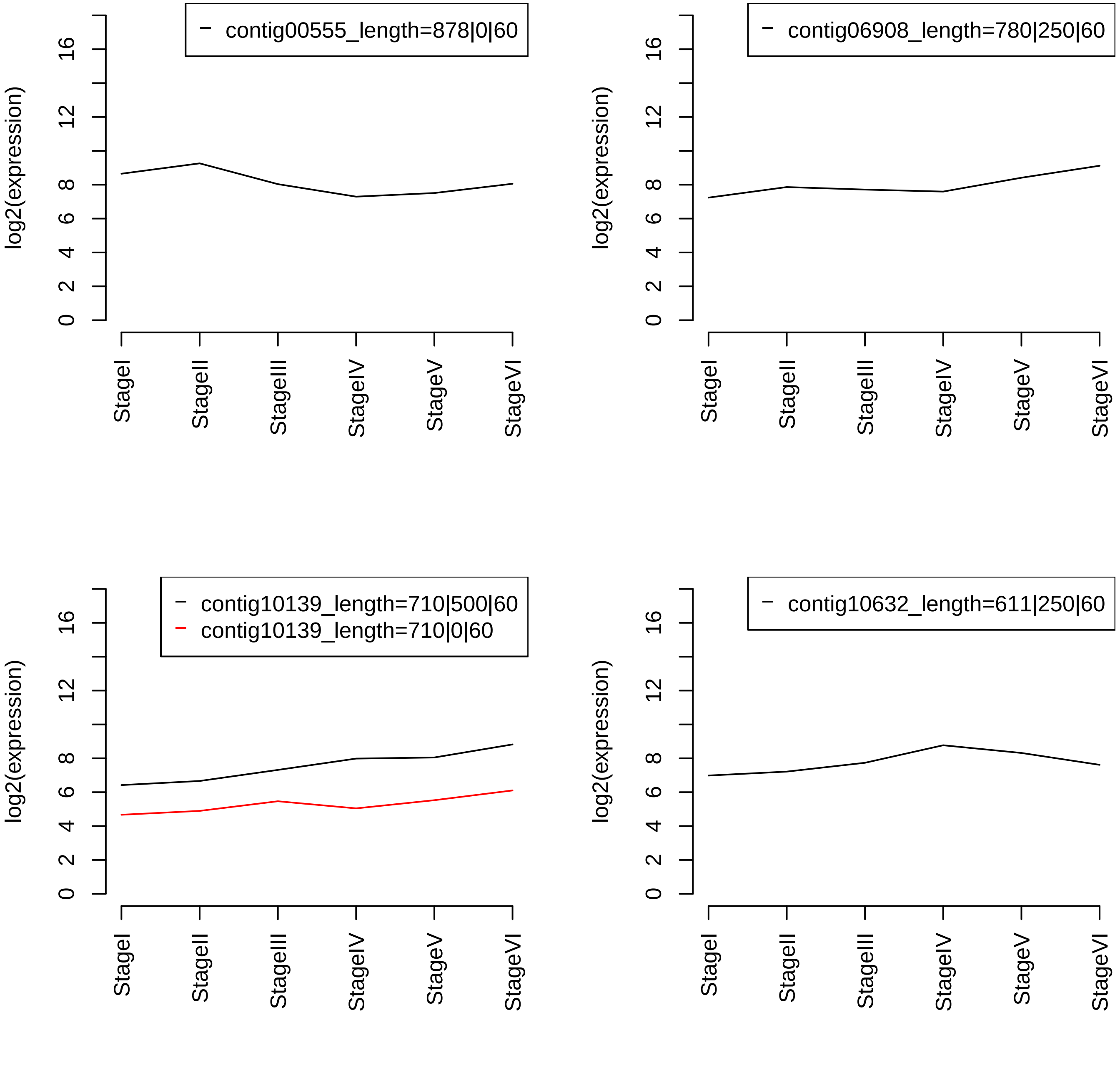
contig00555_length=878|0|60
contig06908_length=780|250|60
contig10139_length=710|500|60
contig10139_length=710|0|60
contig10632_length=611|250|60
log2(expression)
0
2
4
6
8
12
16
StageI
StageII
StageIII
StageIV
StageV
StageVI

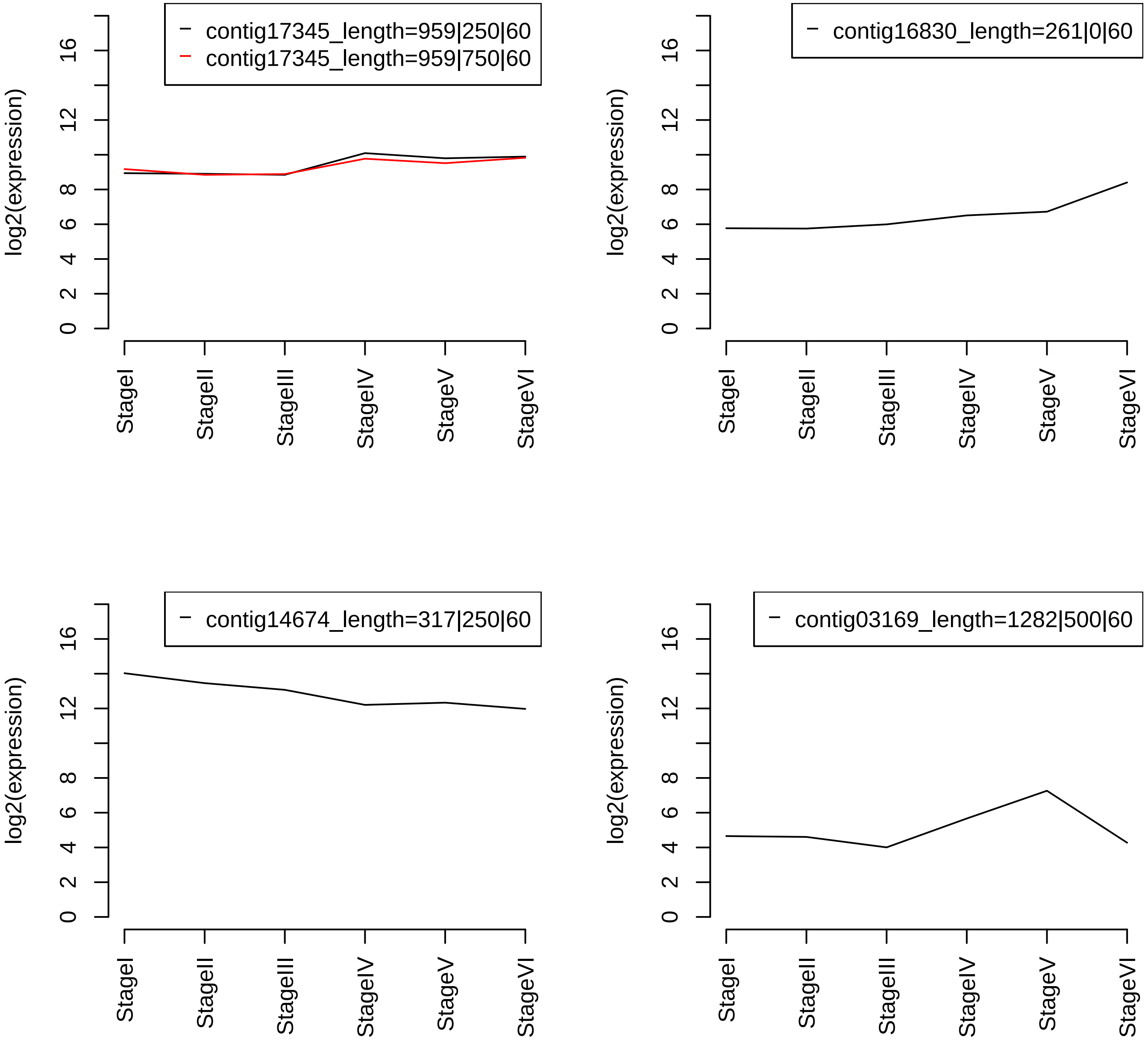

contig17345_length=959|250|60
contig17345_length=959|750|60
contig16830_length=261|0|60
contig14674_length=317|250|60
contig03169_length=1282|500|60
log2(expression)
0
2
4
6
8
12
16
StageI
StageII
StageIII
StageIV
StageV
StageVI

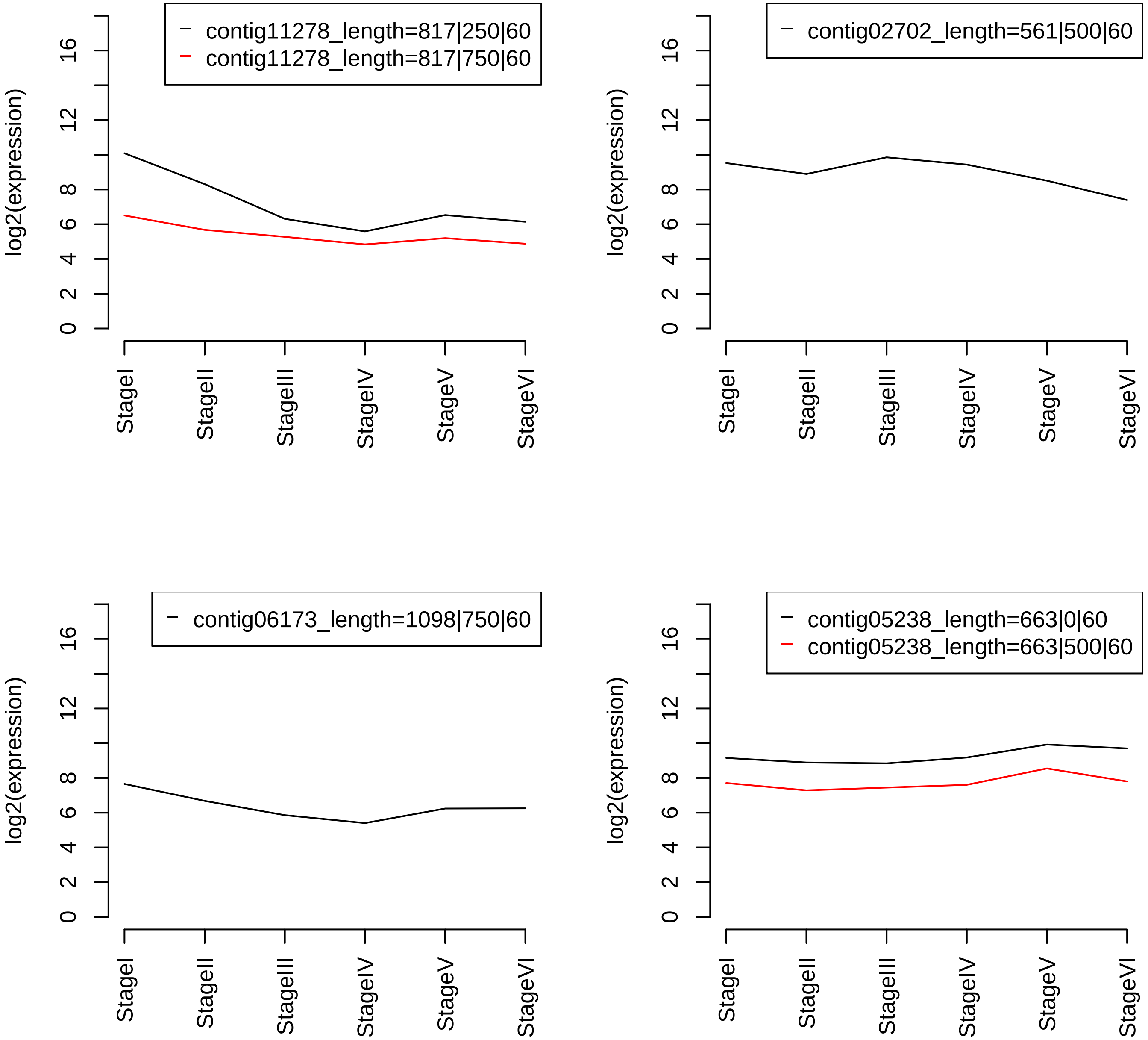
contig11278_length=817|250|60
contig11278_length=817|750|60
contig02702_length=561|500|60
contig06173_length=1098|750|60
contig05238_length=663|0|60
contig05238_length=663|500|60
log2(expression)
0
2
4
6
8
12
16
StageI
StageII
StageIII
StageIV
StageV
StageVI

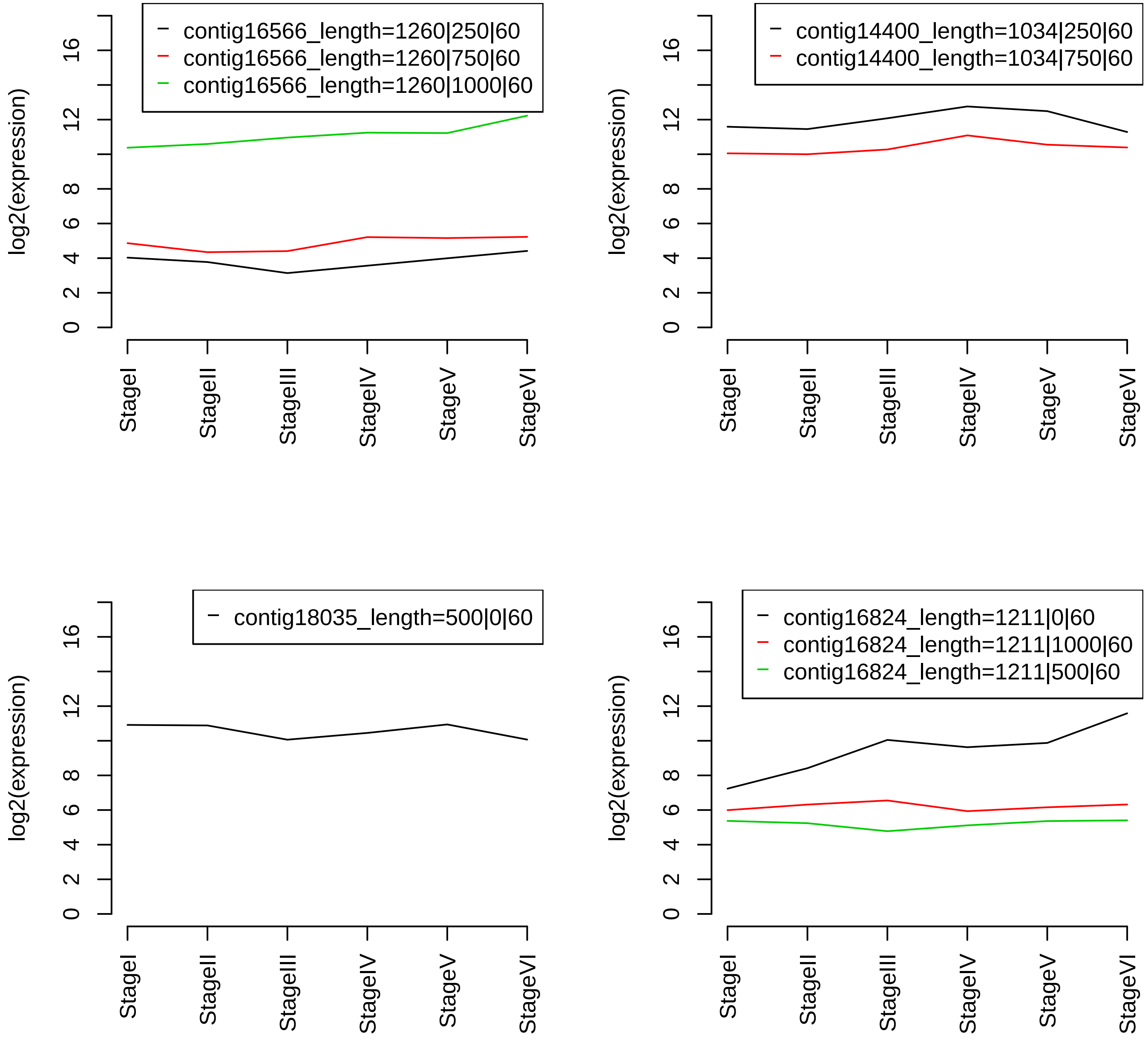

contig16566_length=1260|250|60
contig16566_length=1260|750|60
contig16566_length=1260|1000|60
log2(expression)
0
2
4
6
8
12
16
StageI
StageII
StageIII
StageIV
StageV
StageVI
contig14400_length=1034|250|60
contig14400_length=1034|750|60
contig18035_length=500|0|60
contig16824_length=1211|0|60
contig16824_length=1211|1000|60
contig16824_length=1211|500|60

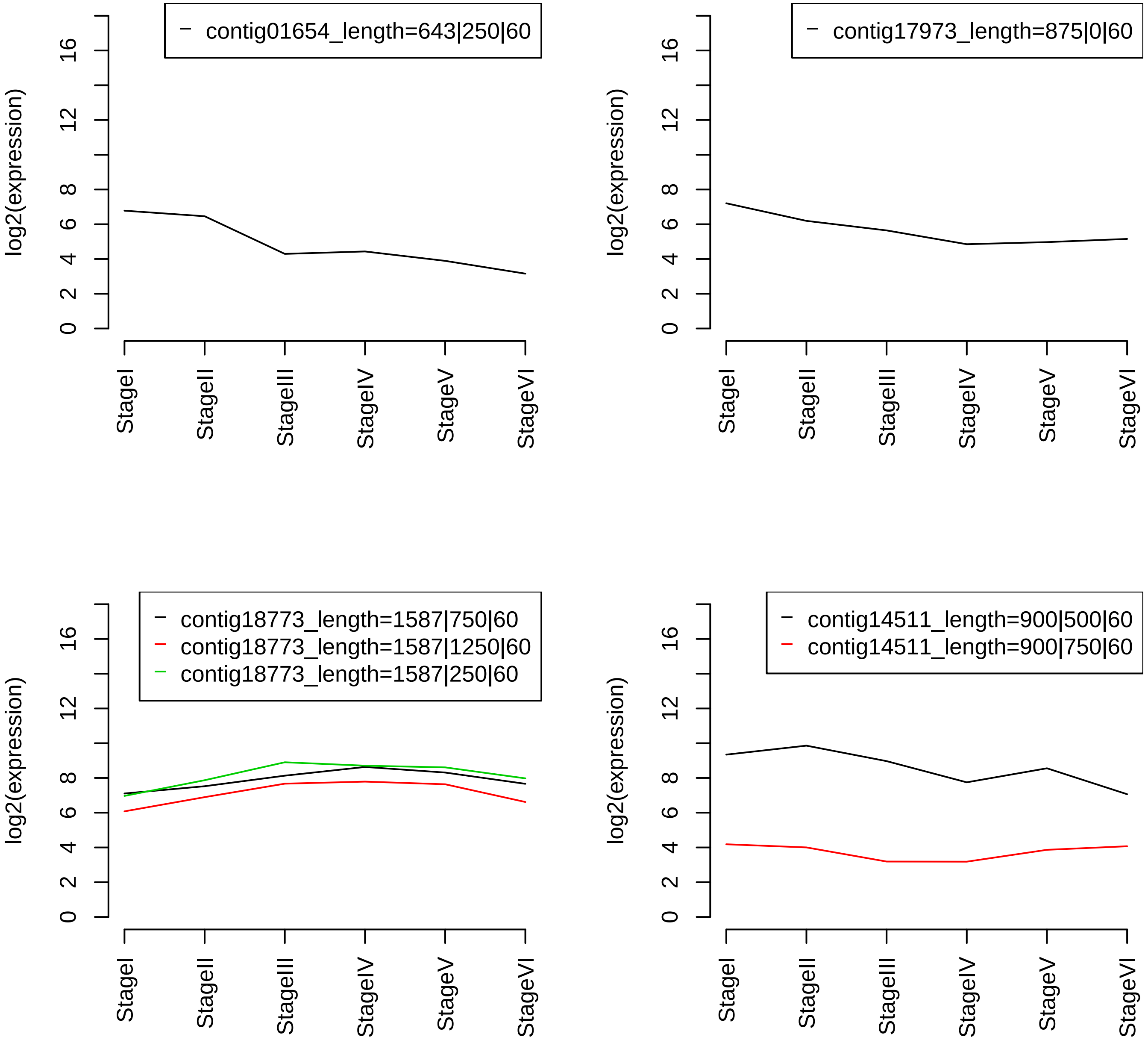

contig01654_length=643|250|60
contig17973_length=875|0|60
contig18773_length=1587|750|60
contig18773_length=1587|1250|60
contig18773_length=1587|250|60
contig14511_length=900|500|60
contig14511_length=900|750|60
log2(expression)
0
2
4
6
8
12
16
StageI
StageII
StageIII
StageIV
StageV
StageVI

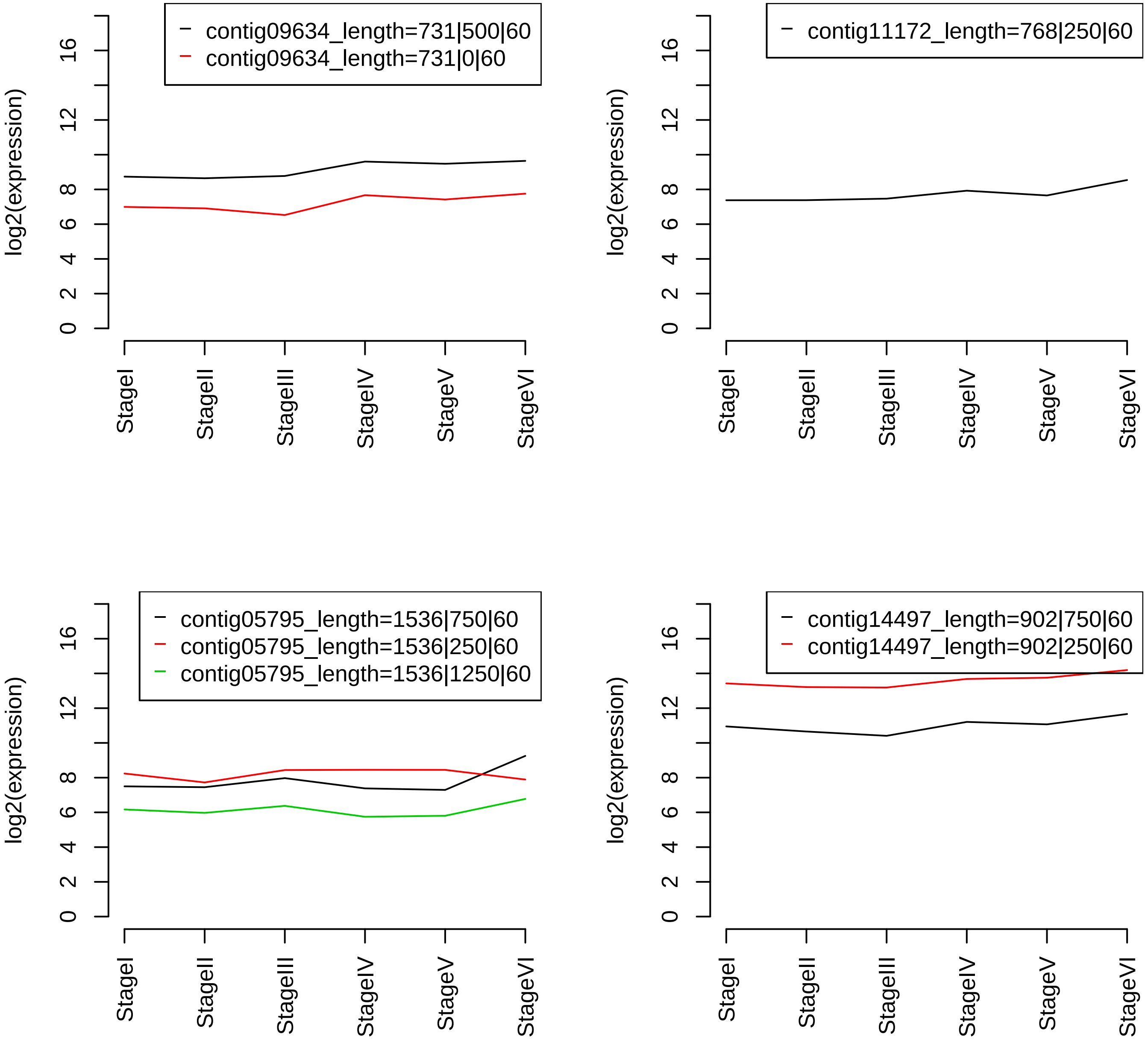
contig09634_length=731|500|60
contig09634_length=731|0|60
contig11172_length=768|250|60
contig05795_length=1536|750|60
contig05795_length=1536|250|60
contig05795_length=1536|1250|60
contig14497_length=902|750|60
contig14497_length=902|250|60
log2(expression)
0
2
4
6
8
12
16
StageI
StageII
StageIII
StageIV
StageV
StageVI

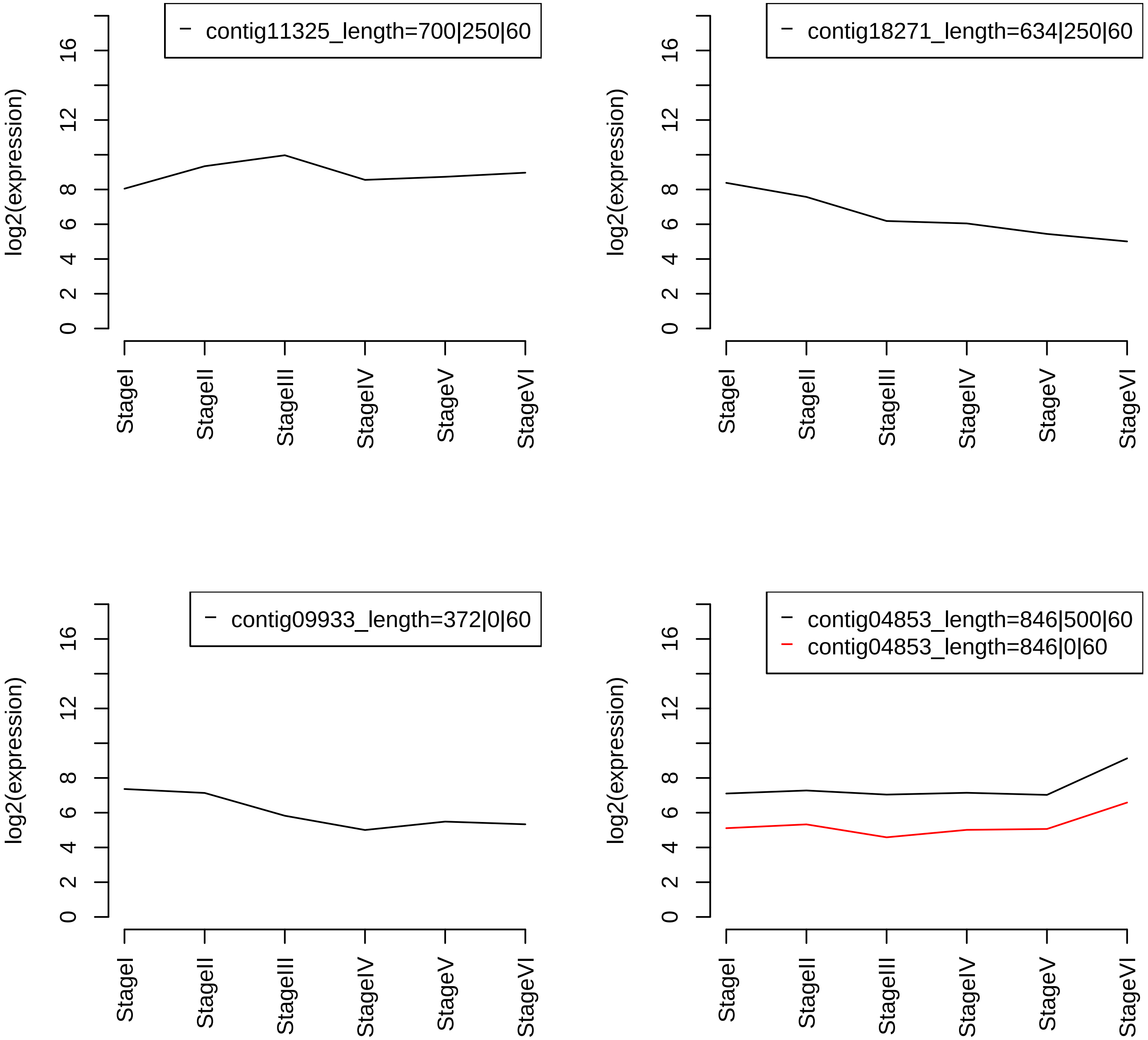
contig11325_length=700|250|60
contig18271_length=634|250|60
contig09933_length=372|0|60
contig04853_length=846|500|60
contig04853_length=846|0|60
log2(expression)
0
2
4
6
8
12
16
StageI
StageII
StageIII
StageIV
StageV
StageVI

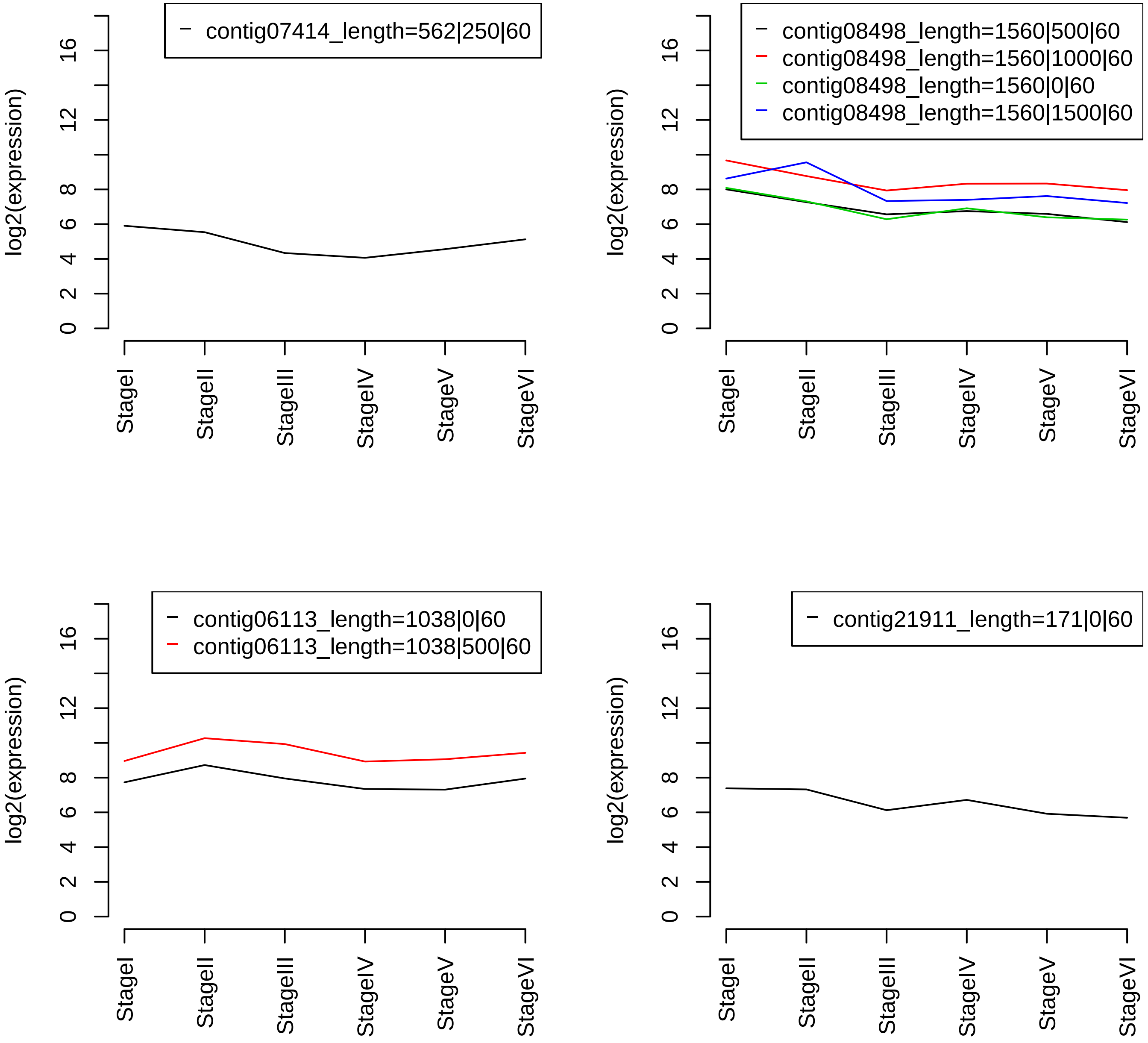

contig07414_length=562|250|60
contig08498_length=1560|500|60
contig08498_length=1560|1000|60
contig08498_length=1560|0|60
contig08498_length=1560|1500|60
contig06113_length=1038|0|60
contig06113_length=1038|500|60
contig21911_length=171|0|60
log2(expression)
0
2
4
6
8
12
16
StageI
StageII
StageIII
StageIV
StageV
StageVI

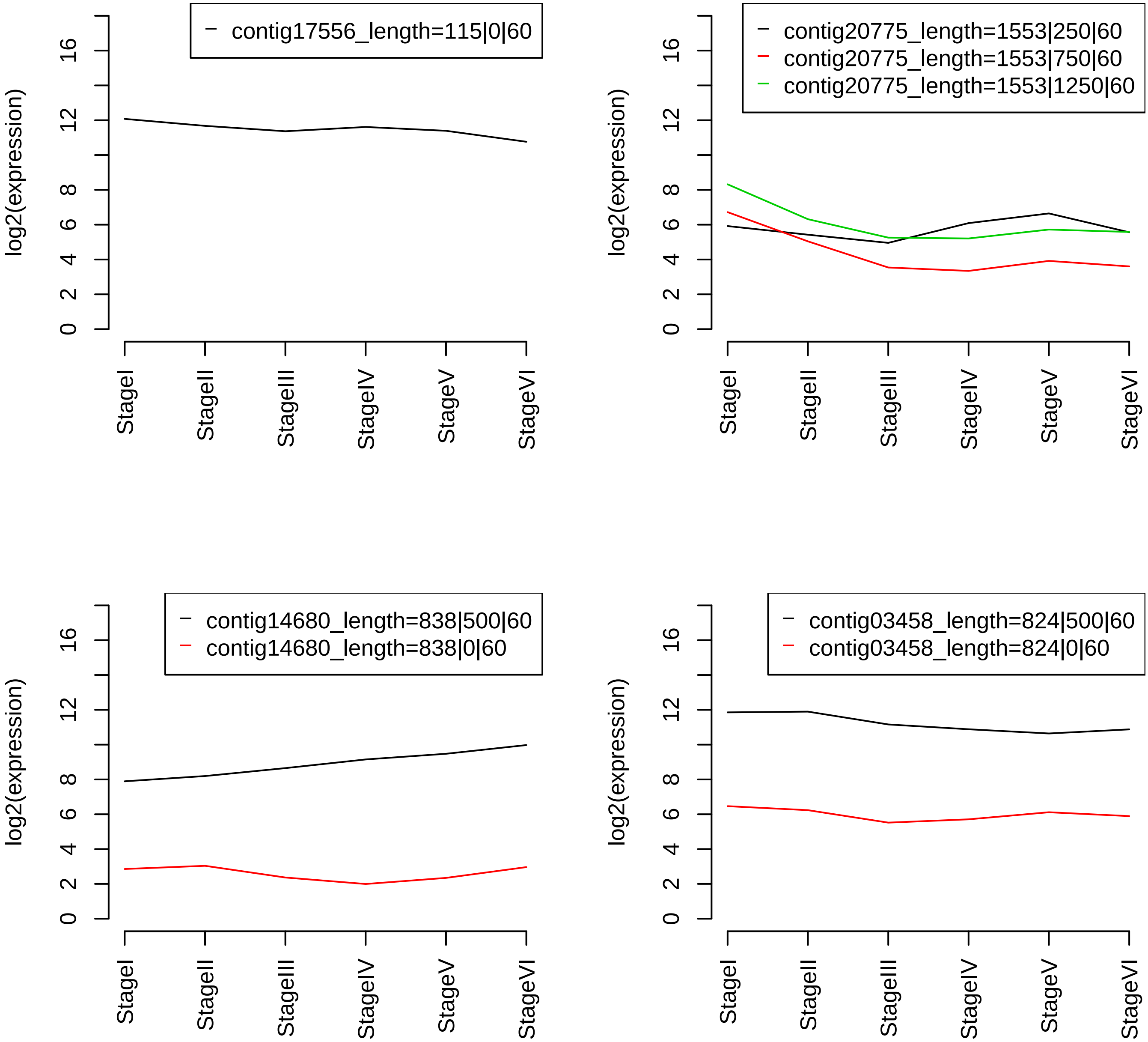
contig17556_length=115|0|60
contig20775_length=1553|250|60
contig20775_length=1553|750|60
contig20775_length=1553|1250|60
contig14680_length=838|500|60
contig14680_length=838|0|60
contig03458_length=824|500|60
contig03458_length=824|0|60
log2(expression)
0
2
4
6
8
12
16
StageI
StageII
StageIII
StageIV
StageV
StageVI

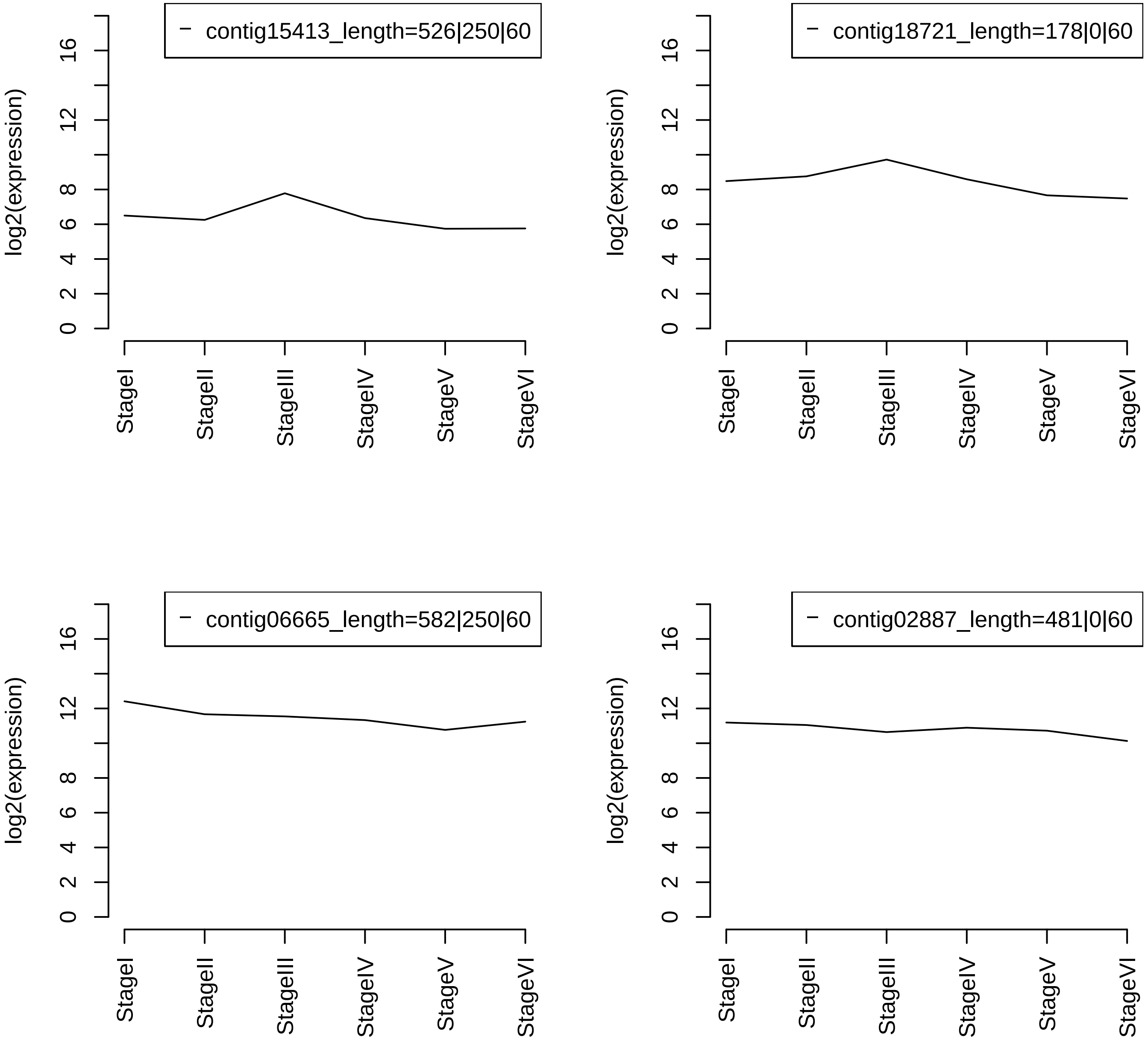

contig15413_length=526|250|60
contig18721_length=178|0|60
contig06665_length=582|250|60
contig02887_length=481|0|60
log2(expression)
0
2
4
6
8
12
16
StageI
StageII
StageIII
StageIV
StageV
StageVI

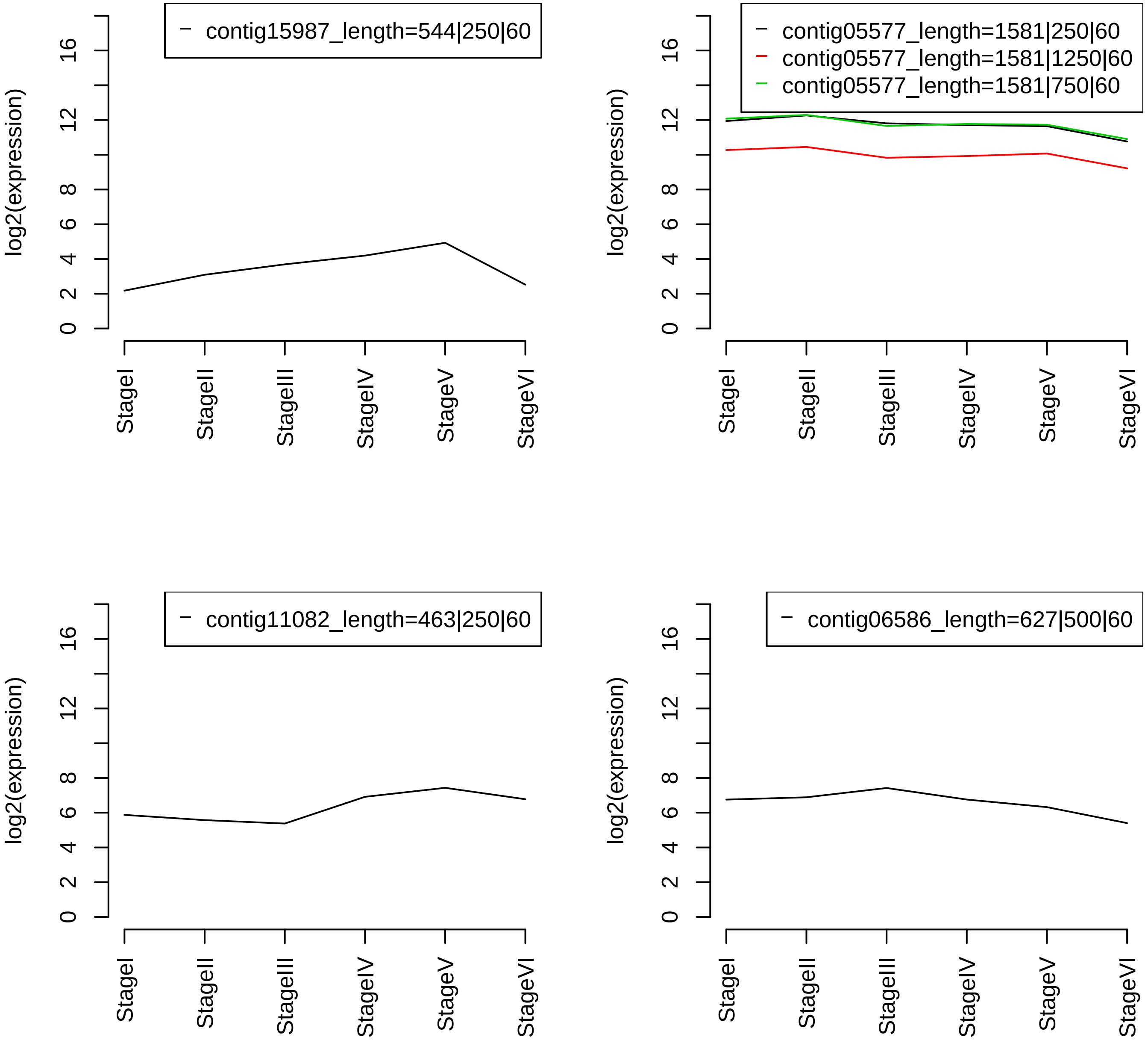

contig15987_length=544|250|60
contig05577_length=1581|250|60
contig05577_length=1581|1250|60
contig05577_length=1581|750|60
contig11082_length=463|250|60
contig06586_length=627|500|60
log2(expression)
0
2
4
6
8
12
16
StageI
StageII
StageIII
StageIV
StageV
StageVI

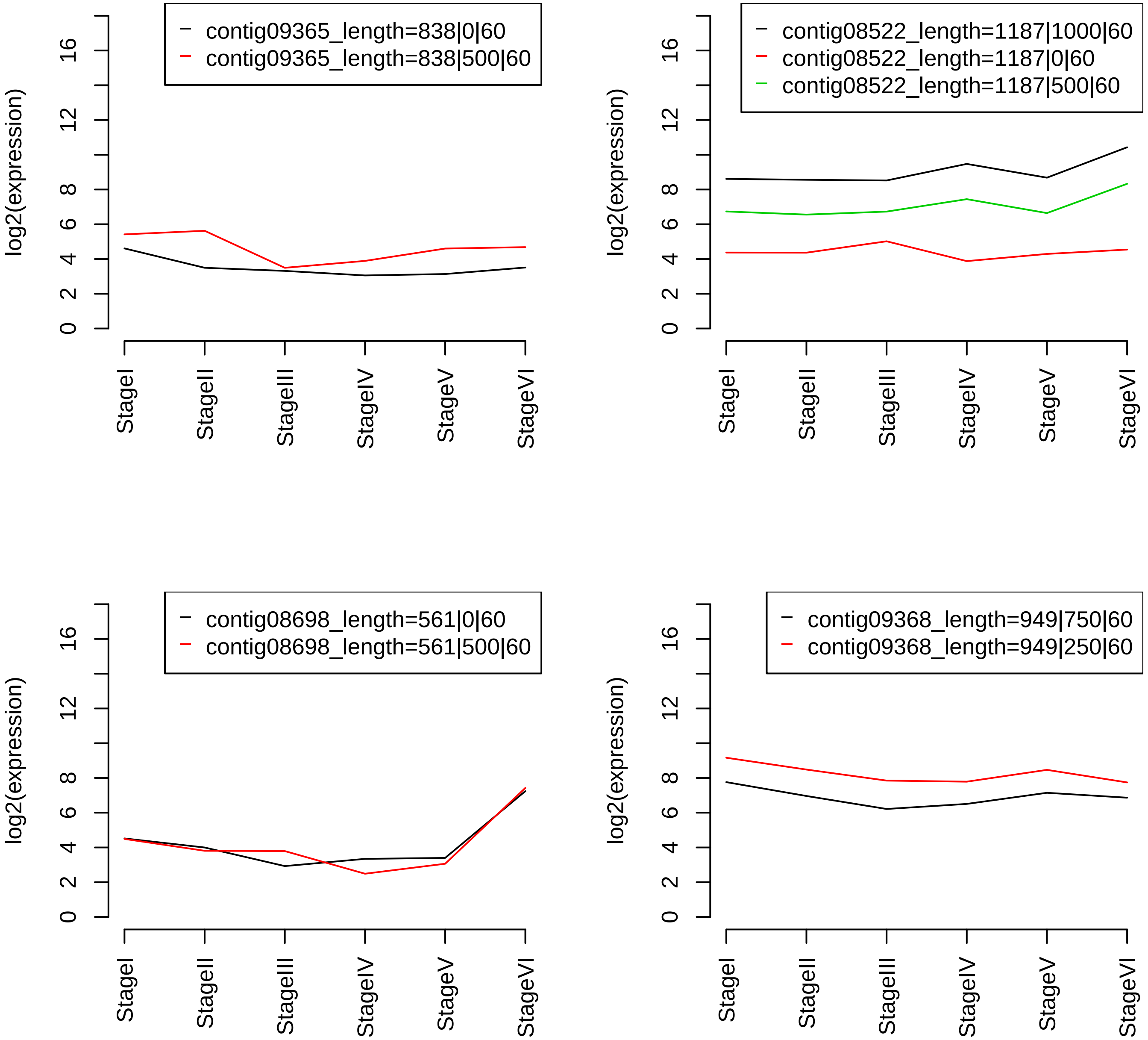

contig09365_length=838|0|60
contig09365_length=838|500|60
contig08522_length=1187|1000|60
contig08522_length=1187|0|60
contig08522_length=1187|500|60
contig08698_length=561|0|60
contig08698_length=561|500|60
contig09368_length=949|750|60
contig09368_length=949|250|60
log2(expression)
0
2
4
6
8
12
16
StageI
StageII
StageIII
StageIV
StageV
StageVI

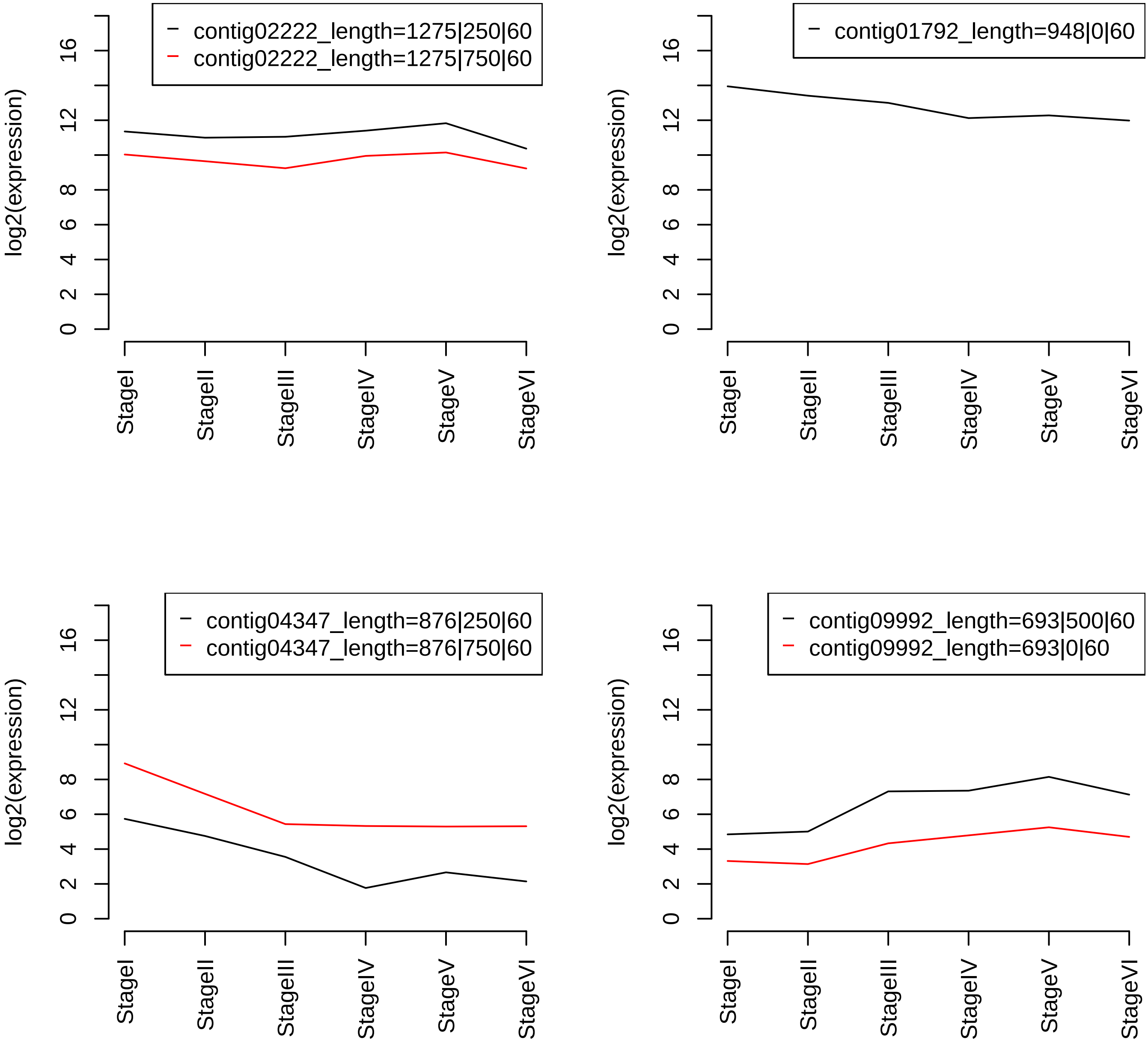
contig02222_length=1275|250|60
contig02222_length=1275|750|60
contig01792_length=948|0|60
contig04347_length=876|250|60
contig04347_length=876|750|60
contig09992_length=693|500|60
contig09992_length=693|0|60
log2(expression)
StageI
StageII
StageIII
StageIV
StageV
StageVI

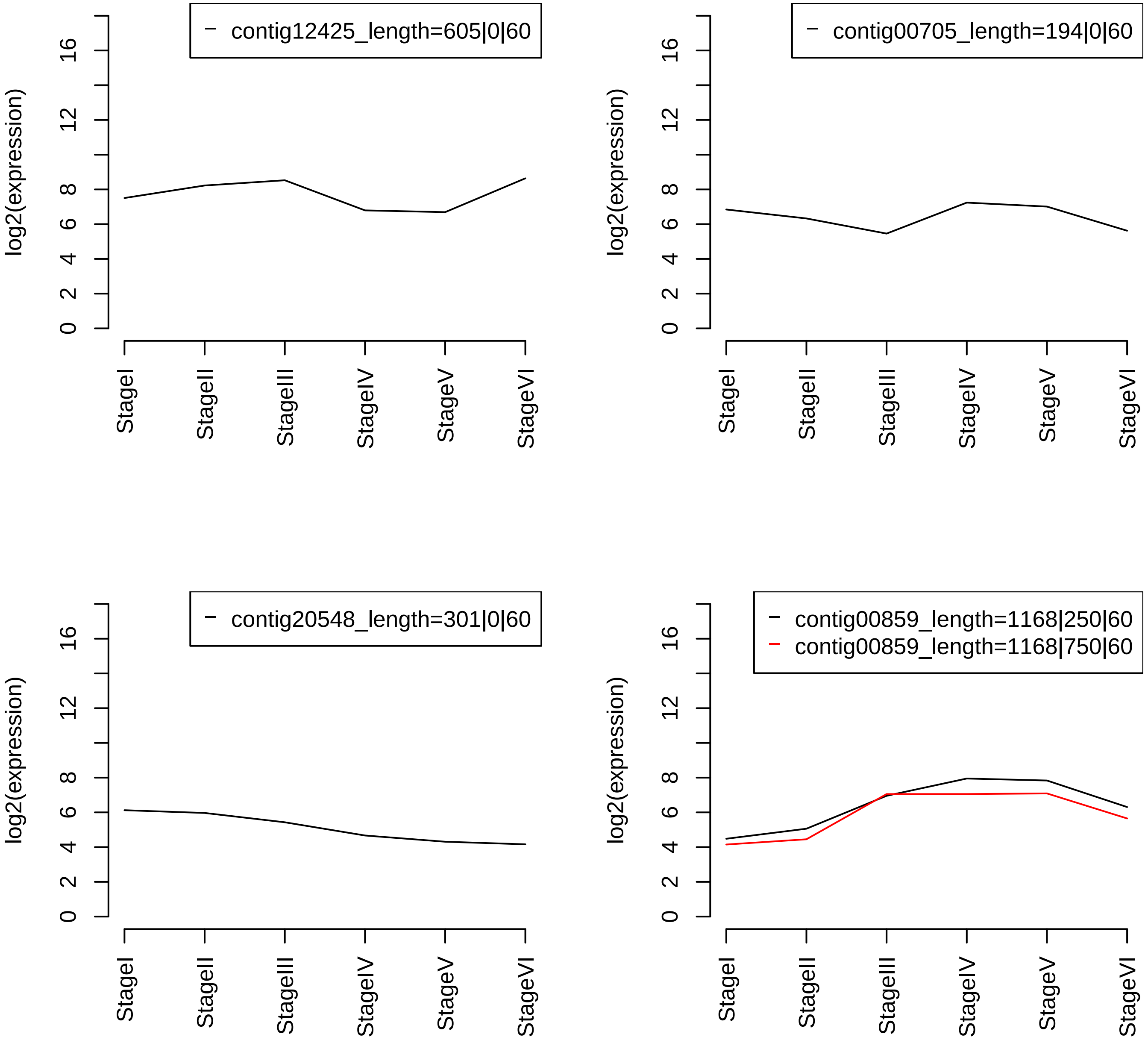
contig12425_length=605|0|60
contig00705_length=194|0|60
contig20548_length=301|0|60
contig00859_length=1168|250|60
contig00859_length=1168|750|60
log2(expression)
0
2
4
6
8
12
16
StageI
StageII
StageIII
StageIV
StageV
StageVI

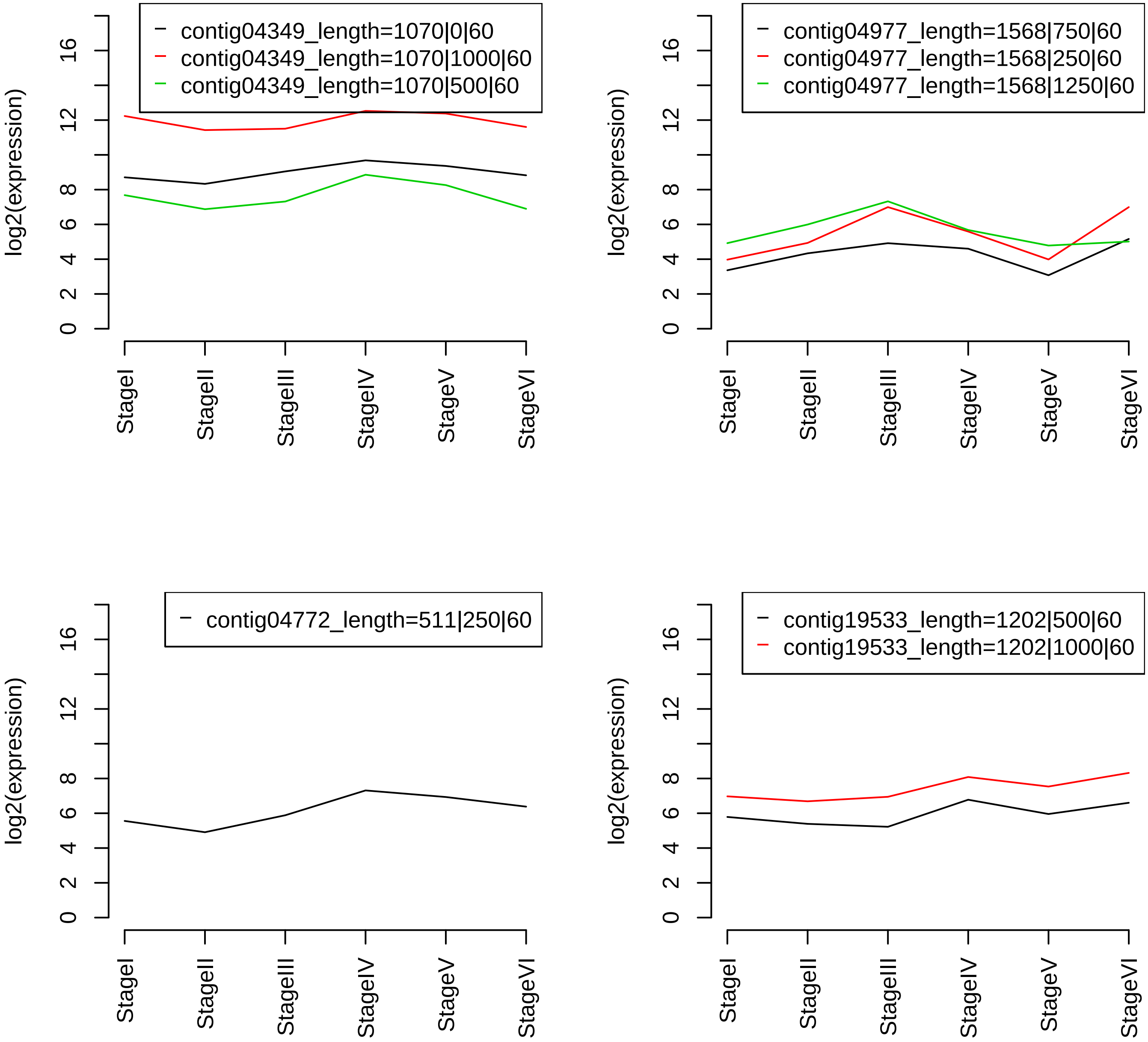
contig04349_length=1070|0|60
contig04349_length=1070|1000|60
contig04349_length=1070|500|60
contig04977_length=1568|750|60
contig04977_length=1568|250|60
contig04977_length=1568|1250|60
contig04772_length=511|250|60
contig19533_length=1202|500|60
contig19533_length=1202|1000|60
log2(expression)
0
2
4
6
8
12
16
StageI
StageII
StageIII
StageIV
StageV
StageVI

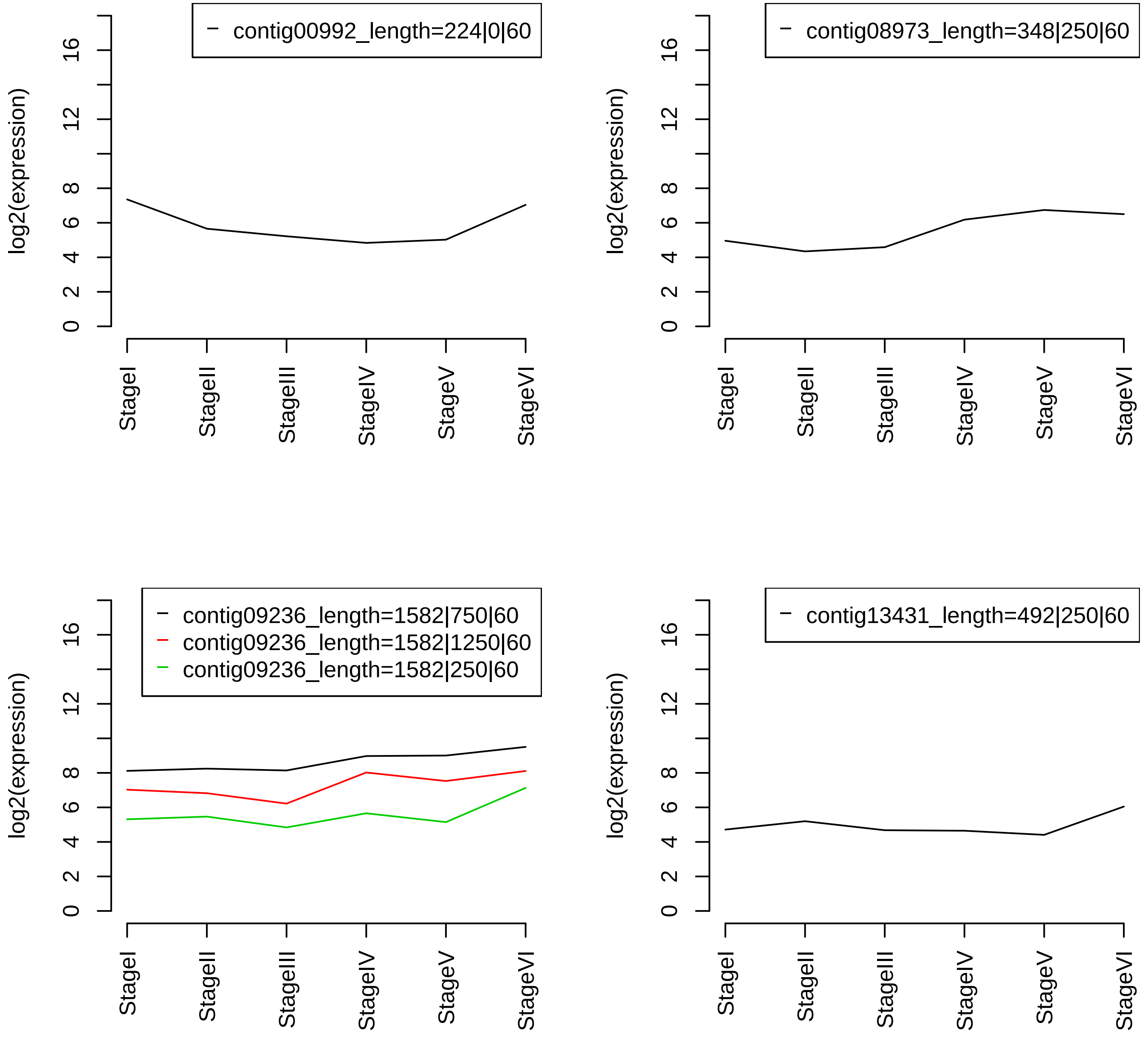

contig00992_length=224|0|60
contig08973_length=348|250|60
contig09236_length=1582|750|60
contig09236_length=1582|1250|60
contig09236_length=1582|250|60
contig13431_length=492|250|60
log2(expression)
0
2
4
6
8
12
16
StageI
StageII
StageIII
StageIV
StageV
StageVI

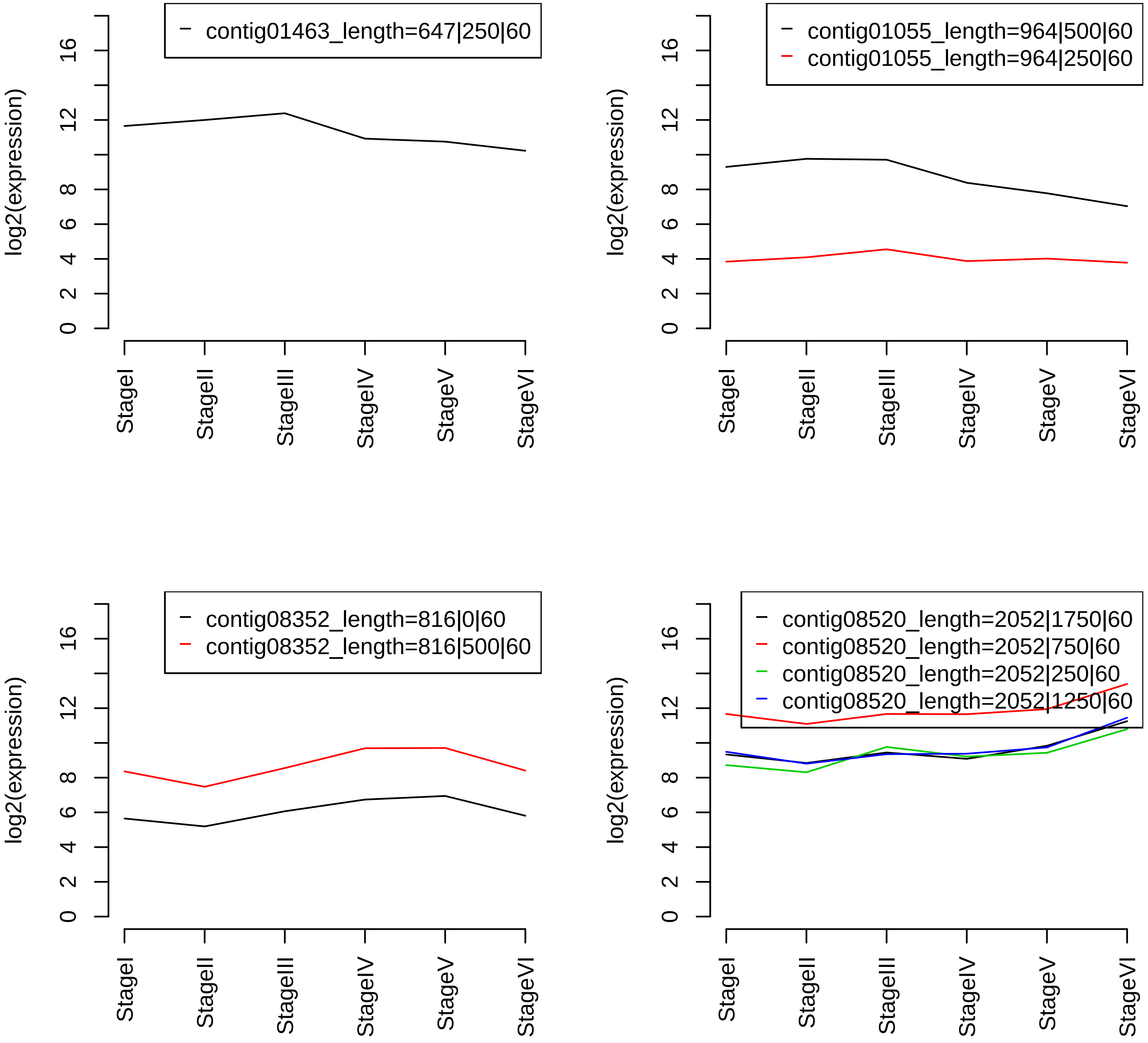

contig01463_length=647|250|60
contig01055_length=964|500|60
contig01055_length=964|250|60
contig08352_length=816|0|60
contig08352_length=816|500|60
contig08520_length=2052|1750|60
contig08520_length=2052|750|60
contig08520_length=2052|250|60
contig08520_length=2052|1250|60
log2(expression)
0
2
4
6
8
12
16
StageI
StageII
StageIII
StageIV
StageV
StageVI

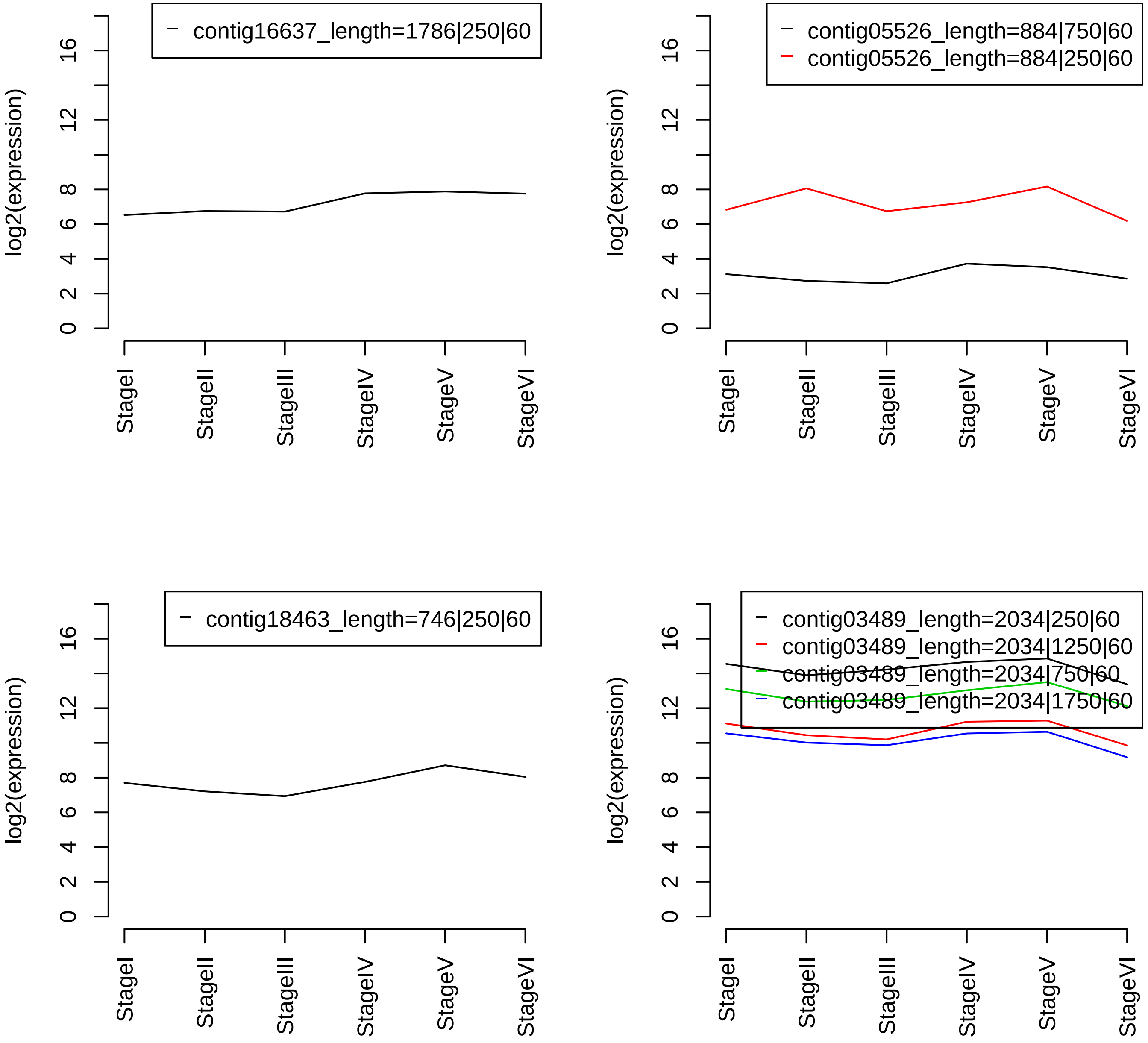
contig16637_length=1786|250|60
contig05526_length=884|750|60
contig05526_length=884|250|60
contig18463_length=746|250|60
contig03489_length=2034|250|60
contig03489_length=2034|1250|60
contig03489_length=2034|750|60
contig03489_length=2034|1750|60
log2(expression)
0
2
4
6
8
12
16
StageI
StageII
StageIII
StageIV
StageV
StageVI

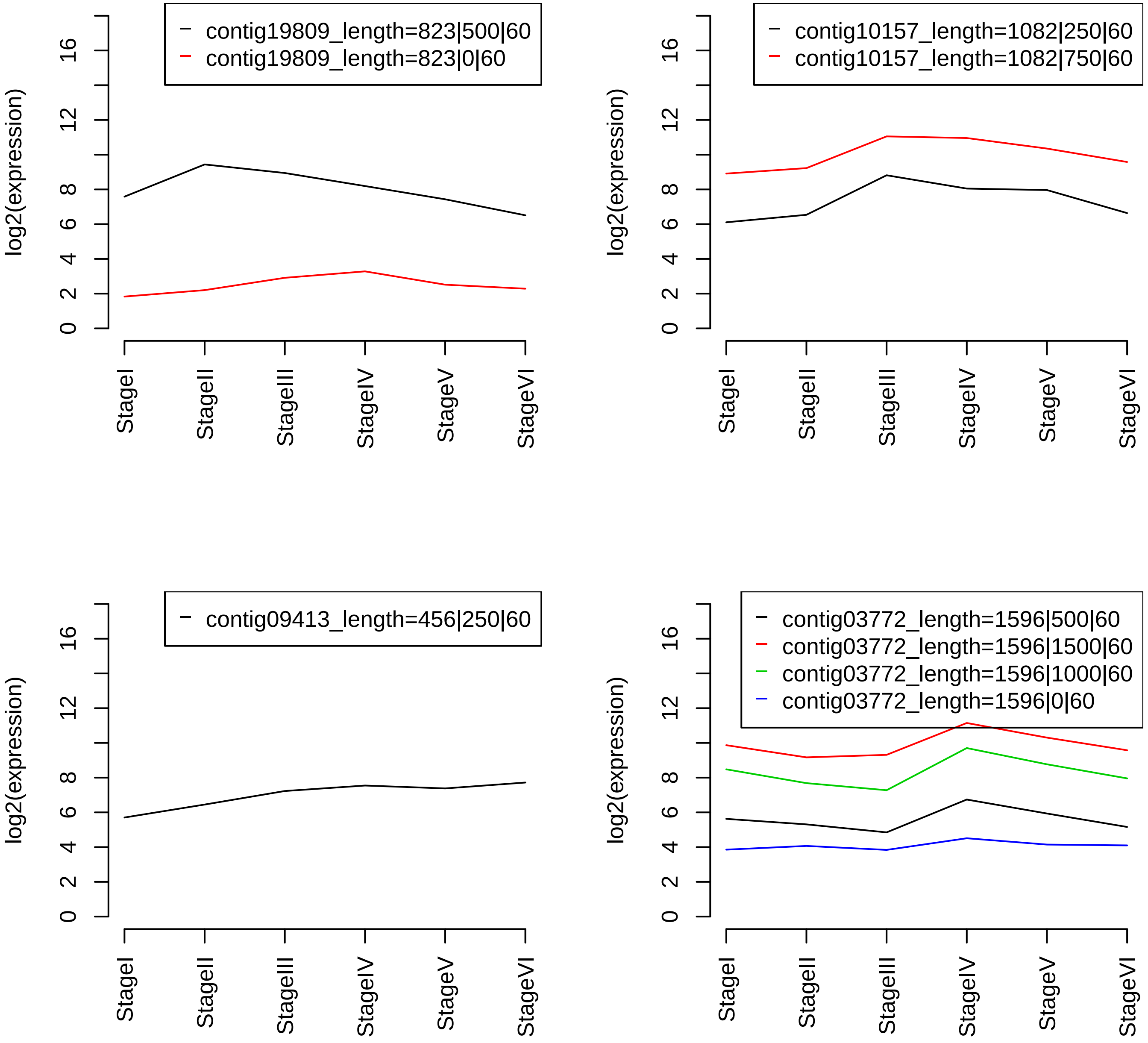
contig19809_length=823|500|60
contig19809_length=823|0|60
contig10157_length=1082|250|60
contig10157_length=1082|750|60
contig09413_length=456|250|60
contig03772_length=1596|500|60
contig03772_length=1596|1500|60
contig03772_length=1596|1000|60
contig03772_length=1596|0|60
log2(expression)
0
2
4
6
8
12
16
StageI
StageII
StageIII
StageIV
StageV
StageVI

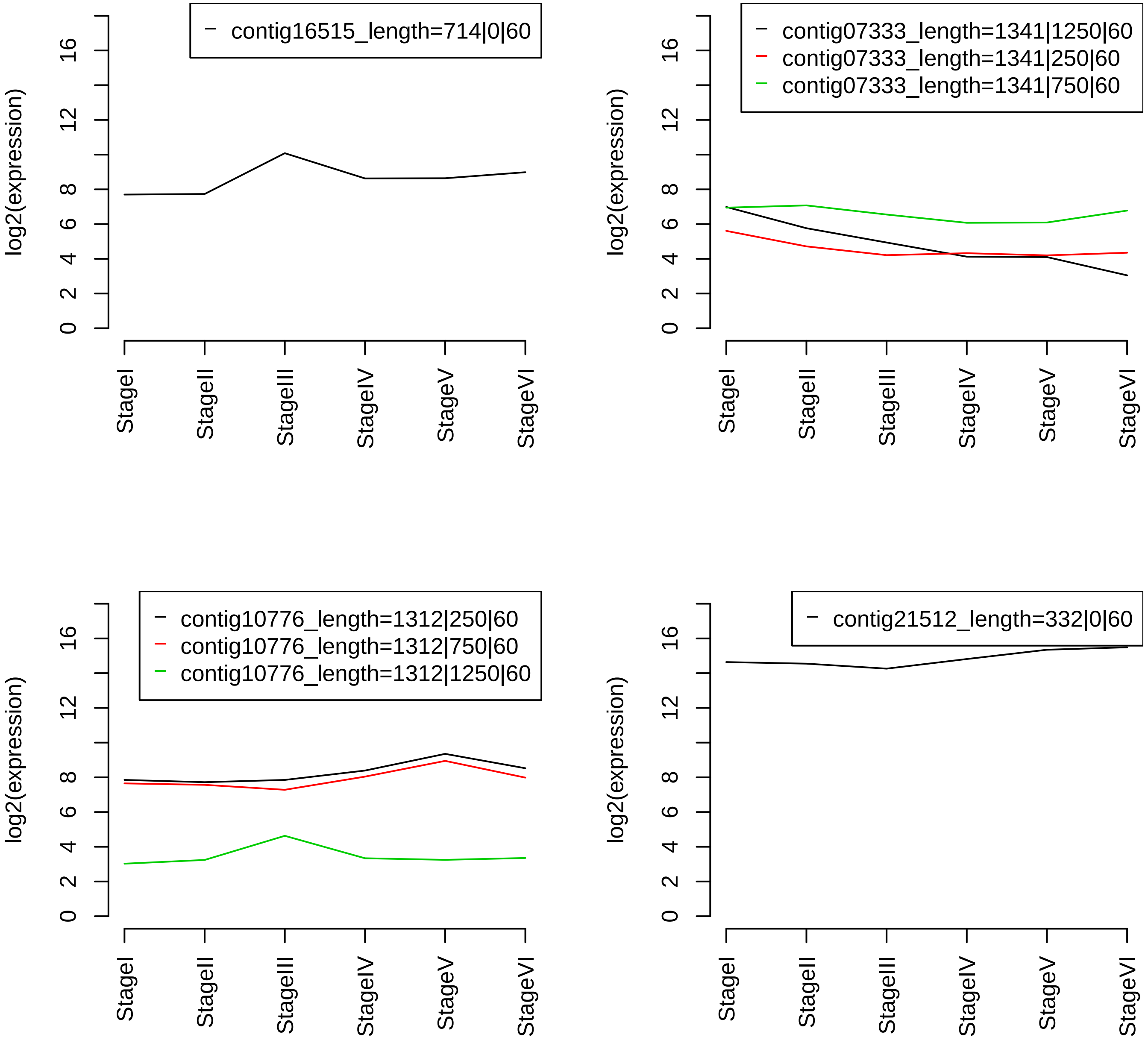

contig16515_length=714|0|60
contig07333_length=1341|1250|60
contig07333_length=1341|250|60
contig07333_length=1341|750|60
contig10776_length=1312|250|60
contig10776_length=1312|750|60
contig10776_length=1312|1250|60
contig21512_length=332|0|60
log2(expression)
0
2
4
6
8
12
16
StageI
StageII
StageIII
StageIV
StageV
StageVI

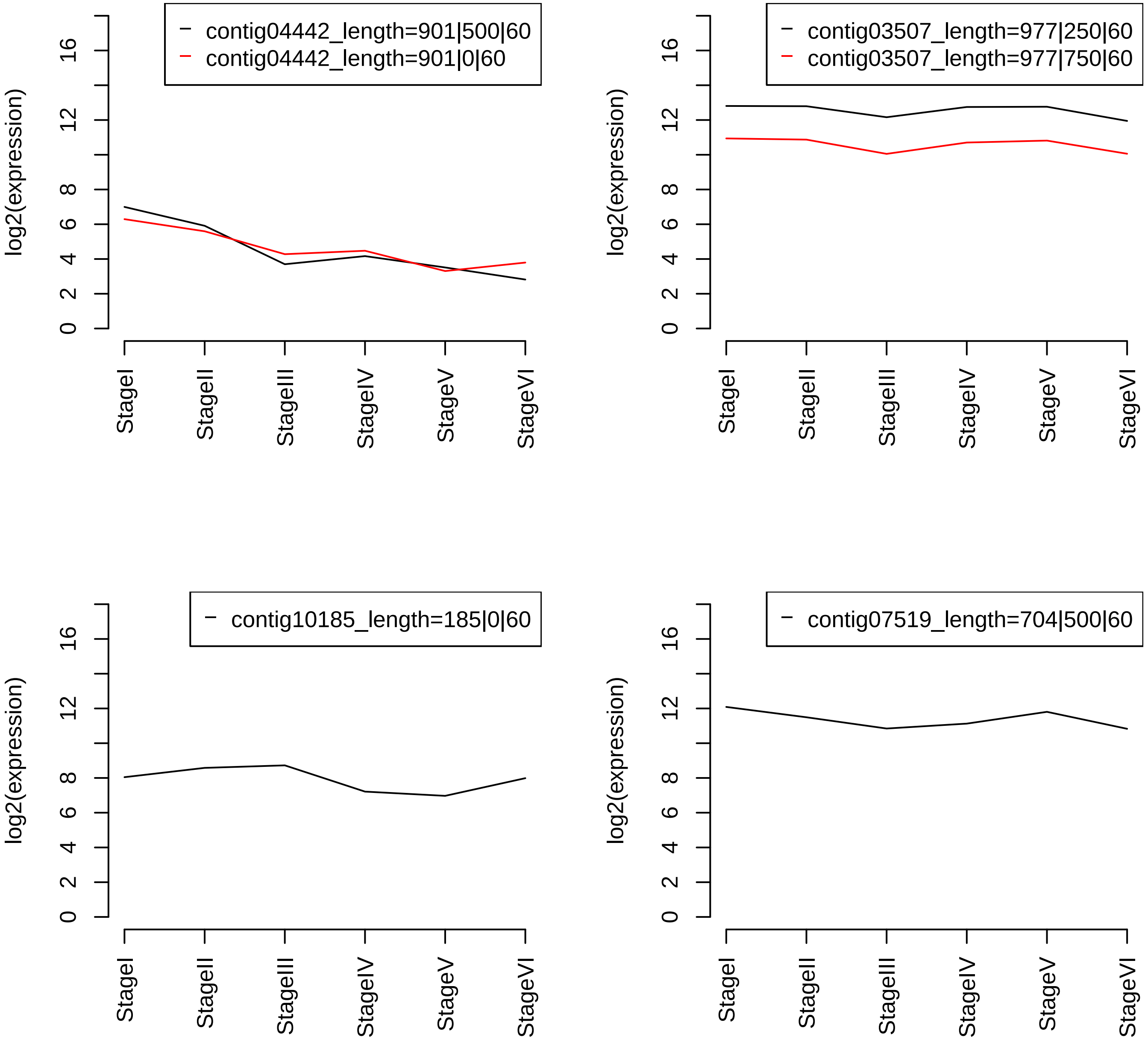

contig04442_length=901|500|60
contig04442_length=901|0|60
contig03507_length=977|250|60
contig03507_length=977|750|60
contig10185_length=185|0|60
contig07519_length=704|500|60
log2(expression)
0
2
4
6
8
12
16
StageI
StageII
StageIII
StageIV
StageV
StageVI

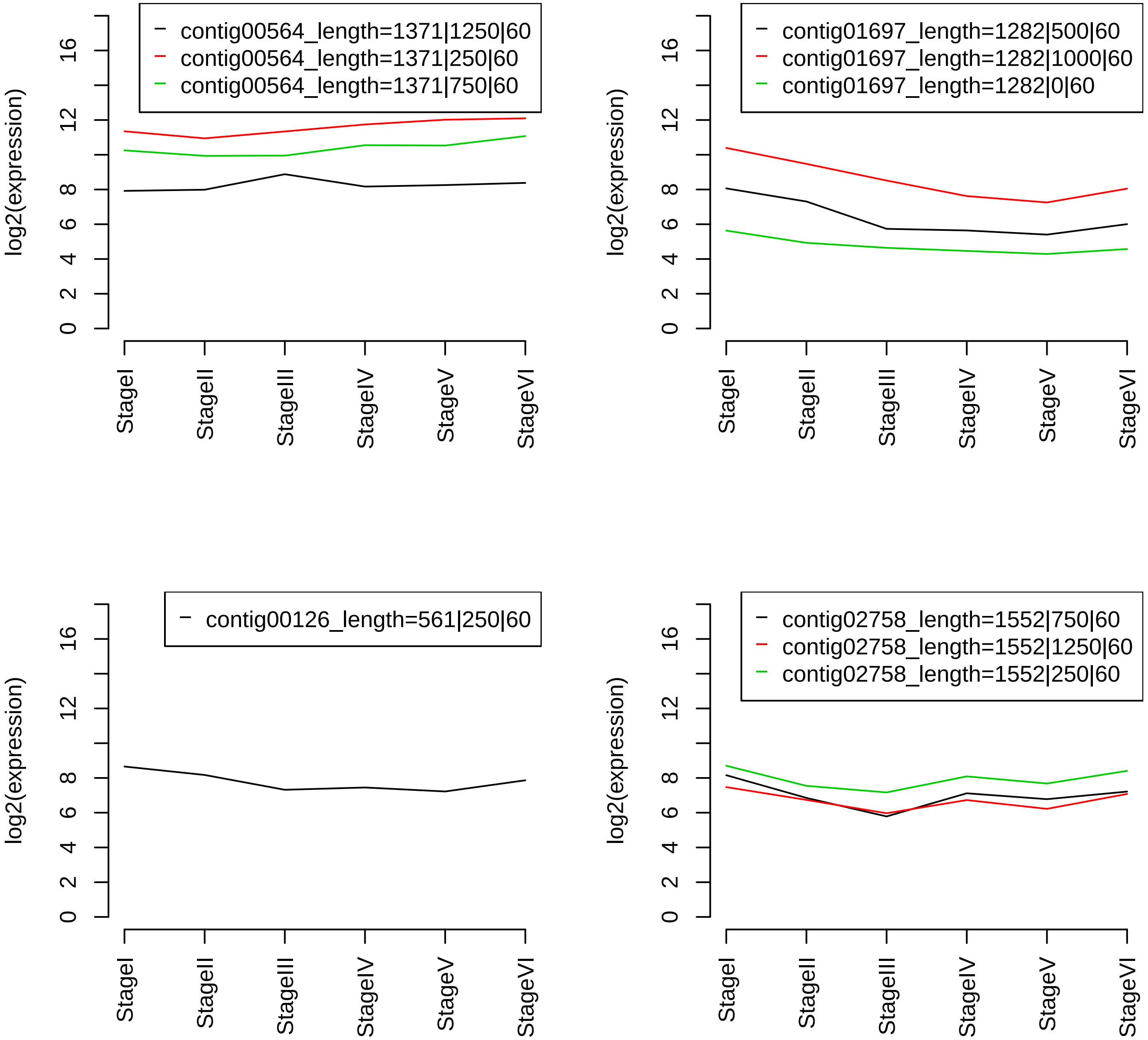
contig00564_length=1371|1250|60
contig00564_length=1371|250|60
contig00564_length=1371|750|60
contig01697_length=1282|500|60
contig01697_length=1282|1000|60
contig01697_length=1282|0|60
contig00126_length=561|250|60
contig02758_length=1552|750|60
contig02758_length=1552|1250|60
contig02758_length=1552|250|60
log2(expression)
0
2
4
6
8
12
16
StageI
StageII
StageIII
StageIV
StageV
StageVI

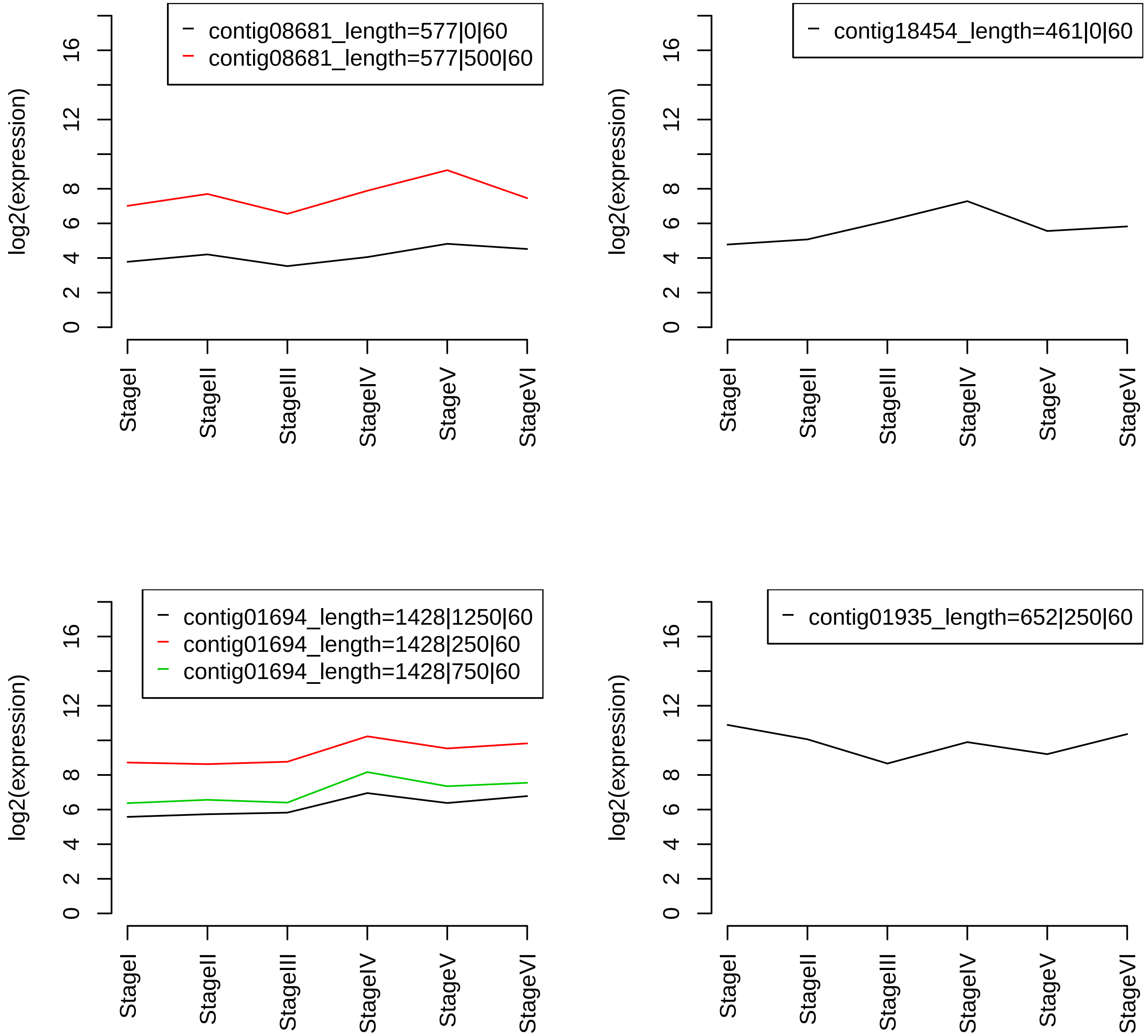
contig08681_length=577|0|60
contig08681_length=577|500|60
contig18454_length=461|0|60
contig01694_length=1428|1250|60
contig01694_length=1428|250|60
contig01694_length=1428|750|60
contig01935_length=652|250|60
log2(expression)
0
2
4
6
8
12
16
StageI
StageII
StageIII
StageIV
StageV
StageVI

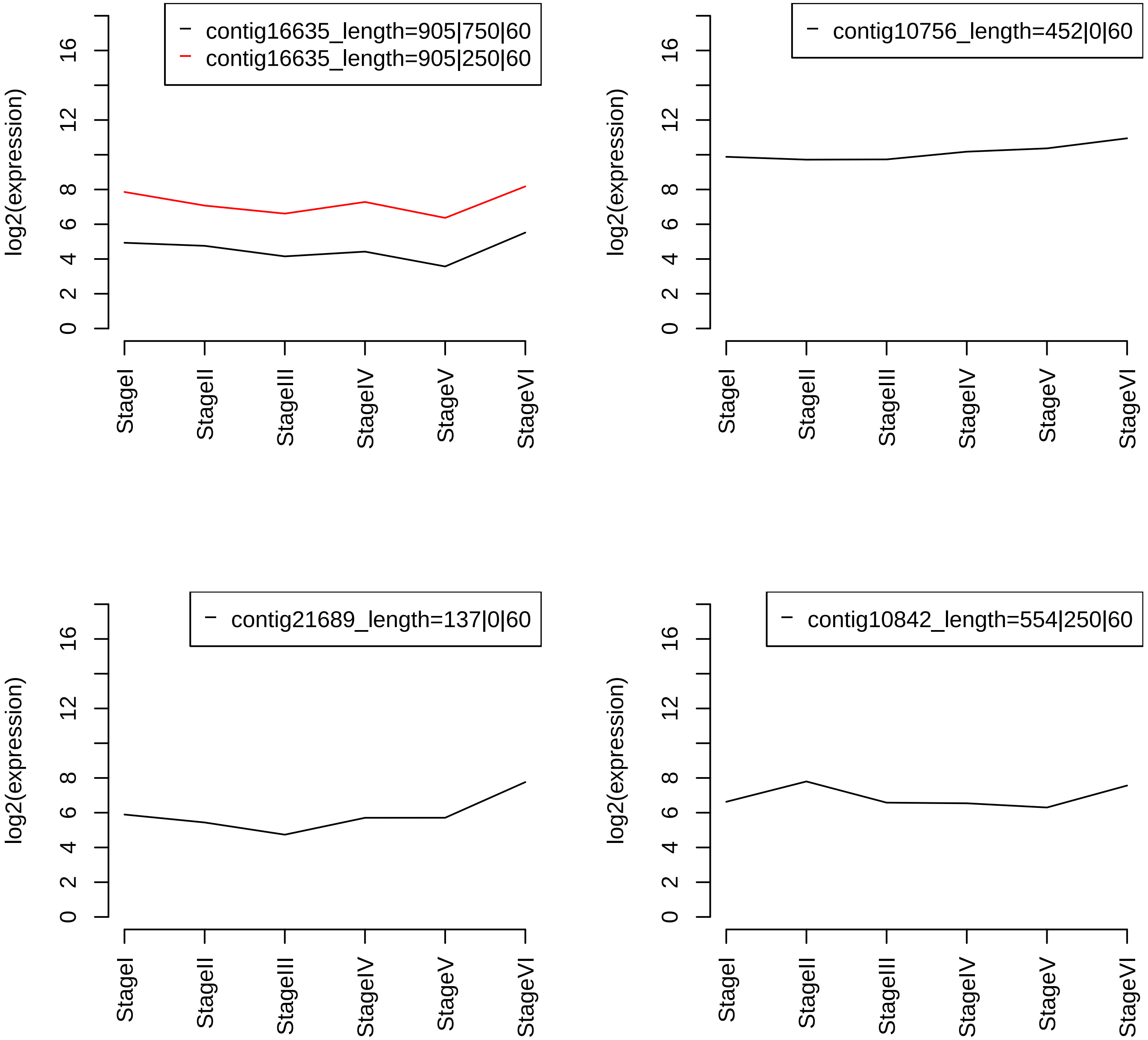
contig16635_length=905|750|60
contig16635_length=905|250|60
contig10756_length=452|0|60
contig21689_length=137|0|60
contig10842_length=554|250|60
log2(expression)
0
2
4
6
8
12
16
StageI
StageII
StageIII
StageIV
StageV
StageVI

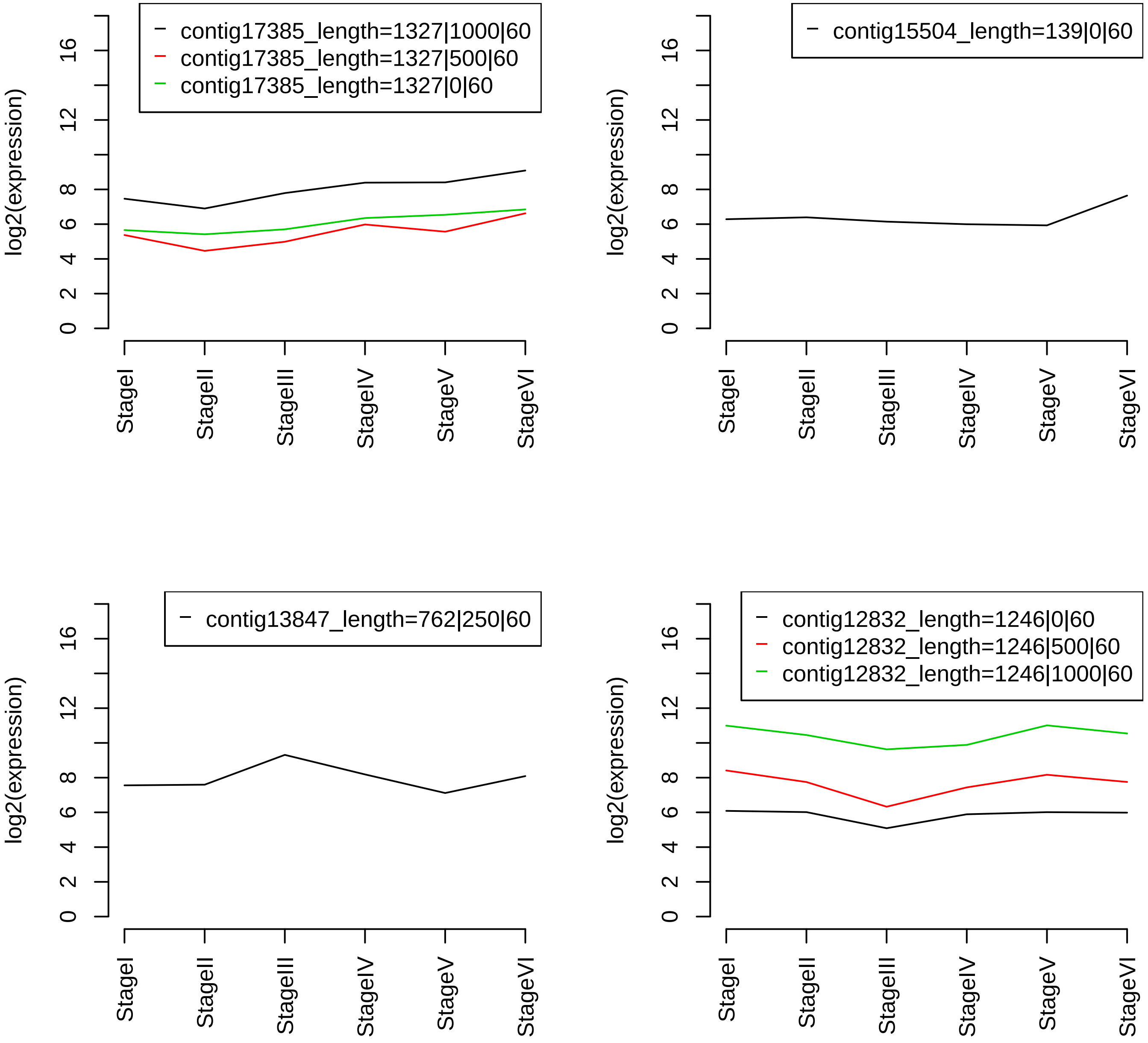

contig17385_length=1327|1000|60
contig17385_length=1327|500|60
contig17385_length=1327|0|60
contig15504_length=139|0|60
contig13847_length=762|250|60
contig12832_length=1246|0|60
contig12832_length=1246|500|60
contig12832_length=1246|1000|60
log2(expression)
0 2 4 6 8 12 16
StageI StageII StageIII StageIV StageV StageVI

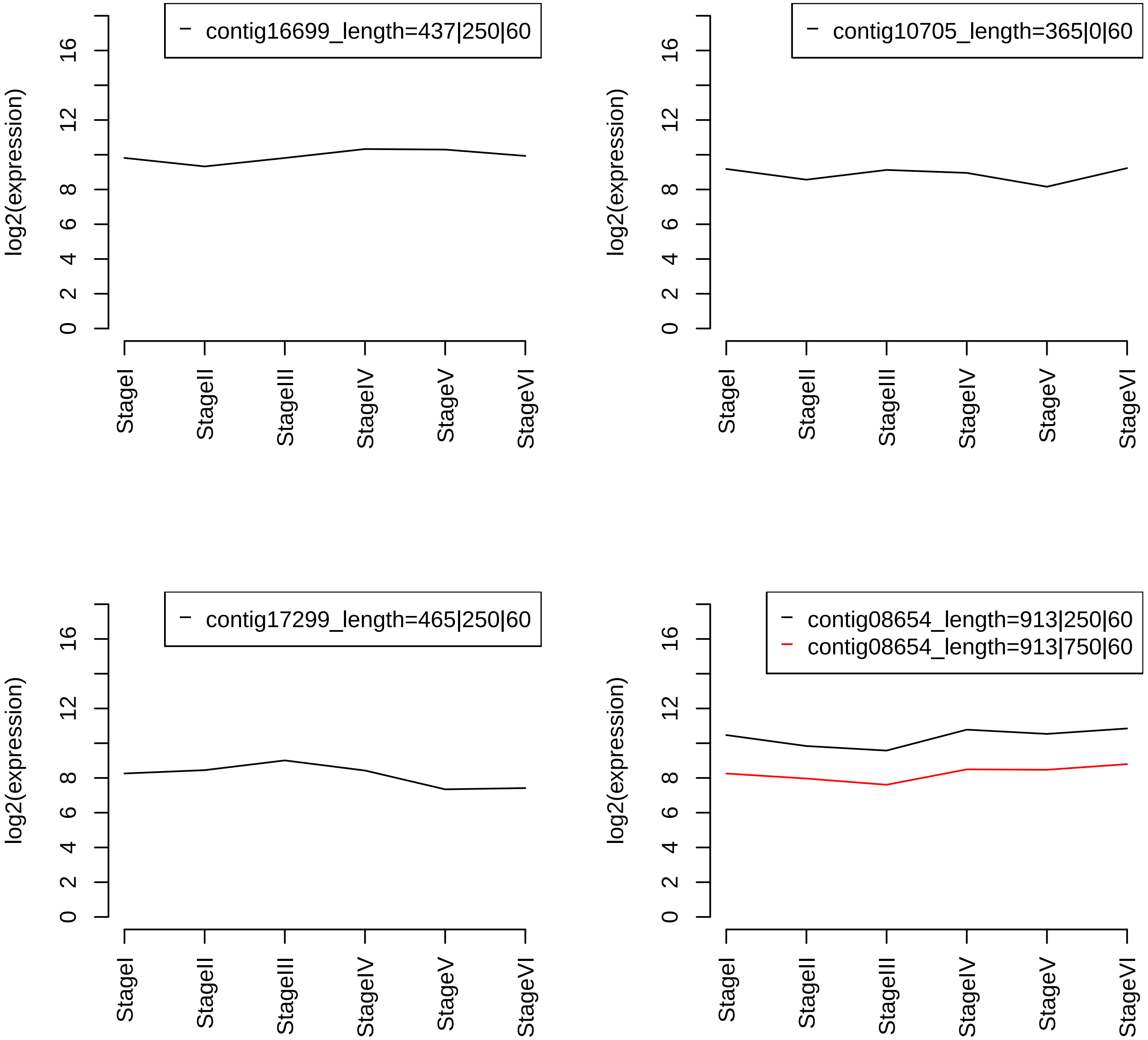
contig16699_length=437|250|60
contig10705_length=365|0|60
contig17299_length=465|250|60
contig08654_length=913|250|60
contig08654_length=913|750|60
log2(expression)
0
2
4
6
8
12
16
StageI
StageII
StageIII
StageIV
StageV
StageVI

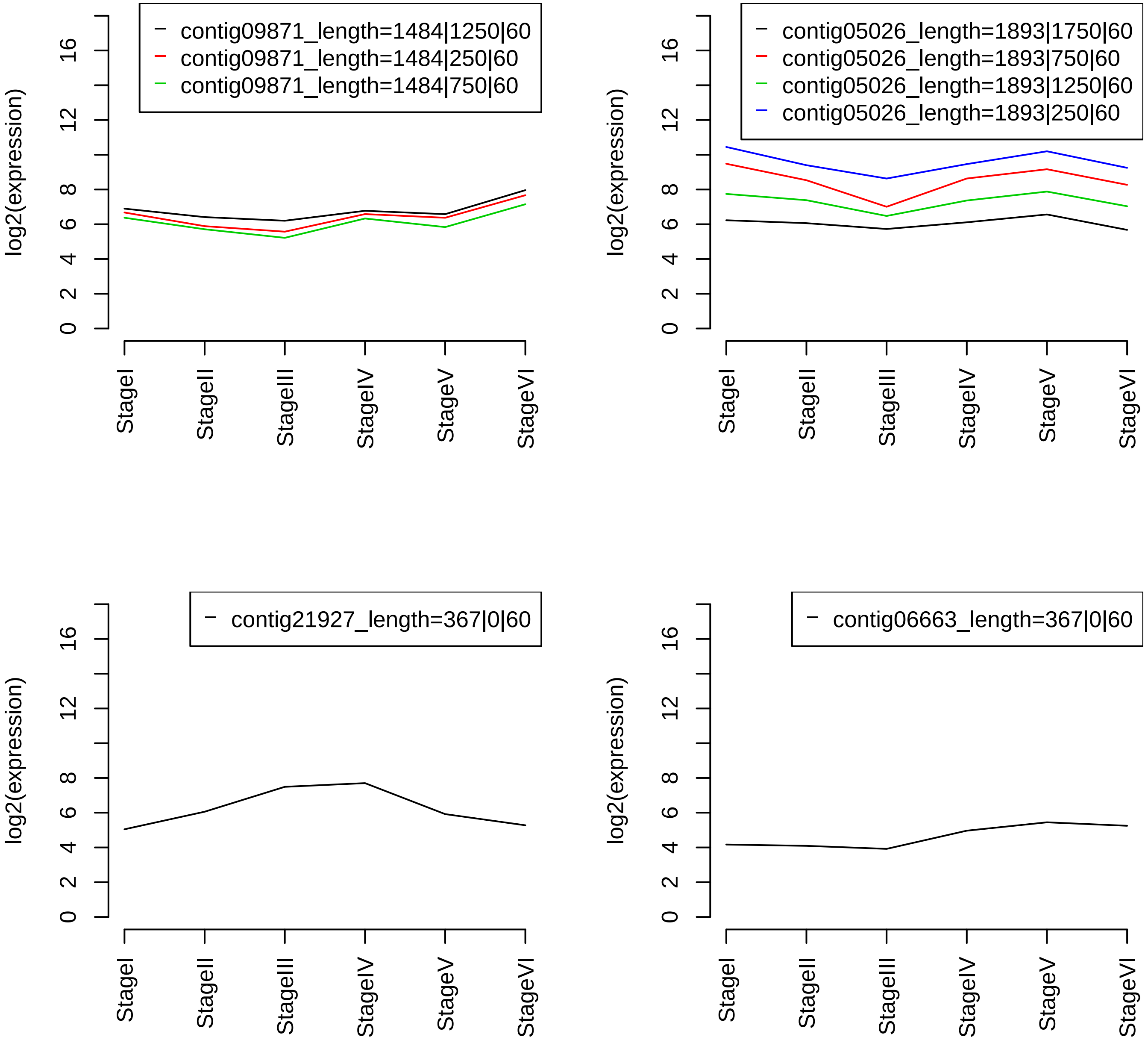

contig09871_length=1484|1250|60
contig09871_length=1484|250|60
contig09871_length=1484|750|60
contig05026_length=1893|1750|60
contig05026_length=1893|750|60
contig05026_length=1893|1250|60
contig05026_length=1893|250|60
contig21927_length=367|0|60
contig06663_length=367|0|60
log2(expression)
0 2 4 6 8 12 16
StageI StageII StageIII StageIV StageV StageVI

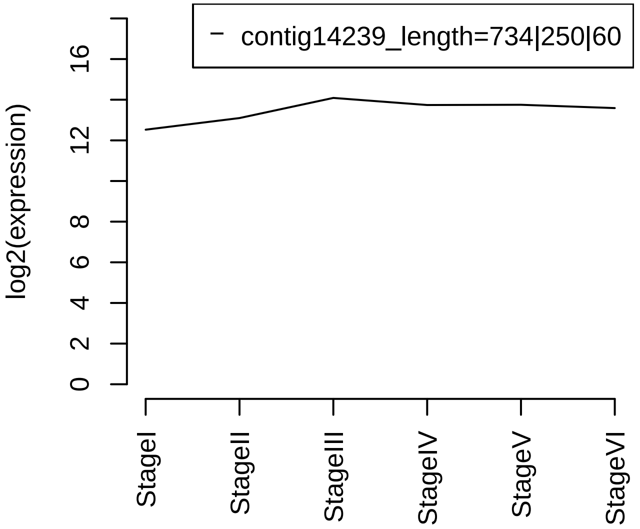
contig14239_length=734|250|60
log2(expression)
0
2
4
6
8
12
16
StageI
StageII
StageIII
StageIV
StageV
StageVI
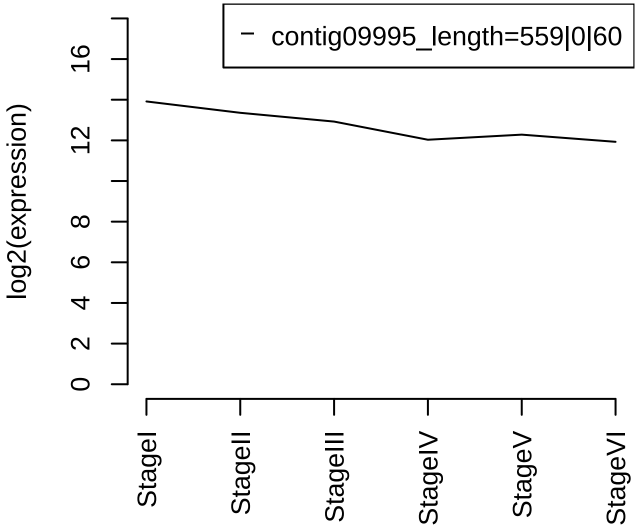
contig09995_length=559|0|60
log2(expression)
0
2
4
6
8
12
16
StageI
StageII
StageIII
StageIV
StageV
StageVI
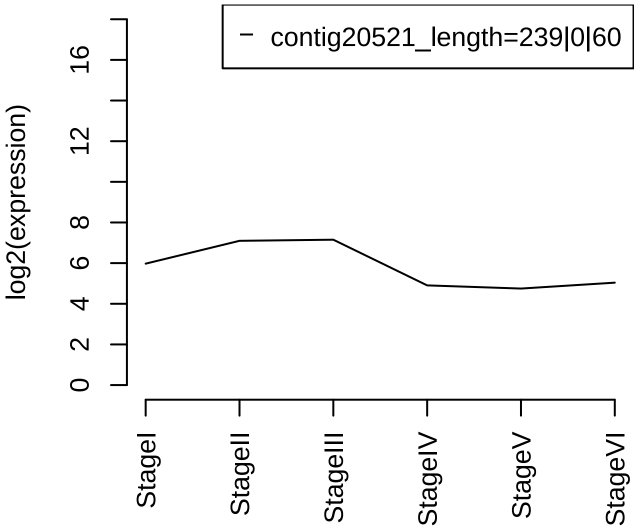
contig20521_length=239|0|60
log2(expression)
0
2
4
6
8
12
16
StageI
StageII
StageIII
StageIV
StageV
StageVI
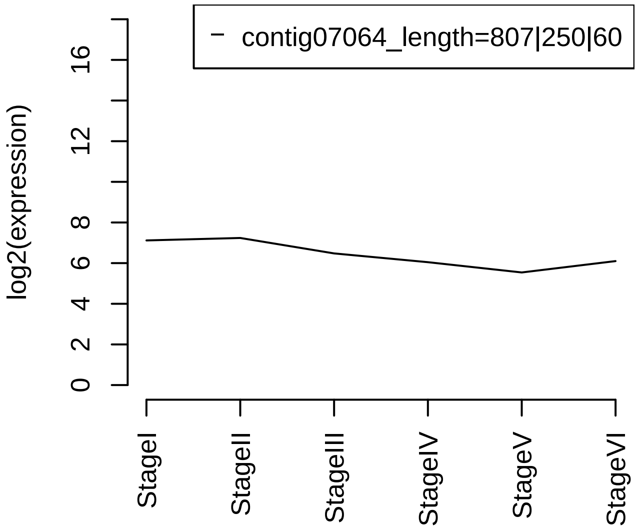
contig07064_length=807|250|60
log2(expression)
0
2
4
6
8
12
16
StageI
StageII
StageIII
StageIV
StageV
StageVI

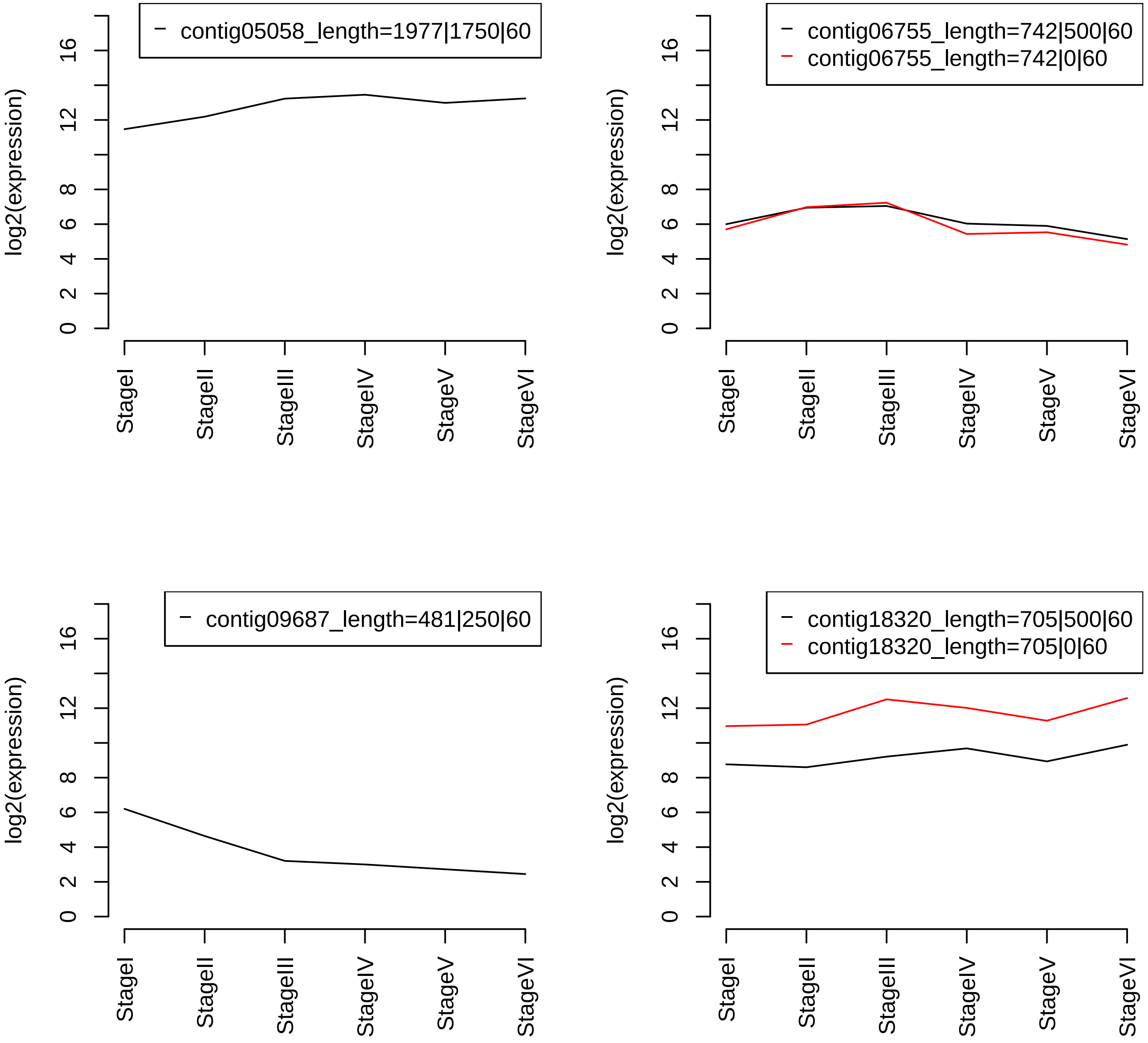

contig05058_length=1977|1750|60
contig06755_length=742|500|60
contig06755_length=742|0|60
contig09687_length=481|250|60
contig18320_length=705|500|60
contig18320_length=705|0|60
log2(expression)
0
2
4
6
8
12
16
StageI
StageII
StageIII
StageIV
StageV
StageVI

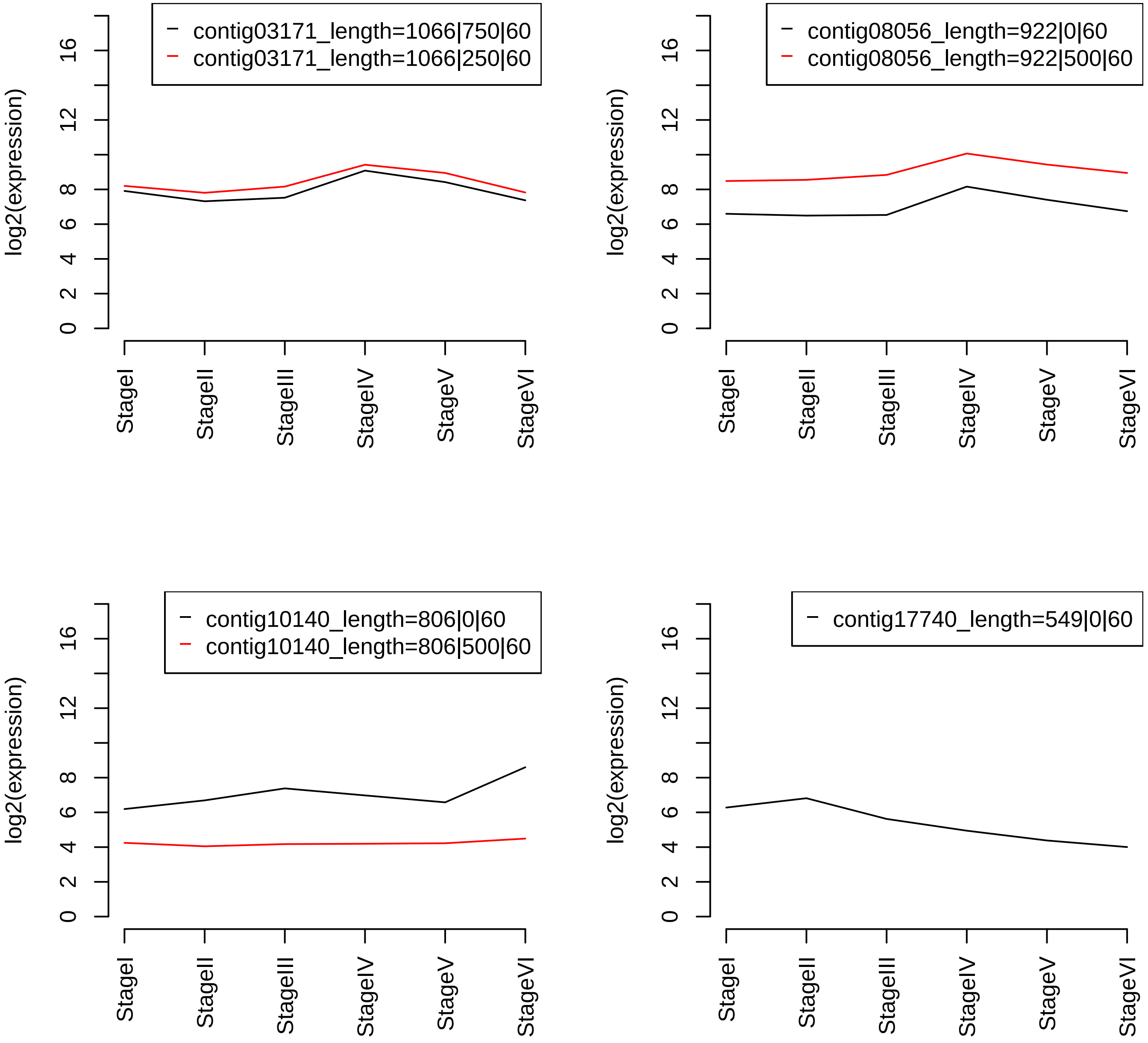

contig03171_length=1066|750|60
contig03171_length=1066|250|60
contig08056_length=922|0|60
contig08056_length=922|500|60
contig10140_length=806|0|60
contig10140_length=806|500|60
contig17740_length=549|0|60
log2(expression)
0
2
4
6
8
12
16
StageI
StageII
StageIII
StageIV
StageV
StageVI

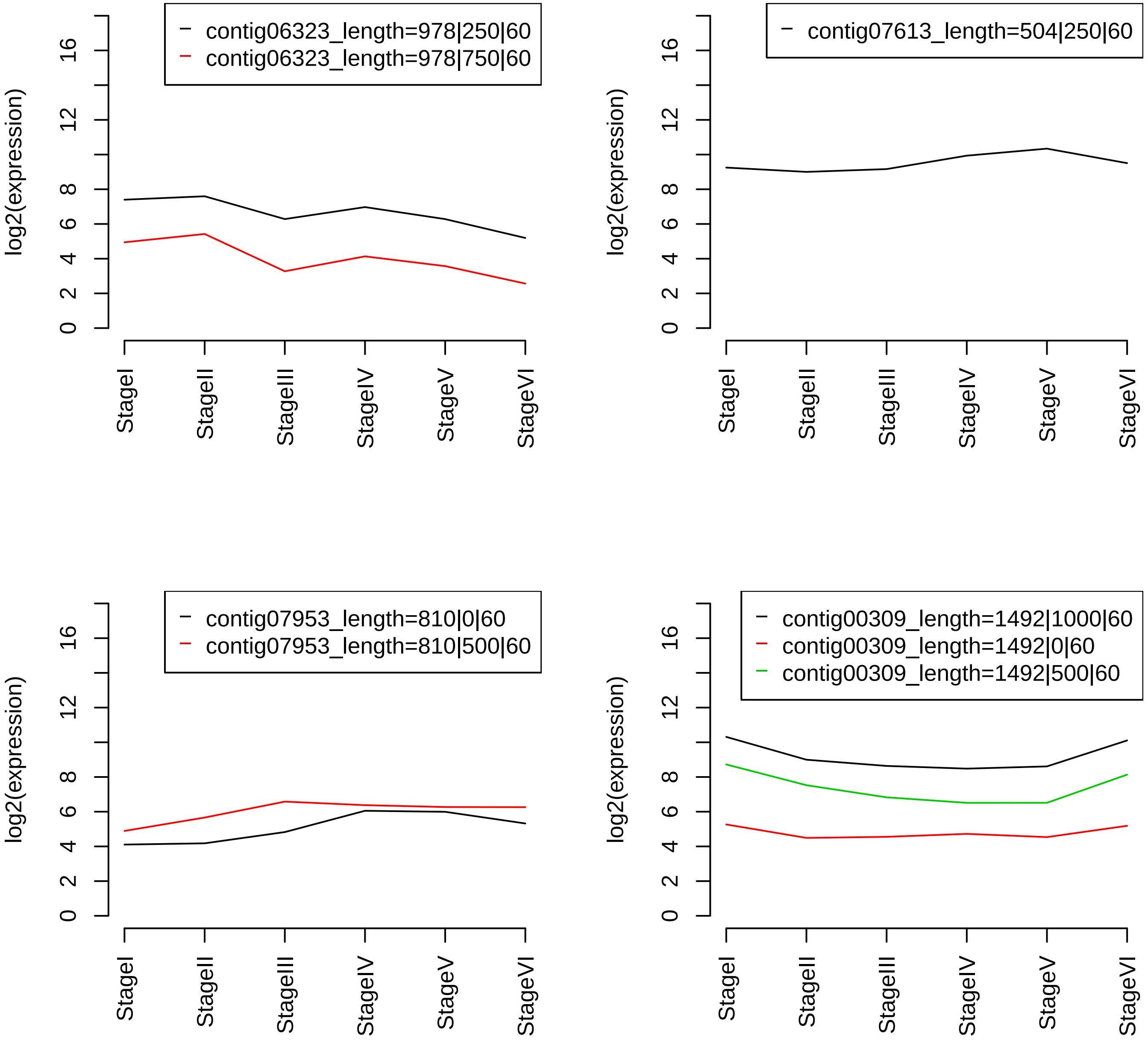

contig06323_length=978|250|60
contig06323_length=978|750|60
contig07613_length=504|250|60
contig07953_length=810|0|60
contig07953_length=810|500|60
contig00309_length=1492|1000|60
contig00309_length=1492|0|60
contig00309_length=1492|500|60
log2(expression)
0 2 4 6 8 12 16
StageI StageII StageIII StageIV StageV StageVI

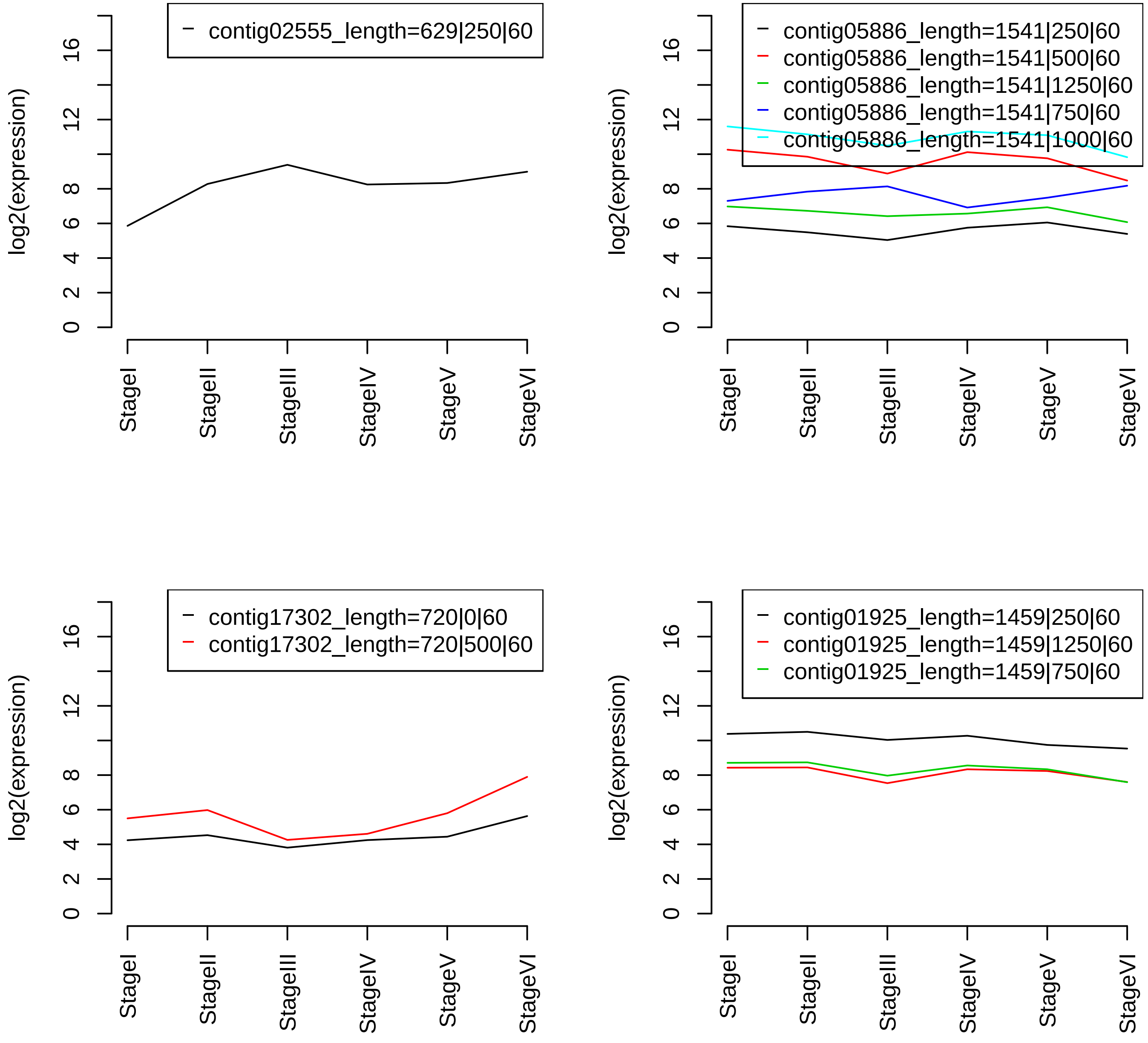

contig02555_length=629|250|60
contig05886_length=1541|250|60
contig05886_length=1541|500|60
contig05886_length=1541|1250|60
contig05886_length=1541|750|60
contig05886_length=1541|1000|60
contig17302_length=720|0|60
contig17302_length=720|500|60
contig01925_length=1459|250|60
contig01925_length=1459|1250|60
contig01925_length=1459|750|60
log2(expression)
0
2
4
6
8
12
16
StageI
StageII
StageIII
StageIV
StageV
StageVI

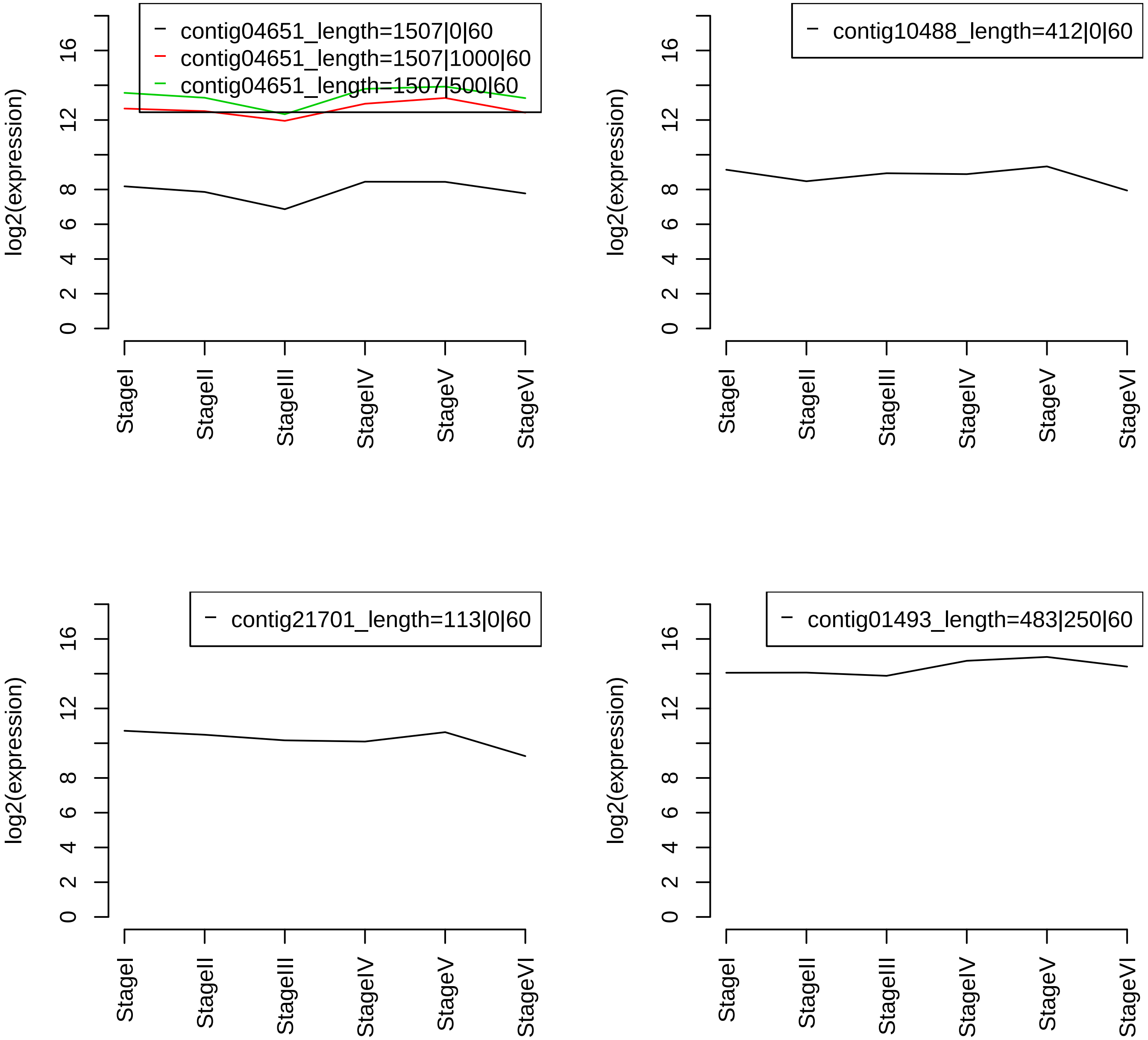

contig04651_length=1507|0|60
contig04651_length=1507|1000|60
contig04651_length=1507|500|60
contig10488_length=412|0|60
contig21701_length=113|0|60
contig01493_length=483|250|60
log2(expression)
0
2
4
6
8
12
16
StageI
StageII
StageIII
StageIV
StageV
StageVI

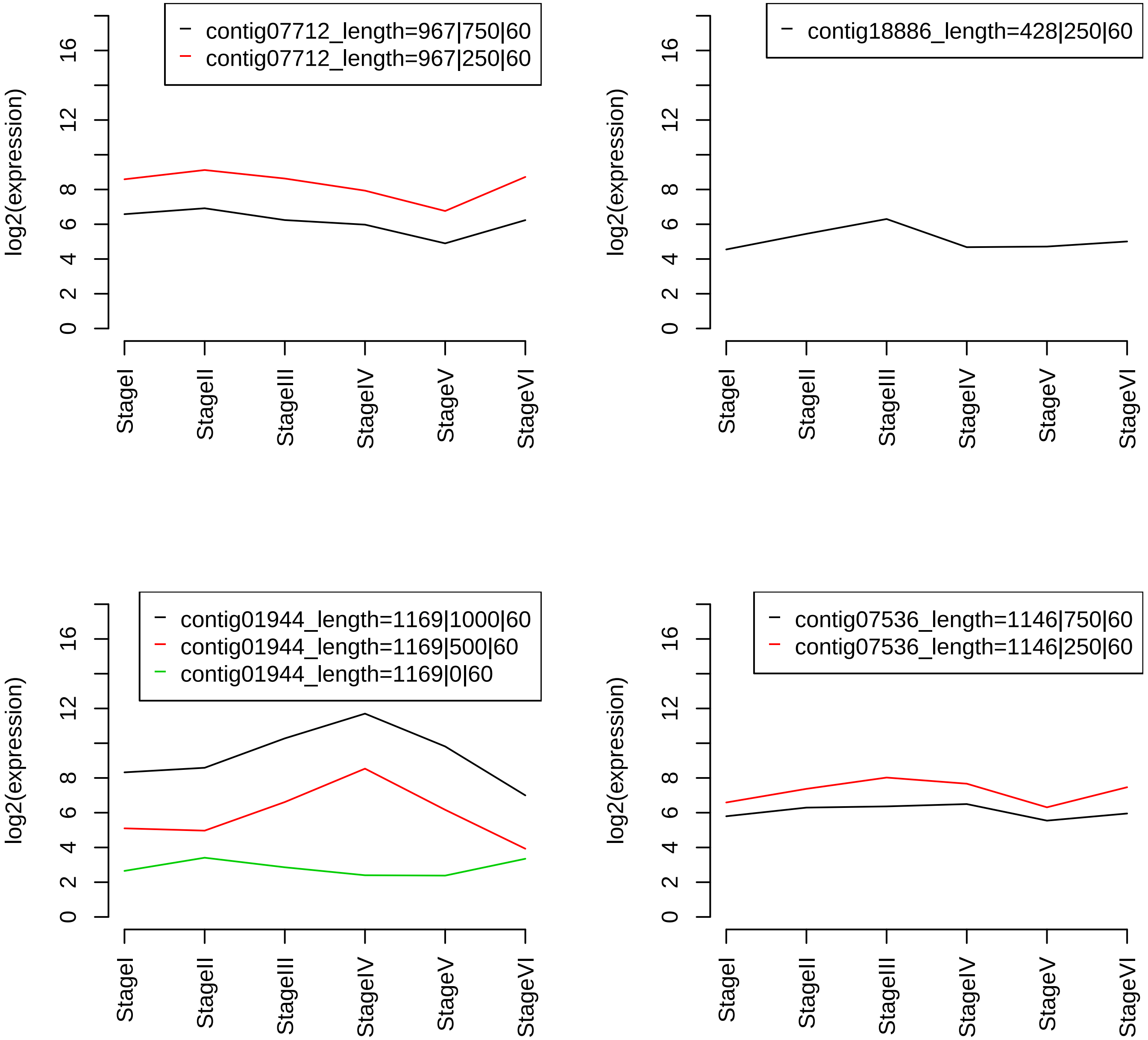

contig07712_length=967|750|60
contig07712_length=967|250|60
contig18886_length=428|250|60
contig01944_length=1169|1000|60
contig01944_length=1169|500|60
contig01944_length=1169|0|60
contig07536_length=1146|750|60
contig07536_length=1146|250|60
log2(expression)
0
2
4
6
8
12
16
StageI
StageII
StageIII
StageIV
StageV
StageVI

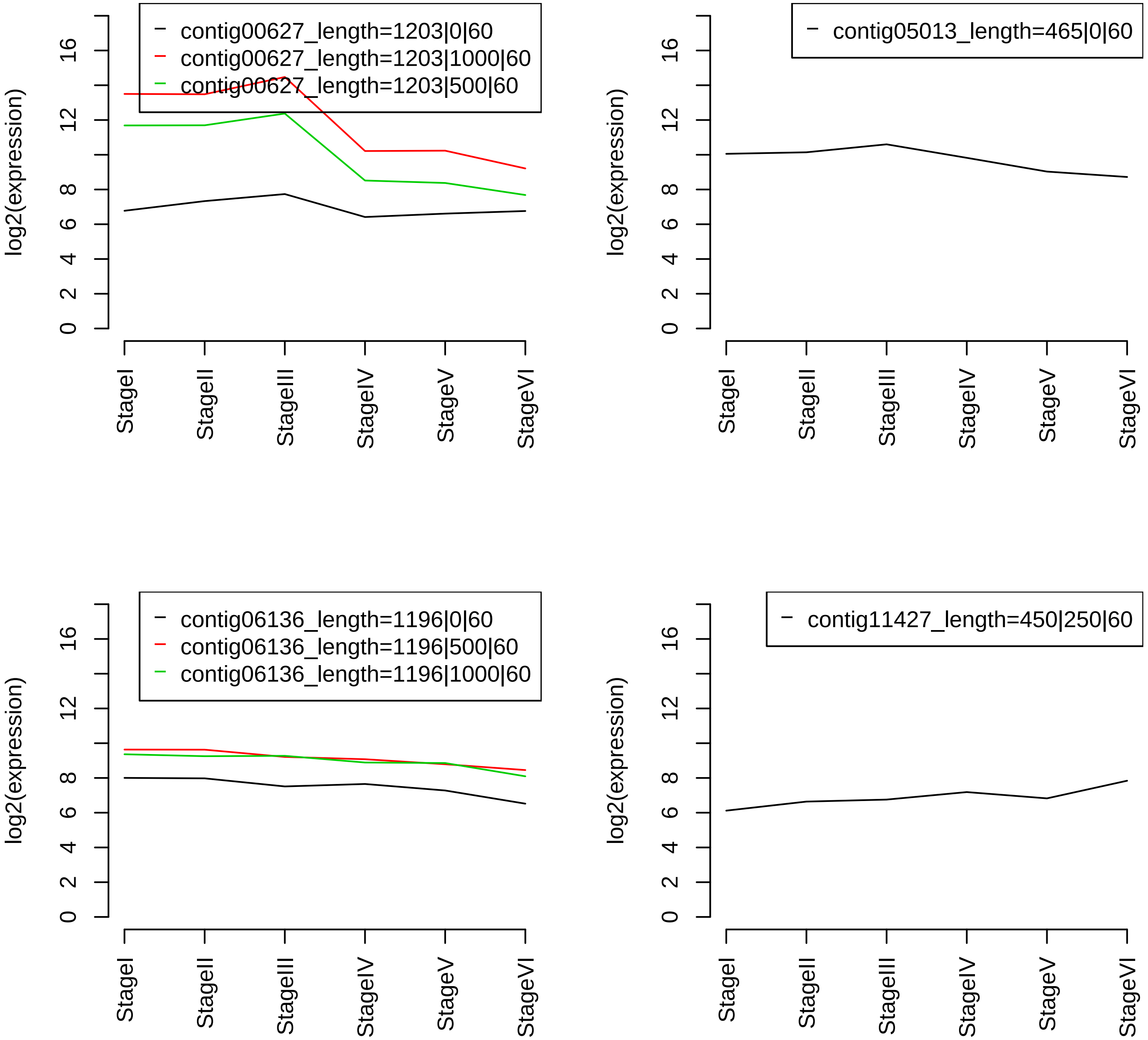

contig00627_length=1203|0|60
contig00627_length=1203|1000|60
contig00627_length=1203|500|60
contig05013_length=465|0|60
contig06136_length=1196|0|60
contig06136_length=1196|500|60
contig06136_length=1196|1000|60
contig11427_length=450|250|60
log2(expression)
0
2
4
6
8
12
16
StageI
StageII
StageIII
StageIV
StageV
StageVI

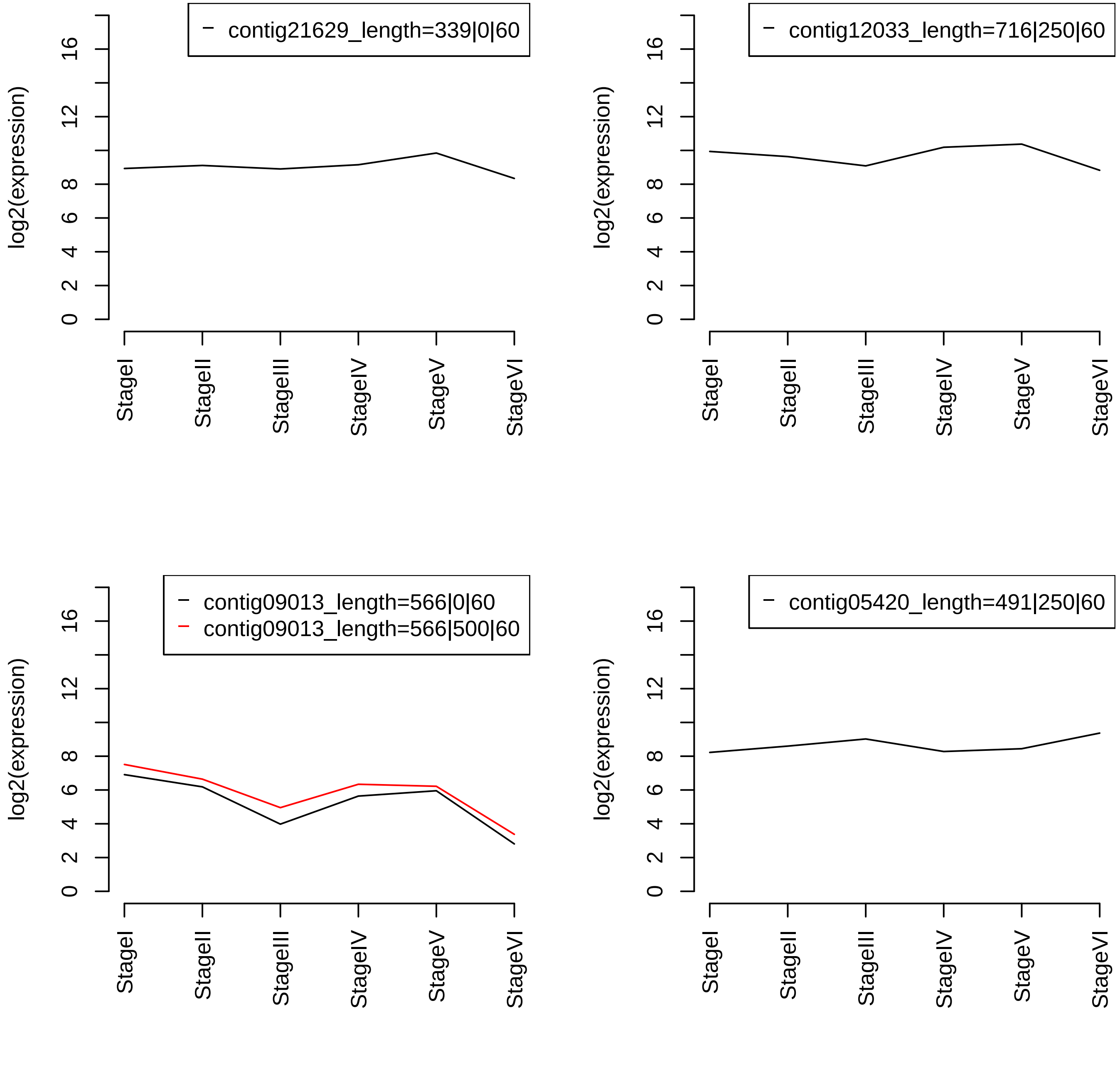
contig21629_length=339|0|60
contig12033_length=716|250|60
contig09013_length=566|0|60
contig09013_length=566|500|60
contig05420_length=491|250|60
log2(expression)
0 2 4 6 8 12 16
StageI StageII StageIII StageIV StageV StageVI

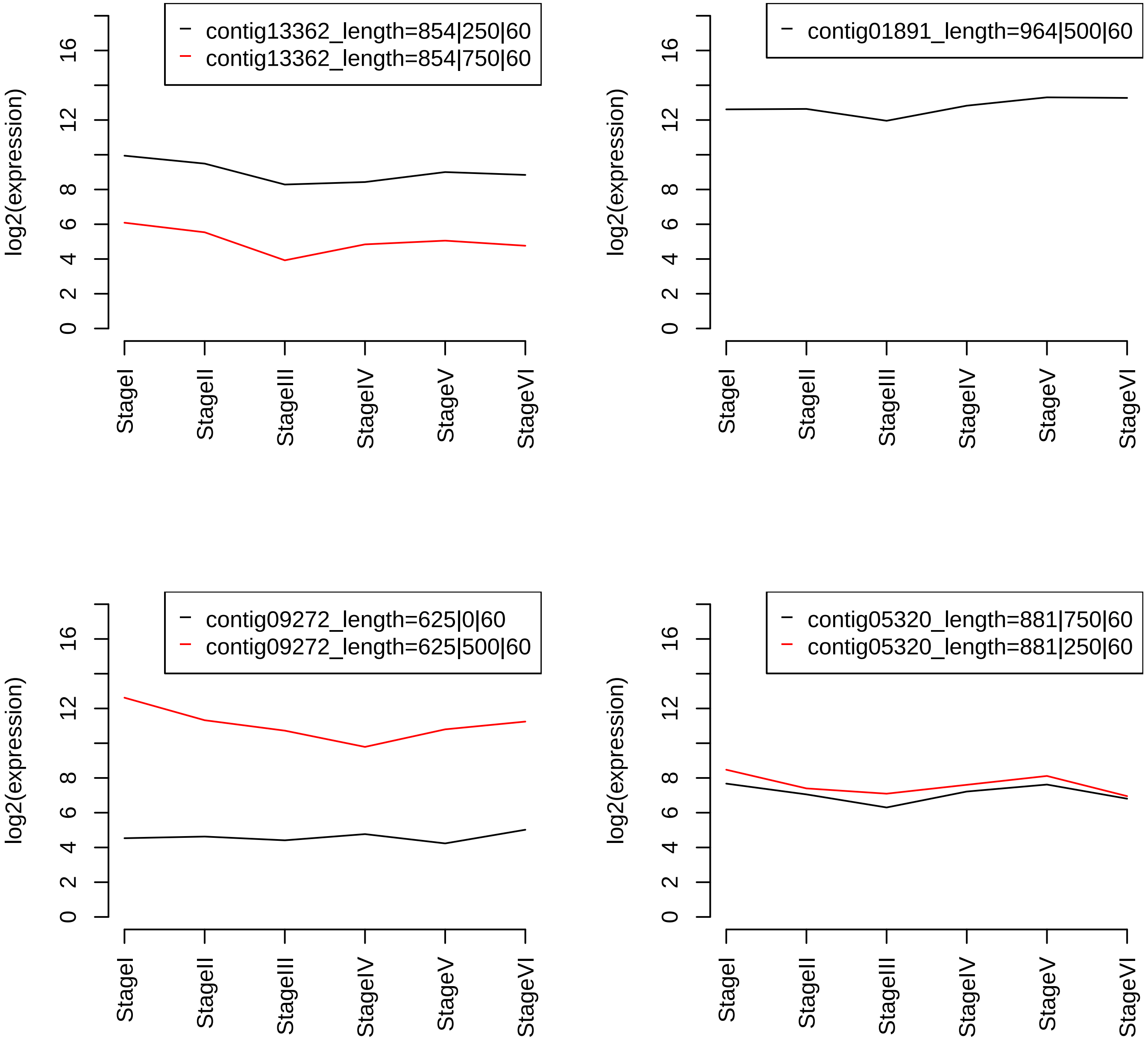

contig13362_length=854|250|60
contig13362_length=854|750|60
contig01891_length=964|500|60
contig09272_length=625|0|60
contig09272_length=625|500|60
contig05320_length=881|750|60
contig05320_length=881|250|60
log2(expression)
0
2
4
6
8
12
16
StageI
StageII
StageIII
StageIV
StageV
StageVI

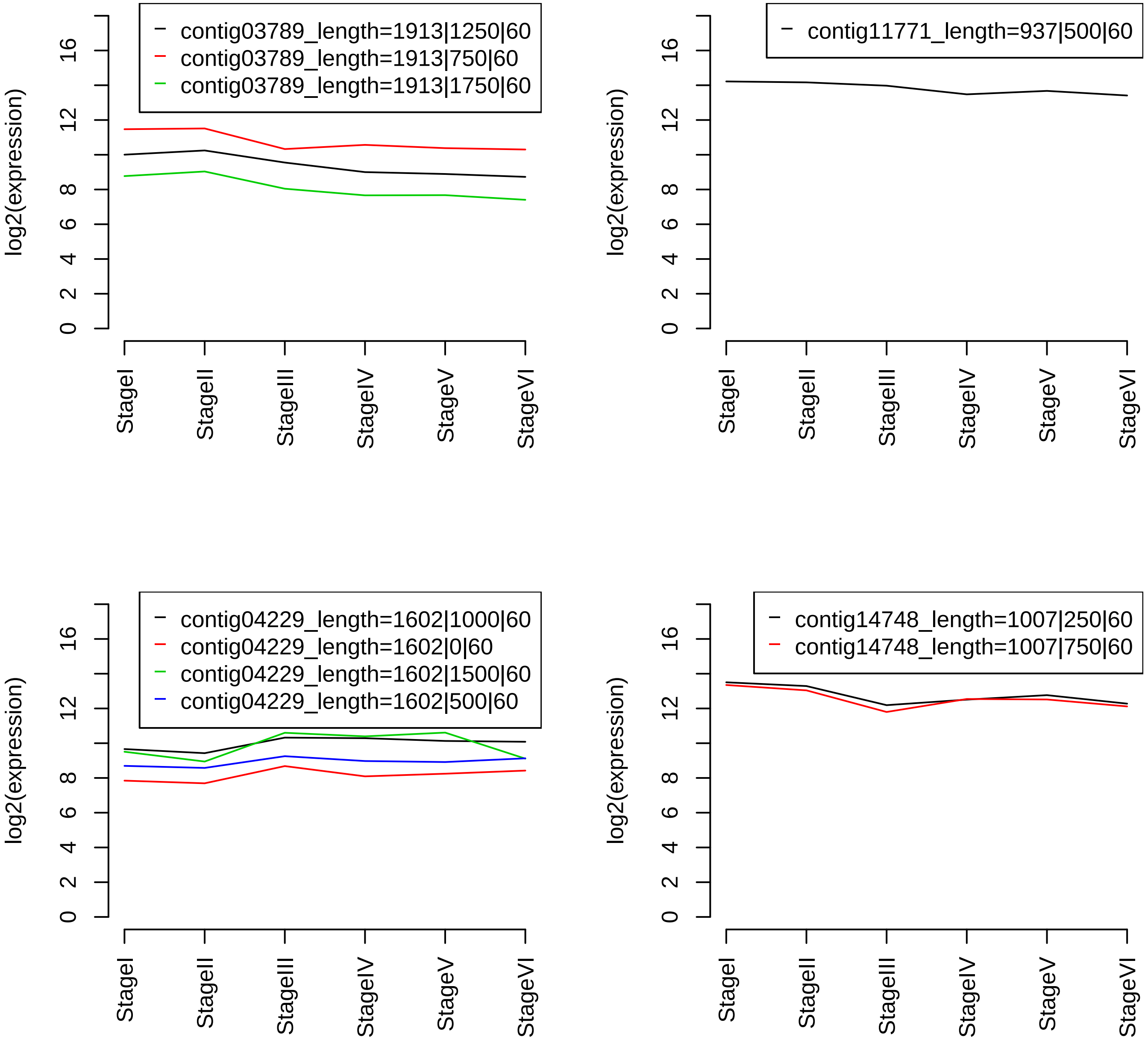

contig03789_length=1913|1250|60
contig03789_length=1913|750|60
contig03789_length=1913|1750|60
contig11771_length=937|500|60
contig04229_length=1602|1000|60
contig04229_length=1602|0|60
contig04229_length=1602|1500|60
contig04229_length=1602|500|60
contig14748_length=1007|250|60
contig14748_length=1007|750|60
log2(expression)
0
2
4
6
8
12
16
StageI
StageII
StageIII
StageIV
StageV
StageVI

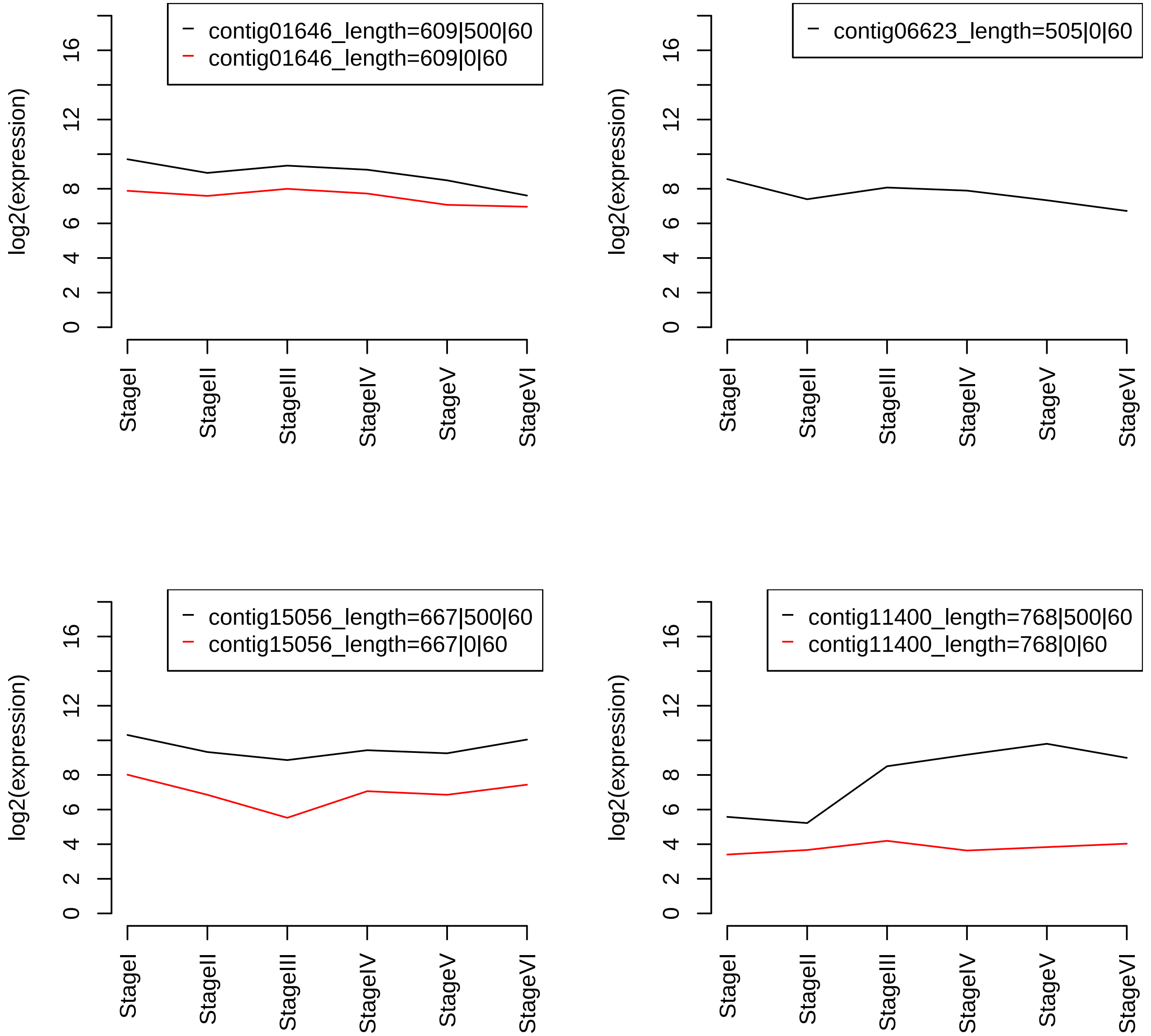
contig01646_length=609|500|60
contig01646_length=609|0|60
log2(expression)
0
2
4
6
8
12
16
StageI
StageII
StageIII
StageIV
StageV
StageVI
contig06623_length=505|0|60
log2(expression)
0
2
4
6
8
12
16
StageI
StageII
StageIII
StageIV
StageV
StageVI
contig15056_length=667|500|60
contig15056_length=667|0|60
log2(expression)
0
2
4
6
8
12
16
StageI
StageII
StageIII
StageIV
StageV
StageVI
contig11400_length=768|500|60
contig11400_length=768|0|60
log2(expression)
0
2
4
6
8
12
16
StageI
StageII
StageIII
StageIV
StageV
StageVI

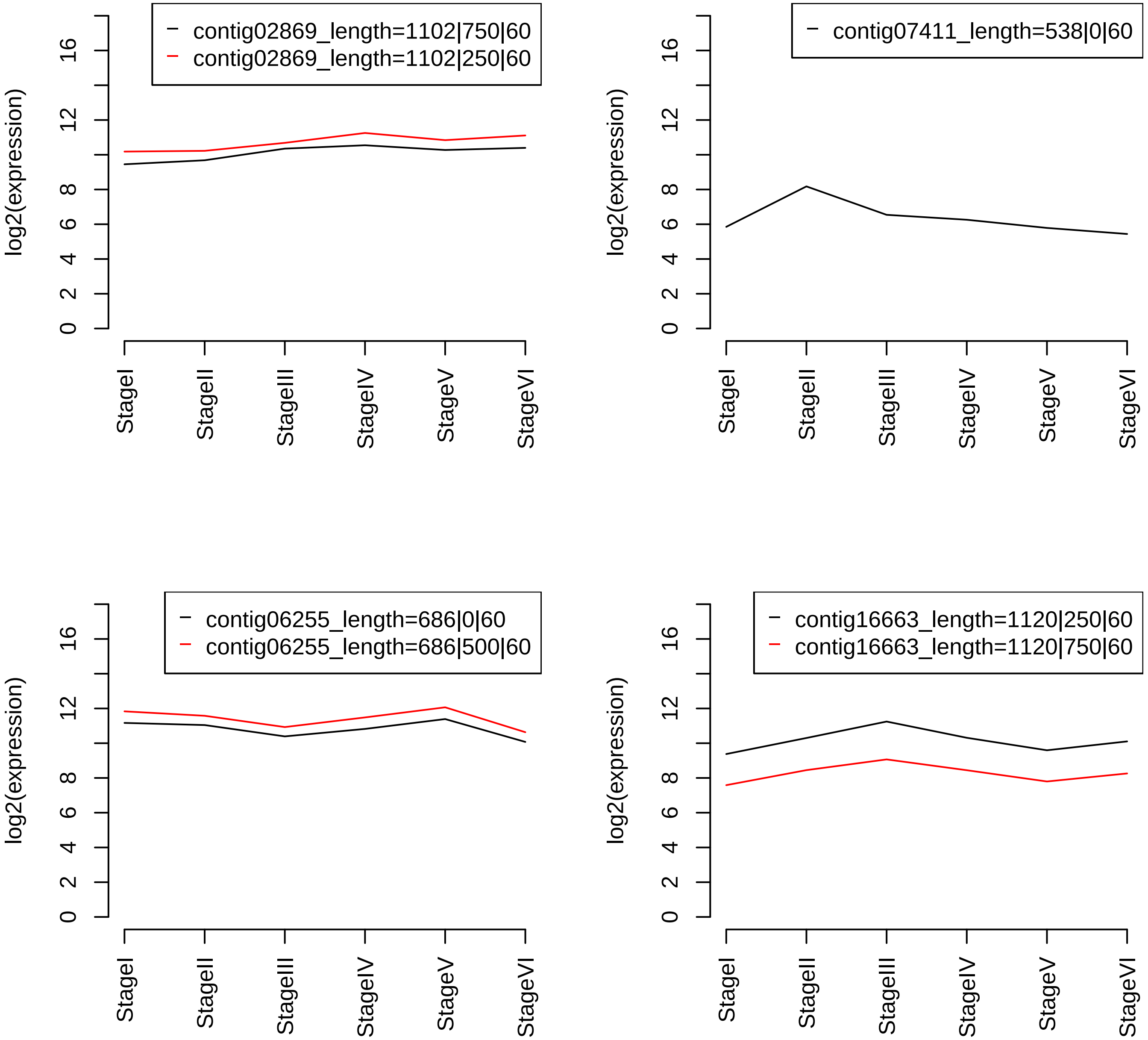
contig02869_length=1102|750|60
contig02869_length=1102|250|60
contig07411_length=538|0|60
contig06255_length=686|0|60
contig06255_length=686|500|60
contig16663_length=1120|250|60
contig16663_length=1120|750|60
log2(expression)
StageI
StageII
StageIII
StageIV
StageV
StageVI

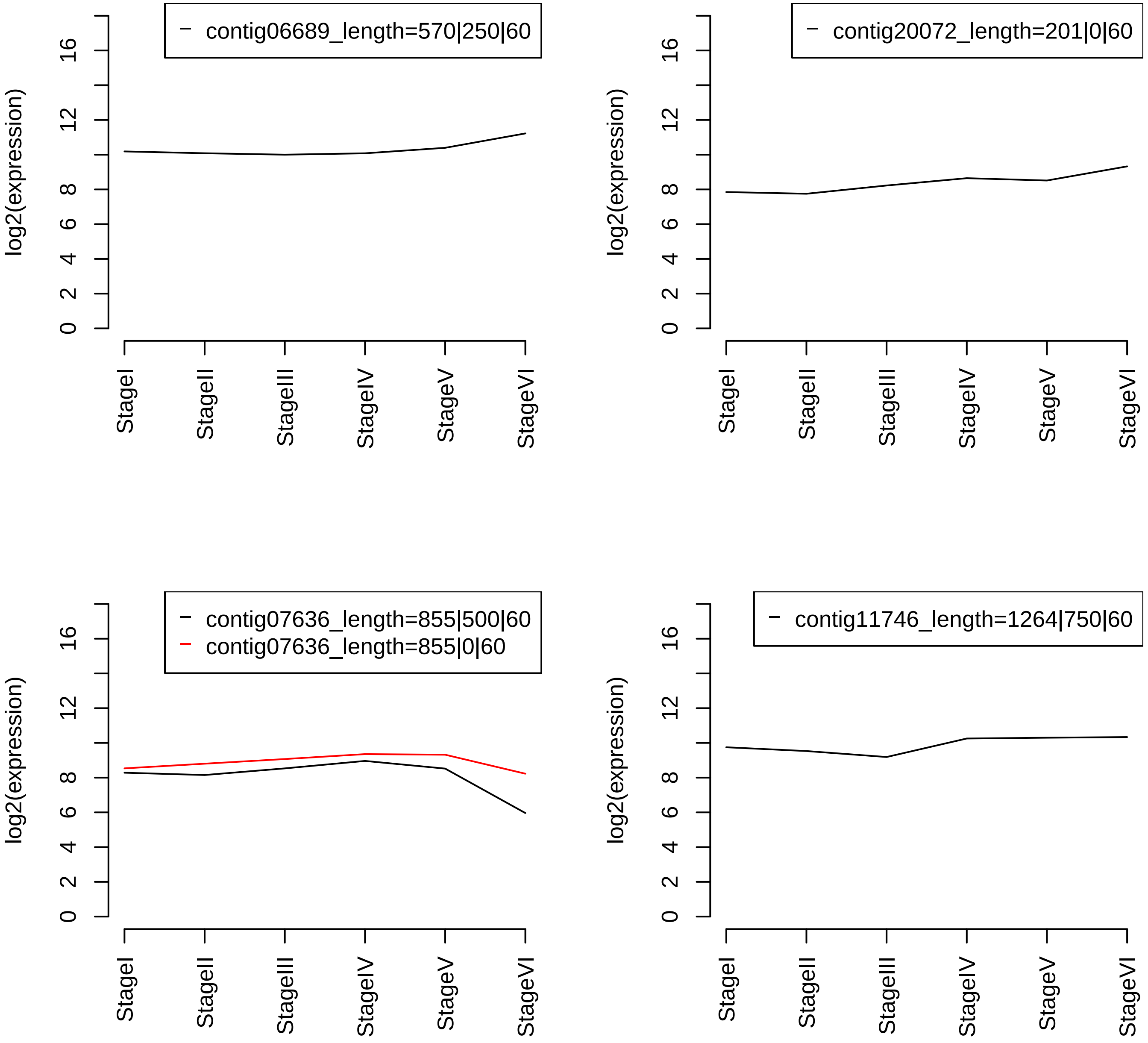

contig06689_length=570|250|60
contig20072_length=201|0|60
contig07636_length=855|500|60
contig07636_length=855|0|60
contig11746_length=1264|750|60
log2(expression)
0
2
4
6
8
12
16
StageI
StageII
StageIII
StageIV
StageV
StageVI

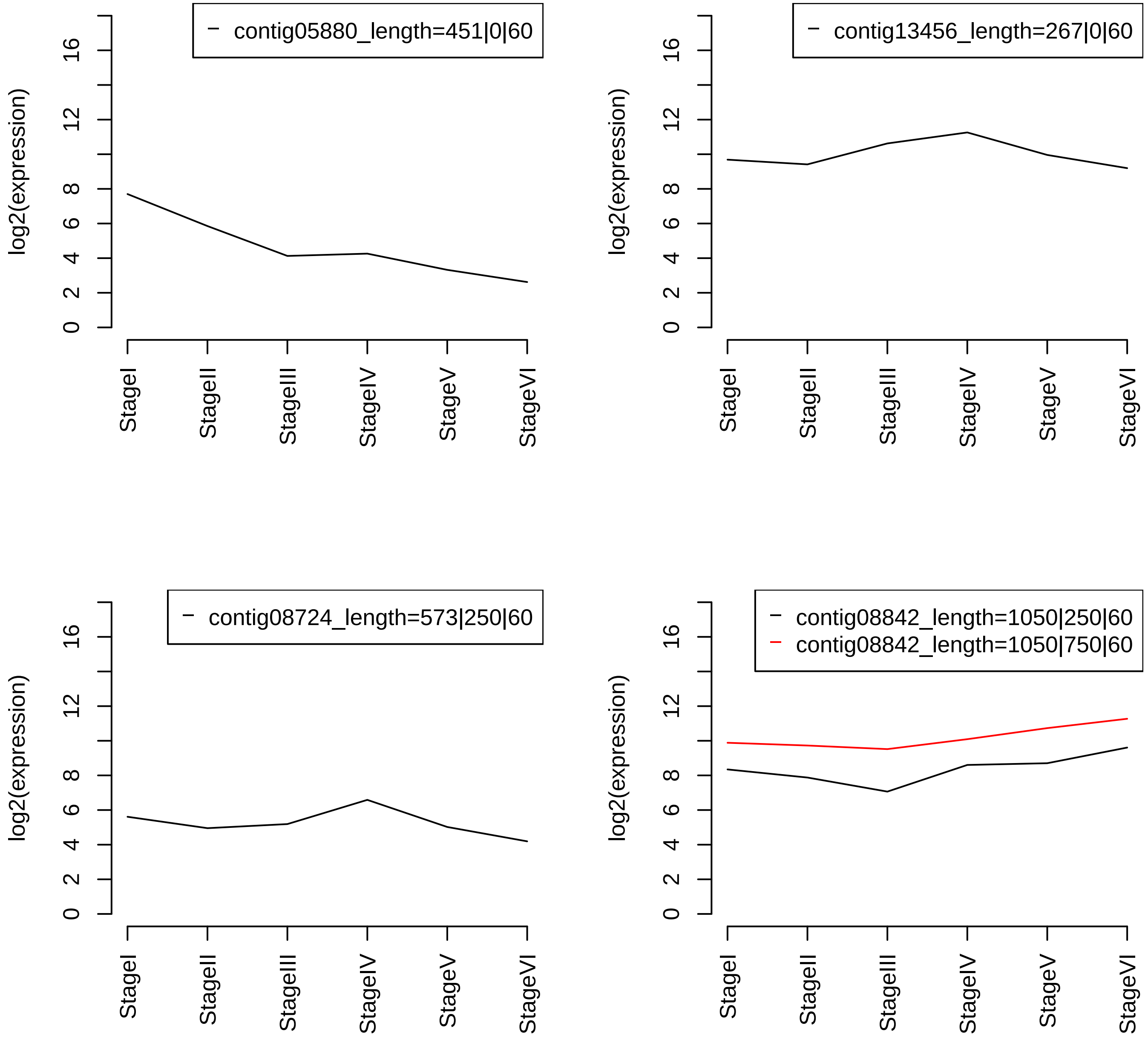

contig05880_length=451|0|60
contig13456_length=267|0|60
contig08724_length=573|250|60
contig08842_length=1050|250|60
contig08842_length=1050|750|60
log2(expression)
0
2
4
6
8
12
16
StageI
StageII
StageIII
StageIV
StageV
StageVI

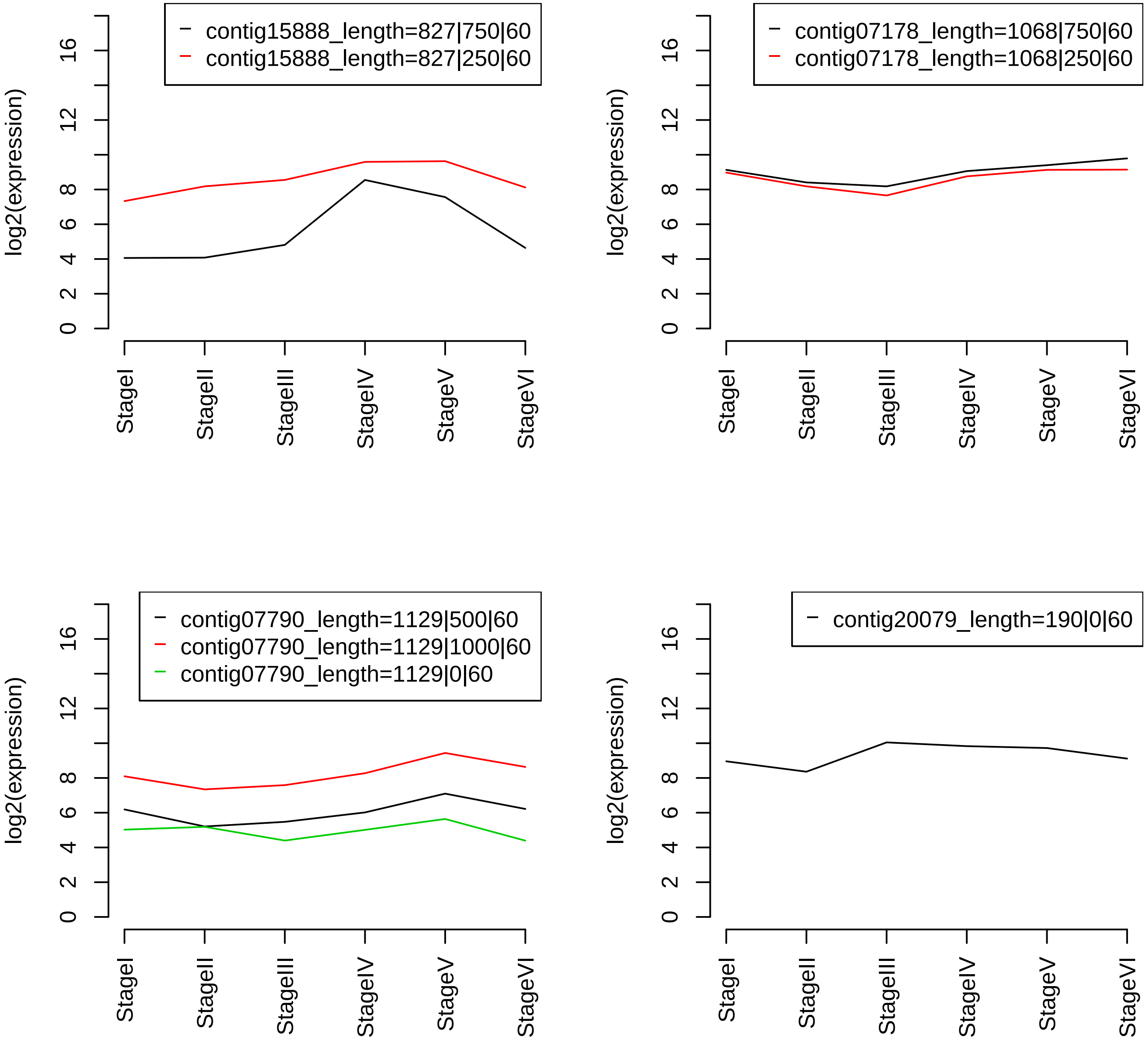
contig15888_length=827|750|60
contig15888_length=827|250|60
contig07178_length=1068|750|60
contig07178_length=1068|250|60
contig07790_length=1129|500|60
contig07790_length=1129|1000|60
contig07790_length=1129|0|60
contig20079_length=190|0|60
log2(expression)
0
2
4
6
8
12
16
StageI
StageII
StageIII
StageIV
StageV
StageVI

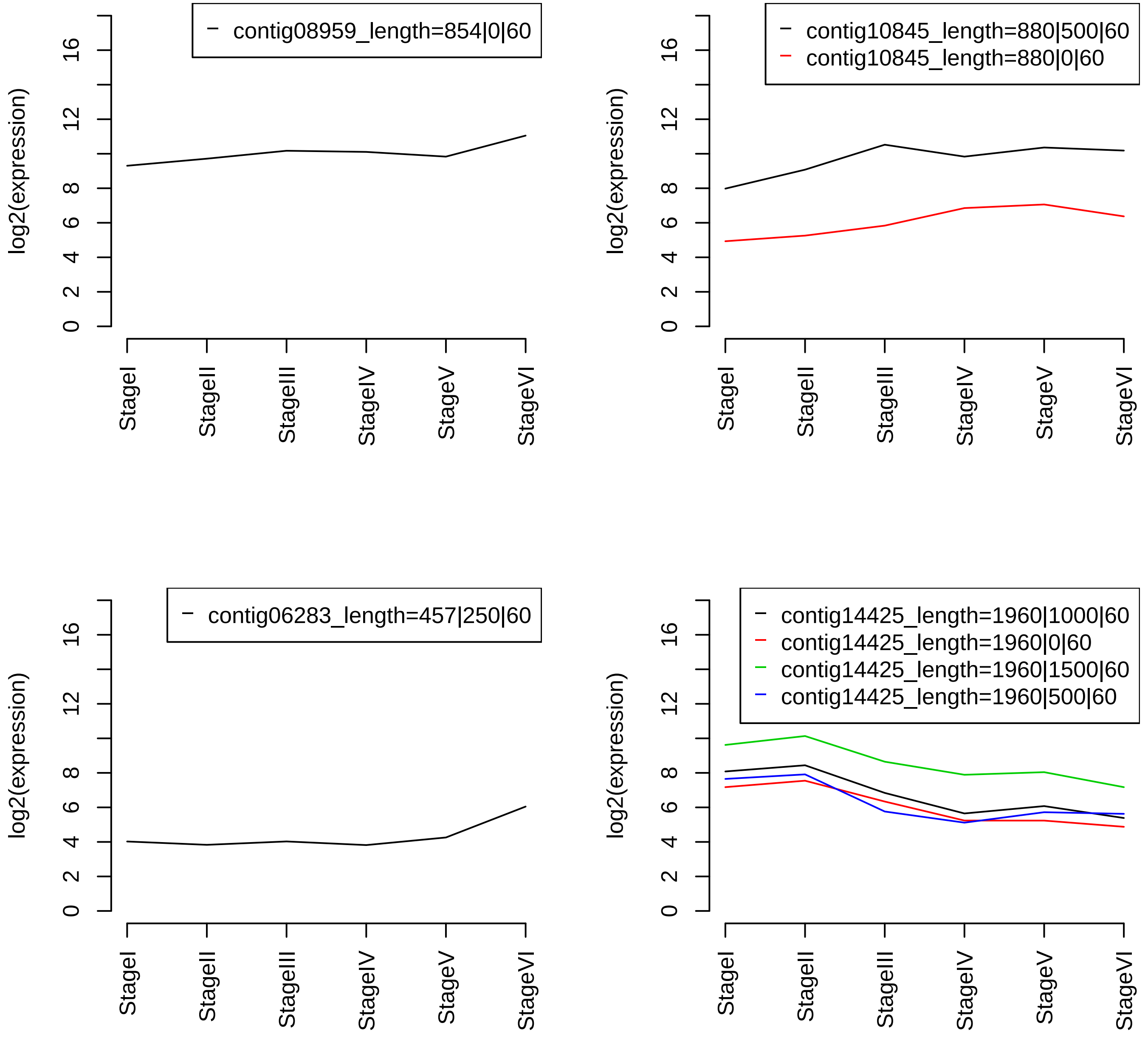
contig08959_length=854|0|60
contig10845_length=880|500|60
contig10845_length=880|0|60
contig06283_length=457|250|60
contig14425_length=1960|1000|60
contig14425_length=1960|0|60
contig14425_length=1960|1500|60
contig14425_length=1960|500|60
log2(expression)
0
2
4
6
8
12
16
StageI
StageII
StageIII
StageIV
StageV
StageVI

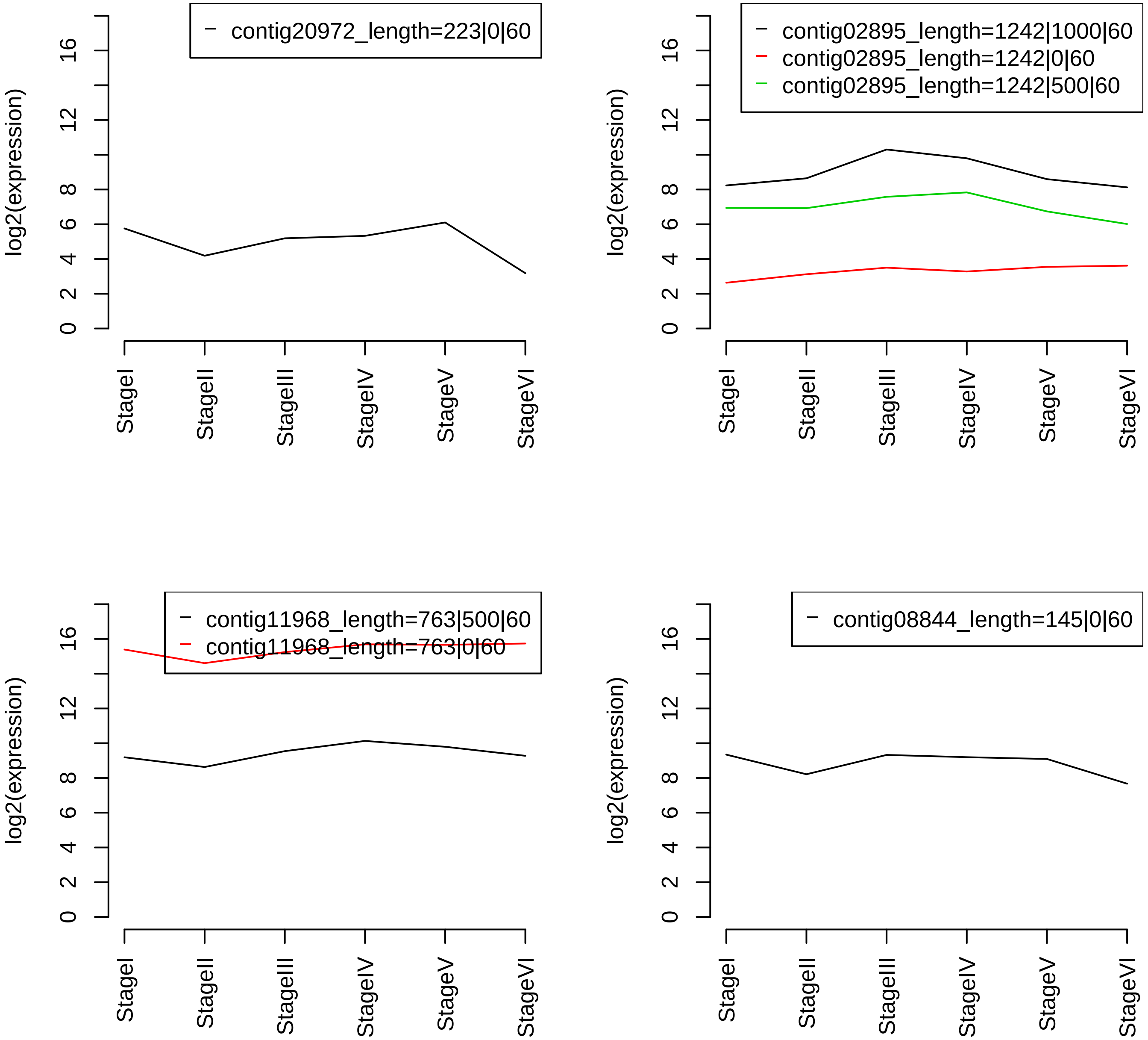
contig20972_length=223|0|60
contig02895_length=1242|1000|60
contig02895_length=1242|0|60
contig02895_length=1242|500|60
contig11968_length=763|500|60
contig11968_length=763|0|60
contig08844_length=145|0|60
log2(expression)
0
2
4
6
8
12
16
StageI
StageII
StageIII
StageIV
StageV
StageVI

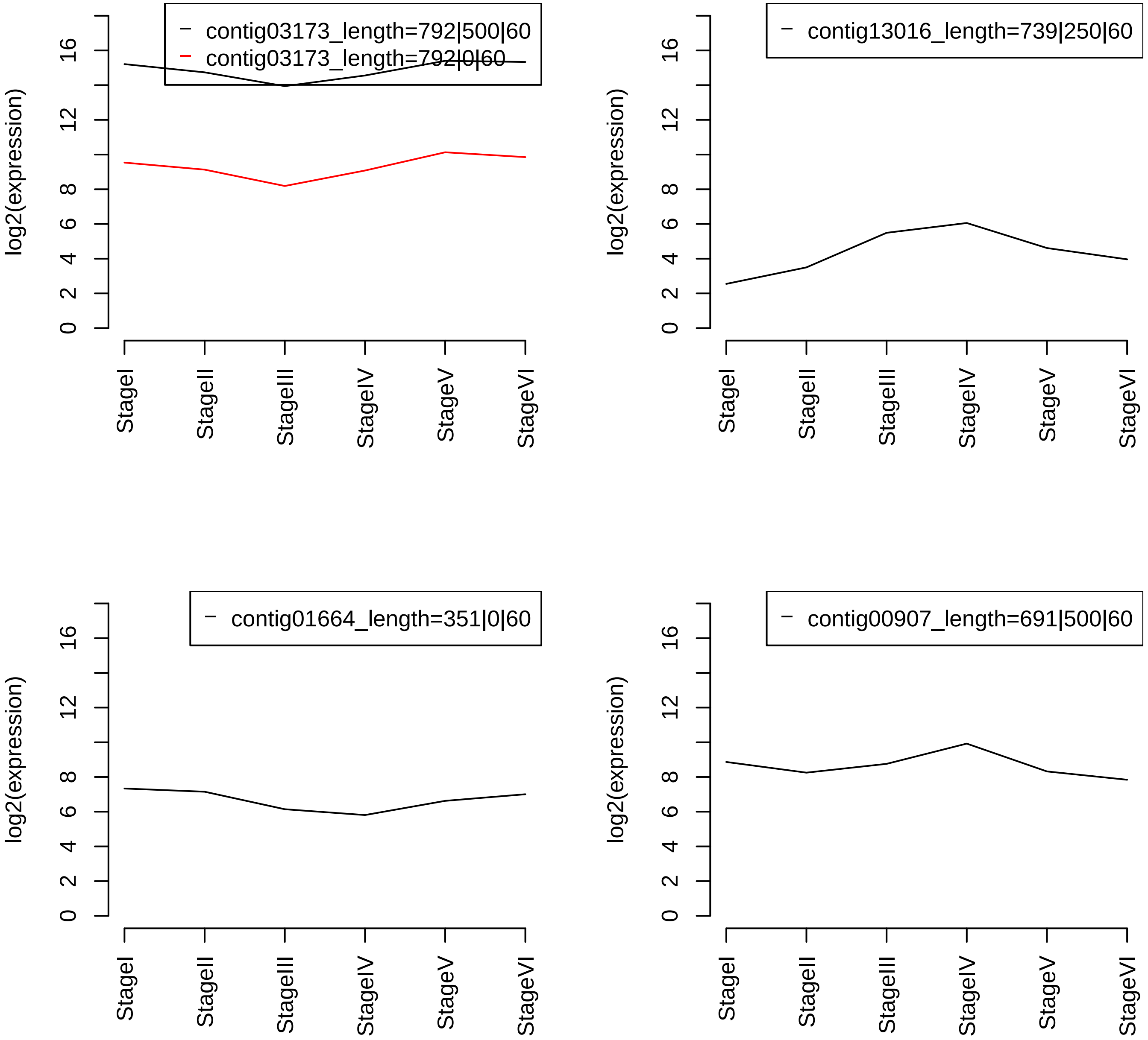
contig03173_length=792|500|60
contig03173_length=792|0|60
log2(expression)
0
2
4
6
8
12
16
StageI
StageII
StageIII
StageIV
StageV
StageVI
contig13016_length=739|250|60
log2(expression)
contig01664_length=351|0|60
log2(expression)
contig00907_length=691|500|60
log2(expression)

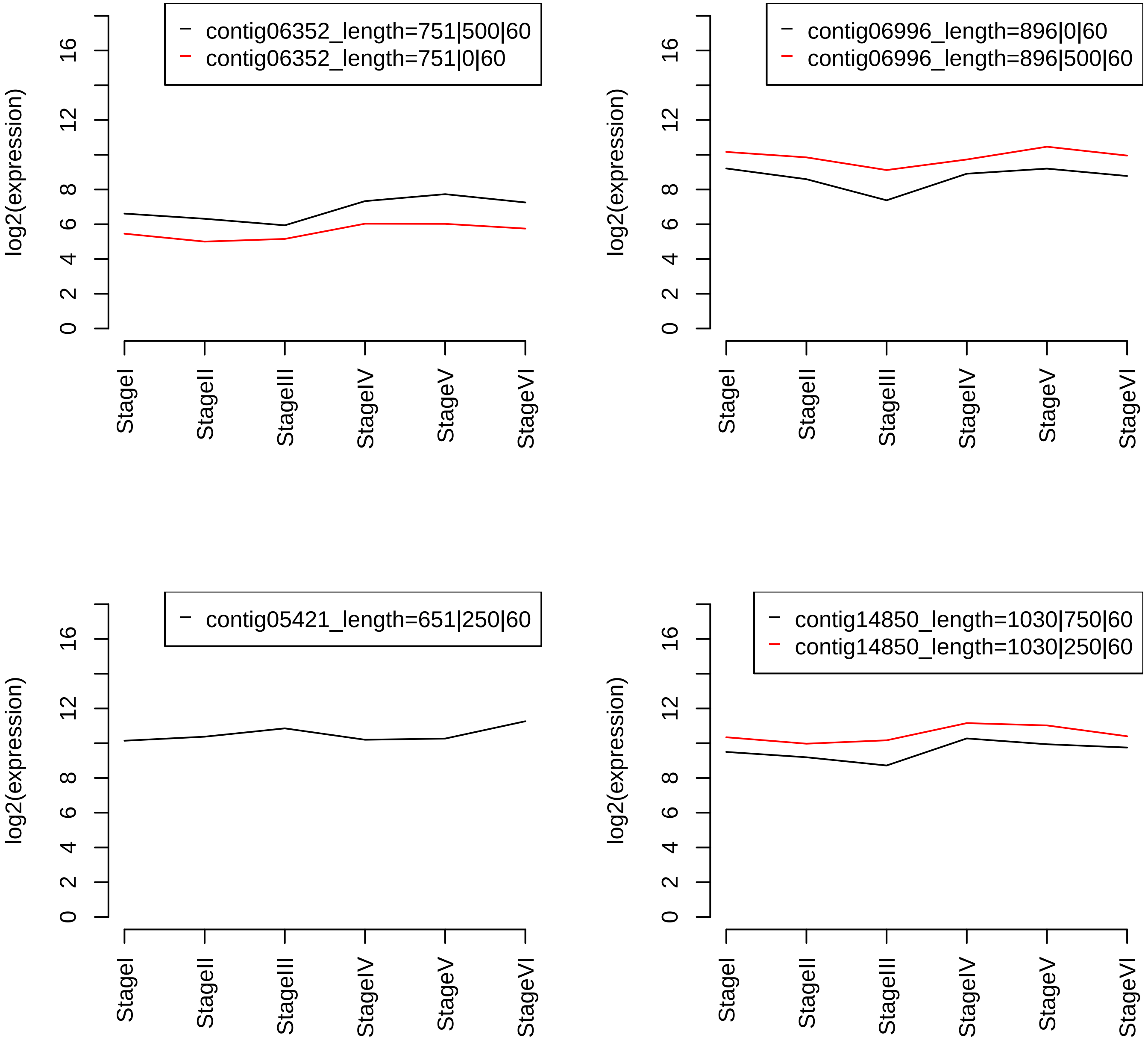

contig06352_length=751|500|60
contig06352_length=751|0|60
contig06996_length=896|0|60
contig06996_length=896|500|60
contig05421_length=651|250|60
contig14850_length=1030|750|60
contig14850_length=1030|250|60
log2(expression)
StageI
StageII
StageIII
StageIV
StageV
StageVI

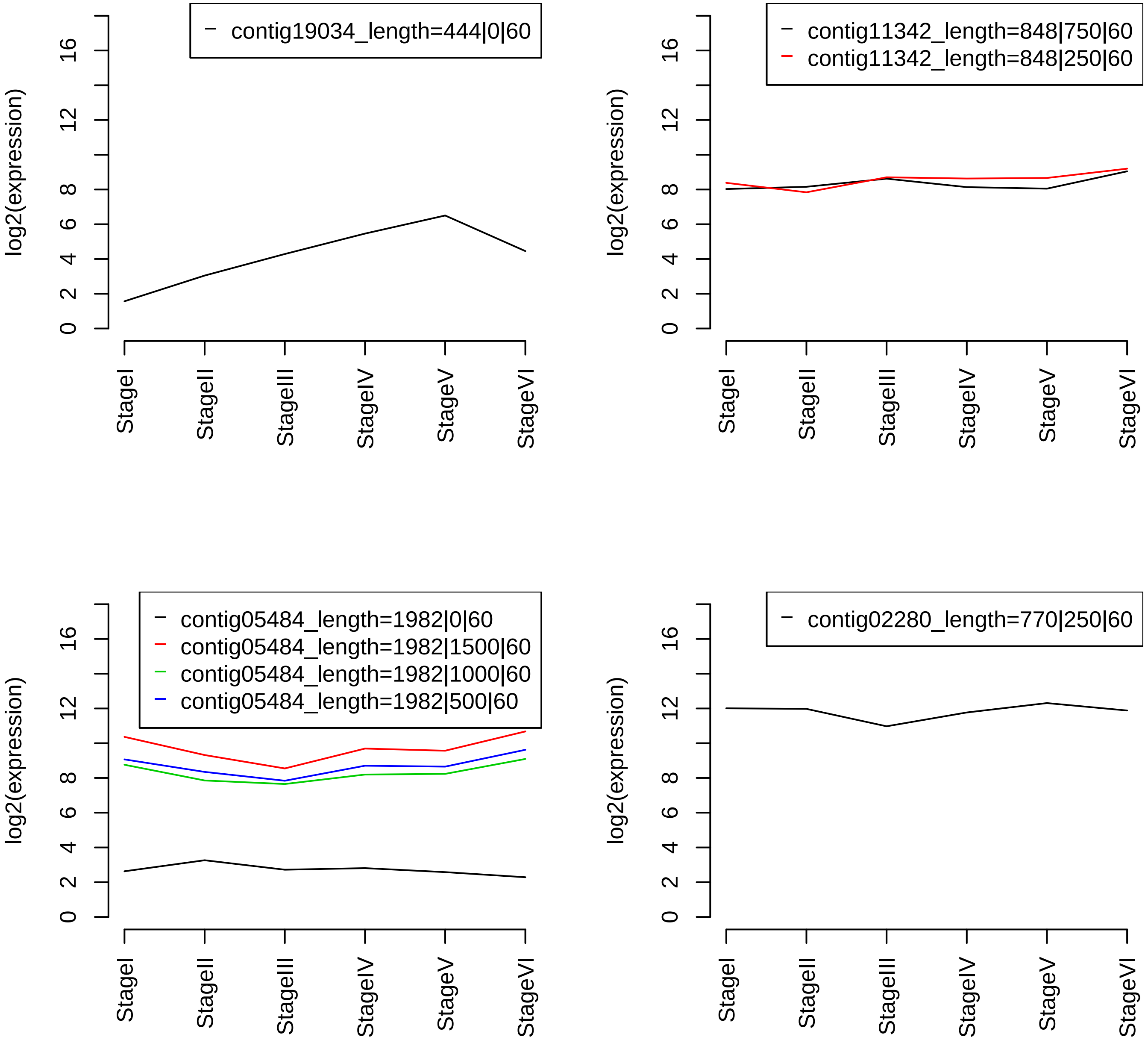
contig19034_length=444|0|60
contig11342_length=848|750|60
contig11342_length=848|250|60
contig05484_length=1982|0|60
contig05484_length=1982|1500|60
contig05484_length=1982|1000|60
contig05484_length=1982|500|60
contig02280_length=770|250|60
log2(expression)
0
2
4
6
8
12
16
StageI
StageII
StageIII
StageIV
StageV
StageVI

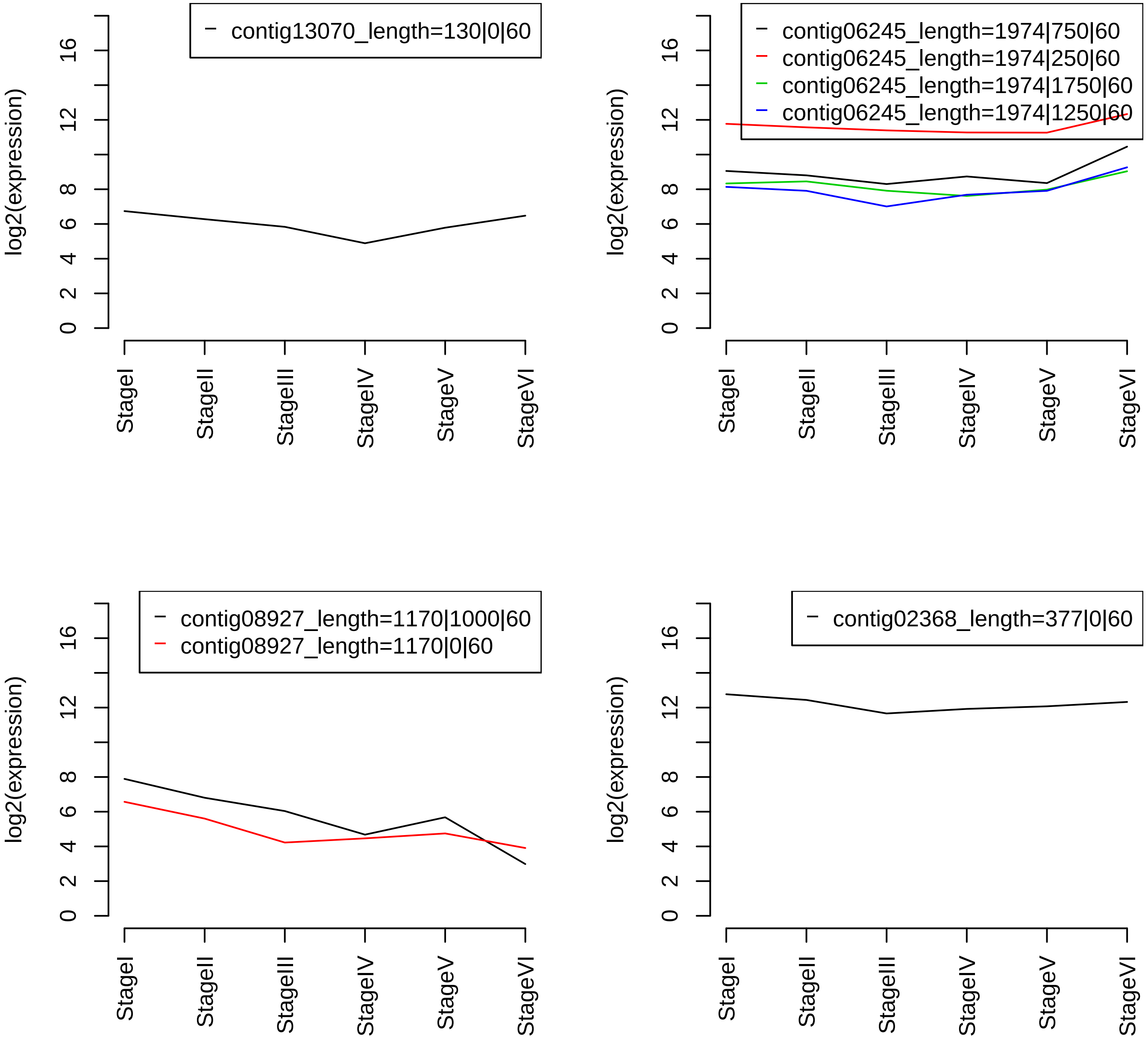

contig13070_length=130|0|60
contig06245_length=1974|750|60
contig06245_length=1974|250|60
contig06245_length=1974|1750|60
contig06245_length=1974|1250|60
contig08927_length=1170|1000|60
contig08927_length=1170|0|60
contig02368_length=377|0|60
log2(expression)
0
2
4
6
8
12
16
StageI
StageII
StageIII
StageIV
StageV
StageVI

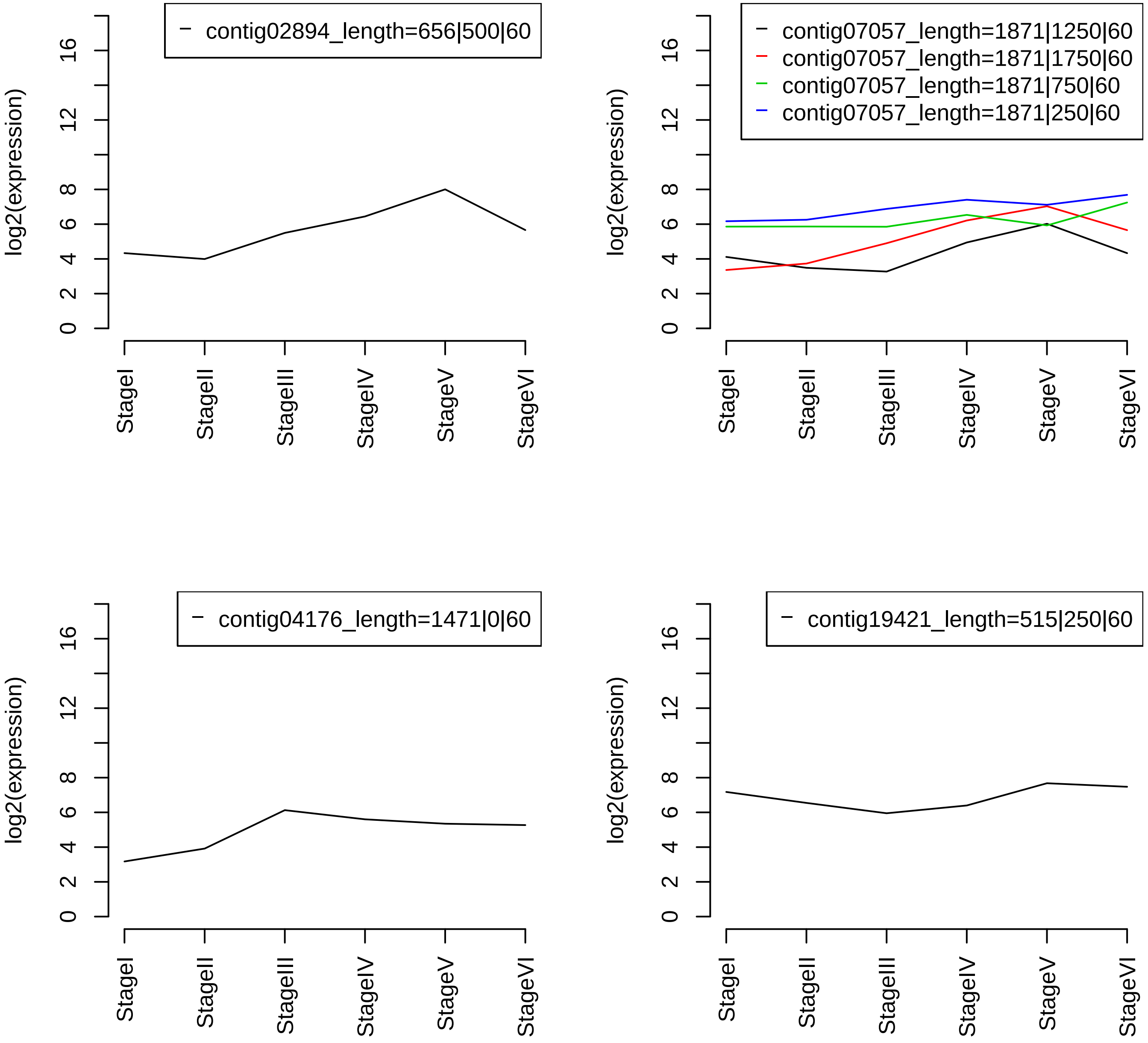

contig02894_length=656|500|60
contig07057_length=1871|1250|60
contig07057_length=1871|1750|60
contig07057_length=1871|750|60
contig07057_length=1871|250|60
contig04176_length=1471|0|60
contig19421_length=515|250|60
log2(expression)
0
2
4
6
8
12
16
StageI
StageII
StageIII
StageIV
StageV
StageVI

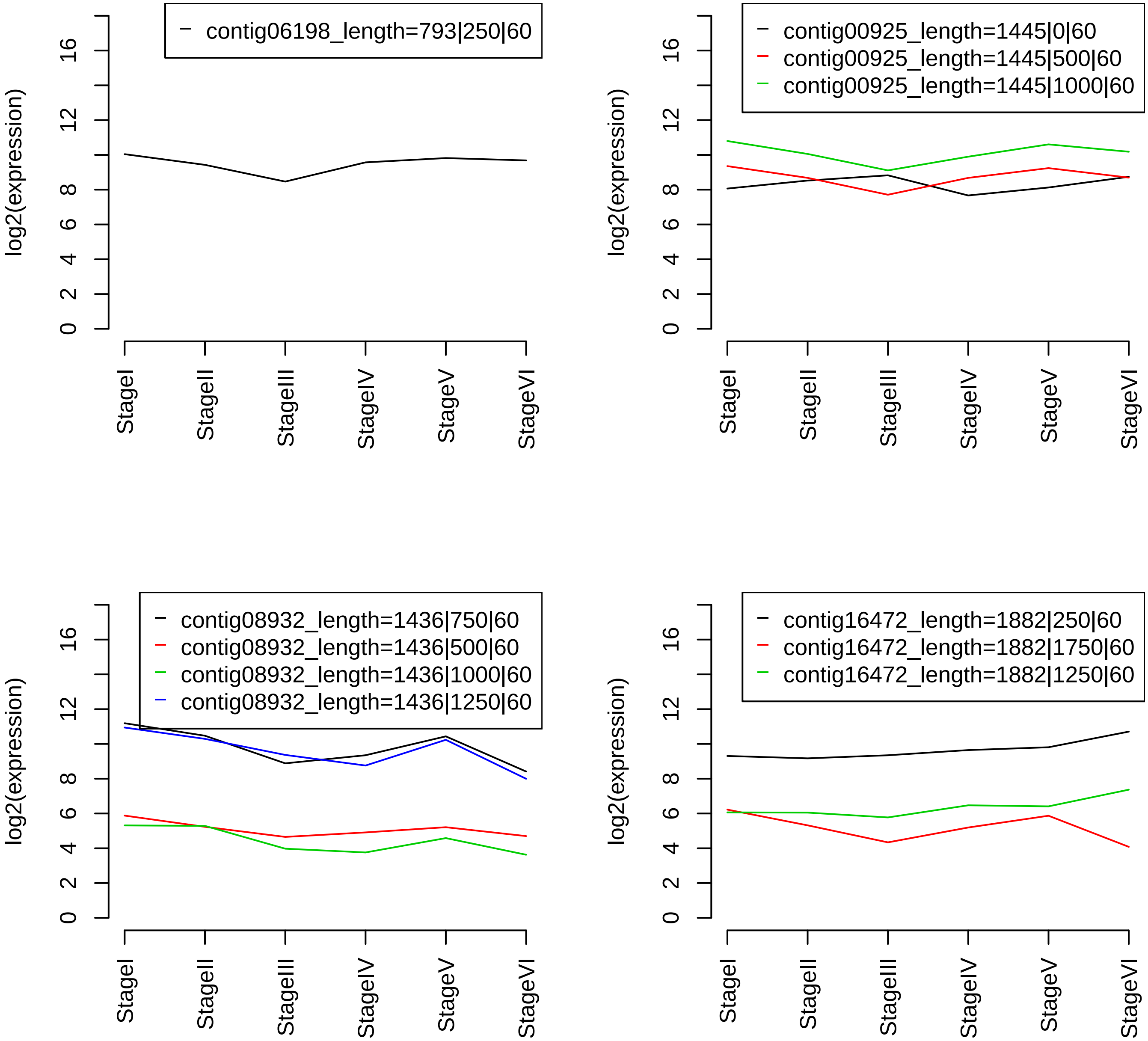

contig06198_length=793|250|60
contig00925_length=1445|0|60
contig00925_length=1445|500|60
contig00925_length=1445|1000|60
contig08932_length=1436|750|60
contig08932_length=1436|500|60
contig08932_length=1436|1000|60
contig08932_length=1436|1250|60
contig16472_length=1882|250|60
contig16472_length=1882|1750|60
contig16472_length=1882|1250|60
log2(expression)
0 2 4 6 8 12 16
StageI StageII StageIII StageIV StageV StageVI

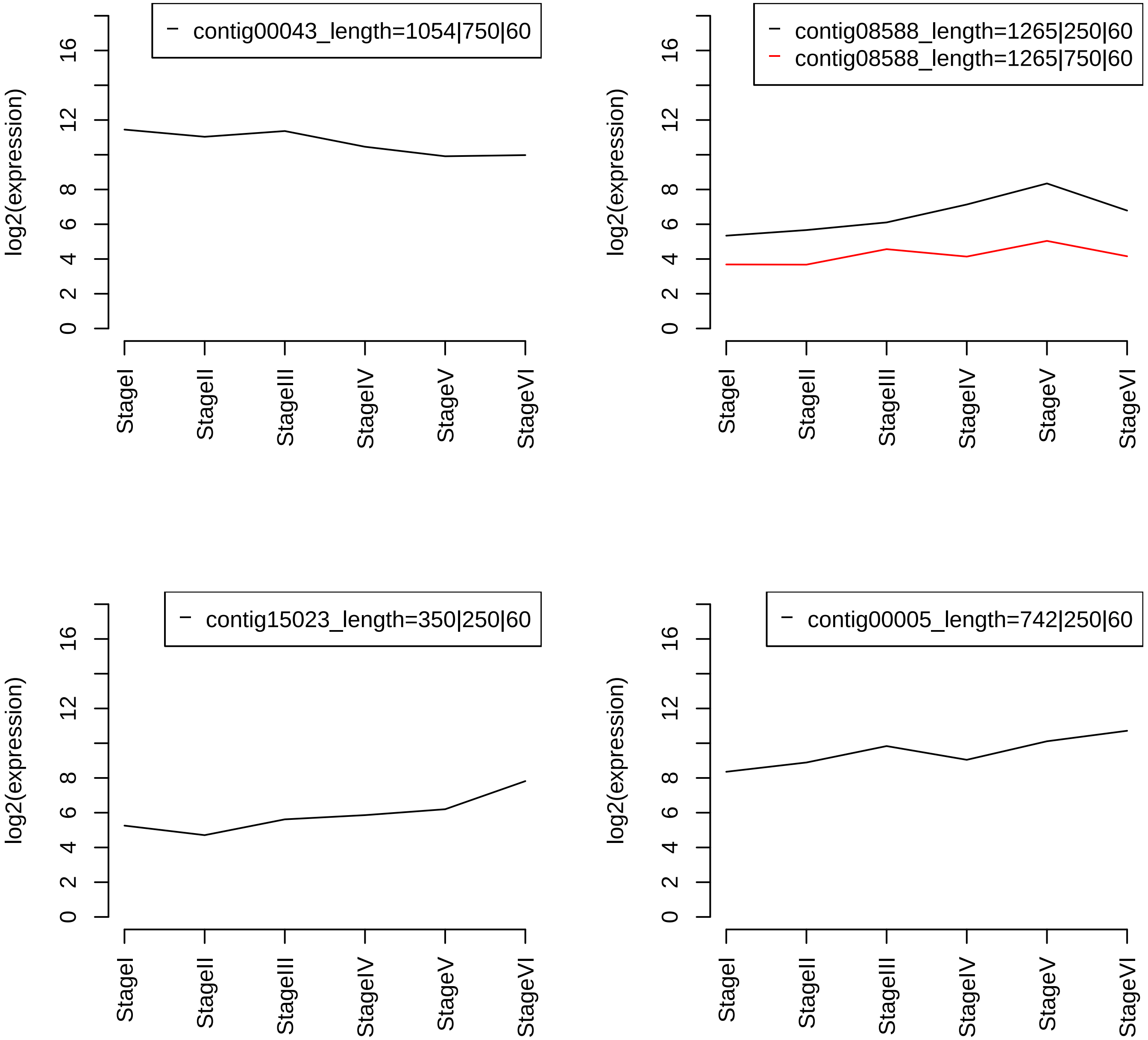

contig00043_length=1054|750|60
contig08588_length=1265|250|60
contig08588_length=1265|750|60
contig15023_length=350|250|60
contig00005_length=742|250|60
log2(expression)
0
2
4
6
8
12
16
StageI
StageII
StageIII
StageIV
StageV
StageVI

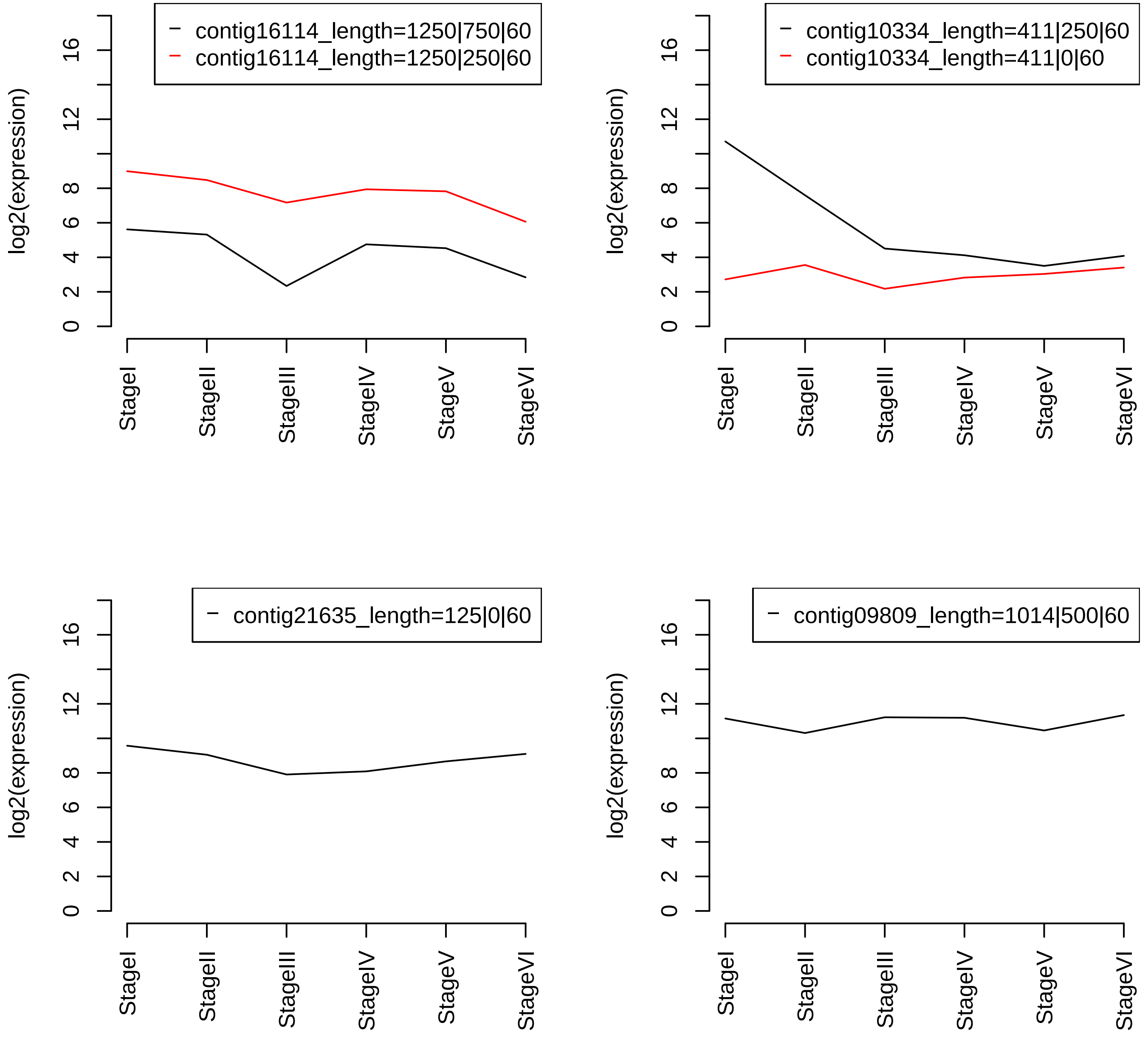
contig16114_length=1250|750|60
contig16114_length=1250|250|60
contig10334_length=411|250|60
contig10334_length=411|0|60
contig21635_length=125|0|60
contig09809_length=1014|500|60
log2(expression)
StageI
StageII
StageIII
StageIV
StageV
StageVI

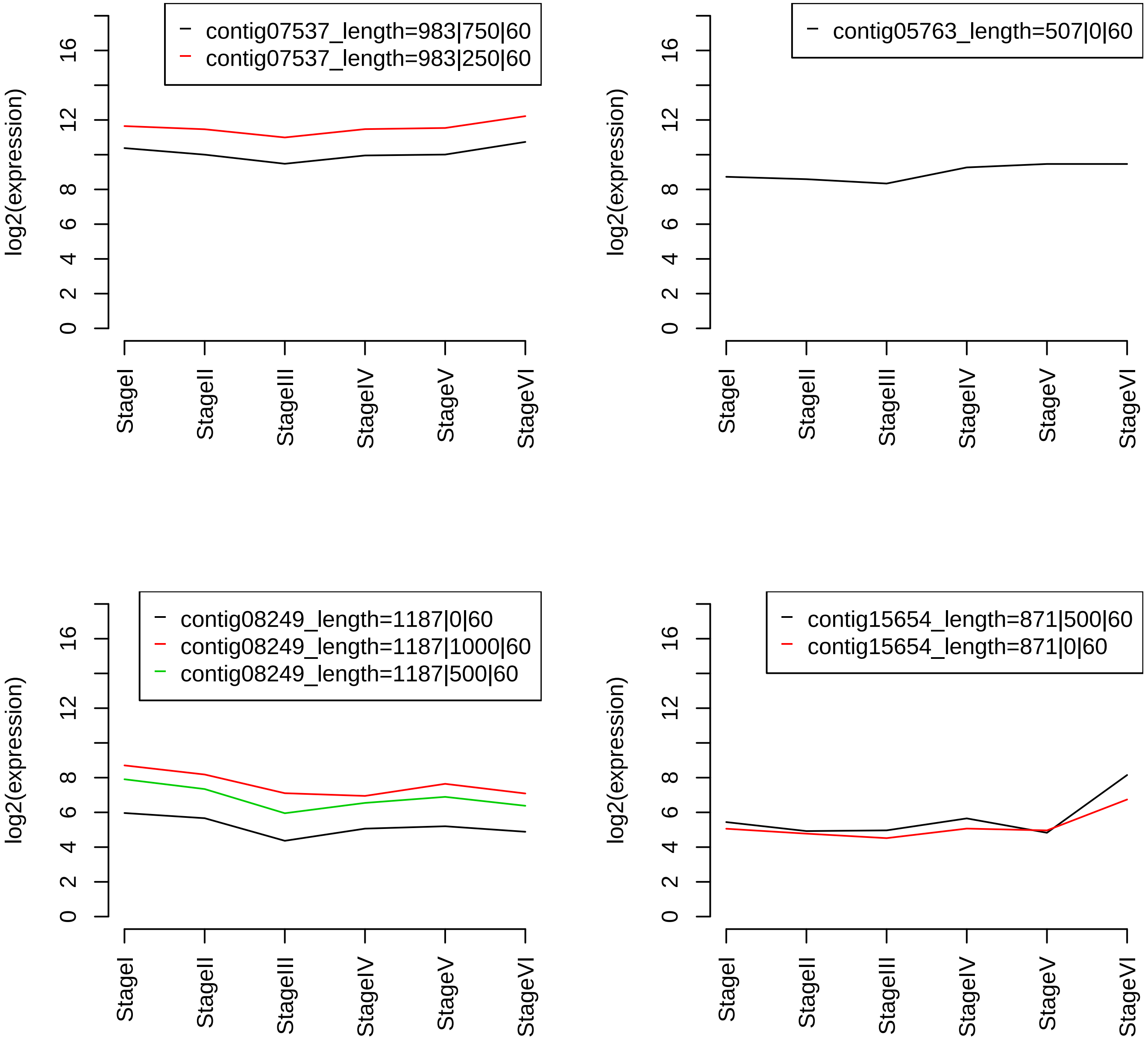
contig07537_length=983|750|60
contig07537_length=983|250|60
contig05763_length=507|0|60
contig08249_length=1187|0|60
contig08249_length=1187|1000|60
contig08249_length=1187|500|60
contig15654_length=871|500|60
contig15654_length=871|0|60
log2(expression)
0
2
4
6
8
12
16
StageI
StageII
StageIII
StageIV
StageV
StageVI

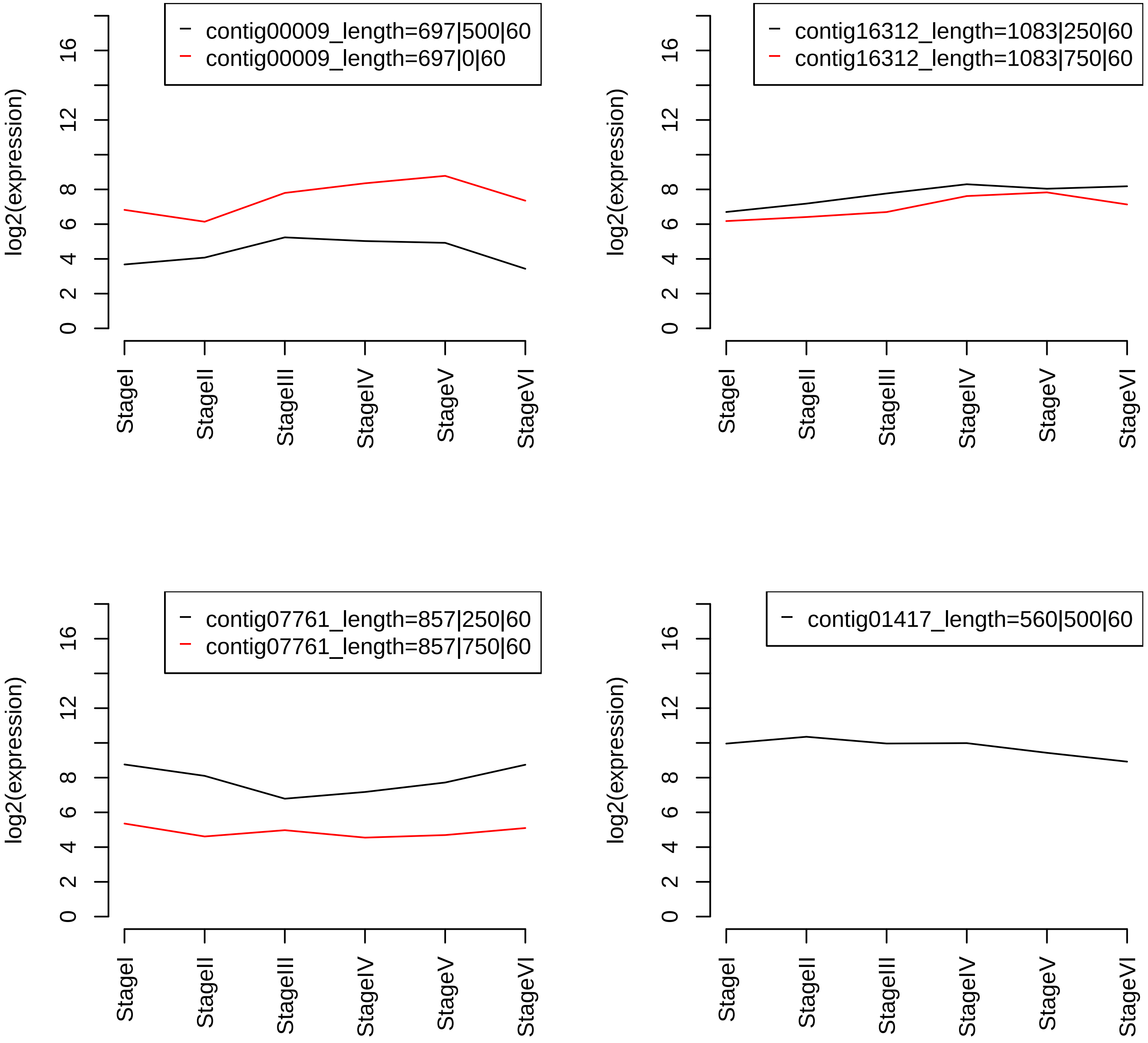

contig00009_length=697|500|60
contig00009_length=697|0|60
log2(expression)
0
2
4
6
8
12
16
StageI
StageII
StageIII
StageIV
StageV
StageVI
contig16312_length=1083|250|60
contig16312_length=1083|750|60
log2(expression)
0
2
4
6
8
12
16
StageI
StageII
StageIII
StageIV
StageV
StageVI
contig07761_length=857|250|60
contig07761_length=857|750|60
log2(expression)
0
2
4
6
8
12
16
StageI
StageII
StageIII
StageIV
StageV
StageVI
contig01417_length=560|500|60
log2(expression)
0
2
4
6
8
12
16
StageI
StageII
StageIII
StageIV
StageV
StageVI

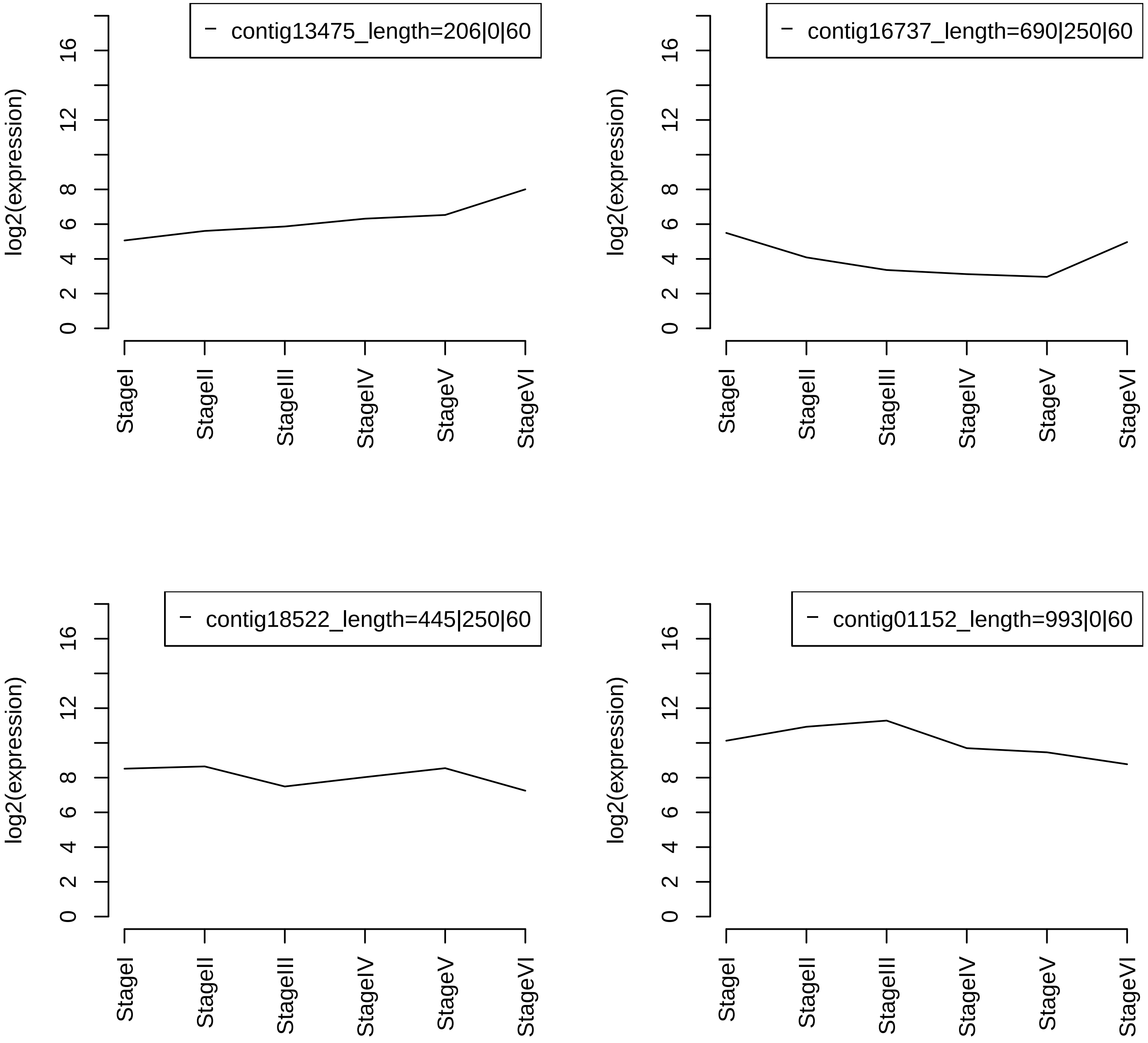
contig13475_length=206|0|60
contig16737_length=690|250|60
contig18522_length=445|250|60
contig01152_length=993|0|60
log2(expression)
0
2
4
6
8
12
16
StageI
StageII
StageIII
StageIV
StageV
StageVI

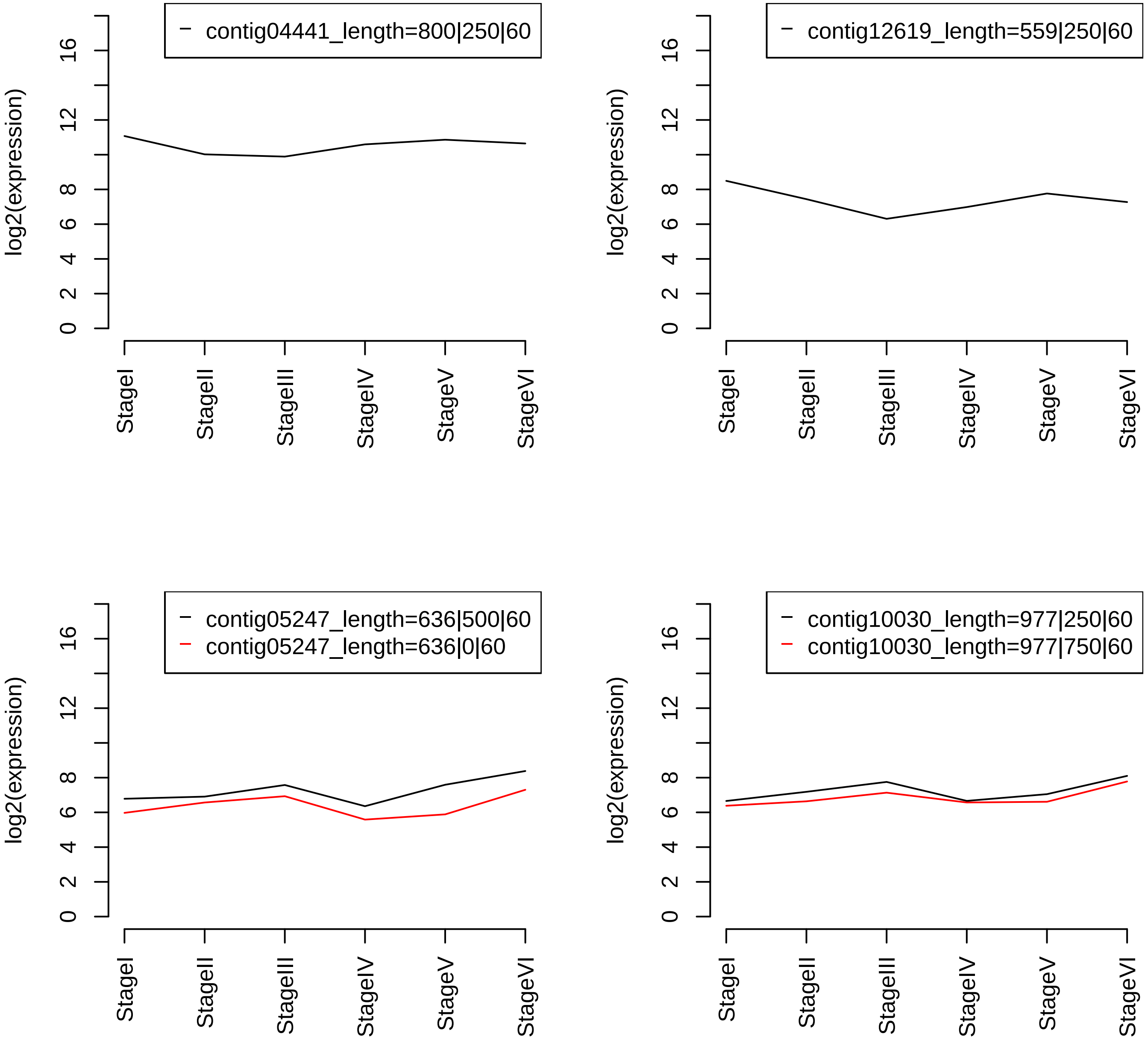
contig04441_length=800|250|60
contig12619_length=559|250|60
contig05247_length=636|500|60
contig05247_length=636|0|60
contig10030_length=977|250|60
contig10030_length=977|750|60
log2(expression)
0
2
4
6
8
12
16
StageI
StageII
StageIII
StageIV
StageV
StageVI

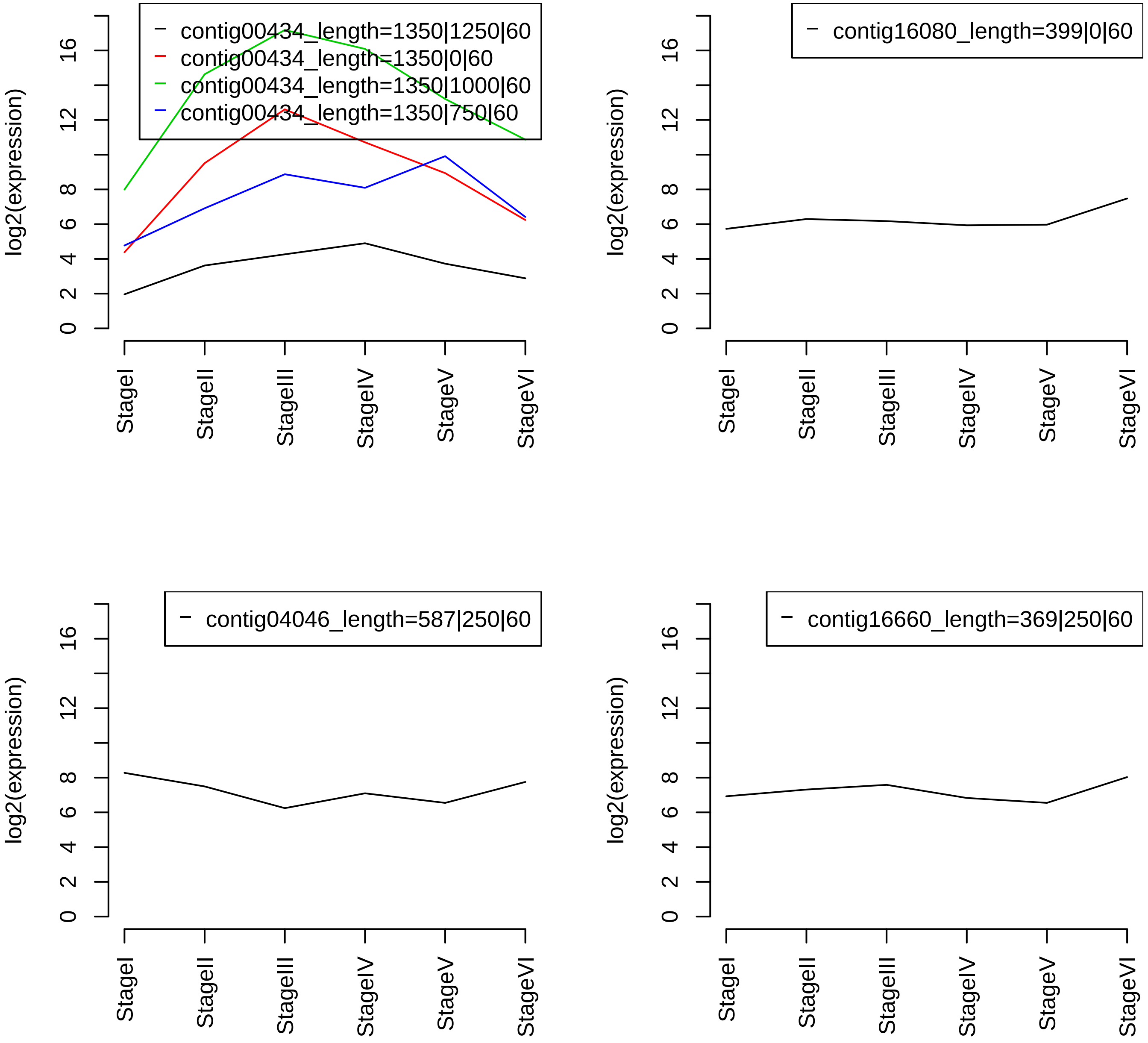
contig00434_length=1350|1250|60
contig00434_length=1350|0|60
contig00434_length=1350|1000|60
contig00434_length=1350|750|60
log2(expression)
0
2
4
6
8
12
16
StageI
StageII
StageIII
StageIV
StageV
StageVI
contig16080_length=399|0|60
contig04046_length=587|250|60
contig16660_length=369|250|60

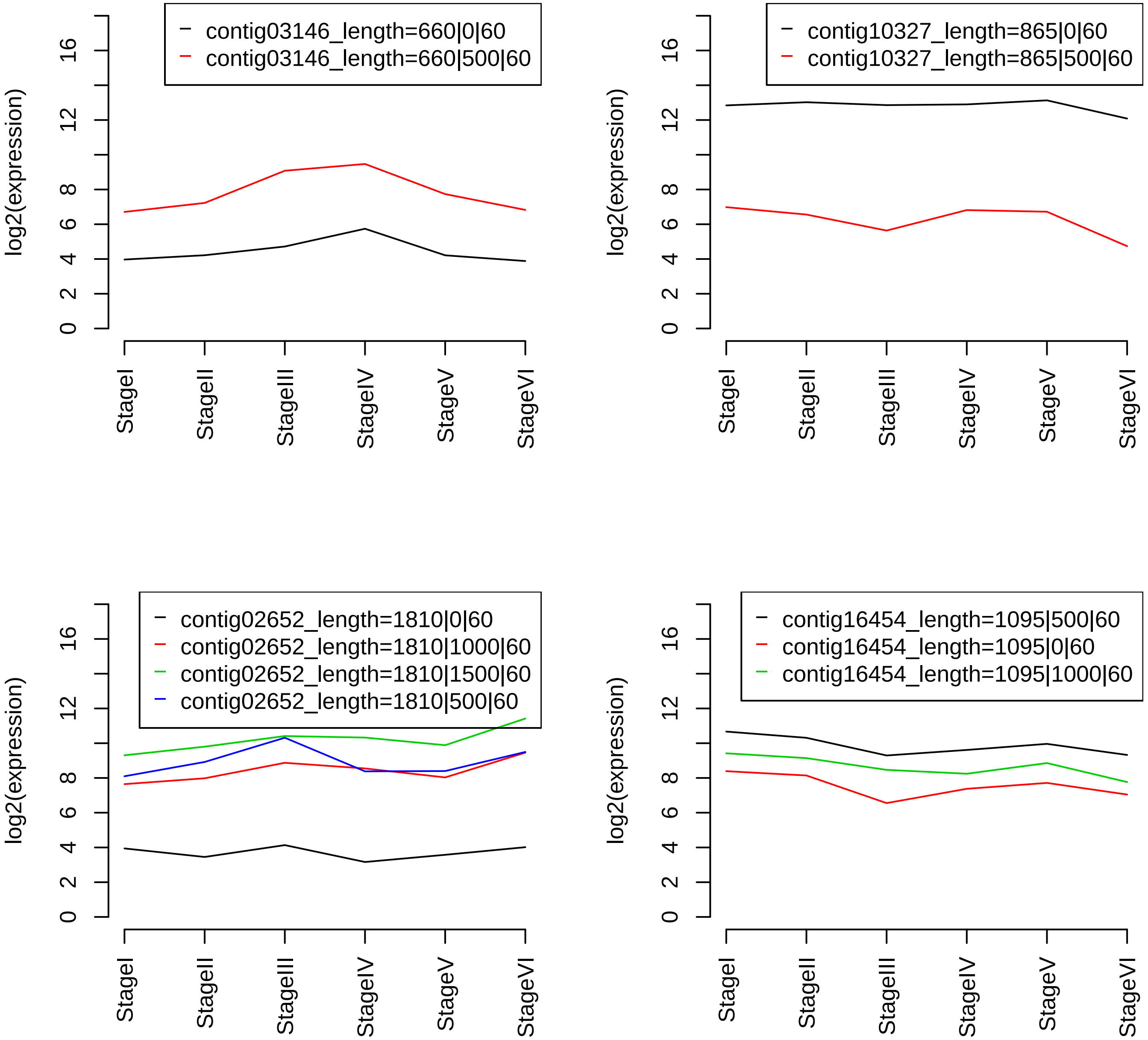

contig03146_length=660|0|60
contig03146_length=660|500|60
log2(expression)
0
2
4
6
8
12
16
StageI
StageII
StageIII
StageIV
StageV
StageVI
contig10327_length=865|0|60
contig10327_length=865|500|60
contig02652_length=1810|0|60
contig02652_length=1810|1000|60
contig02652_length=1810|1500|60
contig02652_length=1810|500|60
contig16454_length=1095|500|60
contig16454_length=1095|0|60
contig16454_length=1095|1000|60

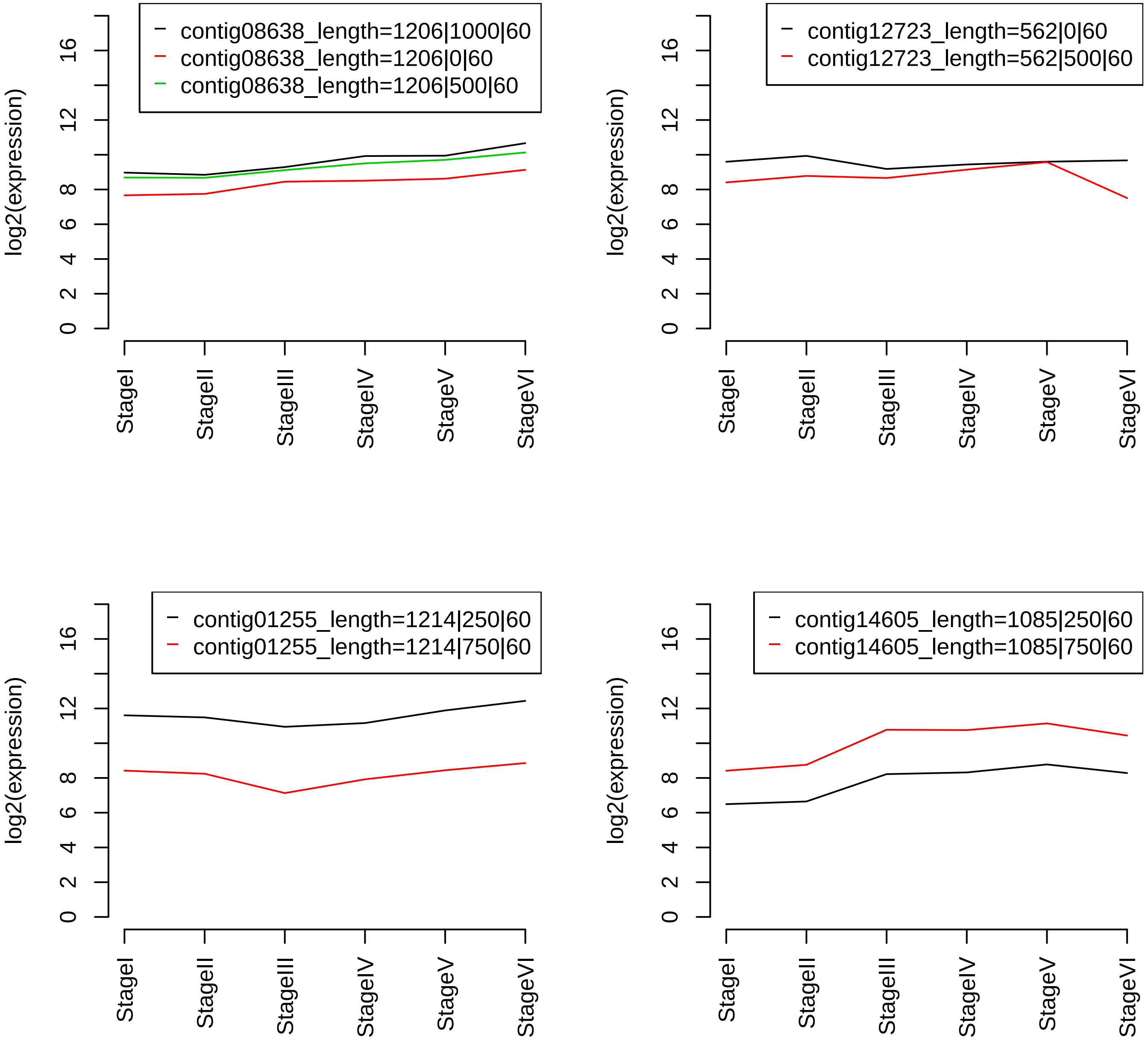
contig08638_length=1206|1000|60
contig08638_length=1206|0|60
contig08638_length=1206|500|60
contig12723_length=562|0|60
contig12723_length=562|500|60
contig01255_length=1214|250|60
contig01255_length=1214|750|60
contig14605_length=1085|250|60
contig14605_length=1085|750|60
log2(expression)
StageI
StageII
StageIII
StageIV
StageV
StageVI

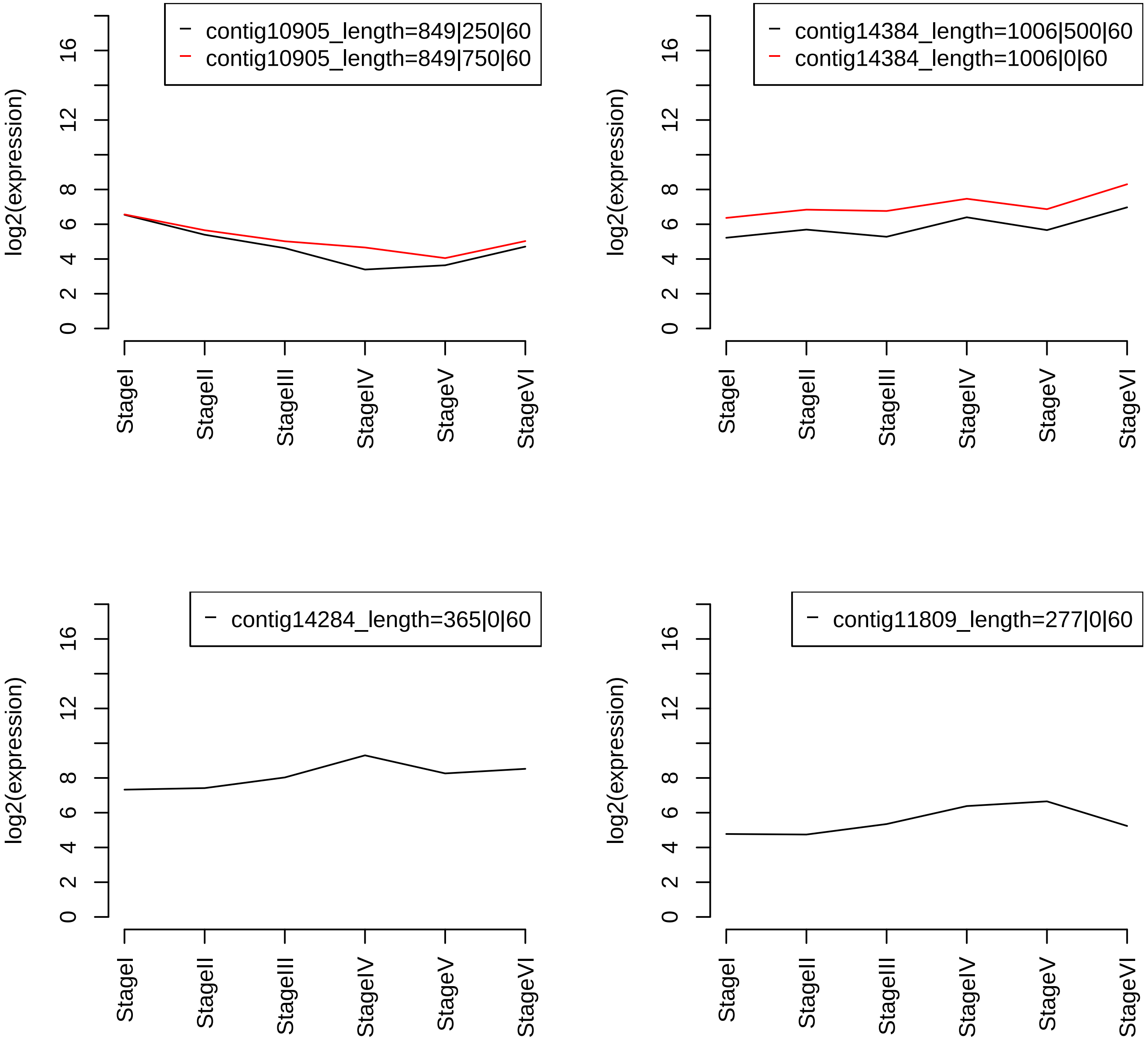

contig10905_length=849|250|60
contig10905_length=849|750|60
contig14384_length=1006|500|60
contig14384_length=1006|0|60
contig14284_length=365|0|60
contig11809_length=277|0|60
log2(expression)
0
2
4
6
8
12
16
StageI
StageII
StageIII
StageIV
StageV
StageVI

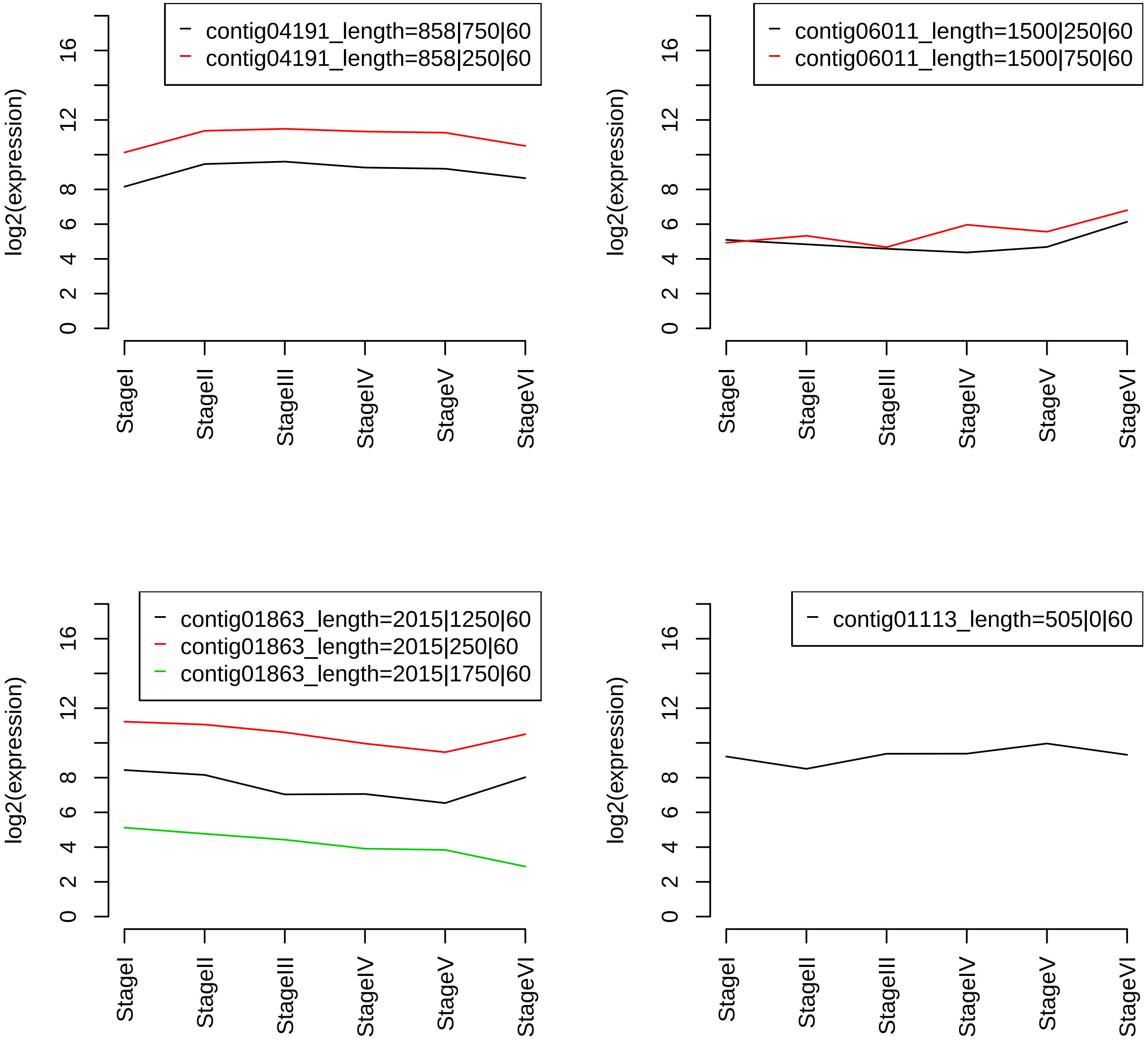
contig04191_length=858|750|60
contig04191_length=858|250|60
contig06011_length=1500|250|60
contig06011_length=1500|750|60
contig01863_length=2015|1250|60
contig01863_length=2015|250|60
contig01863_length=2015|1750|60
contig01113_length=505|0|60
log2(expression)
0
2
4
6
8
12
16
StageI
StageII
StageIII
StageIV
StageV
StageVI

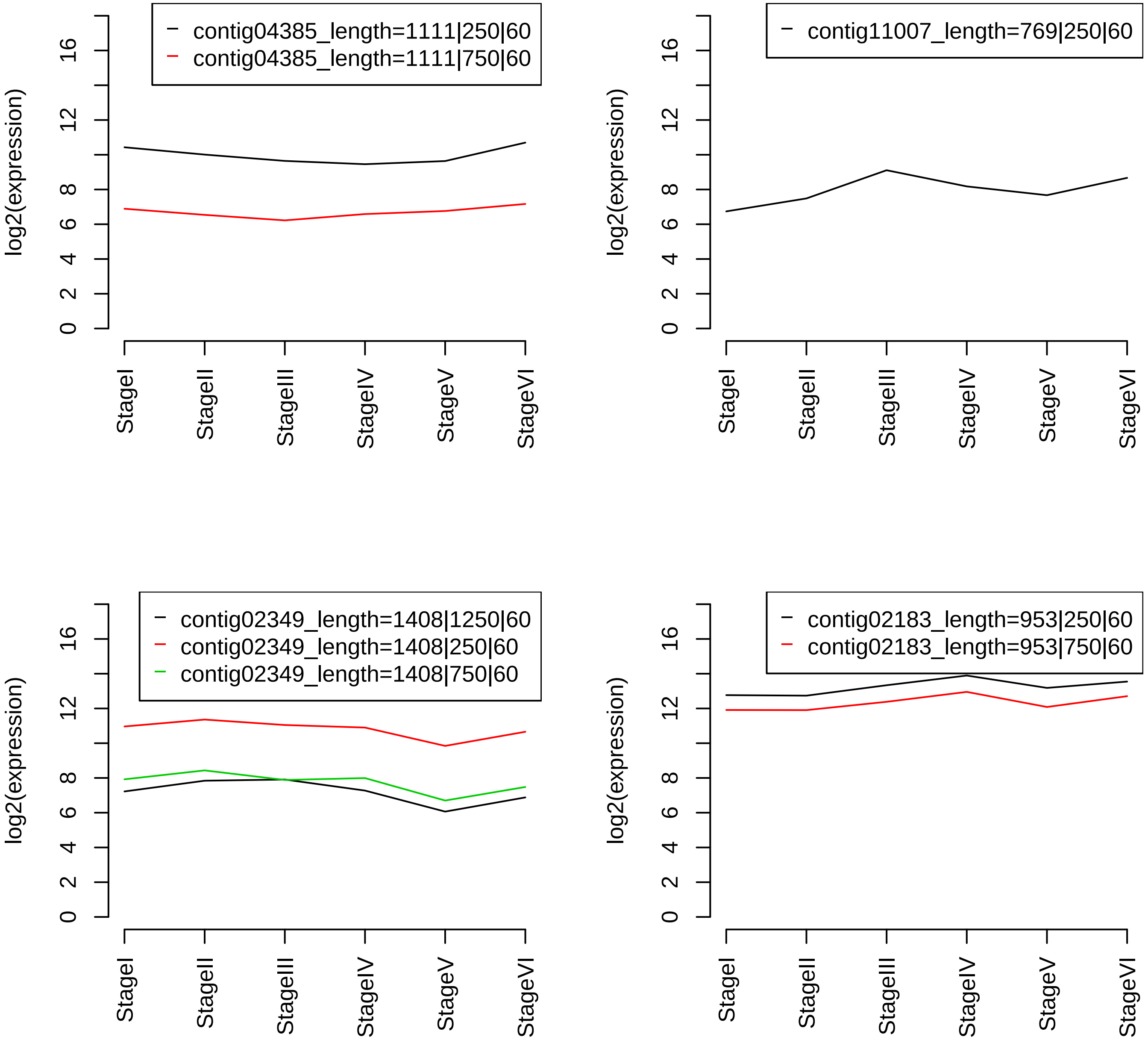

contig04385_length=1111|250|60
contig04385_length=1111|750|60
log2(expression)
0
2
4
6
8
12
16
StageI
StageII
StageIII
StageIV
StageV
StageVI
contig11007_length=769|250|60
log2(expression)
0
2
4
6
8
12
16
StageI
StageII
StageIII
StageIV
StageV
StageVI
contig02349_length=1408|1250|60
contig02349_length=1408|250|60
contig02349_length=1408|750|60
log2(expression)
0
2
4
6
8
12
16
StageI
StageII
StageIII
StageIV
StageV
StageVI
contig02183_length=953|250|60
contig02183_length=953|750|60
log2(expression)
0
2
4
6
8
12
16
StageI
StageII
StageIII
StageIV
StageV
StageVI

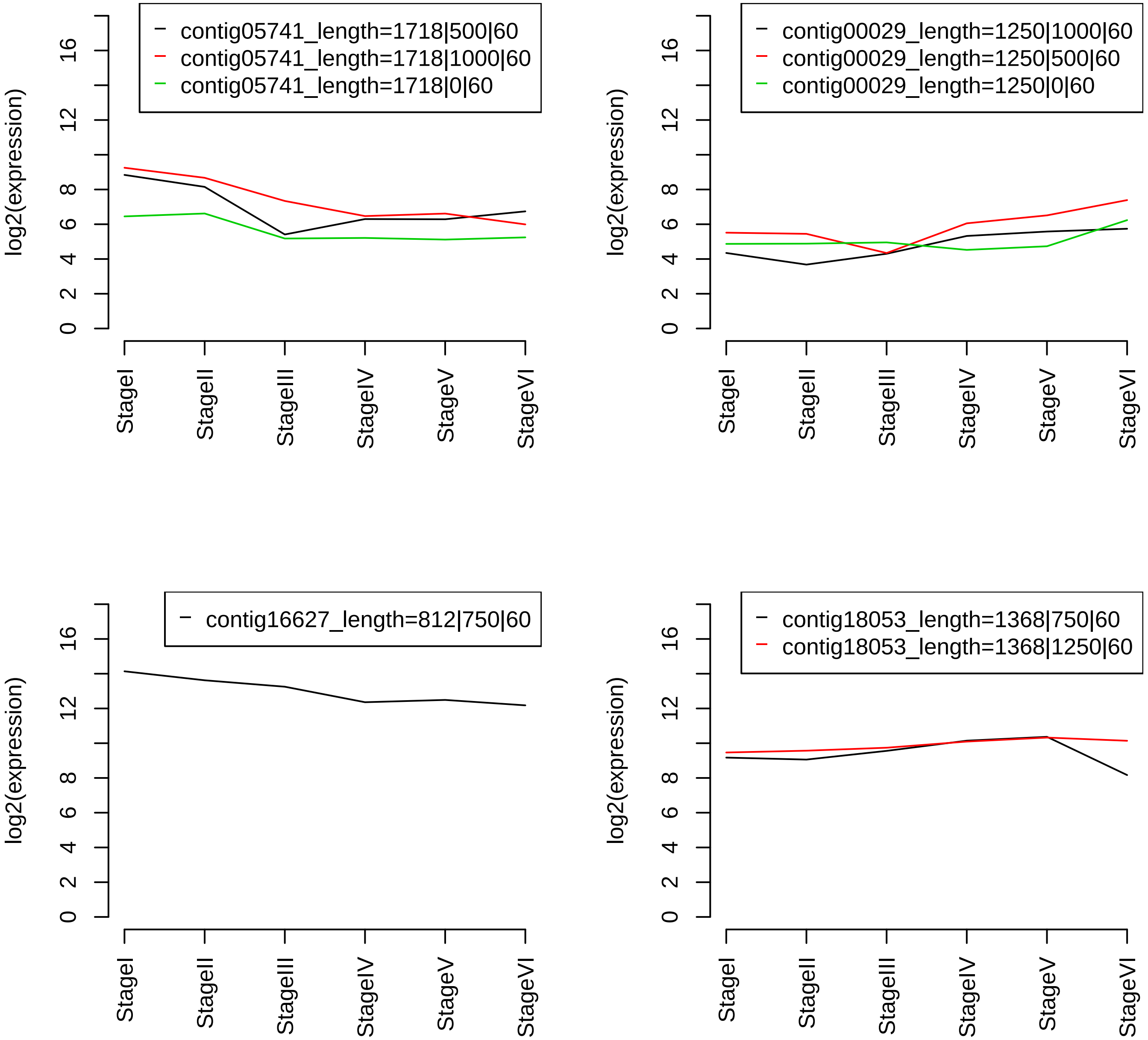
contig05741_length=1718|500|60
contig05741_length=1718|1000|60
contig05741_length=1718|0|60
log2(expression)
0
2
4
6
8
12
16
StageI
StageII
StageIII
StageIV
StageV
StageVI
contig00029_length=1250|1000|60
contig00029_length=1250|500|60
contig00029_length=1250|0|60
contig16627_length=812|750|60
contig18053_length=1368|750|60
contig18053_length=1368|1250|60

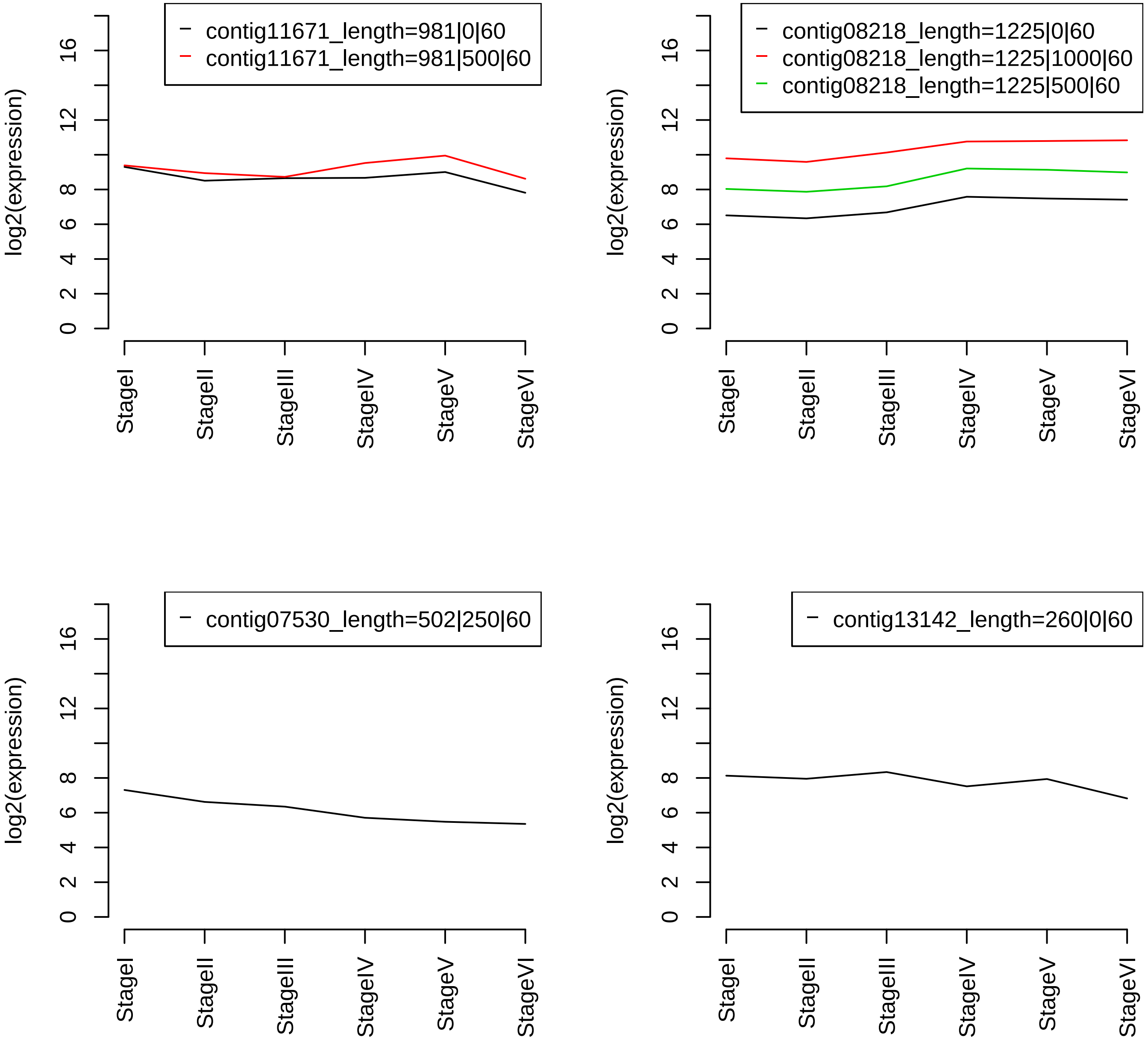
contig11671_length=981|0|60
contig11671_length=981|500|60
contig08218_length=1225|0|60
contig08218_length=1225|1000|60
contig08218_length=1225|500|60
contig07530_length=502|250|60
contig13142_length=260|0|60
log2(expression)
0 2 4 6 8 12 16
StageI
StageII
StageIII
StageIV
StageV
StageVI

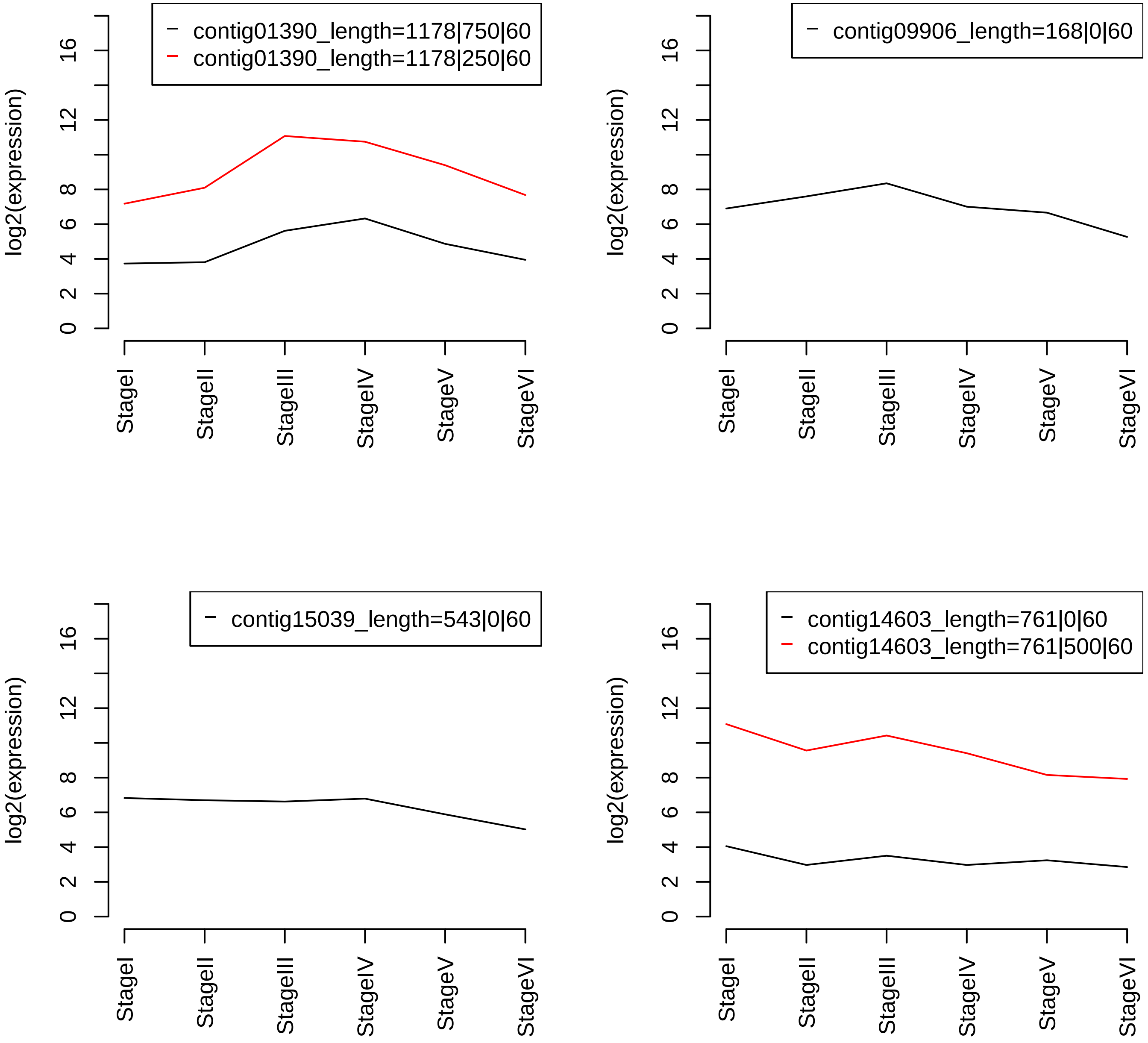
contig01390_length=1178|750|60
contig01390_length=1178|250|60
contig09906_length=168|0|60
contig15039_length=543|0|60
contig14603_length=761|0|60
contig14603_length=761|500|60
log2(expression)
0
2
4
6
8
12
16
StageI
StageII
StageIII
StageIV
StageV
StageVI

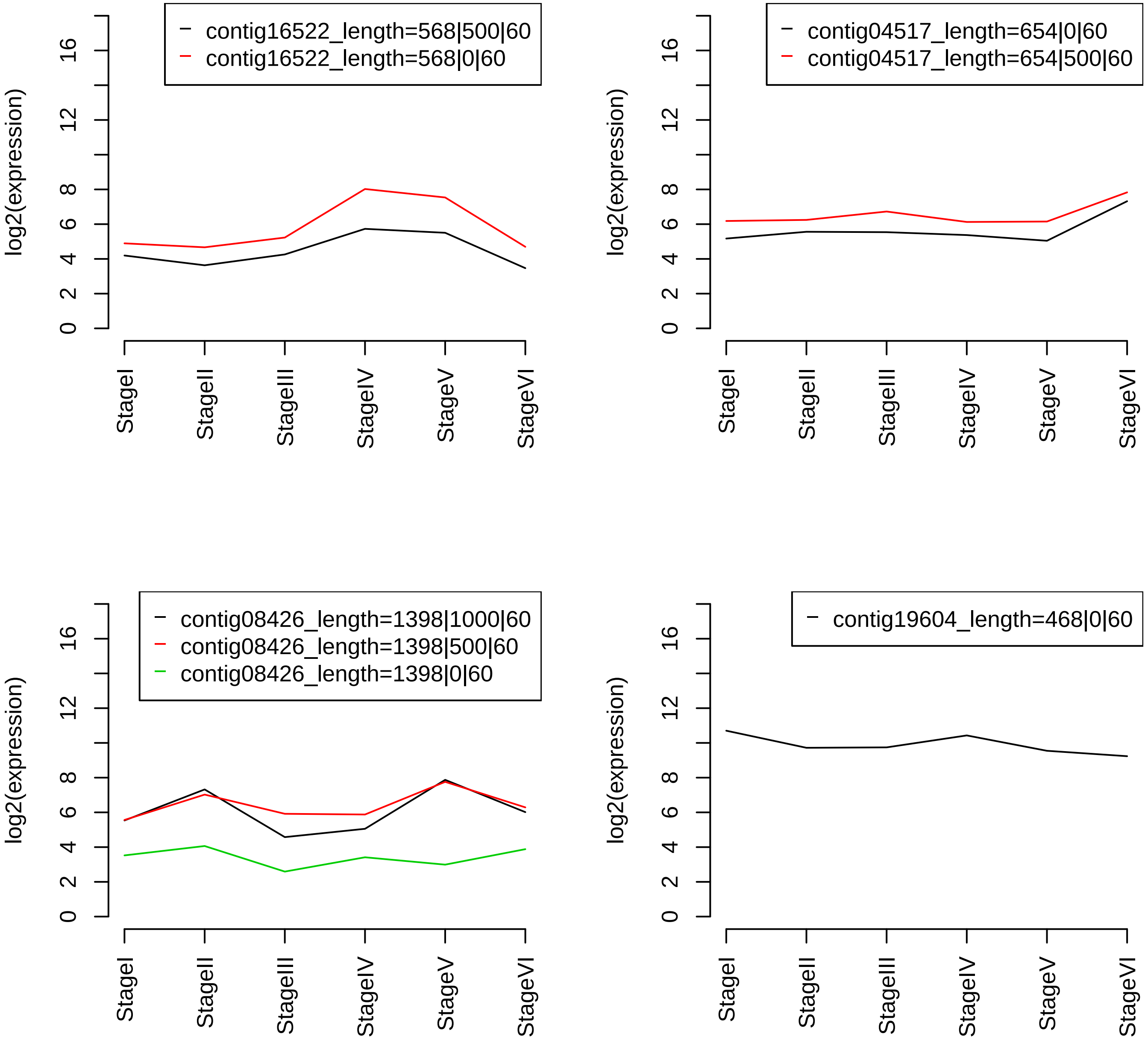
contig16522_length=568|500|60
contig16522_length=568|0|60
contig04517_length=654|0|60
contig04517_length=654|500|60
contig08426_length=1398|1000|60
contig08426_length=1398|500|60
contig08426_length=1398|0|60
contig19604_length=468|0|60
log2(expression)
0
2
4
6
8
12
16
StageI
StageII
StageIII
StageIV
StageV
StageVI

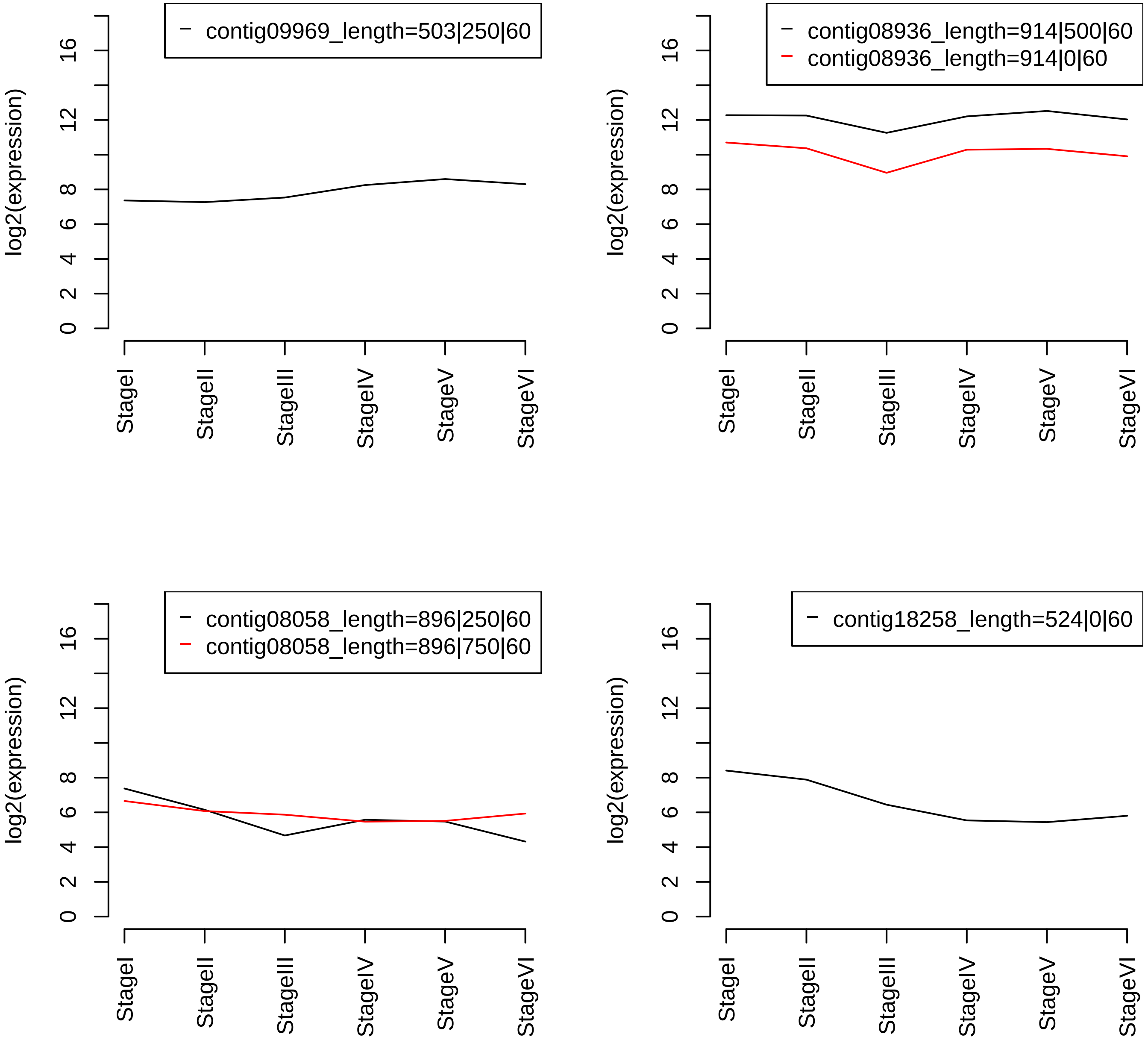

contig09969_length=503|250|60
contig08936_length=914|500|60
contig08936_length=914|0|60
contig08058_length=896|250|60
contig08058_length=896|750|60
contig18258_length=524|0|60
log2(expression)
0
2
4
6
8
12
16
StageI
StageII
StageIII
StageIV
StageV
StageVI

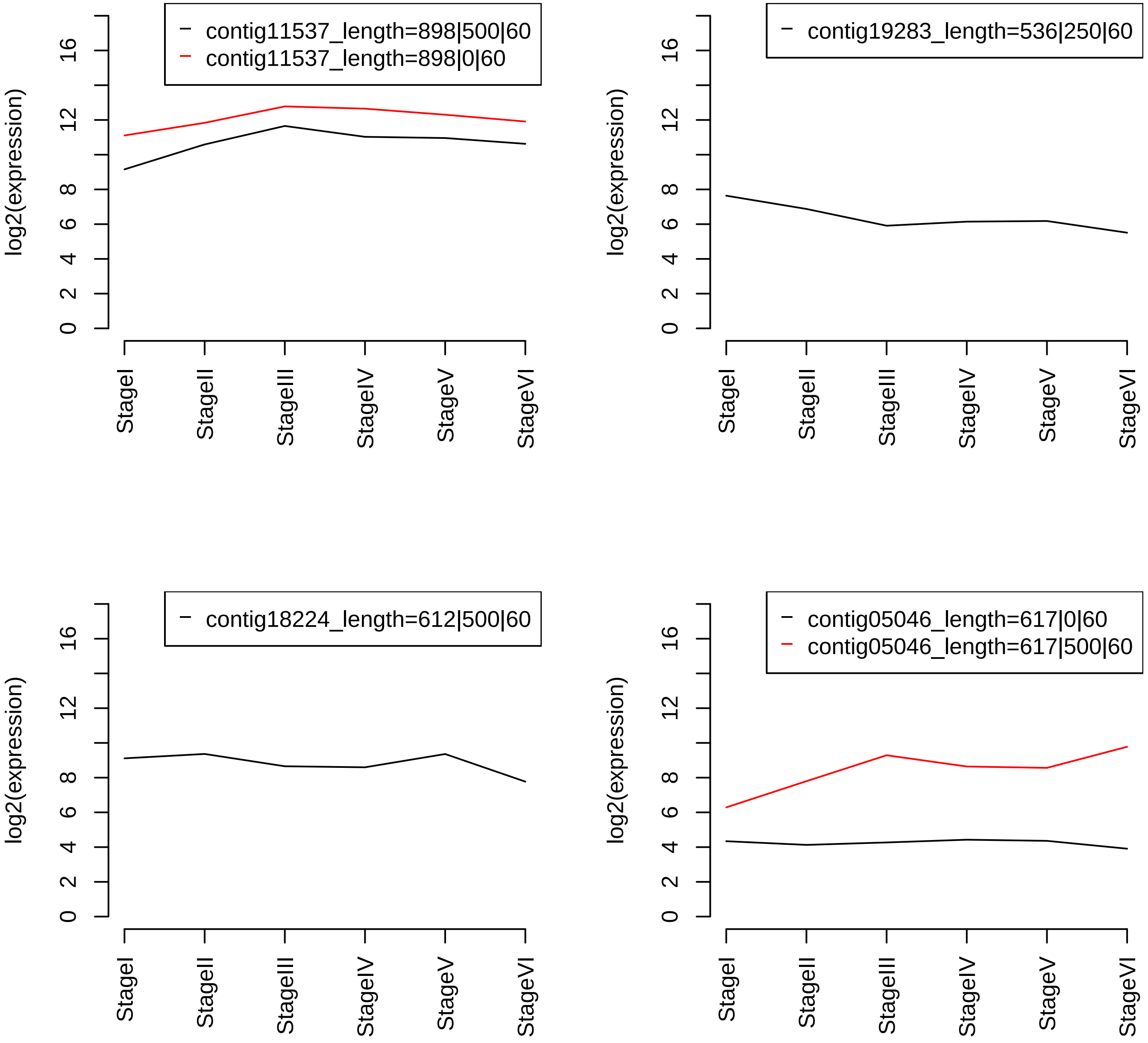

contig11537_length=898|500|60
contig11537_length=898|0|60
contig19283_length=536|250|60
contig18224_length=612|500|60
contig05046_length=617|0|60
contig05046_length=617|500|60
log2(expression)
0
2
4
6
8
12
16
StageI
StageII
StageIII
StageIV
StageV
StageVI

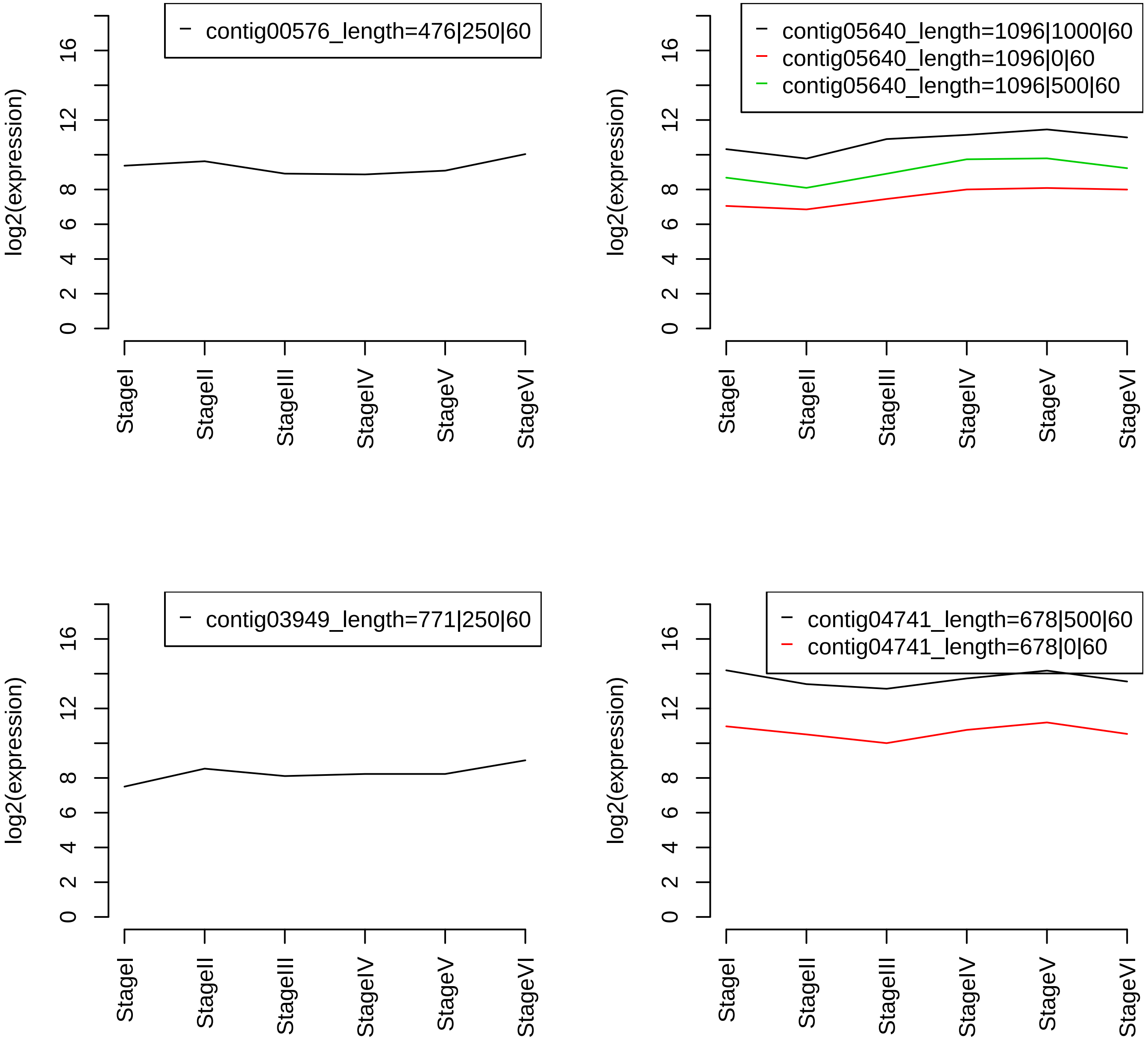

contig00576_length=476|250|60
contig05640_length=1096|1000|60
contig05640_length=1096|0|60
contig05640_length=1096|500|60
contig03949_length=771|250|60
contig04741_length=678|500|60
contig04741_length=678|0|60
log2(expression)
0
2
4
6
8
12
16
StageI
StageII
StageIII
StageIV
StageV
StageVI

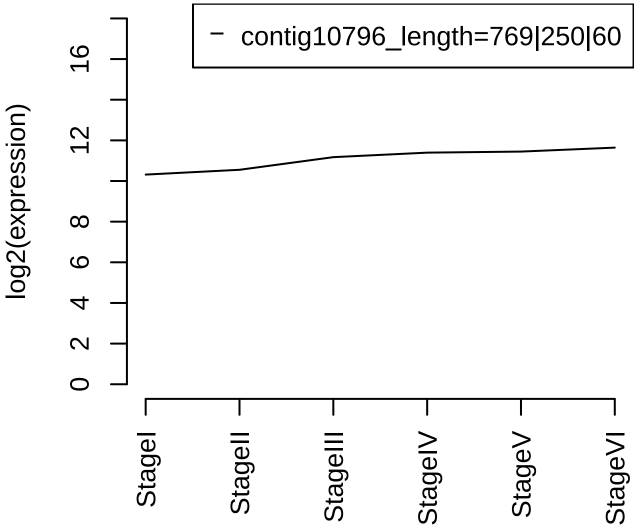

contig10796_length=769|250|60
log2(expression)
0
2
4
6
8
12
16
StageI
StageII
StageIII
StageIV
StageV
StageVI

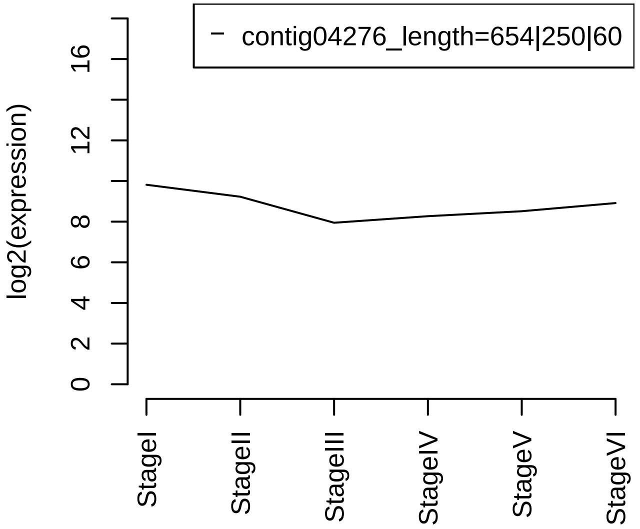

contig04276_length=654|250|60
log2(expression)
0
2
4
6
8
12
16
StageI
StageII
StageIII
StageIV
StageV
StageVI

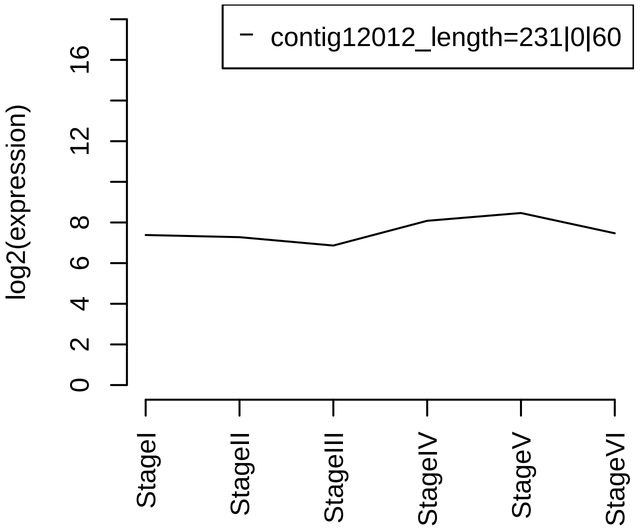

contig12012_length=231|0|60
log2(expression)
0
2
4
6
8
12
16
StageI
StageII
StageIII
StageIV
StageV
StageVI

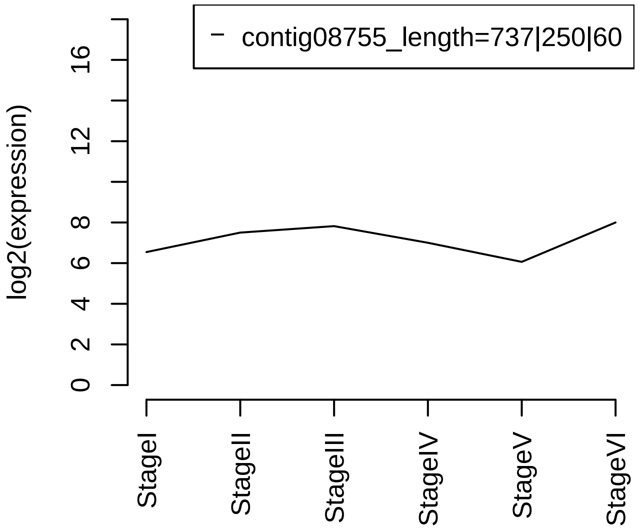

contig08755_length=737|250|60
log2(expression)
0
2
4
6
8
12
16
StageI
StageII
StageIII
StageIV
StageV
StageVI

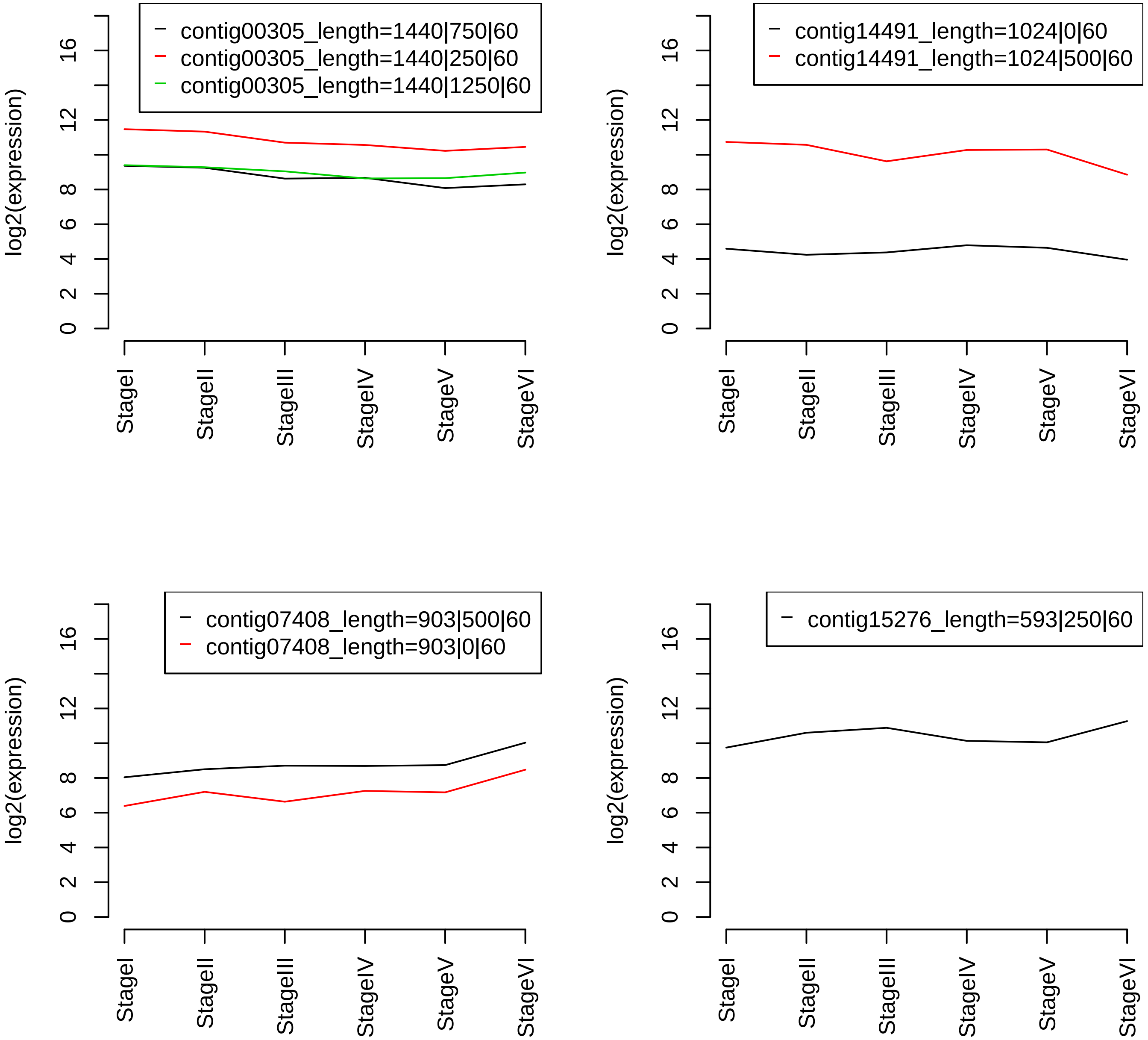

contig00305_length=1440|750|60
contig00305_length=1440|250|60
contig00305_length=1440|1250|60
contig14491_length=1024|0|60
contig14491_length=1024|500|60
contig07408_length=903|500|60
contig07408_length=903|0|60
contig15276_length=593|250|60
log2(expression)
0
2
4
6
8
12
16
StageI
StageII
StageIII
StageIV
StageV
StageVI

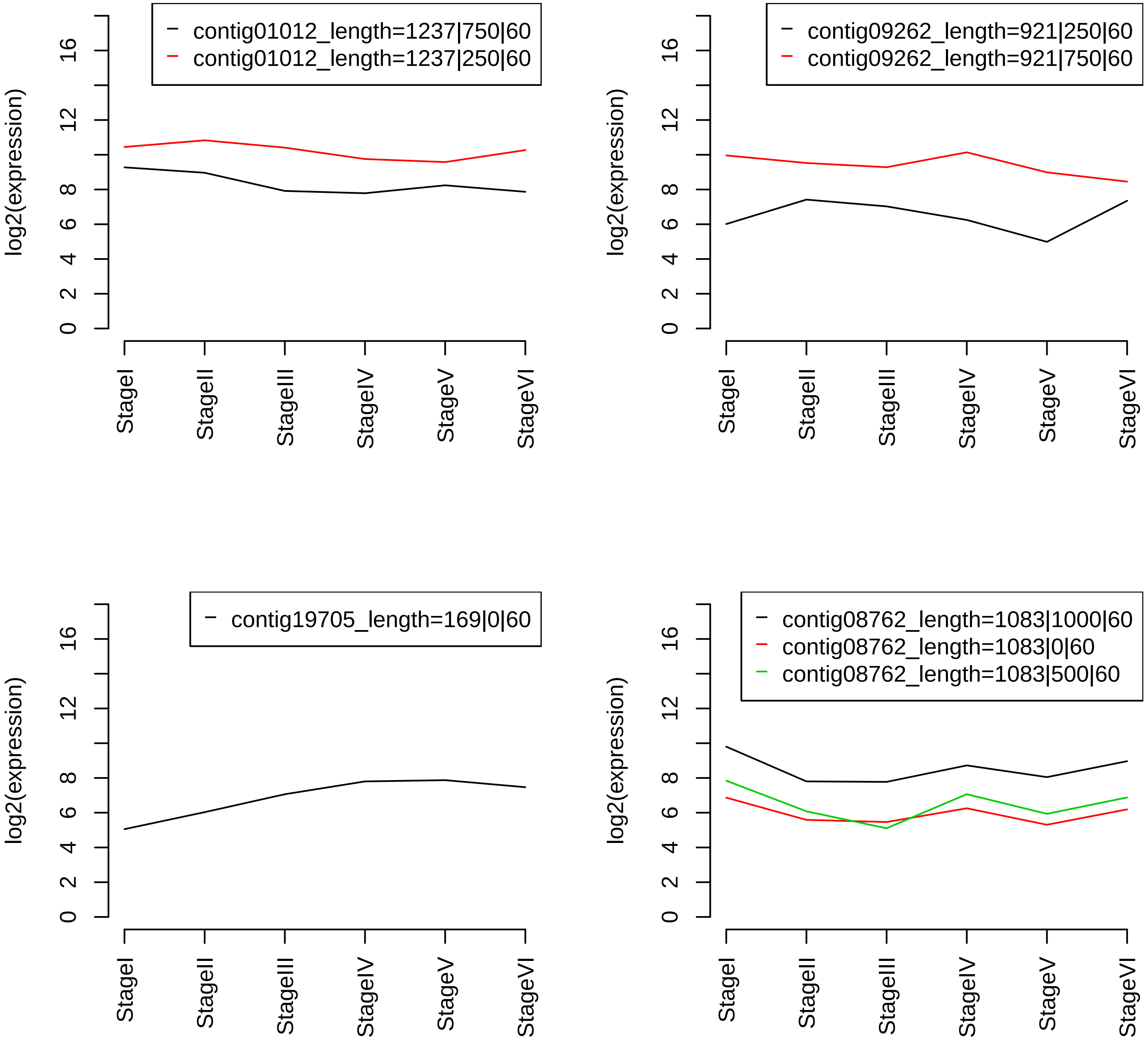
contig01012_length=1237|750|60
contig01012_length=1237|250|60
contig09262_length=921|250|60
contig09262_length=921|750|60
contig19705_length=169|0|60
contig08762_length=1083|1000|60
contig08762_length=1083|0|60
contig08762_length=1083|500|60
log2(expression)
0
2
4
6
8
12
16
StageI
StageII
StageIII
StageIV
StageV
StageVI

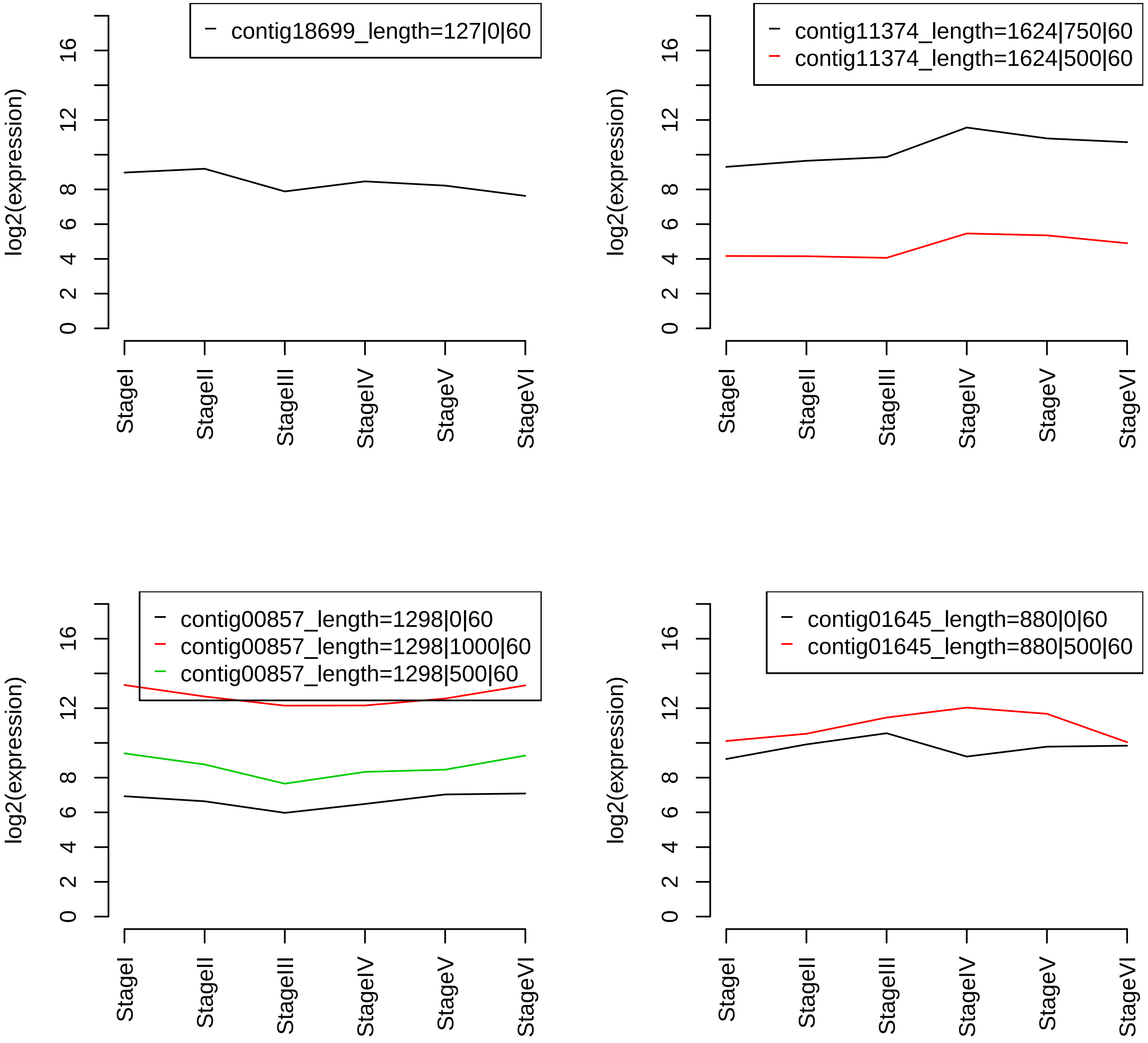
contig18699_length=127|0|60
contig11374_length=1624|750|60
contig11374_length=1624|500|60
contig00857_length=1298|0|60
contig00857_length=1298|1000|60
contig00857_length=1298|500|60
contig01645_length=880|0|60
contig01645_length=880|500|60
log2(expression)
0
2
4
6
8
12
16
StageI
StageII
StageIII
StageIV
StageV
StageVI

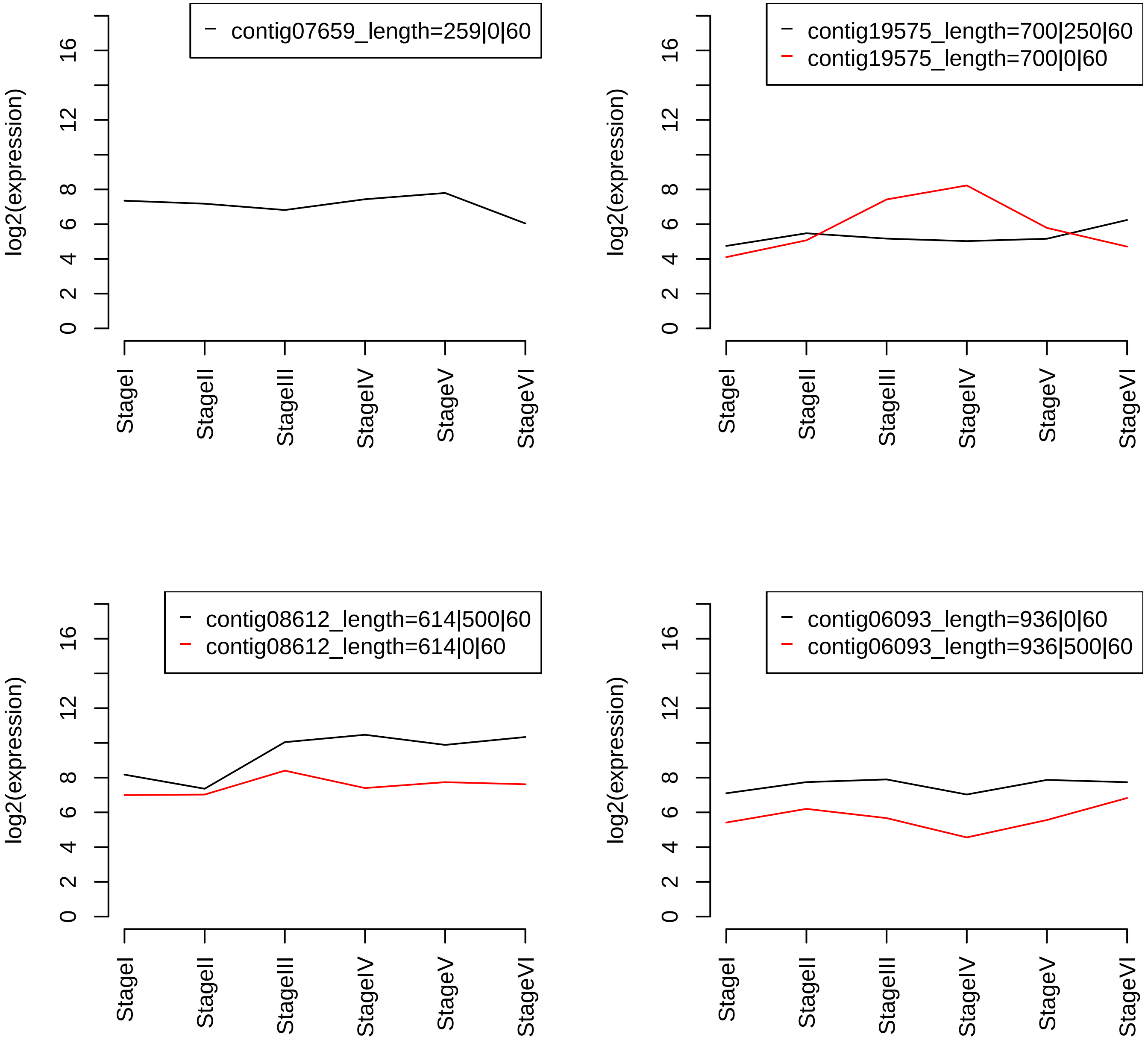
contig07659_length=259|0|60
contig19575_length=700|250|60
contig19575_length=700|0|60
contig08612_length=614|500|60
contig08612_length=614|0|60
contig06093_length=936|0|60
contig06093_length=936|500|60
log2(expression)
0
2
4
6
8
12
16
StageI
StageII
StageIII
StageIV
StageV
StageVI

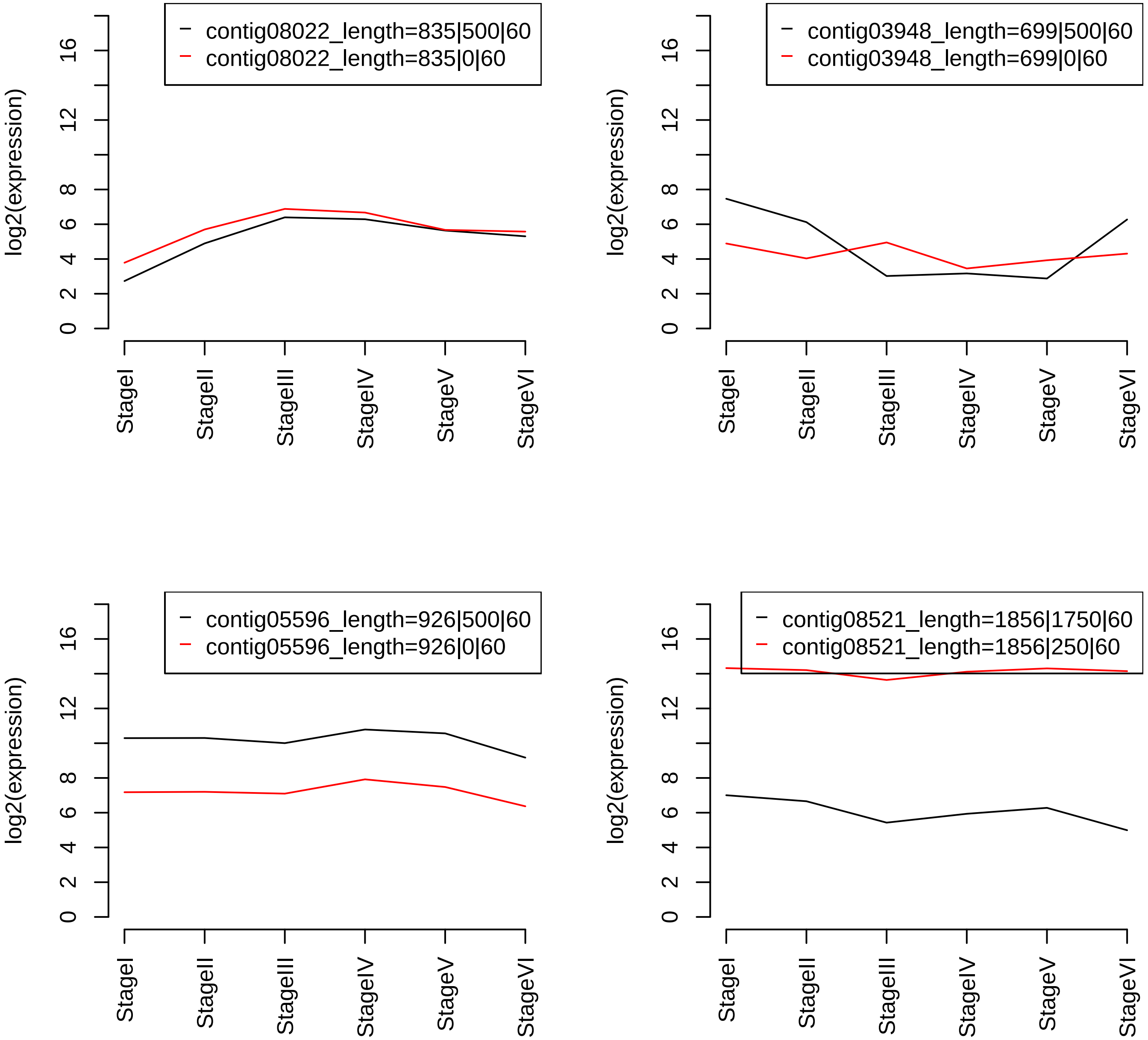
contig08022_length=835|500|60
contig08022_length=835|0|60
contig03948_length=699|500|60
contig03948_length=699|0|60
contig05596_length=926|500|60
contig05596_length=926|0|60
contig08521_length=1856|1750|60
contig08521_length=1856|250|60
log2(expression)
0 2 4 6 8 12 16
StageI StageII StageIII StageIV StageV StageVI

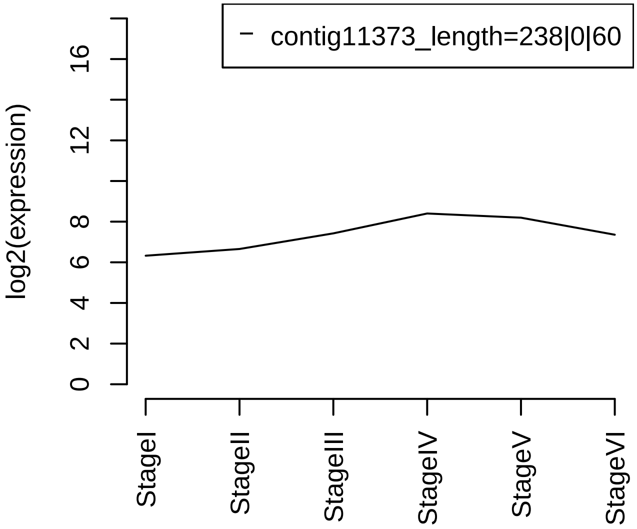
contig11373_length=238|0|60
log2(expression)
0
2
4
6
8
12
16
StageI
StageII
StageIII
StageIV
StageV
StageVI

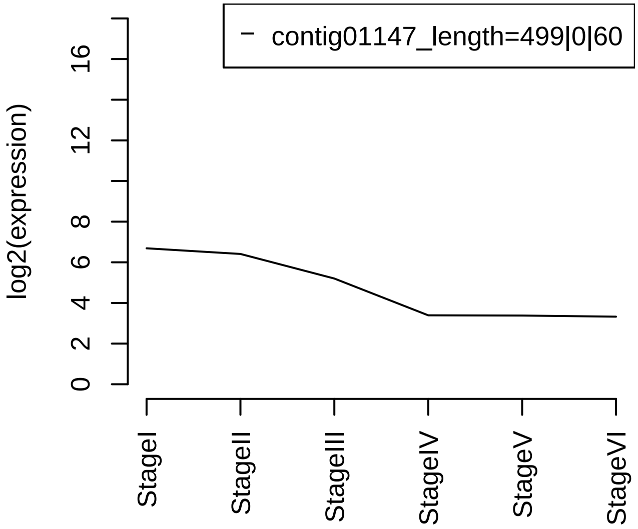
contig01147_length=499|0|60
log2(expression)
0
2
4
6
8
12
16
StageI
StageII
StageIII
StageIV
StageV
StageVI

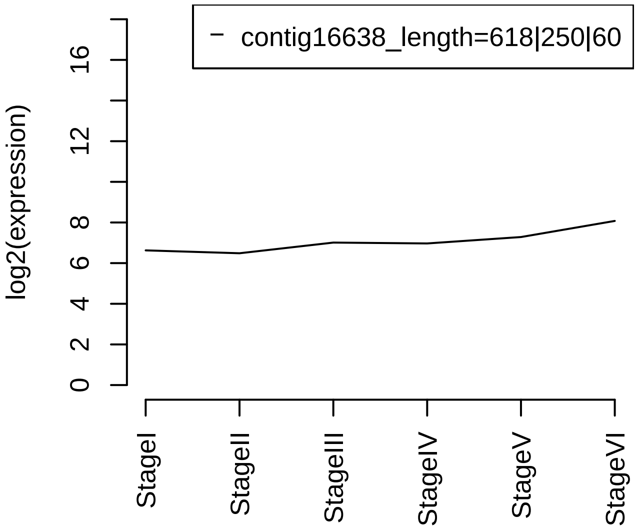
contig16638_length=618|250|60
log2(expression)
0
2
4
6
8
12
16
StageI
StageII
StageIII
StageIV
StageV
StageVI

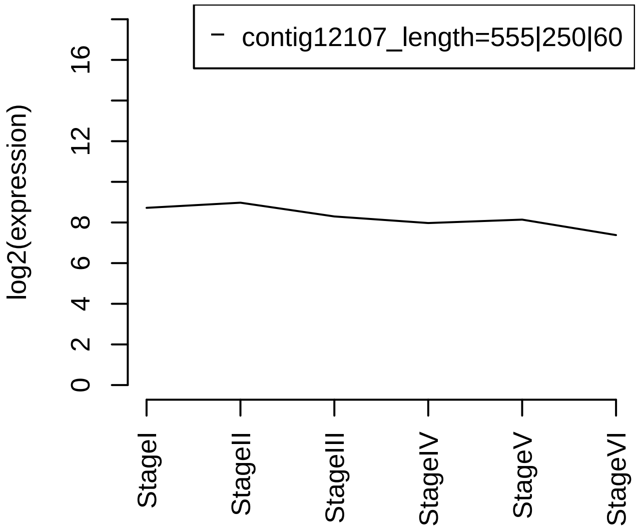
contig12107_length=555|250|60
log2(expression)
0
2
4
6
8
12
16
StageI
StageII
StageIII
StageIV
StageV
StageVI

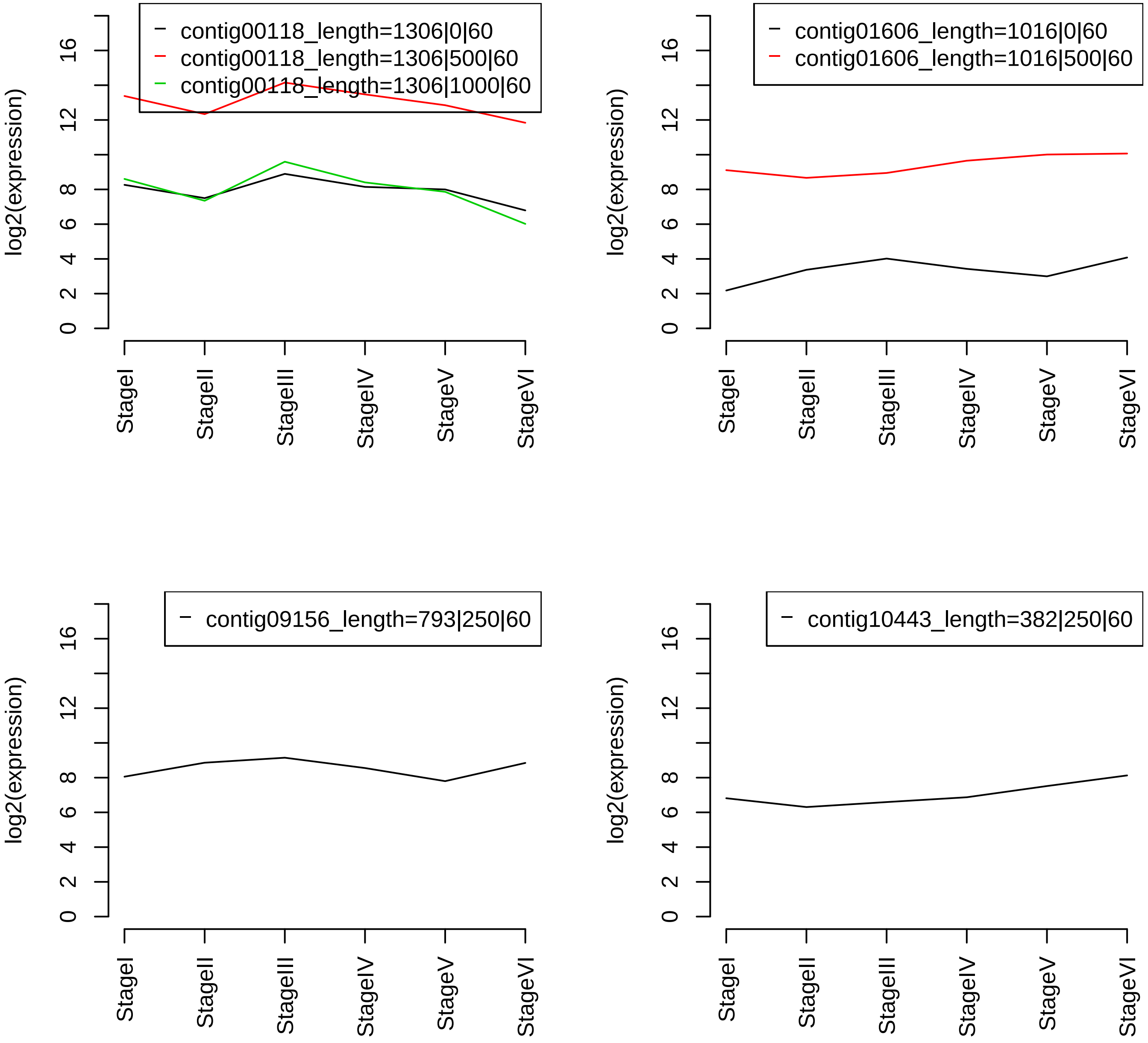

contig00118_length=1306|0|60
contig00118_length=1306|500|60
contig00118_length=1306|1000|60
contig01606_length=1016|0|60
contig01606_length=1016|500|60
contig09156_length=793|250|60
contig10443_length=382|250|60
log2(expression)
0
2
4
6
8
12
16
StageI
StageII
StageIII
StageIV
StageV
StageVI

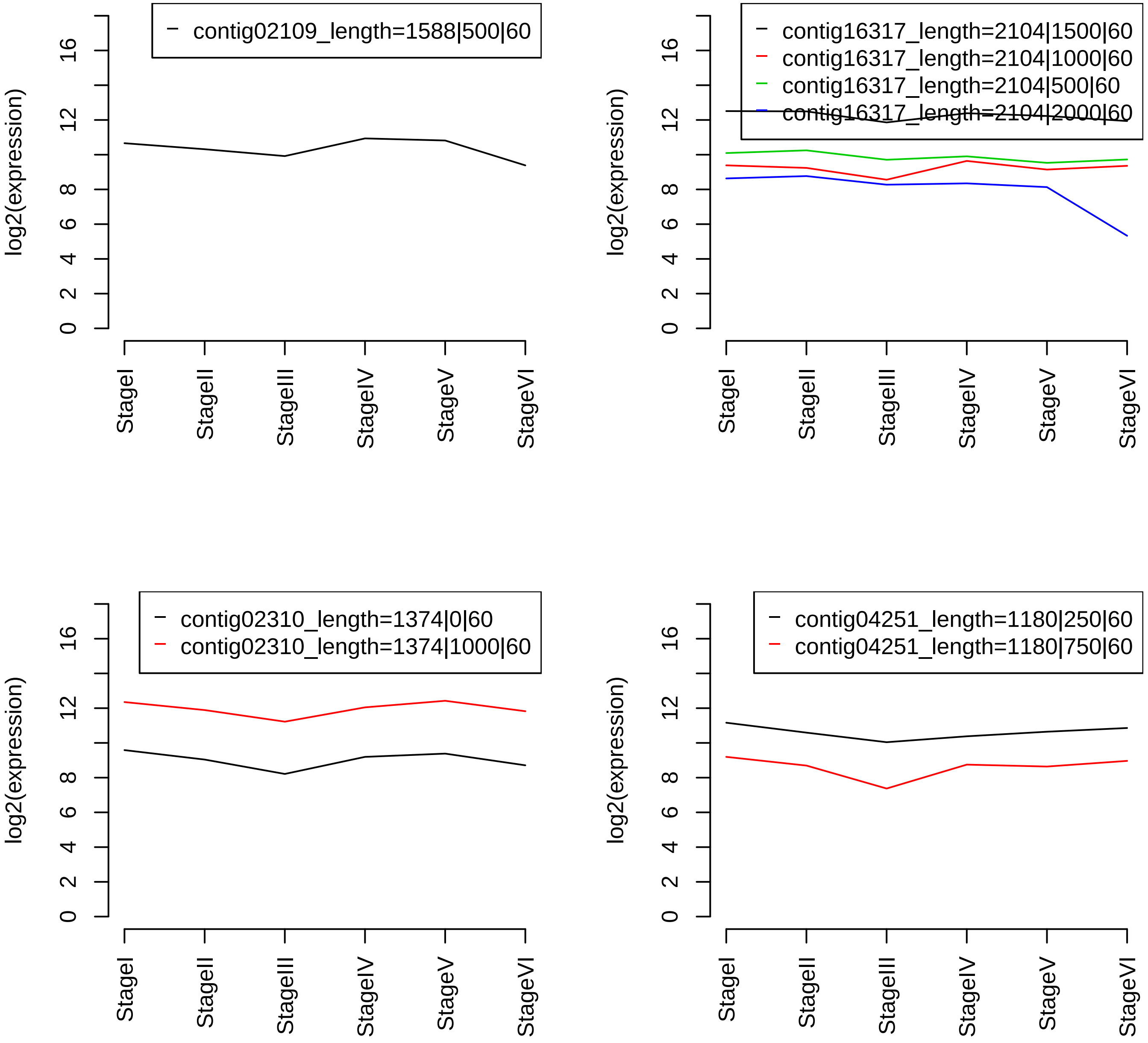
contig02109_length=1588|500|60
contig16317_length=2104|1500|60
contig16317_length=2104|1000|60
contig16317_length=2104|500|60
contig16317_length=2104|2000|60
contig02310_length=1374|0|60
contig02310_length=1374|1000|60
contig04251_length=1180|250|60
contig04251_length=1180|750|60
log2(expression)
0
2
4
6
8
12
16
StageI
StageII
StageIII
StageIV
StageV
StageVI

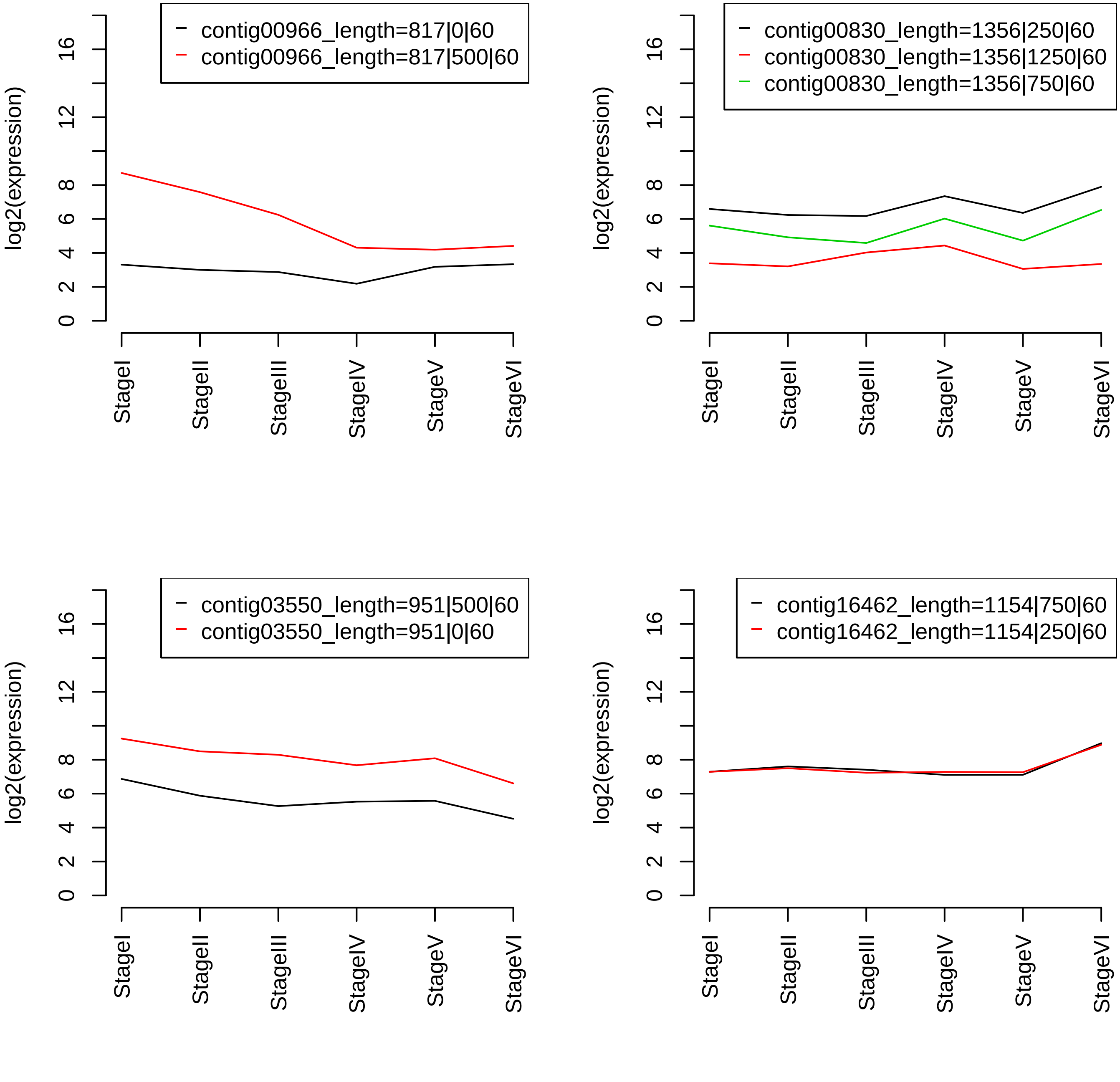
contig00966_length=817|0|60
contig00966_length=817|500|60
contig00830_length=1356|250|60
contig00830_length=1356|1250|60
contig00830_length=1356|750|60
contig03550_length=951|500|60
contig03550_length=951|0|60
contig16462_length=1154|750|60
contig16462_length=1154|250|60
log2(expression)
0
2
4
6
8
12
16
StageI
StageII
StageIII
StageIV
StageV
StageVI

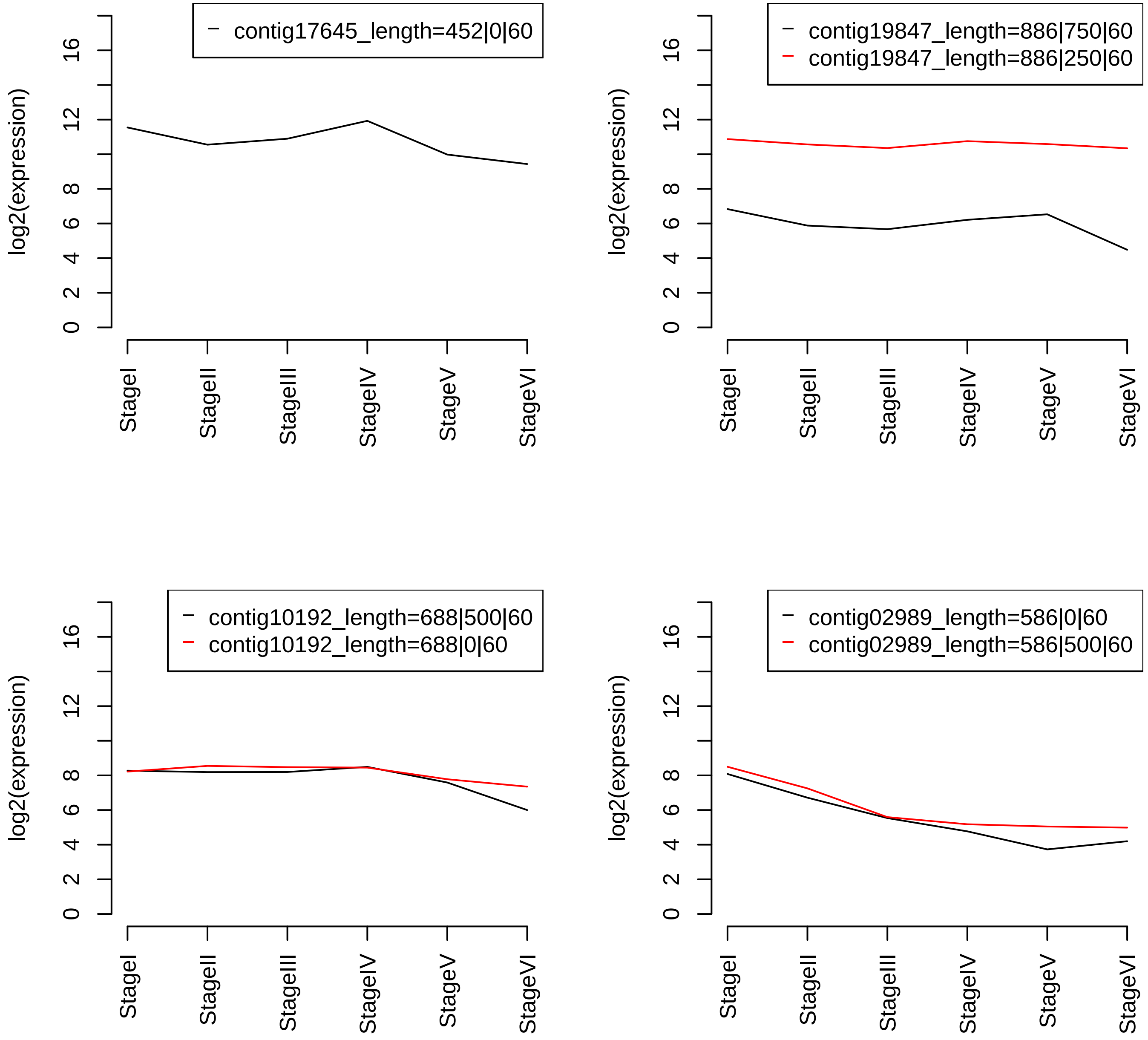

contig17645_length=452|0|60
contig19847_length=886|750|60
contig19847_length=886|250|60
contig10192_length=688|500|60
contig10192_length=688|0|60
contig02989_length=586|0|60
contig02989_length=586|500|60
log2(expression)
0
2
4
6
8
12
16
StageI
StageII
StageIII
StageIV
StageV
StageVI

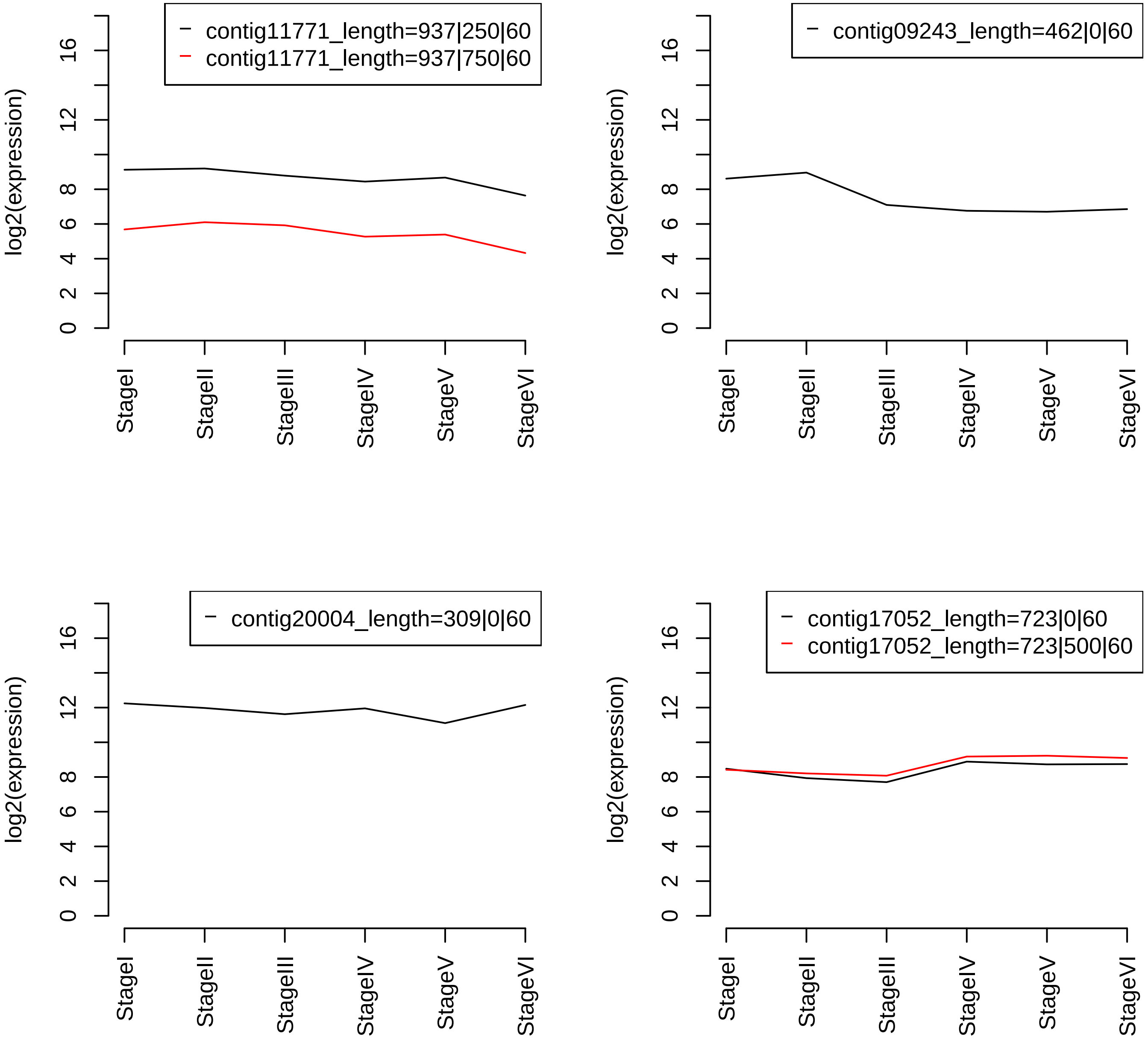
contig11771_length=937|250|60
contig11771_length=937|750|60
contig09243_length=462|0|60
contig20004_length=309|0|60
contig17052_length=723|0|60
contig17052_length=723|500|60
log2(expression)
0
2
4
6
8
12
16
StageI
StageII
StageIII
StageIV
StageV
StageVI

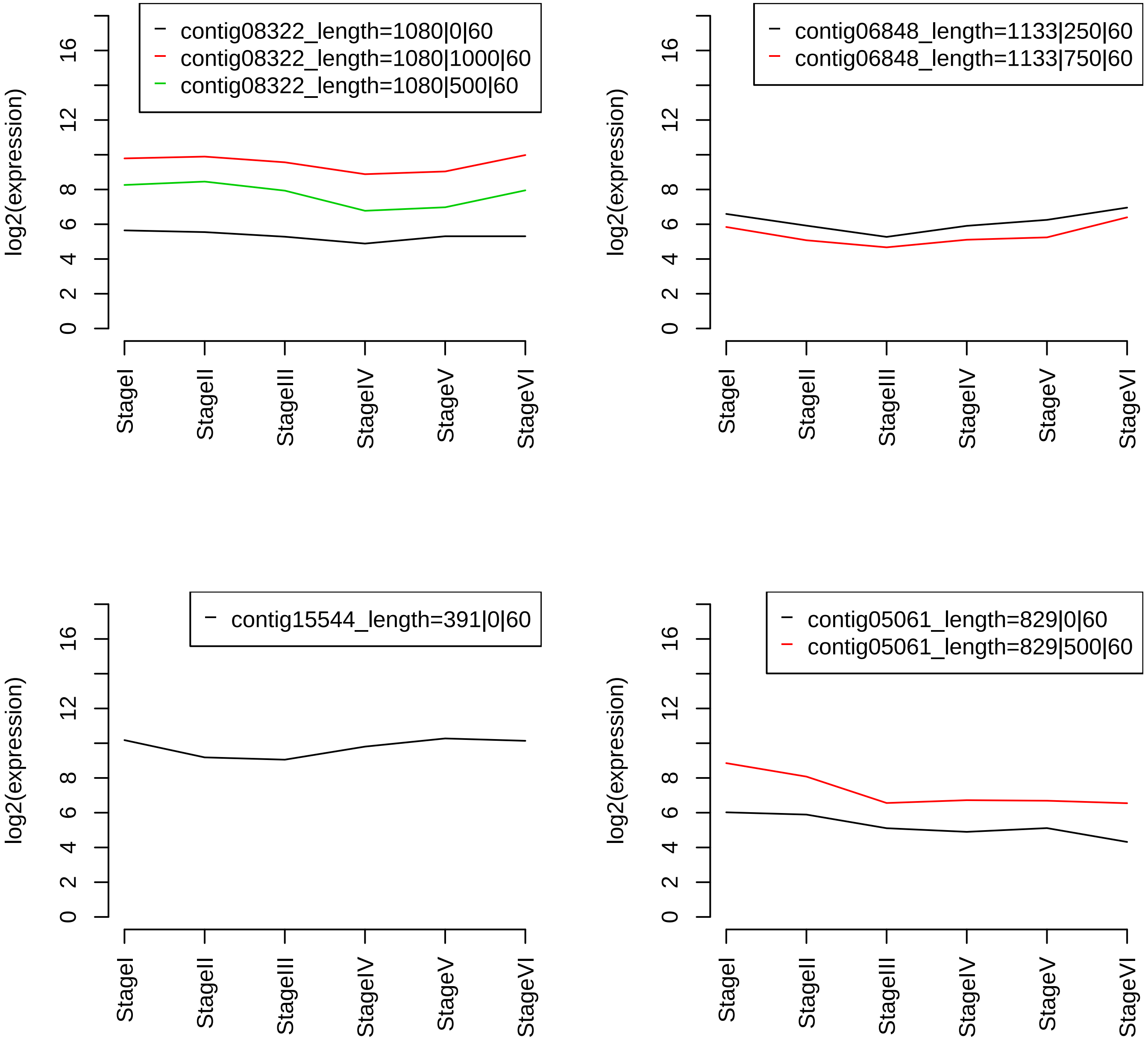

contig08322_length=1080|0|60
contig08322_length=1080|1000|60
contig08322_length=1080|500|60
contig06848_length=1133|250|60
contig06848_length=1133|750|60
contig15544_length=391|0|60
contig05061_length=829|0|60
contig05061_length=829|500|60
log2(expression)
0
2
4
6
8
12
16
StageI
StageII
StageIII
StageIV
StageV
StageVI

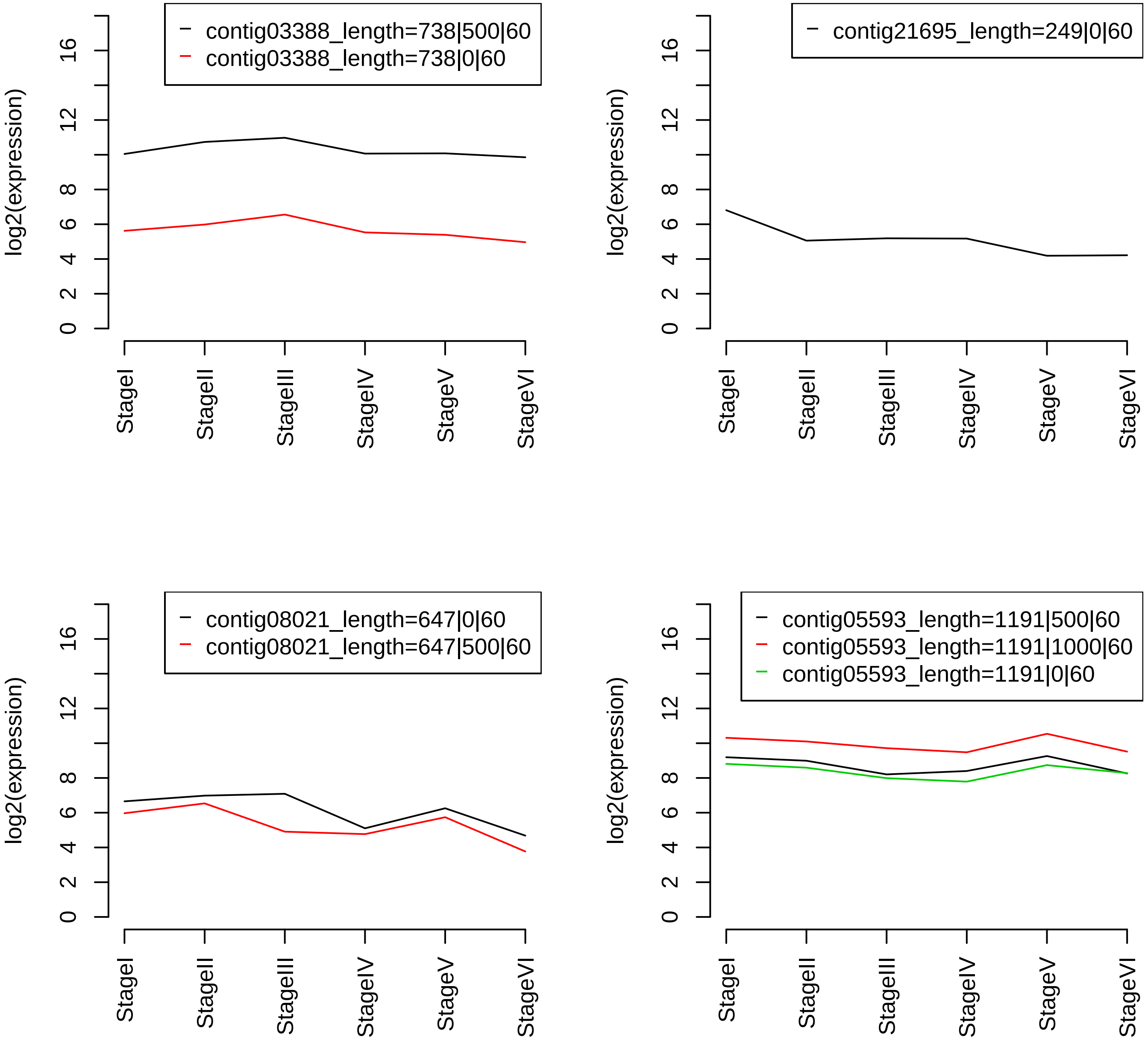
contig03388_length=738|500|60
contig03388_length=738|0|60
contig21695_length=249|0|60
contig08021_length=647|0|60
contig08021_length=647|500|60
contig05593_length=1191|500|60
contig05593_length=1191|1000|60
contig05593_length=1191|0|60
log2(expression)
0
2
4
6
8
12
16
StageI
StageII
StageIII
StageIV
StageV
StageVI

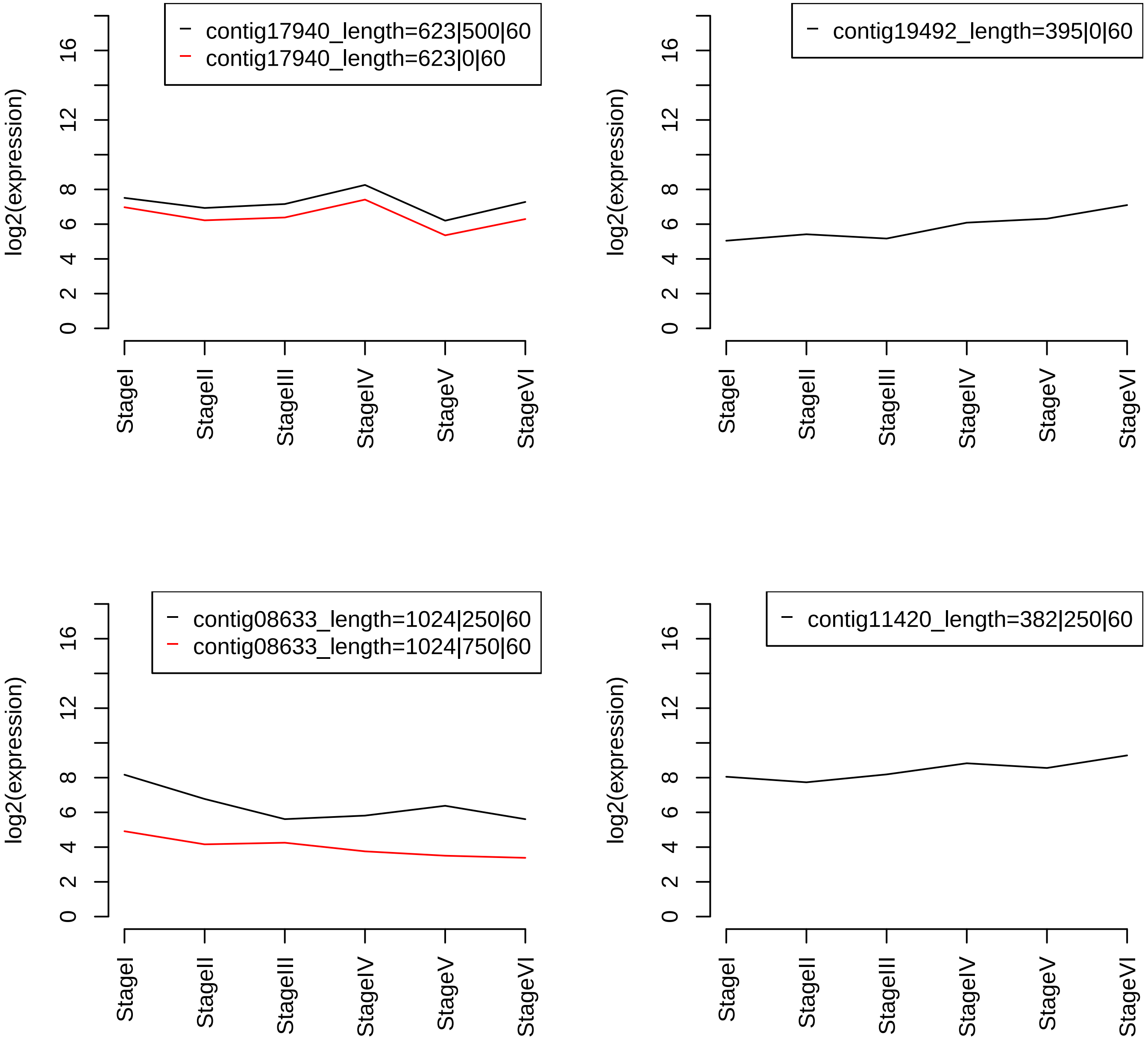
contig17940_length=623|500|60
contig17940_length=623|0|60
contig19492_length=395|0|60
contig08633_length=1024|250|60
contig08633_length=1024|750|60
contig11420_length=382|250|60
log2(expression)
0
2
4
6
8
12
16
StageI
StageII
StageIII
StageIV
StageV
StageVI

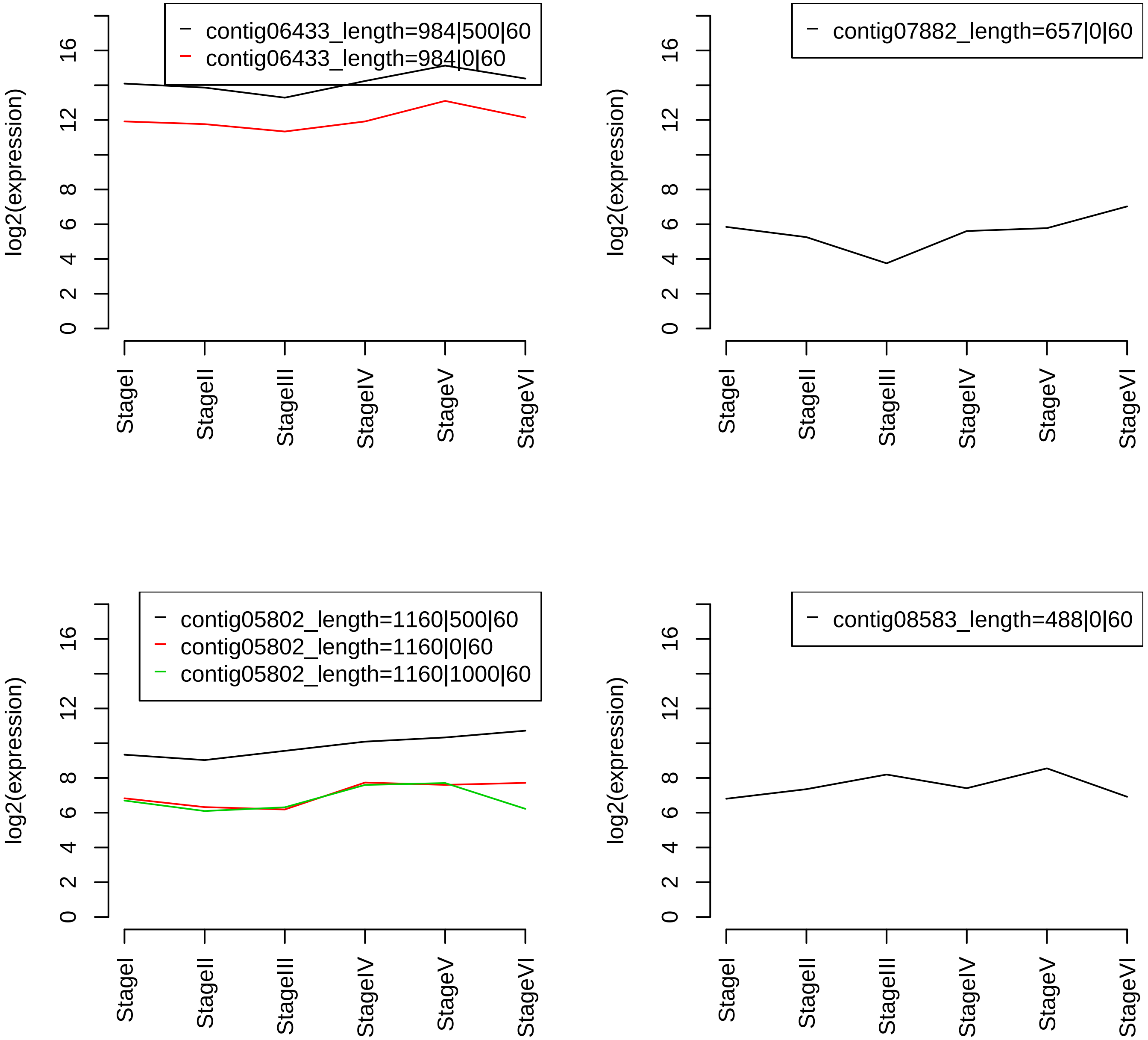

contig06433_length=984|500|60
contig06433_length=984|0|60
contig07882_length=657|0|60
contig05802_length=1160|500|60
contig05802_length=1160|0|60
contig05802_length=1160|1000|60
contig08583_length=488|0|60
log2(expression)
0
2
4
6
8
12
16
StageI
StageII
StageIII
StageIV
StageV
StageVI

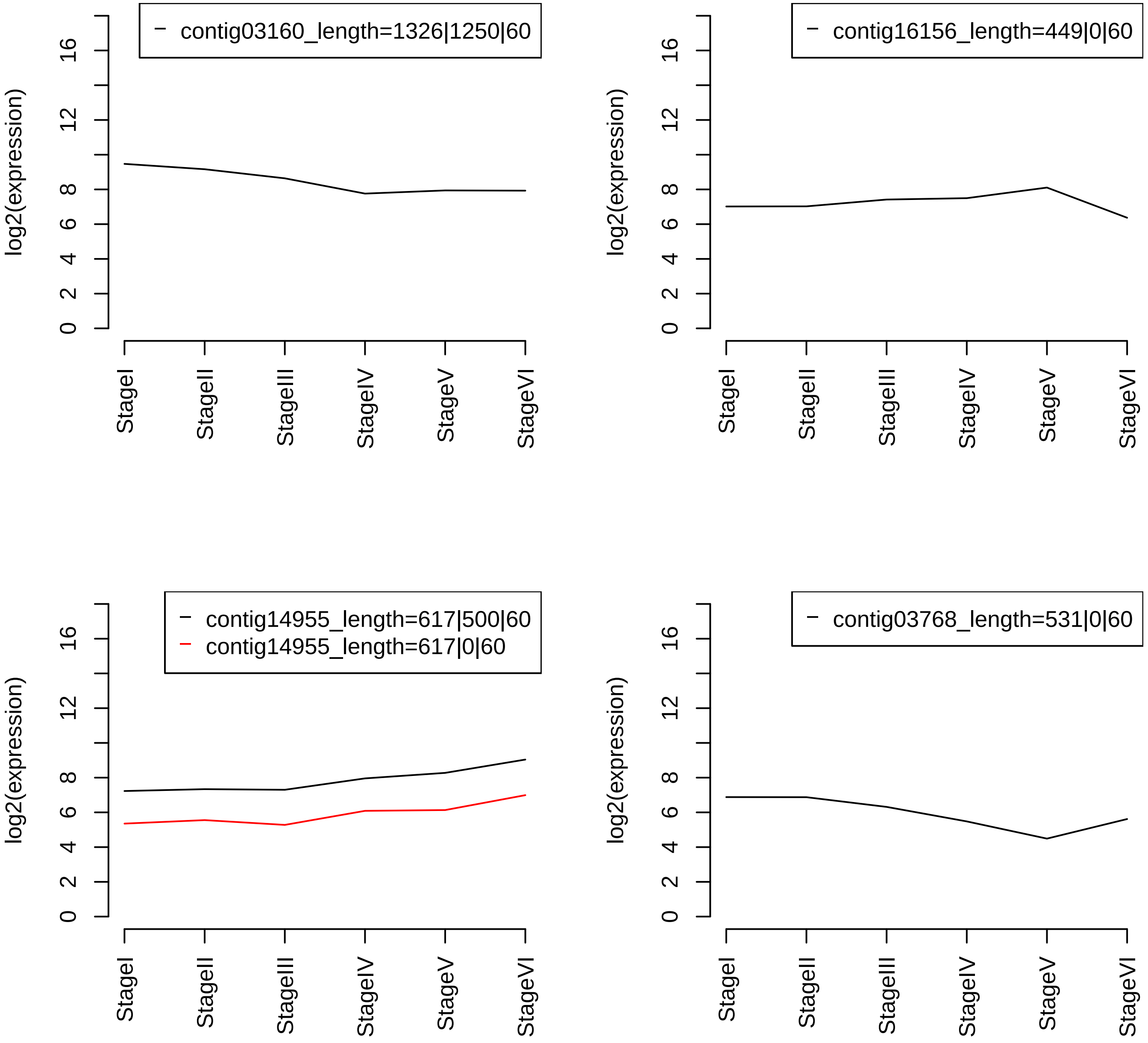

contig03160_length=1326|1250|60
contig16156_length=449|0|60
contig14955_length=617|500|60
contig14955_length=617|0|60
contig03768_length=531|0|60
log2(expression)
0
2
4
6
8
12
16
StageI
StageII
StageIII
StageIV
StageV
StageVI

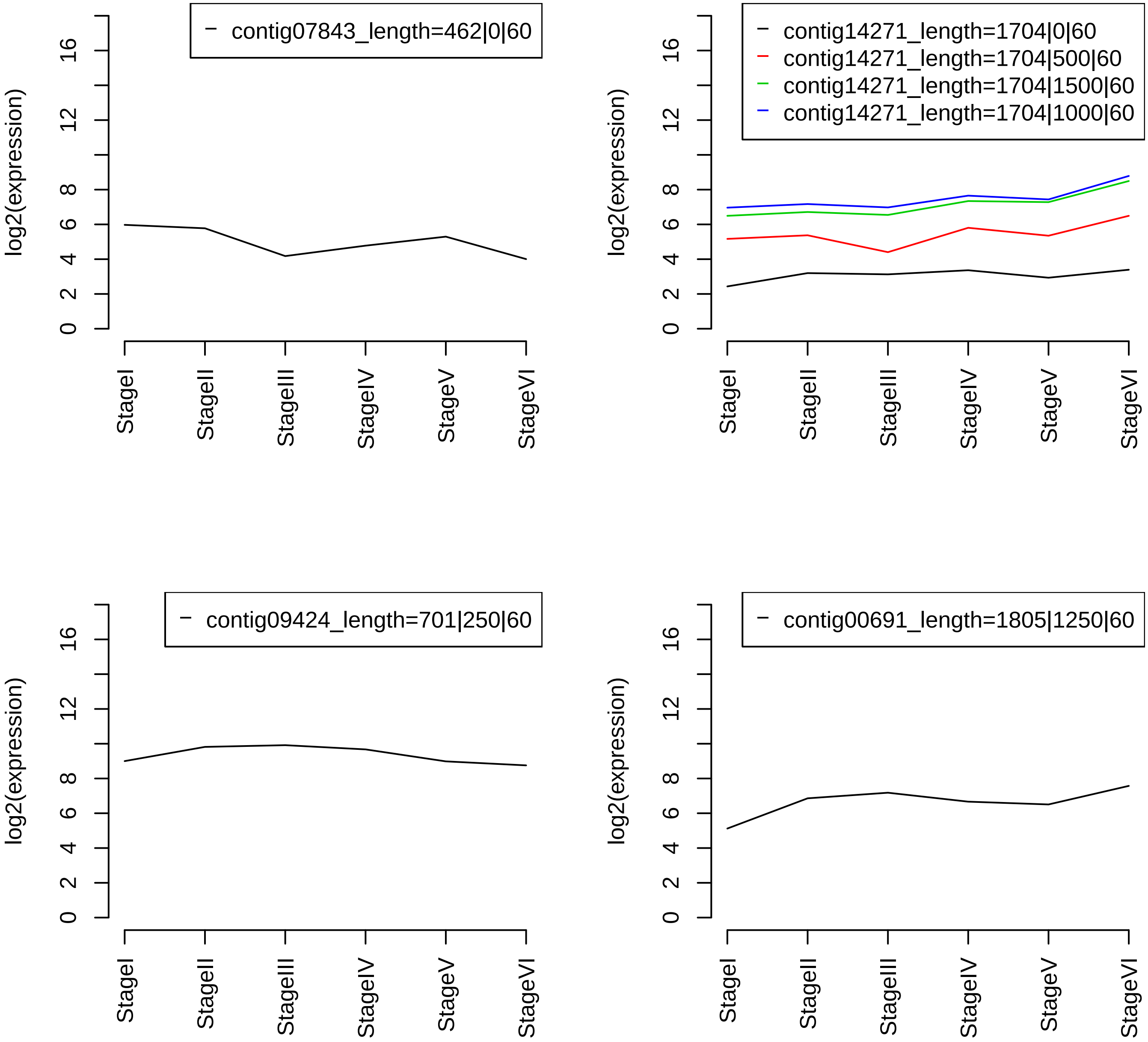

contig07843_length=462|0|60
contig14271_length=1704|0|60
contig14271_length=1704|500|60
contig14271_length=1704|1500|60
contig14271_length=1704|1000|60
contig09424_length=701|250|60
contig00691_length=1805|1250|60
log2(expression)
0
2
4
6
8
12
16
StageI
StageII
StageIII
StageIV
StageV
StageVI

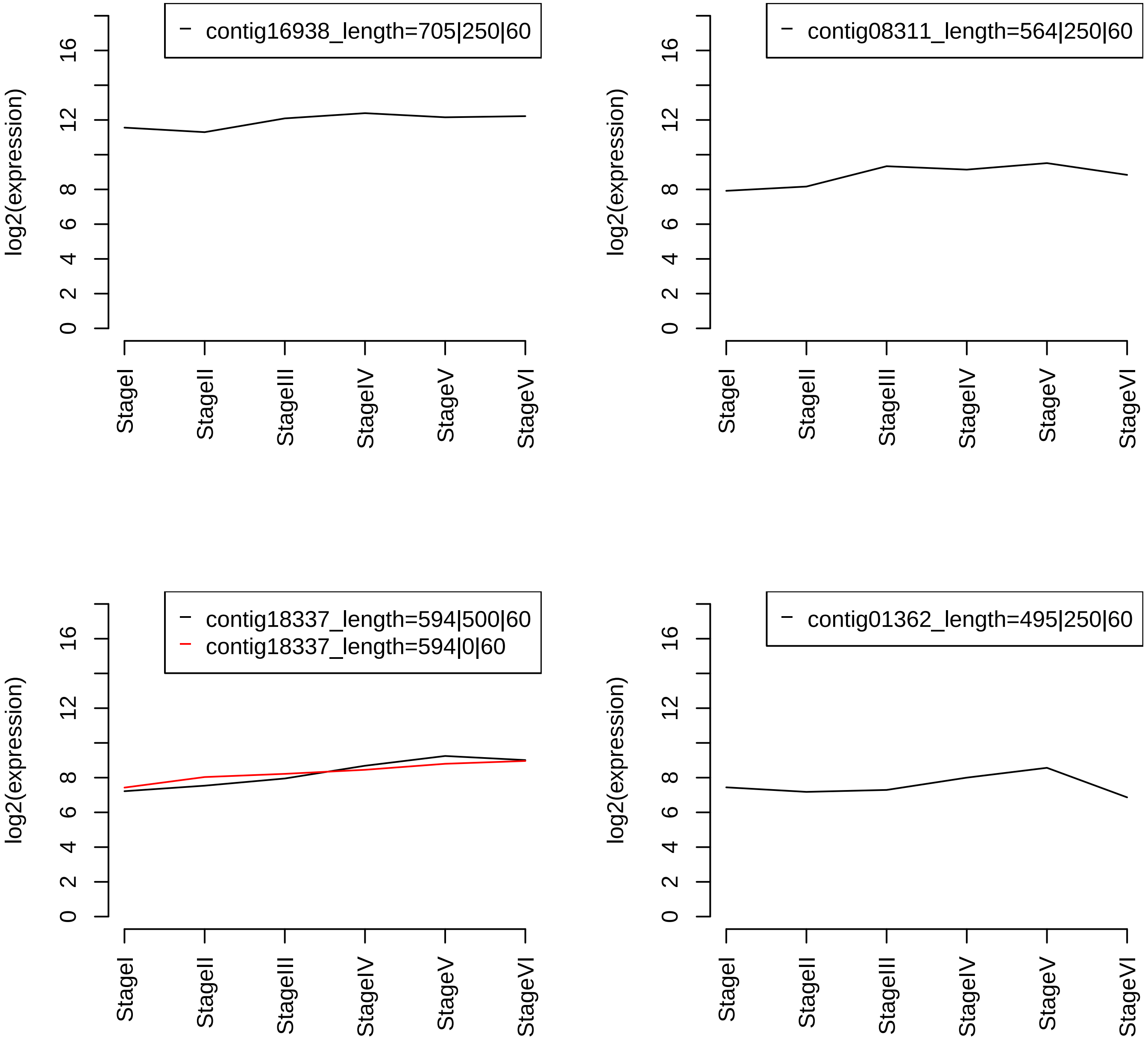

contig16938_length=705|250|60
contig08311_length=564|250|60
contig18337_length=594|500|60
contig18337_length=594|0|60
contig01362_length=495|250|60
log2(expression)
0
2
4
6
8
12
16
StageI
StageII
StageIII
StageIV
StageV
StageVI

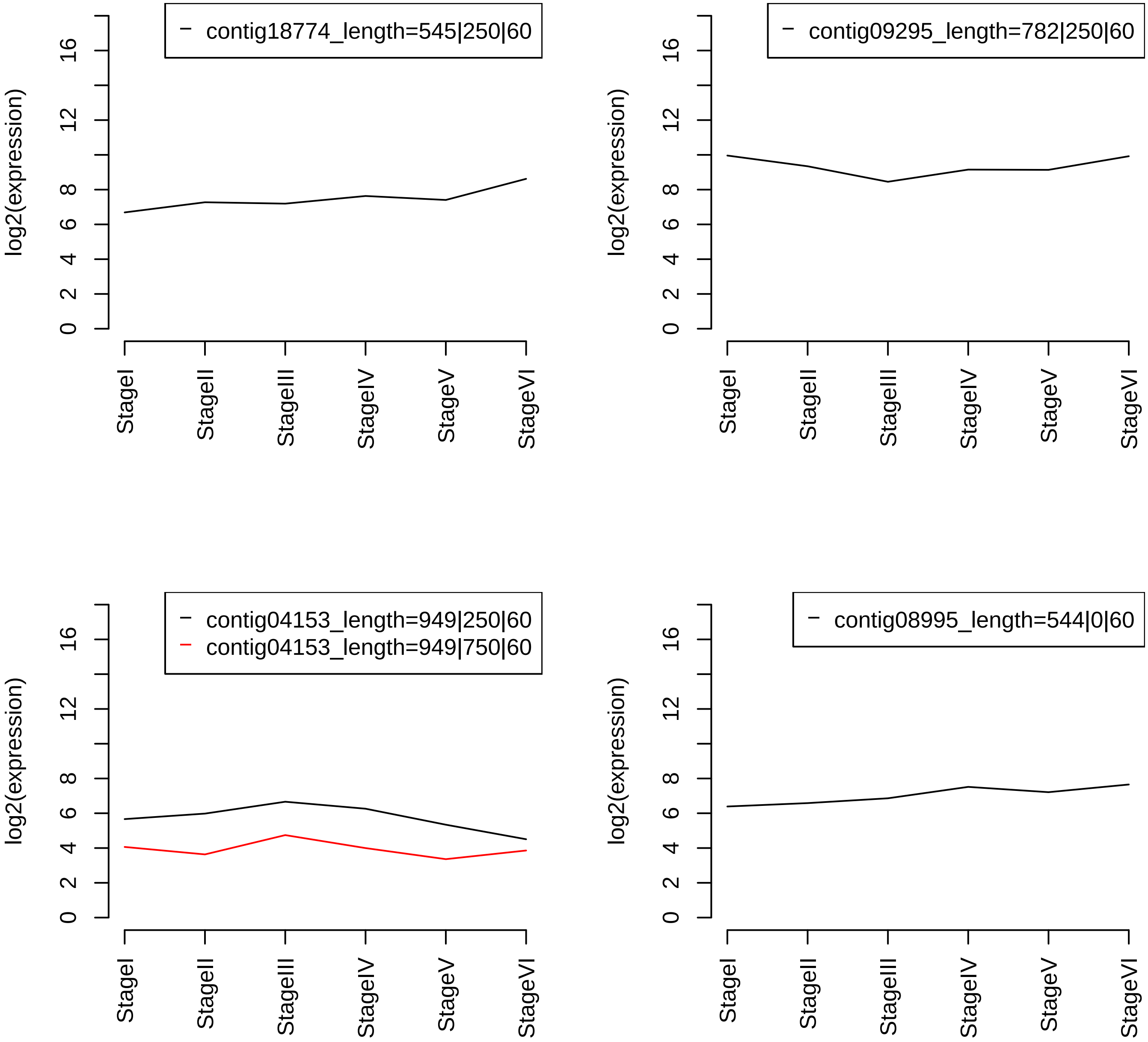
contig18774_length=545|250|60
contig09295_length=782|250|60
contig04153_length=949|250|60
contig04153_length=949|750|60
contig08995_length=544|0|60
log2(expression)
0
2
4
6
8
12
16
StageI
StageII
StageIII
StageIV
StageV
StageVI

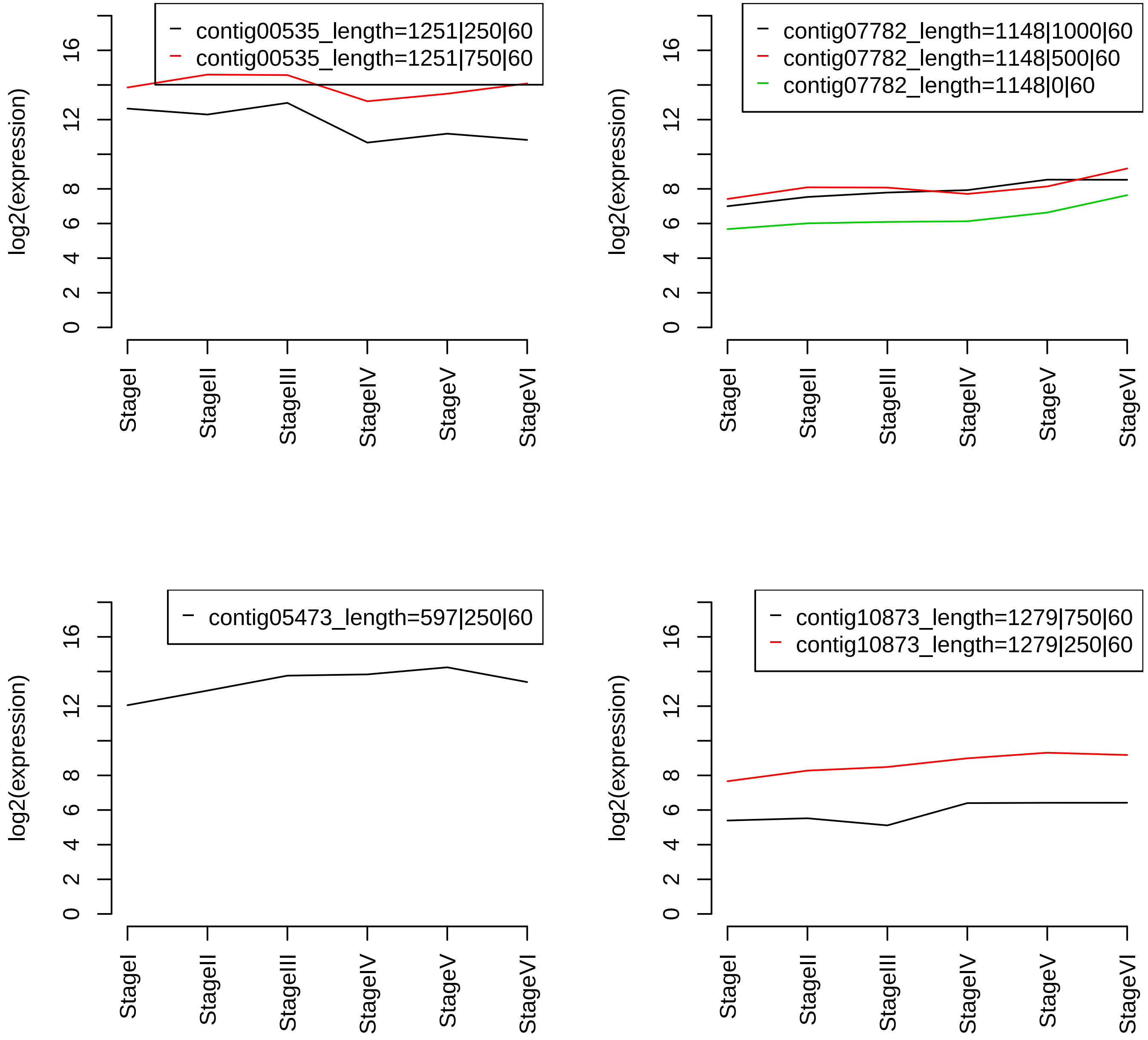

contig00535_length=1251|250|60
contig00535_length=1251|750|60
contig07782_length=1148|1000|60
contig07782_length=1148|500|60
contig07782_length=1148|0|60
contig05473_length=597|250|60
contig10873_length=1279|750|60
contig10873_length=1279|250|60
log2(expression)
0
2
4
6
8
12
16
StageI
StageII
StageIII
StageIV
StageV
StageVI

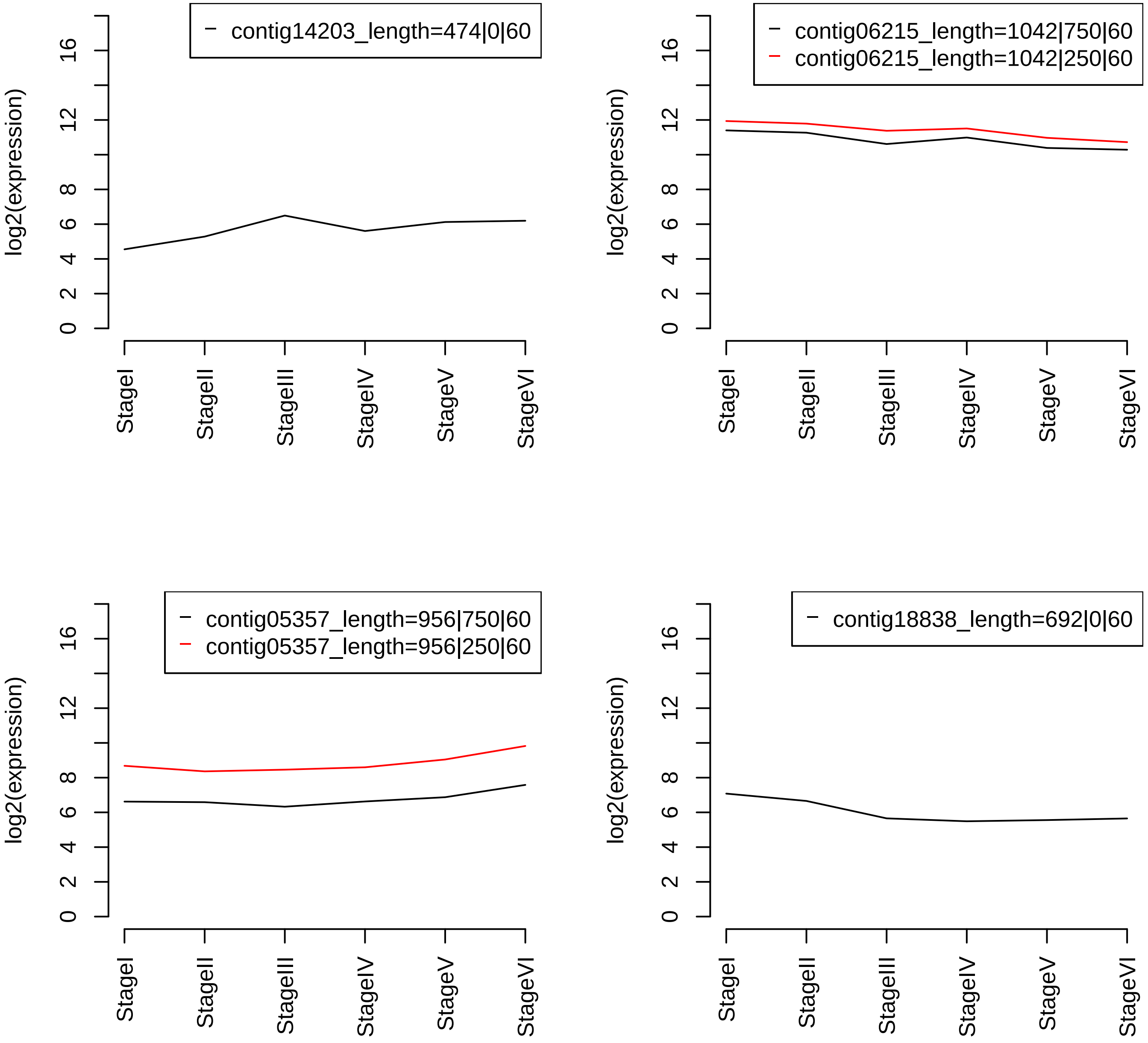

contig14203_length=474|0|60
contig06215_length=1042|750|60
contig06215_length=1042|250|60
contig05357_length=956|750|60
contig05357_length=956|250|60
contig18838_length=692|0|60
log2(expression)
0
2
4
6
8
12
16
StageI
StageII
StageIII
StageIV
StageV
StageVI

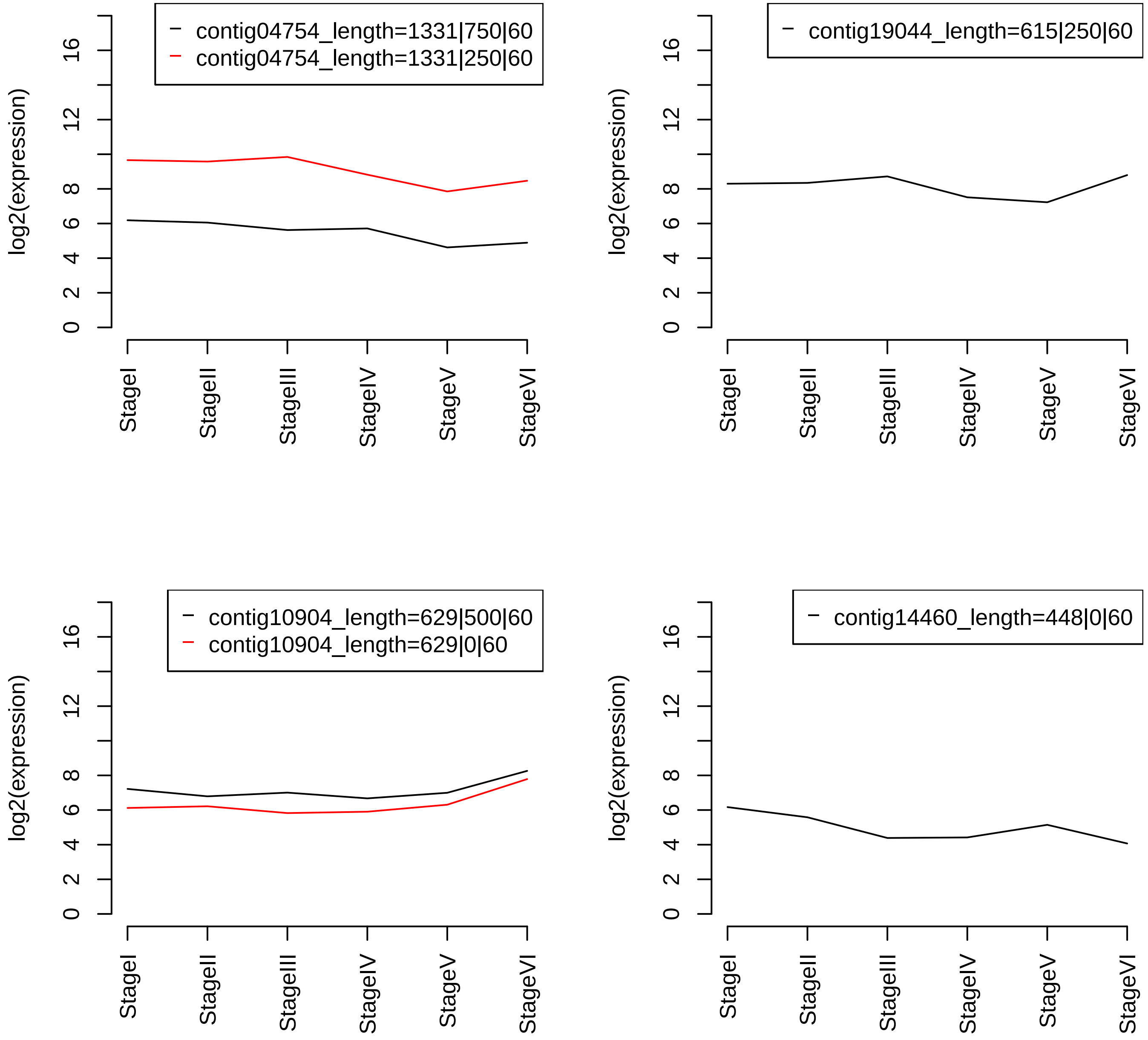

contig04754_length=1331|750|60
contig04754_length=1331|250|60
contig19044_length=615|250|60
contig10904_length=629|500|60
contig10904_length=629|0|60
contig14460_length=448|0|60
log2(expression)
0
2
4
6
8
12
16
StageI
StageII
StageIII
StageIV
StageV
StageVI

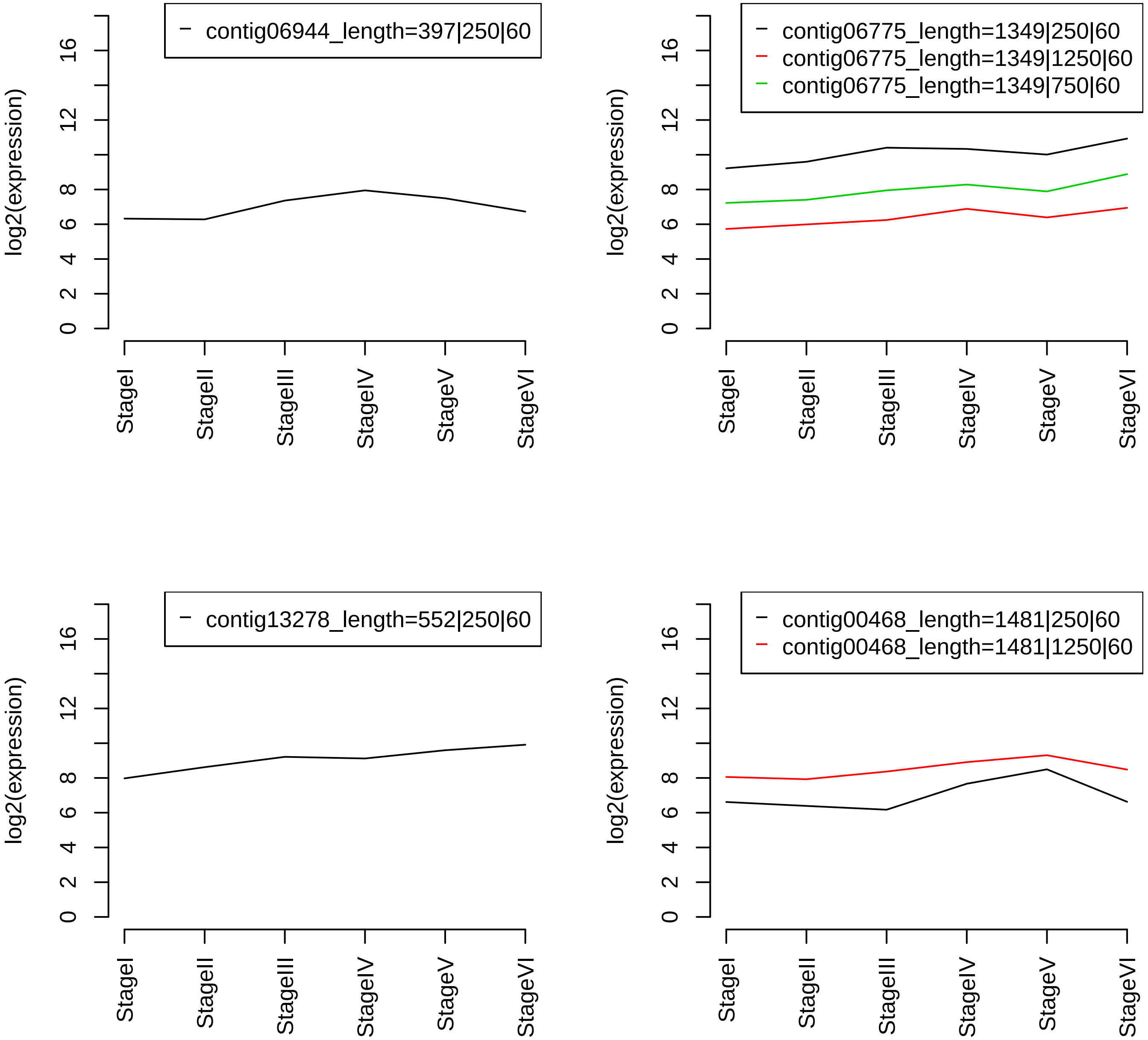

contig06944_length=397|250|60
log2(expression)
0
2
4
6
8
12
16
StageI
StageII
StageIII
StageIV
StageV
StageVI
contig06775_length=1349|250|60
contig06775_length=1349|1250|60
contig06775_length=1349|750|60
log2(expression)
contig13278_length=552|250|60
log2(expression)
contig00468_length=1481|250|60
contig00468_length=1481|1250|60
log2(expression)

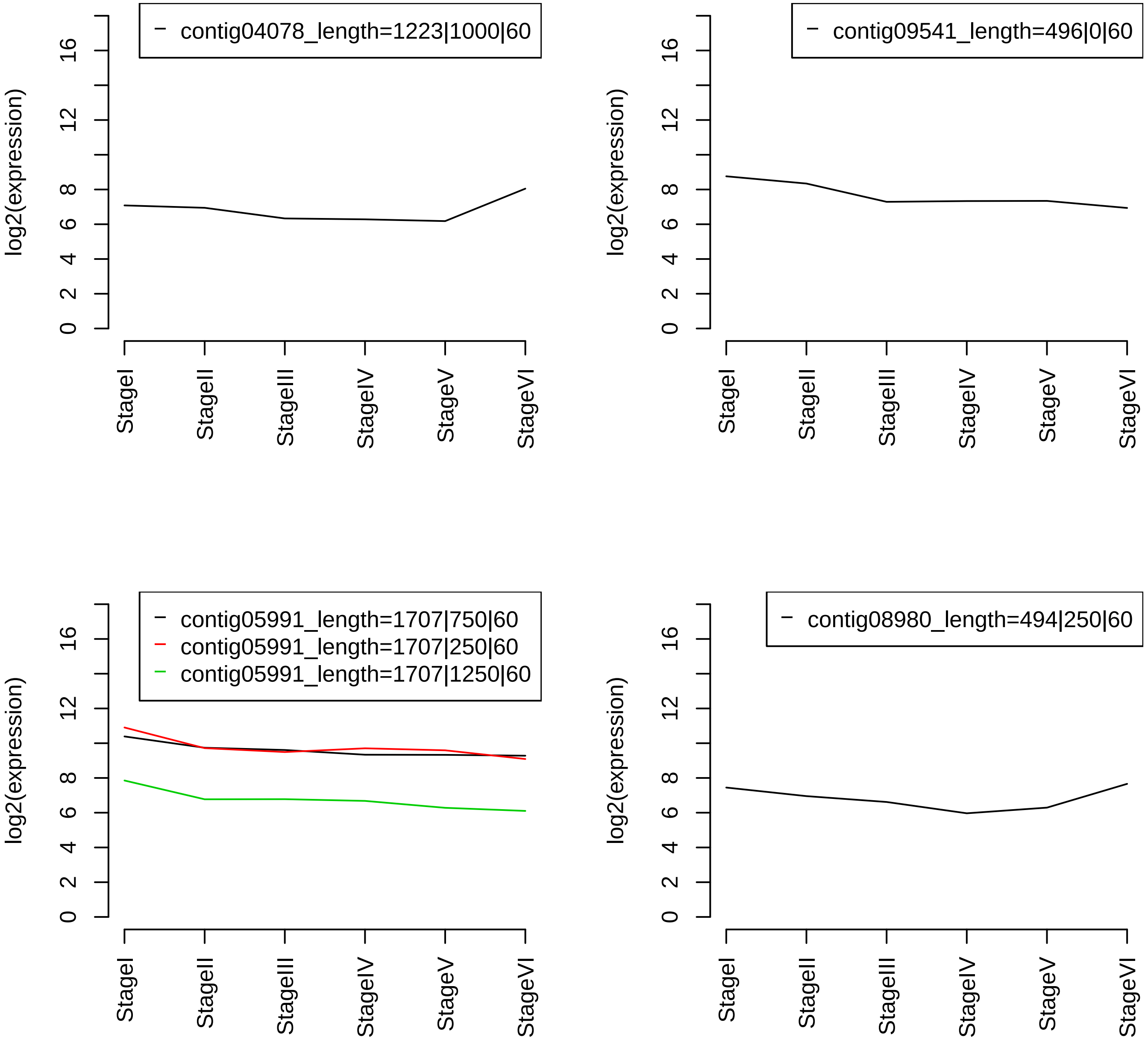

contig04078_length=1223|1000|60
log2(expression)
0
2
4
6
8
12
16
StageI
StageII
StageIII
StageIV
StageV
StageVI
contig09541_length=496|0|60
log2(expression)
0
2
4
6
8
12
16
StageI
StageII
StageIII
StageIV
StageV
StageVI
contig05991_length=1707|750|60
contig05991_length=1707|250|60
contig05991_length=1707|1250|60
log2(expression)
0
2
4
6
8
12
16
StageI
StageII
StageIII
StageIV
StageV
StageVI
contig08980_length=494|250|60
log2(expression)
0
2
4
6
8
12
16
StageI
StageII
StageIII
StageIV
StageV
StageVI

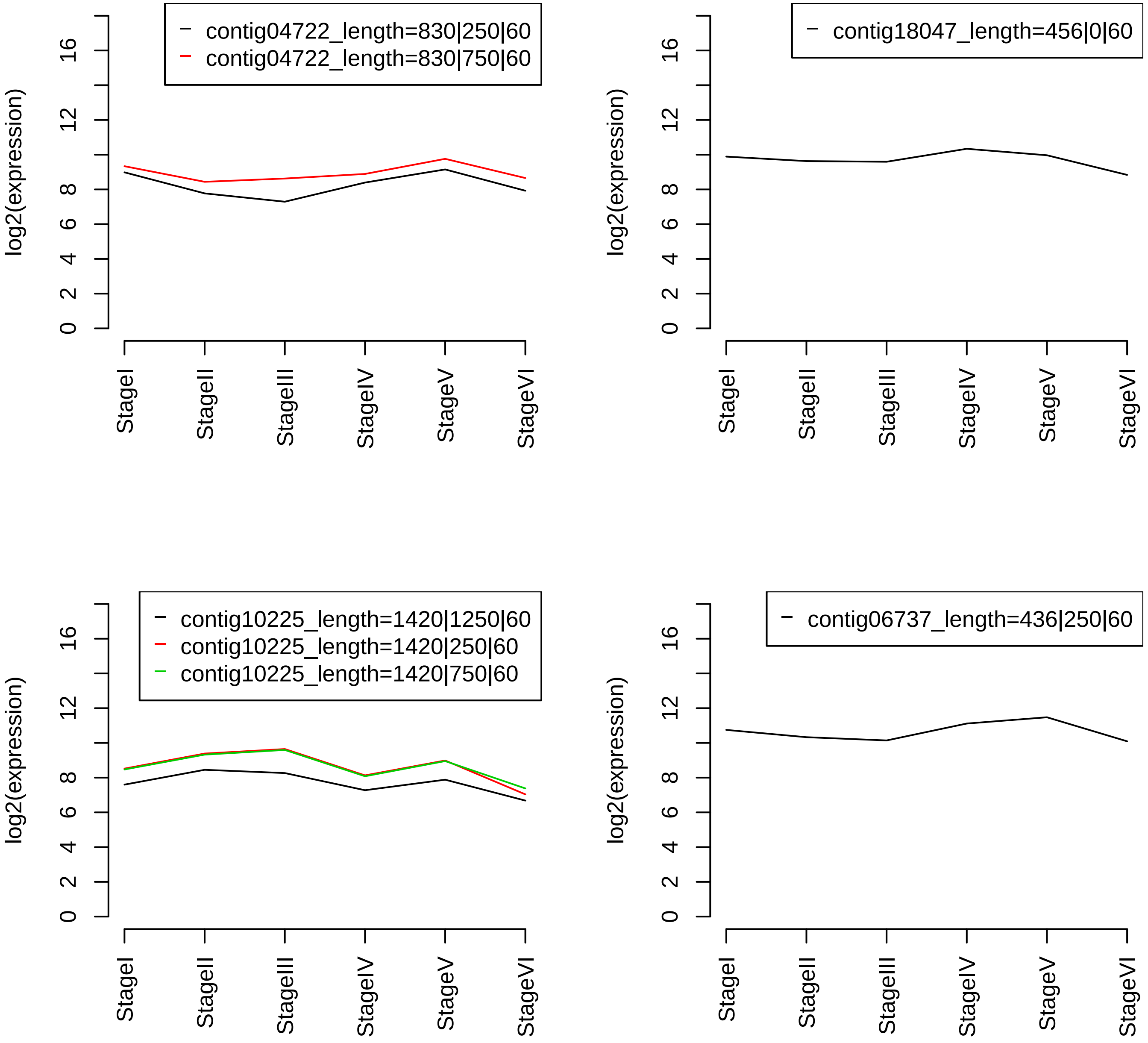
contig04722_length=830|250|60
contig04722_length=830|750|60
log2(expression)
0 2 4 6 8 12 16
StageI StageII StageIII StageIV StageV StageVI
contig18047_length=456|0|60
log2(expression)
0 2 4 6 8 12 16
StageI StageII StageIII StageIV StageV StageVI
contig10225_length=1420|1250|60
contig10225_length=1420|250|60
contig10225_length=1420|750|60
log2(expression)
0 2 4 6 8 12 16
StageI StageII StageIII StageIV StageV StageVI
contig06737_length=436|250|60
log2(expression)
0 2 4 6 8 12 16
StageI StageII StageIII StageIV StageV StageVI

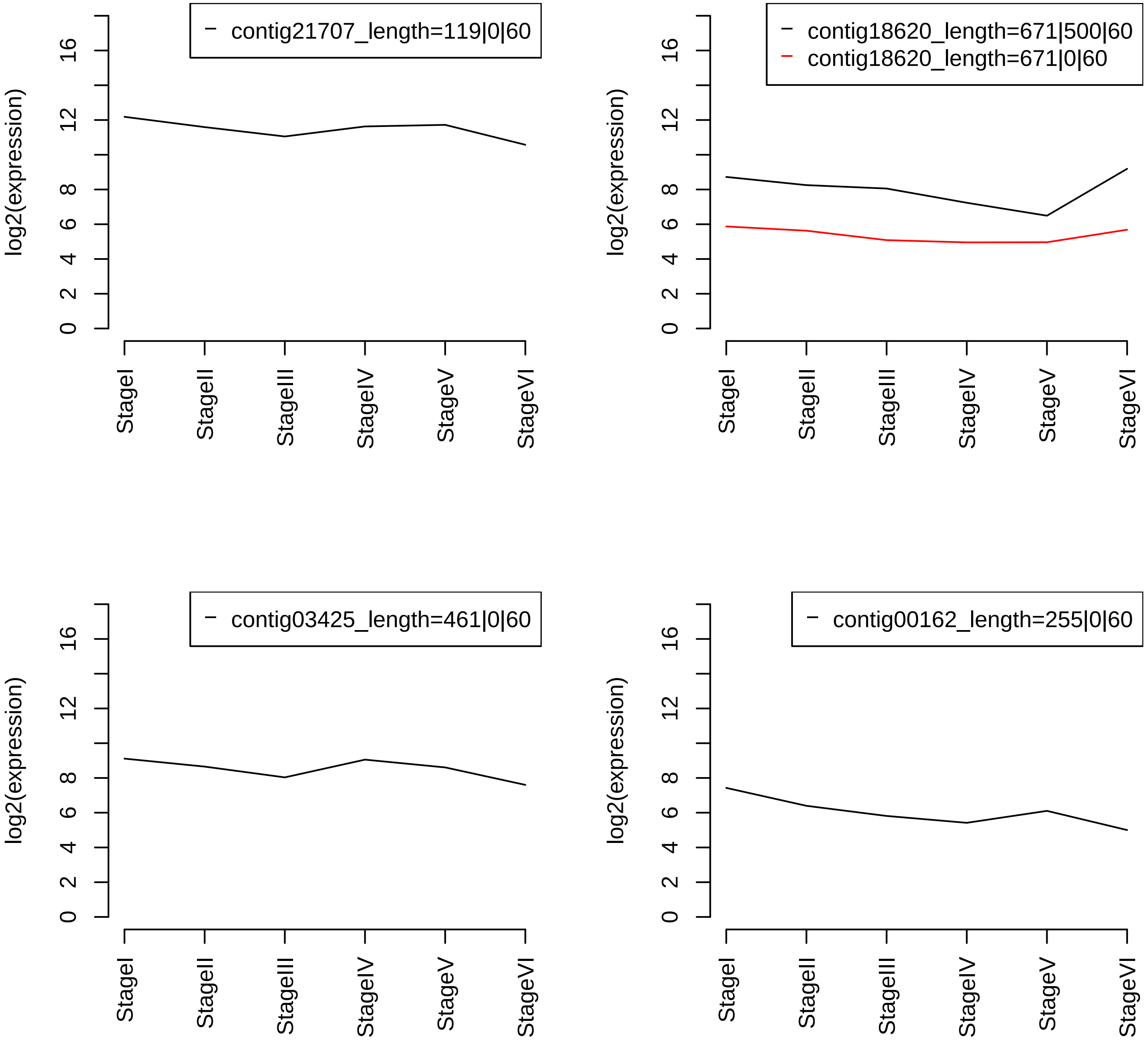
contig21707_length=119|0|60
contig18620_length=671|500|60
contig18620_length=671|0|60
contig03425_length=461|0|60
contig00162_length=255|0|60
log2(expression)
0
2
4
6
8
12
16
StageI
StageII
StageIII
StageIV
StageV
StageVI

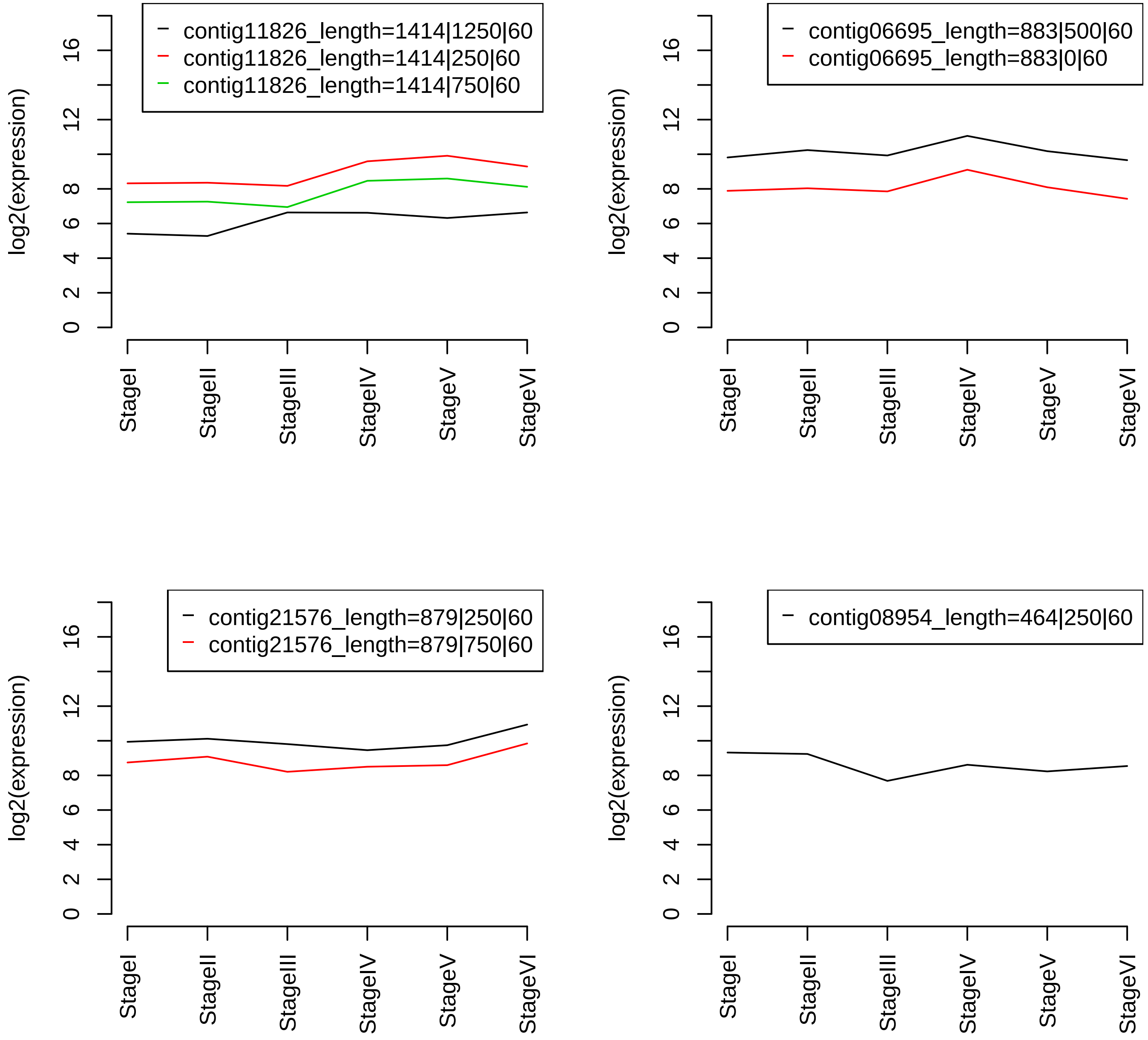

contig11826_length=1414|1250|60
contig11826_length=1414|250|60
contig11826_length=1414|750|60
contig06695_length=883|500|60
contig06695_length=883|0|60
contig21576_length=879|250|60
contig21576_length=879|750|60
contig08954_length=464|250|60
log2(expression)
0 2 4 6 8 12 16
StageI StageII StageIII StageIV StageV StageVI

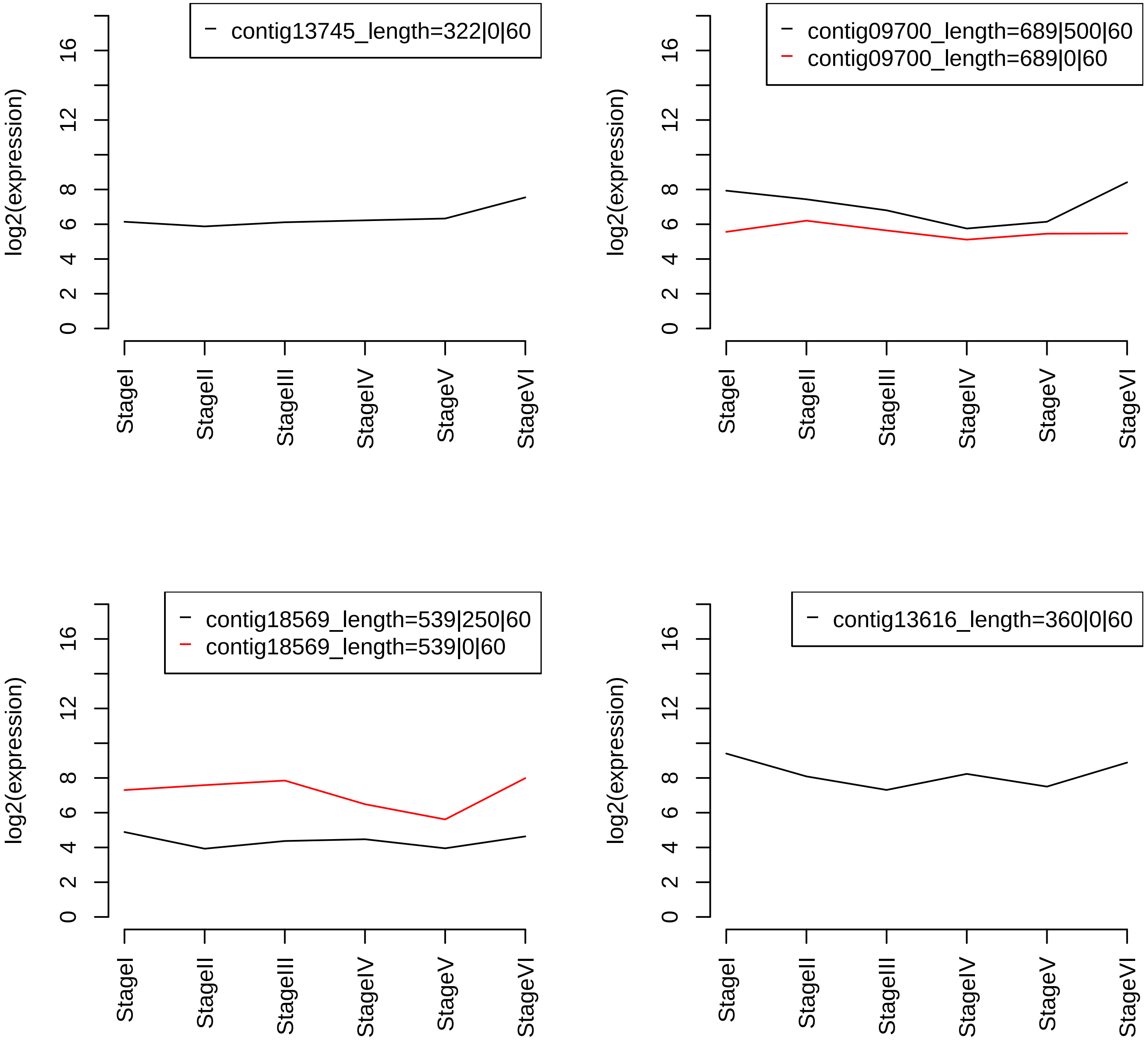

contig13745_length=322|0|60
contig09700_length=689|500|60
contig09700_length=689|0|60
contig18569_length=539|250|60
contig18569_length=539|0|60
contig13616_length=360|0|60
log2(expression)
0
2
4
6
8
12
16
StageI
StageII
StageIII
StageIV
StageV
StageVI

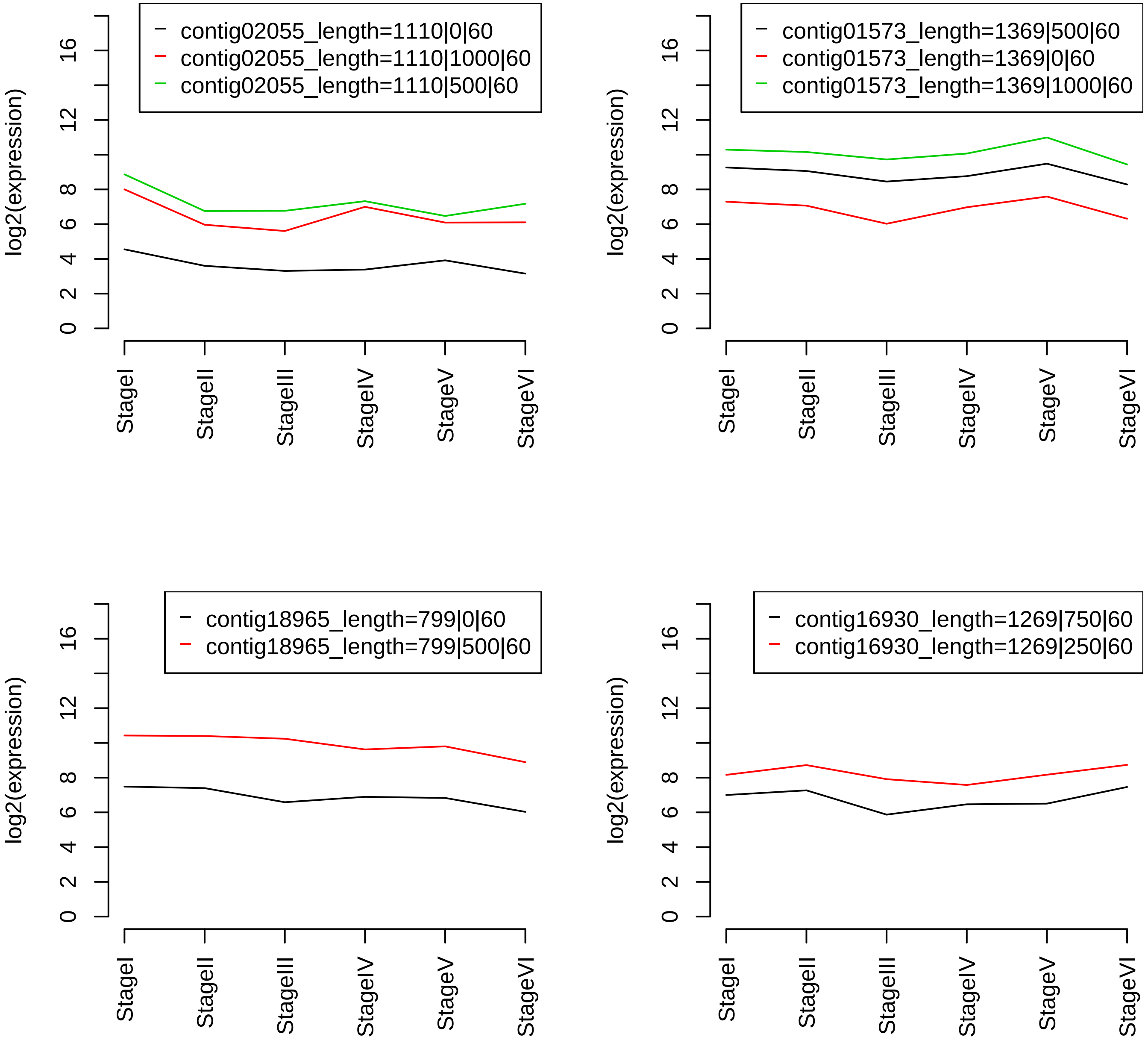
contig02055_length=1110|0|60
contig02055_length=1110|1000|60
contig02055_length=1110|500|60
contig01573_length=1369|500|60
contig01573_length=1369|0|60
contig01573_length=1369|1000|60
contig18965_length=799|0|60
contig18965_length=799|500|60
contig16930_length=1269|750|60
contig16930_length=1269|250|60
log2(expression)
0
2
4
6
8
12
16
StageI
StageII
StageIII
StageIV
StageV
StageVI

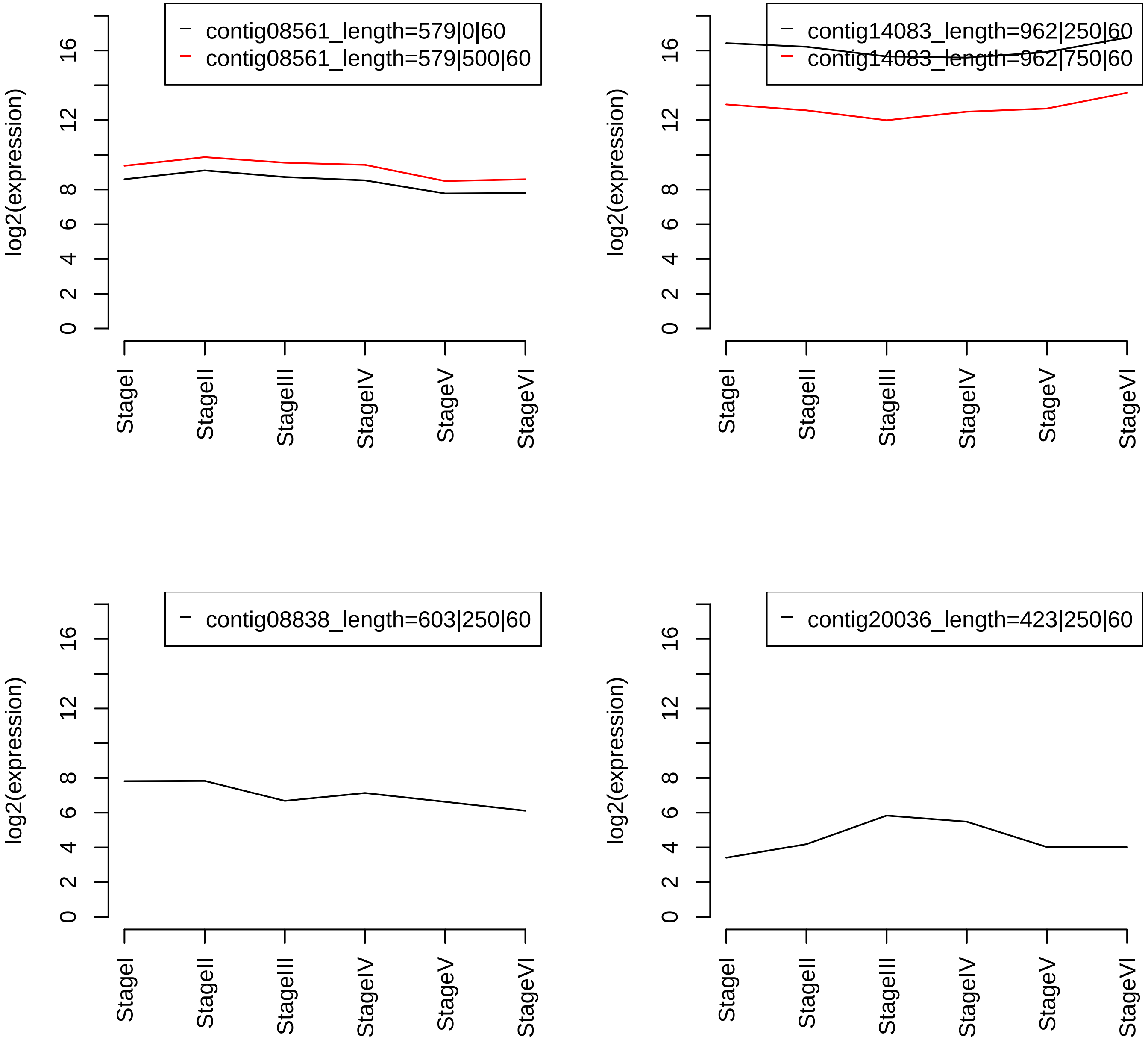
contig08561_length=579|0|60
contig08561_length=579|500|60
contig14083_length=962|250|60
contig14083_length=962|750|60
contig08838_length=603|250|60
contig20036_length=423|250|60
log2(expression)
0 2 4 6 8 12 16
StageI StageII StageIII StageIV StageV StageVI

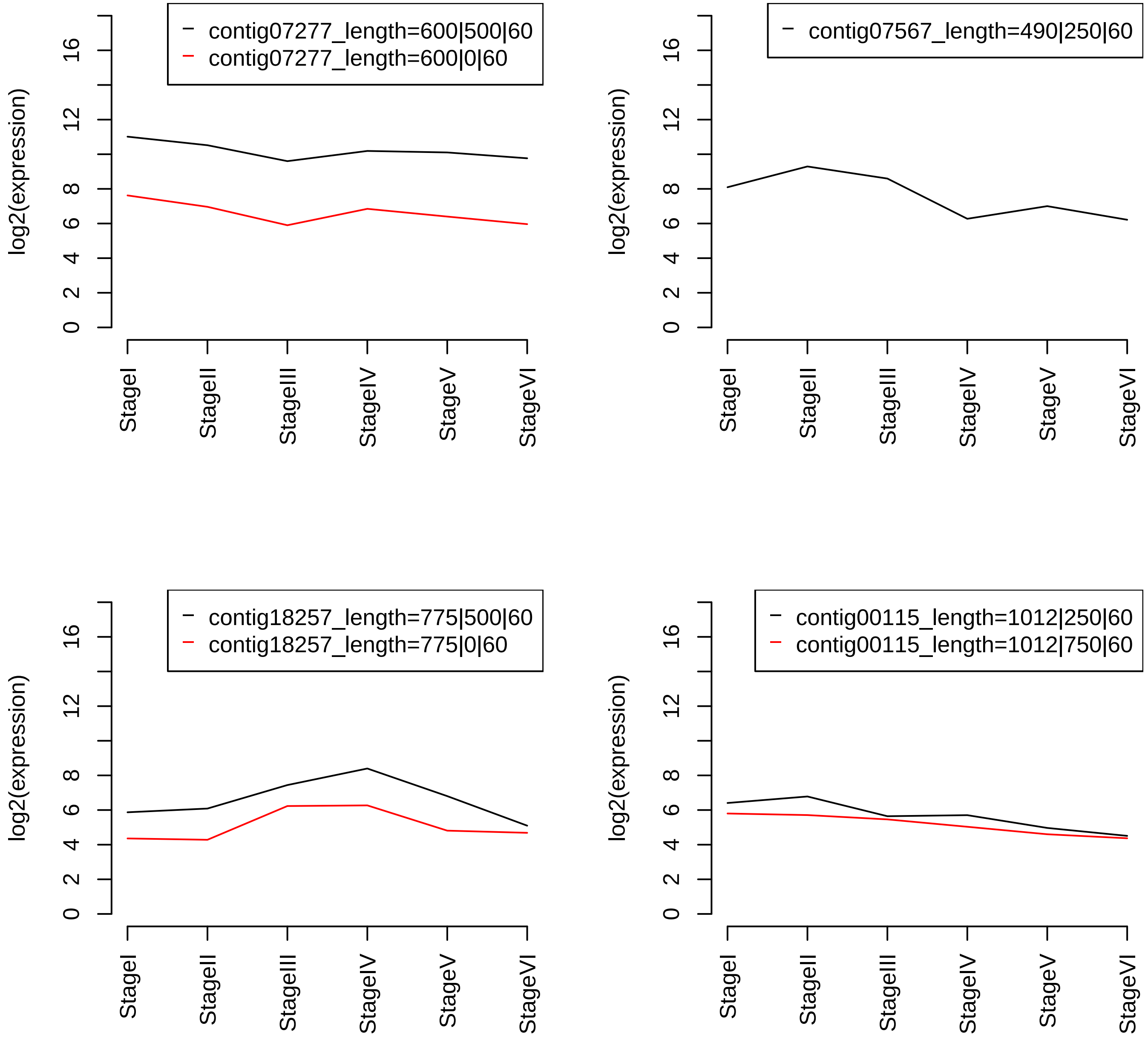
contig07277_length=600|500|60
contig07277_length=600|0|60
contig07567_length=490|250|60
contig18257_length=775|500|60
contig18257_length=775|0|60
contig00115_length=1012|250|60
contig00115_length=1012|750|60
log2(expression)
StageI
StageII
StageIII
StageIV
StageV
StageVI

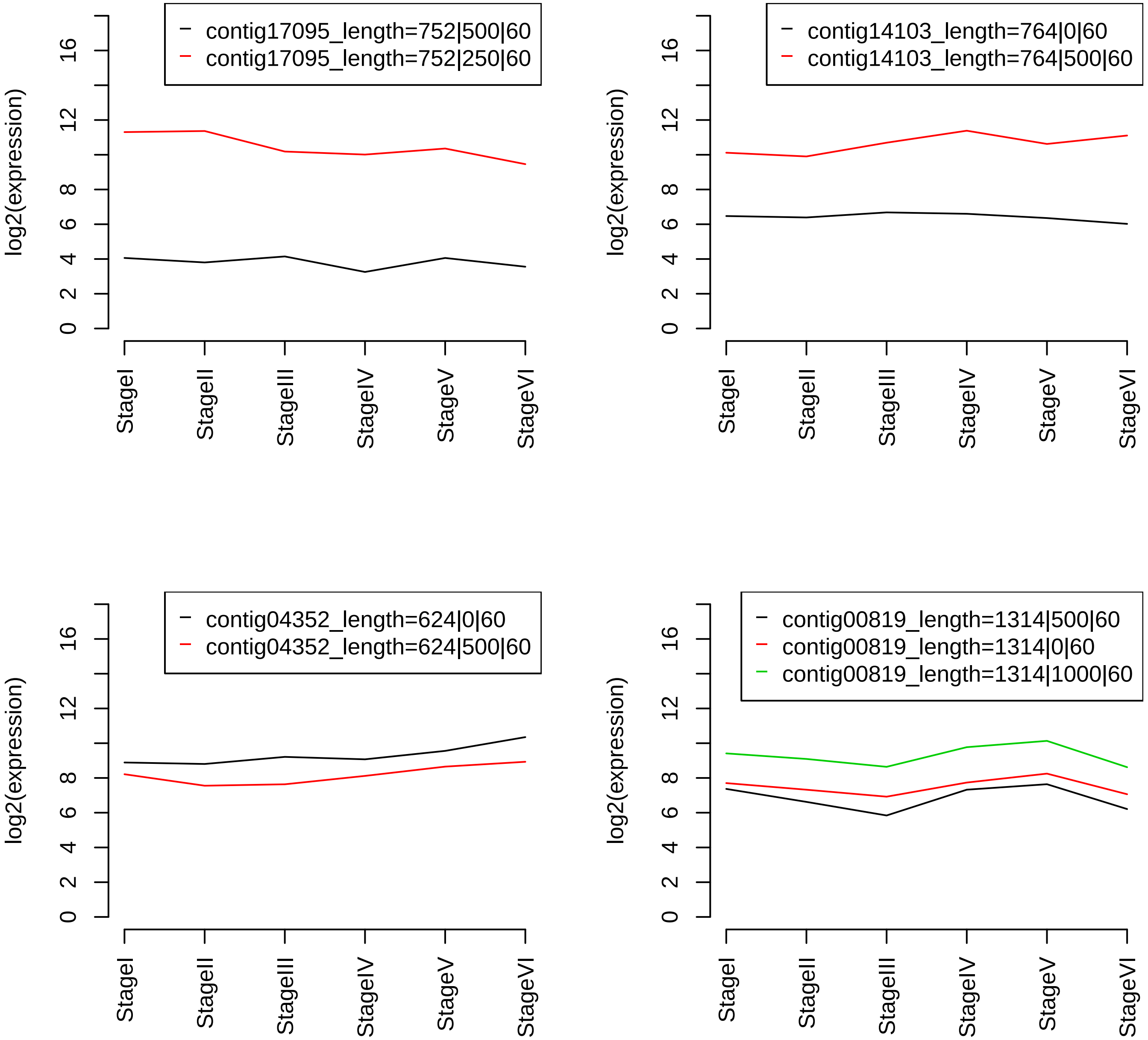

contig17095_length=752|500|60
contig17095_length=752|250|60
contig14103_length=764|0|60
contig14103_length=764|500|60
contig04352_length=624|0|60
contig04352_length=624|500|60
contig00819_length=1314|500|60
contig00819_length=1314|0|60
contig00819_length=1314|1000|60
log2(expression)
0
2
4
6
8
12
16
StageI
StageII
StageIII
StageIV
StageV
StageVI

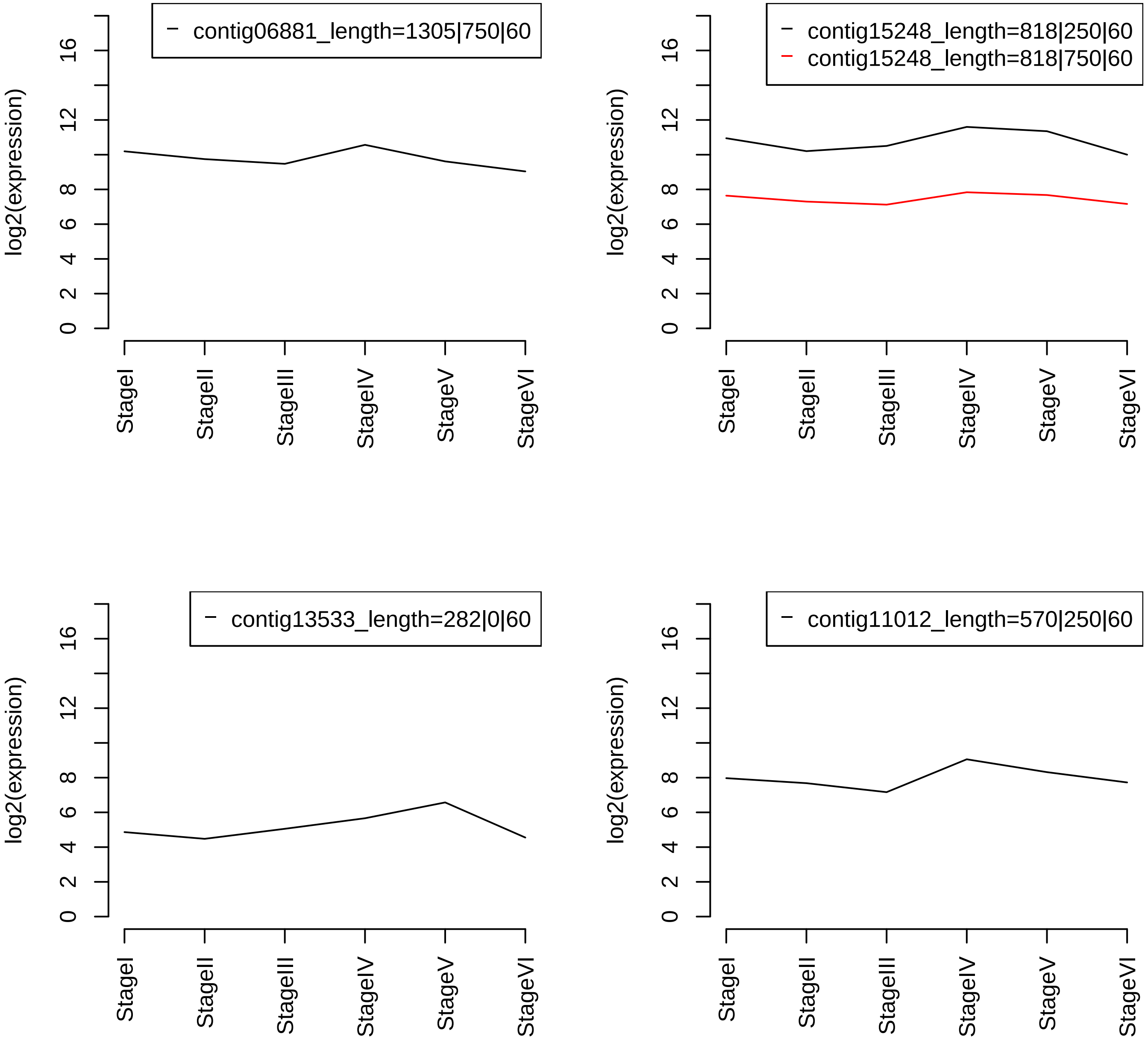

contig06881_length=1305|750|60
contig15248_length=818|250|60
contig15248_length=818|750|60
contig13533_length=282|0|60
contig11012_length=570|250|60
log2(expression)
0
2
4
6
8
12
16
StageI
StageII
StageIII
StageIV
StageV
StageVI

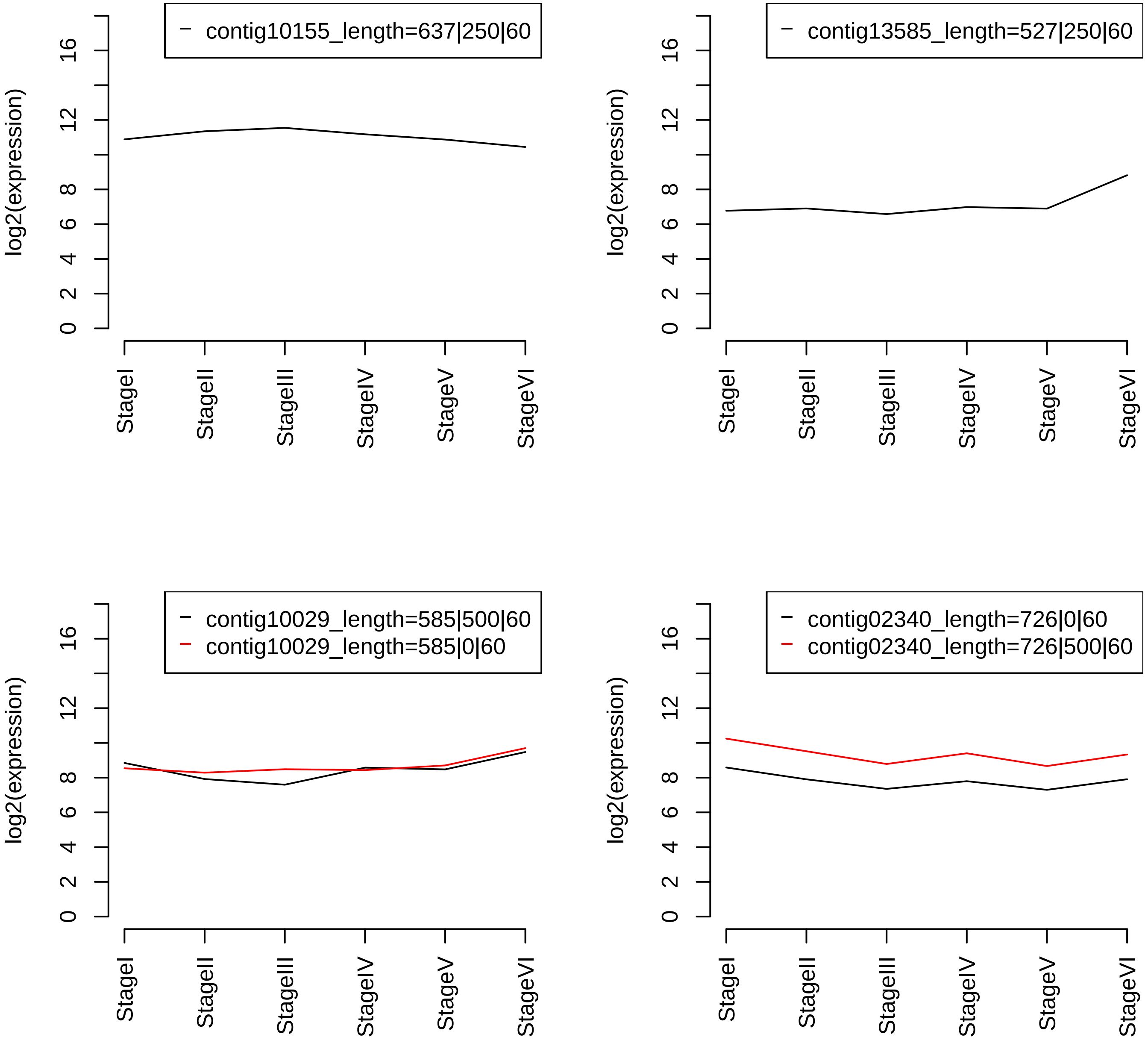

contig10155_length=637|250|60
contig13585_length=527|250|60
contig10029_length=585|500|60
contig10029_length=585|0|60
contig02340_length=726|0|60
contig02340_length=726|500|60
log2(expression)
0
2
4
6
8
12
16
StageI
StageII
StageIII
StageIV
StageV
StageVI

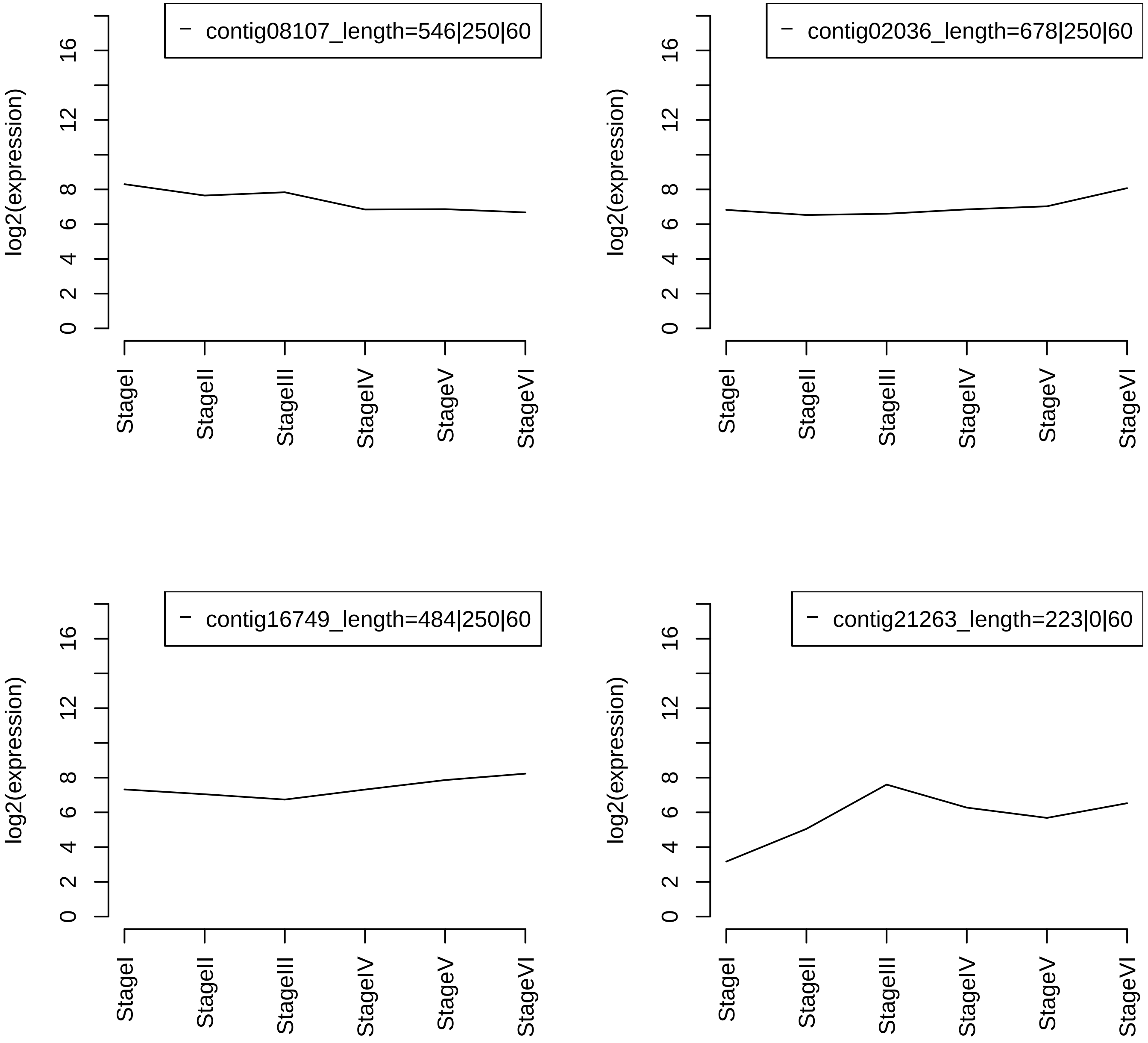
contig08107_length=546|250|60
contig02036_length=678|250|60
contig16749_length=484|250|60
contig21263_length=223|0|60
log2(expression)
0
2
4
6
8
12
16
StageI
StageII
StageIII
StageIV
StageV
StageVI

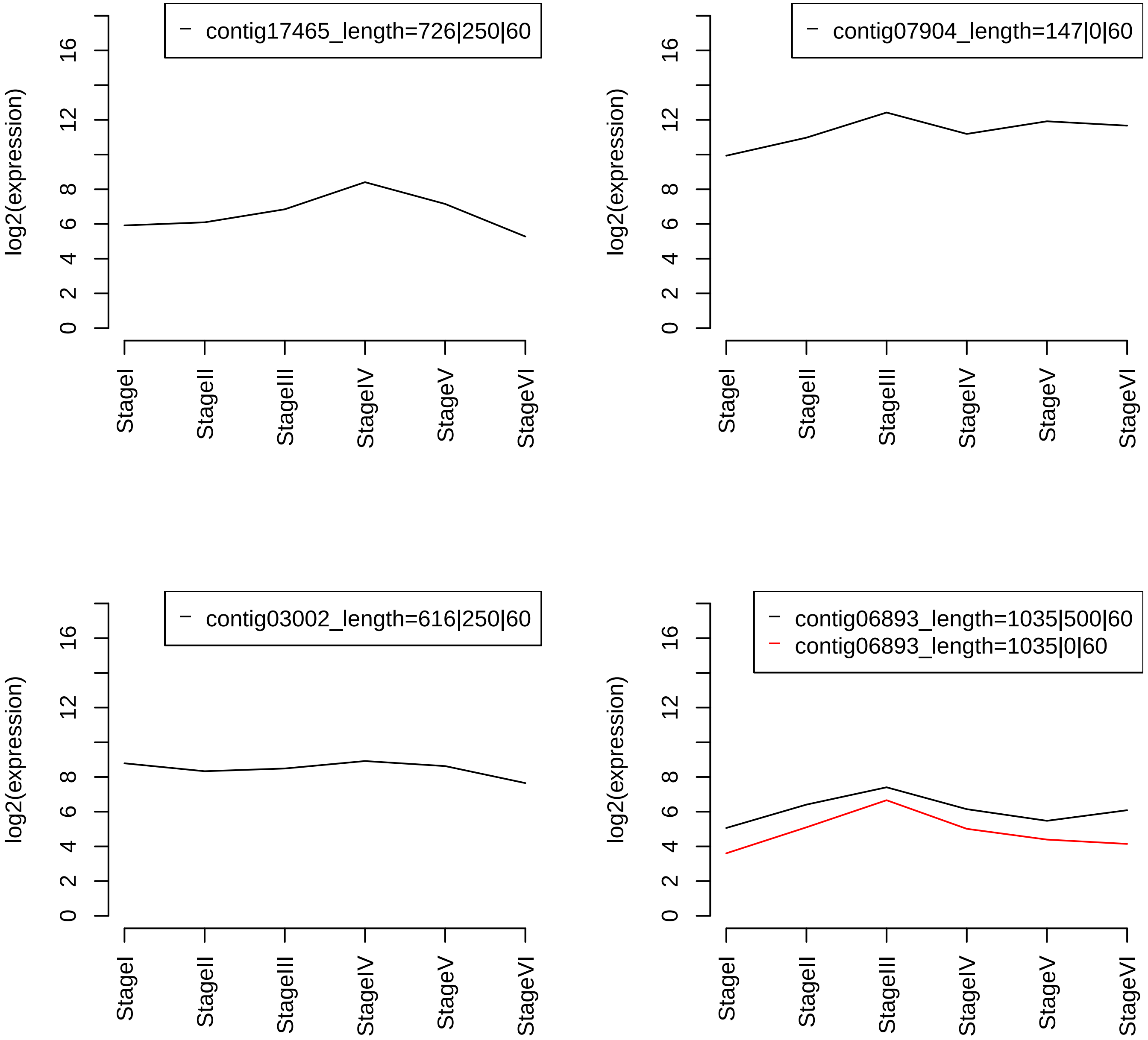
contig17465_length=726|250|60
contig07904_length=147|0|60
contig03002_length=616|250|60
contig06893_length=1035|500|60
contig06893_length=1035|0|60
log2(expression)
0
2
4
6
8
12
16
StageI
StageII
StageIII
StageIV
StageV
StageVI

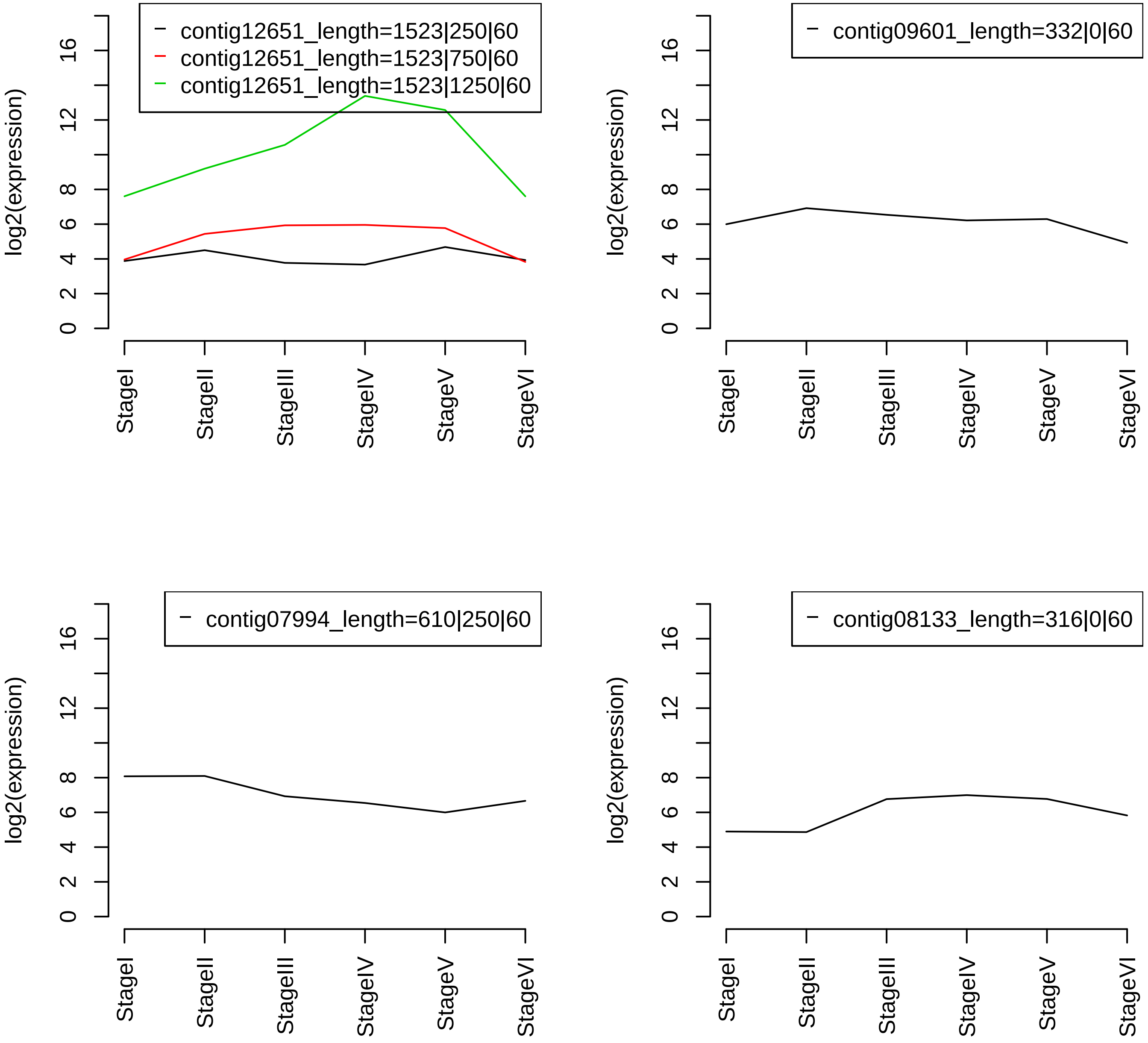
contig12651_length=1523|250|60
contig12651_length=1523|750|60
contig12651_length=1523|1250|60
contig09601_length=332|0|60
contig07994_length=610|250|60
contig08133_length=316|0|60
log2(expression)
0
2
4
6
8
12
16
StageI
StageII
StageIII
StageIV
StageV
StageVI

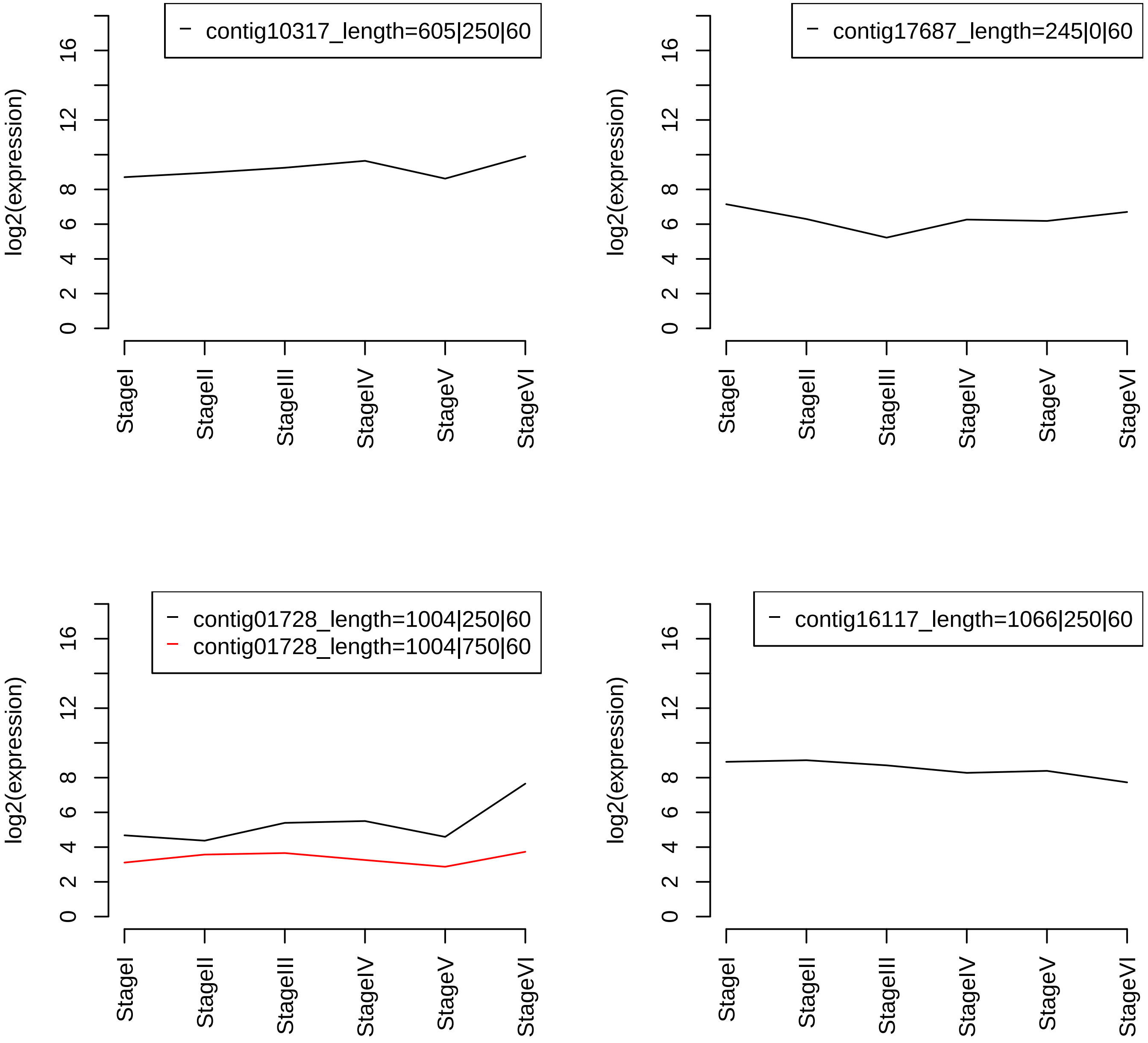
contig10317_length=605|250|60
contig17687_length=245|0|60
contig01728_length=1004|250|60
contig01728_length=1004|750|60
contig16117_length=1066|250|60
log2(expression)
0
2
4
6
8
12
16
StageI
StageII
StageIII
StageIV
StageV
StageVI

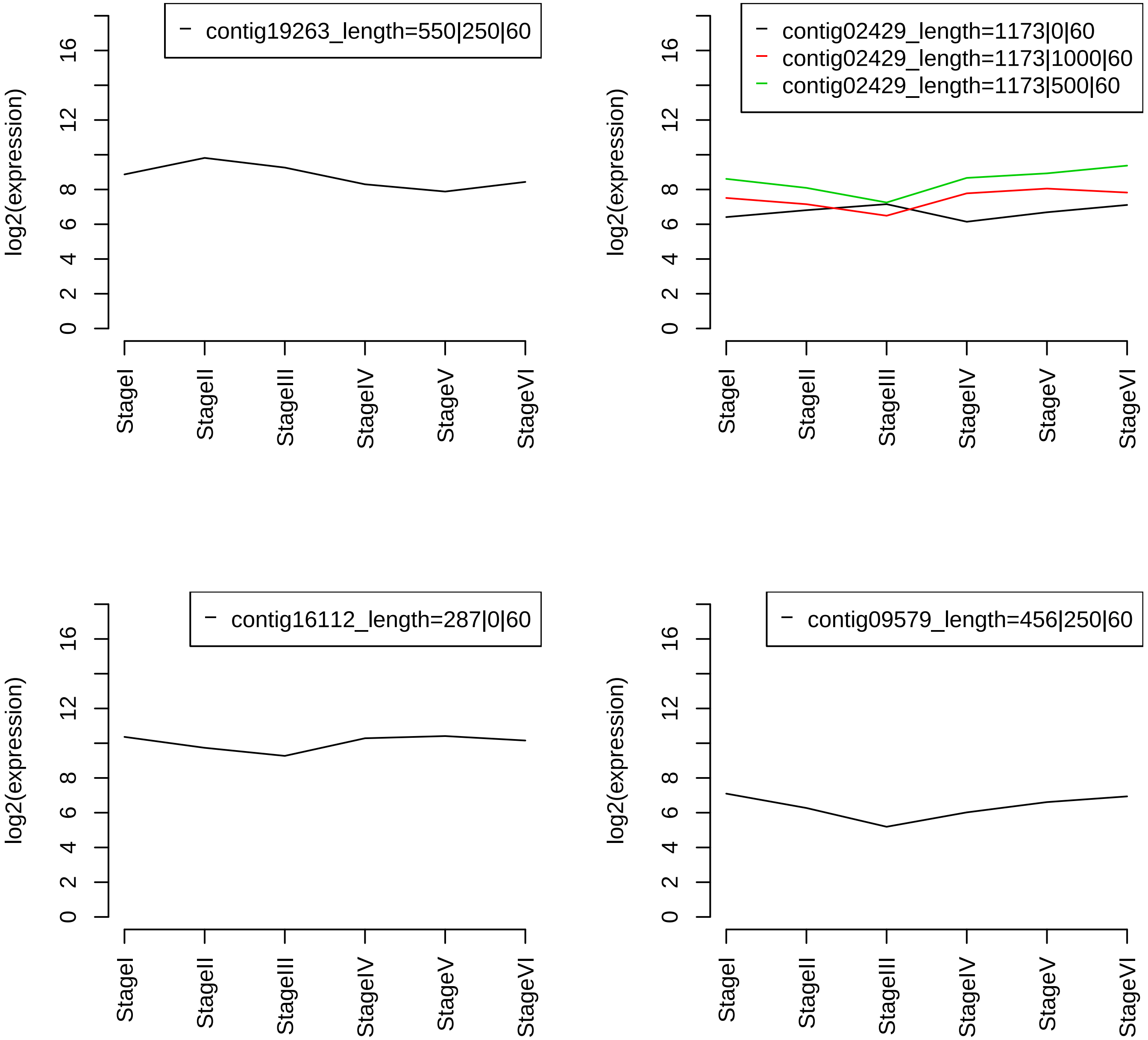

contig19263_length=550|250|60
contig02429_length=1173|0|60
contig02429_length=1173|1000|60
contig02429_length=1173|500|60
contig16112_length=287|0|60
contig09579_length=456|250|60
log2(expression)
0
2
4
6
8
12
16
StageI
StageII
StageIII
StageIV
StageV
StageVI

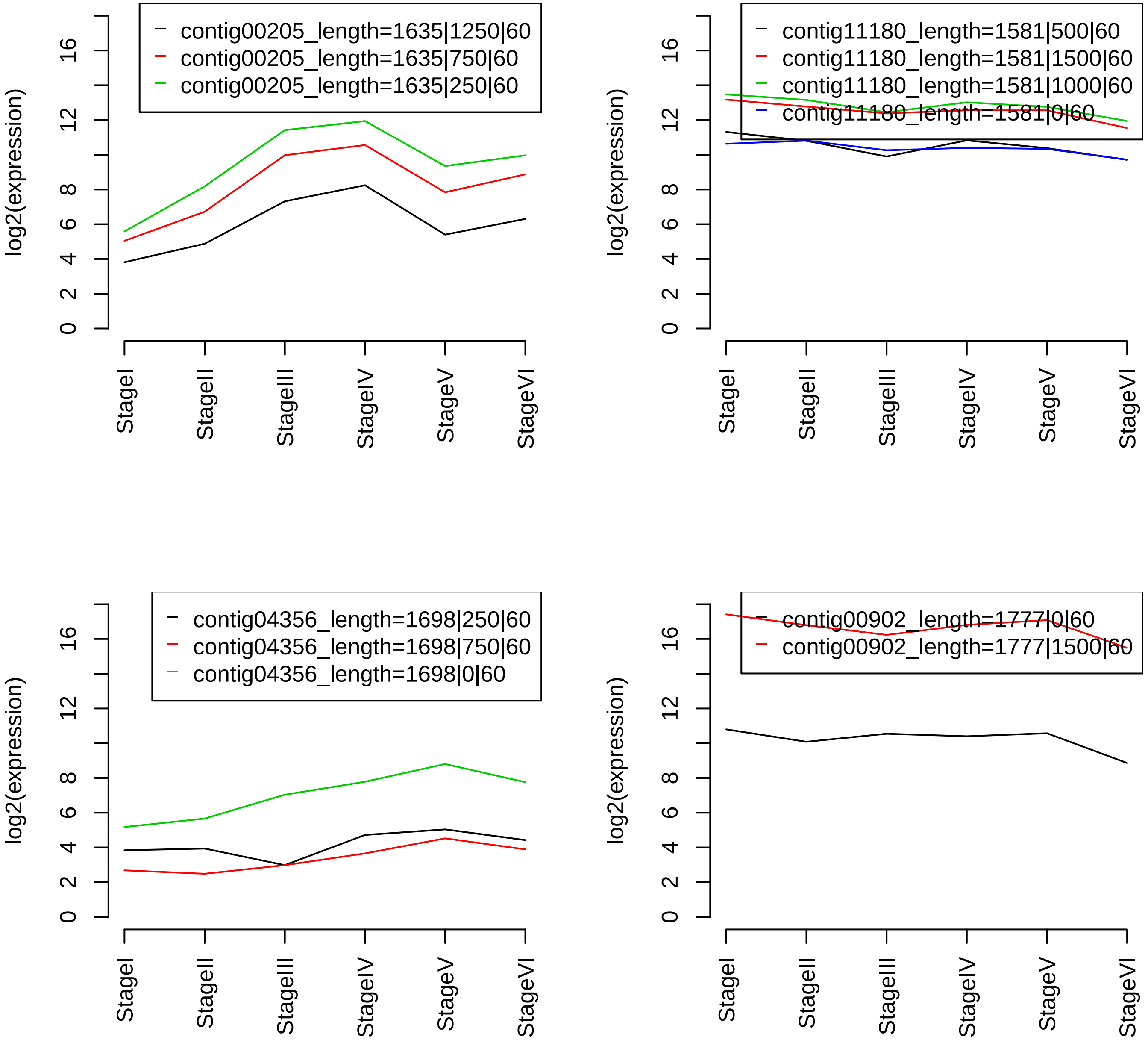
contig00205_length=1635|1250|60
contig00205_length=1635|750|60
contig00205_length=1635|250|60
log2(expression)
0
2
4
6
8
12
16
StageI
StageII
StageIII
StageIV
StageV
StageVI
contig11180_length=1581|500|60
contig11180_length=1581|1500|60
contig11180_length=1581|1000|60
contig11180_length=1581|0|60
contig04356_length=1698|250|60
contig04356_length=1698|750|60
contig04356_length=1698|0|60
contig00902_length=1777|0|60
contig00902_length=1777|1500|60

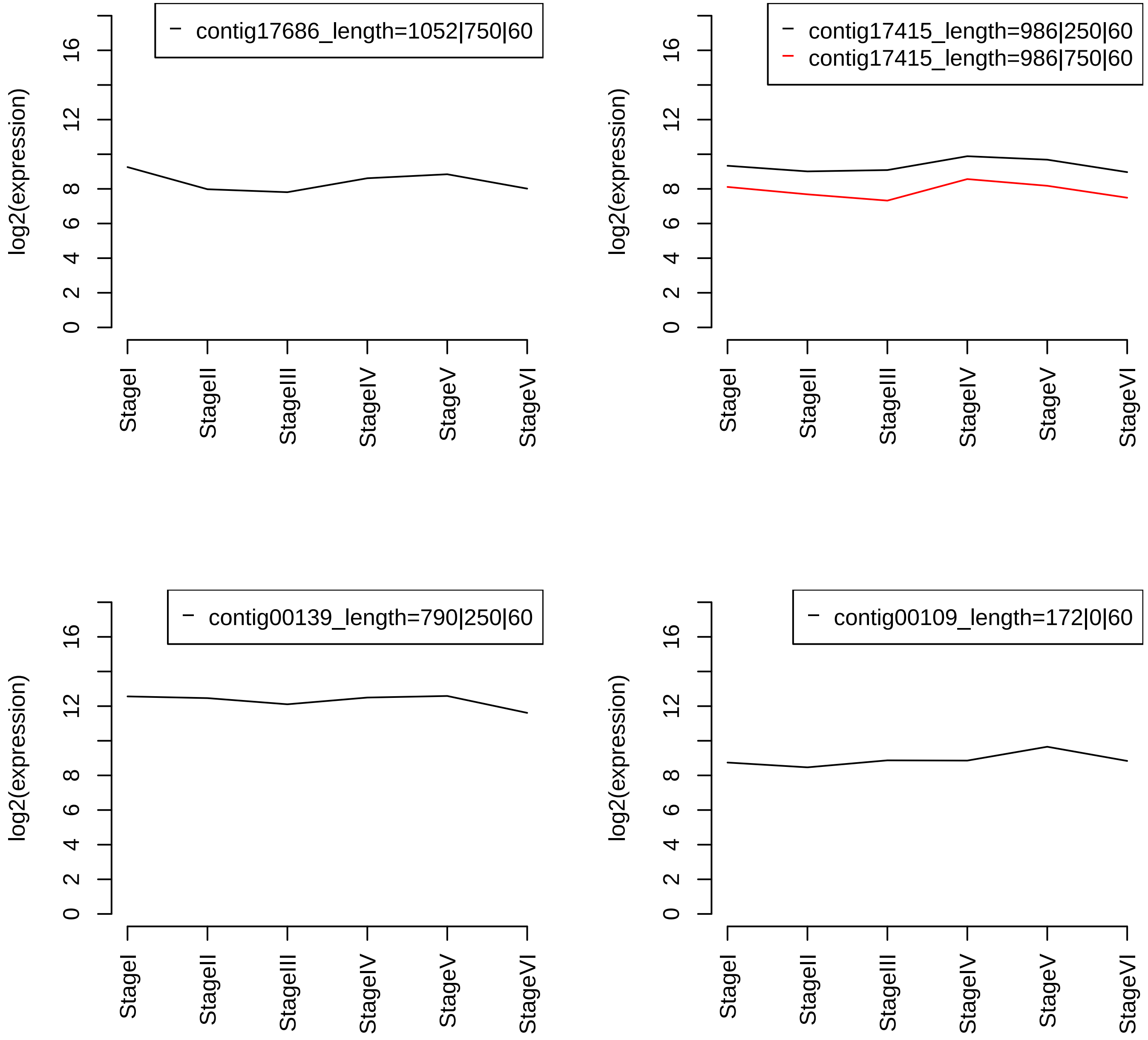
contig17686_length=1052|750|60
contig17415_length=986|250|60
contig17415_length=986|750|60
contig00139_length=790|250|60
contig00109_length=172|0|60
log2(expression)
0
2
4
6
8
12
16
StageI
StageII
StageIII
StageIV
StageV
StageVI

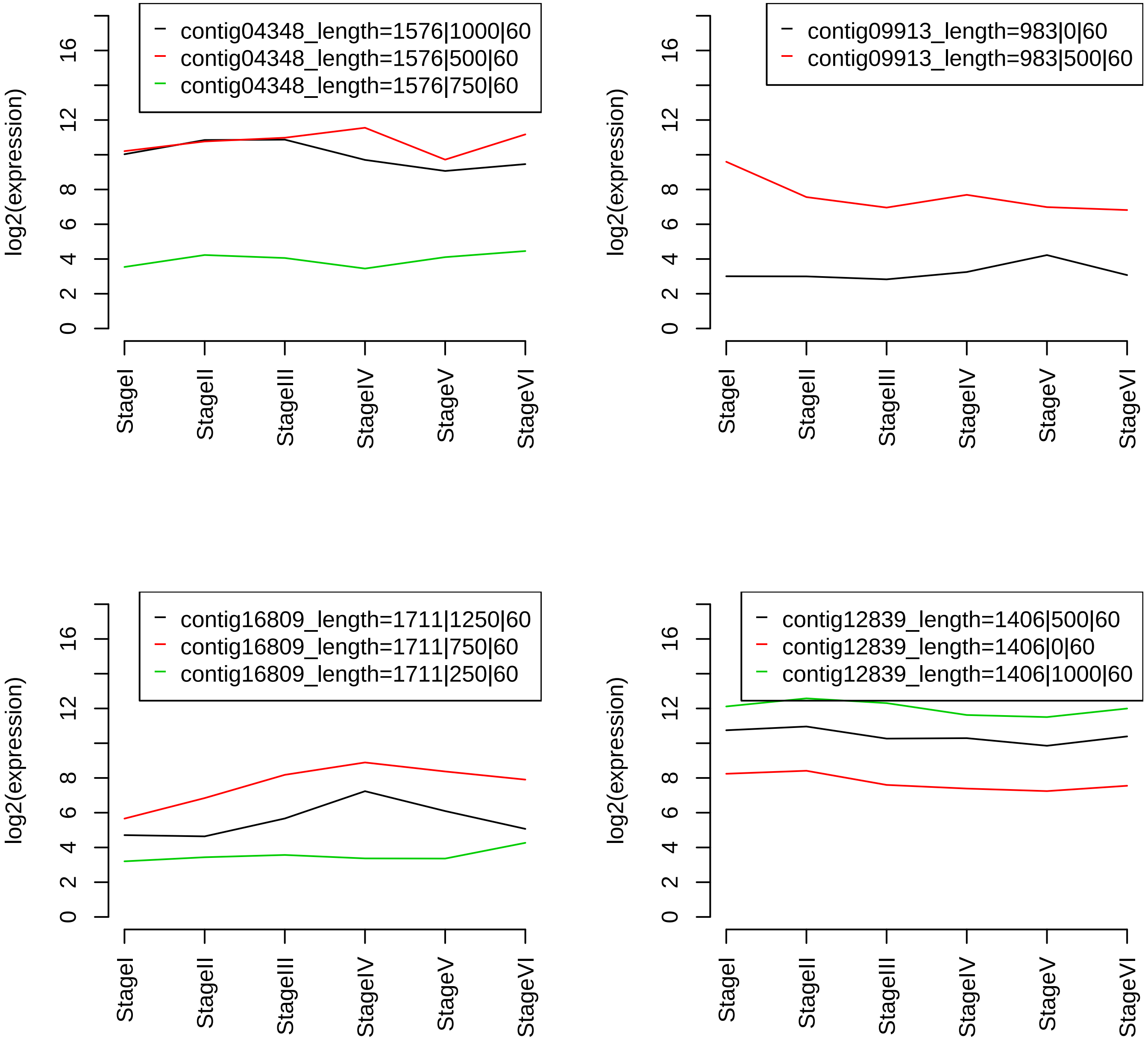

contig04348_length=1576|1000|60
contig04348_length=1576|500|60
contig04348_length=1576|750|60
contig09913_length=983|0|60
contig09913_length=983|500|60
contig16809_length=1711|1250|60
contig16809_length=1711|750|60
contig16809_length=1711|250|60
contig12839_length=1406|500|60
contig12839_length=1406|0|60
contig12839_length=1406|1000|60
log2(expression)
0
2
4
6
8
12
16
StageI
StageII
StageIII
StageIV
StageV
StageVI

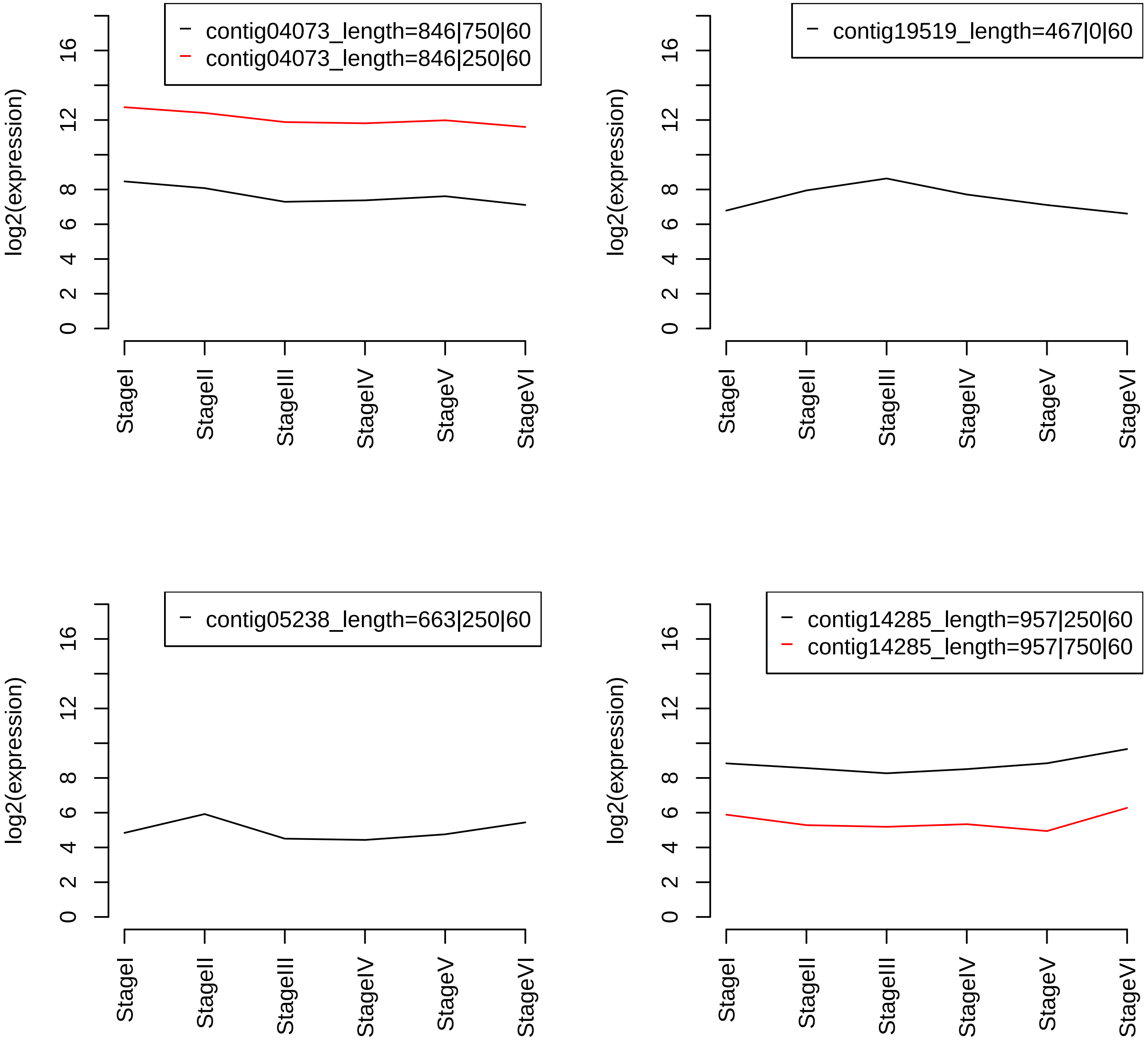
contig04073_length=846|750|60
contig04073_length=846|250|60
log2(expression)
0
2
4
6
8
12
16
StageI
StageII
StageIII
StageIV
StageV
StageVI
contig19519_length=467|0|60
contig05238_length=663|250|60
contig14285_length=957|250|60
contig14285_length=957|750|60

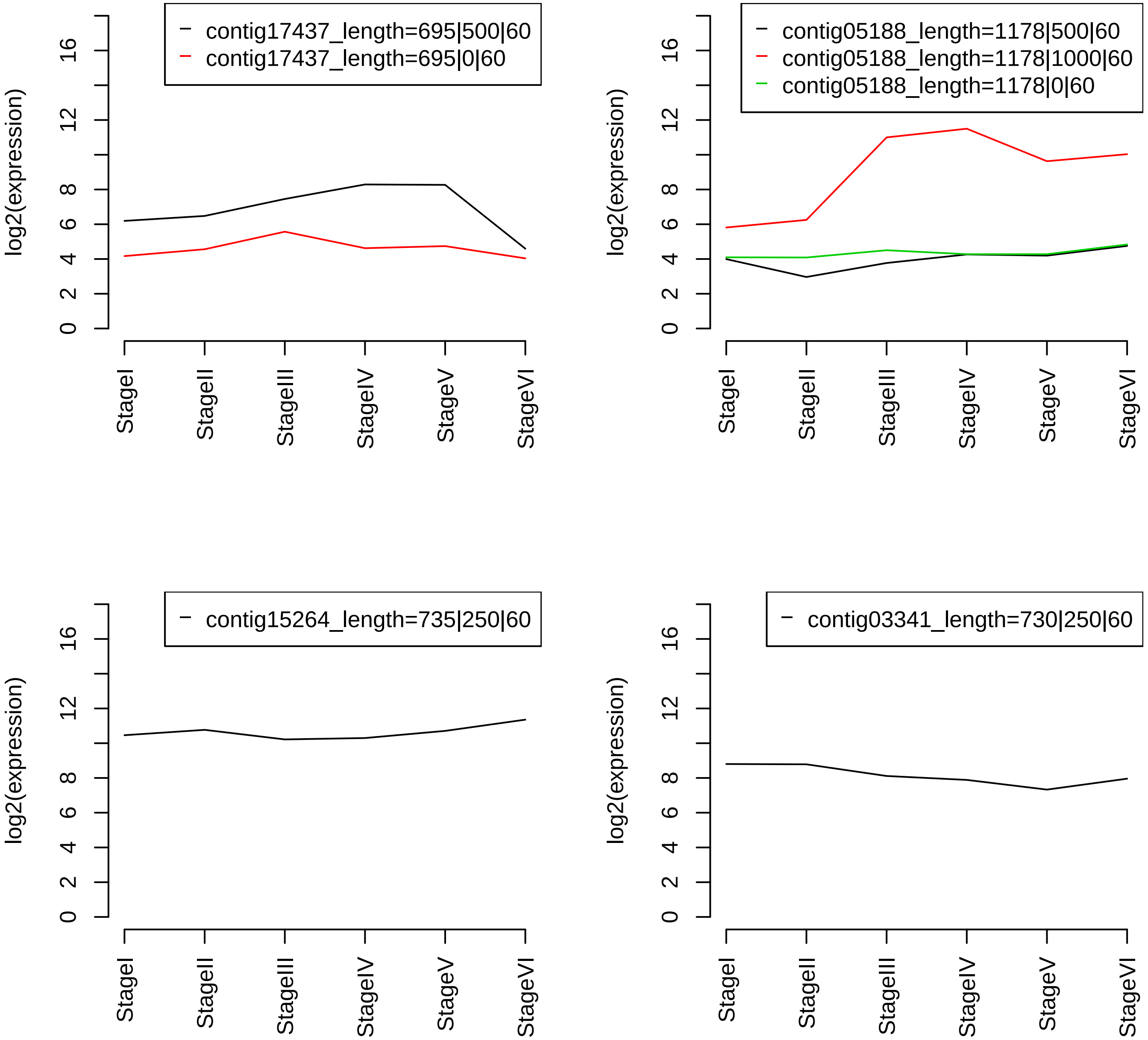
contig17437_length=695|500|60
contig17437_length=695|0|60
contig05188_length=1178|500|60
contig05188_length=1178|1000|60
contig05188_length=1178|0|60
contig15264_length=735|250|60
contig03341_length=730|250|60
log2(expression)
0
2
4
6
8
12
16
StageI
StageII
StageIII
StageIV
StageV
StageVI

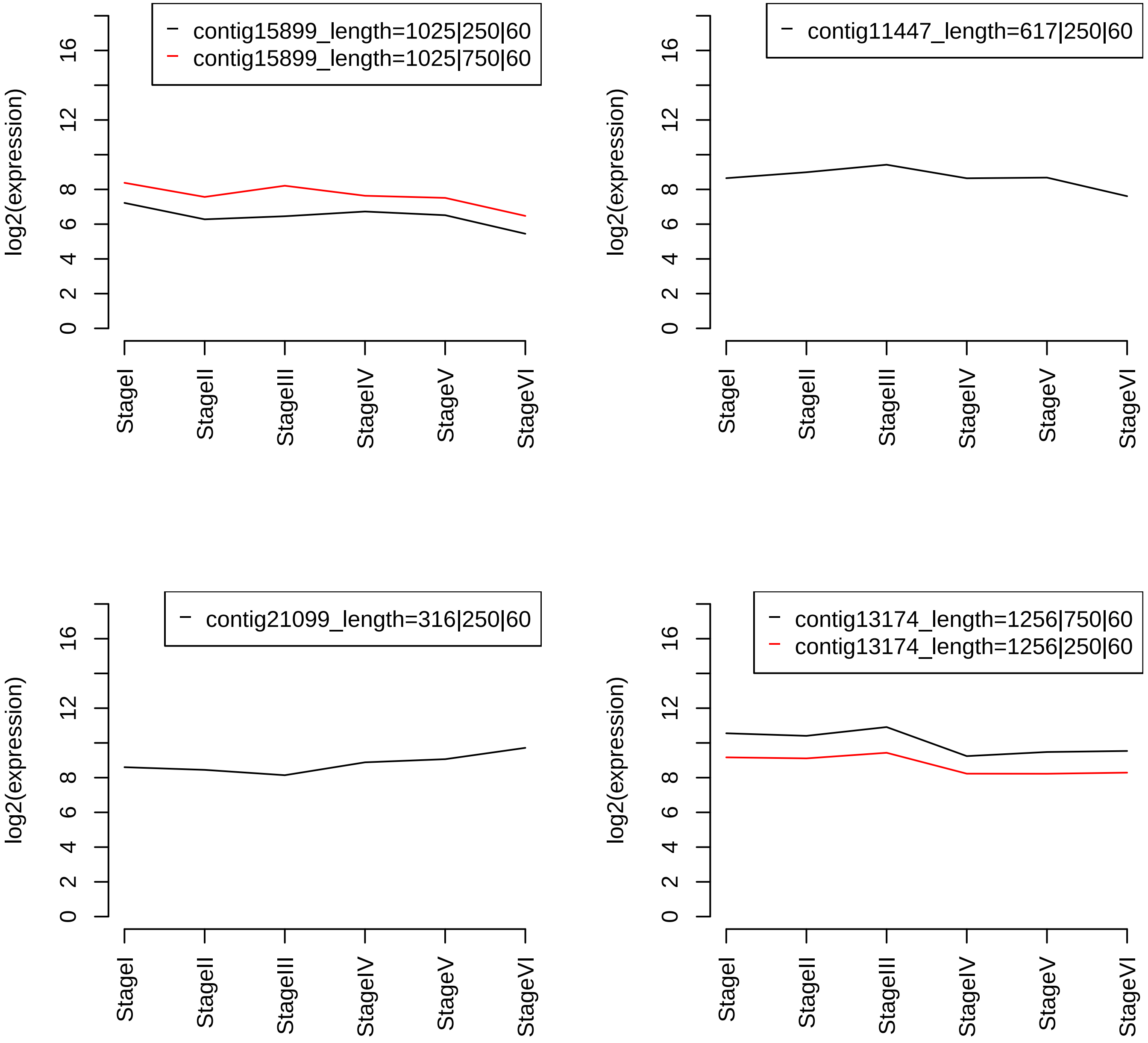

contig15899_length=1025|250|60
contig15899_length=1025|750|60
contig11447_length=617|250|60
contig21099_length=316|250|60
contig13174_length=1256|750|60
contig13174_length=1256|250|60
log2(expression)
0
2
4
6
8
12
16
StageI
StageII
StageIII
StageIV
StageV
StageVI

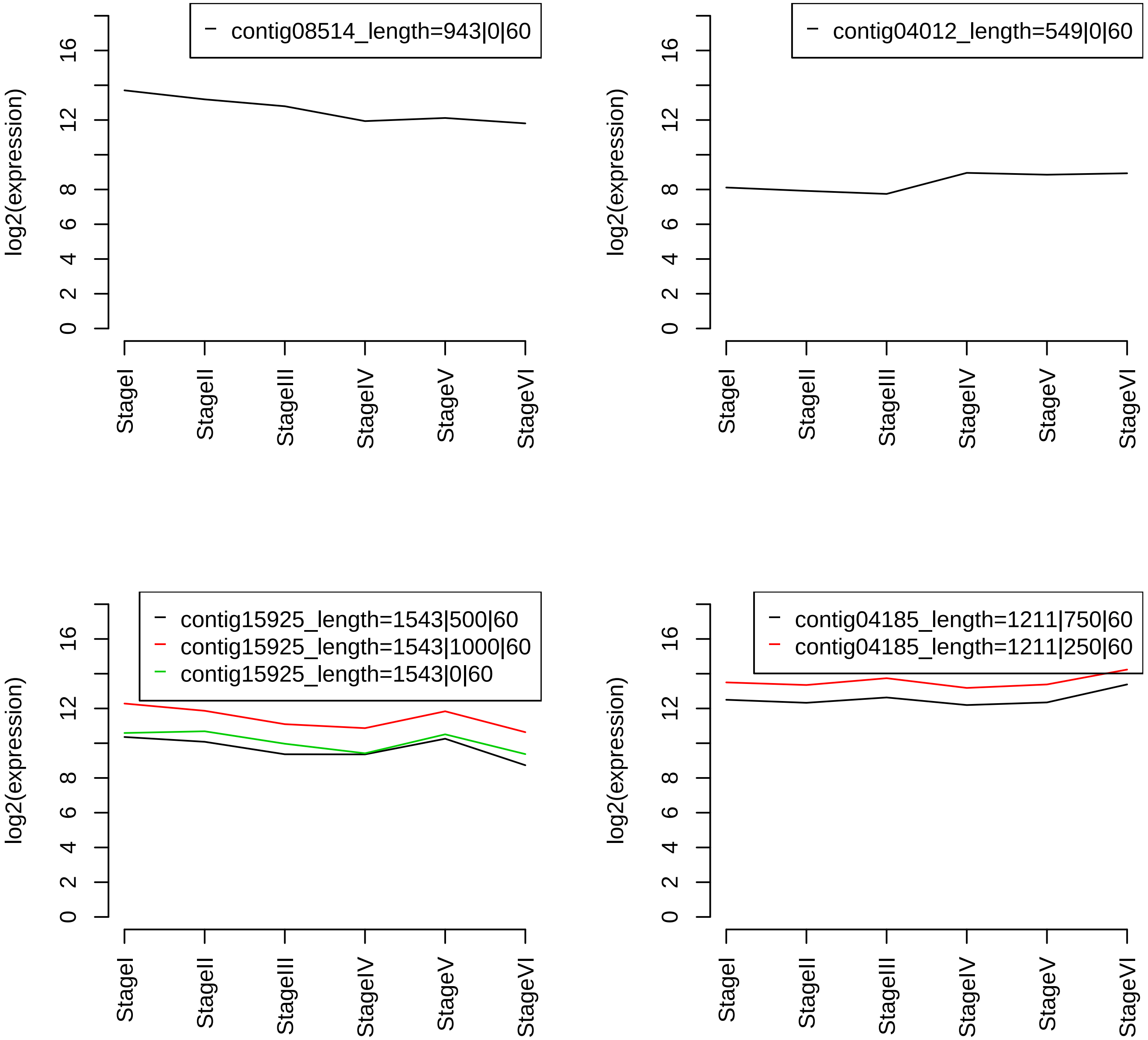

contig08514_length=943|0|60
contig04012_length=549|0|60
contig15925_length=1543|500|60
contig15925_length=1543|1000|60
contig15925_length=1543|0|60
contig04185_length=1211|750|60
contig04185_length=1211|250|60
log2(expression)
0
2
4
6
8
12
16
StageI
StageII
StageIII
StageIV
StageV
StageVI

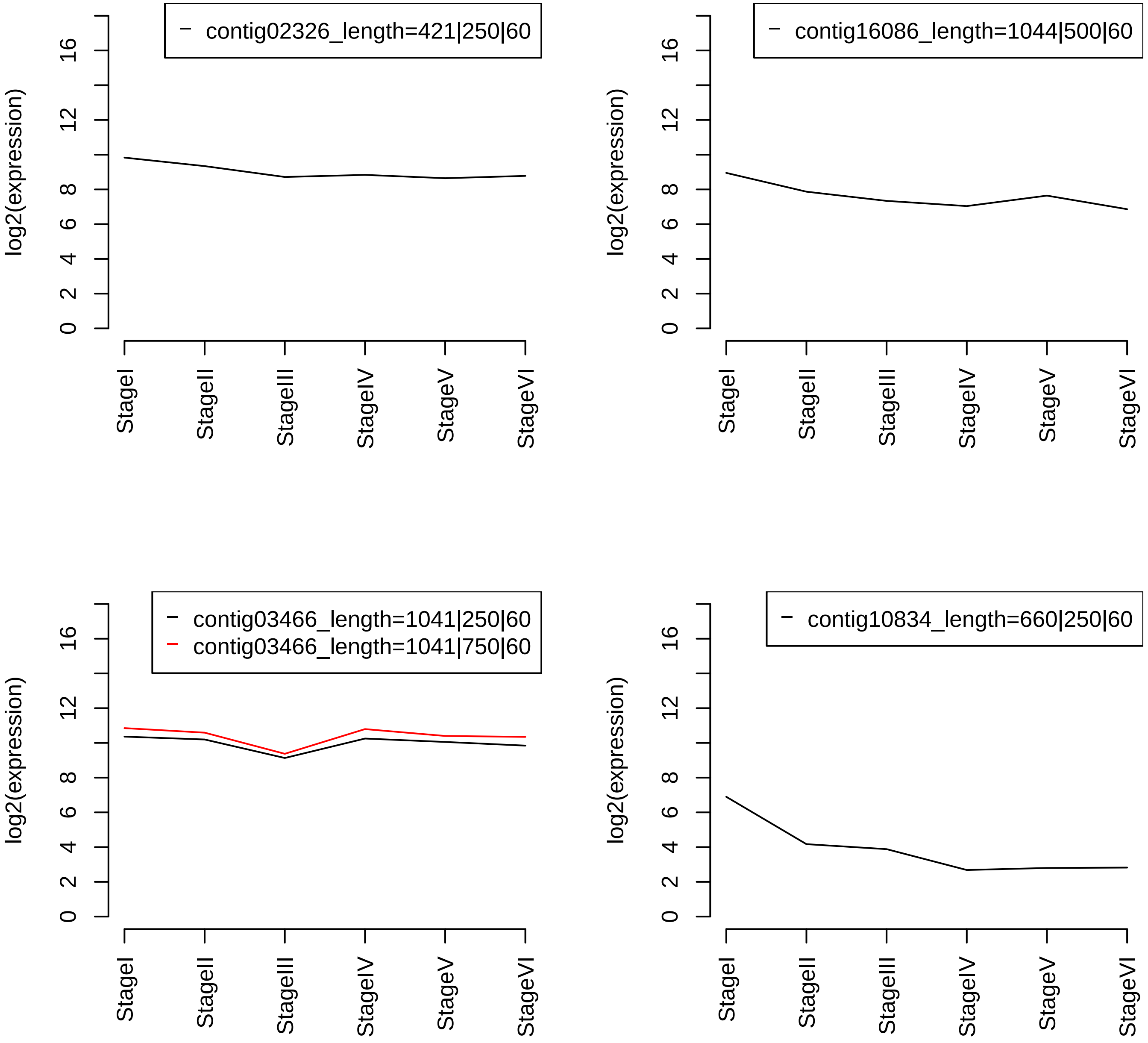
contig02326_length=421|250|60
contig16086_length=1044|500|60
contig03466_length=1041|250|60
contig03466_length=1041|750|60
contig10834_length=660|250|60
log2(expression)
0
2
4
6
8
12
16
StageI
StageII
StageIII
StageIV
StageV
StageVI

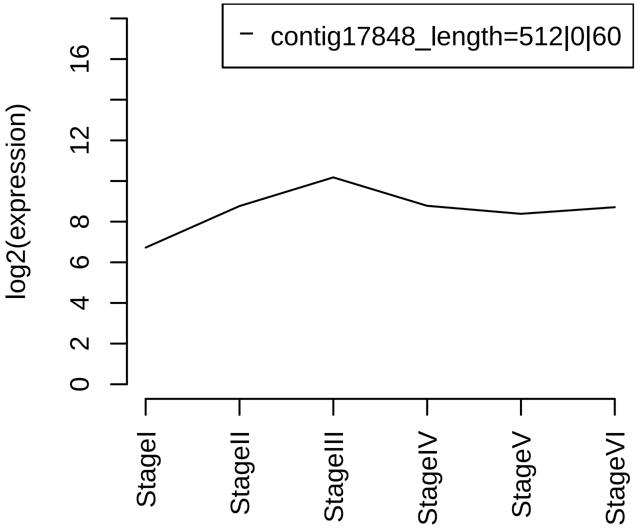

contig17848_length=512|0|60
log2(expression)
0
2
4
6
8
12
16
StageI
StageII
StageIII
StageIV
StageV
StageVI

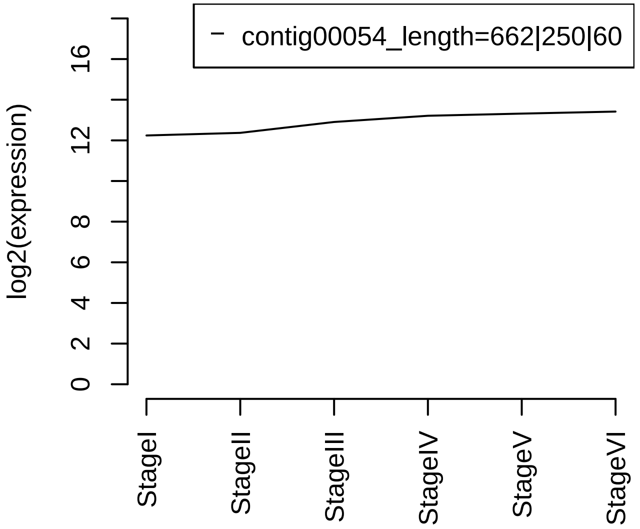

contig00054_length=662|250|60
log2(expression)
0
2
4
6
8
12
16
StageI
StageII
StageIII
StageIV
StageV
StageVI

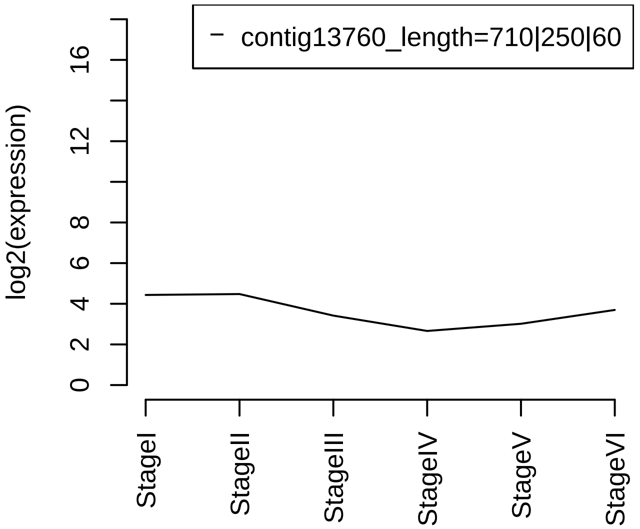

contig13760_length=710|250|60
log2(expression)
0
2
4
6
8
12
16
StageI
StageII
StageIII
StageIV
StageV
StageVI

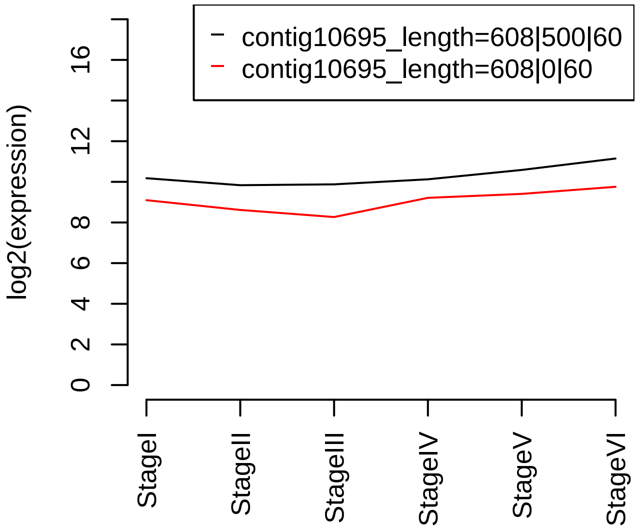

contig10695_length=608|500|60
contig10695_length=608|0|60
log2(expression)
0
2
4
6
8
12
16
StageI
StageII
StageIII
StageIV
StageV
StageVI

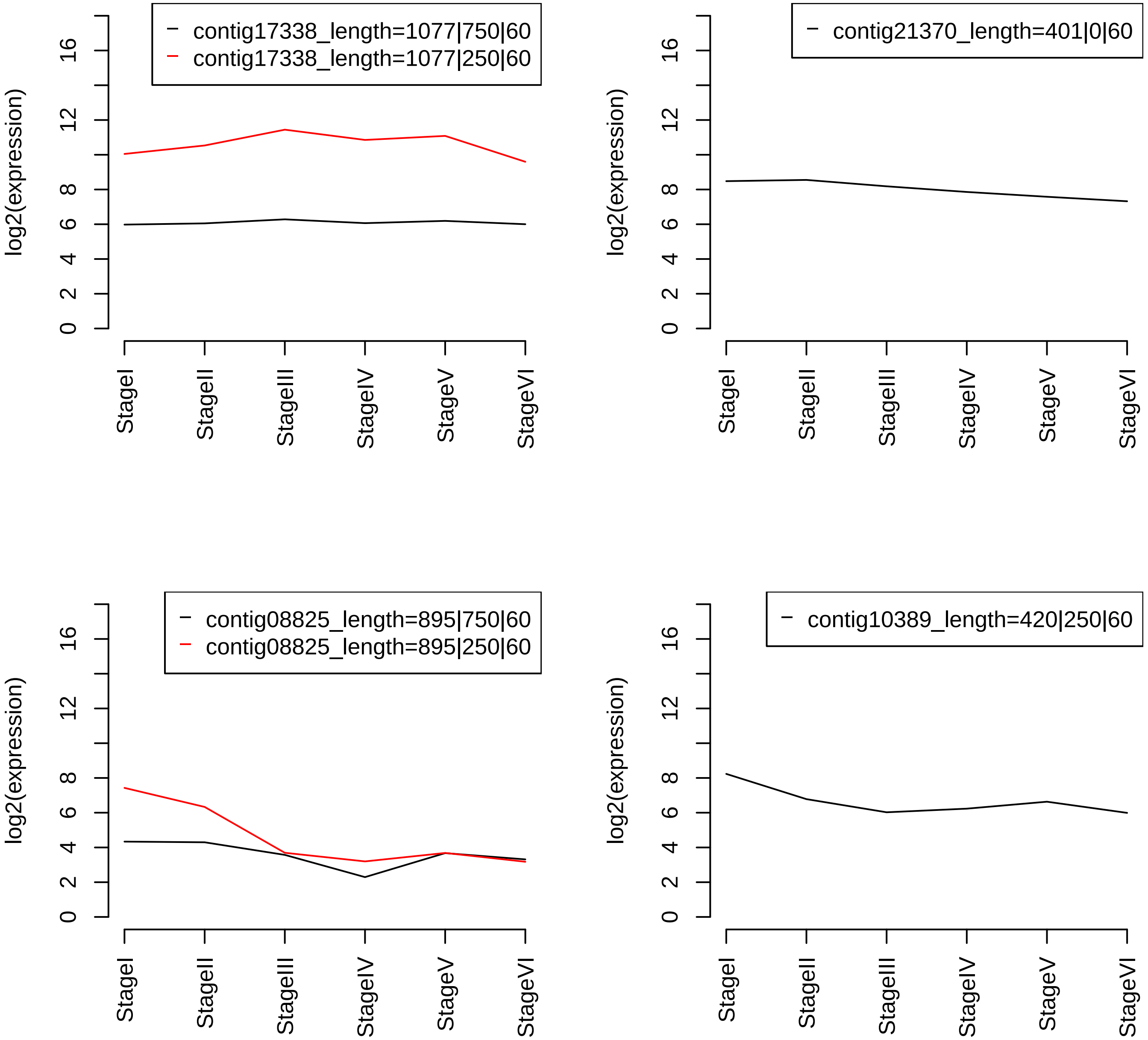
contig17338_length=1077|750|60
contig17338_length=1077|250|60
contig21370_length=401|0|60
contig08825_length=895|750|60
contig08825_length=895|250|60
contig10389_length=420|250|60
log2(expression)
0
2
4
6
8
12
16
StageI
StageII
StageIII
StageIV
StageV
StageVI

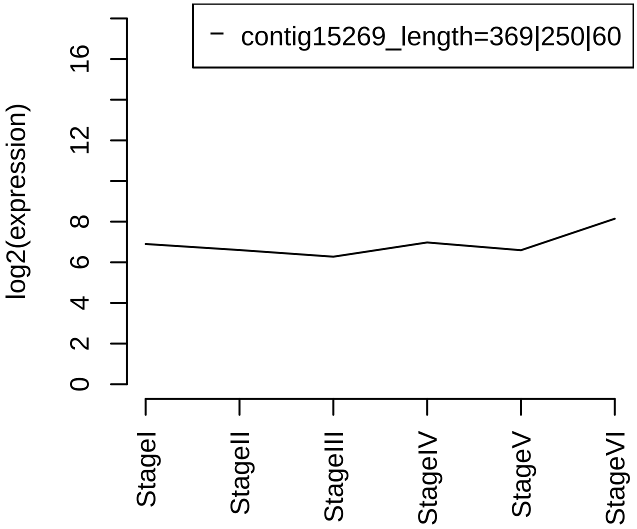

contig15269_length=369|250|60
log2(expression)
0
2
4
6
8
12
16
StageI
StageII
StageIII
StageIV
StageV
StageVI

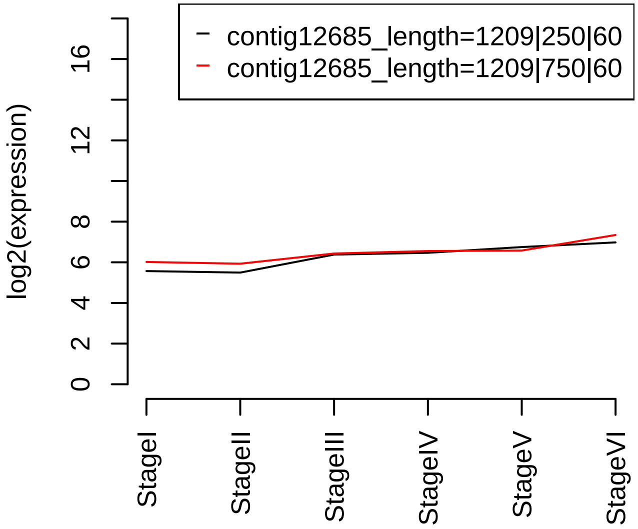

contig12685_length=1209|250|60
contig12685_length=1209|750|60
log2(expression)
0
2
4
6
8
12
16
StageI
StageII
StageIII
StageIV
StageV
StageVI

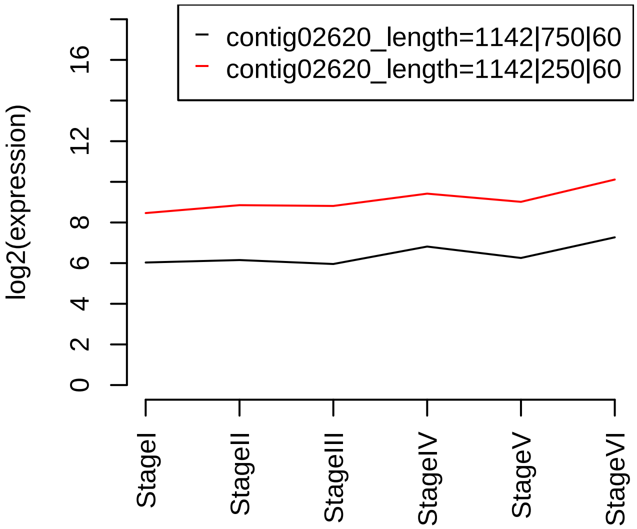

contig02620_length=1142|750|60
contig02620_length=1142|250|60
log2(expression)
0
2
4
6
8
12
16
StageI
StageII
StageIII
StageIV
StageV
StageVI

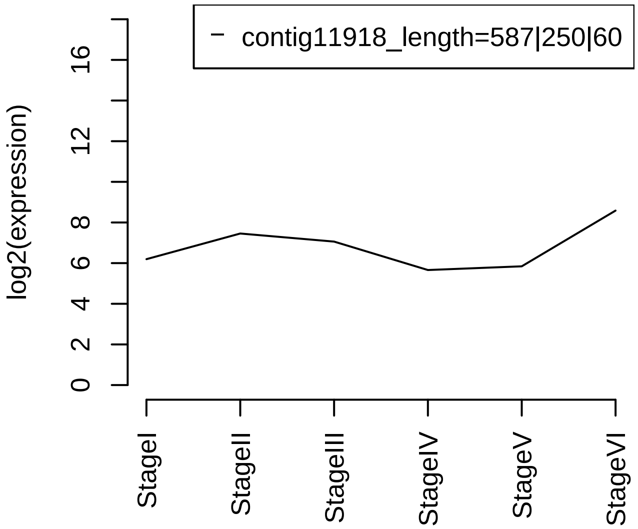

contig11918_length=587|250|60
log2(expression)
0
2
4
6
8
12
16
StageI
StageII
StageIII
StageIV
StageV
StageVI

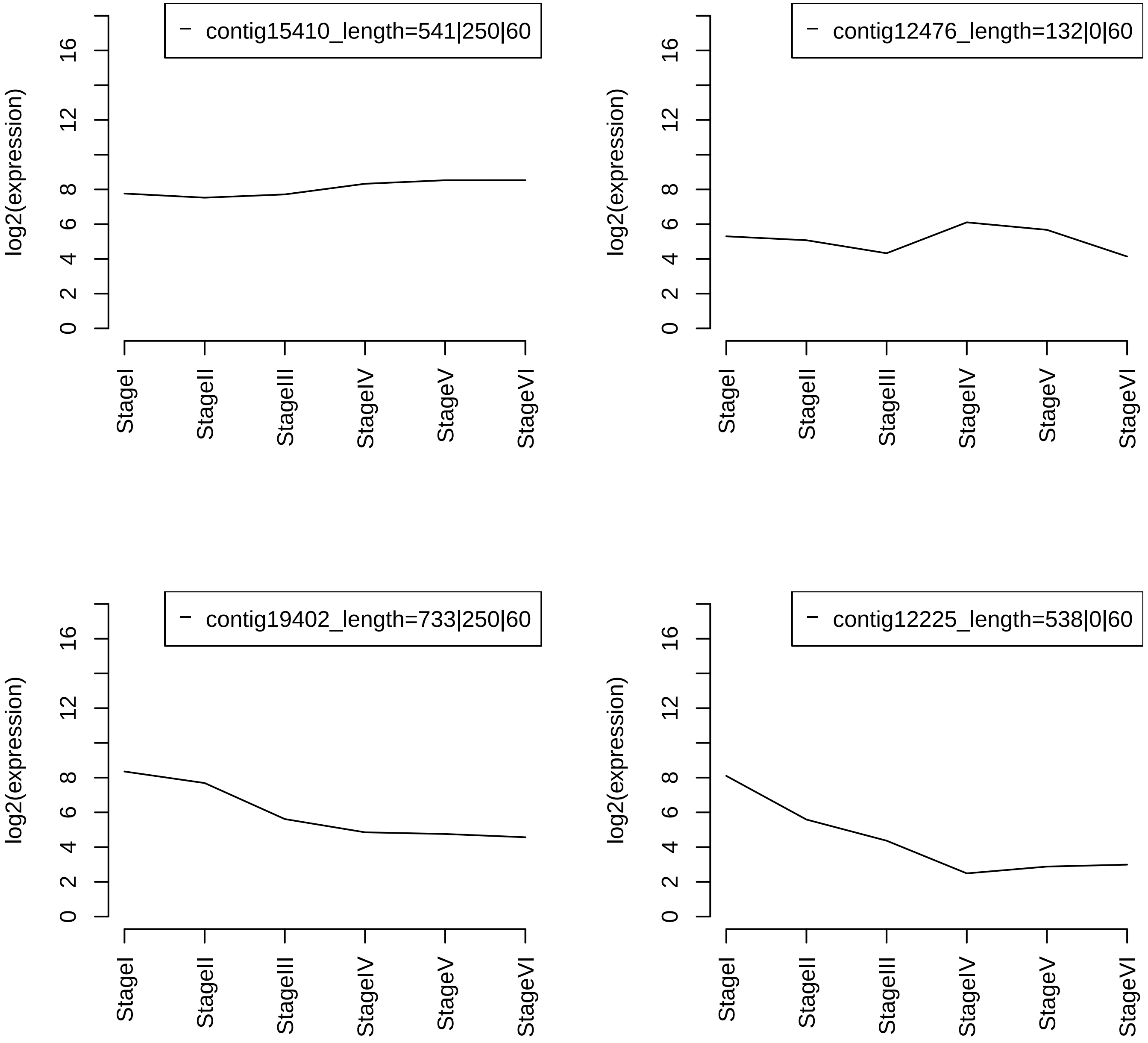
contig15410_length=541|250|60
contig12476_length=132|0|60
contig19402_length=733|250|60
contig12225_length=538|0|60
log2(expression)
0
2
4
6
8
12
16
StageI
StageII
StageIII
StageIV
StageV
StageVI

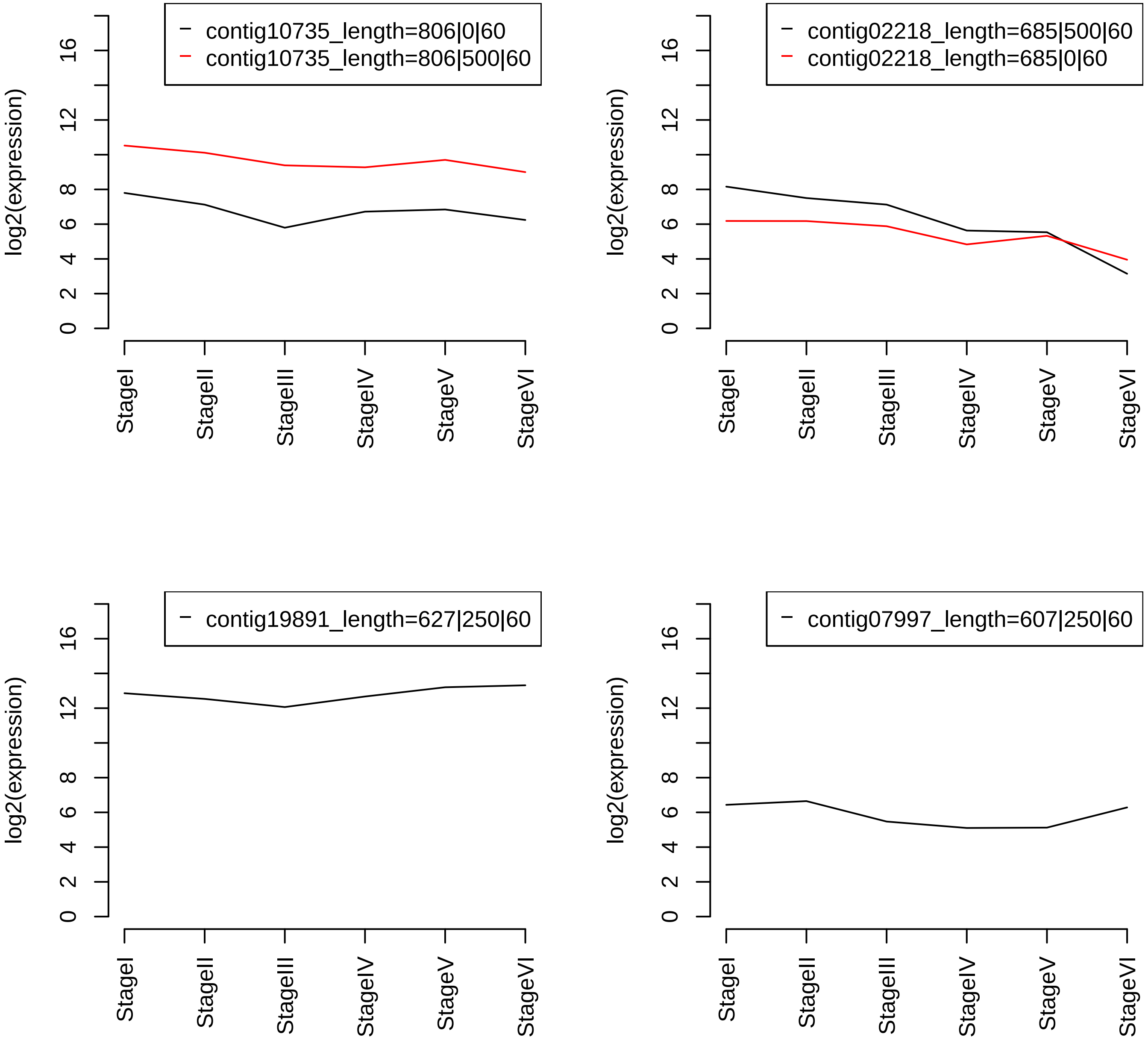

contig10735_length=806|0|60
contig10735_length=806|500|60
log2(expression)
0 2 4 6 8 12 16
StageI
StageII
StageIII
StageIV
StageV
StageVI
contig02218_length=685|500|60
contig02218_length=685|0|60
log2(expression)
0 2 4 6 8 12 16
StageI
StageII
StageIII
StageIV
StageV
StageVI
contig19891_length=627|250|60
log2(expression)
0 2 4 6 8 12 16
StageI
StageII
StageIII
StageIV
StageV
StageVI
contig07997_length=607|250|60
log2(expression)
0 2 4 6 8 12 16
StageI
StageII
StageIII
StageIV
StageV
StageVI

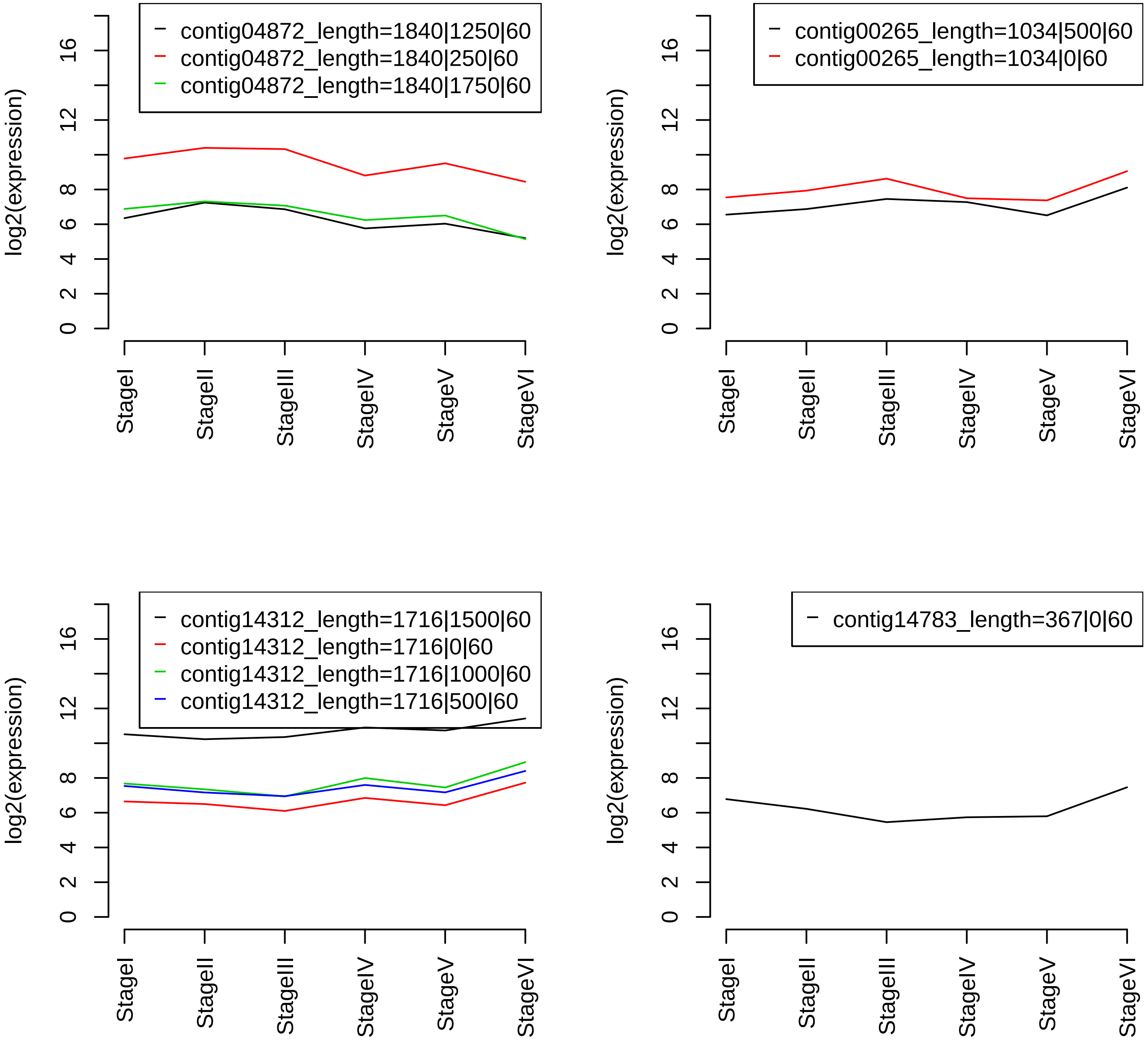
contig04872_length=1840|1250|60
contig04872_length=1840|250|60
contig04872_length=1840|1750|60
log2(expression)
0
2
4
6
8
12
16
StageI
StageII
StageIII
StageIV
StageV
StageVI
contig00265_length=1034|500|60
contig00265_length=1034|0|60
contig14312_length=1716|1500|60
contig14312_length=1716|0|60
contig14312_length=1716|1000|60
contig14312_length=1716|500|60
contig14783_length=367|0|60

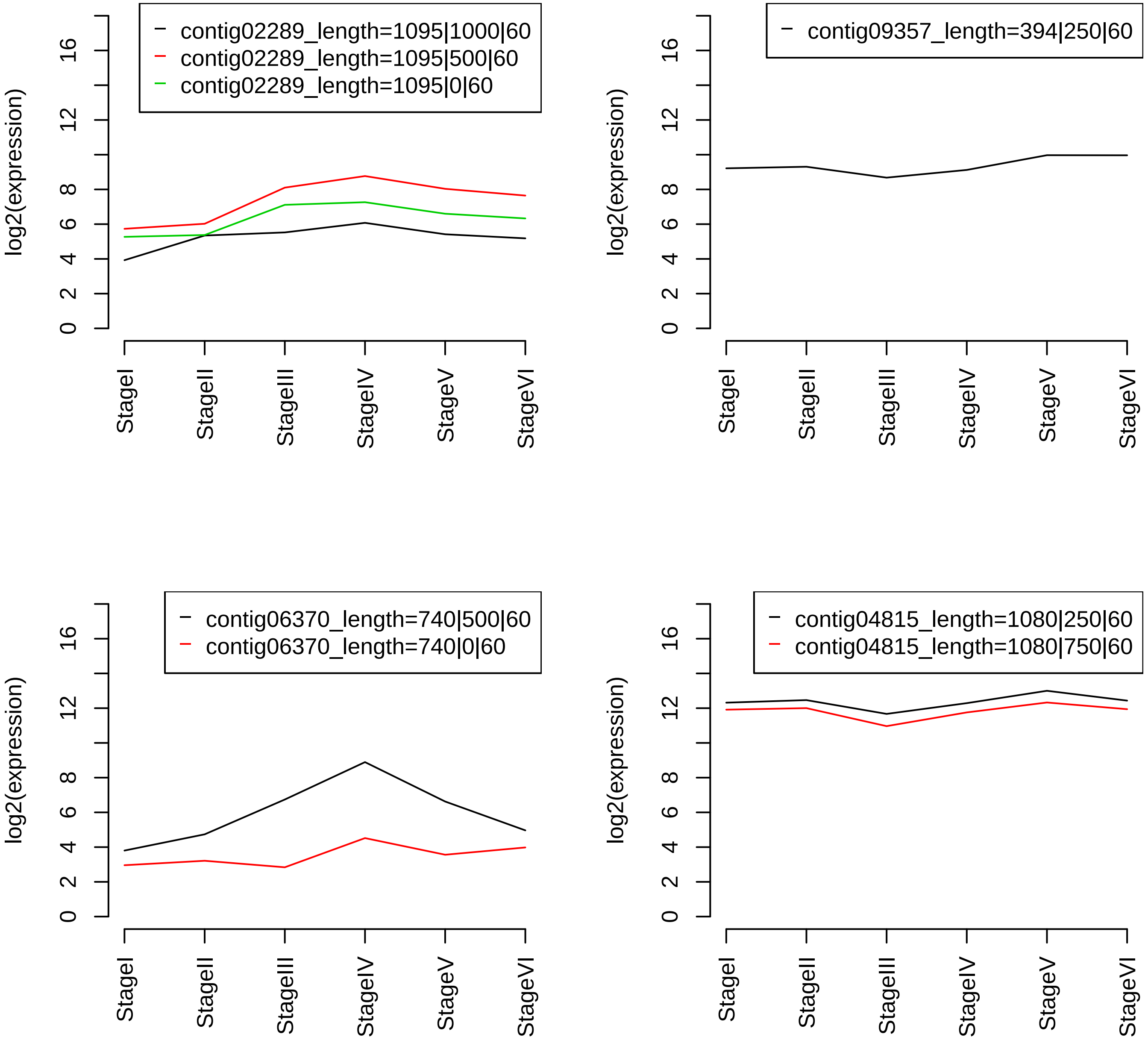
contig02289_length=1095|1000|60
contig02289_length=1095|500|60
contig02289_length=1095|0|60
log2(expression)
0
2
4
6
8
12
16
StageI
StageII
StageIII
StageIV
StageV
StageVI
contig09357_length=394|250|60
contig06370_length=740|500|60
contig06370_length=740|0|60
contig04815_length=1080|250|60
contig04815_length=1080|750|60

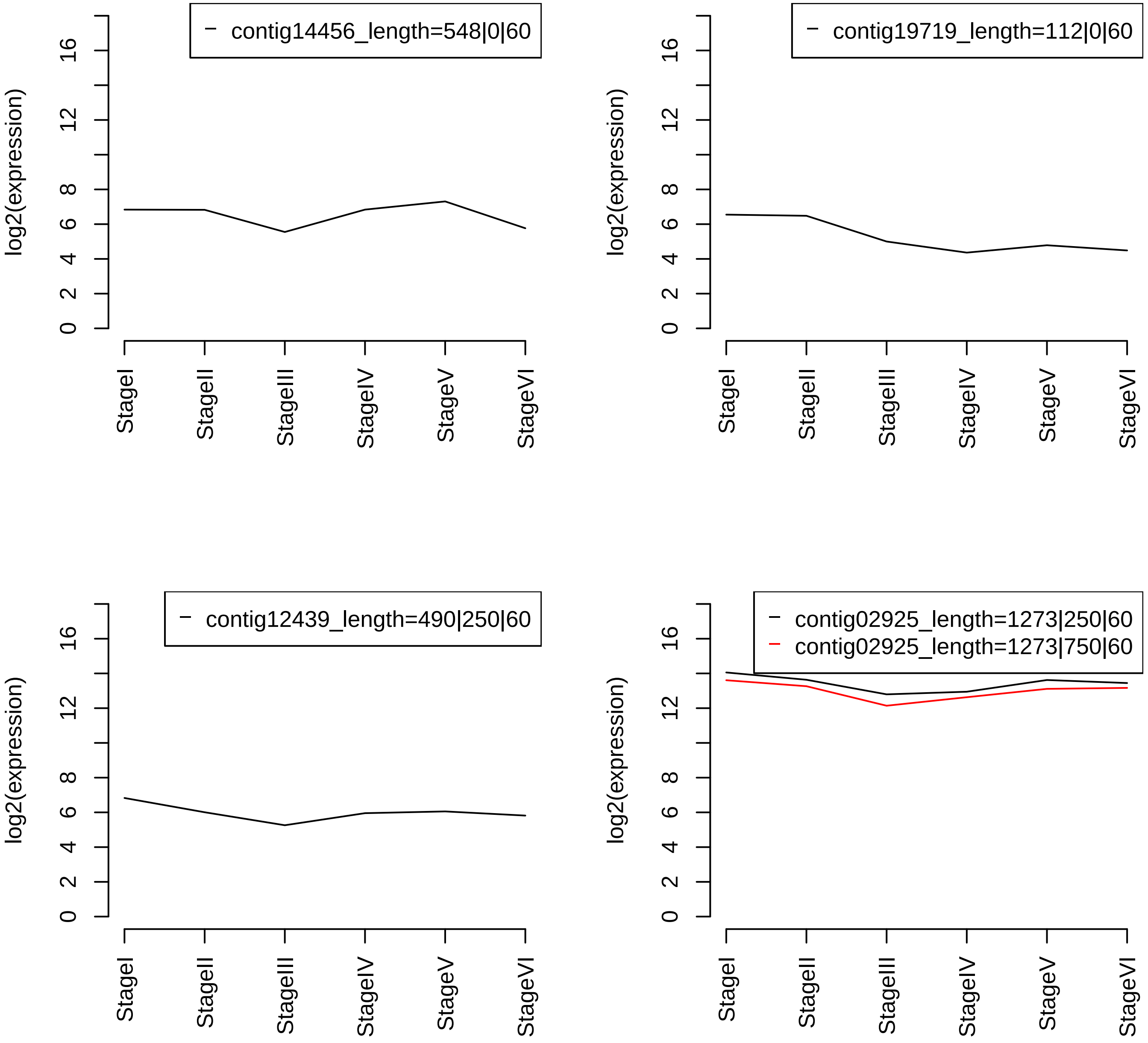
contig14456_length=548|0|60
contig19719_length=112|0|60
contig12439_length=490|250|60
contig02925_length=1273|250|60
contig02925_length=1273|750|60
log2(expression)
0
2
4
6
8
12
16
StageI
StageII
StageIII
StageIV
StageV
StageVI

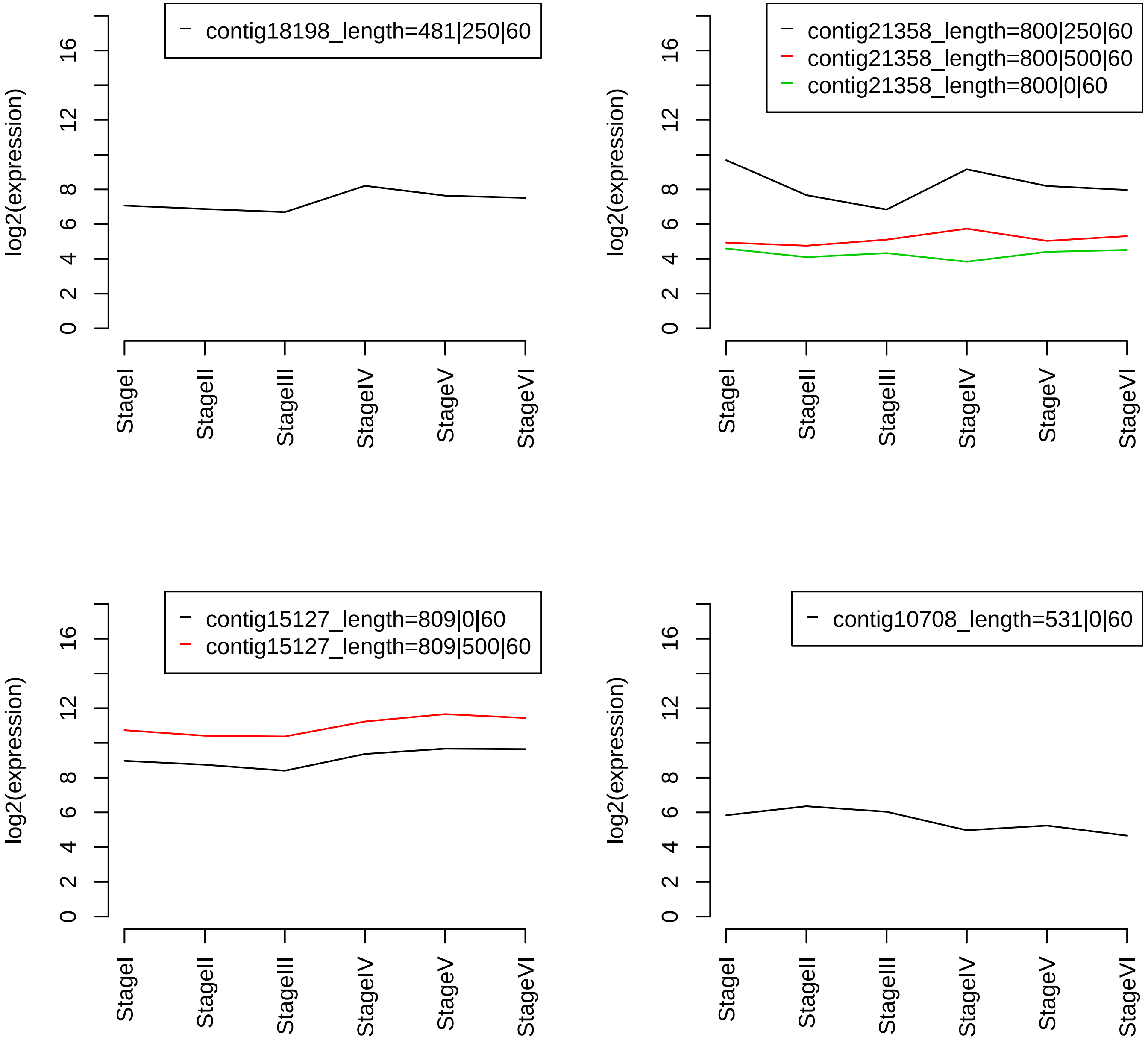

contig18198_length=481|250|60
contig21358_length=800|250|60
contig21358_length=800|500|60
contig21358_length=800|0|60
contig15127_length=809|0|60
contig15127_length=809|500|60
contig10708_length=531|0|60
log2(expression)
0 2 4 6 8 12 16
StageI StageII StageIII StageIV StageV StageVI

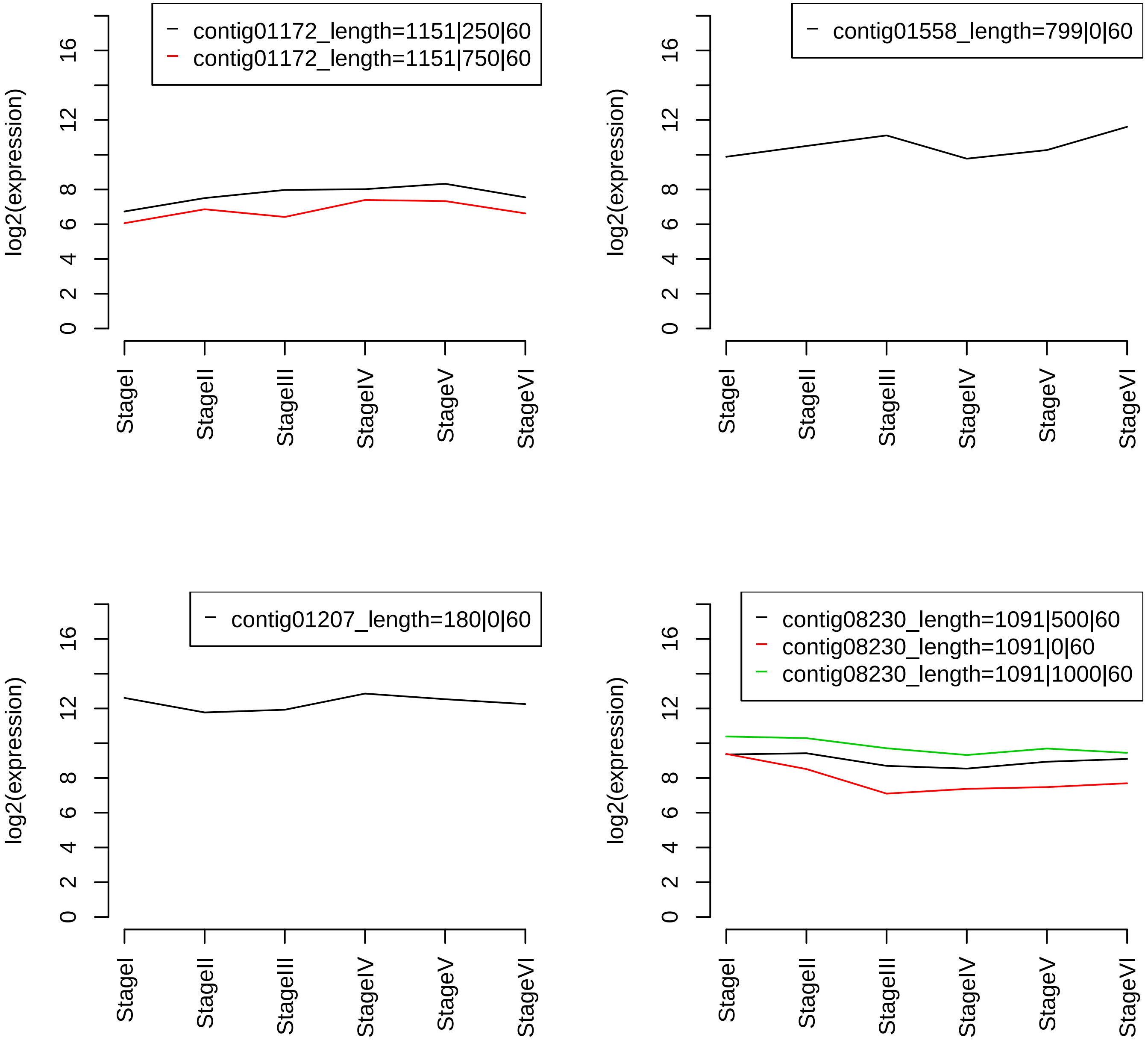

contig01172_length=1151|250|60
contig01172_length=1151|750|60
contig01558_length=799|0|60
contig01207_length=180|0|60
contig08230_length=1091|500|60
contig08230_length=1091|0|60
contig08230_length=1091|1000|60
log2(expression)
0
2
4
6
8
12
16
StageI
StageII
StageIII
StageIV
StageV
StageVI

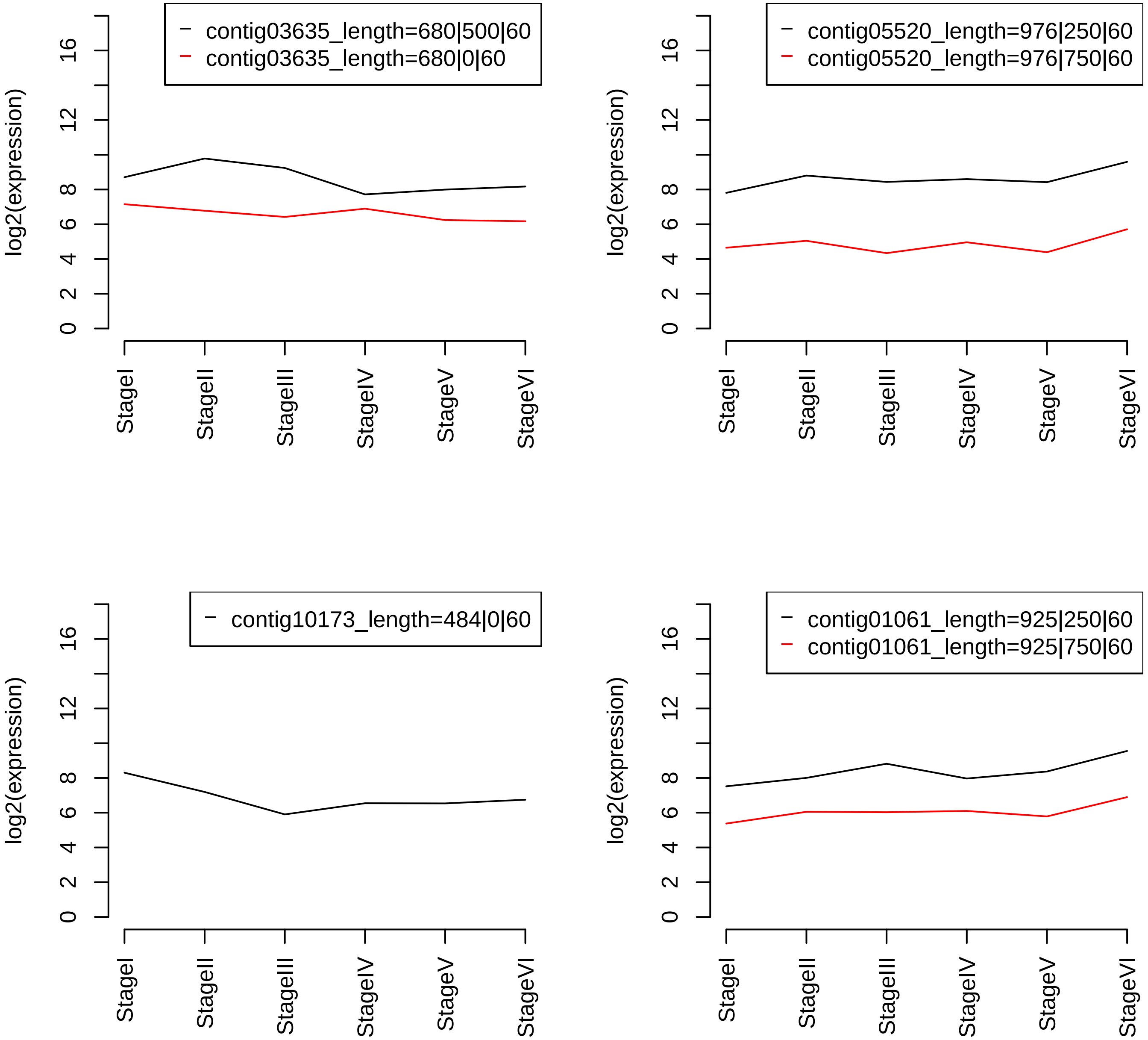

contig03635_length=680|500|60
contig03635_length=680|0|60
contig05520_length=976|250|60
contig05520_length=976|750|60
contig10173_length=484|0|60
contig01061_length=925|250|60
contig01061_length=925|750|60
log2(expression)
0
2
4
6
8
12
16
StageI
StageII
StageIII
StageIV
StageV
StageVI

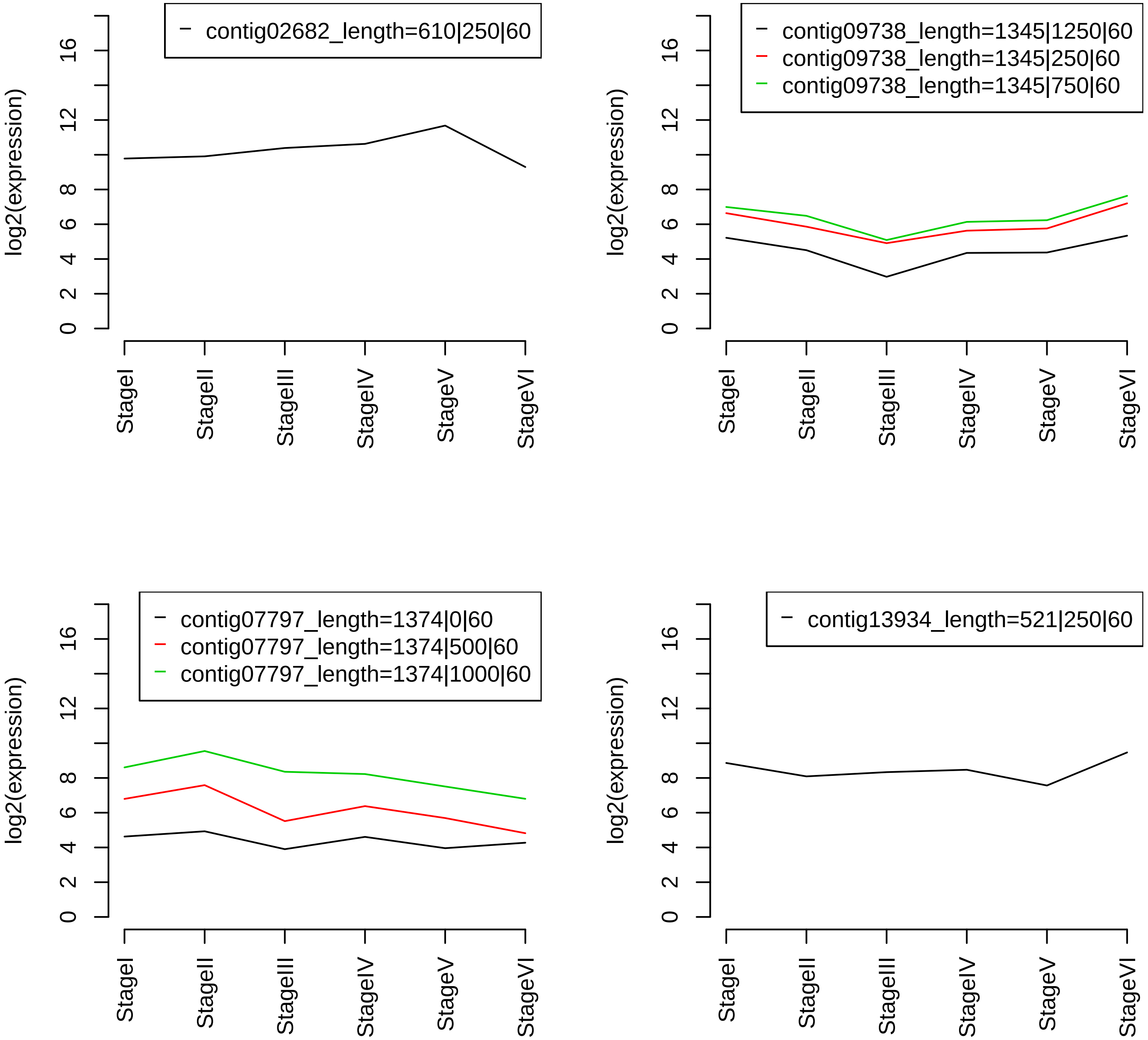

contig02682_length=610|250|60
contig09738_length=1345|1250|60
contig09738_length=1345|250|60
contig09738_length=1345|750|60
contig07797_length=1374|0|60
contig07797_length=1374|500|60
contig07797_length=1374|1000|60
contig13934_length=521|250|60
log2(expression)
0
2
4
6
8
12
16
StageI
StageII
StageIII
StageIV
StageV
StageVI

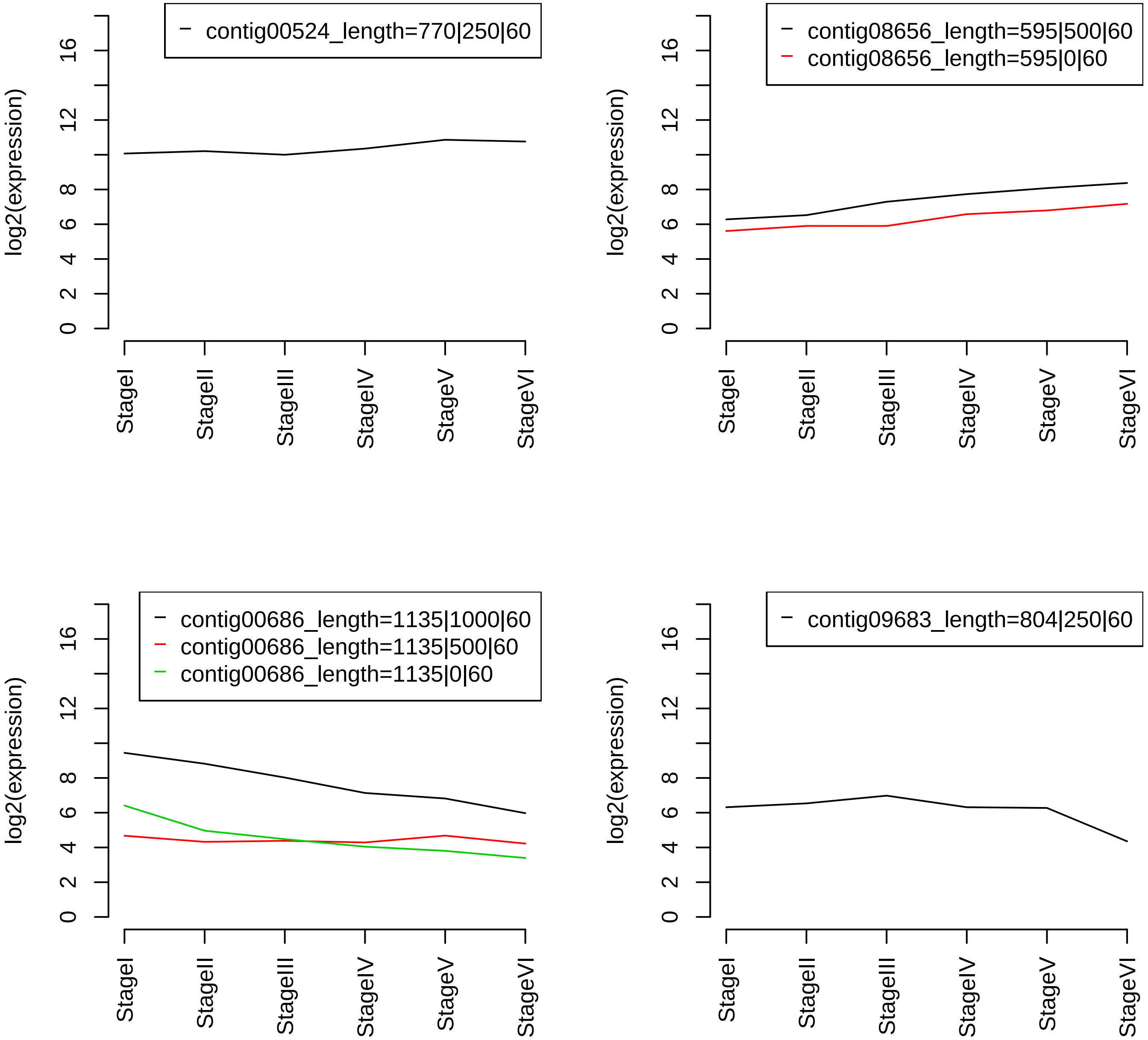
contig00524_length=770|250|60
contig08656_length=595|500|60
contig08656_length=595|0|60
contig00686_length=1135|1000|60
contig00686_length=1135|500|60
contig00686_length=1135|0|60
contig09683_length=804|250|60
log2(expression)
0
2
4
6
8
12
16
StageI
StageII
StageIII
StageIV
StageV
StageVI

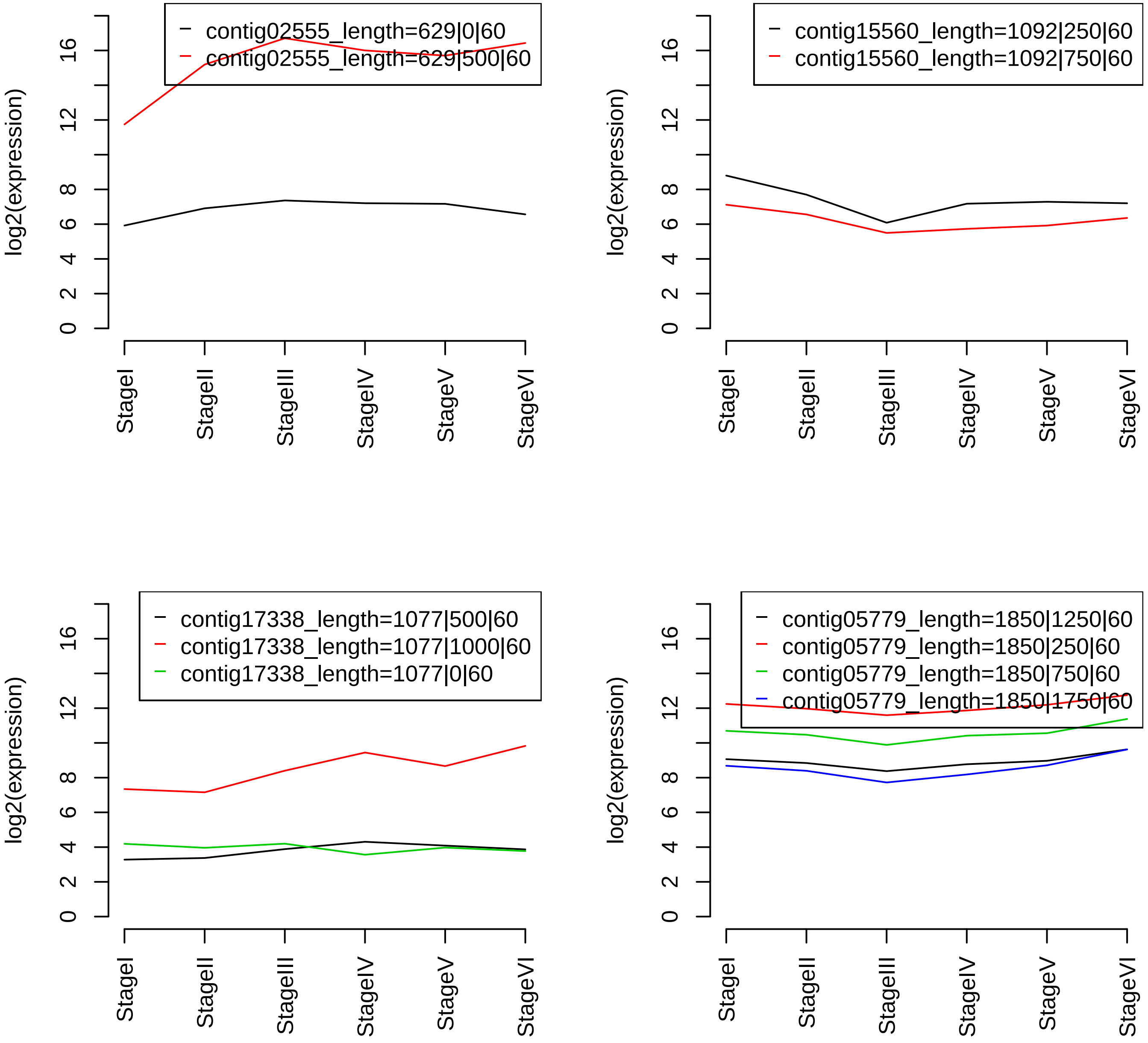

contig02555_length=629|0|60
contig02555_length=629|500|60
contig15560_length=1092|250|60
contig15560_length=1092|750|60
contig17338_length=1077|500|60
contig17338_length=1077|1000|60
contig17338_length=1077|0|60
contig05779_length=1850|1250|60
contig05779_length=1850|250|60
contig05779_length=1850|750|60
contig05779_length=1850|1750|60
log2(expression)
0 2 4 6 8 12 16
StageI StageII StageIII StageIV StageV StageVI

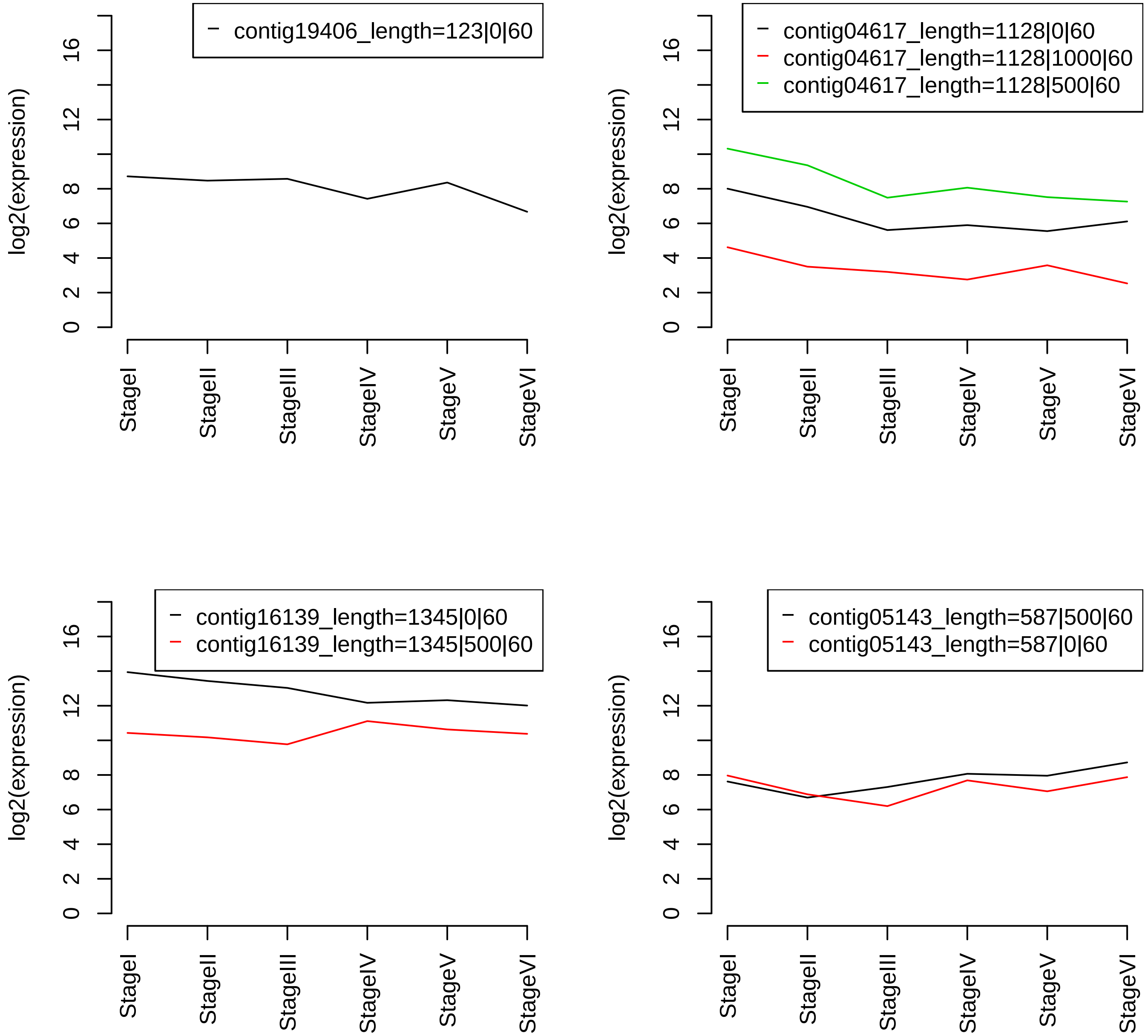

contig19406_length=123|0|60
log2(expression)
0
2
4
6
8
12
16
StageI
StageII
StageIII
StageIV
StageV
StageVI
contig04617_length=1128|0|60
contig04617_length=1128|1000|60
contig04617_length=1128|500|60
contig16139_length=1345|0|60
contig16139_length=1345|500|60
contig05143_length=587|500|60
contig05143_length=587|0|60

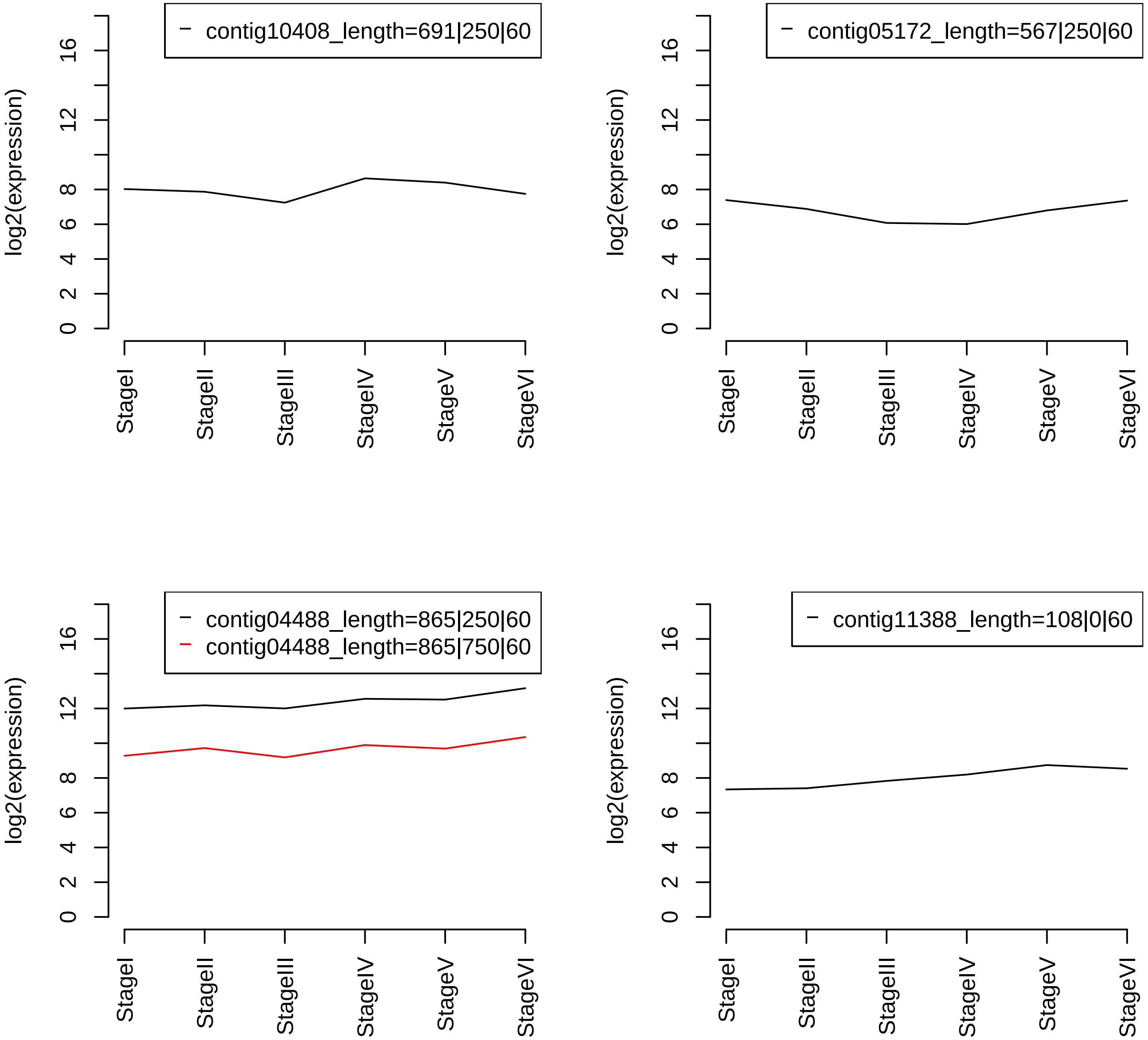
contig10408_length=691|250|60
contig05172_length=567|250|60
contig04488_length=865|250|60
contig04488_length=865|750|60
contig11388_length=108|0|60
log2(expression)
0
2
4
6
8
12
16
StageI
StageII
StageIII
StageIV
StageV
StageVI

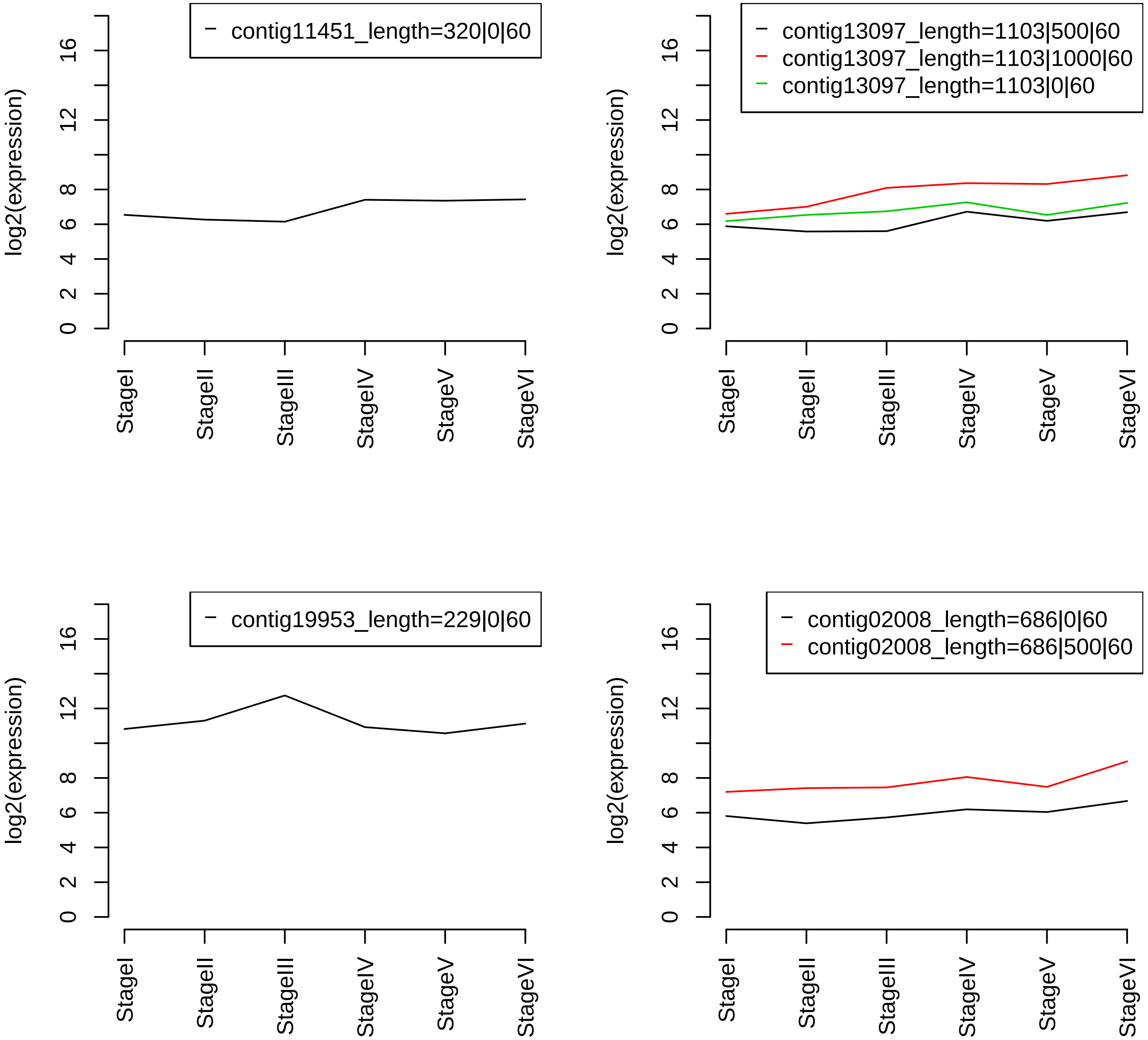

contig11451_length=320|0|60
contig13097_length=1103|500|60
contig13097_length=1103|1000|60
contig13097_length=1103|0|60
contig19953_length=229|0|60
contig02008_length=686|0|60
contig02008_length=686|500|60
log2(expression)
0
2
4
6
8
12
16
StageI
StageII
StageIII
StageIV
StageV
StageVI

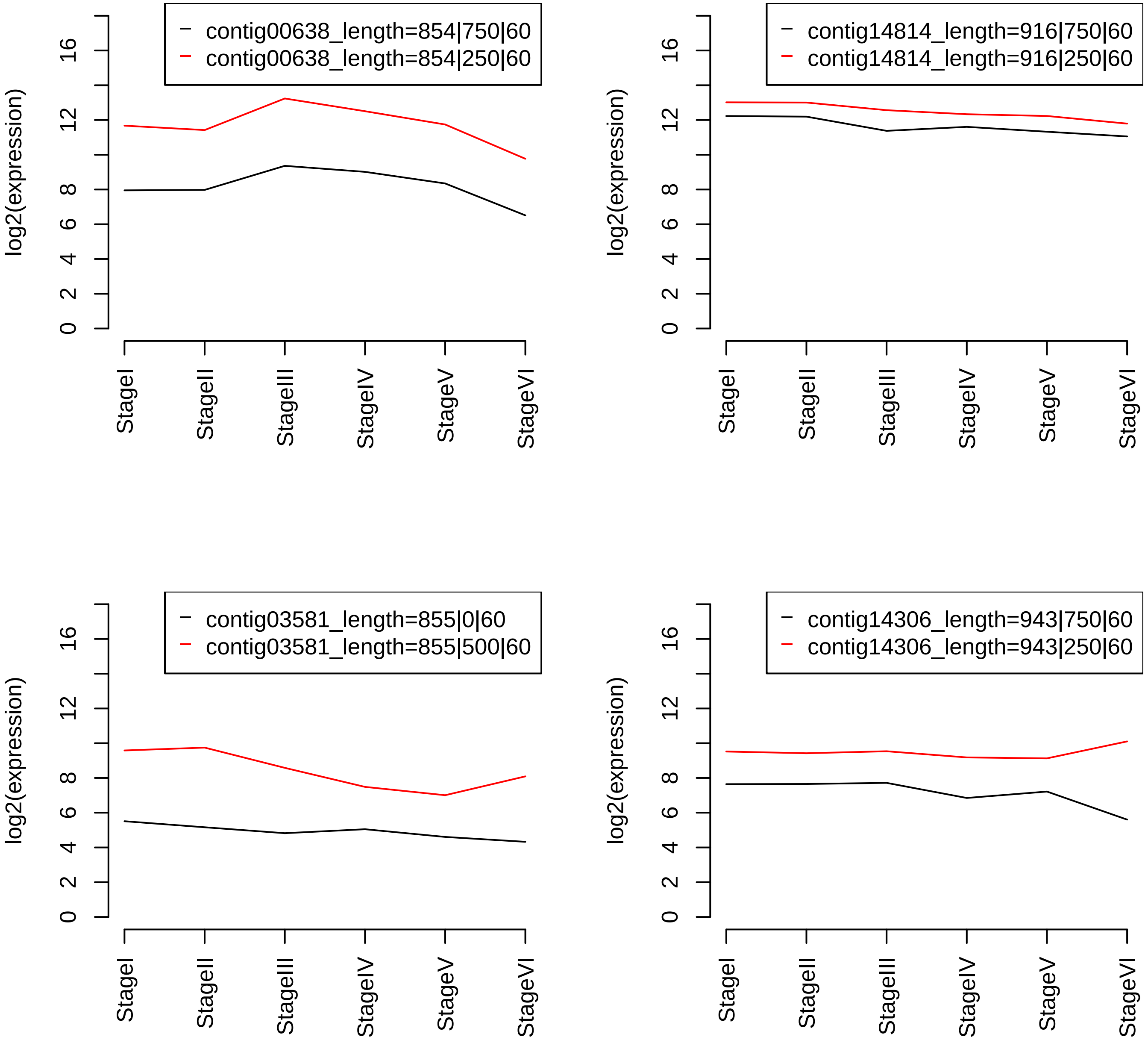

contig00638_length=854|750|60
contig00638_length=854|250|60
contig14814_length=916|750|60
contig14814_length=916|250|60
contig03581_length=855|0|60
contig03581_length=855|500|60
contig14306_length=943|750|60
contig14306_length=943|250|60
log2(expression)
0
2
4
6
8
12
16
StageI
StageII
StageIII
StageIV
StageV
StageVI

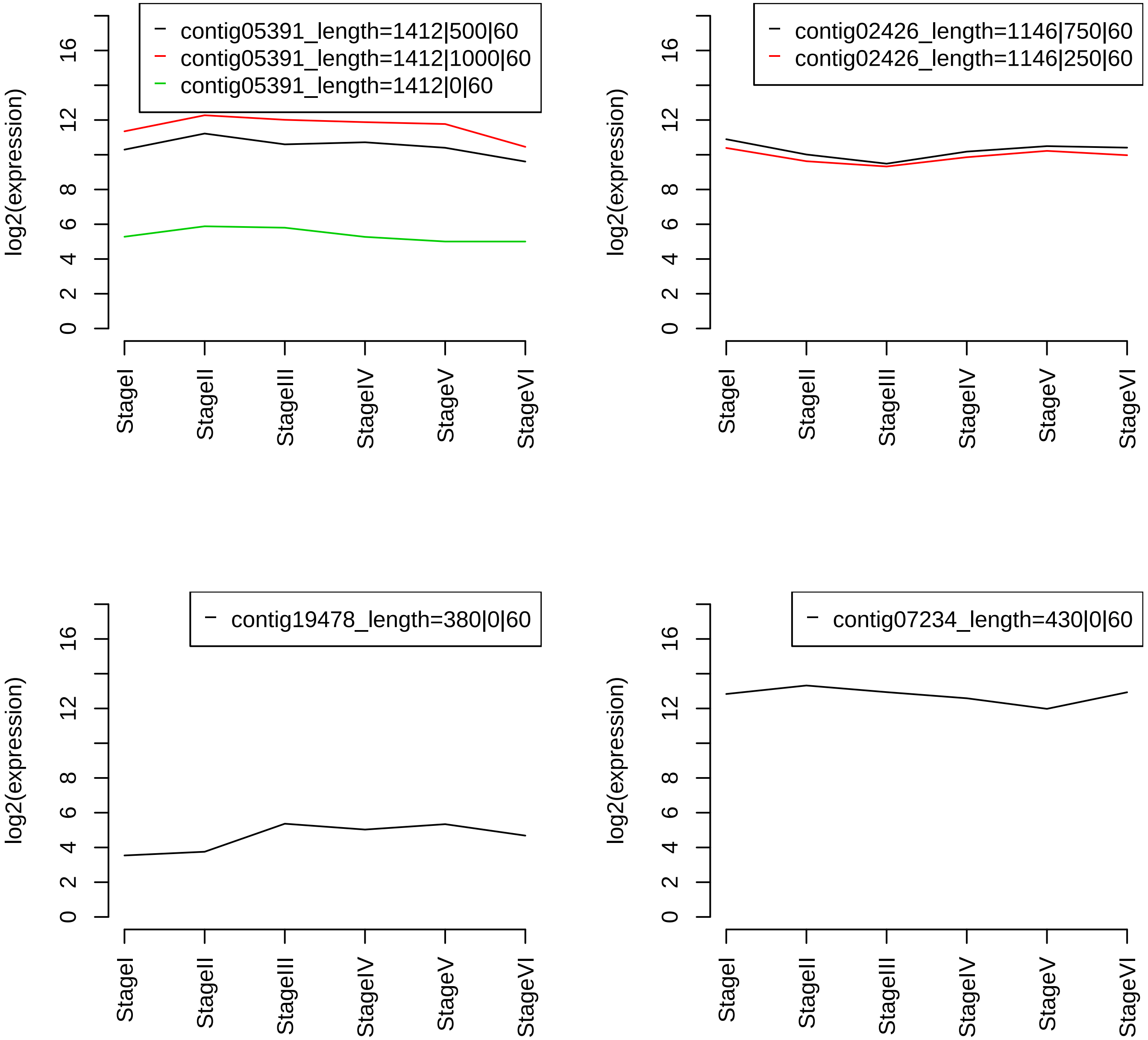
contig05391_length=1412|500|60
contig05391_length=1412|1000|60
contig05391_length=1412|0|60
contig02426_length=1146|750|60
contig02426_length=1146|250|60
contig19478_length=380|0|60
contig07234_length=430|0|60
log2(expression)
0
2
4
6
8
12
16
StageI
StageII
StageIII
StageIV
StageV
StageVI

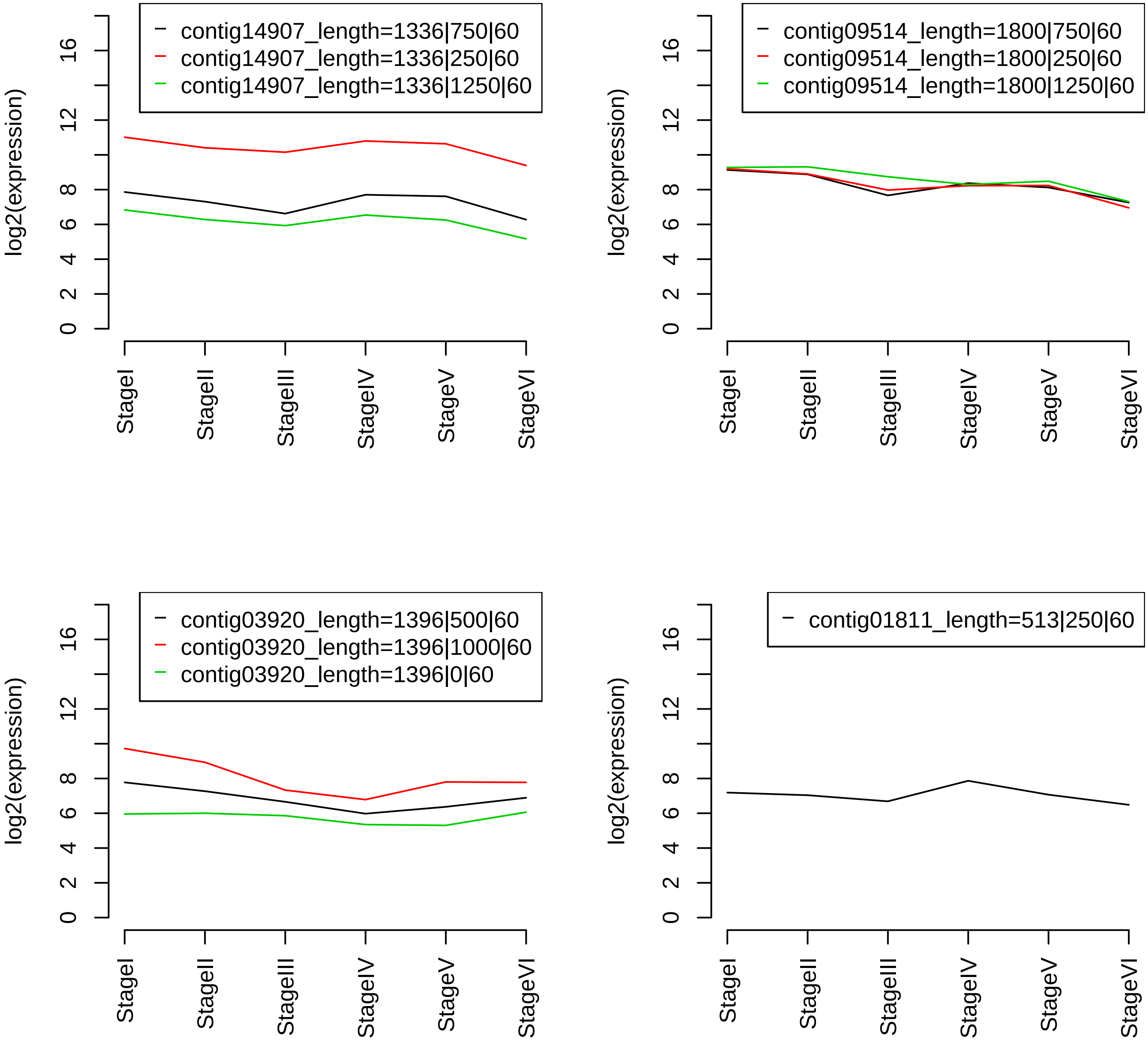
contig14907_length=1336|750|60
contig14907_length=1336|250|60
contig14907_length=1336|1250|60
contig09514_length=1800|750|60
contig09514_length=1800|250|60
contig09514_length=1800|1250|60
contig03920_length=1396|500|60
contig03920_length=1396|1000|60
contig03920_length=1396|0|60
contig01811_length=513|250|60
log2(expression)
0
2
4
6
8
12
16
StageI
StageII
StageIII
StageIV
StageV
StageVI

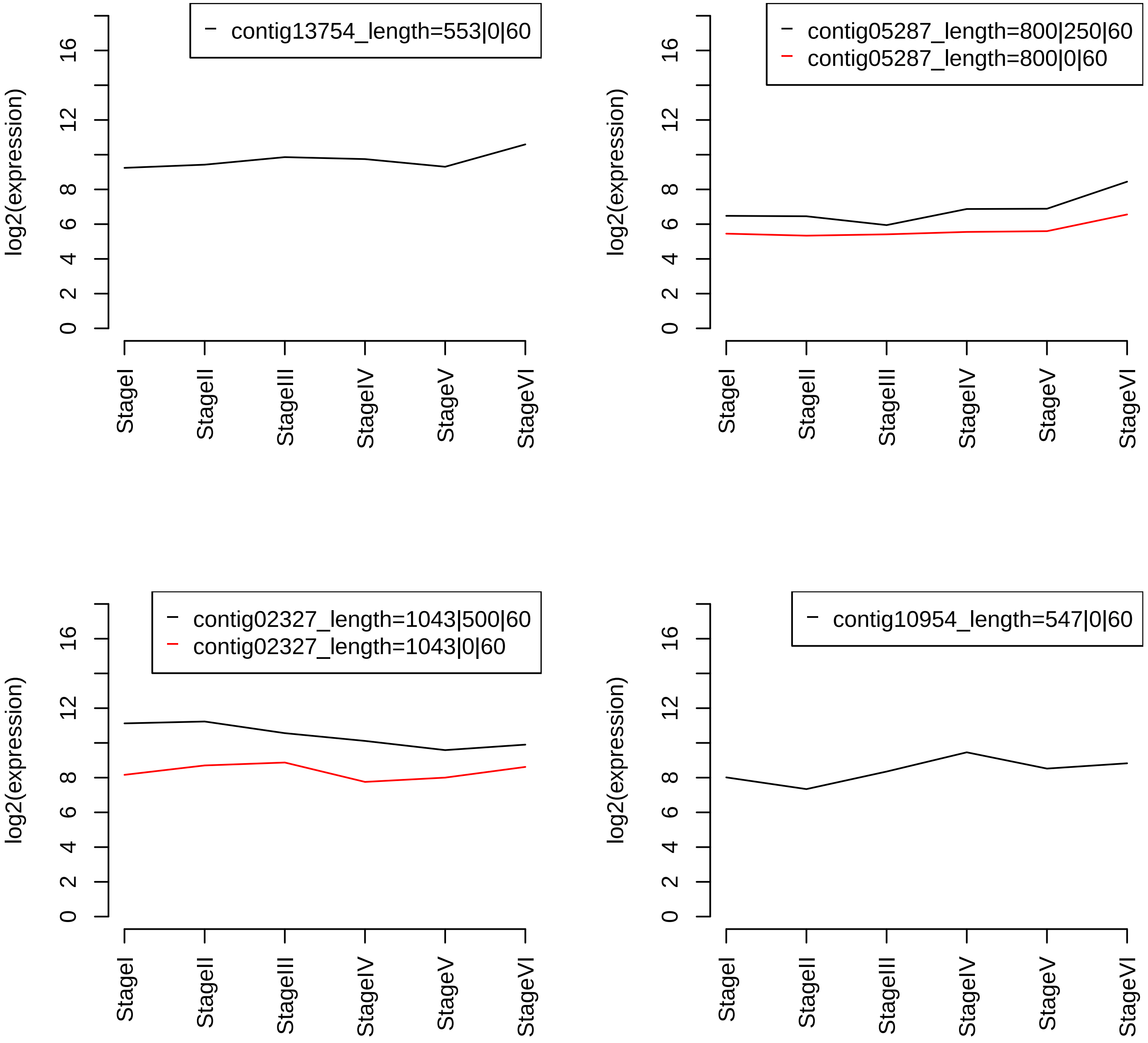
contig13754_length=553|0|60
contig05287_length=800|250|60
contig05287_length=800|0|60
contig02327_length=1043|500|60
contig02327_length=1043|0|60
contig10954_length=547|0|60
log2(expression)
StageI
StageII
StageIII
StageIV
StageV
StageVI
0
2
4
6
8
12
16

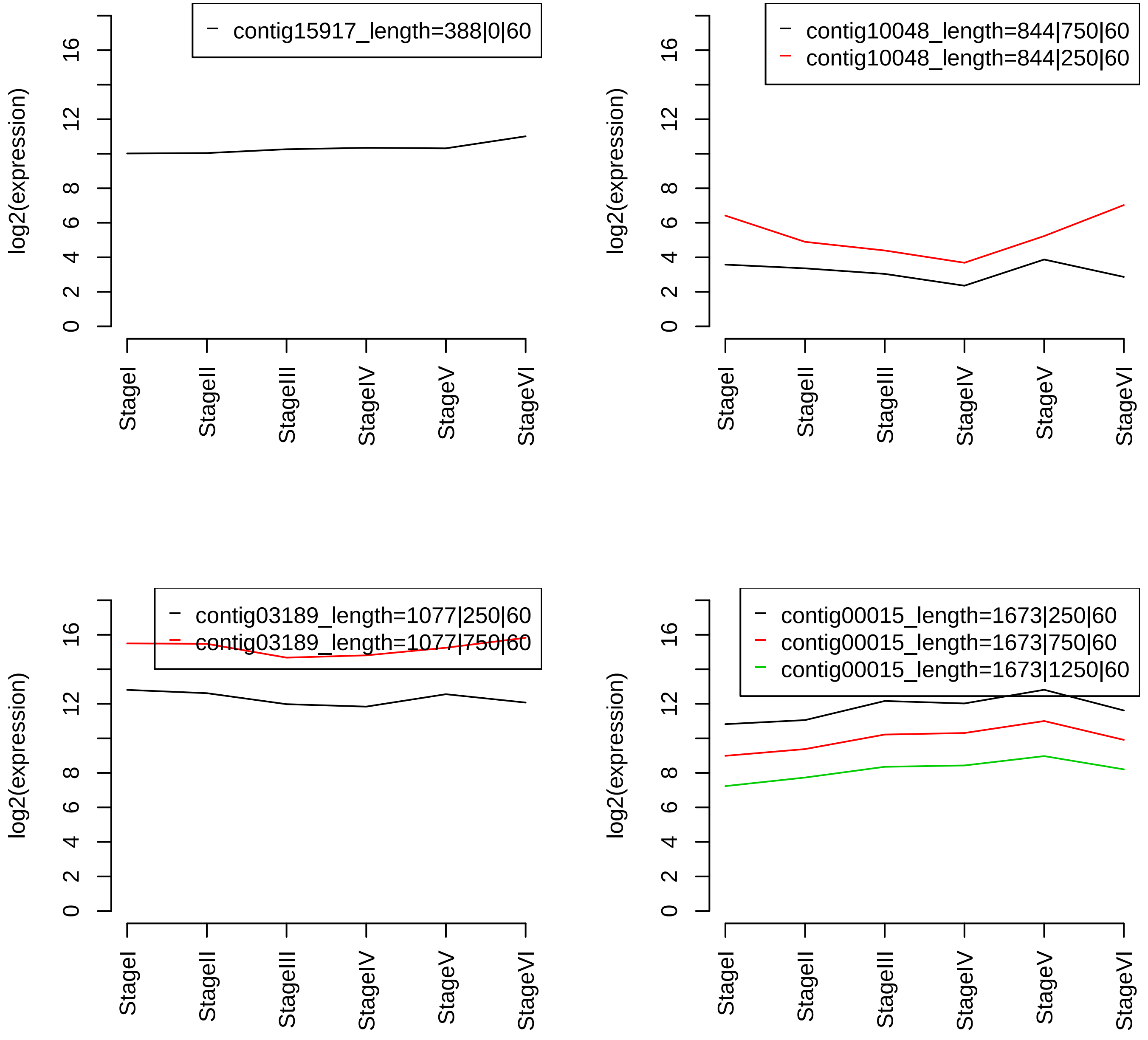
contig15917_length=388|0|60
contig10048_length=844|750|60
contig10048_length=844|250|60
contig03189_length=1077|250|60
contig03189_length=1077|750|60
contig00015_length=1673|250|60
contig00015_length=1673|750|60
contig00015_length=1673|1250|60
log2(expression)
0
2
4
6
8
12
16
StageI
StageII
StageIII
StageIV
StageV
StageVI

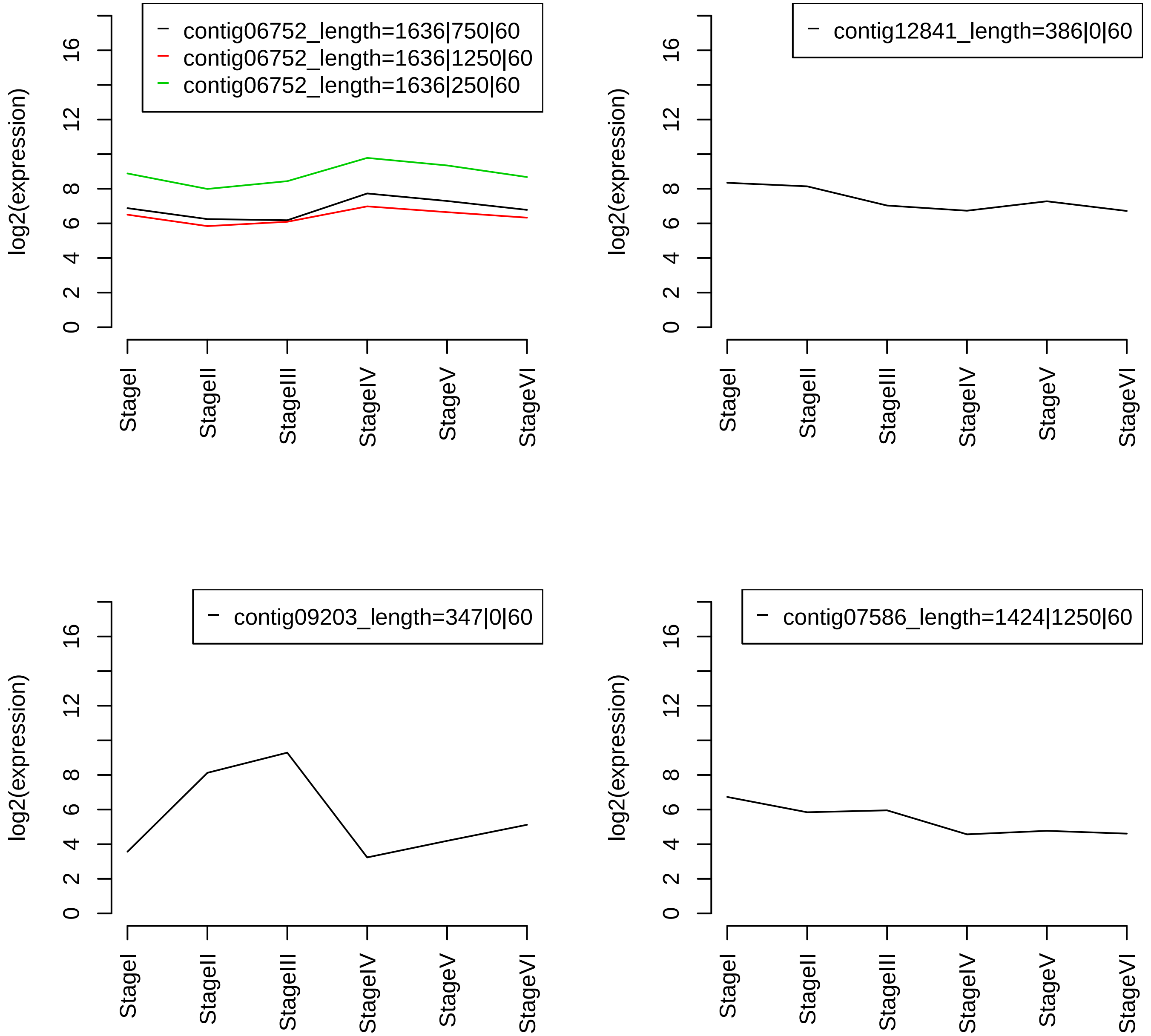
contig06752_length=1636|750|60
contig06752_length=1636|1250|60
contig06752_length=1636|250|60
contig12841_length=386|0|60
contig09203_length=347|0|60
contig07586_length=1424|1250|60
log2(expression)
0
2
4
6
8
12
16
StageI
StageII
StageIII
StageIV
StageV
StageVI

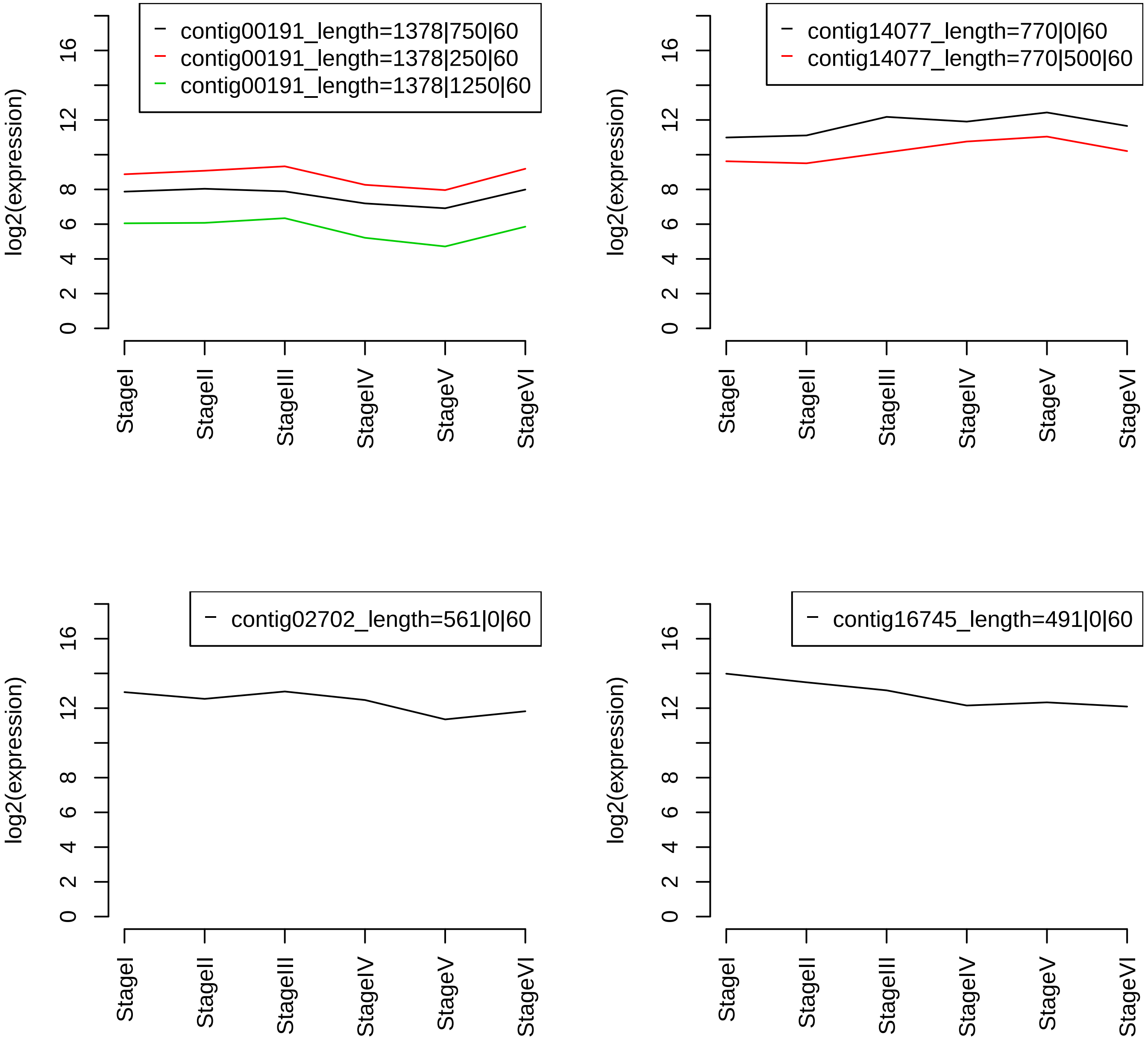
contig00191_length=1378|750|60
contig00191_length=1378|250|60
contig00191_length=1378|1250|60
log2(expression)
0
2
4
6
8
12
16
StageI
StageII
StageIII
StageIV
StageV
StageVI
contig14077_length=770|0|60
contig14077_length=770|500|60
contig02702_length=561|0|60
contig16745_length=491|0|60

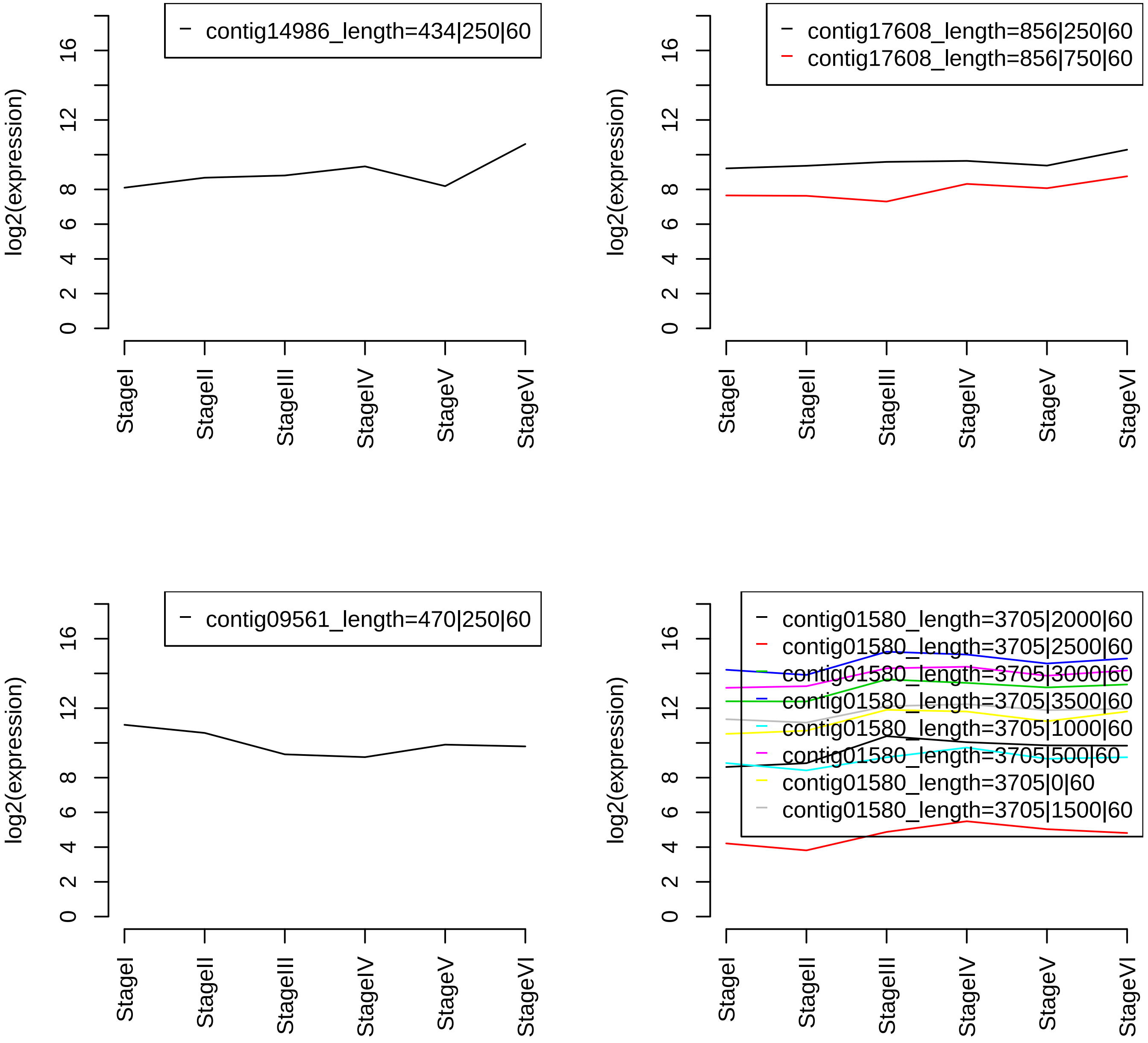
contig14986_length=434|250|60
log2(expression)
0
2
4
6
8
12
16
StageI
StageII
StageIII
StageIV
StageV
StageVI
contig17608_length=856|250|60
contig17608_length=856|750|60
log2(expression)
0
2
4
6
8
12
16
StageI
StageII
StageIII
StageIV
StageV
StageVI
contig09561_length=470|250|60
log2(expression)
0
2
4
6
8
12
16
StageI
StageII
StageIII
StageIV
StageV
StageVI
contig01580_length=3705|2000|60
contig01580_length=3705|2500|60
contig01580_length=3705|3000|60
contig01580_length=3705|3500|60
contig01580_length=3705|1000|60
contig01580_length=3705|500|60
contig01580_length=3705|0|60
contig01580_length=3705|1500|60
log2(expression)
0
2
4
6
8
12
16
StageI
StageII
StageIII
StageIV
StageV
StageVI

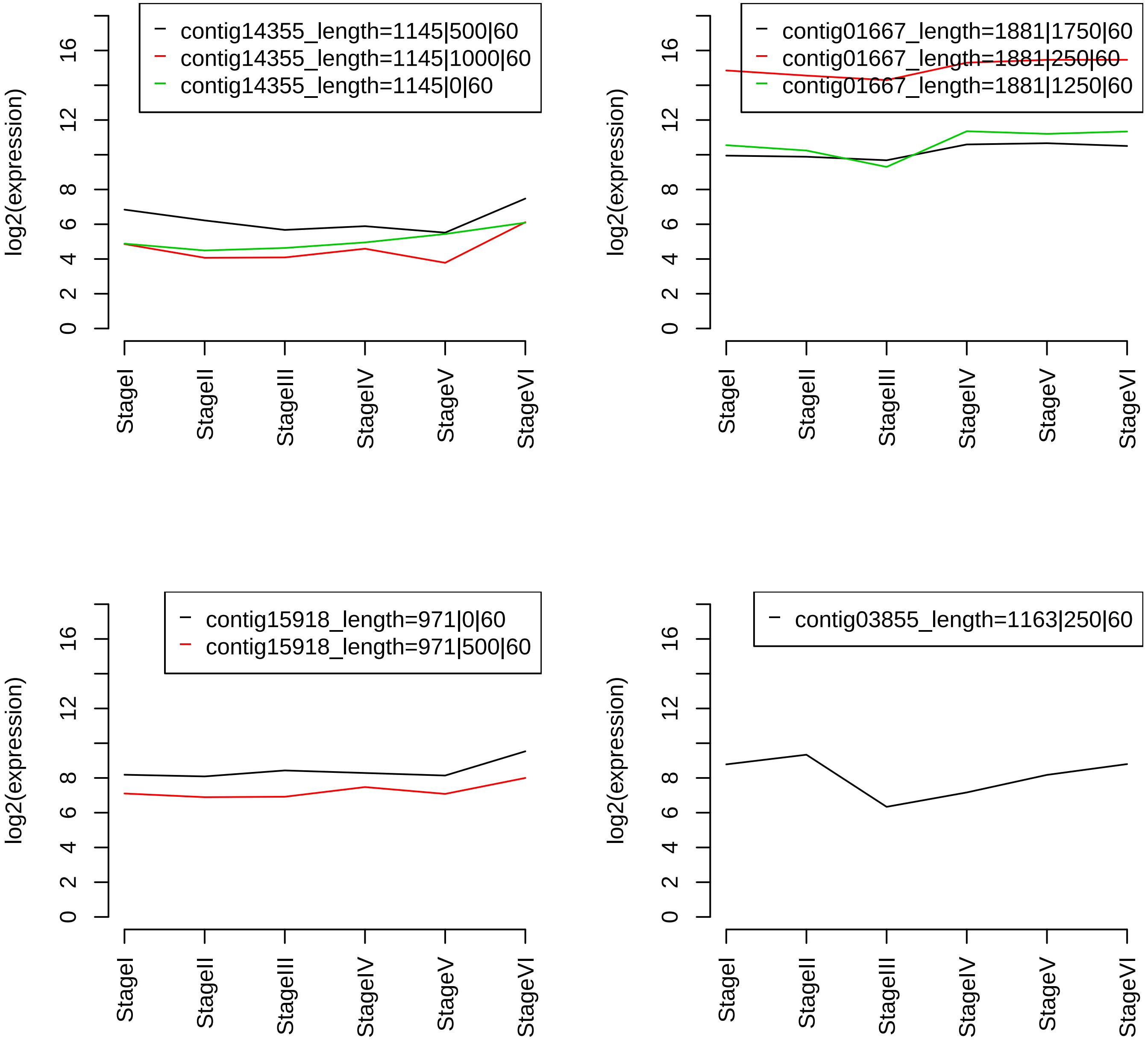
contig14355_length=1145|500|60
contig14355_length=1145|1000|60
contig14355_length=1145|0|60
contig01667_length=1881|1750|60
contig01667_length=1881|250|60
contig01667_length=1881|1250|60
contig15918_length=971|0|60
contig15918_length=971|500|60
contig03855_length=1163|250|60
log2(expression)
0
2
4
6
8
12
16
StageI
StageII
StageIII
StageIV
StageV
StageVI

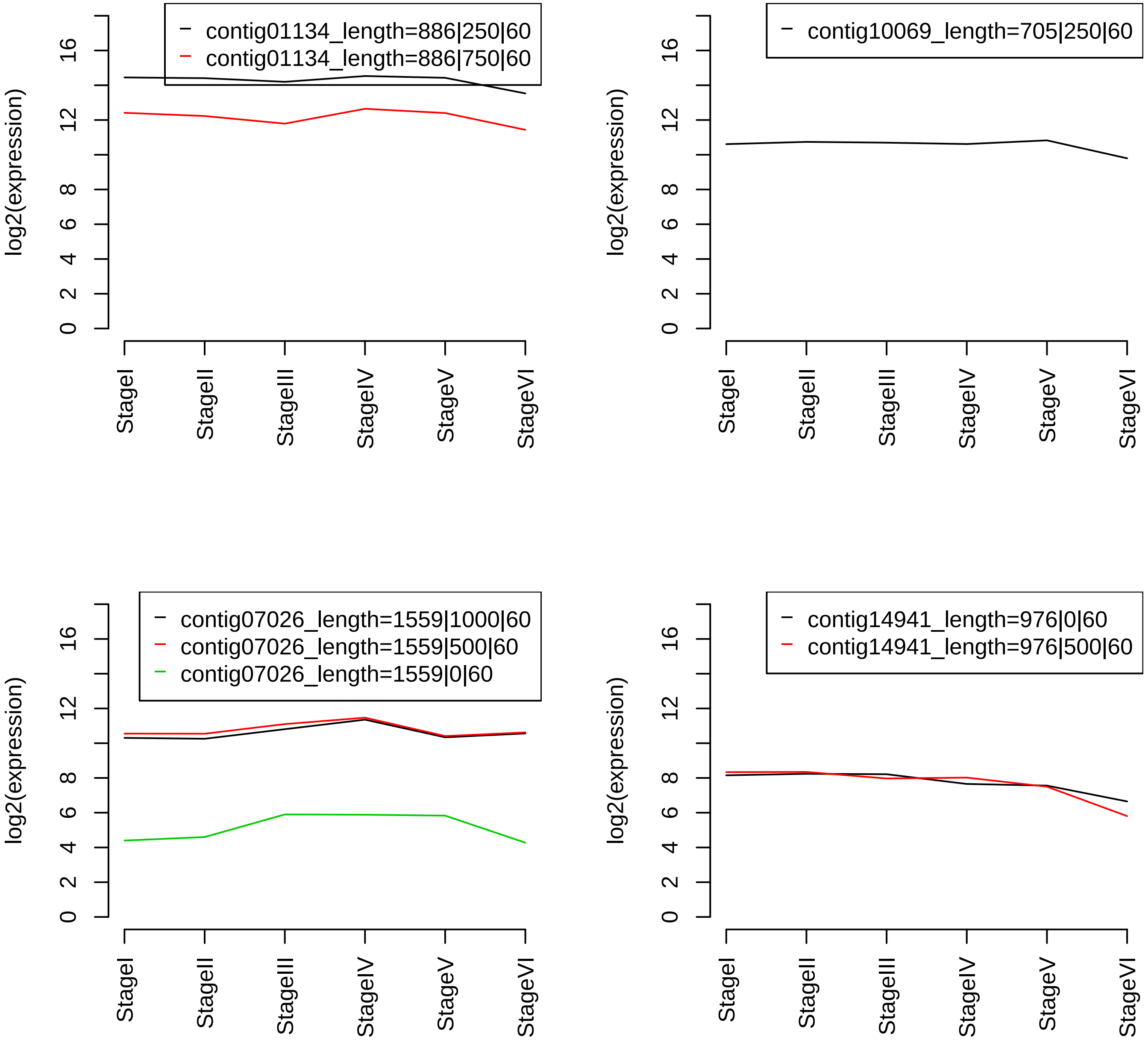

contig01134_length=886|250|60
contig01134_length=886|750|60
log2(expression)
0 2 4 6 8 12 16
StageI StageII StageIII StageIV StageV StageVI
contig10069_length=705|250|60
log2(expression)
0 2 4 6 8 12 16
StageI StageII StageIII StageIV StageV StageVI
contig07026_length=1559|1000|60
contig07026_length=1559|500|60
contig07026_length=1559|0|60
log2(expression)
0 2 4 6 8 12 16
StageI StageII StageIII StageIV StageV StageVI
contig14941_length=976|0|60
contig14941_length=976|500|60
log2(expression)
0 2 4 6 8 12 16
StageI StageII StageIII StageIV StageV StageVI

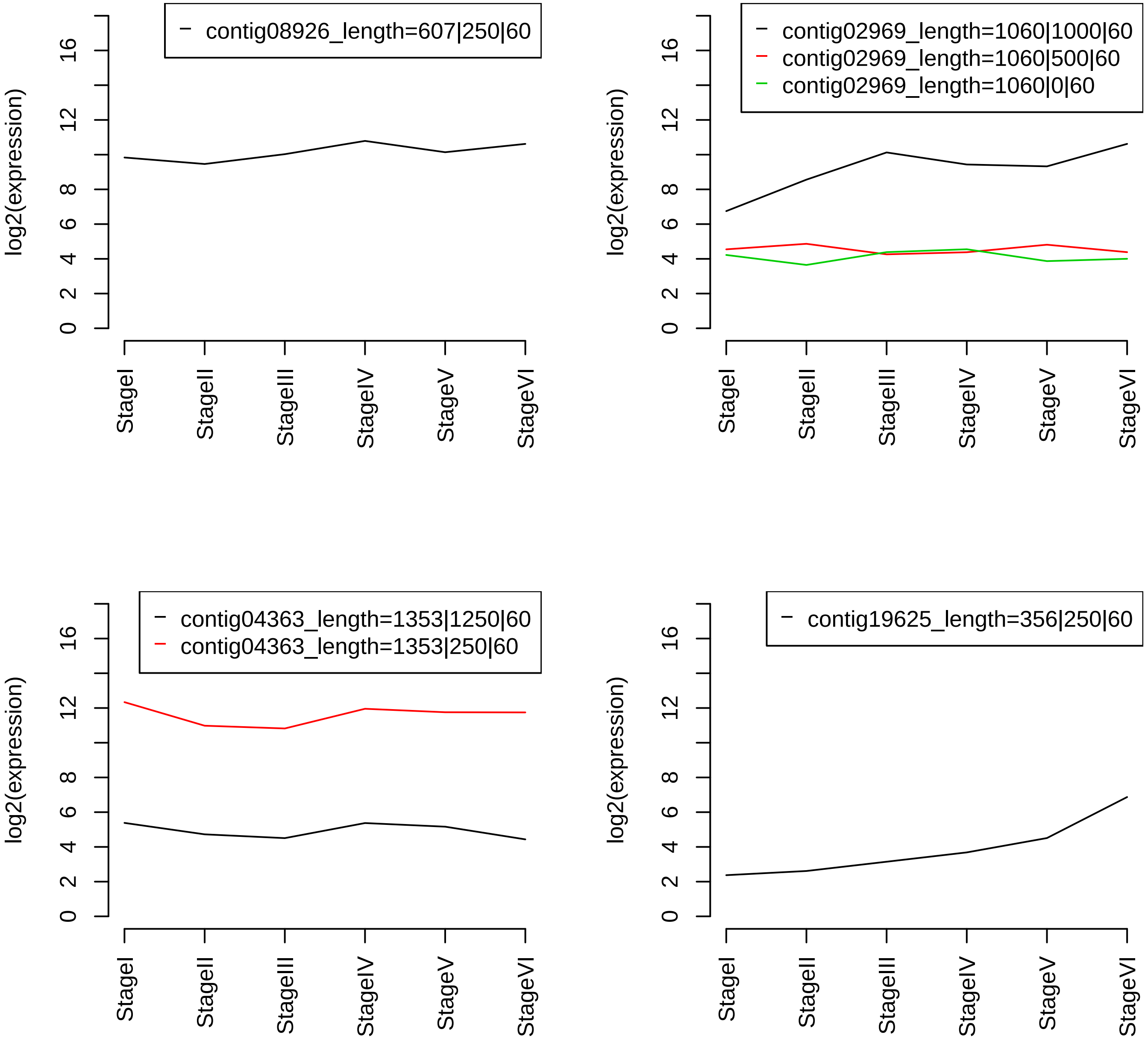

contig08926_length=607|250|60
contig02969_length=1060|1000|60
contig02969_length=1060|500|60
contig02969_length=1060|0|60
contig04363_length=1353|1250|60
contig04363_length=1353|250|60
contig19625_length=356|250|60
log2(expression)
0
2
4
6
8
12
16
StageI
StageII
StageIII
StageIV
StageV
StageVI

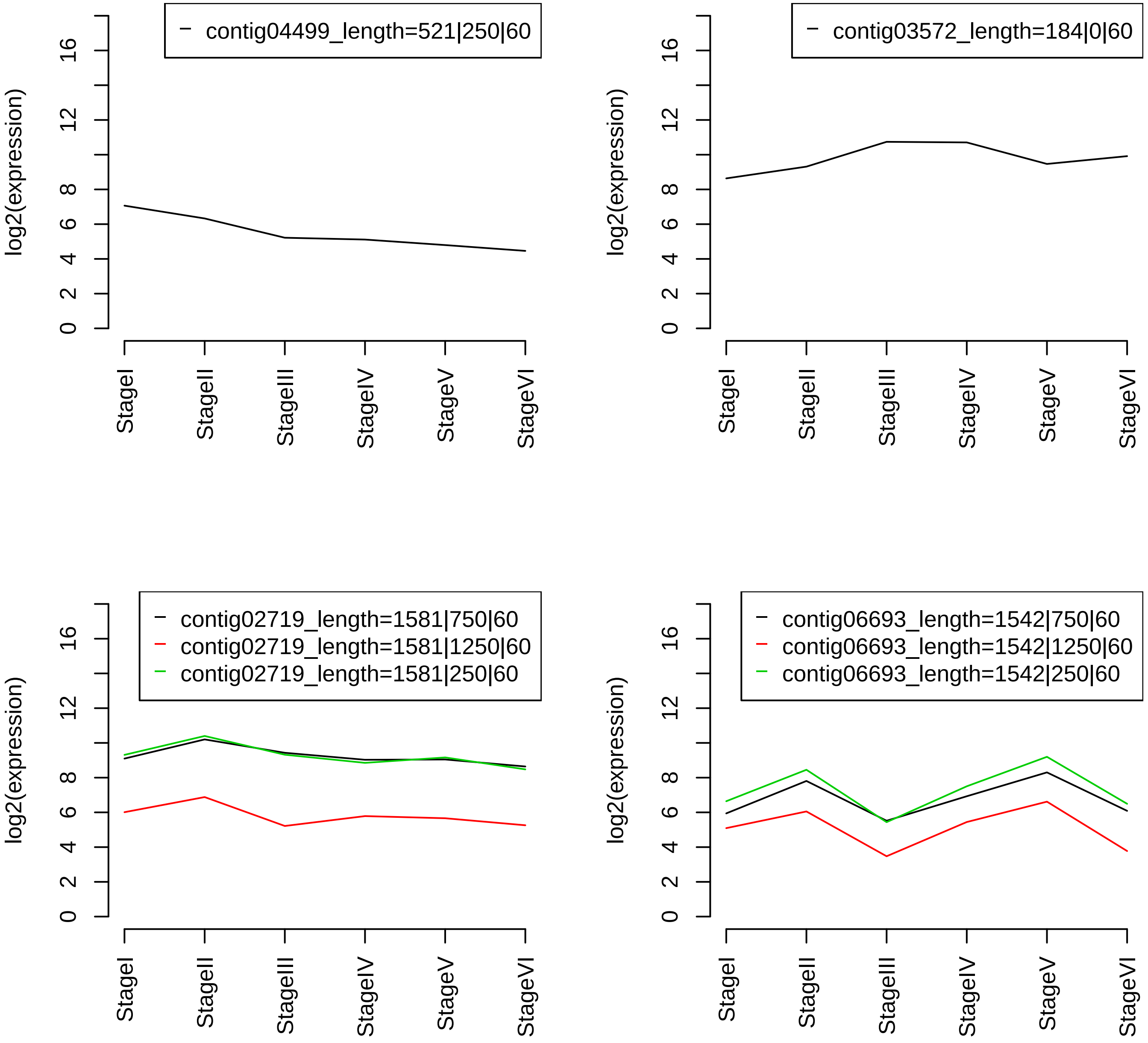
contig04499_length=521|250|60
contig03572_length=184|0|60
contig02719_length=1581|750|60
contig02719_length=1581|1250|60
contig02719_length=1581|250|60
contig06693_length=1542|750|60
contig06693_length=1542|1250|60
contig06693_length=1542|250|60
log2(expression)
0
2
4
6
8
12
16
StageI
StageII
StageIII
StageIV
StageV
StageVI

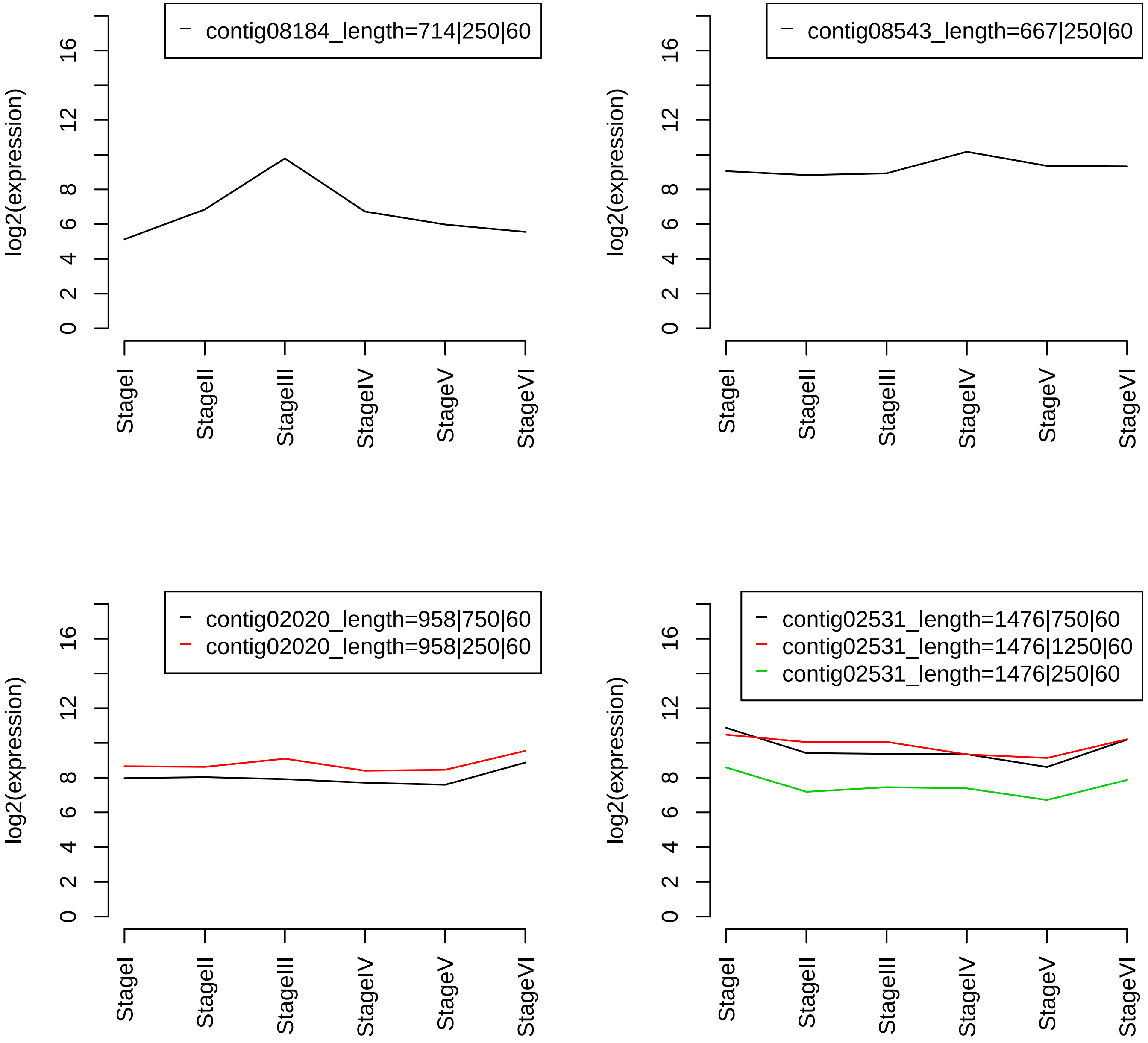
contig08184_length=714|250|60
contig08543_length=667|250|60
contig02020_length=958|750|60
contig02020_length=958|250|60
contig02531_length=1476|750|60
contig02531_length=1476|1250|60
contig02531_length=1476|250|60
log2(expression)
0
2
4
6
8
12
16
StageI
StageII
StageIII
StageIV
StageV
StageVI

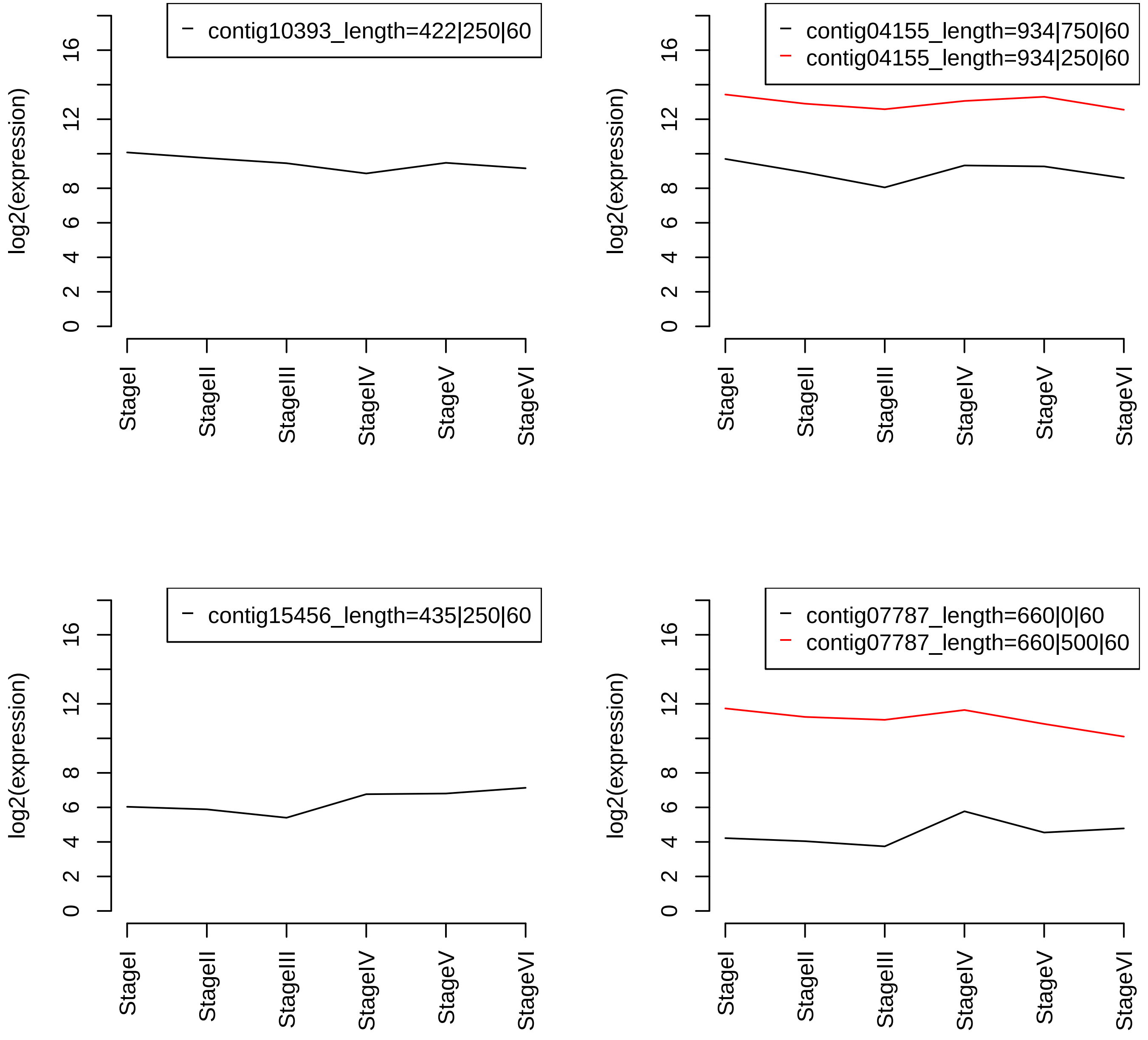

contig10393_length=422|250|60
contig04155_length=934|750|60
contig04155_length=934|250|60
contig15456_length=435|250|60
contig07787_length=660|0|60
contig07787_length=660|500|60
log2(expression)
0
2
4
6
8
12
16
StageI
StageII
StageIII
StageIV
StageV
StageVI

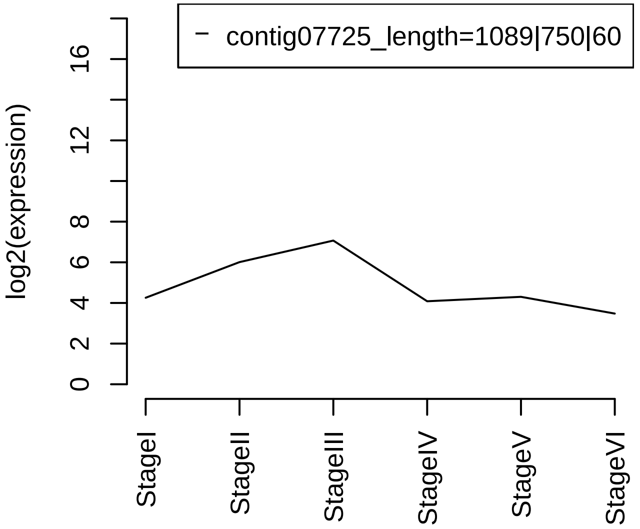

contig07725_length=1089|750|60
log2(expression)
0
2
4
6
8
12
16
StageI
StageII
StageIII
StageIV
StageV
StageVI

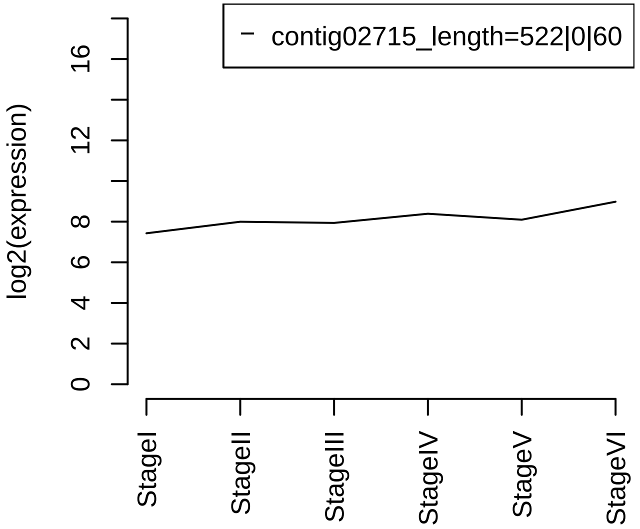

contig02715_length=522|0|60
log2(expression)
0
2
4
6
8
12
16
StageI
StageII
StageIII
StageIV
StageV
StageVI

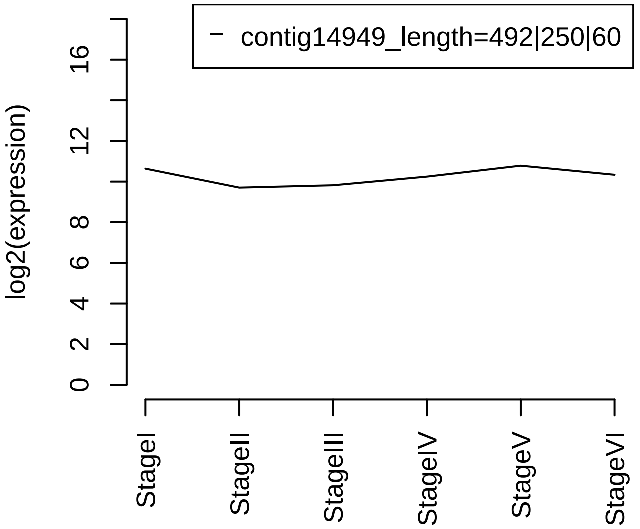

contig14949_length=492|250|60
log2(expression)
0
2
4
6
8
12
16
StageI
StageII
StageIII
StageIV
StageV
StageVI

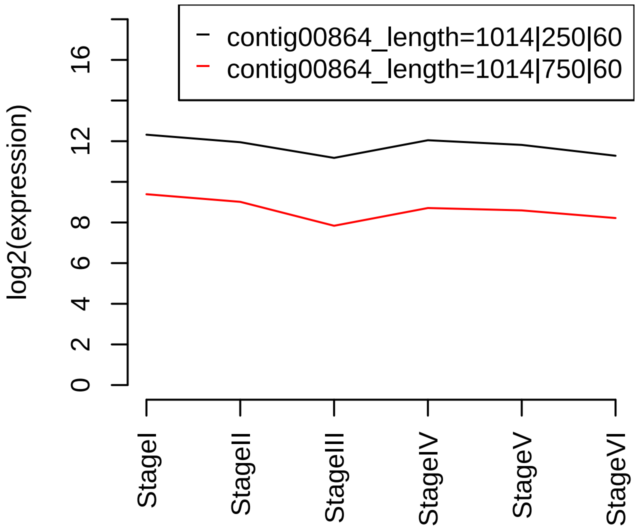

contig00864_length=1014|250|60
contig00864_length=1014|750|60
log2(expression)
0
2
4
6
8
12
16
StageI
StageII
StageIII
StageIV
StageV
StageVI

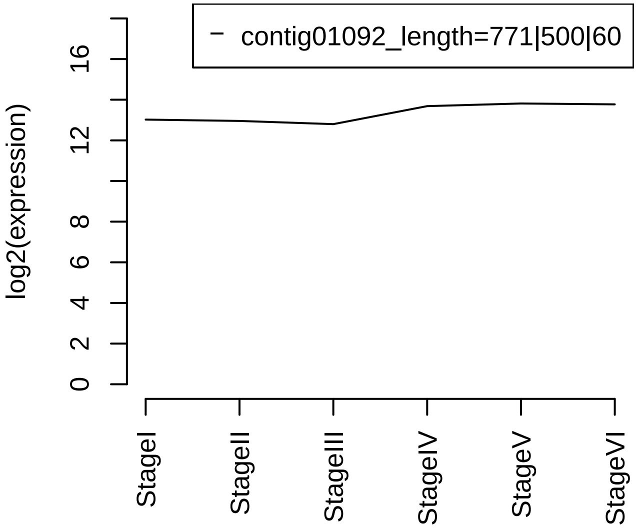
contig01092_length=771|500|60
log2(expression)
0
2
4
6
8
12
16
StageI
StageII
StageIII
StageIV
StageV
StageVI

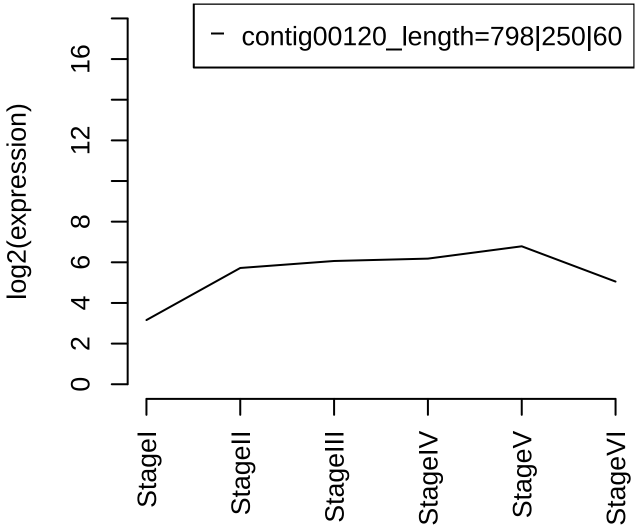
contig00120_length=798|250|60
log2(expression)
0
2
4
6
8
12
16
StageI
StageII
StageIII
StageIV
StageV
StageVI

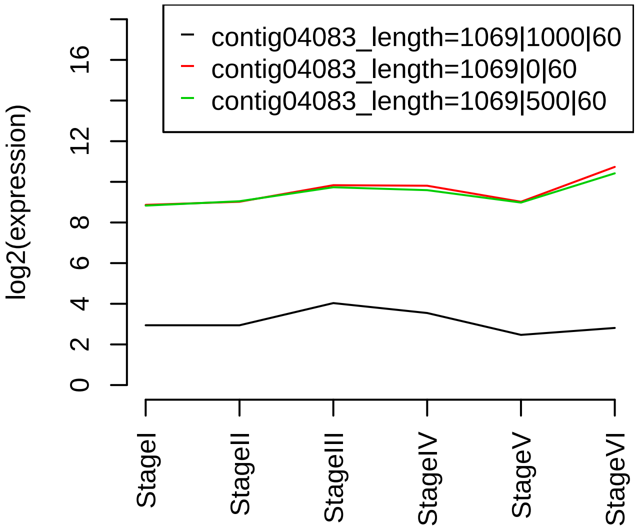
contig04083_length=1069|1000|60
contig04083_length=1069|0|60
contig04083_length=1069|500|60
log2(expression)
0
2
4
6
8
12
16
StageI
StageII
StageIII
StageIV
StageV
StageVI

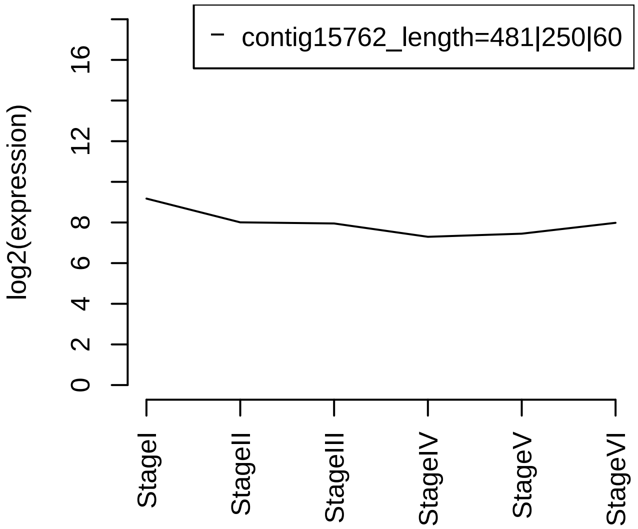
contig15762_length=481|250|60
log2(expression)
0
2
4
6
8
12
16
StageI
StageII
StageIII
StageIV
StageV
StageVI

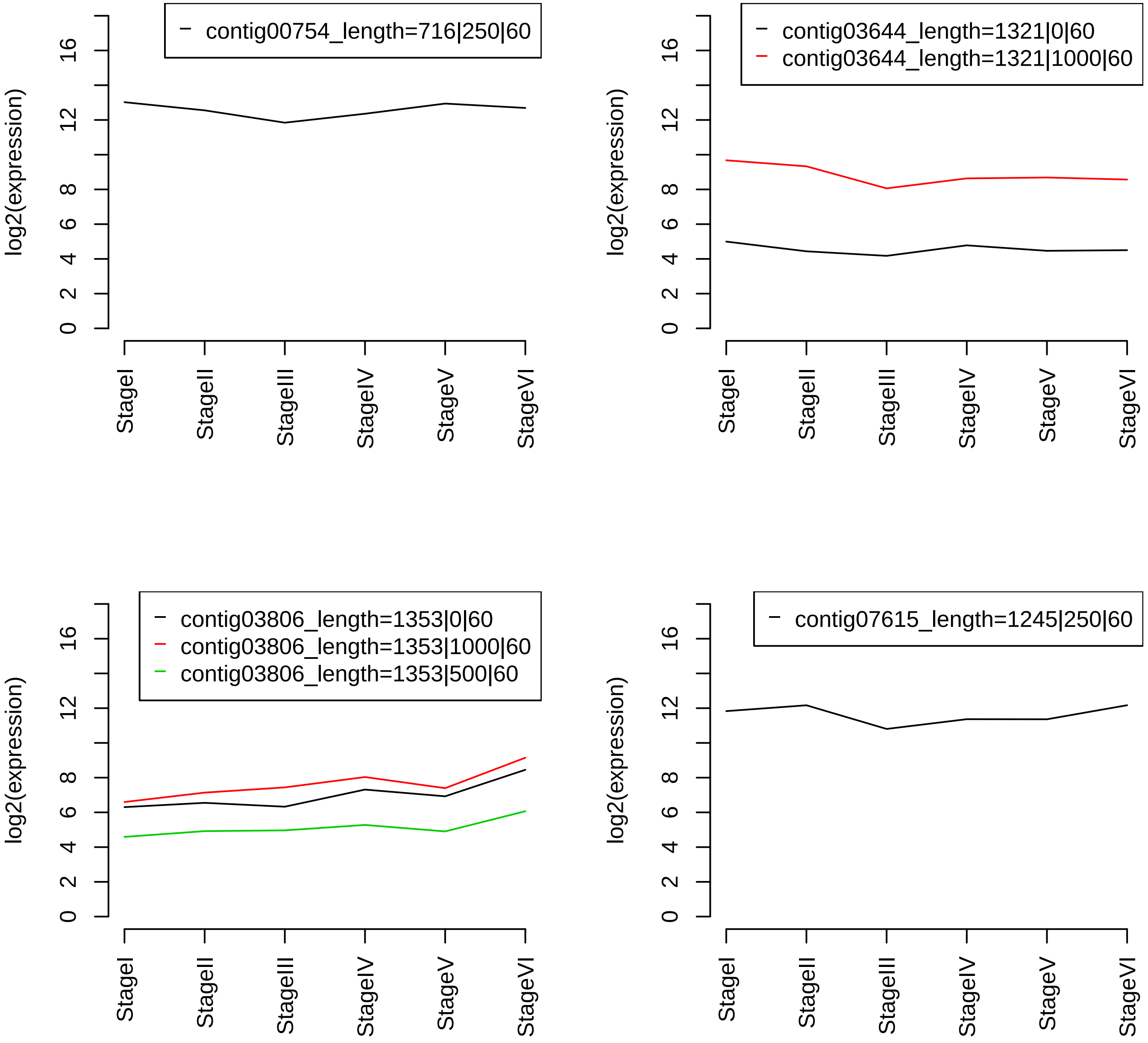

contig00754_length=716|250|60
contig03644_length=1321|0|60
contig03644_length=1321|1000|60
contig03806_length=1353|0|60
contig03806_length=1353|1000|60
contig03806_length=1353|500|60
contig07615_length=1245|250|60
log2(expression)
0 2 4 6 8 12 16
StageI StageII StageIII StageIV StageV StageVI

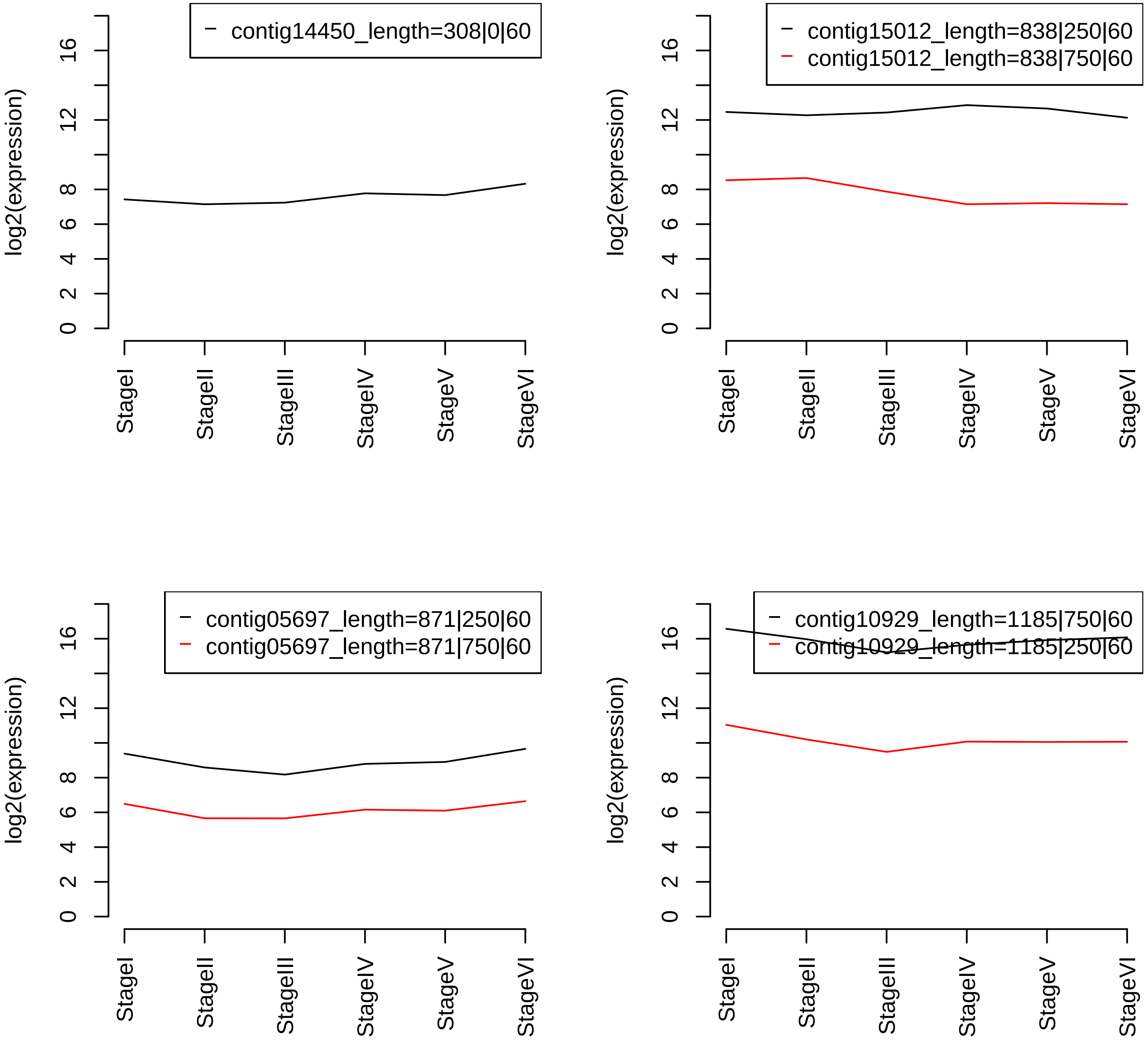
contig14450_length=308|0|60
contig15012_length=838|250|60
contig15012_length=838|750|60
contig05697_length=871|250|60
contig05697_length=871|750|60
contig10929_length=1185|750|60
contig10929_length=1185|250|60
log2(expression)
0
2
4
6
8
12
16
StageI
StageII
StageIII
StageIV
StageV
StageVI

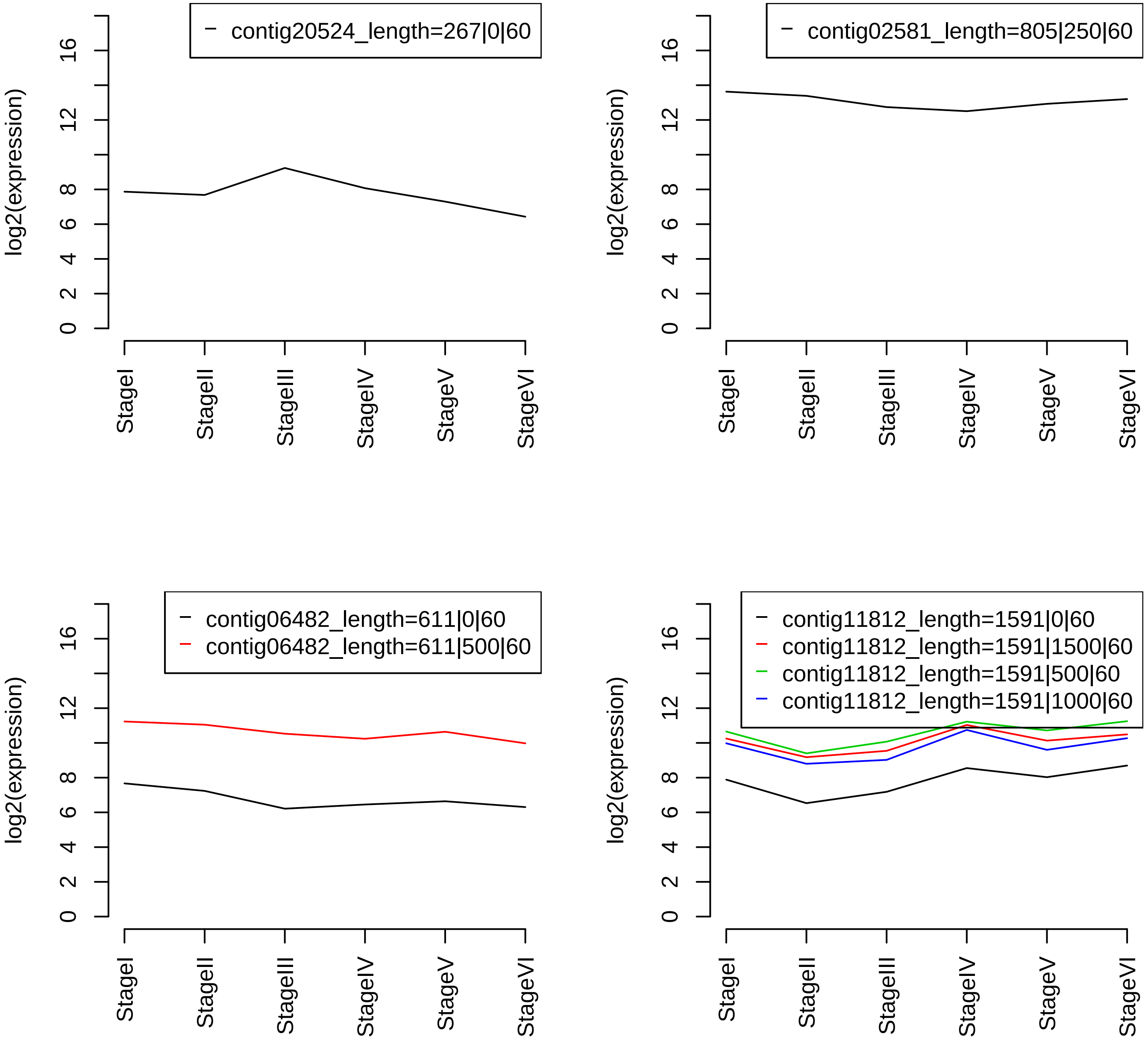
contig20524_length=267|0|60
contig02581_length=805|250|60
contig06482_length=611|0|60
contig06482_length=611|500|60
contig11812_length=1591|0|60
contig11812_length=1591|1500|60
contig11812_length=1591|500|60
contig11812_length=1591|1000|60
log2(expression)
0
2
4
6
8
12
16
StageI
StageII
StageIII
StageIV
StageV
StageVI

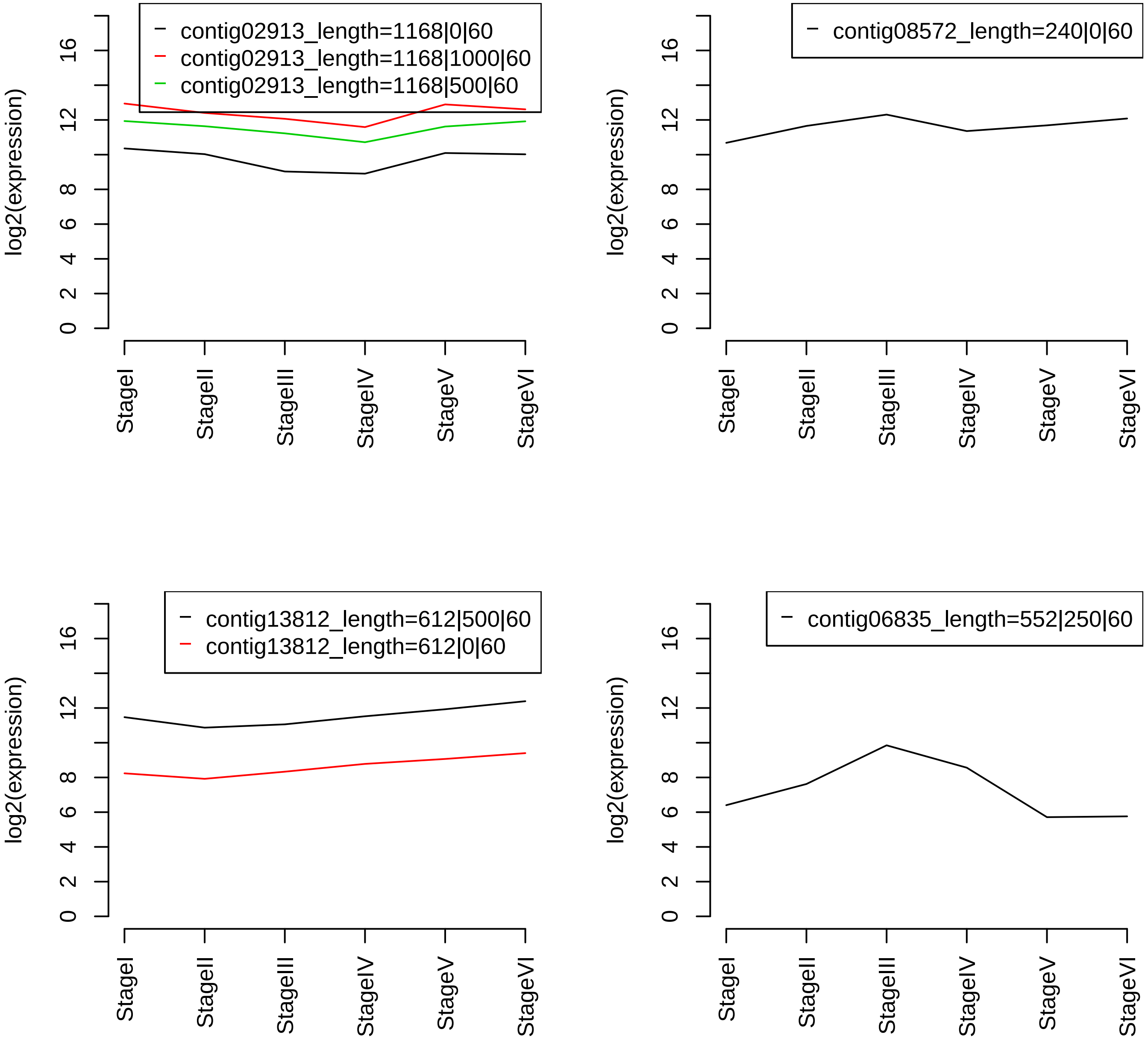
contig02913_length=1168|0|60
contig02913_length=1168|1000|60
contig02913_length=1168|500|60
log2(expression)
0
2
4
6
8
12
16
StageI
StageII
StageIII
StageIV
StageV
StageVI
contig08572_length=240|0|60
log2(expression)
0
2
4
6
8
12
16
StageI
StageII
StageIII
StageIV
StageV
StageVI
contig13812_length=612|500|60
contig13812_length=612|0|60
log2(expression)
0
2
4
6
8
12
16
StageI
StageII
StageIII
StageIV
StageV
StageVI
contig06835_length=552|250|60
log2(expression)
0
2
4
6
8
12
16
StageI
StageII
StageIII
StageIV
StageV
StageVI

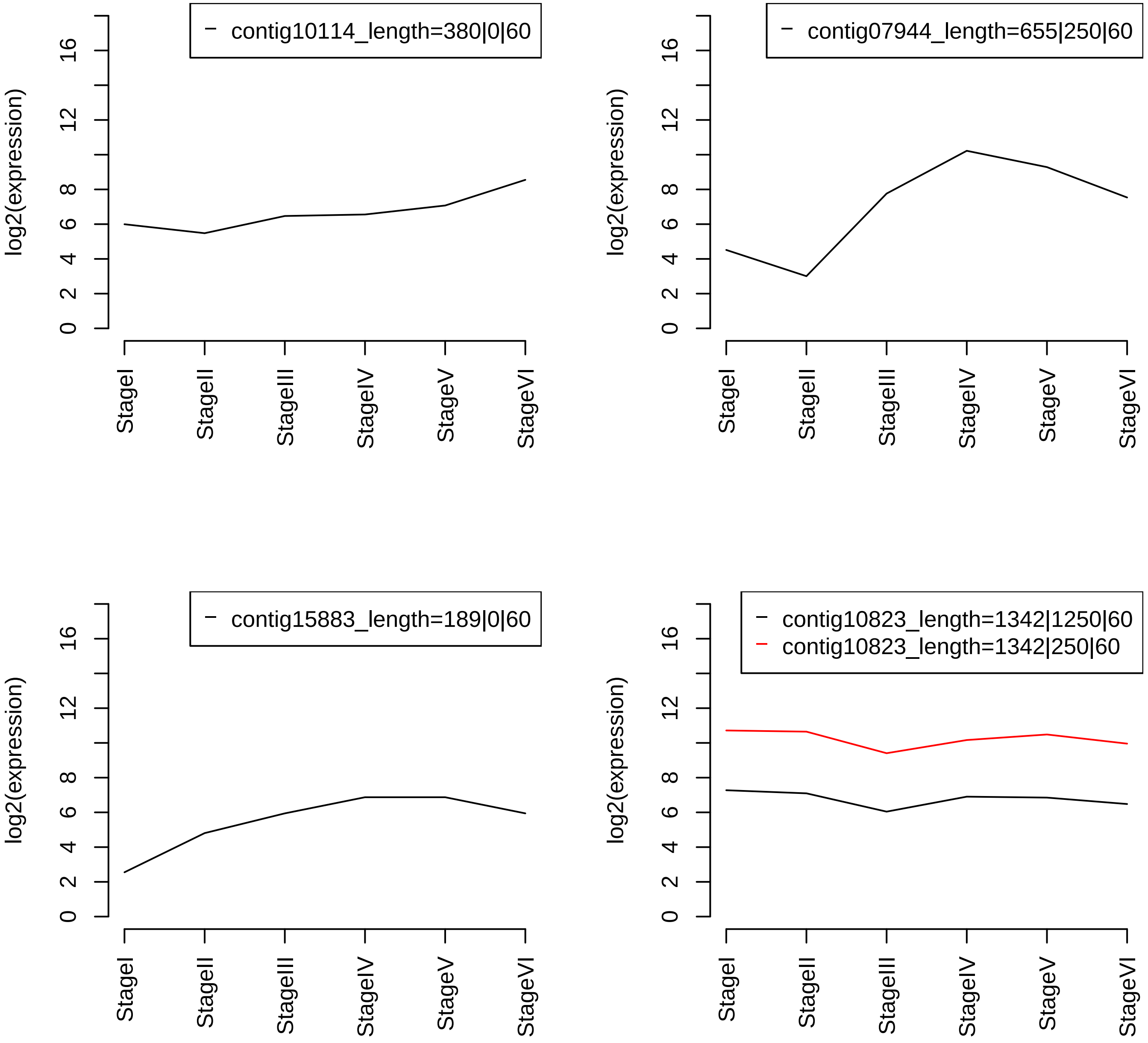
contig10114_length=380|0|60
contig07944_length=655|250|60
contig15883_length=189|0|60
contig10823_length=1342|1250|60
contig10823_length=1342|250|60
log2(expression)
0
2
4
6
8
12
16
StageI
StageII
StageIII
StageIV
StageV
StageVI

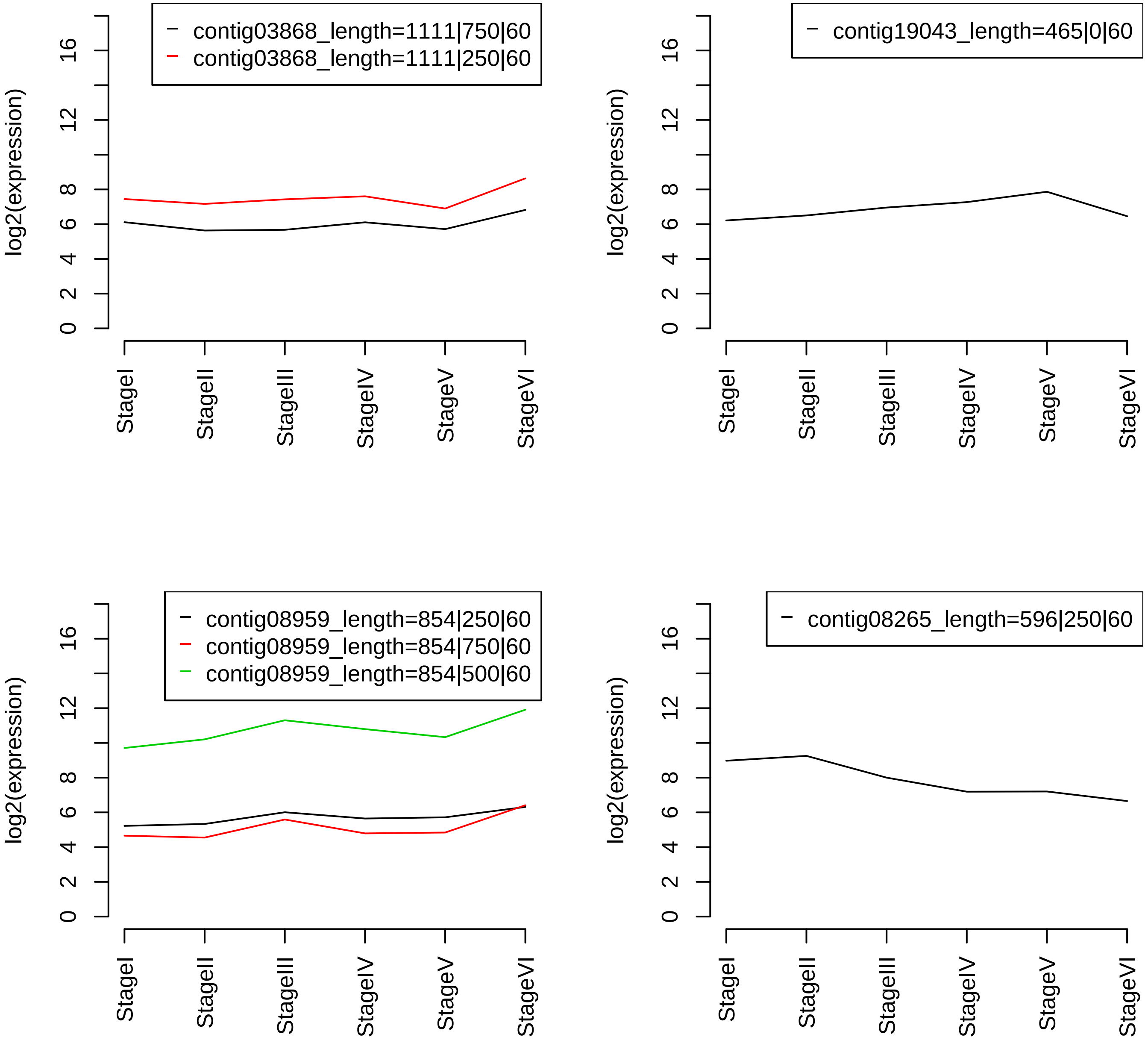
contig03868_length=1111|750|60
contig03868_length=1111|250|60
contig19043_length=465|0|60
contig08959_length=854|250|60
contig08959_length=854|750|60
contig08959_length=854|500|60
contig08265_length=596|250|60
log2(expression)
0
2
4
6
8
12
16
StageI
StageII
StageIII
StageIV
StageV
StageVI

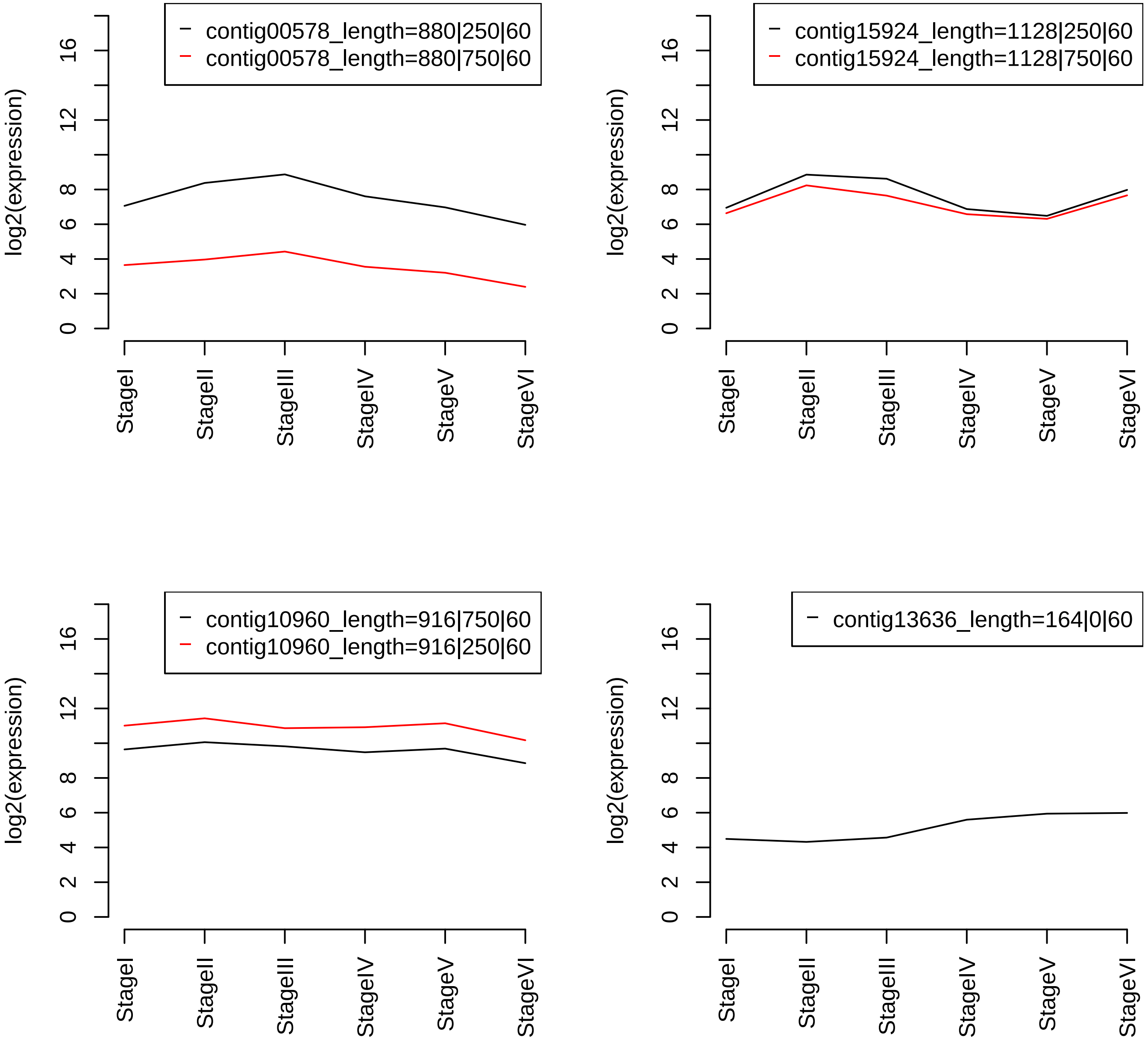
contig00578_length=880|250|60
contig00578_length=880|750|60
contig15924_length=1128|250|60
contig15924_length=1128|750|60
contig10960_length=916|750|60
contig10960_length=916|250|60
contig13636_length=164|0|60
log2(expression)
0
2
4
6
8
12
16
StageI
StageII
StageIII
StageIV
StageV
StageVI

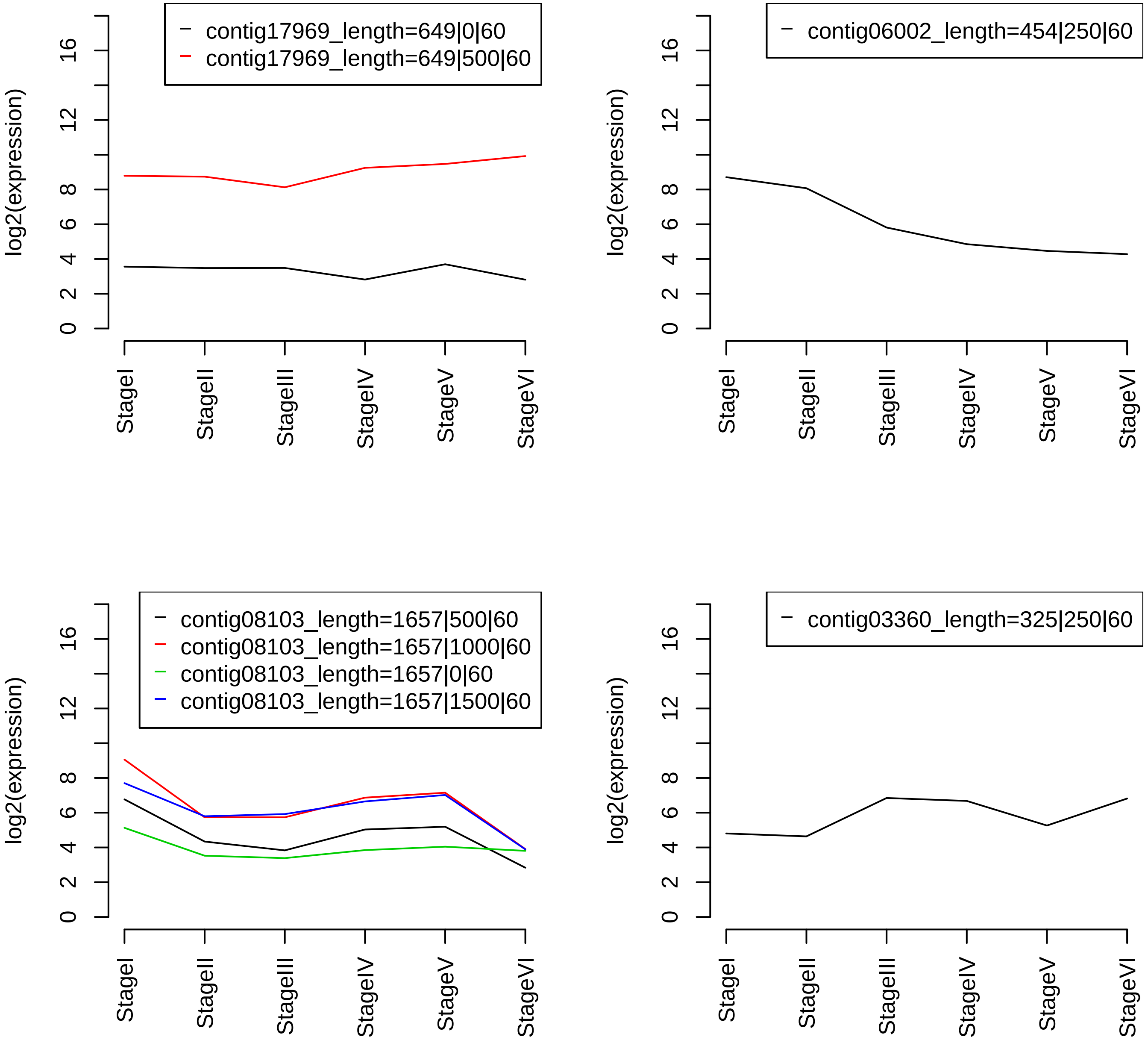

contig17969_length=649|0|60
contig17969_length=649|500|60
contig06002_length=454|250|60
contig08103_length=1657|500|60
contig08103_length=1657|1000|60
contig08103_length=1657|0|60
contig08103_length=1657|1500|60
contig03360_length=325|250|60
log2(expression)
0
2
4
6
8
12
16
StageI
StageII
StageIII
StageIV
StageV
StageVI

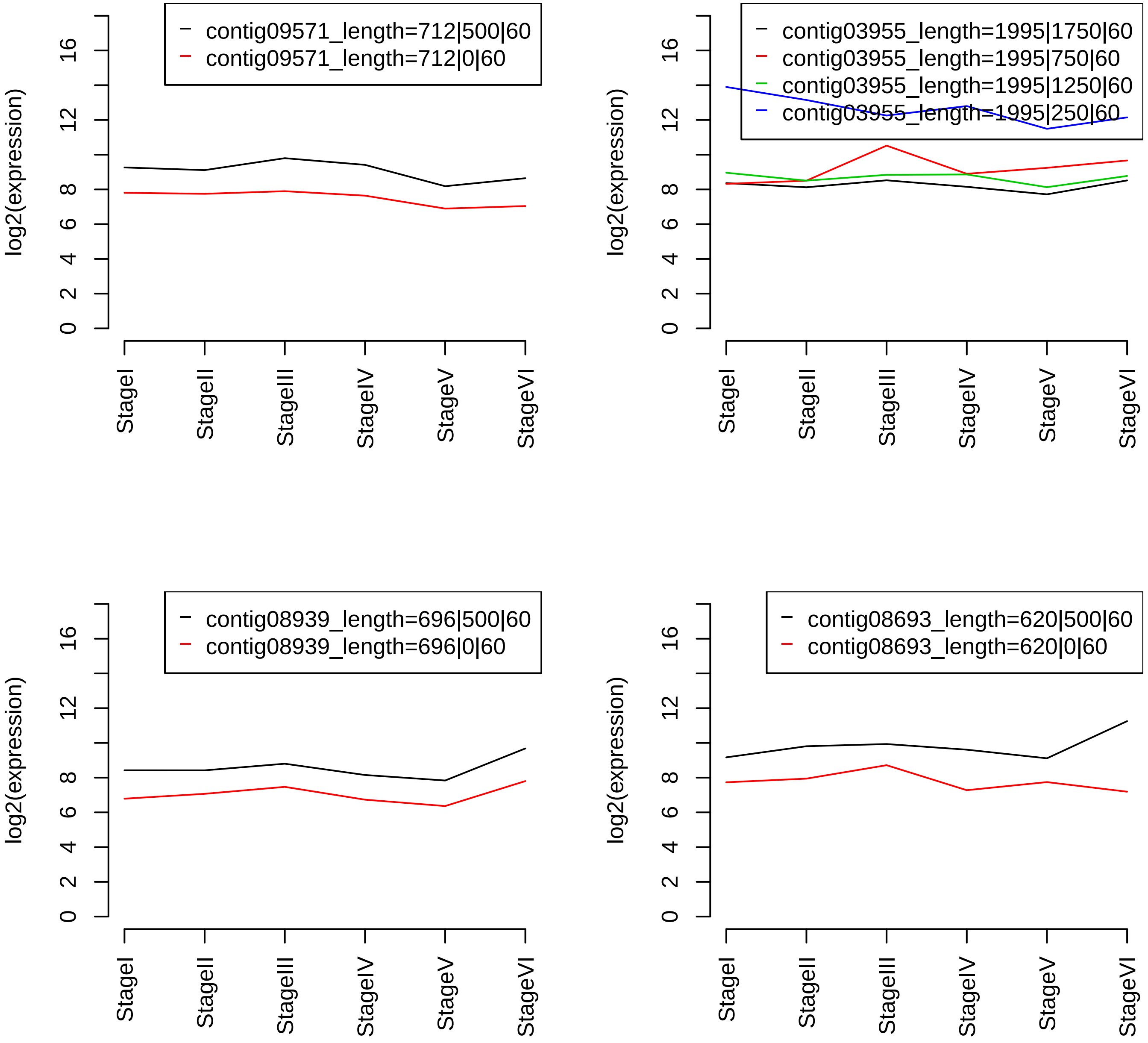

contig09571_length=712|500|60
contig09571_length=712|0|60
contig03955_length=1995|1750|60
contig03955_length=1995|750|60
contig03955_length=1995|1250|60
contig03955_length=1995|250|60
contig08939_length=696|500|60
contig08939_length=696|0|60
contig08693_length=620|500|60
contig08693_length=620|0|60
log2(expression)
0
2
4
6
8
12
16
StageI
StageII
StageIII
StageIV
StageV
StageVI

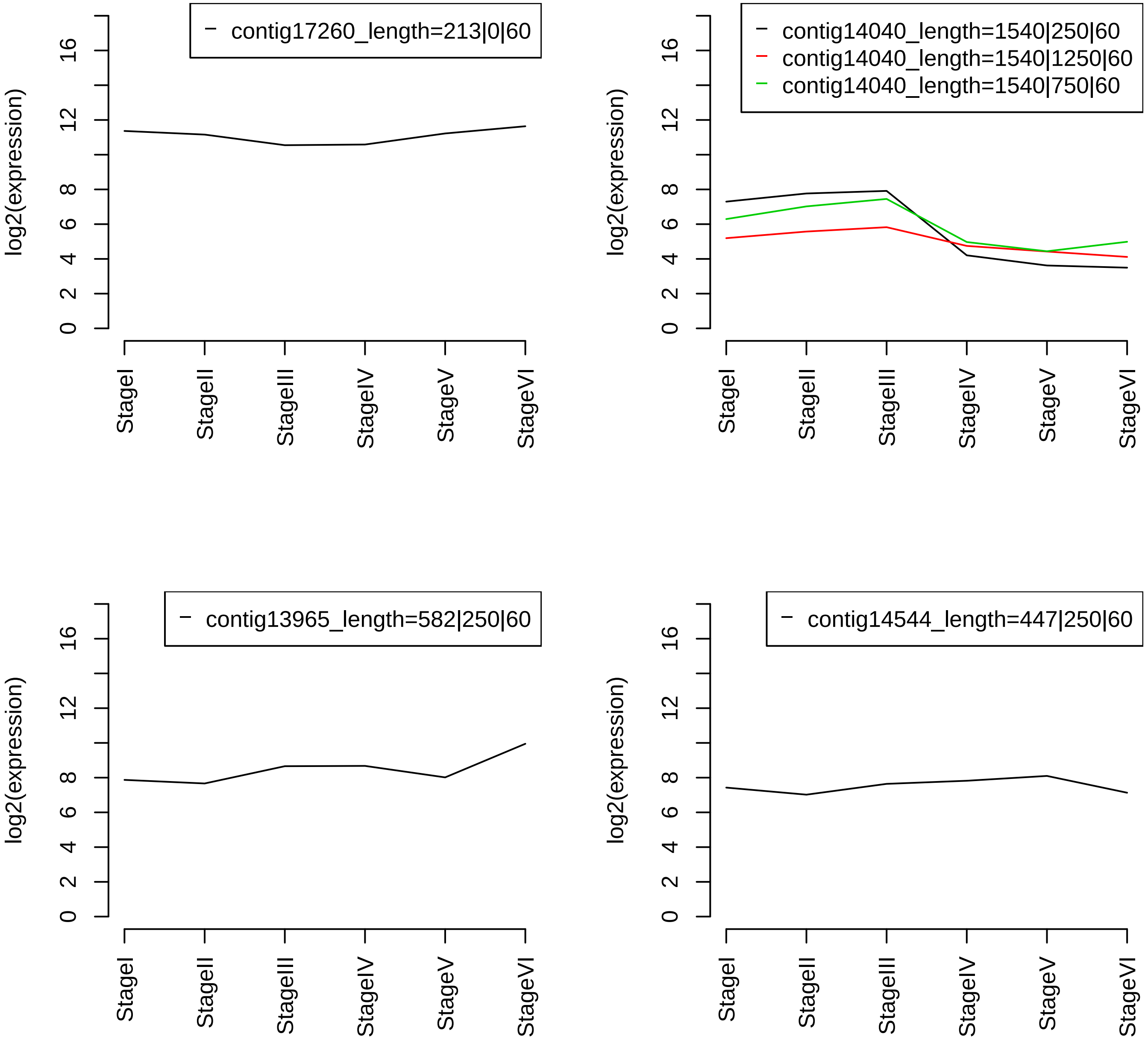
contig17260_length=213|0|60
contig14040_length=1540|250|60
contig14040_length=1540|1250|60
contig14040_length=1540|750|60
contig13965_length=582|250|60
contig14544_length=447|250|60
log2(expression)
0
2
4
6
8
12
16
StageI
StageII
StageIII
StageIV
StageV
StageVI

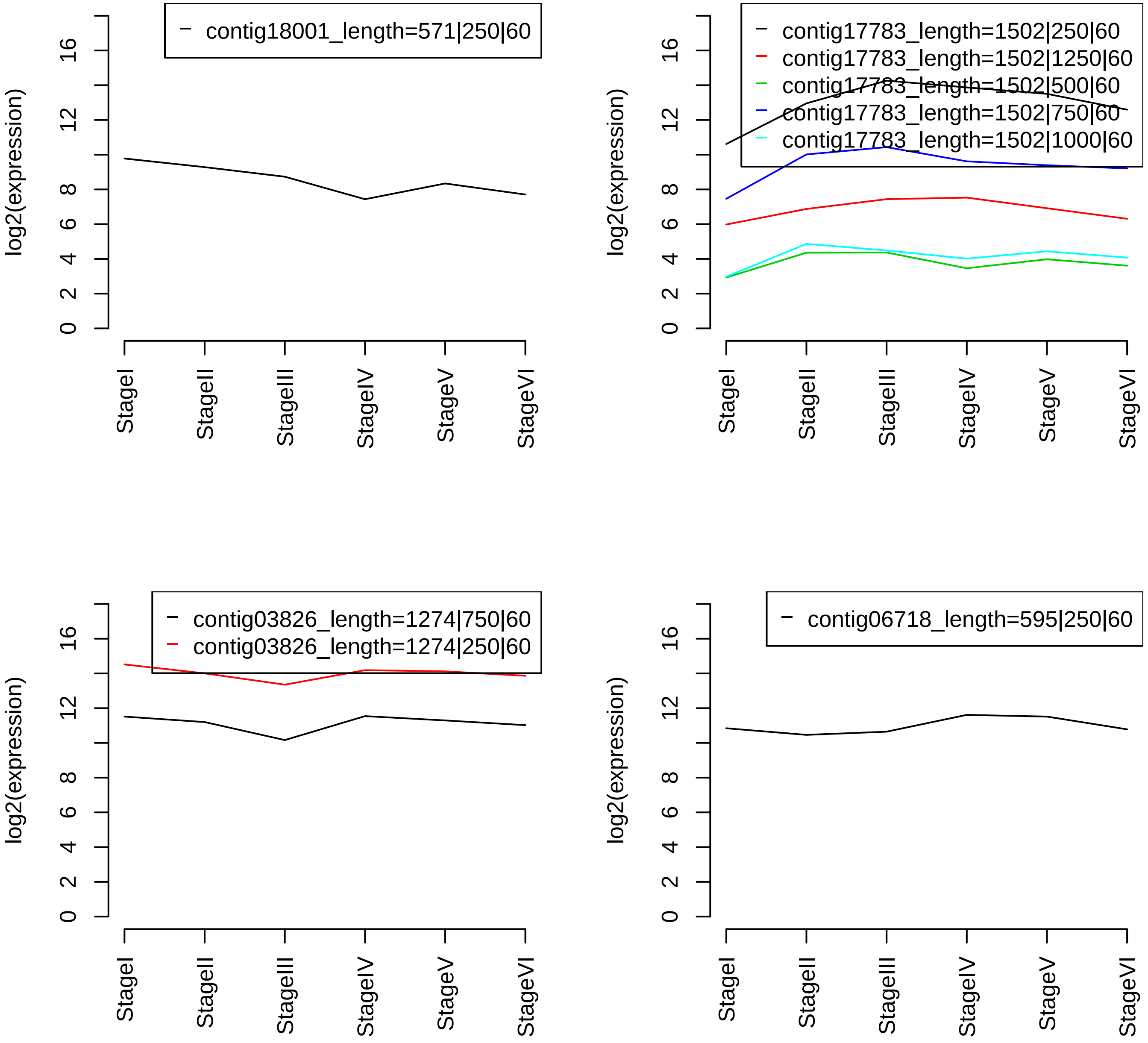
contig18001_length=571|250|60
contig17783_length=1502|250|60
contig17783_length=1502|1250|60
contig17783_length=1502|500|60
contig17783_length=1502|750|60
contig17783_length=1502|1000|60
contig03826_length=1274|750|60
contig03826_length=1274|250|60
contig06718_length=595|250|60
log2(expression)
0
2
4
6
8
12
16
StageI
StageII
StageIII
StageIV
StageV
StageVI

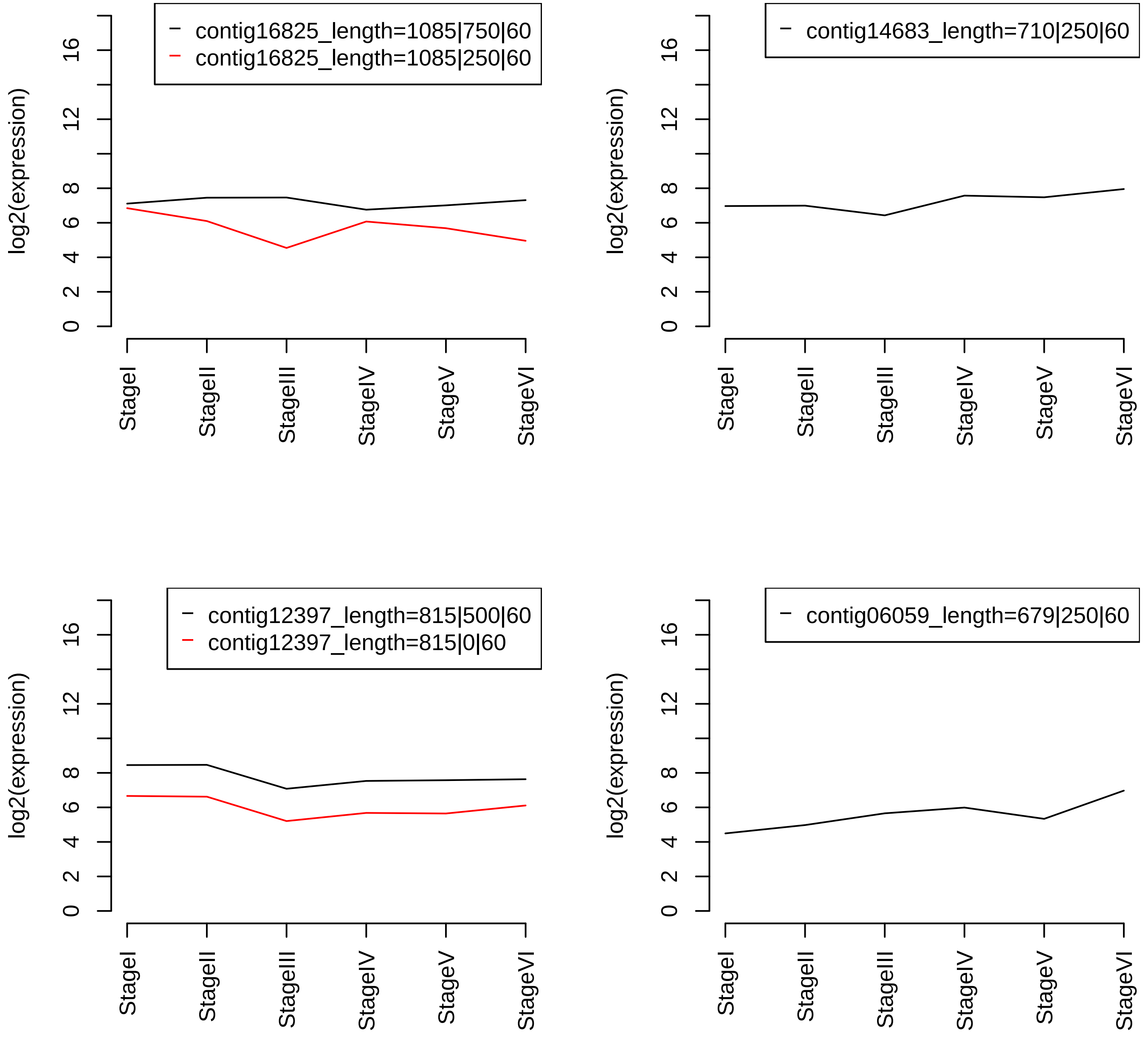
contig16825_length=1085|750|60
contig16825_length=1085|250|60
log2(expression)
0
2
4
6
8
12
16
StageI
StageII
StageIII
StageIV
StageV
StageVI
contig14683_length=710|250|60
log2(expression)
contig12397_length=815|500|60
contig12397_length=815|0|60
log2(expression)
contig06059_length=679|250|60
log2(expression)

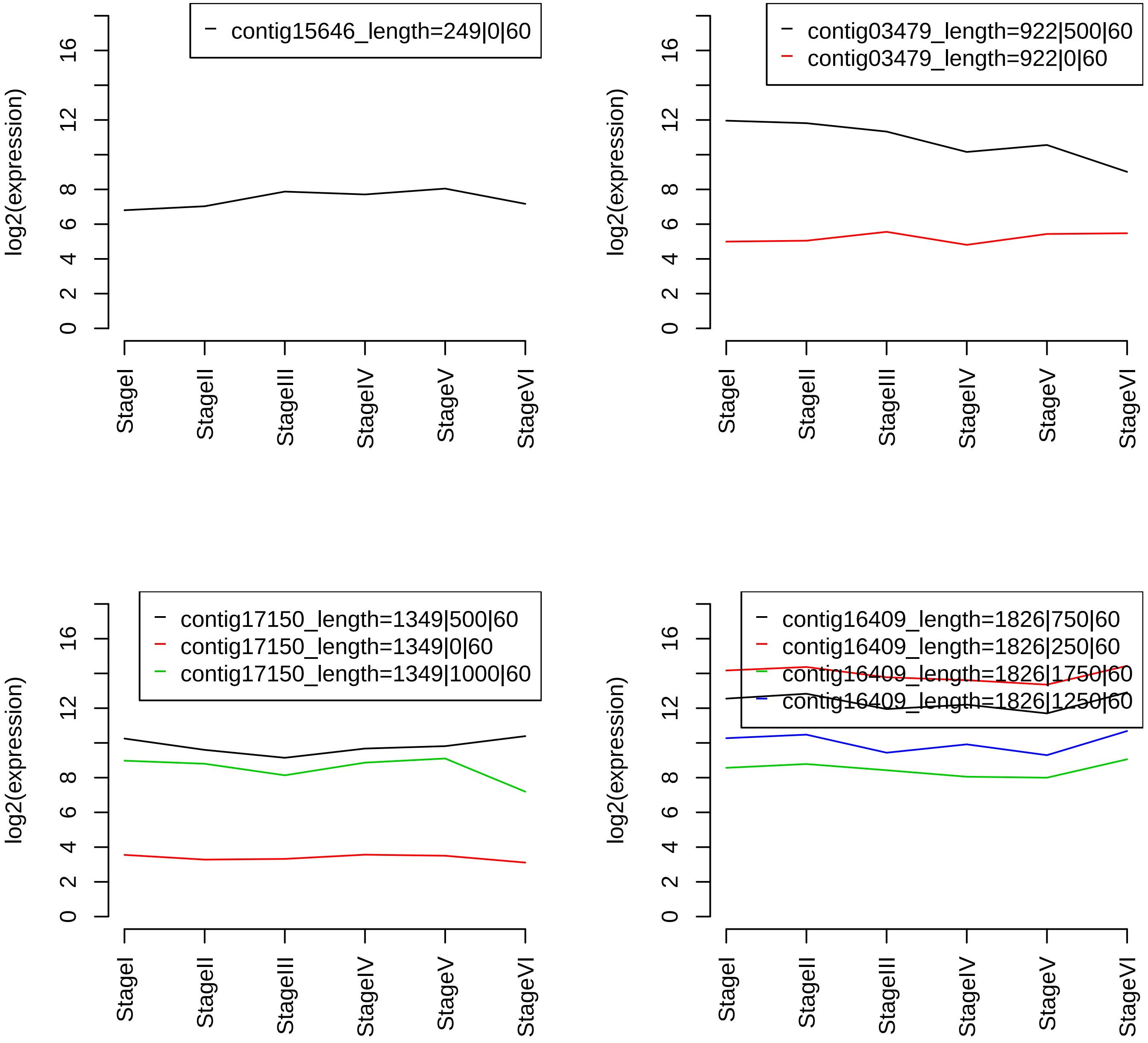
contig15646_length=249|0|60
contig03479_length=922|500|60
contig03479_length=922|0|60
contig17150_length=1349|500|60
contig17150_length=1349|0|60
contig17150_length=1349|1000|60
contig16409_length=1826|750|60
contig16409_length=1826|250|60
contig16409_length=1826|1750|60
contig16409_length=1826|1250|60
log2(expression)
0
2
4
6
8
12
16
StageI
StageII
StageIII
StageIV
StageV
StageVI

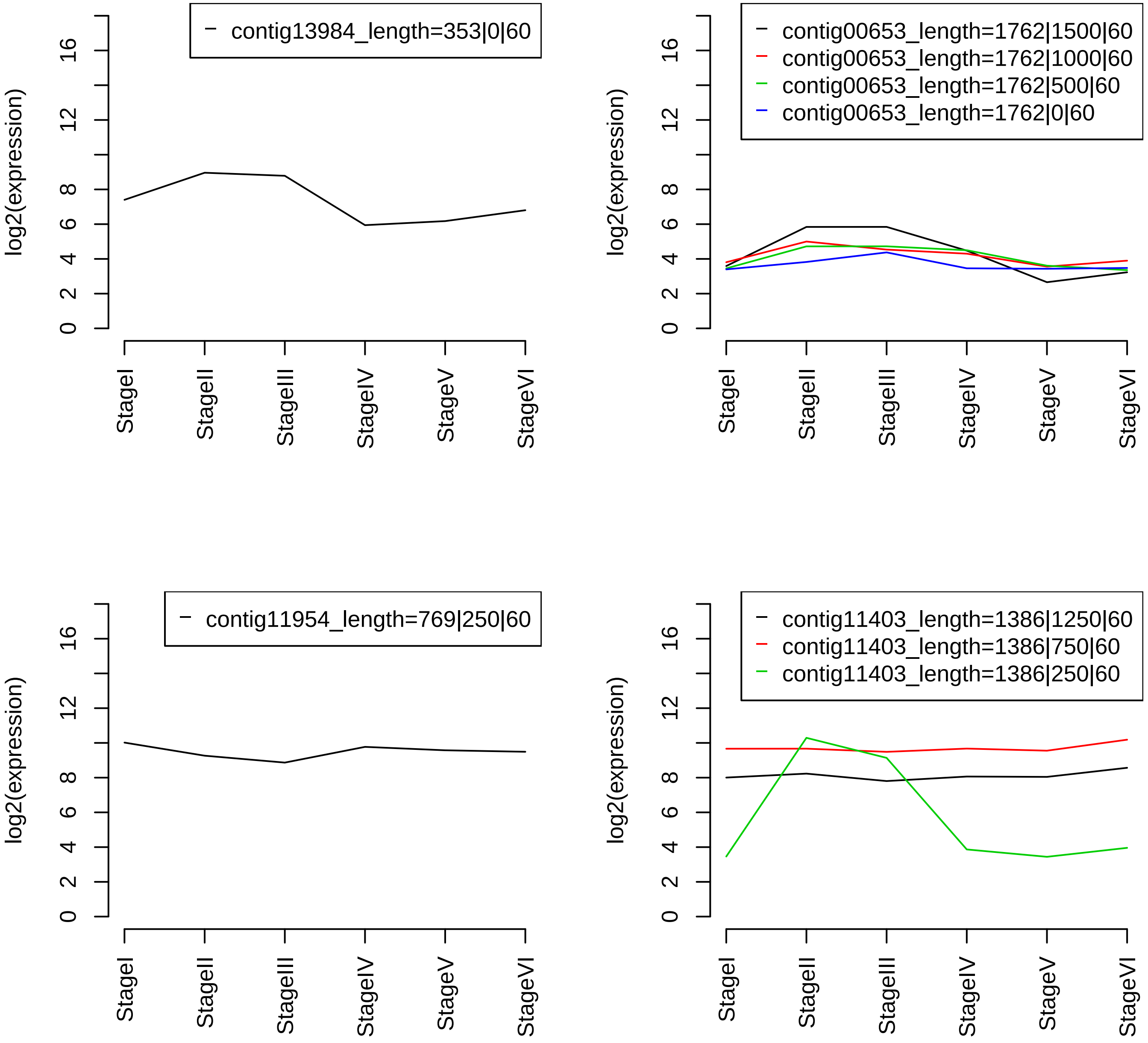

contig13984_length=353|0|60
contig00653_length=1762|1500|60
contig00653_length=1762|1000|60
contig00653_length=1762|500|60
contig00653_length=1762|0|60
contig11954_length=769|250|60
contig11403_length=1386|1250|60
contig11403_length=1386|750|60
contig11403_length=1386|250|60
log2(expression)
0
2
4
6
8
12
16
StageI
StageII
StageIII
StageIV
StageV
StageVI

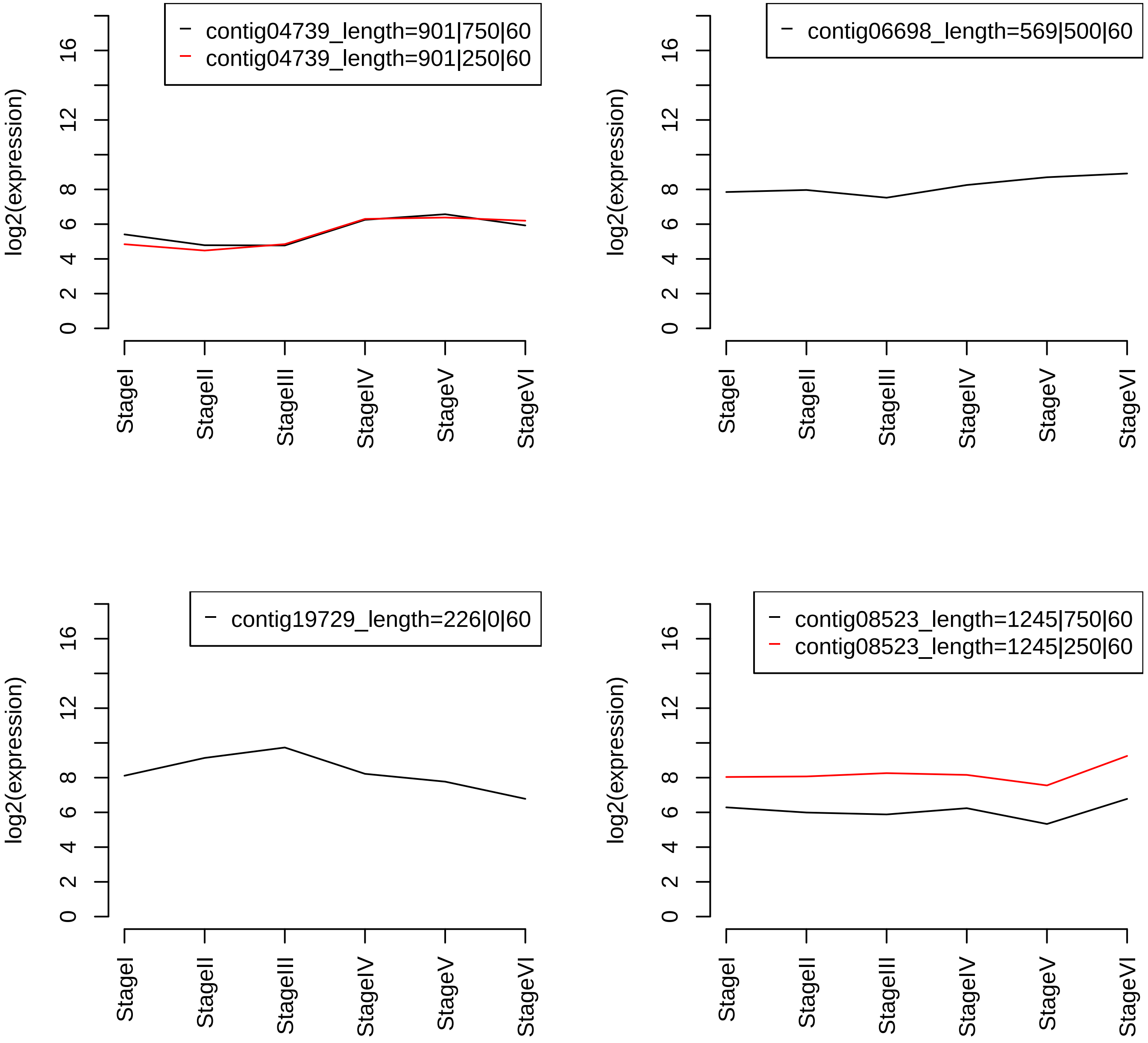
contig04739_length=901|750|60
contig04739_length=901|250|60
contig06698_length=569|500|60
contig19729_length=226|0|60
contig08523_length=1245|750|60
contig08523_length=1245|250|60
log2(expression)
0
2
4
6
8
12
16
StageI
StageII
StageIII
StageIV
StageV
StageVI

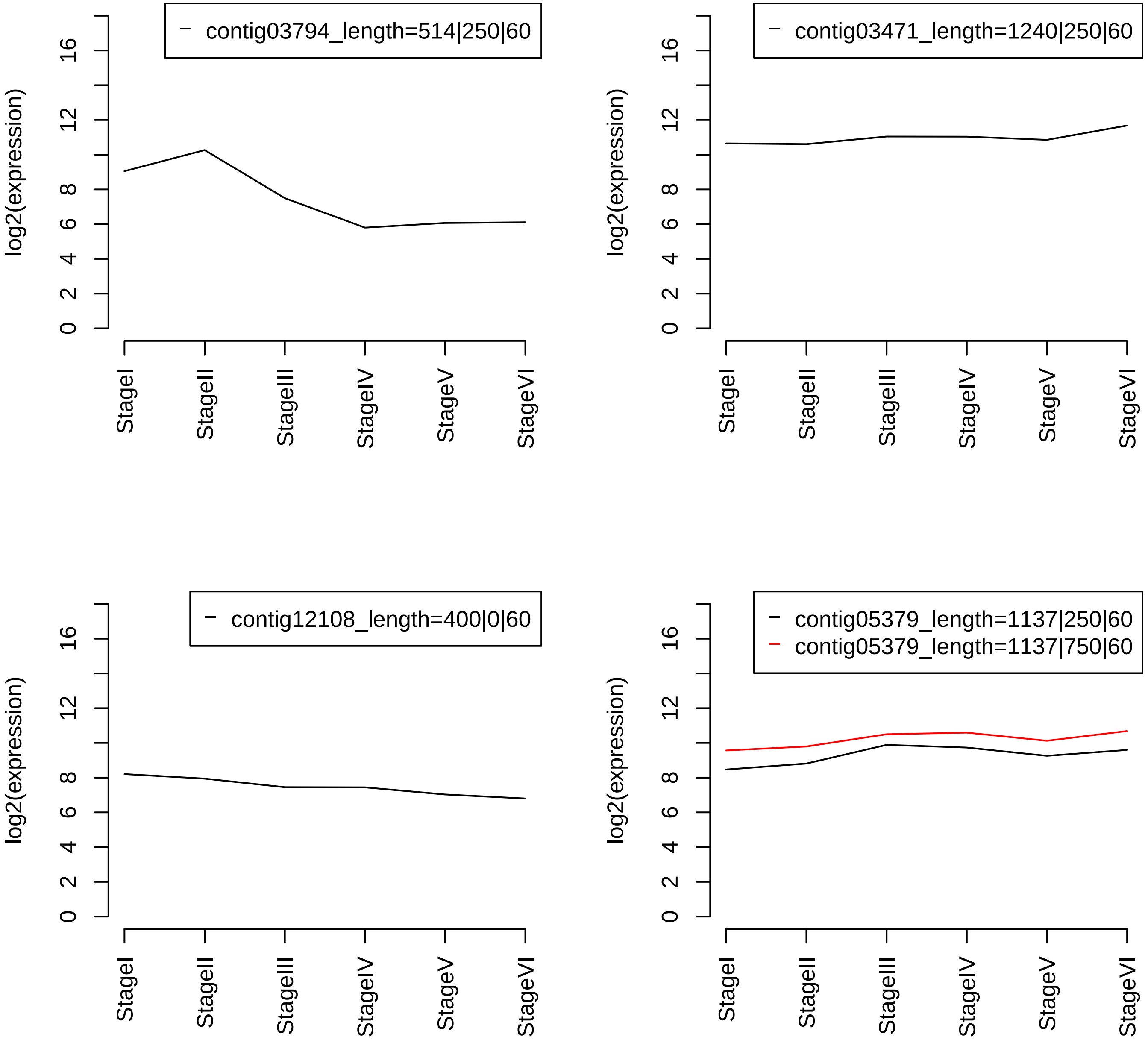
contig03794_length=514|250|60
contig03471_length=1240|250|60
contig12108_length=400|0|60
contig05379_length=1137|250|60
contig05379_length=1137|750|60
log2(expression)
0
2
4
6
8
12
16
StageI
StageII
StageIII
StageIV
StageV
StageVI

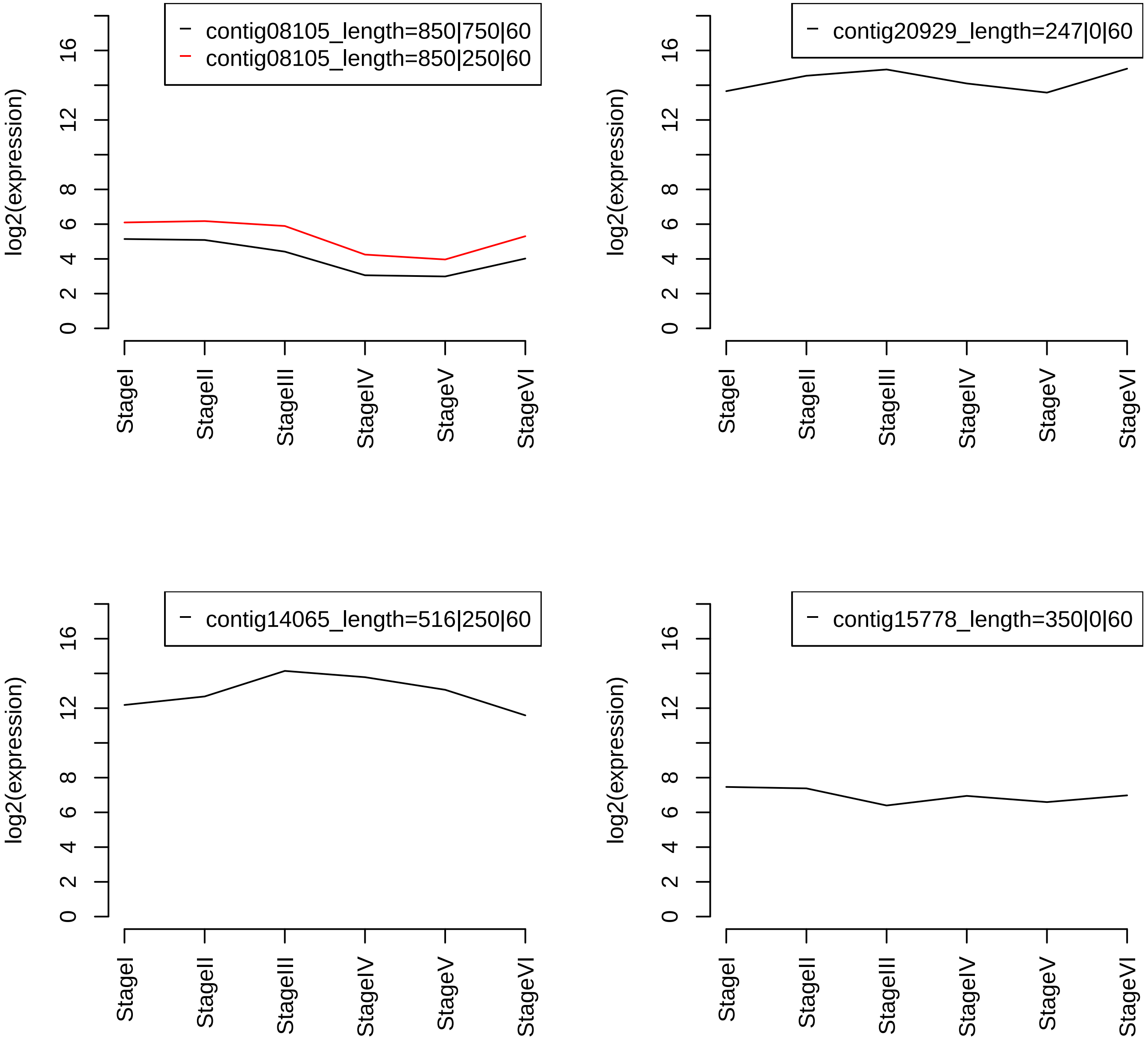
contig08105_length=850|750|60
contig08105_length=850|250|60
contig20929_length=247|0|60
contig14065_length=516|250|60
contig15778_length=350|0|60
log2(expression)
0
2
4
6
8
12
16
StageI
StageII
StageIII
StageIV
StageV
StageVI

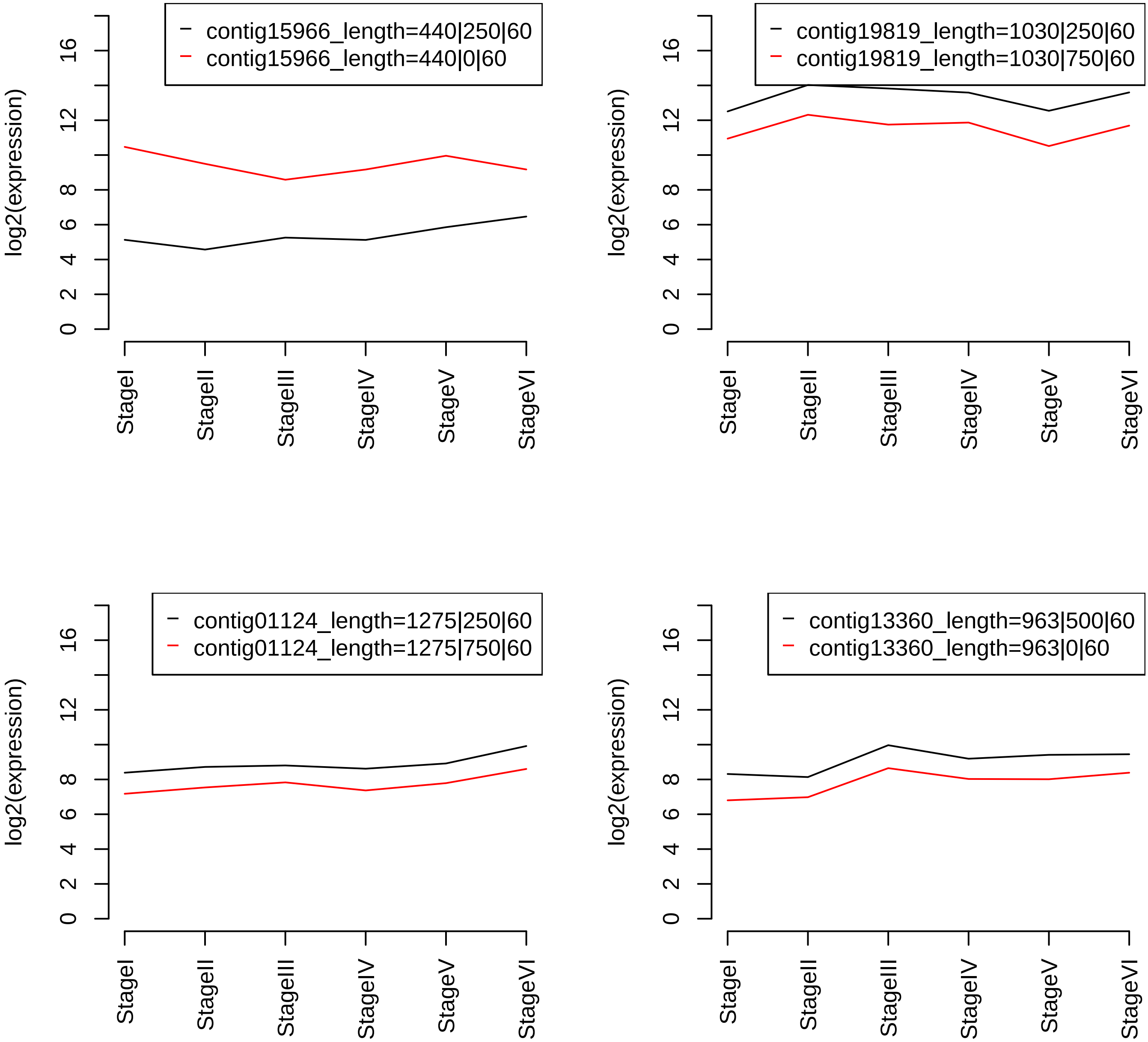
contig15966_length=440|250|60
contig15966_length=440|0|60
contig19819_length=1030|250|60
contig19819_length=1030|750|60
contig01124_length=1275|250|60
contig01124_length=1275|750|60
contig13360_length=963|500|60
contig13360_length=963|0|60
log2(expression)
0
2
4
6
8
12
16
StageI
StageII
StageIII
StageIV
StageV
StageVI

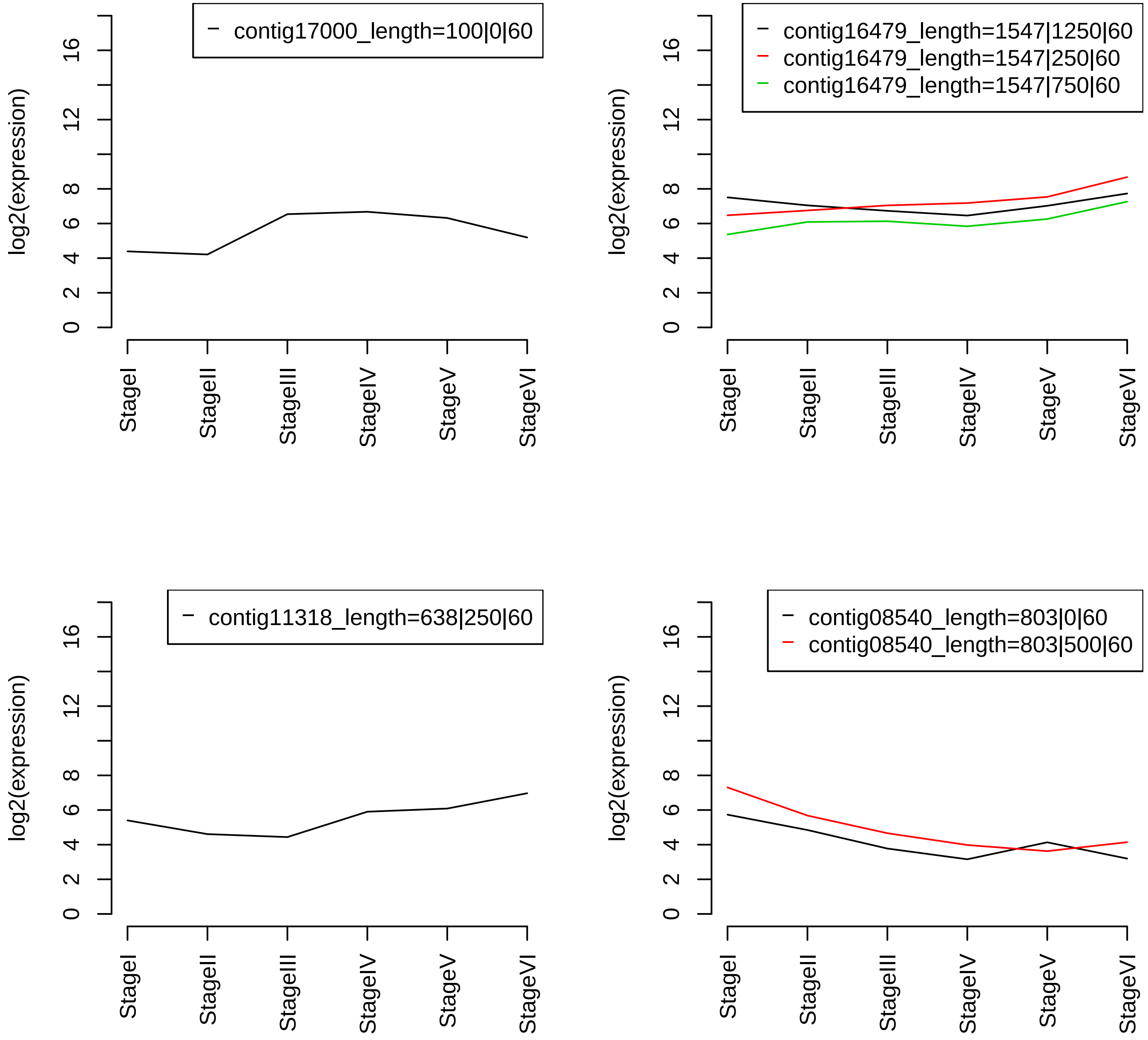

contig17000_length=100|0|60
contig16479_length=1547|1250|60
contig16479_length=1547|250|60
contig16479_length=1547|750|60
contig11318_length=638|250|60
contig08540_length=803|0|60
contig08540_length=803|500|60
log2(expression)
0
2
4
6
8
12
16
StageI
StageII
StageIII
StageIV
StageV
StageVI

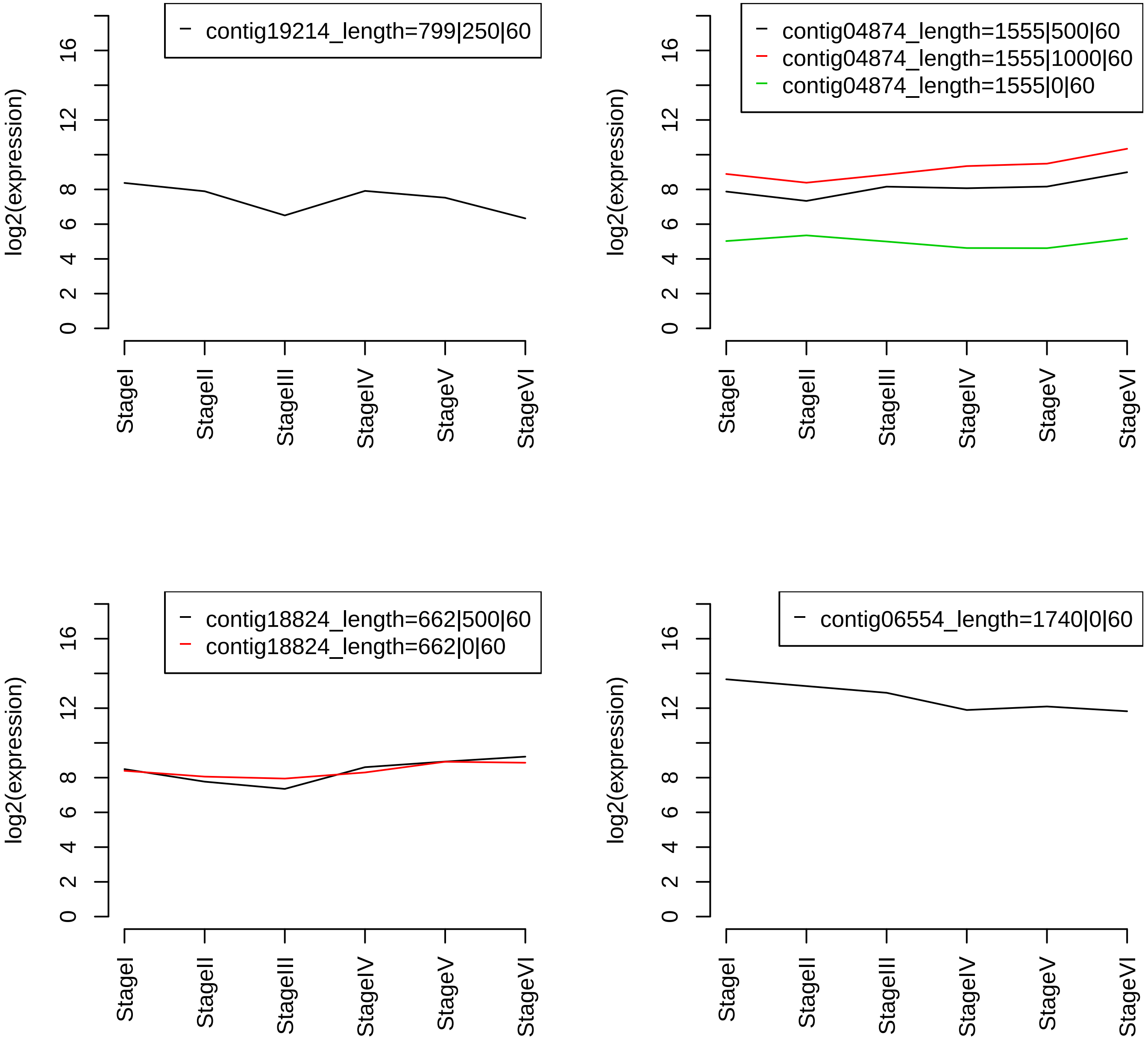

contig19214_length=799|250|60
contig04874_length=1555|500|60
contig04874_length=1555|1000|60
contig04874_length=1555|0|60
contig18824_length=662|500|60
contig18824_length=662|0|60
contig06554_length=1740|0|60
log2(expression)
0
2
4
6
8
12
16
StageI
StageII
StageIII
StageIV
StageV
StageVI

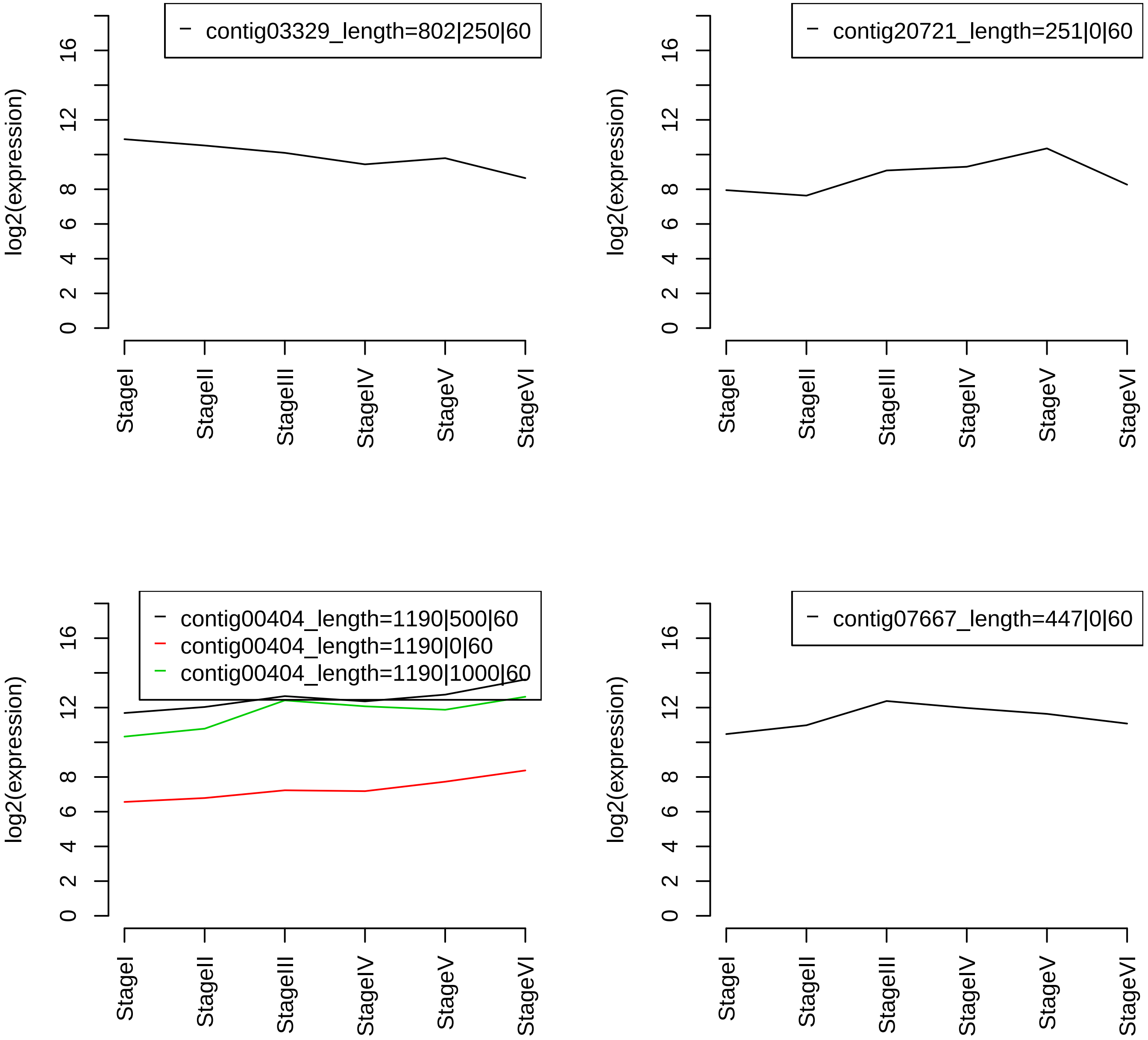
contig03329_length=802|250|60
contig20721_length=251|0|60
contig00404_length=1190|500|60
contig00404_length=1190|0|60
contig00404_length=1190|1000|60
contig07667_length=447|0|60
log2(expression)
0 2 4 6 8 12 16
StageI StageII StageIII StageIV StageV StageVI

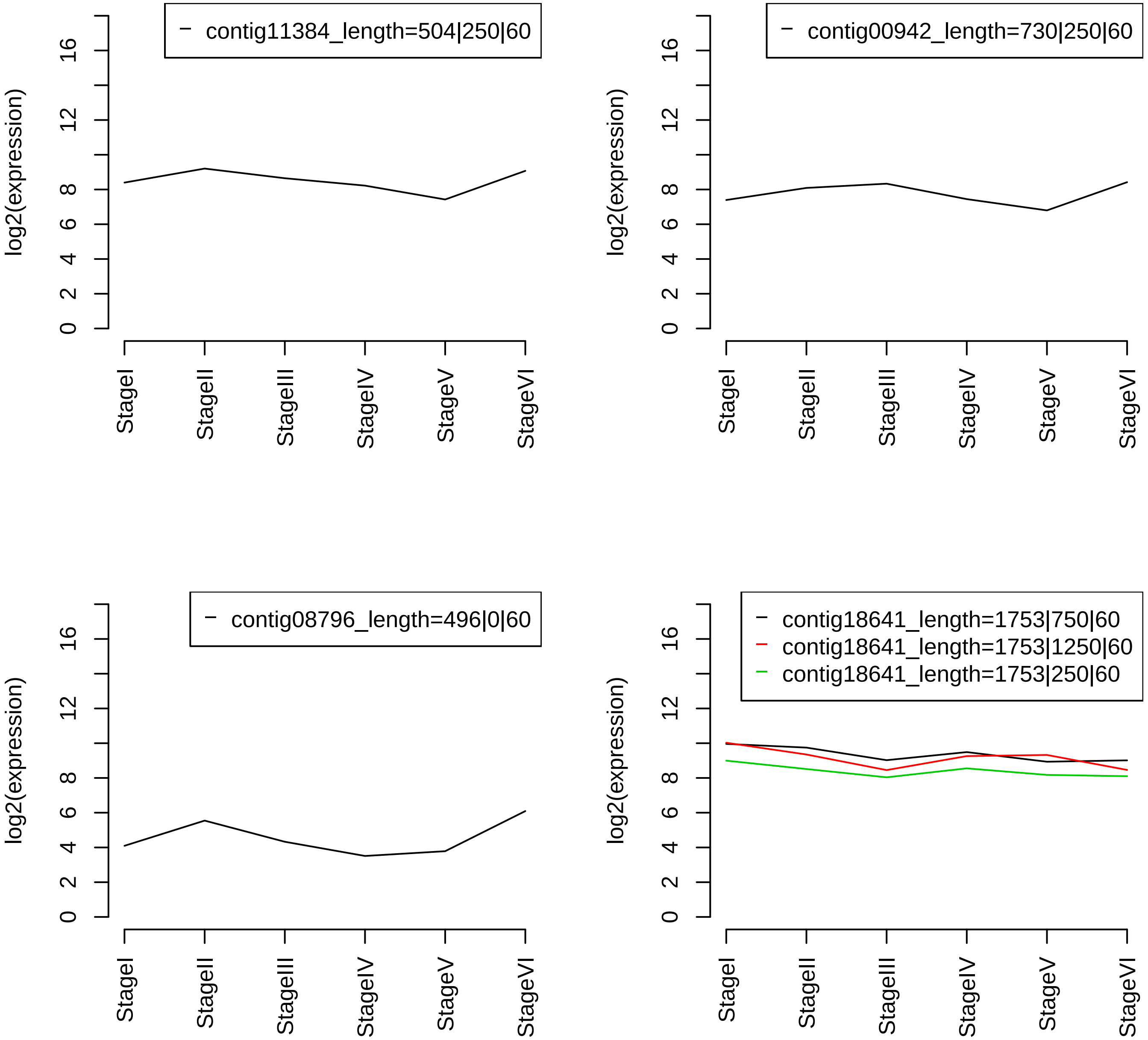

contig11384_length=504|250|60
contig00942_length=730|250|60
contig08796_length=496|0|60
contig18641_length=1753|750|60
contig18641_length=1753|1250|60
contig18641_length=1753|250|60
log2(expression)
0
2
4
6
8
12
16
StageI
StageII
StageIII
StageIV
StageV
StageVI

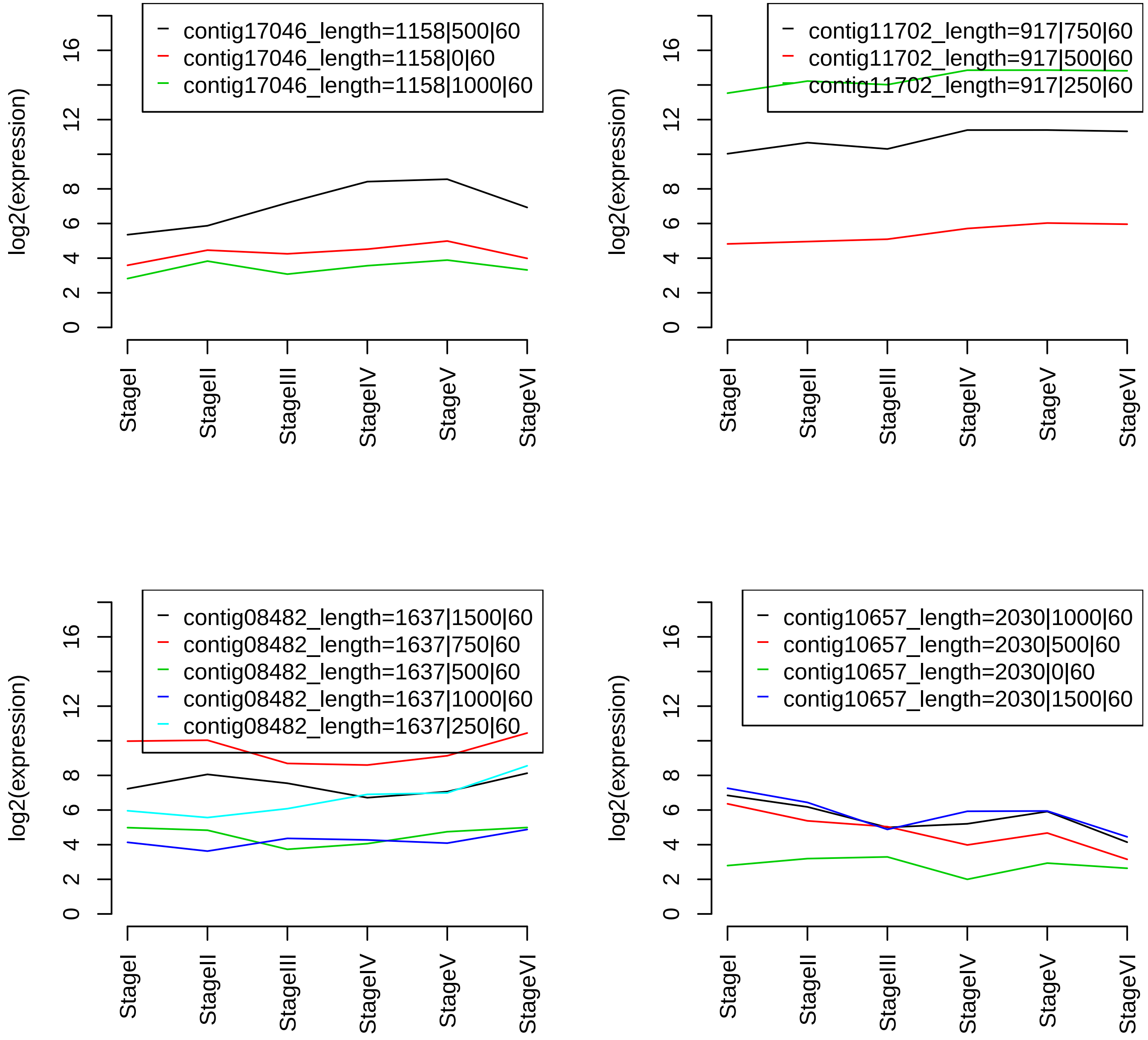

contig17046_length=1158|500|60
contig17046_length=1158|0|60
contig17046_length=1158|1000|60
contig11702_length=917|750|60
contig11702_length=917|500|60
contig11702_length=917|250|60
contig08482_length=1637|1500|60
contig08482_length=1637|750|60
contig08482_length=1637|500|60
contig08482_length=1637|1000|60
contig08482_length=1637|250|60
contig10657_length=2030|1000|60
contig10657_length=2030|500|60
contig10657_length=2030|0|60
contig10657_length=2030|1500|60
log2(expression)
0
2
4
6
8
12
16
StageI
StageII
StageIII
StageIV
StageV
StageVI

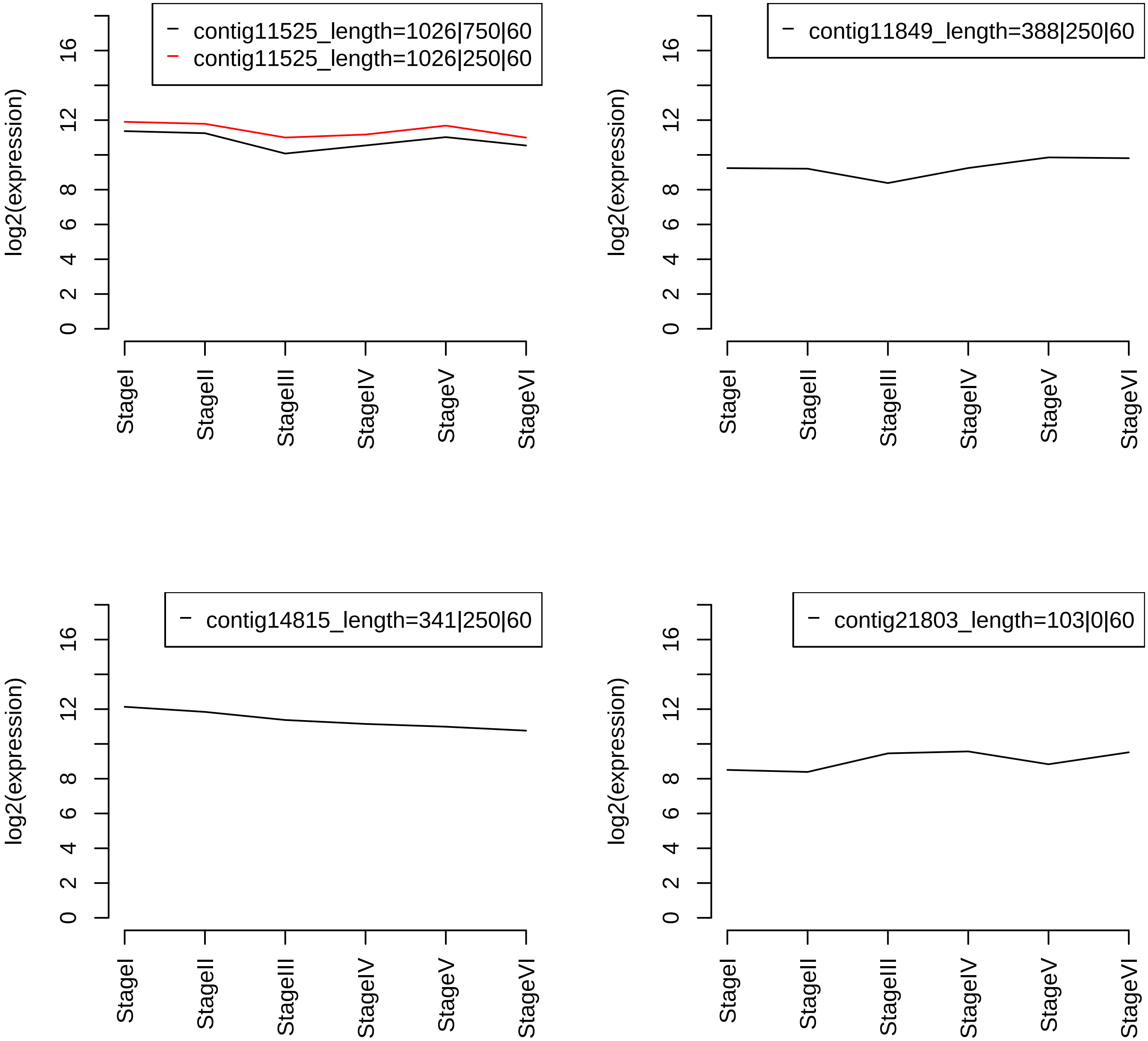
contig11525_length=1026|750|60
contig11525_length=1026|250|60
contig11849_length=388|250|60
contig14815_length=341|250|60
contig21803_length=103|0|60
log2(expression)
0
2
4
6
8
12
16
StageI
StageII
StageIII
StageIV
StageV
StageVI

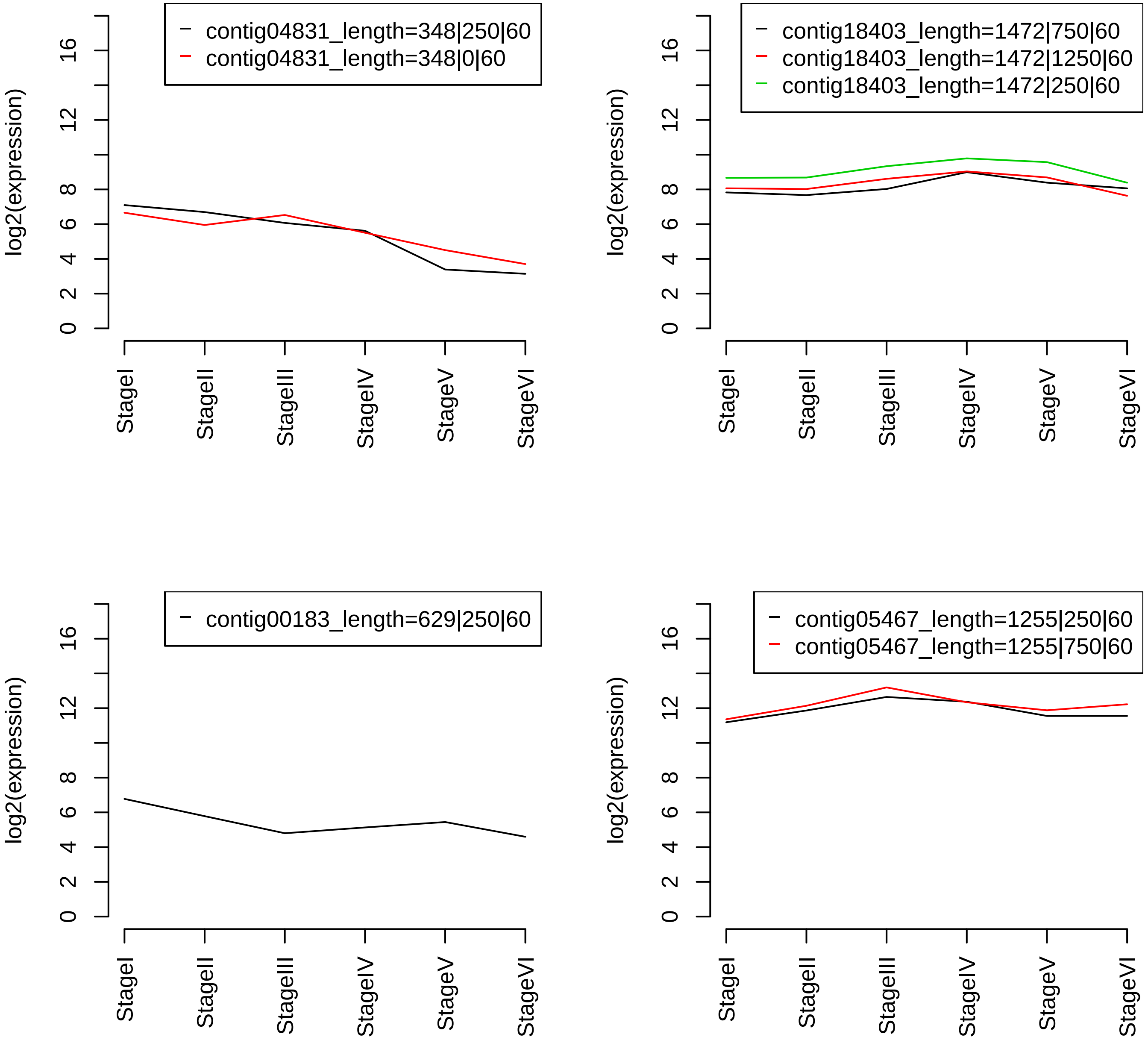

contig04831_length=348|250|60
contig04831_length=348|0|60
contig18403_length=1472|750|60
contig18403_length=1472|1250|60
contig18403_length=1472|250|60
contig00183_length=629|250|60
contig05467_length=1255|250|60
contig05467_length=1255|750|60
log2(expression)
0
2
4
6
8
12
16
StageI
StageII
StageIII
StageIV
StageV
StageVI

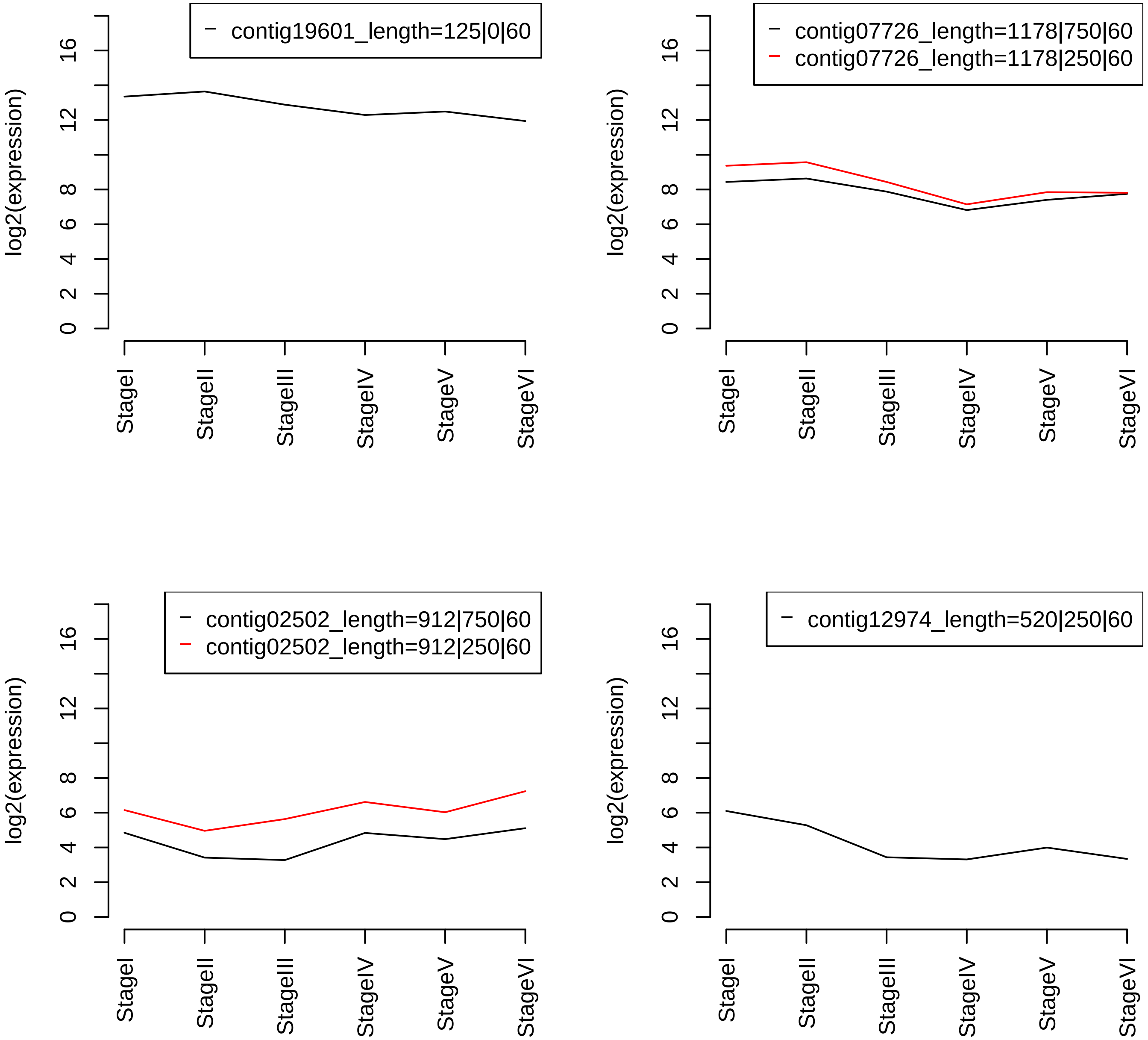
contig19601_length=125|0|60
contig07726_length=1178|750|60
contig07726_length=1178|250|60
contig02502_length=912|750|60
contig02502_length=912|250|60
contig12974_length=520|250|60
log2(expression)
0
2
4
6
8
12
16
StageI
StageII
StageIII
StageIV
StageV
StageVI

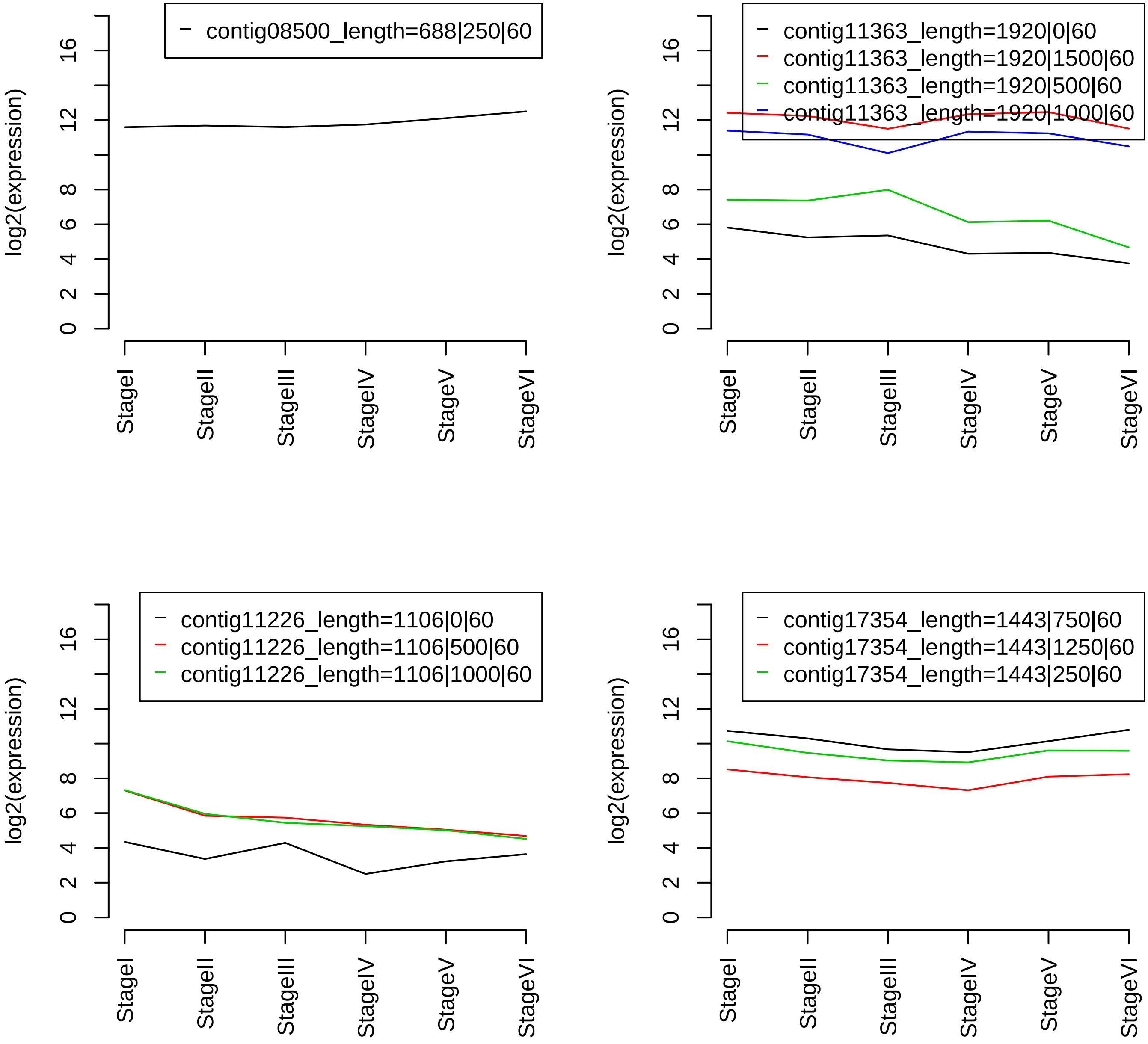
contig08500_length=688|250|60
contig11363_length=1920|0|60
contig11363_length=1920|1500|60
contig11363_length=1920|500|60
contig11363_length=1920|1000|60
contig11226_length=1106|0|60
contig11226_length=1106|500|60
contig11226_length=1106|1000|60
contig17354_length=1443|750|60
contig17354_length=1443|1250|60
contig17354_length=1443|250|60
log2(expression)
0
2
4
6
8
12
16
StageI
StageII
StageIII
StageIV
StageV
StageVI

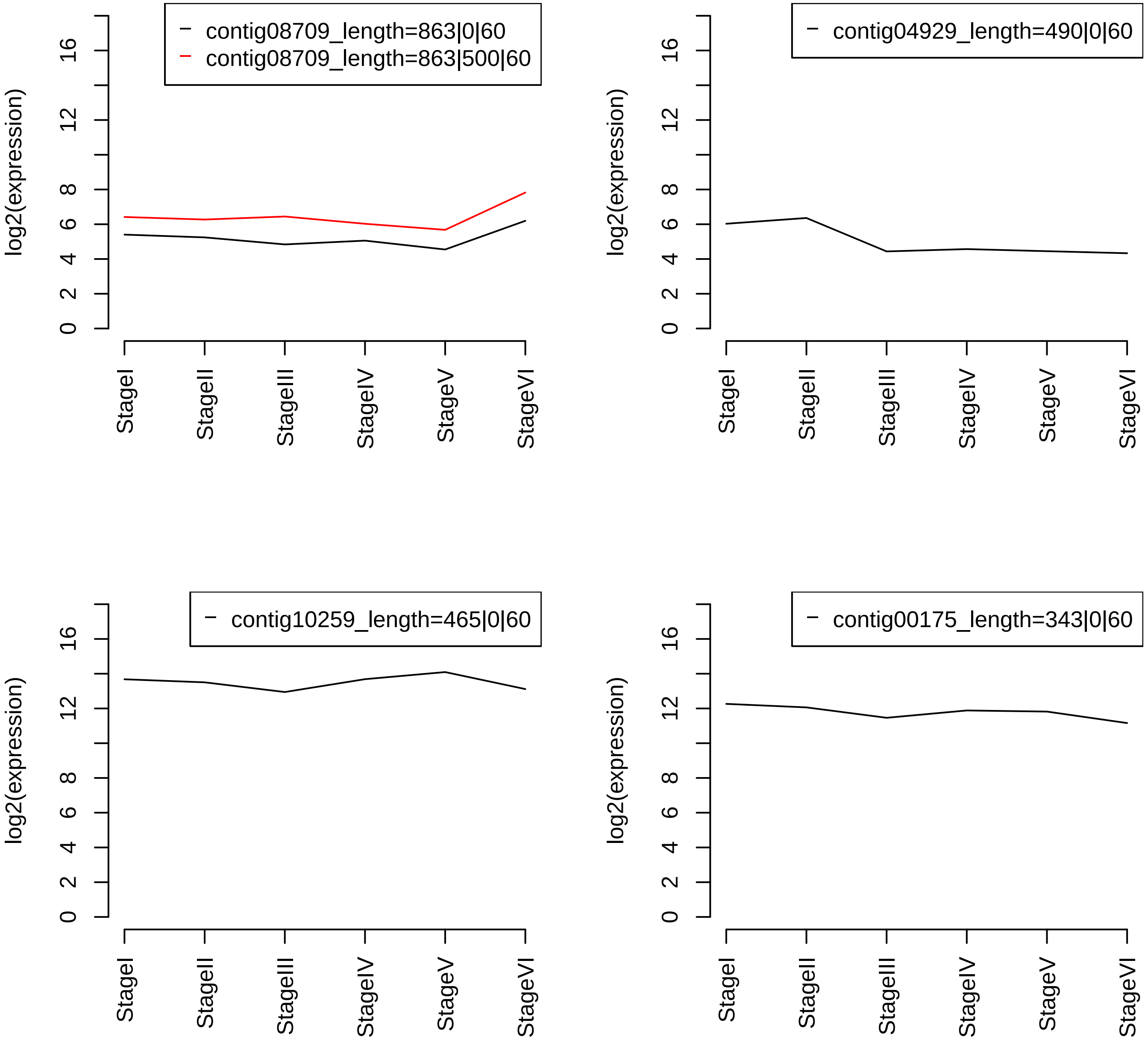

contig08709_length=863|0|60
contig08709_length=863|500|60
contig04929_length=490|0|60
contig10259_length=465|0|60
contig00175_length=343|0|60
log2(expression)
0
2
4
6
8
12
16
StageI
StageII
StageIII
StageIV
StageV
StageVI

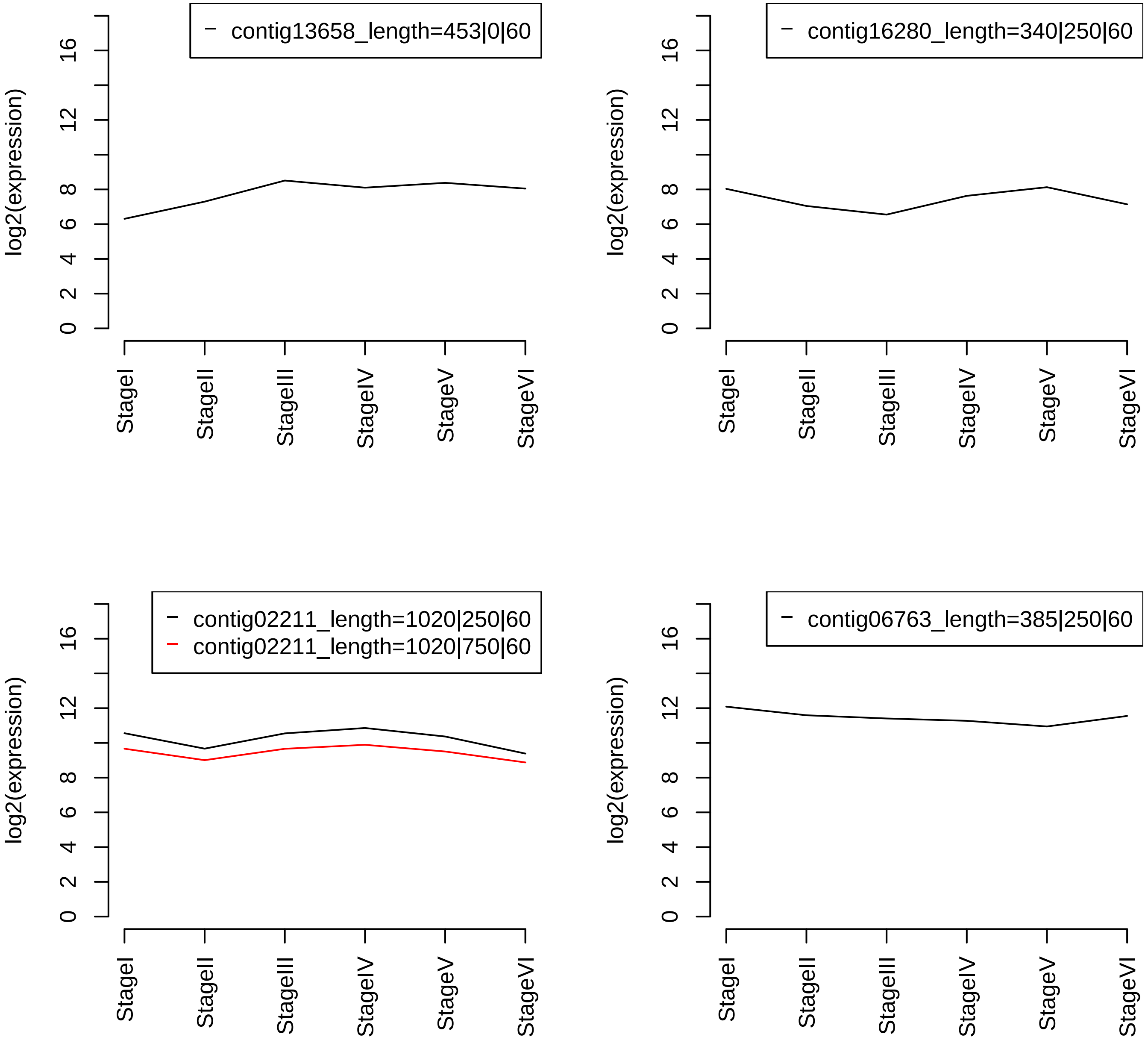
contig13658_length=453|0|60
contig16280_length=340|250|60
contig02211_length=1020|250|60
contig02211_length=1020|750|60
contig06763_length=385|250|60
log2(expression)
0
2
4
6
8
12
16
StageI
StageII
StageIII
StageIV
StageV
StageVI

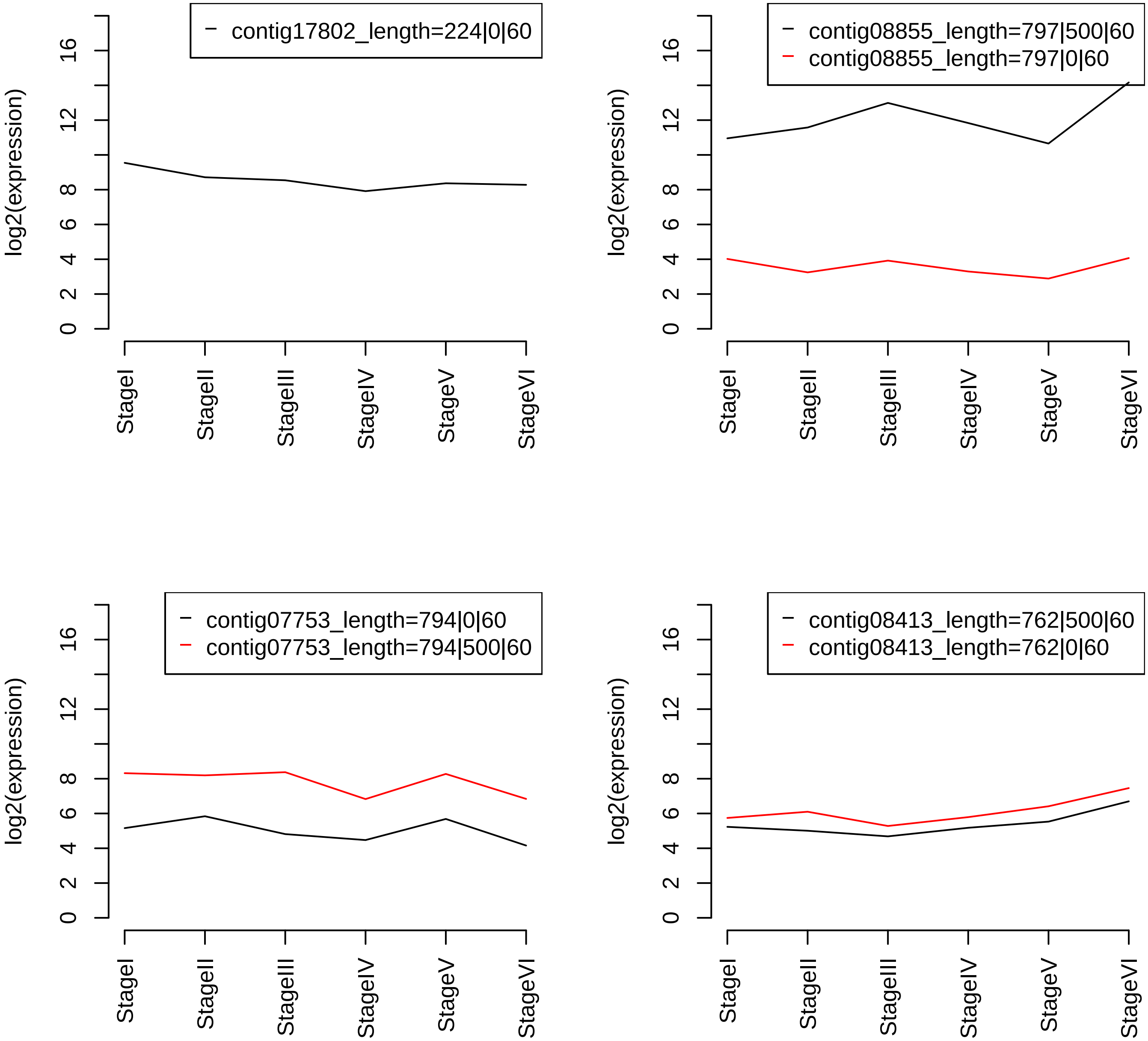

contig17802_length=224|0|60
contig08855_length=797|500|60
contig08855_length=797|0|60
contig07753_length=794|0|60
contig07753_length=794|500|60
contig08413_length=762|500|60
contig08413_length=762|0|60
log2(expression)
0
2
4
6
8
12
16
StageI
StageII
StageIII
StageIV
StageV
StageVI

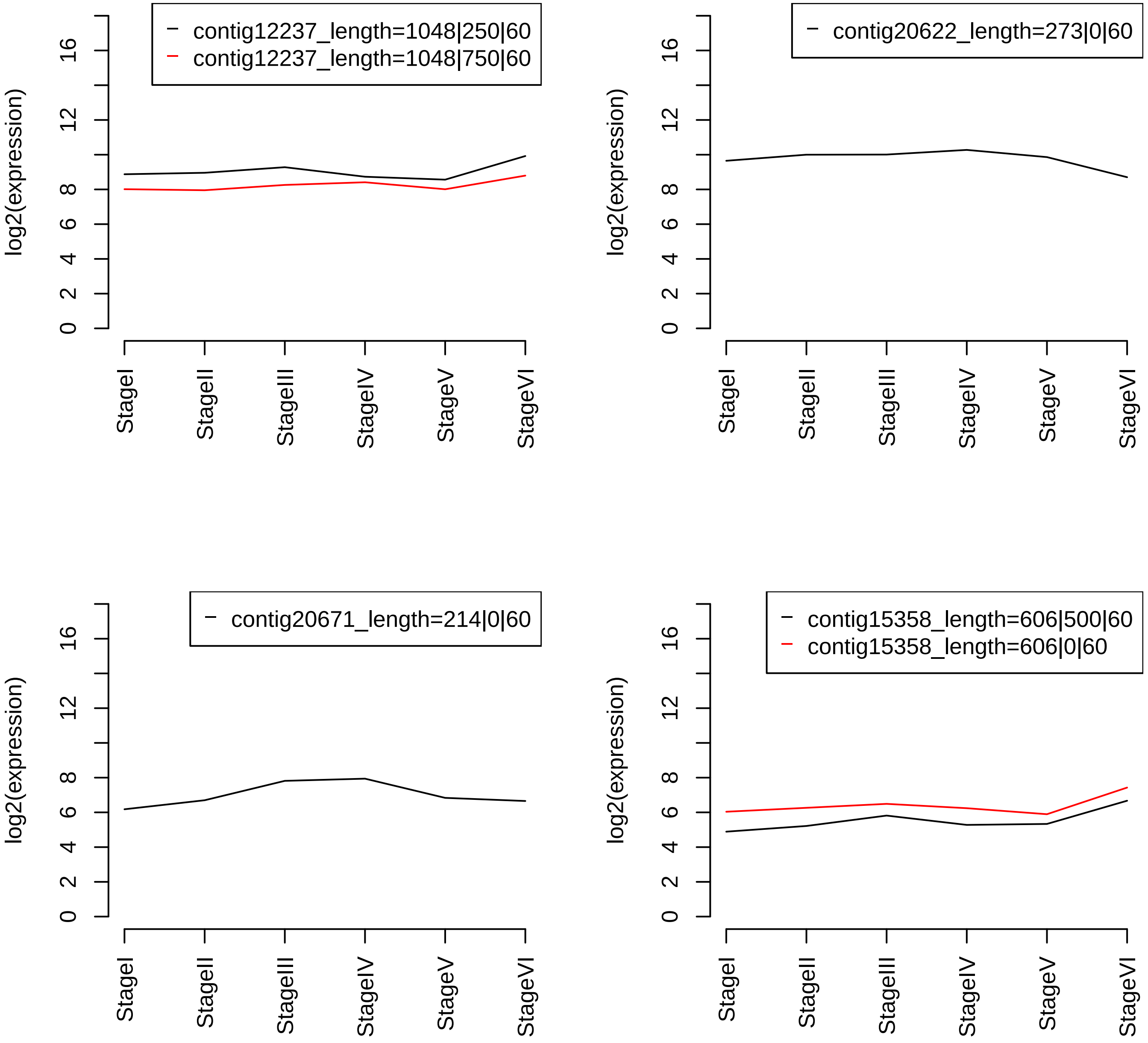
contig12237_length=1048|250|60
contig12237_length=1048|750|60
contig20622_length=273|0|60
contig20671_length=214|0|60
contig15358_length=606|500|60
contig15358_length=606|0|60
log2(expression)
0 2 4 6 8 12 16
StageI StageII StageIII StageIV StageV StageVI

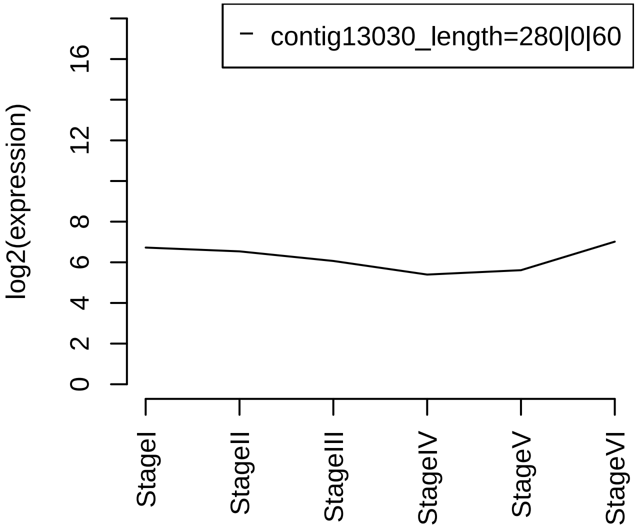
contig13030_length=280|0|60
log2(expression)
0
2
4
6
8
12
16
StageI
StageII
StageIII
StageIV
StageV
StageVI

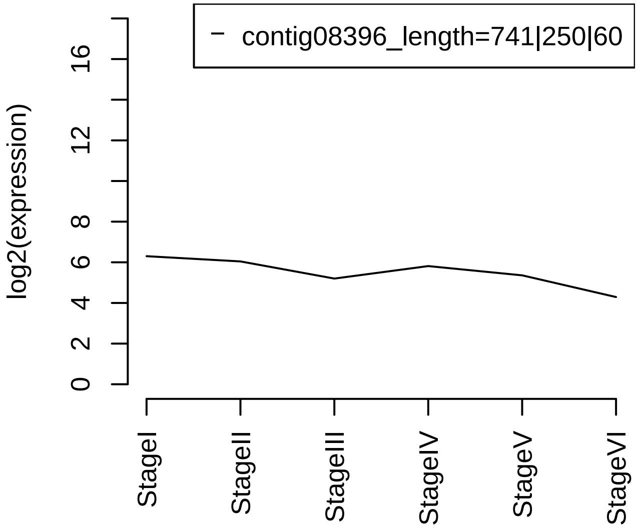
contig08396_length=741|250|60
log2(expression)
0
2
4
6
8
12
16
StageI
StageII
StageIII
StageIV
StageV
StageVI

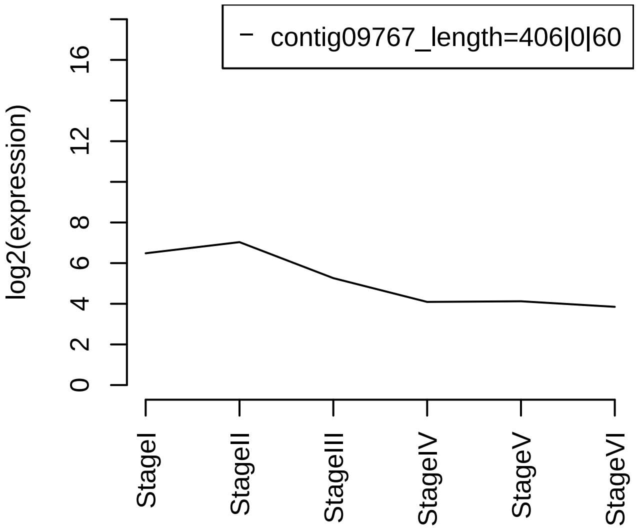
contig09767_length=406|0|60
log2(expression)
0
2
4
6
8
12
16
StageI
StageII
StageIII
StageIV
StageV
StageVI

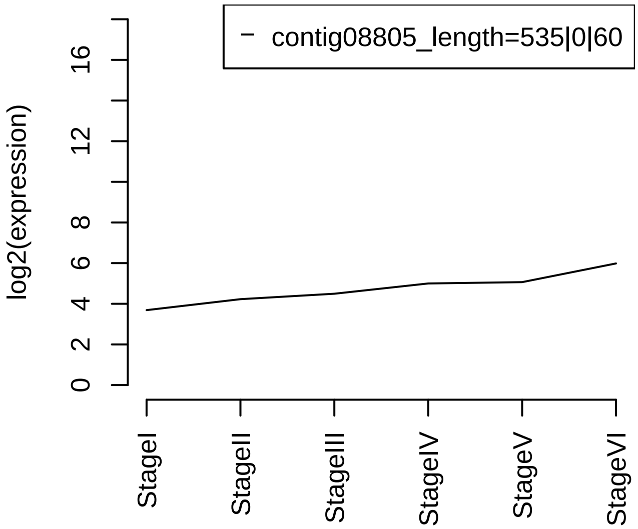
contig08805_length=535|0|60
log2(expression)
0
2
4
6
8
12
16
StageI
StageII
StageIII
StageIV
StageV
StageVI

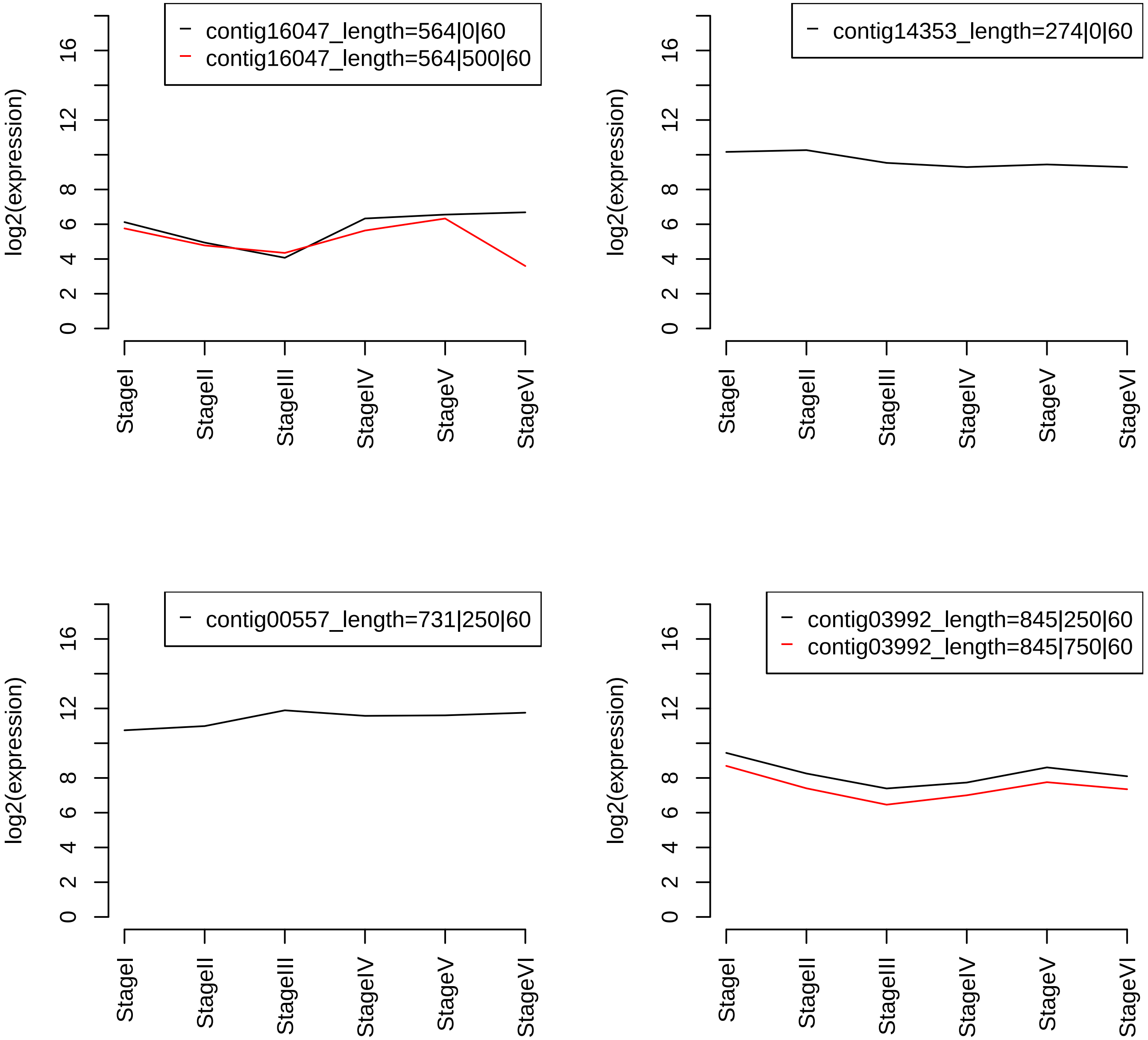
contig16047_length=564|0|60
contig16047_length=564|500|60
contig14353_length=274|0|60
contig00557_length=731|250|60
contig03992_length=845|250|60
contig03992_length=845|750|60
log2(expression)
0
2
4
6
8
12
16
StageI
StageII
StageIII
StageIV
StageV
StageVI

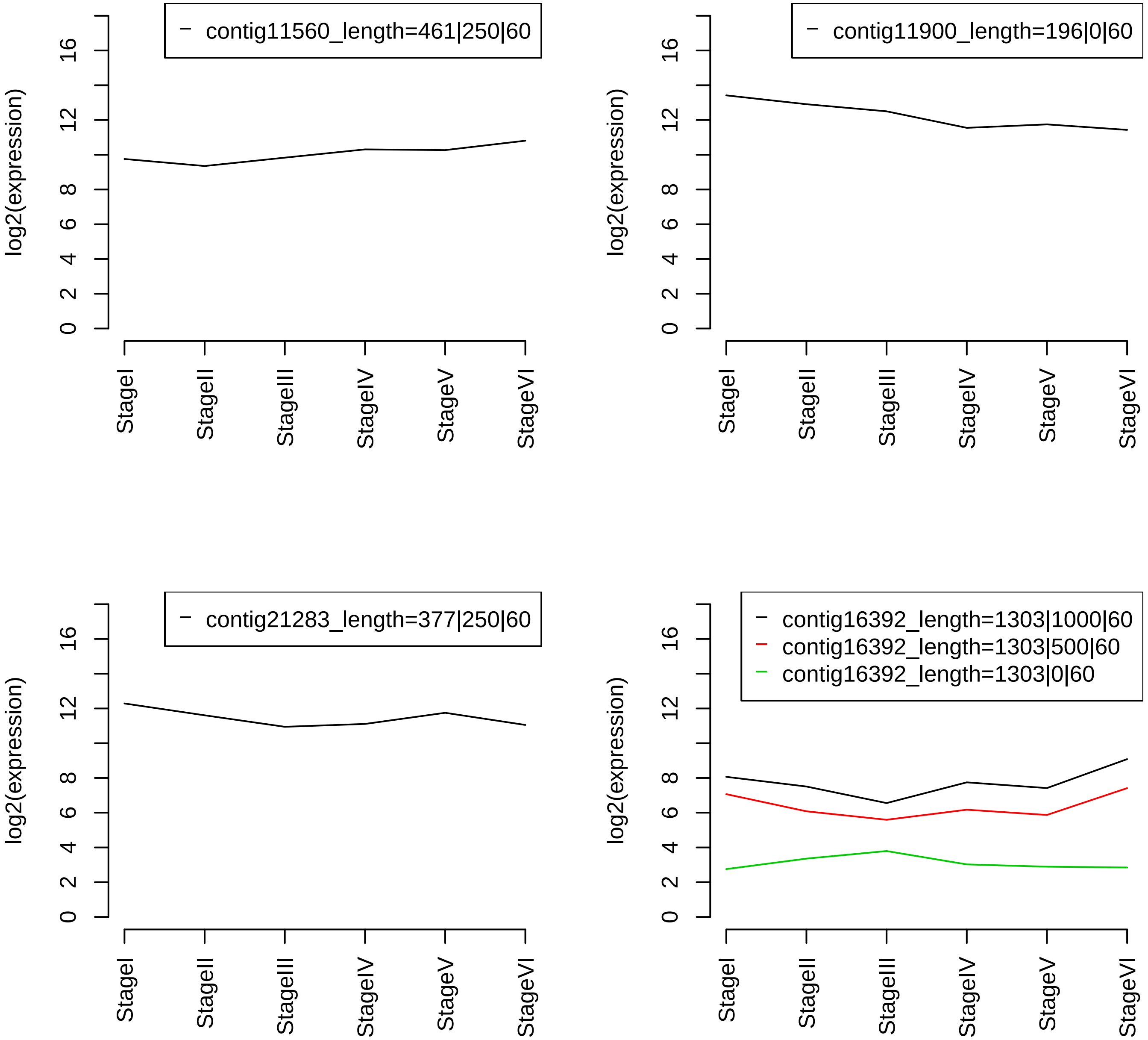

contig11560_length=461|250|60
contig11900_length=196|0|60
contig21283_length=377|250|60
contig16392_length=1303|1000|60
contig16392_length=1303|500|60
contig16392_length=1303|0|60
log2(expression)
0
2
4
6
8
12
16
StageI
StageII
StageIII
StageIV
StageV
StageVI

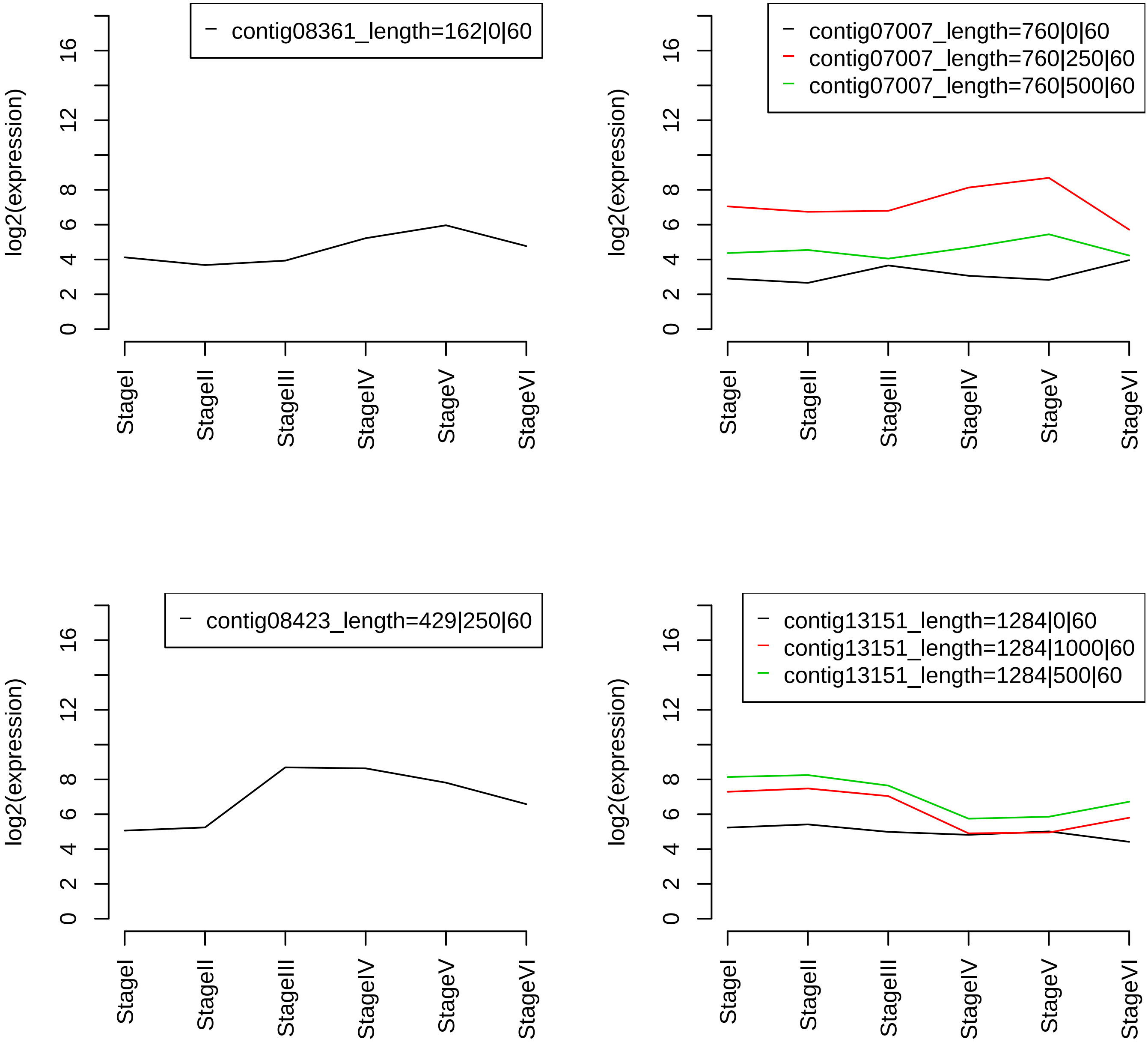
contig08361_length=162|0|60
contig07007_length=760|0|60
contig07007_length=760|250|60
contig07007_length=760|500|60
contig08423_length=429|250|60
contig13151_length=1284|0|60
contig13151_length=1284|1000|60
contig13151_length=1284|500|60
log2(expression)
0
2
4
6
8
12
16
StageI
StageII
StageIII
StageIV
StageV
StageVI

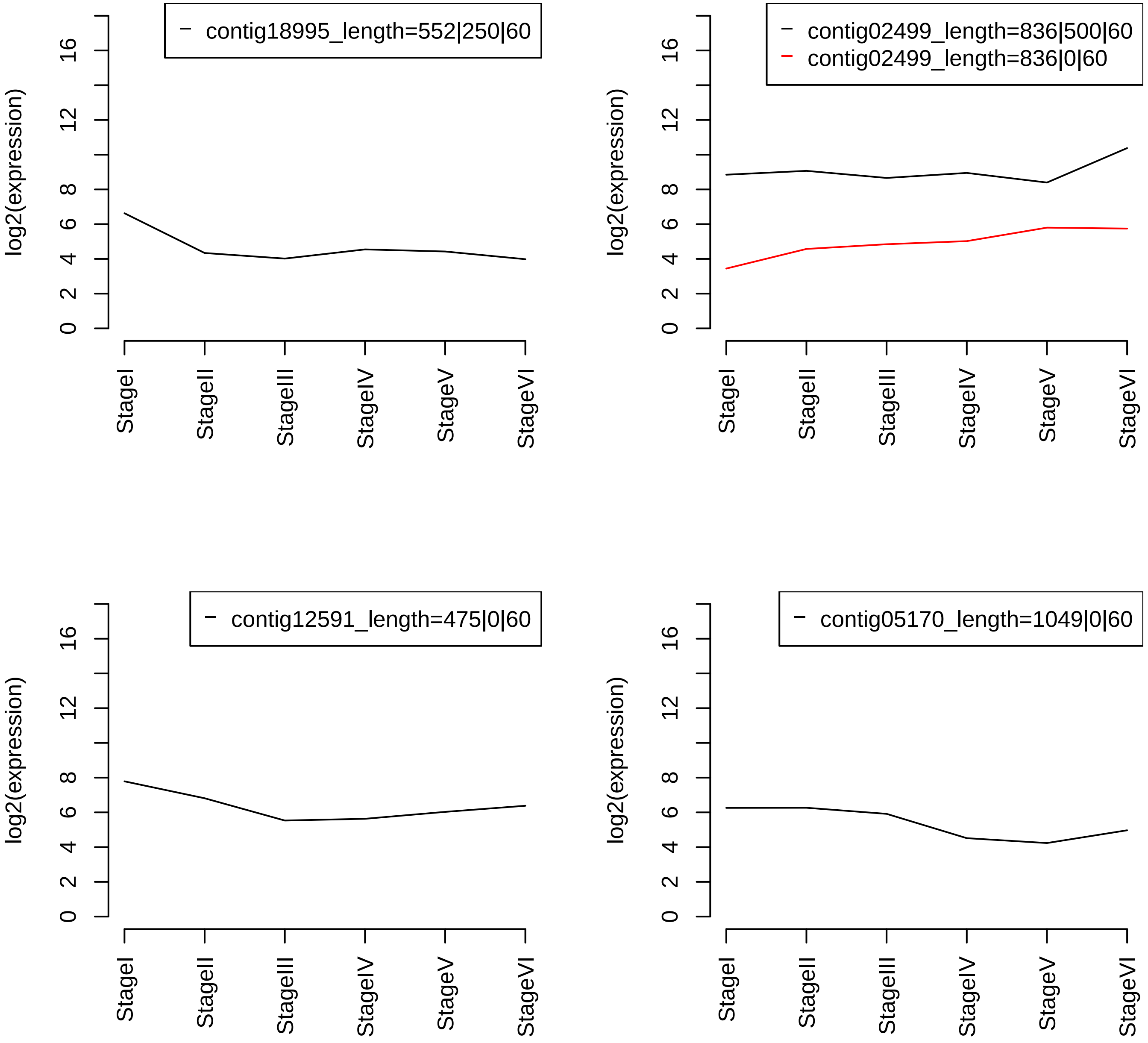
contig18995_length=552|250|60
contig02499_length=836|500|60
contig02499_length=836|0|60
contig12591_length=475|0|60
contig05170_length=1049|0|60
log2(expression)
0
2
4
6
8
12
16
StageI
StageII
StageIII
StageIV
StageV
StageVI

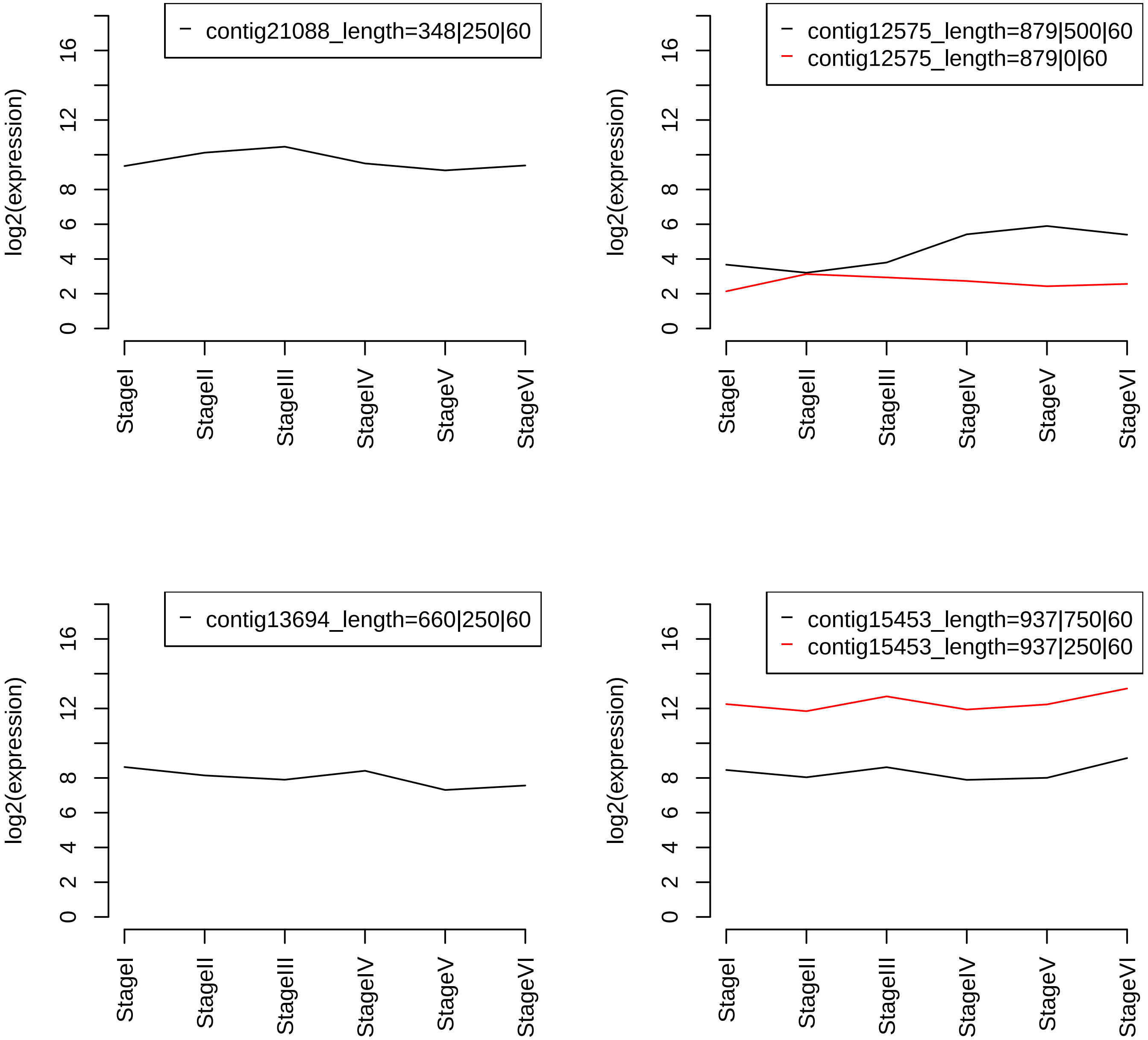
contig21088_length=348|250|60
contig12575_length=879|500|60
contig12575_length=879|0|60
contig13694_length=660|250|60
contig15453_length=937|750|60
contig15453_length=937|250|60
log2(expression)
0
2
4
6
8
12
16
StageI
StageII
StageIII
StageIV
StageV
StageVI

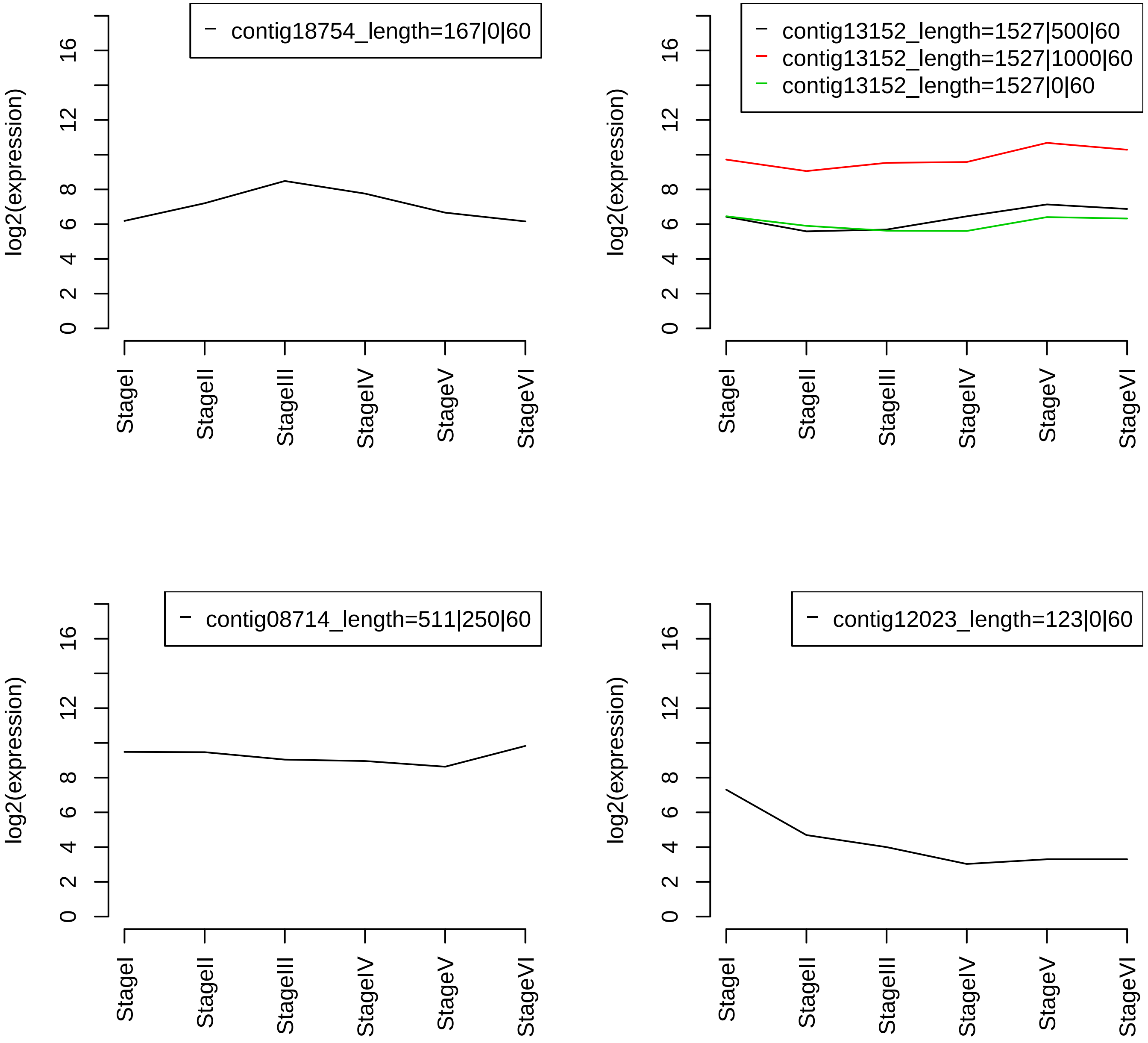

contig18754_length=167|0|60
contig13152_length=1527|500|60
contig13152_length=1527|1000|60
contig13152_length=1527|0|60
contig08714_length=511|250|60
contig12023_length=123|0|60
log2(expression)
0
2
4
6
8
12
16
StageI
StageII
StageIII
StageIV
StageV
StageVI

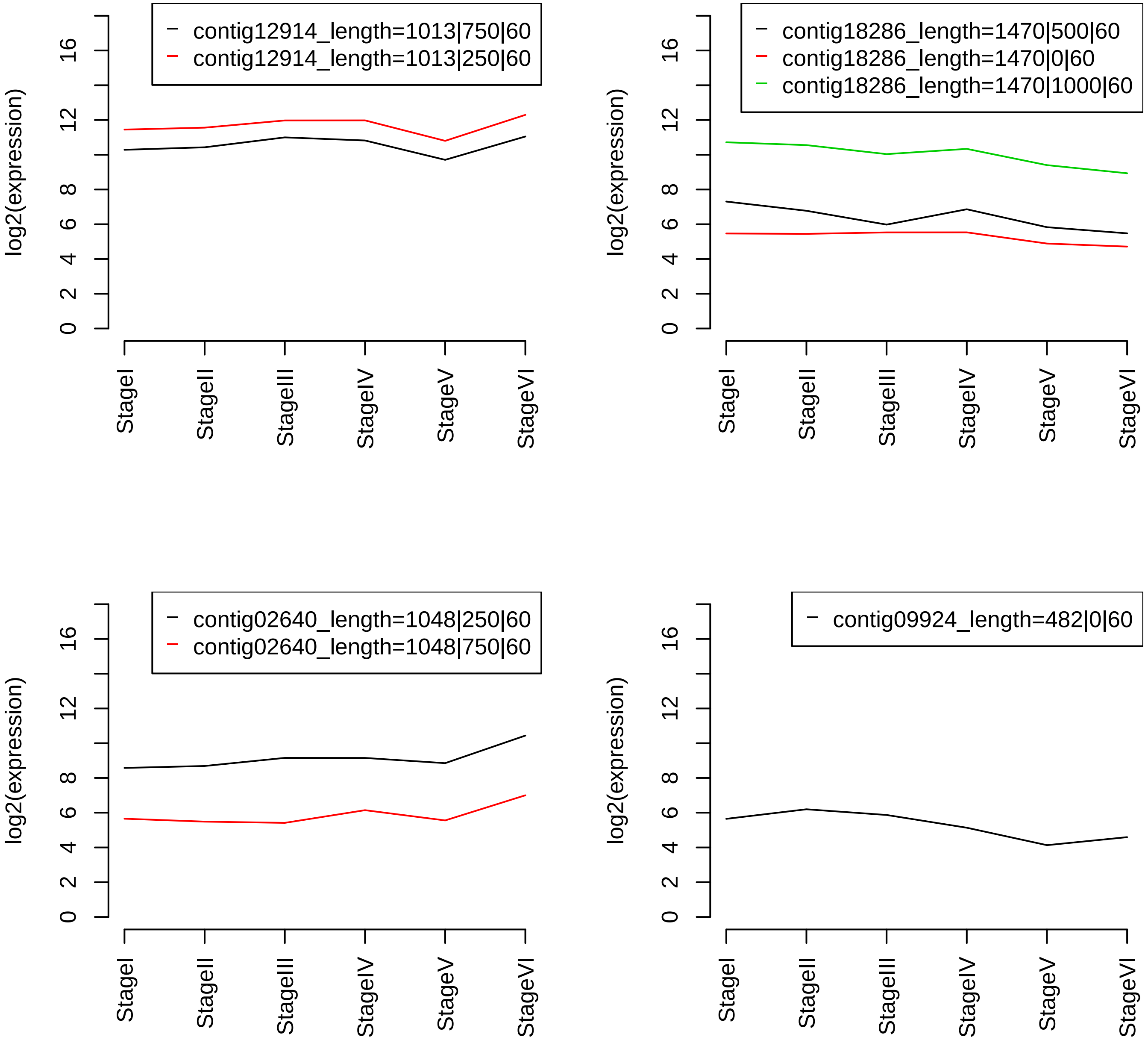
contig12914_length=1013|750|60
contig12914_length=1013|250|60
contig18286_length=1470|500|60
contig18286_length=1470|0|60
contig18286_length=1470|1000|60
contig02640_length=1048|250|60
contig02640_length=1048|750|60
contig09924_length=482|0|60
log2(expression)
0
2
4
6
8
12
16
StageI
StageII
StageIII
StageIV
StageV
StageVI

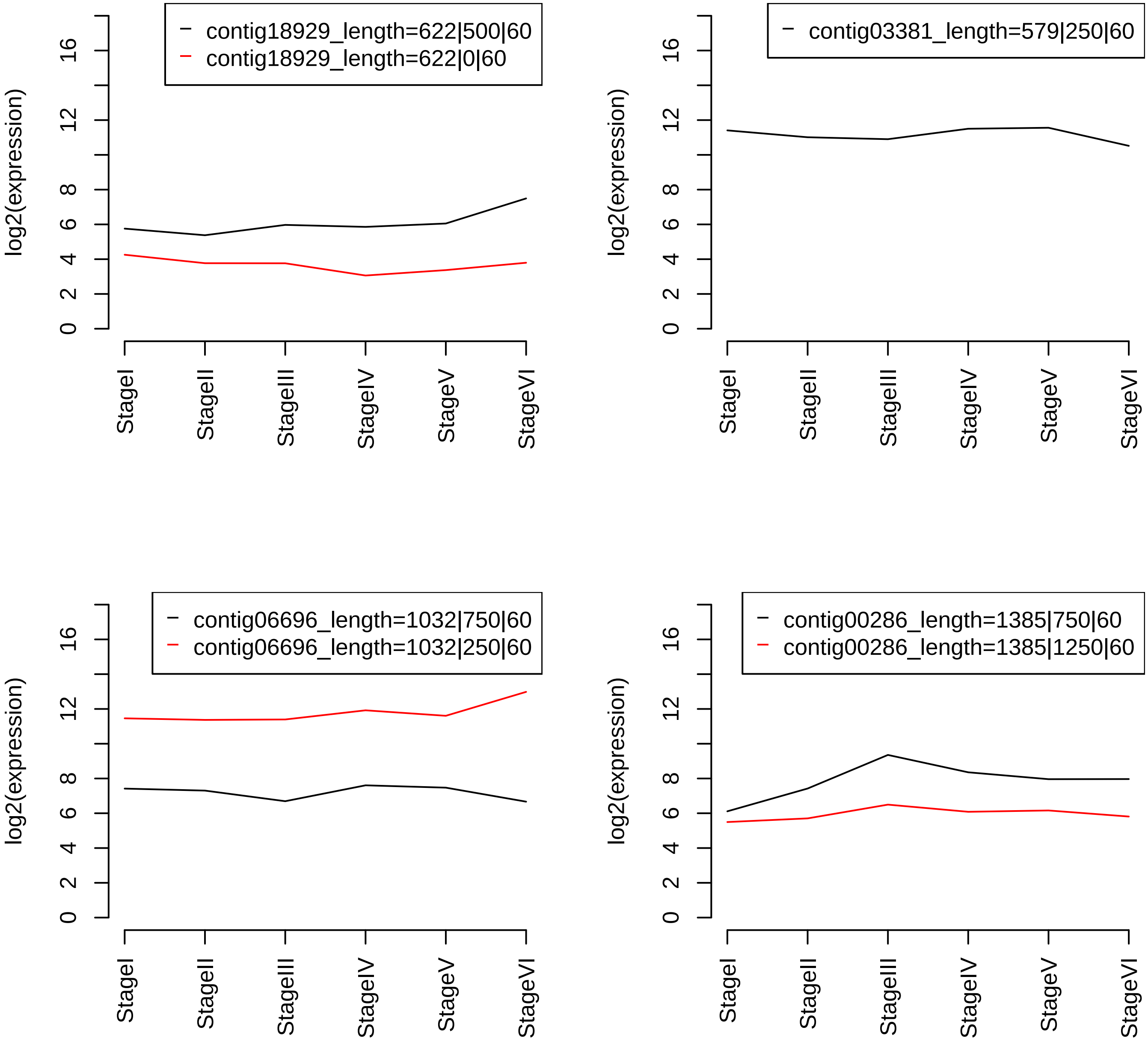
contig18929_length=622|500|60
contig18929_length=622|0|60
contig03381_length=579|250|60
contig06696_length=1032|750|60
contig06696_length=1032|250|60
contig00286_length=1385|750|60
contig00286_length=1385|1250|60
log2(expression)
0
2
4
6
8
12
16
StageI
StageII
StageIII
StageIV
StageV
StageVI

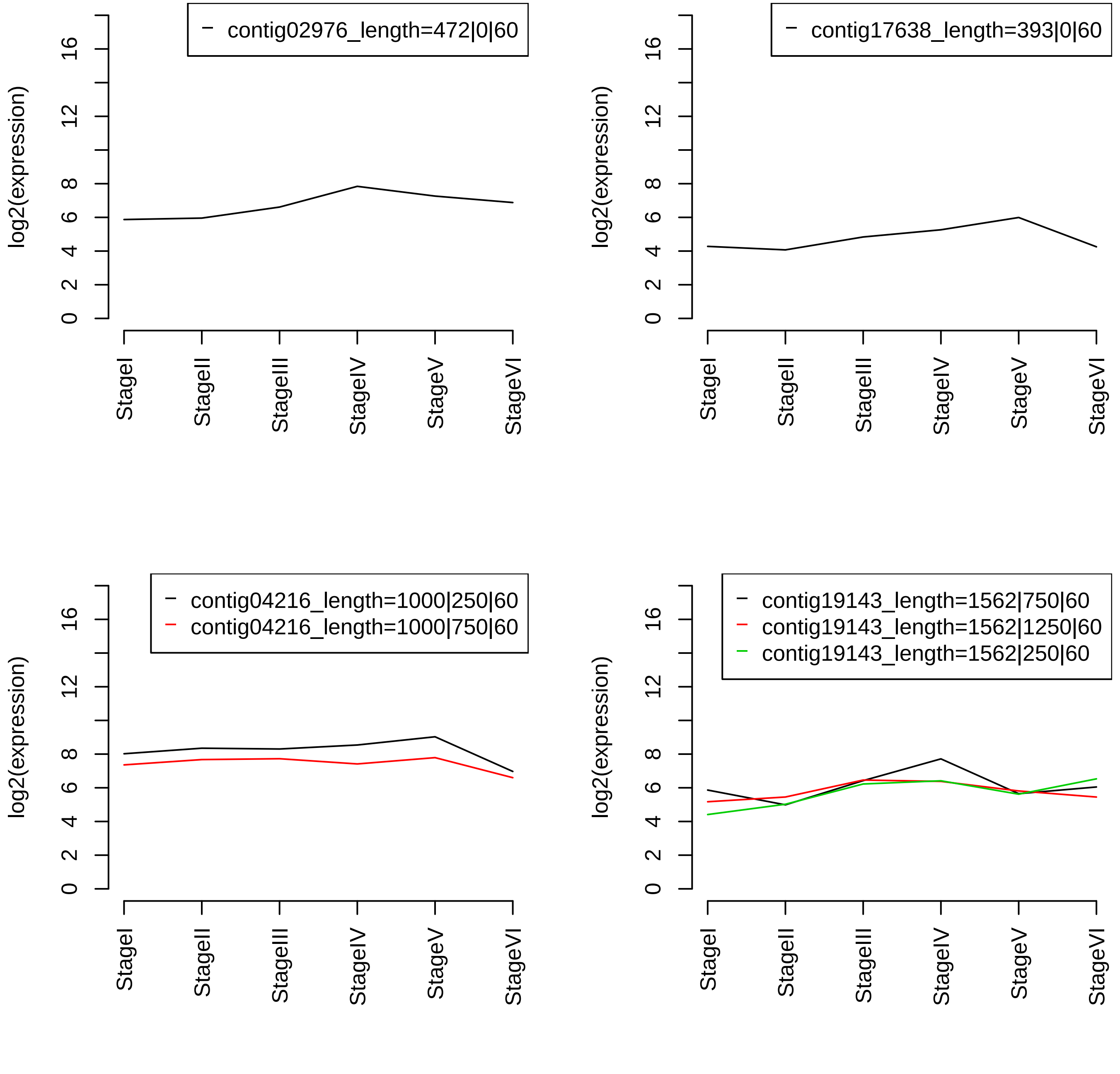

contig02976_length=472|0|60
contig17638_length=393|0|60
contig04216_length=1000|250|60
contig04216_length=1000|750|60
contig19143_length=1562|750|60
contig19143_length=1562|1250|60
contig19143_length=1562|250|60
log2(expression)
0
2
4
6
8
12
16
StageI
StageII
StageIII
StageIV
StageV
StageVI

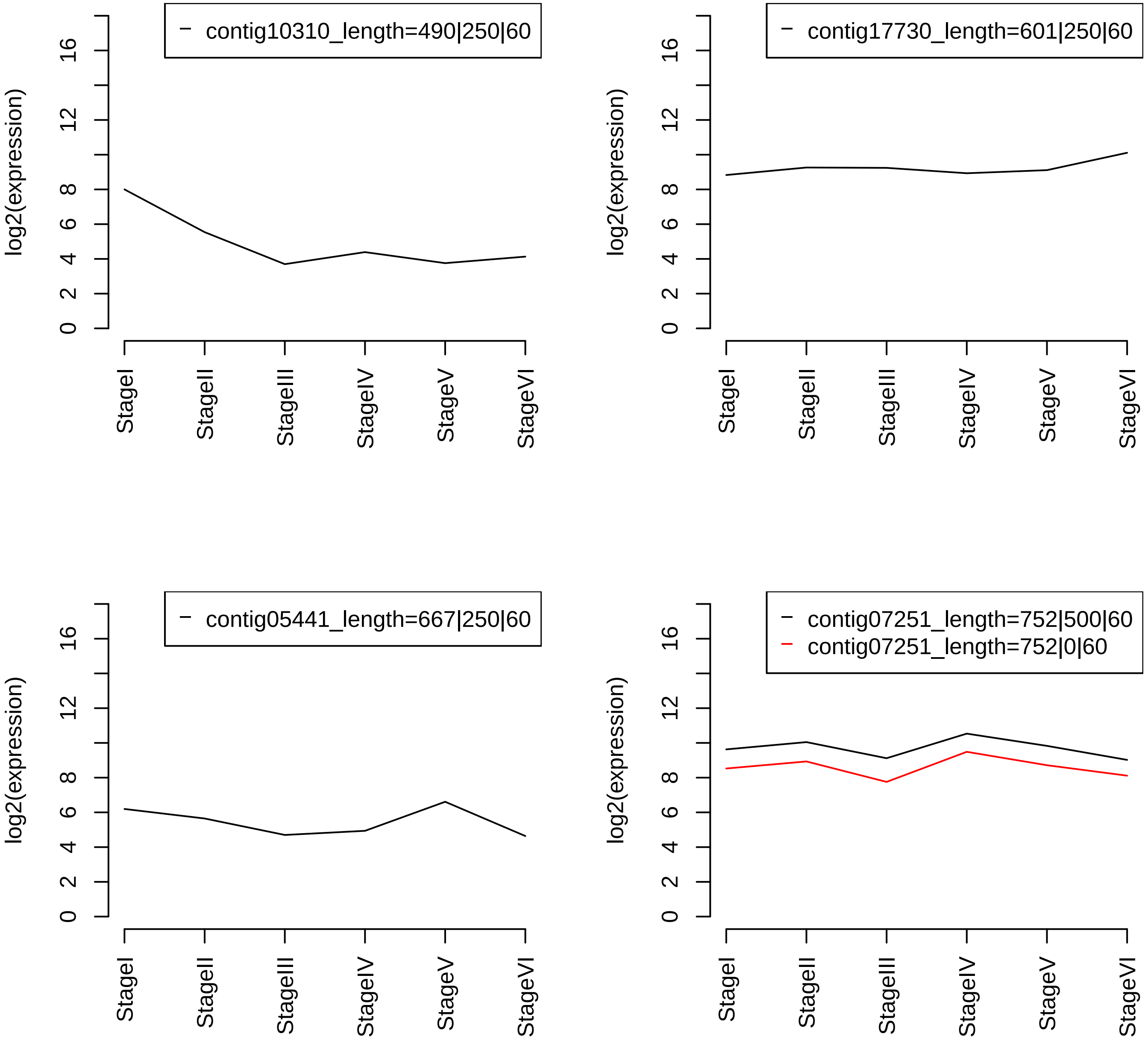

contig10310_length=490|250|60
contig17730_length=601|250|60
contig05441_length=667|250|60
contig07251_length=752|500|60
contig07251_length=752|0|60
log2(expression)
0
2
4
6
8
12
16
StageI
StageII
StageIII
StageIV
StageV
StageVI

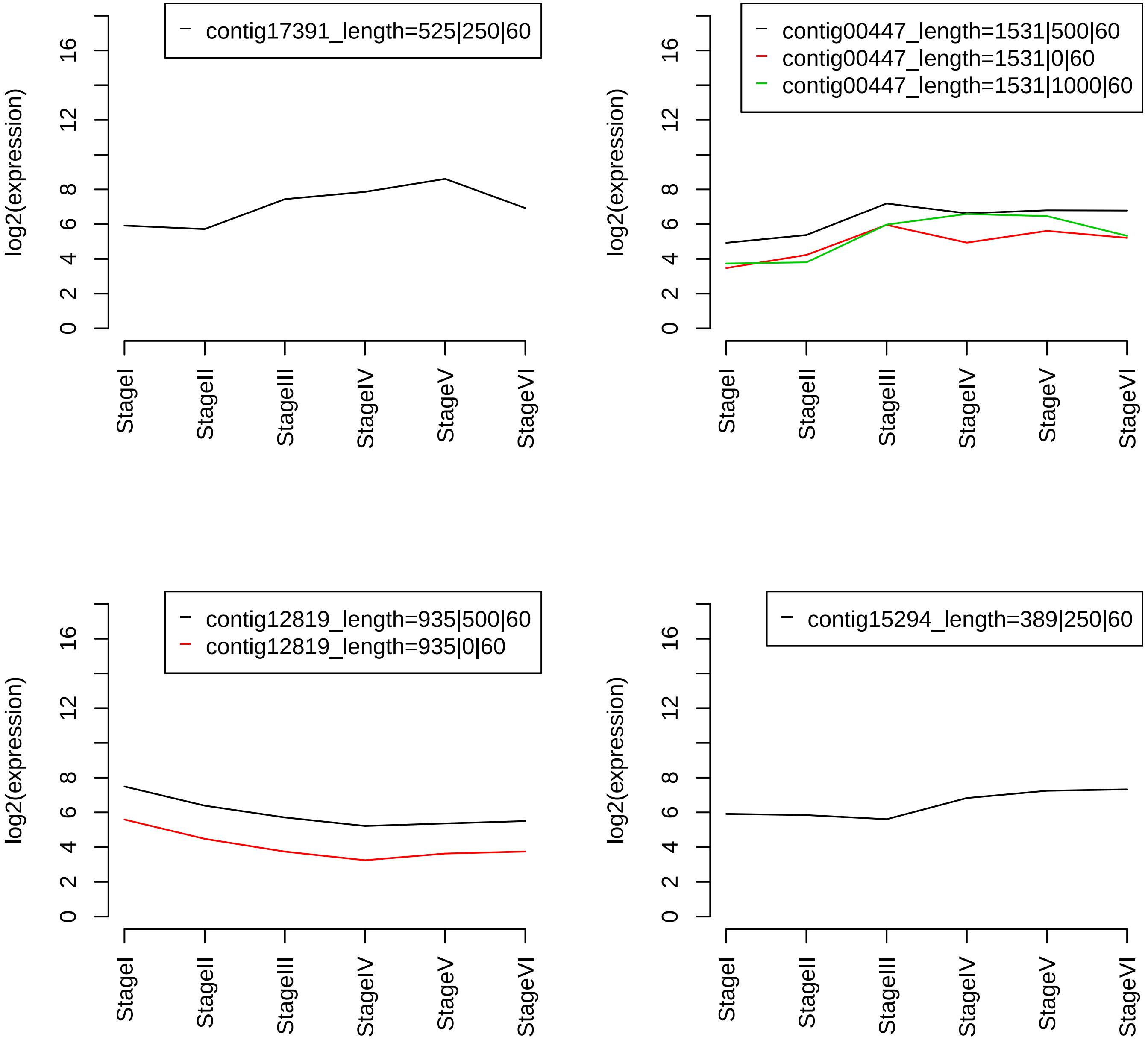
contig17391_length=525|250|60
contig00447_length=1531|500|60
contig00447_length=1531|0|60
contig00447_length=1531|1000|60
contig12819_length=935|500|60
contig12819_length=935|0|60
contig15294_length=389|250|60
log2(expression)
0
2
4
6
8
12
16
StageI
StageII
StageIII
StageIV
StageV
StageVI

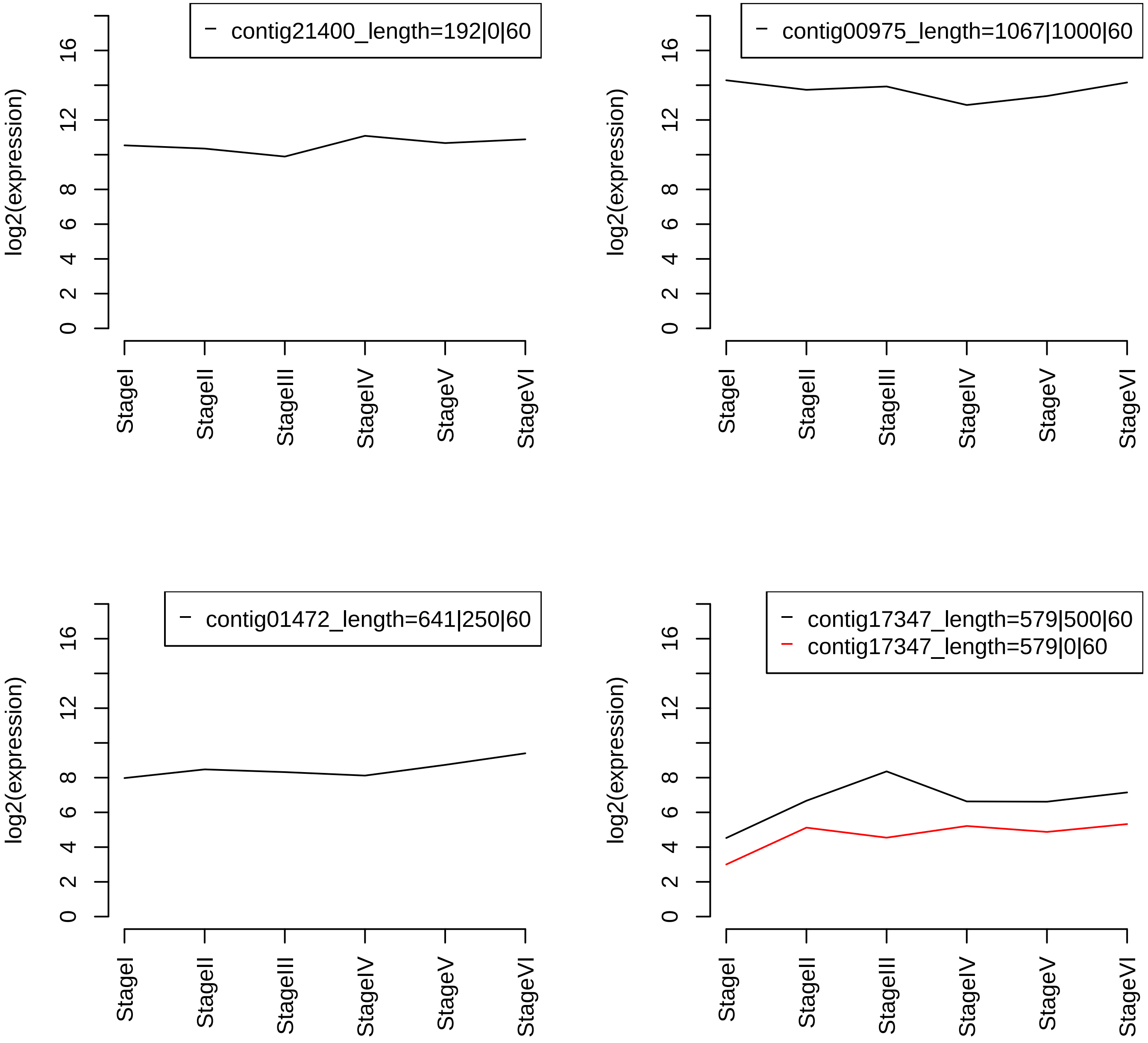

contig21400_length=192|0|60
contig00975_length=1067|1000|60
contig01472_length=641|250|60
contig17347_length=579|500|60
contig17347_length=579|0|60
log2(expression)
0
2
4
6
8
12
16
StageI
StageII
StageIII
StageIV
StageV
StageVI

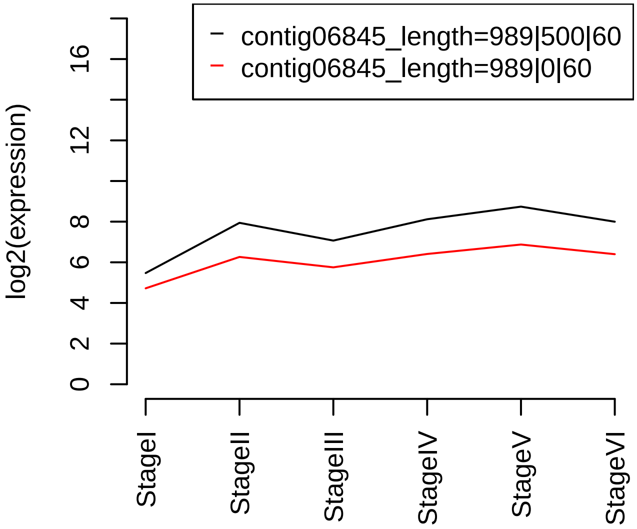
contig06845_length=989|500|60
contig06845_length=989|0|60
log2(expression)
0
2
4
6
8
12
16
StageI
StageII
StageIII
StageIV
StageV
StageVI

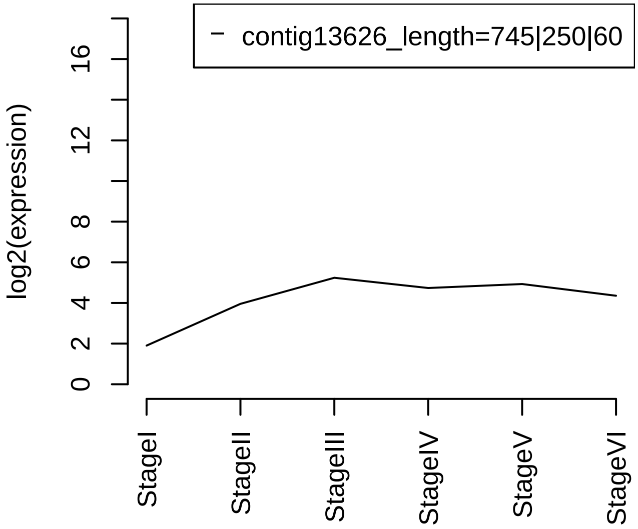
contig13626_length=745|250|60
log2(expression)
0
2
4
6
8
12
16
StageI
StageII
StageIII
StageIV
StageV
StageVI

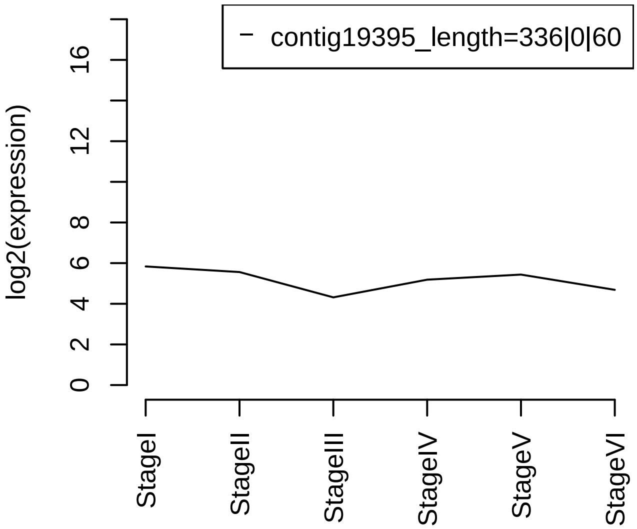
contig19395_length=336|0|60
log2(expression)
0
2
4
6
8
12
16
StageI
StageII
StageIII
StageIV
StageV
StageVI

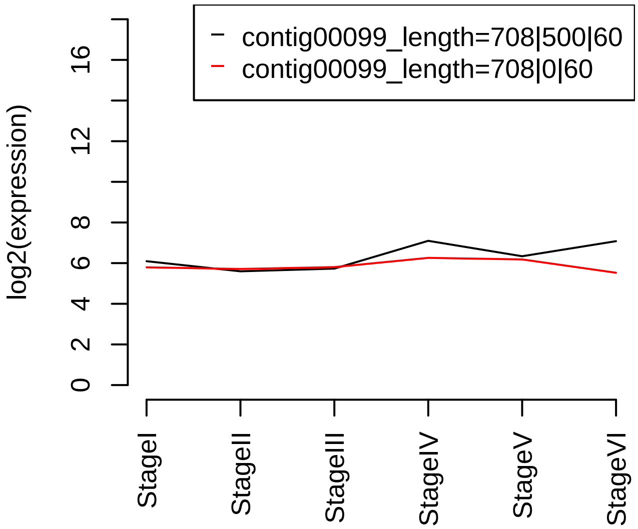
contig00099_length=708|500|60
contig00099_length=708|0|60
log2(expression)
0
2
4
6
8
12
16
StageI
StageII
StageIII
StageIV
StageV
StageVI

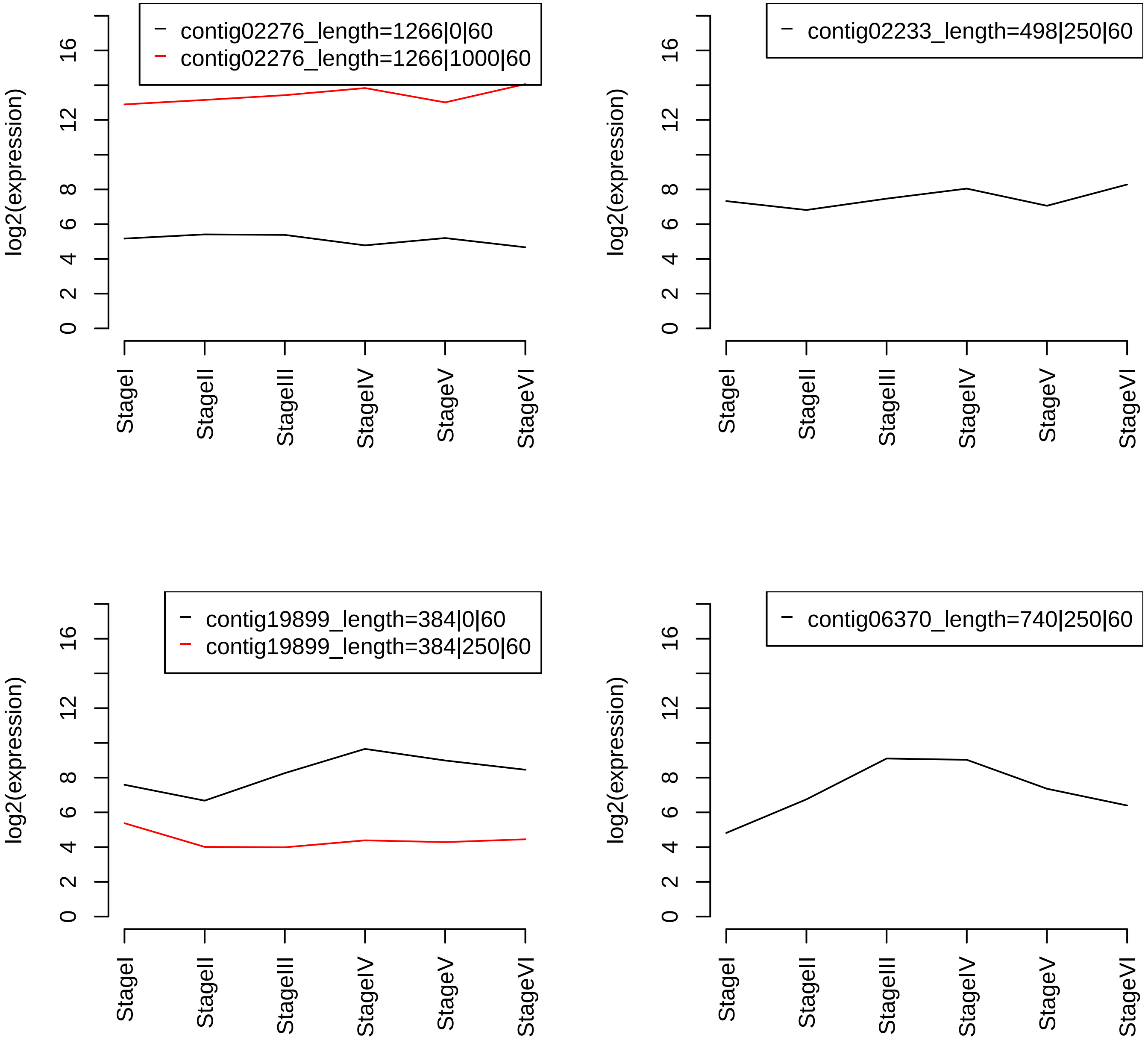

contig02276_length=1266|0|60
contig02276_length=1266|1000|60
contig02233_length=498|250|60
contig19899_length=384|0|60
contig19899_length=384|250|60
contig06370_length=740|250|60
log2(expression)
0
2
4
6
8
12
16
StageI
StageII
StageIII
StageIV
StageV
StageVI

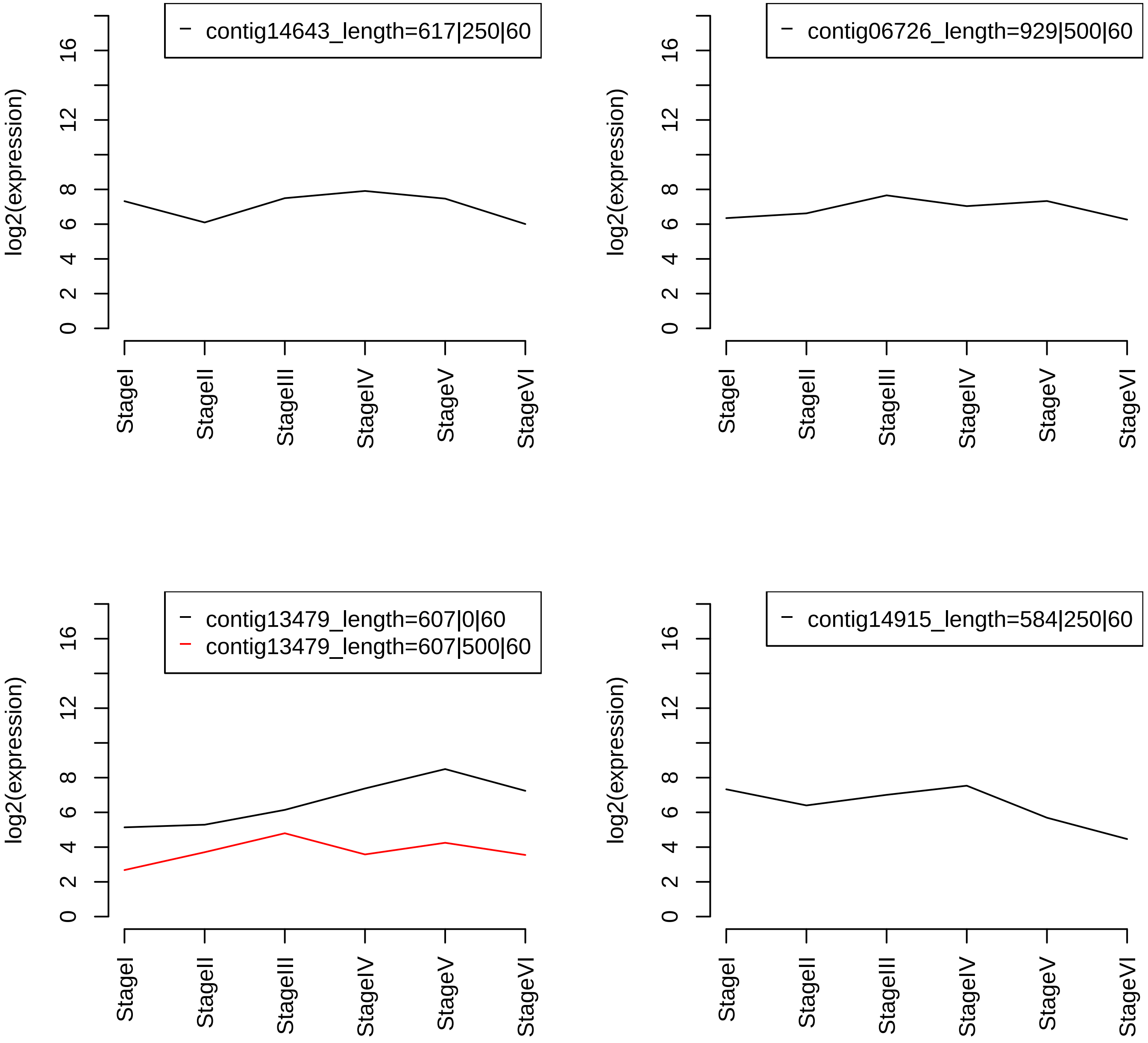

contig14643_length=617|250|60
contig06726_length=929|500|60
contig13479_length=607|0|60
contig13479_length=607|500|60
contig14915_length=584|250|60
log2(expression)
0
2
4
6
8
12
16
StageI
StageII
StageIII
StageIV
StageV
StageVI

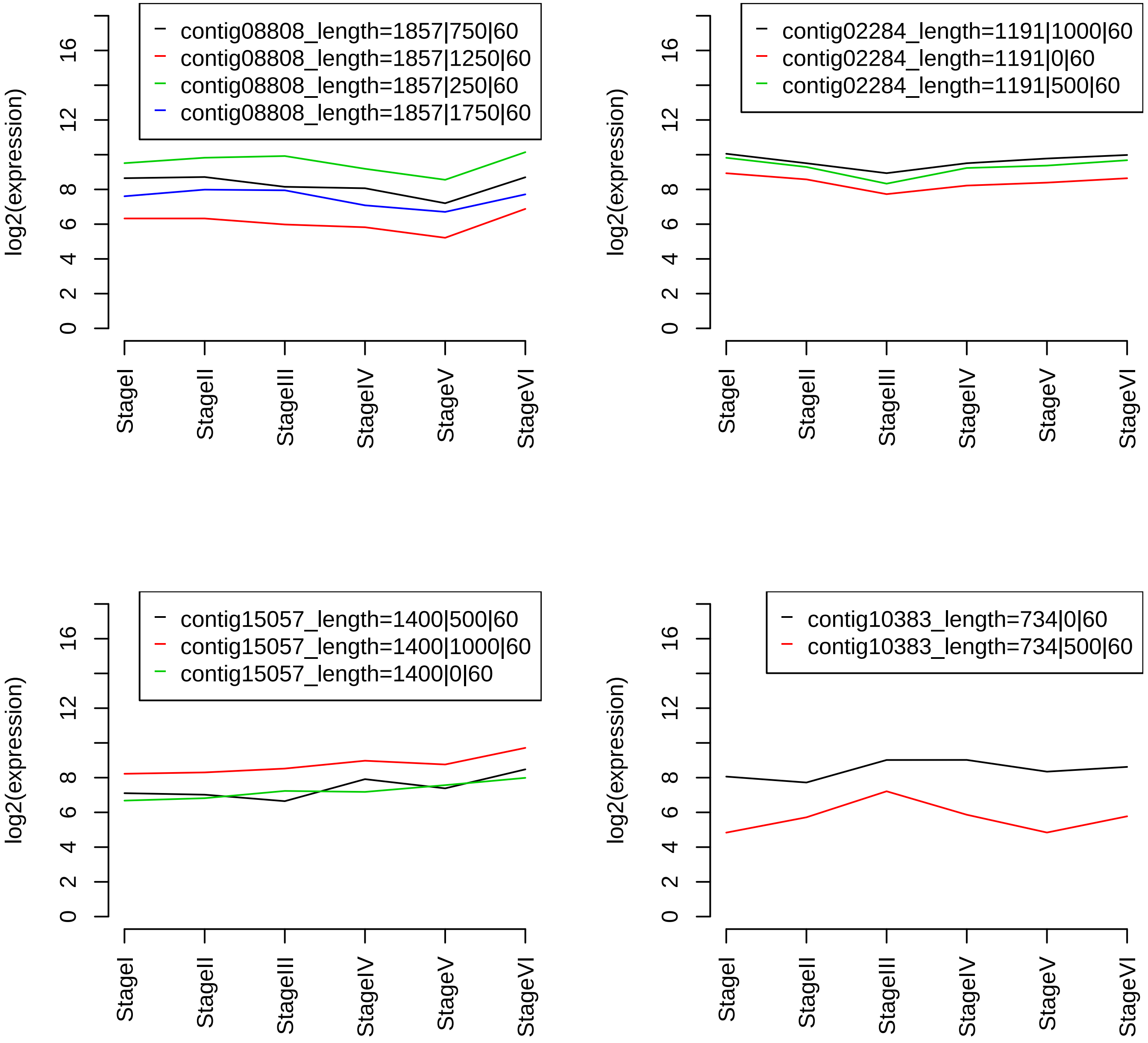
contig08808_length=1857|750|60
contig08808_length=1857|1250|60
contig08808_length=1857|250|60
contig08808_length=1857|1750|60
contig02284_length=1191|1000|60
contig02284_length=1191|0|60
contig02284_length=1191|500|60
contig15057_length=1400|500|60
contig15057_length=1400|1000|60
contig15057_length=1400|0|60
contig10383_length=734|0|60
contig10383_length=734|500|60
log2(expression)
0 2 4 6 8 12 16
StageI StageII StageIII StageIV StageV StageVI

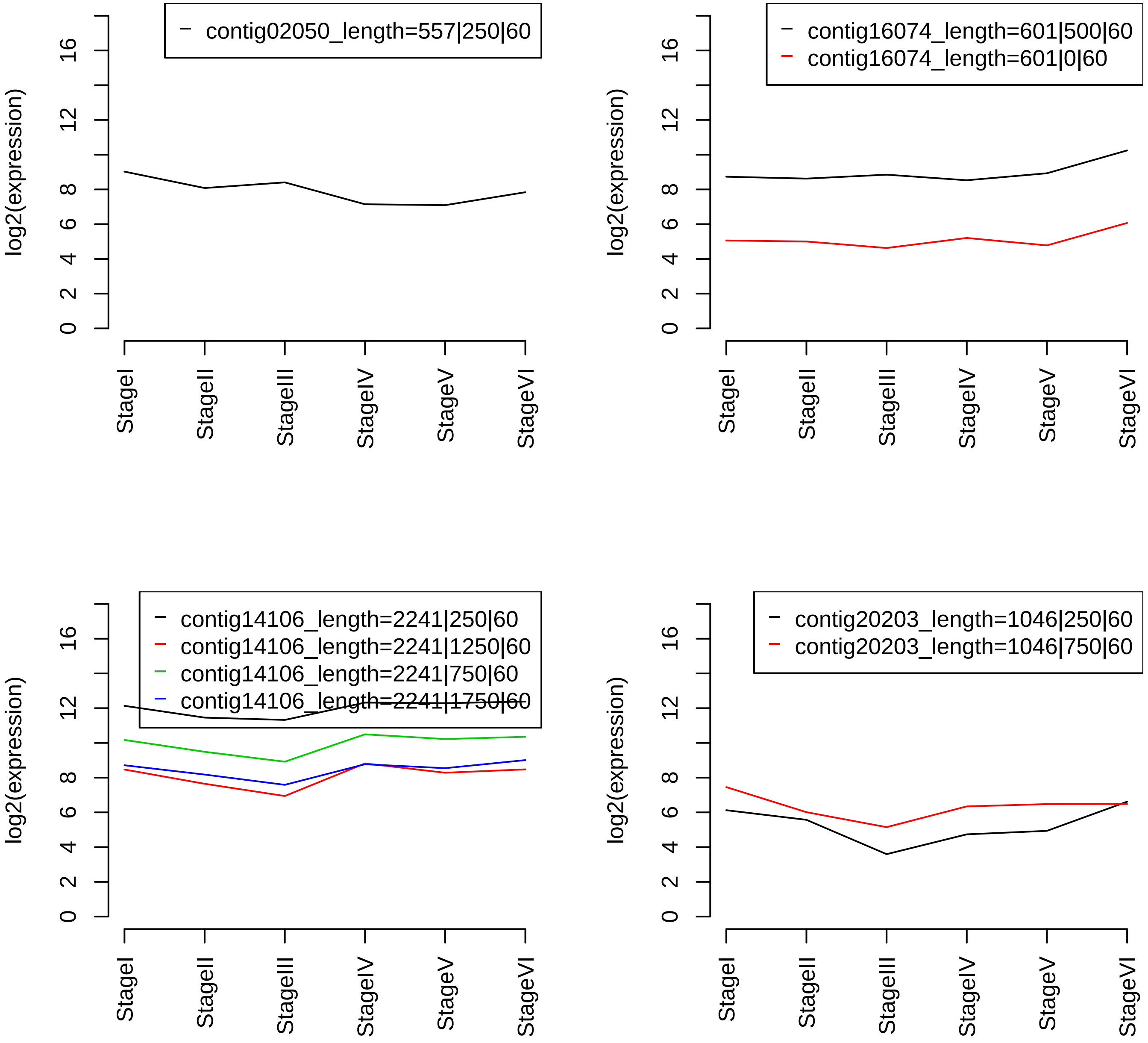

contig02050_length=557|250|60
contig16074_length=601|500|60
contig16074_length=601|0|60
contig14106_length=2241|250|60
contig14106_length=2241|1250|60
contig14106_length=2241|750|60
contig14106_length=2241|1750|60
contig20203_length=1046|250|60
contig20203_length=1046|750|60
log2(expression)
0
2
4
6
8
12
16
StageI
StageII
StageIII
StageIV
StageV
StageVI

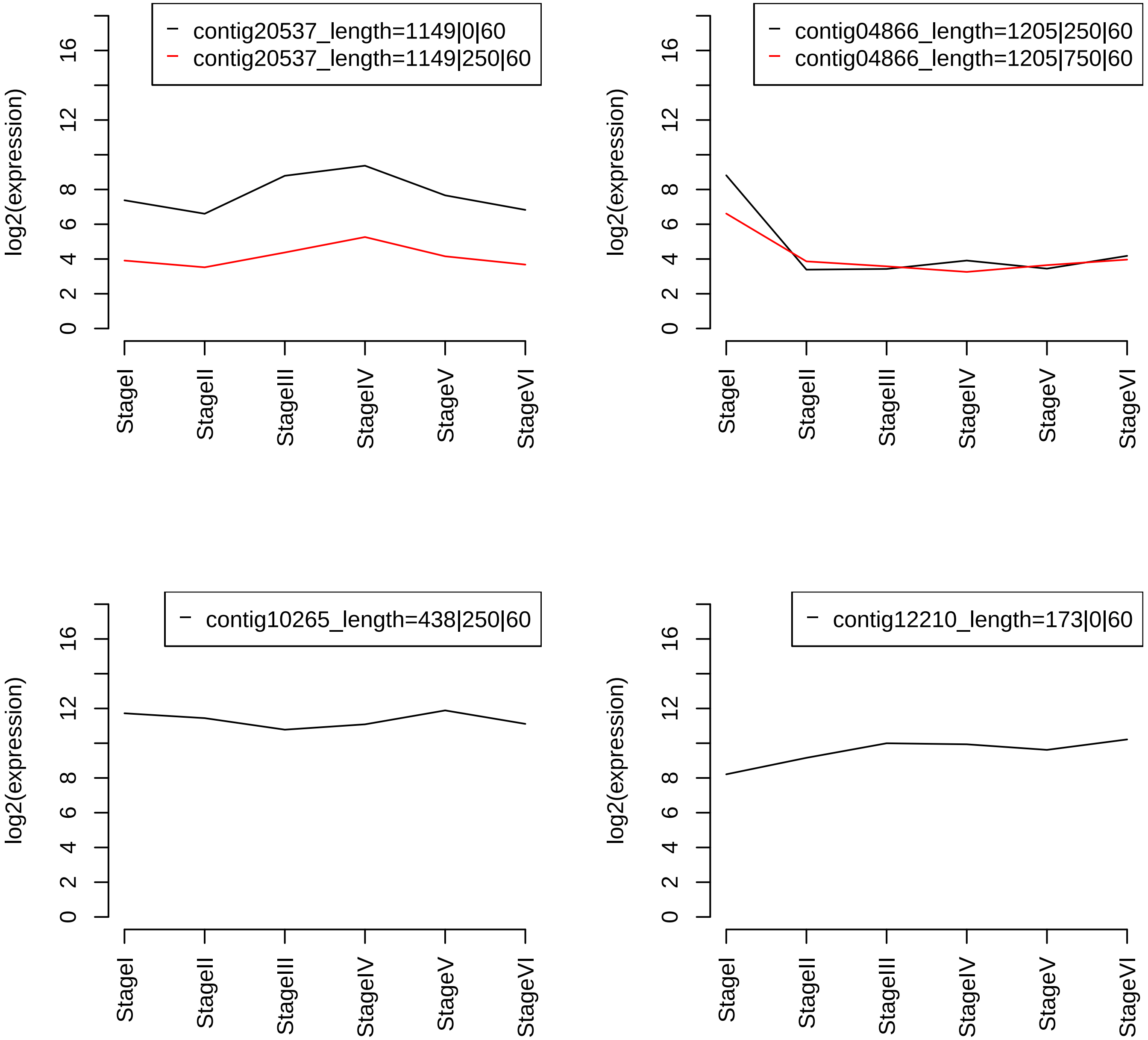
contig20537_length=1149|0|60
contig20537_length=1149|250|60
contig04866_length=1205|250|60
contig04866_length=1205|750|60
contig10265_length=438|250|60
contig12210_length=173|0|60
log2(expression)
StageI
StageII
StageIII
StageIV
StageV
StageVI

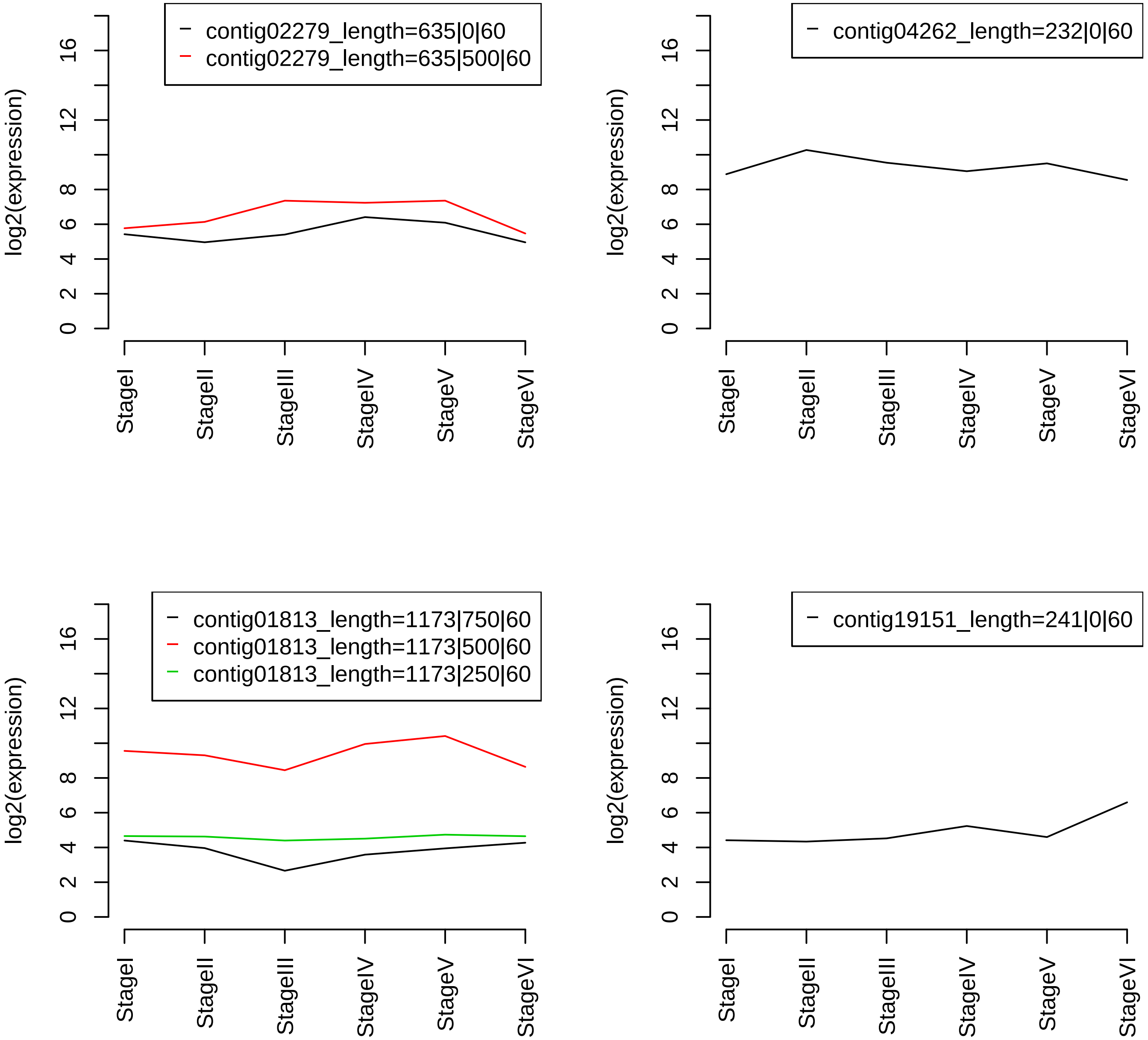
contig02279_length=635|0|60
contig02279_length=635|500|60
contig04262_length=232|0|60
contig01813_length=1173|750|60
contig01813_length=1173|500|60
contig01813_length=1173|250|60
contig19151_length=241|0|60
log2(expression)
0
2
4
6
8
12
16
StageI
StageII
StageIII
StageIV
StageV
StageVI

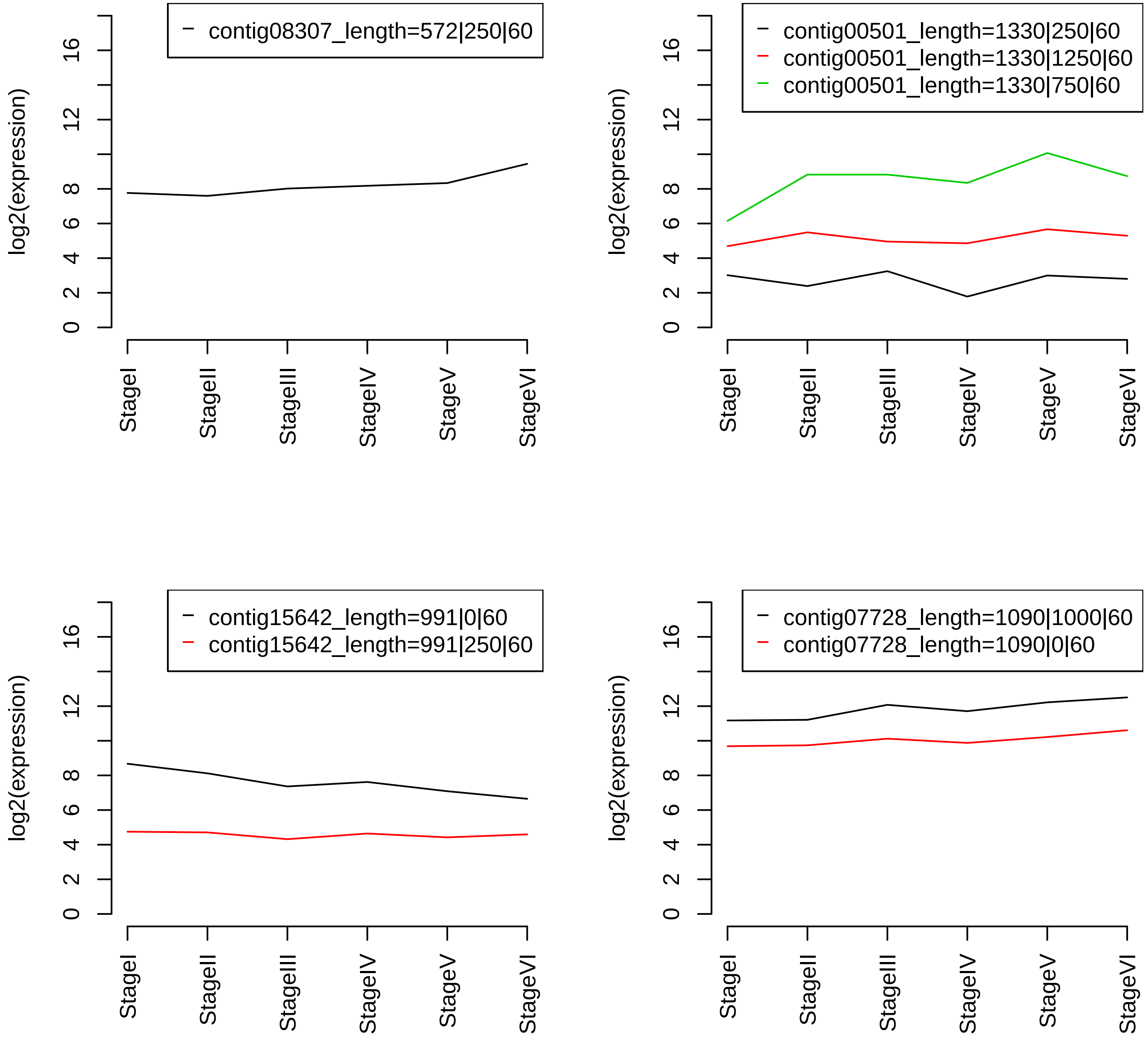
contig08307_length=572|250|60
contig00501_length=1330|250|60
contig00501_length=1330|1250|60
contig00501_length=1330|750|60
contig15642_length=991|0|60
contig15642_length=991|250|60
contig07728_length=1090|1000|60
contig07728_length=1090|0|60
log2(expression)
StageI
StageII
StageIII
StageIV
StageV
StageVI

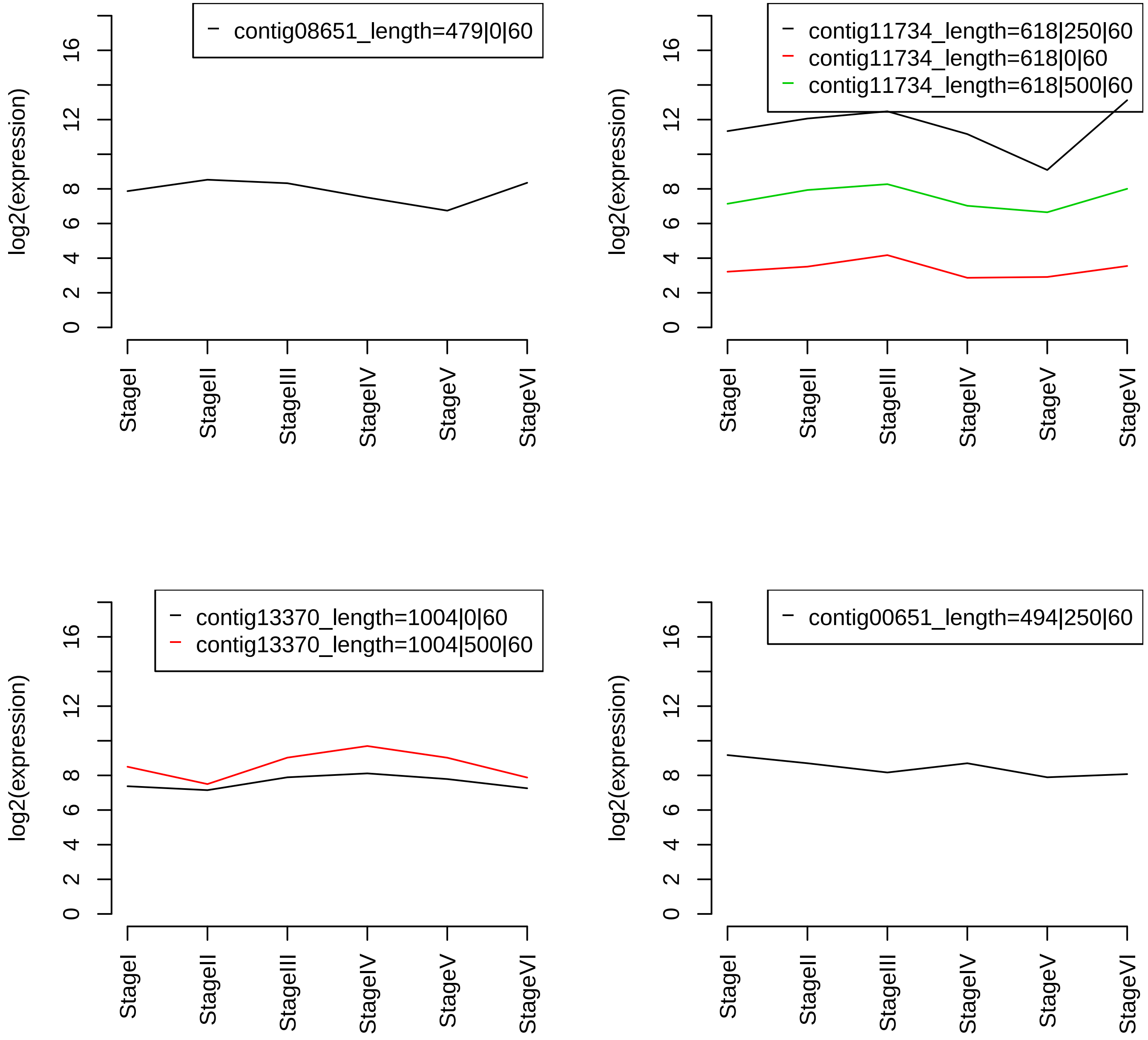

contig08651_length=479|0|60
contig11734_length=618|250|60
contig11734_length=618|0|60
contig11734_length=618|500|60
contig13370_length=1004|0|60
contig13370_length=1004|500|60
contig00651_length=494|250|60
log2(expression)
0
2
4
6
8
12
16
StageI
StageII
StageIII
StageIV
StageV
StageVI

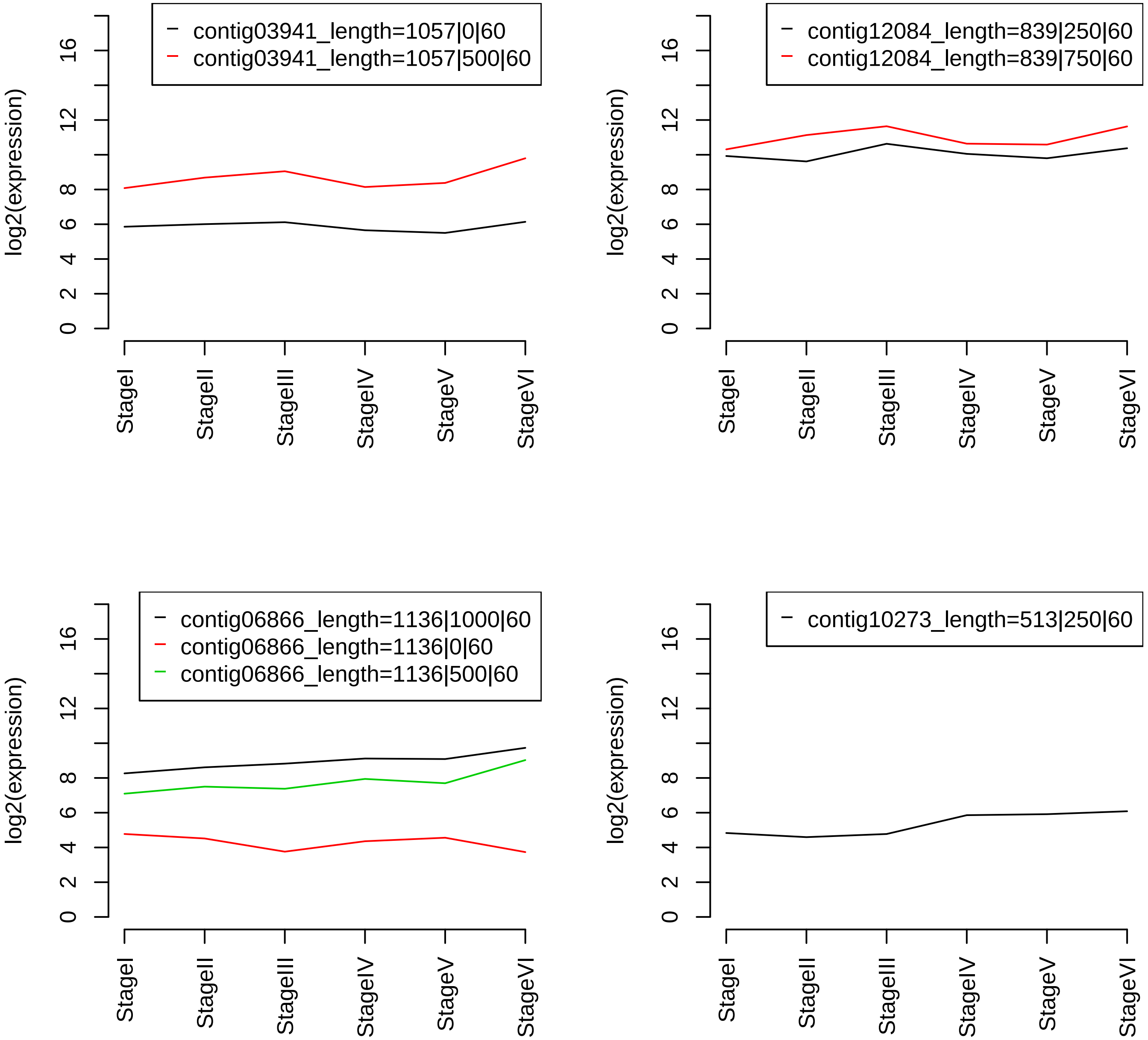
contig03941_length=1057|0|60
contig03941_length=1057|500|60
contig12084_length=839|250|60
contig12084_length=839|750|60
contig06866_length=1136|1000|60
contig06866_length=1136|0|60
contig06866_length=1136|500|60
contig10273_length=513|250|60
log2(expression)
0
2
4
6
8
12
16
StageI
StageII
StageIII
StageIV
StageV
StageVI

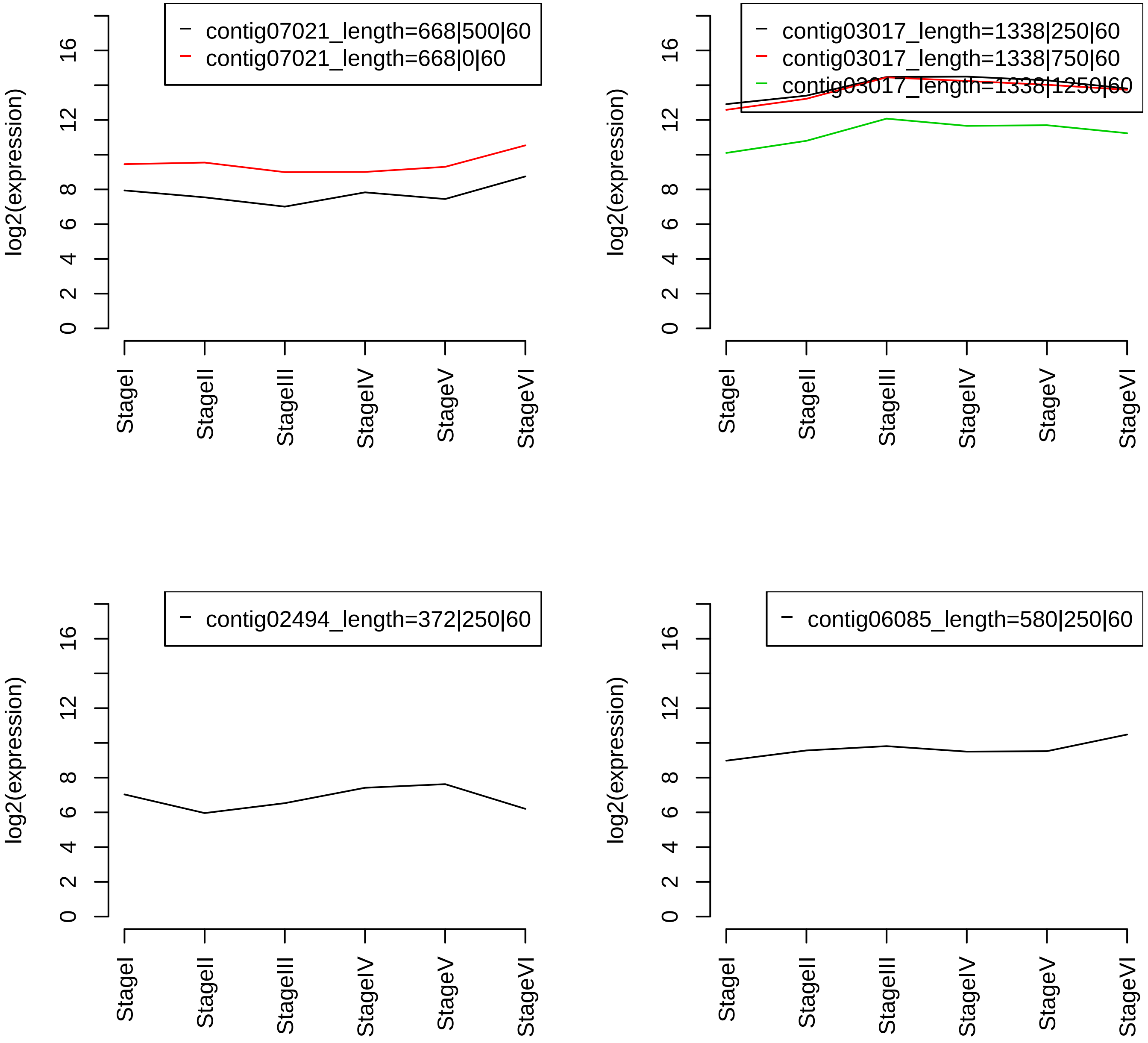
contig07021_length=668|500|60
contig07021_length=668|0|60
contig03017_length=1338|250|60
contig03017_length=1338|750|60
contig03017_length=1338|1250|60
contig02494_length=372|250|60
contig06085_length=580|250|60
log2(expression)
0
2
4
6
8
12
16
StageI
StageII
StageIII
StageIV
StageV
StageVI

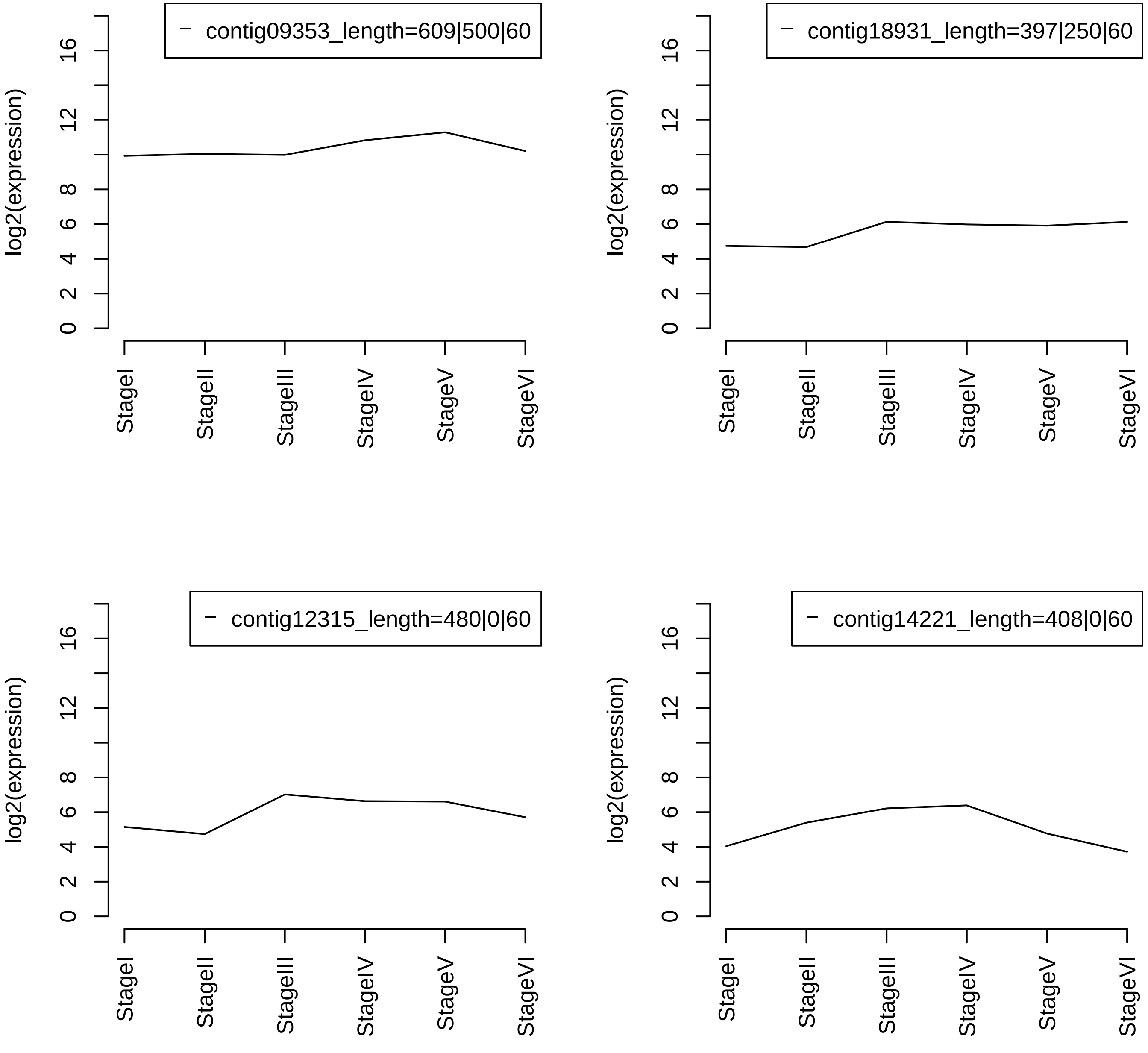
contig09353_length=609|500|60
contig18931_length=397|250|60
contig12315_length=480|0|60
contig14221_length=408|0|60
log2(expression)
0
2
4
6
8
12
16
StageI
StageII
StageIII
StageIV
StageV
StageVI

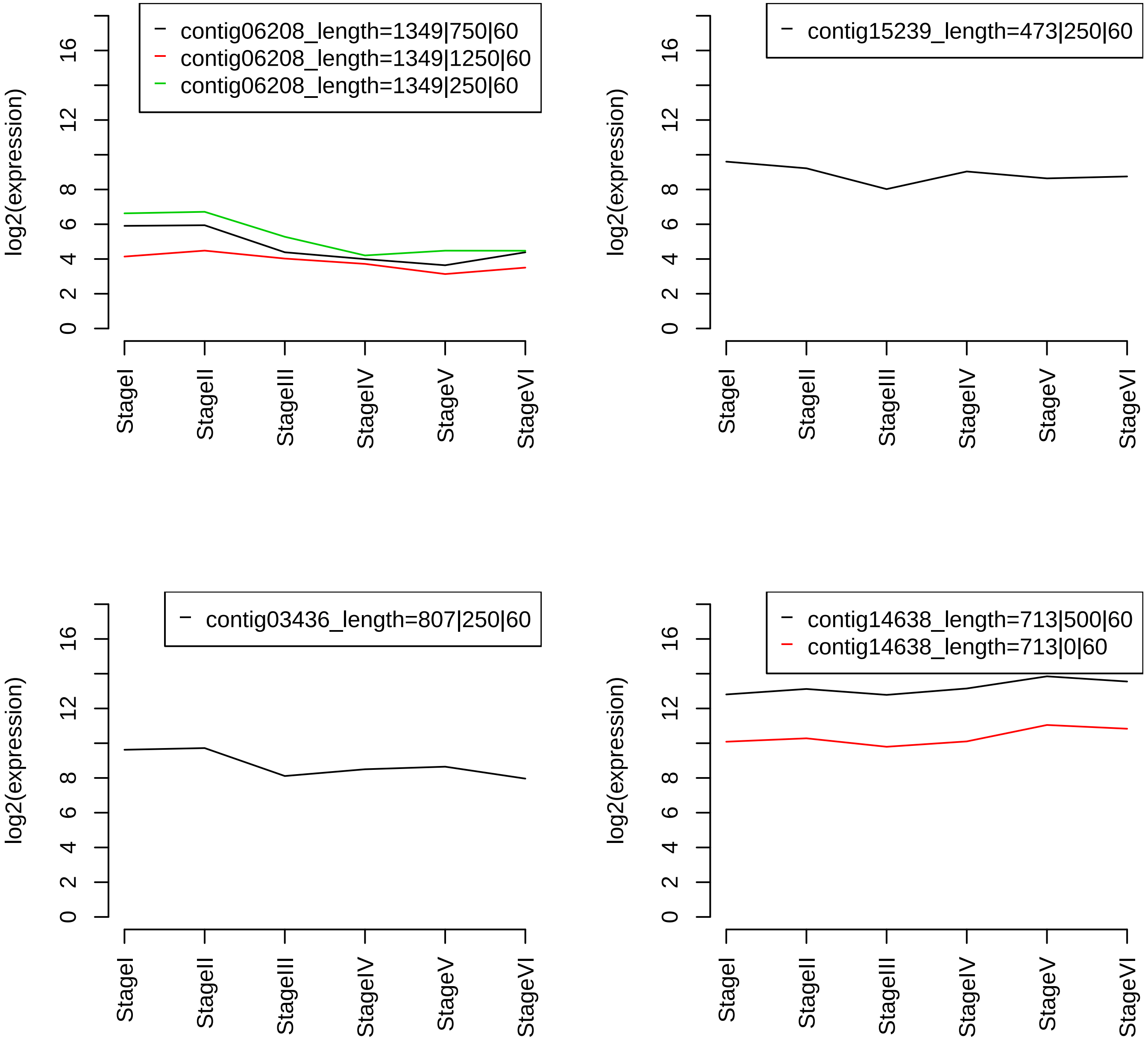

contig06208_length=1349|750|60
contig06208_length=1349|1250|60
contig06208_length=1349|250|60
contig15239_length=473|250|60
contig03436_length=807|250|60
contig14638_length=713|500|60
contig14638_length=713|0|60
log2(expression)
0
2
4
6
8
12
16
StageI
StageII
StageIII
StageIV
StageV
StageVI

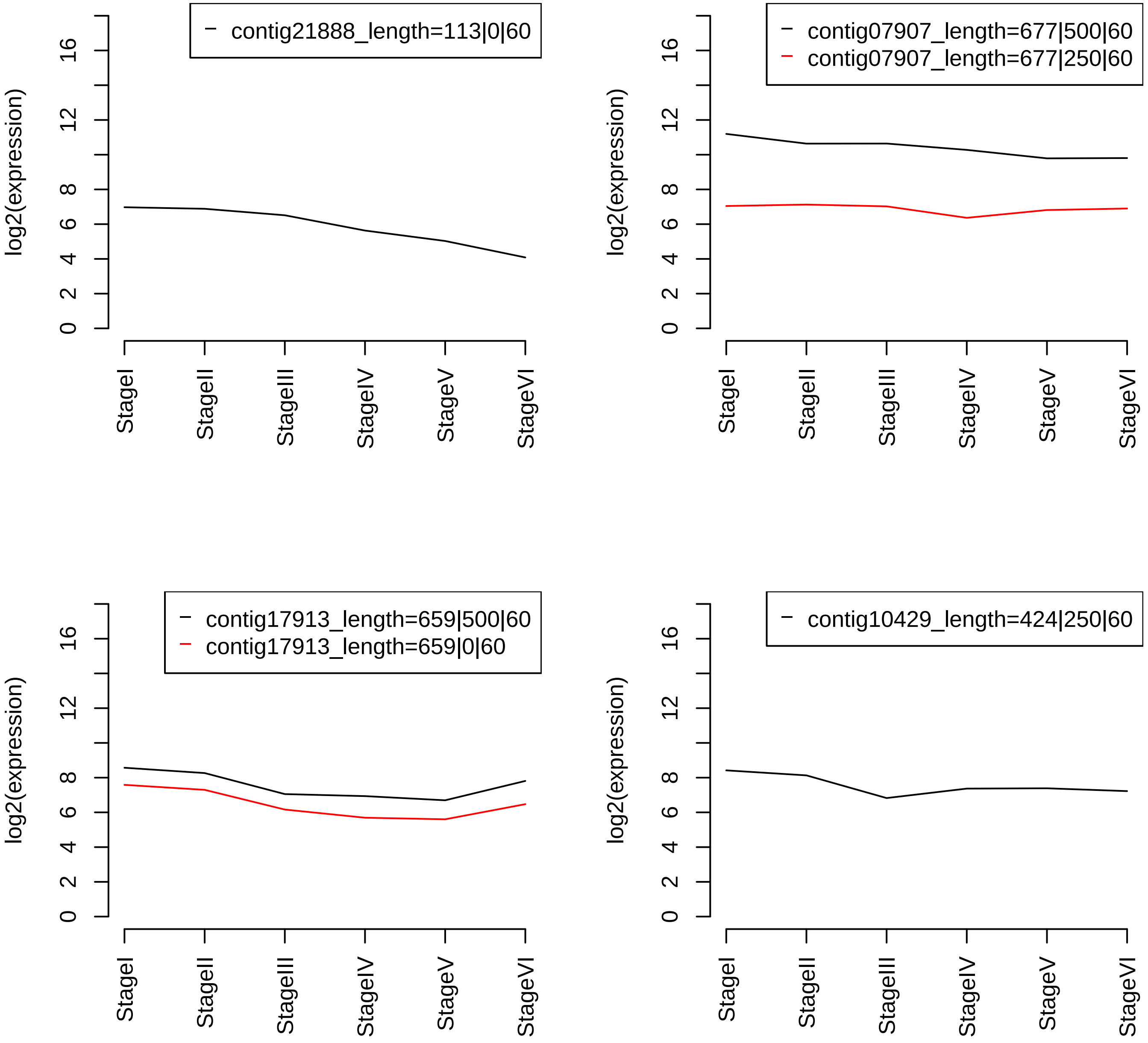

contig21888_length=113|0|60
contig07907_length=677|500|60
contig07907_length=677|250|60
contig17913_length=659|500|60
contig17913_length=659|0|60
contig10429_length=424|250|60
log2(expression)
0
2
4
6
8
12
16
StageI
StageII
StageIII
StageIV
StageV
StageVI

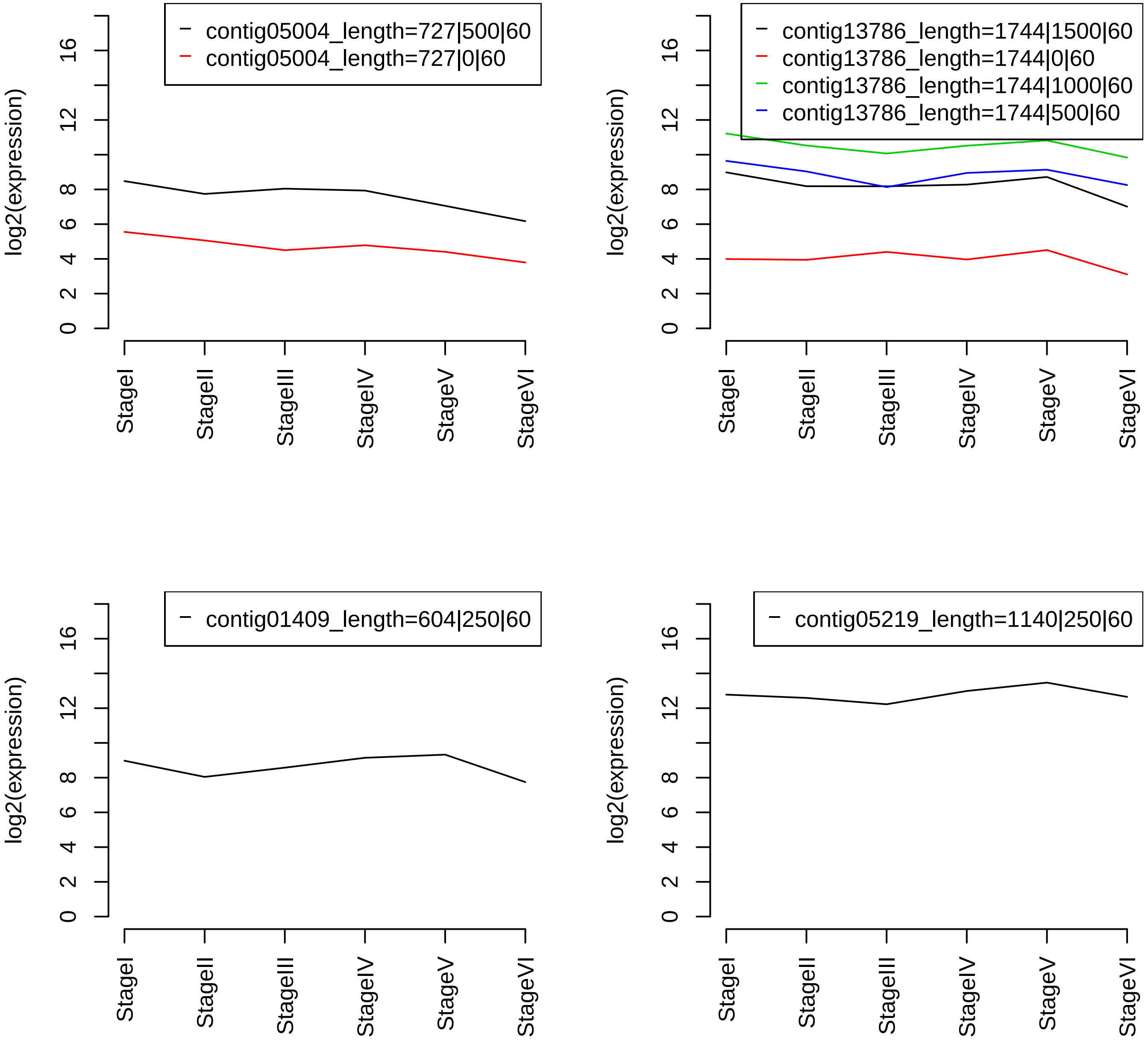
contig05004_length=727|500|60
contig05004_length=727|0|60
log2(expression)
0
2
4
6
8
12
16
StageI
StageII
StageIII
StageIV
StageV
StageVI
contig13786_length=1744|1500|60
contig13786_length=1744|0|60
contig13786_length=1744|1000|60
contig13786_length=1744|500|60
contig01409_length=604|250|60
contig05219_length=1140|250|60

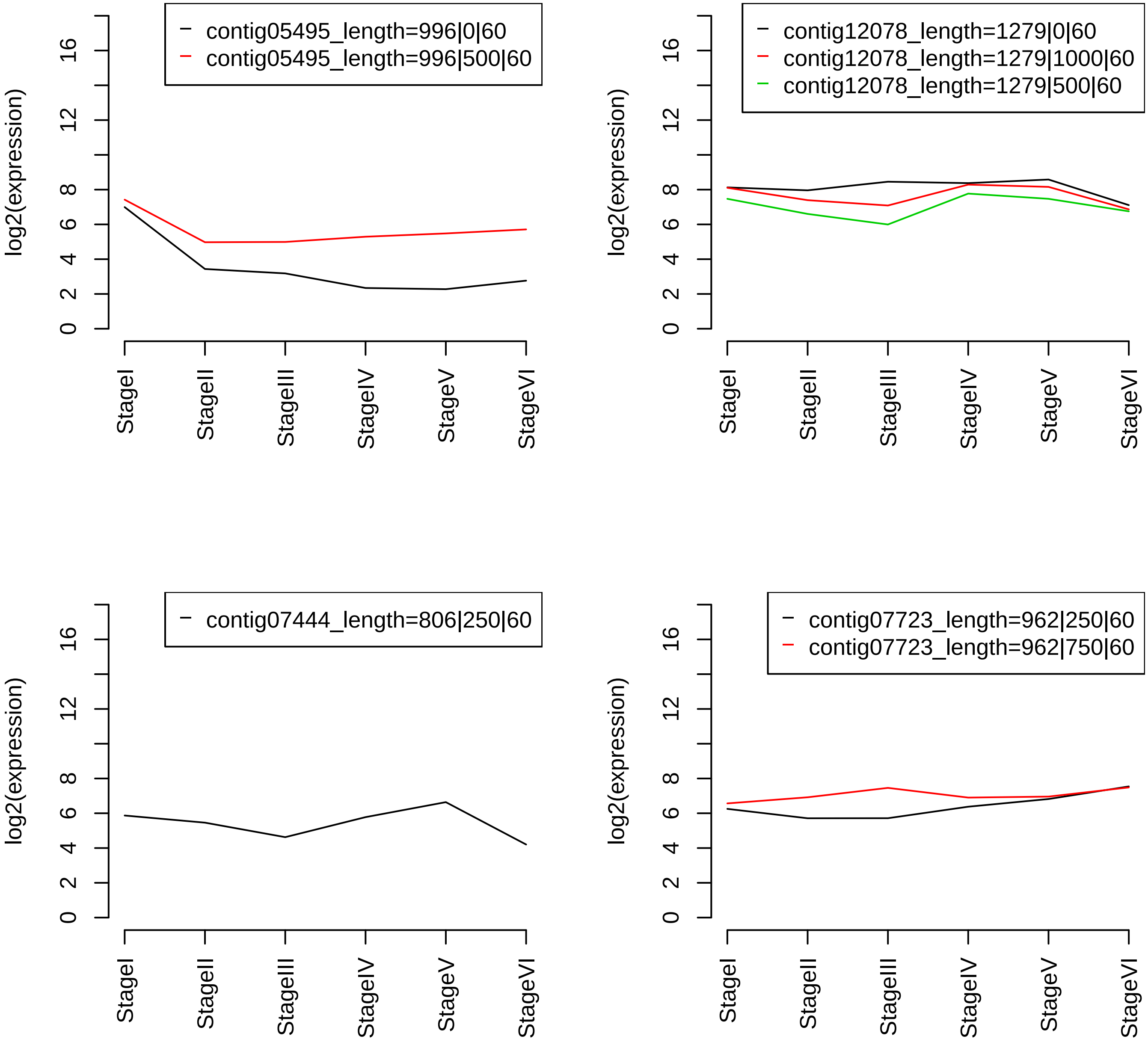
contig05495_length=996|0|60
contig05495_length=996|500|60
log2(expression)
0
2
4
6
8
12
16
StageI
StageII
StageIII
StageIV
StageV
StageVI
contig12078_length=1279|0|60
contig12078_length=1279|1000|60
contig12078_length=1279|500|60
log2(expression)
0
2
4
6
8
12
16
StageI
StageII
StageIII
StageIV
StageV
StageVI
contig07444_length=806|250|60
log2(expression)
0
2
4
6
8
12
16
StageI
StageII
StageIII
StageIV
StageV
StageVI
contig07723_length=962|250|60
contig07723_length=962|750|60
log2(expression)
0
2
4
6
8
12
16
StageI
StageII
StageIII
StageIV
StageV
StageVI

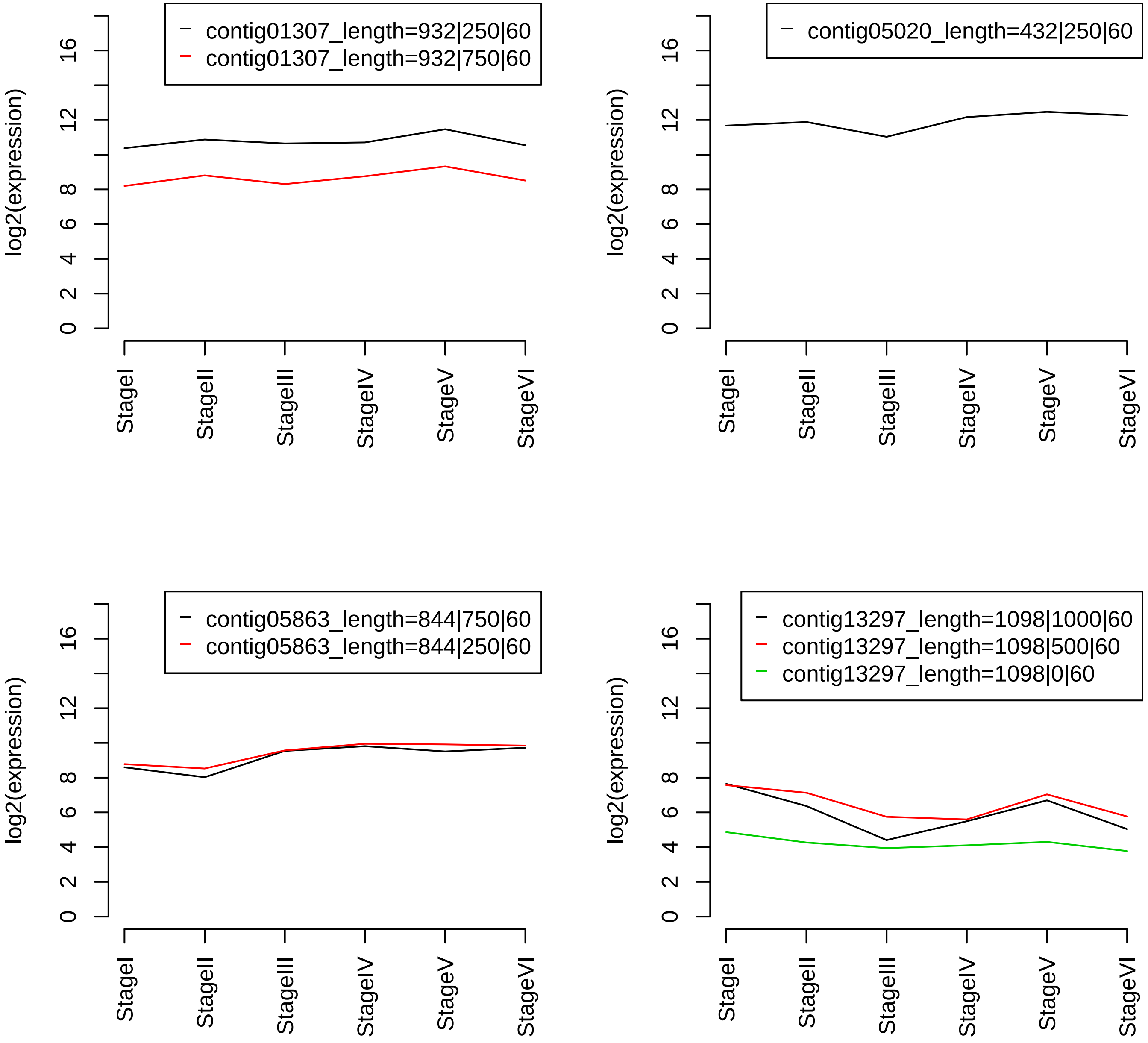
contig01307_length=932|250|60
contig01307_length=932|750|60
log2(expression)
0
2
4
6
8
12
16
StageI
StageII
StageIII
StageIV
StageV
StageVI
contig05020_length=432|250|60
log2(expression)
0
2
4
6
8
12
16
StageI
StageII
StageIII
StageIV
StageV
StageVI
contig05863_length=844|750|60
contig05863_length=844|250|60
log2(expression)
0
2
4
6
8
12
16
StageI
StageII
StageIII
StageIV
StageV
StageVI
contig13297_length=1098|1000|60
contig13297_length=1098|500|60
contig13297_length=1098|0|60
log2(expression)
0
2
4
6
8
12
16
StageI
StageII
StageIII
StageIV
StageV
StageVI

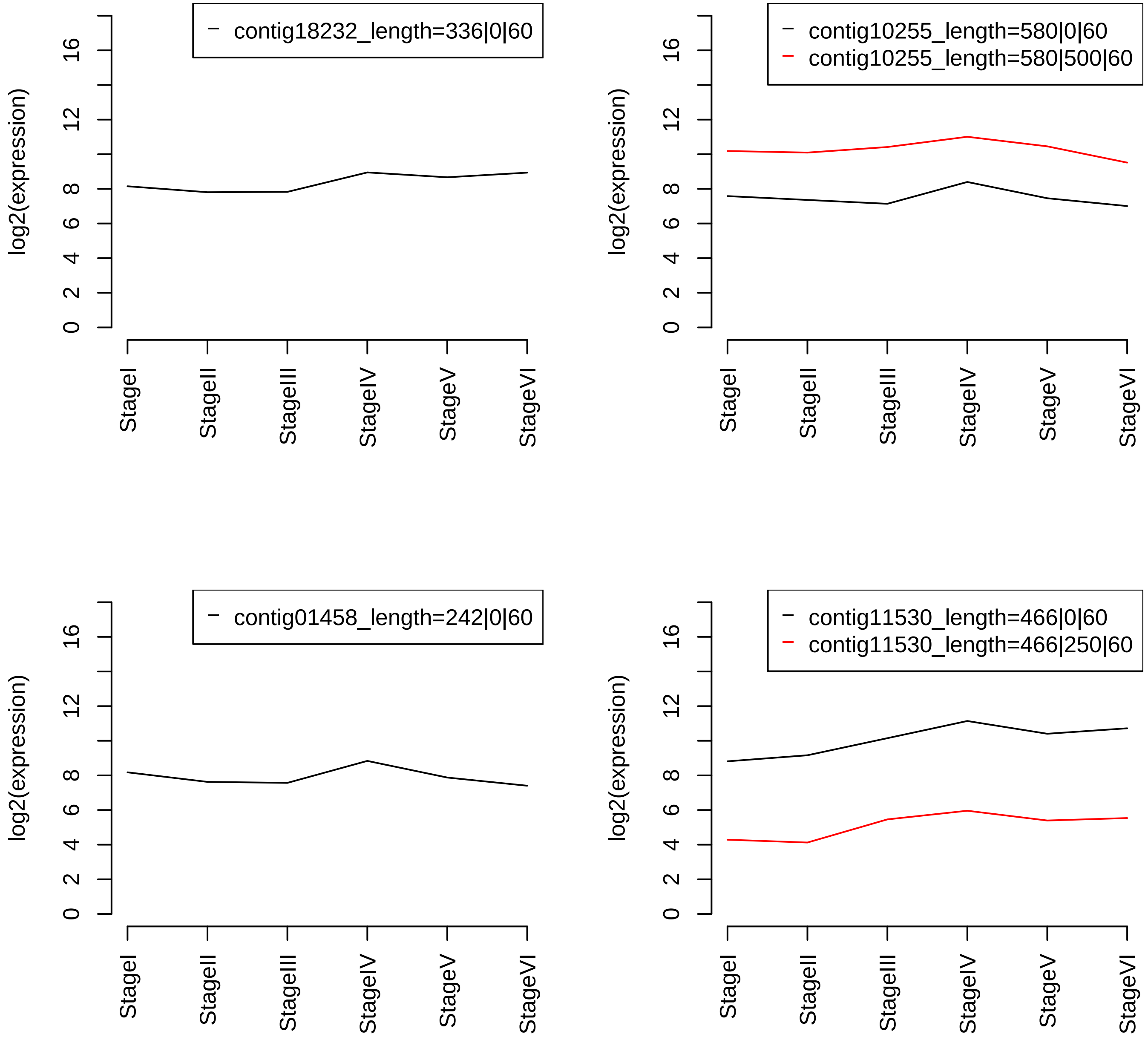
contig18232_length=336|0|60
contig10255_length=580|0|60
contig10255_length=580|500|60
contig01458_length=242|0|60
contig11530_length=466|0|60
contig11530_length=466|250|60
log2(expression)
0
2
4
6
8
12
16
StageI
StageII
StageIII
StageIV
StageV
StageVI

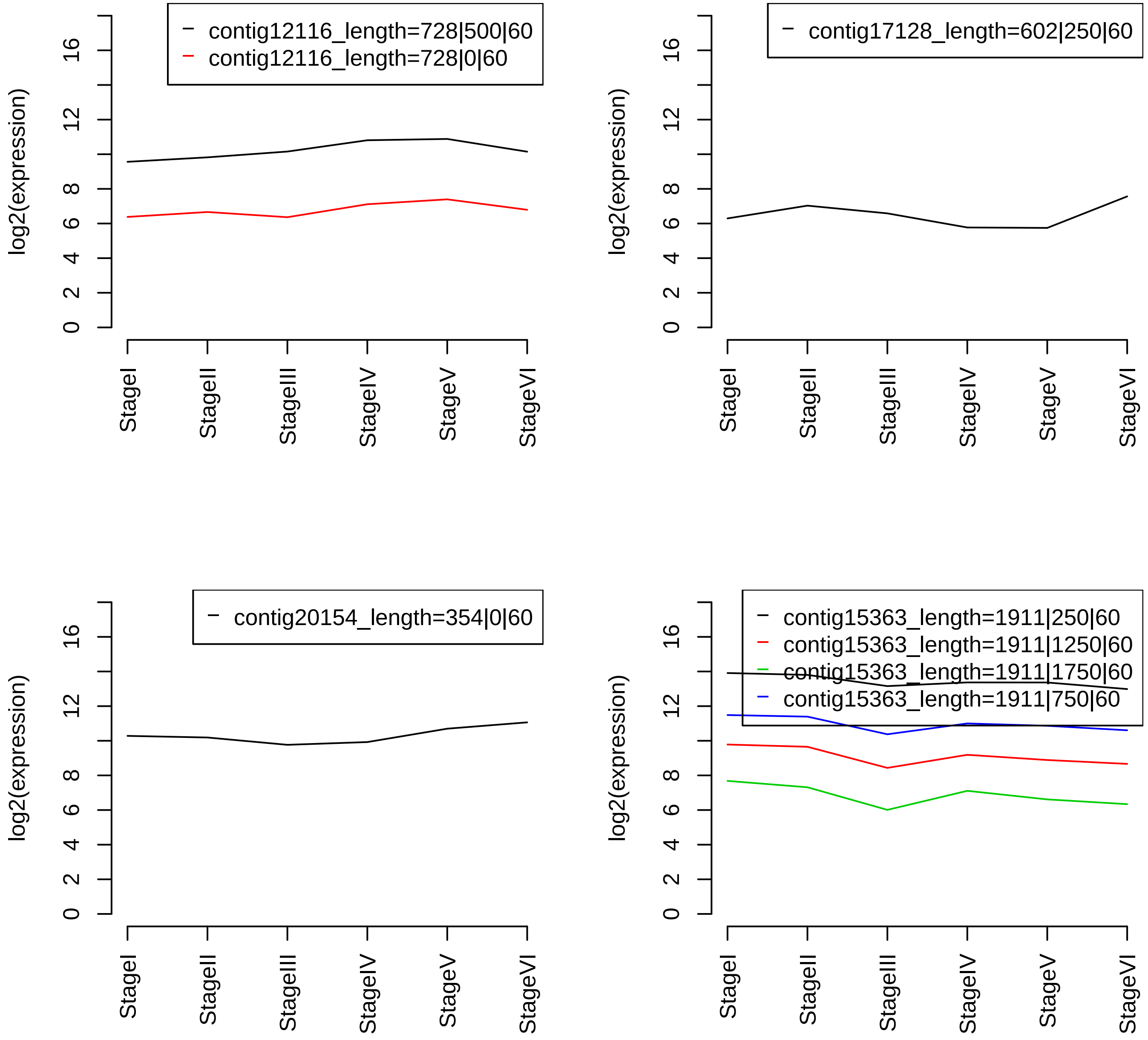
contig12116_length=728|500|60
contig12116_length=728|0|60
log2(expression)
0
2
4
6
8
12
16
StageI
StageII
StageIII
StageIV
StageV
StageVI
contig17128_length=602|250|60
log2(expression)
contig20154_length=354|0|60
log2(expression)
contig15363_length=1911|250|60
contig15363_length=1911|1250|60
contig15363_length=1911|1750|60
contig15363_length=1911|750|60
log2(expression)

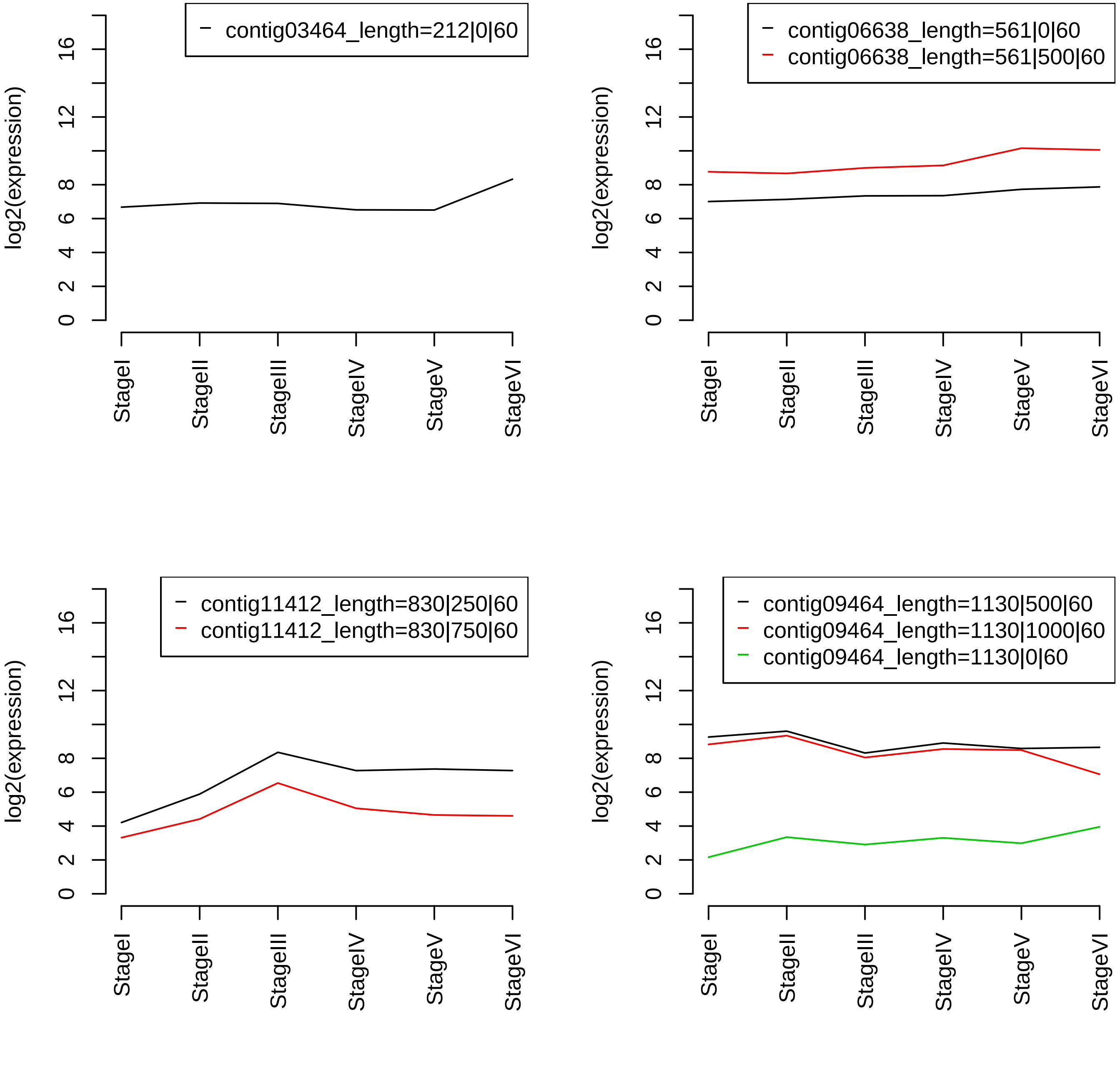

contig03464_length=212|0|60
contig06638_length=561|0|60
contig06638_length=561|500|60
contig11412_length=830|250|60
contig11412_length=830|750|60
contig09464_length=1130|500|60
contig09464_length=1130|1000|60
contig09464_length=1130|0|60
log2(expression)
0 2 4 6 8 12 16
StageI StageII StageIII StageIV StageV StageVI

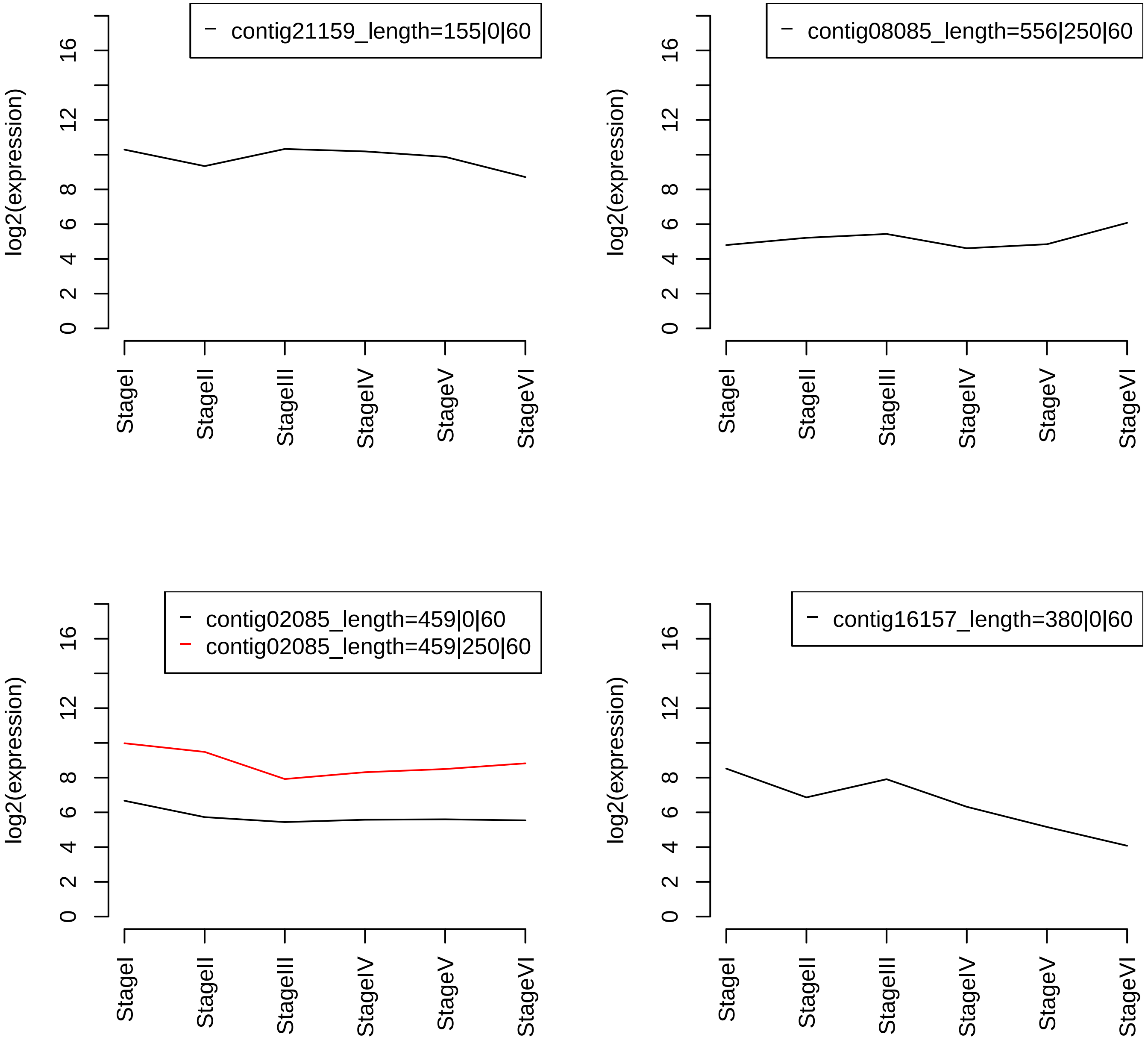

contig21159_length=155|0|60
contig08085_length=556|250|60
contig02085_length=459|0|60
contig02085_length=459|250|60
contig16157_length=380|0|60
log2(expression)
0
2
4
6
8
12
16
StageI
StageII
StageIII
StageIV
StageV
StageVI

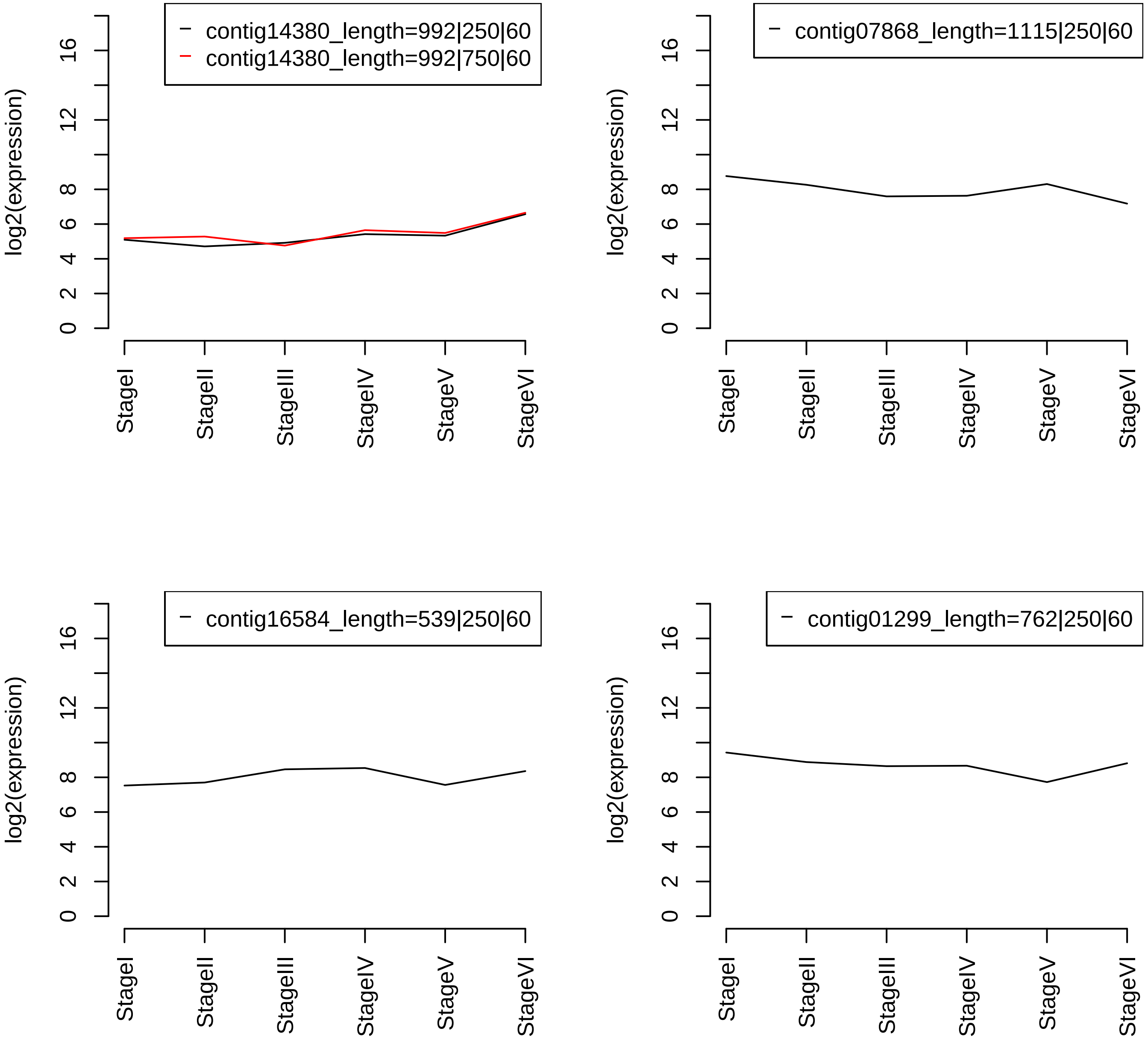

contig14380_length=992|250|60
contig14380_length=992|750|60
contig07868_length=1115|250|60
contig16584_length=539|250|60
contig01299_length=762|250|60
log2(expression)
0
2
4
6
8
12
16
StageI
StageII
StageIII
StageIV
StageV
StageVI

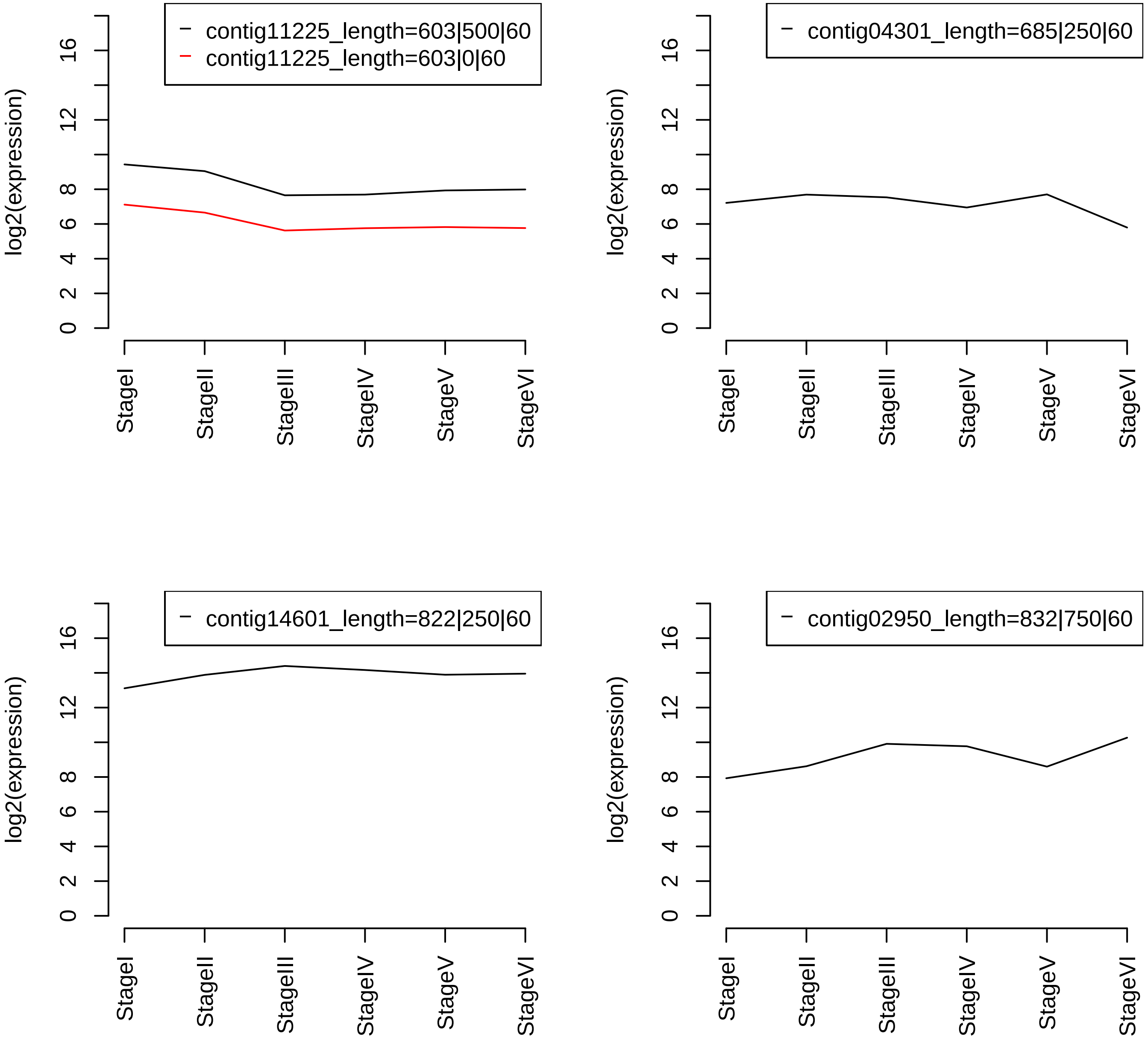

contig11225_length=603|500|60
contig11225_length=603|0|60
contig04301_length=685|250|60
contig14601_length=822|250|60
contig02950_length=832|750|60
log2(expression)
0
2
4
6
8
12
16
StageI
StageII
StageIII
StageIV
StageV
StageVI

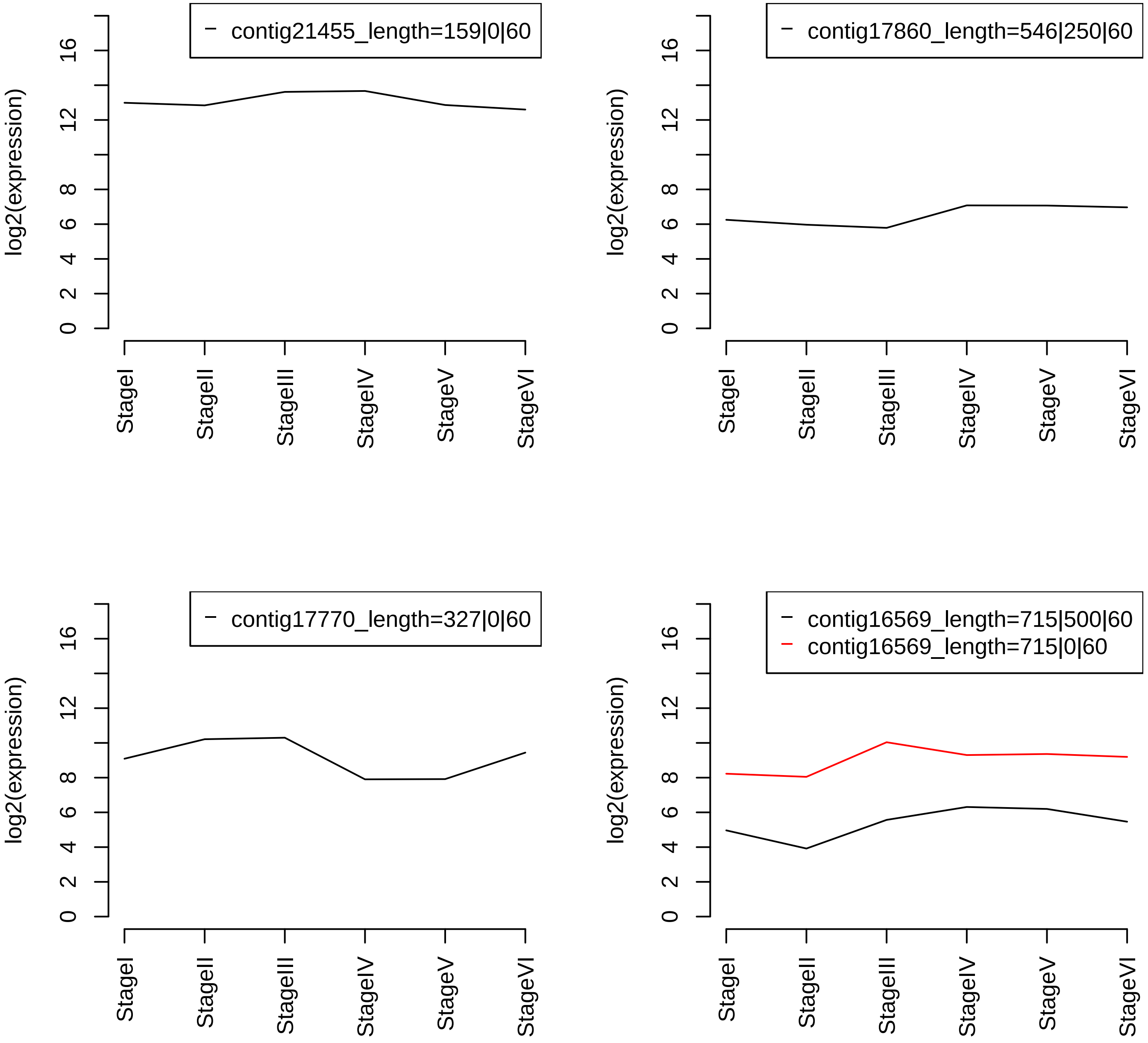
contig21455_length=159|0|60
contig17860_length=546|250|60
contig17770_length=327|0|60
contig16569_length=715|500|60
contig16569_length=715|0|60
log2(expression)
0
2
4
6
8
12
16
StageI
StageII
StageIII
StageIV
StageV
StageVI

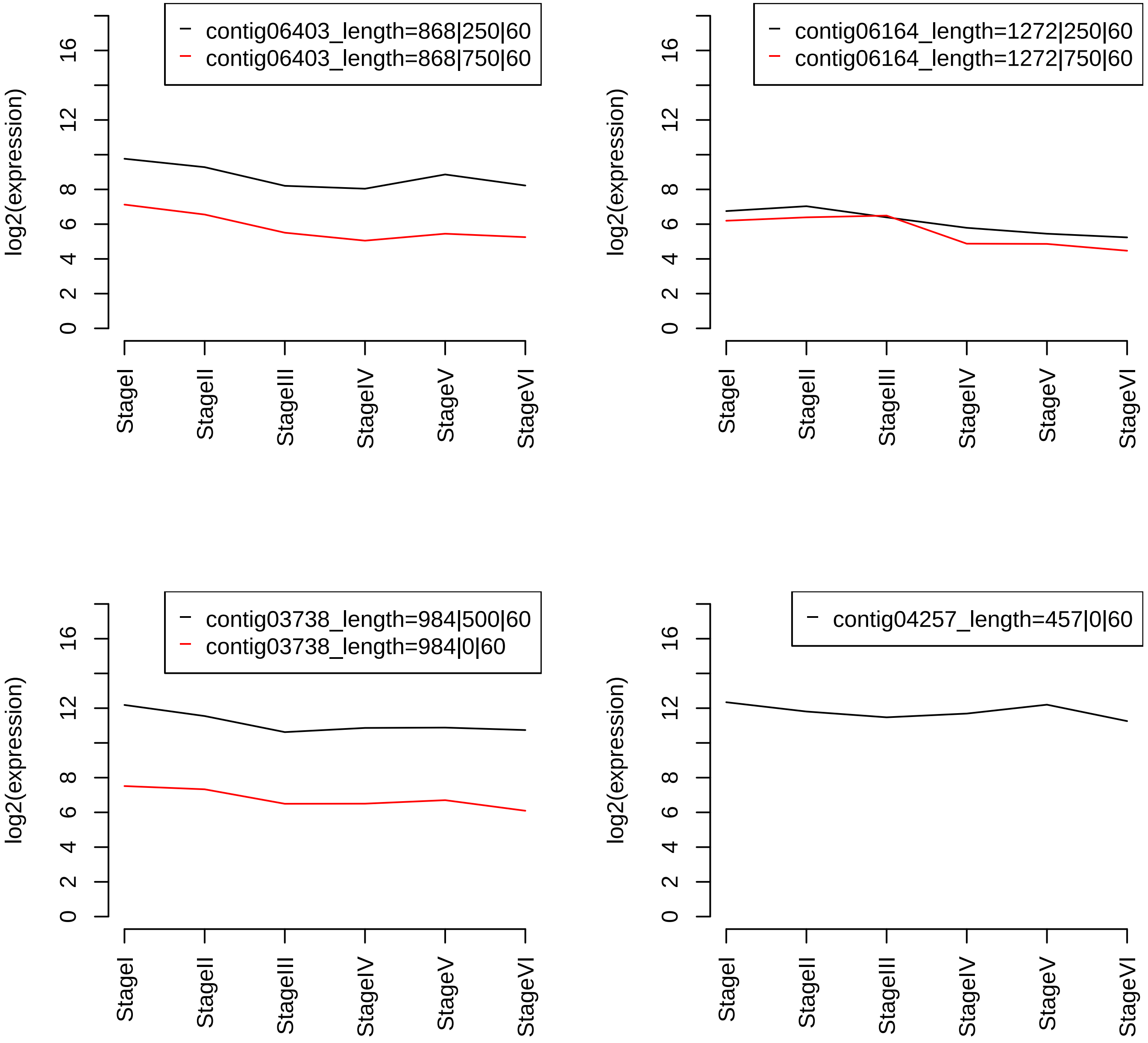
contig06403_length=868|250|60
contig06403_length=868|750|60
contig06164_length=1272|250|60
contig06164_length=1272|750|60
contig03738_length=984|500|60
contig03738_length=984|0|60
contig04257_length=457|0|60
log2(expression)
0
2
4
6
8
12
16
StageI
StageII
StageIII
StageIV
StageV
StageVI

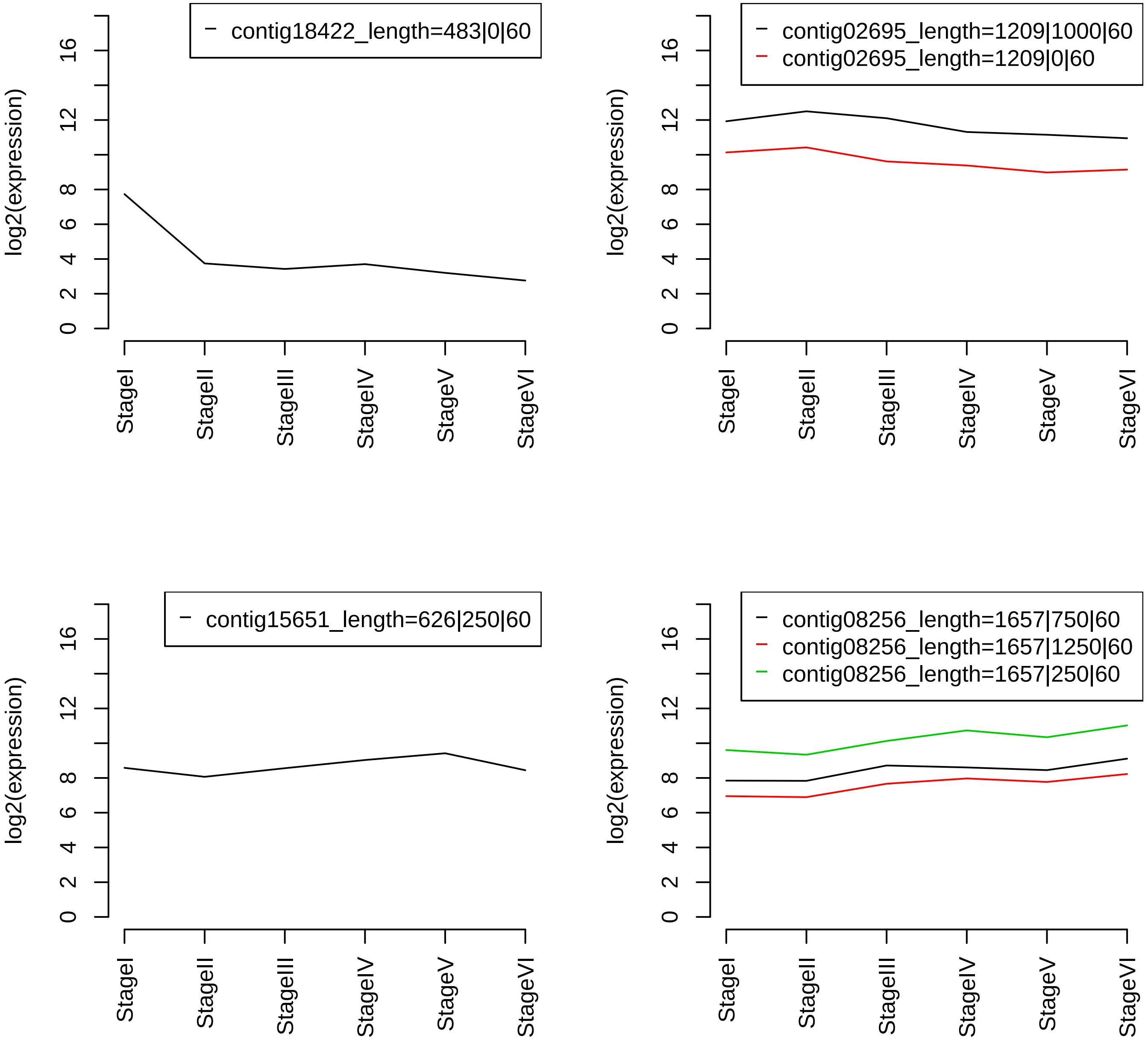
contig18422_length=483|0|60
contig02695_length=1209|1000|60
contig02695_length=1209|0|60
contig15651_length=626|250|60
contig08256_length=1657|750|60
contig08256_length=1657|1250|60
contig08256_length=1657|250|60
log2(expression)
StageI
StageII
StageIII
StageIV
StageV
StageVI

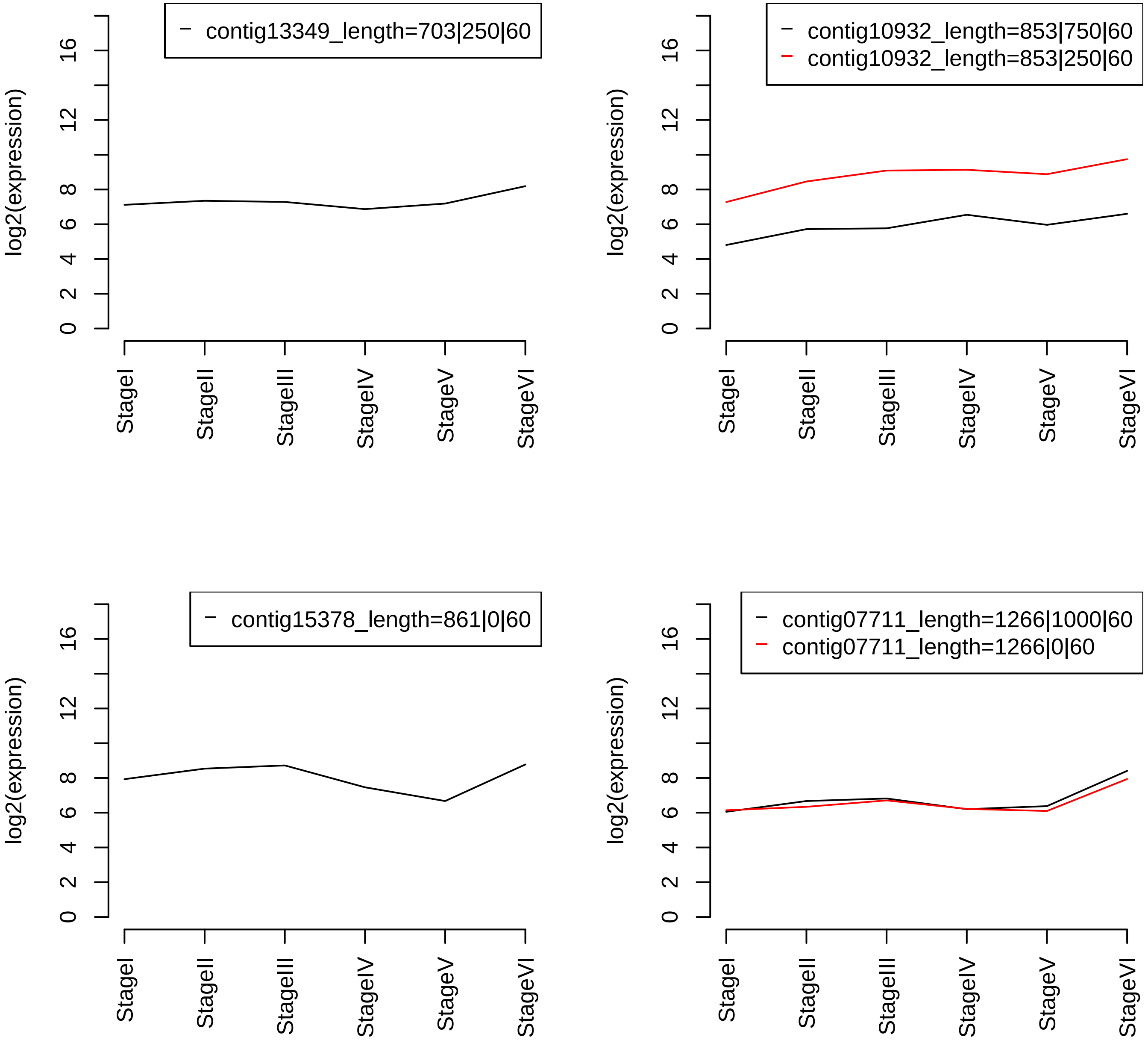
contig13349_length=703|250|60
contig10932_length=853|750|60
contig10932_length=853|250|60
contig15378_length=861|0|60
contig07711_length=1266|1000|60
contig07711_length=1266|0|60
log2(expression)
0
2
4
6
8
12
16
StageI
StageII
StageIII
StageIV
StageV
StageVI

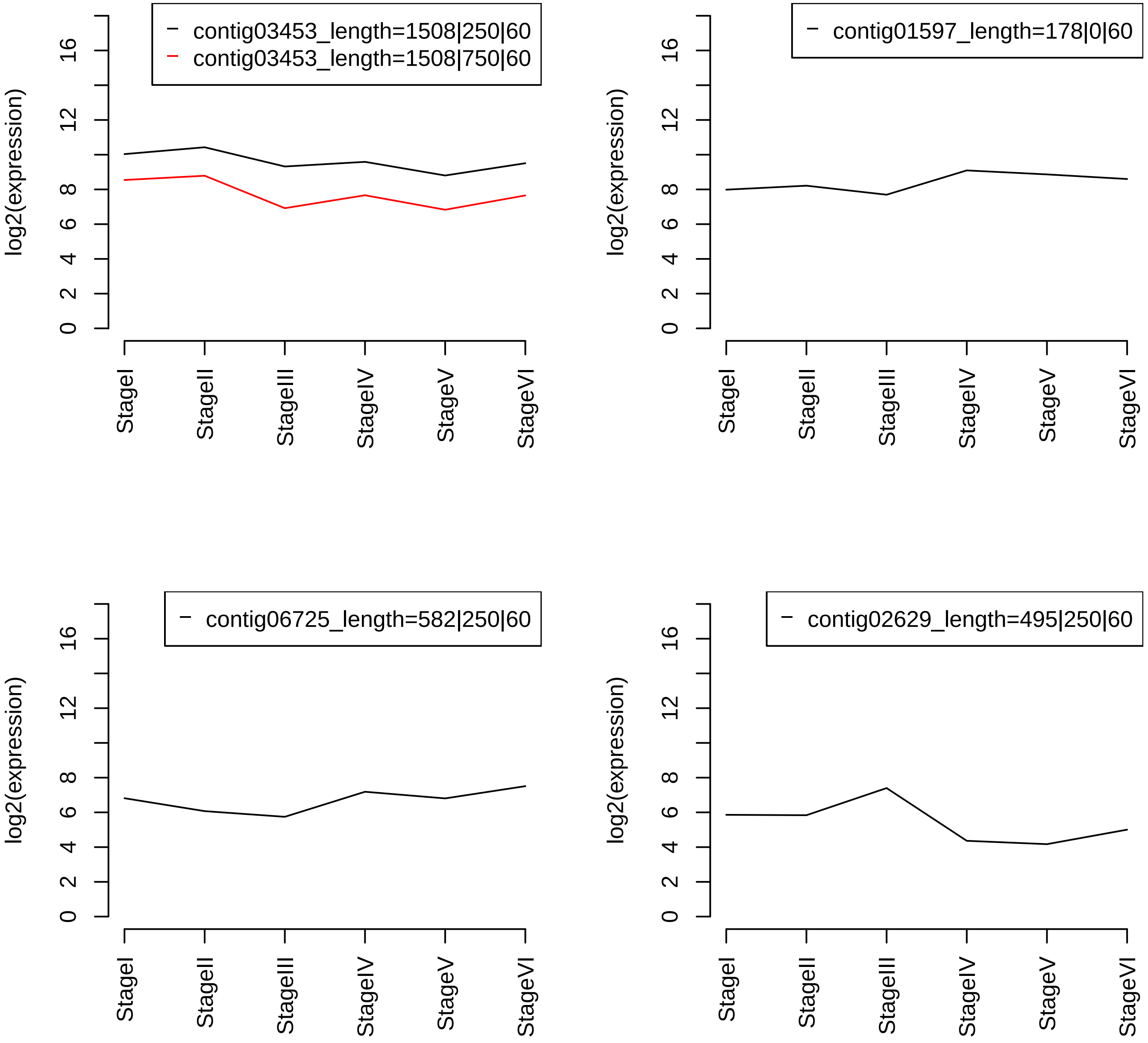

contig03453_length=1508|250|60
contig03453_length=1508|750|60
contig01597_length=178|0|60
contig06725_length=582|250|60
contig02629_length=495|250|60
log2(expression)
0
2
4
6
8
12
16
StageI
StageII
StageIII
StageIV
StageV
StageVI

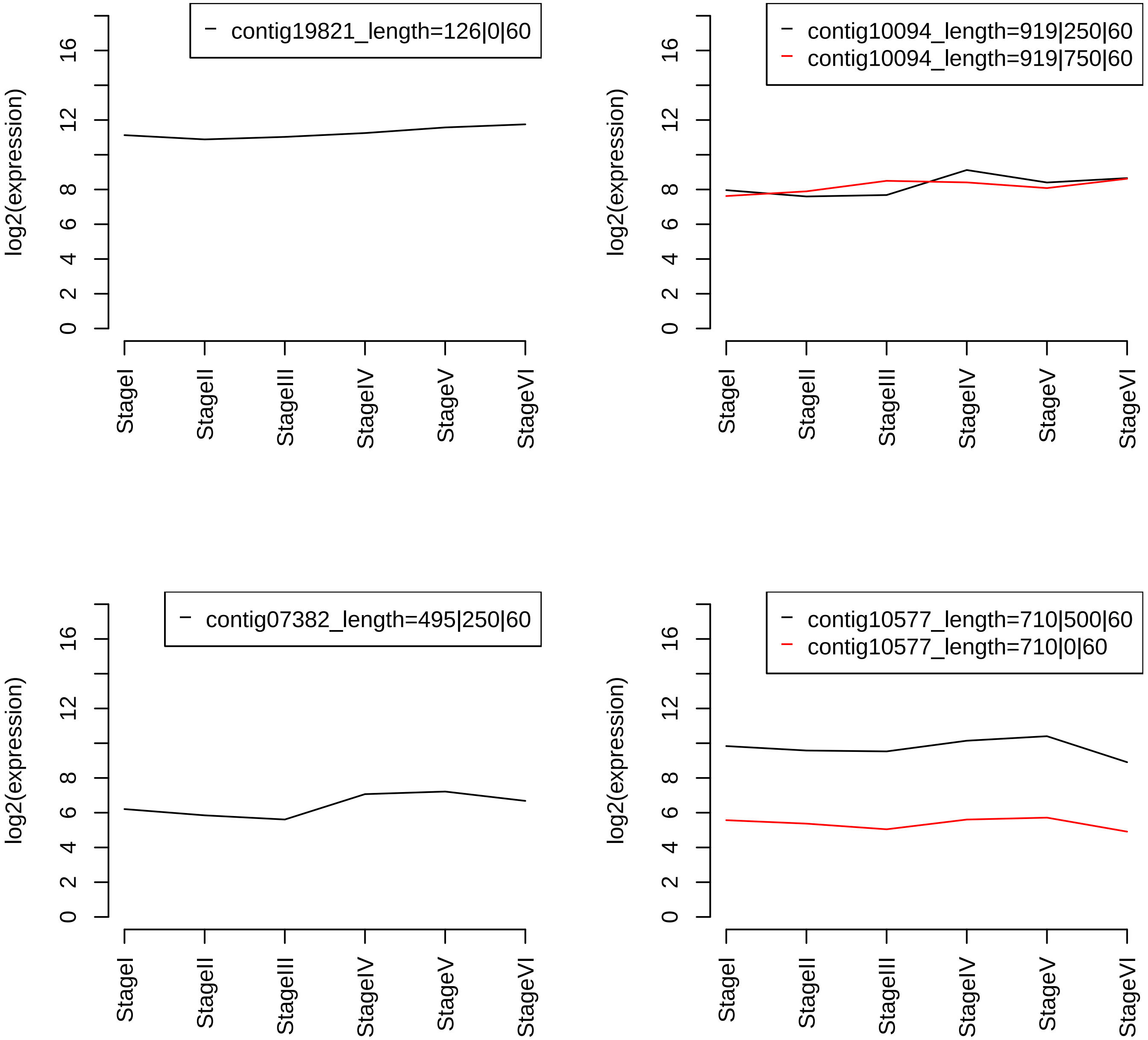
contig19821_length=126|0|60
contig10094_length=919|250|60
contig10094_length=919|750|60
contig07382_length=495|250|60
contig10577_length=710|500|60
contig10577_length=710|0|60
log2(expression)
0
2
4
6
8
12
16
StageI
StageII
StageIII
StageIV
StageV
StageVI

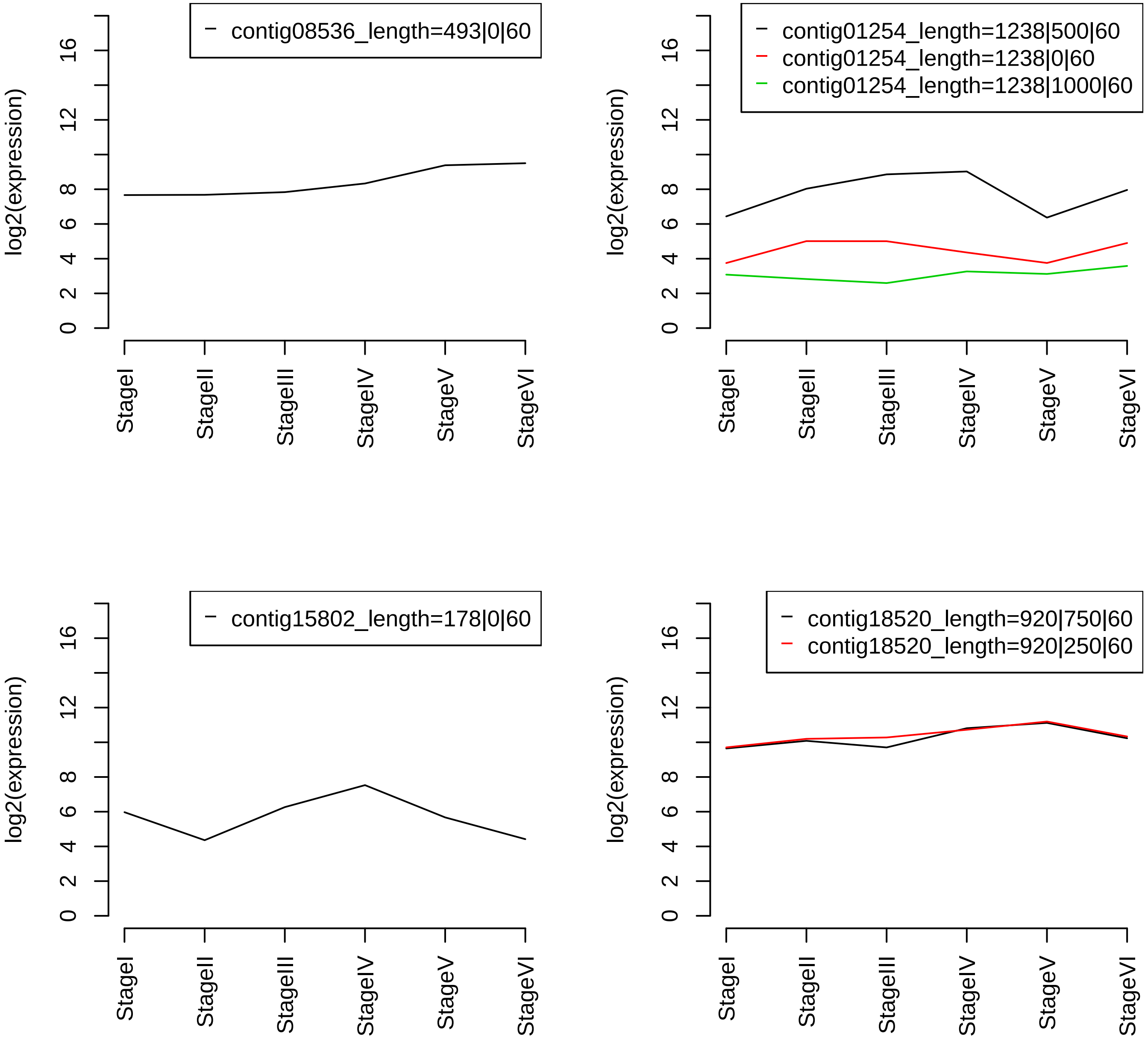

contig08536_length=493|0|60
contig01254_length=1238|500|60
contig01254_length=1238|0|60
contig01254_length=1238|1000|60
contig15802_length=178|0|60
contig18520_length=920|750|60
contig18520_length=920|250|60
log2(expression)
0
2
4
6
8
12
16
StageI
StageII
StageIII
StageIV
StageV
StageVI

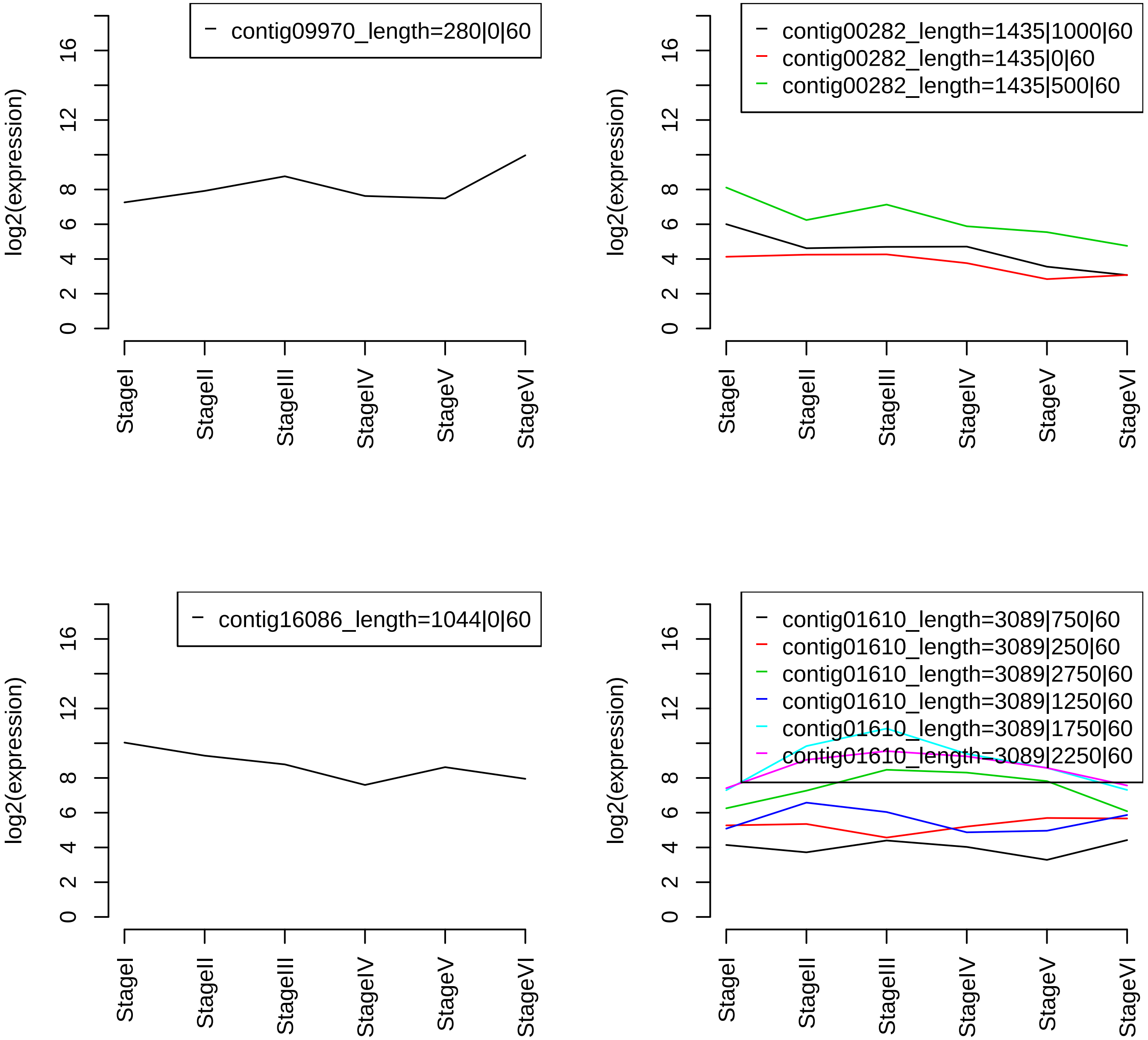

contig09970_length=280|0|60
log2(expression)
0
2
4
6
8
12
16
StageI
StageII
StageIII
StageIV
StageV
StageVI
contig00282_length=1435|1000|60
contig00282_length=1435|0|60
contig00282_length=1435|500|60
contig16086_length=1044|0|60
contig01610_length=3089|750|60
contig01610_length=3089|250|60
contig01610_length=3089|2750|60
contig01610_length=3089|1250|60
contig01610_length=3089|1750|60
contig01610_length=3089|2250|60

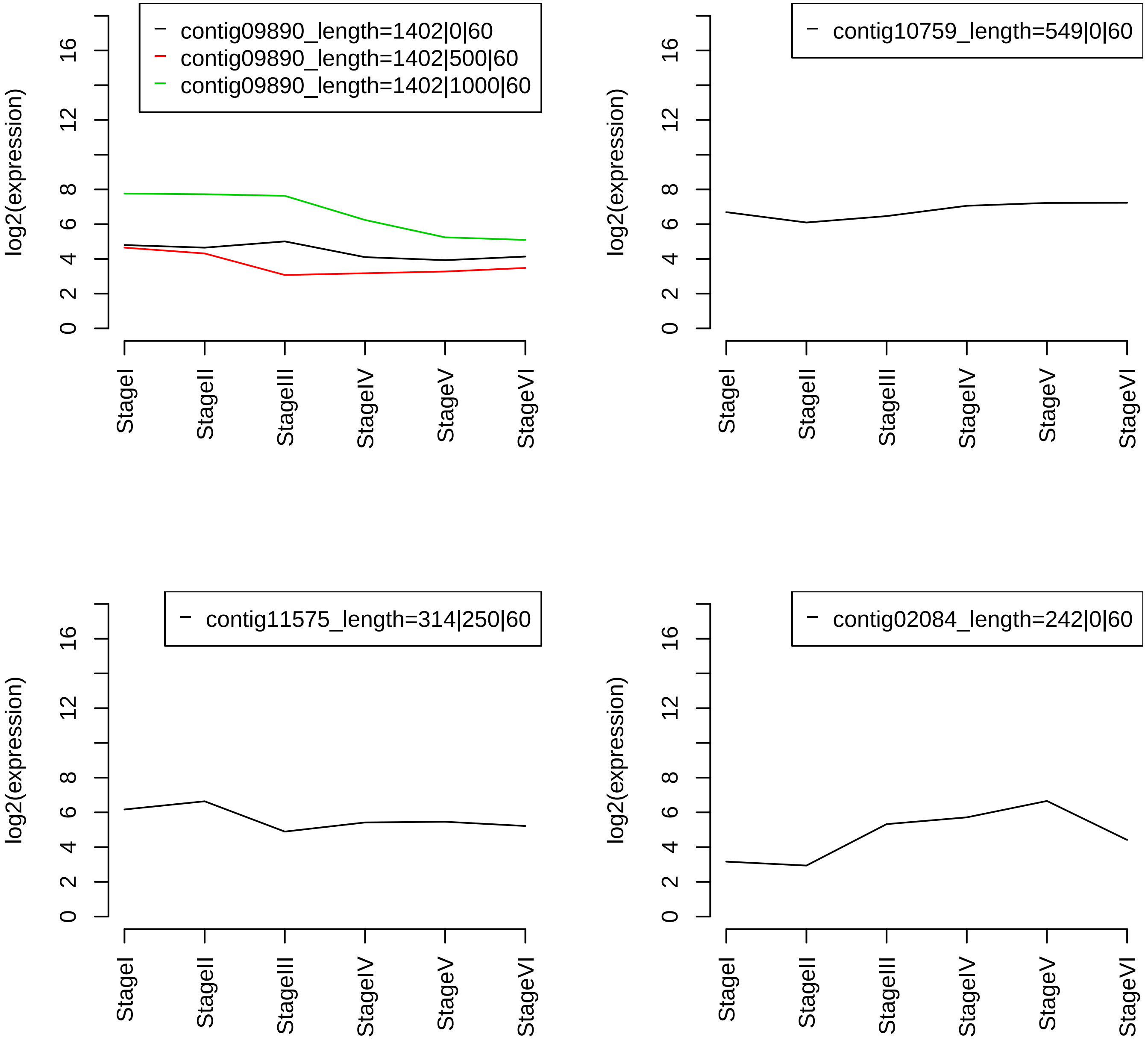

contig09890_length=1402|0|60
contig09890_length=1402|500|60
contig09890_length=1402|1000|60
contig10759_length=549|0|60
contig11575_length=314|250|60
contig02084_length=242|0|60
log2(expression)
0
2
4
6
8
12
16
StageI
StageII
StageIII
StageIV
StageV
StageVI

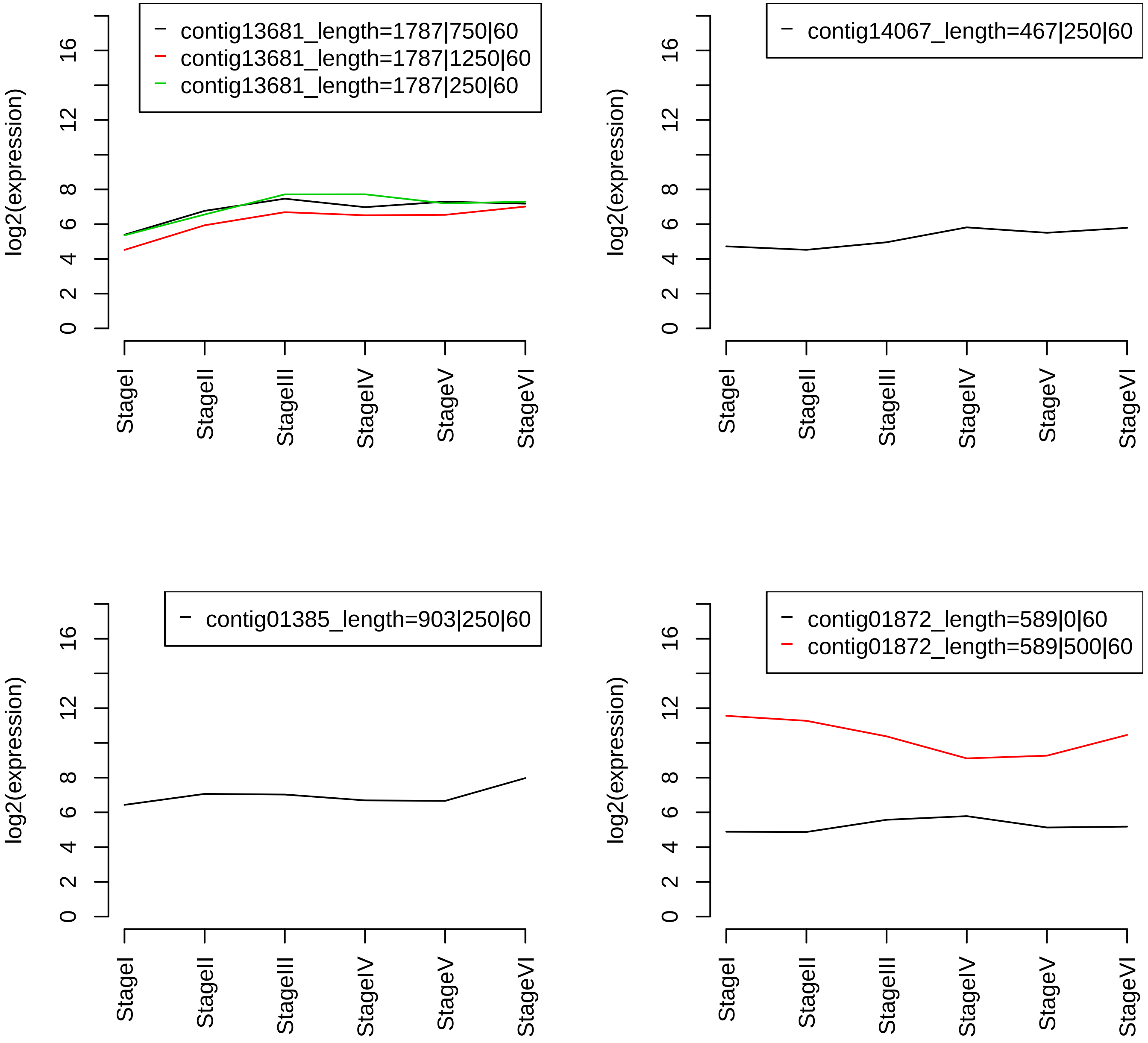
contig13681_length=1787|750|60
contig13681_length=1787|1250|60
contig13681_length=1787|250|60
contig14067_length=467|250|60
contig01385_length=903|250|60
contig01872_length=589|0|60
contig01872_length=589|500|60
log2(expression)
0
2
4
6
8
12
16
StageI
StageII
StageIII
StageIV
StageV
StageVI

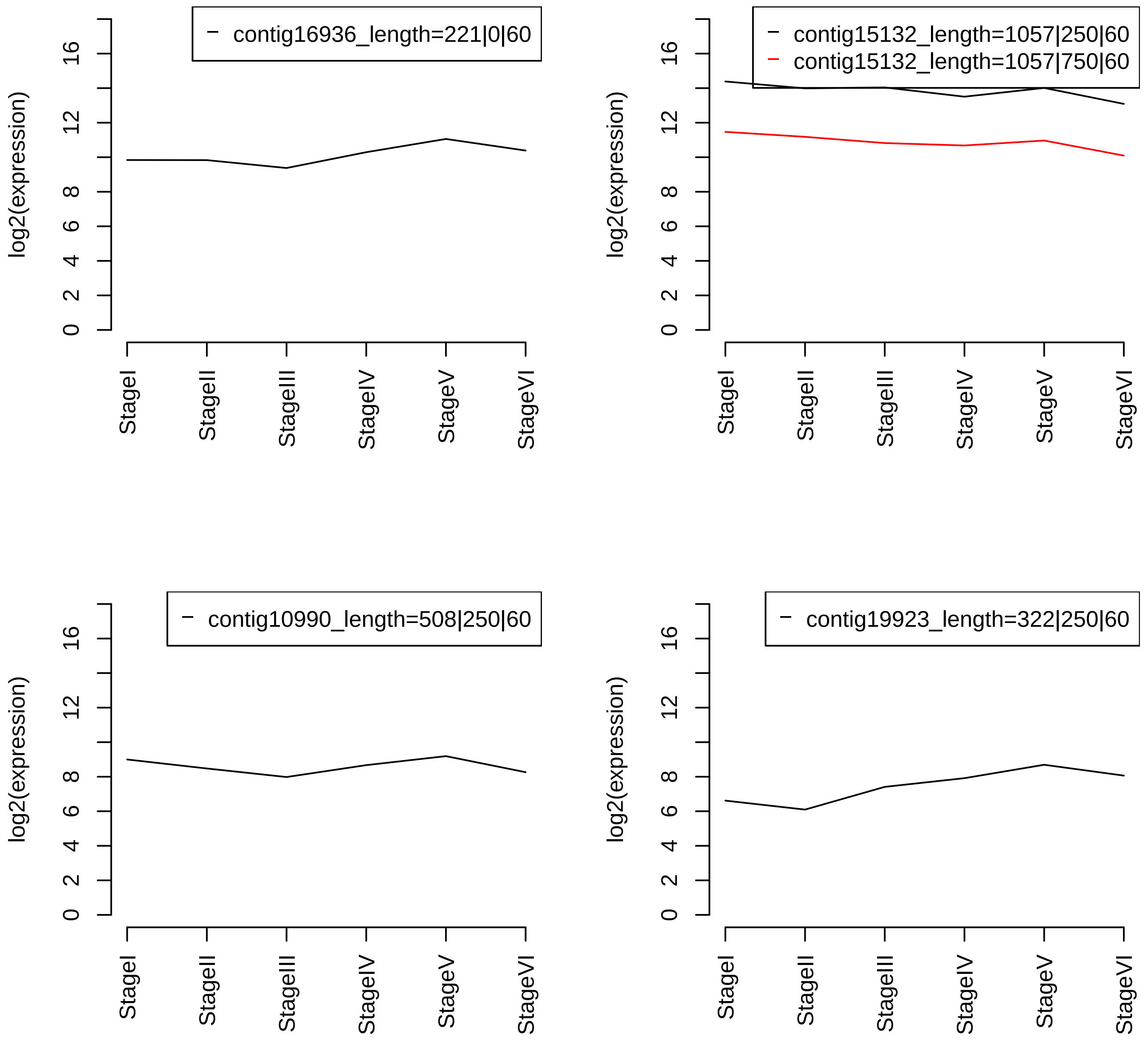

contig16936_length=221|0|60
contig15132_length=1057|250|60
contig15132_length=1057|750|60
contig10990_length=508|250|60
contig19923_length=322|250|60
log2(expression)
0
2
4
6
8
12
16
StageI
StageII
StageIII
StageIV
StageV
StageVI

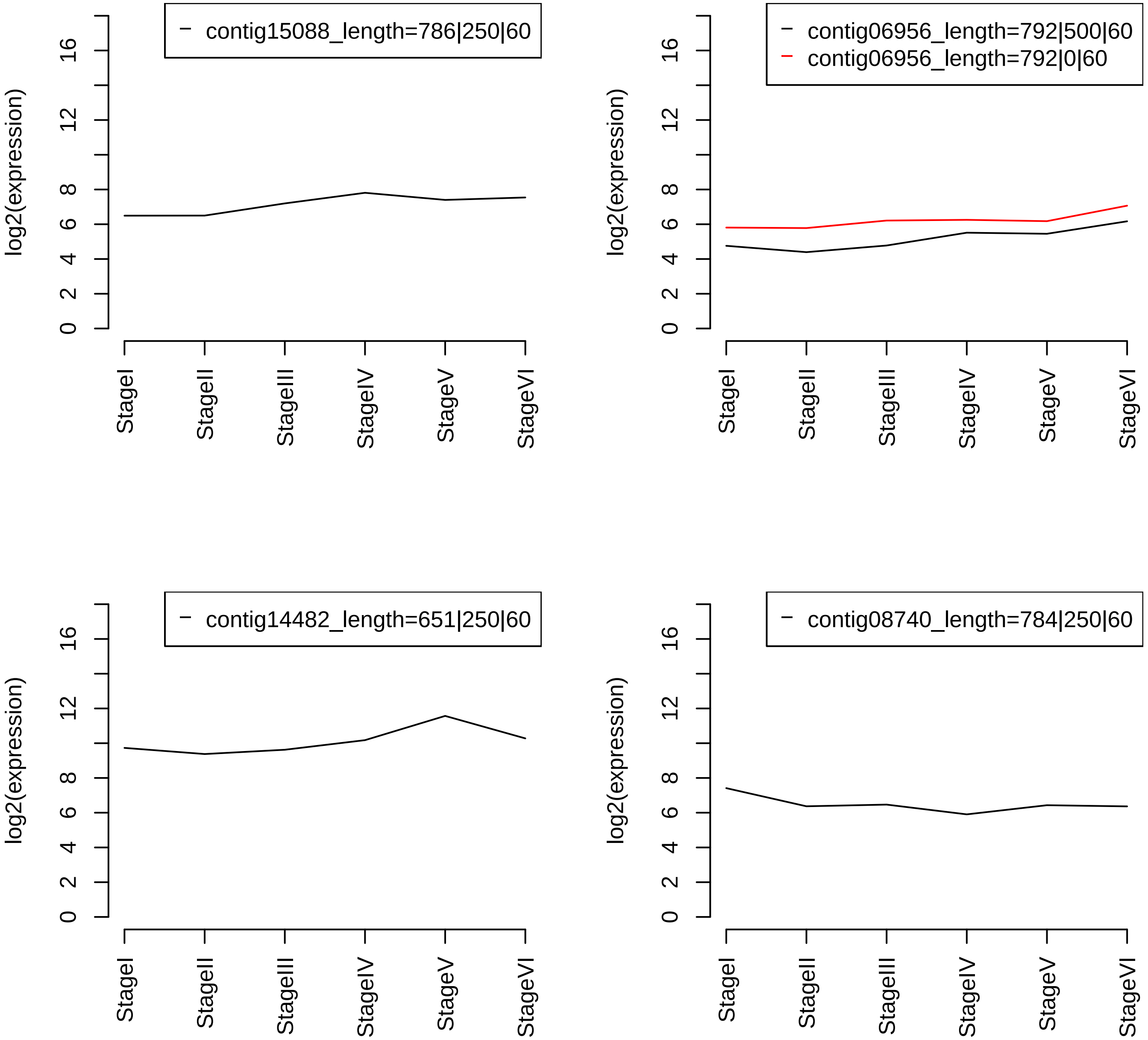

contig15088_length=786|250|60
contig06956_length=792|500|60
contig06956_length=792|0|60
contig14482_length=651|250|60
contig08740_length=784|250|60
log2(expression)
0
2
4
6
8
12
16
StageI
StageII
StageIII
StageIV
StageV
StageVI

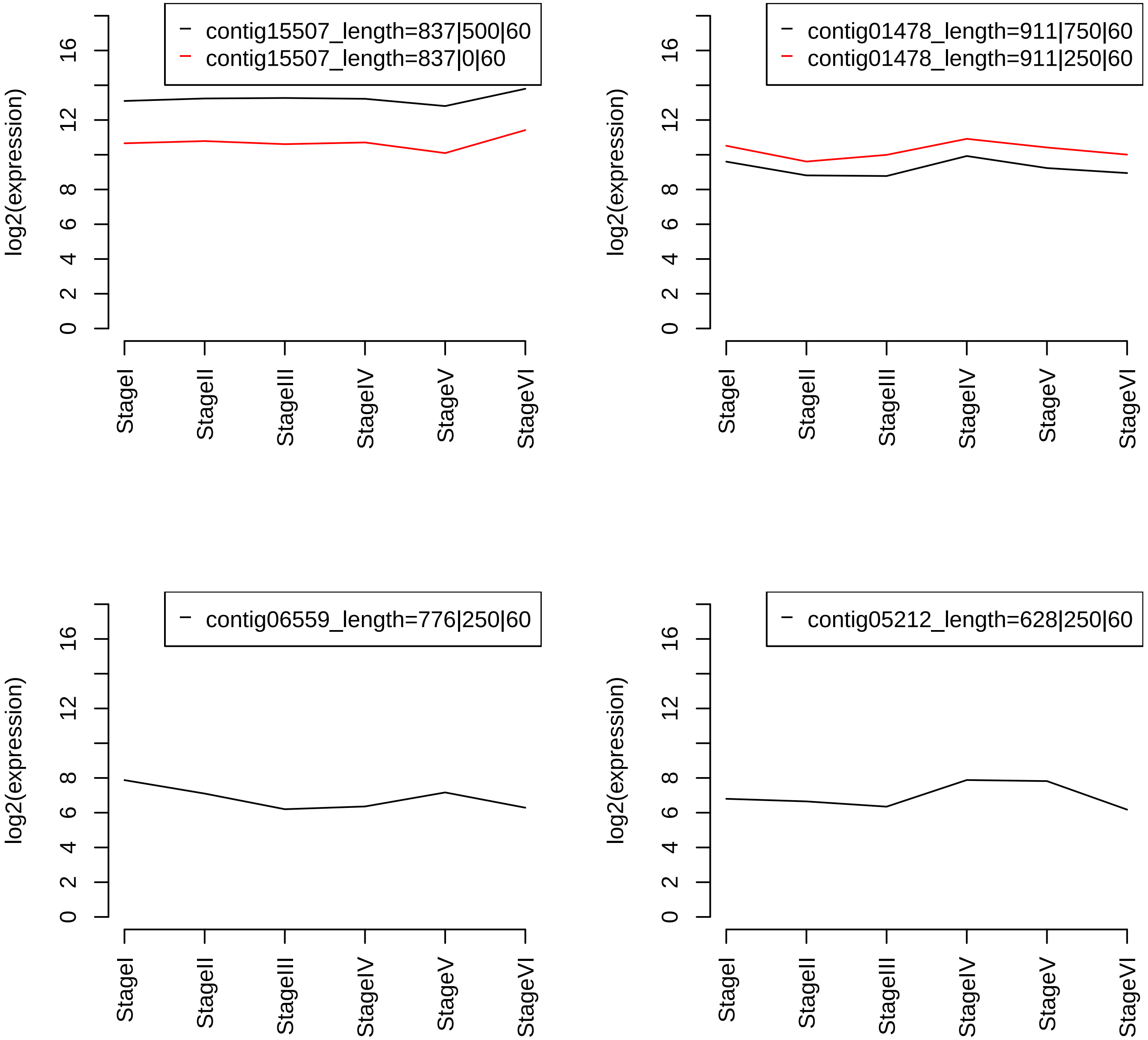

contig15507_length=837|500|60
contig15507_length=837|0|60
contig01478_length=911|750|60
contig01478_length=911|250|60
contig06559_length=776|250|60
contig05212_length=628|250|60
log2(expression)
0 2 4 6 8 12 16
StageI StageII StageIII StageIV StageV StageVI

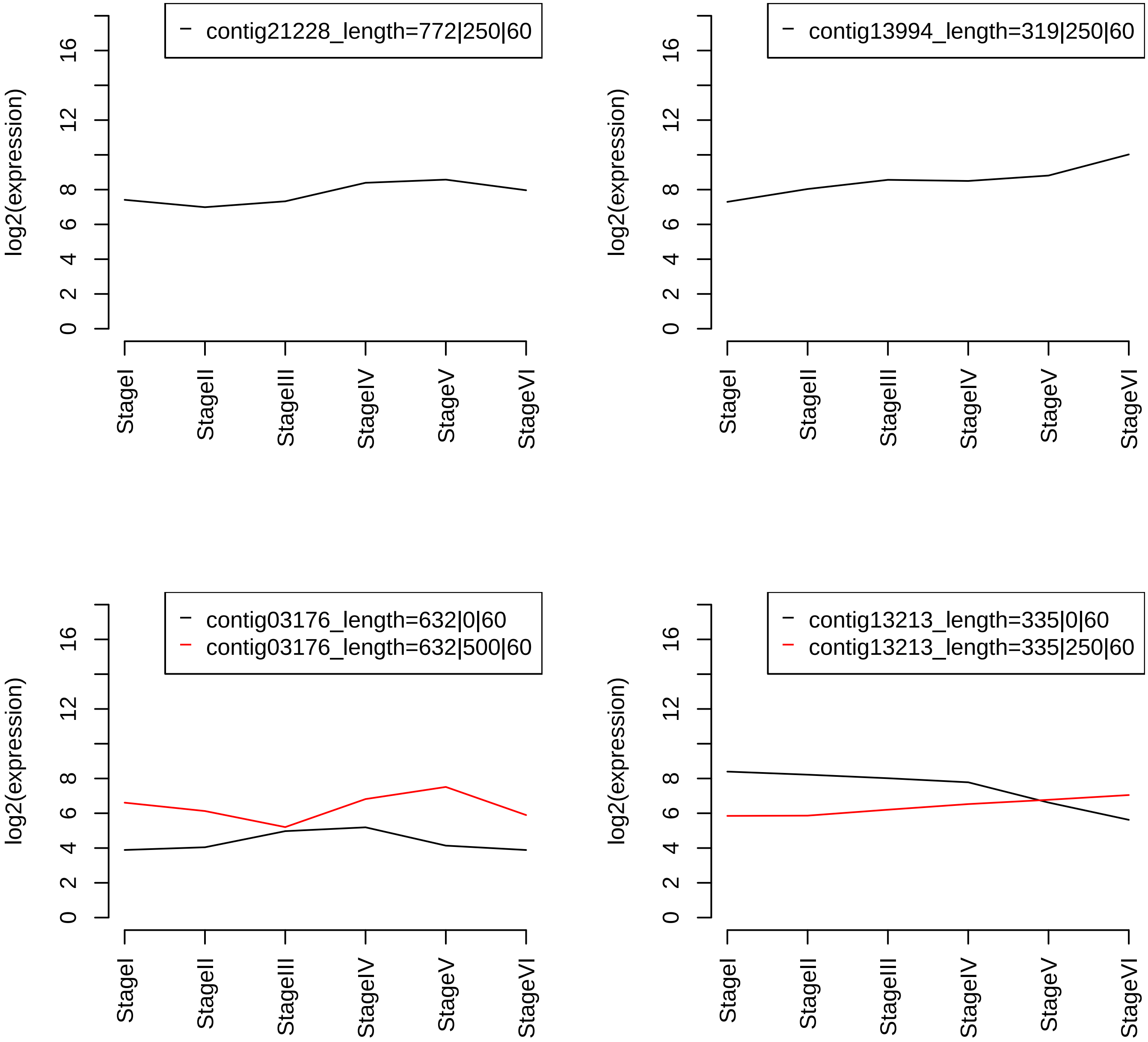
contig21228_length=772|250|60
contig13994_length=319|250|60
contig03176_length=632|0|60
contig03176_length=632|500|60
contig13213_length=335|0|60
contig13213_length=335|250|60
log2(expression)
0
2
4
6
8
12
16
StageI
StageII
StageIII
StageIV
StageV
StageVI

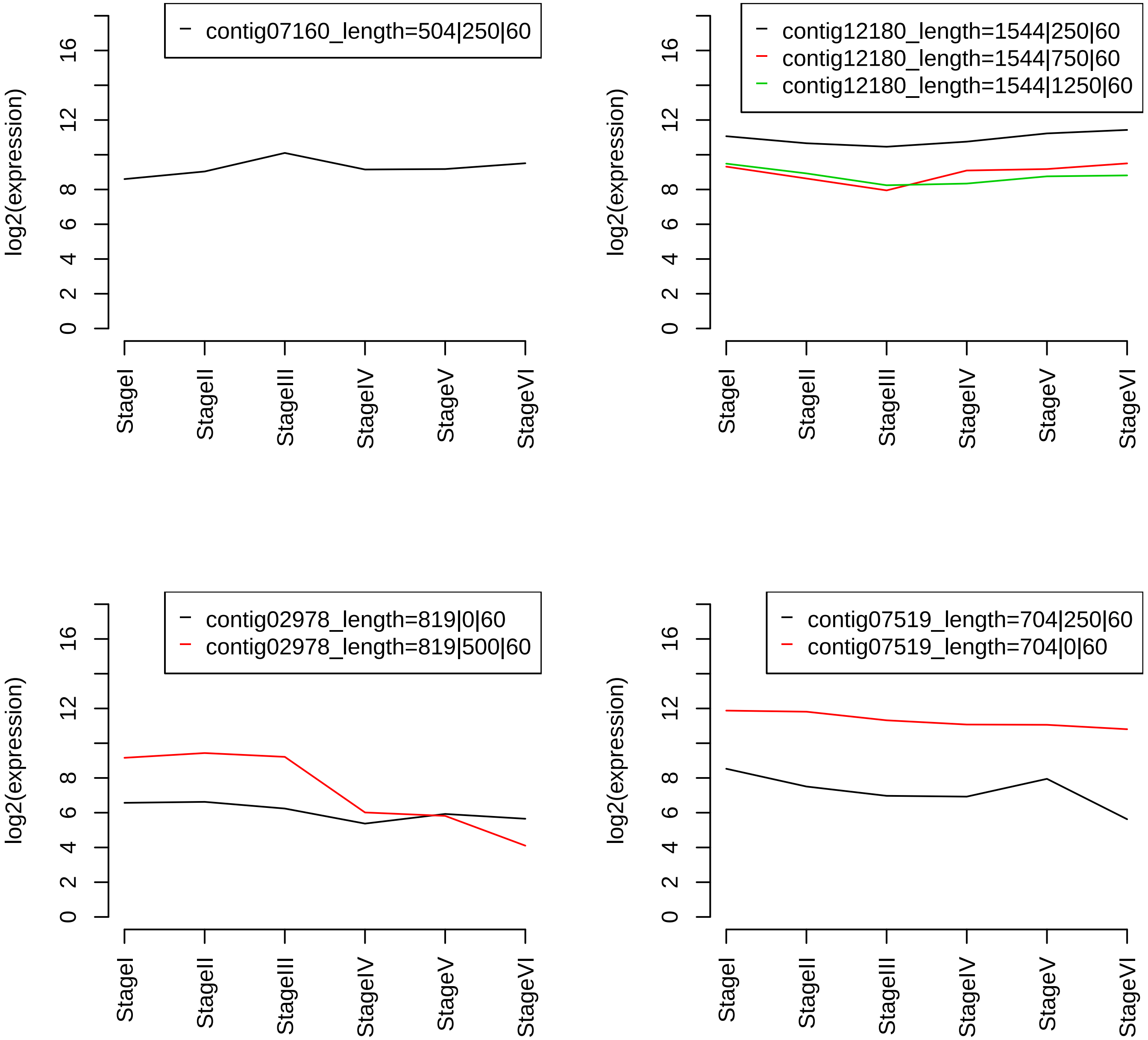
contig07160_length=504|250|60
contig12180_length=1544|250|60
contig12180_length=1544|750|60
contig12180_length=1544|1250|60
contig02978_length=819|0|60
contig02978_length=819|500|60
contig07519_length=704|250|60
contig07519_length=704|0|60
log2(expression)
0
2
4
6
8
12
16
StageI
StageII
StageIII
StageIV
StageV
StageVI

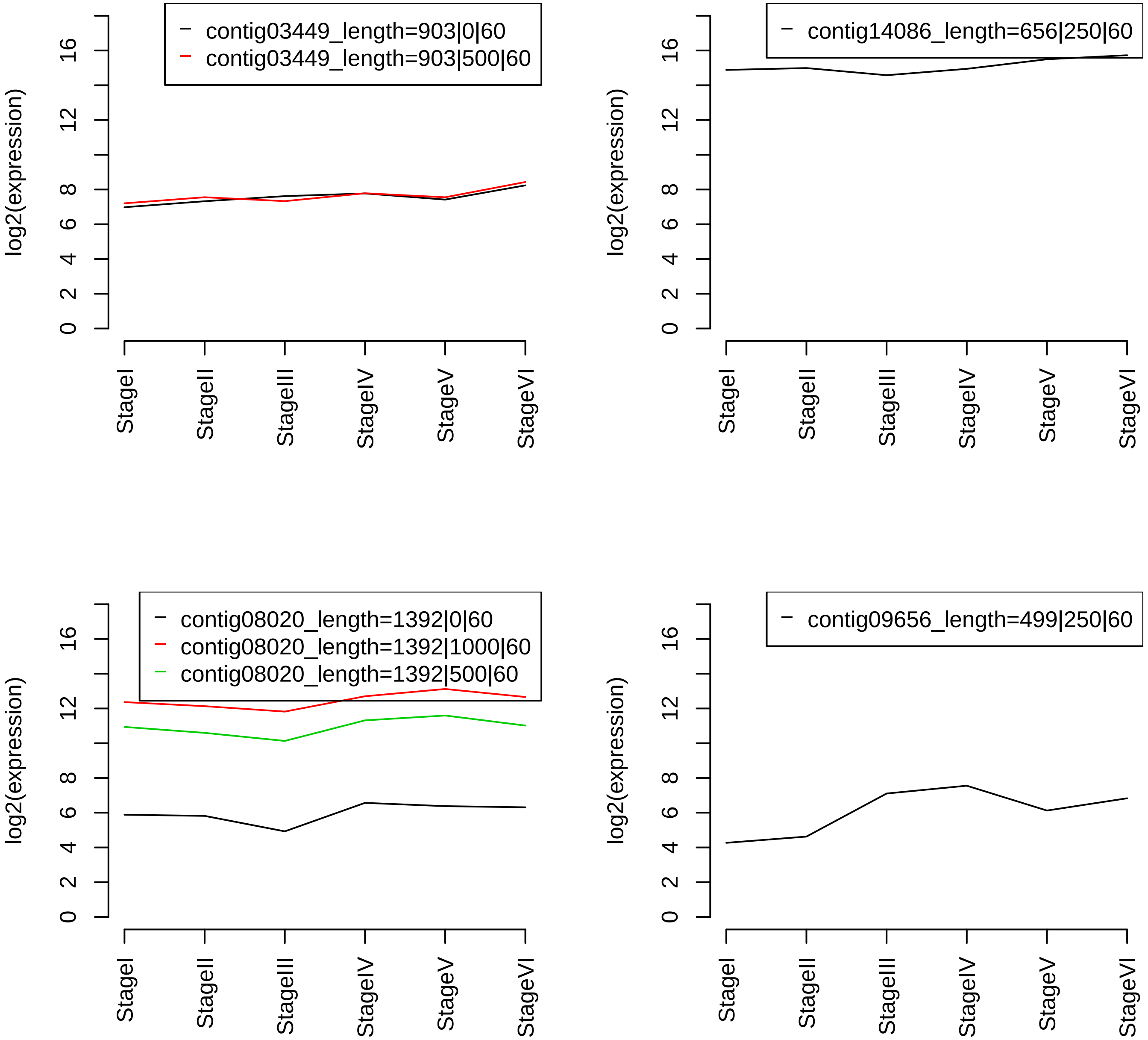

contig03449_length=903|0|60
contig03449_length=903|500|60
contig14086_length=656|250|60
contig08020_length=1392|0|60
contig08020_length=1392|1000|60
contig08020_length=1392|500|60
contig09656_length=499|250|60
log2(expression)
0
2
4
6
8
12
16
StageI
StageII
StageIII
StageIV
StageV
StageVI

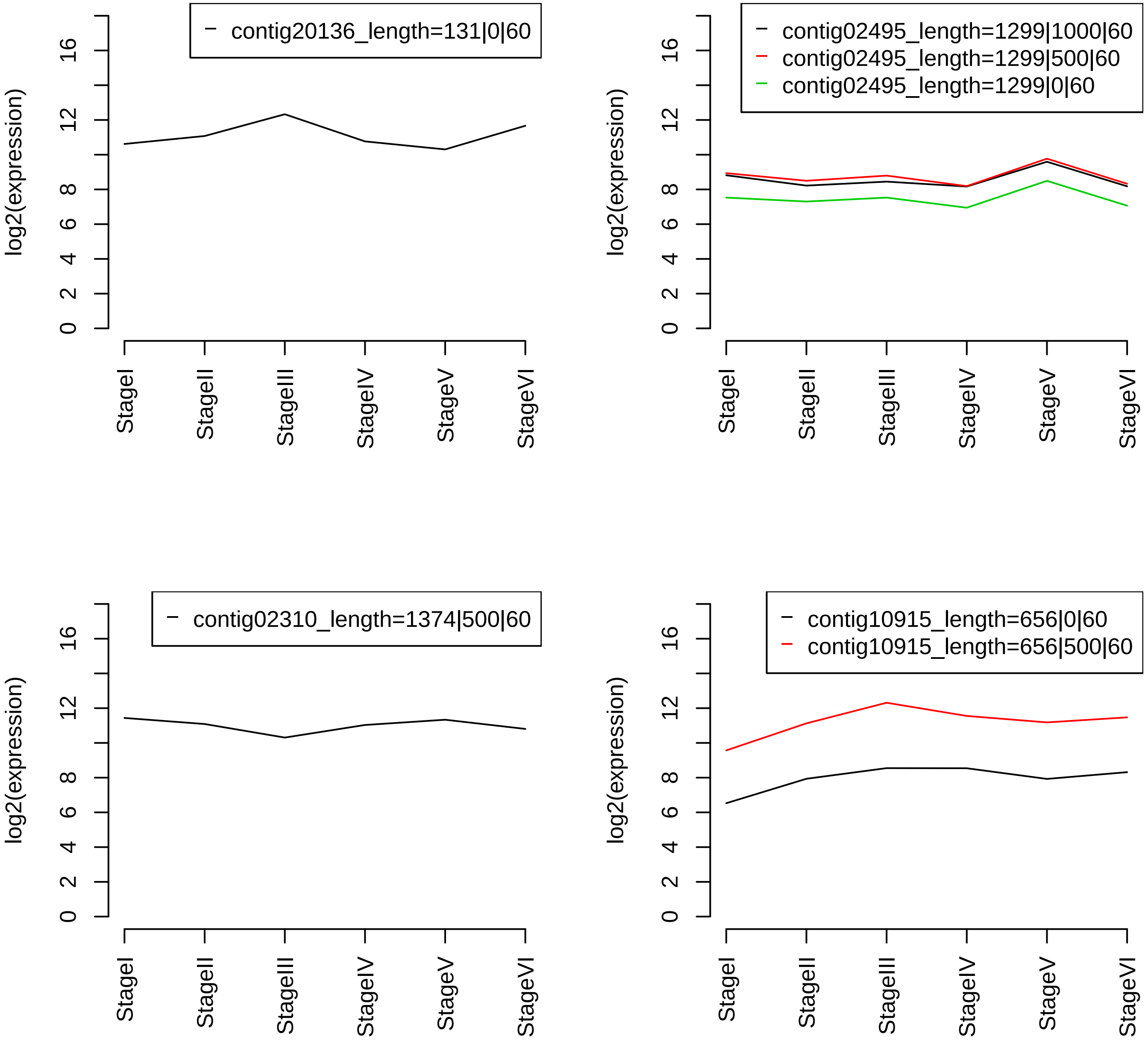

contig20136_length=131|0|60
contig02495_length=1299|1000|60
contig02495_length=1299|500|60
contig02495_length=1299|0|60
contig02310_length=1374|500|60
contig10915_length=656|0|60
contig10915_length=656|500|60
log2(expression)
0
2
4
6
8
12
16
StageI
StageII
StageIII
StageIV
StageV
StageVI

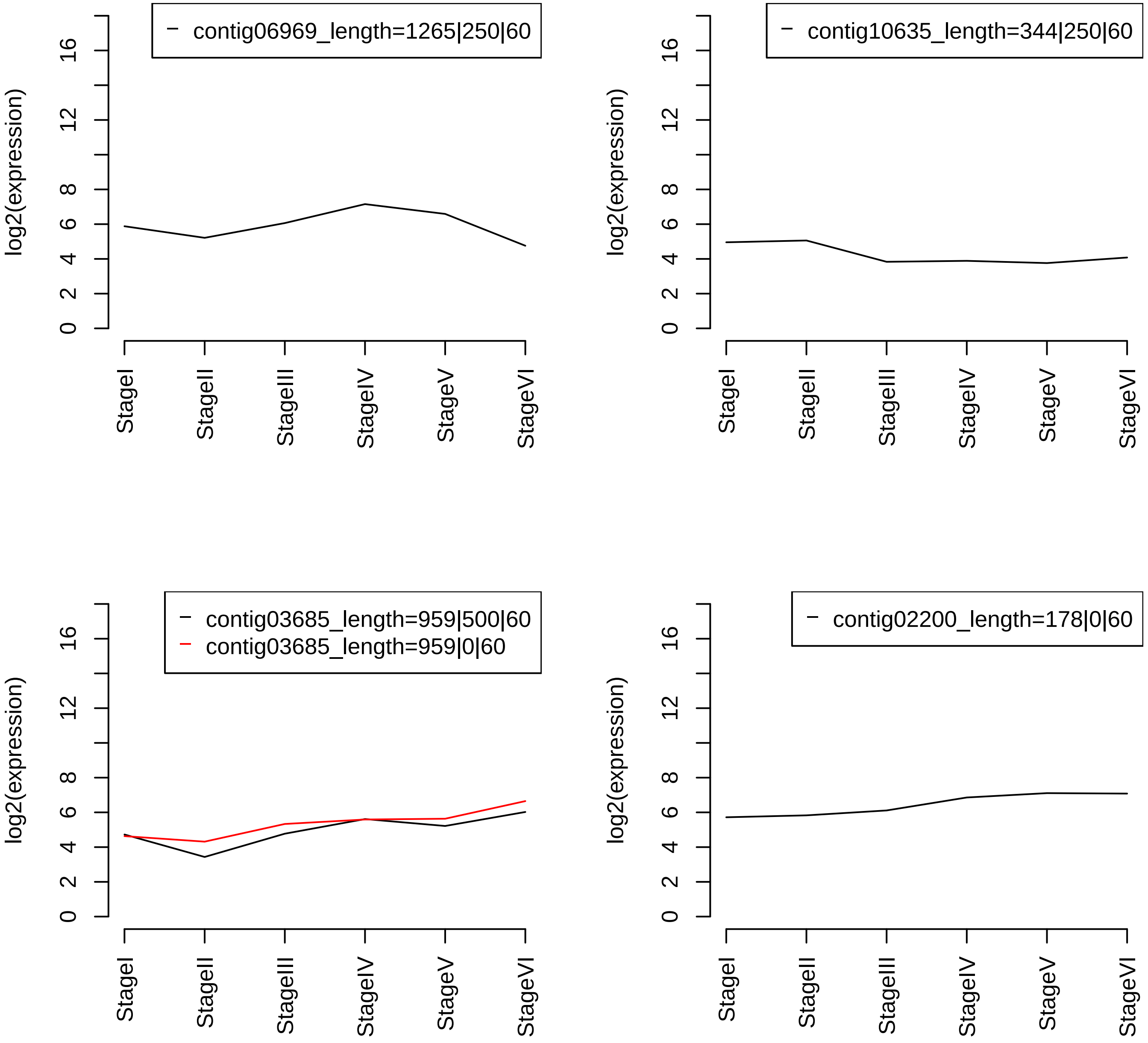

contig06969_length=1265|250|60
contig10635_length=344|250|60
contig03685_length=959|500|60
contig03685_length=959|0|60
contig02200_length=178|0|60
log2(expression)
0
2
4
6
8
12
16
StageI
StageII
StageIII
StageIV
StageV
StageVI

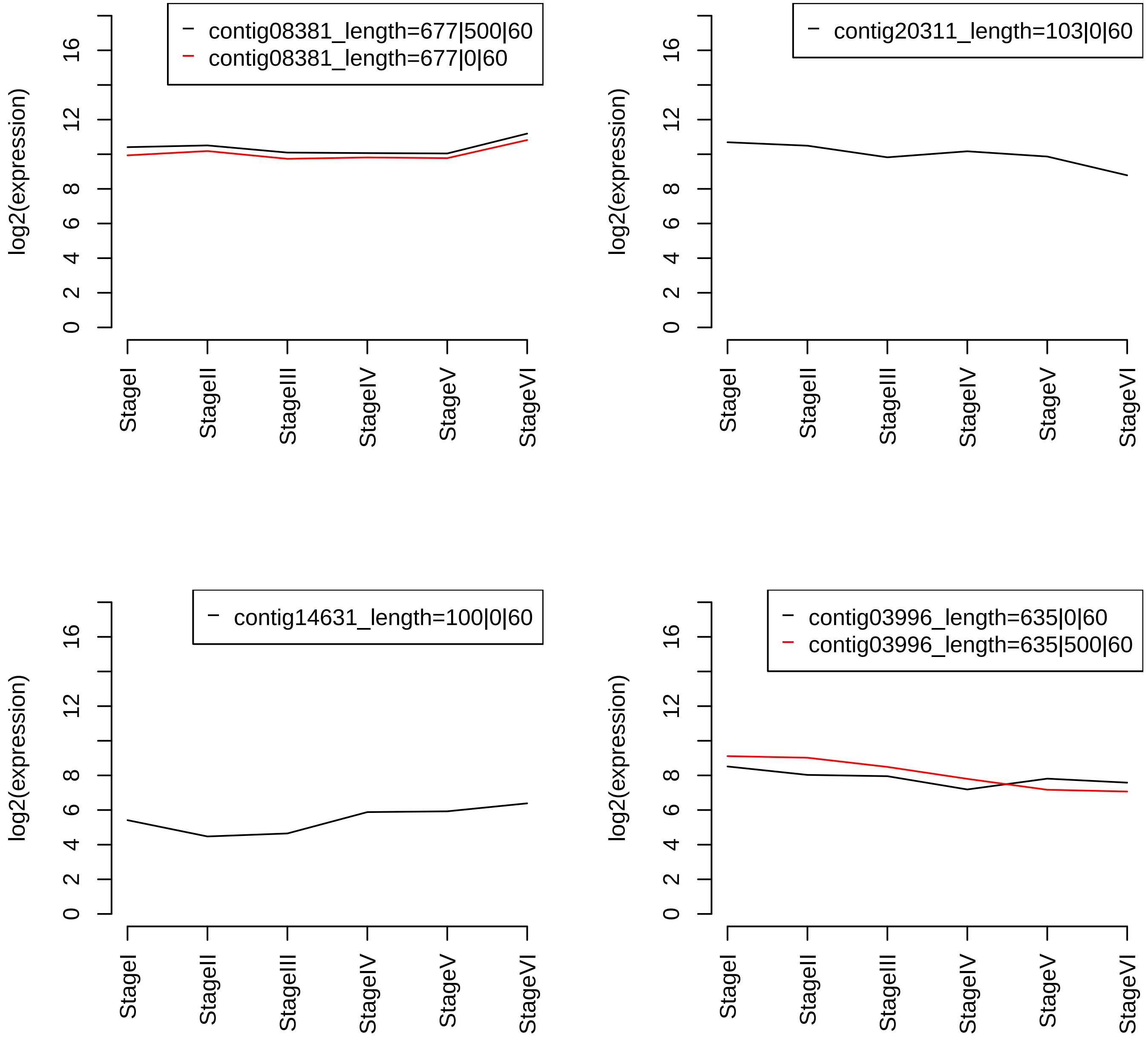

contig08381_length=677|500|60
contig08381_length=677|0|60
contig20311_length=103|0|60
contig14631_length=100|0|60
contig03996_length=635|0|60
contig03996_length=635|500|60
log2(expression)
0
2
4
6
8
12
16
StageI
StageII
StageIII
StageIV
StageV
StageVI

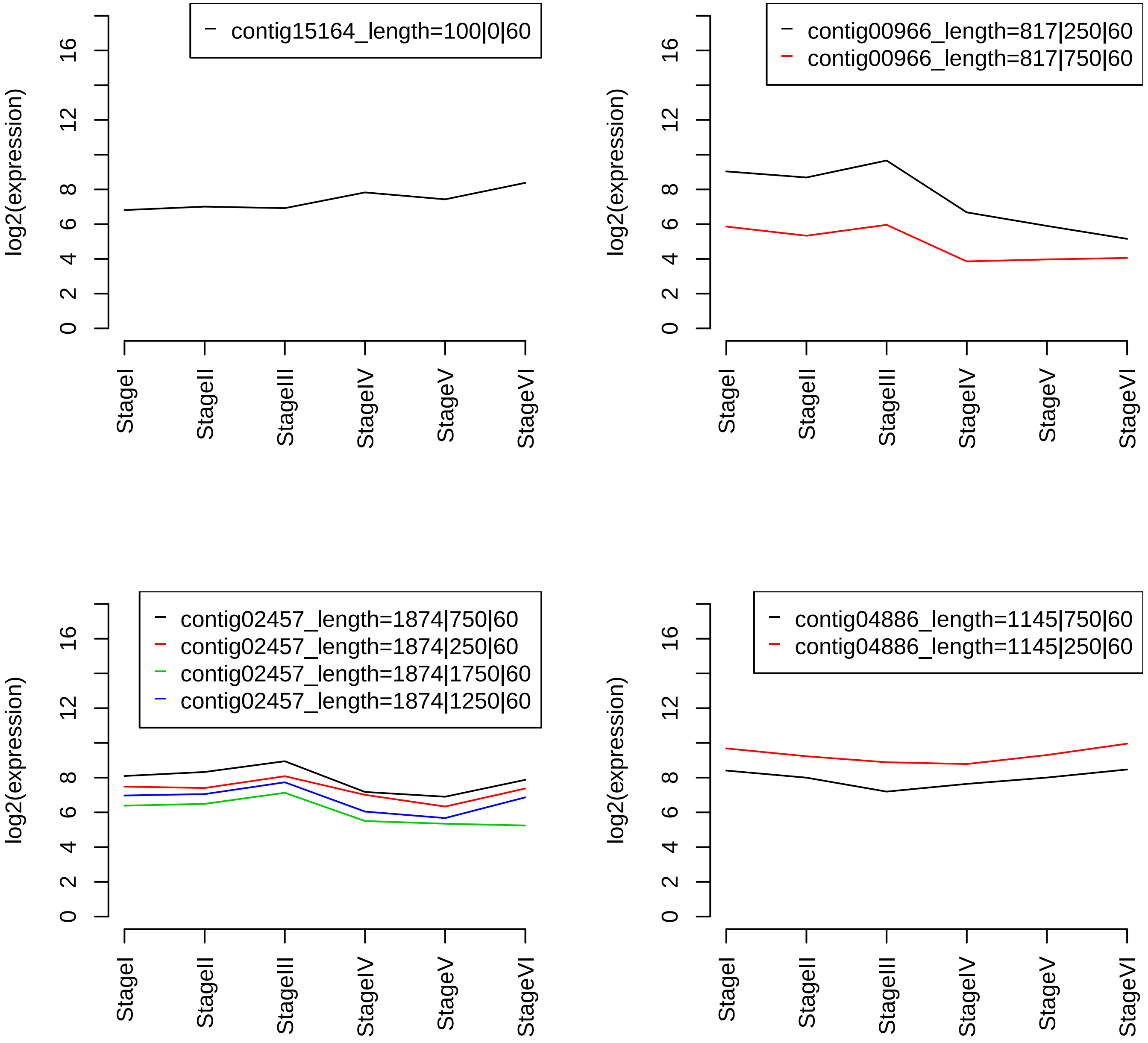

contig15164_length=100|0|60
contig00966_length=817|250|60
contig00966_length=817|750|60
contig02457_length=1874|750|60
contig02457_length=1874|250|60
contig02457_length=1874|1750|60
contig02457_length=1874|1250|60
contig04886_length=1145|750|60
contig04886_length=1145|250|60
log2(expression)
0
2
4
6
8
12
16
StageI
StageII
StageIII
StageIV
StageV
StageVI

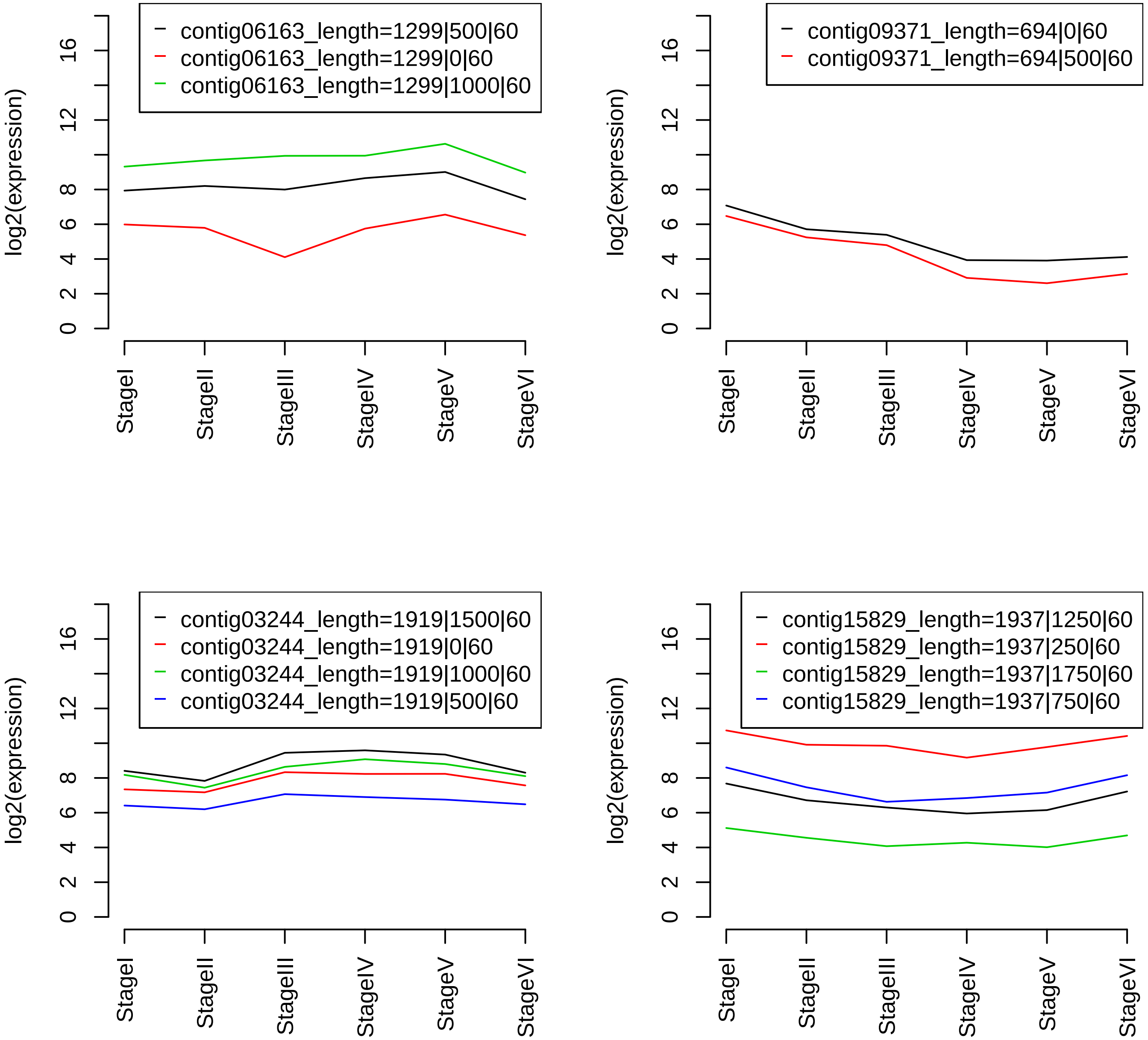

contig06163_length=1299|500|60
contig06163_length=1299|0|60
contig06163_length=1299|1000|60
contig09371_length=694|0|60
contig09371_length=694|500|60
contig03244_length=1919|1500|60
contig03244_length=1919|0|60
contig03244_length=1919|1000|60
contig03244_length=1919|500|60
contig15829_length=1937|1250|60
contig15829_length=1937|250|60
contig15829_length=1937|1750|60
contig15829_length=1937|750|60
log2(expression)
0
2
4
6
8
12
16
StageI
StageII
StageIII
StageIV
StageV
StageVI

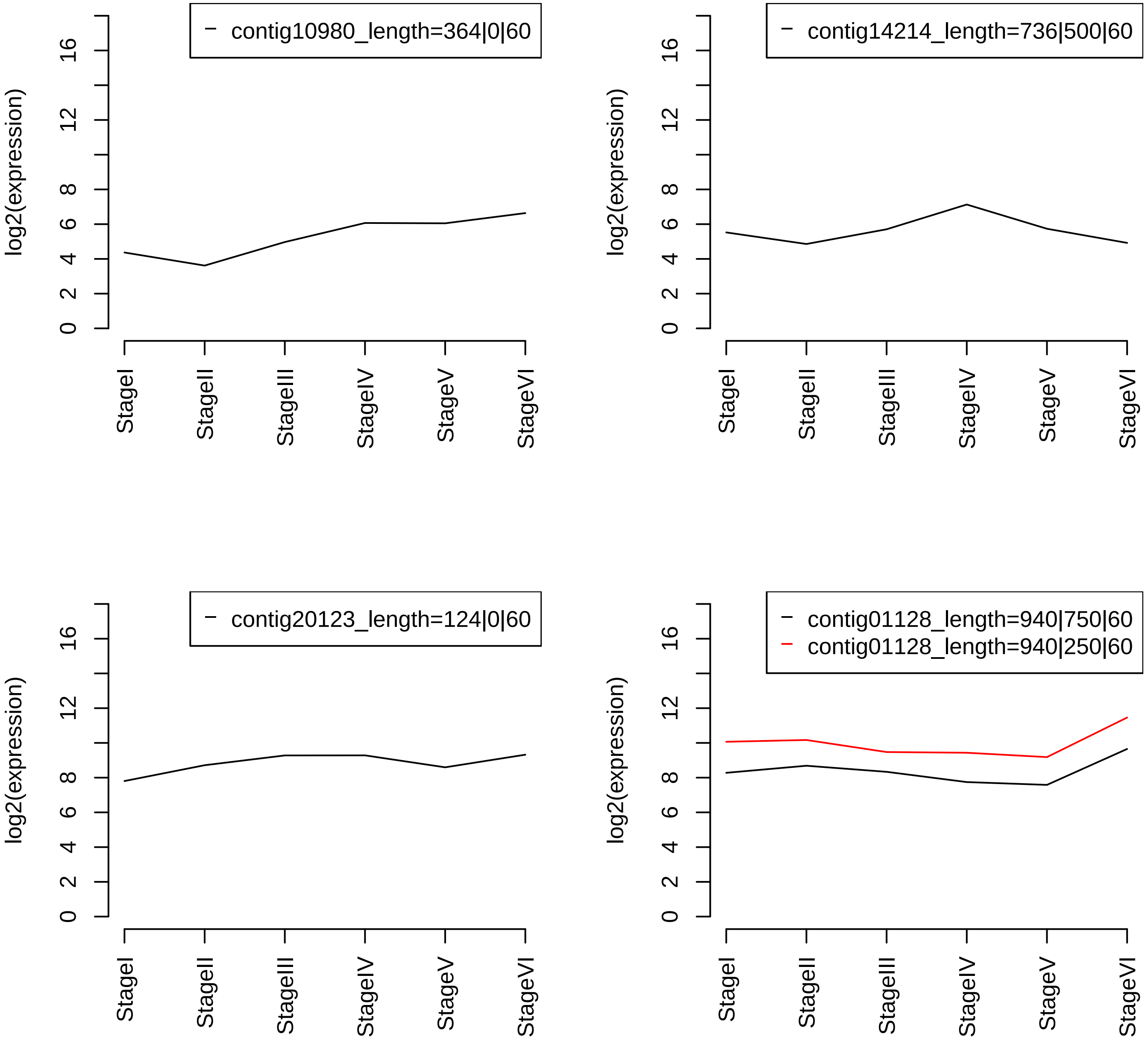
contig10980_length=364|0|60
contig14214_length=736|500|60
contig20123_length=124|0|60
contig01128_length=940|750|60
contig01128_length=940|250|60
log2(expression)
0
2
4
6
8
12
16
StageI
StageII
StageIII
StageIV
StageV
StageVI

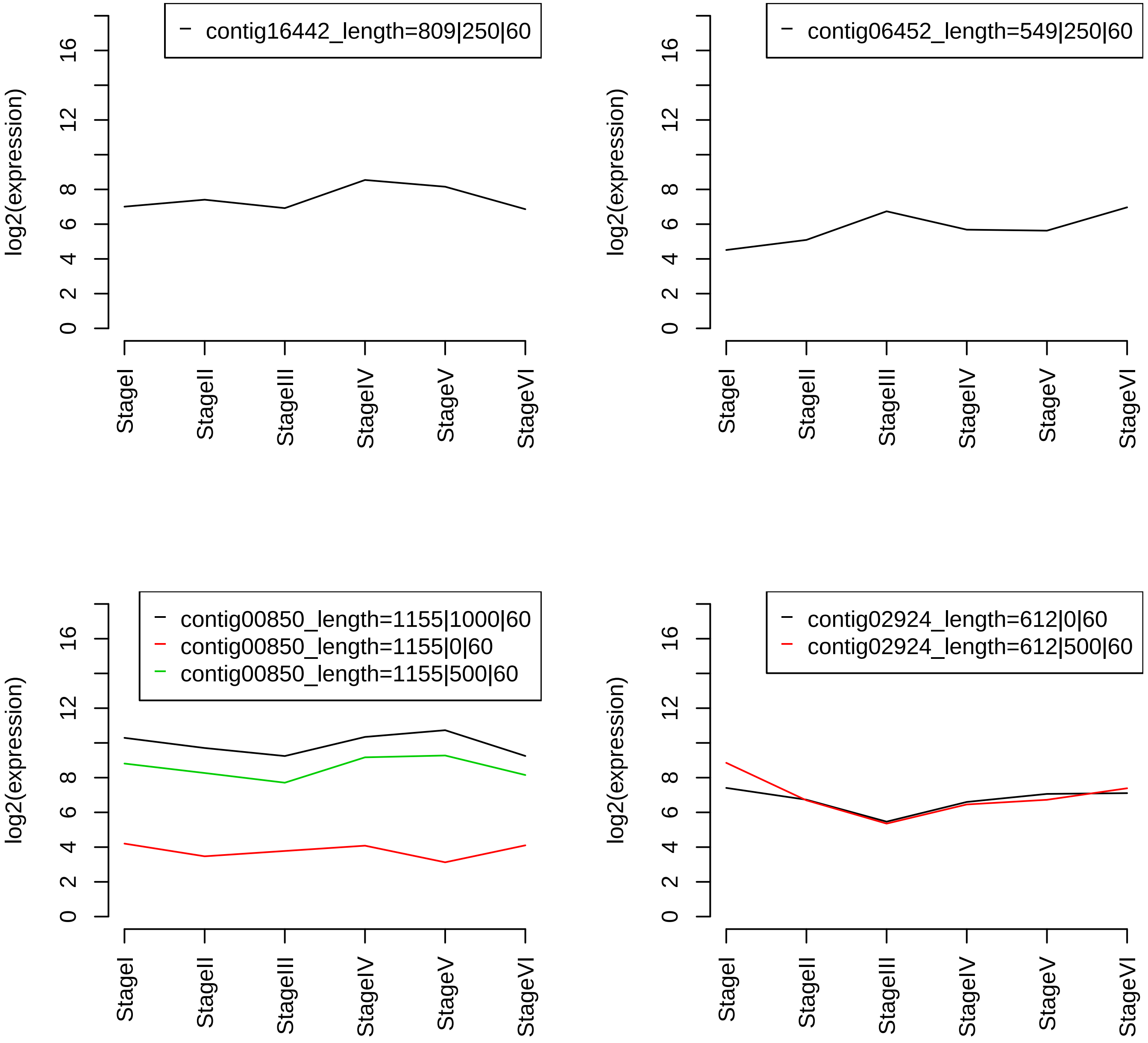
contig16442_length=809|250|60
contig06452_length=549|250|60
contig00850_length=1155|1000|60
contig00850_length=1155|0|60
contig00850_length=1155|500|60
contig02924_length=612|0|60
contig02924_length=612|500|60
log2(expression)
0
2
4
6
8
12
16
StageI
StageII
StageIII
StageIV
StageV
StageVI

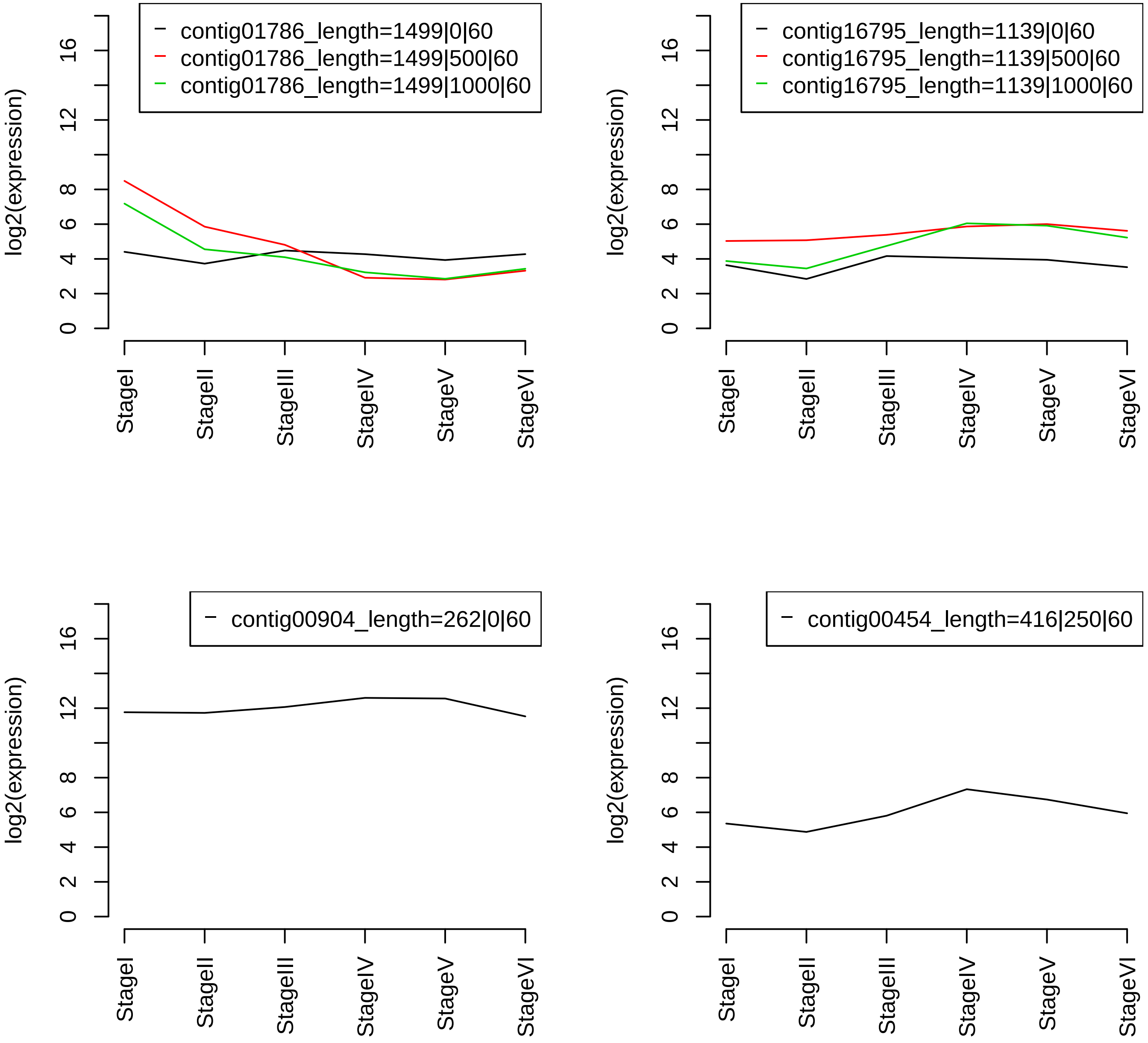
contig01786_length=1499|0|60
contig01786_length=1499|500|60
contig01786_length=1499|1000|60
contig16795_length=1139|0|60
contig16795_length=1139|500|60
contig16795_length=1139|1000|60
contig00904_length=262|0|60
contig00454_length=416|250|60
log2(expression)
0
2
4
6
8
12
16
StageI
StageII
StageIII
StageIV
StageV
StageVI

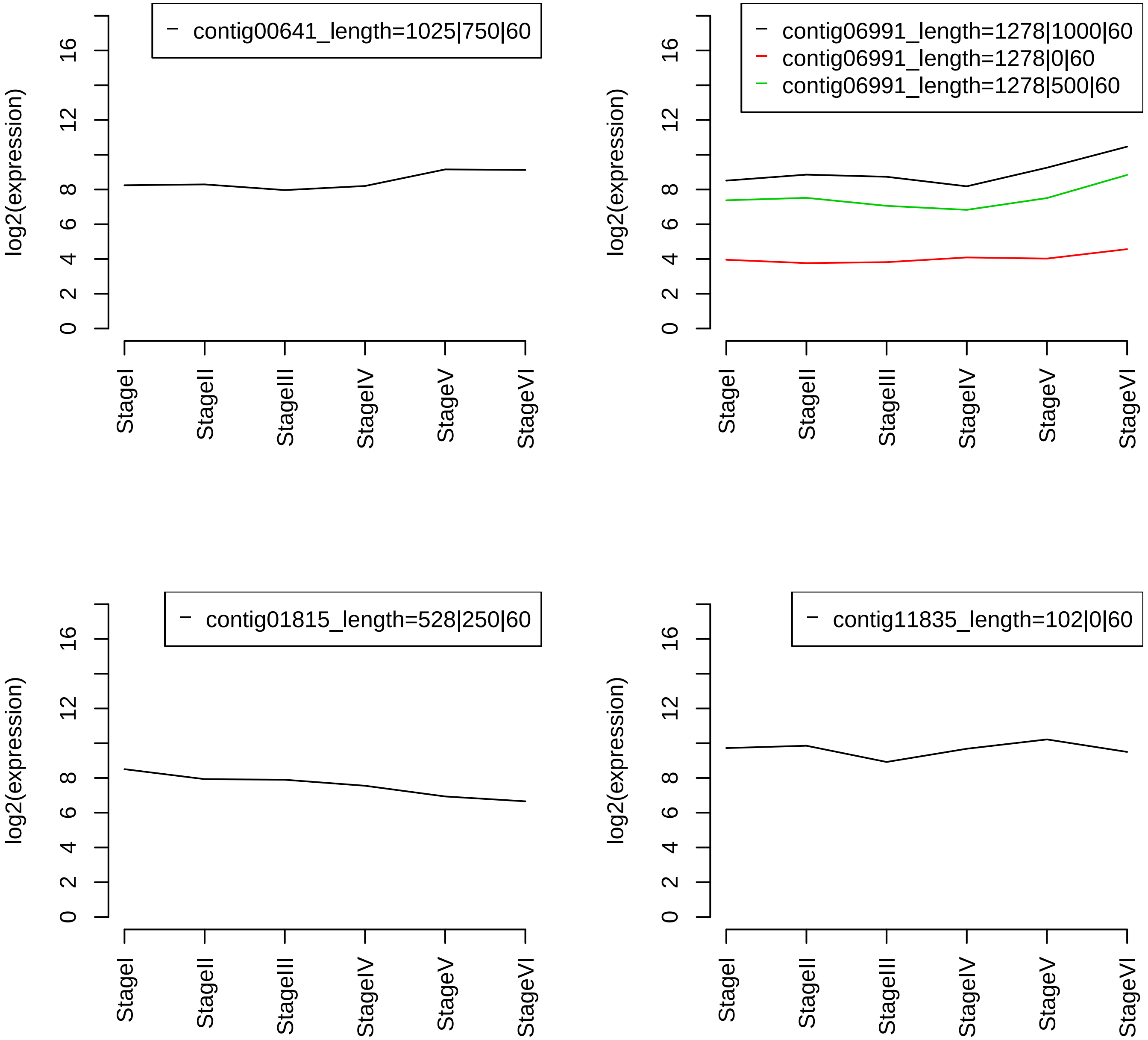

contig00641_length=1025|750|60
contig06991_length=1278|1000|60
contig06991_length=1278|0|60
contig06991_length=1278|500|60
contig01815_length=528|250|60
contig11835_length=102|0|60
log2(expression)
0
2
4
6
8
12
16
StageI
StageII
StageIII
StageIV
StageV
StageVI

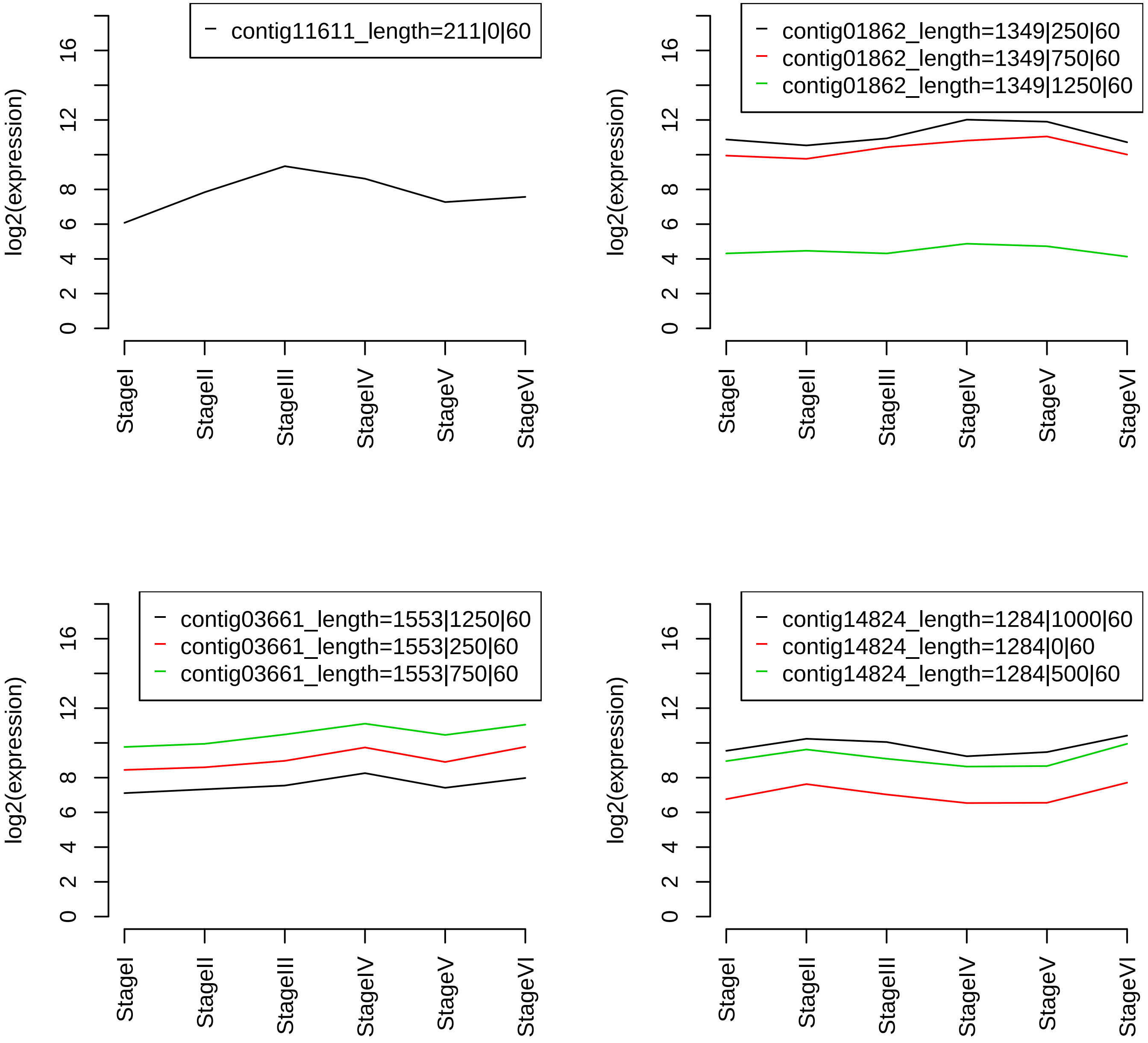
contig11611_length=211|0|60
contig01862_length=1349|250|60
contig01862_length=1349|750|60
contig01862_length=1349|1250|60
contig03661_length=1553|1250|60
contig03661_length=1553|250|60
contig03661_length=1553|750|60
contig14824_length=1284|1000|60
contig14824_length=1284|0|60
contig14824_length=1284|500|60
log2(expression)
0
2
4
6
8
12
16
StageI
StageII
StageIII
StageIV
StageV
StageVI

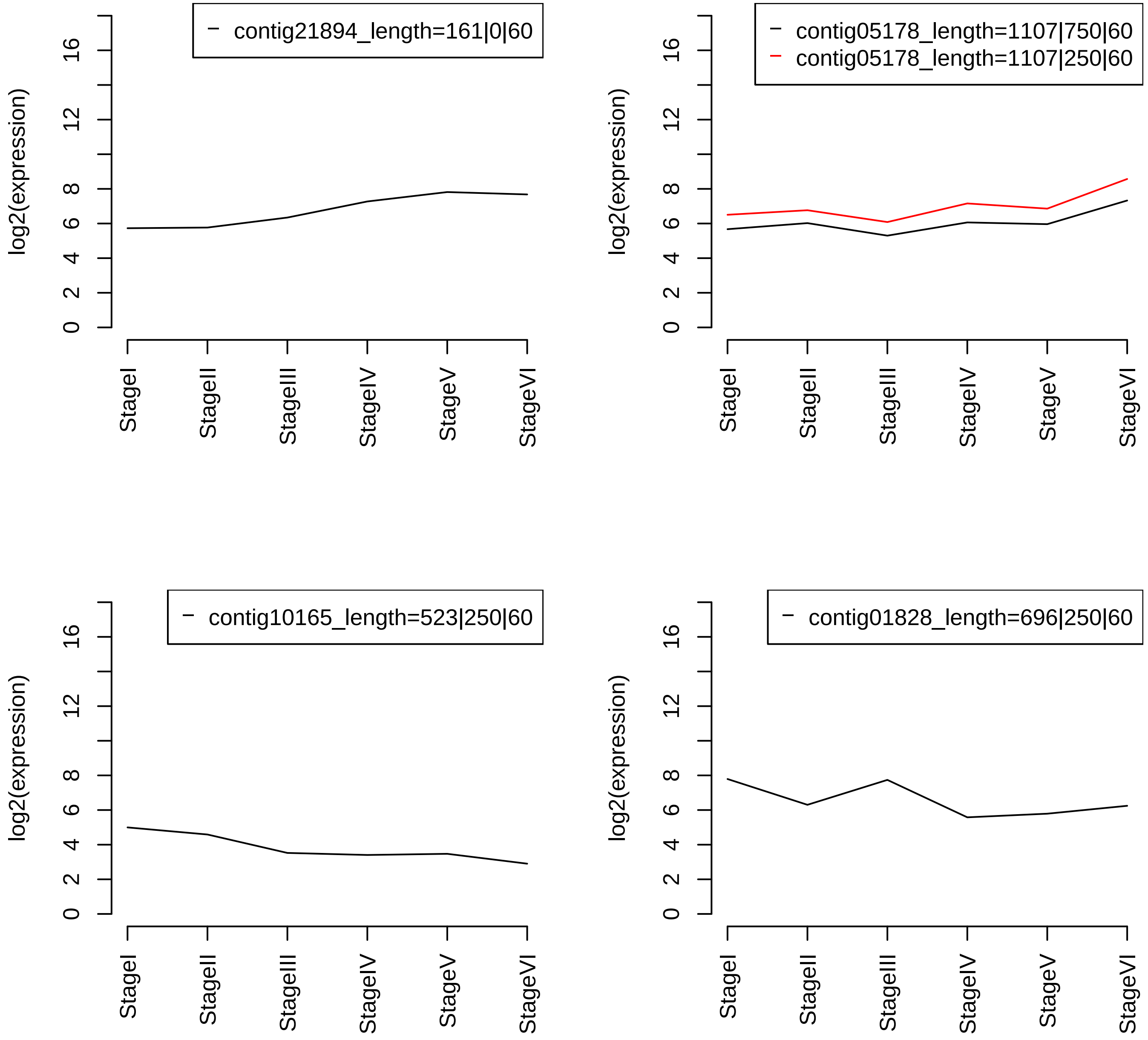
contig21894_length=161|0|60
contig05178_length=1107|750|60
contig05178_length=1107|250|60
contig10165_length=523|250|60
contig01828_length=696|250|60
log2(expression)
0
2
4
6
8
12
16
StageI
StageII
StageIII
StageIV
StageV
StageVI

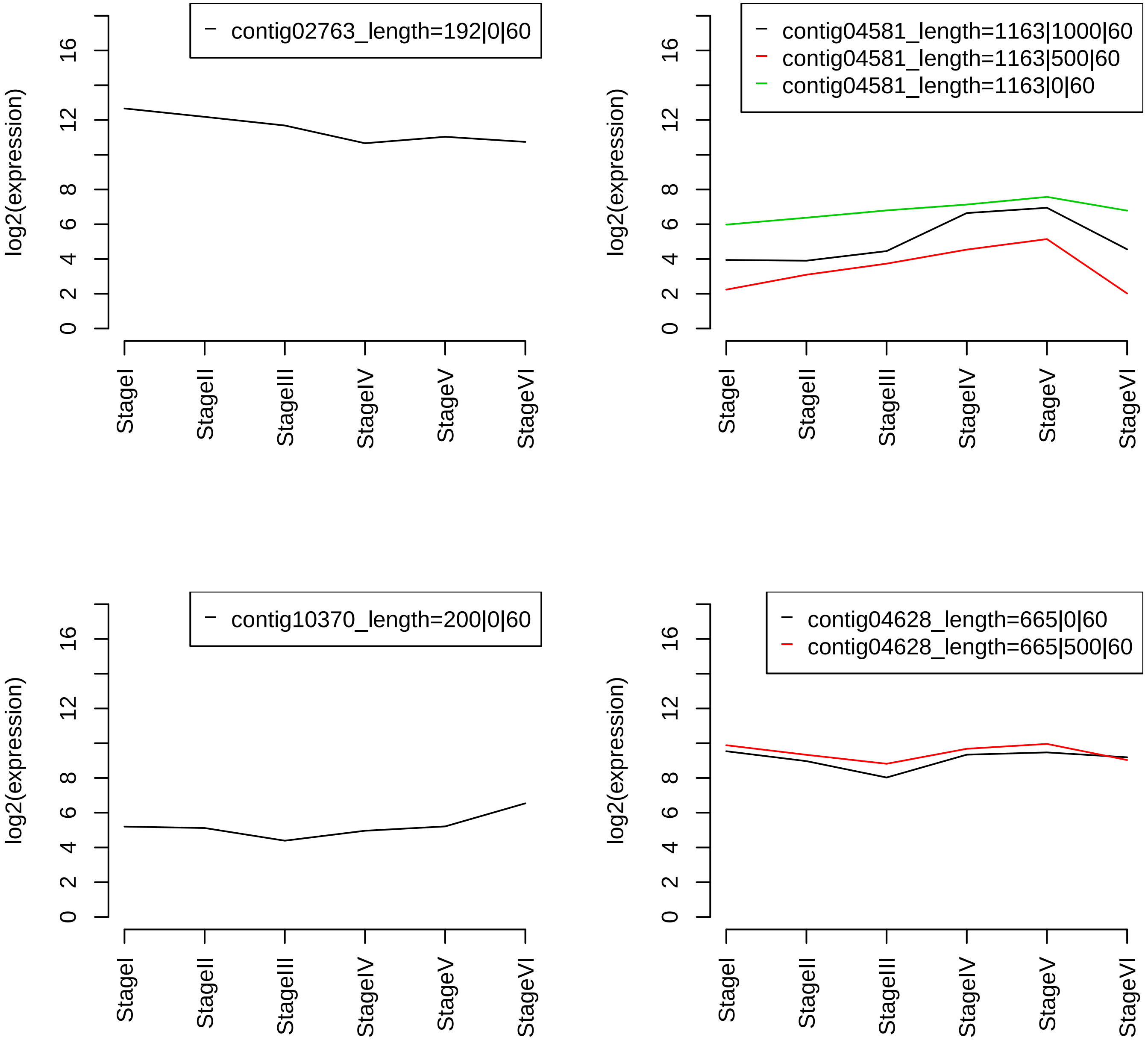

contig02763_length=192|0|60
log2(expression)
0
2
4
6
8
12
16
StageI
StageII
StageIII
StageIV
StageV
StageVI
contig04581_length=1163|1000|60
contig04581_length=1163|500|60
contig04581_length=1163|0|60
log2(expression)
0
2
4
6
8
12
16
StageI
StageII
StageIII
StageIV
StageV
StageVI
contig10370_length=200|0|60
log2(expression)
0
2
4
6
8
12
16
StageI
StageII
StageIII
StageIV
StageV
StageVI
contig04628_length=665|0|60
contig04628_length=665|500|60
log2(expression)
0
2
4
6
8
12
16
StageI
StageII
StageIII
StageIV
StageV
StageVI

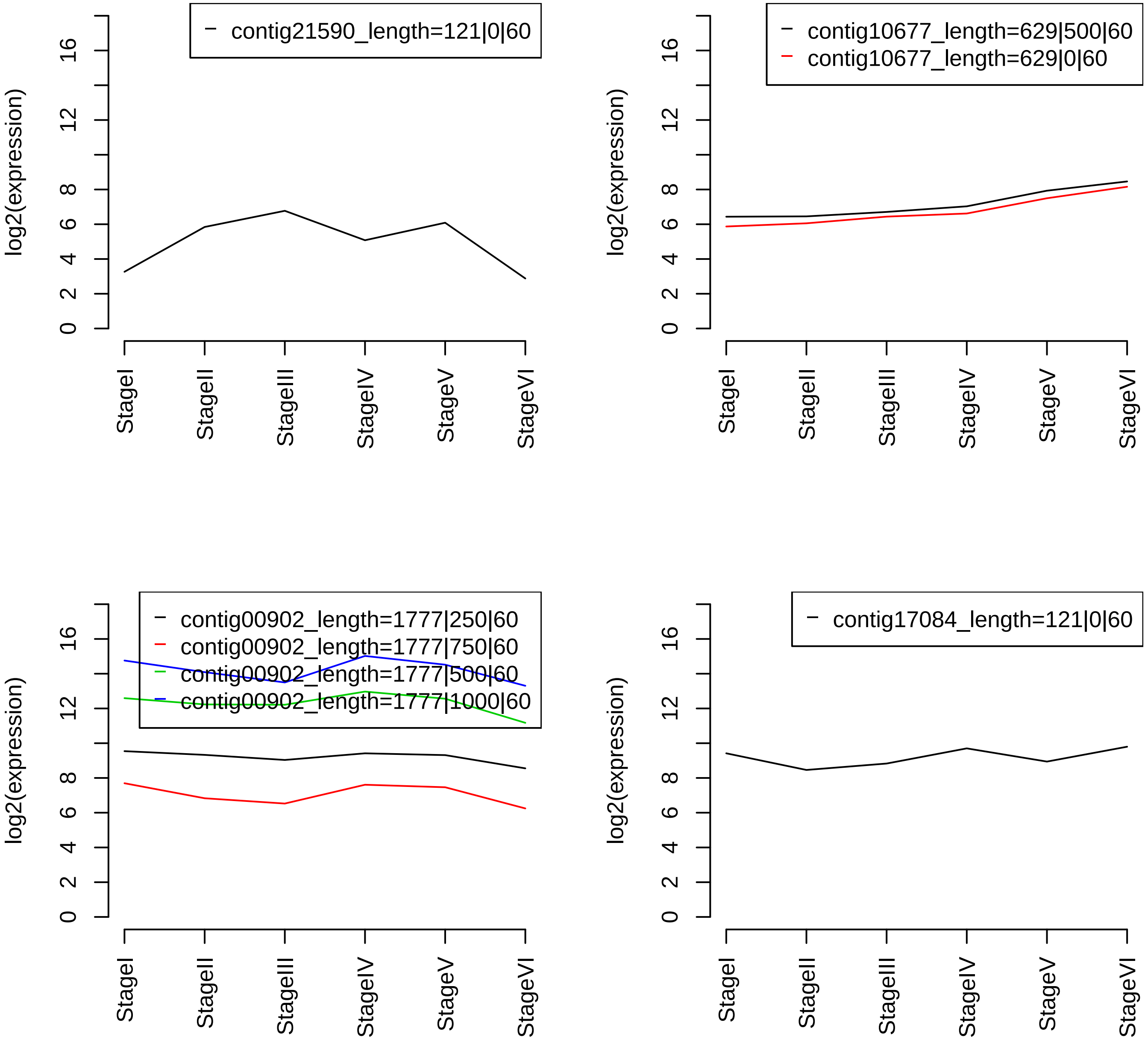

contig21590_length=121|0|60
contig10677_length=629|500|60
contig10677_length=629|0|60
contig00902_length=1777|250|60
contig00902_length=1777|750|60
contig00902_length=1777|500|60
contig00902_length=1777|1000|60
contig17084_length=121|0|60
log2(expression)
0
2
4
6
8
12
16
StageI
StageII
StageIII
StageIV
StageV
StageVI

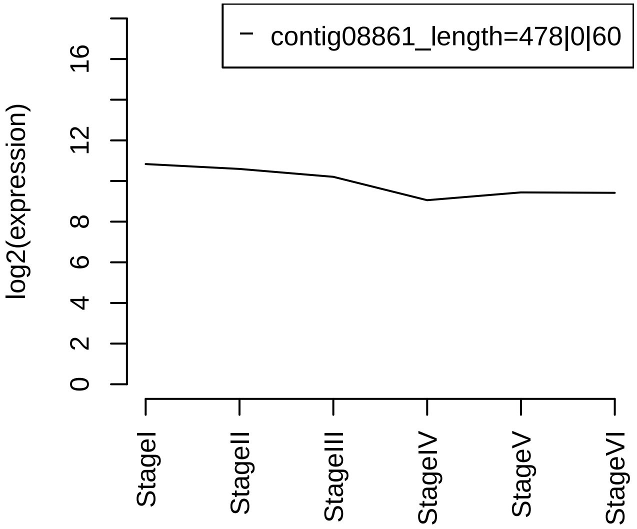

contig08861_length=478|0|60
log2(expression)
0
2
4
6
8
12
16
StageI
StageII
StageIII
StageIV
StageV
StageVI

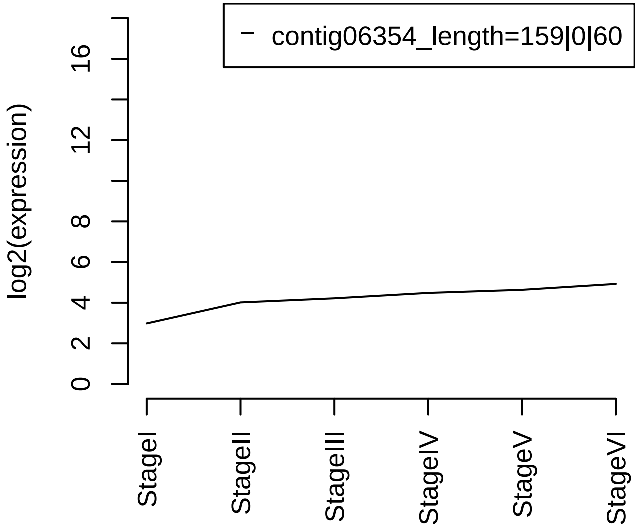

contig06354_length=159|0|60
log2(expression)
0
2
4
6
8
12
16
StageI
StageII
StageIII
StageIV
StageV
StageVI

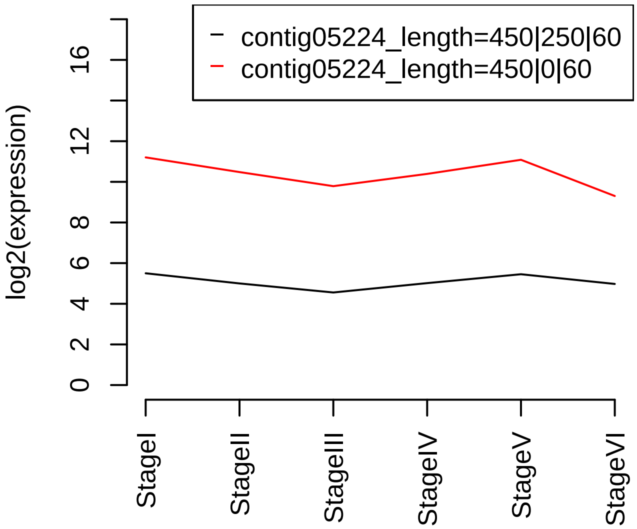

contig05224_length=450|250|60
contig05224_length=450|0|60
log2(expression)
0
2
4
6
8
12
16
StageI
StageII
StageIII
StageIV
StageV
StageVI

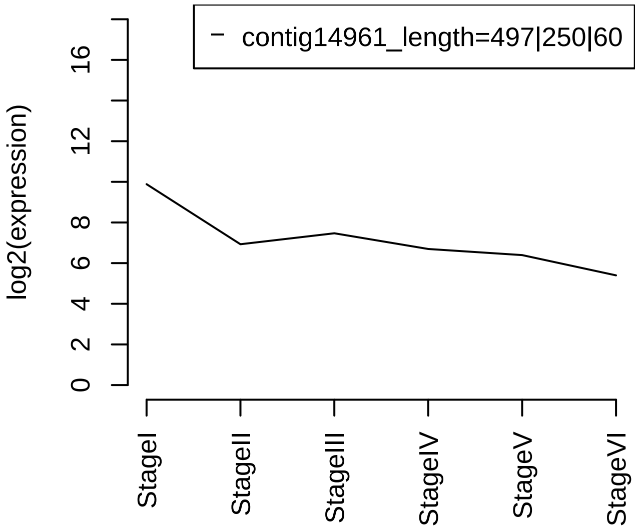

contig14961_length=497|250|60
log2(expression)
0
2
4
6
8
12
16
StageI
StageII
StageIII
StageIV
StageV
StageVI

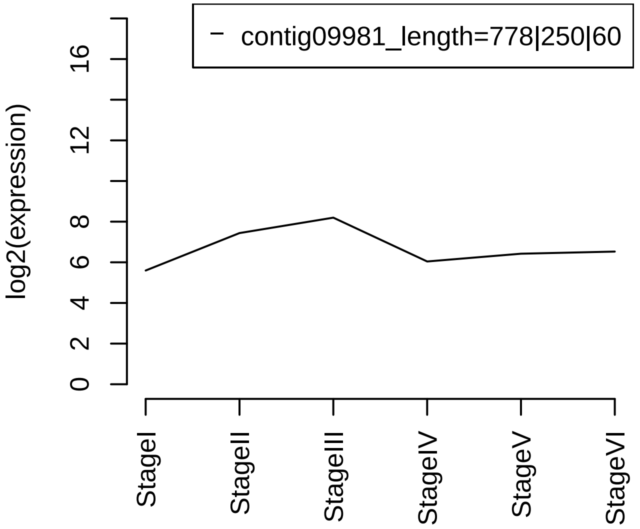
contig09981_length=778|250|60
log2(expression)
0
2
4
6
8
12
16
StageI
StageII
StageIII
StageIV
StageV
StageVI

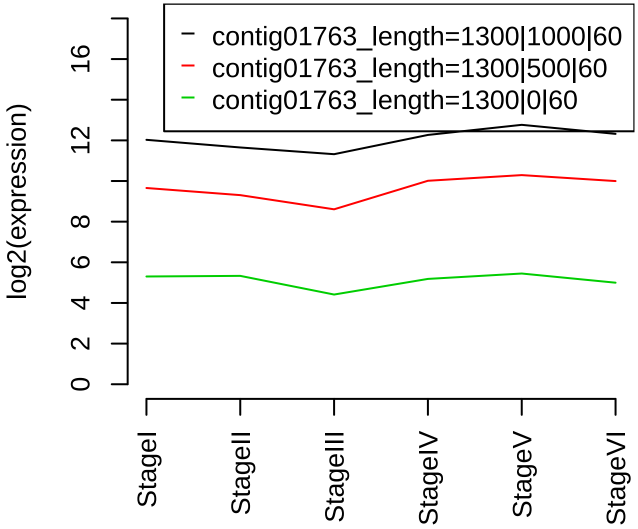
contig01763_length=1300|1000|60
contig01763_length=1300|500|60
contig01763_length=1300|0|60
log2(expression)
0
2
4
6
8
12
16
StageI
StageII
StageIII
StageIV
StageV
StageVI

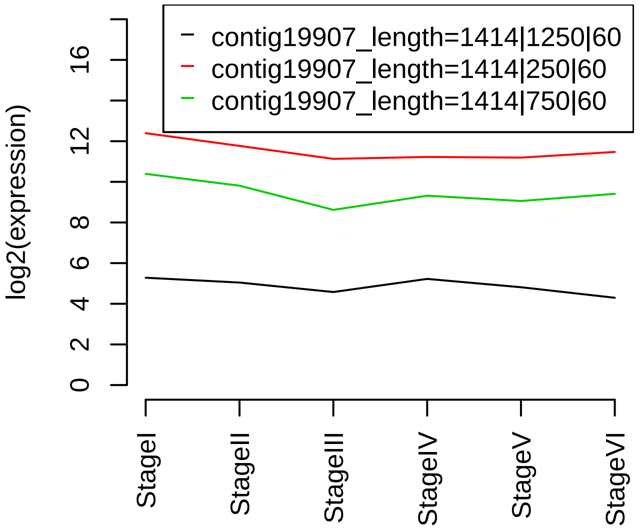
contig19907_length=1414|1250|60
contig19907_length=1414|250|60
contig19907_length=1414|750|60
log2(expression)
0
2
4
6
8
12
16
StageI
StageII
StageIII
StageIV
StageV
StageVI

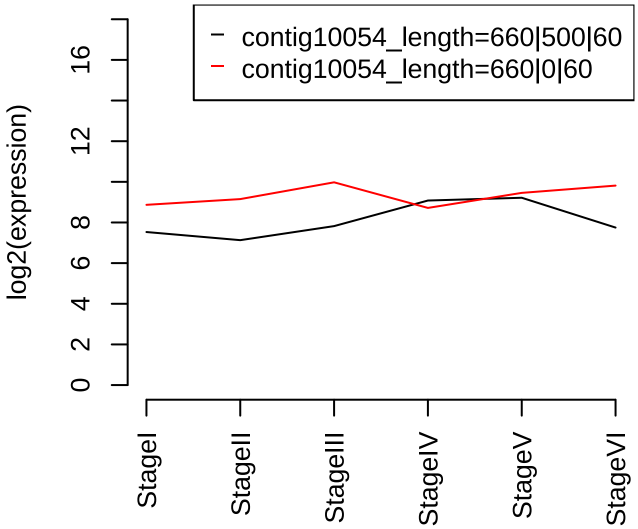
contig10054_length=660|500|60
contig10054_length=660|0|60
log2(expression)
0
2
4
6
8
12
16
StageI
StageII
StageIII
StageIV
StageV
StageVI

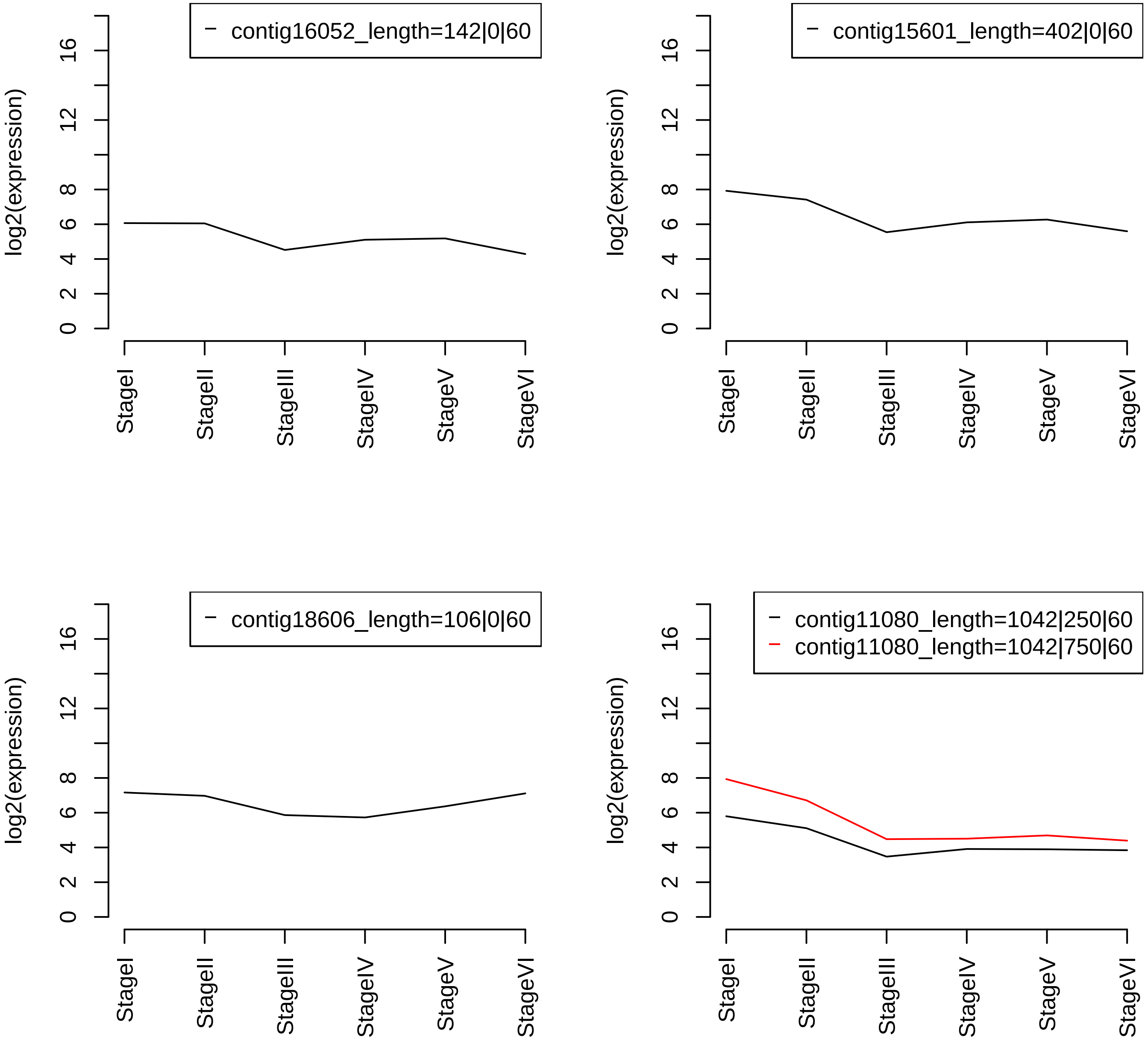

contig16052_length=142|0|60
contig15601_length=402|0|60
contig18606_length=106|0|60
contig11080_length=1042|250|60
contig11080_length=1042|750|60
log2(expression)
0
2
4
6
8
12
16
StageI
StageII
StageIII
StageIV
StageV
StageVI

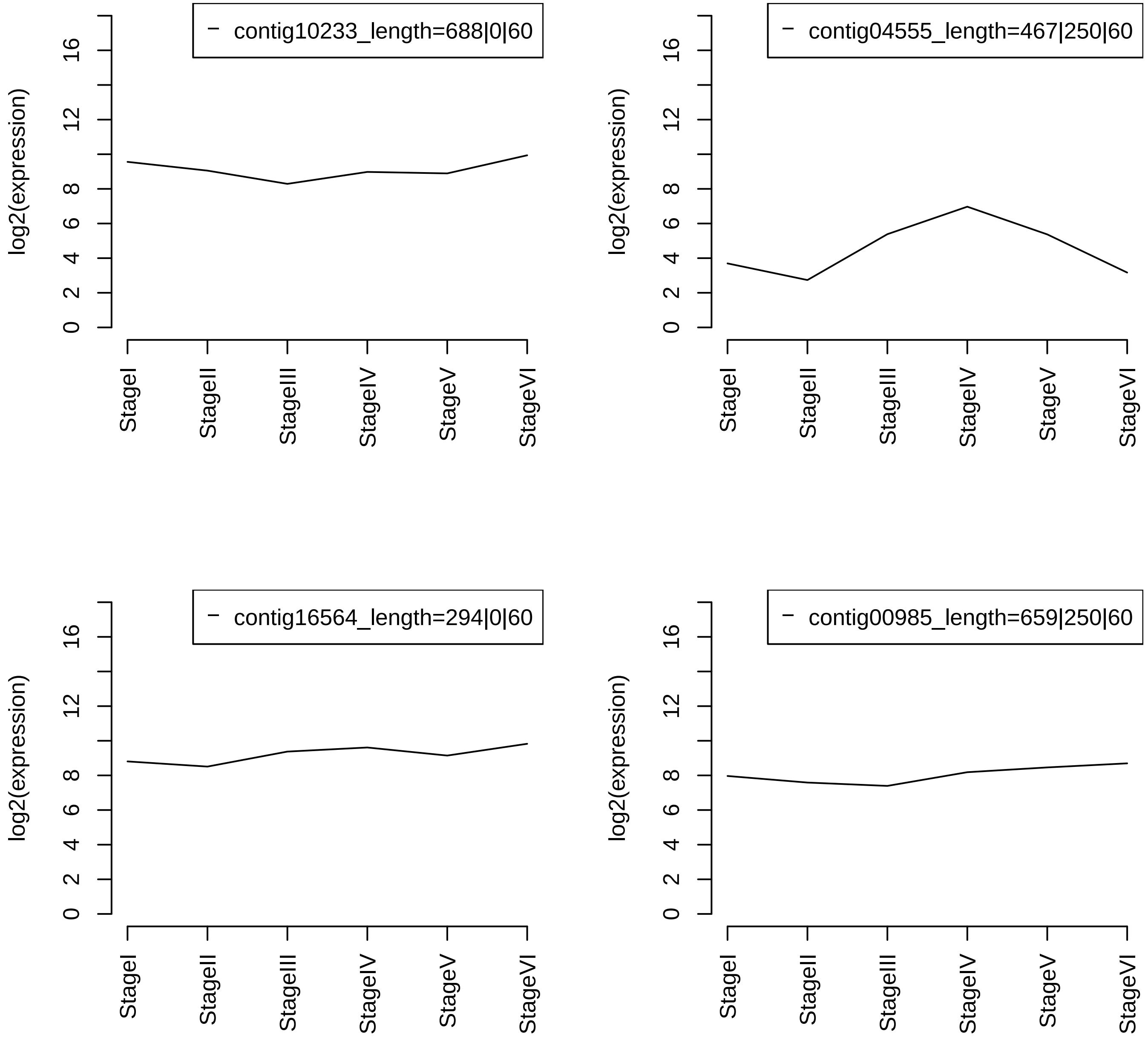
contig10233_length=688|0|60
contig04555_length=467|250|60
contig16564_length=294|0|60
contig00985_length=659|250|60
log2(expression)
0
2
4
6
8
12
16
StageI
StageII
StageIII
StageIV
StageV
StageVI

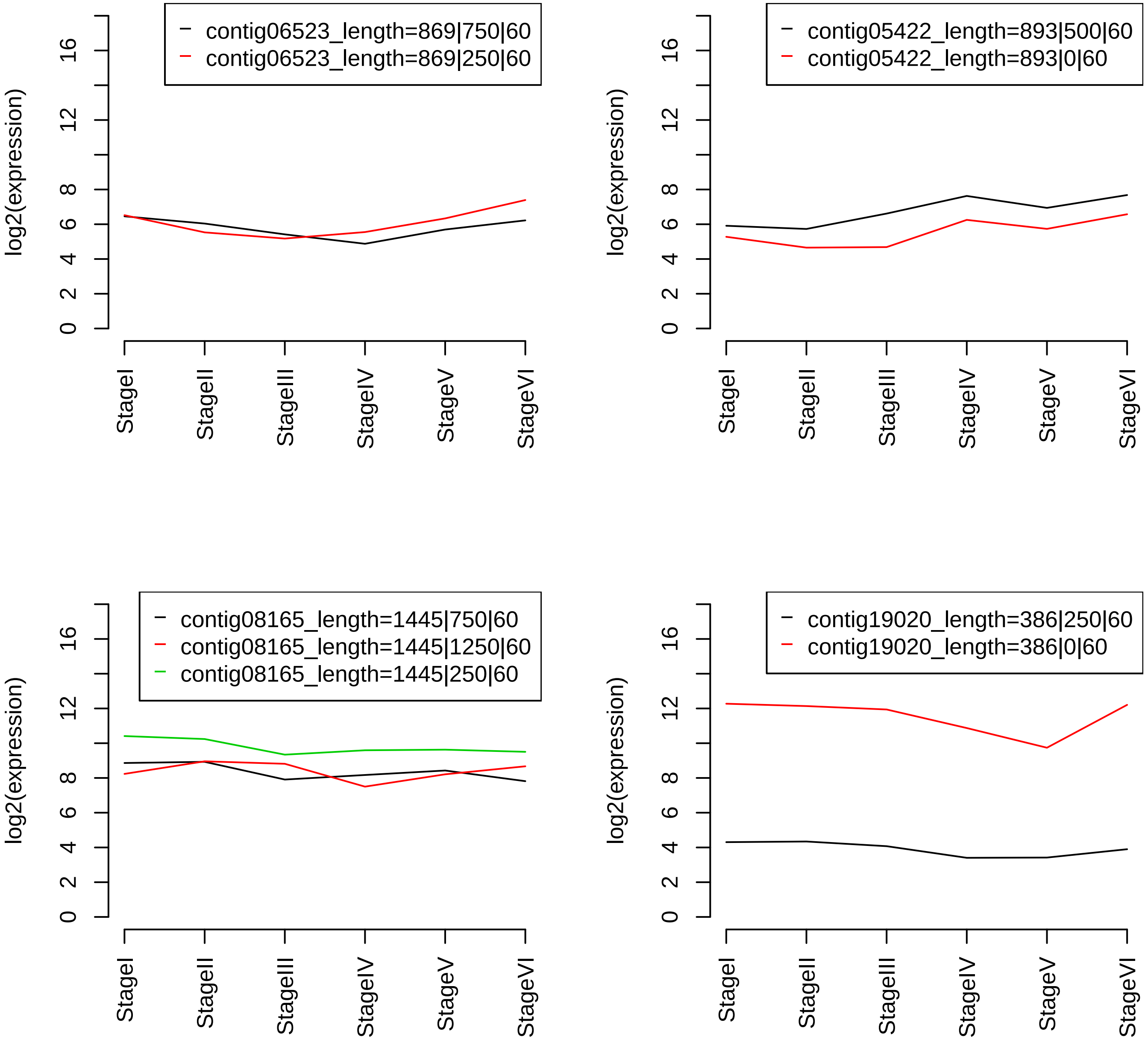
contig06523_length=869|750|60
contig06523_length=869|250|60
contig05422_length=893|500|60
contig05422_length=893|0|60
contig08165_length=1445|750|60
contig08165_length=1445|1250|60
contig08165_length=1445|250|60
contig19020_length=386|250|60
contig19020_length=386|0|60
log2(expression)
0
2
4
6
8
12
16
StageI
StageII
StageIII
StageIV
StageV
StageVI

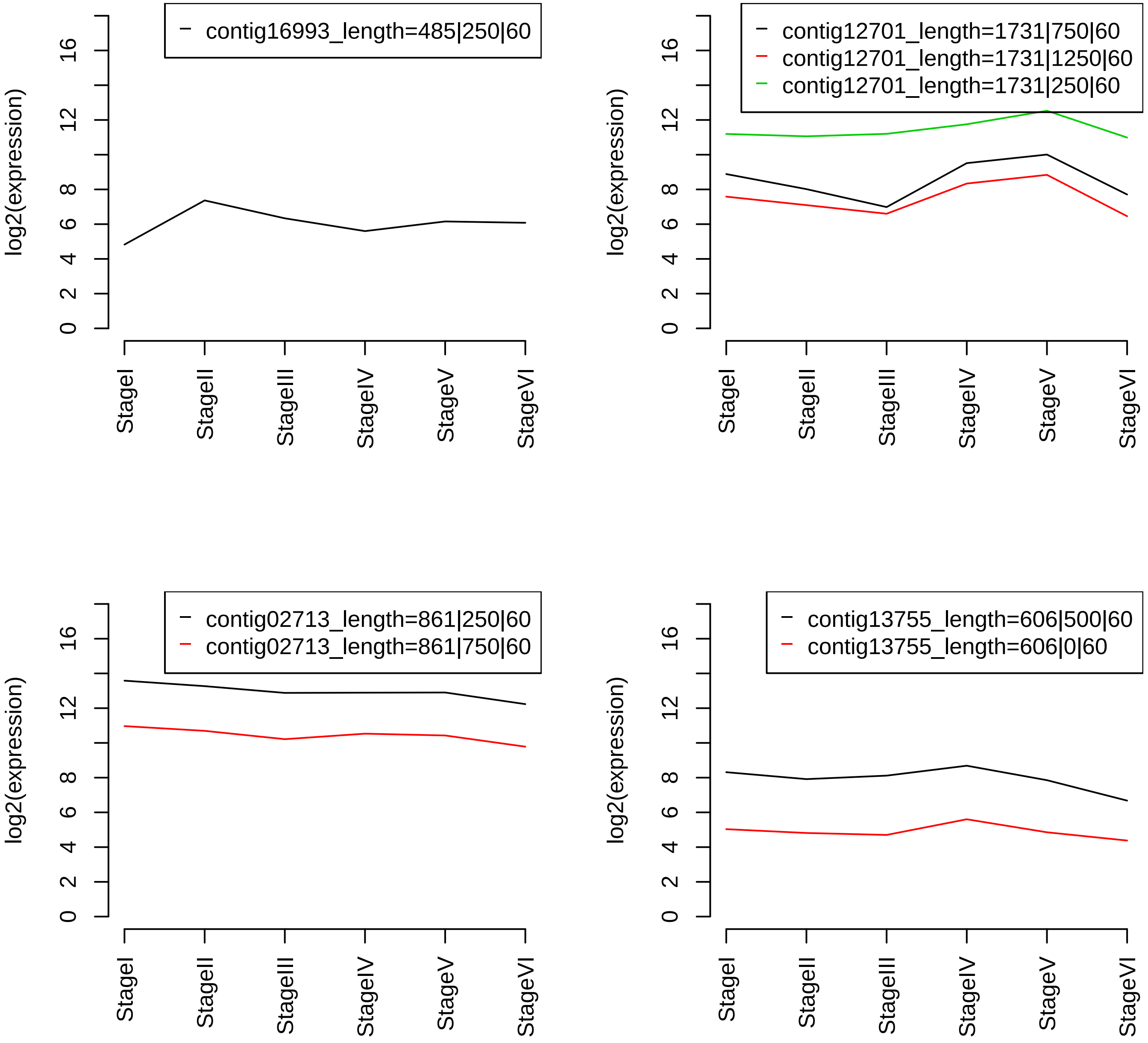
contig16993_length=485|250|60
contig12701_length=1731|750|60
contig12701_length=1731|1250|60
contig12701_length=1731|250|60
contig02713_length=861|250|60
contig02713_length=861|750|60
contig13755_length=606|500|60
contig13755_length=606|0|60
log2(expression)
0
2
4
6
8
12
16
StageI
StageII
StageIII
StageIV
StageV
StageVI

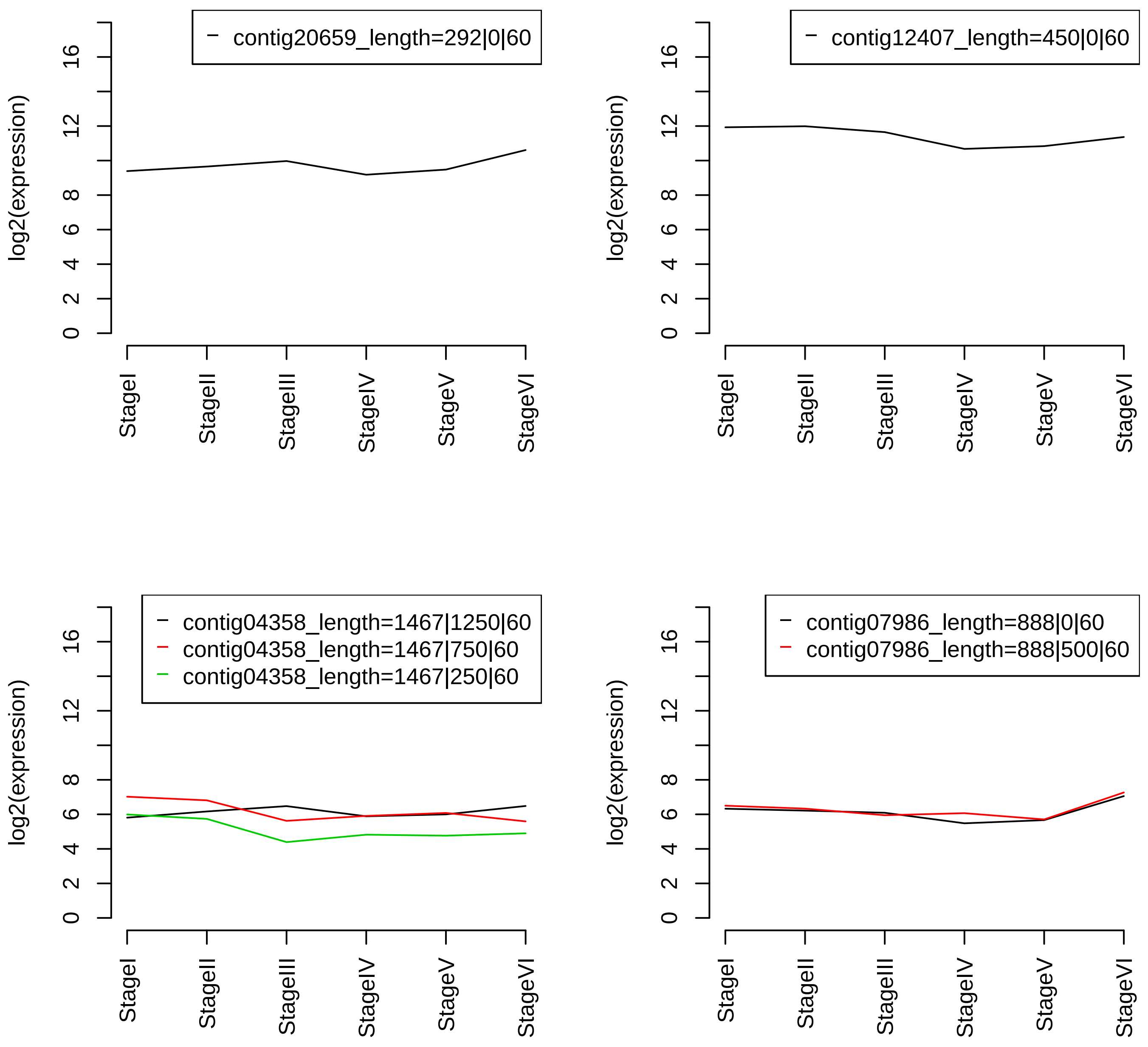

contig20659_length=292|0|60
contig12407_length=450|0|60
contig04358_length=1467|1250|60
contig04358_length=1467|750|60
contig04358_length=1467|250|60
contig07986_length=888|0|60
contig07986_length=888|500|60
log2(expression)
0
2
4
6
8
12
16
StageI
StageII
StageIII
StageIV
StageV
StageVI

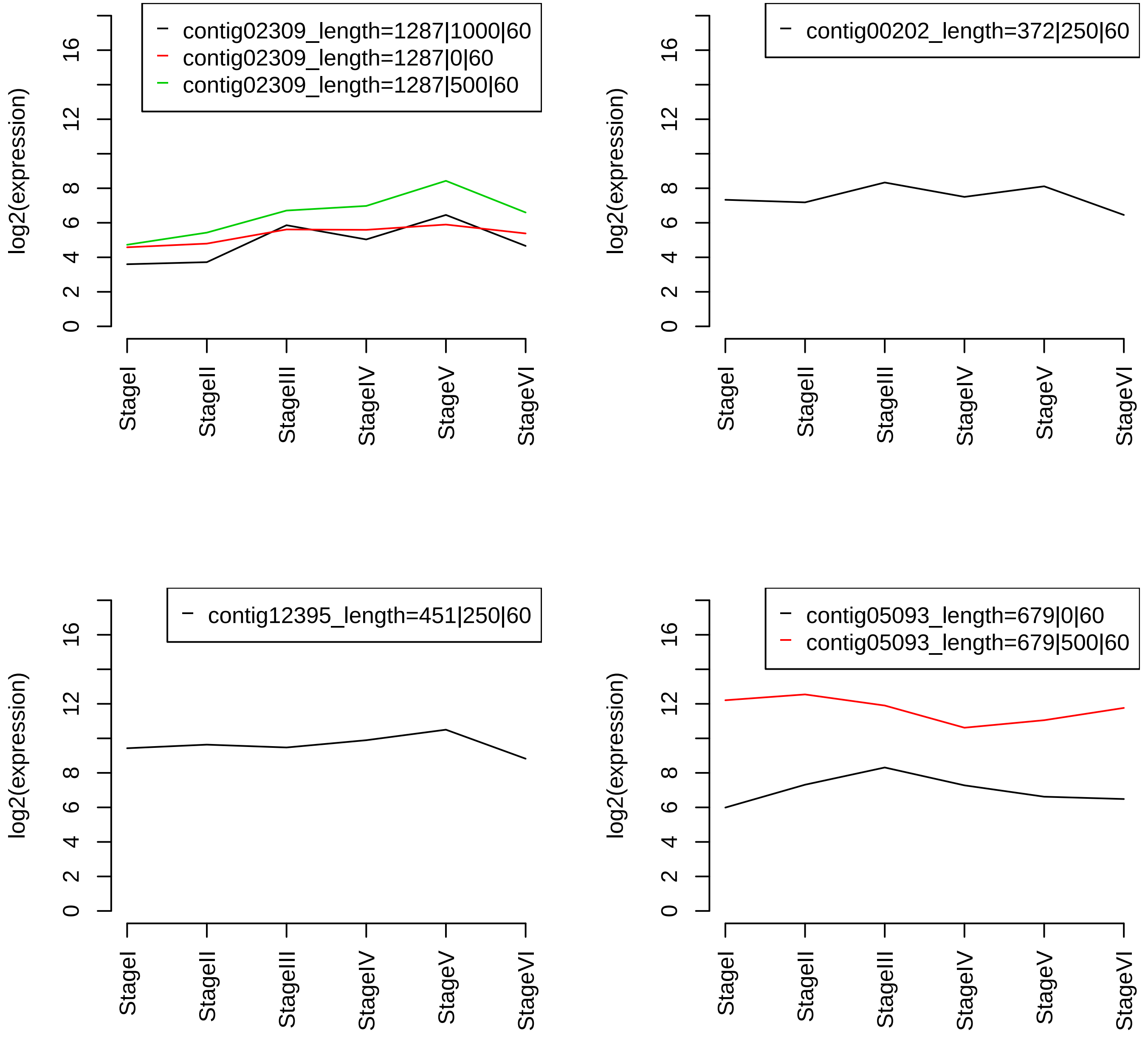
contig02309_length=1287|1000|60
contig02309_length=1287|0|60
contig02309_length=1287|500|60
contig00202_length=372|250|60
contig12395_length=451|250|60
contig05093_length=679|0|60
contig05093_length=679|500|60
log2(expression)
0
2
4
6
8
12
16
StageI
StageII
StageIII
StageIV
StageV
StageVI

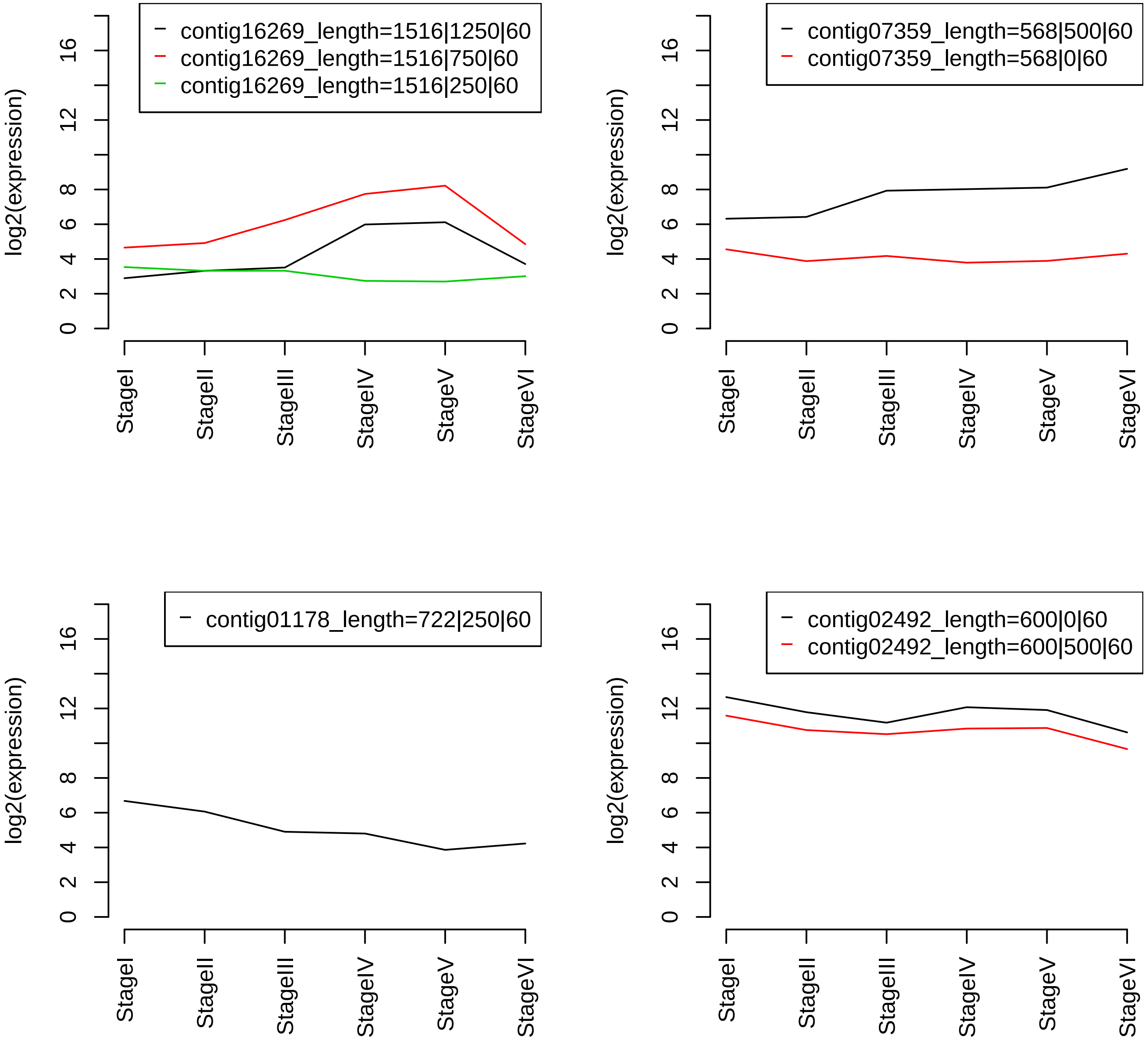

contig16269_length=1516|1250|60
contig16269_length=1516|750|60
contig16269_length=1516|250|60
contig07359_length=568|500|60
contig07359_length=568|0|60
contig01178_length=722|250|60
contig02492_length=600|0|60
contig02492_length=600|500|60
log2(expression)
0
2
4
6
8
12
16
StageI
StageII
StageIII
StageIV
StageV
StageVI

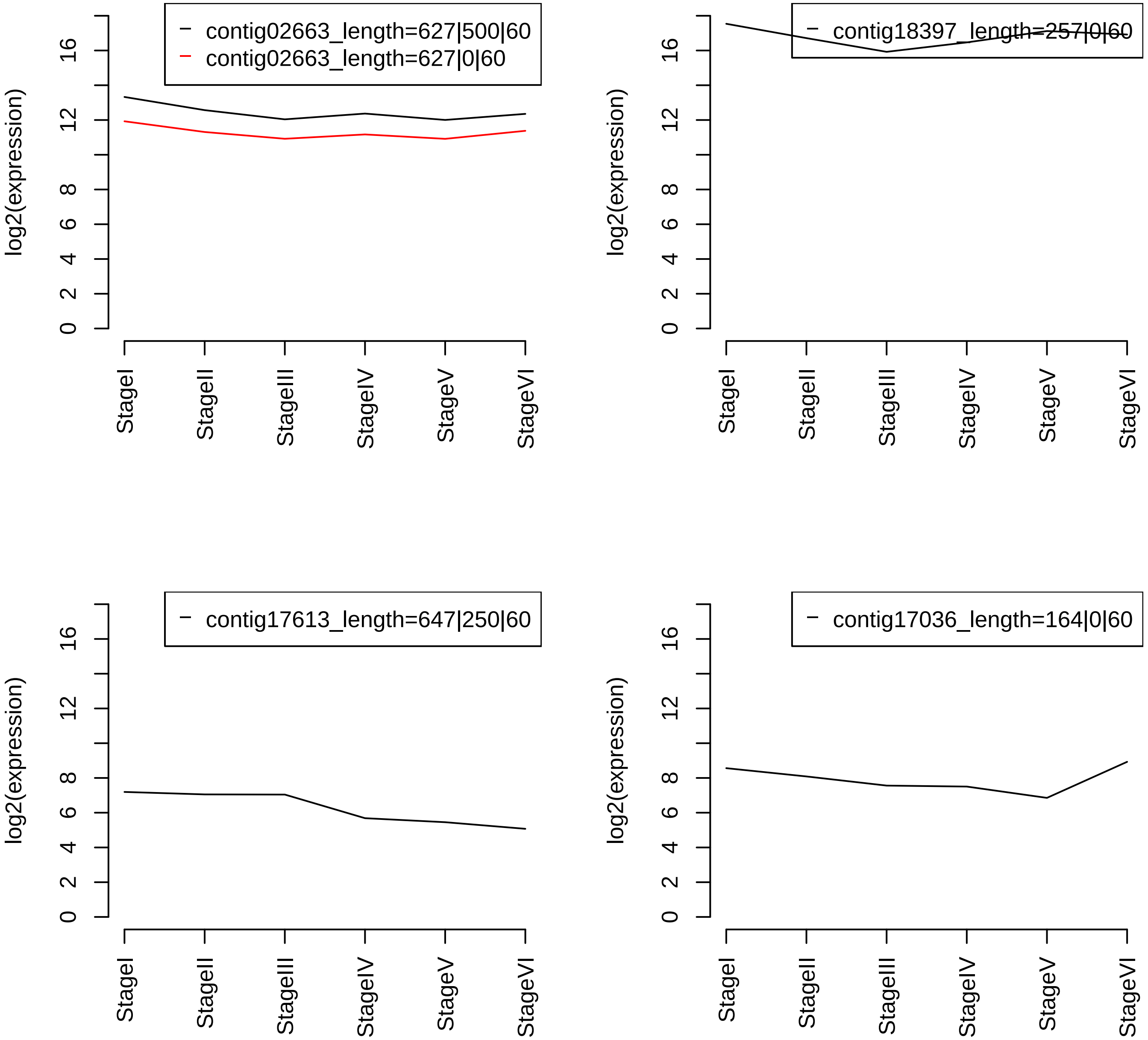
contig02663_length=627|500|60
contig02663_length=627|0|60
contig18397_length=257|0|60
contig17613_length=647|250|60
contig17036_length=164|0|60
log2(expression)
0
2
4
6
8
12
16
StageI
StageII
StageIII
StageIV
StageV
StageVI

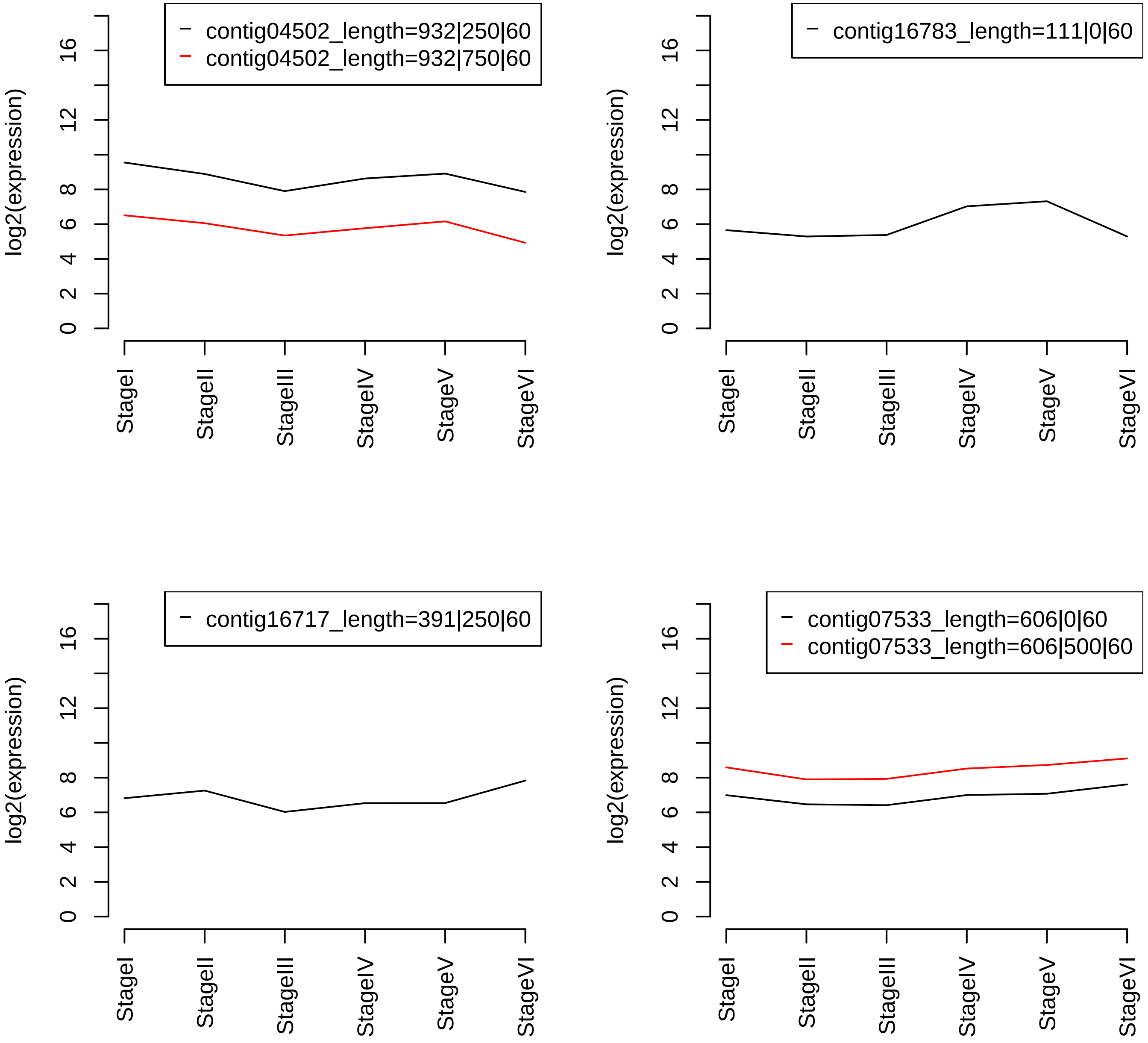

contig04502_length=932|250|60
contig04502_length=932|750|60
contig16783_length=111|0|60
contig16717_length=391|250|60
contig07533_length=606|0|60
contig07533_length=606|500|60
log2(expression)
0
2
4
6
8
12
16
StageI
StageII
StageIII
StageIV
StageV
StageVI

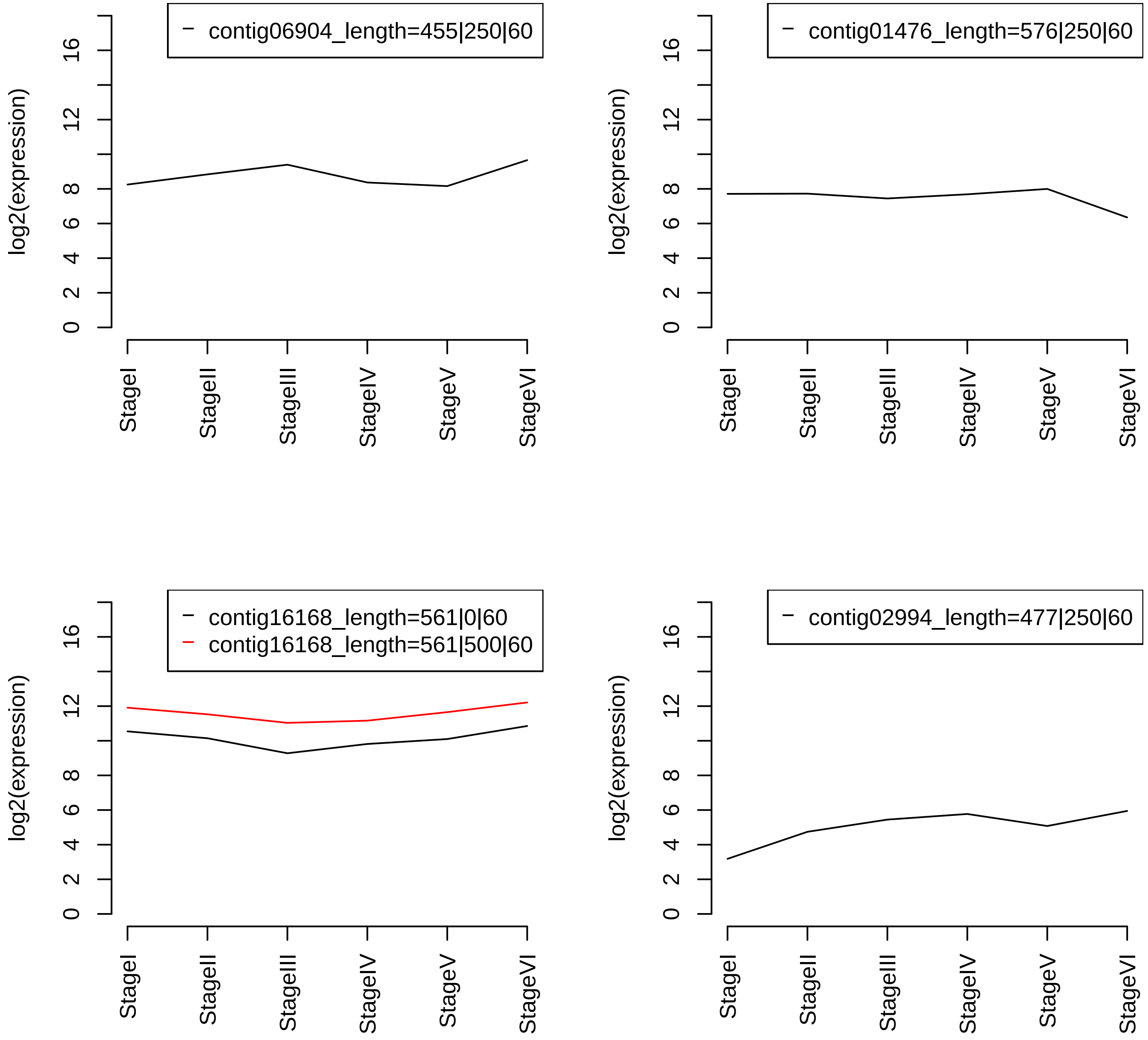

contig06904_length=455|250|60
contig01476_length=576|250|60
contig16168_length=561|0|60
contig16168_length=561|500|60
contig02994_length=477|250|60
log2(expression)
0
2
4
6
8
12
16
StageI
StageII
StageIII
StageIV
StageV
StageVI

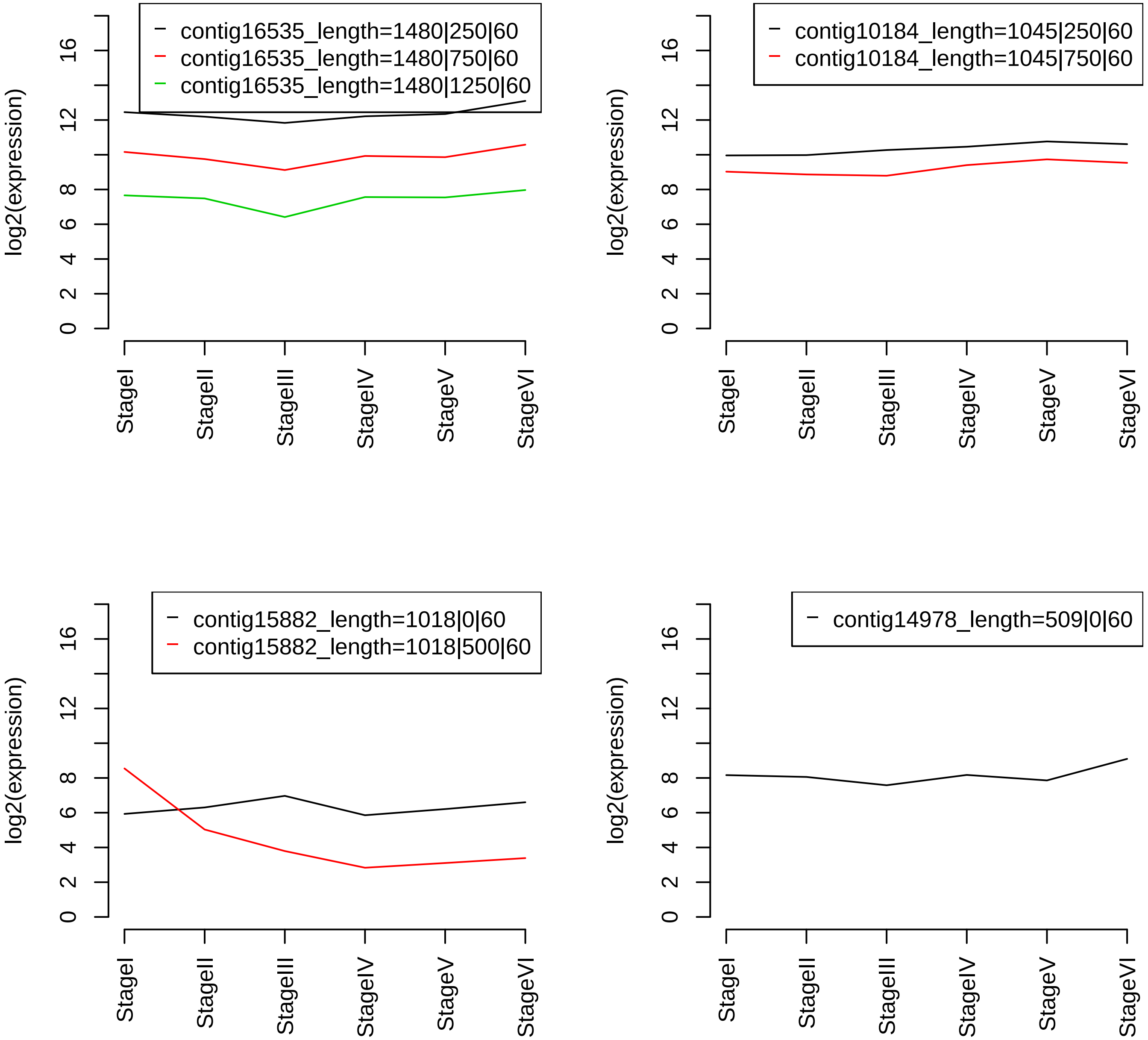

contig16535_length=1480|250|60
contig16535_length=1480|750|60
contig16535_length=1480|1250|60
contig10184_length=1045|250|60
contig10184_length=1045|750|60
contig15882_length=1018|0|60
contig15882_length=1018|500|60
contig14978_length=509|0|60
log2(expression)
0 2 4 6 8 12 16
StageI StageII StageIII StageIV StageV StageVI

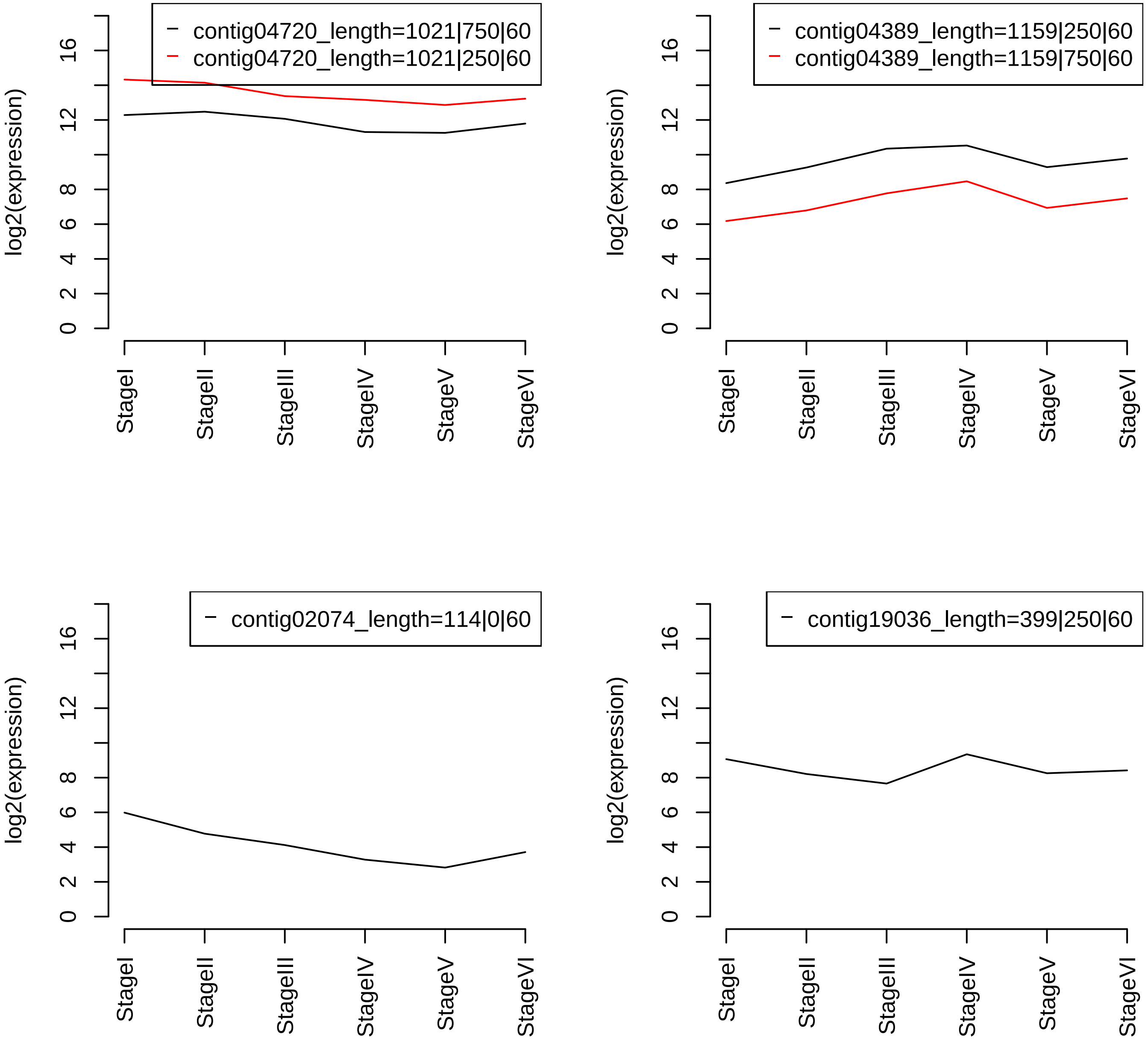

contig04720_length=1021|750|60
contig04720_length=1021|250|60
contig04389_length=1159|250|60
contig04389_length=1159|750|60
contig02074_length=114|0|60
contig19036_length=399|250|60
log2(expression)
0
2
4
6
8
12
16
StageI
StageII
StageIII
StageIV
StageV
StageVI

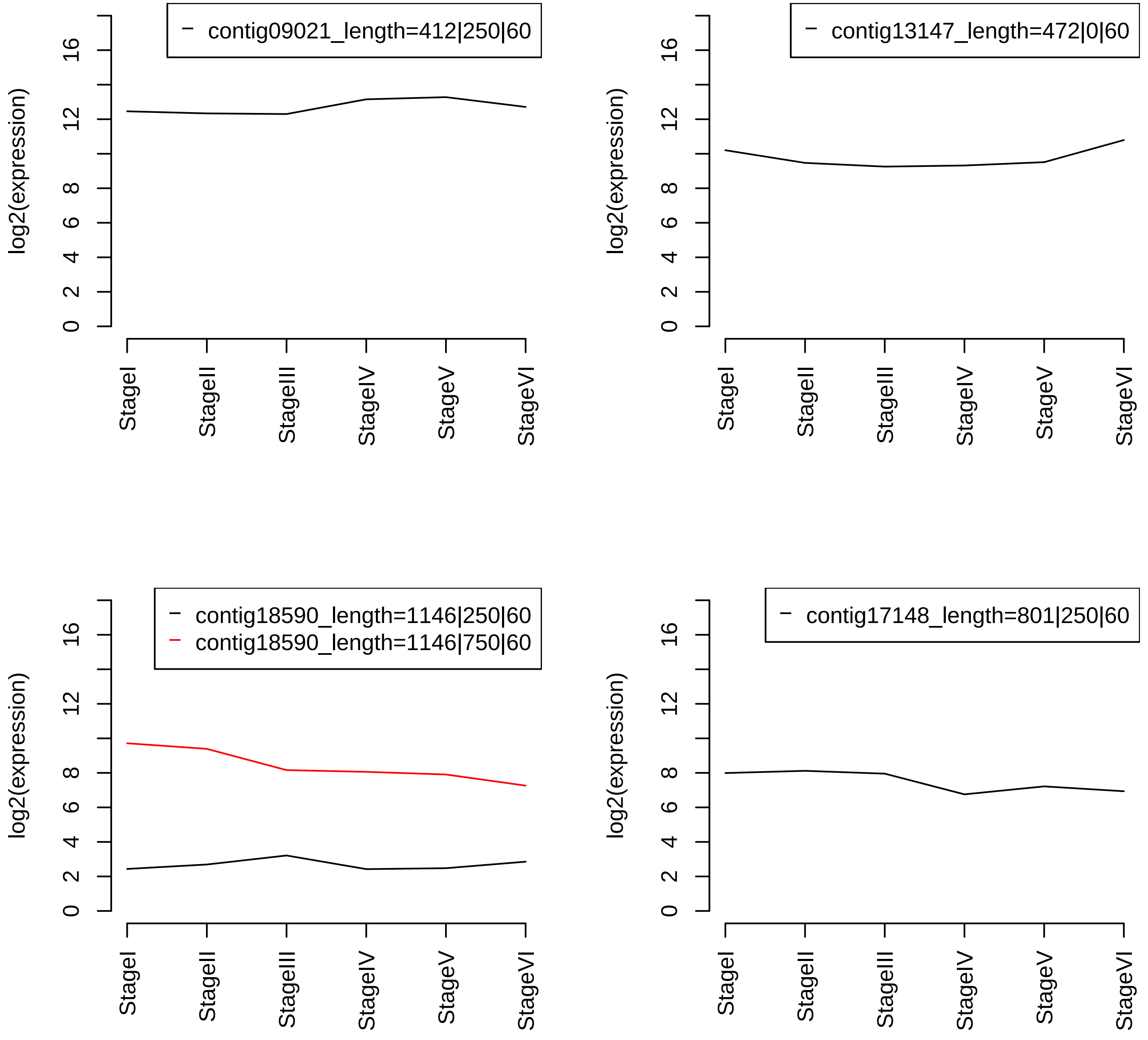

contig09021_length=412|250|60
contig13147_length=472|0|60
contig18590_length=1146|250|60
contig18590_length=1146|750|60
contig17148_length=801|250|60
log2(expression)
0
2
4
6
8
12
16
StageI
StageII
StageIII
StageIV
StageV
StageVI

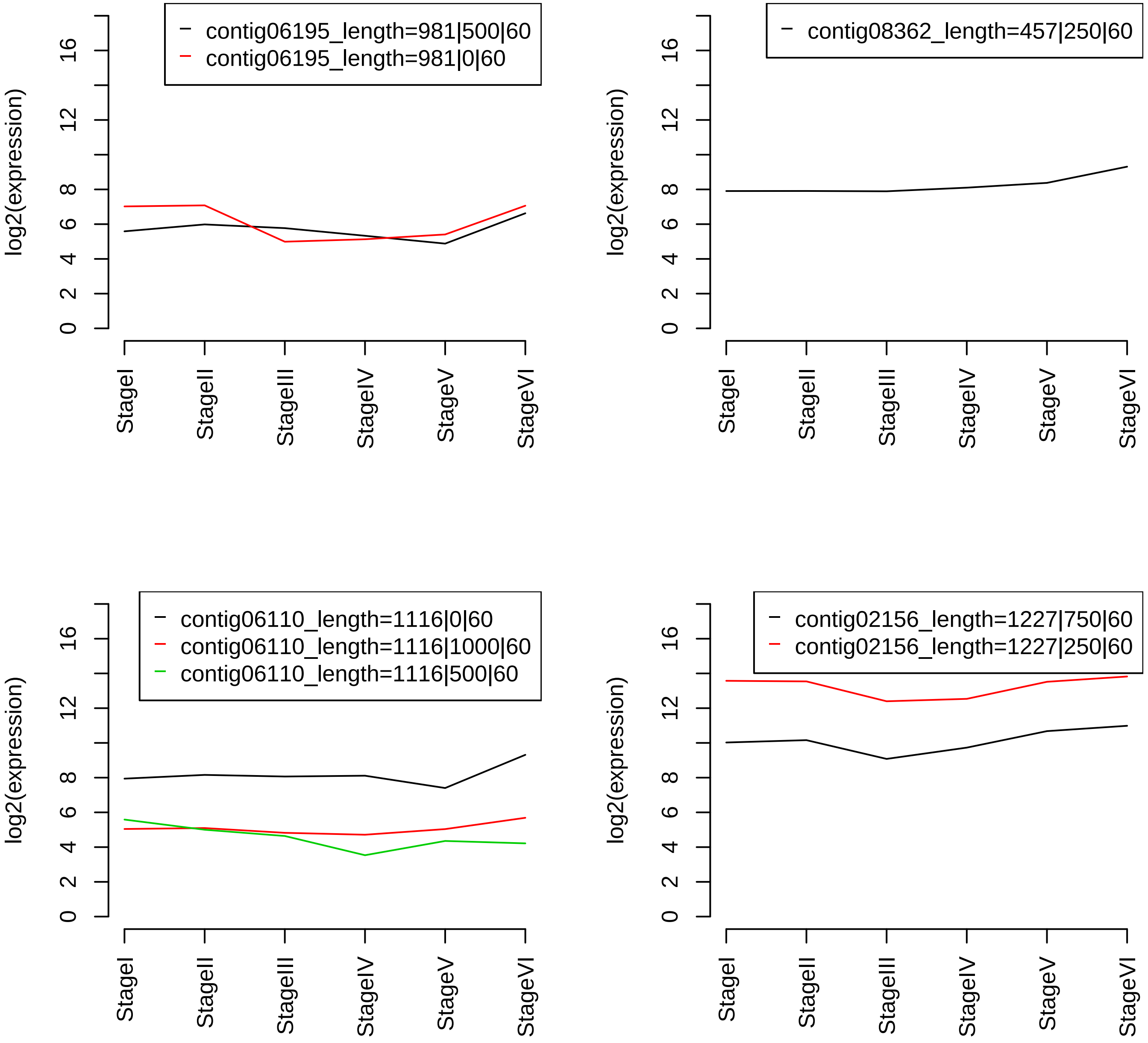
contig06195_length=981|500|60
contig06195_length=981|0|60
contig08362_length=457|250|60
contig06110_length=1116|0|60
contig06110_length=1116|1000|60
contig06110_length=1116|500|60
contig02156_length=1227|750|60
contig02156_length=1227|250|60
log2(expression)
StageI
StageII
StageIII
StageIV
StageV
StageVI

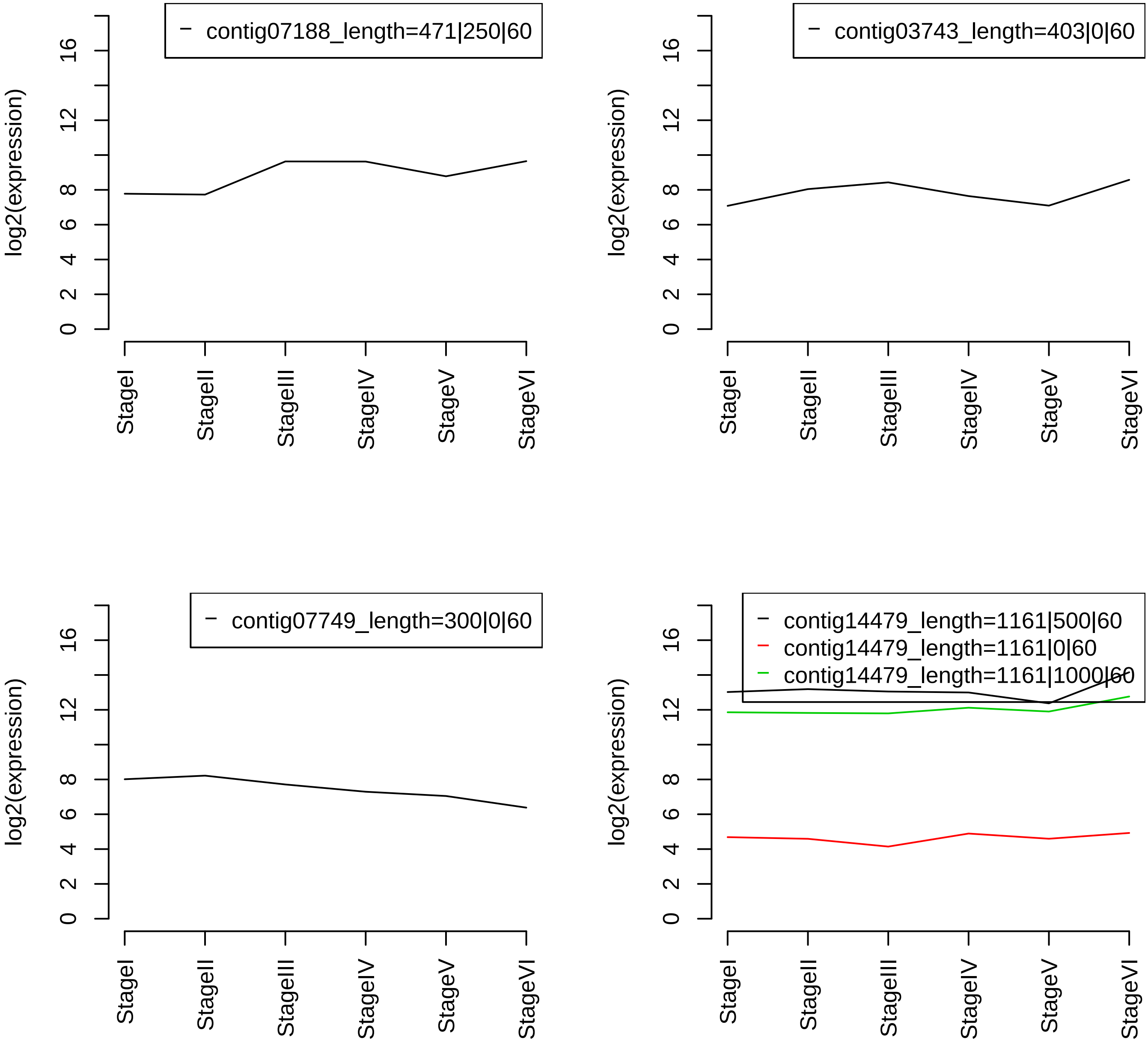
contig07188_length=471|250|60
contig03743_length=403|0|60
contig07749_length=300|0|60
contig14479_length=1161|500|60
contig14479_length=1161|0|60
contig14479_length=1161|1000|60
log2(expression)
0
2
4
6
8
12
16
StageI
StageII
StageIII
StageIV
StageV
StageVI

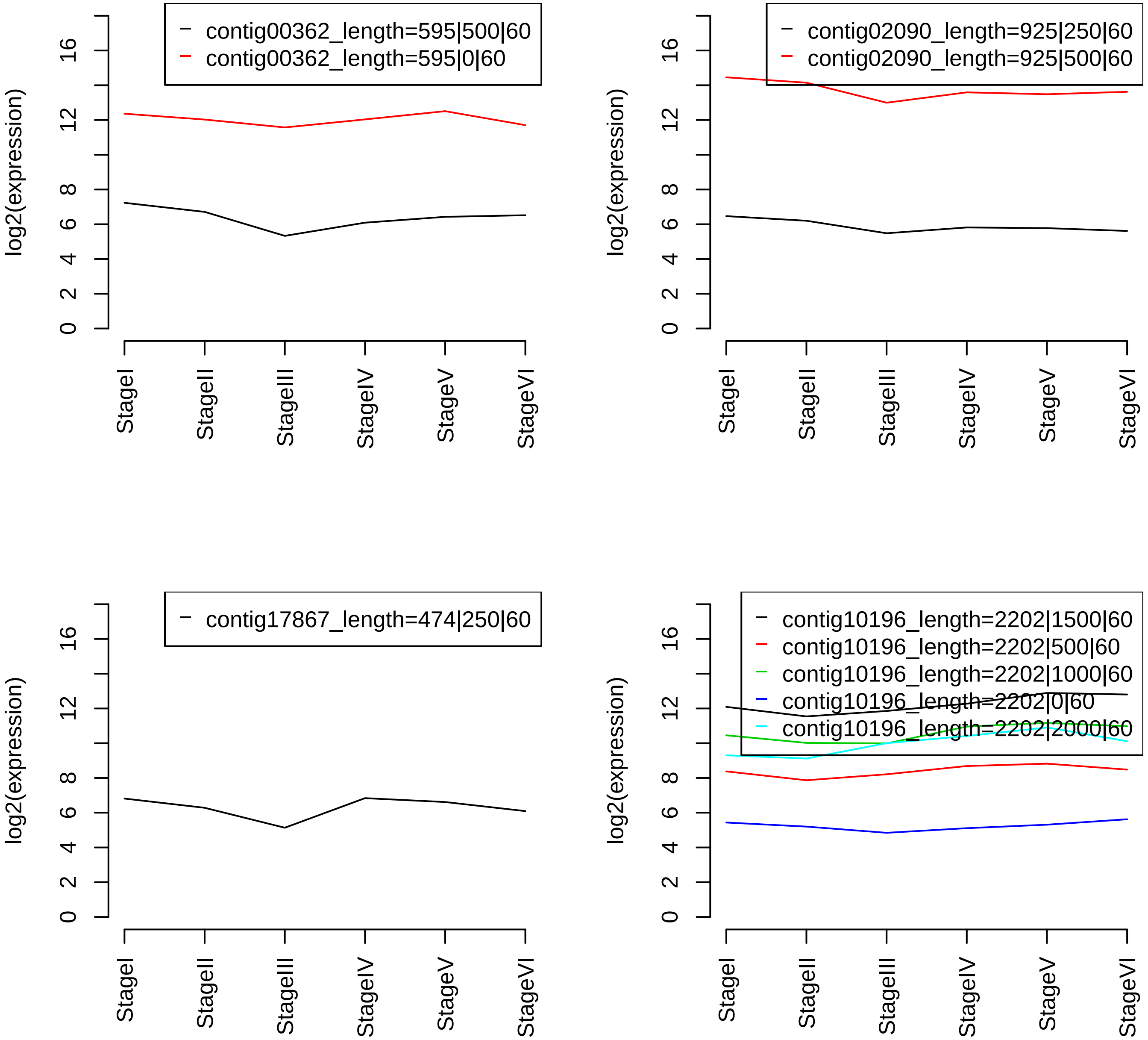
contig00362_length=595|500|60
contig00362_length=595|0|60
contig02090_length=925|250|60
contig02090_length=925|500|60
contig17867_length=474|250|60
contig10196_length=2202|1500|60
contig10196_length=2202|500|60
contig10196_length=2202|1000|60
contig10196_length=2202|0|60
contig10196_length=2202|2000|60
log2(expression)
0
2
4
6
8
12
16
StageI
StageII
StageIII
StageIV
StageV
StageVI

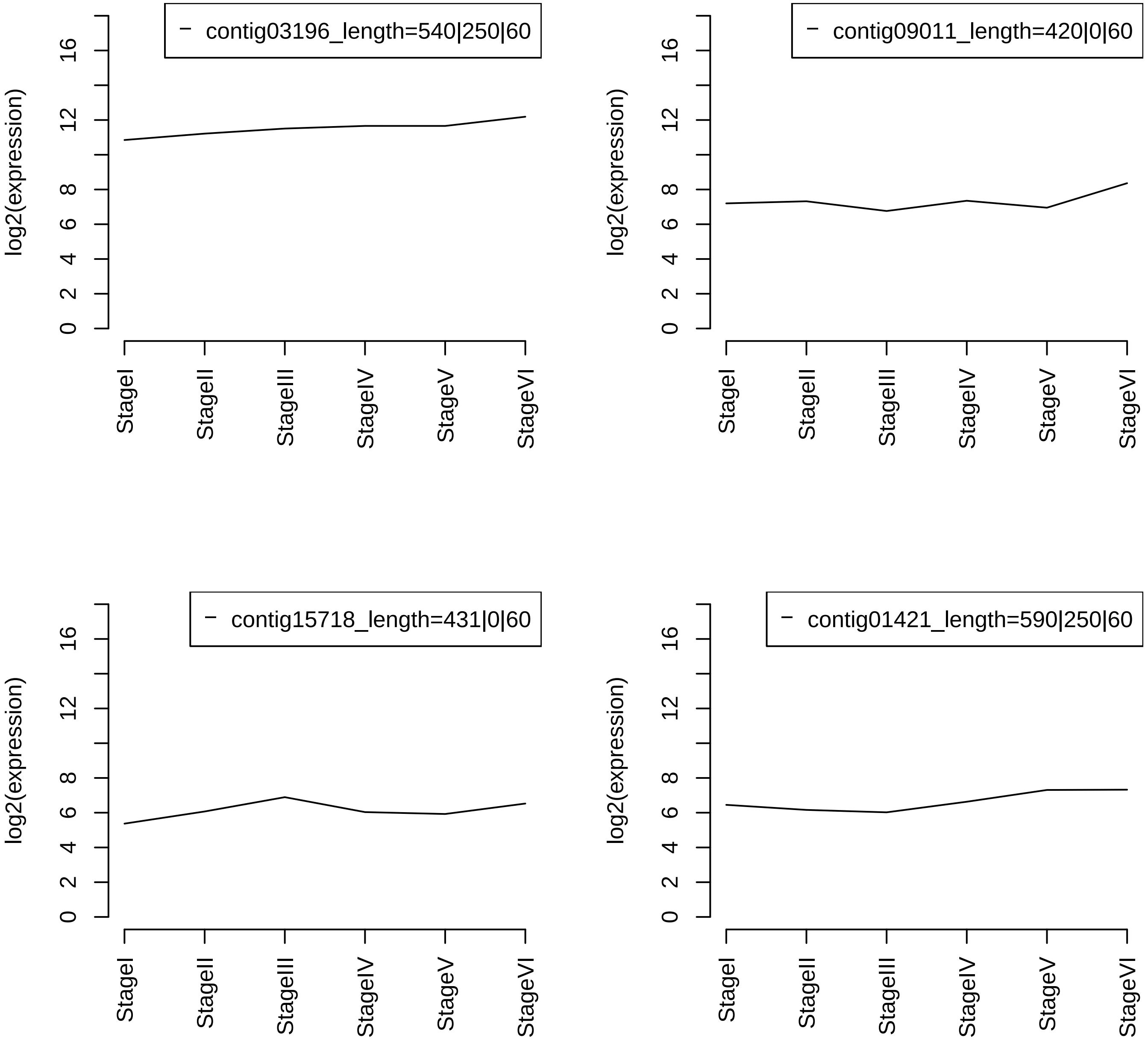
contig03196_length=540|250|60
log2(expression)
0
2
4
6
8
12
16
StageI
StageII
StageIII
StageIV
StageV
StageVI
contig09011_length=420|0|60
log2(expression)
0
2
4
6
8
12
16
StageI
StageII
StageIII
StageIV
StageV
StageVI
contig15718_length=431|0|60
log2(expression)
0
2
4
6
8
12
16
StageI
StageII
StageIII
StageIV
StageV
StageVI
contig01421_length=590|250|60
log2(expression)
0
2
4
6
8
12
16
StageI
StageII
StageIII
StageIV
StageV
StageVI

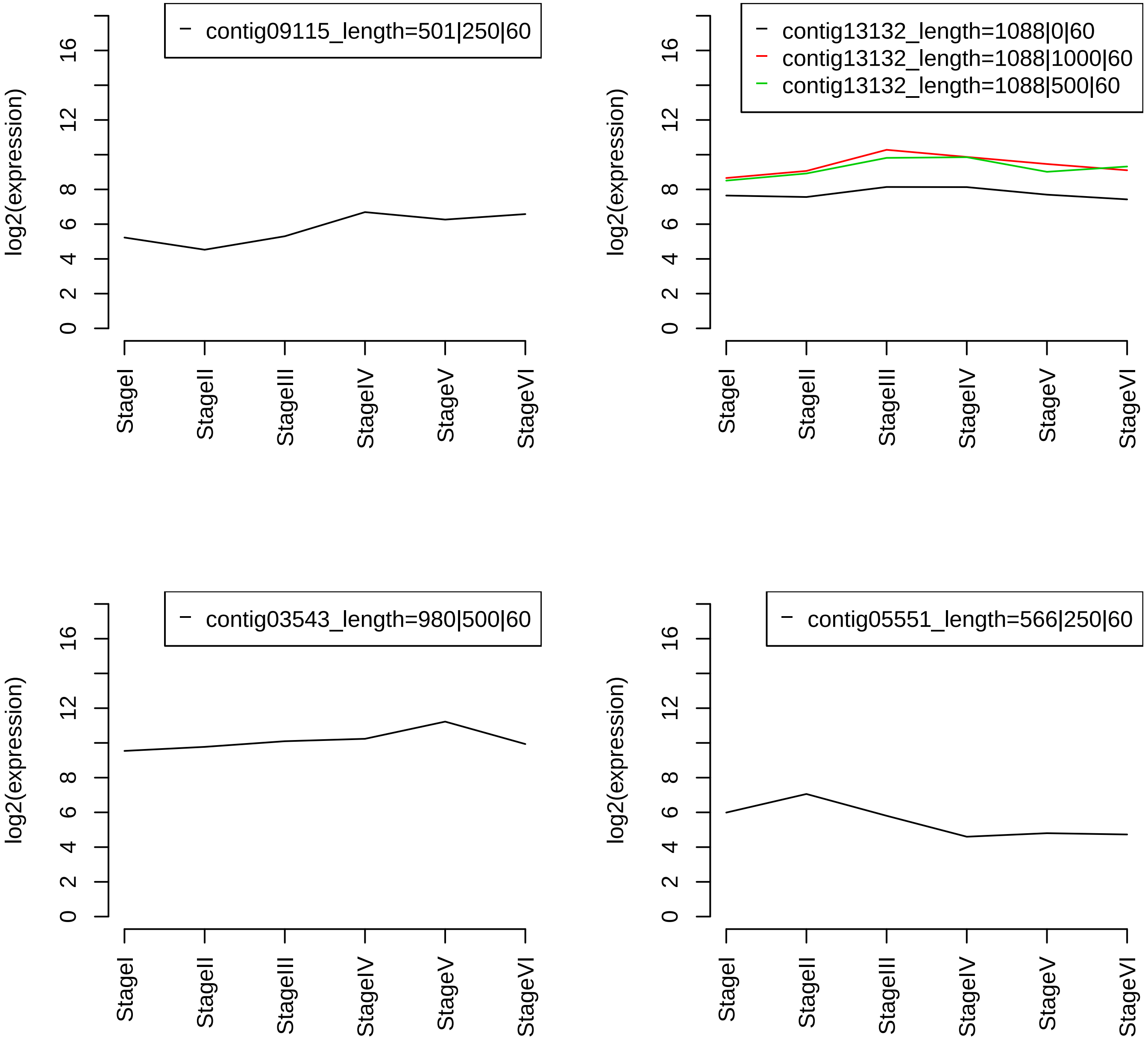
contig09115_length=501|250|60
contig13132_length=1088|0|60
contig13132_length=1088|1000|60
contig13132_length=1088|500|60
contig03543_length=980|500|60
contig05551_length=566|250|60
log2(expression)
0
2
4
6
8
12
16
StageI
StageII
StageIII
StageIV
StageV
StageVI

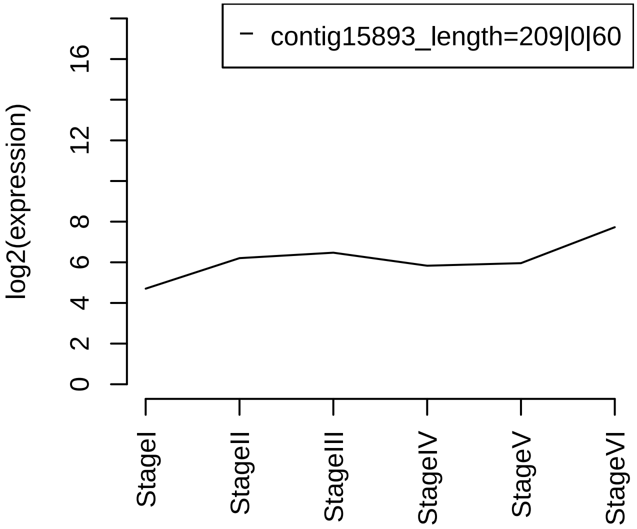
contig15893_length=209|0|60
log2(expression)
0
2
4
6
8
12
16
StageI
StageII
StageIII
StageIV
StageV
StageVI

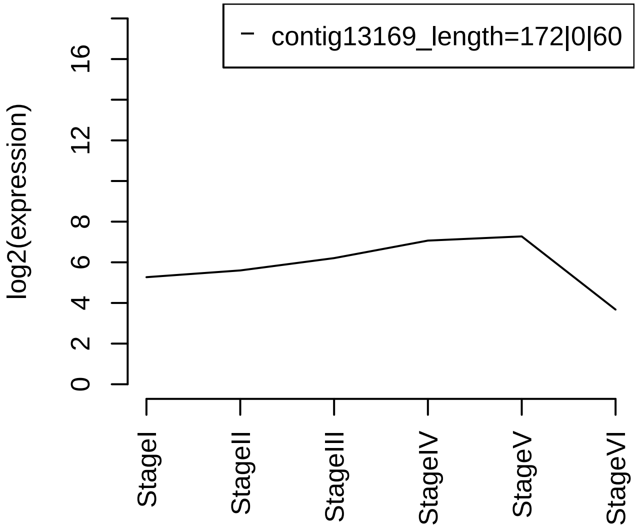
contig13169_length=172|0|60
log2(expression)
0
2
4
6
8
12
16
StageI
StageII
StageIII
StageIV
StageV
StageVI

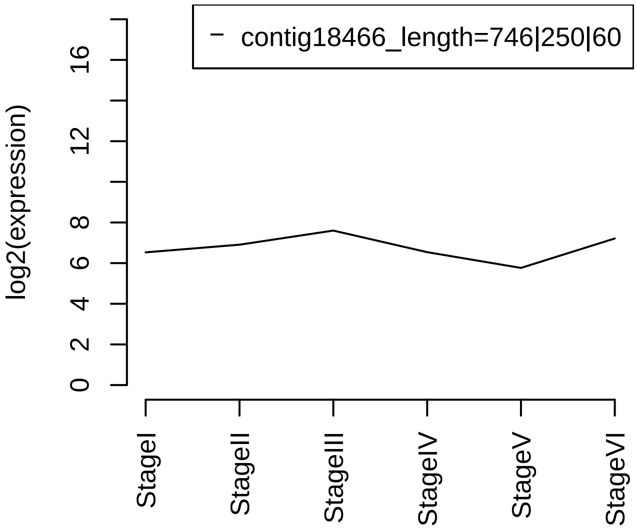
contig18466_length=746|250|60
log2(expression)
0
2
4
6
8
12
16
StageI
StageII
StageIII
StageIV
StageV
StageVI

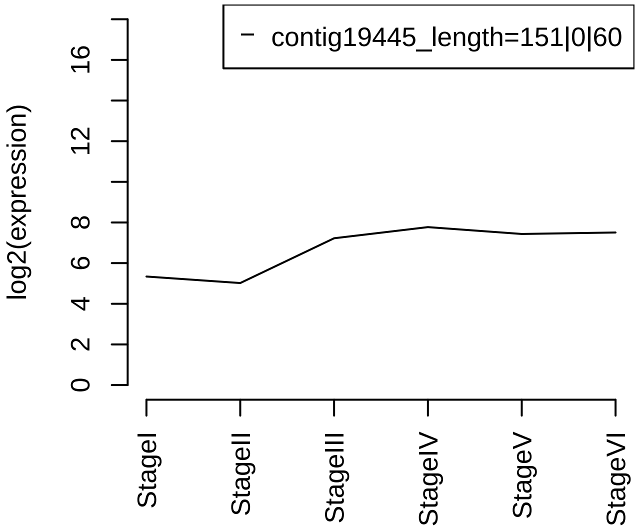
contig19445_length=151|0|60
log2(expression)
0
2
4
6
8
12
16
StageI
StageII
StageIII
StageIV
StageV
StageVI

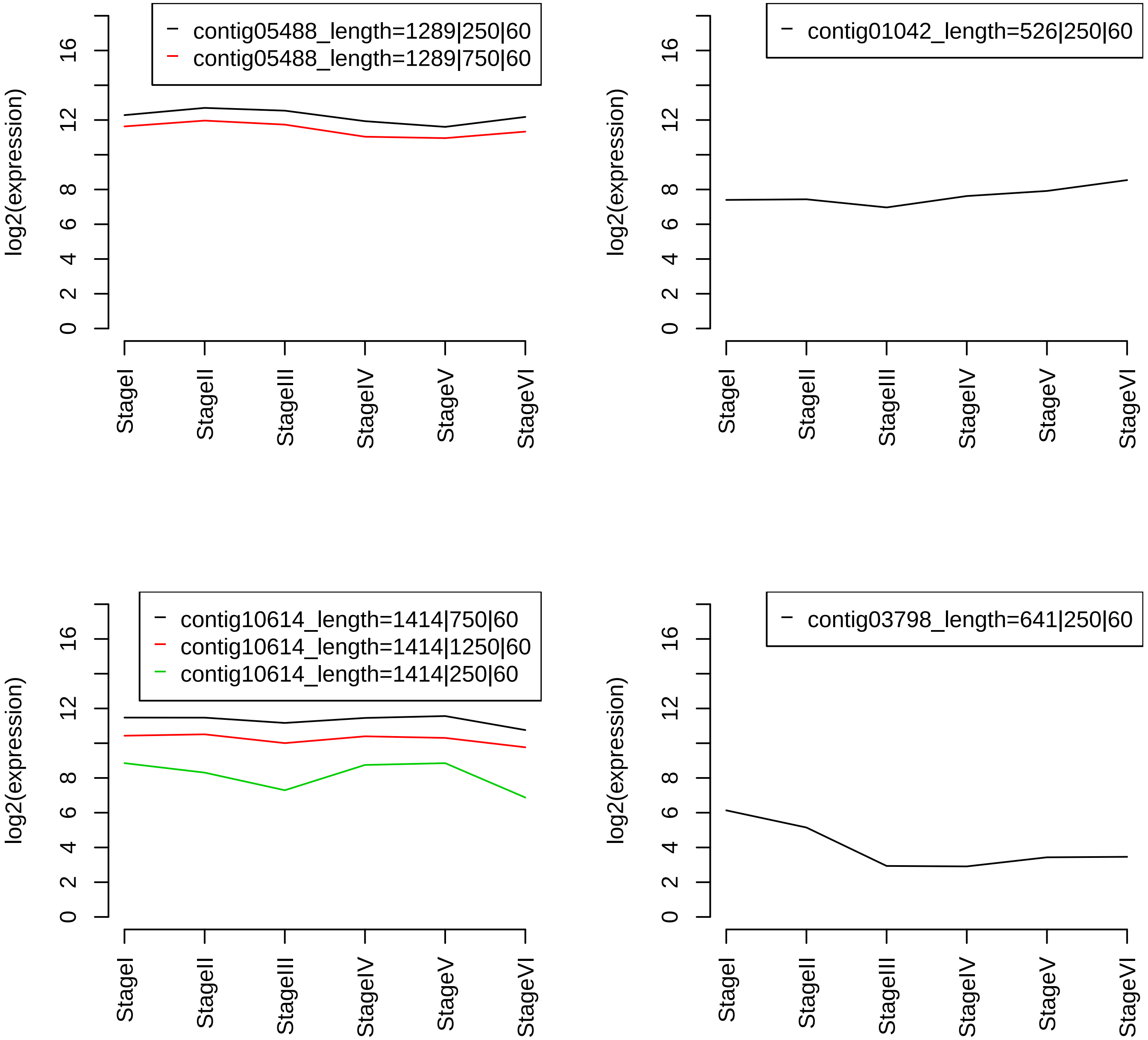

contig05488_length=1289|250|60
contig05488_length=1289|750|60
contig01042_length=526|250|60
contig10614_length=1414|750|60
contig10614_length=1414|1250|60
contig10614_length=1414|250|60
contig03798_length=641|250|60
log2(expression)
0
2
4
6
8
12
16
StageI
StageII
StageIII
StageIV
StageV
StageVI

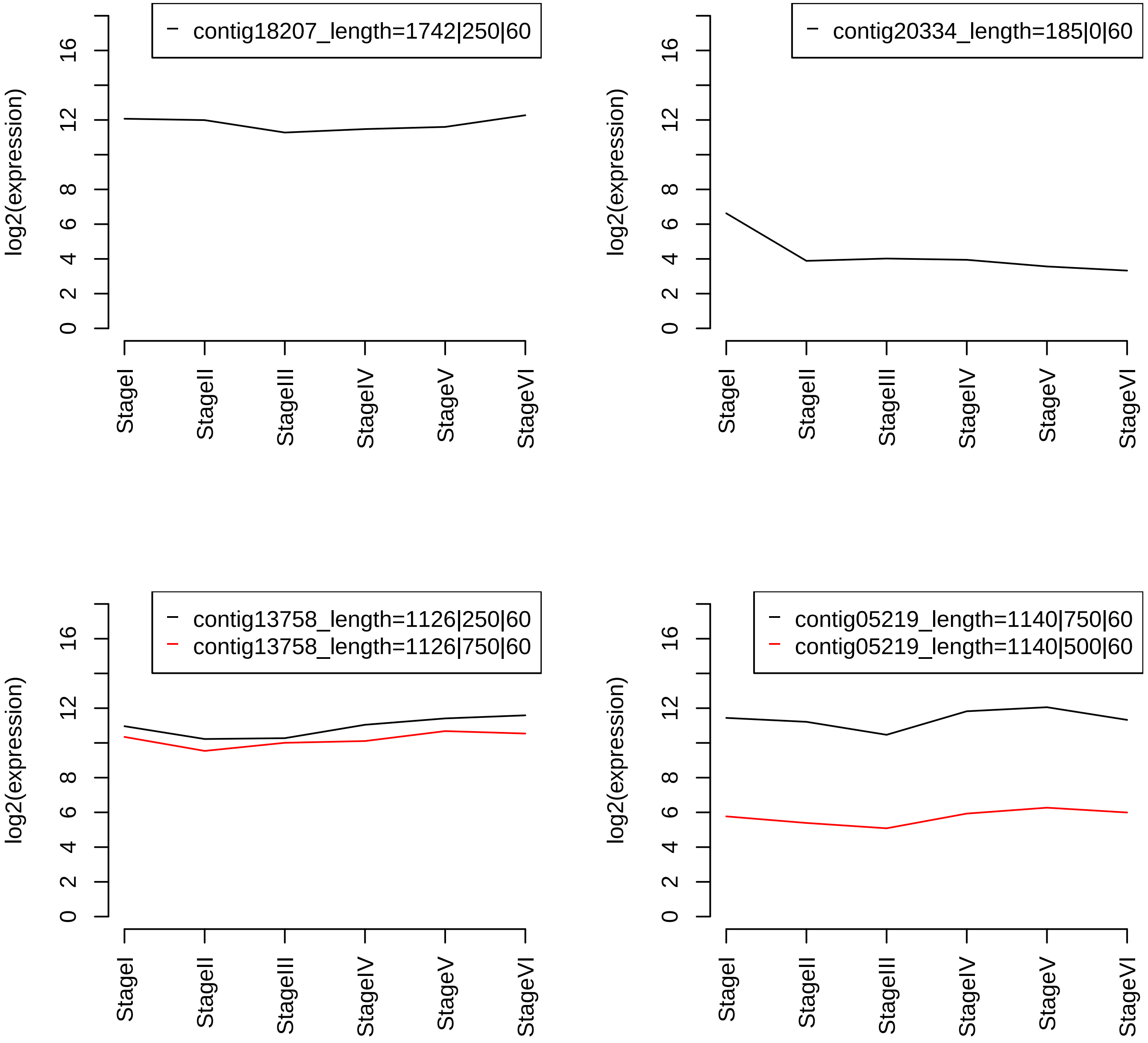
contig18207_length=1742|250|60
contig20334_length=185|0|60
contig13758_length=1126|250|60
contig13758_length=1126|750|60
contig05219_length=1140|750|60
contig05219_length=1140|500|60
log2(expression)
0
2
4
6
8
12
16
StageI
StageII
StageIII
StageIV
StageV
StageVI

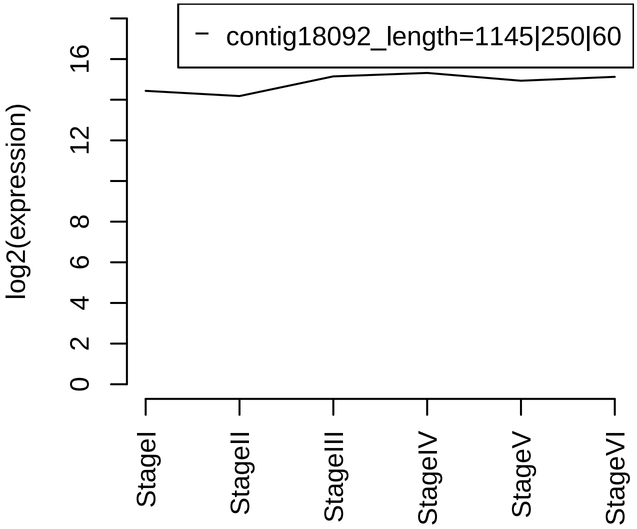

contig18092_length=1145|250|60
log2(expression)
0
2
4
6
8
12
16
StageI
StageII
StageIII
StageIV
StageV
StageVI

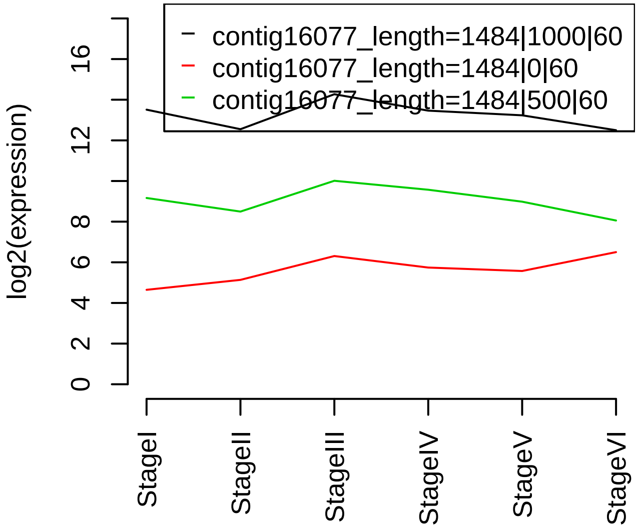

contig16077_length=1484|1000|60
contig16077_length=1484|0|60
contig16077_length=1484|500|60
log2(expression)
0
2
4
6
8
12
16
StageI
StageII
StageIII
StageIV
StageV
StageVI

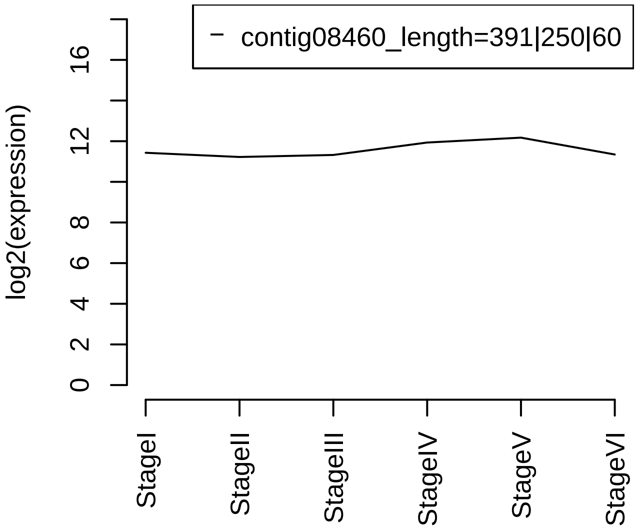

contig08460_length=391|250|60
log2(expression)
0
2
4
6
8
12
16
StageI
StageII
StageIII
StageIV
StageV
StageVI

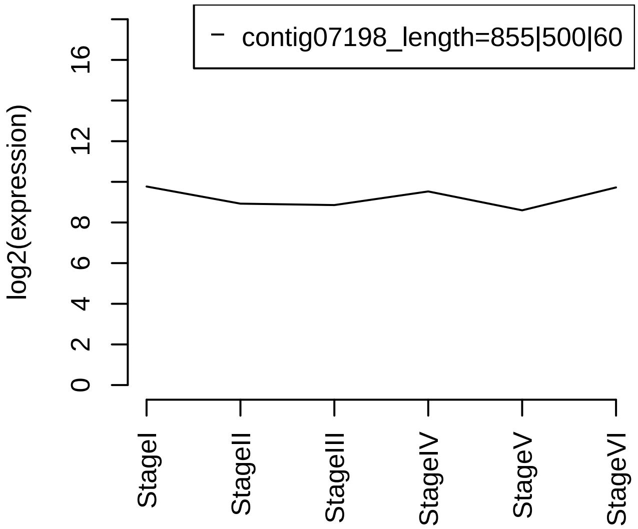

contig07198_length=855|500|60
log2(expression)
0
2
4
6
8
12
16
StageI
StageII
StageIII
StageIV
StageV
StageVI

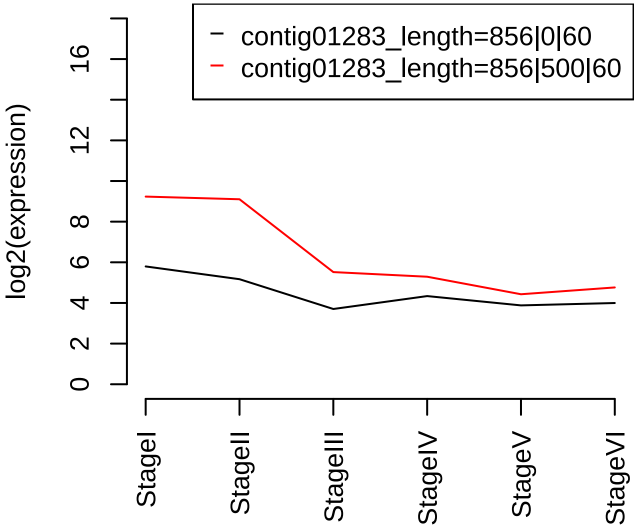

contig01283_length=856|0|60
contig01283_length=856|500|60
log2(expression)
0
2
4
6
8
12
16
StageI
StageII
StageIII
StageIV
StageV
StageVI

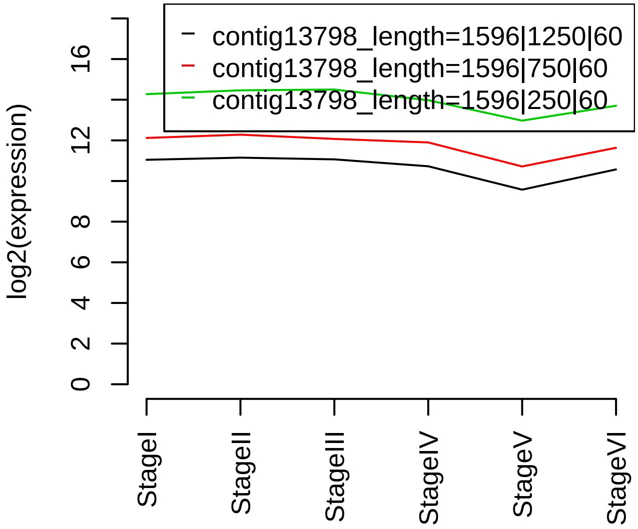

contig13798_length=1596|1250|60
contig13798_length=1596|750|60
contig13798_length=1596|250|60
log2(expression)
0
2
4
6
8
12
16
StageI
StageII
StageIII
StageIV
StageV
StageVI

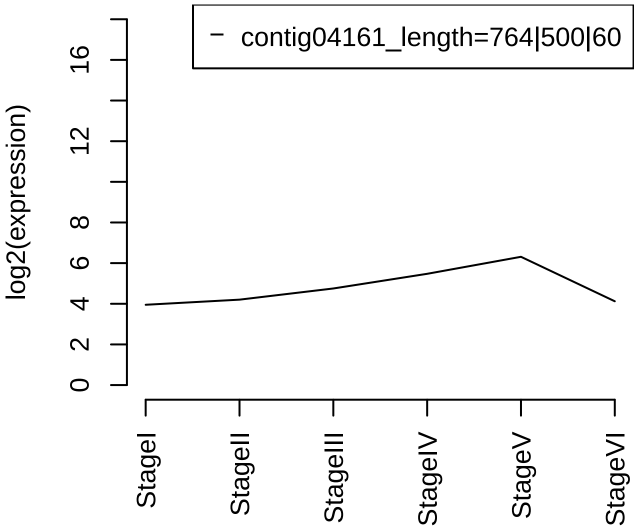

contig04161_length=764|500|60
log2(expression)
0
2
4
6
8
12
16
StageI
StageII
StageIII
StageIV
StageV
StageVI

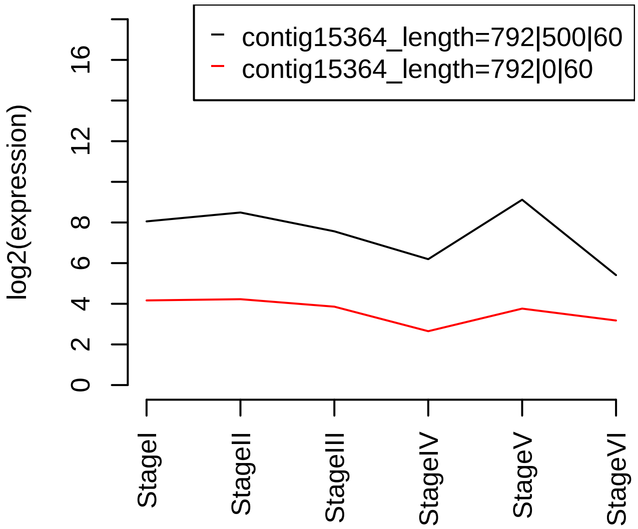

contig15364_length=792|500|60
contig15364_length=792|0|60
log2(expression)
0
2
4
6
8
12
16
StageI
StageII
StageIII
StageIV
StageV
StageVI

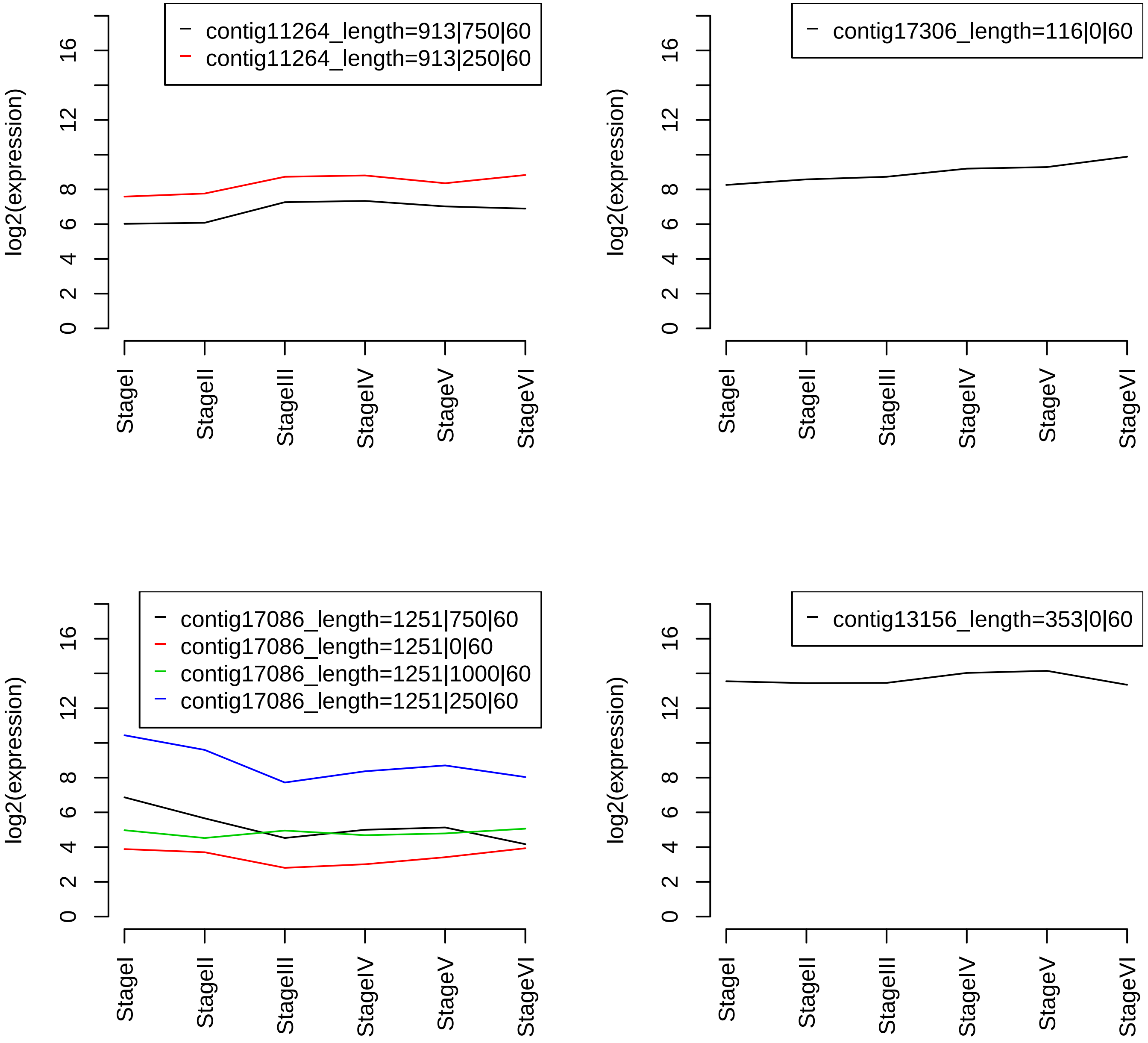
contig11264_length=913|750|60
contig11264_length=913|250|60
contig17306_length=116|0|60
contig17086_length=1251|750|60
contig17086_length=1251|0|60
contig17086_length=1251|1000|60
contig17086_length=1251|250|60
contig13156_length=353|0|60
log2(expression)
StageI
StageII
StageIII
StageIV
StageV
StageVI

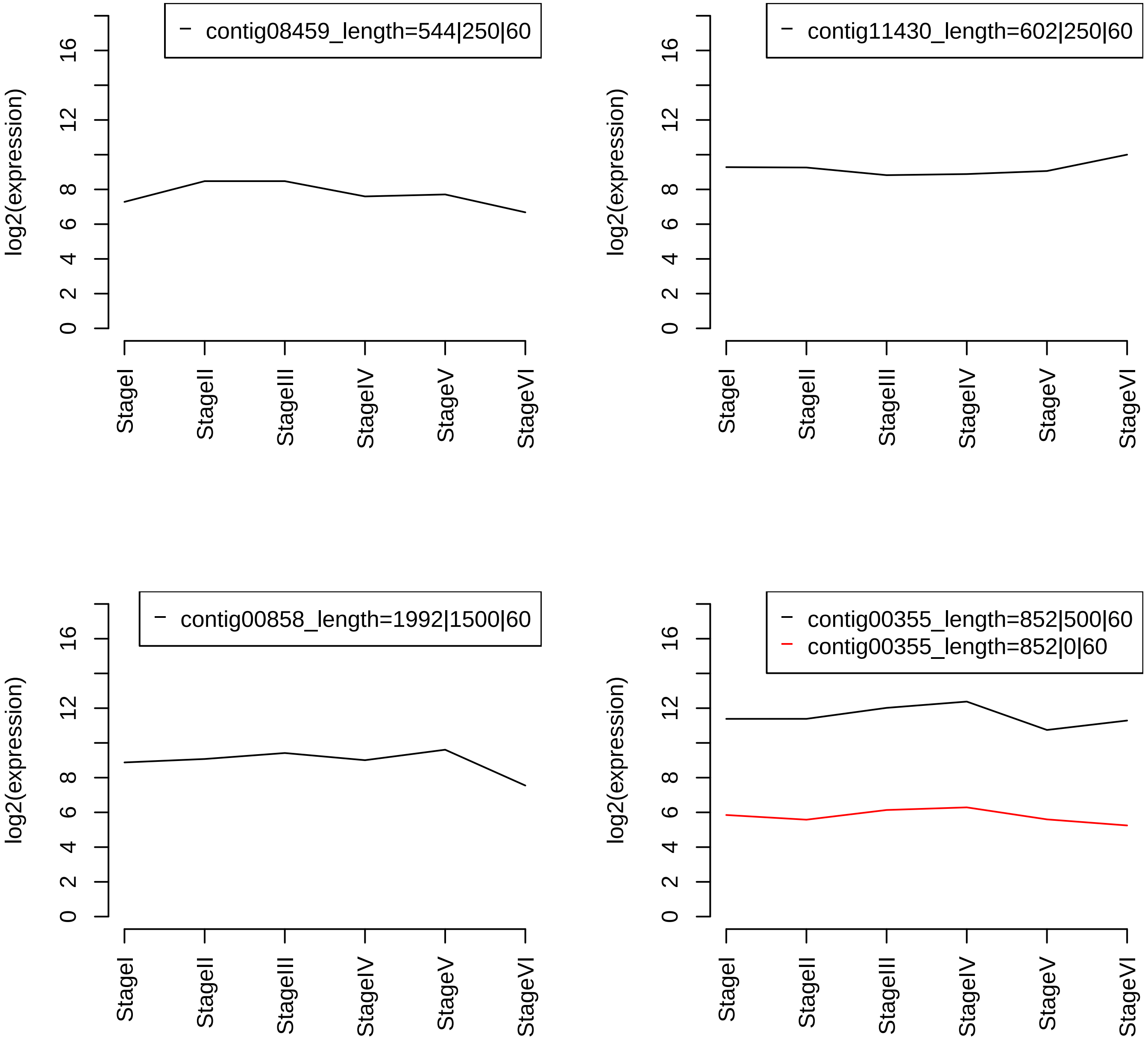
contig08459_length=544|250|60
contig11430_length=602|250|60
contig00858_length=1992|1500|60
contig00355_length=852|500|60
contig00355_length=852|0|60
log2(expression)
0
2
4
6
8
12
16
StageI
StageII
StageIII
StageIV
StageV
StageVI

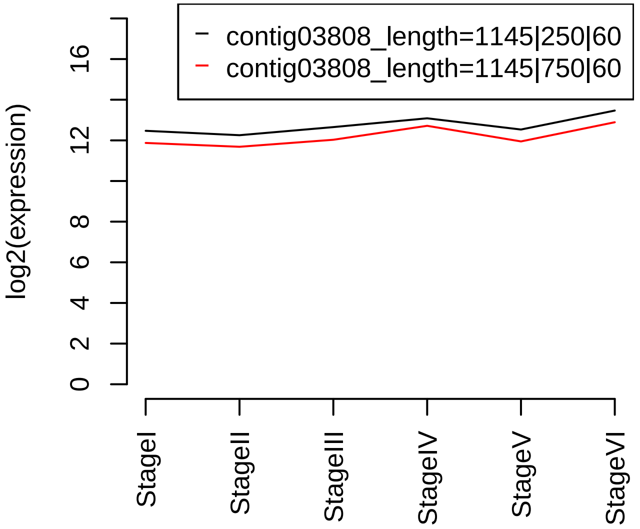

contig03808_length=1145|250|60
contig03808_length=1145|750|60
log2(expression)
0
2
4
6
8
12
16
StageI
StageII
StageIII
StageIV
StageV
StageVI

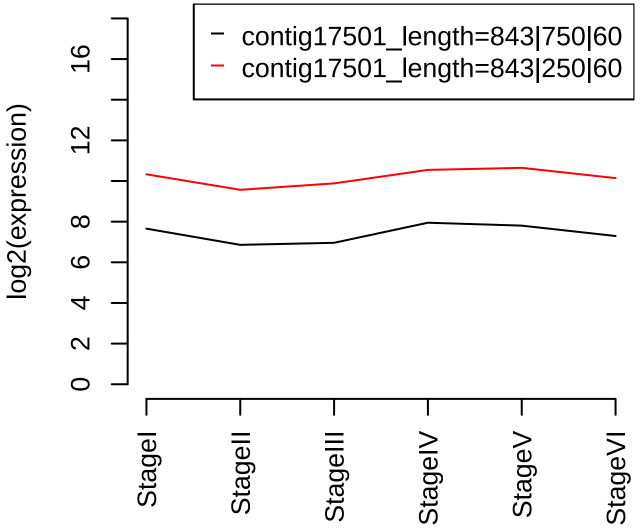

contig17501_length=843|750|60
contig17501_length=843|250|60
log2(expression)
0
2
4
6
8
12
16
StageI
StageII
StageIII
StageIV
StageV
StageVI

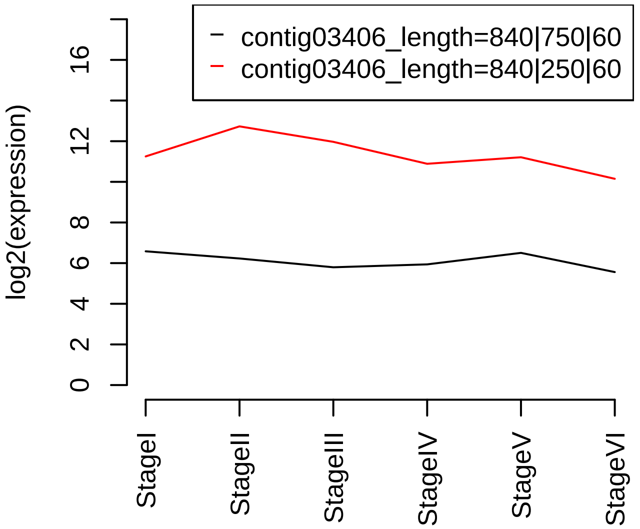

contig03406_length=840|750|60
contig03406_length=840|250|60
log2(expression)
0
2
4
6
8
12
16
StageI
StageII
StageIII
StageIV
StageV
StageVI

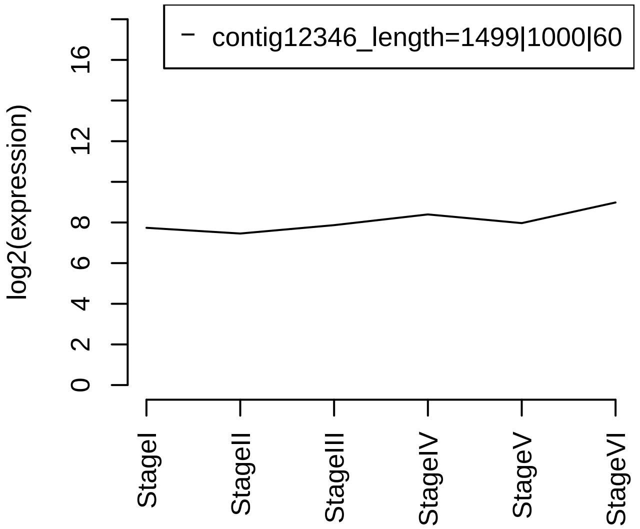

contig12346_length=1499|1000|60
log2(expression)
0
2
4
6
8
12
16
StageI
StageII
StageIII
StageIV
StageV
StageVI

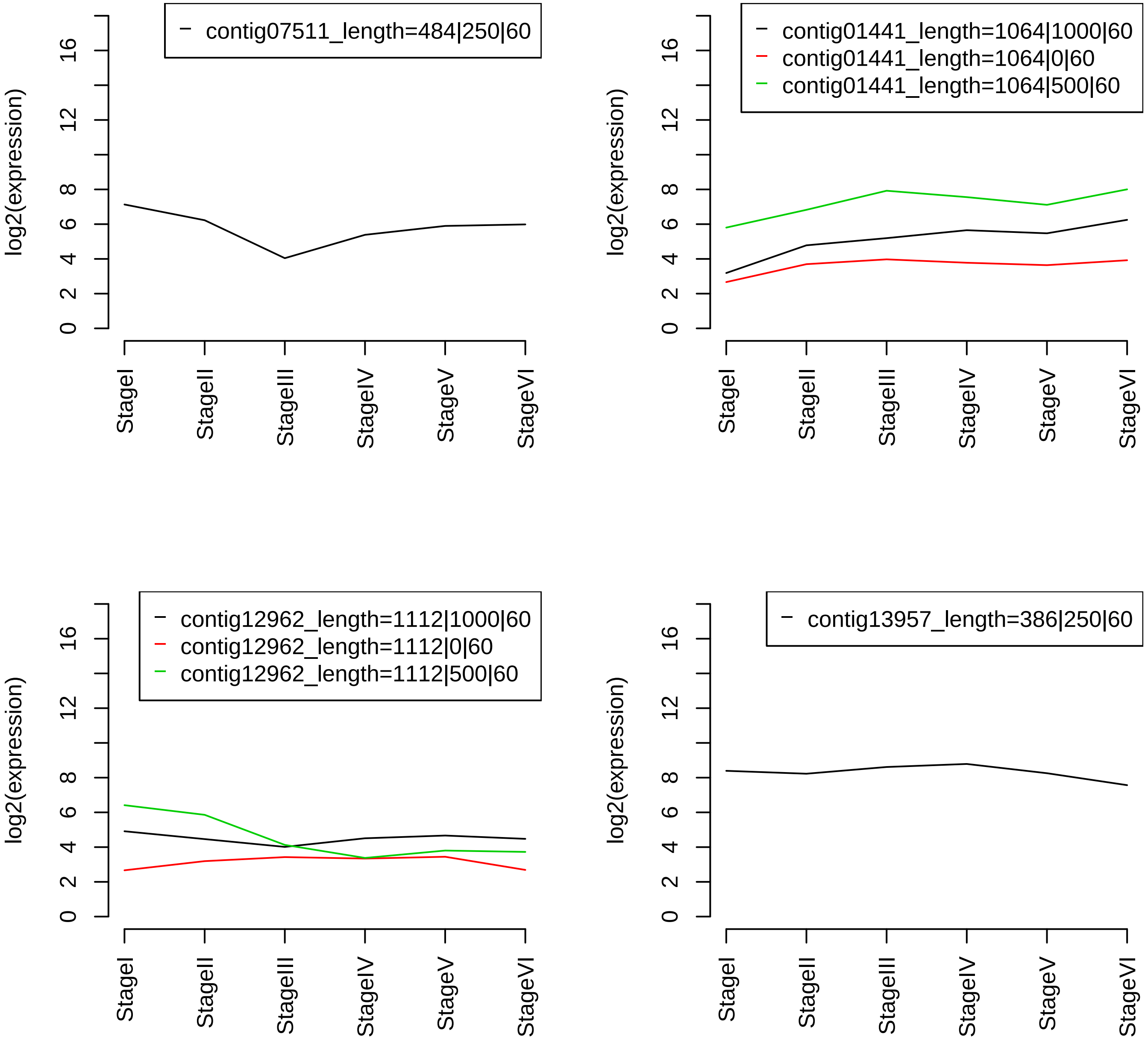
contig07511_length=484|250|60
contig01441_length=1064|1000|60
contig01441_length=1064|0|60
contig01441_length=1064|500|60
contig12962_length=1112|1000|60
contig12962_length=1112|0|60
contig12962_length=1112|500|60
contig13957_length=386|250|60
log2(expression)
0 2 4 6 8 12 16
StageI
StageII
StageIII
StageIV
StageV
StageVI

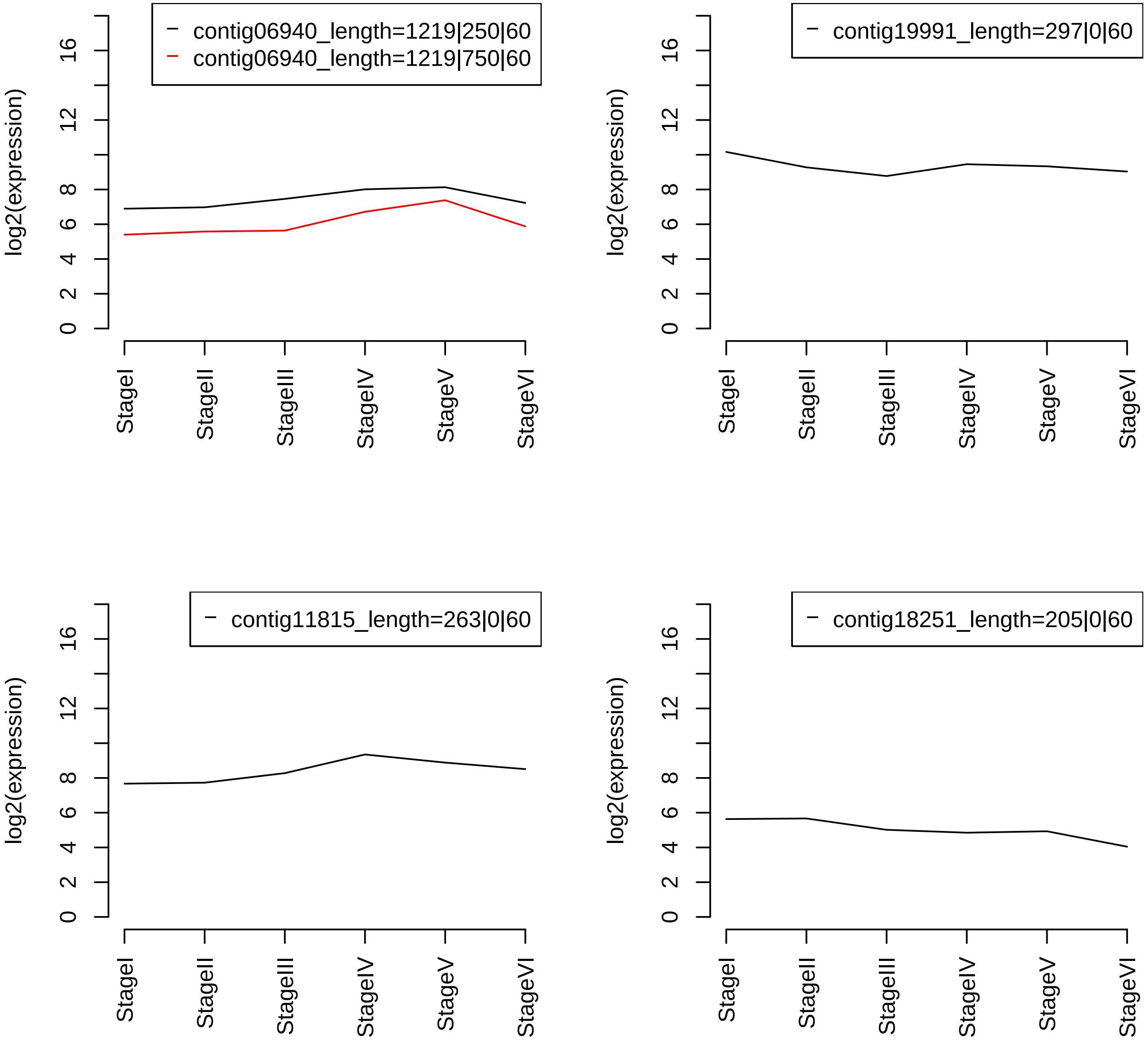
contig06940_length=1219|250|60
contig06940_length=1219|750|60
contig19991_length=297|0|60
contig11815_length=263|0|60
contig18251_length=205|0|60
log2(expression)
0
2
4
6
8
12
16
StageI
StageII
StageIII
StageIV
StageV
StageVI

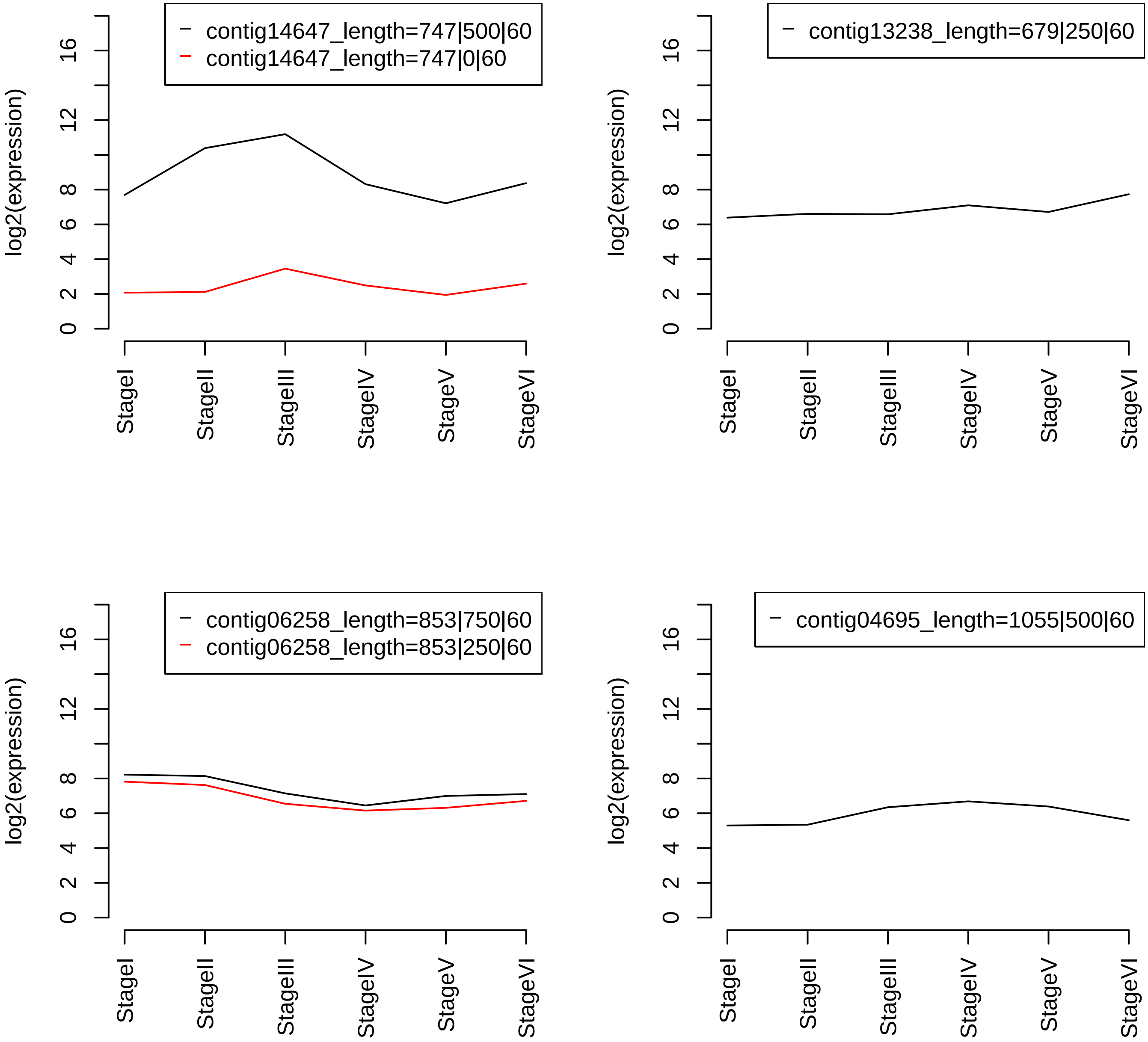

contig14647_length=747|500|60
contig14647_length=747|0|60
log2(expression)
0
2
4
6
8
12
16
StageI
StageII
StageIII
StageIV
StageV
StageVI
contig13238_length=679|250|60
log2(expression)
0
2
4
6
8
12
16
StageI
StageII
StageIII
StageIV
StageV
StageVI
contig06258_length=853|750|60
contig06258_length=853|250|60
log2(expression)
0
2
4
6
8
12
16
StageI
StageII
StageIII
StageIV
StageV
StageVI
contig04695_length=1055|500|60
log2(expression)
0
2
4
6
8
12
16
StageI
StageII
StageIII
StageIV
StageV
StageVI

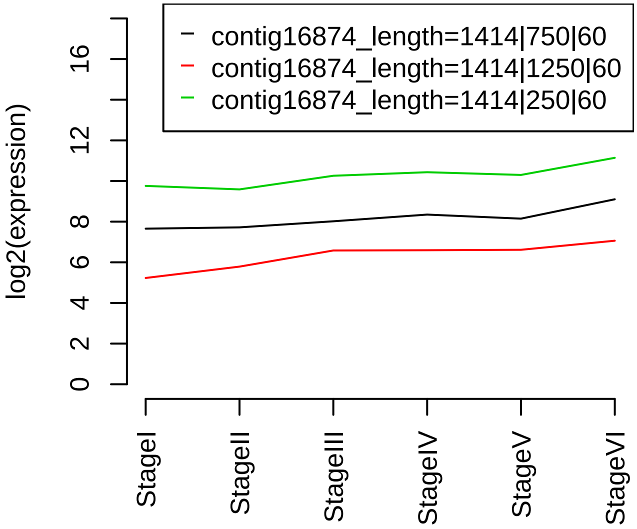

contig16874_length=1414|750|60
contig16874_length=1414|1250|60
contig16874_length=1414|250|60
log2(expression)
0
2
4
6
8
12
16
StageI
StageII
StageIII
StageIV
StageV
StageVI

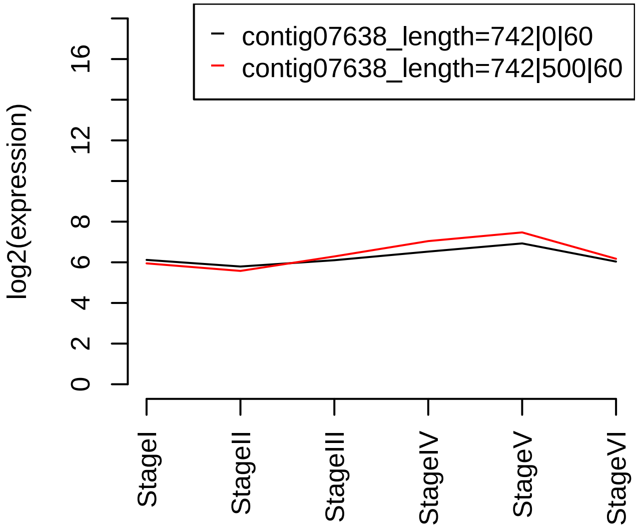

contig07638_length=742|0|60
contig07638_length=742|500|60
log2(expression)
0
2
4
6
8
12
16
StageI
StageII
StageIII
StageIV
StageV
StageVI

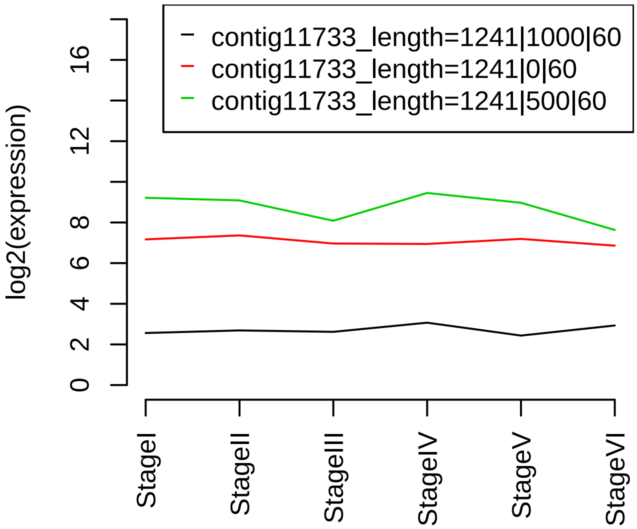

contig11733_length=1241|1000|60
contig11733_length=1241|0|60
contig11733_length=1241|500|60
log2(expression)
0
2
4
6
8
12
16
StageI
StageII
StageIII
StageIV
StageV
StageVI

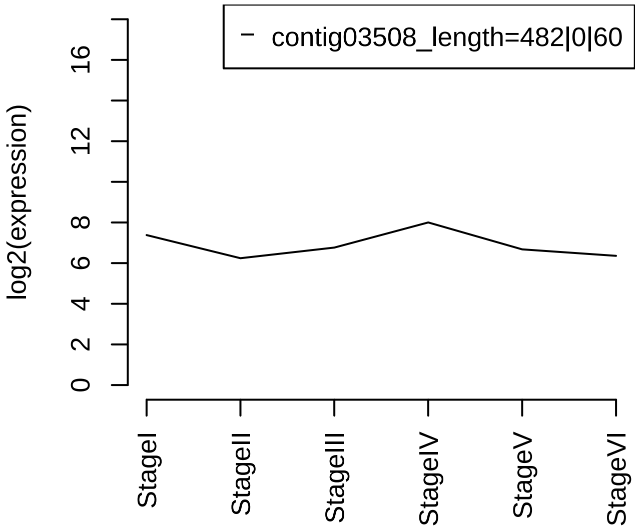

contig03508_length=482|0|60
log2(expression)
0
2
4
6
8
12
16
StageI
StageII
StageIII
StageIV
StageV
StageVI

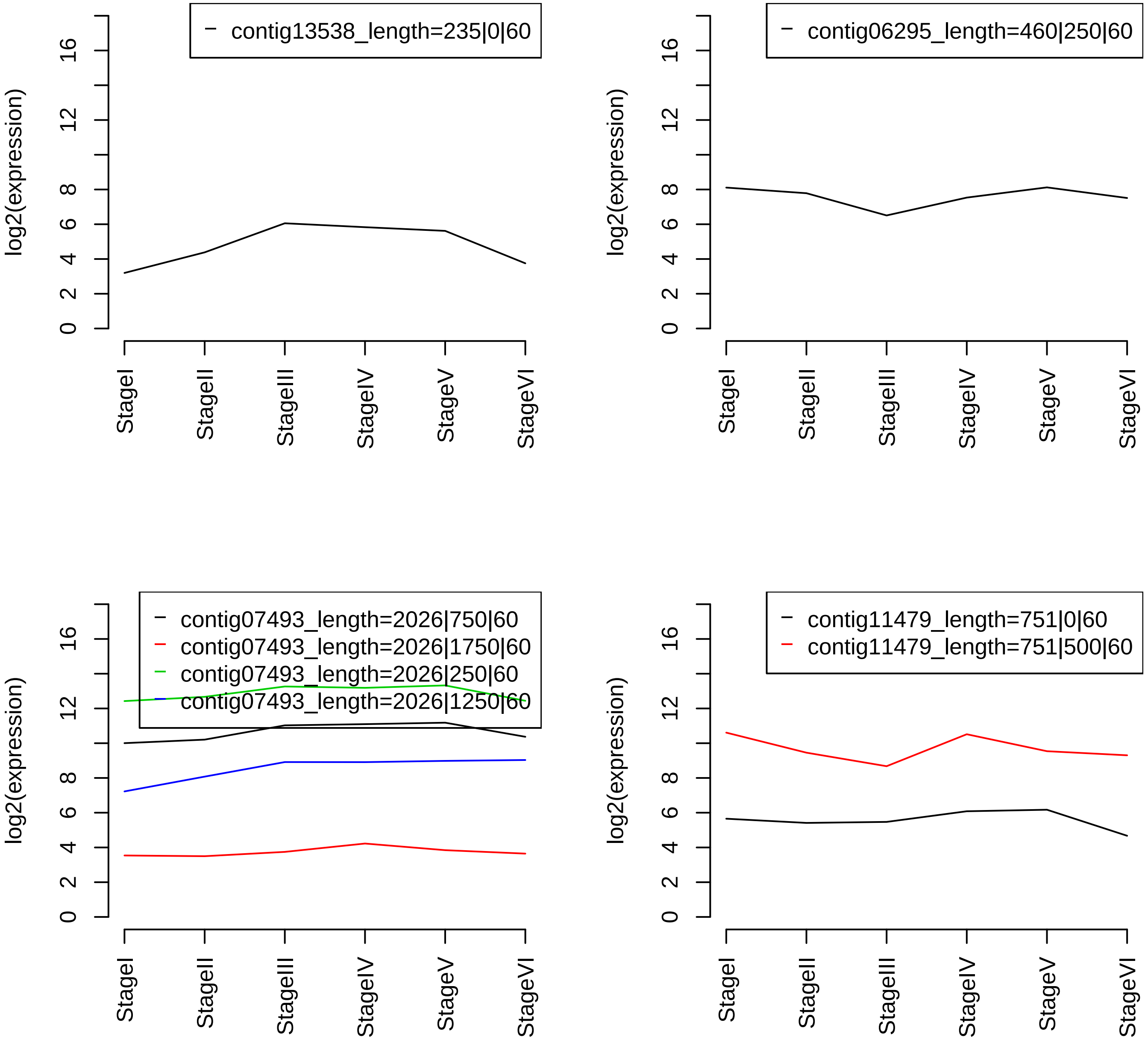

contig13538_length=235|0|60
contig06295_length=460|250|60
contig07493_length=2026|750|60
contig07493_length=2026|1750|60
contig07493_length=2026|250|60
contig07493_length=2026|1250|60
contig11479_length=751|0|60
contig11479_length=751|500|60
log2(expression)
0
2
4
6
8
12
16
StageI
StageII
StageIII
StageIV
StageV
StageVI

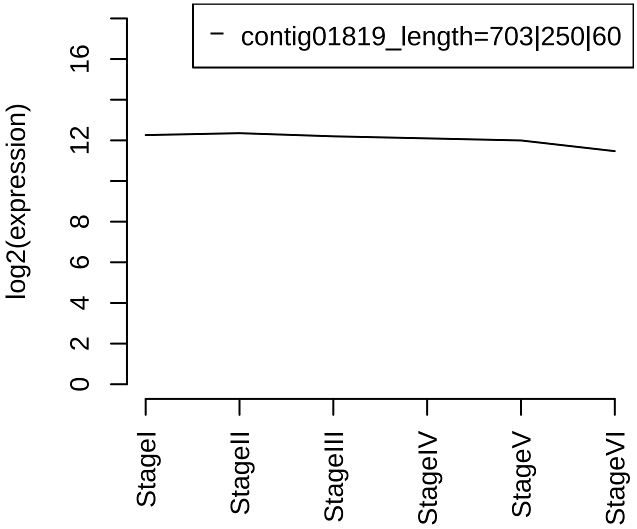
contig01819_length=703|250|60
log2(expression)
0
2
4
6
8
12
16
StageI
StageII
StageIII
StageIV
StageV
StageVI

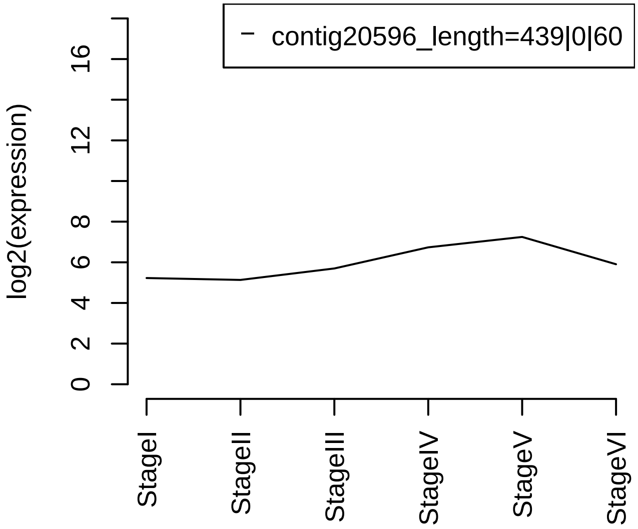
contig20596_length=439|0|60
log2(expression)
0
2
4
6
8
12
16
StageI
StageII
StageIII
StageIV
StageV
StageVI

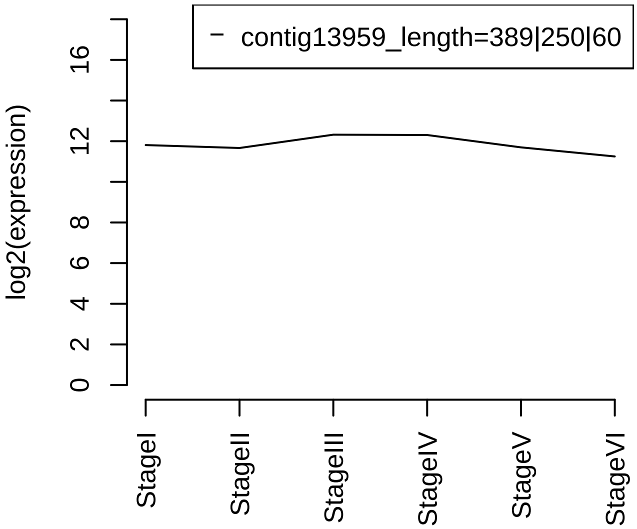
contig13959_length=389|250|60
log2(expression)
0
2
4
6
8
12
16
StageI
StageII
StageIII
StageIV
StageV
StageVI

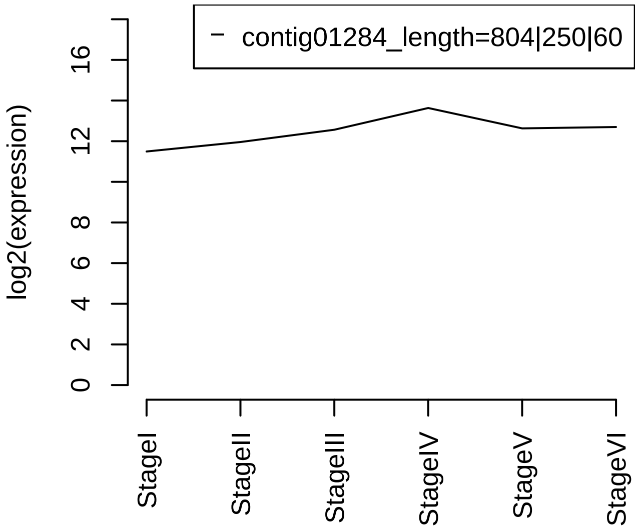
contig01284_length=804|250|60
log2(expression)
0
2
4
6
8
12
16
StageI
StageII
StageIII
StageIV
StageV
StageVI

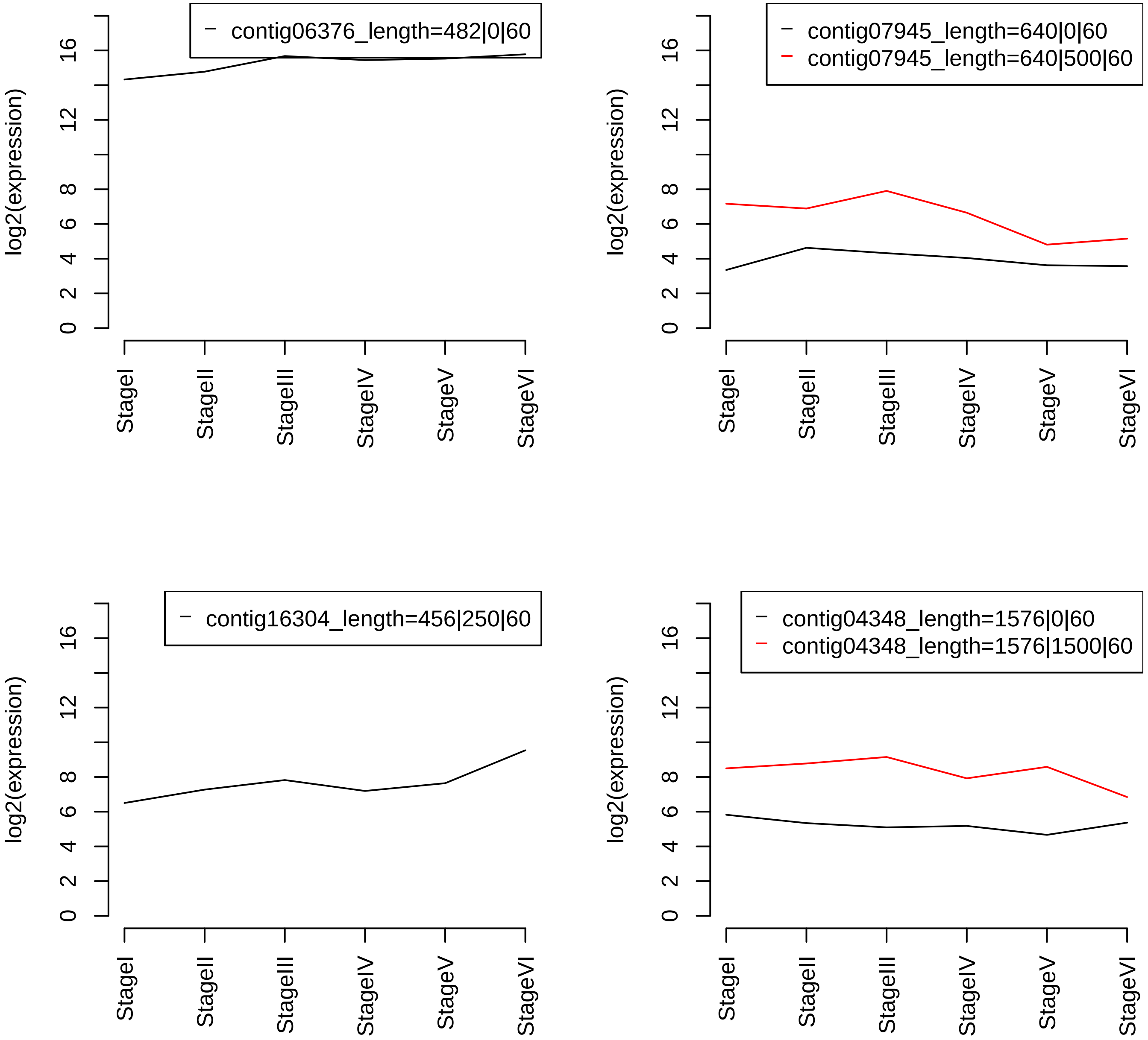
contig06376_length=482|0|60
contig07945_length=640|0|60
contig07945_length=640|500|60
contig16304_length=456|250|60
contig04348_length=1576|0|60
contig04348_length=1576|1500|60
log2(expression)
0
2
4
6
8
12
16
StageI
StageII
StageIII
StageIV
StageV
StageVI

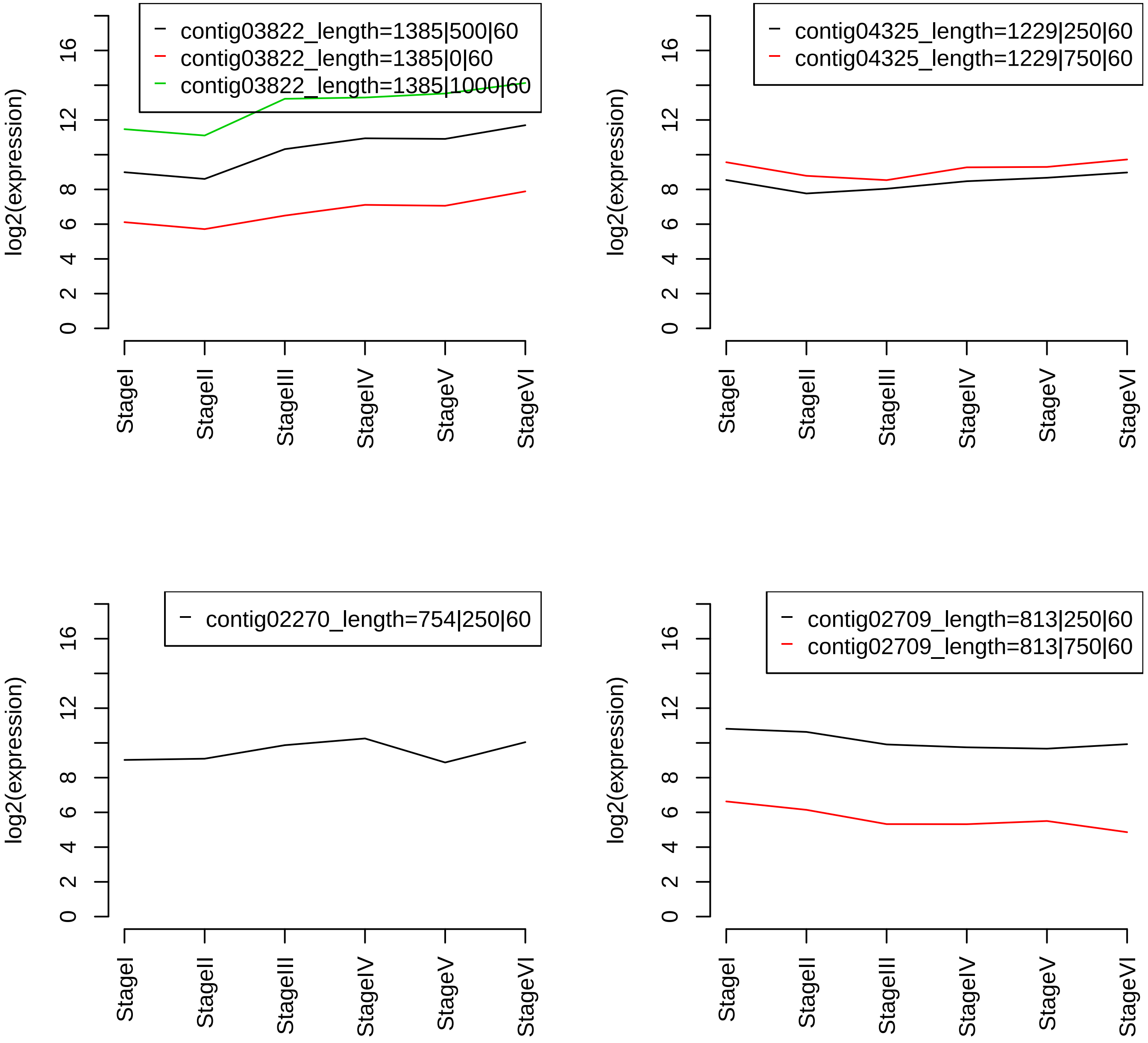
contig03822_length=1385|500|60
contig03822_length=1385|0|60
contig03822_length=1385|1000|60
contig04325_length=1229|250|60
contig04325_length=1229|750|60
contig02270_length=754|250|60
contig02709_length=813|250|60
contig02709_length=813|750|60
log2(expression)
0
2
4
6
8
12
16
StageI
StageII
StageIII
StageIV
StageV
StageVI

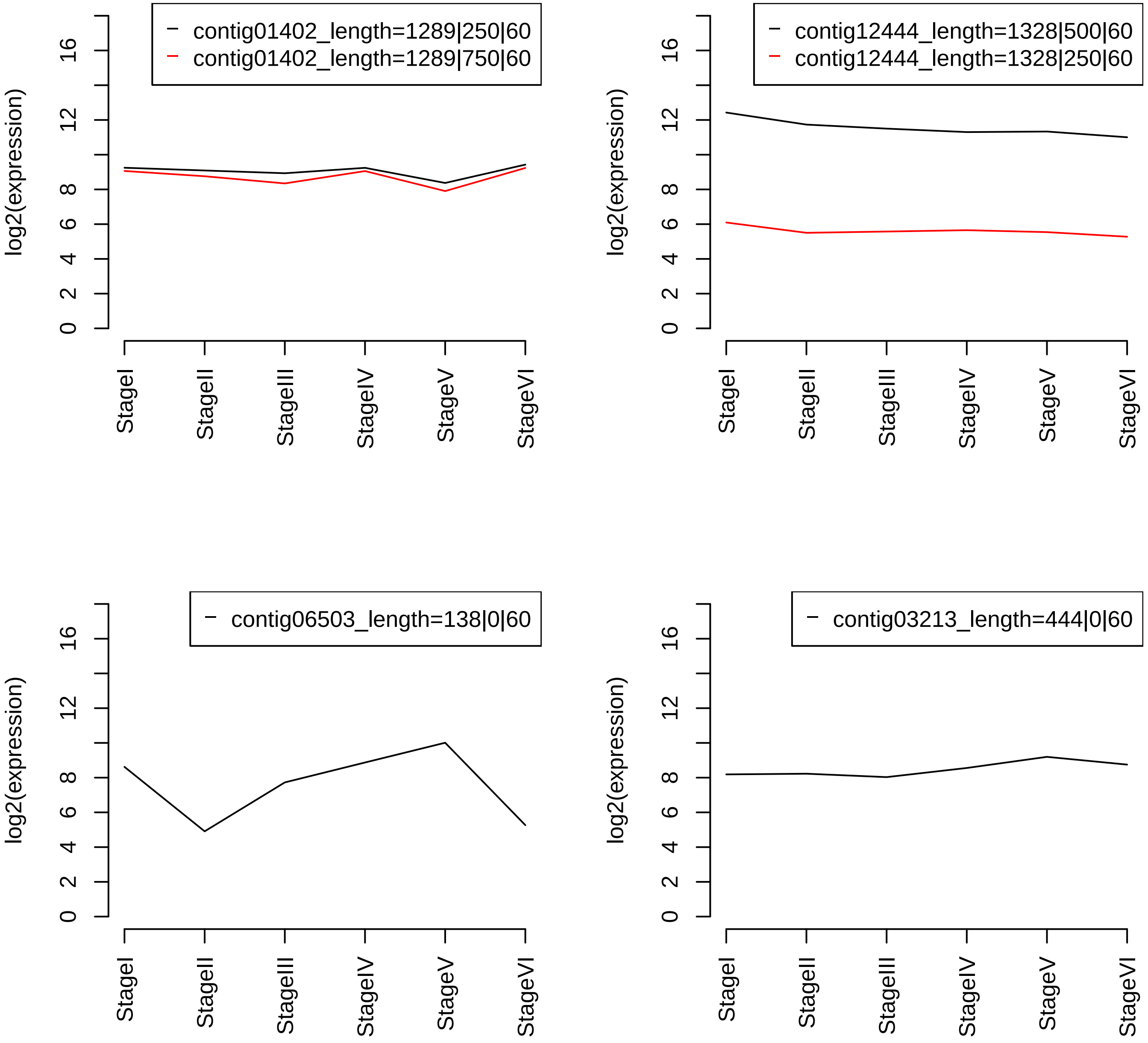
contig01402_length=1289|250|60
contig01402_length=1289|750|60
contig12444_length=1328|500|60
contig12444_length=1328|250|60
contig06503_length=138|0|60
contig03213_length=444|0|60
log2(expression)
0
2
4
6
8
12
16
StageI
StageII
StageIII
StageIV
StageV
StageVI

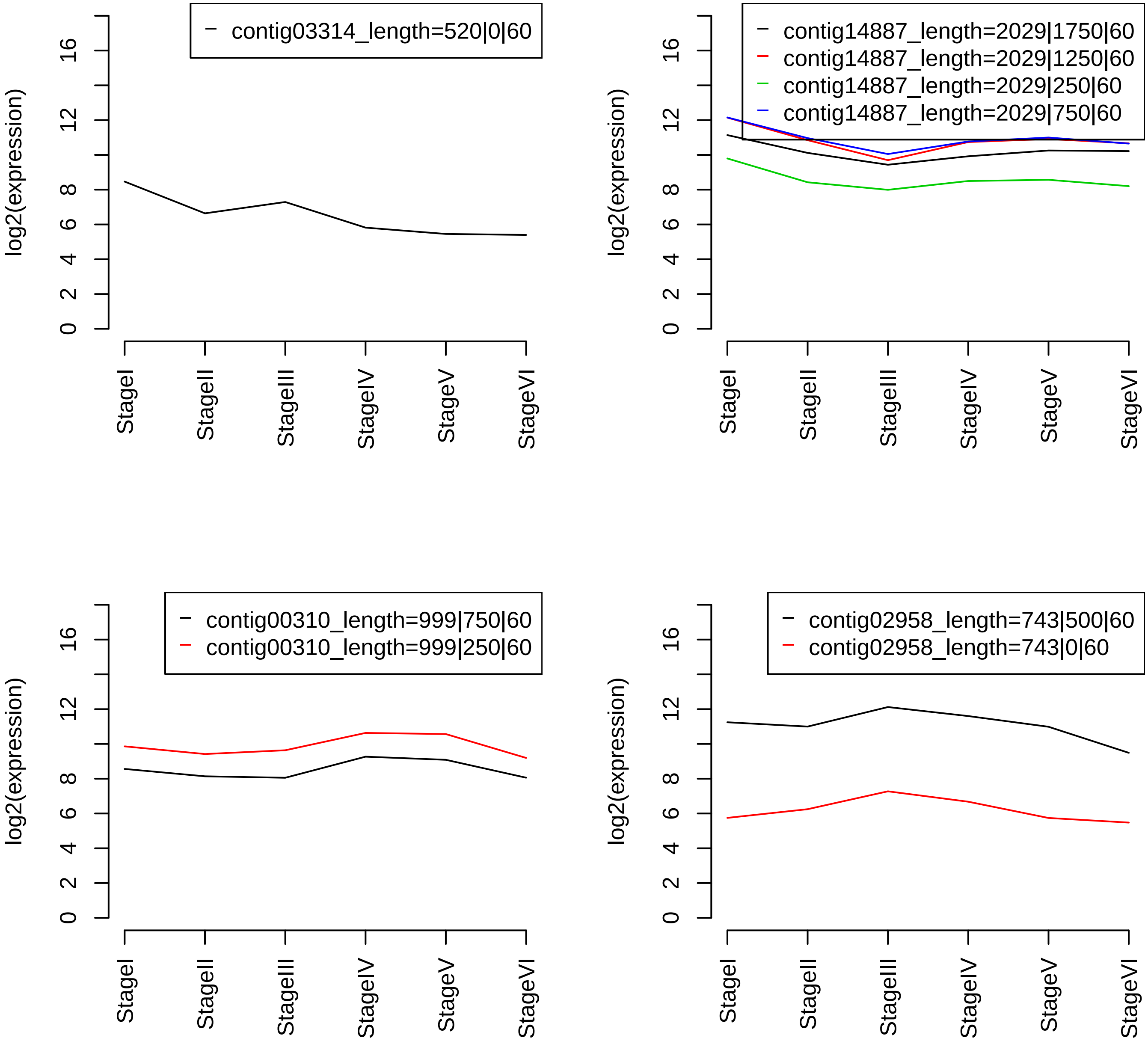

contig03314_length=520|0|60
contig14887_length=2029|1750|60
contig14887_length=2029|1250|60
contig14887_length=2029|250|60
contig14887_length=2029|750|60
contig00310_length=999|750|60
contig00310_length=999|250|60
contig02958_length=743|500|60
contig02958_length=743|0|60
log2(expression)
0
2
4
6
8
12
16
StageI
StageII
StageIII
StageIV
StageV
StageVI

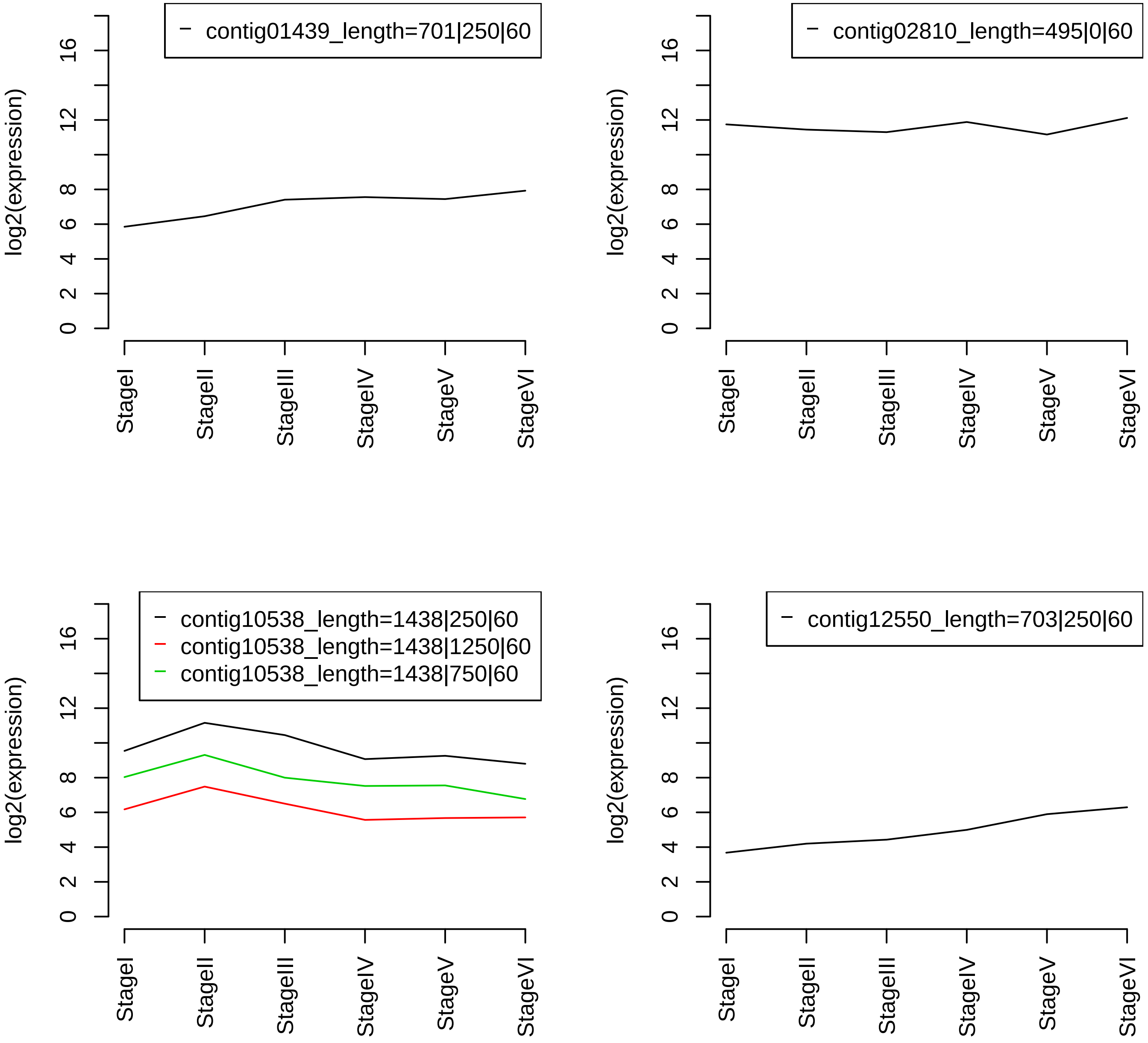

contig01439_length=701|250|60
contig02810_length=495|0|60
contig10538_length=1438|250|60
contig10538_length=1438|1250|60
contig10538_length=1438|750|60
contig12550_length=703|250|60
log2(expression)
0
2
4
6
8
12
16
StageI
StageII
StageIII
StageIV
StageV
StageVI

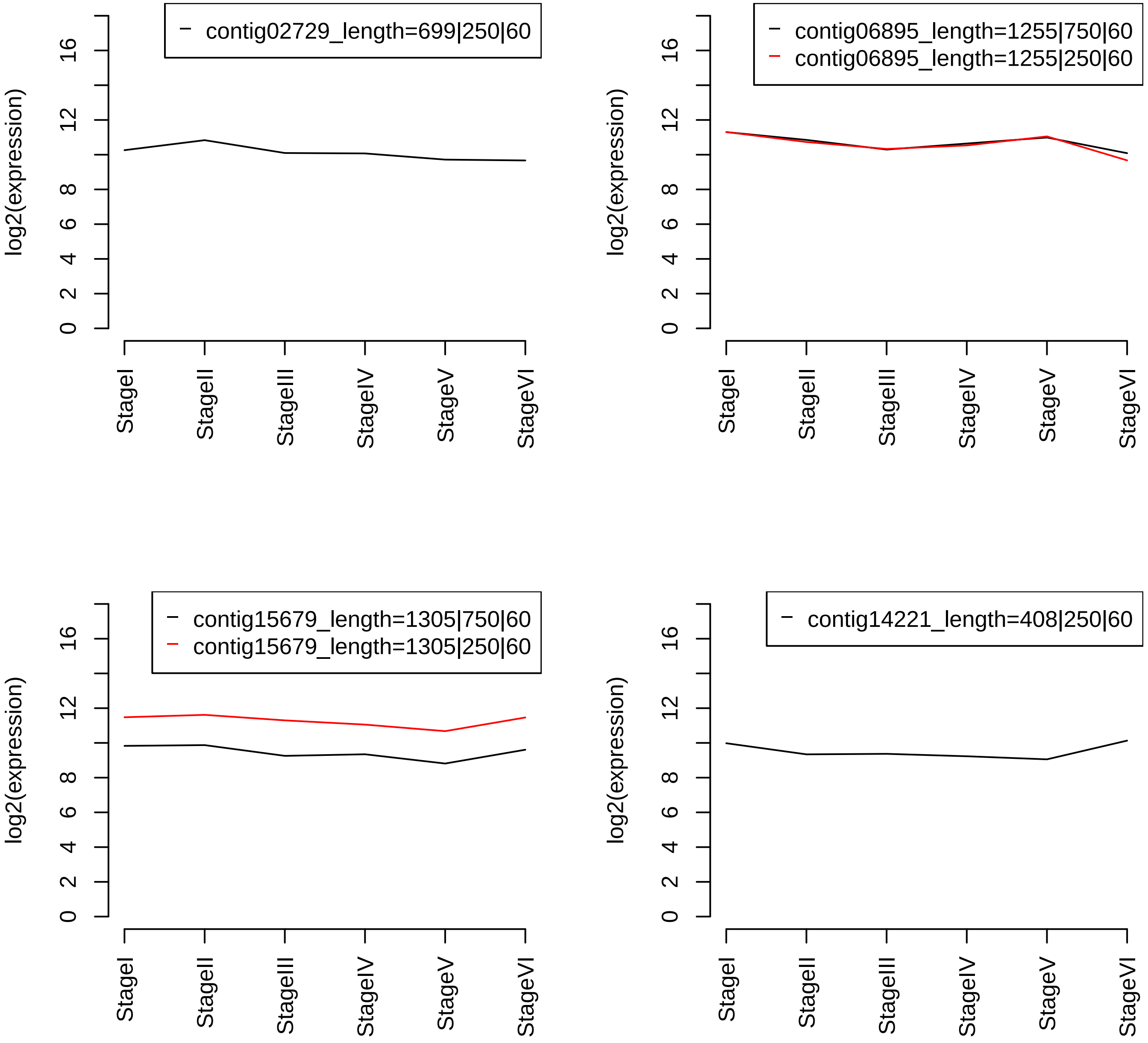
contig02729_length=699|250|60
contig06895_length=1255|750|60
contig06895_length=1255|250|60
contig15679_length=1305|750|60
contig15679_length=1305|250|60
contig14221_length=408|250|60
log2(expression)
0
2
4
6
8
12
16
StageI
StageII
StageIII
StageIV
StageV
StageVI

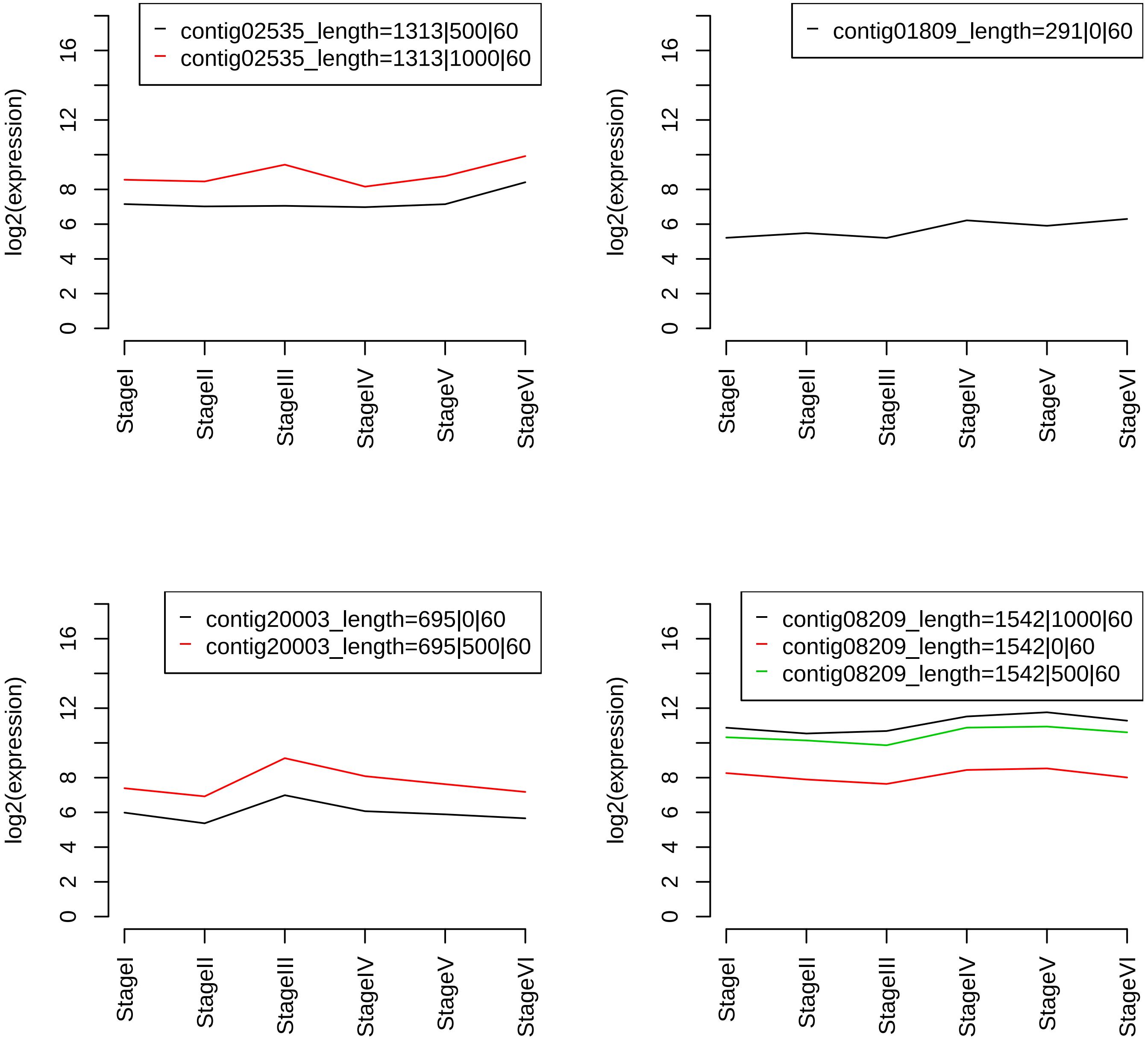
contig02535_length=1313|500|60
contig02535_length=1313|1000|60
log2(expression)
0
2
4
6
8
12
16
StageI
StageII
StageIII
StageIV
StageV
StageVI
contig01809_length=291|0|60
log2(expression)
0
2
4
6
8
12
16
StageI
StageII
StageIII
StageIV
StageV
StageVI
contig20003_length=695|0|60
contig20003_length=695|500|60
log2(expression)
0
2
4
6
8
12
16
StageI
StageII
StageIII
StageIV
StageV
StageVI
contig08209_length=1542|1000|60
contig08209_length=1542|0|60
contig08209_length=1542|500|60
log2(expression)
0
2
4
6
8
12
16
StageI
StageII
StageIII
StageIV
StageV
StageVI

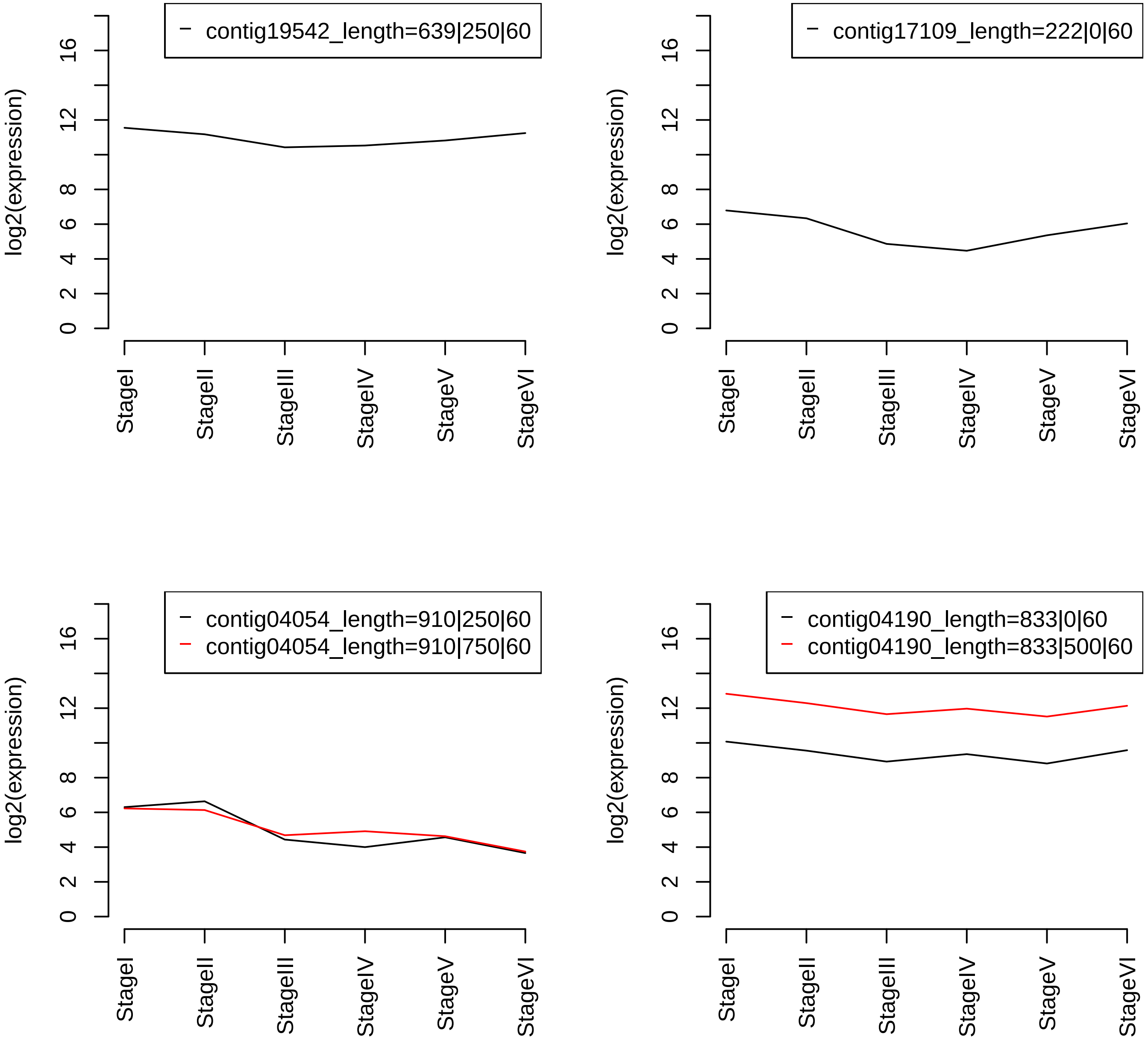

contig19542_length=639|250|60
log2(expression)
0
2
4
6
8
12
16
StageI
StageII
StageIII
StageIV
StageV
StageVI
contig17109_length=222|0|60
log2(expression)
0
2
4
6
8
12
16
StageI
StageII
StageIII
StageIV
StageV
StageVI
contig04054_length=910|250|60
contig04054_length=910|750|60
log2(expression)
0
2
4
6
8
12
16
StageI
StageII
StageIII
StageIV
StageV
StageVI
contig04190_length=833|0|60
contig04190_length=833|500|60
log2(expression)
0
2
4
6
8
12
16
StageI
StageII
StageIII
StageIV
StageV
StageVI

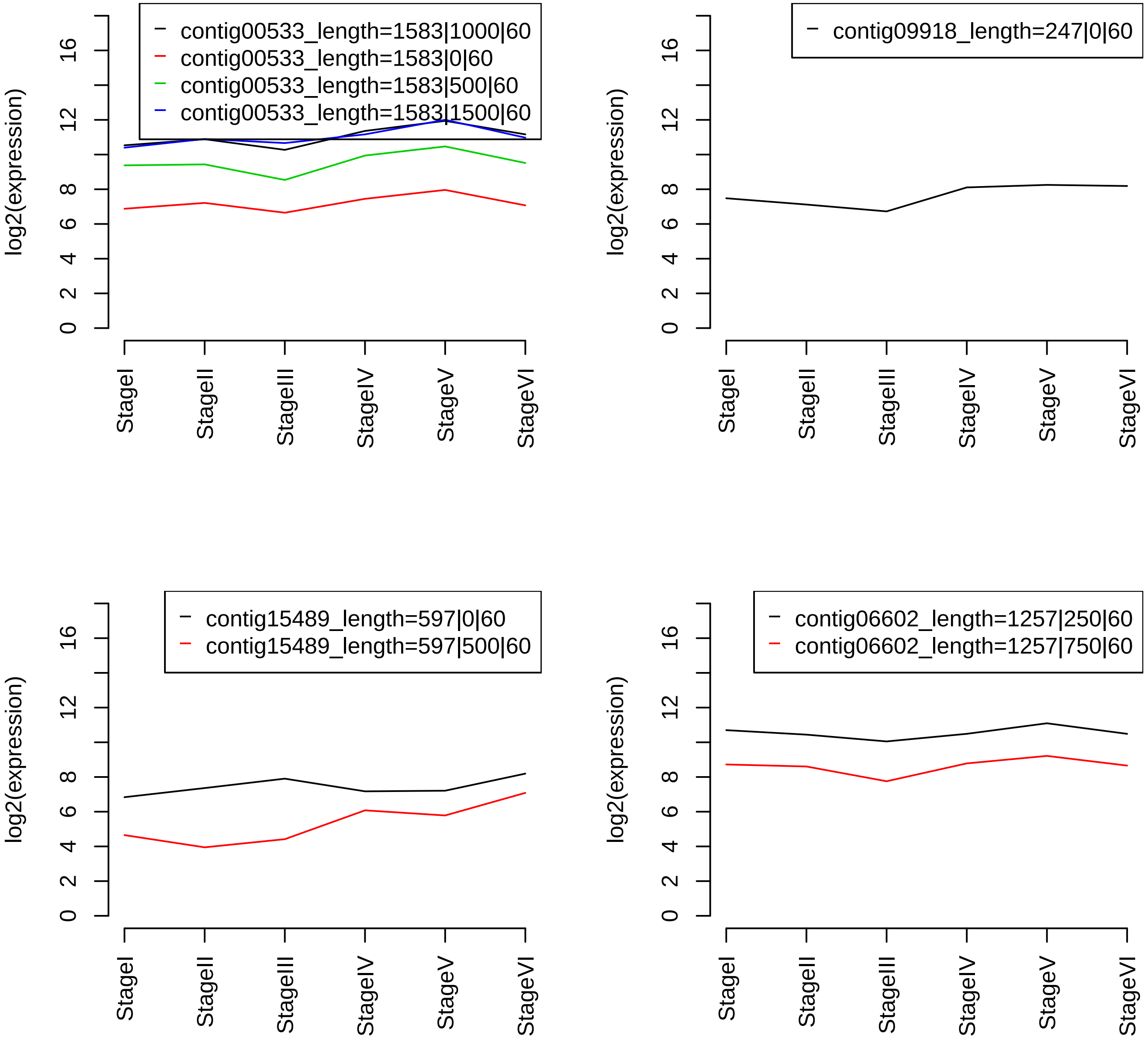
contig00533_length=1583|1000|60
contig00533_length=1583|0|60
contig00533_length=1583|500|60
contig00533_length=1583|1500|60
contig09918_length=247|0|60
contig15489_length=597|0|60
contig15489_length=597|500|60
contig06602_length=1257|250|60
contig06602_length=1257|750|60
log2(expression)
0 2 4 6 8 12 16
StageI StageII StageIII StageIV StageV StageVI

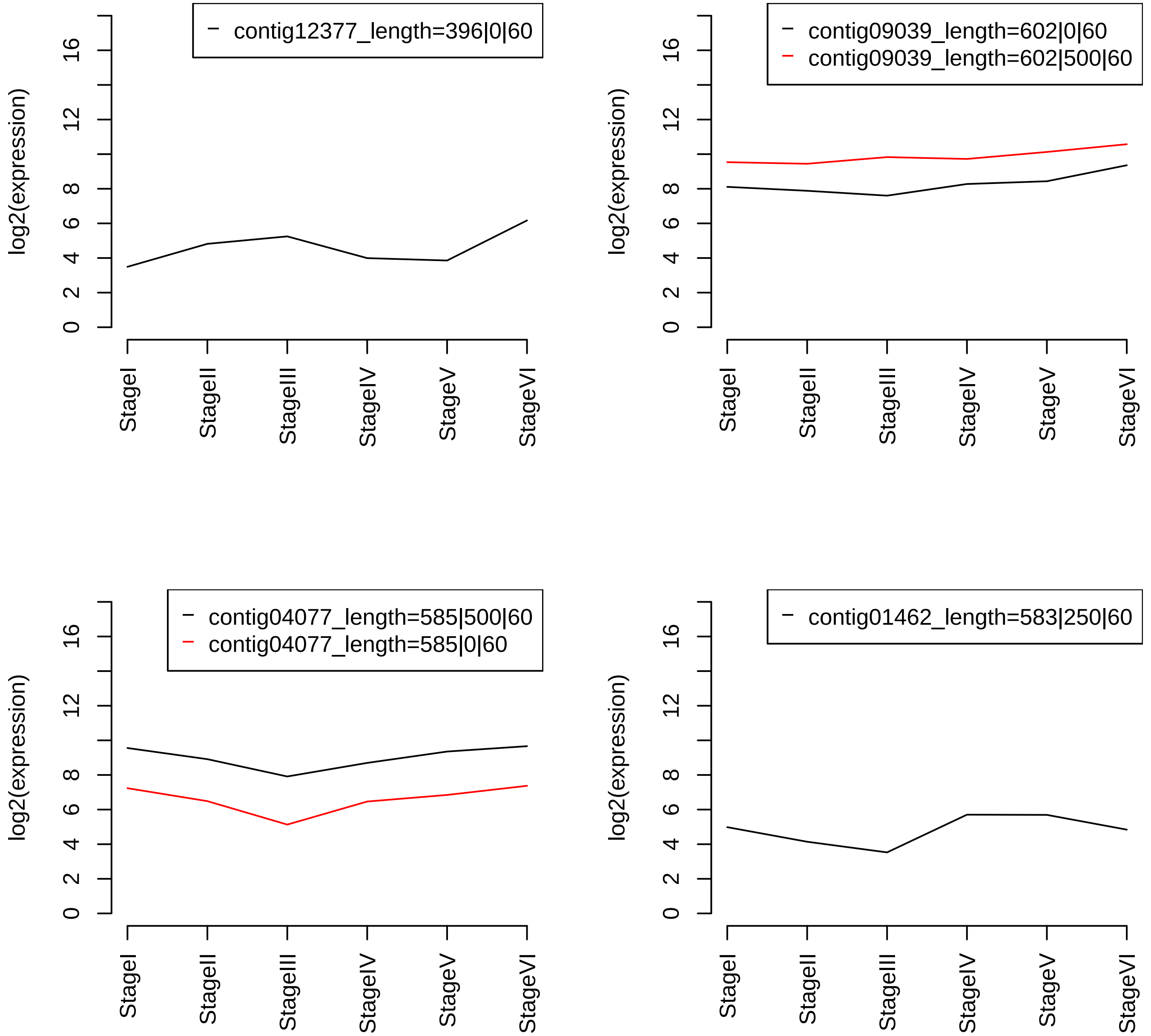
contig12377_length=396|0|60
contig09039_length=602|0|60
contig09039_length=602|500|60
contig04077_length=585|500|60
contig04077_length=585|0|60
contig01462_length=583|250|60
log2(expression)
0
2
4
6
8
12
16
StageI
StageII
StageIII
StageIV
StageV
StageVI

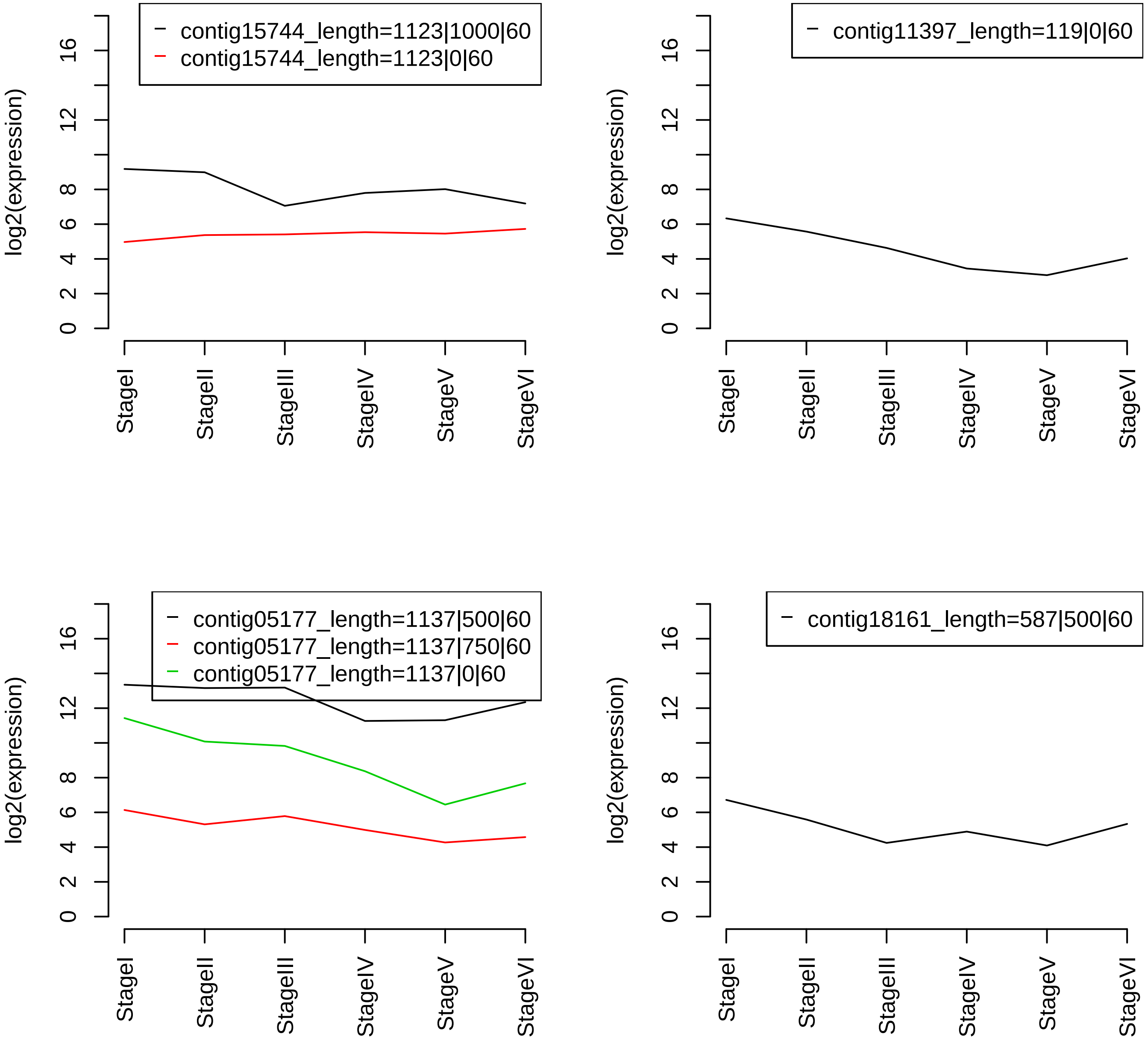
contig15744_length=1123|1000|60
contig15744_length=1123|0|60
contig11397_length=119|0|60
contig05177_length=1137|500|60
contig05177_length=1137|750|60
contig05177_length=1137|0|60
contig18161_length=587|500|60
log2(expression)
0
2
4
6
8
12
16
StageI
StageII
StageIII
StageIV
StageV
StageVI

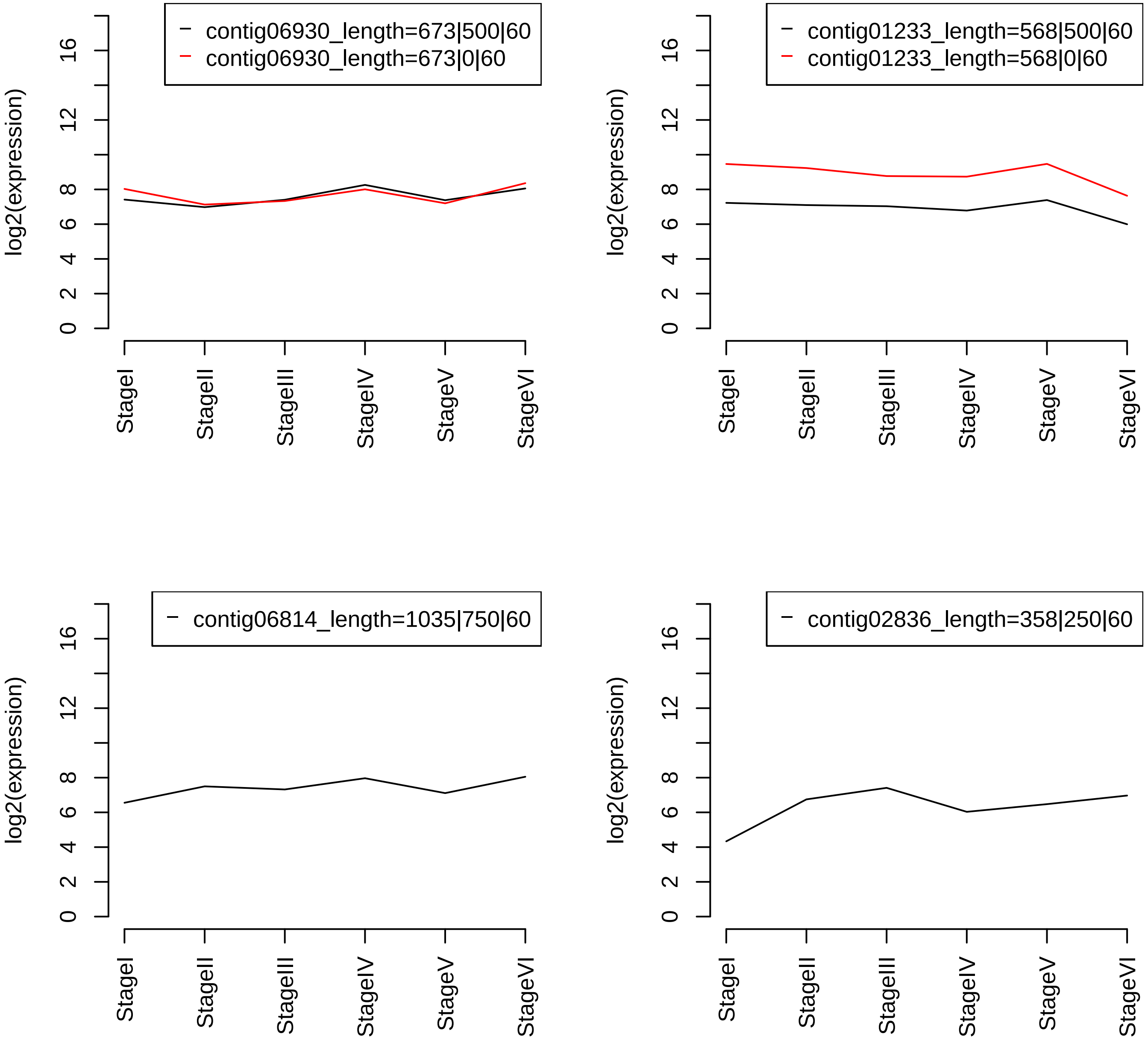

contig06930_length=673|500|60
contig06930_length=673|0|60
contig01233_length=568|500|60
contig01233_length=568|0|60
contig06814_length=1035|750|60
contig02836_length=358|250|60
log2(expression)
0 2 4 6 8 12 16
StageI StageII StageIII StageIV StageV StageVI

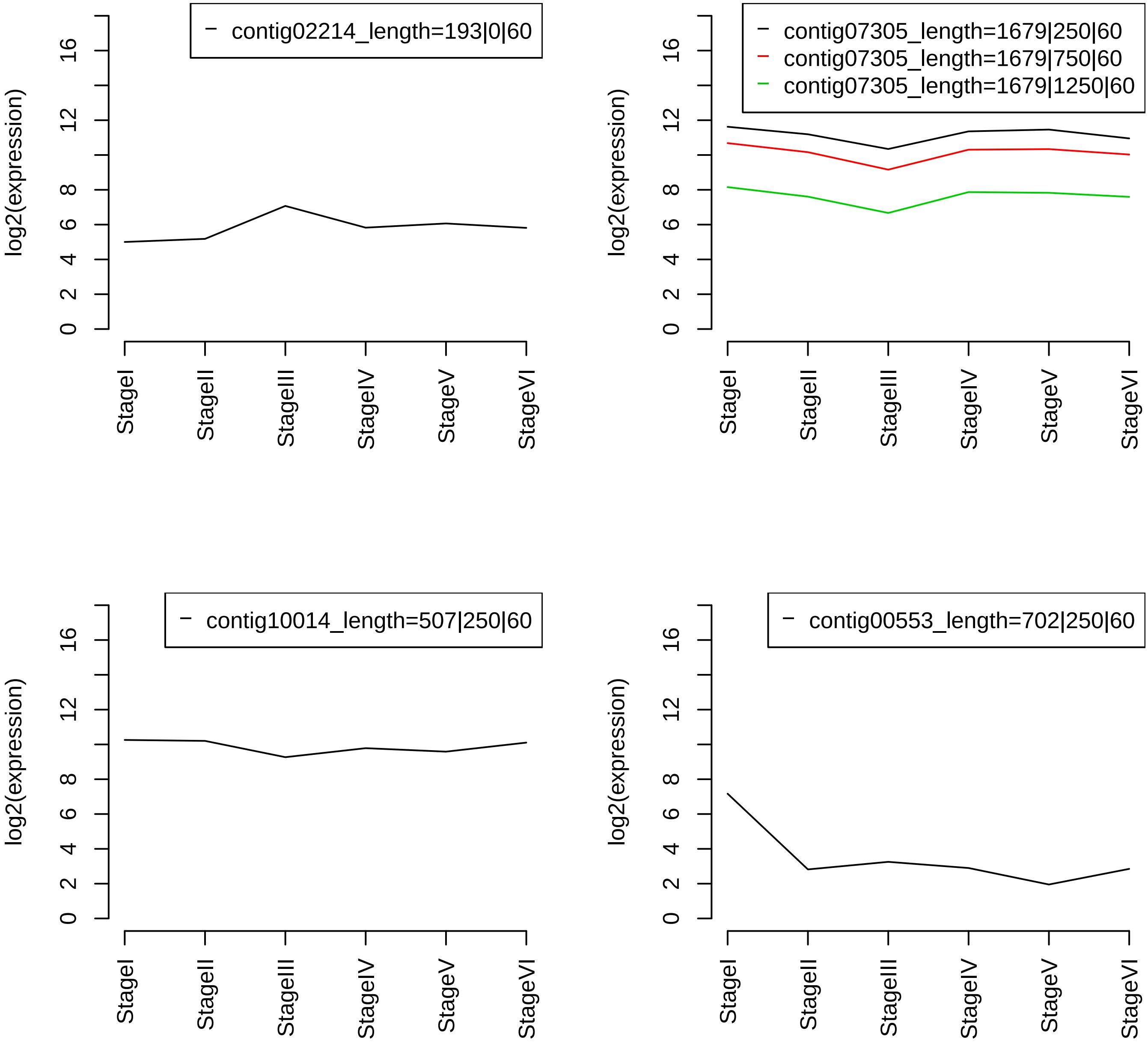
contig02214_length=193|0|60
log2(expression)
0 2 4 6 8 12 16
StageI StageII StageIII StageIV StageV StageVI
contig07305_length=1679|250|60
contig07305_length=1679|750|60
contig07305_length=1679|1250|60
log2(expression)
0 2 4 6 8 12 16
StageI StageII StageIII StageIV StageV StageVI
contig10014_length=507|250|60
log2(expression)
0 2 4 6 8 12 16
StageI StageII StageIII StageIV StageV StageVI
contig00553_length=702|250|60
log2(expression)
0 2 4 6 8 12 16
StageI StageII StageIII StageIV StageV StageVI

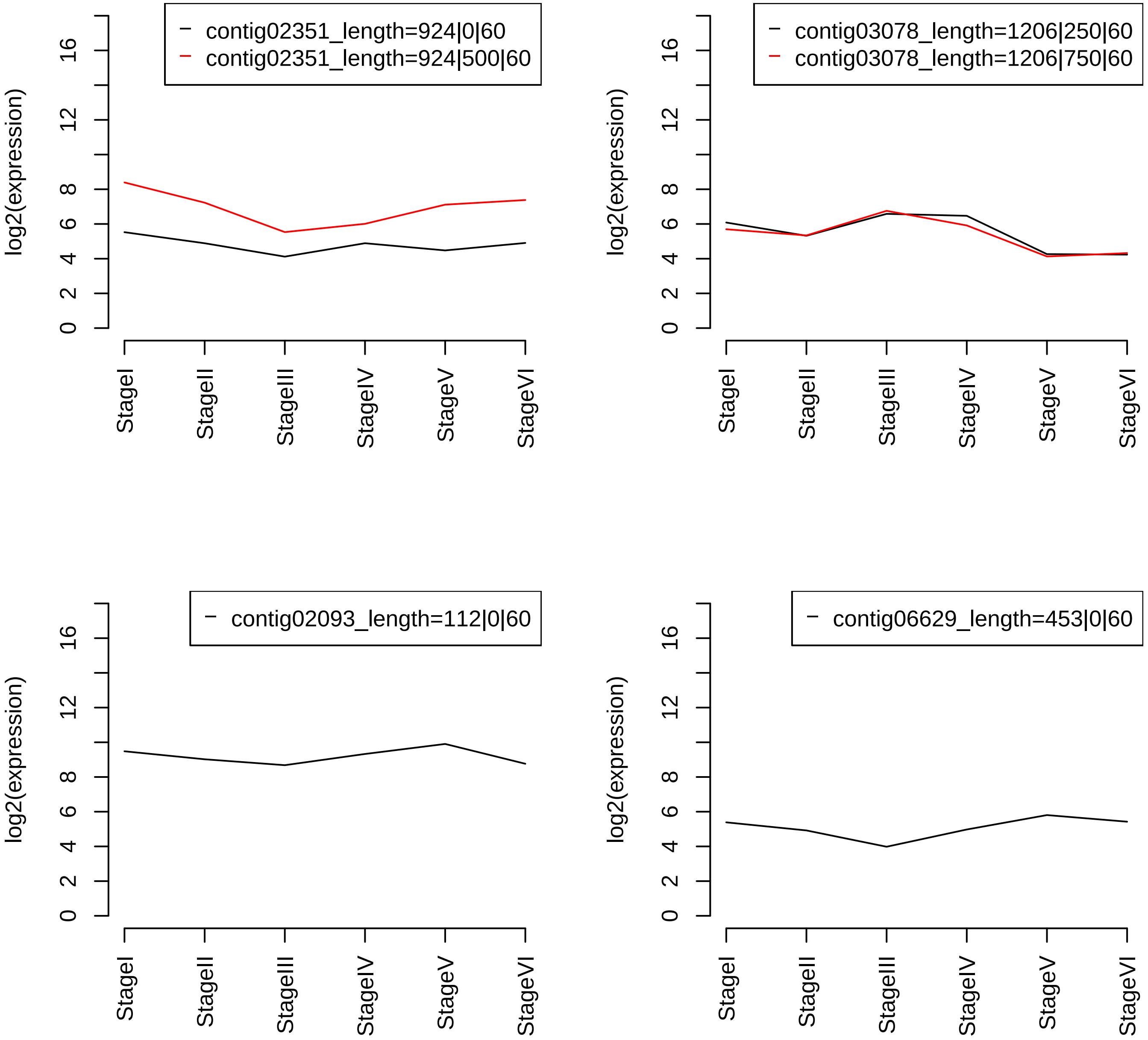
contig02351_length=924|0|60
contig02351_length=924|500|60
contig03078_length=1206|250|60
contig03078_length=1206|750|60
contig02093_length=112|0|60
contig06629_length=453|0|60
log2(expression)
0
2
4
6
8
12
16
StageI
StageII
StageIII
StageIV
StageV
StageVI

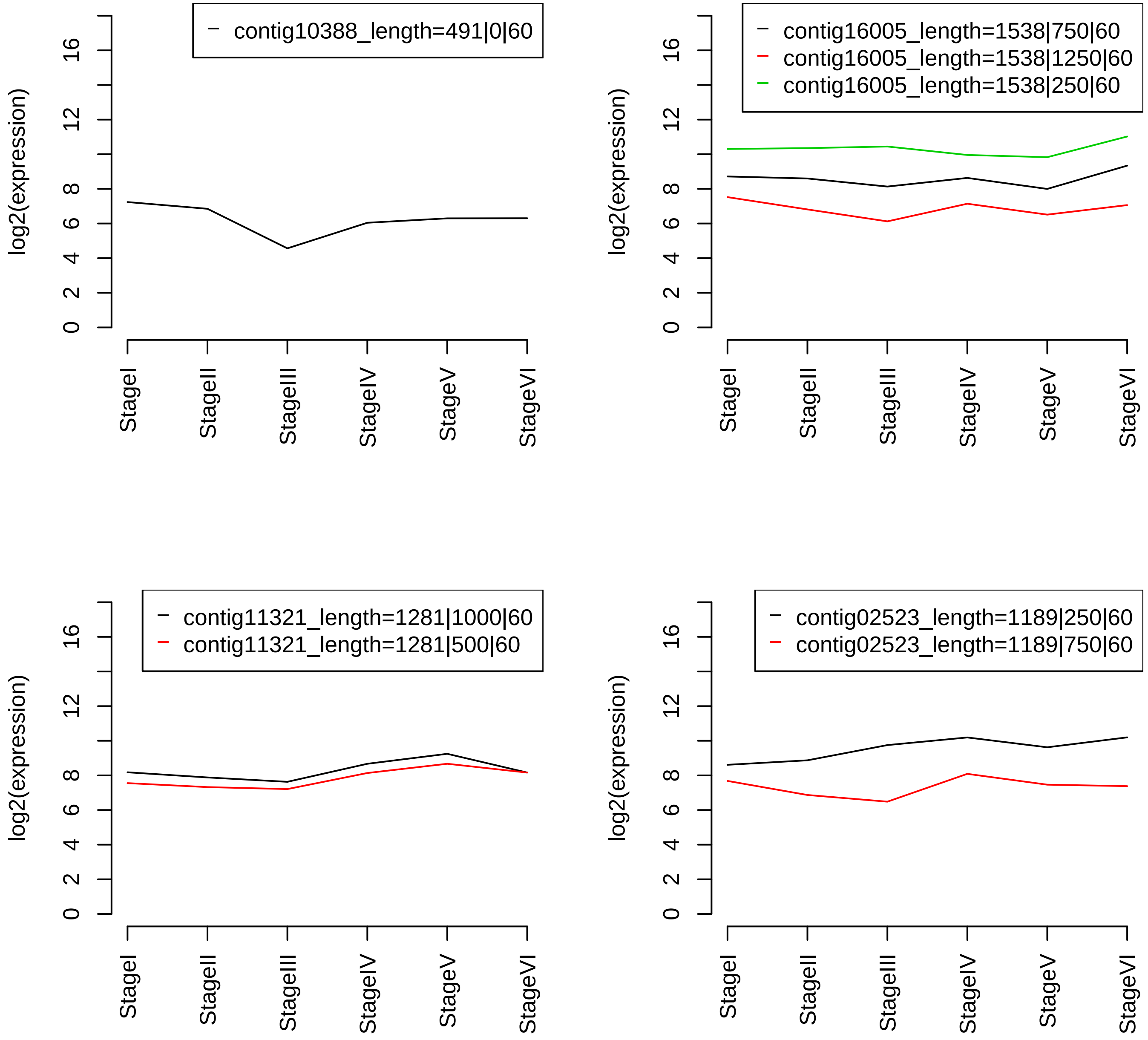

contig10388_length=491|0|60
contig16005_length=1538|750|60
contig16005_length=1538|1250|60
contig16005_length=1538|250|60
contig11321_length=1281|1000|60
contig11321_length=1281|500|60
contig02523_length=1189|250|60
contig02523_length=1189|750|60
log2(expression)
0 2 4 6 8 12 16
StageI StageII StageIII StageIV StageV StageVI

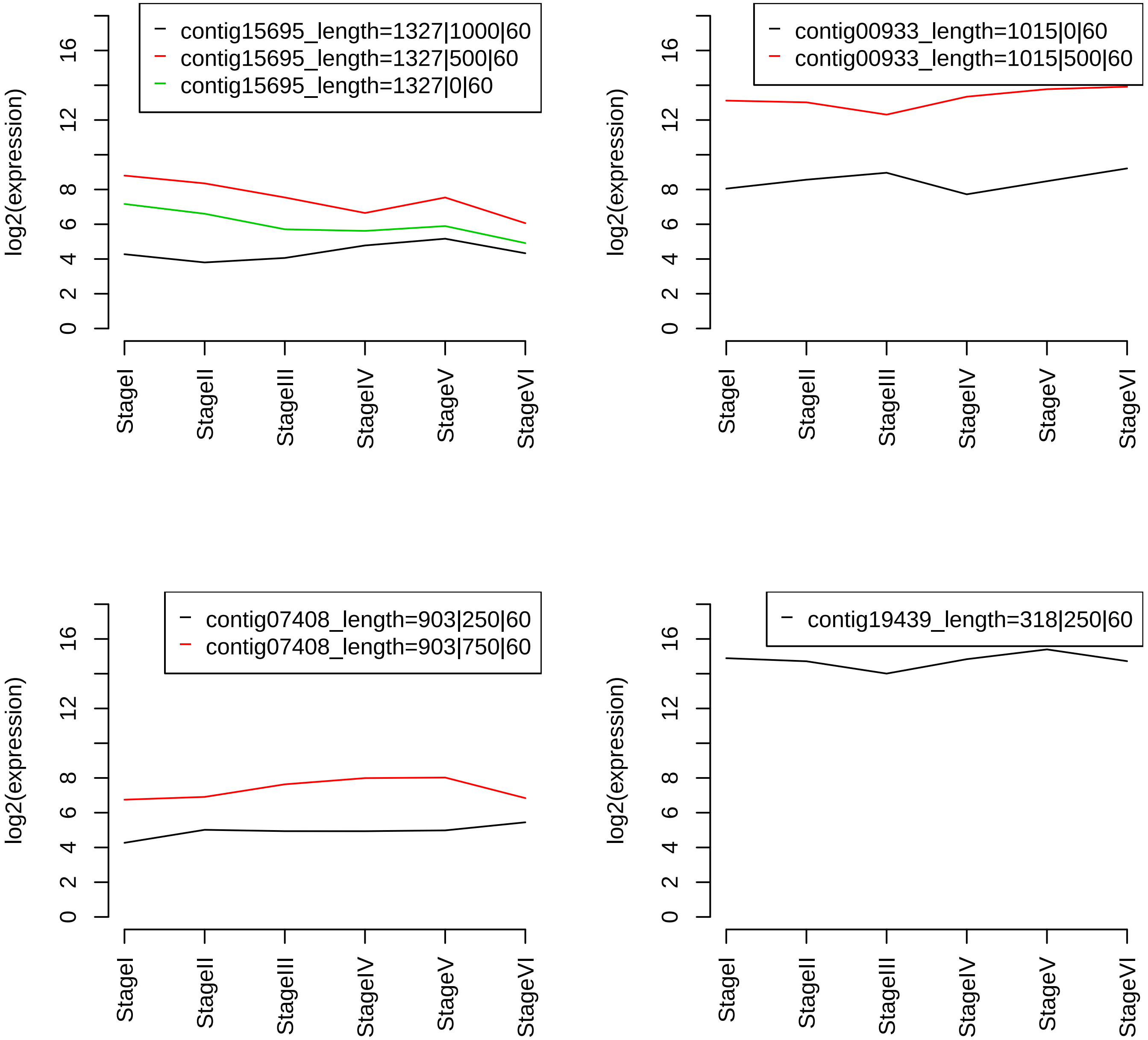

contig15695_length=1327|1000|60
contig15695_length=1327|500|60
contig15695_length=1327|0|60
contig00933_length=1015|0|60
contig00933_length=1015|500|60
contig07408_length=903|250|60
contig07408_length=903|750|60
contig19439_length=318|250|60
log2(expression)
0
2
4
6
8
12
16
StageI
StageII
StageIII
StageIV
StageV
StageVI

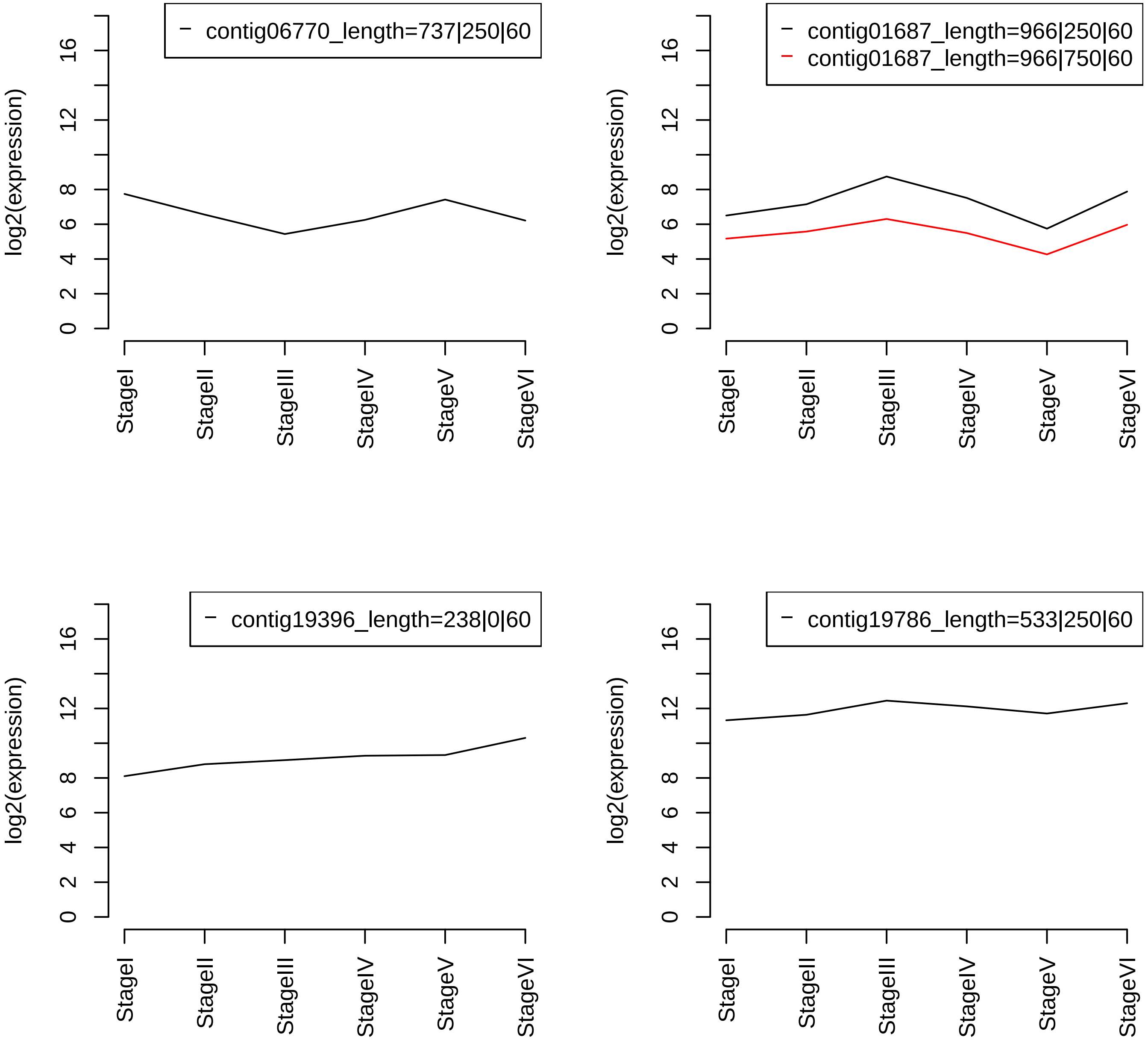
contig06770_length=737|250|60
contig01687_length=966|250|60
contig01687_length=966|750|60
contig19396_length=238|0|60
contig19786_length=533|250|60
log2(expression)
0
2
4
6
8
12
16
StageI
StageII
StageIII
StageIV
StageV
StageVI

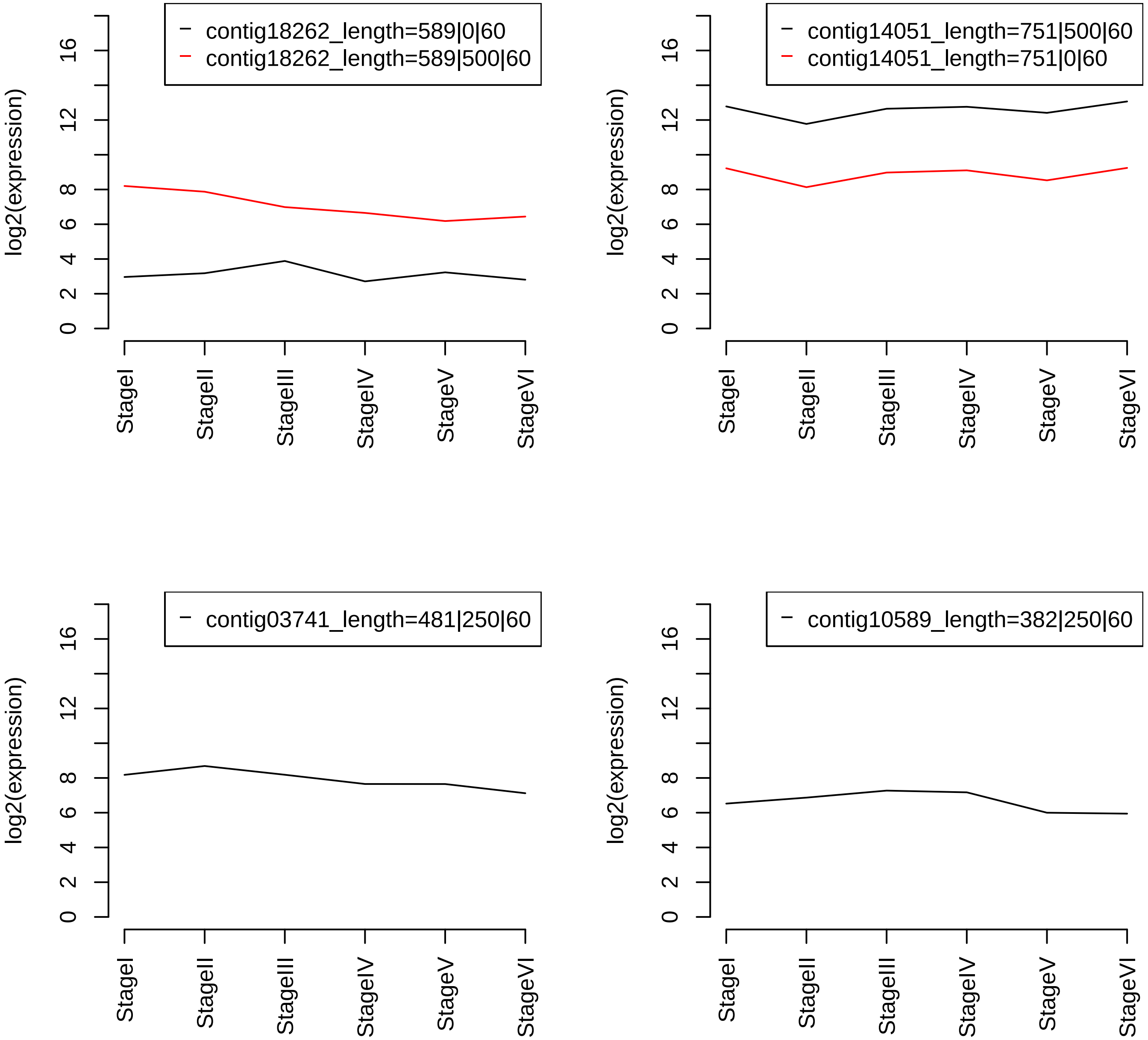
contig18262_length=589|0|60
contig18262_length=589|500|60
contig14051_length=751|500|60
contig14051_length=751|0|60
contig03741_length=481|250|60
contig10589_length=382|250|60
log2(expression)
0
2
4
6
8
12
16
StageI
StageII
StageIII
StageIV
StageV
StageVI

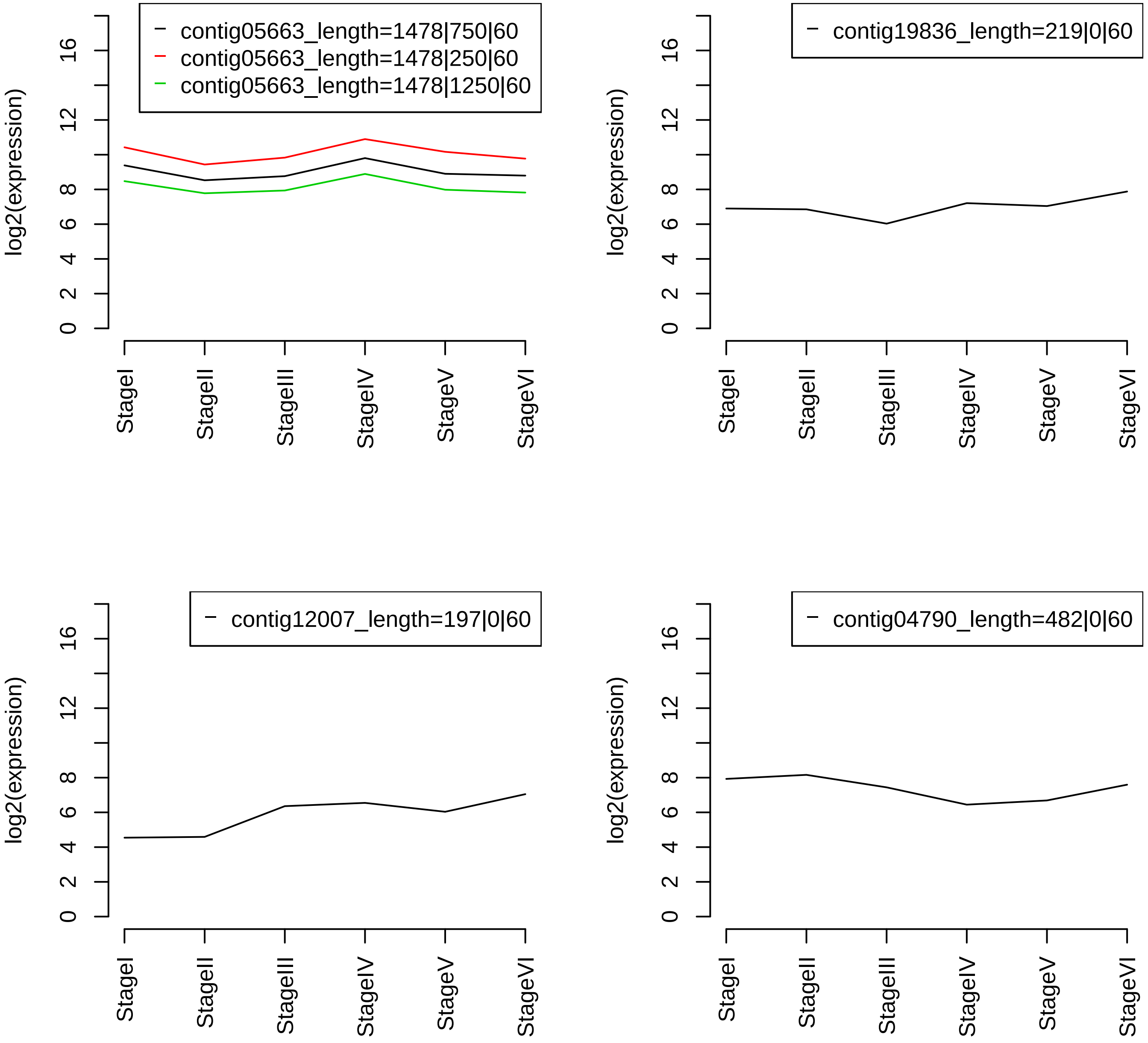

contig05663_length=1478|750|60
contig05663_length=1478|250|60
contig05663_length=1478|1250|60
contig19836_length=219|0|60
contig12007_length=197|0|60
contig04790_length=482|0|60
log2(expression)
0
2
4
6
8
12
16
StageI
StageII
StageIII
StageIV
StageV
StageVI

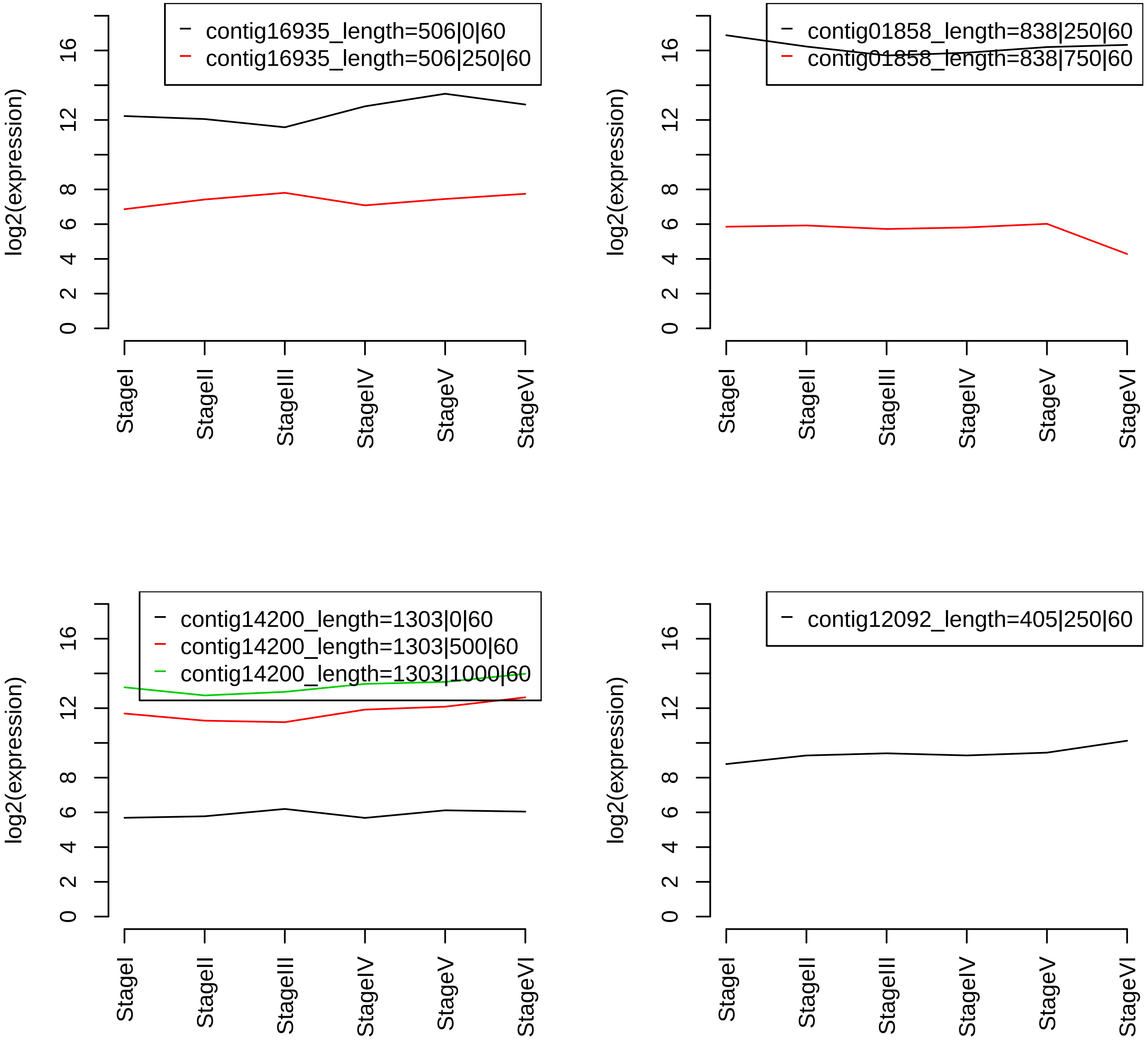
contig16935_length=506|0|60
contig16935_length=506|250|60
contig01858_length=838|250|60
contig01858_length=838|750|60
contig14200_length=1303|0|60
contig14200_length=1303|500|60
contig14200_length=1303|1000|60
contig12092_length=405|250|60
log2(expression)
0 2 4 6 8 12 16
StageI StageII StageIII StageIV StageV StageVI

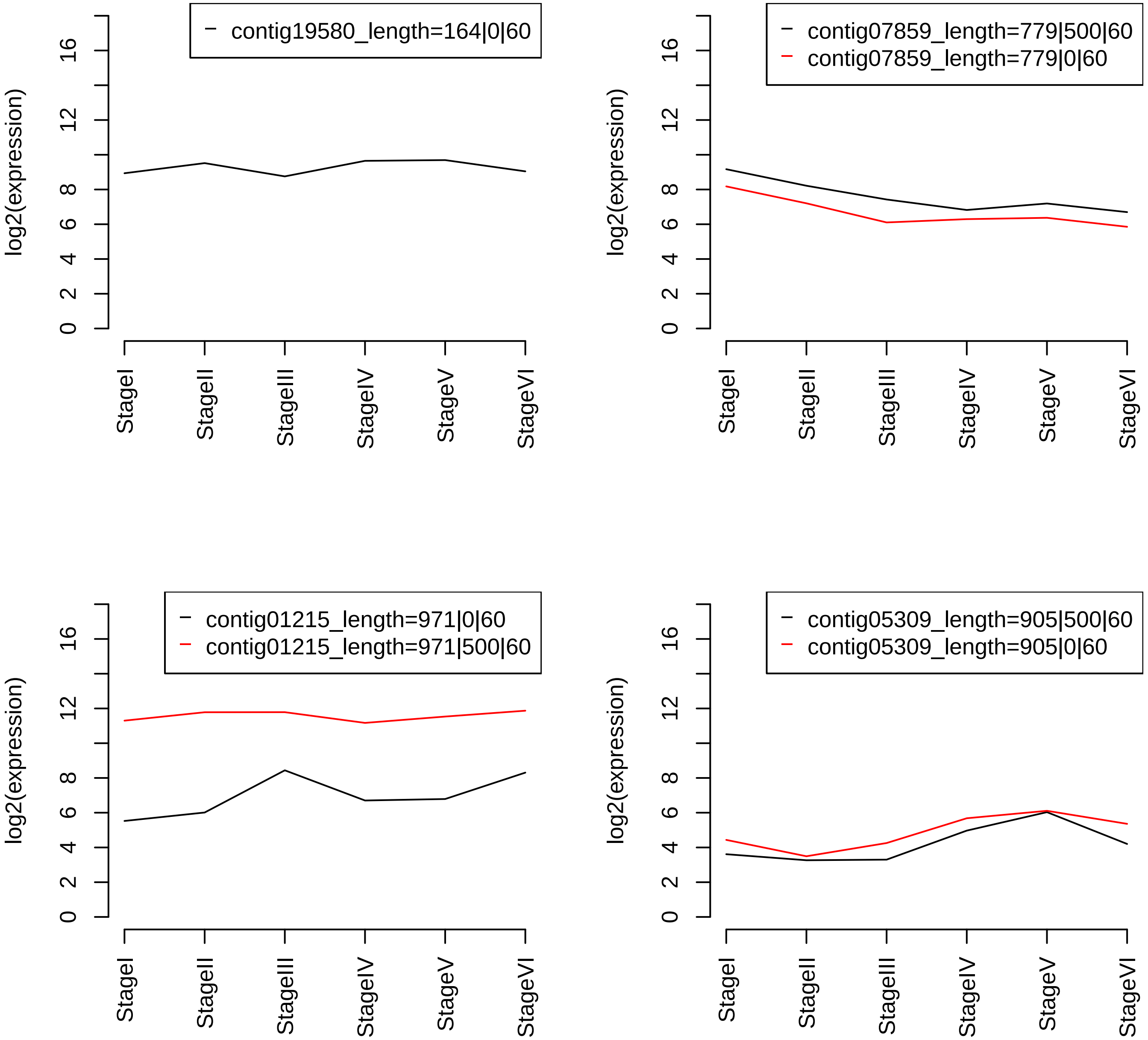

contig19580_length=164|0|60
contig07859_length=779|500|60
contig07859_length=779|0|60
contig01215_length=971|0|60
contig01215_length=971|500|60
contig05309_length=905|500|60
contig05309_length=905|0|60
log2(expression)
0
2
4
6
8
12
16
StageI
StageII
StageIII
StageIV
StageV
StageVI

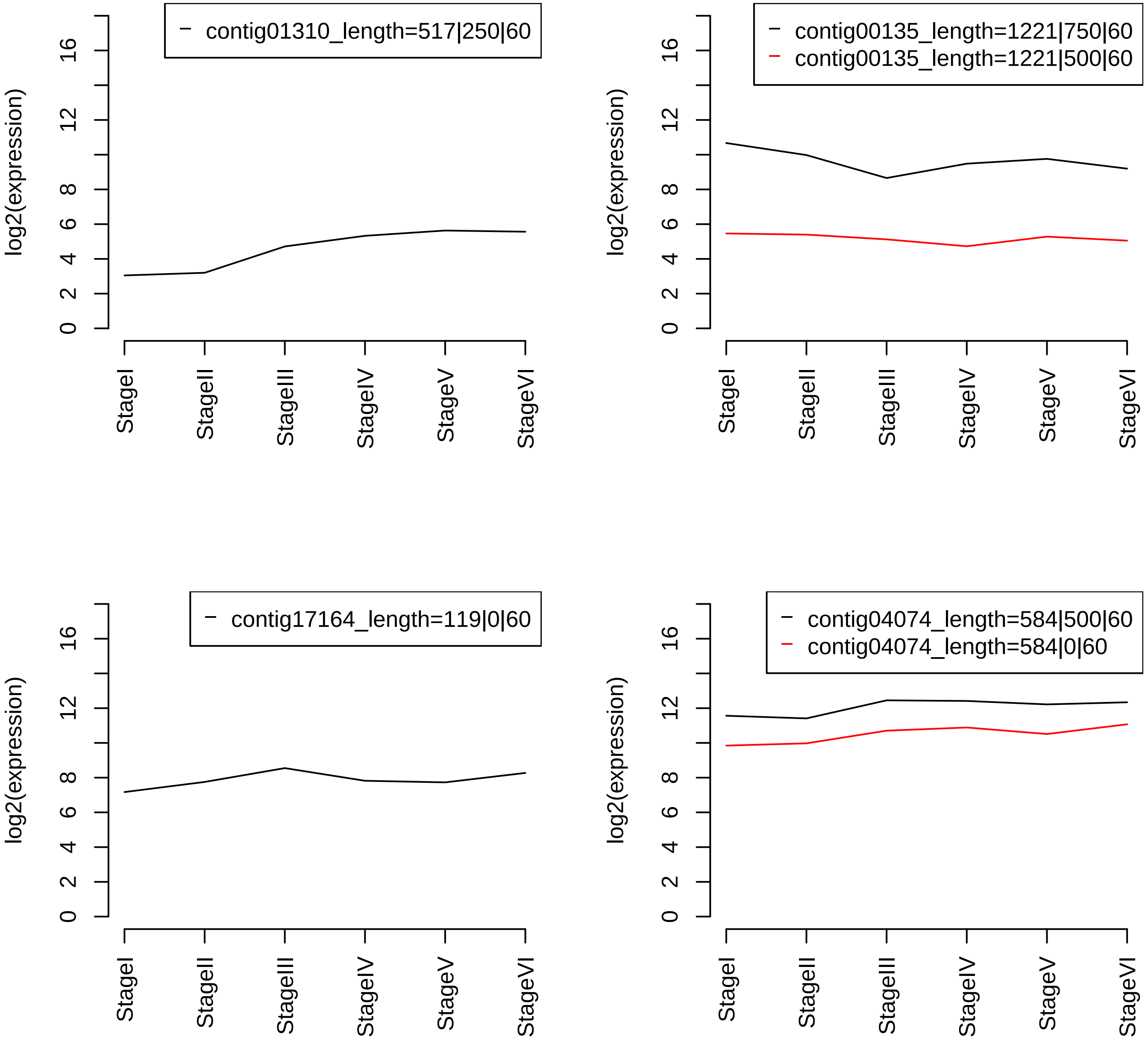

contig01310_length=517|250|60
contig00135_length=1221|750|60
contig00135_length=1221|500|60
contig17164_length=119|0|60
contig04074_length=584|500|60
contig04074_length=584|0|60
log2(expression)
0
2
4
6
8
12
16
StageI
StageII
StageIII
StageIV
StageV
StageVI

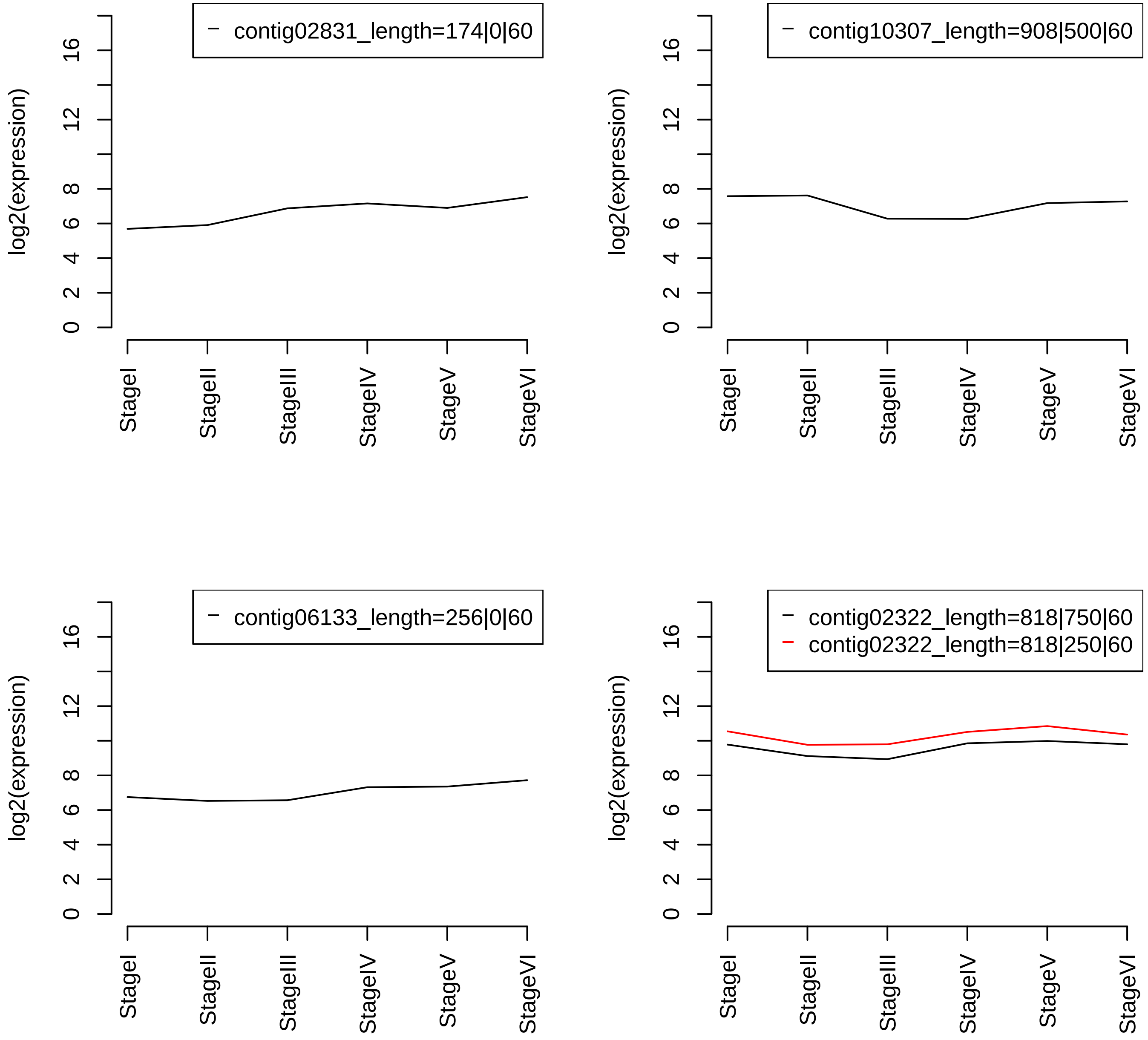
contig02831_length=174|0|60
contig10307_length=908|500|60
contig06133_length=256|0|60
contig02322_length=818|750|60
contig02322_length=818|250|60
log2(expression)
0
2
4
6
8
12
16
StageI
StageII
StageIII
StageIV
StageV
StageVI

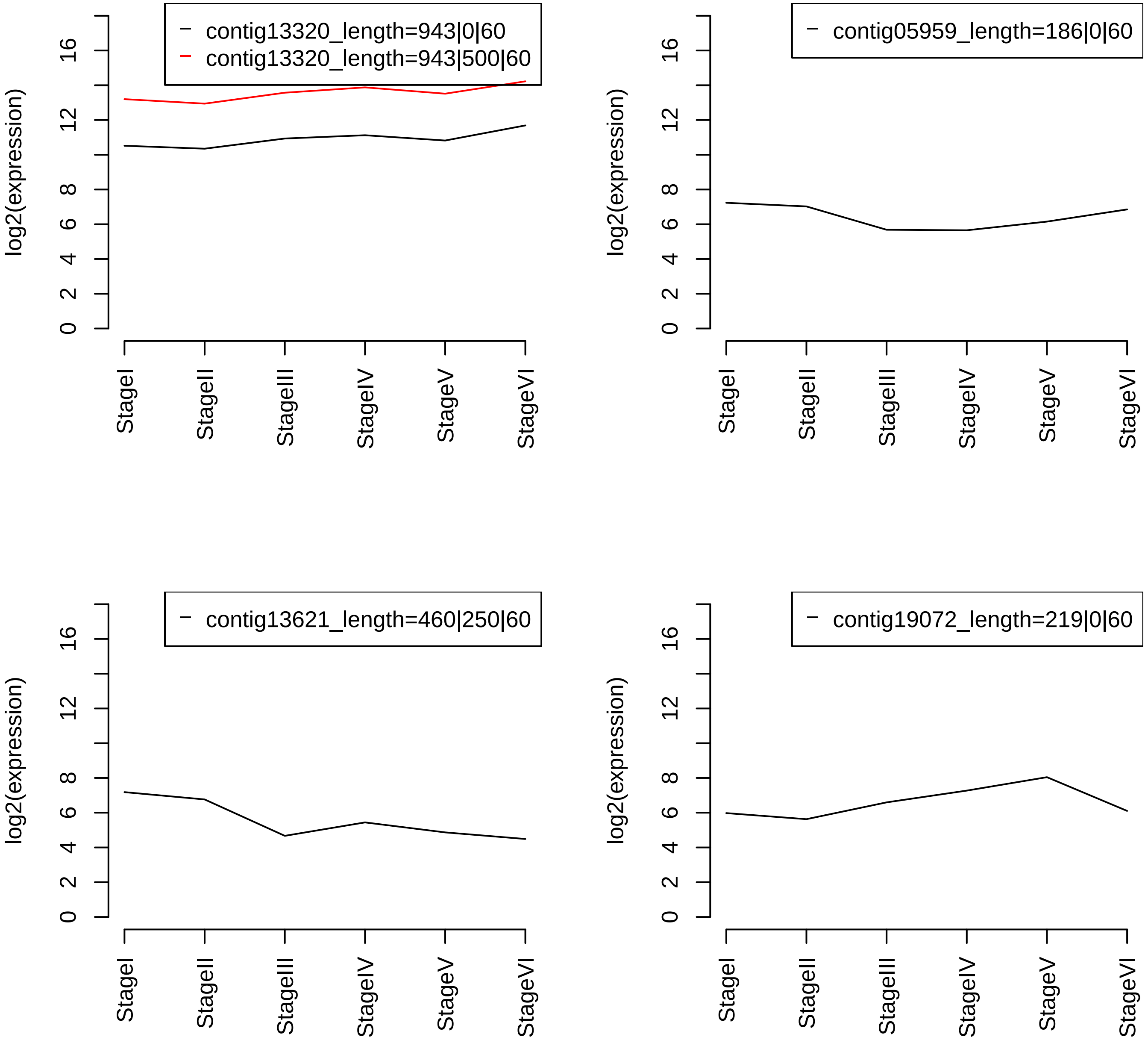
contig13320_length=943|0|60
contig13320_length=943|500|60
contig05959_length=186|0|60
contig13621_length=460|250|60
contig19072_length=219|0|60
log2(expression)
0 2 4 6 8 12 16
StageI
StageII
StageIII
StageIV
StageV
StageVI

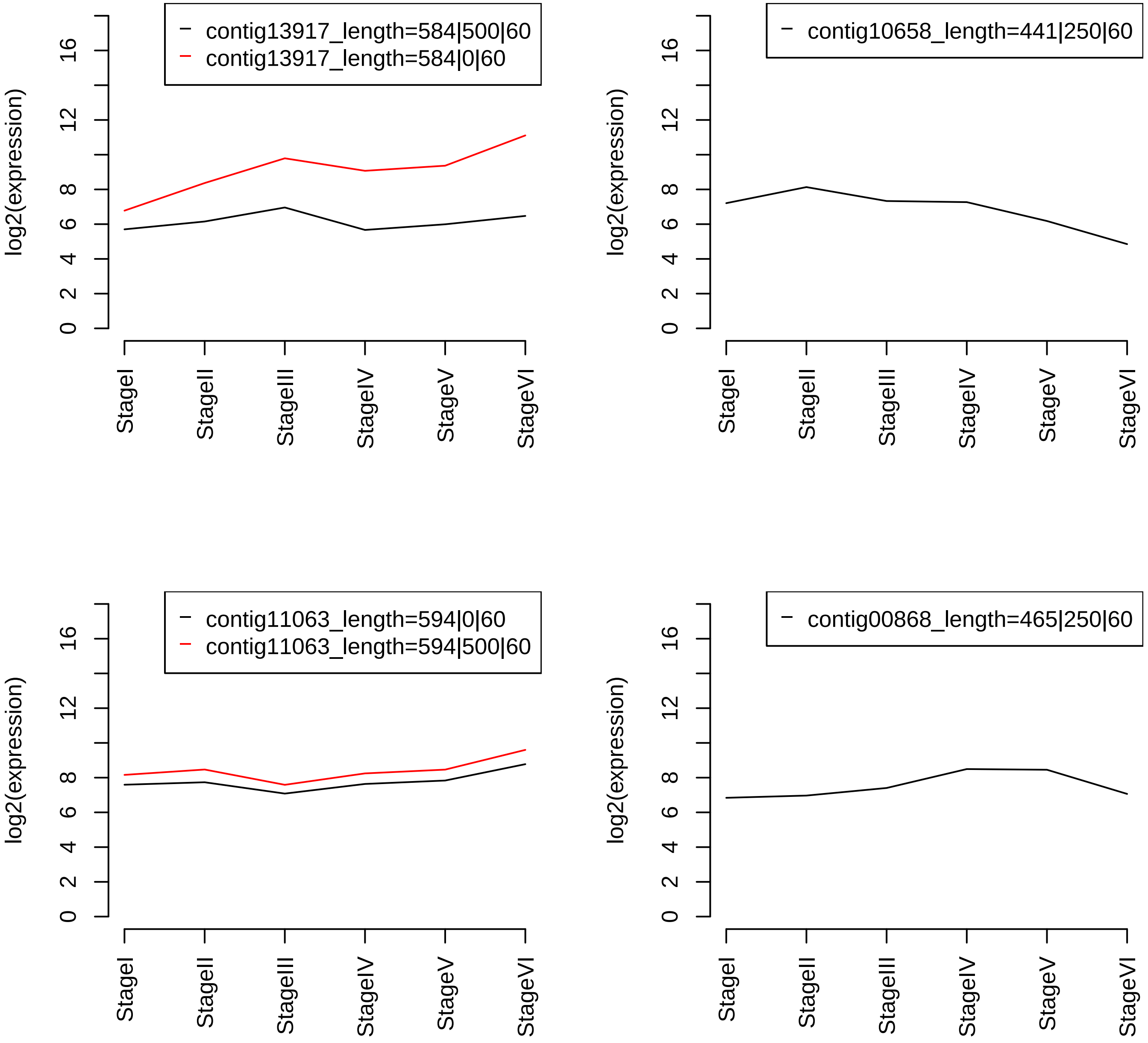
contig13917_length=584|500|60
contig13917_length=584|0|60
contig10658_length=441|250|60
contig11063_length=594|0|60
contig11063_length=594|500|60
contig00868_length=465|250|60
log2(expression)
0
2
4
6
8
12
16
StageI
StageII
StageIII
StageIV
StageV
StageVI

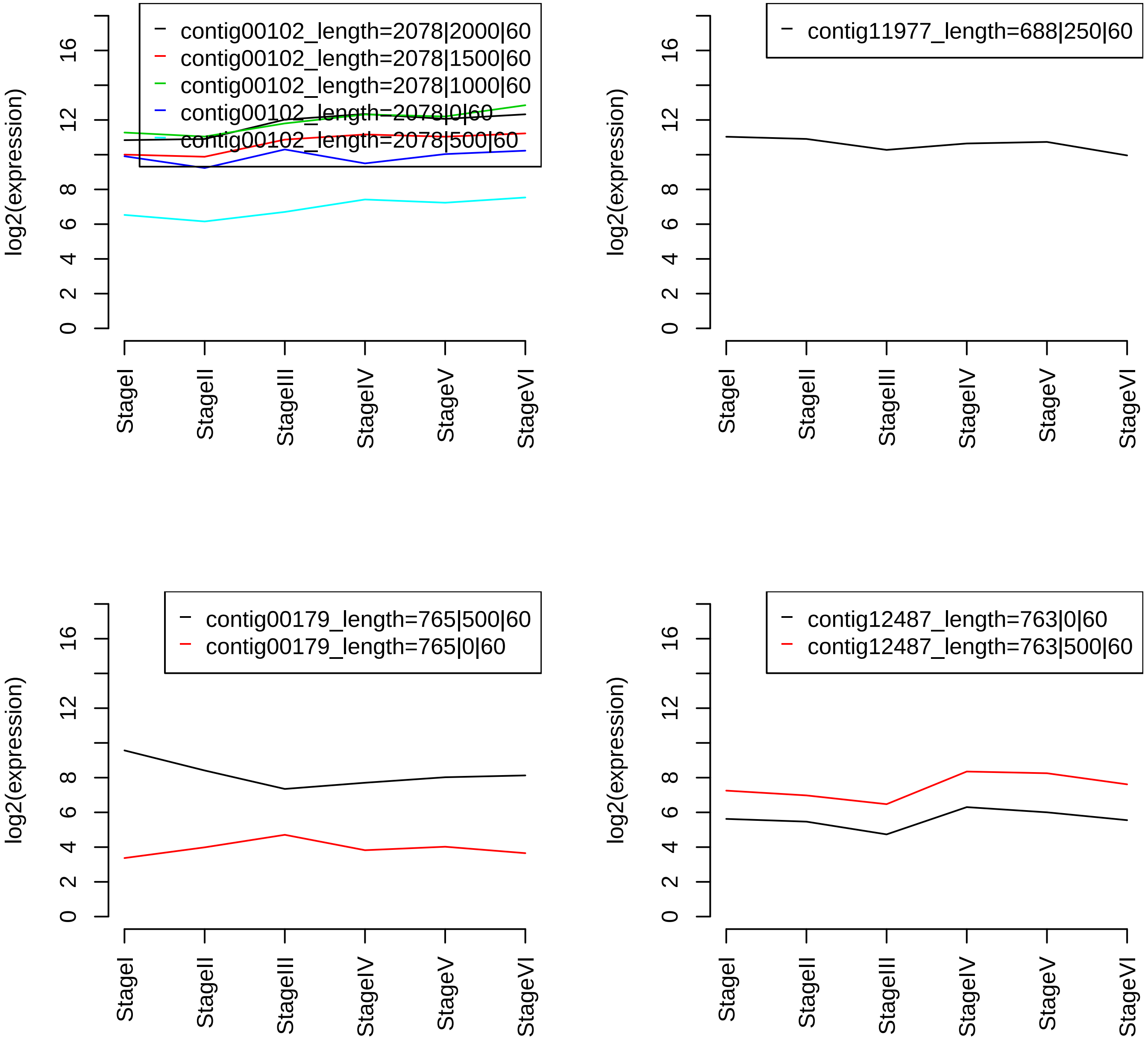

contig00102_length=2078|2000|60
contig00102_length=2078|1500|60
contig00102_length=2078|1000|60
contig00102_length=2078|0|60
contig00102_length=2078|500|60
contig11977_length=688|250|60
contig00179_length=765|500|60
contig00179_length=765|0|60
contig12487_length=763|0|60
contig12487_length=763|500|60
log2(expression)
0
2
4
6
8
12
16
StageI
StageII
StageIII
StageIV
StageV
StageVI

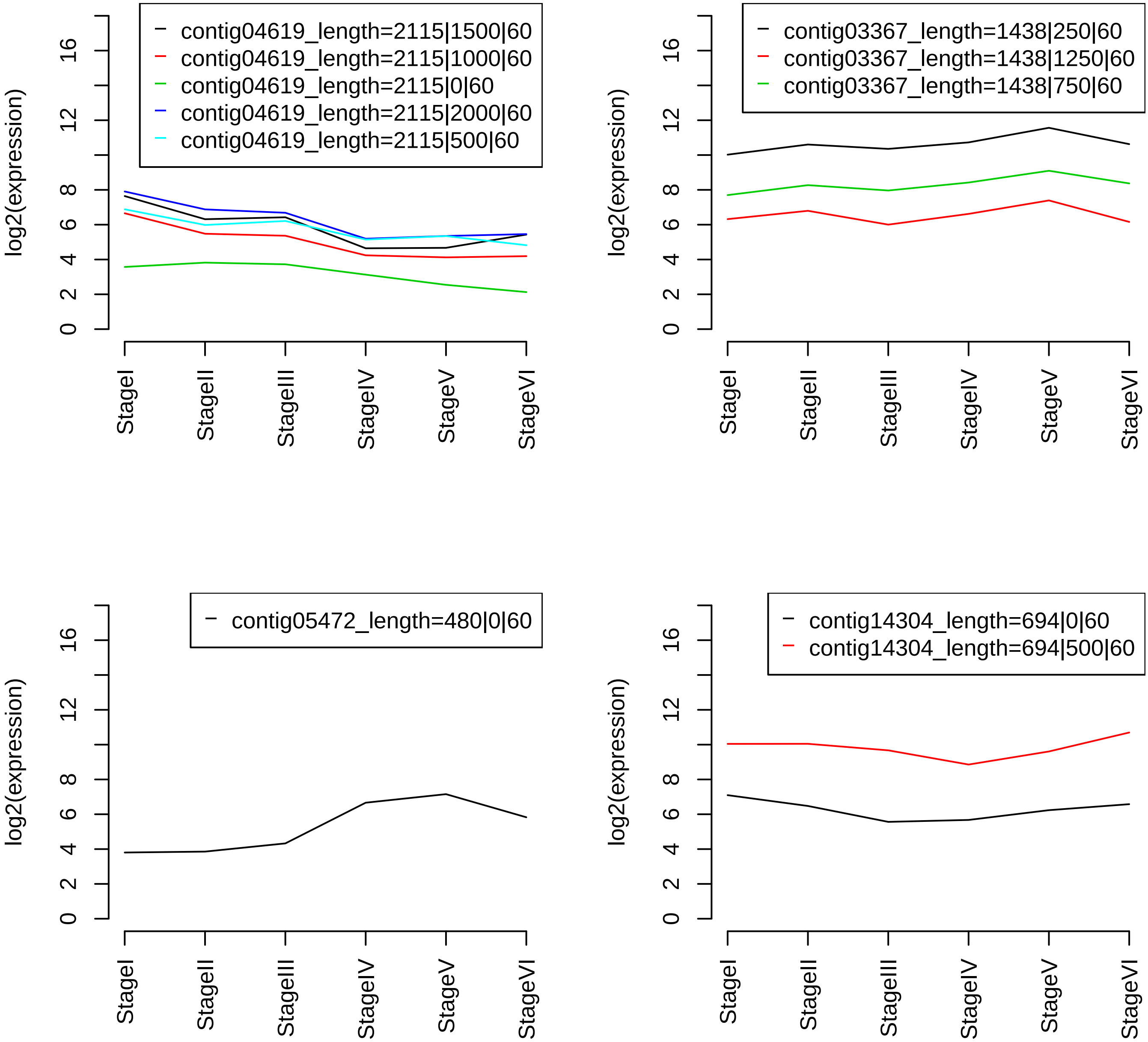

contig04619_length=2115|1500|60
contig04619_length=2115|1000|60
contig04619_length=2115|0|60
contig04619_length=2115|2000|60
contig04619_length=2115|500|60
contig03367_length=1438|250|60
contig03367_length=1438|1250|60
contig03367_length=1438|750|60
contig05472_length=480|0|60
contig14304_length=694|0|60
contig14304_length=694|500|60
log2(expression)
0
2
4
6
8
12
16
StageI
StageII
StageIII
StageIV
StageV
StageVI

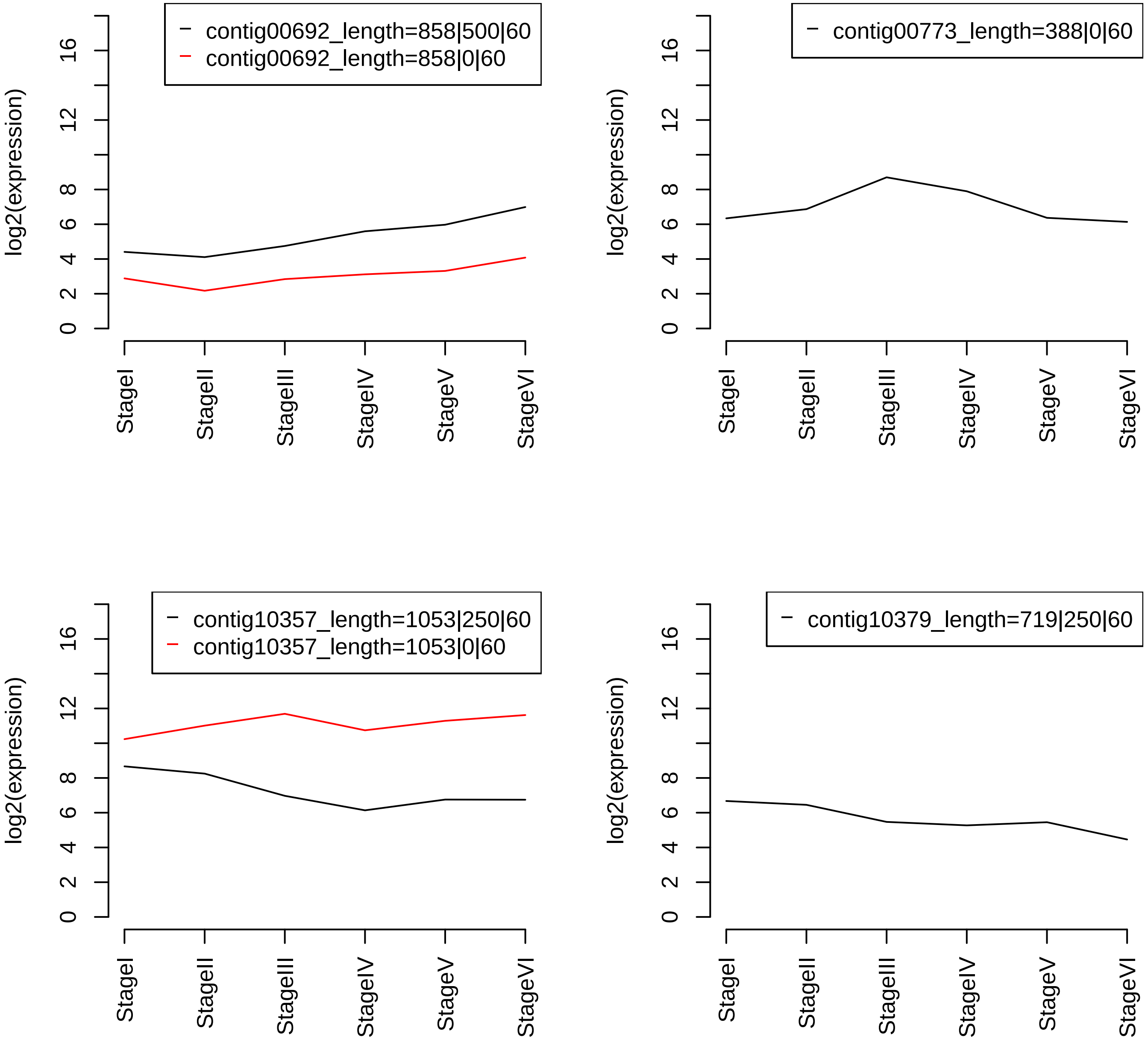

contig00692_length=858|500|60
contig00692_length=858|0|60
contig00773_length=388|0|60
contig10357_length=1053|250|60
contig10357_length=1053|0|60
contig10379_length=719|250|60
log2(expression)
0
2
4
6
8
12
16
StageI
StageII
StageIII
StageIV
StageV
StageVI

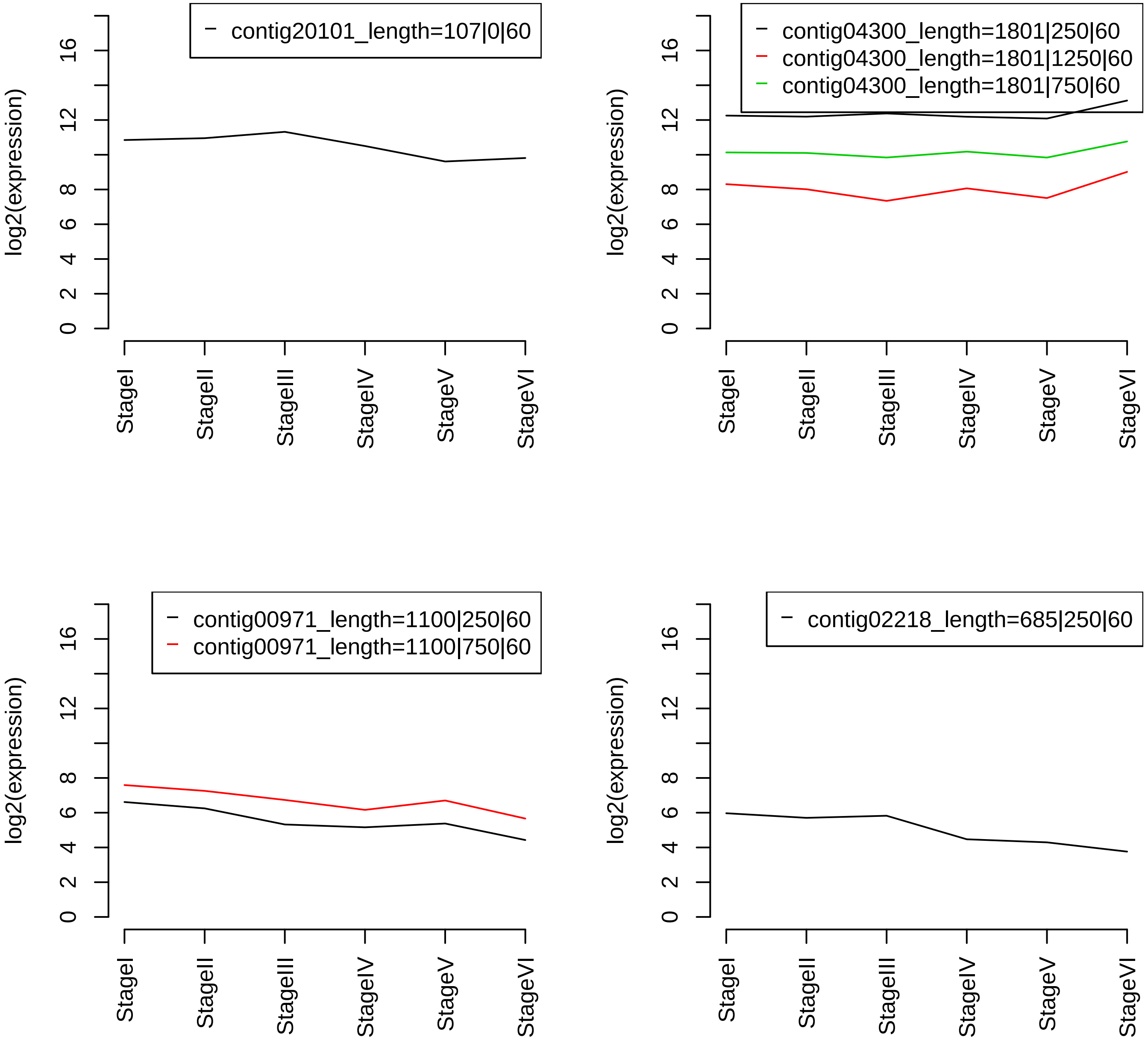
contig20101_length=107|0|60
contig04300_length=1801|250|60
contig04300_length=1801|1250|60
contig04300_length=1801|750|60
contig00971_length=1100|250|60
contig00971_length=1100|750|60
contig02218_length=685|250|60
log2(expression)
0
2
4
6
8
12
16
StageI
StageII
StageIII
StageIV
StageV
StageVI

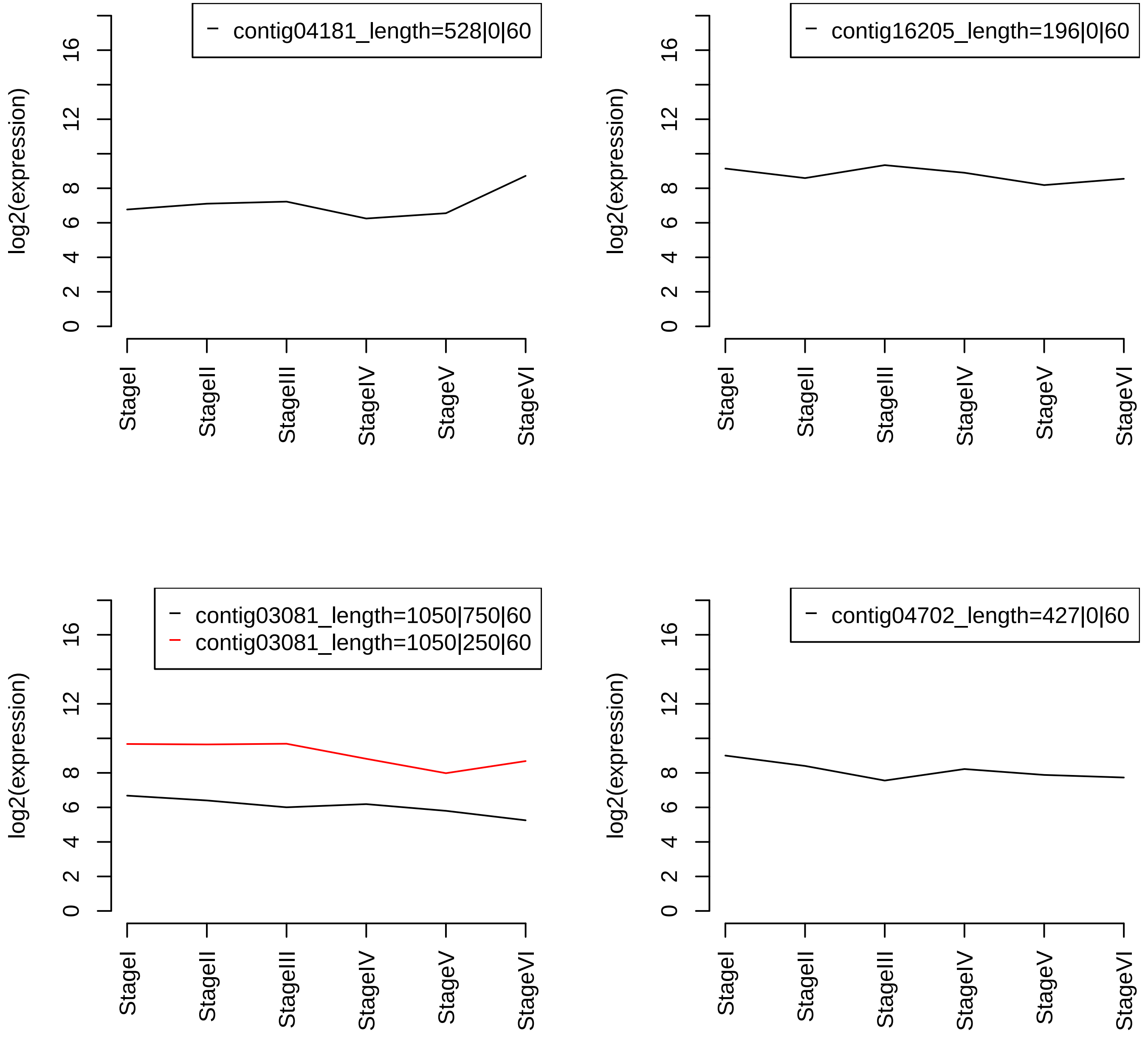
contig04181_length=528|0|60
contig16205_length=196|0|60
contig03081_length=1050|750|60
contig03081_length=1050|250|60
contig04702_length=427|0|60
log2(expression)
0
2
4
6
8
12
16
StageI
StageII
StageIII
StageIV
StageV
StageVI

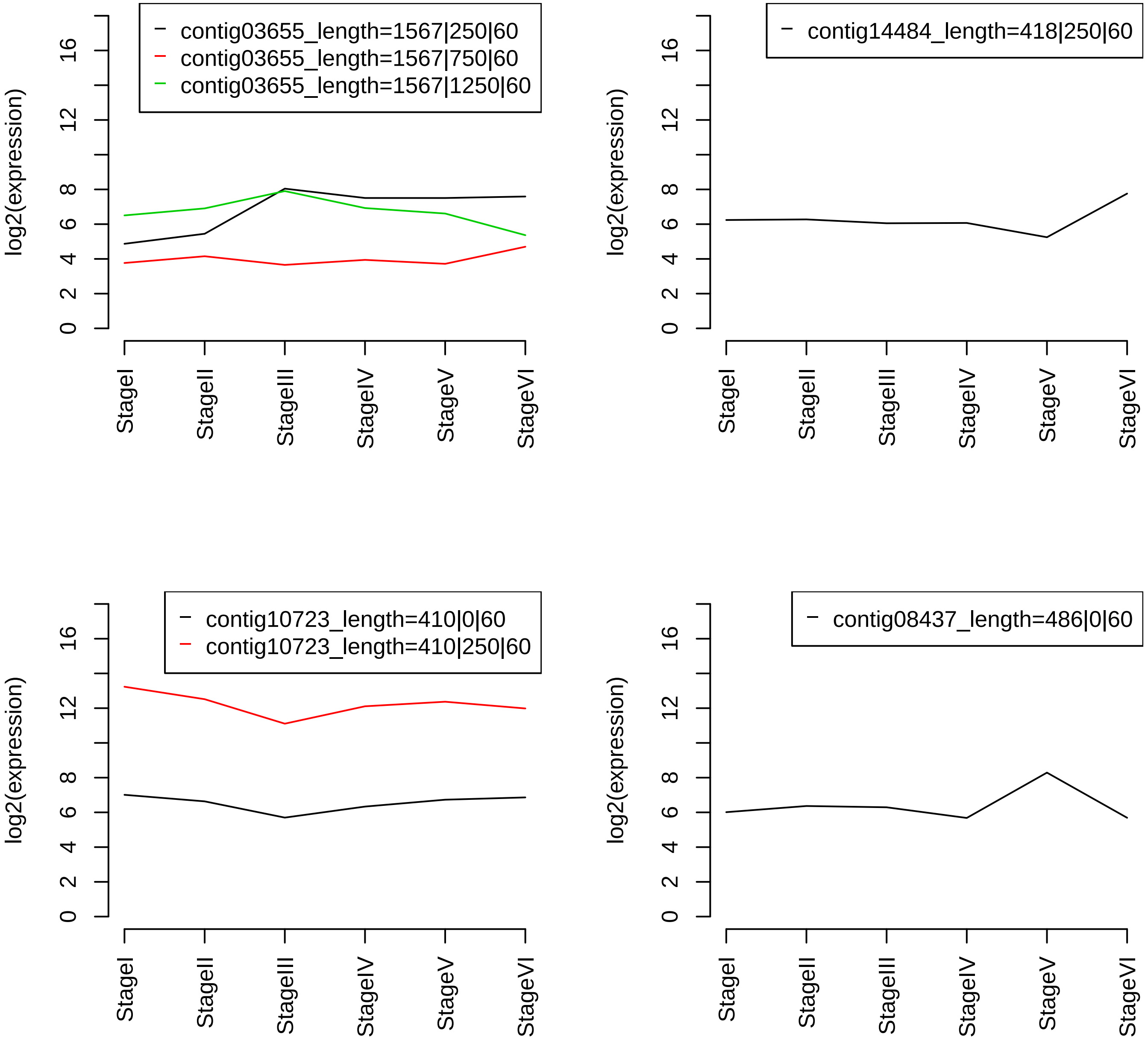

contig03655_length=1567|250|60
contig03655_length=1567|750|60
contig03655_length=1567|1250|60
contig14484_length=418|250|60
contig10723_length=410|0|60
contig10723_length=410|250|60
contig08437_length=486|0|60
log2(expression)
0
2
4
6
8
12
16
StageI
StageII
StageIII
StageIV
StageV
StageVI

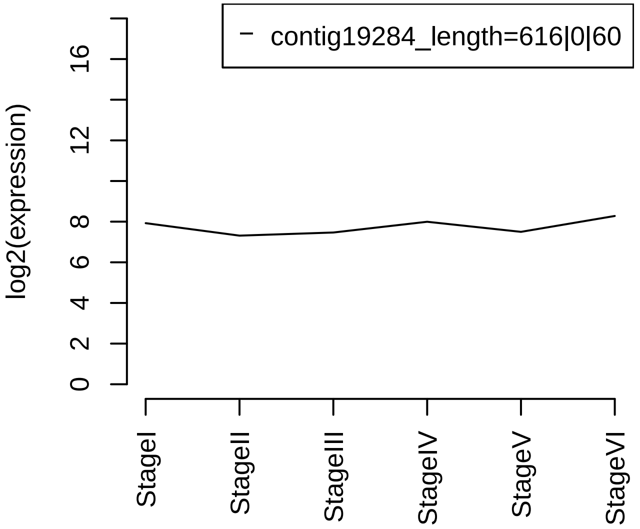
contig19284_length=616|0|60
log2(expression)
0
2
4
6
8
12
16
StageI
StageII
StageIII
StageIV
StageV
StageVI

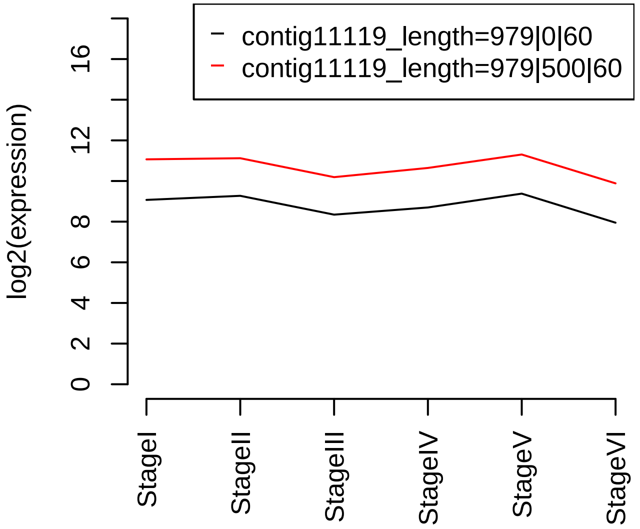
contig11119_length=979|0|60
contig11119_length=979|500|60
log2(expression)
0
2
4
6
8
12
16
StageI
StageII
StageIII
StageIV
StageV
StageVI

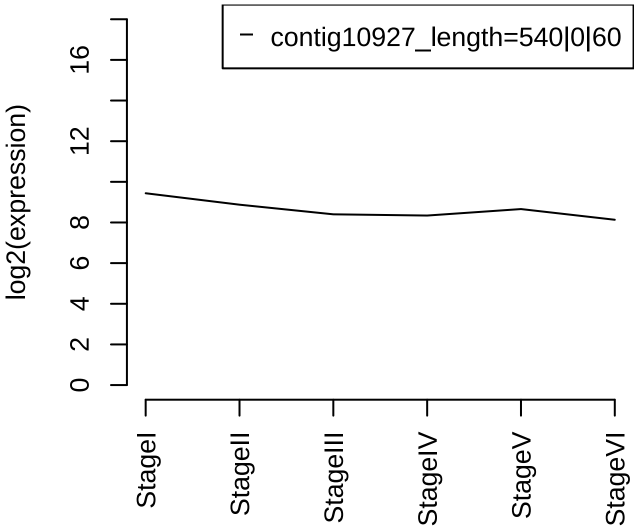
contig10927_length=540|0|60
log2(expression)
0
2
4
6
8
12
16
StageI
StageII
StageIII
StageIV
StageV
StageVI

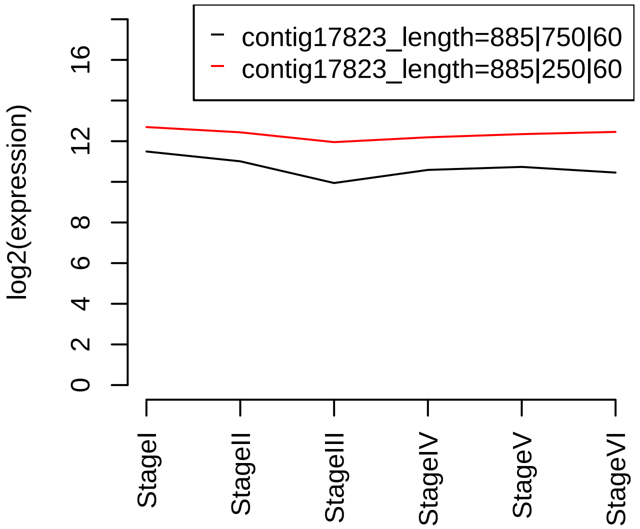
contig17823_length=885|750|60
contig17823_length=885|250|60
log2(expression)
0
2
4
6
8
12
16
StageI
StageII
StageIII
StageIV
StageV
StageVI

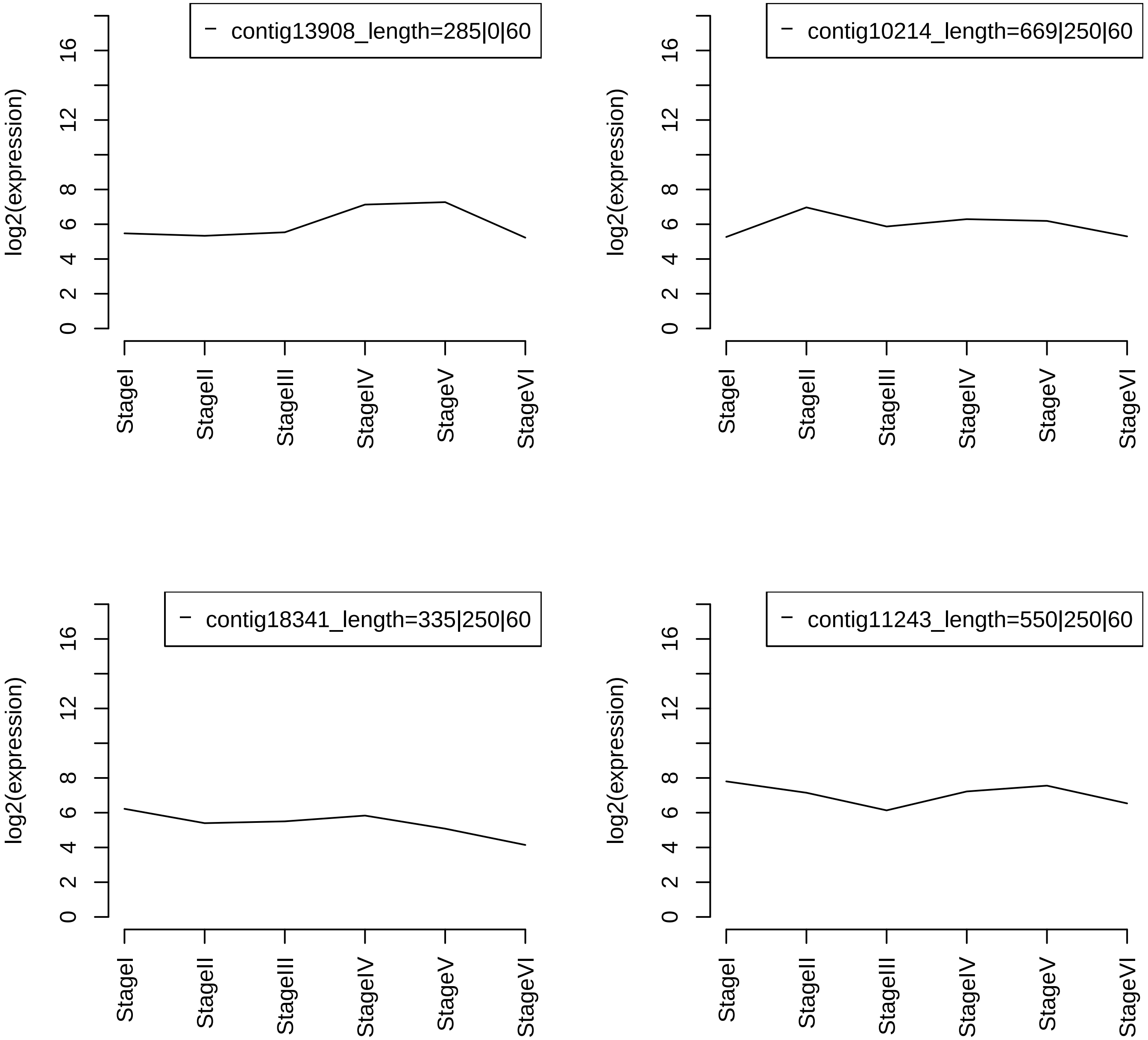

contig13908_length=285|0|60
contig10214_length=669|250|60
contig18341_length=335|250|60
contig11243_length=550|250|60
log2(expression)
0
2
4
6
8
12
16
StageI
StageII
StageIII
StageIV
StageV
StageVI

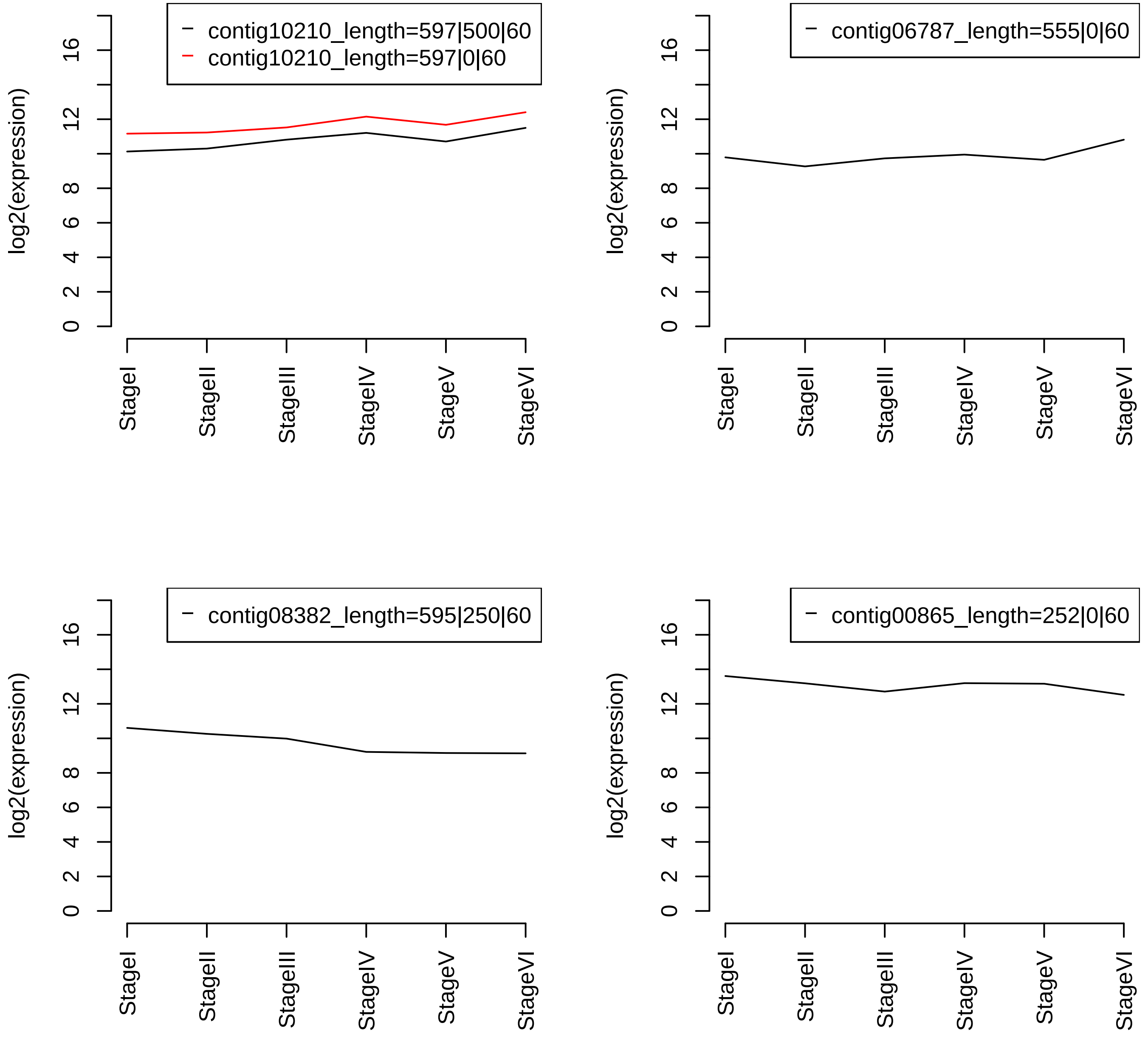
contig10210_length=597|500|60
contig10210_length=597|0|60
contig06787_length=555|0|60
contig08382_length=595|250|60
contig00865_length=252|0|60
log2(expression)
0
2
4
6
8
12
16
StageI
StageII
StageIII
StageIV
StageV
StageVI

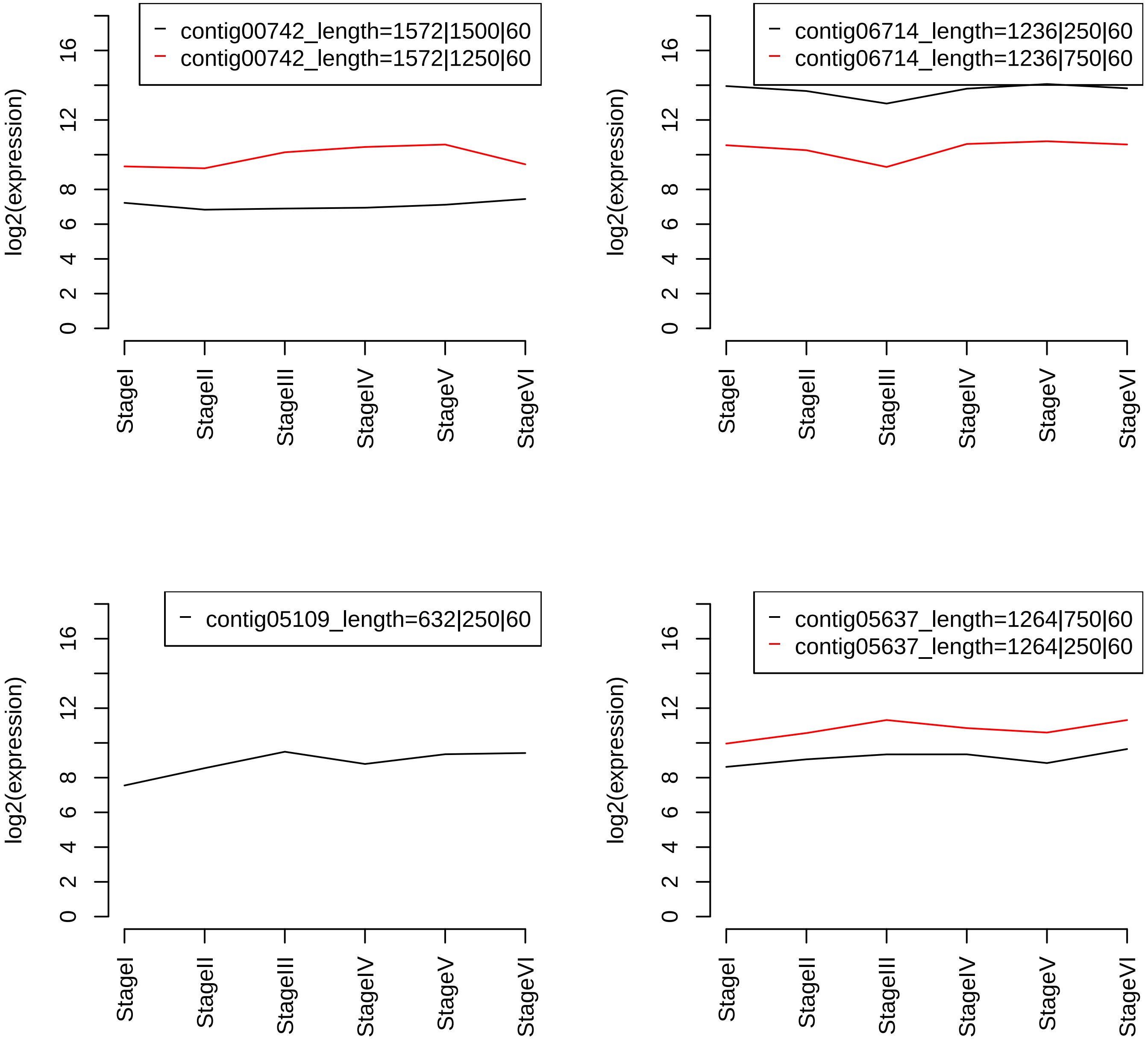

contig00742_length=1572|1500|60
contig00742_length=1572|1250|60
contig06714_length=1236|250|60
contig06714_length=1236|750|60
contig05109_length=632|250|60
contig05637_length=1264|750|60
contig05637_length=1264|250|60
log2(expression)
0
2
4
6
8
12
16
StageI
StageII
StageIII
StageIV
StageV
StageVI

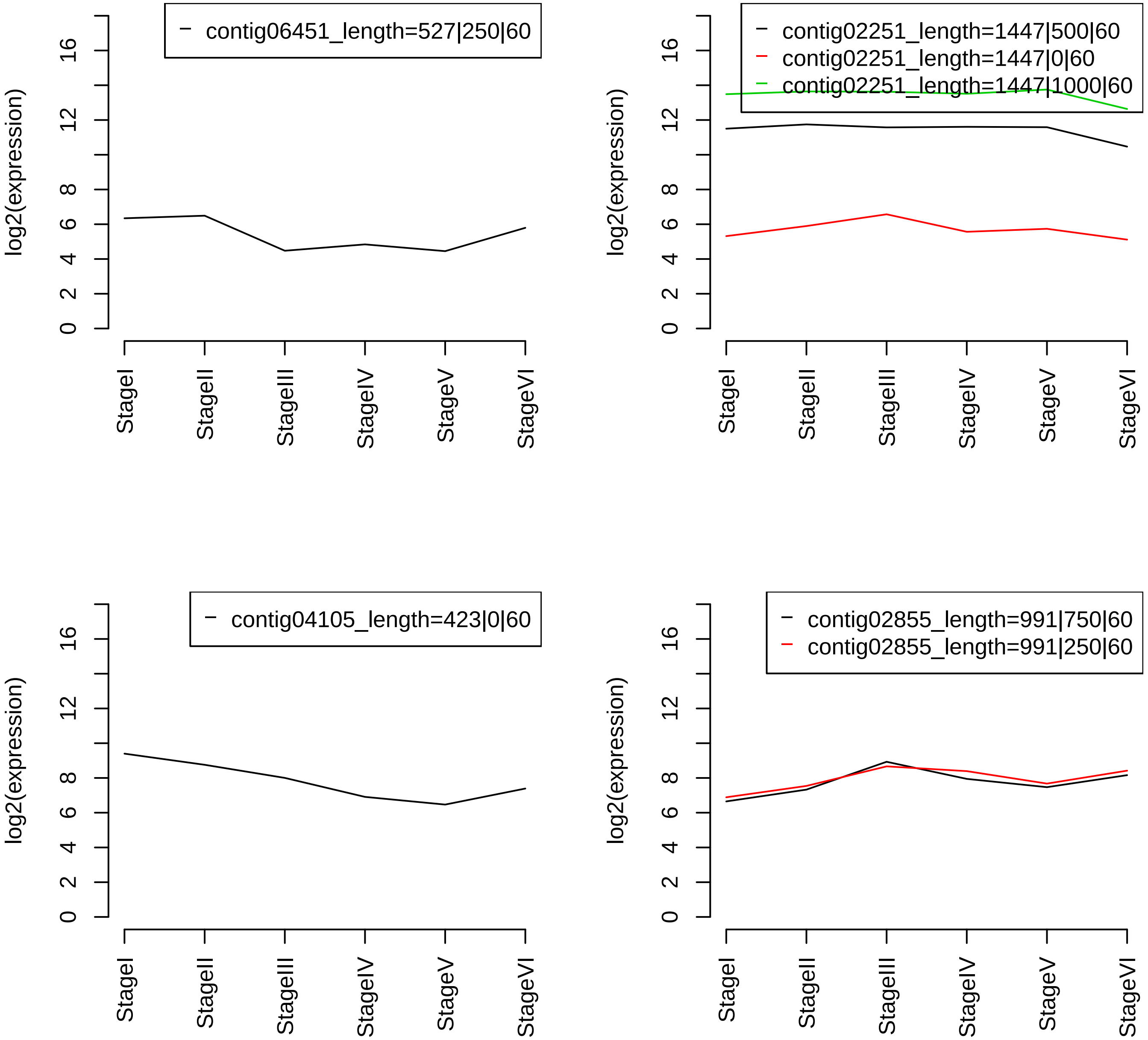
contig06451_length=527|250|60
contig02251_length=1447|500|60
contig02251_length=1447|0|60
contig02251_length=1447|1000|60
contig04105_length=423|0|60
contig02855_length=991|750|60
contig02855_length=991|250|60
log2(expression)
0
2
4
6
8
12
16
StageI
StageII
StageIII
StageIV
StageV
StageVI

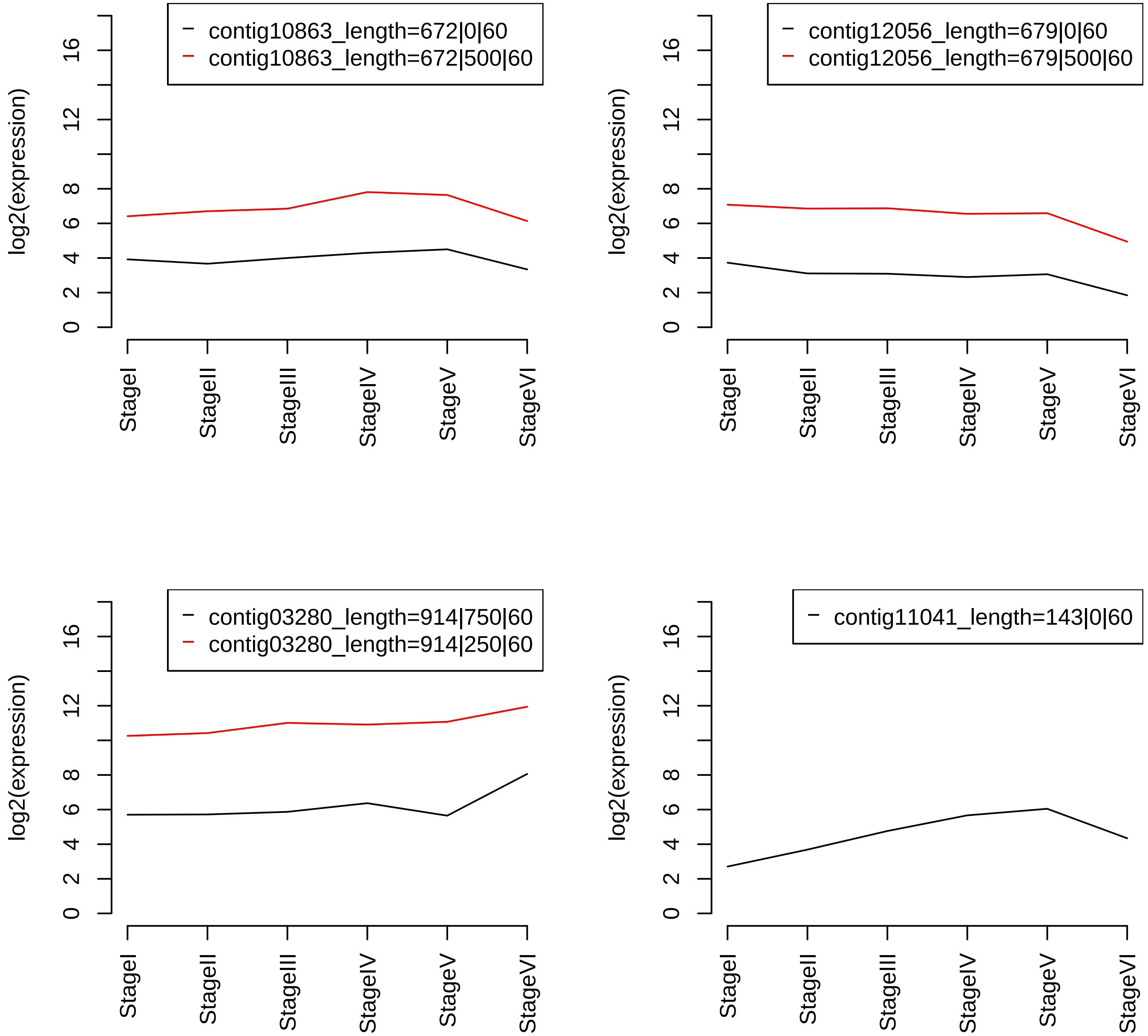
contig10863_length=672|0|60
contig10863_length=672|500|60
contig12056_length=679|0|60
contig12056_length=679|500|60
contig03280_length=914|750|60
contig03280_length=914|250|60
contig11041_length=143|0|60
log2(expression)
0
2
4
6
8
12
16
StageI
StageII
StageIII
StageIV
StageV
StageVI

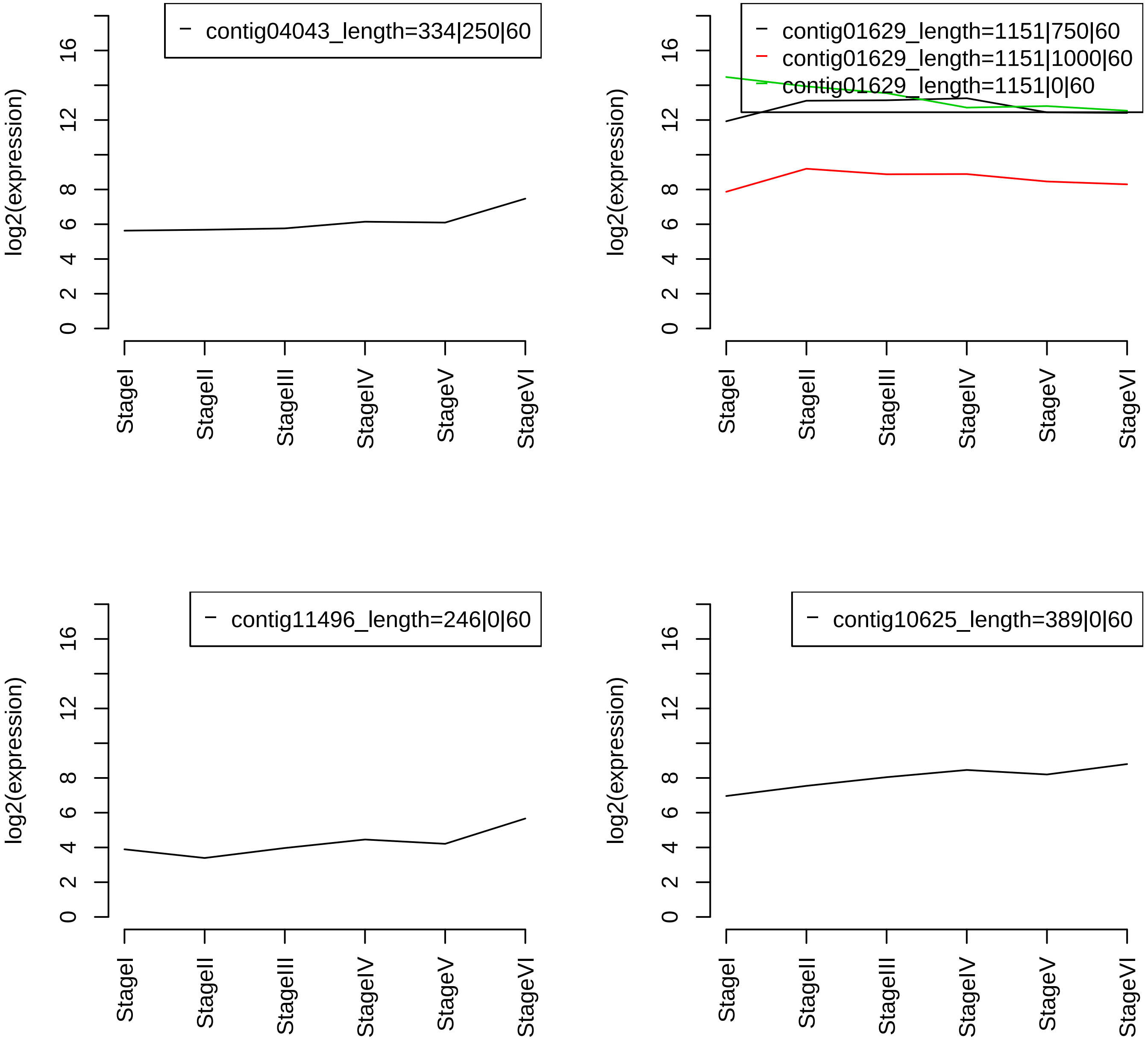

contig04043_length=334|250|60
contig01629_length=1151|750|60
contig01629_length=1151|1000|60
contig01629_length=1151|0|60
contig11496_length=246|0|60
contig10625_length=389|0|60
log2(expression)
0
2
4
6
8
12
16
StageI
StageII
StageIII
StageIV
StageV
StageVI

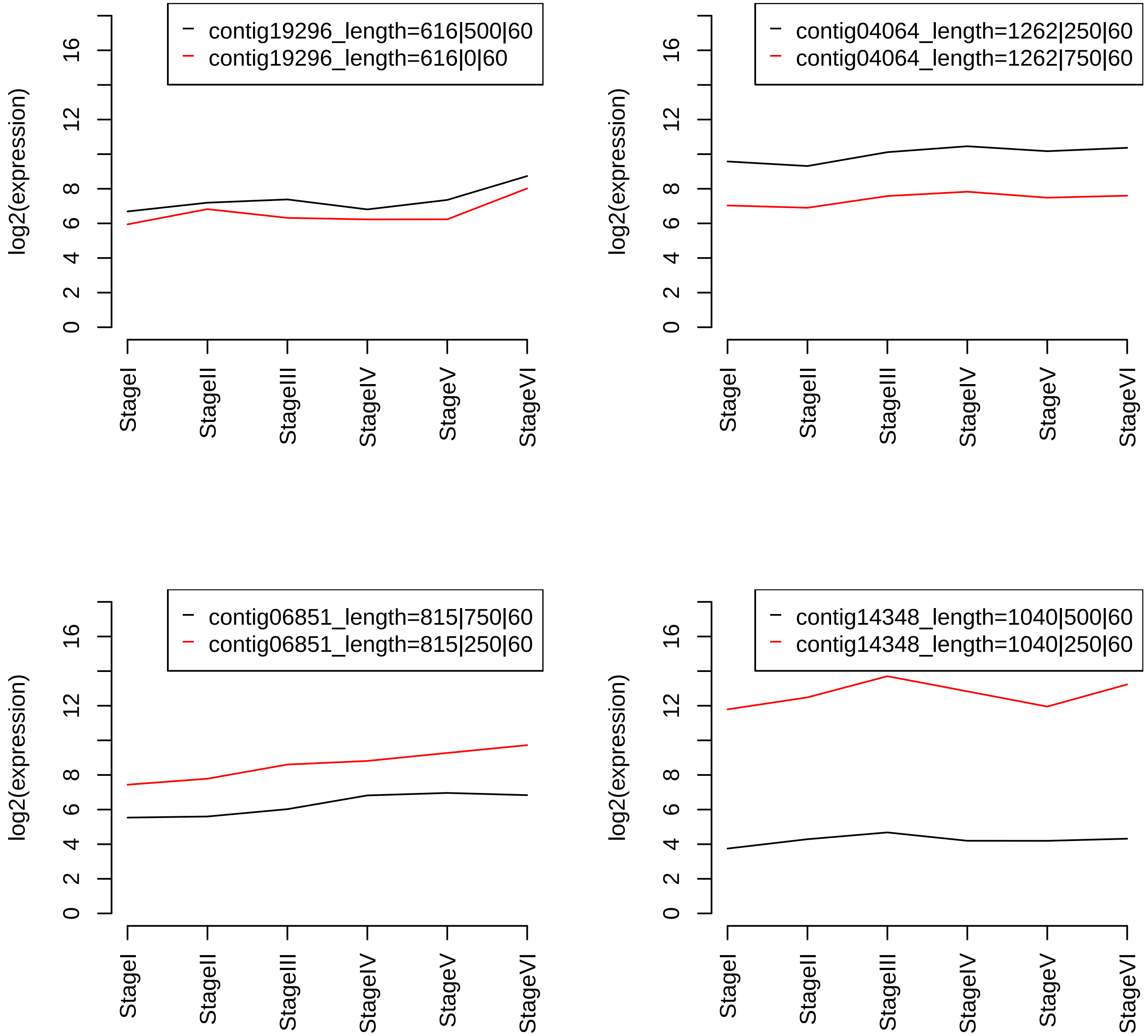

contig19296_length=616|500|60
contig19296_length=616|0|60
contig04064_length=1262|250|60
contig04064_length=1262|750|60
contig06851_length=815|750|60
contig06851_length=815|250|60
contig14348_length=1040|500|60
contig14348_length=1040|250|60
log2(expression)
0 2 4 6 8 12 16
StageI StageII StageIII StageIV StageV StageVI

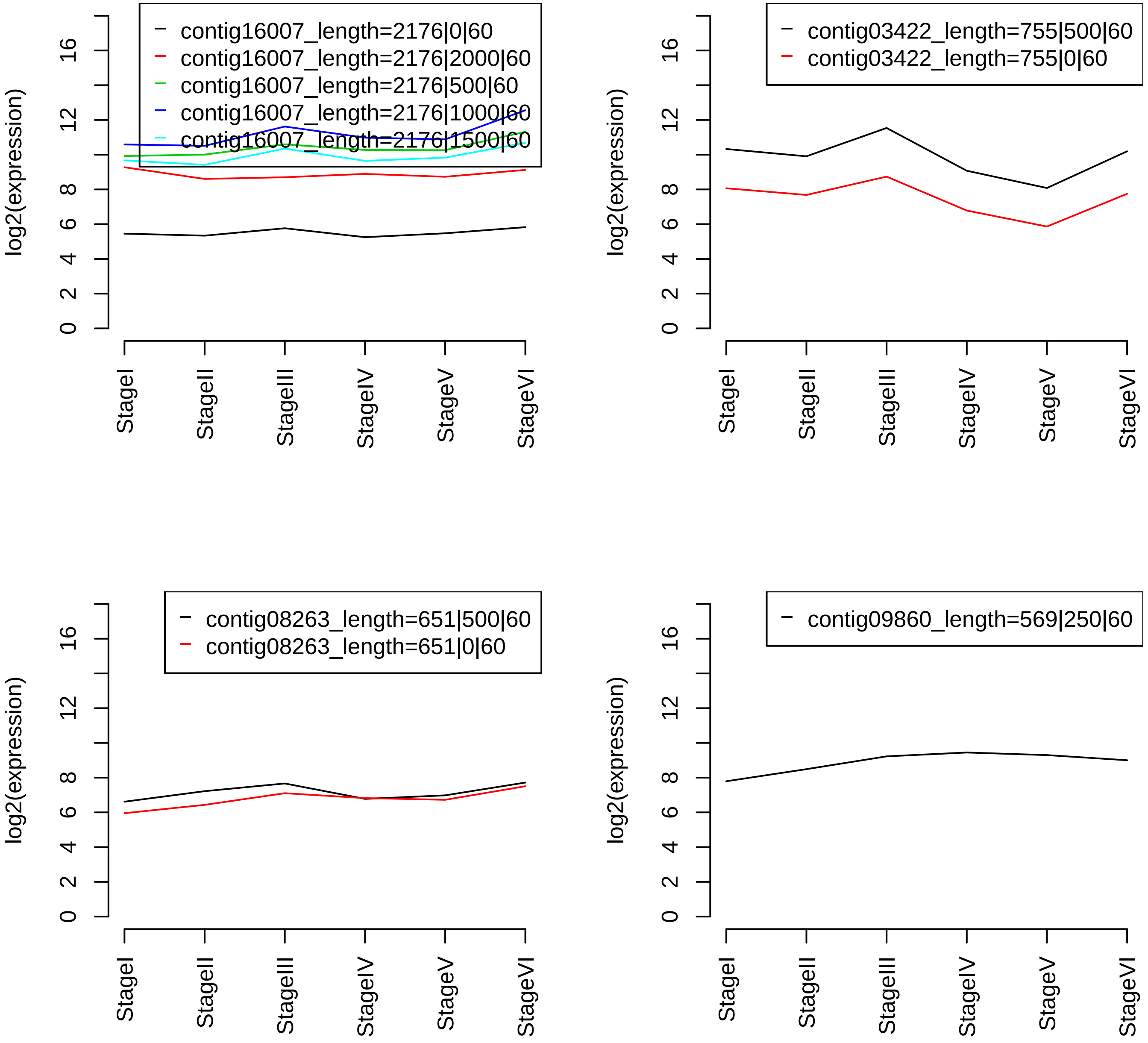

contig16007_length=2176|0|60
contig16007_length=2176|2000|60
contig16007_length=2176|500|60
contig16007_length=2176|1000|60
contig16007_length=2176|1500|60
contig03422_length=755|500|60
contig03422_length=755|0|60
contig08263_length=651|500|60
contig08263_length=651|0|60
contig09860_length=569|250|60
log2(expression)
0
2
4
6
8
12
16
StageI
StageII
StageIII
StageIV
StageV
StageVI

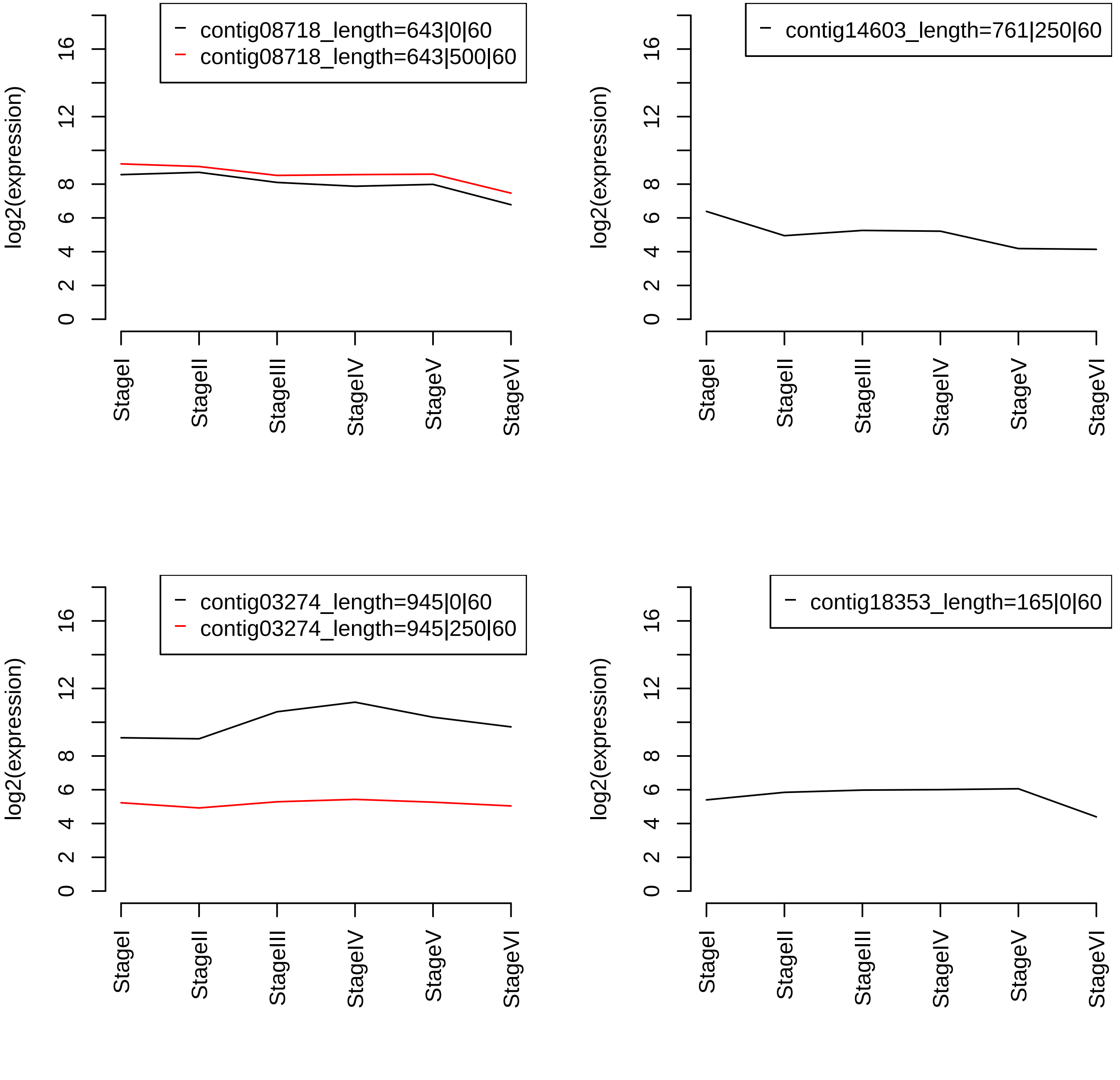

contig08718_length=643|0|60
contig08718_length=643|500|60
contig14603_length=761|250|60
contig03274_length=945|0|60
contig03274_length=945|250|60
contig18353_length=165|0|60
log2(expression)
0
2
4
6
8
12
16
StageI
StageII
StageIII
StageIV
StageV
StageVI

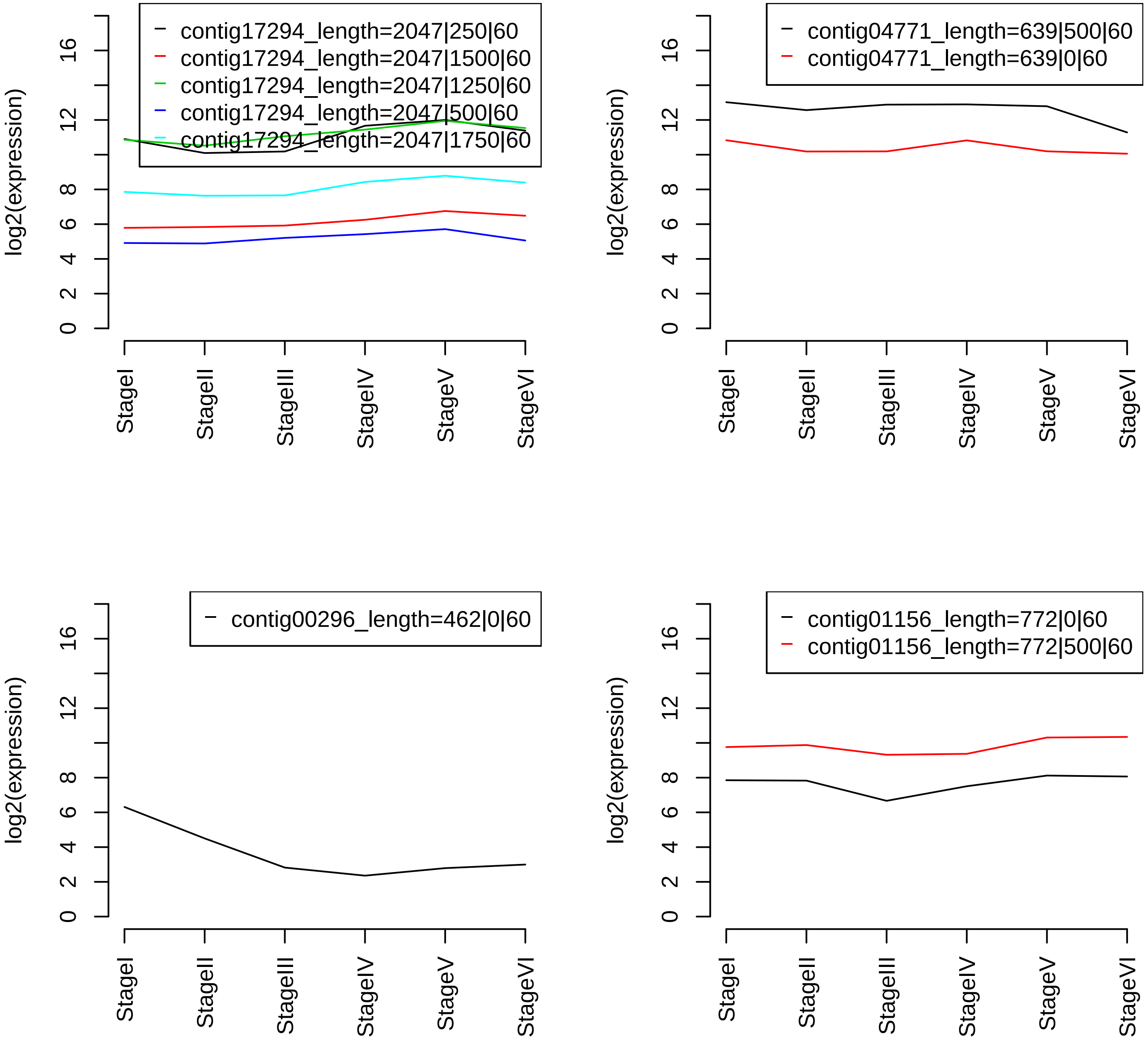
contig17294_length=2047|250|60
contig17294_length=2047|1500|60
contig17294_length=2047|1250|60
contig17294_length=2047|500|60
contig17294_length=2047|1750|60
contig04771_length=639|500|60
contig04771_length=639|0|60
contig00296_length=462|0|60
contig01156_length=772|0|60
contig01156_length=772|500|60
log2(expression)
StageI
StageII
StageIII
StageIV
StageV
StageVI

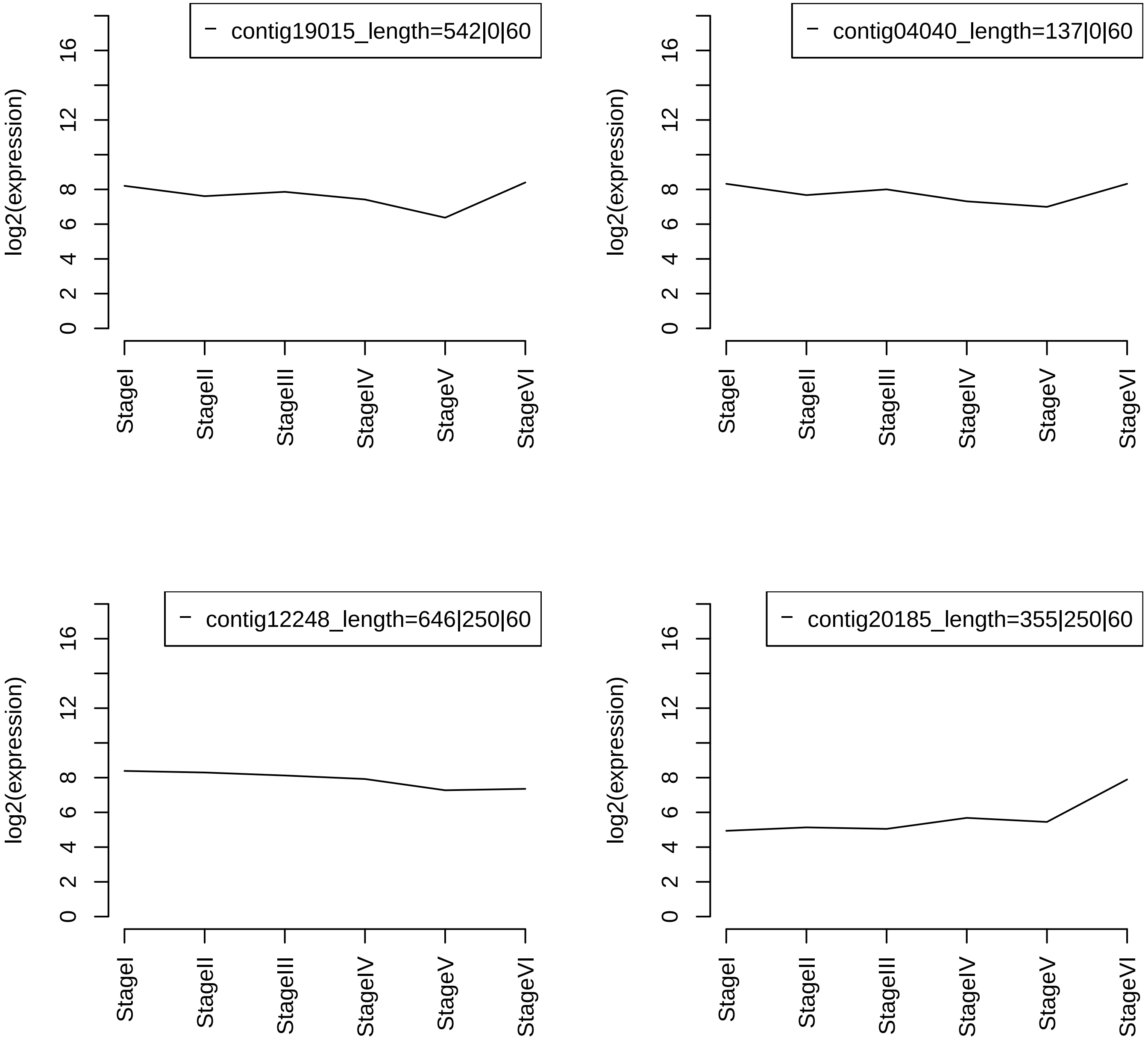

contig19015_length=542|0|60
contig04040_length=137|0|60
contig12248_length=646|250|60
contig20185_length=355|250|60
log2(expression)
0
2
4
6
8
12
16
StageI
StageII
StageIII
StageIV
StageV
StageVI

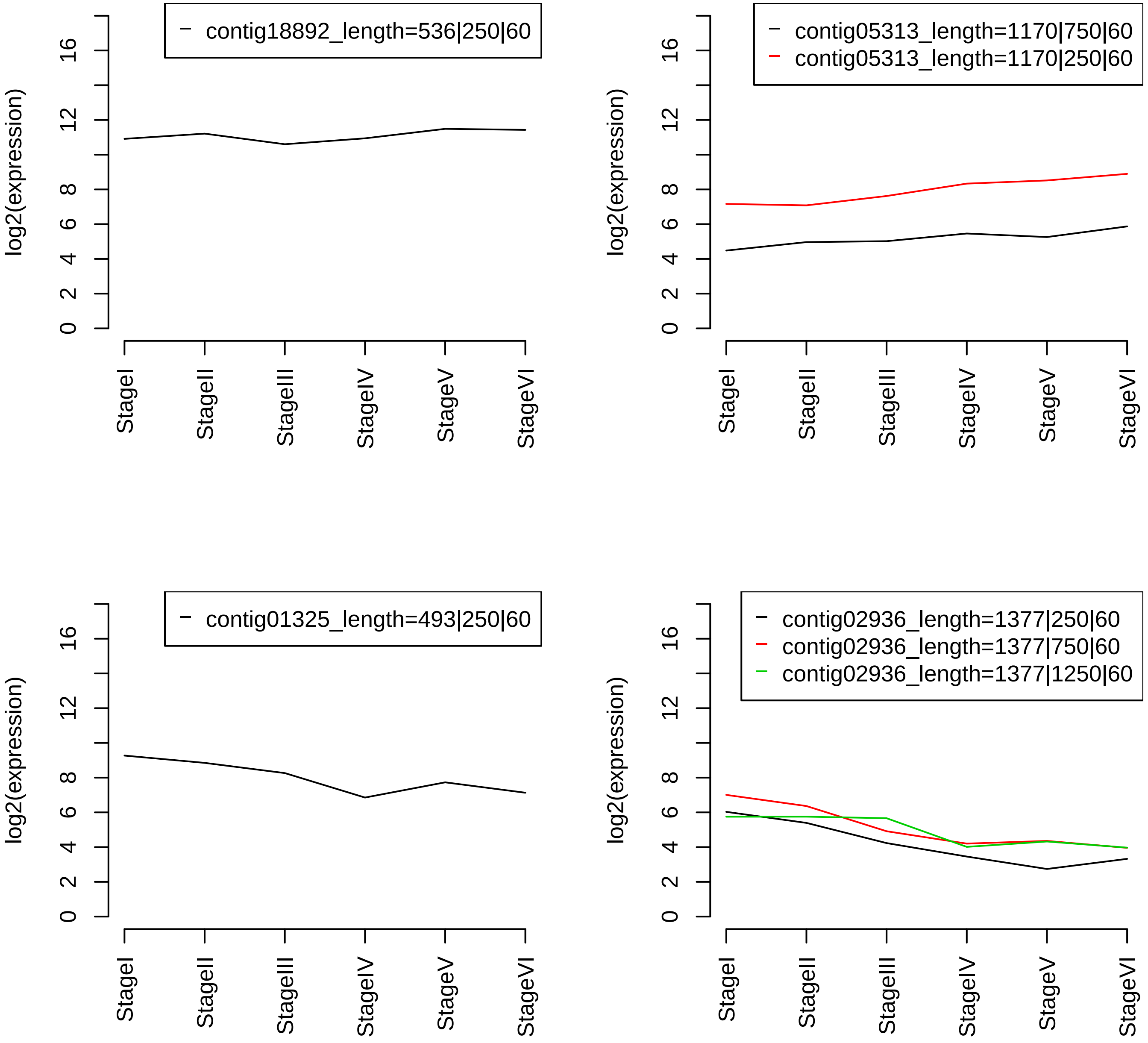

contig18892_length=536|250|60
contig05313_length=1170|750|60
contig05313_length=1170|250|60
contig01325_length=493|250|60
contig02936_length=1377|250|60
contig02936_length=1377|750|60
contig02936_length=1377|1250|60
log2(expression)
0 2 4 6 8 12 16
StageI StageII StageIII StageIV StageV StageVI

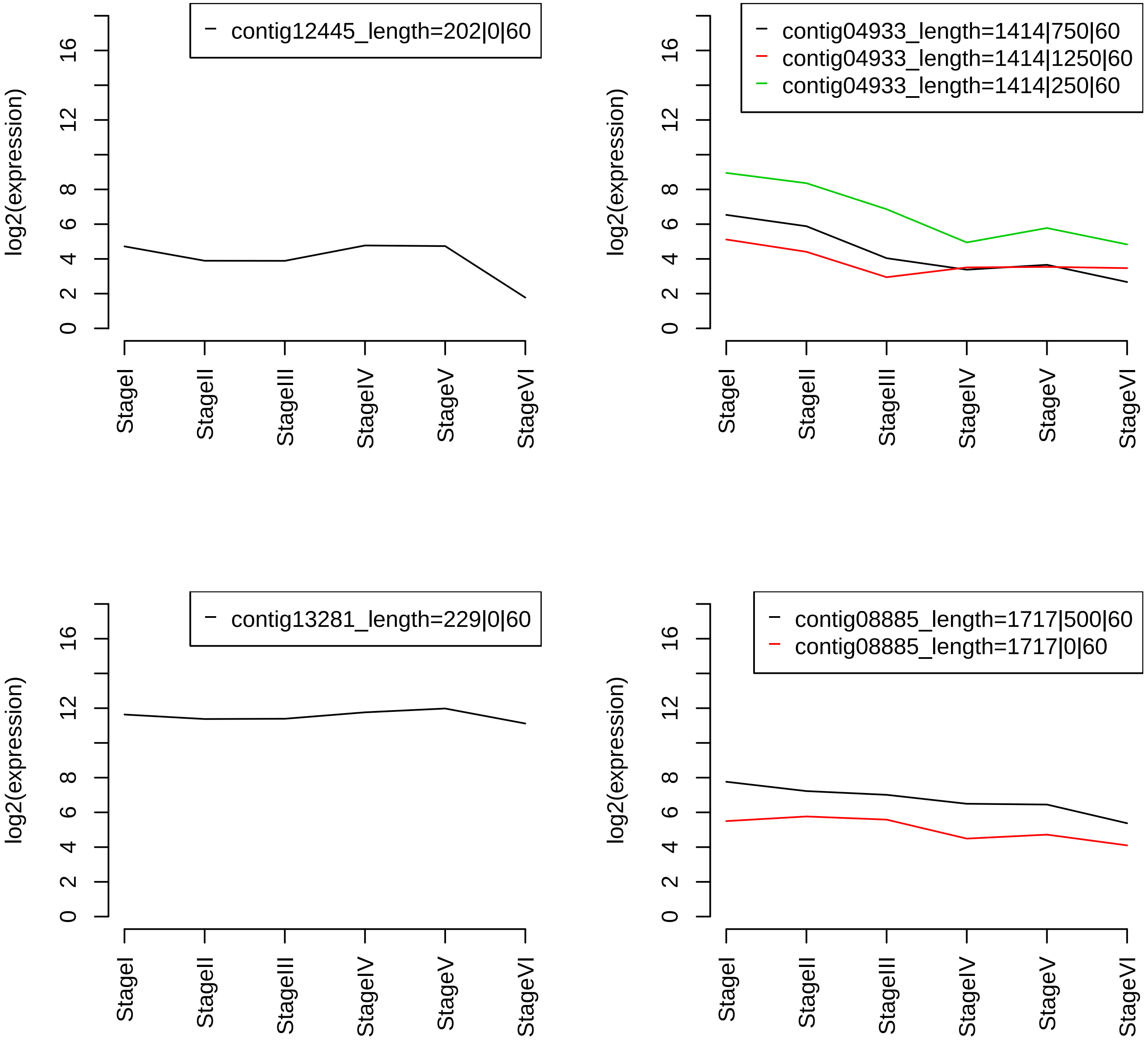

contig12445_length=202|0|60
log2(expression)
0
2
4
6
8
12
16
StageI
StageII
StageIII
StageIV
StageV
StageVI
contig04933_length=1414|750|60
contig04933_length=1414|1250|60
contig04933_length=1414|250|60
log2(expression)
0
2
4
6
8
12
16
StageI
StageII
StageIII
StageIV
StageV
StageVI
contig13281_length=229|0|60
log2(expression)
0
2
4
6
8
12
16
StageI
StageII
StageIII
StageIV
StageV
StageVI
contig08885_length=1717|500|60
contig08885_length=1717|0|60
log2(expression)
0
2
4
6
8
12
16
StageI
StageII
StageIII
StageIV
StageV
StageVI

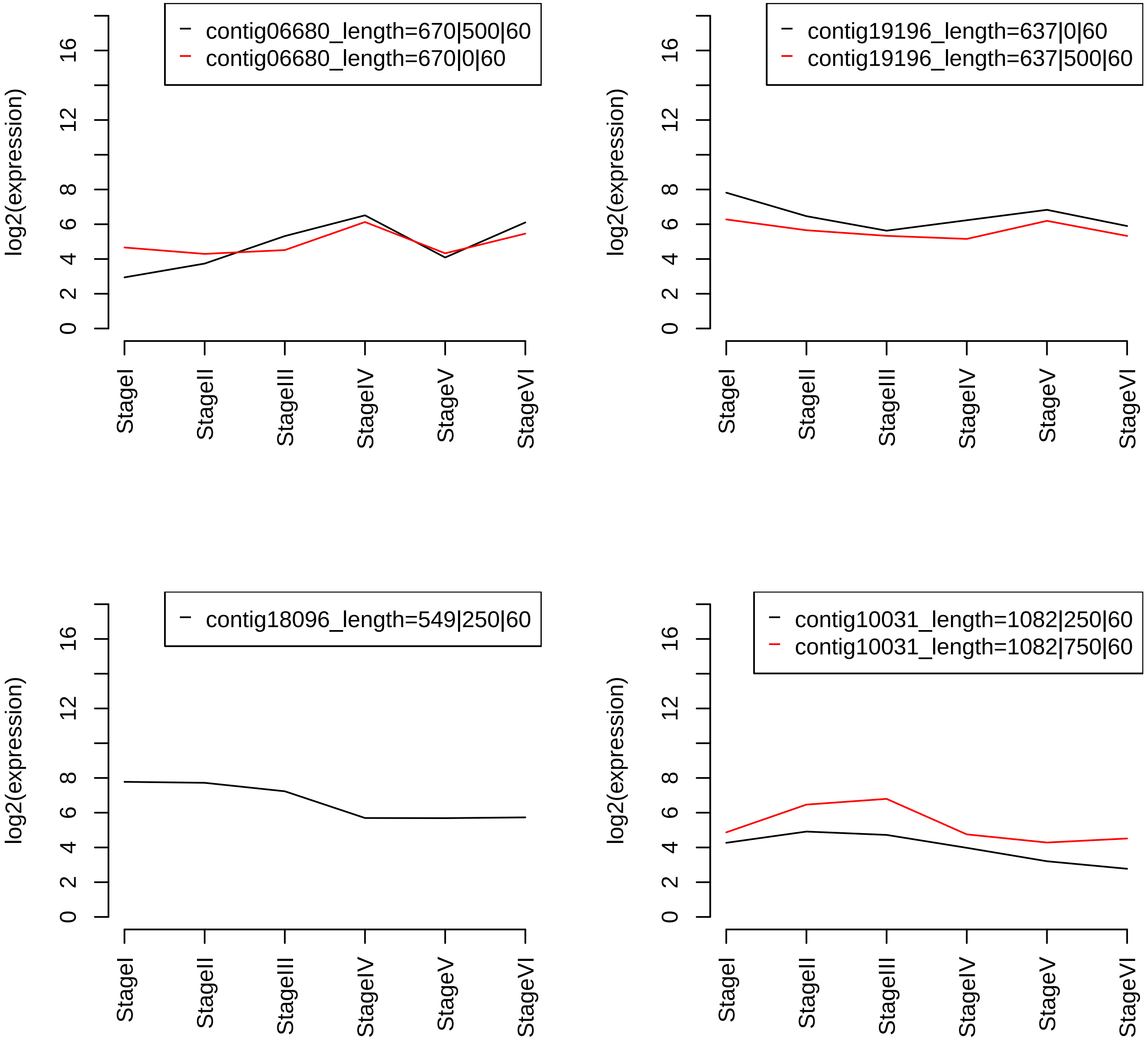
contig06680_length=670|500|60
contig06680_length=670|0|60
contig19196_length=637|0|60
contig19196_length=637|500|60
contig18096_length=549|250|60
contig10031_length=1082|250|60
contig10031_length=1082|750|60
log2(expression)
0
2
4
6
8
12
16
StageI
StageII
StageIII
StageIV
StageV
StageVI

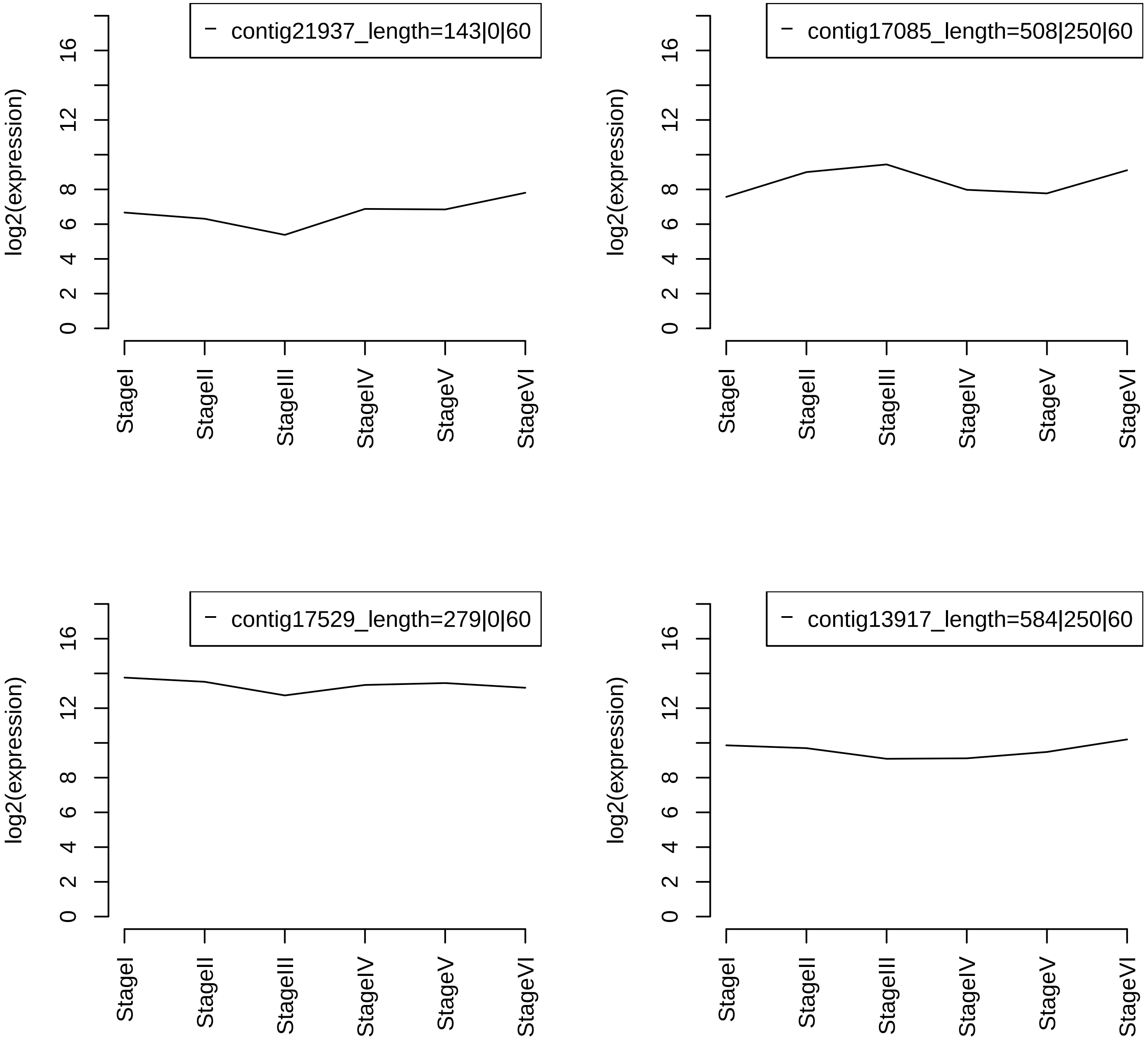
contig21937_length=143|0|60
contig17085_length=508|250|60
contig17529_length=279|0|60
contig13917_length=584|250|60
log2(expression)
0
2
4
6
8
12
16
StageI
StageII
StageIII
StageIV
StageV
StageVI

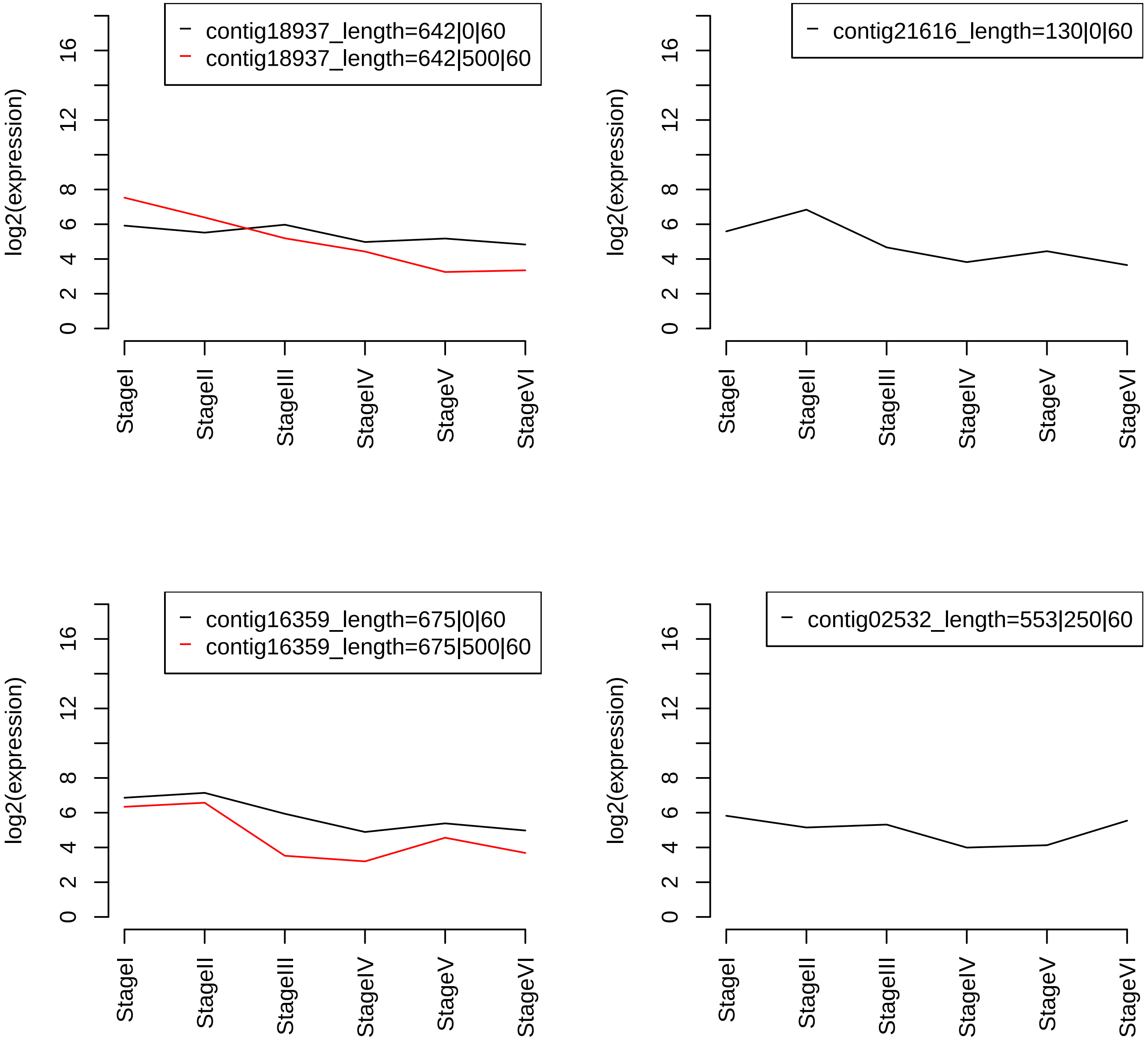
contig18937_length=642|0|60
contig18937_length=642|500|60
log2(expression)
0
2
4
6
8
12
16
StageI
StageII
StageIII
StageIV
StageV
StageVI
contig21616_length=130|0|60
log2(expression)
0
2
4
6
8
12
16
StageI
StageII
StageIII
StageIV
StageV
StageVI
contig16359_length=675|0|60
contig16359_length=675|500|60
log2(expression)
0
2
4
6
8
12
16
StageI
StageII
StageIII
StageIV
StageV
StageVI
contig02532_length=553|250|60
log2(expression)
0
2
4
6
8
12
16
StageI
StageII
StageIII
StageIV
StageV
StageVI

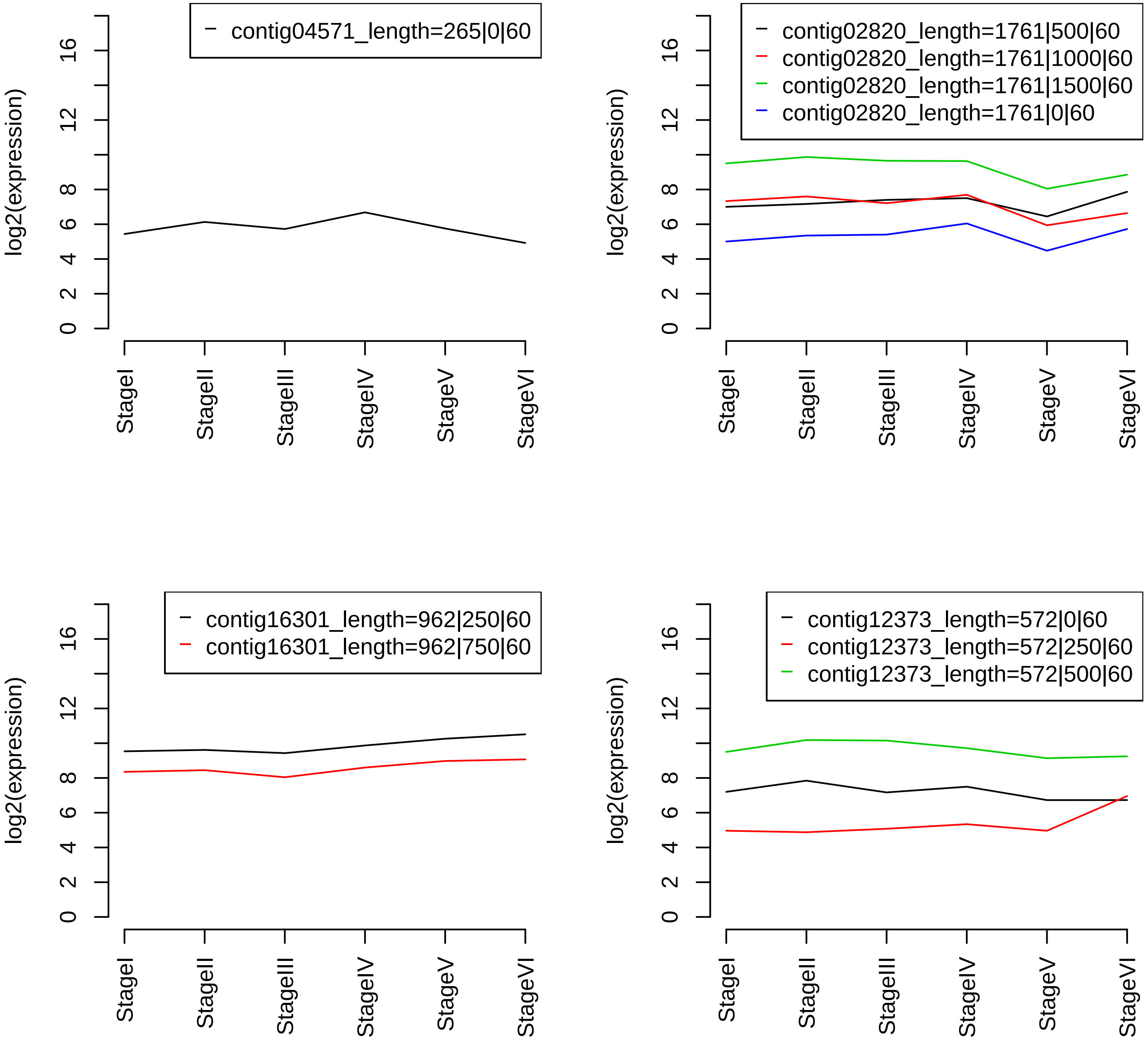
contig04571_length=265|0|60
contig02820_length=1761|500|60
contig02820_length=1761|1000|60
contig02820_length=1761|1500|60
contig02820_length=1761|0|60
contig16301_length=962|250|60
contig16301_length=962|750|60
contig12373_length=572|0|60
contig12373_length=572|250|60
contig12373_length=572|500|60
log2(expression)
0 2 4 6 8 12 16
StageI StageII StageIII StageIV StageV StageVI

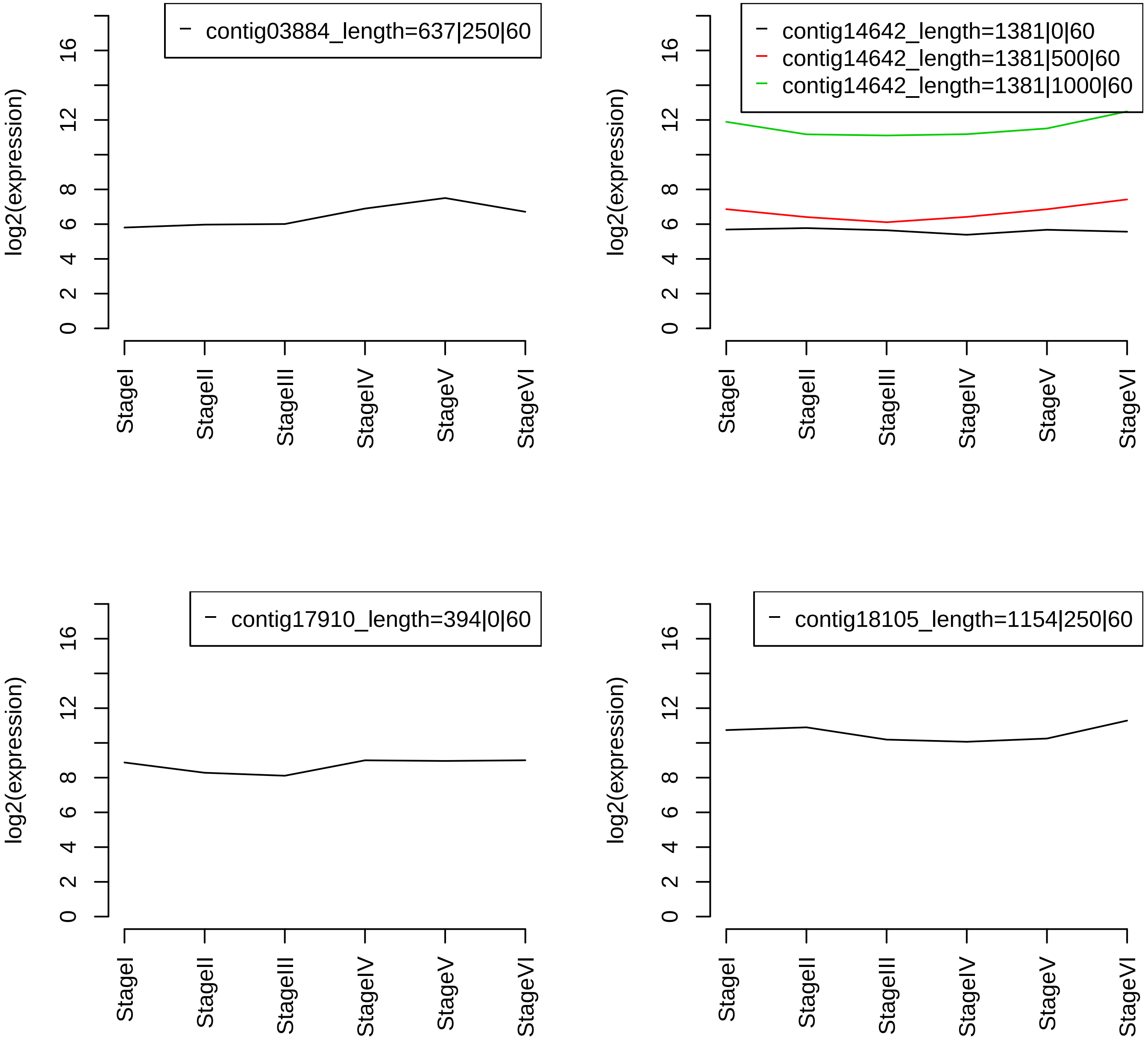
contig03884_length=637|250|60
contig14642_length=1381|0|60
contig14642_length=1381|500|60
contig14642_length=1381|1000|60
contig17910_length=394|0|60
contig18105_length=1154|250|60
log2(expression)
0
2
4
6
8
12
16
StageI
StageII
StageIII
StageIV
StageV
StageVI

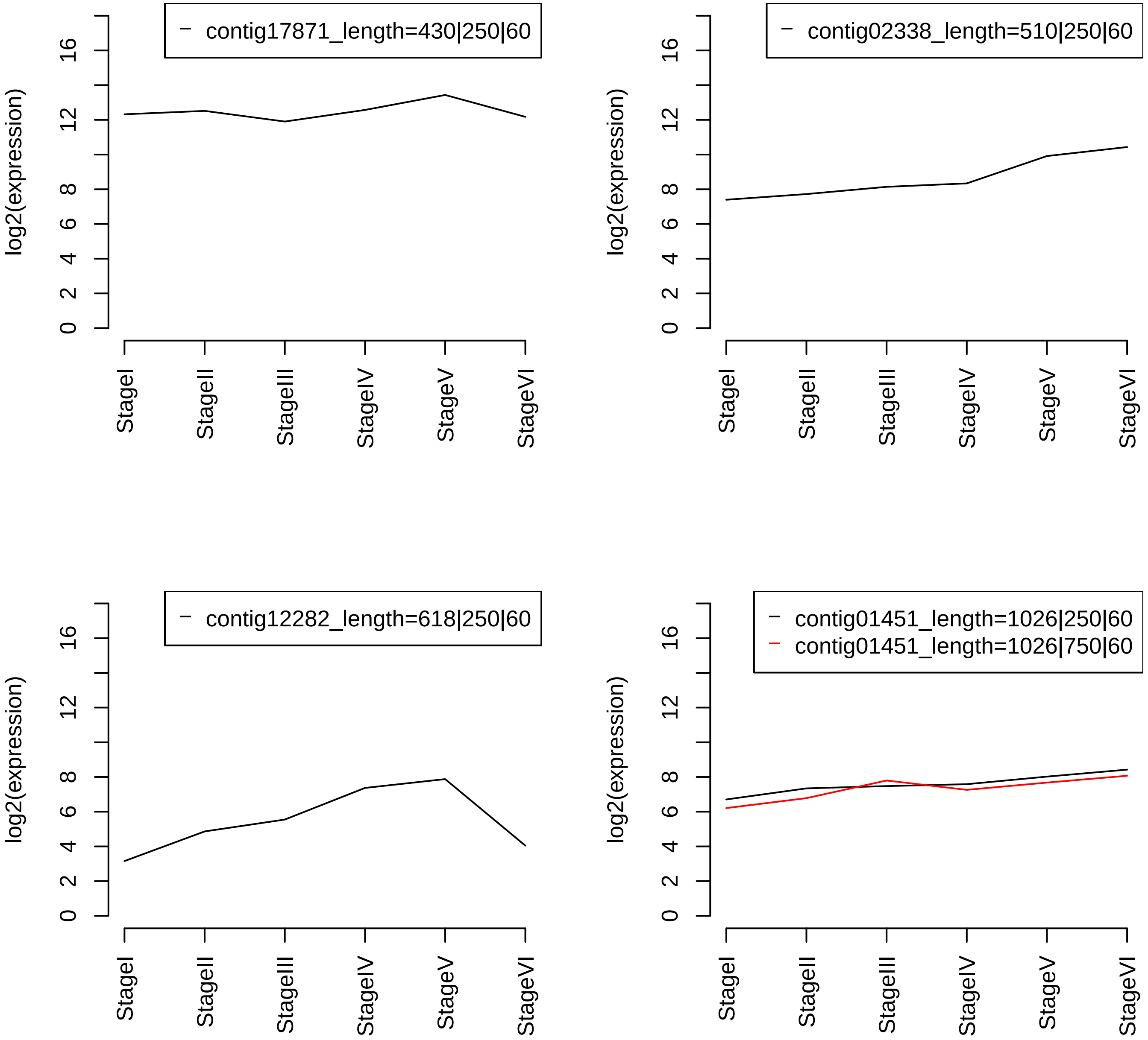
contig17871_length=430|250|60
contig02338_length=510|250|60
contig12282_length=618|250|60
contig01451_length=1026|250|60
contig01451_length=1026|750|60
log2(expression)
0
2
4
6
8
12
16
StageI
StageII
StageIII
StageIV
StageV
StageVI

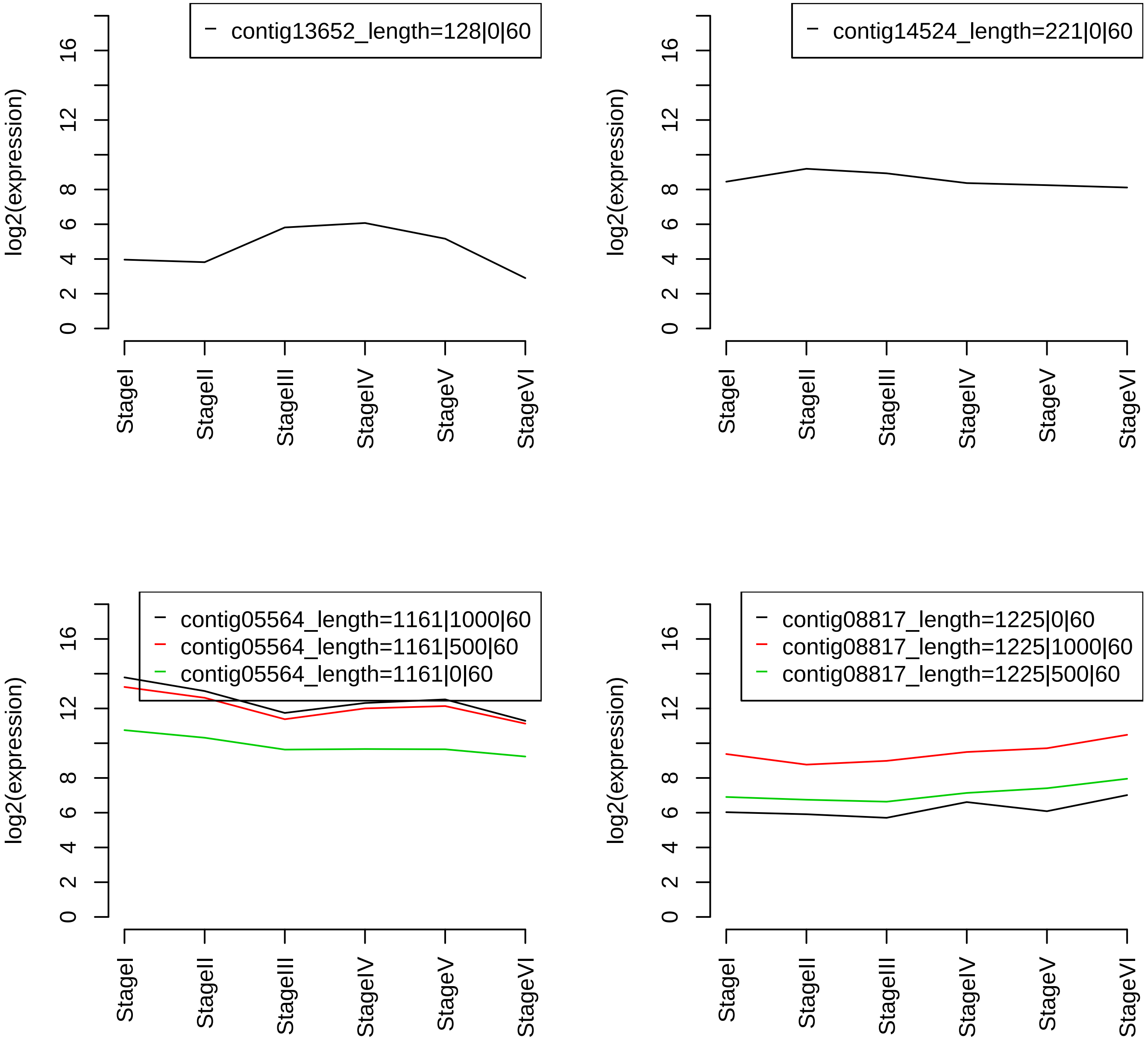
contig13652_length=128|0|60
contig14524_length=221|0|60
contig05564_length=1161|1000|60
contig05564_length=1161|500|60
contig05564_length=1161|0|60
contig08817_length=1225|0|60
contig08817_length=1225|1000|60
contig08817_length=1225|500|60
log2(expression)
0
2
4
6
8
12
16
StageI
StageII
StageIII
StageIV
StageV
StageVI

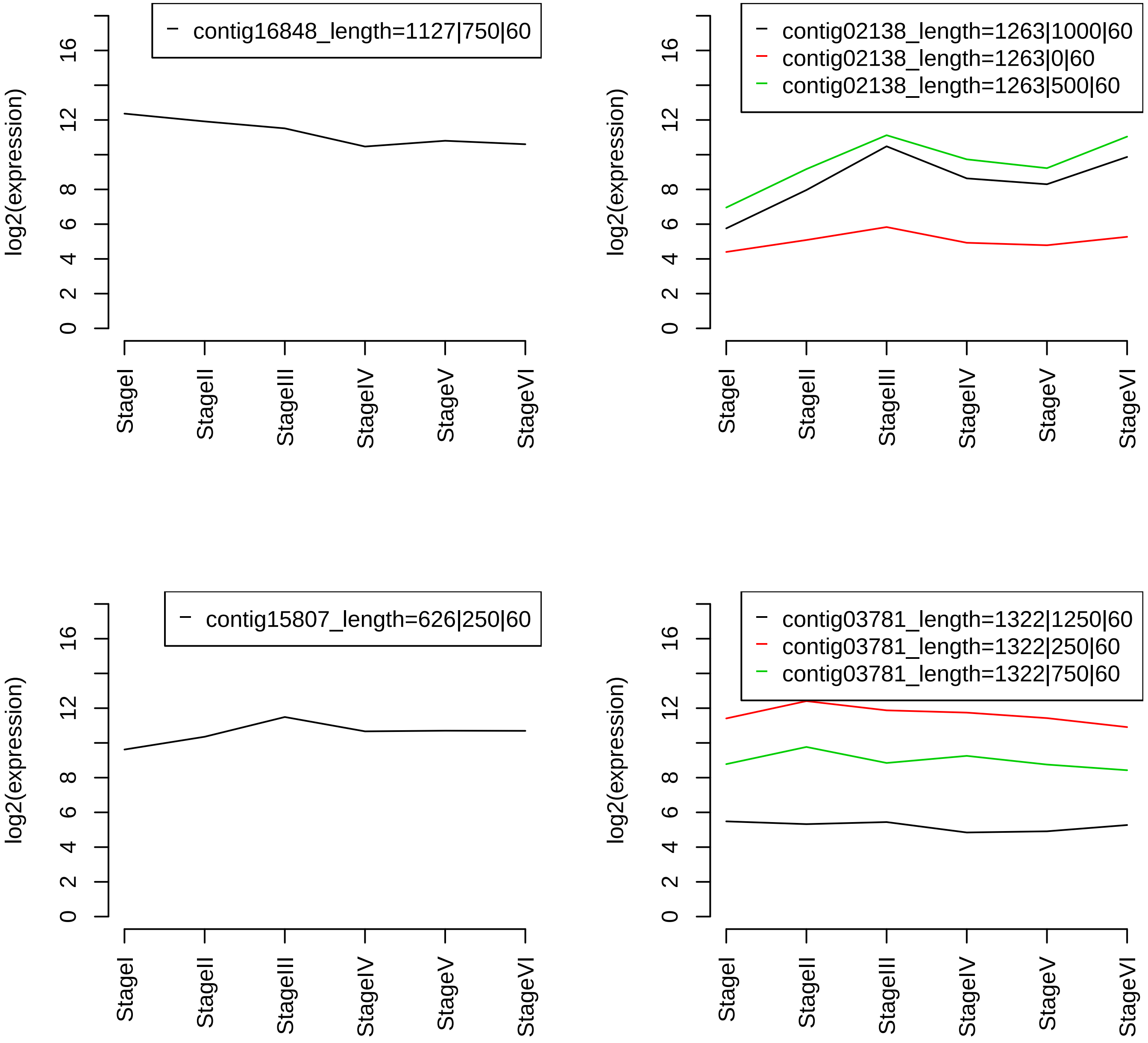
contig16848_length=1127|750|60
contig02138_length=1263|1000|60
contig02138_length=1263|0|60
contig02138_length=1263|500|60
contig15807_length=626|250|60
contig03781_length=1322|1250|60
contig03781_length=1322|250|60
contig03781_length=1322|750|60
log2(expression)
0
2
4
6
8
12
16
StageI
StageII
StageIII
StageIV
StageV
StageVI

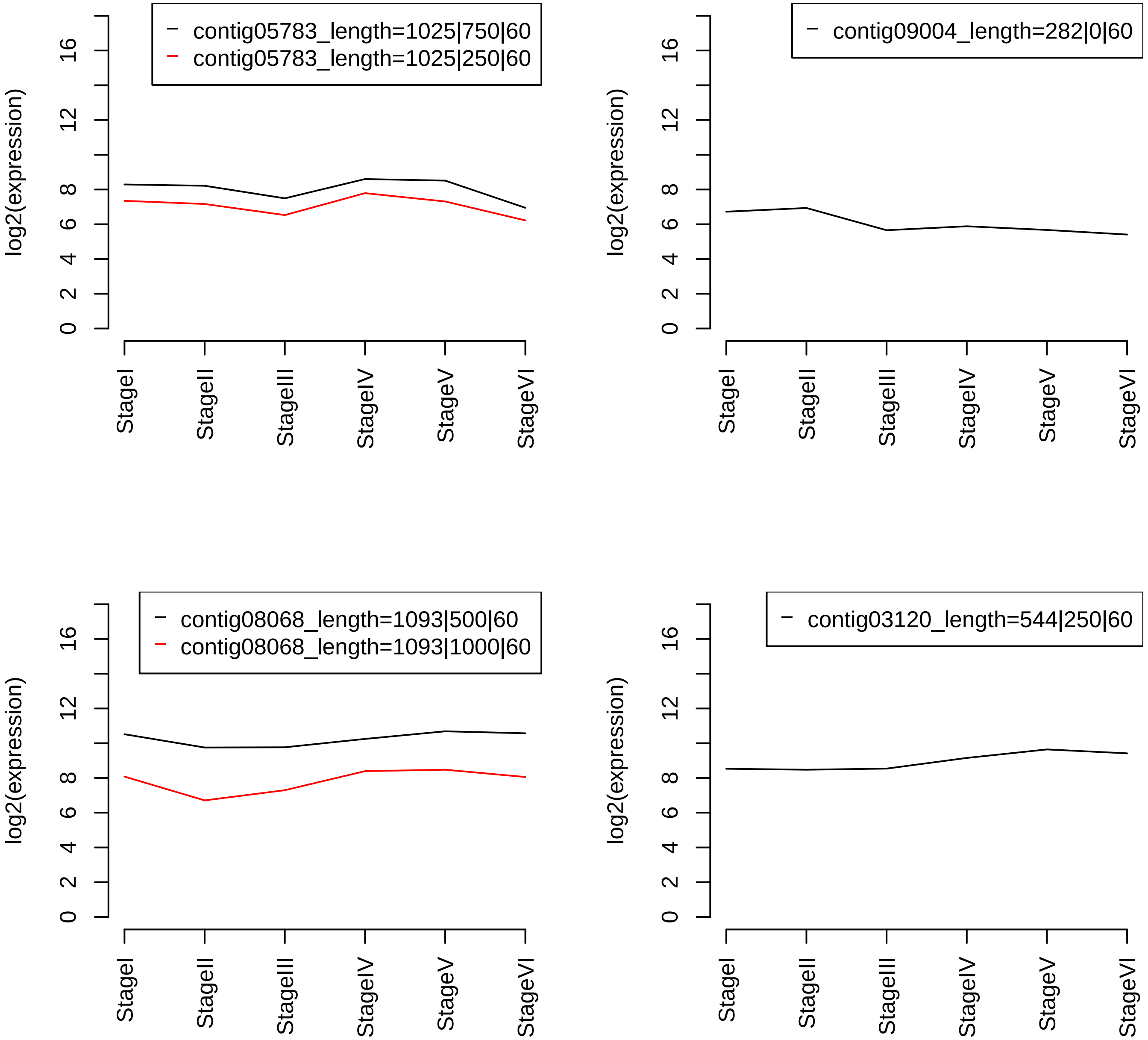

contig05783_length=1025|750|60
contig05783_length=1025|250|60
contig09004_length=282|0|60
contig08068_length=1093|500|60
contig08068_length=1093|1000|60
contig03120_length=544|250|60
log2(expression)
0 2 4 6 8 12 16
StageI StageII StageIII StageIV StageV StageVI

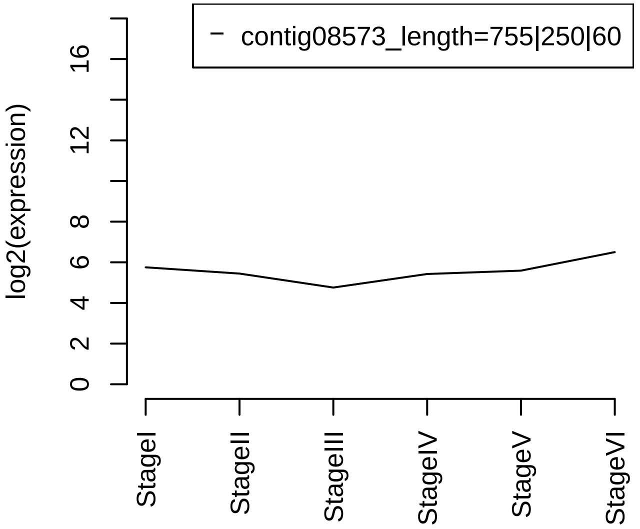

contig08573_length=755|250|60
log2(expression)
0
2
4
6
8
12
16
StageI
StageII
StageIII
StageIV
StageV
StageVI

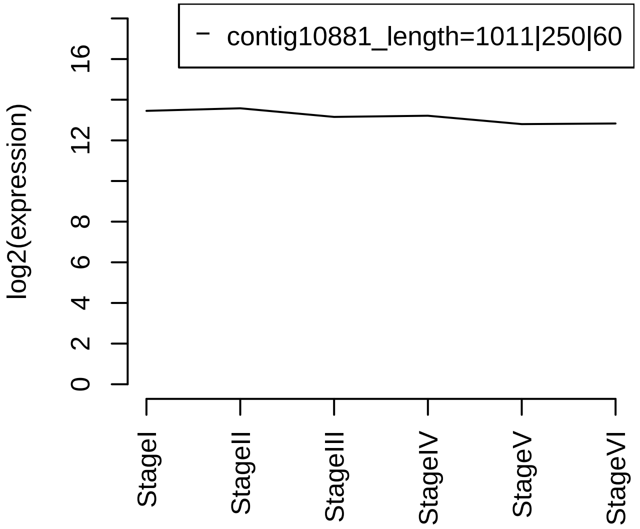

contig10881_length=1011|250|60
log2(expression)
0
2
4
6
8
12
16
StageI
StageII
StageIII
StageIV
StageV
StageVI

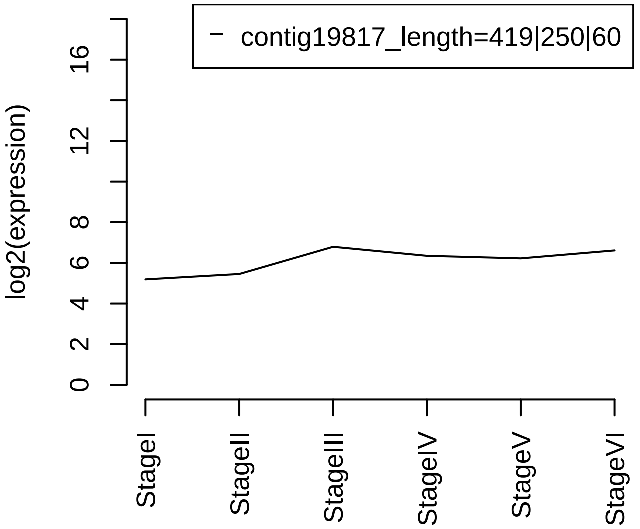

contig19817_length=419|250|60
log2(expression)
0
2
4
6
8
12
16
StageI
StageII
StageIII
StageIV
StageV
StageVI

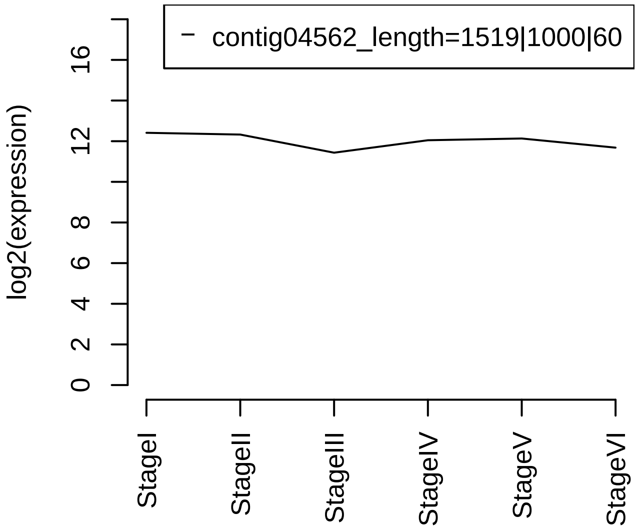

contig04562_length=1519|1000|60
log2(expression)
0
2
4
6
8
12
16
StageI
StageII
StageIII
StageIV
StageV
StageVI

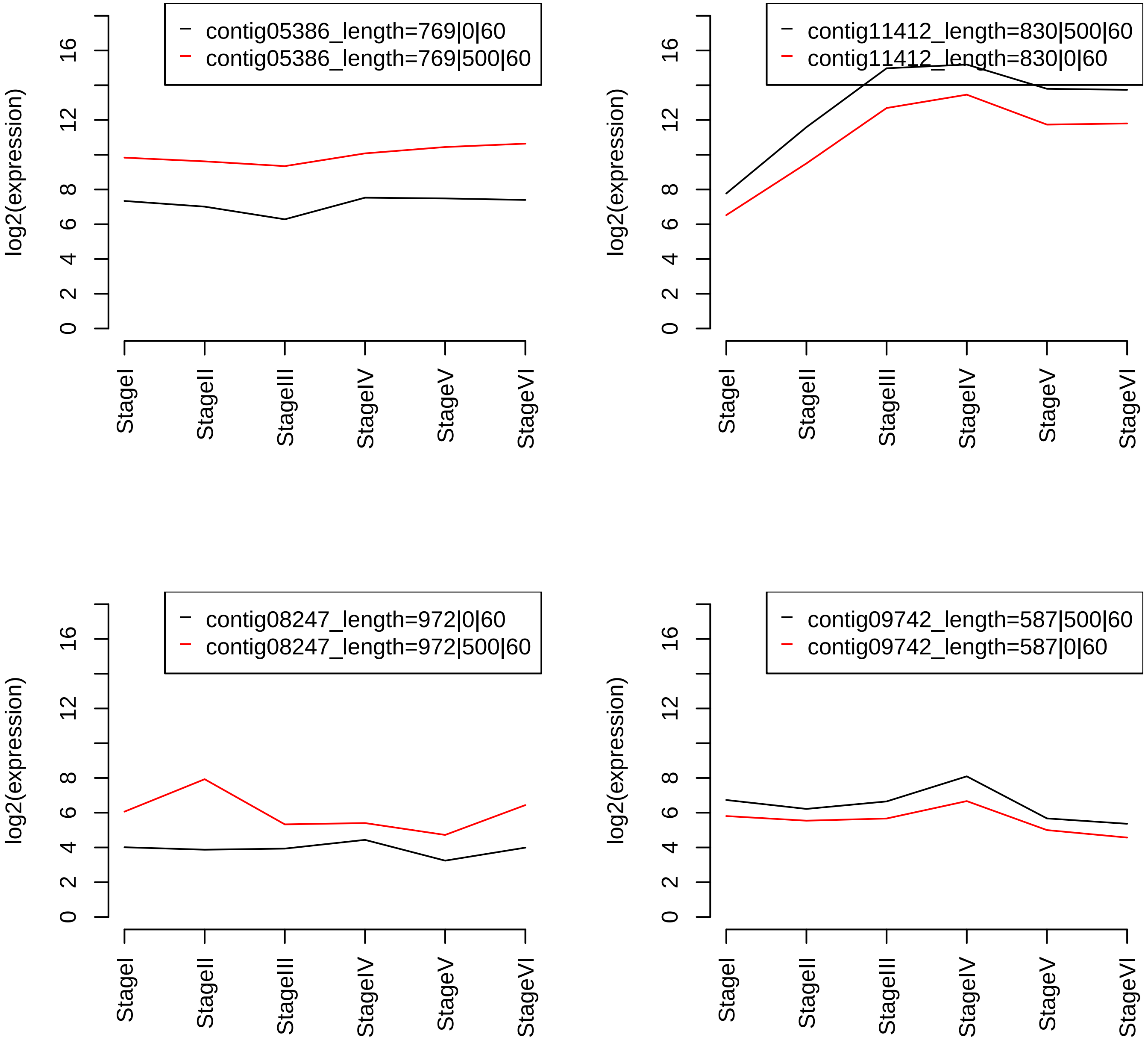
contig05386_length=769|0|60
contig05386_length=769|500|60
contig11412_length=830|500|60
contig11412_length=830|0|60
contig08247_length=972|0|60
contig08247_length=972|500|60
contig09742_length=587|500|60
contig09742_length=587|0|60
log2(expression)
0
2
4
6
8
12
16
StageI
StageII
StageIII
StageIV
StageV
StageVI

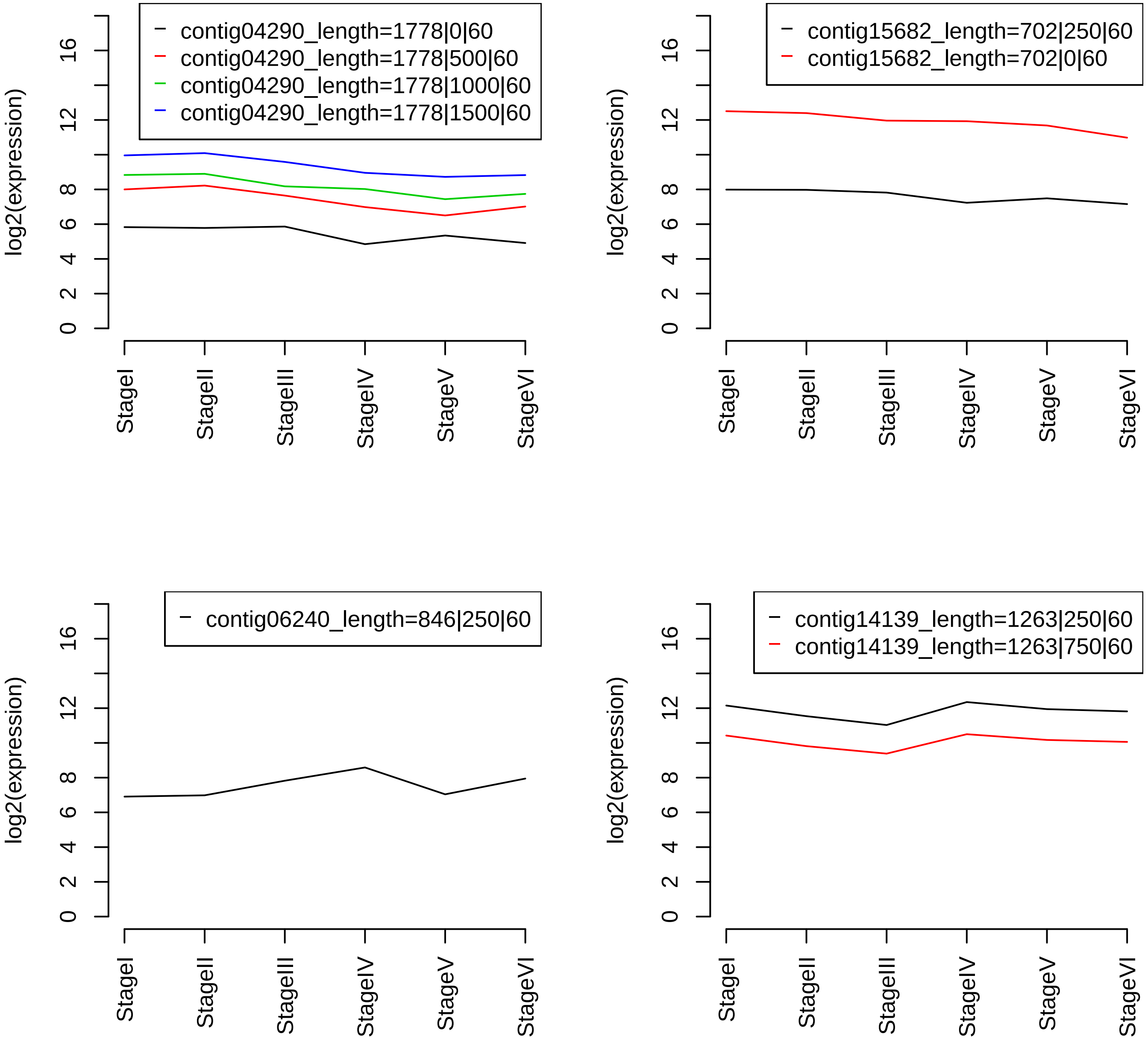
contig04290_length=1778|0|60
contig04290_length=1778|500|60
contig04290_length=1778|1000|60
contig04290_length=1778|1500|60
contig15682_length=702|250|60
contig15682_length=702|0|60
contig06240_length=846|250|60
contig14139_length=1263|250|60
contig14139_length=1263|750|60
log2(expression)
0
2
4
6
8
12
16
StageI
StageII
StageIII
StageIV
StageV
StageVI

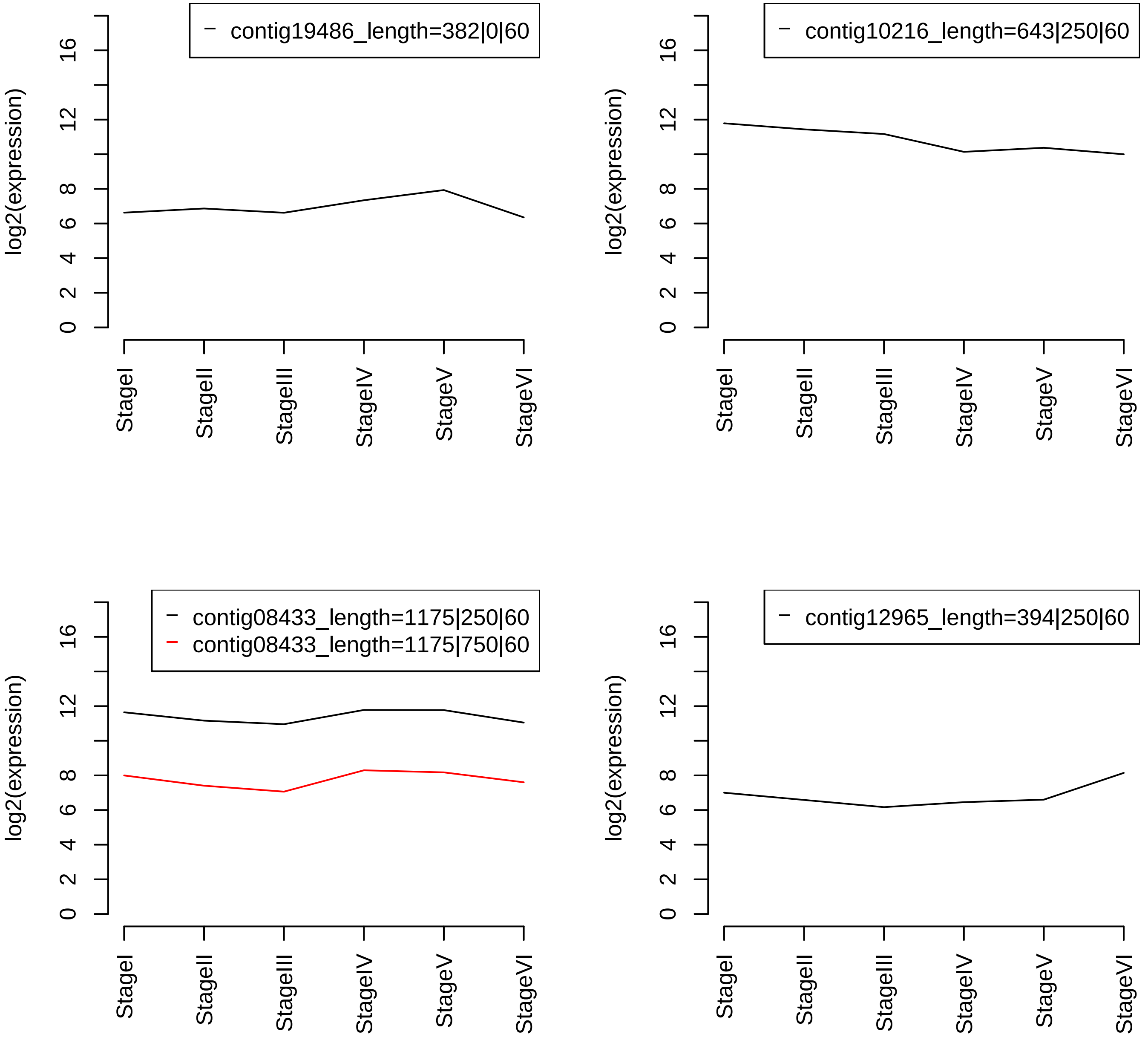
contig19486_length=382|0|60
contig10216_length=643|250|60
contig08433_length=1175|250|60
contig08433_length=1175|750|60
contig12965_length=394|250|60
log2(expression)
0
2
4
6
8
12
16
StageI
StageII
StageIII
StageIV
StageV
StageVI

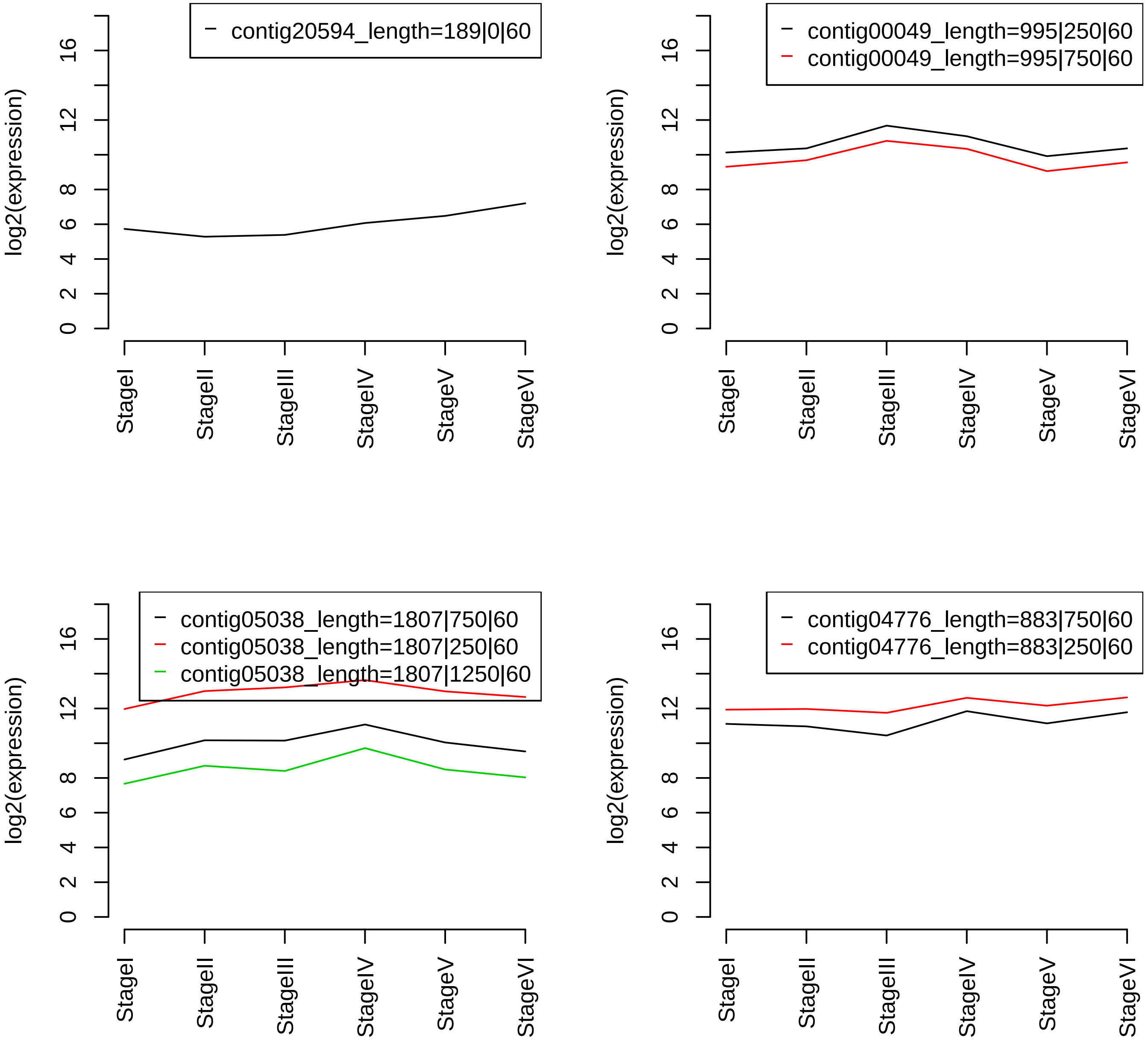
contig20594_length=189|0|60
contig00049_length=995|250|60
contig00049_length=995|750|60
contig05038_length=1807|750|60
contig05038_length=1807|250|60
contig05038_length=1807|1250|60
contig04776_length=883|750|60
contig04776_length=883|250|60
log2(expression)
StageI
StageII
StageIII
StageIV
StageV
StageVI

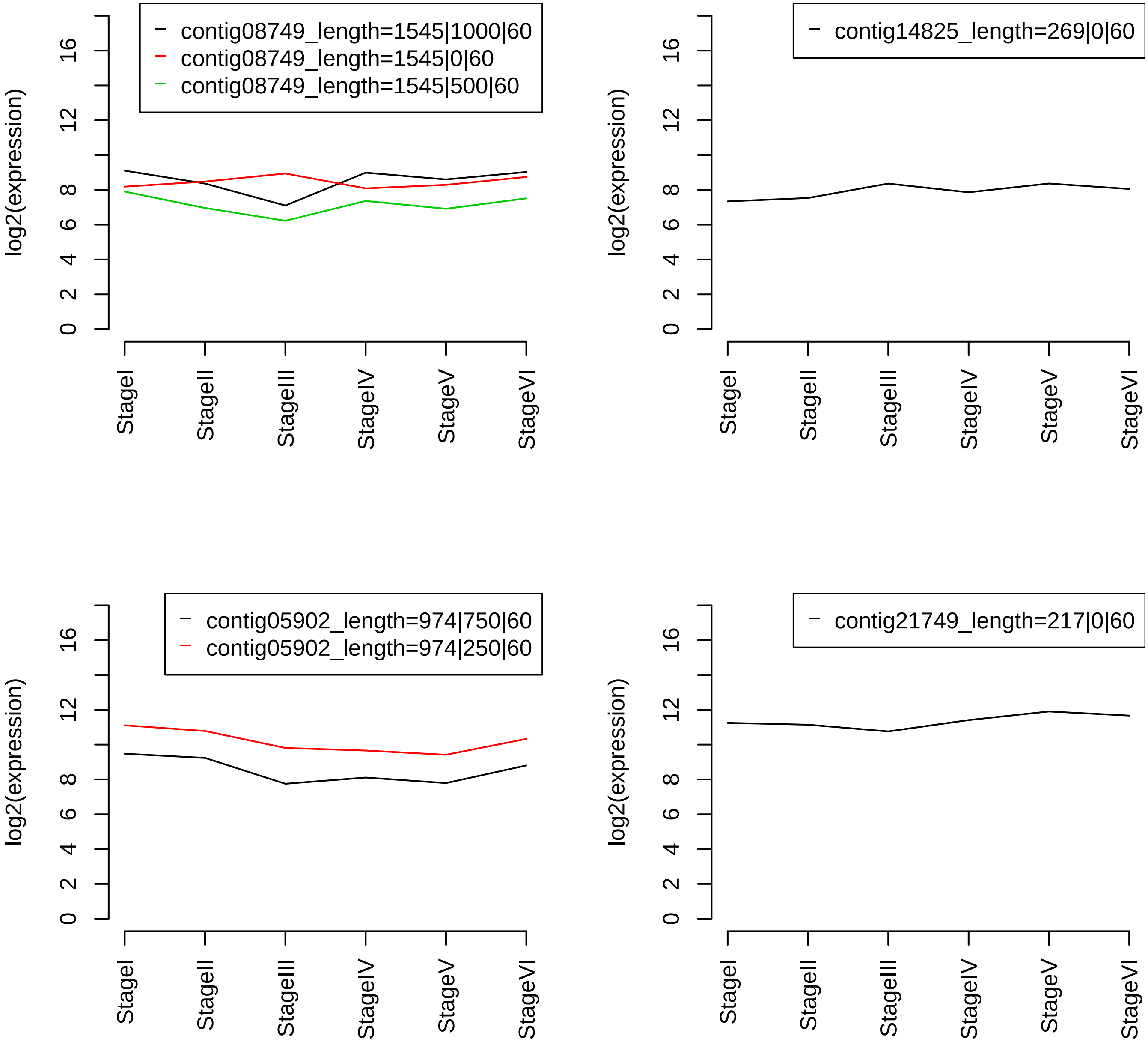
contig08749_length=1545|1000|60
contig08749_length=1545|0|60
contig08749_length=1545|500|60
contig14825_length=269|0|60
contig05902_length=974|750|60
contig05902_length=974|250|60
contig21749_length=217|0|60
log2(expression)
0
2
4
6
8
12
16
StageI
StageII
StageIII
StageIV
StageV
StageVI

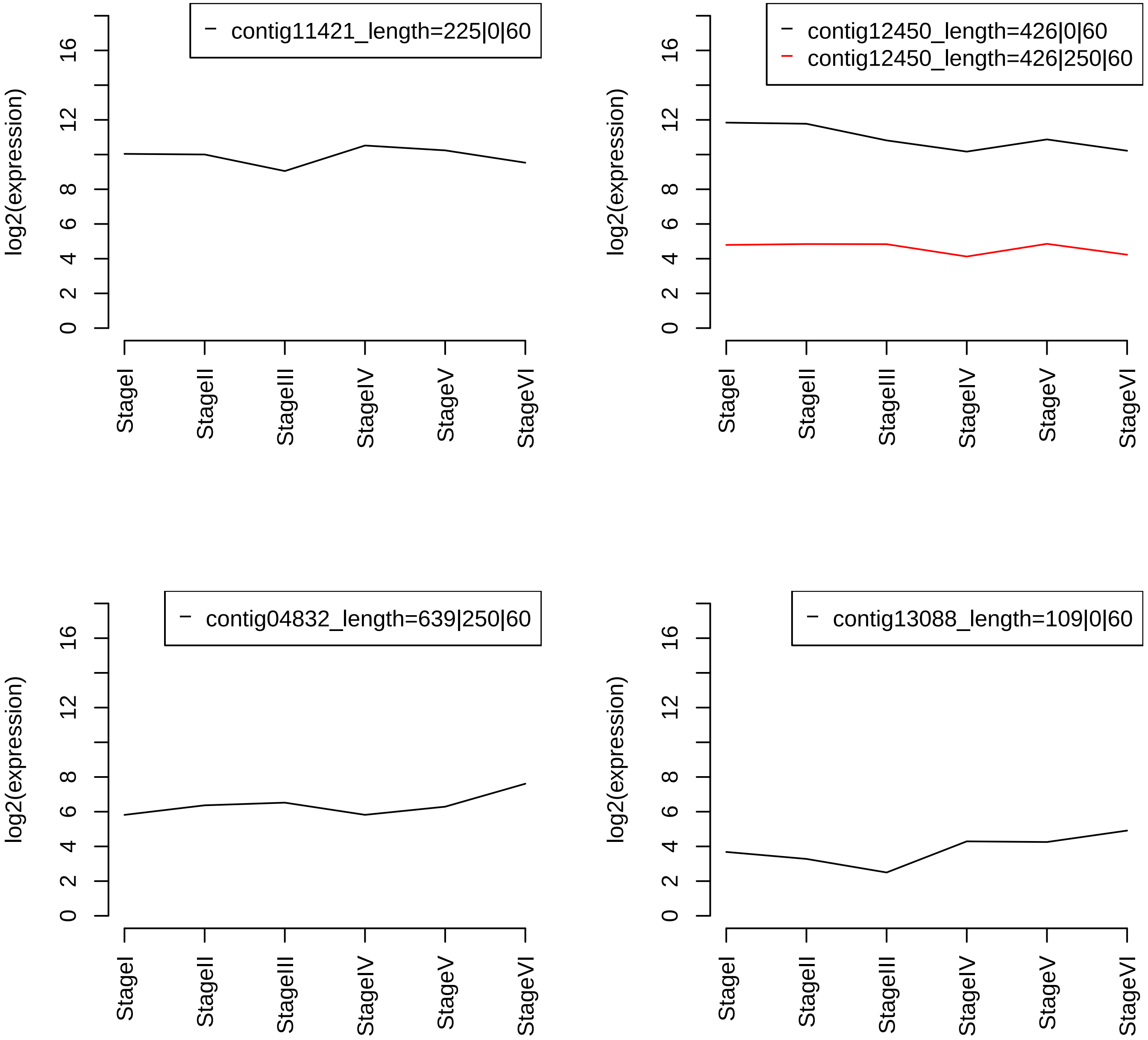
contig11421_length=225|0|60
contig12450_length=426|0|60
contig12450_length=426|250|60
contig04832_length=639|250|60
contig13088_length=109|0|60
log2(expression)
0
2
4
6
8
12
16
StageI
StageII
StageIII
StageIV
StageV
StageVI

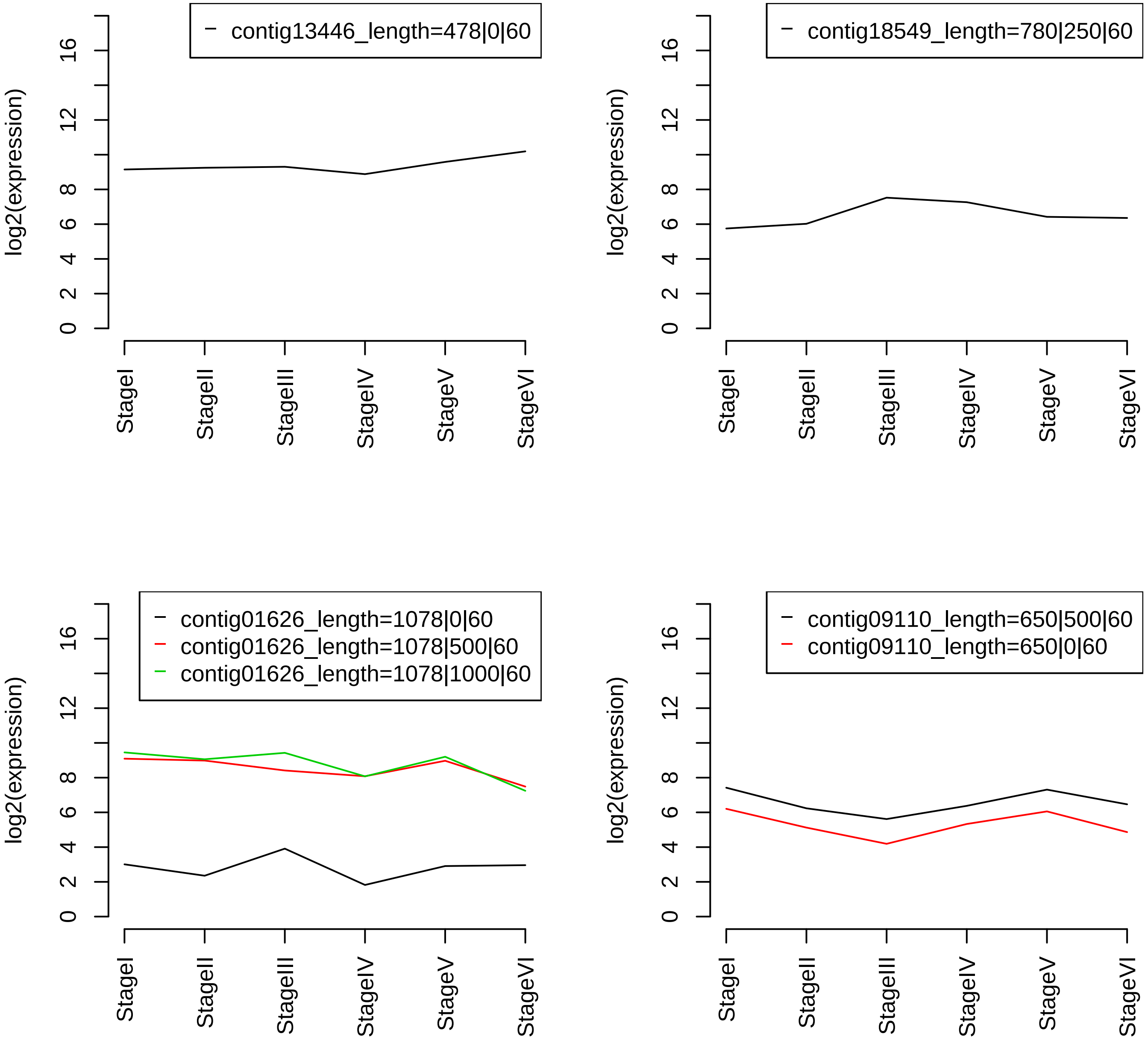
contig13446_length=478|0|60
contig18549_length=780|250|60
contig01626_length=1078|0|60
contig01626_length=1078|500|60
contig01626_length=1078|1000|60
contig09110_length=650|500|60
contig09110_length=650|0|60
log2(expression)
0
2
4
6
8
12
16
StageI
StageII
StageIII
StageIV
StageV
StageVI

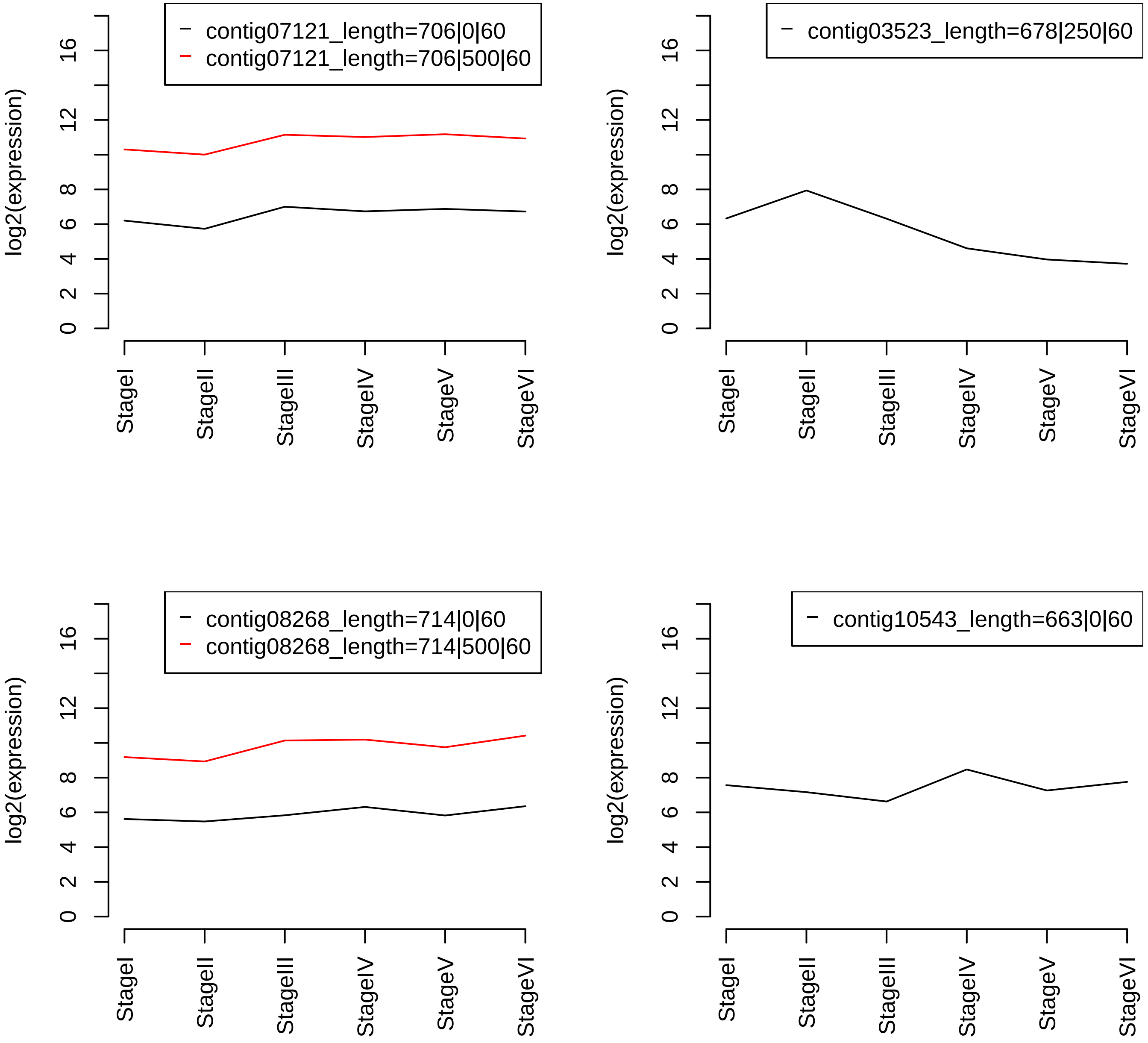
contig07121_length=706|0|60
contig07121_length=706|500|60
contig03523_length=678|250|60
contig08268_length=714|0|60
contig08268_length=714|500|60
contig10543_length=663|0|60
log2(expression)
0
2
4
6
8
12
16
StageI
StageII
StageIII
StageIV
StageV
StageVI

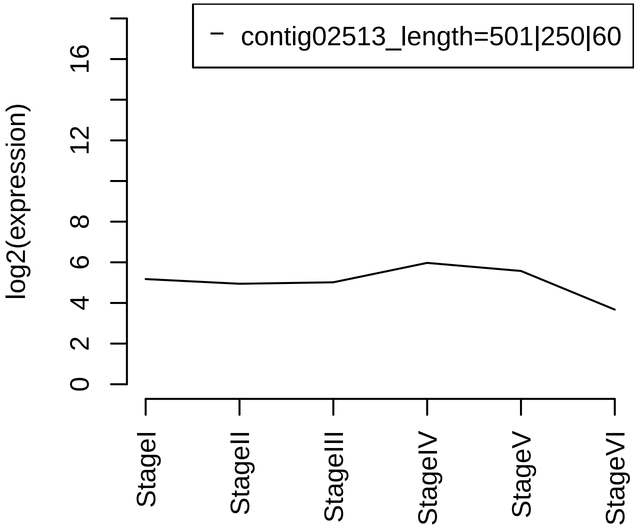
contig02513_length=501|250|60
log2(expression)
0
2
4
6
8
12
16
StageI
StageII
StageIII
StageIV
StageV
StageVI

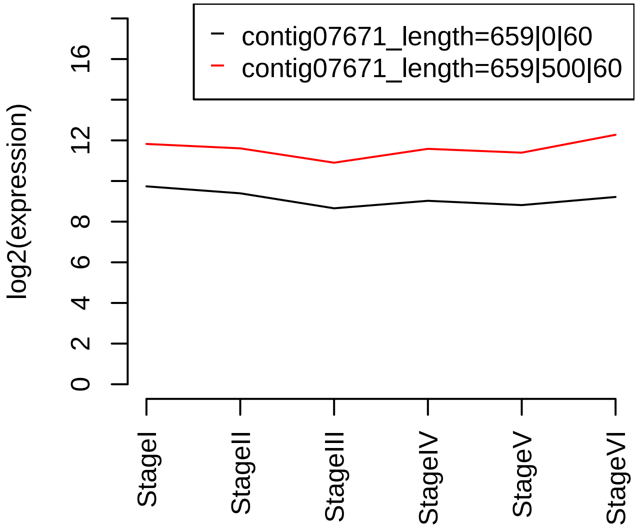
contig07671_length=659|0|60
contig07671_length=659|500|60
log2(expression)
0
2
4
6
8
12
16
StageI
StageII
StageIII
StageIV
StageV
StageVI

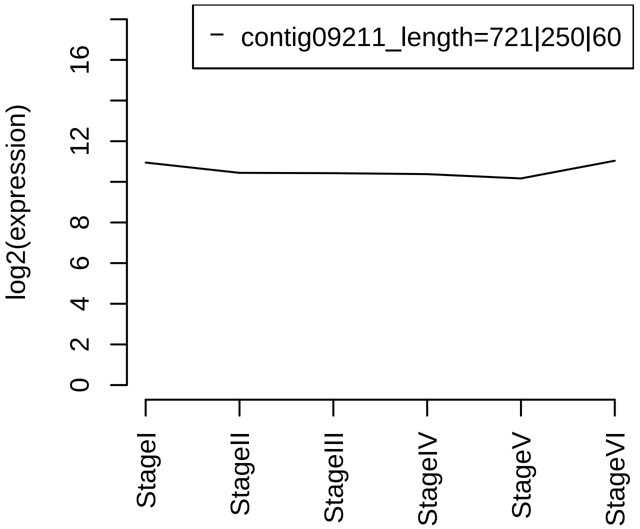
contig09211_length=721|250|60
log2(expression)
0
2
4
6
8
12
16
StageI
StageII
StageIII
StageIV
StageV
StageVI

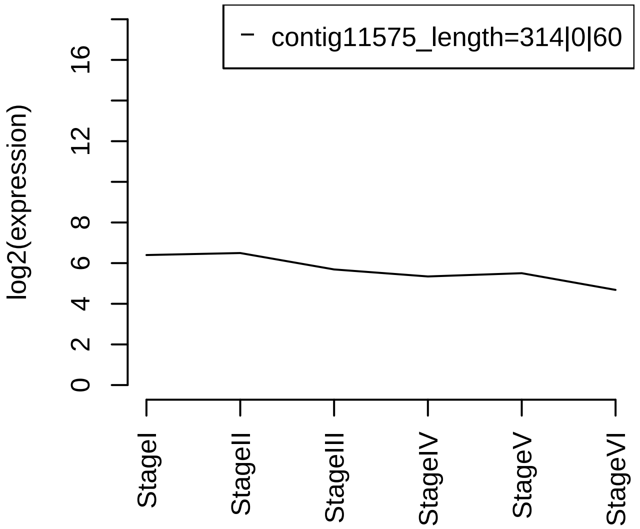
contig11575_length=314|0|60
log2(expression)
0
2
4
6
8
12
16
StageI
StageII
StageIII
StageIV
StageV
StageVI

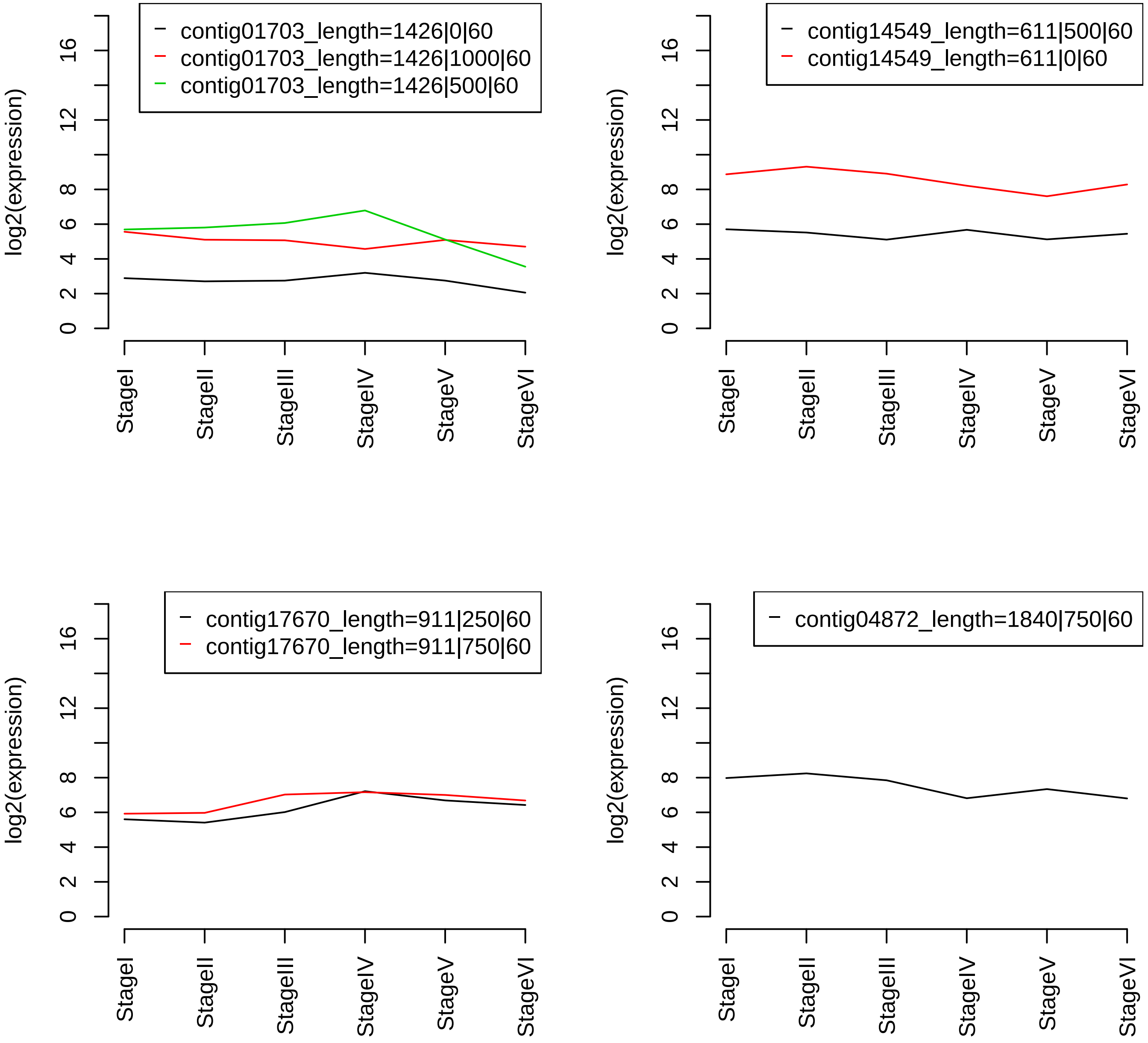
contig01703_length=1426|0|60
contig01703_length=1426|1000|60
contig01703_length=1426|500|60
contig14549_length=611|500|60
contig14549_length=611|0|60
contig17670_length=911|250|60
contig17670_length=911|750|60
contig04872_length=1840|750|60
log2(expression)
0
2
4
6
8
12
16
StageI
StageII
StageIII
StageIV
StageV
StageVI

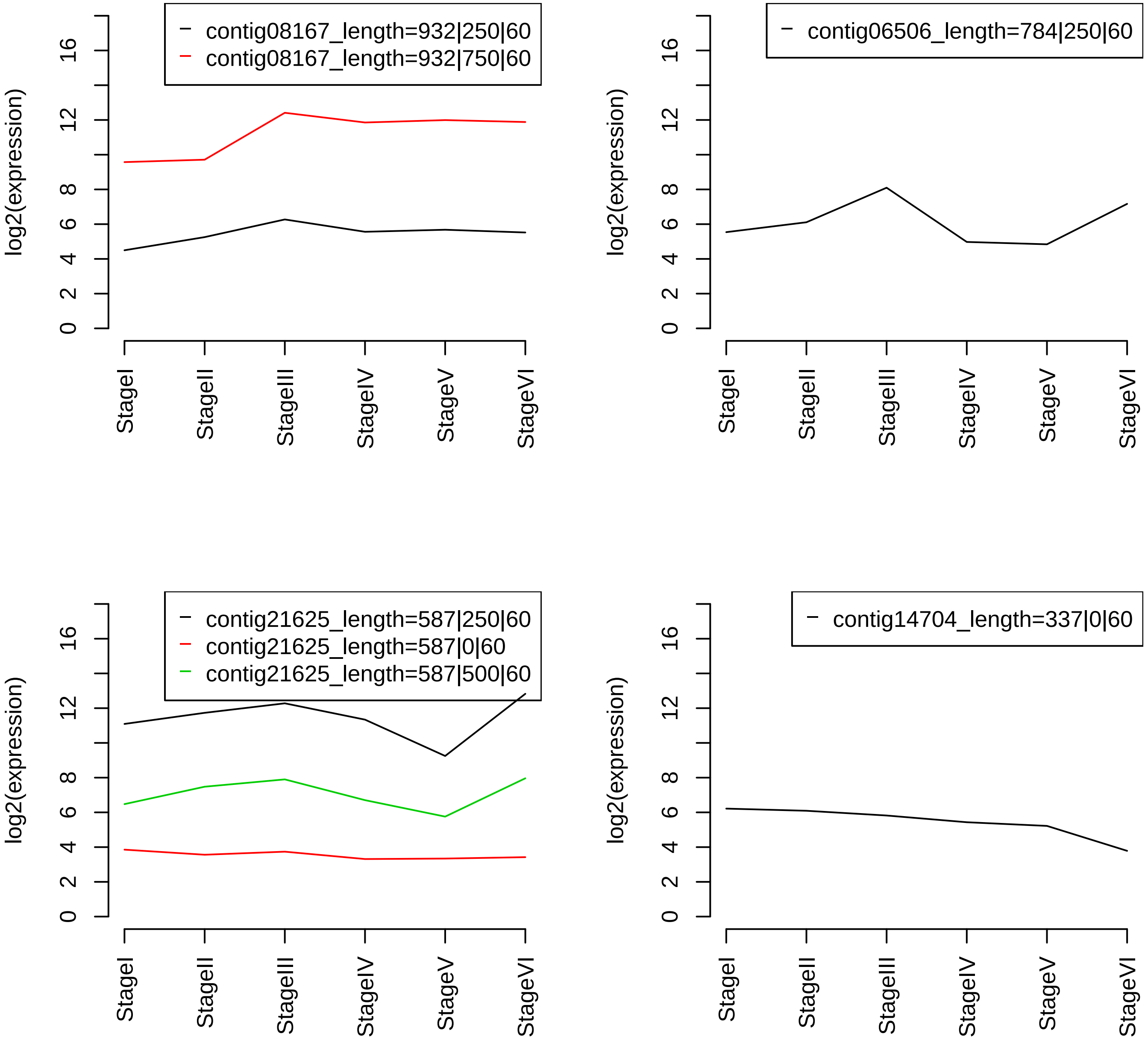
contig08167_length=932|250|60
contig08167_length=932|750|60
contig06506_length=784|250|60
contig21625_length=587|250|60
contig21625_length=587|0|60
contig21625_length=587|500|60
contig14704_length=337|0|60
log2(expression)
0
2
4
6
8
12
16
StageI
StageII
StageIII
StageIV
StageV
StageVI

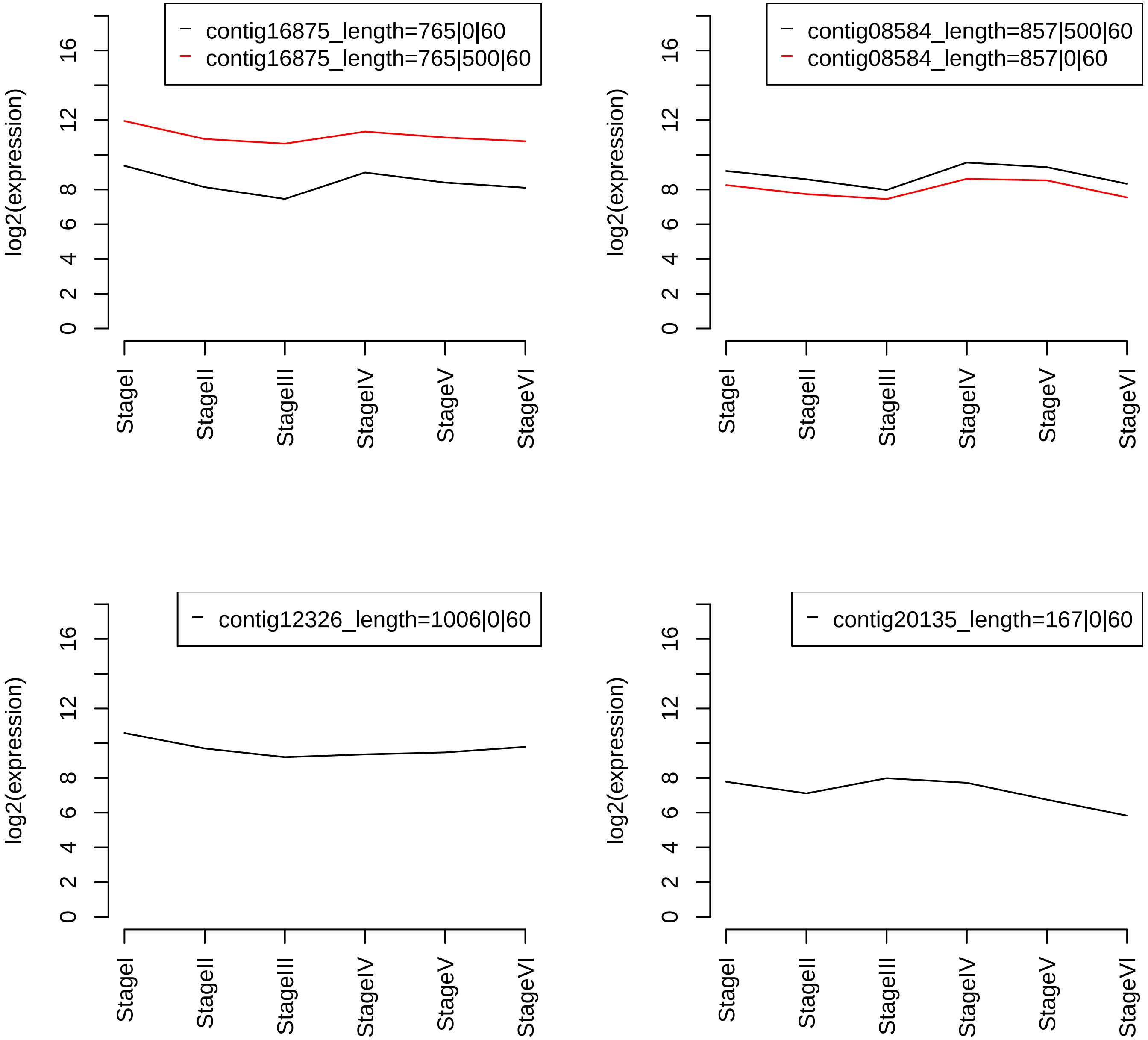

contig16875_length=765|0|60
contig16875_length=765|500|60
contig08584_length=857|500|60
contig08584_length=857|0|60
contig12326_length=1006|0|60
contig20135_length=167|0|60
log2(expression)
0 2 4 6 8 12 16
StageI StageII StageIII StageIV StageV StageVI

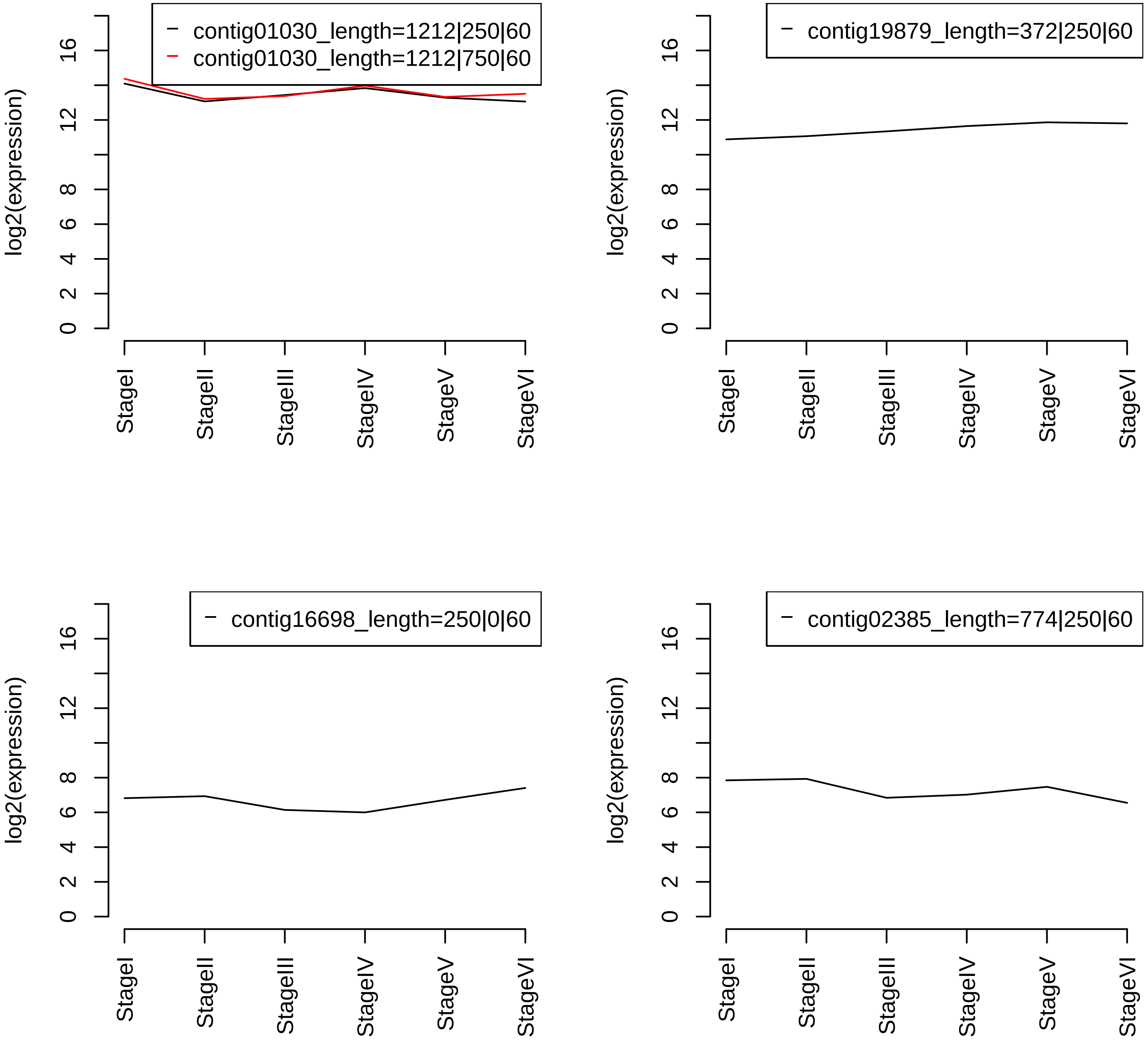

contig01030_length=1212|250|60
contig01030_length=1212|750|60
contig19879_length=372|250|60
contig16698_length=250|0|60
contig02385_length=774|250|60
log2(expression)
0
2
4
6
8
12
16
StageI
StageII
StageIII
StageIV
StageV
StageVI

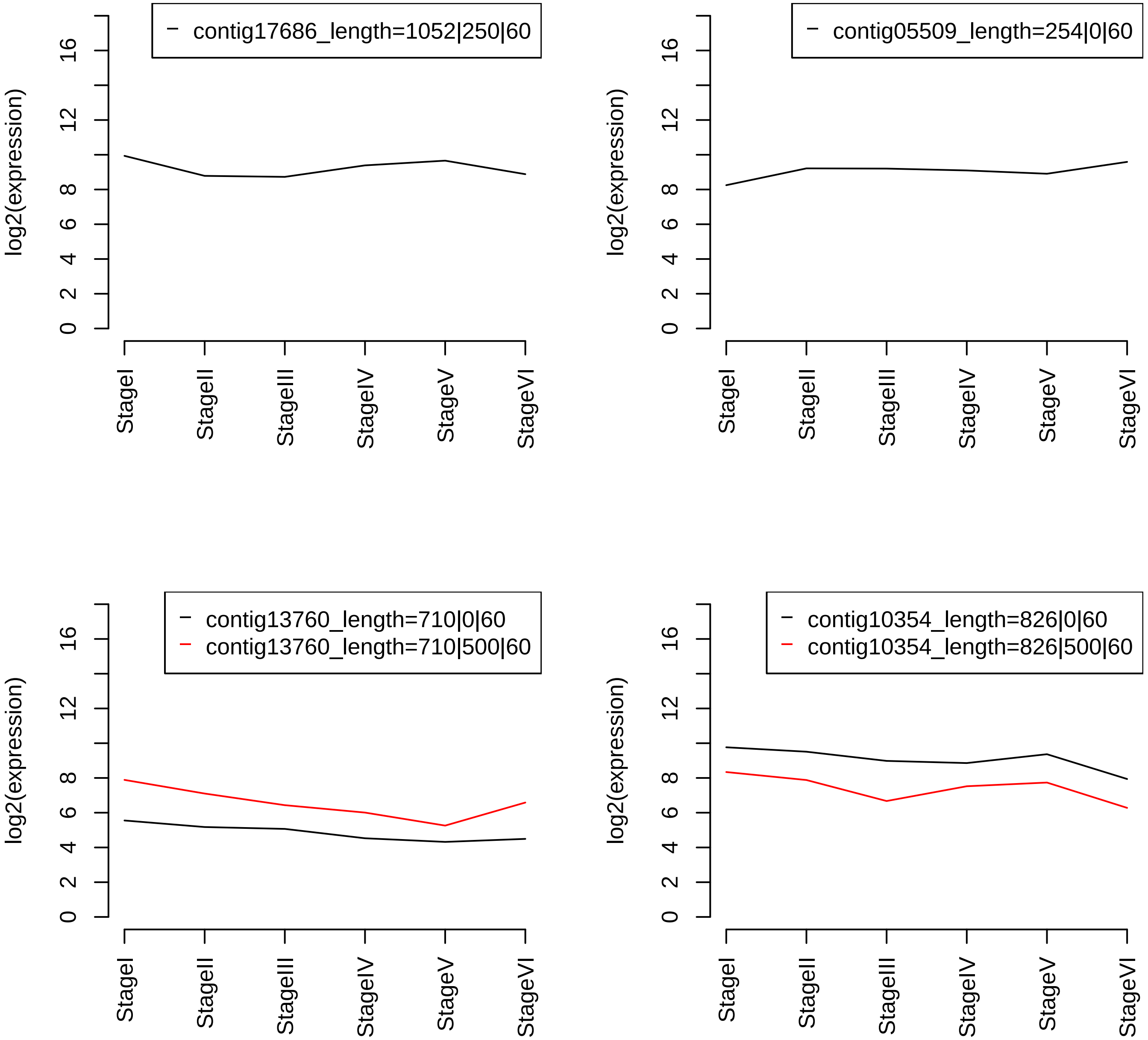
contig17686_length=1052|250|60
contig05509_length=254|0|60
contig13760_length=710|0|60
contig13760_length=710|500|60
contig10354_length=826|0|60
contig10354_length=826|500|60
log2(expression)
0
2
4
6
8
12
16
StageI
StageII
StageIII
StageIV
StageV
StageVI

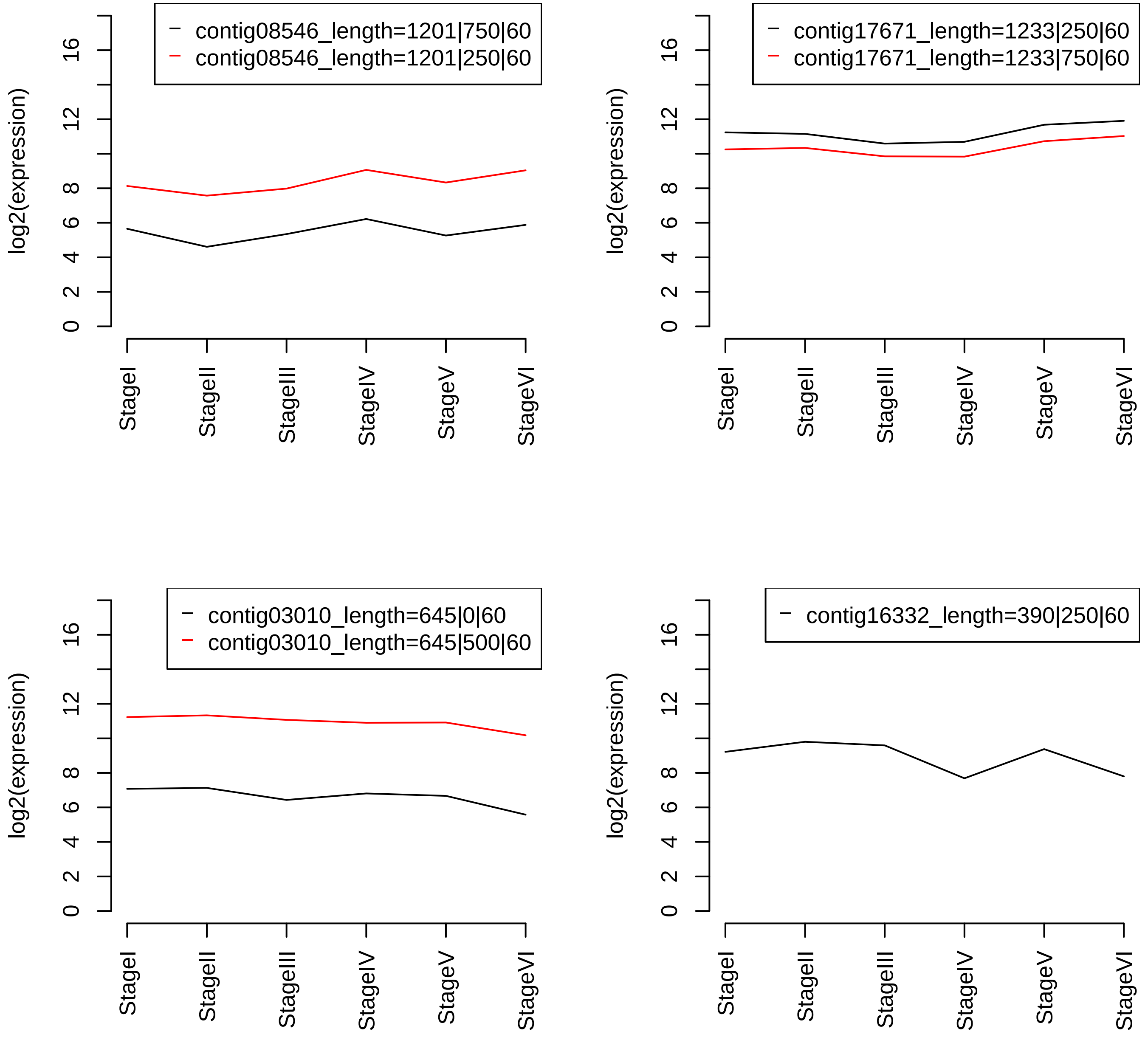

contig08546_length=1201|750|60
contig08546_length=1201|250|60
contig17671_length=1233|250|60
contig17671_length=1233|750|60
contig03010_length=645|0|60
contig03010_length=645|500|60
contig16332_length=390|250|60
log2(expression)
0
2
4
6
8
12
16
StageI
StageII
StageIII
StageIV
StageV
StageVI

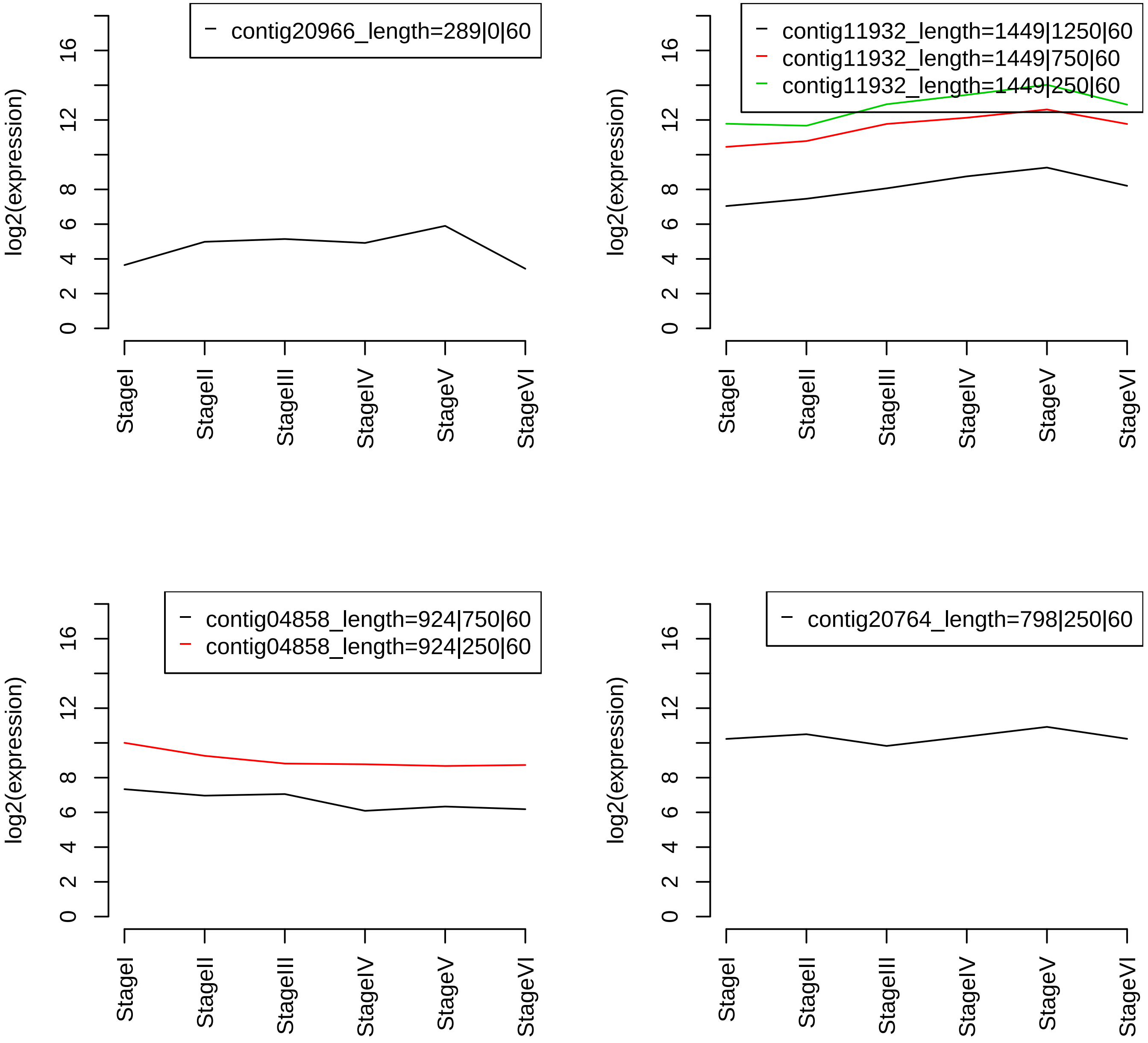
contig20966_length=289|0|60
log2(expression)
0
2
4
6
8
12
16
StageI
StageII
StageIII
StageIV
StageV
StageVI
contig11932_length=1449|1250|60
contig11932_length=1449|750|60
contig11932_length=1449|250|60
log2(expression)
0
2
4
6
8
12
16
StageI
StageII
StageIII
StageIV
StageV
StageVI
contig04858_length=924|750|60
contig04858_length=924|250|60
log2(expression)
0
2
4
6
8
12
16
StageI
StageII
StageIII
StageIV
StageV
StageVI
contig20764_length=798|250|60
log2(expression)
0
2
4
6
8
12
16
StageI
StageII
StageIII
StageIV
StageV
StageVI

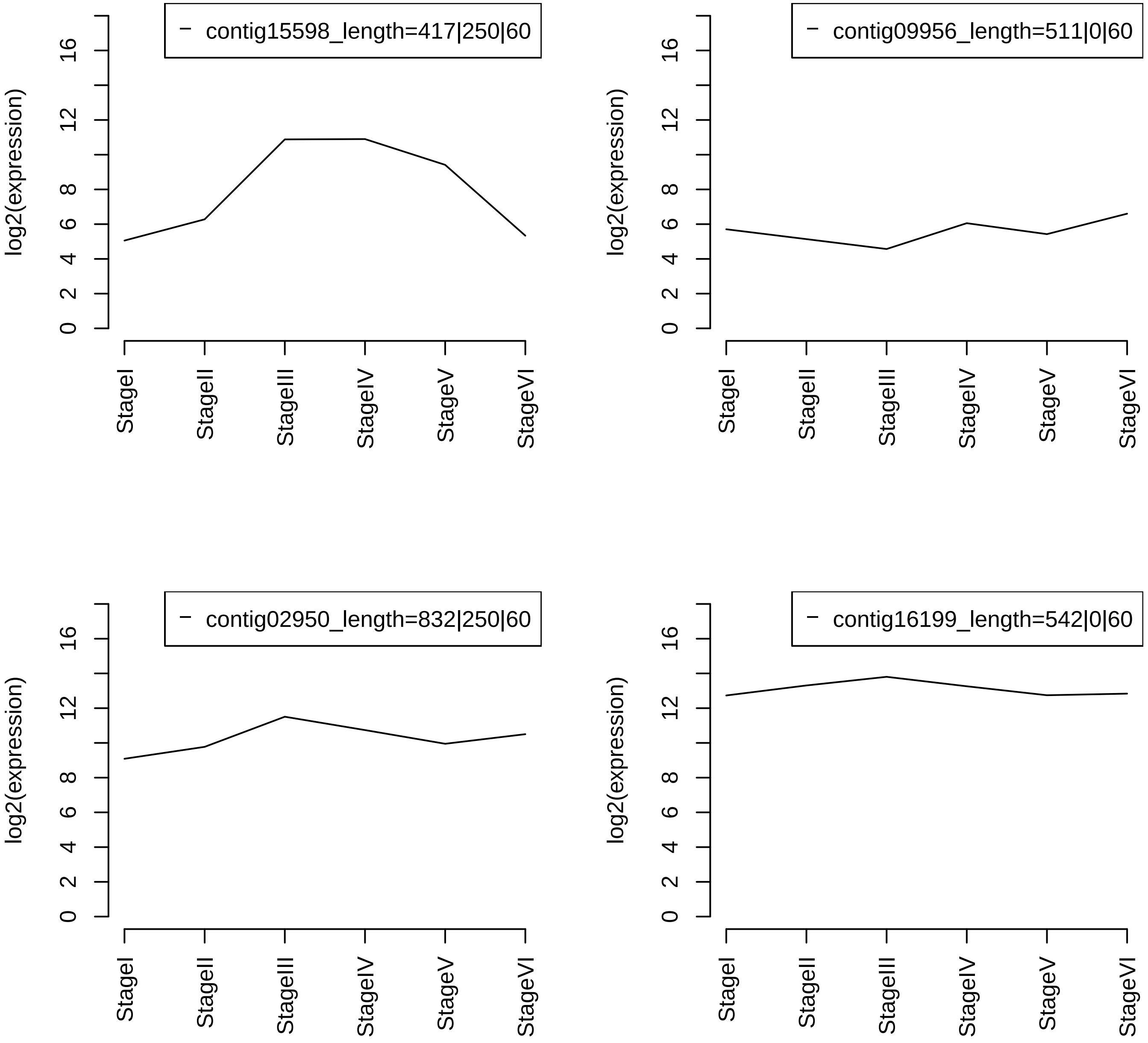

contig15598_length=417|250|60
contig09956_length=511|0|60
contig02950_length=832|250|60
contig16199_length=542|0|60
log2(expression)
0
2
4
6
8
12
16
StageI
StageII
StageIII
StageIV
StageV
StageVI

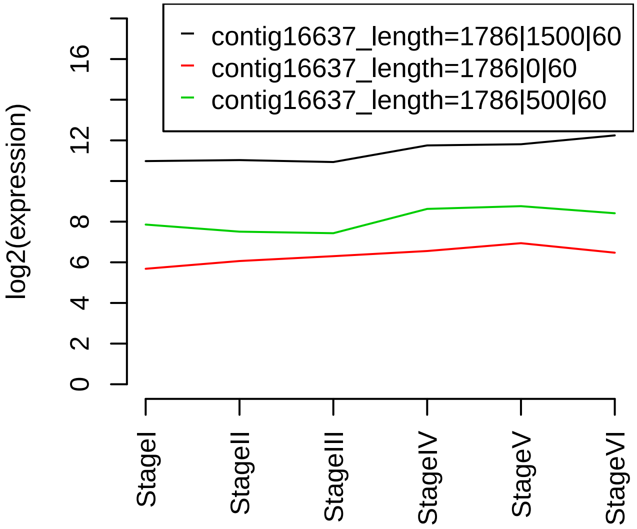
contig16637_length=1786|1500|60
contig16637_length=1786|0|60
contig16637_length=1786|500|60
log2(expression)
0
2
4
6
8
12
16
StageI
StageII
StageIII
StageIV
StageV
StageVI

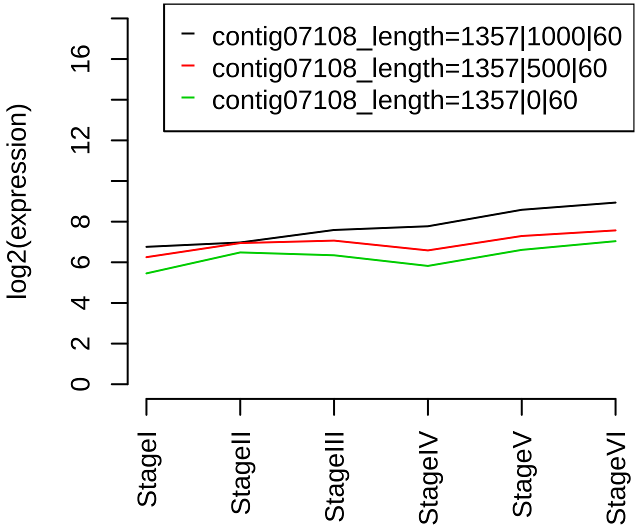
contig07108_length=1357|1000|60
contig07108_length=1357|500|60
contig07108_length=1357|0|60
log2(expression)
0
2
4
6
8
12
16
StageI
StageII
StageIII
StageIV
StageV
StageVI

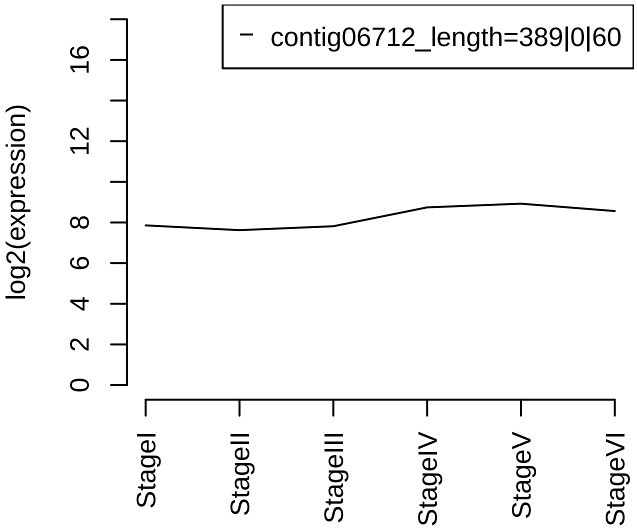
contig06712_length=389|0|60
log2(expression)
0
2
4
6
8
12
16
StageI
StageII
StageIII
StageIV
StageV
StageVI

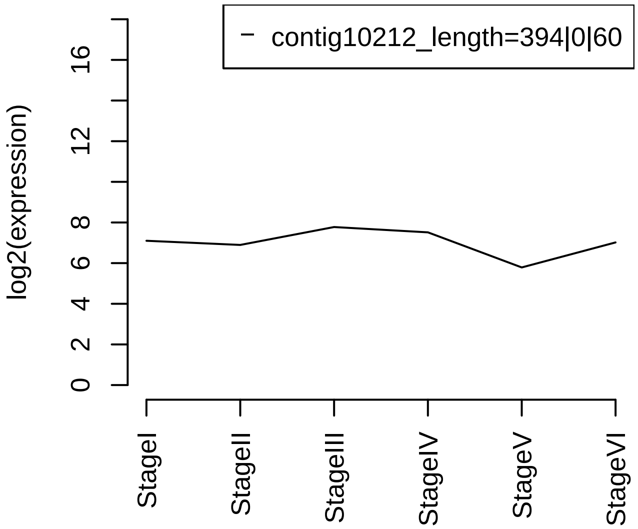
contig10212_length=394|0|60
log2(expression)
0
2
4
6
8
12
16
StageI
StageII
StageIII
StageIV
StageV
StageVI

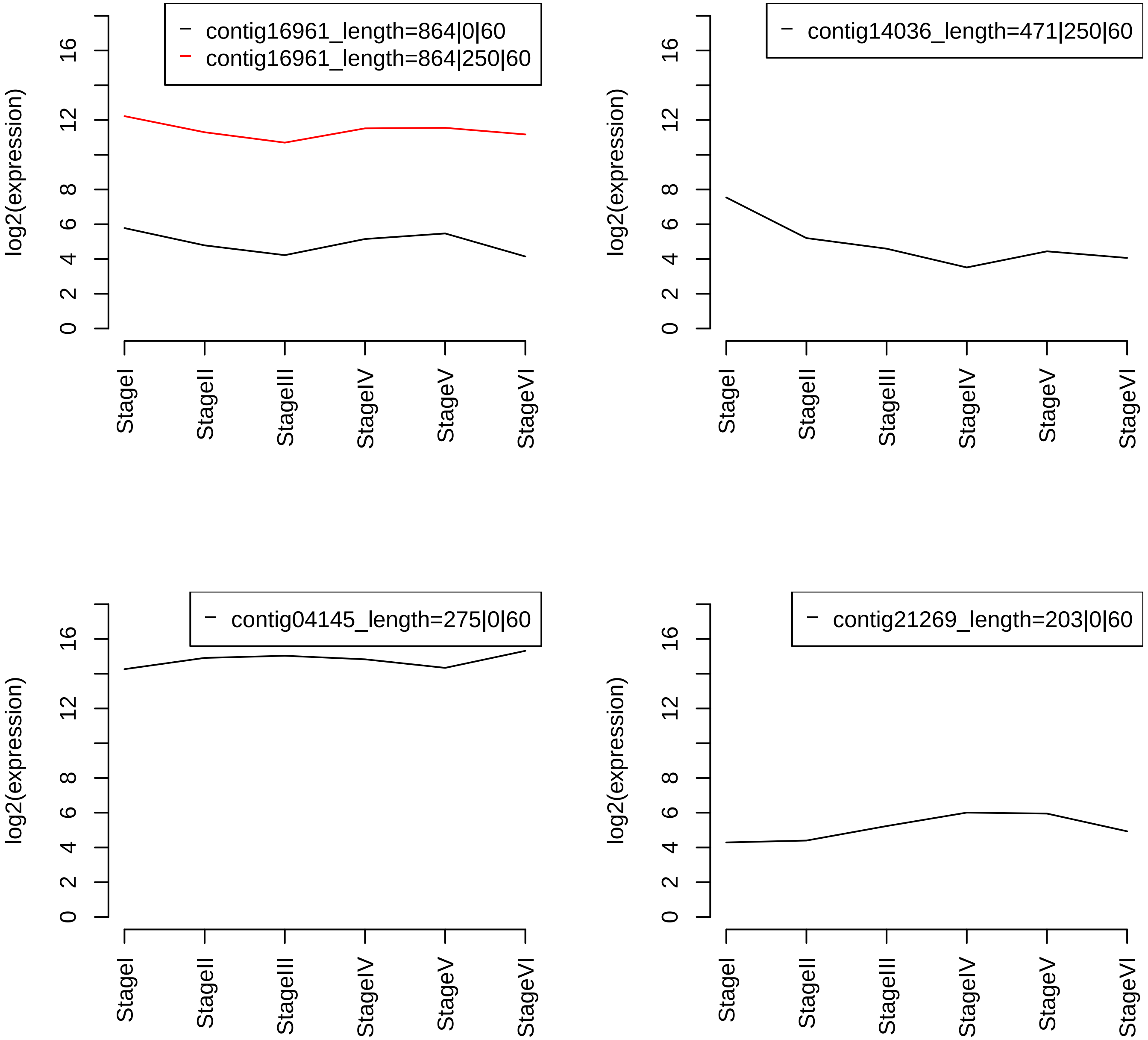
contig16961_length=864|0|60
contig16961_length=864|250|60
contig14036_length=471|250|60
contig04145_length=275|0|60
contig21269_length=203|0|60
log2(expression)
0
2
4
6
8
12
16
StageI
StageII
StageIII
StageIV
StageV
StageVI

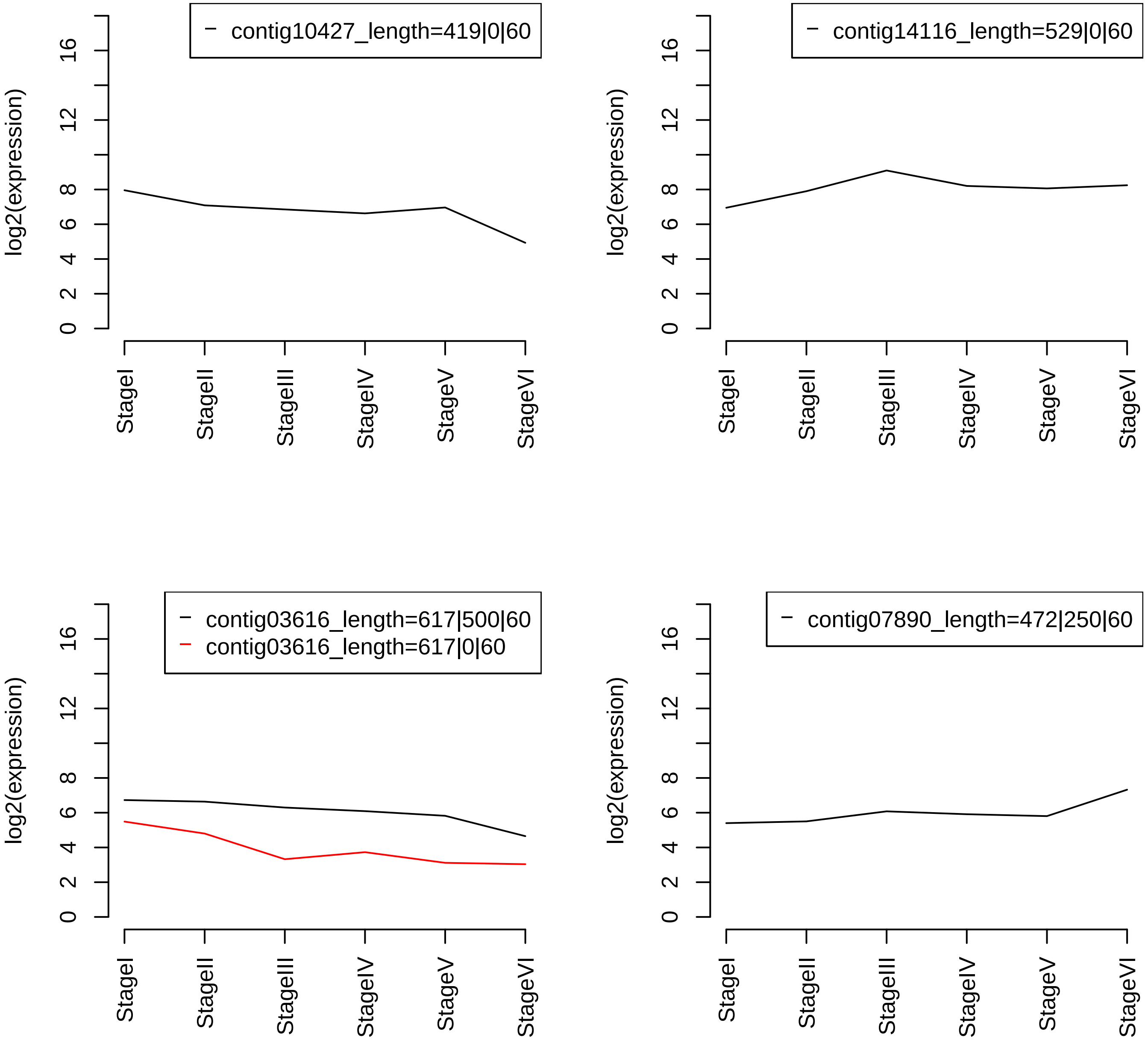
contig10427_length=419|0|60
contig14116_length=529|0|60
contig03616_length=617|500|60
contig03616_length=617|0|60
contig07890_length=472|250|60
log2(expression)
0
2
4
6
8
12
16
StageI
StageII
StageIII
StageIV
StageV
StageVI

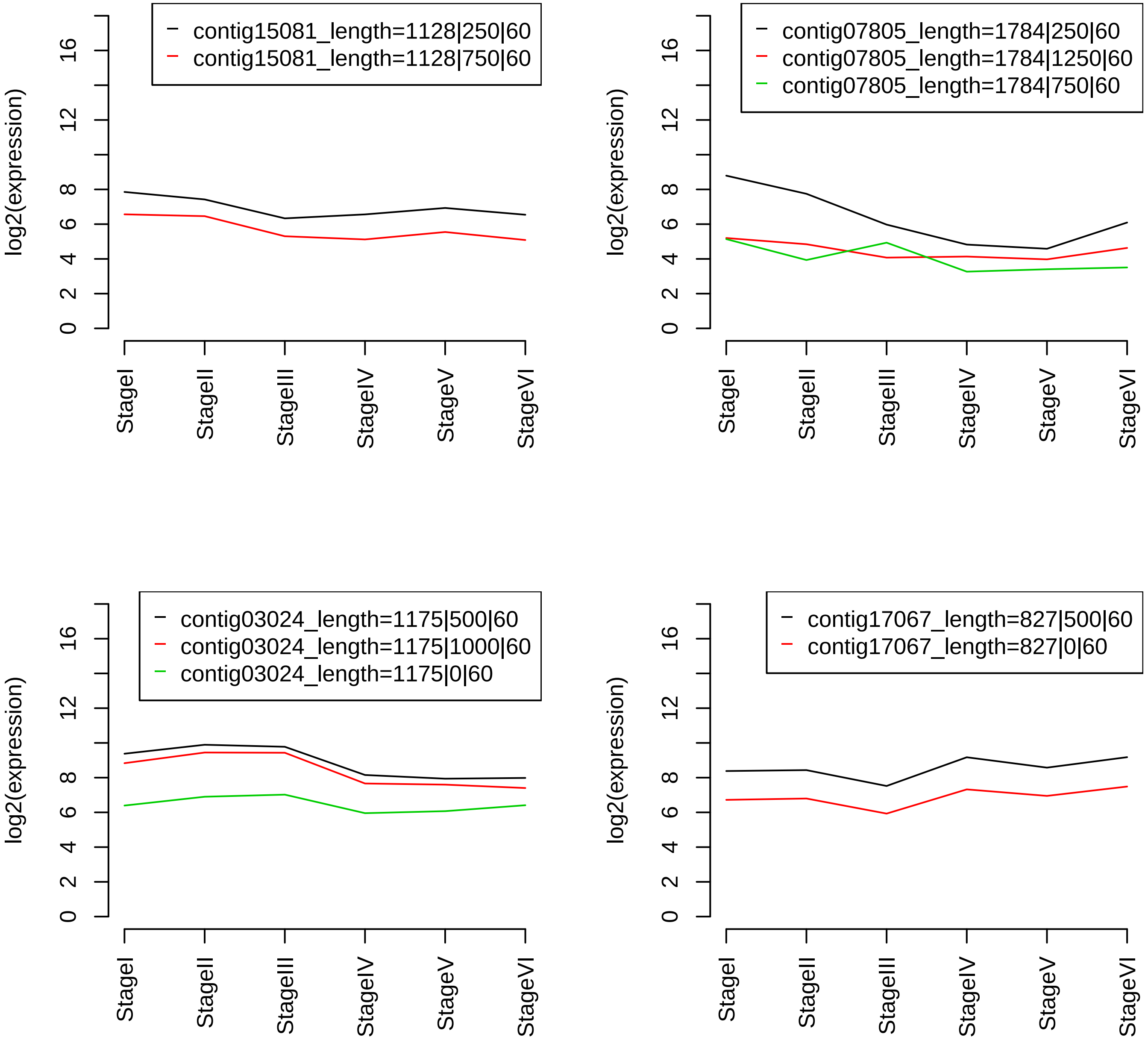

contig15081_length=1128|250|60
contig15081_length=1128|750|60
log2(expression)
0
2
4
6
8
12
16
StageI
StageII
StageIII
StageIV
StageV
StageVI
contig07805_length=1784|250|60
contig07805_length=1784|1250|60
contig07805_length=1784|750|60
log2(expression)
StageI
StageII
StageIII
StageIV
StageV
StageVI
contig03024_length=1175|500|60
contig03024_length=1175|1000|60
contig03024_length=1175|0|60
log2(expression)
StageI
StageII
StageIII
StageIV
StageV
StageVI
contig17067_length=827|500|60
contig17067_length=827|0|60
log2(expression)
StageI
StageII
StageIII
StageIV
StageV
StageVI

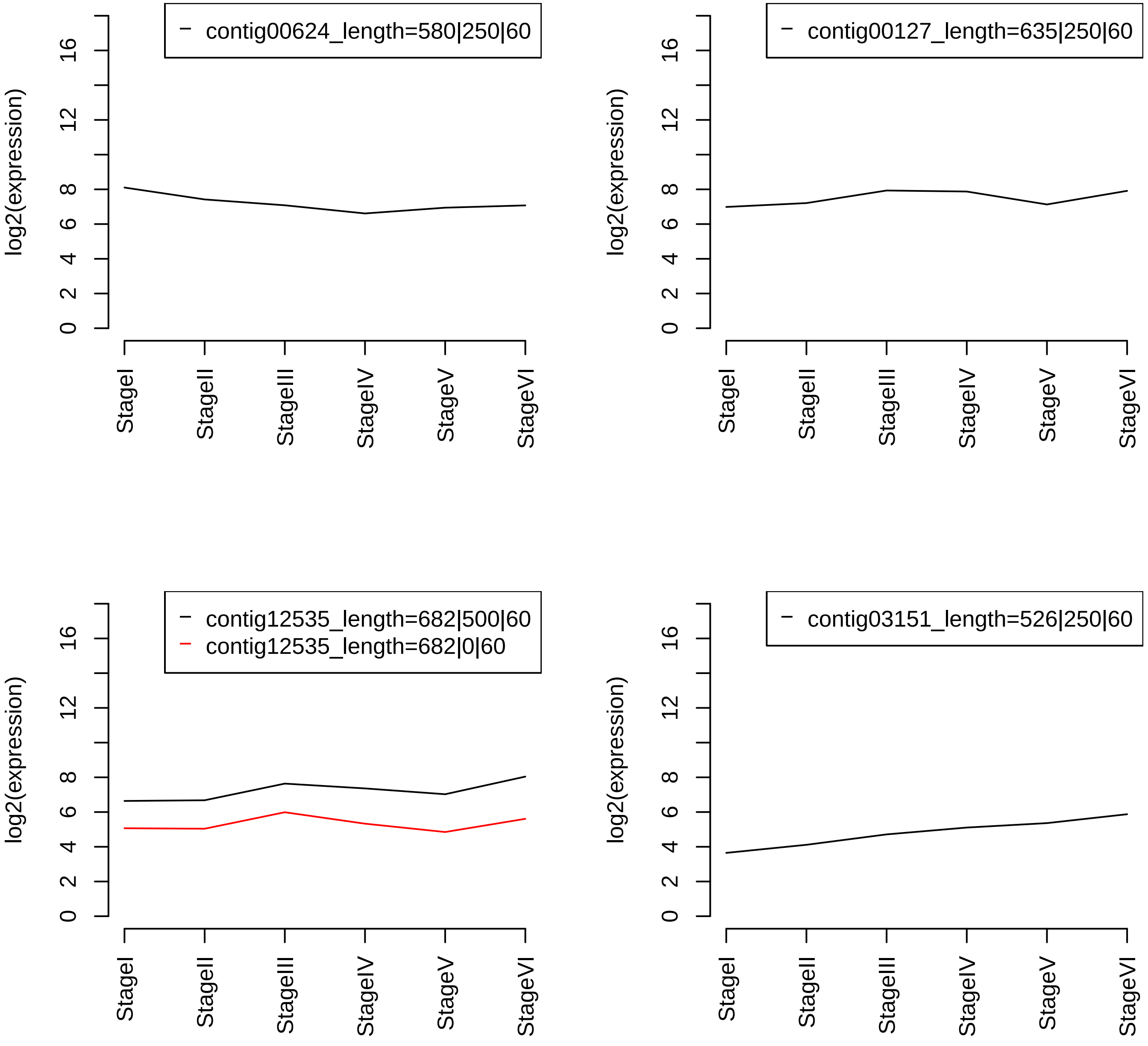

contig00624_length=580|250|60
log2(expression)
0
2
4
6
8
12
16
StageI
StageII
StageIII
StageIV
StageV
StageVI
contig00127_length=635|250|60
log2(expression)
0
2
4
6
8
12
16
StageI
StageII
StageIII
StageIV
StageV
StageVI
contig12535_length=682|500|60
contig12535_length=682|0|60
log2(expression)
0
2
4
6
8
12
16
StageI
StageII
StageIII
StageIV
StageV
StageVI
contig03151_length=526|250|60
log2(expression)
0
2
4
6
8
12
16
StageI
StageII
StageIII
StageIV
StageV
StageVI

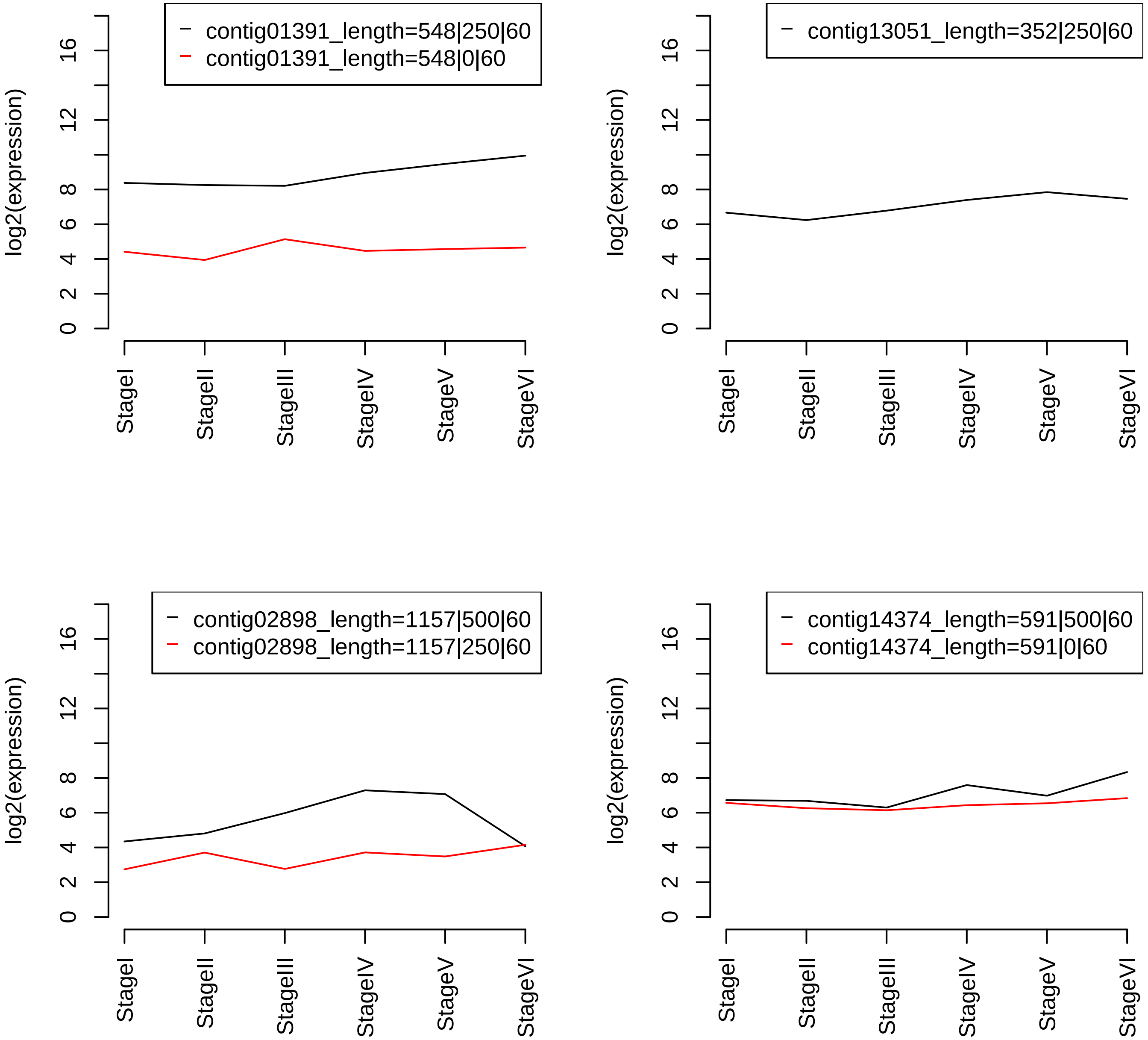
contig01391_length=548|250|60
contig01391_length=548|0|60
contig13051_length=352|250|60
contig02898_length=1157|500|60
contig02898_length=1157|250|60
contig14374_length=591|500|60
contig14374_length=591|0|60
log2(expression)
0
2
4
6
8
12
16
StageI
StageII
StageIII
StageIV
StageV
StageVI

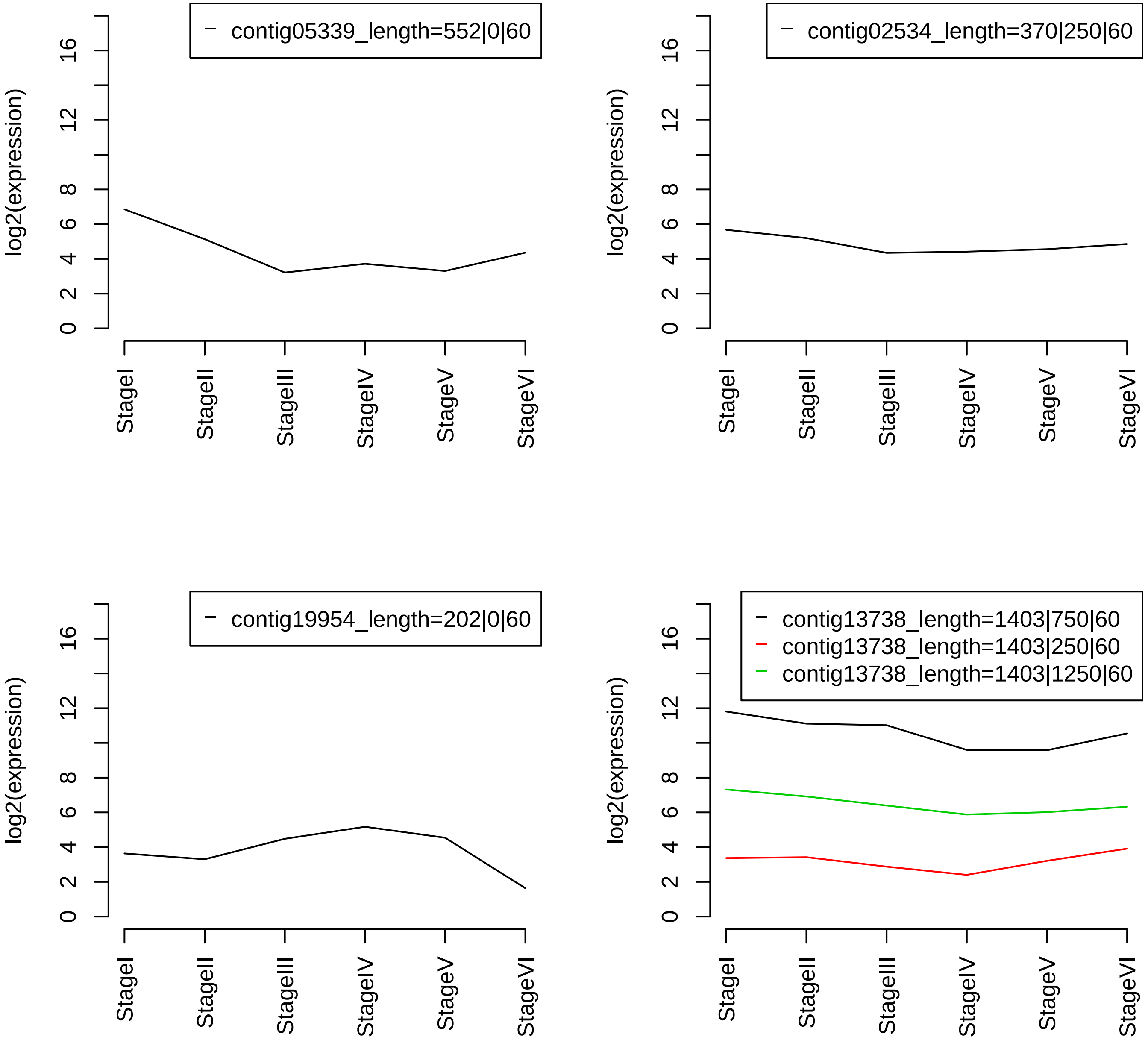
contig05339_length=552|0|60
contig02534_length=370|250|60
contig19954_length=202|0|60
contig13738_length=1403|750|60
contig13738_length=1403|250|60
contig13738_length=1403|1250|60
log2(expression)
0
2
4
6
8
12
16
StageI
StageII
StageIII
StageIV
StageV
StageVI

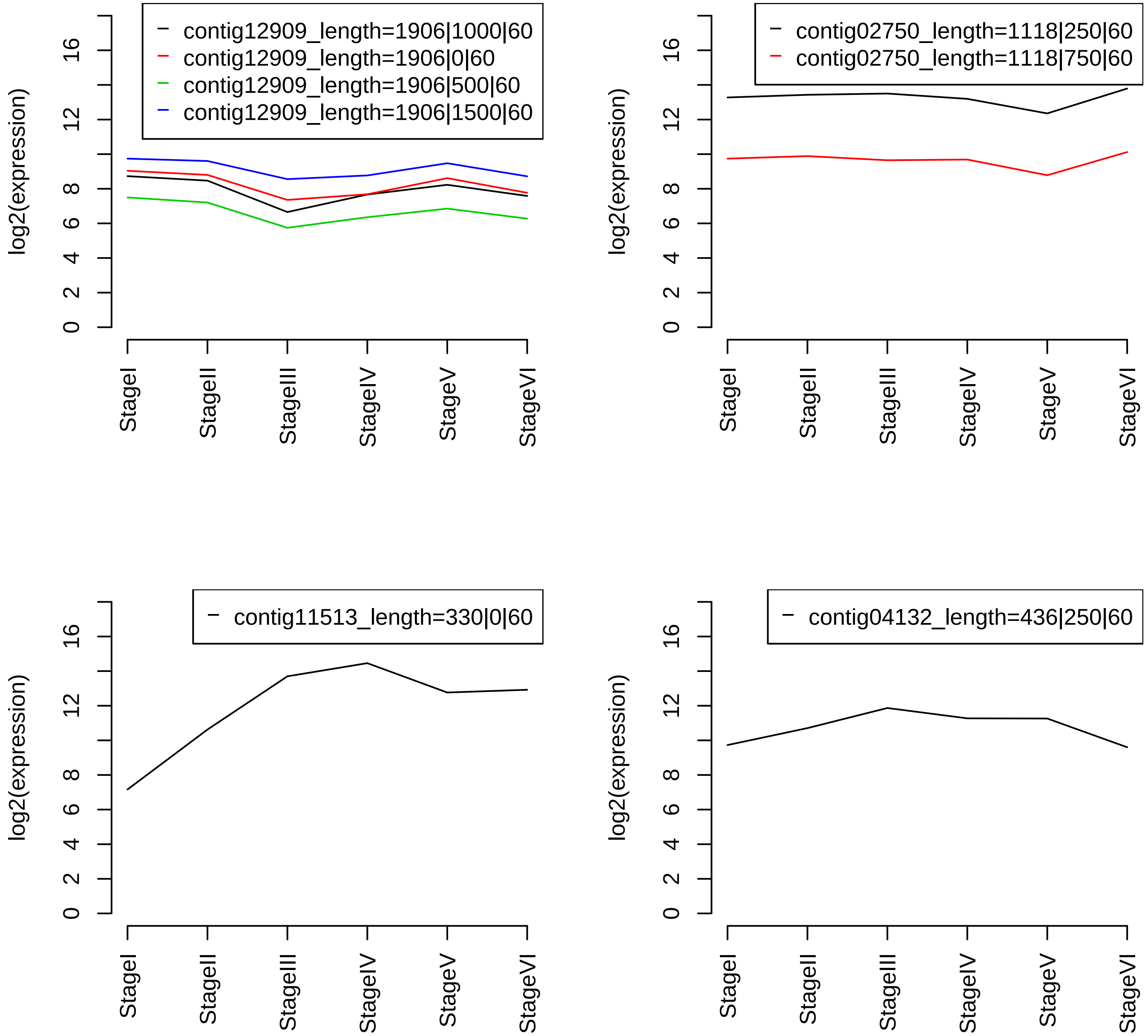

contig12909_length=1906|1000|60
contig12909_length=1906|0|60
contig12909_length=1906|500|60
contig12909_length=1906|1500|60
contig02750_length=1118|250|60
contig02750_length=1118|750|60
contig11513_length=330|0|60
contig04132_length=436|250|60
log2(expression)
0
2
4
6
8
12
16
StageI
StageII
StageIII
StageIV
StageV
StageVI

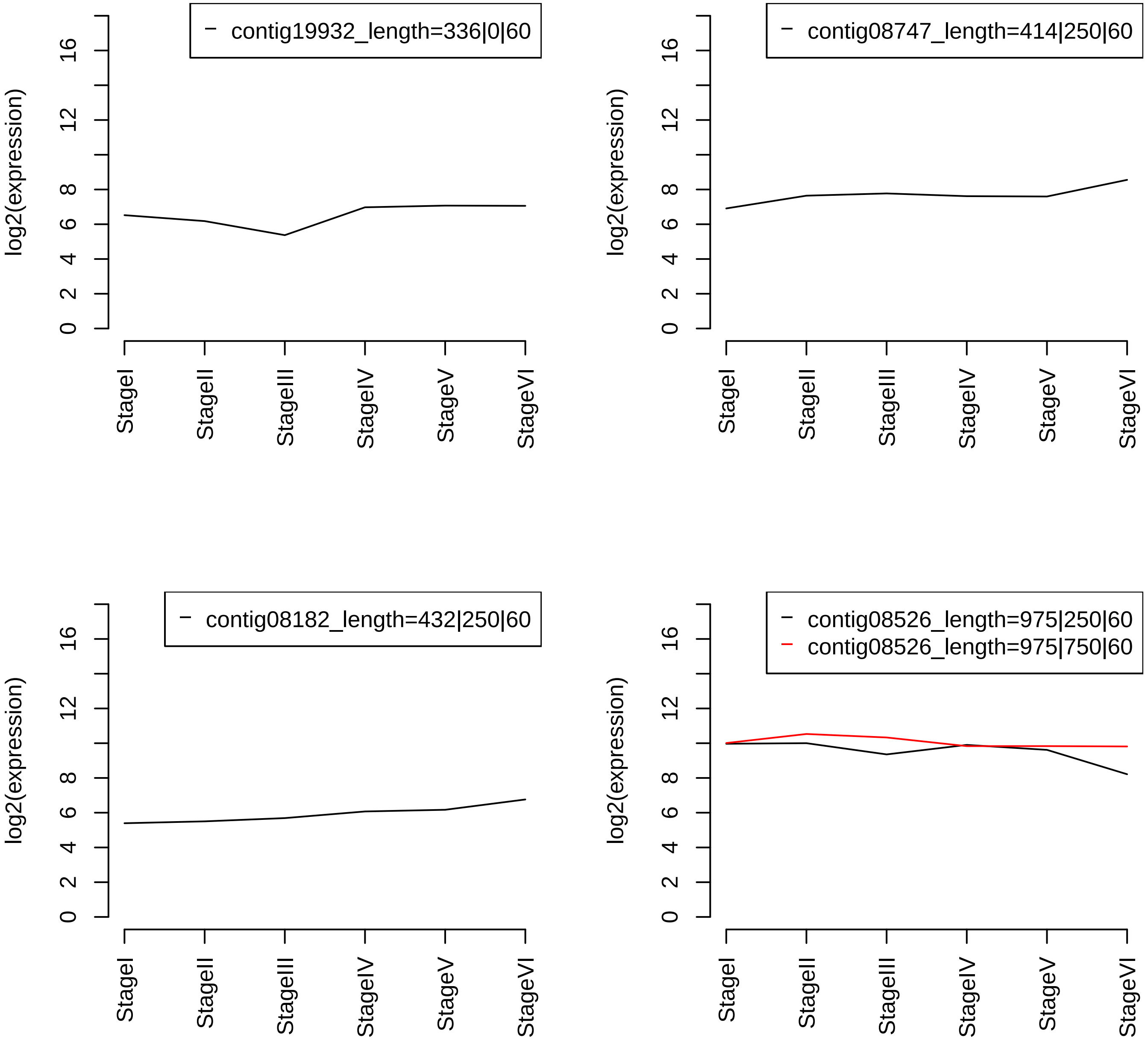
contig19932_length=336|0|60
contig08747_length=414|250|60
contig08182_length=432|250|60
contig08526_length=975|250|60
contig08526_length=975|750|60
log2(expression)
0
2
4
6
8
12
16
StageI
StageII
StageIII
StageIV
StageV
StageVI

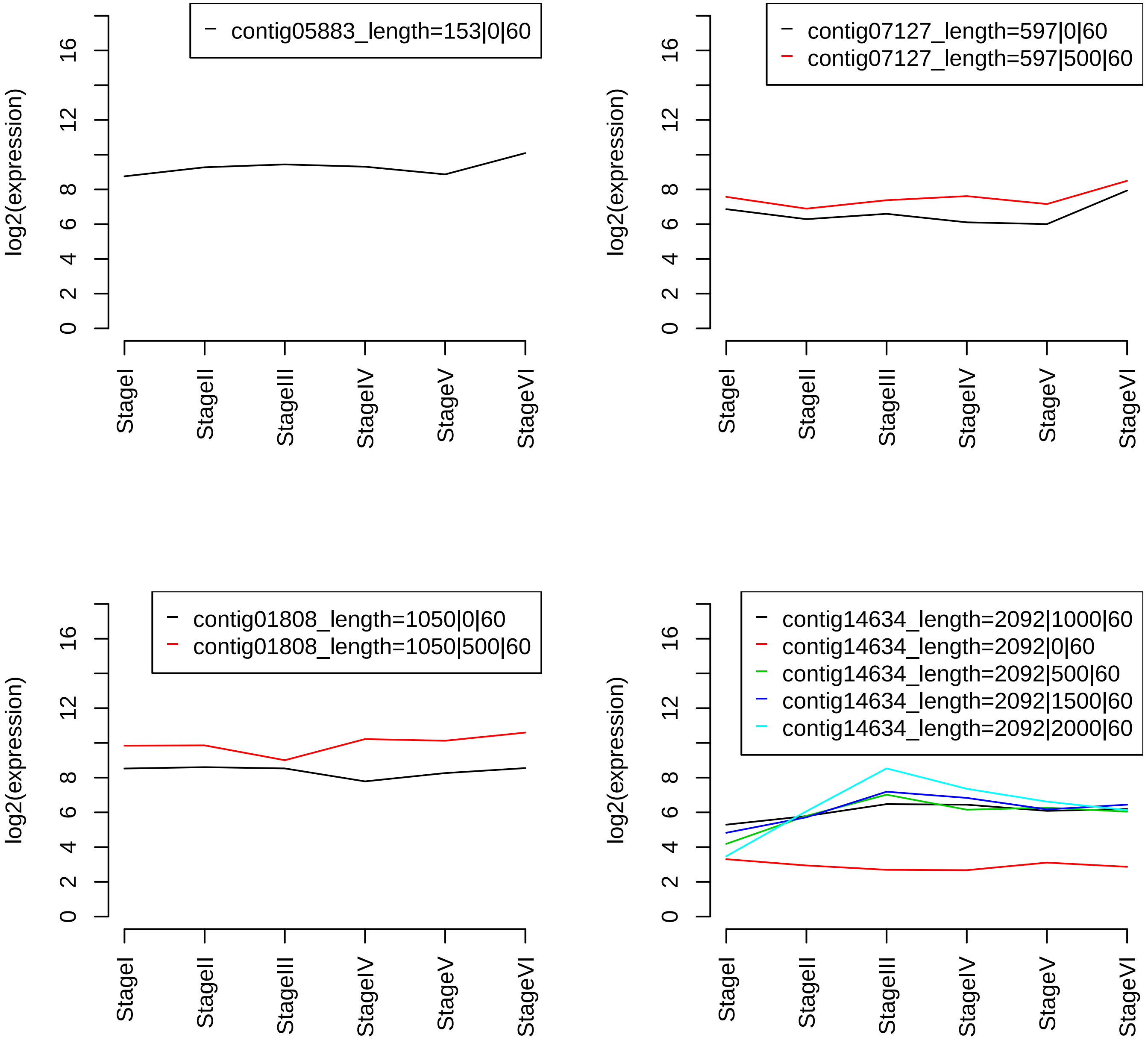
contig05883_length=153|0|60
contig07127_length=597|0|60
contig07127_length=597|500|60
contig01808_length=1050|0|60
contig01808_length=1050|500|60
contig14634_length=2092|1000|60
contig14634_length=2092|0|60
contig14634_length=2092|500|60
contig14634_length=2092|1500|60
contig14634_length=2092|2000|60
log2(expression)
0 2 4 6 8 12 16
StageI StageII StageIII StageIV StageV StageVI

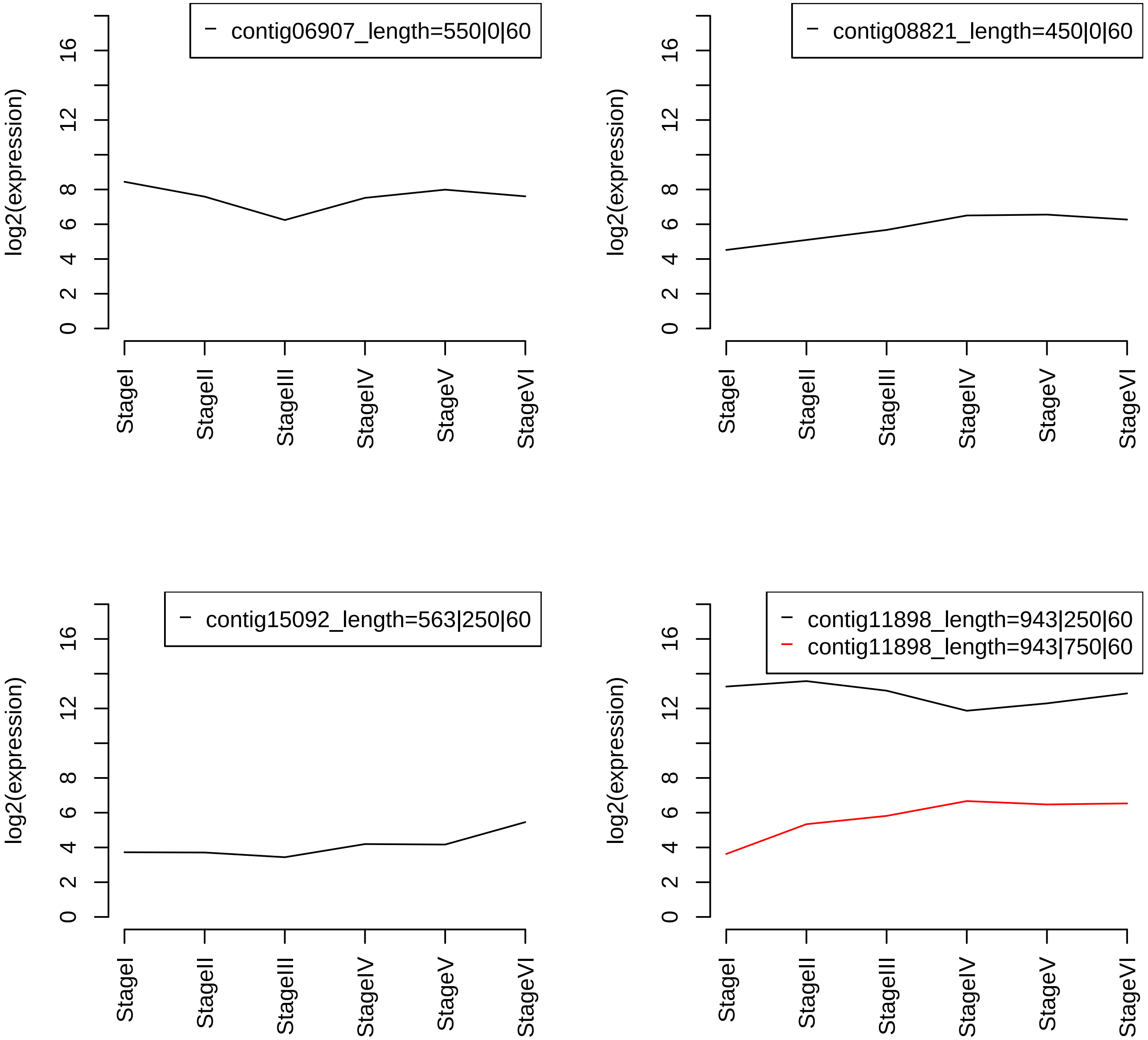
contig06907_length=550|0|60
contig08821_length=450|0|60
contig15092_length=563|250|60
contig11898_length=943|250|60
contig11898_length=943|750|60
log2(expression)
0
2
4
6
8
12
16
StageI
StageII
StageIII
StageIV
StageV
StageVI

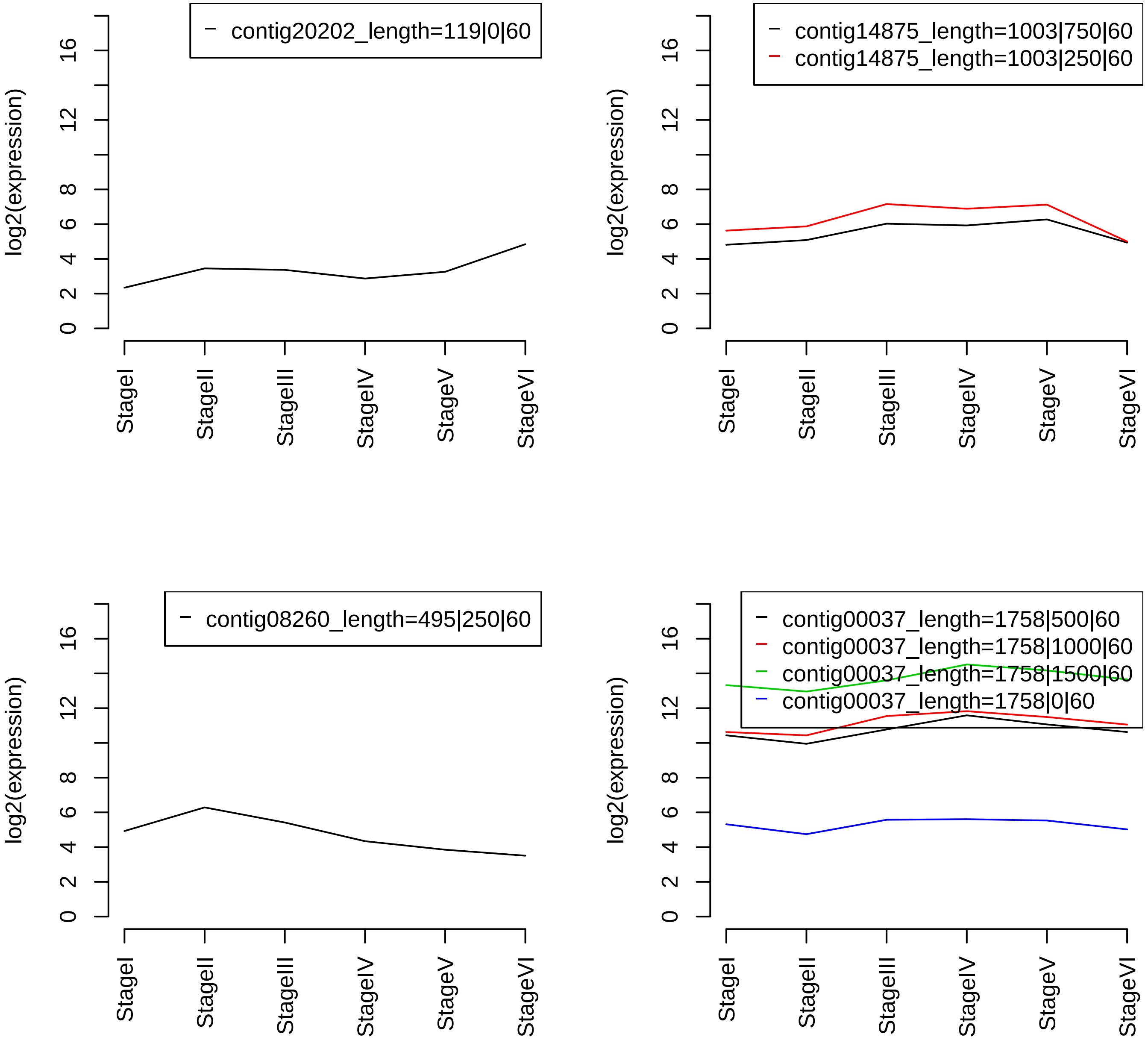

contig20202_length=119|0|60
contig14875_length=1003|750|60
contig14875_length=1003|250|60
contig08260_length=495|250|60
contig00037_length=1758|500|60
contig00037_length=1758|1000|60
contig00037_length=1758|1500|60
contig00037_length=1758|0|60
log2(expression)
0
2
4
6
8
12
16
StageI
StageII
StageIII
StageIV
StageV
StageVI

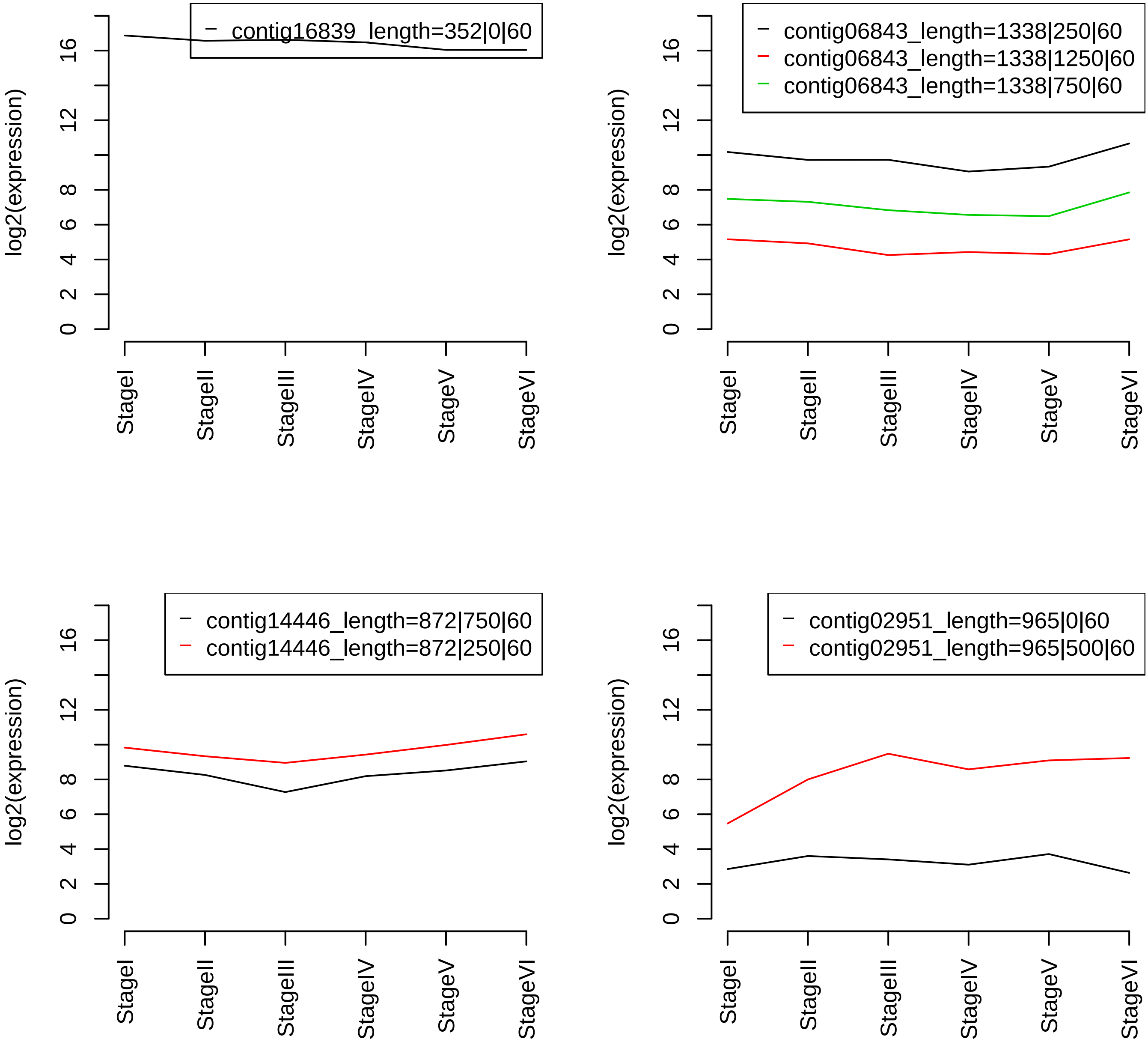

contig16839_length=352|0|60
contig06843_length=1338|250|60
contig06843_length=1338|1250|60
contig06843_length=1338|750|60
contig14446_length=872|750|60
contig14446_length=872|250|60
contig02951_length=965|0|60
contig02951_length=965|500|60
log2(expression)
0 2 4 6 8 12 16
StageI StageII StageIII StageIV StageV StageVI

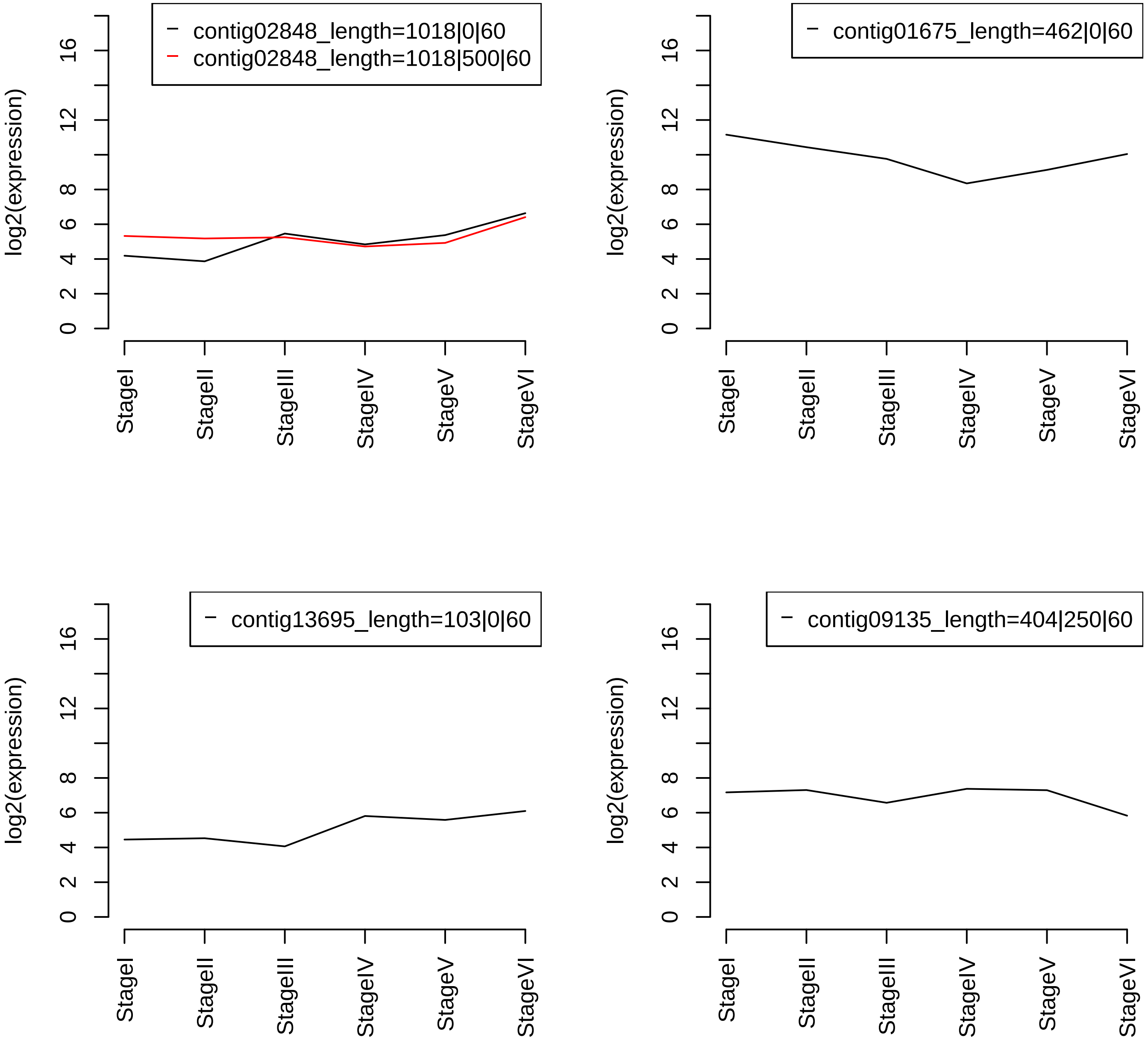
contig02848_length=1018|0|60
contig02848_length=1018|500|60
contig01675_length=462|0|60
contig13695_length=103|0|60
contig09135_length=404|250|60
log2(expression)
0
2
4
6
8
12
16
StageI
StageII
StageIII
StageIV
StageV
StageVI

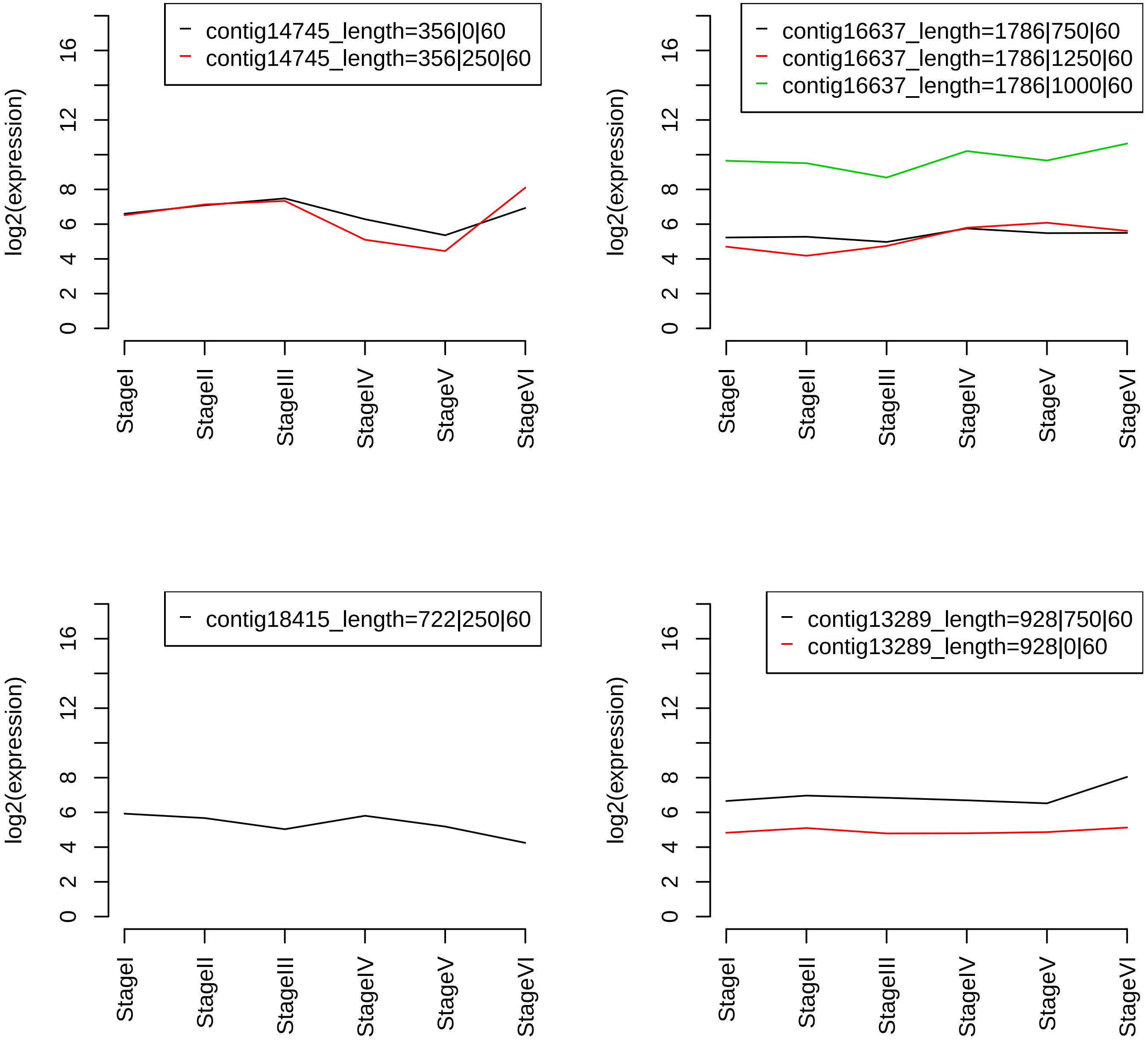
contig14745_length=356|0|60
contig14745_length=356|250|60
contig16637_length=1786|750|60
contig16637_length=1786|1250|60
contig16637_length=1786|1000|60
contig18415_length=722|250|60
contig13289_length=928|750|60
contig13289_length=928|0|60
log2(expression)
0 2 4 6 8 12 16
StageI
StageII
StageIII
StageIV
StageV
StageVI

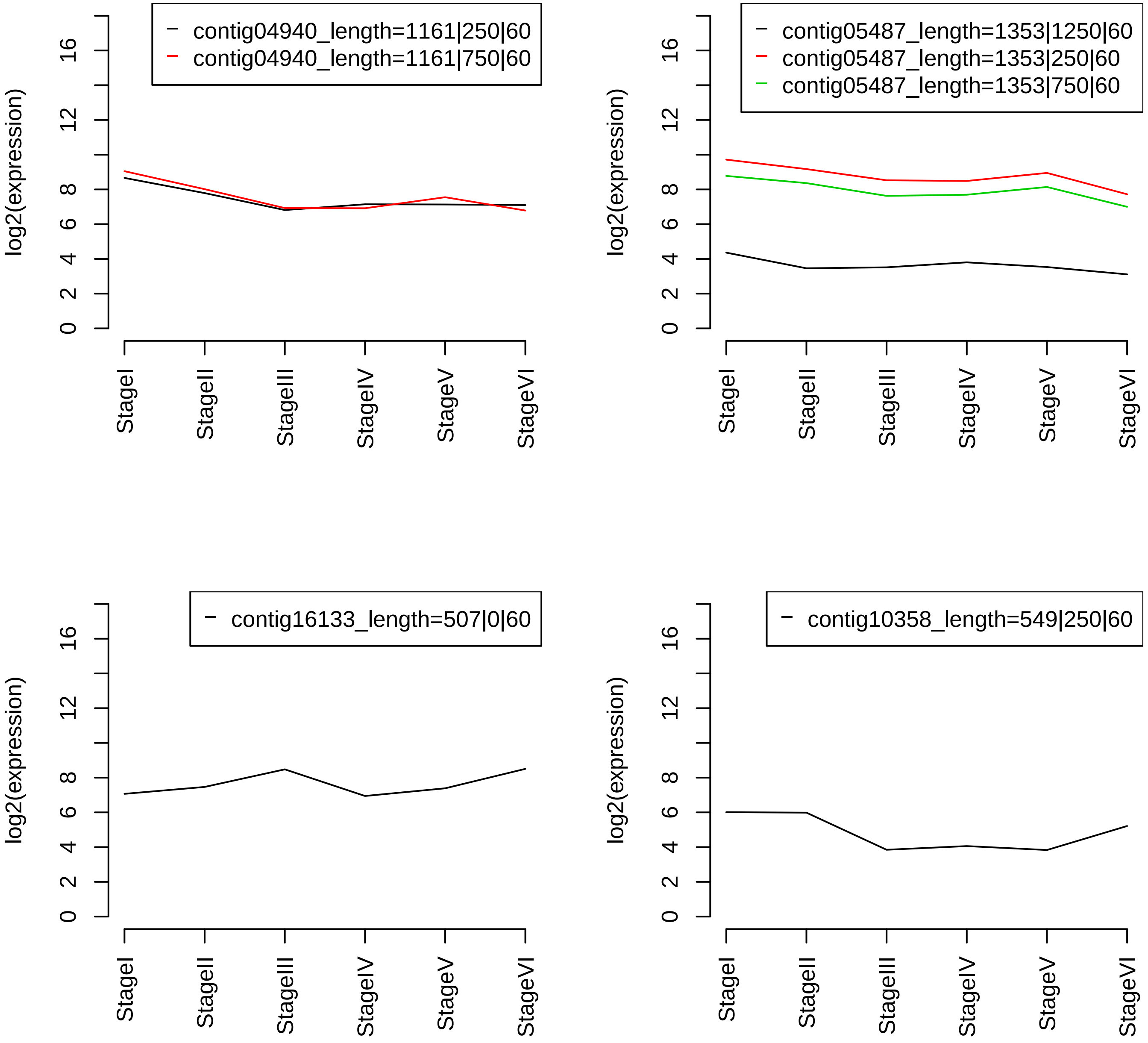
contig04940_length=1161|250|60
contig04940_length=1161|750|60
contig05487_length=1353|1250|60
contig05487_length=1353|250|60
contig05487_length=1353|750|60
contig16133_length=507|0|60
contig10358_length=549|250|60
log2(expression)
0 2 4 6 8 12 16
StageI StageII StageIII StageIV StageV StageVI

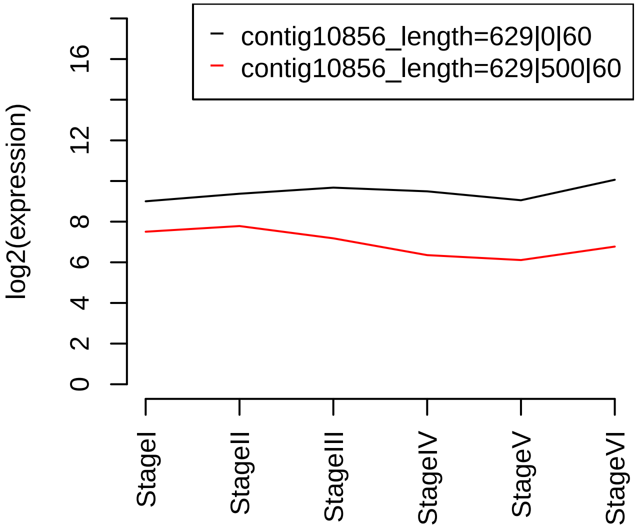
contig10856_length=629|0|60
contig10856_length=629|500|60
log2(expression)
0 2 4 6 8 12 16
StageI StageII StageIII StageIV StageV StageVI

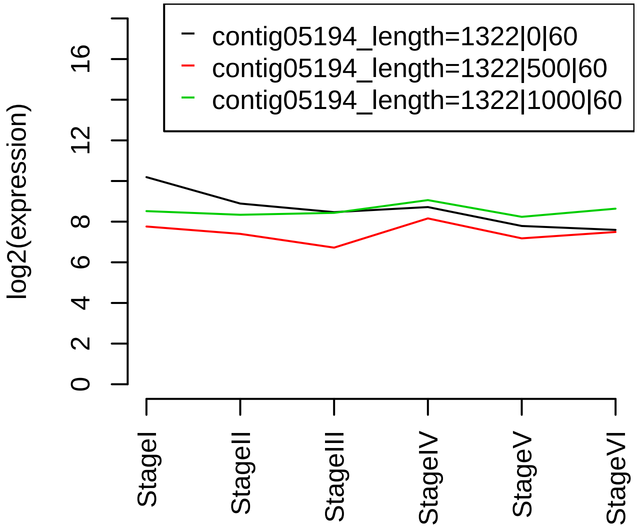
contig05194_length=1322|0|60
contig05194_length=1322|500|60
contig05194_length=1322|1000|60
log2(expression)
0 2 4 6 8 12 16
StageI StageII StageIII StageIV StageV StageVI

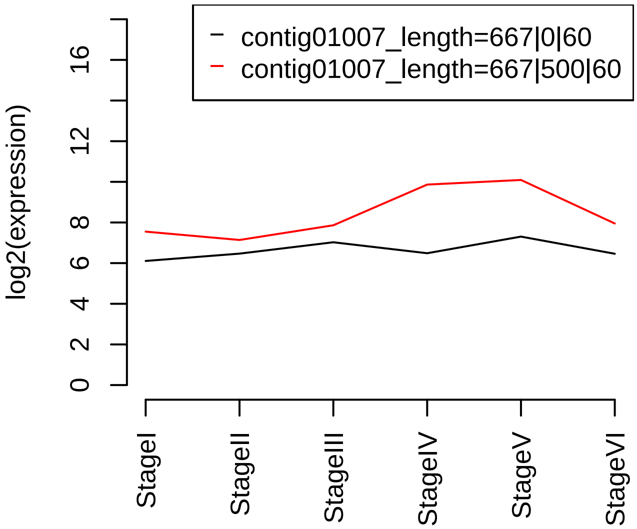
contig01007_length=667|0|60
contig01007_length=667|500|60
log2(expression)
0 2 4 6 8 12 16
StageI StageII StageIII StageIV StageV StageVI

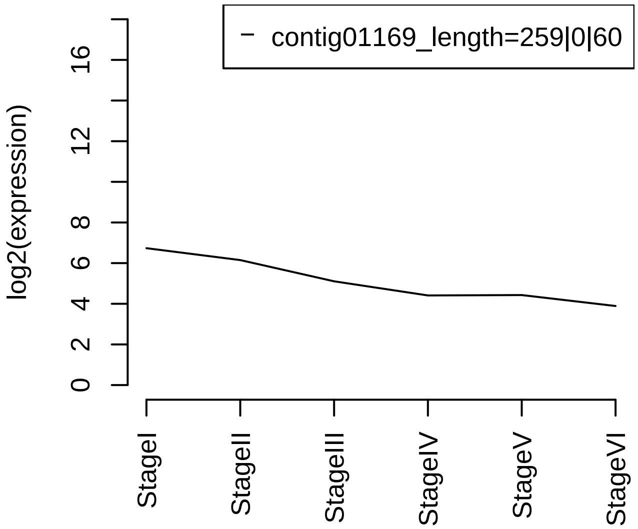
contig01169_length=259|0|60
log2(expression)
0 2 4 6 8 12 16
StageI StageII StageIII StageIV StageV StageVI

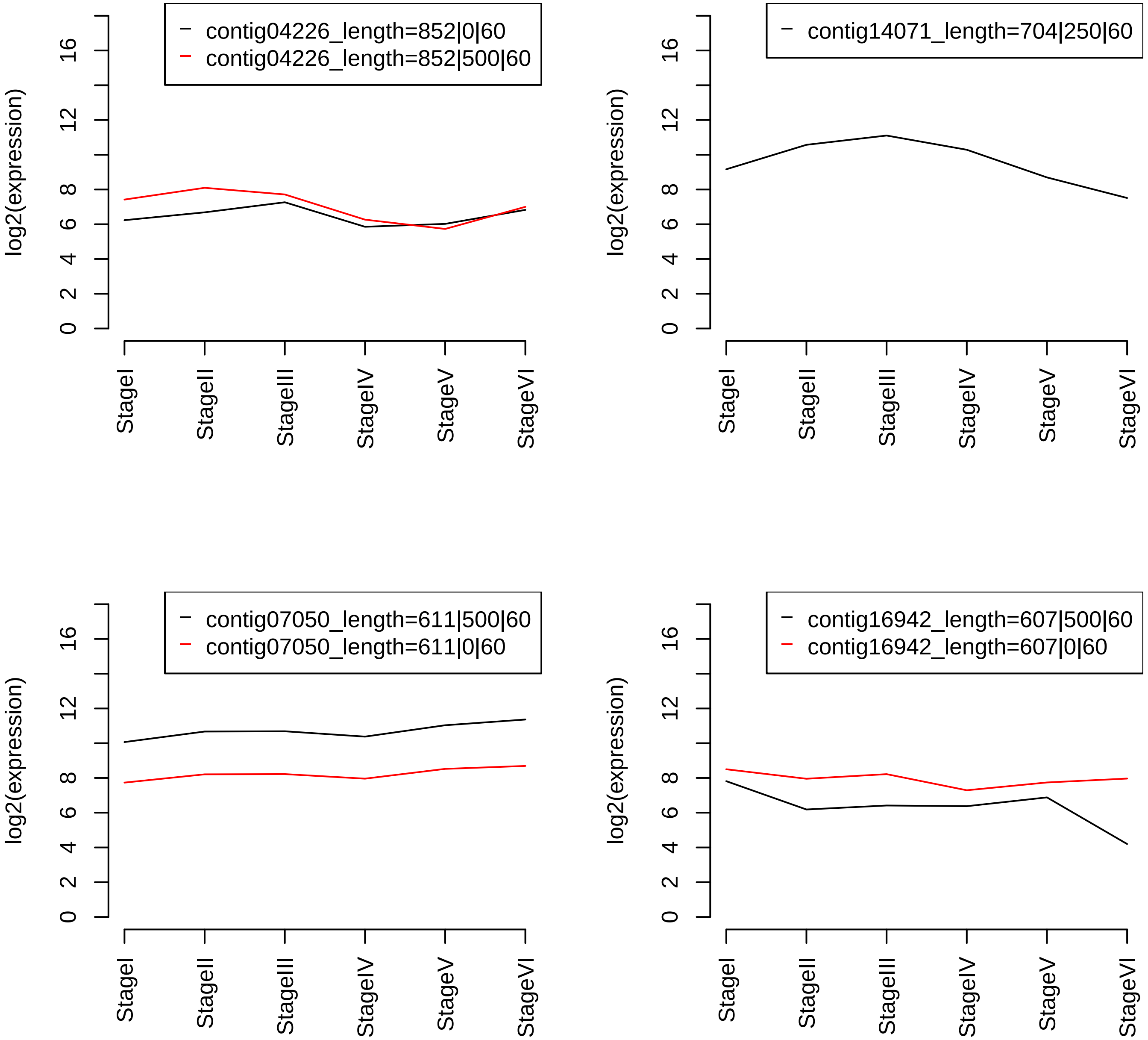
contig04226_length=852|0|60
contig04226_length=852|500|60
contig14071_length=704|250|60
contig07050_length=611|500|60
contig07050_length=611|0|60
contig16942_length=607|500|60
contig16942_length=607|0|60
log2(expression)
StageI
StageII
StageIII
StageIV
StageV
StageVI

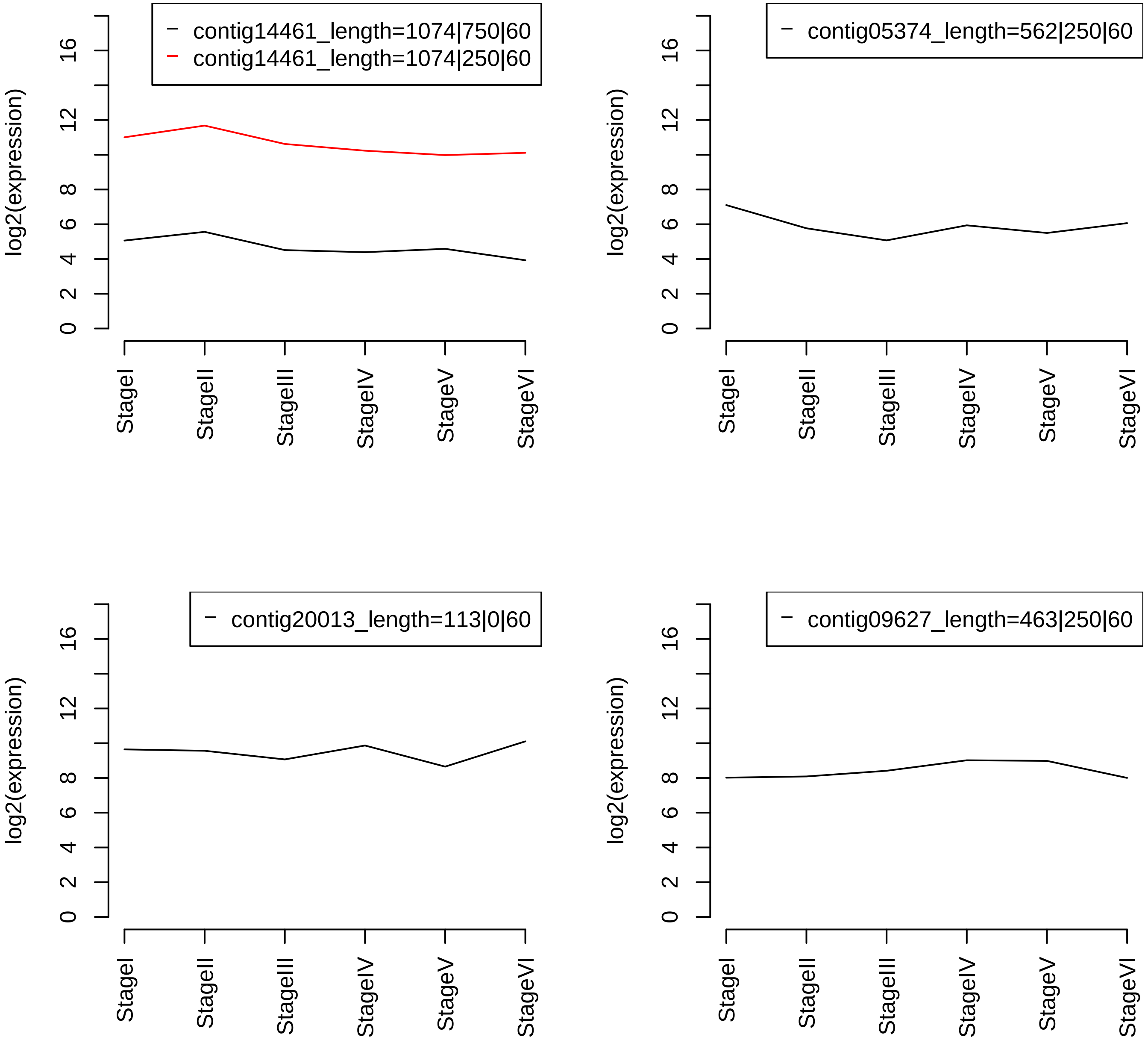
contig14461_length=1074|750|60
contig14461_length=1074|250|60
contig05374_length=562|250|60
contig20013_length=113|0|60
contig09627_length=463|250|60
log2(expression)
0
2
4
6
8
12
16
StageI
StageII
StageIII
StageIV
StageV
StageVI

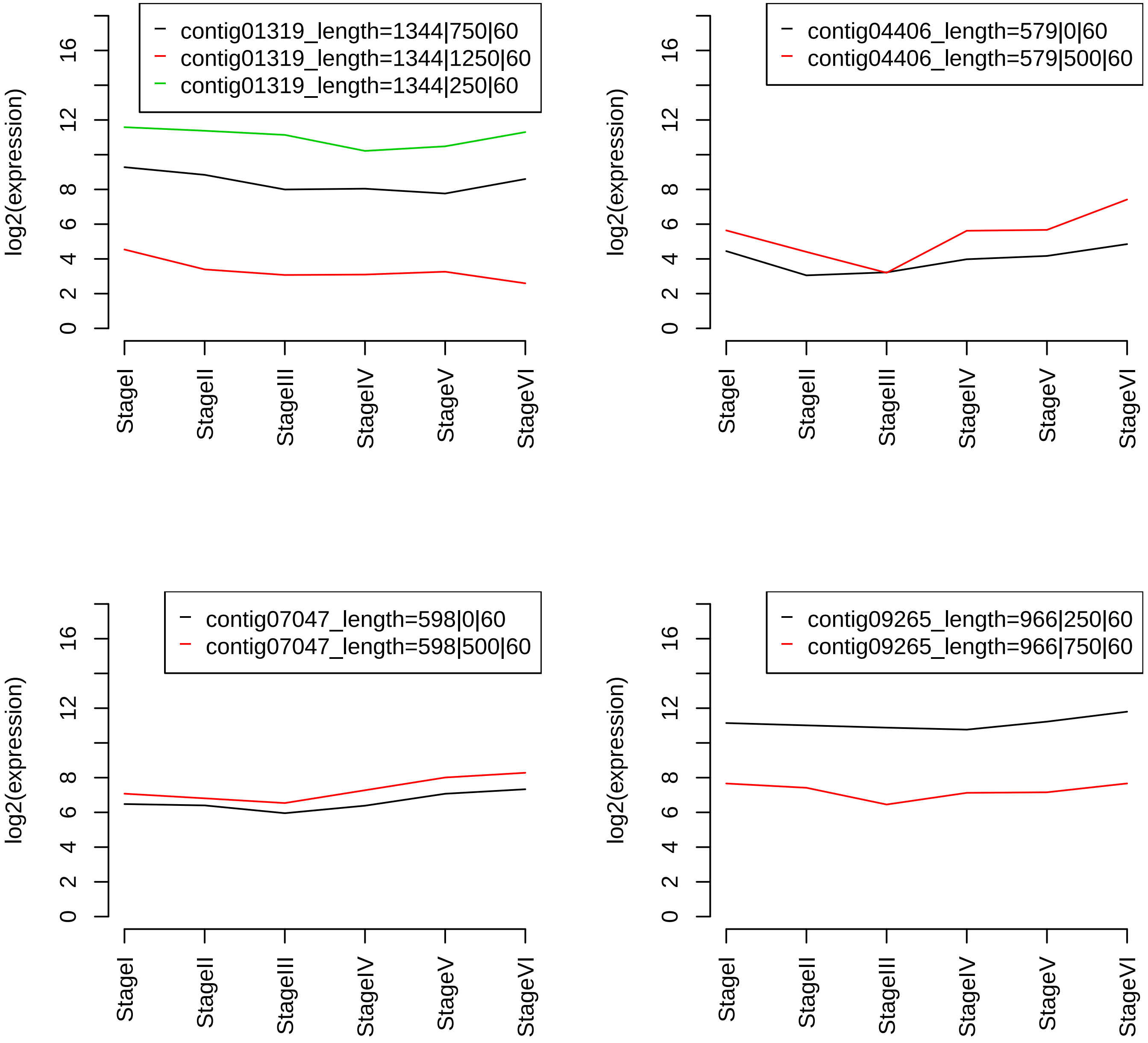
contig01319_length=1344|750|60
contig01319_length=1344|1250|60
contig01319_length=1344|250|60
contig04406_length=579|0|60
contig04406_length=579|500|60
contig07047_length=598|0|60
contig07047_length=598|500|60
contig09265_length=966|250|60
contig09265_length=966|750|60
log2(expression)
0
2
4
6
8
12
16
StageI
StageII
StageIII
StageIV
StageV
StageVI

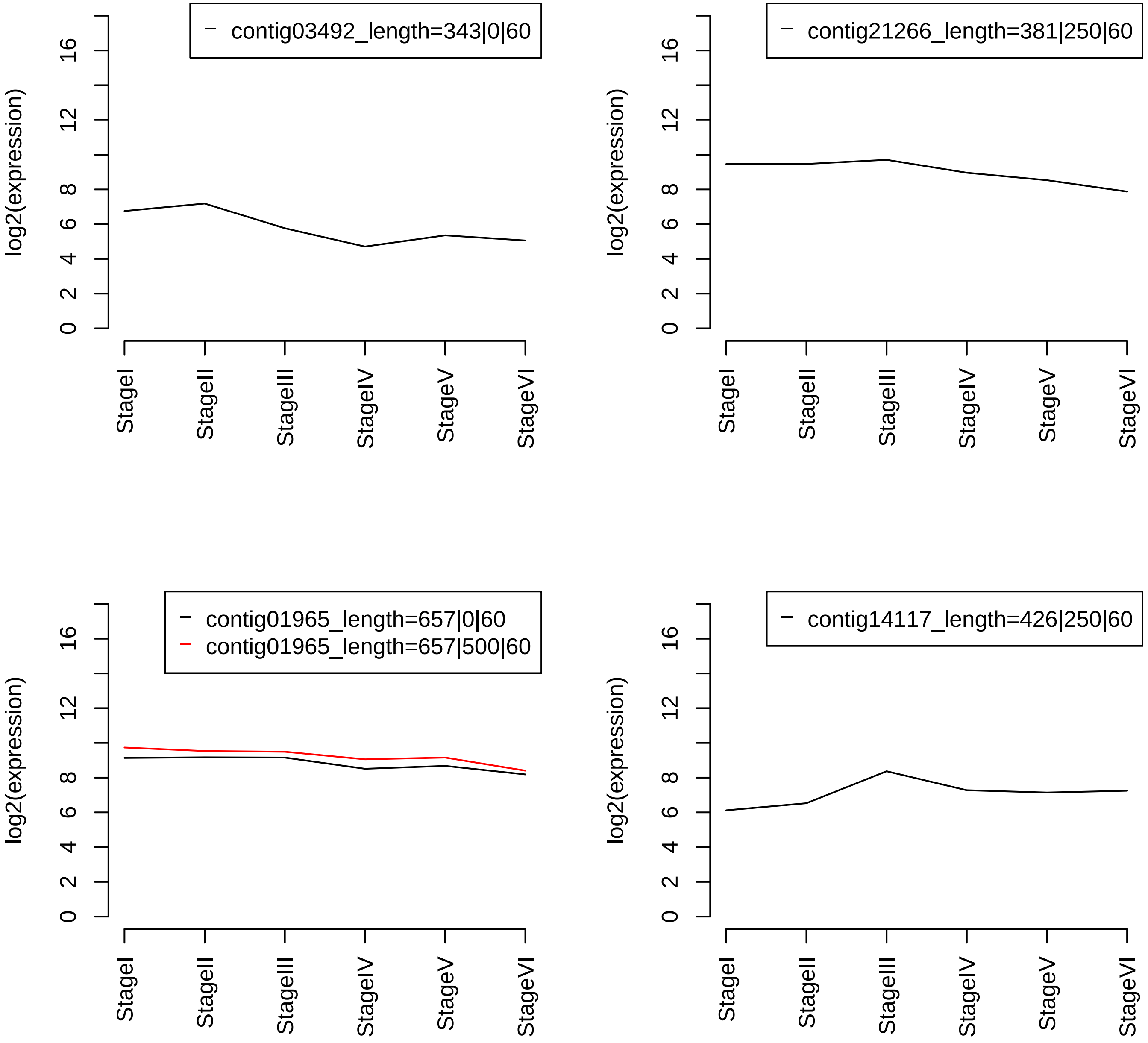

contig03492_length=343|0|60
contig21266_length=381|250|60
contig01965_length=657|0|60
contig01965_length=657|500|60
contig14117_length=426|250|60
log2(expression)
StageI
StageII
StageIII
StageIV
StageV
StageVI

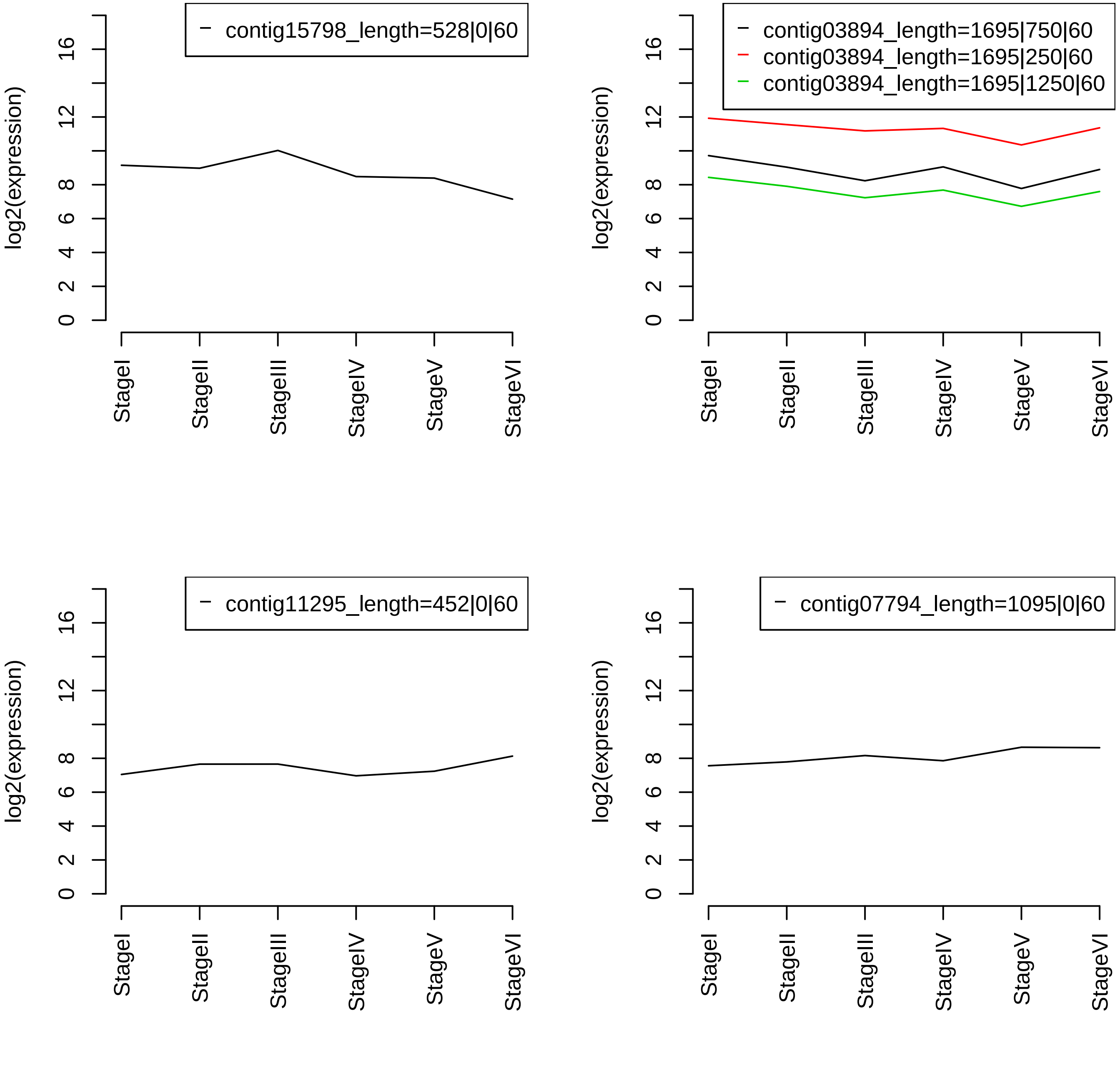
contig15798_length=528|0|60
contig03894_length=1695|750|60
contig03894_length=1695|250|60
contig03894_length=1695|1250|60
contig11295_length=452|0|60
contig07794_length=1095|0|60
log2(expression)
0
2
4
6
8
12
16
StageI
StageII
StageIII
StageIV
StageV
StageVI

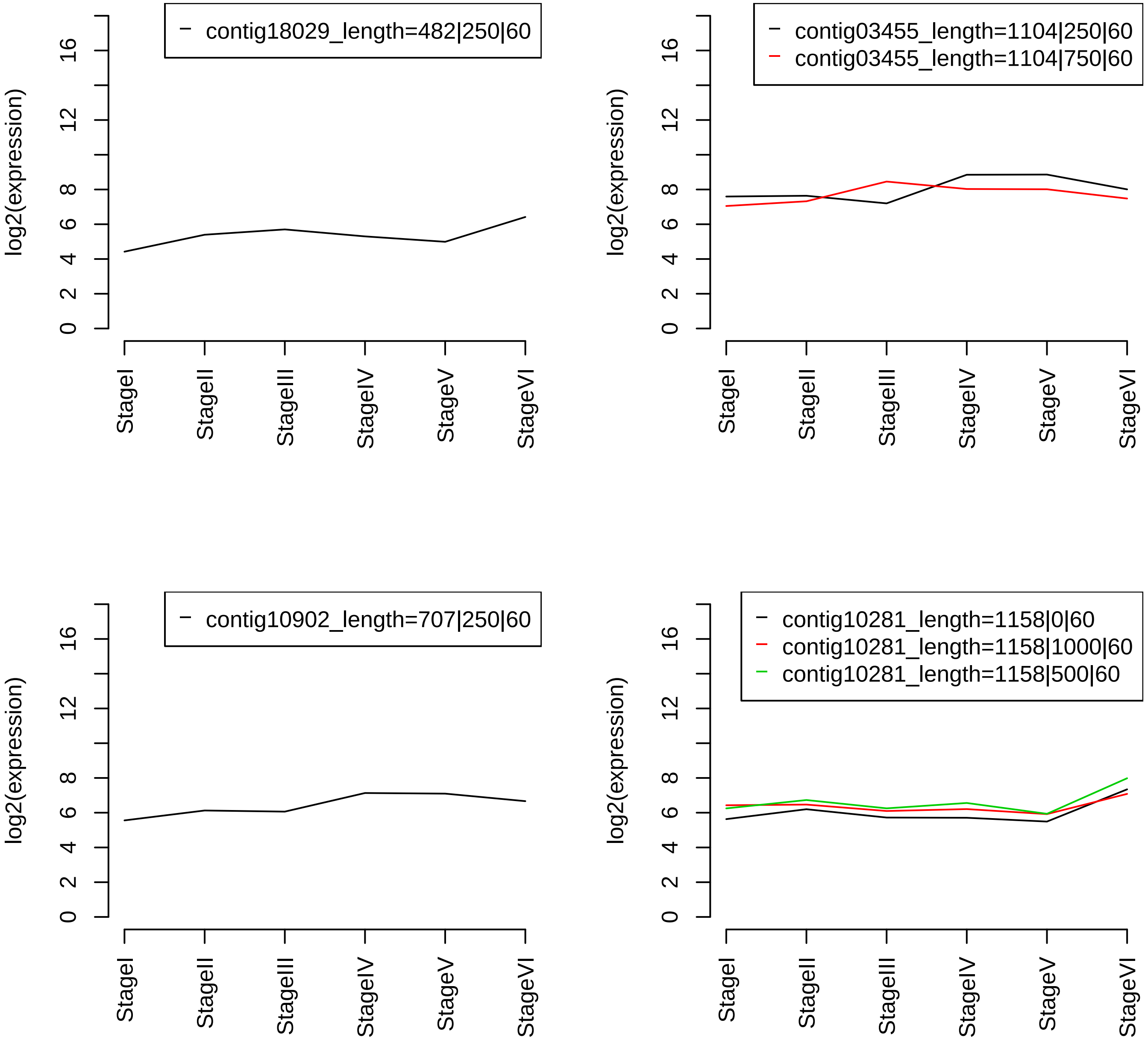

contig18029_length=482|250|60
contig03455_length=1104|250|60
contig03455_length=1104|750|60
contig10902_length=707|250|60
contig10281_length=1158|0|60
contig10281_length=1158|1000|60
contig10281_length=1158|500|60
log2(expression)
0
2
4
6
8
12
16
StageI
StageII
StageIII
StageIV
StageV
StageVI

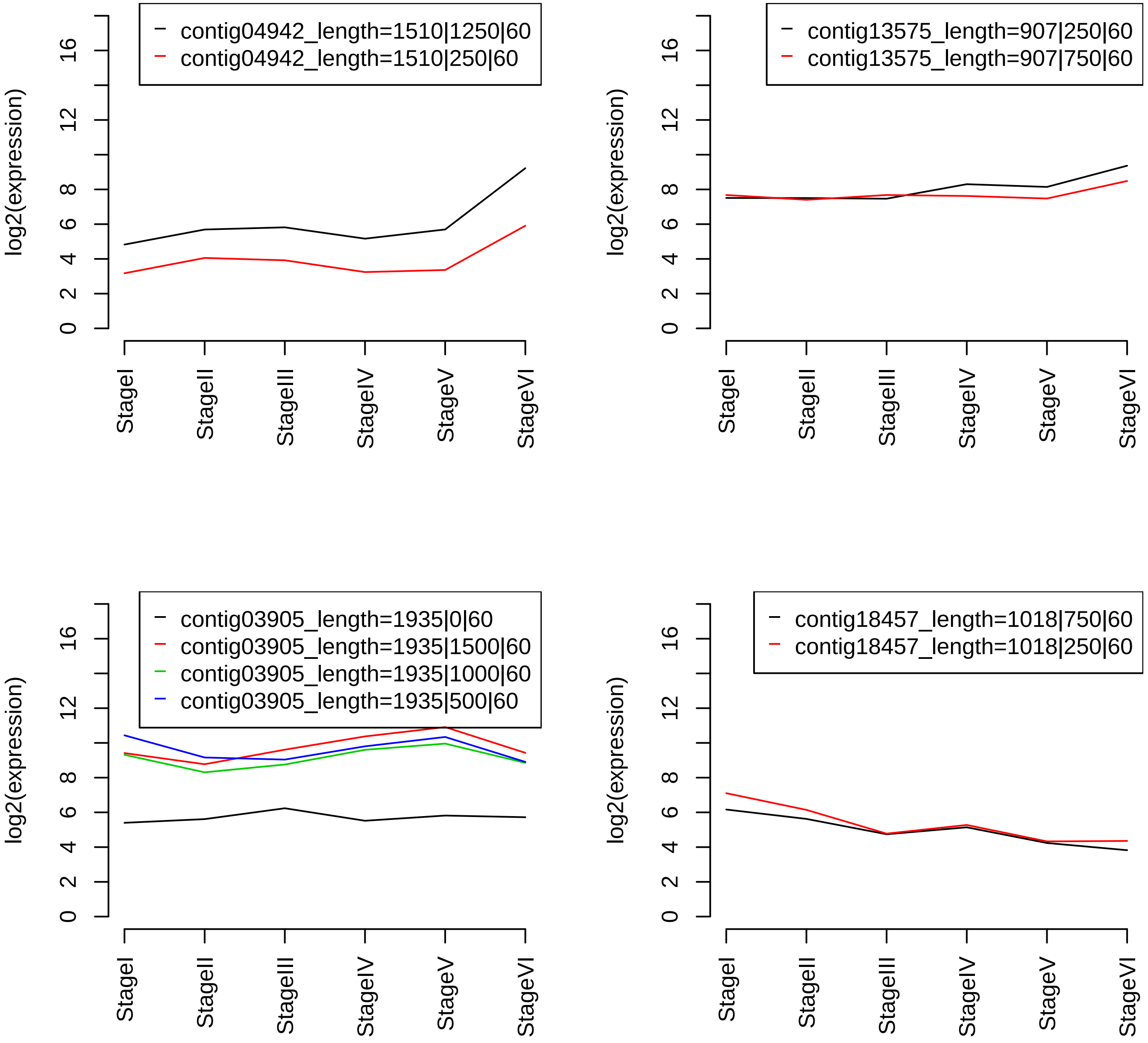

contig04942_length=1510|1250|60
contig04942_length=1510|250|60
contig13575_length=907|250|60
contig13575_length=907|750|60
contig03905_length=1935|0|60
contig03905_length=1935|1500|60
contig03905_length=1935|1000|60
contig03905_length=1935|500|60
contig18457_length=1018|750|60
contig18457_length=1018|250|60
log2(expression)
0
2
4
6
8
12
16
StageI
StageII
StageIII
StageIV
StageV
StageVI

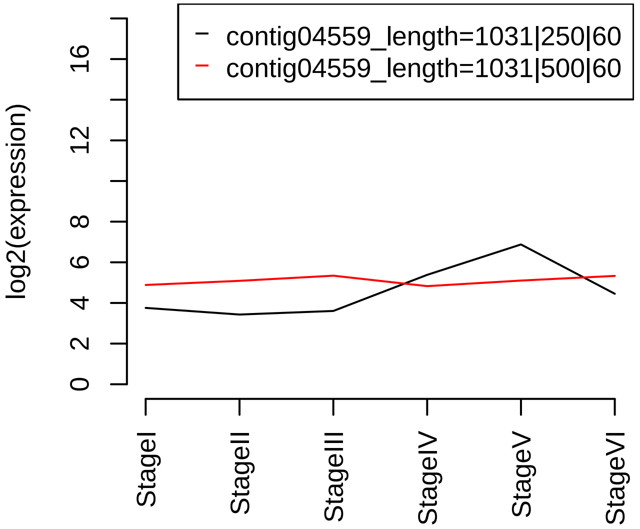
contig04559_length=1031|250|60
contig04559_length=1031|500|60
log2(expression)
0
2
4
6
8
12
16
StageI
StageII
StageIII
StageIV
StageV
StageVI

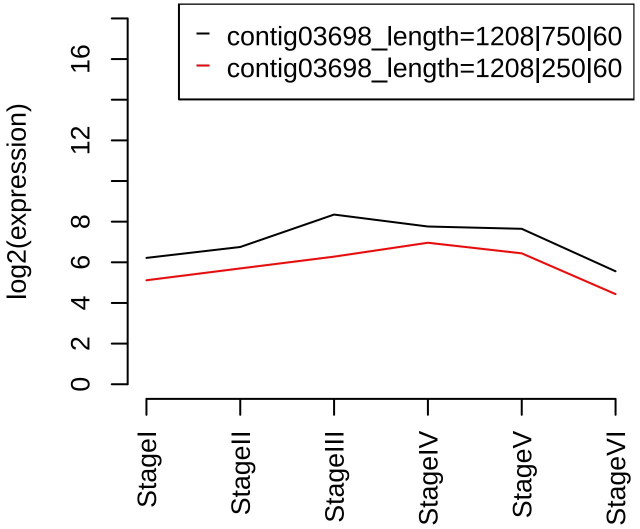
contig03698_length=1208|750|60
contig03698_length=1208|250|60
log2(expression)
0
2
4
6
8
12
16
StageI
StageII
StageIII
StageIV
StageV
StageVI

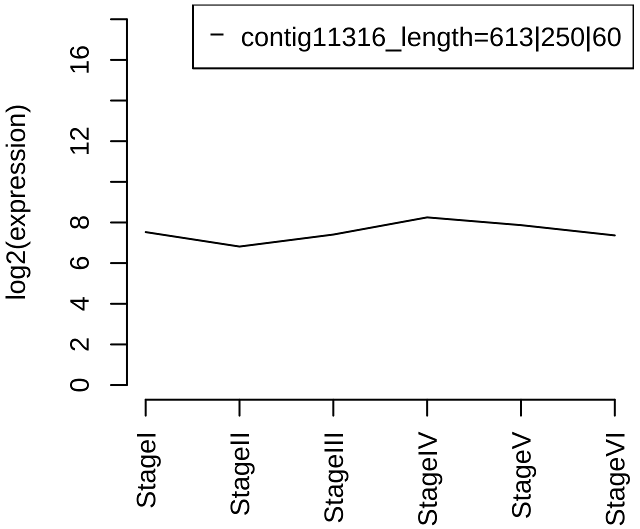
contig11316_length=613|250|60
log2(expression)
0
2
4
6
8
12
16
StageI
StageII
StageIII
StageIV
StageV
StageVI

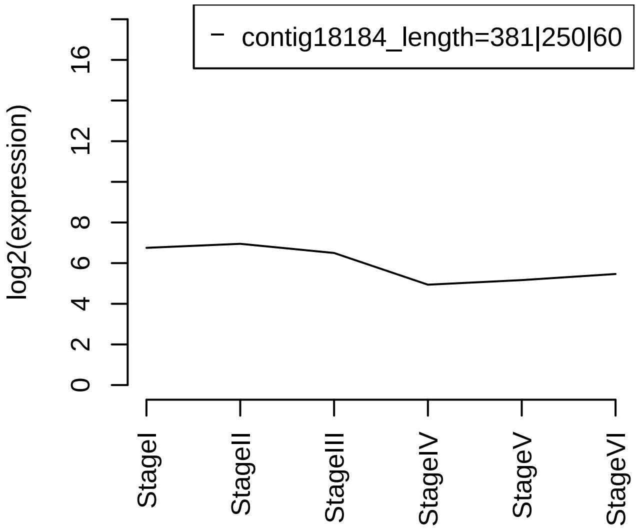
contig18184_length=381|250|60
log2(expression)
0
2
4
6
8
12
16
StageI
StageII
StageIII
StageIV
StageV
StageVI

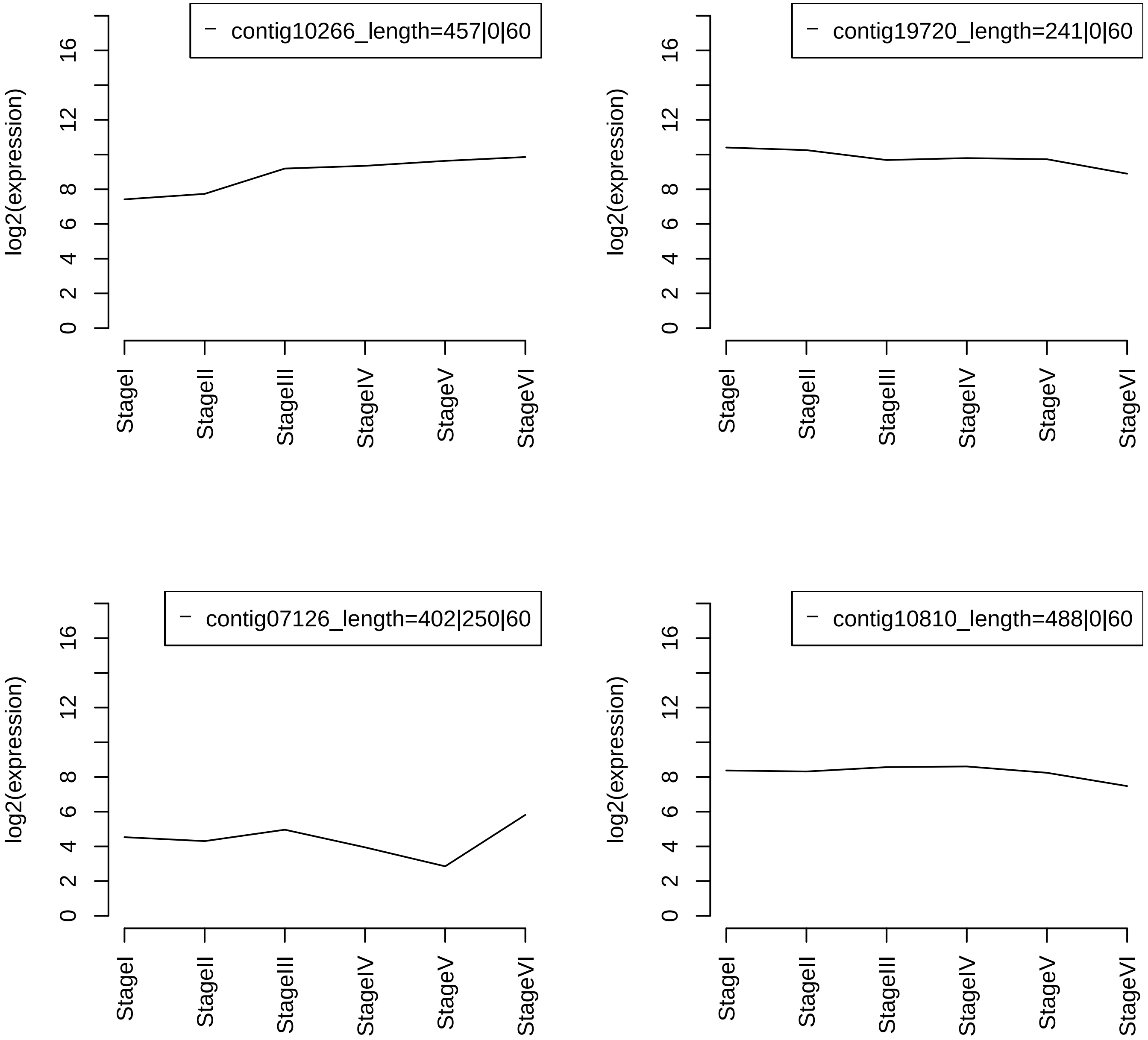

contig10266_length=457|0|60
log2(expression)
0
2
4
6
8
12
16
StageI
StageII
StageIII
StageIV
StageV
StageVI
contig19720_length=241|0|60
log2(expression)
0
2
4
6
8
12
16
StageI
StageII
StageIII
StageIV
StageV
StageVI
contig07126_length=402|250|60
log2(expression)
0
2
4
6
8
12
16
StageI
StageII
StageIII
StageIV
StageV
StageVI
contig10810_length=488|0|60
log2(expression)
0
2
4
6
8
12
16
StageI
StageII
StageIII
StageIV
StageV
StageVI

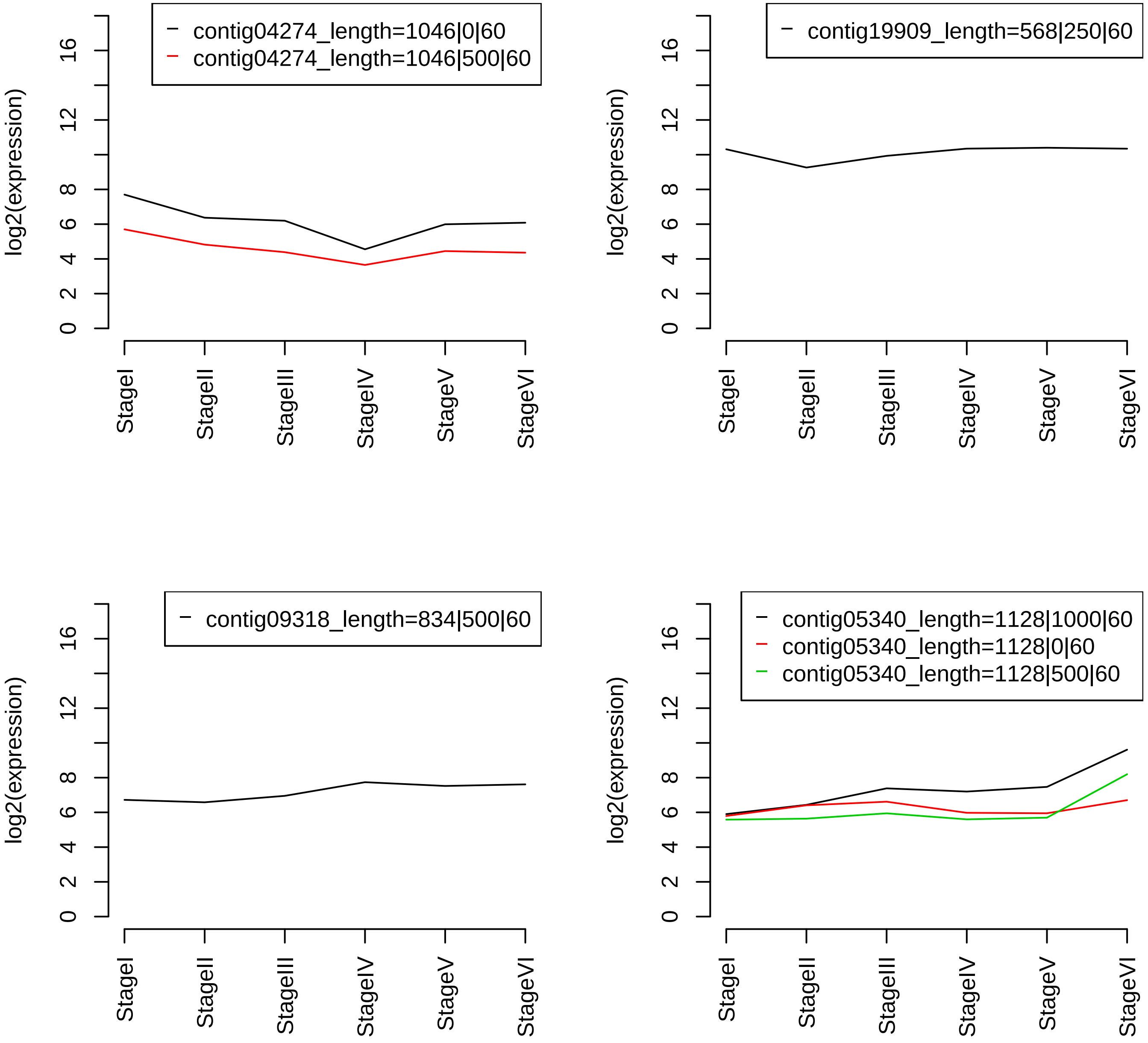

contig04274_length=1046|0|60
contig04274_length=1046|500|60
log2(expression)
0 2 4 6 8 12 16
StageI StageII StageIII StageIV StageV StageVI
contig19909_length=568|250|60
log2(expression)
0 2 4 6 8 12 16
StageI StageII StageIII StageIV StageV StageVI
contig09318_length=834|500|60
log2(expression)
0 2 4 6 8 12 16
StageI StageII StageIII StageIV StageV StageVI
contig05340_length=1128|1000|60
contig05340_length=1128|0|60
contig05340_length=1128|500|60
log2(expression)
0 2 4 6 8 12 16
StageI StageII StageIII StageIV StageV StageVI

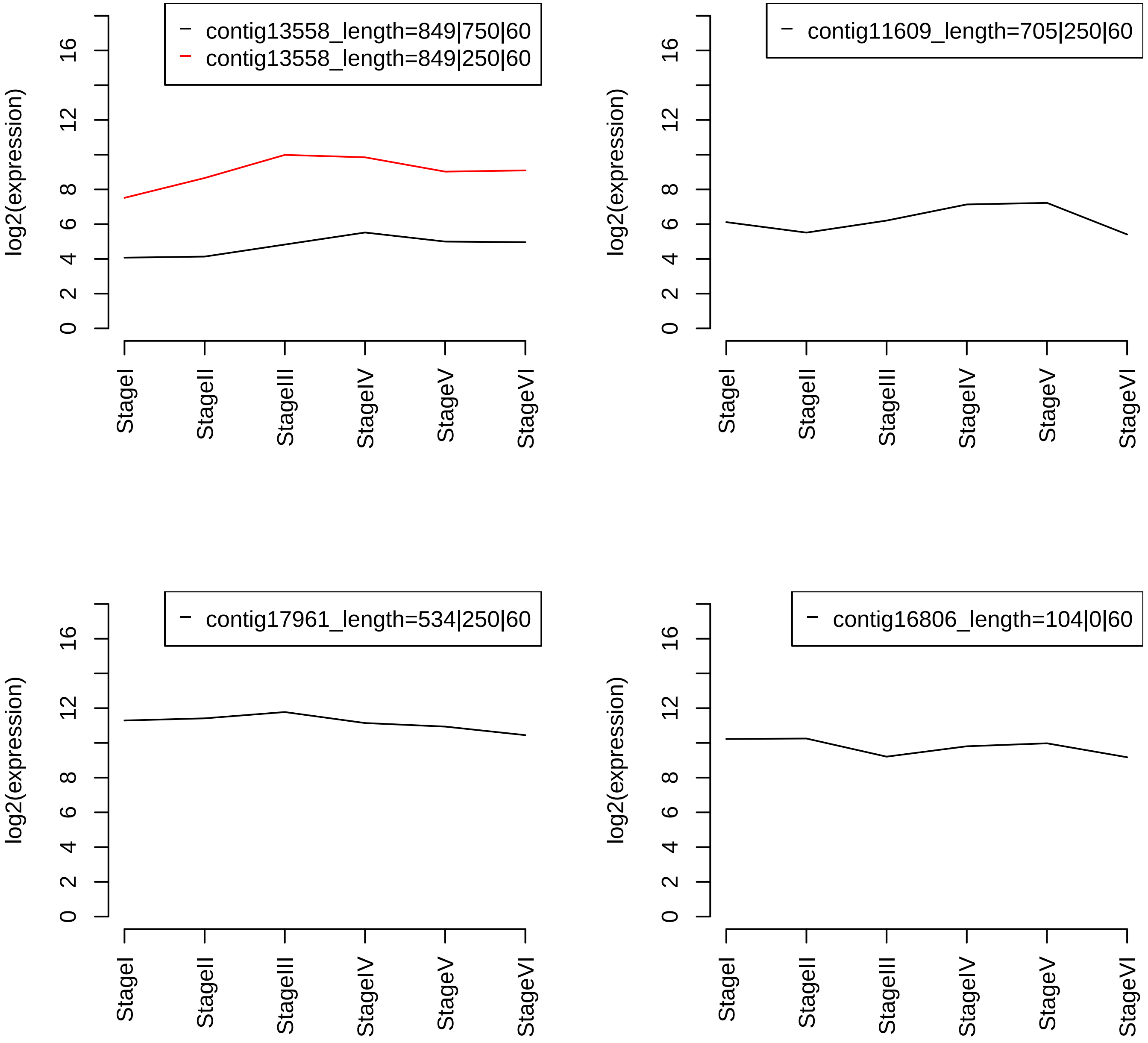

contig13558_length=849|750|60
contig13558_length=849|250|60
contig11609_length=705|250|60
contig17961_length=534|250|60
contig16806_length=104|0|60
log2(expression)
0
2
4
6
8
12
16
StageI
StageII
StageIII
StageIV
StageV
StageVI

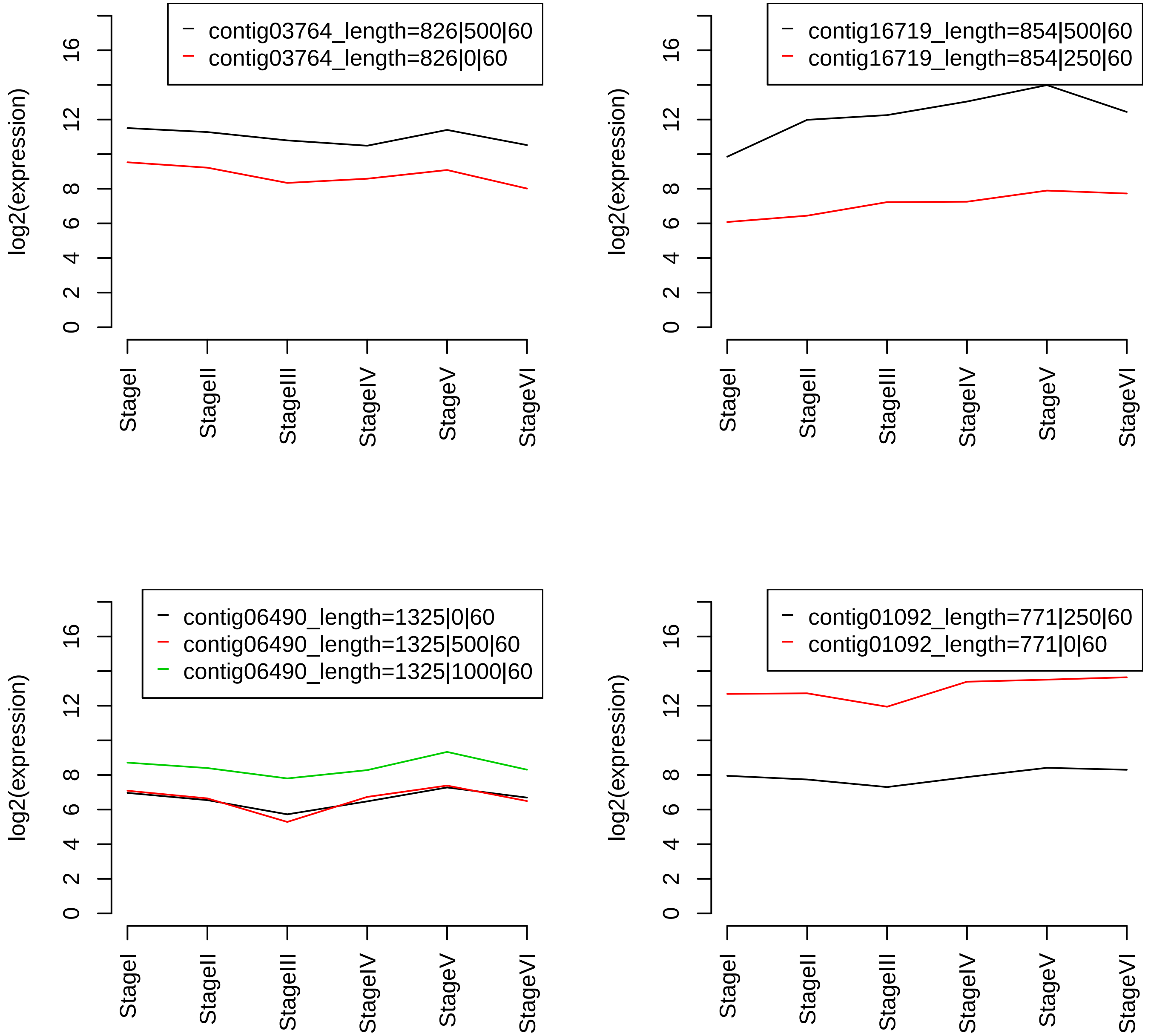
contig03764_length=826|500|60
contig03764_length=826|0|60
log2(expression)
0
2
4
6
8
12
16
StageI
StageII
StageIII
StageIV
StageV
StageVI
contig16719_length=854|500|60
contig16719_length=854|250|60
contig06490_length=1325|0|60
contig06490_length=1325|500|60
contig06490_length=1325|1000|60
contig01092_length=771|250|60
contig01092_length=771|0|60

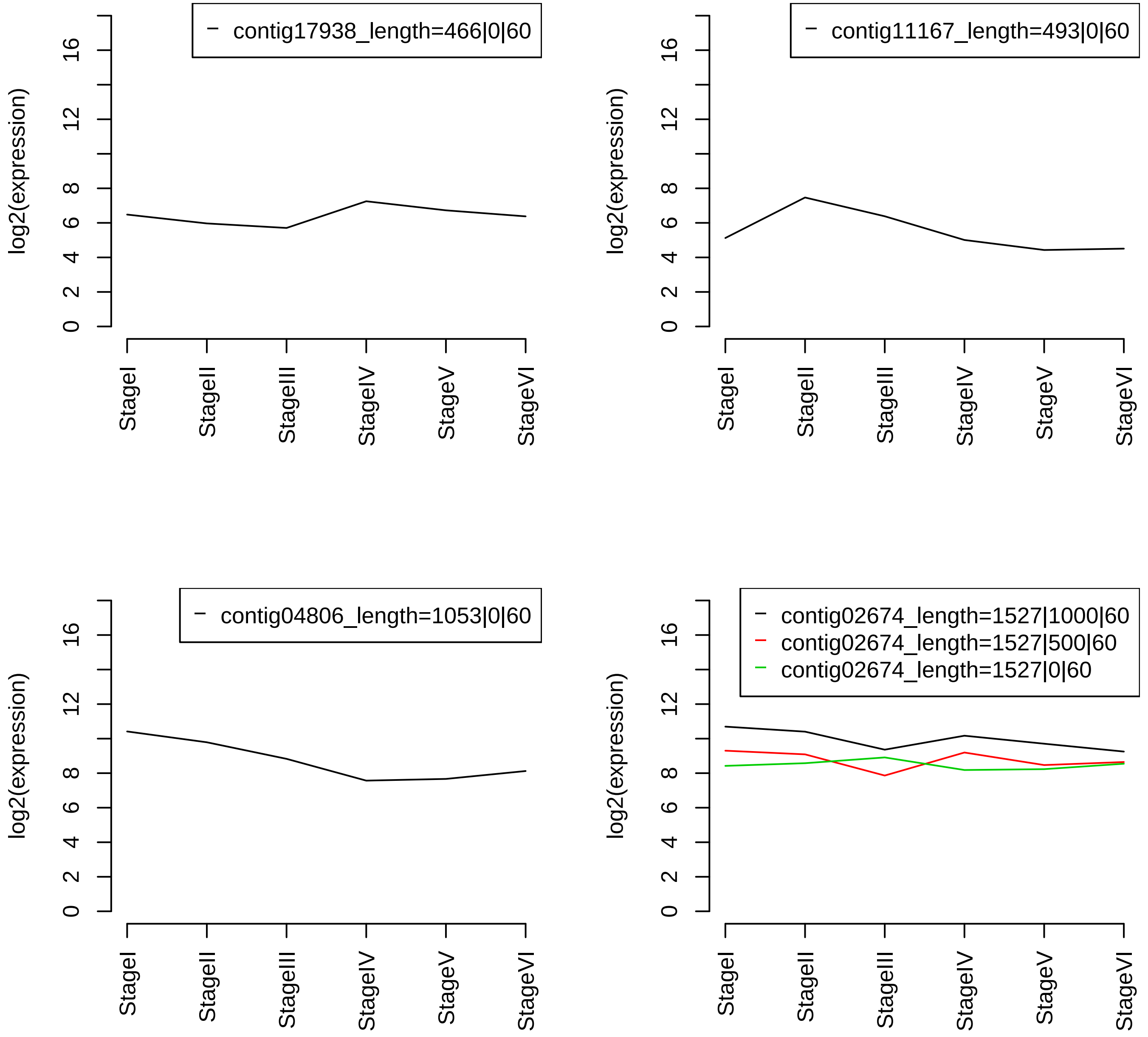
contig17938_length=466|0|60
contig11167_length=493|0|60
contig04806_length=1053|0|60
contig02674_length=1527|1000|60
contig02674_length=1527|500|60
contig02674_length=1527|0|60
log2(expression)
0
2
4
6
8
12
16
StageI
StageII
StageIII
StageIV
StageV
StageVI

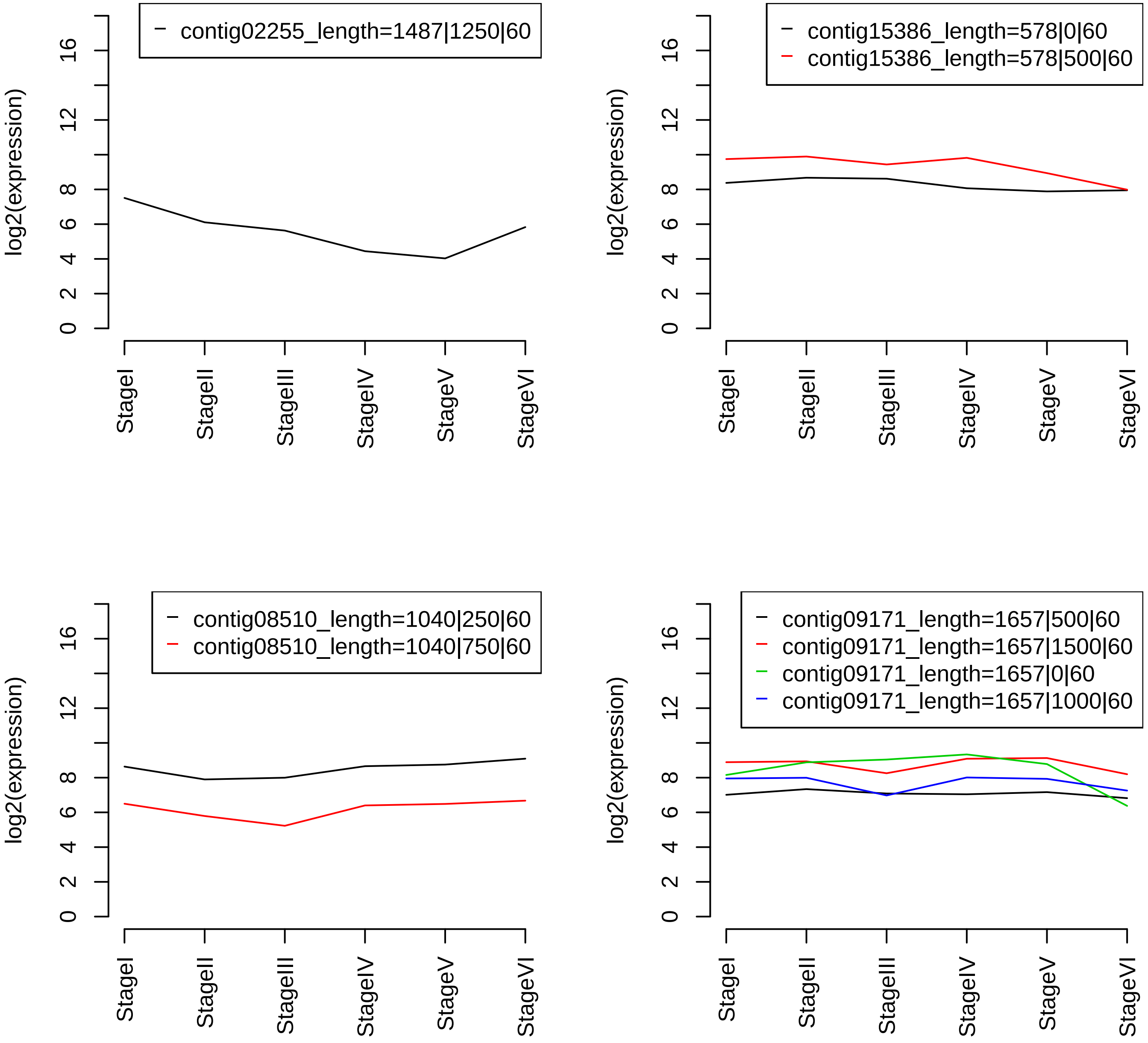
contig02255_length=1487|1250|60
contig15386_length=578|0|60
contig15386_length=578|500|60
contig08510_length=1040|250|60
contig08510_length=1040|750|60
contig09171_length=1657|500|60
contig09171_length=1657|1500|60
contig09171_length=1657|0|60
contig09171_length=1657|1000|60
log2(expression)
0
2
4
6
8
12
16
StageI
StageII
StageIII
StageIV
StageV
StageVI

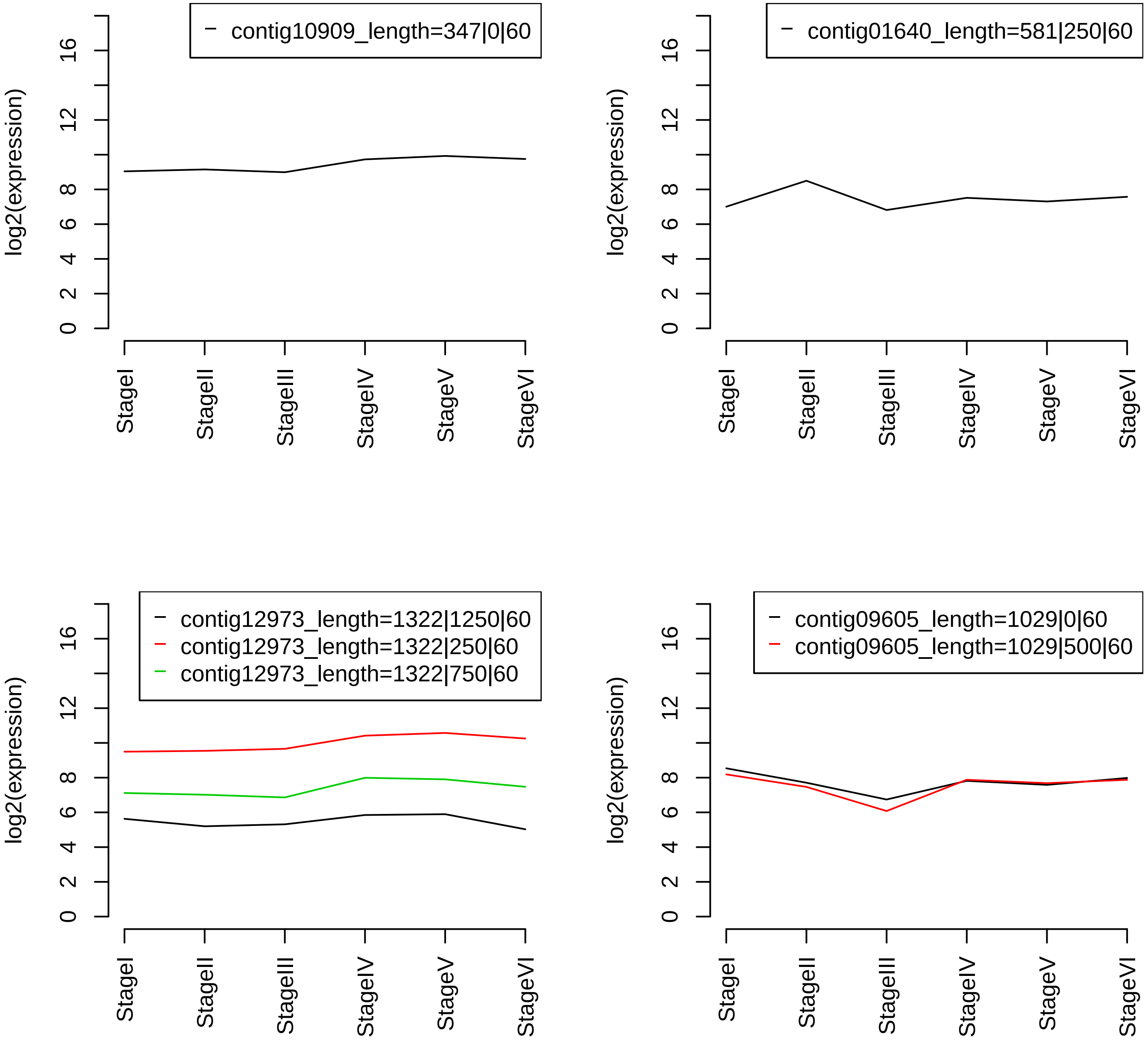

contig10909_length=347|0|60
contig01640_length=581|250|60
contig12973_length=1322|1250|60
contig12973_length=1322|250|60
contig12973_length=1322|750|60
contig09605_length=1029|0|60
contig09605_length=1029|500|60
log2(expression)
0
2
4
6
8
12
16
StageI
StageII
StageIII
StageIV
StageV
StageVI

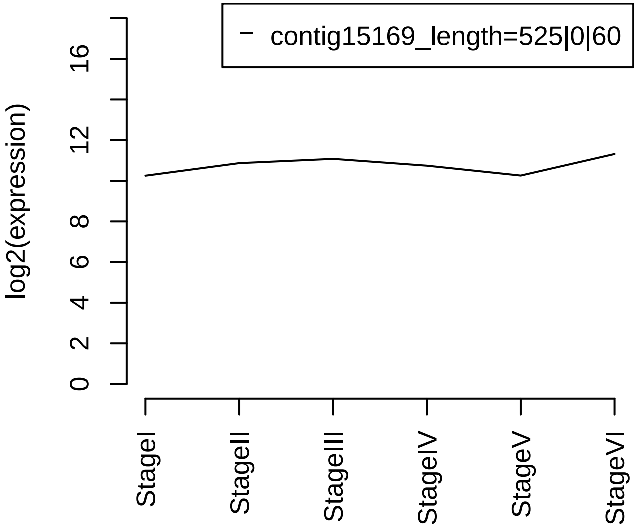

contig15169_length=525|0|60
log2(expression)
0 2 4 6 8 12 16
StageI StageII StageIII StageIV StageV StageVI

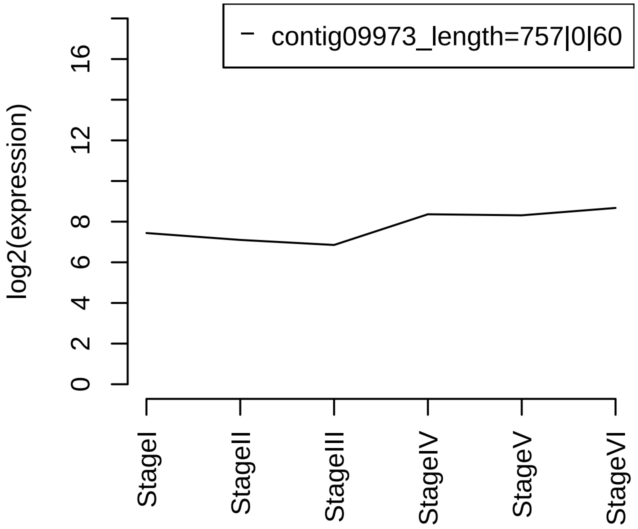

contig09973_length=757|0|60
log2(expression)
0 2 4 6 8 12 16
StageI StageII StageIII StageIV StageV StageVI

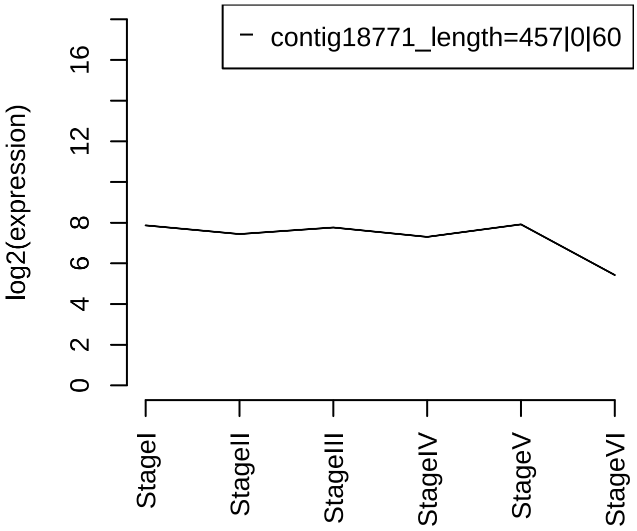

contig18771_length=457|0|60
log2(expression)
0 2 4 6 8 12 16
StageI StageII StageIII StageIV StageV StageVI

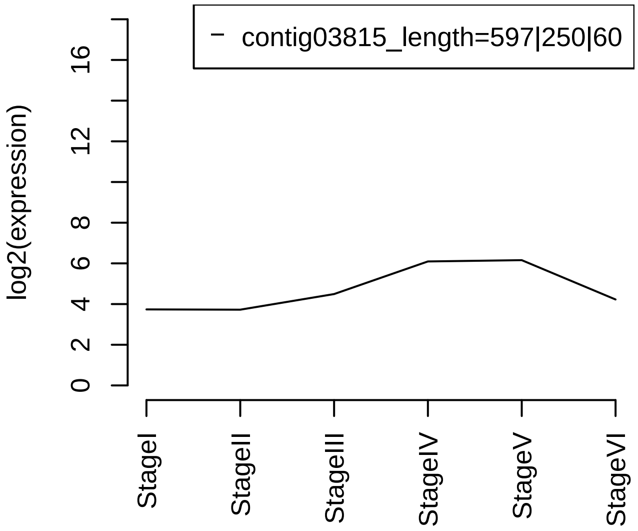

contig03815_length=597|250|60
log2(expression)
0 2 4 6 8 12 16
StageI StageII StageIII StageIV StageV StageVI

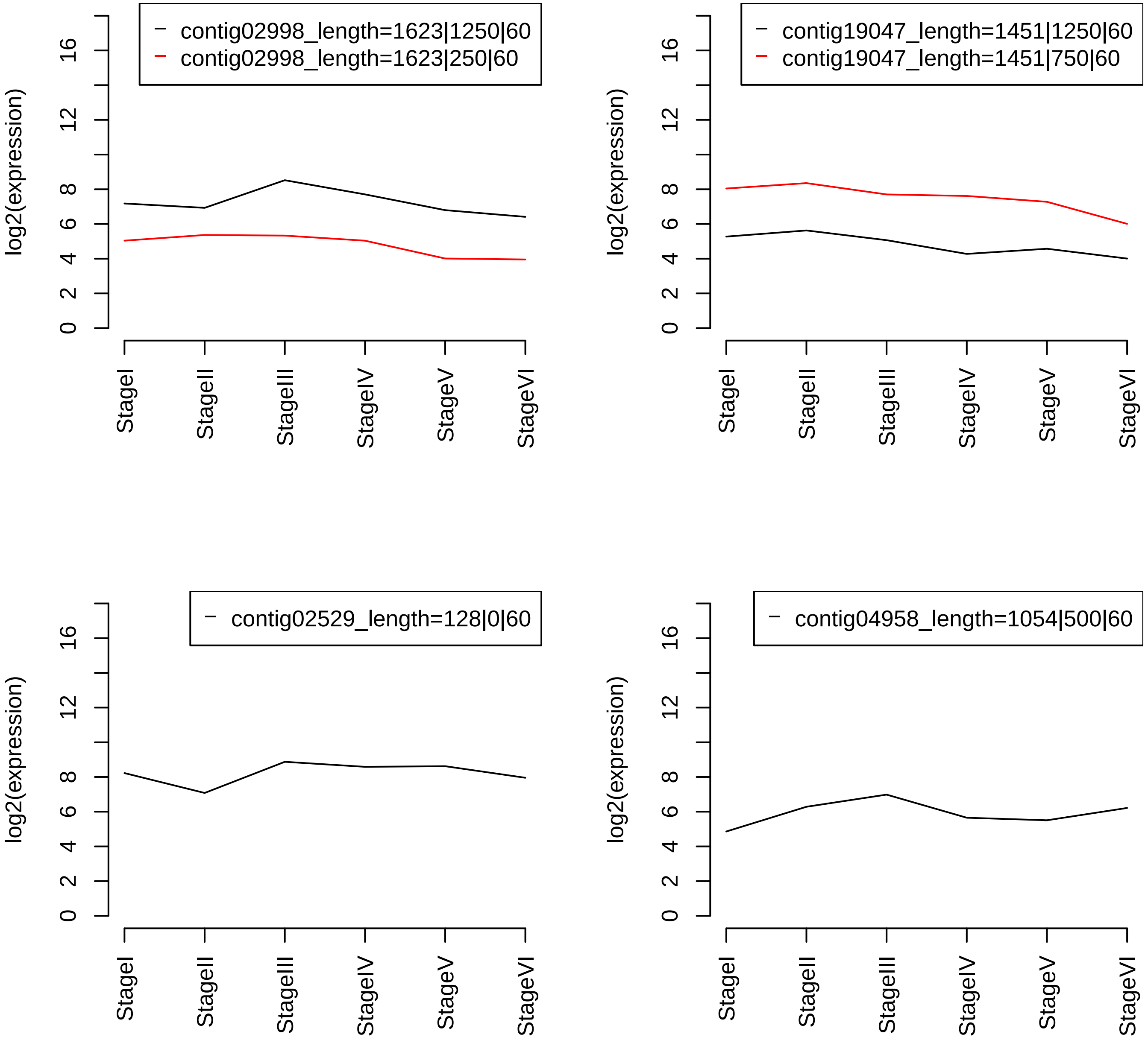
contig02998_length=1623|1250|60
contig02998_length=1623|250|60
contig19047_length=1451|1250|60
contig19047_length=1451|750|60
contig02529_length=128|0|60
contig04958_length=1054|500|60
log2(expression)
0
2
4
6
8
12
16
StageI
StageII
StageIII
StageIV
StageV
StageVI

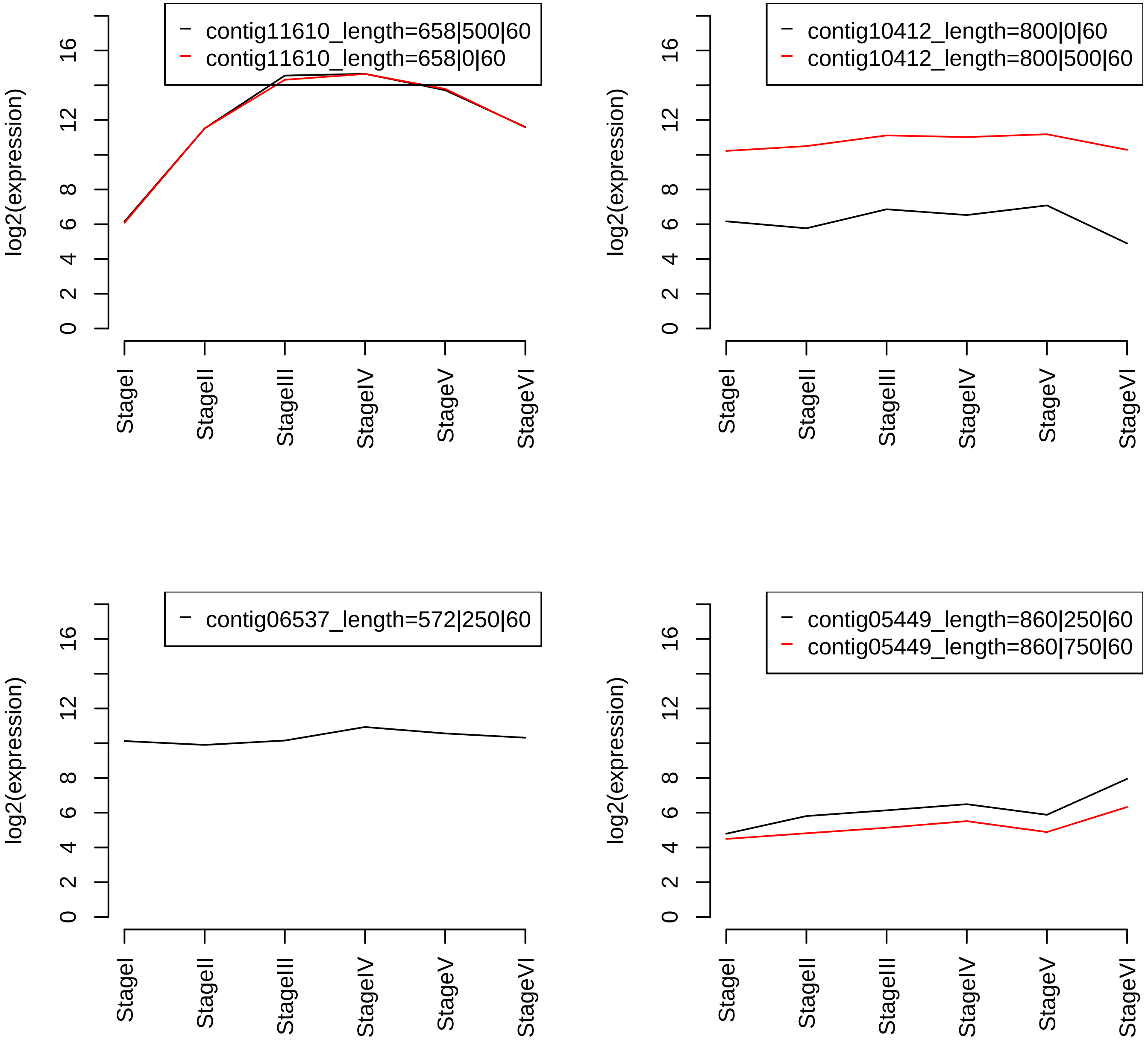

contig11610_length=658|500|60
contig11610_length=658|0|60
contig10412_length=800|0|60
contig10412_length=800|500|60
contig06537_length=572|250|60
contig05449_length=860|250|60
contig05449_length=860|750|60
log2(expression)
0
2
4
6
8
12
16
StageI
StageII
StageIII
StageIV
StageV
StageVI

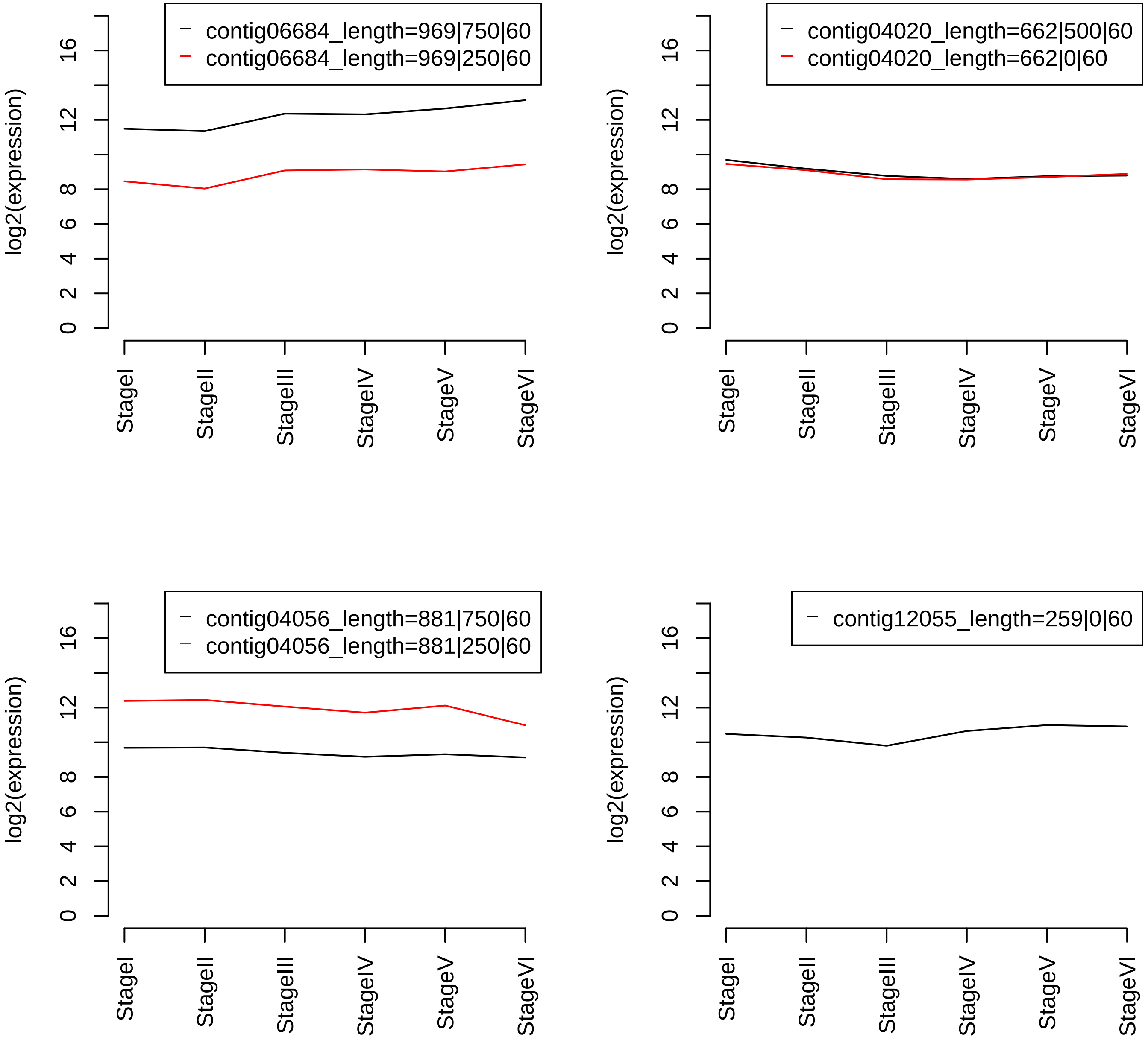
contig06684_length=969|750|60
contig06684_length=969|250|60
log2(expression)
0
2
4
6
8
12
16
StageI
StageII
StageIII
StageIV
StageV
StageVI
contig04020_length=662|500|60
contig04020_length=662|0|60
log2(expression)
0
2
4
6
8
12
16
StageI
StageII
StageIII
StageIV
StageV
StageVI
contig04056_length=881|750|60
contig04056_length=881|250|60
log2(expression)
0
2
4
6
8
12
16
StageI
StageII
StageIII
StageIV
StageV
StageVI
contig12055_length=259|0|60
log2(expression)
0
2
4
6
8
12
16
StageI
StageII
StageIII
StageIV
StageV
StageVI

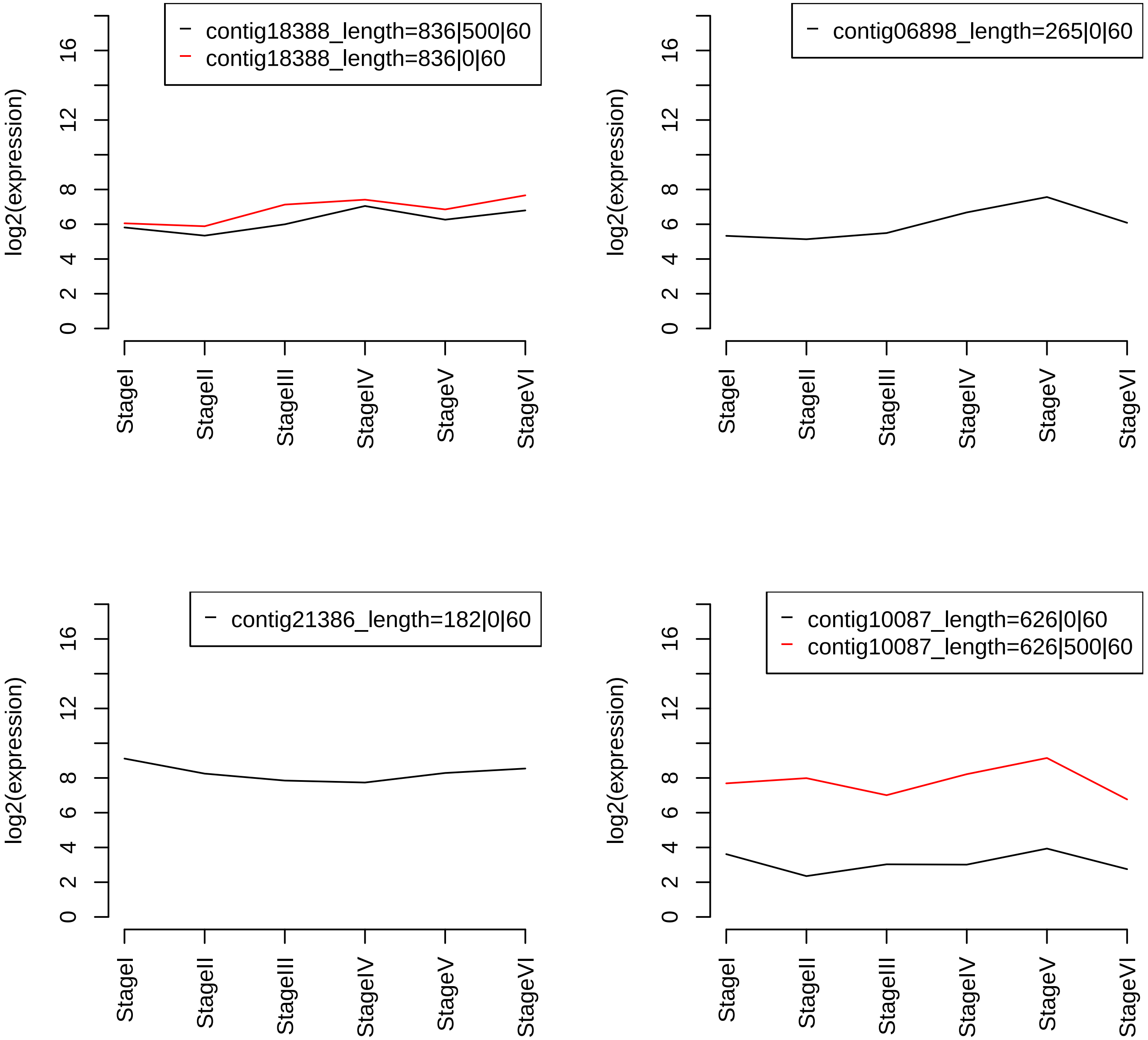
contig18388_length=836|500|60
contig18388_length=836|0|60
contig06898_length=265|0|60
contig21386_length=182|0|60
contig10087_length=626|0|60
contig10087_length=626|500|60
log2(expression)
0
2
4
6
8
12
16
StageI
StageII
StageIII
StageIV
StageV
StageVI

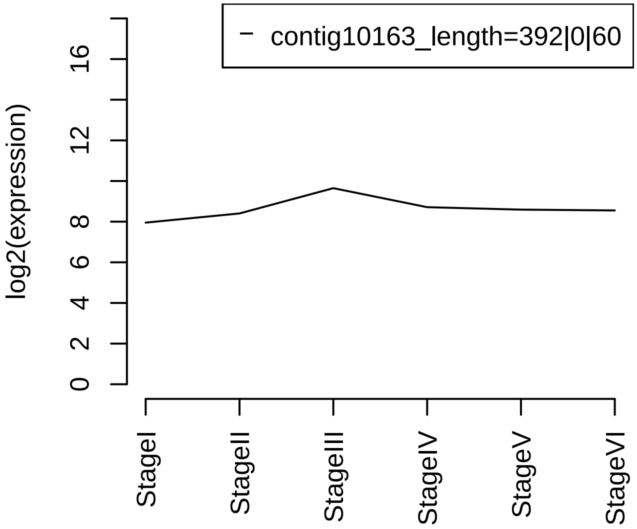

contig10163_length=392|0|60
log2(expression)
0
2
4
6
8
12
16
StageI
StageII
StageIII
StageIV
StageV
StageVI

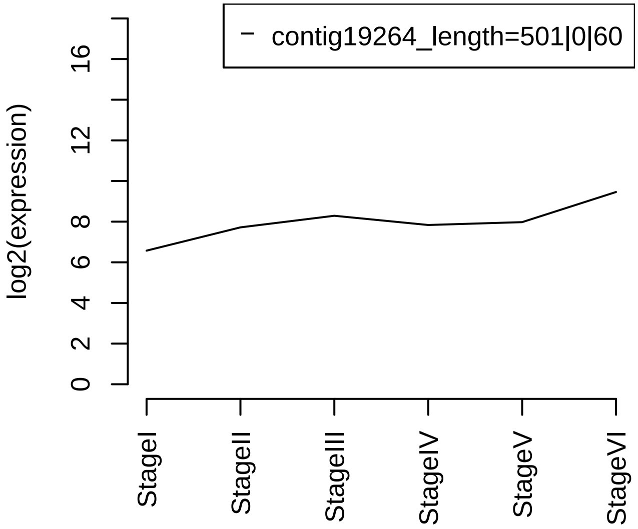

contig19264_length=501|0|60
log2(expression)
0
2
4
6
8
12
16
StageI
StageII
StageIII
StageIV
StageV
StageVI

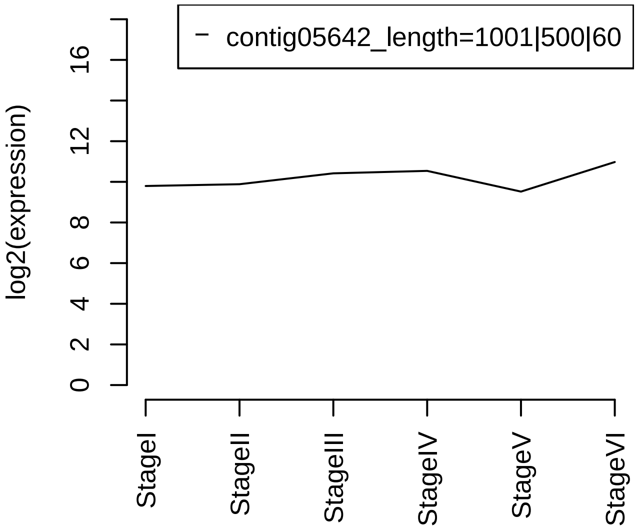

contig05642_length=1001|500|60
log2(expression)
0
2
4
6
8
12
16
StageI
StageII
StageIII
StageIV
StageV
StageVI

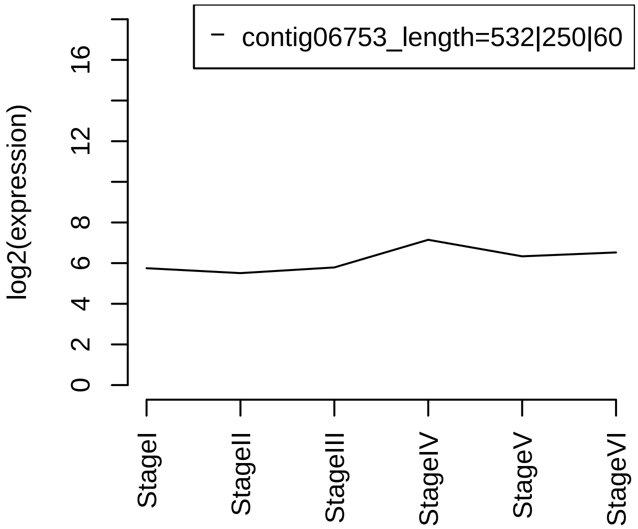

contig06753_length=532|250|60
log2(expression)
0
2
4
6
8
12
16
StageI
StageII
StageIII
StageIV
StageV
StageVI

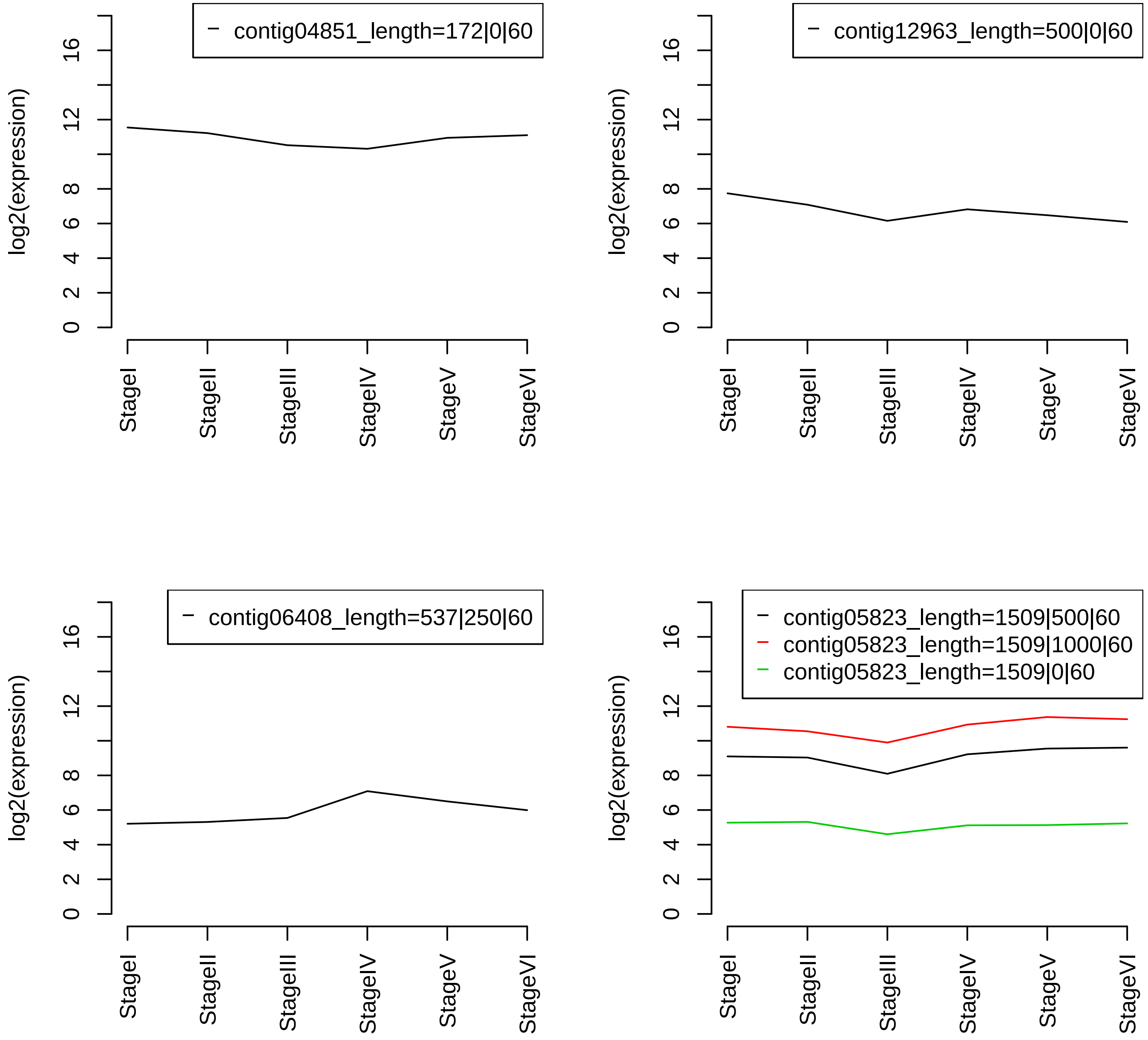
contig04851_length=172|0|60
contig12963_length=500|0|60
contig06408_length=537|250|60
contig05823_length=1509|500|60
contig05823_length=1509|1000|60
contig05823_length=1509|0|60
log2(expression)
0
2
4
6
8
12
16
StageI
StageII
StageIII
StageIV
StageV
StageVI

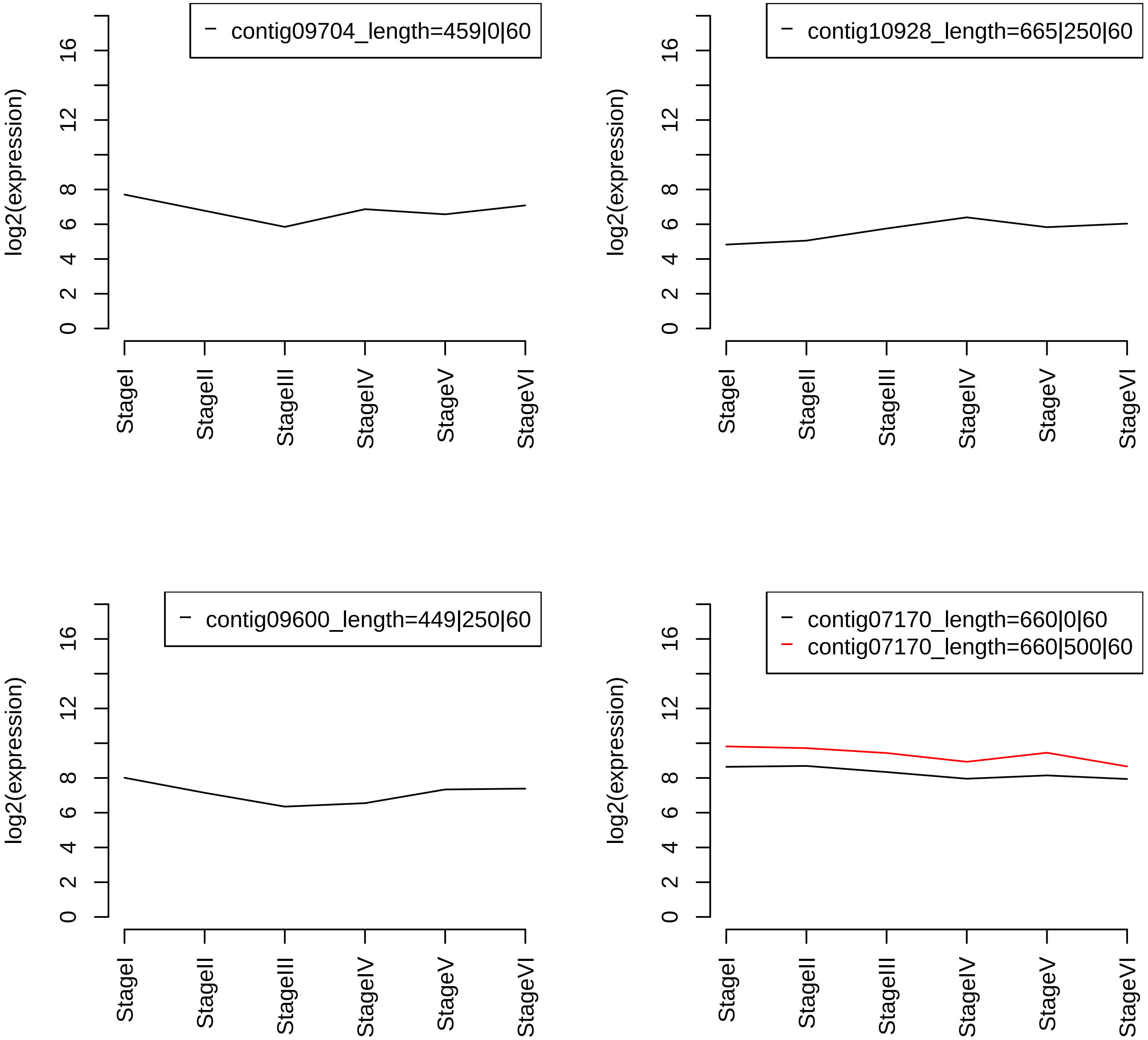

contig09704_length=459|0|60
contig10928_length=665|250|60
contig09600_length=449|250|60
contig07170_length=660|0|60
contig07170_length=660|500|60
log2(expression)
0
2
4
6
8
12
16
StageI
StageII
StageIII
StageIV
StageV
StageVI

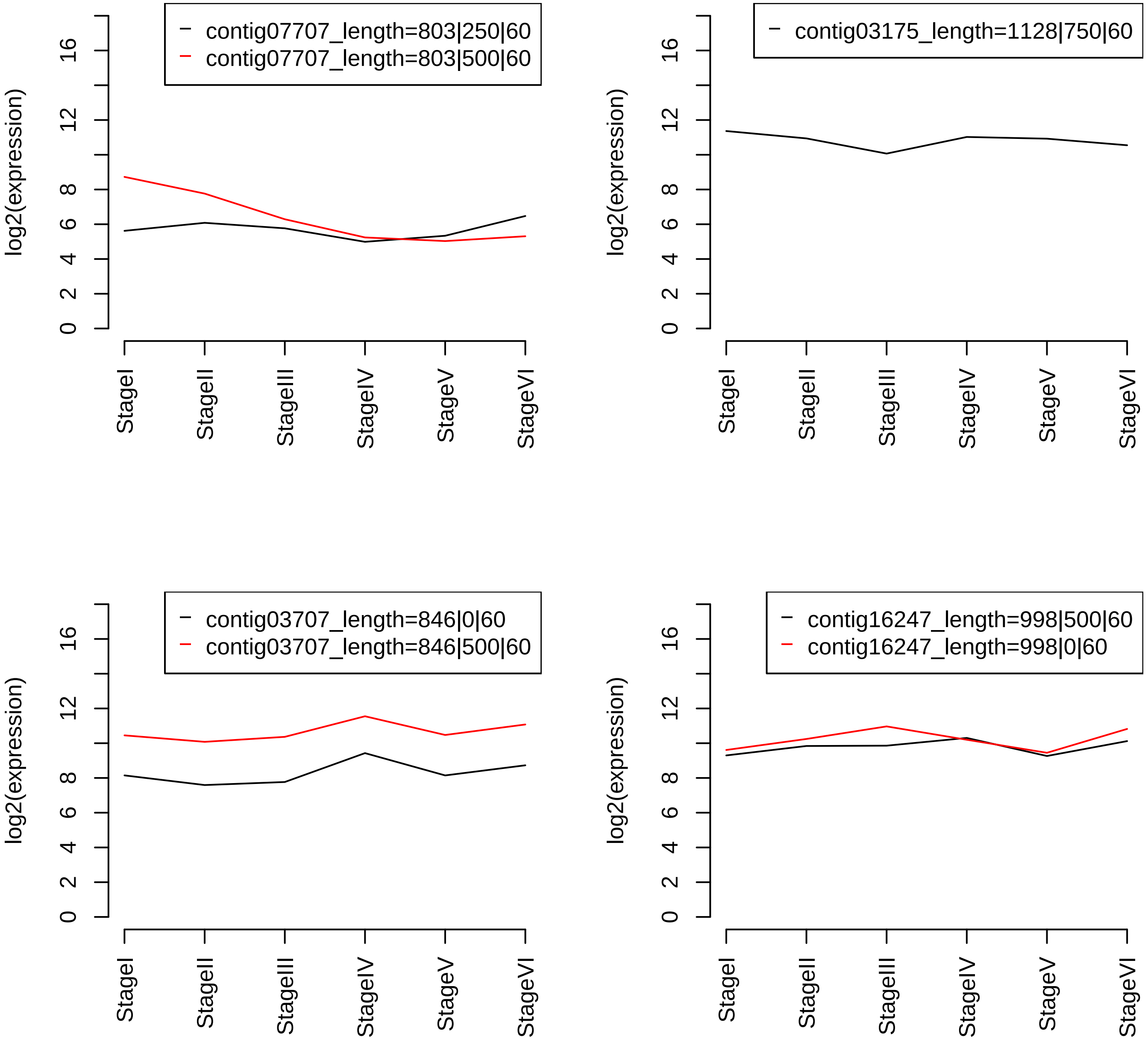
contig07707_length=803|250|60
contig07707_length=803|500|60
log2(expression)
0
2
4
6
8
12
16
StageI
StageII
StageIII
StageIV
StageV
StageVI
contig03175_length=1128|750|60
log2(expression)
contig03707_length=846|0|60
contig03707_length=846|500|60
log2(expression)
contig16247_length=998|500|60
contig16247_length=998|0|60
log2(expression)

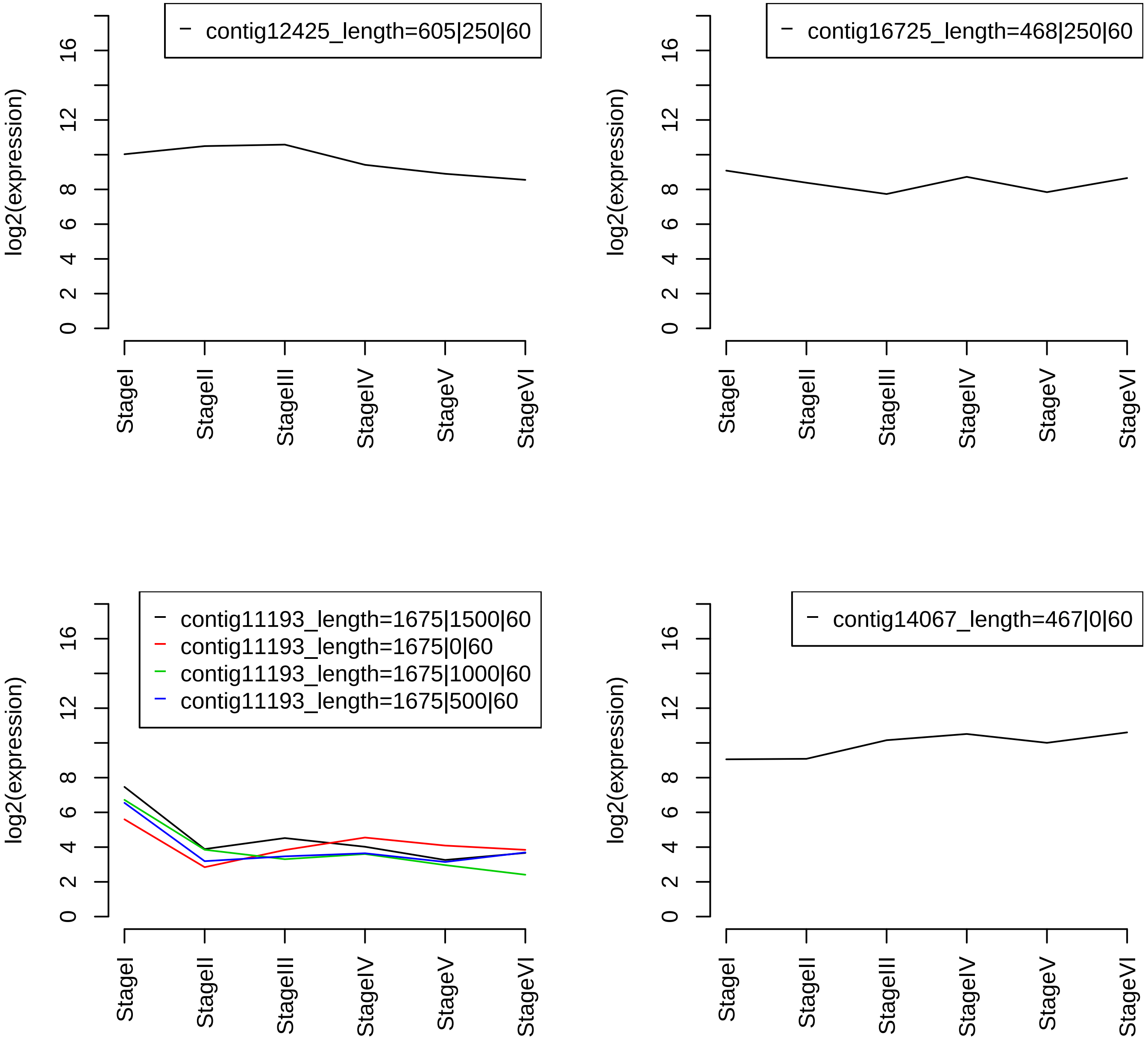
contig12425_length=605|250|60
contig16725_length=468|250|60
contig11193_length=1675|1500|60
contig11193_length=1675|0|60
contig11193_length=1675|1000|60
contig11193_length=1675|500|60
contig14067_length=467|0|60
log2(expression)
0
2
4
6
8
12
16
StageI
StageII
StageIII
StageIV
StageV
StageVI

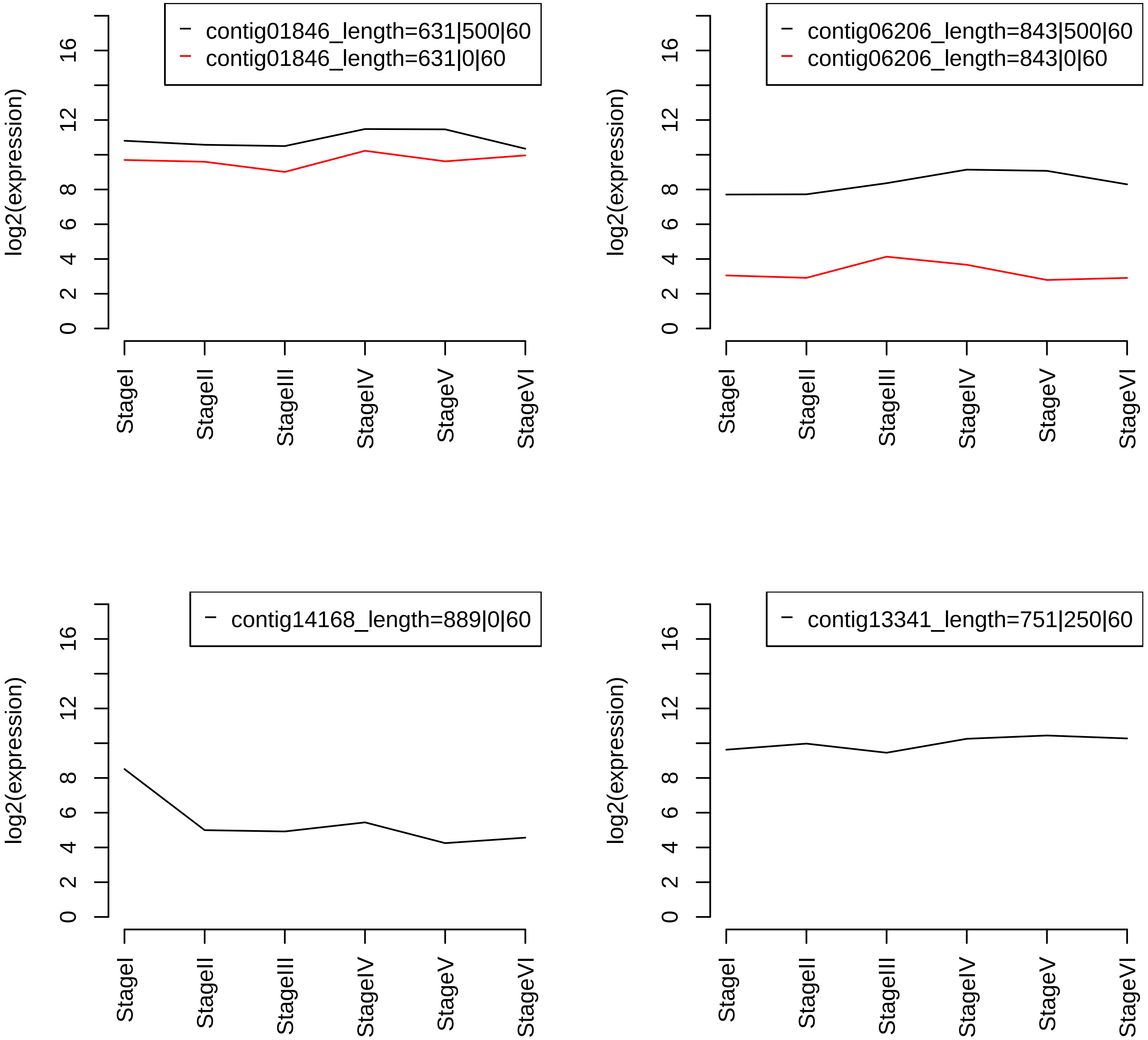

contig01846_length=631|500|60
contig01846_length=631|0|60
log2(expression)
0
2
4
6
8
12
16
StageI
StageII
StageIII
StageIV
StageV
StageVI
contig06206_length=843|500|60
contig06206_length=843|0|60
log2(expression)
contig14168_length=889|0|60
log2(expression)
contig13341_length=751|250|60
log2(expression)

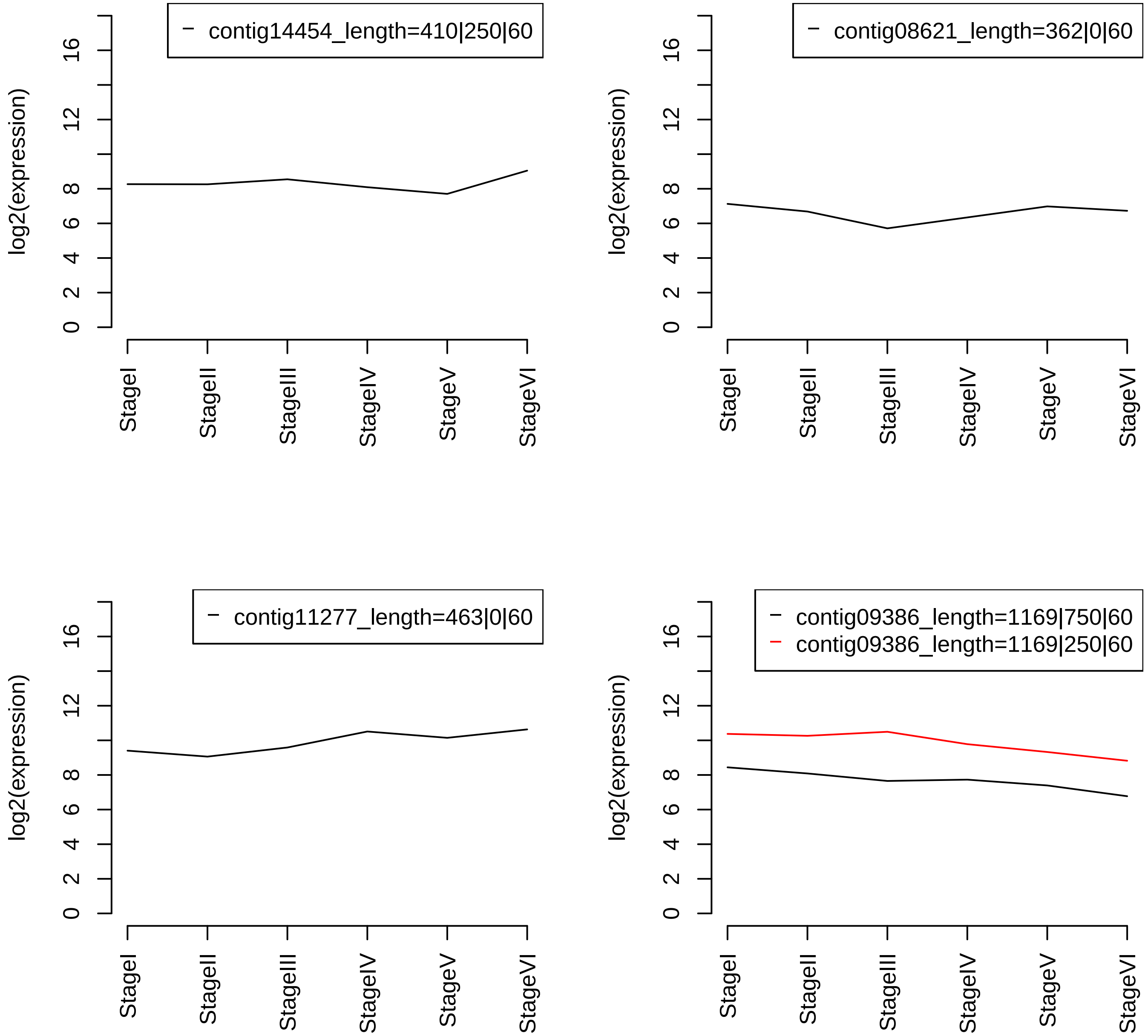

contig14454_length=410|250|60
contig08621_length=362|0|60
contig11277_length=463|0|60
contig09386_length=1169|750|60
contig09386_length=1169|250|60
log2(expression)
0
2
4
6
8
12
16
StageI
StageII
StageIII
StageIV
StageV
StageVI

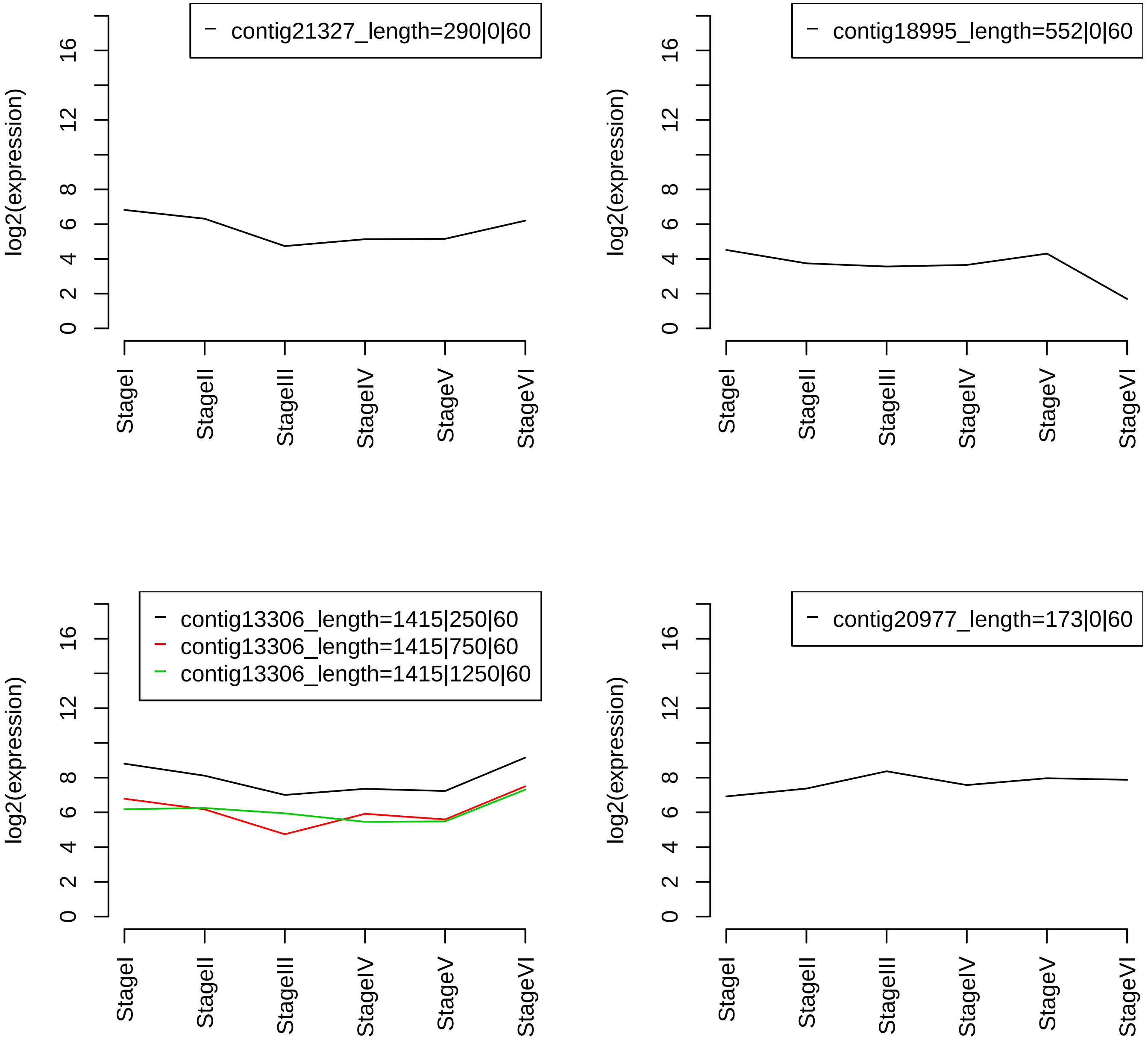
contig21327_length=290|0|60
contig18995_length=552|0|60
contig13306_length=1415|250|60
contig13306_length=1415|750|60
contig13306_length=1415|1250|60
contig20977_length=173|0|60
log2(expression)
0
2
4
6
8
12
16
StageI
StageII
StageIII
StageIV
StageV
StageVI

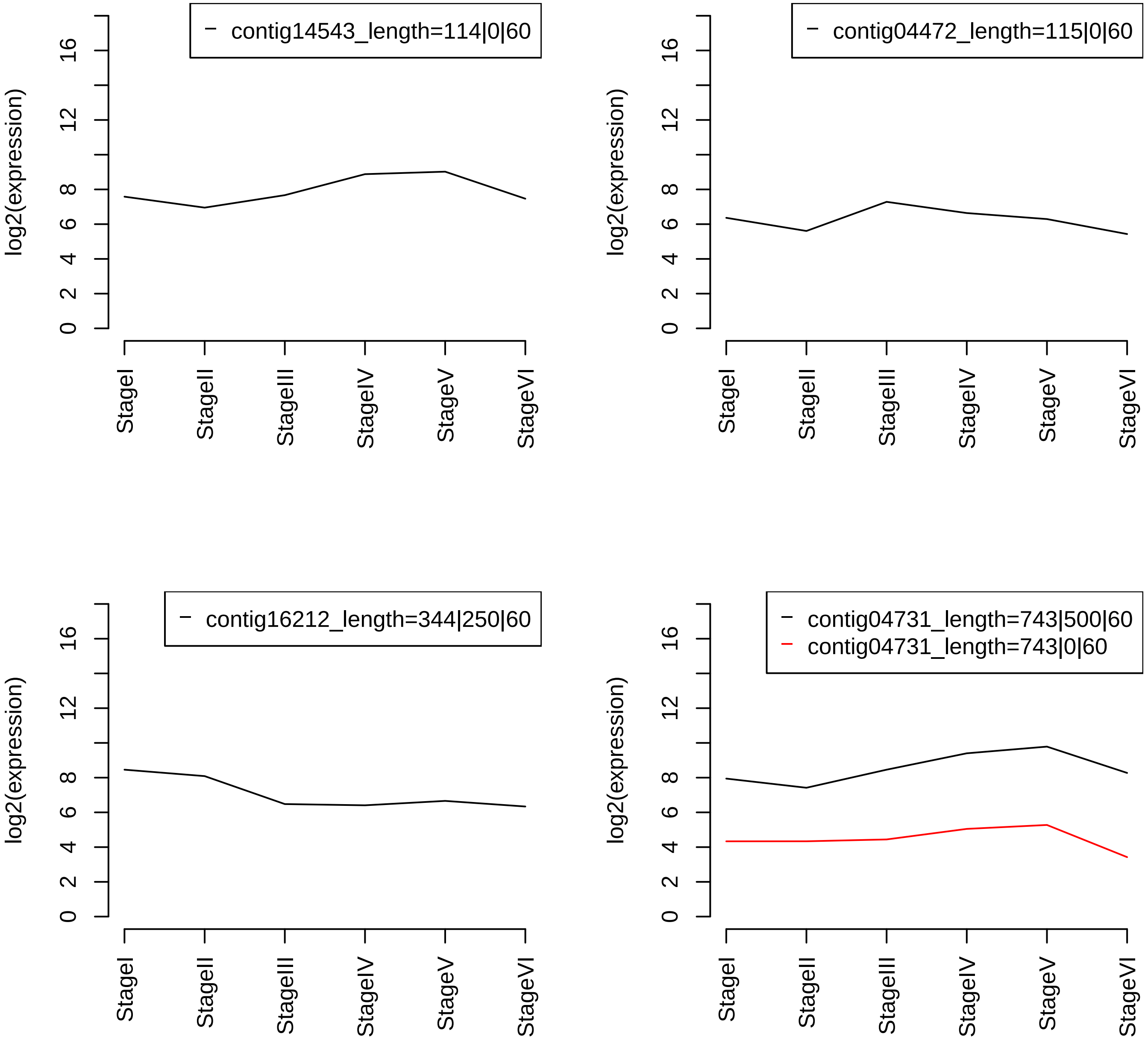

contig14543_length=114|0|60
contig04472_length=115|0|60
contig16212_length=344|250|60
contig04731_length=743|500|60
contig04731_length=743|0|60
log2(expression)
0
2
4
6
8
12
16
StageI
StageII
StageIII
StageIV
StageV
StageVI

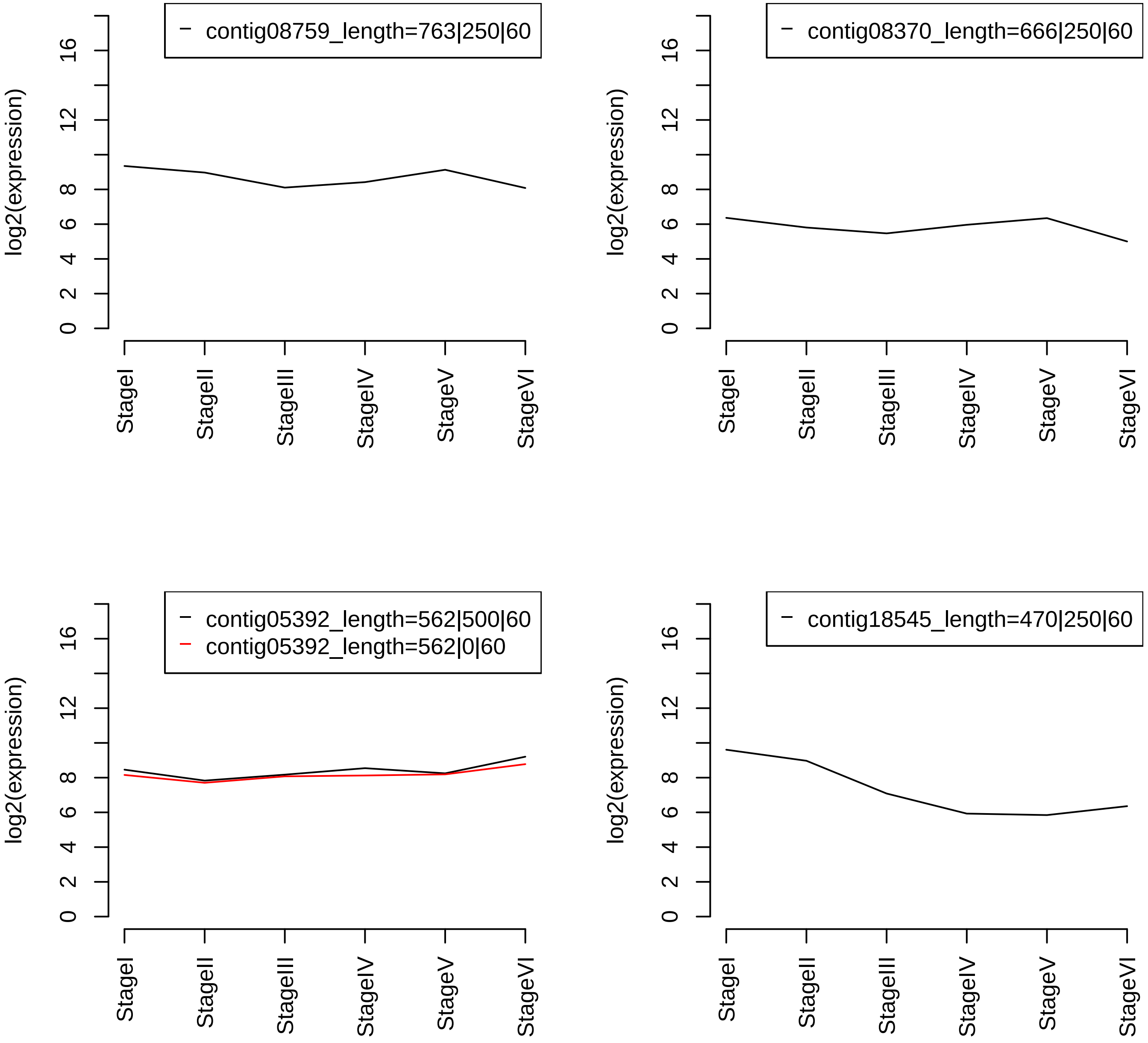

contig08759_length=763|250|60
contig08370_length=666|250|60
contig05392_length=562|500|60
contig05392_length=562|0|60
contig18545_length=470|250|60
log2(expression)
0
2
4
6
8
12
16
StageI
StageII
StageIII
StageIV
StageV
StageVI

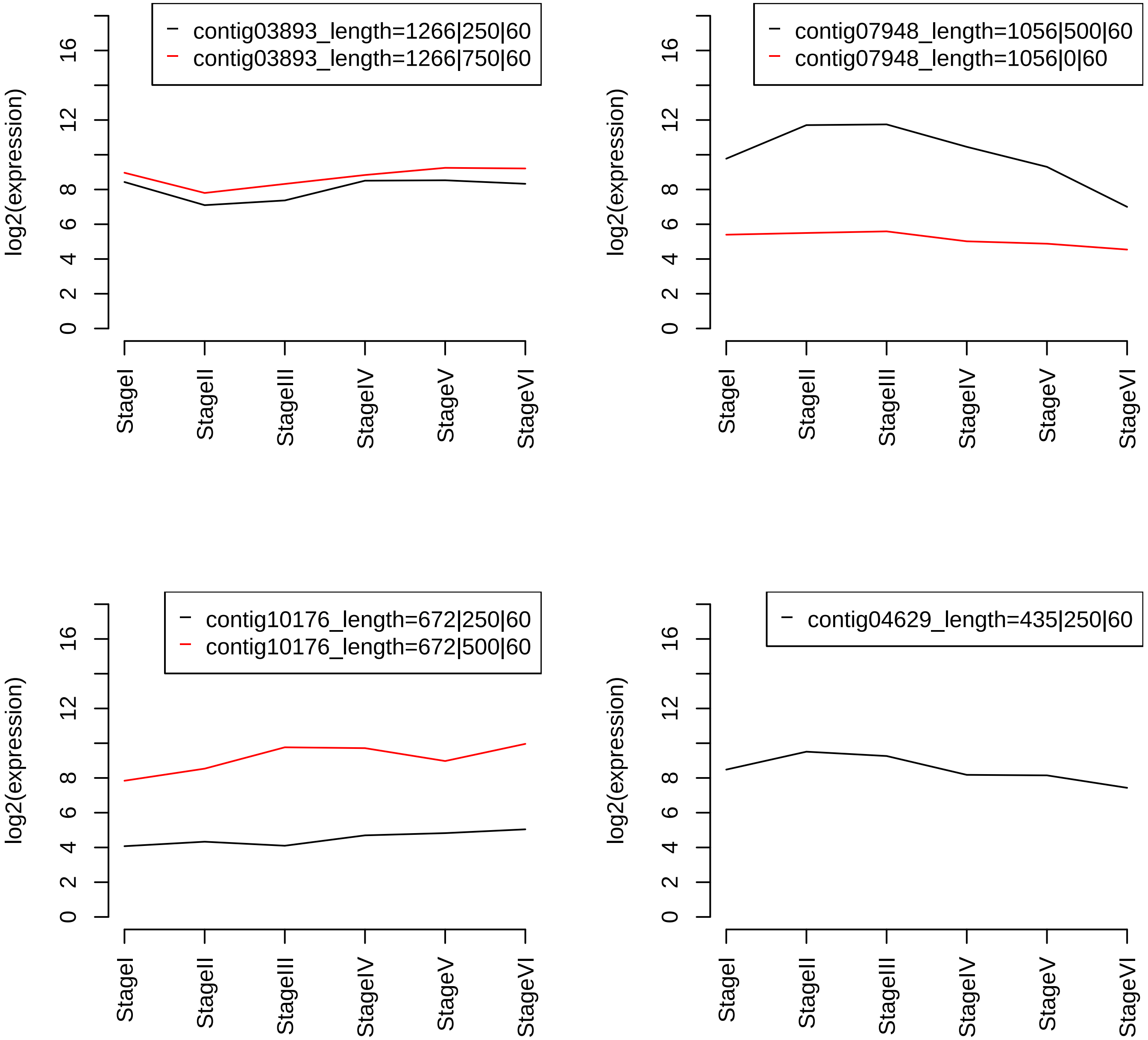

contig03893_length=1266|250|60
contig03893_length=1266|750|60
contig07948_length=1056|500|60
contig07948_length=1056|0|60
contig10176_length=672|250|60
contig10176_length=672|500|60
contig04629_length=435|250|60
log2(expression)
0
2
4
6
8
12
16
StageI
StageII
StageIII
StageIV
StageV
StageVI

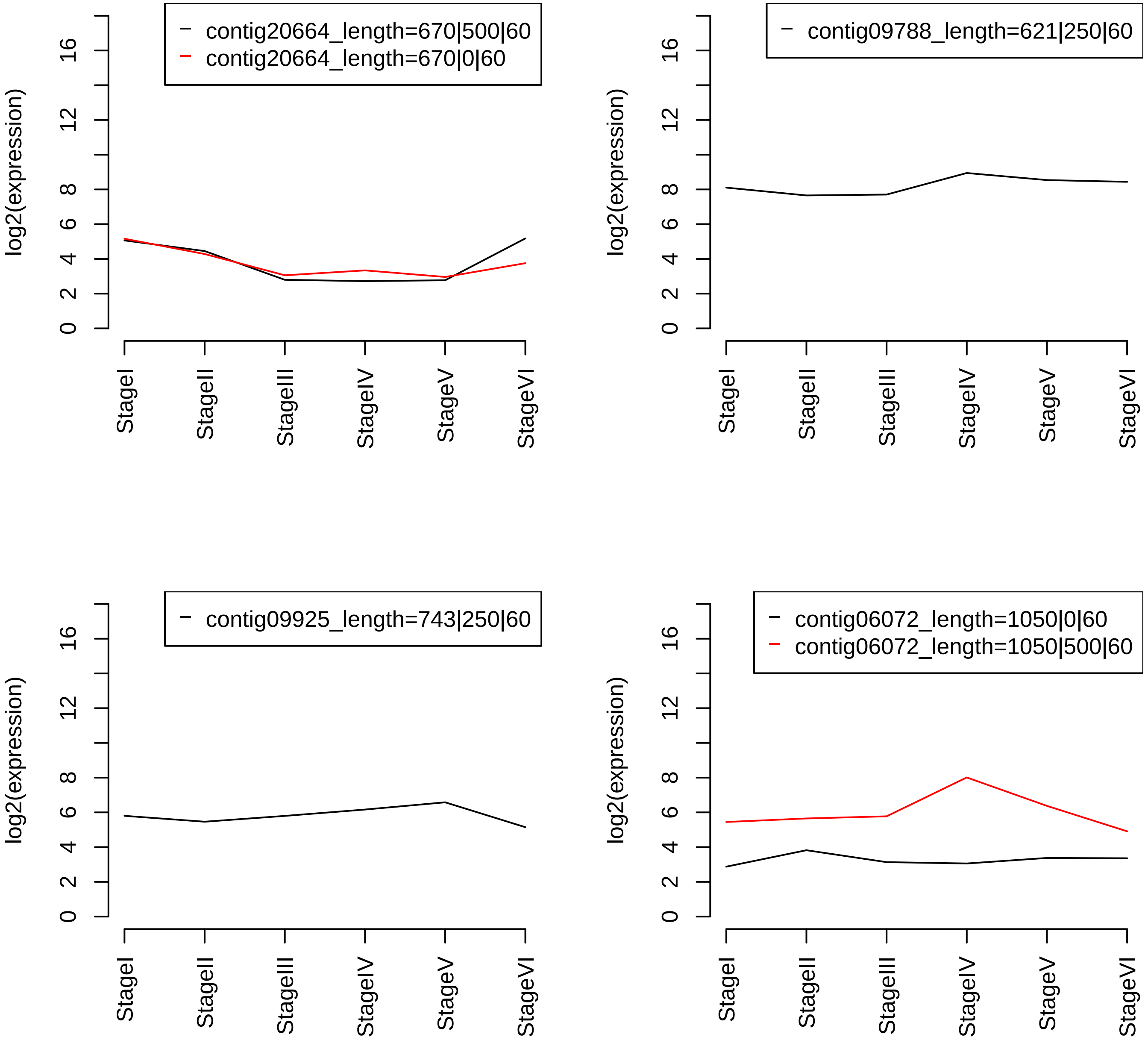

contig20664_length=670|500|60
contig20664_length=670|0|60
contig09788_length=621|250|60
contig09925_length=743|250|60
contig06072_length=1050|0|60
contig06072_length=1050|500|60
log2(expression)
0
2
4
6
8
12
16
StageI
StageII
StageIII
StageIV
StageV
StageVI

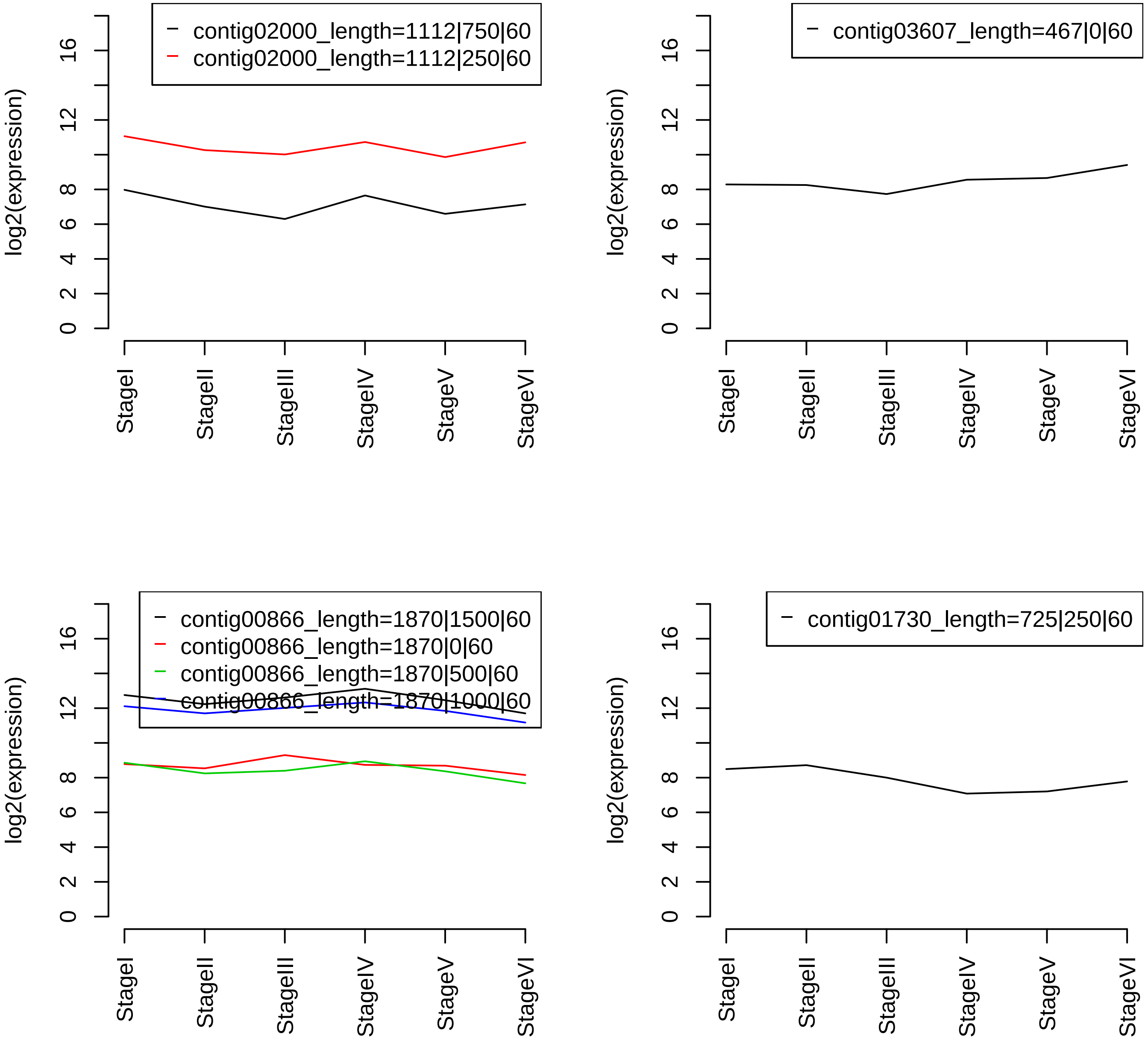
contig02000_length=1112|750|60
contig02000_length=1112|250|60
contig03607_length=467|0|60
contig00866_length=1870|1500|60
contig00866_length=1870|0|60
contig00866_length=1870|500|60
contig00866_length=1870|1000|60
contig01730_length=725|250|60
log2(expression)
0
2
4
6
8
12
16
StageI
StageII
StageIII
StageIV
StageV
StageVI

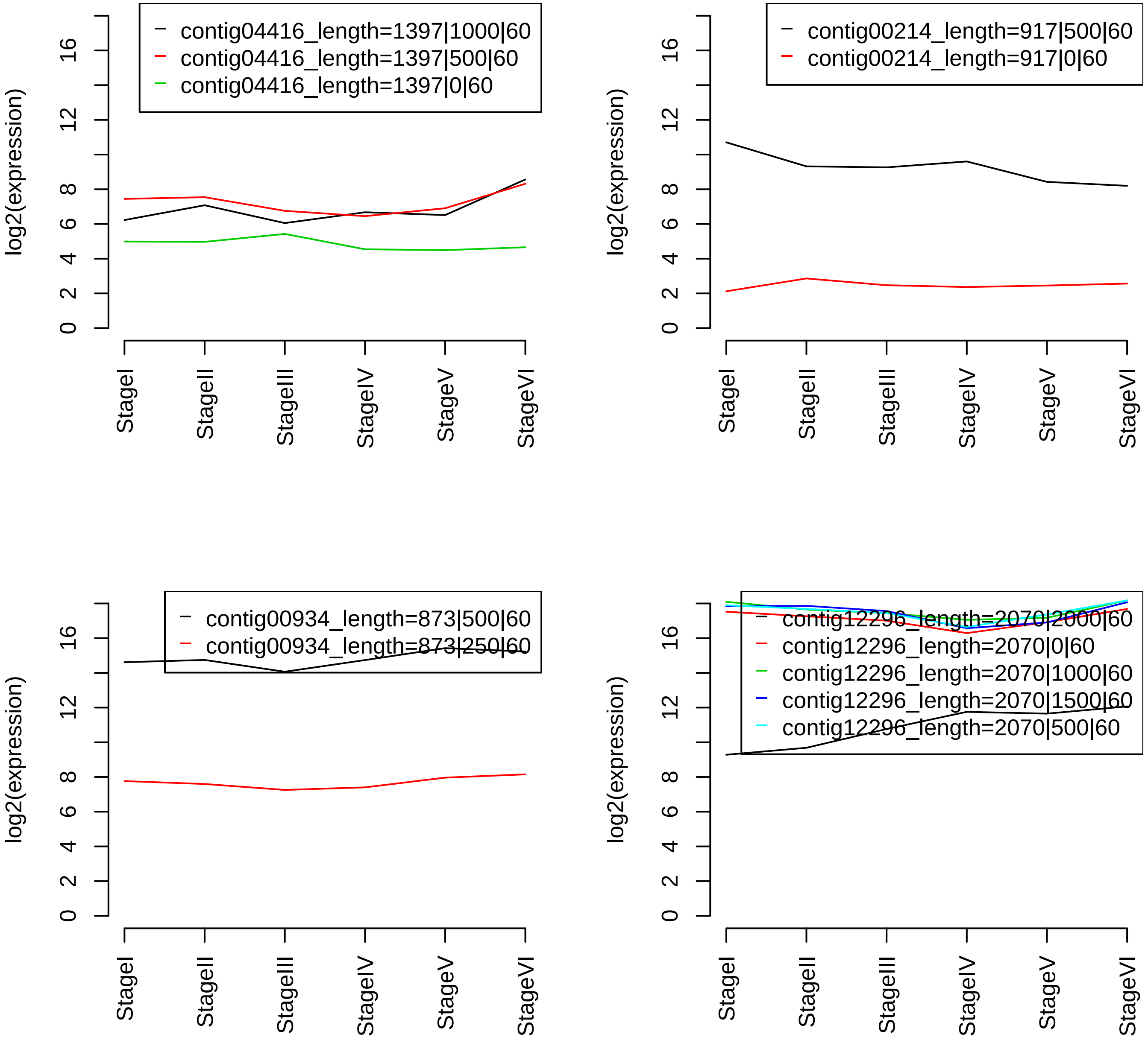
contig04416_length=1397|1000|60
contig04416_length=1397|500|60
contig04416_length=1397|0|60
contig00214_length=917|500|60
contig00214_length=917|0|60
contig00934_length=873|500|60
contig00934_length=873|250|60
contig12296_length=2070|2000|60
contig12296_length=2070|0|60
contig12296_length=2070|1000|60
contig12296_length=2070|1500|60
contig12296_length=2070|500|60
log2(expression)
0
2
4
6
8
12
16
StageI
StageII
StageIII
StageIV
StageV
StageVI

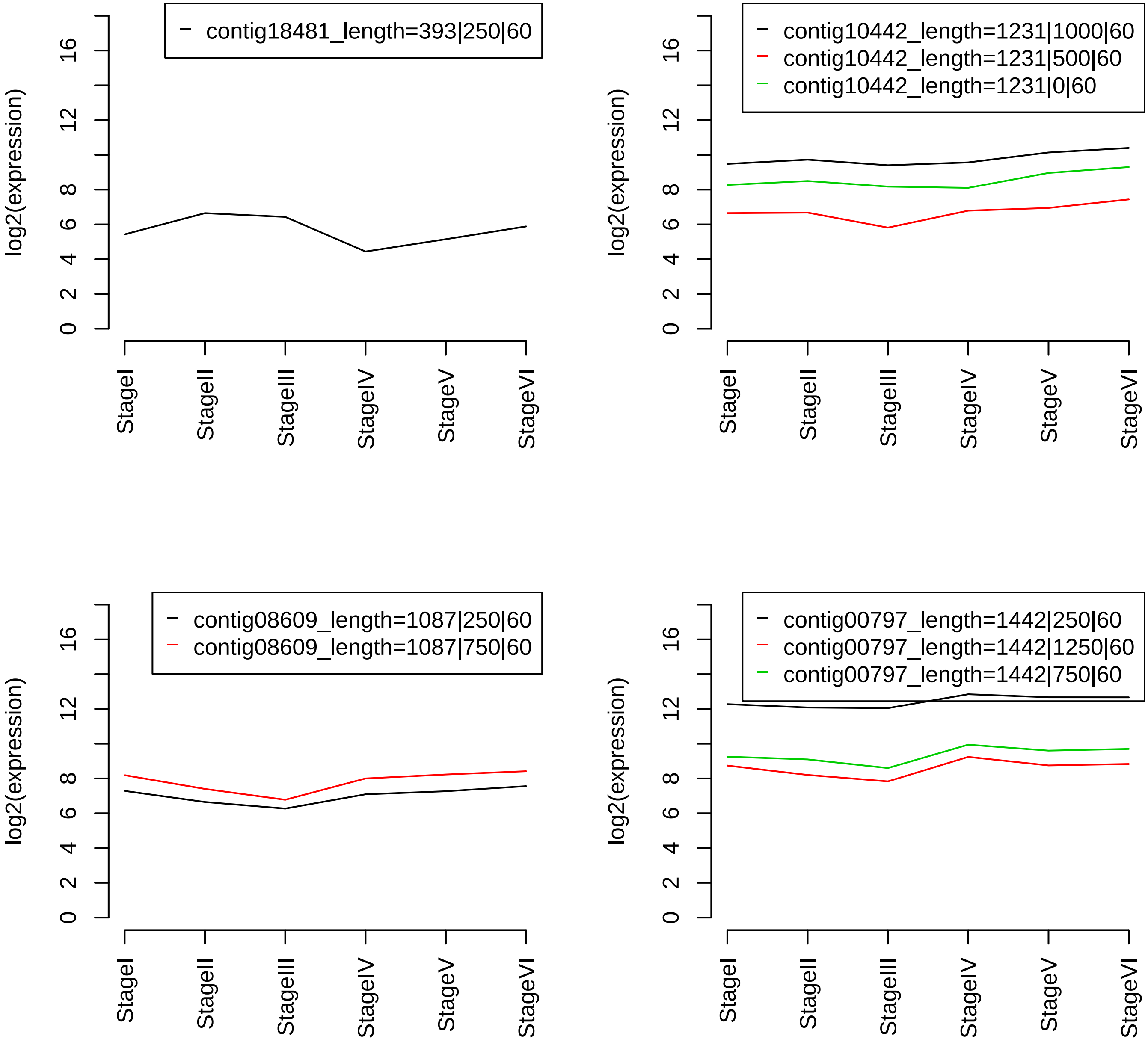

contig18481_length=393|250|60
contig10442_length=1231|1000|60
contig10442_length=1231|500|60
contig10442_length=1231|0|60
contig08609_length=1087|250|60
contig08609_length=1087|750|60
contig00797_length=1442|250|60
contig00797_length=1442|1250|60
contig00797_length=1442|750|60
log2(expression)
0 2 4 6 8 12 16
StageI StageII StageIII StageIV StageV StageVI

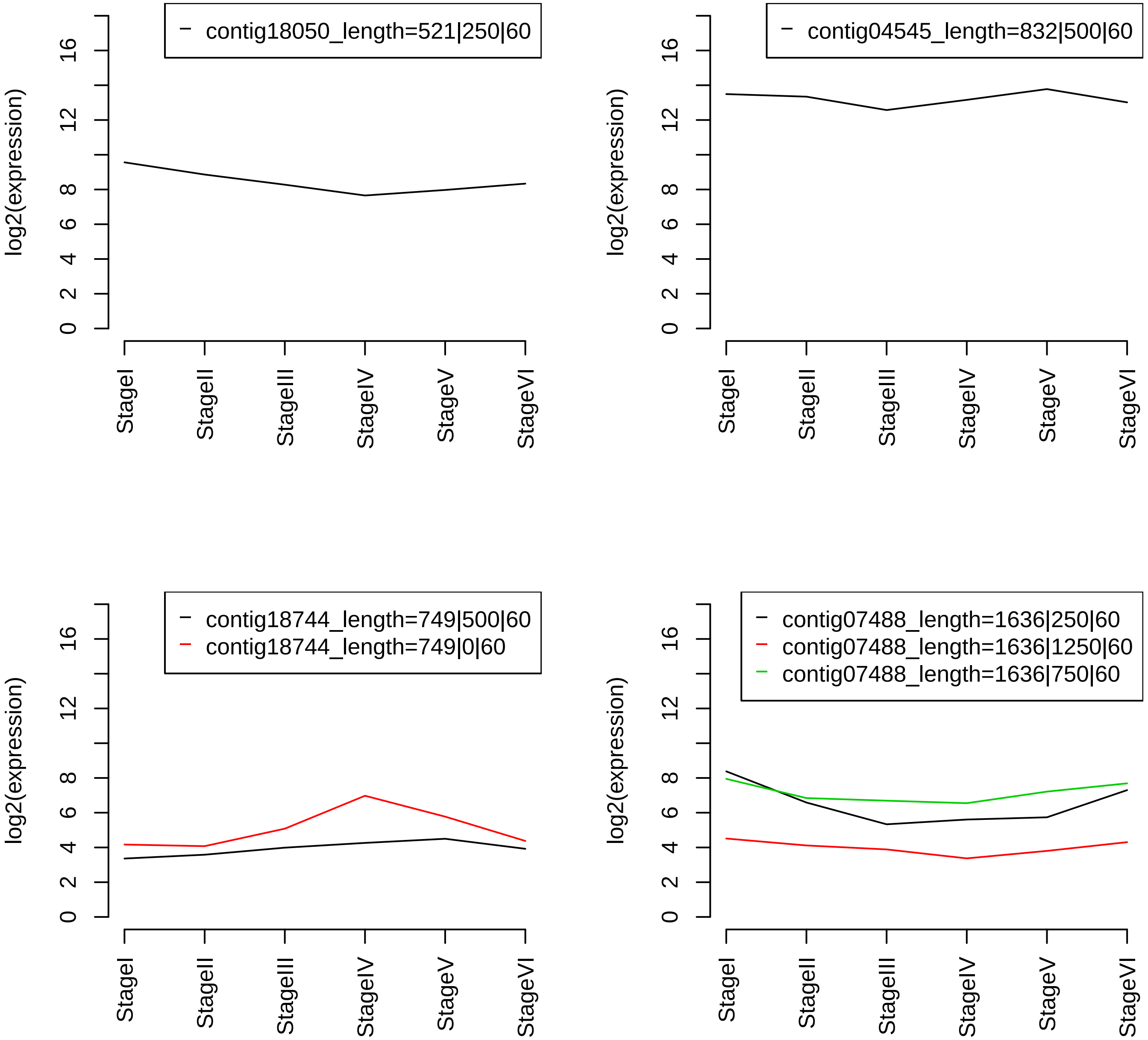

contig18050_length=521|250|60
contig04545_length=832|500|60
contig18744_length=749|500|60
contig18744_length=749|0|60
contig07488_length=1636|250|60
contig07488_length=1636|1250|60
contig07488_length=1636|750|60
log2(expression)
0
2
4
6
8
12
16
StageI
StageII
StageIII
StageIV
StageV
StageVI

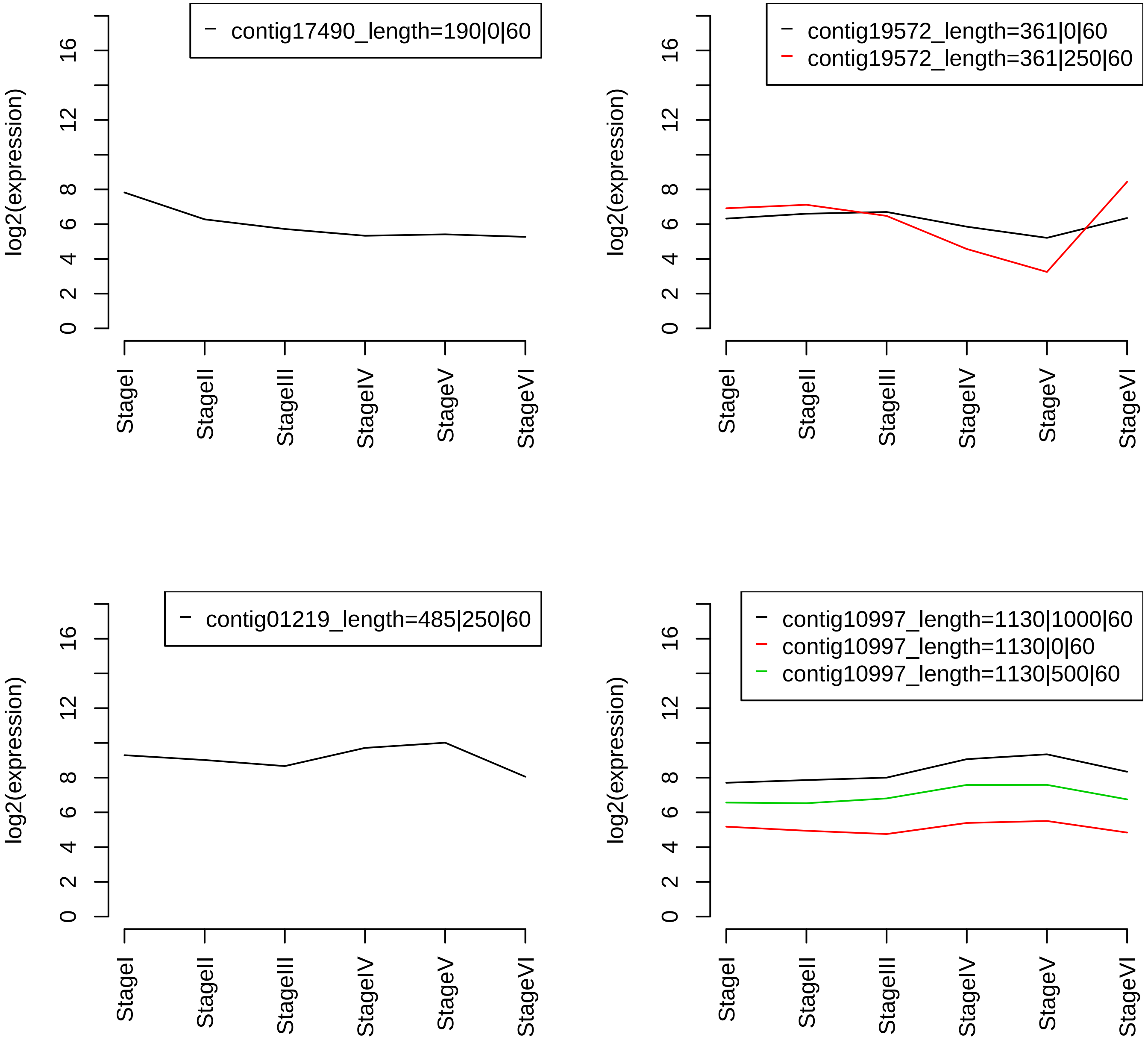

contig17490_length=190|0|60
contig19572_length=361|0|60
contig19572_length=361|250|60
contig01219_length=485|250|60
contig10997_length=1130|1000|60
contig10997_length=1130|0|60
contig10997_length=1130|500|60
log2(expression)
0
2
4
6
8
12
16
StageI
StageII
StageIII
StageIV
StageV
StageVI

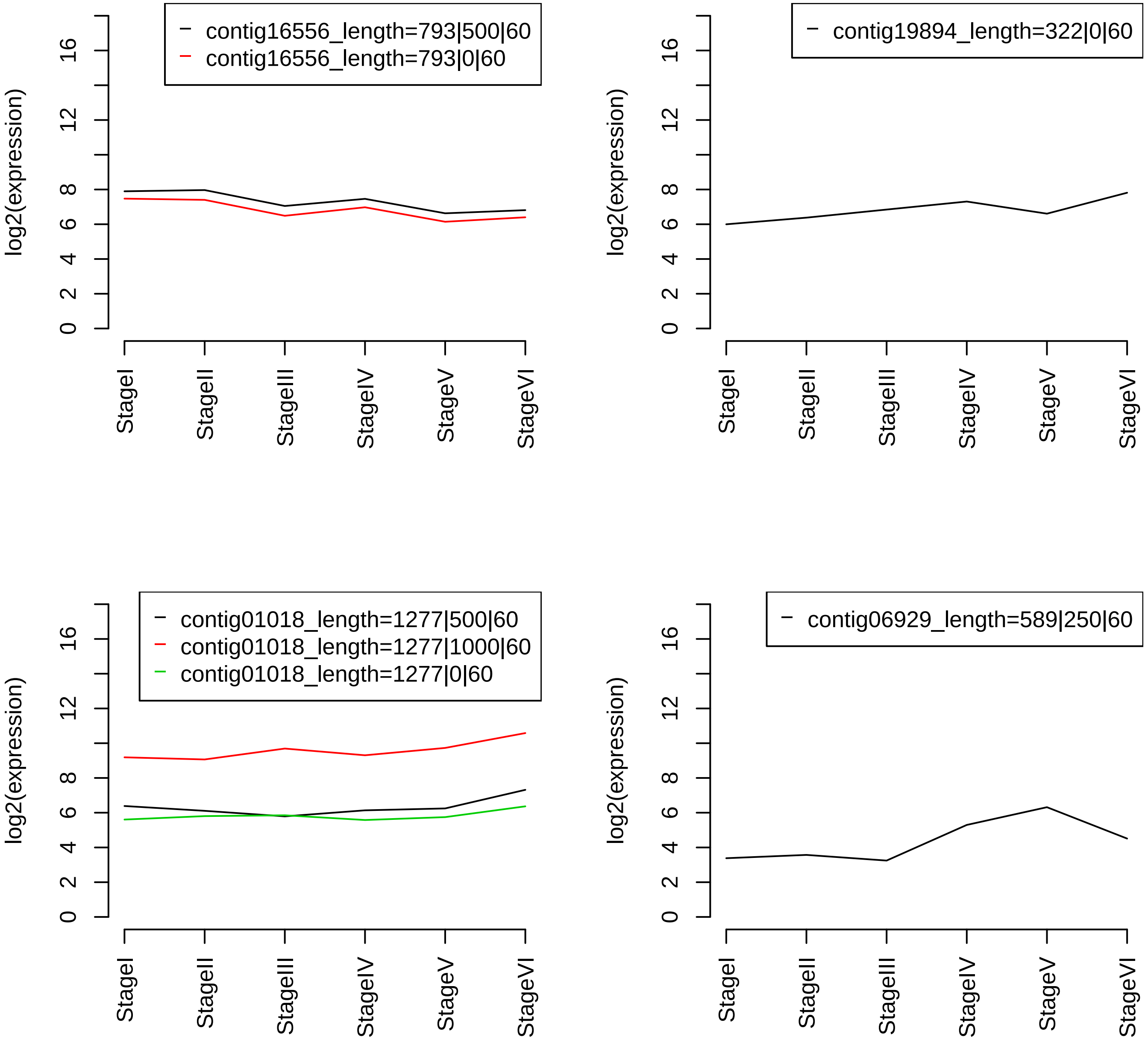

contig16556_length=793|500|60
contig16556_length=793|0|60
contig19894_length=322|0|60
contig01018_length=1277|500|60
contig01018_length=1277|1000|60
contig01018_length=1277|0|60
contig06929_length=589|250|60
log2(expression)
StageI
StageII
StageIII
StageIV
StageV
StageVI

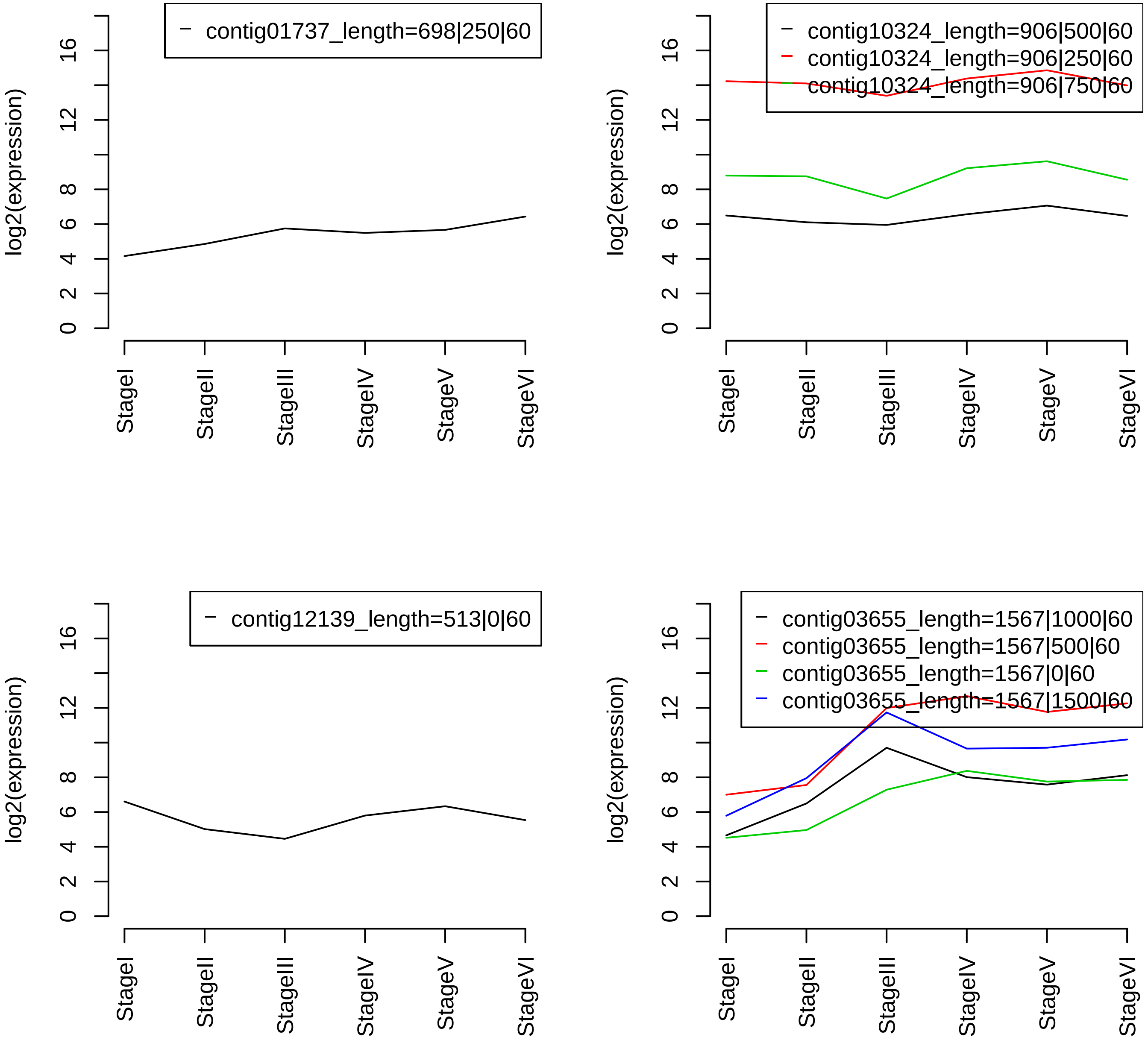

contig01737_length=698|250|60
contig10324_length=906|500|60
contig10324_length=906|250|60
contig10324_length=906|750|60
contig12139_length=513|0|60
contig03655_length=1567|1000|60
contig03655_length=1567|500|60
contig03655_length=1567|0|60
contig03655_length=1567|1500|60
log2(expression)
0
2
4
6
8
12
16
StageI
StageII
StageIII
StageIV
StageV
StageVI

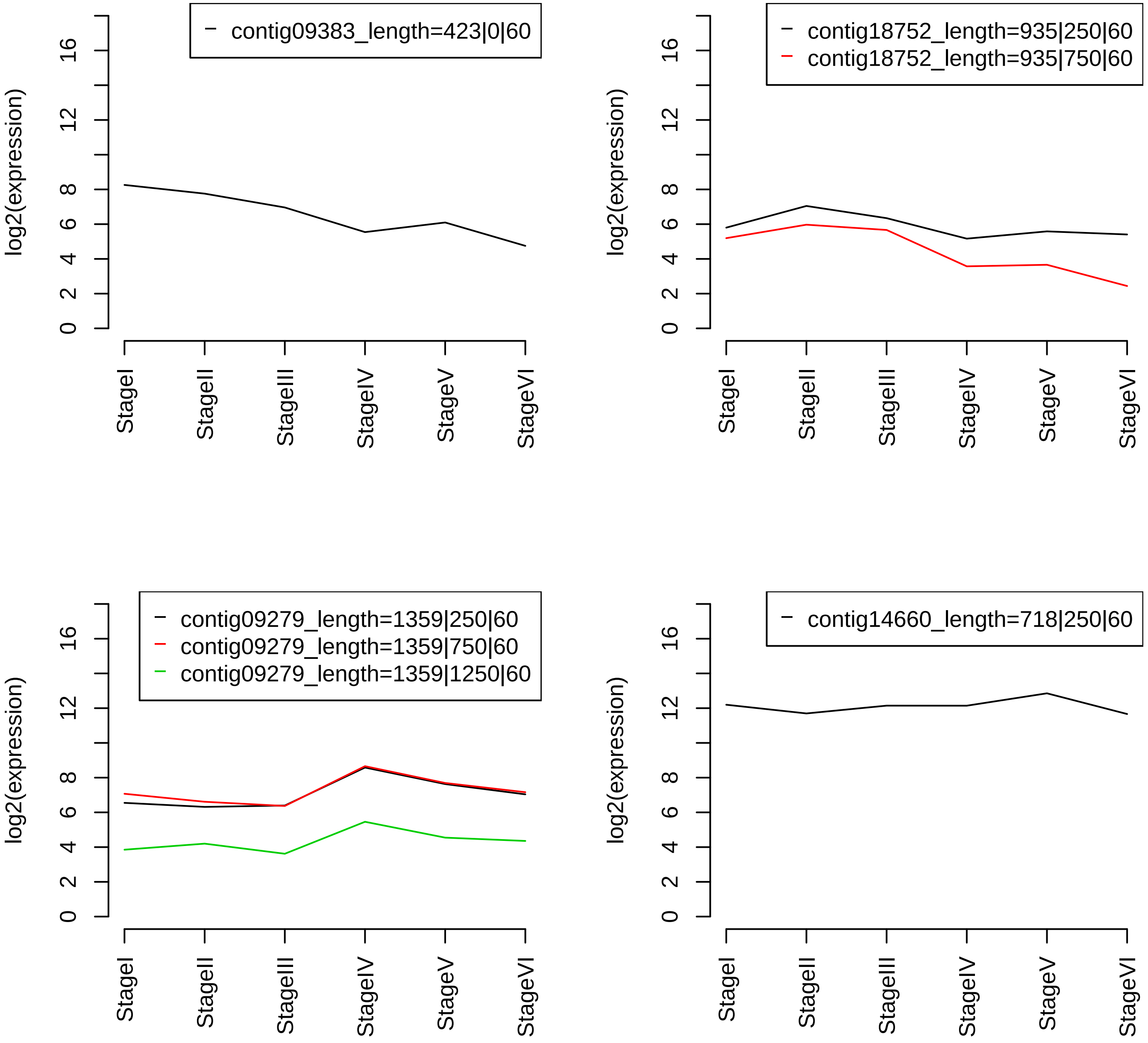
contig09383_length=423|0|60
contig18752_length=935|250|60
contig18752_length=935|750|60
contig09279_length=1359|250|60
contig09279_length=1359|750|60
contig09279_length=1359|1250|60
contig14660_length=718|250|60
log2(expression)
0
2
4
6
8
12
16
StageI
StageII
StageIII
StageIV
StageV
StageVI

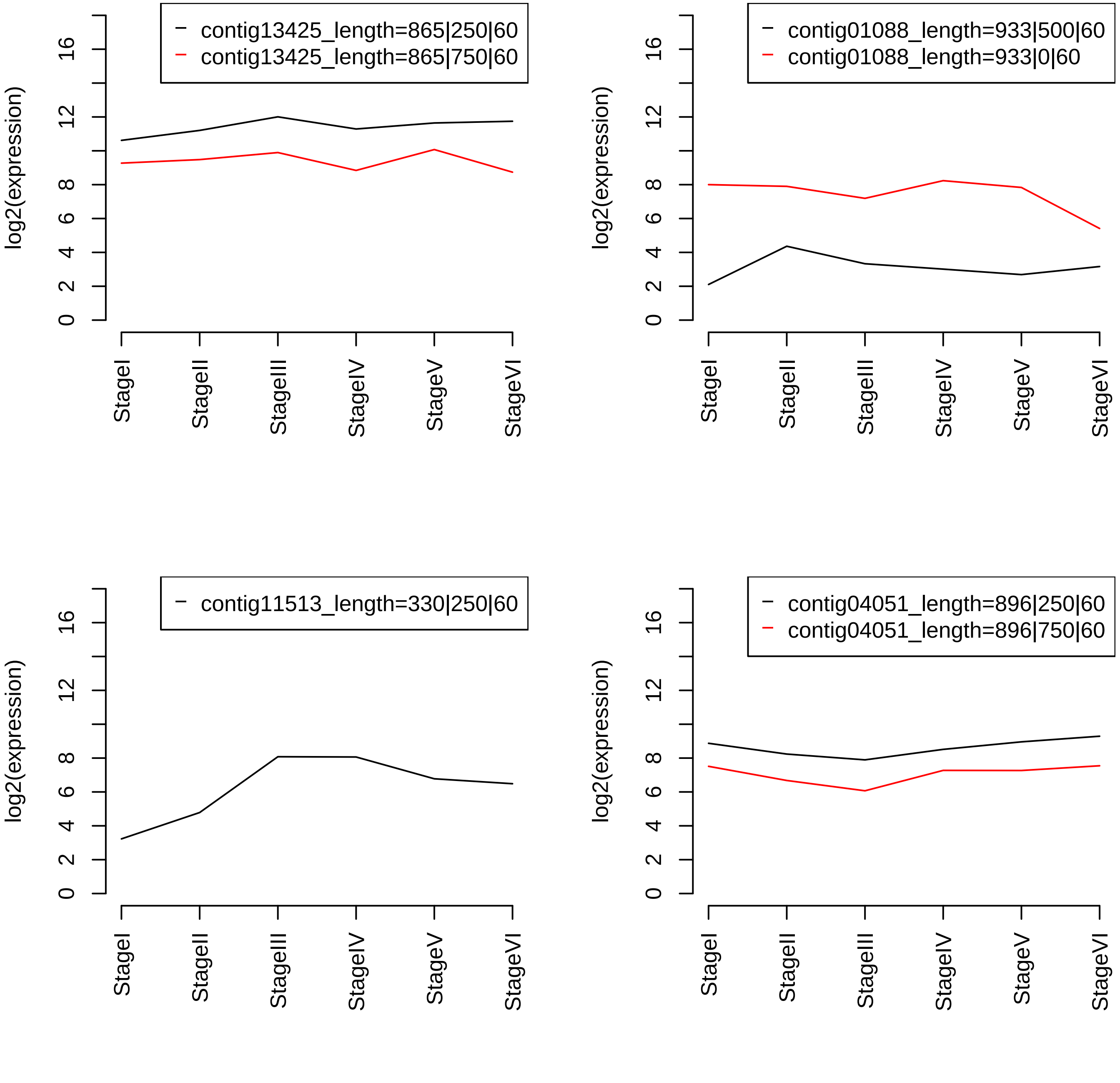

contig13425_length=865|250|60
contig13425_length=865|750|60
contig01088_length=933|500|60
contig01088_length=933|0|60
contig11513_length=330|250|60
contig04051_length=896|250|60
contig04051_length=896|750|60
log2(expression)
0
2
4
6
8
12
16
StageI
StageII
StageIII
StageIV
StageV
StageVI

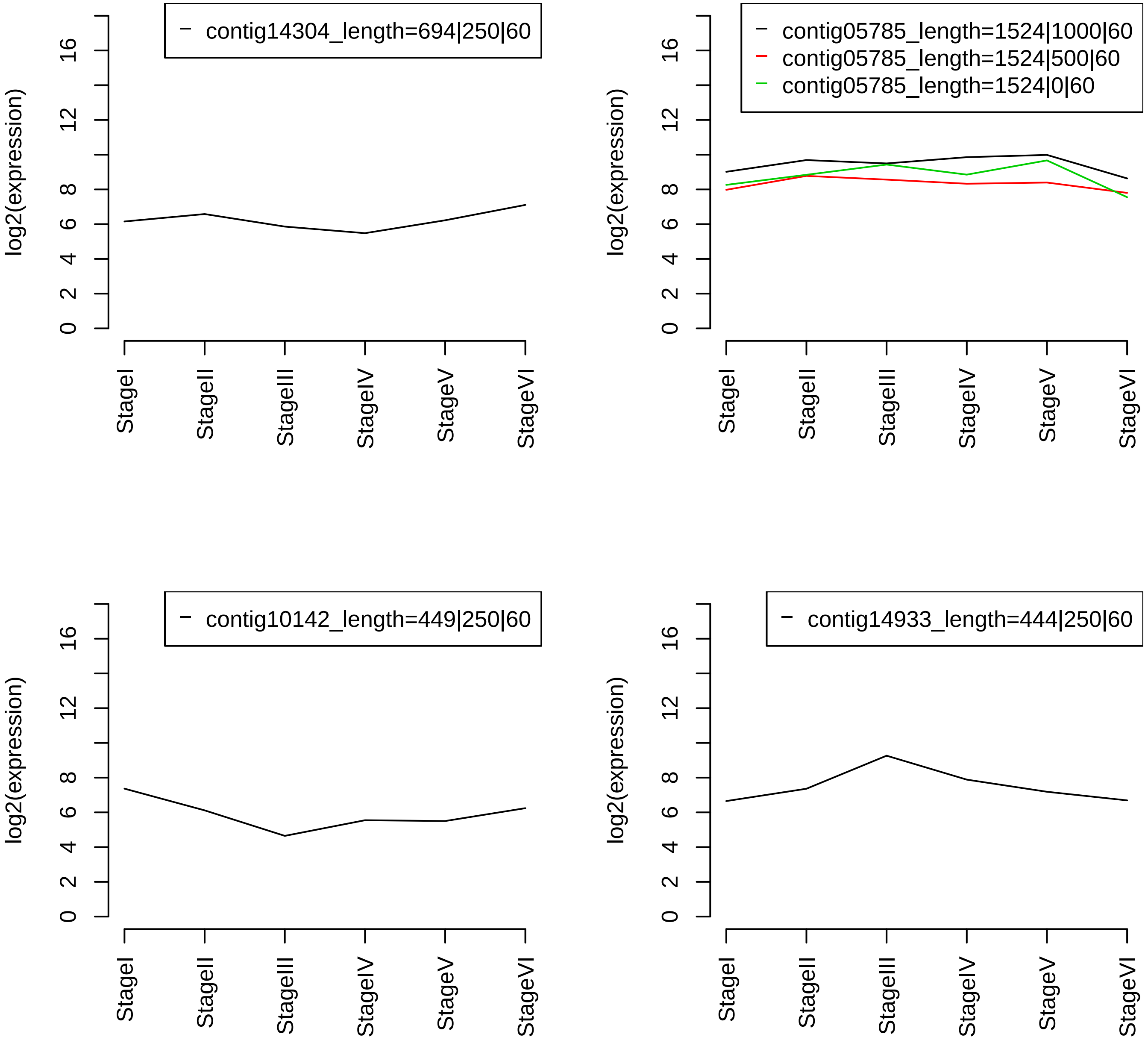
contig14304_length=694|250|60
contig05785_length=1524|1000|60
contig05785_length=1524|500|60
contig05785_length=1524|0|60
contig10142_length=449|250|60
contig14933_length=444|250|60
log2(expression)
0
2
4
6
8
12
16
StageI
StageII
StageIII
StageIV
StageV
StageVI

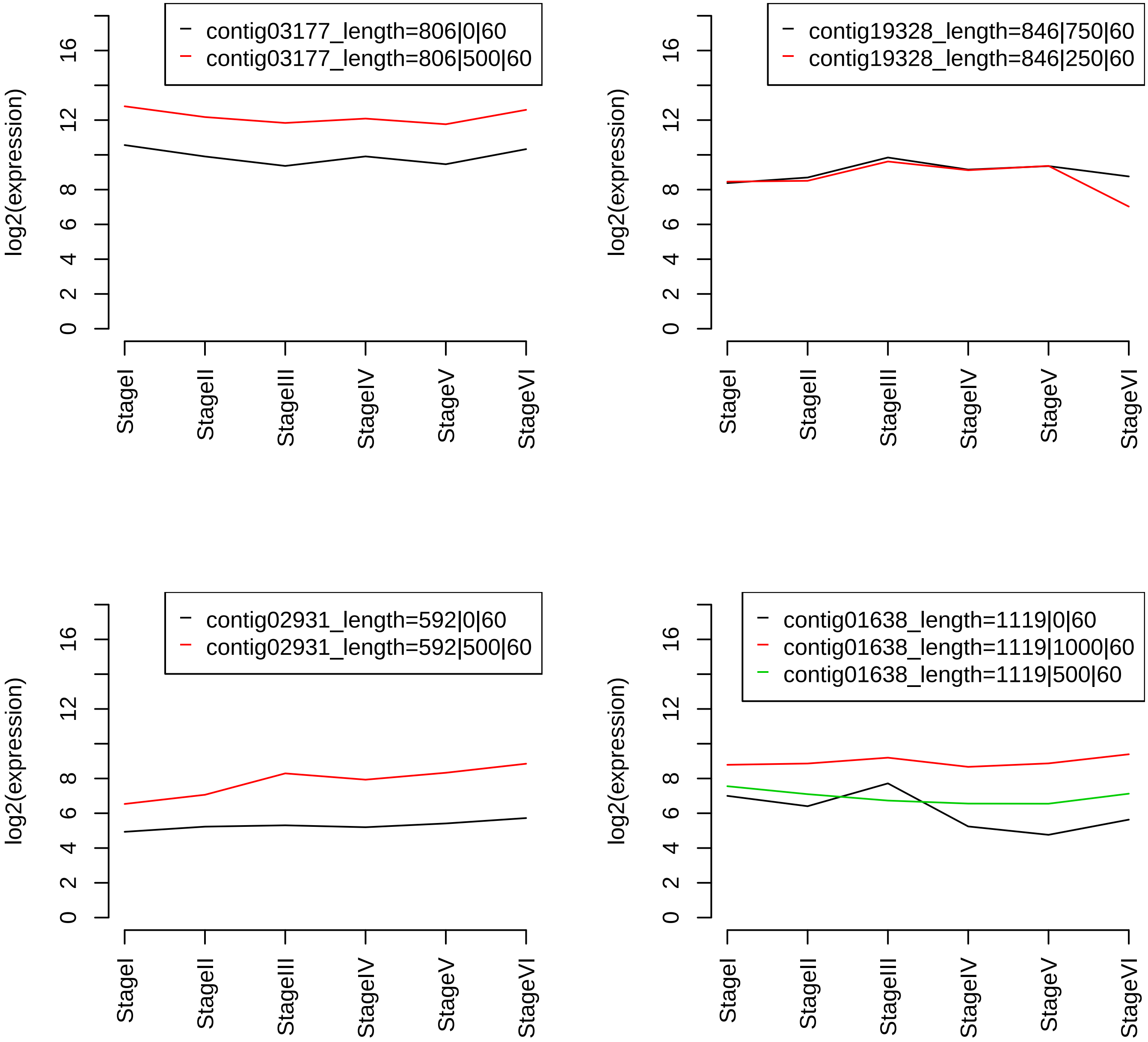
contig03177_length=806|0|60
contig03177_length=806|500|60
contig19328_length=846|750|60
contig19328_length=846|250|60
contig02931_length=592|0|60
contig02931_length=592|500|60
contig01638_length=1119|0|60
contig01638_length=1119|1000|60
contig01638_length=1119|500|60
log2(expression)
0 2 4 6 8 12 16
StageI
StageII
StageIII
StageIV
StageV
StageVI

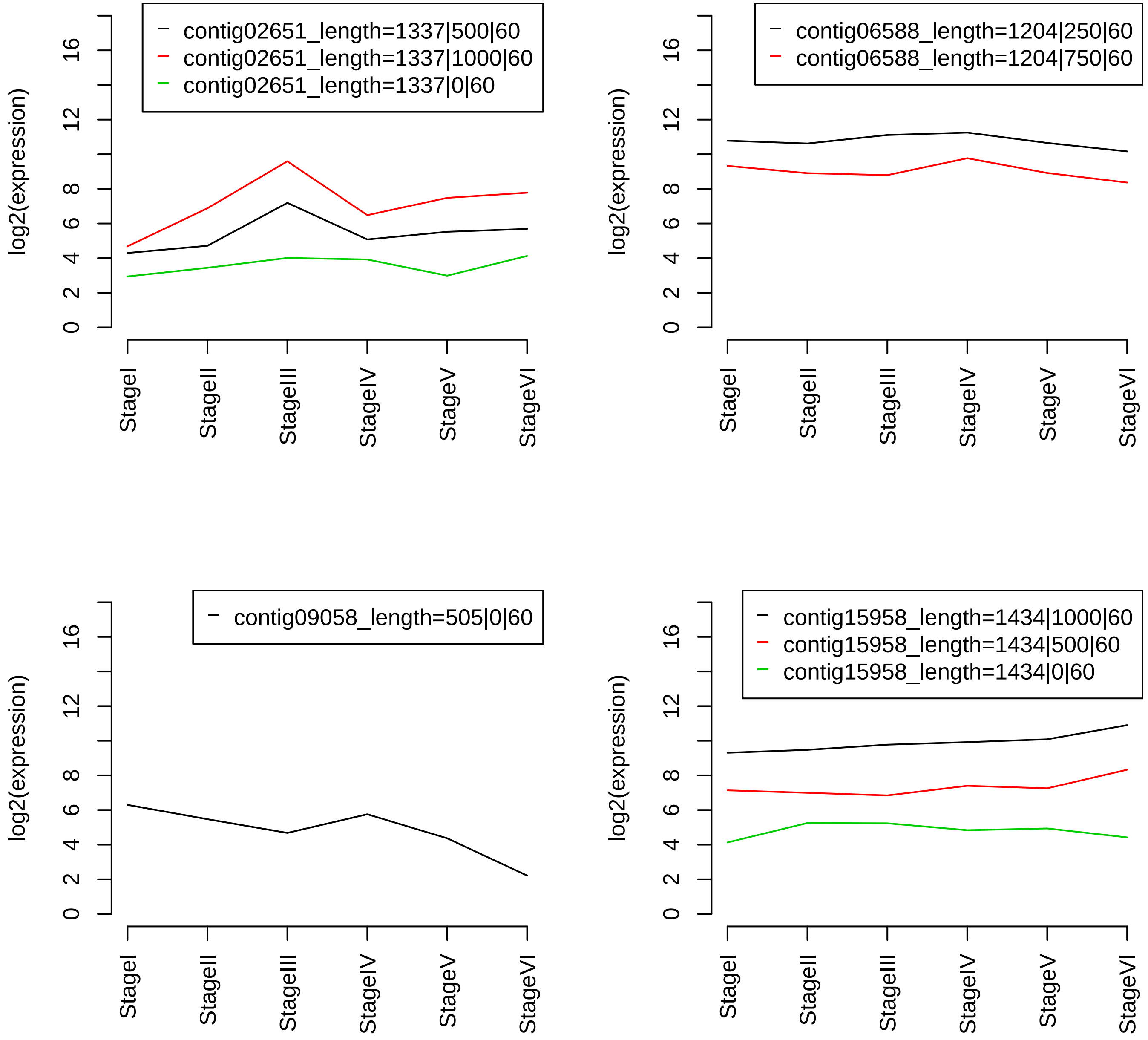

contig02651_length=1337|500|60
contig02651_length=1337|1000|60
contig02651_length=1337|0|60
contig06588_length=1204|250|60
contig06588_length=1204|750|60
contig09058_length=505|0|60
contig15958_length=1434|1000|60
contig15958_length=1434|500|60
contig15958_length=1434|0|60
log2(expression)
0
2
4
6
8
12
16
StageI
StageII
StageIII
StageIV
StageV
StageVI

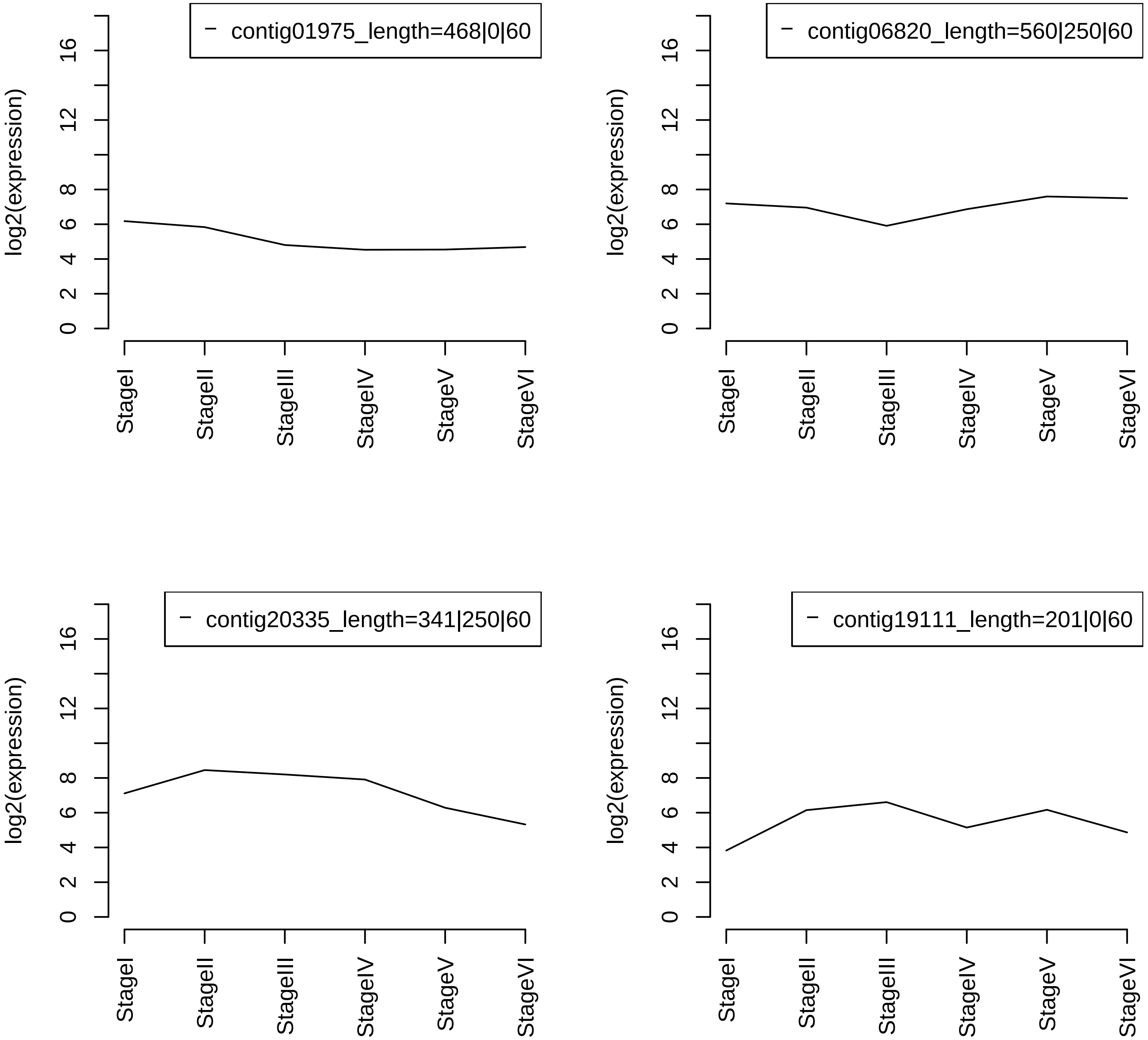
contig01975_length=468|0|60
contig06820_length=560|250|60
contig20335_length=341|250|60
contig19111_length=201|0|60
log2(expression)
0
2
4
6
8
12
16
StageI
StageII
StageIII
StageIV
StageV
StageVI

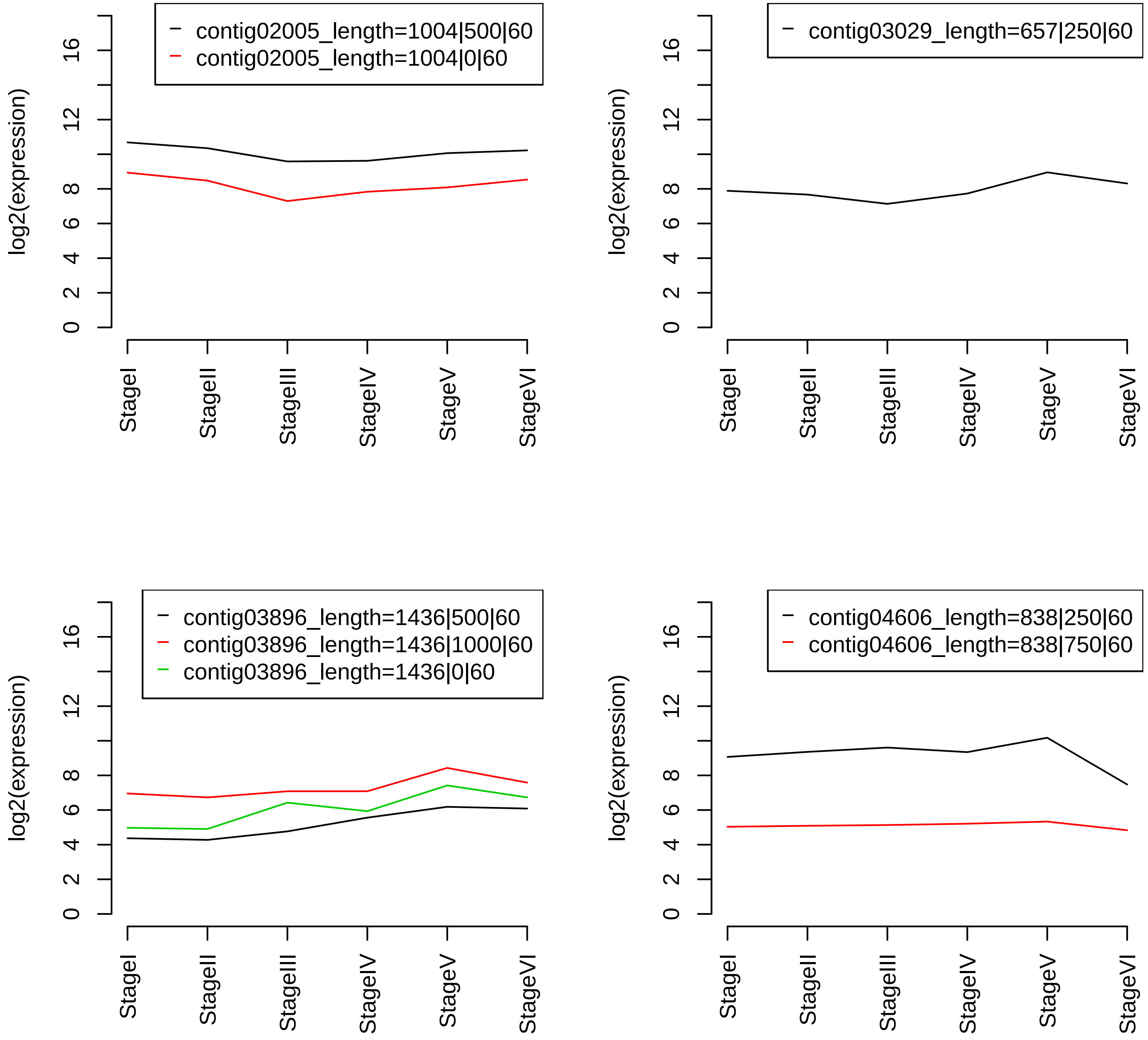

contig02005_length=1004|500|60
contig02005_length=1004|0|60
contig03029_length=657|250|60
contig03896_length=1436|500|60
contig03896_length=1436|1000|60
contig03896_length=1436|0|60
contig04606_length=838|250|60
contig04606_length=838|750|60
log2(expression)
0
2
4
6
8
12
16
StageI
StageII
StageIII
StageIV
StageV
StageVI

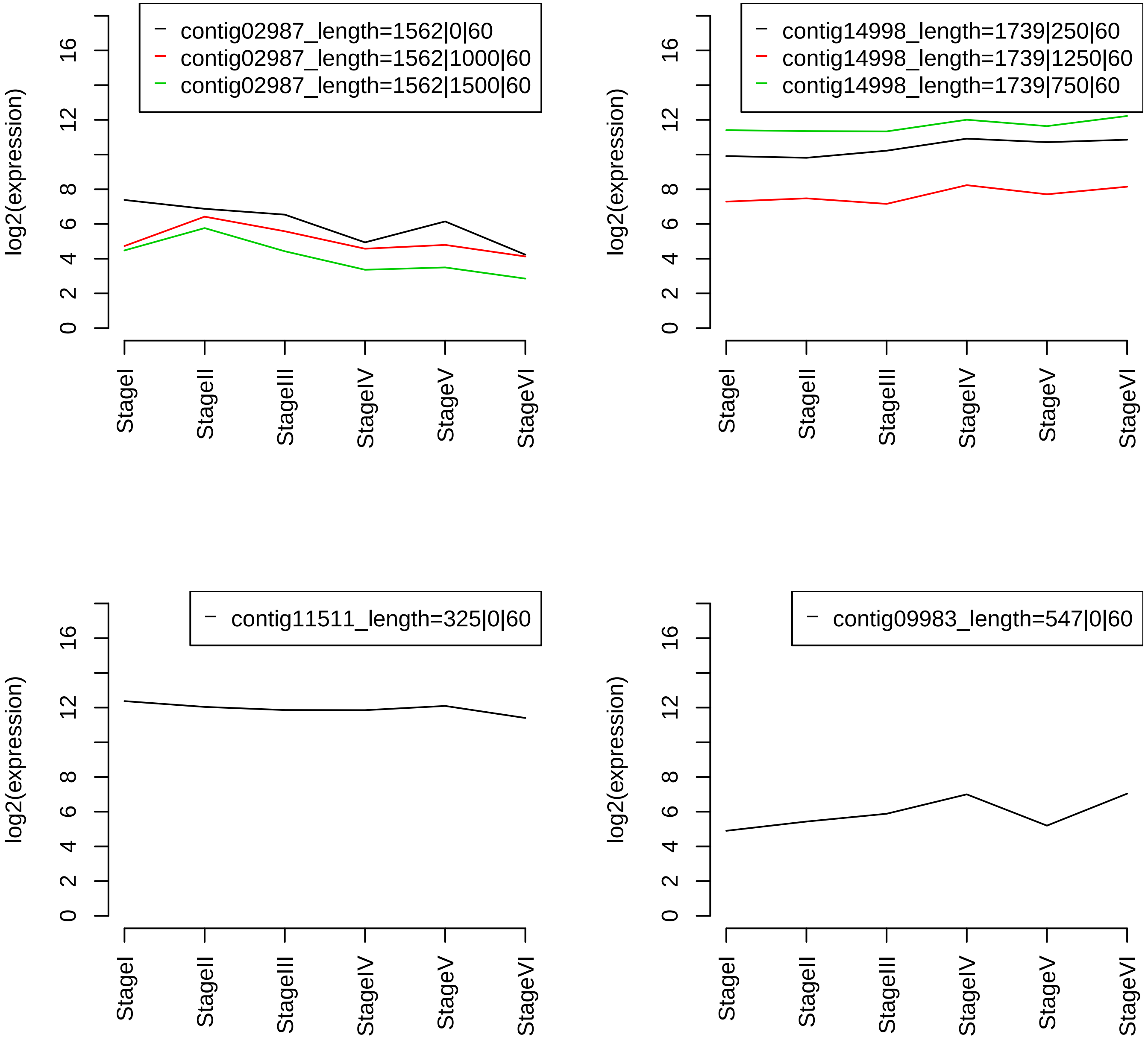

contig02987_length=1562|0|60
contig02987_length=1562|1000|60
contig02987_length=1562|1500|60
contig14998_length=1739|250|60
contig14998_length=1739|1250|60
contig14998_length=1739|750|60
contig11511_length=325|0|60
contig09983_length=547|0|60
log2(expression)
0
2
4
6
8
12
16
StageI
StageII
StageIII
StageIV
StageV
StageVI

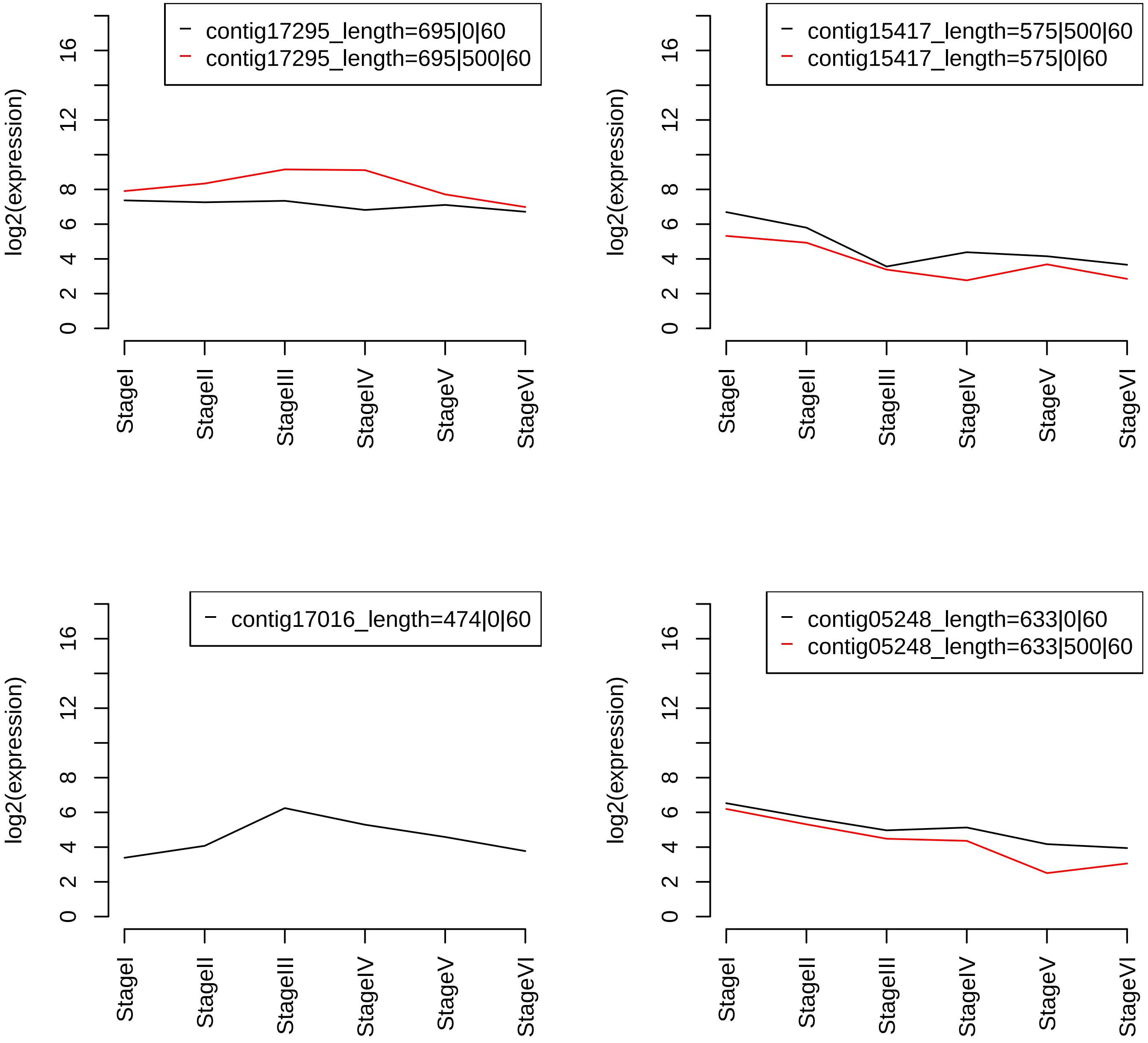

contig17295_length=695|0|60
contig17295_length=695|500|60
log2(expression)
0
2
4
6
8
12
16
StageI
StageII
StageIII
StageIV
StageV
StageVI
contig15417_length=575|500|60
contig15417_length=575|0|60
contig17016_length=474|0|60
contig05248_length=633|0|60
contig05248_length=633|500|60

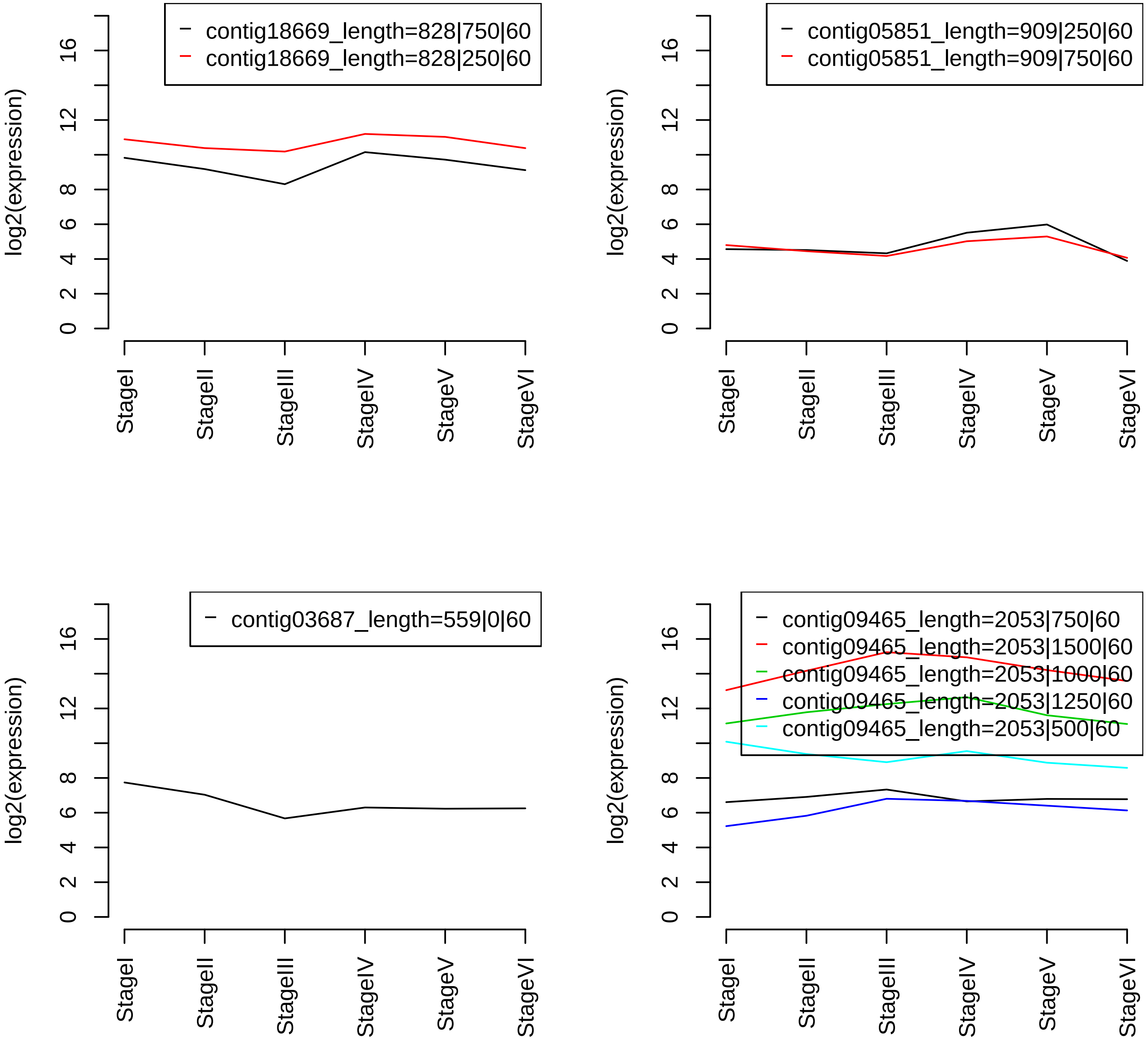

contig18669_length=828|750|60
contig18669_length=828|250|60
contig05851_length=909|250|60
contig05851_length=909|750|60
contig03687_length=559|0|60
contig09465_length=2053|750|60
contig09465_length=2053|1500|60
contig09465_length=2053|1000|60
contig09465_length=2053|1250|60
contig09465_length=2053|500|60
log2(expression)
0
2
4
6
8
12
16
StageI
StageII
StageIII
StageIV
StageV
StageVI

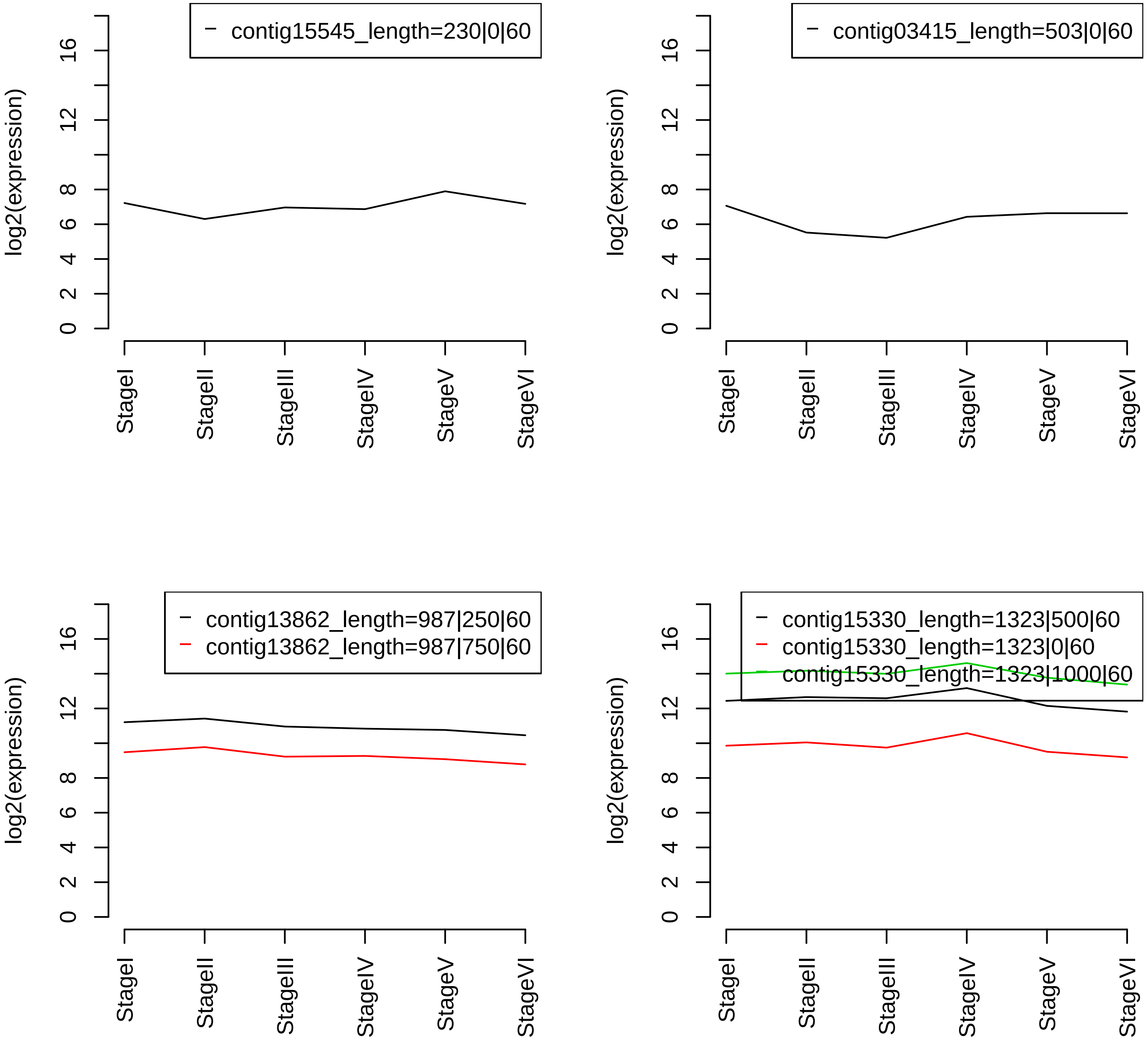

contig15545_length=230|0|60
contig03415_length=503|0|60
contig13862_length=987|250|60
contig13862_length=987|750|60
contig15330_length=1323|500|60
contig15330_length=1323|0|60
contig15330_length=1323|1000|60
log2(expression)
0 2 4 6 8 12 16
StageI
StageII
StageIII
StageIV
StageV
StageVI

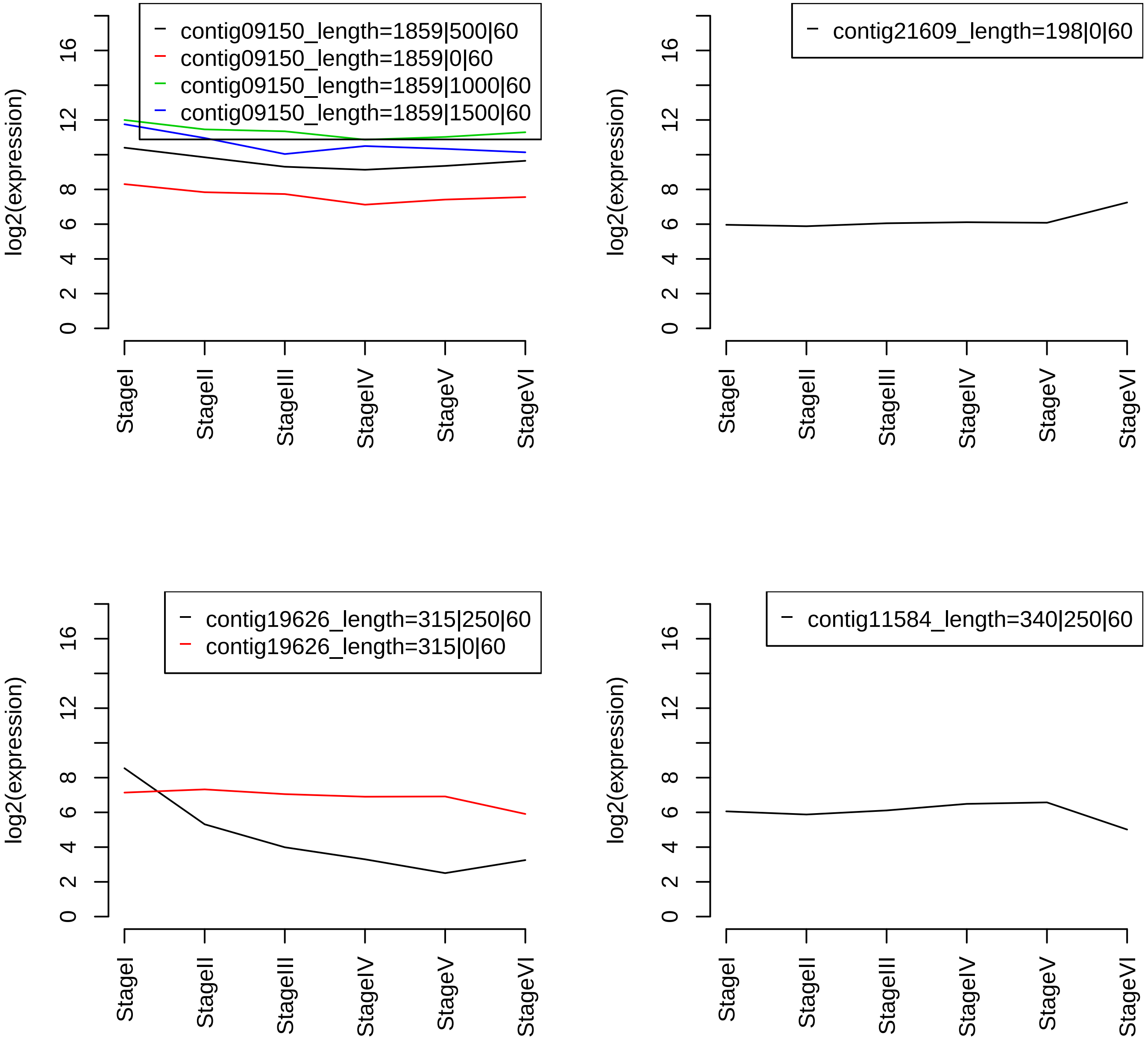
contig09150_length=1859|500|60
contig09150_length=1859|0|60
contig09150_length=1859|1000|60
contig09150_length=1859|1500|60
log2(expression)
0
2
4
6
8
12
16
StageI
StageII
StageIII
StageIV
StageV
StageVI
contig21609_length=198|0|60
contig19626_length=315|250|60
contig19626_length=315|0|60
contig11584_length=340|250|60

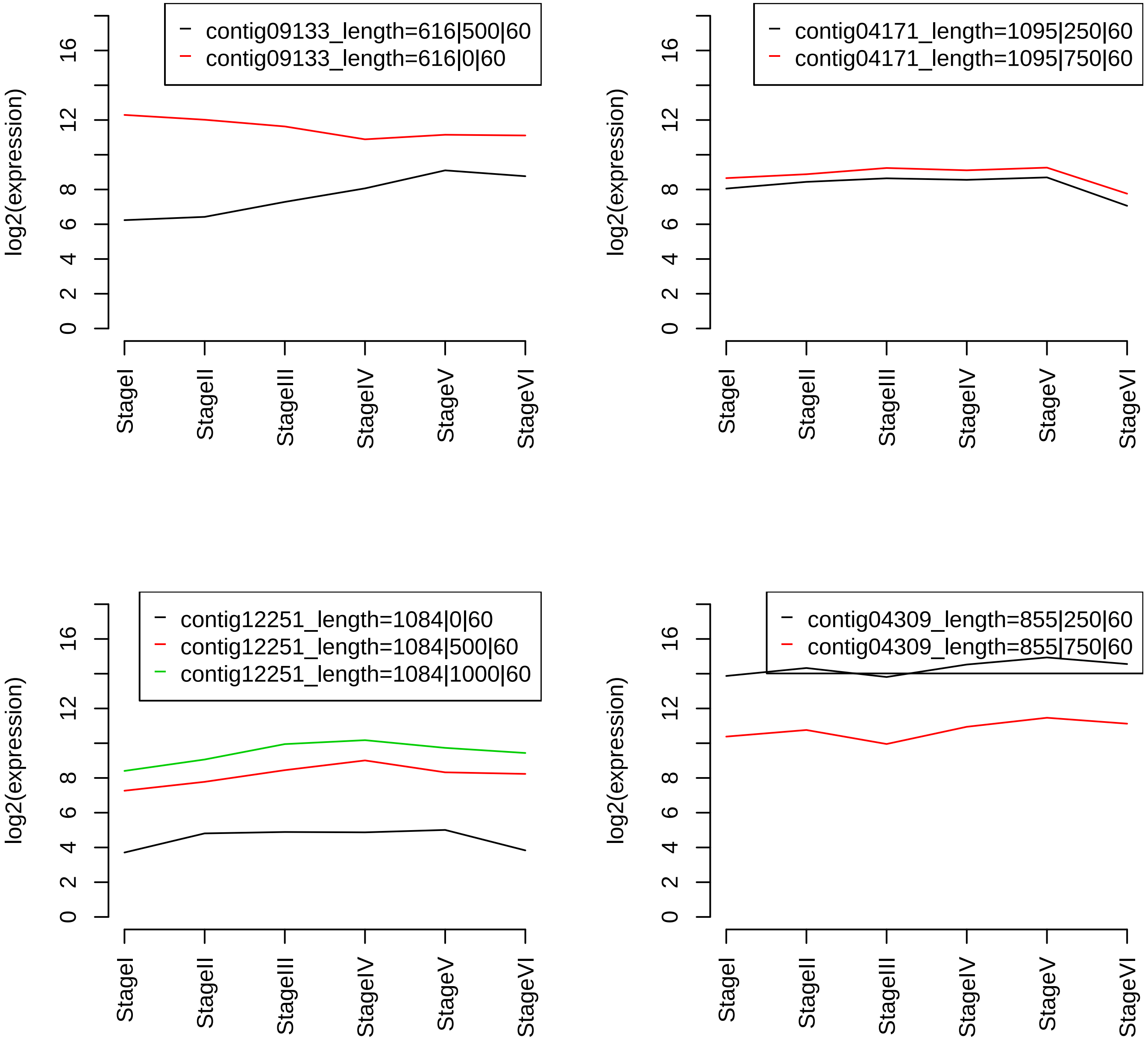

contig09133_length=616|500|60
contig09133_length=616|0|60
contig04171_length=1095|250|60
contig04171_length=1095|750|60
contig12251_length=1084|0|60
contig12251_length=1084|500|60
contig12251_length=1084|1000|60
contig04309_length=855|250|60
contig04309_length=855|750|60
log2(expression)
0 2 4 6 8 12 16
StageI StageII StageIII StageIV StageV StageVI

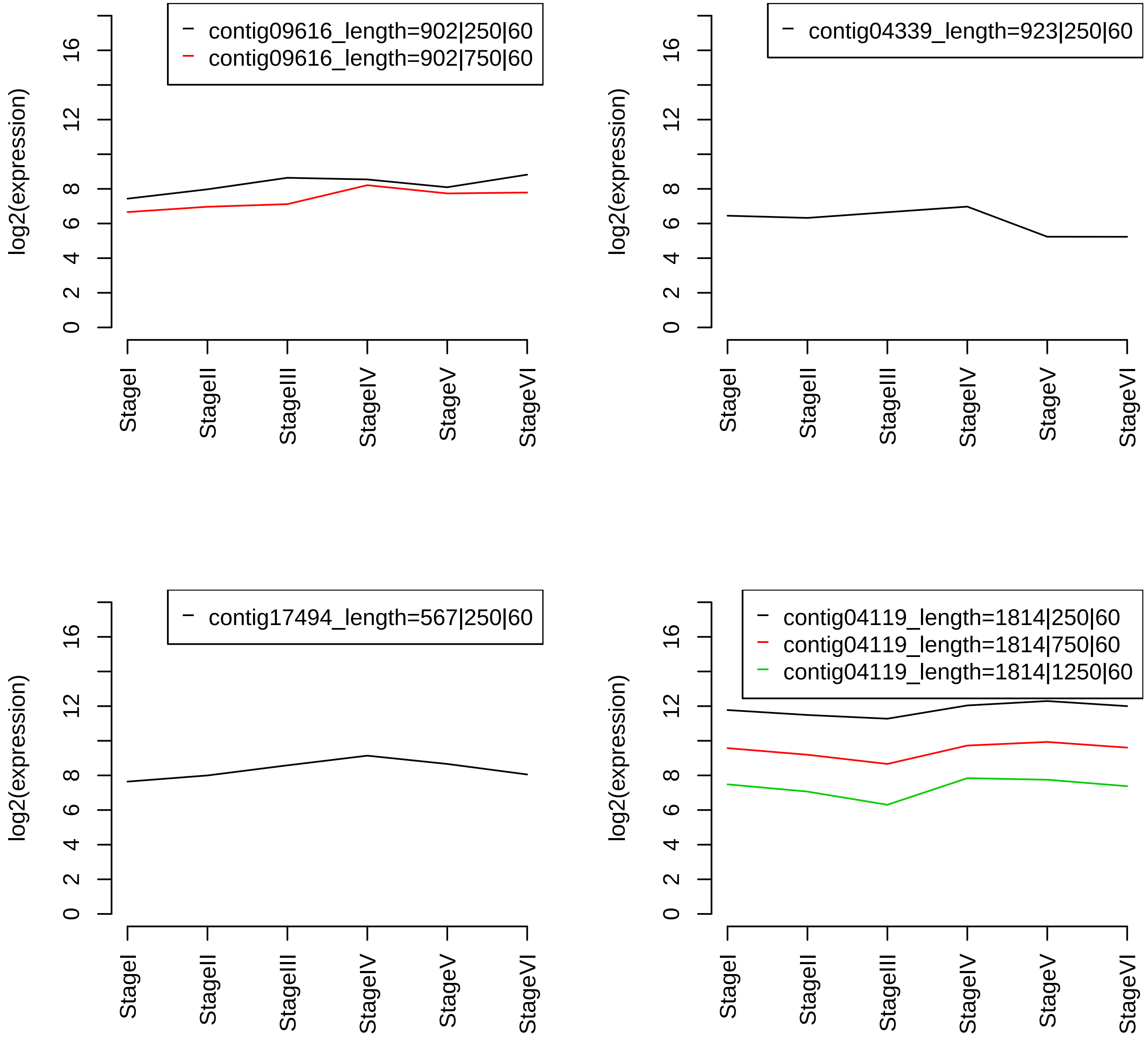
contig09616_length=902|250|60
contig09616_length=902|750|60
log2(expression)
0
2
4
6
8
12
16
StageI
StageII
StageIII
StageIV
StageV
StageVI
contig04339_length=923|250|60
contig17494_length=567|250|60
contig04119_length=1814|250|60
contig04119_length=1814|750|60
contig04119_length=1814|1250|60

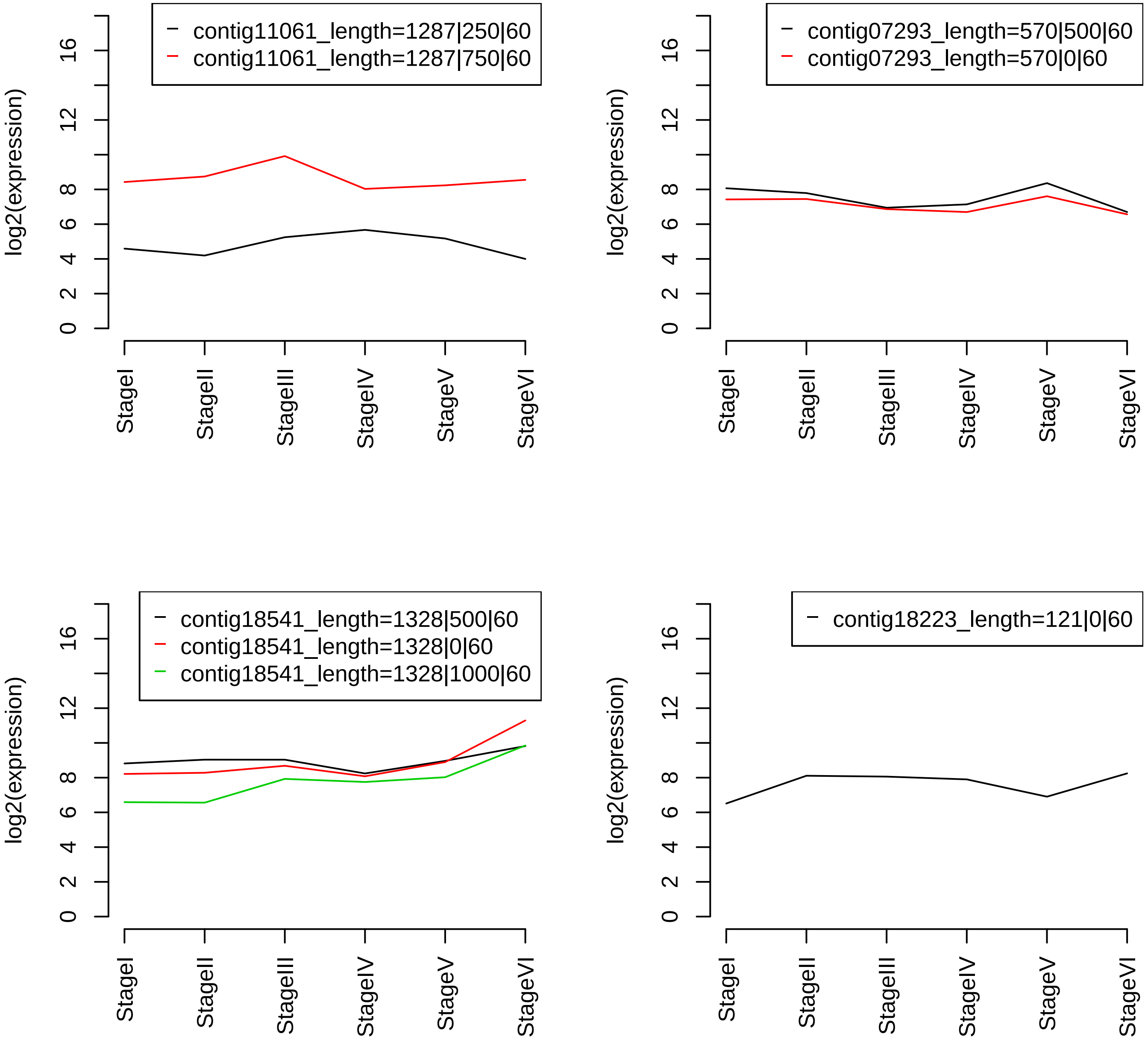

contig11061_length=1287|250|60
contig11061_length=1287|750|60
contig07293_length=570|500|60
contig07293_length=570|0|60
contig18541_length=1328|500|60
contig18541_length=1328|0|60
contig18541_length=1328|1000|60
contig18223_length=121|0|60
log2(expression)
0
2
4
6
8
12
16
StageI
StageII
StageIII
StageIV
StageV
StageVI

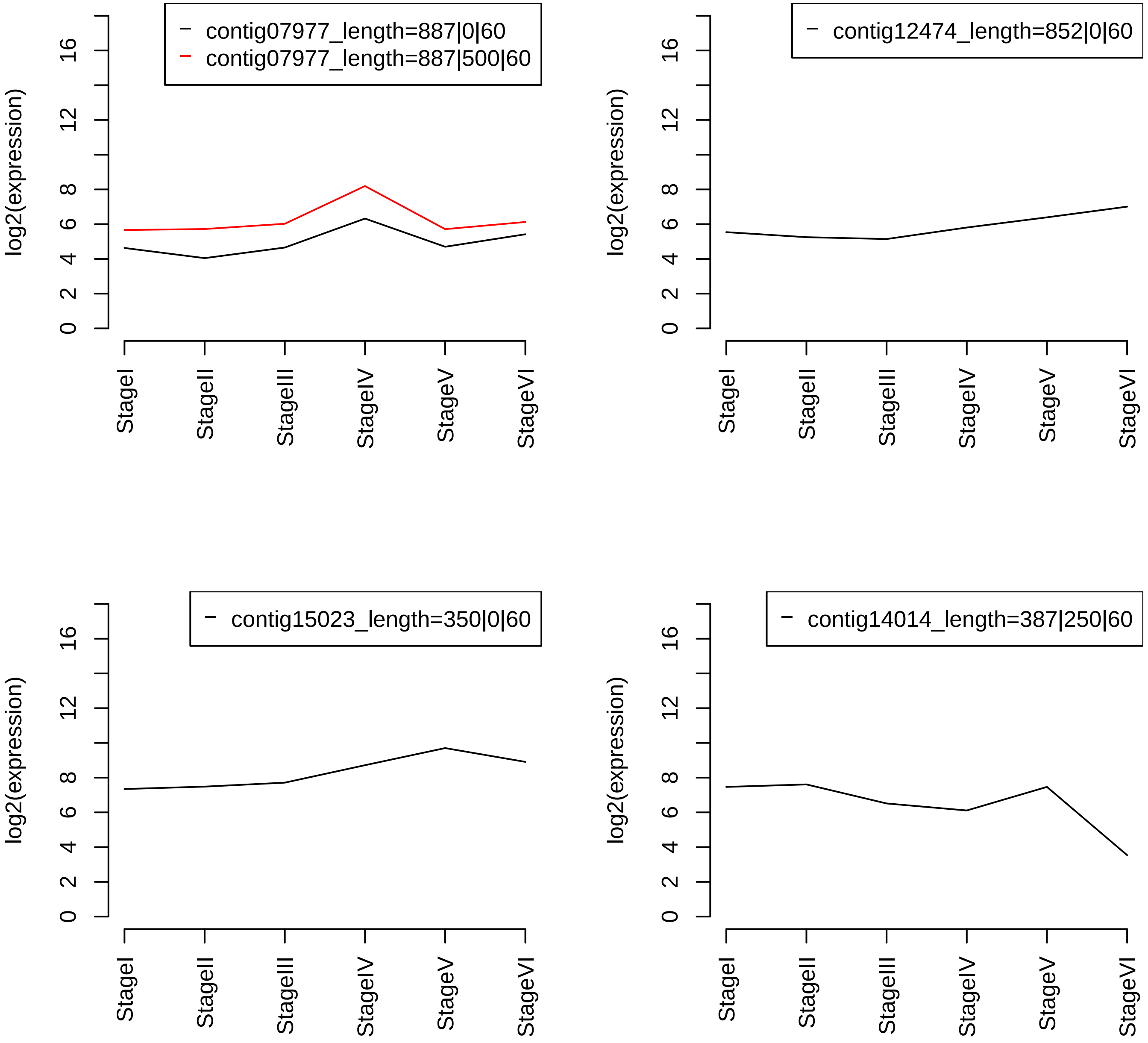
contig07977_length=887|0|60
contig07977_length=887|500|60
contig12474_length=852|0|60
contig15023_length=350|0|60
contig14014_length=387|250|60
log2(expression)
0
2
4
6
8
12
16
StageI
StageII
StageIII
StageIV
StageV
StageVI

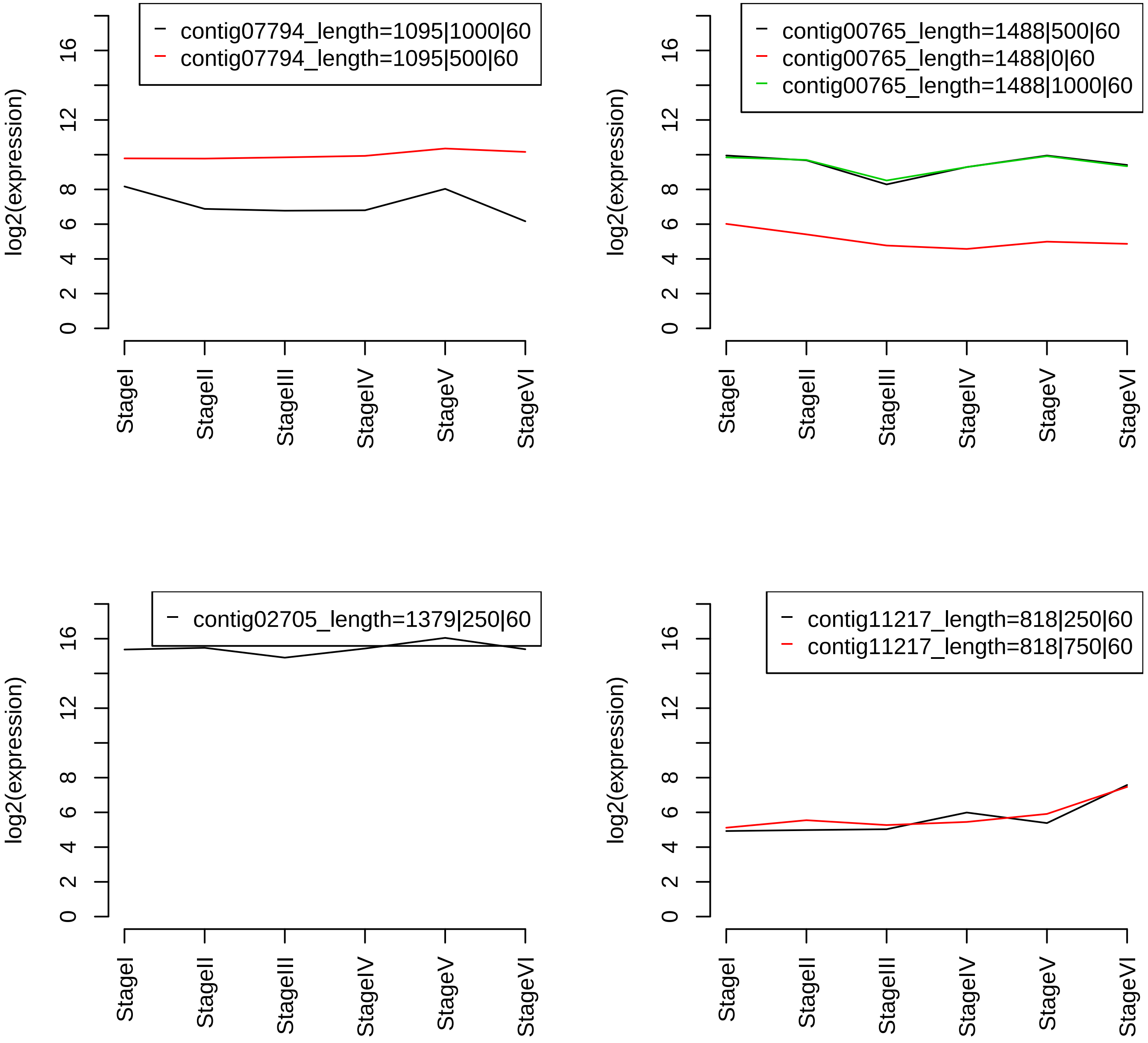
contig07794_length=1095|1000|60
contig07794_length=1095|500|60
contig00765_length=1488|500|60
contig00765_length=1488|0|60
contig00765_length=1488|1000|60
contig02705_length=1379|250|60
contig11217_length=818|250|60
contig11217_length=818|750|60
log2(expression)
0 2 4 6 8 12 16
StageI StageII StageIII StageIV StageV StageVI

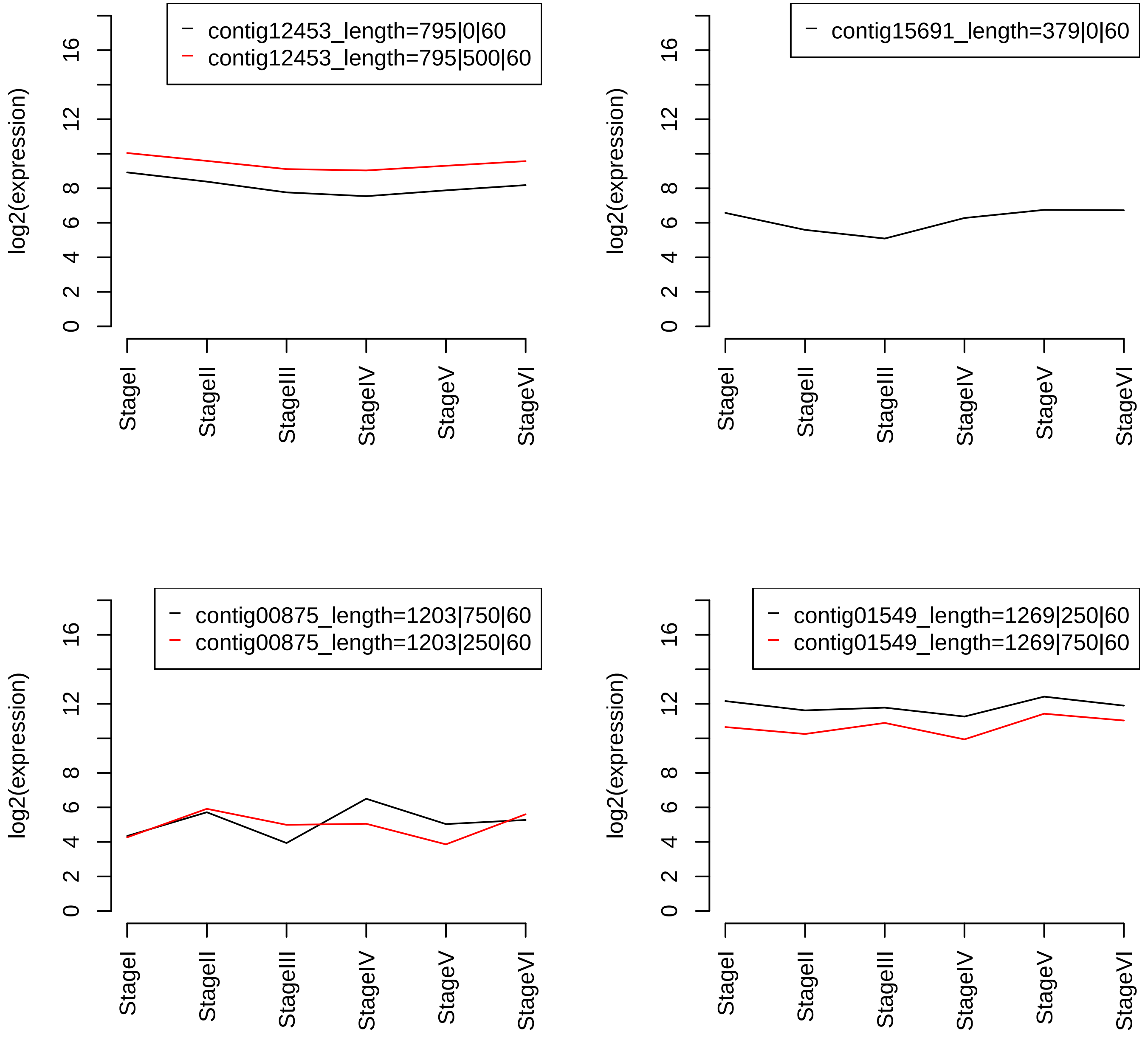
contig12453_length=795|0|60
contig12453_length=795|500|60
contig15691_length=379|0|60
contig00875_length=1203|750|60
contig00875_length=1203|250|60
contig01549_length=1269|250|60
contig01549_length=1269|750|60
log2(expression)
0
2
4
6
8
12
16
StageI
StageII
StageIII
StageIV
StageV
StageVI

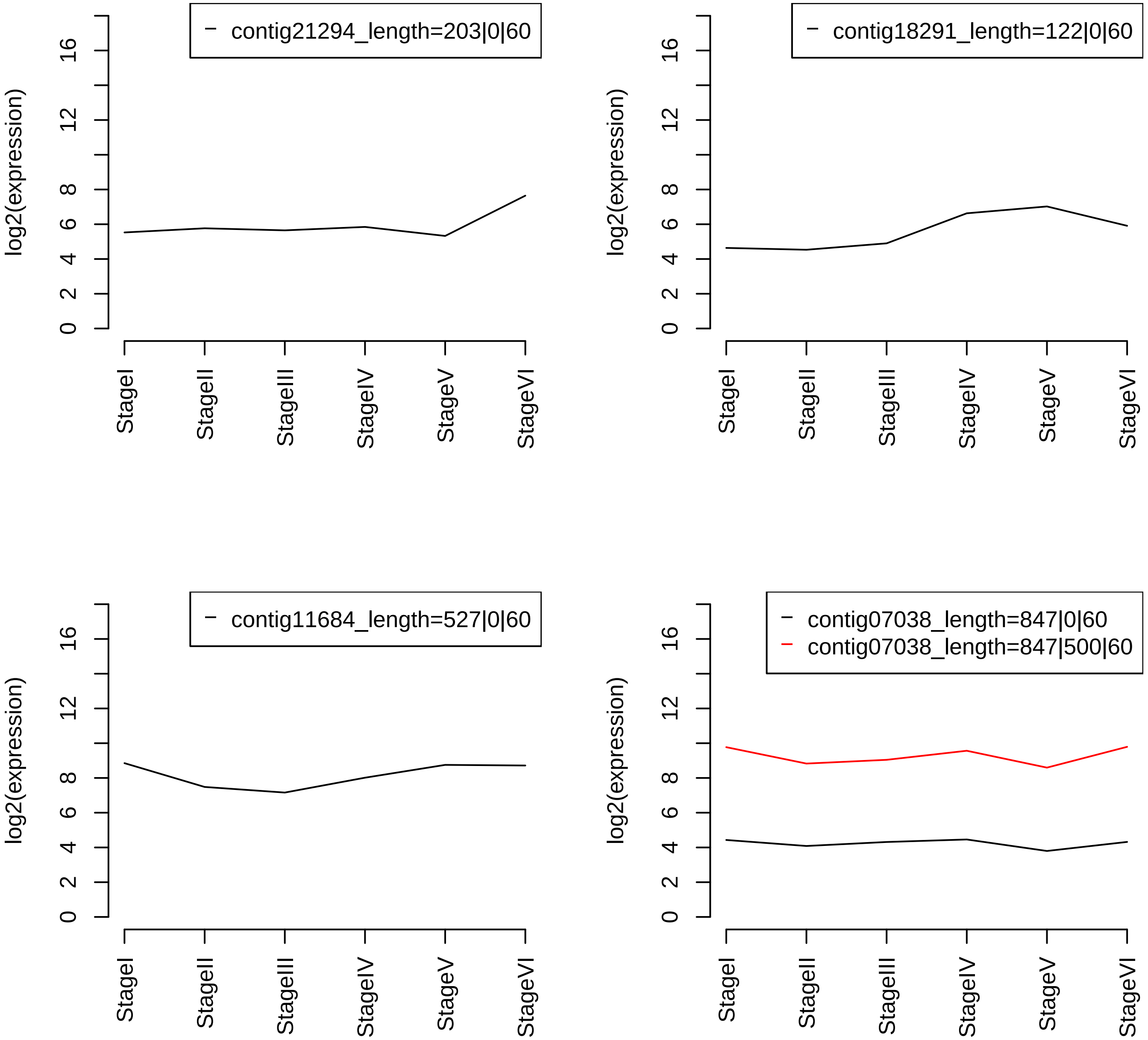
contig21294_length=203|0|60
contig18291_length=122|0|60
contig11684_length=527|0|60
contig07038_length=847|0|60
contig07038_length=847|500|60
log2(expression)
0
2
4
6
8
12
16
StageI
StageII
StageIII
StageIV
StageV
StageVI

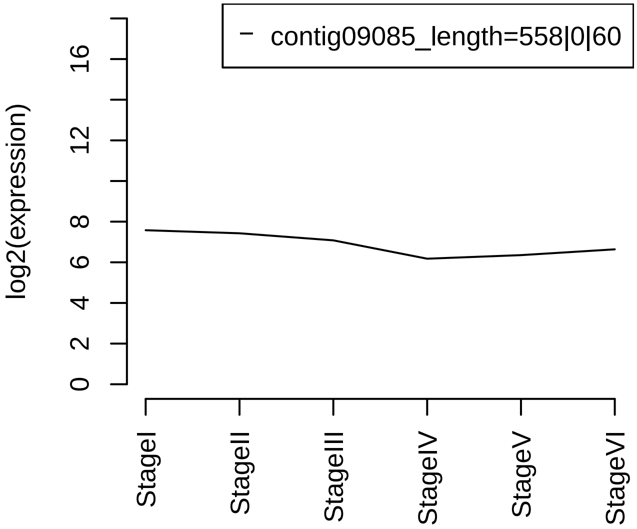

contig09085_length=558|0|60
log2(expression)
0 2 4 6 8 12 16
StageI StageII StageIII StageIV StageV StageVI

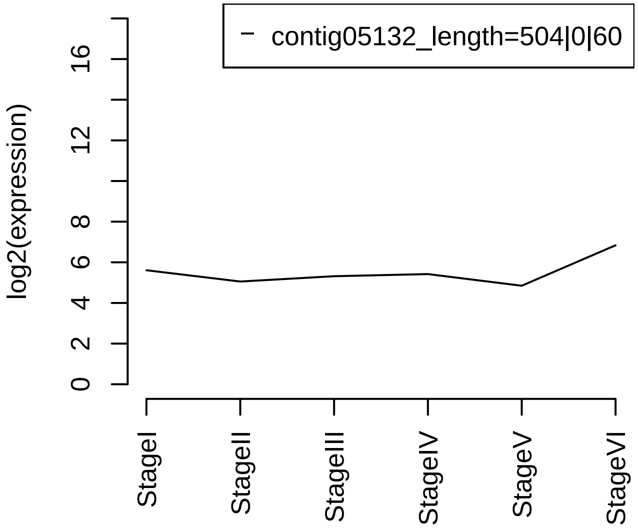

contig05132_length=504|0|60
log2(expression)
0 2 4 6 8 12 16
StageI StageII StageIII StageIV StageV StageVI

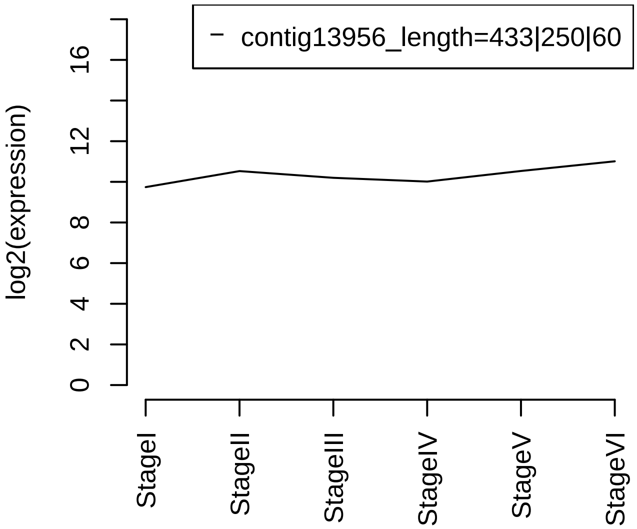

contig13956_length=433|250|60
log2(expression)
0 2 4 6 8 12 16
StageI StageII StageIII StageIV StageV StageVI

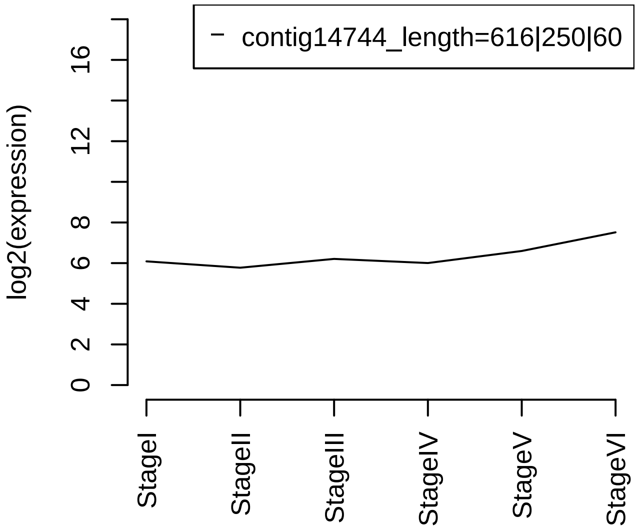

contig14744_length=616|250|60
log2(expression)
0 2 4 6 8 12 16
StageI StageII StageIII StageIV StageV StageVI

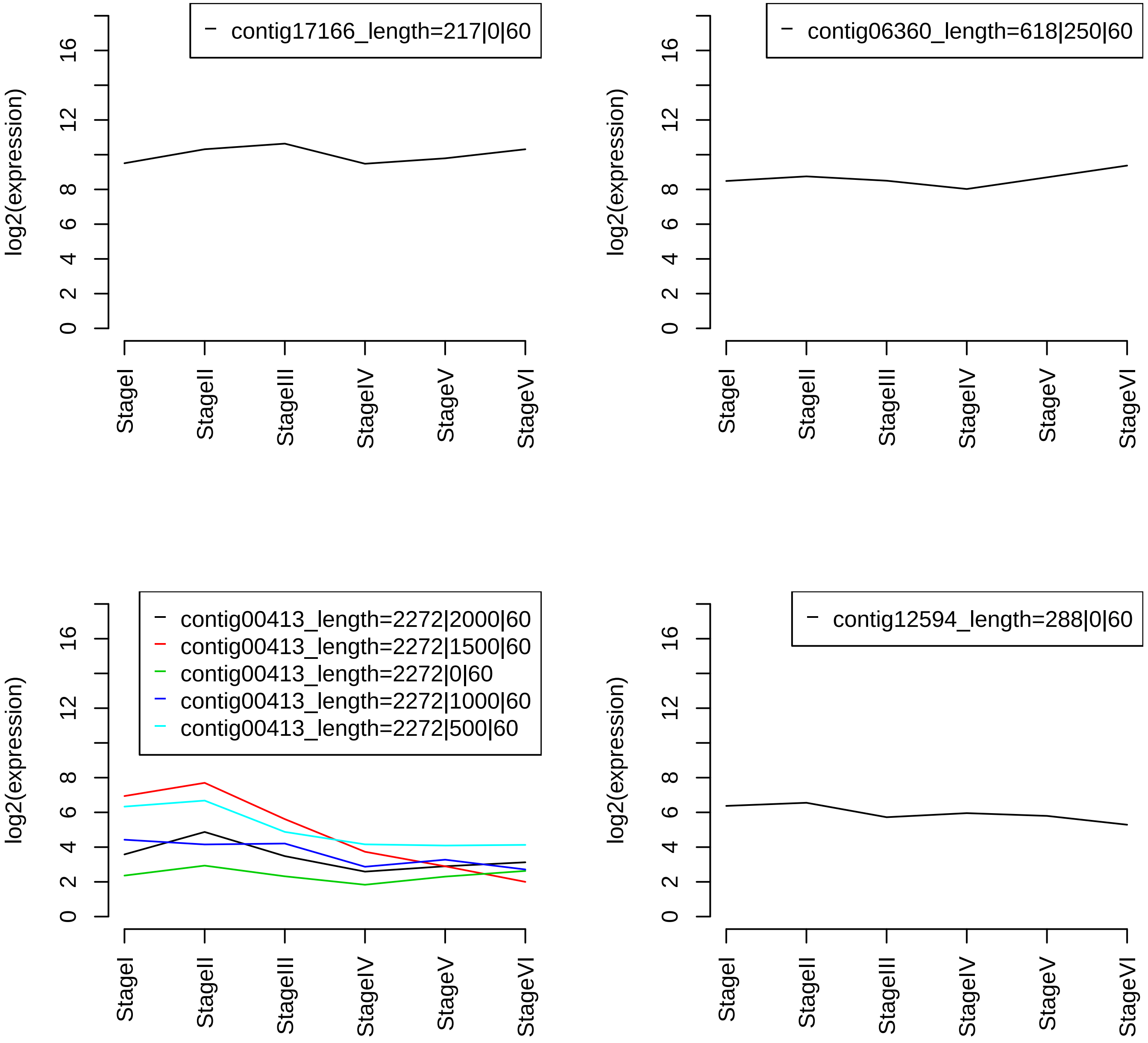
contig17166_length=217|0|60
contig06360_length=618|250|60
contig00413_length=2272|2000|60
contig00413_length=2272|1500|60
contig00413_length=2272|0|60
contig00413_length=2272|1000|60
contig00413_length=2272|500|60
contig12594_length=288|0|60
log2(expression)
0
2
4
6
8
12
16
StageI
StageII
StageIII
StageIV
StageV
StageVI

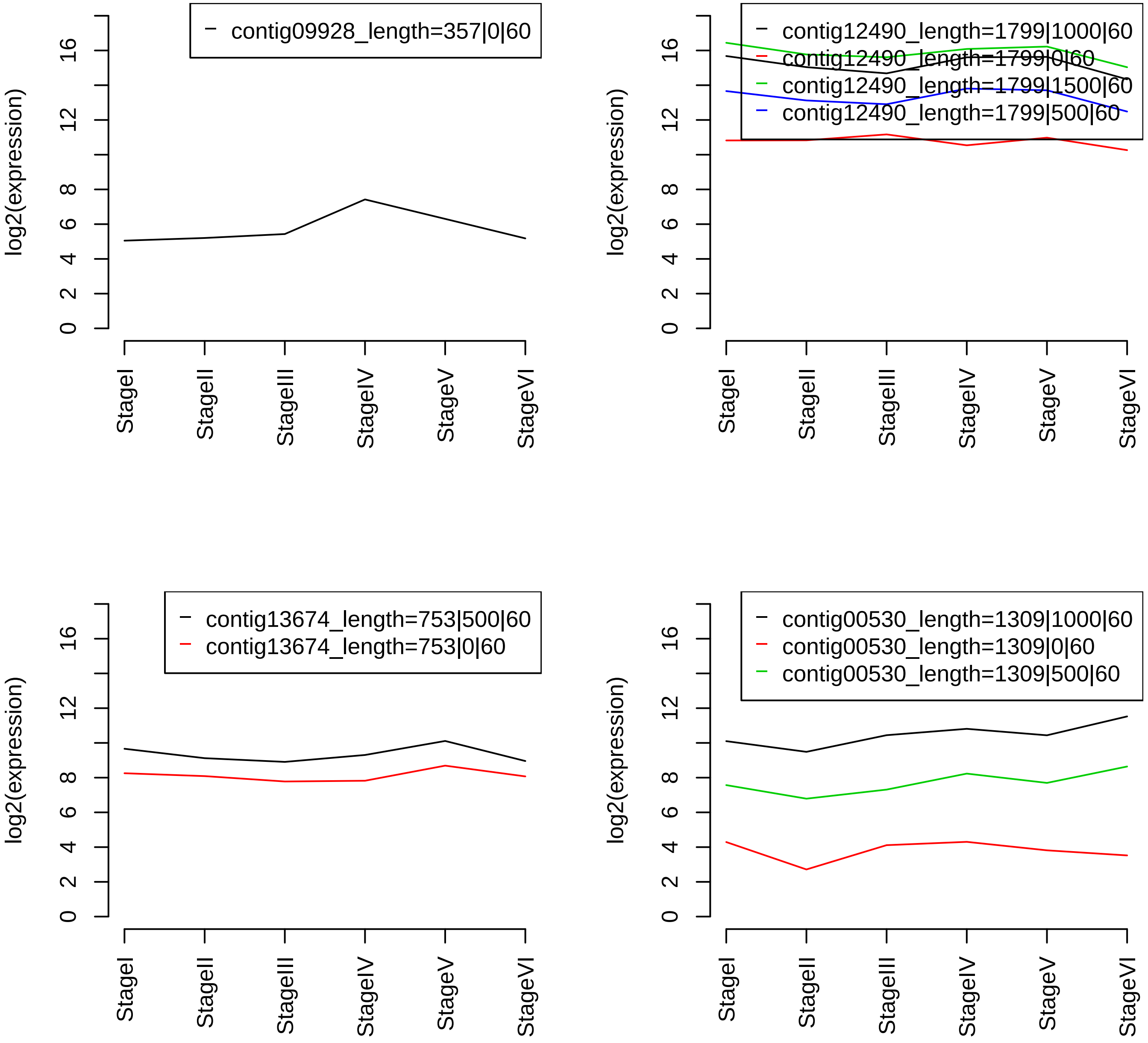
contig09928_length=357|0|60
contig12490_length=1799|1000|60
contig12490_length=1799|0|60
contig12490_length=1799|1500|60
contig12490_length=1799|500|60
contig13674_length=753|500|60
contig13674_length=753|0|60
contig00530_length=1309|1000|60
contig00530_length=1309|0|60
contig00530_length=1309|500|60
log2(expression)
0
2
4
6
8
12
16
StageI
StageII
StageIII
StageIV
StageV
StageVI

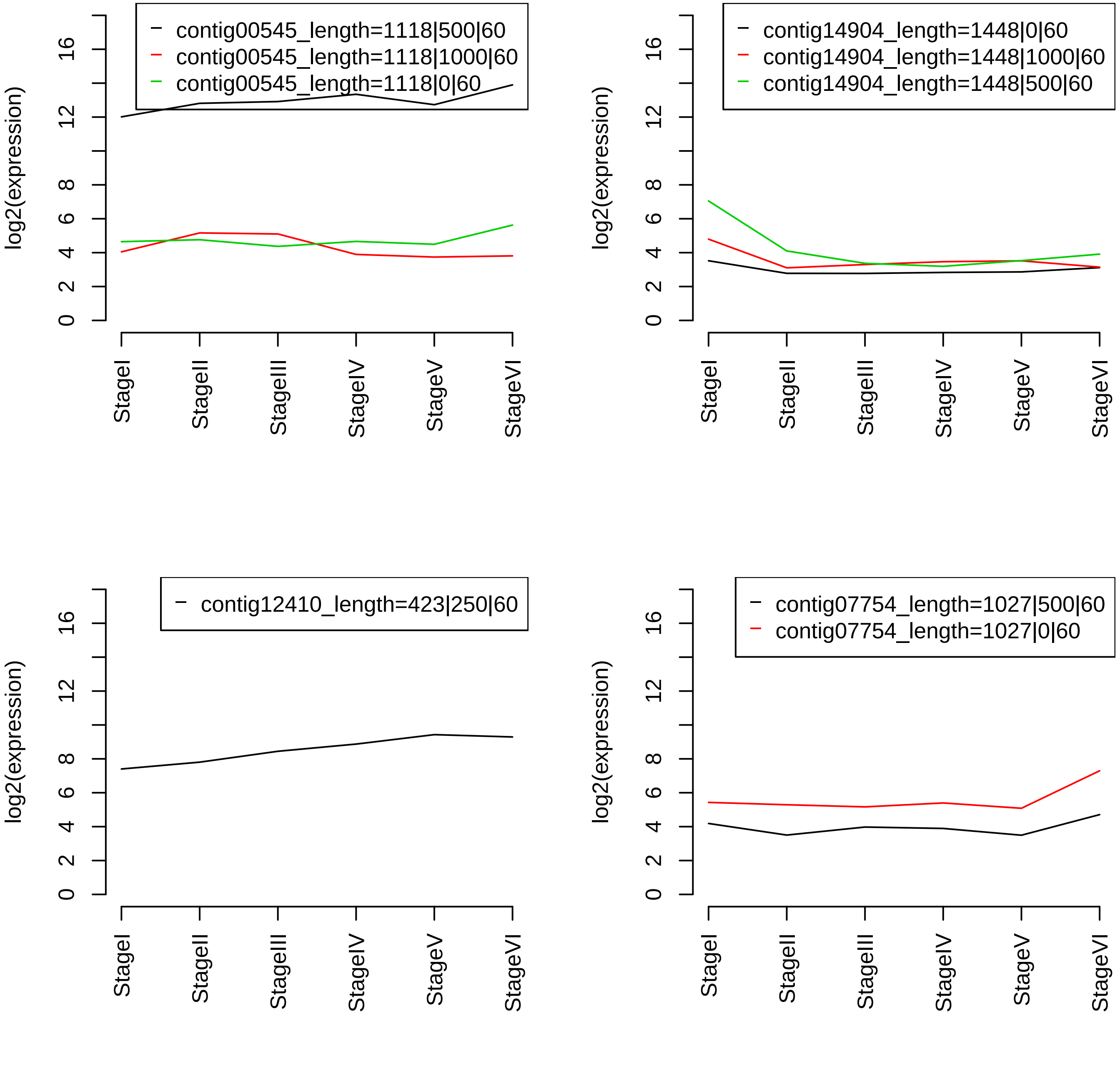
contig00545_length=1118|500|60
contig00545_length=1118|1000|60
contig00545_length=1118|0|60
contig14904_length=1448|0|60
contig14904_length=1448|1000|60
contig14904_length=1448|500|60
contig12410_length=423|250|60
contig07754_length=1027|500|60
contig07754_length=1027|0|60
log2(expression)
0
2
4
6
8
12
16
StageI
StageII
StageIII
StageIV
StageV
StageVI

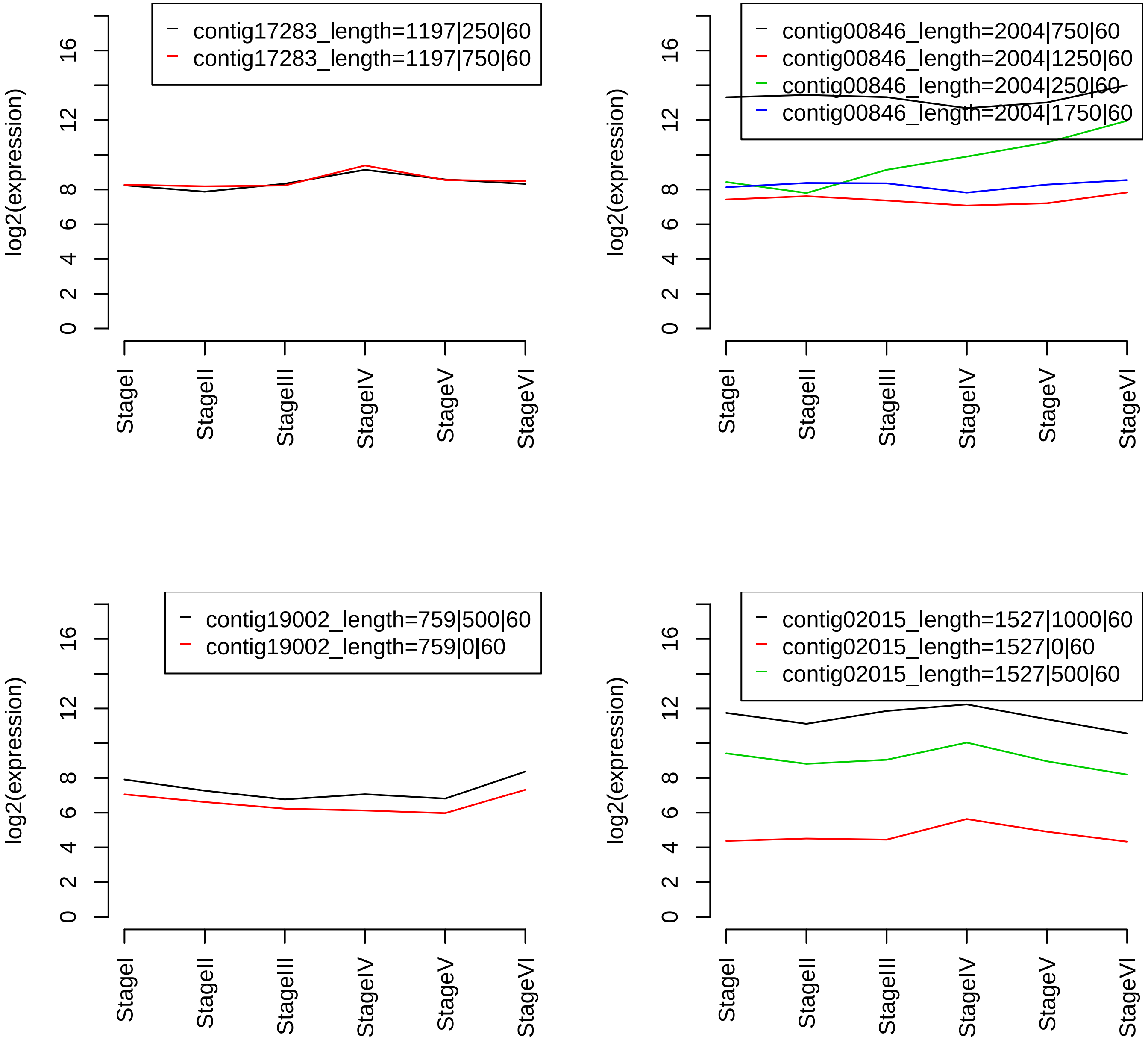
contig17283_length=1197|250|60
contig17283_length=1197|750|60
contig00846_length=2004|750|60
contig00846_length=2004|1250|60
contig00846_length=2004|250|60
contig00846_length=2004|1750|60
contig19002_length=759|500|60
contig19002_length=759|0|60
contig02015_length=1527|1000|60
contig02015_length=1527|0|60
contig02015_length=1527|500|60
log2(expression)
0
2
4
6
8
12
16
StageI
StageII
StageIII
StageIV
StageV
StageVI

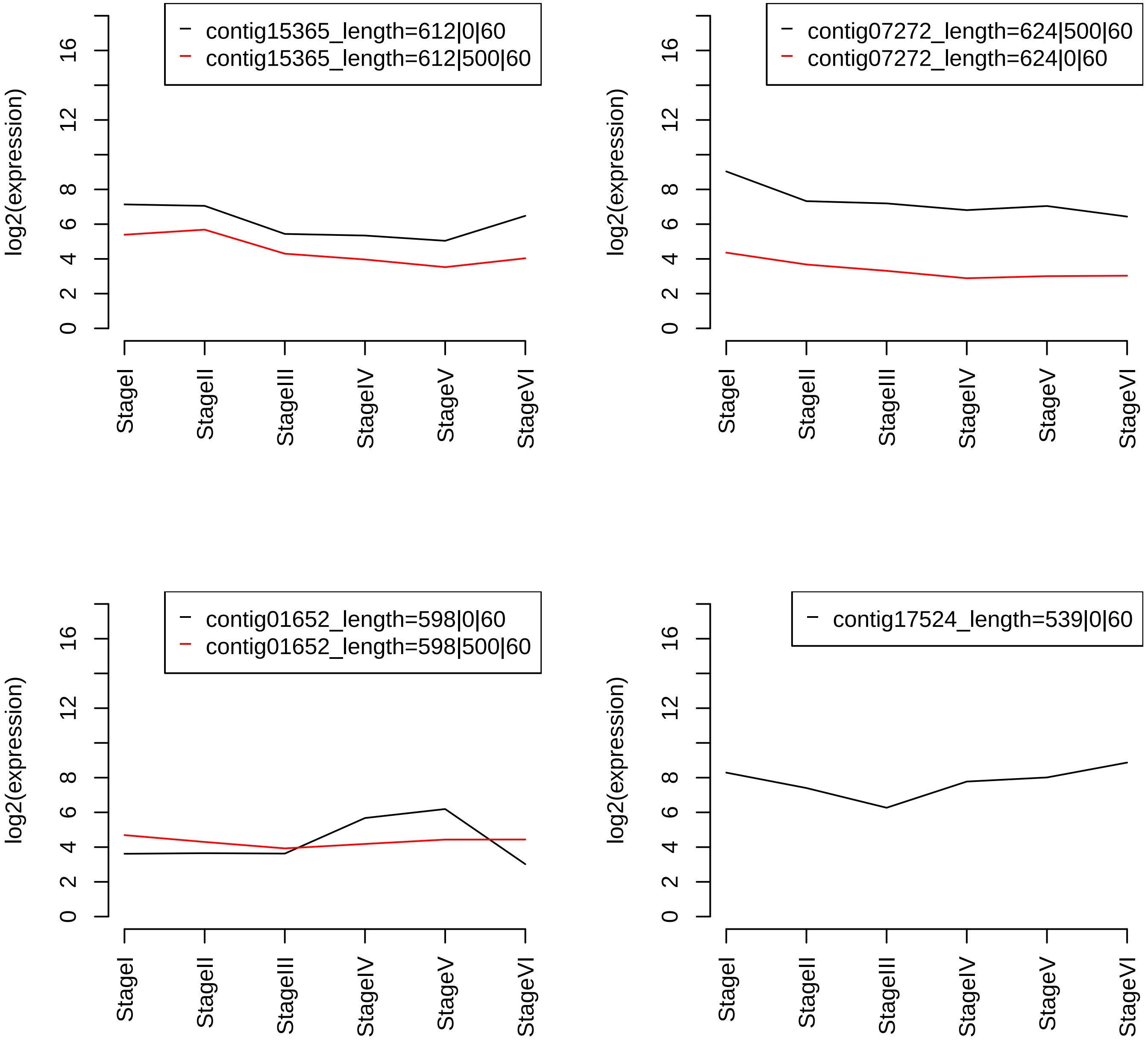

contig15365_length=612|0|60
contig15365_length=612|500|60
contig07272_length=624|500|60
contig07272_length=624|0|60
contig01652_length=598|0|60
contig01652_length=598|500|60
contig17524_length=539|0|60
log2(expression)
0
2
4
6
8
12
16
StageI
StageII
StageIII
StageIV
StageV
StageVI

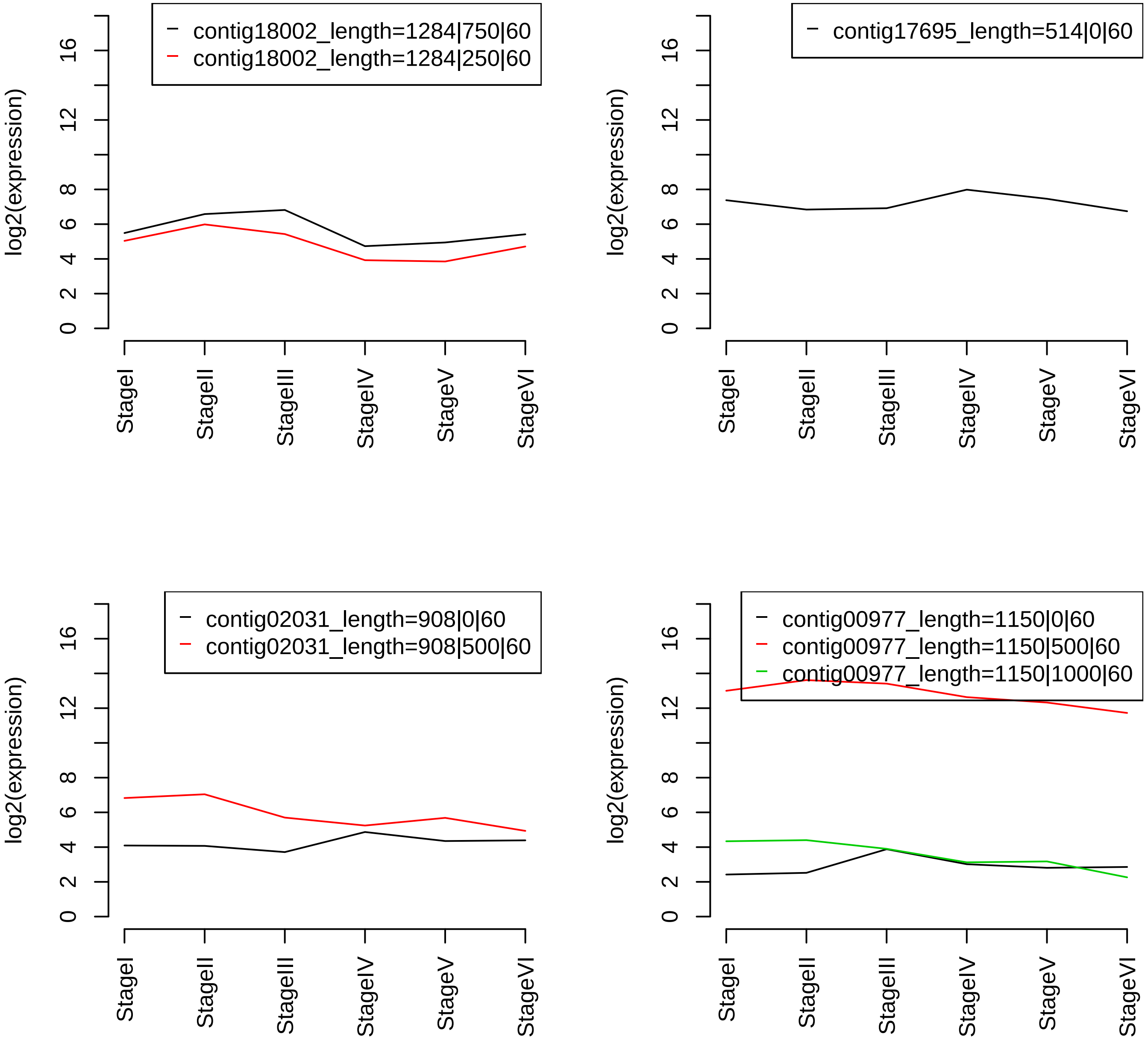
contig18002_length=1284|750|60
contig18002_length=1284|250|60
contig17695_length=514|0|60
contig02031_length=908|0|60
contig02031_length=908|500|60
contig00977_length=1150|0|60
contig00977_length=1150|500|60
contig00977_length=1150|1000|60
log2(expression)
0
2
4
6
8
12
16
StageI
StageII
StageIII
StageIV
StageV
StageVI

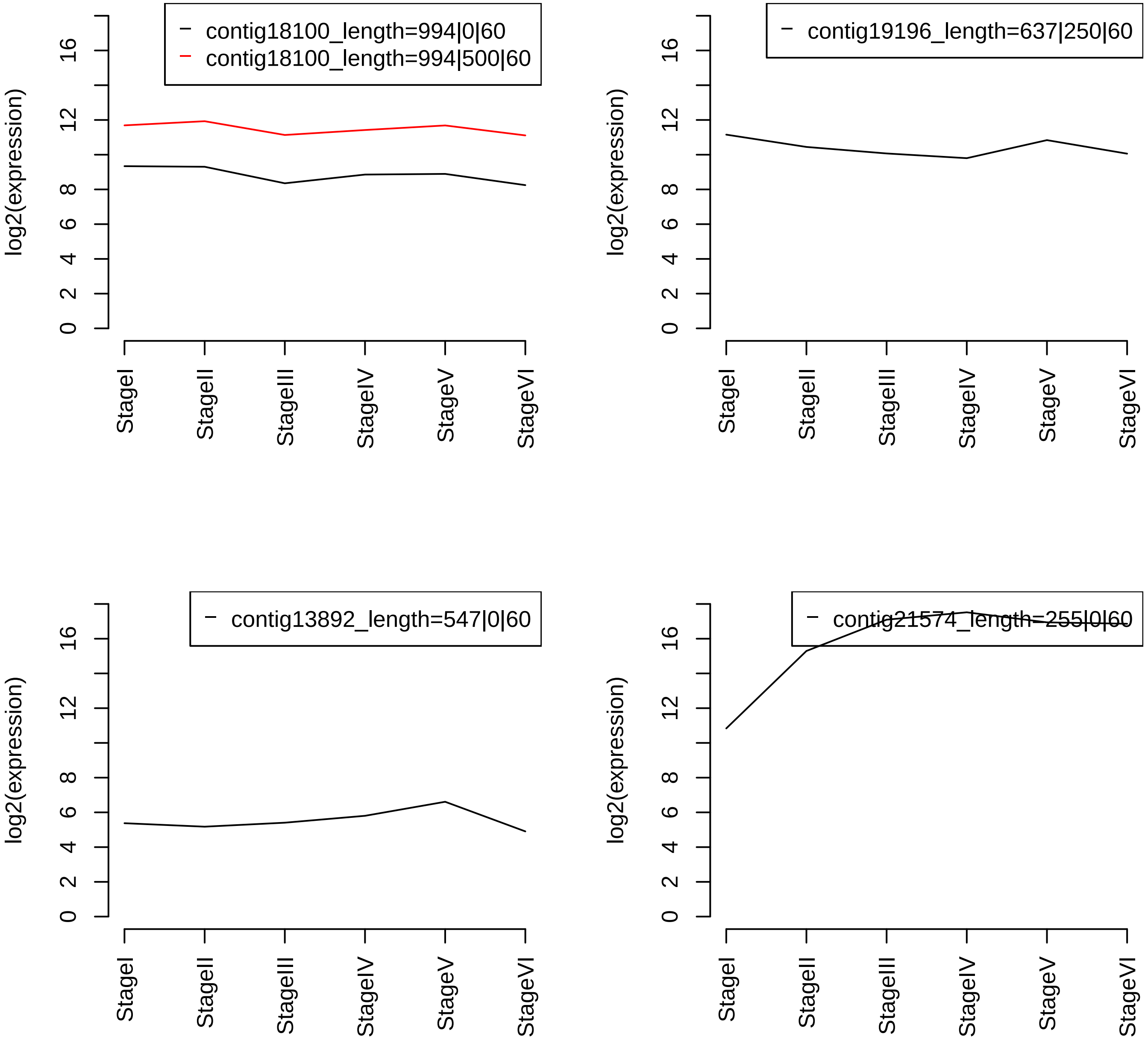
contig18100_length=994|0|60
contig18100_length=994|500|60
contig19196_length=637|250|60
contig13892_length=547|0|60
contig21574_length=255|0|60
log2(expression)
StageI
StageII
StageIII
StageIV
StageV
StageVI

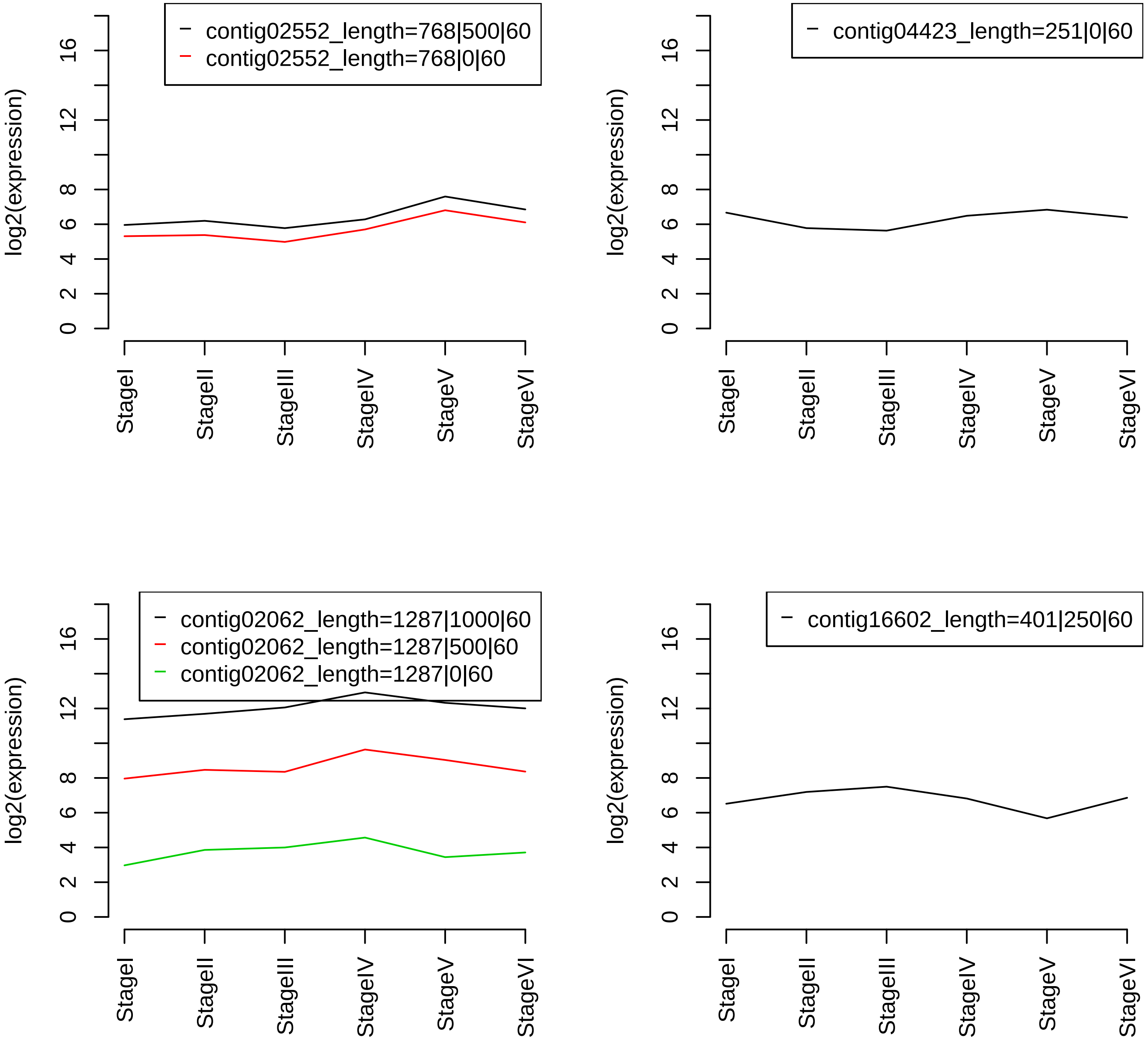
contig02552_length=768|500|60
contig02552_length=768|0|60
contig04423_length=251|0|60
contig02062_length=1287|1000|60
contig02062_length=1287|500|60
contig02062_length=1287|0|60
contig16602_length=401|250|60
log2(expression)
0 2 4 6 8 12 16
StageI
StageII
StageIII
StageIV
StageV
StageVI

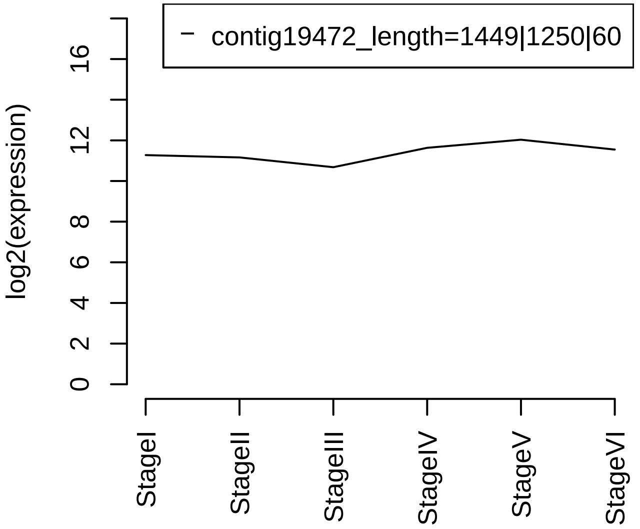

contig19472_length=1449|1250|60
log2(expression)
0
2
4
6
8
12
16
StageI
StageII
StageIII
StageIV
StageV
StageVI

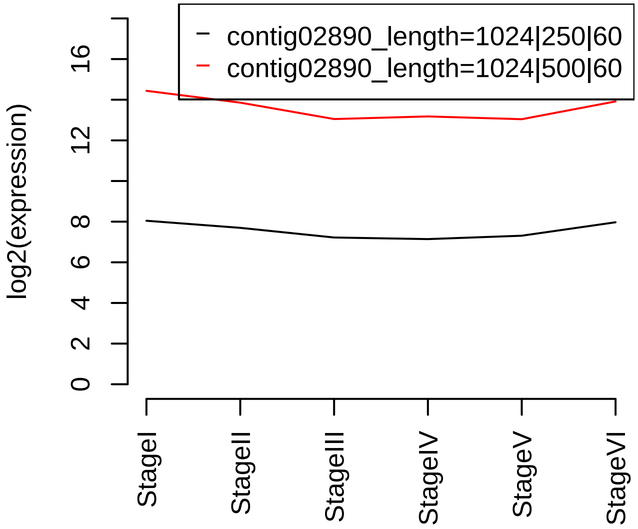

contig02890_length=1024|250|60
contig02890_length=1024|500|60
log2(expression)
0
2
4
6
8
12
16
StageI
StageII
StageIII
StageIV
StageV
StageVI

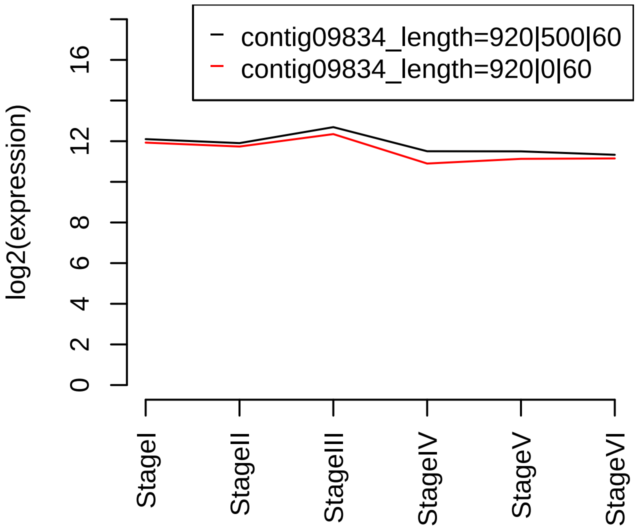

contig09834_length=920|500|60
contig09834_length=920|0|60
log2(expression)
0
2
4
6
8
12
16
StageI
StageII
StageIII
StageIV
StageV
StageVI

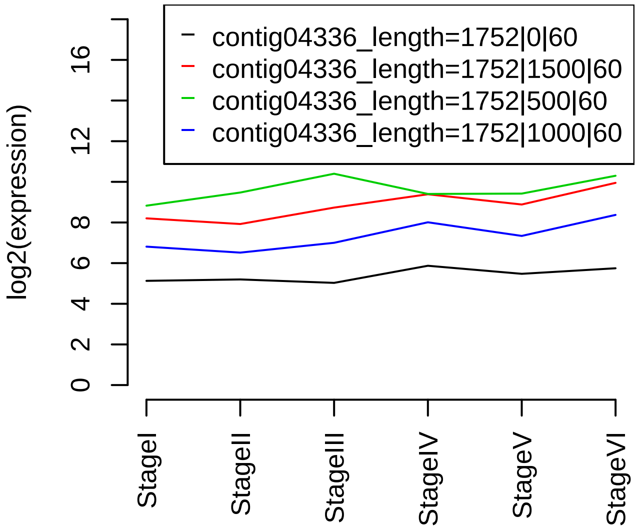

contig04336_length=1752|0|60
contig04336_length=1752|1500|60
contig04336_length=1752|500|60
contig04336_length=1752|1000|60
log2(expression)
0
2
4
6
8
12
16
StageI
StageII
StageIII
StageIV
StageV
StageVI

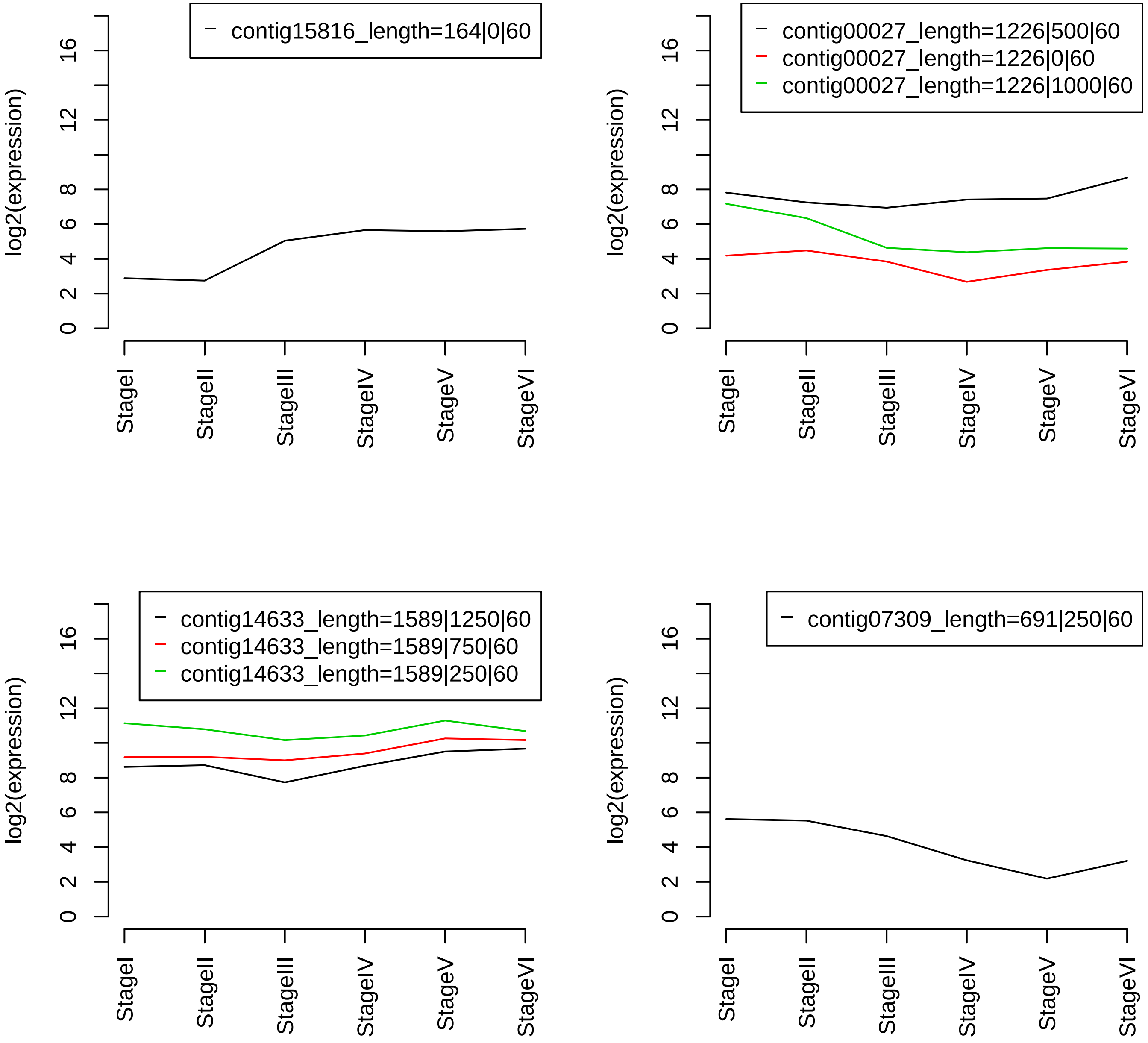

contig15816_length=164|0|60
contig00027_length=1226|500|60
contig00027_length=1226|0|60
contig00027_length=1226|1000|60
contig14633_length=1589|1250|60
contig14633_length=1589|750|60
contig14633_length=1589|250|60
contig07309_length=691|250|60
log2(expression)
0
2
4
6
8
12
16
StageI
StageII
StageIII
StageIV
StageV
StageVI

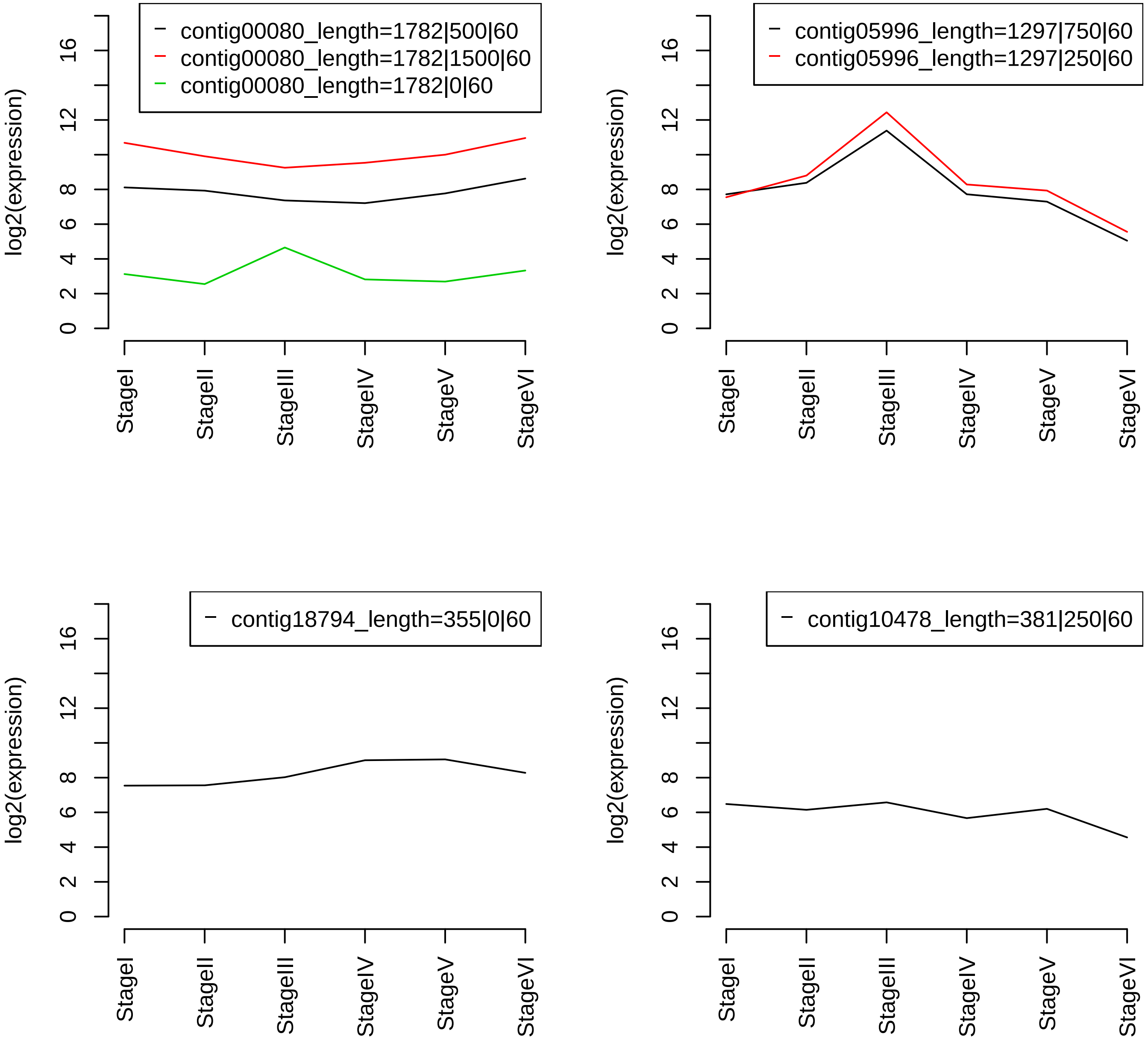

contig00080_length=1782|500|60
contig00080_length=1782|1500|60
contig00080_length=1782|0|60
contig05996_length=1297|750|60
contig05996_length=1297|250|60
contig18794_length=355|0|60
contig10478_length=381|250|60
log2(expression)
0
2
4
6
8
12
16
StageI
StageII
StageIII
StageIV
StageV
StageVI

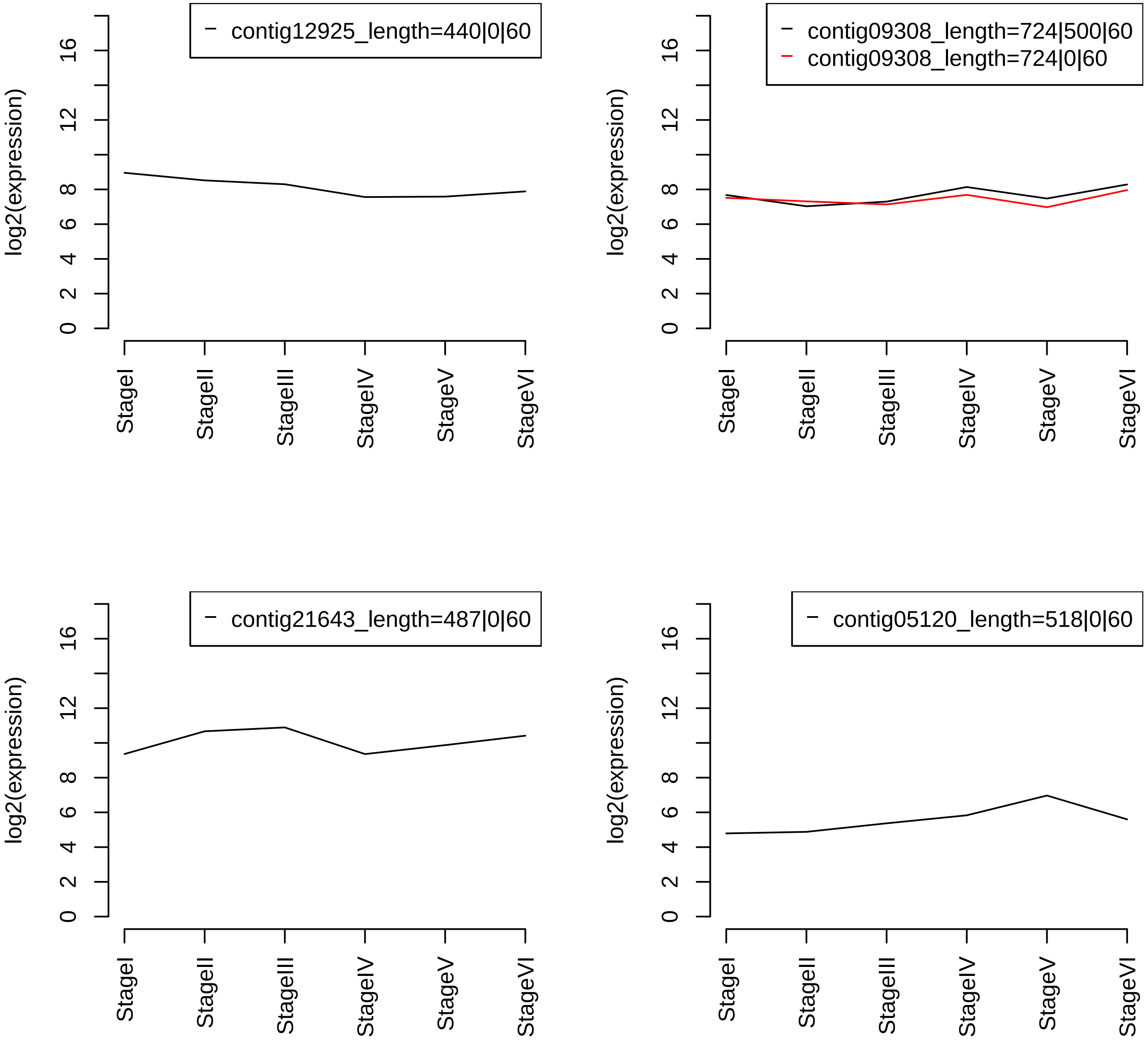
contig12925_length=440|0|60
contig09308_length=724|500|60
contig09308_length=724|0|60
contig21643_length=487|0|60
contig05120_length=518|0|60
log2(expression)
0 2 4 6 8 12 16
StageI
StageII
StageIII
StageIV
StageV
StageVI

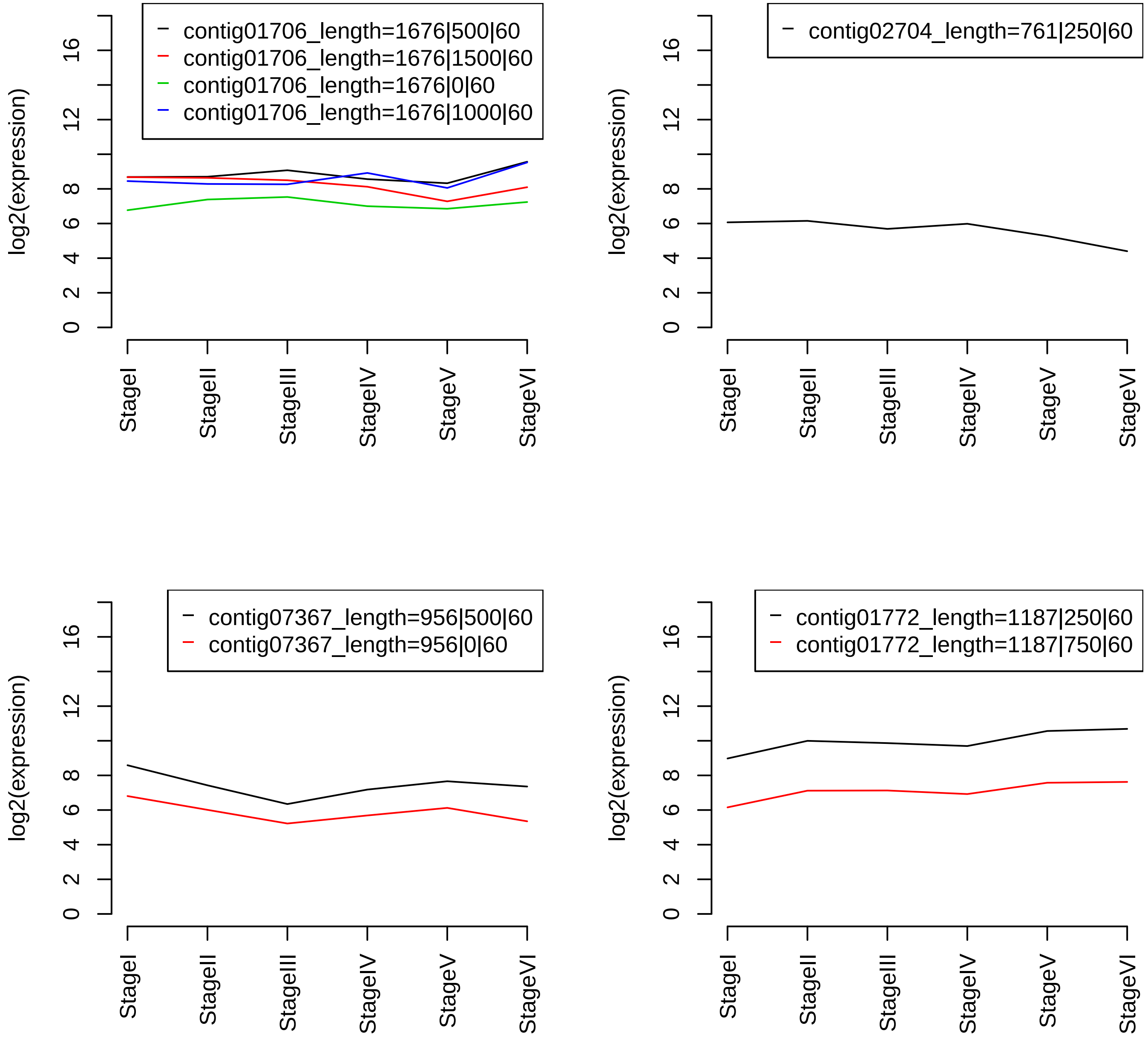

contig01706_length=1676|500|60
contig01706_length=1676|1500|60
contig01706_length=1676|0|60
contig01706_length=1676|1000|60
contig02704_length=761|250|60
contig07367_length=956|500|60
contig07367_length=956|0|60
contig01772_length=1187|250|60
contig01772_length=1187|750|60
log2(expression)
0
2
4
6
8
12
16
StageI
StageII
StageIII
StageIV
StageV
StageVI

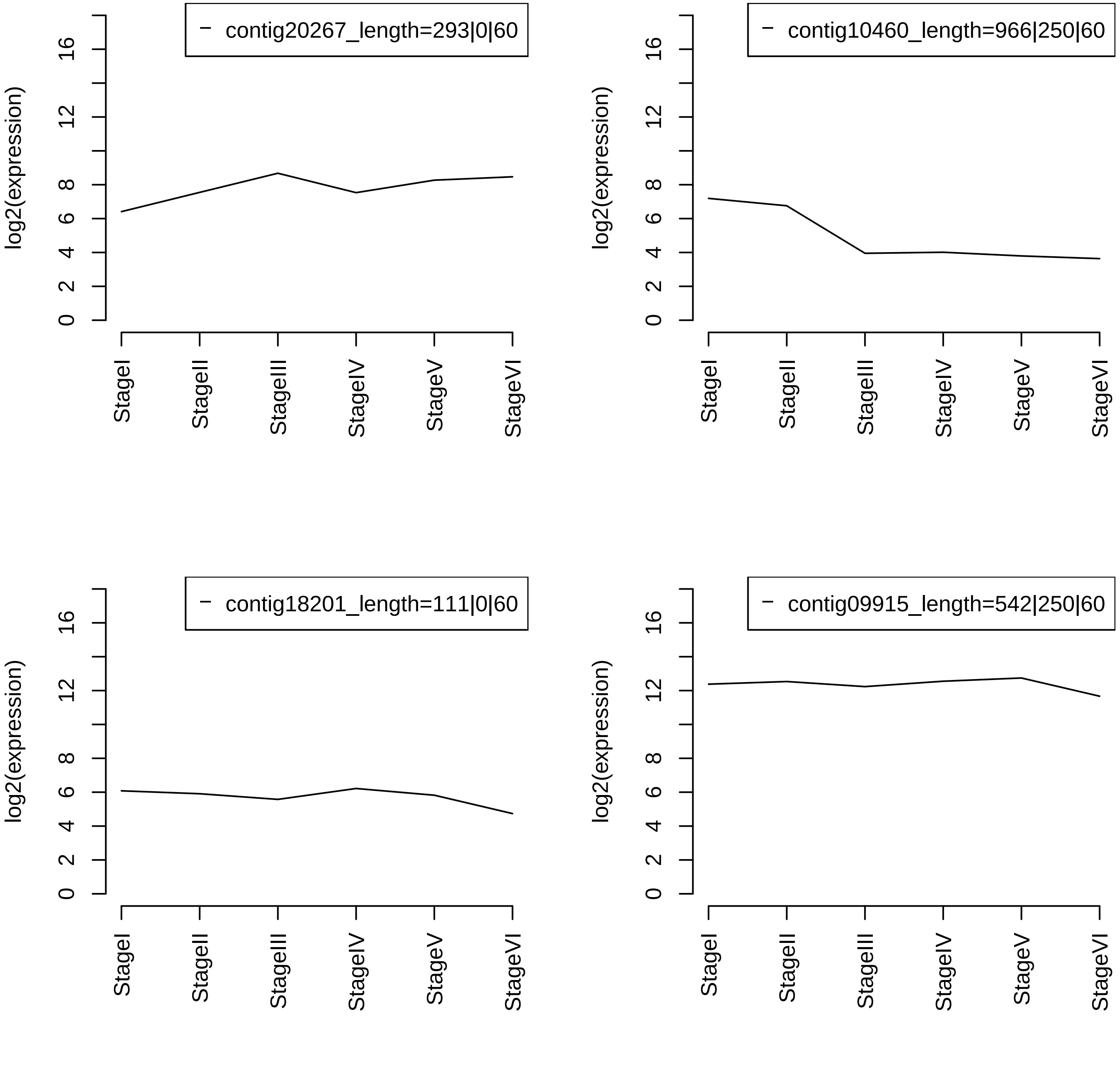

contig20267_length=293|0|60
contig10460_length=966|250|60
contig18201_length=111|0|60
contig09915_length=542|250|60
log2(expression)
0
2
4
6
8
12
16
StageI
StageII
StageIII
StageIV
StageV
StageVI

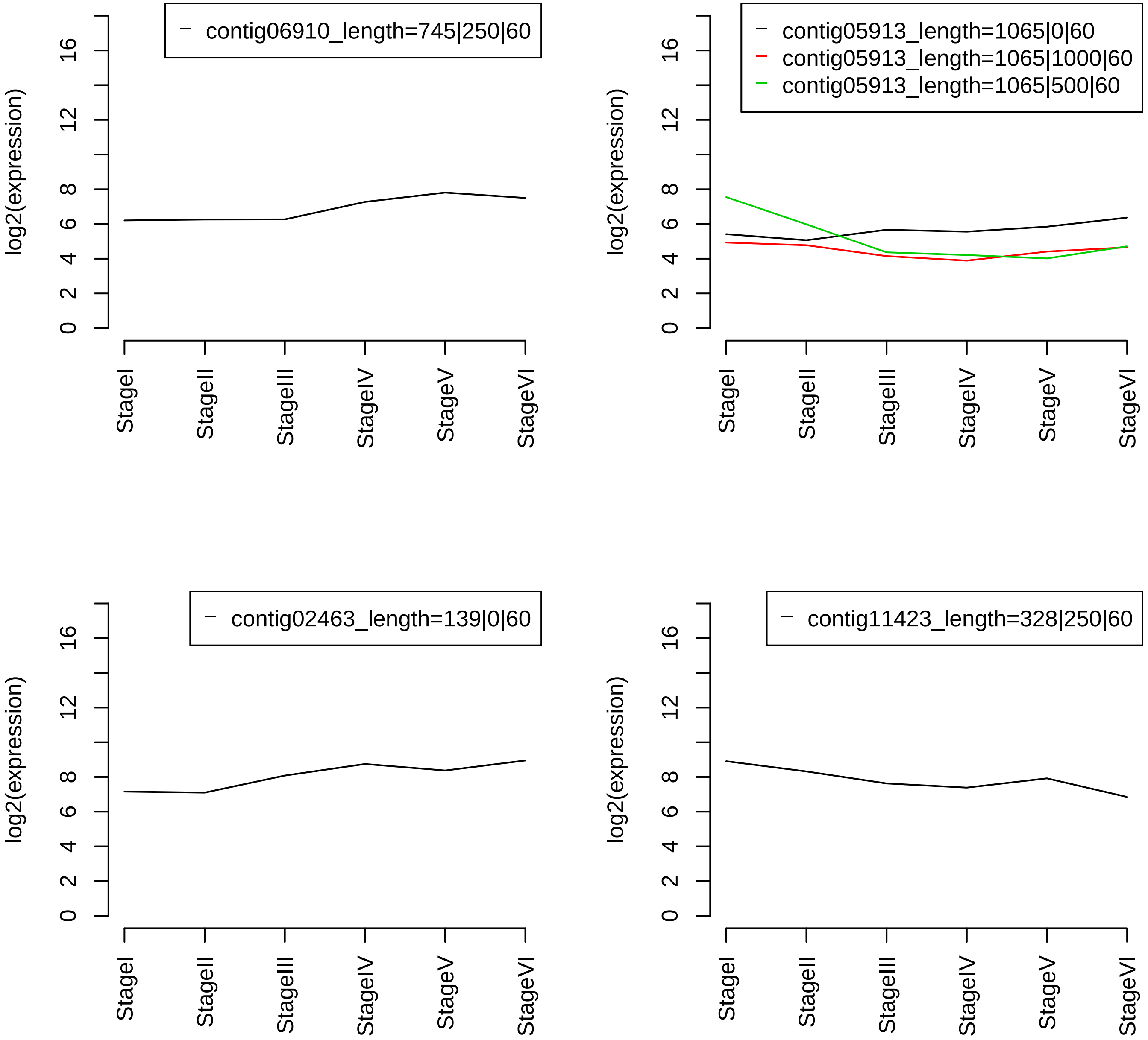
contig06910_length=745|250|60
contig05913_length=1065|0|60
contig05913_length=1065|1000|60
contig05913_length=1065|500|60
contig02463_length=139|0|60
contig11423_length=328|250|60
log2(expression)
0
2
4
6
8
12
16
StageI
StageII
StageIII
StageIV
StageV
StageVI

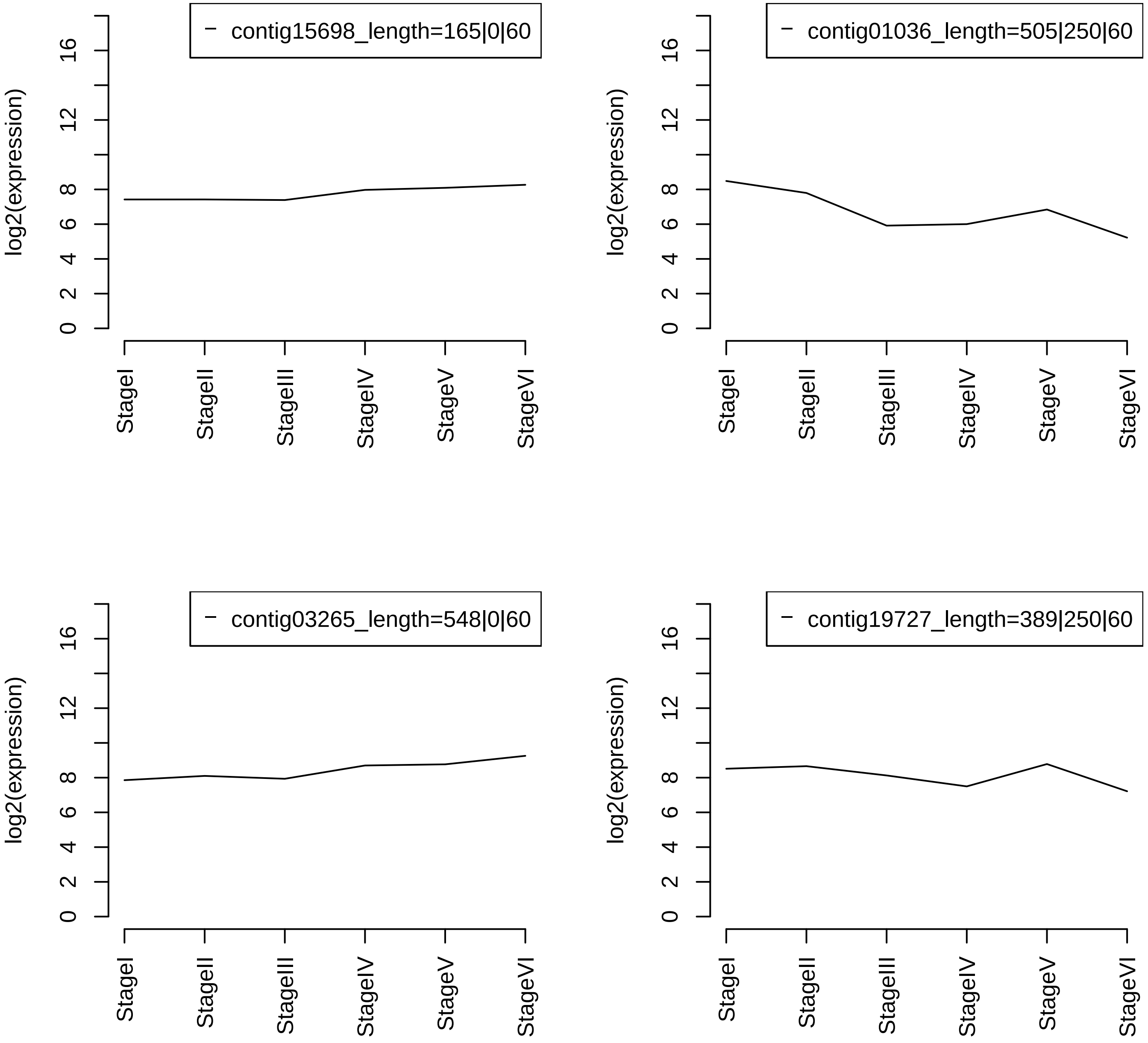
contig15698_length=165|0|60
contig01036_length=505|250|60
contig03265_length=548|0|60
contig19727_length=389|250|60
log2(expression)
0
2
4
6
8
12
16
StageI
StageII
StageIII
StageIV
StageV
StageVI

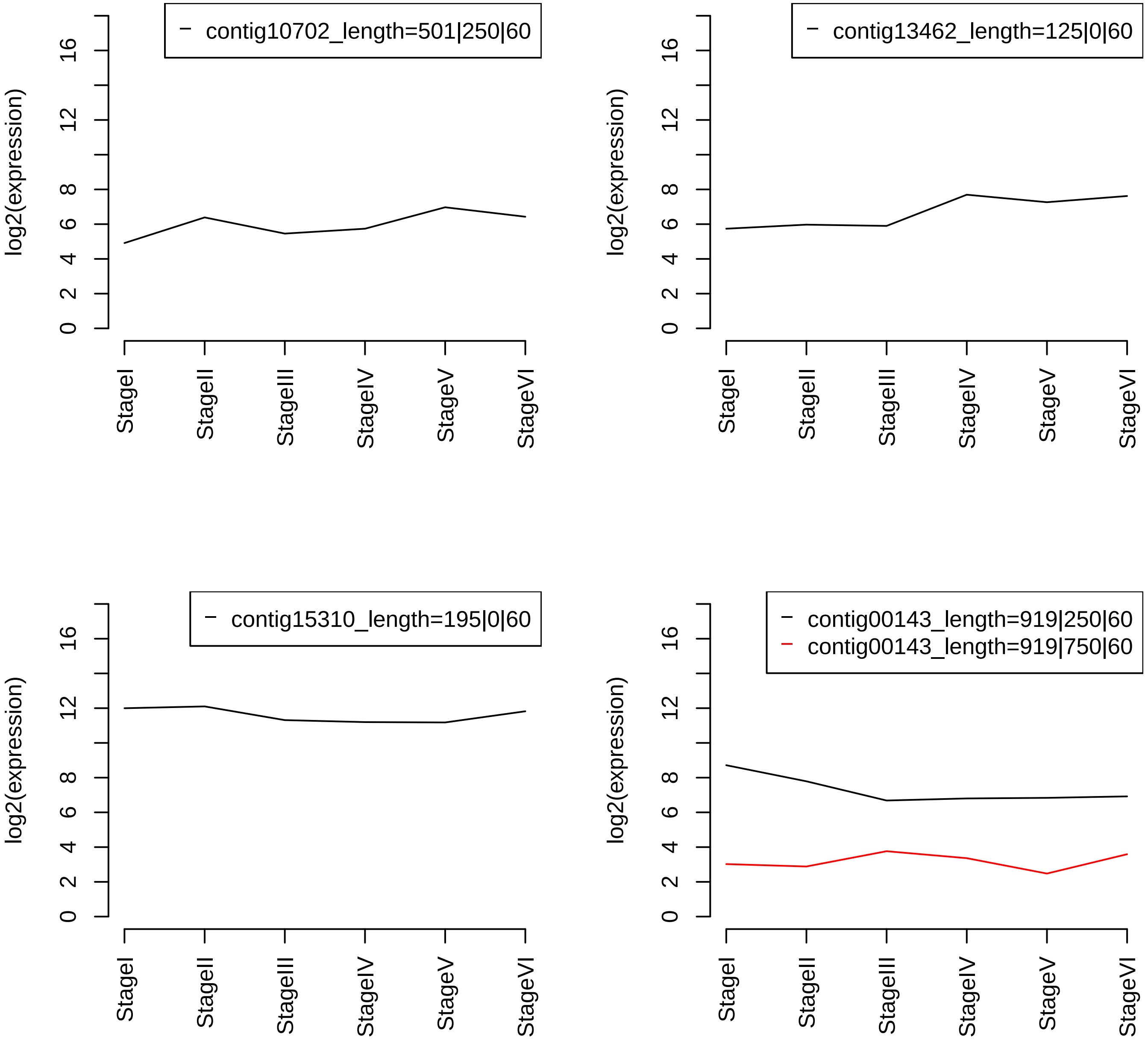
contig10702_length=501|250|60
contig13462_length=125|0|60
contig15310_length=195|0|60
contig00143_length=919|250|60
contig00143_length=919|750|60
log2(expression)
0
2
4
6
8
12
16
StageI
StageII
StageIII
StageIV
StageV
StageVI

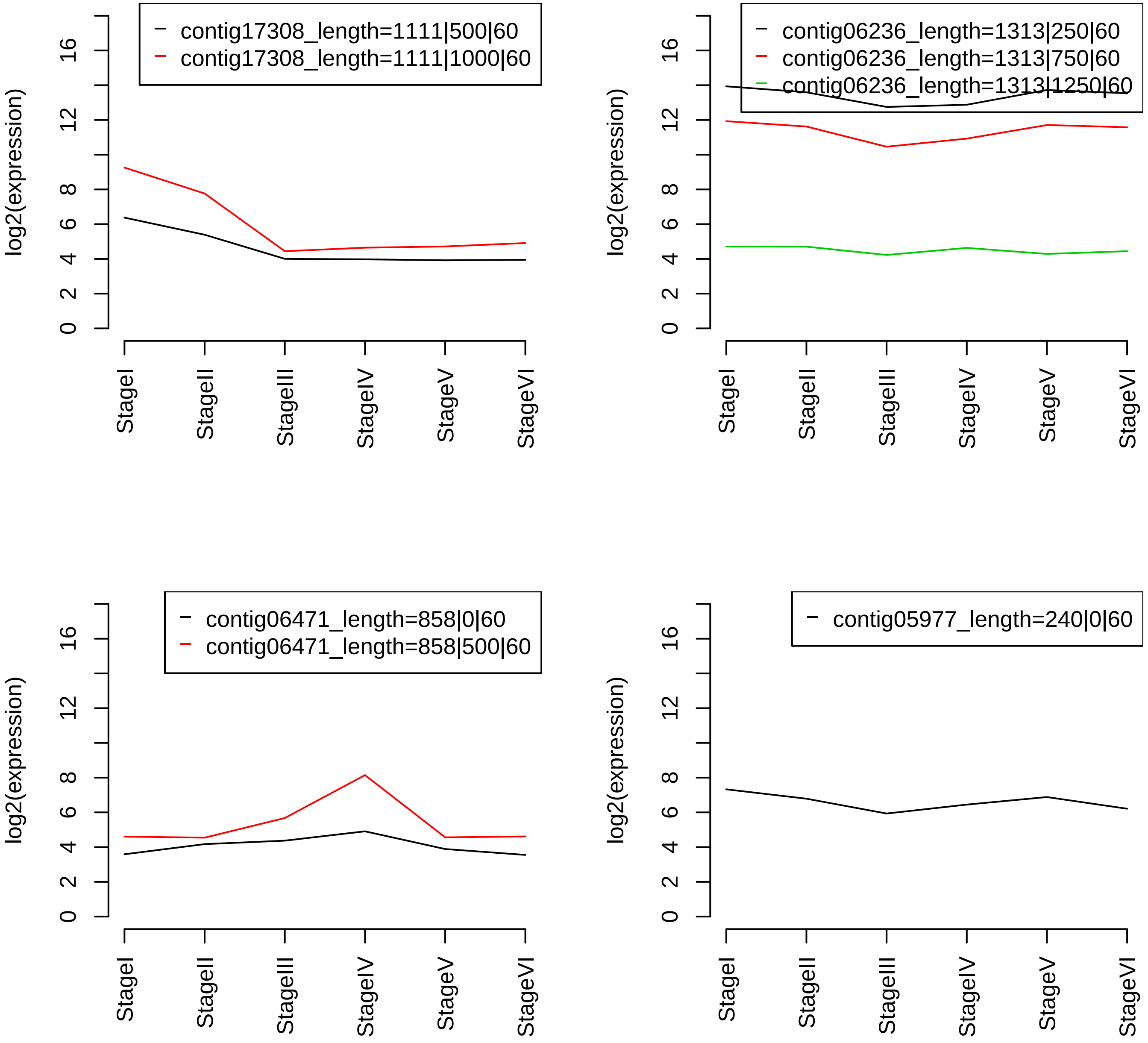

contig17308_length=1111|500|60
contig17308_length=1111|1000|60
contig06236_length=1313|250|60
contig06236_length=1313|750|60
contig06236_length=1313|1250|60
contig06471_length=858|0|60
contig06471_length=858|500|60
contig05977_length=240|0|60
log2(expression)
0 2 4 6 8 12 16
StageI StageII StageIII StageIV StageV StageVI

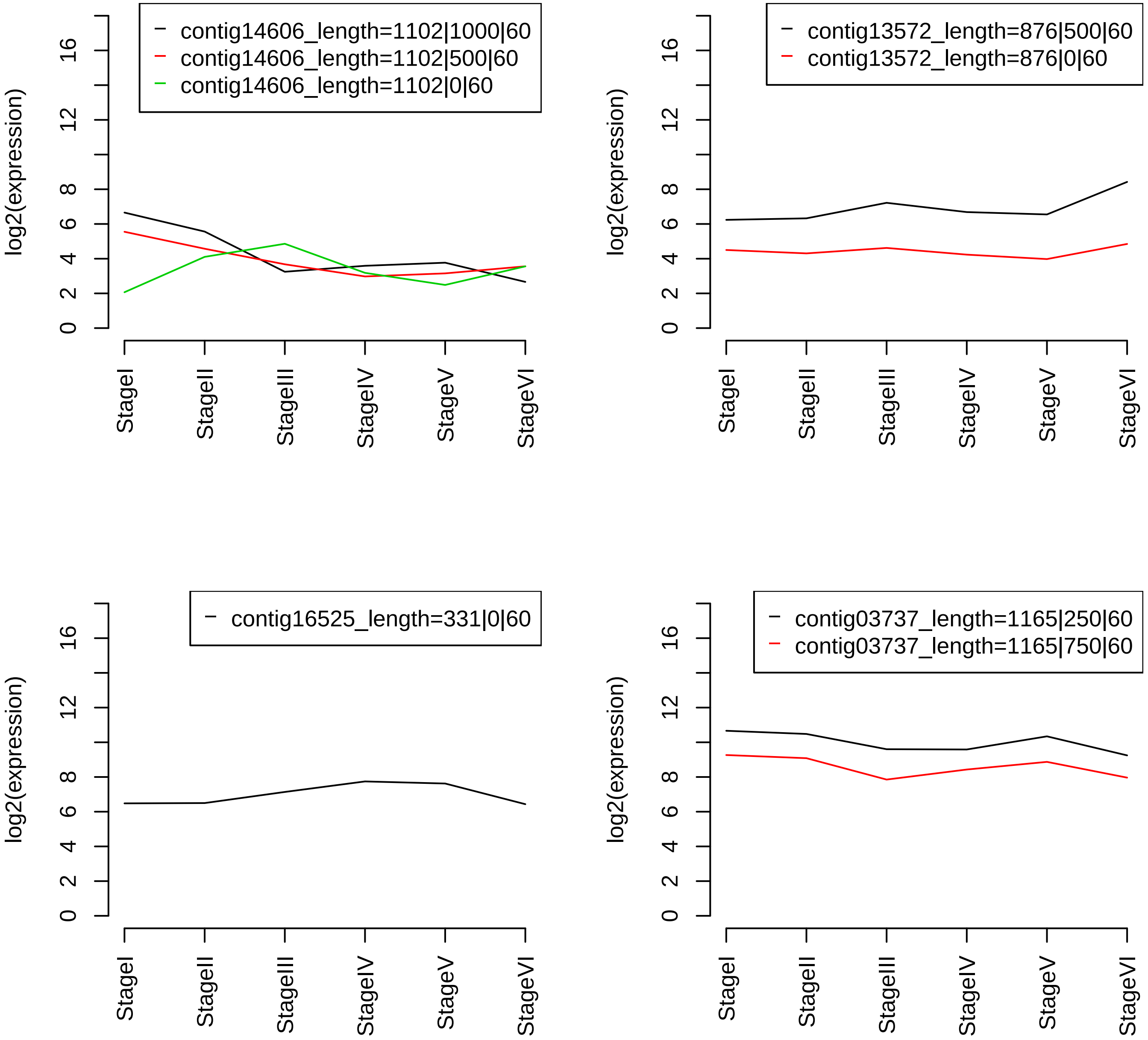
contig14606_length=1102|1000|60
contig14606_length=1102|500|60
contig14606_length=1102|0|60
contig13572_length=876|500|60
contig13572_length=876|0|60
contig16525_length=331|0|60
contig03737_length=1165|250|60
contig03737_length=1165|750|60
log2(expression)
0
2
4
6
8
12
16
StageI
StageII
StageIII
StageIV
StageV
StageVI

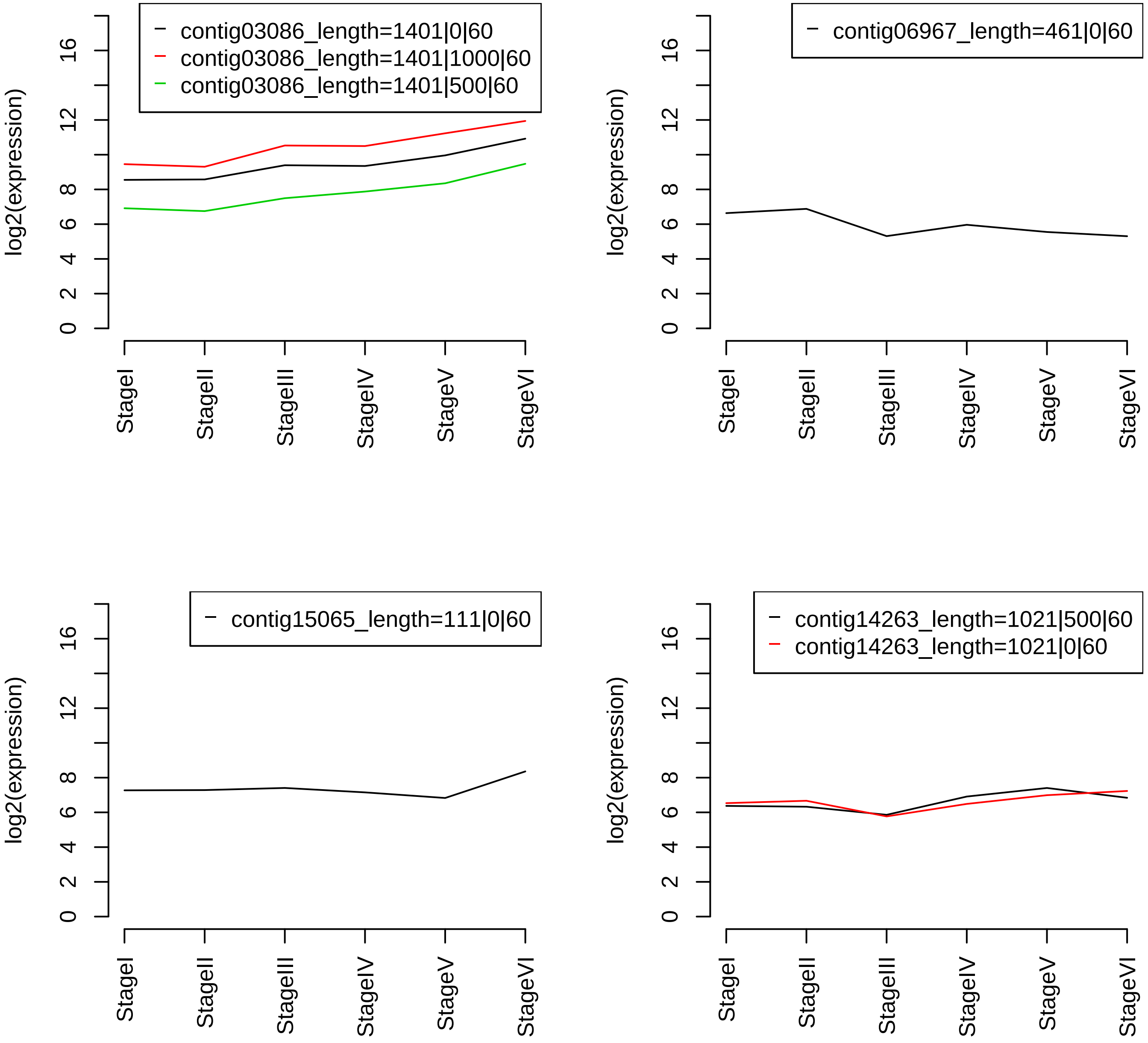

contig03086_length=1401|0|60
contig03086_length=1401|1000|60
contig03086_length=1401|500|60
contig06967_length=461|0|60
contig15065_length=111|0|60
contig14263_length=1021|500|60
contig14263_length=1021|0|60
log2(expression)
0
2
4
6
8
12
16
StageI
StageII
StageIII
StageIV
StageV
StageVI

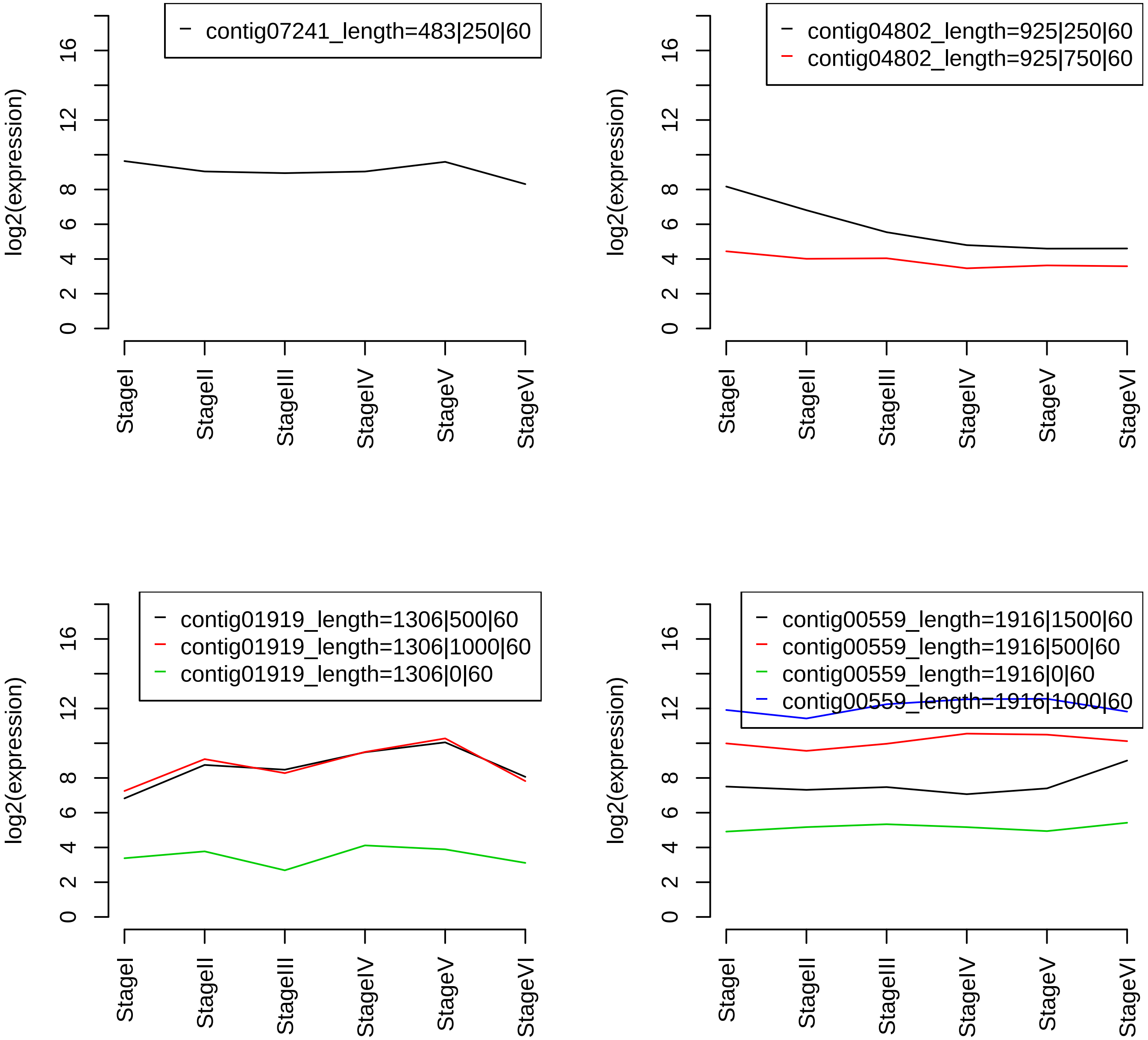
contig07241_length=483|250|60
contig04802_length=925|250|60
contig04802_length=925|750|60
contig01919_length=1306|500|60
contig01919_length=1306|1000|60
contig01919_length=1306|0|60
contig00559_length=1916|1500|60
contig00559_length=1916|500|60
contig00559_length=1916|0|60
contig00559_length=1916|1000|60
log2(expression)
0
2
4
6
8
12
16
StageI
StageII
StageIII
StageIV
StageV
StageVI

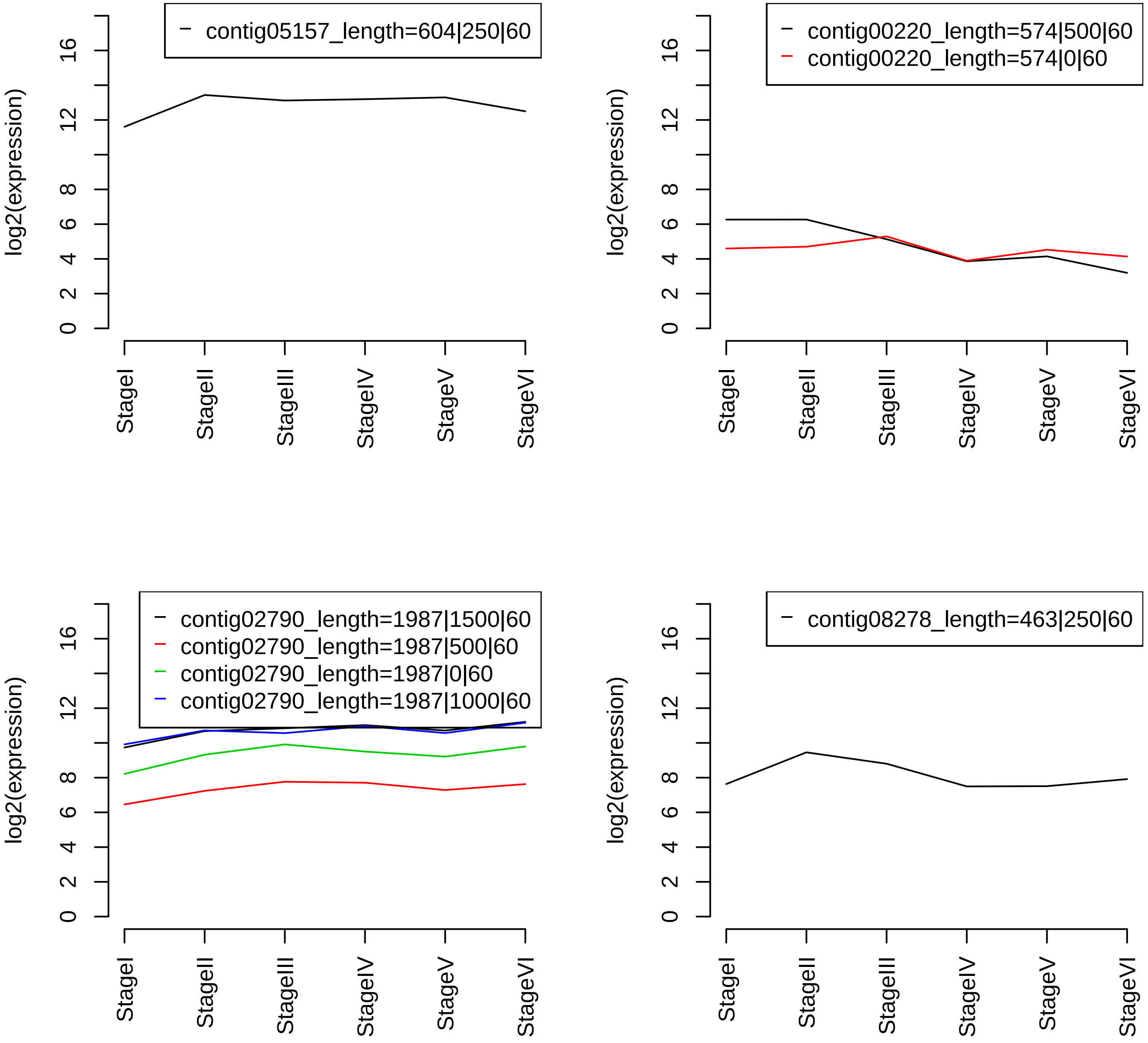

contig05157_length=604|250|60
contig00220_length=574|500|60
contig00220_length=574|0|60
contig02790_length=1987|1500|60
contig02790_length=1987|500|60
contig02790_length=1987|0|60
contig02790_length=1987|1000|60
contig08278_length=463|250|60
log2(expression)
0
2
4
6
8
12
16
StageI
StageII
StageIII
StageIV
StageV
StageVI

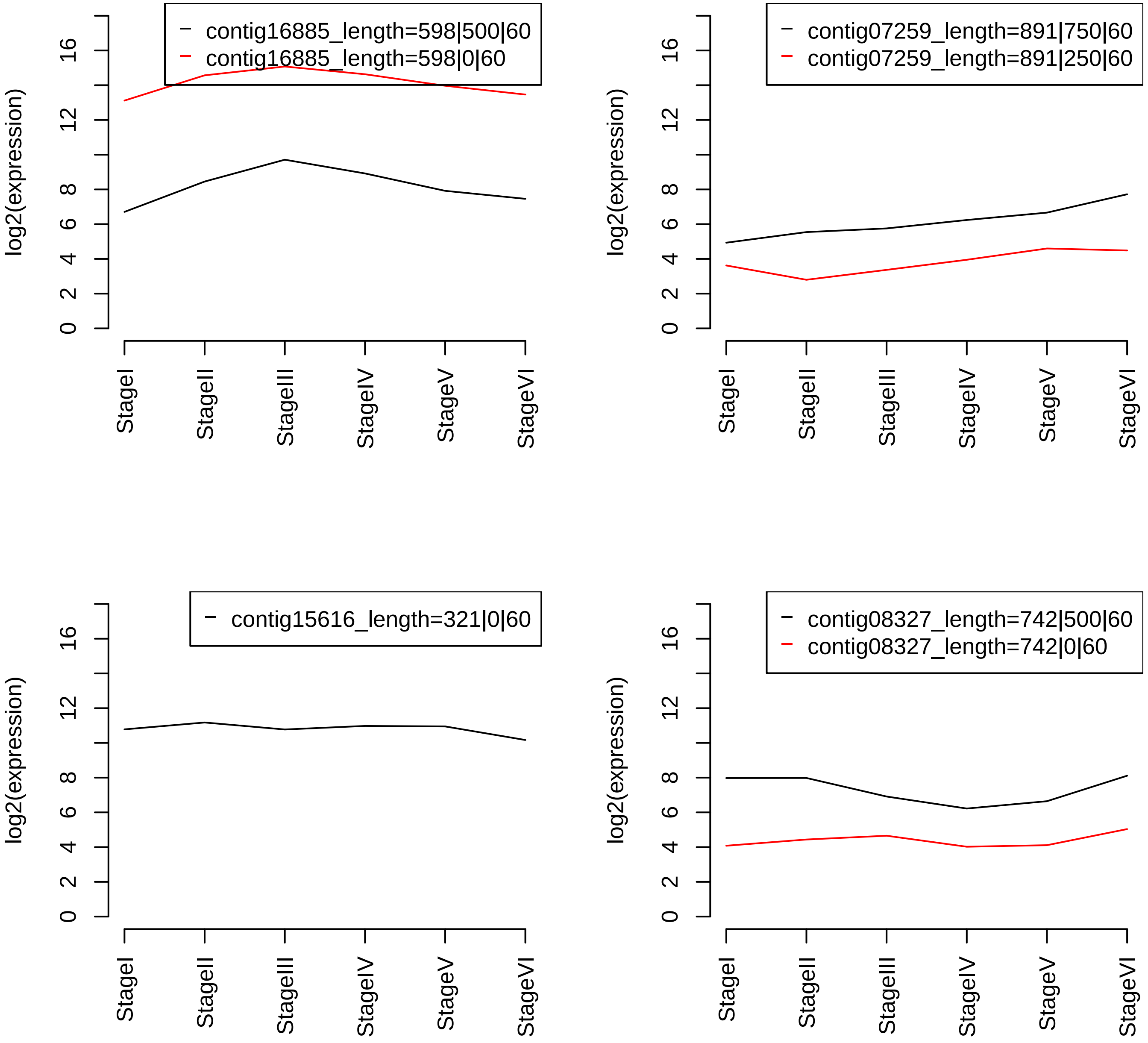

contig16885_length=598|500|60
contig16885_length=598|0|60
contig07259_length=891|750|60
contig07259_length=891|250|60
contig15616_length=321|0|60
contig08327_length=742|500|60
contig08327_length=742|0|60
log2(expression)
0
2
4
6
8
12
16
StageI
StageII
StageIII
StageIV
StageV
StageVI

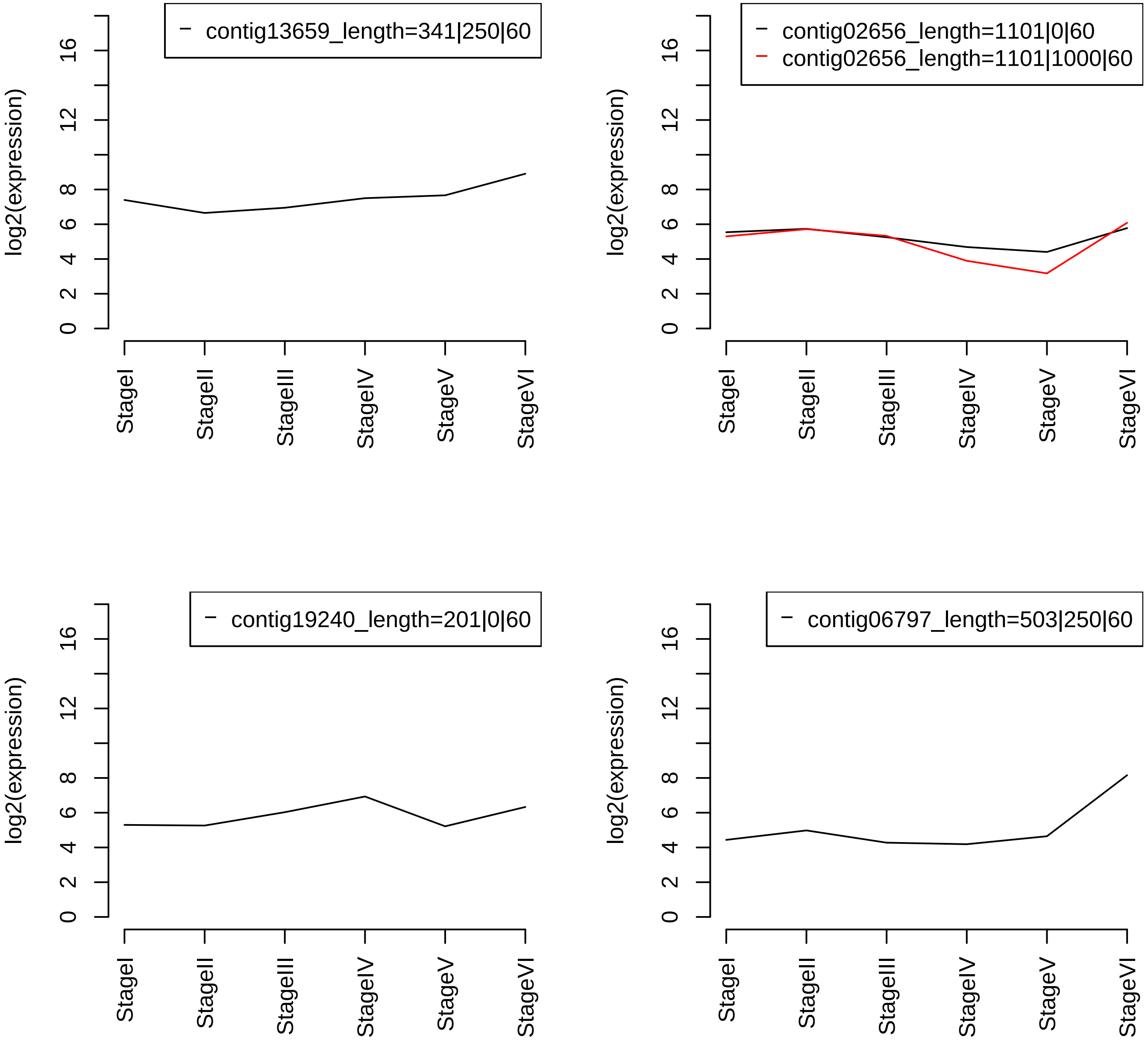
contig13659_length=341|250|60
contig02656_length=1101|0|60
contig02656_length=1101|1000|60
contig19240_length=201|0|60
contig06797_length=503|250|60
log2(expression)
0
2
4
6
8
12
16
StageI
StageII
StageIII
StageIV
StageV
StageVI

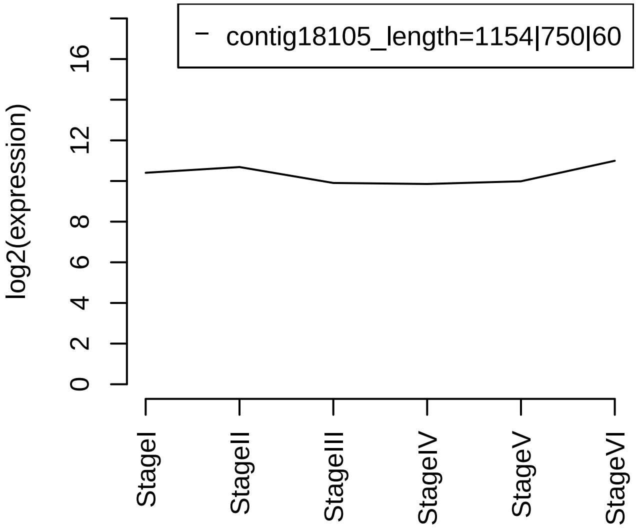
contig18105_length=1154|750|60
log2(expression)
0
2
4
6
8
12
16
StageI
StageII
StageIII
StageIV
StageV
StageVI

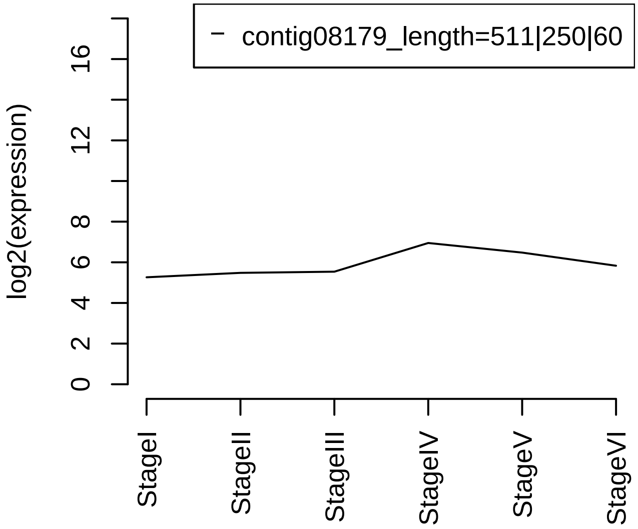
contig08179_length=511|250|60
log2(expression)
0
2
4
6
8
12
16
StageI
StageII
StageIII
StageIV
StageV
StageVI

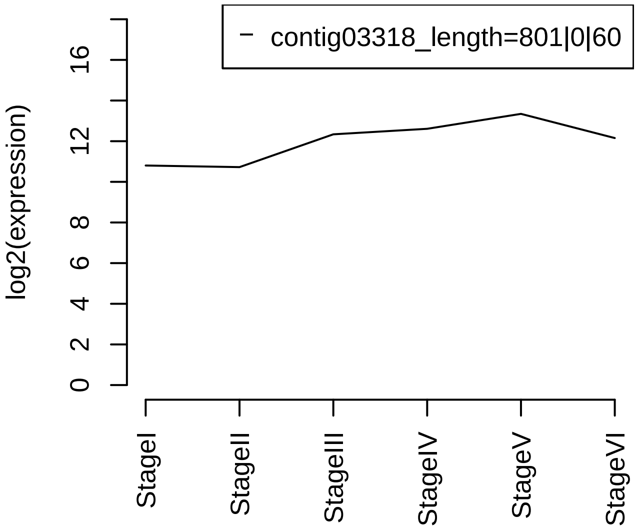
contig03318_length=801|0|60
log2(expression)
0
2
4
6
8
12
16
StageI
StageII
StageIII
StageIV
StageV
StageVI

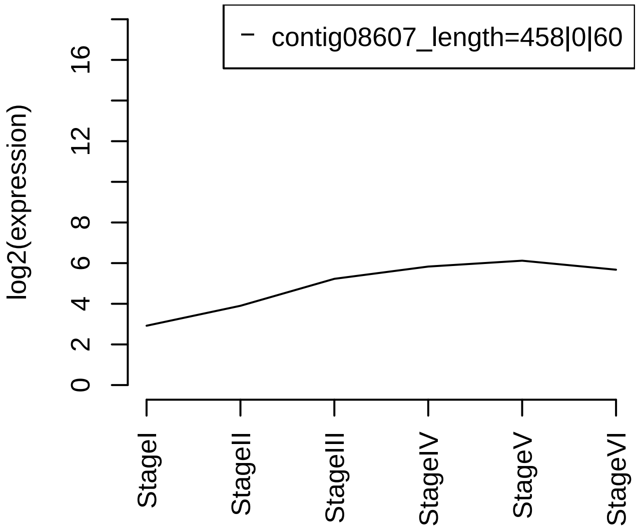
contig08607_length=458|0|60
log2(expression)
0
2
4
6
8
12
16
StageI
StageII
StageIII
StageIV
StageV
StageVI
